# Structural color in the bacterial domain: The ecogenomics of a 2-dimensional optical phenotype

**DOI:** 10.1073/pnas.2309757121

**Published:** 2024-07-11

**Authors:** Aldert Zomer, Colin J. Ingham, F. A. Bastiaan von Meijenfeldt, Álvaro Escobar Doncel, Gea T. van de Kerkhof, Raditijo Hamidjaja, Sanne Schouten, Lukas Schertel, Karin H. Müller, Laura Catón, Richard L. Hahnke, Henk Bolhuis, Silvia Vignolini, Bas E. Dutilh

**Affiliations:** ^a^Division of Infectious Diseases and Immunology, Utrecht University, Utrecht 3584 CL, the Netherlands; ^b^Hoekmine Besloten Vennootschap, Utrecht 3515 GJ, the Netherlands; ^c^Theoretical Biology and Bioinformatics, Department of Biology, Science for Life, Utrecht University, Utrecht 3584 CH, the Netherlands; ^d^Department of Marine Microbiology & Biogeochemistry, Royal Netherlands Institute for Sea Research, ‘t Horntje 1797 SZ, The Netherlands; ^e^Yusuf Hamied Department of Chemistry, University of Cambridge, Cambridge CB2 1EW, United Kingdom; ^f^Department of Physics, University of Fribourg, Fribourg CH-1700, Switzerland; ^g^Department of Physiology, Development and Neuroscience, Cambridge Advanced Imaging Centre, University of Cambridge, Cambridge CB2 3DY, United Kingdom; ^h^Leibniz Institute, German Collection of Microorganisms and Cell Cultures, Braunschweig 38124, Germany; ^i^Sustainable and Bio-inspired Materials, Max Planck Institute of Colloids and Interfaces, Potsdam 14476, Germany; ^j^Institute of Biodiversity, Faculty of Biological Sciences, Cluster of Excellence Balance of the Microverse, Friedrich Schiller University Jena, Jena 07743, Germany

**Keywords:** bacterial genomics, optics, photonic crystal, iridescence, living nanostructures

## Abstract

Structural color is an optical phenomenon resulting from light interacting with nanostructured materials. Although structural color (SC) is widespread in the tree of life, the underlying genetics and genomics are not well understood. Here, we collected and sequenced a set of 87 structurally colored bacterial isolates and 30 related strains lacking SC. Optical analysis of colonies indicated that diverse bacteria from at least two different phyla (*Bacteroidetes* and *Proteobacteria*) can create two-dimensional packing of cells capable of producing SC. A pan-genome-wide association approach was used to identify genes associated with SC. The biosynthesis of uroporphyrin and pterins, as well as carbohydrate utilization and metabolism, was found to be involved. Using this information, we constructed a classifier to predict SC directly from bacterial genome sequences and validated it by cultivating and scoring 100 strains that were not part of the training set. We predicted that SCr is widely distributed within gram-negative bacteria. Analysis of over 13,000 assembled metagenomes suggested that SC is nearly absent from most habitats associated with multicellular organisms except macroalgae and is abundant in marine waters and surface/air interfaces. This work provides a large-scale ecogenomics view of SC in bacteria and identifies microbial pathways and evolutionary relationships that underlie this optical phenomenon.

## Significance

Structural color (SC), created by light interacting with ordered nanostructures, is widespread in nature and has diverse functions: from display to camouflage, light harvesting, and photoprotection. While the interdisciplinary field of structural coloration in living organisms is growing, little is known about the genes involved and its evolution. Here, we focus on bacteria, cultivating new strains with vividly iridescent colonies. By comparing the genomes of structurally colored and nonstructurally colored colonies, we identified the genes responsible and developed a machine learning tool that predicts from sequence data where structurally colored bacteria may be found. Our investigation revealed widespread SC across environments including deep ocean waters and *Gammaproteobacteria* with similar optical structures as were previously identified in the *Flavobacteriia*.

Besides pigmentation, nature’s palette of colors is broadened through the so-called structural color (SC), stemming from nanostructures reflecting light at specific wavelengths and angles (1, 2). SC allows organisms to modify their appearance from striking displays of color to near invisibility (1–3). SC is common within the animal kingdom (in birds, cephalopods, shellfish and other marine invertebrates, insects, fish, arachnids, and a few mammals) in which it is involved in inter- and intraspecies interactions and camouflage (4–13) and in plants, where it is used for light management and signaling (14–18). In the case of unicellular organisms, SC is produced as a collective behavior. For example, SC has been observed in the slime mold *Myxomycetes* (19) and in the bacterial phylum *Bacteroidetes* (*Bacteroidota*), notably within the *Flavobacteriia*, from marine and littoral environments as well as brackish waters and soils (20–22). Microbial SC may be then considered as an optical phenotype, for example, playing a role in photoprotection, but it could also be a side effect of optimizing cellular organization for defense, intermicrobial competition, or nutrient uptake. It is fundamentally a population phenotype since it can only be achieved by colonies and not by individual cells.

In *Flavobacteriaceae,* the SC of *Cellulophaga lytica* and *Flavobacterium* IR1 colonies has been studied in detail and explained by the organization of the cells in a periodic hexagonal lattice (22, 23). While the optical response of such systems can vary in terms of angular dependence and intensity (not only from species to species but also depending on the growing condition of the colony), the physical principles defining the optical response in these types of structures allow them to be considered as a two-dimensional photonic crystal. In addition to the *Flavobacteriia*, several *Gammaproteobacteria* have shown a similar reflective, metallic, iridescent colony appearance that indicate the presence of SC, for example in *Pseudomonas aeruginosa* (24) SC was reported although the underlying optical structures have not been analyzed. It remains challenging to identify cases of SC in the literature, as the language used to describe the optical structures of bacterial colonies is often ambiguous and colony phenotypes are imprecisely addressed (25). For example, some *Listeria* colonies have frequently been described as iridescent (26), but it is not known whether *Listeria* colonies display a periodic organization of the cells or whether the iridescence is due to a periodic modulation in the surface of the colony as is the case for a diffraction grating (27). Therefore, it remains unclear how widely distributed bacterial SC is in the tree of life and in different environments, to what extent there is an evolutionary function, and whether the cellular organization mechanism is similar in different bacteria. To date, the only experimentally supported role for SC is indirect: Highly organized colonies of *Flavobacterium* IR1 appear to predate on other bacteria more effectively than disorganized colonies (28). This is in sharp contrast to bacterial pigments, which are known to have important ecological roles including light harvesting and photoprotection.

Our knowledge of the genetics and genomics of SC is surprisingly limited for such a striking effect. Within eukaryotes, only in butterflies have a small number of genes been identified controlling structural effects in wing patterning (29–34). Some of the *Flavobacteriia* offer an accessible genetic system to study SC (22), and 25 genes involved in a number of pathways have been identified by transposon mutagenesis in *Flavobacterium* IR1, demonstrating that gliding motility is important, but not essential, for the formation of SC (22, 35). In addition, other genes, such as those coding for tRNA modification enzymes, and many with no previously assigned function have been identified as relevant to SC in *Flavobacterium* IR1 (22).

SC implies a formidable capacity for cells to organize. A deeper understanding of SC in bacteria would therefore facilitate our understanding of the evolution and mechanisms behind SC. In addition, SC may form the basis of industrial processes to create sustainable colorants to replace conventional pigments.

Here, we have curated a collection of bacteria, largely gram-negatives, scored for the presence or absence of SC. SC was initially identified in colonies as metallic, angle-dependent, saturated color when illuminated with white light (22, 35) on an opaque background, where the SC is due to periodic organization of the cells in the entire volume of the colony. We excluded colonies displaying only iridescence due to surface grating structures, which are very common and can be formed by many disordered aggregates of bacteria (28), with a very poor correlation to function. The genome sequences of SC strains and non-SC strains were examined to identify pathways common to SC in gram-negative bacteria and create a computational tool that predicts SC from gene content. This predictive tool was used to find SC throughout the bacterial domain of life and in metagenomics datasets to identify likely SC-rich biomes.

## Materials and Methods

### Strains, Culturing, and Phenotypic Testing.

Unless stated otherwise, environmental samples from soil or freshwater sources, including *Flavobacterium* IR1, were cultivated on ASWBC or ASWBLow agar plates (22) at 20 °C with 10 g/L sea salts (Sel Marin, Portugal). Samples of marine origin were cultivated on Rich Marine (RMAR) plates [10 g/L peptone, 4 g/L yeast extract (both Sigma, NL), 30 g/L, sea salts, 200 mg/L nigrosine (Sigma, NL), and 10 g/L agar (Life Technologies, NL)], unless stated otherwise. The strains isolated in this study and optimal growth conditions are listed in *SI Appendix*, Table S1. Screening for new structurally colored isolates was performed on the same media, plating dilutions of the source material and incubating from 3 to 10 d at 20 °C. Microcolonies showing angle-dependent coloration, suggestive of SC, were tested under multiple growth conditions on agar plate (supplemented with nigrosine for optical contrast) before scoring for SC, as described in *SI Appendix*, Supplementary Methods. Swimming of marine bacteria was tested by bacterial spreading within 0.2% (w/v) RMAR agar plates (36).

### Microscopy and Optical Testing.

Surface motility was judged by direct observation of the colony edge by microscopy and observing spread over several days, with visualization of SC when necessary using side illumination with a 50 W white light-emitting diode (LED) lamp, as previously described (22). Optical analysis of angle-dependent spectra was obtained using a custom-built goniometer (37, 38), and cryo-scanning electron microscopy (cryo-SEM) was used to understand the geometry of the SC colonies, both as described in *SI Appendix*, Supplementary Methods.

### Genome Analysis: Pan-GWAS Approach and Phylogeny.

Illumina NextSeq genome sequencing, assembly, quality assessment, genome annotation, and ortholog inference were performed as described in *SI Appendix*, Supplementary Methods. A pan-genome wide association study (pan-GWAS) was performed using Scoary (39), and functional enrichment analysis was performed using STRING (40). Phylogenetic trees were constructed using RaxML 8.2.4 (41) and visualized using iTOL (42).

### Identification of Genes Related to SC Using Transposon Mutagenesis.

Genes inferred to be involved in SC were identified by transposon mutagenesis of *Flavobacterium* IR1 (22). This dataset included those previously identified (22, 43) and six additional genes identified in a subsequent round of random transposon mutagenesis and screening (*SI Appendix*, Table S2).

### Machine Learning Model Construction.

Proteins associated with SC were extracted from the respective genomes and used to build hidden Markov models (HMMs) using HMMer 3.1b2 (http://hmmer.org/). Presence–absence data of the HMM profiles were used as input for randomForest (44). A script in bash was constructed that automates the HMM profile searches and predicts SC using the random forest (RF) function. The script and the associated RF model are available on GitHub (https://github.com/aldertzomer/structuralcolor). An online version of the prediction method is available at http://klif.uu.nl/structuralcolorweb/.

### Public Genome and Metagenome Classification.

All bacterial genomes with a valid species annotation (240,981 in total) were downloaded from the PATRIC genome database (45) on 14 November 2019. Assembled metagenomes (13,873 unique assemblies) and associated metadata were obtained from MGnify (46) on 3 July 2019. In addition, the metagenomes from sinking particulate organic matter (POM) (47) were downloaded from the Sequence Read Archive and individually assembled with MEGAHIT v1.2.9 (48). Proteins were predicted on all genomes and metagenomes using Prodigal v.2.60 (49) using either default settings or the metagenome settings, respectively.

## Results

### Selection of SC and Non-SC Bacterial Strains.

**Fig. 1.**
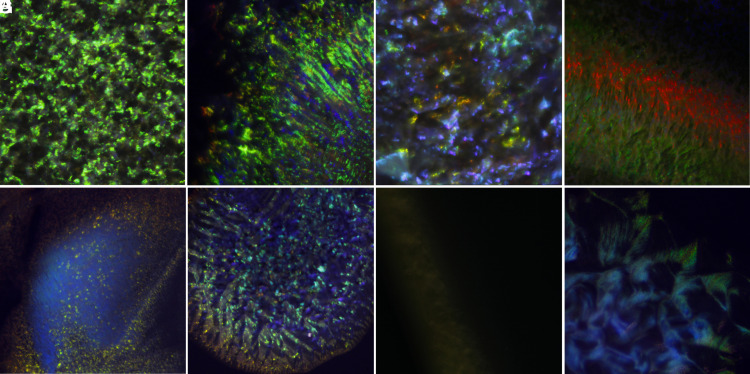
Examples of colonies from selected strains contributing genomes to the SC classifier. Each panel shows a 2 × 2 mm area of a colony on nigrosine containing agar, illuminated at the optimal angle. (*A*) *Flavobacterium* IR1; (*B*) *Cellulophaga lytica* HM-52; (*C*) *Cellulophaga fucicola* strain HM-74; (*D*) *M. algicola* HM-28; (*E*) *Muricauda ruestringensis* HM-37; (*F*) *Tenacibaculum gallaicum* HM-45; (*G*) *Flavobacterium succinicans* DD5b, this strain does not show SC and is less reflective than the other strains in this figure that all display SC; (*H*) *Virgibacillus dokdonensis* HM-38.

To sequence and compare the genomes of SC and non-SC bacterial strains, we created a collection of bacteria showing SC by screening environmental samples on agar plates and scoring for SC or sourcing strains from microbial culture collections (*SI Appendix*, Tables S1 and S3). Strains were cultivated on plates containing nigrosine to enhance optical contrast and therefore the detection of SC (*Materials and Methods* and *SI Appendix*, Table S1). SC was considered to be present in a colony if metallic, angle-dependent, saturated color was visible upon illumination with a broad spectrum white LED (Fig. 1). A full spectrum of SCs was obtained within the collection. Strains showing SC contained a complex mix of pointillistic color when viewed under an optical microscope in dark-field configuration (Fig. 1) and showed iridescence, i.e., change of color when viewed from different angles (*SI Appendix*, Fig. S1). SC was confirmed by mechanical disruption of colonies, demonstrating that such mixing reduced or eliminated SC. The collection was supplemented with strains from the same taxonomic order as those showing SC, but which did not show SC under any growth condition. It was notable that the most intense SC was found in *Flavobacteriia*, and when mechanically disrupted on an agar plate, these gliding bacteria could reform SC rapidly, over a period of 10 to 30 min. SC was generally duller of isolates outside the class *Flavobacteriia*, although there were exceptions within the *Gammaproteobacteria,* notably *Marinobacter algicola* HM-28 (Fig. 1*D* and *SI Appendix*, Fig. S1).

### Identification of Genes Associated with Bacterial SC Using a Pan-GWAS Approach.

**Fig. 2.**
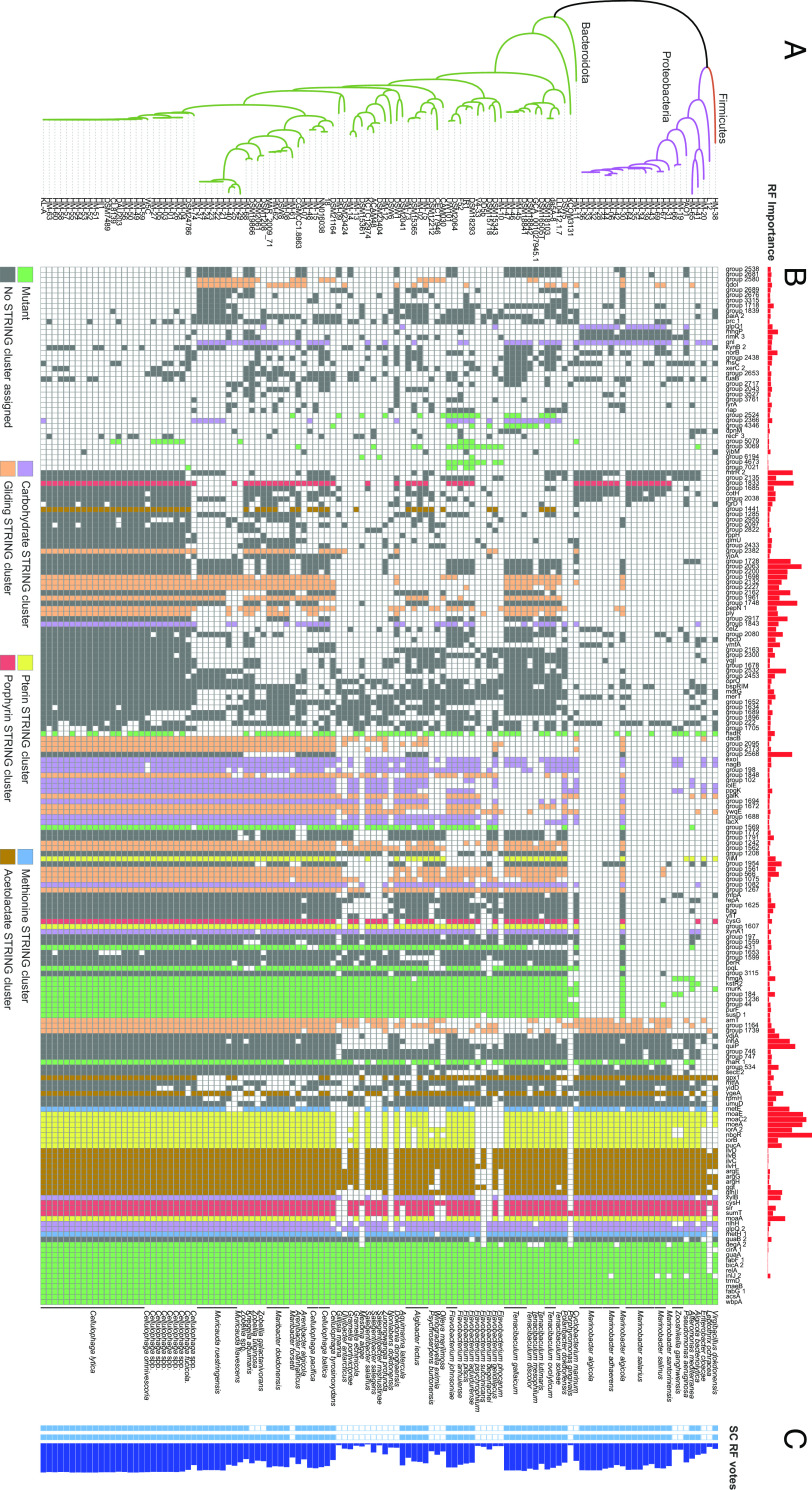
Genes identified by pan-GWAS and mutagenesis (*A*) Phylogenetic tree of the 16S ribosomal RNA gene, showing 117 strains included in this study segregated by phylum (*SI Appendix*, Table S1). (*B*) Gene presence/absence matrix of 199 proteins associated with SC based on both the pan-GWAS and earlier knockout studies (22, 43). Proteins are clustered using Ward’s method. Proteins in green were found using transposon mutagenesis. Proteins from the pan-GWAS analysis were clustered by STRING (*SI Appendix*, Fig. S3), and their clusters are indicated using the color legend displayed below. The importance of each gene for predicting SC in the RF model is given as vertical red bars at the *Top* of each cluster (Gini importance score). (*C*) The SC phenotype is displayed in the two columns in light blue, with the first bar displaying the initial RF model input and the second column the final RF model input. The horizontal blue bars labeled “RF votes” display the fraction of decision trees in the second corrected RF model supporting classification as a strain with SC. Source data for this figure can be found in Dataset S1.

We hypothesized that SC may be genetically determined. To compare the genetic content of non-SC and SC bacterial strains, we selected 69 strains from our own collection (both with and without SC) and sequenced their genomes. Additionally, 48 genomes were selected from GenBank (50), from isolated strains that had previously been shown to display SC. Of the 117 genomes, 86 were initially determined to have SC either by the authors or from literature data, 33 strains from the same genera did not. Their characteristics are listed in *SI Appendix*, Table S1. Our genome set included 93 strains from the phylum *Bacteroidetes* (*Bacteroidota*), 23 from the phylum *Proteobacteria* (*Pseudomonadota*), and one from the phylum *Firmicutes* (*Bacillota)* and may therefore be biased toward *Bacteroidetes*. A phylogenetic tree of the isolates is shown in Fig. 2*A*. Based on the pan-genome of the 117 isolates, an ortholog table of 29,850 protein-coding genes was constructed using Roary (51), revealing a relevant gene set of 366 orthologs. To associate specific orthologs with SC, we used a pan-GWAS approach on the ortholog table using Scoary (39). We found a total of 199 orthologs associated with the SC phenotype (Dataset S1); 31 were detected using the Fisher exact method after Bonferroni correction, 100 using the permutation method, 79 using the phylogenetically informed pairwise comparison test in Scoary, and 31 from mutagenesis (22, 43). A complete list of the proteins and their presence and absence can be found in Dataset S1. Interestingly, some of the SC-associated orthologs that are common in the *Bacteroidetes* are shared by a member of *Proteobacteria* (*M. algicola* HM-30, Fig. 2). We hypothesize that at least some of these genes were transferred from *Bacteroidetes* as the concatenated sequence of 17 of the orthologs from *M. algicola* HM-30 have a high sequence similarity to *Muricauda ruestringensis* HM-25, a member of the phylum *Bacteroidetes* (*SI Appendix*, Fig. S2), although contamination of the isolate cannot be ruled out until the genome sequence is closed.

### Functional Annotation of Phylum Crossing Marker Genes Predicts Key Processes Involved in Bacterial SC.

To predict functional associations between the genes, most of which were annotated as hypothetical proteins, we uploaded the protein sequences of the orthologs selected by the pan-GWAS approach to the STRING database, which integrates diverse sources of evidence for functional interactions between proteins (30). We detected six large clusters of proteins, which were clustered by their closeness in the STRING graph but not always the genomic organization of their genes, which we named after the functions that were encoded by some of their members (Fig. 2*B* and *SI Appendix*, Fig. S3). These were clusters associated with pterin, porphyrin, carbohydrate, methionine, acetolactate biosynthesis, and gliding motility. The latter has previously been shown to facilitate the formation of SC in *Flavobacteriia* (22, 35), the other categories have not been previously associated with bacterial SC.

Some of the identified clusters of proteins were linked to SC in other organisms. Notably, genes encoding proteins that had the highest Gini importance for predicting the SC phenotype in the RF model were those linked to pterin metabolism. Pterins are widespread cofactors that have previously been shown to play a role in SC in insects and crustaceans (52–54), affecting light scattering and selective absorption in the wing scales of pierid butterflies in which they are present as granules, which increase light reflection and amplify iridescent ultraviolet signaling (55–57). In bacteria, pteridine molecules act as enzymatic cofactors and they produce various pigments (58). Pterins have been implicated in phenotypes related to UV protection and phototaxis in *Cyanobacteria* (58), accumulate in some photosynthetic bacteria when they are exposed to light (59) and some can form structurally reflective biomaterials, e.g., in the shrimp eye (60). Although speculative, a link between SC and pterin metabolism might indicate that pterins could enhance the optical response of the colonies. This could be through modulating the refractive index contrasts that are needed for SC (61), or pterin-derived pigments could absorb randomly scattered light, increasing the visibility of the colors, as pigments can do for other organisms displaying SC (62). Alternatively, rather than a direct optical mechanism, pterins could be involved indirectly through the organization of the cells, as pteridines can act as sensors of environmental stress, and are involved in changes in multicellular organization, such as biofilms (58).

The porphyrin cluster includes uroporphyrin biosynthesis proteins. Porphyrins strongly absorb light, which is then converted to energy and heat in the illuminated areas, and they are responsible for the pigmentation of bird eggs (63) and certain bird feathers (64). In bacteria, accumulation of porphyrins causes photosensitivity. In a *vis*A mutant of *Escherichia coli*, accumulation of protoporphyrin IX and subsequent exposure to visible light produces reactive oxygen species that are harmful (65). In addition, porphyrins have been used in artificial systems to create SC (66). Bacteria observed to have SC frequently live in environments exposed to light, such as in air–water interfaces in tidal flats (20, 21). Porphyrins may function as photoprotectants or light-sensitive switches, although any direct role in bacterial SC has not been previously demonstrated (67). Similarly, as for pterins, the link between porphyrin pigmentation and bacterial SC could be an indication that these compounds are involved in the optical response of the colonies.

The presence of a shared carbohydrate cluster is in agreement with observations from *Flavobacterium* IR1 that iridescence induction depends on the selected carbon source, particularly algal polysaccharides such as fucoidan and *k*-carrageenan (22, 43). The organization of the *Flavobacterium* IR1 colony is strongly regulated by cultivation on fucoidan, and transposon knockouts have implicated a specific polymer utilization locus in mediating both the uptake and metabolism of fucoidan and linking this process to the SC displayed by the colony (43). In addition, methionine and acetolactate clusters are part of the gene set and have links to amino acid metabolism, the latter being a precursor in the synthesis of branched-chain amino acids (68). A link with the stringent response, the response to amino acid starvation, may be possible, in which both acetolactate and branched-chain amino acid metabolism are relevant. In support of this hypothesis, we observed that a *spo*T mutant, this gene being responsible for regulation of the stringent response, lacks SC in *Flavobacterium* IR1 (22).

In addition, we identified the *qui*P gene as relevant for SC, which encodes an acyl-homoserine lactone acetylase (Fig. 2 and Dataset S1) (69). This is an indication of any involvement of quorum sensing in the formation of SC colonies and may help explain how colonies organize so effectively. This is interesting given that the “metallic/iridescent” phenotype of *P. aeruginosa* colonies appears complicated and has been suggested to be related both to SC (25) and to quorum sensing (70). As we show in *SI Appendix*, Fig. S4, *P. aeruginosa* does indeed appear to show SC, growing as colonies with punctuate, bright focal points of color that are lost by mechanically disrupting the ordering of cells within a colony. Finally, some of the strongest predictive genes had no assigned function, suggesting that SC involves currently unknown pathways.

### Prediction and Confirmation of Structurally Colored Strains Using Machine Learning.

**Fig. 3.**
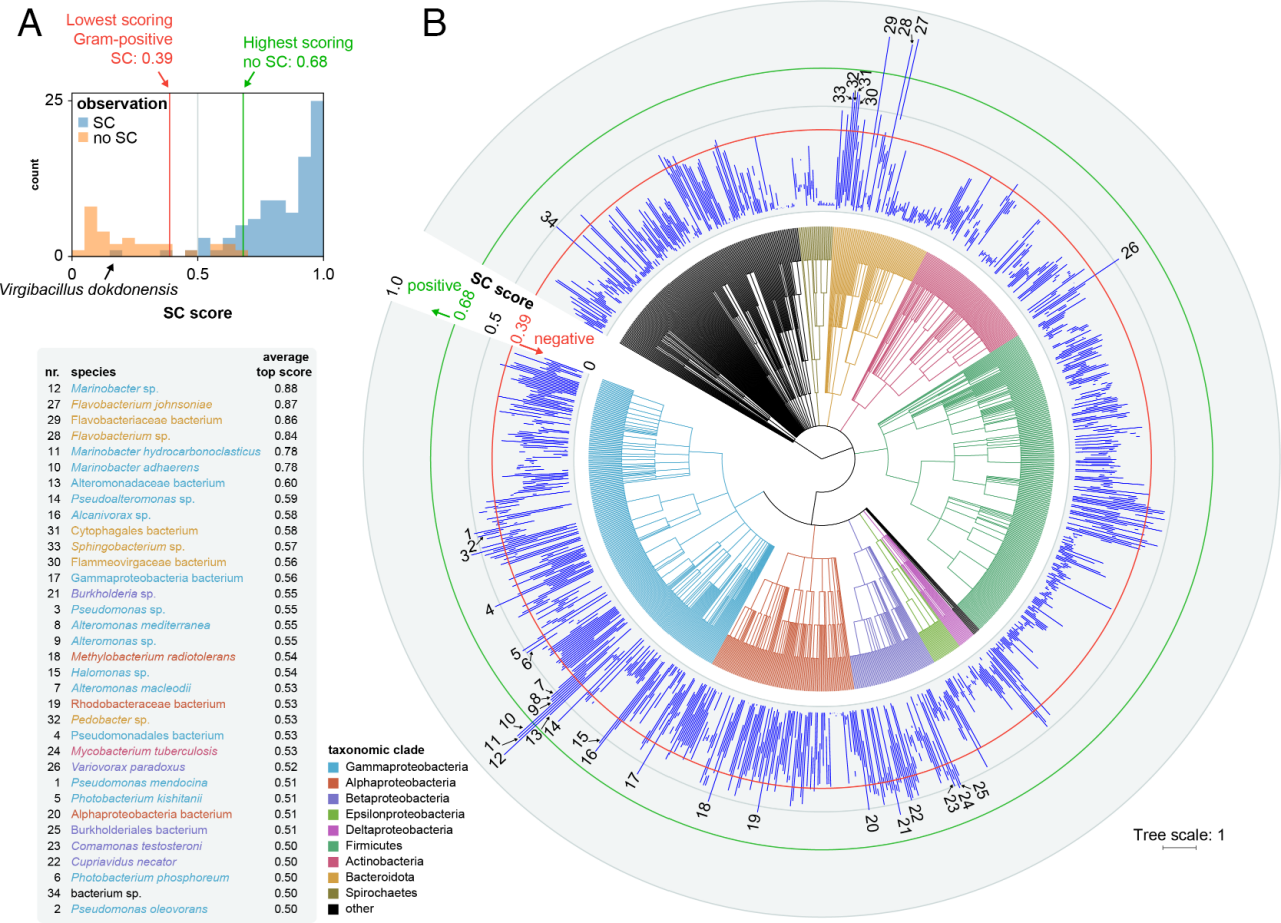
Range of SC scores per species as predicted using the RF model. (*A*) Distribution of 117 SC scores in the training set. The single structurally colored gram-positive bacterium *Virgibacillus dokdonensis* has a low classifier score. (*B*) SC was predicted for 240,981 bacterial genomes from the PATRIC database. Species with at least 10 genomes are displayed (1,120 species) as incidental high scores may not be genuine and these need to be investigated experimentally (Dataset S2). Both ends of each blue line indicate the mean of the five minimum and maximum SC scores (min = 0.03, max = 0.88). The tree indicates the NCBI taxonomy as annotated by PATRIC. High-scoring species are indicated by numbers. Colored rings show the relevant boundaries of the training set in panel a for reference. For details, see Datasets S2 and S3.

Profile HMMs were constructed from the sequence alignments of the 199 orthologs that were associated with the SC phenotype. A machine learning model was constructed using RF (71) with the 117 bacterial genomes as the training set, in which 2/3 of the data were used for training and 1/3 for testing the RF model. An out-of-bag prediction error of 6.8% was observed, suggesting that the model is indeed capable of predicting SC from genome sequences. Unexpectedly, five genomes that were considered SC-negative in the past displayed a score above 0.6 in the classifier. These included *P. aeruginosa* and four *Bacteroidetes*, including *Cyclobacterium marinum*, *Zobellia galactanivorans*, *Zobellia uliginosa*, and *Kriegella aquimaris*. These strains were tested for SC. In *P. aeruginosa*, metallic, angle-dependent, color was observed that was lost when the colony was mixed with a loop, suggesting SC (*SI Appendix*, Fig. S4 *A*–*C*). The *Bacteroidetes* strains similarly revealed similar SC. An updated RF model adding these five strains to the positive set was constructed with an out-of-bag error of 3%. All strains from the training dataset with an RF score above 0.68 display SC, while none of the gram-negative strains with scores below 0.39 display SC (Fig. 3*A*).

The SC classifier was validated by scoring a collection of strains for SC that were not part of the initial classifier group, followed by in vitro cultivation and determining SC (see Table 1 and discussion below). Based on these observations, we calculated the area under the curve (AUC) of the output of the model versus their SC phenotype. The overall AUC was 0.91 for gram-negative bacteria (n = 93), which includes 55 *Bacteroidota* (AUC = 0.92) and 38 *Proteobacteria* (AUC = 0.90). Performance on the seven gram-positive strains was low with scores all below 0.25 and a nonsignificant AUC. Taken together, this suggests good predictive accuracy for the two phyla that comprise most of the strains found that show SC.

**Table 1.** Validation strains

Taxa	Habitat	Score	SC	Src	Species	Habitat	Score	SC	Src
***Bacteroides***					***Proteobacteria***				
***Bacteroidea***					***Alphaproteobacteria***				
*Lentimicrobium saccharophilum*	Bioreactor	0.23	N	P	*Agrobacterium species*	Freshwater Plant	0.41	Y	73
***Chitinophagia***					*Hoeflea alexandrii*	Littoral	0.48	Y	T
*Chitinophaga filiformis*	Soil	0.60	Y	P	*Hoeflea sp*	Littoral	0.48	Y	T
*Sediminibacterium goheungense*	Fresh Water	0.37	N	P	*Rhodobacter capsulatus*	Freshwater	0.26	N	P
*Arachidicoccus ginsenosidivorans*	Soil	0.25	N	P	*Rhodobacter sp.*	Freshwater	0.28	N	P
*Hydrotalea sandarakina*	Hot Spring	0.29	Y	P	*Rhodobacter sp.*	Freshwater	0.28	N	P
*Hydrotalea sandarakina*	Hot Spring	0.29	Y	P	*Rhodospirillum rubrum*	Brackish Water	0.03	N	P
*Arachidicoccus sp*	Soil	0.21		T	*Sphingomonas*	Littoral	0.42	Y	T
***Flavobacteriia***					*Sphingomonas herbicidivorans*	Soil	0.42	Y	P
*C. marinum*	Littoral	0.60	Y	T	*Sphingomonas pruni*	Soil	0.42	Y	T
*Zobellia galactanivorans*	Red algae	0.61	Y	R	*Sulfitobacter porphyrae*	Littoral	0.43	Y	T
*Z. uliginosa*	Seawater	0.59	Y	R	***Betaproteobacteria***				
*Flavobacterium psychrophilum*	Fish pathogen	0.04	Y	P	*Acidovorax delafieldii*	Soil	0.51	Y	P
*Flavobacterium psychrophilum*	Fish pathogen	0.03	Y	P	*Chitinimonas koreensis*	Soil	0.38	Y	P
*Arenibacter catalasegens*	Littoral	0.78	Y	T	*Cupriavidus basilensis*	Fixed bed reactor	0.57	Y	(72)
*Tenacibaculum gallaicum*	Marine	0.96	Y	T	*Dechloromonas sp*	Mine	0.37	Y	P
*Flavobacterium anhuiense*	Soil	0.85	Y	T	*Janthinobacterium svalbardensis*	Glacier	0.49	Y	P
*Tenacibaculum lutimaris*	Littoral	0.75	Y	P	*Paraburkholderia madseniana*	Soil	0.56	Y	P
*Algibacter pectinovorans*	Marine	0.58	Y	T	*Pseudogulbenkiania sp*	Freshwater sediment	0.30	Y	P
*Winogradskyella sediminis*	Marine Sediment	0.13	N	P	*Thauera aromatica*	Sewage	0.39	Y	P
*Dokdonia sp.*	Marine	0.07	N	(73)	***Deltaproteobacteria***				
*Flavobacterium saccharophilum*	Fresh Water	0.83	Y	P	*Anaeromyxobacter dehalogenans*	Freshwater sediment	0.27	N	P
*Muricauda antarctica*	Marine Antarctic	0.91	Y	P	***Gammaproteobacteria***				
*Arenibacter troitsensis*	Marine Sediment	0.88	Y	P	*Alcanivorax balearicus*	Saline groundwater	0.18	Y	(74)
*Tenacibaculum ovolyticum*	Marine Fish	0.90	Y	P	*E. coli*	Fecal	0.25	N	T
*Aquamarina latercula*	Littoral	0.87	Y	15	*Haemophilus influenza*	Human pathogen	0.16	N	T
*Capnocytophaga sputigena*	Human Mouth	0.22	N	P	*Halomonas campisalis*	Alkaline salt flats	0.49	Y	(75)
*Tenacibaculum mesophilum*	Littoral	0.90	Y	(76)	*Kangiella aquimarina*	Littoral	0.11	Y	P
*Flexibacter aurantiacus*	Littoral	0.64	Y	15	*Kangiella koreensis*	Littoral	0.24	Y	P
*Olleya aquimaris*	Marine	0.22	N	P	*Kangiella spongicola*	Marine sponge	0.09	Y	P
*Dokdonia pacifica*	Marine	0.11	N	P	*Klebsiella pneumoniae*	Clinical	0.21	N	P
*Cellulophaga fucicola*	Littoral	0.97	Y	R	*Legionella taurensis*	Hospital water	0.18	Y	P
*Flagellimonas pacifica*	Marine	0.80	Y	T	*Marinobacter spp TK36*	Marine	0.88	Y	P
*Muricauda amoyensis*	Marine	0.82	Y	R	*Marinobacter spp TT-1*	Deep water plume	0.84	Y	P
*Muricauda sp*	Marine	0.89	Y	P	*Marinobacter subterrani*	Deep geosphere	0.71	Y	(77)
*Muricauda sp*	Marine	0.87	Y	R	*Microbulbifer arenaceous*	Littoral	0.53	Y	P
*Cellulophaga sp.*	Marine	0.94	Y	P	*Microbulbifer arenaceous*	Littoral	0.53	Y	T
*Dokdonia pacifica*	Marine	0.11	N	P	*Oceanospirillum beijerinckii*	Marine	0.37	N	P
*Winogradskyella sp*	Marine	0.12	N	T	*Oceanospirillum maris*	Marine	0.35	N	P
***Sphingobacteriia***					*Proteus mirabilis*	Human pathogen	0.22	N	(78)
*Solitalea canadensis*	Soil	0.37	Y	P	*Pseudomonas azotoformans*	Littoral	0.60	Y	T
***Actinomycetota***					*Pseudomonas benzenivorans*	Littoral	0.60	Y	T
***Actinomycetia***					*Pseudomonas composti*	Littoral	0.52	Y	P
*Rhodococcus sp*	Soil	0.25	N	22	*Pseudomonas extremaustralis*	Littoral	0.60	Y	T
*Streptomyces coelicolor*	Soil	0.03	N	P	*Pseudomonas extremorientalis*	Littoral	0.60	Y	T
*Streptomyses lividans*	Soil	0.03	N	P	*Pseudomonas fluorescens*	Littoral	0.60	Y	T
***Firmicutes***					*Pseudomonas lurida*	Littoral	0.60	Y	T
***Bacilli***					*Pseudomonas moorei*	Littoral	0.37	Y	P
*Bacillus subtilis*	Soil	0.22	N	P	*Pseudomonas paracarnis*	Food	0.60	Y	(79)
*Listeria goaensis sp*	Brackish Water	0.24	Y	(80)	*Pseudomonas poae*	Littoral	0.60	Y	T
*Listeria monocytogenes*	Food	0.24	Y	(81)	*Pseudomonas sagittaria*	Littoral	0.50	Y	P
*Paenibacillus glucanolyticus*	Soil	0.26	Y	(82)	*Pseudomonas salomonii*	Littoral	0.60	Y	T
					*Pseudomonas stutzeri*	Littoral	0.51	Y	P
					*Pseudomonas syringae*	Littoral	0.51	Y	P
					*Pseudomonas veronii*	Littoral	0.60	Y	T
					*Pseudoxanthomonas sp*	Littoral	0.42	Y	T
					*Vibrio splendidus*	Marine	0.31	N	P
					*Xylella fastidiosa subsp. multiplex*	Plant	0.10	N	P

A total of 100 bacterial strains were cultivated to validate the SC classifier. SC is scored as yes (Y) or no (N). The source (Src) of the isolates is given in the final column as abbreviation (T: This study, R: Roscoff, D: DSMZ, P: Plymouth) or reference (number). Complete information including strain names is given in *SI Appendix*, Table S3.

### SC throughout the Bacterial Domain of Life.

With our RF-based machine learning model, we set out to predict SC-positive strains throughout the bacterial domain, by calculating the SC score for 240,981 bacterial genome sequences downloaded from the PATRIC database (45) (Dataset S2). We considered genomes scoring above 0.68 as likely SC-positives, and genomes scoring below 0.39 as likely SC-negative, based on the score boundaries of the non-SC and gram-negative SC species in the training set of the classifier (Fig. 3*A*). The other genomes were considered putatively SC-positive.

Although the model was built using a taxonomically biased selection of organisms, we found strong support for SC in many different phyla (Fig. 3*B*). Moreover, predicted SC does not seem to be highly taxonomically biased, i.e., there are high- and low-scoring genomes in many of the taxa. This is an interesting prediction given that to date, SC has only been demonstrated within the phylum *Bacteroidetes*. Within *Bacteroidetes*, the class *Flavobacteriia* shows the highest SC scores, and *Chitinophagia*, *Cytophagia*, *Saprospiria*, and *Sphingobacteriia* had members that were putatively SC-positive (*SI Appendix*, Fig. S5*A*). Even though some members of the class *Bacteroidia* were putatively SC-positive, most had low SC scores, suggesting that they do not form ordered biofilms and in line with their anaerobic lifestyle. SC in the *Proteobacteria* is novel, and was particularly well represented in cultured isolates as discussed below.

Predicted SC in the *Proteobacteria* scored particularly strongly in the classes *Gammaproteobacteria*, *Alphaproteobacteria*, and *Betaproteobacteria* (*SI Appendix*, Fig. S5*B*). An absence of high SC scores in other *Proteobacteria* classes may be due to undersampling, but no *Epsilonproteobacteria* were predicted to show SC even though they were abundant in the PATRIC database (n = 6,268). *Deltaproteobacteria* (n = 1,505) and *Oligoflexia* (n = 166) also had members that were putatively SC-positive.

Host-associated bacteria including members of the order *Enterobacteriales*, which contains the human pathogens *Klebsiella pneumoniae* and *E. coli*, scored poorly within *Gammaproteobacteria* and did not show SC in our experiments. In contrast, SC colonies were developed by *P. aeruginosa* and other pseudomonads, *Kangiella sediminilitoris*, *Pseudoxanthomonas* spp., *M. algicola* and *Microbulbifer arenaceous* (Table 1 and *SI Appendix*, Figs. S1 and S4 and Tables S1 and S3). *Alcanivorax balearicus* has been described as forming iridescent colonies (74). Generally, SC within the *Gammaproteobacteria* appeared to have a less strong reflective component than of many of the *Flavobacteriia*. This may explain the results of previous studies that used plates with a translucent background without nigrosine, which make it harder to detect the presence of weak SC, when the reflected intensity is not very high with respect to the scattering coming from pigmentation. Thus, some studies reported iridescent colonies from *Flavobacteriia*, which exhibit SC, but not from other types of bacteria (20, 21). Subsequently, we studied how the intensity of reflection caused by the organization of the cell can be optimized on diverse cultivation media (*SI Appendix*, Tables S1 and S3), leading to intense coloration, e.g., in *M. algicola* HM-32 and several *Kangiella* and *Hoeflea spp.* (Fig. 1*D*, Table 1, and *SI Appendix*, Figs. S1 and S4*D*).

Iridescent colonies in *Alphaproteobacteria* have only been observed in *Agrobacterium* species (order *Rhizobiales*) isolated from an aquatic fern (83). Our RF classifier predicted that SC may be more widespread within the *Alphaproteobacteria* (Fig. 3), even though no genomes from this class were used to construct the RF classifier. An SC score of 0.85 was predicted for *Rhodobacteraceae* bacterium 4F10 (order *Rhodobacterales*) from coastal ocean surface water (84) (Dataset S2). We screened environmental samples from littoral locations in the Zeeland province (the Netherlands), resulting in the isolation of three *Alphaproteobacteria* displaying weak SC, including two *Hoeflea* species (order *Rhizobiales*) and one *Sulfitobacter* (order *Rhodobacterales*) (Table 1 and *SI Appendix*, Fig. S4).

Although the number of predicted SC strains within the *Betaproteobacteria* were low, *Cupriavidus basilensis RK1* (*Burkholderiales*) showed a metallic, angle-dependent color. Colonies of other Burkholderiales, *Acidovorax delafieldii*, *Janthinobacterium svalbardensis,* and *Chitinomonas koreensis* appeared to show SC (Table 1). In addition, colonies of other *Betaproteobacteria* showed similar metallic, angle-dependent color, e.g., *Sphingomonas pruni* and *Sphingobium herbicidovorans* (both order *Sphingomonadales*). Within order *Neisseriales*, a species of *Pseudogulbenkiana* also showed colonies with metallic and angle-dependent appearance, as did the members of the order *Rhodocylales*; *Dechloromonas* sp. and *Thauera aromatica* (Table 1). In all cases, the optical response suggested the presence of SC.

### Gram-Positive Bacteria with SC.

One gram-positive genome in the SC classifier training set, *Virgibacillus dokdonensis*, showed green/blue SC when grown on RMAR agar with high salinity (6% w/v sea salt) at 50 °C (Fig. 1*H*). The purple/green coloration was visible during vigorous motility over agar, with the colony spreading up to 5 mm/h. Thus, there may be other groups of SC bacteria that are not captured by our classifier, within the gram-positive strains or other phyla. Gram-positive bacteria may create SC using different genes than those in our classifier. However, the current dataset is too small to reliably predict these genes. In the validation study, we found apparent SC in swarming *Paenibacillus vortex* (Table 1). Representative genomes of these species have SC scores of <0.25, again suggesting SC in gram-positive bacteria is not scored highly by the classifier developed based on gram-negative bacteria.

### Optical Analysis of *Gammaproteobacteria* Shows a Two-Dimensional Photonic Crystal Arrangement that Is Similar to the *Flavobacteriia*.

Most of the isolates showing SC appeared to spread over agar, suggesting active surface translocation or motility. Gliding by *Flavobacteria* facilitates the formation of SC (22, 35) and this is supported by the identification of gliding-related genes in this work (Fig. 2*B*). However, we did not find a motility mechanism shared by all SC strains studied. Within the *Gammaproteobacteria*, multiple mechanisms of motility (e.g., flagella and various pili-related) are known (85). To test the role of flagella-based motility, the formation of SC was tested in *Marinobacter subterrani* by comparing colonies of the WT strain with a *fla*BG knockout mutant (77). Despite the loss of flagella motility, as indicated by the mutant strain displaying a nonspreading phenotype when inoculated into 0.2% (w/v) sloppy RMAR plates (*SI Appendix*, Fig. S6 and Table S4), SC was not impaired when cultivated on RMAR plates (0.8% w/v agar). The *fla*BG strain still could spread over the hard agar RMAR plates at 0.6 cm/d, i.e., at the same rate as the WT, suggesting a flagella-independent mechanism of surface translocation, possibly involving Type IV pili. Also, other *Gammaproteobacteria*, *Kangiella sediminilitoris,* and *Hoeflea* sp., were also shown to have SC and displayed surface translocation on RMAR agar (0.8% w/v) plates at rates of up to 1 cm/d (*SI Appendix*, Table S4).

**Fig. 4.**
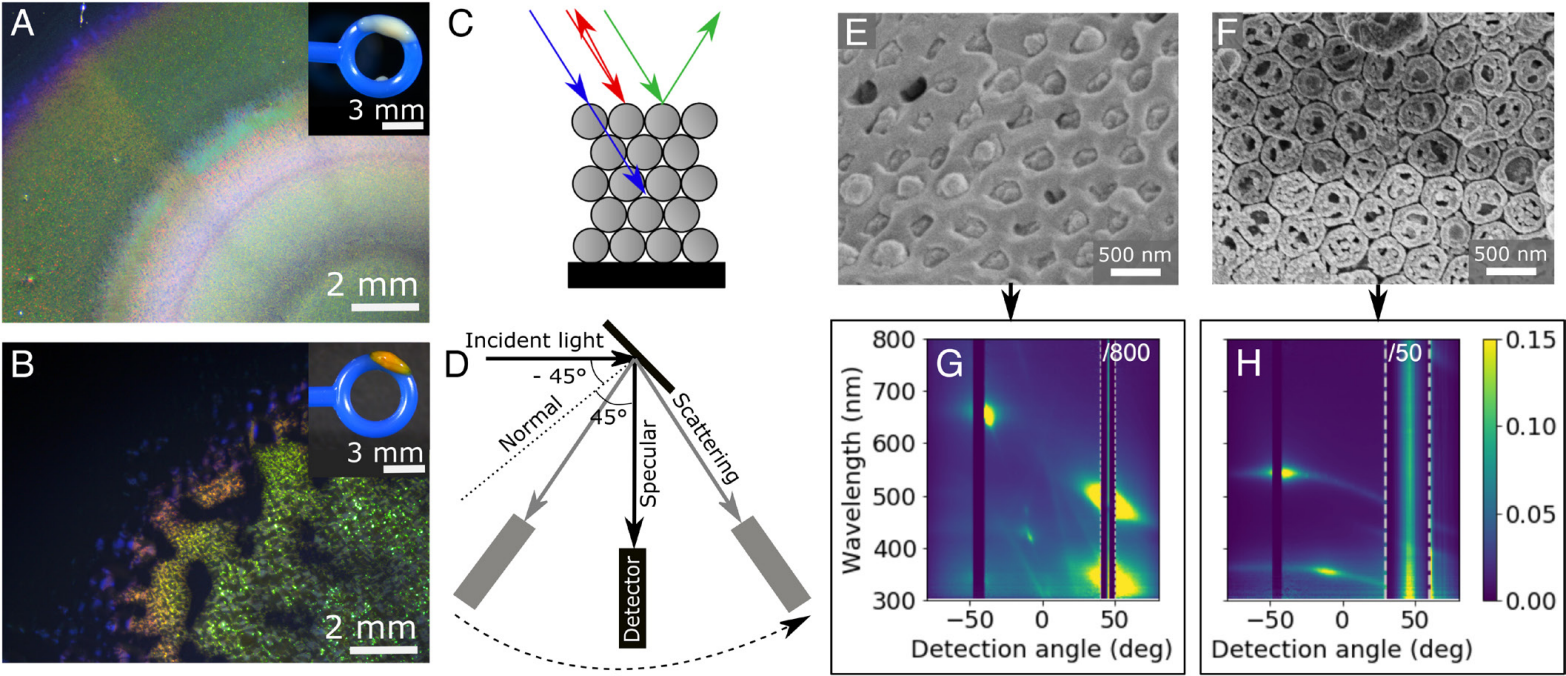
Comparison of the cell arrangement and optical properties of representative strains from the *Marinobacteria* (*M. algicola* HM-28) and *Flavobacteriia* (*Flavobacterium* IR1). (*A*) Colony of *M. algicola* HM-28 showing SC; the *Inset* shows material from the colony losing SC after mixing with an inoculation loop. The inset shows loss of SC after mixing the colony with an inoculation loop. (*B*) As panel *A*, but showing *Flavobacterium* IR1. (*C*) Schematic showing a hexagonally packed photonic crystal in cross-section. When white light reaches the crystal structure, some wavelengths can pass through it (blue arrow), but others cannot (green and red arrow). (*D*) Schematic of the goniometer setup used to capture the spectra shown in panels *E* and *F*. (*E*) Cryogenic SEM image of an *M. algicola* HM-28 colony in cross-section. (*F*) Cryogenic SEM image of a *Flavobacterium* IR1 colony in cross-section. (*G*) Angle-resolved spectra for the *M. algicola* HM-28 colony. (*H*) Similar angle-resolved spectra for the *Flavobacterium* IR1 colony. Both panels show the intensity of the reflected light on a blue to yellow color scale. The incident light angle is kept at −45°, and due to a limitation in the setup no spectra can be taken at this angle. Because the mirror-like reflection (specular) around 45° is far brighter than the scattered light at other angles, and can therefore not be shown on the same scale without saturating the signal, the reflected light intensity between the dashed lines has been divided by a high number (800 for *M. algicola* HM-28 and 50 for *Flavobacterium* IR1, shown at the *Top*). The cryo-SEM image and angle-resolved spectra for *Flavobacterium* IR1 (panels *F* and *H*) are reproduced from ref. 37.

In order to validate that SC was present in the *Gammaproteobacteria*, the underlying optical structure was investigated. We observe that the hexagonal packing of the cells is a feature in both *Flavobacteriia* and *Gammaproteobacteria* with SC (Fig. 4 and *SI Appendix*, Fig. S7). To compare the optical response produced by these structures, we measure angle-resolved reflectance spectra, using an optical goniometer. The obtained spectra show intensity spots at specific wavelengths and angles that are characteristic for these types of structures (Fig. 4 and *SI Appendix*, Fig. S7) (22, 23, 37, 43). The angular dependency of these diffraction spots can be fitted to the grating equation, which reveals the period *d* of each colony (37). We found a lattice constant of the photonic crystal of *d* = 490 nm for *M. algicola* HM-28, *d* = 395 nm for *Flavobacterium* IR1, *d* = 440 nm for *M. subterrani* JG233 (77). Therefore, the different colors observed by the different bacteria strains do not stem from different forms of cell packing, but from variations by differences in the interbacterial distances or in cell size (43).

Another interesting feature to notice is the different colors diffracted by the two strains, which indicate differences in the angular range over which the diffraction spots are spread (goniometer plots in Fig. 4 *E* and *F*). This type of spreading of the reflected light over a wider angular range is typically caused by local variations in orientation of crystalline domains (23, 37, 43). Wherever the crystal structure appears in a tilted orientation, either because the surface of the colony is not flat, or because of imperfections in the structure, light will be reflected under a slightly different angle. However, this feature is again strain-specific, and cannot be attributed as phylum-specific, as the angular range over which the light is reflected varies between both of the *Gammaproteobacteria* strains investigated here (Fig. 4*E* and *SI Appendix*, Fig. S7*C*).

Thus, we conclude that all strains, both *Gammaproteobacteria* and *Flavobacteriia*, follow the same principle mechanism of a hexagonally packed photonic crystal, but with strain-specific variations in the interplay between order and disorder. The two classes of gram-negative bacteria form the same type of SC, despite being from distinct phylogenetic groups and with different mechanisms of motility.

### Identification of Environments that Contain Bacteria that Display Evidence for SC.

**Fig. 5.**
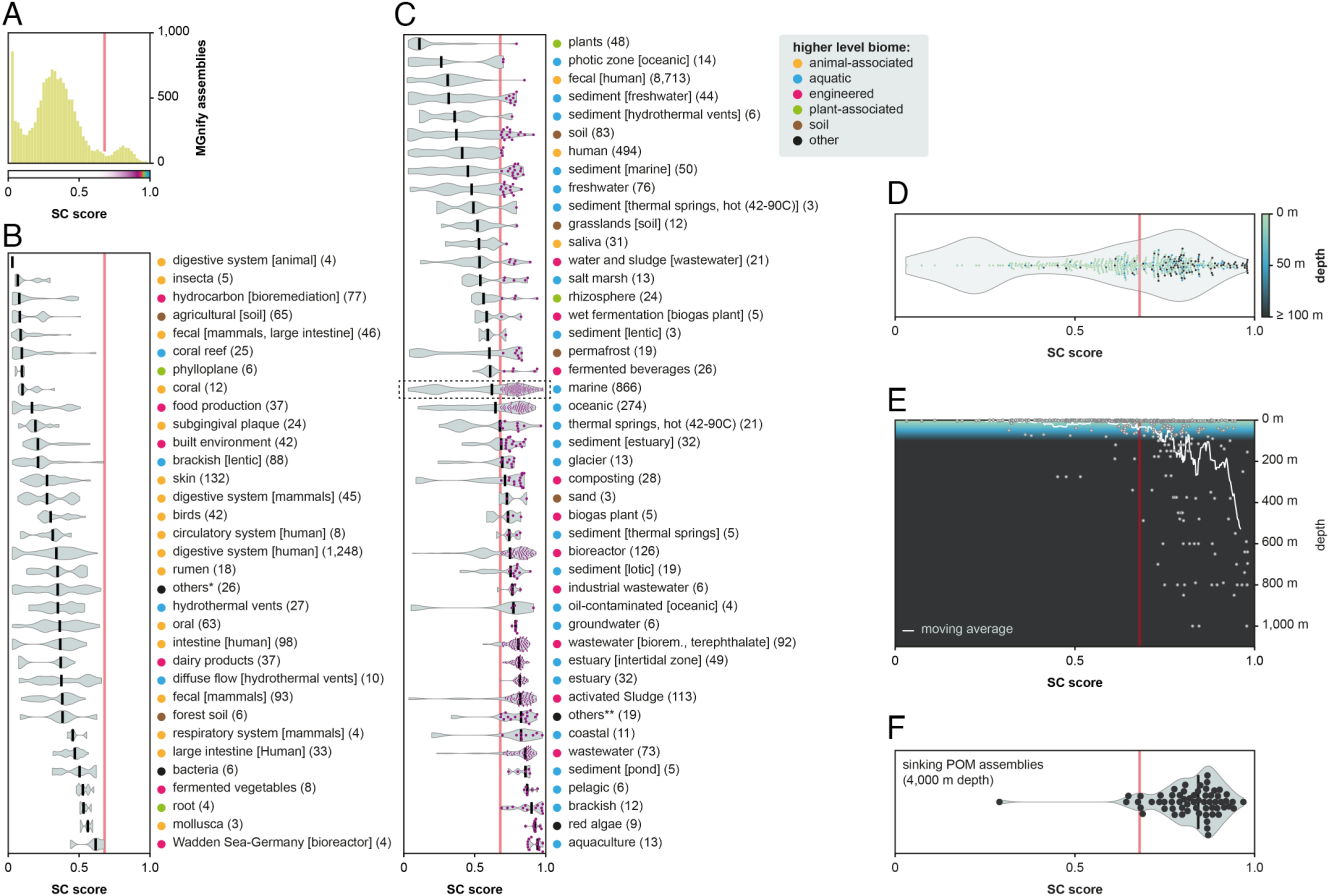
Classifier scores for assembled metagenomes from diverse environments. (*A*) The distribution of SC scores of all metagenomes (n = 13,873) reveals a valley at 0.68 (red line), which we used as a cutoff score. (*B*) Biomes containing no metagenome scoring >0.68. “Others” (indicated with *) is the combined set of biomes with less than three associated datasets each (n = 19). (*C*) Biomes containing at least one high-scoring metagenome above the threshold of 0.68. High-scoring metagenomes are indicated with purple dots. Others (indicated with **) is the combined set of biomes with less than three associated datasets each (n = 13). Black vertical lines in panels *B* and *C* show the median for each biome. (*D*) Distribution of SC scores in the marine biome (see dashed rectangle in panel *C*) with associated water depth metadata. Only assemblies with specific depth information are plotted (n = 450). (*E*) The same figure as panel *D* but with depth in the water column on the *y*-axis. The white line is the moving average with a window size of 20 data points. (*F*) Distribution of the SC score in 62 assembled metagenomes from sinking POM collected at 4 km depth (47).

To investigate the ecological distribution of SC, we downloaded 13,873 assembled metagenomes containing microbial sequences from 108 different biomes (46) and applied the classifier to score each for SC (Fig. 5 *A*–*C* and Dataset S4). As these predictions await further validation in vitro, they serve to prioritize biomes of interest for SC. Animal- and plant-associated bacterial microbiomes scored consistently low for SC (maximum SC score <=0.68, Fig. 5*B*). The notable exceptions were the metagenomes from red macroalgae and aquaculture (maximum SC score >0.68, Fig. 5*C*). This is consistent with previous studies, as bacteria showing SC have been isolated from macroalgae, carry genes for algal polysaccharide metabolism, and regulate their SC in response to these polysaccharides (21, 22, 43). The role of SC in strains in aquaculture has not been investigated, although SC bacteria are frequently isolated from fish (86–88), but might emerge from the rapid and organized colonization of surfaces rich in nutrients. We also predict SC in aquatic and engineered biomes, which include aerobic and light-exposed habitats (biomes with a high median SC score in Fig. 5*C*). SC bacteria were also found in nonilluminated biomes such as pond sediment and groundwater (Fig. 5*C*) (77). Strikingly, from marine metagenomic studies we observed that many of the assembled metagenomes with the highest SC scores were from depths with limited light (Fig. 5 *D* and *E* and Dataset S5), i.e., below the photic zone which extends to 200 m depth [Spearman rank correlation coefficient between SC score and depth: 0.41 (*P* value: 2.1e-19)]. One possible explanation for the depth profile seen is that SC is found on “marine snow” which may permit assembly into highly organized groups of cells. It has been suggested that highly organized groups of *Flavobacteriia*, perceived as structurally colored colonies, have a competitive advantage against other bacteria (28). It is possible that such competition for space is occurring on marine snow and explains the high SC scores in the ocean depths. To test this hypothesis, we downloaded and assembled 62 metagenomes from sinking POM captured at 4,000 m water depth (47) and confirmed that most had high SC scores (Fig. 5*F* and Dataset S6). Future studies may analyze marine snow at shallower depths or ultimately identify SC on marine snow for further confirmation.

## Conclusions

SC is found in many living organisms. In Eukaryotes, SC is intensively studied in the field of optics, but there is little genomics. This work has studied bacteria, in which individual cells coordinate to form colonies showing SC. We created a curated collection of genomes, largely from gram-negative bacteria, both with and without SC, and used this to generate a predictive, genome-based classifier. This work suggests that SC is present in a much more diverse group of bacteria than previously thought, particularly within the phylum *Proteobacteria*. We showed that members of the *Gammaproteobacteria* can form two-dimensional photonic crystals that are essentially the same as previously determined for the *Flavobacteriia.* The classifier was validated with the genomes from additional SC-positive and -negative strains. We identified clusters of genes involved in pterin, porphyrin, carbohydrate, methionine, and acetolactate metabolism that appeared to be common signifiers. The pterin/pteridine-associated cluster was particularly interesting as the most predictive set of genes and because SC can be enhanced by pterin-related pigments in some eukaryotes (55–57). In addition, genes associated with gliding motility were associated with SC in *Flavobacteriia*. *Gammaproteobacteria* also show SC but do not require the flagella to organize. The classifier was applied to metagenomics datasets. Bacterial SC was predicted to be common in aquatic and engineered biomes, but rarely associated with microbiomes of multicellular organisms, with the notable exception of macroalgae and possibly fish. This supports previous observations on the ecological distribution and metabolic properties of bacteria showing SC (20–22, 43, 86). Interestingly, bacteria capable of SC were predicted to be common in the deep ocean which, taken with an apparent illumination-independent role of the cell organization of SC *Flavobacterium* IR1 underlying SC in interbacterial competition, suggests SC may be a side effect of colony organization. In addition, our screen of the bacterial domain predicted SC in the *Alpha-* and *Betaproteobacteria* which we confirmed by isolating SC strains from these taxonomic orders. Our goniometry experiments clearly demonstrated the existence of a two-dimensional photonic crystalline colony structure in *Gammaproteobacteria*, similar to that already demonstrated in the *Flavobacteriia*. Finally, our study suggests the existence of SC within the gram-positive bacteria.

This is a large-scale, genomic-based analysis of SC. Bacterial colonies with SC are living nanostructures that manipulate light, and we have identified several molecular pathways that are linked to the process of generating them. The identified genetic signature of SC may also contribute to answering the question whether SC is selective as an optical phenotype or a side effect of structural organization of the colonies for a different reason. The pathways identified in our work may lead to an understanding of the function of SC in bacteria and the evolutionary relationships and processes that have created this distinctive population phenotype.

## Supplementary Material

Appendix 01 (PDF)

Appendix 02 (PDF)

Appendix 03 (PDF)

Appendix 04 (PDF)

Appendix 05 (PDF)

Appendix 06 (PDF)

Dataset S01 (PDF)

Dataset S02 (PDF)

Dataset S03 (PDF)

Dataset S04 (PDF)

Dataset S05 (PDF)

Dataset S06 (PDF)

Movie S1. Reflectance microscopy of groups of gliding cells of ‘*Cellulophaga lytica HM-52*’ cultivated on RMAR plates without peptone (1 % w/v agar) showing the active formation and rearrangement of structural color over a 6.5 h period. Imaging was at the colony edge with side illumination with a white LED to reveal structural color.

## Data Availability

Genomic sequence data are available under accession PRJEB56913 in the National Center for Biotechnology Information database (89). The aligned sequence data, HMMs, and scripts for classifying sequence data for SC are available at DOI: 10.5281/zenodo.7859454 (90). A web-based interface to the classifier is available at http://klif.uu.nl/structuralcolorweb/.
