# Supplementary material for: Structural color in the bacterial domain: The ecogenomics of a 2-dimensional optical phenotype
Source: Proc Natl Acad Sci U S A. 2024 Jul 11;121(29):e2309757121. doi: 10.1073/pnas.2309757121 (PMC11260094; doi:10.1073/pnas.2309757121)
Supplement: Supplementary file 1 — Appendix 01 (PDF) [file pnas.2309757121.sapp1.pdf]

Supplementary Methods

Optical analysis of structural color

Angle-dependent spectra were taken using a custom-built goniometer setup (1). On this setup, the sample was mounted on a rotating stage so that the angle of incident light could be varied. The incident light from a Ocean Optics HPX-2000 xenon lamp was collimated, with a spot size of 5 mm diameter. Light reflected or scattered from the sample was then collected by an optical fibre connected to a spectrometer (AvaSpec-HS2048, Avantes). This optical fibre was mounted on a rotating arm so that the angle of detection could be varied. At the detection angle which equals the negative of the incident angle, the detection arm blocked the incident light so that no signal could be collected. All the spectra reported here were normalized against a white diffuser (labsphere SRS-99-010).

Phenotypic testing and curation of structural color

Isolates were considered as having SC if colonies showed metallic, angle-dependent, saturated color on plates with a dark background (with nigrosine to enhance optical contrast and therefore the detection of SC) when illuminated from the side with a broad-spectrum white LED. In addition, if this coloration was disrupted by mixing the colony with an inoculation loop then this was considered confirmatory. Strains were scored as negative if they failed these criteria under all conditions tested. The nutrient agar formulations used were based around RMAR medium under aerobic conditions, varying sea salt from 0 to 6% (w/v), cultivation temperature from 20 to 45 °C, with or without peptone, and using agar concentrations of 0.8, 1.5 and 2.5% (w/v). Most tests were conducted on plates containing nigrosine, but this was omitted if the dye appeared to inhibit growth. In addition, strains obtained from the DSMZ (Supplementary Tables 1 and 3) were cultivated on the recommended medium supplemented with 200 mg/l nigrosine to give optical contrast.

Structural analysis of SC colonies by electron microscopy

Cryo-SEM was performed on a FEI Verios 460 scanning electron microscope at the Cambridge Advanced Imaging Centre (University of Cambridge). A piece of agar (approximately 0.5 x 0.2 cm) with bacteria was cut and placed on a small piece of filter paper, which was then placed in a shuttle well containing colloidal graphite paste to hold the agar slice in place during freezing and fracturing and to provide conductivity. Care was taken to not cover the upper part of the sample with graphite paste so as to not contaminate the fracture plane. Next, the shuttle containing the sample was plunge-frozen in liquid ethane and subsequently transferred to a cryo-transfer system (Quorum PP3010T) that was cooled down to approximately -140℃. The samples were then fractured with a blade, sublimed at -90℃ and sputter-coated with platinum at 10mA for 60 seconds. The images were taken at 2.00 keV acceleration voltage with 6.3 pA probe current using the EDT detector in field-free mode for low magnification images and the TLD detector in immersion-mode for high magnification images.

Genome sequencing

DNA was isolated using the Qiagen UltraClean Microbial DNA isolation kit (Qiagen, Venlo, NL). DNA libraries were prepared with the Illumina Nextera kit according to manufacturer's instructions and sequenced using NextSeq sequencing with 150 base pairs reads (Illumina, San Diego, CA, USA). Reads-quality-check and adapter trimming was performed with Trim Galore v0.4.4 (https://www.bioinformatics.babraham.ac.uk/projects/trim_galore/). The genomes were assembled with SPAdes v3.10.1 (2), and contigs smaller than 200 base pairs and with a kmer coverage lower than 10

were removed. Genome quality was assessed with CheckM v1.1.2 in the lineage workflow for completeness (>95%) and contamination (<5%) (3). Reads were submitted to the SRA under accession PRJEB47515. Genes on the genomes were predicted using Prokka version 1.13 (4), followed by orthology clustering using Roary 3.12.0 (5) using a 20% amino acid identity cut-off and a relaxed MCL granularity parameter "-iv 1.3" to allow for orthologous clustering across phyla. Orthologs from Roary were associated with the structural color phenotype using Scoary (6) using default settings including permutation testing (1,000x). Orthologs were considered significantly associated with SC when the Fisher Exact algorithm in Scoary showed a Benjamini Hochberg adjusted p<0.05, or when the contrasting pair algorithm in Scoary showed p<0.05, or when the permutation testing p<0.001. In all cases, only orthologs with an odds ratio >1 were selected. Functional enrichment analysis was performed using STRING (7) using the genomes of *Cellulophaga lytica* DSM 7489, *Flavobacterium johnsoniae* UW101, *Marinobacter algicola* DG893 and *Allomuricauda ruestringensis* DSM 13258 as reference and genes were manually assigned to clusters; "gliding", "carbohydrate", "pterin", "porphyrin", "acetolactate" and "methionine" based on gene functions and interactions between proteins (Supplementary Table 5, Supplementary Figure 3). Phylogenetic trees were constructed using RaxML 8.2.4 (8), using the BINCAT model for gene presence/absence and the GTR-gamma model for full length 16S genes, and visualised using iTOL (9). The potential HGT event between *Proteobacteria* HM25 and *Bacteroides* was investigated by extracting the genes of SC-associated orthologs group_1082, group_1236, group_1559, group_1599, group_1625, group_1653, group_1672, group_1694, group_1772, group_1848, group_1954, group_2568, group_3115, hmgA, lpqL, purF, ywqE, aligning these and generating a phylogenetic tree from the concatenated aligned sequences using RaxML 8.2.4 using the GTR-gamma model.

Machine learning model construction

All proteins from the 117 isolates assigned to the orthologs selected based on the pan-GWAS and the transposon mutagenesis data were extracted from the respective genomes using roary-query_pan_genome (5) and aligned per orthologous group using MAFFT v7.407 (10) with default settings. Hidden Markov models were constructed using HMMer 3.1b2 (http://hmmer.org/) using default settings. HMM profiles were aligned against all proteins with an e-value cut-off of 1E-30. Presence absence data of the HMM profiles was used as input for randomForest 4.6-14 (11) with 5,000 trees and by making use of the sampsize=(c(20, 20)) option to handle class imbalances to generate a prediction model. Feature importance was determined by extracting the Gini importances (11) from the random Forest object. A script in bash was constructed that automates the HMM profile searches and predicts SC using the random forest (RF) function. The script and the associated RF model are available on Github (https://github.com/aldertzomer/structuralcolor). An online version of the prediction method is available on http://klif.uu.nl/structuralcolorweb/.

Supplementary figures, legends to supplementary figures and movie

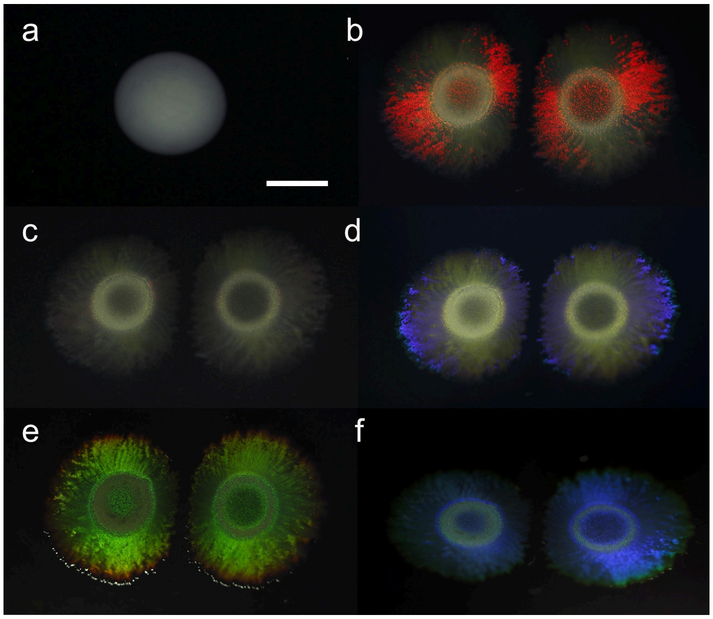

Supplementary Figure 1. Images of colonies '*Marinobacter algicola* HM-28' showing photonic properties. (**a**) Colony of this bacterium mixed to show pigmented coloration with the mechanically disrupted structural color; (**b-f**) Images of the same colonies, illuminated by white light, taken from different angles to show the range of structural colors. Scale bar indicates 1 cm for all panels.

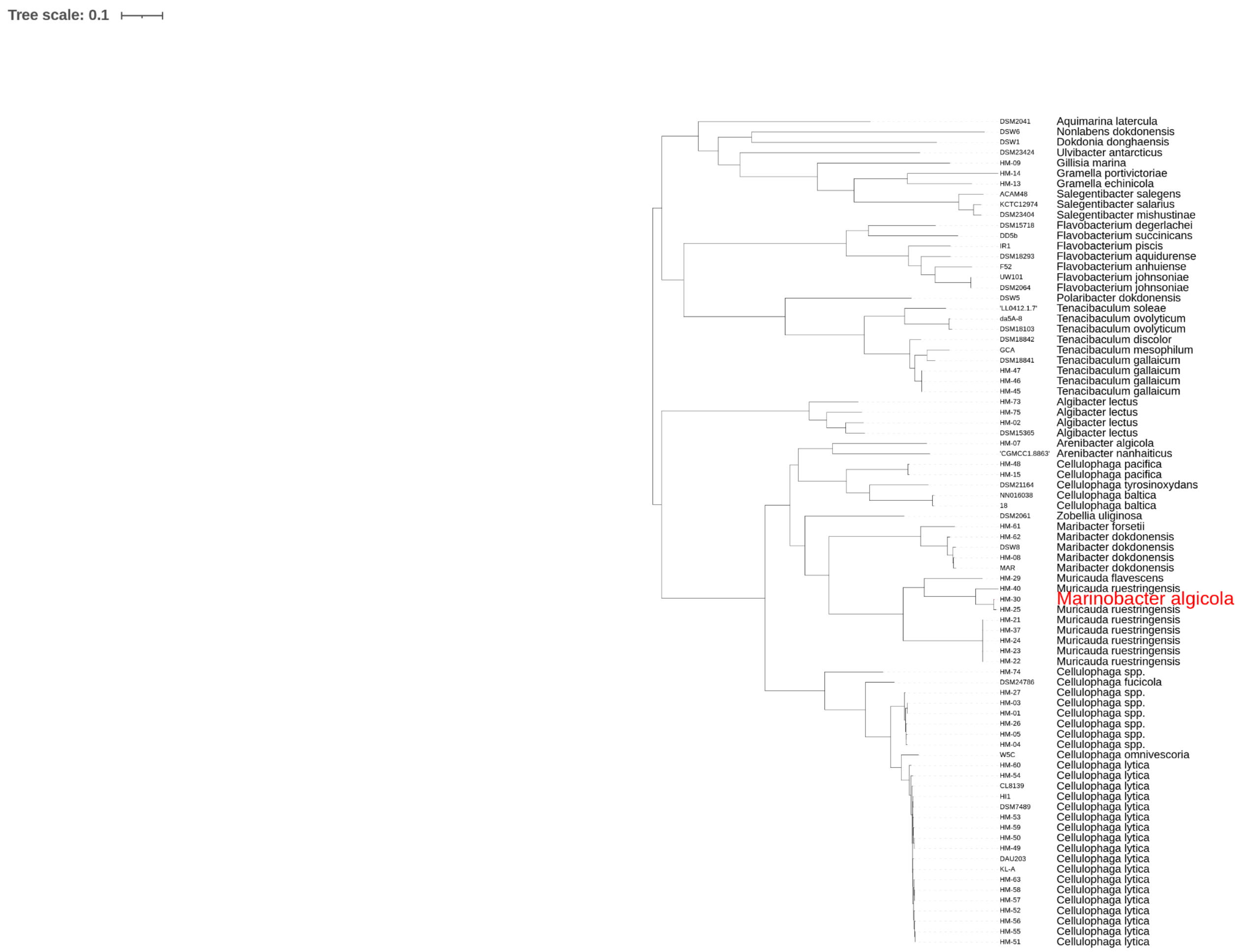

Supplementary Figure 2.

Maximum likelihood phylogenetic tree of a superalignment of SC associated orthologs group_1082, group_1236, group_1559, group_1599, group_1625, group_1653, group_1672, group_1694, group_1772, group_1848, group_1954, group_2568, group_3115, hmgA, lpqL, purF, ywqE. In red the *Proteobacteria* strain *M. algicola* HM25.

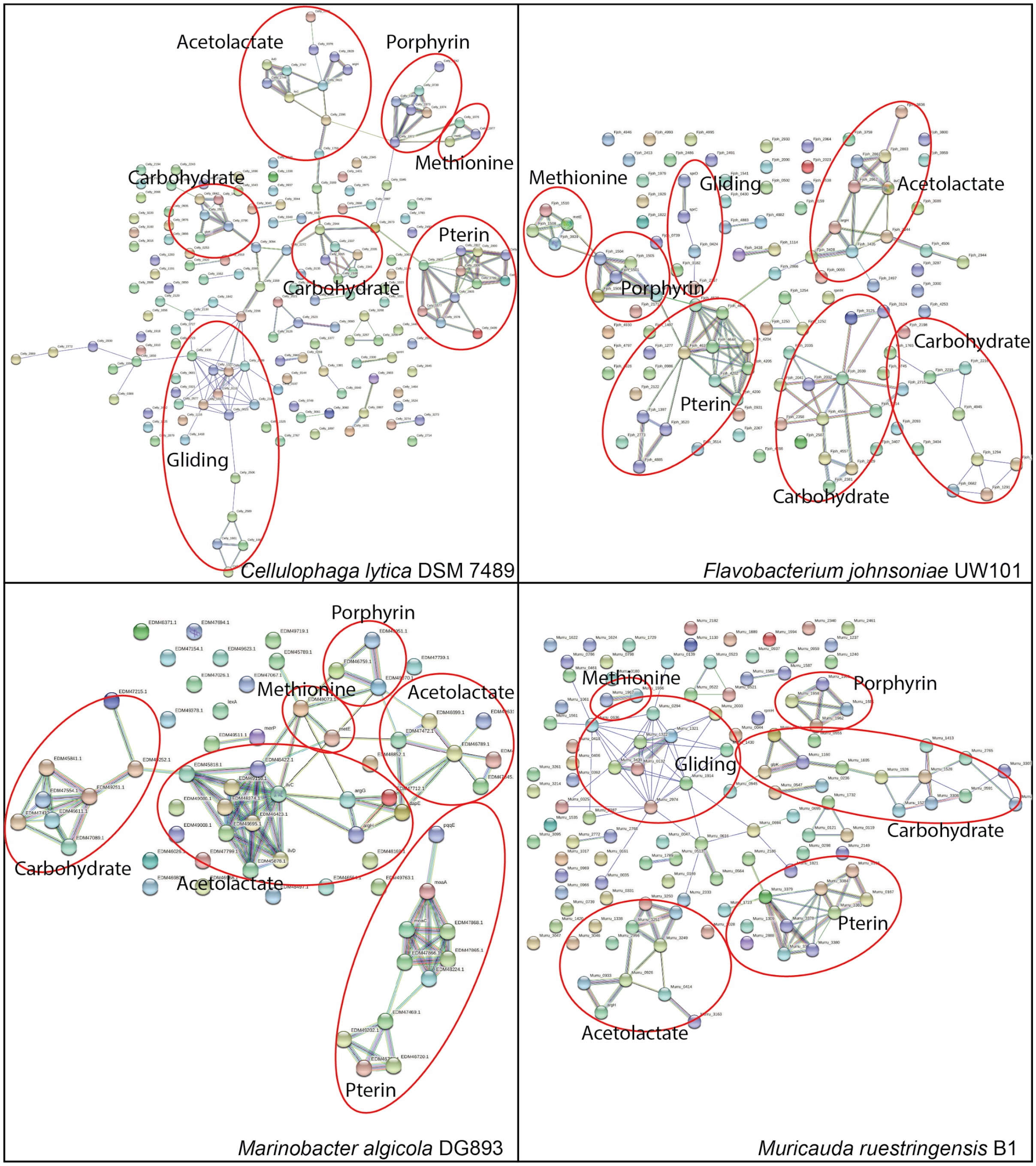

Supplementary Figure 3. STRING analysis showing functional clusters. STRING (7) image of associated genes '*Cellulophaga lytica*', '*Flavobacterium johnsoniae*', '*Marinobacter algicola*', and '*Muricauda ruestringensis*' showing the six functional clusters: pterin, porphyrin, carbohydrate, methionine, acetolactate biosynthesis, and gliding motility.

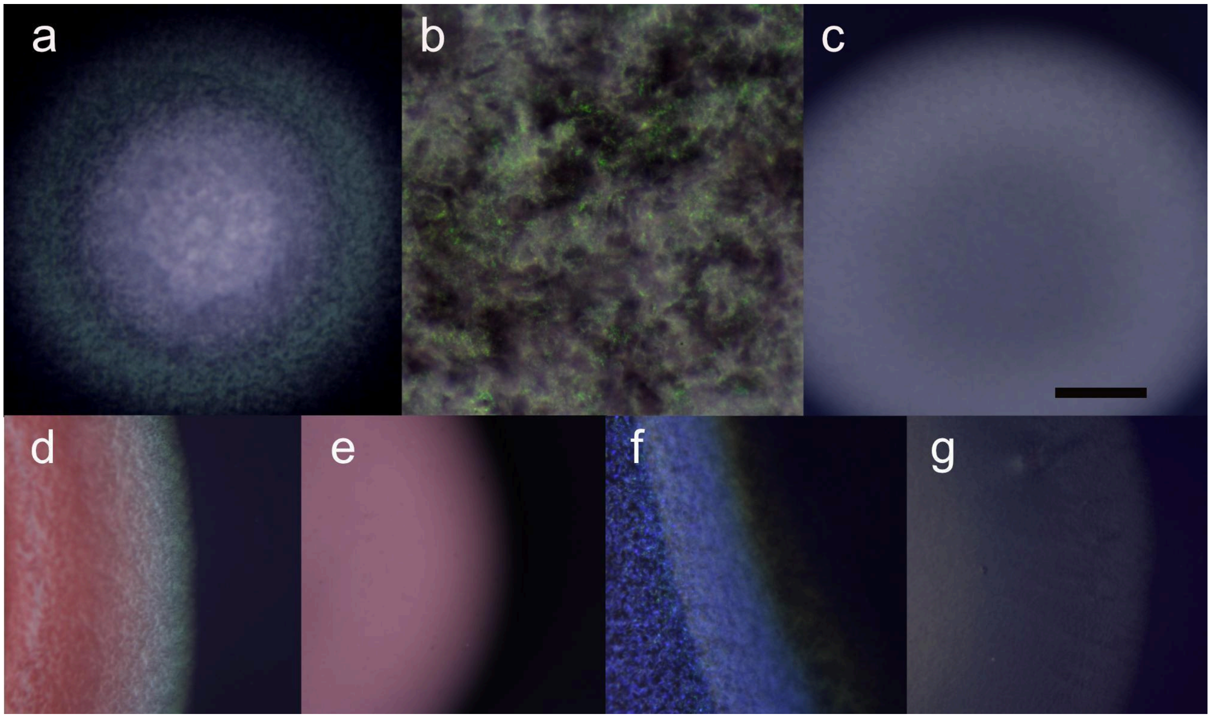

Supplementary Figure 4. Examples of structural color in bacteria from strains used in validation. (**a**) Pinpoint colony of '*Pseudomonas aeruginosa*' cultured on TSA agar (0.8% w/v) plates at 30 °C showing green coloration at the edges when illuminated from the side; (**b**) Microscopy image from the edge of the colony from the previous panel, showing pointillistic, saturated motes of green. (**c**) Colony from panel a, mixed and redeposited on agar, showing loss of green coloration by mechanical disruption. (**d, e**) Images of a colony of '*Hoeflea alexandrii* HHA1' grown on RMAR agar (0.8% w/v) plates at 30 °C. Panel d shows the intact colony with green SC and red pigmentation, panel e shows, after mixing the colony, that the SC but not the pigmentation is lost. Panels (**f**) and (**g**) show an intact colony (grown on RMAR agar (0.8% w/v) plates at 30 °C) and a mixed colony of '*Microbulbifer* sp.', respectively. Scale bar indicates 100 μm for panels except b, 20 μm when applied to panel b. All plates contained 0.05% nigrosin to give contrast and were imaged by microscopy (x40 magnification) with illumination from the side using a 50W white LED.

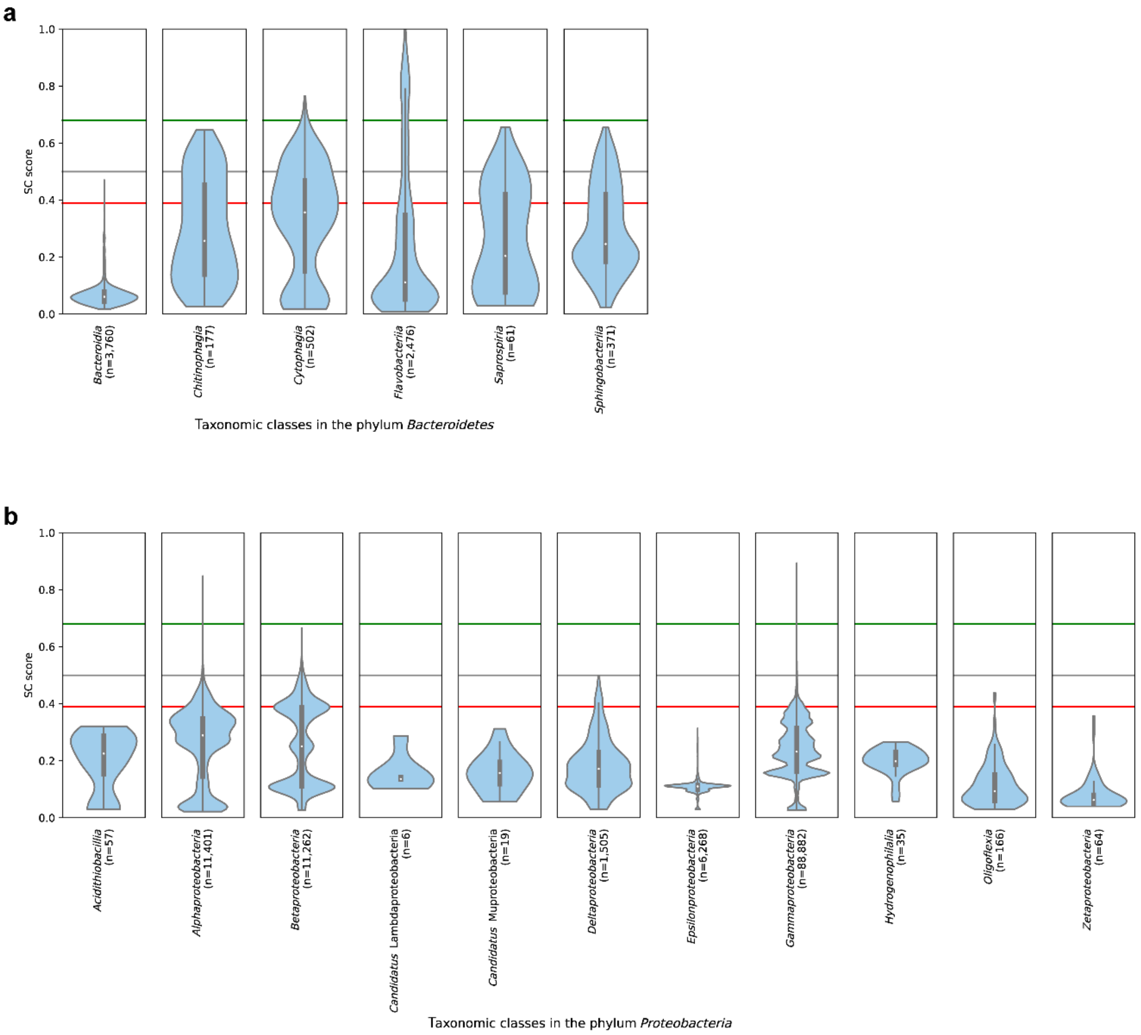

Supplementary Figure 5. Distribution of SC scores in the PATRIC database within *Bacteroidetes* and *Proteobacteria*. (**a**) Taxonomic classes within the phylum *Bacteroidetes*.(**b**) Taxonomic classes within the phylum *Proteobacteria*. Violin plots depict the distribution of SC scores of all genomes within the class, box plots show median and interquartile range. Violins are cut off at maximum and minimum values, whiskers of the box plots extend to 1.5 times the interquartile range.

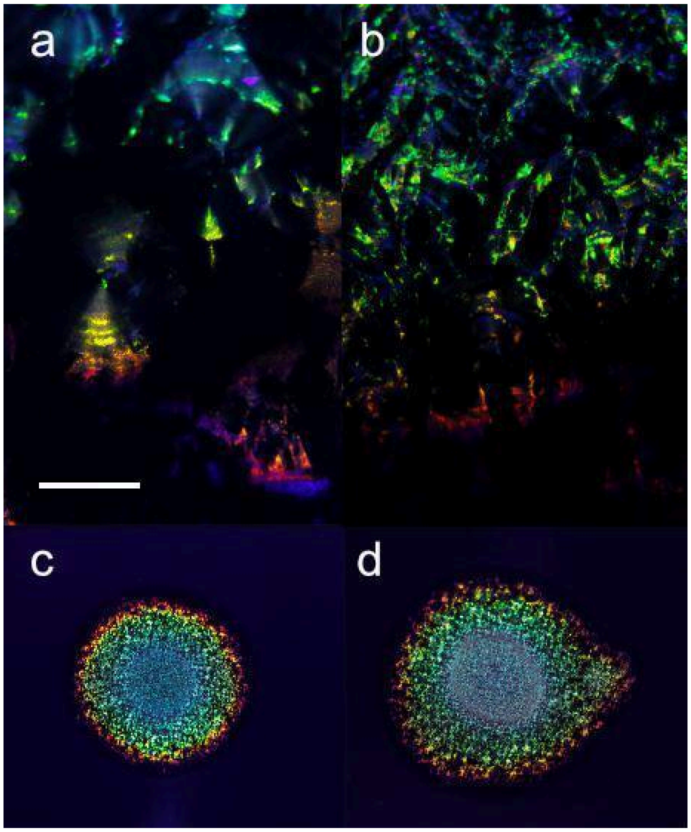

Supplementary Figure 6. The effect of a flagellar motility gene disruption on structural color in '*Marinobacter subterrani* JG233'. Panels (**a**) and (**c**) are the WT strain, panels (**b**) and (**d**) a *fla*BG knockout which is not capable of flagellar motility. Panels a and b are taken by low power microscopy with side illumination. Panels c and d show colonies after 5 days incubation. All images show growth on RMAR plates (0.8% w/v agar) at 29 °C. The scale bar in panel a indicates 0.4 mm for panels a,b and 12 mm for panels c,d.

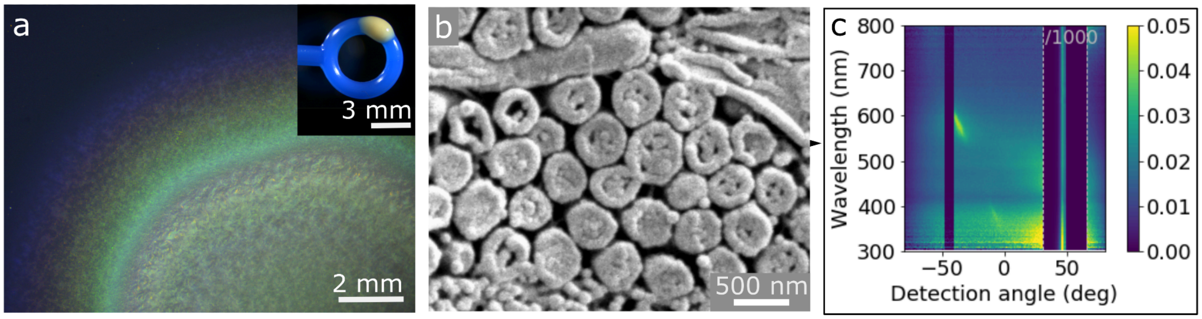

Supplementary Figure 7. Cell ordering and optical response of a colony of '*Marinobacter subterrani*'. (**a**) Colony of '*M. subterrani'* showing SC, inset shows material from the colony losing SC after mixing with an inoculation loop. (**b**) Cryo-SEM image of '*M. subterrani* JG233' in cross section. (**c**) Angle-dependent spectra showing the optical

response of the colony. The intensity of the reflected light is given on a blue to yellow heat map, with yellow high intensity and blue low intensity. The incident light angle is kept at -45°, and due to a limitation in the setup no spectra can be recorded at the incident light angle. Because the mirror-like reflection (specular) around 45° is far brighter than the scattered light at other angles, and can therefore not be shown on the same scale without saturating the signal, the reflected light intensity between the dashed lines has been divided by the number shown at the top.

Supplementary Movie 1. Still from Movie 1. Reflectance microscopy of groups of gliding cells of '*Cellulophaga lytica* HM-52' cultivated on RMAR plates without peptone (1 % w/v agar) showing the active formation and rearrangement of structural color over a 6.5 h period. Imaging was at the colony edge with side illumination with a white LED to reveal structural color.

Legends to SI Tables

Supplementary Table 1. In total 117 bacterial genome sequences were used to create the Random Forest classifier for structural color. The table lists their taxonomic affiliation, structural color phenotype (our observations), accession number for the genome sequence, source of the strain (DSMZ, the German Strain Collection of the Leibniz Institute, this work, or as cited), and the cultivation media. All strains were cultivated on agar plates under aerobic conditions with media: ASWB, Artificial Seawater Agar Black; RMAR, Rich Marine Agar; DSMZ, as recommended for this strain in the culture collection (https://www.dsmz.de/collection/catalogue/microorganisms/catalogue).

Supplementary Table 2. Proteins implicated in structural color in *Flavobacterium* IR1 were identified by transposon mutagenesis (12) with NCBI annotation. The table lists the mutant identifier, independent isolate identifiers, NCBI annotation of the protein function, NCBI accession identifier, and the associated citation. This dataset was used to create the structural color classifier.

Supplementary Table 3. Strains used in validation study. The table lists the species and strain name, taxonomic classification (class and phylum), Gram staining, source and habitat of the strains (either as part of this work from the DSMZ strain collection or otherwise obtained as cited), predicted SC score, and the SC phenotype (YES for structural color under at least one growth condition on agar plates and NO if structural color was not observed under any condition).

Supplementary Table 4. Proteobacterial strains tested for swimming in sloppy agar plates. The table lists the species and strain name, the agar percentage of the plates, culture medium (Rich Marine Agar; LA, Luria Agar; DSMZ), and the colony expansion rate in millimeters per day. Spreading can only occur beyond a few mm if the bacteria use flagella motility to swim through the sloppy agar.

Supplementary Table 5. Sequence analysis output by https://string.embl.de of proteins of *Cellulophaga lytica* DSM 7489, *Flavobacterium johnsoniae* UW101, *Marinobacter algicola* DG893 and *Muricauda ruestringensis* B1 that were orthologs of the 199 marker proteins. Species: species from the ortholog table from this study. queryItem: locus_tag of protein. Orthologname: name of ortholog in ortholog table. stringId: Name of protein in STRING database. Identity: Sequence identity between uploaded protein and protein in STRING database. Bitscore: bitscore of BLAST alignment of uploaded protein and protein in STRING database. preferredName: Protein name given in STRING database. Annotation: Domains present in protein in STRING database. Cluster: cluster assigned based on manual inspection of interaction networks produced by STRING (Supplementary Figure 3).

Legends to SI Data Sets

Supplementary Data 1. Quantitative details displayed as Figure 2.

Supplementary Data 2. The RF-based machine learning model was used to assign a SC score to 240,981 sequences downloaded from the PATRIC database. The table contains the PATRIC identifier, SC score, the complete taxonomic lineage, completeness and contamination information according to CheckM and PATRIC, and the status of the sequence (genome, plasmid, or whole genome shotgun).

Supplementary Data 3. Range of RF-based SC scores of 1,120 species containing at least ten genomes, as shown by the blue lines in Figure 3b. The listed values are the mean of the five lowest and the five highest SC scores.

Supplementary Data 4. List of 13,873 assembled metagenomes from the MGnify database (13). The table lists the assembly file, identifier of the MGnify analysis and the assembly, the biome of the sample, and the SC score.

Supplementary Data 5. List of 866 MGnify metagenomes from the root:Environmental:Aquatic:Marine biome lineage. Columns are identical to Supplementary Data 4, plus the depth in the water column, number of contigs of at least 500 bp, number of predicted proteins on all contigs, and the SC score.

Supplementary Data 6. List of 62 metagenomes from sinking particulate organic matter (marine snow) that were assembled with Megahit (14). The table lists the run identifier, collection date, water depth, number of contigs of at least 500 bp, number of predicted proteins on all contigs, and the SC score.

Supplementary Methods References

1. L. Schertel, et al., Complex photonic response reveals three-dimensional self-organization of structural coloured bacterial colonies. *J Roy Soc Interface* **17**, 20200196 (2020).

2. Prjibelski, et al. Using SPAdes De Novo Assembler. *Curr Protoc Bioinformatics* **70** (2020)

3. Parks DH et al. CheckM: assessing the quality of microbial genomes recovered from isolates, single cells, and metagenomes. *Genome Res* **25** (2015)

4. T. Seemann, Prokka: rapid prokaryotic genome annotation. *Bioinformatics* **30**, 2068–2069 (2014).

5. A. J. Page, et al., Roary: rapid large-scale prokaryote pan genome analysis. *Bioinformatics* **31**, 3691–3693 (2015)

6. O. Brynildsrud, J. Bohlin, L. Scheffer, V. Eldholm, Rapid scoring of genes in microbial pan-genome-wide association studies with Scoary. *Genome Biol* **17**, 238 (2016).

7. D. Szklarczyk, et al., The STRING database in 2021: customizable protein–protein networks, and functional characterization of user-uploaded gene/measurement sets. *Nucleic Acids Res* **49**, (D1): D605-D612 (2021)

8. A. Stamatakis, RAxML version 8: a tool for phylogenetic analysis and post-analysis of large phylogenies. *Bioinformatics* **30**, 1312–1313 (2014).

9. I. Letunic, P. Bork, Interactive Tree Of Life (iTOL) v5: an online tool for phylogenetic tree display and annotation. *Nucleic Acids Res* **49**, W293-W296 (2021).

10. K. Katoh, D. M. Standley, MAFFT Multiple Sequence Alignment Software Version 7: Improvements in Performance and Usability. *Mol Biol Evol* **30**, 772–780 (2013).

11. A. Liaw, M. Wiener, Classification and regression by Random Forest. *R News* **2**, 18–22 (2002).

12. V. E. Johansen, et al., Genetic manipulation of structural color in bacterial colonies. *Proc Natl Acad Sci USA* **115**, 2652–2657 (2018).

13. MGnify: the microbiome analysis resource in 2020. *Nucleic Acids Res* **48**, D570–D578 (2020).

14. Li et al. MEGAHIT: an ultra-fast single-node solution for large and complex metagenomics assembly via succinct de Bruijn graph. Bioinformatics 15 (2015).

References for Supplementary Table 1.

Banach, Artur, Agnieszka Kuźniar, Radosław Mencfel, and Agnieszka Wolińska. "The study on the cultivable microbiome of the aquatic fern *Azolla filiculoides* L. as a new source of beneficial microorganisms." *Applied Sciences* 9, no. 10 (2019): 2143.

Bierne, Hélène, Christophe Sabet, Nicolas Personnic, and Pascale Cossart. "Internalins: a complex family of leucine-rich repeat-containing proteins in *Listeria monocytogenes*." *Microbes and Infection* 9, no. 10 (2007): 1156-1166.

Bonis, Benjamin M., and Jeffrey A. Gralnick. "*Marinobacter subterrani*, a genetically tractable neutrophilic Fe (II)-oxidizing strain isolated from the Soudan Iron Mine." *Frontiers in Microbiology* 6 (2015): 719.

Budding, A. E., C. J. Ingham, W. Bitter, C. M. Vandenbroucke-Grauls, and P. M. Schneeberger. "The Dienes phenomenon: competition and territoriality in swarming *Proteus mirabilis*." *Journal of bacteriology* 191, no. 12 (2009): 3892-3900.

Cousin, Sylvie, Orsola Päuker, and Erko Stackebrandt. "Flavobacterium aquidurense sp. nov. and *Flavobacterium hercynium* sp. nov., from a hard-water creek." *International journal of systematic and evolutionary microbiology* 57, no. 2 (2007): 243-249.

Doijad, Swapnil P., Krupali V. Poharkar, Satyajit B. Kale, Savita Kerkar, Dewanand R. Kalorey, Nitin V. Kurkure, Deepak B. Rawool et al. "*Listeria goaensis* sp. nov." *International Journal of Systematic and Evolutionary Microbiology* 68, no. 10 (2018): 3285-3291.

González, José M., Jarone Pinhassi, Beatriz Fernández-Gómez, Montserrat Coll-Lladó, Mónica González-Velázquez, Pere Puigbò, Sebastian Jaenicke et al. "Genomics of the proteorhodopsin-containing marine flavobacterium *Dokdonia* sp. strain MED134." *Applied and environmental microbiology* 77, no. 24 (2011): 8676-8686.

Hahnke, Richard L., and Jens Harder. "Phylogenetic diversity of *Flavobacteria* isolated from the North Sea on solid media." *Systematic and Applied Microbiology* 36, no. 7 (2013): 497-504.

Hamidjaja, Raditijo, Jérémie Capoulade, Laura Catón, and Colin J. Ingham. "The cell organization underlying structural color is involved in *Flavobacterium* IR1 predation." *The ISME journal* 14, no. 11 (2020): 2890-2900.

Holmfeldt, Karin, Cristina Howard Varona, Natalie Solonenko, and Matthew B. Sullivan. "Contrasting genomic patterns and infection strategies of two co-existing *Bacteroidetes podovirus* genera." *Environmental microbiology* 16, no. 8 (2014): 2501-2513.

Ingham, Colin J., and Eshel Ben Jacob. "Swarming and complex pattern formation in *Paenibacillus vortex* studied by imaging and tracking cells." *BMC microbiology* 8, no. 1 (2008): 1-16.

Kientz, Betty, Adrien Ducret, Stephen Luke, Peter Vukusic, Tâm Mignot, and Eric Rosenfeld. "Glitter-like iridescence within the bacteroidetes, especially *Cellulophaga* spp.: optical properties and correlation with gliding motility." *PLoS One* 7, no. 12 (2012): e52900.

Kientz, Betty, Hélène Agogué, Céline Lavergne, Pauline Marié, and Eric Rosenfeld. "Isolation and distribution of iridescent *Cellulophaga* and other iridescent marine bacteria from the Charente-Maritime coast, French Atlantic." *Systematic and applied microbiology* 36, no. 4 (2013): 244-251.

Kolton, Max, Stefan J. Green, Yael Meller Harel, Noa Sela, Yigal Elad, and Eddie Cytryn. "Draft genome sequence of *Flavobacterium* sp. strain F52, isolated from the rhizosphere of bell pepper (Capsicum annuum L. cv. Maccabi)." (2012): 5462-5463.

Kwak, Min-Jung, Jidam Lee, Soon-Kyeong Kwon, and Jihyun F. Kim. "Genome information of *Maribacter dokdonensis* DSW-8 and comparative analysis with other Maribacter genomes." *Journal of microbiology and biotechnology* 27, no. 3 (2017): 591-597.

Lick, Sonja, Daniel Wibberg, Annika Winkler, Jochen Blom, Christina Grimmler, Alexander Goesmann, Jörn Kalinowski, and Lothar Kröckel. "*Pseudomonas paraversuta* sp. nov. isolated from refrigerated dry-aged beef." *International journal of systematic and evolutionary microbiology* 71, no. 6 (2021): 004822.

McBride, Mark J., and Timothy F. Braun. "GldI is a lipoprotein that is required for *Flavobacterium johnsoniae* gliding motility and chitin utilization." *Journal of bacteriology* 186, no. 8 (2004): 2295-2302.

Mickol, Rebecca L., Artemis S. Louyakis, H. Lynn Kee, Lisa K. Johnson, Scott C. Dawson, Katherine R. Hargreaves, Grayson L. Chadwick, Dianne K. Newman, Jared R. Leadbetter, and C. Titus Brown. "Draft Genome Sequence of the Free-Living, Iridescent Bacterium *Tenacibaculum mesophilum* Strain ECR." *Microbiology resource announcements* 10, no. 1 (2021): e01302-20.

Mormile, Melanie R., Margaret F. Romine, M. Teresa Garcia, Antonio Ventosa, Thomas J. Bailey, and Brent M. Peyton. "*Halomonas campisalis* sp. nov., a denitrifying, moderately haloalkaliphilic bacterium." *Systematic and applied microbiology* 22, no. 4 (1999): 551-558.

Poehlein, Anja, Hristo Najdenski, and Diliana D. Simeonova. "Draft Genome Sequence of *Flavobacterium succinicans* Strain DD5b." *Genome Announcements* 5, no. 2 (2017): e01492-16.

Rivas, Raul, Paula García-Fraile, Alvaro Peix, Pedro F. Mateos, Eustoquio Martínez-Molina, and Encarna Velazquez. "*Alcanivorax balearicus* sp. nov., isolated from Lake Martel." *International journal of systematic and evolutionary microbiology* 57, no. 6 (2007): 1331-1335.

Schertel, L., van de Kerkhof, G.T., Jacucci, G., Catón, L., Ogawa, Y., Wilts, B.D., Ingham, C.J., Vignolini, S. and Johansen, V.E., 2020. Complex photonic response reveals three-dimensional self-organization of structural coloured bacterial colonies. *Journal of the Royal Society Interface*, *17*(166), p.20200196.

Steinle, P., G. Stucki, R. Stettler, and K. W. Hanselmann. "*Ralstonia basilensis* sp. nov." *Validation of publication of new names and new combinations previously effectively published outside the IJSB, List* 71 (1999): 1325-1326.

Stover, AL, CK Pham XQ Erwin. "Mizoguchi SD Warrener P 2000 Complete genome sequence of *Pseudomonas aeruginosa* PA01, an opportunistic pathogen." *Nature* 406: 959964.

Thomas, François, Philippe Bordron, Damien Eveillard, and Gurvan Michel. "Gene expression analysis of *Zobellia galactanivorans* during the degradation of algal polysaccharides reveals both substrate-specific and shared transcriptome-wide responses." *Frontiers in microbiology* 8 (2017): 1808.

Valdehuesa, Kris Niño G., Kristine Rose M. Ramos, Llewelyn S. Moron, Imchang Lee, Grace M. Nisola, Won-keun Lee, and Wook-jin Chung. "Draft genome sequence of newly isolated agarolytic bacteria *Cellulophaga omnivescoria* sp. nov. W5C carrying several gene loci for marine polysaccharide degradation." *Current microbiology* 75, no. 7 (2018): 925-933.
