# Supplementary material for: Structural color in the bacterial domain: The ecogenomics of a 2-dimensional optical phenotype
Source: Proc Natl Acad Sci U S A. 2024 Jul 11;121(29):e2309757121. doi: 10.1073/pnas.2309757121 (PMC11260094; doi:10.1073/pnas.2309757121)
Supplement: Supplementary file 2 — Appendix 02 (PDF) [file pnas.2309757121.sapp2.pdf]

Strain	Species	Phylum	Class	Structural Colour	Accession	Source	Cultivation media
HM-02	Algibacter lectus	Bacteroidetes	Flavobacteriia	yes	PRJEB56913	This study	RMAR
HM-73	Algibacter lectus	Bacteroidetes	Flavobacteriia	yes	PRJEB56913	This study	RMAR
HM-75	Algibacter lectus	Bacteroidetes	Flavobacteriia	yes	PRJEB56913	This study	RMAR
HM-41	Algicola bacteriolytica	Proteobacteria	Gammaproteobacteria	yes	PRJEB56913	This study	RMAR
HM-65	Alteromonas halophila	Proteobacteria	Gammaproteobacteria	yes	PRJEB56913	This study	RMAR
HM-74	Cellulophaga fucicola	Bacteroidetes	Flavobacteriia	yes	PRJEB56913	This study	RMAR
HM-01	Cellulophaga lytica	Bacteroidetes	Flavobacteriia	yes	PRJEB56913	This study	RMAR
HM-03	Cellulophaga lytica	Bacteroidetes	Flavobacteriia	yes	PRJEB56913	This study	RMAR
HM-04	Cellulophaga lytica	Bacteroidetes	Flavobacteriia	yes	PRJEB56913	This study	RMAR
HM-05	Cellulophaga lytica	Bacteroidetes	Flavobacteriia	yes	PRJEB56913	This study	RMAR
HM-26	Cellulophaga lytica	Bacteroidetes	Flavobacteriia	yes	PRJEB56913	This study	RMAR
HM-27	Cellulophaga lytica	Bacteroidetes	Flavobacteriia	yes	PRJEB56913	This study	RMAR
HM-49	Cellulophaga lytica	Bacteroidetes	Flavobacteriia	yes	PRJEB56913	This study	RMAR
HM-50	Cellulophaga lytica	Bacteroidetes	Flavobacteriia	yes	PRJEB56913	This study	RMAR
HM-51	Cellulophaga lytica	Bacteroidetes	Flavobacteriia	yes	PRJEB56913	This study	RMAR
HM-52	Cellulophaga lytica	Bacteroidetes	Flavobacteriia	yes	PRJEB56913	This study	RMAR
HM-53	Cellulophaga lytica	Bacteroidetes	Flavobacteriia	yes	PRJEB56913	This study	RMAR
HM-54	Cellulophaga lytica	Bacteroidetes	Flavobacteriia	yes	PRJEB56913	This study	RMAR
HM-55	Cellulophaga lytica	Bacteroidetes	Flavobacteriia	yes	PRJEB56913	This study	RMAR
HM-56	Cellulophaga lytica	Bacteroidetes	Flavobacteriia	yes	PRJEB56913	This study	RMAR
HM-57	Cellulophaga lytica	Bacteroidetes	Flavobacteriia	yes	PRJEB56913	This study	RMAR
HM-58	Cellulophaga lytica	Bacteroidetes	Flavobacteriia	yes	PRJEB56913	This study	RMAR
HM-59	Cellulophaga lytica	Bacteroidetes	Flavobacteriia	yes	PRJEB56913	This study	RMAR
HM-60	Cellulophaga lytica	Bacteroidetes	Flavobacteriia	yes	PRJEB56913	This study	RMAR
HM-63	Cellulophaga lytica	Bacteroidetes	Flavobacteriia	yes	PRJEB56913	This study	RMAR
HM-15	Cellulophaga pacifica	Bacteroidetes	Flavobacteriia	yes	PRJEB56913	This study	RMAR
HM-48	Cellulophaga pacifica	Bacteroidetes	Flavobacteriia	yes	PRJEB56913	This study	RMAR
HM-11	Cyclobacterium marinum	Bacteroidetes	Cytophagia	no	PRJEB56913	This study	RMAR
HM-20	Enterobacter asburiae	Proteobacteria	Gammaproteobacteria	no	PRJEB56913	Hamidjaja et al 2020	Luria Agar
HM-10	Flavobacterium antarcticum	Bacteroidetes	Flavobacteriia	no	PRJEB56913	This study	RMAR
DSM18293	Flavobacterium aquidurense	Bacteroidetes	Flavobacteriia	yes	GCA_900107365.1	Cousin et al 2007	ASW
DSM15718	Flavobacterium degerlachei	Bacteroidetes	Flavobacteriia	no	GCA_900106645.1	DSMZ Strain Collection	DSMZ
DSM15343	Flavobacterium gelidilacus	Bacteroidetes	Flavobacteriia	no	GCA_900142355.1	DSMZ Strain Collection	DSMZ
DSM2064	Flavobacterium johnsoniae	Bacteroidetes	Flavobacteriia	yes	GCA_000422685.1	McBride and Braun 2004	ASW
UW101	Flavobacterium johnsoniae	Bacteroidetes	Flavobacteriia	yes	GCA_000016645.1	McBride and Braun 2004	ASW
V4-33	Flavobacterium psychrophilum	Bacteroidetes	Flavobacteriia	no	GCA_000831205.1	DSMZ Strain Collection	RMAR
F52	Flavobacterium sp. F52	Bacteroidetes	Flavobacteriia	yes	GCA_000278705.1	Kolton et al 2012	ASW
IR1	Flavobacterium sp. IR1	Bacteroidetes	Flavobacteriia	yes	GCA_002277835.1	Johansen et al 2018	ASW
DD5b	Flavobacterium succinicans	Bacteroidetes	Flavobacteriia	no	GCA_001662485.1	Poehlein et al 2017	RMAR
HM-07	Flexithrix dorotheae	Bacteroidetes	Cytophagia	yes	PRJEB56913	This study	RMAR
HM-09	Gillisia mitskevichiae	Bacteroidetes	Flavobacteriia	no	PRJEB56913	This study	RMAR
HM-13	Gramella echinicola	Bacteroidetes	Flavobacteriia	no	PRJEB56913	This study	RMAR
HM-14	Gramella forsetii	Bacteroidetes	Flavobacteriia	no	PRJEB56913	This study	RMAR
HM-68	Kriegella aquimaris	Bacteroidetes	Flavobacteriia	yes	PRJEB56913	This study	RMAR
HM-08	Maribacter dokdonensis	Bacteroidetes	Flavobacteriia	yes	PRJEB56913	This study	RMAR
HM-62	Maribacter dokdonensis	Bacteroidetes	Flavobacteriia	yes	PRJEB56913	This study	RMAR
HM-61	Maribacter forsetii	Bacteroidetes	Flavobacteriia	no	PRJEB56913	This study	RMAR
HM-06	Marinobacter adhaerens	Proteobacteria	Gammaproteobacteria	yes	PRJEB56913	This study	RMAR
HM-42	Marinobacter adhaerens	Proteobacteria	Gammaproteobacteria	yes	PRJEB56913	This study	RMAR
HM-44	Marinobacter adhaerens	Proteobacteria	Gammaproteobacteria	yes	PRJEB56913	This study	RMAR
HM-28	Marinobacter algicola	Proteobacteria	Gammaproteobacteria	yes	PRJEB56913	This study	RMAR
HM-30	Marinobacter algicola	Proteobacteria	Gammaproteobacteria	yes	PRJEB56913	This study	RMAR
HM-32	Marinobacter algicola	Proteobacteria	Gammaproteobacteria	yes	PRJEB56913	This study	RMAR
HM-33	Marinobacter algicola	Proteobacteria	Gammaproteobacteria	yes	PRJEB56913	This study	RMAR
HM-34	Marinobacter algicola	Proteobacteria	Gammaproteobacteria	yes	PRJEB56913	This study	RMAR
HM-35	Marinobacter algicola	Proteobacteria	Gammaproteobacteria	yes	PRJEB56913	This study	RMAR
HM-36	Marinobacter algicola	Proteobacteria	Gammaproteobacteria	yes	PRJEB56913	This study	RMAR
HM-39	Marinobacter algicola	Proteobacteria	Gammaproteobacteria	yes	PRJEB56913	This study	RMAR
HM-43	Marinobacter algicola	Proteobacteria	Gammaproteobacteria	yes	PRJEB56913	This study	RMAR
HM-64	Marinobacter algicola	Proteobacteria	Gammaproteobacteria	yes	PRJEB56913	This study	RMAR
HM-31	Marinobacter pelagius	Proteobacteria	Gammaproteobacteria	yes	PRJEB56913	This study	RMAR
HM-67	Marinobacter sediminum	Proteobacteria	Gammaproteobacteria	yes	PRJEB56913	This study	RMAR
HM-69	Marinobacter sediminum	Proteobacteria	Gammaproteobacteria	yes	PRJEB56913	This study	RMAR
DSM15361	Mesonia algae	Bacteroidetes	Flavobacteriia	no	GCA_003253545.1	DSMZ Strain Collection	RMAR
HM-29	Muricauda flavescens	Bacteroidetes	Flavobacteriia	yes	PRJEB56913	This study	RMAR
HM-21	Muricauda ruestringensis	Bacteroidetes	Flavobacteriia	yes	PRJEB56913	This study	RMAR
HM-22	Muricauda ruestringensis	Bacteroidetes	Flavobacteriia	yes	PRJEB56913	This study	RMAR
HM-23	Muricauda ruestringensis	Bacteroidetes	Flavobacteriia	yes	PRJEB56913	This study	RMAR
HM-24	Muricauda ruestringensis	Bacteroidetes	Flavobacteriia	yes	PRJEB56913	This study	RMAR
HM-25	Muricauda ruestringensis	Bacteroidetes	Flavobacteriia	yes	PRJEB56913	This study	RMAR
HM-37	Muricauda ruestringensis	Bacteroidetes	Flavobacteriia	yes	PRJEB56913	This study	RMAR
HM-40	Muricauda ruestringensis	Bacteroidetes	Flavobacteriia	yes	PRJEB56913	This study	RMAR
PAO1	Pseudomonas aeruginosa	Proteobacteria	Gammaproteobacteria	no	GCA_000006765.1	Stover et al 2000	RMAR
HM-45	Tenacibaculum gallaicum	Proteobacteria	Gammaproteobacteria	yes	PRJEB56913	This study	RMAR
HM-46	Tenacibaculum gallaicum	Proteobacteria	Gammaproteobacteria	yes	PRJEB56913	This study	RMAR
HM-47	Tenacibaculum gallaicum	Proteobacteria	Gammaproteobacteria	yes	PRJEB56913	This study	RMAR
HM-48	Tenacibaculum mesophilum	Proteobacteria	Gammaproteobacteria	yes	PRJEB56913	This study	RMAR
HM-38	Virgibacillus dokdonensis	Firmicutes	Bacilli	yes	PRJEB56913	This study	RMAR
HM-19	Zooshikella ganghwensis	Proteobacteria	Gammaproteobacteria	no	PRJEB56913	This study	RMAR
HM-66	Zooshikella ganghwensis	Proteobacteria	Gammaproteobacteria	no	PRJEB56913	This study	RMAR
HM-12	Zunongwangia profunda	Proteobacteria	Gammaproteobacteria	no	PRJEB56913	This study	RMAR
18	Cellulophaga baltica	Bacteroidetes	Flavobacteriia	yes	GCA_000468615.2	Hahnke and Harder 2013	RMAR
ACAM48	Salegentibacter salegens	Bacteroidetes	Flavobacteriia	no	GCA_900142975.1	This study	RMAR
CAM030	Olleya marilimosa	Bacteroidetes	Flavobacteriia	no	GCA_000518485.1	This study	RMAR
CECT7946	Winogradskyella eximia	Bacteroidetes	Flavobacteriia	no	GCA_003386165.1	This study	RMAR
CGMCC1.8863	Arenibacter nanhaiticus	Bacteroidetes	Flavobacteriia	yes	GCA_900141935.1	This study	RMAR
CL8139	Cellulophaga lytica	Bacteroidetes	Flavobacteriia	yes	GCA_900197635.1	Kientz et al 2012	RMAR
da5A-8	Taenibaculum ovolyticum	Bacteroidetes	Flavobacteriia	yes	GCA_001641405.1	This study	RMAR
DAU203	Cellulophaga lytica	Bacteroidetes	Flavobacteriia	yes	GCA_001941605.1	Hahnke and Harder 2013	RMAR
DSM1208	Zobellia galactanivorans	Bacteroidetes	Flavobacteriia	no	GCA_000973105.1	DSMZ Strain Collection	DSMZ
DSM12212	Psychroserpens burtonensis	Bacteroidetes	Flavobacteriia	no	GCA_000425305.1	DSMZ Strain Collection	DSMZ
DSM15365	Algibacter lectus	Bacteroidetes	Flavobacteriia	yes	GCA_900112395.1	Hahnke and Harder 2013	RMAR
DSM16505T	Tenacibaculum lutimaris	Bacteroidetes	Flavobacteriia	yes	GCA_003610735.1	DSMZ Strain Collection	DSMZ
DSM18103	Taenibaculum ovolyticum	Bacteroidetes	Flavobacteriia	yes	GCA_003664185.1	DSMZ Strain Collection	DSMZ
DSM18841	Tenacibaculum gallaicum	Bacteroidetes	Flavobacteriia	yes	GCA_003387615.1	DSMZ Strain Collection	DSMZ
DSM18842	Tenacibaculum discolor	Bacteroidetes	Flavobacteriia	yes	GCA_003664185.1	DSMZ Strain Collection	DSMZ
DSM19866	Rhizomicrobium palustre	Proteobacteria	Gammaproteobacteria	no	GCA_900103215.1	DSMZ Strain Collection	DSMZ
DSM2041	Aquimarina latercula	Bacteroidetes	Flavobacteriia	yes	GCA_000430645.1	DSMZ Strain Collection	DSMZ
DSM2061	Zobellia uliginosa Zobell	Bacteroidetes	Flavobacteriia	no	GCA_000744555.1	DSMZ Strain Collection, Thomas et al 2017	DSMZ
DSM21164	Cellulophaga tyrosinoxydans	Bacteroidetes	Flavobacteriia	yes	GCA_900176415.1	DSMZ Strain Collection	DSMZ
DSM23404	Salegentibacter mishustinae	Bacteroidetes	Flavobacteriia	no	GCA_003254095.1	DSMZ Strain Collection	DSMZ
DSM23424	Ulvibacter antarcticus	Bacteroidetes	Flavobacteriia	no	GCA_003688405.1	DSMZ Strain Collection	DSMZ
DSM24786	Cellulophaga fucicola	Bacteroidetes	Flavobacteriia	yes	GCA_900119145.1	Hahnke and Harder 2013	DSMZ
DSM7489	Cellulophaga lytica	Bacteroidetes	Flavobacteriia	yes	GCA_000190595.1	DSMZ Strain Collection	DSMZ
DSW1	Dokdonia donghaensis	Bacteroidetes	Flavobacteriia	no	GCA_001653755.1	This study	RMAR
DSW5	Polaribacter dokdonensis	Bacteroidetes	Flavobacteriia	yes	GCA_001280865.1	Hahnke and Harder 2013	RMAR
DSW6	Nonlabens dokdonensis	Bacteroidetes	Flavobacteriia	no	GCA_000332115.1	This study	RMAR
DSW8	Maribacter dokdonensis	Bacteroidetes	Flavobacteriia	yes	GCA_001447995.1	Hahnke and Harder 2013, Kwak et al 2007	RMAR
HI1	Cellulophaga lytica	Bacteroidetes	Flavobacteriia	yes	GCA_000750195.1	Hahnke and Harder 2013	RMAR
KCOM3131	Porphyromonas gingivalis	Bacteroidetes	Flavobacteriia	no	GCA_002753935.1	This study	RMAR
KCTC12974	Salegentibacter salarius	Bacteroidetes	Flavobacteriia	no	GCA_001752425.1	This study	RMAR
KL-A	Cellulophaga geojensis	Bacteroidetes	Flavobacteriia	yes	GCA_000568425.1	This study	RMAR
L12	Enterobacter cloacae	Proteobacteria	Gammaproteobacteria	no	GCA_000262525.1	This study	Luria Agar
LL0412.1.7	Tenacibaculum soleae	Bacteroidetes	Flavobacteriia	yes	GCA_001693415.1	This study	RMAR
MAR_2009_71	Maribacter dokdonensis	Bacteroidetes	Flavobacteriia	yes	GCA_900105545.1	Hahnke and Harder 2013	RMAR
NN016038	Cellulophaga baltica	Bacteroidetes	Flavobacteriia	yes	GCA_000477035.2	Hahnke and Harder 2013	RMAR
W5C	Cellulophaga omnivescoria	Bacteroidetes	Flavobacteriia	yes	GCA_001999725.1	Valdehuesa et al 2018	RMAR
