# Supplementary material for: Structural color in the bacterial domain: The ecogenomics of a 2-dimensional optical phenotype
Source: Proc Natl Acad Sci U S A. 2024 Jul 11;121(29):e2309757121. doi: 10.1073/pnas.2309757121 (PMC11260094; doi:10.1073/pnas.2309757121)
Supplement: Supplementary file 3 — Appendix 03 (PDF) [file pnas.2309757121.sapp3.pdf]

Mutant	Independent Isolates	NCBI Annotation	NCBI Accession	Citation
M1		Glutamine-hydrolyzing GMP synthase	WP_095382249.1	Johansen et al (2018)
M5	F7	pyrophosphohydrolase	WP_095383901.1	Johansen et al (2018)
M6	M17	Hypothetical protein	WP_095381450.1	Johansen et al (2018)
M8	M16, M43, M54, M70, M79, M84, M103, M108, M115, M130	SRPBCC family protein	WP_095382498.1	Johansen et al (2018)
M9		TetR/AcrR family transcriptional regulator	WP_095385583.1	Johansen et al (2018)
M10		NADP-dependent malic enzyme	WP_095385104.1	Johansen et al (2018)
M12		Type IX secretion system membrane protein PorP/SprF	WP_095384566.1	Johansen et al (2018)
M19		Homogentisate 1,2-dioxygenase	WP_095381596.1	Johansen et al (2018)
M22		SulP family inorganic anion transporter	WP_095382490.1	Johansen et al (2018)
M23	M140, M147, M150	T9SS type B sorting domain-containing protein SprB	WP_095384565.1	Johansen et al (2018)
M40	M45	GH3 auxin-responsive promoter family protein	WP_095385038.1	Johansen et al (2018)
M41		DUF4271 domain-containing protein	WP_095384729.1	Johansen et al (2018)
M47	M61, M73	Non-ribosomal peptide synthetase	WP_095381100.1	Johansen et al (2018)
M49	M64	tRNA (guanosine(37)-N1)-methyltransferase TrmD	WP_089051010.1	Johansen et al (2018)
M51		MFS transporter	WP_095385407.1	Johansen et al (2018)
M52	F12, F19	Hypothetical protein, tetratricopeptide repeat protein.	WP_095384806.1	Johansen et al (2018)
M65		helix-turn-helix domain-containing protein	WP_095385553.1	Johansen et al (2018)
M74		helix-turn-helix transcriptional regulator	WP_095381751.1	Johansen et al (2018)
M75	F2	Amidophosphoribosyl transferase	WP_095385721.1	Johansen et al (2018)
M76	M77, M142	nucleotide sugar dehydrogenase	WP_095383024.1	Johansen et al (2018)
M86	M88	endoxylanase	WP_095382241.1	Johansen et al (2018)
M116		DUF2971 domain-containing protein	WP_095382843.1	Johansen et al (2018)
M141		hypothetical protein	WP_095383019.1	Johansen et al (2018)
M148		restriction endonuclease subunit R	WP_095384051.1	Johansen et al (2018)
F1	F27*	N-acetylglucosamine kinase *F27 hit is in the intergenic region upstream of F1	WP_095383483.1	This work
F5	F14, F21	SusC family, TonB-linked outer membrane protein	WP_095385405.1	Kerkhof et al (2022)
F13		SusD family	WP_095385763.1	Kerkhof et al (2022)
F20		Hypothetical protein, GH3 auxin-responsive promoter family protein	WP_095384466.1	This work
F23		LacI family transcriptional regulator	WP_089049467.1	Kerkhof et al (2022)
F26.2		Hypothetical protein, SH3, type 3 domain protein	WP_095382705.1	This work
D-F160		M28 family peptidase	WP_095381440.1	This work
D-F161		SDR family NAD(P)-dependent oxidoreductase	WP_095381089.1	This work
D-F162		Galactokinase	WP_095385733.1	This work
