# Supplementary material for: Structural color in the bacterial domain: The ecogenomics of a 2-dimensional optical phenotype
Source: Proc Natl Acad Sci U S A. 2024 Jul 11;121(29):e2309757121. doi: 10.1073/pnas.2309757121 (PMC11260094; doi:10.1073/pnas.2309757121)
Supplement: Supplementary file 4 — Appendix 04 (PDF) [file pnas.2309757121.sapp4.pdf]

Species	Strain name	Class	Phylum	Gram	Source/Reference	Habitat	SC score	SC phenotype
Cyclobacterium marinum	HM-11	Flavobacteriia	Bacteroides	Gram negative	This study	Littoral	0.6	Yes
Zobellia galactinovorans	DSM1208	Flavobacteriia	Bacteroides	Gram negative	Roscoff	Red algae	0.61	Yes
Zobellia uliginosa	DSM2061	Flavobacteriia	Bacteroides	Gram negative	Roscoff	Seawater	0.59	Yes
Flavobacterium psychrophilum	DSM 21280	Flavobacteriia	Bacteroides	Gram negative	DSMZ	Fish pathogen	0.04	Yes
Flavobacterium psychrophilum	DSM 3660	Flavobacteriia	Bacteroides	Gram negative	DSMZ	Fish pathogen	0.03	Yes
Arenibacter catalasegens	HACA1	Flavobacteriia	Bacteroides	Gram negative	This study	Littoral	0.78	Yes
Tenacibaculum gallaicum	HTGA1	Flavobacteriia	Bacteroides	Gram negative	This study	Marine	0.96	Yes
Flavobacterium anhuiense	HFAN1	Flavobacteriia	Bacteroides	Gram negative	This study	Soil	0.85	Yes
Tenacibaculum lutimaris	DSM16505	Flavobacteriia	Bacteroides	Gram negative	DSMZ	Littoral	0.75	Yes
Algibacter pectinovorans	HAPE1	Flavobacteriia	Bacteroides	Gram negative	This study	Marine	0.58	Yes
Winogradskyella sediminis	DSM 28134	Flavobacteriia	Bacteroides	Gram negative	DSMZ	Marine Sediment	0.13	No
Dokdonia sp.	MED134	Flavobacteriia	Bacteroides	Gram negative	González et al 2011	Marine	0.07	No
Flavobacterium saccharophilum	DSM 1811	Flavobacteriia	Bacteroides	Gram negative	DSMZ	Fresh Water	0.83	Yes
Muricauda antarctica	DSM 26351	Flavobacteriia	Bacteroides	Gram negative	DSMZ	Marine Antarctica	0.91	Yes
Arenibacter troitsensis	DSM 19835	Flavobacteriia	Bacteroides	Gram negative	DSMZ	Marine Sediment	0.88	Yes
Tenacibaculum ovolyticum	DSM 18103	Flavobacteriia	Bacteroides	Gram negative	DSMZ	Marine Fish	0.9	Yes
Aquamarina latercula	NK	Flavobacteriia	Bacteroides	Gram negative	Kientz et al 2013	Littoral	0.87	Yes
Capnocytophaga sputigena	DSM 7273	Flavobacteriia	Bacteroides	Gram negative	DSMZ	Human Mouth	0.22	No
Tenacibaculum mesophilum	ECR	Flavobacteriia	Bacteroides	Gram negative	Mickol et al 2021	Littoral	0.9	Yes
Flexibacter aurantiacus	ATCC35103	Flavobacteriia	Bacteroides	Gram negative	Kientz et al 2013	Littoral	0.64	Yes
Olleya aquimaris	DSM 24464	Flavobacteriia	Bacteroides	Gram negative	DSMZ	Marine	0.22	No
Dokdonia pacifica	NK	Flavobacteriia	Bacteroides	Gram negative	Plymouth	Marine	0.11	No
Lentimicrobium saccharophilum	DSM 100618	Bacteroidea	Bacteroides	Gram negative	DSMZ	Bioreactor	0.23	No
Cellulophaga fucicola	HKSE1	Flavobacteriia	Bacteroides	Gram negative	Roscoff	Littoral	0.97	Yes
Flagellimonas pacifica	HOEK 21	Flavobacteriia	Bacteroides	Gram negative	This study	Marine	0.8	Yes
Muricauda amoyensis	HKSE3	Flavobacteriia	Bacteroides	Gram negative	Roscoff	Marine	0.82	Yes
Muricauda sp	NK	Flavobacteriia	Bacteroides	Gram negative	Plymouth	Marine	0.89	Yes
Muricauda sp	NK	Flavobacteriia	Bacteroides	Gram negative	Roscoff	Marine	0.87	Yes
Cellulophaga sp.	KL-A	Flavobacteriia	Bacteroides	Gram negative	Plymouth	Marine	0.94	Yes
Dokdonia pacifica	Roscoff	Flavobacteriia	Bacteroides	Gram negative	Plymouth	Marine	0.11	No
Chitinophaga filiformis	DSM 527	Chitinophagia	Bacteroides	Gram negative	DSMZ	Soil	0.6	Yes
Sediminibacterium goheungense	DSM 28323	Chitinophagia	Bacteroides	Gram negative	DSMZ	Fresh Water	0.37	No
Arachidicoccus ginsenosidivorans	DSM 22792	Chitinophagia	Bacteroides	Gram negative	DSMZ	Soil	0.25	No
Hydrotalea sandarakina	DSM 23241	Chitinophagia	Bacteroides	Gram negative	DSMZ	Hot Spring	0.29	Yes
Hydrotalea sandarakina	DSM 23242	Chitinophagia	Bacteroides	Gram negative	DSMZ	Hot Spring	0.29	Yes
Arachidicoccus sp	NK	Chitinophagia	Bacteroides	Gram negative	This study	Soil	0.21	No
Solitalea canadensis	DSM 3403	Sphingobacteriia	Bacteroides	Gram negative	DSMZ	Soil	0.37	Yes
Winogradskyella sp	Roscoff	Flavobacteriia	Bacteroides	Gram negative	This study	Marine	0.12	No
Sulfitobacter porphyrae	HSP1	Alphaproteobacteria	Proteobacteria	Gram negative	This study	Littoral	0.43	Yes
Hoeflea alexandrii	HHA1	Alphaproteobacteria	Proteobacteria	Gram negative	This study	Littoral	0.48	Yes
Hoeflea sp	K57	Alphaproteobacteria	Proteobacteria	Gram negative	This study	Littoral	0.48	Yes
Sphingomonas	HSP1	Alphaproteobacteria	Proteobacteria	Gram negative	This study	Littoral	0.42	Yes
Sphingomonas herbicidivorans	DSM 11019	Alphaproteobacteria	Proteobacteria	Gram negative	DSMZ	Soil	0.42	Yes
Sphingomonas pruni	DSM 10566	Alphaproteobacteria	Proteobacteria	Gram negative	This study	Soil	0.42	Yes
Agrobacterium species	NA	Alphaproteobacteria	Proteobacteria	Gram negative	Banach et al 2019	Freshwater Plant	0.41	Yes
Rhodobacter capsulatus	DSM156	Alphaproteobacteria	Proteobacteria	Gram negative	DSMZ	Freshwater	0.26	No
Rhodospirillum rubrum	HA	Alphaproteobacteria	Proteobacteria	Gram negative	DSMZ	Brackish Water	0.03	No
Rhodobacter sp.	DSM5864	Alphaproteobacteria	Proteobacteria	Gram negative	DSMZ	Freshwater	0.28	No
Janthinobacterium svalbardensis	DSM 25734	Betaproteobacteria	Proteobacteria	Gram negative	DSMZ	Glacier	0.49	Yes
Paraburkholderia madseniana	DSM 110123	Betaproteobacteria	Proteobacteria	Gram negative	DSMZ	Soil	0.56	Yes
Dechloromonas sp	DSM 23276	Betaproteobacteria	Proteobacteria	Gram negative	DSMZ	Mine	0.37	Yes
Pseudogulbenkiania sp	DSM 18807	Betaproteobacteria	Proteobacteria	Gram negative	DSMZ	Freshwater sediment	0.3	Yes
Chitinimonas koreensis	DSM 17726	Betaproteobacteria	Proteobacteria	Gram negative	DSMZ	Soil	0.38	Yes
Thauera aromatica	DSM 6984	Betaproteobacteria	Proteobacteria	Gram negative	DSMZ	Sewage	0.39	Yes
Acidovorax delafieldii	DSM64	Betaproteobacteria	Proteobacteria	Gram negative	DSMZ	Soil	0.51	Yes
Cupriavidus basilensis	DSM 6708	Betaproteobacteria	Proteobacteria	Gram negative	Steinle et al. 1999	Fixed bed reactor	0.57	Yes
Anaeromyxobacter dehalogenans	2CP-1	Deltaproteobacteria	Proteobacteria	Gram negative	DSMZ	Freshwater sediment	0.27	No
Vibrio splendidus	DSM 19640	Gammaproteobacteria	Proteobacteria	Gram negative	DSMZ	Marine	0.31	No
Kangiella koreensis	DSM 16069	Gammaproteobacteria	Proteobacteria	Gram negative	DSMZ	Littoral	0.24	Yes
Kangiella spongicola	DSM 23219	Gammaproteobacteria	Proteobacteria	Gram negative	DSMZ	Marine sponge	0.09	Yes
Kangiella aquimarina	DSM 16071	Gammaproteobacteria	Proteobacteria	Gram negative	DSMZ	Littoral	0.11	Yes
Microbulbifer arenaceous	HMBU2	Gammaproteobacteria	Proteobacteria	Gram negative	DSMZ	Littoral	0.53	Yes
Rhodobacter sp.	Plymouth	Alphaproteobacteria	Proteobacteria	Gram negative	DSMZ	Freshwater	0.28	No
Proteus mirabilis	R1	Gammaproteobacteria	Proteobacteria	Gram negative	Budding et al 2009	Human pathogen	0.22	No
Microbulbifer arenaceous	HMBU1	Gammaproteobacteria	Proteobacteria	Gram negative	This study	Littoral	0.53	Yes
Pseudomonas azotoformans	HPA1	Gammaproteobacteria	Proteobacteria	Gram negative	This study	Littoral	0.6	Yes
Pseudomonas extremorientalis	HPEX1	Gammaproteobacteria	Proteobacteria	Gram negative	This study	Littoral	0.6	Yes
Pseudomonas benzenivorans	HPBE1	Gammaproteobacteria	Proteobacteria	Gram negative	This study	Littoral	0.6	Yes
Pseudomonas lurida	HPL1	Gammaproteobacteria	Proteobacteria	Gram negative	This study	Littoral	0.6	Yes
Pseudomonas fluorescens	HPF1	Gammaproteobacteria	Proteobacteria	Gram negative	This study	Littoral	0.6	Yes
Pseudomonas extremaustralis	HPE1	Gammaproteobacteria	Proteobacteria	Gram negative	This study	Littoral	0.6	Yes
Pseudomonas veronii	HPV1	Gammaproteobacteria	Proteobacteria	Gram negative	This study	Littoral	0.6	Yes
Pseudomonas salomonii	HPS1	Gammaproteobacteria	Proteobacteria	Gram negative	This study	Littoral	0.6	Yes
Pseudomonas poae	HPPO1	Gammaproteobacteria	Proteobacteria	Gram negative	This study	Littoral	0.6	Yes
Legionella taurensis	DSM 21897	Gammaproteobacteria	Proteobacteria	Gram negative	DSMZ	Hospital water	0.18	Yes
Haemophilus influenza	DSM 11121	Gammaproteobacteria	Proteobacteria	Gram negative	This study	Human pathogen	0.16	No
Escherichia coli	K12	Gammaproteobacteria	Proteobacteria	Gram negative	This study	Faecal	0.25	No
Klebsiella pneumoniae	HS11286	Gammaproteobacteria	Proteobacteria	Gram negative	DSMZ	Clinical	0.21	No
Marinobacter spp TT-1	DSM 26291	Gammaproteobacteria	Proteobacteria	Gram negative	DSMZ	Deep water plume	0.84	Yes
Marinobacter spp TK36	DSM 26671	Gammaproteobacteria	Proteobacteria	Gram negative	DSMZ	Marine	0.88	Yes
Marinobacter subterrani	JG233	Gammaproteobacteria	Proteobacteria	Gram negative	Bonis et al 2015	Deep geosphere	0.71	Yes
Pseudoxanthomonas sp	HPSP1	Gammaproteobacteria	Proteobacteria	Gram negative	This study	Littoral	0.42	Yes
Halomonas campisalis	NA	Gammaproteobacteria	Proteobacteria	Gram negative	Mormille et al 1999	Alkaline salt flats	0.49	Yes
Alcanivorax balearicus	MACL04(T)	Gammaproteobacteria	Proteobacteria	Gram negative	Rivas et al 2007	Saline groundwater	0.18	Yes
Pseudomonas composti	DSM 25649	Gammaproteobacteria	Proteobacteria	Gram negative	DSMZ	Littoral	0.52	Yes
Pseudomonas syringae	DSM 50272	Gammaproteobacteria	Proteobacteria	Gram negative	DSMZ	Littoral	0.51	Yes
Pseudomonas sagittaria	DSM 27945	Gammaproteobacteria	Proteobacteria	Gram negative	DSMZ	Littoral	0.5	Yes
Pseudomonas moorei	DSM 12647	Gammaproteobacteria	Proteobacteria	Gram negative	DSMZ	Littoral	0.37	Yes
Pseudomonas stutzeri	DSM 13592	Gammaproteobacteria	Proteobacteria	Gram negative	DSMZ	Littoral	0.51	Yes
Pseudomonas paracarnis	V5/DAB/2/5T	Gammaproteobacteria	Proteobacteria	Gram negative	Lick et al 2021	Food	0.6	Yes
Xylella fastidiosa subsp. multiplex	103418	Gammaproteobacteria	Proteobacteria	Gram negative	DSMZ	Plant	0.1	No
Oceanospirillum maris	DSM6286	Gammaproteobacteria	Proteobacteria	Gram negative	DSMZ	Marine	0.35	No
Oceanospirillum beijerinckii	DSM6288	Gammaproteobacteria	Proteobacteria	Gram negative	DSMZ	Marine	0.37	No
Listeria goaensis sp	DSM 29886	Bacilli	Firmicutes	Gram positive	Doijad et al 2018	Brackish Water	0.24	Yes
Listeria monocytogenes	EGDe	Bacilli	Firmicutes	Gram positive	Bierne et al 2007	Food	0.24	Yes
Paenibacillus glucanolyticus	Vortex	Bacilli	Firmicutes	Gram positive	Ingham and Ben Jacob	Soil	0.26	Yes
Rhodococcus sp	PIR4	Actinobacteria	Actinobacteria	Gram positive	Hamidjaja et al, 2020	Soil	0.25	No
Bacillus subtilis	168	Bacilli	Firmicutes	Gram positive	DSMZ	Soil	0.22	No
Streptomyces coelicolor	A3(2)	Actinobacteria	Actinobacteria	Gram positive	DSMZ	Soil	0.03	No
Streptomyses lividans	ZX7	Actinobacteria	Actinobacteria	Gram positive	DSMZ	Soil	0.03	No
