# Supplementary material for: Structural color in the bacterial domain: The ecogenomics of a 2-dimensional optical phenotype
Source: Proc Natl Acad Sci U S A. 2024 Jul 11;121(29):e2309757121. doi: 10.1073/pnas.2309757121 (PMC11260094; doi:10.1073/pnas.2309757121)
Supplement: Supplementary file 5 — Appendix 05 (PDF) [file pnas.2309757121.sapp5.pdf]

Species	Strain number	% agar (w/v)	Medium Type	Spreading rate (mm/day)
Microbulbifer sp.	HMBU2	0.2	RMAR	0.2 ± 0.1
Microbulbifer sp.	HMBU2	0.25	RMAR	0.2 ± 0.1
Pseudoxanthomonas sp.	HPSP1	0.2	RMAR	2.3 ± 0.9
Pseudoxanthomonas sp.	HPSP1	0.25	RMAR	0.9 ± 0.03
Hoeflea sp.	HMBU1	0.2	RMAR	3.0 ± 0.1
Hoeflea sp.	HMBU1	0.25	RMAR	3.0 ± 0.03
Hoeflea sp.	HMBU1	0.8	RMAR	10.0 ± 0.05
Hoeflea alexandrii	DSM 16655	0.2	RMAR	8.7 ± 0.3
Hoeflea alexandrii	DSM 16655	0.25	RMAR	3.1 ± 0.5
Sulfitobacter sp.	SAN1	0.2	RMAR	0.2 ± 0.1
Sulfitobacter sp.	SAN1	0.25	RMAR	0.3 ± 0.2
Marinobacter algicola	HM28	0.2	RMAR	6.2
Marinobacter algicola	HM28	0.25	RMAR	1.9
Marinobacter subterrani	JG322	0.2	RMAR	8.0 ± 0.4
Marinobacter subterrani	JG322	0.25	RMAR	4.7
Marinobacter subterrani	JG322 ΔflaBG	0.2	RMAR	0.03 ± 0.03
Marinobacter subterrani	JG322 ΔflaBG	0.25	RMAR	0.06 ± 0.1
Escherichia coli	DH5α	0.2	LA	10.5 ± 1.2
Escherichia coli	DH5α	0.25	LA	10.4 ± 2.7
Kangiella sediminilitoralis		0.2	RMAR	0.2 ± 0.2
Kangiella sediminilitoralis		0.8	RMAR	9 ± 0.4
