# Supplementary figures and images for: Structural color in the bacterial domain: The ecogenomics of a 2-dimensional optical phenotype
Source: Proc Natl Acad Sci U S A. 2024 Jul 11;121(29):e2309757121. doi: 10.1073/pnas.2309757121 (PMC11260094; doi:10.1073/pnas.2309757121)

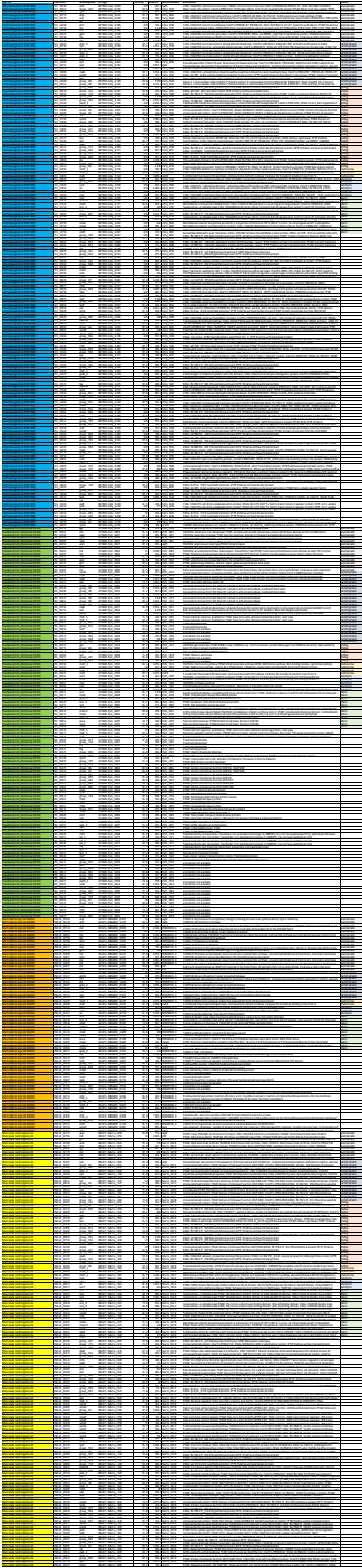

Supplement: Supplementary file 6 — Appendix 06 (PDF) [file pnas.2309757121.sapp6.pdf]
