# Supplementary material for: Structural color in the bacterial domain: The ecogenomics of a 2-dimensional optical phenotype
Source: Proc Natl Acad Sci U S A. 2024 Jul 11;121(29):e2309757121. doi: 10.1073/pnas.2309757121 (PMC11260094; doi:10.1073/pnas.2309757121)
Supplement: Supplementary file 10 — Dataset S04 (PDF) [file pnas.2309757121.sd04.pdf]

Assembly	Analysis accession	Analysis assembly accessio	Sample biome lineage	SC score
MGYA00002187/ERP000673_SL_FASTA_nt_reads.fasta	MGYA00002187	ERR034495	root:Host-associated:Animal:Digestive system	0.0294
MGYA00002188/ERR263549_FASTA_nt_reads.fasta	MGYA00002188	ERR263549	root:Environmental:Terrestrial:Soil:Forest soil	0.0848
MGYA00002318/ERR034496_SL_FASTA_nt_reads.fasta	MGYA00002318	ERR034496	root:Host-associated:Animal:Digestive system	0.0294
MGYA00002319/ERR034497_SL_FASTA_nt_reads.fasta	MGYA00002319	ERR034497	root:Host-associated:Animal:Digestive system	0.0294
MGYA00002320/ERR034498_SL_FASTA_nt_reads.fasta	MGYA00002320	ERR034498	root:Host-associated:Animal:Digestive system	0.0294
MGYA00003147/ERR577137_FASTA_nt_reads.fasta	MGYA00003147	ERR577137_ASSEMBLY	root:Host-associated	0.0342
MGYA00003148/ERR577138_FASTA_nt_reads.fasta	MGYA00003148	ERR577138_ASSEMBLY	root:Host-associated	0.052
MGYA00004354/ERR700144_FASTA_nt_reads.fasta	MGYA00004354	ERR700144	root:Environmental:Aquatic:Freshwater	0.6604
MGYA00004355/ERR700145_FASTA_nt_reads.fasta	MGYA00004355	ERR700145	root:Environmental:Aquatic:Freshwater	0.565
MGYA00004356/ERR700146_FASTA_nt_reads.fasta	MGYA00004356	ERR700146	root:Environmental:Aquatic:Freshwater	0.322
MGYA00004357/ERR700147_FASTA_nt_reads.fasta	MGYA00004357	ERR700147	root:Environmental:Aquatic:Freshwater	0.4754
MGYA00004358/ERR700148_FASTA_nt_reads.fasta	MGYA00004358	ERR700148	root:Environmental:Aquatic:Marine	0.3572
MGYA00004359/ERR700149_FASTA_nt_reads.fasta	MGYA00004359	ERR700149	root:Environmental:Terrestrial:Soil	0.556
MGYA00004360/ERR700150_FASTA_nt_reads.fasta	MGYA00004360	ERR700150	root:Environmental:Aquatic:Freshwater	0.4086
MGYA00004361/ERR700151_FASTA_nt_reads.fasta	MGYA00004361	ERR700151	root:Environmental:Terrestrial:Soil	0.5648
MGYA00004362/ERR700152_FASTA_nt_reads.fasta	MGYA00004362	ERR700152	root:Environmental:Aquatic:Freshwater	0.4114
MGYA00004363/ERR700153_FASTA_nt_reads.fasta	MGYA00004363	ERR700153	root:Environmental:Aquatic:Marine	0.3812
MGYA00004364/ERR700154_FASTA_nt_reads.fasta	MGYA00004364	ERR700154	root:Environmental:Aquatic:Freshwater	0.7032
MGYA00004365/ERR700155_FASTA_nt_reads.fasta	MGYA00004365	ERR700155	root:Environmental:Aquatic:Freshwater	0.4844
MGYA00004366/ERR700156_FASTA_nt_reads.fasta	MGYA00004366	ERR700156	root:Environmental:Aquatic:Freshwater	0.644
MGYA00004367/ERR700157_FASTA_nt_reads.fasta	MGYA00004367	ERR700157	root:Environmental:Aquatic:Freshwater	0.3288
MGYA00004368/ERR700158_FASTA_nt_reads.fasta	MGYA00004368	ERR700158	root:Environmental:Terrestrial:Soil	0.665
MGYA00004369/ERR700159_FASTA_nt_reads.fasta	MGYA00004369	ERR700159	root:Environmental:Terrestrial:Soil	0.7288
MGYA00004370/ERR700160_FASTA_nt_reads.fasta	MGYA00004370	ERR700160	root:Environmental:Aquatic:Freshwater	0.3188
MGYA00004371/ERR700161_FASTA_nt_reads.fasta	MGYA00004371	ERR700161	root:Environmental:Aquatic:Freshwater	0.4814
MGYA00004372/ERR700162_FASTA_nt_reads.fasta	MGYA00004372	ERR700162	root:Environmental:Aquatic:Freshwater	0.4852
MGYA00004373/ERR700163_FASTA_nt_reads.fasta	MGYA00004373	ERR700163	root:Environmental:Aquatic:Freshwater	0.2854
MGYA00004374/ERR700164_FASTA_nt_reads.fasta	MGYA00004374	ERR700164	root:Environmental:Aquatic:Freshwater	0.2922
MGYA00004375/ERR700165_FASTA_nt_reads.fasta	MGYA00004375	ERR700165	root:Environmental:Terrestrial:Soil	0.4646
MGYA00004376/ERR700166_FASTA_nt_reads.fasta	MGYA00004376	ERR700166	root:Environmental:Terrestrial:Soil	0.6248
MGYA00004377/ERR700167_FASTA_nt_reads.fasta	MGYA00004377	ERR700167	root:Environmental:Aquatic:Freshwater	0.6716
MGYA00004378/ERR700168_FASTA_nt_reads.fasta	MGYA00004378	ERR700168	root:Environmental:Terrestrial:Soil	0.4968
MGYA00004379/ERR700169_FASTA_nt_reads.fasta	MGYA00004379	ERR700169	root:Environmental:Terrestrial:Soil	0.6202
MGYA00004380/ERR700170_FASTA_nt_reads.fasta	MGYA00004380	ERR700170	root:Environmental:Aquatic:Freshwater	0.6778
MGYA00004381/ERR700171_FASTA_nt_reads.fasta	MGYA00004381	ERR700171	root:Environmental:Terrestrial:Soil	0.2218
MGYA00004382/ERR700172_FASTA_nt_reads.fasta	MGYA00004382	ERR700172	root:Environmental:Terrestrial:Soil	0.5598
MGYA00004383/ERR700173_FASTA_nt_reads.fasta	MGYA00004383	ERR700173	root:Environmental:Terrestrial:Soil	0.202
MGYA00004384/ERR700174_FASTA_nt_reads.fasta	MGYA00004384	ERR700174	root:Environmental:Terrestrial:Soil	0.5166
MGYA00004385/ERR700175_FASTA_nt_reads.fasta	MGYA00004385	ERR700175	root:Environmental:Aquatic:Freshwater	0.058
MGYA00004386/ERR700176_FASTA_nt_reads.fasta	MGYA00004386	ERR700176	root:Environmental:Aquatic:Freshwater	0.0646
MGYA00004389/ERR700179_FASTA_nt_reads.fasta	MGYA00004389	ERR700179	root:Environmental:Terrestrial:Soil	0.2886
MGYA00004390/ERR700180_FASTA_nt_reads.fasta	MGYA00004390	ERR700180	root:Environmental:Terrestrial:Soil	0.2244
MGYA00004391/ERR700181_FASTA_nt_reads.fasta	MGYA00004391	ERR700181	root:Environmental:Terrestrial:Soil	0.3834
MGYA00004392/ERR700182_FASTA_nt_reads.fasta	MGYA00004392	ERR700182	root:Environmental:Terrestrial:Soil	0.2184
MGYA00004393/ERR700183_FASTA_nt_reads.fasta	MGYA00004393	ERR700183	root:Environmental:Terrestrial:Soil	0.2488
MGYA00004394/ERR700184_FASTA_nt_reads.fasta	MGYA00004394	ERR700184	root:Environmental:Terrestrial:Soil	0.2326
MGYA00004395/ERR700185_FASTA_nt_reads.fasta	MGYA00004395	ERR700185	root:Environmental:Terrestrial:Soil	0.4166
MGYA00004396/ERR700186_FASTA_nt_reads.fasta	MGYA00004396	ERR700186	root:Environmental:Terrestrial:Soil	0.1868
MGYA00004397/ERR700187_FASTA_nt_reads.fasta	MGYA00004397	ERR700187	root:Environmental:Terrestrial:Soil	0.9142
MGYA00004398/ERR700188_FASTA_nt_reads.fasta	MGYA00004398	ERR700188	root:Environmental:Terrestrial:Soil	0.6384
MGYA00004399/ERR700189_FASTA_nt_reads.fasta	MGYA00004399	ERR700189	root:Environmental:Terrestrial:Soil	0.6384
MGYA00004400/ERR700190_FASTA_nt_reads.fasta	MGYA00004400	ERR700190	root:Environmental:Terrestrial:Soil	0.7438
MGYA00004401/ERR700191_FASTA_nt_reads.fasta	MGYA00004401	ERR700191	root:Environmental:Terrestrial:Soil	0.749
MGYA00004402/ERR700192_FASTA_nt_reads.fasta	MGYA00004402	ERR700192	root:Environmental:Aquatic:Freshwater	0.7
MGYA00004403/ERR700193_FASTA_nt_reads.fasta	MGYA00004403	ERR700193	root:Environmental:Terrestrial:Soil	0.8522
MGYA00004404/ERR700194_FASTA_nt_reads.fasta	MGYA00004404	ERR700194	root:Environmental:Aquatic:Freshwater	0.7196
MGYA00004405/ERR700195_FASTA_nt_reads.fasta	MGYA00004405	ERR700195	root:Environmental:Aquatic:Freshwater	0.7096
MGYA00004406/ERR700196_FASTA_nt_reads.fasta	MGYA00004406	ERR700196	root:Environmental:Aquatic:Freshwater	0.6818
MGYA00004407/ERR700197_FASTA_nt_reads.fasta	MGYA00004407	ERR700197	root:Environmental:Terrestrial:Soil	0.694
MGYA00004408/ERR700198_FASTA_nt_reads.fasta	MGYA00004408	ERR700198	root:Environmental:Aquatic:Freshwater	0.0522
MGYA00004409/ERR700199_FASTA_nt_reads.fasta	MGYA00004409	ERR700199	root:Environmental:Aquatic:Freshwater	0.6208
MGYA00004410/ERR700200_FASTA_nt_reads.fasta	MGYA00004410	ERR700200	root:Environmental:Aquatic:Freshwater	0.6878
MGYA00012323/FAUS01000001-FAUS01000008_FASTA.fa	MGYA00012323	ERZ225354	root:Host-associated:Mammals:Circulatory system:Blooc	0.124
MGYA00069120/ERZ312747_FASTA.fasta	MGYA00069120	ERZ312747	root:Host-associated:Mammals:Digestive system:Stoma	0.1676
MGYA00069121/ERZ312748_FASTA.fasta	MGYA00069121	ERZ312748	root:Host-associated:Mammals:Digestive system:Stoma	0.1646
MGYA00069123/ERZ312750_FASTA.fasta	MGYA00069123	ERZ312750	root:Host-associated:Mammals:Digestive system:Stoma	0.2228
MGYA00069124/ERZ312751_FASTA.fasta	MGYA00069124	ERZ312751	root:Host-associated:Mammals:Digestive system:Stoma	0.2266
MGYA00069125/ERZ312752_FASTA.fasta	MGYA00069125	ERZ312752	root:Host-associated:Mammals:Digestive system:Stoma	0.34
MGYA00069130/ERZ312749_FASTA.fasta	MGYA00069130	ERZ312749	root:Host-associated:Mammals:Digestive system:Stoma	0.1298
MGYA00071196/SAMN02954284_FASTA.fasta	MGYA00071196	SAMN02954284	root:Environmental:Terrestrial:Soil:Permafrost	0.1206
MGYA00096440/ERZ376968_FASTA_1.fasta	MGYA00096440	ERZ376968	root:Environmental:Aquatic:Marine	0.7208
MGYA00096440/ERZ376968_FASTA_2.fasta	MGYA00096440	ERZ376968	root:Environmental:Aquatic:Marine	0.7118
MGYA00112337/ERZ404940_FASTA.fasta	MGYA00112337	ERZ404940	root:Environmental:Aquatic:Thermal springs:Hot (42-90	0.8054
MGYA00112338/ERZ404941_FASTA.fasta	MGYA00112338	ERZ404941	root:Environmental:Aquatic:Thermal springs:Hot (42-90	0.7996
MGYA00112339/ERZ404943_FASTA.fasta	MGYA00112339	ERZ404943	root:Environmental:Aquatic:Thermal springs:Hot (42-90	0.8482
MGYA00112340/ERZ404942_FASTA.fasta	MGYA00112340	ERZ404942	root:Environmental:Aquatic:Thermal springs:Hot (42-90	0.807
MGYA00131957/ERZ116324_FASTA.fasta	MGYA00131957	ERZ116324	root:Host-associated:Mammals:Digestive system:Large	0.0652
MGYA00131958/ERZ116327_FASTA.fasta	MGYA00131958	ERZ116327	root:Host-associated:Mammals:Digestive system:Large	0.2066
MGYA00131959/ERZ116342_FASTA.fasta	MGYA00131959	ERZ116342	root:Host-associated:Mammals:Digestive system:Large	0.154
MGYA00131960/ERZ116339_FASTA.fasta	MGYA00131960	ERZ116339	root:Host-associated:Mammals:Digestive system:Large	0.1794
MGYA00131961/ERZ116336_FASTA.fasta	MGYA00131961	ERZ116336	root:Host-associated:Mammals:Digestive system:Large	0.318
MGYA00131962/ERZ116326_FASTA.fasta	MGYA00131962	ERZ116326	root:Host-associated:Mammals:Digestive system:Large	0.2066
MGYA00131963/ERZ116322_FASTA.fasta	MGYA00131963	ERZ116322	root:Host-associated:Mammals:Digestive system:Large	0.0734
MGYA00131964/ERZ116321_FASTA.fasta	MGYA00131964	ERZ116321	root:Host-associated:Mammals:Digestive system:Large	0.0788
MGYA00131965/ERZ116332_FASTA.fasta	MGYA00131965	ERZ116332	root:Host-associated:Mammals:Digestive system:Large	0.178
MGYA00131966/ERZ116340_FASTA.fasta	MGYA00131966	ERZ116340	root:Host-associated:Mammals:Digestive system:Large	0.178
MGYA00131967/ERZ116328_FASTA.fasta	MGYA00131967	ERZ116328	root:Host-associated:Mammals:Digestive system:Large	0.0964
MGYA00131968/ERZ116331_FASTA.fasta	MGYA00131968	ERZ116331	root:Host-associated:Mammals:Digestive system:Large	0.2304
MGYA00131969/ERZ116325_FASTA.fasta	MGYA00131969	ERZ116325	root:Host-associated:Mammals:Digestive system:Large	0.0648
MGYA00131970/ERZ116343_FASTA.fasta	MGYA00131970	ERZ116343	root:Host-associated:Mammals:Digestive system:Large	0.3374
MGYA00131971/ERZ116323_FASTA.fasta	MGYA00131971	ERZ116323	root:Host-associated:Mammals:Digestive system:Large	0.0648
MGYA00131972/ERZ116330_FASTA.fasta	MGYA00131972	ERZ116330	root:Host-associated:Mammals:Digestive system:Large	0.097
MGYA00131973/ERZ116333_FASTA.fasta	MGYA00131973	ERZ116333	root:Host-associated:Mammals:Digestive system:Large	0.0802
MGYA00131974/ERZ116329_FASTA.fasta	MGYA00131974	ERZ116329	root:Host-associated:Mammals:Digestive system:Large	0.0684
MGYA00131975/ERZ116319_FASTA.fasta	MGYA00131975	ERZ116319	root:Host-associated:Mammals:Digestive system:Large	0.0638
MGYA00131976/ERZ116338_FASTA.fasta	MGYA00131976	ERZ116338	root:Host-associated:Mammals:Digestive system:Large	0.2328
MGYA00131977/ERZ116337_FASTA.fasta	MGYA00131977	ERZ116337	root:Host-associated:Mammals:Digestive system:Large	0.2046
MGYA00131978/ERZ116335_FASTA.fasta	MGYA00131978	ERZ116335	root:Host-associated:Mammals:Digestive system:Large	0.1386
MGYA00131979/ERZ116320_FASTA.fasta	MGYA00131979	ERZ116320	root:Host-associated:Mammals:Digestive system:Large	0.068
MGYA00131980/ERZ116334_FASTA.fasta	MGYA00131980	ERZ116334	root:Host-associated:Mammals:Digestive system:Large	0.2682
MGYA00131981/ERZ116341_FASTA.fasta	MGYA00131981	ERZ116341	root:Host-associated:Mammals:Digestive system:Large	0.0446
MGYA00132144/ERZ405284_FASTA.fasta	MGYA00132144	ERZ405284	root:Environmental:Aquatic:Thermal springs:Sediment	0.7956
MGYA00132145/ERZ405283_FASTA.fasta	MGYA00132145	ERZ405283	root:Environmental:Aquatic:Thermal springs:Sediment	0.654
MGYA00132146/ERZ312764_FASTA.fasta	MGYA00132146	ERZ312764	root:Environmental:Aquatic:Marine	0.7794
MGYA00133502/ERZ468417_FASTA.fasta	MGYA00133502	ERZ468417	root:Environmental:Aquatic:Freshwater:Ice:Glacier	0.316
MGYA00136566/ERZ478770_FASTA.fasta	MGYA00136566	ERZ478770	root:Engineered:Wastewater:Industrial wastewater:Agri	0.3764
MGYA00136612/ERZ475228_FASTA.fasta	MGYA00136612	ERZ475228	root:Environmental:Aquatic:Marine	0.626
MGYA00136613/ERZ475168_FASTA.fasta	MGYA00136613	ERZ475168	root:Environmental:Aquatic:Marine	0.11
MGYA00136614/ERZ475162_FASTA.fasta	MGYA00136614	ERZ475162	root:Environmental:Aquatic:Marine	0.4802
MGYA00136615/ERZ475227_FASTA.fasta	MGYA00136615	ERZ475227	root:Environmental:Aquatic:Marine	0.4852
MGYA00136616/ERZ475214_FASTA.fasta	MGYA00136616	ERZ475214	root:Environmental:Aquatic:Marine	0.6934
MGYA00136617/ERZ475247_FASTA.fasta	MGYA00136617	ERZ475247	root:Environmental:Aquatic:Marine	0.4784
MGYA00136618/ERZ475185_FASTA.fasta	MGYA00136618	ERZ475185	root:Environmental:Aquatic:Marine	0.7204
MGYA00136619/ERZ475225_FASTA.fasta	MGYA00136619	ERZ475225	root:Environmental:Aquatic:Marine	0.3532
MGYA00136620/ERZ475211_FASTA.fasta	MGYA00136620	ERZ475211	root:Environmental:Aquatic:Marine	0.5742
MGYA00136621/ERZ475178_FASTA.fasta	MGYA00136621	ERZ475178	root:Environmental:Aquatic:Marine	0.6602
MGYA00136622/ERZ475181_FASTA.fasta	MGYA00136622	ERZ475181	root:Environmental:Aquatic:Marine	0.5532
MGYA00136623/ERZ475158_FASTA.fasta	MGYA00136623	ERZ475158	root:Environmental:Aquatic:Marine	0.5052
MGYA00136624/ERZ475248_FASTA.fasta	MGYA00136624	ERZ475248	root:Environmental:Aquatic:Marine	0.3462
MGYA00136625/ERZ475222_FASTA.fasta	MGYA00136625	ERZ475222	root:Environmental:Aquatic:Marine	0.5754
MGYA00136626/ERZ475161_FASTA.fasta	MGYA00136626	ERZ475161	root:Environmental:Aquatic:Marine	0.4184
MGYA00136627/ERZ475149_FASTA.fasta	MGYA00136627	ERZ475149	root:Environmental:Aquatic:Marine	0.5542
MGYA00136628/ERZ475127_FASTA.fasta	MGYA00136628	ERZ475127	root:Environmental:Aquatic:Marine	0.6782
MGYA00136629/ERZ475182_FASTA.fasta	MGYA00136629	ERZ475182	root:Environmental:Aquatic:Marine	0.594
MGYA00136630/ERZ475254_FASTA.fasta	MGYA00136630	ERZ475254	root:Environmental:Aquatic:Marine	0.5644
MGYA00136631/ERZ475131_FASTA.fasta	MGYA00136631	ERZ475131	root:Environmental:Aquatic:Marine	0.4904
MGYA00136632/ERZ475229_FASTA.fasta	MGYA00136632	ERZ475229	root:Environmental:Aquatic:Marine	0.4756
MGYA00136633/ERZ475200_FASTA.fasta	MGYA00136633	ERZ475200	root:Environmental:Aquatic:Marine	0.5444
MGYA00136634/ERZ475164_FASTA.fasta	MGYA00136634	ERZ475164	root:Environmental:Aquatic:Marine	0.8264
MGYA00136635/ERZ475212_FASTA.fasta	MGYA00136635	ERZ475212	root:Environmental:Aquatic:Marine	0.583
MGYA00136636/ERZ473095_FASTA.fasta	MGYA00136636	ERZ473095	root:Environmental:Aquatic:Marine	0.5262
MGYA00136637/ERZ475128_FASTA.fasta	MGYA00136637	ERZ475128	root:Environmental:Aquatic:Marine	0.2334
MGYA00136638/ERZ475255_FASTA.fasta	MGYA00136638	ERZ475255	root:Environmental:Aquatic:Marine	0.3496
MGYA00136639/ERZ475253_FASTA.fasta	MGYA00136639	ERZ475253	root:Environmental:Aquatic:Marine	0.3996
MGYA00136640/ERZ475221_FASTA.fasta	MGYA00136640	ERZ475221	root:Environmental:Aquatic:Marine	0.4294
MGYA00136641/ERZ475205_FASTA.fasta	MGYA00136641	ERZ475205	root:Environmental:Aquatic:Marine	0.5616
MGYA00136642/ERZ475152_FASTA.fasta	MGYA00136642	ERZ475152	root:Environmental:Aquatic:Marine	0.5772
MGYA00136643/ERZ475261_FASTA.fasta	MGYA00136643	ERZ475261	root:Environmental:Aquatic:Marine	0.6154
MGYA00136644/ERZ475194_FASTA.fasta	MGYA00136644	ERZ475194	root:Environmental:Aquatic:Marine	0.478
MGYA00136645/ERZ475199_FASTA.fasta	MGYA00136645	ERZ475199	root:Environmental:Aquatic:Marine	0.573
MGYA00136646/ERZ475230_FASTA.fasta	MGYA00136646	ERZ475230	root:Environmental:Aquatic:Marine	0.6318
MGYA00136647/ERZ475192_FASTA.fasta	MGYA00136647	ERZ475192	root:Environmental:Aquatic:Marine	0.697
MGYA00136648/ERZ475190_FASTA.fasta	MGYA00136648	ERZ475190	root:Environmental:Aquatic:Marine	0.6698
MGYA00136649/ERZ475179_FASTA.fasta	MGYA00136649	ERZ475179	root:Environmental:Aquatic:Marine	0.9084
MGYA00136650/ERZ475176_FASTA.fasta	MGYA00136650	ERZ475176	root:Environmental:Aquatic:Marine	0.6376
MGYA00136651/ERZ475238_FASTA.fasta	MGYA00136651	ERZ475238	root:Environmental:Aquatic:Marine	0.4104
MGYA00136652/ERZ475240_FASTA.fasta	MGYA00136652	ERZ475240	root:Environmental:Aquatic:Marine	0.444
MGYA00136653/ERZ475150_FASTA.fasta	MGYA00136653	ERZ475150	root:Environmental:Aquatic:Marine	0.6162
MGYA00136654/ERZ475136_FASTA.fasta	MGYA00136654	ERZ475136	root:Environmental:Aquatic:Marine	0.5374
MGYA00136655/ERZ475186_FASTA.fasta	MGYA00136655	ERZ475186	root:Environmental:Aquatic:Marine	0.5956
MGYA00136656/ERZ475206_FASTA.fasta	MGYA00136656	ERZ475206	root:Environmental:Aquatic:Marine	0.484
MGYA00136657/ERZ475146_FASTA.fasta	MGYA00136657	ERZ475146	root:Environmental:Aquatic:Marine	0.5164
MGYA00136658/ERZ475249_FASTA.fasta	MGYA00136658	ERZ475249	root:Environmental:Aquatic:Marine	0.3704
MGYA00136659/ERZ475195_FASTA.fasta	MGYA00136659	ERZ475195	root:Environmental:Aquatic:Marine	0.396
MGYA00136660/ERZ475143_FASTA.fasta	MGYA00136660	ERZ475143	root:Environmental:Aquatic:Marine	0.533
MGYA00136661/ERZ475197_FASTA.fasta	MGYA00136661	ERZ475197	root:Environmental:Aquatic:Marine	0.2858
MGYA00136662/ERZ475166_FASTA.fasta	MGYA00136662	ERZ475166	root:Environmental:Aquatic:Marine	0.7752
MGYA00136663/ERZ475138_FASTA.fasta	MGYA00136663	ERZ475138	root:Environmental:Aquatic:Marine	0.4392
MGYA00136664/ERZ475258_FASTA.fasta	MGYA00136664	ERZ475258	root:Environmental:Aquatic:Marine	0.4644
MGYA00136665/ERZ475174_FASTA.fasta	MGYA00136665	ERZ475174	root:Environmental:Aquatic:Marine	0.8786
MGYA00136666/ERZ475148_FASTA.fasta	MGYA00136666	ERZ475148	root:Environmental:Aquatic:Marine	0.6202
MGYA00136667/ERZ475172_FASTA.fasta	MGYA00136667	ERZ475172	root:Environmental:Aquatic:Marine	0.5642
MGYA00136668/ERZ475133_FASTA.fasta	MGYA00136668	ERZ475133	root:Environmental:Aquatic:Marine	0.3412
MGYA00136669/ERZ475163_FASTA.fasta	MGYA00136669	ERZ475163	root:Environmental:Aquatic:Marine	0.6188
MGYA00136670/ERZ475189_FASTA.fasta	MGYA00136670	ERZ475189	root:Environmental:Aquatic:Marine	0.5004
MGYA00136671/ERZ475234_FASTA.fasta	MGYA00136671	ERZ475234	root:Environmental:Aquatic:Marine	0.7402
MGYA00136672/ERZ475147_FASTA.fasta	MGYA00136672	ERZ475147	root:Environmental:Aquatic:Marine	0.729
MGYA00136673/ERZ475132_FASTA.fasta	MGYA00136673	ERZ475132	root:Environmental:Aquatic:Marine	0.699
MGYA00136674/ERZ475224_FASTA.fasta	MGYA00136674	ERZ475224	root:Environmental:Aquatic:Marine	0.2624
MGYA00136675/ERZ475208_FASTA.fasta	MGYA00136675	ERZ475208	root:Environmental:Aquatic:Marine	0.47
MGYA00136676/ERZ475196_FASTA.fasta	MGYA00136676	ERZ475196	root:Environmental:Aquatic:Marine	0.5314
MGYA00136677/ERZ475260_FASTA.fasta	MGYA00136677	ERZ475260	root:Environmental:Aquatic:Marine	0.5682
MGYA00136678/ERZ475177_FASTA.fasta	MGYA00136678	ERZ475177	root:Environmental:Aquatic:Marine	0.4998
MGYA00136679/ERZ475256_FASTA.fasta	MGYA00136679	ERZ475256	root:Environmental:Aquatic:Marine	0.4892
MGYA00136680/ERZ475135_FASTA.fasta	MGYA00136680	ERZ475135	root:Environmental:Aquatic:Marine	0.408
MGYA00136681/ERZ475203_FASTA.fasta	MGYA00136681	ERZ475203	root:Environmental:Aquatic:Marine	0.5952
MGYA00136682/ERZ475142_FASTA.fasta	MGYA00136682	ERZ475142	root:Environmental:Aquatic:Marine	0.3874
MGYA00136683/ERZ475218_FASTA.fasta	MGYA00136683	ERZ475218	root:Environmental:Aquatic:Marine	0.5458
MGYA00136684/ERZ475235_FASTA.fasta	MGYA00136684	ERZ475235	root:Environmental:Aquatic:Marine	0.7326
MGYA00136685/ERZ475257_FASTA.fasta	MGYA00136685	ERZ475257	root:Environmental:Aquatic:Marine	0.5408
MGYA00136686/ERZ475123_FASTA.fasta	MGYA00136686	ERZ475123	root:Environmental:Aquatic:Marine	0.2994
MGYA00136687/ERZ475202_FASTA.fasta	MGYA00136687	ERZ475202	root:Environmental:Aquatic:Marine	0.5974
MGYA00136688/ERZ475232_FASTA.fasta	MGYA00136688	ERZ475232	root:Environmental:Aquatic:Marine	0.5192
MGYA00136689/ERZ475204_FASTA.fasta	MGYA00136689	ERZ475204	root:Environmental:Aquatic:Marine	0.6478
MGYA00136690/ERZ475231_FASTA.fasta	MGYA00136690	ERZ475231	root:Environmental:Aquatic:Marine	0.5526
MGYA00136691/ERZ475250_FASTA.fasta	MGYA00136691	ERZ475250	root:Environmental:Aquatic:Marine	0.372
MGYA00136692/ERZ475180_FASTA.fasta	MGYA00136692	ERZ475180	root:Environmental:Aquatic:Marine	0.5678
MGYA00136693/ERZ475155_FASTA.fasta	MGYA00136693	ERZ475155	root:Environmental:Aquatic:Marine	0.5322
MGYA00136694/ERZ475223_FASTA.fasta	MGYA00136694	ERZ475223	root:Environmental:Aquatic:Marine	0.6276
MGYA00136695/ERZ475209_FASTA.fasta	MGYA00136695	ERZ475209	root:Environmental:Aquatic:Marine	0.711
MGYA00136696/ERZ475210_FASTA.fasta	MGYA00136696	ERZ475210	root:Environmental:Aquatic:Marine	0.7716
MGYA00136697/ERZ475198_FASTA.fasta	MGYA00136697	ERZ475198	root:Environmental:Aquatic:Marine	0.4544
MGYA00136698/ERZ475243_FASTA.fasta	MGYA00136698	ERZ475243	root:Environmental:Aquatic:Marine	0.3234
MGYA00136699/ERZ475144_FASTA.fasta	MGYA00136699	ERZ475144	root:Environmental:Aquatic:Marine	0.4906
MGYA00136700/ERZ475242_FASTA.fasta	MGYA00136700	ERZ475242	root:Environmental:Aquatic:Marine	0.43
MGYA00136701/ERZ475216_FASTA.fasta	MGYA00136701	ERZ475216	root:Environmental:Aquatic:Marine	0.394
MGYA00136702/ERZ475188_FASTA.fasta	MGYA00136702	ERZ475188	root:Environmental:Aquatic:Marine	0.3286
MGYA00136703/ERZ475125_FASTA.fasta	MGYA00136703	ERZ475125	root:Environmental:Aquatic:Marine	0.4762
MGYA00136704/ERZ475184_FASTA.fasta	MGYA00136704	ERZ475184	root:Environmental:Aquatic:Marine	0.6736
MGYA00136705/ERZ475159_FASTA.fasta	MGYA00136705	ERZ475159	root:Environmental:Aquatic:Marine	0.6728
MGYA00136706/ERZ475244_FASTA.fasta	MGYA00136706	ERZ475244	root:Environmental:Aquatic:Marine	0.5128
MGYA00136707/ERZ475215_FASTA.fasta	MGYA00136707	ERZ475215	root:Environmental:Aquatic:Marine	0.3634
MGYA00136708/ERZ475259_FASTA.fasta	MGYA00136708	ERZ475259	root:Environmental:Aquatic:Marine	0.4532
MGYA00136709/ERZ475145_FASTA.fasta	MGYA00136709	ERZ475145	root:Environmental:Aquatic:Marine	0.662
MGYA00136710/ERZ475193_FASTA.fasta	MGYA00136710	ERZ475193	root:Environmental:Aquatic:Marine	0.487
MGYA00136711/ERZ475156_FASTA.fasta	MGYA00136711	ERZ475156	root:Environmental:Aquatic:Marine	0.6576
MGYA00136712/ERZ475237_FASTA.fasta	MGYA00136712	ERZ475237	root:Environmental:Aquatic:Marine	0.372
MGYA00136713/ERZ475245_FASTA.fasta	MGYA00136713	ERZ475245	root:Environmental:Aquatic:Marine	0.3166
MGYA00136714/ERZ475134_FASTA.fasta	MGYA00136714	ERZ475134	root:Environmental:Aquatic:Marine	0.4468
MGYA00136715/ERZ475173_FASTA.fasta	MGYA00136715	ERZ475173	root:Environmental:Aquatic:Marine	0.6114
MGYA00136716/ERZ475130_FASTA.fasta	MGYA00136716	ERZ475130	root:Environmental:Aquatic:Marine	0.497
MGYA00136717/ERZ475239_FASTA.fasta	MGYA00136717	ERZ475239	root:Environmental:Aquatic:Marine	0.366
MGYA00136718/ERZ475129_FASTA.fasta	MGYA00136718	ERZ475129	root:Environmental:Aquatic:Marine	0.6808
MGYA00136719/ERZ475167_FASTA.fasta	MGYA00136719	ERZ475167	root:Environmental:Aquatic:Marine	0.5564
MGYA00136720/ERZ475187_FASTA.fasta	MGYA00136720	ERZ475187	root:Environmental:Aquatic:Marine	0.6128
MGYA00136721/ERZ475160_FASTA.fasta	MGYA00136721	ERZ475160	root:Environmental:Aquatic:Marine	0.3786
MGYA00136722/ERZ475126_FASTA.fasta	MGYA00136722	ERZ475126	root:Environmental:Aquatic:Marine	0.6472
MGYA00136723/ERZ475220_FASTA.fasta	MGYA00136723	ERZ475220	root:Environmental:Aquatic:Marine	0.5152
MGYA00136724/ERZ475241_FASTA.fasta	MGYA00136724	ERZ475241	root:Environmental:Aquatic:Marine	0.3998
MGYA00136725/ERZ475217_FASTA.fasta	MGYA00136725	ERZ475217	root:Environmental:Aquatic:Marine	0.4938
MGYA00136726/ERZ475154_FASTA.fasta	MGYA00136726	ERZ475154	root:Environmental:Aquatic:Marine	0.5544
MGYA00136727/ERZ475251_FASTA.fasta	MGYA00136727	ERZ475251	root:Environmental:Aquatic:Marine	0.2726
MGYA00136728/ERZ475213_FASTA.fasta	MGYA00136728	ERZ475213	root:Environmental:Aquatic:Marine	0.4738
MGYA00136729/ERZ475183_FASTA.fasta	MGYA00136729	ERZ475183	root:Environmental:Aquatic:Marine	0.5472
MGYA00136730/ERZ475191_FASTA.fasta	MGYA00136730	ERZ475191	root:Environmental:Aquatic:Marine	0.3348
MGYA00136731/ERZ475226_FASTA.fasta	MGYA00136731	ERZ475226	root:Environmental:Aquatic:Marine	0.3538
MGYA00136732/ERZ475151_FASTA.fasta	MGYA00136732	ERZ475151	root:Environmental:Aquatic:Marine	0.6258
MGYA00136733/ERZ475236_FASTA.fasta	MGYA00136733	ERZ475236	root:Environmental:Aquatic:Marine	0.6678
MGYA00136735/ERZ475175_FASTA.fasta	MGYA00136735	ERZ475175	root:Environmental:Aquatic:Marine	0.6336
MGYA00136736/ERZ475201_FASTA.fasta	MGYA00136736	ERZ475201	root:Environmental:Aquatic:Marine	0.612
MGYA00136737/ERZ475165_FASTA.fasta	MGYA00136737	ERZ475165	root:Environmental:Aquatic:Marine	0.3354
MGYA00136738/ERZ475252_FASTA.fasta	MGYA00136738	ERZ475252	root:Environmental:Aquatic:Marine	0.3234
MGYA00136739/ERZ475233_FASTA.fasta	MGYA00136739	ERZ475233	root:Environmental:Aquatic:Marine	0.698
MGYA00136740/ERZ475207_FASTA.fasta	MGYA00136740	ERZ475207	root:Environmental:Aquatic:Marine	0.5052
MGYA00136741/ERZ475219_FASTA.fasta	MGYA00136741	ERZ475219	root:Environmental:Aquatic:Marine	0.4036
MGYA00136742/ERZ475140_FASTA.fasta	MGYA00136742	ERZ475140	root:Environmental:Aquatic:Marine	0.3996
MGYA00136743/ERZ475153_FASTA.fasta	MGYA00136743	ERZ475153	root:Environmental:Aquatic:Marine	0.7202
MGYA00136744/ERZ475141_FASTA.fasta	MGYA00136744	ERZ475141	root:Environmental:Aquatic:Marine	0.5878
MGYA00136745/ERZ475157_FASTA.fasta	MGYA00136745	ERZ475157	root:Environmental:Aquatic:Marine	0.6718
MGYA00136746/ERZ475246_FASTA.fasta	MGYA00136746	ERZ475246	root:Environmental:Aquatic:Marine	0.5034
MGYA00136747/ERZ475137_FASTA.fasta	MGYA00136747	ERZ475137	root:Environmental:Aquatic:Marine	0.587
MGYA00136748/ERZ475170_FASTA.fasta	MGYA00136748	ERZ475170	root:Environmental:Aquatic:Marine	0.655
MGYA00136749/ERZ475124_FASTA.fasta	MGYA00136749	ERZ475124	root:Environmental:Aquatic:Marine	0.3254
MGYA00136750/ERZ475139_FASTA.fasta	MGYA00136750	ERZ475139	root:Environmental:Aquatic:Marine	0.5926
MGYA00136751/ERZ475169_FASTA.fasta	MGYA00136751	ERZ475169	root:Environmental:Aquatic:Marine	0.6304
MGYA00136752/ERZ475171_FASTA.fasta	MGYA00136752	ERZ475171	root:Environmental:Aquatic:Marine	0.575
MGYA00136753/ERZ475865_FASTA.fasta	MGYA00136753	ERZ475865	root:Host-associated:Human	0.2362
MGYA00136754/ERZ475766_FASTA.fasta	MGYA00136754	ERZ475766	root:Host-associated:Human	0.6704
MGYA00136755/ERZ474927_FASTA.fasta	MGYA00136755	ERZ474927	root:Host-associated:Human	0.1194
MGYA00136756/ERZ474577_FASTA.fasta	MGYA00136756	ERZ474577	root:Host-associated:Human	0.6026
MGYA00136757/ERZ475815_FASTA.fasta	MGYA00136757	ERZ475815	root:Host-associated:Human	0.0778
MGYA00136758/ERZ475734_FASTA.fasta	MGYA00136758	ERZ475734	root:Host-associated:Human	0.658
MGYA00136759/ERZ475923_FASTA.fasta	MGYA00136759	ERZ475923	root:Host-associated:Human	0.4508
MGYA00136760/ERZ476009_FASTA.fasta	MGYA00136760	ERZ476009	root:Host-associated:Human	0.0768
MGYA00136766/ERZ474612_FASTA.fasta	MGYA00136766	ERZ474612	root:Host-associated:Human	0.0644
MGYA00136767/ERZ475713_FASTA.fasta	MGYA00136767	ERZ475713	root:Host-associated:Human	0.4378
MGYA00136768/ERZ474610_FASTA.fasta	MGYA00136768	ERZ474610	root:Host-associated:Human	0.6458
MGYA00136769/ERZ475668_FASTA.fasta	MGYA00136769	ERZ475668	root:Host-associated:Human	0.1514
MGYA00136770/ERZ475952_FASTA.fasta	MGYA00136770	ERZ475952	root:Host-associated:Human	0.5224
MGYA00136771/ERZ475891_FASTA.fasta	MGYA00136771	ERZ475891	root:Host-associated:Human	0.59
MGYA00136772/ERZ475873_FASTA.fasta	MGYA00136772	ERZ475873	root:Host-associated:Human	0.4332
MGYA00136775/ERZ475970_FASTA.fasta	MGYA00136775	ERZ475970	root:Host-associated:Human	0.4162
MGYA00136779/ERZ474626_FASTA.fasta	MGYA00136779	ERZ474626	root:Host-associated:Human	0.3602
MGYA00136781/ERZ475690_FASTA.fasta	MGYA00136781	ERZ475690	root:Host-associated:Human	0.3558
MGYA00136782/ERZ476000_FASTA.fasta	MGYA00136782	ERZ476000	root:Host-associated:Human	0.2722
MGYA00136783/ERZ475691_FASTA.fasta	MGYA00136783	ERZ475691	root:Host-associated:Human	0.35
MGYA00136784/ERZ476014_FASTA.fasta	MGYA00136784	ERZ476014	root:Host-associated:Human	0.45
MGYA00136787/ERZ475808_FASTA.fasta	MGYA00136787	ERZ475808	root:Host-associated:Human	0.6236
MGYA00136788/ERZ475794_FASTA.fasta	MGYA00136788	ERZ475794	root:Host-associated:Human	0.5806
MGYA00136791/ERZ475803_FASTA.fasta	MGYA00136791	ERZ475803	root:Host-associated:Human	0.3848
MGYA00136795/ERZ475824_FASTA.fasta	MGYA00136795	ERZ475824	root:Host-associated:Human	0.3658
MGYA00136801/ERZ475913_FASTA.fasta	MGYA00136801	ERZ475913	root:Host-associated:Human	0.5594
MGYA00136802/ERZ475682_FASTA.fasta	MGYA00136802	ERZ475682	root:Host-associated:Human	0.4504
MGYA00136803/ERZ475814_FASTA.fasta	MGYA00136803	ERZ475814	root:Host-associated:Human	0.1202
MGYA00136804/ERZ474581_FASTA.fasta	MGYA00136804	ERZ474581	root:Host-associated:Human	0.4876
MGYA00136806/ERZ475940_FASTA.fasta	MGYA00136806	ERZ475940	root:Host-associated:Human	0.6382
MGYA00136807/ERZ475753_FASTA.fasta	MGYA00136807	ERZ475753	root:Host-associated:Human	0.3606
MGYA00136810/ERZ475904_FASTA.fasta	MGYA00136810	ERZ475904	root:Host-associated:Human	0.3684
MGYA00136811/ERZ475976_FASTA.fasta	MGYA00136811	ERZ475976	root:Host-associated:Human	0.613
MGYA00136812/ERZ475959_FASTA.fasta	MGYA00136812	ERZ475959	root:Host-associated:Human	0.064
MGYA00136813/ERZ475980_FASTA.fasta	MGYA00136813	ERZ475980	root:Host-associated:Human	0.1842
MGYA00136814/ERZ475933_FASTA.fasta	MGYA00136814	ERZ475933	root:Host-associated:Human	0.5562
MGYA00136817/ERZ475812_FASTA.fasta	MGYA00136817	ERZ475812	root:Host-associated:Human	0.474
MGYA00136818/ERZ475787_FASTA.fasta	MGYA00136818	ERZ475787	root:Host-associated:Human	0.1182
MGYA00136821/ERZ475974_FASTA.fasta	MGYA00136821	ERZ475974	root:Host-associated:Human	0.1868
MGYA00136822/ERZ474624_FASTA.fasta	MGYA00136822	ERZ474624	root:Host-associated:Human	0.1074
MGYA00136823/ERZ475991_FASTA.fasta	MGYA00136823	ERZ475991	root:Host-associated:Human	0.6692
MGYA00136826/ERZ475773_FASTA.fasta	MGYA00136826	ERZ475773	root:Host-associated:Human	0.4586
MGYA00136828/ERZ475727_FASTA.fasta	MGYA00136828	ERZ475727	root:Host-associated:Human	0.161
MGYA00136829/ERZ475729_FASTA.fasta	MGYA00136829	ERZ475729	root:Host-associated:Human	0.5534
MGYA00136830/ERZ475825_FASTA.fasta	MGYA00136830	ERZ475825	root:Host-associated:Human	0.6348
MGYA00136831/ERZ475673_FASTA.fasta	MGYA00136831	ERZ475673	root:Host-associated:Human	0.6644
MGYA00136832/ERZ475655_FASTA.fasta	MGYA00136832	ERZ475655	root:Host-associated:Human	0.4808
MGYA00136833/ERZ474596_FASTA.fasta	MGYA00136833	ERZ474596	root:Host-associated:Human	0.5536
MGYA00136834/ERZ475798_FASTA.fasta	MGYA00136834	ERZ475798	root:Host-associated:Human	0.2158
MGYA00136835/ERZ474647_FASTA.fasta	MGYA00136835	ERZ474647	root:Host-associated:Human	0.0768
MGYA00136836/ERZ475805_FASTA.fasta	MGYA00136836	ERZ475805	root:Host-associated:Human	0.6216
MGYA00136837/ERZ476003_FASTA.fasta	MGYA00136837	ERZ476003	root:Host-associated:Human	0.6398
MGYA00136838/ERZ475692_FASTA.fasta	MGYA00136838	ERZ475692	root:Host-associated:Human	0.4264
MGYA00136840/ERZ475946_FASTA.fasta	MGYA00136840	ERZ475946	root:Host-associated:Human	0.6596
MGYA00136841/ERZ475752_FASTA.fasta	MGYA00136841	ERZ475752	root:Host-associated:Human	0.079
MGYA00136842/ERZ475774_FASTA.fasta	MGYA00136842	ERZ475774	root:Host-associated:Human	0.3278
MGYA00136843/ERZ475921_FASTA.fasta	MGYA00136843	ERZ475921	root:Host-associated:Human	0.0304
MGYA00136846/ERZ475783_FASTA.fasta	MGYA00136846	ERZ475783	root:Host-associated:Human	0.3428
MGYA00136847/ERZ474637_FASTA.fasta	MGYA00136847	ERZ474637	root:Host-associated:Human	0.6524
MGYA00136848/ERZ475770_FASTA.fasta	MGYA00136848	ERZ475770	root:Host-associated:Human	0.3422
MGYA00136851/ERZ475912_FASTA.fasta	MGYA00136851	ERZ475912	root:Host-associated:Human	0.6572
MGYA00136852/ERZ475987_FASTA.fasta	MGYA00136852	ERZ475987	root:Host-associated:Human	0.4954
MGYA00136853/ERZ475790_FASTA.fasta	MGYA00136853	ERZ475790	root:Host-associated:Human	0.5998
MGYA00136854/ERZ475918_FASTA.fasta	MGYA00136854	ERZ475918	root:Host-associated:Human	0.4032
MGYA00136856/ERZ475830_FASTA.fasta	MGYA00136856	ERZ475830	root:Host-associated:Human	0.2272
MGYA00136857/ERZ476010_FASTA.fasta	MGYA00136857	ERZ476010	root:Host-associated:Human	0.1068
MGYA00136858/ERZ474599_FASTA.fasta	MGYA00136858	ERZ474599	root:Host-associated:Human	0.2684
MGYA00136859/ERZ475733_FASTA.fasta	MGYA00136859	ERZ475733	root:Host-associated:Human	0.0764
MGYA00136860/ERZ475903_FASTA.fasta	MGYA00136860	ERZ475903	root:Host-associated:Human	0.2348
MGYA00136861/ERZ475838_FASTA.fasta	MGYA00136861	ERZ475838	root:Host-associated:Human	0.6034
MGYA00136863/ERZ474589_FASTA.fasta	MGYA00136863	ERZ474589	root:Host-associated:Human	0.0836
MGYA00136867/ERZ475707_FASTA.fasta	MGYA00136867	ERZ475707	root:Host-associated:Human	0.484
MGYA00136868/ERZ475833_FASTA.fasta	MGYA00136868	ERZ475833	root:Host-associated:Human	0.5962
MGYA00136870/ERZ474591_FASTA.fasta	MGYA00136870	ERZ474591	root:Host-associated:Human	0.678
MGYA00136871/ERZ474642_FASTA.fasta	MGYA00136871	ERZ474642	root:Host-associated:Human	0.4758
MGYA00136875/ERZ475771_FASTA.fasta	MGYA00136875	ERZ475771	root:Host-associated:Human	0.6886
MGYA00136877/ERZ475911_FASTA.fasta	MGYA00136877	ERZ475911	root:Host-associated:Human	0.6146
MGYA00136878/ERZ475897_FASTA.fasta	MGYA00136878	ERZ475897	root:Host-associated:Human	0.5784
MGYA00136882/ERZ475665_FASTA.fasta	MGYA00136882	ERZ475665	root:Host-associated:Human	0.0294
MGYA00136883/ERZ474625_FASTA.fasta	MGYA00136883	ERZ474625	root:Host-associated:Human	0.072
MGYA00136885/ERZ475772_FASTA.fasta	MGYA00136885	ERZ475772	root:Host-associated:Human	0.2054
MGYA00136886/ERZ475925_FASTA.fasta	MGYA00136886	ERZ475925	root:Host-associated:Human	0.202
MGYA00136887/ERZ475708_FASTA.fasta	MGYA00136887	ERZ475708	root:Host-associated:Human	0.5038
MGYA00136889/ERZ474636_FASTA.fasta	MGYA00136889	ERZ474636	root:Host-associated:Human	0.5746
MGYA00136890/ERZ475802_FASTA.fasta	MGYA00136890	ERZ475802	root:Host-associated:Human	0.6572
MGYA00136891/ERZ475792_FASTA.fasta	MGYA00136891	ERZ475792	root:Host-associated:Human	0.6956
MGYA00136892/ERZ475935_FASTA.fasta	MGYA00136892	ERZ475935	root:Host-associated:Human	0.0294
MGYA00136893/ERZ475926_FASTA.fasta	MGYA00136893	ERZ475926	root:Host-associated:Human	0.4412
MGYA00136894/ERZ475738_FASTA.fasta	MGYA00136894	ERZ475738	root:Host-associated:Human	0.1796
MGYA00136895/ERZ475731_FASTA.fasta	MGYA00136895	ERZ475731	root:Host-associated:Human	0.269
MGYA00136896/ERZ474623_FASTA.fasta	MGYA00136896	ERZ474623	root:Host-associated:Human	0.5304
MGYA00136897/ERZ475875_FASTA.fasta	MGYA00136897	ERZ475875	root:Host-associated:Human	0.434
MGYA00136899/ERZ474645_FASTA.fasta	MGYA00136899	ERZ474645	root:Host-associated:Human	0.4878
MGYA00136902/ERZ475662_FASTA.fasta	MGYA00136902	ERZ475662	root:Host-associated:Human	0.5202
MGYA00136904/ERZ476016_FASTA.fasta	MGYA00136904	ERZ476016	root:Host-associated:Human	0.0774
MGYA00136905/ERZ475651_FASTA.fasta	MGYA00136905	ERZ475651	root:Host-associated:Human	0.2352
MGYA00136906/ERZ475999_FASTA.fasta	MGYA00136906	ERZ475999	root:Host-associated:Human	0.3346
MGYA00136908/ERZ475650_FASTA.fasta	MGYA00136908	ERZ475650	root:Host-associated:Human	0.389
MGYA00136910/ERZ474595_FASTA.fasta	MGYA00136910	ERZ474595	root:Host-associated:Human	0.1042
MGYA00136912/ERZ475877_FASTA.fasta	MGYA00136912	ERZ475877	root:Host-associated:Human	0.4734
MGYA00136915/ERZ475882_FASTA.fasta	MGYA00136915	ERZ475882	root:Host-associated:Human	0.2766
MGYA00136916/ERZ475797_FASTA.fasta	MGYA00136916	ERZ475797	root:Host-associated:Human	0.1902
MGYA00136919/ERZ475835_FASTA.fasta	MGYA00136919	ERZ475835	root:Host-associated:Human	0.6256
MGYA00136920/ERZ475748_FASTA.fasta	MGYA00136920	ERZ475748	root:Host-associated:Human	0.4546
MGYA00136923/ERZ475870_FASTA.fasta	MGYA00136923	ERZ475870	root:Host-associated:Human	0.3978
MGYA00136931/ERZ475736_FASTA.fasta	MGYA00136931	ERZ475736	root:Host-associated:Human	0.0664
MGYA00136933/ERZ475704_FASTA.fasta	MGYA00136933	ERZ475704	root:Host-associated:Human	0.654
MGYA00136934/ERZ475789_FASTA.fasta	MGYA00136934	ERZ475789	root:Host-associated:Human	0.5902
MGYA00136936/ERZ475725_FASTA.fasta	MGYA00136936	ERZ475725	root:Host-associated:Human	0.1046
MGYA00136937/ERZ474620_FASTA.fasta	MGYA00136937	ERZ474620	root:Host-associated:Human	0.488
MGYA00136938/ERZ475779_FASTA.fasta	MGYA00136938	ERZ475779	root:Host-associated:Human	0.4694
MGYA00136939/ERZ475674_FASTA.fasta	MGYA00136939	ERZ475674	root:Host-associated:Human	0.2298
MGYA00136941/ERZ475997_FASTA.fasta	MGYA00136941	ERZ475997	root:Host-associated:Human	0.649
MGYA00136942/ERZ475754_FASTA.fasta	MGYA00136942	ERZ475754	root:Host-associated:Human	0.6334
MGYA00136943/ERZ475881_FASTA.fasta	MGYA00136943	ERZ475881	root:Host-associated:Human	0.1886
MGYA00136945/ERZ474634_FASTA.fasta	MGYA00136945	ERZ474634	root:Host-associated:Human	0.2168
MGYA00136946/ERZ474614_FASTA.fasta	MGYA00136946	ERZ474614	root:Host-associated:Human	0.4602
MGYA00136947/ERZ474629_FASTA.fasta	MGYA00136947	ERZ474629	root:Host-associated:Human	0.6458
MGYA00136948/ERZ475961_FASTA.fasta	MGYA00136948	ERZ475961	root:Host-associated:Human	0.6522
MGYA00136949/ERZ475796_FASTA.fasta	MGYA00136949	ERZ475796	root:Host-associated:Human	0.4758
MGYA00136950/ERZ475892_FASTA.fasta	MGYA00136950	ERZ475892	root:Host-associated:Human	0.1674
MGYA00136951/ERZ475958_FASTA.fasta	MGYA00136951	ERZ475958	root:Host-associated:Human	0.44
MGYA00136953/ERZ475916_FASTA.fasta	MGYA00136953	ERZ475916	root:Host-associated:Human	0.4616
MGYA00136954/ERZ475944_FASTA.fasta	MGYA00136954	ERZ475944	root:Host-associated:Human	0.0612
MGYA00136957/ERZ474579_FASTA.fasta	MGYA00136957	ERZ474579	root:Host-associated:Human	0.2116
MGYA00136958/ERZ475986_FASTA.fasta	MGYA00136958	ERZ475986	root:Host-associated:Human	0.5814
MGYA00136959/ERZ474580_FASTA.fasta	MGYA00136959	ERZ474580	root:Host-associated:Human	0.0858
MGYA00136960/ERZ475860_FASTA.fasta	MGYA00136960	ERZ475860	root:Host-associated:Human	0.2182
MGYA00136962/ERZ475702_FASTA.fasta	MGYA00136962	ERZ475702	root:Host-associated:Human	0.663
MGYA00136963/ERZ475981_FASTA.fasta	MGYA00136963	ERZ475981	root:Host-associated:Human	0.5508
MGYA00136967/ERZ475701_FASTA.fasta	MGYA00136967	ERZ475701	root:Host-associated:Human	0.4056
MGYA00136968/ERZ474635_FASTA.fasta	MGYA00136968	ERZ474635	root:Host-associated:Human	0.106
MGYA00136970/ERZ474600_FASTA.fasta	MGYA00136970	ERZ474600	root:Host-associated:Human	0.6244
MGYA00136972/ERZ474611_FASTA.fasta	MGYA00136972	ERZ474611	root:Host-associated:Human	0.3488
MGYA00136974/ERZ475786_FASTA.fasta	MGYA00136974	ERZ475786	root:Host-associated:Human	0.6366
MGYA00136975/ERZ475710_FASTA.fasta	MGYA00136975	ERZ475710	root:Host-associated:Human	0.6418
MGYA00136982/ERZ474632_FASTA.fasta	MGYA00136982	ERZ474632	root:Host-associated:Human	0.4556
MGYA00136984/ERZ475730_FASTA.fasta	MGYA00136984	ERZ475730	root:Host-associated:Human	0.3002
MGYA00136985/ERZ475902_FASTA.fasta	MGYA00136985	ERZ475902	root:Host-associated:Human	0.6554
MGYA00136987/ERZ474578_FASTA.fasta	MGYA00136987	ERZ474578	root:Host-associated:Human	0.3662
MGYA00136988/ERZ475761_FASTA.fasta	MGYA00136988	ERZ475761	root:Host-associated:Human	0.0328
MGYA00136989/ERZ475858_FASTA.fasta	MGYA00136989	ERZ475858	root:Host-associated:Human	0.2814
MGYA00136990/ERZ475898_FASTA.fasta	MGYA00136990	ERZ475898	root:Host-associated:Human	0.7008
MGYA00136991/ERZ474618_FASTA.fasta	MGYA00136991	ERZ474618	root:Host-associated:Human	0.5544
MGYA00136992/ERZ475652_FASTA.fasta	MGYA00136992	ERZ475652	root:Host-associated:Human	0.1724
MGYA00136995/ERZ475929_FASTA.fasta	MGYA00136995	ERZ475929	root:Host-associated:Human	0.491
MGYA00136996/ERZ475851_FASTA.fasta	MGYA00136996	ERZ475851	root:Host-associated:Human	0.5582
MGYA00136998/ERZ475968_FASTA.fasta	MGYA00136998	ERZ475968	root:Host-associated:Human	0.6374
MGYA00136999/ERZ475677_FASTA.fasta	MGYA00136999	ERZ475677	root:Host-associated:Human	0.666
MGYA00137000/ERZ475681_FASTA.fasta	MGYA00137000	ERZ475681	root:Host-associated:Human	0.577
MGYA00137002/ERZ475688_FASTA.fasta	MGYA00137002	ERZ475688	root:Host-associated:Human	0.3954
MGYA00137003/ERZ475703_FASTA.fasta	MGYA00137003	ERZ475703	root:Host-associated:Human	0.639
MGYA00137006/ERZ476005_FASTA.fasta	MGYA00137006	ERZ476005	root:Host-associated:Human	0.0294
MGYA00137007/ERZ475890_FASTA.fasta	MGYA00137007	ERZ475890	root:Host-associated:Human	0.0574
MGYA00137008/ERZ475978_FASTA.fasta	MGYA00137008	ERZ475978	root:Host-associated:Human	0.4946
MGYA00137009/ERZ475880_FASTA.fasta	MGYA00137009	ERZ475880	root:Host-associated:Human	0.3008
MGYA00137010/ERZ475719_FASTA.fasta	MGYA00137010	ERZ475719	root:Host-associated:Human	0.5074
MGYA00137014/ERZ474593_FASTA.fasta	MGYA00137014	ERZ474593	root:Host-associated:Human	0.632
MGYA00137016/ERZ475712_FASTA.fasta	MGYA00137016	ERZ475712	root:Host-associated:Human	0.165
MGYA00137018/ERZ475742_FASTA.fasta	MGYA00137018	ERZ475742	root:Host-associated:Human	0.1402
MGYA00137019/ERZ475893_FASTA.fasta	MGYA00137019	ERZ475893	root:Host-associated:Human	0.3522
MGYA00137021/ERZ475919_FASTA.fasta	MGYA00137021	ERZ475919	root:Host-associated:Human	0.6538
MGYA00137022/ERZ475675_FASTA.fasta	MGYA00137022	ERZ475675	root:Host-associated:Human	0.0864
MGYA00137023/ERZ474649_FASTA.fasta	MGYA00137023	ERZ474649	root:Host-associated:Human	0.439
MGYA00137024/ERZ475720_FASTA.fasta	MGYA00137024	ERZ475720	root:Host-associated:Human	0.0864
MGYA00137027/ERZ476001_FASTA.fasta	MGYA00137027	ERZ476001	root:Host-associated:Human	0.5248
MGYA00137028/ERZ475901_FASTA.fasta	MGYA00137028	ERZ475901	root:Host-associated:Human	0.2098
MGYA00137029/ERZ475939_FASTA.fasta	MGYA00137029	ERZ475939	root:Host-associated:Human	0.6574
MGYA00137031/ERZ475785_FASTA.fasta	MGYA00137031	ERZ475785	root:Host-associated:Human	0.6666
MGYA00137032/ERZ475788_FASTA.fasta	MGYA00137032	ERZ475788	root:Host-associated:Human	0.0768
MGYA00137033/ERZ475965_FASTA.fasta	MGYA00137033	ERZ475965	root:Host-associated:Human	0.1674
MGYA00137035/ERZ474615_FASTA.fasta	MGYA00137035	ERZ474615	root:Host-associated:Human	0.5824
MGYA00137036/ERZ475889_FASTA.fasta	MGYA00137036	ERZ475889	root:Host-associated:Human	0.6334
MGYA00137037/ERZ475846_FASTA.fasta	MGYA00137037	ERZ475846	root:Host-associated:Human	0.6208
MGYA00137038/ERZ475778_FASTA.fasta	MGYA00137038	ERZ475778	root:Host-associated:Human	0.4692
MGYA00137039/ERZ475949_FASTA.fasta	MGYA00137039	ERZ475949	root:Host-associated:Human	0.3798
MGYA00137040/ERZ475679_FASTA.fasta	MGYA00137040	ERZ475679	root:Host-associated:Human	0.3438
MGYA00137041/ERZ475876_FASTA.fasta	MGYA00137041	ERZ475876	root:Host-associated:Human	0.6576
MGYA00137042/ERZ475694_FASTA.fasta	MGYA00137042	ERZ475694	root:Host-associated:Human	0.3224
MGYA00137043/ERZ475686_FASTA.fasta	MGYA00137043	ERZ475686	root:Host-associated:Human	0.6196
MGYA00137044/ERZ474607_FASTA.fasta	MGYA00137044	ERZ474607	root:Host-associated:Human	0.2258
MGYA00137046/ERZ475799_FASTA.fasta	MGYA00137046	ERZ475799	root:Host-associated:Human	0.3506
MGYA00137047/ERZ475975_FASTA.fasta	MGYA00137047	ERZ475975	root:Host-associated:Human	0.1734
MGYA00137048/ERZ475762_FASTA.fasta	MGYA00137048	ERZ475762	root:Host-associated:Human	0.39
MGYA00137049/ERZ475979_FASTA.fasta	MGYA00137049	ERZ475979	root:Host-associated:Human	0.6564
MGYA00137050/ERZ475711_FASTA.fasta	MGYA00137050	ERZ475711	root:Host-associated:Human:Digestive system:Oral:Sali	0.5294
MGYA00137052/ERZ475971_FASTA.fasta	MGYA00137052	ERZ475971	root:Host-associated:Human	0.5898
MGYA00137055/ERZ474584_FASTA.fasta	MGYA00137055	ERZ474584	root:Host-associated:Human	0.523
MGYA00137057/ERZ475658_FASTA.fasta	MGYA00137057	ERZ475658	root:Host-associated:Human	0.4684
MGYA00137059/ERZ475724_FASTA.fasta	MGYA00137059	ERZ475724	root:Host-associated:Human	0.494
MGYA00137061/ERZ475917_FASTA.fasta	MGYA00137061	ERZ475917	root:Host-associated:Human	0.6158
MGYA00137062/ERZ475769_FASTA.fasta	MGYA00137062	ERZ475769	root:Host-associated:Human	0.0808
MGYA00137063/ERZ475834_FASTA.fasta	MGYA00137063	ERZ475834	root:Host-associated:Human	0.2224
MGYA00137065/ERZ475817_FASTA.fasta	MGYA00137065	ERZ475817	root:Host-associated:Human	0.511
MGYA00137067/ERZ475819_FASTA.fasta	MGYA00137067	ERZ475819	root:Host-associated:Human	0.5648
MGYA00137071/ERZ475995_FASTA.fasta	MGYA00137071	ERZ475995	root:Host-associated:Human	0.0764
MGYA00137074/ERZ475709_FASTA.fasta	MGYA00137074	ERZ475709	root:Host-associated:Human	0.3604
MGYA00137075/ERZ475945_FASTA.fasta	MGYA00137075	ERZ475945	root:Host-associated:Human	0.0574
MGYA00137077/ERZ475914_FASTA.fasta	MGYA00137077	ERZ475914	root:Host-associated:Human	0.6632
MGYA00137078/ERZ475698_FASTA.fasta	MGYA00137078	ERZ475698	root:Host-associated:Human	0.559
MGYA00137081/ERZ476017_FASTA.fasta	MGYA00137081	ERZ476017	root:Host-associated:Human	0.4388
MGYA00137082/ERZ475993_FASTA.fasta	MGYA00137082	ERZ475993	root:Host-associated:Human	0.4812
MGYA00137083/ERZ475990_FASTA.fasta	MGYA00137083	ERZ475990	root:Host-associated:Human	0.2788
MGYA00137084/ERZ475746_FASTA.fasta	MGYA00137084	ERZ475746	root:Host-associated:Human	0.2088
MGYA00137085/ERZ475758_FASTA.fasta	MGYA00137085	ERZ475758	root:Host-associated:Human	0.5774
MGYA00137086/ERZ474640_FASTA.fasta	MGYA00137086	ERZ474640	root:Host-associated:Human	0.4502
MGYA00137087/ERZ475973_FASTA.fasta	MGYA00137087	ERZ475973	root:Host-associated:Human	0.357
MGYA00137088/ERZ475983_FASTA.fasta	MGYA00137088	ERZ475983	root:Host-associated:Human	0.1964
MGYA00137090/ERZ475687_FASTA.fasta	MGYA00137090	ERZ475687	root:Host-associated:Human	0.633
MGYA00137094/ERZ475768_FASTA.fasta	MGYA00137094	ERZ475768	root:Host-associated:Human	0.6114
MGYA00137095/ERZ474648_FASTA.fasta	MGYA00137095	ERZ474648	root:Host-associated:Human	0.0784
MGYA00137096/ERZ476007_FASTA.fasta	MGYA00137096	ERZ476007	root:Host-associated:Human	0.572
MGYA00137097/ERZ475856_FASTA.fasta	MGYA00137097	ERZ475856	root:Host-associated:Human	0.3148
MGYA00137098/ERZ475809_FASTA.fasta	MGYA00137098	ERZ475809	root:Host-associated:Human	0.624
MGYA00137099/ERZ475864_FASTA.fasta	MGYA00137099	ERZ475864	root:Host-associated:Human	0.177
MGYA00137101/ERZ475823_FASTA.fasta	MGYA00137101	ERZ475823	root:Host-associated:Human	0.5376
MGYA00137102/ERZ475879_FASTA.fasta	MGYA00137102	ERZ475879	root:Host-associated:Human	0.0764
MGYA00137103/ERZ474575_FASTA.fasta	MGYA00137103	ERZ474575	root:Host-associated:Human	0.511
MGYA00137105/ERZ474598_FASTA.fasta	MGYA00137105	ERZ474598	root:Host-associated:Human	0.4548
MGYA00137106/ERZ476019_FASTA.fasta	MGYA00137106	ERZ476019	root:Host-associated:Human	0.078
MGYA00137110/ERZ475737_FASTA.fasta	MGYA00137110	ERZ475737	root:Host-associated:Human	0.5606
MGYA00137111/ERZ475924_FASTA.fasta	MGYA00137111	ERZ475924	root:Host-associated:Human	0.3936
MGYA00137113/ERZ475872_FASTA.fasta	MGYA00137113	ERZ475872	root:Host-associated:Human	0.088
MGYA00137115/ERZ475849_FASTA.fasta	MGYA00137115	ERZ475849	root:Host-associated:Human	0.495
MGYA00137116/ERZ475721_FASTA.fasta	MGYA00137116	ERZ475721	root:Host-associated:Human	0.083
MGYA00137117/ERZ474621_FASTA.fasta	MGYA00137117	ERZ474621	root:Host-associated:Human	0.3252
MGYA00137120/ERZ475732_FASTA.fasta	MGYA00137120	ERZ475732	root:Host-associated:Human	0.5458
MGYA00137124/ERZ475826_FASTA.fasta	MGYA00137124	ERZ475826	root:Host-associated:Human	0.6586
MGYA00137125/ERZ475861_FASTA.fasta	MGYA00137125	ERZ475861	root:Host-associated:Human	0.0772
MGYA00137129/ERZ475801_FASTA.fasta	MGYA00137129	ERZ475801	root:Host-associated:Human	0.5244
MGYA00137132/ERZ475765_FASTA.fasta	MGYA00137132	ERZ475765	root:Host-associated:Human	0.2836
MGYA00137133/ERZ475804_FASTA.fasta	MGYA00137133	ERZ475804	root:Host-associated:Human	0.5412
MGYA00137136/ERZ475716_FASTA.fasta	MGYA00137136	ERZ475716	root:Host-associated:Human	0.573
MGYA00137137/ERZ475937_FASTA.fasta	MGYA00137137	ERZ475937	root:Host-associated:Human	0.5858
MGYA00137138/ERZ475793_FASTA.fasta	MGYA00137138	ERZ475793	root:Host-associated:Human	0.0788
MGYA00137140/ERZ475660_FASTA.fasta	MGYA00137140	ERZ475660	root:Host-associated:Human	0.2146
MGYA00137141/ERZ475869_FASTA.fasta	MGYA00137141	ERZ475869	root:Host-associated:Human	0.1742
MGYA00137142/ERZ475953_FASTA.fasta	MGYA00137142	ERZ475953	root:Host-associated:Human	0.5124
MGYA00137144/ERZ475669_FASTA.fasta	MGYA00137144	ERZ475669	root:Host-associated:Human	0.3346
MGYA00137146/ERZ474613_FASTA.fasta	MGYA00137146	ERZ474613	root:Host-associated:Human	0.31
MGYA00137148/ERZ474571_FASTA.fasta	MGYA00137148	ERZ474571	root:Host-associated:Human	0.3752
MGYA00137149/ERZ475837_FASTA.fasta	MGYA00137149	ERZ475837	root:Host-associated:Human	0.4928
MGYA00137150/ERZ474573_FASTA.fasta	MGYA00137150	ERZ474573	root:Host-associated:Human	0.0794
MGYA00137151/ERZ475751_FASTA.fasta	MGYA00137151	ERZ475751	root:Host-associated:Human	0.4456
MGYA00137154/ERZ475906_FASTA.fasta	MGYA00137154	ERZ475906	root:Host-associated:Human	0.6438
MGYA00137155/ERZ474601_FASTA.fasta	MGYA00137155	ERZ474601	root:Host-associated:Human	0.3392
MGYA00137156/ERZ475874_FASTA.fasta	MGYA00137156	ERZ475874	root:Host-associated:Human	0.653
MGYA00137158/ERZ475715_FASTA.fasta	MGYA00137158	ERZ475715	root:Host-associated:Human	0.1976
MGYA00137159/ERZ474630_FASTA.fasta	MGYA00137159	ERZ474630	root:Host-associated:Human	0.0398
MGYA00137160/ERZ475840_FASTA.fasta	MGYA00137160	ERZ475840	root:Host-associated:Human	0.5796
MGYA00137161/ERZ475896_FASTA.fasta	MGYA00137161	ERZ475896	root:Host-associated:Human	0.1846
MGYA00137162/ERZ475950_FASTA.fasta	MGYA00137162	ERZ475950	root:Host-associated:Human	0.1398
MGYA00137163/ERZ474583_FASTA.fasta	MGYA00137163	ERZ474583	root:Host-associated:Human	0.6148
MGYA00137164/ERZ475693_FASTA.fasta	MGYA00137164	ERZ475693	root:Host-associated:Human	0.4128
MGYA00137165/ERZ475671_FASTA.fasta	MGYA00137165	ERZ475671	root:Host-associated:Human	0.6642
MGYA00137168/ERZ475951_FASTA.fasta	MGYA00137168	ERZ475951	root:Host-associated:Human	0.4832
MGYA00137170/ERZ475938_FASTA.fasta	MGYA00137170	ERZ475938	root:Host-associated:Human	0.4438
MGYA00137172/ERZ475782_FASTA.fasta	MGYA00137172	ERZ475782	root:Host-associated:Human	0.1624
MGYA00137174/ERZ475936_FASTA.fasta	MGYA00137174	ERZ475936	root:Host-associated:Human	0.1046
MGYA00137175/ERZ475941_FASTA.fasta	MGYA00137175	ERZ475941	root:Host-associated:Human	0.453
MGYA00137176/ERZ476011_FASTA.fasta	MGYA00137176	ERZ476011	root:Host-associated:Human	0.411
MGYA00137177/ERZ475985_FASTA.fasta	MGYA00137177	ERZ475985	root:Host-associated:Human	0.0294
MGYA00137178/ERZ475871_FASTA.fasta	MGYA00137178	ERZ475871	root:Host-associated:Human	0.5798
MGYA00137181/ERZ475836_FASTA.fasta	MGYA00137181	ERZ475836	root:Host-associated:Human	0.5414
MGYA00137183/ERZ475661_FASTA.fasta	MGYA00137183	ERZ475661	root:Host-associated:Human	0.2766
MGYA00137185/ERZ474622_FASTA.fasta	MGYA00137185	ERZ474622	root:Host-associated:Human	0.5848
MGYA00137186/ERZ475726_FASTA.fasta	MGYA00137186	ERZ475726	root:Host-associated:Human	0.427
MGYA00137187/ERZ475791_FASTA.fasta	MGYA00137187	ERZ475791	root:Host-associated:Human	0.2084
MGYA00137188/ERZ475920_FASTA.fasta	MGYA00137188	ERZ475920	root:Host-associated:Human	0.0966
MGYA00137195/ERZ474616_FASTA.fasta	MGYA00137195	ERZ474616	root:Host-associated:Human	0.4698
MGYA00137196/ERZ474606_FASTA.fasta	MGYA00137196	ERZ474606	root:Host-associated:Human	0.6116
MGYA00137199/ERZ475969_FASTA.fasta	MGYA00137199	ERZ475969	root:Host-associated:Human	0.6078
MGYA00137200/ERZ475963_FASTA.fasta	MGYA00137200	ERZ475963	root:Host-associated:Human	0.509
MGYA00137202/ERZ475934_FASTA.fasta	MGYA00137202	ERZ475934	root:Host-associated:Human	0.0896
MGYA00137203/ERZ475684_FASTA.fasta	MGYA00137203	ERZ475684	root:Host-associated:Human	0.5856
MGYA00137207/ERZ475866_FASTA.fasta	MGYA00137207	ERZ475866	root:Host-associated:Human	0.4782
MGYA00137209/ERZ475905_FASTA.fasta	MGYA00137209	ERZ475905	root:Host-associated:Human	0.6512
MGYA00137211/ERZ475907_FASTA.fasta	MGYA00137211	ERZ475907	root:Host-associated:Human	0.201
MGYA00137212/ERZ474633_FASTA.fasta	MGYA00137212	ERZ474633	root:Host-associated:Human	0.5652
MGYA00137214/ERZ475839_FASTA.fasta	MGYA00137214	ERZ475839	root:Host-associated:Human	0.3162
MGYA00137215/ERZ475680_FASTA.fasta	MGYA00137215	ERZ475680	root:Host-associated:Human	0.3014
MGYA00137217/ERZ475930_FASTA.fasta	MGYA00137217	ERZ475930	root:Host-associated:Human	0.636
MGYA00137218/ERZ476008_FASTA.fasta	MGYA00137218	ERZ476008	root:Host-associated:Human	0.5454
MGYA00137219/ERZ475928_FASTA.fasta	MGYA00137219	ERZ475928	root:Host-associated:Human	0.3606
MGYA00137221/ERZ475810_FASTA.fasta	MGYA00137221	ERZ475810	root:Host-associated:Human	0.3718
MGYA00137224/ERZ475706_FASTA.fasta	MGYA00137224	ERZ475706	root:Host-associated:Human	0.195
MGYA00137225/ERZ475848_FASTA.fasta	MGYA00137225	ERZ475848	root:Host-associated:Human	0.484
MGYA00137226/ERZ475654_FASTA.fasta	MGYA00137226	ERZ475654	root:Host-associated:Human	0.641
MGYA00137227/ERZ475784_FASTA.fasta	MGYA00137227	ERZ475784	root:Host-associated:Human	0.6152
MGYA00137228/ERZ475683_FASTA.fasta	MGYA00137228	ERZ475683	root:Host-associated:Human:Digestive system:Oral:Sali	0.4302
MGYA00137229/ERZ474602_FASTA.fasta	MGYA00137229	ERZ474602	root:Host-associated:Human	0.0808
MGYA00137230/ERZ474641_FASTA.fasta	MGYA00137230	ERZ474641	root:Host-associated:Human	0.3372
MGYA00137233/ERZ474586_FASTA.fasta	MGYA00137233	ERZ474586	root:Host-associated:Human	0.3132
MGYA00137234/ERZ475723_FASTA.fasta	MGYA00137234	ERZ475723	root:Host-associated:Human	0.0764
MGYA00137236/ERZ475957_FASTA.fasta	MGYA00137236	ERZ475957	root:Host-associated:Human	0.4564
MGYA00137237/ERZ475955_FASTA.fasta	MGYA00137237	ERZ475955	root:Host-associated:Human	0.6318
MGYA00137239/ERZ475807_FASTA.fasta	MGYA00137239	ERZ475807	root:Host-associated:Human	0.6478
MGYA00137241/ERZ475653_FASTA.fasta	MGYA00137241	ERZ475653	root:Host-associated:Human	0.2428
MGYA00137242/ERZ474572_FASTA.fasta	MGYA00137242	ERZ474572	root:Host-associated:Human	0.3844
MGYA00137243/ERZ475828_FASTA.fasta	MGYA00137243	ERZ475828	root:Host-associated:Human	0.1382
MGYA00137244/ERZ474628_FASTA.fasta	MGYA00137244	ERZ474628	root:Host-associated:Human	0.385
MGYA00137245/ERZ474638_FASTA.fasta	MGYA00137245	ERZ474638	root:Host-associated:Human	0.0294
MGYA00137247/ERZ475705_FASTA.fasta	MGYA00137247	ERZ475705	root:Host-associated:Human	0.4028
MGYA00137249/ERZ474582_FASTA.fasta	MGYA00137249	ERZ474582	root:Host-associated:Human	0.648
MGYA00137250/ERZ475885_FASTA.fasta	MGYA00137250	ERZ475885	root:Host-associated:Human	0.609
MGYA00137251/ERZ475857_FASTA.fasta	MGYA00137251	ERZ475857	root:Host-associated:Human	0.6182
MGYA00137252/ERZ475775_FASTA.fasta	MGYA00137252	ERZ475775	root:Host-associated:Human	0.4716
MGYA00137253/ERZ474604_FASTA.fasta	MGYA00137253	ERZ474604	root:Host-associated:Human	0.3826
MGYA00137255/ERZ475847_FASTA.fasta	MGYA00137255	ERZ475847	root:Host-associated:Human	0.5082
MGYA00137256/ERZ475755_FASTA.fasta	MGYA00137256	ERZ475755	root:Host-associated:Human	0.4332
MGYA00137257/ERZ475855_FASTA.fasta	MGYA00137257	ERZ475855	root:Host-associated:Human	0.535

MGYA00137258/ERZ475811_FASTA.fasta	MGYA00137258	ERZ475811	root:Host-associated:Human	0.5378
MGYA00137263/ERZ475988_FASTA.fasta	MGYA00137263	ERZ475988	root:Host-associated:Human	0.6624
MGYA00137264/ERZ475862_FASTA.fasta	MGYA00137264	ERZ475862	root:Host-associated:Human	0.5882
MGYA00137266/ERZ475910_FASTA.fasta	MGYA00137266	ERZ475910	root:Host-associated:Human	0.4886
MGYA00137268/ERZ475942_FASTA.fasta	MGYA00137268	ERZ475942	root:Host-associated:Human	0.4886
MGYA00137270/ERZ475666_FASTA.fasta	MGYA00137270	ERZ475666	root:Host-associated:Human	0.0556
MGYA00137271/ERZ475843_FASTA.fasta	MGYA00137271	ERZ475843	root:Host-associated:Human	0.57
MGYA00137272/ERZ474590_FASTA.fasta	MGYA00137272	ERZ474590	root:Host-associated:Human	0.3816
MGYA00137273/ERZ475962_FASTA.fasta	MGYA00137273	ERZ475962	root:Host-associated:Human	0.5932
MGYA00137274/ERZ475780_FASTA.fasta	MGYA00137274	ERZ475780	root:Host-associated:Human	0.5658
MGYA00137275/ERZ475685_FASTA.fasta	MGYA00137275	ERZ475685	root:Host-associated:Human	0.493
MGYA00137276/ERZ475967_FASTA.fasta	MGYA00137276	ERZ475967	root:Host-associated:Human	0.3672
MGYA00137277/ERZ476015_FASTA.fasta	MGYA00137277	ERZ476015	root:Host-associated:Human	0.6304
MGYA00137278/ERZ475883_FASTA.fasta	MGYA00137278	ERZ475883	root:Host-associated:Human	0.1292
MGYA00137279/ERZ475821_FASTA.fasta	MGYA00137279	ERZ475821	root:Host-associated:Human	0.311
MGYA00137281/ERZ475984_FASTA.fasta	MGYA00137281	ERZ475984	root:Host-associated:Human	0.5078
MGYA00137282/ERZ475827_FASTA.fasta	MGYA00137282	ERZ475827	root:Host-associated:Human	0.4406
MGYA00137283/ERZ475850_FASTA.fasta	MGYA00137283	ERZ475850	root:Host-associated:Human	0.1676
MGYA00137285/ERZ475696_FASTA.fasta	MGYA00137285	ERZ475696	root:Host-associated:Human	0.0816
MGYA00137286/ERZ475972_FASTA.fasta	MGYA00137286	ERZ475972	root:Host-associated:Human	0.6314
MGYA00137287/ERZ475868_FASTA.fasta	MGYA00137287	ERZ475868	root:Host-associated:Human	0.6392
MGYA00137288/ERZ476004_FASTA.fasta	MGYA00137288	ERZ476004	root:Host-associated:Human	0.116
MGYA00137290/ERZ475831_FASTA.fasta	MGYA00137290	ERZ475831	root:Host-associated:Human	0.1014
MGYA00137291/ERZ474617_FASTA.fasta	MGYA00137291	ERZ474617	root:Host-associated:Human	0.282
MGYA00137293/ERZ476006_FASTA.fasta	MGYA00137293	ERZ476006	root:Host-associated:Human	0.077
MGYA00137294/ERZ475750_FASTA.fasta	MGYA00137294	ERZ475750	root:Host-associated:Human	0.6204
MGYA00137295/ERZ475745_FASTA.fasta	MGYA00137295	ERZ475745	root:Host-associated:Human	0.6324
MGYA00137296/ERZ475992_FASTA.fasta	MGYA00137296	ERZ475992	root:Host-associated:Human	0.089
MGYA00137298/ERZ475947_FASTA.fasta	MGYA00137298	ERZ475947	root:Host-associated:Human	0.1474
MGYA00137300/ERZ474643_FASTA.fasta	MGYA00137300	ERZ474643	root:Host-associated:Human	0.2332
MGYA00137301/ERZ475996_FASTA.fasta	MGYA00137301	ERZ475996	root:Host-associated:Human	0.6092
MGYA00137305/ERZ475977_FASTA.fasta	MGYA00137305	ERZ475977	root:Host-associated:Human	0.3732
MGYA00137306/ERZ475813_FASTA.fasta	MGYA00137306	ERZ475813	root:Host-associated:Human	0.602
MGYA00137307/ERZ475795_FASTA.fasta	MGYA00137307	ERZ475795	root:Host-associated:Human	0.5292
MGYA00137309/ERZ474631_FASTA.fasta	MGYA00137309	ERZ474631	root:Host-associated:Human	0.3628
MGYA00137311/ERZ475842_FASTA.fasta	MGYA00137311	ERZ475842	root:Host-associated:Human	0.4224
MGYA00137312/ERZ475989_FASTA.fasta	MGYA00137312	ERZ475989	root:Host-associated:Human	0.327
MGYA00137313/ERZ474592_FASTA.fasta	MGYA00137313	ERZ474592	root:Host-associated:Human	0.3664
MGYA00137316/ERZ475820_FASTA.fasta	MGYA00137316	ERZ475820	root:Host-associated:Human	0.1192
MGYA00137317/ERZ475678_FASTA.fasta	MGYA00137317	ERZ475678	root:Host-associated:Human	0.5472
MGYA00137318/ERZ475760_FASTA.fasta	MGYA00137318	ERZ475760	root:Host-associated:Human	0.6164
MGYA00137319/ERZ475764_FASTA.fasta	MGYA00137319	ERZ475764	root:Host-associated:Human	0.5456
MGYA00137320/ERZ475695_FASTA.fasta	MGYA00137320	ERZ475695	root:Host-associated:Human	0.0704
MGYA00137321/ERZ475670_FASTA.fasta	MGYA00137321	ERZ475670	root:Host-associated:Human	0.5602
MGYA00137322/ERZ474644_FASTA.fasta	MGYA00137322	ERZ474644	root:Host-associated:Human	0.0326
MGYA00137325/ERZ475867_FASTA.fasta	MGYA00137325	ERZ475867	root:Host-associated:Human	0.1182
MGYA00137328/ERZ474588_FASTA.fasta	MGYA00137328	ERZ474588	root:Host-associated:Human	0.1662
MGYA00137329/ERZ474594_FASTA.fasta	MGYA00137329	ERZ474594	root:Host-associated:Human	0.6234
MGYA00137330/ERZ475816_FASTA.fasta	MGYA00137330	ERZ475816	root:Host-associated:Human	0.3422
MGYA00137332/ERZ475664_FASTA.fasta	MGYA00137332	ERZ475664	root:Host-associated:Human	0.6442
MGYA00137333/ERZ475659_FASTA.fasta	MGYA00137333	ERZ475659	root:Host-associated:Human	0.3974
MGYA00137336/ERZ475747_FASTA.fasta	MGYA00137336	ERZ475747	root:Host-associated:Human	0.6144
MGYA00137338/ERZ475822_FASTA.fasta	MGYA00137338	ERZ475822	root:Host-associated:Human	0.1478
MGYA00137339/ERZ475757_FASTA.fasta	MGYA00137339	ERZ475757	root:Host-associated:Human	0.655
MGYA00137344/ERZ474608_FASTA.fasta	MGYA00137344	ERZ474608	root:Host-associated:Human	0.2138
MGYA00137345/ERZ475672_FASTA.fasta	MGYA00137345	ERZ475672	root:Host-associated:Human	0.5382
MGYA00137346/ERZ475689_FASTA.fasta	MGYA00137346	ERZ475689	root:Host-associated:Human	0.0654
MGYA00137347/ERZ475844_FASTA.fasta	MGYA00137347	ERZ475844	root:Host-associated:Human	0.048
MGYA00137348/ERZ475756_FASTA.fasta	MGYA00137348	ERZ475756	root:Host-associated:Human	0.181
MGYA00137349/ERZ475776_FASTA.fasta	MGYA00137349	ERZ475776	root:Host-associated:Human	0.6232
MGYA00137351/ERZ475718_FASTA.fasta	MGYA00137351	ERZ475718	root:Host-associated:Human	0.5648
MGYA00137352/ERZ474576_FASTA.fasta	MGYA00137352	ERZ474576	root:Host-associated:Human	0.2118
MGYA00137354/ERZ474597_FASTA.fasta	MGYA00137354	ERZ474597	root:Host-associated:Human	0.4728
MGYA00137355/ERZ474603_FASTA.fasta	MGYA00137355	ERZ474603	root:Host-associated:Human	0.454
MGYA00137356/ERZ475956_FASTA.fasta	MGYA00137356	ERZ475956	root:Host-associated:Human	0.4276
MGYA00137358/ERZ475982_FASTA.fasta	MGYA00137358	ERZ475982	root:Host-associated:Human	0.5716
MGYA00137359/ERZ474585_FASTA.fasta	MGYA00137359	ERZ474585	root:Host-associated:Human	0.662
MGYA00137360/ERZ475657_FASTA.fasta	MGYA00137360	ERZ475657	root:Host-associated:Human	0.688
MGYA00137363/ERZ475777_FASTA.fasta	MGYA00137363	ERZ475777	root:Host-associated:Human	0.1674
MGYA00137365/ERZ475697_FASTA.fasta	MGYA00137365	ERZ475697	root:Host-associated:Human	0.0842
MGYA00137366/ERZ475888_FASTA.fasta	MGYA00137366	ERZ475888	root:Host-associated:Human	0.666
MGYA00137367/ERZ474605_FASTA.fasta	MGYA00137367	ERZ474605	root:Host-associated:Human	0.3326
MGYA00137368/ERZ475895_FASTA.fasta	MGYA00137368	ERZ475895	root:Host-associated:Human	0.0728
MGYA00137369/ERZ475998_FASTA.fasta	MGYA00137369	ERZ475998	root:Host-associated:Human	0.2692
MGYA00137372/ERZ475863_FASTA.fasta	MGYA00137372	ERZ475863	root:Host-associated:Human	0.3586
MGYA00137373/ERZ474574_FASTA.fasta	MGYA00137373	ERZ474574	root:Host-associated:Human	0.401
MGYA00137374/ERZ475908_FASTA.fasta	MGYA00137374	ERZ475908	root:Host-associated:Human	0.587
MGYA00137376/ERZ475859_FASTA.fasta	MGYA00137376	ERZ475859	root:Host-associated:Human	0.68
MGYA00137377/ERZ475743_FASTA.fasta	MGYA00137377	ERZ475743	root:Host-associated:Human	0.3744
MGYA00137378/ERZ475852_FASTA.fasta	MGYA00137378	ERZ475852	root:Host-associated:Human	0.3998
MGYA00137379/ERZ475900_FASTA.fasta	MGYA00137379	ERZ475900	root:Host-associated:Human	0.6474
MGYA00137380/ERZ475699_FASTA.fasta	MGYA00137380	ERZ475699	root:Host-associated:Human	0.208
MGYA00137381/ERZ475948_FASTA.fasta	MGYA00137381	ERZ475948	root:Host-associated:Human	0.339
MGYA00137383/ERZ475845_FASTA.fasta	MGYA00137383	ERZ475845	root:Host-associated:Human	0.1434
MGYA00137385/ERZ475749_FASTA.fasta	MGYA00137385	ERZ475749	root:Host-associated:Human	0.6328
MGYA00137386/ERZ475922_FASTA.fasta	MGYA00137386	ERZ475922	root:Host-associated:Human	0.3672
MGYA00137387/ERZ475663_FASTA.fasta	MGYA00137387	ERZ475663	root:Host-associated:Human	0.1818
MGYA00137388/ERZ475966_FASTA.fasta	MGYA00137388	ERZ475966	root:Host-associated:Human	0.6204
MGYA00137389/ERZ475884_FASTA.fasta	MGYA00137389	ERZ475884	root:Host-associated:Human	0.452
MGYA00137390/ERZ476018_FASTA.fasta	MGYA00137390	ERZ476018	root:Host-associated:Human	0.534
MGYA00137391/ERZ475767_FASTA.fasta	MGYA00137391	ERZ475767	root:Host-associated:Human	0.1108
MGYA00137392/ERZ474587_FASTA.fasta	MGYA00137392	ERZ474587	root:Host-associated:Human	0.5628
MGYA00137393/ERZ475932_FASTA.fasta	MGYA00137393	ERZ475932	root:Host-associated:Human	0.4554
MGYA00137394/ERZ475763_FASTA.fasta	MGYA00137394	ERZ475763	root:Host-associated:Human	0.1026
MGYA00137396/ERZ475886_FASTA.fasta	MGYA00137396	ERZ475886	root:Host-associated:Human	0.3376
MGYA00137397/ERZ475894_FASTA.fasta	MGYA00137397	ERZ475894	root:Host-associated:Human	0.245
MGYA00137398/ERZ474619_FASTA.fasta	MGYA00137398	ERZ474619	root:Host-associated:Human	0.4736
MGYA00137399/ERZ475714_FASTA.fasta	MGYA00137399	ERZ475714	root:Host-associated:Human	0.1288
MGYA00137400/ERZ475739_FASTA.fasta	MGYA00137400	ERZ475739	root:Host-associated:Human	0.5696
MGYA00137401/ERZ476002_FASTA.fasta	MGYA00137401	ERZ476002	root:Host-associated:Human	0.49
MGYA00137403/ERZ475728_FASTA.fasta	MGYA00137403	ERZ475728	root:Host-associated:Human	0.4342
MGYA00137406/ERZ475806_FASTA.fasta	MGYA00137406	ERZ475806	root:Host-associated:Human	0.347
MGYA00137407/ERZ475759_FASTA.fasta	MGYA00137407	ERZ475759	root:Host-associated:Human	0.3468
MGYA00137408/ERZ475832_FASTA.fasta	MGYA00137408	ERZ475832	root:Host-associated:Human	0.6576
MGYA00137409/ERZ475915_FASTA.fasta	MGYA00137409	ERZ475915	root:Host-associated:Human	0.6086
MGYA00137414/ERZ476013_FASTA.fasta	MGYA00137414	ERZ476013	root:Host-associated:Human	0.5948
MGYA00137415/ERZ475878_FASTA.fasta	MGYA00137415	ERZ475878	root:Host-associated:Human	0.5884
MGYA00137416/ERZ475656_FASTA.fasta	MGYA00137416	ERZ475656	root:Host-associated:Human	0.257
MGYA00137420/ERZ475818_FASTA.fasta	MGYA00137420	ERZ475818	root:Host-associated:Human	0.403
MGYA00137421/ERZ475700_FASTA.fasta	MGYA00137421	ERZ475700	root:Host-associated:Human	0.522
MGYA00137423/ERZ475781_FASTA.fasta	MGYA00137423	ERZ475781	root:Host-associated:Human	0.6474
MGYA00137425/ERZ475841_FASTA.fasta	MGYA00137425	ERZ475841	root:Host-associated:Human	0.3292
MGYA00137426/ERZ475909_FASTA.fasta	MGYA00137426	ERZ475909	root:Host-associated:Human	0.51
MGYA00137428/ERZ475829_FASTA.fasta	MGYA00137428	ERZ475829	root:Host-associated:Human	0.2098
MGYA00137430/ERZ475722_FASTA.fasta	MGYA00137430	ERZ475722	root:Host-associated:Human	0.4628
MGYA00137431/ERZ475960_FASTA.fasta	MGYA00137431	ERZ475960	root:Host-associated:Human	0.0854
MGYA00137432/ERZ475964_FASTA.fasta	MGYA00137432	ERZ475964	root:Host-associated:Human	0.5978
MGYA00137433/ERZ475744_FASTA.fasta	MGYA00137433	ERZ475744	root:Host-associated:Human	0.417
MGYA00137437/ERZ475740_FASTA.fasta	MGYA00137437	ERZ475740	root:Host-associated:Human	0.6672
MGYA00137439/ERZ475931_FASTA.fasta	MGYA00137439	ERZ475931	root:Host-associated:Human	0.6466
MGYA00137442/ERZ476012_FASTA.fasta	MGYA00137442	ERZ476012	root:Host-associated:Human	0.372
MGYA00137443/ERZ475735_FASTA.fasta	MGYA00137443	ERZ475735	root:Host-associated:Human	0.4746
MGYA00137444/ERZ475853_FASTA.fasta	MGYA00137444	ERZ475853	root:Host-associated:Human	0.3852
MGYA00137446/ERZ475667_FASTA.fasta	MGYA00137446	ERZ475667	root:Host-associated:Human	0.1254
MGYA00137448/ERZ475676_FASTA.fasta	MGYA00137448	ERZ475676	root:Host-associated:Human	0.398
MGYA00137449/ERZ474609_FASTA.fasta	MGYA00137449	ERZ474609	root:Host-associated:Human	0.53
MGYA00137450/ERZ475800_FASTA.fasta	MGYA00137450	ERZ475800	root:Host-associated:Human	0.5998
MGYA00137452/ERZ474646_FASTA.fasta	MGYA00137452	ERZ474646	root:Host-associated:Human	0.6114
MGYA00137453/ERZ475899_FASTA.fasta	MGYA00137453	ERZ475899	root:Host-associated:Human	0.3468
MGYA00137455/ERZ475854_FASTA.fasta	MGYA00137455	ERZ475854	root:Host-associated:Human	0.1454
MGYA00137457/ERZ475994_FASTA.fasta	MGYA00137457	ERZ475994	root:Host-associated:Human	0.6772
MGYA00137458/ERZ474627_FASTA.fasta	MGYA00137458	ERZ474627	root:Host-associated:Human	0.1868
MGYA00137460/ERZ475717_FASTA.fasta	MGYA00137460	ERZ475717	root:Host-associated:Human	0.1522
MGYA00137461/ERZ475887_FASTA.fasta	MGYA00137461	ERZ475887	root:Host-associated:Human	0.4108
MGYA00137462/ERZ475927_FASTA.fasta	MGYA00137462	ERZ475927	root:Host-associated:Human	0.0638
MGYA00137463/ERZ475741_FASTA.fasta	MGYA00137463	ERZ475741	root:Host-associated:Human	0.4416
MGYA00137465/ERZ474639_FASTA.fasta	MGYA00137465	ERZ474639	root:Host-associated:Human	0.0292
MGYA00137466/ERZ475954_FASTA.fasta	MGYA00137466	ERZ475954	root:Host-associated:Human	0.6536
MGYA00137469/ERZ475943_FASTA.fasta	MGYA00137469	ERZ475943	root:Host-associated:Human	0.0556
MGYA00137470/ERZ477417_FASTA.fasta	MGYA00137470	ERZ477417	root:Host-associated:Human:Digestive system	0.3132
MGYA00137471/ERZ477422_FASTA.fasta	MGYA00137471	ERZ477422	root:Host-associated:Human:Digestive system	0.63
MGYA00137472/ERZ477425_FASTA.fasta	MGYA00137472	ERZ477425	root:Host-associated:Human:Digestive system	0.63
MGYA00137473/ERZ477418_FASTA.fasta	MGYA00137473	ERZ477418	root:Host-associated:Human:Digestive system	0.4668
MGYA00137474/ERZ477429_FASTA.fasta	MGYA00137474	ERZ477429	root:Host-associated:Human:Digestive system	0.63
MGYA00137475/ERZ477416_FASTA.fasta	MGYA00137475	ERZ477416	root:Host-associated:Human:Digestive system	0.3294
MGYA00137476/ERZ477427_FASTA.fasta	MGYA00137476	ERZ477427	root:Host-associated:Human:Digestive system	0.63
MGYA00137477/ERZ477426_FASTA.fasta	MGYA00137477	ERZ477426	root:Host-associated:Human:Digestive system	0.63
MGYA00137478/ERZ477419_FASTA.fasta	MGYA00137478	ERZ477419	root:Host-associated:Human:Digestive system	0.4668
MGYA00137479/ERZ477424_FASTA.fasta	MGYA00137479	ERZ477424	root:Host-associated:Human:Digestive system	0.63
MGYA00137480/ERZ477428_FASTA.fasta	MGYA00137480	ERZ477428	root:Host-associated:Human:Digestive system	0.63
MGYA00137481/ERZ477420_FASTA.fasta	MGYA00137481	ERZ477420	root:Host-associated:Human:Digestive system	0.433
MGYA00137482/ERZ477421_FASTA.fasta	MGYA00137482	ERZ477421	root:Host-associated:Human:Digestive system	0.4366
MGYA00137483/ERZ477423_FASTA.fasta	MGYA00137483	ERZ477423	root:Host-associated:Human:Digestive system	0.63
MGYA00137484/ERZ476800_FASTA.fasta	MGYA00137484	ERZ476800	root:Host-associated:Human:Digestive system	0.0294
MGYA00137485/ERZ476845_FASTA.fasta	MGYA00137485	ERZ476845	root:Host-associated:Human:Digestive system	0.0294
MGYA00137486/ERZ476885_FASTA.fasta	MGYA00137486	ERZ476885	root:Host-associated:Human:Digestive system	0.0294
MGYA00137487/ERZ476727_FASTA.fasta	MGYA00137487	ERZ476727	root:Host-associated:Human:Digestive system	0.0294
MGYA00137488/ERZ476886_FASTA.fasta	MGYA00137488	ERZ476886	root:Host-associated:Human:Digestive system	0.0294
MGYA00137489/ERZ476799_FASTA.fasta	MGYA00137489	ERZ476799	root:Host-associated:Human:Digestive system	0.0294
MGYA00137490/ERZ476734_FASTA.fasta	MGYA00137490	ERZ476734	root:Host-associated:Human:Digestive system	0.0294
MGYA00137491/ERZ476741_FASTA.fasta	MGYA00137491	ERZ476741	root:Host-associated:Human:Digestive system	0.0294
MGYA00137492/ERZ476785_FASTA.fasta	MGYA00137492	ERZ476785	root:Host-associated:Human:Digestive system	0.0294
MGYA00137493/ERZ476775_FASTA.fasta	MGYA00137493	ERZ476775	root:Host-associated:Human:Digestive system	0.0294
MGYA00137494/ERZ476735_FASTA.fasta	MGYA00137494	ERZ476735	root:Host-associated:Human:Digestive system	0.0294
MGYA00137495/ERZ476836_FASTA.fasta	MGYA00137495	ERZ476836	root:Host-associated:Human:Digestive system	0.0294
MGYA00137496/ERZ476780_FASTA.fasta	MGYA00137496	ERZ476780	root:Host-associated:Human:Digestive system	0.0294
MGYA00137497/ERZ476841_FASTA.fasta	MGYA00137497	ERZ476841	root:Host-associated:Human:Digestive system	0.0294
MGYA00137498/ERZ476894_FASTA.fasta	MGYA00137498	ERZ476894	root:Host-associated:Human:Digestive system	0.0294
MGYA00137499/ERZ476725_FASTA.fasta	MGYA00137499	ERZ476725	root:Host-associated:Human:Digestive system	0.0294
MGYA00137500/ERZ476890_FASTA.fasta	MGYA00137500	ERZ476890	root:Host-associated:Human:Digestive system	0.0294
MGYA00137501/ERZ476764_FASTA.fasta	MGYA00137501	ERZ476764	root:Host-associated:Human:Digestive system	0.0294
MGYA00137502/ERZ476825_FASTA.fasta	MGYA00137502	ERZ476825	root:Host-associated:Human:Digestive system	0.0294
MGYA00137503/ERZ476832_FASTA.fasta	MGYA00137503	ERZ476832	root:Host-associated:Human:Digestive system	0.0294
MGYA00137504/ERZ476861_FASTA.fasta	MGYA00137504	ERZ476861	root:Host-associated:Human:Digestive system	0.0294
MGYA00137505/ERZ476773_FASTA.fasta	MGYA00137505	ERZ476773	root:Host-associated:Human:Digestive system	0.0294
MGYA00137506/ERZ476874_FASTA.fasta	MGYA00137506	ERZ476874	root:Host-associated:Human:Digestive system	0.0294
MGYA00137507/ERZ476868_FASTA.fasta	MGYA00137507	ERZ476868	root:Host-associated:Human:Digestive system	0.0294
MGYA00137508/ERZ476911_FASTA.fasta	MGYA00137508	ERZ476911	root:Host-associated:Human:Digestive system	0.0294
MGYA00137509/ERZ476781_FASTA.fasta	MGYA00137509	ERZ476781	root:Host-associated:Human:Digestive system	0.0294
MGYA00137510/ERZ476765_FASTA.fasta	MGYA00137510	ERZ476765	root:Host-associated:Human:Digestive system	0.0294
MGYA00137511/ERZ476776_FASTA.fasta	MGYA00137511	ERZ476776	root:Host-associated:Human:Digestive system	0.0294
MGYA00137512/ERZ476794_FASTA.fasta	MGYA00137512	ERZ476794	root:Host-associated:Human:Digestive system	0.0294
MGYA00137513/ERZ476771_FASTA.fasta	MGYA00137513	ERZ476771	root:Host-associated:Human:Digestive system	0.0294
MGYA00137514/ERZ476731_FASTA.fasta	MGYA00137514	ERZ476731	root:Host-associated:Human:Digestive system	0.0294
MGYA00137515/ERZ476824_FASTA.fasta	MGYA00137515	ERZ476824	root:Host-associated:Human:Digestive system	0.0294
MGYA00137516/ERZ476910_FASTA.fasta	MGYA00137516	ERZ476910	root:Host-associated:Human:Digestive system	0.0294
MGYA00137517/ERZ476856_FASTA.fasta	MGYA00137517	ERZ476856	root:Host-associated:Human:Digestive system	0.0294
MGYA00137518/ERZ476752_FASTA.fasta	MGYA00137518	ERZ476752	root:Host-associated:Human:Digestive system	0.0294
MGYA00137519/ERZ476782_FASTA.fasta	MGYA00137519	ERZ476782	root:Host-associated:Human:Digestive system	0.0294
MGYA00137520/ERZ476766_FASTA.fasta	MGYA00137520	ERZ476766	root:Host-associated:Human:Digestive system	0.0294
MGYA00137521/ERZ476749_FASTA.fasta	MGYA00137521	ERZ476749	root:Host-associated:Human:Digestive system	0.0294
MGYA00137522/ERZ476778_FASTA.fasta	MGYA00137522	ERZ476778	root:Host-associated:Human:Digestive system	0.0294
MGYA00137523/ERZ476792_FASTA.fasta	MGYA00137523	ERZ476792	root:Host-associated:Human:Digestive system	0.0294
MGYA00137524/ERZ476757_FASTA.fasta	MGYA00137524	ERZ476757	root:Host-associated:Human:Digestive system	0.0294
MGYA00137525/ERZ476881_FASTA.fasta	MGYA00137525	ERZ476881	root:Host-associated:Human:Digestive system	0.0294
MGYA00137526/ERZ476904_FASTA.fasta	MGYA00137526	ERZ476904	root:Host-associated:Human:Digestive system	0.0294
MGYA00137527/ERZ476889_FASTA.fasta	MGYA00137527	ERZ476889	root:Host-associated:Human:Digestive system	0.0294
MGYA00137528/ERZ476830_FASTA.fasta	MGYA00137528	ERZ476830	root:Host-associated:Human:Digestive system	0.0294
MGYA00137529/ERZ476915_FASTA.fasta	MGYA00137529	ERZ476915	root:Host-associated:Human:Digestive system	0.0294
MGYA00137530/ERZ476884_FASTA.fasta	MGYA00137530	ERZ476884	root:Host-associated:Human:Digestive system	0.0294
MGYA00137531/ERZ476850_FASTA.fasta	MGYA00137531	ERZ476850	root:Host-associated:Human:Digestive system	0.0294
MGYA00137532/ERZ476791_FASTA.fasta	MGYA00137532	ERZ476791	root:Host-associated:Human:Digestive system	0.0294
MGYA00137533/ERZ476877_FASTA.fasta	MGYA00137533	ERZ476877	root:Host-associated:Human:Digestive system	0.0294
MGYA00137534/ERZ476895_FASTA.fasta	MGYA00137534	ERZ476895	root:Host-associated:Human:Digestive system	0.0294
MGYA00137535/ERZ476849_FASTA.fasta	MGYA00137535	ERZ476849	root:Host-associated:Human:Digestive system	0.0294
MGYA00137536/ERZ476777_FASTA.fasta	MGYA00137536	ERZ476777	root:Host-associated:Human:Digestive system	0.0294
MGYA00137537/ERZ476833_FASTA.fasta	MGYA00137537	ERZ476833	root:Host-associated:Human:Digestive system	0.0294
MGYA00137538/ERZ476838_FASTA.fasta	MGYA00137538	ERZ476838	root:Host-associated:Human:Digestive system	0.0294
MGYA00137539/ERZ476908_FASTA.fasta	MGYA00137539	ERZ476908	root:Host-associated:Human:Digestive system	0.0294
MGYA00137540/ERZ476744_FASTA.fasta	MGYA00137540	ERZ476744	root:Host-associated:Human:Digestive system	0.0294
MGYA00137541/ERZ476796_FASTA.fasta	MGYA00137541	ERZ476796	root:Host-associated:Human:Digestive system	0.0294
MGYA00137542/ERZ476769_FASTA.fasta	MGYA00137542	ERZ476769	root:Host-associated:Human:Digestive system	0.0294
MGYA00137543/ERZ476902_FASTA.fasta	MGYA00137543	ERZ476902	root:Host-associated:Human:Digestive system	0.0294
MGYA00137544/ERZ476860_FASTA.fasta	MGYA00137544	ERZ476860	root:Host-associated:Human:Digestive system	0.0294
MGYA00137545/ERZ476846_FASTA.fasta	MGYA00137545	ERZ476846	root:Host-associated:Human:Digestive system	0.0294
MGYA00137546/ERZ476801_FASTA.fasta	MGYA00137546	ERZ476801	root:Host-associated:Human:Digestive system	0.0294
MGYA00137547/ERZ476809_FASTA.fasta	MGYA00137547	ERZ476809	root:Host-associated:Human:Digestive system	0.0294
MGYA00137548/ERZ476815_FASTA.fasta	MGYA00137548	ERZ476815	root:Host-associated:Human:Digestive system	0.0294
MGYA00137549/ERZ476858_FASTA.fasta	MGYA00137549	ERZ476858	root:Host-associated:Human:Digestive system	0.0294
MGYA00137550/ERZ476882_FASTA.fasta	MGYA00137550	ERZ476882	root:Host-associated:Human:Digestive system	0.0294
MGYA00137551/ERZ476888_FASTA.fasta	MGYA00137551	ERZ476888	root:Host-associated:Human:Digestive system	0.0294
MGYA00137552/ERZ476901_FASTA.fasta	MGYA00137552	ERZ476901	root:Host-associated:Human:Digestive system	0.0294
MGYA00137553/ERZ476724_FASTA.fasta	MGYA00137553	ERZ476724	root:Host-associated:Human:Digestive system	0.0294
MGYA00137554/ERZ476761_FASTA.fasta	MGYA00137554	ERZ476761	root:Host-associated:Human:Digestive system	0.0294
MGYA00137555/ERZ476740_FASTA.fasta	MGYA00137555	ERZ476740	root:Host-associated:Human:Digestive system	0.0294
MGYA00137556/ERZ476837_FASTA.fasta	MGYA00137556	ERZ476837	root:Host-associated:Human:Digestive system	0.0294
MGYA00137557/ERZ476746_FASTA.fasta	MGYA00137557	ERZ476746	root:Host-associated:Human:Digestive system	0.0294
MGYA00137558/ERZ476742_FASTA.fasta	MGYA00137558	ERZ476742	root:Host-associated:Human:Digestive system	0.0294
MGYA00137559/ERZ476783_FASTA.fasta	MGYA00137559	ERZ476783	root:Host-associated:Human:Digestive system	0.0294
MGYA00137560/ERZ476865_FASTA.fasta	MGYA00137560	ERZ476865	root:Host-associated:Human:Digestive system	0.0294
MGYA00137561/ERZ476754_FASTA.fasta	MGYA00137561	ERZ476754	root:Host-associated:Human:Digestive system	0.0294
MGYA00137562/ERZ476768_FASTA.fasta	MGYA00137562	ERZ476768	root:Host-associated:Human:Digestive system	0.0294
MGYA00137563/ERZ476883_FASTA.fasta	MGYA00137563	ERZ476883	root:Host-associated:Human:Digestive system	0.0294
MGYA00137564/ERZ476843_FASTA.fasta	MGYA00137564	ERZ476843	root:Host-associated:Human:Digestive system	0.0294
MGYA00137565/ERZ476847_FASTA.fasta	MGYA00137565	ERZ476847	root:Host-associated:Human:Digestive system	0.0294
MGYA00137566/ERZ476820_FASTA.fasta	MGYA00137566	ERZ476820	root:Host-associated:Human:Digestive system	0.0294
MGYA00137567/ERZ476732_FASTA.fasta	MGYA00137567	ERZ476732	root:Host-associated:Human:Digestive system	0.0294
MGYA00137568/ERZ476818_FASTA.fasta	MGYA00137568	ERZ476818	root:Host-associated:Human:Digestive system	0.0294
MGYA00137569/ERZ476772_FASTA.fasta	MGYA00137569	ERZ476772	root:Host-associated:Human:Digestive system	0.0294
MGYA00137570/ERZ476786_FASTA.fasta	MGYA00137570	ERZ476786	root:Host-associated:Human:Digestive system	0.0294
MGYA00137571/ERZ476852_FASTA.fasta	MGYA00137571	ERZ476852	root:Host-associated:Human:Digestive system	0.0294
MGYA00137572/ERZ476903_FASTA.fasta	MGYA00137572	ERZ476903	root:Host-associated:Human:Digestive system	0.0294
MGYA00137573/ERZ476743_FASTA.fasta	MGYA00137573	ERZ476743	root:Host-associated:Human:Digestive system	0.0294
MGYA00137574/ERZ476822_FASTA.fasta	MGYA00137574	ERZ476822	root:Host-associated:Human:Digestive system	0.0294
MGYA00137575/ERZ476859_FASTA.fasta	MGYA00137575	ERZ476859	root:Host-associated:Human:Digestive system	0.0294
MGYA00137576/ERZ476839_FASTA.fasta	MGYA00137576	ERZ476839	root:Host-associated:Human:Digestive system	0.0294
MGYA00137577/ERZ476834_FASTA.fasta	MGYA00137577	ERZ476834	root:Host-associated:Human:Digestive system	0.0294
MGYA00137578/ERZ476808_FASTA.fasta	MGYA00137578	ERZ476808	root:Host-associated:Human:Digestive system	0.0294
MGYA00137579/ERZ476787_FASTA.fasta	MGYA00137579	ERZ476787	root:Host-associated:Human:Digestive system	0.0294
MGYA00137580/ERZ476851_FASTA.fasta	MGYA00137580	ERZ476851	root:Host-associated:Human:Digestive system	0.0294
MGYA00137581/ERZ476875_FASTA.fasta	MGYA00137581	ERZ476875	root:Host-associated:Human:Digestive system	0.0294
MGYA00137582/ERZ476896_FASTA.fasta	MGYA00137582	ERZ476896	root:Host-associated:Human:Digestive system	0.0294
MGYA00137583/ERZ476813_FASTA.fasta	MGYA00137583	ERZ476813	root:Host-associated:Human:Digestive system	0.0294
MGYA00137584/ERZ476793_FASTA.fasta	MGYA00137584	ERZ476793	root:Host-associated:Human:Digestive system	0.0294
MGYA00137585/ERZ476759_FASTA.fasta	MGYA00137585	ERZ476759	root:Host-associated:Human:Digestive system	0.0294
MGYA00137586/ERZ476880_FASTA.fasta	MGYA00137586	ERZ476880	root:Host-associated:Human:Digestive system	0.0294
MGYA00137587/ERZ476823_FASTA.fasta	MGYA00137587	ERZ476823	root:Host-associated:Human:Digestive system	0.0294
MGYA00137588/ERZ476802_FASTA.fasta	MGYA00137588	ERZ476802	root:Host-associated:Human:Digestive system	0.0294
MGYA00137589/ERZ476887_FASTA.fasta	MGYA00137589	ERZ476887	root:Host-associated:Human:Digestive system	0.0294
MGYA00137590/ERZ476803_FASTA.fasta	MGYA00137590	ERZ476803	root:Host-associated:Human:Digestive system	0.0294
MGYA00137591/ERZ476840_FASTA.fasta	MGYA00137591	ERZ476840	root:Host-associated:Human:Digestive system	0.0294
MGYA00137592/ERZ476755_FASTA.fasta	MGYA00137592	ERZ476755	root:Host-associated:Human:Digestive system	0.0294
MGYA00137593/ERZ476819_FASTA.fasta	MGYA00137593	ERZ476819	root:Host-associated:Human:Digestive system	0.0294
MGYA00137594/ERZ476873_FASTA.fasta	MGYA00137594	ERZ476873	root:Host-associated:Human:Digestive system	0.0294
MGYA00137595/ERZ476811_FASTA.fasta	MGYA00137595	ERZ476811	root:Host-associated:Human:Digestive system	0.0294
MGYA00137596/ERZ476872_FASTA.fasta	MGYA00137596	ERZ476872	root:Host-associated:Human:Digestive system	0.0294
MGYA00137597/ERZ476753_FASTA.fasta	MGYA00137597	ERZ476753	root:Host-associated:Human:Digestive system	0.0294
MGYA00137598/ERZ476816_FASTA.fasta	MGYA00137598	ERZ476816	root:Host-associated:Human:Digestive system	0.0294
MGYA00137599/ERZ476905_FASTA.fasta	MGYA00137599	ERZ476905	root:Host-associated:Human:Digestive system	0.0294
MGYA00137600/ERZ476853_FASTA.fasta	MGYA00137600	ERZ476853	root:Host-associated:Human:Digestive system	0.0294
MGYA00137601/ERZ476774_FASTA.fasta	MGYA00137601	ERZ476774	root:Host-associated:Human:Digestive system	0.0294
MGYA00137602/ERZ476848_FASTA.fasta	MGYA00137602	ERZ476848	root:Host-associated:Human:Digestive system	0.0294
MGYA00137603/ERZ476857_FASTA.fasta	MGYA00137603	ERZ476857	root:Host-associated:Human:Digestive system	0.0294
MGYA00137604/ERZ476817_FASTA.fasta	MGYA00137604	ERZ476817	root:Host-associated:Human:Digestive system	0.0294
MGYA00137605/ERZ476862_FASTA.fasta	MGYA00137605	ERZ476862	root:Host-associated:Human:Digestive system	0.0294
MGYA00137606/ERZ476763_FASTA.fasta	MGYA00137606	ERZ476763	root:Host-associated:Human:Digestive system	0.0294
MGYA00137607/ERZ476909_FASTA.fasta	MGYA00137607	ERZ476909	root:Host-associated:Human:Digestive system	0.0294
MGYA00137608/ERZ476738_FASTA.fasta	MGYA00137608	ERZ476738	root:Host-associated:Human:Digestive system	0.0294
MGYA00137609/ERZ476784_FASTA.fasta	MGYA00137609	ERZ476784	root:Host-associated:Human:Digestive system	0.0294
MGYA00137610/ERZ476729_FASTA.fasta	MGYA00137610	ERZ476729	root:Host-associated:Human:Digestive system	0.0294
MGYA00137611/ERZ476871_FASTA.fasta	MGYA00137611	ERZ476871	root:Host-associated:Human:Digestive system	0.0294
MGYA00137612/ERZ476842_FASTA.fasta	MGYA00137612	ERZ476842	root:Host-associated:Human:Digestive system	0.0294
MGYA00137613/ERZ476728_FASTA.fasta	MGYA00137613	ERZ476728	root:Host-associated:Human:Digestive system	0.0294
MGYA00137614/ERZ476855_FASTA.fasta	MGYA00137614	ERZ476855	root:Host-associated:Human:Digestive system	0.0294
MGYA00137615/ERZ476863_FASTA.fasta	MGYA00137615	ERZ476863	root:Host-associated:Human:Digestive system	0.0294
MGYA00137616/ERZ476879_FASTA.fasta	MGYA00137616	ERZ476879	root:Host-associated:Human:Digestive system	0.0294
MGYA00137617/ERZ476867_FASTA.fasta	MGYA00137617	ERZ476867	root:Host-associated:Human:Digestive system	0.0294
MGYA00137618/ERZ476844_FASTA.fasta	MGYA00137618	ERZ476844	root:Host-associated:Human:Digestive system	0.0294
MGYA00137619/ERZ476906_FASTA.fasta	MGYA00137619	ERZ476906	root:Host-associated:Human:Digestive system	0.0294
MGYA00137620/ERZ476812_FASTA.fasta	MGYA00137620	ERZ476812	root:Host-associated:Human:Digestive system	0.0294
MGYA00137621/ERZ476826_FASTA.fasta	MGYA00137621	ERZ476826	root:Host-associated:Human:Digestive system	0.0294
MGYA00137622/ERZ476827_FASTA.fasta	MGYA00137622	ERZ476827	root:Host-associated:Human:Digestive system	0.0294
MGYA00137623/ERZ476779_FASTA.fasta	MGYA00137623	ERZ476779	root:Host-associated:Human:Digestive system	0.0294
MGYA00137624/ERZ476739_FASTA.fasta	MGYA00137624	ERZ476739	root:Host-associated:Human:Digestive system	0.0294
MGYA00137625/ERZ476750_FASTA.fasta	MGYA00137625	ERZ476750	root:Host-associated:Human:Digestive system	0.0294
MGYA00137626/ERZ476758_FASTA.fasta	MGYA00137626	ERZ476758	root:Host-associated:Human:Digestive system	0.0294
MGYA00137627/ERZ476748_FASTA.fasta	MGYA00137627	ERZ476748	root:Host-associated:Human:Digestive system	0.0294
MGYA00137628/ERZ476829_FASTA.fasta	MGYA00137628	ERZ476829	root:Host-associated:Human:Digestive system	0.0294
MGYA00137629/ERZ476806_FASTA.fasta	MGYA00137629	ERZ476806	root:Host-associated:Human:Digestive system	0.0294
MGYA00137630/ERZ476893_FASTA.fasta	MGYA00137630	ERZ476893	root:Host-associated:Human:Digestive system	0.0294
MGYA00137631/ERZ476736_FASTA.fasta	MGYA00137631	ERZ476736	root:Host-associated:Human:Digestive system	0.0294
MGYA00137632/ERZ476899_FASTA.fasta	MGYA00137632	ERZ476899	root:Host-associated:Human:Digestive system	0.0294
MGYA00137633/ERZ476870_FASTA.fasta	MGYA00137633	ERZ476870	root:Host-associated:Human:Digestive system	0.0294
MGYA00137634/ERZ476747_FASTA.fasta	MGYA00137634	ERZ476747	root:Host-associated:Human:Digestive system	0.0294
MGYA00137635/ERZ476821_FASTA.fasta	MGYA00137635	ERZ476821	root:Host-associated:Human:Digestive system	0.0294
MGYA00137636/ERZ476762_FASTA.fasta	MGYA00137636	ERZ476762	root:Host-associated:Human:Digestive system	0.0294
MGYA00137637/ERZ476767_FASTA.fasta	MGYA00137637	ERZ476767	root:Host-associated:Human:Digestive system	0.0294
MGYA00137638/ERZ476878_FASTA.fasta	MGYA00137638	ERZ476878	root:Host-associated:Human:Digestive system	0.0294
MGYA00137639/ERZ476795_FASTA.fasta	MGYA00137639	ERZ476795	root:Host-associated:Human:Digestive system	0.0294
MGYA00137640/ERZ476831_FASTA.fasta	MGYA00137640	ERZ476831	root:Host-associated:Human:Digestive system	0.0294
MGYA00137641/ERZ476913_FASTA.fasta	MGYA00137641	ERZ476913	root:Host-associated:Human:Digestive system	0.0294
MGYA00137642/ERZ476805_FASTA.fasta	MGYA00137642	ERZ476805	root:Host-associated:Human:Digestive system	0.0294
MGYA00137643/ERZ476900_FASTA.fasta	MGYA00137643	ERZ476900	root:Host-associated:Human:Digestive system	0.0294
MGYA00137644/ERZ476733_FASTA.fasta	MGYA00137644	ERZ476733	root:Host-associated:Human:Digestive system	0.0294
MGYA00137645/ERZ476788_FASTA.fasta	MGYA00137645	ERZ476788	root:Host-associated:Human:Digestive system	0.0294
MGYA00137646/ERZ476804_FASTA.fasta	MGYA00137646	ERZ476804	root:Host-associated:Human:Digestive system	0.0294
MGYA00137647/ERZ476876_FASTA.fasta	MGYA00137647	ERZ476876	root:Host-associated:Human:Digestive system	0.0294
MGYA00137648/ERZ476891_FASTA.fasta	MGYA00137648	ERZ476891	root:Host-associated:Human:Digestive system	0.0294
MGYA00137649/ERZ476737_FASTA.fasta	MGYA00137649	ERZ476737	root:Host-associated:Human:Digestive system	0.0294
MGYA00137650/ERZ476835_FASTA.fasta	MGYA00137650	ERZ476835	root:Host-associated:Human:Digestive system	0.0294
MGYA00137651/ERZ476912_FASTA.fasta	MGYA00137651	ERZ476912	root:Host-associated:Human:Digestive system	0.0294
MGYA00137652/ERZ476866_FASTA.fasta	MGYA00137652	ERZ476866	root:Host-associated:Human:Digestive system	0.0294
MGYA00137653/ERZ476770_FASTA.fasta	MGYA00137653	ERZ476770	root:Host-associated:Human:Digestive system	0.0294
MGYA00137654/ERZ476907_FASTA.fasta	MGYA00137654	ERZ476907	root:Host-associated:Human:Digestive system	0.0294
MGYA00137655/ERZ476828_FASTA.fasta	MGYA00137655	ERZ476828	root:Host-associated:Human:Digestive system	0.0294
MGYA00137656/ERZ476790_FASTA.fasta	MGYA00137656	ERZ476790	root:Host-associated:Human:Digestive system	0.0294
MGYA00137657/ERZ476810_FASTA.fasta	MGYA00137657	ERZ476810	root:Host-associated:Human:Digestive system	0.0294
MGYA00137658/ERZ476869_FASTA.fasta	MGYA00137658	ERZ476869	root:Host-associated:Human:Digestive system	0.0294
MGYA00137659/ERZ476745_FASTA.fasta	MGYA00137659	ERZ476745	root:Host-associated:Human:Digestive system	0.0294
MGYA00137660/ERZ476760_FASTA.fasta	MGYA00137660	ERZ476760	root:Host-associated:Human:Digestive system	0.0294
MGYA00137661/ERZ476814_FASTA.fasta	MGYA00137661	ERZ476814	root:Host-associated:Human:Digestive system	0.0294
MGYA00137662/ERZ476807_FASTA.fasta	MGYA00137662	ERZ476807	root:Host-associated:Human:Digestive system	0.0294
MGYA00137663/ERZ476864_FASTA.fasta	MGYA00137663	ERZ476864	root:Host-associated:Human:Digestive system	0.0294
MGYA00137664/ERZ476726_FASTA.fasta	MGYA00137664	ERZ476726	root:Host-associated:Human:Digestive system	0.0294
MGYA00137665/ERZ476897_FASTA.fasta	MGYA00137665	ERZ476897	root:Host-associated:Human:Digestive system	0.0294
MGYA00137666/ERZ476797_FASTA.fasta	MGYA00137666	ERZ476797	root:Host-associated:Human:Digestive system	0.0294
MGYA00137667/ERZ476854_FASTA.fasta	MGYA00137667	ERZ476854	root:Host-associated:Human:Digestive system	0.0294
MGYA00137668/ERZ476892_FASTA.fasta	MGYA00137668	ERZ476892	root:Host-associated:Human:Digestive system	0.0294
MGYA00137669/ERZ476756_FASTA.fasta	MGYA00137669	ERZ476756	root:Host-associated:Human:Digestive system	0.0294
MGYA00137670/ERZ476914_FASTA.fasta	MGYA00137670	ERZ476914	root:Host-associated:Human:Digestive system	0.0294
MGYA00137671/ERZ476898_FASTA.fasta	MGYA00137671	ERZ476898	root:Host-associated:Human:Digestive system	0.0294
MGYA00137672/ERZ476789_FASTA.fasta	MGYA00137672	ERZ476789	root:Host-associated:Human:Digestive system	0.0294
MGYA00137673/ERZ476723_FASTA.fasta	MGYA00137673	ERZ476723	root:Host-associated:Human:Digestive system	0.0294
MGYA00137674/ERZ476798_FASTA.fasta	MGYA00137674	ERZ476798	root:Host-associated:Human:Digestive system	0.0294
MGYA00137675/ERZ476730_FASTA.fasta	MGYA00137675	ERZ476730	root:Host-associated:Human:Digestive system	0.0294
MGYA00137676/ERZ476751_FASTA.fasta	MGYA00137676	ERZ476751	root:Host-associated:Human:Digestive system	0.0294
MGYA00137677/ERZ477003_FASTA.fasta	MGYA00137677	ERZ477003	root:Host-associated:Human:Digestive system:Large int	0.0294
MGYA00137678/ERZ476946_FASTA.fasta	MGYA00137678	ERZ476946	root:Host-associated:Human:Digestive system:Large int	0.0292
MGYA00137679/ERZ477053_FASTA.fasta	MGYA00137679	ERZ477053	root:Host-associated:Human:Digestive system:Large int	0.0294
MGYA00137680/ERZ477011_FASTA.fasta	MGYA00137680	ERZ477011	root:Host-associated:Human:Digestive system:Large int	0.0464
MGYA00137681/ERZ477055_FASTA.fasta	MGYA00137681	ERZ477055	root:Host-associated:Human:Digestive system:Large int	0.0294
MGYA00137682/ERZ476917_FASTA.fasta	MGYA00137682	ERZ476917	root:Host-associated:Human:Digestive system:Large int	0.0258
MGYA00137683/ERZ477010_FASTA.fasta	MGYA00137683	ERZ477010	root:Host-associated:Human:Digestive system:Large int	0.0326
MGYA00137684/ERZ476972_FASTA.fasta	MGYA00137684	ERZ476972	root:Host-associated:Human:Digestive system:Large int	0.0328
MGYA00137685/ERZ476992_FASTA.fasta	MGYA00137685	ERZ476992	root:Host-associated:Human:Digestive system:Large int	0.0294
MGYA00137686/ERZ477068_FASTA.fasta	MGYA00137686	ERZ477068	root:Host-associated:Human:Digestive system:Large int	0.044
MGYA00137687/ERZ477070_FASTA.fasta	MGYA00137687	ERZ477070	root:Host-associated:Human:Digestive system:Large int	0.0294
MGYA00137688/ERZ477074_FASTA.fasta	MGYA00137688	ERZ477074	root:Host-associated:Human:Digestive system:Large int	0.0294
MGYA00137689/ERZ476963_FASTA.fasta	MGYA00137689	ERZ476963	root:Host-associated:Human:Digestive system:Large int	0.1102
MGYA00137690/ERZ476955_FASTA.fasta	MGYA00137690	ERZ476955	root:Host-associated:Human:Digestive system:Large int	0.0284
MGYA00137691/ERZ476926_FASTA.fasta	MGYA00137691	ERZ476926	root:Host-associated:Human:Digestive system:Large int	0.0322
MGYA00137692/ERZ477043_FASTA.fasta	MGYA00137692	ERZ477043	root:Host-associated:Human:Digestive system:Large int	0.0532
MGYA00137693/ERZ477006_FASTA.fasta	MGYA00137693	ERZ477006	root:Host-associated:Human:Digestive system:Large int	0.0294
MGYA00137694/ERZ477001_FASTA.fasta	MGYA00137694	ERZ477001	root:Host-associated:Human:Digestive system:Large int	0.0294
MGYA00137695/ERZ476945_FASTA.fasta	MGYA00137695	ERZ476945	root:Host-associated:Human:Digestive system:Large int	0.0328
MGYA00137696/ERZ477050_FASTA.fasta	MGYA00137696	ERZ477050	root:Host-associated:Human:Digestive system:Large int	0.025
MGYA00137697/ERZ476996_FASTA.fasta	MGYA00137697	ERZ476996	root:Host-associated:Human:Digestive system:Large int	0.0298
MGYA00137698/ERZ476994_FASTA.fasta	MGYA00137698	ERZ476994	root:Host-associated:Human:Digestive system:Large int	0.0444
MGYA00137699/ERZ476919_FASTA.fasta	MGYA00137699	ERZ476919	root:Host-associated:Human:Digestive system:Large int	0.0312
MGYA00137700/ERZ476956_FASTA.fasta	MGYA00137700	ERZ476956	root:Host-associated:Human:Digestive system:Large int	0.0322
MGYA00137701/ERZ476936_FASTA.fasta	MGYA00137701	ERZ476936	root:Host-associated:Human:Digestive system:Large int	0.0296
MGYA00137702/ERZ476986_FASTA.fasta	MGYA00137702	ERZ476986	root:Host-associated:Human:Digestive system:Large int	0.0294
MGYA00137703/ERZ476984_FASTA.fasta	MGYA00137703	ERZ476984	root:Host-associated:Human:Digestive system:Large int	0.0294
MGYA00137704/ERZ477033_FASTA.fasta	MGYA00137704	ERZ477033	root:Host-associated:Human:Digestive system:Large int	0.0442
MGYA00137705/ERZ477058_FASTA.fasta	MGYA00137705	ERZ477058	root:Host-associated:Human:Digestive system:Large int	0.0388
MGYA00137706/ERZ476977_FASTA.fasta	MGYA00137706	ERZ476977	root:Host-associated:Human:Digestive system:Large int	0.0294
MGYA00137707/ERZ477048_FASTA.fasta	MGYA00137707	ERZ477048	root:Host-associated:Human:Digestive system:Large int	0.0294
MGYA00137708/ERZ476952_FASTA.fasta	MGYA00137708	ERZ476952	root:Host-associated:Human:Digestive system:Large int	0.0468
MGYA00137709/ERZ477037_FASTA.fasta	MGYA00137709	ERZ477037	root:Host-associated:Human:Digestive system:Large int	0.03
MGYA00137710/ERZ476925_FASTA.fasta	MGYA00137710	ERZ476925	root:Host-associated:Human:Digestive system:Large int	0.0294
MGYA00137711/ERZ476978_FASTA.fasta	MGYA00137711	ERZ476978	root:Host-associated:Human:Digestive system:Large int	0.0294
MGYA00137712/ERZ476959_FASTA.fasta	MGYA00137712	ERZ476959	root:Host-associated:Human:Digestive system:Large int	0.0294
MGYA00137713/ERZ476940_FASTA.fasta	MGYA00137713	ERZ476940	root:Host-associated:Human:Digestive system:Large int	0.0332
MGYA00137714/ERZ477013_FASTA.fasta	MGYA00137714	ERZ477013	root:Host-associated:Human:Digestive system:Large int	0.0294
MGYA00137715/ERZ477066_FASTA.fasta	MGYA00137715	ERZ477066	root:Host-associated:Human:Digestive system:Large int	0.2138
MGYA00137716/ERZ476934_FASTA.fasta	MGYA00137716	ERZ476934	root:Host-associated:Human:Digestive system:Large int	0.0294
MGYA00137717/ERZ477021_FASTA.fasta	MGYA00137717	ERZ477021	root:Host-associated:Human:Digestive system:Large int	0.0296
MGYA00137718/ERZ477057_FASTA.fasta	MGYA00137718	ERZ477057	root:Host-associated:Human:Digestive system:Large int	0.0626
MGYA00137719/ERZ477030_FASTA.fasta	MGYA00137719	ERZ477030	root:Host-associated:Human:Digestive system:Large int	0.0296
MGYA00137720/ERZ477029_FASTA.fasta	MGYA00137720	ERZ477029	root:Host-associated:Human:Digestive system:Large int	0.0296
MGYA00137721/ERZ477017_FASTA.fasta	MGYA00137721	ERZ477017	root:Host-associated:Human:Digestive system:Large int	0.0294
MGYA00137722/ERZ476950_FASTA.fasta	MGYA00137722	ERZ476950	root:Host-associated:Human:Digestive system:Large int	0.0294
MGYA00137723/ERZ477014_FASTA.fasta	MGYA00137723	ERZ477014	root:Host-associated:Human:Digestive system:Large int	0.0294
MGYA00137724/ERZ477008_FASTA.fasta	MGYA00137724	ERZ477008	root:Host-associated:Human:Digestive system:Large int	0.0294
MGYA00137725/ERZ477025_FASTA.fasta	MGYA00137725	ERZ477025	root:Host-associated:Human:Digestive system:Large int	0.0294
MGYA00137726/ERZ476939_FASTA.fasta	MGYA00137726	ERZ476939	root:Host-associated:Human:Digestive system:Large int	0.0284
MGYA00137727/ERZ477035_FASTA.fasta	MGYA00137727	ERZ477035	root:Host-associated:Human:Digestive system:Large int	0.0294
MGYA00137728/ERZ476995_FASTA.fasta	MGYA00137728	ERZ476995	root:Host-associated:Human:Digestive system:Large int	0.0294
MGYA00137729/ERZ477061_FASTA.fasta	MGYA00137729	ERZ477061	root:Host-associated:Human:Digestive system:Large int	0.0446
MGYA00137730/ERZ477040_FASTA.fasta	MGYA00137730	ERZ477040	root:Host-associated:Human:Digestive system:Large int	0.0294
MGYA00137731/ERZ477020_FASTA.fasta	MGYA00137731	ERZ477020	root:Host-associated:Human:Digestive system:Large int	0.0294
MGYA00137732/ERZ477038_FASTA.fasta	MGYA00137732	ERZ477038	root:Host-associated:Human:Digestive system:Large int	0.0294
MGYA00137733/ERZ476998_FASTA.fasta	MGYA00137733	ERZ476998	root:Host-associated:Human:Digestive system:Large int	0.0564
MGYA00137734/ERZ476967_FASTA.fasta	MGYA00137734	ERZ476967	root:Host-associated:Human:Digestive system:Large int	0.0294
MGYA00137735/ERZ477044_FASTA.fasta	MGYA00137735	ERZ477044	root:Host-associated:Human:Digestive system:Large int	0.0318
MGYA00137736/ERZ476927_FASTA.fasta	MGYA00137736	ERZ476927	root:Host-associated:Human:Digestive system:Large int	0.0464
MGYA00137737/ERZ476924_FASTA.fasta	MGYA00137737	ERZ476924	root:Host-associated:Human:Digestive system:Large int	0.0378
MGYA00137738/ERZ476987_FASTA.fasta	MGYA00137738	ERZ476987	root:Host-associated:Human:Digestive system:Large int	0.0288
MGYA00137739/ERZ477015_FASTA.fasta	MGYA00137739	ERZ477015	root:Host-associated:Human:Digestive system:Large int	0.0444
MGYA00137740/ERZ476965_FASTA.fasta	MGYA00137740	ERZ476965	root:Host-associated:Human:Digestive system:Large int	0.0294
MGYA00137741/ERZ476982_FASTA.fasta	MGYA00137741	ERZ476982	root:Host-associated:Human:Digestive system:Large int	0.0294
MGYA00137742/ERZ476957_FASTA.fasta	MGYA00137742	ERZ476957	root:Host-associated:Human:Digestive system:Large int	0.032
MGYA00137743/ERZ476935_FASTA.fasta	MGYA00137743	ERZ476935	root:Host-associated:Human:Digestive system:Large int	0.0294
MGYA00137744/ERZ477069_FASTA.fasta	MGYA00137744	ERZ477069	root:Host-associated:Human:Digestive system:Large int	0.064
MGYA00137745/ERZ477047_FASTA.fasta	MGYA00137745	ERZ477047	root:Host-associated:Human:Digestive system:Large int	0.0476
MGYA00137746/ERZ477063_FASTA.fasta	MGYA00137746	ERZ477063	root:Host-associated:Human:Digestive system:Large int	0.0294
MGYA00137747/ERZ476993_FASTA.fasta	MGYA00137747	ERZ476993	root:Host-associated:Human:Digestive system:Large int	0.0766
MGYA00137748/ERZ476968_FASTA.fasta	MGYA00137748	ERZ476968	root:Host-associated:Human:Digestive system:Large int	0.0294
MGYA00137749/ERZ477060_FASTA.fasta	MGYA00137749	ERZ477060	root:Host-associated:Human:Digestive system:Large int	0.1124
MGYA00137750/ERZ476941_FASTA.fasta	MGYA00137750	ERZ476941	root:Host-associated:Human:Digestive system:Large int	0.0364
MGYA00137751/ERZ476937_FASTA.fasta	MGYA00137751	ERZ476937	root:Host-associated:Human:Digestive system:Large int	0.0294
MGYA00137752/ERZ477027_FASTA.fasta	MGYA00137752	ERZ477027	root:Host-associated:Human:Digestive system:Large int	0.0292
MGYA00137753/ERZ477024_FASTA.fasta	MGYA00137753	ERZ477024	root:Host-associated:Human:Digestive system:Large int	0.0312
MGYA00137754/ERZ477041_FASTA.fasta	MGYA00137754	ERZ477041	root:Host-associated:Human:Digestive system:Large int	0.0294
MGYA00137755/ERZ477042_FASTA.fasta	MGYA00137755	ERZ477042	root:Host-associated:Human:Digestive system:Large int	0.0722
MGYA00137756/ERZ476930_FASTA.fasta	MGYA00137756	ERZ476930	root:Host-associated:Human:Digestive system:Large int	0.0356
MGYA00137757/ERZ476969_FASTA.fasta	MGYA00137757	ERZ476969	root:Host-associated:Human:Digestive system:Large int	0.0332
MGYA00137758/ERZ476997_FASTA.fasta	MGYA00137758	ERZ476997	root:Host-associated:Human:Digestive system:Large int	0.0288
MGYA00137759/ERZ477071_FASTA.fasta	MGYA00137759	ERZ477071	root:Host-associated:Human:Digestive system:Large int	0.0294
MGYA00137760/ERZ477023_FASTA.fasta	MGYA00137760	ERZ477023	root:Host-associated:Human:Digestive system:Large int	0.0292
MGYA00137761/ERZ477054_FASTA.fasta	MGYA00137761	ERZ477054	root:Host-associated:Human:Digestive system:Large int	0.0626
MGYA00137762/ERZ476928_FASTA.fasta	MGYA00137762	ERZ476928	root:Host-associated:Human:Digestive system:Large int	0.029
MGYA00137763/ERZ477012_FASTA.fasta	MGYA00137763	ERZ477012	root:Host-associated:Human:Digestive system:Large int	0.0284
MGYA00137764/ERZ476999_FASTA.fasta	MGYA00137764	ERZ476999	root:Host-associated:Human:Digestive system:Large int	0.031
MGYA00137765/ERZ476970_FASTA.fasta	MGYA00137765	ERZ476970	root:Host-associated:Human:Digestive system:Large int	0.0294
MGYA00137766/ERZ476960_FASTA.fasta	MGYA00137766	ERZ476960	root:Host-associated:Human:Digestive system:Large int	0.0298
MGYA00137767/ERZ477032_FASTA.fasta	MGYA00137767	ERZ477032	root:Host-associated:Human:Digestive system:Large int	0.0302
MGYA00137768/ERZ476954_FASTA.fasta	MGYA00137768	ERZ476954	root:Host-associated:Human:Digestive system:Large int	0.03
MGYA00137769/ERZ476981_FASTA.fasta	MGYA00137769	ERZ476981	root:Host-associated:Human:Digestive system:Large int	0.0452
MGYA00137770/ERZ476991_FASTA.fasta	MGYA00137770	ERZ476991	root:Host-associated:Human:Digestive system:Large int	0.0362
MGYA00137771/ERZ477026_FASTA.fasta	MGYA00137771	ERZ477026	root:Host-associated:Human:Digestive system:Large int	0.0294
MGYA00137772/ERZ476931_FASTA.fasta	MGYA00137772	ERZ476931	root:Host-associated:Human:Digestive system:Large int	0.0298
MGYA00137773/ERZ477067_FASTA.fasta	MGYA00137773	ERZ477067	root:Host-associated:Human:Digestive system:Large int	0.11
MGYA00137774/ERZ477002_FASTA.fasta	MGYA00137774	ERZ477002	root:Host-associated:Human:Digestive system:Large int	0.0294
MGYA00137775/ERZ477065_FASTA.fasta	MGYA00137775	ERZ477065	root:Host-associated:Human:Digestive system:Large int	0.0342
MGYA00137776/ERZ477059_FASTA.fasta	MGYA00137776	ERZ477059	root:Host-associated:Human:Digestive system:Large int	0.1548
MGYA00137777/ERZ477007_FASTA.fasta	MGYA00137777	ERZ477007	root:Host-associated:Human:Digestive system:Large int	0.0294
MGYA00137778/ERZ477064_FASTA.fasta	MGYA00137778	ERZ477064	root:Host-associated:Human:Digestive system:Large int	0.0294
MGYA00137779/ERZ477018_FASTA.fasta	MGYA00137779	ERZ477018	root:Host-associated:Human:Digestive system:Large int	0.029
MGYA00137780/ERZ476975_FASTA.fasta	MGYA00137780	ERZ476975	root:Host-associated:Human:Digestive system:Large int	0.0294
MGYA00137781/ERZ477009_FASTA.fasta	MGYA00137781	ERZ477009	root:Host-associated:Human:Digestive system:Large int	0.0392
MGYA00137782/ERZ477039_FASTA.fasta	MGYA00137782	ERZ477039	root:Host-associated:Human:Digestive system:Large int	0.0292
MGYA00137783/ERZ476964_FASTA.fasta	MGYA00137783	ERZ476964	root:Host-associated:Human:Digestive system:Large int	0.03
MGYA00137784/ERZ476920_FASTA.fasta	MGYA00137784	ERZ476920	root:Host-associated:Human:Digestive system:Large int	0.0294
MGYA00137785/ERZ476943_FASTA.fasta	MGYA00137785	ERZ476943	root:Host-associated:Human:Digestive system:Large int	0.0352
MGYA00137786/ERZ476951_FASTA.fasta	MGYA00137786	ERZ476951	root:Host-associated:Human:Digestive system:Large int	0.03
MGYA00137787/ERZ476979_FASTA.fasta	MGYA00137787	ERZ476979	root:Host-associated:Human:Digestive system:Large int	0.0294
MGYA00137788/ERZ477046_FASTA.fasta	MGYA00137788	ERZ477046	root:Host-associated:Human:Digestive system:Large int	0.0294
MGYA00137789/ERZ477004_FASTA.fasta	MGYA00137789	ERZ477004	root:Host-associated:Human:Digestive system:Large int	0.0294
MGYA00137790/ERZ476976_FASTA.fasta	MGYA00137790	ERZ476976	root:Host-associated:Human:Digestive system:Large int	0.0294
MGYA00137791/ERZ477049_FASTA.fasta	MGYA00137791	ERZ477049	root:Host-associated:Human:Digestive system:Large int	0.0228
MGYA00137792/ERZ476942_FASTA.fasta	MGYA00137792	ERZ476942	root:Host-associated:Human:Digestive system:Large int	0.0346
MGYA00137793/ERZ477072_FASTA.fasta	MGYA00137793	ERZ477072	root:Host-associated:Human:Digestive system:Large int	0.0412
MGYA00137794/ERZ476949_FASTA.fasta	MGYA00137794	ERZ476949	root:Host-associated:Human:Digestive system:Large int	0.0294
MGYA00137795/ERZ477034_FASTA.fasta	MGYA00137795	ERZ477034	root:Host-associated:Human:Digestive system:Large int	0.0316
MGYA00137796/ERZ476922_FASTA.fasta	MGYA00137796	ERZ476922	root:Host-associated:Human:Digestive system:Large int	0.0302
MGYA00137797/ERZ477005_FASTA.fasta	MGYA00137797	ERZ477005	root:Host-associated:Human:Digestive system:Large int	0.0294
MGYA00137798/ERZ477019_FASTA.fasta	MGYA00137798	ERZ477019	root:Host-associated:Human:Digestive system:Large int	0.0286
MGYA00137799/ERZ477051_FASTA.fasta	MGYA00137799	ERZ477051	root:Host-associated:Human:Digestive system:Large int	0.0372
MGYA00137800/ERZ476921_FASTA.fasta	MGYA00137800	ERZ476921	root:Host-associated:Human:Digestive system:Large int	0.031
MGYA00137801/ERZ477022_FASTA.fasta	MGYA00137801	ERZ477022	root:Host-associated:Human:Digestive system:Large int	0.029
MGYA00137802/ERZ476958_FASTA.fasta	MGYA00137802	ERZ476958	root:Host-associated:Human:Digestive system:Large int	0.0294
MGYA00137803/ERZ476962_FASTA.fasta	MGYA00137803	ERZ476962	root:Host-associated:Human:Digestive system:Large int	0.0294
MGYA00137804/ERZ476966_FASTA.fasta	MGYA00137804	ERZ476966	root:Host-associated:Human:Digestive system:Large int	0.0294
MGYA00137805/ERZ477031_FASTA.fasta	MGYA00137805	ERZ477031	root:Host-associated:Human:Digestive system:Large int	0.0296
MGYA00137806/ERZ477062_FASTA.fasta	MGYA00137806	ERZ477062	root:Host-associated:Human:Digestive system:Large int	0.0314
MGYA00137807/ERZ477016_FASTA.fasta	MGYA00137807	ERZ477016	root:Host-associated:Human:Digestive system:Large int	0.0294
MGYA00137808/ERZ476932_FASTA.fasta	MGYA00137808	ERZ476932	root:Host-associated:Human:Digestive system:Large int	0.0294
MGYA00137809/ERZ477045_FASTA.fasta	MGYA00137809	ERZ477045	root:Host-associated:Human:Digestive system:Large int	0.0294
MGYA00137810/ERZ477000_FASTA.fasta	MGYA00137810	ERZ477000	root:Host-associated:Human:Digestive system:Large int	0.0294
MGYA00137811/ERZ476933_FASTA.fasta	MGYA00137811	ERZ476933	root:Host-associated:Human:Digestive system:Large int	0.0294
MGYA00137812/ERZ476944_FASTA.fasta	MGYA00137812	ERZ476944	root:Host-associated:Human:Digestive system:Large int	0.0304
MGYA00137813/ERZ476948_FASTA.fasta	MGYA00137813	ERZ476948	root:Host-associated:Human:Digestive system:Large int	0.0294
MGYA00137814/ERZ476974_FASTA.fasta	MGYA00137814	ERZ476974	root:Host-associated:Human:Digestive system:Large int	0.0294
MGYA00137815/ERZ476947_FASTA.fasta	MGYA00137815	ERZ476947	root:Host-associated:Human:Digestive system:Large int	0.0806
MGYA00137816/ERZ477056_FASTA.fasta	MGYA00137816	ERZ477056	root:Host-associated:Human:Digestive system:Large int	0.057
MGYA00137817/ERZ477036_FASTA.fasta	MGYA00137817	ERZ477036	root:Host-associated:Human:Digestive system:Large int	0.0294
MGYA00137818/ERZ476985_FASTA.fasta	MGYA00137818	ERZ476985	root:Host-associated:Human:Digestive system:Large int	0.0322
MGYA00137819/ERZ477052_FASTA.fasta	MGYA00137819	ERZ477052	root:Host-associated:Human:Digestive system:Large int	0.0294
MGYA00137820/ERZ476988_FASTA.fasta	MGYA00137820	ERZ476988	root:Host-associated:Human:Digestive system:Large int	0.0294
MGYA00137821/ERZ477028_FASTA.fasta	MGYA00137821	ERZ477028	root:Host-associated:Human:Digestive system:Large int	0.031
MGYA00137822/ERZ476961_FASTA.fasta	MGYA00137822	ERZ476961	root:Host-associated:Human:Digestive system:Large int	0.0294
MGYA00137823/ERZ476916_FASTA.fasta	MGYA00137823	ERZ476916	root:Host-associated:Human:Digestive system:Large int	0.0302
MGYA00137824/ERZ476918_FASTA.fasta	MGYA00137824	ERZ476918	root:Host-associated:Human:Digestive system:Large int	0.0298
MGYA00137825/ERZ476971_FASTA.fasta	MGYA00137825	ERZ476971	root:Host-associated:Human:Digestive system:Large int	0.0294
MGYA00137826/ERZ476989_FASTA.fasta	MGYA00137826	ERZ476989	root:Host-associated:Human:Digestive system:Large int	0.0432
MGYA00137827/ERZ476980_FASTA.fasta	MGYA00137827	ERZ476980	root:Host-associated:Human:Digestive system:Large int	0.051
MGYA00137828/ERZ476953_FASTA.fasta	MGYA00137828	ERZ476953	root:Host-associated:Human:Digestive system:Large int	0.0294
MGYA00137829/ERZ476938_FASTA.fasta	MGYA00137829	ERZ476938	root:Host-associated:Human:Digestive system:Large int	0.0292
MGYA00137830/ERZ476923_FASTA.fasta	MGYA00137830	ERZ476923	root:Host-associated:Human:Digestive system:Large int	0.0294
MGYA00137831/ERZ477073_FASTA.fasta	MGYA00137831	ERZ477073	root:Host-associated:Human:Digestive system:Large int	0.0294
MGYA00137832/ERZ476929_FASTA.fasta	MGYA00137832	ERZ476929	root:Host-associated:Human:Digestive system:Large int	0.0294
MGYA00137833/ERZ476973_FASTA.fasta	MGYA00137833	ERZ476973	root:Host-associated:Human:Digestive system:Large int	0.0556
MGYA00137834/ERZ476990_FASTA.fasta	MGYA00137834	ERZ476990	root:Host-associated:Human:Digestive system:Large int	0.0294
MGYA00137835/ERZ476983_FASTA.fasta	MGYA00137835	ERZ476983	root:Host-associated:Human:Digestive system:Large int	0.0294
MGYA00137836/ERZ477082_FASTA.fasta	MGYA00137836	ERZ477082	root:Engineered:Wastewater:Activated Sludge	0.095
MGYA00137837/ERZ477081_FASTA.fasta	MGYA00137837	ERZ477081	root:Environmental:Terrestrial:Soil:Agricultural	0.2458
MGYA00137838/ERZ477170_FASTA.fasta	MGYA00137838	ERZ477170	root:Environmental:Terrestrial:Soil:Agricultural	0.0294
MGYA00137839/ERZ477158_FASTA.fasta	MGYA00137839	ERZ477158	root:Environmental:Terrestrial:Soil:Agricultural	0.0282
MGYA00137840/ERZ477151_FASTA.fasta	MGYA00137840	ERZ477151	root:Environmental:Terrestrial:Soil:Agricultural	0.0314
MGYA00137841/ERZ477096_FASTA.fasta	MGYA00137841	ERZ477096	root:Environmental:Terrestrial:Soil:Agricultural	0.0712
MGYA00137842/ERZ477156_FASTA.fasta	MGYA00137842	ERZ477156	root:Environmental:Terrestrial:Soil	0.076
MGYA00137843/ERZ477148_FASTA.fasta	MGYA00137843	ERZ477148	root:Engineered:Wastewater:Activated Sludge	0.0358
MGYA00137844/ERZ477099_FASTA.fasta	MGYA00137844	ERZ477099	root:Environmental:Terrestrial:Soil:Agricultural	0.2012
MGYA00137845/ERZ477154_FASTA.fasta	MGYA00137845	ERZ477154	root:Environmental:Terrestrial:Soil:Agricultural	0.295
MGYA00137846/ERZ477078_FASTA.fasta	MGYA00137846	ERZ477078	root:Environmental:Terrestrial:Soil:Agricultural	0.0348
MGYA00137847/ERZ477126_FASTA.fasta	MGYA00137847	ERZ477126	root:Environmental:Terrestrial:Soil:Agricultural	0.0388
MGYA00137848/ERZ477116_FASTA.fasta	MGYA00137848	ERZ477116	root:Environmental:Terrestrial:Soil:Agricultural	0.108
MGYA00137849/ERZ477133_FASTA.fasta	MGYA00137849	ERZ477133	root:Environmental:Terrestrial:Soil:Agricultural	0.056
MGYA00137850/ERZ477137_FASTA.fasta	MGYA00137850	ERZ477137	root:Environmental:Terrestrial:Soil:Agricultural	0.068
MGYA00137851/ERZ477112_FASTA.fasta	MGYA00137851	ERZ477112	root:Engineered:Wastewater:Activated Sludge	0.079
MGYA00137852/ERZ477168_FASTA.fasta	MGYA00137852	ERZ477168	root:Host-associated:Human:Digestive system:Large int	0.377
MGYA00137853/ERZ477159_FASTA.fasta	MGYA00137853	ERZ477159	root:Environmental:Terrestrial:Soil:Agricultural	0.1442
MGYA00137854/ERZ477139_FASTA.fasta	MGYA00137854	ERZ477139	root:Environmental:Terrestrial:Soil:Agricultural	0.2458
MGYA00137855/ERZ477124_FASTA.fasta	MGYA00137855	ERZ477124	root:Environmental:Terrestrial:Soil:Agricultural	0.068
MGYA00137856/ERZ477125_FASTA.fasta	MGYA00137856	ERZ477125	root:Environmental:Terrestrial:Soil:Agricultural	0.2458
MGYA00137857/ERZ477140_FASTA.fasta	MGYA00137857	ERZ477140	root:Environmental:Terrestrial:Soil:Agricultural	0.2458
MGYA00137858/ERZ477105_FASTA.fasta	MGYA00137858	ERZ477105	root:Environmental:Terrestrial:Soil:Agricultural	0.2336
MGYA00137859/ERZ477123_FASTA.fasta	MGYA00137859	ERZ477123	root:Environmental:Terrestrial:Soil:Agricultural	0.2458
MGYA00137860/ERZ477120_FASTA.fasta	MGYA00137860	ERZ477120	root:Environmental:Terrestrial:Soil	0.086
MGYA00137861/ERZ477110_FASTA.fasta	MGYA00137861	ERZ477110	root:Environmental:Terrestrial:Soil:Agricultural	0.2458
MGYA00137862/ERZ477113_FASTA.fasta	MGYA00137862	ERZ477113	root:Environmental:Terrestrial:Soil:Agricultural	0.0954
MGYA00137863/ERZ477085_FASTA.fasta	MGYA00137863	ERZ477085	root:Environmental:Terrestrial:Soil:Agricultural	0.045
MGYA00137864/ERZ477130_FASTA.fasta	MGYA00137864	ERZ477130	root:Environmental:Terrestrial:Soil	0.0284
MGYA00137865/ERZ477165_FASTA.fasta	MGYA00137865	ERZ477165	root:Environmental:Terrestrial:Soil:Agricultural	0.2458
MGYA00137866/ERZ477107_FASTA.fasta	MGYA00137866	ERZ477107	root:Environmental:Terrestrial:Soil:Agricultural	0.2458
MGYA00137867/ERZ477111_FASTA.fasta	MGYA00137867	ERZ477111	root:Environmental:Terrestrial:Soil	0.0568
MGYA00137868/ERZ477102_FASTA.fasta	MGYA00137868	ERZ477102	root:Engineered:Wastewater:Activated Sludge	0.043
MGYA00137869/ERZ477138_FASTA.fasta	MGYA00137869	ERZ477138	root:Environmental:Terrestrial:Soil	0.2476
MGYA00137870/ERZ477136_FASTA.fasta	MGYA00137870	ERZ477136	root:Environmental:Terrestrial:Soil	0.0446
MGYA00137871/ERZ477152_FASTA.fasta	MGYA00137871	ERZ477152	root:Environmental:Terrestrial:Soil:Agricultural	0.2458
MGYA00137872/ERZ477089_FASTA.fasta	MGYA00137872	ERZ477089	root:Environmental:Terrestrial:Soil:Agricultural	0.246
MGYA00137873/ERZ477094_FASTA.fasta	MGYA00137873	ERZ477094	root:Environmental:Terrestrial:Soil:Agricultural	0.036
MGYA00137874/ERZ477121_FASTA.fasta	MGYA00137874	ERZ477121	root:Environmental:Terrestrial:Soil:Agricultural	0.041
MGYA00137875/ERZ477128_FASTA.fasta	MGYA00137875	ERZ477128	root:Environmental:Terrestrial:Soil:Agricultural	0.246
MGYA00137876/ERZ477141_FASTA.fasta	MGYA00137876	ERZ477141	root:Engineered:Wastewater:Activated Sludge	0.04
MGYA00137877/ERZ477167_FASTA.fasta	MGYA00137877	ERZ477167	root:Environmental:Terrestrial:Soil:Agricultural	0.0398
MGYA00137878/ERZ477149_FASTA.fasta	MGYA00137878	ERZ477149	root:Environmental:Terrestrial:Soil:Agricultural	0.0766
MGYA00137879/ERZ477100_FASTA.fasta	MGYA00137879	ERZ477100	root:Environmental:Terrestrial:Soil:Agricultural	0.2458
MGYA00137880/ERZ477095_FASTA.fasta	MGYA00137880	ERZ477095	root:Environmental:Terrestrial:Soil:Agricultural	0.0524
MGYA00137881/ERZ477091_FASTA.fasta	MGYA00137881	ERZ477091	root:Engineered:Wastewater:Activated Sludge	0.0976
MGYA00137882/ERZ477147_FASTA.fasta	MGYA00137882	ERZ477147	root:Environmental:Terrestrial:Soil:Agricultural	0.2458
MGYA00137883/ERZ477144_FASTA.fasta	MGYA00137883	ERZ477144	root:Environmental:Terrestrial:Soil:Agricultural	0.0896
MGYA00137884/ERZ477092_FASTA.fasta	MGYA00137884	ERZ477092	root:Environmental:Terrestrial:Soil:Agricultural	0.0612
MGYA00137885/ERZ477075_FASTA.fasta	MGYA00137885	ERZ477075	root:Environmental:Terrestrial	0.3484
MGYA00137886/ERZ477115_FASTA.fasta	MGYA00137886	ERZ477115	root:Environmental:Terrestrial:Soil:Agricultural	0.0318
MGYA00137887/ERZ477117_FASTA.fasta	MGYA00137887	ERZ477117	root:Environmental:Terrestrial:Soil:Agricultural	0.0342
MGYA00137888/ERZ477127_FASTA.fasta	MGYA00137888	ERZ477127	root:Environmental:Terrestrial:Soil:Agricultural	0.0282
MGYA00137889/ERZ477079_FASTA.fasta	MGYA00137889	ERZ477079	root:Environmental:Terrestrial:Soil	0.2476
MGYA00137890/ERZ477119_FASTA.fasta	MGYA00137890	ERZ477119	root:Environmental:Terrestrial:Soil:Agricultural	0.2458
MGYA00137891/ERZ477129_FASTA.fasta	MGYA00137891	ERZ477129	root:Environmental:Terrestrial:Soil:Agricultural	0.245
MGYA00137892/ERZ477157_FASTA.fasta	MGYA00137892	ERZ477157	root:Environmental:Terrestrial:Soil:Agricultural	0.0448
MGYA00137893/ERZ477088_FASTA.fasta	MGYA00137893	ERZ477088	root:Host-associated:Human:Digestive system:Large int	0.1246
MGYA00137894/ERZ477084_FASTA.fasta	MGYA00137894	ERZ477084	root:Environmental:Terrestrial:Soil:Agricultural	0.0338

MGYA00137895/ERZ477171_FASTA.fasta	MGYA00137895	ERZ477171	root:Environmental:Terrestrial:Soil:Agricultural	0.0294
MGYA00137896/ERZ477080_FASTA.fasta	MGYA00137896	ERZ477080	root:Environmental:Terrestrial:Soil:Agricultural	0.2484
MGYA00137897/ERZ477087_FASTA.fasta	MGYA00137897	ERZ477087	root:Environmental:Terrestrial:Soil:Agricultural	0.1826
MGYA00137898/ERZ477146_FASTA.fasta	MGYA00137898	ERZ477146	root:Environmental:Terrestrial:Soil:Agricultural	0.0622
MGYA00137899/ERZ477090_FASTA.fasta	MGYA00137899	ERZ477090	root:Engineered:Wastewater:Activated Sludge	0.0594
MGYA00137900/ERZ477162_FASTA.fasta	MGYA00137900	ERZ477162	root:Environmental:Terrestrial:Soil:Agricultural	0.2458
MGYA00137901/ERZ477101_FASTA.fasta	MGYA00137901	ERZ477101	root:Environmental:Terrestrial:Soil	0.0884
MGYA00137902/ERZ477164_FASTA.fasta	MGYA00137902	ERZ477164	root:Engineered:Wastewater:Activated Sludge	0.046
MGYA00137903/ERZ477118_FASTA.fasta	MGYA00137903	ERZ477118	root:Environmental:Terrestrial:Soil:Agricultural	0.245
MGYA00137904/ERZ477145_FASTA.fasta	MGYA00137904	ERZ477145	root:Environmental:Terrestrial:Soil:Agricultural	0.0486
MGYA00137905/ERZ477161_FASTA.fasta	MGYA00137905	ERZ477161	root:Environmental:Terrestrial:Soil	0.0362
MGYA00137906/ERZ477108_FASTA.fasta	MGYA00137906	ERZ477108	root:Environmental:Terrestrial:Soil:Agricultural	0.082
MGYA00137907/ERZ477135_FASTA.fasta	MGYA00137907	ERZ477135	root:Environmental:Terrestrial:Soil:Agricultural	0.0536
MGYA00137908/ERZ477143_FASTA.fasta	MGYA00137908	ERZ477143	root:Environmental:Terrestrial:Soil	0.038
MGYA00137909/ERZ477132_FASTA.fasta	MGYA00137909	ERZ477132	root:Environmental:Terrestrial:Soil	0.0392
MGYA00137910/ERZ477106_FASTA.fasta	MGYA00137910	ERZ477106	root:Environmental:Terrestrial:Soil:Agricultural	0.0416
MGYA00137911/ERZ477077_FASTA.fasta	MGYA00137911	ERZ477077	root:Environmental:Terrestrial:Soil:Agricultural	0.0484
MGYA00137912/ERZ477103_FASTA.fasta	MGYA00137912	ERZ477103	root:Environmental:Terrestrial:Soil	0.2476
MGYA00137913/ERZ477169_FASTA.fasta	MGYA00137913	ERZ477169	root:Host-associated:Human:Digestive system:Large in	0.3246
MGYA00137914/ERZ477155_FASTA.fasta	MGYA00137914	ERZ477155	root:Environmental:Terrestrial:Soil	0.0542
MGYA00137915/ERZ477142_FASTA.fasta	MGYA00137915	ERZ477142	root:Environmental:Terrestrial:Soil:Agricultural	0.0326
MGYA00137916/ERZ477098_FASTA.fasta	MGYA00137916	ERZ477098	root:Environmental:Terrestrial:Soil:Agricultural	0.2458
MGYA00137917/ERZ477166_FASTA.fasta	MGYA00137917	ERZ477166	root:Environmental:Terrestrial:Soil:Agricultural	0.2458
MGYA00137918/ERZ477160_FASTA.fasta	MGYA00137918	ERZ477160	root:Environmental:Terrestrial:Soil:Agricultural	0.2652
MGYA00137919/ERZ477150_FASTA.fasta	MGYA00137919	ERZ477150	root:Environmental:Terrestrial:Soil:Agricultural	0.0292
MGYA00137920/ERZ477114_FASTA.fasta	MGYA00137920	ERZ477114	root:Environmental:Terrestrial:Soil:Agricultural	0.0442
MGYA00137921/ERZ477104_FASTA.fasta	MGYA00137921	ERZ477104	root:Environmental:Terrestrial:Soil:Agricultural	0.093
MGYA00137922/ERZ477086_FASTA.fasta	MGYA00137922	ERZ477086	root:Engineered:Wastewater:Activated Sludge	0.0416
MGYA00137923/ERZ477122_FASTA.fasta	MGYA00137923	ERZ477122	root:Environmental:Terrestrial:Soil	0.0282
MGYA00137924/ERZ477076_FASTA.fasta	MGYA00137924	ERZ477076	root:Environmental:Terrestrial:Soil	0.0338
MGYA00137925/ERZ477131_FASTA.fasta	MGYA00137925	ERZ477131	root:Environmental:Terrestrial:Soil	0.0528
MGYA00137926/ERZ477153_FASTA.fasta	MGYA00137926	ERZ477153	root:Environmental:Terrestrial:Soil:Agricultural	0.2458
MGYA00137927/ERZ477083_FASTA.fasta	MGYA00137927	ERZ477083	root:Environmental:Terrestrial:Soil	0.0574
MGYA00137928/ERZ477163_FASTA.fasta	MGYA00137928	ERZ477163	root:Environmental:Terrestrial:Soil	0.0406
MGYA00137929/ERZ477093_FASTA.fasta	MGYA00137929	ERZ477093	root:Environmental:Terrestrial:Soil:Agricultural	0.0436
MGYA00137930/ERZ477134_FASTA.fasta	MGYA00137930	ERZ477134	root:Environmental:Terrestrial:Soil:Agricultural	0.071
MGYA00137931/ERZ477198_FASTA.fasta	MGYA00137931	ERZ477198	root:Host-associated:Human:Digestive system:Intestine	0.417
MGYA00137932/ERZ477184_FASTA.fasta	MGYA00137932	ERZ477184	root:Host-associated:Human:Digestive system:Intestine	0.3822
MGYA00137933/ERZ477194_FASTA.fasta	MGYA00137933	ERZ477194	root:Host-associated:Human:Digestive system:Intestine	0.471
MGYA00137934/ERZ477172_FASTA.fasta	MGYA00137934	ERZ477172	root:Host-associated:Human:Digestive system:Intestine	0.4472
MGYA00137935/ERZ477203_FASTA.fasta	MGYA00137935	ERZ477203	root:Host-associated:Human:Digestive system:Intestine	0.448
MGYA00137936/ERZ477175_FASTA.fasta	MGYA00137936	ERZ477175	root:Host-associated:Human:Digestive system:Intestine	0.553
MGYA00137937/ERZ477215_FASTA.fasta	MGYA00137937	ERZ477215	root:Host-associated:Human:Digestive system:Intestine	0.3024
MGYA00137938/ERZ477202_FASTA.fasta	MGYA00137938	ERZ477202	root:Host-associated:Human:Digestive system:Intestine	0.4416
MGYA00137939/ERZ477176_FASTA.fasta	MGYA00137939	ERZ477176	root:Host-associated:Human:Digestive system:Intestine	0.5322
MGYA00137940/ERZ477206_FASTA.fasta	MGYA00137940	ERZ477206	root:Host-associated:Human:Digestive system:Intestine	0.4562
MGYA00137941/ERZ477173_FASTA.fasta	MGYA00137941	ERZ477173	root:Host-associated:Human:Digestive system:Intestine	0.4372
MGYA00137942/ERZ477227_FASTA.fasta	MGYA00137942	ERZ477227	root:Host-associated:Human:Digestive system:Intestine	0.3098
MGYA00137943/ERZ477182_FASTA.fasta	MGYA00137943	ERZ477182	root:Host-associated:Human:Digestive system:Intestine	0.5306
MGYA00137944/ERZ477228_FASTA.fasta	MGYA00137944	ERZ477228	root:Host-associated:Human:Digestive system:Intestine	0.2856
MGYA00137945/ERZ477211_FASTA.fasta	MGYA00137945	ERZ477211	root:Host-associated:Human:Digestive system:Intestine	0.406
MGYA00137946/ERZ477240_FASTA.fasta	MGYA00137946	ERZ477240	root:Host-associated:Human:Digestive system:Intestine	0.3476
MGYA00137947/ERZ477230_FASTA.fasta	MGYA00137947	ERZ477230	root:Host-associated:Human:Digestive system:Intestine	0.4024
MGYA00137948/ERZ477189_FASTA.fasta	MGYA00137948	ERZ477189	root:Host-associated:Human:Digestive system:Intestine	0.4564
MGYA00137949/ERZ477208_FASTA.fasta	MGYA00137949	ERZ477208	root:Host-associated:Human:Digestive system:Intestine	0.3826
MGYA00137950/ERZ477185_FASTA.fasta	MGYA00137950	ERZ477185	root:Host-associated:Human:Digestive system:Intestine	0.4402
MGYA00137951/ERZ477197_FASTA.fasta	MGYA00137951	ERZ477197	root:Host-associated:Human:Digestive system:Intestine	0.4688
MGYA00137952/ERZ477229_FASTA.fasta	MGYA00137952	ERZ477229	root:Host-associated:Human:Digestive system:Intestine	0.3208
MGYA00137953/ERZ477201_FASTA.fasta	MGYA00137953	ERZ477201	root:Host-associated:Human:Digestive system:Intestine	0.4354
MGYA00137954/ERZ477238_FASTA.fasta	MGYA00137954	ERZ477238	root:Host-associated:Human:Digestive system:Intestine	0.3596
MGYA00137955/ERZ477237_FASTA.fasta	MGYA00137955	ERZ477237	root:Host-associated:Human:Digestive system:Intestine	0.363
MGYA00137956/ERZ477220_FASTA.fasta	MGYA00137956	ERZ477220	root:Host-associated:Human:Digestive system:Intestine	0.365
MGYA00137957/ERZ477178_FASTA.fasta	MGYA00137957	ERZ477178	root:Host-associated:Human:Digestive system:Intestine	0.5158
MGYA00137958/ERZ477239_FASTA.fasta	MGYA00137958	ERZ477239	root:Host-associated:Human:Digestive system:Intestine	0.4022
MGYA00137959/ERZ477207_FASTA.fasta	MGYA00137959	ERZ477207	root:Host-associated:Human:Digestive system:Intestine	0.4094
MGYA00137960/ERZ477204_FASTA.fasta	MGYA00137960	ERZ477204	root:Host-associated:Human:Digestive system:Intestine	0.4442
MGYA00137961/ERZ477205_FASTA.fasta	MGYA00137961	ERZ477205	root:Host-associated:Human:Digestive system:Intestine	0.4152
MGYA00137962/ERZ477179_FASTA.fasta	MGYA00137962	ERZ477179	root:Host-associated:Human:Digestive system:Intestine	0.362
MGYA00137963/ERZ477209_FASTA.fasta	MGYA00137963	ERZ477209	root:Host-associated:Human:Digestive system:Intestine	0.3624
MGYA00137964/ERZ477200_FASTA.fasta	MGYA00137964	ERZ477200	root:Host-associated:Human:Digestive system:Intestine	0.38
MGYA00137965/ERZ477214_FASTA.fasta	MGYA00137965	ERZ477214	root:Host-associated:Human:Digestive system:Intestine	0.4232
MGYA00137966/ERZ477188_FASTA.fasta	MGYA00137966	ERZ477188	root:Host-associated:Human:Digestive system:Intestine	0.4968
MGYA00137967/ERZ477193_FASTA.fasta	MGYA00137967	ERZ477193	root:Host-associated:Human:Digestive system:Intestine	0.4392
MGYA00137968/ERZ477236_FASTA.fasta	MGYA00137968	ERZ477236	root:Host-associated:Human:Digestive system:Intestine	0.3324
MGYA00137969/ERZ477180_FASTA.fasta	MGYA00137969	ERZ477180	root:Host-associated:Human:Digestive system:Intestine	0.4282
MGYA00137970/ERZ477177_FASTA.fasta	MGYA00137970	ERZ477177	root:Host-associated:Human:Digestive system:Intestine	0.5144
MGYA00137971/ERZ477199_FASTA.fasta	MGYA00137971	ERZ477199	root:Host-associated:Human:Digestive system:Intestine	0.3334
MGYA00137972/ERZ477221_FASTA.fasta	MGYA00137972	ERZ477221	root:Host-associated:Human:Digestive system:Intestine	0.349
MGYA00137973/ERZ477196_FASTA.fasta	MGYA00137973	ERZ477196	root:Host-associated:Human:Digestive system:Intestine	0.4336
MGYA00137974/ERZ477174_FASTA.fasta	MGYA00137974	ERZ477174	root:Host-associated:Human:Digestive system:Intestine	0.4546
MGYA00137975/ERZ477232_FASTA.fasta	MGYA00137975	ERZ477232	root:Host-associated:Human:Digestive system:Intestine	0.3252
MGYA00137976/ERZ477235_FASTA.fasta	MGYA00137976	ERZ477235	root:Host-associated:Human:Digestive system:Intestine	0.4392
MGYA00137977/ERZ477225_FASTA.fasta	MGYA00137977	ERZ477225	root:Host-associated:Human:Digestive system:Intestine	0.4396
MGYA00137978/ERZ477181_FASTA.fasta	MGYA00137978	ERZ477181	root:Host-associated:Human:Digestive system:Intestine	0.5036
MGYA00137979/ERZ477231_FASTA.fasta	MGYA00137979	ERZ477231	root:Host-associated:Human:Digestive system:Intestine	0.3218
MGYA00137980/ERZ477187_FASTA.fasta	MGYA00137980	ERZ477187	root:Host-associated:Human:Digestive system:Intestine	0.5292
MGYA00137981/ERZ477212_FASTA.fasta	MGYA00137981	ERZ477212	root:Host-associated:Human:Digestive system:Intestine	0.4268
MGYA00137982/ERZ477183_FASTA.fasta	MGYA00137982	ERZ477183	root:Host-associated:Human:Digestive system:Intestine	0.432
MGYA00137983/ERZ477233_FASTA.fasta	MGYA00137983	ERZ477233	root:Host-associated:Human:Digestive system:Intestine	0.3588
MGYA00137984/ERZ477242_FASTA.fasta	MGYA00137984	ERZ477242	root:Host-associated:Human:Digestive system:Intestine	0.5448
MGYA00137985/ERZ477222_FASTA.fasta	MGYA00137985	ERZ477222	root:Host-associated:Human:Digestive system:Intestine	0.3542
MGYA00137986/ERZ477186_FASTA.fasta	MGYA00137986	ERZ477186	root:Host-associated:Human:Digestive system:Intestine	0.4836
MGYA00137987/ERZ477195_FASTA.fasta	MGYA00137987	ERZ477195	root:Host-associated:Human:Digestive system:Intestine	0.4638
MGYA00137988/ERZ477191_FASTA.fasta	MGYA00137988	ERZ477191	root:Host-associated:Human:Digestive system:Intestine	0.4822
MGYA00137989/ERZ477210_FASTA.fasta	MGYA00137989	ERZ477210	root:Host-associated:Human:Digestive system:Intestine	0.4452
MGYA00137990/ERZ477216_FASTA.fasta	MGYA00137990	ERZ477216	root:Host-associated:Human:Digestive system:Intestine	0.3142
MGYA00137991/ERZ477213_FASTA.fasta	MGYA00137991	ERZ477213	root:Host-associated:Human:Digestive system:Intestine	0.465
MGYA00137992/ERZ477241_FASTA.fasta	MGYA00137992	ERZ477241	root:Host-associated:Human:Digestive system:Intestine	0.3056
MGYA00137993/ERZ477226_FASTA.fasta	MGYA00137993	ERZ477226	root:Host-associated:Human:Digestive system:Intestine	0.4042
MGYA00137994/ERZ477223_FASTA.fasta	MGYA00137994	ERZ477223	root:Host-associated:Human:Digestive system:Intestine	0.369
MGYA00137995/ERZ477192_FASTA.fasta	MGYA00137995	ERZ477192	root:Host-associated:Human:Digestive system:Intestine	0.4824
MGYA00137996/ERZ477218_FASTA.fasta	MGYA00137996	ERZ477218	root:Host-associated:Human:Digestive system:Intestine	0.2938
MGYA00137997/ERZ477224_FASTA.fasta	MGYA00137997	ERZ477224	root:Host-associated:Human:Digestive system:Intestine	0.2964
MGYA00137998/ERZ477217_FASTA.fasta	MGYA00137998	ERZ477217	root:Host-associated:Human:Digestive system:Intestine	0.338
MGYA00137999/ERZ477190_FASTA.fasta	MGYA00137999	ERZ477190	root:Host-associated:Human:Digestive system:Intestine	0.466
MGYA00138000/ERZ477234_FASTA.fasta	MGYA00138000	ERZ477234	root:Host-associated:Human:Digestive system:Intestine	0.3764
MGYA00138001/ERZ477219_FASTA.fasta	MGYA00138001	ERZ477219	root:Host-associated:Human:Digestive system:Intestine	0.3848
MGYA00138002/ERZ477277_FASTA.fasta	MGYA00138002	ERZ477277	root:Host-associated:Human:Digestive system	0.3784
MGYA00138003/ERZ477300_FASTA.fasta	MGYA00138003	ERZ477300	root:Host-associated:Human:Digestive system	0.3596
MGYA00138004/ERZ477288_FASTA.fasta	MGYA00138004	ERZ477288	root:Host-associated:Human:Digestive system	0.3374
MGYA00138005/ERZ477297_FASTA.fasta	MGYA00138005	ERZ477297	root:Host-associated:Human:Digestive system	0.2544
MGYA00138006/ERZ477290_FASTA.fasta	MGYA00138006	ERZ477290	root:Host-associated:Human:Digestive system	0.2214
MGYA00138007/ERZ477293_FASTA.fasta	MGYA00138007	ERZ477293	root:Host-associated:Human:Digestive system	0.3268
MGYA00138008/ERZ477273_FASTA.fasta	MGYA00138008	ERZ477273	root:Host-associated:Human:Digestive system	0.315
MGYA00138009/ERZ477251_FASTA.fasta	MGYA00138009	ERZ477251	root:Host-associated:Human:Digestive system	0.3154
MGYA00138010/ERZ477250_FASTA.fasta	MGYA00138010	ERZ477250	root:Host-associated:Human:Digestive system	0.3356
MGYA00138011/ERZ477302_FASTA.fasta	MGYA00138011	ERZ477302	root:Host-associated:Human:Digestive system	0.3674
MGYA00138012/ERZ477274_FASTA.fasta	MGYA00138012	ERZ477274	root:Host-associated:Human:Digestive system	0.331
MGYA00138013/ERZ477285_FASTA.fasta	MGYA00138013	ERZ477285	root:Host-associated:Human:Digestive system	0.3088
MGYA00138014/ERZ477257_FASTA.fasta	MGYA00138014	ERZ477257	root:Host-associated:Human:Digestive system	0.329
MGYA00138015/ERZ477289_FASTA.fasta	MGYA00138015	ERZ477289	root:Host-associated:Human:Digestive system	0.2608
MGYA00138016/ERZ477268_FASTA.fasta	MGYA00138016	ERZ477268	root:Host-associated:Human:Digestive system	0.1838
MGYA00138017/ERZ477280_FASTA.fasta	MGYA00138017	ERZ477280	root:Host-associated:Human:Digestive system	0.3248
MGYA00138018/ERZ477296_FASTA.fasta	MGYA00138018	ERZ477296	root:Host-associated:Human:Digestive system	0.4094
MGYA00138019/ERZ477270_FASTA.fasta	MGYA00138019	ERZ477270	root:Host-associated:Human:Digestive system	0.3372
MGYA00138020/ERZ477252_FASTA.fasta	MGYA00138020	ERZ477252	root:Host-associated:Human:Digestive system	0.3276
MGYA00138021/ERZ477272_FASTA.fasta	MGYA00138021	ERZ477272	root:Host-associated:Human:Digestive system	0.2556
MGYA00138022/ERZ477279_FASTA.fasta	MGYA00138022	ERZ477279	root:Host-associated:Human:Digestive system	0.2708
MGYA00138023/ERZ477275_FASTA.fasta	MGYA00138023	ERZ477275	root:Host-associated:Human:Digestive system	0.2822
MGYA00138024/ERZ477284_FASTA.fasta	MGYA00138024	ERZ477284	root:Host-associated:Human:Digestive system	0.226
MGYA00138025/ERZ477262_FASTA.fasta	MGYA00138025	ERZ477262	root:Host-associated:Human:Digestive system	0.427
MGYA00138026/ERZ477291_FASTA.fasta	MGYA00138026	ERZ477291	root:Host-associated:Human:Digestive system	0.3338
MGYA00138027/ERZ477267_FASTA.fasta	MGYA00138027	ERZ477267	root:Host-associated:Human:Digestive system	0.3606
MGYA00138028/ERZ477253_FASTA.fasta	MGYA00138028	ERZ477253	root:Host-associated:Human:Digestive system	0.3368
MGYA00138029/ERZ477281_FASTA.fasta	MGYA00138029	ERZ477281	root:Host-associated:Human:Digestive system	0.2516
MGYA00138030/ERZ477295_FASTA.fasta	MGYA00138030	ERZ477295	root:Host-associated:Human:Digestive system	0.1582
MGYA00138031/ERZ477265_FASTA.fasta	MGYA00138031	ERZ477265	root:Host-associated:Human:Digestive system	0.337
MGYA00138032/ERZ477286_FASTA.fasta	MGYA00138032	ERZ477286	root:Host-associated:Human:Digestive system	0.3864
MGYA00138033/ERZ477282_FASTA.fasta	MGYA00138033	ERZ477282	root:Host-associated:Human:Digestive system	0.3778
MGYA00138034/ERZ477260_FASTA.fasta	MGYA00138034	ERZ477260	root:Host-associated:Human:Digestive system	0.3172
MGYA00138035/ERZ477278_FASTA.fasta	MGYA00138035	ERZ477278	root:Host-associated:Human:Digestive system	0.358
MGYA00138036/ERZ477283_FASTA.fasta	MGYA00138036	ERZ477283	root:Host-associated:Human:Digestive system	0.3348
MGYA00138037/ERZ477301_FASTA.fasta	MGYA00138037	ERZ477301	root:Host-associated:Human:Digestive system	0.261
MGYA00138038/ERZ477247_FASTA.fasta	MGYA00138038	ERZ477247	root:Host-associated:Human:Digestive system	0.2794
MGYA00138039/ERZ477287_FASTA.fasta	MGYA00138039	ERZ477287	root:Host-associated:Human:Digestive system	0.3408
MGYA00138040/ERZ477249_FASTA.fasta	MGYA00138040	ERZ477249	root:Host-associated:Human:Digestive system	0.2442
MGYA00138041/ERZ477259_FASTA.fasta	MGYA00138041	ERZ477259	root:Host-associated:Human:Digestive system	0.2746
MGYA00138042/ERZ477292_FASTA.fasta	MGYA00138042	ERZ477292	root:Host-associated:Human:Digestive system	0.3256
MGYA00138043/ERZ477269_FASTA.fasta	MGYA00138043	ERZ477269	root:Host-associated:Human:Digestive system	0.3412
MGYA00138044/ERZ477263_FASTA.fasta	MGYA00138044	ERZ477263	root:Host-associated:Human:Digestive system	0.2286
MGYA00138045/ERZ477276_FASTA.fasta	MGYA00138045	ERZ477276	root:Host-associated:Human:Digestive system	0.3654
MGYA00138046/ERZ477264_FASTA.fasta	MGYA00138046	ERZ477264	root:Host-associated:Human:Digestive system	0.2936
MGYA00138047/ERZ477294_FASTA.fasta	MGYA00138047	ERZ477294	root:Host-associated:Human:Digestive system	0.2316
MGYA00138048/ERZ477299_FASTA.fasta	MGYA00138048	ERZ477299	root:Host-associated:Human:Digestive system	0.264
MGYA00138049/ERZ477261_FASTA.fasta	MGYA00138049	ERZ477261	root:Host-associated:Human:Digestive system	0.2344
MGYA00138050/ERZ477303_FASTA.fasta	MGYA00138050	ERZ477303	root:Host-associated:Human:Digestive system	0.1988
MGYA00138051/ERZ477248_FASTA.fasta	MGYA00138051	ERZ477248	root:Host-associated:Human:Digestive system	0.3278
MGYA00138052/ERZ477256_FASTA.fasta	MGYA00138052	ERZ477256	root:Host-associated:Human:Digestive system	0.3558
MGYA00138053/ERZ477271_FASTA.fasta	MGYA00138053	ERZ477271	root:Host-associated:Human:Digestive system	0.225
MGYA00138054/ERZ477266_FASTA.fasta	MGYA00138054	ERZ477266	root:Host-associated:Human:Digestive system	0.2926
MGYA00138055/ERZ477255_FASTA.fasta	MGYA00138055	ERZ477255	root:Host-associated:Human:Digestive system	0.3718
MGYA00138056/ERZ477254_FASTA.fasta	MGYA00138056	ERZ477254	root:Host-associated:Human:Digestive system	0.3558
MGYA00138057/ERZ477304_FASTA.fasta	MGYA00138057	ERZ477304	root:Host-associated:Human:Digestive system	0.3724
MGYA00138058/ERZ477258_FASTA.fasta	MGYA00138058	ERZ477258	root:Host-associated:Human:Digestive system	0.2842
MGYA00138059/ERZ477298_FASTA.fasta	MGYA00138059	ERZ477298	root:Host-associated:Human:Digestive system	0.272
MGYA00138060/ERZ477362_FASTA.fasta	MGYA00138060	ERZ477362	root:Host-associated:Human:Digestive system:Intestine	0.0294
MGYA00138061/ERZ477376_FASTA.fasta	MGYA00138061	ERZ477376	root:Host-associated:Human:Digestive system:Intestine	0.0294
MGYA00138062/ERZ477354_FASTA.fasta	MGYA00138062	ERZ477354	root:Host-associated:Human:Digestive system:Intestine	0.0294
MGYA00138063/ERZ477355_FASTA.fasta	MGYA00138063	ERZ477355	root:Host-associated:Human:Digestive system:Intestine	0.0294
MGYA00138064/ERZ477375_FASTA.fasta	MGYA00138064	ERZ477375	root:Host-associated:Human:Digestive system:Intestine	0.0294
MGYA00138065/ERZ477371_FASTA.fasta	MGYA00138065	ERZ477371	root:Host-associated:Human:Digestive system:Intestine	0.0324
MGYA00138066/ERZ477352_FASTA.fasta	MGYA00138066	ERZ477352	root:Host-associated:Human:Digestive system:Intestine	0.0294
MGYA00138067/ERZ477358_FASTA.fasta	MGYA00138067	ERZ477358	root:Host-associated:Human:Digestive system:Intestine	0.0294
MGYA00138068/ERZ477373_FASTA.fasta	MGYA00138068	ERZ477373	root:Host-associated:Human:Digestive system:Intestine	0.0294
MGYA00138069/ERZ477368_FASTA.fasta	MGYA00138069	ERZ477368	root:Host-associated:Human:Digestive system:Intestine	0.0294
MGYA00138070/ERZ477353_FASTA.fasta	MGYA00138070	ERZ477353	root:Host-associated:Human:Digestive system:Intestine	0.0294
MGYA00138071/ERZ477370_FASTA.fasta	MGYA00138071	ERZ477370	root:Host-associated:Human:Digestive system:Intestine	0.0294
MGYA00138072/ERZ477357_FASTA.fasta	MGYA00138072	ERZ477357	root:Host-associated:Human:Digestive system:Intestine	0.0294
MGYA00138073/ERZ477359_FASTA.fasta	MGYA00138073	ERZ477359	root:Host-associated:Human:Digestive system:Intestine	0.0294
MGYA00138074/ERZ477367_FASTA.fasta	MGYA00138074	ERZ477367	root:Host-associated:Human:Digestive system:Intestine	0.0294
MGYA00138075/ERZ477369_FASTA.fasta	MGYA00138075	ERZ477369	root:Host-associated:Human:Digestive system:Intestine	0.0294
MGYA00138076/ERZ477364_FASTA.fasta	MGYA00138076	ERZ477364	root:Host-associated:Human:Digestive system:Intestine	0.0504
MGYA00138077/ERZ477361_FASTA.fasta	MGYA00138077	ERZ477361	root:Host-associated:Human:Digestive system:Intestine	0.0294
MGYA00138078/ERZ477356_FASTA.fasta	MGYA00138078	ERZ477356	root:Host-associated:Human:Digestive system:Intestine	0.0298
MGYA00138079/ERZ477372_FASTA.fasta	MGYA00138079	ERZ477372	root:Host-associated:Human:Digestive system:Intestine	0.0294
MGYA00138080/ERZ477374_FASTA.fasta	MGYA00138080	ERZ477374	root:Host-associated:Human:Digestive system:Intestine	0.0948
MGYA00138081/ERZ477366_FASTA.fasta	MGYA00138081	ERZ477366	root:Host-associated:Human:Digestive system:Intestine	0.0294
MGYA00138082/ERZ477360_FASTA.fasta	MGYA00138082	ERZ477360	root:Host-associated:Human:Digestive system:Intestine	0.0294
MGYA00138083/ERZ477351_FASTA.fasta	MGYA00138083	ERZ477351	root:Host-associated:Human:Digestive system:Intestine	0.0294
MGYA00138084/ERZ477350_FASTA.fasta	MGYA00138084	ERZ477350	root:Host-associated:Human:Digestive system:Intestine	0.0294
MGYA00138085/ERZ477363_FASTA.fasta	MGYA00138085	ERZ477363	root:Host-associated:Human:Digestive system:Intestine	0.0294
MGYA00138086/ERZ477365_FASTA.fasta	MGYA00138086	ERZ477365	root:Host-associated:Human:Digestive system:Intestine	0.0294
MGYA00138087/ERZ476297_FASTA.fasta	MGYA00138087	ERZ476297	root:Host-associated:Human:Digestive system:Large in	0.0294
MGYA00138088/ERZ476243_FASTA.fasta	MGYA00138088	ERZ476243	root:Host-associated:Human:Digestive system:Large in	0.0294
MGYA00138089/ERZ476299_FASTA.fasta	MGYA00138089	ERZ476299	root:Host-associated:Human:Digestive system:Large in	0.0294
MGYA00138090/ERZ476215_FASTA.fasta	MGYA00138090	ERZ476215	root:Host-associated:Human:Digestive system:Large in	0.0294
MGYA00138091/ERZ476340_FASTA.fasta	MGYA00138091	ERZ476340	root:Host-associated:Human:Digestive system:Large in	0.0294
MGYA00138092/ERZ476315_FASTA.fasta	MGYA00138092	ERZ476315	root:Host-associated:Human:Digestive system:Large in	0.0294
MGYA00138093/ERZ476267_FASTA.fasta	MGYA00138093	ERZ476267	root:Host-associated:Human:Digestive system:Large in	0.2544
MGYA00138094/ERZ476251_FASTA.fasta	MGYA00138094	ERZ476251	root:Host-associated:Human:Digestive system:Large in	0.5394
MGYA00138095/ERZ476240_FASTA.fasta	MGYA00138095	ERZ476240	root:Host-associated:Human:Digestive system:Large in	0.0294
MGYA00138096/ERZ476368_FASTA.fasta	MGYA00138096	ERZ476368	root:Host-associated:Human:Digestive system:Large in	0.0294
MGYA00138097/ERZ476291_FASTA.fasta	MGYA00138097	ERZ476291	root:Host-associated:Human:Digestive system:Large in	0.2316
MGYA00138098/ERZ476421_FASTA.fasta	MGYA00138098	ERZ476421	root:Host-associated:Human:Digestive system:Large in	0.0294
MGYA00138099/ERZ476189_FASTA.fasta	MGYA00138099	ERZ476189	root:Host-associated:Human:Digestive system:Large in	0.0294
MGYA00138100/ERZ476346_FASTA.fasta	MGYA00138100	ERZ476346	root:Host-associated:Human:Digestive system:Large in	0.0294
MGYA00138101/ERZ476364_FASTA.fasta	MGYA00138101	ERZ476364	root:Host-associated:Human:Digestive system:Large in	0.0294
MGYA00138102/ERZ476270_FASTA.fasta	MGYA00138102	ERZ476270	root:Host-associated:Human:Digestive system:Large in	0.3822
MGYA00138103/ERZ476281_FASTA.fasta	MGYA00138103	ERZ476281	root:Host-associated:Human:Digestive system:Large in	0.2038
MGYA00138104/ERZ476294_FASTA.fasta	MGYA00138104	ERZ476294	root:Host-associated:Human:Digestive system:Large in	0.0294
MGYA00138105/ERZ476424_FASTA.fasta	MGYA00138105	ERZ476424	root:Host-associated:Human:Digestive system:Large in	0.0294
MGYA00138106/ERZ476382_FASTA.fasta	MGYA00138106	ERZ476382	root:Host-associated:Human:Digestive system:Large in	0.0294
MGYA00138107/ERZ476356_FASTA.fasta	MGYA00138107	ERZ476356	root:Host-associated:Human:Digestive system:Large in	0.0294
MGYA00138108/ERZ476207_FASTA.fasta	MGYA00138108	ERZ476207	root:Host-associated:Human:Digestive system:Large in	0.0294
MGYA00138109/ERZ476379_FASTA.fasta	MGYA00138109	ERZ476379	root:Host-associated:Human:Digestive system:Large in	0.0294
MGYA00138110/ERZ476301_FASTA.fasta	MGYA00138110	ERZ476301	root:Host-associated:Human:Digestive system:Large in	0.0294
MGYA00138111/ERZ476412_FASTA.fasta	MGYA00138111	ERZ476412	root:Host-associated:Human:Digestive system:Large in	0.0294
MGYA00138112/ERZ476197_FASTA.fasta	MGYA00138112	ERZ476197	root:Host-associated:Human:Digestive system:Large in	0.0294
MGYA00138113/ERZ476219_FASTA.fasta	MGYA00138113	ERZ476219	root:Host-associated:Human:Digestive system:Large in	0.0294
MGYA00138114/ERZ476290_FASTA.fasta	MGYA00138114	ERZ476290	root:Host-associated:Human:Digestive system:Large in	0.3114
MGYA00138115/ERZ476256_FASTA.fasta	MGYA00138115	ERZ476256	root:Host-associated:Human:Digestive system:Large in	0.3192
MGYA00138116/ERZ476179_FASTA.fasta	MGYA00138116	ERZ476179	root:Host-associated:Human:Digestive system:Large in	0.0294
MGYA00138117/ERZ476283_FASTA.fasta	MGYA00138117	ERZ476283	root:Host-associated:Human:Digestive system:Large in	0.297
MGYA00138118/ERZ476238_FASTA.fasta	MGYA00138118	ERZ476238	root:Host-associated:Human:Digestive system:Large in	0.0294
MGYA00138119/ERZ476181_FASTA.fasta	MGYA00138119	ERZ476181	root:Host-associated:Human:Digestive system:Large in	0.0294
MGYA00138120/ERZ476194_FASTA.fasta	MGYA00138120	ERZ476194	root:Host-associated:Human:Digestive system:Large in	0.0294
MGYA00138121/ERZ476242_FASTA.fasta	MGYA00138121	ERZ476242	root:Host-associated:Human:Digestive system:Large in	0.0294
MGYA00138122/ERZ476262_FASTA.fasta	MGYA00138122	ERZ476262	root:Host-associated:Human:Digestive system:Large in	0.5646
MGYA00138123/ERZ476205_FASTA.fasta	MGYA00138123	ERZ476205	root:Host-associated:Human:Digestive system:Large in	0.0294
MGYA00138124/ERZ476176_FASTA.fasta	MGYA00138124	ERZ476176	root:Host-associated:Human:Digestive system:Large in	0.0294
MGYA00138125/ERZ476378_FASTA.fasta	MGYA00138125	ERZ476378	root:Host-associated:Human:Digestive system:Large in	0.0294
MGYA00138126/ERZ476321_FASTA.fasta	MGYA00138126	ERZ476321	root:Host-associated:Human:Digestive system:Large in	0.0294
MGYA00138127/ERZ476182_FASTA.fasta	MGYA00138127	ERZ476182	root:Host-associated:Human:Digestive system:Large in	0.0294
MGYA00138128/ERZ476386_FASTA.fasta	MGYA00138128	ERZ476386	root:Host-associated:Human:Digestive system:Large in	0.0294
MGYA00138129/ERZ476345_FASTA.fasta	MGYA00138129	ERZ476345	root:Host-associated:Human:Digestive system:Large in	0.0294
MGYA00138130/ERZ476367_FASTA.fasta	MGYA00138130	ERZ476367	root:Host-associated:Human:Digestive system:Large in	0.0294
MGYA00138131/ERZ476335_FASTA.fasta	MGYA00138131	ERZ476335	root:Host-associated:Human:Digestive system:Large in	0.0294
MGYA00138132/ERZ476407_FASTA.fasta	MGYA00138132	ERZ476407	root:Host-associated:Human:Digestive system:Large in	0.0294
MGYA00138133/ERZ476252_FASTA.fasta	MGYA00138133	ERZ476252	root:Host-associated:Human:Digestive system:Large in	0.3268
MGYA00138134/ERZ476183_FASTA.fasta	MGYA00138134	ERZ476183	root:Host-associated:Human:Digestive system:Large in	0.0294
MGYA00138135/ERZ476212_FASTA.fasta	MGYA00138135	ERZ476212	root:Host-associated:Human:Digestive system:Large in	0.0294
MGYA00138136/ERZ476327_FASTA.fasta	MGYA00138136	ERZ476327	root:Host-associated:Human:Digestive system:Large in	0.0294
MGYA00138137/ERZ476336_FASTA.fasta	MGYA00138137	ERZ476336	root:Host-associated:Human:Digestive system:Large in	0.0294
MGYA00138138/ERZ476209_FASTA.fasta	MGYA00138138	ERZ476209	root:Host-associated:Human:Digestive system:Large in	0.0294
MGYA00138139/ERZ476416_FASTA.fasta	MGYA00138139	ERZ476416	root:Host-associated:Human:Digestive system:Large in	0.0294
MGYA00138140/ERZ476230_FASTA.fasta	MGYA00138140	ERZ476230	root:Host-associated:Human:Digestive system:Large in	0.0294
MGYA00138141/ERZ476287_FASTA.fasta	MGYA00138141	ERZ476287	root:Host-associated:Human:Digestive system:Large in	0.2656
MGYA00138142/ERZ476409_FASTA.fasta	MGYA00138142	ERZ476409	root:Host-associated:Human:Digestive system:Large in	0.0294
MGYA00138143/ERZ476383_FASTA.fasta	MGYA00138143	ERZ476383	root:Host-associated:Human:Digestive system:Large in	0.0294
MGYA00138144/ERZ476403_FASTA.fasta	MGYA00138144	ERZ476403	root:Host-associated:Human:Digestive system:Large in	0.0294
MGYA00138145/ERZ476234_FASTA.fasta	MGYA00138145	ERZ476234	root:Host-associated:Human:Digestive system:Large in	0.0294
MGYA00138146/ERZ476390_FASTA.fasta	MGYA00138146	ERZ476390	root:Host-associated:Human:Digestive system:Large in	0.0294
MGYA00138147/ERZ476193_FASTA.fasta	MGYA00138147	ERZ476193	root:Host-associated:Human:Digestive system:Large in	0.0294
MGYA00138148/ERZ476235_FASTA.fasta	MGYA00138148	ERZ476235	root:Host-associated:Human:Digestive system:Large in	0.0294
MGYA00138149/ERZ476204_FASTA.fasta	MGYA00138149	ERZ476204	root:Host-associated:Human:Digestive system:Large in	0.0294
MGYA00138150/ERZ476398_FASTA.fasta	MGYA00138150	ERZ476398	root:Host-associated:Human:Digestive system:Large in	0.0294
MGYA00138151/ERZ476419_FASTA.fasta	MGYA00138151	ERZ476419	root:Host-associated:Human:Digestive system:Large in	0.0294
MGYA00138152/ERZ476233_FASTA.fasta	MGYA00138152	ERZ476233	root:Host-associated:Human:Digestive system:Large in	0.0294
MGYA00138153/ERZ476286_FASTA.fasta	MGYA00138153	ERZ476286	root:Host-associated:Human:Digestive system:Large in	0.2184
MGYA00138154/ERZ476196_FASTA.fasta	MGYA00138154	ERZ476196	root:Host-associated:Human:Digestive system:Large in	0.0294
MGYA00138155/ERZ476338_FASTA.fasta	MGYA00138155	ERZ476338	root:Host-associated:Human:Digestive system:Large in	0.0294
MGYA00138156/ERZ476331_FASTA.fasta	MGYA00138156	ERZ476331	root:Host-associated:Human:Digestive system:Large in	0.0294
MGYA00138157/ERZ476391_FASTA.fasta	MGYA00138157	ERZ476391	root:Host-associated:Human:Digestive system:Large in	0.0294
MGYA00138158/ERZ476295_FASTA.fasta	MGYA00138158	ERZ476295	root:Host-associated:Human:Digestive system:Large in	0.0294
MGYA00138159/ERZ476245_FASTA.fasta	MGYA00138159	ERZ476245	root:Host-associated:Human:Digestive system:Large in	0.0294
MGYA00138160/ERZ476249_FASTA.fasta	MGYA00138160	ERZ476249	root:Host-associated:Human:Digestive system:Large in	0.0294
MGYA00138161/ERZ476428_FASTA.fasta	MGYA00138161	ERZ476428	root:Host-associated:Human:Digestive system:Large in	0.0294
MGYA00138162/ERZ476303_FASTA.fasta	MGYA00138162	ERZ476303	root:Host-associated:Human:Digestive system:Large in	0.0294
MGYA00138163/ERZ476415_FASTA.fasta	MGYA00138163	ERZ476415	root:Host-associated:Human:Digestive system:Large in	0.0294
MGYA00138164/ERZ476413_FASTA.fasta	MGYA00138164	ERZ476413	root:Host-associated:Human:Digestive system:Large in	0.0294
MGYA00138165/ERZ476427_FASTA.fasta	MGYA00138165	ERZ476427	root:Host-associated:Human:Digestive system:Large in	0.0294
MGYA00138166/ERZ476288_FASTA.fasta	MGYA00138166	ERZ476288	root:Host-associated:Human:Digestive system:Large in	0.1778
MGYA00138167/ERZ476203_FASTA.fasta	MGYA00138167	ERZ476203	root:Host-associated:Human:Digestive system:Large in	0.0294
MGYA00138168/ERZ476381_FASTA.fasta	MGYA00138168	ERZ476381	root:Host-associated:Human:Digestive system:Large in	0.0294
MGYA00138169/ERZ476210_FASTA.fasta	MGYA00138169	ERZ476210	root:Host-associated:Human:Digestive system:Large in	0.0294
MGYA00138170/ERZ476370_FASTA.fasta	MGYA00138170	ERZ476370	root:Host-associated:Human:Digestive system:Large in	0.0294
MGYA00138171/ERZ476195_FASTA.fasta	MGYA00138171	ERZ476195	root:Host-associated:Human:Digestive system:Large in	0.0294
MGYA00138172/ERZ476347_FASTA.fasta	MGYA00138172	ERZ476347	root:Host-associated:Human:Digestive system:Large in	0.0294
MGYA00138173/ERZ476213_FASTA.fasta	MGYA00138173	ERZ476213	root:Host-associated:Human:Digestive system:Large in	0.0294
MGYA00138174/ERZ476358_FASTA.fasta	MGYA00138174	ERZ476358	root:Host-associated:Human:Digestive system:Large in	0.0294
MGYA00138175/ERZ476200_FASTA.fasta	MGYA00138175	ERZ476200	root:Host-associated:Human:Digestive system:Large in	0.0294
MGYA00138176/ERZ476202_FASTA.fasta	MGYA00138176	ERZ476202	root:Host-associated:Human:Digestive system:Large in	0.0294
MGYA00138177/ERZ476268_FASTA.fasta	MGYA00138177	ERZ476268	root:Host-associated:Human:Digestive system:Large in	0.0304
MGYA00138178/ERZ476186_FASTA.fasta	MGYA00138178	ERZ476186	root:Host-associated:Human:Digestive system:Large in	0.0294
MGYA00138179/ERZ476337_FASTA.fasta	MGYA00138179	ERZ476337	root:Host-associated:Human:Digestive system:Large in	0.0294
MGYA00138180/ERZ476187_FASTA.fasta	MGYA00138180	ERZ476187	root:Host-associated:Human:Digestive system:Large in	0.0294
MGYA00138181/ERZ476226_FASTA.fasta	MGYA00138181	ERZ476226	root:Host-associated:Human:Digestive system:Large in	0.0294
MGYA00138182/ERZ476293_FASTA.fasta	MGYA00138182	ERZ476293	root:Host-associated:Human:Digestive system:Large in	0.0294
MGYA00138183/ERZ476420_FASTA.fasta	MGYA00138183	ERZ476420	root:Host-associated:Human:Digestive system:Large in	0.0294
MGYA00138184/ERZ476423_FASTA.fasta	MGYA00138184	ERZ476423	root:Host-associated:Human:Digestive system:Large in	0.0294
MGYA00138185/ERZ476284_FASTA.fasta	MGYA00138185	ERZ476284	root:Host-associated:Human:Digestive system:Large in	0.1988
MGYA00138186/ERZ476278_FASTA.fasta	MGYA00138186	ERZ476278	root:Host-associated:Human:Digestive system:Large in	0.0838
MGYA00138187/ERZ476323_FASTA.fasta	MGYA00138187	ERZ476323	root:Host-associated:Human:Digestive system:Large in	0.0294
MGYA00138188/ERZ476362_FASTA.fasta	MGYA00138188	ERZ476362	root:Host-associated:Human:Digestive system:Large in	0.0294
MGYA00138189/ERZ476343_FASTA.fasta	MGYA00138189	ERZ476343	root:Host-associated:Human:Digestive system:Large in	0.0294
MGYA00138190/ERZ476232_FASTA.fasta	MGYA00138190	ERZ476232	root:Host-associated:Human:Digestive system:Large in	0.0294
MGYA00138191/ERZ476292_FASTA.fasta	MGYA00138191	ERZ476292	root:Host-associated:Human:Digestive system:Large in	0.0294
MGYA00138192/ERZ476198_FASTA.fasta	MGYA00138192	ERZ476198	root:Host-associated:Human:Digestive system:Large in	0.0294
MGYA00138193/ERZ476341_FASTA.fasta	MGYA00138193	ERZ476341	root:Host-associated:Human:Digestive system:Large in	0.0294
MGYA00138194/ERZ476369_FASTA.fasta	MGYA00138194	ERZ476369	root:Host-associated:Human:Digestive system:Large in	0.0294
MGYA00138195/ERZ476273_FASTA.fasta	MGYA00138195	ERZ476273	root:Host-associated:Human:Digestive system:Large in	0.298
MGYA00138196/ERZ476302_FASTA.fasta	MGYA00138196	ERZ476302	root:Host-associated:Human:Digestive system:Large in	0.0294
MGYA00138197/ERZ476266_FASTA.fasta	MGYA00138197	ERZ476266	root:Host-associated:Human:Digestive system:Large in	0.2174
MGYA00138198/ERZ476332_FASTA.fasta	MGYA00138198	ERZ476332	root:Host-associated:Human:Digestive system:Large in	0.0294
MGYA00138199/ERZ476363_FASTA.fasta	MGYA00138199	ERZ476363	root:Host-associated:Human:Digestive system:Large in	0.0294
MGYA00138200/ERZ476316_FASTA.fasta	MGYA00138200	ERZ476316	root:Host-associated:Human:Digestive system:Large in	0.0294
MGYA00138201/ERZ476222_FASTA.fasta	MGYA00138201	ERZ476222	root:Host-associated:Human:Digestive system:Large in	0.0294
MGYA00138202/ERZ476401_FASTA.fasta	MGYA00138202	ERZ476401	root:Host-associated:Human:Digestive system:Large in	0.0294
MGYA00138203/ERZ476406_FASTA.fasta	MGYA00138203	ERZ476406	root:Host-associated:Human:Digestive system:Large in	0.0294
MGYA00138204/ERZ476214_FASTA.fasta	MGYA00138204	ERZ476214	root:Host-associated:Human:Digestive system:Large in	0.0294
MGYA00138205/ERZ476376_FASTA.fasta	MGYA00138205	ERZ476376	root:Host-associated:Human:Digestive system:Large in	0.0294
MGYA00138206/ERZ476250_FASTA.fasta	MGYA00138206	ERZ476250	root:Host-associated:Human:Digestive system:Large in	0.4086
MGYA00138207/ERZ476384_FASTA.fasta	MGYA00138207	ERZ476384	root:Host-associated:Human:Digestive system:Large in	0.0294
MGYA00138208/ERZ476280_FASTA.fasta	MGYA00138208	ERZ476280	root:Host-associated:Human:Digestive system:Large in	0.3648
MGYA00138209/ERZ476178_FASTA.fasta	MGYA00138209	ERZ476178	root:Host-associated:Human:Digestive system:Large in	0.0294
MGYA00138210/ERZ476201_FASTA.fasta	MGYA00138210	ERZ476201	root:Host-associated:Human:Digestive system:Large in	0.0294
MGYA00138211/ERZ476241_FASTA.fasta	MGYA00138211	ERZ476241	root:Host-associated:Human:Digestive system:Large in	0.0294
MGYA00138212/ERZ476350_FASTA.fasta	MGYA00138212	ERZ476350	root:Host-associated:Human:Digestive system:Large in	0.0294
MGYA00138213/ERZ476274_FASTA.fasta	MGYA00138213	ERZ476274	root:Host-associated:Human:Digestive system:Large in	0.2418
MGYA00138214/ERZ476206_FASTA.fasta	MGYA00138214	ERZ476206	root:Host-associated:Human:Digestive system:Large in	0.0294
MGYA00138215/ERZ476188_FASTA.fasta	MGYA00138215	ERZ476188	root:Host-associated:Human:Digestive system:Large in	0.0294
MGYA00138216/ERZ476374_FASTA.fasta	MGYA00138216	ERZ476374	root:Host-associated:Human:Digestive system:Large in	0.0294
MGYA00138217/ERZ476255_FASTA.fasta	MGYA00138217	ERZ476255	root:Host-associated:Human:Digestive system:Large in	0.1978
MGYA00138218/ERZ476317_FASTA.fasta	MGYA00138218	ERZ476317	root:Host-associated:Human:Digestive system:Large in	0.0294
MGYA00138219/ERZ476306_FASTA.fasta	MGYA00138219	ERZ476306	root:Host-associated:Human:Digestive system:Large in	0.0294
MGYA00138220/ERZ476387_FASTA.fasta	MGYA00138220	ERZ476387	root:Host-associated:Human:Digestive system:Large in	0.0294
MGYA00138221/ERZ476177_FASTA.fasta	MGYA00138221	ERZ476177	root:Host-associated:Human:Digestive system:Large in	0.0294
MGYA00138222/ERZ476422_FASTA.fasta	MGYA00138222	ERZ476422	root:Host-associated:Human:Digestive system:Large in	0.0294
MGYA00138223/ERZ476236_FASTA.fasta	MGYA00138223	ERZ476236	root:Host-associated:Human:Digestive system:Large in	0.0294
MGYA00138224/ERZ476425_FASTA.fasta	MGYA00138224	ERZ476425	root:Host-associated:Human:Digestive system:Large in	0.0294
MGYA00138225/ERZ476184_FASTA.fasta	MGYA00138225	ERZ476184	root:Host-associated:Human:Digestive system:Large in	0.0294
MGYA00138226/ERZ476320_FASTA.fasta	MGYA00138226	ERZ476320	root:Host-associated:Human:Digestive system:Large in	0.0294
MGYA00138227/ERZ476276_FASTA.fasta	MGYA00138227	ERZ476276	root:Host-associated:Human:Digestive system:Large in	0.0768
MGYA00138228/ERZ476218_FASTA.fasta	MGYA00138228	ERZ476218	root:Host-associated:Human:Digestive system:Large in	0.0294
MGYA00138229/ERZ476417_FASTA.fasta	MGYA00138229	ERZ476417	root:Host-associated:Human:Digestive system:Large in	0.0294
MGYA00138230/ERZ476377_FASTA.fasta	MGYA00138230	ERZ476377	root:Host-associated:Human:Digestive system:Large in	0.0294
MGYA00138231/ERZ476408_FASTA.fasta	MGYA00138231	ERZ476408	root:Host-associated:Human:Digestive system:Large in	0.0294
MGYA00138232/ERZ476247_FASTA.fasta	MGYA00138232	ERZ476247	root:Host-associated:Human:Digestive system:Large in	0.0294
MGYA00138233/ERZ476411_FASTA.fasta	MGYA00138233	ERZ476411	root:Host-associated:Human:Digestive system:Large in	0.0294
MGYA00138234/ERZ476239_FASTA.fasta	MGYA00138234	ERZ476239	root:Host-associated:Human:Digestive system:Large in	0.0294
MGYA00138235/ERZ476371_FASTA.fasta	MGYA00138235	ERZ476371	root:Host-associated:Human:Digestive system:Large in	0.0294
MGYA00138236/ERZ476359_FASTA.fasta	MGYA00138236	ERZ476359	root:Host-associated:Human:Digestive system:Large in	0.0294
MGYA00138237/ERZ476396_FASTA.fasta	MGYA00138237	ERZ476396	root:Host-associated:Human:Digestive system:Large in	0.0294
MGYA00138238/ERZ476351_FASTA.fasta	MGYA00138238	ERZ476351	root:Host-associated:Human:Digestive system:Large in	0.0294
MGYA00138239/ERZ476282_FASTA.fasta	MGYA00138239	ERZ476282	root:Host-associated:Human:Digestive system:Large in	0.1936
MGYA00138240/ERZ476393_FASTA.fasta	MGYA00138240	ERZ476393	root:Host-associated:Human:Digestive system:Large in	0.0294
MGYA00138241/ERZ476326_FASTA.fasta	MGYA00138241	ERZ476326	root:Host-associated:Human:Digestive system:Large in	0.0294
MGYA00138242/ERZ476228_FASTA.fasta	MGYA00138242	ERZ476228	root:Host-associated:Human:Digestive system:Large in	0.0294
MGYA00138243/ERZ476208_FASTA.fasta	MGYA00138243	ERZ476208	root:Host-associated:Human:Digestive system:Large in	0.0294
MGYA00138244/ERZ476275_FASTA.fasta	MGYA00138244	ERZ476275	root:Host-associated:Human:Digestive system:Large in	0.323
MGYA00138245/ERZ476375_FASTA.fasta	MGYA00138245	ERZ476375	root:Host-associated:Human:Digestive system:Large in	0.0294
MGYA00138246/ERZ476380_FASTA.fasta	MGYA00138246	ERZ476380	root:Host-associated:Human:Digestive system:Large in	0.0294
MGYA00138247/ERZ476227_FASTA.fasta	MGYA00138247	ERZ476227	root:Host-associated:Human:Digestive system:Large in	0.0294
MGYA00138248/ERZ476259_FASTA.fasta	MGYA00138248	ERZ476259	root:Host-associated:Human:Digestive system:Large in	0.3564
MGYA00138249/ERZ476248_FASTA.fasta	MGYA00138249	ERZ476248	root:Host-associated:Human:Digestive system:Large in	0.0294
MGYA00138250/ERZ476272_FASTA.fasta	MGYA00138250	ERZ476272	root:Host-associated:Human:Digestive system:Large in	0.1382
MGYA00138251/ERZ476304_FASTA.fasta	MGYA00138251	ERZ476304	root:Host-associated:Human:Digestive system:Large in	0.0294
MGYA00138252/ERZ476265_FASTA.fasta	MGYA00138252	ERZ476265	root:Host-associated:Human:Digestive system:Large in	0.2094
MGYA00138253/ERZ476307_FASTA.fasta	MGYA00138253	ERZ476307	root:Host-associated:Human:Digestive system:Large in	0.0294
MGYA00138254/ERZ476231_FASTA.fasta	MGYA00138254	ERZ476231	root:Host-associated:Human:Digestive system:Large in	0.0294
MGYA00138255/ERZ476220_FASTA.fasta	MGYA00138255	ERZ476220	root:Host-associated:Human:Digestive system:Large in	0.0294
MGYA00138256/ERZ476402_FASTA.fasta	MGYA00138256	ERZ476402	root:Host-associated:Human:Digestive system:Large in	0.0294
MGYA00138257/ERZ476312_FASTA.fasta	MGYA00138257	ERZ476312	root:Host-associated:Human:Digestive system:Large in	0.0294
MGYA00138258/ERZ476414_FASTA.fasta	MGYA00138258	ERZ476414	root:Host-associated:Human:Digestive system:Large in	0.0294
MGYA00138259/ERZ476325_FASTA.fasta	MGYA00138259	ERZ476325	root:Host-associated:Human:Digestive system:Large in	0.0294
MGYA00138260/ERZ476385_FASTA.fasta	MGYA00138260	ERZ476385	root:Host-associated:Human:Digestive system:Large in	0.0294
MGYA00138261/ERZ476353_FASTA.fasta	MGYA00138261	ERZ476353	root:Host-associated:Human:Digestive system:Large in	0.0294
MGYA00138262/ERZ476289_FASTA.fasta	MGYA00138262	ERZ476289	root:Host-associated:Human:Digestive system:Large in	0.0898
MGYA00138263/ERZ476192_FASTA.fasta	MGYA00138263	ERZ476192	root:Host-associated:Human:Digestive system:Large in	0.0294
MGYA00138264/ERZ476269_FASTA.fasta	MGYA00138264	ERZ476269	root:Host-associated:Human:Digestive system:Large in	0.0294
MGYA00138265/ERZ476389_FASTA.fasta	MGYA00138265	ERZ476389	root:Host-associated:Human:Digestive system:Large in	0.0294
MGYA00138266/ERZ476279_FASTA.fasta	MGYA00138266	ERZ476279	root:Host-associated:Human:Digestive system:Large in	0.3198
MGYA00138267/ERZ476254_FASTA.fasta	MGYA00138267	ERZ476254	root:Host-associated:Human:Digestive system:Large in	0.4298
MGYA00138268/ERZ476244_FASTA.fasta	MGYA00138268	ERZ476244	root:Host-associated:Human:Digestive system:Large in	0.0294
MGYA00138269/ERZ476361_FASTA.fasta	MGYA00138269	ERZ476361	root:Host-associated:Human:Digestive system:Large in	0.0294
MGYA00138270/ERZ476355_FASTA.fasta	MGYA00138270	ERZ476355	root:Host-associated:Human:Digestive system:Large in	0.0294
MGYA00138271/ERZ476223_FASTA.fasta	MGYA00138271	ERZ476223	root:Host-associated:Human:Digestive system:Large in	0.0294
MGYA00138273/ERZ476298_FASTA.fasta	MGYA00138273	ERZ476298	root:Host-associated:Human:Digestive system:Large in	0.0294
MGYA00138274/ERZ476372_FASTA.fasta	MGYA00138274	ERZ476372	root:Host-associated:Human:Digestive system:Large in	0.0294
MGYA00138275/ERZ476319_FASTA.fasta	MGYA00138275	ERZ476319	root:Host-associated:Human:Digestive system:Large in	0.0294
MGYA00138276/ERZ476330_FASTA.fasta	MGYA00138276	ERZ476330	root:Host-associated:Human:Digestive system:Large in	0.0294
MGYA00138277/ERZ476300_FASTA.fasta	MGYA00138277	ERZ476300	root:Host-associated:Human:Digestive system:Large in	0.0294
MGYA00138278/ERZ476392_FASTA.fasta	MGYA00138278	ERZ476392	root:Host-associated:Human:Digestive system:Large in	0.0294
MGYA00138279/ERZ476237_FASTA.fasta	MGYA00138279	ERZ476237	root:Host-associated:Human:Digestive system:Large in	0.0294
MGYA00138280/ERZ476311_FASTA.fasta	MGYA00138280	ERZ476311	root:Host-associated:Human:Digestive system:Large in	0.0294
MGYA00138281/ERZ476229_FASTA.fasta	MGYA00138281	ERZ476229	root:Host-associated:Human:Digestive system:Large in	0.0294
MGYA00138282/ERZ476308_FASTA.fasta	MGYA00138282	ERZ476308	root:Host-associated:Human:Digestive system:Large in	0.0294
MGYA00138283/ERZ476328_FASTA.fasta	MGYA00138283	ERZ476328	root:Host-associated:Human:Digestive system:Large in	0.0294
MGYA00138284/ERZ476305_FASTA.fasta	MGYA00138284	ERZ476305	root:Host-associated:Human:Digestive system:Large in	0.0294
MGYA00138285/ERZ476310_FASTA.fasta	MGYA00138285	ERZ476310	root:Host-associated:Human:Digestive system:Large in	0.0294
MGYA00138286/ERZ476400_FASTA.fasta	MGYA00138286	ERZ476400	root:Host-associated:Human:Digestive system:Large in	0.0294
MGYA00138287/ERZ476277_FASTA.fasta	MGYA00138287	ERZ476277	root:Host-associated:Human:Digestive system:Large in	0.0362
MGYA00138288/ERZ476373_FASTA.fasta	MGYA00138288	ERZ476373	root:Host-associated:Human:Digestive system:Large in	0.0294
MGYA00138289/ERZ476349_FASTA.fasta	MGYA00138289	ERZ476349	root:Host-associated:Human:Digestive system:Large in	0.0294
MGYA00138290/ERZ476216_FASTA.fasta	MGYA00138290	ERZ476216	root:Host-associated:Human:Digestive system:Large in	0.0294
MGYA00138291/ERZ476221_FASTA.fasta	MGYA00138291	ERZ476221	root:Host-associated:Human:Digestive system:Large in	0.0294
MGYA00138292/ERZ476357_FASTA.fasta	MGYA00138292	ERZ476357	root:Host-associated:Human:Digestive system:Large in	0.0294
MGYA00138293/ERZ476260_FASTA.fasta	MGYA00138293	ERZ476260	root:Host-associated:Human:Digestive system:Large in	0.506
MGYA00138294/ERZ476257_FASTA.fasta	MGYA00138294	ERZ476257	root:Host-associated:Human:Digestive system:Large in	0.145
MGYA00138295/ERZ476261_FASTA.fasta	MGYA00138295	ERZ476261	root:Host-associated:Human:Digestive system:Large in	0.3064
MGYA00138296/ERZ476190_FASTA.fasta	MGYA00138296	ERZ476190	root:Host-associated:Human:Digestive system:Large in	0.0294
MGYA00138297/ERZ476309_FASTA.fasta	MGYA00138297	ERZ476309	root:Host-associated:Human:Digestive system:Large in	0.0294
MGYA00138298/ERZ476180_FASTA.fasta	MGYA00138298	ERZ476180	root:Host-associated:Human:Digestive system:Large in	0.0294
MGYA00138299/ERZ476365_FASTA.fasta	MGYA00138299	ERZ476365	root:Host-associated:Human:Digestive system:Large in	0.0294
MGYA00138300/ERZ476313_FASTA.fasta	MGYA00138300	ERZ476313	root:Host-associated:Human:Digestive system:Large in	0.0294
MGYA00138301/ERZ476404_FASTA.fasta	MGYA00138301	ERZ476404	root:Host-associated:Human:Digestive system:Large in	0.0294
MGYA00138302/ERZ476344_FASTA.fasta	MGYA00138302	ERZ476344	root:Host-associated:Human:Digestive system:Large in	0.0294
MGYA00138303/ERZ476410_FASTA.fasta	MGYA00138303	ERZ476410	root:Host-associated:Human:Digestive system:Large in	0.0294
MGYA00138304/ERZ476394_FASTA.fasta	MGYA00138304	ERZ476394	root:Host-associated:Human:Digestive system:Large in	0.0294
MGYA00138305/ERZ476264_FASTA.fasta	MGYA00138305	ERZ476264	root:Host-associated:Human:Digestive system:Large in	0.1018
MGYA00138306/ERZ476418_FASTA.fasta	MGYA00138306	ERZ476418	root:Host-associated:Human:Digestive system:Large in	0.0294
MGYA00138307/ERZ476366_FASTA.fasta	MGYA00138307	ERZ476366	root:Host-associated:Human:Digestive system:Large in	0.0294
MGYA00138308/ERZ476324_FASTA.fasta	MGYA00138308	ERZ476324	root:Host-associated:Human:Digestive system:Large in	0.0294
MGYA00138309/ERZ476258_FASTA.fasta	MGYA00138309	ERZ476258	root:Host-associated:Human:Digestive system:Large in	0.1836
MGYA00138310/ERZ476314_FASTA.fasta	MGYA00138310	ERZ476314	root:Host-associated:Human:Digestive system:Large in	0.0294
MGYA00138311/ERZ476354_FASTA.fasta	MGYA00138311	ERZ476354	root:Host-associated:Human:Digestive system:Large in	0.0294
MGYA00138312/ERZ476253_FASTA.fasta	MGYA00138312	ERZ476253	root:Host-associated:Human:Digestive system:Large in	0.2942
MGYA00138313/ERZ476185_FASTA.fasta	MGYA00138313	ERZ476185	root:Host-associated:Human:Digestive system:Large in	0.0294
MGYA00138314/ERZ476296_FASTA.fasta	MGYA00138314	ERZ476296	root:Host-associated:Human:Digestive system:Large in	0.0294
MGYA00138315/ERZ476348_FASTA.fasta	MGYA00138315	ERZ476348	root:Host-associated:Human:Digestive system:Large in	0.0294
MGYA00138316/ERZ476318_FASTA.fasta	MGYA00138316	ERZ476318	root:Host-associated:Human:Digestive system:Large in	0.0294
MGYA00138317/ERZ476199_FASTA.fasta	MGYA00138317	ERZ476199	root:Host-associated:Human:Digestive system:Large in	0.0294
MGYA00138318/ERZ476360_FASTA.fasta	MGYA00138318	ERZ476360	root:Host-associated:Human:Digestive system:Large in	0.0294
MGYA00138319/ERZ476399_FASTA.fasta	MGYA00138319	ERZ476399	root:Host-associated:Human:Digestive system:Large in	0.0294
MGYA00138320/ERZ476333_FASTA.fasta	MGYA00138320	ERZ476333	root:Host-associated:Human:Digestive system:Large in	0.0294
MGYA00138321/ERZ476342_FASTA.fasta	MGYA00138321	ERZ476342	root:Host-associated:Human:Digestive system:Large in	0.0294
MGYA00138322/ERZ476397_FASTA.fasta	MGYA00138322	ERZ476397	root:Host-associated:Human:Digestive system:Large in	0.0294
MGYA00138323/ERZ476329_FASTA.fasta	MGYA00138323	ERZ476329	root:Host-associated:Human:Digestive system:Large in	0.0294
MGYA00138324/ERZ476224_FASTA.fasta	MGYA00138324	ERZ476224	root:Host-associated:Human:Digestive system:Large in	0.0294
MGYA00138325/ERZ476285_FASTA.fasta	MGYA00138325	ERZ476285	root:Host-associated:Human:Digestive system:Large in	0.0294
MGYA00138326/ERZ476395_FASTA.fasta	MGYA00138326	ERZ476395	root:Host-associated:Human:Digestive system:Large in	0.0294
MGYA00138327/ERZ476334_FASTA.fasta	MGYA00138327	ERZ476334	root:Host-associated:Human:Digestive system:Large in	0.0294
MGYA00138328/ERZ476405_FASTA.fasta	MGYA00138328	ERZ476405	root:Host-associated:Human:Digestive system:Large in	0.0294
MGYA00138329/ERZ476217_FASTA.fasta	MGYA00138329	ERZ476217	root:Host-associated:Human:Digestive system:Large in	0.0294
MGYA00138330/ERZ476322_FASTA.fasta	MGYA00138330	ERZ476322	root:Host-associated:Human:Digestive system:Large in	0.0294
MGYA00138331/ERZ476352_FASTA.fasta	MGYA00138331	ERZ476352	root:Host-associated:Human:Digestive system:Large in	0.0294
MGYA00138332/ERZ476388_FASTA.fasta	MGYA00138332	ERZ476388	root:Host-associated:Human:Digestive system:Large in	0.0294
MGYA00138333/ERZ476211_FASTA.fasta	MGYA00138333	ERZ476211	root:Host-associated:Human:Digestive system:Large in	0.0294
MGYA00138334/ERZ476263_FASTA.fasta	MGYA00138334	ERZ476263	root:Host-associated:Human:Digestive system:Large in	0.2548
MGYA00138335/ERZ476191_FASTA.fasta	MGYA00138335	ERZ476191	root:Host-associated:Human:Digestive system:Large in	0.0294
MGYA00138336/ERZ476246_FASTA.fasta	MGYA00138336	ERZ476246	root:Host-associated:Human:Digestive system:Large in	0.0294
MGYA00138337/ERZ476225_FASTA.fasta	MGYA00138337	ERZ476225	root:Host-associated:Human:Digestive system:Large in	0.0294
MGYA00138338/ERZ476339_FASTA.fasta	MGYA00138338	ERZ476339	root:Host-associated:Human:Digestive system:Large in	0.0294
MGYA00138339/ERZ476426_FASTA.fasta	MGYA00138339	ERZ476426	root:Host-associated:Human:Digestive system:Large in	0.0294
MGYA00139071/ERZ477433_FASTA.fasta	MGYA00139071	ERZ477433	root:Environmental:Aquatic:Marine:Oceanic:Photic zon	0.0294
MGYA00139073/ERZ477435_FASTA.fasta	MGYA00139073	ERZ477435	root:Environmental:Aquatic:Marine:Oceanic:Photic zon	0.08
MGYA00139074/ERZ477432_FASTA.fasta	MGYA00139074	ERZ477432	root:Environmental:Aquatic:Marine:Oceanic:Photic zon	0.1332
MGYA00139075/ERZ477440_FASTA.fasta	MGYA00139075	ERZ477440	root:Environmental:Aquatic:Marine:Oceanic:Photic zon	0.295
MGYA00139076/ERZ477434_FASTA.fasta	MGYA00139076	ERZ477434	root:Environmental:Aquatic:Marine:Oceanic:Photic zon	0.0294
MGYA00139078/ERZ477437_FASTA.fasta	MGYA00139078	ERZ477437	root:Environmental:Aquatic:Marine:Oceanic:Photic zon	0.232
MGYA00139079/ERZ477441_FASTA.fasta	MGYA00139079	ERZ477441	root:Environmental:Aquatic:Marine:Oceanic:Photic zon	0.0938
MGYA00139080/ERZ477436_FASTA.fasta	MGYA00139080	ERZ477436	root:Environmental:Aquatic:Marine:Oceanic:Photic zon	0.2156
MGYA00139082/ERZ477453_FASTA.fasta	MGYA00139082	ERZ477453	root:Host-associated:Human:Digestive system	0.2332
MGYA00139083/ERZ477445_FASTA.fasta	MGYA00139083	ERZ477445	root:Host-associated:Human:Digestive system	0.2988
MGYA00139084/ERZ477452_FASTA.fasta	MGYA00139084	ERZ477452	root:Host-associated:Human:Digestive system	0.4594
MGYA00139085/ERZ477447_FASTA.fasta	MGYA00139085	ERZ477447	root:Host-associated:Human:Digestive system	0.3154
MGYA00139086/ERZ477450_FASTA.fasta	MGYA00139086	ERZ477450	root:Host-associated:Human:Digestive system	0.3196
MGYA00139087/ERZ477448_FASTA.fasta	MGYA00139087	ERZ477448	root:Host-associated:Human:Digestive system	0.238
MGYA00139088/ERZ477442_FASTA.fasta	MGYA00139088	ERZ477442	root:Host-associated:Human:Digestive system	0.4628
MGYA00139089/ERZ477451_FASTA.fasta	MGYA00139089	ERZ477451	root:Host-associated:Human:Digestive system	0.3478
MGYA00139090/ERZ477454_FASTA.fasta	MGYA00139090	ERZ477454	root:Host-associated:Human:Digestive system	0.3454
MGYA00139091/ERZ477446_FASTA.fasta	MGYA00139091	ERZ477446	root:Host-associated:Human:Digestive system	0.3958
MGYA00139092/ERZ477449_FASTA.fasta	MGYA00139092	ERZ477449	root:Host-associated:Human:Digestive system	0.4052
MGYA00139093/ERZ477444_FASTA.fasta	MGYA00139093	ERZ477444	root:Host-associated:Human:Digestive system	0.2718
MGYA00139094/ERZ477443_FASTA.fasta	MGYA00139094	ERZ477443	root:Host-associated:Human:Digestive system	0.4862
MGYA00139095/ERZ477575_FASTA.fasta	MGYA00139095	ERZ477575	root:Host-associated:Human:Digestive system:Large in	0.2306
MGYA00139096/ERZ477576_FASTA.fasta	MGYA00139096	ERZ477576	root:Environmental:Aquatic:Marine	0.6366
MGYA00139097/ERZ477577_FASTA.fasta	MGYA00139097	ERZ477577	root:Environmental:Aquatic:Marine:Intertidal zone:Salt	0.873
MGYA00139098/ERZ477582_FASTA.fasta	MGYA00139098	ERZ477582	root:Environmental:Aquatic:Marine:Hydrothermal vents:	0.4194
MGYA00139099/ERZ477581_FASTA.fasta	MGYA00139099	ERZ477581	root:Environmental:Aquatic:Marine:Hydrothermal vents:	0.1262
MGYA00139100/ERZ477584_FASTA.fasta	MGYA00139100	ERZ477584	root:Environmental:Aquatic:Marine:Hydrothermal vents:	0.2966
MGYA00139101/ERZ477583_FASTA.fasta	MGYA00139101	ERZ477583	root:Environmental:Aquatic:Marine:Hydrothermal vents:	0.1094
MGYA00139102/ERZ477580_FASTA.fasta	MGYA00139102	ERZ477580	root:Environmental:Aquatic:Marine:Hydrothermal vents:	0.4678
MGYA00139103/ERZ477585_FASTA.fasta	MGYA00139103	ERZ477585	root:Environmental:Terrestrial:Soil:Grasslands	0.7976
MGYA00139104/ERZ477586_FASTA.fasta	MGYA00139104	ERZ477586	root:Environmental:Terrestrial:Soil:Grasslands	0.6738
MGYA00139105/ERZ477589_FASTA.fasta	MGYA00139105	ERZ477589	root:Host-associated:Human:Digestive system:Large in	0.3164
MGYA00139106/ERZ477587_FASTA.fasta	MGYA00139106	ERZ477587	root:Host-associated:Human:Digestive system:Large in	0.4328
MGYA00139107/ERZ477590_FASTA.fasta	MGYA00139107	ERZ477590	root:Host-associated:Human:Digestive system:Large in	0.3658
MGYA00139108/ERZ477588_FASTA.fasta	MGYA00139108	ERZ477588	root:Host-associated:Human:Digestive system:Large in	0.4188
MGYA00139109/ERZ477591_FASTA.fasta	MGYA00139109	ERZ477591	root:Environmental:Aquatic:Freshwater:Lake	0.8706
MGYA00139110/ERZ477314_FASTA.fasta	MGYA00139110	ERZ477314	root:Host-associated:Human	0.3848
MGYA00139111/ERZ477331_FASTA.fasta	MGYA00139111	ERZ477331	root:Host-associated:Human	0.4264
MGYA00139112/ERZ477319_FASTA.fasta	MGYA00139112	ERZ477319	root:Host-associated:Human	0.1978
MGYA00139113/ERZ477334_FASTA.fasta	MGYA00139113	ERZ477334	root:Host-associated:Human	0.3904
MGYA00139114/ERZ477342_FASTA.fasta	MGYA00139114	ERZ477342	root:Host-associated:Human	0.3934
MGYA00139115/ERZ477338_FASTA.fasta	MGYA00139115	ERZ477338	root:Host-associated:Human	0.3934
MGYA00139116/ERZ477336_FASTA.fasta	MGYA00139116	ERZ477336	root:Host-associated:Human	0.452
MGYA00139117/ERZ477343_FASTA.fasta	MGYA00139117	ERZ477343	root:Host-associated:Human	0.3904
MGYA00139118/ERZ477317_FASTA.fasta	MGYA00139118	ERZ477317	root:Host-associated:Human	0.285
MGYA00139119/ERZ477320_FASTA.fasta	MGYA00139119	ERZ477320	root:Host-associated:Human	0.1054
MGYA00139120/ERZ477339_FASTA.fasta	MGYA00139120	ERZ477339	root:Host-associated:Human	0.2702
MGYA00139121/ERZ477337_FASTA.fasta	MGYA00139121	ERZ477337	root:Host-associated:Human	0.3938
MGYA00139122/ERZ477333_FASTA.fasta	MGYA00139122	ERZ477333	root:Host-associated:Human	0.3604
MGYA00139123/ERZ477309_FASTA.fasta	MGYA00139123	ERZ477309	root:Host-associated:Human	0.3848
MGYA00139124/ERZ477324_FASTA.fasta	MGYA00139124	ERZ477324	root:Host-associated:Human	0.4444
MGYA00139125/ERZ477306_FASTA.fasta	MGYA00139125	ERZ477306	root:Host-associated:Human	0.182
MGYA00139126/ERZ477344_FASTA.fasta	MGYA00139126	ERZ477344	root:Host-associated:Human	0.3462
MGYA00139127/ERZ477307_FASTA.fasta	MGYA00139127	ERZ477307	root:Host-associated:Human	0.3562
MGYA00139128/ERZ477327_FASTA.fasta	MGYA00139128	ERZ477327	root:Host-associated:Human	0.4534
MGYA00139129/ERZ477308_FASTA.fasta	MGYA00139129	ERZ477308	root:Host-associated:Human	0.4034
MGYA00139130/ERZ477345_FASTA.fasta	MGYA00139130	ERZ477345	root:Host-associated:Human	0.3704
MGYA00139131/ERZ477312_FASTA.fasta	MGYA00139131	ERZ477312	root:Host-associated:Human	0.3244
MGYA00139132/ERZ477349_FASTA.fasta	MGYA00139132	ERZ477349	root:Host-associated:Human	0.3848
MGYA00139133/ERZ477346_FASTA.fasta	MGYA00139133	ERZ477346	root:Host-associated:Human	0.3848
MGYA00139134/ERZ477313_FASTA.fasta	MGYA00139134	ERZ477313	root:Host-associated:Human	0.2232
MGYA00139135/ERZ477340_FASTA.fasta	MGYA00139135	ERZ477340	root:Host-associated:Human	0.3934
MGYA00139136/ERZ477321_FASTA.fasta	MGYA00139136	ERZ477321	root:Host-associated:Human	0.3184
MGYA00139137/ERZ477348_FASTA.fasta	MGYA00139137	ERZ477348	root:Host-associated:Human	0.3848
MGYA00139138/ERZ477310_FASTA.fasta	MGYA00139138	ERZ477310	root:Host-associated:Human	0.3554
MGYA00139139/ERZ477328_FASTA.fasta	MGYA00139139	ERZ477328	root:Host-associated:Human	0.2066
MGYA00139140/ERZ477305_FASTA.fasta	MGYA00139140	ERZ477305	root:Host-associated:Human	0.2502
MGYA00139141/ERZ477347_FASTA.fasta	MGYA00139141	ERZ477347	root:Host-associated:Human	0.3878
MGYA00139142/ERZ477316_FASTA.fasta	MGYA00139142	ERZ477316	root:Host-associated:Human	0.446
MGYA00139143/ERZ477341_FASTA.fasta	MGYA00139143	ERZ477341	root:Host-associated:Human	0.3904
MGYA00139144/ERZ477311_FASTA.fasta	MGYA00139144	ERZ477311	root:Host-associated:Human	0.4034
MGYA00139145/ERZ477325_FASTA.fasta	MGYA00139145	ERZ477325	root:Host-associated:Human	0.4534
MGYA00139146/ERZ477329_FASTA.fasta	MGYA00139146	ERZ477329	root:Host-associated:Human	0.3178
MGYA00139147/ERZ477318_FASTA.fasta	MGYA00139147	ERZ477318	root:Host-associated:Human	0.4318
MGYA00139148/ERZ477330_FASTA.fasta	MGYA00139148	ERZ477330	root:Host-associated:Human	0.3294
MGYA00139149/ERZ477326_FASTA.fasta	MGYA00139149	ERZ477326	root:Host-associated:Human	0.456
MGYA00139150/ERZ477315_FASTA.fasta	MGYA00139150	ERZ477315	root:Host-associated:Human	0.4318
MGYA00139151/ERZ477322_FASTA.fasta	MGYA00139151	ERZ477322	root:Host-associated:Human	0.3112
MGYA00139152/ERZ477335_FASTA.fasta	MGYA00139152	ERZ477335	root:Host-associated:Human	0.4528
MGYA00139153/ERZ477332_FASTA.fasta	MGYA00139153	ERZ477332	root:Host-associated:Human	0.3594
MGYA00139154/ERZ477323_FASTA.fasta	MGYA00139154	ERZ477323	root:Host-associated:Human	0.4574
MGYA00139155/ERZ477592_FASTA.fasta	MGYA00139155	ERZ477592	root:Environmental:Terrestrial:Soil	0.4874
MGYA00139156/ERZ477593_FASTA.fasta	MGYA00139156	ERZ477593	root:Environmental:Terrestrial:Soil	0.5808
MGYA00139588/ERZ476618_FASTA.fasta	MGYA00139588	ERZ476618	root:Host-associated:Human:Digestive system:Large in	0.3126
MGYA00139589/ERZ476700_FASTA.fasta	MGYA00139589	ERZ476700	root:Host-associated:Human:Digestive system:Large in	0.576
MGYA00139590/ERZ476489_FASTA.fasta	MGYA00139590	ERZ476489	root:Host-associated:Human:Digestive system:Large in	0.411
MGYA00139591/ERZ476469_FASTA.fasta	MGYA00139591	ERZ476469	root:Host-associated:Human:Digestive system:Large in	0.5016
MGYA00139592/ERZ476598_FASTA.fasta	MGYA00139592	ERZ476598	root:Host-associated:Human:Digestive system:Large in	0.4298
MGYA00139593/ERZ476542_FASTA.fasta	MGYA00139593	ERZ476542	root:Host-associated:Human:Digestive system:Large in	0.4524
MGYA00139594/ERZ476639_FASTA.fasta	MGYA00139594	ERZ476639	root:Host-associated:Human:Digestive system:Large in	0.2938
MGYA00139595/ERZ476562_FASTA.fasta	MGYA00139595	ERZ476562	root:Host-associated:Human:Digestive system:Large in	0.4306
MGYA00139596/ERZ476475_FASTA.fasta	MGYA00139596	ERZ476475	root:Host-associated:Human:Digestive system:Large in	0.5478
MGYA00139597/ERZ476701_FASTA.fasta	MGYA00139597	ERZ476701	root:Host-associated:Human:Digestive system:Large in	0.4516
MGYA00139598/ERZ476619_FASTA.fasta	MGYA00139598	ERZ476619	root:Host-associated:Human:Digestive system:Large in	0.5434
MGYA00139599/ERZ476708_FASTA.fasta	MGYA00139599	ERZ476708	root:Host-associated:Human:Digestive system:Large in	0.4676
MGYA00139600/ERZ476534_FASTA.fasta	MGYA00139600	ERZ476534	root:Host-associated:Human:Digestive system:Large in	0.3754
MGYA00139601/ERZ476606_FASTA.fasta	MGYA00139601	ERZ476606	root:Host-associated:Human:Digestive system:Large in	0.5092
MGYA00139602/ERZ476675_FASTA.fasta	MGYA00139602	ERZ476675	root:Host-associated:Human:Digestive system:Large in	0.3376
MGYA00139603/ERZ476718_FASTA.fasta	MGYA00139603	ERZ476718	root:Host-associated:Human:Digestive system:Large in	0.4238
MGYA00139604/ERZ476699_FASTA.fasta	MGYA00139604	ERZ476699	root:Host-associated:Human:Digestive system:Large in	0.4004
MGYA00139605/ERZ476583_FASTA.fasta	MGYA00139605	ERZ476583	root:Host-associated:Human:Digestive system:Large in	0.356
MGYA00139606/ERZ476627_FASTA.fasta	MGYA00139606	ERZ476627	root:Host-associated:Human:Digestive system:Large in	0.4294
MGYA00139607/ERZ476540_FASTA.fasta	MGYA00139607	ERZ476540	root:Host-associated:Human:Digestive system:Large in	0.3672
MGYA00139608/ERZ476706_FASTA.fasta	MGYA00139608	ERZ476706	root:Host-associated:Human:Digestive system:Large in	0.5174
MGYA00139609/ERZ476608_FASTA.fasta	MGYA00139609	ERZ476608	root:Host-associated:Human:Digestive system:Large in	0.25
MGYA00139610/ERZ476605_FASTA.fasta	MGYA00139610	ERZ476605	root:Host-associated:Human:Digestive system:Large in	0.32
MGYA00139611/ERZ476564_FASTA.fasta	MGYA00139611	ERZ476564	root:Host-associated:Human:Digestive system:Large in	0.437
MGYA00139612/ERZ476459_FASTA.fasta	MGYA00139612	ERZ476459	root:Host-associated:Human:Digestive system:Large in	0.258
MGYA00139613/ERZ476703_FASTA.fasta	MGYA00139613	ERZ476703	root:Host-associated:Human:Digestive system:Large in	0.4268
MGYA00139614/ERZ476678_FASTA.fasta	MGYA00139614	ERZ476678	root:Host-associated:Human:Digestive system:Large in	0.2568
MGYA00139615/ERZ476642_FASTA.fasta	MGYA00139615	ERZ476642	root:Host-associated:Human:Digestive system:Large in	0.3588
MGYA00139616/ERZ476457_FASTA.fasta	MGYA00139616	ERZ476457	root:Host-associated:Human:Digestive system:Large in	0.4256
MGYA00139617/ERZ476458_FASTA.fasta	MGYA00139617	ERZ476458	root:Host-associated:Human:Digestive system:Large in	0.4208
MGYA00139618/ERZ476506_FASTA.fasta	MGYA00139618	ERZ476506	root:Host-associated:Human:Digestive system:Large in	0.4114
MGYA00139619/ERZ476552_FASTA.fasta	MGYA00139619	ERZ476552	root:Host-associated:Human:Digestive system:Large in	0.3964
MGYA00139620/ERZ476687_FASTA.fasta	MGYA00139620	ERZ476687	root:Host-associated:Human:Digestive system:Large in	0.4082
MGYA00139621/ERZ476670_FASTA.fasta	MGYA00139621	ERZ476670	root:Host-associated:Human:Digestive system:Large in	0.4294
MGYA00139622/ERZ476499_FASTA.fasta	MGYA00139622	ERZ476499	root:Host-associated:Human:Digestive system:Large in	0.2544
MGYA00139623/ERZ476547_FASTA.fasta	MGYA00139623	ERZ476547	root:Host-associated:Human:Digestive system:Large in	0.4418
MGYA00139624/ERZ476500_FASTA.fasta	MGYA00139624	ERZ476500	root:Host-associated:Human:Digestive system:Large in	0.3154

MGYA00139625/ERZ476440_FASTA.fasta	MGYA00139625	ERZ476440	root:Host-associated:Human:Digestive system:Large in	0.5422
MGYA00139626/ERZ476673_FASTA.fasta	MGYA00139626	ERZ476673	root:Host-associated:Human:Digestive system:Large in	0.4364
MGYA00139627/ERZ476690_FASTA.fasta	MGYA00139627	ERZ476690	root:Host-associated:Human:Digestive system:Large in	0.3936
MGYA00139628/ERZ476430_FASTA.fasta	MGYA00139628	ERZ476430	root:Host-associated:Human:Digestive system:Large in	0.398
MGYA00139629/ERZ476714_FASTA.fasta	MGYA00139629	ERZ476714	root:Host-associated:Human:Digestive system:Large in	0.378
MGYA00139630/ERZ476635_FASTA.fasta	MGYA00139630	ERZ476635	root:Host-associated:Human:Digestive system:Large in	0.376
MGYA00139631/ERZ476503_FASTA.fasta	MGYA00139631	ERZ476503	root:Host-associated:Human:Digestive system:Large in	0.4682
MGYA00139632/ERZ476482_FASTA.fasta	MGYA00139632	ERZ476482	root:Host-associated:Human:Digestive system:Large in	0.4956
MGYA00139633/ERZ476692_FASTA.fasta	MGYA00139633	ERZ476692	root:Host-associated:Human:Digestive system:Large in	0.4112
MGYA00139634/ERZ476532_FASTA.fasta	MGYA00139634	ERZ476532	root:Host-associated:Human:Digestive system:Large in	0.4476
MGYA00139635/ERZ476637_FASTA.fasta	MGYA00139635	ERZ476637	root:Host-associated:Human:Digestive system:Large in	0.2896
MGYA00139636/ERZ476667_FASTA.fasta	MGYA00139636	ERZ476667	root:Host-associated:Human:Digestive system:Large in	0.432
MGYA00139637/ERZ476603_FASTA.fasta	MGYA00139637	ERZ476603	root:Host-associated:Human:Digestive system:Large in	0.3296
MGYA00139638/ERZ476616_FASTA.fasta	MGYA00139638	ERZ476616	root:Host-associated:Human:Digestive system:Large in	0.3816
MGYA00139639/ERZ476657_FASTA.fasta	MGYA00139639	ERZ476657	root:Host-associated:Human:Digestive system:Large in	0.399
MGYA00139640/ERZ476551_FASTA.fasta	MGYA00139640	ERZ476551	root:Host-associated:Human:Digestive system:Large in	0.3646
MGYA00139641/ERZ476509_FASTA.fasta	MGYA00139641	ERZ476509	root:Host-associated:Human:Digestive system:Large in	0.289
MGYA00139642/ERZ476505_FASTA.fasta	MGYA00139642	ERZ476505	root:Host-associated:Human:Digestive system:Large in	0.4182
MGYA00139643/ERZ476688_FASTA.fasta	MGYA00139643	ERZ476688	root:Host-associated:Human:Digestive system:Large in	0.3048
MGYA00139644/ERZ476597_FASTA.fasta	MGYA00139644	ERZ476597	root:Host-associated:Human:Digestive system:Large in	0.4458
MGYA00139645/ERZ476495_FASTA.fasta	MGYA00139645	ERZ476495	root:Host-associated:Human:Digestive system:Large in	0.3678
MGYA00139646/ERZ476558_FASTA.fasta	MGYA00139646	ERZ476558	root:Host-associated:Human:Digestive system:Large in	0.4402
MGYA00139647/ERZ476453_FASTA.fasta	MGYA00139647	ERZ476453	root:Host-associated:Human:Digestive system:Large in	0.4478
MGYA00139648/ERZ476566_FASTA.fasta	MGYA00139648	ERZ476566	root:Host-associated:Human:Digestive system:Large in	0.426
MGYA00139649/ERZ476602_FASTA.fasta	MGYA00139649	ERZ476602	root:Host-associated:Human:Digestive system:Large in	0.569
MGYA00139650/ERZ476433_FASTA.fasta	MGYA00139650	ERZ476433	root:Host-associated:Human:Digestive system:Large in	0.4568
MGYA00139651/ERZ476631_FASTA.fasta	MGYA00139651	ERZ476631	root:Host-associated:Human:Digestive system:Large in	0.3898
MGYA00139652/ERZ476513_FASTA.fasta	MGYA00139652	ERZ476513	root:Host-associated:Human:Digestive system:Large in	0.441
MGYA00139653/ERZ476644_FASTA.fasta	MGYA00139653	ERZ476644	root:Host-associated:Human:Digestive system:Large in	0.4702
MGYA00139654/ERZ476717_FASTA.fasta	MGYA00139654	ERZ476717	root:Host-associated:Human:Digestive system:Large in	0.3696
MGYA00139655/ERZ476686_FASTA.fasta	MGYA00139655	ERZ476686	root:Host-associated:Human:Digestive system:Large in	0.4592
MGYA00139656/ERZ476560_FASTA.fasta	MGYA00139656	ERZ476560	root:Host-associated:Human:Digestive system:Large in	0.4386
MGYA00139657/ERZ476528_FASTA.fasta	MGYA00139657	ERZ476528	root:Host-associated:Human:Digestive system:Large in	0.3116
MGYA00139658/ERZ476625_FASTA.fasta	MGYA00139658	ERZ476625	root:Host-associated:Human:Digestive system:Large in	0.4692
MGYA00139659/ERZ476620_FASTA.fasta	MGYA00139659	ERZ476620	root:Host-associated:Human:Digestive system:Large in	0.4448
MGYA00139660/ERZ476468_FASTA.fasta	MGYA00139660	ERZ476468	root:Host-associated:Human:Digestive system:Large in	0.4732
MGYA00139661/ERZ476684_FASTA.fasta	MGYA00139661	ERZ476684	root:Host-associated:Human:Digestive system:Large in	0.4572
MGYA00139662/ERZ476668_FASTA.fasta	MGYA00139662	ERZ476668	root:Host-associated:Human:Digestive system:Large in	0.3816
MGYA00139663/ERZ476610_FASTA.fasta	MGYA00139663	ERZ476610	root:Host-associated:Human:Digestive system:Large in	0.3778
MGYA00139664/ERZ476511_FASTA.fasta	MGYA00139664	ERZ476511	root:Host-associated:Human:Digestive system:Large in	0.4128
MGYA00139665/ERZ476443_FASTA.fasta	MGYA00139665	ERZ476443	root:Host-associated:Human:Digestive system:Large in	0.1968
MGYA00139666/ERZ476441_FASTA.fasta	MGYA00139666	ERZ476441	root:Host-associated:Human:Digestive system:Large in	0.536
MGYA00139667/ERZ476609_FASTA.fasta	MGYA00139667	ERZ476609	root:Host-associated:Human:Digestive system:Large in	0.4822
MGYA00139668/ERZ476472_FASTA.fasta	MGYA00139668	ERZ476472	root:Host-associated:Human:Digestive system:Large in	0.302
MGYA00139669/ERZ476636_FASTA.fasta	MGYA00139669	ERZ476636	root:Host-associated:Human:Digestive system:Large in	0.4494
MGYA00139670/ERZ476553_FASTA.fasta	MGYA00139670	ERZ476553	root:Host-associated:Human:Digestive system:Large in	0.4432
MGYA00139671/ERZ476507_FASTA.fasta	MGYA00139671	ERZ476507	root:Host-associated:Human:Digestive system:Large in	0.4234
MGYA00139672/ERZ476623_FASTA.fasta	MGYA00139672	ERZ476623	root:Host-associated:Human:Digestive system:Large in	0.3466
MGYA00139673/ERZ476721_FASTA.fasta	MGYA00139673	ERZ476721	root:Host-associated:Human:Digestive system:Large in	0.4924
MGYA00139674/ERZ476680_FASTA.fasta	MGYA00139674	ERZ476680	root:Host-associated:Human:Digestive system:Large in	0.4718
MGYA00139675/ERZ476491_FASTA.fasta	MGYA00139675	ERZ476491	root:Host-associated:Human:Digestive system:Large in	0.4
MGYA00139676/ERZ476446_FASTA.fasta	MGYA00139676	ERZ476446	root:Host-associated:Human:Digestive system:Large in	0.2656
MGYA00139677/ERZ476436_FASTA.fasta	MGYA00139677	ERZ476436	root:Host-associated:Human:Digestive system:Large in	0.2058
MGYA00139678/ERZ476653_FASTA.fasta	MGYA00139678	ERZ476653	root:Host-associated:Human:Digestive system:Large in	0.3646
MGYA00139679/ERZ476456_FASTA.fasta	MGYA00139679	ERZ476456	root:Host-associated:Human:Digestive system:Large in	0.1032
MGYA00139680/ERZ476544_FASTA.fasta	MGYA00139680	ERZ476544	root:Host-associated:Human:Digestive system:Large in	0.5042
MGYA00139681/ERZ476535_FASTA.fasta	MGYA00139681	ERZ476535	root:Host-associated:Human:Digestive system:Large in	0.4234
MGYA00139682/ERZ476645_FASTA.fasta	MGYA00139682	ERZ476645	root:Host-associated:Human:Digestive system:Large in	0.4496
MGYA00139683/ERZ476621_FASTA.fasta	MGYA00139683	ERZ476621	root:Host-associated:Human:Digestive system:Large in	0.4188
MGYA00139684/ERZ476536_FASTA.fasta	MGYA00139684	ERZ476536	root:Host-associated:Human:Digestive system:Large in	0.5334
MGYA00139685/ERZ476626_FASTA.fasta	MGYA00139685	ERZ476626	root:Host-associated:Human:Digestive system:Large in	0.469
MGYA00139686/ERZ476604_FASTA.fasta	MGYA00139686	ERZ476604	root:Host-associated:Human:Digestive system:Large in	0.376
MGYA00139687/ERZ476454_FASTA.fasta	MGYA00139687	ERZ476454	root:Host-associated:Human:Digestive system:Large in	0.3742
MGYA00139688/ERZ476486_FASTA.fasta	MGYA00139688	ERZ476486	root:Host-associated:Human:Digestive system:Large in	0.2502
MGYA00139689/ERZ476549_FASTA.fasta	MGYA00139689	ERZ476549	root:Host-associated:Human:Digestive system:Large in	0.3572
MGYA00139690/ERZ476641_FASTA.fasta	MGYA00139690	ERZ476641	root:Host-associated:Human:Digestive system:Large in	0.3788
MGYA00139691/ERZ476662_FASTA.fasta	MGYA00139691	ERZ476662	root:Host-associated:Human:Digestive system:Large in	0.44
MGYA00139692/ERZ476431_FASTA.fasta	MGYA00139692	ERZ476431	root:Host-associated:Human:Digestive system:Large in	0.3976
MGYA00139693/ERZ476677_FASTA.fasta	MGYA00139693	ERZ476677	root:Host-associated:Human:Digestive system:Large in	0.539
MGYA00139694/ERZ476656_FASTA.fasta	MGYA00139694	ERZ476656	root:Host-associated:Human:Digestive system:Large in	0.4738
MGYA00139695/ERZ476569_FASTA.fasta	MGYA00139695	ERZ476569	root:Host-associated:Human:Digestive system:Large in	0.3194
MGYA00139696/ERZ476508_FASTA.fasta	MGYA00139696	ERZ476508	root:Host-associated:Human:Digestive system:Large in	0.3678
MGYA00139697/ERZ476545_FASTA.fasta	MGYA00139697	ERZ476545	root:Host-associated:Human:Digestive system:Large in	0.443
MGYA00139698/ERZ476450_FASTA.fasta	MGYA00139698	ERZ476450	root:Host-associated:Human:Digestive system:Large in	0.5472
MGYA00139699/ERZ476483_FASTA.fasta	MGYA00139699	ERZ476483	root:Host-associated:Human:Digestive system:Large in	0.3716
MGYA00139700/ERZ476570_FASTA.fasta	MGYA00139700	ERZ476570	root:Host-associated:Human:Digestive system:Large in	0.5134
MGYA00139701/ERZ476546_FASTA.fasta	MGYA00139701	ERZ476546	root:Host-associated:Human:Digestive system:Large in	0.3626
MGYA00139702/ERZ476439_FASTA.fasta	MGYA00139702	ERZ476439	root:Host-associated:Human:Digestive system:Large in	0.5238
MGYA00139703/ERZ476445_FASTA.fasta	MGYA00139703	ERZ476445	root:Host-associated:Human:Digestive system:Large in	0.3406
MGYA00139704/ERZ476630_FASTA.fasta	MGYA00139704	ERZ476630	root:Host-associated:Human:Digestive system:Large in	0.3462
MGYA00139705/ERZ476525_FASTA.fasta	MGYA00139705	ERZ476525	root:Host-associated:Human:Digestive system:Large in	0.457
MGYA00139706/ERZ476471_FASTA.fasta	MGYA00139706	ERZ476471	root:Host-associated:Human:Digestive system:Large in	0.4554
MGYA00139707/ERZ476600_FASTA.fasta	MGYA00139707	ERZ476600	root:Host-associated:Human:Digestive system:Large in	0.5034
MGYA00139708/ERZ476493_FASTA.fasta	MGYA00139708	ERZ476493	root:Host-associated:Human:Digestive system:Large in	0.3278
MGYA00139709/ERZ476557_FASTA.fasta	MGYA00139709	ERZ476557	root:Host-associated:Human:Digestive system:Large in	0.1886
MGYA00139710/ERZ476466_FASTA.fasta	MGYA00139710	ERZ476466	root:Host-associated:Human:Digestive system:Large in	0.4552
MGYA00139711/ERZ476661_FASTA.fasta	MGYA00139711	ERZ476661	root:Host-associated:Human:Digestive system:Large in	0.4948
MGYA00139712/ERZ476461_FASTA.fasta	MGYA00139712	ERZ476461	root:Host-associated:Human:Digestive system:Large in	0.4584
MGYA00139713/ERZ476654_FASTA.fasta	MGYA00139713	ERZ476654	root:Host-associated:Human:Digestive system:Large in	0.4736
MGYA00139714/ERZ476581_FASTA.fasta	MGYA00139714	ERZ476581	root:Host-associated:Human:Digestive system:Large in	0.4922
MGYA00139715/ERZ476579_FASTA.fasta	MGYA00139715	ERZ476579	root:Host-associated:Human:Digestive system:Large in	0.302
MGYA00139716/ERZ476485_FASTA.fasta	MGYA00139716	ERZ476485	root:Host-associated:Human:Digestive system:Large in	0.2886
MGYA00139717/ERZ476488_FASTA.fasta	MGYA00139717	ERZ476488	root:Host-associated:Human:Digestive system:Large in	0.4018
MGYA00139718/ERZ476685_FASTA.fasta	MGYA00139718	ERZ476685	root:Host-associated:Human:Digestive system:Large in	0.369
MGYA00139719/ERZ476444_FASTA.fasta	MGYA00139719	ERZ476444	root:Host-associated:Human:Digestive system:Large in	0.4774
MGYA00139720/ERZ476438_FASTA.fasta	MGYA00139720	ERZ476438	root:Host-associated:Human:Digestive system:Large in	0.4918
MGYA00139721/ERZ476697_FASTA.fasta	MGYA00139721	ERZ476697	root:Host-associated:Human:Digestive system:Large in	0.414
MGYA00139722/ERZ476437_FASTA.fasta	MGYA00139722	ERZ476437	root:Host-associated:Human:Digestive system:Large in	0.3102
MGYA00139723/ERZ476467_FASTA.fasta	MGYA00139723	ERZ476467	root:Host-associated:Human:Digestive system:Large in	0.4386
MGYA00139724/ERZ476531_FASTA.fasta	MGYA00139724	ERZ476531	root:Host-associated:Human:Digestive system:Large in	0.3242
MGYA00139725/ERZ476665_FASTA.fasta	MGYA00139725	ERZ476665	root:Host-associated:Human:Digestive system:Large in	0.3652
MGYA00139726/ERZ476709_FASTA.fasta	MGYA00139726	ERZ476709	root:Host-associated:Human:Digestive system:Large in	0.3174
MGYA00139727/ERZ476710_FASTA.fasta	MGYA00139727	ERZ476710	root:Host-associated:Human:Digestive system:Large in	0.4358
MGYA00139728/ERZ476648_FASTA.fasta	MGYA00139728	ERZ476648	root:Host-associated:Human:Digestive system:Large in	0.4364
MGYA00139729/ERZ476629_FASTA.fasta	MGYA00139729	ERZ476629	root:Host-associated:Human:Digestive system:Large in	0.4968
MGYA00139730/ERZ476611_FASTA.fasta	MGYA00139730	ERZ476611	root:Host-associated:Human:Digestive system:Large in	0.358
MGYA00139731/ERZ476484_FASTA.fasta	MGYA00139731	ERZ476484	root:Host-associated:Human:Digestive system:Large in	0.3752
MGYA00139732/ERZ476638_FASTA.fasta	MGYA00139732	ERZ476638	root:Host-associated:Human:Digestive system:Large in	0.4466
MGYA00139733/ERZ476655_FASTA.fasta	MGYA00139733	ERZ476655	root:Host-associated:Human:Digestive system:Large in	0.4238
MGYA00139734/ERZ476539_FASTA.fasta	MGYA00139734	ERZ476539	root:Host-associated:Human:Digestive system:Large in	0.3512
MGYA00139735/ERZ476567_FASTA.fasta	MGYA00139735	ERZ476567	root:Host-associated:Human:Digestive system:Large in	0.4702
MGYA00139736/ERZ476580_FASTA.fasta	MGYA00139736	ERZ476580	root:Host-associated:Human:Digestive system:Large in	0.27
MGYA00139737/ERZ476449_FASTA.fasta	MGYA00139737	ERZ476449	root:Host-associated:Human:Digestive system:Large in	0.4502
MGYA00139738/ERZ476473_FASTA.fasta	MGYA00139738	ERZ476473	root:Host-associated:Human:Digestive system:Large in	0.3676
MGYA00139739/ERZ476573_FASTA.fasta	MGYA00139739	ERZ476573	root:Host-associated:Human:Digestive system:Large in	0.319
MGYA00139740/ERZ476432_FASTA.fasta	MGYA00139740	ERZ476432	root:Host-associated:Human:Digestive system:Large in	0.343
MGYA00139741/ERZ476643_FASTA.fasta	MGYA00139741	ERZ476643	root:Host-associated:Human:Digestive system:Large in	0.0294
MGYA00139742/ERZ476595_FASTA.fasta	MGYA00139742	ERZ476595	root:Host-associated:Human:Digestive system:Large in	0.4466
MGYA00139743/ERZ476712_FASTA.fasta	MGYA00139743	ERZ476712	root:Host-associated:Human:Digestive system:Large in	0.3412
MGYA00139744/ERZ476496_FASTA.fasta	MGYA00139744	ERZ476496	root:Host-associated:Human:Digestive system:Large in	0.088
MGYA00139745/ERZ476563_FASTA.fasta	MGYA00139745	ERZ476563	root:Host-associated:Human:Digestive system:Large in	0.3806
MGYA00139746/ERZ476510_FASTA.fasta	MGYA00139746	ERZ476510	root:Host-associated:Human:Digestive system:Large in	0.3782
MGYA00139747/ERZ476664_FASTA.fasta	MGYA00139747	ERZ476664	root:Host-associated:Human:Digestive system:Large in	0.4478
MGYA00139748/ERZ476554_FASTA.fasta	MGYA00139748	ERZ476554	root:Host-associated:Human:Digestive system:Large in	0.5036
MGYA00139749/ERZ476707_FASTA.fasta	MGYA00139749	ERZ476707	root:Host-associated:Human:Digestive system:Large in	0.3764
MGYA00139750/ERZ476455_FASTA.fasta	MGYA00139750	ERZ476455	root:Host-associated:Human:Digestive system:Large in	0.3708
MGYA00139751/ERZ476498_FASTA.fasta	MGYA00139751	ERZ476498	root:Host-associated:Human:Digestive system:Large in	0.4146
MGYA00139752/ERZ476613_FASTA.fasta	MGYA00139752	ERZ476613	root:Host-associated:Human:Digestive system:Large in	0.3762
MGYA00139753/ERZ476713_FASTA.fasta	MGYA00139753	ERZ476713	root:Host-associated:Human:Digestive system:Large in	0.3442
MGYA00139754/ERZ476652_FASTA.fasta	MGYA00139754	ERZ476652	root:Host-associated:Human:Digestive system:Large in	0.481
MGYA00139755/ERZ476660_FASTA.fasta	MGYA00139755	ERZ476660	root:Host-associated:Human:Digestive system:Large in	0.31
MGYA00139756/ERZ476522_FASTA.fasta	MGYA00139756	ERZ476522	root:Host-associated:Human:Digestive system:Large in	0.3794
MGYA00139757/ERZ476634_FASTA.fasta	MGYA00139757	ERZ476634	root:Host-associated:Human:Digestive system:Large in	0.3536
MGYA00139758/ERZ476589_FASTA.fasta	MGYA00139758	ERZ476589	root:Host-associated:Human:Digestive system:Large in	0.5068
MGYA00139759/ERZ476683_FASTA.fasta	MGYA00139759	ERZ476683	root:Host-associated:Human:Digestive system:Large in	0.4456
MGYA00139760/ERZ476669_FASTA.fasta	MGYA00139760	ERZ476669	root:Host-associated:Human:Digestive system:Large in	0.3932
MGYA00139761/ERZ476462_FASTA.fasta	MGYA00139761	ERZ476462	root:Host-associated:Human:Digestive system:Large in	0.4538
MGYA00139762/ERZ476550_FASTA.fasta	MGYA00139762	ERZ476550	root:Host-associated:Human:Digestive system:Large in	0.3726
MGYA00139763/ERZ476512_FASTA.fasta	MGYA00139763	ERZ476512	root:Host-associated:Human:Digestive system:Large in	0.3206
MGYA00139764/ERZ476715_FASTA.fasta	MGYA00139764	ERZ476715	root:Host-associated:Human:Digestive system:Large in	0.5058
MGYA00139765/ERZ476704_FASTA.fasta	MGYA00139765	ERZ476704	root:Host-associated:Human:Digestive system:Large in	0.4564
MGYA00139766/ERZ476695_FASTA.fasta	MGYA00139766	ERZ476695	root:Host-associated:Human:Digestive system:Large in	0.4782
MGYA00139767/ERZ476527_FASTA.fasta	MGYA00139767	ERZ476527	root:Host-associated:Human:Digestive system:Large in	0.4042
MGYA00139768/ERZ476494_FASTA.fasta	MGYA00139768	ERZ476494	root:Host-associated:Human:Digestive system:Large in	0.4466
MGYA00139769/ERZ476698_FASTA.fasta	MGYA00139769	ERZ476698	root:Host-associated:Human:Digestive system:Large in	0.4946
MGYA00139770/ERZ476666_FASTA.fasta	MGYA00139770	ERZ476666	root:Host-associated:Human:Digestive system:Large in	0.2992
MGYA00139771/ERZ476647_FASTA.fasta	MGYA00139771	ERZ476647	root:Host-associated:Human:Digestive system:Large in	0.4878
MGYA00139772/ERZ476612_FASTA.fasta	MGYA00139772	ERZ476612	root:Host-associated:Human:Digestive system:Large in	0.4198
MGYA00139773/ERZ476565_FASTA.fasta	MGYA00139773	ERZ476565	root:Host-associated:Human:Digestive system:Large in	0.4674
MGYA00139774/ERZ476696_FASTA.fasta	MGYA00139774	ERZ476696	root:Host-associated:Human:Digestive system:Large in	0.5032
MGYA00139775/ERZ476640_FASTA.fasta	MGYA00139775	ERZ476640	root:Host-associated:Human:Digestive system:Large in	0.4684
MGYA00139776/ERZ476516_FASTA.fasta	MGYA00139776	ERZ476516	root:Host-associated:Human:Digestive system:Large in	0.3142
MGYA00139777/ERZ476448_FASTA.fasta	MGYA00139777	ERZ476448	root:Host-associated:Human:Digestive system:Large in	0.48
MGYA00139778/ERZ476559_FASTA.fasta	MGYA00139778	ERZ476559	root:Host-associated:Human:Digestive system:Large in	0.3674
MGYA00139779/ERZ476479_FASTA.fasta	MGYA00139779	ERZ476479	root:Host-associated:Human:Digestive system:Large in	0.2998
MGYA00139780/ERZ476464_FASTA.fasta	MGYA00139780	ERZ476464	root:Host-associated:Human:Digestive system:Large in	0.4598
MGYA00139781/ERZ476672_FASTA.fasta	MGYA00139781	ERZ476672	root:Host-associated:Human:Digestive system:Large in	0.3614
MGYA00139782/ERZ476720_FASTA.fasta	MGYA00139782	ERZ476720	root:Host-associated:Human:Digestive system:Large in	0.4686
MGYA00139783/ERZ476593_FASTA.fasta	MGYA00139783	ERZ476593	root:Host-associated:Human:Digestive system:Large in	0.391
MGYA00139784/ERZ476541_FASTA.fasta	MGYA00139784	ERZ476541	root:Host-associated:Human:Digestive system:Large in	0.414
[illegible]	[illegible]	[illegible]	[illegible]	[illegible]

MGYA00153755/OFNQ01_FASTA.fasta	MGYA00153755	ERZ484820	root:Engineered:Bioremediation:Hydrocarbon	0.0888
MGYA00153756/OFNT01_FASTA.fasta	MGYA00153756	ERZ484822	root:Engineered:Bioremediation:Hydrocarbon	0.032
MGYA00153757/OFOP01_FASTA.fasta	MGYA00153757	ERZ484849	root:Engineered:Bioremediation:Hydrocarbon	0.0316
MGYA00153758/OFPG01_FASTA.fasta	MGYA00153758	ERZ484859	root:Engineered:Bioremediation:Hydrocarbon	0.4816
MGYA00153759/OFPB01_FASTA.fasta	MGYA00153759	ERZ484862	root:Engineered:Bioremediation:Hydrocarbon	0.4336
MGYA00153760/OFNH01_FASTA.fasta	MGYA00153760	ERZ484809	root:Engineered:Bioremediation:Hydrocarbon	0.444
MGYA00153761/OFMZ01_FASTA.fasta	MGYA00153761	ERZ484802	root:Engineered:Bioremediation:Hydrocarbon	0.1242
MGYA00153762/OFSK01_FASTA.fasta	MGYA00153762	ERZ484818	root:Engineered:Bioremediation:Hydrocarbon	0.0294
MGYA00153763/OFOT01_FASTA.fasta	MGYA00153763	ERZ484851	root:Engineered:Bioremediation:Hydrocarbon	0.1056
MGYA00153764/OFNZ01_FASTA.fasta	MGYA00153764	ERZ484835	root:Engineered:Bioremediation:Hydrocarbon	0.0574
MGYA00153765/OFNM01_FASTA.fasta	MGYA00153765	ERZ484815	root:Engineered:Bioremediation:Hydrocarbon	0.103
MGYA00153766/OFOV01_FASTA.fasta	MGYA00153766	ERZ484847	root:Engineered:Bioremediation:Hydrocarbon	0.1468
MGYA00153767/OFRP01_FASTA.fasta	MGYA00153767	ERZ484812	root:Engineered:Bioremediation:Hydrocarbon	0.036
MGYA00153768/OFOJ01_FASTA.fasta	MGYA00153768	ERZ484837	root:Engineered:Bioremediation:Hydrocarbon	0.0964
MGYA00153769/OFOL01_FASTA.fasta	MGYA00153769	ERZ484843	root:Engineered:Bioremediation:Hydrocarbon	0.0946
MGYA00153770/OFSJ01_FASTA.fasta	MGYA00153770	ERZ484825	root:Engineered:Bioremediation:Hydrocarbon	0.0294
MGYA00153771/OFOO01_FASTA.fasta	MGYA00153771	ERZ484846	root:Engineered:Bioremediation:Hydrocarbon	0.0344
MGYA00153772/OFNP01_FASTA.fasta	MGYA00153772	ERZ484823	root:Engineered:Bioremediation:Hydrocarbon	0.0316
MGYA00153773/OFOX01_FASTA.fasta	MGYA00153773	ERZ484854	root:Engineered:Bioremediation:Hydrocarbon	0.036
MGYA00153774/OFND01_FASTA.fasta	MGYA00153774	ERZ484808	root:Engineered:Bioremediation:Hydrocarbon	0.0294
MGYA00153775/OFPF01_FASTA.fasta	MGYA00153775	ERZ484864	root:Engineered:Bioremediation:Hydrocarbon	0.1282
MGYA00153776/OFMW01_FASTA.fasta	MGYA00153776	ERZ484801	root:Engineered:Bioremediation:Hydrocarbon	0.0782
MGYA00153777/OFNN01_FASTA.fasta	MGYA00153777	ERZ484814	root:Engineered:Bioremediation:Hydrocarbon	0.1534
MGYA00153778/OFPI01_FASTA.fasta	MGYA00153778	ERZ484870	root:Engineered:Bioremediation:Hydrocarbon	0.4402
MGYA00153779/OFOE01_FASTA.fasta	MGYA00153779	ERZ484841	root:Engineered:Bioremediation:Hydrocarbon	0.032
MGYA00153780/OFPP01_FASTA.fasta	MGYA00153780	ERZ484875	root:Engineered:Bioremediation:Hydrocarbon	0.226
MGYA00153781/OFPJ01_FASTA.fasta	MGYA00153781	ERZ484869	root:Engineered:Bioremediation:Hydrocarbon	0.4492
MGYA00153782/OFPD01_FASTA.fasta	MGYA00153782	ERZ484861	root:Engineered:Bioremediation:Hydrocarbon	0.391
MGYA00153783/OFNG01_FASTA.fasta	MGYA00153783	ERZ484810	root:Engineered:Bioremediation:Hydrocarbon	0.0918
MGYA00153784/OFOI01_FASTA.fasta	MGYA00153784	ERZ484844	root:Engineered:Bioremediation:Hydrocarbon	0.1206
MGYA00153785/OFNC01_FASTA.fasta	MGYA00153785	ERZ484806	root:Engineered:Bioremediation:Hydrocarbon	0.0492
MGYA00153786/OFOY01_FASTA.fasta	MGYA00153786	ERZ484858	root:Engineered:Bioremediation:Hydrocarbon	0.4486
MGYA00153787/OFOR01_FASTA.fasta	MGYA00153787	ERZ484853	root:Engineered:Bioremediation:Hydrocarbon	0.0552
MGYA00153788/OFNV01_FASTA.fasta	MGYA00153788	ERZ484830	root:Engineered:Bioremediation:Hydrocarbon	0.0294
MGYA00153789/OFOD01_FASTA.fasta	MGYA00153789	ERZ484838	root:Engineered:Bioremediation:Hydrocarbon	0.0294
MGYA00153790/OFSI01_FASTA.fasta	MGYA00153790	ERZ484832	root:Engineered:Bioremediation:Hydrocarbon	0.0294
MGYA00153791/OFOC01_FASTA.fasta	MGYA00153791	ERZ484836	root:Engineered:Bioremediation:Hydrocarbon	0.0644
MGYA00153792/OFPC01_FASTA.fasta	MGYA00153792	ERZ484863	root:Engineered:Bioremediation:Hydrocarbon	0.1192
MGYA00153793/OFNI01_FASTA.fasta	MGYA00153793	ERZ484813	root:Engineered:Bioremediation:Hydrocarbon	0.0298
MGYA00153794/OFPM01_FASTA.fasta	MGYA00153794	ERZ484867	root:Engineered:Bioremediation:Hydrocarbon	0.25
MGYA00153795/OFPN01_FASTA.fasta	MGYA00153795	ERZ484872	root:Engineered:Bioremediation:Hydrocarbon	0.4612
MGYA00153796/OFOA01_FASTA.fasta	MGYA00153796	ERZ484834	root:Engineered:Bioremediation:Hydrocarbon	0.032
MGYA00153797/OFPH01_FASTA.fasta	MGYA00153797	ERZ484866	root:Engineered:Bioremediation:Hydrocarbon	0.434
MGYA00153798/OFNF01_FASTA.fasta	MGYA00153798	ERZ484811	root:Engineered:Bioremediation:Hydrocarbon	0.059
MGYA00153799/OFPK01_FASTA.fasta	MGYA00153799	ERZ484871	root:Engineered:Bioremediation:Hydrocarbon	0.4832
MGYA00153800/OFOQ01_FASTA.fasta	MGYA00153800	ERZ484850	root:Engineered:Bioremediation:Hydrocarbon	0.0316
MGYA00153801/OFNE01_FASTA.fasta	MGYA00153801	ERZ484805	root:Engineered:Bioremediation:Hydrocarbon	0.2046
MGYA00153802/OFON01_FASTA.fasta	MGYA00153802	ERZ484848	root:Engineered:Bioremediation:Hydrocarbon	0.0294
MGYA00153803/OFNY01_FASTA.fasta	MGYA00153803	ERZ484828	root:Engineered:Bioremediation:Hydrocarbon	0.1354
MGYA00153804/OFPR01_FASTA.fasta	MGYA00153804	ERZ484873	root:Engineered:Bioremediation:Hydrocarbon	0.4164
MGYA00153805/OFOF01_FASTA.fasta	MGYA00153805	ERZ484839	root:Engineered:Bioremediation:Hydrocarbon	0.0316
MGYA00153806/OFOH01_FASTA.fasta	MGYA00153806	ERZ484842	root:Engineered:Bioremediation:Hydrocarbon	0.0954
MGYA00153807/OFPA01_FASTA.fasta	MGYA00153807	ERZ484860	root:Engineered:Bioremediation:Hydrocarbon	0.4514
MGYA00153808/OFSH01_FASTA.fasta	MGYA00153808	ERZ484865	root:Engineered:Bioremediation:Hydrocarbon	0.0294
MGYA00153809/OFNR01_FASTA.fasta	MGYA00153809	ERZ484827	root:Engineered:Bioremediation:Hydrocarbon	0.0372
MGYA00153810/OFSL01_FASTA.fasta	MGYA00153810	ERZ484874	root:Engineered:Bioremediation:Hydrocarbon	0.0294
MGYA00153811/OFNL01_FASTA.fasta	MGYA00153811	ERZ484816	root:Engineered:Bioremediation:Hydrocarbon	0.0666
MGYA00153812/OFNB01_FASTA.fasta	MGYA00153812	ERZ484807	root:Engineered:Bioremediation:Hydrocarbon	0.0596
MGYA00153813/OFNJ01_FASTA.fasta	MGYA00153813	ERZ484817	root:Engineered:Bioremediation:Hydrocarbon	0.0294
MGYA00153814/OFNW01_FASTA.fasta	MGYA00153814	ERZ484831	root:Engineered:Bioremediation:Hydrocarbon	0.0314
MGYA00153815/OFOB01_FASTA.fasta	MGYA00153815	ERZ484833	root:Engineered:Bioremediation:Hydrocarbon	0.0598
MGYA00153816/OFNU01_FASTA.fasta	MGYA00153816	ERZ484824	root:Engineered:Bioremediation:Hydrocarbon	0.0318
MGYA00153817/OFOM01_FASTA.fasta	MGYA00153817	ERZ484845	root:Engineered:Bioremediation:Hydrocarbon	0.0716
MGYA00153818/OFMY01_FASTA.fasta	MGYA00153818	ERZ484803	root:Engineered:Bioremediation:Hydrocarbon	0.0324
MGYA00153819/OFNK01_FASTA.fasta	MGYA00153819	ERZ484819	root:Engineered:Bioremediation:Hydrocarbon	0.0328
MGYA00153820/OFOG01_FASTA.fasta	MGYA00153820	ERZ484840	root:Engineered:Bioremediation:Hydrocarbon	0.0376
MGYA00153821/OFOW01_FASTA.fasta	MGYA00153821	ERZ484852	root:Engineered:Bioremediation:Hydrocarbon	0.422
MGYA00153822/OFPE01_FASTA.fasta	MGYA00153822	ERZ484856	root:Engineered:Bioremediation:Hydrocarbon	0.415
MGYA00153823/OFNS01_FASTA.fasta	MGYA00153823	ERZ484826	root:Engineered:Bioremediation:Hydrocarbon	0.063
MGYA00153824/OFNA01_FASTA.fasta	MGYA00153824	ERZ484804	root:Engineered:Bioremediation:Hydrocarbon	0.0812
MGYA00153825/OFOZ01_FASTA.fasta	MGYA00153825	ERZ484857	root:Engineered:Bioremediation:Hydrocarbon	0.4504
MGYA00153826/OFNX01_FASTA.fasta	MGYA00153826	ERZ484829	root:Engineered:Bioremediation:Hydrocarbon	0.0978
MGYA00153827/OFPL01_FASTA.fasta	MGYA00153827	ERZ484868	root:Engineered:Bioremediation:Hydrocarbon	0.496
MGYA00153828/OFOS01_FASTA.fasta	MGYA00153828	ERZ484855	root:Engineered:Bioremediation:Hydrocarbon	0.4698
MGYA00153829/OFNO01_FASTA.fasta	MGYA00153829	ERZ484821	root:Engineered:Bioremediation:Hydrocarbon	0.0298
MGYA00153830/OFQE01_FASTA.fasta	MGYA00153830	ERZ484894	root:Environmental:Aquatic:Freshwater:Sediment	0.156
MGYA00153831/OFSB01_FASTA.fasta	MGYA00153831	ERZ484901	root:Environmental:Aquatic:Freshwater:Sediment	0.47
MGYA00153832/OFQN01_FASTA.fasta	MGYA00153832	ERZ484892	root:Environmental:Aquatic:Freshwater:Sediment	0.4424
MGYA00153833/OFRR01_FASTA.fasta	MGYA00153833	ERZ484885	root:Environmental:Aquatic:Freshwater:Sediment	0.0794
MGYA00153834/OFPY01_FASTA.fasta	MGYA00153834	ERZ484891	root:Environmental:Aquatic:Freshwater:Sediment	0.03
MGYA00153835/OFQK01_FASTA.fasta	MGYA00153835	ERZ484900	root:Environmental:Aquatic:Freshwater:Sediment	0.316
MGYA00153836/OFQC01_FASTA.fasta	MGYA00153836	ERZ484887	root:Environmental:Aquatic:Freshwater:Sediment	0.3948
MGYA00153837/OFRU01_FASTA.fasta	MGYA00153837	ERZ484895	root:Environmental:Aquatic:Freshwater:Sediment	0.261
MGYA00153838/OFQH01_FASTA.fasta	MGYA00153838	ERZ484904	root:Environmental:Aquatic:Freshwater:Sediment	0.0556
MGYA00153839/OFRS01_FASTA.fasta	MGYA00153839	ERZ484886	root:Environmental:Aquatic:Freshwater:Sediment	0.2464
MGYA00153840/OFSA01_FASTA.fasta	MGYA00153840	ERZ484899	root:Environmental:Aquatic:Freshwater:Sediment	0.3148
MGYA00153841/OFQB01_FASTA.fasta	MGYA00153841	ERZ484879	root:Environmental:Aquatic:Freshwater:Sediment	0.297
MGYA00153842/OFPT01_FASTA.fasta	MGYA00153842	ERZ484882	root:Environmental:Aquatic:Freshwater:Sediment	0.0342
MGYA00153843/OFRV01_FASTA.fasta	MGYA00153843	ERZ484896	root:Environmental:Aquatic:Freshwater:Sediment	0.289
MGYA00153844/OFQS01_FASTA.fasta	MGYA00153844	ERZ484902	root:Environmental:Aquatic:Freshwater:Sediment	0.5198
MGYA00153845/OFQD01_FASTA.fasta	MGYA00153845	ERZ484883	root:Environmental:Aquatic:Freshwater:Sediment	0.3004
MGYA00153846/OFQG01_FASTA.fasta	MGYA00153846	ERZ484898	root:Environmental:Aquatic:Freshwater:Sediment	0.1656
MGYA00153847/OFQI01_FASTA.fasta	MGYA00153847	ERZ484906	root:Environmental:Aquatic:Freshwater:Sediment	0.0376
MGYA00153848/OFQR01_FASTA.fasta	MGYA00153848	ERZ484903	root:Environmental:Aquatic:Freshwater:Sediment	0.3594
MGYA00153849/OFQP01_FASTA.fasta	MGYA00153849	ERZ484889	root:Environmental:Aquatic:Freshwater:Sediment	0.4696
MGYA00153850/OFQL01_FASTA.fasta	MGYA00153850	ERZ484890	root:Environmental:Aquatic:Freshwater:Sediment	0.469
MGYA00153851/OFPV01_FASTA.fasta	MGYA00153851	ERZ484881	root:Environmental:Aquatic:Freshwater:Sediment	0.0294
MGYA00153852/OFPZ01_FASTA.fasta	MGYA00153852	ERZ484884	root:Environmental:Aquatic:Freshwater:Sediment	0.1686
MGYA00153853/OFPU01_FASTA.fasta	MGYA00153853	ERZ484880	root:Environmental:Aquatic:Freshwater:Sediment	0.0538
MGYA00153854/OFQA01_FASTA.fasta	MGYA00153854	ERZ484893	root:Environmental:Aquatic:Freshwater:Sediment	0.0892
MGYA00153855/OFQM01_FASTA.fasta	MGYA00153855	ERZ484907	root:Environmental:Aquatic:Freshwater:Sediment	0.1282
MGYA00153856/OFQT01_FASTA.fasta	MGYA00153856	ERZ484909	root:Environmental:Aquatic:Freshwater:Sediment	0.4608
MGYA00153857/OFPW01_FASTA.fasta	MGYA00153857	ERZ484878	root:Environmental:Aquatic:Freshwater:Sediment	0.3144
MGYA00153858/OFQJ01_FASTA.fasta	MGYA00153858	ERZ484908	root:Environmental:Aquatic:Freshwater:Sediment	0.0294
MGYA00153859/OFQO01_FASTA.fasta	MGYA00153859	ERZ484905	root:Environmental:Aquatic:Freshwater:Sediment	0.4336
MGYA00153860/OFPX01_FASTA.fasta	MGYA00153860	ERZ484888	root:Environmental:Aquatic:Freshwater:Sediment	0.0698
MGYA00153861/OFQF01_FASTA.fasta	MGYA00153861	ERZ484897	root:Environmental:Aquatic:Freshwater:Sediment	0.0294
MGYA00153862/OFQW01_FASTA.fasta	MGYA00153862	ERZ484910	root:Engineered:Wastewater:Water and sludge	0.3762
MGYA00153863/OFRG01_FASTA.fasta	MGYA00153863	ERZ484925	root:Engineered:Wastewater:Water and sludge	0.1182
MGYA00153864/OFRI01_FASTA.fasta	MGYA00153864	ERZ484922	root:Engineered:Wastewater:Water and sludge	0.891
MGYA00153865/OFRB01_FASTA.fasta	MGYA00153865	ERZ484919	root:Engineered:Wastewater:Water and sludge	0.5328
MGYA00153866/OFQU01_FASTA.fasta	MGYA00153866	ERZ484915	root:Engineered:Wastewater:Water and sludge	0.5534
MGYA00153867/OFRD01_FASTA.fasta	MGYA00153867	ERZ484920	root:Engineered:Wastewater:Water and sludge	0.7774
MGYA00153868/OFRC01_FASTA.fasta	MGYA00153868	ERZ484921	root:Engineered:Wastewater:Water and sludge	0.544
MGYA00153869/OFRA01_FASTA.fasta	MGYA00153869	ERZ484917	root:Engineered:Wastewater:Water and sludge	0.5732
MGYA00153870/OFRH01_FASTA.fasta	MGYA00153870	ERZ484930	root:Engineered:Wastewater:Water and sludge	0.7856
MGYA00153871/OFQZ01_FASTA.fasta	MGYA00153871	ERZ484918	root:Engineered:Wastewater:Water and sludge	0.4724
MGYA00153872/OFSE01_FASTA.fasta	MGYA00153872	ERZ484914	root:Engineered:Wastewater:Water and sludge	0.7448
MGYA00153873/OFQX01_FASTA.fasta	MGYA00153873	ERZ484911	root:Engineered:Wastewater:Water and sludge	0.3014
MGYA00153874/OFQV01_FASTA.fasta	MGYA00153874	ERZ484913	root:Engineered:Wastewater:Water and sludge	0.5266
MGYA00153875/OFRO01_FASTA.fasta	MGYA00153875	ERZ484927	root:Engineered:Wastewater:Water and sludge	0.4338
MGYA00153876/OFRL01_FASTA.fasta	MGYA00153876	ERZ484929	root:Engineered:Wastewater:Water and sludge	0.5408
MGYA00153877/OFRM01_FASTA.fasta	MGYA00153877	ERZ484928	root:Engineered:Wastewater:Water and sludge	0.4516
MGYA00153878/OFRE01_FASTA.fasta	MGYA00153878	ERZ484923	root:Engineered:Wastewater:Water and sludge	0.3972
MGYA00153879/OFQY01_FASTA.fasta	MGYA00153879	ERZ484916	root:Engineered:Wastewater:Water and sludge	0.4206
MGYA00153880/OFSF01_FASTA.fasta	MGYA00153880	ERZ484924	root:Engineered:Wastewater:Water and sludge	0.7502
MGYA00153881/OFRF01_FASTA.fasta	MGYA00153881	ERZ484926	root:Engineered:Wastewater:Water and sludge	0.534
MGYA00153882/OFQQ01_FASTA.fasta	MGYA00153882	ERZ484912	root:Engineered:Wastewater:Water and sludge	0.1418
MGYA00153883/OGCH01_FASTA.fasta	MGYA00153883	ERZ485336	root:Environmental:Aquatic:Thermal springs:Hot (42-90	0.4902
MGYA00153884/OGCB01_FASTA.fasta	MGYA00153884	ERZ485337	root:Environmental:Aquatic:Thermal springs:Hot (42-90	0.3872
MGYA00153885/OGCK01_FASTA.fasta	MGYA00153885	ERZ485338	root:Environmental:Aquatic:Thermal springs:Hot (42-90	0.7936
MGYA00153886/OGCI01_FASTA.fasta	MGYA00153886	ERZ485340	root:Environmental:Aquatic:Thermal springs:Hot (42-90	0.7126
MGYA00153887/OGBZ01_FASTA.fasta	MGYA00153887	ERZ485341	root:Environmental:Aquatic:Thermal springs:Hot (42-90	0.233
MGYA00153888/OGCE01_FASTA.fasta	MGYA00153888	ERZ485342	root:Environmental:Aquatic:Thermal springs:Hot (42-90	0.4072
MGYA00153889/OGCG01_FASTA.fasta	MGYA00153889	ERZ485343	root:Environmental:Aquatic:Thermal springs:Hot (42-90	0.5034
MGYA00153890/OGCA01_FASTA.fasta	MGYA00153890	ERZ485344	root:Environmental:Aquatic:Thermal springs:Hot (42-90	0.3252
MGYA00153891/OGDF01_FASTA.fasta	MGYA00153891	ERZ485444	root:Host-associated:Human:Digestive system:Large in	0.514
MGYA00153892/OGCQ01_FASTA.fasta	MGYA00153892	ERZ485447	root:Host-associated:Human:Digestive system:Large in	0.5216
MGYA00153893/OGCN01_FASTA.fasta	MGYA00153893	ERZ485443	root:Host-associated:Human:Digestive system:Large in	0.5068
MGYA00153894/OGDN01_FASTA.fasta	MGYA00153894	ERZ485448	root:Host-associated:Human:Digestive system:Large in	0.5008
MGYA00153895/OGCO01_FASTA.fasta	MGYA00153895	ERZ485445	root:Host-associated:Human:Digestive system:Large in	0.4608
MGYA00153896/OGCM01_FASTA.fasta	MGYA00153896	ERZ485446	root:Host-associated:Human:Digestive system:Large in	0.4272
MGYA00153897/OGVY01_FASTA.fasta	MGYA00153897	ERZ486668	root:Environmental:Aquatic:Freshwater	0.342
MGYA00153898/OGVL01_FASTA.fasta	MGYA00153898	ERZ486653	root:Environmental:Aquatic:Freshwater	0.1654
MGYA00153899/OGWL01_FASTA.fasta	MGYA00153899	ERZ486683	root:Environmental:Aquatic:Freshwater	0.3082
MGYA00153900/OGVT01_FASTA.fasta	MGYA00153900	ERZ486666	root:Environmental:Aquatic:Freshwater	0.505
MGYA00153901/OGVM01_FASTA.fasta	MGYA00153901	ERZ486656	root:Environmental:Aquatic:Freshwater	0.334
MGYA00153902/OGVJ01_FASTA.fasta	MGYA00153902	ERZ486660	root:Environmental:Aquatic:Freshwater	0.539
MGYA00153903/OGVZ01_FASTA.fasta	MGYA00153903	ERZ486667	root:Environmental:Aquatic:Freshwater	0.4024
MGYA00153904/OGVG01_FASTA.fasta	MGYA00153904	ERZ486652	root:Environmental:Aquatic:Freshwater	0.2374
MGYA00153905/OKRQ01_FASTA.fasta	MGYA00153905	ERZ486650	root:Environmental:Aquatic:Freshwater	0.3676
MGYA00153906/OGWD01_FASTA.fasta	MGYA00153906	ERZ486681	root:Environmental:Aquatic:Freshwater	0.0444
MGYA00153907/OGVQ01_FASTA.fasta	MGYA00153907	ERZ486659	root:Environmental:Aquatic:Freshwater	0.215
MGYA00153908/OGVN01_FASTA.fasta	MGYA00153908	ERZ486657	root:Environmental:Aquatic:Freshwater	0.525
MGYA00153909/OGVW01_FASTA.fasta	MGYA00153909	ERZ486658	root:Environmental:Aquatic:Freshwater	0.5788
MGYA00153910/OGVR01_FASTA.fasta	MGYA00153910	ERZ486665	root:Environmental:Aquatic:Freshwater	0.603
MGYA00153911/OGWJ01_FASTA.fasta	MGYA00153911	ERZ486670	root:Environmental:Aquatic:Freshwater	0.3296
MGYA00153912/OGWK01_FASTA.fasta	MGYA00153912	ERZ486677	root:Environmental:Aquatic:Freshwater	0.2788
MGYA00153913/OGVP01_FASTA.fasta	MGYA00153913	ERZ486655	root:Environmental:Aquatic:Freshwater	0.3336
MGYA00153914/OGWM01_FASTA.fasta	MGYA00153914	ERZ486684	root:Environmental:Aquatic:Freshwater	0.3774
MGYA00153915/OGWP01_FASTA.fasta	MGYA00153915	ERZ486685	root:Environmental:Aquatic:Freshwater	0.163
MGYA00153916/OGWE01_FASTA.fasta	MGYA00153916	ERZ486674	root:Environmental:Aquatic:Freshwater	0.2558
MGYA00153917/OGWN01_FASTA.fasta	MGYA00153917	ERZ486671	root:Environmental:Aquatic:Freshwater	0.3114
MGYA00153918/OGWC01_FASTA.fasta	MGYA00153918	ERZ486675	root:Environmental:Aquatic:Freshwater	0.282
MGYA00153919/OGVU01_FASTA.fasta	MGYA00153919	ERZ486662	root:Environmental:Aquatic:Freshwater	0.5576
MGYA00153920/OGWG01_FASTA.fasta	MGYA00153920	ERZ486676	root:Environmental:Aquatic:Freshwater	0.3138
MGYA00153921/OGWO01_FASTA.fasta	MGYA00153921	ERZ486682	root:Environmental:Aquatic:Freshwater	0.5312
MGYA00153922/OGWB01_FASTA.fasta	MGYA00153922	ERZ486672	root:Environmental:Aquatic:Freshwater	0.0632
MGYA00153923/OGVK01_FASTA.fasta	MGYA00153923	ERZ486649	root:Environmental:Aquatic:Freshwater	0.6784
MGYA00153924/OGVO01_FASTA.fasta	MGYA00153924	ERZ486651	root:Environmental:Aquatic:Freshwater	0.586
MGYA00153925/OGVH01_FASTA.fasta	MGYA00153925	ERZ486654	root:Environmental:Aquatic:Freshwater	0.2066
MGYA00153926/OGWA01_FASTA.fasta	MGYA00153926	ERZ486673	root:Environmental:Aquatic:Freshwater	0.047
MGYA00153927/OGVS01_FASTA.fasta	MGYA00153927	ERZ486664	root:Environmental:Aquatic:Freshwater	0.2512
MGYA00153928/OGVV01_FASTA.fasta	MGYA00153928	ERZ486669	root:Environmental:Aquatic:Freshwater	0.5978
MGYA00153929/OGWF01_FASTA.fasta	MGYA00153929	ERZ486678	root:Environmental:Aquatic:Freshwater	0.2462
MGYA00153930/OGWH01_FASTA.fasta	MGYA00153930	ERZ486680	root:Environmental:Aquatic:Freshwater	0.2624
MGYA00153931/OGWI01_FASTA.fasta	MGYA00153931	ERZ486679	root:Environmental:Aquatic:Freshwater	0.2772
MGYA00153932/OGVX01_FASTA.fasta	MGYA00153932	ERZ486663	root:Environmental:Aquatic:Freshwater	0.3048
MGYA00153933/OGVI01_FASTA.fasta	MGYA00153933	ERZ486661	root:Environmental:Aquatic:Freshwater	0.3914
MGYA00153934/OFEI01_FASTA.fasta	MGYA00153934	ERZ483294	root:Host-associated:Insecta	0.2958
MGYA00153935/OFEL01_FASTA.fasta	MGYA00153935	ERZ483293	root:Host-associated:Insecta	0.2086
MGYA00154943/OJNX01_FASTA.fasta	MGYA00154943	ERZ490643	root:Host-associated:Human:Digestive system	0.397
MGYA00154952/OJOK01_FASTA.fasta	MGYA00154952	ERZ490647	root:Host-associated:Human:Digestive system	0.3942
MGYA00154957/OJNV01_FASTA.fasta	MGYA00154957	ERZ490639	root:Host-associated:Human:Digestive system	0.3724
MGYA00154961/OJNW01_FASTA.fasta	MGYA00154961	ERZ490637	root:Host-associated:Human:Digestive system	0.3992
MGYA00154962/OJOL01_FASTA.fasta	MGYA00154962	ERZ490649	root:Host-associated:Human:Digestive system	0.4382
MGYA00154967/OJNN01_FASTA.fasta	MGYA00154967	ERZ490631	root:Host-associated:Human:Digestive system	0.31
MGYA00154969/OJOP01_FASTA.fasta	MGYA00154969	ERZ490650	root:Host-associated:Human:Digestive system	0.4138
MGYA00154981/OJOO01_FASTA.fasta	MGYA00154981	ERZ490645	root:Host-associated:Human:Digestive system	0.379
MGYA00154986/OJNP01_FASTA.fasta	MGYA00154986	ERZ490633	root:Host-associated:Human:Digestive system	0.3838
MGYA00154990/OJOI01_FASTA.fasta	MGYA00154990	ERZ490648	root:Host-associated:Human:Digestive system	0.3524
MGYA00154991/OJNS01_FASTA.fasta	MGYA00154991	ERZ490634	root:Host-associated:Human:Digestive system	0.412
MGYA00154993/OLJA01_FASTA.fasta	MGYA00154993	ERZ490636	root:Host-associated:Human:Digestive system	0.4334
MGYA00154999/OJNZ01_FASTA.fasta	MGYA00154999	ERZ490642	root:Host-associated:Human:Digestive system	0.3532
MGYA00155000/OJOA01_FASTA.fasta	MGYA00155000	ERZ490635	root:Host-associated:Human:Digestive system	0.37
MGYA00155001/OJNU01_FASTA.fasta	MGYA00155001	ERZ490638	root:Host-associated:Human:Digestive system	0.4038
MGYA00155005/OJOF01_FASTA.fasta	MGYA00155005	ERZ490646	root:Host-associated:Human:Digestive system	0.4384
MGYA00155010/OJOJ01_FASTA.fasta	MGYA00155010	ERZ490651	root:Host-associated:Human:Digestive system	0.4106
MGYA00155011/OJNT01_FASTA.fasta	MGYA00155011	ERZ490640	root:Host-associated:Human:Digestive system	0.4384
MGYA00155013/OJNY01_FASTA.fasta	MGYA00155013	ERZ490641	root:Host-associated:Human:Digestive system	0.4734
MGYA00155014/OLJB01_FASTA.fasta	MGYA00155014	ERZ490632	root:Host-associated:Human:Digestive system	0.3676
MGYA00155015/OJON01_FASTA.fasta	MGYA00155015	ERZ490644	root:Host-associated:Human:Digestive system	0.3868
MGYA00155016/OJNR01_FASTA.fasta	MGYA00155016	ERZ490630	root:Host-associated:Human:Digestive system	0.3158
MGYA00155069/OLKG01_FASTA.fasta	MGYA00155069	ERZ490752	root:Host-associated:Human:Digestive system	0.47
MGYA00155071/OJSK01_FASTA.fasta	MGYA00155071	ERZ490756	root:Host-associated:Human:Digestive system	0.4718
MGYA00155072/OJRX01_FASTA.fasta	MGYA00155072	ERZ490747	root:Host-associated:Human:Digestive system	0.4418
MGYA00155073/OJRV01_FASTA.fasta	MGYA00155073	ERZ490745	root:Host-associated:Human:Digestive system	0.454
MGYA00155074/OJSM01_FASTA.fasta	MGYA00155074	ERZ490762	root:Host-associated:Human:Digestive system:Large in	0.5112
MGYA00155075/OLIY01_FASTA.fasta	MGYA00155075	ERZ490764	root:Host-associated:Human:Digestive system:Large in	0.4644
MGYA00155076/OJSF01_FASTA.fasta	MGYA00155076	ERZ490760	root:Host-associated:Human:Digestive system	0.5022
MGYA00155078/OJSJ01_FASTA.fasta	MGYA00155078	ERZ490759	root:Host-associated:Human:Digestive system	0.4718
MGYA00155079/OJSN01_FASTA.fasta	MGYA00155079	ERZ490763	root:Host-associated:Human:Digestive system:Large in	0.4792
MGYA00155081/OJSQ01_FASTA.fasta	MGYA00155081	ERZ490767	root:Host-associated:Human:Digestive system:Large in	0.5206
MGYA00155082/OJSD01_FASTA.fasta	MGYA00155082	ERZ490749	root:Host-associated:Human:Digestive system	0.45
MGYA00155083/OJUG01_FASTA.fasta	MGYA00155083	ERZ490768	root:Host-associated:Human:Digestive system:Large in	0.517
MGYA00155084/OJSA01_FASTA.fasta	MGYA00155084	ERZ490754	root:Host-associated:Human:Digestive system	0.477
MGYA00155085/OJRZ01_FASTA.fasta	MGYA00155085	ERZ490750	root:Host-associated:Human:Digestive system	0.4736
MGYA00155086/OJRY01_FASTA.fasta	MGYA00155086	ERZ490751	root:Host-associated:Human:Digestive system	0.4876
MGYA00155088/OJSL01_FASTA.fasta	MGYA00155088	ERZ490755	root:Host-associated:Human:Digestive system	0.482
MGYA00155089/OJSE01_FASTA.fasta	MGYA00155089	ERZ490748	root:Host-associated:Human:Digestive system	0.4736
MGYA00155090/OJSG01_FASTA.fasta	MGYA00155090	ERZ490757	root:Host-associated:Human:Digestive system	0.4498
MGYA00155091/OJSH01_FASTA.fasta	MGYA00155091	ERZ490761	root:Host-associated:Human:Digestive system	0.4988
MGYA00155094/OJSB01_FASTA.fasta	MGYA00155094	ERZ490753	root:Host-associated:Human:Digestive system	0.4706
MGYA00155096/OJSP01_FASTA.fasta	MGYA00155096	ERZ490766	root:Host-associated:Human:Digestive system:Large in	0.515
MGYA00155099/OJSO01_FASTA.fasta	MGYA00155099	ERZ490765	root:Host-associated:Human:Digestive system:Large in	0.4762
MGYA00155101/OJRW01_FASTA.fasta	MGYA00155101	ERZ490744	root:Host-associated:Human:Digestive system	0.435
MGYA00155102/OJSC01_FASTA.fasta	MGYA00155102	ERZ490746	root:Host-associated:Human:Digestive system	0.4504
MGYA00155105/OLJW01_FASTA.fasta	MGYA00155105	ERZ490769	root:Host-associated:Human:Digestive system:Large in	0.5202
MGYA00155108/OJSI01_FASTA.fasta	MGYA00155108	ERZ490758	root:Host-associated:Human:Digestive system	0.4736
MGYA00155113/OJNO01_FASTA.fasta	MGYA00155113	ERZ490628	root:Host-associated:Human:Digestive system:Large in	0.4212
MGYA00155114/OJNK01_FASTA.fasta	MGYA00155114	ERZ490629	root:Host-associated:Human:Digestive system:Large in	0.3544
MGYA00155120/OFMK01_FASTA.fasta	MGYA00155120	ERZ484748	root:Host-associated:Human:Digestive system:Large in	0.4916
MGYA00155121/OFMH01_FASTA.fasta	MGYA00155121	ERZ484749	root:Host-associated:Human:Digestive system:Large in	0.3608
MGYA00155122/OFMO01_FASTA.fasta	MGYA00155122	ERZ484756	root:Host-associated:Human:Digestive system:Large in	0.3732
MGYA00155123/OFMJ01_FASTA.fasta	MGYA00155123	ERZ484753	root:Host-associated:Human:Digestive system:Large in	0.5134
MGYA00155124/OFLY01_FASTA.fasta	MGYA00155124	ERZ484739	root:Host-associated:Human:Digestive system:Large in	0.379
MGYA00155125/OFMM01_FASTA.fasta	MGYA00155125	ERZ484754	root:Host-associated:Human:Digestive system:Large in	0.3764
MGYA00155126/OFMC01_FASTA.fasta	MGYA00155126	ERZ484741	root:Host-associated:Human:Digestive system:Large in	0.3906
MGYA00155128/OFMF01_FASTA.fasta	MGYA00155128	ERZ484750	root:Host-associated:Human:Digestive system:Large in	0.4576
MGYA00155130/OFLZ01_FASTA.fasta	MGYA00155130	ERZ484742	root:Host-associated:Human:Digestive system:Large in	0.4054
MGYA00155131/OFMT01_FASTA.fasta	MGYA00155131	ERZ484761	root:Host-associated:Human:Digestive system:Large in	0.3778
MGYA00155133/OFMN01_FASTA.fasta	MGYA00155133	ERZ484755	root:Host-associated:Human:Digestive system:Large in	0.4052
MGYA00155134/OFSD01_FASTA.fasta	MGYA00155134	ERZ484746	root:Host-associated:Human:Digestive system:Large in	0.4542
MGYA00155135/OFMP01_FASTA.fasta	MGYA00155135	ERZ484757	root:Host-associated:Human:Digestive system:Large in	0.3904
MGYA00155136/OFMU01_FASTA.fasta	MGYA00155136	ERZ484764	root:Host-associated:Human:Digestive system:Large in	0.4146
MGYA00155137/OFMR01_FASTA.fasta	MGYA00155137	ERZ484759	root:Host-associated:Human:Digestive system:Large in	0.463
MGYA00155138/OFMI01_FASTA.fasta	MGYA00155138	ERZ484752	root:Host-associated:Human:Digestive system:Large in	0.3612
MGYA00155139/OFMS01_FASTA.fasta	MGYA00155139	ERZ484758	root:Host-associated:Human:Digestive system:Large in	0.4412
MGYA00155140/OFRZ01_FASTA.fasta	MGYA00155140	ERZ484743	root:Host-associated:Human:Digestive system:Large in	0.4188
MGYA00155141/OFMD01_FASTA.fasta	MGYA00155141	ERZ484740	root:Host-associated:Human:Digestive system:Large in	0.4034
MGYA00155142/OFOK01_FASTA.fasta	MGYA00155142	ERZ484767	root:Host-associated:Human:Digestive system:Large in	0.3994
MGYA00155143/OFRY01_FASTA.fasta	MGYA00155143	ERZ484744	root:Host-associated:Human:Digestive system:Large in	0.4024
MGYA00155144/OFMQ01_FASTA.fasta	MGYA00155144	ERZ484762	root:Host-associated:Human:Digestive system:Large in	0.388
MGYA00155145/OFSC01_FASTA.fasta	MGYA00155145	ERZ484768	root:Host-associated:Human:Digestive system:Large in	0.4162
MGYA00155146/OFOU01_FASTA.fasta	MGYA00155146	ERZ484766	root:Host-associated:Human:Digestive system:Large in	0.4882
MGYA00155148/OFPS01_FASTA.fasta	MGYA00155148	ERZ484765	root:Host-associated:Human:Digestive system:Large in	0.5212
MGYA00155150/OFMX01_FASTA.fasta	MGYA00155150	ERZ484760	root:Host-associated:Human:Digestive system:Large in	0.4106
MGYA00155151/OFMA01_FASTA.fasta	MGYA00155151	ERZ484737	root:Host-associated:Human:Digestive system:Large in	0.5004
MGYA00155152/OFME01_FASTA.fasta	MGYA00155152	ERZ484745	root:Host-associated:Human:Digestive system:Large in	0.375
MGYA00155153/OFMV01_FASTA.fasta	MGYA00155153	ERZ484763	root:Host-associated:Human:Digestive system:Large in	0.4418
MGYA00155154/OFMB01_FASTA.fasta	MGYA00155154	ERZ484738	root:Host-associated:Human:Digestive system:Large in	0.3972
MGYA00155155/OFML01_FASTA.fasta	MGYA00155155	ERZ484751	root:Host-associated:Human:Digestive system:Large in	0.4946
MGYA00155156/OFMG01_FASTA.fasta	MGYA00155156	ERZ484747	root:Host-associated:Human:Digestive system:Large in	0.5264
MGYA00155165/OGSA01_FASTA.fasta	MGYA00155165	ERZ485871	root:Host-associated:Human:Digestive system:Large in	0.353
MGYA00155166/OGTJ01_FASTA.fasta	MGYA00155166	ERZ485903	root:Host-associated:Human:Digestive system:Large in	0.286
MGYA00155167/OGSB01_FASTA.fasta	MGYA00155167	ERZ485868	root:Host-associated:Human:Digestive system:Large in	0.4472
MGYA00155168/OGTA01_FASTA.fasta	MGYA00155168	ERZ485892	root:Host-associated:Human:Digestive system:Large in	0.4712
MGYA00155169/OGTE01_FASTA.fasta	MGYA00155169	ERZ485894	root:Host-associated:Human:Digestive system:Large in	0.436
MGYA00155170/OGRZ01_FASTA.fasta	MGYA00155170	ERZ485864	root:Host-associated:Human:Digestive system:Large in	0.421
MGYA00155171/OGUE01_FASTA.fasta	MGYA00155171	ERZ485888	root:Host-associated:Human:Digestive system:Large in	0.4792
MGYA00155172/OGSV01_FASTA.fasta	MGYA00155172	ERZ485889	root:Host-associated:Human:Digestive system:Large in	0.4472
MGYA00155173/OGTN01_FASTA.fasta	MGYA00155173	ERZ485899	root:Host-associated:Human:Digestive system:Large in	0.473
MGYA00155174/OGSQ01_FASTA.fasta	MGYA00155174	ERZ485885	root:Host-associated:Human:Digestive system:Large in	0.4514
MGYA00155175/OGSL01_FASTA.fasta	MGYA00155175	ERZ485879	root:Host-associated:Human:Digestive system:Large in	0.4422
MGYA00155176/OGSN01_FASTA.fasta	MGYA00155176	ERZ485878	root:Host-associated:Human:Digestive system:Large in	0.3776
MGYA00155177/OGRV01_FASTA.fasta	MGYA00155177	ERZ485867	root:Host-associated:Human:Digestive system:Large in	0.3882
MGYA00155178/OGTK01_FASTA.fasta	MGYA00155178	ERZ485897	root:Host-associated:Human:Digestive system:Large in	0.413
MGYA00155179/OGSI01_FASTA.fasta	MGYA00155179	ERZ485874	root:Host-associated:Human:Digestive system:Large in	0.4374
MGYA00155180/OGTM01_FASTA.fasta	MGYA00155180	ERZ485901	root:Host-associated:Human:Digestive system:Large in	0.3622
MGYA00155181/OGTR01_FASTA.fasta	MGYA00155181	ERZ485907	root:Host-associated:Human:Digestive system:Large in	0.3352
MGYA00155182/OIEX01_FASTA.fasta	MGYA00155182	ERZ488699	root:Host-associated:Human:Digestive system:Large in	0.4392
MGYA00155183/OGSR01_FASTA.fasta	MGYA00155183	ERZ485882	root:Host-associated:Human:Digestive system:Large in	0.4462
MGYA00155184/OGSO01_FASTA.fasta	MGYA00155184	ERZ485883	root:Host-associated:Human:Digestive system:Large in	0.4234
MGYA00155185/OGTQ01_FASTA.fasta	MGYA00155185	ERZ485910	root:Host-associated:Human:Digestive system:Large in	0.3092
MGYA00155186/OGTG01_FASTA.fasta	MGYA00155186	ERZ485898	root:Host-associated:Human:Digestive system:Large in	0.3822
MGYA00155187/OGRX01_FASTA.fasta	MGYA00155187	ERZ485863	root:Host-associated:Human:Digestive system:Large in	0.4346
MGYA00155188/OGSF01_FASTA.fasta	MGYA00155188	ERZ485870	root:Host-associated:Human:Digestive system:Large in	0.4878
MGYA00155189/OGTI01_FASTA.fasta	MGYA00155189	ERZ485900	root:Host-associated:Human:Digestive system:Large in	0.4564
MGYA00155190/OGTU01_FASTA.fasta	MGYA00155190	ERZ485905	root:Host-associated:Human:Digestive system:Large in	0.4232
MGYA00155191/OGTO01_FASTA.fasta	MGYA00155191	ERZ485908	root:Host-associated:Human:Digestive system:Large in	0.3608
MGYA00155192/OGTC01_FASTA.fasta	MGYA00155192	ERZ485896	root:Host-associated:Human:Digestive system:Large in	0.295
MGYA00155193/OIEW01_FASTA.fasta	MGYA00155193	ERZ488700	root:Host-associated:Human:Digestive system:Large in	0.3534
MGYA00155194/OGSH01_FASTA.fasta	MGYA00155194	ERZ485872	root:Host-associated:Human:Digestive system:Large in	0.4358
MGYA00155195/OGSC01_FASTA.fasta	MGYA00155195	ERZ485866	root:Host-associated:Human:Digestive system:Large in	0.4922
MGYA00155196/OGTD01_FASTA.fasta	MGYA00155196	ERZ485893	root:Host-associated:Human:Digestive system:Large in	0.4784
MGYA00155197/OGSK01_FASTA.fasta	MGYA00155197	ERZ485876	root:Host-associated:Human:Digestive system:Large in	0.4878
MGYA00155198/OGTS01_FASTA.fasta	MGYA00155198	ERZ485904	root:Host-associated:Human:Digestive system:Large in	0.4686
MGYA00155199/OGTF01_FASTA.fasta	MGYA00155199	ERZ485895	root:Host-associated:Human:Digestive system:Large in	0.3408
MGYA00155200/OGSZ01_FASTA.fasta	MGYA00155200	ERZ485891	root:Host-associated:Human:Digestive system:Large in	0.4774
MGYA00155201/OGSY01_FASTA.fasta	MGYA00155201	ERZ485884	root:Host-associated:Human:Digestive system:Large in	0.4378
MGYA00155202/OGRW01_FASTA.fasta	MGYA00155202	ERZ485865	root:Host-associated:Human:Digestive system:Large in	0.2964
MGYA00155203/OGTH01_FASTA.fasta	MGYA00155203	ERZ485902	root:Host-associated:Human:Digestive system:Large in	0.434
MGYA00155204/OGTT01_FASTA.fasta	MGYA00155204	ERZ485909	root:Host-associated:Human:Digestive system:Large in	0.4298
MGYA00155205/OGUH01_FASTA.fasta	MGYA00155205	ERZ485906	root:Host-associated:Human:Digestive system:Large in	0.4472
MGYA00155206/OGSG01_FASTA.fasta	MGYA00155206	ERZ485873	root:Host-associated:Human:Digestive system:Large in	0.3748
MGYA00155207/OGSX01_FASTA.fasta	MGYA00155207	ERZ485887	root:Host-associated:Human:Digestive system:Large in	0.421
MGYA00155208/OGSE01_FASTA.fasta	MGYA00155208	ERZ485869	root:Host-associated:Human:Digestive system:Large in	0.4192
MGYA00155209/OGST01_FASTA.fasta	MGYA00155209	ERZ485886	root:Host-associated:Human:Digestive system:Large in	0.472
MGYA00155210/OGSM01_FASTA.fasta	MGYA00155210	ERZ485877	root:Host-associated:Human:Digestive system:Large in	0.3086
MGYA00155211/OGSP01_FASTA.fasta	MGYA00155211	ERZ485880	root:Host-associated:Human:Digestive system:Large in	0.3702
MGYA00155212/OGSU01_FASTA.fasta	MGYA00155212	ERZ485881	root:Host-associated:Human:Digestive system:Large in	0.5382
MGYA00155213/OGSJ01_FASTA.fasta	MGYA00155213	ERZ485875	root:Host-associated:Human:Digestive system:Large in	0.4354
MGYA00155214/OGSW01_FASTA.fasta	MGYA00155214	ERZ485890	root:Host-associated:Human:Digestive system:Large in	0.3278
MGYA00155215/OFGM01_FASTA.fasta	MGYA00155215	ERZ483435	root:Host-associated:Mammals:Digestive system:Fecal	0.5046
MGYA00155216/OFFQ01_FASTA.fasta	MGYA00155216	ERZ483399	root:Host-associated:Mammals:Digestive system:Fecal	0.4838
MGYA00155217/OFFT01_FASTA.fasta	MGYA00155217	ERZ483406	root:Host-associated:Mammals:Digestive system:Fecal	0.3818
MGYA00155218/OFGB01_FASTA.fasta	MGYA00155218	ERZ483411	root:Host-associated:Mammals:Digestive system:Fecal	0.3984
MGYA00155219/OFGE01_FASTA.fasta	MGYA00155219	ERZ483420	root:Host-associated:Mammals:Digestive system:Fecal	0.4734
MGYA00155220/OFFX01_FASTA.fasta	MGYA00155220	ERZ483414	root:Host-associated:Mammals:Digestive system:Fecal	0.4474
MGYA00155221/OFGC01_FASTA.fasta	MGYA00155221	ERZ483419	root:Host-associated:Mammals:Digestive system:Fecal	0.4278
MGYA00155222/OFFK01_FASTA.fasta	MGYA00155222	ERZ483397	root:Host-associated:Mammals:Digestive system:Fecal	0.4288
MGYA00155223/OFHA01_FASTA.fasta	MGYA00155223	ERZ483436	root:Host-associated:Mammals:Digestive system:Fecal	0.5096
MGYA00155224/OFGF01_FASTA.fasta	MGYA00155224	ERZ483425	root:Host-associated:Mammals:Digestive system:Fecal	0.4106
MGYA00155225/OFFB01_FASTA.fasta	MGYA00155225	ERZ483388	root:Host-associated:Mammals:Digestive system:Fecal	0.4056
MGYA00155226/OFGN01_FASTA.fasta	MGYA00155226	ERZ483432	root:Host-associated:Mammals:Digestive system:Fecal	0.4352
MGYA00155227/OFFU01_FASTA.fasta	MGYA00155227	ERZ483407	root:Host-associated:Mammals:Digestive system:Fecal	0.4132
MGYA00155228/OFHB01_FASTA.fasta	MGYA00155228	ERZ483438	root:Host-associated:Mammals:Digestive system:Fecal	0.3614
MGYA00155229/OFGQ01_FASTA.fasta	MGYA00155229	ERZ483426	root:Host-associated:Mammals:Digestive system:Fecal	0.4496
MGYA00155230/OFGX01_FASTA.fasta	MGYA00155230	ERZ483431	root:Host-associated:Mammals:Digestive system:Fecal	0.4584
MGYA00155231/OFHN01_FASTA.fasta	MGYA00155231	ERZ483393	root:Host-associated:Mammals:Digestive system:Fecal	0.4918
MGYA00155232/OFGK01_FASTA.fasta	MGYA00155232	ERZ483434	root:Host-associated:Mammals:Digestive system:Fecal	0.379
MGYA00155233/OFFF01_FASTA.fasta	MGYA00155233	ERZ483389	root:Host-associated:Mammals:Digestive system:Fecal	0.4262
MGYA00155234/OFGR01_FASTA.fasta	MGYA00155234	ERZ483422	root:Host-associated:Mammals:Digestive system:Fecal	0.383
MGYA00155235/OFFI01_FASTA.fasta	MGYA00155235	ERZ483390	root:Host-associated:Mammals:Digestive system:Fecal	0.405
MGYA00155236/OFFP01_FASTA.fasta	MGYA00155236	ERZ483404	root:Host-associated:Mammals:Digestive system:Fecal	0.5266
MGYA00155237/OFGL01_FASTA.fasta	MGYA00155237	ERZ483427	root:Host-associated:Mammals:Digestive system:Fecal	0.4578
MGYA00155238/OFFJ01_FASTA.fasta	MGYA00155238	ERZ483395	root:Host-associated:Mammals:Digestive system:Fecal	0.391
MGYA00155239/OFFO01_FASTA.fasta	MGYA00155239	ERZ483405	root:Host-associated:Mammals:Digestive system:Fecal	0.4048
MGYA00155240/OFGA01_FASTA.fasta	MGYA00155240	ERZ483410	root:Host-associated:Mammals:Digestive system:Fecal	0.4446
MGYA00155241/OFHM01_FASTA.fasta	MGYA00155241	ERZ483391	root:Host-associated:Mammals:Digestive system:Fecal	0.4706
MGYA00155242/OFGO01_FASTA.fasta	MGYA00155242	ERZ483428	root:Host-associated:Mammals:Digestive system:Fecal	0.4378
MGYA00155243/OFFS01_FASTA.fasta	MGYA00155243	ERZ483412	root:Host-associated:Mammals:Digestive system:Fecal	0.4652
MGYA00155244/OFFE01_FASTA.fasta	MGYA00155244	ERZ483398	root:Host-associated:Mammals:Digestive system:Fecal	0.3706
MGYA00155245/OFFL01_FASTA.fasta	MGYA00155245	ERZ483401	root:Host-associated:Mammals:Digestive system:Fecal	0.3972
MGYA00155246/OFFN01_FASTA.fasta	MGYA00155246	ERZ483400	root:Host-associated:Mammals:Digestive system:Fecal	0.3576
MGYA00155247/OFHP01_FASTA.fasta	MGYA00155247	ERZ483416	root:Host-associated:Mammals:Digestive system:Fecal	0.389
MGYA00155248/OFEZ01_FASTA.fasta	MGYA00155248	ERZ483387	root:Host-associated:Mammals:Digestive system:Fecal	0.3698
MGYA00155249/OFGI01_FASTA.fasta	MGYA00155249	ERZ483430	root:Host-associated:Mammals:Digestive system:Fecal	0.3998
MGYA00155250/OFFH01_FASTA.fasta	MGYA00155250	ERZ483392	root:Host-associated:Mammals:Digestive system:Fecal	0.3894
MGYA00155251/OFGG01_FASTA.fasta	MGYA00155251	ERZ483423	root:Host-associated:Mammals:Digestive system:Fecal	0.405
MGYA00155252/OFGD01_FASTA.fasta	MGYA00155252	ERZ483418	root:Host-associated:Mammals:Digestive system:Fecal	0.4062
MGYA00155253/OFFV01_FASTA.fasta	MGYA00155253	ERZ483409	root:Host-associated:Mammals:Digestive system:Fecal	0.4826
MGYA00155254/OFFM01_FASTA.fasta	MGYA00155254	ERZ483402	root:Host-associated:Mammals:Digestive system:Fecal	0.4522
MGYA00155255/OFFW01_FASTA.fasta	MGYA00155255	ERZ483413	root:Host-associated:Mammals:Digestive system:Fecal	0.4192
MGYA00155256/OFGP01_FASTA.fasta	MGYA00155256	ERZ483429	root:Host-associated:Mammals:Digestive system:Fecal	0.437
MGYA00155257/OFFC01_FASTA.fasta	MGYA00155257	ERZ483385	root:Host-associated:Mammals:Digestive system:Fecal	0.3578
MGYA00155258/OFFZ01_FASTA.fasta	MGYA00155258	ERZ483417	root:Host-associated:Mammals:Digestive system:Fecal	0.3944
MGYA00155259/OFGH01_FASTA.fasta	MGYA00155259	ERZ483421	root:Host-associated:Mammals:Digestive system:Fecal	0.4494
MGYA00155260/OFGZ01_FASTA.fasta	MGYA00155260	ERZ483437	root:Host-associated:Mammals:Digestive system:Fecal	0.358
MGYA00155261/OFFY01_FASTA.fasta	MGYA00155261	ERZ483415	root:Host-associated:Mammals:Digestive system:Fecal	0.4308
MGYA00155262/OFHO01_FASTA.fasta	MGYA00155262	ERZ483408	root:Host-associated:Mammals:Digestive system:Fecal	0.4576
MGYA00155263/OFEY01_FASTA.fasta	MGYA00155263	ERZ483386	root:Host-associated:Mammals:Digestive system:Fecal	0.3762
MGYA00155264/OFGJ01_FASTA.fasta	MGYA00155264	ERZ483433	root:Host-associated:Mammals:Digestive system:Fecal	0.3858
MGYA00155265/OFFR01_FASTA.fasta	MGYA00155265	ERZ483403	root:Host-associated:Mammals:Digestive system:Fecal	0.396
MGYA00155266/OFFD01_FASTA.fasta	MGYA00155266	ERZ483394	root:Host-associated:Mammals:Digestive system:Fecal	0.3744
MGYA00155267/OFFG01_FASTA.fasta	MGYA00155267	ERZ483396	root:Host-associated:Mammals:Digestive system:Fecal	0.4164
MGYA00155268/OGCF01_FASTA.fasta	MGYA00155268	ERZ485333	root:Host-associated:Insecta	0.0484
MGYA00155269/OGCD01_FASTA.fasta	MGYA00155269	ERZ485335	root:Host-associated:Insecta	0.066
MGYA00155270/OGCC01_FASTA.fasta	MGYA00155270	ERZ485334	root:Host-associated:Insecta	0.0676
MGYA00155271/OFEV01_FASTA.fasta	MGYA00155271	ERZ483306	root:Host-associated:Invertebrates:Cnidaria:Coral	0.087
MGYA00155272/OFES01_FASTA.fasta	MGYA00155272	ERZ483302	root:Host-associated:Invertebrates:Cnidaria:Coral	0.327
MGYA00155273/OFEP01_FASTA.fasta	MGYA00155273	ERZ483296	root:Host-associated:Invertebrates:Cnidaria:Coral	0.1798
MGYA00155274/OFEQ01_FASTA.fasta	MGYA00155274	ERZ483299	root:Host-associated:Invertebrates:Cnidaria:Coral	0.0704
MGYA00155275/OFHR01_FASTA.fasta	MGYA00155275	ERZ483304	root:Host-associated:Invertebrates:Cnidaria:Coral	0.201
MGYA00155276/OFEN01_FASTA.fasta	MGYA00155276	ERZ483298	root:Host-associated:Invertebrates:Cnidaria:Coral	0.2034
MGYA00155277/OFEO01_FASTA.fasta	MGYA00155277	ERZ483295	root:Host-associated:Invertebrates:Cnidaria:Coral	0.0984
MGYA00155278/OFFA01_FASTA.fasta	MGYA00155278	ERZ483305	root:Host-associated:Invertebrates:Cnidaria:Coral	0.0998
MGYA00155279/OFEM01_FASTA.fasta	MGYA00155279	ERZ483301	root:Host-associated:Invertebrates:Cnidaria:Coral	0.0978
MGYA00155280/OFEW01_FASTA.fasta	MGYA00155280	ERZ483303	root:Host-associated:Invertebrates:Cnidaria:Coral	0.213
MGYA00155281/OFER01_FASTA.fasta	MGYA00155281	ERZ483297	root:Host-associated:Invertebrates:Cnidaria:Coral	0.0984
MGYA00155282/OFHQ01_FASTA.fasta	MGYA00155282	ERZ483300	root:Host-associated:Invertebrates:Cnidaria:Coral	0.1024
MGYA00156053/OGNI01_FASTA.fasta	MGYA00156053	ERZ485741	root:Host-associated:Human:Digestive system:Large in	0.4096
MGYA00156054/OGKZ01_FASTA.fasta	MGYA00156054	ERZ485664	root:Host-associated:Human:Digestive system:Large in	0.501
MGYA00156055/OGMT01_FASTA.fasta	MGYA00156055	ERZ485713	root:Host-associated:Human:Digestive system:Large in	0.3752
MGYA00156056/OGGW01_FASTA.fasta	MGYA00156056	ERZ485556	root:Host-associated:Human:Digestive system:Large in	0.5102
MGYA00156057/OGEZ01_FASTA.fasta	MGYA00156057	ERZ485505	root:Host-associated:Human:Digestive system:Large in	0.4634
MGYA00156058/OGDB01_FASTA.fasta	MGYA00156058	ERZ485465	root:Host-associated:Human:Digestive system:Large in	0.2428
MGYA00156059/OGDL01_FASTA.fasta	MGYA00156059	ERZ485476	root:Host-associated:Human:Digestive system:Large in	0.2682
MGYA00156060/OGLV01_FASTA.fasta	MGYA00156060	ERZ485690	root:Host-associated:Human:Digestive system:Large in	0.4354
MGYA00156061/OGMF01_FASTA.fasta	MGYA00156061	ERZ485685	root:Host-associated:Human:Digestive system:Large in	0.5284
MGYA00156062/OGGT01_FASTA.fasta	MGYA00156062	ERZ485557	root:Host-associated:Human:Digestive system:Large in	0.3446
MGYA00156063/OGGR01_FASTA.fasta	MGYA00156063	ERZ485558	root:Host-associated:Human:Digestive system:Large in	0.5574
MGYA00156064/OGDU01_FASTA.fasta	MGYA00156064	ERZ485468	root:Host-associated:Human:Digestive system:Large in	0.429
MGYA00156065/OGCR01_FASTA.fasta	MGYA00156065	ERZ485459	root:Host-associated:Human:Digestive system:Large in	0.0728
MGYA00156066/OGIU01_FASTA.fasta	MGYA00156066	ERZ485604	root:Host-associated:Human:Digestive system:Large in	0.4748
MGYA00156067/OGHA01_FASTA.fasta	MGYA00156067	ERZ485566	root:Host-associated:Human:Digestive system:Large in	0.363
MGYA00156068/OGLH01_FASTA.fasta	MGYA00156068	ERZ485681	root:Host-associated:Human:Digestive system:Large in	0.3444
MGYA00156069/OGFZ01_FASTA.fasta	MGYA00156069	ERZ485538	root:Host-associated:Human:Digestive system:Large in	0.345
MGYA00156070/OGIJ01_FASTA.fasta	MGYA00156070	ERZ485579	root:Host-associated:Human:Digestive system:Large in	0.4282
MGYA00156071/OGIR01_FASTA.fasta	MGYA00156071	ERZ485612	root:Host-associated:Human:Digestive system:Large in	0.4648
MGYA00156072/OGCP01_FASTA.fasta	MGYA00156072	ERZ485456	root:Host-associated:Human:Digestive system:Large in	0.5156
MGYA00156073/OGGM01_FASTA.fasta	MGYA00156073	ERZ485551	root:Host-associated:Human:Digestive system:Large in	0.469
MGYA00156074/OGGL01_FASTA.fasta	MGYA00156074	ERZ485548	root:Host-associated:Human:Digestive system:Large in	0.2738
MGYA00156075/OGMK01_FASTA.fasta	MGYA00156075	ERZ485711	root:Host-associated:Human:Digestive system:Large in	0.3652
MGYA00156076/OGCU01_FASTA.fasta	MGYA00156076	ERZ485454	root:Host-associated:Human:Digestive system:Large in	0.4524
MGYA00156077/OGKP01_FASTA.fasta	MGYA00156077	ERZ485668	root:Host-associated:Human:Digestive system:Large in	0.0926
MGYA00156078/OGEM01_FASTA.fasta	MGYA00156078	ERZ485503	root:Host-associated:Human:Digestive system:Large in	0.548
MGYA00156079/OGNV01_FASTA.fasta	MGYA00156079	ERZ485746	root:Host-associated:Human:Digestive system:Large in	0.4052
MGYA00156080/OGME01_FASTA.fasta	MGYA00156080	ERZ485697	root:Host-associated:Human:Digestive system:Large in	0.3738
MGYA00156081/OGMJ01_FASTA.fasta	MGYA00156081	ERZ485708	root:Host-associated:Human:Digestive system:Large in	0.4402
MGYA00156082/OGHB01_FASTA.fasta	MGYA00156082	ERZ485570	root:Host-associated:Human:Digestive system:Large in	0.4298
MGYA00156083/OGUQ01_FASTA.fasta	MGYA00156083	ERZ485639	root:Host-associated:Human:Digestive system:Large in	0.626
MGYA00156084/OGGI01_FASTA.fasta	MGYA00156084	ERZ485533	root:Host-associated:Human:Digestive system:Large in	0.5006
MGYA00156085/OGKB01_FASTA.fasta	MGYA00156085	ERZ485645	root:Host-associated:Human:Digestive system:Large in	0.5498
MGYA00156086/OGIS01_FASTA.fasta	MGYA00156086	ERZ485603	root:Host-associated:Human:Digestive system:Large in	0.4592
MGYA00156087/OGFV01_FASTA.fasta	MGYA00156087	ERZ485542	root:Host-associated:Human:Digestive system:Large in	0.2502
MGYA00156088/OGCY01_FASTA.fasta	MGYA00156088	ERZ485460	root:Host-associated:Human:Digestive system:Large in	0.4184
MGYA00156089/OGHV01_FASTA.fasta	MGYA00156089	ERZ485583	root:Host-associated:Human:Digestive system:Large in	0.4182
MGYA00156090/OGJH01_FASTA.fasta	MGYA00156090	ERZ485625	root:Host-associated:Human:Digestive system:Large in	0.5108
MGYA00156091/OGFO01_FASTA.fasta	MGYA00156091	ERZ485527	root:Host-associated:Human:Digestive system:Large in	0.3718
MGYA00156092/OGNL01_FASTA.fasta	MGYA00156092	ERZ485739	root:Host-associated:Human:Digestive system:Large in	0.3088
MGYA00156093/OGJE01_FASTA.fasta	MGYA00156093	ERZ485618	root:Host-associated:Human:Digestive system:Large in	0.5494
MGYA00156094/OGFQ01_FASTA.fasta	MGYA00156094	ERZ485528	root:Host-associated:Human:Digestive system:Large in	0.3006
MGYA00156095/OGKF01_FASTA.fasta	MGYA00156095	ERZ485648	root:Host-associated:Human:Digestive system:Large in	0.4058
MGYA00156096/OGMQ01_FASTA.fasta	MGYA00156096	ERZ485716	root:Host-associated:Human:Digestive system:Large in	0.3596
MGYA00156097/OGJS01_FASTA.fasta	MGYA00156097	ERZ485636	root:Host-associated:Human:Digestive system:Large in	0.5126
MGYA00156098/OGMX01_FASTA.fasta	MGYA00156098	ERZ485725	root:Host-associated:Human:Digestive system:Large in	0.4556
MGYA00156099/OGKK01_FASTA.fasta	MGYA00156099	ERZ485654	root:Host-associated:Human:Digestive system:Large in	0.4058
MGYA00156100/OGGB01_FASTA.fasta	MGYA00156100	ERZ485537	root:Host-associated:Human:Digestive system:Large in	0.444
MGYA00156101/OGIO01_FASTA.fasta	MGYA00156101	ERZ485605	root:Host-associated:Human:Digestive system:Large in	0.5718
MGYA00156102/OGFC01_FASTA.fasta	MGYA00156102	ERZ485504	root:Host-associated:Human:Digestive system:Large in	0.551
MGYA00156103/OGGJ01_FASTA.fasta	MGYA00156103	ERZ485532	root:Host-associated:Human:Digestive system:Large in	0.4442
MGYA00156104/OGDG01_FASTA.fasta	MGYA00156104	ERZ485469	root:Host-associated:Human:Digestive system:Large in	0.4378
MGYA00156105/OGCS01_FASTA.fasta	MGYA00156105	ERZ485450	root:Host-associated:Human:Digestive system:Large in	0.1796
MGYA00156106/OGUA01_FASTA.fasta	MGYA00156106	ERZ485699	root:Host-associated:Human:Digestive system:Large in	0.2852
MGYA00156107/OGHD01_FASTA.fasta	MGYA00156107	ERZ485563	root:Host-associated:Human:Digestive system:Large in	0.4494
MGYA00156108/OGGA01_FASTA.fasta	MGYA00156108	ERZ485534	root:Host-associated:Human:Digestive system:Large in	0.504
MGYA00156109/OGNX01_FASTA.fasta	MGYA00156109	ERZ485751	root:Host-associated:Human:Digestive system:Large in	0.2356
MGYA00156110/OGNO01_FASTA.fasta	MGYA00156110	ERZ485738	root:Host-associated:Human:Digestive system:Large in	0.4604
MGYA00156111/OGIV01_FASTA.fasta	MGYA00156111	ERZ485608	root:Host-associated:Human:Digestive system:Large in	0.517
MGYA00156112/OGGK01_FASTA.fasta	MGYA00156112	ERZ485544	root:Host-associated:Human:Digestive system:Large in	0.4262
MGYA00156113/OGKX01_FASTA.fasta	MGYA00156113	ERZ485665	root:Host-associated:Human:Digestive system:Large in	0.4076
MGYA00156114/OGGV01_FASTA.fasta	MGYA00156114	ERZ485554	root:Host-associated:Human:Digestive system:Large in	0.4838
MGYA00156115/OGDI01_FASTA.fasta	MGYA00156115	ERZ485474	root:Host-associated:Human:Digestive system:Large in	0.1082
MGYA00156116/OGKW01_FASTA.fasta	MGYA00156116	ERZ485663	root:Host-associated:Human:Digestive system:Large in	0.425
MGYA00156117/OGKD01_FASTA.fasta	MGYA00156117	ERZ485647	root:Host-associated:Human:Digestive system:Large in	0.422
MGYA00156118/OGDD01_FASTA.fasta	MGYA00156118	ERZ485458	root:Host-associated:Human:Digestive system:Large in	0.5484
MGYA00156119/OGEW01_FASTA.fasta	MGYA00156119	ERZ485513	root:Host-associated:Human:Digestive system:Large in	0.4728
MGYA00156120/OGJM01_FASTA.fasta	MGYA00156120	ERZ485634	root:Host-associated:Human:Digestive system:Large in	0.5034
MGYA00156121/OGKO01_FASTA.fasta	MGYA00156121	ERZ485653	root:Host-associated:Human:Digestive system:Large in	0.4024
MGYA00156122/OGGZ01_FASTA.fasta	MGYA00156122	ERZ485559	root:Host-associated:Human:Digestive system:Large in	0.4818
MGYA00156123/OGLL01_FASTA.fasta	MGYA00156123	ERZ485687	root:Host-associated:Human:Digestive system:Large in	0.1856
MGYA00156124/OGFG01_FASTA.fasta	MGYA00156124	ERZ485509	root:Host-associated:Human:Digestive system:Large in	0.5036
MGYA00156125/OGHG01_FASTA.fasta	MGYA00156125	ERZ485573	root:Host-associated:Human:Digestive system:Large in	0.1626
MGYA00156126/OGIH01_FASTA.fasta	MGYA00156126	ERZ485599	root:Host-associated:Human:Digestive system:Large in	0.3306
MGYA00156127/OGOD01_FASTA.fasta	MGYA00156127	ERZ485754	root:Host-associated:Human:Digestive system:Large in	0.4264
MGYA00156128/OGLP01_FASTA.fasta	MGYA00156128	ERZ485688	root:Host-associated:Human:Digestive system:Large in	0.2756
MGYA00156129/OGEX01_FASTA.fasta	MGYA00156129	ERZ485510	root:Host-associated:Human:Digestive system:Large in	0.1568
MGYA00156130/OGHK01_FASTA.fasta	MGYA00156130	ERZ485560	root:Host-associated:Human:Digestive system:Large in	0.55
MGYA00156131/OGFK01_FASTA.fasta	MGYA00156131	ERZ485520	root:Host-associated:Human:Digestive system:Large in	0.5588
MGYA00156132/OGHX01_FASTA.fasta	MGYA00156132	ERZ485596	root:Host-associated:Human:Digestive system:Large in	0.4232
MGYA00156133/OGGN01_FASTA.fasta	MGYA00156133	ERZ485549	root:Host-associated:Human:Digestive system:Large in	0.1868
MGYA00156134/OGHC01_FASTA.fasta	MGYA00156134	ERZ485564	root:Host-associated:Human:Digestive system:Large in	0.324
MGYA00156135/OGUC01_FASTA.fasta	MGYA00156135	ERZ485703	root:Host-associated:Human:Digestive system:Large in	0.3506
MGYA00156136/OGCX01_FASTA.fasta	MGYA00156136	ERZ485451	root:Host-associated:Human:Digestive system:Large in	0.4246
MGYA00156137/OGHE01_FASTA.fasta	MGYA00156137	ERZ485567	root:Host-associated:Human:Digestive system:Large in	0.4178
MGYA00156138/OGNZ01_FASTA.fasta	MGYA00156138	ERZ485734	root:Host-associated:Human:Digestive system:Large in	0.3416
MGYA00156139/OGDR01_FASTA.fasta	MGYA00156139	ERZ485479	root:Host-associated:Human:Digestive system:Large in	0.3788
MGYA00156140/OGIL01_FASTA.fasta	MGYA00156140	ERZ485597	root:Host-associated:Human:Digestive system:Large in	0.4126
MGYA00156141/OGLM01_FASTA.fasta	MGYA00156141	ERZ485669	root:Host-associated:Human:Digestive system:Large in	0.4834
MGYA00156142/OGDQ01_FASTA.fasta	MGYA00156142	ERZ485473	root:Host-associated:Human:Digestive system:Large in	0.433
MGYA00156144/OGMY01_FASTA.fasta	MGYA00156144	ERZ485726	root:Host-associated:Human:Digestive system:Large in	0.2504
MGYA00156145/OGJV01_FASTA.fasta	MGYA00156145	ERZ485629	root:Host-associated:Human:Digestive system:Large in	0.4918
MGYA00156146/OGUF01_FASTA.fasta	MGYA00156146	ERZ485602	root:Host-associated:Human:Digestive system:Large in	0.4246
MGYA00156147/OGJL01_FASTA.fasta	MGYA00156147	ERZ485640	root:Host-associated:Human:Digestive system:Large in	0.337
MGYA00156148/OGHW01_FASTA.fasta	MGYA00156148	ERZ485591	root:Host-associated:Human:Digestive system:Large in	0.4892
MGYA00156149/OGKQ01_FASTA.fasta	MGYA00156149	ERZ485667	root:Host-associated:Human:Digestive system:Large in	0.1626
MGYA00156150/OGJU01_FASTA.fasta	MGYA00156150	ERZ485632	root:Host-associated:Human:Digestive system:Large in	0.4792
MGYA00156151/OGMB01_FASTA.fasta	MGYA00156151	ERZ485702	root:Host-associated:Human:Digestive system:Large in	0.4968
MGYA00156152/OGFI01_FASTA.fasta	MGYA00156152	ERZ485522	root:Host-associated:Human:Digestive system:Large in	0.472
MGYA00156153/OGHQ01_FASTA.fasta	MGYA00156153	ERZ485582	root:Host-associated:Human:Digestive system:Large in	0.2388
MGYA00156154/OGLC01_FASTA.fasta	MGYA00156154	ERZ485680	root:Host-associated:Human:Digestive system:Large in	0.438
MGYA00156155/OGEB01_FASTA.fasta	MGYA00156155	ERZ485484	root:Host-associated:Human:Digestive system:Large in	0.5284
MGYA00156156/OGNT01_FASTA.fasta	MGYA00156156	ERZ485732	root:Host-associated:Human:Digestive system:Large in	0.3258
MGYA00156157/OGIC01_FASTA.fasta	MGYA00156157	ERZ485595	root:Host-associated:Human:Digestive system:Large in	0.5082
MGYA00156158/OGUJ01_FASTA.fasta	MGYA00156158	ERZ485577	root:Host-associated:Human:Digestive system:Large in	0.5598
MGYA00156159/OGIX01_FASTA.fasta	MGYA00156159	ERZ485616	root:Host-associated:Human:Digestive system:Large in	0.3744
MGYA00156160/OGNK01_FASTA.fasta	MGYA00156160	ERZ485744	root:Host-associated:Human:Digestive system:Large in	0.1356
MGYA00156161/OGDC01_FASTA.fasta	MGYA00156161	ERZ485463	root:Host-associated:Human:Digestive system:Large in	0.3016
MGYA00156162/OGFN01_FASTA.fasta	MGYA00156162	ERZ485519	root:Host-associated:Human:Digestive system:Large in	0.4014
MGYA00156163/OGMC01_FASTA.fasta	MGYA00156163	ERZ485701	root:Host-associated:Human:Digestive system:Large in	0.4192
MGYA00156164/OGFE01_FASTA.fasta	MGYA00156164	ERZ485511	root:Host-associated:Human:Digestive system:Large in	0.464
MGYA00156165/OGDY01_FASTA.fasta	MGYA00156165	ERZ485464	root:Host-associated:Human:Digestive system:Large in	0.4934
MGYA00156166/OGCZ01_FASTA.fasta	MGYA00156166	ERZ485449	root:Host-associated:Human:Digestive system:Large in	0.4652
MGYA00156167/OGLF01_FASTA.fasta	MGYA00156167	ERZ485679	root:Host-associated:Human:Digestive system:Large in	0.3678
MGYA00156168/OGFW01_FASTA.fasta	MGYA00156168	ERZ485529	root:Host-associated:Human:Digestive system:Large in	0.4908
MGYA00156169/OGDZ01_FASTA.fasta	MGYA00156169	ERZ485486	root:Host-associated:Human:Digestive system:Large in	0.2452
MGYA00156170/OGIA01_FASTA.fasta	MGYA00156170	ERZ485593	root:Host-associated:Human:Digestive system:Large in	0.3968
MGYA00156171/OGEC01_FASTA.fasta	MGYA00156171	ERZ485487	root:Host-associated:Human:Digestive system:Large in	0.4374
MGYA00156172/OGOB01_FASTA.fasta	MGYA00156172	ERZ485752	root:Host-associated:Human:Digestive system:Large in	0.3384
MGYA00156173/OGES01_FASTA.fasta	MGYA00156173	ERZ485499	root:Host-associated:Human:Digestive system:Large in	0.5304
MGYA00156174/OGHU01_FASTA.fasta	MGYA00156174	ERZ485576	root:Host-associated:Human:Digestive system:Large in	0.3718
MGYA00156175/OGNF01_FASTA.fasta	MGYA00156175	ERZ485720	root:Host-associated:Human:Digestive system:Large in	0.5054
MGYA00156176/OGDS01_FASTA.fasta	MGYA00156176	ERZ485462	root:Host-associated:Human:Digestive system:Large in	0.4718
MGYA00156177/OGJJ01_FASTA.fasta	MGYA00156177	ERZ485627	root:Host-associated:Human:Digestive system:Large in	0.5118
MGYA00156178/OGMM01_FASTA.fasta	MGYA00156178	ERZ485721	root:Host-associated:Human:Digestive system:Large in	0.3048
MGYA00156179/OGKU01_FASTA.fasta	MGYA00156179	ERZ485671	root:Host-associated:Human:Digestive system:Large in	0.3762
MGYA00156180/OGJA01_FASTA.fasta	MGYA00156180	ERZ485624	root:Host-associated:Human:Digestive system:Large in	0.2812
MGYA00156181/OGJW01_FASTA.fasta	MGYA00156181	ERZ485646	root:Host-associated:Human:Digestive system:Large in	0.3004
MGYA00156182/OGFY01_FASTA.fasta	MGYA00156182	ERZ485523	root:Host-associated:Human:Digestive system:Large in	0.4356
MGYA00156183/OGLA01_FASTA.fasta	MGYA00156183	ERZ485677	root:Host-associated:Human:Digestive system:Large in	0.2608
MGYA00156184/OGGE01_FASTA.fasta	MGYA00156184	ERZ485540	root:Host-associated:Human:Digestive system:Large in	0.4334
MGYA00156185/OGEN01_FASTA.fasta	MGYA00156185	ERZ485491	root:Host-associated:Human:Digestive system:Large in	0.5206
MGYA00156186/OGEQ01_FASTA.fasta	MGYA00156186	ERZ485502	root:Host-associated:Human:Digestive system:Large in	0.544
MGYA00156187/OGLT01_FASTA.fasta	MGYA00156187	ERZ485684	root:Host-associated:Human:Digestive system:Large in	0.389
MGYA00156188/OGGY01_FASTA.fasta	MGYA00156188	ERZ485568	root:Host-associated:Human:Digestive system:Large in	0.0726
MGYA00156189/OGGP01_FASTA.fasta	MGYA00156189	ERZ485555	root:Host-associated:Human:Digestive system:Large in	0.4642
MGYA00156190/OGTZ01_FASTA.fasta	MGYA00156190	ERZ485467	root:Host-associated:Human:Digestive system:Large in	0.4692
MGYA00156191/OGKR01_FASTA.fasta	MGYA00156191	ERZ485651	root:Host-associated:Human:Digestive system:Large in	0.5242
MGYA00156192/OGDA01_FASTA.fasta	MGYA00156192	ERZ485457	root:Host-associated:Human:Digestive system:Large in	0.4512
MGYA00156193/OGDO01_FASTA.fasta	MGYA00156193	ERZ485475	root:Host-associated:Human:Digestive system:Large in	0.3834
MGYA00156194/OGLU01_FASTA.fasta	MGYA00156194	ERZ485696	root:Host-associated:Human:Digestive system:Large in	0.2226
MGYA00156195/OGLQ01_FASTA.fasta	MGYA00156195	ERZ485692	root:Host-associated:Human:Digestive system:Large in	0.3996
MGYA00156196/OGND01_FASTA.fasta	MGYA00156196	ERZ485730	root:Host-associated:Human:Digestive system:Large in	0.333
MGYA00156197/OGEK01_FASTA.fasta	MGYA00156197	ERZ485501	root:Host-associated:Human:Digestive system:Large in	0.4834
MGYA00156198/OGMZ01_FASTA.fasta	MGYA00156198	ERZ485727	root:Host-associated:Human:Digestive system:Large in	0.3994
MGYA00156199/OGMV01_FASTA.fasta	MGYA00156199	ERZ485723	root:Host-associated:Human:Digestive system:Large in	0.4032
MGYA00156200/OGNC01_FASTA.fasta	MGYA00156200	ERZ485719	root:Host-associated:Human:Digestive system:Large in	0.5208
MGYA00156201/OGNR01_FASTA.fasta	MGYA00156201	ERZ485740	root:Host-associated:Human:Digestive system:Large in	0.353
MGYA00156202/OGGF01_FASTA.fasta	MGYA00156202	ERZ485547	root:Host-associated:Human:Digestive system:Large in	0.4996
MGYA00156203/OGIB01_FASTA.fasta	MGYA00156203	ERZ485589	root:Host-associated:Human:Digestive system:Large in	0.5052
MGYA00156204/OGDX01_FASTA.fasta	MGYA00156204	ERZ485478	root:Host-associated:Human:Digestive system:Large in	0.4658
MGYA00156205/OGNY01_FASTA.fasta	MGYA00156205	ERZ485743	root:Host-associated:Human:Digestive system:Large in	0.4136
MGYA00156206/OGIM01_FASTA.fasta	MGYA00156206	ERZ485610	root:Host-associated:Human:Digestive system:Large in	0.402
MGYA00156207/OGKJ01_FASTA.fasta	MGYA00156207	ERZ485650	root:Host-associated:Human:Digestive system:Large in	0.3758
MGYA00156208/OGFH01_FASTA.fasta	MGYA00156208	ERZ485524	root:Host-associated:Human:Digestive system:Large in	0.3364
MGYA00156209/OGHT01_FASTA.fasta	MGYA00156209	ERZ485590	root:Host-associated:Human:Digestive system:Large in	0.2384
MGYA00156210/OGGQ01_FASTA.fasta	MGYA00156210	ERZ485545	root:Host-associated:Human:Digestive system:Large in	0.5538
MGYA00156211/OGLY01_FASTA.fasta	MGYA00156211	ERZ485706	root:Host-associated:Human:Digestive system:Large in	0.2296
MGYA00156212/OGFD01_FASTA.fasta	MGYA00156212	ERZ485517	root:Host-associated:Human:Digestive system:Large in	0.3768
MGYA00156213/OGKI01_FASTA.fasta	MGYA00156213	ERZ485656	root:Host-associated:Human:Digestive system:Large in	0.1612
MGYA00156214/OGER01_FASTA.fasta	MGYA00156214	ERZ485492	root:Host-associated:Human:Digestive system:Large in	0.5996
MGYA00156215/OGLW01_FASTA.fasta	MGYA00156215	ERZ485700	root:Host-associated:Human:Digestive system:Large in	0.2568
MGYA00156216/OGHY01_FASTA.fasta	MGYA00156216	ERZ485585	root:Host-associated:Human:Digestive system:Large in	0.4816
MGYA00156217/OGKG01_FASTA.fasta	MGYA00156217	ERZ485633	root:Host-associated:Human:Digestive system:Large in	0.5084
MGYA00156218/OGED01_FASTA.fasta	MGYA00156218	ERZ485488	root:Host-associated:Human:Digestive system:Large in	0.3798
MGYA00156219/OGNP01_FASTA.fasta	MGYA00156219	ERZ485747	root:Host-associated:Human:Digestive system:Large in	0.339
MGYA00156220/OGLZ01_FASTA.fasta	MGYA00156220	ERZ485698	root:Host-associated:Human:Digestive system:Large in	0.553
MGYA00156221/OGHF01_FASTA.fasta	MGYA00156221	ERZ485572	root:Host-associated:Human:Digestive system:Large in	0.3856
MGYA00156222/OGLI01_FASTA.fasta	MGYA00156222	ERZ485672	root:Host-associated:Human:Digestive system:Large in	0.4524
MGYA00156223/OGIQ01_FASTA.fasta	MGYA00156223	ERZ485606	root:Host-associated:Human:Digestive system:Large in	0.4428
MGYA00156224/OGEE01_FASTA.fasta	MGYA00156224	ERZ485485	root:Host-associated:Human:Digestive system:Large in	0.3048
MGYA00156225/OGMI01_FASTA.fasta	MGYA00156225	ERZ485715	root:Host-associated:Human:Digestive system:Large in	0.345
MGYA00156226/OGCV01_FASTA.fasta	MGYA00156226	ERZ485455	root:Host-associated:Human:Digestive system:Large in	0.399
MGYA00156227/OGHR01_FASTA.fasta	MGYA00156227	ERZ485587	root:Host-associated:Human:Digestive system:Large in	0.5058
MGYA00156228/OGHO01_FASTA.fasta	MGYA00156228	ERZ485569	root:Host-associated:Human:Digestive system:Large in	0.5144
MGYA00156229/OGEU01_FASTA.fasta	MGYA00156229	ERZ485494	root:Host-associated:Human:Digestive system:Large in	0.558
MGYA00156230/OGLX01_FASTA.fasta	MGYA00156230	ERZ485686	root:Host-associated:Human:Digestive system:Large in	0.4568
MGYA00156231/OGLN01_FASTA.fasta	MGYA00156231	ERZ485674	root:Host-associated:Human:Digestive system:Large in	0.5034
MGYA00156232/OGHZ01_FASTA.fasta	MGYA00156232	ERZ485594	root:Host-associated:Human:Digestive system:Large in	0.3618
MGYA00156233/OGLJ01_FASTA.fasta	MGYA00156233	ERZ485682	root:Host-associated:Human:Digestive system:Large in	0.409
MGYA00156234/OGLS01_FASTA.fasta	MGYA00156234	ERZ485683	root:Host-associated:Human:Digestive system:Large in	0.5146
MGYA00156235/OGFJ01_FASTA.fasta	MGYA00156235	ERZ485516	root:Host-associated:Human:Digestive system:Large in	0.426
MGYA00156236/OGFB01_FASTA.fasta	MGYA00156236	ERZ485512	root:Host-associated:Human:Digestive system:Large in	0.4076

MGYA00156237/OGKL01_FASTA.fasta	MGYA00156237	ERZ485657	root:Host-associated:Human:Digestive system:Large in	0.36
MGYA00156239/OGEO01_FASTA.fasta	MGYA00156239	ERZ485500	root:Host-associated:Human:Digestive system:Large in	0.502
MGYA00156240/OGGH01_FASTA.fasta	MGYA00156240	ERZ485541	root:Host-associated:Human:Digestive system:Large in	0.461
MGYA00156241/OGUB01_FASTA.fasta	MGYA00156241	ERZ485755	root:Host-associated:Human:Digestive system:Large in	0.2502
MGYA00156242/OGGG01_FASTA.fasta	MGYA00156242	ERZ485546	root:Host-associated:Human:Digestive system:Large in	0.3862
MGYA00156243/OGET01_FASTA.fasta	MGYA00156243	ERZ485508	root:Host-associated:Human:Digestive system:Large in	0.4246
MGYA00156244/OGNM01_FASTA.fasta	MGYA00156244	ERZ485736	root:Host-associated:Human:Digestive system:Large in	0.3822
MGYA00156245/OGHP01_FASTA.fasta	MGYA00156245	ERZ485584	root:Host-associated:Human:Digestive system:Large in	0.3176
MGYA00156246/OGFX01_FASTA.fasta	MGYA00156246	ERZ485531	root:Host-associated:Human:Digestive system:Large in	0.416
MGYA00156247/OGNE01_FASTA.fasta	MGYA00156247	ERZ485731	root:Host-associated:Human:Digestive system:Large in	0.2978
MGYA00156248/OGNS01_FASTA.fasta	MGYA00156248	ERZ485748	root:Host-associated:Human:Digestive system:Large in	0.3966
MGYA00156249/OGHL01_FASTA.fasta	MGYA00156249	ERZ485561	root:Host-associated:Human:Digestive system:Large in	0.496
MGYA00156250/OGHN01_FASTA.fasta	MGYA00156250	ERZ485575	root:Host-associated:Human:Digestive system:Large in	0.3122
MGYA00156251/OGMP01_FASTA.fasta	MGYA00156251	ERZ485714	root:Host-associated:Human:Digestive system:Large in	0.4354
MGYA00156252/OGJT01_FASTA.fasta	MGYA00156252	ERZ485643	root:Host-associated:Human:Digestive system:Large in	0.506
MGYA00156253/OGLG01_FASTA.fasta	MGYA00156253	ERZ485675	root:Host-associated:Human:Digestive system:Large in	0.3892
MGYA00156254/OGMS01_FASTA.fasta	MGYA00156254	ERZ485724	root:Host-associated:Human:Digestive system:Large in	0.1036
MGYA00156255/OGJC01_FASTA.fasta	MGYA00156255	ERZ485623	root:Host-associated:Human:Digestive system:Large in	0.5482
MGYA00156256/OGDP01_FASTA.fasta	MGYA00156256	ERZ485472	root:Host-associated:Human:Digestive system:Large in	0.4616
MGYA00156257/OGML01_FASTA.fasta	MGYA00156257	ERZ485694	root:Host-associated:Human:Digestive system:Large in	0.561
MGYA00156258/OGEP01_FASTA.fasta	MGYA00156258	ERZ485495	root:Host-associated:Human:Digestive system:Large in	0.5394
MGYA00156259/OGCT01_FASTA.fasta	MGYA00156259	ERZ485453	root:Host-associated:Human:Digestive system:Large in	0.1994
MGYA00156260/OGMN01_FASTA.fasta	MGYA00156260	ERZ485717	root:Host-associated:Human:Digestive system:Large in	0.4686
MGYA00156261/OGKH01_FASTA.fasta	MGYA00156261	ERZ485652	root:Host-associated:Human:Digestive system:Large in	0.4014
MGYA00156262/OGJI01_FASTA.fasta	MGYA00156262	ERZ485626	root:Host-associated:Human:Digestive system:Large in	0.6036
MGYA00156263/OGMD01_FASTA.fasta	MGYA00156263	ERZ485705	root:Host-associated:Human:Digestive system:Large in	0.3602
MGYA00156264/OGFS01_FASTA.fasta	MGYA00156264	ERZ485535	root:Host-associated:Human:Digestive system:Large in	0.0972
MGYA00156265/OGFF01_FASTA.fasta	MGYA00156265	ERZ485507	root:Host-associated:Human:Digestive system:Large in	0.4218
MGYA00156266/OGNA01_FASTA.fasta	MGYA00156266	ERZ485728	root:Host-associated:Human:Digestive system:Large in	0.3666
MGYA00156267/OGDT01_FASTA.fasta	MGYA00156267	ERZ485482	root:Host-associated:Human:Digestive system:Large in	0.4092
MGYA00156268/OGNJ01_FASTA.fasta	MGYA00156268	ERZ485745	root:Host-associated:Human:Digestive system:Large in	0.0922
MGYA00156269/OGKM01_FASTA.fasta	MGYA00156269	ERZ485660	root:Host-associated:Human:Digestive system:Large in	0.4752
MGYA00156270/OGGS01_FASTA.fasta	MGYA00156270	ERZ485550	root:Host-associated:Human:Digestive system:Large in	0.514
MGYA00156271/OGDV01_FASTA.fasta	MGYA00156271	ERZ485480	root:Host-associated:Human:Digestive system:Large in	0.4456
MGYA00156272/OGNB01_FASTA.fasta	MGYA00156272	ERZ485718	root:Host-associated:Human:Digestive system:Large in	0.4078
MGYA00156273/OGIE01_FASTA.fasta	MGYA00156273	ERZ485581	root:Host-associated:Human:Digestive system:Large in	0.523
MGYA00156274/OGNH01_FASTA.fasta	MGYA00156274	ERZ485742	root:Host-associated:Human:Digestive system:Large in	0.2514
MGYA00156275/OGKV01_FASTA.fasta	MGYA00156275	ERZ485673	root:Host-associated:Human:Digestive system:Large in	0.129
MGYA00156276/OGLB01_FASTA.fasta	MGYA00156276	ERZ485666	root:Host-associated:Human:Digestive system:Large in	0.4522
MGYA00156277/OGUG01_FASTA.fasta	MGYA00156277	ERZ485735	root:Host-associated:Human:Digestive system:Large in	0.3414
MGYA00156278/OGLR01_FASTA.fasta	MGYA00156278	ERZ485693	root:Host-associated:Human:Digestive system:Large in	0.303
MGYA00156279/OGEY01_FASTA.fasta	MGYA00156279	ERZ485514	root:Host-associated:Human:Digestive system:Large in	0.1546
MGYA00156280/OGJK01_FASTA.fasta	MGYA00156280	ERZ485615	root:Host-associated:Human:Digestive system:Large in	0.58
MGYA00156281/OGEH01_FASTA.fasta	MGYA00156281	ERZ485493	root:Host-associated:Human:Digestive system:Large in	0.5418
MGYA00156282/OGFP01_FASTA.fasta	MGYA00156282	ERZ485525	root:Host-associated:Human:Digestive system:Large in	0.4438
MGYA00156283/OGNQ01_FASTA.fasta	MGYA00156283	ERZ485737	root:Host-associated:Human:Digestive system:Large in	0.3848
MGYA00156284/OGGX01_FASTA.fasta	MGYA00156284	ERZ485562	root:Host-associated:Human:Digestive system:Large in	0.4452
MGYA00156285/OGUR01_FASTA.fasta	MGYA00156285	ERZ485662	root:Host-associated:Human:Digestive system:Large in	0.368
MGYA00156286/OGDE01_FASTA.fasta	MGYA00156286	ERZ485461	root:Host-associated:Human:Digestive system:Large in	0.4284
MGYA00156287/OGGC01_FASTA.fasta	MGYA00156287	ERZ485543	root:Host-associated:Human:Digestive system:Large in	0.3068
MGYA00156288/OGHJ01_FASTA.fasta	MGYA00156288	ERZ485565	root:Host-associated:Human:Digestive system:Large in	0.5688
MGYA00156289/OGEV01_FASTA.fasta	MGYA00156289	ERZ485506	root:Host-associated:Human:Digestive system:Large in	0.1262
MGYA00156290/OGEI01_FASTA.fasta	MGYA00156290	ERZ485498	root:Host-associated:Human:Digestive system:Large in	0.486
MGYA00156291/OGDJ01_FASTA.fasta	MGYA00156291	ERZ485470	root:Host-associated:Human:Digestive system:Large in	0.487
MGYA00156292/OGMG01_FASTA.fasta	MGYA00156292	ERZ485704	root:Host-associated:Human:Digestive system:Large in	0.3342
MGYA00156293/OGGU01_FASTA.fasta	MGYA00156293	ERZ485552	root:Host-associated:Human:Digestive system:Large in	0.4408
MGYA00156294/OGIF01_FASTA.fasta	MGYA00156294	ERZ485598	root:Host-associated:Human:Digestive system:Large in	0.2416
MGYA00156295/OGID01_FASTA.fasta	MGYA00156295	ERZ485600	root:Host-associated:Human:Digestive system:Large in	0.3214
MGYA00156296/OGMU01_FASTA.fasta	MGYA00156296	ERZ485722	root:Host-associated:Human:Digestive system:Large in	0.3274
MGYA00156297/OGJO01_FASTA.fasta	MGYA00156297	ERZ485638	root:Host-associated:Human:Digestive system:Large in	0.4582
MGYA00156298/OGIT01_FASTA.fasta	MGYA00156298	ERZ485607	root:Host-associated:Human:Digestive system:Large in	0.5292
MGYA00156299/OGNW01_FASTA.fasta	MGYA00156299	ERZ485749	root:Host-associated:Human:Digestive system:Large in	0.3908
MGYA00156300/OGDM01_FASTA.fasta	MGYA00156300	ERZ485481	root:Host-associated:Human:Digestive system:Large in	0.468
MGYA00156301/OGNU01_FASTA.fasta	MGYA00156301	ERZ485750	root:Host-associated:Human:Digestive system:Large in	0.3514
MGYA00156302/OGJG01_FASTA.fasta	MGYA00156302	ERZ485621	root:Host-associated:Human:Digestive system:Large in	0.4144
MGYA00156303/OGMW01_FASTA.fasta	MGYA00156303	ERZ485712	root:Host-associated:Human:Digestive system:Large in	0.492
MGYA00156304/OGUD01_FASTA.fasta	MGYA00156304	ERZ485578	root:Host-associated:Human:Digestive system:Large in	0.4504
MGYA00156305/OGHH01_FASTA.fasta	MGYA00156305	ERZ485571	root:Host-associated:Human:Digestive system:Large in	0.3698
MGYA00156306/OGHM01_FASTA.fasta	MGYA00156306	ERZ485580	root:Host-associated:Human:Digestive system:Large in	0.529
MGYA00156307/OGIY01_FASTA.fasta	MGYA00156307	ERZ485617	root:Host-associated:Human:Digestive system:Large in	0.3338
MGYA00156308/OGIG01_FASTA.fasta	MGYA00156308	ERZ485586	root:Host-associated:Human:Digestive system:Large in	0.444
MGYA00156309/OGHS01_FASTA.fasta	MGYA00156309	ERZ485588	root:Host-associated:Human:Digestive system:Large in	0.322
MGYA00156310/OGLO01_FASTA.fasta	MGYA00156310	ERZ485691	root:Host-associated:Human:Digestive system:Large in	0.3462
MGYA00156311/OGJQ01_FASTA.fasta	MGYA00156311	ERZ485631	root:Host-associated:Human:Digestive system:Large in	0.4448
MGYA00156312/OGOA01_FASTA.fasta	MGYA00156312	ERZ485753	root:Host-associated:Human:Digestive system:Large in	0.4786
MGYA00156313/OGKC01_FASTA.fasta	MGYA00156313	ERZ485655	root:Host-associated:Human:Digestive system:Large in	0.2118
MGYA00156314/OGFU01_FASTA.fasta	MGYA00156314	ERZ485536	root:Host-associated:Human:Digestive system:Large in	0.3326
MGYA00156315/OGMA01_FASTA.fasta	MGYA00156315	ERZ485695	root:Host-associated:Human:Digestive system:Large in	0.3136
MGYA00156316/OGEA01_FASTA.fasta	MGYA00156316	ERZ485483	root:Host-associated:Human:Digestive system:Large in	0.4858
MGYA00156317/OGKT01_FASTA.fasta	MGYA00156317	ERZ485670	root:Host-associated:Human:Digestive system:Large in	0.2954
MGYA00156318/OGHI01_FASTA.fasta	MGYA00156318	ERZ485574	root:Host-associated:Human:Digestive system:Large in	0.3708
MGYA00156319/OGKE01_FASTA.fasta	MGYA00156319	ERZ485644	root:Host-associated:Human:Digestive system:Large in	0.5008
MGYA00156320/OGIZ01_FASTA.fasta	MGYA00156320	ERZ485613	root:Host-associated:Human:Digestive system:Large in	0.474
MGYA00156321/OGKN01_FASTA.fasta	MGYA00156321	ERZ485661	root:Host-associated:Human:Digestive system:Large in	0.367
MGYA00156322/OGJN01_FASTA.fasta	MGYA00156322	ERZ485635	root:Host-associated:Human:Digestive system:Large in	0.502
MGYA00156323/OGMR01_FASTA.fasta	MGYA00156323	ERZ485707	root:Host-associated:Human:Digestive system:Large in	0.4062
MGYA00156324/OGKA01_FASTA.fasta	MGYA00156324	ERZ485630	root:Host-associated:Human:Digestive system:Large in	0.623
MGYA00156325/OGCW01_FASTA.fasta	MGYA00156325	ERZ485452	root:Host-associated:Human:Digestive system:Large in	0.5356
MGYA00156326/OGEF01_FASTA.fasta	MGYA00156326	ERZ485496	root:Host-associated:Human:Digestive system:Large in	0.1568
MGYA00156327/OGDH01_FASTA.fasta	MGYA00156327	ERZ485466	root:Host-associated:Human:Digestive system:Large in	0.2712
MGYA00156328/OGFM01_FASTA.fasta	MGYA00156328	ERZ485515	root:Host-associated:Human:Digestive system:Large in	0.4112
MGYA00156329/OGFA01_FASTA.fasta	MGYA00156329	ERZ485518	root:Host-associated:Human:Digestive system:Large in	0.2914
MGYA00156330/OGIK01_FASTA.fasta	MGYA00156330	ERZ485601	root:Host-associated:Human:Digestive system:Large in	0.458
MGYA00156331/OGLK01_FASTA.fasta	MGYA00156331	ERZ485689	root:Host-associated:Human:Digestive system:Large in	0.3622
MGYA00156332/OGMO01_FASTA.fasta	MGYA00156332	ERZ485710	root:Host-associated:Human:Digestive system:Large in	0.4454
MGYA00156333/OGGD01_FASTA.fasta	MGYA00156333	ERZ485539	root:Host-associated:Human:Digestive system:Large in	0.5396
MGYA00156334/OGNN01_FASTA.fasta	MGYA00156334	ERZ485733	root:Host-associated:Human:Digestive system:Large in	0.386
MGYA00156335/OGJY01_FASTA.fasta	MGYA00156335	ERZ485628	root:Host-associated:Human:Digestive system:Large in	0.5198
MGYA00156336/OGFR01_FASTA.fasta	MGYA00156336	ERZ485526	root:Host-associated:Human:Digestive system:Large in	0.4572
MGYA00156337/OGJD01_FASTA.fasta	MGYA00156337	ERZ485619	root:Host-associated:Human:Digestive system:Large in	0.5548
MGYA00156338/OGKS01_FASTA.fasta	MGYA00156338	ERZ485658	root:Host-associated:Human:Digestive system:Large in	0.3254
MGYA00156339/OGJF01_FASTA.fasta	MGYA00156339	ERZ485622	root:Host-associated:Human:Digestive system:Large in	0.3712
MGYA00156340/OGMH01_FASTA.fasta	MGYA00156340	ERZ485709	root:Host-associated:Human:Digestive system:Large in	0.3172
MGYA00156341/OGII01_FASTA.fasta	MGYA00156341	ERZ485592	root:Host-associated:Human:Digestive system:Large in	0.5046
MGYA00156342/OGKY01_FASTA.fasta	MGYA00156342	ERZ485678	root:Host-associated:Human:Digestive system:Large in	0.25
MGYA00156343/OGIP01_FASTA.fasta	MGYA00156343	ERZ485609	root:Host-associated:Human:Digestive system:Large in	0.2568
MGYA00156344/OGLE01_FASTA.fasta	MGYA00156344	ERZ485676	root:Host-associated:Human:Digestive system:Large in	0.5866
MGYA00156345/OGJX01_FASTA.fasta	MGYA00156345	ERZ485649	root:Host-associated:Human:Digestive system:Large in	0.4938
MGYA00156346/OGJB01_FASTA.fasta	MGYA00156346	ERZ485620	root:Host-associated:Human:Digestive system:Large in	0.3368
MGYA00156347/OGJZ01_FASTA.fasta	MGYA00156347	ERZ485642	root:Host-associated:Human:Digestive system:Large in	0.5976
MGYA00156348/OGEJ01_FASTA.fasta	MGYA00156348	ERZ485497	root:Host-associated:Human:Digestive system:Large in	0.384
MGYA00156349/OGFT01_FASTA.fasta	MGYA00156349	ERZ485530	root:Host-associated:Human:Digestive system:Large in	0.334
MGYA00156350/OGGO01_FASTA.fasta	MGYA00156350	ERZ485553	root:Host-associated:Human:Digestive system:Large in	0.5016
MGYA00156351/OGEG01_FASTA.fasta	MGYA00156351	ERZ485490	root:Host-associated:Human:Digestive system:Large in	0.488
MGYA00156352/OGLD01_FASTA.fasta	MGYA00156352	ERZ485659	root:Host-associated:Human:Digestive system:Large in	0.369
MGYA00156353/OGJR01_FASTA.fasta	MGYA00156353	ERZ485637	root:Host-associated:Human:Digestive system:Large in	0.4482
MGYA00156354/OGIN01_FASTA.fasta	MGYA00156354	ERZ485611	root:Host-associated:Human:Digestive system:Large in	0.4708
MGYA00156355/OGDK01_FASTA.fasta	MGYA00156355	ERZ485477	root:Host-associated:Human:Digestive system:Large in	0.1026
MGYA00156356/OGJP01_FASTA.fasta	MGYA00156356	ERZ485641	root:Host-associated:Human:Digestive system:Large in	0.422
MGYA00156357/OGIW01_FASTA.fasta	MGYA00156357	ERZ485614	root:Host-associated:Human:Digestive system:Large in	0.4816
MGYA00156358/OGNG01_FASTA.fasta	MGYA00156358	ERZ485729	root:Host-associated:Human:Digestive system:Large in	0.4556
MGYA00156359/OGFL01_FASTA.fasta	MGYA00156359	ERZ485521	root:Host-associated:Human:Digestive system:Large in	0.444
MGYA00156360/OFCM01_FASTA.fasta	MGYA00156360	ERZ483238	root:Host-associated:Human:Digestive system:Large in	0.4246
MGYA00156361/OFCG01_FASTA.fasta	MGYA00156361	ERZ483241	root:Host-associated:Human:Digestive system:Large in	0.4154
MGYA00156362/OFDA01_FASTA.fasta	MGYA00156362	ERZ483251	root:Host-associated:Human:Digestive system:Large in	0.3634
MGYA00156363/OFCO01_FASTA.fasta	MGYA00156363	ERZ483239	root:Host-associated:Human:Digestive system:Large in	0.4276
MGYA00156364/OFCP01_FASTA.fasta	MGYA00156364	ERZ483245	root:Host-associated:Human:Digestive system:Large in	0.4814
MGYA00156365/OFCQ01_FASTA.fasta	MGYA00156365	ERZ483248	root:Host-associated:Human:Digestive system:Large in	0.4076
MGYA00156366/OFCH01_FASTA.fasta	MGYA00156366	ERZ483240	root:Host-associated:Human:Digestive system:Large in	0.3686
MGYA00156367/OFCJ01_FASTA.fasta	MGYA00156367	ERZ483242	root:Host-associated:Human:Digestive system:Large in	0.4016
MGYA00156368/OFCV01_FASTA.fasta	MGYA00156368	ERZ483252	root:Host-associated:Human:Digestive system:Large in	0.367
MGYA00156369/OFCL01_FASTA.fasta	MGYA00156369	ERZ483244	root:Host-associated:Human:Digestive system:Large in	0.4482
MGYA00156370/OFCN01_FASTA.fasta	MGYA00156370	ERZ483243	root:Host-associated:Human:Digestive system:Large in	0.4362
MGYA00156371/OFDJ01_FASTA.fasta	MGYA00156371	ERZ483250	root:Host-associated:Human:Digestive system:Large in	0.3986
MGYA00156372/OFCS01_FASTA.fasta	MGYA00156372	ERZ483247	root:Host-associated:Human:Digestive system:Large in	0.3998
MGYA00156373/OFCU01_FASTA.fasta	MGYA00156373	ERZ483249	root:Host-associated:Human:Digestive system:Large in	0.3916
MGYA00156374/OFCR01_FASTA.fasta	MGYA00156374	ERZ483246	root:Host-associated:Human:Digestive system:Large in	0.3422
MGYA00169689/OOCH01_FASTA.fasta	MGYA00169689	ERZ485399	root:Host-associated:Human:Digestive system:Large in	0.4758
MGYA00169690/OODE01_FASTA.fasta	MGYA00169690	ERZ485425	root:Host-associated:Human:Digestive system:Large in	0.493
MGYA00169691/ONZY01_FASTA.fasta	MGYA00169691	ERZ485359	root:Host-associated:Human:Digestive system:Large in	0.4512
MGYA00169692/OODI01_FASTA.fasta	MGYA00169692	ERZ485427	root:Host-associated:Human:Digestive system:Large in	0.3362
MGYA00169693/OOCW01_FASTA.fasta	MGYA00169693	ERZ485416	root:Host-associated:Human:Digestive system:Large in	0.5078
MGYA00169694/OOBO01_FASTA.fasta	MGYA00169694	ERZ485383	root:Host-associated:Human:Digestive system:Large in	0.425
MGYA00169695/OOAH01_FASTA.fasta	MGYA00169695	ERZ485360	root:Host-associated:Human:Digestive system:Large in	0.536
MGYA00169696/OODC01_FASTA.fasta	MGYA00169696	ERZ485423	root:Host-associated:Human:Digestive system:Large in	0.4476
MGYA00169697/OODU01_FASTA.fasta	MGYA00169697	ERZ485437	root:Host-associated:Human:Digestive system:Large in	0.5204
MGYA00169698/OOCQ01_FASTA.fasta	MGYA00169698	ERZ485410	root:Host-associated:Human:Digestive system:Large in	0.3982
MGYA00169699/OOBS01_FASTA.fasta	MGYA00169699	ERZ485389	root:Host-associated:Human:Digestive system:Large in	0.3252
MGYA00169700/OODK01_FASTA.fasta	MGYA00169700	ERZ485434	root:Host-associated:Human:Digestive system:Large in	0.478
MGYA00169701/OOBN01_FASTA.fasta	MGYA00169701	ERZ485372	root:Host-associated:Human:Digestive system:Large in	0.5092
MGYA00169702/OOCT01_FASTA.fasta	MGYA00169702	ERZ485418	root:Host-associated:Human:Digestive system:Large in	0.4934
MGYA00169703/ONZV01_FASTA.fasta	MGYA00169703	ERZ485347	root:Host-associated:Human:Digestive system:Large in	0.4712
MGYA00169704/ONZX01_FASTA.fasta	MGYA00169704	ERZ485355	root:Host-associated:Human:Digestive system:Large in	0.3858
MGYA00169705/OOAC01_FASTA.fasta	MGYA00169705	ERZ485351	root:Host-associated:Human:Digestive system:Large in	0.4704
MGYA00169706/OODS01_FASTA.fasta	MGYA00169706	ERZ485438	root:Host-associated:Human:Digestive system:Large in	0.476
MGYA00169707/OOAG01_FASTA.fasta	MGYA00169707	ERZ485346	root:Host-associated:Human:Digestive system:Large in	0.487
MGYA00169708/ONZT01_FASTA.fasta	MGYA00169708	ERZ485352	root:Host-associated:Human:Digestive system:Large in	0.4324
MGYA00169709/OOBM01_FASTA.fasta	MGYA00169709	ERZ485381	root:Host-associated:Human:Digestive system:Large in	0.423
MGYA00169710/OOAL01_FASTA.fasta	MGYA00169710	ERZ485369	root:Host-associated:Human:Digestive system:Large in	0.2578
MGYA00169711/OOBZ01_FASTA.fasta	MGYA00169711	ERZ485390	root:Host-associated:Human:Digestive system:Large in	0.4792
MGYA00169712/ONZU01_FASTA.fasta	MGYA00169712	ERZ485348	root:Host-associated:Human:Digestive system:Large in	0.4404
MGYA00169713/OOBQ01_FASTA.fasta	MGYA00169713	ERZ485394	root:Host-associated:Human:Digestive system:Large in	0.1456
MGYA00169714/OODN01_FASTA.fasta	MGYA00169714	ERZ485435	root:Host-associated:Human:Digestive system:Large in	0.4618
MGYA00169715/OOBX01_FASTA.fasta	MGYA00169715	ERZ485396	root:Host-associated:Human:Digestive system:Large in	0.4486
MGYA00169716/OODQ01_FASTA.fasta	MGYA00169716	ERZ485440	root:Host-associated:Human:Digestive system:Large in	0.4178
MGYA00169717/OOAT01_FASTA.fasta	MGYA00169717	ERZ485374	root:Host-associated:Human:Digestive system:Large in	0.3802
MGYA00169718/OODH01_FASTA.fasta	MGYA00169718	ERZ485429	root:Host-associated:Human:Digestive system:Large in	0.4248
MGYA00169719/OOAE01_FASTA.fasta	MGYA00169719	ERZ485356	root:Host-associated:Human:Digestive system:Large in	0.469
MGYA00169720/OOCU01_FASTA.fasta	MGYA00169720	ERZ485419	root:Host-associated:Human:Digestive system:Large in	0.3844
MGYA00169721/OOAD01_FASTA.fasta	MGYA00169721	ERZ485349	root:Host-associated:Human:Digestive system:Large in	0.4896
MGYA00169722/OOAQ01_FASTA.fasta	MGYA00169722	ERZ485362	root:Host-associated:Human:Digestive system:Large in	0.4224
MGYA00169723/OOAU01_FASTA.fasta	MGYA00169723	ERZ485376	root:Host-associated:Human:Digestive system:Large in	0.459
MGYA00169724/OOBU01_FASTA.fasta	MGYA00169724	ERZ485391	root:Host-associated:Human:Digestive system:Large in	0.3464
MGYA00169725/OOCA01_FASTA.fasta	MGYA00169725	ERZ485400	root:Host-associated:Human:Digestive system:Large in	0.3928
MGYA00169726/OOCG01_FASTA.fasta	MGYA00169726	ERZ485404	root:Host-associated:Human:Digestive system:Large in	0.388
MGYA00169727/OODB01_FASTA.fasta	MGYA00169727	ERZ485420	root:Host-associated:Human:Digestive system:Large in	0.4004
MGYA00169728/OOCS01_FASTA.fasta	MGYA00169728	ERZ485413	root:Host-associated:Human:Digestive system:Large in	0.3764
MGYA00169729/OODO01_FASTA.fasta	MGYA00169729	ERZ485432	root:Host-associated:Human:Digestive system:Large in	0.5062
MGYA00169730/OOCR01_FASTA.fasta	MGYA00169730	ERZ485411	root:Host-associated:Human:Digestive system:Large in	0.4042
MGYA00169731/OOCN01_FASTA.fasta	MGYA00169731	ERZ485408	root:Host-associated:Human:Digestive system:Large in	0.4338
MGYA00169732/OODP01_FASTA.fasta	MGYA00169732	ERZ485436	root:Host-associated:Human:Digestive system:Large in	0.4056
MGYA00169733/ONZW01_FASTA.fasta	MGYA00169733	ERZ485353	root:Host-associated:Human:Digestive system:Large in	0.4768
MGYA00169734/OOBB01_FASTA.fasta	MGYA00169734	ERZ485377	root:Host-associated:Human:Digestive system:Large in	0.3674
MGYA00169735/OOAW01_FASTA.fasta	MGYA00169735	ERZ485379	root:Host-associated:Human:Digestive system:Large in	0.5582
MGYA00169736/OOCL01_FASTA.fasta	MGYA00169736	ERZ485406	root:Host-associated:Human:Digestive system:Large in	0.4448
MGYA00169737/OOCP01_FASTA.fasta	MGYA00169737	ERZ485409	root:Host-associated:Human:Digestive system:Large in	0.5042
MGYA00169738/OOAZ01_FASTA.fasta	MGYA00169738	ERZ485375	root:Host-associated:Human:Digestive system:Large in	0.3708
MGYA00169739/OOAI01_FASTA.fasta	MGYA00169739	ERZ485364	root:Host-associated:Human:Digestive system:Large in	0.349
MGYA00169740/OOCM01_FASTA.fasta	MGYA00169740	ERZ485403	root:Host-associated:Human:Digestive system:Large in	0.4988
MGYA00169741/OOAJ01_FASTA.fasta	MGYA00169741	ERZ485365	root:Host-associated:Human:Digestive system:Large in	0.401
MGYA00169742/OOCJ01_FASTA.fasta	MGYA00169742	ERZ485405	root:Host-associated:Human:Digestive system:Large in	0.389
MGYA00169743/OOIG01_FASTA.fasta	MGYA00169743	ERZ485442	root:Host-associated:Human:Digestive system:Large in	0.492
MGYA00169744/OOAP01_FASTA.fasta	MGYA00169744	ERZ485373	root:Host-associated:Human:Digestive system:Large in	0.0572
MGYA00169745/OOAY01_FASTA.fasta	MGYA00169745	ERZ485380	root:Host-associated:Human:Digestive system:Large in	0.4074
MGYA00169746/OODM01_FASTA.fasta	MGYA00169746	ERZ485433	root:Host-associated:Human:Digestive system:Large in	0.4512
MGYA00169747/OODG01_FASTA.fasta	MGYA00169747	ERZ485430	root:Host-associated:Human:Digestive system:Large in	0.4552
MGYA00169748/OOBW01_FASTA.fasta	MGYA00169748	ERZ485393	root:Host-associated:Human:Digestive system:Large in	0.494
MGYA00169749/OOCZ01_FASTA.fasta	MGYA00169749	ERZ485415	root:Host-associated:Human:Digestive system:Large in	0.4328
MGYA00169750/OODF01_FASTA.fasta	MGYA00169750	ERZ485431	root:Host-associated:Human:Digestive system:Large in	0.3454
MGYA00169751/OOCO01_FASTA.fasta	MGYA00169751	ERZ485412	root:Host-associated:Human:Digestive system:Large in	0.3154
MGYA00169752/OOCK01_FASTA.fasta	MGYA00169752	ERZ485407	root:Host-associated:Human:Digestive system:Large in	0.3714
MGYA00169753/OOAF01_FASTA.fasta	MGYA00169753	ERZ485361	root:Host-associated:Human:Digestive system:Large in	0.5176
MGYA00169754/OOCV01_FASTA.fasta	MGYA00169754	ERZ485417	root:Host-associated:Human:Digestive system:Large in	0.4334
MGYA00169755/OOCI01_FASTA.fasta	MGYA00169755	ERZ485398	root:Host-associated:Human:Digestive system:Large in	0.46
MGYA00169756/OOBT01_FASTA.fasta	MGYA00169756	ERZ485386	root:Host-associated:Human:Digestive system:Large in	0.3532
MGYA00169757/OOBP01_FASTA.fasta	MGYA00169757	ERZ485385	root:Host-associated:Human:Digestive system:Large in	0.4632
MGYA00169758/OOAK01_FASTA.fasta	MGYA00169758	ERZ485368	root:Host-associated:Human:Digestive system:Large in	0.4078
MGYA00169759/OOCC01_FASTA.fasta	MGYA00169759	ERZ485402	root:Host-associated:Human:Digestive system:Large in	0.3322
MGYA00169760/OODL01_FASTA.fasta	MGYA00169760	ERZ485428	root:Host-associated:Human:Digestive system:Large in	0.5846
MGYA00169761/OOAM01_FASTA.fasta	MGYA00169761	ERZ485366	root:Host-associated:Human:Digestive system:Large in	0.4856
MGYA00169762/OOCX01_FASTA.fasta	MGYA00169762	ERZ485414	root:Host-associated:Human:Digestive system:Large in	0.4136
MGYA00169763/OODD01_FASTA.fasta	MGYA00169763	ERZ485426	root:Host-associated:Human:Digestive system:Large in	0.5178
MGYA00169764/ONZR01_FASTA.fasta	MGYA00169764	ERZ485357	root:Host-associated:Human:Digestive system:Large in	0.398
MGYA00169765/OOCD01_FASTA.fasta	MGYA00169765	ERZ485395	root:Host-associated:Human:Digestive system:Large in	0.429
MGYA00169766/OOBV01_FASTA.fasta	MGYA00169766	ERZ485384	root:Host-associated:Human:Digestive system:Large in	0.4298
MGYA00169767/OOAN01_FASTA.fasta	MGYA00169767	ERZ485367	root:Host-associated:Human:Digestive system:Large in	0.3742
MGYA00169768/OOCE01_FASTA.fasta	MGYA00169768	ERZ485397	root:Host-associated:Human:Digestive system:Large in	0.3344
MGYA00169769/OODA01_FASTA.fasta	MGYA00169769	ERZ485421	root:Host-associated:Human:Digestive system:Large in	0.384
MGYA00169770/OOBK01_FASTA.fasta	MGYA00169770	ERZ485382	root:Host-associated:Human:Digestive system:Large in	0.4748
MGYA00169771/OOBI01_FASTA.fasta	MGYA00169771	ERZ485378	root:Host-associated:Human:Digestive system:Large in	0.4636
MGYA00169772/OODJ01_FASTA.fasta	MGYA00169772	ERZ485424	root:Host-associated:Human:Digestive system:Large in	0.4628
MGYA00169773/OODT01_FASTA.fasta	MGYA00169773	ERZ485441	root:Host-associated:Human:Digestive system:Large in	0.4546
MGYA00169774/ONZS01_FASTA.fasta	MGYA00169774	ERZ485358	root:Host-associated:Human:Digestive system:Large in	0.3616
MGYA00169775/OOAO01_FASTA.fasta	MGYA00169775	ERZ485370	root:Host-associated:Human:Digestive system:Large in	0.4116
MGYA00169776/OOCF01_FASTA.fasta	MGYA00169776	ERZ485401	root:Host-associated:Human:Digestive system:Large in	0.3628
MGYA00169777/OOBJ01_FASTA.fasta	MGYA00169777	ERZ485371	root:Host-associated:Human:Digestive system:Large in	0.5038
MGYA00169778/OOAA01_FASTA.fasta	MGYA00169778	ERZ485350	root:Host-associated:Human:Digestive system:Large in	0.41
MGYA00169779/OOCB01_FASTA.fasta	MGYA00169779	ERZ485388	root:Host-associated:Human:Digestive system:Large in	0.518
MGYA00169780/OOCY01_FASTA.fasta	MGYA00169780	ERZ485422	root:Host-associated:Human:Digestive system:Large in	0.466
MGYA00169781/ONZZ01_FASTA.fasta	MGYA00169781	ERZ485354	root:Host-associated:Human:Digestive system:Large in	0.5014
MGYA00169782/OOBR01_FASTA.fasta	MGYA00169782	ERZ485387	root:Host-associated:Human:Digestive system:Large in	0.3752
MGYA00169783/OODR01_FASTA.fasta	MGYA00169783	ERZ485439	root:Host-associated:Human:Digestive system:Large in	0.4334
MGYA00169784/OOBY01_FASTA.fasta	MGYA00169784	ERZ485392	root:Host-associated:Human:Digestive system:Large in	0.4628
MGYA00169785/OOIF01_FASTA.fasta	MGYA00169785	ERZ485363	root:Host-associated:Human:Digestive system:Large in	0.4722
MGYA00169959/OGXD01_FASTA.fasta	MGYA00169959	ERZ486712	root:Host-associated:Human:Digestive system:Large in	0.5262
MGYA00169960/OGYD01_FASTA.fasta	MGYA00169960	ERZ486740	root:Host-associated:Human:Digestive system:Large in	0.5126
MGYA00169961/OGWT01_FASTA.fasta	MGYA00169961	ERZ486696	root:Host-associated:Human:Digestive system:Large in	0.505
MGYA00169962/OGYX01_FASTA.fasta	MGYA00169962	ERZ486754	root:Host-associated:Human:Digestive system:Large in	0.4884
MGYA00169963/OGWW01_FASTA.fasta	MGYA00169963	ERZ486704	root:Host-associated:Human:Digestive system:Large in	0.3978
MGYA00169964/OKSH01_FASTA.fasta	MGYA00169964	ERZ486787	root:Host-associated:Human:Digestive system:Large in	0.5586
MGYA00169965/OKRX01_FASTA.fasta	MGYA00169965	ERZ486782	root:Host-associated:Human:Digestive system:Large in	0.4538
MGYA00169966/OHAY01_FASTA.fasta	MGYA00169966	ERZ486811	root:Host-associated:Human:Digestive system:Large in	0.499
MGYA00169967/OGYJ01_FASTA.fasta	MGYA00169967	ERZ486747	root:Host-associated:Human:Digestive system:Large in	0.4446
MGYA00169968/OGXX01_FASTA.fasta	MGYA00169968	ERZ486723	root:Host-associated:Human:Digestive system:Large in	0.5814
MGYA00169969/OHAQ01_FASTA.fasta	MGYA00169969	ERZ486816	root:Host-associated:Human:Digestive system:Large in	0.54
MGYA00169970/OGXR01_FASTA.fasta	MGYA00169970	ERZ486724	root:Host-associated:Human:Digestive system:Large in	0.5094
MGYA00169971/OGZE01_FASTA.fasta	MGYA00169971	ERZ486772	root:Host-associated:Human:Digestive system:Large in	0.6108
MGYA00169972/OHAH01_FASTA.fasta	MGYA00169972	ERZ486802	root:Host-associated:Human:Digestive system:Large in	0.534
MGYA00169973/OGWZ01_FASTA.fasta	MGYA00169973	ERZ486703	root:Host-associated:Human:Digestive system:Large in	0.4022
MGYA00169974/OKSB01_FASTA.fasta	MGYA00169974	ERZ486716	root:Host-associated:Human:Digestive system:Large in	0.5566
MGYA00169975/OHAN01_FASTA.fasta	MGYA00169975	ERZ486817	root:Host-associated:Human:Digestive system:Large in	0.447
MGYA00169976/OGYP01_FASTA.fasta	MGYA00169976	ERZ486751	root:Host-associated:Human:Digestive system:Large in	0.4692
MGYA00169977/OKSE01_FASTA.fasta	MGYA00169977	ERZ486785	root:Host-associated:Human:Digestive system:Large in	0.512
MGYA00169978/OHBM01_FASTA.fasta	MGYA00169978	ERZ486804	root:Host-associated:Human:Digestive system:Large in	0.4016
MGYA00169979/OGZV01_FASTA.fasta	MGYA00169979	ERZ486790	root:Host-associated:Human:Digestive system:Large in	0.4996
MGYA00169980/OGWQ01_FASTA.fasta	MGYA00169980	ERZ486695	root:Host-associated:Human:Digestive system:Large in	0.4352
MGYA00169981/OGZI01_FASTA.fasta	MGYA00169981	ERZ486767	root:Host-associated:Human:Digestive system:Large in	0.5978
MGYA00169982/OGXH01_FASTA.fasta	MGYA00169982	ERZ486711	root:Host-associated:Human:Digestive system:Large in	0.5768
MGYA00169983/OGXK01_FASTA.fasta	MGYA00169983	ERZ486721	root:Host-associated:Human:Digestive system:Large in	0.4214
MGYA00169984/OGWY01_FASTA.fasta	MGYA00169984	ERZ486700	root:Host-associated:Human:Digestive system:Large in	0.464
MGYA00169985/OGZY01_FASTA.fasta	MGYA00169985	ERZ486801	root:Host-associated:Human:Digestive system:Large in	0.5748
MGYA00169986/OGYL01_FASTA.fasta	MGYA00169986	ERZ486746	root:Host-associated:Human:Digestive system:Large in	0.4298
MGYA00169987/OHAI01_FASTA.fasta	MGYA00169987	ERZ486805	root:Host-associated:Human:Digestive system:Large in	0.549
MGYA00169988/OGYV01_FASTA.fasta	MGYA00169988	ERZ486755	root:Host-associated:Human:Digestive system:Large in	0.5796
MGYA00169989/OKRV01_FASTA.fasta	MGYA00169989	ERZ486744	root:Host-associated:Human:Digestive system:Large in	0.4952
MGYA00169990/OGZW01_FASTA.fasta	MGYA00169990	ERZ486781	root:Host-associated:Human:Digestive system:Large in	0.4062
MGYA00169991/OGXF01_FASTA.fasta	MGYA00169991	ERZ486718	root:Host-associated:Human:Digestive system:Large in	0.459
MGYA00169992/OGZQ01_FASTA.fasta	MGYA00169992	ERZ486786	root:Host-associated:Human:Digestive system:Large in	0.3896
MGYA00169993/OGYA01_FASTA.fasta	MGYA00169993	ERZ486730	root:Host-associated:Human:Digestive system:Large in	0.4938
MGYA00169994/OHAA01_FASTA.fasta	MGYA00169994	ERZ486789	root:Host-associated:Human:Digestive system:Large in	0.5108
MGYA00169995/OGXM01_FASTA.fasta	MGYA00169995	ERZ486714	root:Host-associated:Human:Digestive system:Large in	0.4566
MGYA00169996/OGZT01_FASTA.fasta	MGYA00169996	ERZ486797	root:Host-associated:Human:Digestive system:Large in	0.4766
MGYA00169997/OGYB01_FASTA.fasta	MGYA00169997	ERZ486741	root:Host-associated:Human:Digestive system:Large in	0.4492
MGYA00169998/OGYY01_FASTA.fasta	MGYA00169998	ERZ486764	root:Host-associated:Human:Digestive system:Large in	0.5092
MGYA00169999/OGZO01_FASTA.fasta	MGYA00169999	ERZ486769	root:Host-associated:Human:Digestive system:Large in	0.5498
MGYA00170000/OGYC01_FASTA.fasta	MGYA00170000	ERZ486737	root:Host-associated:Human:Digestive system:Large in	0.4682
MGYA00170001/OGYF01_FASTA.fasta	MGYA00170001	ERZ486738	root:Host-associated:Human:Digestive system:Large in	0.4192
MGYA00170002/OGYN01_FASTA.fasta	MGYA00170002	ERZ486743	root:Host-associated:Human:Digestive system:Large in	0.5814
MGYA00170003/OGZL01_FASTA.fasta	MGYA00170003	ERZ486775	root:Host-associated:Human:Digestive system:Large in	0.3886
MGYA00170004/OHMN01_FASTA.fasta	MGYA00170004	ERZ486691	root:Host-associated:Human:Digestive system:Large in	0.5588
MGYA00170005/OKRU01_FASTA.fasta	MGYA00170005	ERZ486701	root:Host-associated:Human:Digestive system:Large in	0.46
MGYA00170006/OKSA01_FASTA.fasta	MGYA00170006	ERZ486731	root:Host-associated:Human:Digestive system:Large in	0.4518
MGYA00170007/OGYE01_FASTA.fasta	MGYA00170007	ERZ486742	root:Host-associated:Human:Digestive system:Large in	0.522
MGYA00170008/OGXC01_FASTA.fasta	MGYA00170008	ERZ486713	root:Host-associated:Human:Digestive system:Large in	0.506
MGYA00170009/OGXN01_FASTA.fasta	MGYA00170009	ERZ486719	root:Host-associated:Human:Digestive system:Large in	0.602
MGYA00170010/OGXJ01_FASTA.fasta	MGYA00170010	ERZ486715	root:Host-associated:Human:Digestive system:Large in	0.4918
MGYA00170011/OGZN01_FASTA.fasta	MGYA00170011	ERZ486779	root:Host-associated:Human:Digestive system:Large in	0.4936
MGYA00170012/OGYH01_FASTA.fasta	MGYA00170012	ERZ486748	root:Host-associated:Human:Digestive system:Large in	0.4756
MGYA00170013/OGXZ01_FASTA.fasta	MGYA00170013	ERZ486736	root:Host-associated:Human:Digestive system:Large in	0.5004
MGYA00170014/OGYS01_FASTA.fasta	MGYA00170014	ERZ486757	root:Host-associated:Human:Digestive system:Large in	0.3518
MGYA00170015/OKSF01_FASTA.fasta	MGYA00170015	ERZ486788	root:Host-associated:Human:Digestive system:Large in	0.4068
MGYA00170016/OKSC01_FASTA.fasta	MGYA00170016	ERZ486706	root:Host-associated:Human:Digestive system:Large in	0.4498
MGYA00170017/OHAE01_FASTA.fasta	MGYA00170017	ERZ486800	root:Host-associated:Human:Digestive system:Large in	0.5508
MGYA00170018/OGXL01_FASTA.fasta	MGYA00170018	ERZ486726	root:Host-associated:Human:Digestive system:Large in	0.41
MGYA00170019/OGWS01_FASTA.fasta	MGYA00170019	ERZ486698	root:Host-associated:Human:Digestive system:Large in	0.495
MGYA00170020/OHAU01_FASTA.fasta	MGYA00170020	ERZ486812	root:Host-associated:Human:Digestive system:Large in	0.587
MGYA00170021/OHAO01_FASTA.fasta	MGYA00170021	ERZ486807	root:Host-associated:Human:Digestive system:Large in	0.4014
MGYA00170022/OHAM01_FASTA.fasta	MGYA00170022	ERZ486813	root:Host-associated:Human:Digestive system:Large in	0.4642
MGYA00170023/OGZX01_FASTA.fasta	MGYA00170023	ERZ486792	root:Host-associated:Human:Digestive system:Large in	0.5074
MGYA00170024/OGYM01_FASTA.fasta	MGYA00170024	ERZ486750	root:Host-associated:Human:Digestive system:Large in	0.5696
MGYA00170025/OHBE01_FASTA.fasta	MGYA00170025	ERZ486809	root:Host-associated:Human:Digestive system:Large in	0.4548
MGYA00170026/OGYG01_FASTA.fasta	MGYA00170026	ERZ486749	root:Host-associated:Human:Digestive system:Large in	0.4552
MGYA00170027/OGWU01_FASTA.fasta	MGYA00170027	ERZ486693	root:Host-associated:Human:Digestive system:Large in	0.5556
MGYA00170028/OHAT01_FASTA.fasta	MGYA00170028	ERZ486818	root:Host-associated:Human:Digestive system:Large in	0.4792
MGYA00170029/OGZK01_FASTA.fasta	MGYA00170029	ERZ486773	root:Host-associated:Human:Digestive system:Large in	0.4872
MGYA00170030/OGYT01_FASTA.fasta	MGYA00170030	ERZ486756	root:Host-associated:Human:Digestive system:Large in	0.6106
MGYA00170031/OGZZ01_FASTA.fasta	MGYA00170031	ERZ486791	root:Host-associated:Human:Digestive system:Large in	0.5234
MGYA00170032/OGXP01_FASTA.fasta	MGYA00170032	ERZ486729	root:Host-associated:Human:Digestive system:Large in	0.4334
MGYA00170033/OGYQ01_FASTA.fasta	MGYA00170033	ERZ486760	root:Host-associated:Human:Digestive system:Large in	0.5432
MGYA00170034/OGXI01_FASTA.fasta	MGYA00170034	ERZ486710	root:Host-associated:Human:Digestive system:Large in	0.5868
MGYA00170035/OGWR01_FASTA.fasta	MGYA00170035	ERZ486692	root:Host-associated:Human:Digestive system:Large in	0.4756
MGYA00170036/OGZD01_FASTA.fasta	MGYA00170036	ERZ486771	root:Host-associated:Human:Digestive system:Large in	0.5156
MGYA00170037/OGZA01_FASTA.fasta	MGYA00170037	ERZ486762	root:Host-associated:Human:Digestive system:Large in	0.546
MGYA00170038/OGYO01_FASTA.fasta	MGYA00170038	ERZ486753	root:Host-associated:Human:Digestive system:Large in	0.506
MGYA00170039/OGZU01_FASTA.fasta	MGYA00170039	ERZ486794	root:Host-associated:Human:Digestive system:Large in	0.367
MGYA00170040/OGZB01_FASTA.fasta	MGYA00170040	ERZ486774	root:Host-associated:Human:Digestive system:Large in	0.375
MGYA00170041/OHAG01_FASTA.fasta	MGYA00170041	ERZ486803	root:Host-associated:Human:Digestive system:Large in	0.44
MGYA00170042/OGXA01_FASTA.fasta	MGYA00170042	ERZ486709	root:Host-associated:Human:Digestive system:Large in	0.485
MGYA00170043/OGXG01_FASTA.fasta	MGYA00170043	ERZ486720	root:Host-associated:Human:Digestive system:Large in	0.4452
MGYA00170044/OHAS01_FASTA.fasta	MGYA00170044	ERZ486810	root:Host-associated:Human:Digestive system:Large in	0.5344
MGYA00170045/OGXV01_FASTA.fasta	MGYA00170045	ERZ486734	root:Host-associated:Human:Digestive system:Large in	0.5104
MGYA00170046/OKRW01_FASTA.fasta	MGYA00170046	ERZ486708	root:Host-associated:Human:Digestive system:Large in	0.5376
MGYA00170047/OGXW01_FASTA.fasta	MGYA00170047	ERZ486733	root:Host-associated:Human:Digestive system:Large in	0.4308
MGYA00170048/OGXS01_FASTA.fasta	MGYA00170048	ERZ486728	root:Host-associated:Human:Digestive system:Large in	0.4992
MGYA00170049/OGYW01_FASTA.fasta	MGYA00170049	ERZ486759	root:Host-associated:Human:Digestive system:Large in	0.4986
MGYA00170050/OGWX01_FASTA.fasta	MGYA00170050	ERZ486707	root:Host-associated:Human:Digestive system:Large in	0.4584
MGYA00170051/OGZM01_FASTA.fasta	MGYA00170051	ERZ486778	root:Host-associated:Human:Digestive system:Large in	0.4956
MGYA00170052/OGZR01_FASTA.fasta	MGYA00170052	ERZ486783	root:Host-associated:Human:Digestive system:Large in	0.4972
MGYA00170053/OGXT01_FASTA.fasta	MGYA00170053	ERZ486722	root:Host-associated:Human:Digestive system:Large in	0.5104
MGYA00170054/OGXY01_FASTA.fasta	MGYA00170054	ERZ486739	root:Host-associated:Human:Digestive system:Large in	0.3922
MGYA00170055/OHAJ01_FASTA.fasta	MGYA00170055	ERZ486806	root:Host-associated:Human:Digestive system:Large in	0.5782
MGYA00170056/OGXU01_FASTA.fasta	MGYA00170056	ERZ486735	root:Host-associated:Human:Digestive system:Large in	0.3254
MGYA00170057/OKRS01_FASTA.fasta	MGYA00170057	ERZ486732	root:Host-associated:Human:Digestive system:Large in	0.4044
MGYA00170058/OGXB01_FASTA.fasta	MGYA00170058	ERZ486699	root:Host-associated:Human:Digestive system:Large in	0.4186
MGYA00170059/OGXE01_FASTA.fasta	MGYA00170059	ERZ486717	root:Host-associated:Human:Digestive system:Large in	0.559
MGYA00170060/OGXQ01_FASTA.fasta	MGYA00170060	ERZ486725	root:Host-associated:Human:Digestive system:Large in	0.4824
MGYA00170061/OKRY01_FASTA.fasta	MGYA00170061	ERZ486705	root:Host-associated:Human:Digestive system:Large in	0.4606
MGYA00170062/OHAC01_FASTA.fasta	MGYA00170062	ERZ486799	root:Host-associated:Human:Digestive system:Large in	0.511
MGYA00170063/OGXO01_FASTA.fasta	MGYA00170063	ERZ486727	root:Host-associated:Human:Digestive system:Large in	0.5386
MGYA00170064/OGZF01_FASTA.fasta	MGYA00170064	ERZ486766	root:Host-associated:Human:Digestive system:Large in	0.4098
MGYA00170065/OHAD01_FASTA.fasta	MGYA00170065	ERZ486793	root:Host-associated:Human:Digestive system:Large in	0.4828
MGYA00170066/OGZP01_FASTA.fasta	MGYA00170066	ERZ486777	root:Host-associated:Human:Digestive system:Large in	0.3844
MGYA00170067/OGZJ01_FASTA.fasta	MGYA00170067	ERZ486780	root:Host-associated:Human:Digestive system:Large in	0.435
MGYA00170068/OGYZ01_FASTA.fasta	MGYA00170068	ERZ486765	root:Host-associated:Human:Digestive system:Large in	0.5356
MGYA00170069/OHAP01_FASTA.fasta	MGYA00170069	ERZ486814	root:Host-associated:Human:Digestive system:Large in	0.3726
MGYA00170070/OGZS01_FASTA.fasta	MGYA00170070	ERZ486798	root:Host-associated:Human:Digestive system:Large in	0.4472
MGYA00170071/OKRZ01_FASTA.fasta	MGYA00170071	ERZ486702	root:Host-associated:Human:Digestive system:Large in	0.5236
MGYA00170072/OGZH01_FASTA.fasta	MGYA00170072	ERZ486768	root:Host-associated:Human:Digestive system:Large in	0.4208
MGYA00170073/OGWV01_FASTA.fasta	MGYA00170073	ERZ486694	root:Host-associated:Human:Digestive system:Large in	0.543
MGYA00170074/OGZG01_FASTA.fasta	MGYA00170074	ERZ486770	root:Host-associated:Human:Digestive system:Large in	0.5358
MGYA00170075/OKSD01_FASTA.fasta	MGYA00170075	ERZ486784	root:Host-associated:Human:Digestive system:Large in	0.4298
MGYA00170076/OKRT01_FASTA.fasta	MGYA00170076	ERZ486697	root:Host-associated:Human:Digestive system:Large in	0.3618
MGYA00170077/OGZC01_FASTA.fasta	MGYA00170077	ERZ486763	root:Host-associated:Human:Digestive system:Large in	0.5382
MGYA00170078/OGYR01_FASTA.fasta	MGYA00170078	ERZ486758	root:Host-associated:Human:Digestive system:Large in	0.3488
MGYA00170079/OKRR01_FASTA.fasta	MGYA00170079	ERZ486776	root:Host-associated:Human:Digestive system:Large in	0.5156
MGYA00170080/OGYK01_FASTA.fasta	MGYA00170080	ERZ486752	root:Host-associated:Human:Digestive system:Large in	0.537
MGYA00170081/OHBJ01_FASTA.fasta	MGYA00170081	ERZ486808	root:Host-associated:Human:Digestive system:Large in	0.3932
MGYA00170082/OHAF01_FASTA.fasta	MGYA00170082	ERZ486796	root:Host-associated:Human:Digestive system:Large in	0.553
MGYA00170083/OGYI01_FASTA.fasta	MGYA00170083	ERZ486745	root:Host-associated:Human:Digestive system:Large in	0.494
MGYA00170084/OHAB01_FASTA.fasta	MGYA00170084	ERZ486795	root:Host-associated:Human:Digestive system:Large in	0.3958
MGYA00170085/OHAX01_FASTA.fasta	MGYA00170085	ERZ486815	root:Host-associated:Human:Digestive system:Large in	0.4076
MGYA00170086/OGYU01_FASTA.fasta	MGYA00170086	ERZ486761	root:Host-associated:Human:Digestive system:Large in	0.5068
MGYA00170087/OHPT01_FASTA.fasta	MGYA00170087	ERZ487346	root:Host-associated:Human:Digestive system:Large in	0.2768
MGYA00170088/OHLU01_FASTA.fasta	MGYA00170088	ERZ487224	root:Host-associated:Human:Digestive system:Large in	0.0286
MGYA00170089/OHUA01_FASTA.fasta	MGYA00170089	ERZ487443	root:Host-associated:Human:Digestive system:Large in	0.3866
MGYA00170090/OHKF01_FASTA.fasta	MGYA00170090	ERZ487182	root:Host-associated:Human:Digestive system:Large in	0.5494
MGYA00170091/OHGX01_FASTA.fasta	MGYA00170091	ERZ487079	root:Host-associated:Human:Digestive system:Large in	0.2642
MGYA00170092/OKTE01_FASTA.fasta	MGYA00170092	ERZ487158	root:Host-associated:Human:Digestive system:Large in	0.29
MGYA00170093/OHQL01_FASTA.fasta	MGYA00170093	ERZ487363	root:Host-associated:Human:Digestive system:Large in	0.2906
MGYA00170094/OKSO01_FASTA.fasta	MGYA00170094	ERZ486990	root:Host-associated:Human:Digestive system:Large in	0.3428
MGYA00170095/OHFN01_FASTA.fasta	MGYA00170095	ERZ487051	root:Host-associated:Human:Digestive system:Large in	0.3996
MGYA00170096/OHUK01_FASTA.fasta	MGYA00170096	ERZ487462	root:Host-associated:Human:Digestive system:Large in	0.4566
MGYA00170097/OHQC01_FASTA.fasta	MGYA00170097	ERZ487340	root:Host-associated:Human:Digestive system:Large in	0.3356
MGYA00170098/OHEI01_FASTA.fasta	MGYA00170098	ERZ487013	root:Host-associated:Human:Digestive system:Large in	0.3636
MGYA00170099/OHRQ01_FASTA.fasta	MGYA00170099	ERZ487392	root:Host-associated:Human:Digestive system:Large in	0.345
MGYA00170100/OHQZ01_FASTA.fasta	MGYA00170100	ERZ487368	root:Host-associated:Human:Digestive system:Large in	0.3882
MGYA00170101/OHMT01_FASTA.fasta	MGYA00170101	ERZ487251	root:Host-associated:Human:Digestive system:Large in	0.355
MGYA00170102/OHRT01_FASTA.fasta	MGYA00170102	ERZ487394	root:Host-associated:Human:Digestive system:Large in	0.4286
MGYA00170103/OHSK01_FASTA.fasta	MGYA00170103	ERZ487408	root:Host-associated:Human:Digestive system:Large in	0.304
MGYA00170104/OHGP01_FASTA.fasta	MGYA00170104	ERZ487089	root:Host-associated:Human:Digestive system:Large in	0.277
MGYA00170105/OHEL01_FASTA.fasta	MGYA00170105	ERZ487011	root:Host-associated:Human:Digestive system:Large in	0.347
MGYA00170106/OHJW01_FASTA.fasta	MGYA00170106	ERZ487186	root:Host-associated:Human:Digestive system:Large in	0.0356
MGYA00170107/OKTC01_FASTA.fasta	MGYA00170107	ERZ487109	root:Host-associated:Human:Digestive system:Large in	0.3698
MGYA00170108/OHTP01_FASTA.fasta	MGYA00170108	ERZ487454	root:Host-associated:Human:Digestive system:Large in	0.0426
MGYA00170109/OHUE01_FASTA.fasta	MGYA00170109	ERZ487455	root:Host-associated:Human:Digestive system:Large in	0.3382
MGYA00170110/OHFE01_FASTA.fasta	MGYA00170110	ERZ487046	root:Host-associated:Human:Digestive system:Large in	0.2862
MGYA00170111/OHQH01_FASTA.fasta	MGYA00170111	ERZ487337	root:Host-associated:Human:Digestive system:Large in	0.3416
MGYA00170112/OHCA01_FASTA.fasta	MGYA00170112	ERZ486952	root:Host-associated:Human:Digestive system:Large in	0.3588
MGYA00170113/OKSK01_FASTA.fasta	MGYA00170113	ERZ486943	root:Host-associated:Human:Digestive system:Large in	0.4652
MGYA00170114/OHFY01_FASTA.fasta	MGYA00170114	ERZ487057	root:Host-associated:Human:Digestive system:Large in	0.3486
MGYA00170115/OHNM01_FASTA.fasta	MGYA00170115	ERZ487276	root:Host-associated:Human:Digestive system:Large in	0.2848
MGYA00170116/OHQI01_FASTA.fasta	MGYA00170116	ERZ487352	root:Host-associated:Human:Digestive system:Large in	0.3172
MGYA00170117/OHSE01_FASTA.fasta	MGYA00170117	ERZ487406	root:Host-associated:Human:Digestive system:Large in	0.3822
MGYA00170118/OHHL01_FASTA.fasta	MGYA00170118	ERZ487113	root:Host-associated:Human:Digestive system:Large in	0.2902
MGYA00170119/OHPG01_FASTA.fasta	MGYA00170119	ERZ487335	root:Host-associated:Human:Digestive system:Large in	0.3664
MGYA00170120/OHNU01_FASTA.fasta	MGYA00170120	ERZ487291	root:Host-associated:Human:Digestive system:Large in	0.0394
MGYA00170121/OKTB01_FASTA.fasta	MGYA00170121	ERZ487152	root:Host-associated:Human:Digestive system:Large in	0.3636
MGYA00170122/OHLV01_FASTA.fasta	MGYA00170122	ERZ487225	root:Host-associated:Human:Digestive system:Large in	0.37
MGYA00170123/OHKS01_FASTA.fasta	MGYA00170123	ERZ487201	root:Host-associated:Human:Digestive system:Large in	0.2968
MGYA00170124/OHNX01_FASTA.fasta	MGYA00170124	ERZ487278	root:Host-associated:Human:Digestive system:Large in	0.3552
MGYA00170125/OHEZ01_FASTA.fasta	MGYA00170125	ERZ487035	root:Host-associated:Human:Digestive system:Large in	0.3476
MGYA00170126/OHNB01_FASTA.fasta	MGYA00170126	ERZ487270	root:Host-associated:Human:Digestive system:Large in	0.0768
MGYA00170127/OHIL01_FASTA.fasta	MGYA00170127	ERZ487125	root:Host-associated:Human:Digestive system:Large in	0.3478
MGYA00170128/OHMA01_FASTA.fasta	MGYA00170128	ERZ487228	root:Host-associated:Human:Digestive system:Large in	0.5586
MGYA00170129/OHKJ01_FASTA.fasta	MGYA00170129	ERZ487188	root:Host-associated:Human:Digestive system:Large in	0.4956
MGYA00170130/OHMV01_FASTA.fasta	MGYA00170130	ERZ487254	root:Host-associated:Human:Digestive system:Large in	0.3488
MGYA00170131/OHTB01_FASTA.fasta	MGYA00170131	ERZ487429	root:Host-associated:Human:Digestive system:Large in	0.3748
MGYA00170132/OHMG01_FASTA.fasta	MGYA00170132	ERZ487245	root:Host-associated:Human:Digestive system:Large in	0.3482
MGYA00170133/OHEE01_FASTA.fasta	MGYA00170133	ERZ487016	root:Host-associated:Human:Digestive system:Large in	0.349
MGYA00170134/OHRM01_FASTA.fasta	MGYA00170134	ERZ487379	root:Host-associated:Human:Digestive system:Large in	0.3906
MGYA00170135/OHFW01_FASTA.fasta	MGYA00170135	ERZ487067	root:Host-associated:Human:Digestive system:Large in	0.3502
MGYA00170136/OHHD01_FASTA.fasta	MGYA00170136	ERZ487090	root:Host-associated:Human:Digestive system:Large in	0.2666
MGYA00170137/OHTX01_FASTA.fasta	MGYA00170137	ERZ487460	root:Host-associated:Human:Digestive system:Large in	0.2434
MGYA00170138/OKSY01_FASTA.fasta	MGYA00170138	ERZ487227	root:Host-associated:Human:Digestive system:Large in	0.0454
MGYA00170139/OHED01_FASTA.fasta	MGYA00170139	ERZ487022	root:Host-associated:Human:Digestive system:Large in	0.0844
MGYA00170140/OHMW01_FASTA.fasta	MGYA00170140	ERZ487243	root:Host-associated:Human:Digestive system:Large in	0.479
MGYA00170141/OHNZ01_FASTA.fasta	MGYA00170141	ERZ487287	root:Host-associated:Human:Digestive system:Large in	0.3742
MGYA00170142/OHOV01_FASTA.fasta	MGYA00170142	ERZ487325	root:Host-associated:Human:Digestive system:Large in	0.0388
MGYA00170143/OHRH01_FASTA.fasta	MGYA00170143	ERZ487380	root:Host-associated:Human:Digestive system:Large in	0.4716
MGYA00170144/OHDO01_FASTA.fasta	MGYA00170144	ERZ487007	root:Host-associated:Human:Digestive system:Large in	0.0382
MGYA00170145/OHKA01_FASTA.fasta	MGYA00170145	ERZ487177	root:Host-associated:Human:Digestive system:Large in	0.4108
MGYA00170146/OKTA01_FASTA.fasta	MGYA00170146	ERZ487162	root:Host-associated:Human:Digestive system:Large in	0.3524
MGYA00170147/OHOH01_FASTA.fasta	MGYA00170147	ERZ487306	root:Host-associated:Human:Digestive system:Large in	0.2322
MGYA00170148/OHMM01_FASTA.fasta	MGYA00170148	ERZ487255	root:Host-associated:Human:Digestive system:Large in	0.0834
MGYA00170149/OHQG01_FASTA.fasta	MGYA00170149	ERZ487339	root:Host-associated:Human:Digestive system:Large in	0.3966
MGYA00170150/OHPI01_FASTA.fasta	MGYA00170150	ERZ487320	root:Host-associated:Human:Digestive system:Large in	0.4276
MGYA00170151/OHNT01_FASTA.fasta	MGYA00170151	ERZ487285	root:Host-associated:Human:Digestive system:Large in	0.0288
MGYA00170152/OHIX01_FASTA.fasta	MGYA00170152	ERZ487141	root:Host-associated:Human:Digestive system:Large in	0.413
MGYA00170153/OHSV01_FASTA.fasta	MGYA00170153	ERZ487433	root:Host-associated:Human:Digestive system:Large in	0.104
MGYA00170154/OHQK01_FASTA.fasta	MGYA00170154	ERZ487361	root:Host-associated:Human:Digestive system:Large in	0.2686
MGYA00170155/OHRG01_FASTA.fasta	MGYA00170155	ERZ487372	root:Host-associated:Human:Digestive system:Large in	0.4302
MGYA00170156/OHTY01_FASTA.fasta	MGYA00170156	ERZ487446	root:Host-associated:Human:Digestive system:Large in	0.4344
MGYA00170157/OHGR01_FASTA.fasta	MGYA00170157	ERZ487088	root:Host-associated:Human:Digestive system:Large in	0.2506
MGYA00170158/OHRA01_FASTA.fasta	MGYA00170158	ERZ487376	root:Host-associated:Human:Digestive system:Large in	0.349
MGYA00170159/OHDC01_FASTA.fasta	MGYA00170159	ERZ486974	root:Host-associated:Human:Digestive system:Large in	0.4032
MGYA00170160/OHFM01_FASTA.fasta	MGYA00170160	ERZ487042	root:Host-associated:Human:Digestive system:Large in	0.3668
MGYA00170161/OHJV01_FASTA.fasta	MGYA00170161	ERZ487175	root:Host-associated:Human:Digestive system:Large in	0.049
MGYA00170162/OHKI01_FASTA.fasta	MGYA00170162	ERZ487178	root:Host-associated:Human:Digestive system:Large in	0.3272
MGYA00170163/OHTJ01_FASTA.fasta	MGYA00170163	ERZ487435	root:Host-associated:Human:Digestive system:Large in	0.3104
MGYA00170164/OHGI01_FASTA.fasta	MGYA00170164	ERZ487072	root:Host-associated:Human:Digestive system:Large in	0.3172
MGYA00170165/OHTU01_FASTA.fasta	MGYA00170165	ERZ487451	root:Host-associated:Human:Digestive system:Large in	0.3258
MGYA00170166/OHEQ01_FASTA.fasta	MGYA00170166	ERZ487027	root:Host-associated:Human:Digestive system:Large in	0.3588
MGYA00170167/OHDX01_FASTA.fasta	MGYA00170167	ERZ486991	root:Host-associated:Human:Digestive system:Large in	0.3076
MGYA00170168/OHHI01_FASTA.fasta	MGYA00170168	ERZ487101	root:Host-associated:Human:Digestive system:Large in	0.3946
MGYA00170169/OHCC01_FASTA.fasta	MGYA00170169	ERZ486954	root:Host-associated:Human:Digestive system:Large in	0.4062
MGYA00170170/OHQB01_FASTA.fasta	MGYA00170170	ERZ487353	root:Host-associated:Human:Digestive system:Large in	0.2476
MGYA00170171/OHCX01_FASTA.fasta	MGYA00170171	ERZ486972	root:Host-associated:Human:Digestive system:Large in	0.281
MGYA00170172/OHIQ01_FASTA.fasta	MGYA00170172	ERZ487140	root:Host-associated:Human:Digestive system:Large in	0.3702
MGYA00170173/OHPD01_FASTA.fasta	MGYA00170173	ERZ487319	root:Host-associated:Human:Digestive system:Large in	0.3388
MGYA00170174/OHFX01_FASTA.fasta	MGYA00170174	ERZ487059	root:Host-associated:Human:Digestive system:Large in	0.2644
MGYA00170175/OHRE01_FASTA.fasta	MGYA00170175	ERZ487384	root:Host-associated:Human:Digestive system:Large in	0.3274
MGYA00170176/OHOS01_FASTA.fasta	MGYA00170176	ERZ487321	root:Host-associated:Human:Digestive system:Large in	0.0524
MGYA00170177/OHDK01_FASTA.fasta	MGYA00170177	ERZ487001	root:Host-associated:Human:Digestive system:Large in	0.0368
MGYA00170178/OHPU01_FASTA.fasta	MGYA00170178	ERZ487341	root:Host-associated:Human:Digestive system:Large in	0.0352
MGYA00170179/OHFT01_FASTA.fasta	MGYA00170179	ERZ487064	root:Host-associated:Human:Digestive system:Large in	0.0598
MGYA00170180/OHFS01_FASTA.fasta	MGYA00170180	ERZ487063	root:Host-associated:Human:Digestive system:Large in	0.0618
MGYA00170181/OHJR01_FASTA.fasta	MGYA00170181	ERZ487169	root:Host-associated:Human:Digestive system:Large in	0.3158
MGYA00170182/OHHU01_FASTA.fasta	MGYA00170182	ERZ487114	root:Host-associated:Human:Digestive system:Large in	0.2872
MGYA00170183/OHIN01_FASTA.fasta	MGYA00170183	ERZ487129	root:Host-associated:Human:Digestive system:Large in	0.4046
MGYA00170184/OHFV01_FASTA.fasta	MGYA00170184	ERZ487068	root:Host-associated:Human:Digestive system:Large in	0.3456
MGYA00170185/OHSG01_FASTA.fasta	MGYA00170185	ERZ487400	root:Host-associated:Human:Digestive system:Large in	0.3522
MGYA00170186/OHRY01_FASTA.fasta	MGYA00170186	ERZ487395	root:Host-associated:Human:Digestive system:Large in	0.3724
MGYA00170187/OHCZ01_FASTA.fasta	MGYA00170187	ERZ486978	root:Host-associated:Human:Digestive system:Large in	0.329
MGYA00170188/OHPW01_FASTA.fasta	MGYA00170188	ERZ487336	root:Host-associated:Human:Digestive system:Large in	0.3716
MGYA00170189/OHEC01_FASTA.fasta	MGYA00170189	ERZ487005	root:Host-associated:Human:Digestive system:Large in	0.4074
MGYA00170190/OHPB01_FASTA.fasta	MGYA00170190	ERZ487305	root:Host-associated:Human:Digestive system:Large in	0.4738
MGYA00170191/OKTJ01_FASTA.fasta	MGYA00170191	ERZ487218	root:Host-associated:Human:Digestive system:Large in	0.4092
MGYA00170192/OHHP01_FASTA.fasta	MGYA00170192	ERZ487118	root:Host-associated:Human:Digestive system:Large in	0.3482
MGYA00170193/OHOL01_FASTA.fasta	MGYA00170193	ERZ487315	root:Host-associated:Human:Digestive system:Large in	0.2782
MGYA00170194/OHCP01_FASTA.fasta	MGYA00170194	ERZ486967	root:Host-associated:Human:Digestive system:Large in	0.3174
MGYA00170195/OHKH01_FASTA.fasta	MGYA00170195	ERZ487189	root:Host-associated:Human:Digestive system:Large in	0.39
MGYA00170196/OHIW01_FASTA.fasta	MGYA00170196	ERZ487151	root:Host-associated:Human:Digestive system:Large in	0.0682
MGYA00170197/OHRK01_FASTA.fasta	MGYA00170197	ERZ487382	root:Host-associated:Human:Digestive system:Large in	0.2534
MGYA00170198/OHDV01_FASTA.fasta	MGYA00170198	ERZ486995	root:Host-associated:Human:Digestive system:Large in	0.4236
MGYA00170199/OHIK01_FASTA.fasta	MGYA00170199	ERZ487123	root:Host-associated:Human:Digestive system:Large in	0.4306
MGYA00170200/OHPF01_FASTA.fasta	MGYA00170200	ERZ487317	root:Host-associated:Human:Digestive system:Large in	0.3218
MGYA00170201/OKTR01_FASTA.fasta	MGYA00170201	ERZ487296	root:Host-associated:Human:Digestive system:Large in	0.3532
MGYA00170202/OKTH01_FASTA.fasta	MGYA00170202	ERZ487274	root:Host-associated:Human:Digestive system:Large in	0.4568
MGYA00170203/OHHW01_FASTA.fasta	MGYA00170203	ERZ487127	root:Host-associated:Human:Digestive system:Large in	0.0306
MGYA00170204/OHLE01_FASTA.fasta	MGYA00170204	ERZ487211	root:Host-associated:Human:Digestive system:Large in	0.3284
MGYA00170205/OHLF01_FASTA.fasta	MGYA00170205	ERZ487208	root:Host-associated:Human:Digestive system:Large in	0.3946
MGYA00170206/OHFO01_FASTA.fasta	MGYA00170206	ERZ487052	root:Host-associated:Human:Digestive system:Large in	0.3488
MGYA00170207/OHGQ01_FASTA.fasta	MGYA00170207	ERZ487080	root:Host-associated:Human:Digestive system:Large in	0.3174
MGYA00170208/OHTG01_FASTA.fasta	MGYA00170208	ERZ487427	root:Host-associated:Human:Digestive system:Large in	0.3752
MGYA00170209/OKTS01_FASTA.fasta	MGYA00170209	ERZ487414	root:Host-associated:Human:Digestive system:Large in	0.3582
MGYA00170210/OHTZ01_FASTA.fasta	MGYA00170210	ERZ487442	root:Host-associated:Human:Digestive system:Large in	0.3542
MGYA00170211/OHNR01_FASTA.fasta	MGYA00170211	ERZ487277	root:Host-associated:Human:Digestive system:Large in	0.3308
MGYA00170212/OHGS01_FASTA.fasta	MGYA00170212	ERZ487083	root:Host-associated:Human:Digestive system:Large in	0.3904
MGYA00170213/OHEP01_FASTA.fasta	MGYA00170213	ERZ487021	root:Host-associated:Human:Digestive system:Large in	0.4518
MGYA00170214/OHJO01_FASTA.fasta	MGYA00170214	ERZ487166	root:Host-associated:Human:Digestive system:Large in	0.3504
MGYA00170215/OHUF01_FASTA.fasta	MGYA00170215	ERZ487458	root:Host-associated:Human:Digestive system:Large in	0.3896
MGYA00170216/OHPK01_FASTA.fasta	MGYA00170216	ERZ487318	root:Host-associated:Human:Digestive system:Large in	0.3688
MGYA00170217/OKSQ01_FASTA.fasta	MGYA00170217	ERZ487017	root:Host-associated:Human:Digestive system:Large in	0.1338
MGYA00170218/OHSO01_FASTA.fasta	MGYA00170218	ERZ487409	root:Host-associated:Human:Digestive system:Large in	0.3346
MGYA00170219/OHRF01_FASTA.fasta	MGYA00170219	ERZ487367	root:Host-associated:Human:Digestive system:Large in	0.2968
MGYA00170220/OHLY01_FASTA.fasta	MGYA00170220	ERZ487230	root:Host-associated:Human:Digestive system:Large in	0.3286
MGYA00170221/OHNW01_FASTA.fasta	MGYA00170221	ERZ487289	root:Host-associated:Human:Digestive system:Large in	0.0918
MGYA00170222/OHNI01_FASTA.fasta	MGYA00170222	ERZ487267	root:Host-associated:Human:Digestive system:Large in	0.295
MGYA00170223/OHNJ01_FASTA.fasta	MGYA00170223	ERZ487271	root:Host-associated:Human:Digestive system:Large in	0.3588
MGYA00170224/OHGL01_FASTA.fasta	MGYA00170224	ERZ487076	root:Host-associated:Human:Digestive system:Large in	0.3594
MGYA00170225/OHLG01_FASTA.fasta	MGYA00170225	ERZ487220	root:Host-associated:Human:Digestive system:Large in	0.2442
MGYA00170226/OHMQ01_FASTA.fasta	MGYA00170226	ERZ487248	root:Host-associated:Human:Digestive system:Large in	0.2762
MGYA00170227/OHRW01_FASTA.fasta	MGYA00170227	ERZ487399	root:Host-associated:Human:Digestive system:Large in	0.3298
MGYA00170228/OHOY01_FASTA.fasta	MGYA00170228	ERZ487326	root:Host-associated:Human:Digestive system:Large in	0.0808
MGYA00170229/OHMC01_FASTA.fasta	MGYA00170229	ERZ487242	root:Host-associated:Human:Digestive system:Large in	0.0398
MGYA00170230/OHBC01_FASTA.fasta	MGYA00170230	ERZ486927	root:Host-associated:Human:Digestive system:Large in	0.3806
MGYA00170231/OHSL01_FASTA.fasta	MGYA00170231	ERZ487410	root:Host-associated:Human:Digestive system:Large in	0.368
MGYA00170232/OHCN01_FASTA.fasta	MGYA00170232	ERZ486959	root:Host-associated:Human:Digestive system:Large in	0.375
MGYA00170233/OHOR01_FASTA.fasta	MGYA00170233	ERZ487313	root:Host-associated:Human:Digestive system:Large in	0.4444
MGYA00170234/OHRR01_FASTA.fasta	MGYA00170234	ERZ487386	root:Host-associated:Human:Digestive system:Large in	0.365
MGYA00170235/OHRJ01_FASTA.fasta	MGYA00170235	ERZ487383	root:Host-associated:Human:Digestive system:Large in	0.4256
MGYA00170236/OHIG01_FASTA.fasta	MGYA00170236	ERZ487137	root:Host-associated:Human:Digestive system:Large in	0.0278
MGYA00170237/OHNP01_FASTA.fasta	MGYA00170237	ERZ487264	root:Host-associated:Human:Digestive system:Large in	0.2968
MGYA00170238/OHCD01_FASTA.fasta	MGYA00170238	ERZ486956	root:Host-associated:Human:Digestive system:Large in	0.3066
MGYA00170239/OHMP01_FASTA.fasta	MGYA00170239	ERZ487238	root:Host-associated:Human:Digestive system:Large in	0.4958
MGYA00170240/OHCG01_FASTA.fasta	MGYA00170240	ERZ486968	root:Host-associated:Human:Digestive system:Large in	0.0342
MGYA00170241/OHRZ01_FASTA.fasta	MGYA00170241	ERZ487401	root:Host-associated:Human:Digestive system:Large in	0.295
MGYA00170242/OHBF01_FASTA.fasta	MGYA00170242	ERZ486928	root:Host-associated:Human:Digestive system:Large in	0.5252
MGYA00170243/OHND01_FASTA.fasta	MGYA00170243	ERZ487275	root:Host-associated:Human:Digestive system:Large in	0.1646
MGYA00170244/OHQU01_FASTA.fasta	MGYA00170244	ERZ487370	root:Host-associated:Human:Digestive system:Large in	0.0404
MGYA00170245/OHIE01_FASTA.fasta	MGYA00170245	ERZ487135	root:Host-associated:Human:Digestive system:Large in	0.0344
MGYA00170246/OHCK01_FASTA.fasta	MGYA00170246	ERZ486965	root:Host-associated:Human:Digestive system:Large in	0.4386
MGYA00170247/OKTO01_FASTA.fasta	MGYA00170247	ERZ487292	root:Host-associated:Human:Digestive system:Large in	0.2832
MGYA00170248/OHDZ01_FASTA.fasta	MGYA00170248	ERZ487008	root:Host-associated:Human:Digestive system:Large in	0.3908
MGYA00170249/OHCB01_FASTA.fasta	MGYA00170249	ERZ486950	root:Host-associated:Human:Digestive system:Large in	0.3174
MGYA00170250/OHRB01_FASTA.fasta	MGYA00170250	ERZ487381	root:Host-associated:Human:Digestive system:Large in	0.4044
MGYA00170251/OHDJ01_FASTA.fasta	MGYA00170251	ERZ486998	root:Host-associated:Human:Digestive system:Large in	0.088
MGYA00170252/OHTD01_FASTA.fasta	MGYA00170252	ERZ487420	root:Host-associated:Human:Digestive system:Large in	0.383
MGYA00170253/OHEJ01_FASTA.fasta	MGYA00170253	ERZ487018	root:Host-associated:Human:Digestive system:Large in	0.437
MGYA00170254/OHQF01_FASTA.fasta	MGYA00170254	ERZ487354	root:Host-associated:Human:Digestive system:Large in	0.3338
MGYA00170255/OHIV01_FASTA.fasta	MGYA00170255	ERZ487149	root:Host-associated:Human:Digestive system:Large in	0.0892
MGYA00170256/OHKN01_FASTA.fasta	MGYA00170256	ERZ487196	root:Host-associated:Human:Digestive system:Large in	0.3574
MGYA00170257/OKTT01_FASTA.fasta	MGYA00170257	ERZ487299	root:Host-associated:Human:Digestive system:Large in	0.3576
MGYA00170258/OHOF01_FASTA.fasta	MGYA00170258	ERZ487308	root:Host-associated:Human:Digestive system:Large in	0.036
MGYA00170259/OHPS01_FASTA.fasta	MGYA00170259	ERZ487344	root:Host-associated:Human:Digestive system:Large in	0.2758
MGYA00170260/OHER01_FASTA.fasta	MGYA00170260	ERZ487034	root:Host-associated:Human:Digestive system:Large in	0.2676
MGYA00170261/OHDW01_FASTA.fasta	MGYA00170261	ERZ487004	root:Host-associated:Human:Digestive system:Large in	0.2672
MGYA00170262/OHDP01_FASTA.fasta	MGYA00170262	ERZ486996	root:Host-associated:Human:Digestive system:Large in	0.341
MGYA00170263/OHPQ01_FASTA.fasta	MGYA00170263	ERZ487348	root:Host-associated:Human:Digestive system:Large in	0.354
MGYA00170264/OHHH01_FASTA.fasta	MGYA00170264	ERZ487094	root:Host-associated:Human:Digestive system:Large in	0.4754
MGYA00170265/OHTE01_FASTA.fasta	MGYA00170265	ERZ487432	root:Host-associated:Human:Digestive system:Large in	0.2986
MGYA00170266/OHEK01_FASTA.fasta	MGYA00170266	ERZ487026	root:Host-associated:Human:Digestive system:Large in	0.2976
MGYA00170267/OHLS01_FASTA.fasta	MGYA00170267	ERZ487233	root:Host-associated:Human:Digestive system:Large in	0.036
MGYA00170268/OHEU01_FASTA.fasta	MGYA00170268	ERZ487033	root:Host-associated:Human:Digestive system:Large in	0.376
MGYA00170269/OHQM01_FASTA.fasta	MGYA00170269	ERZ487351	root:Host-associated:Human:Digestive system:Large in	0.3942
MGYA00170270/OHGD01_FASTA.fasta	MGYA00170270	ERZ487074	root:Host-associated:Human:Digestive system:Large in	0.0606
MGYA00170271/OHGJ01_FASTA.fasta	MGYA00170271	ERZ487070	root:Host-associated:Human:Digestive system:Large in	0.348
MGYA00170272/OHID01_FASTA.fasta	MGYA00170272	ERZ487130	root:Host-associated:Human:Digestive system:Large in	0.047
MGYA00170273/OHDL01_FASTA.fasta	MGYA00170273	ERZ487000	root:Host-associated:Human:Digestive system:Large in	0.1092
MGYA00170274/OHNF01_FASTA.fasta	MGYA00170274	ERZ487266	root:Host-associated:Human:Digestive system:Large in	0.4812
MGYA00170275/OHOI01_FASTA.fasta	MGYA00170275	ERZ487293	root:Host-associated:Human:Digestive system:Large in	0.2958
MGYA00170276/OKSP01_FASTA.fasta	MGYA00170276	ERZ487014	root:Host-associated:Human:Digestive system:Large in	0.381
MGYA00170277/OHHR01_FASTA.fasta	MGYA00170277	ERZ487112	root:Host-associated:Human:Digestive system:Large in	0.3904
MGYA00170278/OHPH01_FASTA.fasta	MGYA00170278	ERZ487322	root:Host-associated:Human:Digestive system:Large in	0.3182
MGYA00170279/OHAR01_FASTA.fasta	MGYA00170279	ERZ486921	root:Host-associated:Human:Digestive system:Large in	0.3342
MGYA00170280/OHTS01_FASTA.fasta	MGYA00170280	ERZ487461	root:Host-associated:Human:Digestive system:Large in	0.0592
MGYA00170281/OKSM01_FASTA.fasta	MGYA00170281	ERZ486985	root:Host-associated:Human:Digestive system:Large in	0.313
MGYA00170282/OHTA01_FASTA.fasta	MGYA00170282	ERZ487424	root:Host-associated:Human:Digestive system:Large in	0.3842
MGYA00170283/OHMY01_FASTA.fasta	MGYA00170283	ERZ487249	root:Host-associated:Human:Digestive system:Large in	0.4244
MGYA00170284/OKTL01_FASTA.fasta	MGYA00170284	ERZ487284	root:Host-associated:Human:Digestive system:Large in	0.3354
MGYA00170285/OHFU01_FASTA.fasta	MGYA00170285	ERZ487058	root:Host-associated:Human:Digestive system:Large in	0.0244
MGYA00170286/OHQJ01_FASTA.fasta	MGYA00170286	ERZ487359	root:Host-associated:Human:Digestive system:Large in	0.3876
MGYA00170287/OHDQ01_FASTA.fasta	MGYA00170287	ERZ486994	root:Host-associated:Human:Digestive system:Large in	0.376
MGYA00170288/OHHN01_FASTA.fasta	MGYA00170288	ERZ487116	root:Host-associated:Human:Digestive system:Large in	0.503

MGYA00170289/OHDM01_FASTA.fasta	MGYA00170289	ERZ487003	root:Host-associated:Human:Digestive system:Large in	0.0338
MGYA00170290/OHTR01_FASTA.fasta	MGYA00170290	ERZ487453	root:Host-associated:Human:Digestive system:Large in	0.0308
MGYA00170291/OHLQ01_FASTA.fasta	MGYA00170291	ERZ487213	root:Host-associated:Human:Digestive system:Large in	0.4028
MGYA00170292/OHOG01_FASTA.fasta	MGYA00170292	ERZ487303	root:Host-associated:Human:Digestive system:Large in	0.2258
MGYA00170293/OHGV01_FASTA.fasta	MGYA00170293	ERZ487095	root:Host-associated:Human:Digestive system:Large in	0.0378
MGYA00170294/OHDY01_FASTA.fasta	MGYA00170294	ERZ487015	root:Host-associated:Human:Digestive system:Large in	0.072
MGYA00170295/OHGN01_FASTA.fasta	MGYA00170295	ERZ487077	root:Host-associated:Human:Digestive system:Large in	0.2508
MGYA00170296/OHDG01_FASTA.fasta	MGYA00170296	ERZ486983	root:Host-associated:Human:Digestive system:Large in	0.2918
MGYA00170297/OHLH01_FASTA.fasta	MGYA00170297	ERZ487205	root:Host-associated:Human:Digestive system:Large in	0.3544
MGYA00170298/OHBW01_FASTA.fasta	MGYA00170298	ERZ486938	root:Host-associated:Human:Digestive system:Large in	0.442
MGYA00170299/OHGE01_FASTA.fasta	MGYA00170299	ERZ487075	root:Host-associated:Human:Digestive system:Large in	0.0592
MGYA00170300/OHCL01_FASTA.fasta	MGYA00170300	ERZ486961	root:Host-associated:Human:Digestive system:Large in	0.3106
MGYA00170301/OHEV01_FASTA.fasta	MGYA00170301	ERZ487030	root:Host-associated:Human:Digestive system:Large in	0.3168
MGYA00170303/OHRU01_FASTA.fasta	MGYA00170303	ERZ487391	root:Host-associated:Human:Digestive system:Large in	0.3582
MGYA00170304/OHFF01_FASTA.fasta	MGYA00170304	ERZ487049	root:Host-associated:Human:Digestive system:Large in	0.0302
MGYA00170305/OHJU01_FASTA.fasta	MGYA00170305	ERZ487173	root:Host-associated:Human:Digestive system:Large in	0.3728
MGYA00170306/OKSW01_FASTA.fasta	MGYA00170306	ERZ487098	root:Host-associated:Human:Digestive system:Large in	0.346
MGYA00170307/OHUC01_FASTA.fasta	MGYA00170307	ERZ487457	root:Host-associated:Human:Digestive system:Large in	0.3458
MGYA00170308/OHFH01_FASTA.fasta	MGYA00170308	ERZ487038	root:Host-associated:Human:Digestive system:Large in	0.2838
MGYA00170309/OKTW01_FASTA.fasta	MGYA00170309	ERZ487449	root:Host-associated:Human:Digestive system:Large in	0.3858
MGYA00170310/OHFL01_FASTA.fasta	MGYA00170310	ERZ487040	root:Host-associated:Human:Digestive system:Large in	0.3182
MGYA00170311/OHBO01_FASTA.fasta	MGYA00170311	ERZ486945	root:Host-associated:Human:Digestive system:Large in	0.2866
MGYA00170312/OHSW01_FASTA.fasta	MGYA00170312	ERZ487422	root:Host-associated:Human:Digestive system:Large in	0.3318
MGYA00170313/OHIC01_FASTA.fasta	MGYA00170313	ERZ487117	root:Host-associated:Human:Digestive system:Large in	0.4052
MGYA00170314/OHLK01_FASTA.fasta	MGYA00170314	ERZ487210	root:Host-associated:Human:Digestive system:Large in	0.2368
MGYA00170315/OHGW01_FASTA.fasta	MGYA00170315	ERZ487082	root:Host-associated:Human:Digestive system:Large in	0.4726
MGYA00170316/OHIM01_FASTA.fasta	MGYA00170316	ERZ487131	root:Host-associated:Human:Digestive system:Large in	0.5156
MGYA00170317/OHSN01_FASTA.fasta	MGYA00170317	ERZ487411	root:Host-associated:Human:Digestive system:Large in	0.3302
MGYA00170318/OHKL01_FASTA.fasta	MGYA00170318	ERZ487179	root:Host-associated:Human:Digestive system:Large in	0.333
MGYA00170319/OHII01_FASTA.fasta	MGYA00170319	ERZ487142	root:Host-associated:Human:Digestive system:Large in	0.3618
MGYA00170320/OHKX01_FASTA.fasta	MGYA00170320	ERZ487194	root:Host-associated:Human:Digestive system:Large in	0.3642
MGYA00170321/OHTM01_FASTA.fasta	MGYA00170321	ERZ487439	root:Host-associated:Human:Digestive system:Large in	0.385
MGYA00170322/OHTN01_FASTA.fasta	MGYA00170322	ERZ487450	root:Host-associated:Human:Digestive system:Large in	0.3866
MGYA00170323/OHDS01_FASTA.fasta	MGYA00170323	ERZ486993	root:Host-associated:Human:Digestive system:Large in	0.3056
MGYA00170324/OHTV01_FASTA.fasta	MGYA00170324	ERZ487440	root:Host-associated:Human:Digestive system:Large in	0.3338
MGYA00170325/OHPE01_FASTA.fasta	MGYA00170325	ERZ487327	root:Host-associated:Human:Digestive system:Large in	0.2682
MGYA00170326/OHKT01_FASTA.fasta	MGYA00170326	ERZ487195	root:Host-associated:Human:Digestive system:Large in	0.3178
MGYA00170327/OHNV01_FASTA.fasta	MGYA00170327	ERZ487290	root:Host-associated:Human:Digestive system:Large in	0.0384
MGYA00170328/OHLN01_FASTA.fasta	MGYA00170328	ERZ487216	root:Host-associated:Human:Digestive system:Large in	0.366
MGYA00170329/OHPC01_FASTA.fasta	MGYA00170329	ERZ487314	root:Host-associated:Human:Digestive system:Large in	0.4328
MGYA00170330/OHPL01_FASTA.fasta	MGYA00170330	ERZ487329	root:Host-associated:Human:Digestive system:Large in	0.3298
MGYA00170331/OHIZ01_FASTA.fasta	MGYA00170331	ERZ487157	root:Host-associated:Human:Digestive system:Large in	0.2952
MGYA00170332/OHJT01_FASTA.fasta	MGYA00170332	ERZ487172	root:Host-associated:Human:Digestive system:Large in	0.4468
MGYA00170333/OHBL01_FASTA.fasta	MGYA00170333	ERZ486937	root:Host-associated:Human:Digestive system:Large in	0.2854
MGYA00170334/OHMD01_FASTA.fasta	MGYA00170334	ERZ487234	root:Host-associated:Human:Digestive system:Large in	0.3054
MGYA00170335/OHNC01_FASTA.fasta	MGYA00170335	ERZ487273	root:Host-associated:Human:Digestive system:Large in	0.029
MGYA00170336/OKTI01_FASTA.fasta	MGYA00170336	ERZ487261	root:Host-associated:Human:Digestive system:Large in	0.2868
MGYA00170337/OHKC01_FASTA.fasta	MGYA00170337	ERZ487174	root:Host-associated:Human:Digestive system:Large in	0.3016
MGYA00170338/OHME01_FASTA.fasta	MGYA00170338	ERZ487229	root:Host-associated:Human:Digestive system:Large in	0.2634
MGYA00170339/OHGU01_FASTA.fasta	MGYA00170339	ERZ487085	root:Host-associated:Human:Digestive system:Large in	0.2778
MGYA00170340/OHMH01_FASTA.fasta	MGYA00170340	ERZ487236	root:Host-associated:Human:Digestive system:Large in	0.3292
MGYA00170341/OKTQ01_FASTA.fasta	MGYA00170341	ERZ487452	root:Host-associated:Human:Digestive system:Large in	0.0326
MGYA00170342/OHBR01_FASTA.fasta	MGYA00170342	ERZ486947	root:Host-associated:Human:Digestive system:Large in	0.3578
MGYA00170343/OKST01_FASTA.fasta	MGYA00170343	ERZ487091	root:Host-associated:Human:Digestive system:Large in	0.3688
MGYA00170344/OHSQ01_FASTA.fasta	MGYA00170344	ERZ487415	root:Host-associated:Human:Digestive system:Large in	0.4572
MGYA00170345/OHGG01_FASTA.fasta	MGYA00170345	ERZ487061	root:Host-associated:Human:Digestive system:Large in	0.3276
MGYA00170346/OKSI01_FASTA.fasta	MGYA00170346	ERZ486929	root:Host-associated:Human:Digestive system:Large in	0.3442
MGYA00170347/OHJH01_FASTA.fasta	MGYA00170347	ERZ487155	root:Host-associated:Human:Digestive system:Large in	0.4484
MGYA00170348/OHQQ01_FASTA.fasta	MGYA00170348	ERZ487365	root:Host-associated:Human:Digestive system:Large in	0.3188
MGYA00170349/OHBA01_FASTA.fasta	MGYA00170349	ERZ486924	root:Host-associated:Human:Digestive system:Large in	0.3576
MGYA00170350/OHPJ01_FASTA.fasta	MGYA00170350	ERZ487324	root:Host-associated:Human:Digestive system:Large in	0.4282
MGYA00170351/OKSU01_FASTA.fasta	MGYA00170351	ERZ487065	root:Host-associated:Human:Digestive system:Large in	0.3194
MGYA00170352/OHON01_FASTA.fasta	MGYA00170352	ERZ487307	root:Host-associated:Human:Digestive system:Large in	0.2836
MGYA00170353/OHSY01_FASTA.fasta	MGYA00170353	ERZ487434	root:Host-associated:Human:Digestive system:Large in	0.0326
MGYA00170354/OHPY01_FASTA.fasta	MGYA00170354	ERZ487350	root:Host-associated:Human:Digestive system:Large in	0.3116
MGYA00170355/OHHZ01_FASTA.fasta	MGYA00170355	ERZ487115	root:Host-associated:Human:Digestive system:Large in	0.3592
MGYA00170356/OHAL01_FASTA.fasta	MGYA00170356	ERZ486920	root:Host-associated:Human:Digestive system:Large in	0.2488
MGYA00170357/OHSF01_FASTA.fasta	MGYA00170357	ERZ487402	root:Host-associated:Human:Digestive system:Large in	0.3346
MGYA00170358/OHFD01_FASTA.fasta	MGYA00170358	ERZ487039	root:Host-associated:Human:Digestive system:Large in	0.2566
MGYA00170359/OKSJ01_FASTA.fasta	MGYA00170359	ERZ487006	root:Host-associated:Human:Digestive system:Large in	0.0668
MGYA00170360/OHDE01_FASTA.fasta	MGYA00170360	ERZ486982	root:Host-associated:Human:Digestive system:Large in	0.3298
MGYA00170361/OHLX01_FASTA.fasta	MGYA00170361	ERZ487223	root:Host-associated:Human:Digestive system:Large in	0.3296
MGYA00170362/OHBS01_FASTA.fasta	MGYA00170362	ERZ486951	root:Host-associated:Human:Digestive system:Large in	0.0476
MGYA00170363/OHLI01_FASTA.fasta	MGYA00170363	ERZ487209	root:Host-associated:Human:Digestive system:Large in	0.3076
MGYA00170364/OHRP01_FASTA.fasta	MGYA00170364	ERZ487390	root:Host-associated:Human:Digestive system:Large in	0.2556
MGYA00170365/OHTK01_FASTA.fasta	MGYA00170365	ERZ487437	root:Host-associated:Human:Digestive system:Large in	0.37
MGYA00170366/OHCV01_FASTA.fasta	MGYA00170366	ERZ486979	root:Host-associated:Human:Digestive system:Large in	0.0286
MGYA00170367/OHCR01_FASTA.fasta	MGYA00170367	ERZ486970	root:Host-associated:Human:Digestive system:Large in	0.404
MGYA00170368/OHSR01_FASTA.fasta	MGYA00170368	ERZ487428	root:Host-associated:Human:Digestive system:Large in	0.0502
MGYA00170369/OHQX01_FASTA.fasta	MGYA00170369	ERZ487377	root:Host-associated:Human:Digestive system:Large in	0.3222
MGYA00170370/OKSL01_FASTA.fasta	MGYA00170370	ERZ486942	root:Host-associated:Human:Digestive system:Large in	0.3626
MGYA00170371/OHBG01_FASTA.fasta	MGYA00170371	ERZ486933	root:Host-associated:Human:Digestive system:Large in	0.338
MGYA00170372/OHMJ01_FASTA.fasta	MGYA00170372	ERZ487252	root:Host-associated:Human:Digestive system:Large in	0.0374
MGYA00170373/OHEX01_FASTA.fasta	MGYA00170373	ERZ487029	root:Host-associated:Human:Digestive system:Large in	0.4574
MGYA00170374/OHJJ01_FASTA.fasta	MGYA00170374	ERZ487160	root:Host-associated:Human:Digestive system:Large in	0.3584
MGYA00170375/OHDN01_FASTA.fasta	MGYA00170375	ERZ486986	root:Host-associated:Human:Digestive system:Large in	0.3338
MGYA00170376/OHEW01_FASTA.fasta	MGYA00170376	ERZ487044	root:Host-associated:Human:Digestive system:Large in	0.089
MGYA00170377/OHIT01_FASTA.fasta	MGYA00170377	ERZ487146	root:Host-associated:Human:Digestive system:Large in	0.1702
MGYA00170378/OHBH01_FASTA.fasta	MGYA00170378	ERZ486930	root:Host-associated:Human:Digestive system:Large in	0.473
MGYA00170379/OHDT01_FASTA.fasta	MGYA00170379	ERZ486997	root:Host-associated:Human:Digestive system:Large in	0.3828
MGYA00170380/OKTF01_FASTA.fasta	MGYA00170380	ERZ487150	root:Host-associated:Human:Digestive system:Large in	0.4322
MGYA00170381/OHHK01_FASTA.fasta	MGYA00170381	ERZ487119	root:Host-associated:Human:Digestive system:Large in	0.3762
MGYA00170382/OHST01_FASTA.fasta	MGYA00170382	ERZ487425	root:Host-associated:Human:Digestive system:Large in	0.3494
MGYA00170383/OHKY01_FASTA.fasta	MGYA00170383	ERZ487204	root:Host-associated:Human:Digestive system:Large in	0.3574
MGYA00170384/OHHQ01_FASTA.fasta	MGYA00170384	ERZ487105	root:Host-associated:Human:Digestive system:Large in	0.3872
MGYA00170385/OHTC01_FASTA.fasta	MGYA00170385	ERZ487419	root:Host-associated:Human:Digestive system:Large in	0.364
MGYA00170386/OHRL01_FASTA.fasta	MGYA00170386	ERZ487371	root:Host-associated:Human:Digestive system:Large in	0.4334
MGYA00170387/OHJC01_FASTA.fasta	MGYA00170387	ERZ487148	root:Host-associated:Human:Digestive system:Large in	0.3892
MGYA00170388/OHQW01_FASTA.fasta	MGYA00170388	ERZ487378	root:Host-associated:Human:Digestive system:Large in	0.0758
MGYA00170389/OHEB01_FASTA.fasta	MGYA00170389	ERZ486999	root:Host-associated:Human:Digestive system:Large in	0.277
MGYA00170390/OHOM01_FASTA.fasta	MGYA00170390	ERZ487310	root:Host-associated:Human:Digestive system:Large in	0.2972
MGYA00170391/OHMR01_FASTA.fasta	MGYA00170391	ERZ487247	root:Host-associated:Human:Digestive system:Large in	0.234
MGYA00170392/OHMZ01_FASTA.fasta	MGYA00170392	ERZ487263	root:Host-associated:Human:Digestive system:Large in	0.0294
MGYA00170393/OHKG01_FASTA.fasta	MGYA00170393	ERZ487171	root:Host-associated:Human:Digestive system:Large in	0.363
MGYA00170394/OHPA01_FASTA.fasta	MGYA00170394	ERZ487333	root:Host-associated:Human:Digestive system:Large in	0.0398
MGYA00170395/OHBU01_FASTA.fasta	MGYA00170395	ERZ486944	root:Host-associated:Human:Digestive system:Large in	0.4452
MGYA00170396/OHQN01_FASTA.fasta	MGYA00170396	ERZ487357	root:Host-associated:Human:Digestive system:Large in	0.2904
MGYA00170397/OHLD01_FASTA.fasta	MGYA00170397	ERZ487214	root:Host-associated:Human:Digestive system:Large in	0.087
MGYA00170398/OKTP01_FASTA.fasta	MGYA00170398	ERZ487295	root:Host-associated:Human:Digestive system:Large in	0.2698
MGYA00170399/OHJI01_FASTA.fasta	MGYA00170399	ERZ487164	root:Host-associated:Human:Digestive system:Large in	0.3352
MGYA00170400/OHQA01_FASTA.fasta	MGYA00170400	ERZ487342	root:Host-associated:Human:Digestive system:Large in	0.3724
MGYA00170401/OHOQ01_FASTA.fasta	MGYA00170401	ERZ487309	root:Host-associated:Human:Digestive system:Large in	0.3874
MGYA00170402/OHNQ01_FASTA.fasta	MGYA00170402	ERZ487272	root:Host-associated:Human:Digestive system:Large in	0.35
MGYA00170403/OKTU01_FASTA.fasta	MGYA00170403	ERZ487297	root:Host-associated:Human:Digestive system:Large in	0.465
MGYA00170404/OKSX01_FASTA.fasta	MGYA00170404	ERZ487103	root:Host-associated:Human:Digestive system:Large in	0.3414
MGYA00170405/OHNH01_FASTA.fasta	MGYA00170405	ERZ487268	root:Host-associated:Human:Digestive system:Large in	0.371
MGYA00170406/OHSI01_FASTA.fasta	MGYA00170406	ERZ487396	root:Host-associated:Human:Digestive system:Large in	0.3878
MGYA00170407/OHEN01_FASTA.fasta	MGYA00170407	ERZ487024	root:Host-associated:Human:Digestive system:Large in	0.2998
MGYA00170408/OHLB01_FASTA.fasta	MGYA00170408	ERZ487206	root:Host-associated:Human:Digestive system:Large in	0.3454
MGYA00170409/OHGB01_FASTA.fasta	MGYA00170409	ERZ487073	root:Host-associated:Human:Digestive system:Large in	0.0518
MGYA00170410/OKTG01_FASTA.fasta	MGYA00170410	ERZ487250	root:Host-associated:Human:Digestive system:Large in	0.3698
MGYA00170411/OHOE01_FASTA.fasta	MGYA00170411	ERZ487304	root:Host-associated:Human:Digestive system:Large in	0.0618
MGYA00170412/OHEH01_FASTA.fasta	MGYA00170412	ERZ487020	root:Host-associated:Human:Digestive system:Large in	0.4198
MGYA00170413/OHSU01_FASTA.fasta	MGYA00170413	ERZ487431	root:Host-associated:Human:Digestive system:Large in	0.0532
MGYA00170414/OHJN01_FASTA.fasta	MGYA00170414	ERZ487167	root:Host-associated:Human:Digestive system:Large in	0.3276
MGYA00170415/OHCT01_FASTA.fasta	MGYA00170415	ERZ486975	root:Host-associated:Human:Digestive system:Large in	0.4118
MGYA00170416/OHRV01_FASTA.fasta	MGYA00170416	ERZ487393	root:Host-associated:Human:Digestive system:Large in	0.4702
MGYA00170417/OHKR01_FASTA.fasta	MGYA00170417	ERZ487197	root:Host-associated:Human:Digestive system:Large in	0.2384
MGYA00170418/OHIB01_FASTA.fasta	MGYA00170418	ERZ487121	root:Host-associated:Human:Digestive system:Large in	0.345
MGYA00170419/OHNS01_FASTA.fasta	MGYA00170419	ERZ487282	root:Host-associated:Human:Digestive system:Large in	0.0846
MGYA00170420/OHCJ01_FASTA.fasta	MGYA00170420	ERZ486971	root:Host-associated:Human:Digestive system:Large in	0.4338
MGYA00170421/OHNE01_FASTA.fasta	MGYA00170421	ERZ487262	root:Host-associated:Human:Digestive system:Large in	0.386
MGYA00170422/OHFZ01_FASTA.fasta	MGYA00170422	ERZ487069	root:Host-associated:Human:Digestive system:Large in	0.0422
MGYA00170423/OHTI01_FASTA.fasta	MGYA00170423	ERZ487436	root:Host-associated:Human:Digestive system:Large in	0.3
MGYA00170424/OHAV01_FASTA.fasta	MGYA00170424	ERZ486923	root:Host-associated:Human:Digestive system:Large in	0.39
MGYA00170425/OHDD01_FASTA.fasta	MGYA00170425	ERZ486984	root:Host-associated:Human:Digestive system:Large in	0.5152
MGYA00170426/OHTL01_FASTA.fasta	MGYA00170426	ERZ487441	root:Host-associated:Human:Digestive system:Large in	0.0296
MGYA00170427/OHBX01_FASTA.fasta	MGYA00170427	ERZ486939	root:Host-associated:Human:Digestive system:Large in	0.327
MGYA00170428/OHKD01_FASTA.fasta	MGYA00170428	ERZ487181	root:Host-associated:Human:Digestive system:Large in	0.4066
MGYA00170429/OHFR01_FASTA.fasta	MGYA00170429	ERZ487053	root:Host-associated:Human:Digestive system:Large in	0.3712
MGYA00170430/OHBZ01_FASTA.fasta	MGYA00170430	ERZ486955	root:Host-associated:Human:Digestive system:Large in	0.3558
MGYA00170431/OHMI01_FASTA.fasta	MGYA00170431	ERZ487244	root:Host-associated:Human:Digestive system:Large in	0.3532
MGYA00170432/OHPP01_FASTA.fasta	MGYA00170432	ERZ487332	root:Host-associated:Human:Digestive system:Large in	0.4088
MGYA00170433/OHKB01_FASTA.fasta	MGYA00170433	ERZ487180	root:Host-associated:Human:Digestive system:Large in	0.5
MGYA00170434/OHOK01_FASTA.fasta	MGYA00170434	ERZ487288	root:Host-associated:Human:Digestive system:Large in	0.377
MGYA00170435/OKSZ01_FASTA.fasta	MGYA00170435	ERZ487108	root:Host-associated:Human:Digestive system:Large in	0.338
MGYA00170436/OHHX01_FASTA.fasta	MGYA00170436	ERZ487126	root:Host-associated:Human:Digestive system:Large in	0.0468
MGYA00170437/OHTF01_FASTA.fasta	MGYA00170437	ERZ487417	root:Host-associated:Human:Digestive system:Large in	0.366
MGYA00170438/OHQT01_FASTA.fasta	MGYA00170438	ERZ487366	root:Host-associated:Human:Digestive system:Large in	0.297
MGYA00170439/OHRC01_FASTA.fasta	MGYA00170439	ERZ487369	root:Host-associated:Human:Digestive system:Large in	0.3742
MGYA00170440/OHCO01_FASTA.fasta	MGYA00170440	ERZ486962	root:Host-associated:Human:Digestive system:Large in	0.4584
MGYA00170441/OHQE01_FASTA.fasta	MGYA00170441	ERZ487355	root:Host-associated:Human:Digestive system:Large in	0.2578
MGYA00170442/OHDB01_FASTA.fasta	MGYA00170442	ERZ486987	root:Host-associated:Human:Digestive system:Large in	0.0528
MGYA00170443/OHBQ01_FASTA.fasta	MGYA00170443	ERZ486941	root:Host-associated:Human:Digestive system:Large in	0.4134
MGYA00170444/OHEY01_FASTA.fasta	MGYA00170444	ERZ487043	root:Host-associated:Human:Digestive system:Large in	0.2448
MGYA00170445/OHMU01_FASTA.fasta	MGYA00170445	ERZ487253	root:Host-associated:Human:Digestive system:Large in	0.2946
MGYA00170446/OHSC01_FASTA.fasta	MGYA00170446	ERZ487407	root:Host-associated:Human:Digestive system:Large in	0.0422
MGYA00170447/OHAW01_FASTA.fasta	MGYA00170447	ERZ486922	root:Host-associated:Human:Digestive system:Large in	0.3968
MGYA00170448/OHHY01_FASTA.fasta	MGYA00170448	ERZ487102	root:Host-associated:Human:Digestive system:Large in	0.4158
MGYA00170449/OHQR01_FASTA.fasta	MGYA00170449	ERZ487364	root:Host-associated:Human:Digestive system:Large in	0.4528
MGYA00170450/OHMF01_FASTA.fasta	MGYA00170450	ERZ487235	root:Host-associated:Human:Digestive system:Large in	0.39
MGYA00170451/OHMK01_FASTA.fasta	MGYA00170451	ERZ487239	root:Host-associated:Human:Digestive system:Large in	0.441
MGYA00170452/OHOA01_FASTA.fasta	MGYA00170452	ERZ487280	root:Host-associated:Human:Digestive system:Large in	0.3372
MGYA00170453/OHGF01_FASTA.fasta	MGYA00170453	ERZ487071	root:Host-associated:Human:Digestive system:Large in	0.1176
MGYA00170454/OHJB01_FASTA.fasta	MGYA00170454	ERZ487144	root:Host-associated:Human:Digestive system:Large in	0.3442
MGYA00170455/OHSP01_FASTA.fasta	MGYA00170455	ERZ487416	root:Host-associated:Human:Digestive system:Large in	0.5092
MGYA00170456/OHIR01_FASTA.fasta	MGYA00170456	ERZ487132	root:Host-associated:Human:Digestive system:Large in	0.325
MGYA00170457/OHBY01_FASTA.fasta	MGYA00170457	ERZ486948	root:Host-associated:Human:Digestive system:Large in	0.3552
MGYA00170458/OHTT01_FASTA.fasta	MGYA00170458	ERZ487444	root:Host-associated:Human:Digestive system:Large in	0.3308
MGYA00170459/OHDU01_FASTA.fasta	MGYA00170459	ERZ487002	root:Host-associated:Human:Digestive system:Large in	0.2466
MGYA00170460/OHKU01_FASTA.fasta	MGYA00170460	ERZ487202	root:Host-associated:Human:Digestive system:Large in	0.3836
MGYA00170461/OHJZ01_FASTA.fasta	MGYA00170461	ERZ487185	root:Host-associated:Human:Digestive system:Large in	0.3838
MGYA00170462/OHTQ01_FASTA.fasta	MGYA00170462	ERZ487456	root:Host-associated:Human:Digestive system:Large in	0.0416
MGYA00170463/OHLT01_FASTA.fasta	MGYA00170463	ERZ487231	root:Host-associated:Human:Digestive system:Large in	0.0384
MGYA00170464/OHSB01_FASTA.fasta	MGYA00170464	ERZ487404	root:Host-associated:Human:Digestive system:Large in	0.2886
MGYA00170465/OHGT01_FASTA.fasta	MGYA00170465	ERZ487081	root:Host-associated:Human:Digestive system:Large in	0.3522
MGYA00170466/OHGM01_FASTA.fasta	MGYA00170466	ERZ487084	root:Host-associated:Human:Digestive system:Large in	0.0716
MGYA00170467/OHDF01_FASTA.fasta	MGYA00170467	ERZ486977	root:Host-associated:Human:Digestive system:Large in	0.3058
MGYA00170468/OHQD01_FASTA.fasta	MGYA00170468	ERZ487356	root:Host-associated:Human:Digestive system:Large in	0.035
MGYA00170469/OHDI01_FASTA.fasta	MGYA00170469	ERZ486988	root:Host-associated:Human:Digestive system:Large in	0.3644
MGYA00170470/OHCY01_FASTA.fasta	MGYA00170470	ERZ486980	root:Host-associated:Human:Digestive system:Large in	0.3734
MGYA00170471/OHMX01_FASTA.fasta	MGYA00170471	ERZ487256	root:Host-associated:Human:Digestive system:Large in	0.298
MGYA00170472/OHOP01_FASTA.fasta	MGYA00170472	ERZ487301	root:Host-associated:Human:Digestive system:Large in	0.2798
MGYA00170473/OHKM01_FASTA.fasta	MGYA00170473	ERZ487200	root:Host-associated:Human:Digestive system:Large in	0.0368
MGYA00170474/OHBP01_FASTA.fasta	MGYA00170474	ERZ486940	root:Host-associated:Human:Digestive system:Large in	0.3366
MGYA00170475/OHML01_FASTA.fasta	MGYA00170475	ERZ487246	root:Host-associated:Human:Digestive system:Large in	0.2232
MGYA00170476/OHLC01_FASTA.fasta	MGYA00170476	ERZ487203	root:Host-associated:Human:Digestive system:Large in	0.331
MGYA00170477/OHJS01_FASTA.fasta	MGYA00170477	ERZ487170	root:Host-associated:Human:Digestive system:Large in	0.368
MGYA00170478/OHCH01_FASTA.fasta	MGYA00170478	ERZ486963	root:Host-associated:Human:Digestive system:Large in	0.2728
MGYA00170479/OHSM01_FASTA.fasta	MGYA00170479	ERZ487412	root:Host-associated:Human:Digestive system:Large in	0.3456
MGYA00170480/OHHF01_FASTA.fasta	MGYA00170480	ERZ487111	root:Host-associated:Human:Digestive system:Large in	0.0274
MGYA00170481/OHEA01_FASTA.fasta	MGYA00170481	ERZ487019	root:Host-associated:Human:Digestive system:Large in	0.0736
MGYA00170482/OHIA01_FASTA.fasta	MGYA00170482	ERZ487128	root:Host-associated:Human:Digestive system:Large in	0.0582
MGYA00170483/OHNL01_FASTA.fasta	MGYA00170483	ERZ487269	root:Host-associated:Human:Digestive system:Large in	0.329
MGYA00170484/OHTO01_FASTA.fasta	MGYA00170484	ERZ487445	root:Host-associated:Human:Digestive system:Large in	0.2322
MGYA00170485/OHTH01_FASTA.fasta	MGYA00170485	ERZ487421	root:Host-associated:Human:Digestive system:Large in	0.3904
MGYA00170486/OHCI01_FASTA.fasta	MGYA00170486	ERZ486969	root:Host-associated:Human:Digestive system:Large in	0.3192
MGYA00170487/OHSA01_FASTA.fasta	MGYA00170487	ERZ487403	root:Host-associated:Human:Digestive system:Large in	0.4356
MGYA00170488/OHGA01_FASTA.fasta	MGYA00170488	ERZ487060	root:Host-associated:Human:Digestive system:Large in	0.3752
MGYA00170489/OHCU01_FASTA.fasta	MGYA00170489	ERZ486973	root:Host-associated:Human:Digestive system:Large in	0.3492
MGYA00170490/OHNK01_FASTA.fasta	MGYA00170490	ERZ487260	root:Host-associated:Human:Digestive system:Large in	0.3756
MGYA00170491/OHUD01_FASTA.fasta	MGYA00170491	ERZ487447	root:Host-associated:Human:Digestive system:Large in	0.355
MGYA00170492/OHFC01_FASTA.fasta	MGYA00170492	ERZ487037	root:Host-associated:Human:Digestive system:Large in	0.3068
MGYA00170493/OHQY01_FASTA.fasta	MGYA00170493	ERZ487373	root:Host-associated:Human:Digestive system:Large in	0.3438
MGYA00170494/OHCM01_FASTA.fasta	MGYA00170494	ERZ486966	root:Host-associated:Human:Digestive system:Large in	0.3642
MGYA00170495/OHNG01_FASTA.fasta	MGYA00170495	ERZ487259	root:Host-associated:Human:Digestive system:Large in	0.3148
MGYA00170496/OHOW01_FASTA.fasta	MGYA00170496	ERZ487328	root:Host-associated:Human:Digestive system:Large in	0.0322
MGYA00170497/OHJG01_FASTA.fasta	MGYA00170497	ERZ487156	root:Host-associated:Human:Digestive system:Large in	0.3368
MGYA00170498/OHPO01_FASTA.fasta	MGYA00170498	ERZ487334	root:Host-associated:Human:Digestive system:Large in	0.3776
MGYA00170499/OHES01_FASTA.fasta	MGYA00170499	ERZ487028	root:Host-associated:Human:Digestive system:Large in	0.456
MGYA00170500/OHQP01_FASTA.fasta	MGYA00170500	ERZ487360	root:Host-associated:Human:Digestive system:Large in	0.2464
MGYA00170501/OHIP01_FASTA.fasta	MGYA00170501	ERZ487143	root:Host-associated:Human:Digestive system:Large in	0.0504
MGYA00170502/OHLJ01_FASTA.fasta	MGYA00170502	ERZ487199	root:Host-associated:Human:Digestive system:Large in	0.3246
MGYA00170503/OHQS01_FASTA.fasta	MGYA00170503	ERZ487358	root:Host-associated:Human:Digestive system:Large in	0.424
MGYA00170504/OHBK01_FASTA.fasta	MGYA00170504	ERZ486931	root:Host-associated:Human:Digestive system:Large in	0.3576
MGYA00170505/OKTN01_FASTA.fasta	MGYA00170505	ERZ487283	root:Host-associated:Human:Digestive system:Large in	0.3484
MGYA00170506/OHFI01_FASTA.fasta	MGYA00170506	ERZ487041	root:Host-associated:Human:Digestive system:Large in	0.2888
MGYA00170507/OHRX01_FASTA.fasta	MGYA00170507	ERZ487398	root:Host-associated:Human:Digestive system:Large in	0.295
MGYA00170508/OHKV01_FASTA.fasta	MGYA00170508	ERZ487193	root:Host-associated:Human:Digestive system:Large in	0.405
MGYA00170509/OHPM01_FASTA.fasta	MGYA00170509	ERZ487330	root:Host-associated:Human:Digestive system:Large in	0.305
MGYA00170510/OHDH01_FASTA.fasta	MGYA00170510	ERZ486981	root:Host-associated:Human:Digestive system:Large in	0.4316
MGYA00170511/OHLW01_FASTA.fasta	MGYA00170511	ERZ487226	root:Host-associated:Human:Digestive system:Large in	0.2862
MGYA00170512/OHJK01_FASTA.fasta	MGYA00170512	ERZ487159	root:Host-associated:Human:Digestive system:Large in	0.3884
MGYA00170513/OHKW01_FASTA.fasta	MGYA00170513	ERZ487191	root:Host-associated:Human:Digestive system:Large in	0.399
MGYA00170514/OHPV01_FASTA.fasta	MGYA00170514	ERZ487343	root:Host-associated:Human:Digestive system:Large in	0.3536
MGYA00170515/OHBN01_FASTA.fasta	MGYA00170515	ERZ486936	root:Host-associated:Human:Digestive system:Large in	0.3934
MGYA00170516/OHPZ01_FASTA.fasta	MGYA00170516	ERZ487349	root:Host-associated:Human:Digestive system:Large in	0.3054
MGYA00170517/OHOZ01_FASTA.fasta	MGYA00170517	ERZ487311	root:Host-associated:Human:Digestive system:Large in	0.5242
MGYA00170518/OHKK01_FASTA.fasta	MGYA00170518	ERZ487187	root:Host-associated:Human:Digestive system:Large in	0.381
MGYA00170519/OHSH01_FASTA.fasta	MGYA00170519	ERZ487397	root:Host-associated:Human:Digestive system:Large in	0.3266
MGYA00170520/OHTW01_FASTA.fasta	MGYA00170520	ERZ487438	root:Host-associated:Human:Digestive system:Large in	0.3562
MGYA00170521/OKTM01_FASTA.fasta	MGYA00170521	ERZ487279	root:Host-associated:Human:Digestive system:Large in	0.2936
MGYA00170522/OHOT01_FASTA.fasta	MGYA00170522	ERZ487323	root:Host-associated:Human:Digestive system:Large in	0.0324
MGYA00170523/OHMS01_FASTA.fasta	MGYA00170523	ERZ487240	root:Host-associated:Human:Digestive system:Large in	0.3824
MGYA00170524/OHHC01_FASTA.fasta	MGYA00170524	ERZ487092	root:Host-associated:Human:Digestive system:Large in	0.3938
MGYA00170525/OHHV01_FASTA.fasta	MGYA00170525	ERZ487110	root:Host-associated:Human:Digestive system:Large in	0.3782
MGYA00170526/OHHA01_FASTA.fasta	MGYA00170526	ERZ487093	root:Host-associated:Human:Digestive system:Large in	0.2578
MGYA00170527/OHIY01_FASTA.fasta	MGYA00170527	ERZ487134	root:Host-associated:Human:Digestive system:Large in	0.2848
MGYA00170528/OHKZ01_FASTA.fasta	MGYA00170528	ERZ487212	root:Host-associated:Human:Digestive system:Large in	0.3848
MGYA00170529/OHOX01_FASTA.fasta	MGYA00170529	ERZ487331	root:Host-associated:Human:Digestive system:Large in	0.0396
MGYA00170530/OHOD01_FASTA.fasta	MGYA00170530	ERZ487300	root:Host-associated:Human:Digestive system:Large in	0.0426
MGYA00170531/OHLO01_FASTA.fasta	MGYA00170531	ERZ487221	root:Host-associated:Human:Digestive system:Large in	0.3638
MGYA00170532/OHFJ01_FASTA.fasta	MGYA00170532	ERZ487050	root:Host-associated:Human:Digestive system:Large in	0.0298
MGYA00170533/OHMO01_FASTA.fasta	MGYA00170533	ERZ487241	root:Host-associated:Human:Digestive system:Large in	0.3576
MGYA00170534/OHKO01_FASTA.fasta	MGYA00170534	ERZ487198	root:Host-associated:Human:Digestive system:Large in	0.3542
MGYA00170535/OHJD01_FASTA.fasta	MGYA00170535	ERZ487163	root:Host-associated:Human:Digestive system:Large in	0.0296
MGYA00170536/OHDR01_FASTA.fasta	MGYA00170536	ERZ486992	root:Host-associated:Human:Digestive system:Large in	0.3942
MGYA00170537/OHFG01_FASTA.fasta	MGYA00170537	ERZ487036	root:Host-associated:Human:Digestive system:Large in	0.3854
MGYA00170538/OHOB01_FASTA.fasta	MGYA00170538	ERZ487298	root:Host-associated:Human:Digestive system:Large in	0.036
MGYA00170539/OHSZ01_FASTA.fasta	MGYA00170539	ERZ487418	root:Host-associated:Human:Digestive system:Large in	0.3142
MGYA00170540/OHFA01_FASTA.fasta	MGYA00170540	ERZ487047	root:Host-associated:Human:Digestive system:Large in	0.3694
MGYA00170541/OHSD01_FASTA.fasta	MGYA00170541	ERZ487413	root:Host-associated:Human:Digestive system:Large in	0.0632
MGYA00170542/OHOJ01_FASTA.fasta	MGYA00170542	ERZ487294	root:Host-associated:Human:Digestive system:Large in	0.299
MGYA00170543/OHRN01_FASTA.fasta	MGYA00170543	ERZ487388	root:Host-associated:Human:Digestive system:Large in	0.301
MGYA00170544/OHRD01_FASTA.fasta	MGYA00170544	ERZ487375	root:Host-associated:Human:Digestive system:Large in	0.3414
MGYA00170545/OHHS01_FASTA.fasta	MGYA00170545	ERZ487122	root:Host-associated:Human:Digestive system:Large in	0.2892
MGYA00170546/OKSR01_FASTA.fasta	MGYA00170546	ERZ487012	root:Host-associated:Human:Digestive system:Large in	0.3656
MGYA00170547/OHUB01_FASTA.fasta	MGYA00170547	ERZ487448	root:Host-associated:Human:Digestive system:Large in	0.3542
MGYA00170548/OKSN01_FASTA.fasta	MGYA00170548	ERZ486989	root:Host-associated:Human:Digestive system:Large in	0.3916
MGYA00170549/OHSX01_FASTA.fasta	MGYA00170549	ERZ487423	root:Host-associated:Human:Digestive system:Large in	0.2806
MGYA00170550/OHCF01_FASTA.fasta	MGYA00170550	ERZ486949	root:Host-associated:Human:Digestive system:Large in	0.3572
MGYA00170551/OHFB01_FASTA.fasta	MGYA00170551	ERZ487045	root:Host-associated:Human:Digestive system:Large in	0.3604
MGYA00170552/OKSV01_FASTA.fasta	MGYA00170552	ERZ487031	root:Host-associated:Human:Digestive system:Large in	0.417
MGYA00170553/OHPX01_FASTA.fasta	MGYA00170553	ERZ487347	root:Host-associated:Human:Digestive system:Large in	0.4634
MGYA00170554/OHCQ01_FASTA.fasta	MGYA00170554	ERZ486960	root:Host-associated:Human:Digestive system:Large in	0.374
MGYA00170555/OHJQ01_FASTA.fasta	MGYA00170555	ERZ487161	root:Host-associated:Human:Digestive system:Large in	0.4604
MGYA00170556/OHLA01_FASTA.fasta	MGYA00170556	ERZ487207	root:Host-associated:Human:Digestive system:Large in	0.2948
MGYA00170557/OHHM01_FASTA.fasta	MGYA00170557	ERZ487104	root:Host-associated:Human:Digestive system:Large in	0.445
MGYA00170558/OHUN01_FASTA.fasta	MGYA00170558	ERZ487463	root:Host-associated:Human:Digestive system:Large in	0.3988
MGYA00170559/OHLZ01_FASTA.fasta	MGYA00170559	ERZ487232	root:Host-associated:Human:Digestive system:Large in	0.377
MGYA00170560/OHCS01_FASTA.fasta	MGYA00170560	ERZ486958	root:Host-associated:Human:Digestive system:Large in	0.4078
MGYA00170561/OHKE01_FASTA.fasta	MGYA00170561	ERZ487176	root:Host-associated:Human:Digestive system:Large in	0.298
MGYA00170562/OHRO01_FASTA.fasta	MGYA00170562	ERZ487387	root:Host-associated:Human:Digestive system:Large in	0.37
MGYA00170563/OHIS01_FASTA.fasta	MGYA00170563	ERZ487138	root:Host-associated:Human:Digestive system:Large in	0.3132
MGYA00170564/OHBD01_FASTA.fasta	MGYA00170564	ERZ486925	root:Host-associated:Human:Digestive system:Large in	0.4652
MGYA00170565/OKTK01_FASTA.fasta	MGYA00170565	ERZ487312	root:Host-associated:Human:Digestive system:Large in	0.0398
MGYA00170566/OHHB01_FASTA.fasta	MGYA00170566	ERZ487096	root:Host-associated:Human:Digestive system:Large in	0.338
MGYA00170567/OHHE01_FASTA.fasta	MGYA00170567	ERZ487097	root:Host-associated:Human:Digestive system:Large in	0.2852
MGYA00170568/OHLR01_FASTA.fasta	MGYA00170568	ERZ487222	root:Host-associated:Human:Digestive system:Large in	0.3758
MGYA00170569/OHMB01_FASTA.fasta	MGYA00170569	ERZ487237	root:Host-associated:Human:Digestive system:Large in	0.026
MGYA00170570/OHLP01_FASTA.fasta	MGYA00170570	ERZ487219	root:Host-associated:Human:Digestive system:Large in	0.3296
MGYA00170571/OHJX01_FASTA.fasta	MGYA00170571	ERZ487183	root:Host-associated:Human:Digestive system:Large in	0.4082
MGYA00170572/OHIF01_FASTA.fasta	MGYA00170572	ERZ487133	root:Host-associated:Human:Digestive system:Large in	0.1656
MGYA00170573/OHOC01_FASTA.fasta	MGYA00170573	ERZ487286	root:Host-associated:Human:Digestive system:Large in	0.2994
MGYA00170574/OHLL01_FASTA.fasta	MGYA00170574	ERZ487215	root:Host-associated:Human:Digestive system:Large in	0.3354
MGYA00170575/OHCE01_FASTA.fasta	MGYA00170575	ERZ486957	root:Host-associated:Human:Digestive system:Large in	0.3364
MGYA00170576/OHIJ01_FASTA.fasta	MGYA00170576	ERZ487139	root:Host-associated:Human:Digestive system:Large in	0.3064
MGYA00170577/OHRS01_FASTA.fasta	MGYA00170577	ERZ487389	root:Host-associated:Human:Digestive system:Large in	0.2868
MGYA00170578/OHPN01_FASTA.fasta	MGYA00170578	ERZ487345	root:Host-associated:Human:Digestive system:Large in	0.0758
MGYA00170579/OHCW01_FASTA.fasta	MGYA00170579	ERZ486964	root:Host-associated:Human:Digestive system:Large in	0.3902
MGYA00170580/OHEF01_FASTA.fasta	MGYA00170580	ERZ487009	root:Host-associated:Human:Digestive system:Large in	0.323
MGYA00170581/OHLM01_FASTA.fasta	MGYA00170581	ERZ487217	root:Host-associated:Human:Digestive system:Large in	0.3284
MGYA00170582/OHHG01_FASTA.fasta	MGYA00170582	ERZ487100	root:Host-associated:Human:Digestive system:Large in	0.266
MGYA00170583/OHEG01_FASTA.fasta	MGYA00170583	ERZ487010	root:Host-associated:Human:Digestive system:Large in	0.4248
MGYA00170584/OHJA01_FASTA.fasta	MGYA00170584	ERZ487147	root:Host-associated:Human:Digestive system:Large in	0.352
MGYA00170585/OHJY01_FASTA.fasta	MGYA00170585	ERZ487184	root:Host-associated:Human:Digestive system:Large in	0.3478
MGYA00170586/OHEO01_FASTA.fasta	MGYA00170586	ERZ487025	root:Host-associated:Human:Digestive system:Large in	0.321
MGYA00170587/OHDA01_FASTA.fasta	MGYA00170587	ERZ486976	root:Host-associated:Human:Digestive system:Large in	0.4336
MGYA00170588/OKTX01_FASTA.fasta	MGYA00170588	ERZ487426	root:Host-associated:Human:Digestive system:Large in	0.3638
MGYA00170589/OKSS01_FASTA.fasta	MGYA00170589	ERZ487054	root:Host-associated:Human:Digestive system:Large in	0.3112
MGYA00170590/OHNA01_FASTA.fasta	MGYA00170590	ERZ487257	root:Host-associated:Human:Digestive system:Large in	0.2996
MGYA00170591/OHJF01_FASTA.fasta	MGYA00170591	ERZ487165	root:Host-associated:Human:Digestive system:Large in	0.3724
MGYA00170592/OHAZ01_FASTA.fasta	MGYA00170592	ERZ486926	root:Host-associated:Human:Digestive system:Large in	0.0684
MGYA00170593/OHBB01_FASTA.fasta	MGYA00170593	ERZ486932	root:Host-associated:Human:Digestive system:Large in	0.0706
MGYA00170594/OHNO01_FASTA.fasta	MGYA00170594	ERZ487258	root:Host-associated:Human:Digestive system:Large in	0.407
MGYA00170595/OHGH01_FASTA.fasta	MGYA00170595	ERZ487078	root:Host-associated:Human:Digestive system:Large in	0.304
MGYA00170596/OHRI01_FASTA.fasta	MGYA00170596	ERZ487385	root:Host-associated:Human:Digestive system:Large in	0.3588
MGYA00170597/OHBV01_FASTA.fasta	MGYA00170597	ERZ486946	root:Host-associated:Human:Digestive system:Large in	0.3624
MGYA00170598/OHJE01_FASTA.fasta	MGYA00170598	ERZ487154	root:Host-associated:Human:Digestive system:Large in	0.327
MGYA00170599/OHHU01_FASTA.fasta	MGYA00170599	ERZ487120	root:Host-associated:Human:Digestive system:Large in	0.4982
MGYA00170600/OHGZ01_FASTA.fasta	MGYA00170600	ERZ487087	root:Host-associated:Human:Digestive system:Large in	0.3854
MGYA00170601/OHHO01_FASTA.fasta	MGYA00170601	ERZ487106	root:Host-associated:Human:Digestive system:Large in	0.3334
MGYA00170602/OHOO01_FASTA.fasta	MGYA00170602	ERZ487302	root:Host-associated:Human:Digestive system:Large in	0.2778
MGYA00170603/OHGY01_FASTA.fasta	MGYA00170603	ERZ487099	root:Host-associated:Human:Digestive system:Large in	0.0308
MGYA00170604/OHKQ01_FASTA.fasta	MGYA00170604	ERZ487192	root:Host-associated:Human:Digestive system:Large in	0.3616
MGYA00170605/OHFP01_FASTA.fasta	MGYA00170605	ERZ487056	root:Host-associated:Human:Digestive system:Large in	0.372
MGYA00170606/OHHT01_FASTA.fasta	MGYA00170606	ERZ487107	root:Host-associated:Human:Digestive system:Large in	0.4482
MGYA00170607/OHJP01_FASTA.fasta	MGYA00170607	ERZ487168	root:Host-associated:Human:Digestive system:Large in	0.4074
MGYA00170608/OHIH01_FASTA.fasta	MGYA00170608	ERZ487124	root:Host-associated:Human:Digestive system:Large in	0.26
MGYA00170609/OHNN01_FASTA.fasta	MGYA00170609	ERZ487265	root:Host-associated:Human:Digestive system:Large in	0.3724
MGYA00170610/OHGK01_FASTA.fasta	MGYA00170610	ERZ487062	root:Host-associated:Human:Digestive system:Large in	0.4946
MGYA00170611/OKSG01_FASTA.fasta	MGYA00170611	ERZ486935	root:Host-associated:Human:Digestive system:Large in	0.373
MGYA00170612/OHQV01_FASTA.fasta	MGYA00170612	ERZ487374	root:Host-associated:Human:Digestive system:Large in	0.0562
MGYA00170613/OKUA01_FASTA.fasta	MGYA00170613	ERZ487459	root:Host-associated:Human:Digestive system:Large in	0.4656
MGYA00170614/OHOU01_FASTA.fasta	MGYA00170614	ERZ487316	root:Host-associated:Human:Digestive system:Large in	0.0724
MGYA00170615/OHBI01_FASTA.fasta	MGYA00170615	ERZ486934	root:Host-associated:Human:Digestive system:Large in	0.324
MGYA00170616/OHSS01_FASTA.fasta	MGYA00170616	ERZ487430	root:Host-associated:Human:Digestive system:Large in	0.2792
MGYA00170617/OHSJ01_FASTA.fasta	MGYA00170617	ERZ487405	root:Host-associated:Human:Digestive system:Large in	0.3754
MGYA00170618/OHPR01_FASTA.fasta	MGYA00170618	ERZ487338	root:Host-associated:Human:Digestive system:Large in	0.4436
MGYA00170619/OHGO01_FASTA.fasta	MGYA00170619	ERZ487066	root:Host-associated:Human:Digestive system:Large in	0.1758
MGYA00170620/OHQO01_FASTA.fasta	MGYA00170620	ERZ487362	root:Host-associated:Human:Digestive system:Large in	0.338
MGYA00170621/OHNY01_FASTA.fasta	MGYA00170621	ERZ487281	root:Host-associated:Human:Digestive system:Large in	0.2894
MGYA00170622/OHFK01_FASTA.fasta	MGYA00170622	ERZ487048	root:Host-associated:Human:Digestive system:Large in	0.151
MGYA00170623/OKTD01_FASTA.fasta	MGYA00170623	ERZ487153	root:Host-associated:Human:Digestive system:Large in	0.3562
MGYA00170624/OHKP01_FASTA.fasta	MGYA00170624	ERZ487190	root:Host-associated:Human:Digestive system:Large in	0.388
MGYA00170625/OHGQ01_FASTA.fasta	MGYA00170625	ERZ487086	root:Host-associated:Human:Digestive system:Large in	0.3222
MGYA00170626/OHFQ01_FASTA.fasta	MGYA00170626	ERZ487055	root:Host-associated:Human:Digestive system:Large in	0.3444
MGYA00170627/OHEM01_FASTA.fasta	MGYA00170627	ERZ487023	root:Host-associated:Human:Digestive system:Large in	0.4196
MGYA00170628/OHBT01_FASTA.fasta	MGYA00170628	ERZ486953	root:Host-associated:Human:Digestive system:Large in	0.0438
MGYA00170629/OHIO01_FASTA.fasta	MGYA00170629	ERZ487145	root:Host-associated:Human:Digestive system:Large in	0.0322
MGYA00170630/OHIU01_FASTA.fasta	MGYA00170630	ERZ487136	root:Host-associated:Human:Digestive system:Large in	0.3016
MGYA00170631/OICS01_FASTA.fasta	MGYA00170631	ERZ488600	root:Host-associated:Human:Digestive system:Large in	0.309
MGYA00170632/OHYS01_FASTA.fasta	MGYA00170632	ERZ488487	root:Host-associated:Human:Digestive system:Large in	0.313
MGYA00170633/OHUM01_FASTA.fasta	MGYA00170633	ERZ488378	root:Host-associated:Human:Digestive system:Large in	0.1732
MGYA00170634/OHVQ01_FASTA.fasta	MGYA00170634	ERZ488415	root:Host-associated:Human:Digestive system:Large in	0.1802
MGYA00170635/OIBH01_FASTA.fasta	MGYA00170635	ERZ488575	root:Host-associated:Human:Digestive system:Large in	0.0442
MGYA00170636/OHVZ01_FASTA.fasta	MGYA00170636	ERZ488414	root:Host-associated:Human:Digestive system:Large in	0.326
MGYA00170637/OHUW01_FASTA.fasta	MGYA00170637	ERZ488388	root:Host-associated:Human:Digestive system:Large in	0.0378
MGYA00170638/OKTZ01_FASTA.fasta	MGYA00170638	ERZ488383	root:Host-associated:Human:Digestive system:Large in	0.3108
MGYA00170639/OIDA01_FASTA.fasta	MGYA00170639	ERZ488628	root:Host-associated:Human:Digestive system:Large in	0.36
MGYA00170640/OIDF01_FASTA.fasta	MGYA00170640	ERZ488613	root:Host-associated:Human:Digestive system:Large in	0.5204
MGYA00170641/OHZC01_FASTA.fasta	MGYA00170641	ERZ488494	root:Host-associated:Human:Digestive system:Large in	0.327
MGYA00170642/OHZK01_FASTA.fasta	MGYA00170642	ERZ488497	root:Host-associated:Human:Digestive system:Large in	0.4242
MGYA00170643/OICW01_FASTA.fasta	MGYA00170643	ERZ488612	root:Host-associated:Human:Digestive system:Large in	0.063
MGYA00170644/OKVB01_FASTA.fasta	MGYA00170644	ERZ488632	root:Host-associated:Human:Digestive system:Large in	0.3174
MGYA00170645/OHYV01_FASTA.fasta	MGYA00170645	ERZ488501	root:Host-associated:Human:Digestive system:Large in	0.2654
MGYA00170646/OHWR01_FASTA.fasta	MGYA00170646	ERZ488430	root:Host-associated:Human:Digestive system:Large in	0.3766
MGYA00170647/OHVN01_FASTA.fasta	MGYA00170647	ERZ488405	root:Host-associated:Human:Digestive system:Large in	0.315
MGYA00170648/OIBJ01_FASTA.fasta	MGYA00170648	ERZ488578	root:Host-associated:Human:Digestive system:Large in	0.0606
MGYA00170649/OKUM01_FASTA.fasta	MGYA00170649	ERZ488556	root:Host-associated:Human:Digestive system:Large in	0.3734
MGYA00170650/OHZA01_FASTA.fasta	MGYA00170650	ERZ488504	root:Host-associated:Human:Digestive system:Large in	0.0834
MGYA00170651/OICN01_FASTA.fasta	MGYA00170651	ERZ488601	root:Host-associated:Human:Digestive system:Large in	0.371
MGYA00170652/OHWF01_FASTA.fasta	MGYA00170652	ERZ488423	root:Host-associated:Human:Digestive system:Large in	0.3518
MGYA00170653/OKUB01_FASTA.fasta	MGYA00170653	ERZ488471	root:Host-associated:Human:Digestive system:Large in	0.2044
MGYA00170654/OHZZ01_FASTA.fasta	MGYA00170654	ERZ488531	root:Host-associated:Human:Digestive system:Large in	0.2556
MGYA00170655/OHZO01_FASTA.fasta	MGYA00170655	ERZ488517	root:Host-associated:Human:Digestive system:Large in	0.0514
MGYA00170656/OIBE01_FASTA.fasta	MGYA00170656	ERZ488568	root:Host-associated:Human:Digestive system:Large in	0.309
MGYA00170657/OIAK01_FASTA.fasta	MGYA00170657	ERZ488538	root:Host-associated:Human:Digestive system:Large in	0.3436
MGYA00170658/OIDR01_FASTA.fasta	MGYA00170658	ERZ488648	root:Host-associated:Human:Digestive system:Large in	0.309
MGYA00170659/OIDP01_FASTA.fasta	MGYA00170659	ERZ488642	root:Host-associated:Human:Digestive system:Large in	0.3788
MGYA00170660/OHZM01_FASTA.fasta	MGYA00170660	ERZ488506	root:Host-associated:Human:Digestive system:Large in	0.3058
MGYA00170661/OIDS01_FASTA.fasta	MGYA00170661	ERZ488621	root:Host-associated:Human:Digestive system:Large in	0.3456
MGYA00170662/OHXG01_FASTA.fasta	MGYA00170662	ERZ488454	root:Host-associated:Human:Digestive system:Large in	0.377
MGYA00170663/OIDI01_FASTA.fasta	MGYA00170663	ERZ488622	root:Host-associated:Human:Digestive system:Large in	0.1926
MGYA00170664/OHYF01_FASTA.fasta	MGYA00170664	ERZ488455	root:Host-associated:Human:Digestive system:Large in	0.38
MGYA00170665/OKUI01_FASTA.fasta	MGYA00170665	ERZ488524	root:Host-associated:Human:Digestive system:Large in	0.3226
MGYA00170666/OIDX01_FASTA.fasta	MGYA00170666	ERZ488649	root:Host-associated:Human:Digestive system:Large in	0.2624
MGYA00170667/OIBD01_FASTA.fasta	MGYA00170667	ERZ488572	root:Host-associated:Human:Digestive system:Large in	0.0344
MGYA00170668/OHYI01_FASTA.fasta	MGYA00170668	ERZ488480	root:Host-associated:Human:Digestive system:Large in	0.2696
MGYA00170669/OICX01_FASTA.fasta	MGYA00170669	ERZ488609	root:Host-associated:Human:Digestive system:Large in	0.087
MGYA00170670/OHWG01_FASTA.fasta	MGYA00170670	ERZ488412	root:Host-associated:Human:Digestive system:Large in	0.4946
MGYA00170671/OIDW01_FASTA.fasta	MGYA00170671	ERZ488646	root:Host-associated:Human:Digestive system:Large in	0.2732
MGYA00170672/OIAL01_FASTA.fasta	MGYA00170672	ERZ488534	root:Host-associated:Human:Digestive system:Large in	0.4388
MGYA00170673/OHXS01_FASTA.fasta	MGYA00170673	ERZ488461	root:Host-associated:Human:Digestive system:Large in	0.2272
MGYA00170674/OHWM01_FASTA.fasta	MGYA00170674	ERZ488428	root:Host-associated:Human:Digestive system:Large in	0.3222
MGYA00170675/OIBF01_FASTA.fasta	MGYA00170675	ERZ488557	root:Host-associated:Human:Digestive system:Large in	0.3056
MGYA00170676/OIDV01_FASTA.fasta	MGYA00170676	ERZ488650	root:Host-associated:Human:Digestive system:Large in	0.2998
MGYA00170677/OHWT01_FASTA.fasta	MGYA00170677	ERZ488436	root:Host-associated:Human:Digestive system:Large in	0.3188
MGYA00170678/OHYC01_FASTA.fasta	MGYA00170678	ERZ488470	root:Host-associated:Human:Digestive system:Large in	0.353
MGYA00170679/OICQ01_FASTA.fasta	MGYA00170679	ERZ488604	root:Host-associated:Human:Digestive system:Large in	0.0826
MGYA00170680/OICA01_FASTA.fasta	MGYA00170680	ERZ488589	root:Host-associated:Human:Digestive system:Large in	0.0482
MGYA00170681/OHVC01_FASTA.fasta	MGYA00170681	ERZ488400	root:Host-associated:Human:Digestive system:Large in	0.1774
MGYA00170682/OHYZ01_FASTA.fasta	MGYA00170682	ERZ488508	root:Host-associated:Human:Digestive system:Large in	0.0354
MGYA00170683/OICU01_FASTA.fasta	MGYA00170683	ERZ488605	root:Host-associated:Human:Digestive system:Large in	0.291
MGYA00170684/OHXL01_FASTA.fasta	MGYA00170684	ERZ488460	root:Host-associated:Human:Digestive system:Large in	0.2294
MGYA00170685/OHWL01_FASTA.fasta	MGYA00170685	ERZ488429	root:Host-associated:Human:Digestive system:Large in	0.2984
MGYA00170686/OIAY01_FASTA.fasta	MGYA00170686	ERZ488553	root:Host-associated:Human:Digestive system:Large in	0.3022
MGYA00170687/OHWC01_FASTA.fasta	MGYA00170687	ERZ488413	root:Host-associated:Human:Digestive system:Large in	0.3346
MGYA00170688/OIBG01_FASTA.fasta	MGYA00170688	ERZ488539	root:Host-associated:Human:Digestive system:Large in	0.3094
MGYA00170689/OICD01_FASTA.fasta	MGYA00170689	ERZ488582	root:Host-associated:Human:Digestive system:Large in	0.3474
MGYA00170690/OHUS01_FASTA.fasta	MGYA00170690	ERZ488385	root:Host-associated:Human:Digestive system:Large in	0.2506
MGYA00170691/OICI01_FASTA.fasta	MGYA00170691	ERZ488585	root:Host-associated:Human:Digestive system:Large in	0.4624
MGYA00170692/OHYA01_FASTA.fasta	MGYA00170692	ERZ488457	root:Host-associated:Human:Digestive system:Large in	0.375
MGYA00170693/OKUU01_FASTA.fasta	MGYA00170693	ERZ488617	root:Host-associated:Human:Digestive system:Large in	0.2972
MGYA00170694/OHXI01_FASTA.fasta	MGYA00170694	ERZ488445	root:Host-associated:Human:Digestive system:Large in	0.329
MGYA00170695/OIBA01_FASTA.fasta	MGYA00170695	ERZ488560	root:Host-associated:Human:Digestive system:Large in	0.3214
MGYA00170696/OHYR01_FASTA.fasta	MGYA00170696	ERZ488491	root:Host-associated:Human:Digestive system:Large in	0.3138
MGYA00170697/OHXN01_FASTA.fasta	MGYA00170697	ERZ488468	root:Host-associated:Human:Digestive system:Large in	0.0418
MGYA00170698/OIBO01_FASTA.fasta	MGYA00170698	ERZ488567	root:Host-associated:Human:Digestive system:Large in	0.3018
MGYA00170699/OHVF01_FASTA.fasta	MGYA00170699	ERZ488403	root:Host-associated:Human:Digestive system:Large in	0.0792
MGYA00170700/OIAJ01_FASTA.fasta	MGYA00170700	ERZ488541	root:Host-associated:Human:Digestive system:Large in	0.1394
MGYA00170701/OHVB01_FASTA.fasta	MGYA00170701	ERZ488387	root:Host-associated:Human:Digestive system:Large in	0.3408
MGYA00170702/OIAM01_FASTA.fasta	MGYA00170702	ERZ488547	root:Host-associated:Human:Digestive system:Large in	0.275
MGYA00170703/OHUU01_FASTA.fasta	MGYA00170703	ERZ488374	root:Host-associated:Human:Digestive system:Large in	0.3528
MGYA00170704/OHZS01_FASTA.fasta	MGYA00170704	ERZ488510	root:Host-associated:Human:Digestive system:Large in	0.3616
MGYA00170705/OHZI01_FASTA.fasta	MGYA00170705	ERZ488509	root:Host-associated:Human:Digestive system:Large in	0.3208
MGYA00170706/OIBW01_FASTA.fasta	MGYA00170706	ERZ488579	root:Host-associated:Human:Digestive system:Large in	0.3842
MGYA00170707/OIBI01_FASTA.fasta	MGYA00170707	ERZ488562	root:Host-associated:Human:Digestive system:Large in	0.311
MGYA00170708/OHZW01_FASTA.fasta	MGYA00170708	ERZ488526	root:Host-associated:Human:Digestive system:Large in	0.2812
MGYA00170709/OHVJ01_FASTA.fasta	MGYA00170709	ERZ488402	root:Host-associated:Human:Digestive system:Large in	0.3274
MGYA00170710/OHYD01_FASTA.fasta	MGYA00170710	ERZ488475	root:Host-associated:Human:Digestive system:Large in	0.3674
MGYA00170711/OHWZ01_FASTA.fasta	MGYA00170711	ERZ488439	root:Host-associated:Human:Digestive system:Large in	0.3596
MGYA00170712/OIAO01_FASTA.fasta	MGYA00170712	ERZ488549	root:Host-associated:Human:Digestive system:Large in	0.0322
MGYA00170713/OIAR01_FASTA.fasta	MGYA00170713	ERZ488551	root:Host-associated:Human:Digestive system:Large in	0.1372
MGYA00170714/OHYN01_FASTA.fasta	MGYA00170714	ERZ488492	root:Host-associated:Human:Digestive system:Large in	0.302
MGYA00170715/OHWJ01_FASTA.fasta	MGYA00170715	ERZ488426	root:Host-associated:Human:Digestive system:Large in	0.2194
MGYA00170716/OIAB01_FASTA.fasta	MGYA00170716	ERZ488529	root:Host-associated:Human:Digestive system:Large in	0.2398
MGYA00170717/OHWS01_FASTA.fasta	MGYA00170717	ERZ488442	root:Host-associated:Human:Digestive system:Large in	0.3202
MGYA00170718/OHXM01_FASTA.fasta	MGYA00170718	ERZ488450	root:Host-associated:Human:Digestive system:Large in	0.3026
MGYA00170719/OHYQ01_FASTA.fasta	MGYA00170719	ERZ488484	root:Host-associated:Human:Digestive system:Large in	0.4312
MGYA00170720/OHWD01_FASTA.fasta	MGYA00170720	ERZ488417	root:Host-associated:Human:Digestive system:Large in	0.3108
MGYA00170721/OHYY01_FASTA.fasta	MGYA00170721	ERZ488507	root:Host-associated:Human:Digestive system:Large in	0.068
MGYA00170722/OHYT01_FASTA.fasta	MGYA00170722	ERZ488496	root:Host-associated:Human:Digestive system:Large in	0.0842
MGYA00170723/OHXT01_FASTA.fasta	MGYA00170723	ERZ488476	root:Host-associated:Human:Digestive system:Large in	0.0422
MGYA00170724/OIAG01_FASTA.fasta	MGYA00170724	ERZ488527	root:Host-associated:Human:Digestive system:Large in	0.3382
MGYA00170725/OIBP01_FASTA.fasta	MGYA00170725	ERZ488564	root:Host-associated:Human:Digestive system:Large in	0.3176
MGYA00170726/OKUN01_FASTA.fasta	MGYA00170726	ERZ488606	root:Host-associated:Human:Digestive system:Large in	0.3306
MGYA00170727/OIBL01_FASTA.fasta	MGYA00170727	ERZ488563	root:Host-associated:Human:Digestive system:Large in	0.2708
MGYA00170728/OKUC01_FASTA.fasta	MGYA00170728	ERZ488404	root:Host-associated:Human:Digestive system:Large in	0.379
MGYA00170729/OHXW01_FASTA.fasta	MGYA00170729	ERZ488464	root:Host-associated:Human:Digestive system:Large in	0.3476
MGYA00170730/OIAE01_FASTA.fasta	MGYA00170730	ERZ488535	root:Host-associated:Human:Digestive system:Large in	0.4332
MGYA00170731/OHZD01_FASTA.fasta	MGYA00170731	ERZ488499	root:Host-associated:Human:Digestive system:Large in	0.311
MGYA00170732/OHYU01_FASTA.fasta	MGYA00170732	ERZ488481	root:Host-associated:Human:Digestive system:Large in	0.3312
MGYA00170733/OHUQ01_FASTA.fasta	MGYA00170733	ERZ488384	root:Host-associated:Human:Digestive system:Large in	0.2424
MGYA00170734/OHZY01_FASTA.fasta	MGYA00170734	ERZ488521	root:Host-associated:Human:Digestive system:Large in	0.3028
MGYA00170735/OIDK01_FASTA.fasta	MGYA00170735	ERZ488638	root:Host-associated:Human:Digestive system:Large in	0.2902
MGYA00170736/OIDJ01_FASTA.fasta	MGYA00170736	ERZ488627	root:Host-associated:Human:Digestive system:Large in	0.203
MGYA00170737/OHWN01_FASTA.fasta	MGYA00170737	ERZ488433	root:Host-associated:Human:Digestive system:Large in	0.1706
MGYA00170738/OIBX01_FASTA.fasta	MGYA00170738	ERZ488581	root:Host-associated:Human:Digestive system:Large in	0.3476
MGYA00170739/OHZE01_FASTA.fasta	MGYA00170739	ERZ488493	root:Host-associated:Human:Digestive system:Large in	0.322
MGYA00170740/OHWE01_FASTA.fasta	MGYA00170740	ERZ488421	root:Host-associated:Human:Digestive system:Large in	0.3752
MGYA00170741/OHYG01_FASTA.fasta	MGYA00170741	ERZ488479	root:Host-associated:Human:Digestive system:Large in	0.3144
MGYA00170742/OIAT01_FASTA.fasta	MGYA00170742	ERZ488542	root:Host-associated:Human:Digestive system:Large in	0.3174
MGYA00170743/OHYJ01_FASTA.fasta	MGYA00170743	ERZ488483	root:Host-associated:Human:Digestive system:Large in	0.0338
MGYA00170744/OIDC01_FASTA.fasta	MGYA00170744	ERZ488619	root:Host-associated:Human:Digestive system:Large in	0.3468
MGYA00170745/OIAI01_FASTA.fasta	MGYA00170745	ERZ488540	root:Host-associated:Human:Digestive system:Large in	0.1046
MGYA00170746/OHZV01_FASTA.fasta	MGYA00170746	ERZ488525	root:Host-associated:Human:Digestive system:Large in	0.283
MGYA00170747/OHVX01_FASTA.fasta	MGYA00170747	ERZ488411	root:Host-associated:Human:Digestive system:Large in	0.3138
MGYA00170748/OKUV01_FASTA.fasta	MGYA00170748	ERZ488618	root:Host-associated:Human:Digestive system:Large in	0.2994
MGYA00170749/OHVE01_FASTA.fasta	MGYA00170749	ERZ488396	root:Host-associated:Human:Digestive system:Large in	0.1372
MGYA00170750/OHZB01_FASTA.fasta	MGYA00170750	ERZ488490	root:Host-associated:Human:Digestive system:Large in	0.347
MGYA00170751/OHUO01_FASTA.fasta	MGYA00170751	ERZ488377	root:Host-associated:Human:Digestive system:Large in	0.0672
MGYA00170752/OIDN01_FASTA.fasta	MGYA00170752	ERZ488641	root:Host-associated:Human:Digestive system:Large in	0.2842
MGYA00170753/OIBC01_FASTA.fasta	MGYA00170753	ERZ488555	root:Host-associated:Human:Digestive system:Large in	0.2998
MGYA00170754/OIAQ01_FASTA.fasta	MGYA00170754	ERZ488554	root:Host-associated:Human:Digestive system:Large in	0.0496
MGYA00170755/OKVA01_FASTA.fasta	MGYA00170755	ERZ488655	root:Host-associated:Human:Digestive system:Large in	0.3462
MGYA00170756/OIAW01_FASTA.fasta	MGYA00170756	ERZ488550	root:Host-associated:Human:Digestive system:Large in	0.2032
MGYA00170757/OIAF01_FASTA.fasta	MGYA00170757	ERZ488537	root:Host-associated:Human:Digestive system:Large in	0.3434
MGYA00170758/OHXB01_FASTA.fasta	MGYA00170758	ERZ488443	root:Host-associated:Human:Digestive system:Large in	0.3164
MGYA00170759/OICP01_FASTA.fasta	MGYA00170759	ERZ488602	root:Host-associated:Human:Digestive system:Large in	0.0998
MGYA00170760/OHVV01_FASTA.fasta	MGYA00170760	ERZ488422	root:Host-associated:Human:Digestive system:Large in	0.0416
MGYA00170761/OHUG01_FASTA.fasta	MGYA00170761	ERZ488371	root:Host-associated:Human:Digestive system:Large in	0.319
MGYA00170762/OKUY01_FASTA.fasta	MGYA00170762	ERZ488634	root:Host-associated:Human:Digestive system:Large in	0.2392
MGYA00170763/OHYH01_FASTA.fasta	MGYA00170763	ERZ488477	root:Host-associated:Human:Digestive system:Large in	0.3358
MGYA00170764/OIBY01_FASTA.fasta	MGYA00170764	ERZ488593	root:Host-associated:Human:Digestive system:Large in	0.2418
MGYA00170765/OHVT01_FASTA.fasta	MGYA00170765	ERZ488390	root:Host-associated:Human:Digestive system:Large in	0.3886
MGYA00170766/OIED01_FASTA.fasta	MGYA00170766	ERZ488653	root:Host-associated:Human:Digestive system:Large in	0.3542
MGYA00170767/OHXV01_FASTA.fasta	MGYA00170767	ERZ488478	root:Host-associated:Human:Digestive system:Large in	0.1004
MGYA00170768/OICE01_FASTA.fasta	MGYA00170768	ERZ488590	root:Host-associated:Human:Digestive system:Large in	0.3842
MGYA00170769/OKUJ01_FASTA.fasta	MGYA00170769	ERZ488614	root:Host-associated:Human:Digestive system:Large in	0.0906
MGYA00170770/OHUJ01_FASTA.fasta	MGYA00170770	ERZ488380	root:Host-associated:Human:Digestive system:Large in	0.3112
MGYA00170771/OHWO01_FASTA.fasta	MGYA00170771	ERZ488432	root:Host-associated:Human:Digestive system:Large in	0.1874
MGYA00170772/OHYL01_FASTA.fasta	MGYA00170772	ERZ488486	root:Host-associated:Human:Digestive system:Large in	0.3242
MGYA00170773/OKTV01_FASTA.fasta	MGYA00170773	ERZ488372	root:Host-associated:Human:Digestive system:Large in	0.2048
MGYA00170774/OHZN01_FASTA.fasta	MGYA00170774	ERZ488518	root:Host-associated:Human:Digestive system:Large in	0.038
MGYA00170775/OICK01_FASTA.fasta	MGYA00170775	ERZ488588	root:Host-associated:Human:Digestive system:Large in	0.3562
MGYA00170776/OIDD01_FASTA.fasta	MGYA00170776	ERZ488620	root:Host-associated:Human:Digestive system:Large in	0.5084
MGYA00170777/OHZJ01_FASTA.fasta	MGYA00170777	ERZ488495	root:Host-associated:Human:Digestive system:Large in	0.3232
MGYA00170778/OIAD01_FASTA.fasta	MGYA00170778	ERZ488520	root:Host-associated:Human:Digestive system:Large in	0.3604
MGYA00170779/OKUX01_FASTA.fasta	MGYA00170779	ERZ488631	root:Host-associated:Human:Digestive system:Large in	0.4206
MGYA00170780/OHZT01_FASTA.fasta	MGYA00170780	ERZ488519	root:Host-associated:Human:Digestive system:Large in	0.2418
MGYA00170781/OHVO01_FASTA.fasta	MGYA00170781	ERZ488399	root:Host-associated:Human:Digestive system:Large in	0.3522
MGYA00170782/OHXJ01_FASTA.fasta	MGYA00170782	ERZ488456	root:Host-associated:Human:Digestive system:Large in	0.24
MGYA00170783/OHWU01_FASTA.fasta	MGYA00170783	ERZ488434	root:Host-associated:Human:Digestive system:Large in	0.3242
MGYA00170784/OHXE01_FASTA.fasta	MGYA00170784	ERZ488453	root:Host-associated:Human:Digestive system:Large in	0.2934
MGYA00170785/OIBZ01_FASTA.fasta	MGYA00170785	ERZ488586	root:Host-associated:Human:Digestive system:Large in	0.1492
MGYA00170786/OIBM01_FASTA.fasta	MGYA00170786	ERZ488580	root:Host-associated:Human:Digestive system:Large in	0.0276
MGYA00170787/OKUO01_FASTA.fasta	MGYA00170787	ERZ488584	root:Host-associated:Human:Digestive system:Large in	0.3374
MGYA00170788/OHXA01_FASTA.fasta	MGYA00170788	ERZ488447	root:Host-associated:Human:Digestive system:Large in	0.2524
MGYA00170789/OIDZ01_FASTA.fasta	MGYA00170789	ERZ488651	root:Host-associated:Human:Digestive system:Large in	0.3296
MGYA00170790/OHYO01_FASTA.fasta	MGYA00170790	ERZ488482	root:Host-associated:Human:Digestive system:Large in	0.3582
MGYA00170791/OIBS01_FASTA.fasta	MGYA00170791	ERZ488571	root:Host-associated:Human:Digestive system:Large in	0.2858
MGYA00170792/OICL01_FASTA.fasta	MGYA00170792	ERZ488595	root:Host-associated:Human:Digestive system:Large in	0.379
MGYA00170793/OIAA01_FASTA.fasta	MGYA00170793	ERZ488516	root:Host-associated:Human:Digestive system:Large in	0.3362
MGYA00170794/OHXC01_FASTA.fasta	MGYA00170794	ERZ488438	root:Host-associated:Human:Digestive system:Large in	0.3786
MGYA00170795/OIDB01_FASTA.fasta	MGYA00170795	ERZ488633	root:Host-associated:Human:Digestive system:Large in	0.2796
MGYA00170796/OIDU01_FASTA.fasta	MGYA00170796	ERZ488636	root:Host-associated:Human:Digestive system:Large in	0.3192
MGYA00170797/OHZQ01_FASTA.fasta	MGYA00170797	ERZ488513	root:Host-associated:Human:Digestive system:Large in	0.3494
MGYA00170798/OKUZ01_FASTA.fasta	MGYA00170798	ERZ488635	root:Host-associated:Human:Digestive system:Large in	0.455
MGYA00170799/OHUY01_FASTA.fasta	MGYA00170799	ERZ488381	root:Host-associated:Human:Digestive system:Large in	0.4464
MGYA00170800/OHZR01_FASTA.fasta	MGYA00170800	ERZ488505	root:Host-associated:Human:Digestive system:Large in	0.3246
MGYA00170801/OHYW01_FASTA.fasta	MGYA00170801	ERZ488498	root:Host-associated:Human:Digestive system:Large in	0.3396
MGYA00170802/OKUE01_FASTA.fasta	MGYA00170802	ERZ488523	root:Host-associated:Human:Digestive system:Large in	0.368
MGYA00170803/OHYB01_FASTA.fasta	MGYA00170803	ERZ488472	root:Host-associated:Human:Digestive system:Large in	0.35
MGYA00170804/OHVH01_FASTA.fasta	MGYA00170804	ERZ488392	root:Host-associated:Human:Digestive system:Large in	0.254
MGYA00170805/OICV01_FASTA.fasta	MGYA00170805	ERZ488603	root:Host-associated:Human:Digestive system:Large in	0.321
MGYA00170806/OKUF01_FASTA.fasta	MGYA00170806	ERZ488408	root:Host-associated:Human:Digestive system:Large in	0.3158
MGYA00170807/OHWQ01_FASTA.fasta	MGYA00170807	ERZ488437	root:Host-associated:Human:Digestive system:Large in	0.111
MGYA00170808/OHWP01_FASTA.fasta	MGYA00170808	ERZ488440	root:Host-associated:Human:Digestive system:Large in	0.1382
MGYA00170809/OIBT01_FASTA.fasta	MGYA00170809	ERZ488574	root:Host-associated:Human:Digestive system:Large in	0.3382
MGYA00170810/OIDT01_FASTA.fasta	MGYA00170810	ERZ488643	root:Host-associated:Human:Digestive system:Large in	0.5212
MGYA00170811/OHVY01_FASTA.fasta	MGYA00170811	ERZ488420	root:Host-associated:Human:Digestive system:Large in	0.333
MGYA00170812/OICC01_FASTA.fasta	MGYA00170812	ERZ488592	root:Host-associated:Human:Digestive system:Large in	0.3056
MGYA00170813/OIDO01_FASTA.fasta	MGYA00170813	ERZ488645	root:Host-associated:Human:Digestive system:Large in	0.0668
MGYA00170814/OHZX01_FASTA.fasta	MGYA00170814	ERZ488530	root:Host-associated:Human:Digestive system:Large in	0.1072
MGYA00170815/OKUK01_FASTA.fasta	MGYA00170815	ERZ488615	root:Host-associated:Human:Digestive system:Large in	0.0412
MGYA00170816/OIAP01_FASTA.fasta	MGYA00170816	ERZ488552	root:Host-associated:Human:Digestive system:Large in	0.0456
MGYA00170817/OKUH01_FASTA.fasta	MGYA00170817	ERZ488607	root:Host-associated:Human:Digestive system:Large in	0.0962
MGYA00170818/OIAC01_FASTA.fasta	MGYA00170818	ERZ488532	root:Host-associated:Human:Digestive system:Large in	0.2684
MGYA00170819/OHZH01_FASTA.fasta	MGYA00170819	ERZ488512	root:Host-associated:Human:Digestive system:Large in	0.2628
MGYA00170820/OICG01_FASTA.fasta	MGYA00170820	ERZ488587	root:Host-associated:Human:Digestive system:Large in	0.2606
MGYA00170821/OHZF01_FASTA.fasta	MGYA00170821	ERZ488502	root:Host-associated:Human:Digestive system:Large in	0.3312
MGYA00170822/OICJ01_FASTA.fasta	MGYA00170822	ERZ488596	root:Host-associated:Human:Digestive system:Large in	0.2396
MGYA00170823/OKUW01_FASTA.fasta	MGYA00170823	ERZ488630	root:Host-associated:Human:Digestive system:Large in	0.3504
MGYA00170824/OHUL01_FASTA.fasta	MGYA00170824	ERZ488382	root:Host-associated:Human:Digestive system:Large in	0.056
MGYA00170825/OIDQ01_FASTA.fasta	MGYA00170825	ERZ488637	root:Host-associated:Human:Digestive system:Large in	0.3434
MGYA00170826/OICT01_FASTA.fasta	MGYA00170826	ERZ488608	root:Host-associated:Human:Digestive system:Large in	0.5148
MGYA00170827/OHWA01_FASTA.fasta	MGYA00170827	ERZ488425	root:Host-associated:Human:Digestive system:Large in	0.3468
MGYA00170828/OHZU01_FASTA.fasta	MGYA00170828	ERZ488533	root:Host-associated:Human:Digestive system:Large in	0.081
MGYA00170829/OHVU01_FASTA.fasta	MGYA00170829	ERZ488410	root:Host-associated:Human:Digestive system:Large in	0.4106
MGYA00170830/OIBQ01_FASTA.fasta	MGYA00170830	ERZ488565	root:Host-associated:Human:Digestive system:Large in	0.2832
MGYA00170831/OHVS01_FASTA.fasta	MGYA00170831	ERZ488406	root:Host-associated:Human:Digestive system:Large in	0.3408
MGYA00170832/OHXD01_FASTA.fasta	MGYA00170832	ERZ488451	root:Host-associated:Human:Digestive system:Large in	0.345
MGYA00170833/OHYM01_FASTA.fasta	MGYA00170833	ERZ488485	root:Host-associated:Human:Digestive system:Large in	0.3174
MGYA00170834/OICR01_FASTA.fasta	MGYA00170834	ERZ488598	root:Host-associated:Human:Digestive system:Large in	0.3642
MGYA00170835/OHUZ01_FASTA.fasta	MGYA00170835	ERZ488391	root:Host-associated:Human:Digestive system:Large in	0.0458
MGYA00170836/OHXO01_FASTA.fasta	MGYA00170836	ERZ488467	root:Host-associated:Human:Digestive system:Large in	0.165
MGYA00170837/OHUR01_FASTA.fasta	MGYA00170837	ERZ488375	root:Host-associated:Human:Digestive system:Large in	0.3592
MGYA00170838/OIDG01_FASTA.fasta	MGYA00170838	ERZ488639	root:Host-associated:Human:Digestive system:Large in	0.1652
MGYA00170839/OIAN01_FASTA.fasta	MGYA00170839	ERZ488546	root:Host-associated:Human:Digestive system:Large in	0.3066
MGYA00170840/OHYP01_FASTA.fasta	MGYA00170840	ERZ488488	root:Host-associated:Human:Digestive system:Large in	0.343
MGYA00170841/OIDY01_FASTA.fasta	MGYA00170841	ERZ488652	root:Host-associated:Human:Digestive system:Large in	0.2494
MGYA00170842/OICY01_FASTA.fasta	MGYA00170842	ERZ488611	root:Host-associated:Human:Digestive system:Large in	0.2968
MGYA00170843/OKUQ01_FASTA.fasta	MGYA00170843	ERZ488570	root:Host-associated:Human:Digestive system:Large in	0.3662
MGYA00170844/OIBK01_FASTA.fasta	MGYA00170844	ERZ488548	root:Host-associated:Human:Digestive system:Large in	0.365
MGYA00170845/OHXF01_FASTA.fasta	MGYA00170845	ERZ488452	root:Host-associated:Human:Digestive system:Large in	0.347
MGYA00170846/OKUS01_FASTA.fasta	MGYA00170846	ERZ488610	root:Host-associated:Human:Digestive system:Large in	0.3274
MGYA00170847/OHWI01_FASTA.fasta	MGYA00170847	ERZ488427	root:Host-associated:Human:Digestive system:Large in	0.24
MGYA00170848/OHWX01_FASTA.fasta	MGYA00170848	ERZ488448	root:Host-associated:Human:Digestive system:Large in	0.1566
MGYA00170849/OHWK01_FASTA.fasta	MGYA00170849	ERZ488431	root:Host-associated:Human:Digestive system:Large in	0.3058
MGYA00170850/OICH01_FASTA.fasta	MGYA00170850	ERZ488583	root:Host-associated:Human:Digestive system:Large in	0.3056
MGYA00170851/OHZL01_FASTA.fasta	MGYA00170851	ERZ488511	root:Host-associated:Human:Digestive system:Large in	0.2984
MGYA00170852/OKUL01_FASTA.fasta	MGYA00170852	ERZ488515	root:Host-associated:Human:Digestive system:Large in	0.33
MGYA00170853/OIAV01_FASTA.fasta	MGYA00170853	ERZ488545	root:Host-associated:Human:Digestive system:Large in	0.2378

MGYA00170854/OIAU01_FASTA.fasta	MGYA00170854	ERZ488561	root:Host-associated:Human:Digestive system:Large int	0.0522
MGYA00170855/OHZP01_FASTA.fasta	MGYA00170855	ERZ488522	root:Host-associated:Human:Digestive system:Large int	0.081
MGYA00170856/OIAS01_FASTA.fasta	MGYA00170856	ERZ488543	root:Host-associated:Human:Digestive system:Large int	0.2956
MGYA00170857/OIAH01_FASTA.fasta	MGYA00170857	ERZ488528	root:Host-associated:Human:Digestive system:Large int	0.343
MGYA00170858/OKTY01_FASTA.fasta	MGYA00170858	ERZ488441	root:Host-associated:Human:Digestive system:Large int	0.1612
MGYA00170859/OHXH01_FASTA.fasta	MGYA00170859	ERZ488463	root:Host-associated:Human:Digestive system:Large int	0.0484
MGYA00170860/OIBR01_FASTA.fasta	MGYA00170860	ERZ488573	root:Host-associated:Human:Digestive system:Large int	0.2966
MGYA00170861/OHVI01_FASTA.fasta	MGYA00170861	ERZ488389	root:Host-associated:Human:Digestive system:Large int	0.257
MGYA00170862/OIEB01_FASTA.fasta	MGYA00170862	ERZ488654	root:Host-associated:Human:Digestive system:Large int	0.3132
MGYA00170863/OIDH01_FASTA.fasta	MGYA00170863	ERZ488624	root:Host-associated:Human:Digestive system:Large int	0.1026
MGYA00170864/OHWW01_FASTA.fasta	MGYA00170864	ERZ488444	root:Host-associated:Human:Digestive system:Large int	0.1246
MGYA00170865/OHVD01_FASTA.fasta	MGYA00170865	ERZ488394	root:Host-associated:Human:Digestive system:Large int	0.065
MGYA00170866/OICZ01_FASTA.fasta	MGYA00170866	ERZ488629	root:Host-associated:Human:Digestive system:Large int	0.323
MGYA00170867/OIBB01_FASTA.fasta	MGYA00170867	ERZ488569	root:Host-associated:Human:Digestive system:Large int	0.0568
MGYA00170868/OHXZ01_FASTA.fasta	MGYA00170868	ERZ488459	root:Host-associated:Human:Digestive system:Large int	0.4872
MGYA00170869/OHZG01_FASTA.fasta	MGYA00170869	ERZ488500	root:Host-associated:Human:Digestive system:Large int	0.3542
MGYA00170870/OKUG01_FASTA.fasta	MGYA00170870	ERZ488536	root:Host-associated:Human:Digestive system:Large int	0.2686
MGYA00170871/OHXP01_FASTA.fasta	MGYA00170871	ERZ488469	root:Host-associated:Human:Digestive system:Large int	0.0852
MGYA00170872/OHVW01_FASTA.fasta	MGYA00170872	ERZ488418	root:Host-associated:Human:Digestive system:Large int	0.3476
MGYA00170873/OHVK01_FASTA.fasta	MGYA00170873	ERZ488395	root:Host-associated:Human:Digestive system:Large int	0.472
MGYA00170874/OIAZ01_FASTA.fasta	MGYA00170874	ERZ488559	root:Host-associated:Human:Digestive system:Large int	0.2488
MGYA00170875/OHVR01_FASTA.fasta	MGYA00170875	ERZ488419	root:Host-associated:Human:Digestive system:Large int	0.0478
MGYA00170876/OHUX01_FASTA.fasta	MGYA00170876	ERZ488398	root:Host-associated:Human:Digestive system:Large int	0.0876
MGYA00170877/OHXX01_FASTA.fasta	MGYA00170877	ERZ488458	root:Host-associated:Human:Digestive system:Large int	0.4366
MGYA00170878/OHXY01_FASTA.fasta	MGYA00170878	ERZ488474	root:Host-associated:Human:Digestive system:Large int	0.3612
MGYA00170879/OKUR01_FASTA.fasta	MGYA00170879	ERZ488566	root:Host-associated:Human:Digestive system:Large int	0.345
MGYA00170880/OHXQ01_FASTA.fasta	MGYA00170880	ERZ488466	root:Host-associated:Human:Digestive system:Large int	0.1808
MGYA00170881/OKVC01_FASTA.fasta	MGYA00170881	ERZ488644	root:Host-associated:Human:Digestive system:Large int	0.3318
MGYA00170882/OIBN01_FASTA.fasta	MGYA00170882	ERZ488558	root:Host-associated:Human:Digestive system:Large int	0.3896
MGYA00170883/OHXK01_FASTA.fasta	MGYA00170883	ERZ488446	root:Host-associated:Human:Digestive system:Large int	0.2678
MGYA00170884/OHXR01_FASTA.fasta	MGYA00170884	ERZ488473	root:Host-associated:Human:Digestive system:Large int	0.0794
MGYA00170885/OHWB01_FASTA.fasta	MGYA00170885	ERZ488416	root:Host-associated:Human:Digestive system:Large int	0.2742
MGYA00170886/OHYK01_FASTA.fasta	MGYA00170886	ERZ488489	root:Host-associated:Human:Digestive system:Large int	0.0788
MGYA00170887/OKUP01_FASTA.fasta	MGYA00170887	ERZ488623	root:Host-associated:Human:Digestive system:Large int	0.037
MGYA00170888/OHVM01_FASTA.fasta	MGYA00170888	ERZ488393	root:Host-associated:Human:Digestive system:Large int	0.3934
MGYA00170889/OHWH01_FASTA.fasta	MGYA00170889	ERZ488424	root:Host-associated:Human:Digestive system:Large int	0.518
MGYA00170890/OKUT01_FASTA.fasta	MGYA00170890	ERZ488626	root:Host-associated:Human:Digestive system:Large int	0.3248
MGYA00170891/OHYX01_FASTA.fasta	MGYA00170891	ERZ488503	root:Host-associated:Human:Digestive system:Large int	0.0374
MGYA00170892/OKUD01_FASTA.fasta	MGYA00170892	ERZ488514	root:Host-associated:Human:Digestive system:Large int	0.281
MGYA00170893/OHVA01_FASTA.fasta	MGYA00170893	ERZ488401	root:Host-associated:Human:Digestive system:Large int	0.1184
MGYA00170894/OHUV01_FASTA.fasta	MGYA00170894	ERZ488379	root:Host-associated:Human:Digestive system:Large int	0.5246
MGYA00170895/OICB01_FASTA.fasta	MGYA00170895	ERZ488576	root:Host-associated:Human:Digestive system:Large int	0.4986
MGYA00170896/OHVP01_FASTA.fasta	MGYA00170896	ERZ488407	root:Host-associated:Human:Digestive system:Large int	0.346
MGYA00170897/OHWV01_FASTA.fasta	MGYA00170897	ERZ488449	root:Host-associated:Human:Digestive system:Large int	0.1342
MGYA00170898/OIEA01_FASTA.fasta	MGYA00170898	ERZ488647	root:Host-associated:Human:Digestive system:Large int	0.352
MGYA00170899/OICF01_FASTA.fasta	MGYA00170899	ERZ488594	root:Host-associated:Human:Digestive system:Large int	0.275
MGYA00170900/OIAX01_FASTA.fasta	MGYA00170900	ERZ488544	root:Host-associated:Human:Digestive system:Large int	0.376
MGYA00170901/OHUT01_FASTA.fasta	MGYA00170901	ERZ488386	root:Host-associated:Human:Digestive system:Large int	0.3178
MGYA00170902/OIDE01_FASTA.fasta	MGYA00170902	ERZ488616	root:Host-associated:Human:Digestive system:Large int	0.0816
MGYA00170903/OHUI01_FASTA.fasta	MGYA00170903	ERZ488373	root:Host-associated:Human:Digestive system:Large int	0.3412
MGYA00170904/OHXJ01_FASTA.fasta	MGYA00170904	ERZ488462	root:Host-associated:Human:Digestive system:Large int	0.095
MGYA00170905/OHYE01_FASTA.fasta	MGYA00170905	ERZ488465	root:Host-associated:Human:Digestive system:Large int	0.372
MGYA00170906/OIBU01_FASTA.fasta	MGYA00170906	ERZ488577	root:Host-associated:Human:Digestive system:Large int	0.3428
MGYA00170907/OHUH01_FASTA.fasta	MGYA00170907	ERZ488376	root:Host-associated:Human:Digestive system:Large int	0.235
MGYA00170908/OIDL01_FASTA.fasta	MGYA00170908	ERZ488640	root:Host-associated:Human:Digestive system:Large int	0.305
MGYA00170909/OHWY01_FASTA.fasta	MGYA00170909	ERZ488435	root:Host-associated:Human:Digestive system:Large int	0.3128
MGYA00170910/OHVL01_FASTA.fasta	MGYA00170910	ERZ488409	root:Host-associated:Human:Digestive system:Large int	0.241
MGYA00170911/OIBV01_FASTA.fasta	MGYA00170911	ERZ488591	root:Host-associated:Human:Digestive system:Large int	0.1728
MGYA00170912/OHUP01_FASTA.fasta	MGYA00170912	ERZ488370	root:Host-associated:Human:Digestive system:Large int	0.303
MGYA00170913/OICM01_FASTA.fasta	MGYA00170913	ERZ488597	root:Host-associated:Human:Digestive system:Large int	0.3022
MGYA00170914/OICO01_FASTA.fasta	MGYA00170914	ERZ488599	root:Host-associated:Human:Digestive system:Large int	0.0378
MGYA00170915/OIDM01_FASTA.fasta	MGYA00170915	ERZ488625	root:Host-associated:Human:Digestive system:Large int	0.3346
MGYA00170916/OHVG01_FASTA.fasta	MGYA00170916	ERZ488397	root:Host-associated:Human:Digestive system:Large int	0.0592
MGYA00170917/OIMR01_FASTA.fasta	MGYA00170917	ERZ489128	root:Host-associated:Human:Digestive system:Large int	0.121
MGYA00170918/OLCW01_FASTA.fasta	MGYA00170918	ERZ489217	root:Host-associated:Human:Digestive system:Large int	0.1908
MGYA00170919/OLAF01_FASTA.fasta	MGYA00170919	ERZ488944	root:Host-associated:Human:Digestive system:Large int	0.2372
MGYA00170920/OKXE01_FASTA.fasta	MGYA00170920	ERZ488813	root:Host-associated:Human:Digestive system:Large int	0.3928
MGYA00170921/OLIU01_FASTA.fasta	MGYA00170921	ERZ489030	root:Host-associated:Human:Digestive system:Large int	0.1548
MGYA00170922/OKYJ01_FASTA.fasta	MGYA00170922	ERZ488875	root:Host-associated:Human:Digestive system:Large int	0.178
MGYA00170923/OLET01_FASTA.fasta	MGYA00170923	ERZ489276	root:Host-associated:Human:Digestive system:Large int	0.2632
MGYA00170924/OIHW01_FASTA.fasta	MGYA00170924	ERZ488880	root:Host-associated:Human:Digestive system:Large int	0.3414
MGYA00170925/OIUY01_FASTA.fasta	MGYA00170925	ERZ489406	root:Host-associated:Human:Digestive system:Large int	0.3068
MGYA00170926/OIGJ01_FASTA.fasta	MGYA00170926	ERZ488819	root:Host-associated:Human:Digestive system:Large int	0.3838
MGYA00170927/OIIH01_FASTA.fasta	MGYA00170927	ERZ488945	root:Host-associated:Human:Digestive system:Large int	0.2232
MGYA00170928/OKXA01_FASTA.fasta	MGYA00170928	ERZ488824	root:Host-associated:Human:Digestive system:Large int	0.2412
MGYA00170929/OLDO01_FASTA.fasta	MGYA00170929	ERZ489227	root:Host-associated:Human:Digestive system:Large int	0.294
MGYA00170930/OLEM01_FASTA.fasta	MGYA00170930	ERZ489258	root:Host-associated:Human:Digestive system:Large int	0.2444
MGYA00170931/OIPF01_FASTA.fasta	MGYA00170931	ERZ489201	root:Host-associated:Human:Digestive system:Large int	0.2286
MGYA00170932/OIGM01_FASTA.fasta	MGYA00170932	ERZ488821	root:Host-associated:Human:Digestive system:Large int	0.3946
MGYA00170933/OIRD01_FASTA.fasta	MGYA00170933	ERZ489312	root:Host-associated:Human:Digestive system:Large int	0.2796
MGYA00170934/OIRO01_FASTA.fasta	MGYA00170934	ERZ489333	root:Host-associated:Human:Digestive system:Large int	0.2624
MGYA00170935/OLBP01_FASTA.fasta	MGYA00170935	ERZ489011	root:Host-associated:Human:Digestive system:Large int	0.3756
MGYA00170936/OIIR01_FASTA.fasta	MGYA00170936	ERZ488965	root:Host-associated:Human:Digestive system:Large int	0.3232
MGYA00170937/OKYS01_FASTA.fasta	MGYA00170937	ERZ488893	root:Host-associated:Human:Digestive system:Large int	0.1536
MGYA00170938/OIPJ01_FASTA.fasta	MGYA00170938	ERZ489210	root:Host-associated:Human:Digestive system:Large int	0.06
MGYA00170939/OLEL01_FASTA.fasta	MGYA00170939	ERZ489263	root:Host-associated:Human:Digestive system:Large int	0.0872
MGYA00170940/OLEO01_FASTA.fasta	MGYA00170940	ERZ489266	root:Host-associated:Human:Digestive system:Large int	0.2422
MGYA00170941/OLCJ01_FASTA.fasta	MGYA00170941	ERZ489057	root:Host-associated:Human:Digestive system:Large int	0.3234
MGYA00170942/OIIL01_FASTA.fasta	MGYA00170942	ERZ488954	root:Host-associated:Human:Digestive system:Large int	0.2358
MGYA00170943/OLBN01_FASTA.fasta	MGYA00170943	ERZ489021	root:Host-associated:Human:Digestive system:Large int	0.0964
MGYA00170944/OKYR01_FASTA.fasta	MGYA00170944	ERZ488887	root:Host-associated:Human:Digestive system:Large int	0.228
MGYA00170945/OITN01_FASTA.fasta	MGYA00170945	ERZ489372	root:Host-associated:Human:Digestive system:Large int	0.3138
MGYA00170946/OLAK01_FASTA.fasta	MGYA00170946	ERZ488974	root:Host-associated:Human:Digestive system:Large int	0.2608
MGYA00170947/OKXG01_FASTA.fasta	MGYA00170947	ERZ488807	root:Host-associated:Human:Digestive system:Large int	0.3894
MGYA00170948/OIGX01_FASTA.fasta	MGYA00170948	ERZ488858	root:Host-associated:Human:Digestive system:Large int	0.3766
MGYA00170949/OILE01_FASTA.fasta	MGYA00170949	ERZ489076	root:Host-associated:Human:Digestive system:Large int	0.3212
MGYA00170950/OIGN01_FASTA.fasta	MGYA00170950	ERZ488817	root:Host-associated:Human:Digestive system:Large int	0.3674
MGYA00170951/OKZL01_FASTA.fasta	MGYA00170951	ERZ488917	root:Host-associated:Human:Digestive system:Large int	0.2288
MGYA00170952/OINU01_FASTA.fasta	MGYA00170952	ERZ489166	root:Host-associated:Human:Digestive system:Large int	0.1454
MGYA00170953/OLIV01_FASTA.fasta	MGYA00170953	ERZ489029	root:Host-associated:Human:Digestive system:Large int	0.2066
MGYA00170954/OIKW01_FASTA.fasta	MGYA00170954	ERZ489074	root:Host-associated:Human:Digestive system:Large int	0.1556
MGYA00170955/OLBQ01_FASTA.fasta	MGYA00170955	ERZ489020	root:Host-associated:Human:Digestive system:Large int	0.2872
MGYA00170956/OLEC01_FASTA.fasta	MGYA00170956	ERZ489249	root:Host-associated:Human:Digestive system:Large int	0.2984
MGYA00170957/OLAI01_FASTA.fasta	MGYA00170957	ERZ488957	root:Host-associated:Human:Digestive system:Large int	0.294
MGYA00170958/OIIA01_FASTA.fasta	MGYA00170958	ERZ488937	root:Host-associated:Human:Digestive system:Large int	0.2944
MGYA00170959/OKYH01_FASTA.fasta	MGYA00170959	ERZ488866	root:Host-associated:Human:Digestive system:Large int	0.267
MGYA00170960/OLCE01_FASTA.fasta	MGYA00170960	ERZ489059	root:Host-associated:Human:Digestive system:Large int	0.1628
MGYA00170961/OKZN01_FASTA.fasta	MGYA00170961	ERZ488904	root:Host-associated:Human:Digestive system:Large int	0.3286
MGYA00170962/OIMB01_FASTA.fasta	MGYA00170962	ERZ489098	root:Host-associated:Human:Digestive system:Large int	0.3302
MGYA00170963/OINB01_FASTA.fasta	MGYA00170963	ERZ489143	root:Host-associated:Human:Digestive system:Large int	0.1088
MGYA00170964/OKXH01_FASTA.fasta	MGYA00170964	ERZ488828	root:Host-associated:Human:Digestive system:Large int	0.2562
MGYA00170965/OISX01_FASTA.fasta	MGYA00170965	ERZ489367	root:Host-associated:Human:Digestive system:Large int	0.1924
MGYA00170966/OITU01_FASTA.fasta	MGYA00170966	ERZ489391	root:Host-associated:Human:Digestive system:Large int	0.1882
MGYA00170967/OLAQ01_FASTA.fasta	MGYA00170967	ERZ488976	root:Host-associated:Human:Digestive system:Large int	0.2138
MGYA00170968/OINR01_FASTA.fasta	MGYA00170968	ERZ489154	root:Host-associated:Human:Digestive system:Large int	0.2146
MGYA00170969/OIIM01_FASTA.fasta	MGYA00170969	ERZ488938	root:Host-associated:Human:Digestive system:Large int	0.2688
MGYA00170970/OISK01_FASTA.fasta	MGYA00170970	ERZ489350	root:Host-associated:Human:Digestive system:Large int	0.2546
MGYA00170971/OIRH01_FASTA.fasta	MGYA00170971	ERZ489315	root:Host-associated:Human:Digestive system:Large int	0.3092
MGYA00170972/OISB01_FASTA.fasta	MGYA00170972	ERZ489339	root:Host-associated:Human:Digestive system:Large int	0.2632
MGYA00170973/OILL01_FASTA.fasta	MGYA00170973	ERZ489094	root:Host-associated:Human:Digestive system:Large int	0.2078
MGYA00170974/OIMW01_FASTA.fasta	MGYA00170974	ERZ489123	root:Host-associated:Human:Digestive system:Large int	0.2114
MGYA00170975/OIQF01_FASTA.fasta	MGYA00170975	ERZ489289	root:Host-associated:Human:Digestive system:Large int	0.3074
MGYA00170976/OKXU01_FASTA.fasta	MGYA00170976	ERZ488848	root:Host-associated:Human:Digestive system:Large int	0.3022
MGYA00170977/OIUZ01_FASTA.fasta	MGYA00170977	ERZ489417	root:Host-associated:Human:Digestive system:Large int	0.3168
MGYA00170978/OIMP01_FASTA.fasta	MGYA00170978	ERZ489135	root:Host-associated:Human:Digestive system:Large int	0.4392
MGYA00170979/OLEQ01_FASTA.fasta	MGYA00170979	ERZ489269	root:Host-associated:Human:Digestive system:Large int	0.2796
MGYA00170980/OIUE01_FASTA.fasta	MGYA00170980	ERZ489396	root:Host-associated:Human:Digestive system:Large int	0.1898
MGYA00170981/OLBX01_FASTA.fasta	MGYA00170981	ERZ489032	root:Host-associated:Human:Digestive system:Large int	0.2874
MGYA00170982/OKYO01_FASTA.fasta	MGYA00170982	ERZ488886	root:Host-associated:Human:Digestive system:Large int	0.211
MGYA00170983/OIKT01_FASTA.fasta	MGYA00170983	ERZ489065	root:Host-associated:Human:Digestive system:Large int	0.3174
MGYA00170984/OIHG01_FASTA.fasta	MGYA00170984	ERZ488874	root:Host-associated:Human:Digestive system:Large int	0.2578
MGYA00170985/OIQE01_FASTA.fasta	MGYA00170985	ERZ489291	root:Host-associated:Human:Digestive system:Large int	0.2998
MGYA00170986/OKZM01_FASTA.fasta	MGYA00170986	ERZ488908	root:Host-associated:Human:Digestive system:Large int	0.2786
MGYA00170987/OKZJ01_FASTA.fasta	MGYA00170987	ERZ488915	root:Host-associated:Human:Digestive system:Large int	0.2922
MGYA00170988/OLED01_FASTA.fasta	MGYA00170988	ERZ489256	root:Host-associated:Human:Digestive system:Large int	0.3732
MGYA00170989/OIUU01_FASTA.fasta	MGYA00170989	ERZ489414	root:Host-associated:Human:Digestive system:Large int	0.2732
MGYA00170990/OLCK01_FASTA.fasta	MGYA00170990	ERZ489100	root:Host-associated:Human:Digestive system:Large int	0.1292
MGYA00170991/OLAM01_FASTA.fasta	MGYA00170991	ERZ488964	root:Host-associated:Human:Digestive system:Large int	0.3322
MGYA00170992/OIHU01_FASTA.fasta	MGYA00170992	ERZ488912	root:Host-associated:Human:Digestive system:Large int	0.2276
MGYA00170993/OITD01_FASTA.fasta	MGYA00170993	ERZ489376	root:Host-associated:Human:Digestive system:Large int	0.2302
MGYA00170994/OLIB01_FASTA.fasta	MGYA00170994	ERZ488970	root:Host-associated:Human:Digestive system:Large int	0.2662
MGYA00170995/OIPK01_FASTA.fasta	MGYA00170995	ERZ489205	root:Host-associated:Human:Digestive system:Large int	0.0972
MGYA00170996/OINY01_FASTA.fasta	MGYA00170996	ERZ489155	root:Host-associated:Human:Digestive system:Large int	0.3266
MGYA00170997/OINI01_FASTA.fasta	MGYA00170997	ERZ489132	root:Host-associated:Human:Digestive system:Large int	0.3706
MGYA00170998/OLDH01_FASTA.fasta	MGYA00170998	ERZ489233	root:Host-associated:Human:Digestive system:Large int	0.2344
MGYA00170999/OINK01_FASTA.fasta	MGYA00170999	ERZ489142	root:Host-associated:Human:Digestive system:Large int	0.3414
MGYA00171000/OIHO01_FASTA.fasta	MGYA00171000	ERZ488906	root:Host-associated:Human:Digestive system:Large int	0.2386
MGYA00171001/OIQZ01_FASTA.fasta	MGYA00171001	ERZ489320	root:Host-associated:Human:Digestive system:Large int	0.3072
MGYA00171002/OIKB01_FASTA.fasta	MGYA00171002	ERZ489047	root:Host-associated:Human:Digestive system:Large int	0.247
MGYA00171003/OLIH01_FASTA.fasta	MGYA00171003	ERZ489004	root:Host-associated:Human:Digestive system:Large int	0.2472
MGYA00171004/OIHM01_FASTA.fasta	MGYA00171004	ERZ488895	root:Host-associated:Human:Digestive system:Large int	0.3132
MGYA00171005/OIIX01_FASTA.fasta	MGYA00171005	ERZ488977	root:Host-associated:Human:Digestive system:Large int	0.3256
MGYA00171006/OLEI01_FASTA.fasta	MGYA00171006	ERZ489253	root:Host-associated:Human:Digestive system:Large int	0.3004
MGYA00171007/OIPW01_FASTA.fasta	MGYA00171007	ERZ489213	root:Host-associated:Human:Digestive system:Large int	0.2746
MGYA00171008/OIIZ01_FASTA.fasta	MGYA00171008	ERZ488999	root:Host-associated:Human:Digestive system:Large int	0.1228
MGYA00171009/OINV01_FASTA.fasta	MGYA00171009	ERZ489164	root:Host-associated:Human:Digestive system:Large int	0.0798
MGYA00171010/OLFR01_FASTA.fasta	MGYA00171010	ERZ489297	root:Host-associated:Human:Digestive system:Large int	0.2346
MGYA00171011/OIOL01_FASTA.fasta	MGYA00171011	ERZ489171	root:Host-associated:Human:Digestive system:Large int	0.246
MGYA00171012/OISN01_FASTA.fasta	MGYA00171012	ERZ489362	root:Host-associated:Human:Digestive system:Large int	0.2334
MGYA00171013/OIQS01_FASTA.fasta	MGYA00171013	ERZ489306	root:Host-associated:Human:Digestive system:Large int	0.376
MGYA00171014/OIIU01_FASTA.fasta	MGYA00171014	ERZ488975	root:Host-associated:Human:Digestive system:Large int	0.1552
MGYA00171015/OISV01_FASTA.fasta	MGYA00171015	ERZ489355	root:Host-associated:Human:Digestive system:Large int	0.25
MGYA00171016/OLBK01_FASTA.fasta	MGYA00171016	ERZ489000	root:Host-associated:Human:Digestive system:Large int	0.152
MGYA00171017/OIHC01_FASTA.fasta	MGYA00171017	ERZ488861	root:Host-associated:Human:Digestive system:Large int	0.3262
MGYA00171018/OIVK01_FASTA.fasta	MGYA00171018	ERZ489424	root:Host-associated:Human:Digestive system:Large int	0.2342
MGYA00171019/OLAD01_FASTA.fasta	MGYA00171019	ERZ488966	root:Host-associated:Human:Digestive system:Large int	0.2358
MGYA00171020/OIIJ01_FASTA.fasta	MGYA00171020	ERZ488943	root:Host-associated:Human:Digestive system:Large int	0.225
MGYA00171021/OIVY01_FASTA.fasta	MGYA00171021	ERZ489430	root:Host-associated:Human:Digestive system:Large int	0.329
MGYA00171022/OKZH01_FASTA.fasta	MGYA00171022	ERZ488926	root:Host-associated:Human:Digestive system:Large int	0.2278
MGYA00171023/OLEA01_FASTA.fasta	MGYA00171023	ERZ489250	root:Host-associated:Human:Digestive system:Large int	0.3196
MGYA00171024/OIME01_FASTA.fasta	MGYA00171024	ERZ489112	root:Host-associated:Human:Digestive system:Large int	0.3382
MGYA00171025/OIKP01_FASTA.fasta	MGYA00171025	ERZ489063	root:Host-associated:Human:Digestive system:Large int	0.1712
MGYA00171026/OIRT01_FASTA.fasta	MGYA00171026	ERZ489340	root:Host-associated:Human:Digestive system:Large int	0.2854
MGYA00171027/OIQD01_FASTA.fasta	MGYA00171027	ERZ489282	root:Host-associated:Human:Digestive system:Large int	0.2522
MGYA00171028/OLFG01_FASTA.fasta	MGYA00171028	ERZ489277	root:Host-associated:Human:Digestive system:Large int	0.3142
MGYA00171029/OIKE01_FASTA.fasta	MGYA00171029	ERZ489013	root:Host-associated:Human:Digestive system:Large int	0.4598
MGYA00171030/OIHF01_FASTA.fasta	MGYA00171030	ERZ488863	root:Host-associated:Human:Digestive system:Large int	0.2452
MGYA00171031/OIPX01_FASTA.fasta	MGYA00171031	ERZ489232	root:Host-associated:Human:Digestive system:Large int	0.2238
MGYA00171032/OLAB01_FASTA.fasta	MGYA00171032	ERZ488947	root:Host-associated:Human:Digestive system:Large int	0.2784
MGYA00171033/OLBW01_FASTA.fasta	MGYA00171033	ERZ489038	root:Host-associated:Human:Digestive system:Large int	0.2106
MGYA00171034/OIGT01_FASTA.fasta	MGYA00171034	ERZ488850	root:Host-associated:Human:Digestive system:Large int	0.297
MGYA00171035/OLAO01_FASTA.fasta	MGYA00171035	ERZ488971	root:Host-associated:Human:Digestive system:Large int	0.312
MGYA00171036/OLEK01_FASTA.fasta	MGYA00171036	ERZ489267	root:Host-associated:Human:Digestive system:Large int	0.2488
MGYA00171037/OINO01_FASTA.fasta	MGYA00171037	ERZ489144	root:Host-associated:Human:Digestive system:Large int	0.2922
MGYA00171038/OLIW01_FASTA.fasta	MGYA00171038	ERZ489034	root:Host-associated:Human:Digestive system:Large int	0.4642
MGYA00171039/OIGF01_FASTA.fasta	MGYA00171039	ERZ488827	root:Host-associated:Human:Digestive system:Large int	0.2206
MGYA00171040/OISF01_FASTA.fasta	MGYA00171040	ERZ489345	root:Host-associated:Human:Digestive system:Large int	0.2976
MGYA00171041/OIMX01_FASTA.fasta	MGYA00171041	ERZ489134	root:Host-associated:Human:Digestive system:Large int	0.1908
MGYA00171042/OITY01_FASTA.fasta	MGYA00171042	ERZ489385	root:Host-associated:Human:Digestive system:Large int	0.3188
MGYA00171043/OLBT01_FASTA.fasta	MGYA00171043	ERZ489033	root:Host-associated:Human:Digestive system:Large int	0.2104
MGYA00171044/OILY01_FASTA.fasta	MGYA00171044	ERZ489095	root:Host-associated:Human:Digestive system:Large int	0.3366
MGYA00171045/OIRY01_FASTA.fasta	MGYA00171045	ERZ489334	root:Host-associated:Human:Digestive system:Large int	0.2588
MGYA00171046/OIIV01_FASTA.fasta	MGYA00171046	ERZ488956	root:Host-associated:Human:Digestive system:Large int	0.2114
MGYA00171047/OLCM01_FASTA.fasta	MGYA00171047	ERZ489075	root:Host-associated:Human:Digestive system:Large int	0.1702
MGYA00171048/OIHJ01_FASTA.fasta	MGYA00171048	ERZ488901	root:Host-associated:Human:Digestive system:Large int	0.1676
MGYA00171049/OLEX01_FASTA.fasta	MGYA00171049	ERZ489280	root:Host-associated:Human:Digestive system:Large int	0.324
MGYA00171050/OIOC01_FASTA.fasta	MGYA00171050	ERZ489162	root:Host-associated:Human:Digestive system:Large int	0.2236
MGYA00171051/OIQI01_FASTA.fasta	MGYA00171051	ERZ489230	root:Host-associated:Human:Digestive system:Large int	0.2724
MGYA00171052/OIUT01_FASTA.fasta	MGYA00171052	ERZ489416	root:Host-associated:Human:Digestive system:Large int	0.2162
MGYA00171053/OIPY01_FASTA.fasta	MGYA00171053	ERZ489238	root:Host-associated:Human:Digestive system:Large int	0.3266
MGYA00171054/OIOD01_FASTA.fasta	MGYA00171054	ERZ489172	root:Host-associated:Human:Digestive system:Large int	0.2586
MGYA00171055/OLIM01_FASTA.fasta	MGYA00171055	ERZ489012	root:Host-associated:Human:Digestive system:Large int	0.2784
MGYA00171056/OIUD01_FASTA.fasta	MGYA00171056	ERZ489393	root:Host-associated:Human:Digestive system:Large int	0.2686
MGYA00171057/OIUA01_FASTA.fasta	MGYA00171057	ERZ489387	root:Host-associated:Human:Digestive system:Large int	0.3108
MGYA00171058/OISW01_FASTA.fasta	MGYA00171058	ERZ489366	root:Host-associated:Human:Digestive system:Large int	0.298
MGYA00171059/OINP01_FASTA.fasta	MGYA00171059	ERZ489157	root:Host-associated:Human:Digestive system:Large int	0.2036
MGYA00171060/OIUS01_FASTA.fasta	MGYA00171060	ERZ489421	root:Host-associated:Human:Digestive system:Large int	0.2336
MGYA00171061/OIHH01_FASTA.fasta	MGYA00171061	ERZ488877	root:Host-associated:Human:Digestive system:Large int	0.2336
MGYA00171062/OLJO01_FASTA.fasta	MGYA00171062	ERZ489014	root:Host-associated:Human:Digestive system:Large int	0.2782
MGYA00171063/OIFZ01_FASTA.fasta	MGYA00171063	ERZ488809	root:Host-associated:Human:Digestive system:Large int	0.3876
MGYA00171064/OIND01_FASTA.fasta	MGYA00171064	ERZ489149	root:Host-associated:Human:Digestive system:Large int	0.421
MGYA00171065/OLIK01_FASTA.fasta	MGYA00171065	ERZ489002	root:Host-associated:Human:Digestive system:Large int	0.3734
MGYA00171066/OIVO01_FASTA.fasta	MGYA00171066	ERZ489431	root:Host-associated:Human:Digestive system:Large int	0.2746
MGYA00171067/OIMG01_FASTA.fasta	MGYA00171067	ERZ489122	root:Host-associated:Human:Digestive system:Large int	0.0936
MGYA00171068/OIRL01_FASTA.fasta	MGYA00171068	ERZ489326	root:Host-associated:Human:Digestive system:Large int	0.3726
MGYA00171069/OKZW01_FASTA.fasta	MGYA00171069	ERZ488927	root:Host-associated:Human:Digestive system:Large int	0.2772
MGYA00171070/OLER01_FASTA.fasta	MGYA00171070	ERZ489270	root:Host-associated:Human:Digestive system:Large int	0.3112
MGYA00171071/OIKK01_FASTA.fasta	MGYA00171071	ERZ489050	root:Host-associated:Human:Digestive system:Large int	0.175
MGYA00171072/OIKV01_FASTA.fasta	MGYA00171072	ERZ489080	root:Host-associated:Human:Digestive system:Large int	0.2298
MGYA00171073/OINM01_FASTA.fasta	MGYA00171073	ERZ489137	root:Host-associated:Human:Digestive system:Large int	0.3462
MGYA00171074/OLCF01_FASTA.fasta	MGYA00171074	ERZ489058	root:Host-associated:Human:Digestive system:Large int	0.3084
MGYA00171075/OLFC01_FASTA.fasta	MGYA00171075	ERZ489285	root:Host-associated:Human:Digestive system:Large int	0.2892
MGYA00171076/OITI01_FASTA.fasta	MGYA00171076	ERZ489380	root:Host-associated:Human:Digestive system:Large int	0.3122
MGYA00171077/OION01_FASTA.fasta	MGYA00171077	ERZ489169	root:Host-associated:Human:Digestive system:Large int	0.2674
MGYA00171078/OKZI01_FASTA.fasta	MGYA00171078	ERZ488903	root:Host-associated:Human:Digestive system:Large int	0.326
MGYA00171079/OKYG01_FASTA.fasta	MGYA00171079	ERZ488871	root:Host-associated:Human:Digestive system:Large int	0.2932
MGYA00171080/OIPG01_FASTA.fasta	MGYA00171080	ERZ489199	root:Host-associated:Human:Digestive system:Large int	0.244
MGYA00171081/OLCS01_FASTA.fasta	MGYA00171081	ERZ489192	root:Host-associated:Human:Digestive system:Large int	0.1232
MGYA00171082/OIIW01_FASTA.fasta	MGYA00171082	ERZ488978	root:Host-associated:Human:Digestive system:Large int	0.3454
MGYA00171083/OIQX01_FASTA.fasta	MGYA00171083	ERZ489305	root:Host-associated:Human:Digestive system:Large int	0.3086
MGYA00171084/OIOZ01_FASTA.fasta	MGYA00171084	ERZ489197	root:Host-associated:Human:Digestive system:Large int	0.112
MGYA00171085/OKXY01_FASTA.fasta	MGYA00171085	ERZ488857	root:Host-associated:Human:Digestive system:Large int	0.2994
MGYA00171086/OIOB01_FASTA.fasta	MGYA00171086	ERZ489167	root:Host-associated:Human:Digestive system:Large int	0.2644
MGYA00171087/OIQU01_FASTA.fasta	MGYA00171087	ERZ489310	root:Host-associated:Human:Digestive system:Large int	0.358
MGYA00171088/OLCY01_FASTA.fasta	MGYA00171088	ERZ489215	root:Host-associated:Human:Digestive system:Large int	0.3256
MGYA00171089/OKZA01_FASTA.fasta	MGYA00171089	ERZ488913	root:Host-associated:Human:Digestive system:Large int	0.2406
MGYA00171090/OIGE01_FASTA.fasta	MGYA00171090	ERZ488826	root:Host-associated:Human:Digestive system:Large int	0.3676
MGYA00171091/OKYU01_FASTA.fasta	MGYA00171091	ERZ488885	root:Host-associated:Human:Digestive system:Large int	0.2714
MGYA00171092/OIPR01_FASTA.fasta	MGYA00171092	ERZ489206	root:Host-associated:Human:Digestive system:Large int	0.252
MGYA00171093/OKZZ01_FASTA.fasta	MGYA00171093	ERZ488958	root:Host-associated:Human:Digestive system:Large int	0.0744
MGYA00171094/OIPN01_FASTA.fasta	MGYA00171094	ERZ489200	root:Host-associated:Human:Digestive system:Large int	0.3386
MGYA00171095/OLFF01_FASTA.fasta	MGYA00171095	ERZ489272	root:Host-associated:Human:Digestive system:Large int	0.2952
MGYA00171096/OLBD01_FASTA.fasta	MGYA00171096	ERZ489003	root:Host-associated:Human:Digestive system:Large int	0.248
MGYA00171097/OLDI01_FASTA.fasta	MGYA00171097	ERZ489231	root:Host-associated:Human:Digestive system:Large int	0.3638
MGYA00171098/OLBM01_FASTA.fasta	MGYA00171098	ERZ489027	root:Host-associated:Human:Digestive system:Large int	0.1076
MGYA00171099/OLBF01_FASTA.fasta	MGYA00171099	ERZ489018	root:Host-associated:Human:Digestive system:Large int	0.2914
MGYA00171100/OLDL01_FASTA.fasta	MGYA00171100	ERZ489226	root:Host-associated:Human:Digestive system:Large int	0.322
MGYA00171101/OIIT01_FASTA.fasta	MGYA00171101	ERZ488950	root:Host-associated:Human:Digestive system:Large int	0.2764
MGYA00171102/OITO01_FASTA.fasta	MGYA00171102	ERZ489390	root:Host-associated:Human:Digestive system:Large int	0.247
MGYA00171103/OIKS01_FASTA.fasta	MGYA00171103	ERZ489049	root:Host-associated:Human:Digestive system:Large int	0.3202
MGYA00171104/OIMZ01_FASTA.fasta	MGYA00171104	ERZ489129	root:Host-associated:Human:Digestive system:Large int	0.2674
MGYA00171105/OKXD01_FASTA.fasta	MGYA00171105	ERZ488815	root:Host-associated:Human:Digestive system:Large int	0.372
MGYA00171106/OILV01_FASTA.fasta	MGYA00171106	ERZ489105	root:Host-associated:Human:Digestive system:Large int	0.11
MGYA00171107/OLCL01_FASTA.fasta	MGYA00171107	ERZ489089	root:Host-associated:Human:Digestive system:Large int	0.204
MGYA00171108/OKXN01_FASTA.fasta	MGYA00171108	ERZ488833	root:Host-associated:Human:Digestive system:Large int	0.3702
MGYA00171109/OKYV01_FASTA.fasta	MGYA00171109	ERZ488896	root:Host-associated:Human:Digestive system:Large int	0.2828
MGYA00171110/OIIG01_FASTA.fasta	MGYA00171110	ERZ488930	root:Host-associated:Human:Digestive system:Large int	0.2458
MGYA00171111/OISU01_FASTA.fasta	MGYA00171111	ERZ489352	root:Host-associated:Human:Digestive system:Large int	0.2402
MGYA00171112/OIUN01_FASTA.fasta	MGYA00171112	ERZ489410	root:Host-associated:Human:Digestive system:Large int	0.2712
MGYA00171113/OIHV01_FASTA.fasta	MGYA00171113	ERZ488925	root:Host-associated:Human:Digestive system:Large int	0.2788
MGYA00171114/OIPH01_FASTA.fasta	MGYA00171114	ERZ489196	root:Host-associated:Human:Digestive system:Large int	0.2584
MGYA00171115/OLCH01_FASTA.fasta	MGYA00171115	ERZ489064	root:Host-associated:Human:Digestive system:Large int	0.2812
MGYA00171116/OIHL01_FASTA.fasta	MGYA00171116	ERZ488869	root:Host-associated:Human:Digestive system:Large int	0.2842
MGYA00171117/OINE01_FASTA.fasta	MGYA00171117	ERZ489148	root:Host-associated:Human:Digestive system:Large int	0.4524
MGYA00171118/OKYN01_FASTA.fasta	MGYA00171118	ERZ488884	root:Host-associated:Human:Digestive system:Large int	0.2972
MGYA00171119/OIPD01_FASTA.fasta	MGYA00171119	ERZ489188	root:Host-associated:Human:Digestive system:Large int	0.3182
MGYA00171120/OLFJ01_FASTA.fasta	MGYA00171120	ERZ489295	root:Host-associated:Human:Digestive system:Large int	0.1652
MGYA00171121/OIKC01_FASTA.fasta	MGYA00171121	ERZ489025	root:Host-associated:Human:Digestive system:Large int	0.2832
MGYA00171122/OLFA01_FASTA.fasta	MGYA00171122	ERZ489283	root:Host-associated:Human:Digestive system:Large int	0.3452
MGYA00171123/OIUR01_FASTA.fasta	MGYA00171123	ERZ489422	root:Host-associated:Human:Digestive system:Large int	0.3416
MGYA00171124/OITZ01_FASTA.fasta	MGYA00171124	ERZ489392	root:Host-associated:Human:Digestive system:Large int	0.3736
MGYA00171125/OLBH01_FASTA.fasta	MGYA00171125	ERZ489001	root:Host-associated:Human:Digestive system:Large int	0.2724
MGYA00171126/OIOW01_FASTA.fasta	MGYA00171126	ERZ489191	root:Host-associated:Human:Digestive system:Large int	0.2254
MGYA00171127/OIKG01_FASTA.fasta	MGYA00171127	ERZ489041	root:Host-associated:Human:Digestive system:Large int	0.3416
MGYA00171128/OLBI01_FASTA.fasta	MGYA00171128	ERZ489019	root:Host-associated:Human:Digestive system:Large int	0.1354
MGYA00171129/OILP01_FASTA.fasta	MGYA00171129	ERZ489111	root:Host-associated:Human:Digestive system:Large int	0.1404
MGYA00171130/OKXZ01_FASTA.fasta	MGYA00171130	ERZ488847	root:Host-associated:Human:Digestive system:Large int	0.2724
MGYA00171131/OLDZ01_FASTA.fasta	MGYA00171131	ERZ489239	root:Host-associated:Human:Digestive system:Large int	0.2634
MGYA00171132/OLDX01_FASTA.fasta	MGYA00171132	ERZ489248	root:Host-associated:Human:Digestive system:Large int	0.2592
MGYA00171133/OIRS01_FASTA.fasta	MGYA00171133	ERZ489330	root:Host-associated:Human:Digestive system:Large int	0.3242
MGYA00171134/OINA01_FASTA.fasta	MGYA00171134	ERZ489146	root:Host-associated:Human:Digestive system:Large int	0.0812
MGYA00171135/OITL01_FASTA.fasta	MGYA00171135	ERZ489373	root:Host-associated:Human:Digestive system:Large int	0.2492
MGYA00171136/OIMD01_FASTA.fasta	MGYA00171136	ERZ489116	root:Host-associated:Human:Digestive system:Large int	0.1454
MGYA00171137/OIQH01_FASTA.fasta	MGYA00171137	ERZ489264	root:Host-associated:Human:Digestive system:Large int	0.2416
MGYA00171138/OIHT01_FASTA.fasta	MGYA00171138	ERZ488907	root:Host-associated:Human:Digestive system:Large int	0.3092
MGYA00171139/OIRK01_FASTA.fasta	MGYA00171139	ERZ489324	root:Host-associated:Human:Digestive system:Large int	0.3678
MGYA00171140/OILA01_FASTA.fasta	MGYA00171140	ERZ489078	root:Host-associated:Human:Digestive system:Large int	0.3204
MGYA00171141/OIUC01_FASTA.fasta	MGYA00171141	ERZ489399	root:Host-associated:Human:Digestive system:Large int	0.3176
MGYA00171142/OLJQ01_FASTA.fasta	MGYA00171142	ERZ489010	root:Host-associated:Human:Digestive system:Large int	0.2622
MGYA00171143/OLCN01_FASTA.fasta	MGYA00171143	ERZ489062	root:Host-associated:Human:Digestive system:Large int	0.3096
MGYA00171144/OKYC01_FASTA.fasta	MGYA00171144	ERZ488855	root:Host-associated:Human:Digestive system:Large int	0.269
MGYA00171145/OILH01_FASTA.fasta	MGYA00171145	ERZ489084	root:Host-associated:Human:Digestive system:Large int	0.2434
MGYA00171146/OLDJ01_FASTA.fasta	MGYA00171146	ERZ489228	root:Host-associated:Human:Digestive system:Large int	0.352
MGYA00171147/OIPB01_FASTA.fasta	MGYA00171147	ERZ489198	root:Host-associated:Human:Digestive system:Large int	0.1776
MGYA00171148/OILM01_FASTA.fasta	MGYA00171148	ERZ489101	root:Host-associated:Human:Digestive system:Large int	0.127
MGYA00171149/OKYP01_FASTA.fasta	MGYA00171149	ERZ488878	root:Host-associated:Human:Digestive system:Large int	0.324
MGYA00171150/OIPS01_FASTA.fasta	MGYA00171150	ERZ489203	root:Host-associated:Human:Digestive system:Large int	0.2952
MGYA00171151/OLDB01_FASTA.fasta	MGYA00171151	ERZ489218	root:Host-associated:Human:Digestive system:Large int	0.1834
MGYA00171152/OIKN01_FASTA.fasta	MGYA00171152	ERZ489061	root:Host-associated:Human:Digestive system:Large int	0.4522
MGYA00171153/OKXK01_FASTA.fasta	MGYA00171153	ERZ488832	root:Host-associated:Human:Digestive system:Large int	0.3164
MGYA00171154/OIPP01_FASTA.fasta	MGYA00171154	ERZ489202	root:Host-associated:Human:Digestive system:Large int	0.206
MGYA00171155/OKYT01_FASTA.fasta	MGYA00171155	ERZ488881	root:Host-associated:Human:Digestive system:Large int	0.286
MGYA00171156/OLBU01_FASTA.fasta	MGYA00171156	ERZ489035	root:Host-associated:Human:Digestive system:Large int	0.198
MGYA00171157/OLAP01_FASTA.fasta	MGYA00171157	ERZ488972	root:Host-associated:Human:Digestive system:Large int	0.2608
MGYA00171158/OKZB01_FASTA.fasta	MGYA00171158	ERZ488914	root:Host-associated:Human:Digestive system:Large int	0.202
MGYA00171159/OIMY01_FASTA.fasta	MGYA00171159	ERZ489138	root:Host-associated:Human:Digestive system:Large int	0.1948
MGYA00171160/OLIF01_FASTA.fasta	MGYA00171160	ERZ489005	root:Host-associated:Human:Digestive system:Large int	0.249
MGYA00171161/OLIZ01_FASTA.fasta	MGYA00171161	ERZ489046	root:Host-associated:Human:Digestive system:Large int	0.2084
MGYA00171162/OKZX01_FASTA.fasta	MGYA00171162	ERZ488942	root:Host-associated:Human:Digestive system:Large int	0.2378
MGYA00171163/OILZ01_FASTA.fasta	MGYA00171163	ERZ489104	root:Host-associated:Human:Digestive system:Large int	0.2532
MGYA00171164/OLCZ01_FASTA.fasta	MGYA00171164	ERZ489222	root:Host-associated:Human:Digestive system:Large int	0.1428
MGYA00171165/OINJ01_FASTA.fasta	MGYA00171165	ERZ489126	root:Host-associated:Human:Digestive system:Large int	0.2962
MGYA00171166/OIQQ01_FASTA.fasta	MGYA00171166	ERZ489303	root:Host-associated:Human:Digestive system:Large int	0.252
MGYA00171167/OLBS01_FASTA.fasta	MGYA00171167	ERZ489024	root:Host-associated:Human:Digestive system:Large int	0.281
MGYA00171168/OILN01_FASTA.fasta	MGYA00171168	ERZ489099	root:Host-associated:Human:Digestive system:Large int	0.3944
MGYA00171169/OIRF01_FASTA.fasta	MGYA00171169	ERZ489319	root:Host-associated:Human:Digestive system:Large int	0.253
MGYA00171170/OINH01_FASTA.fasta	MGYA00171170	ERZ489130	root:Host-associated:Human:Digestive system:Large int	0.3124
MGYA00171171/OIGU01_FASTA.fasta	MGYA00171171	ERZ488843	root:Host-associated:Human:Digestive system:Large int	0.2802
MGYA00171172/OIGP01_FASTA.fasta	MGYA00171172	ERZ488829	root:Host-associated:Human:Digestive system:Large int	0.2256
MGYA00171173/OIQY01_FASTA.fasta	MGYA00171173	ERZ489317	root:Host-associated:Human:Digestive system:Large int	0.2926
MGYA00171174/OLFK01_FASTA.fasta	MGYA00171174	ERZ489294	root:Host-associated:Human:Digestive system:Large int	0.3078
MGYA00171175/OIHQ01_FASTA.fasta	MGYA00171175	ERZ488897	root:Host-associated:Human:Digestive system:Large int	0.2634
MGYA00171176/OITS01_FASTA.fasta	MGYA00171176	ERZ489389	root:Host-associated:Human:Digestive system:Large int	0.217
MGYA00171177/OIQN01_FASTA.fasta	MGYA00171177	ERZ489308	root:Host-associated:Human:Digestive system:Large int	0.1718
MGYA00171178/OLAY01_FASTA.fasta	MGYA00171178	ERZ488982	root:Host-associated:Human:Digestive system:Large int	0.2134
MGYA00171179/OLEH01_FASTA.fasta	MGYA00171179	ERZ489254	root:Host-associated:Human:Digestive system:Large int	0.2892
MGYA00171180/OISM01_FASTA.fasta	MGYA00171180	ERZ489358	root:Host-associated:Human:Digestive system:Large int	0.1522
MGYA00171181/OLCX01_FASTA.fasta	MGYA00171181	ERZ489214	root:Host-associated:Human:Digestive system:Large int	0.23
MGYA00171182/OILO01_FASTA.fasta	MGYA00171182	ERZ489102	root:Host-associated:Human:Digestive system:Large int	0.1994
MGYA00171183/OIKU01_FASTA.fasta	MGYA00171183	ERZ489067	root:Host-associated:Human:Digestive system:Large int	0.271
MGYA00171184/OLDE01_FASTA.fasta	MGYA00171184	ERZ489225	root:Host-associated:Human:Digestive system:Large int	0.213
MGYA00171185/OIOQ01_FASTA.fasta	MGYA00171185	ERZ489186	root:Host-associated:Human:Digestive system:Large int	0.1858
MGYA00171186/OIKF01_FASTA.fasta	MGYA00171186	ERZ489036	root:Host-associated:Human:Digestive system:Large int	0.2562
MGYA00171187/OIRE01_FASTA.fasta	MGYA00171187	ERZ489313	root:Host-associated:Human:Digestive system:Large int	0.3188
MGYA00171188/OILQ01_FASTA.fasta	MGYA00171188	ERZ489109	root:Host-associated:Human:Digestive system:Large int	0.192
MGYA00171189/OLDM01_FASTA.fasta	MGYA00171189	ERZ489236	root:Host-associated:Human:Digestive system:Large int	0.2304
MGYA00171190/OIID01_FASTA.fasta	MGYA00171190	ERZ488918	root:Host-associated:Human:Digestive system:Large int	0.319
MGYA00171191/OIQM01_FASTA.fasta	MGYA00171191	ERZ489299	root:Host-associated:Human:Digestive system:Large int	0.3354
MGYA00171192/OIKX01_FASTA.fasta	MGYA00171192	ERZ489081	root:Host-associated:Human:Digestive system:Large int	0.2162
MGYA00171193/OKYQ01_FASTA.fasta	MGYA00171193	ERZ488882	root:Host-associated:Human:Digestive system:Large int	0.3002
MGYA00171194/OLDV01_FASTA.fasta	MGYA00171194	ERZ489251	root:Host-associated:Human:Digestive system:Large int	0.2044
MGYA00171195/OLBO01_FASTA.fasta	MGYA00171195	ERZ488998	root:Host-associated:Human:Digestive system:Large int	0.3238
MGYA00171196/OLCB01_FASTA.fasta	MGYA00171196	ERZ489043	root:Host-associated:Human:Digestive system:Large int	0.209
MGYA00171197/OILT01_FASTA.fasta	MGYA00171197	ERZ489097	root:Host-associated:Human:Digestive system:Large int	0.2532
MGYA00171198/OIMH01_FASTA.fasta	MGYA00171198	ERZ489120	root:Host-associated:Human:Digestive system:Large int	0.0746
MGYA00171199/OIKJ01_FASTA.fasta	MGYA00171199	ERZ489052	root:Host-associated:Human:Digestive system:Large int	0.2784
MGYA00171200/OKYX01_FASTA.fasta	MGYA00171200	ERZ488891	root:Host-associated:Human:Digestive system:Large int	0.2512
MGYA00171201/OLBL01_FASTA.fasta	MGYA00171201	ERZ488991	root:Host-associated:Human:Digestive system:Large int	0.3268
MGYA00171202/OIQG01_FASTA.fasta	MGYA00171202	ERZ489298	root:Host-associated:Human:Digestive system:Large int	0.2012
MGYA00171203/OIUX01_FASTA.fasta	MGYA00171203	ERZ489404	root:Host-associated:Human:Digestive system:Large int	0.2816
MGYA00171204/OIRG01_FASTA.fasta	MGYA00171204	ERZ489328	root:Host-associated:Human:Digestive system:Large int	0.1914
MGYA00171205/OIVM01_FASTA.fasta	MGYA00171205	ERZ489423	root:Host-associated:Human:Digestive system:Large int	0.3312
MGYA00171206/OKZG01_FASTA.fasta	MGYA00171206	ERZ488916	root:Host-associated:Human:Digestive system:Large int	0.2208
MGYA00171207/OLAH01_FASTA.fasta	MGYA00171207	ERZ488969	root:Host-associated:Human:Digestive system:Large int	0.2162
MGYA00171208/OLFL01_FASTA.fasta	MGYA00171208	ERZ489292	root:Host-associated:Human:Digestive system:Large int	0.255
MGYA00171209/OISY01_FASTA.fasta	MGYA00171209	ERZ489360	root:Host-associated:Human:Digestive system:Large int	0.3074
MGYA00171210/OLAR01_FASTA.fasta	MGYA00171210	ERZ488959	root:Host-associated:Human:Digestive system:Large int	0.3218
MGYA00171211/OIMQ01_FASTA.fasta	MGYA00171211	ERZ489136	root:Host-associated:Human:Digestive system:Large int	0.0886
MGYA00171212/OKZY01_FASTA.fasta	MGYA00171212	ERZ488949	root:Host-associated:Human:Digestive system:Large int	0.2048
MGYA00171213/OIUK01_FASTA.fasta	MGYA00171213	ERZ489408	root:Host-associated:Human:Digestive system:Large int	0.2504
MGYA00171214/OKYY01_FASTA.fasta	MGYA00171214	ERZ488889	root:Host-associated:Human:Digestive system:Large int	0.2718
MGYA00171215/OIPL01_FASTA.fasta	MGYA00171215	ERZ489209	root:Host-associated:Human:Digestive system:Large int	0.4784
MGYA00171216/OIMK01_FASTA.fasta	MGYA00171216	ERZ489119	root:Host-associated:Human:Digestive system:Large int	0.302
MGYA00171217/OIVA01_FASTA.fasta	MGYA00171217	ERZ489413	root:Host-associated:Human:Digestive system:Large int	0.2288
MGYA00171218/OLBJ01_FASTA.fasta	MGYA00171218	ERZ489007	root:Host-associated:Human:Digestive system:Large int	0.2304
MGYA00171219/OIHE01_FASTA.fasta	MGYA00171219	ERZ488859	root:Host-associated:Human:Digestive system:Large int	0.3132
MGYA00171220/OIHZ01_FASTA.fasta	MGYA00171220	ERZ488924	root:Host-associated:Human:Digestive system:Large int	0.3194
MGYA00171221/OLDT01_FASTA.fasta	MGYA00171221	ERZ489241	root:Host-associated:Human:Digestive system:Large int	0.2408
MGYA00171222/OIQJ01_FASTA.fasta	MGYA00171222	ERZ489300	root:Host-associated:Human:Digestive system:Large int	0.2558
MGYA00171223/OILB01_FASTA.fasta	MGYA00171223	ERZ489086	root:Host-associated:Human:Digestive system:Large int	0.2858
MGYA00171224/OIPO01_FASTA.fasta	MGYA00171224	ERZ489204	root:Host-associated:Human:Digestive system:Large int	0.2088
MGYA00171225/OLIE01_FASTA.fasta	MGYA00171225	ERZ488996	root:Host-associated:Human:Digestive system:Large int	0.2992
MGYA00171226/OIOE01_FASTA.fasta	MGYA00171226	ERZ489175	root:Host-associated:Human:Digestive system:Large int	0.0998
MGYA00171227/OIGG01_FASTA.fasta	MGYA00171227	ERZ488823	root:Host-associated:Human:Digestive system:Large int	0.3784
MGYA00171228/OIVH01_FASTA.fasta	MGYA00171228	ERZ489428	root:Host-associated:Human:Digestive system:Large int	0.3134
MGYA00171229/OISH01_FASTA.fasta	MGYA00171229	ERZ489354	root:Host-associated:Human:Digestive system:Large int	0.3652
MGYA00171230/OIHD01_FASTA.fasta	MGYA00171230	ERZ488876	root:Host-associated:Human:Digestive system:Large int	0.2036
MGYA00171231/OIMI01_FASTA.fasta	MGYA00171231	ERZ489114	root:Host-associated:Human:Digestive system:Large int	0.2694
MGYA00171232/OITH01_FASTA.fasta	MGYA00171232	ERZ489371	root:Host-associated:Human:Digestive system:Large int	0.2734
MGYA00171233/OIMU01_FASTA.fasta	MGYA00171233	ERZ489141	root:Host-associated:Human:Digestive system:Large int	0.153
MGYA00171234/OLIC01_FASTA.fasta	MGYA00171234	ERZ488990	root:Host-associated:Human:Digestive system:Large int	0.2024
MGYA00171235/OLDF01_FASTA.fasta	MGYA00171235	ERZ489221	root:Host-associated:Human:Digestive system:Large int	0.3834
MGYA00171236/OIHI01_FASTA.fasta	MGYA00171236	ERZ488883	root:Host-associated:Human:Digestive system:Large int	0.1776
MGYA00171237/OIRU01_FASTA.fasta	MGYA00171237	ERZ489337	root:Host-associated:Human:Digestive system:Large int	0.3732
MGYA00171238/OIRB01_FASTA.fasta	MGYA00171238	ERZ489314	root:Host-associated:Human:Digestive system:Large int	0.372
MGYA00171239/OIOM01_FASTA.fasta	MGYA00171239	ERZ489177	root:Host-associated:Human:Digestive system:Large int	0.2968
MGYA00171240/OIIS01_FASTA.fasta	MGYA00171240	ERZ488968	root:Host-associated:Human:Digestive system:Large int	0.181
MGYA00171241/OIRX01_FASTA.fasta	MGYA00171241	ERZ489343	root:Host-associated:Human:Digestive system:Large int	0.2758
MGYA00171242/OIVP01_FASTA.fasta	MGYA00171242	ERZ489427	root:Host-associated:Human:Digestive system:Large int	0.317
MGYA00171243/OIHS01_FASTA.fasta	MGYA00171243	ERZ488911	root:Host-associated:Human:Digestive system:Large int	0.3078
MGYA00171244/OIOT01_FASTA.fasta	MGYA00171244	ERZ489187	root:Host-associated:Human:Digestive system:Large int	0.1784
MGYA00171245/OISO01_FASTA.fasta	MGYA00171245	ERZ489363	root:Host-associated:Human:Digestive system:Large int	0.063
MGYA00171246/OIIC01_FASTA.fasta	MGYA00171246	ERZ488919	root:Host-associated:Human:Digestive system:Large int	0.2482
MGYA00171247/OIGR01_FASTA.fasta	MGYA00171247	ERZ488849	root:Host-associated:Human:Digestive system:Large int	0.3226
MGYA00171248/OKZT01_FASTA.fasta	MGYA00171248	ERZ488941	root:Host-associated:Human:Digestive system:Large int	0.0662
MGYA00171249/OIOJ01_FASTA.fasta	MGYA00171249	ERZ489181	root:Host-associated:Human:Digestive system:Large int	0.2988
MGYA00171250/OIIK01_FASTA.fasta	MGYA00171250	ERZ488952	root:Host-associated:Human:Digestive system:Large int	0.2236
MGYA00171251/OIOG01_FASTA.fasta	MGYA00171251	ERZ489158	root:Host-associated:Human:Digestive system:Large int	0.2322
MGYA00171252/OIUQ01_FASTA.fasta	MGYA00171252	ERZ489415	root:Host-associated:Human:Digestive system:Large int	0.25
MGYA00171253/OKXT01_FASTA.fasta	MGYA00171253	ERZ488842	root:Host-associated:Human:Digestive system:Large int	0.2652
MGYA00171254/OLAC01_FASTA.fasta	MGYA00171254	ERZ488948	root:Host-associated:Human:Digestive system:Large int	0.2658
MGYA00171255/OLAE01_FASTA.fasta	MGYA00171255	ERZ488940	root:Host-associated:Human:Digestive system:Large int	0.2338
MGYA00171256/OKYD01_FASTA.fasta	MGYA00171256	ERZ488870	root:Host-associated:Human:Digestive system:Large int	0.2406
MGYA00171257/OLEE01_FASTA.fasta	MGYA00171257	ERZ489242	root:Host-associated:Human:Digestive system:Large int	0.2578
MGYA00171258/OKYF01_FASTA.fasta	MGYA00171258	ERZ488864	root:Host-associated:Human:Digestive system:Large int	0.309
MGYA00171259/OIKZ01_FASTA.fasta	MGYA00171259	ERZ489070	root:Host-associated:Human:Digestive system:Large int	0.3656
MGYA00171260/OIHK01_FASTA.fasta	MGYA00171260	ERZ488888	root:Host-associated:Human:Digestive system:Large int	0.215
MGYA00171261/OKXC01_FASTA.fasta	MGYA00171261	ERZ488812	root:Host-associated:Human:Digestive system:Large int	0.3734
MGYA00171262/OIIN01_FASTA.fasta	MGYA00171262	ERZ488955	root:Host-associated:Human:Digestive system:Large int	0.1598
MGYA00171263/OILG01_FASTA.fasta	MGYA00171263	ERZ489096	root:Host-associated:Human:Digestive system:Large int	0.064
MGYA00171264/OLEN01_FASTA.fasta	MGYA00171264	ERZ489259	root:Host-associated:Human:Digestive system:Large int	0.357
MGYA00171265/OLFP01_FASTA.fasta	MGYA00171265	ERZ489411	root:Host-associated:Human:Digestive system:Large int	0.2752
MGYA00171266/OIUV01_FASTA.fasta	MGYA00171266	ERZ489401	root:Host-associated:Human:Digestive system:Large int	0.326
MGYA00171267/OLDD01_FASTA.fasta	MGYA00171267	ERZ489220	root:Host-associated:Human:Digestive system:Large int	0.2734
MGYA00171268/OIOU01_FASTA.fasta	MGYA00171268	ERZ489185	root:Host-associated:Human:Digestive system:Large int	0.1688
MGYA00171269/OITA01_FASTA.fasta	MGYA00171269	ERZ489357	root:Host-associated:Human:Digestive system:Large int	0.2494
MGYA00171270/OIPC01_FASTA.fasta	MGYA00171270	ERZ489195	root:Host-associated:Human:Digestive system:Large int	0.1766
MGYA00171271/OLID01_FASTA.fasta	MGYA00171271	ERZ488984	root:Host-associated:Human:Digestive system:Large int	0.2506
MGYA00171272/OLEB01_FASTA.fasta	MGYA00171272	ERZ489255	root:Host-associated:Human:Digestive system:Large int	0.2558
MGYA00171273/OITB01_FASTA.fasta	MGYA00171273	ERZ489369	root:Host-associated:Human:Digestive system:Large int	0.308
MGYA00171274/OIOP01_FASTA.fasta	MGYA00171274	ERZ489184	root:Host-associated:Human:Digestive system:Large int	0.4522
MGYA00171275/OISR01_FASTA.fasta	MGYA00171275	ERZ489364	root:Host-associated:Human:Digestive system:Large int	0.2264
MGYA00171276/OIRA01_FASTA.fasta	MGYA00171276	ERZ489321	root:Host-associated:Human:Digestive system:Large int	0.2008
MGYA00171277/OIPZ01_FASTA.fasta	MGYA00171277	ERZ489216	root:Host-associated:Human:Digestive system:Large int	0.211
MGYA00171278/OLDN01_FASTA.fasta	MGYA00171278	ERZ489234	root:Host-associated:Human:Digestive system:Large int	0.2904
MGYA00171279/OIQW01_FASTA.fasta	MGYA00171279	ERZ489304	root:Host-associated:Human:Digestive system:Large int	0.3552
MGYA00171280/OIMN01_FASTA.fasta	MGYA00171280	ERZ489124	root:Host-associated:Human:Digestive system:Large int	0.3426
MGYA00171281/OLDU01_FASTA.fasta	MGYA00171281	ERZ489247	root:Host-associated:Human:Digestive system:Large int	0.287
MGYA00171282/OITM01_FASTA.fasta	MGYA00171282	ERZ489365	root:Host-associated:Human:Digestive system:Large int	0.252
MGYA00171283/OLDY01_FASTA.fasta	MGYA00171283	ERZ489245	root:Host-associated:Human:Digestive system:Large int	0.3296
MGYA00171284/OIQT01_FASTA.fasta	MGYA00171284	ERZ489302	root:Host-associated:Human:Digestive system:Large int	0.208
MGYA00171285/OIQO01_FASTA.fasta	MGYA00171285	ERZ489301	root:Host-associated:Human:Digestive system:Large int	0.2346
MGYA00171286/OIRC01_FASTA.fasta	MGYA00171286	ERZ489318	root:Host-associated:Human:Digestive system:Large int	0.2696
MGYA00171287/OKXF01_FASTA.fasta	MGYA00171287	ERZ488825	root:Host-associated:Human:Digestive system:Large int	0.2262
MGYA00171288/OLBZ01_FASTA.fasta	MGYA00171288	ERZ489045	root:Host-associated:Human:Digestive system:Large int	0.1648
MGYA00171289/OLCV01_FASTA.fasta	MGYA00171289	ERZ489131	root:Host-associated:Human:Digestive system:Large int	0.3476
MGYA00171290/OLEP01_FASTA.fasta	MGYA00171290	ERZ489268	root:Host-associated:Human:Digestive system:Large int	0.2878
MGYA00171291/OIOH01_FASTA.fasta	MGYA00171291	ERZ489165	root:Host-associated:Human:Digestive system:Large int	0.294
MGYA00171292/OILU01_FASTA.fasta	MGYA00171292	ERZ489108	root:Host-associated:Human:Digestive system:Large int	0.2724
MGYA00171293/OINF01_FASTA.fasta	MGYA00171293	ERZ489151	root:Host-associated:Human:Digestive system:Large int	0.1758
MGYA00171294/OIGH01_FASTA.fasta	MGYA00171294	ERZ488822	root:Host-associated:Human:Digestive system:Large int	0.379
MGYA00171295/OIKH01_FASTA.fasta	MGYA00171295	ERZ489053	root:Host-associated:Human:Digestive system:Large int	0.0914
MGYA00171296/OIMF01_FASTA.fasta	MGYA00171296	ERZ489107	root:Host-associated:Human:Digestive system:Large int	0.2614
MGYA00171297/OIKM01_FASTA.fasta	MGYA00171297	ERZ489055	root:Host-associated:Human:Digestive system:Large int	0.0804
MGYA00171298/OLIR01_FASTA.fasta	MGYA00171298	ERZ489026	root:Host-associated:Human:Digestive system:Large int	0.1676
MGYA00171299/OITT01_FASTA.fasta	MGYA00171299	ERZ489370	root:Host-associated:Human:Digestive system:Large int	0.3112
MGYA00171300/OKZU01_FASTA.fasta	MGYA00171300	ERZ488934	root:Host-associated:Human:Digestive system:Large int	0.254
MGYA00171301/OLDS01_FASTA.fasta	MGYA00171301	ERZ489240	root:Host-associated:Human:Digestive system:Large int	0.2194
MGYA00171302/OIHA01_FASTA.fasta	MGYA00171302	ERZ488854	root:Host-associated:Human:Digestive system:Large int	0.2642
MGYA00171303/OIIY01_FASTA.fasta	MGYA00171303	ERZ488985	root:Host-associated:Human:Digestive system:Large int	0.2776
MGYA00171304/OLDQ01_FASTA.fasta	MGYA00171304	ERZ489243	root:Host-associated:Human:Digestive system:Large int	0.0616
MGYA00171305/OIHR01_FASTA.fasta	MGYA00171305	ERZ488909	root:Host-associated:Human:Digestive system:Large int	0.157
MGYA00171306/OLAU01_FASTA.fasta	MGYA00171306	ERZ488980	root:Host-associated:Human:Digestive system:Large int	0.2082
MGYA00171307/OIPA01_FASTA.fasta	MGYA00171307	ERZ489189	root:Host-associated:Human:Digestive system:Large int	0.3962
MGYA00171308/OIRR01_FASTA.fasta	MGYA00171308	ERZ489325	root:Host-associated:Human:Digestive system:Large int	0.2452
MGYA00171309/OITR01_FASTA.fasta	MGYA00171309	ERZ489384	root:Host-associated:Human:Digestive system:Large int	0.2332
MGYA00171310/OIUJ01_FASTA.fasta	MGYA00171310	ERZ489407	root:Host-associated:Human:Digestive system:Large int	0.2518
MGYA00171311/OIRM01_FASTA.fasta	MGYA00171311	ERZ489331	root:Host-associated:Human:Digestive system:Large int	0.213
MGYA00171312/OIUG01_FASTA.fasta	MGYA00171312	ERZ489409	root:Host-associated:Human:Digestive system:Large int	0.1942
MGYA00171313/OIQB01_FASTA.fasta	MGYA00171313	ERZ489260	root:Host-associated:Human:Digestive system:Large int	0.235
MGYA00171314/OINW01_FASTA.fasta	MGYA00171314	ERZ489160	root:Host-associated:Human:Digestive system:Large int	0.1662
MGYA00171315/OISG01_FASTA.fasta	MGYA00171315	ERZ489346	root:Host-associated:Human:Digestive system:Large int	0.3012
MGYA00171316/OLDA01_FASTA.fasta	MGYA00171316	ERZ489219	root:Host-associated:Human:Digestive system:Large int	0.2726
MGYA00171317/OKZE01_FASTA.fasta	MGYA00171317	ERZ488905	root:Host-associated:Human:Digestive system:Large int	0.3704
MGYA00171318/OLDR01_FASTA.fasta	MGYA00171318	ERZ489229	root:Host-associated:Human:Digestive system:Large int	0.2932
MGYA00171319/OLIY01_FASTA.fasta	MGYA00171319	ERZ489042	root:Host-associated:Human:Digestive system:Large int	0.2104
MGYA00171320/OIIP01_FASTA.fasta	MGYA00171320	ERZ488946	root:Host-associated:Human:Digestive system:Large int	0.3246
MGYA00171321/OIKL01_FASTA.fasta	MGYA00171321	ERZ489056	root:Host-associated:Human:Digestive system:Large int	0.1846
MGYA00171322/OIKO01_FASTA.fasta	MGYA00171322	ERZ489060	root:Host-associated:Human:Digestive system:Large int	0.427
MGYA00171323/OKYZ01_FASTA.fasta	MGYA00171323	ERZ488902	root:Host-associated:Human:Digestive system:Large int	0.3116
MGYA00171324/OIPT01_FASTA.fasta	MGYA00171324	ERZ489212	root:Host-associated:Human:Digestive system:Large int	0.26
MGYA00171325/OLFB01_FASTA.fasta	MGYA00171325	ERZ489273	root:Host-associated:Human:Digestive system:Large int	0.3316
MGYA00171326/OIQV01_FASTA.fasta	MGYA00171326	ERZ489307	root:Host-associated:Human:Digestive system:Large int	0.2186
MGYA00171327/OIUH01_FASTA.fasta	MGYA00171327	ERZ489403	root:Host-associated:Human:Digestive system:Large int	0.3072
MGYA00171328/OLCD01_FASTA.fasta	MGYA00171328	ERZ489039	root:Host-associated:Human:Digestive system:Large int	0.3478
MGYA00171329/OISL01_FASTA.fasta	MGYA00171329	ERZ489349	root:Host-associated:Human:Digestive system:Large int	0.2734
MGYA00171330/OLAV01_FASTA.fasta	MGYA00171330	ERZ488983	root:Host-associated:Human:Digestive system:Large int	0.3866
MGYA00171331/OIVJ01_FASTA.fasta	MGYA00171331	ERZ489426	root:Host-associated:Human:Digestive system:Large int	0.1762
MGYA00171332/OIPV01_FASTA.fasta	MGYA00171332	ERZ489207	root:Host-associated:Human:Digestive system:Large int	0.3442
MGYA00171333/OINX01_FASTA.fasta	MGYA00171333	ERZ489156	root:Host-associated:Human:Digestive system:Large int	0.3362
MGYA00171334/OIOA01_FASTA.fasta	MGYA00171334	ERZ489163	root:Host-associated:Human:Digestive system:Large int	0.3566
MGYA00171335/OIMJ01_FASTA.fasta	MGYA00171335	ERZ489121	root:Host-associated:Human:Digestive system:Large int	0.1936
MGYA00171336/OIGO01_FASTA.fasta	MGYA00171336	ERZ488818	root:Host-associated:Human:Digestive system:Large int	0.3862
MGYA00171337/OIKD01_FASTA.fasta	MGYA00171337	ERZ489044	root:Host-associated:Human:Digestive system:Large int	0.1674
MGYA00171338/OIGK01_FASTA.fasta	MGYA00171338	ERZ488820	root:Host-associated:Human:Digestive system:Large int	0.3802
MGYA00171339/OLCT01_FASTA.fasta	MGYA00171339	ERZ489068	root:Host-associated:Human:Digestive system:Large int	0.354
MGYA00171340/OLIP01_FASTA.fasta	MGYA00171340	ERZ489016	root:Host-associated:Human:Digestive system:Large int	0.4092
MGYA00171341/OLDG01_FASTA.fasta	MGYA00171341	ERZ489223	root:Host-associated:Human:Digestive system:Large int	0.265
MGYA00171342/OIRN01_FASTA.fasta	MGYA00171342	ERZ489323	root:Host-associated:Human:Digestive system:Large int	0.28
MGYA00171343/OIHP01_FASTA.fasta	MGYA00171343	ERZ488899	root:Host-associated:Human:Digestive system:Large int	0.3298
MGYA00171344/OKZR01_FASTA.fasta	MGYA00171344	ERZ488931	root:Host-associated:Human:Digestive system:Large int	0.316
MGYA00171345/OKYE01_FASTA.fasta	MGYA00171345	ERZ488868	root:Host-associated:Human:Digestive system:Large int	0.2366
MGYA00171346/OINQ01_FASTA.fasta	MGYA00171346	ERZ489153	root:Host-associated:Human:Digestive system:Large int	0.3512
MGYA00171347/OISZ01_FASTA.fasta	MGYA00171347	ERZ489356	root:Host-associated:Human:Digestive system:Large int	0.2434
MGYA00171348/OIMM01_FASTA.fasta	MGYA00171348	ERZ489115	root:Host-associated:Human:Digestive system:Large int	0.3362
MGYA00171349/OILF01_FASTA.fasta	MGYA00171349	ERZ489091	root:Host-associated:Human:Digestive system:Large int	0.2352
MGYA00171350/OIOO01_FASTA.fasta	MGYA00171350	ERZ489161	root:Host-associated:Human:Digestive system:Large int	0.4124
MGYA00171351/OKZF01_FASTA.fasta	MGYA00171351	ERZ488910	root:Host-associated:Human:Digestive system:Large int	0.1896
MGYA00171352/OIVC01_FASTA.fasta	MGYA00171352	ERZ489420	root:Host-associated:Human:Digestive system:Large int	0.3496
MGYA00171353/OLCQ01_FASTA.fasta	MGYA00171353	ERZ489085	root:Host-associated:Human:Digestive system:Large int	0.3014
MGYA00171354/OILS01_FASTA.fasta	MGYA00171354	ERZ489090	root:Host-associated:Human:Digestive system:Large int	0.2838
MGYA00171355/OILI01_FASTA.fasta	MGYA00171355	ERZ489079	root:Host-associated:Human:Digestive system:Large int	0.3472
MGYA00171356/OKXP01_FASTA.fasta	MGYA00171356	ERZ488839	root:Host-associated:Human:Digestive system:Large int	0.1948
MGYA00171357/OLCO01_FASTA.fasta	MGYA00171357	ERZ489077	root:Host-associated:Human:Digestive system:Large int	0.3188
MGYA00171358/OIGQ01_FASTA.fasta	MGYA00171358	ERZ488835	root:Host-associated:Human:Digestive system:Large int	0.2404
MGYA00171359/OITJ01_FASTA.fasta	MGYA00171359	ERZ489386	root:Host-associated:Human:Digestive system:Large int	0.059
MGYA00171360/OKXW01_FASTA.fasta	MGYA00171360	ERZ488837	root:Host-associated:Human:Digestive system:Large int	0.2676
MGYA00171361/OIGW01_FASTA.fasta	MGYA00171361	ERZ488853	root:Host-associated:Human:Digestive system:Large int	0.241
MGYA00171362/OIRZ01_FASTA.fasta	MGYA00171362	ERZ489344	root:Host-associated:Human:Digestive system:Large int	0.3296
MGYA00171363/OKZS01_FASTA.fasta	MGYA00171363	ERZ488935	root:Host-associated:Human:Digestive system:Large int	0.1942
MGYA00171364/OILR01_FASTA.fasta	MGYA00171364	ERZ489106	root:Host-associated:Human:Digestive system:Large int	0.1706
MGYA00171365/OIMA01_FASTA.fasta	MGYA00171365	ERZ489103	root:Host-associated:Human:Digestive system:Large int	0.1472
MGYA00171366/OIKQ01_FASTA.fasta	MGYA00171366	ERZ489073	root:Host-associated:Human:Digestive system:Large int	0.4624
MGYA00171367/OLDK01_FASTA.fasta	MGYA00171367	ERZ489235	root:Host-associated:Human:Digestive system:Large int	0.2856
MGYA00171368/OIUP01_FASTA.fasta	MGYA00171368	ERZ489418	root:Host-associated:Human:Digestive system:Large int	0.202
MGYA00171369/OISI01_FASTA.fasta	MGYA00171369	ERZ489347	root:Host-associated:Human:Digestive system:Large int	0.2708
MGYA00171370/OINT01_FASTA.fasta	MGYA00171370	ERZ489168	root:Host-associated:Human:Digestive system:Large int	0.1168
MGYA00171371/OIIO01_FASTA.fasta	MGYA00171371	ERZ488961	root:Host-associated:Human:Digestive system:Large int	0.2662
MGYA00171372/OIHN01_FASTA.fasta	MGYA00171372	ERZ488892	root:Host-associated:Human:Digestive system:Large int	0.3382
MGYA00171373/OIUB01_FASTA.fasta	MGYA00171373	ERZ489397	root:Host-associated:Human:Digestive system:Large int	0.2448
MGYA00171374/OIRW01_FASTA.fasta	MGYA00171374	ERZ489341	root:Host-associated:Human:Digestive system:Large int	0.2654
MGYA00171375/OIIF01_FASTA.fasta	MGYA00171375	ERZ488928	root:Host-associated:Human:Digestive system:Large int	0.2794
MGYA00171376/OIMC01_FASTA.fasta	MGYA00171376	ERZ489117	root:Host-associated:Human:Digestive system:Large int	0.2142
MGYA00171377/OLBG01_FASTA.fasta	MGYA00171377	ERZ488988	root:Host-associated:Human:Digestive system:Large int	0.3016
MGYA00171378/OLCC01_FASTA.fasta	MGYA00171378	ERZ489054	root:Host-associated:Human:Digestive system:Large int	0.1498
MGYA00171379/OLJA01_FASTA.fasta	MGYA00171379	ERZ488997	root:Host-associated:Human:Digestive system:Large int	0.2182
MGYA00171380/OKYB01_FASTA.fasta	MGYA00171380	ERZ488846	root:Host-associated:Human:Digestive system:Large int	0.2318
MGYA00171381/OKZD01_FASTA.fasta	MGYA00171381	ERZ488894	root:Host-associated:Human:Digestive system:Large int	0.2764
MGYA00171382/OISE01_FASTA.fasta	MGYA00171382	ERZ489342	root:Host-associated:Human:Digestive system:Large int	0.2588
MGYA00171383/OIOR01_FASTA.fasta	MGYA00171383	ERZ489176	root:Host-associated:Human:Digestive system:Large int	0.3174
MGYA00171384/OIKR01_FASTA.fasta	MGYA00171384	ERZ489069	root:Host-associated:Human:Digestive system:Large int	0.211
MGYA00171385/OLJL01_FASTA.fasta	MGYA00171385	ERZ489009	root:Host-associated:Human:Digestive system:Large int	0.4184
MGYA00171386/OIUL01_FASTA.fasta	MGYA00171386	ERZ489405	root:Host-associated:Human:Digestive system:Large int	0.3138
MGYA00171387/OIPQ01_FASTA.fasta	MGYA00171387	ERZ489208	root:Host-associated:Human:Digestive system:Large int	0.261
MGYA00171388/OIIE01_FASTA.fasta	MGYA00171388	ERZ488932	root:Host-associated:Human:Digestive system:Large int	0.2872
MGYA00171389/OITQ01_FASTA.fasta	MGYA00171389	ERZ489382	root:Host-associated:Human:Digestive system:Large int	0.1858
MGYA00171390/OLFN01_FASTA.fasta	MGYA00171390	ERZ489388	root:Host-associated:Human:Digestive system:Large int	0.3616
MGYA00171391/OLBV01_FASTA.fasta	MGYA00171391	ERZ489028	root:Host-associated:Human:Digestive system:Large int	0.2712
MGYA00171392/OLDW01_FASTA.fasta	MGYA00171392	ERZ489244	root:Host-associated:Human:Digestive system:Large int	0.3344
MGYA00171393/OIGY01_FASTA.fasta	MGYA00171393	ERZ488856	root:Host-associated:Human:Digestive system:Large int	0.295
MGYA00171394/OLAG01_FASTA.fasta	MGYA00171394	ERZ488953	root:Host-associated:Human:Digestive system:Large int	0.2848
MGYA00171395/OLFE01_FASTA.fasta	MGYA00171395	ERZ489287	root:Host-associated:Human:Digestive system:Large int	0.287
MGYA00171396/OINS01_FASTA.fasta	MGYA00171396	ERZ489150	root:Host-associated:Human:Digestive system:Large int	0.3756
MGYA00171397/OLEW01_FASTA.fasta	MGYA00171397	ERZ489284	root:Host-associated:Human:Digestive system:Large int	0.1744
MGYA00171398/OIOI01_FASTA.fasta	MGYA00171398	ERZ489178	root:Host-associated:Human:Digestive system:Large int	0.1816
MGYA00171399/OIRJ01_FASTA.fasta	MGYA00171399	ERZ489322	root:Host-associated:Human:Digestive system:Large int	0.2826
MGYA00171400/OIGB01_FASTA.fasta	MGYA00171400	ERZ488808	root:Host-associated:Human:Digestive system:Large int	0.3948
MGYA00171401/OKYW01_FASTA.fasta	MGYA00171401	ERZ488900	root:Host-associated:Human:Digestive system:Large int	0.2218
MGYA00171402/OIUO01_FASTA.fasta	MGYA00171402	ERZ489400	root:Host-associated:Human:Digestive system:Large int	0.2546
MGYA00171403/OKYI01_FASTA.fasta	MGYA00171403	ERZ488862	root:Host-associated:Human:Digestive system:Large int	0.288
MGYA00171404/OITV01_FASTA.fasta	MGYA00171404	ERZ489394	root:Host-associated:Human:Digestive system:Large int	0.1532
MGYA00171405/OLEF01_FASTA.fasta	MGYA00171405	ERZ489246	root:Host-associated:Human:Digestive system:Large int	0.2878
MGYA00171406/OLIX01_FASTA.fasta	MGYA00171406	ERZ489031	root:Host-associated:Human:Digestive system:Large int	0.3192
MGYA00171407/OIQR01_FASTA.fasta	MGYA00171407	ERZ489309	root:Host-associated:Human:Digestive system:Large int	0.3078
MGYA00171408/OISP01_FASTA.fasta	MGYA00171408	ERZ489359	root:Host-associated:Human:Digestive system:Large int	0.2248
MGYA00171409/OISA01_FASTA.fasta	MGYA00171409	ERZ489336	root:Host-associated:Human:Digestive system:Large int	0.3048
MGYA00171410/OITP01_FASTA.fasta	MGYA00171410	ERZ489379	root:Host-associated:Human:Digestive system:Large int	0.209
MGYA00171411/OLEJ01_FASTA.fasta	MGYA00171411	ERZ489261	root:Host-associated:Human:Digestive system:Large int	0.3166
MGYA00171412/OISS01_FASTA.fasta	MGYA00171412	ERZ489351	root:Host-associated:Human:Digestive system:Large int	0.287
MGYA00171413/OKYM01_FASTA.fasta	MGYA00171413	ERZ488872	root:Host-associated:Human:Digestive system:Large int	0.3148
MGYA00171414/OIOX01_FASTA.fasta	MGYA00171414	ERZ489180	root:Host-associated:Human:Digestive system:Large int	0.2792
MGYA00171415/OIVL01_FASTA.fasta	MGYA00171415	ERZ489429	root:Host-associated:Human:Digestive system:Large int	0.302
MGYA00171416/OIOS01_FASTA.fasta	MGYA00171416	ERZ489182	root:Host-associated:Human:Digestive system:Large int	0.2838
MGYA00171417/OKZV01_FASTA.fasta	MGYA00171417	ERZ488936	root:Host-associated:Human:Digestive system:Large int	0.2882

MGYA00171418/OLAL01_FASTA.fasta	MGYA00171418	ERZ488963	root:Host-associated:Human:Digestive system:Large int	0.3844
MGYA00171419/OILC01_FASTA.fasta	MGYA00171419	ERZ489082	root:Host-associated:Human:Digestive system:Large int	0.1832
MGYA00171420/OLEV01_FASTA.fasta	MGYA00171420	ERZ489279	root:Host-associated:Human:Digestive system:Large int	0.2946
MGYA00171421/OILK01_FASTA.fasta	MGYA00171421	ERZ489093	root:Host-associated:Human:Digestive system:Large int	0.1452
MGYA00171422/OLCI01_FASTA.fasta	MGYA00171422	ERZ489066	root:Host-associated:Human:Digestive system:Large int	0.3078
MGYA00171423/OITW01_FASTA.fasta	MGYA00171423	ERZ489383	root:Host-associated:Human:Digestive system:Large int	0.2782
MGYA00171424/OLAW01_FASTA.fasta	MGYA00171424	ERZ488989	root:Host-associated:Human:Digestive system:Large int	0.1062
MGYA00171425/OLDC01_FASTA.fasta	MGYA00171425	ERZ489224	root:Host-associated:Human:Digestive system:Large int	0.228
MGYA00171426/OIPE01_FASTA.fasta	MGYA00171426	ERZ489190	root:Host-associated:Human:Digestive system:Large int	0.3428
MGYA00171427/OISD01_FASTA.fasta	MGYA00171427	ERZ489335	root:Host-associated:Human:Digestive system:Large int	0.2614
MGYA00171428/OIIB01_FASTA.fasta	MGYA00171428	ERZ488922	root:Host-associated:Human:Digestive system:Large int	0.297
MGYA00171429/OIQL01_FASTA.fasta	MGYA00171429	ERZ489262	root:Host-associated:Human:Digestive system:Large int	0.2524
MGYA00171430/OITK01_FASTA.fasta	MGYA00171430	ERZ489381	root:Host-associated:Human:Digestive system:Large int	0.2816
MGYA00171431/OLBY01_FASTA.fasta	MGYA00171431	ERZ489040	root:Host-associated:Human:Digestive system:Large int	0.2728
MGYA00171432/OIOY01_FASTA.fasta	MGYA00171432	ERZ489183	root:Host-associated:Human:Digestive system:Large int	0.2716
MGYA00171433/OIJI01_FASTA.fasta	MGYA00171433	ERZ488993	root:Host-associated:Human:Digestive system:Large int	0.328
MGYA00171434/OKYL01_FASTA.fasta	MGYA00171434	ERZ488879	root:Host-associated:Human:Digestive system:Large int	0.222
MGYA00171435/OLBB01_FASTA.fasta	MGYA00171435	ERZ488987	root:Host-associated:Human:Digestive system:Large int	0.3204
MGYA00171436/OIGZ01_FASTA.fasta	MGYA00171436	ERZ488851	root:Host-associated:Human:Digestive system:Large int	0.2362
MGYA00171437/OILW01_FASTA.fasta	MGYA00171437	ERZ489113	root:Host-associated:Human:Digestive system:Large int	0.2998
MGYA00171438/OLFM01_FASTA.fasta	MGYA00171438	ERZ489293	root:Host-associated:Human:Digestive system:Large int	0.2506
MGYA00171439/OIVQ01_FASTA.fasta	MGYA00171439	ERZ489425	root:Host-associated:Human:Digestive system:Large int	0.2786
MGYA00171440/OIPU01_FASTA.fasta	MGYA00171440	ERZ489211	root:Host-associated:Human:Digestive system:Large int	0.3766
MGYA00171441/OILD01_FASTA.fasta	MGYA00171441	ERZ489092	root:Host-associated:Human:Digestive system:Large int	0.0772
MGYA00171442/OLFO01_FASTA.fasta	MGYA00171442	ERZ489378	root:Host-associated:Human:Digestive system:Large int	0.3128
MGYA00171443/OLEY01_FASTA.fasta	MGYA00171443	ERZ489281	root:Host-associated:Human:Digestive system:Large int	0.3072
MGYA00171444/OIRV01_FASTA.fasta	MGYA00171444	ERZ489338	root:Host-associated:Human:Digestive system:Large int	0.2822
MGYA00171445/OIKI01_FASTA.fasta	MGYA00171445	ERZ489048	root:Host-associated:Human:Digestive system:Large int	0.4022
MGYA00171446/OLEG01_FASTA.fasta	MGYA00171446	ERZ489257	root:Host-associated:Human:Digestive system:Large int	0.2308
MGYA00171447/OIMS01_FASTA.fasta	MGYA00171447	ERZ489133	root:Host-associated:Human:Digestive system:Large int	0.1834
MGYA00171448/OIGC01_FASTA.fasta	MGYA00171448	ERZ488810	root:Host-associated:Human:Digestive system:Large int	0.3734
MGYA00171449/OLES01_FASTA.fasta	MGYA00171449	ERZ489271	root:Host-associated:Human:Digestive system:Large int	0.2598
MGYA00171450/OIMV01_FASTA.fasta	MGYA00171450	ERZ489139	root:Host-associated:Human:Digestive system:Large int	0.1802
MGYA00171451/OKYA01_FASTA.fasta	MGYA00171451	ERZ488867	root:Host-associated:Human:Digestive system:Large int	0.2356
MGYA00171452/OKXS01_FASTA.fasta	MGYA00171452	ERZ488841	root:Host-associated:Human:Digestive system:Large int	0.2696
MGYA00171453/OLCA01_FASTA.fasta	MGYA00171453	ERZ489051	root:Host-associated:Human:Digestive system:Large int	0.1478
MGYA00171454/OISQ01_FASTA.fasta	MGYA00171454	ERZ489348	root:Host-associated:Human:Digestive system:Large int	0.312
MGYA00171455/OKZP01_FASTA.fasta	MGYA00171455	ERZ488933	root:Host-associated:Human:Digestive system:Large int	0.2234
MGYA00171456/OIHB01_FASTA.fasta	MGYA00171456	ERZ488860	root:Host-associated:Human:Digestive system:Large int	0.2468
MGYA00171457/OLAZ01_FASTA.fasta	MGYA00171457	ERZ488994	root:Host-associated:Human:Digestive system:Large int	0.4674
MGYA00171458/OIGA01_FASTA.fasta	MGYA00171458	ERZ488811	root:Host-associated:Human:Digestive system:Large int	0.3864
MGYA00171459/OIJF01_FASTA.fasta	MGYA00171459	ERZ489412	root:Host-associated:Human:Digestive system:Large int	0.272
MGYA00171460/OIGL01_FASTA.fasta	MGYA00171460	ERZ488814	root:Host-associated:Human:Digestive system:Large int	0.3892
MGYA00171461/OITX01_FASTA.fasta	MGYA00171461	ERZ489395	root:Host-associated:Human:Digestive system:Large int	0.1612
MGYA00171462/OLCU01_FASTA.fasta	MGYA00171462	ERZ489088	root:Host-associated:Human:Digestive system:Large int	0.3502
MGYA00171463/OLAT01_FASTA.fasta	MGYA00171463	ERZ488973	root:Host-associated:Human:Digestive system:Large int	0.2684
MGYA00171464/OIIQ01_FASTA.fasta	MGYA00171464	ERZ488962	root:Host-associated:Human:Digestive system:Large int	0.1796
MGYA00171465/OIQK01_FASTA.fasta	MGYA00171465	ERZ489311	root:Host-associated:Human:Digestive system:Large int	0.082
MGYA00171466/OLBE01_FASTA.fasta	MGYA00171466	ERZ489008	root:Host-associated:Human:Digestive system:Large int	0.3954
MGYA00171467/OLFI01_FASTA.fasta	MGYA00171467	ERZ489286	root:Host-associated:Human:Digestive system:Large int	0.2166
MGYA00171468/OKXM01_FASTA.fasta	MGYA00171468	ERZ488834	root:Host-associated:Human:Digestive system:Large int	0.1936
MGYA00171469/OINL01_FASTA.fasta	MGYA00171469	ERZ489152	root:Host-associated:Human:Digestive system:Large int	0.111
MGYA00171470/OKXL01_FASTA.fasta	MGYA00171470	ERZ488831	root:Host-associated:Human:Digestive system:Large int	0.2818
MGYA00171471/OIQC01_FASTA.fasta	MGYA00171471	ERZ489252	root:Host-associated:Human:Digestive system:Large int	0.3286
MGYA00171472/OITF01_FASTA.fasta	MGYA00171472	ERZ489377	root:Host-associated:Human:Digestive system:Large int	0.191
MGYA00171473/OISC01_FASTA.fasta	MGYA00171473	ERZ489329	root:Host-associated:Human:Digestive system:Large int	0.2994
MGYA00171474/OIRQ01_FASTA.fasta	MGYA00171474	ERZ489327	root:Host-associated:Human:Digestive system:Large int	0.2444
MGYA00171475/OIII01_FASTA.fasta	MGYA00171475	ERZ488939	root:Host-associated:Human:Digestive system:Large int	0.2834
MGYA00171476/OLFH01_FASTA.fasta	MGYA00171476	ERZ489290	root:Host-associated:Human:Digestive system:Large int	0.1912
MGYA00171477/OIJG01_FASTA.fasta	MGYA00171477	ERZ489006	root:Host-associated:Human:Digestive system:Large int	0.3558
MGYA00171478/OITE01_FASTA.fasta	MGYA00171478	ERZ489374	root:Host-associated:Human:Digestive system:Large int	0.3014
MGYA00171479/OITG01_FASTA.fasta	MGYA00171479	ERZ489368	root:Host-associated:Human:Digestive system:Large int	0.2428
MGYA00171480/OIRP01_FASTA.fasta	MGYA00171480	ERZ489332	root:Host-associated:Human:Digestive system:Large int	0.2048
MGYA00171481/OIJJ01_FASTA.fasta	MGYA00171481	ERZ488992	root:Host-associated:Human:Digestive system:Large int	0.3854
MGYA00171482/OLAA01_FASTA.fasta	MGYA00171482	ERZ488951	root:Host-associated:Human:Digestive system:Large int	0.194
MGYA00171483/OLCP01_FASTA.fasta	MGYA00171483	ERZ489159	root:Host-associated:Human:Digestive system:Large int	0.2194
MGYA00171484/OISJ01_FASTA.fasta	MGYA00171484	ERZ489353	root:Host-associated:Human:Digestive system:Large int	0.28
MGYA00171485/OIQA01_FASTA.fasta	MGYA00171485	ERZ489265	root:Host-associated:Human:Digestive system:Large int	0.313
MGYA00171486/OLBA01_FASTA.fasta	MGYA00171486	ERZ488986	root:Host-associated:Human:Digestive system:Large int	0.3006
MGYA00171487/OIJN01_FASTA.fasta	MGYA00171487	ERZ489015	root:Host-associated:Human:Digestive system:Large int	0.1948
MGYA00171488/OIJT01_FASTA.fasta	MGYA00171488	ERZ489017	root:Host-associated:Human:Digestive system:Large int	0.2372
MGYA00171489/OKXX01_FASTA.fasta	MGYA00171489	ERZ488865	root:Host-associated:Human:Digestive system:Large int	0.0442
MGYA00171490/OKXJ01_FASTA.fasta	MGYA00171490	ERZ488830	root:Host-associated:Human:Digestive system:Large int	0.2692
MGYA00171491/OILJ01_FASTA.fasta	MGYA00171491	ERZ489083	root:Host-associated:Human:Digestive system:Large int	0.2628
MGYA00171492/OILI01_FASTA.fasta	MGYA00171492	ERZ489402	root:Host-associated:Human:Digestive system:Large int	0.3412
MGYA00171493/OLDP01_FASTA.fasta	MGYA00171493	ERZ489237	root:Host-associated:Human:Digestive system:Large int	0.2796
MGYA00171494/OIKY01_FASTA.fasta	MGYA00171494	ERZ489072	root:Host-associated:Human:Digestive system:Large int	0.3878
MGYA00171495/OIPI01_FASTA.fasta	MGYA00171495	ERZ489193	root:Host-associated:Human:Digestive system:Large int	0.3248
MGYA00171496/OIHX01_FASTA.fasta	MGYA00171496	ERZ488923	root:Host-associated:Human:Digestive system:Large int	0.2152
MGYA00171497/OIGV01_FASTA.fasta	MGYA00171497	ERZ488838	root:Host-associated:Human:Digestive system:Large int	0.3058
MGYA00171498/OINZ01_FASTA.fasta	MGYA00171498	ERZ489170	root:Host-associated:Human:Digestive system:Large int	0.0966
MGYA00171499/OKZQ01_FASTA.fasta	MGYA00171499	ERZ488921	root:Host-associated:Human:Digestive system:Large int	0.3022
MGYA00171500/OLAS01_FASTA.fasta	MGYA00171500	ERZ488981	root:Host-associated:Human:Digestive system:Large int	0.1202
MGYA00171501/OLAX01_FASTA.fasta	MGYA00171501	ERZ488979	root:Host-associated:Human:Digestive system:Large int	0.2702
MGYA00171502/OIOK01_FASTA.fasta	MGYA00171502	ERZ489173	root:Host-associated:Human:Digestive system:Large int	0.233
MGYA00171503/OKZC01_FASTA.fasta	MGYA00171503	ERZ488890	root:Host-associated:Human:Digestive system:Large int	0.2528
MGYA00171504/OIRI01_FASTA.fasta	MGYA00171504	ERZ489316	root:Host-associated:Human:Digestive system:Large int	0.2872
MGYA00171505/OING01_FASTA.fasta	MGYA00171505	ERZ489145	root:Host-associated:Human:Digestive system:Large int	0.1828
MGYA00171506/OIUW01_FASTA.fasta	MGYA00171506	ERZ489419	root:Host-associated:Human:Digestive system:Large int	0.3732
MGYA00171507/OKXR01_FASTA.fasta	MGYA00171507	ERZ488845	root:Host-associated:Human:Digestive system:Large int	0.3646
MGYA00171508/OIPM01_FASTA.fasta	MGYA00171508	ERZ489194	root:Host-associated:Human:Digestive system:Large int	0.3112
MGYA00171509/OIJS01_FASTA.fasta	MGYA00171509	ERZ489022	root:Host-associated:Human:Digestive system:Large int	0.194
MGYA00171510/OLAN01_FASTA.fasta	MGYA00171510	ERZ488967	root:Host-associated:Human:Digestive system:Large int	0.258
MGYA00171511/OIOV01_FASTA.fasta	MGYA00171511	ERZ489179	root:Host-associated:Human:Digestive system:Large int	0.2494
MGYA00171512/OIGI01_FASTA.fasta	MGYA00171512	ERZ488816	root:Host-associated:Human:Digestive system:Large int	0.364
MGYA00171513/OIUM01_FASTA.fasta	MGYA00171513	ERZ489398	root:Host-associated:Human:Digestive system:Large int	0.2336
MGYA00171514/OKXV01_FASTA.fasta	MGYA00171514	ERZ488852	root:Host-associated:Human:Digestive system:Large int	0.2018
MGYA00171515/OIMD01_FASTA.fasta	MGYA00171515	ERZ489125	root:Host-associated:Human:Digestive system:Large int	0.2756
MGYA00171516/OKYK01_FASTA.fasta	MGYA00171516	ERZ488873	root:Host-associated:Human:Digestive system:Large int	0.2484
MGYA00171517/OLEZ01_FASTA.fasta	MGYA00171517	ERZ489275	root:Host-associated:Human:Digestive system:Large int	0.1856
MGYA00171518/OKZK01_FASTA.fasta	MGYA00171518	ERZ488898	root:Host-associated:Human:Digestive system:Large int	0.3604
MGYA00171519/OIHY01_FASTA.fasta	MGYA00171519	ERZ488920	root:Host-associated:Human:Digestive system:Large int	0.2546
MGYA00171520/OINC01_FASTA.fasta	MGYA00171520	ERZ489127	root:Host-associated:Human:Digestive system:Large int	0.2382
MGYA00171521/OIGS01_FASTA.fasta	MGYA00171521	ERZ488840	root:Host-associated:Human:Digestive system:Large int	0.2098
MGYA00171522/OIST01_FASTA.fasta	MGYA00171522	ERZ489361	root:Host-associated:Human:Digestive system:Large int	0.312
MGYA00171523/OIKA01_FASTA.fasta	MGYA00171523	ERZ489023	root:Host-associated:Human:Digestive system:Large int	0.3736
MGYA00171524/OKXO01_FASTA.fasta	MGYA00171524	ERZ488836	root:Host-associated:Human:Digestive system:Large int	0.3438
MGYA00171525/OITC01_FASTA.fasta	MGYA00171525	ERZ489375	root:Host-associated:Human:Digestive system:Large int	0.313
MGYA00171526/OKZO01_FASTA.fasta	MGYA00171526	ERZ488929	root:Host-associated:Human:Digestive system:Large int	0.3002
MGYA00171527/OKXQ01_FASTA.fasta	MGYA00171527	ERZ488844	root:Host-associated:Human:Digestive system:Large int	0.3286
MGYA00171528/OIMT01_FASTA.fasta	MGYA00171528	ERZ489140	root:Host-associated:Human:Digestive system:Large int	0.1516
MGYA00171529/OIOF01_FASTA.fasta	MGYA00171529	ERZ489174	root:Host-associated:Human:Digestive system:Large int	0.1448
MGYA00171530/OLAJ01_FASTA.fasta	MGYA00171530	ERZ488960	root:Host-associated:Human:Digestive system:Large int	0.2754
MGYA00171531/OLEU01_FASTA.fasta	MGYA00171531	ERZ489278	root:Host-associated:Human:Digestive system:Large int	0.2826
MGYA00171532/OLCG01_FASTA.fasta	MGYA00171532	ERZ489071	root:Host-associated:Human:Digestive system:Large int	0.1022
MGYA00171533/OLBR01_FASTA.fasta	MGYA00171533	ERZ489037	root:Host-associated:Human:Digestive system:Large int	0.0942
MGYA00171534/OLBC01_FASTA.fasta	MGYA00171534	ERZ488995	root:Host-associated:Human:Digestive system:Large int	0.311
MGYA00171535/OLKF01_FASTA.fasta	MGYA00171535	ERZ489274	root:Host-associated:Human:Digestive system:Large int	0.275
MGYA00171536/OLFD01_FASTA.fasta	MGYA00171536	ERZ489288	root:Host-associated:Human:Digestive system:Large int	0.2714
MGYA00171537/OIML01_FASTA.fasta	MGYA00171537	ERZ489118	root:Host-associated:Human:Digestive system:Large int	0.3564
MGYA00171538/OLCR01_FASTA.fasta	MGYA00171538	ERZ489087	root:Host-associated:Human:Digestive system:Large int	0.3156
MGYA00171539/OINN01_FASTA.fasta	MGYA00171539	ERZ489147	root:Host-associated:Human:Digestive system:Large int	0.244
MGYA00171540/OIQP01_FASTA.fasta	MGYA00171540	ERZ489296	root:Host-associated:Human:Digestive system:Large int	0.3166
MGYA00171541/OILX01_FASTA.fasta	MGYA00171541	ERZ489110	root:Host-associated:Human:Digestive system:Large int	0.2828
MGYA00171542/OLHZ01_FASTA.fasta	MGYA00171542	ERZ490315	root:Environmental:Aquatic:Marine:Oceanic	0.7768
MGYA00171543/OJAZ01_FASTA.fasta	MGYA00171543	ERZ490155	root:Environmental:Aquatic:Marine:Oceanic	0.8276
MGYA00171544/OJAT01_FASTA.fasta	MGYA00171544	ERZ490159	root:Environmental:Aquatic:Marine:Oceanic	0.4774
MGYA00171545/OJGJ01_FASTA.fasta	MGYA00171545	ERZ490335	root:Environmental:Aquatic:Marine:Oceanic	0.0992
MGYA00171546/OJGZ01_FASTA.fasta	MGYA00171546	ERZ490351	root:Environmental:Aquatic:Marine:Oceanic	0.7958
MGYA00171547/OJDF01_FASTA.fasta	MGYA00171547	ERZ490232	root:Environmental:Aquatic:Marine:Oceanic	0.6324
MGYA00171548/OJEE01_FASTA.fasta	MGYA00171548	ERZ490255	root:Environmental:Aquatic:Marine:Oceanic	0.5694
MGYA00171549/OJBM01_FASTA.fasta	MGYA00171549	ERZ490185	root:Environmental:Aquatic:Marine:Oceanic	0.706
MGYA00171550/OJHW01_FASTA.fasta	MGYA00171550	ERZ490372	root:Environmental:Aquatic:Marine:Oceanic	0.5736
MGYA00171551/OJFS01_FASTA.fasta	MGYA00171551	ERZ490303	root:Environmental:Aquatic:Marine:Oceanic	0.7746
MGYA00171552/OJBN01_FASTA.fasta	MGYA00171552	ERZ490170	root:Environmental:Aquatic:Marine:Oceanic	0.6528
MGYA00171553/OJIQ01_FASTA.fasta	MGYA00171553	ERZ490395	root:Environmental:Aquatic:Marine:Oceanic	0.4472
MGYA00171554/OJBZ01_FASTA.fasta	MGYA00171554	ERZ490188	root:Environmental:Aquatic:Marine:Oceanic	0.8234
MGYA00171555/OJAP01_FASTA.fasta	MGYA00171555	ERZ490157	root:Environmental:Aquatic:Marine:Oceanic	0.6076
MGYA00171556/OJHV01_FASTA.fasta	MGYA00171556	ERZ490374	root:Environmental:Aquatic:Marine:Oceanic	0.4622
MGYA00171557/OJCU01_FASTA.fasta	MGYA00171557	ERZ490210	root:Environmental:Aquatic:Marine:Oceanic	0.4016
MGYA00171558/OLHO01_FASTA.fasta	MGYA00171558	ERZ490311	root:Environmental:Aquatic:Marine:Oceanic	0.181
MGYA00171559/OJEN01_FASTA.fasta	MGYA00171559	ERZ490267	root:Environmental:Aquatic:Marine:Oceanic	0.811
MGYA00171560/OJFP01_FASTA.fasta	MGYA00171560	ERZ490297	root:Environmental:Aquatic:Marine:Oceanic	0.8928
MGYA00171561/OJAV01_FASTA.fasta	MGYA00171561	ERZ490156	root:Environmental:Aquatic:Marine:Oceanic	0.7942
MGYA00171562/OJAN01_FASTA.fasta	MGYA00171562	ERZ490162	root:Environmental:Aquatic:Marine:Oceanic	0.223
MGYA00171563/OJEP01_FASTA.fasta	MGYA00171563	ERZ490276	root:Environmental:Aquatic:Marine:Oceanic	0.8386
MGYA00171564/OJHB01_FASTA.fasta	MGYA00171564	ERZ490346	root:Environmental:Aquatic:Marine:Oceanic	0.562
MGYA00171565/OJHN01_FASTA.fasta	MGYA00171565	ERZ490362	root:Environmental:Aquatic:Marine:Oceanic	0.4872
MGYA00171566/OLHK01_FASTA.fasta	MGYA00171566	ERZ490265	root:Environmental:Aquatic:Marine:Oceanic	0.8352
MGYA00171567/OLHH01_FASTA.fasta	MGYA00171567	ERZ490258	root:Environmental:Aquatic:Marine:Oceanic	0.6292
MGYA00171568/OJFV01_FASTA.fasta	MGYA00171568	ERZ490313	root:Environmental:Aquatic:Marine:Oceanic	0.7764
MGYA00171569/OJAY01_FASTA.fasta	MGYA00171569	ERZ490163	root:Environmental:Aquatic:Marine:Oceanic	0.8052
MGYA00171570/OJIT01_FASTA.fasta	MGYA00171570	ERZ490397	root:Environmental:Aquatic:Marine:Oceanic	0.6336
MGYA00171571/OJCB01_FASTA.fasta	MGYA00171571	ERZ490174	root:Environmental:Aquatic:Marine:Oceanic	0.8448
MGYA00171572/OLHB01_FASTA.fasta	MGYA00171572	ERZ490259	root:Environmental:Aquatic:Marine:Oceanic	0.558
MGYA00171573/OJCT01_FASTA.fasta	MGYA00171573	ERZ490198	root:Environmental:Aquatic:Marine:Oceanic	0.5218
MGYA00171574/OJIA01_FASTA.fasta	MGYA00171574	ERZ490387	root:Environmental:Aquatic:Marine:Oceanic	0.2598
MGYA00171575/OJER01_FASTA.fasta	MGYA00171575	ERZ490288	root:Environmental:Aquatic:Marine:Oceanic	0.3702
MGYA00171576/OJBG01_FASTA.fasta	MGYA00171576	ERZ490166	root:Environmental:Aquatic:Marine:Oceanic	0.3388
MGYA00171577/OJIB01_FASTA.fasta	MGYA00171577	ERZ490386	root:Environmental:Aquatic:Marine:Oceanic	0.394
MGYA00171578/OJDY01_FASTA.fasta	MGYA00171578	ERZ490250	root:Environmental:Aquatic:Marine:Oceanic	0.5902
MGYA00171579/OJFX01_FASTA.fasta	MGYA00171579	ERZ490293	root:Environmental:Aquatic:Marine:Oceanic	0.8514
MGYA00171580/OLGP01_FASTA.fasta	MGYA00171580	ERZ490154	root:Environmental:Aquatic:Marine:Oceanic	0.1932
MGYA00171581/OJDV01_FASTA.fasta	MGYA00171581	ERZ490240	root:Environmental:Aquatic:Marine:Oceanic	0.884
MGYA00171582/OJAO01_FASTA.fasta	MGYA00171582	ERZ490160	root:Environmental:Aquatic:Marine:Oceanic	0.3946
MGYA00171583/OJBL01_FASTA.fasta	MGYA00171583	ERZ490184	root:Environmental:Aquatic:Marine:Oceanic	0.212
MGYA00171584/OJCE01_FASTA.fasta	MGYA00171584	ERZ490199	root:Environmental:Aquatic:Marine:Oceanic	0.3334
MGYA00171585/OLHF01_FASTA.fasta	MGYA00171585	ERZ490263	root:Environmental:Aquatic:Marine:Oceanic	0.765
MGYA00171586/OJHT01_FASTA.fasta	MGYA00171586	ERZ490376	root:Environmental:Aquatic:Marine:Oceanic	0.304
MGYA00171587/OJCW01_FASTA.fasta	MGYA00171587	ERZ490217	root:Environmental:Aquatic:Marine:Oceanic	0.7386
MGYA00171588/OJGA01_FASTA.fasta	MGYA00171588	ERZ490323	root:Environmental:Aquatic:Marine:Oceanic	0.1022
MGYA00171589/OLHM01_FASTA.fasta	MGYA00171589	ERZ490264	root:Environmental:Aquatic:Marine:Oceanic	0.754
MGYA00171590/OJGT01_FASTA.fasta	MGYA00171590	ERZ490348	root:Environmental:Aquatic:Marine:Oceanic	0.7016
MGYA00171591/OJEG01_FASTA.fasta	MGYA00171591	ERZ490270	root:Environmental:Aquatic:Marine:Oceanic	0.6538
MGYA00171592/OLII01_FASTA.fasta	MGYA00171592	ERZ490358	root:Environmental:Aquatic:Marine:Oceanic	0.8978
MGYA00171593/OJDP01_FASTA.fasta	MGYA00171593	ERZ490242	root:Environmental:Aquatic:Marine:Oceanic	0.6902
MGYA00171594/OJDL01_FASTA.fasta	MGYA00171594	ERZ490229	root:Environmental:Aquatic:Marine:Oceanic	0.8178
MGYA00171595/OJIH01_FASTA.fasta	MGYA00171595	ERZ490373	root:Environmental:Aquatic:Marine:Oceanic	0.6212
MGYA00171596/OJBW01_FASTA.fasta	MGYA00171596	ERZ490192	root:Environmental:Aquatic:Marine:Oceanic	0.4058
MGYA00171597/OJHS01_FASTA.fasta	MGYA00171597	ERZ490377	root:Environmental:Aquatic:Marine:Oceanic	0.212
MGYA00171598/OJFI01_FASTA.fasta	MGYA00171598	ERZ490292	root:Environmental:Aquatic:Marine:Oceanic	0.1562
MGYA00171599/OJEJ01_FASTA.fasta	MGYA00171599	ERZ490257	root:Environmental:Aquatic:Marine:Oceanic	0.7528
MGYA00171600/OJAQ01_FASTA.fasta	MGYA00171600	ERZ490153	root:Environmental:Aquatic:Marine:Oceanic	0.5944
MGYA00171601/OJBY01_FASTA.fasta	MGYA00171601	ERZ490195	root:Environmental:Aquatic:Marine:Oceanic	0.8464
MGYA00171602/OJHZ01_FASTA.fasta	MGYA00171602	ERZ490378	root:Environmental:Aquatic:Marine:Oceanic	0.103
MGYA00171603/OJFE01_FASTA.fasta	MGYA00171603	ERZ490301	root:Environmental:Aquatic:Marine:Oceanic	0.4496
MGYA00171604/OJDA01_FASTA.fasta	MGYA00171604	ERZ490226	root:Environmental:Aquatic:Marine:Oceanic	0.7532
MGYA00171605/OJET01_FASTA.fasta	MGYA00171605	ERZ490279	root:Environmental:Aquatic:Marine:Oceanic	0.7126
MGYA00171606/OJDK01_FASTA.fasta	MGYA00171606	ERZ490236	root:Environmental:Aquatic:Marine:Oceanic	0.8492
MGYA00171607/OJCA01_FASTA.fasta	MGYA00171607	ERZ490191	root:Environmental:Aquatic:Marine:Oceanic	0.6588
MGYA00171608/OJDH01_FASTA.fasta	MGYA00171608	ERZ490237	root:Environmental:Aquatic:Marine:Oceanic	0.7554
MGYA00171609/OJHH01_FASTA.fasta	MGYA00171609	ERZ490343	root:Environmental:Aquatic:Marine:Oceanic	0.8668
MGYA00171610/OJGG01_FASTA.fasta	MGYA00171610	ERZ490328	root:Environmental:Aquatic:Marine:Oceanic	0.5402
MGYA00171611/OJIG01_FASTA.fasta	MGYA00171611	ERZ490370	root:Environmental:Aquatic:Marine:Oceanic	0.5546
MGYA00171612/OLHS01_FASTA.fasta	MGYA00171612	ERZ490320	root:Environmental:Aquatic:Marine:Oceanic	0.4084
MGYA00171613/OJGE01_FASTA.fasta	MGYA00171613	ERZ490322	root:Environmental:Aquatic:Marine:Oceanic	0.5222
MGYA00171614/OJIR01_FASTA.fasta	MGYA00171614	ERZ490384	root:Environmental:Aquatic:Marine:Oceanic	0.8138
MGYA00171615/OJBQ01_FASTA.fasta	MGYA00171615	ERZ490183	root:Environmental:Aquatic:Marine:Oceanic	0.3358
MGYA00171616/OJGF01_FASTA.fasta	MGYA00171616	ERZ490329	root:Environmental:Aquatic:Marine:Oceanic	0.6452
MGYA00171617/OMOM01_FASTA.fasta	MGYA00171617	ERZ490238	root:Environmental:Aquatic:Marine:Oceanic	0.8154
MGYA00171618/OJFL01_FASTA.fasta	MGYA00171618	ERZ490289	root:Environmental:Aquatic:Marine:Oceanic	0.8676
MGYA00171619/OJCY01_FASTA.fasta	MGYA00171619	ERZ490211	root:Environmental:Aquatic:Marine:Oceanic	0.822
MGYA00171620/OJEB01_FASTA.fasta	MGYA00171620	ERZ490253	root:Environmental:Aquatic:Marine:Oceanic	0.5692
MGYA00171621/OJFA01_FASTA.fasta	MGYA00171621	ERZ490298	root:Environmental:Aquatic:Marine:Oceanic	0.2248
MGYA00171622/OJIS01_FASTA.fasta	MGYA00171622	ERZ490383	root:Environmental:Aquatic:Marine:Oceanic	0.7634
MGYA00171623/OJDZ01_FASTA.fasta	MGYA00171623	ERZ490246	root:Environmental:Aquatic:Marine:Oceanic	0.8034
MGYA00171624/OJGD01_FASTA.fasta	MGYA00171624	ERZ490325	root:Environmental:Aquatic:Marine:Oceanic	0.7894
MGYA00171625/OLHU01_FASTA.fasta	MGYA00171625	ERZ490314	root:Environmental:Aquatic:Marine:Oceanic	0.5778
MGYA00171626/OJED01_FASTA.fasta	MGYA00171626	ERZ490256	root:Environmental:Aquatic:Marine:Oceanic	0.7442
MGYA00171627/OJDQ01_FASTA.fasta	MGYA00171627	ERZ490241	root:Environmental:Aquatic:Marine:Oceanic	0.8288
MGYA00171628/OJGS01_FASTA.fasta	MGYA00171628	ERZ490321	root:Environmental:Aquatic:Marine:Oceanic	0.7916
MGYA00171629/OJFN01_FASTA.fasta	MGYA00171629	ERZ490310	root:Environmental:Aquatic:Marine:Oceanic	0.1978
MGYA00171630/OJBA01_FASTA.fasta	MGYA00171630	ERZ490161	root:Environmental:Aquatic:Marine:Oceanic	0.671
MGYA00171631/OJEK01_FASTA.fasta	MGYA00171631	ERZ490272	root:Environmental:Aquatic:Marine:Oceanic	0.131
MGYA00171632/OJDO01_FASTA.fasta	MGYA00171632	ERZ490235	root:Environmental:Aquatic:Marine:Oceanic	0.7352
MGYA00171633/OJIJ01_FASTA.fasta	MGYA00171633	ERZ490390	root:Environmental:Aquatic:Marine:Oceanic	0.7094
MGYA00171634/OJBO01_FASTA.fasta	MGYA00171634	ERZ490168	root:Environmental:Aquatic:Marine:Oceanic	0.8274
MGYA00171635/OJHK01_FASTA.fasta	MGYA00171635	ERZ490354	root:Environmental:Aquatic:Marine:Oceanic	0.875
MGYA00171636/OJEO01_FASTA.fasta	MGYA00171636	ERZ490275	root:Environmental:Aquatic:Marine:Oceanic	0.8694
MGYA00171637/OLGS01_FASTA.fasta	MGYA00171637	ERZ490215	root:Environmental:Aquatic:Marine:Oceanic	0.7092
MGYA00171638/OLID01_FASTA.fasta	MGYA00171638	ERZ490379	root:Environmental:Aquatic:Marine:Oceanic	0.91
MGYA00171639/OJDD01_FASTA.fasta	MGYA00171639	ERZ490224	root:Environmental:Aquatic:Marine:Oceanic	0.8038
MGYA00171640/OLHP01_FASTA.fasta	MGYA00171640	ERZ490296	root:Environmental:Aquatic:Marine:Oceanic	0.4934
MGYA00171641/OJHE01_FASTA.fasta	MGYA00171641	ERZ490359	root:Environmental:Aquatic:Marine:Oceanic	0.814
MGYA00171642/OLGW01_FASTA.fasta	MGYA00171642	ERZ490227	root:Environmental:Aquatic:Marine:Oceanic	0.3044
MGYA00171643/OJIE01_FASTA.fasta	MGYA00171643	ERZ490391	root:Environmental:Aquatic:Marine:Oceanic	0.2638
MGYA00171644/OJGC01_FASTA.fasta	MGYA00171644	ERZ490318	root:Environmental:Aquatic:Marine:Oceanic	0.7602
MGYA00171645/OJHO01_FASTA.fasta	MGYA00171645	ERZ490360	root:Environmental:Aquatic:Marine:Oceanic	0.8378
MGYA00171646/OJDX01_FASTA.fasta	MGYA00171646	ERZ490252	root:Environmental:Aquatic:Marine:Oceanic	0.4354
MGYA00171647/OJCV01_FASTA.fasta	MGYA00171647	ERZ490221	root:Environmental:Aquatic:Marine:Oceanic	0.1284
MGYA00171648/OJBI01_FASTA.fasta	MGYA00171648	ERZ490175	root:Environmental:Aquatic:Marine:Oceanic	0.7168
MGYA00171649/OLHI01_FASTA.fasta	MGYA00171649	ERZ490260	root:Environmental:Aquatic:Marine:Oceanic	0.8032
MGYA00171650/OJFF01_FASTA.fasta	MGYA00171650	ERZ490294	root:Environmental:Aquatic:Marine:Oceanic	0.479
MGYA00171651/OJGU01_FASTA.fasta	MGYA00171651	ERZ490334	root:Environmental:Aquatic:Marine:Oceanic	0.88
MGYA00171652/OJCC01_FASTA.fasta	MGYA00171652	ERZ490194	root:Environmental:Aquatic:Marine:Oceanic	0.5212
MGYA00171653/OJDN01_FASTA.fasta	MGYA00171653	ERZ490230	root:Environmental:Aquatic:Marine:Oceanic	0.8592
MGYA00171654/OJDJ01_FASTA.fasta	MGYA00171654	ERZ490220	root:Environmental:Aquatic:Marine:Oceanic	0.1994
MGYA00171655/OJGP01_FASTA.fasta	MGYA00171655	ERZ490331	root:Environmental:Aquatic:Marine:Oceanic	0.8264
MGYA00171656/OJCX01_FASTA.fasta	MGYA00171656	ERZ490218	root:Environmental:Aquatic:Marine:Oceanic	0.395
MGYA00171657/OJHG01_FASTA.fasta	MGYA00171657	ERZ490339	root:Environmental:Aquatic:Marine:Oceanic	0.8818
MGYA00171658/OJGM01_FASTA.fasta	MGYA00171658	ERZ490330	root:Environmental:Aquatic:Marine:Oceanic	0.552
MGYA00171659/OJEW01_FASTA.fasta	MGYA00171659	ERZ490278	root:Environmental:Aquatic:Marine:Oceanic	0.7338
MGYA00171660/OJFC01_FASTA.fasta	MGYA00171660	ERZ490280	root:Environmental:Aquatic:Marine:Oceanic	0.7158
MGYA00171661/OJHX01_FASTA.fasta	MGYA00171661	ERZ490382	root:Environmental:Aquatic:Marine:Oceanic	0.141
MGYA00171662/OJCI01_FASTA.fasta	MGYA00171662	ERZ490205	root:Environmental:Aquatic:Marine:Oceanic	0.225
MGYA00171663/OJCR01_FASTA.fasta	MGYA00171663	ERZ490189	root:Environmental:Aquatic:Marine:Oceanic	0.832
MGYA00171664/OLHY01_FASTA.fasta	MGYA00171664	ERZ490304	root:Environmental:Aquatic:Marine:Oceanic	0.843
MGYA00171665/OJGL01_FASTA.fasta	MGYA00171665	ERZ490340	root:Environmental:Aquatic:Marine:Oceanic	0.2354
MGYA00171666/OJEF01_FASTA.fasta	MGYA00171666	ERZ490247	root:Environmental:Aquatic:Marine:Oceanic	0.8456
MGYA00171667/OJBJ01_FASTA.fasta	MGYA00171667	ERZ490179	root:Environmental:Aquatic:Marine:Oceanic	0.6666
MGYA00171668/OJBK01_FASTA.fasta	MGYA00171668	ERZ490177	root:Environmental:Aquatic:Marine:Oceanic	0.642
MGYA00171669/OJBS01_FASTA.fasta	MGYA00171669	ERZ490176	root:Environmental:Aquatic:Marine:Oceanic	0.831
MGYA00171670/OJEX01_FASTA.fasta	MGYA00171670	ERZ490287	root:Environmental:Aquatic:Marine:Oceanic	0.8096
MGYA00171671/OJDU01_FASTA.fasta	MGYA00171671	ERZ490233	root:Environmental:Aquatic:Marine:Oceanic	0.8306
MGYA00171672/OJFT01_FASTA.fasta	MGYA00171672	ERZ490317	root:Environmental:Aquatic:Marine:Oceanic	0.2682
MGYA00171673/OJGO01_FASTA.fasta	MGYA00171673	ERZ490342	root:Environmental:Aquatic:Marine:Oceanic	0.8182
MGYA00171674/OJEL01_FASTA.fasta	MGYA00171674	ERZ490274	root:Environmental:Aquatic:Marine:Oceanic	0.4064
MGYA00171675/OJID01_FASTA.fasta	MGYA00171675	ERZ490381	root:Environmental:Aquatic:Marine:Oceanic	0.455
MGYA00171676/OJCQ01_FASTA.fasta	MGYA00171676	ERZ490201	root:Environmental:Aquatic:Marine:Oceanic	0.471
MGYA00171677/OJBV01_FASTA.fasta	MGYA00171677	ERZ490182	root:Environmental:Aquatic:Marine:Oceanic	0.6914
MGYA00171678/OJBR01_FASTA.fasta	MGYA00171678	ERZ490187	root:Environmental:Aquatic:Marine:Oceanic	0.7898
MGYA00171679/OJCF01_FASTA.fasta	MGYA00171679	ERZ490204	root:Environmental:Aquatic:Marine:Oceanic	0.3302
MGYA00171680/OJIC01_FASTA.fasta	MGYA00171680	ERZ490389	root:Environmental:Aquatic:Marine:Oceanic	0.4058
MGYA00171681/OJIM01_FASTA.fasta	MGYA00171681	ERZ490396	root:Environmental:Aquatic:Marine:Oceanic	0.2754
MGYA00171682/OJGW01_FASTA.fasta	MGYA00171682	ERZ490324	root:Environmental:Aquatic:Marine:Oceanic	0.862
MGYA00171683/OJEI01_FASTA.fasta	MGYA00171683	ERZ490273	root:Environmental:Aquatic:Marine:Oceanic	0.715
MGYA00171684/OLGQ01_FASTA.fasta	MGYA00171684	ERZ490207	root:Environmental:Aquatic:Marine:Oceanic	0.2786
MGYA00171685/OJCZ01_FASTA.fasta	MGYA00171685	ERZ490228	root:Environmental:Aquatic:Marine:Oceanic	0.4528
MGYA00171686/OLGU01_FASTA.fasta	MGYA00171686	ERZ490149	root:Environmental:Aquatic:Marine:Oceanic	0.788
MGYA00171687/OJGY01_FASTA.fasta	MGYA00171687	ERZ490355	root:Environmental:Aquatic:Marine:Oceanic	0.3944
MGYA00171688/OJEC01_FASTA.fasta	MGYA00171688	ERZ490245	root:Environmental:Aquatic:Marine:Oceanic	0.626
MGYA00171689/OJBU01_FASTA.fasta	MGYA00171689	ERZ490186	root:Environmental:Aquatic:Marine:Oceanic	0.6414
MGYA00171690/OLHT01_FASTA.fasta	MGYA00171690	ERZ490369	root:Environmental:Aquatic:Marine:Oceanic	0.2726
MGYA00171691/OJGB01_FASTA.fasta	MGYA00171691	ERZ490332	root:Environmental:Aquatic:Marine:Oceanic	0.3516
MGYA00171692/OLHC01_FASTA.fasta	MGYA00171692	ERZ490262	root:Environmental:Aquatic:Marine:Oceanic	0.3126
MGYA00171693/OJES01_FASTA.fasta	MGYA00171693	ERZ490285	root:Environmental:Aquatic:Marine:Oceanic	0.2942
MGYA00171694/OJGR01_FASTA.fasta	MGYA00171694	ERZ490349	root:Environmental:Aquatic:Marine:Oceanic	0.134
MGYA00171695/OJAE01_FASTA.fasta	MGYA00171695	ERZ490148	root:Environmental:Aquatic:Marine:Oceanic	0.1854
MGYA00171696/OJFD01_FASTA.fasta	MGYA00171696	ERZ490300	root:Environmental:Aquatic:Marine:Oceanic	0.2154
MGYA00171697/OJBH01_FASTA.fasta	MGYA00171697	ERZ490181	root:Environmental:Aquatic:Marine:Oceanic	0.1418
MGYA00171698/OLHW01_FASTA.fasta	MGYA00171698	ERZ490347	root:Environmental:Aquatic:Marine:Oceanic	0.5526
MGYA00171699/OJAH01_FASTA.fasta	MGYA00171699	ERZ490150	root:Environmental:Aquatic:Marine:Oceanic	0.2578
MGYA00171700/OJGI01_FASTA.fasta	MGYA00171700	ERZ490333	root:Environmental:Aquatic:Marine:Oceanic	0.5576
MGYA00171701/OJBP01_FASTA.fasta	MGYA00171701	ERZ490180	root:Environmental:Aquatic:Marine:Oceanic	0.8736
MGYA00171702/OJCK01_FASTA.fasta	MGYA00171702	ERZ490197	root:Environmental:Aquatic:Marine:Oceanic	0.7552
MGYA00171703/OLGX01_FASTA.fasta	MGYA00171703	ERZ490222	root:Environmental:Aquatic:Marine:Oceanic	0.8098
MGYA00171704/OLHD01_FASTA.fasta	MGYA00171704	ERZ490261	root:Environmental:Aquatic:Marine:Oceanic	0.563
MGYA00171705/OJFG01_FASTA.fasta	MGYA00171705	ERZ490295	root:Environmental:Aquatic:Marine:Oceanic	0.7428
MGYA00171706/OJFK01_FASTA.fasta	MGYA00171706	ERZ490291	root:Environmental:Aquatic:Marine:Oceanic	0.6852
MGYA00171707/OJIV01_FASTA.fasta	MGYA00171707	ERZ490394	root:Environmental:Aquatic:Marine:Oceanic	0.8256
MGYA00171708/OJDT01_FASTA.fasta	MGYA00171708	ERZ490243	root:Environmental:Aquatic:Marine:Oceanic	0.6666
MGYA00171709/OJIL01_FASTA.fasta	MGYA00171709	ERZ490385	root:Environmental:Aquatic:Marine:Oceanic	0.6314
MGYA00171710/OJIF01_FASTA.fasta	MGYA00171710	ERZ490393	root:Environmental:Aquatic:Marine:Oceanic	0.3064
MGYA00171711/OJHJ01_FASTA.fasta	MGYA00171711	ERZ490357	root:Environmental:Aquatic:Marine:Oceanic	0.8578
MGYA00171712/OJHL01_FASTA.fasta	MGYA00171712	ERZ490361	root:Environmental:Aquatic:Marine:Oceanic	0.4102
MGYA00171713/OJBF01_FASTA.fasta	MGYA00171713	ERZ490171	root:Environmental:Aquatic:Marine:Oceanic	0.254
MGYA00171714/OJHA01_FASTA.fasta	MGYA00171714	ERZ490350	root:Environmental:Aquatic:Marine:Oceanic	0.4874
MGYA00171715/OJDE01_FASTA.fasta	MGYA00171715	ERZ490234	root:Environmental:Aquatic:Marine:Oceanic	0.139
MGYA00171716/OJCM01_FASTA.fasta	MGYA00171716	ERZ490208	root:Environmental:Aquatic:Marine:Oceanic	0.2058
MGYA00171717/OJGK01_FASTA.fasta	MGYA00171717	ERZ490337	root:Environmental:Aquatic:Marine:Oceanic	0.342
MGYA00171718/OJGH01_FASTA.fasta	MGYA00171718	ERZ490326	root:Environmental:Aquatic:Marine:Oceanic	0.761
MGYA00171719/OLHX01_FASTA.fasta	MGYA00171719	ERZ490365	root:Environmental:Aquatic:Marine:Oceanic	0.5566
MGYA00171720/OJGV01_FASTA.fasta	MGYA00171720	ERZ490353	root:Environmental:Aquatic:Marine:Oceanic	0.5146
MGYA00171721/OLHV01_FASTA.fasta	MGYA00171721	ERZ490341	root:Environmental:Aquatic:Marine:Oceanic	0.8074
MGYA00171722/OJIK01_FASTA.fasta	MGYA00171722	ERZ490368	root:Environmental:Aquatic:Marine:Oceanic	0.8262
MGYA00171723/OLHN01_FASTA.fasta	MGYA00171723	ERZ490269	root:Environmental:Aquatic:Marine:Oceanic	0.7568
MGYA00171724/OJBT01_FASTA.fasta	MGYA00171724	ERZ490190	root:Environmental:Aquatic:Marine:Oceanic	0.81
MGYA00171725/OJAS01_FASTA.fasta	MGYA00171725	ERZ490164	root:Environmental:Aquatic:Marine:Oceanic	0.6026
MGYA00171726/OLGR01_FASTA.fasta	MGYA00171726	ERZ490151	root:Environmental:Aquatic:Marine:Oceanic	0.3906
MGYA00171727/OLHJ01_FASTA.fasta	MGYA00171727	ERZ490249	root:Environmental:Aquatic:Marine:Oceanic	0.9046
MGYA00171728/OJEY01_FASTA.fasta	MGYA00171728	ERZ490284	root:Environmental:Aquatic:Marine:Oceanic	0.5528
MGYA00171729/OJHC01_FASTA.fasta	MGYA00171729	ERZ490352	root:Environmental:Aquatic:Marine:Oceanic	0.7962
MGYA00171730/OJDS01_FASTA.fasta	MGYA00171730	ERZ490219	root:Environmental:Aquatic:Marine:Oceanic	0.1542
MGYA00171731/OJII01_FASTA.fasta	MGYA00171731	ERZ490388	root:Environmental:Aquatic:Marine:Oceanic	0.3858
MGYA00171732/OJEZ01_FASTA.fasta	MGYA00171732	ERZ490283	root:Environmental:Aquatic:Marine:Oceanic	0.505
MGYA00171733/OJFO01_FASTA.fasta	MGYA00171733	ERZ490306	root:Environmental:Aquatic:Marine:Oceanic	0.5436
MGYA00171734/OJFQ01_FASTA.fasta	MGYA00171734	ERZ490308	root:Environmental:Aquatic:Marine:Oceanic	0.4372
MGYA00171735/OJFM01_FASTA.fasta	MGYA00171735	ERZ490302	root:Environmental:Aquatic:Marine:Oceanic	0.738
MGYA00171736/OLHQ01_FASTA.fasta	MGYA00171736	ERZ490299	root:Environmental:Aquatic:Marine:Oceanic	0.5924
MGYA00171737/OJCD01_FASTA.fasta	MGYA00171737	ERZ490202	root:Environmental:Aquatic:Marine:Oceanic	0.2208
MGYA00171738/OJDI01_FASTA.fasta	MGYA00171738	ERZ490225	root:Environmental:Aquatic:Marine:Oceanic	0.7736
MGYA00171739/OJEV01_FASTA.fasta	MGYA00171739	ERZ490281	root:Environmental:Aquatic:Marine:Oceanic	0.545
MGYA00171740/OJCJ01_FASTA.fasta	MGYA00171740	ERZ490193	root:Environmental:Aquatic:Marine:Oceanic	0.6098
MGYA00171741/OJHP01_FASTA.fasta	MGYA00171741	ERZ490366	root:Environmental:Aquatic:Marine:Oceanic	0.2504
MGYA00171742/OJAU01_FASTA.fasta	MGYA00171742	ERZ490152	root:Environmental:Aquatic:Marine:Oceanic	0.8374
MGYA00171743/OJDG01_FASTA.fasta	MGYA00171743	ERZ490213	root:Environmental:Aquatic:Marine:Oceanic	0.6826
MGYA00171744/OJAJ01_FASTA.fasta	MGYA00171744	ERZ490147	root:Environmental:Aquatic:Marine:Oceanic	0.296
MGYA00171745/OLHL01_FASTA.fasta	MGYA00171745	ERZ490268	root:Environmental:Aquatic:Marine:Oceanic	0.8142
MGYA00171746/OJBE01_FASTA.fasta	MGYA00171746	ERZ490173	root:Environmental:Aquatic:Marine:Oceanic	0.7558
MGYA00171747/OJEA01_FASTA.fasta	MGYA00171747	ERZ490248	root:Environmental:Aquatic:Marine:Oceanic	0.5788
MGYA00171748/OJDM01_FASTA.fasta	MGYA00171748	ERZ490239	root:Environmental:Aquatic:Marine:Oceanic	0.4656
MGYA00171749/OJBC01_FASTA.fasta	MGYA00171749	ERZ490167	root:Environmental:Aquatic:Marine:Oceanic	0.7536
MGYA00171750/OJHM01_FASTA.fasta	MGYA00171750	ERZ490364	root:Environmental:Aquatic:Marine:Oceanic	0.8168
MGYA00171751/OJCS01_FASTA.fasta	MGYA00171751	ERZ490216	root:Environmental:Aquatic:Marine:Oceanic	0.8146
MGYA00171752/OJDR01_FASTA.fasta	MGYA00171752	ERZ490251	root:Environmental:Aquatic:Marine:Oceanic	0.676
MGYA00171753/OJHD01_FASTA.fasta	MGYA00171753	ERZ490345	root:Environmental:Aquatic:Marine:Oceanic	0.6316
MGYA00171754/OJCP01_FASTA.fasta	MGYA00171754	ERZ490209	root:Environmental:Aquatic:Marine:Oceanic	0.447
MGYA00171755/OJDB01_FASTA.fasta	MGYA00171755	ERZ490231	root:Environmental:Aquatic:Marine:Oceanic	0.239
MGYA00171756/OJGN01_FASTA.fasta	MGYA00171756	ERZ490338	root:Environmental:Aquatic:Marine:Oceanic	0.2806
MGYA00171757/OJBB01_FASTA.fasta	MGYA00171757	ERZ490169	root:Environmental:Aquatic:Marine:Oceanic	0.8188
MGYA00171758/OJCL01_FASTA.fasta	MGYA00171758	ERZ490212	root:Environmental:Aquatic:Marine:Oceanic	0.1092
MGYA00171759/OJGX01_FASTA.fasta	MGYA00171759	ERZ490327	root:Environmental:Aquatic:Marine:Oceanic	0.9084
MGYA00171760/OJFJ01_FASTA.fasta	MGYA00171760	ERZ490309	root:Environmental:Aquatic:Marine:Oceanic	0.2376
MGYA00171761/OJCN01_FASTA.fasta	MGYA00171761	ERZ490206	root:Environmental:Aquatic:Marine:Oceanic	0.3556
MGYA00171762/OJAX01_FASTA.fasta	MGYA00171762	ERZ490165	root:Environmental:Aquatic:Marine:Oceanic	0.1214
MGYA00171763/OJHI01_FASTA.fasta	MGYA00171763	ERZ490356	root:Environmental:Aquatic:Marine:Oceanic	0.8434
MGYA00171764/OJDC01_FASTA.fasta	MGYA00171764	ERZ490223	root:Environmental:Aquatic:Marine:Oceanic	0.3904
MGYA00171765/OJHY01_FASTA.fasta	MGYA00171765	ERZ490363	root:Environmental:Aquatic:Marine:Oceanic	0.853
MGYA00171766/OJHQ01_FASTA.fasta	MGYA00171766	ERZ490367	root:Environmental:Aquatic:Marine:Oceanic	0.2406
MGYA00171767/OJEQ01_FASTA.fasta	MGYA00171767	ERZ490286	root:Environmental:Aquatic:Marine:Oceanic	0.3826
MGYA00171768/OJHR01_FASTA.fasta	MGYA00171768	ERZ490375	root:Environmental:Aquatic:Marine:Oceanic	0.5606
MGYA00171769/OJHF01_FASTA.fasta	MGYA00171769	ERZ490336	root:Environmental:Aquatic:Marine:Oceanic	0.7016
MGYA00171770/OLGT01_FASTA.fasta	MGYA00171770	ERZ490200	root:Environmental:Aquatic:Marine:Oceanic	0.4098
MGYA00171771/OJGQ01_FASTA.fasta	MGYA00171771	ERZ490344	root:Environmental:Aquatic:Marine:Oceanic	0.2492
MGYA00171772/OJEM01_FASTA.fasta	MGYA00171772	ERZ490271	root:Environmental:Aquatic:Marine:Oceanic	0.81
MGYA00171773/OLHR01_FASTA.fasta	MGYA00171773	ERZ490266	root:Environmental:Aquatic:Marine:Oceanic	0.699
MGYA00171774/OJFB01_FASTA.fasta	MGYA00171774	ERZ490277	root:Environmental:Aquatic:Marine:Oceanic	0.6598
MGYA00171775/OJFZ01_FASTA.fasta	MGYA00171775	ERZ490319	root:Environmental:Aquatic:Marine:Oceanic	0.1938
MGYA00171776/OJFW01_FASTA.fasta	MGYA00171776	ERZ490316	root:Environmental:Aquatic:Marine:Oceanic	0.4152
MGYA00171777/OJBD01_FASTA.fasta	MGYA00171777	ERZ490172	root:Environmental:Aquatic:Marine:Oceanic	0.6738
MGYA00171778/OJBX01_FASTA.fasta	MGYA00171778	ERZ490178	root:Environmental:Aquatic:Marine:Oceanic	0.8732
MGYA00171779/OJFR01_FASTA.fasta	MGYA00171779	ERZ490290	root:Environmental:Aquatic:Marine:Oceanic	0.841
MGYA00171780/OJFY01_FASTA.fasta	MGYA00171780	ERZ490312	root:Environmental:Aquatic:Marine:Oceanic	0.6178
MGYA00171781/OJAK01_FASTA.fasta	MGYA00171781	ERZ490158	root:Environmental:Aquatic:Marine:Oceanic	0.2362
MGYA00171782/OJEU01_FASTA.fasta	MGYA00171782	ERZ490282	root:Environmental:Aquatic:Marine:Oceanic	0.3264
MGYA00171783/OJIO01_FASTA.fasta	MGYA00171783	ERZ490380	root:Environmental:Aquatic:Marine:Oceanic	0.9302
MGYA00171784/OJCO01_FASTA.fasta	MGYA00171784	ERZ490214	root:Environmental:Aquatic:Marine:Oceanic	0.4292
MGYA00171785/OJIP01_FASTA.fasta	MGYA00171785	ERZ490392	root:Environmental:Aquatic:Marine:Oceanic	0.146
MGYA00171786/OJFU01_FASTA.fasta	MGYA00171786	ERZ490307	root:Environmental:Aquatic:Marine:Oceanic	0.7308
MGYA00171787/OJAF01_FASTA.fasta	MGYA00171787	ERZ490146	root:Environmental:Aquatic:Marine:Oceanic	0.6402
MGYA00171788/OJCG01_FASTA.fasta	MGYA00171788	ERZ490203	root:Environmental:Aquatic:Marine:Oceanic	0.2994
MGYA00171789/OJCH01_FASTA.fasta	MGYA00171789	ERZ490196	root:Environmental:Aquatic:Marine:Oceanic	0.2782
MGYA00171790/OJEH01_FASTA.fasta	MGYA00171790	ERZ490244	root:Environmental:Aquatic:Marine:Oceanic	0.836
MGYA00171791/OJDW01_FASTA.fasta	MGYA00171791	ERZ490254	root:Environmental:Aquatic:Marine:Oceanic	0.7836
MGYA00171792/OJFH01_FASTA.fasta	MGYA00171792	ERZ490305	root:Environmental:Aquatic:Marine:Oceanic	0.3572
MGYA00171793/OJHU01_FASTA.fasta	MGYA00171793	ERZ490371	root:Environmental:Aquatic:Marine:Oceanic	0.6802
MGYA00172070/OLJD01_FASTA.fasta	MGYA00172070	ERZ490786	root:Host-associated:Human:Digestive system:Large int	0.3276
MGYA00172071/OJYO01_FASTA.fasta	MGYA00172071	ERZ490938	root:Host-associated:Human:Digestive system:Large int	0.2868
MGYA00172072/OKCF01_FASTA.fasta	MGYA00172072	ERZ491031	root:Host-associated:Human:Digestive system:Large int	0.3542
MGYA00172073/OJVV01_FASTA.fasta	MGYA00172073	ERZ490857	root:Host-associated:Human:Digestive system:Large int	0.4346
MGYA00172074/OKEY01_FASTA.fasta	MGYA00172074	ERZ491115	root:Host-associated:Human:Digestive system:Large int	0.1388
MGYA00172075/OJXM01_FASTA.fasta	MGYA00172075	ERZ490918	root:Host-associated:Human:Digestive system:Large int	0.2708
MGYA00172076/OJUI01_FASTA.fasta	MGYA00172076	ERZ490817	root:Host-associated:Human:Digestive system:Large int	0.2994
MGYA00172077/OKDW01_FASTA.fasta	MGYA00172077	ERZ491087	root:Host-associated:Human:Digestive system:Large int	0.3756
MGYA00172078/OKPM01_FASTA.fasta	MGYA00172078	ERZ491390	root:Host-associated:Human:Digestive system:Large int	0.3326
MGYA00172079/OKIE01_FASTA.fasta	MGYA00172079	ERZ491193	root:Host-associated:Human:Digestive system:Large int	0.313
MGYA00172080/OLJI01_FASTA.fasta	MGYA00172080	ERZ490838	root:Host-associated:Human:Digestive system:Large int	0.2
MGYA00172081/OJUN01_FASTA.fasta	MGYA00172081	ERZ490820	root:Host-associated:Human:Digestive system:Large int	0.3152
MGYA00172082/OKOL01_FASTA.fasta	MGYA00172082	ERZ491360	root:Host-associated:Human:Digestive system:Large int	0.3622
MGYA00172083/OKNT01_FASTA.fasta	MGYA00172083	ERZ491345	root:Host-associated:Human:Digestive system:Large int	0.2352
MGYA00172084/OKDD01_FASTA.fasta	MGYA00172084	ERZ491063	root:Host-associated:Human:Digestive system:Large int	0.1594
MGYA00172085/OKOF01_FASTA.fasta	MGYA00172085	ERZ491364	root:Host-associated:Human:Digestive system:Large int	0.3764
MGYA00172086/OKNZ01_FASTA.fasta	MGYA00172086	ERZ491347	root:Host-associated:Human:Digestive system:Large int	0.2002
MGYA00172087/OKEL01_FASTA.fasta	MGYA00172087	ERZ491100	root:Host-associated:Human:Digestive system:Large int	0.3582
MGYA00172088/OKCS01_FASTA.fasta	MGYA00172088	ERZ491053	root:Host-associated:Human:Digestive system:Large int	0.1426
MGYA00172089/OKFW01_FASTA.fasta	MGYA00172089	ERZ491136	root:Host-associated:Human:Digestive system:Large int	0.3268
MGYA00172090/OJZN01_FASTA.fasta	MGYA00172090	ERZ490961	root:Host-associated:Human:Digestive system:Large int	0.2928
MGYA00172091/OKBV01_FASTA.fasta	MGYA00172091	ERZ491026	root:Host-associated:Human:Digestive system:Large int	0.3634
MGYA00172092/OJUC01_FASTA.fasta	MGYA00172092	ERZ490804	root:Host-associated:Human:Digestive system:Large int	0.2196
MGYA00172093/OKCP01_FASTA.fasta	MGYA00172093	ERZ491040	root:Host-associated:Human:Digestive system:Large int	0.4034
MGYA00172094/OJXA01_FASTA.fasta	MGYA00172094	ERZ490894	root:Host-associated:Human:Digestive system:Large int	0.3764
MGYA00172095/OKDI01_FASTA.fasta	MGYA00172095	ERZ491068	root:Host-associated:Human:Digestive system:Large int	0.1682
MGYA00172096/OKAB01_FASTA.fasta	MGYA00172096	ERZ490981	root:Host-associated:Human:Digestive system:Large int	0.164
MGYA00172097/OKKJ01_FASTA.fasta	MGYA00172097	ERZ491259	root:Host-associated:Human:Digestive system:Large int	0.3118
MGYA00172098/OKHM01_FASTA.fasta	MGYA00172098	ERZ491176	root:Host-associated:Human:Digestive system:Large int	0.3598
MGYA00172099/OKKF01_FASTA.fasta	MGYA00172099	ERZ491246	root:Host-associated:Human:Digestive system:Large int	0.3038
MGYA00172100/OKFM01_FASTA.fasta	MGYA00172100	ERZ491122	root:Host-associated:Human:Digestive system:Large int	0.3254
MGYA00172101/OKNV01_FASTA.fasta	MGYA00172101	ERZ491348	root:Host-associated:Human:Digestive system:Large int	0.3094
MGYA00172102/OKIQ01_FASTA.fasta	MGYA00172102	ERZ491206	root:Host-associated:Human:Digestive system:Large int	0.2522
MGYA00172103/OKBZ01_FASTA.fasta	MGYA00172103	ERZ491037	root:Host-associated:Human:Digestive system:Large int	0.3002
MGYA00172104/OJXE01_FASTA.fasta	MGYA00172104	ERZ490903	root:Host-associated:Human:Digestive system:Large int	0.2998
MGYA00172105/OKHA01_FASTA.fasta	MGYA00172105	ERZ491169	root:Host-associated:Human:Digestive system:Large int	0.3214
MGYA00172106/OKKP01_FASTA.fasta	MGYA00172106	ERZ491249	root:Host-associated:Human:Digestive system:Large int	0.3602
MGYA00172107/OKKB01_FASTA.fasta	MGYA00172107	ERZ491248	root:Host-associated:Human:Digestive system:Large int	0.367
MGYA00172108/OJUD01_FASTA.fasta	MGYA00172108	ERZ490814	root:Host-associated:Human:Digestive system:Large int	0.2626
MGYA00172109/OJWA01_FASTA.fasta	MGYA00172109	ERZ490871	root:Host-associated:Human:Digestive system:Large int	0.3128
MGYA00172110/OKHW01_FASTA.fasta	MGYA00172110	ERZ491189	root:Host-associated:Human:Digestive system:Large int	0.3578
MGYA00172111/OJZM01_FASTA.fasta	MGYA00172111	ERZ490963	root:Host-associated:Human:Digestive system:Large int	0.4338
MGYA00172112/OKNW01_FASTA.fasta	MGYA00172112	ERZ491346	root:Host-associated:Human:Digestive system:Large int	0.2988
MGYA00172113/OKMD01_FASTA.fasta	MGYA00172113	ERZ491314	root:Host-associated:Human:Digestive system:Large int	0.27
MGYA00172114/OJZR01_FASTA.fasta	MGYA00172114	ERZ490967	root:Host-associated:Human:Digestive system:Large int	0.2002
MGYA00172115/OJVB01_FASTA.fasta	MGYA00172115	ERZ490836	root:Host-associated:Human:Digestive system:Large int	0.2862
MGYA00172116/OKMZ01_FASTA.fasta	MGYA00172116	ERZ491325	root:Host-associated:Human:Digestive system:Large int	0.239
MGYA00172117/OLKA01_FASTA.fasta	MGYA00172117	ERZ491059	root:Host-associated:Human:Digestive system:Large int	0.375
MGYA00172118/OKBE01_FASTA.fasta	MGYA00172118	ERZ491007	root:Host-associated:Human:Digestive system:Large int	0.2714
MGYA00172119/OKHS01_FASTA.fasta	MGYA00172119	ERZ491182	root:Host-associated:Human:Digestive system:Large int	0.2824
MGYA00172120/OKIS01_FASTA.fasta	MGYA00172120	ERZ491207	root:Host-associated:Human:Digestive system:Large int	0.2008
MGYA00172121/OKLQ01_FASTA.fasta	MGYA00172121	ERZ491271	root:Host-associated:Human:Digestive system:Large int	0.2846
MGYA00172122/OKPG01_FASTA.fasta	MGYA00172122	ERZ491387	root:Host-associated:Human:Digestive system:Large int	0.16
MGYA00172123/OJZI01_FASTA.fasta	MGYA00172123	ERZ490956	root:Host-associated:Human:Digestive system:Large int	0.3062
MGYA00172124/OKQI01_FASTA.fasta	MGYA00172124	ERZ491399	root:Host-associated:Human:Digestive system:Large int	0.4032
MGYA00172125/OKBY01_FASTA.fasta	MGYA00172125	ERZ491028	root:Host-associated:Human:Digestive system:Large int	0.3546
MGYA00172126/OKDU01_FASTA.fasta	MGYA00172126	ERZ491079	root:Host-associated:Human:Digestive system:Large int	0.1836
MGYA00172127/OJUR01_FASTA.fasta	MGYA00172127	ERZ490825	root:Host-associated:Human:Digestive system:Large int	0.2752
MGYA00172128/OJWJ01_FASTA.fasta	MGYA00172128	ERZ490892	root:Host-associated:Human:Digestive system:Large int	0.3532
MGYA00172129/OKFD01_FASTA.fasta	MGYA00172129	ERZ491113	root:Host-associated:Human:Digestive system:Large int	0.2138
MGYA00172130/OKLE01_FASTA.fasta	MGYA00172130	ERZ491273	root:Host-associated:Human:Digestive system:Large int	0.3222
MGYA00172131/OKCT01_FASTA.fasta	MGYA00172131	ERZ491060	root:Host-associated:Human:Digestive system:Large int	0.3686
MGYA00172132/OKMI01_FASTA.fasta	MGYA00172132	ERZ491310	root:Host-associated:Human:Digestive system:Large int	0.2834
MGYA00172133/OLJY01_FASTA.fasta	MGYA00172133	ERZ491121	root:Host-associated:Human:Digestive system:Large int	0.3894
MGYA00172134/OKIA01_FASTA.fasta	MGYA00172134	ERZ491190	root:Host-associated:Human:Digestive system:Large int	0.3078
MGYA00172135/OKKE01_FASTA.fasta	MGYA00172135	ERZ491251	root:Host-associated:Human:Digestive system:Large int	0.435
MGYA00172136/OJTV01_FASTA.fasta	MGYA00172136	ERZ490802	root:Host-associated:Human:Digestive system:Large int	0.3978
MGYA00172137/OKAS01_FASTA.fasta	MGYA00172137	ERZ490997	root:Host-associated:Human:Digestive system:Large int	0.4088
MGYA00172138/OJYT01_FASTA.fasta	MGYA00172138	ERZ490943	root:Host-associated:Human:Digestive system:Large int	0.3992
MGYA00172139/OKIR01_FASTA.fasta	MGYA00172139	ERZ491204	root:Host-associated:Human:Digestive system:Large int	0.4596
MGYA00172140/OKBA01_FASTA.fasta	MGYA00172140	ERZ491004	root:Host-associated:Human:Digestive system:Large int	0.323
MGYA00172141/OJSX01_FASTA.fasta	MGYA00172141	ERZ490774	root:Host-associated:Human:Digestive system:Large int	0.2334
MGYA00172142/OKNU01_FASTA.fasta	MGYA00172142	ERZ491349	root:Host-associated:Human:Digestive system:Large int	0.3078
MGYA00172143/OLJP01_FASTA.fasta	MGYA00172143	ERZ490877	root:Host-associated:Human:Digestive system:Large int	0.3144
MGYA00172144/OKPF01_FASTA.fasta	MGYA00172144	ERZ491375	root:Host-associated:Human:Digestive system:Large int	0.391
MGYA00172145/OKMD01_FASTA.fasta	MGYA00172145	ERZ491301	root:Host-associated:Human:Digestive system:Large int	0.3548
MGYA00172146/OJWL01_FASTA.fasta	MGYA00172146	ERZ490886	root:Host-associated:Human:Digestive system:Large int	0.161
MGYA00172147/OKJW01_FASTA.fasta	MGYA00172147	ERZ491243	root:Host-associated:Human:Digestive system:Large int	0.3922
MGYA00172148/OJZL01_FASTA.fasta	MGYA00172148	ERZ490964	root:Host-associated:Human:Digestive system:Large int	0.335
MGYA00172149/OKJK01_FASTA.fasta	MGYA00172149	ERZ491224	root:Host-associated:Human:Digestive system:Large int	0.2542
MGYA00172150/OKGR01_FASTA.fasta	MGYA00172150	ERZ491153	root:Host-associated:Human:Digestive system:Large int	0.1994
MGYA00172151/OKHU01_FASTA.fasta	MGYA00172151	ERZ491180	root:Host-associated:Human:Digestive system:Large int	0.3122
MGYA00172152/OJYZ01_FASTA.fasta	MGYA00172152	ERZ490949	root:Host-associated:Human:Digestive system:Large int	0.1834
MGYA00172153/OKMN01_FASTA.fasta	MGYA00172153	ERZ491313	root:Host-associated:Human:Digestive system:Large int	0.3236
MGYA00172154/OKMK01_FASTA.fasta	MGYA00172154	ERZ491304	root:Host-associated:Human:Digestive system:Large int	0.3396
MGYA00172155/OKIN01_FASTA.fasta	MGYA00172155	ERZ491209	root:Host-associated:Human:Digestive system:Large int	0.2884
MGYA00172156/OKBB01_FASTA.fasta	MGYA00172156	ERZ491003	root:Host-associated:Human:Digestive system:Large int	0.313
MGYA00172157/OKNE01_FASTA.fasta	MGYA00172157	ERZ491326	root:Host-associated:Human:Digestive system:Large int	0.3556
MGYA00172158/OKMG01_FASTA.fasta	MGYA00172158	ERZ491307	root:Host-associated:Human:Digestive system:Large int	0.1852
MGYA00172159/OKLR01_FASTA.fasta	MGYA00172159	ERZ491290	root:Host-associated:Human:Digestive system:Large int	0.3988
MGYA00172160/OJTO01_FASTA.fasta	MGYA00172160	ERZ490798	root:Host-associated:Human:Digestive system:Large int	0.2786
MGYA00172161/OJWR01_FASTA.fasta	MGYA00172161	ERZ490879	root:Host-associated:Human:Digestive system:Large int	0.3514
MGYA00172162/OKKI01_FASTA.fasta	MGYA00172162	ERZ491261	root:Host-associated:Human:Digestive system:Large int	0.331
MGYA00172163/OKGE01_FASTA.fasta	MGYA00172163	ERZ491144	root:Host-associated:Human:Digestive system:Large int	0.186
MGYA00172164/OJUY01_FASTA.fasta	MGYA00172164	ERZ490812	root:Host-associated:Human:Digestive system:Large int	0.3198
MGYA00172165/OKKL01_FASTA.fasta	MGYA00172165	ERZ491257	root:Host-associated:Human:Digestive system:Large int	0.2754
MGYA00172166/OJWV01_FASTA.fasta	MGYA00172166	ERZ490890	root:Host-associated:Human:Digestive system:Large int	0.1638
MGYA00172167/OKLA01_FASTA.fasta	MGYA00172167	ERZ491266	root:Host-associated:Human:Digestive system:Large int	0.3384
MGYA00172168/OKBH01_FASTA.fasta	MGYA00172168	ERZ491014	root:Host-associated:Human:Digestive system:Large int	0.145
MGYA00172169/OJVG01_FASTA.fasta	MGYA00172169	ERZ490846	root:Host-associated:Human:Digestive system:Large int	0.2062
MGYA00172170/OKFO01_FASTA.fasta	MGYA00172170	ERZ491127	root:Host-associated:Human:Digestive system:Large int	0.3456
MGYA00172171/OKCR01_FASTA.fasta	MGYA00172171	ERZ491051	root:Host-associated:Human:Digestive system:Large int	0.1834
MGYA00172172/OKBX01_FASTA.fasta	MGYA00172172	ERZ491032	root:Host-associated:Human:Digestive system:Large int	0.289
MGYA00172173/OKJI01_FASTA.fasta	MGYA00172173	ERZ491231	root:Host-associated:Human:Digestive system:Large int	0.2606
MGYA00172174/OJYC01_FASTA.fasta	MGYA00172174	ERZ490930	root:Host-associated:Human:Digestive system:Large int	0.3064
MGYA00172175/OKFI01_FASTA.fasta	MGYA00172175	ERZ491120	root:Host-associated:Human:Digestive system:Large int	0.2178
MGYA00172176/OKAF01_FASTA.fasta	MGYA00172176	ERZ490982	root:Host-associated:Human:Digestive system:Large int	0.3258
MGYA00172177/OKAI01_FASTA.fasta	MGYA00172177	ERZ490989	root:Host-associated:Human:Digestive system:Large int	0.3546
MGYA00172178/OJXV01_FASTA.fasta	MGYA00172178	ERZ490920	root:Host-associated:Human:Digestive system:Large int	0.2338
MGYA00172179/OKOJ01_FASTA.fasta	MGYA00172179	ERZ491361	root:Host-associated:Human:Digestive system:Large int	0.1054
MGYA00172180/OKGQ01_FASTA.fasta	MGYA00172180	ERZ491157	root:Host-associated:Human:Digestive system:Large int	0.246
MGYA00172181/OKAM01_FASTA.fasta	MGYA00172181	ERZ490987	root:Host-associated:Human:Digestive system:Large int	0.3044
MGYA00172182/OKKZ01_FASTA.fasta	MGYA00172182	ERZ491276	root:Host-associated:Human:Digestive system:Large int	0.2792
MGYA00172183/OJUH01_FASTA.fasta	MGYA00172183	ERZ490811	root:Host-associated:Human:Digestive system:Large int	0.3304
MGYA00172184/OJVD01_FASTA.fasta	MGYA00172184	ERZ490839	root:Host-associated:Human:Digestive system:Large int	0.3138
MGYA00172185/OKLU01_FASTA.fasta	MGYA00172185	ERZ491293	root:Host-associated:Human:Digestive system:Large int	0.2668
MGYA00172186/OJYD01_FASTA.fasta	MGYA00172186	ERZ490929	root:Host-associated:Human:Digestive system:Large int	0.27
MGYA00172187/OKIH01_FASTA.fasta	MGYA00172187	ERZ491201	root:Host-associated:Human:Digestive system:Large int	0.2888
MGYA00172188/OKCM01_FASTA.fasta	MGYA00172188	ERZ491043	root:Host-associated:Human:Digestive system:Large int	0.2596
MGYA00172189/OJSS01_FASTA.fasta	MGYA00172189	ERZ490775	root:Host-associated:Human:Digestive system:Large int	0.2152
MGYA00172190/OKMC01_FASTA.fasta	MGYA00172190	ERZ491300	root:Host-associated:Human:Digestive system:Large int	0.29
MGYA00172191/OKAV01_FASTA.fasta	MGYA00172191	ERZ491000	root:Host-associated:Human:Digestive system:Large int	0.3158
MGYA00172192/OKJE01_FASTA.fasta	MGYA00172192	ERZ491227	root:Host-associated:Human:Digestive system:Large int	0.3184
MGYA00172193/OJVJ01_FASTA.fasta	MGYA00172193	ERZ490853	root:Host-associated:Human:Digestive system:Large int	0.3304
MGYA00172194/OJTD01_FASTA.fasta	MGYA00172194	ERZ490785	root:Host-associated:Human:Digestive system:Large int	0.2662
MGYA00172195/OKCH01_FASTA.fasta	MGYA00172195	ERZ491048	root:Host-associated:Human:Digestive system:Large int	0.324
MGYA00172196/OKLK01_FASTA.fasta	MGYA00172196	ERZ491289	root:Host-associated:Human:Digestive system:Large int	0.2794
MGYA00172197/OKHG01_FASTA.fasta	MGYA00172197	ERZ491175	root:Host-associated:Human:Digestive system:Large int	0.177
MGYA00172198/OKLO01_FASTA.fasta	MGYA00172198	ERZ491291	root:Host-associated:Human:Digestive system:Large int	0.2206
MGYA00172199/OLJR01_FASTA.fasta	MGYA00172199	ERZ490958	root:Host-associated:Human:Digestive system:Large int	0.3352
MGYA00172200/OJYL01_FASTA.fasta	MGYA00172200	ERZ490937	root:Host-associated:Human:Digestive system:Large int	0.1708
MGYA00172201/OJTP01_FASTA.fasta	MGYA00172201	ERZ490788	root:Host-associated:Human:Digestive system:Large int	0.3236
MGYA00172202/OKLS01_FASTA.fasta	MGYA00172202	ERZ491288	root:Host-associated:Human:Digestive system:Large int	0.147
MGYA00172203/OLJQ01_FASTA.fasta	MGYA00172203	ERZ490875	root:Host-associated:Human:Digestive system:Large int	0.3474
MGYA00172204/OKKW01_FASTA.fasta	MGYA00172204	ERZ491267	root:Host-associated:Human:Digestive system:Large int	0.3842
MGYA00172205/OKEQ01_FASTA.fasta	MGYA00172205	ERZ491093	root:Host-associated:Human:Digestive system:Large int	0.1694
MGYA00172206/OKLB01_FASTA.fasta	MGYA00172206	ERZ491272	root:Host-associated:Human:Digestive system:Large int	0.2502
MGYA00172207/OJWI01_FASTA.fasta	MGYA00172207	ERZ490878	root:Host-associated:Human:Digestive system:Large int	0.3394
MGYA00172208/OKER01_FASTA.fasta	MGYA00172208	ERZ491102	root:Host-associated:Human:Digestive system:Large int	0.3808
MGYA00172209/OKHD01_FASTA.fasta	MGYA00172209	ERZ491171	root:Host-associated:Human:Digestive system:Large int	0.224
MGYA00172210/OKKC01_FASTA.fasta	MGYA00172210	ERZ491254	root:Host-associated:Human:Digestive system:Large int	0.2686
MGYA00172211/OKDO01_FASTA.fasta	MGYA00172211	ERZ491077	root:Host-associated:Human:Digestive system:Large int	0.1954
MGYA00172212/OJUQ01_FASTA.fasta	MGYA00172212	ERZ490828	root:Host-associated:Human:Digestive system:Large int	0.2538
MGYA00172213/OJTS01_FASTA.fasta	MGYA00172213	ERZ490791	root:Host-associated:Human:Digestive system:Large int	0.2168
MGYA00172214/OJYM01_FASTA.fasta	MGYA00172214	ERZ490935	root:Host-associated:Human:Digestive system:Large int	0.2376
MGYA00172215/OKLM01_FASTA.fasta	MGYA00172215	ERZ491285	root:Host-associated:Human:Digestive system:Large int	0.0616
MGYA00172216/OJSV01_FASTA.fasta	MGYA00172216	ERZ490771	root:Host-associated:Human:Digestive system:Large int	0.2146
MGYA00172217/OKFK01_FASTA.fasta	MGYA00172217	ERZ491119	root:Host-associated:Human:Digestive system:Large int	0.3658
MGYA00172218/OKGJ01_FASTA.fasta	MGYA00172218	ERZ491150	root:Host-associated:Human:Digestive system:Large int	0.2628
MGYA00172219/OKMQ01_FASTA.fasta	MGYA00172219	ERZ491284	root:Host-associated:Human:Digestive system:Large int	0.317
MGYA00172220/OKAG01_FASTA.fasta	MGYA00172220	ERZ490984	root:Host-associated:Human:Digestive system:Large int	0.2066
MGYA00172221/OJVY01_FASTA.fasta	MGYA00172221	ERZ490865	root:Host-associated:Human:Digestive system:Large int	0.418
MGYA00172222/OKIZ01_FASTA.fasta	MGYA00172222	ERZ491221	root:Host-associated:Human:Digestive system:Large int	0.305
MGYA00172223/OKEA01_FASTA.fasta	MGYA00172223	ERZ491083	root:Host-associated:Human:Digestive system:Large int	0.3154
MGYA00172224/OKQK01_FASTA.fasta	MGYA00172224	ERZ491410	root:Host-associated:Human:Digestive system:Large int	0.4504
MGYA00172225/OKPE01_FASTA.fasta	MGYA00172225	ERZ491374	root:Host-associated:Human:Digestive system:Large int	0.189
MGYA00172226/OKHQ01_FASTA.fasta	MGYA00172226	ERZ491185	root:Host-associated:Human:Digestive system:Large int	0.29
MGYA00172227/OJSR01_FASTA.fasta	MGYA00172227	ERZ490776	root:Host-associated:Human:Digestive system:Large int	0.3126
MGYA00172228/OJXJ01_FASTA.fasta	MGYA00172228	ERZ490909	root:Host-associated:Human:Digestive system:Large int	0.3048
MGYA00172229/OKJY01_FASTA.fasta	MGYA00172229	ERZ491218	root:Host-associated:Human:Digestive system:Large int	0.2842
MGYA00172230/OJZE01_FASTA.fasta	MGYA00172230	ERZ490959	root:Host-associated:Human:Digestive system:Large int	0.2874
MGYA00172231/OKFZ01_FASTA.fasta	MGYA00172231	ERZ491141	root:Host-associated:Human:Digestive system:Large int	0.2898
MGYA00172232/OKEU01_FASTA.fasta	MGYA00172232	ERZ491109	root:Host-associated:Human:Digestive system:Large int	0.3418
MGYA00172233/OKAN01_FASTA.fasta	MGYA00172233	ERZ490975	root:Host-associated:Human:Digestive system:Large int	0.1832
MGYA00172234/OKOA01_FASTA.fasta	MGYA00172234	ERZ491350	root:Host-associated:Human:Digestive system:Large int	0.2014
MGYA00172235/OKBG01_FASTA.fasta	MGYA00172235	ERZ491017	root:Host-associated:Human:Digestive system:Large int	0.362
MGYA00172236/OKIW01_FASTA.fasta	MGYA00172236	ERZ491212	root:Host-associated:Human:Digestive system:Large int	0.3006
MGYA00172237/OJTT01_FASTA.fasta	MGYA00172237	ERZ490799	root:Host-associated:Human:Digestive system:Large int	0.3856
MGYA00172238/OJUE01_FASTA.fasta	MGYA00172238	ERZ490813	root:Host-associated:Human:Digestive system:Large int	0.3032
MGYA00172239/OJXP01_FASTA.fasta	MGYA00172239	ERZ490911	root:Host-associated:Human:Digestive system:Large int	0.1798
MGYA00172240/OKEE01_FASTA.fasta	MGYA00172240	ERZ491092	root:Host-associated:Human:Digestive system:Large int	0.4018
MGYA00172241/OKLG01_FASTA.fasta	MGYA00172241	ERZ491274	root:Host-associated:Human:Digestive system:Large int	0.2538
MGYA00172242/OKLI01_FASTA.fasta	MGYA00172242	ERZ491280	root:Host-associated:Human:Digestive system:Large int	0.3206
MGYA00172243/OJVE01_FASTA.fasta	MGYA00172243	ERZ490841	root:Host-associated:Human:Digestive system:Large int	0.397
MGYA00172244/OKPC01_FASTA.fasta	MGYA00172244	ERZ491388	root:Host-associated:Human:Digestive system:Large int	0.0676
MGYA00172245/OKGS01_FASTA.fasta	MGYA00172245	ERZ491154	root:Host-associated:Human:Digestive system:Large int	0.3726
MGYA00172246/OJTN01_FASTA.fasta	MGYA00172246	ERZ490797	root:Host-associated:Human:Digestive system:Large int	0.3442
MGYA00172247/OJWD01_FASTA.fasta	MGYA00172247	ERZ490868	root:Host-associated:Human:Digestive system:Large int	0.2352
MGYA00172248/OJWK01_FASTA.fasta	MGYA00172248	ERZ490883	root:Host-associated:Human:Digestive system:Large int	0.2012
MGYA00172249/OKPS01_FASTA.fasta	MGYA00172249	ERZ491395	root:Host-associated:Human:Digestive system:Large int	0.3988
MGYA00172250/OKBP01_FASTA.fasta	MGYA00172250	ERZ491012	root:Host-associated:Human:Digestive system:Large int	0.2422
MGYA00172251/OKDE01_FASTA.fasta	MGYA00172251	ERZ491064	root:Host-associated:Human:Digestive system:Large int	0.4406
MGYA00172252/OLJC01_FASTA.fasta	MGYA00172252	ERZ490790	root:Host-associated:Human:Digestive system:Large int	0.321
MGYA00172253/OKFV01_FASTA.fasta	MGYA00172253	ERZ491133	root:Host-associated:Human:Digestive system:Large int	0.2808
MGYA00172254/OJVN01_FASTA.fasta	MGYA00172254	ERZ490854	root:Host-associated:Human:Digestive system:Large int	0.22
MGYA00172255/OJXF01_FASTA.fasta	MGYA00172255	ERZ490900	root:Host-associated:Human:Digestive system:Large int	0.3862
MGYA00172256/OKGM01_FASTA.fasta	MGYA00172256	ERZ491156	root:Host-associated:Human:Digestive system:Large int	0.3546
MGYA00172257/OKNS01_FASTA.fasta	MGYA00172257	ERZ491327	root:Host-associated:Human:Digestive system:Large int	0.2942

MGYA00172258/OKAP01_FASTA.fasta	MGYA00172258	ERZ490993	root:Host-associated:Human:Digestive system:Large in	0.3304
MGYA00172259/OKEG01_FASTA.fasta	MGYA00172259	ERZ491104	root:Host-associated:Human:Digestive system:Large in	0.3644
MGYA00172260/OJWH01_FASTA.fasta	MGYA00172260	ERZ490870	root:Host-associated:Human:Digestive system:Large in	0.3662
MGYA00172261/OJYN01_FASTA.fasta	MGYA00172261	ERZ490936	root:Host-associated:Human:Digestive system:Large in	0.374
MGYA00172262/OKKO01_FASTA.fasta	MGYA00172262	ERZ491258	root:Host-associated:Human:Digestive system:Large in	0.2826
MGYA00172263/OKGU01_FASTA.fasta	MGYA00172263	ERZ491167	root:Host-associated:Human:Digestive system:Large in	0.1868
MGYA00172264/OKKK01_FASTA.fasta	MGYA00172264	ERZ491256	root:Host-associated:Human:Digestive system:Large in	0.3076
MGYA00172265/OJXH01_FASTA.fasta	MGYA00172265	ERZ490902	root:Host-associated:Human:Digestive system:Large in	0.4326
MGYA00172266/OKCZ01_FASTA.fasta	MGYA00172266	ERZ491052	root:Host-associated:Human:Digestive system:Large in	0.2764
MGYA00172267/OJWY01_FASTA.fasta	MGYA00172267	ERZ490899	root:Host-associated:Human:Digestive system:Large in	0.2202
MGYA00172268/OKBT01_FASTA.fasta	MGYA00172268	ERZ491006	root:Host-associated:Human:Digestive system:Large in	0.4076
MGYA00172269/OKCA01_FASTA.fasta	MGYA00172269	ERZ491034	root:Host-associated:Human:Digestive system:Large in	0.193
MGYA00172270/OKIY01_FASTA.fasta	MGYA00172270	ERZ491214	root:Host-associated:Human:Digestive system:Large in	0.307
MGYA00172271/OKMM01_FASTA.fasta	MGYA00172271	ERZ491302	root:Host-associated:Human:Digestive system:Large in	0.3918
MGYA00172272/OJYK01_FASTA.fasta	MGYA00172272	ERZ490934	root:Host-associated:Human:Digestive system:Large in	0.4164
MGYA00172273/OKPZ01_FASTA.fasta	MGYA00172273	ERZ491406	root:Host-associated:Human:Digestive system:Large in	0.271
MGYA00172274/OKIX01_FASTA.fasta	MGYA00172274	ERZ491217	root:Host-associated:Human:Digestive system:Large in	0.406
MGYA00172275/OKOV01_FASTA.fasta	MGYA00172275	ERZ491372	root:Host-associated:Human:Digestive system:Large in	0.2676
MGYA00172276/OKDK01_FASTA.fasta	MGYA00172276	ERZ491071	root:Host-associated:Human:Digestive system:Large in	0.1568
MGYA00172277/OJWG01_FASTA.fasta	MGYA00172277	ERZ490876	root:Host-associated:Human:Digestive system:Large in	0.4404
MGYA00172278/OKOQ01_FASTA.fasta	MGYA00172278	ERZ491358	root:Host-associated:Human:Digestive system:Large in	0.313
MGYA00172279/OKON01_FASTA.fasta	MGYA00172279	ERZ491365	root:Host-associated:Human:Digestive system:Large in	0.2518
MGYA00172280/OJXQ01_FASTA.fasta	MGYA00172280	ERZ490915	root:Host-associated:Human:Digestive system:Large in	0.2486
MGYA00172281/OKKV01_FASTA.fasta	MGYA00172281	ERZ491277	root:Host-associated:Human:Digestive system:Large in	0.3866
MGYA00172282/OKGZ01_FASTA.fasta	MGYA00172282	ERZ491164	root:Host-associated:Human:Digestive system:Large in	0.3426
MGYA00172283/OJZA01_FASTA.fasta	MGYA00172283	ERZ490951	root:Host-associated:Human:Digestive system:Large in	0.421
MGYA00172284/OLJH01_FASTA.fasta	MGYA00172284	ERZ490832	root:Host-associated:Human:Digestive system:Large in	0.2228
MGYA00172285/OKDN01_FASTA.fasta	MGYA00172285	ERZ491073	root:Host-associated:Human:Digestive system:Large in	0.1522
MGYA00172286/OJYR01_FASTA.fasta	MGYA00172286	ERZ490941	root:Host-associated:Human:Digestive system:Large in	0.3564
MGYA00172287/OKAL01_FASTA.fasta	MGYA00172287	ERZ490988	root:Host-associated:Human:Digestive system:Large in	0.391
MGYA00172288/OJSW01_FASTA.fasta	MGYA00172288	ERZ490773	root:Host-associated:Human:Digestive system:Large in	0.2606
MGYA00172289/OKDA01_FASTA.fasta	MGYA00172289	ERZ491061	root:Host-associated:Human:Digestive system:Large in	0.2792
MGYA00172290/OJZV01_FASTA.fasta	MGYA00172290	ERZ490979	root:Host-associated:Human:Digestive system:Large in	0.253
MGYA00172291/OKDR01_FASTA.fasta	MGYA00172291	ERZ491081	root:Host-associated:Human:Digestive system:Large in	0.4914
MGYA00172292/OKIT01_FASTA.fasta	MGYA00172292	ERZ491213	root:Host-associated:Human:Digestive system:Large in	0.3102
MGYA00172293/OKIV01_FASTA.fasta	MGYA00172293	ERZ491215	root:Host-associated:Human:Digestive system:Large in	0.3266
MGYA00172294/OKCL01_FASTA.fasta	MGYA00172294	ERZ491049	root:Host-associated:Human:Digestive system:Large in	0.324
MGYA00172295/OLJE01_FASTA.fasta	MGYA00172295	ERZ490806	root:Host-associated:Human:Digestive system:Large in	0.39
MGYA00172296/OJVP01_FASTA.fasta	MGYA00172296	ERZ490837	root:Host-associated:Human:Digestive system:Large in	0.2008
MGYA00172297/OJUP01_FASTA.fasta	MGYA00172297	ERZ490830	root:Host-associated:Human:Digestive system:Large in	0.174
MGYA00172298/OKBO01_FASTA.fasta	MGYA00172298	ERZ491023	root:Host-associated:Human:Digestive system:Large in	0.3124
MGYA00172299/OJYJ01_FASTA.fasta	MGYA00172299	ERZ490933	root:Host-associated:Human:Digestive system:Large in	0.3332
MGYA00172300/OKMS01_FASTA.fasta	MGYA00172300	ERZ491322	root:Host-associated:Human:Digestive system:Large in	0.3078
MGYA00172301/OKCN01_FASTA.fasta	MGYA00172301	ERZ491039	root:Host-associated:Human:Digestive system:Large in	0.1626
MGYA00172302/OJSY01_FASTA.fasta	MGYA00172302	ERZ490772	root:Host-associated:Human:Digestive system:Large in	0.3222
MGYA00172303/OJVZ01_FASTA.fasta	MGYA00172303	ERZ490866	root:Host-associated:Human:Digestive system:Large in	0.2382
MGYA00172304/OJVM01_FASTA.fasta	MGYA00172304	ERZ490847	root:Host-associated:Human:Digestive system:Large in	0.3674
MGYA00172305/OJVF01_FASTA.fasta	MGYA00172305	ERZ490845	root:Host-associated:Human:Digestive system:Large in	0.2214
MGYA00172306/OKCB01_FASTA.fasta	MGYA00172306	ERZ491033	root:Host-associated:Human:Digestive system:Large in	0.311
MGYA00172307/OKEN01_FASTA.fasta	MGYA00172307	ERZ491105	root:Host-associated:Human:Digestive system:Large in	0.2174
MGYA00172308/OKNF01_FASTA.fasta	MGYA00172308	ERZ491329	root:Host-associated:Human:Digestive system:Large in	0.3034
MGYA00172309/OKND01_FASTA.fasta	MGYA00172309	ERZ491328	root:Host-associated:Human:Digestive system:Large in	0.0414
MGYA00172310/OKQH01_FASTA.fasta	MGYA00172310	ERZ491405	root:Host-associated:Human:Digestive system:Large in	0.294
MGYA00172311/OJZK01_FASTA.fasta	MGYA00172311	ERZ490953	root:Host-associated:Human:Digestive system:Large in	0.2576
MGYA00172312/OKPB01_FASTA.fasta	MGYA00172312	ERZ491381	root:Host-associated:Human:Digestive system:Large in	0.2936
MGYA00172313/OJZU01_FASTA.fasta	MGYA00172313	ERZ490978	root:Host-associated:Human:Digestive system:Large in	0.29
MGYA00172314/OKPR01_FASTA.fasta	MGYA00172314	ERZ491394	root:Host-associated:Human:Digestive system:Large in	0.1792
MGYA00172315/OJVS01_FASTA.fasta	MGYA00172315	ERZ490859	root:Host-associated:Human:Digestive system:Large in	0.363
MGYA00172316/OKBI01_FASTA.fasta	MGYA00172316	ERZ491010	root:Host-associated:Human:Digestive system:Large in	0.3138
MGYA00172317/OJUK01_FASTA.fasta	MGYA00172317	ERZ490822	root:Host-associated:Human:Digestive system:Large in	0.2488
MGYA00172318/OKJD01_FASTA.fasta	MGYA00172318	ERZ491220	root:Host-associated:Human:Digestive system:Large in	0.29
MGYA00172319/OKII01_FASTA.fasta	MGYA00172319	ERZ491198	root:Host-associated:Human:Digestive system:Large in	0.1602
MGYA00172320/OKNG01_FASTA.fasta	MGYA00172320	ERZ491333	root:Host-associated:Human:Digestive system:Large in	0.4088
MGYA00172321/OKPQ01_FASTA.fasta	MGYA00172321	ERZ491384	root:Host-associated:Human:Digestive system:Large in	0.4322
MGYA00172322/OKED01_FASTA.fasta	MGYA00172322	ERZ491089	root:Host-associated:Human:Digestive system:Large in	0.2454
MGYA00172323/OKKT01_FASTA.fasta	MGYA00172323	ERZ491263	root:Host-associated:Human:Digestive system:Large in	0.3442
MGYA00172324/OKJG01_FASTA.fasta	MGYA00172324	ERZ491222	root:Host-associated:Human:Digestive system:Large in	0.2694
MGYA00172325/OKBF01_FASTA.fasta	MGYA00172325	ERZ491008	root:Host-associated:Human:Digestive system:Large in	0.3228
MGYA00172326/OKAU01_FASTA.fasta	MGYA00172326	ERZ490995	root:Host-associated:Human:Digestive system:Large in	0.2158
MGYA00172327/OJXU01_FASTA.fasta	MGYA00172327	ERZ490917	root:Host-associated:Human:Digestive system:Large in	0.3078
MGYA00172328/OJST01_FASTA.fasta	MGYA00172328	ERZ490777	root:Host-associated:Human:Digestive system:Large in	0.451
MGYA00172329/OKHH01_FASTA.fasta	MGYA00172329	ERZ491178	root:Host-associated:Human:Digestive system:Large in	0.2134
MGYA00172330/OKGN01_FASTA.fasta	MGYA00172330	ERZ491158	root:Host-associated:Human:Digestive system:Large in	0.374
MGYA00172331/OKKU01_FASTA.fasta	MGYA00172331	ERZ491269	root:Host-associated:Human:Digestive system:Large in	0.4104
MGYA00172332/OJTJ01_FASTA.fasta	MGYA00172332	ERZ490789	root:Host-associated:Human:Digestive system:Large in	0.1802
MGYA00172333/OKDH01_FASTA.fasta	MGYA00172333	ERZ491069	root:Host-associated:Human:Digestive system:Large in	0.4116
MGYA00172334/OJVU01_FASTA.fasta	MGYA00172334	ERZ490862	root:Host-associated:Human:Digestive system:Large in	0.2088
MGYA00172335/OKEP01_FASTA.fasta	MGYA00172335	ERZ491106	root:Host-associated:Human:Digestive system:Large in	0.4306
MGYA00172336/OJVQ01_FASTA.fasta	MGYA00172336	ERZ490856	root:Host-associated:Human:Digestive system:Large in	0.2954
MGYA00172337/OKLP01_FASTA.fasta	MGYA00172337	ERZ491286	root:Host-associated:Human:Digestive system:Large in	0.3238
MGYA00172338/OKJO01_FASTA.fasta	MGYA00172338	ERZ491235	root:Host-associated:Human:Digestive system:Large in	0.3202
MGYA00172339/OJXY01_FASTA.fasta	MGYA00172339	ERZ490923	root:Host-associated:Human:Digestive system:Large in	0.1774
MGYA00172340/OKIJ01_FASTA.fasta	MGYA00172340	ERZ491202	root:Host-associated:Human:Digestive system:Large in	0.2076
MGYA00172341/OJYY01_FASTA.fasta	MGYA00172341	ERZ490948	root:Host-associated:Human:Digestive system:Large in	0.3066
MGYA00172342/OKKX01_FASTA.fasta	MGYA00172342	ERZ491275	root:Host-associated:Human:Digestive system:Large in	0.3296
MGYA00172343/OKAQ01_FASTA.fasta	MGYA00172343	ERZ490994	root:Host-associated:Human:Digestive system:Large in	0.2954
MGYA00172344/OKQG01_FASTA.fasta	MGYA00172344	ERZ491412	root:Host-associated:Human:Digestive system:Large in	0.151
MGYA00172345/OKCX01_FASTA.fasta	MGYA00172345	ERZ491058	root:Host-associated:Human:Digestive system:Large in	0.402
MGYA00172346/OKOX01_FASTA.fasta	MGYA00172346	ERZ491368	root:Host-associated:Human:Digestive system:Large in	0.4362
MGYA00172347/OKJZ01_FASTA.fasta	MGYA00172347	ERZ491244	root:Host-associated:Human:Digestive system:Large in	0.257
MGYA00172348/OJUZ01_FASTA.fasta	MGYA00172348	ERZ490843	root:Host-associated:Human:Digestive system:Large in	0.2052
MGYA00172349/OJXG01_FASTA.fasta	MGYA00172349	ERZ490906	root:Host-associated:Human:Digestive system:Large in	0.2998
MGYA00172350/OKJP01_FASTA.fasta	MGYA00172350	ERZ491236	root:Host-associated:Human:Digestive system:Large in	0.1414
MGYA00172351/OKOU01_FASTA.fasta	MGYA00172351	ERZ491371	root:Host-associated:Human:Digestive system:Large in	0.2174
MGYA00172352/OKCI01_FASTA.fasta	MGYA00172352	ERZ491042	root:Host-associated:Human:Digestive system:Large in	0.2698
MGYA00172353/OKDY01_FASTA.fasta	MGYA00172353	ERZ491088	root:Host-associated:Human:Digestive system:Large in	0.2884
MGYA00172354/OKOH01_FASTA.fasta	MGYA00172354	ERZ491359	root:Host-associated:Human:Digestive system:Large in	0.2614
MGYA00172355/OKNI01_FASTA.fasta	MGYA00172355	ERZ491334	root:Host-associated:Human:Digestive system:Large in	0.3414
MGYA00172356/OKGG01_FASTA.fasta	MGYA00172356	ERZ491139	root:Host-associated:Human:Digestive system:Large in	0.2362
MGYA00172357/OKHO01_FASTA.fasta	MGYA00172357	ERZ491181	root:Host-associated:Human:Digestive system:Large in	0.3046
MGYA00172358/OKMB01_FASTA.fasta	MGYA00172358	ERZ491306	root:Host-associated:Human:Digestive system:Large in	0.2036
MGYA00172359/OJUL01_FASTA.fasta	MGYA00172359	ERZ490818	root:Host-associated:Human:Digestive system:Large in	0.4264
MGYA00172360/OKJX01_FASTA.fasta	MGYA00172360	ERZ491242	root:Host-associated:Human:Digestive system:Large in	0.2696
MGYA00172361/OJVT01_FASTA.fasta	MGYA00172361	ERZ490860	root:Host-associated:Human:Digestive system:Large in	0.3158
MGYA00172362/OJVK01_FASTA.fasta	MGYA00172362	ERZ490852	root:Host-associated:Human:Digestive system:Large in	0.3198
MGYA00172363/OKJR01_FASTA.fasta	MGYA00172363	ERZ491234	root:Host-associated:Human:Digestive system:Large in	0.2428
MGYA00172364/OKPN01_FASTA.fasta	MGYA00172364	ERZ491391	root:Host-associated:Human:Digestive system:Large in	0.3688
MGYA00172365/OJYG01_FASTA.fasta	MGYA00172365	ERZ490926	root:Host-associated:Human:Digestive system:Large in	0.3768
MGYA00172366/OJXX01_FASTA.fasta	MGYA00172366	ERZ490919	root:Host-associated:Human:Digestive system:Large in	0.2646
MGYA00172367/OKFA01_FASTA.fasta	MGYA00172367	ERZ491112	root:Host-associated:Human:Digestive system:Large in	0.3866
MGYA00172368/OKFO01_FASTA.fasta	MGYA00172368	ERZ491117	root:Host-associated:Human:Digestive system:Large in	0.3956
MGYA00172369/OKCJ01_FASTA.fasta	MGYA00172369	ERZ491041	root:Host-associated:Human:Digestive system:Large in	0.3096
MGYA00172370/OJUX01_FASTA.fasta	MGYA00172370	ERZ490821	root:Host-associated:Human:Digestive system:Large in	0.4048
MGYA00172371/OJTE01_FASTA.fasta	MGYA00172371	ERZ490783	root:Host-associated:Human:Digestive system:Large in	0.3636
MGYA00172372/OJUB01_FASTA.fasta	MGYA00172372	ERZ490816	root:Host-associated:Human:Digestive system:Large in	0.3292
MGYA00172373/OKEB01_FASTA.fasta	MGYA00172373	ERZ491085	root:Host-associated:Human:Digestive system:Large in	0.2216
MGYA00172374/OKBS01_FASTA.fasta	MGYA00172374	ERZ491020	root:Host-associated:Human:Digestive system:Large in	0.4024
MGYA00172375/OKLT01_FASTA.fasta	MGYA00172375	ERZ491294	root:Host-associated:Human:Digestive system:Large in	0.3196
MGYA00172376/OLJU01_FASTA.fasta	MGYA00172376	ERZ491045	root:Host-associated:Human:Digestive system:Large in	0.149
MGYA00172377/OKJS01_FASTA.fasta	MGYA00172377	ERZ491239	root:Host-associated:Human:Digestive system:Large in	0.1972
MGYA00172378/OJXI01_FASTA.fasta	MGYA00172378	ERZ490908	root:Host-associated:Human:Digestive system:Large in	0.2852
MGYA00172379/OKPL01_FASTA.fasta	MGYA00172379	ERZ491386	root:Host-associated:Human:Digestive system:Large in	0.3602
MGYA00172380/OKPY01_FASTA.fasta	MGYA00172380	ERZ491408	root:Host-associated:Human:Digestive system:Large in	0.2128
MGYA00172381/OKBK01_FASTA.fasta	MGYA00172381	ERZ491009	root:Host-associated:Human:Digestive system:Large in	0.2148
MGYA00172382/OJWO01_FASTA.fasta	MGYA00172382	ERZ490880	root:Host-associated:Human:Digestive system:Large in	0.3268
MGYA00172383/OKMR01_FASTA.fasta	MGYA00172383	ERZ491316	root:Host-associated:Human:Digestive system:Large in	0.2096
MGYA00172384/OKIB01_FASTA.fasta	MGYA00172384	ERZ491199	root:Host-associated:Human:Digestive system:Large in	0.4004
MGYA00172385/OKPU01_FASTA.fasta	MGYA00172385	ERZ491396	root:Host-associated:Human:Digestive system:Large in	0.4186
MGYA00172386/OKKA01_FASTA.fasta	MGYA00172386	ERZ491252	root:Host-associated:Human:Digestive system:Large in	0.2518
MGYA00172387/OKOC01_FASTA.fasta	MGYA00172387	ERZ491352	root:Host-associated:Human:Digestive system:Large in	0.4136
MGYA00172388/OKPO01_FASTA.fasta	MGYA00172388	ERZ491392	root:Host-associated:Human:Digestive system:Large in	0.1812
MGYA00172389/OKMT01_FASTA.fasta	MGYA00172389	ERZ491319	root:Host-associated:Human:Digestive system:Large in	0.268
MGYA00172390/OJWX01_FASTA.fasta	MGYA00172390	ERZ490896	root:Host-associated:Human:Digestive system:Large in	0.2398
MGYA00172391/OKJV01_FASTA.fasta	MGYA00172391	ERZ491238	root:Host-associated:Human:Digestive system:Large in	0.336
MGYA00172392/OJZZ01_FASTA.fasta	MGYA00172392	ERZ490972	root:Host-associated:Human:Digestive system:Large in	0.3098
MGYA00172393/OKNK01_FASTA.fasta	MGYA00172393	ERZ491337	root:Host-associated:Human:Digestive system:Large in	0.2
MGYA00172394/OJZQ01_FASTA.fasta	MGYA00172394	ERZ490965	root:Host-associated:Human:Digestive system:Large in	0.4462
MGYA00172395/OJTK01_FASTA.fasta	MGYA00172395	ERZ490795	root:Host-associated:Human:Digestive system:Large in	0.3442
MGYA00172396/OJYE01_FASTA.fasta	MGYA00172396	ERZ490928	root:Host-associated:Human:Digestive system:Large in	0.3538
MGYA00172397/OKAE01_FASTA.fasta	MGYA00172397	ERZ490969	root:Host-associated:Human:Digestive system:Large in	0.306
MGYA00172398/OKJN01_FASTA.fasta	MGYA00172398	ERZ491230	root:Host-associated:Human:Digestive system:Large in	0.2008
MGYA00172399/OKMY01_FASTA.fasta	MGYA00172399	ERZ491317	root:Host-associated:Human:Digestive system:Large in	0.401
MGYA00172400/OJWT01_FASTA.fasta	MGYA00172400	ERZ490888	root:Host-associated:Human:Digestive system:Large in	0.183
MGYA00172401/OJXO01_FASTA.fasta	MGYA00172401	ERZ490914	root:Host-associated:Human:Digestive system:Large in	0.359
MGYA00172402/OKBL01_FASTA.fasta	MGYA00172402	ERZ491018	root:Host-associated:Human:Digestive system:Large in	0.3544
MGYA00172403/OKDT01_FASTA.fasta	MGYA00172403	ERZ491072	root:Host-associated:Human:Digestive system:Large in	0.3272
MGYA00172404/OJTU01_FASTA.fasta	MGYA00172404	ERZ490803	root:Host-associated:Human:Digestive system:Large in	0.3096
MGYA00172405/OKQD01_FASTA.fasta	MGYA00172405	ERZ491402	root:Host-associated:Human:Digestive system:Large in	0.3552
MGYA00172406/OJUA01_FASTA.fasta	MGYA00172406	ERZ490807	root:Host-associated:Human:Digestive system:Large in	0.3872
MGYA00172407/OLIZ01_FASTA.fasta	MGYA00172407	ERZ490781	root:Host-associated:Human:Digestive system:Large in	0.2732
MGYA00172408/OKOG01_FASTA.fasta	MGYA00172408	ERZ491356	root:Host-associated:Human:Digestive system:Large in	0.3394
MGYA00172409/OKHE01_FASTA.fasta	MGYA00172409	ERZ491162	root:Host-associated:Human:Digestive system:Large in	0.437
MGYA00172410/OKJU01_FASTA.fasta	MGYA00172410	ERZ491240	root:Host-associated:Human:Digestive system:Large in	0.3668
MGYA00172411/OKMF01_FASTA.fasta	MGYA00172411	ERZ491308	root:Host-associated:Human:Digestive system:Large in	0.3304
MGYA00172412/OKGX01_FASTA.fasta	MGYA00172412	ERZ491163	root:Host-associated:Human:Digestive system:Large in	0.2526
MGYA00172413/OJZO01_FASTA.fasta	MGYA00172413	ERZ490968	root:Host-associated:Human:Digestive system:Large in	0.3146
MGYA00172414/OLJZ01_FASTA.fasta	MGYA00172414	ERZ491107	root:Host-associated:Human:Digestive system:Large in	0.1992
MGYA00172415/OKLC01_FASTA.fasta	MGYA00172415	ERZ491279	root:Host-associated:Human:Digestive system:Large in	0.3016
MGYA00172416/OKFE01_FASTA.fasta	MGYA00172416	ERZ491116	root:Host-associated:Human:Digestive system:Large in	0.4062
MGYA00172417/OKKN01_FASTA.fasta	MGYA00172417	ERZ491250	root:Host-associated:Human:Digestive system:Large in	0.405
MGYA00172418/OJYQ01_FASTA.fasta	MGYA00172418	ERZ490939	root:Host-associated:Human:Digestive system:Large in	0.1992
MGYA00172419/OKQJ01_FASTA.fasta	MGYA00172419	ERZ491411	root:Host-associated:Human:Digestive system:Large in	0.3752
MGYA00172420/OKEK01_FASTA.fasta	MGYA00172420	ERZ491098	root:Host-associated:Human:Digestive system:Large in	0.217
MGYA00172421/OKGP01_FASTA.fasta	MGYA00172421	ERZ491155	root:Host-associated:Human:Digestive system:Large in	0.2096
MGYA00172422/OKDP01_FASTA.fasta	MGYA00172422	ERZ491078	root:Host-associated:Human:Digestive system:Large in	0.3132
MGYA00172423/OKNO01_FASTA.fasta	MGYA00172423	ERZ491332	root:Host-associated:Human:Digestive system:Large in	0.31
MGYA00172424/OKCC01_FASTA.fasta	MGYA00172424	ERZ491030	root:Host-associated:Human:Digestive system:Large in	0.301
MGYA00172425/OKHB01_FASTA.fasta	MGYA00172425	ERZ491170	root:Host-associated:Human:Digestive system:Large in	0.247
MGYA00172426/OKJJ01_FASTA.fasta	MGYA00172426	ERZ491233	root:Host-associated:Human:Digestive system:Large in	0.3186
MGYA00172427/OLJS01_FASTA.fasta	MGYA00172427	ERZ491011	root:Host-associated:Human:Digestive system:Large in	0.2178
MGYA00172428/OKFN01_FASTA.fasta	MGYA00172428	ERZ491129	root:Host-associated:Human:Digestive system:Large in	0.2124
MGYA00172429/OKDQ01_FASTA.fasta	MGYA00172429	ERZ491082	root:Host-associated:Human:Digestive system:Large in	0.3374
MGYA00172430/OKOM01_FASTA.fasta	MGYA00172430	ERZ491366	root:Host-associated:Human:Digestive system:Large in	0.286
MGYA00172431/OJXT01_FASTA.fasta	MGYA00172431	ERZ490912	root:Host-associated:Human:Digestive system:Large in	0.3194
MGYA00172432/OJVO01_FASTA.fasta	MGYA00172432	ERZ490849	root:Host-associated:Human:Digestive system:Large in	0.3262
MGYA00172433/OKET01_FASTA.fasta	MGYA00172433	ERZ491094	root:Host-associated:Human:Digestive system:Large in	0.33
MGYA00172434/OKBC01_FASTA.fasta	MGYA00172434	ERZ491015	root:Host-associated:Human:Digestive system:Large in	0.3588
MGYA00172435/OKLN01_FASTA.fasta	MGYA00172435	ERZ491268	root:Host-associated:Human:Digestive system:Large in	0.303
MGYA00172436/OKFL01_FASTA.fasta	MGYA00172436	ERZ491125	root:Host-associated:Human:Digestive system:Large in	0.2534
MGYA00172437/OJXS01_FASTA.fasta	MGYA00172437	ERZ490916	root:Host-associated:Human:Digestive system:Large in	0.4194
MGYA00172438/OKPV01_FASTA.fasta	MGYA00172438	ERZ491409	root:Host-associated:Human:Digestive system:Large in	0.3318
MGYA00172439/OJTY01_FASTA.fasta	MGYA00172439	ERZ490809	root:Host-associated:Human:Digestive system:Large in	0.4028
MGYA00172440/OKQC01_FASTA.fasta	MGYA00172440	ERZ491407	root:Host-associated:Human:Digestive system:Large in	0.3834
MGYA00172441/OKEJ01_FASTA.fasta	MGYA00172441	ERZ491091	root:Host-associated:Human:Digestive system:Large in	0.2444
MGYA00172442/OKKM01_FASTA.fasta	MGYA00172442	ERZ491260	root:Host-associated:Human:Digestive system:Large in	0.2996
MGYA00172443/OJXK01_FASTA.fasta	MGYA00172443	ERZ490897	root:Host-associated:Human:Digestive system:Large in	0.3998
MGYA00172444/OKGI01_FASTA.fasta	MGYA00172444	ERZ491148	root:Host-associated:Human:Digestive system:Large in	0.2056
MGYA00172445/OJZT01_FASTA.fasta	MGYA00172445	ERZ490971	root:Host-associated:Human:Digestive system:Large in	0.2102
MGYA00172446/OKPI01_FASTA.fasta	MGYA00172446	ERZ491380	root:Host-associated:Human:Digestive system:Large in	0.317
MGYA00172447/OKLJ01_FASTA.fasta	MGYA00172447	ERZ491281	root:Host-associated:Human:Digestive system:Large in	0.25
MGYA00172448/OKFY01_FASTA.fasta	MGYA00172448	ERZ491143	root:Host-associated:Human:Digestive system:Large in	0.3218
MGYA00172449/OJZC01_FASTA.fasta	MGYA00172449	ERZ490960	root:Host-associated:Human:Digestive system:Large in	0.2412
MGYA00172450/OKOT01_FASTA.fasta	MGYA00172450	ERZ491367	root:Host-associated:Human:Digestive system:Large in	0.1416
MGYA00172451/OKOI01_FASTA.fasta	MGYA00172451	ERZ491353	root:Host-associated:Human:Digestive system:Large in	0.2214
MGYA00172452/OJZF01_FASTA.fasta	MGYA00172452	ERZ490957	root:Host-associated:Human:Digestive system:Large in	0.1838
MGYA00172453/OKAT01_FASTA.fasta	MGYA00172453	ERZ490999	root:Host-associated:Human:Digestive system:Large in	0.3426
MGYA00172454/OJUJ01_FASTA.fasta	MGYA00172454	ERZ490819	root:Host-associated:Human:Digestive system:Large in	0.3914
MGYA00172455/OKBD01_FASTA.fasta	MGYA00172455	ERZ491016	root:Host-associated:Human:Digestive system:Large in	0.3522
MGYA00172456/OJUF01_FASTA.fasta	MGYA00172456	ERZ490815	root:Host-associated:Human:Digestive system:Large in	0.4022
MGYA00172457/OKOD01_FASTA.fasta	MGYA00172457	ERZ491363	root:Host-associated:Human:Digestive system:Large in	0.065
MGYA00172458/OJWM01_FASTA.fasta	MGYA00172458	ERZ490885	root:Host-associated:Human:Digestive system:Large in	0.2882
MGYA00172459/OKOY01_FASTA.fasta	MGYA00172459	ERZ491370	root:Host-associated:Human:Digestive system:Large in	0.2014
MGYA00172460/OJVL01_FASTA.fasta	MGYA00172460	ERZ490851	root:Host-associated:Human:Digestive system:Large in	0.331
MGYA00172461/OKNB01_FASTA.fasta	MGYA00172461	ERZ491324	root:Host-associated:Human:Digestive system:Large in	0.3092
MGYA00172462/OKDC01_FASTA.fasta	MGYA00172462	ERZ491067	root:Host-associated:Human:Digestive system:Large in	0.2754
MGYA00172463/OJUW01_FASTA.fasta	MGYA00172463	ERZ490833	root:Host-associated:Human:Digestive system:Large in	0.4034
MGYA00172464/OKHK01_FASTA.fasta	MGYA00172464	ERZ491173	root:Host-associated:Human:Digestive system:Large in	0.3668
MGYA00172465/OKJT01_FASTA.fasta	MGYA00172465	ERZ491241	root:Host-associated:Human:Digestive system:Large in	0.2326
MGYA00172466/OJTM01_FASTA.fasta	MGYA00172466	ERZ490796	root:Host-associated:Human:Digestive system:Large in	0.3212
MGYA00172467/OKBU01_FASTA.fasta	MGYA00172467	ERZ491027	root:Host-associated:Human:Digestive system:Large in	0.304
MGYA00172468/OJWF01_FASTA.fasta	MGYA00172468	ERZ490872	root:Host-associated:Human:Digestive system:Large in	0.1916
MGYA00172469/OKME01_FASTA.fasta	MGYA00172469	ERZ491303	root:Host-associated:Human:Digestive system:Large in	0.1096
MGYA00172470/OLJG01_FASTA.fasta	MGYA00172470	ERZ490823	root:Host-associated:Human:Digestive system:Large in	0.3514
MGYA00172471/OJXD01_FASTA.fasta	MGYA00172471	ERZ490905	root:Host-associated:Human:Digestive system:Large in	0.3566
MGYA00172472/OLJX01_FASTA.fasta	MGYA00172472	ERZ491253	root:Host-associated:Human:Digestive system:Large in	0.3038
MGYA00172473/OKGW01_FASTA.fasta	MGYA00172473	ERZ491161	root:Host-associated:Human:Digestive system:Large in	0.2042
MGYA00172474/OJYP01_FASTA.fasta	MGYA00172474	ERZ490940	root:Host-associated:Human:Digestive system:Large in	0.2728
MGYA00172475/OKIG01_FASTA.fasta	MGYA00172475	ERZ491194	root:Host-associated:Human:Digestive system:Large in	0.2836
MGYA00172476/OJTR01_FASTA.fasta	MGYA00172476	ERZ490800	root:Host-associated:Human:Digestive system:Large in	0.2572
MGYA00172477/OJWU01_FASTA.fasta	MGYA00172477	ERZ490882	root:Host-associated:Human:Digestive system:Large in	0.3356
MGYA00172478/OKDS01_FASTA.fasta	MGYA00172478	ERZ491074	root:Host-associated:Human:Digestive system:Large in	0.2554
MGYA00172479/OKBR01_FASTA.fasta	MGYA00172479	ERZ491024	root:Host-associated:Human:Digestive system:Large in	0.2586
MGYA00172480/OLJL01_FASTA.fasta	MGYA00172480	ERZ490864	root:Host-associated:Human:Digestive system:Large in	0.1536
MGYA00172481/OKNL01_FASTA.fasta	MGYA00172481	ERZ491339	root:Host-associated:Human:Digestive system:Large in	0.2102
MGYA00172482/OJZB01_FASTA.fasta	MGYA00172482	ERZ490950	root:Host-associated:Human:Digestive system:Large in	0.3222
MGYA00172483/OJXB01_FASTA.fasta	MGYA00172483	ERZ490904	root:Host-associated:Human:Digestive system:Large in	0.3568
MGYA00172484/OKCO01_FASTA.fasta	MGYA00172484	ERZ491046	root:Host-associated:Human:Digestive system:Large in	0.3912
MGYA00172485/OKKD01_FASTA.fasta	MGYA00172485	ERZ491247	root:Host-associated:Human:Digestive system:Large in	0.3316
MGYA00172486/OKQA01_FASTA.fasta	MGYA00172486	ERZ491397	root:Host-associated:Human:Digestive system:Large in	0.2662
MGYA00172487/OKES01_FASTA.fasta	MGYA00172487	ERZ491097	root:Host-associated:Human:Digestive system:Large in	0.2176
MGYA00172488/OKBQ01_FASTA.fasta	MGYA00172488	ERZ491013	root:Host-associated:Human:Digestive system:Large in	0.0998
MGYA00172489/OKPW01_FASTA.fasta	MGYA00172489	ERZ491401	root:Host-associated:Human:Digestive system:Large in	0.2902
MGYA00172490/OKLL01_FASTA.fasta	MGYA00172490	ERZ491287	root:Host-associated:Human:Digestive system:Large in	0.4152
MGYA00172491/OKPX01_FASTA.fasta	MGYA00172491	ERZ491398	root:Host-associated:Human:Digestive system:Large in	0.5234
MGYA00172492/OKHR01_FASTA.fasta	MGYA00172492	ERZ491188	root:Host-associated:Human:Digestive system:Large in	0.3096
MGYA00172493/OKMU01_FASTA.fasta	MGYA00172493	ERZ491321	root:Host-associated:Human:Digestive system:Large in	0.2038
MGYA00172494/OKOO01_FASTA.fasta	MGYA00172494	ERZ491357	root:Host-associated:Human:Digestive system:Large in	0.2116
MGYA00172495/OJWB01_FASTA.fasta	MGYA00172495	ERZ490873	root:Host-associated:Human:Digestive system:Large in	0.3446
MGYA00172496/OKDG01_FASTA.fasta	MGYA00172496	ERZ491066	root:Host-associated:Human:Digestive system:Large in	0.1564
MGYA00172497/OKHX01_FASTA.fasta	MGYA00172497	ERZ491192	root:Host-associated:Human:Digestive system:Large in	0.2042
MGYA00172498/OLJT01_FASTA.fasta	MGYA00172498	ERZ490996	root:Host-associated:Human:Digestive system:Large in	0.208
MGYA00172499/OJXR01_FASTA.fasta	MGYA00172499	ERZ490913	root:Host-associated:Human:Digestive system:Large in	0.2206
MGYA00172500/OJTB01_FASTA.fasta	MGYA00172500	ERZ490779	root:Host-associated:Human:Digestive system:Large in	0.3206
MGYA00172501/OKDB01_FASTA.fasta	MGYA00172501	ERZ491056	root:Host-associated:Human:Digestive system:Large in	0.3594
MGYA00172502/OKIC01_FASTA.fasta	MGYA00172502	ERZ491187	root:Host-associated:Human:Digestive system:Large in	0.3856
MGYA00172503/OKGO01_FASTA.fasta	MGYA00172503	ERZ491152	root:Host-associated:Human:Digestive system:Large in	0.4632
MGYA00172504/OKLW01_FASTA.fasta	MGYA00172504	ERZ491297	root:Host-associated:Human:Digestive system:Large in	0.3536
MGYA00172505/OKMW01_FASTA.fasta	MGYA00172505	ERZ491318	root:Host-associated:Human:Digestive system:Large in	0.3924
MGYA00172506/OKKG01_FASTA.fasta	MGYA00172506	ERZ491255	root:Host-associated:Human:Digestive system:Large in	0.4156
MGYA00172507/OKOW01_FASTA.fasta	MGYA00172507	ERZ491369	root:Host-associated:Human:Digestive system:Large in	0.2548
MGYA00172508/OJWC01_FASTA.fasta	MGYA00172508	ERZ490869	root:Host-associated:Human:Digestive system:Large in	0.3672
MGYA00172509/OKLV01_FASTA.fasta	MGYA00172509	ERZ491299	root:Host-associated:Human:Digestive system:Large in	0.3128
MGYA00172510/OKFU01_FASTA.fasta	MGYA00172510	ERZ491135	root:Host-associated:Human:Digestive system:Large in	0.4496
MGYA00172511/OKCD01_FASTA.fasta	MGYA00172511	ERZ491038	root:Host-associated:Human:Digestive system:Large in	0.3948
MGYA00172512/OKDV01_FASTA.fasta	MGYA00172512	ERZ491086	root:Host-associated:Human:Digestive system:Large in	0.1952
MGYA00172513/OLJN01_FASTA.fasta	MGYA00172513	ERZ490895	root:Host-associated:Human:Digestive system:Large in	0.0716
MGYA00172514/OKAH01_FASTA.fasta	MGYA00172514	ERZ490985	root:Host-associated:Human:Digestive system:Large in	0.292
MGYA00172515/OKEZ01_FASTA.fasta	MGYA00172515	ERZ491110	root:Host-associated:Human:Digestive system:Large in	0.3366
MGYA00172516/OKBJ01_FASTA.fasta	MGYA00172516	ERZ491019	root:Host-associated:Human:Digestive system:Large in	0.1956
MGYA00172517/OLJK01_FASTA.fasta	MGYA00172517	ERZ490893	root:Host-associated:Human:Digestive system:Large in	0.2832
MGYA00172518/OKNX01_FASTA.fasta	MGYA00172518	ERZ491343	root:Host-associated:Human:Digestive system:Large in	0.3212
MGYA00172519/OJZW01_FASTA.fasta	MGYA00172519	ERZ490977	root:Host-associated:Human:Digestive system:Large in	0.2178
MGYA00172520/OKGH01_FASTA.fasta	MGYA00172520	ERZ491147	root:Host-associated:Human:Digestive system:Large in	0.2554
MGYA00172521/OKJA01_FASTA.fasta	MGYA00172521	ERZ491219	root:Host-associated:Human:Digestive system:Large in	0.342
MGYA00172522/OLKB01_FASTA.fasta	MGYA00172522	ERZ491196	root:Host-associated:Human:Digestive system:Large in	0.1344
MGYA00172523/OKKR01_FASTA.fasta	MGYA00172523	ERZ491265	root:Host-associated:Human:Digestive system:Large in	0.344
MGYA00172524/OJWZ01_FASTA.fasta	MGYA00172524	ERZ490898	root:Host-associated:Human:Digestive system:Large in	0.387
MGYA00172525/OJZG01_FASTA.fasta	MGYA00172525	ERZ490955	root:Host-associated:Human:Digestive system:Large in	0.1678
MGYA00172526/OKPK01_FASTA.fasta	MGYA00172526	ERZ491389	root:Host-associated:Human:Digestive system:Large in	0.342
MGYA00172527/OJUT01_FASTA.fasta	MGYA00172527	ERZ490831	root:Host-associated:Human:Digestive system:Large in	0.3282
MGYA00172528/OKGV01_FASTA.fasta	MGYA00172528	ERZ491159	root:Host-associated:Human:Digestive system:Large in	0.1962
MGYA00172529/OKKS01_FASTA.fasta	MGYA00172529	ERZ491264	root:Host-associated:Human:Digestive system:Large in	0.251
MGYA00172530/OKJQ01_FASTA.fasta	MGYA00172530	ERZ491237	root:Host-associated:Human:Digestive system:Large in	0.2408
MGYA00172531/OKHY01_FASTA.fasta	MGYA00172531	ERZ491191	root:Host-associated:Human:Digestive system:Large in	0.3722
MGYA00172532/OKBN01_FASTA.fasta	MGYA00172532	ERZ491022	root:Host-associated:Human:Digestive system:Large in	0.3144
MGYA00172533/OKAA01_FASTA.fasta	MGYA00172533	ERZ490974	root:Host-associated:Human:Digestive system:Large in	0.3808
MGYA00172534/OKDM01_FASTA.fasta	MGYA00172534	ERZ491070	root:Host-associated:Human:Digestive system:Large in	0.2502
MGYA00172535/OKPP01_FASTA.fasta	MGYA00172535	ERZ491382	root:Host-associated:Human:Digestive system:Large in	0.4384
MGYA00172536/OJXL01_FASTA.fasta	MGYA00172536	ERZ490907	root:Host-associated:Human:Digestive system:Large in	0.4284
MGYA00172537/OKCU01_FASTA.fasta	MGYA00172537	ERZ491047	root:Host-associated:Human:Digestive system:Large in	0.3112
MGYA00172538/OKAY01_FASTA.fasta	MGYA00172538	ERZ490998	root:Host-associated:Human:Digestive system:Large in	0.2364
MGYA00172539/OKDZ01_FASTA.fasta	MGYA00172539	ERZ491090	root:Host-associated:Human:Digestive system:Large in	0.2932
MGYA00172540/OJXZ01_FASTA.fasta	MGYA00172540	ERZ490922	root:Host-associated:Human:Digestive system:Large in	0.3098
MGYA00172541/OKLZ01_FASTA.fasta	MGYA00172541	ERZ491292	root:Host-associated:Human:Digestive system:Large in	0.3746
MGYA00172542/OJSU01_FASTA.fasta	MGYA00172542	ERZ490770	root:Host-associated:Human:Digestive system:Large in	0.2744
MGYA00172543/OKKH01_FASTA.fasta	MGYA00172543	ERZ491245	root:Host-associated:Human:Digestive system:Large in	0.2352
MGYA00172544/OLJO01_FASTA.fasta	MGYA00172544	ERZ490863	root:Host-associated:Human:Digestive system:Large in	0.289
MGYA00172545/OKKY01_FASTA.fasta	MGYA00172545	ERZ491270	root:Host-associated:Human:Digestive system:Large in	0.3318
MGYA00172546/OJXC01_FASTA.fasta	MGYA00172546	ERZ490901	root:Host-associated:Human:Digestive system:Large in	0.2544
MGYA00172547/OKQB01_FASTA.fasta	MGYA00172547	ERZ491404	root:Host-associated:Human:Digestive system:Large in	0.2388
MGYA00172548/OKAO01_FASTA.fasta	MGYA00172548	ERZ490991	root:Host-associated:Human:Digestive system:Large in	0.3364
MGYA00172549/OJZD01_FASTA.fasta	MGYA00172549	ERZ490954	root:Host-associated:Human:Digestive system:Large in	0.2712
MGYA00172550/OJVI01_FASTA.fasta	MGYA00172550	ERZ490848	root:Host-associated:Human:Digestive system:Large in	0.1916
MGYA00172551/OKFR01_FASTA.fasta	MGYA00172551	ERZ491130	root:Host-associated:Human:Digestive system:Large in	0.2456
MGYA00172552/OJZP01_FASTA.fasta	MGYA00172552	ERZ490966	root:Host-associated:Human:Digestive system:Large in	0.343
MGYA00172553/OJYX01_FASTA.fasta	MGYA00172553	ERZ490944	root:Host-associated:Human:Digestive system:Large in	0.3494
MGYA00172554/OKDX01_FASTA.fasta	MGYA00172554	ERZ491075	root:Host-associated:Human:Digestive system:Large in	0.3806
MGYA00172555/OJYF01_FASTA.fasta	MGYA00172555	ERZ490924	root:Host-associated:Human:Digestive system:Large in	0.318
MGYA00172556/OKGF01_FASTA.fasta	MGYA00172556	ERZ491142	root:Host-associated:Human:Digestive system:Large in	0.3896
MGYA00172557/OKGC01_FASTA.fasta	MGYA00172557	ERZ491146	root:Host-associated:Human:Digestive system:Large in	0.2582
MGYA00172558/OKOR01_FASTA.fasta	MGYA00172558	ERZ491373	root:Host-associated:Human:Digestive system:Large in	0.2848
MGYA00172559/OKGT01_FASTA.fasta	MGYA00172559	ERZ491166	root:Host-associated:Human:Digestive system:Large in	0.3078
MGYA00172560/OJXW01_FASTA.fasta	MGYA00172560	ERZ490921	root:Host-associated:Human:Digestive system:Large in	0.2458
MGYA00172561/OKPD01_FASTA.fasta	MGYA00172561	ERZ491377	root:Host-associated:Human:Digestive system:Large in	0.3066
MGYA00172562/OKCQ01_FASTA.fasta	MGYA00172562	ERZ491050	root:Host-associated:Human:Digestive system:Large in	0.3854
MGYA00172563/OLJM01_FASTA.fasta	MGYA00172563	ERZ490884	root:Host-associated:Human:Digestive system:Large in	0.3164
MGYA00172564/OKPH01_FASTA.fasta	MGYA00172564	ERZ491383	root:Host-associated:Human:Digestive system:Large in	0.4424
MGYA00172565/OKLF01_FASTA.fasta	MGYA00172565	ERZ491278	root:Host-associated:Human:Digestive system:Large in	0.3788
MGYA00172566/OJWQ01_FASTA.fasta	MGYA00172566	ERZ490881	root:Host-associated:Human:Digestive system:Large in	0.2714
MGYA00172567/OKEI01_FASTA.fasta	MGYA00172567	ERZ491099	root:Host-associated:Human:Digestive system:Large in	0.3736
MGYA00172568/OKHT01_FASTA.fasta	MGYA00172568	ERZ491186	root:Host-associated:Human:Digestive system:Large in	0.2118
MGYA00172569/OJYI01_FASTA.fasta	MGYA00172569	ERZ490927	root:Host-associated:Human:Digestive system:Large in	0.1516
MGYA00172570/OJUU01_FASTA.fasta	MGYA00172570	ERZ490834	root:Host-associated:Human:Digestive system:Large in	0.2624
MGYA00172571/OKNH01_FASTA.fasta	MGYA00172571	ERZ491338	root:Host-associated:Human:Digestive system:Large in	0.2984
MGYA00172572/OKEO01_FASTA.fasta	MGYA00172572	ERZ491096	root:Host-associated:Human:Digestive system:Large in	0.3854
MGYA00172573/OKAR01_FASTA.fasta	MGYA00172573	ERZ490992	root:Host-associated:Human:Digestive system:Large in	0.3468
MGYA00172574/OKHV01_FASTA.fasta	MGYA00172574	ERZ491184	root:Host-associated:Human:Digestive system:Large in	0.3236
MGYA00172575/OKAW01_FASTA.fasta	MGYA00172575	ERZ491005	root:Host-associated:Human:Digestive system:Large in	0.0846
MGYA00172576/OJZJ01_FASTA.fasta	MGYA00172576	ERZ490962	root:Host-associated:Human:Digestive system:Large in	0.2052
MGYA00172577/OKFS01_FASTA.fasta	MGYA00172577	ERZ491132	root:Host-associated:Human:Digestive system:Large in	0.29
MGYA00172578/OKEW01_FASTA.fasta	MGYA00172578	ERZ491108	root:Host-associated:Human:Digestive system:Large in	0.389
MGYA00172579/OKJH01_FASTA.fasta	MGYA00172579	ERZ491228	root:Host-associated:Human:Digestive system:Large in	0.292
MGYA00172580/OJZH01_FASTA.fasta	MGYA00172580	ERZ490952	root:Host-associated:Human:Digestive system:Large in	0.3542
MGYA00172581/OKEH01_FASTA.fasta	MGYA00172581	ERZ491103	root:Host-associated:Human:Digestive system:Large in	0.1884
MGYA00172582/OJZX01_FASTA.fasta	MGYA00172582	ERZ490973	root:Host-associated:Human:Digestive system:Large in	0.1932
MGYA00172583/OJVH01_FASTA.fasta	MGYA00172583	ERZ490850	root:Host-associated:Human:Digestive system:Large in	0.2326
MGYA00172584/OKOP01_FASTA.fasta	MGYA00172584	ERZ491362	root:Host-associated:Human:Digestive system:Large in	0.3782
MGYA00172585/OJTW01_FASTA.fasta	MGYA00172585	ERZ490805	root:Host-associated:Human:Digestive system:Large in	0.3678
MGYA00172586/OKHF01_FASTA.fasta	MGYA00172586	ERZ491160	root:Host-associated:Human:Digestive system:Large in	0.4982
MGYA00172587/OKBM01_FASTA.fasta	MGYA00172587	ERZ491021	root:Host-associated:Human:Digestive system:Large in	0.2606
MGYA00172588/OJYH01_FASTA.fasta	MGYA00172588	ERZ490932	root:Host-associated:Human:Digestive system:Large in	0.4046
MGYA00172589/OJZY01_FASTA.fasta	MGYA00172589	ERZ490976	root:Host-associated:Human:Digestive system:Large in	0.3128
MGYA00172590/OKGK01_FASTA.fasta	MGYA00172590	ERZ491151	root:Host-associated:Human:Digestive system:Large in	0.1928
MGYA00172591/OKDJ01_FASTA.fasta	MGYA00172591	ERZ491065	root:Host-associated:Human:Digestive system:Large in	0.3782
MGYA00172592/OKNP01_FASTA.fasta	MGYA00172592	ERZ491336	root:Host-associated:Human:Digestive system:Large in	0.387
MGYA00172593/OJUV01_FASTA.fasta	MGYA00172593	ERZ490835	root:Host-associated:Human:Digestive system:Large in	0.3764
MGYA00172594/OKEF01_FASTA.fasta	MGYA00172594	ERZ491080	root:Host-associated:Human:Digestive system:Large in	0.2082
MGYA00172595/OKCV01_FASTA.fasta	MGYA00172595	ERZ491057	root:Host-associated:Human:Digestive system:Large in	0.3022
MGYA00172596/OKQE01_FASTA.fasta	MGYA00172596	ERZ491403	root:Host-associated:Human:Digestive system:Large in	0.411
MGYA00172597/OKOE01_FASTA.fasta	MGYA00172597	ERZ491354	root:Host-associated:Human:Digestive system:Large in	0.2642
MGYA00172598/OKIO01_FASTA.fasta	MGYA00172598	ERZ491208	root:Host-associated:Human:Digestive system:Large in	0.4124
MGYA00172599/OJXN01_FASTA.fasta	MGYA00172599	ERZ490910	root:Host-associated:Human:Digestive system:Large in	0.2752
MGYA00172600/OKIL01_FASTA.fasta	MGYA00172600	ERZ491205	root:Host-associated:Human:Digestive system:Large in	0.293
MGYA00172601/OJTO01_FASTA.fasta	MGYA00172601	ERZ490782	root:Host-associated:Human:Digestive system:Large in	0.1562
MGYA00172602/OKCW01_FASTA.fasta	MGYA00172602	ERZ491054	root:Host-associated:Human:Digestive system:Large in	0.393
MGYA00172603/OJVC01_FASTA.fasta	MGYA00172603	ERZ490842	root:Host-associated:Human:Digestive system:Large in	0.2808
MGYA00172604/OKEM01_FASTA.fasta	MGYA00172604	ERZ491101	root:Host-associated:Human:Digestive system:Large in	0.3508
MGYA00172605/OJTA01_FASTA.fasta	MGYA00172605	ERZ490778	root:Host-associated:Human:Digestive system:Large in	0.3092
MGYA00172606/OJTL01_FASTA.fasta	MGYA00172606	ERZ490794	root:Host-associated:Human:Digestive system:Large in	0.1756
MGYA00172607/OKJM01_FASTA.fasta	MGYA00172607	ERZ491232	root:Host-associated:Human:Digestive system:Large in	0.0986
MGYA00172608/OKGA01_FASTA.fasta	MGYA00172608	ERZ491140	root:Host-associated:Human:Digestive system:Large in	0.3602
MGYA00172609/OKCY01_FASTA.fasta	MGYA00172609	ERZ491055	root:Host-associated:Human:Digestive system:Large in	0.1526
MGYA00172610/OJWE01_FASTA.fasta	MGYA00172610	ERZ490874	root:Host-associated:Human:Digestive system:Large in	0.3058
MGYA00172611/OJTQ01_FASTA.fasta	MGYA00172611	ERZ490801	root:Host-associated:Human:Digestive system:Large in	0.341
MGYA00172612/OJWS01_FASTA.fasta	MGYA00172612	ERZ490889	root:Host-associated:Human:Digestive system:Large in	0.2638
MGYA00172613/OKLY01_FASTA.fasta	MGYA00172613	ERZ491298	root:Host-associated:Human:Digestive system:Large in	0.2696
MGYA00172614/OKAC01_FASTA.fasta	MGYA00172614	ERZ490980	root:Host-associated:Human:Digestive system:Large in	0.3584
MGYA00172615/OKNM01_FASTA.fasta	MGYA00172615	ERZ491330	root:Host-associated:Human:Digestive system:Large in	0.2408
MGYA00172616/OKQF01_FASTA.fasta	MGYA00172616	ERZ491400	root:Host-associated:Human:Digestive system:Large in	0.3914
MGYA00172617/OJVX01_FASTA.fasta	MGYA00172617	ERZ490858	root:Host-associated:Human:Digestive system:Large in	0.307
MGYA00172618/OJUM01_FASTA.fasta	MGYA00172618	ERZ490824	root:Host-associated:Human:Digestive system:Large in	0.3074
MGYA00172619/OKOS01_FASTA.fasta	MGYA00172619	ERZ491376	root:Host-associated:Human:Digestive system:Large in	0.058
MGYA00172620/OJWN01_FASTA.fasta	MGYA00172620	ERZ490887	root:Host-associated:Human:Digestive system:Large in	0.3314
MGYA00172621/OJYW01_FASTA.fasta	MGYA00172621	ERZ490947	root:Host-associated:Human:Digestive system:Large in	0.1586
MGYA00172622/OKMX01_FASTA.fasta	MGYA00172622	ERZ491315	root:Host-associated:Human:Digestive system:Large in	0.343
MGYA00172623/OJZS01_FASTA.fasta	MGYA00172623	ERZ490970	root:Host-associated:Human:Digestive system:Large in	0.3694
MGYA00172624/OKFG01_FASTA.fasta	MGYA00172624	ERZ491124	root:Host-associated:Human:Digestive system:Large in	0.394
MGYA00172625/OKNR01_FASTA.fasta	MGYA00172625	ERZ491344	root:Host-associated:Human:Digestive system:Large in	0.3398
MGYA00172626/OKIF01_FASTA.fasta	MGYA00172626	ERZ491195	root:Host-associated:Human:Digestive system:Large in	0.2746
MGYA00172627/OLJV01_FASTA.fasta	MGYA00172627	ERZ491036	root:Host-associated:Human:Digestive system:Large in	0.2124
MGYA00172628/OKJB01_FASTA.fasta	MGYA00172628	ERZ491226	root:Host-associated:Human:Digestive system:Large in	0.3222
MGYA00172629/OKCE01_FASTA.fasta	MGYA00172629	ERZ491029	root:Host-associated:Human:Digestive system:Large in	0.3088
MGYA00172630/OKNC01_FASTA.fasta	MGYA00172630	ERZ491323	root:Host-associated:Human:Digestive system:Large in	0.2424
MGYA00172631/OKHC01_FASTA.fasta	MGYA00172631	ERZ491165	root:Host-associated:Human:Digestive system:Large in	0.3078
MGYA00172632/OJTZ01_FASTA.fasta	MGYA00172632	ERZ490810	root:Host-associated:Human:Digestive system:Large in	0.323
MGYA00172633/OKLX01_FASTA.fasta	MGYA00172633	ERZ491295	root:Host-associated:Human:Digestive system:Large in	0.1358
MGYA00172634/OKMJ01_FASTA.fasta	MGYA00172634	ERZ491305	root:Host-associated:Human:Digestive system:Large in	0.3228
MGYA00172635/OKEV01_FASTA.fasta	MGYA00172635	ERZ491095	root:Host-associated:Human:Digestive system:Large in	0.4024
MGYA00172636/OKBW01_FASTA.fasta	MGYA00172636	ERZ491025	root:Host-associated:Human:Digestive system:Large in	0.2326
MGYA00172637/OKMH01_FASTA.fasta	MGYA00172637	ERZ491309	root:Host-associated:Human:Digestive system:Large in	0.203
MGYA00172638/OKHP01_FASTA.fasta	MGYA00172638	ERZ491183	root:Host-associated:Human:Digestive system:Large in	0.3088
MGYA00172639/OKHU01_FASTA.fasta	MGYA00172639	ERZ491177	root:Host-associated:Human:Digestive system:Large in	0.255
MGYA00172640/OKFB01_FASTA.fasta	MGYA00172640	ERZ491111	root:Host-associated:Human:Digestive system:Large in	0.2774
MGYA00172641/OKFQ01_FASTA.fasta	MGYA00172641	ERZ491131	root:Host-associated:Human:Digestive system:Large in	0.2108
MGYA00172642/OKNJ01_FASTA.fasta	MGYA00172642	ERZ491335	root:Host-associated:Human:Digestive system:Large in	0.3256
MGYA00172643/OKMA01_FASTA.fasta	MGYA00172643	ERZ491296	root:Host-associated:Human:Digestive system:Large in	0.2394
MGYA00172644/OKGD01_FASTA.fasta	MGYA00172644	ERZ491138	root:Host-associated:Human:Digestive system:Large in	0.1368
MGYA00172645/OKLD01_FASTA.fasta	MGYA00172645	ERZ491283	root:Host-associated:Human:Digestive system:Large in	0.196
MGYA00172646/OKHN01_FASTA.fasta	MGYA00172646	ERZ491179	root:Host-associated:Human:Digestive system:Large in	0.3828
MGYA00172647/OJYS01_FASTA.fasta	MGYA00172647	ERZ490942	root:Host-associated:Human:Digestive system:Large in	0.2036
MGYA00172648/OKOK01_FASTA.fasta	MGYA00172648	ERZ491355	root:Host-associated:Human:Digestive system:Large in	0.1836
MGYA00172649/OKEC01_FASTA.fasta	MGYA00172649	ERZ491084	root:Host-associated:Human:Digestive system:Large in	0.2642
MGYA00172650/OKHI01_FASTA.fasta	MGYA00172650	ERZ491172	root:Host-associated:Human:Digestive system:Large in	0.1296
MGYA00172651/OKDF01_FASTA.fasta	MGYA00172651	ERZ491062	root:Host-associated:Human:Digestive system:Large in	0.4864
MGYA00172652/OJSZ01_FASTA.fasta	MGYA00172652	ERZ490780	root:Host-associated:Human:Digestive system:Large in	0.3038
MGYA00172653/OKID01_FASTA.fasta	MGYA00172653	ERZ491197	root:Host-associated:Human:Digestive system:Large in	0.4128
MGYA00172654/OKJC01_FASTA.fasta	MGYA00172654	ERZ491223	root:Host-associated:Human:Digestive system:Large in	0.3582
MGYA00172655/OKGL01_FASTA.fasta	MGYA00172655	ERZ491149	root:Host-associated:Human:Digestive system:Large in	0.251
MGYA00172656/OKIP01_FASTA.fasta	MGYA00172656	ERZ491211	root:Host-associated:Human:Digestive system:Large in	0.2004
MGYA00172657/OJYV01_FASTA.fasta	MGYA00172657	ERZ490945	root:Host-associated:Human:Digestive system:Large in	0.2818
MGYA00172658/OKAX01_FASTA.fasta	MGYA00172658	ERZ491002	root:Host-associated:Human:Digestive system:Large in	0.3614
MGYA00172659/OKIU01_FASTA.fasta	MGYA00172659	ERZ491216	root:Host-associated:Human:Digestive system:Large in	0.2066
MGYA00172660/OKAK01_FASTA.fasta	MGYA00172660	ERZ490990	root:Host-associated:Human:Digestive system:Large in	0.252
MGYA00172661/OJVA01_FASTA.fasta	MGYA00172661	ERZ490840	root:Host-associated:Human:Digestive system:Large in	0.277
MGYA00172662/OKEX01_FASTA.fasta	MGYA00172662	ERZ491114	root:Host-associated:Human:Digestive system:Large in	0.2084
MGYA00172663/OKGB01_FASTA.fasta	MGYA00172663	ERZ491145	root:Host-associated:Human:Digestive system:Large in	0.2478
MGYA00172664/OKNN01_FASTA.fasta	MGYA00172664	ERZ491342	root:Host-associated:Human:Digestive system:Large in	0.2932
MGYA00172665/OKNY01_FASTA.fasta	MGYA00172665	ERZ491340	root:Host-associated:Human:Digestive system:Large in	0.3234
MGYA00172666/OKJL01_FASTA.fasta	MGYA00172666	ERZ491229	root:Host-associated:Human:Digestive system:Large in	0.3646
MGYA00172667/OLKD01_FASTA.fasta	MGYA00172667	ERZ490826	root:Host-associated:Human:Digestive system:Large in	0.271
MGYA00172668/OKFF01_FASTA.fasta	MGYA00172668	ERZ491118	root:Host-associated:Human:Digestive system:Large in	0.3326
MGYA00172669/OKML01_FASTA.fasta	MGYA00172669	ERZ491311	root:Host-associated:Human:Digestive system:Large in	0.1896
MGYA00172670/OKCK01_FASTA.fasta	MGYA00172670	ERZ491044	root:Host-associated:Human:Digestive system:Large in	0.2998
MGYA00172671/OKFT01_FASTA.fasta	MGYA00172671	ERZ491134	root:Host-associated:Human:Digestive system:Large in	0.165
MGYA00172672/OKNA01_FASTA.fasta	MGYA00172672	ERZ491331	root:Host-associated:Human:Digestive system:Large in	0.3046
MGYA00172673/OKMP01_FASTA.fasta	MGYA00172673	ERZ491312	root:Host-associated:Human:Digestive system:Large in	0.3026
MGYA00172674/OKGY01_FASTA.fasta	MGYA00172674	ERZ491168	root:Host-associated:Human:Digestive system:Large in	0.3082
MGYA00172675/OKIM01_FASTA.fasta	MGYA00172675	ERZ491210	root:Host-associated:Human:Digestive system:Large in	0.2874
MGYA00172676/OJUS01_FASTA.fasta	MGYA00172676	ERZ490827	root:Host-associated:Human:Digestive system:Large in	0.3848
MGYA00172677/OKIK01_FASTA.fasta	MGYA00172677	ERZ491203	root:Host-associated:Human:Digestive system:Large in	0.4518
MGYA00172678/OJTI01_FASTA.fasta	MGYA00172678	ERZ490793	root:Host-associated:Human:Digestive system:Large in	0.2062
MGYA00172679/OKPA01_FASTA.fasta	MGYA00172679	ERZ491378	root:Host-associated:Human:Digestive system:Large in	0.3038
MGYA00172680/OJUO01_FASTA.fasta	MGYA00172680	ERZ490829	root:Host-associated:Human:Digestive system:Large in	0.3804
MGYA00172681/OKJF01_FASTA.fasta	MGYA00172681	ERZ491225	root:Host-associated:Human:Digestive system:Large in	0.325
MGYA00172682/OKFP01_FASTA.fasta	MGYA00172682	ERZ491128	root:Host-associated:Human:Digestive system:Large in	0.2786
MGYA00172683/OJWP01_FASTA.fasta	MGYA00172683	ERZ490867	root:Host-associated:Human:Digestive system:Large in	0.3208
MGYA00172684/OJVW01_FASTA.fasta	MGYA00172684	ERZ490861	root:Host-associated:Human:Digestive system:Large in	0.3462
MGYA00172685/OKFH01_FASTA.fasta	MGYA00172685	ERZ491126	root:Host-associated:Human:Digestive system:Large in	0.3014
MGYA00172686/OJTF01_FASTA.fasta	MGYA00172686	ERZ490787	root:Host-associated:Human:Digestive system:Large in	0.1996
MGYA00172687/OKFJ01_FASTA.fasta	MGYA00172687	ERZ491123	root:Host-associated:Human:Digestive system:Large in	0.4044
MGYA00172688/OJTX01_FASTA.fasta	MGYA00172688	ERZ490808	root:Host-associated:Human:Digestive system:Large in	0.2078
MGYA00172689/OKOB01_FASTA.fasta	MGYA00172689	ERZ491351	root:Host-associated:Human:Digestive system:Large in	0.3788
MGYA00172690/OKKQ01_FASTA.fasta	MGYA00172690	ERZ491262	root:Host-associated:Human:Digestive system:Large in	0.2966
MGYA00172691/OJWW01_FASTA.fasta	MGYA00172691	ERZ490891	root:Host-associated:Human:Digestive system:Large in	0.2676
MGYA00172692/OKNQ01_FASTA.fasta	MGYA00172692	ERZ491341	root:Host-associated:Human:Digestive system:Large in	0.3554
MGYA00172693/OKOZ01_FASTA.fasta	MGYA00172693	ERZ491385	root:Host-associated:Human:Digestive system:Large in	0.2978
MGYA00172694/OJVR01_FASTA.fasta	MGYA00172694	ERZ490855	root:Host-associated:Human:Digestive system:Large in	0.3292
MGYA00172695/OJYA01_FASTA.fasta	MGYA00172695	ERZ490925	root:Host-associated:Human:Digestive system:Large in	0.2764
MGYA00172696/OKPT01_FASTA.fasta	MGYA00172696	ERZ491393	root:Host-associated:Human:Digestive system:Large in	0.3872
MGYA00172697/OKHL01_FASTA.fasta	MGYA00172697	ERZ491174	root:Host-associated:Human:Digestive system:Large in	0.1774
MGYA00172698/OJTG01_FASTA.fasta	MGYA00172698	ERZ490784	root:Host-associated:Human:Digestive system:Large in	0.2834
MGYA00172699/OKPJ01_FASTA.fasta	MGYA00172699	ERZ491379	root:Host-associated:Human:Digestive system:Large in	0.4116
MGYA00172700/OKLH01_FASTA.fasta	MGYA00172700	ERZ491282	root:Host-associated:Human:Digestive system:Large in	0.3082
MGYA00172701/OJTH01_FASTA.fasta	MGYA00172701	ERZ490792	root:Host-associated:Human:Digestive system:Large in	0.4026
MGYA00172702/OKAZ01_FASTA.fasta	MGYA00172702	ERZ491001	root:Host-associated:Human:Digestive system:Large in	0.224
MGYA00172703/OKMV01_FASTA.fasta	MGYA00172703	ERZ491320	root:Host-associated:Human:Digestive system:Large in	0.3124
MGYA00172704/OKCG01_FASTA.fasta	MGYA00172704	ERZ491035	root:Host-associated:Human:Digestive system:Large in	0.4636
MGYA00172705/OJYB01_FASTA.fasta	MGYA00172705	ERZ490931	root:Host-associated:Human:Digestive system:Large in	0.1756
MGYA00172706/OKFX01_FASTA.fasta	MGYA00172706	ERZ491137	root:Host-associated:Human:Digestive system:Large in	0.382
MGYA00172707/OKAD01_FASTA.fasta	MGYA00172707	ERZ490983	root:Host-associated:Human:Digestive system:Large in	0.2554
MGYA00172708/OLJJ01_FASTA.fasta	MGYA00172708	ERZ490844	root:Host-associated:Human:Digestive system:Large in	0.1766
MGYA00172709/OJYU01_FASTA.fasta	MGYA00172709	ERZ490946	root:Host-associated:Human:Digestive system:Large in	0.2012
MGYA00172710/OKDL01_FASTA.fasta	MGYA00172710	ERZ491076	root:Host-associated:Human:Digestive system:Large in	0.3044
MGYA00172711/OKHZ01_FASTA.fasta	MGYA00172711	ERZ491200	root:Host-associated:Human:Digestive system:Large in	0.3064
MGYA00172712/OKAJ01_FASTA.fasta	MGYA00172712	ERZ490986	root:Host-associated:Human:Digestive system:Large in	0.1472
MGYA00172871/OLRX01_FASTA.fasta	MGYA00172871	ERZ493428	root:Host-associated:Human:Digestive system:Large in	0.5038
MGYA00172872/OLVD01_FASTA.fasta	MGYA00172872	ERZ493514	root:Host-associated:Human:Digestive system:Large in	0.1924
MGYA00172873/OLVN01_FASTA.fasta	MGYA00172873	ERZ493523	root:Host-associated:Human:Digestive system:Large in	0.2448
MGYA00172874/OLUC01_FASTA.fasta	MGYA00172874	ERZ493484	root:Host-associated:Human:Digestive system:Large in	0.25
MGYA00172875/OLSI01_FASTA.fasta	MGYA00172875	ERZ493439	root:Host-associated:Human:Digestive system:Large in	0.3642
MGYA00172876/OLQR01_FASTA.fasta	MGYA00172876	ERZ493395	root:Host-associated:Human:Digestive system:Large in	0.334
MGYA00172877/OLNG01_FASTA.fasta	MGYA00172877	ERZ493310	root:Host-associated:Human:Digestive system:Large in	0.3438
MGYA00172878/OLNQ01_FASTA.fasta	MGYA00172878	ERZ493316	root:Host-associated:Human:Digestive system:Large in	0.4216
MGYA00172879/OLPW01_FASTA.fasta	MGYA00172879	ERZ493367	root:Host-associated:Human:Digestive system:Large in	0.4474
MGYA00172880/OLVO01_FASTA.fasta	MGYA00172880	ERZ493450	root:Host-associated:Human:Digestive system:Large in	0.4198
MGYA00172881/OLRC01_FASTA.fasta	MGYA00172881	ERZ493406	root:Host-associated:Human:Digestive system:Large in	0.3956
MGYA00172882/OLVR01_FASTA.fasta	MGYA00172882	ERZ493524	root:Host-associated:Human:Digestive system:Large in	0.4194
MGYA00172883/OLPP01_FASTA.fasta	MGYA00172883	ERZ493368	root:Host-associated:Human:Digestive system:Large in	0.48
MGYA00172884/OLOZ01_FASTA.fasta	MGYA00172884	ERZ493350	root:Host-associated:Human:Digestive system:Large in	0.3808
MGYA00172885/OLNW01_FASTA.fasta	MGYA00172885	ERZ493318	root:Host-associated:Human:Digestive system:Large in	0.4178
MGYA00172886/OLTX01_FASTA.fasta	MGYA00172886	ERZ493479	root:Host-associated:Human:Digestive system:Large in	0.0754
MGYA00172887/OLQF01_FASTA.fasta	MGYA00172887	ERZ493386	root:Host-associated:Human:Digestive system:Large in	0.4558
MGYA00172888/OLUA01_FASTA.fasta	MGYA00172888	ERZ493481	root:Host-associated:Human:Digestive system:Large in	0.041
MGYA00172889/OLUR01_FASTA.fasta	MGYA00172889	ERZ493494	root:Host-associated:Human:Digestive system:Large in	0.2288
MGYA00172890/OLUW01_FASTA.fasta	MGYA00172890	ERZ493507	root:Host-associated:Human:Digestive system:Large in	0.0324
MGYA00172891/OLNJ01_FASTA.fasta	MGYA00172891	ERZ493309	root:Host-associated:Human:Digestive system:Large in	0.3732
MGYA00172892/OLTD01_FASTA.fasta	MGYA00172892	ERZ493448	root:Host-associated:Human:Digestive system:Large in	0.3246
MGYA00172893/OLOP01_FASTA.fasta	MGYA00172893	ERZ493342	root:Host-associated:Human:Digestive system:Large in	0.41
MGYA00172894/OLTQ01_FASTA.fasta	MGYA00172894	ERZ493472	root:Host-associated:Human:Digestive system:Large in	0.1392
MGYA00172895/OLRJ01_FASTA.fasta	MGYA00172895	ERZ493417	root:Host-associated:Human:Digestive system:Large in	0.4758
MGYA00172896/OLUE01_FASTA.fasta	MGYA00172896	ERZ493487	root:Host-associated:Human:Digestive system:Large in	0.2032
MGYA00172897/OLQX01_FASTA.fasta	MGYA00172897	ERZ493401	root:Host-associated:Human:Digestive system:Large in	0.5406
MGYA00172898/OLVM01_FASTA.fasta	MGYA00172898	ERZ493519	root:Host-associated:Human:Digestive system:Large in	0.0734
MGYA00172899/OLNS01_FASTA.fasta	MGYA00172899	ERZ493323	root:Host-associated:Human:Digestive system:Large in	0.4278
MGYA00172900/OLNT01_FASTA.fasta	MGYA00172900	ERZ493326	root:Host-associated:Human:Digestive system:Large in	0.328
MGYA00172901/OLOB01_FASTA.fasta	MGYA00172901	ERZ493327	root:Host-associated:Human:Digestive system:Large in	0.4178
MGYA00172902/OLRL01_FASTA.fasta	MGYA00172902	ERZ493405	root:Host-associated:Human:Digestive system:Large in	0.3924
MGYA00172903/OLTS01_FASTA.fasta	MGYA00172903	ERZ493477	root:Host-associated:Human:Digestive system:Large in	0.196
MGYA00172904/OLOT01_FASTA.fasta	MGYA00172904	ERZ493341	root:Host-associated:Human:Digestive system:Large in	0.4864
MGYA00172905/OLSM01_FASTA.fasta	MGYA00172905	ERZ493449	root:Host-associated:Human:Digestive system:Large in	0.4246
MGYA00172906/OLTB01_FASTA.fasta	MGYA00172906	ERZ493457	root:Host-associated:Human:Digestive system:Large in	0.2058
MGYA00172907/OLWB01_FASTA.fasta	MGYA00172907	ERZ493529	root:Host-associated:Human:Digestive system:Large in	0.4584
MGYA00172908/OLTZ01_FASTA.fasta	MGYA00172908	ERZ493485	root:Host-associated:Human:Digestive system:Large in	0.0504
MGYA00172909/OLUB01_FASTA.fasta	MGYA00172909	ERZ493489	root:Host-associated:Human:Digestive system:Large in	0.301
MGYA00172910/OLPE01_FASTA.fasta	MGYA00172910	ERZ493357	root:Host-associated:Human:Digestive system:Large in	0.3632
MGYA00172911/OLRM01_FASTA.fasta	MGYA00172911	ERZ493418	root:Host-associated:Human:Digestive system:Large in	0.4206
MGYA00172912/OLOA01_FASTA.fasta	MGYA00172912	ERZ493320	root:Host-associated:Human:Digestive system:Large in	0.5418
MGYA00172913/OLUK01_FASTA.fasta	MGYA00172913	ERZ493493	root:Host-associated:Human:Digestive system:Large in	0.1384
MGYA00172914/OLUT01_FASTA.fasta	MGYA00172914	ERZ493504	root:Host-associated:Human:Digestive system:Large in	0.041
MGYA00172915/OLPS01_FASTA.fasta	MGYA00172915	ERZ493361	root:Host-associated:Human:Digestive system:Large in	0.4584
MGYA00172916/OLND01_FASTA.fasta	MGYA00172916	ERZ493315	root:Host-associated:Human:Digestive system:Large in	0.4504
MGYA00172917/OLPL01_FASTA.fasta	MGYA00172917	ERZ493362	root:Host-associated:Human:Digestive system:Large in	0.4322
MGYA00172918/OLOJ01_FASTA.fasta	MGYA00172918	ERZ493337	root:Host-associated:Human:Digestive system:Large in	0.4036
MGYA00172919/OLNE01_FASTA.fasta	MGYA00172919	ERZ493304	root:Host-associated:Human:Digestive system:Large in	0.3778
MGYA00172920/OLSZ01_FASTA.fasta	MGYA00172920	ERZ493458	root:Host-associated:Human:Digestive system:Large in	0.0638
MGYA00172921/OLNK01_FASTA.fasta	MGYA00172921	ERZ493306	root:Host-associated:Human:Digestive system:Large in	0.5434
MGYA00172922/OLSB01_FASTA.fasta	MGYA00172922	ERZ493433	root:Host-associated:Human:Digestive system:Large in	0.426
MGYA00172923/OLVA01_FASTA.fasta	MGYA00172923	ERZ493510	root:Host-associated:Human:Digestive system:Large in	0.2544
MGYA00172924/OLRF01_FASTA.fasta	MGYA00172924	ERZ493415	root:Host-associated:Human:Digestive system:Large in	0.4564
MGYA00172925/OLRK01_FASTA.fasta	MGYA00172925	ERZ493416	root:Host-associated:Human:Digestive system:Large in	0.5502
MGYA00172926/OLNR01_FASTA.fasta	MGYA00172926	ERZ493324	root:Host-associated:Human:Digestive system:Large in	0.3462
MGYA00172927/OLVE01_FASTA.fasta	MGYA00172927	ERZ493511	root:Host-associated:Human:Digestive system:Large in	0.2106
MGYA00172928/OLTJ01_FASTA.fasta	MGYA00172928	ERZ493467	root:Host-associated:Human:Digestive system:Large in	0.158
MGYA00172929/OLTG01_FASTA.fasta	MGYA00172929	ERZ493466	root:Host-associated:Human:Digestive system:Large in	0.0682
MGYA00172930/OLQE01_FASTA.fasta	MGYA00172930	ERZ493385	root:Host-associated:Human:Digestive system:Large in	0.398
MGYA00172931/OLTP01_FASTA.fasta	MGYA00172931	ERZ493473	root:Host-associated:Human:Digestive system:Large in	0.115
MGYA00172932/OLQO01_FASTA.fasta	MGYA00172932	ERZ493391	root:Host-associated:Human:Digestive system:Large in	0.4354
MGYA00172933/OLVQ01_FASTA.fasta	MGYA00172933	ERZ493531	root:Host-associated:Human:Digestive system:Large in	0.0332
MGYA00172934/OLRV01_FASTA.fasta	MGYA00172934	ERZ493422	root:Host-associated:Human:Digestive system:Large in	0.505
MGYA00172935/OLRI01_FASTA.fasta	MGYA00172935	ERZ493407	root:Host-associated:Human:Digestive system:Large in	0.4612
MGYA00172936/OLRQ01_FASTA.fasta	MGYA00172936	ERZ493419	root:Host-associated:Human:Digestive system:Large in	0.3692
MGYA00172937/OLOE01_FASTA.fasta	MGYA00172937	ERZ493328	root:Host-associated:Human:Digestive system:Large in	0.4802
MGYA00172938/OLQW01_FASTA.fasta	MGYA00172938	ERZ493402	root:Host-associated:Human:Digestive system:Large in	0.3332
MGYA00172939/OLVH01_FASTA.fasta	MGYA00172939	ERZ493520	root:Host-associated:Human:Digestive system:Large in	0.1888
MGYA00172940/OLNP01_FASTA.fasta	MGYA00172940	ERZ493314	root:Host-associated:Human:Digestive system:Large in	0.5452
MGYA00172941/OLPA01_FASTA.fasta	MGYA00172941	ERZ493352	root:Host-associated:Human:Digestive system:Large in	0.48
MGYA00172942/OLPU01_FASTA.fasta	MGYA00172942	ERZ493374	root:Host-associated:Human:Digestive system:Large in	0.395
MGYA00172943/OLUY01_FASTA.fasta	MGYA00172943	ERZ493505	root:Host-associated:Human:Digestive system:Large in	0.2034
MGYA00172944/OLSR01_FASTA.fasta	MGYA00172944	ERZ493454	root:Host-associated:Human:Digestive system:Large in	0.0382
MGYA00172945/OLTC01_FASTA.fasta	MGYA00172945	ERZ493461	root:Host-associated:Human:Digestive system:Large in	0.138
MGYA00172946/OLNF01_FASTA.fasta	MGYA00172946	ERZ493302	root:Host-associated:Human:Digestive system:Large in	0.406
MGYA00172947/OLPY01_FASTA.fasta	MGYA00172947	ERZ493378	root:Host-associated:Human:Digestive system:Large in	0.3638
MGYA00172948/OLRZ01_FASTA.fasta	MGYA00172948	ERZ493434	root:Host-associated:Human:Digestive system:Large in	0.503
MGYA00172949/OLQV01_FASTA.fasta	MGYA00172949	ERZ493396	root:Host-associated:Human:Digestive system:Large in	0.3828
MGYA00172950/OLSP01_FASTA.fasta	MGYA00172950	ERZ493446	root:Host-associated:Human:Digestive system:Large in	0.5144
MGYA00172951/OLVI01_FASTA.fasta	MGYA00172951	ERZ493521	root:Host-associated:Human:Digestive system:Large in	0.0898
MGYA00172952/OLVW01_FASTA.fasta	MGYA00172952	ERZ493532	root:Host-associated:Human:Digestive system:Large in	0.3812
MGYA00172953/OLSF01_FASTA.fasta	MGYA00172953	ERZ493435	root:Host-associated:Human:Digestive system:Large in	0.455
MGYA00172954/OLRH01_FASTA.fasta	MGYA00172954	ERZ493408	root:Host-associated:Human:Digestive system:Large in	0.3656
MGYA00172955/OLQM01_FASTA.fasta	MGYA00172955	ERZ493389	root:Host-associated:Human:Digestive system:Large in	0.6076
MGYA00172956/OLOK01_FASTA.fasta	MGYA00172956	ERZ493336	root:Host-associated:Human:Digestive system:Large in	0.429
MGYA00172957/OLNV01_FASTA.fasta	MGYA00172957	ERZ493317	root:Host-associated:Human:Digestive system:Large in	0.535
MGYA00172958/OLUS01_FASTA.fasta	MGYA00172958	ERZ493502	root:Host-associated:Human:Digestive system:Large in	0.0412
MGYA00172959/OLNX01_FASTA.fasta	MGYA00172959	ERZ493325	root:Host-associated:Human:Digestive system:Large in	0.4168
MGYA00172960/OLPM01_FASTA.fasta	MGYA00172960	ERZ493372	root:Host-associated:Human:Digestive system:Large in	0.0594
MGYA00172961/OLRT01_FASTA.fasta	MGYA00172961	ERZ493420	root:Host-associated:Human:Digestive system:Large in	0.4002
MGYA00172962/OLVT01_FASTA.fasta	MGYA00172962	ERZ493533	root:Host-associated:Human:Digestive system:Large in	0.4078
MGYA00172963/OLOW01_FASTA.fasta	MGYA00172963	ERZ493347	root:Host-associated:Human:Digestive system:Large in	0.4706
MGYA00172964/OLVX01_FASTA.fasta	MGYA00172964	ERZ493525	root:Host-associated:Human:Digestive system:Large in	0.5376
MGYA00172965/OLPG01_FASTA.fasta	MGYA00172965	ERZ493358	root:Host-associated:Human:Digestive system:Large in	0.3404
MGYA00172966/OLVK01_FASTA.fasta	MGYA00172966	ERZ493516	root:Host-associated:Human:Digestive system:Large in	0.0908
MGYA00172967/OLTA01_FASTA.fasta	MGYA00172967	ERZ493460	root:Host-associated:Human:Digestive system:Large in	0.0702
MGYA00172968/OLTR01_FASTA.fasta	MGYA00172968	ERZ493476	root:Host-associated:Human:Digestive system:Large in	0.0334
MGYA00172969/OLVC01_FASTA.fasta	MGYA00172969	ERZ493513	root:Host-associated:Human:Digestive system:Large in	0.27
MGYA00172970/OLON01_FASTA.fasta	MGYA00172970	ERZ493348	root:Host-associated:Human:Digestive system:Large in	0.3888
MGYA00172971/OLVB01_FASTA.fasta	MGYA00172971	ERZ493515	root:Host-associated:Human:Digestive system:Large in	0.0678
MGYA00172972/OLRW01_FASTA.fasta	MGYA00172972	ERZ493432	root:Host-associated:Human:Digestive system:Large in	0.3838
MGYA00172973/OLQT01_FASTA.fasta	MGYA00172973	ERZ493399	root:Host-associated:Human:Digestive system:Large in	0.3782
MGYA00172974/OLSK01_FASTA.fasta	MGYA00172974	ERZ493447	root:Host-associated:Human:Digestive system:Large in	0.5124
MGYA00172975/OLSX01_FASTA.fasta	MGYA00172975	ERZ493441	root:Host-associated:Human:Digestive system:Large in	0.4534
MGYA00172976/OLVP01_FASTA.fasta	MGYA00172976	ERZ493528	root:Host-associated:Human:Digestive system:Large in	0.4148
MGYA00172977/OLNZ01_FASTA.fasta	MGYA00172977	ERZ493319	root:Host-associated:Human:Digestive system:Large in	0.4046
MGYA00172978/OLOG01_FASTA.fasta	MGYA00172978	ERZ493333	root:Host-associated:Human:Digestive system:Large in	0.3664
MGYA00172979/OLPH01_FASTA.fasta	MGYA00172979	ERZ493359	root:Host-associated:Human:Digestive system:Large in	0.611

MGYA00172980/OLPZ01_FASTA.fasta	MGYA00172980	ERZ493380	root:Host-associated:Human:Digestive system:Large in	0.4184
MGYA00172981/OLUJ01_FASTA.fasta	MGYA00172981	ERZ493497	root:Host-associated:Human:Digestive system:Large in	0.069
MGYA00172982/OLNL01_FASTA.fasta	MGYA00172982	ERZ493313	root:Host-associated:Human:Digestive system:Large in	0.396
MGYA00172983/OLNC01_FASTA.fasta	MGYA00172983	ERZ493303	root:Host-associated:Human:Digestive system:Large in	0.4502
MGYA00172984/OLPO01_FASTA.fasta	MGYA00172984	ERZ493365	root:Host-associated:Human:Digestive system:Large in	0.41
MGYA00172985/OLRP01_FASTA.fasta	MGYA00172985	ERZ493423	root:Host-associated:Human:Digestive system:Large in	0.4752
MGYA00172986/OLTK01_FASTA.fasta	MGYA00172986	ERZ493470	root:Host-associated:Human:Digestive system:Large in	0.2068
MGYA00172987/OLQZ01_FASTA.fasta	MGYA00172987	ERZ493411	root:Host-associated:Human:Digestive system:Large in	0.367
MGYA00172988/OLSD01_FASTA.fasta	MGYA00172988	ERZ493436	root:Host-associated:Human:Digestive system:Large in	0.5708
MGYA00172989/OLPC01_FASTA.fasta	MGYA00172989	ERZ493360	root:Host-associated:Human:Digestive system:Large in	0.3784
MGYA00172990/OLND01_FASTA.fasta	MGYA00172990	ERZ493305	root:Host-associated:Human:Digestive system:Large in	0.4076
MGYA00172991/OLSL01_FASTA.fasta	MGYA00172991	ERZ493438	root:Host-associated:Human:Digestive system:Large in	0.3988
MGYA00172992/OLSC01_FASTA.fasta	MGYA00172992	ERZ493431	root:Host-associated:Human:Digestive system:Large in	0.4876
MGYA00172993/OLUD01_FASTA.fasta	MGYA00172993	ERZ493488	root:Host-associated:Human:Digestive system:Large in	0.1098
MGYA00172994/OLVF01_FASTA.fasta	MGYA00172994	ERZ493512	root:Host-associated:Human:Digestive system:Large in	0.1136
MGYA00172995/OLTE01_FASTA.fasta	MGYA00172995	ERZ493465	root:Host-associated:Human:Digestive system:Large in	0.2718
MGYA00172996/OLUX01_FASTA.fasta	MGYA00172996	ERZ493508	root:Host-associated:Human:Digestive system:Large in	0.0884
MGYA00172997/OLRO01_FASTA.fasta	MGYA00172997	ERZ493413	root:Host-associated:Human:Digestive system:Large in	0.4238
MGYA00172998/OLSN01_FASTA.fasta	MGYA00172998	ERZ493442	root:Host-associated:Human:Digestive system:Large in	0.5156
MGYA00172999/OLPK01_FASTA.fasta	MGYA00172999	ERZ493364	root:Host-associated:Human:Digestive system:Large in	0.5014
MGYA00173000/OLOR01_FASTA.fasta	MGYA00173000	ERZ493344	root:Host-associated:Human:Digestive system:Large in	0.367
MGYA00173001/OLVL01_FASTA.fasta	MGYA00173001	ERZ493522	root:Host-associated:Human:Digestive system:Large in	0.0662
MGYA00173002/OLUG01_FASTA.fasta	MGYA00173002	ERZ493490	root:Host-associated:Human:Digestive system:Large in	0.1752
MGYA00173003/OLQQ01_FASTA.fasta	MGYA00173003	ERZ493398	root:Host-associated:Human:Digestive system:Large in	0.3652
MGYA00173004/OLUI01_FASTA.fasta	MGYA00173004	ERZ493492	root:Host-associated:Human:Digestive system:Large in	0.07
MGYA00173005/OLST01_FASTA.fasta	MGYA00173005	ERZ493451	root:Host-associated:Human:Digestive system:Large in	0.2032
MGYA00173006/OLUL01_FASTA.fasta	MGYA00173006	ERZ493499	root:Host-associated:Human:Digestive system:Large in	0.0994
MGYA00173007/OLTM01_FASTA.fasta	MGYA00173007	ERZ493469	root:Host-associated:Human:Digestive system:Large in	0.094
MGYA00173008/OLUP01_FASTA.fasta	MGYA00173008	ERZ493501	root:Host-associated:Human:Digestive system:Large in	0.0954
MGYA00173009/OLPX01_FASTA.fasta	MGYA00173009	ERZ493379	root:Host-associated:Human:Digestive system:Large in	0.5102
MGYA00173010/OLPF01_FASTA.fasta	MGYA00173010	ERZ493355	root:Host-associated:Human:Digestive system:Large in	0.397
MGYA00173011/OLTF01_FASTA.fasta	MGYA00173011	ERZ493462	root:Host-associated:Human:Digestive system:Large in	0.2084
MGYA00173013/OLUO01_FASTA.fasta	MGYA00173013	ERZ493500	root:Host-associated:Human:Digestive system:Large in	0.197
MGYA00173014/OMNT01_FASTA.fasta	MGYA00173014	ERZ493376	root:Host-associated:Human:Digestive system:Large in	0.4468
MGYA00173015/OLUZ01_FASTA.fasta	MGYA00173015	ERZ493503	root:Host-associated:Human:Digestive system:Large in	0.1312
MGYA00173016/OLOS01_FASTA.fasta	MGYA00173016	ERZ493338	root:Host-associated:Human:Digestive system:Large in	0.6182
MGYA00173017/OLOD01_FASTA.fasta	MGYA00173017	ERZ493329	root:Host-associated:Human:Digestive system:Large in	0.3806
MGYA00173018/OLQP01_FASTA.fasta	MGYA00173018	ERZ493397	root:Host-associated:Human:Digestive system:Large in	0.3374
MGYA00173019/OLQK01_FASTA.fasta	MGYA00173019	ERZ493388	root:Host-associated:Human:Digestive system:Large in	0.3832
MGYA00173020/OLRR01_FASTA.fasta	MGYA00173020	ERZ493424	root:Host-associated:Human:Digestive system:Large in	0.517
MGYA00173021/OLQY01_FASTA.fasta	MGYA00173021	ERZ493404	root:Host-associated:Human:Digestive system:Large in	0.5156
MGYA00173022/OLSV01_FASTA.fasta	MGYA00173022	ERZ493452	root:Host-associated:Human:Digestive system:Large in	0.0578
MGYA00173023/OLQJ01_FASTA.fasta	MGYA00173023	ERZ493387	root:Host-associated:Human:Digestive system:Large in	0.4738
MGYA00173024/OLUH01_FASTA.fasta	MGYA00173024	ERZ493491	root:Host-associated:Human:Digestive system:Large in	0.1842
MGYA00173025/OLUN01_FASTA.fasta	MGYA00173025	ERZ493495	root:Host-associated:Human:Digestive system:Large in	0.095
MGYA00173026/OLOO01_FASTA.fasta	MGYA00173026	ERZ493349	root:Host-associated:Human:Digestive system:Large in	0.3652
MGYA00173027/OLOX01_FASTA.fasta	MGYA00173027	ERZ493340	root:Host-associated:Human:Digestive system:Large in	0.4798
MGYA00173028/OLOV01_FASTA.fasta	MGYA00173028	ERZ493345	root:Host-associated:Human:Digestive system:Large in	0.4802
MGYA00173029/OLPI01_FASTA.fasta	MGYA00173029	ERZ493354	root:Host-associated:Human:Digestive system:Large in	0.4448
MGYA00173030/OLPB01_FASTA.fasta	MGYA00173030	ERZ493356	root:Host-associated:Human:Digestive system:Large in	0.416
MGYA00173031/OLRB01_FASTA.fasta	MGYA00173031	ERZ493414	root:Host-associated:Human:Digestive system:Large in	0.3682
MGYA00173032/OLOH01_FASTA.fasta	MGYA00173032	ERZ493331	root:Host-associated:Human:Digestive system:Large in	0.5732
MGYA00173033/OLUF01_FASTA.fasta	MGYA00173033	ERZ493486	root:Host-associated:Human:Digestive system:Large in	0.159
MGYA00173034/OLTY01_FASTA.fasta	MGYA00173034	ERZ493480	root:Host-associated:Human:Digestive system:Large in	0.1718
MGYA00173035/OLRE01_FASTA.fasta	MGYA00173035	ERZ493409	root:Host-associated:Human:Digestive system:Large in	0.4046
MGYA00173036/OLSO01_FASTA.fasta	MGYA00173036	ERZ493445	root:Host-associated:Human:Digestive system:Large in	0.4804
MGYA00173037/OLTU01_FASTA.fasta	MGYA00173037	ERZ493475	root:Host-associated:Human:Digestive system:Large in	0.122
MGYA00173038/OLSW01_FASTA.fasta	MGYA00173038	ERZ493456	root:Host-associated:Human:Digestive system:Large in	0.0514
MGYA00173039/OLTL01_FASTA.fasta	MGYA00173039	ERZ493468	root:Host-associated:Human:Digestive system:Large in	0.1308
MGYA00173040/OLNU01_FASTA.fasta	MGYA00173040	ERZ493322	root:Host-associated:Human:Digestive system:Large in	0.4264
MGYA00173041/OLTT01_FASTA.fasta	MGYA00173041	ERZ493478	root:Host-associated:Human:Digestive system:Large in	0.1776
MGYA00173042/OLPD01_FASTA.fasta	MGYA00173042	ERZ493353	root:Host-associated:Human:Digestive system:Large in	0.5072
MGYA00173043/OLSE01_FASTA.fasta	MGYA00173043	ERZ493427	root:Host-associated:Human:Digestive system:Large in	0.5158
MGYA00173044/OLSS01_FASTA.fasta	MGYA00173044	ERZ493453	root:Host-associated:Human:Digestive system:Large in	0.0564
MGYA00173045/OLTO01_FASTA.fasta	MGYA00173045	ERZ493471	root:Host-associated:Human:Digestive system:Large in	0.0396
MGYA00173046/OLPV01_FASTA.fasta	MGYA00173046	ERZ493366	root:Host-associated:Human:Digestive system:Large in	0.4222
MGYA00173047/OLQB01_FASTA.fasta	MGYA00173047	ERZ493377	root:Host-associated:Human:Digestive system:Large in	0.5066
MGYA00173048/OLNH01_FASTA.fasta	MGYA00173048	ERZ493312	root:Host-associated:Human:Digestive system:Large in	0.3572
MGYA00173049/OLSY01_FASTA.fasta	MGYA00173049	ERZ493459	root:Host-associated:Human:Digestive system:Large in	0.0322
MGYA00173050/OLVG01_FASTA.fasta	MGYA00173050	ERZ493518	root:Host-associated:Human:Digestive system:Large in	0.0796
MGYA00173051/OLQN01_FASTA.fasta	MGYA00173051	ERZ493390	root:Host-associated:Human:Digestive system:Large in	0.5214
MGYA00173052/OLUV01_FASTA.fasta	MGYA00173052	ERZ493506	root:Host-associated:Human:Digestive system:Large in	0.0532
MGYA00173053/OLTH01_FASTA.fasta	MGYA00173053	ERZ493463	root:Host-associated:Human:Digestive system:Large in	0.1504
MGYA00173054/OLRA01_FASTA.fasta	MGYA00173054	ERZ493403	root:Host-associated:Human:Digestive system:Large in	0.5868
MGYA00173055/OLUU01_FASTA.fasta	MGYA00173055	ERZ493509	root:Host-associated:Human:Digestive system:Large in	0.2658
MGYA00173056/OLTV01_FASTA.fasta	MGYA00173056	ERZ493482	root:Host-associated:Human:Digestive system:Large in	0.123
MGYA00173057/OLOF01_FASTA.fasta	MGYA00173057	ERZ493332	root:Host-associated:Human:Digestive system:Large in	0.3836
MGYA00173058/OLQL01_FASTA.fasta	MGYA00173058	ERZ493384	root:Host-associated:Human:Digestive system:Large in	0.4742
MGYA00173059/OLQG01_FASTA.fasta	MGYA00173059	ERZ493383	root:Host-associated:Human:Digestive system:Large in	0.4308
MGYA00173060/OLRG01_FASTA.fasta	MGYA00173060	ERZ493410	root:Host-associated:Human:Digestive system:Large in	0.4254
MGYA00173061/OLOC01_FASTA.fasta	MGYA00173061	ERZ493330	root:Host-associated:Human:Digestive system:Large in	0.5186
MGYA00173062/OLPT01_FASTA.fasta	MGYA00173062	ERZ493373	root:Host-associated:Human:Digestive system:Large in	0.358
MGYA00173063/OLQI01_FASTA.fasta	MGYA00173063	ERZ493392	root:Host-associated:Human:Digestive system:Large in	0.4092
MGYA00173064/OLQU01_FASTA.fasta	MGYA00173064	ERZ493400	root:Host-associated:Human:Digestive system:Large in	0.4392
MGYA00173065/OLTN01_FASTA.fasta	MGYA00173065	ERZ493474	root:Host-associated:Human:Digestive system:Large in	0.0912
MGYA00173066/OLSQ01_FASTA.fasta	MGYA00173066	ERZ493444	root:Host-associated:Human:Digestive system:Large in	0.4592
MGYA00173067/OLTW01_FASTA.fasta	MGYA00173067	ERZ493483	root:Host-associated:Human:Digestive system:Large in	0.1052
MGYA00173068/OLWA01_FASTA.fasta	MGYA00173068	ERZ493526	root:Host-associated:Human:Digestive system:Large in	0.4124
MGYA00173069/OLRY01_FASTA.fasta	MGYA00173069	ERZ493429	root:Host-associated:Human:Digestive system:Large in	0.4344
MGYA00173070/OLQC01_FASTA.fasta	MGYA00173070	ERZ493381	root:Host-associated:Human:Digestive system:Large in	0.512
MGYA00173071/OLUQ01_FASTA.fasta	MGYA00173071	ERZ493496	root:Host-associated:Human:Digestive system:Large in	0.1606
MGYA00173072/OLPJ01_FASTA.fasta	MGYA00173072	ERZ493370	root:Host-associated:Human:Digestive system:Large in	0.034
MGYA00173073/OLSU01_FASTA.fasta	MGYA00173073	ERZ493455	root:Host-associated:Human:Digestive system:Large in	0.2036
MGYA00173074/OLRU01_FASTA.fasta	MGYA00173074	ERZ493426	root:Host-associated:Human:Digestive system:Large in	0.517
MGYA00173075/OLOL01_FASTA.fasta	MGYA00173075	ERZ493339	root:Host-associated:Human:Digestive system:Large in	0.5188
MGYA00173076/OLNN01_FASTA.fasta	MGYA00173076	ERZ493308	root:Host-associated:Human:Digestive system:Large in	0.5342
MGYA00173077/OLRN01_FASTA.fasta	MGYA00173077	ERZ493421	root:Host-associated:Human:Digestive system:Large in	0.4096
MGYA00173078/OLQH01_FASTA.fasta	MGYA00173078	ERZ493393	root:Host-associated:Human:Digestive system:Large in	0.3968
MGYA00173079/OLVJ01_FASTA.fasta	MGYA00173079	ERZ493517	root:Host-associated:Human:Digestive system:Large in	0.0814
MGYA00173080/OLNM01_FASTA.fasta	MGYA00173080	ERZ493311	root:Host-associated:Human:Digestive system:Large in	0.4606
MGYA00173081/OLPR01_FASTA.fasta	MGYA00173081	ERZ493371	root:Host-associated:Human:Digestive system:Large in	0.4172
MGYA00173082/OLQD01_FASTA.fasta	MGYA00173082	ERZ493382	root:Host-associated:Human:Digestive system:Large in	0.479
MGYA00173083/OLVZ01_FASTA.fasta	MGYA00173083	ERZ493527	root:Host-associated:Human:Digestive system:Large in	0.42
MGYA00173084/OLOU01_FASTA.fasta	MGYA00173084	ERZ493343	root:Host-associated:Human:Digestive system:Large in	0.4708
MGYA00173085/OLUM01_FASTA.fasta	MGYA00173085	ERZ493498	root:Host-associated:Human:Digestive system:Large in	0.2364
MGYA00173086/OLRD01_FASTA.fasta	MGYA00173086	ERZ493412	root:Host-associated:Human:Digestive system:Large in	0.371
MGYA00173087/OLSG01_FASTA.fasta	MGYA00173087	ERZ493437	root:Host-associated:Human:Digestive system:Large in	0.4586
MGYA00173088/OLSJ01_FASTA.fasta	MGYA00173088	ERZ493443	root:Host-associated:Human:Digestive system:Large in	0.4988
MGYA00173089/OLRS01_FASTA.fasta	MGYA00173089	ERZ493425	root:Host-associated:Human:Digestive system:Large in	0.3778
MGYA00173090/OLPQ01_FASTA.fasta	MGYA00173090	ERZ493369	root:Host-associated:Human:Digestive system:Large in	0.3874
MGYA00173091/OLSA01_FASTA.fasta	MGYA00173091	ERZ493430	root:Host-associated:Human:Digestive system:Large in	0.4066
MGYA00173092/OLOY01_FASTA.fasta	MGYA00173092	ERZ493351	root:Host-associated:Human:Digestive system:Large in	0.4438
MGYA00173093/OLOM01_FASTA.fasta	MGYA00173093	ERZ493334	root:Host-associated:Human:Digestive system:Large in	0.4248
MGYA00173094/OLSH01_FASTA.fasta	MGYA00173094	ERZ493440	root:Host-associated:Human:Digestive system:Large in	0.5432
MGYA00173095/OLOQ01_FASTA.fasta	MGYA00173095	ERZ493346	root:Host-associated:Human:Digestive system:Large in	0.3956
MGYA00173096/OLVU01_FASTA.fasta	MGYA00173096	ERZ493530	root:Host-associated:Human:Digestive system:Large in	0.388
MGYA00173097/OLQA01_FASTA.fasta	MGYA00173097	ERZ493375	root:Host-associated:Human:Digestive system:Large in	0.4238
MGYA00173098/OLTI01_FASTA.fasta	MGYA00173098	ERZ493464	root:Host-associated:Human:Digestive system:Large in	0.1918
MGYA00173099/OLNY01_FASTA.fasta	MGYA00173099	ERZ493321	root:Host-associated:Human:Digestive system:Large in	0.5212
MGYA00173100/OLOI01_FASTA.fasta	MGYA00173100	ERZ493335	root:Host-associated:Human:Digestive system:Large in	0.3928
MGYA00173101/OLNI01_FASTA.fasta	MGYA00173101	ERZ493307	root:Host-associated:Human:Digestive system:Large in	0.3692
MGYA00173102/OLQS01_FASTA.fasta	MGYA00173102	ERZ493394	root:Host-associated:Human:Digestive system:Large in	0.5286
MGYA00173103/OMDV01_FASTA.fasta	MGYA00173103	ERZ493741	root:Host-associated:Human:Digestive system:Large in	0.3982
MGYA00173104/OMCP01_FASTA.fasta	MGYA00173104	ERZ493704	root:Host-associated:Human:Digestive system:Large in	0.3468
MGYA00173105/OLZM01_FASTA.fasta	MGYA00173105	ERZ493629	root:Host-associated:Human:Digestive system:Large in	0.1918
MGYA00173106/OMCK01_FASTA.fasta	MGYA00173106	ERZ493710	root:Host-associated:Human:Digestive system:Large in	0.1376
MGYA00173107/OLZP01_FASTA.fasta	MGYA00173107	ERZ493637	root:Host-associated:Human:Digestive system:Large in	0.1894
MGYA00173108/OLZH01_FASTA.fasta	MGYA00173108	ERZ493622	root:Host-associated:Human:Digestive system:Large in	0.3316
MGYA00173109/OMFG01_FASTA.fasta	MGYA00173109	ERZ493780	root:Host-associated:Human:Digestive system:Large in	0.1788
MGYA00173110/OMCJ01_FASTA.fasta	MGYA00173110	ERZ493706	root:Host-associated:Human:Digestive system:Large in	0.2796
MGYA00173111/OLZG01_FASTA.fasta	MGYA00173111	ERZ493623	root:Host-associated:Human:Digestive system:Large in	0.3636
MGYA00173112/OLWP01_FASTA.fasta	MGYA00173112	ERZ493551	root:Host-associated:Human:Digestive system:Large in	0.3254
MGYA00173113/OLYS01_FASTA.fasta	MGYA00173113	ERZ493609	root:Host-associated:Human:Digestive system:Large in	0.115
MGYA00173114/OMFD01_FASTA.fasta	MGYA00173114	ERZ493773	root:Host-associated:Human:Digestive system:Large in	0.393
MGYA00173115/OLWE01_FASTA.fasta	MGYA00173115	ERZ493537	root:Host-associated:Human:Digestive system:Large in	0.5062
MGYA00173116/OLZT01_FASTA.fasta	MGYA00173116	ERZ493627	root:Host-associated:Human:Digestive system:Large in	0.3914
MGYA00173117/OMGD01_FASTA.fasta	MGYA00173117	ERZ493801	root:Host-associated:Human:Digestive system:Large in	0.0498
MGYA00173118/OLZU01_FASTA.fasta	MGYA00173118	ERZ493638	root:Host-associated:Human:Digestive system:Large in	0.383
MGYA00173119/OLVV01_FASTA.fasta	MGYA00173119	ERZ493535	root:Host-associated:Human:Digestive system:Large in	0.4782
MGYA00173120/OMEB01_FASTA.fasta	MGYA00173120	ERZ493748	root:Host-associated:Human:Digestive system:Large in	0.3488
MGYA00173121/OLXG01_FASTA.fasta	MGYA00173121	ERZ493562	root:Host-associated:Human:Digestive system:Large in	0.3888
MGYA00173122/OLZX01_FASTA.fasta	MGYA00173122	ERZ493641	root:Host-associated:Human:Digestive system:Large in	0.0632
MGYA00173123/OMFU01_FASTA.fasta	MGYA00173123	ERZ493795	root:Host-associated:Human:Digestive system:Large in	0.0356
MGYA00173124/OMEX01_FASTA.fasta	MGYA00173124	ERZ493772	root:Host-associated:Human:Digestive system:Large in	0.1976
MGYA00173125/OMCV01_FASTA.fasta	MGYA00173125	ERZ493717	root:Host-associated:Human:Digestive system:Large in	0.035
MGYA00173126/OMDX01_FASTA.fasta	MGYA00173126	ERZ493738	root:Host-associated:Human:Digestive system:Large in	0.3548
MGYA00173127/OLZS01_FASTA.fasta	MGYA00173127	ERZ493631	root:Host-associated:Human:Digestive system:Large in	0.1296
MGYA00173128/OMDH01_FASTA.fasta	MGYA00173128	ERZ493727	root:Host-associated:Human:Digestive system:Large in	0.1862
MGYA00173129/OLYL01_FASTA.fasta	MGYA00173129	ERZ493594	root:Host-associated:Human:Digestive system:Large in	0.3452
MGYA00173130/OLZI01_FASTA.fasta	MGYA00173130	ERZ493620	root:Host-associated:Human:Digestive system:Large in	0.3912
MGYA00173131/OLWD01_FASTA.fasta	MGYA00173131	ERZ493539	root:Host-associated:Human:Digestive system:Large in	0.3284
MGYA00173132/OMAV01_FASTA.fasta	MGYA00173132	ERZ493668	root:Host-associated:Human:Digestive system:Large in	0.2134
MGYA00173133/OLXU01_FASTA.fasta	MGYA00173133	ERZ493589	root:Host-associated:Human:Digestive system:Large in	0.1232
MGYA00173134/OLXP01_FASTA.fasta	MGYA00173134	ERZ493573	root:Host-associated:Human:Digestive system:Large in	0.2652
MGYA00173135/OMFF01_FASTA.fasta	MGYA00173135	ERZ493776	root:Host-associated:Human:Digestive system:Large in	0.4036
MGYA00173136/OMCC01_FASTA.fasta	MGYA00173136	ERZ493698	root:Host-associated:Human:Digestive system:Large in	0.439
MGYA00173137/OLWX01_FASTA.fasta	MGYA00173137	ERZ493568	root:Host-associated:Human:Digestive system:Large in	0.3302
MGYA00173138/OMNQ01_FASTA.fasta	MGYA00173138	ERZ493547	root:Host-associated:Human:Digestive system:Large in	0.3172
MGYA00173139/OMFI01_FASTA.fasta	MGYA00173139	ERZ493778	root:Host-associated:Human:Digestive system:Large in	0.357
MGYA00173140/OLZZ01_FASTA.fasta	MGYA00173140	ERZ493645	root:Host-associated:Human:Digestive system:Large in	0.3292
MGYA00173141/OMAP01_FASTA.fasta	MGYA00173141	ERZ493651	root:Host-associated:Human:Digestive system:Large in	0.212
MGYA00173142/OLXB01_FASTA.fasta	MGYA00173142	ERZ493561	root:Host-associated:Human:Digestive system:Large in	0.3974
MGYA00173143/OMBV01_FASTA.fasta	MGYA00173143	ERZ493692	root:Host-associated:Human:Digestive system:Large in	0.0708
MGYA00173144/OMCY01_FASTA.fasta	MGYA00173144	ERZ493720	root:Host-associated:Human:Digestive system:Large in	0.1158
MGYA00173145/OMDK01_FASTA.fasta	MGYA00173145	ERZ493732	root:Host-associated:Human:Digestive system:Large in	0.3016
MGYA00173146/OLXQ01_FASTA.fasta	MGYA00173146	ERZ493575	root:Host-associated:Human:Digestive system:Large in	0.3022
MGYA00173147/OLZJ01_FASTA.fasta	MGYA00173147	ERZ493625	root:Host-associated:Human:Digestive system:Large in	0.3608
MGYA00173148/OMAU01_FASTA.fasta	MGYA00173148	ERZ493663	root:Host-associated:Human:Digestive system:Large in	0.1904
MGYA00173149/OMDG01_FASTA.fasta	MGYA00173149	ERZ493728	root:Host-associated:Human:Digestive system:Large in	0.3452
MGYA00173150/OMAD01_FASTA.fasta	MGYA00173150	ERZ493639	root:Host-associated:Human:Digestive system:Large in	0.3738
MGYA00173151/OLWI01_FASTA.fasta	MGYA00173151	ERZ493543	root:Host-associated:Human:Digestive system:Large in	0.3756
MGYA00173152/OMBB01_FASTA.fasta	MGYA00173152	ERZ493669	root:Host-associated:Human:Digestive system:Large in	0.3182
MGYA00173153/OLYM01_FASTA.fasta	MGYA00173153	ERZ493595	root:Host-associated:Human:Digestive system:Large in	0.3314
MGYA00173154/OLZN01_FASTA.fasta	MGYA00173154	ERZ493632	root:Host-associated:Human:Digestive system:Large in	0.3356
MGYA00173155/OLWS01_FASTA.fasta	MGYA00173155	ERZ493548	root:Host-associated:Human:Digestive system:Large in	0.4286
MGYA00173156/OMBH01_FASTA.fasta	MGYA00173156	ERZ493675	root:Host-associated:Human:Digestive system:Large in	0.041
MGYA00173157/OMBQ01_FASTA.fasta	MGYA00173157	ERZ493674	root:Host-associated:Human:Digestive system:Large in	0.5522
MGYA00173158/OMEM01_FASTA.fasta	MGYA00173158	ERZ493762	root:Host-associated:Human:Digestive system:Large in	0.1022
MGYA00173159/OMBL01_FASTA.fasta	MGYA00173159	ERZ493676	root:Host-associated:Human:Digestive system:Large in	0.3362
MGYA00173160/OLWZ01_FASTA.fasta	MGYA00173160	ERZ493563	root:Host-associated:Human:Digestive system:Large in	0.4194
MGYA00173161/OMEL01_FASTA.fasta	MGYA00173161	ERZ493761	root:Host-associated:Human:Digestive system:Large in	0.1346
MGYA00173162/OLYN01_FASTA.fasta	MGYA00173162	ERZ493592	root:Host-associated:Human:Digestive system:Large in	0.3962
MGYA00173163/OMAF01_FASTA.fasta	MGYA00173163	ERZ493655	root:Host-associated:Human:Digestive system:Large in	0.0428
MGYA00173164/OLYO01_FASTA.fasta	MGYA00173164	ERZ493606	root:Host-associated:Human:Digestive system:Large in	0.1856
MGYA00173165/OMDA01_FASTA.fasta	MGYA00173165	ERZ493718	root:Host-associated:Human:Digestive system:Large in	0.3258
MGYA00173166/OMDP01_FASTA.fasta	MGYA00173166	ERZ493739	root:Host-associated:Human:Digestive system:Large in	0.0986
MGYA00173167/OMAT01_FASTA.fasta	MGYA00173167	ERZ493662	root:Host-associated:Human:Digestive system:Large in	0.1596
MGYA00173168/OMBO01_FASTA.fasta	MGYA00173168	ERZ493680	root:Host-associated:Human:Digestive system:Large in	0.493
MGYA00173169/OMES01_FASTA.fasta	MGYA00173169	ERZ493766	root:Host-associated:Human:Digestive system:Large in	0.308
MGYA00173170/OMDE01_FASTA.fasta	MGYA00173170	ERZ493733	root:Host-associated:Human:Digestive system:Large in	0.4214
MGYA00173171/OMCW01_FASTA.fasta	MGYA00173171	ERZ493716	root:Host-associated:Human:Digestive system:Large in	0.0516
MGYA00173172/OLZL01_FASTA.fasta	MGYA00173172	ERZ493635	root:Host-associated:Human:Digestive system:Large in	0.1104
MGYA00173173/OMCL01_FASTA.fasta	MGYA00173173	ERZ493702	root:Host-associated:Human:Digestive system:Large in	0.2574
MGYA00173174/OMDY01_FASTA.fasta	MGYA00173174	ERZ493744	root:Host-associated:Human:Digestive system:Large in	0.3576
MGYA00173175/OMAA01_FASTA.fasta	MGYA00173175	ERZ493640	root:Host-associated:Human:Digestive system:Large in	0.39
MGYA00173176/OLYH01_FASTA.fasta	MGYA00173176	ERZ493599	root:Host-associated:Human:Digestive system:Large in	0.2278
MGYA00173177/OMEF01_FASTA.fasta	MGYA00173177	ERZ493755	root:Host-associated:Human:Digestive system:Large in	0.1524
MGYA00173178/OLXT01_FASTA.fasta	MGYA00173178	ERZ493581	root:Host-associated:Human:Digestive system:Large in	0.346
MGYA00173179/OMCN01_FASTA.fasta	MGYA00173179	ERZ493712	root:Host-associated:Human:Digestive system:Large in	0.3238
MGYA00173180/OLXF01_FASTA.fasta	MGYA00173180	ERZ493560	root:Host-associated:Human:Digestive system:Large in	0.5252
MGYA00173181/OMGM01_FASTA.fasta	MGYA00173181	ERZ493803	root:Host-associated:Human:Digestive system:Large in	0.4056
MGYA00173182/OMEU01_FASTA.fasta	MGYA00173182	ERZ493774	root:Host-associated:Human:Digestive system:Large in	0.0252
MGYA00173183/OMBA01_FASTA.fasta	MGYA00173183	ERZ493664	root:Host-associated:Human:Digestive system:Large in	0.3768
MGYA00173184/OMEI01_FASTA.fasta	MGYA00173184	ERZ493754	root:Host-associated:Human:Digestive system:Large in	0.3342
MGYA00173185/OLXM01_FASTA.fasta	MGYA00173185	ERZ493574	root:Host-associated:Human:Digestive system:Large in	0.3102
MGYA00173186/OLZA01_FASTA.fasta	MGYA00173186	ERZ493607	root:Host-associated:Human:Digestive system:Large in	0.389
MGYA00173187/OMGC01_FASTA.fasta	MGYA00173187	ERZ493799	root:Host-associated:Human:Digestive system:Large in	0.2708
MGYA00173188/OLYI01_FASTA.fasta	MGYA00173188	ERZ493596	root:Host-associated:Human:Digestive system:Large in	0.3968
MGYA00173189/OMBN01_FASTA.fasta	MGYA00173189	ERZ493682	root:Host-associated:Human:Digestive system:Large in	0.5008
MGYA00173190/OLWT01_FASTA.fasta	MGYA00173190	ERZ493555	root:Host-associated:Human:Digestive system:Large in	0.4144
MGYA00173191/OMGE01_FASTA.fasta	MGYA00173191	ERZ493802	root:Host-associated:Human:Digestive system:Large in	0.1504
MGYA00173192/OMDS01_FASTA.fasta	MGYA00173192	ERZ493743	root:Host-associated:Human:Digestive system:Large in	0.0516
MGYA00173193/OLXR01_FASTA.fasta	MGYA00173193	ERZ493582	root:Host-associated:Human:Digestive system:Large in	0.386
MGYA00173194/OMAZ01_FASTA.fasta	MGYA00173194	ERZ493667	root:Host-associated:Human:Digestive system:Large in	0.5836
MGYA00173195/OMBD01_FASTA.fasta	MGYA00173195	ERZ493672	root:Host-associated:Human:Digestive system:Large in	0.2348
MGYA00173196/OMFY01_FASTA.fasta	MGYA00173196	ERZ493793	root:Host-associated:Human:Digestive system:Large in	0.4244
MGYA00173197/OLYA01_FASTA.fasta	MGYA00173197	ERZ493591	root:Host-associated:Human:Digestive system:Large in	0.3208
MGYA00173198/OLXE01_FASTA.fasta	MGYA00173198	ERZ493571	root:Host-associated:Human:Digestive system:Large in	0.478
MGYA00173199/OMFC01_FASTA.fasta	MGYA00173199	ERZ493767	root:Host-associated:Human:Digestive system:Large in	0.4162
MGYA00173200/OMBG01_FASTA.fasta	MGYA00173200	ERZ493661	root:Host-associated:Human:Digestive system:Large in	0.4242
MGYA00173201/OLYF01_FASTA.fasta	MGYA00173201	ERZ493593	root:Host-associated:Human:Digestive system:Large in	0.136
MGYA00173202/OMDM01_FASTA.fasta	MGYA00173202	ERZ493724	root:Host-associated:Human:Digestive system:Large in	0.3362
MGYA00173203/OMEY01_FASTA.fasta	MGYA00173203	ERZ493770	root:Host-associated:Human:Digestive system:Large in	0.1882
MGYA00173204/OMCE01_FASTA.fasta	MGYA00173204	ERZ493694	root:Host-associated:Human:Digestive system:Large in	0.2956
MGYA00173205/OMCX01_FASTA.fasta	MGYA00173205	ERZ493719	root:Host-associated:Human:Digestive system:Large in	0.1408
MGYA00173206/OMBY01_FASTA.fasta	MGYA00173206	ERZ493696	root:Host-associated:Human:Digestive system:Large in	0.0548
MGYA00173207/OMFB01_FASTA.fasta	MGYA00173207	ERZ493777	root:Host-associated:Human:Digestive system:Large in	0.1548
MGYA00173208/OLZE01_FASTA.fasta	MGYA00173208	ERZ493615	root:Host-associated:Human:Digestive system:Large in	0.424
MGYA00173209/OMDU01_FASTA.fasta	MGYA00173209	ERZ493745	root:Host-associated:Human:Digestive system:Large in	0.242
MGYA00173210/OLYX01_FASTA.fasta	MGYA00173210	ERZ493616	root:Host-associated:Human:Digestive system:Large in	0.2862
MGYA00173211/OMAQ01_FASTA.fasta	MGYA00173211	ERZ493654	root:Host-associated:Human:Digestive system:Large in	0.4592
MGYA00173212/OMEH01_FASTA.fasta	MGYA00173212	ERZ493756	root:Host-associated:Human:Digestive system:Large in	0.3322
MGYA00173213/OMDR01_FASTA.fasta	MGYA00173213	ERZ493737	root:Host-associated:Human:Digestive system:Large in	0.3902
MGYA00173214/OLZF01_FASTA.fasta	MGYA00173214	ERZ493613	root:Host-associated:Human:Digestive system:Large in	0.4796
MGYA00173215/OMAW01_FASTA.fasta	MGYA00173215	ERZ493660	root:Host-associated:Human:Digestive system:Large in	0.3474
MGYA00173216/OMNU01_FASTA.fasta	MGYA00173216	ERZ493564	root:Host-associated:Human:Digestive system:Large in	0.5162
MGYA00173217/OMAN01_FASTA.fasta	MGYA00173217	ERZ493646	root:Host-associated:Human:Digestive system:Large in	0.2982
MGYA00173218/OLZR01_FASTA.fasta	MGYA00173218	ERZ493634	root:Host-associated:Human:Digestive system:Large in	0.4308
MGYA00173219/OMBI01_FASTA.fasta	MGYA00173219	ERZ493678	root:Host-associated:Human:Digestive system:Large in	0.1932
MGYA00173220/OMBK01_FASTA.fasta	MGYA00173220	ERZ493679	root:Host-associated:Human:Digestive system:Large in	0.4066
MGYA00173221/OMEJ01_FASTA.fasta	MGYA00173221	ERZ493753	root:Host-associated:Human:Digestive system:Large in	0.3706
MGYA00173222/OMDC01_FASTA.fasta	MGYA00173222	ERZ493722	root:Host-associated:Human:Digestive system:Large in	0.3768
MGYA00173223/OMAH01_FASTA.fasta	MGYA00173223	ERZ493652	root:Host-associated:Human:Digestive system:Large in	0.1216
MGYA00173224/OMAX01_FASTA.fasta	MGYA00173224	ERZ493666	root:Host-associated:Human:Digestive system:Large in	0.0254
MGYA00173225/OLYU01_FASTA.fasta	MGYA00173225	ERZ493617	root:Host-associated:Human:Digestive system:Large in	0.1656
MGYA00173226/OMDO01_FASTA.fasta	MGYA00173226	ERZ493731	root:Host-associated:Human:Digestive system:Large in	0.4508
MGYA00173227/OMFX01_FASTA.fasta	MGYA00173227	ERZ493798	root:Host-associated:Human:Digestive system:Large in	0.1174
MGYA00173228/OMFT01_FASTA.fasta	MGYA00173228	ERZ493794	root:Host-associated:Human:Digestive system:Large in	0.0856
MGYA00173229/OLWO01_FASTA.fasta	MGYA00173229	ERZ493556	root:Host-associated:Human:Digestive system:Large in	0.3348
MGYA00173230/OLWW01_FASTA.fasta	MGYA00173230	ERZ493557	root:Host-associated:Human:Digestive system:Large in	0.361
MGYA00173231/OMEZ01_FASTA.fasta	MGYA00173231	ERZ493771	root:Host-associated:Human:Digestive system:Large in	0.191
MGYA00173232/OLYR01_FASTA.fasta	MGYA00173232	ERZ493612	root:Host-associated:Human:Digestive system:Large in	0.2206
MGYA00173233/OMAJ01_FASTA.fasta	MGYA00173233	ERZ493650	root:Host-associated:Human:Digestive system:Large in	0.2956
MGYA00173234/OMEV01_FASTA.fasta	MGYA00173234	ERZ493768	root:Host-associated:Human:Digestive system:Large in	0.3952
MGYA00173235/OLWV01_FASTA.fasta	MGYA00173235	ERZ493558	root:Host-associated:Human:Digestive system:Large in	0.283
MGYA00173236/OMEP01_FASTA.fasta	MGYA00173236	ERZ493757	root:Host-associated:Human:Digestive system:Large in	0.2528
MGYA00173237/OMCZ01_FASTA.fasta	MGYA00173237	ERZ493715	root:Host-associated:Human:Digestive system:Large in	0.1446
MGYA00173238/OLWG01_FASTA.fasta	MGYA00173238	ERZ493541	root:Host-associated:Human:Digestive system:Large in	0.3704
MGYA00173239/OMGA01_FASTA.fasta	MGYA00173239	ERZ493791	root:Host-associated:Human:Digestive system:Large in	0.3944
MGYA00173240/OMAK01_FASTA.fasta	MGYA00173240	ERZ493656	root:Host-associated:Human:Digestive system:Large in	0.3088
MGYA00173241/OMCS01_FASTA.fasta	MGYA00173241	ERZ493705	root:Host-associated:Human:Digestive system:Large in	0.4018
MGYA00173242/OMNP01_FASTA.fasta	MGYA00173242	ERZ493697	root:Host-associated:Human:Digestive system:Large in	0.3542
MGYA00173243/OMCI01_FASTA.fasta	MGYA00173243	ERZ493711	root:Host-associated:Human:Digestive system:Large in	0.2126
MGYA00173244/OMAC01_FASTA.fasta	MGYA00173244	ERZ493636	root:Host-associated:Human:Digestive system:Large in	0.4996
MGYA00173245/OLZC01_FASTA.fasta	MGYA00173245	ERZ493614	root:Host-associated:Human:Digestive system:Large in	0.3898
MGYA00173246/OMDQ01_FASTA.fasta	MGYA00173246	ERZ493736	root:Host-associated:Human:Digestive system:Large in	0.2144
MGYA00173247/OLXD01_FASTA.fasta	MGYA00173247	ERZ493565	root:Host-associated:Human:Digestive system:Large in	0.367
MGYA00173248/OMBZ01_FASTA.fasta	MGYA00173248	ERZ493687	root:Host-associated:Human:Digestive system:Large in	0.4114
MGYA00173249/OMAI01_FASTA.fasta	MGYA00173249	ERZ493657	root:Host-associated:Human:Digestive system:Large in	0.0842
MGYA00173250/OMAE01_FASTA.fasta	MGYA00173250	ERZ493647	root:Host-associated:Human:Digestive system:Large in	0.3492
MGYA00173251/OMED01_FASTA.fasta	MGYA00173251	ERZ493747	root:Host-associated:Human:Digestive system:Large in	0.37
MGYA00173252/OLXJ01_FASTA.fasta	MGYA00173252	ERZ493576	root:Host-associated:Human:Digestive system:Large in	0.3638
MGYA00173253/OLZY01_FASTA.fasta	MGYA00173253	ERZ493642	root:Host-associated:Human:Digestive system:Large in	0.0638
MGYA00173254/OMAG01_FASTA.fasta	MGYA00173254	ERZ493644	root:Host-associated:Human:Digestive system:Large in	0.3246
MGYA00173255/OMEO01_FASTA.fasta	MGYA00173255	ERZ493765	root:Host-associated:Human:Digestive system:Large in	0.1466
MGYA00173256/OMCG01_FASTA.fasta	MGYA00173256	ERZ493691	root:Host-associated:Human:Digestive system:Large in	0.461
MGYA00173257/OMCH01_FASTA.fasta	MGYA00173257	ERZ493699	root:Host-associated:Human:Digestive system:Large in	0.4118
MGYA00173258/OMET01_FASTA.fasta	MGYA00173258	ERZ493759	root:Host-associated:Human:Digestive system:Large in	0.4578
MGYA00173259/OMFM01_FASTA.fasta	MGYA00173259	ERZ493785	root:Host-associated:Human:Digestive system:Large in	0.2972
MGYA00173260/OMBW01_FASTA.fasta	MGYA00173260	ERZ493693	root:Host-associated:Human:Digestive system:Large in	0.0318
MGYA00173261/OMAY01_FASTA.fasta	MGYA00173261	ERZ493665	root:Host-associated:Human:Digestive system:Large in	0.459
MGYA00173262/OLYB01_FASTA.fasta	MGYA00173262	ERZ493585	root:Host-associated:Human:Digestive system:Large in	0.4352
MGYA00173263/OMDF01_FASTA.fasta	MGYA00173263	ERZ493734	root:Host-associated:Human:Digestive system:Large in	0.5614
MGYA00173264/OMBE01_FASTA.fasta	MGYA00173264	ERZ493670	root:Host-associated:Human:Digestive system:Large in	0.2588
MGYA00173265/OMCR01_FASTA.fasta	MGYA00173265	ERZ493714	root:Host-associated:Human:Digestive system:Large in	0.2064
MGYA00173266/OMCO01_FASTA.fasta	MGYA00173266	ERZ493703	root:Host-associated:Human:Digestive system:Large in	0.32
MGYA00173267/OMEC01_FASTA.fasta	MGYA00173267	ERZ493746	root:Host-associated:Human:Digestive system:Large in	0.3962
MGYA00173268/OLWF01_FASTA.fasta	MGYA00173268	ERZ493540	root:Host-associated:Human:Digestive system:Large in	0.3146
MGYA00173269/OLXK01_FASTA.fasta	MGYA00173269	ERZ493572	root:Host-associated:Human:Digestive system:Large in	0.6116
MGYA00173270/OMFZ01_FASTA.fasta	MGYA00173270	ERZ493790	root:Host-associated:Human:Digestive system:Large in	0.4856
MGYA00173271/OLWH01_FASTA.fasta	MGYA00173271	ERZ493542	root:Host-associated:Human:Digestive system:Large in	0.3766
MGYA00173272/OMBU01_FASTA.fasta	MGYA00173272	ERZ493690	root:Host-associated:Human:Digestive system:Large in	0.248
MGYA00173274/OMBF01_FASTA.fasta	MGYA00173274	ERZ493673	root:Host-associated:Human:Digestive system:Large in	0.2606
MGYA00173275/OMDL01_FASTA.fasta	MGYA00173275	ERZ493725	root:Host-associated:Human:Digestive system:Large in	0.383
MGYA00173276/OMBJ01_FASTA.fasta	MGYA00173276	ERZ493683	root:Host-associated:Human:Digestive system:Large in	0.0948
MGYA00173277/OLXA01_FASTA.fasta	MGYA00173277	ERZ493566	root:Host-associated:Human:Digestive system:Large in	0.3902
MGYA00173278/OLVY01_FASTA.fasta	MGYA00173278	ERZ493534	root:Host-associated:Human:Digestive system:Large in	0.5798
MGYA00173279/OMFE01_FASTA.fasta	MGYA00173279	ERZ493782	root:Host-associated:Human:Digestive system:Large in	0.0316
MGYA00173280/OLXW01_FASTA.fasta	MGYA00173280	ERZ493584	root:Host-associated:Human:Digestive system:Large in	0.36
MGYA00173281/OMDB01_FASTA.fasta	MGYA00173281	ERZ493721	root:Host-associated:Human:Digestive system:Large in	0.3176
MGYA00173282/OLYG01_FASTA.fasta	MGYA00173282	ERZ493598	root:Host-associated:Human:Digestive system:Large in	0.2446
MGYA00173283/OMDW01_FASTA.fasta	MGYA00173283	ERZ493740	root:Host-associated:Human:Digestive system:Large in	0.3566
MGYA00173284/OMEK01_FASTA.fasta	MGYA00173284	ERZ493750	root:Host-associated:Human:Digestive system:Large in	0.3214
MGYA00173285/OMCQ01_FASTA.fasta	MGYA00173285	ERZ493709	root:Host-associated:Human:Digestive system:Large in	0.4126
MGYA00173286/OMGN01_FASTA.fasta	MGYA00173286	ERZ493804	root:Host-associated:Human:Digestive system:Large in	0.4402
MGYA00173287/OMFR01_FASTA.fasta	MGYA00173287	ERZ493789	root:Host-associated:Human:Digestive system:Large in	0.4726
MGYA00173288/OMAR01_FASTA.fasta	MGYA00173288	ERZ493659	root:Host-associated:Human:Digestive system:Large in	0.2664
MGYA00173289/OMCA01_FASTA.fasta	MGYA00173289	ERZ493688	root:Host-associated:Human:Digestive system:Large in	0.5032
MGYA00173290/OMDN01_FASTA.fasta	MGYA00173290	ERZ493726	root:Host-associated:Human:Digestive system:Large in	0.3886
MGYA00173291/OMFL01_FASTA.fasta	MGYA00173291	ERZ493788	root:Host-associated:Human:Digestive system:Large in	0.216
MGYA00173292/OLWJ01_FASTA.fasta	MGYA00173292	ERZ493546	root:Host-associated:Human:Digestive system:Large in	0.3658
MGYA00173293/OLXV01_FASTA.fasta	MGYA00173293	ERZ493578	root:Host-associated:Human:Digestive system:Large in	0.527
MGYA00173294/OMAS01_FASTA.fasta	MGYA00173294	ERZ493658	root:Host-associated:Human:Digestive system:Large in	0.4638
MGYA00173295/OMFA01_FASTA.fasta	MGYA00173295	ERZ493769	root:Host-associated:Human:Digestive system:Large in	0.1604
MGYA00173296/OMER01_FASTA.fasta	MGYA00173296	ERZ493758	root:Host-associated:Human:Digestive system:Large in	0.4644
MGYA00173297/OLYW01_FASTA.fasta	MGYA00173297	ERZ493619	root:Host-associated:Human:Digestive system:Large in	0.3084
MGYA00173298/OLXN01_FASTA.fasta	MGYA00173298	ERZ493580	root:Host-associated:Human:Digestive system:Large in	0.437
MGYA00173299/OLXS01_FASTA.fasta	MGYA00173299	ERZ493583	root:Host-associated:Human:Digestive system:Large in	0.3516
MGYA00173300/OLXO01_FASTA.fasta	MGYA00173300	ERZ493577	root:Host-associated:Human:Digestive system:Large in	0.32
MGYA00173301/OLWY01_FASTA.fasta	MGYA00173301	ERZ493569	root:Host-associated:Human:Digestive system:Large in	0.421
MGYA00173302/OLWR01_FASTA.fasta	MGYA00173302	ERZ493554	root:Host-associated:Human:Digestive system:Large in	0.3124
MGYA00173303/OMDD01_FASTA.fasta	MGYA00173303	ERZ493723	root:Host-associated:Human:Digestive system:Large in	0.3372
MGYA00173304/OLZQ01_FASTA.fasta	MGYA00173304	ERZ493628	root:Host-associated:Human:Digestive system:Large in	0.4112
MGYA00173305/OLWK01_FASTA.fasta	MGYA00173305	ERZ493544	root:Host-associated:Human:Digestive system:Large in	0.5046
MGYA00173306/OMDT01_FASTA.fasta	MGYA00173306	ERZ493735	root:Host-associated:Human:Digestive system:Large in	0.2726
MGYA00173307/OLVS01_FASTA.fasta	MGYA00173307	ERZ493536	root:Host-associated:Human:Digestive system:Large in	0.397
MGYA00173308/OMBR01_FASTA.fasta	MGYA00173308	ERZ493681	root:Host-associated:Human:Digestive system:Large in	0.467
MGYA00173309/OMFO01_FASTA.fasta	MGYA00173309	ERZ493779	root:Host-associated:Human:Digestive system:Large in	0.4526
MGYA00173310/OMBM01_FASTA.fasta	MGYA00173310	ERZ493677	root:Host-associated:Human:Digestive system:Large in	0.3304
MGYA00173311/OMDJ01_FASTA.fasta	MGYA00173311	ERZ493729	root:Host-associated:Human:Digestive system:Large in	0.3514
MGYA00173312/OMEQ01_FASTA.fasta	MGYA00173312	ERZ493760	root:Host-associated:Human:Digestive system:Large in	0.3904
MGYA00173313/OLXC01_FASTA.fasta	MGYA00173313	ERZ493567	root:Host-associated:Human:Digestive system:Large in	0.41
MGYA00173314/OMBT01_FASTA.fasta	MGYA00173314	ERZ493686	root:Host-associated:Human:Digestive system:Large in	0.3346
MGYA00173315/OLZD01_FASTA.fasta	MGYA00173315	ERZ493618	root:Host-associated:Human:Digestive system:Large in	0.4158
MGYA00173316/OMEW01_FASTA.fasta	MGYA00173316	ERZ493764	root:Host-associated:Human:Digestive system:Large in	0.3966
MGYA00173317/OMAL01_FASTA.fasta	MGYA00173317	ERZ493653	root:Host-associated:Human:Digestive system:Large in	0.3152
MGYA00173318/OLXY01_FASTA.fasta	MGYA00173318	ERZ493587	root:Host-associated:Human:Digestive system:Large in	0.3702
MGYA00173319/OLYD01_FASTA.fasta	MGYA00173319	ERZ493597	root:Host-associated:Human:Digestive system:Large in	0.0322
MGYA00173320/OLYQ01_FASTA.fasta	MGYA00173320	ERZ493605	root:Host-associated:Human:Digestive system:Large in	0.0536
MGYA00173321/OMBS01_FASTA.fasta	MGYA00173321	ERZ493685	root:Host-associated:Human:Digestive system:Large in	0.2518
MGYA00173322/OLYT01_FASTA.fasta	MGYA00173322	ERZ493608	root:Host-associated:Human:Digestive system:Large in	0.2482
MGYA00173323/OLZB01_FASTA.fasta	MGYA00173323	ERZ493611	root:Host-associated:Human:Digestive system:Large in	0.433
MGYA00173324/OLXI01_FASTA.fasta	MGYA00173324	ERZ493570	root:Host-associated:Human:Digestive system:Large in	0.4892
MGYA00173325/OMAO01_FASTA.fasta	MGYA00173325	ERZ493648	root:Host-associated:Human:Digestive system:Large in	0.4084
MGYA00173326/OLZW01_FASTA.fasta	MGYA00173326	ERZ493630	root:Host-associated:Human:Digestive system:Large in	0.4348
MGYA00173327/OMAB01_FASTA.fasta	MGYA00173327	ERZ493643	root:Host-associated:Human:Digestive system:Large in	0.2712
MGYA00173328/OMBX01_FASTA.fasta	MGYA00173328	ERZ493684	root:Host-associated:Human:Digestive system:Large in	0.323
MGYA00173329/OMFN01_FASTA.fasta	MGYA00173329	ERZ493784	root:Host-associated:Human:Digestive system:Large in	0.413
MGYA00173330/OMCD01_FASTA.fasta	MGYA00173330	ERZ493701	root:Host-associated:Human:Digestive system:Large in	0.4658
MGYA00173331/OLXH01_FASTA.fasta	MGYA00173331	ERZ493559	root:Host-associated:Human:Digestive system:Large in	0.3512
MGYA00173332/OLYZ01_FASTA.fasta	MGYA00173332	ERZ493621	root:Host-associated:Human:Digestive system:Large in	0.1594
MGYA00173333/OLWC01_FASTA.fasta	MGYA00173333	ERZ493538	root:Host-associated:Human:Digestive system:Large in	0.3372
MGYA00173334/OMCB01_FASTA.fasta	MGYA00173334	ERZ493695	root:Host-associated:Human:Digestive system:Large in	0.2338
MGYA00173335/OMCU01_FASTA.fasta	MGYA00173335	ERZ493713	root:Host-associated:Human:Digestive system:Large in	0.3372
MGYA00173336/OLXZ01_FASTA.fasta	MGYA00173336	ERZ493586	root:Host-associated:Human:Digestive system:Large in	0.404
MGYA00173337/OLXX01_FASTA.fasta	MGYA00173337	ERZ493588	root:Host-associated:Human:Digestive system:Large in	0.2758
MGYA00173338/OLYJ01_FASTA.fasta	MGYA00173338	ERZ493601	root:Host-associated:Human:Digestive system:Large in	0.3548
MGYA00173339/OMCM01_FASTA.fasta	MGYA00173339	ERZ493708	root:Host-associated:Human:Digestive system:Large in	0.3922
MGYA00173340/OMEG01_FASTA.fasta	MGYA00173340	ERZ493752	root:Host-associated:Human:Digestive system:Large in	0.2018
MGYA00173341/OMFP01_FASTA.fasta	MGYA00173341	ERZ493783	root:Host-associated:Human:Digestive system:Large in	0.4016
MGYA00173342/OMFW01_FASTA.fasta	MGYA00173342	ERZ493796	root:Host-associated:Human:Digestive system:Large in	0.2058
MGYA00173343/OMEA01_FASTA.fasta	MGYA00173343	ERZ493749	root:Host-associated:Human:Digestive system:Large in	0.3072
MGYA00173344/OLWQ01_FASTA.fasta	MGYA00173344	ERZ493552	root:Host-associated:Human:Digestive system:Large in	0.3968
MGYA00173345/OLWU01_FASTA.fasta	MGYA00173345	ERZ493549	root:Host-associated:Human:Digestive system:Large in	0.3606
MGYA00173346/OMFK01_FASTA.fasta	MGYA00173346	ERZ493786	root:Host-associated:Human:Digestive system:Large in	0.2714
MGYA00173347/OMAM01_FASTA.fasta	MGYA00173347	ERZ493649	root:Host-associated:Human:Digestive system:Large in	0.4652
MGYA00173348/OMFS01_FASTA.fasta	MGYA00173348	ERZ493792	root:Host-associated:Human:Digestive system:Large in	0.1926
MGYA00173349/OMFJ01_FASTA.fasta	MGYA00173349	ERZ493775	root:Host-associated:Human:Digestive system:Large in	0.3848
MGYA00173350/OMFH01_FASTA.fasta	MGYA00173350	ERZ493781	root:Host-associated:Human:Digestive system:Large in	0.1402
MGYA00173351/OLWM01_FASTA.fasta	MGYA00173351	ERZ493553	root:Host-associated:Human:Digestive system:Large in	0.3816
MGYA00173352/OLYY01_FASTA.fasta	MGYA00173352	ERZ493604	root:Host-associated:Human:Digestive system:Large in	0.3358
MGYA00173353/OMBC01_FASTA.fasta	MGYA00173353	ERZ493671	root:Host-associated:Human:Digestive system:Large in	0.2228
MGYA00173354/OLYE01_FASTA.fasta	MGYA00173354	ERZ493600	root:Host-associated:Human:Digestive system:Large in	0.052
MGYA00173355/OMEN01_FASTA.fasta	MGYA00173355	ERZ493763	root:Host-associated:Human:Digestive system:Large in	0.122
MGYA00173356/OMFV01_FASTA.fasta	MGYA00173356	ERZ493797	root:Host-associated:Human:Digestive system:Large in	0.1978
MGYA00173357/OLYC01_FASTA.fasta	MGYA00173357	ERZ493590	root:Host-associated:Human:Digestive system:Large in	0.528
MGYA00173358/OLWN01_FASTA.fasta	MGYA00173358	ERZ493550	root:Host-associated:Human:Digestive system:Large in	0.4056
MGYA00173359/OLZK01_FASTA.fasta	MGYA00173359	ERZ493626	root:Host-associated:Human:Digestive system:Large in	0.0376
MGYA00173360/OMEE01_FASTA.fasta	MGYA00173360	ERZ493751	root:Host-associated:Human:Digestive system:Large in	0.1014
MGYA00173361/OLYK01_FASTA.fasta	MGYA00173361	ERZ493602	root:Host-associated:Human:Digestive system:Large in	0.3738
MGYA00173362/OMGB01_FASTA.fasta	MGYA00173362	ERZ493800	root:Host-associated:Human:Digestive system:Large in	0.392
MGYA00173363/OMDZ01_FASTA.fasta	MGYA00173363	ERZ493742	root:Host-associated:Human:Digestive system:Large in	0.497
MGYA00173364/OLZO01_FASTA.fasta	MGYA00173364	ERZ493624	root:Host-associated:Human:Digestive system:Large in	0.3192
MGYA00173365/OLYV01_FASTA.fasta	MGYA00173365	ERZ493610	root:Host-associated:Human:Digestive system:Large in	0.2698
MGYA00173366/OLYP01_FASTA.fasta	MGYA00173366	ERZ493603	root:Host-associated:Human:Digestive system:Large in	0.1206
MGYA00173367/OMCT01_FASTA.fasta	MGYA00173367	ERZ493707	root:Host-associated:Human:Digestive system:Large in	0.4996
MGYA00173368/OLXL01_FASTA.fasta	MGYA00173368	ERZ493579	root:Host-associated:Human:Digestive system:Large in	0.3488
MGYA00173369/OMDI01_FASTA.fasta	MGYA00173369	ERZ493730	root:Host-associated:Human:Digestive system:Large in	0.256
MGYA00173370/OMFQ01_FASTA.fasta	MGYA00173370	ERZ493787	root:Host-associated:Human:Digestive system:Large in	0.371
MGYA00173371/OLZV01_FASTA.fasta	MGYA00173371	ERZ493633	root:Host-associated:Human:Digestive system:Large in	0.3576
MGYA00173372/OMBP01_FASTA.fasta	MGYA00173372	ERZ493689	root:Host-associated:Human:Digestive system:Large in	0.3416
MGYA00173373/OLWL01_FASTA.fasta	MGYA00173373	ERZ493545	root:Host-associated:Human:Digestive system:Large in	0.5056
MGYA00173728/OJOT01_FASTA.fasta	MGYA00173728	ERZ490662	root:Host-associated:Human:Digestive system:Large in	0.4434
MGYA00173729/OJQP01_FASTA.fasta	MGYA00173729	ERZ490720	root:Host-associated:Human:Digestive system:Large in	0.3282
MGYA00173730/OJPD01_FASTA.fasta	MGYA00173730	ERZ490673	root:Host-associated:Human:Digestive system:Large in	0.4652
MGYA00173731/OJPC01_FASTA.fasta	MGYA00173731	ERZ490666	root:Host-associated:Human:Digestive system:Large in	0.28
MGYA00173732/OJQQ01_FASTA.fasta	MGYA00173732	ERZ490721	root:Host-associated:Human:Digestive system:Large in	0.3516
MGYA00173733/OJOZ01_FASTA.fasta	MGYA00173733	ERZ490671	root:Host-associated:Human:Digestive system:Large in	0.2788
MGYA00173734/OJPU01_FASTA.fasta	MGYA00173734	ERZ490698	root:Host-associated:Human:Digestive system:Large in	0.4338
MGYA00173735/OJRU01_FASTA.fasta	MGYA00173735	ERZ490741	root:Host-associated:Human:Digestive system:Large in	0.2778
MGYA00173736/OJOR01_FASTA.fasta	MGYA00173736	ERZ490661	root:Host-associated:Human:Digestive system:Large in	0.279
MGYA00173737/OJRE01_FASTA.fasta	MGYA00173737	ERZ490731	root:Host-associated:Human:Digestive system:Large in	0.4114
MGYA00173738/OJRI01_FASTA.fasta	MGYA00173738	ERZ490729	root:Host-associated:Human:Digestive system:Large in	0.3308
MGYA00173739/OJPZ01_FASTA.fasta	MGYA00173739	ERZ490691	root:Host-associated:Human:Digestive system:Large in	0.4104
MGYA00173740/OJQS01_FASTA.fasta	MGYA00173740	ERZ490715	root:Host-associated:Human:Digestive system:Large in	0.3498
MGYA00173741/OJRS01_FASTA.fasta	MGYA00173741	ERZ490732	root:Host-associated:Human:Digestive system:Large in	0.469
MGYA00173742/OJQB01_FASTA.fasta	MGYA00173742	ERZ490693	root:Host-associated:Human:Digestive system:Large in	0.4184
MGYA00173743/OJQC01_FASTA.fasta	MGYA00173743	ERZ490700	root:Host-associated:Human:Digestive system:Large in	0.2706
MGYA00173744/OJRK01_FASTA.fasta	MGYA00173744	ERZ490736	root:Host-associated:Human:Digestive system:Large in	0.2696
MGYA00173745/OJPR01_FASTA.fasta	MGYA00173745	ERZ490686	root:Host-associated:Human:Digestive system:Large in	0.2528
MGYA00173746/OJRA01_FASTA.fasta	MGYA00173746	ERZ490726	root:Host-associated:Human:Digestive system:Large in	0.2948
MGYA00173747/OJRM01_FASTA.fasta	MGYA00173747	ERZ490739	root:Host-associated:Human:Digestive system:Large in	0.3822
MGYA00173748/OJRH01_FASTA.fasta	MGYA00173748	ERZ490727	root:Host-associated:Human:Digestive system:Large in	0.3542
MGYA00173749/OJOM01_FASTA.fasta	MGYA00173749	ERZ490659	root:Host-associated:Human:Digestive system:Large in	0.331
MGYA00173750/OJPH01_FASTA.fasta	MGYA00173750	ERZ490678	root:Host-associated:Human:Digestive system:Large in	0.3438
MGYA00173751/OJPA01_FASTA.fasta	MGYA00173751	ERZ490672	root:Host-associated:Human:Digestive system:Large in	0.3592
MGYA00173752/OLIU01_FASTA.fasta	MGYA00173752	ERZ490706	root:Host-associated:Human:Digestive system:Large in	0.3596
MGYA00173753/OJOW01_FASTA.fasta	MGYA00173753	ERZ490677	root:Host-associated:Human:Digestive system:Large in	0.286
MGYA00173754/OJPT01_FASTA.fasta	MGYA00173754	ERZ490692	root:Host-associated:Human:Digestive system:Large in	0.376
MGYA00173755/OJPB01_FASTA.fasta	MGYA00173755	ERZ490674	root:Host-associated:Human:Digestive system:Large in	0.4456
MGYA00173756/OJQO01_FASTA.fasta	MGYA00173756	ERZ490714	root:Host-associated:Human:Digestive system:Large in	0.2878
MGYA00173757/OJQZ01_FASTA.fasta	MGYA00173757	ERZ490725	root:Host-associated:Human:Digestive system:Large in	0.352
MGYA00173758/OJPW01_FASTA.fasta	MGYA00173758	ERZ490690	root:Host-associated:Human:Digestive system:Large in	0.407
MGYA00173759/OJPO01_FASTA.fasta	MGYA00173759	ERZ490685	root:Host-associated:Human:Digestive system:Large in	0.3622
MGYA00173760/OJOB01_FASTA.fasta	MGYA00173760	ERZ490655	root:Host-associated:Human:Digestive system:Large in	0.3144
MGYA00173761/OJQG01_FASTA.fasta	MGYA00173761	ERZ490702	root:Host-associated:Human:Digestive system:Large in	0.2838
MGYA00173762/OJQW01_FASTA.fasta	MGYA00173762	ERZ490717	root:Host-associated:Human:Digestive system:Large in	0.3554
MGYA00173763/OJPM01_FASTA.fasta	MGYA00173763	ERZ490680	root:Host-associated:Human:Digestive system:Large in	0.4268
MGYA00173764/OJPJ01_FASTA.fasta	MGYA00173764	ERZ490675	root:Host-associated:Human:Digestive system:Large in	0.2968
MGYA00173765/OJRR01_FASTA.fasta	MGYA00173765	ERZ490737	root:Host-associated:Human:Digestive system:Large in	0.3198
MGYA00173766/OJQN01_FASTA.fasta	MGYA00173766	ERZ490711	root:Host-associated:Human:Digestive system:Large in	0.3026
MGYA00173767/OLIV01_FASTA.fasta	MGYA00173767	ERZ490652	root:Host-associated:Human:Digestive system:Large in	0.4214
MGYA00173768/OJPL01_FASTA.fasta	MGYA00173768	ERZ490682	root:Host-associated:Human:Digestive system:Large in	0.318
MGYA00173769/OJOE01_FASTA.fasta	MGYA00173769	ERZ490657	root:Host-associated:Human:Digestive system:Large in	0.371
MGYA00173770/OJQV01_FASTA.fasta	MGYA00173770	ERZ490708	root:Host-associated:Human:Digestive system:Large in	0.4326
MGYA00173771/OJPF01_FASTA.fasta	MGYA00173771	ERZ490669	root:Host-associated:Human:Digestive system:Large in	0.3502
MGYA00173772/OJQA01_FASTA.fasta	MGYA00173772	ERZ490696	root:Host-associated:Human:Digestive system:Large in	0.4784
MGYA00173773/OJOG01_FASTA.fasta	MGYA00173773	ERZ490654	root:Host-associated:Human:Digestive system:Large in	0.351
MGYA00173774/OJQI01_FASTA.fasta	MGYA00173774	ERZ490703	root:Host-associated:Human:Digestive system:Large in	0.3802
MGYA00173775/OJOS01_FASTA.fasta	MGYA00173775	ERZ490663	root:Host-associated:Human:Digestive system:Large in	0.2962
MGYA00173776/OJQU01_FASTA.fasta	MGYA00173776	ERZ490713	root:Host-associated:Human:Digestive system:Large in	0.3832
MGYA00173777/OJOQ01_FASTA.fasta	MGYA00173777	ERZ490660	root:Host-associated:Human:Digestive system:Large in	0.3314
MGYA00173778/OJRP01_FASTA.fasta	MGYA00173778	ERZ490735	root:Host-associated:Human:Digestive system:Large in	0.3688
MGYA00173779/OJQX01_FASTA.fasta	MGYA00173779	ERZ490712	root:Host-associated:Human:Digestive system:Large in	0.4178
MGYA00173780/OJQJ01_FASTA.fasta	MGYA00173780	ERZ490697	root:Host-associated:Human:Digestive system:Large in	0.3978
MGYA00173781/OJRL01_FASTA.fasta	MGYA00173781	ERZ490738	root:Host-associated:Human:Digestive system:Large in	0.3858
MGYA00173782/OJRF01_FASTA.fasta	MGYA00173782	ERZ490730	root:Host-associated:Human:Digestive system:Large in	0.331
MGYA00173783/OJQK01_FASTA.fasta	MGYA00173783	ERZ490710	root:Host-associated:Human:Digestive system:Large in	0.3346
MGYA00173784/OJOU01_FASTA.fasta	MGYA00173784	ERZ490665	root:Host-associated:Human:Digestive system:Large in	0.3462
MGYA00173785/OJPK01_FASTA.fasta	MGYA00173785	ERZ490683	root:Host-associated:Human:Digestive system:Large in	0.4672
MGYA00173786/OJPI01_FASTA.fasta	MGYA00173786	ERZ490679	root:Host-associated:Human:Digestive system:Large in	0.3982
MGYA00173787/OJQE01_FASTA.fasta	MGYA00173787	ERZ490695	root:Host-associated:Human:Digestive system:Large in	0.2694
MGYA00173788/OJRG01_FASTA.fasta	MGYA00173788	ERZ490728	root:Host-associated:Human:Digestive system:Large in	0.3738
MGYA00173789/OJOC01_FASTA.fasta	MGYA00173789	ERZ490653	root:Host-associated:Human:Digestive system:Large in	0.3294
MGYA00173790/OJRQ01_FASTA.fasta	MGYA00173790	ERZ490734	root:Host-associated:Human:Digestive system:Large in	0.3692
MGYA00173791/OJQH01_FASTA.fasta	MGYA00173791	ERZ490701	root:Host-associated:Human:Digestive system:Large in	0.4346
MGYA00173792/OJOX01_FASTA.fasta	MGYA00173792	ERZ490676	root:Host-associated:Human:Digestive system:Large in	0.3084
MGYA00173793/OJPE01_FASTA.fasta	MGYA00173793	ERZ490670	root:Host-associated:Human:Digestive system:Large in	0.3558
MGYA00173794/OJRC01_FASTA.fasta	MGYA00173794	ERZ490719	root:Host-associated:Human:Digestive system:Large in	0.3664
MGYA00173795/OJRD01_FASTA.fasta	MGYA00173795	ERZ490723	root:Host-associated:Human:Digestive system:Large in	0.3816
MGYA00173796/OJRN01_FASTA.fasta	MGYA00173796	ERZ490733	root:Host-associated:Human:Digestive system:Large in	0.308
MGYA00173797/OJOY01_FASTA.fasta	MGYA00173797	ERZ490667	root:Host-associated:Human:Digestive system:Large in	0.4504
MGYA00173798/OJPV01_FASTA.fasta	MGYA00173798	ERZ490699	root:Host-associated:Human:Digestive system:Large in	0.385
MGYA00173799/OJQM01_FASTA.fasta	MGYA00173799	ERZ490707	root:Host-associated:Human:Digestive system:Large in	0.3436
MGYA00173800/OJPX01_FASTA.fasta	MGYA00173800	ERZ490694	root:Host-associated:Human:Digestive system:Large in	0.2954
MGYA00173801/OJQY01_FASTA.fasta	MGYA00173801	ERZ490724	root:Host-associated:Human:Digestive system:Large in	0.357
MGYA00173802/OJQR01_FASTA.fasta	MGYA00173802	ERZ490718	root:Host-associated:Human:Digestive system:Large in	0.4134
MGYA00173803/OJRB01_FASTA.fasta	MGYA00173803	ERZ490722	root:Host-associated:Human:Digestive system:Large in	0.4064
MGYA00173804/OJQD01_FASTA.fasta	MGYA00173804	ERZ490705	root:Host-associated:Human:Digestive system:Large in	0.3244
MGYA00173805/OJOD01_FASTA.fasta	MGYA00173805	ERZ490658	root:Host-associated:Human:Digestive system:Large in	0.3348
MGYA00173806/OJPQ01_FASTA.fasta	MGYA00173806	ERZ490688	root:Host-associated:Human:Digestive system:Large in	0.3108
MGYA00173807/OJPS01_FASTA.fasta	MGYA00173807	ERZ490684	root:Host-associated:Human:Digestive system:Large in	0.444
MGYA00173808/OJPY01_FASTA.fasta	MGYA00173808	ERZ490689	root:Host-associated:Human:Digestive system:Large in	0.3842
MGYA00173809/OJRT01_FASTA.fasta	MGYA00173809	ERZ490740	root:Host-associated:Human:Digestive system:Large in	0.3676
MGYA00173810/OJPP01_FASTA.fasta	MGYA00173810	ERZ490687	root:Host-associated:Human:Digestive system:Large in	0.404
MGYA00173811/OJOV01_FASTA.fasta	MGYA00173811	ERZ490664	root:Host-associated:Human:Digestive system:Large in	0.433
MGYA00173812/OJRO01_FASTA.fasta	MGYA00173812	ERZ490743	root:Host-associated:Human:Digestive system:Large in	0.417
MGYA00173813/OJPG01_FASTA.fasta	MGYA00173813	ERZ490668	root:Host-associated:Human:Digestive system:Large in	0.4292
MGYA00173814/OJRJ01_FASTA.fasta	MGYA00173814	ERZ490742	root:Host-associated:Human:Digestive system:Large in	0.3702
MGYA00173815/OJQT01_FASTA.fasta	MGYA00173815	ERZ490716	root:Host-associated:Human:Digestive system:Large in	0.353
MGYA00173816/OJQL01_FASTA.fasta	MGYA00173816	ERZ490709	root:Host-associated:Human:Digestive system:Large in	0.4336
MGYA00173817/OJPN01_FASTA.fasta	MGYA00173817	ERZ490681	root:Host-associated:Human:Digestive system:Large in	0.4886
MGYA00173818/OJOH01_FASTA.fasta	MGYA00173818	ERZ490656	root:Host-associated:Human:Digestive system:Large in	0.3932
MGYA00173819/OJQF01_FASTA.fasta	MGYA00173819	ERZ490704	root:Host-associated:Human:Digestive system:Large in	0.3928
MGYA00174916/OBLU01_FASTA_1.fasta	MGYA00174916	ERZ477456	root:Environmental:Terrestrial:Soil:Permafrost	0.8308
MGYA00174916/OBLU01_FASTA_2.fasta	MGYA00174916	ERZ477456	root:Environmental:Terrestrial:Soil:Permafrost	0.6614
MGYA00174917/OBLW01_FASTA_1.fasta	MGYA00174917	ERZ477460	root:Environmental:Terrestrial:Soil:Permafrost	0.7954
MGYA00174917/OBLW01_FASTA_2.fasta	MGYA00174917	ERZ477460	root:Environmental:Terrestrial:Soil:Permafrost	0.4426
MGYA00174918/ODAK01_FASTA_1.fasta	MGYA00174918	ERZ477455	root:Environmental:Terrestrial:Soil:Permafrost	0.8132
MGYA00174918/ODAK01_FASTA_2.fasta	MGYA00174918	ERZ477455	root:Environmental:Terrestrial:Soil:Permafrost	0.7002
MGYA00174919/OCZT01_FASTA_1.fasta	MGYA00174919	ERZ477459	root:Environmental:Terrestrial:Soil:Permafrost	0.8322
MGYA00174919/OCZT01_FASTA_2.fasta	MGYA00174919	ERZ477459	root:Environmental:Terrestrial:Soil:Permafrost	0.6026
MGYA00174920/OBLT01_FASTA_1.fasta	MGYA00174920	ERZ477461	root:Environmental:Terrestrial:Soil:Permafrost	0.786
MGYA00174920/OBLT01_FASTA_2.fasta	MGYA00174920	ERZ477461	root:Environmental:Terrestrial:Soil:Permafrost	0.3024
MGYA00174921/OCZU01_FASTA_1.fasta	MGYA00174921	ERZ477457	root:Environmental:Terrestrial:Soil:Permafrost	0.8042
MGYA00174921/OCZU01_FASTA_2.fasta	MGYA00174921	ERZ477457	root:Environmental:Terrestrial:Soil:Permafrost	0.098
MGYA00174922/OCTA01_FASTA_1.fasta	MGYA00174922	ERZ477458	root:Environmental:Terrestrial:Soil:Permafrost	0.8118
MGYA00174922/OCTA01_FASTA_2.fasta	MGYA00174922	ERZ477458	root:Environmental:Terrestrial:Soil:Permafrost	0.5394
MGYA00197452/OMJG01_FASTA.fasta	MGYA00197452	ERZ493870	root:Engineered:Food production:Dairy products	0.4716
MGYA00197453/OMHQ01_FASTA.fasta	MGYA00197453	ERZ493865	root:Host-associated:Human:Digestive system:Oral:Sub	0.2
MGYA00197454/OMHA01_FASTA.fasta	MGYA00197454	ERZ493851	root:Host-associated:Human:Digestive system:Oral:Sub	0.0934
MGYA00197455/OMHU01_FASTA.fasta	MGYA00197455	ERZ493848	root:Host-associated:Human:Digestive system:Oral:Sub	0.1888
MGYA00197456/OMHD01_FASTA.fasta	MGYA00197456	ERZ493852	root:Host-associated:Human:Digestive system:Oral:Sub	0.2028
MGYA00197457/OMHO01_FASTA.fasta	MGYA00197457	ERZ493857	root:Host-associated:Human:Digestive system:Oral:Sub	0.234
MGYA00197458/OMHM01_FASTA.fasta	MGYA00197458	ERZ493856	root:Host-associated:Human:Digestive system:Oral:Sub	0.2952
MGYA00197459/OMHH01_FASTA.fasta	MGYA00197459	ERZ493845	root:Host-associated:Human:Digestive system:Oral:Sub	0.3372
MGYA00197460/OMHB01_FASTA.fasta	MGYA00197460	ERZ493847	root:Host-associated:Human:Digestive system:Oral:Sub	0.1742
MGYA00197461/OMIB01_FASTA.fasta	MGYA00197461	ERZ493866	root:Host-associated:Human:Digestive system:Oral:Sub	0.1656
MGYA00197462/OMHC01_FASTA.fasta	MGYA00197462	ERZ493853	root:Host-associated:Human:Digestive system:Oral:Sub	0.3584
MGYA00197463/OMHP01_FASTA.fasta	MGYA00197463	ERZ493864	root:Host-associated:Human:Digestive system:Oral:Sub	0.1696
MGYA00197464/OMHG01_FASTA.fasta	MGYA00197464	ERZ493854	root:Host-associated:Human:Digestive system:Oral:Sub	0.1952
MGYA00197465/OMHR01_FASTA.fasta	MGYA00197465	ERZ493862	root:Host-associated:Human:Digestive system:Oral:Sub	0.0718
MGYA00197466/OMHS01_FASTA.fasta	MGYA00197466	ERZ493861	root:Host-associated:Human:Digestive system:Oral:Sub	0.1578
MGYA00197467/OMHL01_FASTA.fasta	MGYA00197467	ERZ493858	root:Host-associated:Human:Digestive system:Oral:Sub	0.1564
MGYA00197468/OMGZ01_FASTA.fasta	MGYA00197468	ERZ493846	root:Host-associated:Human:Digestive system:Oral:Sub	0.0722
MGYA00197469/OMHK01_FASTA.fasta	MGYA00197469	ERZ493859	root:Host-associated:Human:Digestive system:Oral:Sub	0.2322
MGYA00197470/OMHE01_FASTA.fasta	MGYA00197470	ERZ493844	root:Host-associated:Human:Digestive system:Oral:Sub	0.2538
MGYA00197471/OMHV01_FASTA.fasta	MGYA00197471	ERZ493855	root:Host-associated:Human:Digestive system:Oral:Sub	0.217
MGYA00197472/OMHI01_FASTA.fasta	MGYA00197472	ERZ493850	root:Host-associated:Human:Digestive system:Oral:Sub	0.294
MGYA00197473/OMHF01_FASTA.fasta	MGYA00197473	ERZ493849	root:Host-associated:Human:Digestive system:Oral:Sub	0.1854
MGYA00197474/OMHN01_FASTA.fasta	MGYA00197474	ERZ493860	root:Host-associated:Human:Digestive system:Oral:Sub	0.1996
MGYA00197475/OMHT01_FASTA.fasta	MGYA00197475	ERZ493863	root:Host-associated:Human:Digestive system:Oral:Sub	0.15
MGYA00197476/OMIA01_FASTA.fasta	MGYA00197476	ERZ493867	root:Host-associated:Human:Digestive system:Oral:Sub	0.1494
MGYA00197477/OLIO01_FASTA.fasta	MGYA00197477	ERZ490619	root:Host-associated:Mammals:Digestive system:Large	0.037
MGYA00197478/OJMQ01_FASTA.fasta	MGYA00197478	ERZ490609	root:Host-associated:Mammals:Digestive system:Large	0.024
MGYA00197479/OJNF01_FASTA.fasta	MGYA00197479	ERZ490623	root:Host-associated:Mammals:Digestive system:Large	0.1114
MGYA00197480/OLIN01_FASTA.fasta	MGYA00197480	ERZ490617	root:Host-associated:Mammals:Digestive system:Large	0.0462
MGYA00197481/OJNA01_FASTA.fasta	MGYA00197481	ERZ490620	root:Host-associated:Mammals:Digestive system:Large	0.1192
MGYA00197482/OJMX01_FASTA.fasta	MGYA00197482	ERZ490608	root:Host-associated:Mammals:Digestive system:Large	0.0292
MGYA00197483/OJNG01_FASTA.fasta	MGYA00197483	ERZ490627	root:Host-associated:Mammals:Digestive system:Large	0.0824
MGYA00197484/OJMS01_FASTA.fasta	MGYA00197484	ERZ490612	root:Host-associated:Mammals:Digestive system:Large	0.0938
MGYA00197485/OJMY01_FASTA.fasta	MGYA00197485	ERZ490618	root:Host-associated:Mammals:Digestive system:Large	0.0822
MGYA00197486/OLIP01_FASTA.fasta	MGYA00197486	ERZ490625	root:Host-associated:Mammals:Digestive system:Large	0.1124
MGYA00197487/OJMT01_FASTA.fasta	MGYA00197487	ERZ490610	root:Host-associated:Mammals:Digestive system:Large	0.0234
MGYA00197488/OJMV01_FASTA.fasta	MGYA00197488	ERZ490614	root:Host-associated:Mammals:Digestive system:Large	0.085
MGYA00197489/OJNE01_FASTA.fasta	MGYA00197489	ERZ490624	root:Host-associated:Mammals:Digestive system:Large	0.1666
MGYA00197490/OJMW01_FASTA.fasta	MGYA00197490	ERZ490616	root:Host-associated:Mammals:Digestive system:Large	0.0878
MGYA00197491/OJMR01_FASTA.fasta	MGYA00197491	ERZ490611	root:Host-associated:Mammals:Digestive system:Large	0.032
MGYA00197492/OJNB01_FASTA.fasta	MGYA00197492	ERZ490621	root:Host-associated:Mammals:Digestive system:Large	0.0786
MGYA00197493/OJMZ01_FASTA.fasta	MGYA00197493	ERZ490615	root:Host-associated:Mammals:Digestive system:Large	0.0754
MGYA00197494/OJNC01_FASTA.fasta	MGYA00197494	ERZ490622	root:Host-associated:Mammals:Digestive system:Large	0.1078
MGYA00197495/OJMU01_FASTA.fasta	MGYA00197495	ERZ490613	root:Host-associated:Mammals:Digestive system:Large	0.0574
MGYA00197496/OJND01_FASTA.fasta	MGYA00197496	ERZ490626	root:Host-associated:Mammals:Digestive system:Large	0.0816
MGYA00197497/OIFN01_FASTA.fasta	MGYA00197497	ERZ488737	root:Host-associated:Human:Digestive system:Large in	0.4438
MGYA00197498/OIFA01_FASTA.fasta	MGYA00197498	ERZ488723	root:Host-associated:Human:Digestive system:Large in	0.3916
MGYA00197499/OKWM01_FASTA.fasta	MGYA00197499	ERZ488729	root:Host-associated:Human:Digestive system:Large in	0.3374
MGYA00197500/OIFK01_FASTA.fasta	MGYA00197500	ERZ488738	root:Host-associated:Human:Digestive system:Large in	0.512
MGYA00197501/OIFM01_FASTA.fasta	MGYA00197501	ERZ488741	root:Host-associated:Human:Digestive system:Large in	0.3454
MGYA00197502/OKWX01_FASTA.fasta	MGYA00197502	ERZ488747	root:Host-associated:Human:Digestive system:Large in	0.4092
MGYA00197503/OIFT01_FASTA.fasta	MGYA00197503	ERZ488754	root:Host-associated:Human:Digestive system:Large in	0.4496
MGYA00197504/OIFI01_FASTA.fasta	MGYA00197504	ERZ488730	root:Host-associated:Human:Digestive system:Large in	0.333
MGYA00197505/OKWP01_FASTA.fasta	MGYA00197505	ERZ488739	root:Host-associated:Human:Digestive system:Large in	0.4788
MGYA00197506/OKWO01_FASTA.fasta	MGYA00197506	ERZ488733	root:Host-associated:Human:Digestive system:Large in	0.4382
MGYA00197507/OKWY01_FASTA.fasta	MGYA00197507	ERZ488753	root:Host-associated:Human:Digestive system:Large in	0.365
MGYA00197508/OIFX01_FASTA.fasta	MGYA00197508	ERZ488748	root:Host-associated:Human:Digestive system:Large in	0.3934
MGYA00197509/OIFJ01_FASTA.fasta	MGYA00197509	ERZ488726	root:Host-associated:Human:Digestive system:Large in	0.5606
MGYA00197510/OKWN01_FASTA.fasta	MGYA00197510	ERZ488732	root:Host-associated:Human:Digestive system:Large in	0.491
MGYA00197511/OIFQ01_FASTA.fasta	MGYA00197511	ERZ488731	root:Host-associated:Human:Digestive system:Large in	0.4246
MGYA00197512/OIFO01_FASTA.fasta	MGYA00197512	ERZ488742	root:Host-associated:Human:Digestive system:Large in	0.2958
MGYA00197513/OKWB01_FASTA.fasta	MGYA00197513	ERZ488720	root:Host-associated:Human:Digestive system:Large in	0.4158
MGYA00197514/OIFU01_FASTA.fasta	MGYA00197514	ERZ488752	root:Host-associated:Human:Digestive system:Large in	0.4872
MGYA00197515/OKWW01_FASTA.fasta	MGYA00197515	ERZ488746	root:Host-associated:Human:Digestive system:Large in	0.4412
MGYA00197516/OIFS01_FASTA.fasta	MGYA00197516	ERZ488751	root:Host-associated:Human:Digestive system:Large in	0.409
MGYA00197517/OKWL01_FASTA.fasta	MGYA00197517	ERZ488725	root:Host-associated:Human:Digestive system:Large in	0.5256

MGYA00197518/OKWI01_FASTA.fasta	MGYA00197518	ERZ488724	root:Host-associated:Human:Digestive system:Large in	0.4838
MGYA00197519/OIFW01_FASTA.fasta	MGYA00197519	ERZ488750	root:Host-associated:Human:Digestive system:Large in	0.39
MGYA00197520/OIFV01_FASTA.fasta	MGYA00197520	ERZ488749	root:Host-associated:Human:Digestive system:Large in	0.3084
MGYA00197521/OIFP01_FASTA.fasta	MGYA00197521	ERZ488735	root:Host-associated:Human:Digestive system:Large in	0.4816
MGYA00197522/OIFF01_FASTA.fasta	MGYA00197522	ERZ488719	root:Host-associated:Human:Digestive system:Large in	0.3976
MGYA00197523/OKWS01_FASTA.fasta	MGYA00197523	ERZ488740	root:Host-associated:Human:Digestive system:Large in	0.4326
MGYA00197524/OIFG01_FASTA.fasta	MGYA00197524	ERZ488728	root:Host-associated:Human:Digestive system:Large in	0.49
MGYA00197525/OKWT01_FASTA.fasta	MGYA00197525	ERZ488736	root:Host-associated:Human:Digestive system:Large in	0.4086
MGYA00197526/OKWV01_FASTA.fasta	MGYA00197526	ERZ488744	root:Host-associated:Human:Digestive system:Large in	0.4906
MGYA00197527/OIFC01_FASTA.fasta	MGYA00197527	ERZ488722	root:Host-associated:Human:Digestive system:Large in	0.3374
MGYA00197528/OKWU01_FASTA.fasta	MGYA00197528	ERZ488743	root:Host-associated:Human:Digestive system:Large in	0.424
MGYA00197529/OKWK01_FASTA.fasta	MGYA00197529	ERZ488721	root:Host-associated:Human:Digestive system:Large in	0.5304
MGYA00197530/OKWZ01_FASTA.fasta	MGYA00197530	ERZ488745	root:Host-associated:Human:Digestive system:Large in	0.4614
MGYA00197531/OKWQ01_FASTA.fasta	MGYA00197531	ERZ488734	root:Host-associated:Human:Digestive system:Large in	0.368
MGYA00197532/OIFD01_FASTA.fasta	MGYA00197532	ERZ488727	root:Host-associated:Human:Digestive system:Large in	0.4336
MGYA00197533/OGCL01_FASTA.fasta	MGYA00197533	ERZ485339	root:Environmental:Aquatic:Thermal springs:Hot (42-90	0.8018
MGYA00197537/OMKS01_FASTA.fasta	MGYA00197537	ERZ493869	root:Environmental:Aquatic:Thermal springs:Hot (42-90	0.6772
MGYA00198709/OMGR01_FASTA.fasta	MGYA00198709	ERZ493816	root:Host-associated:Human:Digestive system:Large in	0.4094
MGYA00198710/OMGT01_FASTA.fasta	MGYA00198710	ERZ493820	root:Host-associated:Human:Digestive system:Large in	0.4022
MGYA00198711/OMGQ01_FASTA.fasta	MGYA00198711	ERZ493818	root:Host-associated:Human:Digestive system:Large in	0.4886
MGYA00198712/OMGV01_FASTA.fasta	MGYA00198712	ERZ493821	root:Host-associated:Human:Digestive system:Large in	0.3768
MGYA00198713/OMGO01_FASTA.fasta	MGYA00198713	ERZ493817	root:Host-associated:Human:Digestive system:Large in	0.5702
MGYA00198714/OMGS01_FASTA.fasta	MGYA00198714	ERZ493819	root:Host-associated:Human:Digestive system:Large in	0.586
MGYA00198715/OMGY01_FASTA.fasta	MGYA00198715	ERZ493810	root:Host-associated:Human:Digestive system:Large in	0.4998
MGYA00198716/OMGP01_FASTA.fasta	MGYA00198716	ERZ493815	root:Host-associated:Human:Digestive system:Large in	0.4222
MGYA00198717/OMGF01_FASTA.fasta	MGYA00198717	ERZ493813	root:Host-associated:Human:Digestive system:Large in	0.4254
MGYA00198718/OMGI01_FASTA.fasta	MGYA00198718	ERZ493812	root:Host-associated:Human:Digestive system:Large in	0.397
MGYA00198719/OMGU01_FASTA.fasta	MGYA00198719	ERZ493822	root:Host-associated:Human:Digestive system:Large in	0.4774
MGYA00198720/OMGJ01_FASTA.fasta	MGYA00198720	ERZ493811	root:Host-associated:Human:Digestive system:Large in	0.5196
MGYA00198721/OMGG01_FASTA.fasta	MGYA00198721	ERZ493809	root:Host-associated:Human:Digestive system:Large in	0.5198
MGYA00198722/OMGX01_FASTA.fasta	MGYA00198722	ERZ493814	root:Host-associated:Human:Digestive system:Large in	0.4226
MGYA00198723/OMGW01_FASTA.fasta	MGYA00198723	ERZ493823	root:Host-associated:Human:Digestive system:Large in	0.4064
MGYA00198724/OJLC01_FASTA.fasta	MGYA00198724	ERZ490479	root:Engineered:Built environment	0.1706
MGYA00198725/OJKR01_FASTA.fasta	MGYA00198725	ERZ490464	root:Engineered:Built environment	0.3932
MGYA00198726/OJLP01_FASTA.fasta	MGYA00198726	ERZ490492	root:Engineered:Built environment	0.2114
MGYA00198727/OJLE01_FASTA.fasta	MGYA00198727	ERZ490475	root:Engineered:Built environment	0.3616
MGYA00198728/OJLO01_FASTA.fasta	MGYA00198728	ERZ490465	root:Engineered:Built environment	0.1344
MGYA00198729/OJKS01_FASTA.fasta	MGYA00198729	ERZ490471	root:Engineered:Built environment	0.389
MGYA00198730/OJLF01_FASTA.fasta	MGYA00198730	ERZ490486	root:Engineered:Built environment	0.2072
MGYA00198731/OJLS01_FASTA.fasta	MGYA00198731	ERZ490499	root:Engineered:Built environment	0.251
MGYA00198732/OLIG01_FASTA.fasta	MGYA00198732	ERZ490462	root:Engineered:Built environment	0.148
MGYA00198733/OJMA01_FASTA.fasta	MGYA00198733	ERZ490483	root:Engineered:Built environment	0.3558
MGYA00198734/OJKN01_FASTA.fasta	MGYA00198734	ERZ490467	root:Engineered:Built environment	0.2402
MGYA00198735/OJMD01_FASTA.fasta	MGYA00198735	ERZ490496	root:Engineered:Built environment	0.2778
MGYA00198736/OJLR01_FASTA.fasta	MGYA00198736	ERZ490470	root:Engineered:Built environment	0.126
MGYA00198737/OJLN01_FASTA.fasta	MGYA00198737	ERZ490491	root:Engineered:Built environment	0.164
MGYA00198738/OJLU01_FASTA.fasta	MGYA00198738	ERZ490498	root:Engineered:Built environment	0.2056
MGYA00198739/OJMB01_FASTA.fasta	MGYA00198739	ERZ490461	root:Engineered:Built environment	0.1888
MGYA00198740/OJLW01_FASTA.fasta	MGYA00198740	ERZ490493	root:Engineered:Built environment	0.4404
MGYA00198741/OJLQ01_FASTA.fasta	MGYA00198741	ERZ490495	root:Engineered:Built environment	0.107
MGYA00198742/OJKM01_FASTA.fasta	MGYA00198742	ERZ490463	root:Engineered:Built environment	0.2164
MGYA00198743/OJLZ01_FASTA.fasta	MGYA00198743	ERZ490500	root:Engineered:Built environment	0.4938
MGYA00198744/OJMC01_FASTA.fasta	MGYA00198744	ERZ490469	root:Engineered:Built environment	0.1718
MGYA00198745/OJLL01_FASTA.fasta	MGYA00198745	ERZ490485	root:Engineered:Built environment	0.2526
MGYA00198746/OJLJ01_FASTA.fasta	MGYA00198746	ERZ490489	root:Engineered:Built environment	0.1928
MGYA00198747/OJKZ01_FASTA.fasta	MGYA00198747	ERZ490472	root:Engineered:Built environment	0.4304
MGYA00198748/OJLK01_FASTA.fasta	MGYA00198748	ERZ490488	root:Engineered:Built environment	0.1952
MGYA00198749/OJNM01_FASTA.fasta	MGYA00198749	ERZ490466	root:Engineered:Built environment	0.4734
MGYA00198750/OJLT01_FASTA.fasta	MGYA00198750	ERZ490478	root:Engineered:Built environment	0.1952
MGYA00198751/OJLX01_FASTA.fasta	MGYA00198751	ERZ490502	root:Engineered:Built environment	0.1582
MGYA00198752/OJLH01_FASTA.fasta	MGYA00198752	ERZ490487	root:Engineered:Built environment	0.1312
MGYA00198753/OJKU01_FASTA.fasta	MGYA00198753	ERZ490474	root:Engineered:Built environment	0.1966
MGYA00198754/OJLY01_FASTA.fasta	MGYA00198754	ERZ490494	root:Engineered:Built environment	0.5372
MGYA00198755/OJKT01_FASTA.fasta	MGYA00198755	ERZ490473	root:Engineered:Built environment	0.1032
MGYA00198756/OLKE01_FASTA.fasta	MGYA00198756	ERZ490501	root:Engineered:Built environment	0.2238
MGYA00198757/OJKV01_FASTA.fasta	MGYA00198757	ERZ490468	root:Engineered:Built environment	0.1644
MGYA00198758/OJLD01_FASTA.fasta	MGYA00198758	ERZ490481	root:Engineered:Built environment	0.2228
MGYA00198759/OJMF01_FASTA.fasta	MGYA00198759	ERZ490482	root:Engineered:Built environment	0.2468
MGYA00198760/OJLI01_FASTA.fasta	MGYA00198760	ERZ490490	root:Engineered:Built environment	0.1406
MGYA00198761/OJKX01_FASTA.fasta	MGYA00198761	ERZ490476	root:Engineered:Built environment	0.1456
MGYA00198762/OJLM01_FASTA.fasta	MGYA00198762	ERZ490477	root:Engineered:Built environment	0.2198
MGYA00198763/OJLB01_FASTA.fasta	MGYA00198763	ERZ490480	root:Engineered:Built environment	0.5764
MGYA00198764/OJLG01_FASTA.fasta	MGYA00198764	ERZ490484	root:Engineered:Built environment	0.1886
MGYA00198765/OJLV01_FASTA.fasta	MGYA00198765	ERZ490497	root:Engineered:Built environment	0.225
MGYA00198766/OIFH01_FASTA.fasta	MGYA00198766	ERZ488714	root:Host-associated:Human:Digestive system:Large in	0.1796
MGYA00198767/OKWC01_FASTA.fasta	MGYA00198767	ERZ488717	root:Host-associated:Human:Digestive system:Large in	0.2924
MGYA00198768/OIEY01_FASTA.fasta	MGYA00198768	ERZ488716	root:Host-associated:Human:Digestive system:Large in	0.0688
MGYA00198769/OKWE01_FASTA.fasta	MGYA00198769	ERZ488710	root:Host-associated:Human:Digestive system:Large in	0.482
MGYA00198770/OIEZ01_FASTA.fasta	MGYA00198770	ERZ488707	root:Host-associated:Human:Digestive system:Large in	0.2258
MGYA00198771/OIFY01_FASTA.fasta	MGYA00198771	ERZ488701	root:Host-associated:Human:Digestive system:Large in	0.266
MGYA00198772/OKWF01_FASTA.fasta	MGYA00198772	ERZ488713	root:Host-associated:Human:Digestive system:Large in	0.1502
MGYA00198773/OKWG01_FASTA.fasta	MGYA00198773	ERZ488702	root:Host-associated:Human:Digestive system:Large in	0.3476
MGYA00198774/OIFL01_FASTA.fasta	MGYA00198774	ERZ488715	root:Host-associated:Human:Digestive system:Large in	0.4086
MGYA00198775/OIGD01_FASTA.fasta	MGYA00198775	ERZ488709	root:Host-associated:Human:Digestive system:Large in	0.4424
MGYA00198776/OIFE01_FASTA.fasta	MGYA00198776	ERZ488704	root:Host-associated:Human:Digestive system:Large in	0.4386
MGYA00198777/OIFB01_FASTA.fasta	MGYA00198777	ERZ488705	root:Host-associated:Human:Digestive system:Large in	0.364
MGYA00198778/OKXI01_FASTA.fasta	MGYA00198778	ERZ488706	root:Host-associated:Human:Digestive system:Large in	0.3728
MGYA00198779/OKWH01_FASTA.fasta	MGYA00198779	ERZ488703	root:Host-associated:Human:Digestive system:Large in	0.346
MGYA00198780/OKWD01_FASTA.fasta	MGYA00198780	ERZ488712	root:Host-associated:Human:Digestive system:Large in	0.1474
MGYA00198781/OKWR01_FASTA.fasta	MGYA00198781	ERZ488711	root:Host-associated:Human:Digestive system:Large in	0.4758
MGYA00198782/OKWJ01_FASTA.fasta	MGYA00198782	ERZ488708	root:Host-associated:Human:Digestive system:Large in	0.3926
MGYA00198783/OKXB01_FASTA.fasta	MGYA00198783	ERZ488718	root:Host-associated:Human:Digestive system:Large in	0.4354
MGYA00198784/OFGV01_FASTA.fasta	MGYA00198784	ERZ483443	root:Environmental:Terrestrial:Soil:Grasslands	0.409
MGYA00198785/OFGU01_FASTA.fasta	MGYA00198785	ERZ483441	root:Environmental:Terrestrial:Soil:Grasslands	0.4476
MGYA00198786/OFGY01_FASTA.fasta	MGYA00198786	ERZ483442	root:Environmental:Terrestrial:Soil:Grasslands	0.5598
MGYA00198787/OFGW01_FASTA.fasta	MGYA00198787	ERZ483440	root:Environmental:Terrestrial:Soil:Grasslands	0.5068
MGYA00198788/OMJW01_FASTA.fasta	MGYA00198788	ERZ493872	root:Host-associated:Arthropoda:Digestive system:Gut	0.0766
MGYA00198789/OMHX01_FASTA.fasta	MGYA00198789	ERZ493873	root:Host-associated:Arthropoda:Digestive system:Gut	0.0294
MGYA00198790/OKVT01_FASTA.fasta	MGYA00198790	ERZ488692	root:Host-associated:Human:Digestive system:Large in	0.3966
MGYA00198791/OKVX01_FASTA.fasta	MGYA00198791	ERZ488685	root:Host-associated:Human:Digestive system:Large in	0.559
MGYA00198792/OIEV01_FASTA.fasta	MGYA00198792	ERZ488696	root:Host-associated:Human:Digestive system:Large in	0.4936
MGYA00198793/OIEK01_FASTA.fasta	MGYA00198793	ERZ488680	root:Host-associated:Human:Digestive system:Large in	0.3966
MGYA00198794/OKVJ01_FASTA.fasta	MGYA00198794	ERZ488674	root:Host-associated:Human:Digestive system:Large in	0.4648
MGYA00198795/OKVI01_FASTA.fasta	MGYA00198795	ERZ488672	root:Host-associated:Human:Digestive system:Large in	0.5566
MGYA00198796/OIEP01_FASTA.fasta	MGYA00198796	ERZ488697	root:Host-associated:Human:Digestive system:Large in	0.3746
MGYA00198797/OKVS01_FASTA.fasta	MGYA00198797	ERZ488684	root:Host-associated:Human:Digestive system:Large in	0.3598
MGYA00198798/OIER01_FASTA.fasta	MGYA00198798	ERZ488678	root:Host-associated:Human:Digestive system:Large in	0.505
MGYA00198799/OKVV01_FASTA.fasta	MGYA00198799	ERZ488686	root:Host-associated:Human:Digestive system:Large in	0.4896
MGYA00198800/OKVZ01_FASTA.fasta	MGYA00198800	ERZ488698	root:Host-associated:Human:Digestive system:Large in	0.4616
MGYA00198801/OIEJ01_FASTA.fasta	MGYA00198801	ERZ488673	root:Host-associated:Human:Digestive system:Large in	0.402
MGYA00198802/OKVU01_FASTA.fasta	MGYA00198802	ERZ488693	root:Host-associated:Human:Digestive system:Large in	0.5238
MGYA00198803/OIET01_FASTA.fasta	MGYA00198803	ERZ488695	root:Host-associated:Human:Digestive system:Large in	0.5034
MGYA00198804/OIES01_FASTA.fasta	MGYA00198804	ERZ488676	root:Host-associated:Human:Digestive system:Large in	0.418
MGYA00198805/OKVO01_FASTA.fasta	MGYA00198805	ERZ488681	root:Host-associated:Human:Digestive system:Large in	0.2662
MGYA00198806/OKWA01_FASTA.fasta	MGYA00198806	ERZ488682	root:Host-associated:Human:Digestive system:Large in	0.5044
MGYA00198807/OIEO01_FASTA.fasta	MGYA00198807	ERZ488690	root:Host-associated:Human:Digestive system:Large in	0.3726
MGYA00198808/OKVG01_FASTA.fasta	MGYA00198808	ERZ488671	root:Host-associated:Human:Digestive system:Large in	0.544
MGYA00198809/OKVR01_FASTA.fasta	MGYA00198809	ERZ488688	root:Host-associated:Human:Digestive system:Large in	0.4006
MGYA00198810/OIEQ01_FASTA.fasta	MGYA00198810	ERZ488691	root:Host-associated:Human:Digestive system:Large in	0.4806
MGYA00198811/OKVM01_FASTA.fasta	MGYA00198811	ERZ488679	root:Host-associated:Human:Digestive system:Large in	0.4752
MGYA00198812/OKVD01_FASTA.fasta	MGYA00198812	ERZ488670	root:Host-associated:Human:Digestive system:Large in	0.4146
MGYA00198813/OKVY01_FASTA.fasta	MGYA00198813	ERZ488694	root:Host-associated:Human:Digestive system:Large in	0.4342
MGYA00198814/OKVL01_FASTA.fasta	MGYA00198814	ERZ488677	root:Host-associated:Human:Digestive system:Large in	0.4896
MGYA00198815/OKVW01_FASTA.fasta	MGYA00198815	ERZ488683	root:Host-associated:Human:Digestive system:Large in	0.509
MGYA00198816/OKVN01_FASTA.fasta	MGYA00198816	ERZ488675	root:Host-associated:Human:Digestive system:Large in	0.4992
MGYA00198817/OIEU01_FASTA.fasta	MGYA00198817	ERZ488689	root:Host-associated:Human:Digestive system:Large in	0.4186
MGYA00198818/OKVQ01_FASTA.fasta	MGYA00198818	ERZ488687	root:Host-associated:Human:Digestive system:Large in	0.3854
MGYA00199410/OLFZ01_FASTA.fasta	MGYA00199410	ERZ489898	root:Host-associated:Human:Digestive system:Large in	0.503
MGYA00199411/OIWL01_FASTA.fasta	MGYA00199411	ERZ489912	root:Host-associated:Human:Digestive system:Large in	0.4124
MGYA00199412/OLGL01_FASTA.fasta	MGYA00199412	ERZ489909	root:Host-associated:Human:Digestive system:Large in	0.4008
MGYA00199413/OLFS01_FASTA.fasta	MGYA00199413	ERZ489882	root:Host-associated:Human:Digestive system:Large in	0.5814
MGYA00199414/OIWF01_FASTA.fasta	MGYA00199414	ERZ489914	root:Host-associated:Human:Digestive system:Large in	0.0282
MGYA00199415/OLGD01_FASTA.fasta	MGYA00199415	ERZ489878	root:Host-associated:Human:Digestive system:Large in	0.4662
MGYA00199417/OIVZ01_FASTA.fasta	MGYA00199417	ERZ489903	root:Host-associated:Human:Digestive system:Large in	0.0436
MGYA00199418/OIVR01_FASTA.fasta	MGYA00199418	ERZ489887	root:Host-associated:Human:Digestive system:Large in	0.06
MGYA00199419/OIXG01_FASTA.fasta	MGYA00199419	ERZ489919	root:Host-associated:Human:Digestive system:Large in	0.55
MGYA00199420/OLFX01_FASTA.fasta	MGYA00199420	ERZ489893	root:Host-associated:Human:Digestive system:Large in	0.6084
MGYA00199421/OLGH01_FASTA.fasta	MGYA00199421	ERZ489900	root:Host-associated:Human:Digestive system:Large in	0.4508
MGYA00199422/OIWI01_FASTA.fasta	MGYA00199422	ERZ489921	root:Host-associated:Human:Digestive system:Large in	0.0644
MGYA00199423/OIWD01_FASTA.fasta	MGYA00199423	ERZ489910	root:Host-associated:Human:Digestive system:Large in	0.0544
MGYA00199424/OIWB01_FASTA.fasta	MGYA00199424	ERZ489904	root:Host-associated:Human:Digestive system:Large in	0.1108
MGYA00199425/OLGF01_FASTA.fasta	MGYA00199425	ERZ489899	root:Host-associated:Human:Digestive system:Large in	0.4688
MGYA00199426/OIWM01_FASTA.fasta	MGYA00199426	ERZ489885	root:Host-associated:Human:Digestive system:Large in	0.5304
MGYA00199427/OIWS01_FASTA.fasta	MGYA00199427	ERZ489922	root:Host-associated:Human:Digestive system:Large in	0.5598
MGYA00199428/OLGJ01_FASTA.fasta	MGYA00199428	ERZ489907	root:Host-associated:Human:Digestive system:Large in	0.3196
MGYA00199429/OLFY01_FASTA.fasta	MGYA00199429	ERZ489886	root:Host-associated:Human:Digestive system:Large in	0.6158
MGYA00199430/OIWN01_FASTA.fasta	MGYA00199430	ERZ489917	root:Host-associated:Human:Digestive system:Large in	0.5428
MGYA00199431/OIWG01_FASTA.fasta	MGYA00199431	ERZ489915	root:Host-associated:Human:Digestive system:Large in	0.0432
MGYA00199432/OLGI01_FASTA.fasta	MGYA00199432	ERZ489902	root:Host-associated:Human:Digestive system:Large in	0.496
MGYA00199433/OLGC01_FASTA.fasta	MGYA00199433	ERZ489913	root:Host-associated:Human:Digestive system:Large in	0.0846
MGYA00199434/OLFV01_FASTA.fasta	MGYA00199434	ERZ489895	root:Host-associated:Human:Digestive system:Large in	0.4758
MGYA00199435/OIXH01_FASTA.fasta	MGYA00199435	ERZ489924	root:Host-associated:Human:Digestive system:Large in	0.571
MGYA00199436/OIXV01_FASTA.fasta	MGYA00199436	ERZ489928	root:Host-associated:Human:Digestive system:Large in	0.5682
MGYA00199437/OIVX01_FASTA.fasta	MGYA00199437	ERZ489894	root:Host-associated:Human:Digestive system:Large in	0.0374
MGYA00199438/OLGB01_FASTA.fasta	MGYA00199438	ERZ489890	root:Host-associated:Human:Digestive system:Large in	0.5636
MGYA00199439/OLFU01_FASTA.fasta	MGYA00199439	ERZ489877	root:Host-associated:Human:Digestive system:Large in	0.6048
MGYA00199440/OIWJ01_FASTA.fasta	MGYA00199440	ERZ489925	root:Host-associated:Human:Digestive system:Large in	0.0772
MGYA00199441/OIWH01_FASTA.fasta	MGYA00199441	ERZ489918	root:Host-associated:Human:Digestive system:Large in	0.0588
MGYA00199442/OIVV01_FASTA.fasta	MGYA00199442	ERZ489883	root:Host-associated:Human:Digestive system:Large in	0.0554
MGYA00199443/OIWK01_FASTA.fasta	MGYA00199443	ERZ489879	root:Host-associated:Human:Digestive system:Large in	0.4426
MGYA00199444/OIWO01_FASTA.fasta	MGYA00199444	ERZ489923	root:Host-associated:Human:Digestive system:Large in	0.544
MGYA00199445/OIWQ01_FASTA.fasta	MGYA00199445	ERZ489920	root:Host-associated:Human:Digestive system:Large in	0.5908
MGYA00199446/OLGA01_FASTA.fasta	MGYA00199446	ERZ489896	root:Host-associated:Human:Digestive system:Large in	0.4292
MGYA00199447/OLFT01_FASTA.fasta	MGYA00199447	ERZ489892	root:Host-associated:Human:Digestive system:Large in	0.5262
MGYA00199448/OIVT01_FASTA.fasta	MGYA00199448	ERZ489884	root:Host-associated:Human:Digestive system:Large in	0.0428
MGYA00199449/OIVU01_FASTA.fasta	MGYA00199449	ERZ489891	root:Host-associated:Human:Digestive system:Large in	0.051
MGYA00199450/OIWE01_FASTA.fasta	MGYA00199450	ERZ489911	root:Host-associated:Human:Digestive system:Large in	0.0856
MGYA00199451/OIWV01_FASTA.fasta	MGYA00199451	ERZ489926	root:Host-associated:Human:Digestive system:Large in	0.5442
MGYA00199452/OIWA01_FASTA.fasta	MGYA00199452	ERZ489901	root:Host-associated:Human:Digestive system:Large in	0.0656
MGYA00199453/OIWP01_FASTA.fasta	MGYA00199453	ERZ489927	root:Host-associated:Human:Digestive system:Large in	0.6148
MGYA00199454/OIWU01_FASTA.fasta	MGYA00199454	ERZ489906	root:Host-associated:Human:Digestive system:Large in	0.5224
MGYA00199455/OIVW01_FASTA.fasta	MGYA00199455	ERZ489881	root:Host-associated:Human:Digestive system:Large in	0.0678
MGYA00199456/OLFQ01_FASTA.fasta	MGYA00199456	ERZ489888	root:Host-associated:Human:Digestive system:Large in	0.043
MGYA00199457/OIVS01_FASTA.fasta	MGYA00199457	ERZ489889	root:Host-associated:Human:Digestive system:Large in	0.03
MGYA00199458/OIXA01_FASTA.fasta	MGYA00199458	ERZ489916	root:Host-associated:Human:Digestive system:Large in	0.5546
MGYA00199459/OLGE01_FASTA.fasta	MGYA00199459	ERZ489880	root:Host-associated:Human:Digestive system:Large in	0.5618
MGYA00199460/OIWT01_FASTA.fasta	MGYA00199460	ERZ489908	root:Host-associated:Human:Digestive system:Large in	0.3894
MGYA00199461/OLFW01_FASTA.fasta	MGYA00199461	ERZ489897	root:Host-associated:Human:Digestive system:Large in	0.3822
MGYA00199462/OIWC01_FASTA.fasta	MGYA00199462	ERZ489905	root:Host-associated:Human:Digestive system:Large in	0.0618
MGYA00237556/ERZ505227_FASTA.fasta	MGYA00237556	ERZ505227	root:Environmental:Aquatic:Marine	0.8174
MGYA00237558/ERZ650671_FASTA.fasta	MGYA00237558	ERZ650671	root:Host-associated:Birds:Digestive system:Ceca	0.2982
MGYA00237559/ERZ493807_FASTA.fasta	MGYA00237559	ERZ493807	root:Host-associated:Human:Digestive system:Large in	0.4892
MGYA00237560/ERZ493806_FASTA.fasta	MGYA00237560	ERZ493806	root:Host-associated:Human:Digestive system:Large in	0.3704
MGYA00237561/ERZ493808_FASTA.fasta	MGYA00237561	ERZ493808	root:Host-associated:Human:Digestive system:Large in	0.3992
MGYA00237636/ERZ504362_FASTA.fasta	MGYA00237636	ERZ504362	root:Host-associated:Insecta:Digestive system	0.2036
MGYA00237637/ERZ505144_FASTA.fasta	MGYA00237637	ERZ505144	root:Environmental:Aquatic:Marine:Intertidal zone:Coral	0.6182
MGYA00237638/ERZ505149_FASTA.fasta	MGYA00237638	ERZ505149	root:Host-associated:Human:Digestive system:Oral	0.3892
MGYA00237639/ERZ505151_FASTA.fasta	MGYA00237639	ERZ505151	root:Host-associated:Human:Digestive system:Oral	0.0322
MGYA00237640/ERZ505153_FASTA.fasta	MGYA00237640	ERZ505153	root:Host-associated:Human:Digestive system:Oral	0.19
MGYA00237641/ERZ505150_FASTA.fasta	MGYA00237641	ERZ505150	root:Host-associated:Human:Digestive system:Oral	0.3636
MGYA00237642/ERZ505152_FASTA.fasta	MGYA00237642	ERZ505152	root:Host-associated:Human:Digestive system:Oral	0.0294
MGYA00237643/ERZ505155_FASTA.fasta	MGYA00237643	ERZ505155	root:Host-associated:Human:Digestive system:Oral	0.084
MGYA00237644/ERZ505207_FASTA.fasta	MGYA00237644	ERZ505207	root:Environmental:Aquatic:Marine	0.534
MGYA00237645/ERZ505209_FASTA.fasta	MGYA00237645	ERZ505209	root:Environmental:Aquatic:Marine	0.812
MGYA00237646/ERZ505213_FASTA.fasta	MGYA00237646	ERZ505213	root:Environmental:Aquatic:Marine	0.8932
MGYA00237647/ERZ505214_FASTA.fasta	MGYA00237647	ERZ505214	root:Environmental:Aquatic:Marine	0.883
MGYA00237648/ERZ505217_FASTA.fasta	MGYA00237648	ERZ505217	root:Environmental:Aquatic:Marine	0.8884
MGYA00237649/ERZ505219_FASTA.fasta	MGYA00237649	ERZ505219	root:Environmental:Aquatic:Marine	0.8274
MGYA00237650/ERZ505221_FASTA.fasta	MGYA00237650	ERZ505221	root:Environmental:Aquatic:Marine	0.8838
MGYA00237651/ERZ505222_FASTA.fasta	MGYA00237651	ERZ505222	root:Environmental:Aquatic:Marine	0.7934
MGYA00237652/ERZ505224_FASTA.fasta	MGYA00237652	ERZ505224	root:Environmental:Aquatic:Marine	0.8242
MGYA00237653/ERZ505226_FASTA.fasta	MGYA00237653	ERZ505226	root:Environmental:Aquatic:Marine	0.7712
MGYA00237654/ERZ505229_FASTA.fasta	MGYA00237654	ERZ505229	root:Environmental:Aquatic:Marine	0.842
MGYA00237655/ERZ505230_FASTA.fasta	MGYA00237655	ERZ505230	root:Environmental:Aquatic:Marine	0.8046
MGYA00237656/ERZ505234_FASTA.fasta	MGYA00237656	ERZ505234	root:Environmental:Aquatic:Marine	0.8668
MGYA00237657/ERZ505235_FASTA.fasta	MGYA00237657	ERZ505235	root:Environmental:Aquatic:Marine	0.5988
MGYA00237658/ERZ505237_FASTA.fasta	MGYA00237658	ERZ505237	root:Environmental:Aquatic:Marine	0.6042
MGYA00237659/ERZ505238_FASTA.fasta	MGYA00237659	ERZ505238	root:Environmental:Aquatic:Marine	0.7992
MGYA00237660/ERZ505240_FASTA.fasta	MGYA00237660	ERZ505240	root:Environmental:Aquatic:Marine	0.8082
MGYA00237661/ERZ505243_FASTA.fasta	MGYA00237661	ERZ505243	root:Environmental:Aquatic:Marine	0.8154
MGYA00237662/ERZ505245_FASTA.fasta	MGYA00237662	ERZ505245	root:Environmental:Aquatic:Marine	0.8598
MGYA00237663/ERZ505246_FASTA.fasta	MGYA00237663	ERZ505246	root:Environmental:Aquatic:Marine	0.7964
MGYA00237664/ERZ505252_FASTA.fasta	MGYA00237664	ERZ505252	root:Environmental:Aquatic:Marine	0.8616
MGYA00237665/ERZ505255_FASTA.fasta	MGYA00237665	ERZ505255	root:Environmental:Aquatic:Marine	0.8504
MGYA00237666/ERZ505259_FASTA.fasta	MGYA00237666	ERZ505259	root:Environmental:Aquatic:Marine	0.8096
MGYA00237667/ERZ505261_FASTA.fasta	MGYA00237667	ERZ505261	root:Environmental:Aquatic:Marine	0.8158
MGYA00237668/ERZ505262_FASTA.fasta	MGYA00237668	ERZ505262	root:Environmental:Aquatic:Marine	0.8468
MGYA00237669/ERZ505265_FASTA.fasta	MGYA00237669	ERZ505265	root:Environmental:Aquatic:Marine	0.7718
MGYA00237670/ERZ505269_FASTA.fasta	MGYA00237670	ERZ505269	root:Environmental:Aquatic:Marine	0.729
MGYA00237671/ERZ505271_FASTA.fasta	MGYA00237671	ERZ505271	root:Environmental:Aquatic:Marine	0.9112
MGYA00237672/ERZ505275_FASTA.fasta	MGYA00237672	ERZ505275	root:Environmental:Aquatic:Marine	0.7912
MGYA00237673/ERZ505277_FASTA.fasta	MGYA00237673	ERZ505277	root:Environmental:Aquatic:Marine	0.8424
MGYA00237674/ERZ505278_FASTA.fasta	MGYA00237674	ERZ505278	root:Environmental:Aquatic:Marine	0.8844
MGYA00237675/ERZ505285_FASTA.fasta	MGYA00237675	ERZ505285	root:Environmental:Aquatic:Marine	0.8288
MGYA00237676/ERZ505291_FASTA.fasta	MGYA00237676	ERZ505291	root:Environmental:Aquatic:Marine	0.8072
MGYA00237677/ERZ505295_FASTA.fasta	MGYA00237677	ERZ505295	root:Environmental:Aquatic:Marine	0.8452
MGYA00237678/ERZ505297_FASTA.fasta	MGYA00237678	ERZ505297	root:Environmental:Aquatic:Marine	0.8274
MGYA00237679/ERZ505431_FASTA.fasta	MGYA00237679	ERZ505431	root:Host-associated:Human:Digestive system	0.3078
MGYA00237680/ERZ505433_FASTA.fasta	MGYA00237680	ERZ505433	root:Host-associated:Human:Digestive system	0.3464
MGYA00237681/ERZ505437_FASTA.fasta	MGYA00237681	ERZ505437	root:Host-associated:Human:Digestive system	0.4636
MGYA00237682/ERZ505439_FASTA.fasta	MGYA00237682	ERZ505439	root:Host-associated:Human:Digestive system	0.4514
MGYA00237683/ERZ505441_FASTA.fasta	MGYA00237683	ERZ505441	root:Host-associated:Human:Digestive system	0.4376
MGYA00237684/ERZ505443_FASTA.fasta	MGYA00237684	ERZ505443	root:Host-associated:Human:Digestive system	0.4808
MGYA00237685/ERZ505445_FASTA.fasta	MGYA00237685	ERZ505445	root:Host-associated:Human:Digestive system	0.4546
MGYA00237686/ERZ505447_FASTA.fasta	MGYA00237686	ERZ505447	root:Host-associated:Human:Digestive system	0.4502
MGYA00237687/ERZ505449_FASTA.fasta	MGYA00237687	ERZ505449	root:Host-associated:Human:Digestive system	0.511
MGYA00237688/ERZ505451_FASTA.fasta	MGYA00237688	ERZ505451	root:Host-associated:Human:Digestive system	0.4006
MGYA00237689/ERZ505453_FASTA.fasta	MGYA00237689	ERZ505453	root:Host-associated:Human:Digestive system	0.5094
MGYA00237690/ERZ505455_FASTA.fasta	MGYA00237690	ERZ505455	root:Host-associated:Human:Digestive system	0.423
MGYA00237691/ERZ505457_FASTA.fasta	MGYA00237691	ERZ505457	root:Host-associated:Human:Digestive system	0.455
MGYA00237692/ERZ505459_FASTA.fasta	MGYA00237692	ERZ505459	root:Host-associated:Human:Digestive system	0.4846
MGYA00237693/ERZ505461_FASTA.fasta	MGYA00237693	ERZ505461	root:Host-associated:Human:Digestive system	0.483
MGYA00237694/ERZ505463_FASTA.fasta	MGYA00237694	ERZ505463	root:Host-associated:Human:Digestive system	0.4448
MGYA00237695/ERZ505465_FASTA.fasta	MGYA00237695	ERZ505465	root:Host-associated:Human:Digestive system	0.3752
MGYA00237696/ERZ505467_FASTA.fasta	MGYA00237696	ERZ505467	root:Host-associated:Human:Digestive system	0.493
MGYA00237697/ERZ505469_FASTA.fasta	MGYA00237697	ERZ505469	root:Host-associated:Human:Digestive system	0.342
MGYA00237698/ERZ505471_FASTA.fasta	MGYA00237698	ERZ505471	root:Host-associated:Human:Digestive system	0.431
MGYA00237699/ERZ505392_FASTA.fasta	MGYA00237699	ERZ505392	root:Host-associated:Human:Digestive system	0.4248
MGYA00237700/ERZ505394_FASTA.fasta	MGYA00237700	ERZ505394	root:Host-associated:Human:Digestive system	0.4498
MGYA00237701/ERZ505395_FASTA.fasta	MGYA00237701	ERZ505395	root:Host-associated:Human:Digestive system	0.4512
MGYA00237702/ERZ505396_FASTA.fasta	MGYA00237702	ERZ505396	root:Host-associated:Human:Digestive system	0.4754
MGYA00237703/ERZ505400_FASTA.fasta	MGYA00237703	ERZ505400	root:Host-associated:Human:Digestive system	0.4936
MGYA00237704/ERZ505402_FASTA.fasta	MGYA00237704	ERZ505402	root:Host-associated:Human:Digestive system	0.4644
MGYA00237705/ERZ505404_FASTA.fasta	MGYA00237705	ERZ505404	root:Host-associated:Human:Digestive system	0.3844
MGYA00237706/ERZ505406_FASTA.fasta	MGYA00237706	ERZ505406	root:Host-associated:Human:Digestive system	0.449
MGYA00237707/ERZ505408_FASTA.fasta	MGYA00237707	ERZ505408	root:Host-associated:Human:Digestive system	0.4486
MGYA00237708/ERZ505410_FASTA.fasta	MGYA00237708	ERZ505410	root:Host-associated:Human:Digestive system	0.3708
MGYA00237709/ERZ505412_FASTA.fasta	MGYA00237709	ERZ505412	root:Host-associated:Human:Digestive system	0.35
MGYA00237710/ERZ505414_FASTA.fasta	MGYA00237710	ERZ505414	root:Host-associated:Human:Digestive system	0.3796
MGYA00237711/ERZ505416_FASTA.fasta	MGYA00237711	ERZ505416	root:Host-associated:Human:Digestive system	0.496
MGYA00237712/ERZ505418_FASTA.fasta	MGYA00237712	ERZ505418	root:Host-associated:Human:Digestive system	0.4116
MGYA00237713/ERZ505473_FASTA.fasta	MGYA00237713	ERZ505473	root:Host-associated:Human:Digestive system	0.3134
MGYA00237714/ERZ505475_FASTA.fasta	MGYA00237714	ERZ505475	root:Host-associated:Human:Digestive system	0.44
MGYA00237715/ERZ505477_FASTA.fasta	MGYA00237715	ERZ505477	root:Host-associated:Human:Digestive system	0.476
MGYA00237716/ERZ505479_FASTA.fasta	MGYA00237716	ERZ505479	root:Host-associated:Human:Digestive system	0.5176
MGYA00237717/ERZ505481_FASTA.fasta	MGYA00237717	ERZ505481	root:Host-associated:Human:Digestive system	0.4722
MGYA00237718/ERZ505483_FASTA.fasta	MGYA00237718	ERZ505483	root:Host-associated:Human:Digestive system	0.537
MGYA00237719/ERZ505487_FASTA.fasta	MGYA00237719	ERZ505487	root:Host-associated:Human:Digestive system	0.4136
MGYA00237720/ERZ505420_FASTA.fasta	MGYA00237720	ERZ505420	root:Host-associated:Human:Digestive system	0.4056
MGYA00237721/ERZ505422_FASTA.fasta	MGYA00237721	ERZ505422	root:Host-associated:Human:Digestive system	0.502
MGYA00237722/ERZ505424_FASTA.fasta	MGYA00237722	ERZ505424	root:Host-associated:Human:Digestive system	0.425
MGYA00237723/ERZ505426_FASTA.fasta	MGYA00237723	ERZ505426	root:Host-associated:Human:Digestive system	0.5014
MGYA00237724/ERZ505428_FASTA.fasta	MGYA00237724	ERZ505428	root:Host-associated:Human:Digestive system	0.4014
MGYA00237725/ERZ505430_FASTA.fasta	MGYA00237725	ERZ505430	root:Host-associated:Human:Digestive system	0.4934
MGYA00237726/ERZ505432_FASTA.fasta	MGYA00237726	ERZ505432	root:Host-associated:Human:Digestive system	0.4218
MGYA00237727/ERZ505434_FASTA.fasta	MGYA00237727	ERZ505434	root:Host-associated:Human:Digestive system	0.4616
MGYA00237728/ERZ505436_FASTA.fasta	MGYA00237728	ERZ505436	root:Host-associated:Human:Digestive system	0.495
MGYA00237729/ERZ505438_FASTA.fasta	MGYA00237729	ERZ505438	root:Host-associated:Human:Digestive system	0.4248
MGYA00237730/ERZ505440_FASTA.fasta	MGYA00237730	ERZ505440	root:Host-associated:Human:Digestive system	0.3742
MGYA00237731/ERZ505442_FASTA.fasta	MGYA00237731	ERZ505442	root:Host-associated:Human:Digestive system	0.3982
MGYA00237732/ERZ505444_FASTA.fasta	MGYA00237732	ERZ505444	root:Host-associated:Human:Digestive system	0.3958
MGYA00237733/ERZ505446_FASTA.fasta	MGYA00237733	ERZ505446	root:Host-associated:Human:Digestive system	0.469
MGYA00237734/ERZ505448_FASTA.fasta	MGYA00237734	ERZ505448	root:Host-associated:Human:Digestive system	0.3984
MGYA00237735/ERZ505450_FASTA.fasta	MGYA00237735	ERZ505450	root:Host-associated:Human:Digestive system	0.4042
MGYA00237736/ERZ505452_FASTA.fasta	MGYA00237736	ERZ505452	root:Host-associated:Human:Digestive system	0.4612
MGYA00237737/ERZ505454_FASTA.fasta	MGYA00237737	ERZ505454	root:Host-associated:Human:Digestive system	0.4532
MGYA00237738/ERZ505456_FASTA.fasta	MGYA00237738	ERZ505456	root:Host-associated:Human:Digestive system	0.4328
MGYA00237739/ERZ505458_FASTA.fasta	MGYA00237739	ERZ505458	root:Host-associated:Human:Digestive system	0.3608
MGYA00237740/ERZ505460_FASTA.fasta	MGYA00237740	ERZ505460	root:Host-associated:Human:Digestive system	0.3784
MGYA00237741/ERZ505462_FASTA.fasta	MGYA00237741	ERZ505462	root:Host-associated:Human:Digestive system	0.349
MGYA00237742/ERZ505464_FASTA.fasta	MGYA00237742	ERZ505464	root:Host-associated:Human:Digestive system	0.5346
MGYA00237743/ERZ505466_FASTA.fasta	MGYA00237743	ERZ505466	root:Host-associated:Human:Digestive system	0.4776
MGYA00237744/ERZ505468_FASTA.fasta	MGYA00237744	ERZ505468	root:Host-associated:Human:Digestive system	0.3912
MGYA00237745/ERZ505470_FASTA.fasta	MGYA00237745	ERZ505470	root:Host-associated:Human:Digestive system	0.4704
MGYA00237746/ERZ505472_FASTA.fasta	MGYA00237746	ERZ505472	root:Host-associated:Human:Digestive system	0.4196
MGYA00237747/ERZ505474_FASTA.fasta	MGYA00237747	ERZ505474	root:Host-associated:Human:Digestive system	0.3252
MGYA00237748/ERZ505476_FASTA.fasta	MGYA00237748	ERZ505476	root:Host-associated:Human:Digestive system	0.401
MGYA00237749/ERZ505478_FASTA.fasta	MGYA00237749	ERZ505478	root:Host-associated:Human:Digestive system	0.4176
MGYA00237750/ERZ505480_FASTA.fasta	MGYA00237750	ERZ505480	root:Host-associated:Human:Digestive system	0.4112
MGYA00237751/ERZ505482_FASTA.fasta	MGYA00237751	ERZ505482	root:Host-associated:Human:Digestive system	0.4298
MGYA00237752/ERZ505484_FASTA.fasta	MGYA00237752	ERZ505484	root:Host-associated:Human:Digestive system	0.422
MGYA00237753/ERZ505486_FASTA.fasta	MGYA00237753	ERZ505486	root:Host-associated:Human:Digestive system	0.494
MGYA00237754/ERZ505488_FASTA.fasta	MGYA00237754	ERZ505488	root:Host-associated:Human:Digestive system	0.4706
MGYA00237755/ERZ505489_FASTA.fasta	MGYA00237755	ERZ505489	root:Host-associated:Human:Digestive system	0.4338
MGYA00237756/ERZ505490_FASTA.fasta	MGYA00237756	ERZ505490	root:Host-associated:Human:Digestive system	0.387
MGYA00237757/ERZ505491_FASTA.fasta	MGYA00237757	ERZ505491	root:Host-associated:Human:Digestive system	0.4988
MGYA00237758/ERZ505393_FASTA.fasta	MGYA00237758	ERZ505393	root:Host-associated:Human:Digestive system	0.4224
MGYA00237759/ERZ505397_FASTA.fasta	MGYA00237759	ERZ505397	root:Host-associated:Human:Digestive system	0.4856
MGYA00237760/ERZ505399_FASTA.fasta	MGYA00237760	ERZ505399	root:Host-associated:Human:Digestive system	0.414
MGYA00237761/ERZ505401_FASTA.fasta	MGYA00237761	ERZ505401	root:Host-associated:Human:Digestive system	0.373
MGYA00237762/ERZ505403_FASTA.fasta	MGYA00237762	ERZ505403	root:Host-associated:Human:Digestive system	0.3532
MGYA00237763/ERZ505405_FASTA.fasta	MGYA00237763	ERZ505405	root:Host-associated:Human:Digestive system	0.3894
MGYA00237764/ERZ505407_FASTA.fasta	MGYA00237764	ERZ505407	root:Host-associated:Human:Digestive system	0.3758
MGYA00237765/ERZ505409_FASTA.fasta	MGYA00237765	ERZ505409	root:Host-associated:Human:Digestive system	0.4322
MGYA00237766/ERZ505411_FASTA.fasta	MGYA00237766	ERZ505411	root:Host-associated:Human:Digestive system	0.4748
MGYA00237767/ERZ505413_FASTA.fasta	MGYA00237767	ERZ505413	root:Host-associated:Human:Digestive system	0.4836
MGYA00237768/ERZ505415_FASTA.fasta	MGYA00237768	ERZ505415	root:Host-associated:Human:Digestive system	0.4792
MGYA00237769/ERZ505417_FASTA.fasta	MGYA00237769	ERZ505417	root:Host-associated:Human:Digestive system	0.5204
MGYA00237770/ERZ505419_FASTA.fasta	MGYA00237770	ERZ505419	root:Host-associated:Human:Digestive system	0.5062
MGYA00237771/ERZ505421_FASTA.fasta	MGYA00237771	ERZ505421	root:Host-associated:Human:Digestive system	0.4712
MGYA00237772/ERZ505423_FASTA.fasta	MGYA00237772	ERZ505423	root:Host-associated:Human:Digestive system	0.3712
MGYA00237773/ERZ505425_FASTA.fasta	MGYA00237773	ERZ505425	root:Host-associated:Human:Digestive system	0.4996
MGYA00237774/ERZ505427_FASTA.fasta	MGYA00237774	ERZ505427	root:Host-associated:Human:Digestive system	0.518
MGYA00237775/ERZ505429_FASTA.fasta	MGYA00237775	ERZ505429	root:Host-associated:Human:Digestive system	0.5806
MGYA00237776/ERZ505498_FASTA.fasta	MGYA00237776	ERZ505498	root:Environmental:Aquatic:Marine	0.8976
MGYA00237777/ERZ505514_FASTA.fasta	MGYA00237777	ERZ505514	root:Environmental:Aquatic:Freshwater:Lake	0.8838
MGYA00237778/ERZ508506_FASTA.fasta	MGYA00237778	ERZ508506	root:Environmental:Aquatic:Marine	0.8502
MGYA00237779/ERZ508509_FASTA.fasta	MGYA00237779	ERZ508509	root:Environmental:Aquatic:Marine	0.8892
MGYA00237780/ERZ508510_FASTA.fasta	MGYA00237780	ERZ508510	root:Environmental:Aquatic:Marine	0.8268
MGYA00237781/ERZ508513_FASTA.fasta	MGYA00237781	ERZ508513	root:Environmental:Aquatic:Marine:Intertidal zone:Salt	0.4456
MGYA00237782/ERZ508514_FASTA.fasta	MGYA00237782	ERZ508514	root:Environmental:Aquatic:Marine:Intertidal zone:Salt	0.4096
MGYA00237783/ERZ508515_FASTA.fasta	MGYA00237783	ERZ508515	root:Environmental:Aquatic:Marine:Intertidal zone:Salt	0.7062
MGYA00237784/ERZ508517_FASTA.fasta	MGYA00237784	ERZ508517	root:Environmental:Aquatic:Marine	0.825
MGYA00237785/ERZ508519_FASTA.fasta	MGYA00237785	ERZ508519	root:Environmental:Aquatic:Marine	0.7332
MGYA00237786/ERZ508520_FASTA.fasta	MGYA00237786	ERZ508520	root:Environmental:Aquatic:Marine	0.892
MGYA00237787/ERZ508521_FASTA.fasta	MGYA00237787	ERZ508521	root:Environmental:Aquatic:Marine	0.7886
MGYA00237788/ERZ508522_FASTA.fasta	MGYA00237788	ERZ508522	root:Environmental:Aquatic:Marine	0.8758
MGYA00237789/ERZ508523_FASTA.fasta	MGYA00237789	ERZ508523	root:Environmental:Aquatic:Marine	0.7872
MGYA00237790/ERZ508526_FASTA.fasta	MGYA00237790	ERZ508526	root:Environmental:Aquatic:Marine	0.8456
MGYA00237791/ERZ508527_FASTA.fasta	MGYA00237791	ERZ508527	root:Environmental:Aquatic:Marine	0.7736
MGYA00237792/ERZ508528_FASTA.fasta	MGYA00237792	ERZ508528	root:Environmental:Aquatic:Marine	0.8342
MGYA00237793/ERZ508529_FASTA.fasta	MGYA00237793	ERZ508529	root:Environmental:Aquatic:Marine	0.7176
MGYA00237794/ERZ508530_FASTA.fasta	MGYA00237794	ERZ508530	root:Environmental:Aquatic:Marine	0.6836
MGYA00237795/ERZ508531_FASTA.fasta	MGYA00237795	ERZ508531	root:Environmental:Aquatic:Marine	0.867
MGYA00237796/ERZ508532_FASTA.fasta	MGYA00237796	ERZ508532	root:Environmental:Aquatic:Marine	0.853
MGYA00237797/ERZ508534_FASTA.fasta	MGYA00237797	ERZ508534	root:Environmental:Aquatic:Marine	0.7528
MGYA00237798/ERZ508535_FASTA.fasta	MGYA00237798	ERZ508535	root:Environmental:Aquatic:Marine	0.7796
MGYA00237799/ERZ508536_FASTA.fasta	MGYA00237799	ERZ508536	root:Environmental:Aquatic:Marine	0.8048
MGYA00237800/ERZ508537_FASTA.fasta	MGYA00237800	ERZ508537	root:Environmental:Aquatic:Marine	0.6842
MGYA00237801/ERZ508541_FASTA.fasta	MGYA00237801	ERZ508541	root:Host-associated:Mollusca	0.5618
MGYA00237802/ERZ508542_FASTA.fasta	MGYA00237802	ERZ508542	root:Host-associated:Mollusca	0.5088
MGYA00237803/ERZ508544_FASTA.fasta	MGYA00237803	ERZ508544	root:Environmental:Aquatic:Marine:Intertidal zone:Salt	0.8562
MGYA00237804/ERZ508545_FASTA.fasta	MGYA00237804	ERZ508545	root:Environmental:Aquatic:Marine	0.8118
MGYA00237805/ERZ508546_FASTA.fasta	MGYA00237805	ERZ508546	root:Environmental:Aquatic:Marine	0.7748
MGYA00237806/ERZ508548_FASTA.fasta	MGYA00237806	ERZ508548	root:Environmental:Aquatic:Marine:Sediment	0.3574
MGYA00237807/ERZ508549_FASTA.fasta	MGYA00237807	ERZ508549	root:Environmental:Aquatic:Marine:Intertidal zone:Estu	0.7874
MGYA00237808/ERZ508550_FASTA.fasta	MGYA00237808	ERZ508550	root:Environmental:Aquatic:Marine:Intertidal zone:Cora	0.2386
MGYA00237809/ERZ508551_FASTA.fasta	MGYA00237809	ERZ508551	root:Environmental:Aquatic:Marine	0.8756
MGYA00237810/ERZ508553_FASTA.fasta	MGYA00237810	ERZ508553	root:Environmental:Aquatic:Marine	0.8468
MGYA00237811/ERZ508554_FASTA.fasta	MGYA00237811	ERZ508554	root:Environmental:Aquatic:Marine	0.8512
MGYA00237812/ERZ508556_FASTA.fasta	MGYA00237812	ERZ508556	root:Environmental:Aquatic:Marine:Intertidal zone:Estu	0.7952
MGYA00237813/ERZ508557_FASTA.fasta	MGYA00237813	ERZ508557	root:Environmental:Aquatic:Marine:Intertidal zone:Estu	0.8072
MGYA00237814/ERZ508559_FASTA.fasta	MGYA00237814	ERZ508559	root:Environmental:Aquatic:Marine:Intertidal zone:Estu	0.8222
MGYA00237815/ERZ508560_FASTA.fasta	MGYA00237815	ERZ508560	root:Environmental:Aquatic:Marine:Intertidal zone:Estu	0.8346
MGYA00237816/ERZ508562_FASTA.fasta	MGYA00237816	ERZ508562	root:Environmental:Aquatic:Marine:Intertidal zone:Estu	0.8212
MGYA00237817/ERZ508563_FASTA.fasta	MGYA00237817	ERZ508563	root:Environmental:Aquatic:Marine:Intertidal zone:Estu	0.8686
MGYA00237818/ERZ508564_FASTA.fasta	MGYA00237818	ERZ508564	root:Environmental:Aquatic:Marine:Intertidal zone:Estu	0.7252
MGYA00237819/ERZ508565_FASTA.fasta	MGYA00237819	ERZ508565	root:Environmental:Aquatic:Marine:Intertidal zone:Estu	0.7926
MGYA00237820/ERZ508566_FASTA.fasta	MGYA00237820	ERZ508566	root:Environmental:Aquatic:Marine:Intertidal zone:Estu	0.8032
MGYA00237821/ERZ508568_FASTA.fasta	MGYA00237821	ERZ508568	root:Environmental:Aquatic:Marine:Intertidal zone:Estu	0.7844
MGYA00237822/ERZ508569_FASTA.fasta	MGYA00237822	ERZ508569	root:Environmental:Aquatic:Marine:Intertidal zone:Estu	0.8124
MGYA00237823/ERZ508570_FASTA.fasta	MGYA00237823	ERZ508570	root:Environmental:Aquatic:Marine:Intertidal zone:Estu	0.7544
MGYA00237824/ERZ508572_FASTA.fasta	MGYA00237824	ERZ508572	root:Environmental:Aquatic:Marine:Intertidal zone:Estu	0.7986
MGYA00237825/ERZ508573_FASTA.fasta	MGYA00237825	ERZ508573	root:Environmental:Aquatic:Marine:Intertidal zone:Estu	0.8168
MGYA00237826/ERZ508574_FASTA.fasta	MGYA00237826	ERZ508574	root:Environmental:Aquatic:Marine:Intertidal zone:Estu	0.7636
MGYA00237827/ERZ508575_FASTA.fasta	MGYA00237827	ERZ508575	root:Environmental:Aquatic:Marine:Intertidal zone:Estu	0.7974
MGYA00237828/ERZ508577_FASTA.fasta	MGYA00237828	ERZ508577	root:Environmental:Aquatic:Marine:Intertidal zone:Estu	0.8134
MGYA00237829/ERZ508578_FASTA.fasta	MGYA00237829	ERZ508578	root:Environmental:Aquatic:Marine:Intertidal zone:Estu	0.844
MGYA00237830/ERZ508579_FASTA.fasta	MGYA00237830	ERZ508579	root:Environmental:Aquatic:Marine:Intertidal zone:Estu	0.842
MGYA00237831/ERZ508580_FASTA.fasta	MGYA00237831	ERZ508580	root:Environmental:Aquatic:Marine:Intertidal zone:Estu	0.814
MGYA00237832/ERZ508581_FASTA.fasta	MGYA00237832	ERZ508581	root:Environmental:Aquatic:Marine:Intertidal zone:Estu	0.8118
MGYA00237833/ERZ508582_FASTA.fasta	MGYA00237833	ERZ508582	root:Environmental:Aquatic:Marine:Intertidal zone:Estu	0.8028
MGYA00237834/ERZ508583_FASTA.fasta	MGYA00237834	ERZ508583	root:Environmental:Aquatic:Marine:Intertidal zone:Estu	0.7754
MGYA00237835/ERZ508584_FASTA.fasta	MGYA00237835	ERZ508584	root:Environmental:Aquatic:Marine:Intertidal zone:Estu	0.7586
MGYA00237836/ERZ508586_FASTA.fasta	MGYA00237836	ERZ508586	root:Environmental:Aquatic:Marine:Intertidal zone:Estu	0.831
MGYA00237837/ERZ508588_FASTA.fasta	MGYA00237837	ERZ508588	root:Environmental:Aquatic:Marine:Intertidal zone:Estu	0.7698
MGYA00237838/ERZ508589_FASTA.fasta	MGYA00237838	ERZ508589	root:Environmental:Aquatic:Marine:Intertidal zone:Estu	0.7676
MGYA00237839/ERZ508590_FASTA.fasta	MGYA00237839	ERZ508590	root:Environmental:Aquatic:Marine:Intertidal zone:Estu	0.8186
MGYA00237840/ERZ508591_FASTA.fasta	MGYA00237840	ERZ508591	root:Environmental:Aquatic:Marine:Intertidal zone:Estu	0.7868
MGYA00237841/ERZ508595_FASTA.fasta	MGYA00237841	ERZ508595	root:Environmental:Aquatic:Thermal springs:Hot (42-90	0.4282
MGYA00237842/ERZ508596_FASTA.fasta	MGYA00237842	ERZ508596	root:Environmental:Aquatic:Thermal springs:Hot (42-90	0.684
MGYA00237843/ERZ508599_FASTA.fasta	MGYA00237843	ERZ508599	root:Environmental:Aquatic:Marine:Intertidal zone:Salt	0.4854
MGYA00237844/ERZ508600_FASTA.fasta	MGYA00237844	ERZ508600	root:Environmental:Aquatic:Marine:Intertidal zone:Salt	0.711
MGYA00237845/ERZ508601_FASTA.fasta	MGYA00237845	ERZ508601	root:Environmental:Aquatic:Marine	0.8096
MGYA00237846/ERZ508604_FASTA.fasta	MGYA00237846	ERZ508604	root:Environmental:Aquatic:Marine	0.6076
MGYA00237847/ERZ508605_FASTA.fasta	MGYA00237847	ERZ508605	root:Environmental:Aquatic:Marine	0.7936
MGYA00237848/ERZ508608_FASTA.fasta	MGYA00237848	ERZ508608	root:Environmental:Aquatic:Marine	0.8438
MGYA00237849/ERZ508657_FASTA.fasta	MGYA00237849	ERZ508657	root:Environmental:Aquatic:Marine:Intertidal zone:Estu	0.8302
MGYA00237850/ERZ508658_FASTA.fasta	MGYA00237850	ERZ508658	root:Environmental:Aquatic:Marine:Intertidal zone:Estu	0.8266
MGYA00237851/ERZ508659_FASTA.fasta	MGYA00237851	ERZ508659	root:Environmental:Aquatic:Marine:Intertidal zone:Estu	0.8132
MGYA00237852/ERZ508660_FASTA.fasta	MGYA00237852	ERZ508660	root:Host-associated:Algae:Red algae	0.9244
MGYA00237853/ERZ510998_FASTA.fasta	MGYA00237853	ERZ510998	root:Environmental:Aquatic:Marine:Oceanic:Oil-contami	0.9146
MGYA00237854/ERZ510997_FASTA.fasta	MGYA00237854	ERZ510997	root:Environmental:Aquatic:Marine:Oceanic:Oil-contami	0.7616
MGYA00237855/ERZ510999_FASTA.fasta	MGYA00237855	ERZ510999	root:Environmental:Aquatic:Marine:Oceanic:Oil-contami	0.7828
MGYA00237856/ERZ511461_FASTA.fasta	MGYA00237856	ERZ511461	root:Environmental:Aquatic:Marine:Intertidal zone:Estu	0.8374
MGYA00237857/ERZ511462_FASTA.fasta	MGYA00237857	ERZ511462	root:Environmental:Aquatic:Marine:Intertidal zone:Estu	0.8392
MGYA00237858/ERZ511464_FASTA.fasta	MGYA00237858	ERZ511464	root:Environmental:Aquatic:Marine:Intertidal zone:Estu	0.7978
MGYA00237859/ERZ511465_FASTA.fasta	MGYA00237859	ERZ511465	root:Environmental:Aquatic:Marine:Sediment	0.7842
MGYA00237860/ERZ511466_FASTA.fasta	MGYA00237860	ERZ511466	root:Environmental:Aquatic:Marine:Sediment	0.7762
MGYA00237861/ERZ511468_FASTA.fasta	MGYA00237861	ERZ511468	root:Environmental:Aquatic:Marine:Sediment	0.791
MGYA00237862/ERZ511471_FASTA.fasta	MGYA00237862	ERZ511471	root:Environmental:Aquatic:Marine:Intertidal zone:Sedi	0.5818
MGYA00237863/ERZ511475_FASTA.fasta	MGYA00237863	ERZ511475	root:Environmental:Aquatic:Thermal springs:Sediment	0.7326
MGYA00237864/ERZ511476_FASTA.fasta	MGYA00237864	ERZ511476	root:Environmental:Aquatic:Marine:Coastal	0.8252
MGYA00237865/ERZ511478_FASTA.fasta	MGYA00237865	ERZ511478	root:Host-associated:Algae:Red algae	0.9694
MGYA00237866/ERZ511479_FASTA.fasta	MGYA00237866	ERZ511479	root:Host-associated:Algae:Red algae	0.9162
MGYA00237867/ERZ511480_FASTA.fasta	MGYA00237867	ERZ511480	root:Host-associated:Algae:Red algae	0.9596
MGYA00237868/ERZ511482_FASTA.fasta	MGYA00237868	ERZ511482	root:Host-associated:Algae:Red algae	0.9176
MGYA00237869/ERZ511484_FASTA.fasta	MGYA00237869	ERZ511484	root:Host-associated:Algae:Red algae	0.9312
MGYA00237870/ERZ529317_FASTA.fasta	MGYA00237870	ERZ529317	root:Environmental:Aquatic:Marine	0.4518
MGYA00237871/ERZ529316_FASTA.fasta	MGYA00237871	ERZ529316	root:Environmental:Aquatic:Marine	0.4758
MGYA00237872/ERZ529318_FASTA.fasta	MGYA00237872	ERZ529318	root:Environmental:Aquatic:Marine	0.5152
MGYA00237873/ERZ529320_FASTA.fasta	MGYA00237873	ERZ529320	root:Environmental:Aquatic:Marine	0.0294
MGYA00237874/ERZ529319_FASTA.fasta	MGYA00237874	ERZ529319	root:Environmental:Aquatic:Marine	0.0294
MGYA00237875/ERZ529327_FASTA.fasta	MGYA00237875	ERZ529327	root:Environmental:Aquatic:Marine	0.7748
MGYA00237876/ERZ529329_FASTA.fasta	MGYA00237876	ERZ529329	root:Environmental:Aquatic:Marine	0.7658
MGYA00237877/ERZ529326_FASTA.fasta	MGYA00237877	ERZ529326	root:Environmental:Aquatic:Marine	0.5012
MGYA00237878/ERZ529328_FASTA.fasta	MGYA00237878	ERZ529328	root:Environmental:Aquatic:Marine	0.7372
MGYA00237879/ERZ529330_FASTA.fasta	MGYA00237879	ERZ529330	root:Environmental:Aquatic:Marine	0.791
MGYA00237880/ERZ529332_FASTA.fasta	MGYA00237880	ERZ529332	root:Environmental:Aquatic:Sediment	0.6826
MGYA00237881/ERZ529331_FASTA.fasta	MGYA00237881	ERZ529331	root:Environmental:Aquatic:Sediment	0.3344
MGYA00237882/ERZ534229_FASTA.fasta	MGYA00237882	ERZ534229	root:Environmental:Aquatic:Marine:Sediment	0.8426
MGYA00237883/ERZ534232_FASTA.fasta	MGYA00237883	ERZ534232	root:Environmental:Aquatic:Freshwater:Lotic:Sediment	0.6304
MGYA00237884/ERZ534233_FASTA.fasta	MGYA00237884	ERZ534233	root:Environmental:Aquatic:Estuary:Sediment	0.8568
MGYA00237885/ERZ534234_FASTA.fasta	MGYA00237885	ERZ534234	root:Environmental:Aquatic:Marine:Coastal	0.7942
MGYA00237886/ERZ534235_FASTA.fasta	MGYA00237886	ERZ534235	root:Host-associated:Algae:Red algae	0.8576
MGYA00237887/ERZ534236_FASTA.fasta	MGYA00237887	ERZ534236	root:Environmental:Aquatic:Freshwater:Sediment	0.551
MGYA00237888/ERZ534237_FASTA.fasta	MGYA00237888	ERZ534237	root:Environmental:Aquatic:Freshwater:Sediment	0.5504
MGYA00237889/ERZ534238_FASTA.fasta	MGYA00237889	ERZ534238	root:Environmental:Aquatic:Estuary:Sediment	0.6304
MGYA00237890/ERZ534239_FASTA.fasta	MGYA00237890	ERZ534239	root:Environmental:Aquatic:Marine	0.7444
MGYA00237891/ERZ534240_FASTA.fasta	MGYA00237891	ERZ534240	root:Environmental:Aquatic:Marine:Sediment	0.576
MGYA00237892/ERZ548782_FASTA.fasta	MGYA00237892	ERZ548782	root:Host-associated:Human:Digestive system:Large in	0.389
MGYA00237893/ERZ548784_FASTA.fasta	MGYA00237893	ERZ548784	root:Host-associated:Human:Digestive system:Large in	0.3732
MGYA00237894/ERZ548786_FASTA.fasta	MGYA00237894	ERZ548786	root:Host-associated:Human:Digestive system:Large in	0.3506
MGYA00237895/ERZ548792_FASTA.fasta	MGYA00237895	ERZ548792	root:Host-associated:Human:Digestive system:Large in	0.3016
MGYA00237896/ERZ548794_FASTA.fasta	MGYA00237896	ERZ548794	root:Host-associated:Human:Digestive system:Large in	0.3164
MGYA00237897/ERZ548800_FASTA.fasta	MGYA00237897	ERZ548800	root:Host-associated:Human:Digestive system:Large in	0.4096
MGYA00237898/ERZ548802_FASTA.fasta	MGYA00237898	ERZ548802	root:Host-associated:Human:Digestive system:Large in	0.373
MGYA00237899/ERZ548804_FASTA.fasta	MGYA00237899	ERZ548804	root:Host-associated:Human:Digestive system:Large in	0.3756
MGYA00237900/ERZ548805_FASTA.fasta	MGYA00237900	ERZ548805	root:Host-associated:Human:Digestive system:Large in	0.4174
MGYA00237901/ERZ548810_FASTA.fasta	MGYA00237901	ERZ548810	root:Host-associated:Human:Digestive system:Large in	0.2748
MGYA00237902/ERZ548812_FASTA.fasta	MGYA00237902	ERZ548812	root:Host-associated:Human:Digestive system:Large in	0.3432
MGYA00237903/ERZ548816_FASTA.fasta	MGYA00237903	ERZ548816	root:Host-associated:Human:Digestive system:Large in	0.3764
MGYA00237904/ERZ548818_FASTA.fasta	MGYA00237904	ERZ548818	root:Host-associated:Human:Digestive system:Large in	0.3448
MGYA00237905/ERZ548820_FASTA.fasta	MGYA00237905	ERZ548820	root:Host-associated:Human:Digestive system:Large in	0.3352
MGYA00237906/ERZ548822_FASTA.fasta	MGYA00237906	ERZ548822	root:Host-associated:Human:Digestive system:Large in	0.3524
MGYA00237907/ERZ548833_FASTA.fasta	MGYA00237907	ERZ548833	root:Host-associated:Human:Digestive system:Large in	0.448
MGYA00237908/ERZ548835_FASTA.fasta	MGYA00237908	ERZ548835	root:Host-associated:Human:Digestive system:Large in	0.3412
MGYA00237909/ERZ548839_FASTA.fasta	MGYA00237909	ERZ548839	root:Host-associated:Human:Digestive system:Large in	0.4066
MGYA00237910/ERZ548843_FASTA.fasta	MGYA00237910	ERZ548843	root:Host-associated:Human:Digestive system:Large in	0.407
MGYA00237911/ERZ548845_FASTA.fasta	MGYA00237911	ERZ548845	root:Host-associated:Human:Digestive system:Large in	0.4252
MGYA00237912/ERZ548777_FASTA.fasta	MGYA00237912	ERZ548777	root:Host-associated:Human:Digestive system:Large in	0.3988
MGYA00237913/ERZ548779_FASTA.fasta	MGYA00237913	ERZ548779	root:Host-associated:Human:Digestive system:Large in	0.2936
MGYA00237914/ERZ548781_FASTA.fasta	MGYA00237914	ERZ548781	root:Host-associated:Human:Digestive system:Large in	0.25
MGYA00237915/ERZ548787_FASTA.fasta	MGYA00237915	ERZ548787	root:Host-associated:Human:Digestive system:Large in	0.3266
MGYA00237916/ERZ548789_FASTA.fasta	MGYA00237916	ERZ548789	root:Host-associated:Human:Digestive system:Large in	0.3446
MGYA00237917/ERZ548791_FASTA.fasta	MGYA00237917	ERZ548791	root:Host-associated:Human:Digestive system:Large in	0.3216
MGYA00237918/ERZ548796_FASTA.fasta	MGYA00237918	ERZ548796	root:Host-associated:Human:Digestive system:Large in	0.438
MGYA00237919/ERZ548797_FASTA.fasta	MGYA00237919	ERZ548797	root:Host-associated:Human:Digestive system:Large in	0.3416
MGYA00237920/ERZ548799_FASTA.fasta	MGYA00237920	ERZ548799	root:Host-associated:Human:Digestive system:Large in	0.4056
MGYA00237921/ERZ548744_FASTA.fasta	MGYA00237921	ERZ548744	root:Host-associated:Human:Digestive system:Large in	0.439
MGYA00237922/ERZ548746_FASTA.fasta	MGYA00237922	ERZ548746	root:Host-associated:Human:Digestive system:Large in	0.4004
MGYA00237923/ERZ548749_FASTA.fasta	MGYA00237923	ERZ548749	root:Host-associated:Human:Digestive system:Large in	0.3514
MGYA00237924/ERZ548750_FASTA.fasta	MGYA00237924	ERZ548750	root:Host-associated:Human:Digestive system:Large in	0.448
MGYA00237925/ERZ548752_FASTA.fasta	MGYA00237925	ERZ548752	root:Host-associated:Human:Digestive system:Large in	0.3516
MGYA00237926/ERZ548754_FASTA.fasta	MGYA00237926	ERZ548754	root:Host-associated:Human:Digestive system:Large in	0.3734
MGYA00237927/ERZ548762_FASTA.fasta	MGYA00237927	ERZ548762	root:Host-associated:Human:Digestive system:Large in	0.2502
MGYA00237928/ERZ548764_FASTA.fasta	MGYA00237928	ERZ548764	root:Host-associated:Human:Digestive system:Large in	0.4004
MGYA00237929/ERZ548773_FASTA.fasta	MGYA00237929	ERZ548773	root:Host-associated:Human:Digestive system:Large in	0.2642
MGYA00237930/ERZ548775_FASTA.fasta	MGYA00237930	ERZ548775	root:Host-associated:Human:Digestive system:Large in	0.2972
MGYA00237931/ERZ548747_FASTA.fasta	MGYA00237931	ERZ548747	root:Host-associated:Human:Digestive system:Large in	0.375
MGYA00237932/ERZ548755_FASTA.fasta	MGYA00237932	ERZ548755	root:Host-associated:Human:Digestive system:Large in	0.3124
MGYA00237933/ERZ548757_FASTA.fasta	MGYA00237933	ERZ548757	root:Host-associated:Human:Digestive system:Large in	0.3998
MGYA00237934/ERZ548759_FASTA.fasta	MGYA00237934	ERZ548759	root:Host-associated:Human:Digestive system:Large in	0.4324
MGYA00237935/ERZ548761_FASTA.fasta	MGYA00237935	ERZ548761	root:Host-associated:Human:Digestive system:Large in	0.278
MGYA00237936/ERZ548765_FASTA.fasta	MGYA00237936	ERZ548765	root:Host-associated:Human:Digestive system:Large in	0.4
MGYA00237937/ERZ548767_FASTA.fasta	MGYA00237937	ERZ548767	root:Host-associated:Human:Digestive system:Large in	0.4022
MGYA00237938/ERZ548769_FASTA.fasta	MGYA00237938	ERZ548769	root:Host-associated:Human:Digestive system:Large in	0.391
MGYA00237939/ERZ548770_FASTA.fasta	MGYA00237939	ERZ548770	root:Host-associated:Human:Digestive system:Large in	0.3738
MGYA00237940/ERZ548809_FASTA.fasta	MGYA00237940	ERZ548809	root:Host-associated:Human:Digestive system:Large in	0.3208
MGYA00237941/ERZ548813_FASTA.fasta	MGYA00237941	ERZ548813	root:Host-associated:Human:Digestive system:Large in	0.366
MGYA00237942/ERZ548815_FASTA.fasta	MGYA00237942	ERZ548815	root:Host-associated:Human:Digestive system:Large in	0.4
MGYA00237943/ERZ548823_FASTA.fasta	MGYA00237943	ERZ548823	root:Host-associated:Human:Digestive system:Large in	0.3852
MGYA00237944/ERZ548825_FASTA.fasta	MGYA00237944	ERZ548825	root:Host-associated:Human:Digestive system:Large in	0.344
MGYA00237945/ERZ548829_FASTA.fasta	MGYA00237945	ERZ548829	root:Host-associated:Human:Digestive system:Large in	0.3498
MGYA00237946/ERZ548807_FASTA.fasta	MGYA00237946	ERZ548807	root:Host-associated:Human:Digestive system:Large in	0.2714
MGYA00237947/ERZ548826_FASTA.fasta	MGYA00237947	ERZ548826	root:Host-associated:Human:Digestive system:Large in	0.3892
MGYA00237948/ERZ548828_FASTA.fasta	MGYA00237948	ERZ548828	root:Host-associated:Human:Digestive system:Large in	0.4084
MGYA00237949/ERZ548830_FASTA.fasta	MGYA00237949	ERZ548830	root:Host-associated:Human:Digestive system:Large in	0.3504
MGYA00237950/ERZ548832_FASTA.fasta	MGYA00237950	ERZ548832	root:Host-associated:Human:Digestive system:Large in	0.3796
MGYA00237951/ERZ548836_FASTA.fasta	MGYA00237951	ERZ548836	root:Host-associated:Human:Digestive system:Large in	0.4238
MGYA00237952/ERZ548838_FASTA.fasta	MGYA00237952	ERZ548838	root:Host-associated:Human:Digestive system:Large in	0.3982
MGYA00237953/ERZ548840_FASTA.fasta	MGYA00237953	ERZ548840	root:Host-associated:Human:Digestive system:Large in	0.3702
MGYA00237954/ERZ548842_FASTA.fasta	MGYA00237954	ERZ548842	root:Host-associated:Human:Digestive system:Large in	0.3756
MGYA00237955/ERZ584523_FASTA.fasta	MGYA00237955	ERZ584523	root:Environmental:Aquatic:Marine	0.1816
MGYA00237956/ERZ584525_FASTA.fasta	MGYA00237956	ERZ584525	root:Environmental:Aquatic:Marine	0.188
MGYA00237957/ERZ584527_FASTA.fasta	MGYA00237957	ERZ584527	root:Environmental:Aquatic:Marine	0.2248
MGYA00237958/ERZ584529_FASTA.fasta	MGYA00237958	ERZ584529	root:Environmental:Aquatic:Marine	0.1918
MGYA00237959/ERZ584531_FASTA.fasta	MGYA00237959	ERZ584531	root:Environmental:Aquatic:Marine	0.2578
MGYA00237960/ERZ584533_FASTA.fasta	MGYA00237960	ERZ584533	root:Environmental:Aquatic:Marine	0.2512
MGYA00237961/ERZ584535_FASTA.fasta	MGYA00237961	ERZ584535	root:Environmental:Aquatic:Marine	0.2496
MGYA00237962/ERZ584537_FASTA.fasta	MGYA00237962	ERZ584537	root:Environmental:Aquatic:Marine	0.2766
MGYA00237963/ERZ584539_FASTA.fasta	MGYA00237963	ERZ584539	root:Environmental:Aquatic:Marine	0.2036
MGYA00237964/ERZ584541_FASTA.fasta	MGYA00237964	ERZ584541	root:Environmental:Aquatic:Marine	0.2454
MGYA00237965/ERZ584543_FASTA.fasta	MGYA00237965	ERZ584543	root:Environmental:Aquatic:Marine	0.187
MGYA00237966/ERZ584545_FASTA.fasta	MGYA00237966	ERZ584545	root:Environmental:Aquatic:Marine	0.1412
MGYA00237967/ERZ584547_FASTA.fasta	MGYA00237967	ERZ584547	root:Environmental:Aquatic:Marine	0.1972
MGYA00237968/ERZ584549_FASTA.fasta	MGYA00237968	ERZ584549	root:Environmental:Aquatic:Marine	0.2276
MGYA00237969/ERZ584551_FASTA.fasta	MGYA00237969	ERZ584551	root:Environmental:Aquatic:Marine	0.2368
MGYA00237970/ERZ584553_FASTA.fasta	MGYA00237970	ERZ584553	root:Environmental:Aquatic:Marine	0.2242
MGYA00237971/ERZ584555_FASTA.fasta	MGYA00237971	ERZ584555	root:Environmental:Aquatic:Marine	0.2384
MGYA00237972/ERZ584557_FASTA.fasta	MGYA00237972	ERZ584557	root:Environmental:Aquatic:Marine	0.2454
MGYA00237973/ERZ584559_FASTA.fasta	MGYA00237973	ERZ584559	root:Environmental:Aquatic:Marine	0.2026
MGYA00237974/ERZ584522_FASTA.fasta	MGYA00237974	ERZ584522	root:Environmental:Aquatic:Marine	0.2466
MGYA00237975/ERZ584526_FASTA.fasta	MGYA00237975	ERZ584526	root:Environmental:Aquatic:Marine	0.265
MGYA00237976/ERZ584528_FASTA.fasta	MGYA00237976	ERZ584528	root:Environmental:Aquatic:Marine	0.241
MGYA00237977/ERZ584530_FASTA.fasta	MGYA00237977	ERZ584530	root:Environmental:Aquatic:Marine	0.2272
MGYA00237978/ERZ584532_FASTA.fasta	MGYA00237978	ERZ584532	root:Environmental:Aquatic:Marine	0.1552
MGYA00237979/ERZ584534_FASTA.fasta	MGYA00237979	ERZ584534	root:Environmental:Aquatic:Marine	0.2128
MGYA00237980/ERZ584536_FASTA.fasta	MGYA00237980	ERZ584536	root:Environmental:Aquatic:Marine	0.2496
MGYA00237981/ERZ584538_FASTA.fasta	MGYA00237981	ERZ584538	root:Environmental:Aquatic:Marine	0.2648
MGYA00237982/ERZ584540_FASTA.fasta	MGYA00237982	ERZ584540	root:Environmental:Aquatic:Marine	0.0932
MGYA00237983/ERZ584542_FASTA.fasta	MGYA00237983	ERZ584542	root:Environmental:Aquatic:Marine	0.25
MGYA00237984/ERZ584544_FASTA.fasta	MGYA00237984	ERZ584544	root:Environmental:Aquatic:Marine	0.2454
MGYA00237985/ERZ584546_FASTA.fasta	MGYA00237985	ERZ584546	root:Environmental:Aquatic:Marine	0.2628
MGYA00237986/ERZ584548_FASTA.fasta	MGYA00237986	ERZ584548	root:Environmental:Aquatic:Marine	0.2274
MGYA00237987/ERZ584550_FASTA.fasta	MGYA00237987	ERZ584550	root:Environmental:Aquatic:Marine	0.2454
MGYA00237988/ERZ584552_FASTA.fasta	MGYA00237988	ERZ584552	root:Environmental:Aquatic:Marine	0.2008
MGYA00237989/ERZ584554_FASTA.fasta	MGYA00237989	ERZ584554	root:Environmental:Aquatic:Marine	0.245
MGYA00237990/ERZ584556_FASTA.fasta	MGYA00237990	ERZ584556	root:Environmental:Aquatic:Marine	0.2496
MGYA00237991/ERZ584558_FASTA.fasta	MGYA00237991	ERZ584558	root:Environmental:Aquatic:Marine	0.2544
MGYA00237992/ERZ584603_FASTA.fasta	MGYA00237992	ERZ584603	root:Environmental:Aquatic:Marine	0.2496
MGYA00237993/ERZ584605_FASTA.fasta	MGYA00237993	ERZ584605	root:Environmental:Aquatic:Marine	0.2398
MGYA00237994/ERZ584607_FASTA.fasta	MGYA00237994	ERZ584607	root:Environmental:Aquatic:Marine	0.2168
MGYA00237995/ERZ584609_FASTA.fasta	MGYA00237995	ERZ584609	root:Environmental:Aquatic:Marine	0.1696
MGYA00237996/ERZ584611_FASTA.fasta	MGYA00237996	ERZ584611	root:Environmental:Aquatic:Marine	0.2294
MGYA00237997/ERZ584613_FASTA.fasta	MGYA00237997	ERZ584613	root:Environmental:Aquatic:Marine	0.1998
MGYA00237998/ERZ584615_FASTA.fasta	MGYA00237998	ERZ584615	root:Environmental:Aquatic:Marine	0.22
MGYA00237999/ERZ584585_FASTA.fasta	MGYA00237999	ERZ584585	root:Environmental:Aquatic:Marine	0.1968
MGYA00238000/ERZ584587_FASTA.fasta	MGYA00238000	ERZ584587	root:Environmental:Aquatic:Marine	0.0888
MGYA00238001/ERZ584589_FASTA.fasta	MGYA00238001	ERZ584589	root:Environmental:Aquatic:Marine	0.211
MGYA00238002/ERZ584591_FASTA.fasta	MGYA00238002	ERZ584591	root:Environmental:Aquatic:Marine	0.2496
MGYA00238003/ERZ584593_FASTA.fasta	MGYA00238003	ERZ584593	root:Environmental:Aquatic:Marine	0.1388
MGYA00238004/ERZ584595_FASTA.fasta	MGYA00238004	ERZ584595	root:Environmental:Aquatic:Marine	0.22
MGYA00238005/ERZ584597_FASTA.fasta	MGYA00238005	ERZ584597	root:Environmental:Aquatic:Marine	0.2508
MGYA00238006/ERZ584599_FASTA.fasta	MGYA00238006	ERZ584599	root:Environmental:Aquatic:Marine	0.2496
MGYA00238007/ERZ584601_FASTA.fasta	MGYA00238007	ERZ584601	root:Environmental:Aquatic:Marine	0.1548
MGYA00238008/ERZ584584_FASTA.fasta	MGYA00238008	ERZ584584	root:Environmental:Aquatic:Marine	0.1852
MGYA00238009/ERZ584586_FASTA.fasta	MGYA00238009	ERZ584586	root:Environmental:Aquatic:Marine	0.2692
MGYA00238010/ERZ584588_FASTA.fasta	MGYA00238010	ERZ584588	root:Environmental:Aquatic:Marine	0.2682
MGYA00238011/ERZ584590_FASTA.fasta	MGYA00238011	ERZ584590	root:Environmental:Aquatic:Marine	0.2492
MGYA00238012/ERZ584592_FASTA.fasta	MGYA00238012	ERZ584592	root:Environmental:Aquatic:Marine	0.2486
MGYA00238013/ERZ584594_FASTA.fasta	MGYA00238013	ERZ584594	root:Environmental:Aquatic:Marine	0.195
MGYA00238014/ERZ584596_FASTA.fasta	MGYA00238014	ERZ584596	root:Environmental:Aquatic:Marine	0.2156
MGYA00238015/ERZ584598_FASTA.fasta	MGYA00238015	ERZ584598	root:Environmental:Aquatic:Marine	0.2462

MGYA00238016/ERZ584600_FASTA.fasta	MGYA00238016	ERZ584600	root:Environmental:Aquatic:Marine	0.1752
MGYA00238017/ERZ584602_FASTA.fasta	MGYA00238017	ERZ584602	root:Environmental:Aquatic:Marine	0.2106
MGYA00238018/ERZ584604_FASTA.fasta	MGYA00238018	ERZ584604	root:Environmental:Aquatic:Marine	0.2286
MGYA00238019/ERZ584606_FASTA.fasta	MGYA00238019	ERZ584606	root:Environmental:Aquatic:Marine	0.1758
MGYA00238020/ERZ584608_FASTA.fasta	MGYA00238020	ERZ584608	root:Environmental:Aquatic:Marine	0.1524
MGYA00238021/ERZ584610_FASTA.fasta	MGYA00238021	ERZ584610	root:Environmental:Aquatic:Marine	0.2454
MGYA00238022/ERZ584612_FASTA.fasta	MGYA00238022	ERZ584612	root:Environmental:Aquatic:Marine	0.2096
MGYA00238023/ERZ584614_FASTA.fasta	MGYA00238023	ERZ584614	root:Environmental:Aquatic:Marine	0.1836
MGYA00238024/ERZ584637_FASTA.fasta	MGYA00238024	ERZ584637	root:Environmental:Aquatic:Marine	0.1278
MGYA00238025/ERZ584638_FASTA.fasta	MGYA00238025	ERZ584638	root:Environmental:Aquatic:Marine	0.1968
MGYA00238026/ERZ584640_FASTA.fasta	MGYA00238026	ERZ584640	root:Environmental:Aquatic:Marine	0.0686
MGYA00238027/ERZ584642_FASTA.fasta	MGYA00238027	ERZ584642	root:Environmental:Aquatic:Marine	0.261
MGYA00238028/ERZ584644_FASTA.fasta	MGYA00238028	ERZ584644	root:Environmental:Aquatic:Marine	0.0328
MGYA00238029/ERZ584646_FASTA.fasta	MGYA00238029	ERZ584646	root:Environmental:Aquatic:Marine	0.1494
MGYA00238030/ERZ584648_FASTA.fasta	MGYA00238030	ERZ584648	root:Environmental:Aquatic:Marine	0.1272
MGYA00238031/ERZ584650_FASTA.fasta	MGYA00238031	ERZ584650	root:Environmental:Aquatic:Marine	0.1932
MGYA00238032/ERZ584652_FASTA.fasta	MGYA00238032	ERZ584652	root:Environmental:Aquatic:Marine	0.0534
MGYA00238033/ERZ584654_FASTA.fasta	MGYA00238033	ERZ584654	root:Environmental:Aquatic:Marine	0.1966
MGYA00238034/ERZ584657_FASTA.fasta	MGYA00238034	ERZ584657	root:Environmental:Aquatic:Marine	0.1564
MGYA00238035/ERZ584616_FASTA.fasta	MGYA00238035	ERZ584616	root:Environmental:Aquatic:Marine	0.2176
MGYA00238036/ERZ584618_FASTA.fasta	MGYA00238036	ERZ584618	root:Environmental:Aquatic:Marine	0.154
MGYA00238037/ERZ584620_FASTA.fasta	MGYA00238037	ERZ584620	root:Environmental:Aquatic:Marine	0.049
MGYA00238038/ERZ584622_FASTA.fasta	MGYA00238038	ERZ584622	root:Environmental:Aquatic:Marine	0.139
MGYA00238039/ERZ584624_FASTA.fasta	MGYA00238039	ERZ584624	root:Environmental:Aquatic:Marine	0.1512
MGYA00238040/ERZ584626_FASTA.fasta	MGYA00238040	ERZ584626	root:Environmental:Aquatic:Marine	0.1536
MGYA00238041/ERZ584628_FASTA.fasta	MGYA00238041	ERZ584628	root:Environmental:Aquatic:Marine	0.2476
MGYA00238042/ERZ584630_FASTA.fasta	MGYA00238042	ERZ584630	root:Environmental:Aquatic:Marine	0.2288
MGYA00238043/ERZ584632_FASTA.fasta	MGYA00238043	ERZ584632	root:Environmental:Aquatic:Marine	0.266
MGYA00238044/ERZ584634_FASTA.fasta	MGYA00238044	ERZ584634	root:Environmental:Aquatic:Marine	0.0548
MGYA00238045/ERZ584635_FASTA.fasta	MGYA00238045	ERZ584635	root:Environmental:Aquatic:Marine	0.233
MGYA00238046/ERZ584617_FASTA.fasta	MGYA00238046	ERZ584617	root:Environmental:Aquatic:Marine	0.1136
MGYA00238047/ERZ584619_FASTA.fasta	MGYA00238047	ERZ584619	root:Environmental:Aquatic:Marine	0.1736
MGYA00238048/ERZ584621_FASTA.fasta	MGYA00238048	ERZ584621	root:Environmental:Aquatic:Marine	0.0814
MGYA00238049/ERZ584623_FASTA.fasta	MGYA00238049	ERZ584623	root:Environmental:Aquatic:Marine	0.0694
MGYA00238050/ERZ584625_FASTA.fasta	MGYA00238050	ERZ584625	root:Environmental:Aquatic:Marine	0.1464
MGYA00238051/ERZ584627_FASTA.fasta	MGYA00238051	ERZ584627	root:Environmental:Aquatic:Marine	0.142
MGYA00238052/ERZ584629_FASTA.fasta	MGYA00238052	ERZ584629	root:Environmental:Aquatic:Marine	0.156
MGYA00238053/ERZ584631_FASTA.fasta	MGYA00238053	ERZ584631	root:Environmental:Aquatic:Marine	0.0566
MGYA00238054/ERZ584633_FASTA.fasta	MGYA00238054	ERZ584633	root:Environmental:Aquatic:Marine	0.0548
MGYA00238055/ERZ584636_FASTA.fasta	MGYA00238055	ERZ584636	root:Environmental:Aquatic:Marine	0.2852
MGYA00238056/ERZ584639_FASTA.fasta	MGYA00238056	ERZ584639	root:Environmental:Aquatic:Marine	0.202
MGYA00238057/ERZ584641_FASTA.fasta	MGYA00238057	ERZ584641	root:Environmental:Aquatic:Marine	0.065
MGYA00238058/ERZ584643_FASTA.fasta	MGYA00238058	ERZ584643	root:Environmental:Aquatic:Marine	0.2008
MGYA00238059/ERZ584645_FASTA.fasta	MGYA00238059	ERZ584645	root:Environmental:Aquatic:Marine	0.043
MGYA00238060/ERZ584647_FASTA.fasta	MGYA00238060	ERZ584647	root:Environmental:Aquatic:Marine	0.0792
MGYA00238061/ERZ584649_FASTA.fasta	MGYA00238061	ERZ584649	root:Environmental:Aquatic:Marine	0.1204
MGYA00238062/ERZ584651_FASTA.fasta	MGYA00238062	ERZ584651	root:Environmental:Aquatic:Marine	0.0662
MGYA00238063/ERZ584653_FASTA.fasta	MGYA00238063	ERZ584653	root:Environmental:Aquatic:Marine	0.234
MGYA00238064/ERZ584655_FASTA.fasta	MGYA00238064	ERZ584655	root:Environmental:Aquatic:Marine	0.2168
MGYA00238065/ERZ584658_FASTA.fasta	MGYA00238065	ERZ584658	root:Environmental:Aquatic:Marine	0.833
MGYA00238066/ERZ650152_FASTA.fasta	MGYA00238066	ERZ650152	root:Environmental:Aquatic:Aquaculture	0.8828
MGYA00238067/ERZ650154_FASTA.fasta	MGYA00238067	ERZ650154	root:Environmental:Aquatic:Aquaculture	0.873
MGYA00238068/ERZ650156_FASTA.fasta	MGYA00238068	ERZ650156	root:Environmental:Aquatic:Aquaculture	0.8852
MGYA00238069/ERZ650153_FASTA.fasta	MGYA00238069	ERZ650153	root:Environmental:Aquatic:Aquaculture	0.9786
MGYA00238070/ERZ650155_FASTA.fasta	MGYA00238070	ERZ650155	root:Environmental:Aquatic:Aquaculture	0.9546
MGYA00238071/ERZ650157_FASTA.fasta	MGYA00238071	ERZ650157	root:Environmental:Aquatic:Aquaculture	0.946
MGYA00238072/ERZ650159_FASTA.fasta	MGYA00238072	ERZ650159	root:Environmental:Aquatic:Aquaculture	0.8752
MGYA00238073/ERZ650161_FASTA.fasta	MGYA00238073	ERZ650161	root:Environmental:Aquatic:Aquaculture	0.9522
MGYA00238074/ERZ650163_FASTA.fasta	MGYA00238074	ERZ650163	root:Environmental:Aquatic:Aquaculture	0.955
MGYA00238075/ERZ650158_FASTA.fasta	MGYA00238075	ERZ650158	root:Environmental:Aquatic:Aquaculture	0.8992
MGYA00238076/ERZ650160_FASTA.fasta	MGYA00238076	ERZ650160	root:Environmental:Aquatic:Aquaculture	0.9428
MGYA00238077/ERZ650162_FASTA.fasta	MGYA00238077	ERZ650162	root:Environmental:Aquatic:Aquaculture	0.8736
MGYA00238078/ERZ650164_FASTA.fasta	MGYA00238078	ERZ650164	root:Environmental:Aquatic:Aquaculture	0.98
MGYA00238079/ERZ650174_FASTA.fasta	MGYA00238079	ERZ650174	root:Environmental:Aquatic:Marine:Oceanic:Oil-contami	0.051
MGYA00238080/ERZ650175_FASTA.fasta	MGYA00238080	ERZ650175	root:Environmental:Aquatic:Marine	0.0294
MGYA00238081/ERZ650194_FASTA.fasta	MGYA00238081	ERZ650194	root:Environmental:Aquatic:Marine:Oceanic:Sediment	0.582
MGYA00238082/ERZ650195_FASTA.fasta	MGYA00238082	ERZ650195	root:Environmental:Aquatic:Marine:Oceanic:Sediment	0.6534
MGYA00238083/ERZ650340_FASTA.fasta	MGYA00238083	ERZ650340	root:Environmental:Aquatic:Marine:Brackish	0.7678
MGYA00238084/ERZ650341_FASTA.fasta	MGYA00238084	ERZ650341	root:Environmental:Aquatic:Marine:Oil-contaminated se	0.547
MGYA00238085/ERZ650343_FASTA.fasta	MGYA00238085	ERZ650343	root:Environmental:Aquatic:Marine:Brackish	0.6892
MGYA00238086/ERZ650342_FASTA.fasta	MGYA00238086	ERZ650342	root:Environmental:Aquatic:Marine:Brackish	0.7306
MGYA00238087/ERZ650344_FASTA.fasta	MGYA00238087	ERZ650344	root:Environmental:Aquatic:Marine:Brackish	0.9752
MGYA00238088/ERZ650347_FASTA.fasta	MGYA00238088	ERZ650347	root:Environmental:Aquatic:Marine:Brackish	0.9708
MGYA00238089/ERZ650640_FASTA.fasta	MGYA00238089	ERZ650640	root:Environmental:Aquatic:Lentic:Brackish	0.0542
MGYA00238090/ERZ650642_FASTA.fasta	MGYA00238090	ERZ650642	root:Environmental:Aquatic:Lentic:Brackish	0.2204
MGYA00238091/ERZ650644_FASTA.fasta	MGYA00238091	ERZ650644	root:Environmental:Aquatic:Lentic:Brackish	0.3852
MGYA00238092/ERZ650639_FASTA.fasta	MGYA00238092	ERZ650639	root:Environmental:Aquatic:Lentic:Brackish	0.0302
MGYA00238093/ERZ650641_FASTA.fasta	MGYA00238093	ERZ650641	root:Environmental:Aquatic:Lentic:Brackish	0.1148
MGYA00238094/ERZ650643_FASTA.fasta	MGYA00238094	ERZ650643	root:Environmental:Aquatic:Lentic:Brackish	0.1582
MGYA00238095/ERZ650645_FASTA.fasta	MGYA00238095	ERZ650645	root:Environmental:Aquatic:Lentic:Brackish	0.1696
MGYA00238096/ERZ650647_FASTA.fasta	MGYA00238096	ERZ650647	root:Environmental:Aquatic:Lentic:Brackish	0.115
MGYA00238097/ERZ650651_FASTA.fasta	MGYA00238097	ERZ650651	root:Environmental:Aquatic:Lentic:Brackish	0.0534
MGYA00238098/ERZ650653_FASTA.fasta	MGYA00238098	ERZ650653	root:Environmental:Aquatic:Lentic:Brackish	0.156
MGYA00238099/ERZ650655_FASTA.fasta	MGYA00238099	ERZ650655	root:Environmental:Aquatic:Lentic:Brackish	0.1218
MGYA00238100/ERZ650657_FASTA.fasta	MGYA00238100	ERZ650657	root:Environmental:Aquatic:Lentic:Brackish	0.2092
MGYA00238101/ERZ650659_FASTA.fasta	MGYA00238101	ERZ650659	root:Environmental:Aquatic:Lentic:Brackish	0.2398
MGYA00238102/ERZ650661_FASTA.fasta	MGYA00238102	ERZ650661	root:Environmental:Aquatic:Lentic:Brackish	0.201
MGYA00238103/ERZ650663_FASTA.fasta	MGYA00238103	ERZ650663	root:Environmental:Aquatic:Lentic:Brackish	0.0768
MGYA00238104/ERZ650646_FASTA.fasta	MGYA00238104	ERZ650646	root:Environmental:Aquatic:Lentic:Brackish	0.2434
MGYA00238105/ERZ650648_FASTA.fasta	MGYA00238105	ERZ650648	root:Environmental:Aquatic:Lentic:Brackish	0.1042
MGYA00238106/ERZ650649_FASTA.fasta	MGYA00238106	ERZ650649	root:Environmental:Aquatic:Lentic:Brackish	0.2292
MGYA00238107/ERZ650650_FASTA.fasta	MGYA00238107	ERZ650650	root:Environmental:Aquatic:Lentic:Brackish	0.0678
MGYA00238108/ERZ650652_FASTA.fasta	MGYA00238108	ERZ650652	root:Environmental:Aquatic:Lentic:Brackish	0.1902
MGYA00238109/ERZ650654_FASTA.fasta	MGYA00238109	ERZ650654	root:Environmental:Aquatic:Lentic:Brackish	0.0438
MGYA00238110/ERZ650656_FASTA.fasta	MGYA00238110	ERZ650656	root:Environmental:Aquatic:Lentic:Brackish	0.066
MGYA00238111/ERZ650658_FASTA.fasta	MGYA00238111	ERZ650658	root:Environmental:Aquatic:Lentic:Brackish	0.1342
MGYA00238112/ERZ650660_FASTA.fasta	MGYA00238112	ERZ650660	root:Environmental:Aquatic:Lentic:Brackish	0.1246
MGYA00238113/ERZ650662_FASTA.fasta	MGYA00238113	ERZ650662	root:Environmental:Aquatic:Lentic:Brackish	0.074
MGYA00238114/ERZ650664_FASTA.fasta	MGYA00238114	ERZ650664	root:Environmental:Aquatic:Marine:Brackish	0.9426
MGYA00238115/ERZ650665_FASTA.fasta	MGYA00238115	ERZ650665	root:Environmental:Aquatic:Marine:Brackish	0.857
MGYA00238116/ERZ650667_FASTA.fasta	MGYA00238116	ERZ650667	root:Environmental:Aquatic:Marine:Brackish	0.9798
MGYA00238117/ERZ650669_FASTA.fasta	MGYA00238117	ERZ650669	root:Environmental:Aquatic:Thermal springs:Near-boilin	0.6848
MGYA00238118/ERZ650670_FASTA.fasta	MGYA00238118	ERZ650670	root:Environmental:Aquatic:Thermal springs:Near-boilin	0.3662
MGYA00238119/ERZ650933_FASTA.fasta	MGYA00238119	ERZ650933	root:Host-associated:Human:Digestive system:Large int	0.4492
MGYA00238120/ERZ650935_FASTA.fasta	MGYA00238120	ERZ650935	root:Host-associated:Human:Digestive system:Large int	0.438
MGYA00238121/ERZ650937_FASTA.fasta	MGYA00238121	ERZ650937	root:Host-associated:Human:Digestive system:Large int	0.5
MGYA00238122/ERZ650939_FASTA.fasta	MGYA00238122	ERZ650939	root:Host-associated:Human:Digestive system:Large int	0.4788
MGYA00238123/ERZ650941_FASTA.fasta	MGYA00238123	ERZ650941	root:Host-associated:Human:Digestive system:Large int	0.4788
MGYA00238124/ERZ650943_FASTA.fasta	MGYA00238124	ERZ650943	root:Host-associated:Human:Digestive system:Large int	0.3872
MGYA00238125/ERZ650945_FASTA.fasta	MGYA00238125	ERZ650945	root:Host-associated:Human:Digestive system:Large int	0.4002
MGYA00238126/ERZ650947_FASTA.fasta	MGYA00238126	ERZ650947	root:Host-associated:Human:Digestive system:Large int	0.3986
MGYA00238127/ERZ650932_FASTA.fasta	MGYA00238127	ERZ650932	root:Host-associated:Human:Digestive system:Large int	0.4536
MGYA00238128/ERZ650934_FASTA.fasta	MGYA00238128	ERZ650934	root:Host-associated:Human:Digestive system:Large int	0.442
MGYA00238129/ERZ650936_FASTA.fasta	MGYA00238129	ERZ650936	root:Host-associated:Human:Digestive system:Large int	0.4386
MGYA00238130/ERZ650938_FASTA.fasta	MGYA00238130	ERZ650938	root:Host-associated:Human:Digestive system:Large int	0.3938
MGYA00238131/ERZ650940_FASTA.fasta	MGYA00238131	ERZ650940	root:Host-associated:Human:Digestive system:Large int	0.469
MGYA00238132/ERZ650942_FASTA.fasta	MGYA00238132	ERZ650942	root:Host-associated:Human:Digestive system:Large int	0.4068
MGYA00238133/ERZ650944_FASTA.fasta	MGYA00238133	ERZ650944	root:Host-associated:Human:Digestive system:Large int	0.5014
MGYA00238134/ERZ650946_FASTA.fasta	MGYA00238134	ERZ650946	root:Host-associated:Human:Digestive system:Large int	0.4396
MGYA00238135/ERZ651158_FASTA.fasta	MGYA00238135	ERZ651158	root:Environmental:Aquatic:Marine	0.8242
MGYA00238136/ERZ651165_FASTA.fasta	MGYA00238136	ERZ651165	root:Environmental:Aquatic:Marine:Coastal	0.9766
MGYA00238137/ERZ651772_FASTA.fasta	MGYA00238137	ERZ651772	root:Environmental:Aquatic:Marine:Intertidal zone:Estu	0.8616
MGYA00238138/ERZ651775_FASTA.fasta	MGYA00238138	ERZ651775	root:Environmental:Aquatic:Marine	0.8576
MGYA00238139/ERZ651803_FASTA.fasta	MGYA00238139	ERZ651803	root:Engineered:Biogas plant:Wet fermentation	0.5272
MGYA00238140/ERZ651804_FASTA.fasta	MGYA00238140	ERZ651804	root:Engineered:Biogas plant:Wet fermentation	0.5004
MGYA00238141/ERZ746978_FASTA.fasta	MGYA00238141	ERZ746978	root:Engineered:Lab Synthesis:Genetic cross	0.3564
MGYA00238142/ERZ746979_FASTA.fasta	MGYA00238142	ERZ746979	root:Engineered:Lab Synthesis:Genetic cross	0.2186
MGYA00238143/ERZ746982_FASTA.fasta	MGYA00238143	ERZ746982	root:Engineered:Solid waste:Composting	0.113
MGYA00238144/ERZ746983_FASTA.fasta	MGYA00238144	ERZ746983	root:Engineered:Solid waste:Composting	0.0996
MGYA00238146/ERZ747223_FASTA.fasta	MGYA00238146	ERZ747223	root:Engineered:Solid waste:Composting	0.1008
MGYA00238147/ERZ761563_FASTA.fasta	MGYA00238147	ERZ761563	root:Engineered:Wastewater:Activated Sludge	0.7962
MGYA00238148/ERZ761564_FASTA.fasta	MGYA00238148	ERZ761564	root:Engineered:Wastewater:Activated Sludge	0.9324
MGYA00238149/ERZ761566_FASTA.fasta	MGYA00238149	ERZ761566	root:Host-associated:Plants	0.2704
MGYA00238150/ERZ761569_FASTA.fasta	MGYA00238150	ERZ761569	root:Host-associated:Plants	0.08
MGYA00238151/ERZ761570_FASTA.fasta	MGYA00238151	ERZ761570	root:Host-associated:Plants	0.2534
MGYA00238152/ERZ761572_FASTA.fasta	MGYA00238152	ERZ761572	root:Host-associated:Plants	0.2586
MGYA00238153/ERZ761573_FASTA.fasta	MGYA00238153	ERZ761573	root:Host-associated:Plants	0.388
MGYA00238154/ERZ761575_FASTA.fasta	MGYA00238154	ERZ761575	root:Environmental:Aquatic:Lentic:Brackish	0.4692
MGYA00238155/ERZ761577_FASTA.fasta	MGYA00238155	ERZ761577	root:Engineered:Solid waste:Composting	0.8426
MGYA00238156/ERZ761611_FASTA.fasta	MGYA00238156	ERZ761611	root:Engineered:Bioreactor	0.774
MGYA00238157/ERZ761612_FASTA.fasta	MGYA00238157	ERZ761612	root:Engineered:Bioreactor	0.8164
MGYA00238158/ERZ761615_FASTA.fasta	MGYA00238158	ERZ761615	root:Engineered:Bioreactor	0.8416
MGYA00238159/ERZ761634_FASTA.fasta	MGYA00238159	ERZ761634	root:Environmental:Aquatic:Lentic:Brackish	0.4778
MGYA00238160/ERZ761638_FASTA.fasta	MGYA00238160	ERZ761638	root:Environmental:Aquatic:Lentic:Brackish	0.6116
MGYA00238161/ERZ761639_FASTA.fasta	MGYA00238161	ERZ761639	root:Environmental:Aquatic:Lentic:Brackish	0.6334
MGYA00238162/ERZ761640_FASTA.fasta	MGYA00238162	ERZ761640	root:Environmental:Aquatic:Lentic:Brackish	0.6724
MGYA00238163/ERZ764804_FASTA.fasta	MGYA00238163	ERZ764804	root:Engineered:Wastewater:Activated Sludge	0.829
MGYA00238164/ERZ764825_FASTA.fasta	MGYA00238164	ERZ764825	root:Engineered:Bioreactor	0.665
MGYA00238165/ERZ766961_FASTA.fasta	MGYA00238165	ERZ766961	root:Environmental:Aquatic:Marine	0.8796
MGYA00238166/ERZ767019_FASTA.fasta	MGYA00238166	ERZ767019	root:Environmental:Aquatic:Marine:Oceanic	0.7612
MGYA00238167/ERZ767053_FASTA.fasta	MGYA00238167	ERZ767053	root:Environmental:Aquatic:Estuary	0.8664
MGYA00238168/ERZ767080_FASTA.fasta	MGYA00238168	ERZ767080	root:Environmental:Aquatic:Estuary	0.849
MGYA00238169/ERZ767097_FASTA.fasta	MGYA00238169	ERZ767097	root:Environmental:Aquatic:Estuary	0.834
MGYA00238170/ERZ767106_FASTA.fasta	MGYA00238170	ERZ767106	root:Environmental:Aquatic:Estuary	0.7862
MGYA00238171/ERZ767123_FASTA.fasta	MGYA00238171	ERZ767123	root:Environmental:Aquatic:Estuary	0.8226
MGYA00238172/ERZ767134_FASTA.fasta	MGYA00238172	ERZ767134	root:Environmental:Aquatic:Estuary	0.7768
MGYA00238173/ERZ767189_FASTA.fasta	MGYA00238173	ERZ767189	root:Environmental:Aquatic:Estuary	0.8168
MGYA00238174/ERZ767213_FASTA.fasta	MGYA00238174	ERZ767213	root:Environmental:Aquatic:Estuary	0.8194
MGYA00238175/ERZ767261_FASTA.fasta	MGYA00238175	ERZ767261	root:Environmental:Aquatic:Estuary	0.8384
MGYA00238176/ERZ767358_FASTA.fasta	MGYA00238176	ERZ767358	root:Environmental:Aquatic:Estuary	0.7942
MGYA00238177/ERZ767431_FASTA.fasta	MGYA00238177	ERZ767431	root:Environmental:Aquatic:Estuary	0.7904
MGYA00238178/ERZ772708_FASTA.fasta	MGYA00238178	ERZ772708	root:Engineered:Wastewater:Activated Sludge	0.8844
MGYA00238179/ERZ772710_FASTA.fasta	MGYA00238179	ERZ772710	root:Engineered:Bioreactor	0.5158
MGYA00238180/ERZ772747_FASTA.fasta	MGYA00238180	ERZ772747	root:Engineered:Bioreactor	0.061
MGYA00238181/ERZ773283_FASTA.fasta	MGYA00238181	ERZ773283	root:Engineered:Wastewater:Activated Sludge	0.8924
MGYA00238182/ERZ773286_FASTA.fasta	MGYA00238182	ERZ773286	root:Engineered:Bioreactor	0.5504
MGYA00238183/ERZ773288_FASTA.fasta	MGYA00238183	ERZ773288	root:Engineered:Bioreactor	0.5908
MGYA00238184/ERZ773361_FASTA.fasta	MGYA00238184	ERZ773361	root:Engineered:Bioreactor	0.8398
MGYA00240992/ERZ505281_FASTA.fasta	MGYA00240992	ERZ505281	root:Environmental:Aquatic:Marine	0.786
MGYA00240993/ERZ508538_FASTA.fasta	MGYA00240993	ERZ508538	root:Environmental:Aquatic:Marine	0.7852
MGYA00240994/ERZ511481_FASTA.fasta	MGYA00240994	ERZ511481	root:Host-associated:Algae:Red algae	0.9218
MGYA00240995/ERZ534231_FASTA.fasta	MGYA00240995	ERZ534231	root:Environmental:Aquatic:Marine	0.7782
MGYA00240996/ERZ650346_FASTA.fasta	MGYA00240996	ERZ650346	root:Environmental:Aquatic:Marine:Brackish	0.8402
MGYA00240997/ERZ746985_FASTA.fasta	MGYA00240997	ERZ746985	root:Engineered:Solid waste:Composting	0.1104
MGYA00240998/ERZ772709_FASTA.fasta	MGYA00240998	ERZ772709	root:Engineered:Wastewater:Activated Sludge	0.863
MGYA00240999/ERZ501732_FASTA.fasta	MGYA00240999	ERZ501732	root:Host-associated:Human:Digestive system:Large int	0.18
MGYA00241000/ERZ501734_FASTA.fasta	MGYA00241000	ERZ501734	root:Host-associated:Human:Digestive system:Large int	0.1464
MGYA00241001/ERZ501736_FASTA.fasta	MGYA00241001	ERZ501736	root:Host-associated:Human:Digestive system:Large int	0.2714
MGYA00241002/ERZ501738_FASTA.fasta	MGYA00241002	ERZ501738	root:Host-associated:Human:Digestive system:Large int	0.113
MGYA00241003/ERZ501740_FASTA.fasta	MGYA00241003	ERZ501740	root:Host-associated:Human:Digestive system:Large int	0.167
MGYA00241004/ERZ501742_FASTA.fasta	MGYA00241004	ERZ501742	root:Host-associated:Human:Digestive system:Large int	0.4038
MGYA00241005/ERZ501744_FASTA.fasta	MGYA00241005	ERZ501744	root:Host-associated:Human:Digestive system:Large int	0.2116
MGYA00241006/ERZ501746_FASTA.fasta	MGYA00241006	ERZ501746	root:Host-associated:Human:Digestive system:Large int	0.2816
MGYA00241007/ERZ501748_FASTA.fasta	MGYA00241007	ERZ501748	root:Host-associated:Human:Digestive system:Large int	0.2018
MGYA00241008/ERZ501750_FASTA.fasta	MGYA00241008	ERZ501750	root:Host-associated:Human:Digestive system:Large int	0.3836
MGYA00241009/ERZ501752_FASTA.fasta	MGYA00241009	ERZ501752	root:Host-associated:Human:Digestive system:Large int	0.3954
MGYA00241010/ERZ501753_FASTA.fasta	MGYA00241010	ERZ501753	root:Host-associated:Human:Digestive system:Large int	0.203
MGYA00241011/ERZ501755_FASTA.fasta	MGYA00241011	ERZ501755	root:Host-associated:Human:Digestive system:Large int	0.2814
MGYA00241012/ERZ501757_FASTA.fasta	MGYA00241012	ERZ501757	root:Host-associated:Human:Digestive system:Large int	0.2218
MGYA00241013/ERZ501760_FASTA.fasta	MGYA00241013	ERZ501760	root:Host-associated:Human:Digestive system:Large int	0.2156
MGYA00241014/ERZ501762_FASTA.fasta	MGYA00241014	ERZ501762	root:Host-associated:Human:Digestive system:Large int	0.4482
MGYA00241015/ERZ501764_FASTA.fasta	MGYA00241015	ERZ501764	root:Host-associated:Human:Digestive system:Large int	0.433
MGYA00241016/ERZ501766_FASTA.fasta	MGYA00241016	ERZ501766	root:Host-associated:Human:Digestive system:Large int	0.3068
MGYA00241017/ERZ501768_FASTA.fasta	MGYA00241017	ERZ501768	root:Host-associated:Human:Digestive system:Large int	0.1726
MGYA00241018/ERZ501770_FASTA.fasta	MGYA00241018	ERZ501770	root:Host-associated:Human:Digestive system:Large int	0.2848
MGYA00241019/ERZ501772_FASTA.fasta	MGYA00241019	ERZ501772	root:Host-associated:Human:Digestive system:Large int	0.1186
MGYA00241020/ERZ501774_FASTA.fasta	MGYA00241020	ERZ501774	root:Host-associated:Human:Digestive system:Large int	0.2624
MGYA00241021/ERZ501776_FASTA.fasta	MGYA00241021	ERZ501776	root:Host-associated:Human:Digestive system:Large int	0.2802
MGYA00241022/ERZ501778_FASTA.fasta	MGYA00241022	ERZ501778	root:Host-associated:Human:Digestive system:Large int	0.406
MGYA00241023/ERZ501780_FASTA.fasta	MGYA00241023	ERZ501780	root:Host-associated:Human:Digestive system:Large int	0.2886
MGYA00241024/ERZ501782_FASTA.fasta	MGYA00241024	ERZ501782	root:Host-associated:Human:Digestive system:Large int	0.2004
MGYA00241025/ERZ501784_FASTA.fasta	MGYA00241025	ERZ501784	root:Host-associated:Human:Digestive system:Large int	0.1536
MGYA00241026/ERZ501786_FASTA.fasta	MGYA00241026	ERZ501786	root:Host-associated:Human:Digestive system:Large int	0.1768
MGYA00241027/ERZ501788_FASTA.fasta	MGYA00241027	ERZ501788	root:Host-associated:Human:Digestive system:Large int	0.3264
MGYA00241028/ERZ501790_FASTA.fasta	MGYA00241028	ERZ501790	root:Host-associated:Human:Digestive system:Large int	0.1908
MGYA00241029/ERZ501792_FASTA.fasta	MGYA00241029	ERZ501792	root:Host-associated:Human:Digestive system:Large int	0.21
MGYA00241030/ERZ501794_FASTA.fasta	MGYA00241030	ERZ501794	root:Host-associated:Human:Digestive system:Large int	0.4446
MGYA00241031/ERZ501796_FASTA.fasta	MGYA00241031	ERZ501796	root:Host-associated:Human:Digestive system:Large int	0.3834
MGYA00241032/ERZ501798_FASTA.fasta	MGYA00241032	ERZ501798	root:Host-associated:Human:Digestive system:Large int	0.3188
MGYA00241033/ERZ501800_FASTA.fasta	MGYA00241033	ERZ501800	root:Host-associated:Human:Digestive system:Large int	0.3584
MGYA00241034/ERZ501802_FASTA.fasta	MGYA00241034	ERZ501802	root:Host-associated:Human:Digestive system:Large int	0.1644
MGYA00241035/ERZ501386_FASTA.fasta	MGYA00241035	ERZ501386	root:Host-associated:Human:Digestive system:Large int	0.2322
MGYA00241036/ERZ501388_FASTA.fasta	MGYA00241036	ERZ501388	root:Host-associated:Human:Digestive system:Large int	0.2678
MGYA00241037/ERZ501390_FASTA.fasta	MGYA00241037	ERZ501390	root:Host-associated:Human:Digestive system:Large int	0.0786
MGYA00241038/ERZ501392_FASTA.fasta	MGYA00241038	ERZ501392	root:Host-associated:Human:Digestive system:Large int	0.3876
MGYA00241039/ERZ501394_FASTA.fasta	MGYA00241039	ERZ501394	root:Host-associated:Human:Digestive system:Large int	0.1314
MGYA00241040/ERZ501396_FASTA.fasta	MGYA00241040	ERZ501396	root:Host-associated:Human:Digestive system:Large int	0.3988
MGYA00241041/ERZ501398_FASTA.fasta	MGYA00241041	ERZ501398	root:Host-associated:Human:Digestive system:Large int	0.2032
MGYA00241042/ERZ501400_FASTA.fasta	MGYA00241042	ERZ501400	root:Host-associated:Human:Digestive system:Large int	0.2938
MGYA00241043/ERZ501402_FASTA.fasta	MGYA00241043	ERZ501402	root:Host-associated:Human:Digestive system:Large int	0.4338
MGYA00241044/ERZ501404_FASTA.fasta	MGYA00241044	ERZ501404	root:Host-associated:Human:Digestive system:Large int	0.2458
MGYA00241045/ERZ501406_FASTA.fasta	MGYA00241045	ERZ501406	root:Host-associated:Human:Digestive system:Large int	0.4612
MGYA00241046/ERZ501408_FASTA.fasta	MGYA00241046	ERZ501408	root:Host-associated:Human:Digestive system:Large int	0.2178
MGYA00241047/ERZ501410_FASTA.fasta	MGYA00241047	ERZ501410	root:Host-associated:Human:Digestive system:Large int	0.533
MGYA00241048/ERZ501412_FASTA.fasta	MGYA00241048	ERZ501412	root:Host-associated:Human:Digestive system:Large int	0.4794
MGYA00241049/ERZ501414_FASTA.fasta	MGYA00241049	ERZ501414	root:Host-associated:Human:Digestive system:Large int	0.47
MGYA00241050/ERZ501416_FASTA.fasta	MGYA00241050	ERZ501416	root:Host-associated:Human:Digestive system:Large int	0.3916
MGYA00241051/ERZ501418_FASTA.fasta	MGYA00241051	ERZ501418	root:Host-associated:Human:Digestive system:Large int	0.198
MGYA00241052/ERZ501420_FASTA.fasta	MGYA00241052	ERZ501420	root:Host-associated:Human:Digestive system:Large int	0.4276
MGYA00241053/ERZ501422_FASTA.fasta	MGYA00241053	ERZ501422	root:Host-associated:Human:Digestive system:Large int	0.4392
MGYA00241054/ERZ501424_FASTA.fasta	MGYA00241054	ERZ501424	root:Host-associated:Human:Digestive system:Large int	0.4812
MGYA00241055/ERZ501426_FASTA.fasta	MGYA00241055	ERZ501426	root:Host-associated:Human:Digestive system:Large int	0.315
MGYA00241056/ERZ501428_FASTA.fasta	MGYA00241056	ERZ501428	root:Host-associated:Human:Digestive system:Large int	0.4634
MGYA00241057/ERZ501430_FASTA.fasta	MGYA00241057	ERZ501430	root:Host-associated:Human:Digestive system:Large int	0.4346
MGYA00241058/ERZ501432_FASTA.fasta	MGYA00241058	ERZ501432	root:Host-associated:Human:Digestive system:Large int	0.41
MGYA00241059/ERZ501434_FASTA.fasta	MGYA00241059	ERZ501434	root:Host-associated:Human:Digestive system:Large int	0.3264
MGYA00241060/ERZ501436_FASTA.fasta	MGYA00241060	ERZ501436	root:Host-associated:Human:Digestive system:Large int	0.4526
MGYA00241061/ERZ501438_FASTA.fasta	MGYA00241061	ERZ501438	root:Host-associated:Human:Digestive system:Large int	0.3652
MGYA00241062/ERZ501440_FASTA.fasta	MGYA00241062	ERZ501440	root:Host-associated:Human:Digestive system:Large int	0.4732
MGYA00241063/ERZ501442_FASTA.fasta	MGYA00241063	ERZ501442	root:Host-associated:Human:Digestive system:Large int	0.4024
MGYA00241064/ERZ501444_FASTA.fasta	MGYA00241064	ERZ501444	root:Host-associated:Human:Digestive system:Large int	0.1654
MGYA00241065/ERZ501446_FASTA.fasta	MGYA00241065	ERZ501446	root:Host-associated:Human:Digestive system:Large int	0.3864
MGYA00241066/ERZ501448_FASTA.fasta	MGYA00241066	ERZ501448	root:Host-associated:Human:Digestive system:Large int	0.3834
MGYA00241067/ERZ501450_FASTA.fasta	MGYA00241067	ERZ501450	root:Host-associated:Human:Digestive system:Large int	0.3728
MGYA00241068/ERZ501452_FASTA.fasta	MGYA00241068	ERZ501452	root:Host-associated:Human:Digestive system:Large int	0.4268
MGYA00241069/ERZ501454_FASTA.fasta	MGYA00241069	ERZ501454	root:Host-associated:Human:Digestive system:Large int	0.4562
MGYA00241070/ERZ501456_FASTA.fasta	MGYA00241070	ERZ501456	root:Host-associated:Human:Digestive system:Large int	0.4134
MGYA00241071/ERZ501458_FASTA.fasta	MGYA00241071	ERZ501458	root:Host-associated:Human:Digestive system:Large int	0.3886
MGYA00241072/ERZ501459_FASTA.fasta	MGYA00241072	ERZ501459	root:Host-associated:Human:Digestive system:Large int	0.3752
MGYA00241073/ERZ501461_FASTA.fasta	MGYA00241073	ERZ501461	root:Host-associated:Human:Digestive system:Large int	0.4654
MGYA00241074/ERZ501463_FASTA.fasta	MGYA00241074	ERZ501463	root:Host-associated:Human:Digestive system:Large int	0.5304
MGYA00241075/ERZ501466_FASTA.fasta	MGYA00241075	ERZ501466	root:Host-associated:Human:Digestive system:Large int	0.4056
MGYA00241076/ERZ501468_FASTA.fasta	MGYA00241076	ERZ501468	root:Host-associated:Human:Digestive system:Large int	0.4142
MGYA00241077/ERZ501470_FASTA.fasta	MGYA00241077	ERZ501470	root:Host-associated:Human:Digestive system:Large int	0.3402
MGYA00241078/ERZ501472_FASTA.fasta	MGYA00241078	ERZ501472	root:Host-associated:Human:Digestive system:Large int	0.0606
MGYA00241079/ERZ501143_FASTA.fasta	MGYA00241079	ERZ501143	root:Host-associated:Human:Digestive system:Large int	0.1196
MGYA00241080/ERZ501145_FASTA.fasta	MGYA00241080	ERZ501145	root:Host-associated:Human:Digestive system:Large int	0.1572
MGYA00241081/ERZ501147_FASTA.fasta	MGYA00241081	ERZ501147	root:Host-associated:Human:Digestive system:Large int	0.188
MGYA00241082/ERZ501149_FASTA.fasta	MGYA00241082	ERZ501149	root:Host-associated:Human:Digestive system:Large int	0.1974
MGYA00241083/ERZ501151_FASTA.fasta	MGYA00241083	ERZ501151	root:Host-associated:Human:Digestive system:Large int	0.1284
MGYA00241084/ERZ501153_FASTA.fasta	MGYA00241084	ERZ501153	root:Host-associated:Human:Digestive system:Large int	0.143
MGYA00241085/ERZ501155_FASTA.fasta	MGYA00241085	ERZ501155	root:Host-associated:Human:Digestive system:Large int	0.2676
MGYA00241086/ERZ501157_FASTA.fasta	MGYA00241086	ERZ501157	root:Host-associated:Human:Digestive system:Large int	0.1052
MGYA00241087/ERZ501160_FASTA.fasta	MGYA00241087	ERZ501160	root:Host-associated:Human:Digestive system:Large int	0.2014
MGYA00241088/ERZ501162_FASTA.fasta	MGYA00241088	ERZ501162	root:Host-associated:Human:Digestive system:Large int	0.3282
MGYA00241089/ERZ501164_FASTA.fasta	MGYA00241089	ERZ501164	root:Host-associated:Human:Digestive system:Large int	0.255
MGYA00241090/ERZ501165_FASTA.fasta	MGYA00241090	ERZ501165	root:Host-associated:Human:Digestive system:Large int	0.0638
MGYA00241091/ERZ501167_FASTA.fasta	MGYA00241091	ERZ501167	root:Host-associated:Human:Digestive system:Large int	0.1526
MGYA00241092/ERZ501169_FASTA.fasta	MGYA00241092	ERZ501169	root:Host-associated:Human:Digestive system:Large int	0.2746
MGYA00241093/ERZ501975_FASTA.fasta	MGYA00241093	ERZ501975	root:Host-associated:Human:Digestive system:Large int	0.3898
MGYA00241094/ERZ501977_FASTA.fasta	MGYA00241094	ERZ501977	root:Host-associated:Human:Digestive system:Large int	0.3772
MGYA00241095/ERZ501979_FASTA.fasta	MGYA00241095	ERZ501979	root:Host-associated:Human:Digestive system:Large int	0.3958
MGYA00241096/ERZ501981_FASTA.fasta	MGYA00241096	ERZ501981	root:Host-associated:Human:Digestive system:Large int	0.4622
MGYA00241097/ERZ501983_FASTA.fasta	MGYA00241097	ERZ501983	root:Host-associated:Human:Digestive system:Large int	0.4402
MGYA00241098/ERZ501985_FASTA.fasta	MGYA00241098	ERZ501985	root:Host-associated:Human:Digestive system:Large int	0.4134
MGYA00241099/ERZ501987_FASTA.fasta	MGYA00241099	ERZ501987	root:Host-associated:Human:Digestive system:Large int	0.4914
MGYA00241100/ERZ501989_FASTA.fasta	MGYA00241100	ERZ501989	root:Host-associated:Human:Digestive system:Large int	0.3028
MGYA00241101/ERZ501991_FASTA.fasta	MGYA00241101	ERZ501991	root:Host-associated:Human:Digestive system:Large int	0.218
MGYA00241102/ERZ501993_FASTA.fasta	MGYA00241102	ERZ501993	root:Host-associated:Human:Digestive system:Large int	0.3808
MGYA00241103/ERZ501995_FASTA.fasta	MGYA00241103	ERZ501995	root:Host-associated:Human:Digestive system:Large int	0.4644
MGYA00241104/ERZ501997_FASTA.fasta	MGYA00241104	ERZ501997	root:Host-associated:Human:Digestive system:Large int	0.4604
MGYA00241105/ERZ501999_FASTA.fasta	MGYA00241105	ERZ501999	root:Host-associated:Human:Digestive system:Large int	0.288
MGYA00241106/ERZ502001_FASTA.fasta	MGYA00241106	ERZ502001	root:Host-associated:Human:Digestive system:Large int	0.4754
MGYA00241107/ERZ502003_FASTA.fasta	MGYA00241107	ERZ502003	root:Host-associated:Human:Digestive system:Large int	0.4716
MGYA00241108/ERZ502005_FASTA.fasta	MGYA00241108	ERZ502005	root:Host-associated:Human:Digestive system:Large int	0.4508
MGYA00241109/ERZ502007_FASTA.fasta	MGYA00241109	ERZ502007	root:Host-associated:Human:Digestive system:Large int	0.1446
MGYA00241110/ERZ502009_FASTA.fasta	MGYA00241110	ERZ502009	root:Host-associated:Human:Digestive system:Large int	0.3218
MGYA00241111/ERZ502011_FASTA.fasta	MGYA00241111	ERZ502011	root:Host-associated:Human:Digestive system:Large int	0.4704
MGYA00241112/ERZ502013_FASTA.fasta	MGYA00241112	ERZ502013	root:Host-associated:Human:Digestive system:Large int	0.2352
MGYA00241113/ERZ502015_FASTA.fasta	MGYA00241113	ERZ502015	root:Host-associated:Human:Digestive system:Large int	0.342
MGYA00241114/ERZ502017_FASTA.fasta	MGYA00241114	ERZ502017	root:Host-associated:Human:Digestive system:Large int	0.191
MGYA00241115/ERZ502019_FASTA.fasta	MGYA00241115	ERZ502019	root:Host-associated:Human:Digestive system:Large int	0.271
MGYA00241116/ERZ502021_FASTA.fasta	MGYA00241116	ERZ502021	root:Host-associated:Human:Digestive system:Large int	0.1552
MGYA00241117/ERZ502023_FASTA.fasta	MGYA00241117	ERZ502023	root:Host-associated:Human:Digestive system:Large int	0.4268
MGYA00241118/ERZ502025_FASTA.fasta	MGYA00241118	ERZ502025	root:Host-associated:Human:Digestive system:Large int	0.2388
MGYA00241119/ERZ502027_FASTA.fasta	MGYA00241119	ERZ502027	root:Host-associated:Human:Digestive system:Large int	0.2666
MGYA00241120/ERZ502029_FASTA.fasta	MGYA00241120	ERZ502029	root:Host-associated:Human:Digestive system:Large int	0.3874
MGYA00241121/ERZ502031_FASTA.fasta	MGYA00241121	ERZ502031	root:Host-associated:Human:Digestive system:Large int	0.3906
MGYA00241122/ERZ502033_FASTA.fasta	MGYA00241122	ERZ502033	root:Host-associated:Human:Digestive system:Large int	0.4088
MGYA00241123/ERZ501142_FASTA.fasta	MGYA00241123	ERZ501142	root:Host-associated:Human:Digestive system:Large int	0.2106
MGYA00241124/ERZ501144_FASTA.fasta	MGYA00241124	ERZ501144	root:Host-associated:Human:Digestive system:Large int	0.3502
MGYA00241125/ERZ501146_FASTA.fasta	MGYA00241125	ERZ501146	root:Host-associated:Human:Digestive system:Large int	0.265
MGYA00241126/ERZ501148_FASTA.fasta	MGYA00241126	ERZ501148	root:Host-associated:Human:Digestive system:Large int	0.2096
MGYA00241127/ERZ501150_FASTA.fasta	MGYA00241127	ERZ501150	root:Host-associated:Human:Digestive system:Large int	0.1006
MGYA00241128/ERZ501152_FASTA.fasta	MGYA00241128	ERZ501152	root:Host-associated:Human:Digestive system:Large int	0.271
MGYA00241129/ERZ501154_FASTA.fasta	MGYA00241129	ERZ501154	root:Host-associated:Human:Digestive system:Large int	0.1324
MGYA00241130/ERZ501156_FASTA.fasta	MGYA00241130	ERZ501156	root:Host-associated:Human:Digestive system:Large int	0.24
MGYA00241131/ERZ501158_FASTA.fasta	MGYA00241131	ERZ501158	root:Host-associated:Human:Digestive system:Large int	0.2444
MGYA00241132/ERZ501159_FASTA.fasta	MGYA00241132	ERZ501159	root:Host-associated:Human:Digestive system:Large int	0.2394
MGYA00241133/ERZ501161_FASTA.fasta	MGYA00241133	ERZ501161	root:Host-associated:Human:Digestive system:Large int	0.1928
MGYA00241134/ERZ501163_FASTA.fasta	MGYA00241134	ERZ501163	root:Host-associated:Human:Digestive system:Large int	0.2574
MGYA00241135/ERZ501166_FASTA.fasta	MGYA00241135	ERZ501166	root:Host-associated:Human:Digestive system:Large int	0.1634
MGYA00241136/ERZ501168_FASTA.fasta	MGYA00241136	ERZ501168	root:Host-associated:Human:Digestive system:Large int	0.0854
MGYA00241137/ERZ501170_FASTA.fasta	MGYA00241137	ERZ501170	root:Host-associated:Human:Digestive system:Large int	0.3012
MGYA00241138/ERZ501172_FASTA.fasta	MGYA00241138	ERZ501172	root:Host-associated:Human:Digestive system:Large int	0.3448
MGYA00241139/ERZ502036_FASTA.fasta	MGYA00241139	ERZ502036	root:Host-associated:Human:Digestive system:Large int	0.3774
MGYA00241140/ERZ502038_FASTA.fasta	MGYA00241140	ERZ502038	root:Host-associated:Human:Digestive system:Large int	0.364
MGYA00241141/ERZ502040_FASTA.fasta	MGYA00241141	ERZ502040	root:Host-associated:Human:Digestive system:Large int	0.151
MGYA00241142/ERZ502041_FASTA.fasta	MGYA00241142	ERZ502041	root:Host-associated:Human:Digestive system:Large int	0.4406
MGYA00241143/ERZ502043_FASTA.fasta	MGYA00241143	ERZ502043	root:Host-associated:Human:Digestive system:Large int	0.3878
MGYA00241144/ERZ502045_FASTA.fasta	MGYA00241144	ERZ502045	root:Host-associated:Human:Digestive system:Large int	0.0336
MGYA00241145/ERZ501360_FASTA.fasta	MGYA00241145	ERZ501360	root:Host-associated:Human:Digestive system:Large int	0.235
MGYA00241146/ERZ501362_FASTA.fasta	MGYA00241146	ERZ501362	root:Host-associated:Human:Digestive system:Large int	0.1626
MGYA00241147/ERZ501364_FASTA.fasta	MGYA00241147	ERZ501364	root:Host-associated:Human:Digestive system:Large int	0.3628
MGYA00241148/ERZ501366_FASTA.fasta	MGYA00241148	ERZ501366	root:Host-associated:Human:Digestive system:Large int	0.2488
MGYA00241149/ERZ501367_FASTA.fasta	MGYA00241149	ERZ501367	root:Host-associated:Human:Digestive system:Large int	0.1396
MGYA00241150/ERZ501369_FASTA.fasta	MGYA00241150	ERZ501369	root:Host-associated:Human:Digestive system:Large int	0.517
MGYA00241151/ERZ501371_FASTA.fasta	MGYA00241151	ERZ501371	root:Host-associated:Human:Digestive system:Large int	0.0998
MGYA00241152/ERZ501373_FASTA.fasta	MGYA00241152	ERZ501373	root:Host-associated:Human:Digestive system:Large int	0.2276
MGYA00241153/ERZ501375_FASTA.fasta	MGYA00241153	ERZ501375	root:Host-associated:Human:Digestive system:Large int	0.4596
MGYA00241154/ERZ501377_FASTA.fasta	MGYA00241154	ERZ501377	root:Host-associated:Human:Digestive system:Large int	0.1286
MGYA00241155/ERZ501379_FASTA.fasta	MGYA00241155	ERZ501379	root:Host-associated:Human:Digestive system:Large int	0.0454
MGYA00241156/ERZ501381_FASTA.fasta	MGYA00241156	ERZ501381	root:Host-associated:Human:Digestive system:Large int	0.3726
MGYA00241157/ERZ501383_FASTA.fasta	MGYA00241157	ERZ501383	root:Host-associated:Human:Digestive system:Large int	0.509
MGYA00241158/ERZ501174_FASTA.fasta	MGYA00241158	ERZ501174	root:Host-associated:Human:Digestive system:Large int	0.102
MGYA00241159/ERZ501176_FASTA.fasta	MGYA00241159	ERZ501176	root:Host-associated:Human:Digestive system:Large int	0.3142
MGYA00241160/ERZ501178_FASTA.fasta	MGYA00241160	ERZ501178	root:Host-associated:Human:Digestive system:Large int	0.26
MGYA00241161/ERZ501180_FASTA.fasta	MGYA00241161	ERZ501180	root:Host-associated:Human:Digestive system:Large int	0.3618
MGYA00241162/ERZ501182_FASTA.fasta	MGYA00241162	ERZ501182	root:Host-associated:Human:Digestive system:Large int	0.1552
MGYA00241163/ERZ501184_FASTA.fasta	MGYA00241163	ERZ501184	root:Host-associated:Human:Digestive system:Large int	0.3106
MGYA00241164/ERZ501186_FASTA.fasta	MGYA00241164	ERZ501186	root:Host-associated:Human:Digestive system:Large int	0.123
MGYA00241165/ERZ501188_FASTA.fasta	MGYA00241165	ERZ501188	root:Host-associated:Human:Digestive system:Large int	0.1778
MGYA00241166/ERZ501190_FASTA.fasta	MGYA00241166	ERZ501190	root:Host-associated:Human:Digestive system:Large int	0.051
MGYA00241167/ERZ501192_FASTA.fasta	MGYA00241167	ERZ501192	root:Host-associated:Human:Digestive system:Large int	0.2366
MGYA00241168/ERZ501194_FASTA.fasta	MGYA00241168	ERZ501194	root:Host-associated:Human:Digestive system:Large int	0.2736
MGYA00241169/ERZ501196_FASTA.fasta	MGYA00241169	ERZ501196	root:Host-associated:Human:Digestive system:Large int	0.2
MGYA00241170/ERZ501198_FASTA.fasta	MGYA00241170	ERZ501198	root:Host-associated:Human:Digestive system:Large int	0.2102
MGYA00241171/ERZ501200_FASTA.fasta	MGYA00241171	ERZ501200	root:Host-associated:Human:Digestive system:Large int	0.3036
MGYA00241172/ERZ501202_FASTA.fasta	MGYA00241172	ERZ501202	root:Host-associated:Human:Digestive system:Large int	0.0828
MGYA00241173/ERZ501204_FASTA.fasta	MGYA00241173	ERZ501204	root:Host-associated:Human:Digestive system:Large int	0.2762
MGYA00241174/ERZ501206_FASTA.fasta	MGYA00241174	ERZ501206	root:Host-associated:Human:Digestive system:Large int	0.2632
MGYA00241175/ERZ501208_FASTA.fasta	MGYA00241175	ERZ501208	root:Host-associated:Human:Digestive system:Large int	0.1354
MGYA00241176/ERZ501210_FASTA.fasta	MGYA00241176	ERZ501210	root:Host-associated:Human:Digestive system:Large int	0.1158
MGYA00241177/ERZ501212_FASTA.fasta	MGYA00241177	ERZ501212	root:Host-associated:Human:Digestive system:Large int	0.2184
MGYA00241178/ERZ501214_FASTA.fasta	MGYA00241178	ERZ501214	root:Host-associated:Human:Digestive system:Large int	0.196
MGYA00241179/ERZ501216_FASTA.fasta	MGYA00241179	ERZ501216	root:Host-associated:Human:Digestive system:Large int	0.3436
MGYA00241180/ERZ501218_FASTA.fasta	MGYA00241180	ERZ501218	root:Host-associated:Human:Digestive system:Large int	0.125
MGYA00241181/ERZ501385_FASTA.fasta	MGYA00241181	ERZ501385	root:Host-associated:Human:Digestive system:Large int	0.1912
MGYA00241182/ERZ501387_FASTA.fasta	MGYA00241182	ERZ501387	root:Host-associated:Human:Digestive system:Large int	0.3942
MGYA00241183/ERZ501389_FASTA.fasta	MGYA00241183	ERZ501389	root:Host-associated:Human:Digestive system:Large int	0.281
MGYA00241184/ERZ501391_FASTA.fasta	MGYA00241184	ERZ501391	root:Host-associated:Human:Digestive system:Large int	0.1926
MGYA00241185/ERZ501393_FASTA.fasta	MGYA00241185	ERZ501393	root:Host-associated:Human:Digestive system:Large int	0.2168
MGYA00241186/ERZ501395_FASTA.fasta	MGYA00241186	ERZ501395	root:Host-associated:Human:Digestive system:Large int	0.3078
MGYA00241187/ERZ501397_FASTA.fasta	MGYA00241187	ERZ501397	root:Host-associated:Human:Digestive system:Large int	0.4172
MGYA00241188/ERZ501399_FASTA.fasta	MGYA00241188	ERZ501399	root:Host-associated:Human:Digestive system:Large int	0.3996
MGYA00241189/ERZ501401_FASTA.fasta	MGYA00241189	ERZ501401	root:Host-associated:Human:Digestive system:Large int	0.2404
MGYA00241190/ERZ501403_FASTA.fasta	MGYA00241190	ERZ501403	root:Host-associated:Human:Digestive system:Large int	0.0968
MGYA00241191/ERZ501405_FASTA.fasta	MGYA00241191	ERZ501405	root:Host-associated:Human:Digestive system:Large int	0.2156
MGYA00241192/ERZ501407_FASTA.fasta	MGYA00241192	ERZ501407	root:Host-associated:Human:Digestive system:Large int	0.3874
MGYA00241193/ERZ501409_FASTA.fasta	MGYA00241193	ERZ501409	root:Host-associated:Human:Digestive system:Large int	0.235
MGYA00241194/ERZ501411_FASTA.fasta	MGYA00241194	ERZ501411	root:Host-associated:Human:Digestive system:Large int	0.443
MGYA00241195/ERZ501413_FASTA.fasta	MGYA00241195	ERZ501413	root:Host-associated:Human:Digestive system:Large int	0.3668
MGYA00241196/ERZ501415_FASTA.fasta	MGYA00241196	ERZ501415	root:Host-associated:Human:Digestive system:Large int	0.3978
MGYA00241197/ERZ501417_FASTA.fasta	MGYA00241197	ERZ501417	root:Host-associated:Human:Digestive system:Large int	0.233
MGYA00241198/ERZ501419_FASTA.fasta	MGYA00241198	ERZ501419	root:Host-associated:Human:Digestive system:Large int	0.3398
MGYA00241199/ERZ501421_FASTA.fasta	MGYA00241199	ERZ501421	root:Host-associated:Human:Digestive system:Large int	0.4738
MGYA00241200/ERZ501423_FASTA.fasta	MGYA00241200	ERZ501423	root:Host-associated:Human:Digestive system:Large int	0.3928
MGYA00241201/ERZ501425_FASTA.fasta	MGYA00241201	ERZ501425	root:Host-associated:Human:Digestive system:Large int	0.2488
MGYA00241202/ERZ501427_FASTA.fasta	MGYA00241202	ERZ501427	root:Host-associated:Human:Digestive system:Large int	0.433
MGYA00241203/ERZ501220_FASTA.fasta	MGYA00241203	ERZ501220	root:Host-associated:Human:Digestive system:Large int	0.2506
MGYA00241204/ERZ501222_FASTA.fasta	MGYA00241204	ERZ501222	root:Host-associated:Human:Digestive system:Large int	0.1394
MGYA00241205/ERZ501224_FASTA.fasta	MGYA00241205	ERZ501224	root:Host-associated:Human:Digestive system:Large int	0.0464
MGYA00241206/ERZ501226_FASTA.fasta	MGYA00241206	ERZ501226	root:Host-associated:Human:Digestive system:Large int	0.2722
MGYA00241207/ERZ501228_FASTA.fasta	MGYA00241207	ERZ501228	root:Host-associated:Human:Digestive system:Large int	0.2748
MGYA00241208/ERZ501230_FASTA.fasta	MGYA00241208	ERZ501230	root:Host-associated:Human:Digestive system:Large int	0.112
MGYA00241209/ERZ501232_FASTA.fasta	MGYA00241209	ERZ501232	root:Host-associated:Human:Digestive system:Large int	0.263
MGYA00241210/ERZ501234_FASTA.fasta	MGYA00241210	ERZ501234	root:Host-associated:Human:Digestive system:Large int	0.1806
MGYA00241211/ERZ501236_FASTA.fasta	MGYA00241211	ERZ501236	root:Host-associated:Human:Digestive system:Large int	0.1052
MGYA00241212/ERZ501238_FASTA.fasta	MGYA00241212	ERZ501238	root:Host-associated:Human:Digestive system:Large int	0.2616
MGYA00241213/ERZ501240_FASTA.fasta	MGYA00241213	ERZ501240	root:Host-associated:Human:Digestive system:Large int	0.1036
MGYA00241214/ERZ501242_FASTA.fasta	MGYA00241214	ERZ501242	root:Host-associated:Human:Digestive system:Large int	0.1
MGYA00241215/ERZ501244_FASTA.fasta	MGYA00241215	ERZ501244	root:Host-associated:Human:Digestive system:Large int	0.0506
MGYA00241216/ERZ501246_FASTA.fasta	MGYA00241216	ERZ501246	root:Host-associated:Human:Digestive system:Large int	0.422
MGYA00241217/ERZ501248_FASTA.fasta	MGYA00241217	ERZ501248	root:Host-associated:Human:Digestive system:Large int	0.3468
MGYA00241218/ERZ501250_FASTA.fasta	MGYA00241218	ERZ501250	root:Host-associated:Human:Digestive system:Large int	0.2338
MGYA00241219/ERZ501252_FASTA.fasta	MGYA00241219	ERZ501252	root:Host-associated:Human:Digestive system:Large int	0.0802
MGYA00241220/ERZ501254_FASTA.fasta	MGYA00241220	ERZ501254	root:Host-associated:Human:Digestive system:Large int	0.0304
MGYA00241221/ERZ501256_FASTA.fasta	MGYA00241221	ERZ501256	root:Host-associated:Human:Digestive system:Large int	0.0776
MGYA00241222/ERZ501258_FASTA.fasta	MGYA00241222	ERZ501258	root:Host-associated:Human:Digestive system:Large int	0.0322
MGYA00241223/ERZ501260_FASTA.fasta	MGYA00241223	ERZ501260	root:Host-associated:Human:Digestive system:Large int	0.076
MGYA00241224/ERZ501261_FASTA.fasta	MGYA00241224	ERZ501261	root:Host-associated:Human:Digestive system:Large int	0.048
MGYA00241225/ERZ501429_FASTA.fasta	MGYA00241225	ERZ501429	root:Host-associated:Human:Digestive system:Large int	0.405
MGYA00241226/ERZ501431_FASTA.fasta	MGYA00241226	ERZ501431	root:Host-associated:Human:Digestive system:Large int	0.4992
MGYA00241227/ERZ501433_FASTA.fasta	MGYA00241227	ERZ501433	root:Host-associated:Human:Digestive system:Large int	0.3948
MGYA00241228/ERZ501435_FASTA.fasta	MGYA00241228	ERZ501435	root:Host-associated:Human:Digestive system:Large int	0.3524
MGYA00241229/ERZ501437_FASTA.fasta	MGYA00241229	ERZ501437	root:Host-associated:Human:Digestive system:Large int	0.4412
MGYA00241230/ERZ501439_FASTA.fasta	MGYA00241230	ERZ501439	root:Host-associated:Human:Digestive system:Large int	0.3586
MGYA00241231/ERZ501441_FASTA.fasta	MGYA00241231	ERZ501441	root:Host-associated:Human:Digestive system:Large int	0.4528
MGYA00241232/ERZ501443_FASTA.fasta	MGYA00241232	ERZ501443	root:Host-associated:Human:Digestive system:Large int	0.3822
MGYA00241233/ERZ501445_FASTA.fasta	MGYA00241233	ERZ501445	root:Host-associated:Human:Digestive system:Large int	0.2274
MGYA00241234/ERZ501447_FASTA.fasta	MGYA00241234	ERZ501447	root:Host-associated:Human:Digestive system:Large int	0.2876
MGYA00241235/ERZ501449_FASTA.fasta	MGYA00241235	ERZ501449	root:Host-associated:Human:Digestive system:Large int	0.4048
MGYA00241236/ERZ501451_FASTA.fasta	MGYA00241236	ERZ501451	root:Host-associated:Human:Digestive system:Large int	0.3234
MGYA00241237/ERZ501453_FASTA.fasta	MGYA00241237	ERZ501453	root:Host-associated:Human:Digestive system:Large int	0.4296
MGYA00241238/ERZ501455_FASTA.fasta	MGYA00241238	ERZ501455	root:Host-associated:Human:Digestive system:Large int	0.3182
MGYA00241239/ERZ501457_FASTA.fasta	MGYA00241239	ERZ501457	root:Host-associated:Human:Digestive system:Large int	0.271
MGYA00241240/ERZ501460_FASTA.fasta	MGYA00241240	ERZ501460	root:Host-associated:Human:Digestive system:Large int	0.4436
MGYA00241241/ERZ501462_FASTA.fasta	MGYA00241241	ERZ501462	root:Host-associated:Human:Digestive system:Large int	0.4404
MGYA00241242/ERZ501464_FASTA.fasta	MGYA00241242	ERZ501464	root:Host-associated:Human:Digestive system:Large int	0.4816
MGYA00241243/ERZ501465_FASTA.fasta	MGYA00241243	ERZ501465	root:Host-associated:Human:Digestive system:Large int	0.2184
MGYA00241244/ERZ501263_FASTA.fasta	MGYA00241244	ERZ501263	root:Host-associated:Human:Digestive system:Large int	0.0318
MGYA00241245/ERZ501265_FASTA.fasta	MGYA00241245	ERZ501265	root:Host-associated:Human:Digestive system:Large int	0.0546
MGYA00241246/ERZ501268_FASTA.fasta	MGYA00241246	ERZ501268	root:Host-associated:Human:Digestive system:Large int	0.0298
MGYA00241247/ERZ501474_FASTA.fasta	MGYA00241247	ERZ501474	root:Host-associated:Human:Digestive system:Large int	0.4416
MGYA00241248/ERZ501476_FASTA.fasta	MGYA00241248	ERZ501476	root:Host-associated:Human:Digestive system:Large int	0.2962
MGYA00241249/ERZ501478_FASTA.fasta	MGYA00241249	ERZ501478	root:Host-associated:Human:Digestive system:Large int	0.3846
MGYA00241250/ERZ501480_FASTA.fasta	MGYA00241250	ERZ501480	root:Host-associated:Human:Digestive system:Large int	0.3688
MGYA00241251/ERZ501482_FASTA.fasta	MGYA00241251	ERZ501482	root:Host-associated:Human:Digestive system:Large int	0.4522
MGYA00241252/ERZ501484_FASTA.fasta	MGYA00241252	ERZ501484	root:Host-associated:Human:Digestive system:Large int	0.475
MGYA00241253/ERZ501486_FASTA.fasta	MGYA00241253	ERZ501486	root:Host-associated:Human:Digestive system:Large int	0.3986
MGYA00241254/ERZ501488_FASTA.fasta	MGYA00241254	ERZ501488	root:Host-associated:Human:Digestive system:Large int	0.3982
MGYA00241255/ERZ501490_FASTA.fasta	MGYA00241255	ERZ501490	root:Host-associated:Human:Digestive system:Large int	0.3052
MGYA00241256/ERZ501492_FASTA.fasta	MGYA00241256	ERZ501492	root:Host-associated:Human:Digestive system:Large int	0.3432
MGYA00241257/ERZ501494_FASTA.fasta	MGYA00241257	ERZ501494	root:Host-associated:Human:Digestive system:Large int	0.414
MGYA00241258/ERZ501496_FASTA.fasta	MGYA00241258	ERZ501496	root:Host-associated:Human:Digestive system:Large int	0.3858
MGYA00241259/ERZ501498_FASTA.fasta	MGYA00241259	ERZ501498	root:Host-associated:Human:Digestive system:Large int	0.3702
MGYA00241260/ERZ501500_FASTA.fasta	MGYA00241260	ERZ501500	root:Host-associated:Human:Digestive system:Large int	0.4506
MGYA00241261/ERZ501502_FASTA.fasta	MGYA00241261	ERZ501502	root:Host-associated:Human:Digestive system:Large int	0.3826
MGYA00241262/ERZ501504_FASTA.fasta	MGYA00241262	ERZ501504	root:Host-associated:Human:Digestive system:Large int	0.41
MGYA00241263/ERZ501506_FASTA.fasta	MGYA00241263	ERZ501506	root:Host-associated:Human:Digestive system:Large int	0.4612
MGYA00241264/ERZ501508_FASTA.fasta	MGYA00241264	ERZ501508	root:Host-associated:Human:Digestive system:Large int	0.446
MGYA00241265/ERZ501510_FASTA.fasta	MGYA00241265	ERZ501510	root:Host-associated:Human:Digestive system:Large int	0.088
MGYA00241266/ERZ501171_FASTA.fasta	MGYA00241266	ERZ501171	root:Host-associated:Human:Digestive system:Large int	0.2342
MGYA00241267/ERZ501173_FASTA.fasta	MGYA00241267	ERZ501173	root:Host-associated:Human:Digestive system:Large int	0.2992
MGYA00241268/ERZ501175_FASTA.fasta	MGYA00241268	ERZ501175	root:Host-associated:Human:Digestive system:Large int	0.1774
MGYA00241269/ERZ501177_FASTA.fasta	MGYA00241269	ERZ501177	root:Host-associated:Human:Digestive system:Large int	0.3336
MGYA00241270/ERZ501179_FASTA.fasta	MGYA00241270	ERZ501179	root:Host-associated:Human:Digestive system:Large int	0.2608
MGYA00241271/ERZ501181_FASTA.fasta	MGYA00241271	ERZ501181	root:Host-associated:Human:Digestive system:Large int	0.3348
MGYA00241272/ERZ501183_FASTA.fasta	MGYA00241272	ERZ501183	root:Host-associated:Human:Digestive system:Large int	0.4374
MGYA00241273/ERZ501185_FASTA.fasta	MGYA00241273	ERZ501185	root:Host-associated:Human:Digestive system:Large int	0.0636
MGYA00241274/ERZ501187_FASTA.fasta	MGYA00241274	ERZ501187	root:Host-associated:Human:Digestive system:Large int	0.2954
MGYA00241275/ERZ501189_FASTA.fasta	MGYA00241275	ERZ501189	root:Host-associated:Human:Digestive system:Large int	0.1318
MGYA00241276/ERZ501191_FASTA.fasta	MGYA00241276	ERZ501191	root:Host-associated:Human:Digestive system:Large int	0.2022
MGYA00241277/ERZ501193_FASTA.fasta	MGYA00241277	ERZ501193	root:Host-associated:Human:Digestive system:Large int	0.2602
MGYA00241278/ERZ501195_FASTA.fasta	MGYA00241278	ERZ501195	root:Host-associated:Human:Digestive system:Large int	0.2268
MGYA00241279/ERZ501197_FASTA.fasta	MGYA00241279	ERZ501197	root:Host-associated:Human:Digestive system:Large int	0.1778
MGYA00241280/ERZ501199_FASTA.fasta	MGYA00241280	ERZ501199	root:Host-associated:Human:Digestive system:Large int	0.2578
MGYA00241281/ERZ501201_FASTA.fasta	MGYA00241281	ERZ501201	root:Host-associated:Human:Digestive system:Large int	0.2386
MGYA00241282/ERZ501203_FASTA.fasta	MGYA00241282	ERZ501203	root:Host-associated:Human:Digestive system:Large int	0.166
MGYA00241283/ERZ501205_FASTA.fasta	MGYA00241283	ERZ501205	root:Host-associated:Human:Digestive system:Large int	0.1994
MGYA00241284/ERZ501207_FASTA.fasta	MGYA00241284	ERZ501207	root:Host-associated:Human:Digestive system:Large int	0.245
MGYA00241285/ERZ501209_FASTA.fasta	MGYA00241285	ERZ501209	root:Host-associated:Human:Digestive system:Large int	0.2272
MGYA00241286/ERZ501211_FASTA.fasta	MGYA00241286	ERZ501211	root:Host-associated:Human:Digestive system:Large int	0.3182
MGYA00241287/ERZ501213_FASTA.fasta	MGYA00241287	ERZ501213	root:Host-associated:Human:Digestive system:Large int	0.121
MGYA00241288/ERZ501215_FASTA.fasta	MGYA00241288	ERZ501215	root:Host-associated:Human:Digestive system:Large int	0.1328
MGYA00241289/ERZ501512_FASTA.fasta	MGYA00241289	ERZ501512	root:Host-associated:Human:Digestive system:Large int	0.0924
MGYA00241290/ERZ501514_FASTA.fasta	MGYA00241290	ERZ501514	root:Host-associated:Human:Digestive system:Large int	0.0396
MGYA00241291/ERZ501516_FASTA.fasta	MGYA00241291	ERZ501516	root:Host-associated:Human:Digestive system:Large int	0.3346
MGYA00241292/ERZ501518_FASTA.fasta	MGYA00241292	ERZ501518	root:Host-associated:Human:Digestive system:Large int	0.2618
MGYA00241293/ERZ501520_FASTA.fasta	MGYA00241293	ERZ501520	root:Host-associated:Human:Digestive system:Large int	0.232
MGYA00241294/ERZ501522_FASTA.fasta	MGYA00241294	ERZ501522	root:Host-associated:Human:Digestive system:Large int	0.2316
MGYA00241295/ERZ501524_FASTA.fasta	MGYA00241295	ERZ501524	root:Host-associated:Human:Digestive system:Large int	0.1526
MGYA00241296/ERZ501526_FASTA.fasta	MGYA00241296	ERZ501526	root:Host-associated:Human:Digestive system:Large int	0.1348
MGYA00241297/ERZ501528_FASTA.fasta	MGYA00241297	ERZ501528	root:Host-associated:Human:Digestive system:Large int	0.1486
MGYA00241298/ERZ501530_FASTA.fasta	MGYA00241298	ERZ501530	root:Host-associated:Human:Digestive system:Large int	0.3132
MGYA00241299/ERZ501532_FASTA.fasta	MGYA00241299	ERZ501532	root:Host-associated:Human:Digestive system:Large int	0.2336
MGYA00241300/ERZ501534_FASTA.fasta	MGYA00241300	ERZ501534	root:Host-associated:Human:Digestive system:Large int	0.0658
MGYA00241301/ERZ501536_FASTA.fasta	MGYA00241301	ERZ501536	root:Host-associated:Human:Digestive system:Large int	0.1138
MGYA00241302/ERZ501538_FASTA.fasta	MGYA00241302	ERZ501538	root:Host-associated:Human:Digestive system:Large int	0.1166
MGYA00241303/ERZ501540_FASTA.fasta	MGYA00241303	ERZ501540	root:Host-associated:Human:Digestive system:Large int	0.1386
MGYA00241304/ERZ501542_FASTA.fasta	MGYA00241304	ERZ501542	root:Host-associated:Human:Digestive system:Large int	0.3716
MGYA00241305/ERZ501544_FASTA.fasta	MGYA00241305	ERZ501544	root:Host-associated:Human:Digestive system:Large int	0.1892
MGYA00241306/ERZ501546_FASTA.fasta	MGYA00241306	ERZ501546	root:Host-associated:Human:Digestive system:Large int	0.1044
MGYA00241307/ERZ501548_FASTA.fasta	MGYA00241307	ERZ501548	root:Host-associated:Human:Digestive system:Large int	0.2594
MGYA00241308/ERZ501550_FASTA.fasta	MGYA00241308	ERZ501550	root:Host-associated:Human:Digestive system:Large int	0.5012
MGYA00241309/ERZ501552_FASTA.fasta	MGYA00241309	ERZ501552	root:Host-associated:Human:Digestive system:Large int	0.1642
MGYA00241310/ERZ501554_FASTA.fasta	MGYA00241310	ERZ501554	root:Host-associated:Human:Digestive system:Large int	0.1612
MGYA00241311/ERZ501217_FASTA.fasta	MGYA00241311	ERZ501217	root:Host-associated:Human:Digestive system:Large int	0.252
MGYA00241312/ERZ501219_FASTA.fasta	MGYA00241312	ERZ501219	root:Host-associated:Human:Digestive system:Large int	0.151
MGYA00241313/ERZ501221_FASTA.fasta	MGYA00241313	ERZ501221	root:Host-associated:Human:Digestive system:Large int	0.1162
MGYA00241314/ERZ501223_FASTA.fasta	MGYA00241314	ERZ501223	root:Host-associated:Human:Digestive system:Large int	0.1038
MGYA00241315/ERZ501225_FASTA.fasta	MGYA00241315	ERZ501225	root:Host-associated:Human:Digestive system:Large int	0.3312
MGYA00241316/ERZ501227_FASTA.fasta	MGYA00241316	ERZ501227	root:Host-associated:Human:Digestive system:Large int	0.2218
MGYA00241317/ERZ501229_FASTA.fasta	MGYA00241317	ERZ501229	root:Host-associated:Human:Digestive system:Large int	0.104
MGYA00241318/ERZ501231_FASTA.fasta	MGYA00241318	ERZ501231	root:Host-associated:Human:Digestive system:Large int	0.2102
MGYA00241319/ERZ501233_FASTA.fasta	MGYA00241319	ERZ501233	root:Host-associated:Human:Digestive system:Large int	0.2422
MGYA00241320/ERZ501235_FASTA.fasta	MGYA00241320	ERZ501235	root:Host-associated:Human:Digestive system:Large int	0.2378
MGYA00241321/ERZ501237_FASTA.fasta	MGYA00241321	ERZ501237	root:Host-associated:Human:Digestive system:Large int	0.3156
MGYA00241322/ERZ501239_FASTA.fasta	MGYA00241322	ERZ501239	root:Host-associated:Human:Digestive system:Large int	0.1314
MGYA00241323/ERZ501241_FASTA.fasta	MGYA00241323	ERZ501241	root:Host-associated:Human:Digestive system:Large int	0.1322
MGYA00241324/ERZ501243_FASTA.fasta	MGYA00241324	ERZ501243	root:Host-associated:Human:Digestive system:Large int	0.3308
MGYA00241325/ERZ501245_FASTA.fasta	MGYA00241325	ERZ501245	root:Host-associated:Human:Digestive system:Large int	0.0646
MGYA00241326/ERZ501247_FASTA.fasta	MGYA00241326	ERZ501247	root:Host-associated:Human:Digestive system:Large int	0.2522
MGYA00241327/ERZ501249_FASTA.fasta	MGYA00241327	ERZ501249	root:Host-associated:Human:Digestive system:Large int	0.0718
MGYA00241328/ERZ501251_FASTA.fasta	MGYA00241328	ERZ501251	root:Host-associated:Human:Digestive system:Large int	0.0352
MGYA00241329/ERZ501253_FASTA.fasta	MGYA00241329	ERZ501253	root:Host-associated:Human:Digestive system:Large int	0.3162
MGYA00241330/ERZ501255_FASTA.fasta	MGYA00241330	ERZ501255	root:Host-associated:Human:Digestive system:Large int	0.0544
MGYA00241331/ERZ501257_FASTA.fasta	MGYA00241331	ERZ501257	root:Host-associated:Human:Digestive system:Large int	0.1782
MGYA00241332/ERZ501259_FASTA.fasta	MGYA00241332	ERZ501259	root:Host-associated:Human:Digestive system:Large int	0.0518
MGYA00241333/ERZ501556_FASTA.fasta	MGYA00241333	ERZ501556	root:Host-associated:Human:Digestive system:Large int	0.0784
MGYA00241334/ERZ501557_FASTA.fasta	MGYA00241334	ERZ501557	root:Host-associated:Human:Digestive system:Large int	0.317
MGYA00241335/ERZ501559_FASTA.fasta	MGYA00241335	ERZ501559	root:Host-associated:Human:Digestive system:Large int	0.2552
MGYA00241336/ERZ501561_FASTA.fasta	MGYA00241336	ERZ501561	root:Host-associated:Human:Digestive system:Large int	0.3124
MGYA00241337/ERZ501564_FASTA.fasta	MGYA00241337	ERZ501564	root:Host-associated:Human:Digestive system:Large int	0.2454
MGYA00241338/ERZ501566_FASTA.fasta	MGYA00241338	ERZ501566	root:Host-associated:Human:Digestive system:Large int	0.209
MGYA00241339/ERZ501568_FASTA.fasta	MGYA00241339	ERZ501568	root:Host-associated:Human:Digestive system:Large int	0.2472
MGYA00241340/ERZ501570_FASTA.fasta	MGYA00241340	ERZ501570	root:Host-associated:Human:Digestive system:Large int	0.0814
MGYA00241341/ERZ501572_FASTA.fasta	MGYA00241341	ERZ501572	root:Host-associated:Human:Digestive system:Large int	0.107
MGYA00241342/ERZ501574_FASTA.fasta	MGYA00241342	ERZ501574	root:Host-associated:Human:Digestive system:Large int	0.0654
MGYA00241343/ERZ501576_FASTA.fasta	MGYA00241343	ERZ501576	root:Host-associated:Human:Digestive system:Large int	0.112
MGYA00241344/ERZ501578_FASTA.fasta	MGYA00241344	ERZ501578	root:Host-associated:Human:Digestive system:Large int	0.0904
MGYA00241345/ERZ501580_FASTA.fasta	MGYA00241345	ERZ501580	root:Host-associated:Human:Digestive system:Large int	0.343
MGYA00241346/ERZ501582_FASTA.fasta	MGYA00241346	ERZ501582	root:Host-associated:Human:Digestive system:Large int	0.309
MGYA00241347/ERZ501584_FASTA.fasta	MGYA00241347	ERZ501584	root:Host-associated:Human:Digestive system:Large int	0.1674
MGYA00241348/ERZ501586_FASTA.fasta	MGYA00241348	ERZ501586	root:Host-associated:Human:Digestive system:Large int	0.1202
MGYA00241349/ERZ501588_FASTA.fasta	MGYA00241349	ERZ501588	root:Host-associated:Human:Digestive system:Large int	0.2652
MGYA00241350/ERZ501590_FASTA.fasta	MGYA00241350	ERZ501590	root:Host-associated:Human:Digestive system:Large int	0.2632
MGYA00241351/ERZ501592_FASTA.fasta	MGYA00241351	ERZ501592	root:Host-associated:Human:Digestive system:Large int	0.2362
MGYA00241352/ERZ501594_FASTA.fasta	MGYA00241352	ERZ501594	root:Host-associated:Human:Digestive system:Large int	0.2644
MGYA00241353/ERZ501596_FASTA.fasta	MGYA00241353	ERZ501596	root:Host-associated:Human:Digestive system:Large int	0.1756
MGYA00241354/ERZ501598_FASTA.fasta	MGYA00241354	ERZ501598	root:Host-associated:Human:Digestive system:Large int	0.2464
MGYA00241355/ERZ501262_FASTA.fasta	MGYA00241355	ERZ501262	root:Host-associated:Human:Digestive system:Large int	0.0354
MGYA00241356/ERZ501264_FASTA.fasta	MGYA00241356	ERZ501264	root:Host-associated:Human:Digestive system:Large int	0.036
MGYA00241357/ERZ501266_FASTA.fasta	MGYA00241357	ERZ501266	root:Host-associated:Human:Digestive system:Large int	0.0906
MGYA00241358/ERZ501267_FASTA.fasta	MGYA00241358	ERZ501267	root:Host-associated:Human:Digestive system:Large int	0.1058
MGYA00241359/ERZ501269_FASTA.fasta	MGYA00241359	ERZ501269	root:Host-associated:Human:Digestive system:Large int	0.0774
MGYA00241360/ERZ501271_FASTA.fasta	MGYA00241360	ERZ501271	root:Host-associated:Human:Digestive system:Large int	0.2214
MGYA00241361/ERZ501273_FASTA.fasta	MGYA00241361	ERZ501273	root:Host-associated:Human:Digestive system:Large int	0.1662
MGYA00241362/ERZ501275_FASTA.fasta	MGYA00241362	ERZ501275	root:Host-associated:Human:Digestive system:Large int	0.2694
MGYA00241363/ERZ501277_FASTA.fasta	MGYA00241363	ERZ501277	root:Host-associated:Human:Digestive system:Large int	0.284
MGYA00241364/ERZ501279_FASTA.fasta	MGYA00241364	ERZ501279	root:Host-associated:Human:Digestive system:Large int	0.2644
MGYA00241365/ERZ501281_FASTA.fasta	MGYA00241365	ERZ501281	root:Host-associated:Human:Digestive system:Large int	0.249
MGYA00241366/ERZ501283_FASTA.fasta	MGYA00241366	ERZ501283	root:Host-associated:Human:Digestive system:Large int	0.2328
MGYA00241367/ERZ501285_FASTA.fasta	MGYA00241367	ERZ501285	root:Host-associated:Human:Digestive system:Large int	0.3456
MGYA00241368/ERZ501287_FASTA.fasta	MGYA00241368	ERZ501287	root:Host-associated:Human:Digestive system:Large int	0.3144
MGYA00241369/ERZ501289_FASTA.fasta	MGYA00241369	ERZ501289	root:Host-associated:Human:Digestive system:Large int	0.3642
MGYA00241370/ERZ501291_FASTA.fasta	MGYA00241370	ERZ501291	root:Host-associated:Human:Digestive system:Large int	0.2042
MGYA00241371/ERZ501293_FASTA.fasta	MGYA00241371	ERZ501293	root:Host-associated:Human:Digestive system:Large int	0.1924
MGYA00241372/ERZ501295_FASTA.fasta	MGYA00241372	ERZ501295	root:Host-associated:Human:Digestive system:Large int	0.2968
MGYA00241373/ERZ501297_FASTA.fasta	MGYA00241373	ERZ501297	root:Host-associated:Human:Digestive system:Large int	0.267
MGYA00241374/ERZ501299_FASTA.fasta	MGYA00241374	ERZ501299	root:Host-associated:Human:Digestive system:Large int	0.327
MGYA00241375/ERZ501301_FASTA.fasta	MGYA00241375	ERZ501301	root:Host-associated:Human:Digestive system:Large int	0.248
MGYA00241376/ERZ501303_FASTA.fasta	MGYA00241376	ERZ501303	root:Host-associated:Human:Digestive system:Large int	0.2646
MGYA00241377/ERZ501600_FASTA.fasta	MGYA00241377	ERZ501600	root:Host-associated:Human:Digestive system:Large int	0.1044
MGYA00241378/ERZ501602_FASTA.fasta	MGYA00241378	ERZ501602	root:Host-associated:Human:Digestive system:Large int	0.1038
MGYA00241379/ERZ501604_FASTA.fasta	MGYA00241379	ERZ501604	root:Host-associated:Human:Digestive system:Large int	0.2522
MGYA00241380/ERZ501606_FASTA.fasta	MGYA00241380	ERZ501606	root:Host-associated:Human:Digestive system:Large int	0.2226
MGYA00241381/ERZ501608_FASTA.fasta	MGYA00241381	ERZ501608	root:Host-associated:Human:Digestive system:Large int	0.1068
MGYA00241382/ERZ501610_FASTA.fasta	MGYA00241382	ERZ501610	root:Host-associated:Human:Digestive system:Large int	0.258
MGYA00241383/ERZ501612_FASTA.fasta	MGYA00241383	ERZ501612	root:Host-associated:Human:Digestive system:Large int	0.0898
MGYA00241384/ERZ501614_FASTA.fasta	MGYA00241384	ERZ501614	root:Host-associated:Human:Digestive system:Large int	0.1204
MGYA00241385/ERZ501616_FASTA.fasta	MGYA00241385	ERZ501616	root:Host-associated:Human:Digestive system:Large int	0.0312
MGYA00241386/ERZ501618_FASTA.fasta	MGYA00241386	ERZ501618	root:Host-associated:Human:Digestive system:Large int	0.1832
MGYA00241387/ERZ501620_FASTA.fasta	MGYA00241387	ERZ501620	root:Host-associated:Human:Digestive system:Large int	0.0454

MGYA00241388/ERZ501622_FASTA.fasta	MGYA00241388	ERZ501622	root:Host-associated:Human:Digestive system:Large in	0.053
MGYA00241389/ERZ501624_FASTA.fasta	MGYA00241389	ERZ501624	root:Host-associated:Human:Digestive system:Large in	0.0652
MGYA00241390/ERZ501626_FASTA.fasta	MGYA00241390	ERZ501626	root:Host-associated:Human:Digestive system:Large in	0.0876
MGYA00241391/ERZ501628_FASTA.fasta	MGYA00241391	ERZ501628	root:Host-associated:Human:Digestive system:Large in	0.3338
MGYA00241392/ERZ501630_FASTA.fasta	MGYA00241392	ERZ501630	root:Host-associated:Human:Digestive system:Large in	0.1282
MGYA00241393/ERZ501632_FASTA.fasta	MGYA00241393	ERZ501632	root:Host-associated:Human:Digestive system:Large in	0.1112
MGYA00241394/ERZ501634_FASTA.fasta	MGYA00241394	ERZ501634	root:Host-associated:Human:Digestive system:Large in	0.1448
MGYA00241395/ERZ501636_FASTA.fasta	MGYA00241395	ERZ501636	root:Host-associated:Human:Digestive system:Large in	0.239
MGYA00241396/ERZ501638_FASTA.fasta	MGYA00241396	ERZ501638	root:Host-associated:Human:Digestive system:Large in	0.1702
MGYA00241397/ERZ501640_FASTA.fasta	MGYA00241397	ERZ501640	root:Host-associated:Human:Digestive system:Large in	0.2778
MGYA00241398/ERZ501642_FASTA.fasta	MGYA00241398	ERZ501642	root:Host-associated:Human:Digestive system:Large in	0.1102
MGYA00241399/ERZ501305_FASTA.fasta	MGYA00241399	ERZ501305	root:Host-associated:Human:Digestive system:Large in	0.092
MGYA00241400/ERZ501307_FASTA.fasta	MGYA00241400	ERZ501307	root:Host-associated:Human:Digestive system:Large in	0.2014
MGYA00241401/ERZ501309_FASTA.fasta	MGYA00241401	ERZ501309	root:Host-associated:Human:Digestive system:Large in	0.2066
MGYA00241402/ERZ501311_FASTA.fasta	MGYA00241402	ERZ501311	root:Host-associated:Human:Digestive system:Large in	0.3448
MGYA00241403/ERZ501313_FASTA.fasta	MGYA00241403	ERZ501313	root:Host-associated:Human:Digestive system:Large in	0.2016
MGYA00241404/ERZ501315_FASTA.fasta	MGYA00241404	ERZ501315	root:Host-associated:Human:Digestive system:Large in	0.2752
MGYA00241405/ERZ501317_FASTA.fasta	MGYA00241405	ERZ501317	root:Host-associated:Human:Digestive system:Large in	0.3834
MGYA00241406/ERZ501319_FASTA.fasta	MGYA00241406	ERZ501319	root:Host-associated:Human:Digestive system:Large in	0.4478
MGYA00241407/ERZ501644_FASTA.fasta	MGYA00241407	ERZ501644	root:Host-associated:Human:Digestive system:Large in	0.0696
MGYA00241408/ERZ501646_FASTA.fasta	MGYA00241408	ERZ501646	root:Host-associated:Human:Digestive system:Large in	0.166
MGYA00241409/ERZ501648_FASTA.fasta	MGYA00241409	ERZ501648	root:Host-associated:Human:Digestive system:Large in	0.1854
MGYA00241410/ERZ501650_FASTA.fasta	MGYA00241410	ERZ501650	root:Host-associated:Human:Digestive system:Large in	0.176
MGYA00241411/ERZ501652_FASTA.fasta	MGYA00241411	ERZ501652	root:Host-associated:Human:Digestive system:Large in	0.1078
MGYA00241412/ERZ501654_FASTA.fasta	MGYA00241412	ERZ501654	root:Host-associated:Human:Digestive system:Large in	0.0774
MGYA00241413/ERZ501656_FASTA.fasta	MGYA00241413	ERZ501656	root:Host-associated:Human:Digestive system:Large in	0.2442
MGYA00241414/ERZ501657_FASTA.fasta	MGYA00241414	ERZ501657	root:Host-associated:Human:Digestive system:Large in	0.2178
MGYA00241415/ERZ501659_FASTA.fasta	MGYA00241415	ERZ501659	root:Host-associated:Human:Digestive system:Large in	0.35
MGYA00241416/ERZ501661_FASTA.fasta	MGYA00241416	ERZ501661	root:Host-associated:Human:Digestive system:Large in	0.0536
MGYA00241417/ERZ501664_FASTA.fasta	MGYA00241417	ERZ501664	root:Host-associated:Human:Digestive system:Large in	0.2502
MGYA00241418/ERZ501666_FASTA.fasta	MGYA00241418	ERZ501666	root:Host-associated:Human:Digestive system:Large in	0.2956
MGYA00241419/ERZ501668_FASTA.fasta	MGYA00241419	ERZ501668	root:Host-associated:Human:Digestive system:Large in	0.099
MGYA00241420/ERZ501670_FASTA.fasta	MGYA00241420	ERZ501670	root:Host-associated:Human:Digestive system:Large in	0.2498
MGYA00241421/ERZ501672_FASTA.fasta	MGYA00241421	ERZ501672	root:Host-associated:Human:Digestive system:Large in	0.1048
MGYA00241422/ERZ501674_FASTA.fasta	MGYA00241422	ERZ501674	root:Host-associated:Human:Digestive system:Large in	0.232
MGYA00241423/ERZ501676_FASTA.fasta	MGYA00241423	ERZ501676	root:Host-associated:Human:Digestive system:Large in	0.175
MGYA00241424/ERZ501678_FASTA.fasta	MGYA00241424	ERZ501678	root:Host-associated:Human:Digestive system:Large in	0.1304
MGYA00241425/ERZ501680_FASTA.fasta	MGYA00241425	ERZ501680	root:Host-associated:Human:Digestive system:Large in	0.1896
MGYA00241426/ERZ501682_FASTA.fasta	MGYA00241426	ERZ501682	root:Host-associated:Human:Digestive system:Large in	0.1662
MGYA00241427/ERZ501684_FASTA.fasta	MGYA00241427	ERZ501684	root:Host-associated:Human:Digestive system:Large in	0.182
MGYA00241428/ERZ501686_FASTA.fasta	MGYA00241428	ERZ501686	root:Host-associated:Human:Digestive system:Large in	0.2022
MGYA00241429/ERZ501688_FASTA.fasta	MGYA00241429	ERZ501688	root:Host-associated:Human:Digestive system:Large in	0.224
MGYA00241430/ERZ501690_FASTA.fasta	MGYA00241430	ERZ501690	root:Host-associated:Human:Digestive system:Large in	0.206
MGYA00241431/ERZ501692_FASTA.fasta	MGYA00241431	ERZ501692	root:Host-associated:Human:Digestive system:Large in	0.154
MGYA00241432/ERZ501694_FASTA.fasta	MGYA00241432	ERZ501694	root:Host-associated:Human:Digestive system:Large in	0.1116
MGYA00241433/ERZ501696_FASTA.fasta	MGYA00241433	ERZ501696	root:Host-associated:Human:Digestive system:Large in	0.3418
MGYA00241434/ERZ501700_FASTA.fasta	MGYA00241434	ERZ501700	root:Host-associated:Human:Digestive system:Large in	0.106
MGYA00241435/ERZ501702_FASTA.fasta	MGYA00241435	ERZ501702	root:Host-associated:Human:Digestive system:Large in	0.1242
MGYA00241436/ERZ501704_FASTA.fasta	MGYA00241436	ERZ501704	root:Host-associated:Human:Digestive system:Large in	0.2022
MGYA00241437/ERZ501706_FASTA.fasta	MGYA00241437	ERZ501706	root:Host-associated:Human:Digestive system:Large in	0.192
MGYA00241438/ERZ501708_FASTA.fasta	MGYA00241438	ERZ501708	root:Host-associated:Human:Digestive system:Large in	0.207
MGYA00241439/ERZ501710_FASTA.fasta	MGYA00241439	ERZ501710	root:Host-associated:Human:Digestive system:Large in	0.2156
MGYA00241440/ERZ501712_FASTA.fasta	MGYA00241440	ERZ501712	root:Host-associated:Human:Digestive system:Large in	0.1496
MGYA00241441/ERZ501714_FASTA.fasta	MGYA00241441	ERZ501714	root:Host-associated:Human:Digestive system:Large in	0.1836
MGYA00241442/ERZ501716_FASTA.fasta	MGYA00241442	ERZ501716	root:Host-associated:Human:Digestive system:Large in	0.3408
MGYA00241443/ERZ501718_FASTA.fasta	MGYA00241443	ERZ501718	root:Host-associated:Human:Digestive system:Large in	0.1612
MGYA00241444/ERZ501720_FASTA.fasta	MGYA00241444	ERZ501720	root:Host-associated:Human:Digestive system:Large in	0.2746
MGYA00241445/ERZ501722_FASTA.fasta	MGYA00241445	ERZ501722	root:Host-associated:Human:Digestive system:Large in	0.2706
MGYA00241446/ERZ501724_FASTA.fasta	MGYA00241446	ERZ501724	root:Host-associated:Human:Digestive system:Large in	0.0494
MGYA00241447/ERZ501726_FASTA.fasta	MGYA00241447	ERZ501726	root:Host-associated:Human:Digestive system:Large in	0.3634
MGYA00241448/ERZ501728_FASTA.fasta	MGYA00241448	ERZ501728	root:Host-associated:Human:Digestive system:Large in	0.1962
MGYA00241449/ERZ501730_FASTA.fasta	MGYA00241449	ERZ501730	root:Host-associated:Human:Digestive system:Large in	0.1958
MGYA00241450/ERZ501892_FASTA.fasta	MGYA00241450	ERZ501892	root:Host-associated:Human:Digestive system:Large in	0.411
MGYA00241451/ERZ501894_FASTA.fasta	MGYA00241451	ERZ501894	root:Host-associated:Human:Digestive system:Large in	0.1466
MGYA00241452/ERZ501896_FASTA.fasta	MGYA00241452	ERZ501896	root:Host-associated:Human:Digestive system:Large in	0.4516
MGYA00241453/ERZ501898_FASTA.fasta	MGYA00241453	ERZ501898	root:Host-associated:Human:Digestive system:Large in	0.4018
MGYA00241454/ERZ501900_FASTA.fasta	MGYA00241454	ERZ501900	root:Host-associated:Human:Digestive system:Large in	0.447
MGYA00241455/ERZ501902_FASTA.fasta	MGYA00241455	ERZ501902	root:Host-associated:Human:Digestive system:Large in	0.3774
MGYA00241456/ERZ501904_FASTA.fasta	MGYA00241456	ERZ501904	root:Host-associated:Human:Digestive system:Large in	0.5582
MGYA00241457/ERZ501906_FASTA.fasta	MGYA00241457	ERZ501906	root:Host-associated:Human:Digestive system:Large in	0.278
MGYA00241458/ERZ501908_FASTA.fasta	MGYA00241458	ERZ501908	root:Host-associated:Human:Digestive system:Large in	0.3712
MGYA00241459/ERZ501910_FASTA.fasta	MGYA00241459	ERZ501910	root:Host-associated:Human:Digestive system:Large in	0.3158
MGYA00241460/ERZ501912_FASTA.fasta	MGYA00241460	ERZ501912	root:Host-associated:Human:Digestive system:Large in	0.4376
MGYA00241461/ERZ501914_FASTA.fasta	MGYA00241461	ERZ501914	root:Host-associated:Human:Digestive system:Large in	0.202
MGYA00241462/ERZ501916_FASTA.fasta	MGYA00241462	ERZ501916	root:Host-associated:Human:Digestive system:Large in	0.409
MGYA00241463/ERZ501918_FASTA.fasta	MGYA00241463	ERZ501918	root:Host-associated:Human:Digestive system:Large in	0.4416
MGYA00241464/ERZ501920_FASTA.fasta	MGYA00241464	ERZ501920	root:Host-associated:Human:Digestive system:Large in	0.3952
MGYA00241465/ERZ501922_FASTA.fasta	MGYA00241465	ERZ501922	root:Host-associated:Human:Digestive system:Large in	0.4076
MGYA00241466/ERZ501924_FASTA.fasta	MGYA00241466	ERZ501924	root:Host-associated:Human:Digestive system:Large in	0.411
MGYA00241467/ERZ501926_FASTA.fasta	MGYA00241467	ERZ501926	root:Host-associated:Human:Digestive system:Large in	0.0562
MGYA00241468/ERZ501928_FASTA.fasta	MGYA00241468	ERZ501928	root:Host-associated:Human:Digestive system:Large in	0.0608
MGYA00241469/ERZ501930_FASTA.fasta	MGYA00241469	ERZ501930	root:Host-associated:Human:Digestive system:Large in	0.0778
MGYA00241470/ERZ501932_FASTA.fasta	MGYA00241470	ERZ501932	root:Host-associated:Human:Digestive system:Large in	0.0324
MGYA00241471/ERZ501934_FASTA.fasta	MGYA00241471	ERZ501934	root:Host-associated:Human:Digestive system:Large in	0.0428
MGYA00241472/ERZ501936_FASTA.fasta	MGYA00241472	ERZ501936	root:Host-associated:Human:Digestive system:Large in	0.0294
MGYA00241473/ERZ501938_FASTA.fasta	MGYA00241473	ERZ501938	root:Host-associated:Human:Digestive system:Large in	0.0622
MGYA00241474/ERZ501939_FASTA.fasta	MGYA00241474	ERZ501939	root:Host-associated:Human:Digestive system:Large in	0.0736
MGYA00241475/ERZ501941_FASTA.fasta	MGYA00241475	ERZ501941	root:Host-associated:Human:Digestive system:Large in	0.0444
MGYA00241476/ERZ501943_FASTA.fasta	MGYA00241476	ERZ501943	root:Host-associated:Human:Digestive system:Large in	0.048
MGYA00241477/ERZ501946_FASTA.fasta	MGYA00241477	ERZ501946	root:Host-associated:Human:Digestive system:Large in	0.0604
MGYA00241478/ERZ501948_FASTA.fasta	MGYA00241478	ERZ501948	root:Host-associated:Human:Digestive system:Large in	0.3184
MGYA00241479/ERZ501950_FASTA.fasta	MGYA00241479	ERZ501950	root:Host-associated:Human:Digestive system:Large in	0.412
MGYA00241480/ERZ501952_FASTA.fasta	MGYA00241480	ERZ501952	root:Host-associated:Human:Digestive system:Large in	0.1032
MGYA00241481/ERZ501954_FASTA.fasta	MGYA00241481	ERZ501954	root:Host-associated:Human:Digestive system:Large in	0.4118
MGYA00241482/ERZ501956_FASTA.fasta	MGYA00241482	ERZ501956	root:Host-associated:Human:Digestive system:Large in	0.4402
MGYA00241483/ERZ501958_FASTA.fasta	MGYA00241483	ERZ501958	root:Host-associated:Human:Digestive system:Large in	0.206
MGYA00241484/ERZ501960_FASTA.fasta	MGYA00241484	ERZ501960	root:Host-associated:Human:Digestive system:Large in	0.2076
MGYA00241485/ERZ501962_FASTA.fasta	MGYA00241485	ERZ501962	root:Host-associated:Human:Digestive system:Large in	0.1976
MGYA00241486/ERZ501964_FASTA.fasta	MGYA00241486	ERZ501964	root:Host-associated:Human:Digestive system:Large in	0.4812
MGYA00241487/ERZ501966_FASTA.fasta	MGYA00241487	ERZ501966	root:Host-associated:Human:Digestive system:Large in	0.3928
MGYA00241488/ERZ501968_FASTA.fasta	MGYA00241488	ERZ501968	root:Host-associated:Human:Digestive system:Large in	0.3576
MGYA00241489/ERZ501970_FASTA.fasta	MGYA00241489	ERZ501970	root:Host-associated:Human:Digestive system:Large in	0.3058
MGYA00241490/ERZ501972_FASTA.fasta	MGYA00241490	ERZ501972	root:Host-associated:Human:Digestive system:Large in	0.3894
MGYA00241491/ERZ501974_FASTA.fasta	MGYA00241491	ERZ501974	root:Host-associated:Human:Digestive system:Large in	0.4292
MGYA00241492/ERZ501976_FASTA.fasta	MGYA00241492	ERZ501976	root:Host-associated:Human:Digestive system:Large in	0.132
MGYA00241493/ERZ501978_FASTA.fasta	MGYA00241493	ERZ501978	root:Host-associated:Human:Digestive system:Large in	0.4014
MGYA00241494/ERZ501980_FASTA.fasta	MGYA00241494	ERZ501980	root:Host-associated:Human:Digestive system:Large in	0.4052
MGYA00241495/ERZ501982_FASTA.fasta	MGYA00241495	ERZ501982	root:Host-associated:Human:Digestive system:Large in	0.39
MGYA00241496/ERZ501984_FASTA.fasta	MGYA00241496	ERZ501984	root:Host-associated:Human:Digestive system:Large in	0.4426
MGYA00241497/ERZ501986_FASTA.fasta	MGYA00241497	ERZ501986	root:Host-associated:Human:Digestive system:Large in	0.479
MGYA00241498/ERZ501988_FASTA.fasta	MGYA00241498	ERZ501988	root:Host-associated:Human:Digestive system:Large in	0.1742
MGYA00241499/ERZ501990_FASTA.fasta	MGYA00241499	ERZ501990	root:Host-associated:Human:Digestive system:Large in	0.36
MGYA00241500/ERZ501992_FASTA.fasta	MGYA00241500	ERZ501992	root:Host-associated:Human:Digestive system:Large in	0.4212
MGYA00241501/ERZ501994_FASTA.fasta	MGYA00241501	ERZ501994	root:Host-associated:Human:Digestive system:Large in	0.2692
MGYA00241502/ERZ501996_FASTA.fasta	MGYA00241502	ERZ501996	root:Host-associated:Human:Digestive system:Large in	0.3946
MGYA00241503/ERZ501998_FASTA.fasta	MGYA00241503	ERZ501998	root:Host-associated:Human:Digestive system:Large in	0.4066
MGYA00241504/ERZ502000_FASTA.fasta	MGYA00241504	ERZ502000	root:Host-associated:Human:Digestive system:Large in	0.4048
MGYA00241505/ERZ502002_FASTA.fasta	MGYA00241505	ERZ502002	root:Host-associated:Human:Digestive system:Large in	0.1574
MGYA00241506/ERZ502004_FASTA.fasta	MGYA00241506	ERZ502004	root:Host-associated:Human:Digestive system:Large in	0.4422
MGYA00241507/ERZ502006_FASTA.fasta	MGYA00241507	ERZ502006	root:Host-associated:Human:Digestive system:Large in	0.4778
MGYA00241508/ERZ502008_FASTA.fasta	MGYA00241508	ERZ502008	root:Host-associated:Human:Digestive system:Large in	0.3658
MGYA00241509/ERZ502010_FASTA.fasta	MGYA00241509	ERZ502010	root:Host-associated:Human:Digestive system:Large in	0.3104
MGYA00241510/ERZ502012_FASTA.fasta	MGYA00241510	ERZ502012	root:Host-associated:Human:Digestive system:Large in	0.3164
MGYA00241511/ERZ502014_FASTA.fasta	MGYA00241511	ERZ502014	root:Host-associated:Human:Digestive system:Large in	0.0442
MGYA00241512/ERZ502016_FASTA.fasta	MGYA00241512	ERZ502016	root:Host-associated:Human:Digestive system:Large in	0.4662
MGYA00241513/ERZ502018_FASTA.fasta	MGYA00241513	ERZ502018	root:Host-associated:Human:Digestive system:Large in	0.3668
MGYA00241514/ERZ502020_FASTA.fasta	MGYA00241514	ERZ502020	root:Host-associated:Human:Digestive system:Large in	0.4278
MGYA00241515/ERZ502022_FASTA.fasta	MGYA00241515	ERZ502022	root:Host-associated:Human:Digestive system:Large in	0.3858
MGYA00241516/ERZ502024_FASTA.fasta	MGYA00241516	ERZ502024	root:Host-associated:Human:Digestive system:Large in	0.4208
MGYA00241517/ERZ502026_FASTA.fasta	MGYA00241517	ERZ502026	root:Host-associated:Human:Digestive system:Large in	0.1708
MGYA00241518/ERZ502028_FASTA.fasta	MGYA00241518	ERZ502028	root:Host-associated:Human:Digestive system:Large in	0.095
MGYA00241519/ERZ502030_FASTA.fasta	MGYA00241519	ERZ502030	root:Host-associated:Human:Digestive system:Large in	0.3946
MGYA00241520/ERZ502032_FASTA.fasta	MGYA00241520	ERZ502032	root:Host-associated:Human:Digestive system:Large in	0.3978
MGYA00241521/ERZ502034_FASTA.fasta	MGYA00241521	ERZ502034	root:Host-associated:Human:Digestive system:Large in	0.4298
MGYA00241522/ERZ501741_FASTA.fasta	MGYA00241522	ERZ501741	root:Host-associated:Human:Digestive system:Large in	0.359
MGYA00241523/ERZ501743_FASTA.fasta	MGYA00241523	ERZ501743	root:Host-associated:Human:Digestive system:Large in	0.3242
MGYA00241524/ERZ501745_FASTA.fasta	MGYA00241524	ERZ501745	root:Host-associated:Human:Digestive system:Large in	0.2958
MGYA00241525/ERZ501747_FASTA.fasta	MGYA00241525	ERZ501747	root:Host-associated:Human:Digestive system:Large in	0.255
MGYA00241526/ERZ501749_FASTA.fasta	MGYA00241526	ERZ501749	root:Host-associated:Human:Digestive system:Large in	0.1784
MGYA00241527/ERZ501751_FASTA.fasta	MGYA00241527	ERZ501751	root:Host-associated:Human:Digestive system:Large in	0.4826
MGYA00241528/ERZ501754_FASTA.fasta	MGYA00241528	ERZ501754	root:Host-associated:Human:Digestive system:Large in	0.454
MGYA00241529/ERZ501756_FASTA.fasta	MGYA00241529	ERZ501756	root:Host-associated:Human:Digestive system:Large in	0.2462
MGYA00241530/ERZ501758_FASTA.fasta	MGYA00241530	ERZ501758	root:Host-associated:Human:Digestive system:Large in	0.277
MGYA00241531/ERZ501759_FASTA.fasta	MGYA00241531	ERZ501759	root:Host-associated:Human:Digestive system:Large in	0.1648
MGYA00241532/ERZ501761_FASTA.fasta	MGYA00241532	ERZ501761	root:Host-associated:Human:Digestive system:Large in	0.2624
MGYA00241533/ERZ501763_FASTA.fasta	MGYA00241533	ERZ501763	root:Host-associated:Human:Digestive system:Large in	0.2274
MGYA00241534/ERZ501765_FASTA.fasta	MGYA00241534	ERZ501765	root:Host-associated:Human:Digestive system:Large in	0.2146
MGYA00241535/ERZ501767_FASTA.fasta	MGYA00241535	ERZ501767	root:Host-associated:Human:Digestive system:Large in	0.247
MGYA00241536/ERZ501769_FASTA.fasta	MGYA00241536	ERZ501769	root:Host-associated:Human:Digestive system:Large in	0.2614
MGYA00241537/ERZ501771_FASTA.fasta	MGYA00241537	ERZ501771	root:Host-associated:Human:Digestive system:Large in	0.3888
MGYA00241538/ERZ501773_FASTA.fasta	MGYA00241538	ERZ501773	root:Host-associated:Human:Digestive system:Large in	0.303
MGYA00241539/ERZ501775_FASTA.fasta	MGYA00241539	ERZ501775	root:Host-associated:Human:Digestive system:Large in	0.2556
MGYA00241540/ERZ501777_FASTA.fasta	MGYA00241540	ERZ501777	root:Host-associated:Human:Digestive system:Large in	0.3922
MGYA00241541/ERZ501779_FASTA.fasta	MGYA00241541	ERZ501779	root:Host-associated:Human:Digestive system:Large in	0.2772
MGYA00241542/ERZ501781_FASTA.fasta	MGYA00241542	ERZ501781	root:Host-associated:Human:Digestive system:Large in	0.4
MGYA00241543/ERZ501783_FASTA.fasta	MGYA00241543	ERZ501783	root:Host-associated:Human:Digestive system:Large in	0.3248
MGYA00241544/ERZ501270_FASTA.fasta	MGYA00241544	ERZ501270	root:Host-associated:Human:Digestive system:Large in	0.1448
MGYA00241545/ERZ501272_FASTA.fasta	MGYA00241545	ERZ501272	root:Host-associated:Human:Digestive system:Large in	0.2042
MGYA00241546/ERZ501274_FASTA.fasta	MGYA00241546	ERZ501274	root:Host-associated:Human:Digestive system:Large in	0.3606
MGYA00241547/ERZ501276_FASTA.fasta	MGYA00241547	ERZ501276	root:Host-associated:Human:Digestive system:Large in	0.141
MGYA00241548/ERZ501278_FASTA.fasta	MGYA00241548	ERZ501278	root:Host-associated:Human:Digestive system:Large in	0.2278
MGYA00241549/ERZ501280_FASTA.fasta	MGYA00241549	ERZ501280	root:Host-associated:Human:Digestive system:Large in	0.227
MGYA00241550/ERZ501282_FASTA.fasta	MGYA00241550	ERZ501282	root:Host-associated:Human:Digestive system:Large in	0.177
MGYA00241551/ERZ501284_FASTA.fasta	MGYA00241551	ERZ501284	root:Host-associated:Human:Digestive system:Large in	0.2224
MGYA00241552/ERZ501286_FASTA.fasta	MGYA00241552	ERZ501286	root:Host-associated:Human:Digestive system:Large in	0.059
MGYA00241553/ERZ501288_FASTA.fasta	MGYA00241553	ERZ501288	root:Host-associated:Human:Digestive system:Large in	0.2826
MGYA00241554/ERZ501290_FASTA.fasta	MGYA00241554	ERZ501290	root:Host-associated:Human:Digestive system:Large in	0.301
MGYA00241555/ERZ501292_FASTA.fasta	MGYA00241555	ERZ501292	root:Host-associated:Human:Digestive system:Large in	0.1838
MGYA00241556/ERZ501294_FASTA.fasta	MGYA00241556	ERZ501294	root:Host-associated:Human:Digestive system:Large in	0.1434
MGYA00241557/ERZ501296_FASTA.fasta	MGYA00241557	ERZ501296	root:Host-associated:Human:Digestive system:Large in	0.327
MGYA00241558/ERZ501467_FASTA.fasta	MGYA00241558	ERZ501467	root:Host-associated:Human:Digestive system:Large in	0.435
MGYA00241559/ERZ501469_FASTA.fasta	MGYA00241559	ERZ501469	root:Host-associated:Human:Digestive system:Large in	0.1252
MGYA00241560/ERZ501471_FASTA.fasta	MGYA00241560	ERZ501471	root:Host-associated:Human:Digestive system:Large in	0.5286
MGYA00241561/ERZ501473_FASTA.fasta	MGYA00241561	ERZ501473	root:Host-associated:Human:Digestive system:Large in	0.4614
MGYA00241562/ERZ501475_FASTA.fasta	MGYA00241562	ERZ501475	root:Host-associated:Human:Digestive system:Large in	0.3662
MGYA00241563/ERZ501477_FASTA.fasta	MGYA00241563	ERZ501477	root:Host-associated:Human:Digestive system:Large in	0.405
MGYA00241564/ERZ501479_FASTA.fasta	MGYA00241564	ERZ501479	root:Host-associated:Human:Digestive system:Large in	0.4434
MGYA00241565/ERZ501481_FASTA.fasta	MGYA00241565	ERZ501481	root:Host-associated:Human:Digestive system:Large in	0.2508
MGYA00241566/ERZ501483_FASTA.fasta	MGYA00241566	ERZ501483	root:Host-associated:Human:Digestive system:Large in	0.3608
MGYA00241567/ERZ501485_FASTA.fasta	MGYA00241567	ERZ501485	root:Host-associated:Human:Digestive system:Large in	0.3988
MGYA00241568/ERZ501487_FASTA.fasta	MGYA00241568	ERZ501487	root:Host-associated:Human:Digestive system:Large in	0.4298
MGYA00241569/ERZ501489_FASTA.fasta	MGYA00241569	ERZ501489	root:Host-associated:Human:Digestive system:Large in	0.2294
MGYA00241570/ERZ501491_FASTA.fasta	MGYA00241570	ERZ501491	root:Host-associated:Human:Digestive system:Large in	0.459
MGYA00241571/ERZ501493_FASTA.fasta	MGYA00241571	ERZ501493	root:Host-associated:Human:Digestive system:Large in	0.2236
MGYA00241572/ERZ501495_FASTA.fasta	MGYA00241572	ERZ501495	root:Host-associated:Human:Digestive system:Large in	0.364
MGYA00241573/ERZ501497_FASTA.fasta	MGYA00241573	ERZ501497	root:Host-associated:Human:Digestive system:Large in	0.301
MGYA00241574/ERZ501499_FASTA.fasta	MGYA00241574	ERZ501499	root:Host-associated:Human:Digestive system:Large in	0.3856
MGYA00241575/ERZ501501_FASTA.fasta	MGYA00241575	ERZ501501	root:Host-associated:Human:Digestive system:Large in	0.376
MGYA00241576/ERZ501321_FASTA.fasta	MGYA00241576	ERZ501321	root:Host-associated:Human:Digestive system:Large in	0.4134
MGYA00241577/ERZ501323_FASTA.fasta	MGYA00241577	ERZ501323	root:Host-associated:Human:Digestive system:Large in	0.1972
MGYA00241578/ERZ501325_FASTA.fasta	MGYA00241578	ERZ501325	root:Host-associated:Human:Digestive system:Large in	0.3706
MGYA00241579/ERZ501327_FASTA.fasta	MGYA00241579	ERZ501327	root:Host-associated:Human:Digestive system:Large in	0.462
MGYA00241580/ERZ501329_FASTA.fasta	MGYA00241580	ERZ501329	root:Host-associated:Human:Digestive system:Large in	0.3818
MGYA00241581/ERZ501331_FASTA.fasta	MGYA00241581	ERZ501331	root:Host-associated:Human:Digestive system:Large in	0.4494
MGYA00241582/ERZ501333_FASTA.fasta	MGYA00241582	ERZ501333	root:Host-associated:Human:Digestive system:Large in	0.2212
MGYA00241583/ERZ501335_FASTA.fasta	MGYA00241583	ERZ501335	root:Host-associated:Human:Digestive system:Large in	0.322
MGYA00241584/ERZ501337_FASTA.fasta	MGYA00241584	ERZ501337	root:Host-associated:Human:Digestive system:Large in	0.3814
MGYA00241585/ERZ501339_FASTA.fasta	MGYA00241585	ERZ501339	root:Host-associated:Human:Digestive system:Large in	0.3936
MGYA00241586/ERZ501341_FASTA.fasta	MGYA00241586	ERZ501341	root:Host-associated:Human:Digestive system:Large in	0.4568
MGYA00241587/ERZ501343_FASTA.fasta	MGYA00241587	ERZ501343	root:Host-associated:Human:Digestive system:Large in	0.5096
MGYA00241588/ERZ501345_FASTA.fasta	MGYA00241588	ERZ501345	root:Host-associated:Human:Digestive system:Large in	0.0468
MGYA00241589/ERZ501347_FASTA.fasta	MGYA00241589	ERZ501347	root:Host-associated:Human:Digestive system:Large in	0.4098
MGYA00241590/ERZ501349_FASTA.fasta	MGYA00241590	ERZ501349	root:Host-associated:Human:Digestive system:Large in	0.3896
MGYA00241591/ERZ501351_FASTA.fasta	MGYA00241591	ERZ501351	root:Host-associated:Human:Digestive system:Large in	0.3624
MGYA00241592/ERZ501353_FASTA.fasta	MGYA00241592	ERZ501353	root:Host-associated:Human:Digestive system:Large in	0.2264
MGYA00241593/ERZ501355_FASTA.fasta	MGYA00241593	ERZ501355	root:Host-associated:Human:Digestive system:Large in	0.3862
MGYA00241594/ERZ501357_FASTA.fasta	MGYA00241594	ERZ501357	root:Host-associated:Human:Digestive system:Large in	0.3466
MGYA00241595/ERZ501359_FASTA.fasta	MGYA00241595	ERZ501359	root:Host-associated:Human:Digestive system:Large in	0.2618
MGYA00241596/ERZ501785_FASTA.fasta	MGYA00241596	ERZ501785	root:Host-associated:Human:Digestive system:Large in	0.3612
MGYA00241597/ERZ501787_FASTA.fasta	MGYA00241597	ERZ501787	root:Host-associated:Human:Digestive system:Large in	0.3688
MGYA00241598/ERZ501298_FASTA.fasta	MGYA00241598	ERZ501298	root:Host-associated:Human:Digestive system:Large in	0.2272
MGYA00241599/ERZ501300_FASTA.fasta	MGYA00241599	ERZ501300	root:Host-associated:Human:Digestive system:Large in	0.211
MGYA00241600/ERZ501302_FASTA.fasta	MGYA00241600	ERZ501302	root:Host-associated:Human:Digestive system:Large in	0.2162
MGYA00241601/ERZ501304_FASTA.fasta	MGYA00241601	ERZ501304	root:Host-associated:Human:Digestive system:Large in	0.0832
MGYA00241602/ERZ501306_FASTA.fasta	MGYA00241602	ERZ501306	root:Host-associated:Human:Digestive system:Large in	0.216
MGYA00241603/ERZ501308_FASTA.fasta	MGYA00241603	ERZ501308	root:Host-associated:Human:Digestive system:Large in	0.2292
MGYA00241604/ERZ501310_FASTA.fasta	MGYA00241604	ERZ501310	root:Host-associated:Human:Digestive system:Large in	0.152
MGYA00241605/ERZ501312_FASTA.fasta	MGYA00241605	ERZ501312	root:Host-associated:Human:Digestive system:Large in	0.3708
MGYA00241606/ERZ501314_FASTA.fasta	MGYA00241606	ERZ501314	root:Host-associated:Human:Digestive system:Large in	0.2064
MGYA00241607/ERZ501316_FASTA.fasta	MGYA00241607	ERZ501316	root:Host-associated:Human:Digestive system:Large in	0.3384
MGYA00241608/ERZ501318_FASTA.fasta	MGYA00241608	ERZ501318	root:Host-associated:Human:Digestive system:Large in	0.3792
MGYA00241609/ERZ501320_FASTA.fasta	MGYA00241609	ERZ501320	root:Host-associated:Human:Digestive system:Large in	0.3792
MGYA00241610/ERZ501322_FASTA.fasta	MGYA00241610	ERZ501322	root:Host-associated:Human:Digestive system:Large in	0.2462
MGYA00241611/ERZ501324_FASTA.fasta	MGYA00241611	ERZ501324	root:Host-associated:Human:Digestive system:Large in	0.2138
MGYA00241612/ERZ501326_FASTA.fasta	MGYA00241612	ERZ501326	root:Host-associated:Human:Digestive system:Large in	0.3372
MGYA00241613/ERZ501328_FASTA.fasta	MGYA00241613	ERZ501328	root:Host-associated:Human:Digestive system:Large in	0.4354
MGYA00241614/ERZ501330_FASTA.fasta	MGYA00241614	ERZ501330	root:Host-associated:Human:Digestive system:Large in	0.2062
MGYA00241615/ERZ501332_FASTA.fasta	MGYA00241615	ERZ501332	root:Host-associated:Human:Digestive system:Large in	0.0904
MGYA00241616/ERZ501334_FASTA.fasta	MGYA00241616	ERZ501334	root:Host-associated:Human:Digestive system:Large in	0.4502
MGYA00241617/ERZ501336_FASTA.fasta	MGYA00241617	ERZ501336	root:Host-associated:Human:Digestive system:Large in	0.3904
MGYA00241618/ERZ501338_FASTA.fasta	MGYA00241618	ERZ501338	root:Host-associated:Human:Digestive system:Large in	0.1426
MGYA00241619/ERZ501340_FASTA.fasta	MGYA00241619	ERZ501340	root:Host-associated:Human:Digestive system:Large in	0.2428
MGYA00241620/ERZ501503_FASTA.fasta	MGYA00241620	ERZ501503	root:Host-associated:Human:Digestive system:Large in	0.2942
MGYA00241621/ERZ501505_FASTA.fasta	MGYA00241621	ERZ501505	root:Host-associated:Human:Digestive system:Large in	0.415
MGYA00241622/ERZ501507_FASTA.fasta	MGYA00241622	ERZ501507	root:Host-associated:Human:Digestive system:Large in	0.3734
MGYA00241623/ERZ501509_FASTA.fasta	MGYA00241623	ERZ501509	root:Host-associated:Human:Digestive system:Large in	0.349
MGYA00241624/ERZ501511_FASTA.fasta	MGYA00241624	ERZ501511	root:Host-associated:Human:Digestive system:Large in	0.0326
MGYA00241625/ERZ501513_FASTA.fasta	MGYA00241625	ERZ501513	root:Host-associated:Human:Digestive system:Large in	0.0508
MGYA00241626/ERZ501515_FASTA.fasta	MGYA00241626	ERZ501515	root:Host-associated:Human:Digestive system:Large in	0.1312
MGYA00241627/ERZ501517_FASTA.fasta	MGYA00241627	ERZ501517	root:Host-associated:Human:Digestive system:Large in	0.3004
MGYA00241628/ERZ501519_FASTA.fasta	MGYA00241628	ERZ501519	root:Host-associated:Human:Digestive system:Large in	0.2902
MGYA00241629/ERZ501521_FASTA.fasta	MGYA00241629	ERZ501521	root:Host-associated:Human:Digestive system:Large in	0.166
MGYA00241630/ERZ501523_FASTA.fasta	MGYA00241630	ERZ501523	root:Host-associated:Human:Digestive system:Large in	0.1038
MGYA00241631/ERZ501525_FASTA.fasta	MGYA00241631	ERZ501525	root:Host-associated:Human:Digestive system:Large in	0.122
MGYA00241632/ERZ501527_FASTA.fasta	MGYA00241632	ERZ501527	root:Host-associated:Human:Digestive system:Large in	0.2596
MGYA00241633/ERZ501529_FASTA.fasta	MGYA00241633	ERZ501529	root:Host-associated:Human:Digestive system:Large in	0.2998
MGYA00241634/ERZ501531_FASTA.fasta	MGYA00241634	ERZ501531	root:Host-associated:Human:Digestive system:Large in	0.3052
MGYA00241635/ERZ501533_FASTA.fasta	MGYA00241635	ERZ501533	root:Host-associated:Human:Digestive system:Large in	0.378
MGYA00241636/ERZ501535_FASTA.fasta	MGYA00241636	ERZ501535	root:Host-associated:Human:Digestive system:Large in	0.2274
MGYA00241637/ERZ501537_FASTA.fasta	MGYA00241637	ERZ501537	root:Host-associated:Human:Digestive system:Large in	0.3782
MGYA00241638/ERZ501539_FASTA.fasta	MGYA00241638	ERZ501539	root:Host-associated:Human:Digestive system:Large in	0.1118
MGYA00241639/ERZ501541_FASTA.fasta	MGYA00241639	ERZ501541	root:Host-associated:Human:Digestive system:Large in	0.3016
MGYA00241640/ERZ501543_FASTA.fasta	MGYA00241640	ERZ501543	root:Host-associated:Human:Digestive system:Large in	0.0924
MGYA00241641/ERZ501545_FASTA.fasta	MGYA00241641	ERZ501545	root:Host-associated:Human:Digestive system:Large in	0.2598
MGYA00241642/ERZ501581_FASTA.fasta	MGYA00241642	ERZ501581	root:Host-associated:Human:Digestive system:Large in	0.272
MGYA00241643/ERZ501583_FASTA.fasta	MGYA00241643	ERZ501583	root:Host-associated:Human:Digestive system:Large in	0.1436
MGYA00241644/ERZ501585_FASTA.fasta	MGYA00241644	ERZ501585	root:Host-associated:Human:Digestive system:Large in	0.3368
MGYA00241645/ERZ501587_FASTA.fasta	MGYA00241645	ERZ501587	root:Host-associated:Human:Digestive system:Large in	0.0454
MGYA00241646/ERZ501589_FASTA.fasta	MGYA00241646	ERZ501589	root:Host-associated:Human:Digestive system:Large in	0.2878
MGYA00241647/ERZ501591_FASTA.fasta	MGYA00241647	ERZ501591	root:Host-associated:Human:Digestive system:Large in	0.2818
MGYA00241648/ERZ501593_FASTA.fasta	MGYA00241648	ERZ501593	root:Host-associated:Human:Digestive system:Large in	0.2498
MGYA00241649/ERZ501595_FASTA.fasta	MGYA00241649	ERZ501595	root:Host-associated:Human:Digestive system:Large in	0.254
MGYA00241650/ERZ501597_FASTA.fasta	MGYA00241650	ERZ501597	root:Host-associated:Human:Digestive system:Large in	0.0786
MGYA00241651/ERZ501599_FASTA.fasta	MGYA00241651	ERZ501599	root:Host-associated:Human:Digestive system:Large in	0.401
MGYA00241652/ERZ501601_FASTA.fasta	MGYA00241652	ERZ501601	root:Host-associated:Human:Digestive system:Large in	0.27
MGYA00241653/ERZ501603_FASTA.fasta	MGYA00241653	ERZ501603	root:Host-associated:Human:Digestive system:Large in	0.1168
MGYA00241654/ERZ501605_FASTA.fasta	MGYA00241654	ERZ501605	root:Host-associated:Human:Digestive system:Large in	0.3538
MGYA00241655/ERZ501607_FASTA.fasta	MGYA00241655	ERZ501607	root:Host-associated:Human:Digestive system:Large in	0.1154
MGYA00241656/ERZ501609_FASTA.fasta	MGYA00241656	ERZ501609	root:Host-associated:Human:Digestive system:Large in	0.049
MGYA00241657/ERZ501611_FASTA.fasta	MGYA00241657	ERZ501611	root:Host-associated:Human:Digestive system:Large in	0.105
MGYA00241658/ERZ501613_FASTA.fasta	MGYA00241658	ERZ501613	root:Host-associated:Human:Digestive system:Large in	0.1788
MGYA00241659/ERZ501615_FASTA.fasta	MGYA00241659	ERZ501615	root:Host-associated:Human:Digestive system:Large in	0.0522
MGYA00241660/ERZ501617_FASTA.fasta	MGYA00241660	ERZ501617	root:Host-associated:Human:Digestive system:Large in	0.0292
MGYA00241661/ERZ501619_FASTA.fasta	MGYA00241661	ERZ501619	root:Host-associated:Human:Digestive system:Large in	0.0372
MGYA00241662/ERZ501621_FASTA.fasta	MGYA00241662	ERZ501621	root:Host-associated:Human:Digestive system:Large in	0.0738
MGYA00241663/ERZ501623_FASTA.fasta	MGYA00241663	ERZ501623	root:Host-associated:Human:Digestive system:Large in	0.0328
MGYA00241664/ERZ501789_FASTA.fasta	MGYA00241664	ERZ501789	root:Host-associated:Human:Digestive system:Large in	0.1828
MGYA00241665/ERZ501791_FASTA.fasta	MGYA00241665	ERZ501791	root:Host-associated:Human:Digestive system:Large in	0.4126
MGYA00241666/ERZ501793_FASTA.fasta	MGYA00241666	ERZ501793	root:Host-associated:Human:Digestive system:Large in	0.5358
MGYA00241667/ERZ501795_FASTA.fasta	MGYA00241667	ERZ501795	root:Host-associated:Human:Digestive system:Large in	0.1534
MGYA00241668/ERZ501797_FASTA.fasta	MGYA00241668	ERZ501797	root:Host-associated:Human:Digestive system:Large in	0.2032
MGYA00241669/ERZ501799_FASTA.fasta	MGYA00241669	ERZ501799	root:Host-associated:Human:Digestive system:Large in	0.1584
MGYA00241670/ERZ501801_FASTA.fasta	MGYA00241670	ERZ501801	root:Host-associated:Human:Digestive system:Large in	0.1886
MGYA00241671/ERZ501803_FASTA.fasta	MGYA00241671	ERZ501803	root:Host-associated:Human:Digestive system:Large in	0.1624
MGYA00241672/ERZ501805_FASTA.fasta	MGYA00241672	ERZ501805	root:Host-associated:Human:Digestive system:Large in	0.2868
MGYA00241673/ERZ501807_FASTA.fasta	MGYA00241673	ERZ501807	root:Host-associated:Human:Digestive system:Large in	0.2234
MGYA00241674/ERZ501809_FASTA.fasta	MGYA00241674	ERZ501809	root:Host-associated:Human:Digestive system:Large in	0.1272
MGYA00241675/ERZ501811_FASTA.fasta	MGYA00241675	ERZ501811	root:Host-associated:Human:Digestive system:Large in	0.1942
MGYA00241676/ERZ501813_FASTA.fasta	MGYA00241676	ERZ501813	root:Host-associated:Human:Digestive system:Large in	0.3812
MGYA00241677/ERZ501815_FASTA.fasta	MGYA00241677	ERZ501815	root:Host-associated:Human:Digestive system:Large in	0.1236
MGYA00241678/ERZ501817_FASTA.fasta	MGYA00241678	ERZ501817	root:Host-associated:Human:Digestive system:Large in	0.219
MGYA00241679/ERZ501819_FASTA.fasta	MGYA00241679	ERZ501819	root:Host-associated:Human:Digestive system:Large in	0.2496
MGYA00241680/ERZ501821_FASTA.fasta	MGYA00241680	ERZ501821	root:Host-associated:Human:Digestive system:Large in	0.1308
MGYA00241681/ERZ501823_FASTA.fasta	MGYA00241681	ERZ501823	root:Host-associated:Human:Digestive system:Large in	0.2368
MGYA00241682/ERZ501825_FASTA.fasta	MGYA00241682	ERZ501825	root:Host-associated:Human:Digestive system:Large in	0.313
MGYA00241683/ERZ501827_FASTA.fasta	MGYA00241683	ERZ501827	root:Host-associated:Human:Digestive system:Large in	0.3226
MGYA00241684/ERZ501829_FASTA.fasta	MGYA00241684	ERZ501829	root:Host-associated:Human:Digestive system:Large in	0.1746
MGYA00241685/ERZ501831_FASTA.fasta	MGYA00241685	ERZ501831	root:Host-associated:Human:Digestive system:Large in	0.3766
MGYA00241686/ERZ501342_FASTA.fasta	MGYA00241686	ERZ501342	root:Host-associated:Human:Digestive system:Large in	0.2266
MGYA00241687/ERZ501344_FASTA.fasta	MGYA00241687	ERZ501344	root:Host-associated:Human:Digestive system:Large in	0.2516
MGYA00241688/ERZ501346_FASTA.fasta	MGYA00241688	ERZ501346	root:Host-associated:Human:Digestive system:Large in	0.2152
MGYA00241689/ERZ501348_FASTA.fasta	MGYA00241689	ERZ501348	root:Host-associated:Human:Digestive system:Large in	0.3208
MGYA00241690/ERZ501350_FASTA.fasta	MGYA00241690	ERZ501350	root:Host-associated:Human:Digestive system:Large in	0.2948
MGYA00241691/ERZ501352_FASTA.fasta	MGYA00241691	ERZ501352	root:Host-associated:Human:Digestive system:Large in	0.197
MGYA00241692/ERZ501354_FASTA.fasta	MGYA00241692	ERZ501354	root:Host-associated:Human:Digestive system:Large in	0.368
MGYA00241693/ERZ501356_FASTA.fasta	MGYA00241693	ERZ501356	root:Host-associated:Human:Digestive system:Large in	0.1842
MGYA00241694/ERZ501358_FASTA.fasta	MGYA00241694	ERZ501358	root:Host-associated:Human:Digestive system:Large in	0.129
MGYA00241695/ERZ501361_FASTA.fasta	MGYA00241695	ERZ501361	root:Host-associated:Human:Digestive system:Large in	0.2854
MGYA00241696/ERZ501363_FASTA.fasta	MGYA00241696	ERZ501363	root:Host-associated:Human:Digestive system:Large in	0.236
MGYA00241697/ERZ501365_FASTA.fasta	MGYA00241697	ERZ501365	root:Host-associated:Human:Digestive system:Large in	0.2376
MGYA00241698/ERZ501368_FASTA.fasta	MGYA00241698	ERZ501368	root:Host-associated:Human:Digestive system:Large in	0.2082
MGYA00241699/ERZ501370_FASTA.fasta	MGYA00241699	ERZ501370	root:Host-associated:Human:Digestive system:Large in	0.2508
MGYA00241700/ERZ501372_FASTA.fasta	MGYA00241700	ERZ501372	root:Host-associated:Human:Digestive system:Large in	0.112
MGYA00241701/ERZ501374_FASTA.fasta	MGYA00241701	ERZ501374	root:Host-associated:Human:Digestive system:Large in	0.3342
MGYA00241702/ERZ501376_FASTA.fasta	MGYA00241702	ERZ501376	root:Host-associated:Human:Digestive system:Large in	0.2724
MGYA00241703/ERZ501378_FASTA.fasta	MGYA00241703	ERZ501378	root:Host-associated:Human:Digestive system:Large in	0.3388
MGYA00241704/ERZ501380_FASTA.fasta	MGYA00241704	ERZ501380	root:Host-associated:Human:Digestive system:Large in	0.446
MGYA00241705/ERZ501382_FASTA.fasta	MGYA00241705	ERZ501382	root:Host-associated:Human:Digestive system:Large in	0.2348
MGYA00241706/ERZ501384_FASTA.fasta	MGYA00241706	ERZ501384	root:Host-associated:Human:Digestive system:Large in	0.3938
MGYA00241707/ERZ502035_FASTA.fasta	MGYA00241707	ERZ502035	root:Host-associated:Human:Digestive system:Large in	0.382
MGYA00241708/ERZ501547_FASTA.fasta	MGYA00241708	ERZ501547	root:Host-associated:Human:Digestive system:Large in	0.2284
MGYA00241709/ERZ501549_FASTA.fasta	MGYA00241709	ERZ501549	root:Host-associated:Human:Digestive system:Large in	0.12
MGYA00241710/ERZ501551_FASTA.fasta	MGYA00241710	ERZ501551	root:Host-associated:Human:Digestive system:Large in	0.2668
MGYA00241711/ERZ501553_FASTA.fasta	MGYA00241711	ERZ501553	root:Host-associated:Human:Digestive system:Large in	0.0882
MGYA00241712/ERZ501555_FASTA.fasta	MGYA00241712	ERZ501555	root:Host-associated:Human:Digestive system:Large in	0.098
MGYA00241713/ERZ501558_FASTA.fasta	MGYA00241713	ERZ501558	root:Host-associated:Human:Digestive system:Large in	0.232
MGYA00241714/ERZ501560_FASTA.fasta	MGYA00241714	ERZ501560	root:Host-associated:Human:Digestive system:Large in	0.408
MGYA00241715/ERZ501562_FASTA.fasta	MGYA00241715	ERZ501562	root:Host-associated:Human:Digestive system:Large in	0.113
MGYA00241716/ERZ501563_FASTA.fasta	MGYA00241716	ERZ501563	root:Host-associated:Human:Digestive system:Large in	0.1202
MGYA00241717/ERZ501565_FASTA.fasta	MGYA00241717	ERZ501565	root:Host-associated:Human:Digestive system:Large in	0.1832
MGYA00241718/ERZ501567_FASTA.fasta	MGYA00241718	ERZ501567	root:Host-associated:Human:Digestive system:Large in	0.2184
MGYA00241719/ERZ501569_FASTA.fasta	MGYA00241719	ERZ501569	root:Host-associated:Human:Digestive system:Large in	0.187
MGYA00241720/ERZ501571_FASTA.fasta	MGYA00241720	ERZ501571	root:Host-associated:Human:Digestive system:Large in	0.0666
MGYA00241721/ERZ501573_FASTA.fasta	MGYA00241721	ERZ501573	root:Host-associated:Human:Digestive system:Large in	0.2524
MGYA00241722/ERZ501575_FASTA.fasta	MGYA00241722	ERZ501575	root:Host-associated:Human:Digestive system:Large in	0.1892
MGYA00241723/ERZ501577_FASTA.fasta	MGYA00241723	ERZ501577	root:Host-associated:Human:Digestive system:Large in	0.0478
MGYA00241724/ERZ501579_FASTA.fasta	MGYA00241724	ERZ501579	root:Host-associated:Human:Digestive system:Large in	0.23
MGYA00241725/ERZ501625_FASTA.fasta	MGYA00241725	ERZ501625	root:Host-associated:Human:Digestive system:Large in	0.0542
MGYA00241726/ERZ501627_FASTA.fasta	MGYA00241726	ERZ501627	root:Host-associated:Human:Digestive system:Large in	0.034
MGYA00241727/ERZ501629_FASTA.fasta	MGYA00241727	ERZ501629	root:Host-associated:Human:Digestive system:Large in	0.137
MGYA00241728/ERZ501631_FASTA.fasta	MGYA00241728	ERZ501631	root:Host-associated:Human:Digestive system:Large in	0.1856
MGYA00241729/ERZ501633_FASTA.fasta	MGYA00241729	ERZ501633	root:Host-associated:Human:Digestive system:Large in	0.1108
MGYA00241730/ERZ501635_FASTA.fasta	MGYA00241730	ERZ501635	root:Host-associated:Human:Digestive system:Large in	0.2172
MGYA00241731/ERZ501637_FASTA.fasta	MGYA00241731	ERZ501637	root:Host-associated:Human:Digestive system:Large in	0.1794
MGYA00241732/ERZ501641_FASTA.fasta	MGYA00241732	ERZ501641	root:Host-associated:Human:Digestive system:Large in	0.2292
MGYA00241733/ERZ501643_FASTA.fasta	MGYA00241733	ERZ501643	root:Host-associated:Human:Digestive system:Large in	0.2052
MGYA00241734/ERZ501645_FASTA.fasta	MGYA00241734	ERZ501645	root:Host-associated:Human:Digestive system:Large in	0.2016
MGYA00241735/ERZ501647_FASTA.fasta	MGYA00241735	ERZ501647	root:Host-associated:Human:Digestive system:Large in	0.211
MGYA00241736/ERZ501649_FASTA.fasta	MGYA00241736	ERZ501649	root:Host-associated:Human:Digestive system:Large in	0.2394
MGYA00241737/ERZ501651_FASTA.fasta	MGYA00241737	ERZ501651	root:Host-associated:Human:Digestive system:Large in	0.2334
MGYA00241738/ERZ501653_FASTA.fasta	MGYA00241738	ERZ501653	root:Host-associated:Human:Digestive system:Large in	0.096
MGYA00241739/ERZ501655_FASTA.fasta	MGYA00241739	ERZ501655	root:Host-associated:Human:Digestive system:Large in	0.256
MGYA00241740/ERZ501658_FASTA.fasta	MGYA00241740	ERZ501658	root:Host-associated:Human:Digestive system:Large in	0.2042
MGYA00241741/ERZ501660_FASTA.fasta	MGYA00241741	ERZ501660	root:Host-associated:Human:Digestive system:Large in	0.244
MGYA00241742/ERZ501663_FASTA.fasta	MGYA00241742	ERZ501663	root:Host-associated:Human:Digestive system:Large in	0.3224
MGYA00241743/ERZ501665_FASTA.fasta	MGYA00241743	ERZ501665	root:Host-associated:Human:Digestive system:Large in	0.3228
MGYA00241744/ERZ501667_FASTA.fasta	MGYA00241744	ERZ501667	root:Host-associated:Human:Digestive system:Large in	0.148
MGYA00241745/ERZ501833_FASTA.fasta	MGYA00241745	ERZ501833	root:Host-associated:Human:Digestive system:Large in	0.1758
MGYA00241746/ERZ501835_FASTA.fasta	MGYA00241746	ERZ501835	root:Host-associated:Human:Digestive system:Large in	0.4006
MGYA00241747/ERZ501837_FASTA.fasta	MGYA00241747	ERZ501837	root:Host-associated:Human:Digestive system:Large in	0.4238
MGYA00241748/ERZ501839_FASTA.fasta	MGYA00241748	ERZ501839	root:Host-associated:Human:Digestive system:Large in	0.447
MGYA00241749/ERZ501841_FASTA.fasta	MGYA00241749	ERZ501841	root:Host-associated:Human:Digestive system:Large in	0.2272
MGYA00241750/ERZ501843_FASTA.fasta	MGYA00241750	ERZ501843	root:Host-associated:Human:Digestive system:Large in	0.4068
MGYA00241751/ERZ501845_FASTA.fasta	MGYA00241751	ERZ501845	root:Host-associated:Human:Digestive system:Large in	0.3876
MGYA00241752/ERZ501848_FASTA.fasta	MGYA00241752	ERZ501848	root:Host-associated:Human:Digestive system:Large in	0.3182
MGYA00241753/ERZ501850_FASTA.fasta	MGYA00241753	ERZ501850	root:Host-associated:Human:Digestive system:Large in	0.4308
MGYA00241754/ERZ501852_FASTA.fasta	MGYA00241754	ERZ501852	root:Host-associated:Human:Digestive system:Large in	0.4472
MGYA00241755/ERZ501853_FASTA.fasta	MGYA00241755	ERZ501853	root:Host-associated:Human:Digestive system:Large in	0.1444
MGYA00241756/ERZ501855_FASTA.fasta	MGYA00241756	ERZ501855	root:Host-associated:Human:Digestive system:Large in	0.1172
MGYA00241757/ERZ501857_FASTA.fasta	MGYA00241757	ERZ501857	root:Host-associated:Human:Digestive system:Large in	0.3858
MGYA00241758/ERZ501859_FASTA.fasta	MGYA00241758	ERZ501859	root:Host-associated:Human:Digestive system:Large in	0.4124
MGYA00241759/ERZ501861_FASTA.fasta	MGYA00241759	ERZ501861	root:Host-associated:Human:Digestive system:Large in	0.2318
MGYA00241760/ERZ501863_FASTA.fasta	MGYA00241760	ERZ501863	root:Host-associated:Human:Digestive system:Large in	0.521
MGYA00241761/ERZ501865_FASTA.fasta	MGYA00241761	ERZ501865	root:Host-associated:Human:Digestive system:Large in	0.5184
MGYA00241762/ERZ501867_FASTA.fasta	MGYA00241762	ERZ501867	root:Host-associated:Human:Digestive system:Large in	0.284
MGYA00241763/ERZ501869_FASTA.fasta	MGYA00241763	ERZ501869	root:Host-associated:Human:Digestive system:Large in	0.4038
MGYA00241764/ERZ501871_FASTA.fasta	MGYA00241764	ERZ501871	root:Host-associated:Human:Digestive system:Large in	0.466
MGYA00241765/ERZ501873_FASTA.fasta	MGYA00241765	ERZ501873	root:Host-associated:Human:Digestive system:Large in	0.4474
MGYA00241766/ERZ501875_FASTA.fasta	MGYA00241766	ERZ501875	root:Host-associated:Human:Digestive system:Large in	0.3638
MGYA00241767/ERZ502037_FASTA.fasta	MGYA00241767	ERZ502037	root:Host-associated:Human:Digestive system:Large in	0.5022
MGYA00241768/ERZ502039_FASTA.fasta	MGYA00241768	ERZ502039	root:Host-associated:Human:Digestive system:Large in	0.2996
MGYA00241769/ERZ502042_FASTA.fasta	MGYA00241769	ERZ502042	root:Host-associated:Human:Digestive system:Large in	0.3892
MGYA00241770/ERZ502044_FASTA.fasta	MGYA00241770	ERZ502044	root:Host-associated:Human:Digestive system:Large in	0.2836
MGYA00241771/ERZ502046_FASTA.fasta	MGYA00241771	ERZ502046	root:Host-associated:Human:Digestive system:Large in	0.4006
MGYA00241772/ERZ501669_FASTA.fasta	MGYA00241772	ERZ501669	root:Host-associated:Human:Digestive system:Large in	0.3144
MGYA00241773/ERZ501671_FASTA.fasta	MGYA00241773	ERZ501671	root:Host-associated:Human:Digestive system:Large in	0.2008
MGYA00241774/ERZ501673_FASTA.fasta	MGYA00241774	ERZ501673	root:Host-associated:Human:Digestive system:Large in	0.2414
MGYA00241775/ERZ501675_FASTA.fasta	MGYA00241775	ERZ501675	root:Host-associated:Human:Digestive system:Large in	0.2284
MGYA00241776/ERZ501677_FASTA.fasta	MGYA00241776	ERZ501677	root:Host-associated:Human:Digestive system:Large in	0.2832
MGYA00241777/ERZ501679_FASTA.fasta	MGYA00241777	ERZ501679	root:Host-associated:Human:Digestive system:Large in	0.1862
MGYA00241778/ERZ501681_FASTA.fasta	MGYA00241778	ERZ501681	root:Host-associated:Human:Digestive system:Large in	0.0812
MGYA00241779/ERZ501685_FASTA.fasta	MGYA00241779	ERZ501685	root:Host-associated:Human:Digestive system:Large in	0.2628
MGYA00241780/ERZ501687_FASTA.fasta	MGYA00241780	ERZ501687	root:Host-associated:Human:Digestive system:Large in	0.2728
MGYA00241781/ERZ501689_FASTA.fasta	MGYA00241781	ERZ501689	root:Host-associated:Human:Digestive system:Large in	0.1298
MGYA00241782/ERZ501691_FASTA.fasta	MGYA00241782	ERZ501691	root:Host-associated:Human:Digestive system:Large in	0.1098
MGYA00241783/ERZ501693_FASTA.fasta	MGYA00241783	ERZ501693	root:Host-associated:Human:Digestive system:Large in	0.248
MGYA00241784/ERZ501695_FASTA.fasta	MGYA00241784	ERZ501695	root:Host-associated:Human:Digestive system:Large in	0.1664
MGYA00241785/ERZ501697_FASTA.fasta	MGYA00241785	ERZ501697	root:Host-associated:Human:Digestive system:Large in	0.387
MGYA00241786/ERZ501699_FASTA.fasta	MGYA00241786	ERZ501699	root:Host-associated:Human:Digestive system:Large in	0.1228
MGYA00241787/ERZ501701_FASTA.fasta	MGYA00241787	ERZ501701	root:Host-associated:Human:Digestive system:Large in	0.3206
MGYA00241788/ERZ501703_FASTA.fasta	MGYA00241788	ERZ501703	root:Host-associated:Human:Digestive system:Large in	0.2394
MGYA00241789/ERZ501705_FASTA.fasta	MGYA00241789	ERZ501705	root:Host-associated:Human:Digestive system:Large in	0.0696
MGYA00241790/ERZ501707_FASTA.fasta	MGYA00241790	ERZ501707	root:Host-associated:Human:Digestive system:Large in	0.0954
MGYA00241791/ERZ501709_FASTA.fasta	MGYA00241791	ERZ501709	root:Host-associated:Human:Digestive system:Large in	0.1806
MGYA00241792/ERZ501711_FASTA.fasta	MGYA00241792	ERZ501711	root:Host-associated:Human:Digestive system:Large in	0.1966
MGYA00241793/ERZ501877_FASTA.fasta	MGYA00241793	ERZ501877	root:Host-associated:Human:Digestive system:Large in	0.3804
MGYA00241794/ERZ501879_FASTA.fasta	MGYA00241794	ERZ501879	root:Host-associated:Human:Digestive system:Large in	0.2384
MGYA00241795/ERZ501881_FASTA.fasta	MGYA00241795	ERZ501881	root:Host-associated:Human:Digestive system:Large in	0.4064
MGYA00241796/ERZ501883_FASTA.fasta	MGYA00241796	ERZ501883	root:Host-associated:Human:Digestive system:Large in	0.4464
MGYA00241797/ERZ501885_FASTA.fasta	MGYA00241797	ERZ501885	root:Host-associated:Human:Digestive system:Large in	0.213
MGYA00241798/ERZ501887_FASTA.fasta	MGYA00241798	ERZ501887	root:Host-associated:Human:Digestive system:Large in	0.176
MGYA00241799/ERZ501889_FASTA.fasta	MGYA00241799	ERZ501889	root:Host-associated:Human:Digestive system:Large in	0.228
MGYA00241800/ERZ501891_FASTA.fasta	MGYA00241800	ERZ501891	root:Host-associated:Human:Digestive system:Large in	0.3564
MGYA00241801/ERZ501893_FASTA.fasta	MGYA00241801	ERZ501893	root:Host-associated:Human:Digestive system:Large in	0.4428
MGYA00241802/ERZ501895_FASTA.fasta	MGYA00241802	ERZ501895	root:Host-associated:Human:Digestive system:Large in	0.362
MGYA00241803/ERZ501897_FASTA.fasta	MGYA00241803	ERZ501897	root:Host-associated:Human:Digestive system:Large in	0.1818
MGYA00241804/ERZ501899_FASTA.fasta	MGYA00241804	ERZ501899	root:Host-associated:Human:Digestive system:Large in	0.1414
MGYA00241805/ERZ501901_FASTA.fasta	MGYA00241805	ERZ501901	root:Host-associated:Human:Digestive system:Large in	0.4318
MGYA00241806/ERZ501903_FASTA.fasta	MGYA00241806	ERZ501903	root:Host-associated:Human:Digestive system:Large in	0.2688
MGYA00241807/ERZ501905_FASTA.fasta	MGYA00241807	ERZ501905	root:Host-associated:Human:Digestive system:Large in	0.4508
MGYA00241809/ERZ501909_FASTA.fasta	MGYA00241809	ERZ501909	root:Host-associated:Human:Digestive system:Large in	0.2628
MGYA00241810/ERZ501911_FASTA.fasta	MGYA00241810	ERZ501911	root:Host-associated:Human:Digestive system:Large in	0.47
MGYA00241811/ERZ501913_FASTA.fasta	MGYA00241811	ERZ501913	root:Host-associated:Human:Digestive system:Large in	0.3658
MGYA00241812/ERZ501915_FASTA.fasta	MGYA00241812	ERZ501915	root:Host-associated:Human:Digestive system:Large in	0.2724
MGYA00241813/ERZ501917_FASTA.fasta	MGYA00241813	ERZ501917	root:Host-associated:Human:Digestive system:Large in	0.3446
MGYA00241814/ERZ501919_FASTA.fasta	MGYA00241814	ERZ501919	root:Host-associated:Human:Digestive system:Large in	0.4194
MGYA00241815/ERZ501715_FASTA.fasta	MGYA00241815	ERZ501715	root:Host-associated:Human:Digestive system:Large in	0.1412
MGYA00241816/ERZ501717_FASTA.fasta	MGYA00241816	ERZ501717	root:Host-associated:Human:Digestive system:Large in	0.1668
MGYA00241817/ERZ501719_FASTA.fasta	MGYA00241817	ERZ501719	root:Host-associated:Human:Digestive system:Large in	0.1492
MGYA00241818/ERZ501721_FASTA.fasta	MGYA00241818	ERZ501721	root:Host-associated:Human:Digestive system:Large in	0.2824
MGYA00241819/ERZ501723_FASTA.fasta	MGYA00241819	ERZ501723	root:Host-associated:Human:Digestive system:Large in	0.2492
MGYA00241820/ERZ501725_FASTA.fasta	MGYA00241820	ERZ501725	root:Host-associated:Human:Digestive system:Large in	0.2374
MGYA00241821/ERZ501727_FASTA.fasta	MGYA00241821	ERZ501727	root:Host-associated:Human:Digestive system:Large in	0.2744
MGYA00241822/ERZ501729_FASTA.fasta	MGYA00241822	ERZ501729	root:Host-associated:Human:Digestive system:Large in	0.2864
MGYA00241823/ERZ501731_FASTA.fasta	MGYA00241823	ERZ501731	root:Host-associated:Human:Digestive system:Large in	0.3816
MGYA00241824/ERZ501733_FASTA.fasta	MGYA00241824	ERZ501733	root:Host-associated:Human:Digestive system:Large in	0.185
MGYA00241825/ERZ501735_FASTA.fasta	MGYA00241825	ERZ501735	root:Host-associated:Human:Digestive system:Large in	0.124
MGYA00241826/ERZ501737_FASTA.fasta	MGYA00241826	ERZ501737	root:Host-associated:Human:Digestive system:Large in	0.2172
MGYA00241827/ERZ501739_FASTA.fasta	MGYA00241827	ERZ501739	root:Host-associated:Human:Digestive system:Large in	0.2268
MGYA00241828/ERZ501921_FASTA.fasta	MGYA00241828	ERZ501921	root:Host-associated:Human:Digestive system:Large in	0.4304
MGYA00241829/ERZ501923_FASTA.fasta	MGYA00241829	ERZ501923	root:Host-associated:Human:Digestive system:Large in	0.1638
MGYA00241830/ERZ501925_FASTA.fasta	MGYA00241830	ERZ501925	root:Host-associated:Human:Digestive system:Large in	0.0462
MGYA00241831/ERZ501927_FASTA.fasta	MGYA00241831	ERZ501927	root:Host-associated:Human:Digestive system:Large in	0.0398
MGYA00241832/ERZ501929_FASTA.fasta	MGYA00241832	ERZ501929	root:Host-associated:Human:Digestive system:Large in	0.0498
MGYA00241833/ERZ501931_FASTA.fasta	MGYA00241833	ERZ501931	root:Host-associated:Human:Digestive system:Large in	0.0652
MGYA00241834/ERZ501933_FASTA.fasta	MGYA00241834	ERZ501933	root:Host-associated:Human:Digestive system:Large in	0.0252
MGYA00241835/ERZ501935_FASTA.fasta	MGYA00241835	ERZ501935	root:Host-associated:Human:Digestive system:Large in	0.0508
MGYA00241836/ERZ501937_FASTA.fasta	MGYA00241836	ERZ501937	root:Host-associated:Human:Digestive system:Large in	0.053
MGYA00241837/ERZ501940_FASTA.fasta	MGYA00241837	ERZ501940	root:Host-associated:Human:Digestive system:Large in	0.0364
MGYA00241838/ERZ501942_FASTA.fasta	MGYA00241838	ERZ501942	root:Host-associated:Human:Digestive system:Large in	0.0984
MGYA00241839/ERZ501944_FASTA.fasta	MGYA00241839	ERZ501944	root:Host-associated:Human:Digestive system:Large in	0.0768
MGYA00241840/ERZ501945_FASTA.fasta	MGYA00241840	ERZ501945	root:Host-associated:Human:Digestive system:Large in	0.0878
MGYA00241841/ERZ501947_FASTA.fasta	MGYA00241841	ERZ501947	root:Host-associated:Human:Digestive system:Large in	0.1964
MGYA00241842/ERZ501949_FASTA.fasta	MGYA00241842	ERZ501949	root:Host-associated:Human:Digestive system:Large in	0.3212
MGYA00241843/ERZ501951_FASTA.fasta	MGYA00241843	ERZ501951	root:Host-associated:Human:Digestive system:Large in	0.3884
MGYA00241844/ERZ501953_FASTA.fasta	MGYA00241844	ERZ501953	root:Host-associated:Human:Digestive system:Large in	0.4382
MGYA00241845/ERZ501955_FASTA.fasta	MGYA00241845	ERZ501955	root:Host-associated:Human:Digestive system:Large in	0.4776
MGYA00241846/ERZ501957_FASTA.fasta	MGYA00241846	ERZ501957	root:Host-associated:Human:Digestive system:Large in	0.4328
MGYA00241847/ERZ501959_FASTA.fasta	MGYA00241847	ERZ501959	root:Host-associated:Human:Digestive system:Large in	0.3206
MGYA00241848/ERZ501961_FASTA.fasta	MGYA00241848	ERZ501961	root:Host-associated:Human:Digestive system:Large in	0.4162
MGYA00241849/ERZ501963_FASTA.fasta	MGYA00241849	ERZ501963	root:Host-associated:Human:Digestive system:Large in	0.2452
MGYA00241850/ERZ501965_FASTA.fasta	MGYA00241850	ERZ501965	root:Host-associated:Human:Digestive system:Large in	0.4232
MGYA00241851/ERZ501967_FASTA.fasta	MGYA00241851	ERZ501967	root:Host-associated:Human:Digestive system:Large in	0.2066
MGYA00241852/ERZ501969_FASTA.fasta	MGYA00241852	ERZ501969	root:Host-associated:Human:Digestive system:Large in	0.288
MGYA00241853/ERZ501971_FASTA.fasta	MGYA00241853	ERZ501971	root:Host-associated:Human:Digestive system:Large in	0.083
MGYA00241854/ERZ501973_FASTA.fasta	MGYA00241854	ERZ501973	root:Host-associated:Human:Digestive system:Large in	0.4552
MGYA00241855/ERZ501804_FASTA.fasta	MGYA00241855	ERZ501804	root:Host-associated:Human:Digestive system:Large in	0.2068
MGYA00241856/ERZ501806_FASTA.fasta	MGYA00241856	ERZ501806	root:Host-associated:Human:Digestive system:Large in	0.1766
MGYA00241857/ERZ501808_FASTA.fasta	MGYA00241857	ERZ501808	root:Host-associated:Human:Digestive system:Large in	0.2378
MGYA00241858/ERZ501810_FASTA.fasta	MGYA00241858	ERZ501810	root:Host-associated:Human:Digestive system:Large in	0.3154
MGYA00241859/ERZ501812_FASTA.fasta	MGYA00241859	ERZ501812	root:Host-associated:Human:Digestive system:Large in	0.1248
MGYA00241860/ERZ501814_FASTA.fasta	MGYA00241860	ERZ501814	root:Host-associated:Human:Digestive system:Large in	0.273
MGYA00241861/ERZ501816_FASTA.fasta	MGYA00241861	ERZ501816	root:Host-associated:Human:Digestive system:Large in	0.1956
MGYA00241862/ERZ501818_FASTA.fasta	MGYA00241862	ERZ501818	root:Host-associated:Human:Digestive system:Large in	0.312
MGYA00241863/ERZ501820_FASTA.fasta	MGYA00241863	ERZ501820	root:Host-associated:Human:Digestive system:Large in	0.1836
MGYA00241864/ERZ501822_FASTA.fasta	MGYA00241864	ERZ501822	root:Host-associated:Human:Digestive system:Large in	0.2828
MGYA00241865/ERZ501824_FASTA.fasta	MGYA00241865	ERZ501824	root:Host-associated:Human:Digestive system:Large in	0.2118
MGYA00241866/ERZ501826_FASTA.fasta	MGYA00241866	ERZ501826	root:Host-associated:Human:Digestive system:Large in	0.4046
MGYA00241867/ERZ501828_FASTA.fasta	MGYA00241867	ERZ501828	root:Host-associated:Human:Digestive system:Large in	0.4432
MGYA00241868/ERZ501830_FASTA.fasta	MGYA00241868	ERZ501830	root:Host-associated:Human:Digestive system:Large in	0.3856
MGYA00241869/ERZ501832_FASTA.fasta	MGYA00241869	ERZ501832	root:Host-associated:Human:Digestive system:Large in	0.2122
MGYA00241870/ERZ501834_FASTA.fasta	MGYA00241870	ERZ501834	root:Host-associated:Human:Digestive system:Large in	0.372
MGYA00241871/ERZ501836_FASTA.fasta	MGYA00241871	ERZ501836	root:Host-associated:Human:Digestive system:Large in	0.3306
MGYA00241872/ERZ501838_FASTA.fasta	MGYA00241872	ERZ501838	root:Host-associated:Human:Digestive system:Large in	0.4156
MGYA00241873/ERZ501840_FASTA.fasta	MGYA00241873	ERZ501840	root:Host-associated:Human:Digestive system:Large in	0.4048
MGYA00241874/ERZ501842_FASTA.fasta	MGYA00241874	ERZ501842	root:Host-associated:Human:Digestive system:Large in	0.4434
MGYA00241875/ERZ501844_FASTA.fasta	MGYA00241875	ERZ501844	root:Host-associated:Human:Digestive system:Large in	0.3484
MGYA00241876/ERZ501846_FASTA.fasta	MGYA00241876	ERZ501846	root:Host-associated:Human:Digestive system:Large in	0.138
MGYA00241877/ERZ501847_FASTA.fasta	MGYA00241877	ERZ501847	root:Host-associated:Human:Digestive system:Large in	0.4874
MGYA00241878/ERZ501849_FASTA.fasta	MGYA00241878	ERZ501849	root:Host-associated:Human:Digestive system:Large in	0.525
MGYA00241879/ERZ501851_FASTA.fasta	MGYA00241879	ERZ501851	root:Host-associated:Human:Digestive system:Large in	0.4232
MGYA00241880/ERZ501854_FASTA.fasta	MGYA00241880	ERZ501854	root:Host-associated:Human:Digestive system:Large in	0.4192
MGYA00241881/ERZ501856_FASTA.fasta	MGYA00241881	ERZ501856	root:Host-associated:Human:Digestive system:Large in	0.118
MGYA00241882/ERZ501858_FASTA.fasta	MGYA00241882	ERZ501858	root:Host-associated:Human:Digestive system:Large in	0.1382
MGYA00241883/ERZ501860_FASTA.fasta	MGYA00241883	ERZ501860	root:Host-associated:Human:Digestive system:Large in	0.3958
MGYA00241884/ERZ501862_FASTA.fasta	MGYA00241884	ERZ501862	root:Host-associated:Human:Digestive system:Large in	0.4398
MGYA00241885/ERZ501864_FASTA.fasta	MGYA00241885	ERZ501864	root:Host-associated:Human:Digestive system:Large in	0.2462
MGYA00241886/ERZ501866_FASTA.fasta	MGYA00241886	ERZ501866	root:Host-associated:Human:Digestive system:Large in	0.2196
MGYA00241887/ERZ501868_FASTA.fasta	MGYA00241887	ERZ501868	root:Host-associated:Human:Digestive system:Large in	0.367
MGYA00241888/ERZ501870_FASTA.fasta	MGYA00241888	ERZ501870	root:Host-associated:Human:Digestive system:Large in	0.3446
MGYA00241889/ERZ501872_FASTA.fasta	MGYA00241889	ERZ501872	root:Host-associated:Human:Digestive system:Large in	0.4598
MGYA00241890/ERZ501874_FASTA.fasta	MGYA00241890	ERZ501874	root:Host-associated:Human:Digestive system:Large in	0.413
MGYA00241891/ERZ501876_FASTA.fasta	MGYA00241891	ERZ501876	root:Host-associated:Human:Digestive system:Large in	0.211
MGYA00241892/ERZ501878_FASTA.fasta	MGYA00241892	ERZ501878	root:Host-associated:Human:Digestive system:Large in	0.2814
MGYA00241893/ERZ501880_FASTA.fasta	MGYA00241893	ERZ501880	root:Host-associated:Human:Digestive system:Large in	0.258
MGYA00241894/ERZ501882_FASTA.fasta	MGYA00241894	ERZ501882	root:Host-associated:Human:Digestive system:Large in	0.1986
MGYA00241895/ERZ501884_FASTA.fasta	MGYA00241895	ERZ501884	root:Host-associated:Human:Digestive system:Large in	0.4424
MGYA00241896/ERZ501886_FASTA.fasta	MGYA00241896	ERZ501886	root:Host-associated:Human:Digestive system:Large in	0.5186
MGYA00241897/ERZ501888_FASTA.fasta	MGYA00241897	ERZ501888	root:Host-associated:Human:Digestive system:Large in	0.4176
MGYA00241898/ERZ501890_FASTA.fasta	MGYA00241898	ERZ501890	root:Host-associated:Human:Digestive system:Large in	0.4112
MGYA00241899/ERZ509162_FASTA.fasta	MGYA00241899	ERZ509162	root:Host-associated:Human:Digestive system:Large in	0.508
MGYA00241900/ERZ509164_FASTA.fasta	MGYA00241900	ERZ509164	root:Host-associated:Human:Digestive system:Large in	0.4112
MGYA00241901/ERZ509166_FASTA.fasta	MGYA00241901	ERZ509166	root:Host-associated:Human:Digestive system:Large in	0.4824
MGYA00241902/ERZ509168_FASTA.fasta	MGYA00241902	ERZ509168	root:Host-associated:Human:Digestive system:Large in	0.3626
MGYA00241903/ERZ509170_FASTA.fasta	MGYA00241903	ERZ509170	root:Host-associated:Human:Digestive system:Large in	0.4378
MGYA00241904/ERZ509172_FASTA.fasta	MGYA00241904	ERZ509172	root:Host-associated:Human:Digestive system:Large in	0.498
MGYA00241905/ERZ509174_FASTA.fasta	MGYA00241905	ERZ509174	root:Host-associated:Human:Digestive system:Large in	0.4456
MGYA00241906/ERZ509176_FASTA.fasta	MGYA00241906	ERZ509176	root:Host-associated:Human:Digestive system:Large in	0.4008
MGYA00241907/ERZ509178_FASTA.fasta	MGYA00241907	ERZ509178	root:Host-associated:Human:Digestive system:Large in	0.3942
MGYA00241908/ERZ509180_FASTA.fasta	MGYA00241908	ERZ509180	root:Host-associated:Human:Digestive system:Large in	0.4282
MGYA00241909/ERZ509182_FASTA.fasta	MGYA00241909	ERZ509182	root:Host-associated:Human:Digestive system:Large in	0.5958
MGYA00241910/ERZ509184_FASTA.fasta	MGYA00241910	ERZ509184	root:Host-associated:Human:Digestive system:Large in	0.4918
MGYA00241911/ERZ509186_FASTA.fasta	MGYA00241911	ERZ509186	root:Host-associated:Human:Digestive system:Large in	0.45
MGYA00241912/ERZ509188_FASTA.fasta	MGYA00241912	ERZ509188	root:Host-associated:Human:Digestive system:Large in	0.44
MGYA00241913/ERZ509190_FASTA.fasta	MGYA00241913	ERZ509190	root:Host-associated:Human:Digestive system:Large in	0.4508
MGYA00241914/ERZ509192_FASTA.fasta	MGYA00241914	ERZ509192	root:Host-associated:Human:Digestive system:Large in	0.5002
MGYA00241915/ERZ509194_FASTA.fasta	MGYA00241915	ERZ509194	root:Host-associated:Human:Digestive system:Large in	0.4436
MGYA00241916/ERZ509196_FASTA.fasta	MGYA00241916	ERZ509196	root:Host-associated:Human:Digestive system:Large in	0.4056
MGYA00241917/ERZ509198_FASTA.fasta	MGYA00241917	ERZ509198	root:Host-associated:Human:Digestive system:Large in	0.4784
MGYA00241918/ERZ509200_FASTA.fasta	MGYA00241918	ERZ509200	root:Host-associated:Human:Digestive system:Large in	0.452
MGYA00241919/ERZ509202_FASTA.fasta	MGYA00241919	ERZ509202	root:Host-associated:Human:Digestive system:Large in	0.4824
MGYA00241920/ERZ509204_FASTA.fasta	MGYA00241920	ERZ509204	root:Host-associated:Human:Digestive system:Large in	0.5092
MGYA00241921/ERZ509206_FASTA.fasta	MGYA00241921	ERZ509206	root:Host-associated:Human:Digestive system:Large in	0.4026
MGYA00241922/ERZ509208_FASTA.fasta	MGYA00241922	ERZ509208	root:Host-associated:Human:Digestive system:Large in	0.4058
MGYA00241923/ERZ509210_FASTA.fasta	MGYA00241923	ERZ509210	root:Host-associated:Human:Digestive system:Large in	0.4884
MGYA00241924/ERZ509212_FASTA.fasta	MGYA00241924	ERZ509212	root:Host-associated:Human:Digestive system:Large in	0.4268
MGYA00241925/ERZ509213_FASTA.fasta	MGYA00241925	ERZ509213	root:Host-associated:Human:Digestive system:Large in	0.5002
MGYA00241926/ERZ509216_FASTA.fasta	MGYA00241926	ERZ509216	root:Host-associated:Human:Digestive system:Large in	0.4788
MGYA00241927/ERZ509218_FASTA.fasta	MGYA00241927	ERZ509218	root:Host-associated:Human:Digestive system:Large in	0.4382
MGYA00241928/ERZ509220_FASTA.fasta	MGYA00241928	ERZ509220	root:Host-associated:Human:Digestive system:Large in	0.545
MGYA00241929/ERZ509222_FASTA.fasta	MGYA00241929	ERZ509222	root:Host-associated:Human:Digestive system:Large in	0.3782
MGYA00241930/ERZ509224_FASTA.fasta	MGYA00241930	ERZ509224	root:Host-associated:Human:Digestive system:Large in	0.4872
MGYA00241931/ERZ509226_FASTA.fasta	MGYA00241931	ERZ509226	root:Host-associated:Human:Digestive system:Large in	0.4754
MGYA00241932/ERZ509228_FASTA.fasta	MGYA00241932	ERZ509228	root:Host-associated:Human:Digestive system:Large in	0.4426
MGYA00241933/ERZ509230_FASTA.fasta	MGYA00241933	ERZ509230	root:Host-associated:Human:Digestive system:Large in	0.4368
MGYA00241934/ERZ509232_FASTA.fasta	MGYA00241934	ERZ509232	root:Host-associated:Human:Digestive system:Large in	0.4342
MGYA00241935/ERZ509235_FASTA.fasta	MGYA00241935	ERZ509235	root:Host-associated:Human:Digestive system:Large in	0.425
MGYA00241936/ERZ509236_FASTA.fasta	MGYA00241936	ERZ509236	root:Host-associated:Human:Digestive system:Large in	0.495
MGYA00241937/ERZ509238_FASTA.fasta	MGYA00241937	ERZ509238	root:Host-associated:Human:Digestive system:Large in	0.4498
MGYA00241938/ERZ509240_FASTA.fasta	MGYA00241938	ERZ509240	root:Host-associated:Human:Digestive system:Large in	0.458
MGYA00241939/ERZ509242_FASTA.fasta	MGYA00241939	ERZ509242	root:Host-associated:Human:Digestive system:Large in	0.5394
MGYA00241940/ERZ509244_FASTA.fasta	MGYA00241940	ERZ509244	root:Host-associated:Human:Digestive system:Large in	0.0628
MGYA00241941/ERZ509246_FASTA.fasta	MGYA00241941	ERZ509246	root:Host-associated:Human:Digestive system:Large in	0.5588
MGYA00241942/ERZ509248_FASTA.fasta	MGYA00241942	ERZ509248	root:Host-associated:Human:Digestive system:Large in	0.4406
MGYA00241943/ERZ509250_FASTA.fasta	MGYA00241943	ERZ509250	root:Host-associated:Human:Digestive system:Large in	0.387
MGYA00241944/ERZ509252_FASTA.fasta	MGYA00241944	ERZ509252	root:Host-associated:Human:Digestive system:Large in	0.4526
MGYA00241945/ERZ509254_FASTA.fasta	MGYA00241945	ERZ509254	root:Host-associated:Human:Digestive system:Large in	0.4688
MGYA00241946/ERZ509256_FASTA.fasta	MGYA00241946	ERZ509256	root:Host-associated:Human:Digestive system:Large in	0.4222
MGYA00241947/ERZ509258_FASTA.fasta	MGYA00241947	ERZ509258	root:Host-associated:Human:Digestive system:Large in	0.6182
MGYA00241948/ERZ509260_FASTA.fasta	MGYA00241948	ERZ509260	root:Host-associated:Human:Digestive system:Large in	0.445
MGYA00241949/ERZ509262_FASTA.fasta	MGYA00241949	ERZ509262	root:Host-associated:Human:Digestive system:Large in	0.4502
MGYA00241950/ERZ509264_FASTA.fasta	MGYA00241950	ERZ509264	root:Host-associated:Human:Digestive system:Large in	0.4188
MGYA00241951/ERZ509266_FASTA.fasta	MGYA00241951	ERZ509266	root:Host-associated:Human:Digestive system:Large in	0.4124
MGYA00241952/ERZ509268_FASTA.fasta	MGYA00241952	ERZ509268	root:Host-associated:Human:Digestive system:Large in	0.2868

MGYA00241953/ERZ509270_FASTA.fasta	MGYA00241953	ERZ509270	root:Host-associated:Human:Digestive system:Large in	0.4652
MGYA00241954/ERZ509272_FASTA.fasta	MGYA00241954	ERZ509272	root:Host-associated:Human:Digestive system:Large in	0.1444
MGYA00241955/ERZ509274_FASTA.fasta	MGYA00241955	ERZ509274	root:Host-associated:Human:Digestive system:Large in	0.3826
MGYA00241956/ERZ509276_FASTA.fasta	MGYA00241956	ERZ509276	root:Host-associated:Human:Digestive system:Large in	0.0468
MGYA00241957/ERZ508977_FASTA.fasta	MGYA00241957	ERZ508977	root:Host-associated:Human:Digestive system:Large in	0.038
MGYA00241958/ERZ508979_FASTA.fasta	MGYA00241958	ERZ508979	root:Host-associated:Human:Digestive system:Large in	0.042
MGYA00241959/ERZ508982_FASTA.fasta	MGYA00241959	ERZ508982	root:Host-associated:Human:Digestive system:Large in	0.3778
MGYA00241960/ERZ508984_FASTA.fasta	MGYA00241960	ERZ508984	root:Host-associated:Human:Digestive system:Large in	0.275
MGYA00241961/ERZ508986_FASTA.fasta	MGYA00241961	ERZ508986	root:Host-associated:Human:Digestive system:Large in	0.4212
MGYA00241962/ERZ508988_FASTA.fasta	MGYA00241962	ERZ508988	root:Host-associated:Human:Digestive system:Large in	0.4178
MGYA00241963/ERZ508990_FASTA.fasta	MGYA00241963	ERZ508990	root:Host-associated:Human:Digestive system:Large in	0.3596
MGYA00241964/ERZ508992_FASTA.fasta	MGYA00241964	ERZ508992	root:Host-associated:Human:Digestive system:Large in	0.4978
MGYA00241965/ERZ508994_FASTA.fasta	MGYA00241965	ERZ508994	root:Host-associated:Human:Digestive system:Large in	0.4656
MGYA00241966/ERZ508996_FASTA.fasta	MGYA00241966	ERZ508996	root:Host-associated:Human:Digestive system:Large in	0.4774
MGYA00241967/ERZ508998_FASTA.fasta	MGYA00241967	ERZ508998	root:Host-associated:Human:Digestive system:Large in	0.5738
MGYA00241968/ERZ509000_FASTA.fasta	MGYA00241968	ERZ509000	root:Host-associated:Human:Digestive system:Large in	0.5318
MGYA00241969/ERZ509002_FASTA.fasta	MGYA00241969	ERZ509002	root:Host-associated:Human:Digestive system:Large in	0.4224
MGYA00241970/ERZ509004_FASTA.fasta	MGYA00241970	ERZ509004	root:Host-associated:Human:Digestive system:Large in	0.4184
MGYA00241971/ERZ509006_FASTA.fasta	MGYA00241971	ERZ509006	root:Host-associated:Human:Digestive system:Large in	0.4448
MGYA00241972/ERZ509008_FASTA.fasta	MGYA00241972	ERZ509008	root:Host-associated:Human:Digestive system:Large in	0.4222
MGYA00241973/ERZ509009_FASTA.fasta	MGYA00241973	ERZ509009	root:Host-associated:Human:Digestive system:Large in	0.5238
MGYA00241974/ERZ509011_FASTA.fasta	MGYA00241974	ERZ509011	root:Host-associated:Human:Digestive system:Large in	0.5274
MGYA00241975/ERZ509013_FASTA.fasta	MGYA00241975	ERZ509013	root:Host-associated:Human:Digestive system:Large in	0.4254
MGYA00241976/ERZ509015_FASTA.fasta	MGYA00241976	ERZ509015	root:Host-associated:Human:Digestive system:Large in	0.4786
MGYA00241977/ERZ509017_FASTA.fasta	MGYA00241977	ERZ509017	root:Host-associated:Human:Digestive system:Large in	0.475
MGYA00241978/ERZ509019_FASTA.fasta	MGYA00241978	ERZ509019	root:Host-associated:Human:Digestive system:Large in	0.5156
MGYA00241979/ERZ509021_FASTA.fasta	MGYA00241979	ERZ509021	root:Host-associated:Human:Digestive system:Large in	0.4122
MGYA00241980/ERZ509023_FASTA.fasta	MGYA00241980	ERZ509023	root:Host-associated:Human:Digestive system:Large in	0.45
MGYA00241981/ERZ509025_FASTA.fasta	MGYA00241981	ERZ509025	root:Host-associated:Human:Digestive system:Large in	0.4192
MGYA00241982/ERZ509027_FASTA.fasta	MGYA00241982	ERZ509027	root:Host-associated:Human:Digestive system:Large in	0.4434
MGYA00241983/ERZ509029_FASTA.fasta	MGYA00241983	ERZ509029	root:Host-associated:Human:Digestive system:Large in	0.419
MGYA00241984/ERZ509031_FASTA.fasta	MGYA00241984	ERZ509031	root:Host-associated:Human:Digestive system:Large in	0.5148
MGYA00241985/ERZ509033_FASTA.fasta	MGYA00241985	ERZ509033	root:Host-associated:Human:Digestive system:Large in	0.4664
MGYA00241986/ERZ509035_FASTA.fasta	MGYA00241986	ERZ509035	root:Host-associated:Human:Digestive system:Large in	0.476
MGYA00241987/ERZ509037_FASTA.fasta	MGYA00241987	ERZ509037	root:Host-associated:Human:Digestive system:Large in	0.4824
MGYA00241988/ERZ509039_FASTA.fasta	MGYA00241988	ERZ509039	root:Host-associated:Human:Digestive system:Large in	0.4676
MGYA00241989/ERZ509041_FASTA.fasta	MGYA00241989	ERZ509041	root:Host-associated:Human:Digestive system:Large in	0.3858
MGYA00241990/ERZ509043_FASTA.fasta	MGYA00241990	ERZ509043	root:Host-associated:Human:Digestive system:Large in	0.4918
MGYA00241991/ERZ509045_FASTA.fasta	MGYA00241991	ERZ509045	root:Host-associated:Human:Digestive system:Large in	0.4748
MGYA00241992/ERZ509047_FASTA.fasta	MGYA00241992	ERZ509047	root:Host-associated:Human:Digestive system:Large in	0.5194
MGYA00241993/ERZ509049_FASTA.fasta	MGYA00241993	ERZ509049	root:Host-associated:Human:Digestive system:Large in	0.4486
MGYA00241994/ERZ509051_FASTA.fasta	MGYA00241994	ERZ509051	root:Host-associated:Human:Digestive system:Large in	0.3734
MGYA00241995/ERZ509053_FASTA.fasta	MGYA00241995	ERZ509053	root:Host-associated:Human:Digestive system:Large in	0.4326
MGYA00241996/ERZ509055_FASTA.fasta	MGYA00241996	ERZ509055	root:Host-associated:Human:Digestive system:Large in	0.5468
MGYA00241997/ERZ509057_FASTA.fasta	MGYA00241997	ERZ509057	root:Host-associated:Human:Digestive system:Large in	0.4004
MGYA00241998/ERZ509061_FASTA.fasta	MGYA00241998	ERZ509061	root:Host-associated:Human:Digestive system:Large in	0.4374
MGYA00241999/ERZ509063_FASTA.fasta	MGYA00241999	ERZ509063	root:Host-associated:Human:Digestive system:Large in	0.4666
MGYA00242000/ERZ509065_FASTA.fasta	MGYA00242000	ERZ509065	root:Host-associated:Human:Digestive system:Large in	0.4452
MGYA00242001/ERZ509067_FASTA.fasta	MGYA00242001	ERZ509067	root:Host-associated:Human:Digestive system:Large in	0.4068
MGYA00242002/ERZ509069_FASTA.fasta	MGYA00242002	ERZ509069	root:Host-associated:Human:Digestive system:Large in	0.48
MGYA00242003/ERZ509071_FASTA.fasta	MGYA00242003	ERZ509071	root:Host-associated:Human:Digestive system:Large in	0.3568
MGYA00242004/ERZ509073_FASTA.fasta	MGYA00242004	ERZ509073	root:Host-associated:Human:Digestive system:Large in	0.519
MGYA00242005/ERZ509075_FASTA.fasta	MGYA00242005	ERZ509075	root:Host-associated:Human:Digestive system:Large in	0.4724
MGYA00242006/ERZ509077_FASTA.fasta	MGYA00242006	ERZ509077	root:Host-associated:Human:Digestive system:Large in	0.4444
MGYA00242007/ERZ509079_FASTA.fasta	MGYA00242007	ERZ509079	root:Host-associated:Human:Digestive system:Large in	0.3968
MGYA00242008/ERZ509081_FASTA.fasta	MGYA00242008	ERZ509081	root:Host-associated:Human:Digestive system:Large in	0.4584
MGYA00242009/ERZ509083_FASTA.fasta	MGYA00242009	ERZ509083	root:Host-associated:Human:Digestive system:Large in	0.4686
MGYA00242010/ERZ509085_FASTA.fasta	MGYA00242010	ERZ509085	root:Host-associated:Human:Digestive system:Large in	0.4216
MGYA00242011/ERZ509087_FASTA.fasta	MGYA00242011	ERZ509087	root:Host-associated:Human:Digestive system:Large in	0.5108
MGYA00242012/ERZ509097_FASTA.fasta	MGYA00242012	ERZ509097	root:Host-associated:Human:Digestive system:Large in	0.4056
MGYA00242013/ERZ509099_FASTA.fasta	MGYA00242013	ERZ509099	root:Host-associated:Human:Digestive system:Large in	0.5724
MGYA00242014/ERZ509101_FASTA.fasta	MGYA00242014	ERZ509101	root:Host-associated:Human:Digestive system:Large in	0.2974
MGYA00242015/ERZ509103_FASTA.fasta	MGYA00242015	ERZ509103	root:Host-associated:Human:Digestive system:Large in	0.4906
MGYA00242016/ERZ509105_FASTA.fasta	MGYA00242016	ERZ509105	root:Host-associated:Human:Digestive system:Large in	0.4834
MGYA00242017/ERZ509107_FASTA.fasta	MGYA00242017	ERZ509107	root:Host-associated:Human:Digestive system:Large in	0.4502
MGYA00242018/ERZ509109_FASTA.fasta	MGYA00242018	ERZ509109	root:Host-associated:Human:Digestive system:Large in	0.5246
MGYA00242019/ERZ509111_FASTA.fasta	MGYA00242019	ERZ509111	root:Host-associated:Human:Digestive system:Large in	0.2996
MGYA00242020/ERZ509113_FASTA.fasta	MGYA00242020	ERZ509113	root:Host-associated:Human:Digestive system:Large in	0.474
MGYA00242021/ERZ509115_FASTA.fasta	MGYA00242021	ERZ509115	root:Host-associated:Human:Digestive system:Large in	0.6184
MGYA00242022/ERZ509117_FASTA.fasta	MGYA00242022	ERZ509117	root:Host-associated:Human:Digestive system:Large in	0.4214
MGYA00242023/ERZ509119_FASTA.fasta	MGYA00242023	ERZ509119	root:Host-associated:Human:Digestive system:Large in	0.5106
MGYA00242024/ERZ509121_FASTA.fasta	MGYA00242024	ERZ509121	root:Host-associated:Human:Digestive system:Large in	0.4926
MGYA00242025/ERZ509123_FASTA.fasta	MGYA00242025	ERZ509123	root:Host-associated:Human:Digestive system:Large in	0.528
MGYA00242026/ERZ509125_FASTA.fasta	MGYA00242026	ERZ509125	root:Host-associated:Human:Digestive system:Large in	0.4858
MGYA00242027/ERZ509127_FASTA.fasta	MGYA00242027	ERZ509127	root:Host-associated:Human:Digestive system:Large in	0.4634
MGYA00242028/ERZ509129_FASTA.fasta	MGYA00242028	ERZ509129	root:Host-associated:Human:Digestive system:Large in	0.2852
MGYA00242029/ERZ509131_FASTA.fasta	MGYA00242029	ERZ509131	root:Host-associated:Human:Digestive system:Large in	0.5118
MGYA00242030/ERZ509133_FASTA.fasta	MGYA00242030	ERZ509133	root:Host-associated:Human:Digestive system:Large in	0.3876
MGYA00242031/ERZ509135_FASTA.fasta	MGYA00242031	ERZ509135	root:Host-associated:Human:Digestive system:Large in	0.5778
MGYA00242032/ERZ509137_FASTA.fasta	MGYA00242032	ERZ509137	root:Host-associated:Human:Digestive system:Large in	0.427
MGYA00242033/ERZ509139_FASTA.fasta	MGYA00242033	ERZ509139	root:Host-associated:Human:Digestive system:Large in	0.3924
MGYA00242034/ERZ509141_FASTA.fasta	MGYA00242034	ERZ509141	root:Host-associated:Human:Digestive system:Large in	0.525
MGYA00242035/ERZ509143_FASTA.fasta	MGYA00242035	ERZ509143	root:Host-associated:Human:Digestive system:Large in	0.3752
MGYA00242036/ERZ509145_FASTA.fasta	MGYA00242036	ERZ509145	root:Host-associated:Human:Digestive system:Large in	0.5052
MGYA00242037/ERZ509147_FASTA.fasta	MGYA00242037	ERZ509147	root:Host-associated:Human:Digestive system:Large in	0.496
MGYA00242038/ERZ509149_FASTA.fasta	MGYA00242038	ERZ509149	root:Host-associated:Human:Digestive system:Large in	0.4552
MGYA00242039/ERZ509237_FASTA.fasta	MGYA00242039	ERZ509237	root:Host-associated:Human:Digestive system:Large in	0.407
MGYA00242040/ERZ509239_FASTA.fasta	MGYA00242040	ERZ509239	root:Host-associated:Human:Digestive system:Large in	0.566
MGYA00242041/ERZ509241_FASTA.fasta	MGYA00242041	ERZ509241	root:Host-associated:Human:Digestive system:Large in	0.3278
MGYA00242042/ERZ509243_FASTA.fasta	MGYA00242042	ERZ509243	root:Host-associated:Human:Digestive system:Large in	0.4846
MGYA00242043/ERZ509245_FASTA.fasta	MGYA00242043	ERZ509245	root:Host-associated:Human:Digestive system:Large in	0.4004
MGYA00242044/ERZ509247_FASTA.fasta	MGYA00242044	ERZ509247	root:Host-associated:Human:Digestive system:Large in	0.457
MGYA00242045/ERZ509249_FASTA.fasta	MGYA00242045	ERZ509249	root:Host-associated:Human:Digestive system:Large in	0.5008
MGYA00242046/ERZ509251_FASTA.fasta	MGYA00242046	ERZ509251	root:Host-associated:Human:Digestive system:Large in	0.597
MGYA00242047/ERZ509253_FASTA.fasta	MGYA00242047	ERZ509253	root:Host-associated:Human:Digestive system:Large in	0.5442
MGYA00242048/ERZ509255_FASTA.fasta	MGYA00242048	ERZ509255	root:Host-associated:Human:Digestive system:Large in	0.5016
MGYA00242049/ERZ509257_FASTA.fasta	MGYA00242049	ERZ509257	root:Host-associated:Human:Digestive system:Large in	0.3992
MGYA00242050/ERZ509259_FASTA.fasta	MGYA00242050	ERZ509259	root:Host-associated:Human:Digestive system:Large in	0.5838
MGYA00242051/ERZ509261_FASTA.fasta	MGYA00242051	ERZ509261	root:Host-associated:Human:Digestive system:Large in	0.469
MGYA00242052/ERZ509263_FASTA.fasta	MGYA00242052	ERZ509263	root:Host-associated:Human:Digestive system:Large in	0.5158
MGYA00242053/ERZ509265_FASTA.fasta	MGYA00242053	ERZ509265	root:Host-associated:Human:Digestive system:Large in	0.442
MGYA00242054/ERZ509267_FASTA.fasta	MGYA00242054	ERZ509267	root:Host-associated:Human:Digestive system:Large in	0.0294
MGYA00242055/ERZ509269_FASTA.fasta	MGYA00242055	ERZ509269	root:Host-associated:Human:Digestive system:Large in	0.3796
MGYA00242056/ERZ509271_FASTA.fasta	MGYA00242056	ERZ509271	root:Host-associated:Human:Digestive system:Large in	0.3194
MGYA00242057/ERZ509273_FASTA.fasta	MGYA00242057	ERZ509273	root:Host-associated:Human:Digestive system:Large in	0.4654
MGYA00242058/ERZ509275_FASTA.fasta	MGYA00242058	ERZ509275	root:Host-associated:Human:Digestive system:Large in	0.4478
MGYA00242059/ERZ509277_FASTA.fasta	MGYA00242059	ERZ509277	root:Host-associated:Human:Digestive system:Large in	0.2192
MGYA00242060/ERZ509169_FASTA.fasta	MGYA00242060	ERZ509169	root:Host-associated:Human:Digestive system:Large in	0.4424
MGYA00242061/ERZ509171_FASTA.fasta	MGYA00242061	ERZ509171	root:Host-associated:Human:Digestive system:Large in	0.438
MGYA00242062/ERZ509173_FASTA.fasta	MGYA00242062	ERZ509173	root:Host-associated:Human:Digestive system:Large in	0.4624
MGYA00242063/ERZ509175_FASTA.fasta	MGYA00242063	ERZ509175	root:Host-associated:Human:Digestive system:Large in	0.0486
MGYA00242064/ERZ509177_FASTA.fasta	MGYA00242064	ERZ509177	root:Host-associated:Human:Digestive system:Large in	0.4496
MGYA00242065/ERZ509179_FASTA.fasta	MGYA00242065	ERZ509179	root:Host-associated:Human:Digestive system:Large in	0.5192
MGYA00242066/ERZ509181_FASTA.fasta	MGYA00242066	ERZ509181	root:Host-associated:Human:Digestive system:Large in	0.457
MGYA00242067/ERZ509183_FASTA.fasta	MGYA00242067	ERZ509183	root:Host-associated:Human:Digestive system:Large in	0.4286
MGYA00242068/ERZ509185_FASTA.fasta	MGYA00242068	ERZ509185	root:Host-associated:Human:Digestive system:Large in	0.4246
MGYA00242069/ERZ509187_FASTA.fasta	MGYA00242069	ERZ509187	root:Host-associated:Human:Digestive system:Large in	0.371
MGYA00242070/ERZ509189_FASTA.fasta	MGYA00242070	ERZ509189	root:Host-associated:Human:Digestive system:Large in	0.3988
MGYA00242071/ERZ509191_FASTA.fasta	MGYA00242071	ERZ509191	root:Host-associated:Human:Digestive system:Large in	0.4678
MGYA00242072/ERZ509193_FASTA.fasta	MGYA00242072	ERZ509193	root:Host-associated:Human:Digestive system:Large in	0.4536
MGYA00242073/ERZ509195_FASTA.fasta	MGYA00242073	ERZ509195	root:Host-associated:Human:Digestive system:Large in	0.46
MGYA00242074/ERZ509197_FASTA.fasta	MGYA00242074	ERZ509197	root:Host-associated:Human:Digestive system:Large in	0.4736
MGYA00242075/ERZ509199_FASTA.fasta	MGYA00242075	ERZ509199	root:Host-associated:Human:Digestive system:Large in	0.473
MGYA00242076/ERZ509201_FASTA.fasta	MGYA00242076	ERZ509201	root:Host-associated:Human:Digestive system:Large in	0.4484
MGYA00242077/ERZ509203_FASTA.fasta	MGYA00242077	ERZ509203	root:Host-associated:Human:Digestive system:Large in	0.4678
MGYA00242078/ERZ509205_FASTA.fasta	MGYA00242078	ERZ509205	root:Host-associated:Human:Digestive system:Large in	0.5378
MGYA00242079/ERZ509207_FASTA.fasta	MGYA00242079	ERZ509207	root:Host-associated:Human:Digestive system:Large in	0.4822
MGYA00242080/ERZ509209_FASTA.fasta	MGYA00242080	ERZ509209	root:Host-associated:Human:Digestive system:Large in	0.4046
MGYA00242081/ERZ509211_FASTA.fasta	MGYA00242081	ERZ509211	root:Host-associated:Human:Digestive system:Large in	0.4022
MGYA00242082/ERZ509215_FASTA.fasta	MGYA00242082	ERZ509215	root:Host-associated:Human:Digestive system:Large in	0.5018
MGYA00242083/ERZ509217_FASTA.fasta	MGYA00242083	ERZ509217	root:Host-associated:Human:Digestive system:Large in	0.4208
MGYA00242084/ERZ509219_FASTA.fasta	MGYA00242084	ERZ509219	root:Host-associated:Human:Digestive system:Large in	0.4552
MGYA00242085/ERZ509221_FASTA.fasta	MGYA00242085	ERZ509221	root:Host-associated:Human:Digestive system:Large in	0.45
MGYA00242086/ERZ509223_FASTA.fasta	MGYA00242086	ERZ509223	root:Host-associated:Human:Digestive system:Large in	0.4206
MGYA00242087/ERZ509225_FASTA.fasta	MGYA00242087	ERZ509225	root:Host-associated:Human:Digestive system:Large in	0.5612
MGYA00242088/ERZ509227_FASTA.fasta	MGYA00242088	ERZ509227	root:Host-associated:Human:Digestive system:Large in	0.4768
MGYA00242089/ERZ509229_FASTA.fasta	MGYA00242089	ERZ509229	root:Host-associated:Human:Digestive system:Large in	0.3312
MGYA00242090/ERZ509231_FASTA.fasta	MGYA00242090	ERZ509231	root:Host-associated:Human:Digestive system:Large in	0.407
MGYA00242091/ERZ509233_FASTA.fasta	MGYA00242091	ERZ509233	root:Host-associated:Human:Digestive system:Large in	0.4672
MGYA00242092/ERZ509234_FASTA.fasta	MGYA00242092	ERZ509234	root:Host-associated:Human:Digestive system:Large in	0.5236
MGYA00242093/ERZ508976_FASTA.fasta	MGYA00242093	ERZ508976	root:Host-associated:Human:Digestive system:Large in	0.215
MGYA00242094/ERZ508978_FASTA.fasta	MGYA00242094	ERZ508978	root:Host-associated:Human:Digestive system:Large in	0.0728
MGYA00242095/ERZ508980_FASTA.fasta	MGYA00242095	ERZ508980	root:Host-associated:Human:Digestive system:Large in	0.2474
MGYA00242096/ERZ508981_FASTA.fasta	MGYA00242096	ERZ508981	root:Host-associated:Human:Digestive system:Large in	0.3362
MGYA00242097/ERZ508983_FASTA.fasta	MGYA00242097	ERZ508983	root:Host-associated:Human:Digestive system:Large in	0.4738
MGYA00242098/ERZ508985_FASTA.fasta	MGYA00242098	ERZ508985	root:Host-associated:Human:Digestive system:Large in	0.2832
MGYA00242099/ERZ508987_FASTA.fasta	MGYA00242099	ERZ508987	root:Host-associated:Human:Digestive system:Large in	0.3696
MGYA00242100/ERZ508989_FASTA.fasta	MGYA00242100	ERZ508989	root:Host-associated:Human:Digestive system:Large in	0.4374
MGYA00242101/ERZ508991_FASTA.fasta	MGYA00242101	ERZ508991	root:Host-associated:Human:Digestive system:Large in	0.2078
MGYA00242102/ERZ508993_FASTA.fasta	MGYA00242102	ERZ508993	root:Host-associated:Human:Digestive system:Large in	0.407
MGYA00242103/ERZ508995_FASTA.fasta	MGYA00242103	ERZ508995	root:Host-associated:Human:Digestive system:Large in	0.5282
MGYA00242104/ERZ508997_FASTA.fasta	MGYA00242104	ERZ508997	root:Host-associated:Human:Digestive system:Large in	0.5532
MGYA00242105/ERZ508999_FASTA.fasta	MGYA00242105	ERZ508999	root:Host-associated:Human:Digestive system:Large in	0.481
MGYA00242106/ERZ509001_FASTA.fasta	MGYA00242106	ERZ509001	root:Host-associated:Human:Digestive system:Large in	0.5638
MGYA00242107/ERZ509003_FASTA.fasta	MGYA00242107	ERZ509003	root:Host-associated:Human:Digestive system:Large in	0.4118
MGYA00242108/ERZ509007_FASTA.fasta	MGYA00242108	ERZ509007	root:Host-associated:Human:Digestive system:Large in	0.4302
MGYA00242109/ERZ509010_FASTA.fasta	MGYA00242109	ERZ509010	root:Host-associated:Human:Digestive system:Large in	0.4158
MGYA00242110/ERZ509012_FASTA.fasta	MGYA00242110	ERZ509012	root:Host-associated:Human:Digestive system:Large in	0.4716
MGYA00242111/ERZ509014_FASTA.fasta	MGYA00242111	ERZ509014	root:Host-associated:Human:Digestive system:Large in	0.4156
MGYA00242112/ERZ509016_FASTA.fasta	MGYA00242112	ERZ509016	root:Host-associated:Human:Digestive system:Large in	0.4704
MGYA00242113/ERZ509018_FASTA.fasta	MGYA00242113	ERZ509018	root:Host-associated:Human:Digestive system:Large in	0.4672
MGYA00242114/ERZ509020_FASTA.fasta	MGYA00242114	ERZ509020	root:Host-associated:Human:Digestive system:Large in	0.466
MGYA00242115/ERZ509022_FASTA.fasta	MGYA00242115	ERZ509022	root:Host-associated:Human:Digestive system:Large in	0.5934
MGYA00242116/ERZ509024_FASTA.fasta	MGYA00242116	ERZ509024	root:Host-associated:Human:Digestive system:Large in	0.428
MGYA00242117/ERZ509026_FASTA.fasta	MGYA00242117	ERZ509026	root:Host-associated:Human:Digestive system:Large in	0.3952
MGYA00242118/ERZ509028_FASTA.fasta	MGYA00242118	ERZ509028	root:Host-associated:Human:Digestive system:Large in	0.3754
MGYA00242119/ERZ509030_FASTA.fasta	MGYA00242119	ERZ509030	root:Host-associated:Human:Digestive system:Large in	0.4646
MGYA00242120/ERZ509032_FASTA.fasta	MGYA00242120	ERZ509032	root:Host-associated:Human:Digestive system:Large in	0.4262
MGYA00242121/ERZ509034_FASTA.fasta	MGYA00242121	ERZ509034	root:Host-associated:Human:Digestive system:Large in	0.5518
MGYA00242122/ERZ509036_FASTA.fasta	MGYA00242122	ERZ509036	root:Host-associated:Human:Digestive system:Large in	0.4788
MGYA00242123/ERZ509038_FASTA.fasta	MGYA00242123	ERZ509038	root:Host-associated:Human:Digestive system:Large in	0.4074
MGYA00242124/ERZ509040_FASTA.fasta	MGYA00242124	ERZ509040	root:Host-associated:Human:Digestive system:Large in	0.501
MGYA00242125/ERZ509042_FASTA.fasta	MGYA00242125	ERZ509042	root:Host-associated:Human:Digestive system:Large in	0.4374
MGYA00242126/ERZ509044_FASTA.fasta	MGYA00242126	ERZ509044	root:Host-associated:Human:Digestive system:Large in	0.4434
MGYA00242127/ERZ509046_FASTA.fasta	MGYA00242127	ERZ509046	root:Host-associated:Human:Digestive system:Large in	0.4688
MGYA00242128/ERZ509048_FASTA.fasta	MGYA00242128	ERZ509048	root:Host-associated:Human:Digestive system:Large in	0.4674
MGYA00242129/ERZ509050_FASTA.fasta	MGYA00242129	ERZ509050	root:Host-associated:Human:Digestive system:Large in	0.4366
MGYA00242130/ERZ509052_FASTA.fasta	MGYA00242130	ERZ509052	root:Host-associated:Human:Digestive system:Large in	0.419
MGYA00242131/ERZ509054_FASTA.fasta	MGYA00242131	ERZ509054	root:Host-associated:Human:Digestive system:Large in	0.435
MGYA00242132/ERZ509056_FASTA.fasta	MGYA00242132	ERZ509056	root:Host-associated:Human:Digestive system:Large in	0.4644
MGYA00242133/ERZ509058_FASTA.fasta	MGYA00242133	ERZ509058	root:Host-associated:Human:Digestive system:Large in	0.5398
MGYA00242134/ERZ509059_FASTA.fasta	MGYA00242134	ERZ509059	root:Host-associated:Human:Digestive system:Large in	0.3842
MGYA00242135/ERZ509060_FASTA.fasta	MGYA00242135	ERZ509060	root:Host-associated:Human:Digestive system:Large in	0.457
MGYA00242136/ERZ509062_FASTA.fasta	MGYA00242136	ERZ509062	root:Host-associated:Human:Digestive system:Large in	0.4794
MGYA00242137/ERZ509064_FASTA.fasta	MGYA00242137	ERZ509064	root:Host-associated:Human:Digestive system:Large in	0.4506
MGYA00242138/ERZ509066_FASTA.fasta	MGYA00242138	ERZ509066	root:Host-associated:Human:Digestive system:Large in	0.484
MGYA00242139/ERZ509068_FASTA.fasta	MGYA00242139	ERZ509068	root:Host-associated:Human:Digestive system:Large in	0.4054
MGYA00242140/ERZ509070_FASTA.fasta	MGYA00242140	ERZ509070	root:Host-associated:Human:Digestive system:Large in	0.3776
MGYA00242141/ERZ509072_FASTA.fasta	MGYA00242141	ERZ509072	root:Host-associated:Human:Digestive system:Large in	0.5038
MGYA00242142/ERZ509074_FASTA.fasta	MGYA00242142	ERZ509074	root:Host-associated:Human:Digestive system:Large in	0.5222
MGYA00242143/ERZ509076_FASTA.fasta	MGYA00242143	ERZ509076	root:Host-associated:Human:Digestive system:Large in	0.4258
MGYA00242144/ERZ509078_FASTA.fasta	MGYA00242144	ERZ509078	root:Host-associated:Human:Digestive system:Large in	0.5294
MGYA00242145/ERZ509080_FASTA.fasta	MGYA00242145	ERZ509080	root:Host-associated:Human:Digestive system:Large in	0.5686
MGYA00242146/ERZ509082_FASTA.fasta	MGYA00242146	ERZ509082	root:Host-associated:Human:Digestive system:Large in	0.522
MGYA00242147/ERZ509084_FASTA.fasta	MGYA00242147	ERZ509084	root:Host-associated:Human:Digestive system:Large in	0.531
MGYA00242148/ERZ509086_FASTA.fasta	MGYA00242148	ERZ509086	root:Host-associated:Human:Digestive system:Large in	0.3256
MGYA00242149/ERZ509088_FASTA.fasta	MGYA00242149	ERZ509088	root:Host-associated:Human:Digestive system:Large in	0.3986
MGYA00242150/ERZ509089_FASTA.fasta	MGYA00242150	ERZ509089	root:Host-associated:Human:Digestive system:Large in	0.2832
MGYA00242151/ERZ509090_FASTA.fasta	MGYA00242151	ERZ509090	root:Host-associated:Human:Digestive system:Large in	0.4468
MGYA00242152/ERZ509091_FASTA.fasta	MGYA00242152	ERZ509091	root:Host-associated:Human:Digestive system:Large in	0.4786
MGYA00242153/ERZ509092_FASTA.fasta	MGYA00242153	ERZ509092	root:Host-associated:Human:Digestive system:Large in	0.4878
MGYA00242154/ERZ509093_FASTA.fasta	MGYA00242154	ERZ509093	root:Host-associated:Human:Digestive system:Large in	0.4194
MGYA00242155/ERZ509094_FASTA.fasta	MGYA00242155	ERZ509094	root:Host-associated:Human:Digestive system:Large in	0.4298
MGYA00242156/ERZ509095_FASTA.fasta	MGYA00242156	ERZ509095	root:Host-associated:Human:Digestive system:Large in	0.4648
MGYA00242157/ERZ509096_FASTA.fasta	MGYA00242157	ERZ509096	root:Host-associated:Human:Digestive system:Large in	0.4192
MGYA00242158/ERZ509098_FASTA.fasta	MGYA00242158	ERZ509098	root:Host-associated:Human:Digestive system:Large in	0.503
MGYA00242159/ERZ509100_FASTA.fasta	MGYA00242159	ERZ509100	root:Host-associated:Human:Digestive system:Large in	0.2834
MGYA00242160/ERZ509102_FASTA.fasta	MGYA00242160	ERZ509102	root:Host-associated:Human:Digestive system:Large in	0.4448
MGYA00242161/ERZ509104_FASTA.fasta	MGYA00242161	ERZ509104	root:Host-associated:Human:Digestive system:Large in	0.395
MGYA00242162/ERZ509106_FASTA.fasta	MGYA00242162	ERZ509106	root:Host-associated:Human:Digestive system:Large in	0.5144
MGYA00242163/ERZ509110_FASTA.fasta	MGYA00242163	ERZ509110	root:Host-associated:Human:Digestive system:Large in	0.4414
MGYA00242164/ERZ509112_FASTA.fasta	MGYA00242164	ERZ509112	root:Host-associated:Human:Digestive system:Large in	0.3566
MGYA00242165/ERZ509114_FASTA.fasta	MGYA00242165	ERZ509114	root:Host-associated:Human:Digestive system:Large in	0.5046
MGYA00242166/ERZ509116_FASTA.fasta	MGYA00242166	ERZ509116	root:Host-associated:Human:Digestive system:Large in	0.4446
MGYA00242167/ERZ509118_FASTA.fasta	MGYA00242167	ERZ509118	root:Host-associated:Human:Digestive system:Large in	0.4552
MGYA00242168/ERZ509120_FASTA.fasta	MGYA00242168	ERZ509120	root:Host-associated:Human:Digestive system:Large in	0.5122
MGYA00242169/ERZ509122_FASTA.fasta	MGYA00242169	ERZ509122	root:Host-associated:Human:Digestive system:Large in	0.5962
MGYA00242170/ERZ509124_FASTA.fasta	MGYA00242170	ERZ509124	root:Host-associated:Human:Digestive system:Large in	0.4676
MGYA00242171/ERZ509126_FASTA.fasta	MGYA00242171	ERZ509126	root:Host-associated:Human:Digestive system:Large in	0.4488
MGYA00242172/ERZ509128_FASTA.fasta	MGYA00242172	ERZ509128	root:Host-associated:Human:Digestive system:Large in	0.3652
MGYA00242173/ERZ509130_FASTA.fasta	MGYA00242173	ERZ509130	root:Host-associated:Human:Digestive system:Large in	0.3324
MGYA00242174/ERZ509132_FASTA.fasta	MGYA00242174	ERZ509132	root:Host-associated:Human:Digestive system:Large in	0.5234
MGYA00242175/ERZ509134_FASTA.fasta	MGYA00242175	ERZ509134	root:Host-associated:Human:Digestive system:Large in	0.45
MGYA00242176/ERZ509136_FASTA.fasta	MGYA00242176	ERZ509136	root:Host-associated:Human:Digestive system:Large in	0.4838
MGYA00242177/ERZ509138_FASTA.fasta	MGYA00242177	ERZ509138	root:Host-associated:Human:Digestive system:Large in	0.4892
MGYA00242178/ERZ509140_FASTA.fasta	MGYA00242178	ERZ509140	root:Host-associated:Human:Digestive system:Large in	0.4234
MGYA00242179/ERZ509142_FASTA.fasta	MGYA00242179	ERZ509142	root:Host-associated:Human:Digestive system:Large in	0.4516
MGYA00242180/ERZ509144_FASTA.fasta	MGYA00242180	ERZ509144	root:Host-associated:Human:Digestive system:Large in	0.5224
MGYA00242181/ERZ509146_FASTA.fasta	MGYA00242181	ERZ509146	root:Host-associated:Human:Digestive system:Large in	0.4742
MGYA00242182/ERZ509148_FASTA.fasta	MGYA00242182	ERZ509148	root:Host-associated:Human:Digestive system:Large in	0.5026
MGYA00242183/ERZ509150_FASTA.fasta	MGYA00242183	ERZ509150	root:Host-associated:Human:Digestive system:Large in	0.5208
MGYA00242184/ERZ509151_FASTA.fasta	MGYA00242184	ERZ509151	root:Host-associated:Human:Digestive system:Large in	0.4264
MGYA00242185/ERZ509152_FASTA.fasta	MGYA00242185	ERZ509152	root:Host-associated:Human:Digestive system:Large in	0.4918
MGYA00242186/ERZ509153_FASTA.fasta	MGYA00242186	ERZ509153	root:Host-associated:Human:Digestive system:Large in	0.2692
MGYA00242187/ERZ509154_FASTA.fasta	MGYA00242187	ERZ509154	root:Host-associated:Human:Digestive system:Large in	0.5022
MGYA00242188/ERZ509155_FASTA.fasta	MGYA00242188	ERZ509155	root:Host-associated:Human:Digestive system:Large in	0.3278
MGYA00242189/ERZ509156_FASTA.fasta	MGYA00242189	ERZ509156	root:Host-associated:Human:Digestive system:Large in	0.4344
MGYA00242190/ERZ509157_FASTA.fasta	MGYA00242190	ERZ509157	root:Host-associated:Human:Digestive system:Large in	0.4286
MGYA00242191/ERZ509158_FASTA.fasta	MGYA00242191	ERZ509158	root:Host-associated:Human:Digestive system:Large in	0.4186
MGYA00242192/ERZ509159_FASTA.fasta	MGYA00242192	ERZ509159	root:Host-associated:Human:Digestive system:Large in	0.4988
MGYA00242193/ERZ509160_FASTA.fasta	MGYA00242193	ERZ509160	root:Host-associated:Human:Digestive system:Large in	0.4332
MGYA00242194/ERZ509161_FASTA.fasta	MGYA00242194	ERZ509161	root:Host-associated:Human:Digestive system:Large in	0.5328
MGYA00242195/ERZ509163_FASTA.fasta	MGYA00242195	ERZ509163	root:Host-associated:Human:Digestive system:Large in	0.3968
MGYA00242196/ERZ509165_FASTA.fasta	MGYA00242196	ERZ509165	root:Host-associated:Human:Digestive system:Large in	0.4858
MGYA00242197/ERZ509167_FASTA.fasta	MGYA00242197	ERZ509167	root:Host-associated:Human:Digestive system:Large in	0.4654
MGYA00242198/ERZ724078_FASTA.fasta	MGYA00242198	ERZ724078	root:Engineered:Bioreactor	0.858
MGYA00242199/ERZ724080_FASTA.fasta	MGYA00242199	ERZ724080	root:Engineered:Bioreactor	0.8384
MGYA00242200/ERZ724082_FASTA.fasta	MGYA00242200	ERZ724082	root:Engineered:Bioreactor	0.8316
MGYA00242201/ERZ724084_FASTA.fasta	MGYA00242201	ERZ724084	root:Engineered:Bioreactor	0.8454
MGYA00242202/ERZ724086_FASTA.fasta	MGYA00242202	ERZ724086	root:Engineered:Bioreactor	0.8248
MGYA00242203/ERZ724088_FASTA.fasta	MGYA00242203	ERZ724088	root:Engineered:Bioreactor	0.9142
MGYA00242204/ERZ724079_FASTA.fasta	MGYA00242204	ERZ724079	root:Engineered:Bioreactor	0.897
MGYA00242205/ERZ724081_FASTA.fasta	MGYA00242205	ERZ724081	root:Engineered:Bioreactor	0.8246
MGYA00242206/ERZ724083_FASTA.fasta	MGYA00242206	ERZ724083	root:Engineered:Bioreactor	0.8156
MGYA00242207/ERZ724085_FASTA.fasta	MGYA00242207	ERZ724085	root:Engineered:Bioreactor	0.8318
MGYA00242208/ERZ724087_FASTA.fasta	MGYA00242208	ERZ724087	root:Engineered:Bioreactor	0.8474
MGYA00242209/ERZ724089_FASTA.fasta	MGYA00242209	ERZ724089	root:Engineered:Bioreactor	0.8946
MGYA00242470/ERZ504477_FASTA.fasta	MGYA00242470	ERZ504477	root:Host-associated:Human:Digestive system	0.21
MGYA00242471/ERZ504479_FASTA.fasta	MGYA00242471	ERZ504479	root:Host-associated:Human:Digestive system	0.2984
MGYA00242472/ERZ504481_FASTA.fasta	MGYA00242472	ERZ504481	root:Host-associated:Human:Digestive system	0.4124
MGYA00242473/ERZ504483_FASTA.fasta	MGYA00242473	ERZ504483	root:Host-associated:Human:Digestive system	0.3138
MGYA00242474/ERZ504485_FASTA.fasta	MGYA00242474	ERZ504485	root:Host-associated:Human:Digestive system	0.2292
MGYA00242475/ERZ504487_FASTA.fasta	MGYA00242475	ERZ504487	root:Host-associated:Human:Digestive system	0.0652
MGYA00242476/ERZ504489_FASTA.fasta	MGYA00242476	ERZ504489	root:Host-associated:Human:Digestive system	0.0394
MGYA00242477/ERZ504491_FASTA.fasta	MGYA00242477	ERZ504491	root:Host-associated:Human:Digestive system	0.1766
MGYA00242478/ERZ504493_FASTA.fasta	MGYA00242478	ERZ504493	root:Host-associated:Human:Digestive system	0.3222
MGYA00242479/ERZ504495_FASTA.fasta	MGYA00242479	ERZ504495	root:Host-associated:Human:Digestive system	0.2414
MGYA00242480/ERZ504497_FASTA.fasta	MGYA00242480	ERZ504497	root:Host-associated:Human:Digestive system	0.121
MGYA00242481/ERZ504499_FASTA.fasta	MGYA00242481	ERZ504499	root:Host-associated:Human:Digestive system	0.0516
MGYA00242482/ERZ504501_FASTA.fasta	MGYA00242482	ERZ504501	root:Host-associated:Human:Digestive system	0.0728
MGYA00242483/ERZ504503_FASTA.fasta	MGYA00242483	ERZ504503	root:Host-associated:Human:Digestive system	0.2838
MGYA00242484/ERZ504505_FASTA.fasta	MGYA00242484	ERZ504505	root:Host-associated:Human:Digestive system	0.1016
MGYA00242485/ERZ504507_FASTA.fasta	MGYA00242485	ERZ504507	root:Host-associated:Human:Digestive system	0.1586
MGYA00242486/ERZ504509_FASTA.fasta	MGYA00242486	ERZ504509	root:Host-associated:Human:Digestive system	0.216
MGYA00242487/ERZ504511_FASTA.fasta	MGYA00242487	ERZ504511	root:Host-associated:Human:Digestive system	0.4876
MGYA00242488/ERZ504513_FASTA.fasta	MGYA00242488	ERZ504513	root:Host-associated:Human:Digestive system	0.2094
MGYA00242489/ERZ504515_FASTA.fasta	MGYA00242489	ERZ504515	root:Host-associated:Human:Digestive system	0.3398
MGYA00242490/ERZ504517_FASTA.fasta	MGYA00242490	ERZ504517	root:Host-associated:Human:Digestive system	0.4258
MGYA00242491/ERZ504519_FASTA.fasta	MGYA00242491	ERZ504519	root:Host-associated:Human:Digestive system	0.1664
MGYA00242492/ERZ504566_FASTA.fasta	MGYA00242492	ERZ504566	root:Host-associated:Human:Digestive system	0.155
MGYA00242493/ERZ504568_FASTA.fasta	MGYA00242493	ERZ504568	root:Host-associated:Human:Digestive system	0.3762
MGYA00242494/ERZ504570_FASTA.fasta	MGYA00242494	ERZ504570	root:Host-associated:Human:Digestive system	0.1042
MGYA00242495/ERZ504572_FASTA.fasta	MGYA00242495	ERZ504572	root:Host-associated:Human:Digestive system	0.3352
MGYA00242496/ERZ504574_FASTA.fasta	MGYA00242496	ERZ504574	root:Host-associated:Human:Digestive system	0.1482
MGYA00242497/ERZ504576_FASTA.fasta	MGYA00242497	ERZ504576	root:Host-associated:Human:Digestive system	0.3268
MGYA00242498/ERZ504578_FASTA.fasta	MGYA00242498	ERZ504578	root:Host-associated:Human:Digestive system	0.3718
MGYA00242499/ERZ504580_FASTA.fasta	MGYA00242499	ERZ504580	root:Host-associated:Human:Digestive system	0.3018
MGYA00242500/ERZ504582_FASTA.fasta	MGYA00242500	ERZ504582	root:Host-associated:Human:Digestive system	0.3212
MGYA00242501/ERZ504584_FASTA.fasta	MGYA00242501	ERZ504584	root:Host-associated:Human:Digestive system	0.2278
MGYA00242502/ERZ504586_FASTA.fasta	MGYA00242502	ERZ504586	root:Host-associated:Human:Digestive system	0.2888
MGYA00242503/ERZ504588_FASTA.fasta	MGYA00242503	ERZ504588	root:Host-associated:Human:Digestive system	0.2258
MGYA00242504/ERZ504590_FASTA.fasta	MGYA00242504	ERZ504590	root:Host-associated:Human:Digestive system	0.286
MGYA00242505/ERZ504594_FASTA.fasta	MGYA00242505	ERZ504594	root:Host-associated:Human:Digestive system	0.2646
MGYA00242506/ERZ504596_FASTA.fasta	MGYA00242506	ERZ504596	root:Host-associated:Human:Digestive system	0.092
MGYA00242507/ERZ504598_FASTA.fasta	MGYA00242507	ERZ504598	root:Host-associated:Human:Digestive system	0.2274
MGYA00242508/ERZ504600_FASTA.fasta	MGYA00242508	ERZ504600	root:Host-associated:Human:Digestive system	0.3182
MGYA00242509/ERZ504602_FASTA.fasta	MGYA00242509	ERZ504602	root:Host-associated:Human:Digestive system	0.2458
MGYA00242510/ERZ504604_FASTA.fasta	MGYA00242510	ERZ504604	root:Host-associated:Human:Digestive system	0.3382
MGYA00242511/ERZ504606_FASTA.fasta	MGYA00242511	ERZ504606	root:Host-associated:Human:Digestive system	0.4058
MGYA00242512/ERZ504608_FASTA.fasta	MGYA00242512	ERZ504608	root:Host-associated:Human:Digestive system	0.4616
MGYA00242513/ERZ504610_FASTA.fasta	MGYA00242513	ERZ504610	root:Host-associated:Human:Digestive system	0.1226
MGYA00242514/ERZ504612_FASTA.fasta	MGYA00242514	ERZ504612	root:Host-associated:Human:Digestive system	0.4232
MGYA00242515/ERZ504614_FASTA.fasta	MGYA00242515	ERZ504614	root:Host-associated:Human:Digestive system	0.2224
MGYA00242516/ERZ504616_FASTA.fasta	MGYA00242516	ERZ504616	root:Host-associated:Human:Digestive system	0.211
MGYA00242517/ERZ504618_FASTA.fasta	MGYA00242517	ERZ504618	root:Host-associated:Human:Digestive system	0.3018
MGYA00242518/ERZ504620_FASTA.fasta	MGYA00242518	ERZ504620	root:Host-associated:Human:Digestive system	0.167
MGYA00242519/ERZ504624_FASTA.fasta	MGYA00242519	ERZ504624	root:Host-associated:Human:Digestive system	0.2538
MGYA00242520/ERZ504626_FASTA.fasta	MGYA00242520	ERZ504626	root:Host-associated:Human:Digestive system	0.204
MGYA00242521/ERZ504628_FASTA.fasta	MGYA00242521	ERZ504628	root:Host-associated:Human:Digestive system	0.3574
MGYA00242522/ERZ504630_FASTA.fasta	MGYA00242522	ERZ504630	root:Host-associated:Human:Digestive system	0.2214
MGYA00242523/ERZ504632_FASTA.fasta	MGYA00242523	ERZ504632	root:Host-associated:Human:Digestive system	0.4076
MGYA00242524/ERZ504634_FASTA.fasta	MGYA00242524	ERZ504634	root:Host-associated:Human:Digestive system	0.1854
MGYA00242525/ERZ504636_FASTA.fasta	MGYA00242525	ERZ504636	root:Host-associated:Human:Digestive system	0.0776
MGYA00242526/ERZ504638_FASTA.fasta	MGYA00242526	ERZ504638	root:Host-associated:Human:Digestive system	0.3786
MGYA00242527/ERZ504640_FASTA.fasta	MGYA00242527	ERZ504640	root:Host-associated:Human:Digestive system	0.2784
MGYA00242528/ERZ504644_FASTA.fasta	MGYA00242528	ERZ504644	root:Host-associated:Human:Digestive system	0.068
MGYA00242529/ERZ504646_FASTA.fasta	MGYA00242529	ERZ504646	root:Host-associated:Human:Digestive system	0.086
MGYA00242530/ERZ504648_FASTA.fasta	MGYA00242530	ERZ504648	root:Host-associated:Human:Digestive system	0.408
MGYA00242531/ERZ504650_FASTA.fasta	MGYA00242531	ERZ504650	root:Host-associated:Human:Digestive system	0.2866
MGYA00242532/ERZ504652_FASTA.fasta	MGYA00242532	ERZ504652	root:Host-associated:Human:Digestive system	0.3584
MGYA00242533/ERZ504654_FASTA.fasta	MGYA00242533	ERZ504654	root:Host-associated:Human:Digestive system	0.2504
MGYA00242534/ERZ504656_FASTA.fasta	MGYA00242534	ERZ504656	root:Host-associated:Human:Digestive system	0.3654
MGYA00242535/ERZ504658_FASTA.fasta	MGYA00242535	ERZ504658	root:Host-associated:Human:Digestive system	0.3952
MGYA00242536/ERZ504660_FASTA.fasta	MGYA00242536	ERZ504660	root:Host-associated:Human:Digestive system	0.3766
MGYA00242537/ERZ504664_FASTA.fasta	MGYA00242537	ERZ504664	root:Host-associated:Human:Digestive system	0.3416
MGYA00242538/ERZ504667_FASTA.fasta	MGYA00242538	ERZ504667	root:Host-associated:Human:Digestive system	0.4336
MGYA00242539/ERZ504669_FASTA.fasta	MGYA00242539	ERZ504669	root:Host-associated:Human:Digestive system	0.314
MGYA00242540/ERZ504671_FASTA.fasta	MGYA00242540	ERZ504671	root:Host-associated:Human:Digestive system	0.3806
MGYA00242541/ERZ504673_FASTA.fasta	MGYA00242541	ERZ504673	root:Host-associated:Human:Digestive system	0.2752
MGYA00242542/ERZ504675_FASTA.fasta	MGYA00242542	ERZ504675	root:Host-associated:Human:Digestive system	0.298
MGYA00242543/ERZ504677_FASTA.fasta	MGYA00242543	ERZ504677	root:Host-associated:Human:Digestive system	0.4336
MGYA00242544/ERZ504679_FASTA.fasta	MGYA00242544	ERZ504679	root:Host-associated:Human:Digestive system	0.3668
MGYA00242545/ERZ504681_FASTA.fasta	MGYA00242545	ERZ504681	root:Host-associated:Human:Digestive system	0.3316
MGYA00242546/ERZ504683_FASTA.fasta	MGYA00242546	ERZ504683	root:Host-associated:Human:Digestive system	0.2492
MGYA00242547/ERZ504685_FASTA.fasta	MGYA00242547	ERZ504685	root:Host-associated:Human:Digestive system	0.3536
MGYA00242548/ERZ504687_FASTA.fasta	MGYA00242548	ERZ504687	root:Host-associated:Human:Digestive system	0.278
MGYA00242549/ERZ504689_FASTA.fasta	MGYA00242549	ERZ504689	root:Host-associated:Human:Digestive system	0.3252
MGYA00242550/ERZ504691_FASTA.fasta	MGYA00242550	ERZ504691	root:Host-associated:Human:Digestive system	0.3822
MGYA00242551/ERZ504693_FASTA.fasta	MGYA00242551	ERZ504693	root:Host-associated:Human:Digestive system	0.3232
MGYA00242552/ERZ504695_FASTA.fasta	MGYA00242552	ERZ504695	root:Host-associated:Human:Digestive system	0.313
MGYA00242553/ERZ504697_FASTA.fasta	MGYA00242553	ERZ504697	root:Host-associated:Human:Digestive system	0.3192
MGYA00242554/ERZ504699_FASTA.fasta	MGYA00242554	ERZ504699	root:Host-associated:Human:Digestive system	0.3642
MGYA00242555/ERZ504701_FASTA.fasta	MGYA00242555	ERZ504701	root:Host-associated:Human:Digestive system	0.3554
MGYA00242556/ERZ504703_FASTA.fasta	MGYA00242556	ERZ504703	root:Host-associated:Human:Digestive system	0.3896
MGYA00242557/ERZ504705_FASTA.fasta	MGYA00242557	ERZ504705	root:Host-associated:Human:Digestive system	0.3454
MGYA00242558/ERZ504707_FASTA.fasta	MGYA00242558	ERZ504707	root:Host-associated:Human:Digestive system	0.2694
MGYA00242559/ERZ504709_FASTA.fasta	MGYA00242559	ERZ504709	root:Host-associated:Human:Digestive system	0.3556
MGYA00242560/ERZ504711_FASTA.fasta	MGYA00242560	ERZ504711	root:Host-associated:Human:Digestive system	0.3024
MGYA00242561/ERZ504713_FASTA.fasta	MGYA00242561	ERZ504713	root:Host-associated:Human:Digestive system	0.372
MGYA00242562/ERZ504715_FASTA.fasta	MGYA00242562	ERZ504715	root:Host-associated:Human:Digestive system	0.356
MGYA00242563/ERZ504717_FASTA.fasta	MGYA00242563	ERZ504717	root:Host-associated:Human:Digestive system	0.2792
MGYA00242564/ERZ504721_FASTA.fasta	MGYA00242564	ERZ504721	root:Host-associated:Human:Digestive system	0.4584
MGYA00242565/ERZ504723_FASTA.fasta	MGYA00242565	ERZ504723	root:Host-associated:Human:Digestive system	0.4142
MGYA00242566/ERZ504725_FASTA.fasta	MGYA00242566	ERZ504725	root:Host-associated:Human:Digestive system	0.3636
MGYA00242567/ERZ504727_FASTA.fasta	MGYA00242567	ERZ504727	root:Host-associated:Human:Digestive system	0.3482
MGYA00242568/ERZ504729_FASTA.fasta	MGYA00242568	ERZ504729	root:Host-associated:Human:Digestive system	0.2726
MGYA00242569/ERZ504731_FASTA.fasta	MGYA00242569	ERZ504731	root:Host-associated:Human:Digestive system	0.3742
MGYA00242570/ERZ504733_FASTA.fasta	MGYA00242570	ERZ504733	root:Host-associated:Human:Digestive system	0.4116
MGYA00242571/ERZ504735_FASTA.fasta	MGYA00242571	ERZ504735	root:Host-associated:Human:Digestive system	0.1966
MGYA00242572/ERZ504736_FASTA.fasta	MGYA00242572	ERZ504736	root:Host-associated:Human:Digestive system	0.314
MGYA00242573/ERZ504737_FASTA.fasta	MGYA00242573	ERZ504737	root:Host-associated:Human:Digestive system	0.3948
MGYA00242574/ERZ504738_FASTA.fasta	MGYA00242574	ERZ504738	root:Host-associated:Human:Digestive system	0.3526
MGYA00242575/ERZ504740_FASTA.fasta	MGYA00242575	ERZ504740	root:Host-associated:Human:Digestive system	0.4014
MGYA00242576/ERZ504741_FASTA.fasta	MGYA00242576	ERZ504741	root:Host-associated:Human:Digestive system	0.4208
MGYA00242577/ERZ504742_FASTA.fasta	MGYA00242577	ERZ504742	root:Host-associated:Human:Digestive system	0.2846
MGYA00242578/ERZ504743_FASTA.fasta	MGYA00242578	ERZ504743	root:Host-associated:Human:Digestive system	0.3866
MGYA00242579/ERZ504744_FASTA.fasta	MGYA00242579	ERZ504744	root:Host-associated:Human:Digestive system	0.3696
MGYA00242580/ERZ504745_FASTA.fasta	MGYA00242580	ERZ504745	root:Host-associated:Human:Digestive system	0.35
MGYA00242581/ERZ504746_FASTA.fasta	MGYA00242581	ERZ504746	root:Host-associated:Human:Digestive system	0.487
MGYA00242582/ERZ504747_FASTA.fasta	MGYA00242582	ERZ504747	root:Host-associated:Human:Digestive system	0.2326
MGYA00242583/ERZ504748_FASTA.fasta	MGYA00242583	ERZ504748	root:Host-associated:Human:Digestive system	0.352
MGYA00242584/ERZ504749_FASTA.fasta	MGYA00242584	ERZ504749	root:Host-associated:Human:Digestive system	0.4854
MGYA00242585/ERZ504750_FASTA.fasta	MGYA00242585	ERZ504750	root:Host-associated:Human:Digestive system	0.2548
MGYA00242586/ERZ504751_FASTA.fasta	MGYA00242586	ERZ504751	root:Host-associated:Human:Digestive system	0.1944
MGYA00242587/ERZ504752_FASTA.fasta	MGYA00242587	ERZ504752	root:Host-associated:Human:Digestive system	0.361
MGYA00242588/ERZ504753_FASTA.fasta	MGYA00242588	ERZ504753	root:Host-associated:Human:Digestive system	0.3214
MGYA00242589/ERZ504754_FASTA.fasta	MGYA00242589	ERZ504754	root:Host-associated:Human:Digestive system	0.3032
MGYA00242590/ERZ504755_FASTA.fasta	MGYA00242590	ERZ504755	root:Host-associated:Human:Digestive system	0.2584
MGYA00242591/ERZ504756_FASTA.fasta	MGYA00242591	ERZ504756	root:Host-associated:Human:Digestive system	0.2514
MGYA00242592/ERZ504757_FASTA.fasta	MGYA00242592	ERZ504757	root:Host-associated:Human:Digestive system	0.185
MGYA00242593/ERZ504758_FASTA.fasta	MGYA00242593	ERZ504758	root:Host-associated:Human:Digestive system	0.562
MGYA00242594/ERZ504760_FASTA.fasta	MGYA00242594	ERZ504760	root:Host-associated:Human:Digestive system	0.2802
MGYA00242595/ERZ504761_FASTA.fasta	MGYA00242595	ERZ504761	root:Host-associated:Human:Digestive system	0.2732
MGYA00242596/ERZ504762_FASTA.fasta	MGYA00242596	ERZ504762	root:Host-associated:Human:Digestive system	0.2556
MGYA00242597/ERZ504763_FASTA.fasta	MGYA00242597	ERZ504763	root:Host-associated:Human:Digestive system	0.2636
MGYA00242598/ERZ504764_FASTA.fasta	MGYA00242598	ERZ504764	root:Host-associated:Human:Digestive system	0.205
MGYA00242599/ERZ504765_FASTA.fasta	MGYA00242599	ERZ504765	root:Host-associated:Human:Digestive system	0.3078
MGYA00242600/ERZ504766_FASTA.fasta	MGYA00242600	ERZ504766	root:Host-associated:Human:Digestive system	0.2714
MGYA00242601/ERZ504767_FASTA.fasta	MGYA00242601	ERZ504767	root:Host-associated:Human:Digestive system	0.2516
MGYA00242602/ERZ504768_FASTA.fasta	MGYA00242602	ERZ504768	root:Host-associated:Human:Digestive system	0.272
MGYA00242603/ERZ504769_FASTA.fasta	MGYA00242603	ERZ504769	root:Host-associated:Human:Digestive system	0.2238
MGYA00242604/ERZ504770_FASTA.fasta	MGYA00242604	ERZ504770	root:Host-associated:Human:Digestive system	0.3296
MGYA00242605/ERZ504771_FASTA.fasta	MGYA00242605	ERZ504771	root:Host-associated:Human:Digestive system	0.3252
MGYA00242606/ERZ504772_FASTA.fasta	MGYA00242606	ERZ504772	root:Host-associated:Human:Digestive system	0.2024
MGYA00242607/ERZ504773_FASTA.fasta	MGYA00242607	ERZ504773	root:Host-associated:Human:Digestive system	0.2394
MGYA00242608/ERZ504774_FASTA.fasta	MGYA00242608	ERZ504774	root:Host-associated:Human:Digestive system	0.3778
MGYA00242609/ERZ504776_FASTA.fasta	MGYA00242609	ERZ504776	root:Host-associated:Human:Digestive system	0.3494
MGYA00242610/ERZ504777_FASTA.fasta	MGYA00242610	ERZ504777	root:Host-associated:Human:Digestive system	0.325
MGYA00242611/ERZ504778_FASTA.fasta	MGYA00242611	ERZ504778	root:Host-associated:Human:Digestive system	0.3216
MGYA00242612/ERZ504779_FASTA.fasta	MGYA00242612	ERZ504779	root:Host-associated:Human:Digestive system	0.234
MGYA00242613/ERZ504780_FASTA.fasta	MGYA00242613	ERZ504780	root:Host-associated:Human:Digestive system	0.2058
MGYA00242614/ERZ504781_FASTA.fasta	MGYA00242614	ERZ504781	root:Host-associated:Human:Digestive system	0.3432
MGYA00242615/ERZ504782_FASTA.fasta	MGYA00242615	ERZ504782	root:Host-associated:Human:Digestive system	0.291
MGYA00242616/ERZ504783_FASTA.fasta	MGYA00242616	ERZ504783	root:Host-associated:Human:Digestive system	0.2412
MGYA00242617/ERZ504784_FASTA.fasta	MGYA00242617	ERZ504784	root:Host-associated:Human:Digestive system	0.5528
MGYA00242618/ERZ504785_FASTA.fasta	MGYA00242618	ERZ504785	root:Host-associated:Human:Digestive system	0.35
MGYA00242619/ERZ504786_FASTA.fasta	MGYA00242619	ERZ504786	root:Host-associated:Human:Digestive system	0.2632
MGYA00242620/ERZ504787_FASTA.fasta	MGYA00242620	ERZ504787	root:Host-associated:Human:Digestive system	0.389
MGYA00242621/ERZ504862_FASTA.fasta	MGYA00242621	ERZ504862	root:Host-associated:Human:Digestive system	0.2562
MGYA00242622/ERZ504866_FASTA.fasta	MGYA00242622	ERZ504866	root:Host-associated:Human:Digestive system	0.2332
MGYA00242623/ERZ504868_FASTA.fasta	MGYA00242623	ERZ504868	root:Host-associated:Human:Digestive system	0.3632
MGYA00242624/ERZ504870_FASTA.fasta	MGYA00242624	ERZ504870	root:Host-associated:Human:Digestive system	0.429
MGYA00242625/ERZ504872_FASTA.fasta	MGYA00242625	ERZ504872	root:Host-associated:Human:Digestive system	0.2148
MGYA00242626/ERZ504874_FASTA.fasta	MGYA00242626	ERZ504874	root:Host-associated:Human:Digestive system	0.24
MGYA00242627/ERZ504876_FASTA.fasta	MGYA00242627	ERZ504876	root:Host-associated:Human:Digestive system	0.4838
MGYA00242628/ERZ504878_FASTA.fasta	MGYA00242628	ERZ504878	root:Host-associated:Human:Digestive system	0.229
MGYA00242629/ERZ504880_FASTA.fasta	MGYA00242629	ERZ504880	root:Host-associated:Human:Digestive system	0.5536
MGYA00242630/ERZ504882_FASTA.fasta	MGYA00242630	ERZ504882	root:Host-associated:Human:Digestive system	0.2332
MGYA00242631/ERZ504884_FASTA.fasta	MGYA00242631	ERZ504884	root:Host-associated:Human:Digestive system	0.231
MGYA00242632/ERZ504886_FASTA.fasta	MGYA00242632	ERZ504886	root:Host-associated:Human:Digestive system	0.3286
MGYA00242633/ERZ504888_FASTA.fasta	MGYA00242633	ERZ504888	root:Host-associated:Human:Digestive system	0.293
MGYA00242634/ERZ504890_FASTA.fasta	MGYA00242634	ERZ504890	root:Host-associated:Human:Digestive system	0.259
MGYA00242635/ERZ504478_FASTA.fasta	MGYA00242635	ERZ504478	root:Host-associated:Human:Digestive system	0.1772
MGYA00242636/ERZ504480_FASTA.fasta	MGYA00242636	ERZ504480	root:Host-associated:Human:Digestive system	0.338
MGYA00242637/ERZ504482_FASTA.fasta	MGYA00242637	ERZ504482	root:Host-associated:Human:Digestive system	0.3378
MGYA00242638/ERZ504484_FASTA.fasta	MGYA00242638	ERZ504484	root:Host-associated:Human:Digestive system	0.1558
MGYA00242639/ERZ504486_FASTA.fasta	MGYA00242639	ERZ504486	root:Host-associated:Human:Digestive system	0.3432
MGYA00242640/ERZ504488_FASTA.fasta	MGYA00242640	ERZ504488	root:Host-associated:Human:Digestive system	0.0598
MGYA00242641/ERZ504732_FASTA.fasta	MGYA00242641	ERZ504732	root:Host-associated:Human:Digestive system	0.3116
MGYA00242642/ERZ504734_FASTA.fasta	MGYA00242642	ERZ504734	root:Host-associated:Human:Digestive system	0.4598
MGYA00242643/ERZ504788_FASTA.fasta	MGYA00242643	ERZ504788	root:Host-associated:Human:Digestive system	0.2194
MGYA00242644/ERZ504789_FASTA.fasta	MGYA00242644	ERZ504789	root:Host-associated:Human:Digestive system	0.289
MGYA00242645/ERZ504791_FASTA.fasta	MGYA00242645	ERZ504791	root:Host-associated:Human:Digestive system	0.3392
MGYA00242646/ERZ504793_FASTA.fasta	MGYA00242646	ERZ504793	root:Host-associated:Human:Digestive system	0.322
MGYA00242647/ERZ504795_FASTA.fasta	MGYA00242647	ERZ504795	root:Host-associated:Human:Digestive system	0.2804
MGYA00242648/ERZ504797_FASTA.fasta	MGYA00242648	ERZ504797	root:Host-associated:Human:Digestive system	0.287
MGYA00242649/ERZ504799_FASTA.fasta	MGYA00242649	ERZ504799	root:Host-associated:Human:Digestive system	0.1928
MGYA00242650/ERZ504801_FASTA.fasta	MGYA00242650	ERZ504801	root:Host-associated:Human:Digestive system	0.2296
MGYA00242651/ERZ504803_FASTA.fasta	MGYA00242651	ERZ504803	root:Host-associated:Human:Digestive system	0.3218
MGYA00242652/ERZ504805_FASTA.fasta	MGYA00242652	ERZ504805	root:Host-associated:Human:Digestive system	0.454
MGYA00242653/ERZ504807_FASTA.fasta	MGYA00242653	ERZ504807	root:Host-associated:Human:Digestive system	0.3536
MGYA00242654/ERZ504810_FASTA.fasta	MGYA00242654	ERZ504810	root:Host-associated:Human:Digestive system	0.371
MGYA00242655/ERZ504811_FASTA.fasta	MGYA00242655	ERZ504811	root:Host-associated:Human:Digestive system	0.3322
MGYA00242656/ERZ504813_FASTA.fasta	MGYA00242656	ERZ504813	root:Host-associated:Human:Digestive system	0.3804
MGYA00242657/ERZ504815_FASTA.fasta	MGYA00242657	ERZ504815	root:Host-associated:Human:Digestive system	0.3034
MGYA00242658/ERZ504817_FASTA.fasta	MGYA00242658	ERZ504817	root:Host-associated:Human:Digestive system	0.2968
MGYA00242659/ERZ504819_FASTA.fasta	MGYA00242659	ERZ504819	root:Host-associated:Human:Digestive system	0.3664
MGYA00242660/ERZ504821_FASTA.fasta	MGYA00242660	ERZ504821	root:Host-associated:Human:Digestive system	0.336
MGYA00242661/ERZ504823_FASTA.fasta	MGYA00242661	ERZ504823	root:Host-associated:Human:Digestive system	0.2974
MGYA00242662/ERZ504490_FASTA.fasta	MGYA00242662	ERZ504490	root:Host-associated:Human:Digestive system	0.0562
MGYA00242663/ERZ504492_FASTA.fasta	MGYA00242663	ERZ504492	root:Host-associated:Human:Digestive system	0.185
MGYA00242664/ERZ504494_FASTA.fasta	MGYA00242664	ERZ504494	root:Host-associated:Human:Digestive system	0.3314
MGYA00242665/ERZ504496_FASTA.fasta	MGYA00242665	ERZ504496	root:Host-associated:Human:Digestive system	0.2418
MGYA00242666/ERZ504498_FASTA.fasta	MGYA00242666	ERZ504498	root:Host-associated:Human:Digestive system	0.3444
MGYA00242667/ERZ504500_FASTA.fasta	MGYA00242667	ERZ504500	root:Host-associated:Human:Digestive system	0.1818
MGYA00242668/ERZ504502_FASTA.fasta	MGYA00242668	ERZ504502	root:Host-associated:Human:Digestive system	0.244
MGYA00242669/ERZ504504_FASTA.fasta	MGYA00242669	ERZ504504	root:Host-associated:Human:Digestive system	0.2516
MGYA00242670/ERZ504506_FASTA.fasta	MGYA00242670	ERZ504506	root:Host-associated:Human:Digestive system	0.2502
MGYA00242671/ERZ504508_FASTA.fasta	MGYA00242671	ERZ504508	root:Host-associated:Human:Digestive system	0.0736
MGYA00242672/ERZ504510_FASTA.fasta	MGYA00242672	ERZ504510	root:Host-associated:Human:Digestive system	0.2058
MGYA00242673/ERZ504512_FASTA.fasta	MGYA00242673	ERZ504512	root:Host-associated:Human:Digestive system	0.2822
MGYA00242674/ERZ504514_FASTA.fasta	MGYA00242674	ERZ504514	root:Host-associated:Human:Digestive system	0.3464
MGYA00242675/ERZ504516_FASTA.fasta	MGYA00242675	ERZ504516	root:Host-associated:Human:Digestive system	0.0728
MGYA00242676/ERZ504518_FASTA.fasta	MGYA00242676	ERZ504518	root:Host-associated:Human:Digestive system	0.3596
MGYA00242677/ERZ504520_FASTA.fasta	MGYA00242677	ERZ504520	root:Host-associated:Human:Digestive system	0.3332
MGYA00242678/ERZ504522_FASTA.fasta	MGYA00242678	ERZ504522	root:Host-associated:Human:Digestive system	0.0882
MGYA00242679/ERZ504524_FASTA.fasta	MGYA00242679	ERZ504524	root:Host-associated:Human:Digestive system	0.0986
MGYA00242680/ERZ504526_FASTA.fasta	MGYA00242680	ERZ504526	root:Host-associated:Human:Digestive system	0.2696
MGYA00242681/ERZ504528_FASTA.fasta	MGYA00242681	ERZ504528	root:Host-associated:Human:Digestive system	0.2462
MGYA00242682/ERZ504530_FASTA.fasta	MGYA00242682	ERZ504530	root:Host-associated:Human:Digestive system	0.3684
MGYA00242683/ERZ504825_FASTA.fasta	MGYA00242683	ERZ504825	root:Host-associated:Human:Digestive system	0.2616
MGYA00242684/ERZ504827_FASTA.fasta	MGYA00242684	ERZ504827	root:Host-associated:Human:Digestive system	0.3594
MGYA00242685/ERZ504829_FASTA.fasta	MGYA00242685	ERZ504829	root:Host-associated:Human:Digestive system	0.2476
MGYA00242686/ERZ504831_FASTA.fasta	MGYA00242686	ERZ504831	root:Host-associated:Human:Digestive system	0.281
MGYA00242687/ERZ504833_FASTA.fasta	MGYA00242687	ERZ504833	root:Host-associated:Human:Digestive system	0.2582
MGYA00242688/ERZ504835_FASTA.fasta	MGYA00242688	ERZ504835	root:Host-associated:Human:Digestive system	0.394
MGYA00242689/ERZ504837_FASTA.fasta	MGYA00242689	ERZ504837	root:Host-associated:Human:Digestive system	0.2966
MGYA00242690/ERZ504839_FASTA.fasta	MGYA00242690	ERZ504839	root:Host-associated:Human:Digestive system	0.3332
MGYA00242691/ERZ504841_FASTA.fasta	MGYA00242691	ERZ504841	root:Host-associated:Human:Digestive system	0.3516
MGYA00242692/ERZ504843_FASTA.fasta	MGYA00242692	ERZ504843	root:Host-associated:Human:Digestive system	0.3352
MGYA00242693/ERZ504845_FASTA.fasta	MGYA00242693	ERZ504845	root:Host-associated:Human:Digestive system	0.4224
MGYA00242694/ERZ504847_FASTA.fasta	MGYA00242694	ERZ504847	root:Host-associated:Human:Digestive system	0.5358
MGYA00242695/ERZ504532_FASTA.fasta	MGYA00242695	ERZ504532	root:Host-associated:Human:Digestive system	0.0672
MGYA00242696/ERZ504534_FASTA.fasta	MGYA00242696	ERZ504534	root:Host-associated:Human:Digestive system	0.3786
MGYA00242697/ERZ504536_FASTA.fasta	MGYA00242697	ERZ504536	root:Host-associated:Human:Digestive system	0.1028
MGYA00242698/ERZ504538_FASTA.fasta	MGYA00242698	ERZ504538	root:Host-associated:Human:Digestive system	0.1734
MGYA00242699/ERZ504540_FASTA.fasta	MGYA00242699	ERZ504540	root:Host-associated:Human:Digestive system	0.2366
MGYA00242700/ERZ504542_FASTA.fasta	MGYA00242700	ERZ504542	root:Host-associated:Human:Digestive system	0.3572
MGYA00242701/ERZ504544_FASTA.fasta	MGYA00242701	ERZ504544	root:Host-associated:Human:Digestive system	0.31
MGYA00242702/ERZ504546_FASTA.fasta	MGYA00242702	ERZ504546	root:Host-associated:Human:Digestive system	0.341
MGYA00242703/ERZ504548_FASTA.fasta	MGYA00242703	ERZ504548	root:Host-associated:Human:Digestive system	0.3692
MGYA00242704/ERZ504550_FASTA.fasta	MGYA00242704	ERZ504550	root:Host-associated:Human:Digestive system	0.2708
MGYA00242705/ERZ504552_FASTA.fasta	MGYA00242705	ERZ504552	root:Host-associated:Human:Digestive system	0.4084
MGYA00242706/ERZ504554_FASTA.fasta	MGYA00242706	ERZ504554	root:Host-associated:Human:Digestive system	0.4278
MGYA00242707/ERZ504556_FASTA.fasta	MGYA00242707	ERZ504556	root:Host-associated:Human:Digestive system	0.0636
MGYA00242708/ERZ504558_FASTA.fasta	MGYA00242708	ERZ504558	root:Host-associated:Human:Digestive system	0.3396
MGYA00242709/ERZ504560_FASTA.fasta	MGYA00242709	ERZ504560	root:Host-associated:Human:Digestive system	0.187
MGYA00242710/ERZ504562_FASTA.fasta	MGYA00242710	ERZ504562	root:Host-associated:Human:Digestive system	0.4992
MGYA00242711/ERZ504564_FASTA.fasta	MGYA00242711	ERZ504564	root:Host-associated:Human:Digestive system	0.3408
MGYA00242712/ERZ504849_FASTA.fasta	MGYA00242712	ERZ504849	root:Host-associated:Human:Digestive system	0.275
MGYA00242713/ERZ504851_FASTA.fasta	MGYA00242713	ERZ504851	root:Host-associated:Human:Digestive system	0.1914
MGYA00242714/ERZ504853_FASTA.fasta	MGYA00242714	ERZ504853	root:Host-associated:Human:Digestive system	0.2646
MGYA00242715/ERZ504855_FASTA.fasta	MGYA00242715	ERZ504855	root:Host-associated:Human:Digestive system	0.1676
MGYA00242716/ERZ504857_FASTA.fasta	MGYA00242716	ERZ504857	root:Host-associated:Human:Digestive system	0.2462
MGYA00242717/ERZ504859_FASTA.fasta	MGYA00242717	ERZ504859	root:Host-associated:Human:Digestive system	0.2568
MGYA00242718/ERZ504863_FASTA.fasta	MGYA00242718	ERZ504863	root:Host-associated:Human:Digestive system	0.2116
MGYA00242719/ERZ504865_FASTA.fasta	MGYA00242719	ERZ504865	root:Host-associated:Human:Digestive system	0.2956
MGYA00242720/ERZ504867_FASTA.fasta	MGYA00242720	ERZ504867	root:Host-associated:Human:Digestive system	0.2398
MGYA00242721/ERZ504869_FASTA.fasta	MGYA00242721	ERZ504869	root:Host-associated:Human:Digestive system	0.485
MGYA00242722/ERZ504871_FASTA.fasta	MGYA00242722	ERZ504871	root:Host-associated:Human:Digestive system	0.3276
MGYA00242723/ERZ504873_FASTA.fasta	MGYA00242723	ERZ504873	root:Host-associated:Human:Digestive system	0.4544
MGYA00242724/ERZ504875_FASTA.fasta	MGYA00242724	ERZ504875	root:Host-associated:Human:Digestive system	0.3034
MGYA00242725/ERZ504877_FASTA.fasta	MGYA00242725	ERZ504877	root:Host-associated:Human:Digestive system	0.428
MGYA00242726/ERZ504879_FASTA.fasta	MGYA00242726	ERZ504879	root:Host-associated:Human:Digestive system	0.2712
MGYA00242727/ERZ504881_FASTA.fasta	MGYA00242727	ERZ504881	root:Host-associated:Human:Digestive system	0.4268
MGYA00242728/ERZ504883_FASTA.fasta	MGYA00242728	ERZ504883	root:Host-associated:Human:Digestive system	0.4456
MGYA00242729/ERZ504885_FASTA.fasta	MGYA00242729	ERZ504885	root:Host-associated:Human:Digestive system	0.4684
MGYA00242730/ERZ504887_FASTA.fasta	MGYA00242730	ERZ504887	root:Host-associated:Human:Digestive system	0.2572
MGYA00242731/ERZ504889_FASTA.fasta	MGYA00242731	ERZ504889	root:Host-associated:Human:Digestive system	0.2672
MGYA00242732/ERZ504891_FASTA.fasta	MGYA00242732	ERZ504891	root:Host-associated:Human:Digestive system	0.2366
MGYA00242733/ERZ504523_FASTA.fasta	MGYA00242733	ERZ504523	root:Host-associated:Human:Digestive system	0.2858
MGYA00242734/ERZ504525_FASTA.fasta	MGYA00242734	ERZ504525	root:Host-associated:Human:Digestive system	0.219
MGYA00242735/ERZ504527_FASTA.fasta	MGYA00242735	ERZ504527	root:Host-associated:Human:Digestive system	0.3428
MGYA00242736/ERZ504529_FASTA.fasta	MGYA00242736	ERZ504529	root:Host-associated:Human:Digestive system	0.301
MGYA00242737/ERZ504531_FASTA.fasta	MGYA00242737	ERZ504531	root:Host-associated:Human:Digestive system	0.3124
MGYA00242738/ERZ504533_FASTA.fasta	MGYA00242738	ERZ504533	root:Host-associated:Human:Digestive system	0.2298
MGYA00242739/ERZ504535_FASTA.fasta	MGYA00242739	ERZ504535	root:Host-associated:Human:Digestive system	0.0752
MGYA00242740/ERZ504537_FASTA.fasta	MGYA00242740	ERZ504537	root:Host-associated:Human:Digestive system	0.303
MGYA00242741/ERZ504539_FASTA.fasta	MGYA00242741	ERZ504539	root:Host-associated:Human:Digestive system	0.223
MGYA00242742/ERZ504543_FASTA.fasta	MGYA00242742	ERZ504543	root:Host-associated:Human:Digestive system	0.3728
MGYA00242743/ERZ504545_FASTA.fasta	MGYA00242743	ERZ504545	root:Host-associated:Human:Digestive system	0.3416
MGYA00242744/ERZ504547_FASTA.fasta	MGYA00242744	ERZ504547	root:Host-associated:Human:Digestive system	0.2034
MGYA00242745/ERZ504549_FASTA.fasta	MGYA00242745	ERZ504549	root:Host-associated:Human:Digestive system	0.1842
MGYA00242746/ERZ504551_FASTA.fasta	MGYA00242746	ERZ504551	root:Host-associated:Human:Digestive system	0.1968
MGYA00242747/ERZ504553_FASTA.fasta	MGYA00242747	ERZ504553	root:Host-associated:Human:Digestive system	0.4052
MGYA00242748/ERZ504555_FASTA.fasta	MGYA00242748	ERZ504555	root:Host-associated:Human:Digestive system	0.1674
MGYA00242749/ERZ504557_FASTA.fasta	MGYA00242749	ERZ504557	root:Host-associated:Human:Digestive system	0.3192
MGYA00242750/ERZ504559_FASTA.fasta	MGYA00242750	ERZ504559	root:Host-associated:Human:Digestive system	0.437
MGYA00242751/ERZ504561_FASTA.fasta	MGYA00242751	ERZ504561	root:Host-associated:Human:Digestive system	0.174
MGYA00242752/ERZ504565_FASTA.fasta	MGYA00242752	ERZ504565	root:Host-associated:Human:Digestive system	0.0914
MGYA00242753/ERZ504567_FASTA.fasta	MGYA00242753	ERZ504567	root:Host-associated:Human:Digestive system	0.1168
MGYA00242754/ERZ504569_FASTA.fasta	MGYA00242754	ERZ504569	root:Host-associated:Human:Digestive system	0.0724
MGYA00242755/ERZ504571_FASTA.fasta	MGYA00242755	ERZ504571	root:Host-associated:Human:Digestive system	0.2094
MGYA00242756/ERZ504573_FASTA.fasta	MGYA00242756	ERZ504573	root:Host-associated:Human:Digestive system	0.2938
MGYA00242757/ERZ504575_FASTA.fasta	MGYA00242757	ERZ504575	root:Host-associated:Human:Digestive system	0.3064
MGYA00242758/ERZ504577_FASTA.fasta	MGYA00242758	ERZ504577	root:Host-associated:Human:Digestive system	0.2054
MGYA00242759/ERZ504579_FASTA.fasta	MGYA00242759	ERZ504579	root:Host-associated:Human:Digestive system	0.147
MGYA00242760/ERZ504583_FASTA.fasta	MGYA00242760	ERZ504583	root:Host-associated:Human:Digestive system	0.3188
MGYA00242761/ERZ504585_FASTA.fasta	MGYA00242761	ERZ504585	root:Host-associated:Human:Digestive system	0.1942
MGYA00242762/ERZ504587_FASTA.fasta	MGYA00242762	ERZ504587	root:Host-associated:Human:Digestive system	0.3836
MGYA00242763/ERZ504589_FASTA.fasta	MGYA00242763	ERZ504589	root:Host-associated:Human:Digestive system	0.2062
MGYA00242764/ERZ504591_FASTA.fasta	MGYA00242764	ERZ504591	root:Host-associated:Human:Digestive system	0.1802
MGYA00242765/ERZ504861_FASTA.fasta	MGYA00242765	ERZ504861	root:Host-associated:Human:Digestive system	0.1952
MGYA00242766/ERZ504592_FASTA.fasta	MGYA00242766	ERZ504592	root:Host-associated:Human:Digestive system	0.2348
MGYA00242767/ERZ504593_FASTA.fasta	MGYA00242767	ERZ504593	root:Host-associated:Human:Digestive system	0.3548
MGYA00242768/ERZ504595_FASTA.fasta	MGYA00242768	ERZ504595	root:Host-associated:Human:Digestive system	0.3512
MGYA00242769/ERZ504597_FASTA.fasta	MGYA00242769	ERZ504597	root:Host-associated:Human:Digestive system	0.219
MGYA00242770/ERZ504601_FASTA.fasta	MGYA00242770	ERZ504601	root:Host-associated:Human:Digestive system	0.414
MGYA00242771/ERZ504603_FASTA.fasta	MGYA00242771	ERZ504603	root:Host-associated:Human:Digestive system	0.3872
MGYA00242772/ERZ504605_FASTA.fasta	MGYA00242772	ERZ504605	root:Host-associated:Human:Digestive system	0.3436
MGYA00242773/ERZ504607_FASTA.fasta	MGYA00242773	ERZ504607	root:Host-associated:Human:Digestive system	0.119
MGYA00242774/ERZ504790_FASTA.fasta	MGYA00242774	ERZ504790	root:Host-associated:Human:Digestive system	0.3896
MGYA00242775/ERZ504792_FASTA.fasta	MGYA00242775	ERZ504792	root:Host-associated:Human:Digestive system	0.3126
MGYA00242776/ERZ504794_FASTA.fasta	MGYA00242776	ERZ504794	root:Host-associated:Human:Digestive system	0.5364

MGYA00242777/ERZ504796_FASTA.fasta	MGYA00242777	ERZ504796	root:Host-associated:Human:Digestive system	0.4218
MGYA00242778/ERZ504798_FASTA.fasta	MGYA00242778	ERZ504798	root:Host-associated:Human:Digestive system	0.2776
MGYA00242779/ERZ504800_FASTA.fasta	MGYA00242779	ERZ504800	root:Host-associated:Human:Digestive system	0.3002
MGYA00242780/ERZ504802_FASTA.fasta	MGYA00242780	ERZ504802	root:Host-associated:Human:Digestive system	0.451
MGYA00242781/ERZ504804_FASTA.fasta	MGYA00242781	ERZ504804	root:Host-associated:Human:Digestive system	0.1756
MGYA00242782/ERZ504609_FASTA.fasta	MGYA00242782	ERZ504609	root:Host-associated:Human:Digestive system	0.342
MGYA00242783/ERZ504611_FASTA.fasta	MGYA00242783	ERZ504611	root:Host-associated:Human:Digestive system	0.173
MGYA00242784/ERZ504613_FASTA.fasta	MGYA00242784	ERZ504613	root:Host-associated:Human:Digestive system	0.3284
MGYA00242785/ERZ504615_FASTA.fasta	MGYA00242785	ERZ504615	root:Host-associated:Human:Digestive system	0.1852
MGYA00242786/ERZ504617_FASTA.fasta	MGYA00242786	ERZ504617	root:Host-associated:Human:Digestive system	0.3874
MGYA00242787/ERZ504619_FASTA.fasta	MGYA00242787	ERZ504619	root:Host-associated:Human:Digestive system	0.3394
MGYA00242788/ERZ504621_FASTA.fasta	MGYA00242788	ERZ504621	root:Host-associated:Human:Digestive system	0.0648
MGYA00242789/ERZ504623_FASTA.fasta	MGYA00242789	ERZ504623	root:Host-associated:Human:Digestive system	0.0926
MGYA00242790/ERZ504625_FASTA.fasta	MGYA00242790	ERZ504625	root:Host-associated:Human:Digestive system	0.3302
MGYA00242791/ERZ504627_FASTA.fasta	MGYA00242791	ERZ504627	root:Host-associated:Human:Digestive system	0.3226
MGYA00242792/ERZ504629_FASTA.fasta	MGYA00242792	ERZ504629	root:Host-associated:Human:Digestive system	0.1394
MGYA00242793/ERZ504631_FASTA.fasta	MGYA00242793	ERZ504631	root:Host-associated:Human:Digestive system	0.1756
MGYA00242794/ERZ504633_FASTA.fasta	MGYA00242794	ERZ504633	root:Host-associated:Human:Digestive system	0.1798
MGYA00242795/ERZ504635_FASTA.fasta	MGYA00242795	ERZ504635	root:Host-associated:Human:Digestive system	0.2124
MGYA00242796/ERZ504637_FASTA.fasta	MGYA00242796	ERZ504637	root:Host-associated:Human:Digestive system	0.2668
MGYA00242797/ERZ504639_FASTA.fasta	MGYA00242797	ERZ504639	root:Host-associated:Human:Digestive system	0.3918
MGYA00242798/ERZ504641_FASTA.fasta	MGYA00242798	ERZ504641	root:Host-associated:Human:Digestive system	0.2908
MGYA00242799/ERZ504643_FASTA.fasta	MGYA00242799	ERZ504643	root:Host-associated:Human:Digestive system	0.1778
MGYA00242800/ERZ504645_FASTA.fasta	MGYA00242800	ERZ504645	root:Host-associated:Human:Digestive system	0.3766
MGYA00242801/ERZ504647_FASTA.fasta	MGYA00242801	ERZ504647	root:Host-associated:Human:Digestive system	0.4206
MGYA00242802/ERZ504649_FASTA.fasta	MGYA00242802	ERZ504649	root:Host-associated:Human:Digestive system	0.251
MGYA00242803/ERZ504806_FASTA.fasta	MGYA00242803	ERZ504806	root:Host-associated:Human:Digestive system	0.3318
MGYA00242804/ERZ504808_FASTA.fasta	MGYA00242804	ERZ504808	root:Host-associated:Human:Digestive system	0.2192
MGYA00242805/ERZ504812_FASTA.fasta	MGYA00242805	ERZ504812	root:Host-associated:Human:Digestive system	0.488
MGYA00242806/ERZ504814_FASTA.fasta	MGYA00242806	ERZ504814	root:Host-associated:Human:Digestive system	0.2826
MGYA00242807/ERZ504816_FASTA.fasta	MGYA00242807	ERZ504816	root:Host-associated:Human:Digestive system	0.186
MGYA00242808/ERZ504818_FASTA.fasta	MGYA00242808	ERZ504818	root:Host-associated:Human:Digestive system	0.2442
MGYA00242809/ERZ504820_FASTA.fasta	MGYA00242809	ERZ504820	root:Host-associated:Human:Digestive system	0.2064
MGYA00242810/ERZ504822_FASTA.fasta	MGYA00242810	ERZ504822	root:Host-associated:Human:Digestive system	0.4298
MGYA00242811/ERZ504824_FASTA.fasta	MGYA00242811	ERZ504824	root:Host-associated:Human:Digestive system	0.2882
MGYA00242812/ERZ504826_FASTA.fasta	MGYA00242812	ERZ504826	root:Host-associated:Human:Digestive system	0.4896
MGYA00242813/ERZ504828_FASTA.fasta	MGYA00242813	ERZ504828	root:Host-associated:Human:Digestive system	0.3246
MGYA00242814/ERZ504830_FASTA.fasta	MGYA00242814	ERZ504830	root:Host-associated:Human:Digestive system	0.3626
MGYA00242815/ERZ504832_FASTA.fasta	MGYA00242815	ERZ504832	root:Host-associated:Human:Digestive system	0.2668
MGYA00242816/ERZ504834_FASTA.fasta	MGYA00242816	ERZ504834	root:Host-associated:Human:Digestive system	0.28
MGYA00242817/ERZ504838_FASTA.fasta	MGYA00242817	ERZ504838	root:Host-associated:Human:Digestive system	0.2318
MGYA00242818/ERZ504840_FASTA.fasta	MGYA00242818	ERZ504840	root:Host-associated:Human:Digestive system	0.3634
MGYA00242819/ERZ504842_FASTA.fasta	MGYA00242819	ERZ504842	root:Host-associated:Human:Digestive system	0.3752
MGYA00242820/ERZ504651_FASTA.fasta	MGYA00242820	ERZ504651	root:Host-associated:Human:Digestive system	0.3836
MGYA00242821/ERZ504653_FASTA.fasta	MGYA00242821	ERZ504653	root:Host-associated:Human:Digestive system	0.4618
MGYA00242822/ERZ504655_FASTA.fasta	MGYA00242822	ERZ504655	root:Host-associated:Human:Digestive system	0.3146
MGYA00242823/ERZ504657_FASTA.fasta	MGYA00242823	ERZ504657	root:Host-associated:Human:Digestive system	0.3802
MGYA00242824/ERZ504659_FASTA.fasta	MGYA00242824	ERZ504659	root:Host-associated:Human:Digestive system	0.3676
MGYA00242825/ERZ504661_FASTA.fasta	MGYA00242825	ERZ504661	root:Host-associated:Human:Digestive system	0.3546
MGYA00242826/ERZ504663_FASTA.fasta	MGYA00242826	ERZ504663	root:Host-associated:Human:Digestive system	0.3186
MGYA00242827/ERZ504665_FASTA.fasta	MGYA00242827	ERZ504665	root:Host-associated:Human:Digestive system	0.3262
MGYA00242828/ERZ504666_FASTA.fasta	MGYA00242828	ERZ504666	root:Host-associated:Human:Digestive system	0.3592
MGYA00242829/ERZ504668_FASTA.fasta	MGYA00242829	ERZ504668	root:Host-associated:Human:Digestive system	0.2664
MGYA00242830/ERZ504670_FASTA.fasta	MGYA00242830	ERZ504670	root:Host-associated:Human:Digestive system	0.3782
MGYA00242831/ERZ504672_FASTA.fasta	MGYA00242831	ERZ504672	root:Host-associated:Human:Digestive system	0.3322
MGYA00242832/ERZ504674_FASTA.fasta	MGYA00242832	ERZ504674	root:Host-associated:Human:Digestive system	0.1902
MGYA00242833/ERZ504676_FASTA.fasta	MGYA00242833	ERZ504676	root:Host-associated:Human:Digestive system	0.4148
MGYA00242834/ERZ504678_FASTA.fasta	MGYA00242834	ERZ504678	root:Host-associated:Human:Digestive system	0.2634
MGYA00242835/ERZ504680_FASTA.fasta	MGYA00242835	ERZ504680	root:Host-associated:Human:Digestive system	0.302
MGYA00242836/ERZ504682_FASTA.fasta	MGYA00242836	ERZ504682	root:Host-associated:Human:Digestive system	0.2962
MGYA00242837/ERZ504684_FASTA.fasta	MGYA00242837	ERZ504684	root:Host-associated:Human:Digestive system	0.3216
MGYA00242838/ERZ504686_FASTA.fasta	MGYA00242838	ERZ504686	root:Host-associated:Human:Digestive system	0.3408
MGYA00242839/ERZ504688_FASTA.fasta	MGYA00242839	ERZ504688	root:Host-associated:Human:Digestive system	0.3892
MGYA00242840/ERZ504690_FASTA.fasta	MGYA00242840	ERZ504690	root:Host-associated:Human:Digestive system	0.2896
MGYA00242841/ERZ504844_FASTA.fasta	MGYA00242841	ERZ504844	root:Host-associated:Human:Digestive system	0.2028
MGYA00242842/ERZ504846_FASTA.fasta	MGYA00242842	ERZ504846	root:Host-associated:Human:Digestive system	0.236
MGYA00242843/ERZ504848_FASTA.fasta	MGYA00242843	ERZ504848	root:Host-associated:Human:Digestive system	0.2584
MGYA00242844/ERZ504850_FASTA.fasta	MGYA00242844	ERZ504850	root:Host-associated:Human:Digestive system	0.4938
MGYA00242845/ERZ504852_FASTA.fasta	MGYA00242845	ERZ504852	root:Host-associated:Human:Digestive system	0.5094
MGYA00242846/ERZ504854_FASTA.fasta	MGYA00242846	ERZ504854	root:Host-associated:Human:Digestive system	0.2528
MGYA00242847/ERZ504856_FASTA.fasta	MGYA00242847	ERZ504856	root:Host-associated:Human:Digestive system	0.2064
MGYA00242848/ERZ504858_FASTA.fasta	MGYA00242848	ERZ504858	root:Host-associated:Human:Digestive system	0.4538
MGYA00242849/ERZ504860_FASTA.fasta	MGYA00242849	ERZ504860	root:Host-associated:Human:Digestive system	0.354
MGYA00242850/ERZ504694_FASTA.fasta	MGYA00242850	ERZ504694	root:Host-associated:Human:Digestive system	0.3078
MGYA00242851/ERZ504696_FASTA.fasta	MGYA00242851	ERZ504696	root:Host-associated:Human:Digestive system	0.306
MGYA00242852/ERZ504698_FASTA.fasta	MGYA00242852	ERZ504698	root:Host-associated:Human:Digestive system	0.2998
MGYA00242853/ERZ504700_FASTA.fasta	MGYA00242853	ERZ504700	root:Host-associated:Human:Digestive system	0.4478
MGYA00242854/ERZ504702_FASTA.fasta	MGYA00242854	ERZ504702	root:Host-associated:Human:Digestive system	0.4014
MGYA00242855/ERZ504704_FASTA.fasta	MGYA00242855	ERZ504704	root:Host-associated:Human:Digestive system	0.336
MGYA00242856/ERZ504706_FASTA.fasta	MGYA00242856	ERZ504706	root:Host-associated:Human:Digestive system	0.3488
MGYA00242857/ERZ504708_FASTA.fasta	MGYA00242857	ERZ504708	root:Host-associated:Human:Digestive system	0.2786
MGYA00242858/ERZ504710_FASTA.fasta	MGYA00242858	ERZ504710	root:Host-associated:Human:Digestive system	0.2852
MGYA00242859/ERZ504712_FASTA.fasta	MGYA00242859	ERZ504712	root:Host-associated:Human:Digestive system	0.407
MGYA00242860/ERZ504714_FASTA.fasta	MGYA00242860	ERZ504714	root:Host-associated:Human:Digestive system	0.3178
MGYA00242861/ERZ504716_FASTA.fasta	MGYA00242861	ERZ504716	root:Host-associated:Human:Digestive system	0.3658
MGYA00242862/ERZ504718_FASTA.fasta	MGYA00242862	ERZ504718	root:Host-associated:Human:Digestive system	0.3224
MGYA00242863/ERZ504720_FASTA.fasta	MGYA00242863	ERZ504720	root:Host-associated:Human:Digestive system	0.3286
MGYA00242864/ERZ504722_FASTA.fasta	MGYA00242864	ERZ504722	root:Host-associated:Human:Digestive system	0.4136
MGYA00242865/ERZ504724_FASTA.fasta	MGYA00242865	ERZ504724	root:Host-associated:Human:Digestive system	0.2774
MGYA00242866/ERZ504726_FASTA.fasta	MGYA00242866	ERZ504726	root:Host-associated:Human:Digestive system	0.4232
MGYA00242867/ERZ504728_FASTA.fasta	MGYA00242867	ERZ504728	root:Host-associated:Human:Digestive system	0.3436
MGYA00242868/ERZ504730_FASTA.fasta	MGYA00242868	ERZ504730	root:Host-associated:Human:Digestive system	0.2994
MGYA00242869/ERZ508010_FASTA.fasta	MGYA00242869	ERZ508010	root:Host-associated:Human:Digestive system	0.5622
MGYA00242870/ERZ508011_FASTA.fasta	MGYA00242870	ERZ508011	root:Host-associated:Human:Digestive system	0.354
MGYA00242871/ERZ508012_FASTA.fasta	MGYA00242871	ERZ508012	root:Host-associated:Human:Digestive system	0.3988
MGYA00242872/ERZ508013_FASTA.fasta	MGYA00242872	ERZ508013	root:Host-associated:Human:Digestive system	0.4568
MGYA00242873/ERZ508014_FASTA.fasta	MGYA00242873	ERZ508014	root:Host-associated:Human:Digestive system	0.5274
MGYA00242874/ERZ508015_FASTA.fasta	MGYA00242874	ERZ508015	root:Host-associated:Human:Digestive system	0.5208
MGYA00242875/ERZ508016_FASTA.fasta	MGYA00242875	ERZ508016	root:Host-associated:Human:Digestive system	0.3812
MGYA00242876/ERZ507913_FASTA.fasta	MGYA00242876	ERZ507913	root:Host-associated:Human:Digestive system	0.375
MGYA00242877/ERZ507915_FASTA.fasta	MGYA00242877	ERZ507915	root:Host-associated:Human:Digestive system	0.4556
MGYA00242878/ERZ507917_FASTA.fasta	MGYA00242878	ERZ507917	root:Host-associated:Human:Digestive system	0.545
MGYA00242879/ERZ507874_FASTA.fasta	MGYA00242879	ERZ507874	root:Host-associated:Human:Digestive system	0.349
MGYA00242880/ERZ507876_FASTA.fasta	MGYA00242880	ERZ507876	root:Host-associated:Human:Digestive system	0.5544
MGYA00242881/ERZ507878_FASTA.fasta	MGYA00242881	ERZ507878	root:Host-associated:Human:Digestive system	0.4166
MGYA00242882/ERZ507880_FASTA.fasta	MGYA00242882	ERZ507880	root:Host-associated:Human:Digestive system	0.4094
MGYA00242883/ERZ507882_FASTA.fasta	MGYA00242883	ERZ507882	root:Host-associated:Human:Digestive system	0.3952
MGYA00242884/ERZ507884_FASTA.fasta	MGYA00242884	ERZ507884	root:Host-associated:Human:Digestive system	0.4118
MGYA00242885/ERZ507886_FASTA.fasta	MGYA00242885	ERZ507886	root:Host-associated:Human:Digestive system	0.3716
MGYA00242886/ERZ507888_FASTA.fasta	MGYA00242886	ERZ507888	root:Host-associated:Human:Digestive system	0.5144
MGYA00242887/ERZ507890_FASTA.fasta	MGYA00242887	ERZ507890	root:Host-associated:Human:Digestive system	0.4218
MGYA00242888/ERZ507892_FASTA.fasta	MGYA00242888	ERZ507892	root:Host-associated:Human:Digestive system	0.39
MGYA00242889/ERZ507894_FASTA.fasta	MGYA00242889	ERZ507894	root:Host-associated:Human:Digestive system	0.3426
MGYA00242890/ERZ507896_FASTA.fasta	MGYA00242890	ERZ507896	root:Host-associated:Human:Digestive system	0.5224
MGYA00242891/ERZ507898_FASTA.fasta	MGYA00242891	ERZ507898	root:Host-associated:Human:Digestive system	0.5448
MGYA00242892/ERZ507900_FASTA.fasta	MGYA00242892	ERZ507900	root:Host-associated:Human:Digestive system	0.3738
MGYA00242893/ERZ507902_FASTA.fasta	MGYA00242893	ERZ507902	root:Host-associated:Human:Digestive system	0.5176
MGYA00242894/ERZ507904_FASTA.fasta	MGYA00242894	ERZ507904	root:Host-associated:Human:Digestive system	0.4526
MGYA00242895/ERZ507906_FASTA.fasta	MGYA00242895	ERZ507906	root:Host-associated:Human:Digestive system	0.4246
MGYA00242896/ERZ507908_FASTA.fasta	MGYA00242896	ERZ507908	root:Host-associated:Human:Digestive system	0.522
MGYA00242897/ERZ507910_FASTA.fasta	MGYA00242897	ERZ507910	root:Host-associated:Human:Digestive system	0.4124
MGYA00242898/ERZ507912_FASTA.fasta	MGYA00242898	ERZ507912	root:Host-associated:Human:Digestive system	0.3754
MGYA00242899/ERZ507914_FASTA.fasta	MGYA00242899	ERZ507914	root:Host-associated:Human:Digestive system	0.5374
MGYA00242900/ERZ507916_FASTA.fasta	MGYA00242900	ERZ507916	root:Host-associated:Human:Digestive system	0.4168
MGYA00242901/ERZ507918_FASTA.fasta	MGYA00242901	ERZ507918	root:Host-associated:Human:Digestive system	0.5276
MGYA00242902/ERZ507920_FASTA.fasta	MGYA00242902	ERZ507920	root:Host-associated:Human:Digestive system	0.4992
MGYA00242903/ERZ507922_FASTA.fasta	MGYA00242903	ERZ507922	root:Host-associated:Human:Digestive system	0.444
MGYA00242904/ERZ507924_FASTA.fasta	MGYA00242904	ERZ507924	root:Host-associated:Human:Digestive system	0.5148
MGYA00242905/ERZ507926_FASTA.fasta	MGYA00242905	ERZ507926	root:Host-associated:Human:Digestive system	0.4918
MGYA00242906/ERZ507928_FASTA.fasta	MGYA00242906	ERZ507928	root:Host-associated:Human:Digestive system	0.558
MGYA00242907/ERZ507875_FASTA.fasta	MGYA00242907	ERZ507875	root:Host-associated:Human:Digestive system	0.491
MGYA00242908/ERZ507877_FASTA.fasta	MGYA00242908	ERZ507877	root:Host-associated:Human:Digestive system	0.3498
MGYA00242909/ERZ507879_FASTA.fasta	MGYA00242909	ERZ507879	root:Host-associated:Human:Digestive system	0.3664
MGYA00242910/ERZ507881_FASTA.fasta	MGYA00242910	ERZ507881	root:Host-associated:Human:Digestive system	0.455
MGYA00242911/ERZ507883_FASTA.fasta	MGYA00242911	ERZ507883	root:Host-associated:Human:Digestive system	0.5008
MGYA00242912/ERZ507887_FASTA.fasta	MGYA00242912	ERZ507887	root:Host-associated:Human:Digestive system	0.3946
MGYA00242913/ERZ507889_FASTA.fasta	MGYA00242913	ERZ507889	root:Host-associated:Human:Digestive system	0.4444
MGYA00242914/ERZ507891_FASTA.fasta	MGYA00242914	ERZ507891	root:Host-associated:Human:Digestive system	0.497
MGYA00242915/ERZ507893_FASTA.fasta	MGYA00242915	ERZ507893	root:Host-associated:Human:Digestive system	0.4174
MGYA00242916/ERZ507895_FASTA.fasta	MGYA00242916	ERZ507895	root:Host-associated:Human:Digestive system	0.5114
MGYA00242917/ERZ507897_FASTA.fasta	MGYA00242917	ERZ507897	root:Host-associated:Human:Digestive system	0.5014
MGYA00242918/ERZ507899_FASTA.fasta	MGYA00242918	ERZ507899	root:Host-associated:Human:Digestive system	0.3846
MGYA00242919/ERZ507901_FASTA.fasta	MGYA00242919	ERZ507901	root:Host-associated:Human:Digestive system	0.3598
MGYA00242920/ERZ507903_FASTA.fasta	MGYA00242920	ERZ507903	root:Host-associated:Human:Digestive system	0.5622
MGYA00242921/ERZ507905_FASTA.fasta	MGYA00242921	ERZ507905	root:Host-associated:Human:Digestive system	0.3492
MGYA00242922/ERZ507907_FASTA.fasta	MGYA00242922	ERZ507907	root:Host-associated:Human:Digestive system	0.5134
MGYA00242923/ERZ507909_FASTA.fasta	MGYA00242923	ERZ507909	root:Host-associated:Human:Digestive system	0.3702
MGYA00242924/ERZ507911_FASTA.fasta	MGYA00242924	ERZ507911	root:Host-associated:Human:Digestive system	0.4624
MGYA00242925/ERZ507919_FASTA.fasta	MGYA00242925	ERZ507919	root:Host-associated:Human:Digestive system	0.5994
MGYA00242926/ERZ507921_FASTA.fasta	MGYA00242926	ERZ507921	root:Host-associated:Human:Digestive system	0.4358
MGYA00242927/ERZ507923_FASTA.fasta	MGYA00242927	ERZ507923	root:Host-associated:Human:Digestive system	0.4806
MGYA00242928/ERZ507927_FASTA.fasta	MGYA00242928	ERZ507927	root:Host-associated:Human:Digestive system	0.3782
MGYA00242929/ERZ507929_FASTA.fasta	MGYA00242929	ERZ507929	root:Host-associated:Human:Digestive system	0.4198
MGYA00242930/ERZ507930_FASTA.fasta	MGYA00242930	ERZ507930	root:Host-associated:Human:Digestive system	0.5602
MGYA00242931/ERZ507931_FASTA.fasta	MGYA00242931	ERZ507931	root:Host-associated:Human:Digestive system	0.405
MGYA00242932/ERZ507932_FASTA.fasta	MGYA00242932	ERZ507932	root:Host-associated:Human:Digestive system	0.5398
MGYA00242933/ERZ507934_FASTA.fasta	MGYA00242933	ERZ507934	root:Host-associated:Human:Digestive system	0.4912
MGYA00242934/ERZ507935_FASTA.fasta	MGYA00242934	ERZ507935	root:Host-associated:Human:Digestive system	0.5928
MGYA00242935/ERZ507936_FASTA.fasta	MGYA00242935	ERZ507936	root:Host-associated:Human:Digestive system	0.3566
MGYA00242936/ERZ507937_FASTA.fasta	MGYA00242936	ERZ507937	root:Host-associated:Human:Digestive system	0.391
MGYA00242937/ERZ507938_FASTA.fasta	MGYA00242937	ERZ507938	root:Host-associated:Human:Digestive system	0.578
MGYA00242938/ERZ507940_FASTA.fasta	MGYA00242938	ERZ507940	root:Host-associated:Human:Digestive system	0.5584
MGYA00242939/ERZ507942_FASTA.fasta	MGYA00242939	ERZ507942	root:Host-associated:Human:Digestive system	0.456
MGYA00242940/ERZ507944_FASTA.fasta	MGYA00242940	ERZ507944	root:Host-associated:Human:Digestive system	0.4692
MGYA00242941/ERZ507946_FASTA.fasta	MGYA00242941	ERZ507946	root:Host-associated:Human:Digestive system	0.5462
MGYA00242942/ERZ507925_FASTA.fasta	MGYA00242942	ERZ507925	root:Host-associated:Human:Digestive system	0.453
MGYA00242943/ERZ507939_FASTA.fasta	MGYA00242943	ERZ507939	root:Host-associated:Human:Digestive system	0.477
MGYA00242944/ERZ507941_FASTA.fasta	MGYA00242944	ERZ507941	root:Host-associated:Human:Digestive system	0.4212
MGYA00242945/ERZ507943_FASTA.fasta	MGYA00242945	ERZ507943	root:Host-associated:Human:Digestive system	0.344
MGYA00242946/ERZ507945_FASTA.fasta	MGYA00242946	ERZ507945	root:Host-associated:Human:Digestive system	0.295
MGYA00242947/ERZ507947_FASTA.fasta	MGYA00242947	ERZ507947	root:Host-associated:Human:Digestive system	0.6014
MGYA00242948/ERZ507948_FASTA.fasta	MGYA00242948	ERZ507948	root:Host-associated:Human:Digestive system	0.4336
MGYA00242949/ERZ507949_FASTA.fasta	MGYA00242949	ERZ507949	root:Host-associated:Human:Digestive system	0.5808
MGYA00242950/ERZ507950_FASTA.fasta	MGYA00242950	ERZ507950	root:Host-associated:Human:Digestive system	0.5146
MGYA00242951/ERZ507951_FASTA.fasta	MGYA00242951	ERZ507951	root:Host-associated:Human:Digestive system	0.433
MGYA00242952/ERZ507952_FASTA.fasta	MGYA00242952	ERZ507952	root:Host-associated:Human:Digestive system	0.501
MGYA00242953/ERZ507953_FASTA.fasta	MGYA00242953	ERZ507953	root:Host-associated:Human:Digestive system	0.416
MGYA00242954/ERZ507954_FASTA.fasta	MGYA00242954	ERZ507954	root:Host-associated:Human:Digestive system	0.524
MGYA00242955/ERZ507955_FASTA.fasta	MGYA00242955	ERZ507955	root:Host-associated:Human:Digestive system	0.4756
MGYA00242956/ERZ507956_FASTA.fasta	MGYA00242956	ERZ507956	root:Host-associated:Human:Digestive system	0.3612
MGYA00242957/ERZ507957_FASTA.fasta	MGYA00242957	ERZ507957	root:Host-associated:Human:Digestive system	0.398
MGYA00242958/ERZ507958_FASTA.fasta	MGYA00242958	ERZ507958	root:Host-associated:Human:Digestive system	0.431
MGYA00242959/ERZ507959_FASTA.fasta	MGYA00242959	ERZ507959	root:Host-associated:Human:Digestive system	0.528
MGYA00242960/ERZ507960_FASTA.fasta	MGYA00242960	ERZ507960	root:Host-associated:Human:Digestive system	0.3504
MGYA00242961/ERZ507961_FASTA.fasta	MGYA00242961	ERZ507961	root:Host-associated:Human:Digestive system	0.3578
MGYA00242962/ERZ507962_FASTA.fasta	MGYA00242962	ERZ507962	root:Host-associated:Human:Digestive system	0.3588
MGYA00242963/ERZ507963_FASTA.fasta	MGYA00242963	ERZ507963	root:Host-associated:Human:Digestive system	0.5604
MGYA00242964/ERZ507964_FASTA.fasta	MGYA00242964	ERZ507964	root:Host-associated:Human:Digestive system	0.331
MGYA00242965/ERZ507965_FASTA.fasta	MGYA00242965	ERZ507965	root:Host-associated:Human:Digestive system	0.5322
MGYA00242966/ERZ507966_FASTA.fasta	MGYA00242966	ERZ507966	root:Host-associated:Human:Digestive system	0.3944
MGYA00242967/ERZ507967_FASTA.fasta	MGYA00242967	ERZ507967	root:Host-associated:Human:Digestive system	0.5176
MGYA00242968/ERZ507968_FASTA.fasta	MGYA00242968	ERZ507968	root:Host-associated:Human:Digestive system	0.3338
MGYA00242969/ERZ507969_FASTA.fasta	MGYA00242969	ERZ507969	root:Host-associated:Human:Digestive system	0.5072
MGYA00242970/ERZ507970_FASTA.fasta	MGYA00242970	ERZ507970	root:Host-associated:Human:Digestive system	0.4758
MGYA00242971/ERZ507971_FASTA.fasta	MGYA00242971	ERZ507971	root:Host-associated:Human:Digestive system	0.3984
MGYA00242972/ERZ507972_FASTA.fasta	MGYA00242972	ERZ507972	root:Host-associated:Human:Digestive system	0.4098
MGYA00242973/ERZ507973_FASTA.fasta	MGYA00242973	ERZ507973	root:Host-associated:Human:Digestive system	0.3914
MGYA00242974/ERZ507974_FASTA.fasta	MGYA00242974	ERZ507974	root:Host-associated:Human:Digestive system	0.605
MGYA00242975/ERZ507975_FASTA.fasta	MGYA00242975	ERZ507975	root:Host-associated:Human:Digestive system	0.4522
MGYA00242976/ERZ507976_FASTA.fasta	MGYA00242976	ERZ507976	root:Host-associated:Human:Digestive system	0.4524
MGYA00242977/ERZ507977_FASTA.fasta	MGYA00242977	ERZ507977	root:Host-associated:Human:Digestive system	0.4122
MGYA00242978/ERZ507978_FASTA.fasta	MGYA00242978	ERZ507978	root:Host-associated:Human:Digestive system	0.4538
MGYA00242979/ERZ507979_FASTA.fasta	MGYA00242979	ERZ507979	root:Host-associated:Human:Digestive system	0.373
MGYA00242980/ERZ507980_FASTA.fasta	MGYA00242980	ERZ507980	root:Host-associated:Human:Digestive system	0.3628
MGYA00242981/ERZ507981_FASTA.fasta	MGYA00242981	ERZ507981	root:Host-associated:Human:Digestive system	0.507
MGYA00242982/ERZ507982_FASTA.fasta	MGYA00242982	ERZ507982	root:Host-associated:Human:Digestive system	0.4948
MGYA00242983/ERZ507983_FASTA.fasta	MGYA00242983	ERZ507983	root:Host-associated:Human:Digestive system	0.5038
MGYA00242984/ERZ507984_FASTA.fasta	MGYA00242984	ERZ507984	root:Host-associated:Human:Digestive system	0.4202
MGYA00242985/ERZ507986_FASTA.fasta	MGYA00242985	ERZ507986	root:Host-associated:Human:Digestive system	0.4416
MGYA00242986/ERZ507987_FASTA.fasta	MGYA00242986	ERZ507987	root:Host-associated:Human:Digestive system	0.4888
MGYA00242987/ERZ507988_FASTA.fasta	MGYA00242987	ERZ507988	root:Host-associated:Human:Digestive system	0.35
MGYA00242988/ERZ507989_FASTA.fasta	MGYA00242988	ERZ507989	root:Host-associated:Human:Digestive system	0.473
MGYA00242989/ERZ507990_FASTA.fasta	MGYA00242989	ERZ507990	root:Host-associated:Human:Digestive system	0.5764
MGYA00242990/ERZ507991_FASTA.fasta	MGYA00242990	ERZ507991	root:Host-associated:Human:Digestive system	0.4422
MGYA00242991/ERZ507992_FASTA.fasta	MGYA00242991	ERZ507992	root:Host-associated:Human:Digestive system	0.4518
MGYA00242992/ERZ507993_FASTA.fasta	MGYA00242992	ERZ507993	root:Host-associated:Human:Digestive system	0.4868
MGYA00242993/ERZ507994_FASTA.fasta	MGYA00242993	ERZ507994	root:Host-associated:Human:Digestive system	0.476
MGYA00242994/ERZ507995_FASTA.fasta	MGYA00242994	ERZ507995	root:Host-associated:Human:Digestive system	0.4126
MGYA00242995/ERZ507996_FASTA.fasta	MGYA00242995	ERZ507996	root:Host-associated:Human:Digestive system	0.4288
MGYA00242996/ERZ507997_FASTA.fasta	MGYA00242996	ERZ507997	root:Host-associated:Human:Digestive system	0.4788
MGYA00242997/ERZ507998_FASTA.fasta	MGYA00242997	ERZ507998	root:Host-associated:Human:Digestive system	0.4756
MGYA00242998/ERZ507999_FASTA.fasta	MGYA00242998	ERZ507999	root:Host-associated:Human:Digestive system	0.4708
MGYA00242999/ERZ508000_FASTA.fasta	MGYA00242999	ERZ508000	root:Host-associated:Human:Digestive system	0.3304
MGYA00243000/ERZ508001_FASTA.fasta	MGYA00243000	ERZ508001	root:Host-associated:Human:Digestive system	0.375
MGYA00243001/ERZ508002_FASTA.fasta	MGYA00243001	ERZ508002	root:Host-associated:Human:Digestive system	0.4016
MGYA00243002/ERZ508003_FASTA.fasta	MGYA00243002	ERZ508003	root:Host-associated:Human:Digestive system	0.408
MGYA00243003/ERZ508004_FASTA.fasta	MGYA00243003	ERZ508004	root:Host-associated:Human:Digestive system	0.4974
MGYA00243004/ERZ508005_FASTA.fasta	MGYA00243004	ERZ508005	root:Host-associated:Human:Digestive system	0.3994
MGYA00243005/ERZ508006_FASTA.fasta	MGYA00243005	ERZ508006	root:Host-associated:Human:Digestive system	0.385
MGYA00243006/ERZ508007_FASTA.fasta	MGYA00243006	ERZ508007	root:Host-associated:Human:Digestive system	0.4186
MGYA00243007/ERZ508008_FASTA.fasta	MGYA00243007	ERZ508008	root:Host-associated:Human:Digestive system	0.5398
MGYA00243008/ERZ508009_FASTA.fasta	MGYA00243008	ERZ508009	root:Host-associated:Human:Digestive system	0.4582
MGYA00243009/ERZ508833_FASTA.fasta	MGYA00243009	ERZ508833	root:Host-associated:Human:Digestive system:Large in	0.4382
MGYA00243010/ERZ508835_FASTA.fasta	MGYA00243010	ERZ508835	root:Host-associated:Human:Digestive system:Large in	0.3544
MGYA00243011/ERZ508837_FASTA.fasta	MGYA00243011	ERZ508837	root:Host-associated:Human:Digestive system:Large in	0.0598
MGYA00243012/ERZ508839_FASTA.fasta	MGYA00243012	ERZ508839	root:Host-associated:Human:Digestive system:Large in	0.1448
MGYA00243013/ERZ508841_FASTA.fasta	MGYA00243013	ERZ508841	root:Host-associated:Human:Digestive system:Large in	0.1748
MGYA00243014/ERZ508843_FASTA.fasta	MGYA00243014	ERZ508843	root:Host-associated:Human:Digestive system:Large in	0.2386
MGYA00243015/ERZ508825_FASTA.fasta	MGYA00243015	ERZ508825	root:Host-associated:Human:Digestive system:Large in	0.2802
MGYA00243016/ERZ508827_FASTA.fasta	MGYA00243016	ERZ508827	root:Host-associated:Human:Digestive system:Large in	0.2204
MGYA00243017/ERZ508829_FASTA.fasta	MGYA00243017	ERZ508829	root:Host-associated:Human:Digestive system:Large in	0.4776
MGYA00243018/ERZ508831_FASTA.fasta	MGYA00243018	ERZ508831	root:Host-associated:Human:Digestive system:Large in	0.0724
MGYA00243019/ERZ508723_FASTA.fasta	MGYA00243019	ERZ508723	root:Host-associated:Human:Digestive system:Large in	0.088
MGYA00243020/ERZ508725_FASTA.fasta	MGYA00243020	ERZ508725	root:Host-associated:Human:Digestive system:Large in	0.1428
MGYA00243021/ERZ508727_FASTA.fasta	MGYA00243021	ERZ508727	root:Host-associated:Human:Digestive system:Large in	0.1294
MGYA00243022/ERZ508730_FASTA.fasta	MGYA00243022	ERZ508730	root:Host-associated:Human:Digestive system:Large in	0.307
MGYA00243023/ERZ508732_FASTA.fasta	MGYA00243023	ERZ508732	root:Host-associated:Human:Digestive system:Large in	0.4584
MGYA00243024/ERZ508734_FASTA.fasta	MGYA00243024	ERZ508734	root:Host-associated:Human:Digestive system:Large in	0.2886
MGYA00243025/ERZ508736_FASTA.fasta	MGYA00243025	ERZ508736	root:Host-associated:Human:Digestive system:Large in	0.1464
MGYA00243026/ERZ508738_FASTA.fasta	MGYA00243026	ERZ508738	root:Host-associated:Human:Digestive system:Large in	0.4028
MGYA00243027/ERZ508740_FASTA.fasta	MGYA00243027	ERZ508740	root:Host-associated:Human:Digestive system:Large in	0.2932
MGYA00243028/ERZ508742_FASTA.fasta	MGYA00243028	ERZ508742	root:Host-associated:Human:Digestive system:Large in	0.1648
MGYA00243029/ERZ508744_FASTA.fasta	MGYA00243029	ERZ508744	root:Host-associated:Human:Digestive system:Large in	0.1938
MGYA00243030/ERZ508746_FASTA.fasta	MGYA00243030	ERZ508746	root:Host-associated:Human:Digestive system:Large in	0.3922
MGYA00243031/ERZ508748_FASTA.fasta	MGYA00243031	ERZ508748	root:Host-associated:Human:Digestive system:Large in	0.1702
MGYA00243032/ERZ508750_FASTA.fasta	MGYA00243032	ERZ508750	root:Host-associated:Human:Digestive system:Large in	0.2502
MGYA00243033/ERZ508754_FASTA.fasta	MGYA00243033	ERZ508754	root:Host-associated:Human:Digestive system:Large in	0.2264
MGYA00243034/ERZ508756_FASTA.fasta	MGYA00243034	ERZ508756	root:Host-associated:Human:Digestive system:Large in	0.1214
MGYA00243035/ERZ508758_FASTA.fasta	MGYA00243035	ERZ508758	root:Host-associated:Human:Digestive system:Large in	0.2968
MGYA00243036/ERZ508760_FASTA.fasta	MGYA00243036	ERZ508760	root:Host-associated:Human:Digestive system:Large in	0.2396
MGYA00243037/ERZ508762_FASTA.fasta	MGYA00243037	ERZ508762	root:Host-associated:Human:Digestive system:Large in	0.1454
MGYA00243038/ERZ508764_FASTA.fasta	MGYA00243038	ERZ508764	root:Host-associated:Human:Digestive system:Large in	0.15
MGYA00243039/ERZ508766_FASTA.fasta	MGYA00243039	ERZ508766	root:Host-associated:Human:Digestive system:Large in	0.233
MGYA00243040/ERZ508768_FASTA.fasta	MGYA00243040	ERZ508768	root:Host-associated:Human:Digestive system:Large in	0.2994
MGYA00243041/ERZ508770_FASTA.fasta	MGYA00243041	ERZ508770	root:Host-associated:Human:Digestive system:Large in	0.4392
MGYA00243042/ERZ508772_FASTA.fasta	MGYA00243042	ERZ508772	root:Host-associated:Human:Digestive system:Large in	0.2664
MGYA00243043/ERZ508774_FASTA.fasta	MGYA00243043	ERZ508774	root:Host-associated:Human:Digestive system:Large in	0.284
MGYA00243044/ERZ508777_FASTA.fasta	MGYA00243044	ERZ508777	root:Host-associated:Human:Digestive system:Large in	0.4194
MGYA00243045/ERZ508779_FASTA.fasta	MGYA00243045	ERZ508779	root:Host-associated:Human:Digestive system:Large in	0.3252
MGYA00243046/ERZ508781_FASTA.fasta	MGYA00243046	ERZ508781	root:Host-associated:Human:Digestive system:Large in	0.4224
MGYA00243047/ERZ508783_FASTA.fasta	MGYA00243047	ERZ508783	root:Host-associated:Human:Digestive system:Large in	0.2328
MGYA00243048/ERZ508785_FASTA.fasta	MGYA00243048	ERZ508785	root:Host-associated:Human:Digestive system:Large in	0.147
MGYA00243049/ERZ508787_FASTA.fasta	MGYA00243049	ERZ508787	root:Host-associated:Human:Digestive system:Large in	0.202
MGYA00243050/ERZ508789_FASTA.fasta	MGYA00243050	ERZ508789	root:Host-associated:Human:Digestive system:Large in	0.4544
MGYA00243051/ERZ508791_FASTA.fasta	MGYA00243051	ERZ508791	root:Host-associated:Human:Digestive system:Large in	0.2384
MGYA00243052/ERZ508793_FASTA.fasta	MGYA00243052	ERZ508793	root:Host-associated:Human:Digestive system:Large in	0.2424
MGYA00243053/ERZ508795_FASTA.fasta	MGYA00243053	ERZ508795	root:Host-associated:Human:Digestive system:Large in	0.1096
MGYA00243054/ERZ508797_FASTA.fasta	MGYA00243054	ERZ508797	root:Host-associated:Human:Digestive system:Large in	0.0924
MGYA00243055/ERZ508799_FASTA.fasta	MGYA00243055	ERZ508799	root:Host-associated:Human:Digestive system:Large in	0.4878
MGYA00243056/ERZ508801_FASTA.fasta	MGYA00243056	ERZ508801	root:Host-associated:Human:Digestive system:Large in	0.2376
MGYA00243057/ERZ508803_FASTA.fasta	MGYA00243057	ERZ508803	root:Host-associated:Human:Digestive system:Large in	0.2426
MGYA00243058/ERZ508805_FASTA.fasta	MGYA00243058	ERZ508805	root:Host-associated:Human:Digestive system:Large in	0.4154
MGYA00243059/ERZ508807_FASTA.fasta	MGYA00243059	ERZ508807	root:Host-associated:Human:Digestive system:Large in	0.2598
MGYA00243060/ERZ508809_FASTA.fasta	MGYA00243060	ERZ508809	root:Host-associated:Human:Digestive system:Large in	0.2792
MGYA00243061/ERZ508811_FASTA.fasta	MGYA00243061	ERZ508811	root:Host-associated:Human:Digestive system:Large in	0.2512
MGYA00243062/ERZ508813_FASTA.fasta	MGYA00243062	ERZ508813	root:Host-associated:Human:Digestive system:Large in	0.3164
MGYA00243063/ERZ508815_FASTA.fasta	MGYA00243063	ERZ508815	root:Host-associated:Human:Digestive system:Large in	0.4114
MGYA00243064/ERZ508817_FASTA.fasta	MGYA00243064	ERZ508817	root:Host-associated:Human:Digestive system:Large in	0.4308
MGYA00243065/ERZ508819_FASTA.fasta	MGYA00243065	ERZ508819	root:Host-associated:Human:Digestive system:Large in	0.1286
MGYA00243066/ERZ508822_FASTA.fasta	MGYA00243066	ERZ508822	root:Host-associated:Human:Digestive system:Large in	0.361
MGYA00243067/ERZ508824_FASTA.fasta	MGYA00243067	ERZ508824	root:Host-associated:Human:Digestive system:Large in	0.2682
MGYA00243068/ERZ508826_FASTA.fasta	MGYA00243068	ERZ508826	root:Host-associated:Human:Digestive system:Large in	0.2528
MGYA00243069/ERZ508828_FASTA.fasta	MGYA00243069	ERZ508828	root:Host-associated:Human:Digestive system:Large in	0.2776
MGYA00243070/ERZ508830_FASTA.fasta	MGYA00243070	ERZ508830	root:Host-associated:Human:Digestive system:Large in	0.2576
MGYA00243071/ERZ508832_FASTA.fasta	MGYA00243071	ERZ508832	root:Host-associated:Human:Digestive system:Large in	0.5244
MGYA00243072/ERZ508834_FASTA.fasta	MGYA00243072	ERZ508834	root:Host-associated:Human:Digestive system:Large in	0.137
MGYA00243073/ERZ508836_FASTA.fasta	MGYA00243073	ERZ508836	root:Host-associated:Human:Digestive system:Large in	0.1866
MGYA00243074/ERZ508838_FASTA.fasta	MGYA00243074	ERZ508838	root:Host-associated:Human:Digestive system:Large in	0.2024
MGYA00243075/ERZ508840_FASTA.fasta	MGYA00243075	ERZ508840	root:Host-associated:Human:Digestive system:Large in	0.3882
MGYA00243076/ERZ508842_FASTA.fasta	MGYA00243076	ERZ508842	root:Host-associated:Human:Digestive system:Large in	0.3038
MGYA00243077/ERZ508724_FASTA.fasta	MGYA00243077	ERZ508724	root:Host-associated:Human:Digestive system:Large in	0.1064
MGYA00243078/ERZ508726_FASTA.fasta	MGYA00243078	ERZ508726	root:Host-associated:Human:Digestive system:Large in	0.1066
MGYA00243079/ERZ508728_FASTA.fasta	MGYA00243079	ERZ508728	root:Host-associated:Human:Digestive system:Large in	0.1212
MGYA00243080/ERZ508729_FASTA.fasta	MGYA00243080	ERZ508729	root:Host-associated:Human:Digestive system:Large in	0.1896
MGYA00243081/ERZ508731_FASTA.fasta	MGYA00243081	ERZ508731	root:Host-associated:Human:Digestive system:Large in	0.1798
MGYA00243082/ERZ508733_FASTA.fasta	MGYA00243082	ERZ508733	root:Host-associated:Human:Digestive system:Large in	0.133
MGYA00243083/ERZ508735_FASTA.fasta	MGYA00243083	ERZ508735	root:Host-associated:Human:Digestive system:Large in	0.3132
MGYA00243084/ERZ508737_FASTA.fasta	MGYA00243084	ERZ508737	root:Host-associated:Human:Digestive system:Large in	0.1262
MGYA00243085/ERZ508739_FASTA.fasta	MGYA00243085	ERZ508739	root:Host-associated:Human:Digestive system:Large in	0.3404
MGYA00243086/ERZ508741_FASTA.fasta	MGYA00243086	ERZ508741	root:Host-associated:Human:Digestive system:Large in	0.2208
MGYA00243087/ERZ508743_FASTA.fasta	MGYA00243087	ERZ508743	root:Host-associated:Human:Digestive system:Large in	0.2038
MGYA00243088/ERZ508745_FASTA.fasta	MGYA00243088	ERZ508745	root:Host-associated:Human:Digestive system:Large in	0.3738
MGYA00243089/ERZ508747_FASTA.fasta	MGYA00243089	ERZ508747	root:Host-associated:Human:Digestive system:Large in	0.3076
MGYA00243090/ERZ508749_FASTA.fasta	MGYA00243090	ERZ508749	root:Host-associated:Human:Digestive system:Large in	0.1772
MGYA00243091/ERZ508751_FASTA.fasta	MGYA00243091	ERZ508751	root:Host-associated:Human:Digestive system:Large in	0.2638
MGYA00243092/ERZ508753_FASTA.fasta	MGYA00243092	ERZ508753	root:Host-associated:Human:Digestive system:Large in	0.2044
MGYA00243093/ERZ508755_FASTA.fasta	MGYA00243093	ERZ508755	root:Host-associated:Human:Digestive system:Large in	0.2392
MGYA00243094/ERZ508757_FASTA.fasta	MGYA00243094	ERZ508757	root:Host-associated:Human:Digestive system:Large in	0.1762
MGYA00243095/ERZ508759_FASTA.fasta	MGYA00243095	ERZ508759	root:Host-associated:Human:Digestive system:Large in	0.239
MGYA00243096/ERZ508761_FASTA.fasta	MGYA00243096	ERZ508761	root:Host-associated:Human:Digestive system:Large in	0.1272
MGYA00243097/ERZ508763_FASTA.fasta	MGYA00243097	ERZ508763	root:Host-associated:Human:Digestive system:Large in	0.2074
MGYA00243098/ERZ508765_FASTA.fasta	MGYA00243098	ERZ508765	root:Host-associated:Human:Digestive system:Large in	0.2362
MGYA00243099/ERZ508767_FASTA.fasta	MGYA00243099	ERZ508767	root:Host-associated:Human:Digestive system:Large in	0.1964
MGYA00243100/ERZ508769_FASTA.fasta	MGYA00243100	ERZ508769	root:Host-associated:Human:Digestive system:Large in	0.1572
MGYA00243101/ERZ508771_FASTA.fasta	MGYA00243101	ERZ508771	root:Host-associated:Human:Digestive system:Large in	0.2274
MGYA00243102/ERZ508773_FASTA.fasta	MGYA00243102	ERZ508773	root:Host-associated:Human:Digestive system:Large in	0.3914
MGYA00243103/ERZ508775_FASTA.fasta	MGYA00243103	ERZ508775	root:Host-associated:Human:Digestive system:Large in	0.5944
MGYA00243104/ERZ508776_FASTA.fasta	MGYA00243104	ERZ508776	root:Host-associated:Human:Digestive system:Large in	0.4574
MGYA00243105/ERZ508778_FASTA.fasta	MGYA00243105	ERZ508778	root:Host-associated:Human:Digestive system:Large in	0.2056
MGYA00243106/ERZ508780_FASTA.fasta	MGYA00243106	ERZ508780	root:Host-associated:Human:Digestive system:Large in	0.254
MGYA00243107/ERZ508782_FASTA.fasta	MGYA00243107	ERZ508782	root:Host-associated:Human:Digestive system:Large in	0.4476
MGYA00243108/ERZ508784_FASTA.fasta	MGYA00243108	ERZ508784	root:Host-associated:Human:Digestive system:Large in	0.4408
MGYA00243109/ERZ508786_FASTA.fasta	MGYA00243109	ERZ508786	root:Host-associated:Human:Digestive system:Large in	0.2626
MGYA00243110/ERZ508788_FASTA.fasta	MGYA00243110	ERZ508788	root:Host-associated:Human:Digestive system:Large in	0.2438
MGYA00243111/ERZ508790_FASTA.fasta	MGYA00243111	ERZ508790	root:Host-associated:Human:Digestive system:Large in	0.1824
MGYA00243112/ERZ508792_FASTA.fasta	MGYA00243112	ERZ508792	root:Host-associated:Human:Digestive system:Large in	0.2006
MGYA00243113/ERZ508794_FASTA.fasta	MGYA00243113	ERZ508794	root:Host-associated:Human:Digestive system:Large in	0.2064
MGYA00243114/ERZ508796_FASTA.fasta	MGYA00243114	ERZ508796	root:Host-associated:Human:Digestive system:Large in	0.17
MGYA00243115/ERZ508798_FASTA.fasta	MGYA00243115	ERZ508798	root:Host-associated:Human:Digestive system:Large in	0.4126
MGYA00243116/ERZ508800_FASTA.fasta	MGYA00243116	ERZ508800	root:Host-associated:Human:Digestive system:Large in	0.3712
MGYA00243117/ERZ508802_FASTA.fasta	MGYA00243117	ERZ508802	root:Host-associated:Human:Digestive system:Large in	0.2418
MGYA00243118/ERZ508804_FASTA.fasta	MGYA00243118	ERZ508804	root:Host-associated:Human:Digestive system:Large in	0.3598
MGYA00243119/ERZ508806_FASTA.fasta	MGYA00243119	ERZ508806	root:Host-associated:Human:Digestive system:Large in	0.2696
MGYA00243120/ERZ508808_FASTA.fasta	MGYA00243120	ERZ508808	root:Host-associated:Human:Digestive system:Large in	0.2354
MGYA00243121/ERZ508810_FASTA.fasta	MGYA00243121	ERZ508810	root:Host-associated:Human:Digestive system:Large in	0.2262
MGYA00243122/ERZ508812_FASTA.fasta	MGYA00243122	ERZ508812	root:Host-associated:Human:Digestive system:Large in	0.2686
MGYA00243123/ERZ508814_FASTA.fasta	MGYA00243123	ERZ508814	root:Host-associated:Human:Digestive system:Large in	0.2774
MGYA00243124/ERZ508816_FASTA.fasta	MGYA00243124	ERZ508816	root:Host-associated:Human:Digestive system:Large in	0.1432
MGYA00243125/ERZ508818_FASTA.fasta	MGYA00243125	ERZ508818	root:Host-associated:Human:Digestive system:Large in	0.1154
MGYA00243126/ERZ508820_FASTA.fasta	MGYA00243126	ERZ508820	root:Host-associated:Human:Digestive system:Large in	0.2828
MGYA00243127/ERZ508821_FASTA.fasta	MGYA00243127	ERZ508821	root:Host-associated:Human:Digestive system:Large in	0.1684
MGYA00243128/ERZ508823_FASTA.fasta	MGYA00243128	ERZ508823	root:Host-associated:Human:Digestive system:Large in	0.4422
MGYA00243129/ERZ490445_FASTA.fasta	MGYA00243129	ERZ490445	root:Host-associated:Human:Digestive system:Large in	0.404
MGYA00243130/ERZ490447_FASTA.fasta	MGYA00243130	ERZ490447	root:Host-associated:Human:Digestive system:Large in	0.304
MGYA00243131/ERZ490449_FASTA.fasta	MGYA00243131	ERZ490449	root:Host-associated:Human:Digestive system:Large in	0.4974
MGYA00243132/ERZ490451_FASTA.fasta	MGYA00243132	ERZ490451	root:Host-associated:Human:Digestive system:Large in	0.4614
MGYA00243133/ERZ490453_FASTA.fasta	MGYA00243133	ERZ490453	root:Host-associated:Human:Digestive system:Large in	0.4024
MGYA00243134/ERZ490455_FASTA.fasta	MGYA00243134	ERZ490455	root:Host-associated:Human:Digestive system:Large in	0.3658
MGYA00243135/ERZ490457_FASTA.fasta	MGYA00243135	ERZ490457	root:Host-associated:Human:Digestive system:Large in	0.4646
MGYA00243136/ERZ490459_FASTA.fasta	MGYA00243136	ERZ490459	root:Host-associated:Human:Digestive system:Large in	0.4292
MGYA00243137/ERZ490400_FASTA.fasta	MGYA00243137	ERZ490400	root:Host-associated:Human:Digestive system:Large in	0.493
MGYA00243138/ERZ490402_FASTA.fasta	MGYA00243138	ERZ490402	root:Host-associated:Human:Digestive system:Large in	0.1356
MGYA00243139/ERZ490404_FASTA.fasta	MGYA00243139	ERZ490404	root:Host-associated:Human:Digestive system:Large in	0.3434
MGYA00243140/ERZ490406_FASTA.fasta	MGYA00243140	ERZ490406	root:Host-associated:Human:Digestive system:Large in	0.4324
MGYA00243141/ERZ490408_FASTA.fasta	MGYA00243141	ERZ490408	root:Host-associated:Human:Digestive system:Large in	0.4912
MGYA00243142/ERZ490410_FASTA.fasta	MGYA00243142	ERZ490410	root:Host-associated:Human:Digestive system:Large in	0.4844
MGYA00243143/ERZ490412_FASTA.fasta	MGYA00243143	ERZ490412	root:Host-associated:Human:Digestive system:Large in	0.459
MGYA00243144/ERZ490414_FASTA.fasta	MGYA00243144	ERZ490414	root:Host-associated:Human:Digestive system:Large in	0.1958
MGYA00243145/ERZ490416_FASTA.fasta	MGYA00243145	ERZ490416	root:Host-associated:Human:Digestive system:Large in	0.325
MGYA00243146/ERZ490418_FASTA.fasta	MGYA00243146	ERZ490418	root:Host-associated:Human:Digestive system:Large in	0.3194
MGYA00243147/ERZ490420_FASTA.fasta	MGYA00243147	ERZ490420	root:Host-associated:Human:Digestive system:Large in	0.4654
MGYA00243148/ERZ490422_FASTA.fasta	MGYA00243148	ERZ490422	root:Host-associated:Human:Digestive system:Large in	0.37
MGYA00243149/ERZ490424_FASTA.fasta	MGYA00243149	ERZ490424	root:Host-associated:Human:Digestive system:Large in	0.2802
MGYA00243150/ERZ490426_FASTA.fasta	MGYA00243150	ERZ490426	root:Host-associated:Human:Digestive system:Large in	0.4404
MGYA00243151/ERZ490399_FASTA.fasta	MGYA00243151	ERZ490399	root:Host-associated:Human:Digestive system:Large in	0.4202
MGYA00243152/ERZ490401_FASTA.fasta	MGYA00243152	ERZ490401	root:Host-associated:Human:Digestive system:Large in	0.4198
MGYA00243153/ERZ490403_FASTA.fasta	MGYA00243153	ERZ490403	root:Host-associated:Human:Digestive system:Large in	0.5064
MGYA00243154/ERZ490405_FASTA.fasta	MGYA00243154	ERZ490405	root:Host-associated:Human:Digestive system:Large in	0.3788
MGYA00243155/ERZ490407_FASTA.fasta	MGYA00243155	ERZ490407	root:Host-associated:Human:Digestive system:Large in	0.3334
MGYA00243156/ERZ490409_FASTA.fasta	MGYA00243156	ERZ490409	root:Host-associated:Human:Digestive system:Large in	0.328
MGYA00243157/ERZ490411_FASTA.fasta	MGYA00243157	ERZ490411	root:Host-associated:Human:Digestive system:Large in	0.4254
MGYA00243158/ERZ490413_FASTA.fasta	MGYA00243158	ERZ490413	root:Host-associated:Human:Digestive system:Large in	0.3332
MGYA00243159/ERZ490415_FASTA.fasta	MGYA00243159	ERZ490415	root:Host-associated:Human:Digestive system:Large in	0.3792
MGYA00243160/ERZ490417_FASTA.fasta	MGYA00243160	ERZ490417	root:Host-associated:Human:Digestive system:Large in	0.314
MGYA00243161/ERZ490419_FASTA.fasta	MGYA00243161	ERZ490419	root:Host-associated:Human:Digestive system:Large in	0.3804
MGYA00243162/ERZ490421_FASTA.fasta	MGYA00243162	ERZ490421	root:Host-associated:Human:Digestive system:Large in	0.3386
MGYA00243163/ERZ490423_FASTA.fasta	MGYA00243163	ERZ490423	root:Host-associated:Human:Digestive system:Large in	0.3644
MGYA00243164/ERZ490425_FASTA.fasta	MGYA00243164	ERZ490425	root:Host-associated:Human:Digestive system:Large in	0.4778
MGYA00243165/ERZ490427_FASTA.fasta	MGYA00243165	ERZ490427	root:Host-associated:Human:Digestive system:Large in	0.3524
MGYA00243166/ERZ490428_FASTA.fasta	MGYA00243166	ERZ490428	root:Host-associated:Human:Digestive system:Large in	0.4486
MGYA00243167/ERZ490431_FASTA.fasta	MGYA00243167	ERZ490431	root:Host-associated:Human:Digestive system:Large in	0.383
MGYA00243168/ERZ490433_FASTA.fasta	MGYA00243168	ERZ490433	root:Host-associated:Human:Digestive system:Large in	0.29
MGYA00243169/ERZ490435_FASTA.fasta	MGYA00243169	ERZ490435	root:Host-associated:Human:Digestive system:Large in	0.2964
MGYA00243170/ERZ490437_FASTA.fasta	MGYA00243170	ERZ490437	root:Host-associated:Human:Digestive system:Large in	0.2818
MGYA00243171/ERZ490439_FASTA.fasta	MGYA00243171	ERZ490439	root:Host-associated:Human:Digestive system:Large in	0.5664
MGYA00243172/ERZ490441_FASTA.fasta	MGYA00243172	ERZ490441	root:Host-associated:Human:Digestive system:Large in	0.4586
MGYA00243173/ERZ490443_FASTA.fasta	MGYA00243173	ERZ490443	root:Host-associated:Human:Digestive system:Large in	0.511
MGYA00243174/ERZ490429_FASTA.fasta	MGYA00243174	ERZ490429	root:Host-associated:Human:Digestive system:Large in	0.3156
MGYA00243175/ERZ490430_FASTA.fasta	MGYA00243175	ERZ490430	root:Host-associated:Human:Digestive system:Large in	0.3652
MGYA00243176/ERZ490432_FASTA.fasta	MGYA00243176	ERZ490432	root:Host-associated:Human:Digestive system:Large in	0.4544
MGYA00243177/ERZ490434_FASTA.fasta	MGYA00243177	ERZ490434	root:Host-associated:Human:Digestive system:Large in	0.293
MGYA00243178/ERZ490436_FASTA.fasta	MGYA00243178	ERZ490436	root:Host-associated:Human:Digestive system:Large in	0.3126
MGYA00243179/ERZ490438_FASTA.fasta	MGYA00243179	ERZ490438	root:Host-associated:Human:Digestive system:Large in	0.5156
MGYA00243180/ERZ490440_FASTA.fasta	MGYA00243180	ERZ490440	root:Host-associated:Human:Digestive system:Large in	0.3546
MGYA00243181/ERZ490442_FASTA.fasta	MGYA00243181	ERZ490442	root:Host-associated:Human:Digestive system:Large in	0.3006
MGYA00243182/ERZ490444_FASTA.fasta	MGYA00243182	ERZ490444	root:Host-associated:Human:Digestive system:Large in	0.4294
MGYA00243183/ERZ490446_FASTA.fasta	MGYA00243183	ERZ490446	root:Host-associated:Human:Digestive system:Large in	0.3192
MGYA00243184/ERZ490448_FASTA.fasta	MGYA00243184	ERZ490448	root:Host-associated:Human:Digestive system:Large in	0.4062
MGYA00243185/ERZ490450_FASTA.fasta	MGYA00243185	ERZ490450	root:Host-associated:Human:Digestive system:Large in	0.3596
MGYA00243186/ERZ490452_FASTA.fasta	MGYA00243186	ERZ490452	root:Host-associated:Human:Digestive system:Large in	0.2548
MGYA00243187/ERZ490454_FASTA.fasta	MGYA00243187	ERZ490454	root:Host-associated:Human:Digestive system:Large in	0.4508
MGYA00243188/ERZ490456_FASTA.fasta	MGYA00243188	ERZ490456	root:Host-associated:Human:Digestive system:Large in	0.4354
MGYA00243189/ERZ490458_FASTA.fasta	MGYA00243189	ERZ490458	root:Host-associated:Human:Digestive system:Large in	0.423
MGYA00243190/ERZ490460_FASTA.fasta	MGYA00243190	ERZ490460	root:Host-associated:Human:Digestive system:Large in	0.4902
MGYA00243191/ERZ505059_FASTA.fasta	MGYA00243191	ERZ505059	root:Host-associated:Human:Digestive system:Large in	0.474
MGYA00243192/ERZ505061_FASTA.fasta	MGYA00243192	ERZ505061	root:Host-associated:Human:Digestive system:Large in	0.3492
MGYA00243193/ERZ505063_FASTA.fasta	MGYA00243193	ERZ505063	root:Host-associated:Human:Digestive system:Large in	0.3652
MGYA00243194/ERZ505065_FASTA.fasta	MGYA00243194	ERZ505065	root:Host-associated:Human:Digestive system:Large in	0.3606
MGYA00243195/ERZ505067_FASTA.fasta	MGYA00243195	ERZ505067	root:Host-associated:Human:Digestive system:Large in	0.406
MGYA00243196/ERZ505069_FASTA.fasta	MGYA00243196	ERZ505069	root:Host-associated:Human:Digestive system:Large in	0.4108
MGYA00243197/ERZ505071_FASTA.fasta	MGYA00243197	ERZ505071	root:Host-associated:Human:Digestive system:Large in	0.4294
MGYA00243198/ERZ505073_FASTA.fasta	MGYA00243198	ERZ505073	root:Host-associated:Human:Digestive system:Large in	0.4144
MGYA00243199/ERZ505075_FASTA.fasta	MGYA00243199	ERZ505075	root:Host-associated:Human:Digestive system:Large in	0.3574
MGYA00243200/ERZ505077_FASTA.fasta	MGYA00243200	ERZ505077	root:Host-associated:Human:Digestive system:Large in	0.4112
MGYA00243201/ERZ505079_FASTA.fasta	MGYA00243201	ERZ505079	root:Host-associated:Human:Digestive system:Large in	0.38
MGYA00243202/ERZ505081_FASTA.fasta	MGYA00243202	ERZ505081	root:Host-associated:Human:Digestive system:Large in	0.4234
MGYA00243203/ERZ505083_FASTA.fasta	MGYA00243203	ERZ505083	root:Host-associated:Human:Digestive system:Large in	0.3482
MGYA00243204/ERZ505085_FASTA.fasta	MGYA00243204	ERZ505085	root:Host-associated:Human:Digestive system:Large in	0.3694
MGYA00243205/ERZ505087_FASTA.fasta	MGYA00243205	ERZ505087	root:Host-associated:Human:Digestive system:Large in	0.4182
MGYA00243206/ERZ505089_FASTA.fasta	MGYA00243206	ERZ505089	root:Host-associated:Human:Digestive system:Large in	0.3576
MGYA00243207/ERZ505091_FASTA.fasta	MGYA00243207	ERZ505091	root:Host-associated:Human:Digestive system:Large in	0.4138
MGYA00243208/ERZ505093_FASTA.fasta	MGYA00243208	ERZ505093	root:Host-associated:Human:Digestive system:Large in	0.4192
MGYA00243209/ERZ505095_FASTA.fasta	MGYA00243209	ERZ505095	root:Host-associated:Human:Digestive system:Large in	0.4096
MGYA00243210/ERZ505097_FASTA.fasta	MGYA00243210	ERZ505097	root:Host-associated:Human:Digestive system:Large in	0.4166
MGYA00243211/ERZ505099_FASTA.fasta	MGYA00243211	ERZ505099	root:Host-associated:Human:Digestive system:Large in	0.3872
MGYA00243212/ERZ505101_FASTA.fasta	MGYA00243212	ERZ505101	root:Host-associated:Human:Digestive system:Large in	0.4454
MGYA00243213/ERZ505103_FASTA.fasta	MGYA00243213	ERZ505103	root:Host-associated:Human:Digestive system:Large in	0.391
MGYA00243214/ERZ505105_FASTA.fasta	MGYA00243214	ERZ505105	root:Host-associated:Human:Digestive system:Large in	0.3708
MGYA00243215/ERZ505060_FASTA.fasta	MGYA00243215	ERZ505060	root:Host-associated:Human:Digestive system:Large in	0.437
MGYA00243216/ERZ505062_FASTA.fasta	MGYA00243216	ERZ505062	root:Host-associated:Human:Digestive system:Large in	0.4004
MGYA00243217/ERZ505064_FASTA.fasta	MGYA00243217	ERZ505064	root:Host-associated:Human:Digestive system:Large in	0.372
MGYA00243218/ERZ505066_FASTA.fasta	MGYA00243218	ERZ505066	root:Host-associated:Human:Digestive system:Large in	0.3574
MGYA00243219/ERZ505068_FASTA.fasta	MGYA00243219	ERZ505068	root:Host-associated:Human:Digestive system:Large in	0.392
MGYA00243220/ERZ505070_FASTA.fasta	MGYA00243220	ERZ505070	root:Host-associated:Human:Digestive system:Large in	0.4308
MGYA00243221/ERZ505072_FASTA.fasta	MGYA00243221	ERZ505072	root:Host-associated:Human:Digestive system:Large in	0.435
MGYA00243222/ERZ505074_FASTA.fasta	MGYA00243222	ERZ505074	root:Host-associated:Human:Digestive system:Large in	0.4162
MGYA00243223/ERZ505076_FASTA.fasta	MGYA00243223	ERZ505076	root:Host-associated:Human:Digestive system:Large in	0.4358
MGYA00243224/ERZ505078_FASTA.fasta	MGYA00243224	ERZ505078	root:Host-associated:Human:Digestive system:Large in	0.3376
MGYA00243225/ERZ505080_FASTA.fasta	MGYA00243225	ERZ505080	root:Host-associated:Human:Digestive system:Large in	0.4586
MGYA00243226/ERZ505082_FASTA.fasta	MGYA00243226	ERZ505082	root:Host-associated:Human:Digestive system:Large in	0.3512
MGYA00243227/ERZ505084_FASTA.fasta	MGYA00243227	ERZ505084	root:Host-associated:Human:Digestive system:Large in	0.4694
MGYA00243228/ERZ505086_FASTA.fasta	MGYA00243228	ERZ505086	root:Host-associated:Human:Digestive system:Large in	0.433
MGYA00243229/ERZ505088_FASTA.fasta	MGYA00243229	ERZ505088	root:Host-associated:Human:Digestive system:Large in	0.3496
MGYA00243230/ERZ505090_FASTA.fasta	MGYA00243230	ERZ505090	root:Host-associated:Human:Digestive system:Large in	0.3456
MGYA00243231/ERZ505092_FASTA.fasta	MGYA00243231	ERZ505092	root:Host-associated:Human:Digestive system:Large in	0.4062
MGYA00243232/ERZ505094_FASTA.fasta	MGYA00243232	ERZ505094	root:Host-associated:Human:Digestive system:Large in	0.4296
MGYA00243233/ERZ505096_FASTA.fasta	MGYA00243233	ERZ505096	root:Host-associated:Human:Digestive system:Large in	0.4326
MGYA00243234/ERZ505098_FASTA.fasta	MGYA00243234	ERZ505098	root:Host-associated:Human:Digestive system:Large in	0.3688
MGYA00243235/ERZ505100_FASTA.fasta	MGYA00243235	ERZ505100	root:Host-associated:Human:Digestive system:Large in	0.4662
MGYA00243236/ERZ505102_FASTA.fasta	MGYA00243236	ERZ505102	root:Host-associated:Human:Digestive system:Large in	0.3744
MGYA00243237/ERZ505104_FASTA.fasta	MGYA00243237	ERZ505104	root:Host-associated:Human:Digestive system:Large in	0.391
MGYA00243238/ERZ505106_FASTA.fasta	MGYA00243238	ERZ505106	root:Host-associated:Human:Digestive system:Large in	0.3532
MGYA00243239/ERZ653427_FASTA.fasta	MGYA00243239	ERZ653427	root:Host-associated:Human:Digestive system:Large in	0.3684
MGYA00243240/ERZ653429_FASTA.fasta	MGYA00243240	ERZ653429	root:Host-associated:Human:Digestive system:Large in	0.367
MGYA00243241/ERZ653431_FASTA.fasta	MGYA00243241	ERZ653431	root:Host-associated:Human:Digestive system:Large in	0.3002
MGYA00243242/ERZ653433_FASTA.fasta	MGYA00243242	ERZ653433	root:Host-associated:Human:Digestive system:Large in	0.299
MGYA00243243/ERZ653435_FASTA.fasta	MGYA00243243	ERZ653435	root:Host-associated:Human:Digestive system:Large in	0.3186
MGYA00243244/ERZ653437_FASTA.fasta	MGYA00243244	ERZ653437	root:Host-associated:Human:Digestive system:Large in	0.3118
MGYA00243245/ERZ653439_FASTA.fasta	MGYA00243245	ERZ653439	root:Host-associated:Human:Digestive system:Large in	0.3618
MGYA00243246/ERZ653441_FASTA.fasta	MGYA00243246	ERZ653441	root:Host-associated:Human:Digestive system:Large in	0.4302
MGYA00243247/ERZ653443_FASTA.fasta	MGYA00243247	ERZ653443	root:Host-associated:Human:Digestive system:Large in	0.3354
MGYA00243248/ERZ653447_FASTA.fasta	MGYA00243248	ERZ653447	root:Host-associated:Human:Digestive system:Large in	0.2998
MGYA00243249/ERZ653449_FASTA.fasta	MGYA00243249	ERZ653449	root:Host-associated:Human:Digestive system:Large in	0.3314
MGYA00243250/ERZ653451_FASTA.fasta	MGYA00243250	ERZ653451	root:Host-associated:Human:Digestive system:Large in	0.301
MGYA00243251/ERZ653453_FASTA.fasta	MGYA00243251	ERZ653453	root:Host-associated:Human:Digestive system:Large in	0.358
MGYA00243252/ERZ653455_FASTA.fasta	MGYA00243252	ERZ653455	root:Host-associated:Human:Digestive system:Large in	0.381
MGYA00243253/ERZ653457_FASTA.fasta	MGYA00243253	ERZ653457	root:Host-associated:Human:Digestive system:Large in	0.227
MGYA00243254/ERZ653459_FASTA.fasta	MGYA00243254	ERZ653459	root:Host-associated:Human:Digestive system:Large in	0.2634
MGYA00243255/ERZ653461_FASTA.fasta	MGYA00243255	ERZ653461	root:Host-associated:Human:Digestive system:Large in	0.3842
MGYA00243256/ERZ653463_FASTA.fasta	MGYA00243256	ERZ653463	root:Host-associated:Human:Digestive system:Large in	0.3544
MGYA00243257/ERZ653465_FASTA.fasta	MGYA00243257	ERZ653465	root:Host-associated:Human:Digestive system:Large in	0.4614
MGYA00243258/ERZ653467_FASTA.fasta	MGYA00243258	ERZ653467	root:Host-associated:Human:Digestive system:Large in	0.404
MGYA00243259/ERZ653469_FASTA.fasta	MGYA00243259	ERZ653469	root:Host-associated:Human:Digestive system:Large in	0.3576
MGYA00243260/ERZ654246_FASTA.fasta	MGYA00243260	ERZ654246	root:Host-associated:Human:Digestive system:Large in	0.3148
MGYA00243261/ERZ654248_FASTA.fasta	MGYA00243261	ERZ654248	root:Host-associated:Human:Digestive system:Large in	0.1462
MGYA00243262/ERZ653931_FASTA.fasta	MGYA00243262	ERZ653931	root:Host-associated:Human:Digestive system:Large in	0.441
MGYA00243263/ERZ653933_FASTA.fasta	MGYA00243263	ERZ653933	root:Host-associated:Human:Digestive system:Large in	0.246
MGYA00243264/ERZ653935_FASTA.fasta	MGYA00243264	ERZ653935	root:Host-associated:Human:Digestive system:Large in	0.2508
MGYA00243265/ERZ653937_FASTA.fasta	MGYA00243265	ERZ653937	root:Host-associated:Human:Digestive system:Large in	0.2932
MGYA00243266/ERZ653939_FASTA.fasta	MGYA00243266	ERZ653939	root:Host-associated:Human:Digestive system:Large in	0.2576
MGYA00243267/ERZ653941_FASTA.fasta	MGYA00243267	ERZ653941	root:Host-associated:Human:Digestive system:Large in	0.3604
MGYA00243268/ERZ653943_FASTA.fasta	MGYA00243268	ERZ653943	root:Host-associated:Human:Digestive system:Large in	0.3556
MGYA00243269/ERZ653945_FASTA.fasta	MGYA00243269	ERZ653945	root:Host-associated:Human:Digestive system:Large in	0.3638
MGYA00243270/ERZ653947_FASTA.fasta	MGYA00243270	ERZ653947	root:Host-associated:Human:Digestive system:Large in	0.2502
MGYA00243271/ERZ653949_FASTA.fasta	MGYA00243271	ERZ653949	root:Host-associated:Human:Digestive system:Large in	0.3094
MGYA00243272/ERZ653951_FASTA.fasta	MGYA00243272	ERZ653951	root:Host-associated:Human:Digestive system:Large in	0.2674
MGYA00243273/ERZ653953_FASTA.fasta	MGYA00243273	ERZ653953	root:Host-associated:Human:Digestive system:Large in	0.2832
MGYA00243274/ERZ653955_FASTA.fasta	MGYA00243274	ERZ653955	root:Host-associated:Human:Digestive system:Large in	0.2776
MGYA00243275/ERZ653959_FASTA.fasta	MGYA00243275	ERZ653959	root:Host-associated:Human:Digestive system:Large in	0.2544
MGYA00243276/ERZ653961_FASTA.fasta	MGYA00243276	ERZ653961	root:Host-associated:Human:Digestive system:Large in	0.2174
MGYA00243277/ERZ653963_FASTA.fasta	MGYA00243277	ERZ653963	root:Host-associated:Human:Digestive system:Large in	0.2586
MGYA00243278/ERZ653965_FASTA.fasta	MGYA00243278	ERZ653965	root:Host-associated:Human:Digestive system:Large in	0.245
MGYA00243279/ERZ653967_FASTA.fasta	MGYA00243279	ERZ653967	root:Host-associated:Human:Digestive system:Large in	0.1312
MGYA00243280/ERZ653969_FASTA.fasta	MGYA00243280	ERZ653969	root:Host-associated:Human:Digestive system:Large in	0.2358
MGYA00243281/ERZ653971_FASTA.fasta	MGYA00243281	ERZ653971	root:Host-associated:Human:Digestive system:Large in	0.2684
MGYA00243282/ERZ653973_FASTA.fasta	MGYA00243282	ERZ653973	root:Host-associated:Human:Digestive system:Large in	0.3078
MGYA00243283/ERZ654497_FASTA.fasta	MGYA00243283	ERZ654497	root:Host-associated:Human:Digestive system:Large in	0.3066
MGYA00243284/ERZ654499_FASTA.fasta	MGYA00243284	ERZ654499	root:Host-associated:Human:Digestive system:Large in	0.3074
MGYA00243285/ERZ654501_FASTA.fasta	MGYA00243285	ERZ654501	root:Host-associated:Human:Digestive system:Large in	0.3428
MGYA00243286/ERZ654505_FASTA.fasta	MGYA00243286	ERZ654505	root:Host-associated:Human:Digestive system:Large in	0.3862
MGYA00243287/ERZ654507_FASTA.fasta	MGYA00243287	ERZ654507	root:Host-associated:Human:Digestive system:Large in	0.3534
MGYA00243288/ERZ653471_FASTA.fasta	MGYA00243288	ERZ653471	root:Host-associated:Human:Digestive system:Large in	0.3474
MGYA00243289/ERZ653473_FASTA.fasta	MGYA00243289	ERZ653473	root:Host-associated:Human:Digestive system:Large in	0.287
MGYA00243290/ERZ653475_FASTA.fasta	MGYA00243290	ERZ653475	root:Host-associated:Human:Digestive system:Large in	0.2834
MGYA00243291/ERZ653477_FASTA.fasta	MGYA00243291	ERZ653477	root:Host-associated:Human:Digestive system:Large in	0.3262
MGYA00243292/ERZ653479_FASTA.fasta	MGYA00243292	ERZ653479	root:Host-associated:Human:Digestive system:Large in	0.345
MGYA00243293/ERZ653481_FASTA.fasta	MGYA00243293	ERZ653481	root:Host-associated:Human:Digestive system:Large in	0.3364
MGYA00243294/ERZ653483_FASTA.fasta	MGYA00243294	ERZ653483	root:Host-associated:Human:Digestive system:Large in	0.3906
MGYA00243295/ERZ653487_FASTA.fasta	MGYA00243295	ERZ653487	root:Host-associated:Human:Digestive system:Large in	0.3594
MGYA00243296/ERZ653489_FASTA.fasta	MGYA00243296	ERZ653489	root:Host-associated:Human:Digestive system:Large in	0.3716
MGYA00243297/ERZ653491_FASTA.fasta	MGYA00243297	ERZ653491	root:Host-associated:Human:Digestive system:Large in	0.3748
MGYA00243298/ERZ653493_FASTA.fasta	MGYA00243298	ERZ653493	root:Host-associated:Human:Digestive system:Large in	0.3424
MGYA00243299/ERZ653495_FASTA.fasta	MGYA00243299	ERZ653495	root:Host-associated:Human:Digestive system:Large in	0.3234
MGYA00243300/ERZ653497_FASTA.fasta	MGYA00243300	ERZ653497	root:Host-associated:Human:Digestive system:Large in	0.2792
MGYA00243301/ERZ653499_FASTA.fasta	MGYA00243301	ERZ653499	root:Host-associated:Human:Digestive system:Large in	0.3622
MGYA00243302/ERZ653501_FASTA.fasta	MGYA00243302	ERZ653501	root:Host-associated:Human:Digestive system:Large in	0.2538
MGYA00243303/ERZ653503_FASTA.fasta	MGYA00243303	ERZ653503	root:Host-associated:Human:Digestive system:Large in	0.3558
MGYA00243304/ERZ653505_FASTA.fasta	MGYA00243304	ERZ653505	root:Host-associated:Human:Digestive system:Large in	0.3762
MGYA00243305/ERZ653507_FASTA.fasta	MGYA00243305	ERZ653507	root:Host-associated:Human:Digestive system:Large in	0.3632
MGYA00243306/ERZ653509_FASTA.fasta	MGYA00243306	ERZ653509	root:Host-associated:Human:Digestive system:Large in	0.3618
MGYA00243307/ERZ653511_FASTA.fasta	MGYA00243307	ERZ653511	root:Host-associated:Human:Digestive system:Large in	0.3422
MGYA00243308/ERZ654319_FASTA.fasta	MGYA00243308	ERZ654319	root:Host-associated:Human:Digestive system:Large in	0.272
MGYA00243309/ERZ653975_FASTA.fasta	MGYA00243309	ERZ653975	root:Host-associated:Human:Digestive system:Large in	0.2906
MGYA00243310/ERZ653977_FASTA.fasta	MGYA00243310	ERZ653977	root:Host-associated:Human:Digestive system:Large in	0.2602
MGYA00243311/ERZ653979_FASTA.fasta	MGYA00243311	ERZ653979	root:Host-associated:Human:Digestive system:Large in	0.2412
MGYA00243312/ERZ653981_FASTA.fasta	MGYA00243312	ERZ653981	root:Host-associated:Human:Digestive system:Large in	0.3624
MGYA00243313/ERZ653983_FASTA.fasta	MGYA00243313	ERZ653983	root:Host-associated:Human:Digestive system:Large in	0.3044
MGYA00243314/ERZ653985_FASTA.fasta	MGYA00243314	ERZ653985	root:Host-associated:Human:Digestive system:Large in	0.2054
MGYA00243315/ERZ653987_FASTA.fasta	MGYA00243315	ERZ653987	root:Host-associated:Human:Digestive system:Large in	0.2226
MGYA00243316/ERZ653989_FASTA.fasta	MGYA00243316	ERZ653989	root:Host-associated:Human:Digestive system:Large in	0.3364
MGYA00243317/ERZ653991_FASTA.fasta	MGYA00243317	ERZ653991	root:Host-associated:Human:Digestive system:Large in	0.303
MGYA00243318/ERZ653993_FASTA.fasta	MGYA00243318	ERZ653993	root:Host-associated:Human:Digestive system:Large in	0.3328
MGYA00243319/ERZ653995_FASTA.fasta	MGYA00243319	ERZ653995	root:Host-associated:Human:Digestive system:Large in	0.2774
MGYA00243320/ERZ653997_FASTA.fasta	MGYA00243320	ERZ653997	root:Host-associated:Human:Digestive system:Large in	0.2882
MGYA00243321/ERZ653999_FASTA.fasta	MGYA00243321	ERZ653999	root:Host-associated:Human:Digestive system:Large in	0.0708
MGYA00243322/ERZ654001_FASTA.fasta	MGYA00243322	ERZ654001	root:Host-associated:Human:Digestive system:Large in	0.3268
MGYA00243323/ERZ654003_FASTA.fasta	MGYA00243323	ERZ654003	root:Host-associated:Human:Digestive system:Large in	0.2748
MGYA00243324/ERZ654005_FASTA.fasta	MGYA00243324	ERZ654005	root:Host-associated:Human:Digestive system:Large in	0.1308
MGYA00243325/ERZ654007_FASTA.fasta	MGYA00243325	ERZ654007	root:Host-associated:Human:Digestive system:Large in	0.2878
MGYA00243326/ERZ654009_FASTA.fasta	MGYA00243326	ERZ654009	root:Host-associated:Human:Digestive system:Large in	0.2402
MGYA00243327/ERZ654011_FASTA.fasta	MGYA00243327	ERZ654011	root:Host-associated:Human:Digestive system:Large in	0.2434
MGYA00243328/ERZ654013_FASTA.fasta	MGYA00243328	ERZ654013	root:Host-associated:Human:Digestive system:Large in	0.267
MGYA00243329/ERZ654015_FASTA.fasta	MGYA00243329	ERZ654015	root:Host-associated:Human:Digestive system:Large in	0.292
MGYA00243330/ERZ654017_FASTA.fasta	MGYA00243330	ERZ654017	root:Host-associated:Human:Digestive system:Large in	0.2736
MGYA00243331/ERZ654509_FASTA.fasta	MGYA00243331	ERZ654509	root:Host-associated:Human:Digestive system:Large in	0.2988
MGYA00243332/ERZ654511_FASTA.fasta	MGYA00243332	ERZ654511	root:Host-associated:Human:Digestive system:Large in	0.378
MGYA00243333/ERZ654513_FASTA.fasta	MGYA00243333	ERZ654513	root:Host-associated:Human:Digestive system:Large in	0.3198
MGYA00243334/ERZ654515_FASTA.fasta	MGYA00243334	ERZ654515	root:Host-associated:Human:Digestive system:Large in	0.295
MGYA00243335/ERZ654517_FASTA.fasta	MGYA00243335	ERZ654517	root:Host-associated:Human:Digestive system:Large in	0.3102
MGYA00243336/ERZ654519_FASTA.fasta	MGYA00243336	ERZ654519	root:Host-associated:Human:Digestive system:Large in	0.279
MGYA00243337/ERZ654521_FASTA.fasta	MGYA00243337	ERZ654521	root:Host-associated:Human:Digestive system:Large in	0.314
MGYA00243338/ERZ654523_FASTA.fasta	MGYA00243338	ERZ654523	root:Host-associated:Human:Digestive system:Large in	0.3164
MGYA00243339/ERZ654525_FASTA.fasta	MGYA00243339	ERZ654525	root:Host-associated:Human:Digestive system:Large in	0.2736
MGYA00243340/ERZ654527_FASTA.fasta	MGYA00243340	ERZ654527	root:Host-associated:Human:Digestive system:Large in	0.4082

MGYA00243341/ERZ654529_FASTA.fasta	MGYA00243341	ERZ654529	root:Host-associated:Human:Digestive system:Large int	0.3768
MGYA00243342/ERZ654531_FASTA.fasta	MGYA00243342	ERZ654531	root:Host-associated:Human:Digestive system:Large int	0.2984
MGYA00243343/ERZ654533_FASTA.fasta	MGYA00243343	ERZ654533	root:Host-associated:Human:Digestive system:Large int	0.312
MGYA00243344/ERZ654535_FASTA.fasta	MGYA00243344	ERZ654535	root:Host-associated:Human:Digestive system:Large int	0.4124
MGYA00243345/ERZ654537_FASTA.fasta	MGYA00243345	ERZ654537	root:Host-associated:Human:Digestive system:Large int	0.265
MGYA00243346/ERZ654539_FASTA.fasta	MGYA00243346	ERZ654539	root:Host-associated:Human:Digestive system:Large int	0.332
MGYA00243347/ERZ654541_FASTA.fasta	MGYA00243347	ERZ654541	root:Host-associated:Human:Digestive system:Large int	0.3214
MGYA00243348/ERZ654543_FASTA.fasta	MGYA00243348	ERZ654543	root:Host-associated:Human:Digestive system:Large int	0.2988
MGYA00243349/ERZ654545_FASTA.fasta	MGYA00243349	ERZ654545	root:Host-associated:Human:Digestive system:Large int	0.3818
MGYA00243350/ERZ654547_FASTA.fasta	MGYA00243350	ERZ654547	root:Host-associated:Human:Digestive system:Large int	0.3322
MGYA00243351/ERZ654549_FASTA.fasta	MGYA00243351	ERZ654549	root:Host-associated:Human:Digestive system:Large int	0.4508
MGYA00243352/ERZ654551_FASTA.fasta	MGYA00243352	ERZ654551	root:Host-associated:Human:Digestive system:Large int	0.2982
MGYA00243353/ERZ653513_FASTA.fasta	MGYA00243353	ERZ653513	root:Host-associated:Human:Digestive system:Large int	0.3112
MGYA00243354/ERZ653515_FASTA.fasta	MGYA00243354	ERZ653515	root:Host-associated:Human:Digestive system:Large int	0.2744
MGYA00243355/ERZ653517_FASTA.fasta	MGYA00243355	ERZ653517	root:Host-associated:Human:Digestive system:Large int	0.2914
MGYA00243356/ERZ653519_FASTA.fasta	MGYA00243356	ERZ653519	root:Host-associated:Human:Digestive system:Large int	0.3742
MGYA00243357/ERZ653521_FASTA.fasta	MGYA00243357	ERZ653521	root:Host-associated:Human:Digestive system:Large int	0.3842
MGYA00243358/ERZ653523_FASTA.fasta	MGYA00243358	ERZ653523	root:Host-associated:Human:Digestive system:Large int	0.4614
MGYA00243359/ERZ653525_FASTA.fasta	MGYA00243359	ERZ653525	root:Host-associated:Human:Digestive system:Large int	0.325
MGYA00243360/ERZ653527_FASTA.fasta	MGYA00243360	ERZ653527	root:Host-associated:Human:Digestive system:Large int	0.3124
MGYA00243361/ERZ653529_FASTA.fasta	MGYA00243361	ERZ653529	root:Host-associated:Human:Digestive system:Large int	0.1796
MGYA00243362/ERZ653531_FASTA.fasta	MGYA00243362	ERZ653531	root:Host-associated:Human:Digestive system:Large int	0.3352
MGYA00243363/ERZ653533_FASTA.fasta	MGYA00243363	ERZ653533	root:Host-associated:Human:Digestive system:Large int	0.4106
MGYA00243364/ERZ653535_FASTA.fasta	MGYA00243364	ERZ653535	root:Host-associated:Human:Digestive system:Large int	0.4484
MGYA00243365/ERZ653537_FASTA.fasta	MGYA00243365	ERZ653537	root:Host-associated:Human:Digestive system:Large int	0.343
MGYA00243366/ERZ653539_FASTA.fasta	MGYA00243366	ERZ653539	root:Host-associated:Human:Digestive system:Large int	0.348
MGYA00243367/ERZ653541_FASTA.fasta	MGYA00243367	ERZ653541	root:Host-associated:Human:Digestive system:Large int	0.3194
MGYA00243368/ERZ653543_FASTA.fasta	MGYA00243368	ERZ653543	root:Host-associated:Human:Digestive system:Large int	0.3158
MGYA00243369/ERZ653545_FASTA.fasta	MGYA00243369	ERZ653545	root:Host-associated:Human:Digestive system:Large int	0.3392
MGYA00243370/ERZ653547_FASTA.fasta	MGYA00243370	ERZ653547	root:Host-associated:Human:Digestive system:Large int	0.3724
MGYA00243371/ERZ653549_FASTA.fasta	MGYA00243371	ERZ653549	root:Host-associated:Human:Digestive system:Large int	0.2594
MGYA00243372/ERZ653551_FASTA.fasta	MGYA00243372	ERZ653551	root:Host-associated:Human:Digestive system:Large int	0.3104
MGYA00243373/ERZ653267_FASTA.fasta	MGYA00243373	ERZ653267	root:Host-associated:Human:Digestive system:Large int	0.1938
MGYA00243374/ERZ653269_FASTA.fasta	MGYA00243374	ERZ653269	root:Host-associated:Human:Digestive system:Large int	0.0456
MGYA00243375/ERZ653271_FASTA.fasta	MGYA00243375	ERZ653271	root:Host-associated:Human:Digestive system:Large int	0.0714
MGYA00243376/ERZ653273_FASTA.fasta	MGYA00243376	ERZ653273	root:Host-associated:Human:Digestive system:Large int	0.0794
MGYA00243377/ERZ653275_FASTA.fasta	MGYA00243377	ERZ653275	root:Host-associated:Human:Digestive system:Large int	0.0548
MGYA00243378/ERZ653277_FASTA.fasta	MGYA00243378	ERZ653277	root:Host-associated:Human:Digestive system:Large int	0.1334
MGYA00243379/ERZ653279_FASTA.fasta	MGYA00243379	ERZ653279	root:Host-associated:Human:Digestive system:Large int	0.1212
MGYA00243380/ERZ653281_FASTA.fasta	MGYA00243380	ERZ653281	root:Host-associated:Human:Digestive system:Large int	0.14
MGYA00243381/ERZ653283_FASTA.fasta	MGYA00243381	ERZ653283	root:Host-associated:Human:Digestive system:Large int	0.1022
MGYA00243382/ERZ653285_FASTA.fasta	MGYA00243382	ERZ653285	root:Host-associated:Human:Digestive system:Large int	0.2402
MGYA00243383/ERZ653287_FASTA.fasta	MGYA00243383	ERZ653287	root:Host-associated:Human:Digestive system:Large int	0.068
MGYA00243384/ERZ653289_FASTA.fasta	MGYA00243384	ERZ653289	root:Host-associated:Human:Digestive system:Large int	0.0624
MGYA00243385/ERZ653291_FASTA.fasta	MGYA00243385	ERZ653291	root:Host-associated:Human:Digestive system:Large int	0.1182
MGYA00243386/ERZ653293_FASTA.fasta	MGYA00243386	ERZ653293	root:Host-associated:Human:Digestive system:Large int	0.1218
MGYA00243387/ERZ653295_FASTA.fasta	MGYA00243387	ERZ653295	root:Host-associated:Human:Digestive system:Large int	0.1296
MGYA00243388/ERZ653297_FASTA.fasta	MGYA00243388	ERZ653297	root:Host-associated:Human:Digestive system:Large int	0.1442
MGYA00243389/ERZ653299_FASTA.fasta	MGYA00243389	ERZ653299	root:Host-associated:Human:Digestive system:Large int	0.1234
MGYA00243390/ERZ653301_FASTA.fasta	MGYA00243390	ERZ653301	root:Host-associated:Human:Digestive system:Large int	0.1308
MGYA00243391/ERZ653303_FASTA.fasta	MGYA00243391	ERZ653303	root:Host-associated:Human:Digestive system:Large int	0.2938
MGYA00243392/ERZ653305_FASTA.fasta	MGYA00243392	ERZ653305	root:Host-associated:Human:Digestive system:Large int	0.2216
MGYA00243393/ERZ653307_FASTA.fasta	MGYA00243393	ERZ653307	root:Host-associated:Human:Digestive system:Large int	0.1712
MGYA00243394/ERZ653309_FASTA.fasta	MGYA00243394	ERZ653309	root:Host-associated:Human:Digestive system:Large int	0.0406
MGYA00243395/ERZ654320_FASTA.fasta	MGYA00243395	ERZ654320	root:Host-associated:Human:Digestive system:Large int	0.3132
MGYA00243396/ERZ654321_FASTA.fasta	MGYA00243396	ERZ654321	root:Host-associated:Human:Digestive system:Large int	0.2762
MGYA00243397/ERZ654322_FASTA.fasta	MGYA00243397	ERZ654322	root:Host-associated:Human:Digestive system:Large int	0.166
MGYA00243398/ERZ654323_FASTA.fasta	MGYA00243398	ERZ654323	root:Host-associated:Human:Digestive system:Large int	0.3428
MGYA00243399/ERZ654324_FASTA.fasta	MGYA00243399	ERZ654324	root:Host-associated:Human:Digestive system:Large int	0.3062
MGYA00243400/ERZ654325_FASTA.fasta	MGYA00243400	ERZ654325	root:Host-associated:Human:Digestive system:Large int	0.2666
MGYA00243401/ERZ654326_FASTA.fasta	MGYA00243401	ERZ654326	root:Host-associated:Human:Digestive system:Large int	0.4062
MGYA00243402/ERZ654327_FASTA.fasta	MGYA00243402	ERZ654327	root:Host-associated:Human:Digestive system:Large int	0.1004
MGYA00243403/ERZ654328_FASTA.fasta	MGYA00243403	ERZ654328	root:Host-associated:Human:Digestive system:Large int	0.2752
MGYA00243404/ERZ654329_FASTA.fasta	MGYA00243404	ERZ654329	root:Host-associated:Human:Digestive system:Large int	0.286
MGYA00243405/ERZ654332_FASTA.fasta	MGYA00243405	ERZ654332	root:Host-associated:Human:Digestive system:Large int	0.2664
MGYA00243406/ERZ654334_FASTA.fasta	MGYA00243406	ERZ654334	root:Host-associated:Human:Digestive system:Large int	0.2818
MGYA00243407/ERZ654336_FASTA.fasta	MGYA00243407	ERZ654336	root:Host-associated:Human:Digestive system:Large int	0.252
MGYA00243408/ERZ654338_FASTA.fasta	MGYA00243408	ERZ654338	root:Host-associated:Human:Digestive system:Large int	0.4058
MGYA00243409/ERZ654340_FASTA.fasta	MGYA00243409	ERZ654340	root:Host-associated:Human:Digestive system:Large int	0.2824
MGYA00243410/ERZ654342_FASTA.fasta	MGYA00243410	ERZ654342	root:Host-associated:Human:Digestive system:Large int	0.2668
MGYA00243411/ERZ654344_FASTA.fasta	MGYA00243411	ERZ654344	root:Host-associated:Human:Digestive system:Large int	0.2946
MGYA00243412/ERZ654346_FASTA.fasta	MGYA00243412	ERZ654346	root:Host-associated:Human:Digestive system:Large int	0.2896
MGYA00243413/ERZ654348_FASTA.fasta	MGYA00243413	ERZ654348	root:Host-associated:Human:Digestive system:Large int	0.2718
MGYA00243414/ERZ654350_FASTA.fasta	MGYA00243414	ERZ654350	root:Host-associated:Human:Digestive system:Large int	0.2228
MGYA00243415/ERZ654352_FASTA.fasta	MGYA00243415	ERZ654352	root:Host-associated:Human:Digestive system:Large int	0.315
MGYA00243416/ERZ654354_FASTA.fasta	MGYA00243416	ERZ654354	root:Host-associated:Human:Digestive system:Large int	0.3336
MGYA00243417/ERZ652798_FASTA.fasta	MGYA00243417	ERZ652798	root:Host-associated:Human:Digestive system:Large int	0.1106
MGYA00243418/ERZ652800_FASTA.fasta	MGYA00243418	ERZ652800	root:Host-associated:Human:Digestive system:Large int	0.3136
MGYA00243419/ERZ652802_FASTA.fasta	MGYA00243419	ERZ652802	root:Host-associated:Human:Digestive system:Large int	0.2528
MGYA00243420/ERZ652804_FASTA.fasta	MGYA00243420	ERZ652804	root:Host-associated:Human:Digestive system:Large int	0.192
MGYA00243421/ERZ652806_FASTA.fasta	MGYA00243421	ERZ652806	root:Host-associated:Human:Digestive system:Large int	0.253
MGYA00243422/ERZ652808_FASTA.fasta	MGYA00243422	ERZ652808	root:Host-associated:Human:Digestive system:Large int	0.2798
MGYA00243423/ERZ652810_FASTA.fasta	MGYA00243423	ERZ652810	root:Host-associated:Human:Digestive system:Large int	0.2798
MGYA00243424/ERZ652812_FASTA.fasta	MGYA00243424	ERZ652812	root:Host-associated:Human:Digestive system:Large int	0.2732
MGYA00243425/ERZ652814_FASTA.fasta	MGYA00243425	ERZ652814	root:Host-associated:Human:Digestive system:Large int	0.2834
MGYA00243426/ERZ652816_FASTA.fasta	MGYA00243426	ERZ652816	root:Host-associated:Human:Digestive system:Large int	0.2604
MGYA00243427/ERZ652818_FASTA.fasta	MGYA00243427	ERZ652818	root:Host-associated:Human:Digestive system:Large int	0.2622
MGYA00243428/ERZ652820_FASTA.fasta	MGYA00243428	ERZ652820	root:Host-associated:Human:Digestive system:Large int	0.2636
MGYA00243429/ERZ652822_FASTA.fasta	MGYA00243429	ERZ652822	root:Host-associated:Human:Digestive system:Large int	0.3004
MGYA00243430/ERZ652824_FASTA.fasta	MGYA00243430	ERZ652824	root:Host-associated:Human:Digestive system:Large int	0.3194
MGYA00243431/ERZ652826_FASTA.fasta	MGYA00243431	ERZ652826	root:Host-associated:Human:Digestive system:Large int	0.351
MGYA00243432/ERZ654019_FASTA.fasta	MGYA00243432	ERZ654019	root:Host-associated:Human:Digestive system:Large int	0.2852
MGYA00243433/ERZ654021_FASTA.fasta	MGYA00243433	ERZ654021	root:Host-associated:Human:Digestive system:Large int	0.2684
MGYA00243434/ERZ654023_FASTA.fasta	MGYA00243434	ERZ654023	root:Host-associated:Human:Digestive system:Large int	0.3862
MGYA00243435/ERZ654025_FASTA.fasta	MGYA00243435	ERZ654025	root:Host-associated:Human:Digestive system:Large int	0.3348
MGYA00243436/ERZ654027_FASTA.fasta	MGYA00243436	ERZ654027	root:Host-associated:Human:Digestive system:Large int	0.3116
MGYA00243437/ERZ654029_FASTA.fasta	MGYA00243437	ERZ654029	root:Host-associated:Human:Digestive system:Large int	0.2626
MGYA00243438/ERZ654031_FASTA.fasta	MGYA00243438	ERZ654031	root:Host-associated:Human:Digestive system:Large int	0.2716
MGYA00243439/ERZ654033_FASTA.fasta	MGYA00243439	ERZ654033	root:Host-associated:Human:Digestive system:Large int	0.343
MGYA00243440/ERZ654035_FASTA.fasta	MGYA00243440	ERZ654035	root:Host-associated:Human:Digestive system:Large int	0.3228
MGYA00243441/ERZ654037_FASTA.fasta	MGYA00243441	ERZ654037	root:Host-associated:Human:Digestive system:Large int	0.2294
MGYA00243442/ERZ654039_FASTA.fasta	MGYA00243442	ERZ654039	root:Host-associated:Human:Digestive system:Large int	0.284
MGYA00243443/ERZ654041_FASTA.fasta	MGYA00243443	ERZ654041	root:Host-associated:Human:Digestive system:Large int	0.29
MGYA00243444/ERZ654043_FASTA.fasta	MGYA00243444	ERZ654043	root:Host-associated:Human:Digestive system:Large int	0.3078
MGYA00243445/ERZ654045_FASTA.fasta	MGYA00243445	ERZ654045	root:Host-associated:Human:Digestive system:Large int	0.3306
MGYA00243446/ERZ654047_FASTA.fasta	MGYA00243446	ERZ654047	root:Host-associated:Human:Digestive system:Large int	0.3754
MGYA00243447/ERZ654049_FASTA.fasta	MGYA00243447	ERZ654049	root:Host-associated:Human:Digestive system:Large int	0.2896
MGYA00243448/ERZ654051_FASTA.fasta	MGYA00243448	ERZ654051	root:Host-associated:Human:Digestive system:Large int	0.2186
MGYA00243449/ERZ654053_FASTA.fasta	MGYA00243449	ERZ654053	root:Host-associated:Human:Digestive system:Large int	0.2468
MGYA00243450/ERZ654055_FASTA.fasta	MGYA00243450	ERZ654055	root:Host-associated:Human:Digestive system:Large int	0.2822
MGYA00243451/ERZ654057_FASTA.fasta	MGYA00243451	ERZ654057	root:Host-associated:Human:Digestive system:Large int	0.2724
MGYA00243452/ERZ654059_FASTA.fasta	MGYA00243452	ERZ654059	root:Host-associated:Human:Digestive system:Large int	0.3144
MGYA00243453/ERZ654061_FASTA.fasta	MGYA00243453	ERZ654061	root:Host-associated:Human:Digestive system:Large int	0.2938
MGYA00243454/ERZ652875_FASTA.fasta	MGYA00243454	ERZ652875	root:Host-associated:Human:Digestive system:Large int	0.307
MGYA00243455/ERZ652877_FASTA.fasta	MGYA00243455	ERZ652877	root:Host-associated:Human:Digestive system:Large int	0.312
MGYA00243456/ERZ652879_FASTA.fasta	MGYA00243456	ERZ652879	root:Host-associated:Human:Digestive system:Large int	0.2656
MGYA00243457/ERZ652881_FASTA.fasta	MGYA00243457	ERZ652881	root:Host-associated:Human:Digestive system:Large int	0.4318
MGYA00243458/ERZ652883_FASTA.fasta	MGYA00243458	ERZ652883	root:Host-associated:Human:Digestive system:Large int	0.26
MGYA00243459/ERZ652885_FASTA.fasta	MGYA00243459	ERZ652885	root:Host-associated:Human:Digestive system:Large int	0.162
MGYA00243460/ERZ652887_FASTA.fasta	MGYA00243460	ERZ652887	root:Host-associated:Human:Digestive system:Large int	0.362
MGYA00243461/ERZ652889_FASTA.fasta	MGYA00243461	ERZ652889	root:Host-associated:Human:Digestive system:Large int	0.246
MGYA00243462/ERZ652891_FASTA.fasta	MGYA00243462	ERZ652891	root:Host-associated:Human:Digestive system:Large int	0.3284
MGYA00243463/ERZ652893_FASTA.fasta	MGYA00243463	ERZ652893	root:Host-associated:Human:Digestive system:Large int	0.2534
MGYA00243464/ERZ652895_FASTA.fasta	MGYA00243464	ERZ652895	root:Host-associated:Human:Digestive system:Large int	0.2604
MGYA00243465/ERZ652897_FASTA.fasta	MGYA00243465	ERZ652897	root:Host-associated:Human:Digestive system:Large int	0.3184
MGYA00243466/ERZ652899_FASTA.fasta	MGYA00243466	ERZ652899	root:Host-associated:Human:Digestive system:Large int	0.221
MGYA00243467/ERZ654553_FASTA.fasta	MGYA00243467	ERZ654553	root:Host-associated:Human:Digestive system:Large int	0.3722
MGYA00243468/ERZ654555_FASTA.fasta	MGYA00243468	ERZ654555	root:Host-associated:Human:Digestive system:Large int	0.3446
MGYA00243469/ERZ654557_FASTA.fasta	MGYA00243469	ERZ654557	root:Host-associated:Human:Digestive system:Large int	0.3326
MGYA00243470/ERZ654559_FASTA.fasta	MGYA00243470	ERZ654559	root:Host-associated:Human:Digestive system:Large int	0.3812
MGYA00243471/ERZ654561_FASTA.fasta	MGYA00243471	ERZ654561	root:Host-associated:Human:Digestive system:Large int	0.428
MGYA00243472/ERZ654562_FASTA.fasta	MGYA00243472	ERZ654562	root:Host-associated:Human:Digestive system:Large int	0.5064
MGYA00243473/ERZ654563_FASTA.fasta	MGYA00243473	ERZ654563	root:Host-associated:Human:Digestive system:Large int	0.3238
MGYA00243474/ERZ654565_FASTA.fasta	MGYA00243474	ERZ654565	root:Host-associated:Human:Digestive system:Large int	0.4038
MGYA00243475/ERZ654567_FASTA.fasta	MGYA00243475	ERZ654567	root:Host-associated:Human:Digestive system:Large int	0.4878
MGYA00243476/ERZ654569_FASTA.fasta	MGYA00243476	ERZ654569	root:Host-associated:Human:Digestive system:Large int	0.3872
MGYA00243477/ERZ654571_FASTA.fasta	MGYA00243477	ERZ654571	root:Host-associated:Human:Digestive system:Large int	0.2954
MGYA00243478/ERZ654573_FASTA.fasta	MGYA00243478	ERZ654573	root:Host-associated:Human:Digestive system:Large int	0.3316
MGYA00243479/ERZ654575_FASTA.fasta	MGYA00243479	ERZ654575	root:Host-associated:Human:Digestive system:Large int	0.3738
MGYA00243480/ERZ654577_FASTA.fasta	MGYA00243480	ERZ654577	root:Host-associated:Human:Digestive system:Large int	0.397
MGYA00243481/ERZ654579_FASTA.fasta	MGYA00243481	ERZ654579	root:Host-associated:Human:Digestive system:Large int	0.3776
MGYA00243482/ERZ654581_FASTA.fasta	MGYA00243482	ERZ654581	root:Host-associated:Human:Digestive system:Large int	0.3188
MGYA00243483/ERZ653553_FASTA.fasta	MGYA00243483	ERZ653553	root:Host-associated:Human:Digestive system:Large int	0.3022
MGYA00243484/ERZ653555_FASTA.fasta	MGYA00243484	ERZ653555	root:Host-associated:Human:Digestive system:Large int	0.368
MGYA00243485/ERZ653557_FASTA.fasta	MGYA00243485	ERZ653557	root:Host-associated:Human:Digestive system:Large int	0.289
MGYA00243486/ERZ653559_FASTA.fasta	MGYA00243486	ERZ653559	root:Host-associated:Human:Digestive system:Large int	0.4292
MGYA00243487/ERZ653561_FASTA.fasta	MGYA00243487	ERZ653561	root:Host-associated:Human:Digestive system:Large int	0.4378
MGYA00243488/ERZ653563_FASTA.fasta	MGYA00243488	ERZ653563	root:Host-associated:Human:Digestive system:Large int	0.272
MGYA00243489/ERZ653564_FASTA.fasta	MGYA00243489	ERZ653564	root:Host-associated:Human:Digestive system:Large int	0.2774
MGYA00243490/ERZ653847_FASTA.fasta	MGYA00243490	ERZ653847	root:Host-associated:Human:Digestive system:Large int	0.236
MGYA00243491/ERZ653848_FASTA.fasta	MGYA00243491	ERZ653848	root:Host-associated:Human:Digestive system:Large int	0.3892
MGYA00243492/ERZ653850_FASTA.fasta	MGYA00243492	ERZ653850	root:Host-associated:Human:Digestive system:Large int	0.2918
MGYA00243493/ERZ653852_FASTA.fasta	MGYA00243493	ERZ653852	root:Host-associated:Human:Digestive system:Large int	0.337
MGYA00243494/ERZ653854_FASTA.fasta	MGYA00243494	ERZ653854	root:Host-associated:Human:Digestive system:Large int	0.1928
MGYA00243495/ERZ653856_FASTA.fasta	MGYA00243495	ERZ653856	root:Host-associated:Human:Digestive system:Large int	0.2536
MGYA00243496/ERZ653858_FASTA.fasta	MGYA00243496	ERZ653858	root:Host-associated:Human:Digestive system:Large int	0.2974
MGYA00243497/ERZ653860_FASTA.fasta	MGYA00243497	ERZ653860	root:Host-associated:Human:Digestive system:Large int	0.2422
MGYA00243498/ERZ653862_FASTA.fasta	MGYA00243498	ERZ653862	root:Host-associated:Human:Digestive system:Large int	0.1174
MGYA00243499/ERZ653864_FASTA.fasta	MGYA00243499	ERZ653864	root:Host-associated:Human:Digestive system:Large int	0.2784
MGYA00243500/ERZ653866_FASTA.fasta	MGYA00243500	ERZ653866	root:Host-associated:Human:Digestive system:Large int	0.1572
MGYA00243501/ERZ653868_FASTA.fasta	MGYA00243501	ERZ653868	root:Host-associated:Human:Digestive system:Large int	0.3262
MGYA00243502/ERZ653870_FASTA.fasta	MGYA00243502	ERZ653870	root:Host-associated:Human:Digestive system:Large int	0.4176
MGYA00243503/ERZ653872_FASTA.fasta	MGYA00243503	ERZ653872	root:Host-associated:Human:Digestive system:Large int	0.252
MGYA00243504/ERZ653874_FASTA.fasta	MGYA00243504	ERZ653874	root:Host-associated:Human:Digestive system:Large int	0.2656
MGYA00243505/ERZ654356_FASTA.fasta	MGYA00243505	ERZ654356	root:Host-associated:Human:Digestive system:Large int	0.2934
MGYA00243506/ERZ654358_FASTA.fasta	MGYA00243506	ERZ654358	root:Host-associated:Human:Digestive system:Large int	0.4768
MGYA00243507/ERZ654360_FASTA.fasta	MGYA00243507	ERZ654360	root:Host-associated:Human:Digestive system:Large int	0.275
MGYA00243508/ERZ654362_FASTA.fasta	MGYA00243508	ERZ654362	root:Host-associated:Human:Digestive system:Large int	0.2048
MGYA00243509/ERZ654364_FASTA.fasta	MGYA00243509	ERZ654364	root:Host-associated:Human:Digestive system:Large int	0.361
MGYA00243510/ERZ654366_FASTA.fasta	MGYA00243510	ERZ654366	root:Host-associated:Human:Digestive system:Large int	0.3382
MGYA00243511/ERZ654368_FASTA.fasta	MGYA00243511	ERZ654368	root:Host-associated:Human:Digestive system:Large int	0.325
MGYA00243512/ERZ654370_FASTA.fasta	MGYA00243512	ERZ654370	root:Host-associated:Human:Digestive system:Large int	0.3504
MGYA00243513/ERZ654372_FASTA.fasta	MGYA00243513	ERZ654372	root:Host-associated:Human:Digestive system:Large int	0.244
MGYA00243514/ERZ654374_FASTA.fasta	MGYA00243514	ERZ654374	root:Host-associated:Human:Digestive system:Large int	0.2724
MGYA00243515/ERZ654376_FASTA.fasta	MGYA00243515	ERZ654376	root:Host-associated:Human:Digestive system:Large int	0.2912
MGYA00243516/ERZ654378_FASTA.fasta	MGYA00243516	ERZ654378	root:Host-associated:Human:Digestive system:Large int	0.3276
MGYA00243517/ERZ654380_FASTA.fasta	MGYA00243517	ERZ654380	root:Host-associated:Human:Digestive system:Large int	0.1372
MGYA00243518/ERZ654382_FASTA.fasta	MGYA00243518	ERZ654382	root:Host-associated:Human:Digestive system:Large int	0.36
MGYA00243519/ERZ654384_FASTA.fasta	MGYA00243519	ERZ654384	root:Host-associated:Human:Digestive system:Large int	0.177
MGYA00243520/ERZ654386_FASTA.fasta	MGYA00243520	ERZ654386	root:Host-associated:Human:Digestive system:Large int	0.275
MGYA00243521/ERZ654388_FASTA.fasta	MGYA00243521	ERZ654388	root:Host-associated:Human:Digestive system:Large int	0.3274
MGYA00243522/ERZ654390_FASTA.fasta	MGYA00243522	ERZ654390	root:Host-associated:Human:Digestive system:Large int	0.2816
MGYA00243523/ERZ654394_FASTA.fasta	MGYA00243523	ERZ654394	root:Host-associated:Human:Digestive system:Large int	0.217
MGYA00243524/ERZ654396_FASTA.fasta	MGYA00243524	ERZ654396	root:Host-associated:Human:Digestive system:Large int	0.2568
MGYA00243525/ERZ654398_FASTA.fasta	MGYA00243525	ERZ654398	root:Host-associated:Human:Digestive system:Large int	0.2282
MGYA00243526/ERZ654063_FASTA.fasta	MGYA00243526	ERZ654063	root:Host-associated:Human:Digestive system:Large int	0.2694
MGYA00243527/ERZ654065_FASTA.fasta	MGYA00243527	ERZ654065	root:Host-associated:Human:Digestive system:Large int	0.2936
MGYA00243528/ERZ654067_FASTA.fasta	MGYA00243528	ERZ654067	root:Host-associated:Human:Digestive system:Large int	0.313
MGYA00243529/ERZ654069_FASTA.fasta	MGYA00243529	ERZ654069	root:Host-associated:Human:Digestive system:Large int	0.2684
MGYA00243530/ERZ654071_FASTA.fasta	MGYA00243530	ERZ654071	root:Host-associated:Human:Digestive system:Large int	0.2566
MGYA00243531/ERZ654073_FASTA.fasta	MGYA00243531	ERZ654073	root:Host-associated:Human:Digestive system:Large int	0.3074
MGYA00243532/ERZ654075_FASTA.fasta	MGYA00243532	ERZ654075	root:Host-associated:Human:Digestive system:Large int	0.2982
MGYA00243533/ERZ654077_FASTA.fasta	MGYA00243533	ERZ654077	root:Host-associated:Human:Digestive system:Large int	0.1704
MGYA00243534/ERZ654079_FASTA.fasta	MGYA00243534	ERZ654079	root:Host-associated:Human:Digestive system:Large int	0.2886
MGYA00243535/ERZ654081_FASTA.fasta	MGYA00243535	ERZ654081	root:Host-associated:Human:Digestive system:Large int	0.247
MGYA00243536/ERZ654085_FASTA.fasta	MGYA00243536	ERZ654085	root:Host-associated:Human:Digestive system:Large int	0.1672
MGYA00243537/ERZ654087_FASTA.fasta	MGYA00243537	ERZ654087	root:Host-associated:Human:Digestive system:Large int	0.2938
MGYA00243538/ERZ654089_FASTA.fasta	MGYA00243538	ERZ654089	root:Host-associated:Human:Digestive system:Large int	0.2884
MGYA00243539/ERZ654091_FASTA.fasta	MGYA00243539	ERZ654091	root:Host-associated:Human:Digestive system:Large int	0.2794
MGYA00243540/ERZ654093_FASTA.fasta	MGYA00243540	ERZ654093	root:Host-associated:Human:Digestive system:Large int	0.2456
MGYA00243541/ERZ654095_FASTA.fasta	MGYA00243541	ERZ654095	root:Host-associated:Human:Digestive system:Large int	0.131
MGYA00243542/ERZ654097_FASTA.fasta	MGYA00243542	ERZ654097	root:Host-associated:Human:Digestive system:Large int	0.291
MGYA00243543/ERZ654099_FASTA.fasta	MGYA00243543	ERZ654099	root:Host-associated:Human:Digestive system:Large int	0.269
MGYA00243544/ERZ654101_FASTA.fasta	MGYA00243544	ERZ654101	root:Host-associated:Human:Digestive system:Large int	0.2636
MGYA00243545/ERZ654103_FASTA.fasta	MGYA00243545	ERZ654103	root:Host-associated:Human:Digestive system:Large int	0.2178
MGYA00243546/ERZ654105_FASTA.fasta	MGYA00243546	ERZ654105	root:Host-associated:Human:Digestive system:Large int	0.2386
MGYA00243547/ERZ654107_FASTA.fasta	MGYA00243547	ERZ654107	root:Host-associated:Human:Digestive system:Large int	0.2108
MGYA00243548/ERZ652901_FASTA.fasta	MGYA00243548	ERZ652901	root:Host-associated:Human:Digestive system:Large int	0.18
MGYA00243549/ERZ652903_FASTA.fasta	MGYA00243549	ERZ652903	root:Host-associated:Human:Digestive system:Large int	0.2404
MGYA00243550/ERZ652905_FASTA.fasta	MGYA00243550	ERZ652905	root:Host-associated:Human:Digestive system:Large int	0.2722
MGYA00243551/ERZ652907_FASTA.fasta	MGYA00243551	ERZ652907	root:Host-associated:Human:Digestive system:Large int	0.2654
MGYA00243552/ERZ652909_FASTA.fasta	MGYA00243552	ERZ652909	root:Host-associated:Human:Digestive system:Large int	0.229
MGYA00243553/ERZ652911_FASTA.fasta	MGYA00243553	ERZ652911	root:Host-associated:Human:Digestive system:Large int	0.3108
MGYA00243554/ERZ652913_FASTA.fasta	MGYA00243554	ERZ652913	root:Host-associated:Human:Digestive system:Large int	0.2184
MGYA00243555/ERZ652915_FASTA.fasta	MGYA00243555	ERZ652915	root:Host-associated:Human:Digestive system:Large int	0.3072
MGYA00243556/ERZ652917_FASTA.fasta	MGYA00243556	ERZ652917	root:Host-associated:Human:Digestive system:Large int	0.2842
MGYA00243557/ERZ652919_FASTA.fasta	MGYA00243557	ERZ652919	root:Host-associated:Human:Digestive system:Large int	0.28
MGYA00243558/ERZ652921_FASTA.fasta	MGYA00243558	ERZ652921	root:Host-associated:Human:Digestive system:Large int	0.327
MGYA00243559/ERZ652923_FASTA.fasta	MGYA00243559	ERZ652923	root:Host-associated:Human:Digestive system:Large int	0.3548
MGYA00243560/ERZ652925_FASTA.fasta	MGYA00243560	ERZ652925	root:Host-associated:Human:Digestive system:Large int	0.3968
MGYA00243561/ERZ652927_FASTA.fasta	MGYA00243561	ERZ652927	root:Host-associated:Human:Digestive system:Large int	0.2688
MGYA00243562/ERZ652929_FASTA.fasta	MGYA00243562	ERZ652929	root:Host-associated:Human:Digestive system:Large int	0.2076
MGYA00243563/ERZ652931_FASTA.fasta	MGYA00243563	ERZ652931	root:Host-associated:Human:Digestive system:Large int	0.3366
MGYA00243564/ERZ652933_FASTA.fasta	MGYA00243564	ERZ652933	root:Host-associated:Human:Digestive system:Large int	0.063
MGYA00243565/ERZ652935_FASTA.fasta	MGYA00243565	ERZ652935	root:Host-associated:Human:Digestive system:Large int	0.0376
MGYA00243566/ERZ653876_FASTA.fasta	MGYA00243566	ERZ653876	root:Host-associated:Human:Digestive system:Large int	0.3008
MGYA00243567/ERZ653878_FASTA.fasta	MGYA00243567	ERZ653878	root:Host-associated:Human:Digestive system:Large int	0.0858
MGYA00243568/ERZ653880_FASTA.fasta	MGYA00243568	ERZ653880	root:Host-associated:Human:Digestive system:Large int	0.167
MGYA00243569/ERZ653882_FASTA.fasta	MGYA00243569	ERZ653882	root:Host-associated:Human:Digestive system:Large int	0.2744
MGYA00243570/ERZ653884_FASTA.fasta	MGYA00243570	ERZ653884	root:Host-associated:Human:Digestive system:Large int	0.1184
MGYA00243571/ERZ653886_FASTA.fasta	MGYA00243571	ERZ653886	root:Host-associated:Human:Digestive system:Large int	0.2768
MGYA00243572/ERZ653888_FASTA.fasta	MGYA00243572	ERZ653888	root:Host-associated:Human:Digestive system:Large int	0.3048
MGYA00243573/ERZ653890_FASTA.fasta	MGYA00243573	ERZ653890	root:Host-associated:Human:Digestive system:Large int	0.2074
MGYA00243574/ERZ653892_FASTA.fasta	MGYA00243574	ERZ653892	root:Host-associated:Human:Digestive system:Large int	0.431
MGYA00243575/ERZ653894_FASTA.fasta	MGYA00243575	ERZ653894	root:Host-associated:Human:Digestive system:Large int	0.3288
MGYA00243576/ERZ653896_FASTA.fasta	MGYA00243576	ERZ653896	root:Host-associated:Human:Digestive system:Large int	0.3048
MGYA00243577/ERZ653898_FASTA.fasta	MGYA00243577	ERZ653898	root:Host-associated:Human:Digestive system:Large int	0.3572
MGYA00243578/ERZ653900_FASTA.fasta	MGYA00243578	ERZ653900	root:Host-associated:Human:Digestive system:Large int	0.283
MGYA00243579/ERZ653902_FASTA.fasta	MGYA00243579	ERZ653902	root:Host-associated:Human:Digestive system:Large int	0.3282
MGYA00243580/ERZ653904_FASTA.fasta	MGYA00243580	ERZ653904	root:Host-associated:Human:Digestive system:Large int	0.2578
MGYA00243581/ERZ653311_FASTA.fasta	MGYA00243581	ERZ653311	root:Host-associated:Human:Digestive system:Large int	0.1016
MGYA00243582/ERZ653344_FASTA.fasta	MGYA00243582	ERZ653344	root:Host-associated:Human:Digestive system:Large int	0.2596
MGYA00243583/ERZ653345_FASTA.fasta	MGYA00243583	ERZ653345	root:Host-associated:Human:Digestive system:Large int	0.0894
MGYA00243584/ERZ653347_FASTA.fasta	MGYA00243584	ERZ653347	root:Host-associated:Human:Digestive system:Large int	0.05
MGYA00243585/ERZ653349_FASTA.fasta	MGYA00243585	ERZ653349	root:Host-associated:Human:Digestive system:Large int	0.1704
MGYA00243586/ERZ653351_FASTA.fasta	MGYA00243586	ERZ653351	root:Host-associated:Human:Digestive system:Large int	0.051
MGYA00243587/ERZ654400_FASTA.fasta	MGYA00243587	ERZ654400	root:Host-associated:Human:Digestive system:Large int	0.164
MGYA00243588/ERZ654402_FASTA.fasta	MGYA00243588	ERZ654402	root:Host-associated:Human:Digestive system:Large int	0.3486
MGYA00243589/ERZ654404_FASTA.fasta	MGYA00243589	ERZ654404	root:Host-associated:Human:Digestive system:Large int	0.2864
MGYA00243590/ERZ654406_FASTA.fasta	MGYA00243590	ERZ654406	root:Host-associated:Human:Digestive system:Large int	0.2676
MGYA00243591/ERZ654408_FASTA.fasta	MGYA00243591	ERZ654408	root:Host-associated:Human:Digestive system:Large int	0.3672
MGYA00243592/ERZ654410_FASTA.fasta	MGYA00243592	ERZ654410	root:Host-associated:Human:Digestive system:Large int	0.2334
MGYA00243593/ERZ654412_FASTA.fasta	MGYA00243593	ERZ654412	root:Host-associated:Human:Digestive system:Large int	0.2698
MGYA00243594/ERZ654414_FASTA.fasta	MGYA00243594	ERZ654414	root:Host-associated:Human:Digestive system:Large int	0.2572
MGYA00243595/ERZ654416_FASTA.fasta	MGYA00243595	ERZ654416	root:Host-associated:Human:Digestive system:Large int	0.3144
MGYA00243596/ERZ654418_FASTA.fasta	MGYA00243596	ERZ654418	root:Host-associated:Human:Digestive system:Large int	0.277
MGYA00243597/ERZ654420_FASTA.fasta	MGYA00243597	ERZ654420	root:Host-associated:Human:Digestive system:Large int	0.2212
MGYA00243598/ERZ654422_FASTA.fasta	MGYA00243598	ERZ654422	root:Host-associated:Human:Digestive system:Large int	0.2088
MGYA00243599/ERZ654424_FASTA.fasta	MGYA00243599	ERZ654424	root:Host-associated:Human:Digestive system:Large int	0.2862
MGYA00243600/ERZ654426_FASTA.fasta	MGYA00243600	ERZ654426	root:Host-associated:Human:Digestive system:Large int	0.27
MGYA00243601/ERZ654428_FASTA.fasta	MGYA00243601	ERZ654428	root:Host-associated:Human:Digestive system:Large int	0.2204
MGYA00243602/ERZ654430_FASTA.fasta	MGYA00243602	ERZ654430	root:Host-associated:Human:Digestive system:Large int	0.3862
MGYA00243603/ERZ654432_FASTA.fasta	MGYA00243603	ERZ654432	root:Host-associated:Human:Digestive system:Large int	0.318
MGYA00243604/ERZ654434_FASTA.fasta	MGYA00243604	ERZ654434	root:Host-associated:Human:Digestive system:Large int	0.5416
MGYA00243605/ERZ654436_FASTA.fasta	MGYA00243605	ERZ654436	root:Host-associated:Human:Digestive system:Large int	0.3708
MGYA00243606/ERZ654438_FASTA.fasta	MGYA00243606	ERZ654438	root:Host-associated:Human:Digestive system:Large int	0.2896
MGYA00243607/ERZ654440_FASTA.fasta	MGYA00243607	ERZ654440	root:Host-associated:Human:Digestive system:Large int	0.385
MGYA00243608/ERZ654442_FASTA.fasta	MGYA00243608	ERZ654442	root:Host-associated:Human:Digestive system:Large int	0.393
MGYA00243609/ERZ652828_FASTA.fasta	MGYA00243609	ERZ652828	root:Host-associated:Human:Digestive system:Large int	0.1994
MGYA00243610/ERZ652830_FASTA.fasta	MGYA00243610	ERZ652830	root:Host-associated:Human:Digestive system:Large int	0.217
MGYA00243611/ERZ652832_FASTA.fasta	MGYA00243611	ERZ652832	root:Host-associated:Human:Digestive system:Large int	0.1496
MGYA00243612/ERZ652834_FASTA.fasta	MGYA00243612	ERZ652834	root:Host-associated:Human:Digestive system:Large int	0.3346
MGYA00243613/ERZ652836_FASTA.fasta	MGYA00243613	ERZ652836	root:Host-associated:Human:Digestive system:Large int	0.3086
MGYA00243614/ERZ652838_FASTA.fasta	MGYA00243614	ERZ652838	root:Host-associated:Human:Digestive system:Large int	0.318
MGYA00243615/ERZ652840_FASTA.fasta	MGYA00243615	ERZ652840	root:Host-associated:Human:Digestive system:Large int	0.2022
MGYA00243616/ERZ652842_FASTA.fasta	MGYA00243616	ERZ652842	root:Host-associated:Human:Digestive system:Large int	0.304
MGYA00243617/ERZ652844_FASTA.fasta	MGYA00243617	ERZ652844	root:Host-associated:Human:Digestive system:Large int	0.1642
MGYA00243618/ERZ652846_FASTA.fasta	MGYA00243618	ERZ652846	root:Host-associated:Human:Digestive system:Large int	0.2848
MGYA00243619/ERZ652848_FASTA.fasta	MGYA00243619	ERZ652848	root:Host-associated:Human:Digestive system:Large int	0.2252
MGYA00243620/ERZ652850_FASTA.fasta	MGYA00243620	ERZ652850	root:Host-associated:Human:Digestive system:Large int	0.2604
MGYA00243621/ERZ652852_FASTA.fasta	MGYA00243621	ERZ652852	root:Host-associated:Human:Digestive system:Large int	0.2744
MGYA00243622/ERZ652854_FASTA.fasta	MGYA00243622	ERZ652854	root:Host-associated:Human:Digestive system:Large int	0.2378
MGYA00243623/ERZ652856_FASTA.fasta	MGYA00243623	ERZ652856	root:Host-associated:Human:Digestive system:Large int	0.2758
MGYA00243624/ERZ652858_FASTA.fasta	MGYA00243624	ERZ652858	root:Host-associated:Human:Digestive system:Large int	0.2548
MGYA00243625/ERZ652860_FASTA.fasta	MGYA00243625	ERZ652860	root:Host-associated:Human:Digestive system:Large int	0.4336
MGYA00243626/ERZ654109_FASTA.fasta	MGYA00243626	ERZ654109	root:Host-associated:Human:Digestive system:Large int	0.307
MGYA00243627/ERZ654111_FASTA.fasta	MGYA00243627	ERZ654111	root:Host-associated:Human:Digestive system:Large int	0.3646
MGYA00243628/ERZ654113_FASTA.fasta	MGYA00243628	ERZ654113	root:Host-associated:Human:Digestive system:Large int	0.2032
MGYA00243629/ERZ654115_FASTA.fasta	MGYA00243629	ERZ654115	root:Host-associated:Human:Digestive system:Large int	0.2006
MGYA00243630/ERZ654117_FASTA.fasta	MGYA00243630	ERZ654117	root:Host-associated:Human:Digestive system:Large int	0.321
MGYA00243631/ERZ654119_FASTA.fasta	MGYA00243631	ERZ654119	root:Host-associated:Human:Digestive system:Large int	0.2744
MGYA00243632/ERZ654121_FASTA.fasta	MGYA00243632	ERZ654121	root:Host-associated:Human:Digestive system:Large int	0.3034
MGYA00243633/ERZ654123_FASTA.fasta	MGYA00243633	ERZ654123	root:Host-associated:Human:Digestive system:Large int	0.2866
MGYA00243634/ERZ654125_FASTA.fasta	MGYA00243634	ERZ654125	root:Host-associated:Human:Digestive system:Large int	0.3136
MGYA00243635/ERZ654127_FASTA.fasta	MGYA00243635	ERZ654127	root:Host-associated:Human:Digestive system:Large int	0.206
MGYA00243636/ERZ654129_FASTA.fasta	MGYA00243636	ERZ654129	root:Host-associated:Human:Digestive system:Large int	0.289
MGYA00243637/ERZ654131_FASTA.fasta	MGYA00243637	ERZ654131	root:Host-associated:Human:Digestive system:Large int	0.2482
MGYA00243638/ERZ654133_FASTA.fasta	MGYA00243638	ERZ654133	root:Host-associated:Human:Digestive system:Large int	0.315
MGYA00243639/ERZ654135_FASTA.fasta	MGYA00243639	ERZ654135	root:Host-associated:Human:Digestive system:Large int	0.2188
MGYA00243640/ERZ654137_FASTA.fasta	MGYA00243640	ERZ654137	root:Host-associated:Human:Digestive system:Large int	0.3258
MGYA00243641/ERZ654139_FASTA.fasta	MGYA00243641	ERZ654139	root:Host-associated:Human:Digestive system:Large int	0.1976
MGYA00243642/ERZ654141_FASTA.fasta	MGYA00243642	ERZ654141	root:Host-associated:Human:Digestive system:Large int	0.2556
MGYA00243643/ERZ654143_FASTA.fasta	MGYA00243643	ERZ654143	root:Host-associated:Human:Digestive system:Large int	0.254
MGYA00243644/ERZ654145_FASTA.fasta	MGYA00243644	ERZ654145	root:Host-associated:Human:Digestive system:Large int	0.295
MGYA00243645/ERZ654147_FASTA.fasta	MGYA00243645	ERZ654147	root:Host-associated:Human:Digestive system:Large int	0.356
MGYA00243646/ERZ654149_FASTA.fasta	MGYA00243646	ERZ654149	root:Host-associated:Human:Digestive system:Large int	0.2866
MGYA00243647/ERZ654151_FASTA.fasta	MGYA00243647	ERZ654151	root:Host-associated:Human:Digestive system:Large int	0.3466
MGYA00243648/ERZ652937_FASTA.fasta	MGYA00243648	ERZ652937	root:Host-associated:Human:Digestive system:Large int	0.0312
MGYA00243649/ERZ652939_FASTA.fasta	MGYA00243649	ERZ652939	root:Host-associated:Human:Digestive system:Large int	0.24
MGYA00243650/ERZ652941_FASTA.fasta	MGYA00243650	ERZ652941	root:Host-associated:Human:Digestive system:Large int	0.0474
MGYA00243651/ERZ652943_FASTA.fasta	MGYA00243651	ERZ652943	root:Host-associated:Human:Digestive system:Large int	0.0686
MGYA00243652/ERZ652945_FASTA.fasta	MGYA00243652	ERZ652945	root:Host-associated:Human:Digestive system:Large int	0.089
MGYA00243653/ERZ652947_FASTA.fasta	MGYA00243653	ERZ652947	root:Host-associated:Human:Digestive system:Large int	0.3188
MGYA00243654/ERZ652951_FASTA.fasta	MGYA00243654	ERZ652951	root:Host-associated:Human:Digestive system:Large int	0.0556
MGYA00243655/ERZ652953_FASTA.fasta	MGYA00243655	ERZ652953	root:Host-associated:Human:Digestive system:Large int	0.1246
MGYA00243656/ERZ652955_FASTA.fasta	MGYA00243656	ERZ652955	root:Host-associated:Human:Digestive system:Large int	0.0286
MGYA00243657/ERZ652957_FASTA.fasta	MGYA00243657	ERZ652957	root:Host-associated:Human:Digestive system:Large int	0.2454
MGYA00243658/ERZ652959_FASTA.fasta	MGYA00243658	ERZ652959	root:Host-associated:Human:Digestive system:Large int	0.146
MGYA00243659/ERZ652961_FASTA.fasta	MGYA00243659	ERZ652961	root:Host-associated:Human:Digestive system:Large int	0.2854
MGYA00243660/ERZ652963_FASTA.fasta	MGYA00243660	ERZ652963	root:Host-associated:Human:Digestive system:Large int	0.2068
MGYA00243661/ERZ652965_FASTA.fasta	MGYA00243661	ERZ652965	root:Host-associated:Human:Digestive system:Large int	0.1366
MGYA00243662/ERZ652967_FASTA.fasta	MGYA00243662	ERZ652967	root:Host-associated:Human:Digestive system:Large int	0.1268
MGYA00243663/ERZ652969_FASTA.fasta	MGYA00243663	ERZ652969	root:Host-associated:Human:Digestive system:Large int	0.2992
MGYA00243664/ERZ652971_FASTA.fasta	MGYA00243664	ERZ652971	root:Host-associated:Human:Digestive system:Large int	0.0924
MGYA00243665/ERZ652973_FASTA.fasta	MGYA00243665	ERZ652973	root:Host-associated:Human:Digestive system:Large int	0.0472
MGYA00243666/ERZ652975_FASTA.fasta	MGYA00243666	ERZ652975	root:Host-associated:Human:Digestive system:Large int	0.0466
MGYA00243667/ERZ652977_FASTA.fasta	MGYA00243667	ERZ652977	root:Host-associated:Human:Digestive system:Large int	0.3006
MGYA00243668/ERZ653906_FASTA.fasta	MGYA00243668	ERZ653906	root:Host-associated:Human:Digestive system:Large int	0.2846
MGYA00243669/ERZ653908_FASTA.fasta	MGYA00243669	ERZ653908	root:Host-associated:Human:Digestive system:Large int	0.2562
MGYA00243670/ERZ653910_FASTA.fasta	MGYA00243670	ERZ653910	root:Host-associated:Human:Digestive system:Large int	0.248
MGYA00243671/ERZ653912_FASTA.fasta	MGYA00243671	ERZ653912	root:Host-associated:Human:Digestive system:Large int	0.297
MGYA00243672/ERZ653914_FASTA.fasta	MGYA00243672	ERZ653914	root:Host-associated:Human:Digestive system:Large int	0.3104
MGYA00243673/ERZ653916_FASTA.fasta	MGYA00243673	ERZ653916	root:Host-associated:Human:Digestive system:Large int	0.4598
MGYA00243674/ERZ653918_FASTA.fasta	MGYA00243674	ERZ653918	root:Host-associated:Human:Digestive system:Large int	0.3298
MGYA00243675/ERZ652878_FASTA.fasta	MGYA00243675	ERZ652878	root:Host-associated:Human:Digestive system:Large int	0.366
MGYA00243676/ERZ652880_FASTA.fasta	MGYA00243676	ERZ652880	root:Host-associated:Human:Digestive system:Large int	0.3168
MGYA00243677/ERZ652882_FASTA.fasta	MGYA00243677	ERZ652882	root:Host-associated:Human:Digestive system:Large int	0.2824
MGYA00243678/ERZ652884_FASTA.fasta	MGYA00243678	ERZ652884	root:Host-associated:Human:Digestive system:Large int	0.138
MGYA00243679/ERZ652886_FASTA.fasta	MGYA00243679	ERZ652886	root:Host-associated:Human:Digestive system:Large int	0.2812
MGYA00243680/ERZ652888_FASTA.fasta	MGYA00243680	ERZ652888	root:Host-associated:Human:Digestive system:Large int	0.3966
MGYA00243681/ERZ652890_FASTA.fasta	MGYA00243681	ERZ652890	root:Host-associated:Human:Digestive system:Large int	0.3178
MGYA00243682/ERZ652892_FASTA.fasta	MGYA00243682	ERZ652892	root:Host-associated:Human:Digestive system:Large int	0.2852
MGYA00243683/ERZ652894_FASTA.fasta	MGYA00243683	ERZ652894	root:Host-associated:Human:Digestive system:Large int	0.1248
MGYA00243684/ERZ652896_FASTA.fasta	MGYA00243684	ERZ652896	root:Host-associated:Human:Digestive system:Large int	0.2916
MGYA00243685/ERZ652898_FASTA.fasta	MGYA00243685	ERZ652898	root:Host-associated:Human:Digestive system:Large int	0.3034
MGYA00243686/ERZ652900_FASTA.fasta	MGYA00243686	ERZ652900	root:Host-associated:Human:Digestive system:Large int	0.2928
MGYA00243687/ERZ652902_FASTA.fasta	MGYA00243687	ERZ652902	root:Host-associated:Human:Digestive system:Large int	0.3396
MGYA00243688/ERZ654444_FASTA.fasta	MGYA00243688	ERZ654444	root:Host-associated:Human:Digestive system:Large int	0.2142
MGYA00243689/ERZ654446_FASTA.fasta	MGYA00243689	ERZ654446	root:Host-associated:Human:Digestive system:Large int	0.285
MGYA00243690/ERZ654448_FASTA.fasta	MGYA00243690	ERZ654448	root:Host-associated:Human:Digestive system:Large int	0.2886
MGYA00243691/ERZ654450_FASTA.fasta	MGYA00243691	ERZ654450	root:Host-associated:Human:Digestive system:Large int	0.3882
MGYA00243692/ERZ654452_FASTA.fasta	MGYA00243692	ERZ654452	root:Host-associated:Human:Digestive system:Large int	0.3412
MGYA00243693/ERZ654454_FASTA.fasta	MGYA00243693	ERZ654454	root:Host-associated:Human:Digestive system:Large int	0.2388
MGYA00243694/ERZ654456_FASTA.fasta	MGYA00243694	ERZ654456	root:Host-associated:Human:Digestive system:Large int	0.303
MGYA00243695/ERZ654458_FASTA.fasta	MGYA00243695	ERZ654458	root:Host-associated:Human:Digestive system:Large int	0.4782
MGYA00243696/ERZ654460_FASTA.fasta	MGYA00243696	ERZ654460	root:Host-associated:Human:Digestive system:Large int	0.3202
MGYA00243697/ERZ654462_FASTA.fasta	MGYA00243697	ERZ654462	root:Host-associated:Human:Digestive system:Large int	0.2798
MGYA00243698/ERZ654464_FASTA.fasta	MGYA00243698	ERZ654464	root:Host-associated:Human:Digestive system:Large int	0.3732
MGYA00243699/ERZ654466_FASTA.fasta	MGYA00243699	ERZ654466	root:Host-associated:Human:Digestive system:Large int	0.3786
MGYA00243700/ERZ654468_FASTA.fasta	MGYA00243700	ERZ654468	root:Host-associated:Human:Digestive system:Large int	0.2832
MGYA00243701/ERZ654470_FASTA.fasta	MGYA00243701	ERZ654470	root:Host-associated:Human:Digestive system:Large int	0.3532
MGYA00243702/ERZ654472_FASTA.fasta	MGYA00243702	ERZ654472	root:Host-associated:Human:Digestive system:Large int	0.3632
MGYA00243703/ERZ654476_FASTA.fasta	MGYA00243703	ERZ654476	root:Host-associated:Human:Digestive system:Large int	0.284
MGYA00243704/ERZ654478_FASTA.fasta	MGYA00243704	ERZ654478	root:Host-associated:Human:Digestive system:Large int	0.3352
MGYA00243705/ERZ654480_FASTA.fasta	MGYA00243705	ERZ654480	root:Host-associated:Human:Digestive system:Large int	0.213
MGYA00243706/ERZ654482_FASTA.fasta	MGYA00243706	ERZ654482	root:Host-associated:Human:Digestive system:Large int	0.3656
MGYA00243707/ERZ654484_FASTA.fasta	MGYA00243707	ERZ654484	root:Host-associated:Human:Digestive system:Large int	0.4232
MGYA00243708/ERZ654486_FASTA.fasta	MGYA00243708	ERZ654486	root:Host-associated:Human:Digestive system:Large int	0.3376
MGYA00243709/ERZ652862_FASTA.fasta	MGYA00243709	ERZ652862	root:Host-associated:Human:Digestive system:Large int	0.2894
MGYA00243710/ERZ652864_FASTA.fasta	MGYA00243710	ERZ652864	root:Host-associated:Human:Digestive system:Large int	0.3296
MGYA00243711/ERZ652866_FASTA.fasta	MGYA00243711	ERZ652866	root:Host-associated:Human:Digestive system:Large int	0.2576
MGYA00243712/ERZ652868_FASTA.fasta	MGYA00243712	ERZ652868	root:Host-associated:Human:Digestive system:Large int	0.2998
MGYA00243713/ERZ652870_FASTA.fasta	MGYA00243713	ERZ652870	root:Host-associated:Human:Digestive system:Large int	0.3148
MGYA00243714/ERZ652873_FASTA.fasta	MGYA00243714	ERZ652873	root:Host-associated:Human:Digestive system:Large int	0.1
MGYA00243715/ERZ652874_FASTA.fasta	MGYA00243715	ERZ652874	root:Host-associated:Human:Digestive system:Large int	0.2906
MGYA00243716/ERZ652876_FASTA.fasta	MGYA00243716	ERZ652876	root:Host-associated:Human:Digestive system:Large int	0.2574
MGYA00243717/ERZ652996_FASTA.fasta	MGYA00243717	ERZ652996	root:Host-associated:Human:Digestive system:Large int	0.0404
MGYA00243718/ERZ652998_FASTA.fasta	MGYA00243718	ERZ652998	root:Host-associated:Human:Digestive system:Large int	0.0554
MGYA00243719/ERZ653000_FASTA.fasta	MGYA00243719	ERZ653000	root:Host-associated:Human:Digestive system:Large int	0.281
MGYA00243720/ERZ653002_FASTA.fasta	MGYA00243720	ERZ653002	root:Host-associated:Human:Digestive system:Large int	0.0968
MGYA00243721/ERZ653004_FASTA.fasta	MGYA00243721	ERZ653004	root:Host-associated:Human:Digestive system:Large int	0.0384
MGYA00243722/ERZ653006_FASTA.fasta	MGYA00243722	ERZ653006	root:Host-associated:Human:Digestive system:Large int	0.0502
MGYA00243723/ERZ653008_FASTA.fasta	MGYA00243723	ERZ653008	root:Host-associated:Human:Digestive system:Large int	0.057
MGYA00243724/ERZ653010_FASTA.fasta	MGYA00243724	ERZ653010	root:Host-associated:Human:Digestive system:Large int	0.0462
MGYA00243725/ERZ653012_FASTA.fasta	MGYA00243725	ERZ653012	root:Host-associated:Human:Digestive system:Large int	0.0402
MGYA00243726/ERZ653014_FASTA.fasta	MGYA00243726	ERZ653014	root:Host-associated:Human:Digestive system:Large int	0.1108
MGYA00243727/ERZ653016_FASTA.fasta	MGYA00243727	ERZ653016	root:Host-associated:Human:Digestive system:Large int	0.035
MGYA00243728/ERZ653018_FASTA.fasta	MGYA00243728	ERZ653018	root:Host-associated:Human:Digestive system:Large int	0.1402
MGYA00243729/ERZ653020_FASTA.fasta	MGYA00243729	ERZ653020	root:Host-associated:Human:Digestive system:Large int	0.2038
MGYA00243730/ERZ653022_FASTA.fasta	MGYA00243730	ERZ653022	root:Host-associated:Human:Digestive system:Large int	0.0368
MGYA00243731/ERZ654153_FASTA.fasta	MGYA00243731	ERZ654153	root:Host-associated:Human:Digestive system:Large int	0.28
MGYA00243732/ERZ654155_FASTA.fasta	MGYA00243732	ERZ654155	root:Host-associated:Human:Digestive system:Large int	0.3062
MGYA00243733/ERZ654157_FASTA.fasta	MGYA00243733	ERZ654157	root:Host-associated:Human:Digestive system:Large int	0.2818
MGYA00243734/ERZ654159_FASTA.fasta	MGYA00243734	ERZ654159	root:Host-associated:Human:Digestive system:Large int	0.284
MGYA00243735/ERZ654161_FASTA.fasta	MGYA00243735	ERZ654161	root:Host-associated:Human:Digestive system:Large int	0.228
MGYA00243736/ERZ654163_FASTA.fasta	MGYA00243736	ERZ654163	root:Host-associated:Human:Digestive system:Large int	0.3232
MGYA00243737/ERZ654167_FASTA.fasta	MGYA00243737	ERZ654167	root:Host-associated:Human:Digestive system:Large int	0.3874
MGYA00243738/ERZ654169_FASTA.fasta	MGYA00243738	ERZ654169	root:Host-associated:Human:Digestive system:Large int	0.2828
MGYA00243739/ERZ654171_FASTA.fasta	MGYA00243739	ERZ654171	root:Host-associated:Human:Digestive system:Large int	0.2662
MGYA00243740/ERZ654173_FASTA.fasta	MGYA00243740	ERZ654173	root:Host-associated:Human:Digestive system:Large int	0.2996
MGYA00243741/ERZ654175_FASTA.fasta	MGYA00243741	ERZ654175	root:Host-associated:Human:Digestive system:Large int	0.2868
MGYA00243742/ERZ654177_FASTA.fasta	MGYA00243742	ERZ654177	root:Host-associated:Human:Digestive system:Large int	0.2238
MGYA00243743/ERZ654179_FASTA.fasta	MGYA00243743	ERZ654179	root:Host-associated:Human:Digestive system:Large int	0.2944
MGYA00243744/ERZ654181_FASTA.fasta	MGYA00243744	ERZ654181	root:Host-associated:Human:Digestive system:Large int	0.3624
MGYA00243745/ERZ654183_FASTA.fasta	MGYA00243745	ERZ654183	root:Host-associated:Human:Digestive system:Large int	0.1318
MGYA00243746/ERZ654185_FASTA.fasta	MGYA00243746	ERZ654185	root:Host-associated:Human:Digestive system:Large int	0.303
MGYA00243747/ERZ654187_FASTA.fasta	MGYA00243747	ERZ654187	root:Host-associated:Human:Digestive system:Large int	0.226
MGYA00243748/ERZ654189_FASTA.fasta	MGYA00243748	ERZ654189	root:Host-associated:Human:Digestive system:Large int	0.2452
MGYA00243749/ERZ654191_FASTA.fasta	MGYA00243749	ERZ654191	root:Host-associated:Human:Digestive system:Large int	0.228
MGYA00243750/ERZ654193_FASTA.fasta	MGYA00243750	ERZ654193	root:Host-associated:Human:Digestive system:Large int	0.2546
MGYA00243751/ERZ654195_FASTA.fasta	MGYA00243751	ERZ654195	root:Host-associated:Human:Digestive system:Large int	0.2462
MGYA00243752/ERZ654197_FASTA.fasta	MGYA00243752	ERZ654197	root:Host-associated:Human:Digestive system:Large int	0.2174
MGYA00243753/ERZ652979_FASTA.fasta	MGYA00243753	ERZ652979	root:Host-associated:Human:Digestive system:Large int	0.1232
MGYA00243754/ERZ652981_FASTA.fasta	MGYA00243754	ERZ652981	root:Host-associated:Human:Digestive system:Large int	0.0546
MGYA00243755/ERZ652983_FASTA.fasta	MGYA00243755	ERZ652983	root:Host-associated:Human:Digestive system:Large int	0.2814
MGYA00243756/ERZ652985_FASTA.fasta	MGYA00243756	ERZ652985	root:Host-associated:Human:Digestive system:Large int	0.056
MGYA00243757/ERZ652987_FASTA.fasta	MGYA00243757	ERZ652987	root:Host-associated:Human:Digestive system:Large int	0.0826
MGYA00243758/ERZ652989_FASTA.fasta	MGYA00243758	ERZ652989	root:Host-associated:Human:Digestive system:Large int	0.0376
MGYA00243759/ERZ652991_FASTA.fasta	MGYA00243759	ERZ652991	root:Host-associated:Human:Digestive system:Large int	0.2946
MGYA00243760/ERZ652993_FASTA.fasta	MGYA00243760	ERZ652993	root:Host-associated:Human:Digestive system:Large int	0.157
MGYA00243761/ERZ652995_FASTA.fasta	MGYA00243761	ERZ652995	root:Host-associated:Human:Digestive system:Large int	0.1424
MGYA00243762/ERZ652997_FASTA.fasta	MGYA00243762	ERZ652997	root:Host-associated:Human:Digestive system:Large int	0.2916
MGYA00243763/ERZ652999_FASTA.fasta	MGYA00243763	ERZ652999	root:Host-associated:Human:Digestive system:Large int	0.1246
MGYA00243764/ERZ653001_FASTA.fasta	MGYA00243764	ERZ653001	root:Host-associated:Human:Digestive system:Large int	0.247
MGYA00243765/ERZ653003_FASTA.fasta	MGYA00243765	ERZ653003	root:Host-associated:Human:Digestive system:Large int	0.0418
MGYA00243766/ERZ653005_FASTA.fasta	MGYA00243766	ERZ653005	root:Host-associated:Human:Digestive system:Large int	0.126
MGYA00243767/ERZ653007_FASTA.fasta	MGYA00243767	ERZ653007	root:Host-associated:Human:Digestive system:Large int	0.0822
MGYA00243768/ERZ653009_FASTA.fasta	MGYA00243768	ERZ653009	root:Host-associated:Human:Digestive system:Large int	0.256
MGYA00243769/ERZ653011_FASTA.fasta	MGYA00243769	ERZ653011	root:Host-associated:Human:Digestive system:Large int	0.078
MGYA00243770/ERZ653013_FASTA.fasta	MGYA00243770	ERZ653013	root:Host-associated:Human:Digestive system:Large int	0.0992
MGYA00243771/ERZ653015_FASTA.fasta	MGYA00243771	ERZ653015	root:Host-associated:Human:Digestive system:Large int	0.1408
MGYA00243772/ERZ653017_FASTA.fasta	MGYA00243772	ERZ653017	root:Host-associated:Human:Digestive system:Large int	0.2188
MGYA00243773/ERZ653019_FASTA.fasta	MGYA00243773	ERZ653019	root:Host-associated:Human:Digestive system:Large int	0.0524
MGYA00243774/ERZ653021_FASTA.fasta	MGYA00243774	ERZ653021	root:Host-associated:Human:Digestive system:Large int	0.0454
MGYA00243775/ERZ653920_FASTA.fasta	MGYA00243775	ERZ653920	root:Host-associated:Human:Digestive system:Large int	0.2278
MGYA00243776/ERZ653922_FASTA.fasta	MGYA00243776	ERZ653922	root:Host-associated:Human:Digestive system:Large int	0.317
MGYA00243777/ERZ653924_FASTA.fasta	MGYA00243777	ERZ653924	root:Host-associated:Human:Digestive system:Large int	0.2934
MGYA00243778/ERZ653926_FASTA.fasta	MGYA00243778	ERZ653926	root:Host-associated:Human:Digestive system:Large int	0.3036
MGYA00243779/ERZ653928_FASTA.fasta	MGYA00243779	ERZ653928	root:Host-associated:Human:Digestive system:Large int	0.2086
MGYA00243780/ERZ653930_FASTA.fasta	MGYA00243780	ERZ653930	root:Host-associated:Human:Digestive system:Large int	0.3738
MGYA00243781/ERZ653932_FASTA.fasta	MGYA00243781	ERZ653932	root:Host-associated:Human:Digestive system:Large int	0.3918
MGYA00243782/ERZ653934_FASTA.fasta	MGYA00243782	ERZ653934	root:Host-associated:Human:Digestive system:Large int	0.287
MGYA00243783/ERZ653936_FASTA.fasta	MGYA00243783	ERZ653936	root:Host-associated:Human:Digestive system:Large int	0.3346
MGYA00243784/ERZ653938_FASTA.fasta	MGYA00243784	ERZ653938	root:Host-associated:Human:Digestive system:Large int	0.3064
MGYA00243785/ERZ653940_FASTA.fasta	MGYA00243785	ERZ653940	root:Host-associated:Human:Digestive system:Large int	0.2962
MGYA00243786/ERZ653942_FASTA.fasta	MGYA00243786	ERZ653942	root:Host-associated:Human:Digestive system:Large int	0.222
MGYA00243787/ERZ653944_FASTA.fasta	MGYA00243787	ERZ653944	root:Host-associated:Human:Digestive system:Large int	0.3508
MGYA00243788/ERZ653946_FASTA.fasta	MGYA00243788	ERZ653946	root:Host-associated:Human:Digestive system:Large int	0.2514
MGYA00243789/ERZ653948_FASTA.fasta	MGYA00243789	ERZ653948	root:Host-associated:Human:Digestive system:Large int	0.0494
MGYA00243790/ERZ653950_FASTA.fasta	MGYA00243790	ERZ653950	root:Host-associated:Human:Digestive system:Large int	0.3296
MGYA00243791/ERZ652703_FASTA.fasta	MGYA00243791	ERZ652703	root:Host-associated:Human:Digestive system:Large int	0.1624
MGYA00243792/ERZ652705_FASTA.fasta	MGYA00243792	ERZ652705	root:Host-associated:Human:Digestive system:Large int	0.3668
MGYA00243793/ERZ652707_FASTA.fasta	MGYA00243793	ERZ652707	root:Host-associated:Human:Digestive system:Large int	0.0938
MGYA00243794/ERZ652709_FASTA.fasta	MGYA00243794	ERZ652709	root:Host-associated:Human:Digestive system:Large int	0.3938
MGYA00243795/ERZ652711_FASTA.fasta	MGYA00243795	ERZ652711	root:Host-associated:Human:Digestive system:Large int	0.4448
MGYA00243796/ERZ652713_FASTA.fasta	MGYA00243796	ERZ652713	root:Host-associated:Human:Digestive system:Large int	0.263
MGYA00243797/ERZ652715_FASTA.fasta	MGYA00243797	ERZ652715	root:Host-associated:Human:Digestive system:Large int	0.3668
MGYA00243798/ERZ652717_FASTA.fasta	MGYA00243798	ERZ652717	root:Host-associated:Human:Digestive system:Large int	0.3002
MGYA00243799/ERZ652719_FASTA.fasta	MGYA00243799	ERZ652719	root:Host-associated:Human:Digestive system:Large int	0.23
MGYA00243800/ERZ652721_FASTA.fasta	MGYA00243800	ERZ652721	root:Host-associated:Human:Digestive system:Large int	0.3234
MGYA00243801/ERZ652904_FASTA.fasta	MGYA00243801	ERZ652904	root:Host-associated:Human:Digestive system:Large int	0.3136
MGYA00243802/ERZ652906_FASTA.fasta	MGYA00243802	ERZ652906	root:Host-associated:Human:Digestive system:Large int	0.2574
MGYA00243803/ERZ652908_FASTA.fasta	MGYA00243803	ERZ652908	root:Host-associated:Human:Digestive system:Large int	0.2828
MGYA00243804/ERZ652910_FASTA.fasta	MGYA00243804	ERZ652910	root:Host-associated:Human:Digestive system:Large int	0.2862
MGYA00243805/ERZ652912_FASTA.fasta	MGYA00243805	ERZ652912	root:Host-associated:Human:Digestive system:Large int	0.2774
MGYA00243806/ERZ652914_FASTA.fasta	MGYA00243806	ERZ652914	root:Host-associated:Human:Digestive system:Large int	0.267
MGYA00243807/ERZ652916_FASTA.fasta	MGYA00243807	ERZ652916	root:Host-associated:Human:Digestive system:Large int	0.3194
MGYA00243808/ERZ652918_FASTA.fasta	MGYA00243808	ERZ652918	root:Host-associated:Human:Digestive system:Large int	0.3404
MGYA00243809/ERZ652920_FASTA.fasta	MGYA00243809	ERZ652920	root:Host-associated:Human:Digestive system:Large int	0.3376
MGYA00243810/ERZ652922_FASTA.fasta	MGYA00243810	ERZ652922	root:Host-associated:Human:Digestive system:Large int	0.2658
MGYA00243811/ERZ652924_FASTA.fasta	MGYA00243811	ERZ652924	root:Host-associated:Human:Digestive system:Large int	0.2734
MGYA00243812/ERZ652926_FASTA.fasta	MGYA00243812	ERZ652926	root:Host-associated:Human:Digestive system:Large int	0.3172
MGYA00243813/ERZ652928_FASTA.fasta	MGYA00243813	ERZ652928	root:Host-associated:Human:Digestive system:Large int	0.2594
MGYA00243814/ERZ652930_FASTA.fasta	MGYA00243814	ERZ652930	root:Host-associated:Human:Digestive system:Large int	0.276
MGYA00243815/ERZ652932_FASTA.fasta	MGYA00243815	ERZ652932	root:Host-associated:Human:Digestive system:Large int	0.0366
MGYA00243816/ERZ652934_FASTA.fasta	MGYA00243816	ERZ652934	root:Host-associated:Human:Digestive system:Large int	0.0488
MGYA00243817/ERZ652936_FASTA.fasta	MGYA00243817	ERZ652936	root:Host-associated:Human:Digestive system:Large int	0.0578
MGYA00243818/ERZ652938_FASTA.fasta	MGYA00243818	ERZ652938	root:Host-associated:Human:Digestive system:Large int	0.2452
MGYA00243819/ERZ652940_FASTA.fasta	MGYA00243819	ERZ652940	root:Host-associated:Human:Digestive system:Large int	0.1604
MGYA00243820/ERZ652942_FASTA.fasta	MGYA00243820	ERZ652942	root:Host-associated:Human:Digestive system:Large int	0.0802
MGYA00243821/ERZ652944_FASTA.fasta	MGYA00243821	ERZ652944	root:Host-associated:Human:Digestive system:Large int	0.1074
MGYA00243822/ERZ652946_FASTA.fasta	MGYA00243822	ERZ652946	root:Host-associated:Human:Digestive system:Large int	0.091
MGYA00243823/ERZ654488_FASTA.fasta	MGYA00243823	ERZ654488	root:Host-associated:Human:Digestive system:Large int	0.1786
MGYA00243824/ERZ654490_FASTA.fasta	MGYA00243824	ERZ654490	root:Host-associated:Human:Digestive system:Large int	0.3712
MGYA00243825/ERZ654492_FASTA.fasta	MGYA00243825	ERZ654492	root:Host-associated:Human:Digestive system:Large int	0.1568
MGYA00243826/ERZ654496_FASTA.fasta	MGYA00243826	ERZ654496	root:Host-associated:Human:Digestive system:Large int	0.2904
MGYA00243827/ERZ654498_FASTA.fasta	MGYA00243827	ERZ654498	root:Host-associated:Human:Digestive system:Large int	0.2894
MGYA00243828/ERZ654504_FASTA.fasta	MGYA00243828	ERZ654504	root:Host-associated:Human:Digestive system:Large int	0.3366
MGYA00243829/ERZ654506_FASTA.fasta	MGYA00243829	ERZ654506	root:Host-associated:Human:Digestive system:Large int	0.3264
MGYA00243830/ERZ654508_FASTA.fasta	MGYA00243830	ERZ654508	root:Host-associated:Human:Digestive system:Large int	0.3158
MGYA00243831/ERZ654510_FASTA.fasta	MGYA00243831	ERZ654510	root:Host-associated:Human:Digestive system:Large int	0.3096
MGYA00243832/ERZ654512_FASTA.fasta	MGYA00243832	ERZ654512	root:Host-associated:Human:Digestive system:Large int	0.3696
MGYA00243833/ERZ654520_FASTA.fasta	MGYA00243833	ERZ654520	root:Host-associated:Human:Digestive system:Large int	0.362
MGYA00243834/ERZ654522_FASTA.fasta	MGYA00243834	ERZ654522	root:Host-associated:Human:Digestive system:Large int	0.3694
MGYA00243835/ERZ654524_FASTA.fasta	MGYA00243835	ERZ654524	root:Host-associated:Human:Digestive system:Large int	0.36
MGYA00243836/ERZ654526_FASTA.fasta	MGYA00243836	ERZ654526	root:Host-associated:Human:Digestive system:Large int	0.3448
MGYA00243837/ERZ654528_FASTA.fasta	MGYA00243837	ERZ654528	root:Host-associated:Human:Digestive system:Large int	0.2748
MGYA00243838/ERZ654530_FASTA.fasta	MGYA00243838	ERZ654530	root:Host-associated:Human:Digestive system:Large int	0.3176
MGYA00243839/ERZ654532_FASTA.fasta	MGYA00243839	ERZ654532	root:Host-associated:Human:Digestive system:Large int	0.407
MGYA00243840/ERZ653024_FASTA.fasta	MGYA00243840	ERZ653024	root:Host-associated:Human:Digestive system:Large int	0.0408
MGYA00243841/ERZ653026_FASTA.fasta	MGYA00243841	ERZ653026	root:Host-associated:Human:Digestive system:Large int	0.053
MGYA00243842/ERZ653028_FASTA.fasta	MGYA00243842	ERZ653028	root:Host-associated:Human:Digestive system:Large int	0.0386
MGYA00243843/ERZ653030_FASTA.fasta	MGYA00243843	ERZ653030	root:Host-associated:Human:Digestive system:Large int	0.0388
MGYA00243844/ERZ653032_FASTA.fasta	MGYA00243844	ERZ653032	root:Host-associated:Human:Digestive system:Large int	0.2676
MGYA00243845/ERZ653034_FASTA.fasta	MGYA00243845	ERZ653034	root:Host-associated:Human:Digestive system:Large int	0.0386
MGYA00243846/ERZ653036_FASTA.fasta	MGYA00243846	ERZ653036	root:Host-associated:Human:Digestive system:Large int	0.0346
MGYA00243847/ERZ653759_FASTA.fasta	MGYA00243847	ERZ653759	root:Host-associated:Human:Digestive system:Large int	0.3834
MGYA00243848/ERZ653761_FASTA.fasta	MGYA00243848	ERZ653761	root:Host-associated:Human:Digestive system:Large int	0.284
MGYA00243849/ERZ653763_FASTA.fasta	MGYA00243849	ERZ653763	root:Host-associated:Human:Digestive system:Large int	0.2766
MGYA00243850/ERZ653765_FASTA.fasta	MGYA00243850	ERZ653765	root:Host-associated:Human:Digestive system:Large int	0.2932
MGYA00243851/ERZ653767_FASTA.fasta	MGYA00243851	ERZ653767	root:Host-associated:Human:Digestive system:Large int	0.4074
MGYA00243852/ERZ653769_FASTA.fasta	MGYA00243852	ERZ653769	root:Host-associated:Human:Digestive system:Large int	0.2782
MGYA00243853/ERZ653771_FASTA.fasta	MGYA00243853	ERZ653771	root:Host-associated:Human:Digestive system:Large int	0.572
MGYA00243854/ERZ653772_FASTA.fasta	MGYA00243854	ERZ653772	root:Host-associated:Human:Digestive system:Large int	0.329
MGYA00243855/ERZ653775_FASTA.fasta	MGYA00243855	ERZ653775	root:Host-associated:Human:Digestive system:Large int	0.4066
MGYA00243856/ERZ653777_FASTA.fasta	MGYA00243856	ERZ653777	root:Host-associated:Human:Digestive system:Large int	0.2814
MGYA00243857/ERZ653779_FASTA.fasta	MGYA00243857	ERZ653779	root:Host-associated:Human:Digestive system:Large int	0.2692
MGYA00243858/ERZ653781_FASTA.fasta	MGYA00243858	ERZ653781	root:Host-associated:Human:Digestive system:Large int	0.2806
MGYA00243859/ERZ653783_FASTA.fasta	MGYA00243859	ERZ653783	root:Host-associated:Human:Digestive system:Large int	0.2952
MGYA00243860/ERZ653785_FASTA.fasta	MGYA00243860	ERZ653785	root:Host-associated:Human:Digestive system:Large int	0.3518
MGYA00243861/ERZ653787_FASTA.fasta	MGYA00243861	ERZ653787	root:Host-associated:Human:Digestive system:Large int	0.0376
MGYA00243862/ERZ653789_FASTA.fasta	MGYA00243862	ERZ653789	root:Host-associated:Human:Digestive system:Large int	0.1544
MGYA00243863/ERZ653791_FASTA.fasta	MGYA00243863	ERZ653791	root:Host-associated:Human:Digestive system:Large int	0.2588
MGYA00243864/ERZ653793_FASTA.fasta	MGYA00243864	ERZ653793	root:Host-associated:Human:Digestive system:Large int	0.3662
MGYA00243865/ERZ653795_FASTA.fasta	MGYA00243865	ERZ653795	root:Host-associated:Human:Digestive system:Large int	0.4004
MGYA00243866/ERZ654199_FASTA.fasta	MGYA00243866	ERZ654199	root:Host-associated:Human:Digestive system:Large int	0.2148
MGYA00243867/ERZ654201_FASTA.fasta	MGYA00243867	ERZ654201	root:Host-associated:Human:Digestive system:Large int	0.3158
MGYA00243868/ERZ654203_FASTA.fasta	MGYA00243868	ERZ654203	root:Host-associated:Human:Digestive system:Large int	0.176
MGYA00243869/ERZ654205_FASTA.fasta	MGYA00243869	ERZ654205	root:Host-associated:Human:Digestive system:Large int	0.4804
MGYA00243870/ERZ654207_FASTA.fasta	MGYA00243870	ERZ654207	root:Host-associated:Human:Digestive system:Large int	0.3332
MGYA00243871/ERZ654209_FASTA.fasta	MGYA00243871	ERZ654209	root:Host-associated:Human:Digestive system:Large int	0.2484
MGYA00243872/ERZ654211_FASTA.fasta	MGYA00243872	ERZ654211	root:Host-associated:Human:Digestive system:Large int	0.196
MGYA00243873/ERZ654213_FASTA.fasta	MGYA00243873	ERZ654213	root:Host-associated:Human:Digestive system:Large int	0.3888
MGYA00243874/ERZ654215_FASTA.fasta	MGYA00243874	ERZ654215	root:Host-associated:Human:Digestive system:Large int	0.271
MGYA00243875/ERZ654217_FASTA.fasta	MGYA00243875	ERZ654217	root:Host-associated:Human:Digestive system:Large int	0.228
MGYA00243876/ERZ654219_FASTA.fasta	MGYA00243876	ERZ654219	root:Host-associated:Human:Digestive system:Large int	0.354
MGYA00243877/ERZ654221_FASTA.fasta	MGYA00243877	ERZ654221	root:Host-associated:Human:Digestive system:Large int	0.3006
MGYA00243878/ERZ654223_FASTA.fasta	MGYA00243878	ERZ654223	root:Host-associated:Human:Digestive system:Large int	0.304
MGYA00243879/ERZ654225_FASTA.fasta	MGYA00243879	ERZ654225	root:Host-associated:Human:Digestive system:Large int	0.164
MGYA00243880/ERZ654227_FASTA.fasta	MGYA00243880	ERZ654227	root:Host-associated:Human:Digestive system:Large int	0.2568
MGYA00243881/ERZ654229_FASTA.fasta	MGYA00243881	ERZ654229	root:Host-associated:Human:Digestive system:Large int	0.26
MGYA00243882/ERZ654231_FASTA.fasta	MGYA00243882	ERZ654231	root:Host-associated:Human:Digestive system:Large int	0.3054
MGYA00243883/ERZ654233_FASTA.fasta	MGYA00243883	ERZ654233	root:Host-associated:Human:Digestive system:Large int	0.2584
MGYA00243884/ERZ654235_FASTA.fasta	MGYA00243884	ERZ654235	root:Host-associated:Human:Digestive system:Large int	0.2692
MGYA00243885/ERZ654237_FASTA.fasta	MGYA00243885	ERZ654237	root:Host-associated:Human:Digestive system:Large int	0.3346
MGYA00243886/ERZ654239_FASTA.fasta	MGYA00243886	ERZ654239	root:Host-associated:Human:Digestive system:Large int	0.2612
MGYA00243887/ERZ653023_FASTA.fasta	MGYA00243887	ERZ653023	root:Host-associated:Human:Digestive system:Large int	0.063
MGYA00243888/ERZ653025_FASTA.fasta	MGYA00243888	ERZ653025	root:Host-associated:Human:Digestive system:Large int	0.2418
MGYA00243889/ERZ653027_FASTA.fasta	MGYA00243889	ERZ653027	root:Host-associated:Human:Digestive system:Large int	0.0488
MGYA00243890/ERZ653029_FASTA.fasta	MGYA00243890	ERZ653029	root:Host-associated:Human:Digestive system:Large int	0.0806
MGYA00243891/ERZ653031_FASTA.fasta	MGYA00243891	ERZ653031	root:Host-associated:Human:Digestive system:Large int	0.134
MGYA00243892/ERZ653033_FASTA.fasta	MGYA00243892	ERZ653033	root:Host-associated:Human:Digestive system:Large int	0.0938
MGYA00243893/ERZ653035_FASTA.fasta	MGYA00243893	ERZ653035	root:Host-associated:Human:Digestive system:Large int	0.0482
MGYA00243894/ERZ653037_FASTA.fasta	MGYA00243894	ERZ653037	root:Host-associated:Human:Digestive system:Large int	0.0896
MGYA00243895/ERZ653039_FASTA.fasta	MGYA00243895	ERZ653039	root:Host-associated:Human:Digestive system:Large int	0.0324
MGYA00243896/ERZ653041_FASTA.fasta	MGYA00243896	ERZ653041	root:Host-associated:Human:Digestive system:Large int	0.1546
MGYA00243897/ERZ653043_FASTA.fasta	MGYA00243897	ERZ653043	root:Host-associated:Human:Digestive system:Large int	0.0686
MGYA00243898/ERZ653045_FASTA.fasta	MGYA00243898	ERZ653045	root:Host-associated:Human:Digestive system:Large int	0.0822
MGYA00243899/ERZ653047_FASTA.fasta	MGYA00243899	ERZ653047	root:Host-associated:Human:Digestive system:Large int	0.0364
MGYA00243900/ERZ653049_FASTA.fasta	MGYA00243900	ERZ653049	root:Host-associated:Human:Digestive system:Large int	0.1342
MGYA00243901/ERZ653051_FASTA.fasta	MGYA00243901	ERZ653051	root:Host-associated:Human:Digestive system:Large int	0.072
MGYA00243902/ERZ653053_FASTA.fasta	MGYA00243902	ERZ653053	root:Host-associated:Human:Digestive system:Large int	0.0472
MGYA00243903/ERZ653055_FASTA.fasta	MGYA00243903	ERZ653055	root:Host-associated:Human:Digestive system:Large int	0.0604
MGYA00243904/ERZ653057_FASTA.fasta	MGYA00243904	ERZ653057	root:Host-associated:Human:Digestive system:Large int	0.1022

MGYA00243905/ERZ653059_FASTA.fasta	MGYA00243905	ERZ653059	root:Host-associated:Human:Digestive system:Large in/	0.0576
MGYA00243906/ERZ653061_FASTA.fasta	MGYA00243906	ERZ653061	root:Host-associated:Human:Digestive system:Large in/	0.0744
MGYA00243907/ERZ653063_FASTA.fasta	MGYA00243907	ERZ653063	root:Host-associated:Human:Digestive system:Large in/	0.06
MGYA00243908/ERZ653428_FASTA.fasta	MGYA00243908	ERZ653428	root:Host-associated:Human:Digestive system:Large in/	0.2922
MGYA00243909/ERZ653952_FASTA.fasta	MGYA00243909	ERZ653952	root:Host-associated:Human:Digestive system:Large in/	0.3454
MGYA00243910/ERZ653954_FASTA.fasta	MGYA00243910	ERZ653954	root:Host-associated:Human:Digestive system:Large in/	0.2044
MGYA00243911/ERZ653313_FASTA.fasta	MGYA00243911	ERZ653313	root:Host-associated:Human:Digestive system:Large in/	0.1394
MGYA00243912/ERZ653315_FASTA.fasta	MGYA00243912	ERZ653315	root:Host-associated:Human:Digestive system:Large in/	0.1168
MGYA00243913/ERZ653317_FASTA.fasta	MGYA00243913	ERZ653317	root:Host-associated:Human:Digestive system:Large in/	0.1634
MGYA00243914/ERZ653319_FASTA.fasta	MGYA00243914	ERZ653319	root:Host-associated:Human:Digestive system:Large in/	0.0592
MGYA00243915/ERZ653321_FASTA.fasta	MGYA00243915	ERZ653321	root:Host-associated:Human:Digestive system:Large in/	0.0446
MGYA00243916/ERZ653323_FASTA.fasta	MGYA00243916	ERZ653323	root:Host-associated:Human:Digestive system:Large in/	0.1886
MGYA00243917/ERZ653325_FASTA.fasta	MGYA00243917	ERZ653325	root:Host-associated:Human:Digestive system:Large in/	0.1048
MGYA00243918/ERZ653327_FASTA.fasta	MGYA00243918	ERZ653327	root:Host-associated:Human:Digestive system:Large in/	0.1044
MGYA00243919/ERZ653329_FASTA.fasta	MGYA00243919	ERZ653329	root:Host-associated:Human:Digestive system:Large in/	0.2368
MGYA00243920/ERZ653331_FASTA.fasta	MGYA00243920	ERZ653331	root:Host-associated:Human:Digestive system:Large in/	0.0744
MGYA00243921/ERZ653333_FASTA.fasta	MGYA00243921	ERZ653333	root:Host-associated:Human:Digestive system:Large in/	0.037
MGYA00243922/ERZ653335_FASTA.fasta	MGYA00243922	ERZ653335	root:Host-associated:Human:Digestive system:Large in/	0.102
MGYA00243923/ERZ653337_FASTA.fasta	MGYA00243923	ERZ653337	root:Host-associated:Human:Digestive system:Large in/	0.1092
MGYA00243924/ERZ653339_FASTA.fasta	MGYA00243924	ERZ653339	root:Host-associated:Human:Digestive system:Large in/	0.12
MGYA00243925/ERZ653341_FASTA.fasta	MGYA00243925	ERZ653341	root:Host-associated:Human:Digestive system:Large in/	0.0768
MGYA00243926/ERZ653343_FASTA.fasta	MGYA00243926	ERZ653343	root:Host-associated:Human:Digestive system:Large in/	0.1106
MGYA00243927/ERZ653506_FASTA.fasta	MGYA00243927	ERZ653506	root:Host-associated:Human:Digestive system:Large in/	0.3546
MGYA00243928/ERZ653508_FASTA.fasta	MGYA00243928	ERZ653508	root:Host-associated:Human:Digestive system:Large in/	0.293
MGYA00243929/ERZ653510_FASTA.fasta	MGYA00243929	ERZ653510	root:Host-associated:Human:Digestive system:Large in/	0.2938
MGYA00243930/ERZ653512_FASTA.fasta	MGYA00243930	ERZ653512	root:Host-associated:Human:Digestive system:Large in/	0.3144
MGYA00243931/ERZ653514_FASTA.fasta	MGYA00243931	ERZ653514	root:Host-associated:Human:Digestive system:Large in/	0.3774
MGYA00243932/ERZ653516_FASTA.fasta	MGYA00243932	ERZ653516	root:Host-associated:Human:Digestive system:Large in/	0.2116
MGYA00243933/ERZ654534_FASTA.fasta	MGYA00243933	ERZ654534	root:Host-associated:Human:Digestive system:Large in/	0.3272
MGYA00243934/ERZ654536_FASTA.fasta	MGYA00243934	ERZ654536	root:Host-associated:Human:Digestive system:Large in/	0.3708
MGYA00243935/ERZ654540_FASTA.fasta	MGYA00243935	ERZ654540	root:Host-associated:Human:Digestive system:Large in/	0.2958
MGYA00243936/ERZ654542_FASTA.fasta	MGYA00243936	ERZ654542	root:Host-associated:Human:Digestive system:Large in/	0.239
MGYA00243937/ERZ654544_FASTA.fasta	MGYA00243937	ERZ654544	root:Host-associated:Human:Digestive system:Large in/	0.2516
MGYA00243938/ERZ654546_FASTA.fasta	MGYA00243938	ERZ654546	root:Host-associated:Human:Digestive system:Large in/	0.319
MGYA00243939/ERZ654548_FASTA.fasta	MGYA00243939	ERZ654548	root:Host-associated:Human:Digestive system:Large in/	0.329
MGYA00243940/ERZ654550_FASTA.fasta	MGYA00243940	ERZ654550	root:Host-associated:Human:Digestive system:Large in/	0.3758
MGYA00243941/ERZ654552_FASTA.fasta	MGYA00243941	ERZ654552	root:Host-associated:Human:Digestive system:Large in/	0.3844
MGYA00243942/ERZ654554_FASTA.fasta	MGYA00243942	ERZ654554	root:Host-associated:Human:Digestive system:Large in/	0.385
MGYA00243943/ERZ654556_FASTA.fasta	MGYA00243943	ERZ654556	root:Host-associated:Human:Digestive system:Large in/	0.3006
MGYA00243944/ERZ654558_FASTA.fasta	MGYA00243944	ERZ654558	root:Host-associated:Human:Digestive system:Large in/	0.3438
MGYA00243945/ERZ654560_FASTA.fasta	MGYA00243945	ERZ654560	root:Host-associated:Human:Digestive system:Large in/	0.21
MGYA00243946/ERZ654564_FASTA.fasta	MGYA00243946	ERZ654564	root:Host-associated:Human:Digestive system:Large in/	0.2406
MGYA00243947/ERZ654566_FASTA.fasta	MGYA00243947	ERZ654566	root:Host-associated:Human:Digestive system:Large in/	0.4024
MGYA00243948/ERZ654568_FASTA.fasta	MGYA00243948	ERZ654568	root:Host-associated:Human:Digestive system:Large in/	0.2042
MGYA00243949/ERZ654570_FASTA.fasta	MGYA00243949	ERZ654570	root:Host-associated:Human:Digestive system:Large in/	0.3482
MGYA00243950/ERZ654572_FASTA.fasta	MGYA00243950	ERZ654572	root:Host-associated:Human:Digestive system:Large in/	0.1508
MGYA00243951/ERZ654574_FASTA.fasta	MGYA00243951	ERZ654574	root:Host-associated:Human:Digestive system:Large in/	0.2634
MGYA00243952/ERZ654576_FASTA.fasta	MGYA00243952	ERZ654576	root:Host-associated:Human:Digestive system:Large in/	0.5212
MGYA00243953/ERZ654578_FASTA.fasta	MGYA00243953	ERZ654578	root:Host-associated:Human:Digestive system:Large in/	0.2494
MGYA00243954/ERZ653038_FASTA.fasta	MGYA00243954	ERZ653038	root:Host-associated:Human:Digestive system:Large in/	0.0682
MGYA00243955/ERZ653040_FASTA.fasta	MGYA00243955	ERZ653040	root:Host-associated:Human:Digestive system:Large in/	0.2836
MGYA00243956/ERZ653042_FASTA.fasta	MGYA00243956	ERZ653042	root:Host-associated:Human:Digestive system:Large in/	0.2338
MGYA00243957/ERZ653044_FASTA.fasta	MGYA00243957	ERZ653044	root:Host-associated:Human:Digestive system:Large in/	0.1474
MGYA00243958/ERZ653046_FASTA.fasta	MGYA00243958	ERZ653046	root:Host-associated:Human:Digestive system:Large in/	0.037
MGYA00243959/ERZ653048_FASTA.fasta	MGYA00243959	ERZ653048	root:Host-associated:Human:Digestive system:Large in/	0.0606
MGYA00243960/ERZ653050_FASTA.fasta	MGYA00243960	ERZ653050	root:Host-associated:Human:Digestive system:Large in/	0.0606
MGYA00243961/ERZ653052_FASTA.fasta	MGYA00243961	ERZ653052	root:Host-associated:Human:Digestive system:Large in/	0.1424
MGYA00243962/ERZ653054_FASTA.fasta	MGYA00243962	ERZ653054	root:Host-associated:Human:Digestive system:Large in/	0.128
MGYA00243963/ERZ653056_FASTA.fasta	MGYA00243963	ERZ653056	root:Host-associated:Human:Digestive system:Large in/	0.0862
MGYA00243964/ERZ653058_FASTA.fasta	MGYA00243964	ERZ653058	root:Host-associated:Human:Digestive system:Large in/	0.1242
MGYA00243965/ERZ653060_FASTA.fasta	MGYA00243965	ERZ653060	root:Host-associated:Human:Digestive system:Large in/	0.1192
MGYA00243966/ERZ653062_FASTA.fasta	MGYA00243966	ERZ653062	root:Host-associated:Human:Digestive system:Large in/	0.106
MGYA00243967/ERZ653064_FASTA.fasta	MGYA00243967	ERZ653064	root:Host-associated:Human:Digestive system:Large in/	0.0348
MGYA00243968/ERZ653135_FASTA.fasta	MGYA00243968	ERZ653135	root:Host-associated:Human:Digestive system:Large in/	0.1226
MGYA00243969/ERZ653136_FASTA.fasta	MGYA00243969	ERZ653136	root:Host-associated:Human:Digestive system:Large in/	0.098
MGYA00243970/ERZ653138_FASTA.fasta	MGYA00243970	ERZ653138	root:Host-associated:Human:Digestive system:Large in/	0.0962
MGYA00243971/ERZ653140_FASTA.fasta	MGYA00243971	ERZ653140	root:Host-associated:Human:Digestive system:Large in/	0.1754
MGYA00243972/ERZ653142_FASTA.fasta	MGYA00243972	ERZ653142	root:Host-associated:Human:Digestive system:Large in/	0.154
MGYA00243973/ERZ653144_FASTA.fasta	MGYA00243973	ERZ653144	root:Host-associated:Human:Digestive system:Large in/	0.0444
MGYA00243974/ERZ653430_FASTA.fasta	MGYA00243974	ERZ653430	root:Host-associated:Human:Digestive system:Large in/	0.4012
MGYA00243975/ERZ653432_FASTA.fasta	MGYA00243975	ERZ653432	root:Host-associated:Human:Digestive system:Large in/	0.3098
MGYA00243976/ERZ653434_FASTA.fasta	MGYA00243976	ERZ653434	root:Host-associated:Human:Digestive system:Large in/	0.306
MGYA00243977/ERZ653436_FASTA.fasta	MGYA00243977	ERZ653436	root:Host-associated:Human:Digestive system:Large in/	0.3314
MGYA00243978/ERZ653518_FASTA.fasta	MGYA00243978	ERZ653518	root:Host-associated:Human:Digestive system:Large in/	0.3236
MGYA00243979/ERZ653520_FASTA.fasta	MGYA00243979	ERZ653520	root:Host-associated:Human:Digestive system:Large in/	0.2746
MGYA00243980/ERZ653522_FASTA.fasta	MGYA00243980	ERZ653522	root:Host-associated:Human:Digestive system:Large in/	0.3664
MGYA00243981/ERZ653524_FASTA.fasta	MGYA00243981	ERZ653524	root:Host-associated:Human:Digestive system:Large in/	0.3502
MGYA00243982/ERZ653526_FASTA.fasta	MGYA00243982	ERZ653526	root:Host-associated:Human:Digestive system:Large in/	0.3212
MGYA00243983/ERZ653528_FASTA.fasta	MGYA00243983	ERZ653528	root:Host-associated:Human:Digestive system:Large in/	0.2076
MGYA00243984/ERZ653530_FASTA.fasta	MGYA00243984	ERZ653530	root:Host-associated:Human:Digestive system:Large in/	0.3088
MGYA00243985/ERZ653532_FASTA.fasta	MGYA00243985	ERZ653532	root:Host-associated:Human:Digestive system:Large in/	0.2438
MGYA00243986/ERZ653536_FASTA.fasta	MGYA00243986	ERZ653536	root:Host-associated:Human:Digestive system:Large in/	0.4202
MGYA00243987/ERZ653538_FASTA.fasta	MGYA00243987	ERZ653538	root:Host-associated:Human:Digestive system:Large in/	0.337
MGYA00243988/ERZ653540_FASTA.fasta	MGYA00243988	ERZ653540	root:Host-associated:Human:Digestive system:Large in/	0.3092
MGYA00243989/ERZ653542_FASTA.fasta	MGYA00243989	ERZ653542	root:Host-associated:Human:Digestive system:Large in/	0.2202
MGYA00243990/ERZ653544_FASTA.fasta	MGYA00243990	ERZ653544	root:Host-associated:Human:Digestive system:Large in/	0.3668
MGYA00243991/ERZ653546_FASTA.fasta	MGYA00243991	ERZ653546	root:Host-associated:Human:Digestive system:Large in/	0.3004
MGYA00243992/ERZ653548_FASTA.fasta	MGYA00243992	ERZ653548	root:Host-associated:Human:Digestive system:Large in/	0.386
MGYA00243993/ERZ653550_FASTA.fasta	MGYA00243993	ERZ653550	root:Host-associated:Human:Digestive system:Large in/	0.3696
MGYA00243994/ERZ653554_FASTA.fasta	MGYA00243994	ERZ653554	root:Host-associated:Human:Digestive system:Large in/	0.3138
MGYA00243995/ERZ653556_FASTA.fasta	MGYA00243995	ERZ653556	root:Host-associated:Human:Digestive system:Large in/	0.3522
MGYA00243996/ERZ653558_FASTA.fasta	MGYA00243996	ERZ653558	root:Host-associated:Human:Digestive system:Large in/	0.431
MGYA00243997/ERZ653560_FASTA.fasta	MGYA00243997	ERZ653560	root:Host-associated:Human:Digestive system:Large in/	0.2414
MGYA00243998/ERZ653066_FASTA.fasta	MGYA00243998	ERZ653066	root:Host-associated:Human:Digestive system:Large in/	0.0892
MGYA00243999/ERZ653068_FASTA.fasta	MGYA00243999	ERZ653068	root:Host-associated:Human:Digestive system:Large in/	0.1324
MGYA00244000/ERZ653070_FASTA.fasta	MGYA00244000	ERZ653070	root:Host-associated:Human:Digestive system:Large in/	0.0918
MGYA00244001/ERZ653072_FASTA.fasta	MGYA00244001	ERZ653072	root:Host-associated:Human:Digestive system:Large in/	0.0356
MGYA00244002/ERZ653074_FASTA.fasta	MGYA00244002	ERZ653074	root:Host-associated:Human:Digestive system:Large in/	0.0322
MGYA00244003/ERZ653076_FASTA.fasta	MGYA00244003	ERZ653076	root:Host-associated:Human:Digestive system:Large in/	0.041
MGYA00244004/ERZ653078_FASTA.fasta	MGYA00244004	ERZ653078	root:Host-associated:Human:Digestive system:Large in/	0.0352
MGYA00244005/ERZ653082_FASTA.fasta	MGYA00244005	ERZ653082	root:Host-associated:Human:Digestive system:Large in/	0.0802
MGYA00244006/ERZ653084_FASTA.fasta	MGYA00244006	ERZ653084	root:Host-associated:Human:Digestive system:Large in/	0.0698
MGYA00244007/ERZ653086_FASTA.fasta	MGYA00244007	ERZ653086	root:Host-associated:Human:Digestive system:Large in/	0.0684
MGYA00244008/ERZ653088_FASTA.fasta	MGYA00244008	ERZ653088	root:Host-associated:Human:Digestive system:Large in/	0.064
MGYA00244009/ERZ653090_FASTA.fasta	MGYA00244009	ERZ653090	root:Host-associated:Human:Digestive system:Large in/	0.0394
MGYA00244010/ERZ653092_FASTA.fasta	MGYA00244010	ERZ653092	root:Host-associated:Human:Digestive system:Large in/	0.0576
MGYA00244011/ERZ653094_FASTA.fasta	MGYA00244011	ERZ653094	root:Host-associated:Human:Digestive system:Large in/	0.189
MGYA00244012/ERZ653096_FASTA.fasta	MGYA00244012	ERZ653096	root:Host-associated:Human:Digestive system:Large in/	0.0596
MGYA00244013/ERZ653098_FASTA.fasta	MGYA00244013	ERZ653098	root:Host-associated:Human:Digestive system:Large in/	0.0384
MGYA00244014/ERZ653100_FASTA.fasta	MGYA00244014	ERZ653100	root:Host-associated:Human:Digestive system:Large in/	0.0462
MGYA00244015/ERZ653102_FASTA.fasta	MGYA00244015	ERZ653102	root:Host-associated:Human:Digestive system:Large in/	0.1202
MGYA00244016/ERZ654582_FASTA.fasta	MGYA00244016	ERZ654582	root:Host-associated:Human:Digestive system:Large in/	0.4134
MGYA00244017/ERZ654600_FASTA.fasta	MGYA00244017	ERZ654600	root:Host-associated:Human:Digestive system:Large in/	0.3994
MGYA00244018/ERZ653146_FASTA.fasta	MGYA00244018	ERZ653146	root:Host-associated:Human:Digestive system:Large in/	0.1248
MGYA00244019/ERZ653148_FASTA.fasta	MGYA00244019	ERZ653148	root:Host-associated:Human:Digestive system:Large in/	0.1872
MGYA00244020/ERZ653150_FASTA.fasta	MGYA00244020	ERZ653150	root:Host-associated:Human:Digestive system:Large in/	0.0654
MGYA00244021/ERZ653152_FASTA.fasta	MGYA00244021	ERZ653152	root:Host-associated:Human:Digestive system:Large in/	0.0952
MGYA00244022/ERZ653154_FASTA.fasta	MGYA00244022	ERZ653154	root:Host-associated:Human:Digestive system:Large in/	0.0792
MGYA00244023/ERZ653156_FASTA.fasta	MGYA00244023	ERZ653156	root:Host-associated:Human:Digestive system:Large in/	0.0666
MGYA00244024/ERZ653158_FASTA.fasta	MGYA00244024	ERZ653158	root:Host-associated:Human:Digestive system:Large in/	0.037
MGYA00244025/ERZ653160_FASTA.fasta	MGYA00244025	ERZ653160	root:Host-associated:Human:Digestive system:Large in/	0.0974
MGYA00244026/ERZ653162_FASTA.fasta	MGYA00244026	ERZ653162	root:Host-associated:Human:Digestive system:Large in/	0.0332
MGYA00244027/ERZ653164_FASTA.fasta	MGYA00244027	ERZ653164	root:Host-associated:Human:Digestive system:Large in/	0.0428
MGYA00244028/ERZ653166_FASTA.fasta	MGYA00244028	ERZ653166	root:Host-associated:Human:Digestive system:Large in/	0.2208
MGYA00244029/ERZ653168_FASTA.fasta	MGYA00244029	ERZ653168	root:Host-associated:Human:Digestive system:Large in/	0.099
MGYA00244030/ERZ653170_FASTA.fasta	MGYA00244030	ERZ653170	root:Host-associated:Human:Digestive system:Large in/	0.0656
MGYA00244031/ERZ653172_FASTA.fasta	MGYA00244031	ERZ653172	root:Host-associated:Human:Digestive system:Large in/	0.057
MGYA00244032/ERZ653174_FASTA.fasta	MGYA00244032	ERZ653174	root:Host-associated:Human:Digestive system:Large in/	0.0638
MGYA00244033/ERZ653176_FASTA.fasta	MGYA00244033	ERZ653176	root:Host-associated:Human:Digestive system:Large in/	0.1882
MGYA00244034/ERZ653178_FASTA.fasta	MGYA00244034	ERZ653178	root:Host-associated:Human:Digestive system:Large in/	0.2004
MGYA00244035/ERZ653180_FASTA.fasta	MGYA00244035	ERZ653180	root:Host-associated:Human:Digestive system:Large in/	0.1678
MGYA00244036/ERZ653182_FASTA.fasta	MGYA00244036	ERZ653182	root:Host-associated:Human:Digestive system:Large in/	0.0384
MGYA00244037/ERZ653184_FASTA.fasta	MGYA00244037	ERZ653184	root:Host-associated:Human:Digestive system:Large in/	0.0972
MGYA00244038/ERZ653186_FASTA.fasta	MGYA00244038	ERZ653186	root:Host-associated:Human:Digestive system:Large in/	0.0442
MGYA00244039/ERZ653188_FASTA.fasta	MGYA00244039	ERZ653188	root:Host-associated:Human:Digestive system:Large in/	0.1028
MGYA00244040/ERZ653674_FASTA.fasta	MGYA00244040	ERZ653674	root:Host-associated:Human:Digestive system:Large in/	0.3654
MGYA00244041/ERZ653676_FASTA.fasta	MGYA00244041	ERZ653676	root:Host-associated:Human:Digestive system:Large in/	0.2886
MGYA00244042/ERZ653678_FASTA.fasta	MGYA00244042	ERZ653678	root:Host-associated:Human:Digestive system:Large in/	0.3684
MGYA00244043/ERZ653680_FASTA.fasta	MGYA00244043	ERZ653680	root:Host-associated:Human:Digestive system:Large in/	0.3698
MGYA00244044/ERZ653682_FASTA.fasta	MGYA00244044	ERZ653682	root:Host-associated:Human:Digestive system:Large in/	0.3534
MGYA00244045/ERZ653684_FASTA.fasta	MGYA00244045	ERZ653684	root:Host-associated:Human:Digestive system:Large in/	0.4436
MGYA00244046/ERZ653686_FASTA.fasta	MGYA00244046	ERZ653686	root:Host-associated:Human:Digestive system:Large in/	0.3964
MGYA00244047/ERZ653688_FASTA.fasta	MGYA00244047	ERZ653688	root:Host-associated:Human:Digestive system:Large in/	0.3536
MGYA00244048/ERZ653690_FASTA.fasta	MGYA00244048	ERZ653690	root:Host-associated:Human:Digestive system:Large in/	0.2112
MGYA00244049/ERZ653692_FASTA.fasta	MGYA00244049	ERZ653692	root:Host-associated:Human:Digestive system:Large in/	0.3666
MGYA00244050/ERZ653694_FASTA.fasta	MGYA00244050	ERZ653694	root:Host-associated:Human:Digestive system:Large in/	0.4506
MGYA00244051/ERZ653696_FASTA.fasta	MGYA00244051	ERZ653696	root:Host-associated:Human:Digestive system:Large in/	0.2618
MGYA00244052/ERZ653698_FASTA.fasta	MGYA00244052	ERZ653698	root:Host-associated:Human:Digestive system:Large in/	0.3022
MGYA00244053/ERZ653699_FASTA.fasta	MGYA00244053	ERZ653699	root:Host-associated:Human:Digestive system:Large in/	0.336
MGYA00244054/ERZ653700_FASTA.fasta	MGYA00244054	ERZ653700	root:Host-associated:Human:Digestive system:Large in/	0.3336
MGYA00244055/ERZ653702_FASTA.fasta	MGYA00244055	ERZ653702	root:Host-associated:Human:Digestive system:Large in/	0.052
MGYA00244056/ERZ653704_FASTA.fasta	MGYA00244056	ERZ653704	root:Host-associated:Human:Digestive system:Large in/	0.346
MGYA00244057/ERZ653706_FASTA.fasta	MGYA00244057	ERZ653706	root:Host-associated:Human:Digestive system:Large in/	0.3574
MGYA00244058/ERZ653438_FASTA.fasta	MGYA00244058	ERZ653438	root:Host-associated:Human:Digestive system:Large in/	0.3332
MGYA00244059/ERZ653440_FASTA.fasta	MGYA00244059	ERZ653440	root:Host-associated:Human:Digestive system:Large in/	0.3324
MGYA00244060/ERZ653442_FASTA.fasta	MGYA00244060	ERZ653442	root:Host-associated:Human:Digestive system:Large in/	0.1514
MGYA00244061/ERZ653444_FASTA.fasta	MGYA00244061	ERZ653444	root:Host-associated:Human:Digestive system:Large in/	0.3932
MGYA00244062/ERZ653446_FASTA.fasta	MGYA00244062	ERZ653446	root:Host-associated:Human:Digestive system:Large in/	0.3212
MGYA00244063/ERZ653448_FASTA.fasta	MGYA00244063	ERZ653448	root:Host-associated:Human:Digestive system:Large in/	0.3616
MGYA00244064/ERZ653450_FASTA.fasta	MGYA00244064	ERZ653450	root:Host-associated:Human:Digestive system:Large in/	0.2034
MGYA00244065/ERZ653452_FASTA.fasta	MGYA00244065	ERZ653452	root:Host-associated:Human:Digestive system:Large in/	0.212
MGYA00244066/ERZ653454_FASTA.fasta	MGYA00244066	ERZ653454	root:Host-associated:Human:Digestive system:Large in/	0.392
MGYA00244067/ERZ653456_FASTA.fasta	MGYA00244067	ERZ653456	root:Host-associated:Human:Digestive system:Large in/	0.3378
MGYA00244068/ERZ653460_FASTA.fasta	MGYA00244068	ERZ653460	root:Host-associated:Human:Digestive system:Large in/	0.3928
MGYA00244069/ERZ653462_FASTA.fasta	MGYA00244069	ERZ653462	root:Host-associated:Human:Digestive system:Large in/	0.2816
MGYA00244070/ERZ653464_FASTA.fasta	MGYA00244070	ERZ653464	root:Host-associated:Human:Digestive system:Large in/	0.2216
MGYA00244071/ERZ653466_FASTA.fasta	MGYA00244071	ERZ653466	root:Host-associated:Human:Digestive system:Large in/	0.3992
MGYA00244072/ERZ653468_FASTA.fasta	MGYA00244072	ERZ653468	root:Host-associated:Human:Digestive system:Large in/	0.2624
MGYA00244073/ERZ653470_FASTA.fasta	MGYA00244073	ERZ653470	root:Host-associated:Human:Digestive system:Large in/	0.384
MGYA00244074/ERZ653472_FASTA.fasta	MGYA00244074	ERZ653472	root:Host-associated:Human:Digestive system:Large in/	0.345
MGYA00244075/ERZ653474_FASTA.fasta	MGYA00244075	ERZ653474	root:Host-associated:Human:Digestive system:Large in/	0.3832
MGYA00244076/ERZ653476_FASTA.fasta	MGYA00244076	ERZ653476	root:Host-associated:Human:Digestive system:Large in/	0.3778
MGYA00244077/ERZ653478_FASTA.fasta	MGYA00244077	ERZ653478	root:Host-associated:Human:Digestive system:Large in/	0.278
MGYA00244078/ERZ653480_FASTA.fasta	MGYA00244078	ERZ653480	root:Host-associated:Human:Digestive system:Large in/	0.2252
MGYA00244079/ERZ653562_FASTA.fasta	MGYA00244079	ERZ653562	root:Host-associated:Human:Digestive system:Large in/	0.2674
MGYA00244080/ERZ653566_FASTA.fasta	MGYA00244080	ERZ653566	root:Host-associated:Human:Digestive system:Large in/	0.3056
MGYA00244081/ERZ653568_FASTA.fasta	MGYA00244081	ERZ653568	root:Host-associated:Human:Digestive system:Large in/	0.235
MGYA00244082/ERZ653570_FASTA.fasta	MGYA00244082	ERZ653570	root:Host-associated:Human:Digestive system:Large in/	0.3318
MGYA00244083/ERZ653572_FASTA.fasta	MGYA00244083	ERZ653572	root:Host-associated:Human:Digestive system:Large in/	0.4034
MGYA00244084/ERZ653574_FASTA.fasta	MGYA00244084	ERZ653574	root:Host-associated:Human:Digestive system:Large in/	0.2912
MGYA00244085/ERZ653576_FASTA.fasta	MGYA00244085	ERZ653576	root:Host-associated:Human:Digestive system:Large in/	0.2586
MGYA00244086/ERZ653578_FASTA.fasta	MGYA00244086	ERZ653578	root:Host-associated:Human:Digestive system:Large in/	0.4758
MGYA00244087/ERZ653580_FASTA.fasta	MGYA00244087	ERZ653580	root:Host-associated:Human:Digestive system:Large in/	0.339
MGYA00244088/ERZ653582_FASTA.fasta	MGYA00244088	ERZ653582	root:Host-associated:Human:Digestive system:Large in/	0.3812
MGYA00244089/ERZ653584_FASTA.fasta	MGYA00244089	ERZ653584	root:Host-associated:Human:Digestive system:Large in/	0.3068
MGYA00244090/ERZ653586_FASTA.fasta	MGYA00244090	ERZ653586	root:Host-associated:Human:Digestive system:Large in/	0.3282
MGYA00244091/ERZ653104_FASTA.fasta	MGYA00244091	ERZ653104	root:Host-associated:Human:Digestive system:Large in/	0.1202
MGYA00244092/ERZ653106_FASTA.fasta	MGYA00244092	ERZ653106	root:Host-associated:Human:Digestive system:Large in/	0.0788
MGYA00244093/ERZ653108_FASTA.fasta	MGYA00244093	ERZ653108	root:Host-associated:Human:Digestive system:Large in/	0.0656
MGYA00244094/ERZ653110_FASTA.fasta	MGYA00244094	ERZ653110	root:Host-associated:Human:Digestive system:Large in/	0.07
MGYA00244095/ERZ653112_FASTA.fasta	MGYA00244095	ERZ653112	root:Host-associated:Human:Digestive system:Large in/	0.2714
MGYA00244096/ERZ653114_FASTA.fasta	MGYA00244096	ERZ653114	root:Host-associated:Human:Digestive system:Large in/	0.0942
MGYA00244097/ERZ653116_FASTA.fasta	MGYA00244097	ERZ653116	root:Host-associated:Human:Digestive system:Large in/	0.0382
MGYA00244098/ERZ653118_FASTA.fasta	MGYA00244098	ERZ653118	root:Host-associated:Human:Digestive system:Large in/	0.141
MGYA00244099/ERZ653120_FASTA.fasta	MGYA00244099	ERZ653120	root:Host-associated:Human:Digestive system:Large in/	0.0914
MGYA00244100/ERZ653122_FASTA.fasta	MGYA00244100	ERZ653122	root:Host-associated:Human:Digestive system:Large in/	0.0582
MGYA00244101/ERZ653124_FASTA.fasta	MGYA00244101	ERZ653124	root:Host-associated:Human:Digestive system:Large in/	0.0666
MGYA00244102/ERZ653126_FASTA.fasta	MGYA00244102	ERZ653126	root:Host-associated:Human:Digestive system:Large in/	0.04
MGYA00244103/ERZ653128_FASTA.fasta	MGYA00244103	ERZ653128	root:Host-associated:Human:Digestive system:Large in/	0.0458
MGYA00244104/ERZ653130_FASTA.fasta	MGYA00244104	ERZ653130	root:Host-associated:Human:Digestive system:Large in/	0.0754
MGYA00244105/ERZ653132_FASTA.fasta	MGYA00244105	ERZ653132	root:Host-associated:Human:Digestive system:Large in/	0.0422
MGYA00244106/ERZ653355_FASTA.fasta	MGYA00244106	ERZ653355	root:Host-associated:Human:Digestive system:Large in/	0.052
MGYA00244107/ERZ653357_FASTA.fasta	MGYA00244107	ERZ653357	root:Host-associated:Human:Digestive system:Large in/	0.0256
MGYA00244108/ERZ653359_FASTA.fasta	MGYA00244108	ERZ653359	root:Host-associated:Human:Digestive system:Large in/	0.0326
MGYA00244109/ERZ653361_FASTA.fasta	MGYA00244109	ERZ653361	root:Host-associated:Human:Digestive system:Large in/	0.1314
MGYA00244110/ERZ653363_FASTA.fasta	MGYA00244110	ERZ653363	root:Host-associated:Human:Digestive system:Large in/	0.0758
MGYA00244111/ERZ653365_FASTA.fasta	MGYA00244111	ERZ653365	root:Host-associated:Human:Digestive system:Large in/	0.069
MGYA00244112/ERZ654658_FASTA.fasta	MGYA00244112	ERZ654658	root:Host-associated:Human:Digestive system:Large in/	0.3
MGYA00244113/ERZ653190_FASTA.fasta	MGYA00244113	ERZ653190	root:Host-associated:Human:Digestive system:Large in/	0.0414
MGYA00244114/ERZ653192_FASTA.fasta	MGYA00244114	ERZ653192	root:Host-associated:Human:Digestive system:Large in/	0.1804
MGYA00244115/ERZ653194_FASTA.fasta	MGYA00244115	ERZ653194	root:Host-associated:Human:Digestive system:Large in/	0.1712
MGYA00244116/ERZ653196_FASTA.fasta	MGYA00244116	ERZ653196	root:Host-associated:Human:Digestive system:Large in/	0.0414
MGYA00244117/ERZ653956_FASTA.fasta	MGYA00244117	ERZ653956	root:Host-associated:Human:Digestive system:Large in/	0.2412
MGYA00244118/ERZ653958_FASTA.fasta	MGYA00244118	ERZ653958	root:Host-associated:Human:Digestive system:Large in/	0.3302
MGYA00244119/ERZ653960_FASTA.fasta	MGYA00244119	ERZ653960	root:Host-associated:Human:Digestive system:Large in/	0.2694
MGYA00244120/ERZ653962_FASTA.fasta	MGYA00244120	ERZ653962	root:Host-associated:Human:Digestive system:Large in/	0.254
MGYA00244121/ERZ653964_FASTA.fasta	MGYA00244121	ERZ653964	root:Host-associated:Human:Digestive system:Large in/	0.2636
MGYA00244122/ERZ653966_FASTA.fasta	MGYA00244122	ERZ653966	root:Host-associated:Human:Digestive system:Large in/	0.154
MGYA00244123/ERZ653968_FASTA.fasta	MGYA00244123	ERZ653968	root:Host-associated:Human:Digestive system:Large in/	0.3512
MGYA00244124/ERZ653970_FASTA.fasta	MGYA00244124	ERZ653970	root:Host-associated:Human:Digestive system:Large in/	0.2814
MGYA00244125/ERZ653972_FASTA.fasta	MGYA00244125	ERZ653972	root:Host-associated:Human:Digestive system:Large in/	0.2684
MGYA00244126/ERZ653974_FASTA.fasta	MGYA00244126	ERZ653974	root:Host-associated:Human:Digestive system:Large in/	0.281
MGYA00244127/ERZ653976_FASTA.fasta	MGYA00244127	ERZ653976	root:Host-associated:Human:Digestive system:Large in/	0.2366
MGYA00244128/ERZ653978_FASTA.fasta	MGYA00244128	ERZ653978	root:Host-associated:Human:Digestive system:Large in/	0.2048
MGYA00244129/ERZ653980_FASTA.fasta	MGYA00244129	ERZ653980	root:Host-associated:Human:Digestive system:Large in/	0.2956
MGYA00244130/ERZ653982_FASTA.fasta	MGYA00244130	ERZ653982	root:Host-associated:Human:Digestive system:Large in/	0.1404
MGYA00244131/ERZ653984_FASTA.fasta	MGYA00244131	ERZ653984	root:Host-associated:Human:Digestive system:Large in/	0.2822
MGYA00244132/ERZ653986_FASTA.fasta	MGYA00244132	ERZ653986	root:Host-associated:Human:Digestive system:Large in/	0.2922
MGYA00244133/ERZ653708_FASTA.fasta	MGYA00244133	ERZ653708	root:Host-associated:Human:Digestive system:Large in/	0.3212
MGYA00244134/ERZ653710_FASTA.fasta	MGYA00244134	ERZ653710	root:Host-associated:Human:Digestive system:Large in/	0.352
MGYA00244135/ERZ653712_FASTA.fasta	MGYA00244135	ERZ653712	root:Host-associated:Human:Digestive system:Large in/	0.3236
MGYA00244136/ERZ653714_FASTA.fasta	MGYA00244136	ERZ653714	root:Host-associated:Human:Digestive system:Large in/	0.3328
MGYA00244137/ERZ653716_FASTA.fasta	MGYA00244137	ERZ653716	root:Host-associated:Human:Digestive system:Large in/	0.3612
MGYA00244138/ERZ653718_FASTA.fasta	MGYA00244138	ERZ653718	root:Host-associated:Human:Digestive system:Large in/	0.282
MGYA00244139/ERZ653720_FASTA.fasta	MGYA00244139	ERZ653720	root:Host-associated:Human:Digestive system:Large in/	0.3896
MGYA00244140/ERZ653722_FASTA.fasta	MGYA00244140	ERZ653722	root:Host-associated:Human:Digestive system:Large in/	0.349
MGYA00244141/ERZ653724_FASTA.fasta	MGYA00244141	ERZ653724	root:Host-associated:Human:Digestive system:Large in/	0.5004
MGYA00244142/ERZ653726_FASTA.fasta	MGYA00244142	ERZ653726	root:Host-associated:Human:Digestive system:Large in/	0.3274
MGYA00244143/ERZ653728_FASTA.fasta	MGYA00244143	ERZ653728	root:Host-associated:Human:Digestive system:Large in/	0.3714
MGYA00244144/ERZ653730_FASTA.fasta	MGYA00244144	ERZ653730	root:Host-associated:Human:Digestive system:Large in/	0.2314
MGYA00244145/ERZ653732_FASTA.fasta	MGYA00244145	ERZ653732	root:Host-associated:Human:Digestive system:Large in/	0.2632
MGYA00244146/ERZ653734_FASTA.fasta	MGYA00244146	ERZ653734	root:Host-associated:Human:Digestive system:Large in/	0.4084
MGYA00244147/ERZ653736_FASTA.fasta	MGYA00244147	ERZ653736	root:Host-associated:Human:Digestive system:Large in/	0.31
MGYA00244148/ERZ653738_FASTA.fasta	MGYA00244148	ERZ653738	root:Host-associated:Human:Digestive system:Large in/	0.3282
MGYA00244149/ERZ653740_FASTA.fasta	MGYA00244149	ERZ653740	root:Host-associated:Human:Digestive system:Large in/	0.5818
MGYA00244150/ERZ653742_FASTA.fasta	MGYA00244150	ERZ653742	root:Host-associated:Human:Digestive system:Large in/	0.3052
MGYA00244151/ERZ653744_FASTA.fasta	MGYA00244151	ERZ653744	root:Host-associated:Human:Digestive system:Large in/	0.3068
MGYA00244152/ERZ653746_FASTA.fasta	MGYA00244152	ERZ653746	root:Host-associated:Human:Digestive system:Large in/	0.3658
MGYA00244153/ERZ653748_FASTA.fasta	MGYA00244153	ERZ653748	root:Host-associated:Human:Digestive system:Large in/	0.2782
MGYA00244154/ERZ653750_FASTA.fasta	MGYA00244154	ERZ653750	root:Host-associated:Human:Digestive system:Large in/	0.3042
MGYA00244155/ERZ653367_FASTA.fasta	MGYA00244155	ERZ653367	root:Host-associated:Human:Digestive system:Large in/	0.1958
MGYA00244156/ERZ653369_FASTA.fasta	MGYA00244156	ERZ653369	root:Host-associated:Human:Digestive system:Large in/	0.0514
MGYA00244157/ERZ653371_FASTA.fasta	MGYA00244157	ERZ653371	root:Host-associated:Human:Digestive system:Large in/	0.526
MGYA00244158/ERZ653373_FASTA.fasta	MGYA00244158	ERZ653373	root:Host-associated:Human:Digestive system:Large in/	0.3392
MGYA00244159/ERZ653375_FASTA.fasta	MGYA00244159	ERZ653375	root:Host-associated:Human:Digestive system:Large in/	0.445
MGYA00244160/ERZ653377_FASTA.fasta	MGYA00244160	ERZ653377	root:Host-associated:Human:Digestive system:Large in/	0.4052
MGYA00244161/ERZ653379_FASTA.fasta	MGYA00244161	ERZ653379	root:Host-associated:Human:Digestive system:Large in/	0.4244
MGYA00244162/ERZ653381_FASTA.fasta	MGYA00244162	ERZ653381	root:Host-associated:Human:Digestive system:Large in/	0.3828
MGYA00244163/ERZ653383_FASTA.fasta	MGYA00244163	ERZ653383	root:Host-associated:Human:Digestive system:Large in/	0.3294
MGYA00244164/ERZ653385_FASTA.fasta	MGYA00244164	ERZ653385	root:Host-associated:Human:Digestive system:Large in/	0.3392
MGYA00244165/ERZ653387_FASTA.fasta	MGYA00244165	ERZ653387	root:Host-associated:Human:Digestive system:Large in/	0.3476
MGYA00244166/ERZ653389_FASTA.fasta	MGYA00244166	ERZ653389	root:Host-associated:Human:Digestive system:Large in/	0.316
MGYA00244167/ERZ653391_FASTA.fasta	MGYA00244167	ERZ653391	root:Host-associated:Human:Digestive system:Large in/	0.2824
MGYA00244168/ERZ653393_FASTA.fasta	MGYA00244168	ERZ653393	root:Host-associated:Human:Digestive system:Large in/	0.3242
MGYA00244169/ERZ653395_FASTA.fasta	MGYA00244169	ERZ653395	root:Host-associated:Human:Digestive system:Large in/	0.3344
MGYA00244170/ERZ653397_FASTA.fasta	MGYA00244170	ERZ653397	root:Host-associated:Human:Digestive system:Large in/	0.436
MGYA00244171/ERZ653399_FASTA.fasta	MGYA00244171	ERZ653399	root:Host-associated:Human:Digestive system:Large in/	0.3534
MGYA00244172/ERZ653401_FASTA.fasta	MGYA00244172	ERZ653401	root:Host-associated:Human:Digestive system:Large in/	0.347
MGYA00244173/ERZ653403_FASTA.fasta	MGYA00244173	ERZ653403	root:Host-associated:Human:Digestive system:Large in/	0.3436
MGYA00244174/ERZ653405_FASTA.fasta	MGYA00244174	ERZ653405	root:Host-associated:Human:Digestive system:Large in/	0.2528
MGYA00244175/ERZ653407_FASTA.fasta	MGYA00244175	ERZ653407	root:Host-associated:Human:Digestive system:Large in/	0.337
MGYA00244176/ERZ653409_FASTA.fasta	MGYA00244176	ERZ653409	root:Host-associated:Human:Digestive system:Large in/	0.3764
MGYA00244177/ERZ653752_FASTA.fasta	MGYA00244177	ERZ653752	root:Host-associated:Human:Digestive system:Large in/	0.32
MGYA00244178/ERZ653754_FASTA.fasta	MGYA00244178	ERZ653754	root:Host-associated:Human:Digestive system:Large in/	0.496
MGYA00244179/ERZ653756_FASTA.fasta	MGYA00244179	ERZ653756	root:Host-associated:Human:Digestive system:Large in/	0.306
MGYA00244180/ERZ653758_FASTA.fasta	MGYA00244180	ERZ653758	root:Host-associated:Human:Digestive system:Large in/	0.4288
MGYA00244181/ERZ653797_FASTA.fasta	MGYA00244181	ERZ653797	root:Host-associated:Human:Digestive system:Large in/	0.2934
MGYA00244182/ERZ653799_FASTA.fasta	MGYA00244182	ERZ653799	root:Host-associated:Human:Digestive system:Large in/	0.255
MGYA00244183/ERZ653801_FASTA.fasta	MGYA00244183	ERZ653801	root:Host-associated:Human:Digestive system:Large in/	0.3206
MGYA00244184/ERZ653803_FASTA.fasta	MGYA00244184	ERZ653803	root:Host-associated:Human:Digestive system:Large in/	0.3076
MGYA00244185/ERZ653805_FASTA.fasta	MGYA00244185	ERZ653805	root:Host-associated:Human:Digestive system:Large in/	0.3184
MGYA00244186/ERZ653807_FASTA.fasta	MGYA00244186	ERZ653807	root:Host-associated:Human:Digestive system:Large in/	0.1724
MGYA00244187/ERZ653809_FASTA.fasta	MGYA00244187	ERZ653809	root:Host-associated:Human:Digestive system:Large in/	0.2692
MGYA00244188/ERZ653811_FASTA.fasta	MGYA00244188	ERZ653811	root:Host-associated:Human:Digestive system:Large in/	0.2698
MGYA00244189/ERZ653813_FASTA.fasta	MGYA00244189	ERZ653813	root:Host-associated:Human:Digestive system:Large in/	0.2508
MGYA00244190/ERZ653815_FASTA.fasta	MGYA00244190	ERZ653815	root:Host-associated:Human:Digestive system:Large in/	0.2522
MGYA00244191/ERZ653817_FASTA.fasta	MGYA00244191	ERZ653817	root:Host-associated:Human:Digestive system:Large in/	0.3316
MGYA00244192/ERZ653819_FASTA.fasta	MGYA00244192	ERZ653819	root:Host-associated:Human:Digestive system:Large in/	0.2492
MGYA00244193/ERZ653821_FASTA.fasta	MGYA00244193	ERZ653821	root:Host-associated:Human:Digestive system:Large in/	0.3928
MGYA00244194/ERZ653823_FASTA.fasta	MGYA00244194	ERZ653823	root:Host-associated:Human:Digestive system:Large in/	0.2648
MGYA00244195/ERZ653825_FASTA.fasta	MGYA00244195	ERZ653825	root:Host-associated:Human:Digestive system:Large in/	0.059
MGYA00244196/ERZ653827_FASTA.fasta	MGYA00244196	ERZ653827	root:Host-associated:Human:Digestive system:Large in/	0.2958
MGYA00244197/ERZ653829_FASTA.fasta	MGYA00244197	ERZ653829	root:Host-associated:Human:Digestive system:Large in/	0.285
MGYA00244198/ERZ653831_FASTA.fasta	MGYA00244198	ERZ653831	root:Host-associated:Human:Digestive system:Large in/	0.3012
MGYA00244199/ERZ653482_FASTA.fasta	MGYA00244199	ERZ653482	root:Host-associated:Human:Digestive system:Large in/	0.327
MGYA00244200/ERZ653484_FASTA.fasta	MGYA00244200	ERZ653484	root:Host-associated:Human:Digestive system:Large in/	0.3258
MGYA00244201/ERZ653486_FASTA.fasta	MGYA00244201	ERZ653486	root:Host-associated:Human:Digestive system:Large in/	0.2622
MGYA00244202/ERZ653488_FASTA.fasta	MGYA00244202	ERZ653488	root:Host-associated:Human:Digestive system:Large in/	0.3428
MGYA00244203/ERZ653490_FASTA.fasta	MGYA00244203	ERZ653490	root:Host-associated:Human:Digestive system:Large in/	0.3212
MGYA00244204/ERZ653492_FASTA.fasta	MGYA00244204	ERZ653492	root:Host-associated:Human:Digestive system:Large in/	0.3864
MGYA00244205/ERZ653494_FASTA.fasta	MGYA00244205	ERZ653494	root:Host-associated:Human:Digestive system:Large in/	0.3614
MGYA00244206/ERZ653496_FASTA.fasta	MGYA00244206	ERZ653496	root:Host-associated:Human:Digestive system:Large in/	0.329
MGYA00244207/ERZ653498_FASTA.fasta	MGYA00244207	ERZ653498	root:Host-associated:Human:Digestive system:Large in/	0.3742
MGYA00244208/ERZ653500_FASTA.fasta	MGYA00244208	ERZ653500	root:Host-associated:Human:Digestive system:Large in/	0.4378
MGYA00244209/ERZ653502_FASTA.fasta	MGYA00244209	ERZ653502	root:Host-associated:Human:Digestive system:Large in/	0.3584
MGYA00244210/ERZ653504_FASTA.fasta	MGYA00244210	ERZ653504	root:Host-associated:Human:Digestive system:Large in/	0.1096
MGYA00244211/ERZ654495_FASTA.fasta	MGYA00244211	ERZ654495	root:Host-associated:Human:Digestive system:Large in/	0.452
MGYA00244212/ERZ653411_FASTA.fasta	MGYA00244212	ERZ653411	root:Host-associated:Human:Digestive system:Large in/	0.3694
MGYA00244213/ERZ653413_FASTA.fasta	MGYA00244213	ERZ653413	root:Host-associated:Human:Digestive system:Large in/	0.257
MGYA00244214/ERZ653415_FASTA.fasta	MGYA00244214	ERZ653415	root:Host-associated:Human:Digestive system:Large in/	0.3368
MGYA00244215/ERZ653417_FASTA.fasta	MGYA00244215	ERZ653417	root:Host-associated:Human:Digestive system:Large in/	0.4174
MGYA00244216/ERZ653419_FASTA.fasta	MGYA00244216	ERZ653419	root:Host-associated:Human:Digestive system:Large in/	0.27
MGYA00244217/ERZ653421_FASTA.fasta	MGYA00244217	ERZ653421	root:Host-associated:Human:Digestive system:Large in/	0.3454
MGYA00244218/ERZ653423_FASTA.fasta	MGYA00244218	ERZ653423	root:Host-associated:Human:Digestive system:Large in/	0.3514
MGYA00244219/ERZ653425_FASTA.fasta	MGYA00244219	ERZ653425	root:Host-associated:Human:Digestive system:Large in/	0.3242
MGYA00244220/ERZ654241_FASTA.fasta	MGYA00244220	ERZ654241	root:Host-associated:Human:Digestive system:Large in/	0.2698
MGYA00244221/ERZ654243_FASTA.fasta	MGYA00244221	ERZ654243	root:Host-associated:Human:Digestive system:Large in/	0.209
MGYA00244222/ERZ654245_FASTA.fasta	MGYA00244222	ERZ654245	root:Host-associated:Human:Digestive system:Large in/	0.2728
MGYA00244223/ERZ654247_FASTA.fasta	MGYA00244223	ERZ654247	root:Host-associated:Human:Digestive system:Large in/	0.3122
MGYA00244224/ERZ654249_FASTA.fasta	MGYA00244224	ERZ654249	root:Host-associated:Human:Digestive system:Large in/	0.233
MGYA00244225/ERZ654250_FASTA.fasta	MGYA00244225	ERZ654250	root:Host-associated:Human:Digestive system:Large in/	0.3006
MGYA00244226/ERZ654251_FASTA.fasta	MGYA00244226	ERZ654251	root:Host-associated:Human:Digestive system:Large in/	0.2676
MGYA00244227/ERZ654252_FASTA.fasta	MGYA00244227	ERZ654252	root:Host-associated:Human:Digestive system:Large in/	0.2252
MGYA00244228/ERZ654716_FASTA.fasta	MGYA00244228	ERZ654716	root:Host-associated:Human:Digestive system:Large in/	0.4148
MGYA00244229/ERZ653988_FASTA.fasta	MGYA00244229	ERZ653988	root:Host-associated:Human:Digestive system:Large in/	0.2998
MGYA00244230/ERZ653990_FASTA.fasta	MGYA00244230	ERZ653990	root:Host-associated:Human:Digestive system:Large in/	0.1894
MGYA00244231/ERZ653992_FASTA.fasta	MGYA00244231	ERZ653992	root:Host-associated:Human:Digestive system:Large in/	0.2636
MGYA00244232/ERZ653994_FASTA.fasta	MGYA00244232	ERZ653994	root:Host-associated:Human:Digestive system:Large in/	0.3432
MGYA00244233/ERZ653996_FASTA.fasta	MGYA00244233	ERZ653996	root:Host-associated:Human:Digestive system:Large in/	0.212
MGYA00244234/ERZ653998_FASTA.fasta	MGYA00244234	ERZ653998	root:Host-associated:Human:Digestive system:Large in/	0.2682
MGYA00244235/ERZ653833_FASTA.fasta	MGYA00244235	ERZ653833	root:Host-associated:Human:Digestive system:Large in/	0.275
MGYA00244236/ERZ653835_FASTA.fasta	MGYA00244236	ERZ653835	root:Host-associated:Human:Digestive system:Large in/	0.0818
MGYA00244237/ERZ653837_FASTA.fasta	MGYA00244237	ERZ653837	root:Host-associated:Human:Digestive system:Large in/	0.2408
MGYA00244238/ERZ653839_FASTA.fasta	MGYA00244238	ERZ653839	root:Host-associated:Human:Digestive system:Large in/	0.2506
MGYA00244239/ERZ653841_FASTA.fasta	MGYA00244239	ERZ653841	root:Host-associated:Human:Digestive system:Large in/	0.2734
MGYA00244240/ERZ653843_FASTA.fasta	MGYA00244240	ERZ653843	root:Host-associated:Human:Digestive system:Large in/	0.2976
MGYA00244241/ERZ653845_FASTA.fasta	MGYA00244241	ERZ653845	root:Host-associated:Human:Digestive system:Large in/	0.2426
MGYA00244242/ERZ653849_FASTA.fasta	MGYA00244242	ERZ653849	root:Host-associated:Human:Digestive system:Large in/	0.2046
MGYA00244243/ERZ653851_FASTA.fasta	MGYA00244243	ERZ653851	root:Host-associated:Human:Digestive system:Large in/	0.2988
MGYA00244244/ERZ653853_FASTA.fasta	MGYA00244244	ERZ653853	root:Host-associated:Human:Digestive system:Large in/	0.2674
MGYA00244245/ERZ653855_FASTA.fasta	MGYA00244245	ERZ653855	root:Host-associated:Human:Digestive system:Large in/	0.2382
MGYA00244246/ERZ653857_FASTA.fasta	MGYA00244246	ERZ653857	root:Host-associated:Human:Digestive system:Large in/	0.2604
MGYA00244247/ERZ653859_FASTA.fasta	MGYA00244247	ERZ653859	root:Host-associated:Human:Digestive system:Large in/	0.3014
MGYA00244248/ERZ653861_FASTA.fasta	MGYA00244248	ERZ653861	root:Host-associated:Human:Digestive system:Large in/	0.322
MGYA00244249/ERZ653863_FASTA.fasta	MGYA00244249	ERZ653863	root:Host-associated:Human:Digestive system:Large in/	0.2214
MGYA00244250/ERZ653865_FASTA.fasta	MGYA00244250	ERZ653865	root:Host-associated:Human:Digestive system:Large in/	0.3098
MGYA00244251/ERZ653867_FASTA.fasta	MGYA00244251	ERZ653867	root:Host-associated:Human:Digestive system:Large in/	0.3138
MGYA00244252/ERZ653869_FASTA.fasta	MGYA00244252	ERZ653869	root:Host-associated:Human:Digestive system:Large in/	0.2512
MGYA00244253/ERZ653871_FASTA.fasta	MGYA00244253	ERZ653871	root:Host-associated:Human:Digestive system:Large in/	0.0494
MGYA00244254/ERZ653873_FASTA.fasta	MGYA00244254	ERZ653873	root:Host-associated:Human:Digestive system:Large in/	0.2936
MGYA00244255/ERZ653875_FASTA.fasta	MGYA00244255	ERZ653875	root:Host-associated:Human:Digestive system:Large in/	0.129
MGYA00244256/ERZ653877_FASTA.fasta	MGYA00244256	ERZ653877	root:Host-associated:Human:Digestive system:Large in/	0.2712
MGYA00244257/ERZ654253_FASTA.fasta	MGYA00244257	ERZ654253	root:Host-associated:Human:Digestive system:Large in/	0.2646
MGYA00244258/ERZ654254_FASTA.fasta	MGYA00244258	ERZ654254	root:Host-associated:Human:Digestive system:Large in/	0.2932
MGYA00244259/ERZ654255_FASTA.fasta	MGYA00244259	ERZ654255	root:Host-associated:Human:Digestive system:Large in/	0.2762
MGYA00244260/ERZ654256_FASTA.fasta	MGYA00244260	ERZ654256	root:Host-associated:Human:Digestive system:Large in/	0.2416
MGYA00244261/ERZ654257_FASTA.fasta	MGYA00244261	ERZ654257	root:Host-associated:Human:Digestive system:Large in/	0.3814
MGYA00244262/ERZ654258_FASTA.fasta	MGYA00244262	ERZ654258	root:Host-associated:Human:Digestive system:Large in/	0.4382
MGYA00244263/ERZ654259_FASTA.fasta	MGYA00244263	ERZ654259	root:Host-associated:Human:Digestive system:Large in/	0.3036
MGYA00244264/ERZ654260_FASTA.fasta	MGYA00244264	ERZ654260	root:Host-associated:Human:Digestive system:Large in/	0.2628
MGYA00244265/ERZ654261_FASTA.fasta	MGYA00244265	ERZ654261	root:Host-associated:Human:Digestive system:Large in/	0.372
MGYA00244266/ERZ654262_FASTA.fasta	MGYA00244266	ERZ654262	root:Host-associated:Human:Digestive system:Large in/	0.3098
MGYA00244267/ERZ654263_FASTA.fasta	MGYA00244267	ERZ654263	root:Host-associated:Human:Digestive system:Large in/	0.2014
MGYA00244268/ERZ654264_FASTA.fasta	MGYA00244268	ERZ654264	root:Host-associated:Human:Digestive system:Large in/	0.2754
MGYA00244269/ERZ654265_FASTA.fasta	MGYA00244269	ERZ654265	root:Host-associated:Human:Digestive system:Large in/	0.163
MGYA00244270/ERZ654266_FASTA.fasta	MGYA00244270	ERZ654266	root:Host-associated:Human:Digestive system:Large in/	0.316
MGYA00244271/ERZ654267_FASTA.fasta	MGYA00244271	ERZ654267	root:Host-associated:Human:Digestive system:Large in/	0.3068
MGYA00244272/ERZ654268_FASTA.fasta	MGYA00244272	ERZ654268	root:Host-associated:Human:Digestive system:Large in/	0.2998
MGYA00244273/ERZ654269_FASTA.fasta	MGYA00244273	ERZ654269	root:Host-associated:Human:Digestive system:Large in/	0.3598
MGYA00244274/ERZ654270_FASTA.fasta	MGYA00244274	ERZ654270	root:Host-associated:Human:Digestive system:Large in/	0.2522
MGYA00244275/ERZ654271_FASTA.fasta	MGYA00244275	ERZ654271	root:Host-associated:Human:Digestive system:Large in/	0.2774
MGYA00244276/ERZ654272_FASTA.fasta	MGYA00244276	ERZ654272	root:Host-associated:Human:Digestive system:Large in/	0.3088
MGYA00244277/ERZ654273_FASTA.fasta	MGYA00244277	ERZ654273	root:Host-associated:Human:Digestive system:Large in/	0.1746
MGYA00244278/ERZ654274_FASTA.fasta	MGYA00244278	ERZ654274	root:Host-associated:Human:Digestive system:Large in/	0.306
MGYA00244279/ERZ654774_FASTA.fasta	MGYA00244279	ERZ654774	root:Host-associated:Human:Digestive system:Large in/	0.4214
MGYA00244280/ERZ654275_FASTA.fasta	MGYA00244280	ERZ654275	root:Host-associated:Human:Digestive system:Large in/	0.306
MGYA00244281/ERZ654276_FASTA.fasta	MGYA00244281	ERZ654276	root:Host-associated:Human:Digestive system:Large in/	0.1838
MGYA00244282/ERZ654277_FASTA.fasta	MGYA00244282	ERZ654277	root:Host-associated:Human:Digestive system:Large in/	0.1962
MGYA00244283/ERZ654278_FASTA.fasta	MGYA00244283	ERZ654278	root:Host-associated:Human:Digestive system:Large in/	0.2882
MGYA00244284/ERZ654279_FASTA.fasta	MGYA00244284	ERZ654279	root:Host-associated:Human:Digestive system:Large in/	0.2334
MGYA00244285/ERZ654280_FASTA.fasta	MGYA00244285	ERZ654280	root:Host-associated:Human:Digestive system:Large in/	0.3972
MGYA00244286/ERZ654281_FASTA.fasta	MGYA00244286	ERZ654281	root:Host-associated:Human:Digestive system:Large in/	0.2786
MGYA00244287/ERZ654282_FASTA.fasta	MGYA00244287	ERZ654282	root:Host-associated:Human:Digestive system:Large in/	0.1804
MGYA00244288/ERZ654283_FASTA.fasta	MGYA00244288	ERZ654283	root:Host-associated:Human:Digestive system:Large in/	0.258
MGYA00244289/ERZ654284_FASTA.fasta	MGYA00244289	ERZ654284	root:Host-associated:Human:Digestive system:Large in/	0.2208
MGYA00244290/ERZ654285_FASTA.fasta	MGYA00244290	ERZ654285	root:Host-associated:Human:Digestive system:Large in/	0.1892
MGYA00244291/ERZ654286_FASTA.fasta	MGYA00244291	ERZ654286	root:Host-associated:Human:Digestive system:Large in/	0.2742
MGYA00244292/ERZ654287_FASTA.fasta	MGYA00244292	ERZ654287	root:Host-associated:Human:Digestive system:Large in/	0.2956
MGYA00244293/ERZ654288_FASTA.fasta	MGYA00244293	ERZ654288	root:Host-associated:Human:Digestive system:Large in/	0.2702
MGYA00244294/ERZ654289_FASTA.fasta	MGYA00244294	ERZ654289	root:Host-associated:Human:Digestive system:Large in/	0.0514
MGYA00244295/ERZ654290_FASTA.fasta	MGYA00244295	ERZ654290	root:Host-associated:Human:Digestive system:Large in/	0.1794
MGYA00244296/ERZ654291_FASTA.fasta	MGYA00244296	ERZ654291	root:Host-associated:Human:Digestive system:Large in/	0.2088
MGYA00244297/ERZ654292_FASTA.fasta	MGYA00244297	ERZ654292	root:Host-associated:Human:Digestive system:Large in/	0.338
MGYA00244298/ERZ654293_FASTA.fasta	MGYA00244298	ERZ654293	root:Host-associated:Human:Digestive system:Large in/	0.1474
MGYA00244299/ERZ654294_FASTA.fasta	MGYA00244299	ERZ654294	root:Host-associated:Human:Digestive system:Large in/	0.2596
MGYA00244300/ERZ654295_FASTA.fasta	MGYA00244300	ERZ654295	root:Host-associated:Human:Digestive system:Large in/	0.2428
MGYA00244301/ERZ654296_FASTA.fasta	MGYA00244301	ERZ654296	root:Host-associated:Human:Digestive system:Large in/	0.2268
MGYA00244302/ERZ653879_FASTA.fasta	MGYA00244302	ERZ653879	root:Host-associated:Human:Digestive system:Large in/	0.2258
MGYA00244303/ERZ653881_FASTA.fasta	MGYA00244303	ERZ653881	root:Host-associated:Human:Digestive system:Large in/	0.2398
MGYA00244304/ERZ653883_FASTA.fasta	MGYA00244304	ERZ653883	root:Host-associated:Human:Digestive system:Large in/	0.2688
MGYA00244305/ERZ653885_FASTA.fasta	MGYA00244305	ERZ653885	root:Host-associated:Human:Digestive system:Large in/	0.2824
MGYA00244306/ERZ654297_FASTA.fasta	MGYA00244306	ERZ654297	root:Host-associated:Human:Digestive system:Large in/	0.2022
MGYA00244307/ERZ654298_FASTA.fasta	MGYA00244307	ERZ654298	root:Host-associated:Human:Digestive system:Large in/	0.222
MGYA00244308/ERZ654299_FASTA.fasta	MGYA00244308	ERZ654299	root:Host-associated:Human:Digestive system:Large in/	0.3464
MGYA00244309/ERZ654300_FASTA.fasta	MGYA00244309	ERZ654300	root:Host-associated:Human:Digestive system:Large in/	0.2634
MGYA00244310/ERZ654301_FASTA.fasta	MGYA00244310	ERZ654301	root:Host-associated:Human:Digestive system:Large in/	0.2518
MGYA00244311/ERZ654302_FASTA.fasta	MGYA00244311	ERZ654302	root:Host-associated:Human:Digestive system:Large in/	0.242
MGYA00244312/ERZ654303_FASTA.fasta	MGYA00244312	ERZ654303	root:Host-associated:Human:Digestive system:Large in/	0.332
MGYA00244313/ERZ654304_FASTA.fasta	MGYA00244313	ERZ654304	root:Host-associated:Human:Digestive system:Large in/	0.2618
MGYA00244314/ERZ654305_FASTA.fasta	MGYA00244314	ERZ654305	root:Host-associated:Human:Digestive system:Large in/	0.2884
MGYA00244315/ERZ654306_FASTA.fasta	MGYA00244315	ERZ654306	root:Host-associated:Human:Digestive system:Large in/	0.2668
MGYA00244316/ERZ654308_FASTA.fasta	MGYA00244316	ERZ654308	root:Host-associated:Human:Digestive system:Large in/	0.2062
MGYA00244317/ERZ654309_FASTA.fasta	MGYA00244317	ERZ654309	root:Host-associated:Human:Digestive system:Large in/	0.2206
MGYA00244318/ERZ654310_FASTA.fasta	MGYA00244318	ERZ654310	root:Host-associated:Human:Digestive system:Large in/	0.3098
MGYA00244319/ERZ654311_FASTA.fasta	MGYA00244319	ERZ654311	root:Host-associated:Human:Digestive system:Large in/	0.2804
MGYA00244320/ERZ654312_FASTA.fasta	MGYA00244320	ERZ654312	root:Host-associated:Human:Digestive system:Large in/	0.381
MGYA00244321/ERZ654313_FASTA.fasta	MGYA00244321	ERZ654313	root:Host-associated:Human:Digestive system:Large in/	0.2688
MGYA00244322/ERZ654314_FASTA.fasta	MGYA00244322	ERZ654314	root:Host-associated:Human:Digestive system:Large in/	0.2728
MGYA00244323/ERZ654315_FASTA.fasta	MGYA00244323	ERZ654315	root:Host-associated:Human:Digestive system:Large in/	0.2704
MGYA00244324/ERZ654316_FASTA.fasta	MGYA00244324	ERZ654316	root:Host-associated:Human:Digestive system:Large in/	0.156
MGYA00244325/ERZ654317_FASTA.fasta	MGYA00244325	ERZ654317	root:Host-associated:Human:Digestive system:Large in/	0.3894
MGYA00244326/ERZ654318_FASTA.fasta	MGYA00244326	ERZ654318	root:Host-associated:Human:Digestive system:Large in/	0.2886
MGYA00244327/ERZ654165_FASTA.fasta	MGYA00244327	ERZ654165	root:Host-associated:Human:Digestive system:Large in/	0.2274
MGYA00244328/ERZ654166_FASTA.fasta	MGYA00244328	ERZ654166	root:Host-associated:Human:Digestive system:Large in/	0.2258
MGYA00244329/ERZ654168_FASTA.fasta	MGYA00244329	ERZ654168	root:Host-associated:Human:Digestive system:Large in/	0.2744
MGYA00244330/ERZ654170_FASTA.fasta	MGYA00244330	ERZ654170	root:Host-associated:Human:Digestive system:Large in/	0.1878
MGYA00244331/ERZ654172_FASTA.fasta	MGYA00244331	ERZ654172	root:Host-associated:Human:Digestive system:Large in/	0.2736
MGYA00244332/ERZ654174_FASTA.fasta	MGYA00244332	ERZ654174	root:Host-associated:Human:Digestive system:Large in/	0.418
MGYA00244333/ERZ654176_FASTA.fasta	MGYA00244333	ERZ654176	root:Host-associated:Human:Digestive system:Large in/	0.1964
MGYA00244334/ERZ654178_FASTA.fasta	MGYA00244334	ERZ654178	root:Host-associated:Human:Digestive system:Large in/	0.2736
MGYA00244335/ERZ654180_FASTA.fasta	MGYA00244335	ERZ654180	root:Host-associated:Human:Digestive system:Large in/	0.1342
MGYA00244336/ERZ654182_FASTA.fasta	MGYA00244336	ERZ654182	root:Host-associated:Human:Digestive system:Large in/	0.2574
MGYA00244337/ERZ654184_FASTA.fasta	MGYA00244337	ERZ654184	root:Host-associated:Human:Digestive system:Large in/	0.2852
MGYA00244338/ERZ654186_FASTA.fasta	MGYA00244338	ERZ654186	root:Host-associated:Human:Digestive system:Large in/	0.2566
MGYA00244339/ERZ654188_FASTA.fasta	MGYA00244339	ERZ654188	root:Host-associated:Human:Digestive system:Large in/	0.3176
MGYA00244340/ERZ654190_FASTA.fasta	MGYA00244340	ERZ654190	root:Host-associated:Human:Digestive system:Large in/	0.331
MGYA00244341/ERZ654192_FASTA.fasta	MGYA00244341	ERZ654192	root:Host-associated:Human:Digestive system:Large in/	0.3098
MGYA00244342/ERZ654194_FASTA.fasta	MGYA00244342	ERZ654194	root:Host-associated:Human:Digestive system:Large in/	0.2252
MGYA00244343/ERZ654196_FASTA.fasta	MGYA00244343	ERZ654196	root:Host-associated:Human:Digestive system:Large in/	0.2224
MGYA00244344/ERZ654198_FASTA.fasta	MGYA00244344	ERZ654198	root:Host-associated:Human:Digestive system:Large in/	0.2554
MGYA00244345/ERZ654200_FASTA.fasta	MGYA00244345	ERZ654200	root:Host-associated:Human:Digestive system:Large in/	0.273
MGYA00244346/ERZ654202_FASTA.fasta	MGYA00244346	ERZ654202	root:Host-associated:Human:Digestive system:Large in/	0.3092
MGYA00244347/ERZ654204_FASTA.fasta	MGYA00244347	ERZ654204	root:Host-associated:Human:Digestive system:Large in/	0.215
MGYA00244348/ERZ654206_FASTA.fasta	MGYA00244348	ERZ654206	root:Host-associated:Human:Digestive system:Large in/	0.3106
MGYA00244349/ERZ653887_FASTA.fasta	MGYA00244349	ERZ653887	root:Host-associated:Human:Digestive system:Large in/	0.2776
MGYA00244350/ERZ653889_FASTA.fasta	MGYA00244350	ERZ653889	root:Host-associated:Human:Digestive system:Large in/	0.4056
MGYA00244351/ERZ653891_FASTA.fasta	MGYA00244351	ERZ653891	root:Host-associated:Human:Digestive system:Large in/	0.3516
MGYA00244352/ERZ653893_FASTA.fasta	MGYA00244352	ERZ653893	root:Host-associated:Human:Digestive system:Large in/	0.3128
MGYA00244353/ERZ653895_FASTA.fasta	MGYA00244353	ERZ653895	root:Host-associated:Human:Digestive system:Large in/	0.3912
MGYA00244354/ERZ653897_FASTA.fasta	MGYA00244354	ERZ653897	root:Host-associated:Human:Digestive system:Large in/	0.3396
MGYA00244355/ERZ653899_FASTA.fasta	MGYA00244355	ERZ653899	root:Host-associated:Human:Digestive system:Large in/	0.3124
MGYA00244356/ERZ653901_FASTA.fasta	MGYA00244356	ERZ653901	root:Host-associated:Human:Digestive system:Large in/	0.3068
MGYA00244357/ERZ653903_FASTA.fasta	MGYA00244357	ERZ653903	root:Host-associated:Human:Digestive system:Large in/	0.2858
MGYA00244358/ERZ653905_FASTA.fasta	MGYA00244358	ERZ653905	root:Host-associated:Human:Digestive system:Large in/	0.2566
MGYA00244359/ERZ653907_FASTA.fasta	MGYA00244359	ERZ653907	root:Host-associated:Human:Digestive system:Large in/	0.2836
MGYA00244360/ERZ653909_FASTA.fasta	MGYA00244360	ERZ653909	root:Host-associated:Human:Digestive system:Large in/	0.308
MGYA00244361/ERZ653911_FASTA.fasta	MGYA00244361	ERZ653911	root:Host-associated:Human:Digestive system:Large in/	0.2224
MGYA00244362/ERZ653913_FASTA.fasta	MGYA00244362	ERZ653913	root:Host-associated:Human:Digestive system:Large in/	0.2842
MGYA00244363/ERZ653915_FASTA.fasta	MGYA00244363	ERZ653915	root:Host-associated:Human:Digestive system:Large in/	0.289
MGYA00244364/ERZ653917_FASTA.fasta	MGYA00244364	ERZ653917	root:Host-associated:Human:Digestive system:Large in/	0.2516
MGYA00244365/ERZ653919_FASTA.fasta	MGYA00244365	ERZ653919	root:Host-associated:Human:Digestive system:Large in/	0.261
MGYA00244366/ERZ653921_FASTA.fasta	MGYA00244366	ERZ653921	root:Host-associated:Human:Digestive system:Large in/	0.2934
MGYA00244367/ERZ653923_FASTA.fasta	MGYA00244367	ERZ653923	root:Host-associated:Human:Digestive system:Large in/	0.207
MGYA00244368/ERZ653925_FASTA.fasta	MGYA00244368	ERZ653925	root:Host-associated:Human:Digestive system:Large in/	0.382
MGYA00244369/ERZ653927_FASTA.fasta	MGYA00244369	ERZ653927	root:Host-associated:Human:Digestive system:Large in/	0.1954
MGYA00244370/ERZ653929_FASTA.fasta	MGYA00244370	ERZ653929	root:Host-associated:Human:Digestive system:Large in/	0.2974
MGYA00244371/ERZ654208_FASTA.fasta	MGYA00244371	ERZ654208	root:Host-associated:Human:Digestive system:Large in/	0.3086
MGYA00244372/ERZ654210_FASTA.fasta	MGYA00244372	ERZ654210	root:Host-associated:Human:Digestive system:Large in/	0.4358
MGYA00244373/ERZ654212_FASTA.fasta	MGYA00244373	ERZ654212	root:Host-associated:Human:Digestive system:Large in/	0.3932
MGYA00244374/ERZ654214_FASTA.fasta	MGYA00244374	ERZ654214	root:Host-associated:Human:Digestive system:Large in/	0.2468
MGYA00244375/ERZ654216_FASTA.fasta	MGYA00244375	ERZ654216	root:Host-associated:Human:Digestive system:Large in/	0.3666
MGYA00244376/ERZ654218_FASTA.fasta	MGYA00244376	ERZ654218	root:Host-associated:Human:Digestive system:Large in/	0.29
MGYA00244377/ERZ654220_FASTA.fasta	MGYA00244377	ERZ654220	root:Host-associated:Human:Digestive system:Large in/	0.2728
MGYA00244378/ERZ654222_FASTA.fasta	MGYA00244378	ERZ654222	root:Host-associated:Human:Digestive system:Large in/	0.3784
MGYA00244379/ERZ654224_FASTA.fasta	MGYA00244379	ERZ654224	root:Host-associated:Human:Digestive system:Large in/	0.2716
MGYA00244380/ERZ654226_FASTA.fasta	MGYA00244380	ERZ654226	root:Host-associated:Human:Digestive system:Large in/	0.2632
MGYA00244381/ERZ654228_FASTA.fasta	MGYA00244381	ERZ654228	root:Host-associated:Human:Digestive system:Large in/	0.3502
MGYA00244382/ERZ654230_FASTA.fasta	MGYA00244382	ERZ654230	root:Host-associated:Human:Digestive system:Large in/	0.2244
MGYA00244383/ERZ654232_FASTA.fasta	MGYA00244383	ERZ654232	root:Host-associated:Human:Digestive system:Large in/	0.2012
MGYA00244384/ERZ654234_FASTA.fasta	MGYA00244384	ERZ654234	root:Host-associated:Human:Digestive system:Large in/	0.3508
MGYA00244385/ERZ654236_FASTA.fasta	MGYA00244385	ERZ654236	root:Host-associated:Human:Digestive system:Large in/	0.3946
MGYA00244386/ERZ654238_FASTA.fasta	MGYA00244386	ERZ654238	root:Host-associated:Human:Digestive system:Large in/	0.2742
MGYA00244387/ERZ654240_FASTA.fasta	MGYA00244387	ERZ654240	root:Host-associated:Human:Digestive system:Large in/	0.358
MGYA00244388/ERZ654242_FASTA.fasta	MGYA00244388	ERZ654242	root:Host-associated:Human:Digestive system:Large in/	0.2286
MGYA00244389/ERZ654244_FASTA.fasta	MGYA00244389	ERZ654244	root:Host-associated:Human:Digestive system:Large in/	0.204
MGYA00244390/ERZ654330_FASTA.fasta	MGYA00244390	ERZ654330	root:Host-associated:Human:Digestive system:Large in/	0.3898
MGYA00244391/ERZ654331_FASTA.fasta	MGYA00244391	ERZ654331	root:Host-associated:Human:Digestive system:Large in/	0.36
MGYA00244392/ERZ654333_FASTA.fasta	MGYA00244392	ERZ654333	root:Host-associated:Human:Digestive system:Large in/	0.2926
MGYA00244393/ERZ654335_FASTA.fasta	MGYA00244393	ERZ654335	root:Host-associated:Human:Digestive system:Large in/	0.2858
MGYA00244394/ERZ654337_FASTA.fasta	MGYA00244394	ERZ654337	root:Host-associated:Human:Digestive system:Large in/	0.2832
MGYA00244395/ERZ654339_FASTA.fasta	MGYA00244395	ERZ654339	root:Host-associated:Human:Digestive system:Large in/	0.2816
MGYA00244396/ERZ654341_FASTA.fasta	MGYA00244396	ERZ654341	root:Host-associated:Human:Digestive system:Large in/	0.3312
MGYA00244397/ERZ654343_FASTA.fasta	MGYA00244397	ERZ654343	root:Host-associated:Human:Digestive system:Large in/	0.2764
MGYA00244398/ERZ654345_FASTA.fasta	MGYA00244398	ERZ654345	root:Host-associated:Human:Digestive system:Large in/	0.3466
MGYA00244399/ERZ654347_FASTA.fasta	MGYA00244399	ERZ654347	root:Host-associated:Human:Digestive system:Large in/	0.365
MGYA00244400/ERZ654349_FASTA.fasta	MGYA00244400	ERZ654349	root:Host-associated:Human:Digestive system:Large in/	0.3404
MGYA00244401/ERZ654351_FASTA.fasta	MGYA00244401	ERZ654351	root:Host-associated:Human:Digestive system:Large in/	0.199
MGYA00244402/ERZ654353_FASTA.fasta	MGYA00244402	ERZ654353	root:Host-associated:Human:Digestive system:Large in/	0.2748
MGYA00244403/ERZ654357_FASTA.fasta	MGYA00244403	ERZ654357	root:Host-associated:Human:Digestive system:Large in/	0.309
MGYA00244404/ERZ654359_FASTA.fasta	MGYA00244404	ERZ654359	root:Host-associated:Human:Digestive system:Large in/	0.3282
MGYA00244405/ERZ654361_FASTA.fasta	MGYA00244405	ERZ654361	root:Host-associated:Human:Digestive system:Large in/	0.0614
MGYA00244406/ERZ654363_FASTA.fasta	MGYA00244406	ERZ654363	root:Host-associated:Human:Digestive system:Large in/	0.2986
MGYA00244407/ERZ654365_FASTA.fasta	MGYA00244407	ERZ654365	root:Host-associated:Human:Digestive system:Large in/	0.2808
MGYA00244408/ERZ654367_FASTA.fasta	MGYA00244408	ERZ654367	root:Host-associated:Human:Digestive system:Large in/	0.2558
MGYA00244409/ERZ654369_FASTA.fasta	MGYA00244409	ERZ654369	root:Host-associated:Human:Digestive system:Large in/	0.275
MGYA00244410/ERZ654371_FASTA.fasta	MGYA00244410	ERZ654371	root:Host-associated:Human:Digestive system:Large in/	0.2704
MGYA00244411/ERZ654373_FASTA.fasta	MGYA00244411	ERZ654373	root:Host-associated:Human:Digestive system:Large in/	0.3122
MGYA00244412/ERZ654375_FASTA.fasta	MGYA00244412	ERZ654375	root:Host-associated:Human:Digestive system:Large in/	0.2444
MGYA00244413/ERZ654377_FASTA.fasta	MGYA00244413	ERZ654377	root:Host-associated:Human:Digestive system:Large in/	0.3048
MGYA00244414/ERZ654379_FASTA.fasta	MGYA00244414	ERZ654379	root:Host-associated:Human:Digestive system:Large in/	0.1244
MGYA00244415/ERZ654381_FASTA.fasta	MGYA00244415	ERZ654381	root:Host-associated:Human:Digestive system:Large in/	0.322
MGYA00244416/ERZ654383_FASTA.fasta	MGYA00244416	ERZ654383	root:Host-associated:Human:Digestive system:Large in/	0.2142
MGYA00244417/ERZ654385_FASTA.fasta	MGYA00244417	ERZ654385	root:Host-associated:Human:Digestive system:Large in/	0.1718
MGYA00244418/ERZ654387_FASTA.fasta	MGYA00244418	ERZ654387	root:Host-associated:Human:Digestive system:Large in/	0.26
MGYA00244419/ERZ654389_FASTA.fasta	MGYA00244419	ERZ654389	root:Host-associated:Human:Digestive system:Large in/	0.1096
MGYA00244420/ERZ654391_FASTA.fasta	MGYA00244420	ERZ654391	root:Host-associated:Human:Digestive system:Large in/	0.2374
MGYA00244421/ERZ654393_FASTA.fasta	MGYA00244421	ERZ654393	root:Host-associated:Human:Digestive system:Large in/	0.2156
MGYA00244422/ERZ654397_FASTA.fasta	MGYA00244422	ERZ654397	root:Host-associated:Human:Digestive system:Large in/	0.2438
MGYA00244423/ERZ654401_FASTA.fasta	MGYA00244423	ERZ654401	root:Host-associated:Human:Digestive system:Large in/	0.237
MGYA00244424/ERZ654403_FASTA.fasta	MGYA00244424	ERZ654403	root:Host-associated:Human:Digestive system:Large in/	0.308
MGYA00244425/ERZ654405_FASTA.fasta	MGYA00244425	ERZ654405	root:Host-associated:Human:Digestive system:Large in/	0.2452
MGYA00244426/ERZ654407_FASTA.fasta	MGYA00244426	ERZ654407	root:Host-associated:Human:Digestive system:Large in/	0.2148
MGYA00244427/ERZ654409_FASTA.fasta	MGYA00244427	ERZ654409	root:Host-associated:Human:Digestive system:Large in/	0.3252
MGYA00244428/ERZ654411_FASTA.fasta	MGYA00244428	ERZ654411	root:Host-associated:Human:Digestive system:Large in/	0.3108
MGYA00244429/ERZ654413_FASTA.fasta	MGYA00244429	ERZ654413	root:Host-associated:Human:Digestive system:Large in/	0.2842
MGYA00244430/ERZ654415_FASTA.fasta	MGYA00244430	ERZ654415	root:Host-associated:Human:Digestive system:Large in/	0.155
MGYA00244431/ERZ654417_FASTA.fasta	MGYA00244431	ERZ654417	root:Host-associated:Human:Digestive system:Large in/	0.152
MGYA00244432/ERZ654421_FASTA.fasta	MGYA00244432	ERZ654421	root:Host-associated:Human:Digestive system:Large in/	0.4498
MGYA00244433/ERZ654423_FASTA.fasta	MGYA00244433	ERZ654423	root:Host-associated:Human:Digestive system:Large in/	0.2816
MGYA00244434/ERZ654425_FASTA.fasta	MGYA00244434	ERZ654425	root:Host-associated:Human:Digestive system:Large in/	0.3414
MGYA00244435/ERZ654427_FASTA.fasta	MGYA00244435	ERZ654427	root:Host-associated:Human:Digestive system:Large in/	0.3604
MGYA00244436/ERZ654429_FASTA.fasta	MGYA00244436	ERZ654429	root:Host-associated:Human:Digestive system:Large in/	0.2568
MGYA00244437/ERZ653197_FASTA.fasta	MGYA00244437	ERZ653197	root:Host-associated:Human:Digestive system:Large in/	0.1354
MGYA00244438/ERZ653199_FASTA.fasta	MGYA00244438	ERZ653199	root:Host-associated:Human:Digestive system:Large in/	0.064
MGYA00244439/ERZ653201_FASTA.fasta	MGYA00244439	ERZ653201	root:Host-associated:Human:Digestive system:Large in/	0.0836
MGYA00244440/ERZ653203_FASTA.fasta	MGYA00244440	ERZ653203	root:Host-associated:Human:Digestive system:Large in/	0.0662
MGYA00244441/ERZ653205_FASTA.fasta	MGYA00244441	ERZ653205	root:Host-associated:Human:Digestive system:Large in/	0.0906
MGYA00244442/ERZ653207_FASTA.fasta	MGYA00244442	ERZ653207	root:Host-associated:Human:Digestive system:Large in/	0.0758
MGYA00244443/ERZ653209_FASTA.fasta	MGYA00244443	ERZ653209	root:Host-associated:Human:Digestive system:Large in/	0.1348
MGYA00244444/ERZ654355_FASTA.fasta	MGYA00244444	ERZ654355	root:Host-associated:Human:Digestive system:Large in/	0.2812
MGYA00244445/ERZ654399_FASTA.fasta	MGYA00244445	ERZ654399	root:Host-associated:Human:Digestive system:Large in/	0.4688
MGYA00244446/ERZ654433_FASTA.fasta	MGYA00244446	ERZ654433	root:Host-associated:Human:Digestive system:Large in/	0.3178
MGYA00244447/ERZ654435_FASTA.fasta	MGYA00244447	ERZ654435	root:Host-associated:Human:Digestive system:Large in/	0.4344
MGYA00244448/ERZ654437_FASTA.fasta	MGYA00244448	ERZ654437	root:Host-associated:Human:Digestive system:Large in/	0.3734
MGYA00244449/ERZ654439_FASTA.fasta	MGYA00244449	ERZ654439	root:Host-associated:Human:Digestive system:Large in/	0.3414
MGYA00244450/ERZ654441_FASTA.fasta	MGYA00244450	ERZ654441	root:Host-associated:Human:Digestive system:Large in/	0.404
MGYA00244451/ERZ654443_FASTA.fasta	MGYA00244451	ERZ654443	root:Host-associated:Human:Digestive system:Large in/	0.4578
MGYA00244452/ERZ654445_FASTA.fasta	MGYA00244452	ERZ654445	root:Host-associated:Human:Digestive system:Large in/	0.3568
MGYA00244453/ERZ654447_FASTA.fasta	MGYA00244453	ERZ654447	root:Host-associated:Human:Digestive system:Large in/	0.448
MGYA00244454/ERZ654449_FASTA.fasta	MGYA00244454	ERZ654449	root:Host-associated:Human:Digestive system:Large in/	0.385
MGYA00244455/ERZ654451_FASTA.fasta	MGYA00244455	ERZ654451	root:Host-associated:Human:Digestive system:Large in/	0.3088
MGYA00244456/ERZ654453_FASTA.fasta	MGYA00244456	ERZ654453	root:Host-associated:Human:Digestive system:Large in/	0.418
MGYA00244457/ERZ654455_FASTA.fasta	MGYA00244457	ERZ654455	root:Host-associated:Human:Digestive system:Large in/	0.3056
MGYA00244458/ERZ654457_FASTA.fasta	MGYA00244458	ERZ654457	root:Host-associated:Human:Digestive system:Large in/	0.5622
MGYA00244459/ERZ654459_FASTA.fasta	MGYA00244459	ERZ654459	root:Host-associated:Human:Digestive system:Large in/	0.4584
MGYA00244460/ERZ654461_FASTA.fasta	MGYA00244460	ERZ654461	root:Host-associated:Human:Digestive system:Large in/	0.2906
MGYA00244461/ERZ654463_FASTA.fasta	MGYA00244461	ERZ654463	root:Host-associated:Human:Digestive system:Large in/	0.488
MGYA00244462/ERZ654467_FASTA.fasta	MGYA00244462	ERZ654467	root:Host-associated:Human:Digestive system:Large in/	0.3726
MGYA00244463/ERZ653211_FASTA.fasta	MGYA00244463	ERZ653211	root:Host-associated:Human:Digestive system:Large in/	0.1638
MGYA00244464/ERZ653213_FASTA.fasta	MGYA00244464	ERZ653213	root:Host-associated:Human:Digestive system:Large in/	0.0344
MGYA00244465/ERZ653215_FASTA.fasta	MGYA00244465	ERZ653215	root:Host-associated:Human:Digestive system:Large in/	0.0688
MGYA00244466/ERZ653217_FASTA.fasta	MGYA00244466	ERZ653217	root:Host-associated:Human:Digestive system:Large in/	0.0942
MGYA00244467/ERZ653219_FASTA.fasta	MGYA00244467	ERZ653219	root:Host-associated:Human:Digestive system:Large in/	0.1198
MGYA00244468/ERZ653221_FASTA.fasta	MGYA00244468	ERZ653221	root:Host-associated:Human:Digestive system:Large in/	0.067

MGYA00244469/ERZ653223_FASTA.fasta	MGYA00244469	ERZ653223	root:Host-associated:Human:Digestive system:Large int	0.1012
MGYA00244470/ERZ653225_FASTA.fasta	MGYA00244470	ERZ653225	root:Host-associated:Human:Digestive system:Large int	0.0672
MGYA00244471/ERZ653227_FASTA.fasta	MGYA00244471	ERZ653227	root:Host-associated:Human:Digestive system:Large int	0.0736
MGYA00244472/ERZ653229_FASTA.fasta	MGYA00244472	ERZ653229	root:Host-associated:Human:Digestive system:Large int	0.081
MGYA00244473/ERZ653231_FASTA.fasta	MGYA00244473	ERZ653231	root:Host-associated:Human:Digestive system:Large int	0.1422
MGYA00244474/ERZ653233_FASTA.fasta	MGYA00244474	ERZ653233	root:Host-associated:Human:Digestive system:Large int	0.1152
MGYA00244475/ERZ653235_FASTA.fasta	MGYA00244475	ERZ653235	root:Host-associated:Human:Digestive system:Large int	0.1286
MGYA00244476/ERZ653237_FASTA.fasta	MGYA00244476	ERZ653237	root:Host-associated:Human:Digestive system:Large int	0.0686
MGYA00244477/ERZ653239_FASTA.fasta	MGYA00244477	ERZ653239	root:Host-associated:Human:Digestive system:Large int	0.1578
MGYA00244478/ERZ654879_FASTA.fasta	MGYA00244478	ERZ654879	root:Host-associated:Human:Digestive system:Large int	0.3162
MGYA00244479/ERZ654881_FASTA.fasta	MGYA00244479	ERZ654881	root:Host-associated:Human:Digestive system:Large int	0.3588
MGYA00244480/ERZ654885_FASTA.fasta	MGYA00244480	ERZ654885	root:Host-associated:Human:Digestive system:Large int	0.3164
MGYA00244481/ERZ654887_FASTA.fasta	MGYA00244481	ERZ654887	root:Host-associated:Human:Digestive system:Large int	0.1844
MGYA00244482/ERZ654891_FASTA.fasta	MGYA00244482	ERZ654891	root:Host-associated:Human:Digestive system:Large int	0.3636
MGYA00244483/ERZ654893_FASTA.fasta	MGYA00244483	ERZ654893	root:Host-associated:Human:Digestive system:Large int	0.3498
MGYA00244484/ERZ654895_FASTA.fasta	MGYA00244484	ERZ654895	root:Host-associated:Human:Digestive system:Large int	0.3416
MGYA00244485/ERZ654899_FASTA.fasta	MGYA00244485	ERZ654899	root:Host-associated:Human:Digestive system:Large int	0.3298
MGYA00244486/ERZ654901_FASTA.fasta	MGYA00244486	ERZ654901	root:Host-associated:Human:Digestive system:Large int	0.2512
MGYA00244487/ERZ654903_FASTA.fasta	MGYA00244487	ERZ654903	root:Host-associated:Human:Digestive system:Large int	0.3418
MGYA00244488/ERZ654905_FASTA.fasta	MGYA00244488	ERZ654905	root:Host-associated:Human:Digestive system:Large int	0.351
MGYA00244489/ERZ652704_FASTA.fasta	MGYA00244489	ERZ652704	root:Host-associated:Human:Digestive system:Large int	0.1648
MGYA00244490/ERZ652706_FASTA.fasta	MGYA00244490	ERZ652706	root:Host-associated:Human:Digestive system:Large int	0.3318
MGYA00244491/ERZ652708_FASTA.fasta	MGYA00244491	ERZ652708	root:Host-associated:Human:Digestive system:Large int	0.158
MGYA00244492/ERZ652710_FASTA.fasta	MGYA00244492	ERZ652710	root:Host-associated:Human:Digestive system:Large int	0.3062
MGYA00244493/ERZ652712_FASTA.fasta	MGYA00244493	ERZ652712	root:Host-associated:Human:Digestive system:Large int	0.309
MGYA00244494/ERZ652714_FASTA.fasta	MGYA00244494	ERZ652714	root:Host-associated:Human:Digestive system:Large int	0.2708
MGYA00244495/ERZ652716_FASTA.fasta	MGYA00244495	ERZ652716	root:Host-associated:Human:Digestive system:Large int	0.3246
MGYA00244496/ERZ652718_FASTA.fasta	MGYA00244496	ERZ652718	root:Host-associated:Human:Digestive system:Large int	0.2828
MGYA00244497/ERZ652720_FASTA.fasta	MGYA00244497	ERZ652720	root:Host-associated:Human:Digestive system:Large int	0.2414
MGYA00244498/ERZ652724_FASTA.fasta	MGYA00244498	ERZ652724	root:Host-associated:Human:Digestive system:Large int	0.2776
MGYA00244499/ERZ652726_FASTA.fasta	MGYA00244499	ERZ652726	root:Host-associated:Human:Digestive system:Large int	0.2936
MGYA00244500/ERZ652728_FASTA.fasta	MGYA00244500	ERZ652728	root:Host-associated:Human:Digestive system:Large int	0.2168
MGYA00244501/ERZ652730_FASTA.fasta	MGYA00244501	ERZ652730	root:Host-associated:Human:Digestive system:Large int	0.2772
MGYA00244502/ERZ652732_FASTA.fasta	MGYA00244502	ERZ652732	root:Host-associated:Human:Digestive system:Large int	0.3242
MGYA00244503/ERZ652734_FASTA.fasta	MGYA00244503	ERZ652734	root:Host-associated:Human:Digestive system:Large int	0.277
MGYA00244504/ERZ652736_FASTA.fasta	MGYA00244504	ERZ652736	root:Host-associated:Human:Digestive system:Large int	0.3198
MGYA00244505/ERZ652738_FASTA.fasta	MGYA00244505	ERZ652738	root:Host-associated:Human:Digestive system:Large int	0.2386
MGYA00244506/ERZ652740_FASTA.fasta	MGYA00244506	ERZ652740	root:Host-associated:Human:Digestive system:Large int	0.2952
MGYA00244507/ERZ652742_FASTA.fasta	MGYA00244507	ERZ652742	root:Host-associated:Human:Digestive system:Large int	0.2824
MGYA00244508/ERZ652801_FASTA.fasta	MGYA00244508	ERZ652801	root:Host-associated:Human:Digestive system:Large int	0.2126
MGYA00244509/ERZ652803_FASTA.fasta	MGYA00244509	ERZ652803	root:Host-associated:Human:Digestive system:Large int	0.161
MGYA00244510/ERZ652805_FASTA.fasta	MGYA00244510	ERZ652805	root:Host-associated:Human:Digestive system:Large int	0.3072
MGYA00244511/ERZ652807_FASTA.fasta	MGYA00244511	ERZ652807	root:Host-associated:Human:Digestive system:Large int	0.4412
MGYA00244512/ERZ652809_FASTA.fasta	MGYA00244512	ERZ652809	root:Host-associated:Human:Digestive system:Large int	0.3436
MGYA00244513/ERZ652811_FASTA.fasta	MGYA00244513	ERZ652811	root:Host-associated:Human:Digestive system:Large int	0.2844
MGYA00244514/ERZ652813_FASTA.fasta	MGYA00244514	ERZ652813	root:Host-associated:Human:Digestive system:Large int	0.2448
MGYA00244515/ERZ652815_FASTA.fasta	MGYA00244515	ERZ652815	root:Host-associated:Human:Digestive system:Large int	0.281
MGYA00244516/ERZ652817_FASTA.fasta	MGYA00244516	ERZ652817	root:Host-associated:Human:Digestive system:Large int	0.3072
MGYA00244517/ERZ652819_FASTA.fasta	MGYA00244517	ERZ652819	root:Host-associated:Human:Digestive system:Large int	0.2912
MGYA00244518/ERZ652821_FASTA.fasta	MGYA00244518	ERZ652821	root:Host-associated:Human:Digestive system:Large int	0.4328
MGYA00244519/ERZ652823_FASTA.fasta	MGYA00244519	ERZ652823	root:Host-associated:Human:Digestive system:Large int	0.3266
MGYA00244520/ERZ652825_FASTA.fasta	MGYA00244520	ERZ652825	root:Host-associated:Human:Digestive system:Large int	0.2804
MGYA00244521/ERZ652827_FASTA.fasta	MGYA00244521	ERZ652827	root:Host-associated:Human:Digestive system:Large int	0.2884
MGYA00244522/ERZ652829_FASTA.fasta	MGYA00244522	ERZ652829	root:Host-associated:Human:Digestive system:Large int	0.3144
MGYA00244523/ERZ653198_FASTA.fasta	MGYA00244523	ERZ653198	root:Host-associated:Human:Digestive system:Large int	0.2224
MGYA00244524/ERZ653200_FASTA.fasta	MGYA00244524	ERZ653200	root:Host-associated:Human:Digestive system:Large int	0.0276
MGYA00244525/ERZ653202_FASTA.fasta	MGYA00244525	ERZ653202	root:Host-associated:Human:Digestive system:Large int	0.043
MGYA00244526/ERZ653204_FASTA.fasta	MGYA00244526	ERZ653204	root:Host-associated:Human:Digestive system:Large int	0.074
MGYA00244527/ERZ653206_FASTA.fasta	MGYA00244527	ERZ653206	root:Host-associated:Human:Digestive system:Large int	0.0642
MGYA00244528/ERZ652948_FASTA.fasta	MGYA00244528	ERZ652948	root:Host-associated:Human:Digestive system:Large int	0.1188
MGYA00244529/ERZ652949_FASTA.fasta	MGYA00244529	ERZ652949	root:Host-associated:Human:Digestive system:Large int	0.0804
MGYA00244530/ERZ652950_FASTA.fasta	MGYA00244530	ERZ652950	root:Host-associated:Human:Digestive system:Large int	0.1354
MGYA00244531/ERZ652952_FASTA.fasta	MGYA00244531	ERZ652952	root:Host-associated:Human:Digestive system:Large int	0.0574
MGYA00244532/ERZ652954_FASTA.fasta	MGYA00244532	ERZ652954	root:Host-associated:Human:Digestive system:Large int	0.126
MGYA00244533/ERZ652956_FASTA.fasta	MGYA00244533	ERZ652956	root:Host-associated:Human:Digestive system:Large int	0.054
MGYA00244534/ERZ652958_FASTA.fasta	MGYA00244534	ERZ652958	root:Host-associated:Human:Digestive system:Large int	0.0496
MGYA00244535/ERZ652960_FASTA.fasta	MGYA00244535	ERZ652960	root:Host-associated:Human:Digestive system:Large int	0.1414
MGYA00244536/ERZ652962_FASTA.fasta	MGYA00244536	ERZ652962	root:Host-associated:Human:Digestive system:Large int	0.129
MGYA00244537/ERZ652964_FASTA.fasta	MGYA00244537	ERZ652964	root:Host-associated:Human:Digestive system:Large int	0.0514
MGYA00244538/ERZ652966_FASTA.fasta	MGYA00244538	ERZ652966	root:Host-associated:Human:Digestive system:Large int	0.2946
MGYA00244539/ERZ652968_FASTA.fasta	MGYA00244539	ERZ652968	root:Host-associated:Human:Digestive system:Large int	0.077
MGYA00244540/ERZ652970_FASTA.fasta	MGYA00244540	ERZ652970	root:Host-associated:Human:Digestive system:Large int	0.2946
MGYA00244541/ERZ652972_FASTA.fasta	MGYA00244541	ERZ652972	root:Host-associated:Human:Digestive system:Large int	0.118
MGYA00244542/ERZ652974_FASTA.fasta	MGYA00244542	ERZ652974	root:Host-associated:Human:Digestive system:Large int	0.0816
MGYA00244543/ERZ652976_FASTA.fasta	MGYA00244543	ERZ652976	root:Host-associated:Human:Digestive system:Large int	0.0684
MGYA00244544/ERZ652978_FASTA.fasta	MGYA00244544	ERZ652978	root:Host-associated:Human:Digestive system:Large int	0.0548
MGYA00244545/ERZ652980_FASTA.fasta	MGYA00244545	ERZ652980	root:Host-associated:Human:Digestive system:Large int	0.108
MGYA00244546/ERZ652982_FASTA.fasta	MGYA00244546	ERZ652982	root:Host-associated:Human:Digestive system:Large int	0.0748
MGYA00244547/ERZ652984_FASTA.fasta	MGYA00244547	ERZ652984	root:Host-associated:Human:Digestive system:Large int	0.0434
MGYA00244548/ERZ652986_FASTA.fasta	MGYA00244548	ERZ652986	root:Host-associated:Human:Digestive system:Large int	0.0802
MGYA00244549/ERZ652988_FASTA.fasta	MGYA00244549	ERZ652988	root:Host-associated:Human:Digestive system:Large int	0.044
MGYA00244550/ERZ654907_FASTA.fasta	MGYA00244550	ERZ654907	root:Host-associated:Human:Digestive system:Large int	0.0794
MGYA00244551/ERZ654909_FASTA.fasta	MGYA00244551	ERZ654909	root:Host-associated:Human:Digestive system:Large int	0.3596
MGYA00244552/ERZ654919_FASTA.fasta	MGYA00244552	ERZ654919	root:Host-associated:Human:Digestive system:Large int	0.4354
MGYA00244553/ERZ652744_FASTA.fasta	MGYA00244553	ERZ652744	root:Host-associated:Human:Digestive system:Large int	0.3958
MGYA00244554/ERZ652746_FASTA.fasta	MGYA00244554	ERZ652746	root:Host-associated:Human:Digestive system:Large int	0.3066
MGYA00244555/ERZ652748_FASTA.fasta	MGYA00244555	ERZ652748	root:Host-associated:Human:Digestive system:Large int	0.36
MGYA00244556/ERZ652750_FASTA.fasta	MGYA00244556	ERZ652750	root:Host-associated:Human:Digestive system:Large int	0.2798
MGYA00244557/ERZ652752_FASTA.fasta	MGYA00244557	ERZ652752	root:Host-associated:Human:Digestive system:Large int	0.3546
MGYA00244558/ERZ653177_FASTA.fasta	MGYA00244558	ERZ653177	root:Host-associated:Human:Digestive system:Large int	0.0758
MGYA00244559/ERZ653179_FASTA.fasta	MGYA00244559	ERZ653179	root:Host-associated:Human:Digestive system:Large int	0.1784
MGYA00244560/ERZ653181_FASTA.fasta	MGYA00244560	ERZ653181	root:Host-associated:Human:Digestive system:Large int	0.0384
MGYA00244561/ERZ653183_FASTA.fasta	MGYA00244561	ERZ653183	root:Host-associated:Human:Digestive system:Large int	0.1968
MGYA00244562/ERZ653185_FASTA.fasta	MGYA00244562	ERZ653185	root:Host-associated:Human:Digestive system:Large int	0.1012
MGYA00244563/ERZ653187_FASTA.fasta	MGYA00244563	ERZ653187	root:Host-associated:Human:Digestive system:Large int	0.1158
MGYA00244564/ERZ653189_FASTA.fasta	MGYA00244564	ERZ653189	root:Host-associated:Human:Digestive system:Large int	0.0356
MGYA00244565/ERZ653191_FASTA.fasta	MGYA00244565	ERZ653191	root:Host-associated:Human:Digestive system:Large int	0.038
MGYA00244566/ERZ653193_FASTA.fasta	MGYA00244566	ERZ653193	root:Host-associated:Human:Digestive system:Large int	0.0618
MGYA00244567/ERZ653195_FASTA.fasta	MGYA00244567	ERZ653195	root:Host-associated:Human:Digestive system:Large int	0.0988
MGYA00244568/ERZ653588_FASTA.fasta	MGYA00244568	ERZ653588	root:Host-associated:Human:Digestive system:Large int	0.3574
MGYA00244569/ERZ653590_FASTA.fasta	MGYA00244569	ERZ653590	root:Host-associated:Human:Digestive system:Large int	0.375
MGYA00244570/ERZ653592_FASTA.fasta	MGYA00244570	ERZ653592	root:Host-associated:Human:Digestive system:Large int	0.2916
MGYA00244571/ERZ653594_FASTA.fasta	MGYA00244571	ERZ653594	root:Host-associated:Human:Digestive system:Large int	0.4468
MGYA00244572/ERZ653596_FASTA.fasta	MGYA00244572	ERZ653596	root:Host-associated:Human:Digestive system:Large int	0.2902
MGYA00244573/ERZ653598_FASTA.fasta	MGYA00244573	ERZ653598	root:Host-associated:Human:Digestive system:Large int	0.3
MGYA00244574/ERZ653600_FASTA.fasta	MGYA00244574	ERZ653600	root:Host-associated:Human:Digestive system:Large int	0.3294
MGYA00244575/ERZ652831_FASTA.fasta	MGYA00244575	ERZ652831	root:Host-associated:Human:Digestive system:Large int	0.3148
MGYA00244576/ERZ652833_FASTA.fasta	MGYA00244576	ERZ652833	root:Host-associated:Human:Digestive system:Large int	0.359
MGYA00244577/ERZ652835_FASTA.fasta	MGYA00244577	ERZ652835	root:Host-associated:Human:Digestive system:Large int	0.3336
MGYA00244578/ERZ652837_FASTA.fasta	MGYA00244578	ERZ652837	root:Host-associated:Human:Digestive system:Large int	0.3286
MGYA00244579/ERZ652839_FASTA.fasta	MGYA00244579	ERZ652839	root:Host-associated:Human:Digestive system:Large int	0.1594
MGYA00244580/ERZ652841_FASTA.fasta	MGYA00244580	ERZ652841	root:Host-associated:Human:Digestive system:Large int	0.4584
MGYA00244581/ERZ652843_FASTA.fasta	MGYA00244581	ERZ652843	root:Host-associated:Human:Digestive system:Large int	0.2124
MGYA00244582/ERZ652845_FASTA.fasta	MGYA00244582	ERZ652845	root:Host-associated:Human:Digestive system:Large int	0.2692
MGYA00244583/ERZ652847_FASTA.fasta	MGYA00244583	ERZ652847	root:Host-associated:Human:Digestive system:Large int	0.343
MGYA00244584/ERZ652849_FASTA.fasta	MGYA00244584	ERZ652849	root:Host-associated:Human:Digestive system:Large int	0.2868
MGYA00244585/ERZ652851_FASTA.fasta	MGYA00244585	ERZ652851	root:Host-associated:Human:Digestive system:Large int	0.288
MGYA00244586/ERZ652853_FASTA.fasta	MGYA00244586	ERZ652853	root:Host-associated:Human:Digestive system:Large int	0.3218
MGYA00244587/ERZ652855_FASTA.fasta	MGYA00244587	ERZ652855	root:Host-associated:Human:Digestive system:Large int	0.2222
MGYA00244588/ERZ652857_FASTA.fasta	MGYA00244588	ERZ652857	root:Host-associated:Human:Digestive system:Large int	0.1758
MGYA00244589/ERZ652859_FASTA.fasta	MGYA00244589	ERZ652859	root:Host-associated:Human:Digestive system:Large int	0.255
MGYA00244590/ERZ653208_FASTA.fasta	MGYA00244590	ERZ653208	root:Host-associated:Human:Digestive system:Large int	0.0704
MGYA00244591/ERZ653210_FASTA.fasta	MGYA00244591	ERZ653210	root:Host-associated:Human:Digestive system:Large int	0.053
MGYA00244592/ERZ653212_FASTA.fasta	MGYA00244592	ERZ653212	root:Host-associated:Human:Digestive system:Large int	0.1428
MGYA00244593/ERZ653214_FASTA.fasta	MGYA00244593	ERZ653214	root:Host-associated:Human:Digestive system:Large int	0.0392
MGYA00244594/ERZ653216_FASTA.fasta	MGYA00244594	ERZ653216	root:Host-associated:Human:Digestive system:Large int	0.0838
MGYA00244595/ERZ653218_FASTA.fasta	MGYA00244595	ERZ653218	root:Host-associated:Human:Digestive system:Large int	0.073
MGYA00244596/ERZ653220_FASTA.fasta	MGYA00244596	ERZ653220	root:Host-associated:Human:Digestive system:Large int	0.0654
MGYA00244597/ERZ653222_FASTA.fasta	MGYA00244597	ERZ653222	root:Host-associated:Human:Digestive system:Large int	0.0538
MGYA00244598/ERZ653224_FASTA.fasta	MGYA00244598	ERZ653224	root:Host-associated:Human:Digestive system:Large int	0.0416
MGYA00244599/ERZ652990_FASTA.fasta	MGYA00244599	ERZ652990	root:Host-associated:Human:Digestive system:Large int	0.054
MGYA00244600/ERZ652992_FASTA.fasta	MGYA00244600	ERZ652992	root:Host-associated:Human:Digestive system:Large int	0.1274
MGYA00244601/ERZ652994_FASTA.fasta	MGYA00244601	ERZ652994	root:Host-associated:Human:Digestive system:Large int	0.1072
MGYA00244602/ERZ653602_FASTA.fasta	MGYA00244602	ERZ653602	root:Host-associated:Human:Digestive system:Large int	0.2646
MGYA00244603/ERZ653604_FASTA.fasta	MGYA00244603	ERZ653604	root:Host-associated:Human:Digestive system:Large int	0.4028
MGYA00244604/ERZ653606_FASTA.fasta	MGYA00244604	ERZ653606	root:Host-associated:Human:Digestive system:Large int	0.4816
MGYA00244605/ERZ653608_FASTA.fasta	MGYA00244605	ERZ653608	root:Host-associated:Human:Digestive system:Large int	0.347
MGYA00244606/ERZ653610_FASTA.fasta	MGYA00244606	ERZ653610	root:Host-associated:Human:Digestive system:Large int	0.4184
MGYA00244607/ERZ653612_FASTA.fasta	MGYA00244607	ERZ653612	root:Host-associated:Human:Digestive system:Large int	0.293
MGYA00244608/ERZ653614_FASTA.fasta	MGYA00244608	ERZ653614	root:Host-associated:Human:Digestive system:Large int	0.293
MGYA00244609/ERZ653616_FASTA.fasta	MGYA00244609	ERZ653616	root:Host-associated:Human:Digestive system:Large int	0.4224
MGYA00244610/ERZ653618_FASTA.fasta	MGYA00244610	ERZ653618	root:Host-associated:Human:Digestive system:Large int	0.262
MGYA00244611/ERZ653620_FASTA.fasta	MGYA00244611	ERZ653620	root:Host-associated:Human:Digestive system:Large int	0.3352
MGYA00244612/ERZ653622_FASTA.fasta	MGYA00244612	ERZ653622	root:Host-associated:Human:Digestive system:Large int	0.3034
MGYA00244613/ERZ653624_FASTA.fasta	MGYA00244613	ERZ653624	root:Host-associated:Human:Digestive system:Large int	0.2848
MGYA00244614/ERZ653626_FASTA.fasta	MGYA00244614	ERZ653626	root:Host-associated:Human:Digestive system:Large int	0.3386
MGYA00244615/ERZ653628_FASTA.fasta	MGYA00244615	ERZ653628	root:Host-associated:Human:Digestive system:Large int	0.3796
MGYA00244616/ERZ653629_FASTA.fasta	MGYA00244616	ERZ653629	root:Host-associated:Human:Digestive system:Large int	0.2292
MGYA00244617/ERZ653632_FASTA.fasta	MGYA00244617	ERZ653632	root:Host-associated:Human:Digestive system:Large int	0.2984
MGYA00244618/ERZ653634_FASTA.fasta	MGYA00244618	ERZ653634	root:Host-associated:Human:Digestive system:Large int	0.1332
MGYA00244619/ERZ653636_FASTA.fasta	MGYA00244619	ERZ653636	root:Host-associated:Human:Digestive system:Large int	0.2614
MGYA00244620/ERZ653638_FASTA.fasta	MGYA00244620	ERZ653638	root:Host-associated:Human:Digestive system:Large int	0.2588
MGYA00244621/ERZ653640_FASTA.fasta	MGYA00244621	ERZ653640	root:Host-associated:Human:Digestive system:Large int	0.3356
MGYA00244622/ERZ653642_FASTA.fasta	MGYA00244622	ERZ653642	root:Host-associated:Human:Digestive system:Large int	0.2908
MGYA00244623/ERZ653644_FASTA.fasta	MGYA00244623	ERZ653644	root:Host-associated:Human:Digestive system:Large int	0.2294
MGYA00244624/ERZ652861_FASTA.fasta	MGYA00244624	ERZ652861	root:Host-associated:Human:Digestive system:Large int	0.3114
MGYA00244625/ERZ652863_FASTA.fasta	MGYA00244625	ERZ652863	root:Host-associated:Human:Digestive system:Large int	0.2478
MGYA00244626/ERZ652865_FASTA.fasta	MGYA00244626	ERZ652865	root:Host-associated:Human:Digestive system:Large int	0.3024
MGYA00244627/ERZ652867_FASTA.fasta	MGYA00244627	ERZ652867	root:Host-associated:Human:Digestive system:Large int	0.1978
MGYA00244628/ERZ653226_FASTA.fasta	MGYA00244628	ERZ653226	root:Host-associated:Human:Digestive system:Large int	0.2086
MGYA00244629/ERZ653228_FASTA.fasta	MGYA00244629	ERZ653228	root:Host-associated:Human:Digestive system:Large int	0.0608
MGYA00244630/ERZ653230_FASTA.fasta	MGYA00244630	ERZ653230	root:Host-associated:Human:Digestive system:Large int	0.0564
MGYA00244631/ERZ653232_FASTA.fasta	MGYA00244631	ERZ653232	root:Host-associated:Human:Digestive system:Large int	0.101
MGYA00244632/ERZ653234_FASTA.fasta	MGYA00244632	ERZ653234	root:Host-associated:Human:Digestive system:Large int	0.0726
MGYA00244633/ERZ653236_FASTA.fasta	MGYA00244633	ERZ653236	root:Host-associated:Human:Digestive system:Large int	0.0452
MGYA00244634/ERZ653238_FASTA.fasta	MGYA00244634	ERZ653238	root:Host-associated:Human:Digestive system:Large int	0.1514
MGYA00244635/ERZ653240_FASTA.fasta	MGYA00244635	ERZ653240	root:Host-associated:Human:Digestive system:Large int	0.0338
MGYA00244636/ERZ653242_FASTA.fasta	MGYA00244636	ERZ653242	root:Host-associated:Human:Digestive system:Large int	0.078
MGYA00244637/ERZ653244_FASTA.fasta	MGYA00244637	ERZ653244	root:Host-associated:Human:Digestive system:Large int	0.0906
MGYA00244638/ERZ653246_FASTA.fasta	MGYA00244638	ERZ653246	root:Host-associated:Human:Digestive system:Large int	0.119
MGYA00244639/ERZ653248_FASTA.fasta	MGYA00244639	ERZ653248	root:Host-associated:Human:Digestive system:Large int	0.086
MGYA00244640/ERZ653250_FASTA.fasta	MGYA00244640	ERZ653250	root:Host-associated:Human:Digestive system:Large int	0.035
MGYA00244641/ERZ653252_FASTA.fasta	MGYA00244641	ERZ653252	root:Host-associated:Human:Digestive system:Large int	0.1456
MGYA00244642/ERZ653254_FASTA.fasta	MGYA00244642	ERZ653254	root:Host-associated:Human:Digestive system:Large int	0.1022
MGYA00244643/ERZ653256_FASTA.fasta	MGYA00244643	ERZ653256	root:Host-associated:Human:Digestive system:Large int	0.0976
MGYA00244644/ERZ653258_FASTA.fasta	MGYA00244644	ERZ653258	root:Host-associated:Human:Digestive system:Large int	0.1078
MGYA00244645/ERZ653260_FASTA.fasta	MGYA00244645	ERZ653260	root:Host-associated:Human:Digestive system:Large int	0.0476
MGYA00244646/ERZ653262_FASTA.fasta	MGYA00244646	ERZ653262	root:Host-associated:Human:Digestive system:Large int	0.091
MGYA00244647/ERZ653264_FASTA.fasta	MGYA00244647	ERZ653264	root:Host-associated:Human:Digestive system:Large int	0.0692
MGYA00244648/ERZ653266_FASTA.fasta	MGYA00244648	ERZ653266	root:Host-associated:Human:Digestive system:Large int	0.194
MGYA00244649/ERZ653268_FASTA.fasta	MGYA00244649	ERZ653268	root:Host-associated:Human:Digestive system:Large int	0.0512
MGYA00244650/ERZ653646_FASTA.fasta	MGYA00244650	ERZ653646	root:Host-associated:Human:Digestive system:Large int	0.2902
MGYA00244651/ERZ653648_FASTA.fasta	MGYA00244651	ERZ653648	root:Host-associated:Human:Digestive system:Large int	0.3894
MGYA00244652/ERZ653650_FASTA.fasta	MGYA00244652	ERZ653650	root:Host-associated:Human:Digestive system:Large int	0.301
MGYA00244653/ERZ653652_FASTA.fasta	MGYA00244653	ERZ653652	root:Host-associated:Human:Digestive system:Large int	0.4218
MGYA00244654/ERZ653654_FASTA.fasta	MGYA00244654	ERZ653654	root:Host-associated:Human:Digestive system:Large int	0.3136
MGYA00244655/ERZ653656_FASTA.fasta	MGYA00244655	ERZ653656	root:Host-associated:Human:Digestive system:Large int	0.346
MGYA00244656/ERZ653658_FASTA.fasta	MGYA00244656	ERZ653658	root:Host-associated:Human:Digestive system:Large int	0.2588
MGYA00244657/ERZ653660_FASTA.fasta	MGYA00244657	ERZ653660	root:Host-associated:Human:Digestive system:Large int	0.3068
MGYA00244658/ERZ653662_FASTA.fasta	MGYA00244658	ERZ653662	root:Host-associated:Human:Digestive system:Large int	0.3932
MGYA00244659/ERZ653664_FASTA.fasta	MGYA00244659	ERZ653664	root:Host-associated:Human:Digestive system:Large int	0.3082
MGYA00244660/ERZ653666_FASTA.fasta	MGYA00244660	ERZ653666	root:Host-associated:Human:Digestive system:Large int	0.3122
MGYA00244661/ERZ653668_FASTA.fasta	MGYA00244661	ERZ653668	root:Host-associated:Human:Digestive system:Large int	0.2438
MGYA00244662/ERZ653670_FASTA.fasta	MGYA00244662	ERZ653670	root:Host-associated:Human:Digestive system:Large int	0.444
MGYA00244663/ERZ653672_FASTA.fasta	MGYA00244663	ERZ653672	root:Host-associated:Human:Digestive system:Large int	0.3482
MGYA00244664/ERZ652869_FASTA.fasta	MGYA00244664	ERZ652869	root:Host-associated:Human:Digestive system:Large int	0.3242
MGYA00244665/ERZ652871_FASTA.fasta	MGYA00244665	ERZ652871	root:Host-associated:Human:Digestive system:Large int	0.3114
MGYA00244666/ERZ652872_FASTA.fasta	MGYA00244666	ERZ652872	root:Host-associated:Human:Digestive system:Large int	0.2336
MGYA00244667/ERZ653270_FASTA.fasta	MGYA00244667	ERZ653270	root:Host-associated:Human:Digestive system:Large int	0.0732
MGYA00244668/ERZ653272_FASTA.fasta	MGYA00244668	ERZ653272	root:Host-associated:Human:Digestive system:Large int	0.1996
MGYA00244669/ERZ653274_FASTA.fasta	MGYA00244669	ERZ653274	root:Host-associated:Human:Digestive system:Large int	0.1728
MGYA00244670/ERZ653276_FASTA.fasta	MGYA00244670	ERZ653276	root:Host-associated:Human:Digestive system:Large int	0.1494
MGYA00244671/ERZ653278_FASTA.fasta	MGYA00244671	ERZ653278	root:Host-associated:Human:Digestive system:Large int	0.1596
MGYA00244672/ERZ653280_FASTA.fasta	MGYA00244672	ERZ653280	root:Host-associated:Human:Digestive system:Large int	0.0518
MGYA00244673/ERZ653282_FASTA.fasta	MGYA00244673	ERZ653282	root:Host-associated:Human:Digestive system:Large int	0.083
MGYA00244674/ERZ653284_FASTA.fasta	MGYA00244674	ERZ653284	root:Host-associated:Human:Digestive system:Large int	0.2482
MGYA00244675/ERZ653286_FASTA.fasta	MGYA00244675	ERZ653286	root:Host-associated:Human:Digestive system:Large int	0.0588
MGYA00244676/ERZ653288_FASTA.fasta	MGYA00244676	ERZ653288	root:Host-associated:Human:Digestive system:Large int	0.145
MGYA00244677/ERZ653290_FASTA.fasta	MGYA00244677	ERZ653290	root:Host-associated:Human:Digestive system:Large int	0.0364
MGYA00244678/ERZ653292_FASTA.fasta	MGYA00244678	ERZ653292	root:Host-associated:Human:Digestive system:Large int	0.1424
MGYA00244679/ERZ653294_FASTA.fasta	MGYA00244679	ERZ653294	root:Host-associated:Human:Digestive system:Large int	0.0796
MGYA00244680/ERZ653296_FASTA.fasta	MGYA00244680	ERZ653296	root:Host-associated:Human:Digestive system:Large int	0.2956
MGYA00244681/ERZ653298_FASTA.fasta	MGYA00244681	ERZ653298	root:Host-associated:Human:Digestive system:Large int	0.048
MGYA00244682/ERZ653300_FASTA.fasta	MGYA00244682	ERZ653300	root:Host-associated:Human:Digestive system:Large int	0.0632
MGYA00244683/ERZ653302_FASTA.fasta	MGYA00244683	ERZ653302	root:Host-associated:Human:Digestive system:Large int	0.1642
MGYA00244684/ERZ653304_FASTA.fasta	MGYA00244684	ERZ653304	root:Host-associated:Human:Digestive system:Large int	0.1836
MGYA00244685/ERZ653306_FASTA.fasta	MGYA00244685	ERZ653306	root:Host-associated:Human:Digestive system:Large int	0.067
MGYA00244686/ERZ653308_FASTA.fasta	MGYA00244686	ERZ653308	root:Host-associated:Human:Digestive system:Large int	0.0374
MGYA00244687/ERZ653310_FASTA.fasta	MGYA00244687	ERZ653310	root:Host-associated:Human:Digestive system:Large int	0.1518
MGYA00244688/ERZ653312_FASTA.fasta	MGYA00244688	ERZ653312	root:Host-associated:Human:Digestive system:Large int	0.0252
MGYA00244689/ERZ652722_FASTA.fasta	MGYA00244689	ERZ652722	root:Host-associated:Human:Digestive system:Large int	0.3356
MGYA00244690/ERZ652723_FASTA.fasta	MGYA00244690	ERZ652723	root:Host-associated:Human:Digestive system:Large int	0.2512
MGYA00244691/ERZ652725_FASTA.fasta	MGYA00244691	ERZ652725	root:Host-associated:Human:Digestive system:Large int	0.2126
MGYA00244692/ERZ652727_FASTA.fasta	MGYA00244692	ERZ652727	root:Host-associated:Human:Digestive system:Large int	0.2458
MGYA00244693/ERZ652729_FASTA.fasta	MGYA00244693	ERZ652729	root:Host-associated:Human:Digestive system:Large int	0.261
MGYA00244694/ERZ652731_FASTA.fasta	MGYA00244694	ERZ652731	root:Host-associated:Human:Digestive system:Large int	0.2708
MGYA00244695/ERZ652733_FASTA.fasta	MGYA00244695	ERZ652733	root:Host-associated:Human:Digestive system:Large int	0.2044
MGYA00244696/ERZ652735_FASTA.fasta	MGYA00244696	ERZ652735	root:Host-associated:Human:Digestive system:Large int	0.3068
MGYA00244697/ERZ652737_FASTA.fasta	MGYA00244697	ERZ652737	root:Host-associated:Human:Digestive system:Large int	0.2856
MGYA00244698/ERZ652739_FASTA.fasta	MGYA00244698	ERZ652739	root:Host-associated:Human:Digestive system:Large int	0.2132
MGYA00244699/ERZ652741_FASTA.fasta	MGYA00244699	ERZ652741	root:Host-associated:Human:Digestive system:Large int	0.1056
MGYA00244700/ERZ653314_FASTA.fasta	MGYA00244700	ERZ653314	root:Host-associated:Human:Digestive system:Large int	0.1166
MGYA00244701/ERZ653316_FASTA.fasta	MGYA00244701	ERZ653316	root:Host-associated:Human:Digestive system:Large int	0.1286
MGYA00244702/ERZ653318_FASTA.fasta	MGYA00244702	ERZ653318	root:Host-associated:Human:Digestive system:Large int	0.0558
MGYA00244703/ERZ653320_FASTA.fasta	MGYA00244703	ERZ653320	root:Host-associated:Human:Digestive system:Large int	0.0976
MGYA00244704/ERZ653322_FASTA.fasta	MGYA00244704	ERZ653322	root:Host-associated:Human:Digestive system:Large int	0.0686
MGYA00244705/ERZ653324_FASTA.fasta	MGYA00244705	ERZ653324	root:Host-associated:Human:Digestive system:Large int	0.097
MGYA00244706/ERZ653326_FASTA.fasta	MGYA00244706	ERZ653326	root:Host-associated:Human:Digestive system:Large int	0.127
MGYA00244707/ERZ653328_FASTA.fasta	MGYA00244707	ERZ653328	root:Host-associated:Human:Digestive system:Large int	0.0942
MGYA00244708/ERZ653330_FASTA.fasta	MGYA00244708	ERZ653330	root:Host-associated:Human:Digestive system:Large int	0.0404
MGYA00244709/ERZ653332_FASTA.fasta	MGYA00244709	ERZ653332	root:Host-associated:Human:Digestive system:Large int	0.0446
MGYA00244710/ERZ653334_FASTA.fasta	MGYA00244710	ERZ653334	root:Host-associated:Human:Digestive system:Large int	0.114
MGYA00244711/ERZ653336_FASTA.fasta	MGYA00244711	ERZ653336	root:Host-associated:Human:Digestive system:Large int	0.0714
MGYA00244712/ERZ653338_FASTA.fasta	MGYA00244712	ERZ653338	root:Host-associated:Human:Digestive system:Large int	0.2694
MGYA00244713/ERZ653340_FASTA.fasta	MGYA00244713	ERZ653340	root:Host-associated:Human:Digestive system:Large int	0.2032
MGYA00244714/ERZ653342_FASTA.fasta	MGYA00244714	ERZ653342	root:Host-associated:Human:Digestive system:Large int	0.091
MGYA00244715/ERZ653346_FASTA.fasta	MGYA00244715	ERZ653346	root:Host-associated:Human:Digestive system:Large int	0.1222
MGYA00244716/ERZ653348_FASTA.fasta	MGYA00244716	ERZ653348	root:Host-associated:Human:Digestive system:Large int	0.177
MGYA00244717/ERZ653350_FASTA.fasta	MGYA00244717	ERZ653350	root:Host-associated:Human:Digestive system:Large int	0.0414
MGYA00244718/ERZ653352_FASTA.fasta	MGYA00244718	ERZ653352	root:Host-associated:Human:Digestive system:Large int	0.259
MGYA00244719/ERZ653354_FASTA.fasta	MGYA00244719	ERZ653354	root:Host-associated:Human:Digestive system:Large int	0.1016
MGYA00244720/ERZ653356_FASTA.fasta	MGYA00244720	ERZ653356	root:Host-associated:Human:Digestive system:Large int	0.072
MGYA00244721/ERZ653358_FASTA.fasta	MGYA00244721	ERZ653358	root:Host-associated:Human:Digestive system:Large int	0.109
MGYA00244722/ERZ652743_FASTA.fasta	MGYA00244722	ERZ652743	root:Host-associated:Human:Digestive system:Large int	0.3314
MGYA00244723/ERZ652745_FASTA.fasta	MGYA00244723	ERZ652745	root:Host-associated:Human:Digestive system:Large int	0.2712
MGYA00244724/ERZ652747_FASTA.fasta	MGYA00244724	ERZ652747	root:Host-associated:Human:Digestive system:Large int	0.3352
MGYA00244725/ERZ652749_FASTA.fasta	MGYA00244725	ERZ652749	root:Host-associated:Human:Digestive system:Large int	0.3224
MGYA00244726/ERZ652751_FASTA.fasta	MGYA00244726	ERZ652751	root:Host-associated:Human:Digestive system:Large int	0.2792
MGYA00244727/ERZ652753_FASTA.fasta	MGYA00244727	ERZ652753	root:Host-associated:Human:Digestive system:Large int	0.2084
MGYA00244728/ERZ652755_FASTA.fasta	MGYA00244728	ERZ652755	root:Host-associated:Human:Digestive system:Large int	0.2658
MGYA00244729/ERZ652757_FASTA.fasta	MGYA00244729	ERZ652757	root:Host-associated:Human:Digestive system:Large int	0.2592
MGYA00244730/ERZ652759_FASTA.fasta	MGYA00244730	ERZ652759	root:Host-associated:Human:Digestive system:Large int	0.2758
MGYA00244731/ERZ652761_FASTA.fasta	MGYA00244731	ERZ652761	root:Host-associated:Human:Digestive system:Large int	0.3078
MGYA00244732/ERZ652763_FASTA.fasta	MGYA00244732	ERZ652763	root:Host-associated:Human:Digestive system:Large int	0.2544
MGYA00244733/ERZ653673_FASTA.fasta	MGYA00244733	ERZ653673	root:Host-associated:Human:Digestive system:Large int	0.3184
MGYA00244734/ERZ653675_FASTA.fasta	MGYA00244734	ERZ653675	root:Host-associated:Human:Digestive system:Large int	0.3086
MGYA00244735/ERZ653677_FASTA.fasta	MGYA00244735	ERZ653677	root:Host-associated:Human:Digestive system:Large int	0.3054
MGYA00244736/ERZ653679_FASTA.fasta	MGYA00244736	ERZ653679	root:Host-associated:Human:Digestive system:Large int	0.2988
MGYA00244737/ERZ653681_FASTA.fasta	MGYA00244737	ERZ653681	root:Host-associated:Human:Digestive system:Large int	0.3386
MGYA00244738/ERZ653683_FASTA.fasta	MGYA00244738	ERZ653683	root:Host-associated:Human:Digestive system:Large int	0.3068
MGYA00244739/ERZ653685_FASTA.fasta	MGYA00244739	ERZ653685	root:Host-associated:Human:Digestive system:Large int	0.2904
MGYA00244740/ERZ653687_FASTA.fasta	MGYA00244740	ERZ653687	root:Host-associated:Human:Digestive system:Large int	0.3482
MGYA00244741/ERZ653689_FASTA.fasta	MGYA00244741	ERZ653689	root:Host-associated:Human:Digestive system:Large int	0.2846
MGYA00244742/ERZ653691_FASTA.fasta	MGYA00244742	ERZ653691	root:Host-associated:Human:Digestive system:Large int	0.3214
MGYA00244743/ERZ653693_FASTA.fasta	MGYA00244743	ERZ653693	root:Host-associated:Human:Digestive system:Large int	0.382
MGYA00244744/ERZ653695_FASTA.fasta	MGYA00244744	ERZ653695	root:Host-associated:Human:Digestive system:Large int	0.3094
MGYA00244745/ERZ653697_FASTA.fasta	MGYA00244745	ERZ653697	root:Host-associated:Human:Digestive system:Large int	0.3812
MGYA00244746/ERZ653701_FASTA.fasta	MGYA00244746	ERZ653701	root:Host-associated:Human:Digestive system:Large int	0.3688
MGYA00244747/ERZ653703_FASTA.fasta	MGYA00244747	ERZ653703	root:Host-associated:Human:Digestive system:Large int	0.237
MGYA00244748/ERZ653360_FASTA.fasta	MGYA00244748	ERZ653360	root:Host-associated:Human:Digestive system:Large int	0.1778
MGYA00244749/ERZ653362_FASTA.fasta	MGYA00244749	ERZ653362	root:Host-associated:Human:Digestive system:Large int	0.1456
MGYA00244750/ERZ653364_FASTA.fasta	MGYA00244750	ERZ653364	root:Host-associated:Human:Digestive system:Large int	0.1352
MGYA00244751/ERZ653366_FASTA.fasta	MGYA00244751	ERZ653366	root:Host-associated:Human:Digestive system:Large int	0.1164
MGYA00244752/ERZ653368_FASTA.fasta	MGYA00244752	ERZ653368	root:Host-associated:Human:Digestive system:Large int	0.0552
MGYA00244753/ERZ652765_FASTA.fasta	MGYA00244753	ERZ652765	root:Host-associated:Human:Digestive system:Large int	0.3136
MGYA00244754/ERZ652767_FASTA.fasta	MGYA00244754	ERZ652767	root:Host-associated:Human:Digestive system:Large int	0.3304
MGYA00244755/ERZ652769_FASTA.fasta	MGYA00244755	ERZ652769	root:Host-associated:Human:Digestive system:Large int	0.2796
MGYA00244756/ERZ652771_FASTA.fasta	MGYA00244756	ERZ652771	root:Host-associated:Human:Digestive system:Large int	0.3348
MGYA00244757/ERZ652773_FASTA.fasta	MGYA00244757	ERZ652773	root:Host-associated:Human:Digestive system:Large int	0.3024
MGYA00244758/ERZ652775_FASTA.fasta	MGYA00244758	ERZ652775	root:Host-associated:Human:Digestive system:Large int	0.2426
MGYA00244759/ERZ652777_FASTA.fasta	MGYA00244759	ERZ652777	root:Host-associated:Human:Digestive system:Large int	0.272
MGYA00244760/ERZ652779_FASTA.fasta	MGYA00244760	ERZ652779	root:Host-associated:Human:Digestive system:Large int	0.2498
MGYA00244761/ERZ652781_FASTA.fasta	MGYA00244761	ERZ652781	root:Host-associated:Human:Digestive system:Large int	0.3314
MGYA00244762/ERZ652783_FASTA.fasta	MGYA00244762	ERZ652783	root:Host-associated:Human:Digestive system:Large int	0.2648
MGYA00244763/ERZ652785_FASTA.fasta	MGYA00244763	ERZ652785	root:Host-associated:Human:Digestive system:Large int	0.3142
MGYA00244764/ERZ652787_FASTA.fasta	MGYA00244764	ERZ652787	root:Host-associated:Human:Digestive system:Large int	0.2506
MGYA00244765/ERZ652789_FASTA.fasta	MGYA00244765	ERZ652789	root:Host-associated:Human:Digestive system:Large int	0.2666
MGYA00244766/ERZ652791_FASTA.fasta	MGYA00244766	ERZ652791	root:Host-associated:Human:Digestive system:Large int	0.1434
MGYA00244767/ERZ652793_FASTA.fasta	MGYA00244767	ERZ652793	root:Host-associated:Human:Digestive system:Large int	0.4302
MGYA00244768/ERZ652795_FASTA.fasta	MGYA00244768	ERZ652795	root:Host-associated:Human:Digestive system:Large int	0.2658
MGYA00244769/ERZ652796_FASTA.fasta	MGYA00244769	ERZ652796	root:Host-associated:Human:Digestive system:Large int	0.2776
MGYA00244770/ERZ652797_FASTA.fasta	MGYA00244770	ERZ652797	root:Host-associated:Human:Digestive system:Large int	0.3654
MGYA00244771/ERZ652799_FASTA.fasta	MGYA00244771	ERZ652799	root:Host-associated:Human:Digestive system:Large int	0.2968
MGYA00244772/ERZ653241_FASTA.fasta	MGYA00244772	ERZ653241	root:Host-associated:Human:Digestive system:Large int	0.0516
MGYA00244773/ERZ653243_FASTA.fasta	MGYA00244773	ERZ653243	root:Host-associated:Human:Digestive system:Large int	0.1706
MGYA00244774/ERZ653245_FASTA.fasta	MGYA00244774	ERZ653245	root:Host-associated:Human:Digestive system:Large int	0.0948
MGYA00244775/ERZ653705_FASTA.fasta	MGYA00244775	ERZ653705	root:Host-associated:Human:Digestive system:Large int	0.3474
MGYA00244776/ERZ653707_FASTA.fasta	MGYA00244776	ERZ653707	root:Host-associated:Human:Digestive system:Large int	0.2564
MGYA00244777/ERZ653709_FASTA.fasta	MGYA00244777	ERZ653709	root:Host-associated:Human:Digestive system:Large int	0.2398
MGYA00244778/ERZ653711_FASTA.fasta	MGYA00244778	ERZ653711	root:Host-associated:Human:Digestive system:Large int	0.405
MGYA00244779/ERZ653713_FASTA.fasta	MGYA00244779	ERZ653713	root:Host-associated:Human:Digestive system:Large int	0.3152
MGYA00244780/ERZ653715_FASTA.fasta	MGYA00244780	ERZ653715	root:Host-associated:Human:Digestive system:Large int	0.281
MGYA00244781/ERZ653717_FASTA.fasta	MGYA00244781	ERZ653717	root:Host-associated:Human:Digestive system:Large int	0.3592
MGYA00244782/ERZ653719_FASTA.fasta	MGYA00244782	ERZ653719	root:Host-associated:Human:Digestive system:Large int	0.3422
MGYA00244783/ERZ653721_FASTA.fasta	MGYA00244783	ERZ653721	root:Host-associated:Human:Digestive system:Large int	0.3376
MGYA00244784/ERZ653723_FASTA.fasta	MGYA00244784	ERZ653723	root:Host-associated:Human:Digestive system:Large int	0.2926
MGYA00244785/ERZ653725_FASTA.fasta	MGYA00244785	ERZ653725	root:Host-associated:Human:Digestive system:Large int	0.4672
MGYA00244786/ERZ653727_FASTA.fasta	MGYA00244786	ERZ653727	root:Host-associated:Human:Digestive system:Large int	0.1516
MGYA00244787/ERZ653729_FASTA.fasta	MGYA00244787	ERZ653729	root:Host-associated:Human:Digestive system:Large int	0.3206
MGYA00244788/ERZ653731_FASTA.fasta	MGYA00244788	ERZ653731	root:Host-associated:Human:Digestive system:Large int	0.2536
MGYA00244789/ERZ653733_FASTA.fasta	MGYA00244789	ERZ653733	root:Host-associated:Human:Digestive system:Large int	0.4008
MGYA00244790/ERZ653735_FASTA.fasta	MGYA00244790	ERZ653735	root:Host-associated:Human:Digestive system:Large int	0.2404
MGYA00244791/ERZ653737_FASTA.fasta	MGYA00244791	ERZ653737	root:Host-associated:Human:Digestive system:Large int	0.3514
MGYA00244792/ERZ653739_FASTA.fasta	MGYA00244792	ERZ653739	root:Host-associated:Human:Digestive system:Large int	0.3692
MGYA00244793/ERZ653741_FASTA.fasta	MGYA00244793	ERZ653741	root:Host-associated:Human:Digestive system:Large int	0.406
MGYA00244794/ERZ653743_FASTA.fasta	MGYA00244794	ERZ653743	root:Host-associated:Human:Digestive system:Large int	0.431
MGYA00244795/ERZ653745_FASTA.fasta	MGYA00244795	ERZ653745	root:Host-associated:Human:Digestive system:Large int	0.3422
MGYA00244796/ERZ653747_FASTA.fasta	MGYA00244796	ERZ653747	root:Host-associated:Human:Digestive system:Large int	0.3022
MGYA00244797/ERZ653370_FASTA.fasta	MGYA00244797	ERZ653370	root:Host-associated:Human:Digestive system:Large int	0.2854
MGYA00244798/ERZ653372_FASTA.fasta	MGYA00244798	ERZ653372	root:Host-associated:Human:Digestive system:Large int	0.3052
MGYA00244799/ERZ653374_FASTA.fasta	MGYA00244799	ERZ653374	root:Host-associated:Human:Digestive system:Large int	0.2564
MGYA00244800/ERZ653376_FASTA.fasta	MGYA00244800	ERZ653376	root:Host-associated:Human:Digestive system:Large int	0.3182
MGYA00244801/ERZ653378_FASTA.fasta	MGYA00244801	ERZ653378	root:Host-associated:Human:Digestive system:Large int	0.3084
MGYA00244802/ERZ653380_FASTA.fasta	MGYA00244802	ERZ653380	root:Host-associated:Human:Digestive system:Large int	0.4192
MGYA00244803/ERZ653382_FASTA.fasta	MGYA00244803	ERZ653382	root:Host-associated:Human:Digestive system:Large int	0.3228
MGYA00244804/ERZ653384_FASTA.fasta	MGYA00244804	ERZ653384	root:Host-associated:Human:Digestive system:Large int	0.4818
MGYA00244805/ERZ653386_FASTA.fasta	MGYA00244805	ERZ653386	root:Host-associated:Human:Digestive system:Large int	0.2302
MGYA00244806/ERZ653388_FASTA.fasta	MGYA00244806	ERZ653388	root:Host-associated:Human:Digestive system:Large int	0.3834
MGYA00244807/ERZ653390_FASTA.fasta	MGYA00244807	ERZ653390	root:Host-associated:Human:Digestive system:Large int	0.3874
MGYA00244808/ERZ653392_FASTA.fasta	MGYA00244808	ERZ653392	root:Host-associated:Human:Digestive system:Large int	0.3454
MGYA00244809/ERZ653394_FASTA.fasta	MGYA00244809	ERZ653394	root:Host-associated:Human:Digestive system:Large int	0.239
MGYA00244810/ERZ653396_FASTA.fasta	MGYA00244810	ERZ653396	root:Host-associated:Human:Digestive system:Large int	0.4102
MGYA00244811/ERZ653398_FASTA.fasta	MGYA00244811	ERZ653398	root:Host-associated:Human:Digestive system:Large int	0.244
MGYA00244812/ERZ653400_FASTA.fasta	MGYA00244812	ERZ653400	root:Host-associated:Human:Digestive system:Large int	0.392
MGYA00244813/ERZ653402_FASTA.fasta	MGYA00244813	ERZ653402	root:Host-associated:Human:Digestive system:Large int	0.3948
MGYA00244814/ERZ653404_FASTA.fasta	MGYA00244814	ERZ653404	root:Host-associated:Human:Digestive system:Large int	0.2986
MGYA00244815/ERZ653406_FASTA.fasta	MGYA00244815	ERZ653406	root:Host-associated:Human:Digestive system:Large int	0.408
MGYA00244816/ERZ653408_FASTA.fasta	MGYA00244816	ERZ653408	root:Host-associated:Human:Digestive system:Large int	0.3354
MGYA00244817/ERZ653410_FASTA.fasta	MGYA00244817	ERZ653410	root:Host-associated:Human:Digestive system:Large int	0.2494
MGYA00244818/ERZ653412_FASTA.fasta	MGYA00244818	ERZ653412	root:Host-associated:Human:Digestive system:Large int	0.3828
MGYA00244819/ERZ653749_FASTA.fasta	MGYA00244819	ERZ653749	root:Host-associated:Human:Digestive system:Large int	0.325
MGYA00244820/ERZ653751_FASTA.fasta	MGYA00244820	ERZ653751	root:Host-associated:Human:Digestive system:Large int	0.2644
MGYA00244821/ERZ653753_FASTA.fasta	MGYA00244821	ERZ653753	root:Host-associated:Human:Digestive system:Large int	0.4872
MGYA00244822/ERZ653755_FASTA.fasta	MGYA00244822	ERZ653755	root:Host-associated:Human:Digestive system:Large int	0.3648
MGYA00244823/ERZ653757_FASTA.fasta	MGYA00244823	ERZ653757	root:Host-associated:Human:Digestive system:Large int	0.3346
MGYA00244824/ERZ653760_FASTA.fasta	MGYA00244824	ERZ653760	root:Host-associated:Human:Digestive system:Large int	0.3254
MGYA00244825/ERZ653762_FASTA.fasta	MGYA00244825	ERZ653762	root:Host-associated:Human:Digestive system:Large int	0.2274
MGYA00244826/ERZ653764_FASTA.fasta	MGYA00244826	ERZ653764	root:Host-associated:Human:Digestive system:Large int	0.2638
MGYA00244827/ERZ653766_FASTA.fasta	MGYA00244827	ERZ653766	root:Host-associated:Human:Digestive system:Large int	0.3838
MGYA00244828/ERZ653768_FASTA.fasta	MGYA00244828	ERZ653768	root:Host-associated:Human:Digestive system:Large int	0.2704
MGYA00244829/ERZ653770_FASTA.fasta	MGYA00244829	ERZ653770	root:Host-associated:Human:Digestive system:Large int	0.4016
MGYA00244830/ERZ653773_FASTA.fasta	MGYA00244830	ERZ653773	root:Host-associated:Human:Digestive system:Large int	0.484
MGYA00244831/ERZ653774_FASTA.fasta	MGYA00244831	ERZ653774	root:Host-associated:Human:Digestive system:Large int	0.31
MGYA00244832/ERZ653776_FASTA.fasta	MGYA00244832	ERZ653776	root:Host-associated:Human:Digestive system:Large int	0.4326
MGYA00244833/ERZ653778_FASTA.fasta	MGYA00244833	ERZ653778	root:Host-associated:Human:Digestive system:Large int	0.2756
MGYA00244834/ERZ653780_FASTA.fasta	MGYA00244834	ERZ653780	root:Host-associated:Human:Digestive system:Large int	0.2466
MGYA00244835/ERZ653782_FASTA.fasta	MGYA00244835	ERZ653782	root:Host-associated:Human:Digestive system:Large int	0.1872
MGYA00244836/ERZ653784_FASTA.fasta	MGYA00244836	ERZ653784	root:Host-associated:Human:Digestive system:Large int	0.2676
MGYA00244837/ERZ653786_FASTA.fasta	MGYA00244837	ERZ653786	root:Host-associated:Human:Digestive system:Large int	0.0978
MGYA00244838/ERZ653788_FASTA.fasta	MGYA00244838	ERZ653788	root:Host-associated:Human:Digestive system:Large int	0.2912
MGYA00244839/ERZ653790_FASTA.fasta	MGYA00244839	ERZ653790	root:Host-associated:Human:Digestive system:Large int	0.2768
MGYA00244840/ERZ653792_FASTA.fasta	MGYA00244840	ERZ653792	root:Host-associated:Human:Digestive system:Large int	0.3066
MGYA00244841/ERZ653414_FASTA.fasta	MGYA00244841	ERZ653414	root:Host-associated:Human:Digestive system:Large int	0.4056
MGYA00244842/ERZ653416_FASTA.fasta	MGYA00244842	ERZ653416	root:Host-associated:Human:Digestive system:Large int	0.3386
MGYA00244843/ERZ653418_FASTA.fasta	MGYA00244843	ERZ653418	root:Host-associated:Human:Digestive system:Large int	0.332
MGYA00244844/ERZ653420_FASTA.fasta	MGYA00244844	ERZ653420	root:Host-associated:Human:Digestive system:Large int	0.2906
MGYA00244845/ERZ653422_FASTA.fasta	MGYA00244845	ERZ653422	root:Host-associated:Human:Digestive system:Large int	0.3028
MGYA00244846/ERZ653424_FASTA.fasta	MGYA00244846	ERZ653424	root:Host-associated:Human:Digestive system:Large int	0.5226
MGYA00244847/ERZ653426_FASTA.fasta	MGYA00244847	ERZ653426	root:Host-associated:Human:Digestive system:Large int	0.3336
MGYA00244848/ERZ654082_FASTA.fasta	MGYA00244848	ERZ654082	root:Host-associated:Human:Digestive system:Large int	0.2832
MGYA00244849/ERZ654083_FASTA.fasta	MGYA00244849	ERZ654083	root:Host-associated:Human:Digestive system:Large int	0.2626
MGYA00244850/ERZ654084_FASTA.fasta	MGYA00244850	ERZ654084	root:Host-associated:Human:Digestive system:Large int	0.2404
MGYA00244851/ERZ654086_FASTA.fasta	MGYA00244851	ERZ654086	root:Host-associated:Human:Digestive system:Large int	0.3222
MGYA00244852/ERZ654088_FASTA.fasta	MGYA00244852	ERZ654088	root:Host-associated:Human:Digestive system:Large int	0.3342
MGYA00244853/ERZ654090_FASTA.fasta	MGYA00244853	ERZ654090	root:Host-associated:Human:Digestive system:Large int	0.3034
MGYA00244854/ERZ654092_FASTA.fasta	MGYA00244854	ERZ654092	root:Host-associated:Human:Digestive system:Large int	0.3762
MGYA00244855/ERZ654094_FASTA.fasta	MGYA00244855	ERZ654094	root:Host-associated:Human:Digestive system:Large int	0.2748
MGYA00244856/ERZ654096_FASTA.fasta	MGYA00244856	ERZ654096	root:Host-associated:Human:Digestive system:Large int	0.205
MGYA00244857/ERZ654098_FASTA.fasta	MGYA00244857	ERZ654098	root:Host-associated:Human:Digestive system:Large int	0.306
MGYA00244858/ERZ654100_FASTA.fasta	MGYA00244858	ERZ654100	root:Host-associated:Human:Digestive system:Large int	0.1872
MGYA00244859/ERZ654102_FASTA.fasta	MGYA00244859	ERZ654102	root:Host-associated:Human:Digestive system:Large int	0.3286
MGYA00244860/ERZ654104_FASTA.fasta	MGYA00244860	ERZ654104	root:Host-associated:Human:Digestive system:Large int	0.2498
MGYA00244861/ERZ654106_FASTA.fasta	MGYA00244861	ERZ654106	root:Host-associated:Human:Digestive system:Large int	0.2704
MGYA00244862/ERZ654108_FASTA.fasta	MGYA00244862	ERZ654108	root:Host-associated:Human:Digestive system:Large int	0.191
MGYA00244863/ERZ653794_FASTA.fasta	MGYA00244863	ERZ653794	root:Host-associated:Human:Digestive system:Large int	0.1546
MGYA00244864/ERZ653796_FASTA.fasta	MGYA00244864	ERZ653796	root:Host-associated:Human:Digestive system:Large int	0.2884
MGYA00244865/ERZ653798_FASTA.fasta	MGYA00244865	ERZ653798	root:Host-associated:Human:Digestive system:Large int	0.2378
MGYA00244866/ERZ653800_FASTA.fasta	MGYA00244866	ERZ653800	root:Host-associated:Human:Digestive system:Large int	0.3048
MGYA00244867/ERZ653802_FASTA.fasta	MGYA00244867	ERZ653802	root:Host-associated:Human:Digestive system:Large int	0.3032
MGYA00244868/ERZ653804_FASTA.fasta	MGYA00244868	ERZ653804	root:Host-associated:Human:Digestive system:Large int	0.3472
MGYA00244869/ERZ653806_FASTA.fasta	MGYA00244869	ERZ653806	root:Host-associated:Human:Digestive system:Large int	0.2082
MGYA00244870/ERZ653808_FASTA.fasta	MGYA00244870	ERZ653808	root:Host-associated:Human:Digestive system:Large int	0.2804
MGYA00244871/ERZ653810_FASTA.fasta	MGYA00244871	ERZ653810	root:Host-associated:Human:Digestive system:Large int	0.3858
MGYA00244872/ERZ653812_FASTA.fasta	MGYA00244872	ERZ653812	root:Host-associated:Human:Digestive system:Large int	0.322
MGYA00244873/ERZ653814_FASTA.fasta	MGYA00244873	ERZ653814	root:Host-associated:Human:Digestive system:Large int	0.33
MGYA00244874/ERZ653816_FASTA.fasta	MGYA00244874	ERZ653816	root:Host-associated:Human:Digestive system:Large int	0.1786
MGYA00244875/ERZ653818_FASTA.fasta	MGYA00244875	ERZ653818	root:Host-associated:Human:Digestive system:Large int	0.1742
MGYA00244876/ERZ653820_FASTA.fasta	MGYA00244876	ERZ653820	root:Host-associated:Human:Digestive system:Large int	0.231
MGYA00244877/ERZ653822_FASTA.fasta	MGYA00244877	ERZ653822	root:Host-associated:Human:Digestive system:Large int	0.2536
MGYA00244878/ERZ653824_FASTA.fasta	MGYA00244878	ERZ653824	root:Host-associated:Human:Digestive system:Large int	0.2152
MGYA00244879/ERZ653826_FASTA.fasta	MGYA00244879	ERZ653826	root:Host-associated:Human:Digestive system:Large int	0.2644
MGYA00244880/ERZ653828_FASTA.fasta	MGYA00244880	ERZ653828	root:Host-associated:Human:Digestive system:Large int	0.1882
MGYA00244881/ERZ653830_FASTA.fasta	MGYA00244881	ERZ653830	root:Host-associated:Human:Digestive system:Large int	0.1692
MGYA00244882/ERZ653832_FASTA.fasta	MGYA00244882	ERZ653832	root:Host-associated:Human:Digestive system:Large int	0.346
MGYA00244883/ERZ653834_FASTA.fasta	MGYA00244883	ERZ653834	root:Host-associated:Human:Digestive system:Large int	0.392
MGYA00244884/ERZ653836_FASTA.fasta	MGYA00244884	ERZ653836	root:Host-associated:Human:Digestive system:Large int	0.386
MGYA00244885/ERZ654469_FASTA.fasta	MGYA00244885	ERZ654469	root:Host-associated:Human:Digestive system:Large int	0.3186
MGYA00244886/ERZ654471_FASTA.fasta	MGYA00244886	ERZ654471	root:Host-associated:Human:Digestive system:Large int	0.4368
MGYA00244887/ERZ654473_FASTA.fasta	MGYA00244887	ERZ654473	root:Host-associated:Human:Digestive system:Large int	0.3242
MGYA00244888/ERZ654477_FASTA.fasta	MGYA00244888	ERZ654477	root:Host-associated:Human:Digestive system:Large int	0.5266
MGYA00244889/ERZ654479_FASTA.fasta	MGYA00244889	ERZ654479	root:Host-associated:Human:Digestive system:Large int	0.2816
MGYA00244890/ERZ654481_FASTA.fasta	MGYA00244890	ERZ654481	root:Host-associated:Human:Digestive system:Large int	0.3116
MGYA00244891/ERZ654483_FASTA.fasta	MGYA00244891	ERZ654483	root:Host-associated:Human:Digestive system:Large int	0.207
MGYA00244892/ERZ654485_FASTA.fasta	MGYA00244892	ERZ654485	root:Host-associated:Human:Digestive system:Large int	0.3376
MGYA00244893/ERZ654487_FASTA.fasta	MGYA00244893	ERZ654487	root:Host-associated:Human:Digestive system:Large int	0.4118
MGYA00244894/ERZ654489_FASTA.fasta	MGYA00244894	ERZ654489	root:Host-associated:Human:Digestive system:Large int	0.3234
MGYA00244895/ERZ654491_FASTA.fasta	MGYA00244895	ERZ654491	root:Host-associated:Human:Digestive system:Large int	0.3684
MGYA00244896/ERZ654493_FASTA.fasta	MGYA00244896	ERZ654493	root:Host-associated:Human:Digestive system:Large int	0.3352
MGYA00244897/ERZ654494_FASTA.fasta	MGYA00244897	ERZ654494	root:Host-associated:Human:Digestive system:Large int	0.3974
MGYA00244898/ERZ653838_FASTA.fasta	MGYA00244898	ERZ653838	root:Host-associated:Human:Digestive system:Large int	0.3384
MGYA00244899/ERZ653840_FASTA.fasta	MGYA00244899	ERZ653840	root:Host-associated:Human:Digestive system:Large int	0.2706
MGYA00244900/ERZ653842_FASTA.fasta	MGYA00244900	ERZ653842	root:Host-associated:Human:Digestive system:Large int	0.3122
MGYA00244901/ERZ653844_FASTA.fasta	MGYA00244901	ERZ653844	root:Host-associated:Human:Digestive system:Large int	0.2508
MGYA00244902/ERZ653846_FASTA.fasta	MGYA00244902	ERZ653846	root:Host-associated:Human:Digestive system:Large int	0.2722
MGYA00244903/ERZ654000_FASTA.fasta	MGYA00244903	ERZ654000	root:Host-associated:Human:Digestive system:Large int	0.3838
MGYA00244904/ERZ654002_FASTA.fasta	MGYA00244904	ERZ654002	root:Host-associated:Human:Digestive system:Large int	0.358
MGYA00244905/ERZ654004_FASTA.fasta	MGYA00244905	ERZ654004	root:Host-associated:Human:Digestive system:Large int	0.231
MGYA00244906/ERZ654006_FASTA.fasta	MGYA00244906	ERZ654006	root:Host-associated:Human:Digestive system:Large int	0.2758
MGYA00244907/ERZ654008_FASTA.fasta	MGYA00244907	ERZ654008	root:Host-associated:Human:Digestive system:Large int	0.2696
MGYA00244908/ERZ654010_FASTA.fasta	MGYA00244908	ERZ654010	root:Host-associated:Human:Digestive system:Large int	0.3056
MGYA00244909/ERZ654012_FASTA.fasta	MGYA00244909	ERZ654012	root:Host-associated:Human:Digestive system:Large int	0.2458
MGYA00244910/ERZ654016_FASTA.fasta	MGYA00244910	ERZ654016	root:Host-associated:Human:Digestive system:Large int	0.2836
MGYA00244911/ERZ654018_FASTA.fasta	MGYA00244911	ERZ654018	root:Host-associated:Human:Digestive system:Large int	0.2802
MGYA00244912/ERZ654020_FASTA.fasta	MGYA00244912	ERZ654020	root:Host-associated:Human:Digestive system:Large int	0.2978
MGYA00244913/ERZ654022_FASTA.fasta	MGYA00244913	ERZ654022	root:Host-associated:Human:Digestive system:Large int	0.3552
MGYA00244914/ERZ654024_FASTA.fasta	MGYA00244914	ERZ654024	root:Host-associated:Human:Digestive system:Large int	0.1778
MGYA00244915/ERZ654026_FASTA.fasta	MGYA00244915	ERZ654026	root:Host-associated:Human:Digestive system:Large int	0.2814
MGYA00244916/ERZ654028_FASTA.fasta	MGYA00244916	ERZ654028	root:Host-associated:Human:Digestive system:Large int	0.2878
MGYA00244917/ERZ654030_FASTA.fasta	MGYA00244917	ERZ654030	root:Host-associated:Human:Digestive system:Large int	0.2716
MGYA00244918/ERZ654032_FASTA.fasta	MGYA00244918	ERZ654032	root:Host-associated:Human:Digestive system:Large int	0.2644
MGYA00244919/ERZ654110_FASTA.fasta	MGYA00244919	ERZ654110	root:Host-associated:Human:Digestive system:Large int	0.3028
MGYA00244920/ERZ654112_FASTA.fasta	MGYA00244920	ERZ654112	root:Host-associated:Human:Digestive system:Large int	0.2544
MGYA00244921/ERZ654114_FASTA.fasta	MGYA00244921	ERZ654114	root:Host-associated:Human:Digestive system:Large int	0.2662
MGYA00244922/ERZ654116_FASTA.fasta	MGYA00244922	ERZ654116	root:Host-associated:Human:Digestive system:Large int	0.2282
MGYA00244923/ERZ654118_FASTA.fasta	MGYA00244923	ERZ654118	root:Host-associated:Human:Digestive system:Large int	0.3434
MGYA00244924/ERZ654120_FASTA.fasta	MGYA00244924	ERZ654120	root:Host-associated:Human:Digestive system:Large int	0.2552
MGYA00244925/ERZ654122_FASTA.fasta	MGYA00244925	ERZ654122	root:Host-associated:Human:Digestive system:Large int	0.2172
MGYA00244926/ERZ654124_FASTA.fasta	MGYA00244926	ERZ654124	root:Host-associated:Human:Digestive system:Large int	0.415
MGYA00244927/ERZ654126_FASTA.fasta	MGYA00244927	ERZ654126	root:Host-associated:Human:Digestive system:Large int	0.3564
MGYA00244928/ERZ654128_FASTA.fasta	MGYA00244928	ERZ654128	root:Host-associated:Human:Digestive system:Large int	0.257
MGYA00244929/ERZ654130_FASTA.fasta	MGYA00244929	ERZ654130	root:Host-associated:Human:Digestive system:Large int	0.348
MGYA00244930/ERZ654132_FASTA.fasta	MGYA00244930	ERZ654132	root:Host-associated:Human:Digestive system:Large int	0.3216
MGYA00244931/ERZ654134_FASTA.fasta	MGYA00244931	ERZ654134	root:Host-associated:Human:Digestive system:Large int	0.2174
MGYA00244932/ERZ654136_FASTA.fasta	MGYA00244932	ERZ654136	root:Host-associated:Human:Digestive system:Large int	0.2548
MGYA00244933/ERZ654138_FASTA.fasta	MGYA00244933	ERZ654138	root:Host-associated:Human:Digestive system:Large int	0.2436
MGYA00244934/ERZ654140_FASTA.fasta	MGYA00244934	ERZ654140	root:Host-associated:Human:Digestive system:Large int	0.2818
MGYA00244935/ERZ654142_FASTA.fasta	MGYA00244935	ERZ654142	root:Host-associated:Human:Digestive system:Large int	0.2916
MGYA00244936/ERZ654144_FASTA.fasta	MGYA00244936	ERZ654144	root:Host-associated:Human:Digestive system:Large int	0.2698
MGYA00244937/ERZ654146_FASTA.fasta	MGYA00244937	ERZ654146	root:Host-associated:Human:Digestive system:Large int	0.4252
MGYA00244938/ERZ654148_FASTA.fasta	MGYA00244938	ERZ654148	root:Host-associated:Human:Digestive system:Large int	0.2414
MGYA00244939/ERZ654150_FASTA.fasta	MGYA00244939	ERZ654150	root:Host-associated:Human:Digestive system:Large int	0.2352
MGYA00244940/ERZ654152_FASTA.fasta	MGYA00244940	ERZ654152	root:Host-associated:Human:Digestive system:Large int	0.263
MGYA00244941/ERZ652754_FASTA.fasta	MGYA00244941	ERZ652754	root:Host-associated:Human:Digestive system:Large int	0.2786
MGYA00244942/ERZ652756_FASTA.fasta	MGYA00244942	ERZ652756	root:Host-associated:Human:Digestive system:Large int	0.2372
MGYA00244943/ERZ652758_FASTA.fasta	MGYA00244943	ERZ652758	root:Host-associated:Human:Digestive system:Large int	0.2272
MGYA00244944/ERZ654876_FASTA.fasta	MGYA00244944	ERZ654876	root:Host-associated:Human:Digestive system:Large int	0.324
MGYA00244945/ERZ654882_FASTA.fasta	MGYA00244945	ERZ654882	root:Host-associated:Human:Digestive system:Large int	0.3972
MGYA00244946/ERZ653065_FASTA.fasta	MGYA00244946	ERZ653065	root:Host-associated:Human:Digestive system:Large int	0.0608
MGYA00244947/ERZ653067_FASTA.fasta	MGYA00244947	ERZ653067	root:Host-associated:Human:Digestive system:Large int	0.252
MGYA00244948/ERZ653069_FASTA.fasta	MGYA00244948	ERZ653069	root:Host-associated:Human:Digestive system:Large int	0.038
MGYA00244949/ERZ653071_FASTA.fasta	MGYA00244949	ERZ653071	root:Host-associated:Human:Digestive system:Large int	0.1294
MGYA00244950/ERZ653073_FASTA.fasta	MGYA00244950	ERZ653073	root:Host-associated:Human:Digestive system:Large int	0.285
MGYA00244951/ERZ653075_FASTA.fasta	MGYA00244951	ERZ653075	root:Host-associated:Human:Digestive system:Large int	0.2174
MGYA00244952/ERZ653077_FASTA.fasta	MGYA00244952	ERZ653077	root:Host-associated:Human:Digestive system:Large int	0.1458
MGYA00244953/ERZ653079_FASTA.fasta	MGYA00244953	ERZ653079	root:Host-associated:Human:Digestive system:Large int	0.1456
MGYA00244954/ERZ653081_FASTA.fasta	MGYA00244954	ERZ653081	root:Host-associated:Human:Digestive system:Large int	0.1318
MGYA00244955/ERZ653083_FASTA.fasta	MGYA00244955	ERZ653083	root:Host-associated:Human:Digestive system:Large int	0.0672
MGYA00244956/ERZ653085_FASTA.fasta	MGYA00244956	ERZ653085	root:Host-associated:Human:Digestive system:Large int	0.1868
MGYA00244957/ERZ653087_FASTA.fasta	MGYA00244957	ERZ653087	root:Host-associated:Human:Digestive system:Large int	0.071
MGYA00244958/ERZ653089_FASTA.fasta	MGYA00244958	ERZ653089	root:Host-associated:Human:Digestive system:Large int	0.101
MGYA00244959/ERZ653091_FASTA.fasta	MGYA00244959	ERZ653091	root:Host-associated:Human:Digestive system:Large int	0.2612
MGYA00244960/ERZ653093_FASTA.fasta	MGYA00244960	ERZ653093	root:Host-associated:Human:Digestive system:Large int	0.0374
MGYA00244961/ERZ653247_FASTA.fasta	MGYA00244961	ERZ653247	root:Host-associated:Human:Digestive system:Large int	0.0986
MGYA00244962/ERZ653249_FASTA.fasta	MGYA00244962	ERZ653249	root:Host-associated:Human:Digestive system:Large int	0.1764
MGYA00244963/ERZ653251_FASTA.fasta	MGYA00244963	ERZ653251	root:Host-associated:Human:Digestive system:Large int	0.131
MGYA00244964/ERZ653253_FASTA.fasta	MGYA00244964	ERZ653253	root:Host-associated:Human:Digestive system:Large int	0.0336
MGYA00244965/ERZ653255_FASTA.fasta	MGYA00244965	ERZ653255	root:Host-associated:Human:Digestive system:Large int	0.0528
MGYA00244966/ERZ653257_FASTA.fasta	MGYA00244966	ERZ653257	root:Host-associated:Human:Digestive system:Large int	0.1048
MGYA00244967/ERZ653259_FASTA.fasta	MGYA00244967	ERZ653259	root:Host-associated:Human:Digestive system:Large int	0.0818
MGYA00244968/ERZ653261_FASTA.fasta	MGYA00244968	ERZ653261	root:Host-associated:Human:Digestive system:Large int	0.1846
MGYA00244969/ERZ653263_FASTA.fasta	MGYA00244969	ERZ653263	root:Host-associated:Human:Digestive system:Large int	0.0524
MGYA00244970/ERZ653265_FASTA.fasta	MGYA00244970	ERZ653265	root:Host-associated:Human:Digestive system:Large int	0.0958
MGYA00244971/ERZ653565_FASTA.fasta	MGYA00244971	ERZ653565	root:Host-associated:Human:Digestive system:Large int	0.4082
MGYA00244972/ERZ653567_FASTA.fasta	MGYA00244972	ERZ653567	root:Host-associated:Human:Digestive system:Large int	0.3722
MGYA00244973/ERZ653569_FASTA.fasta	MGYA00244973	ERZ653569	root:Host-associated:Human:Digestive system:Large int	0.3152
MGYA00244974/ERZ653571_FASTA.fasta	MGYA00244974	ERZ653571	root:Host-associated:Human:Digestive system:Large int	0.2302
MGYA00244975/ERZ653573_FASTA.fasta	MGYA00244975	ERZ653573	root:Host-associated:Human:Digestive system:Large int	0.4298
MGYA00244976/ERZ653575_FASTA.fasta	MGYA00244976	ERZ653575	root:Host-associated:Human:Digestive system:Large int	0.265
MGYA00244977/ERZ653577_FASTA.fasta	MGYA00244977	ERZ653577	root:Host-associated:Human:Digestive system:Large int	0.2962
MGYA00244978/ERZ653579_FASTA.fasta	MGYA00244978	ERZ653579	root:Host-associated:Human:Digestive system:Large int	0.3128
MGYA00244979/ERZ653581_FASTA.fasta	MGYA00244979	ERZ653581	root:Host-associated:Human:Digestive system:Large int	0.346
MGYA00244980/ERZ653583_FASTA.fasta	MGYA00244980	ERZ653583	root:Host-associated:Human:Digestive system:Large int	0.3448
MGYA00244981/ERZ654034_FASTA.fasta	MGYA00244981	ERZ654034	root:Host-associated:Human:Digestive system:Large int	0.2998
MGYA00244982/ERZ654036_FASTA.fasta	MGYA00244982	ERZ654036	root:Host-associated:Human:Digestive system:Large int	0.3486
MGYA00244983/ERZ654038_FASTA.fasta	MGYA00244983	ERZ654038	root:Host-associated:Human:Digestive system:Large int	0.2596
MGYA00244984/ERZ654040_FASTA.fasta	MGYA00244984	ERZ654040	root:Host-associated:Human:Digestive system:Large int	0.3014
MGYA00244985/ERZ654042_FASTA.fasta	MGYA00244985	ERZ654042	root:Host-associated:Human:Digestive system:Large int	0.2924
MGYA00244986/ERZ654044_FASTA.fasta	MGYA00244986	ERZ654044	root:Host-associated:Human:Digestive system:Large int	0.3442
MGYA00244987/ERZ654046_FASTA.fasta	MGYA00244987	ERZ654046	root:Host-associated:Human:Digestive system:Large int	0.3272
MGYA00244988/ERZ654048_FASTA.fasta	MGYA00244988	ERZ654048	root:Host-associated:Human:Digestive system:Large int	0.2696
MGYA00244989/ERZ654050_FASTA.fasta	MGYA00244989	ERZ654050	root:Host-associated:Human:Digestive system:Large int	0.275
MGYA00244990/ERZ654052_FASTA.fasta	MGYA00244990	ERZ654052	root:Host-associated:Human:Digestive system:Large int	0.2316
MGYA00244991/ERZ654054_FASTA.fasta	MGYA00244991	ERZ654054	root:Host-associated:Human:Digestive system:Large int	0.4166
MGYA00244992/ERZ654056_FASTA.fasta	MGYA00244992	ERZ654056	root:Host-associated:Human:Digestive system:Large int	0.227
MGYA00244993/ERZ654058_FASTA.fasta	MGYA00244993	ERZ654058	root:Host-associated:Human:Digestive system:Large int	0.1646
MGYA00244994/ERZ654060_FASTA.fasta	MGYA00244994	ERZ654060	root:Host-associated:Human:Digestive system:Large int	0.2624
MGYA00244995/ERZ654062_FASTA.fasta	MGYA00244995	ERZ654062	root:Host-associated:Human:Digestive system:Large int	0.2846
MGYA00244996/ERZ654064_FASTA.fasta	MGYA00244996	ERZ654064	root:Host-associated:Human:Digestive system:Large int	0.2648
MGYA00244997/ERZ654066_FASTA.fasta	MGYA00244997	ERZ654066	root:Host-associated:Human:Digestive system:Large int	0.1888
MGYA00244998/ERZ654068_FASTA.fasta	MGYA00244998	ERZ654068	root:Host-associated:Human:Digestive system:Large int	0.1578
MGYA00244999/ERZ654070_FASTA.fasta	MGYA00244999	ERZ654070	root:Host-associated:Human:Digestive system:Large int	0.2666
MGYA00245000/ERZ654072_FASTA.fasta	MGYA00245000	ERZ654072	root:Host-associated:Human:Digestive system:Large int	0.3928
MGYA00245001/ERZ654074_FASTA.fasta	MGYA00245001	ERZ654074	root:Host-associated:Human:Digestive system:Large int	0.4072
MGYA00245002/ERZ654076_FASTA.fasta	MGYA00245002	ERZ654076	root:Host-associated:Human:Digestive system:Large int	0.2646
MGYA00245003/ERZ654154_FASTA.fasta	MGYA00245003	ERZ654154	root:Host-associated:Human:Digestive system:Large int	0.294
MGYA00245004/ERZ654156_FASTA.fasta	MGYA00245004	ERZ654156	root:Host-associated:Human:Digestive system:Large int	0.1112
MGYA00245005/ERZ654158_FASTA.fasta	MGYA00245005	ERZ654158	root:Host-associated:Human:Digestive system:Large int	0.2116
MGYA00245006/ERZ654160_FASTA.fasta	MGYA00245006	ERZ654160	root:Host-associated:Human:Digestive system:Large int	0.2934
MGYA00245007/ERZ654162_FASTA.fasta	MGYA00245007	ERZ654162	root:Host-associated:Human:Digestive system:Large int	0.3198
MGYA00245008/ERZ654164_FASTA.fasta	MGYA00245008	ERZ654164	root:Host-associated:Human:Digestive system:Large int	0.2512
MGYA00245009/ERZ652760_FASTA.fasta	MGYA00245009	ERZ652760	root:Host-associated:Human:Digestive system:Large int	0.2948
MGYA00245010/ERZ652762_FASTA.fasta	MGYA00245010	ERZ652762	root:Host-associated:Human:Digestive system:Large int	0.2394
MGYA00245011/ERZ652764_FASTA.fasta	MGYA00245011	ERZ652764	root:Host-associated:Human:Digestive system:Large int	0.2082
MGYA00245012/ERZ652766_FASTA.fasta	MGYA00245012	ERZ652766	root:Host-associated:Human:Digestive system:Large int	0.2594
MGYA00245013/ERZ652768_FASTA.fasta	MGYA00245013	ERZ652768	root:Host-associated:Human:Digestive system:Large int	0.2278
MGYA00245014/ERZ652770_FASTA.fasta	MGYA00245014	ERZ652770	root:Host-associated:Human:Digestive system:Large int	0.246
MGYA00245015/ERZ652772_FASTA.fasta	MGYA00245015	ERZ652772	root:Host-associated:Human:Digestive system:Large int	0.2914
MGYA00245016/ERZ652774_FASTA.fasta	MGYA00245016	ERZ652774	root:Host-associated:Human:Digestive system:Large int	0.2288
MGYA00245017/ERZ652776_FASTA.fasta	MGYA00245017	ERZ652776	root:Host-associated:Human:Digestive system:Large int	0.2448
MGYA00245018/ERZ652778_FASTA.fasta	MGYA00245018	ERZ652778	root:Host-associated:Human:Digestive system:Large int	0.2758
MGYA00245019/ERZ652780_FASTA.fasta	MGYA00245019	ERZ652780	root:Host-associated:Human:Digestive system:Large int	0.3326
MGYA00245020/ERZ652782_FASTA.fasta	MGYA00245020	ERZ652782	root:Host-associated:Human:Digestive system:Large int	0.2802
MGYA00245021/ERZ652784_FASTA.fasta	MGYA00245021	ERZ652784	root:Host-associated:Human:Digestive system:Large int	0.0902
MGYA00245022/ERZ652786_FASTA.fasta	MGYA00245022	ERZ652786	root:Host-associated:Human:Digestive system:Large int	0.289
MGYA00245023/ERZ652788_FASTA.fasta	MGYA00245023	ERZ652788	root:Host-associated:Human:Digestive system:Large int	0.42
MGYA00245024/ERZ652790_FASTA.fasta	MGYA00245024	ERZ652790	root:Host-associated:Human:Digestive system:Large int	0.2774
MGYA00245025/ERZ652792_FASTA.fasta	MGYA00245025	ERZ652792	root:Host-associated:Human:Digestive system:Large int	0.2884
MGYA00245026/ERZ652794_FASTA.fasta	MGYA00245026	ERZ652794	root:Host-associated:Human:Digestive system:Large int	0.2256
MGYA00245027/ERZ654886_FASTA.fasta	MGYA00245027	ERZ654886	root:Host-associated:Human:Digestive system:Large int	0.3382
MGYA00245028/ERZ654890_FASTA.fasta	MGYA00245028	ERZ654890	root:Host-associated:Human:Digestive system:Large int	0.384
MGYA00245029/ERZ654892_FASTA.fasta	MGYA00245029	ERZ654892	root:Host-associated:Human:Digestive system:Large int	0.4164
MGYA00245030/ERZ654894_FASTA.fasta	MGYA00245030	ERZ654894	root:Host-associated:Human:Digestive system:Large int	0.3666
MGYA00245031/ERZ654896_FASTA.fasta	MGYA00245031	ERZ654896	root:Host-associated:Human:Digestive system:Large int	0.3092
MGYA00245032/ERZ654900_FASTA.fasta	MGYA00245032	ERZ654900	root:Host-associated:Human:Digestive system:Large int	0.3286

MGYA00245033/ERZ654902_FASTA.fasta	MGYA00245033	ERZ654902	root:Host-associated:Human:Digestive system:Large int	0.3998
MGYA00245034/ERZ654904_FASTA.fasta	MGYA00245034	ERZ654904	root:Host-associated:Human:Digestive system:Large int	0.4052
MGYA00245035/ERZ654906_FASTA.fasta	MGYA00245035	ERZ654906	root:Host-associated:Human:Digestive system:Large int	0.3114
MGYA00245036/ERZ654910_FASTA.fasta	MGYA00245036	ERZ654910	root:Host-associated:Human:Digestive system:Large int	0.4968
MGYA00245037/ERZ654920_FASTA.fasta	MGYA00245037	ERZ654920	root:Host-associated:Human:Digestive system:Large int	0.3912
MGYA00245038/ERZ653095_FASTA.fasta	MGYA00245038	ERZ653095	root:Host-associated:Human:Digestive system:Large int	0.0634
MGYA00245039/ERZ653097_FASTA.fasta	MGYA00245039	ERZ653097	root:Host-associated:Human:Digestive system:Large int	0.0444
MGYA00245040/ERZ653099_FASTA.fasta	MGYA00245040	ERZ653099	root:Host-associated:Human:Digestive system:Large int	0.0586
MGYA00245041/ERZ653101_FASTA.fasta	MGYA00245041	ERZ653101	root:Host-associated:Human:Digestive system:Large int	0.0732
MGYA00245042/ERZ653103_FASTA.fasta	MGYA00245042	ERZ653103	root:Host-associated:Human:Digestive system:Large int	0.0858
MGYA00245043/ERZ653105_FASTA.fasta	MGYA00245043	ERZ653105	root:Host-associated:Human:Digestive system:Large int	0.1492
MGYA00245044/ERZ653107_FASTA.fasta	MGYA00245044	ERZ653107	root:Host-associated:Human:Digestive system:Large int	0.2152
MGYA00245045/ERZ653109_FASTA.fasta	MGYA00245045	ERZ653109	root:Host-associated:Human:Digestive system:Large int	0.1016
MGYA00245046/ERZ653111_FASTA.fasta	MGYA00245046	ERZ653111	root:Host-associated:Human:Digestive system:Large int	0.0542
MGYA00245047/ERZ653113_FASTA.fasta	MGYA00245047	ERZ653113	root:Host-associated:Human:Digestive system:Large int	0.3266
MGYA00245048/ERZ653115_FASTA.fasta	MGYA00245048	ERZ653115	root:Host-associated:Human:Digestive system:Large int	0.0786
MGYA00245049/ERZ653117_FASTA.fasta	MGYA00245049	ERZ653117	root:Host-associated:Human:Digestive system:Large int	0.1408
MGYA00245050/ERZ653119_FASTA.fasta	MGYA00245050	ERZ653119	root:Host-associated:Human:Digestive system:Large int	0.2434
MGYA00245051/ERZ653121_FASTA.fasta	MGYA00245051	ERZ653121	root:Host-associated:Human:Digestive system:Large int	0.1324
MGYA00245052/ERZ653123_FASTA.fasta	MGYA00245052	ERZ653123	root:Host-associated:Human:Digestive system:Large int	0.0606
MGYA00245053/ERZ653125_FASTA.fasta	MGYA00245053	ERZ653125	root:Host-associated:Human:Digestive system:Large int	0.121
MGYA00245054/ERZ653127_FASTA.fasta	MGYA00245054	ERZ653127	root:Host-associated:Human:Digestive system:Large int	0.1566
MGYA00245055/ERZ653129_FASTA.fasta	MGYA00245055	ERZ653129	root:Host-associated:Human:Digestive system:Large int	0.04
MGYA00245056/ERZ653585_FASTA.fasta	MGYA00245056	ERZ653585	root:Host-associated:Human:Digestive system:Large int	0.301
MGYA00245057/ERZ653587_FASTA.fasta	MGYA00245057	ERZ653587	root:Host-associated:Human:Digestive system:Large int	0.2898
MGYA00245058/ERZ653589_FASTA.fasta	MGYA00245058	ERZ653589	root:Host-associated:Human:Digestive system:Large int	0.33
MGYA00245059/ERZ653591_FASTA.fasta	MGYA00245059	ERZ653591	root:Host-associated:Human:Digestive system:Large int	0.3582
MGYA00245060/ERZ653593_FASTA.fasta	MGYA00245060	ERZ653593	root:Host-associated:Human:Digestive system:Large int	0.351
MGYA00245061/ERZ653595_FASTA.fasta	MGYA00245061	ERZ653595	root:Host-associated:Human:Digestive system:Large int	0.1292
MGYA00245062/ERZ653597_FASTA.fasta	MGYA00245062	ERZ653597	root:Host-associated:Human:Digestive system:Large int	0.339
MGYA00245063/ERZ653599_FASTA.fasta	MGYA00245063	ERZ653599	root:Host-associated:Human:Digestive system:Large int	0.3246
MGYA00245064/ERZ653601_FASTA.fasta	MGYA00245064	ERZ653601	root:Host-associated:Human:Digestive system:Large int	0.3654
MGYA00245065/ERZ653603_FASTA.fasta	MGYA00245065	ERZ653603	root:Host-associated:Human:Digestive system:Large int	0.3474
MGYA00245066/ERZ653605_FASTA.fasta	MGYA00245066	ERZ653605	root:Host-associated:Human:Digestive system:Large int	0.2966
MGYA00245067/ERZ653607_FASTA.fasta	MGYA00245067	ERZ653607	root:Host-associated:Human:Digestive system:Large int	0.2866
MGYA00245068/ERZ653609_FASTA.fasta	MGYA00245068	ERZ653609	root:Host-associated:Human:Digestive system:Large int	0.3644
MGYA00245069/ERZ653611_FASTA.fasta	MGYA00245069	ERZ653611	root:Host-associated:Human:Digestive system:Large int	0.3774
MGYA00245070/ERZ653613_FASTA.fasta	MGYA00245070	ERZ653613	root:Host-associated:Human:Digestive system:Large int	0.2742
MGYA00245071/ERZ653615_FASTA.fasta	MGYA00245071	ERZ653615	root:Host-associated:Human:Digestive system:Large int	0.3746
MGYA00245072/ERZ653617_FASTA.fasta	MGYA00245072	ERZ653617	root:Host-associated:Human:Digestive system:Large int	0.337
MGYA00245073/ERZ653619_FASTA.fasta	MGYA00245073	ERZ653619	root:Host-associated:Human:Digestive system:Large int	0.3696
MGYA00245074/ERZ653621_FASTA.fasta	MGYA00245074	ERZ653621	root:Host-associated:Human:Digestive system:Large int	0.3204
MGYA00245075/ERZ653623_FASTA.fasta	MGYA00245075	ERZ653623	root:Host-associated:Human:Digestive system:Large int	0.3156
MGYA00245076/ERZ653625_FASTA.fasta	MGYA00245076	ERZ653625	root:Host-associated:Human:Digestive system:Large int	0.3602
MGYA00245077/ERZ653627_FASTA.fasta	MGYA00245077	ERZ653627	root:Host-associated:Human:Digestive system:Large int	0.2542
MGYA00245078/ERZ654078_FASTA.fasta	MGYA00245078	ERZ654078	root:Host-associated:Human:Digestive system:Large int	0.3338
MGYA00245079/ERZ654080_FASTA.fasta	MGYA00245079	ERZ654080	root:Host-associated:Human:Digestive system:Large int	0.267
MGYA00245080/ERZ653131_FASTA.fasta	MGYA00245080	ERZ653131	root:Host-associated:Human:Digestive system:Large int	0.0594
MGYA00245081/ERZ653133_FASTA.fasta	MGYA00245081	ERZ653133	root:Host-associated:Human:Digestive system:Large int	0.2296
MGYA00245082/ERZ653137_FASTA.fasta	MGYA00245082	ERZ653137	root:Host-associated:Human:Digestive system:Large int	0.1122
MGYA00245083/ERZ653139_FASTA.fasta	MGYA00245083	ERZ653139	root:Host-associated:Human:Digestive system:Large int	0.0514
MGYA00245084/ERZ653141_FASTA.fasta	MGYA00245084	ERZ653141	root:Host-associated:Human:Digestive system:Large int	0.0646
MGYA00245085/ERZ653143_FASTA.fasta	MGYA00245085	ERZ653143	root:Host-associated:Human:Digestive system:Large int	0.033
MGYA00245086/ERZ653145_FASTA.fasta	MGYA00245086	ERZ653145	root:Host-associated:Human:Digestive system:Large int	0.1642
MGYA00245087/ERZ653147_FASTA.fasta	MGYA00245087	ERZ653147	root:Host-associated:Human:Digestive system:Large int	0.0666
MGYA00245088/ERZ653149_FASTA.fasta	MGYA00245088	ERZ653149	root:Host-associated:Human:Digestive system:Large int	0.0688
MGYA00245089/ERZ653151_FASTA.fasta	MGYA00245089	ERZ653151	root:Host-associated:Human:Digestive system:Large int	0.0456
MGYA00245090/ERZ653153_FASTA.fasta	MGYA00245090	ERZ653153	root:Host-associated:Human:Digestive system:Large int	0.1048
MGYA00245091/ERZ653155_FASTA.fasta	MGYA00245091	ERZ653155	root:Host-associated:Human:Digestive system:Large int	0.133
MGYA00245092/ERZ653157_FASTA.fasta	MGYA00245092	ERZ653157	root:Host-associated:Human:Digestive system:Large int	0.0758
MGYA00245093/ERZ653159_FASTA.fasta	MGYA00245093	ERZ653159	root:Host-associated:Human:Digestive system:Large int	0.0812
MGYA00245094/ERZ653161_FASTA.fasta	MGYA00245094	ERZ653161	root:Host-associated:Human:Digestive system:Large int	0.1014
MGYA00245095/ERZ653163_FASTA.fasta	MGYA00245095	ERZ653163	root:Host-associated:Human:Digestive system:Large int	0.0498
MGYA00245096/ERZ653165_FASTA.fasta	MGYA00245096	ERZ653165	root:Host-associated:Human:Digestive system:Large int	0.1978
MGYA00245097/ERZ653167_FASTA.fasta	MGYA00245097	ERZ653167	root:Host-associated:Human:Digestive system:Large int	0.1304
MGYA00245098/ERZ653169_FASTA.fasta	MGYA00245098	ERZ653169	root:Host-associated:Human:Digestive system:Large int	0.0928
MGYA00245099/ERZ653171_FASTA.fasta	MGYA00245099	ERZ653171	root:Host-associated:Human:Digestive system:Large int	0.1494
MGYA00245100/ERZ653173_FASTA.fasta	MGYA00245100	ERZ653173	root:Host-associated:Human:Digestive system:Large int	0.092
MGYA00245101/ERZ653175_FASTA.fasta	MGYA00245101	ERZ653175	root:Host-associated:Human:Digestive system:Large int	0.1334
MGYA00245102/ERZ653630_FASTA.fasta	MGYA00245102	ERZ653630	root:Host-associated:Human:Digestive system:Large int	0.346
MGYA00245103/ERZ653631_FASTA.fasta	MGYA00245103	ERZ653631	root:Host-associated:Human:Digestive system:Large int	0.3364
MGYA00245104/ERZ653633_FASTA.fasta	MGYA00245104	ERZ653633	root:Host-associated:Human:Digestive system:Large int	0.2994
MGYA00245105/ERZ653635_FASTA.fasta	MGYA00245105	ERZ653635	root:Host-associated:Human:Digestive system:Large int	0.3298
MGYA00245106/ERZ653637_FASTA.fasta	MGYA00245106	ERZ653637	root:Host-associated:Human:Digestive system:Large int	0.379
MGYA00245107/ERZ653639_FASTA.fasta	MGYA00245107	ERZ653639	root:Host-associated:Human:Digestive system:Large int	0.3112
MGYA00245108/ERZ653641_FASTA.fasta	MGYA00245108	ERZ653641	root:Host-associated:Human:Digestive system:Large int	0.3052
MGYA00245109/ERZ653643_FASTA.fasta	MGYA00245109	ERZ653643	root:Host-associated:Human:Digestive system:Large int	0.496
MGYA00245110/ERZ653645_FASTA.fasta	MGYA00245110	ERZ653645	root:Host-associated:Human:Digestive system:Large int	0.3488
MGYA00245111/ERZ653647_FASTA.fasta	MGYA00245111	ERZ653647	root:Host-associated:Human:Digestive system:Large int	0.3028
MGYA00245112/ERZ653649_FASTA.fasta	MGYA00245112	ERZ653649	root:Host-associated:Human:Digestive system:Large int	0.4242
MGYA00245113/ERZ653651_FASTA.fasta	MGYA00245113	ERZ653651	root:Host-associated:Human:Digestive system:Large int	0.3966
MGYA00245114/ERZ653653_FASTA.fasta	MGYA00245114	ERZ653653	root:Host-associated:Human:Digestive system:Large int	0.421
MGYA00245115/ERZ653655_FASTA.fasta	MGYA00245115	ERZ653655	root:Host-associated:Human:Digestive system:Large int	0.3046
MGYA00245116/ERZ653657_FASTA.fasta	MGYA00245116	ERZ653657	root:Host-associated:Human:Digestive system:Large int	0.3654
MGYA00245117/ERZ653659_FASTA.fasta	MGYA00245117	ERZ653659	root:Host-associated:Human:Digestive system:Large int	0.3382
MGYA00245118/ERZ653661_FASTA.fasta	MGYA00245118	ERZ653661	root:Host-associated:Human:Digestive system:Large int	0.3266
MGYA00245119/ERZ653663_FASTA.fasta	MGYA00245119	ERZ653663	root:Host-associated:Human:Digestive system:Large int	0.0392
MGYA00245120/ERZ653665_FASTA.fasta	MGYA00245120	ERZ653665	root:Host-associated:Human:Digestive system:Large int	0.3776
MGYA00245121/ERZ653667_FASTA.fasta	MGYA00245121	ERZ653667	root:Host-associated:Human:Digestive system:Large int	0.3024
MGYA00245122/ERZ653669_FASTA.fasta	MGYA00245122	ERZ653669	root:Host-associated:Human:Digestive system:Large int	0.3218
MGYA00245123/ERZ653671_FASTA.fasta	MGYA00245123	ERZ653671	root:Host-associated:Human:Digestive system:Large int	0.2588
MGYA00245256/ERZ501121_FASTA.fasta	MGYA00245256	ERZ501121	root:Host-associated:Human:Digestive system	0.034
MGYA00245257/ERZ501123_FASTA.fasta	MGYA00245257	ERZ501123	root:Host-associated:Human:Digestive system	0.0324
MGYA00245258/ERZ501125_FASTA.fasta	MGYA00245258	ERZ501125	root:Host-associated:Human:Digestive system	0.0712
MGYA00245259/ERZ501127_FASTA.fasta	MGYA00245259	ERZ501127	root:Host-associated:Human:Digestive system	0.0712
MGYA00245260/ERZ501129_FASTA.fasta	MGYA00245260	ERZ501129	root:Host-associated:Human:Digestive system	0.1876
MGYA00245261/ERZ501131_FASTA.fasta	MGYA00245261	ERZ501131	root:Host-associated:Human:Digestive system	0.0666
MGYA00245262/ERZ501133_FASTA.fasta	MGYA00245262	ERZ501133	root:Host-associated:Human:Digestive system	0.09
MGYA00245263/ERZ501135_FASTA.fasta	MGYA00245263	ERZ501135	root:Host-associated:Human:Digestive system	0.0532
MGYA00245264/ERZ501137_FASTA.fasta	MGYA00245264	ERZ501137	root:Host-associated:Human:Digestive system	0.1532
MGYA00245265/ERZ501139_FASTA.fasta	MGYA00245265	ERZ501139	root:Host-associated:Human:Digestive system	0.0506
MGYA00245266/ERZ501141_FASTA.fasta	MGYA00245266	ERZ501141	root:Host-associated:Human:Digestive system	0.0424
MGYA00245267/ERZ501069_FASTA.fasta	MGYA00245267	ERZ501069	root:Host-associated:Human:Digestive system	0.03
MGYA00245268/ERZ501071_FASTA.fasta	MGYA00245268	ERZ501071	root:Host-associated:Human:Digestive system	0.039
MGYA00245269/ERZ501073_FASTA.fasta	MGYA00245269	ERZ501073	root:Host-associated:Human:Digestive system	0.0572
MGYA00245270/ERZ501075_FASTA.fasta	MGYA00245270	ERZ501075	root:Host-associated:Human:Digestive system	0.0418
MGYA00245271/ERZ501077_FASTA.fasta	MGYA00245271	ERZ501077	root:Host-associated:Human:Digestive system	0.0318
MGYA00245272/ERZ501079_FASTA.fasta	MGYA00245272	ERZ501079	root:Host-associated:Human:Digestive system	0.0328
MGYA00245273/ERZ501081_FASTA.fasta	MGYA00245273	ERZ501081	root:Host-associated:Human:Digestive system	0.0464
MGYA00245274/ERZ501083_FASTA.fasta	MGYA00245274	ERZ501083	root:Host-associated:Human:Digestive system	0.0412
MGYA00245275/ERZ501085_FASTA.fasta	MGYA00245275	ERZ501085	root:Host-associated:Human:Digestive system	0.0538
MGYA00245276/ERZ501087_FASTA.fasta	MGYA00245276	ERZ501087	root:Host-associated:Human:Digestive system	0.0298
MGYA00245277/ERZ501089_FASTA.fasta	MGYA00245277	ERZ501089	root:Host-associated:Human:Digestive system	0.0322
MGYA00245278/ERZ501091_FASTA.fasta	MGYA00245278	ERZ501091	root:Host-associated:Human:Digestive system	0.0296
MGYA00245279/ERZ501093_FASTA.fasta	MGYA00245279	ERZ501093	root:Host-associated:Human:Digestive system	0.0486
MGYA00245280/ERZ501095_FASTA.fasta	MGYA00245280	ERZ501095	root:Host-associated:Human:Digestive system	0.0384
MGYA00245281/ERZ501097_FASTA.fasta	MGYA00245281	ERZ501097	root:Host-associated:Human:Digestive system	0.0362
MGYA00245282/ERZ501099_FASTA.fasta	MGYA00245282	ERZ501099	root:Host-associated:Human:Digestive system	0.0594
MGYA00245283/ERZ501101_FASTA.fasta	MGYA00245283	ERZ501101	root:Host-associated:Human:Digestive system	0.0412
MGYA00245284/ERZ501103_FASTA.fasta	MGYA00245284	ERZ501103	root:Host-associated:Human:Digestive system	0.0368
MGYA00245285/ERZ501105_FASTA.fasta	MGYA00245285	ERZ501105	root:Host-associated:Human:Digestive system	0.0332
MGYA00245286/ERZ501107_FASTA.fasta	MGYA00245286	ERZ501107	root:Host-associated:Human:Digestive system	0.0304
MGYA00245287/ERZ501109_FASTA.fasta	MGYA00245287	ERZ501109	root:Host-associated:Human:Digestive system	0.047
MGYA00245288/ERZ501111_FASTA.fasta	MGYA00245288	ERZ501111	root:Host-associated:Human:Digestive system	0.0294
MGYA00245289/ERZ501113_FASTA.fasta	MGYA00245289	ERZ501113	root:Host-associated:Human:Digestive system	0.0304
MGYA00245290/ERZ501122_FASTA.fasta	MGYA00245290	ERZ501122	root:Host-associated:Human:Digestive system	0.028
MGYA00245291/ERZ501124_FASTA.fasta	MGYA00245291	ERZ501124	root:Host-associated:Human:Digestive system	0.118
MGYA00245292/ERZ501126_FASTA.fasta	MGYA00245292	ERZ501126	root:Host-associated:Human:Digestive system	0.0904
MGYA00245293/ERZ501128_FASTA.fasta	MGYA00245293	ERZ501128	root:Host-associated:Human:Digestive system	0.055
MGYA00245294/ERZ501130_FASTA.fasta	MGYA00245294	ERZ501130	root:Host-associated:Human:Digestive system	0.0392
MGYA00245295/ERZ501132_FASTA.fasta	MGYA00245295	ERZ501132	root:Host-associated:Human:Digestive system	0.0946
MGYA00245296/ERZ501134_FASTA.fasta	MGYA00245296	ERZ501134	root:Host-associated:Human:Digestive system	0.0314
MGYA00245297/ERZ501136_FASTA.fasta	MGYA00245297	ERZ501136	root:Host-associated:Human:Digestive system	0.0328
MGYA00245298/ERZ501138_FASTA.fasta	MGYA00245298	ERZ501138	root:Host-associated:Human:Digestive system	0.048
MGYA00245299/ERZ501140_FASTA.fasta	MGYA00245299	ERZ501140	root:Host-associated:Human:Digestive system	0.1076
MGYA00245300/ERZ501115_FASTA.fasta	MGYA00245300	ERZ501115	root:Host-associated:Human:Digestive system	0.0772
MGYA00245301/ERZ501117_FASTA.fasta	MGYA00245301	ERZ501117	root:Host-associated:Human:Digestive system	0.0694
MGYA00245302/ERZ501119_FASTA.fasta	MGYA00245302	ERZ501119	root:Host-associated:Human:Digestive system	0.0646
MGYA00245303/ERZ501068_FASTA.fasta	MGYA00245303	ERZ501068	root:Host-associated:Human:Digestive system	0.0312
MGYA00245304/ERZ501070_FASTA.fasta	MGYA00245304	ERZ501070	root:Host-associated:Human:Digestive system	0.0298
MGYA00245305/ERZ501072_FASTA.fasta	MGYA00245305	ERZ501072	root:Host-associated:Human:Digestive system	0.0386
MGYA00245306/ERZ501074_FASTA.fasta	MGYA00245306	ERZ501074	root:Host-associated:Human:Digestive system	0.037
MGYA00245307/ERZ501076_FASTA.fasta	MGYA00245307	ERZ501076	root:Host-associated:Human:Digestive system	0.0706
MGYA00245308/ERZ501078_FASTA.fasta	MGYA00245308	ERZ501078	root:Host-associated:Human:Digestive system	0.0356
MGYA00245309/ERZ501080_FASTA.fasta	MGYA00245309	ERZ501080	root:Host-associated:Human:Digestive system	0.1072
MGYA00245310/ERZ501082_FASTA.fasta	MGYA00245310	ERZ501082	root:Host-associated:Human:Digestive system	0.0578
MGYA00245311/ERZ501084_FASTA.fasta	MGYA00245311	ERZ501084	root:Host-associated:Human:Digestive system	0.0348
MGYA00245312/ERZ501086_FASTA.fasta	MGYA00245312	ERZ501086	root:Host-associated:Human:Digestive system	0.0476
MGYA00245313/ERZ501088_FASTA.fasta	MGYA00245313	ERZ501088	root:Host-associated:Human:Digestive system	0.03
MGYA00245314/ERZ501090_FASTA.fasta	MGYA00245314	ERZ501090	root:Host-associated:Human:Digestive system	0.0602
MGYA00245315/ERZ501092_FASTA.fasta	MGYA00245315	ERZ501092	root:Host-associated:Human:Digestive system	0.0422
MGYA00245316/ERZ501094_FASTA.fasta	MGYA00245316	ERZ501094	root:Host-associated:Human:Digestive system	0.0624
MGYA00245317/ERZ501096_FASTA.fasta	MGYA00245317	ERZ501096	root:Host-associated:Human:Digestive system	0.0298
MGYA00245318/ERZ501098_FASTA.fasta	MGYA00245318	ERZ501098	root:Host-associated:Human:Digestive system	0.0524
MGYA00245319/ERZ501100_FASTA.fasta	MGYA00245319	ERZ501100	root:Host-associated:Human:Digestive system	0.0564
MGYA00245320/ERZ501102_FASTA.fasta	MGYA00245320	ERZ501102	root:Host-associated:Human:Digestive system	0.0686
MGYA00245321/ERZ501104_FASTA.fasta	MGYA00245321	ERZ501104	root:Host-associated:Human:Digestive system	0.0378
MGYA00245322/ERZ501106_FASTA.fasta	MGYA00245322	ERZ501106	root:Host-associated:Human:Digestive system	0.0648
MGYA00245323/ERZ501108_FASTA.fasta	MGYA00245323	ERZ501108	root:Host-associated:Human:Digestive system	0.0316
MGYA00245324/ERZ501110_FASTA.fasta	MGYA00245324	ERZ501110	root:Host-associated:Human:Digestive system	0.0294
MGYA00245325/ERZ501112_FASTA.fasta	MGYA00245325	ERZ501112	root:Host-associated:Human:Digestive system	0.041
MGYA00245326/ERZ501114_FASTA.fasta	MGYA00245326	ERZ501114	root:Host-associated:Human:Digestive system	0.0422
MGYA00245327/ERZ501116_FASTA.fasta	MGYA00245327	ERZ501116	root:Host-associated:Human:Digestive system	0.0544
MGYA00245328/ERZ501118_FASTA.fasta	MGYA00245328	ERZ501118	root:Host-associated:Human:Digestive system	0.059
MGYA00245329/ERZ501120_FASTA.fasta	MGYA00245329	ERZ501120	root:Host-associated:Human:Digestive system	0.1708
MGYA00245330/ERZ651093_FASTA.fasta	MGYA00245330	ERZ651093	root:Host-associated:Human:Digestive system:Oral:Sali	0.6344
MGYA00245331/ERZ651095_FASTA.fasta	MGYA00245331	ERZ651095	root:Host-associated:Human:Digestive system:Oral:Sali	0.6198
MGYA00245332/ERZ651097_FASTA.fasta	MGYA00245332	ERZ651097	root:Host-associated:Human:Digestive system:Oral:Sali	0.296
MGYA00245333/ERZ651099_FASTA.fasta	MGYA00245333	ERZ651099	root:Host-associated:Human:Digestive system:Oral:Sali	0.6238
MGYA00245334/ERZ651103_FASTA.fasta	MGYA00245334	ERZ651103	root:Host-associated:Human:Digestive system:Oral:Sali	0.6076
MGYA00245335/ERZ651105_FASTA.fasta	MGYA00245335	ERZ651105	root:Host-associated:Human:Digestive system:Oral:Sali	0.5792
MGYA00245336/ERZ651107_FASTA.fasta	MGYA00245336	ERZ651107	root:Host-associated:Human:Digestive system:Oral:Sali	0.4842
MGYA00245337/ERZ651109_FASTA.fasta	MGYA00245337	ERZ651109	root:Host-associated:Human:Digestive system:Oral:Sali	0.6162
MGYA00245338/ERZ651111_FASTA.fasta	MGYA00245338	ERZ651111	root:Host-associated:Human:Digestive system:Oral:Sali	0.632
MGYA00245339/ERZ651113_FASTA.fasta	MGYA00245339	ERZ651113	root:Host-associated:Human:Digestive system:Oral:Sali	0.5108
MGYA00245340/ERZ651115_FASTA.fasta	MGYA00245340	ERZ651115	root:Host-associated:Human:Digestive system:Oral:Sali	0.3568
MGYA00245341/ERZ651117_FASTA.fasta	MGYA00245341	ERZ651117	root:Host-associated:Human:Digestive system:Oral:Sali	0.5202
MGYA00245342/ERZ651119_FASTA.fasta	MGYA00245342	ERZ651119	root:Host-associated:Human:Digestive system:Oral:Sali	0.6602
MGYA00245343/ERZ651121_FASTA.fasta	MGYA00245343	ERZ651121	root:Host-associated:Human:Digestive system:Oral:Sali	0.637
MGYA00245344/ERZ651092_FASTA.fasta	MGYA00245344	ERZ651092	root:Host-associated:Human:Digestive system:Oral:Sali	0.5252
MGYA00245345/ERZ651094_FASTA.fasta	MGYA00245345	ERZ651094	root:Host-associated:Human:Digestive system:Oral:Sali	0.5392
MGYA00245346/ERZ651096_FASTA.fasta	MGYA00245346	ERZ651096	root:Host-associated:Human:Digestive system:Oral:Sali	0.399
MGYA00245347/ERZ651098_FASTA.fasta	MGYA00245347	ERZ651098	root:Host-associated:Human:Digestive system:Oral:Sali	0.5634
MGYA00245348/ERZ651100_FASTA.fasta	MGYA00245348	ERZ651100	root:Host-associated:Human:Digestive system:Oral:Sali	0.2926
MGYA00245349/ERZ651102_FASTA.fasta	MGYA00245349	ERZ651102	root:Host-associated:Human:Digestive system:Oral:Sali	0.7246
MGYA00245350/ERZ651104_FASTA.fasta	MGYA00245350	ERZ651104	root:Host-associated:Human:Digestive system:Oral:Sali	0.3018
MGYA00245351/ERZ651106_FASTA.fasta	MGYA00245351	ERZ651106	root:Host-associated:Human:Digestive system:Oral:Sali	0.654
MGYA00245352/ERZ651110_FASTA.fasta	MGYA00245352	ERZ651110	root:Host-associated:Human:Digestive system:Oral:Sali	0.4486
MGYA00245353/ERZ651112_FASTA.fasta	MGYA00245353	ERZ651112	root:Host-associated:Human:Digestive system:Oral:Sali	0.6456
MGYA00245354/ERZ651114_FASTA.fasta	MGYA00245354	ERZ651114	root:Host-associated:Human:Digestive system:Oral:Sali	0.4512
MGYA00245355/ERZ651116_FASTA.fasta	MGYA00245355	ERZ651116	root:Host-associated:Human:Digestive system:Oral:Sali	0.3528
MGYA00245356/ERZ651118_FASTA.fasta	MGYA00245356	ERZ651118	root:Host-associated:Human:Digestive system:Oral:Sali	0.4232
MGYA00245357/ERZ651120_FASTA.fasta	MGYA00245357	ERZ651120	root:Host-associated:Human:Digestive system:Oral:Sali	0.4002
MGYA00245358/ERZ651122_FASTA.fasta	MGYA00245358	ERZ651122	root:Host-associated:Human:Digestive system:Oral:Sali	0.5692
MGYA00245776/ERZ724021_FASTA.fasta	MGYA00245776	ERZ724021	root:Engineered:Food production:Fermented beverage	0.5716
MGYA00245777/ERZ724023_FASTA.fasta	MGYA00245777	ERZ724023	root:Engineered:Food production:Fermented beverage	0.6398
MGYA00245778/ERZ724025_FASTA.fasta	MGYA00245778	ERZ724025	root:Engineered:Food production:Fermented beverage	0.618
MGYA00245779/ERZ724027_FASTA.fasta	MGYA00245779	ERZ724027	root:Engineered:Food production:Fermented beverage	0.6
MGYA00245780/ERZ724029_FASTA.fasta	MGYA00245780	ERZ724029	root:Engineered:Food production:Fermented beverage	0.6496
MGYA00245781/ERZ724031_FASTA.fasta	MGYA00245781	ERZ724031	root:Engineered:Food production:Fermented beverage	0.6104
MGYA00245782/ERZ724033_FASTA.fasta	MGYA00245782	ERZ724033	root:Engineered:Food production:Fermented beverage	0.6084
MGYA00245783/ERZ724035_FASTA.fasta	MGYA00245783	ERZ724035	root:Engineered:Food production:Fermented beverage	0.5682
MGYA00245784/ERZ724037_FASTA.fasta	MGYA00245784	ERZ724037	root:Engineered:Food production:Fermented beverage	0.597
MGYA00245785/ERZ724039_FASTA.fasta	MGYA00245785	ERZ724039	root:Engineered:Food production:Fermented beverage	0.6088
MGYA00245786/ERZ724041_FASTA.fasta	MGYA00245786	ERZ724041	root:Engineered:Food production:Fermented beverage	0.5852
MGYA00245787/ERZ724043_FASTA.fasta	MGYA00245787	ERZ724043	root:Engineered:Food production:Fermented beverage	0.5418
MGYA00245788/ERZ724022_FASTA.fasta	MGYA00245788	ERZ724022	root:Engineered:Food production:Fermented beverage	0.654
MGYA00245789/ERZ724024_FASTA.fasta	MGYA00245789	ERZ724024	root:Engineered:Food production:Fermented beverage	0.6134
MGYA00245790/ERZ724026_FASTA.fasta	MGYA00245790	ERZ724026	root:Engineered:Food production:Fermented beverage	0.7082
MGYA00245791/ERZ724028_FASTA.fasta	MGYA00245791	ERZ724028	root:Engineered:Food production:Fermented beverage	0.7682
MGYA00245792/ERZ724030_FASTA.fasta	MGYA00245792	ERZ724030	root:Engineered:Food production:Fermented beverage	0.7724
MGYA00245793/ERZ724032_FASTA.fasta	MGYA00245793	ERZ724032	root:Engineered:Food production:Fermented beverage	0.9398
MGYA00245794/ERZ724034_FASTA.fasta	MGYA00245794	ERZ724034	root:Engineered:Food production:Fermented beverage	0.9218
MGYA00245795/ERZ724036_FASTA.fasta	MGYA00245795	ERZ724036	root:Engineered:Food production:Fermented beverage	0.6056
MGYA00245796/ERZ724038_FASTA.fasta	MGYA00245796	ERZ724038	root:Engineered:Food production:Fermented beverage	0.5874
MGYA00245797/ERZ724040_FASTA.fasta	MGYA00245797	ERZ724040	root:Engineered:Food production:Fermented beverage	0.6092
MGYA00245798/ERZ724042_FASTA.fasta	MGYA00245798	ERZ724042	root:Engineered:Food production:Fermented beverage	0.5952
MGYA00245799/ERZ724044_FASTA.fasta	MGYA00245799	ERZ724044	root:Engineered:Food production:Fermented beverage	0.5482
MGYA00248238/ERZ651777_FASTA.fasta	MGYA00248238	ERZ651777	root:Host-associated:Human:Digestive system:Large int	0.3598
MGYA00248239/ERZ651779_FASTA.fasta	MGYA00248239	ERZ651779	root:Host-associated:Human:Digestive system:Large int	0.3872
MGYA00248240/ERZ651781_FASTA.fasta	MGYA00248240	ERZ651781	root:Host-associated:Human:Digestive system:Large int	0.4882
MGYA00248241/ERZ651783_FASTA.fasta	MGYA00248241	ERZ651783	root:Host-associated:Human:Digestive system:Large int	0.49
MGYA00248242/ERZ651785_FASTA.fasta	MGYA00248242	ERZ651785	root:Host-associated:Human:Digestive system:Large int	0.3286
MGYA00248243/ERZ651787_FASTA.fasta	MGYA00248243	ERZ651787	root:Host-associated:Human:Digestive system:Large int	0.3858
MGYA00248244/ERZ651789_FASTA.fasta	MGYA00248244	ERZ651789	root:Host-associated:Human:Digestive system:Large int	0.5028
MGYA00248245/ERZ651791_FASTA.fasta	MGYA00248245	ERZ651791	root:Host-associated:Human:Digestive system:Large int	0.4746
MGYA00248246/ERZ651795_FASTA.fasta	MGYA00248246	ERZ651795	root:Host-associated:Human:Digestive system:Large int	0.4156
MGYA00248247/ERZ651799_FASTA.fasta	MGYA00248247	ERZ651799	root:Host-associated:Human:Digestive system:Large int	0.3544
MGYA00248248/ERZ651801_FASTA.fasta	MGYA00248248	ERZ651801	root:Host-associated:Human:Digestive system:Large int	0.5222
MGYA00248249/ERZ651793_FASTA.fasta	MGYA00248249	ERZ651793	root:Host-associated:Human:Digestive system:Large int	0.5014
MGYA00248250/ERZ651778_FASTA.fasta	MGYA00248250	ERZ651778	root:Host-associated:Human:Digestive system:Large int	0.4984
MGYA00248251/ERZ651780_FASTA.fasta	MGYA00248251	ERZ651780	root:Host-associated:Human:Digestive system:Large int	0.4046
MGYA00248252/ERZ651782_FASTA.fasta	MGYA00248252	ERZ651782	root:Host-associated:Human:Digestive system:Large int	0.5658
MGYA00248253/ERZ651784_FASTA.fasta	MGYA00248253	ERZ651784	root:Host-associated:Human:Digestive system:Large int	0.4436
MGYA00248254/ERZ651788_FASTA.fasta	MGYA00248254	ERZ651788	root:Host-associated:Human:Digestive system:Large int	0.4856
MGYA00248255/ERZ651790_FASTA.fasta	MGYA00248255	ERZ651790	root:Host-associated:Human:Digestive system:Large int	0.3186
MGYA00248256/ERZ651792_FASTA.fasta	MGYA00248256	ERZ651792	root:Host-associated:Human:Digestive system:Large int	0.459
MGYA00248257/ERZ651798_FASTA.fasta	MGYA00248257	ERZ651798	root:Host-associated:Human:Digestive system:Large int	0.4804
MGYA00248258/ERZ651800_FASTA.fasta	MGYA00248258	ERZ651800	root:Host-associated:Human:Digestive system:Large int	0.4488
MGYA00248259/ERZ651802_FASTA.fasta	MGYA00248259	ERZ651802	root:Host-associated:Human:Digestive system:Large int	0.4206
MGYA00248260/ERZ651838_FASTA.fasta	MGYA00248260	ERZ651838	root:Host-associated:Human:Digestive system:Large int	0.3932
MGYA00248261/ERZ651840_FASTA.fasta	MGYA00248261	ERZ651840	root:Host-associated:Human:Digestive system:Large int	0.5212
MGYA00248262/ERZ651842_FASTA.fasta	MGYA00248262	ERZ651842	root:Host-associated:Human:Digestive system:Large int	0.4242
MGYA00248263/ERZ651844_FASTA.fasta	MGYA00248263	ERZ651844	root:Host-associated:Human:Digestive system:Large int	0.574
MGYA00248264/ERZ651846_FASTA.fasta	MGYA00248264	ERZ651846	root:Host-associated:Human:Digestive system:Large int	0.5238
MGYA00248265/ERZ651848_FASTA.fasta	MGYA00248265	ERZ651848	root:Host-associated:Human:Digestive system:Large int	0.546
MGYA00248266/ERZ651850_FASTA.fasta	MGYA00248266	ERZ651850	root:Host-associated:Human:Digestive system:Large int	0.5272
MGYA00248267/ERZ651852_FASTA.fasta	MGYA00248267	ERZ651852	root:Host-associated:Human:Digestive system:Large int	0.4852
MGYA00248268/ERZ651895_FASTA.fasta	MGYA00248268	ERZ651895	root:Host-associated:Human:Digestive system:Large int	0.503
MGYA00248269/ERZ651897_FASTA.fasta	MGYA00248269	ERZ651897	root:Host-associated:Human:Digestive system:Large int	0.48
MGYA00248270/ERZ651899_FASTA.fasta	MGYA00248270	ERZ651899	root:Host-associated:Human:Digestive system:Large int	0.4478
MGYA00248271/ERZ651901_FASTA.fasta	MGYA00248271	ERZ651901	root:Host-associated:Human:Digestive system:Large int	0.453
MGYA00248272/ERZ651903_FASTA.fasta	MGYA00248272	ERZ651903	root:Host-associated:Human:Digestive system:Large int	0.4058
MGYA00248273/ERZ651905_FASTA.fasta	MGYA00248273	ERZ651905	root:Host-associated:Human:Digestive system:Large int	0.5108
MGYA00248274/ERZ651907_FASTA.fasta	MGYA00248274	ERZ651907	root:Host-associated:Human:Digestive system:Large int	0.5198
MGYA00248275/ERZ651909_FASTA.fasta	MGYA00248275	ERZ651909	root:Host-associated:Human:Digestive system:Large int	0.46
MGYA00248276/ERZ651911_FASTA.fasta	MGYA00248276	ERZ651911	root:Host-associated:Human:Digestive system:Large int	0.4826
MGYA00248277/ERZ651913_FASTA.fasta	MGYA00248277	ERZ651913	root:Host-associated:Human:Digestive system:Large int	0.5336
MGYA00248278/ERZ651915_FASTA.fasta	MGYA00248278	ERZ651915	root:Host-associated:Human:Digestive system:Large int	0.4792
MGYA00248279/ERZ651917_FASTA.fasta	MGYA00248279	ERZ651917	root:Host-associated:Human:Digestive system:Large int	0.5652
MGYA00248280/ERZ651919_FASTA.fasta	MGYA00248280	ERZ651919	root:Host-associated:Human:Digestive system:Large int	0.5494
MGYA00248281/ERZ651921_FASTA.fasta	MGYA00248281	ERZ651921	root:Host-associated:Human:Digestive system:Large int	0.4934
MGYA00248282/ERZ651923_FASTA.fasta	MGYA00248282	ERZ651923	root:Host-associated:Human:Digestive system:Large int	0.4306
MGYA00248283/ERZ651925_FASTA.fasta	MGYA00248283	ERZ651925	root:Host-associated:Human:Digestive system:Large int	0.5602
MGYA00248284/ERZ651927_FASTA.fasta	MGYA00248284	ERZ651927	root:Host-associated:Human:Digestive system:Large int	0.5652
MGYA00248285/ERZ651929_FASTA.fasta	MGYA00248285	ERZ651929	root:Host-associated:Human:Digestive system:Large int	0.489
MGYA00248286/ERZ651931_FASTA.fasta	MGYA00248286	ERZ651931	root:Host-associated:Human:Digestive system:Large int	0.487
MGYA00248287/ERZ651933_FASTA.fasta	MGYA00248287	ERZ651933	root:Host-associated:Human:Digestive system:Large int	0.418
MGYA00248288/ERZ651935_FASTA.fasta	MGYA00248288	ERZ651935	root:Host-associated:Human:Digestive system:Large int	0.5252
MGYA00248289/ERZ651937_FASTA.fasta	MGYA00248289	ERZ651937	root:Host-associated:Human:Digestive system:Large int	0.4784
MGYA00248290/ERZ651847_FASTA.fasta	MGYA00248290	ERZ651847	root:Host-associated:Human:Digestive system:Large int	0.4794
MGYA00248291/ERZ651849_FASTA.fasta	MGYA00248291	ERZ651849	root:Host-associated:Human:Digestive system:Large int	0.5146
MGYA00248292/ERZ651853_FASTA.fasta	MGYA00248292	ERZ651853	root:Host-associated:Human:Digestive system:Large int	0.4516
MGYA00248293/ERZ651857_FASTA.fasta	MGYA00248293	ERZ651857	root:Host-associated:Human:Digestive system:Large int	0.569
MGYA00248294/ERZ651859_FASTA.fasta	MGYA00248294	ERZ651859	root:Host-associated:Human:Digestive system:Large int	0.5696
MGYA00248295/ERZ651861_FASTA.fasta	MGYA00248295	ERZ651861	root:Host-associated:Human:Digestive system:Large int	0.4846
MGYA00248296/ERZ651863_FASTA.fasta	MGYA00248296	ERZ651863	root:Host-associated:Human:Digestive system:Large int	0.4466
MGYA00248297/ERZ651865_FASTA.fasta	MGYA00248297	ERZ651865	root:Host-associated:Human:Digestive system:Large int	0.5014
MGYA00248298/ERZ651867_FASTA.fasta	MGYA00248298	ERZ651867	root:Host-associated:Human:Digestive system:Large int	0.5592
MGYA00248299/ERZ651869_FASTA.fasta	MGYA00248299	ERZ651869	root:Host-associated:Human:Digestive system:Large int	0.4424
MGYA00248300/ERZ651871_FASTA.fasta	MGYA00248300	ERZ651871	root:Host-associated:Human:Digestive system:Large int	0.51
MGYA00248301/ERZ651873_FASTA.fasta	MGYA00248301	ERZ651873	root:Host-associated:Human:Digestive system:Large int	0.5486
MGYA00248302/ERZ651875_FASTA.fasta	MGYA00248302	ERZ651875	root:Host-associated:Human:Digestive system:Large int	0.4194
MGYA00248303/ERZ651877_FASTA.fasta	MGYA00248303	ERZ651877	root:Host-associated:Human:Digestive system:Large int	0.5084
MGYA00248304/ERZ651879_FASTA.fasta	MGYA00248304	ERZ651879	root:Host-associated:Human:Digestive system:Large int	0.5002
MGYA00248305/ERZ651881_FASTA.fasta	MGYA00248305	ERZ651881	root:Host-associated:Human:Digestive system:Large int	0.4816
MGYA00248306/ERZ651883_FASTA.fasta	MGYA00248306	ERZ651883	root:Host-associated:Human:Digestive system:Large int	0.4168
MGYA00248307/ERZ651885_FASTA.fasta	MGYA00248307	ERZ651885	root:Host-associated:Human:Digestive system:Large int	0.6008
MGYA00248308/ERZ651887_FASTA.fasta	MGYA00248308	ERZ651887	root:Host-associated:Human:Digestive system:Large int	0.5232
MGYA00248309/ERZ651889_FASTA.fasta	MGYA00248309	ERZ651889	root:Host-associated:Human:Digestive system:Large int	0.5408
MGYA00248310/ERZ651891_FASTA.fasta	MGYA00248310	ERZ651891	root:Host-associated:Human:Digestive system:Large int	0.4668
MGYA00248311/ERZ651893_FASTA.fasta	MGYA00248311	ERZ651893	root:Host-associated:Human:Digestive system:Large int	0.5318
MGYA00248312/ERZ651854_FASTA.fasta	MGYA00248312	ERZ651854	root:Host-associated:Human:Digestive system:Large int	0.4186
MGYA00248313/ERZ651856_FASTA.fasta	MGYA00248313	ERZ651856	root:Host-associated:Human:Digestive system:Large int	0.5304
MGYA00248314/ERZ651858_FASTA.fasta	MGYA00248314	ERZ651858	root:Host-associated:Human:Digestive system:Large int	0.435
MGYA00248315/ERZ651860_FASTA.fasta	MGYA00248315	ERZ651860	root:Host-associated:Human:Digestive system:Large int	0.5666
MGYA00248316/ERZ651862_FASTA.fasta	MGYA00248316	ERZ651862	root:Host-associated:Human:Digestive system:Large int	0.5738
MGYA00248317/ERZ651864_FASTA.fasta	MGYA00248317	ERZ651864	root:Host-associated:Human:Digestive system:Large int	0.4872
MGYA00248318/ERZ651866_FASTA.fasta	MGYA00248318	ERZ651866	root:Host-associated:Human:Digestive system:Large int	0.5858
MGYA00248319/ERZ651868_FASTA.fasta	MGYA00248319	ERZ651868	root:Host-associated:Human:Digestive system:Large int	0.5296
MGYA00248320/ERZ651870_FASTA.fasta	MGYA00248320	ERZ651870	root:Host-associated:Human:Digestive system:Large int	0.5834
MGYA00248321/ERZ651872_FASTA.fasta	MGYA00248321	ERZ651872	root:Host-associated:Human:Digestive system:Large int	0.4468
MGYA00248322/ERZ651874_FASTA.fasta	MGYA00248322	ERZ651874	root:Host-associated:Human:Digestive system:Large int	0.4726
MGYA00248323/ERZ651876_FASTA.fasta	MGYA00248323	ERZ651876	root:Host-associated:Human:Digestive system:Large int	0.46
MGYA00248324/ERZ651878_FASTA.fasta	MGYA00248324	ERZ651878	root:Host-associated:Human:Digestive system:Large int	0.5598
MGYA00248325/ERZ651880_FASTA.fasta	MGYA00248325	ERZ651880	root:Host-associated:Human:Digestive system:Large int	0.4926
MGYA00248326/ERZ651882_FASTA.fasta	MGYA00248326	ERZ651882	root:Host-associated:Human:Digestive system:Large int	0.485
MGYA00248327/ERZ651884_FASTA.fasta	MGYA00248327	ERZ651884	root:Host-associated:Human:Digestive system:Large int	0.5654
MGYA00248328/ERZ651886_FASTA.fasta	MGYA00248328	ERZ651886	root:Host-associated:Human:Digestive system:Large int	0.4498
MGYA00248329/ERZ651888_FASTA.fasta	MGYA00248329	ERZ651888	root:Host-associated:Human:Digestive system:Large int	0.5458
MGYA00248330/ERZ651892_FASTA.fasta	MGYA00248330	ERZ651892	root:Host-associated:Human:Digestive system:Large int	0.4364
MGYA00248331/ERZ651894_FASTA.fasta	MGYA00248331	ERZ651894	root:Host-associated:Human:Digestive system:Large int	0.4798
MGYA00248332/ERZ651896_FASTA.fasta	MGYA00248332	ERZ651896	root:Host-associated:Human:Digestive system:Large int	0.5884
MGYA00248333/ERZ651898_FASTA.fasta	MGYA00248333	ERZ651898	root:Host-associated:Human:Digestive system:Large int	0.4962
MGYA00248334/ERZ651900_FASTA.fasta	MGYA00248334	ERZ651900	root:Host-associated:Human:Digestive system:Large int	0.497
MGYA00248335/ERZ651902_FASTA.fasta	MGYA00248335	ERZ651902	root:Host-associated:Human:Digestive system:Large int	0.4438
MGYA00248336/ERZ651904_FASTA.fasta	MGYA00248336	ERZ651904	root:Host-associated:Human:Digestive system:Large int	0.4728
MGYA00248337/ERZ651906_FASTA.fasta	MGYA00248337	ERZ651906	root:Host-associated:Human:Digestive system:Large int	0.5256
MGYA00248338/ERZ651908_FASTA.fasta	MGYA00248338	ERZ651908	root:Host-associated:Human:Digestive system:Large int	0.5238
MGYA00248339/ERZ651910_FASTA.fasta	MGYA00248339	ERZ651910	root:Host-associated:Human:Digestive system:Large int	0.5148
MGYA00248340/ERZ651912_FASTA.fasta	MGYA00248340	ERZ651912	root:Host-associated:Human:Digestive system:Large int	0.5448
MGYA00248341/ERZ651837_FASTA.fasta	MGYA00248341	ERZ651837	root:Host-associated:Human:Digestive system:Large int	0.54
MGYA00248342/ERZ651839_FASTA.fasta	MGYA00248342	ERZ651839	root:Host-associated:Human:Digestive system:Large int	0.4788
MGYA00248343/ERZ651841_FASTA.fasta	MGYA00248343	ERZ651841	root:Host-associated:Human:Digestive system:Large int	0.505
MGYA00248344/ERZ651843_FASTA.fasta	MGYA00248344	ERZ651843	root:Host-associated:Human:Digestive system:Large int	0.5058
MGYA00248345/ERZ651914_FASTA.fasta	MGYA00248345	ERZ651914	root:Host-associated:Human:Digestive system:Large int	0.4756
MGYA00248346/ERZ651916_FASTA.fasta	MGYA00248346	ERZ651916	root:Host-associated:Human:Digestive system:Large int	0.3984
MGYA00248347/ERZ651918_FASTA.fasta	MGYA00248347	ERZ651918	root:Host-associated:Human:Digestive system:Large int	0.5474
MGYA00248348/ERZ651920_FASTA.fasta	MGYA00248348	ERZ651920	root:Host-associated:Human:Digestive system:Large int	0.5808
MGYA00248349/ERZ651922_FASTA.fasta	MGYA00248349	ERZ651922	root:Host-associated:Human:Digestive system:Large int	0.6302
MGYA00248350/ERZ651924_FASTA.fasta	MGYA00248350	ERZ651924	root:Host-associated:Human:Digestive system:Large int	0.4592
MGYA00248351/ERZ651926_FASTA.fasta	MGYA00248351	ERZ651926	root:Host-associated:Human:Digestive system:Large int	0.5232
MGYA00248352/ERZ651928_FASTA.fasta	MGYA00248352	ERZ651928	root:Host-associated:Human:Digestive system:Large int	0.6098
MGYA00248353/ERZ651930_FASTA.fasta	MGYA00248353	ERZ651930	root:Host-associated:Human:Digestive system:Large int	0.5318
MGYA00248354/ERZ651932_FASTA.fasta	MGYA00248354	ERZ651932	root:Host-associated:Human:Digestive system:Large int	0.4244
MGYA00248355/ERZ651934_FASTA.fasta	MGYA00248355	ERZ651934	root:Host-associated:Human:Digestive system:Large int	0.549
MGYA00248356/ERZ651936_FASTA.fasta	MGYA00248356	ERZ651936	root:Host-associated:Human:Digestive system:Large int	0.5626
MGYA00248357/ERZ724020_FASTA.fasta	MGYA00248357	ERZ724020	root:Engineered:Wastewater:Industrial wastewater	0.823
MGYA00248358/ERZ724071_FASTA.fasta	MGYA00248358	ERZ724071	root:Engineered:Bioreactor	0.3064
MGYA00248359/ERZ724073_FASTA.fasta	MGYA00248359	ERZ724073	root:Engineered:Bioreactor	0.4384
MGYA00248360/ERZ724075_FASTA.fasta	MGYA00248360	ERZ724075	root:Engineered:Bioreactor	0.4682
MGYA00248361/ERZ724077_FASTA.fasta	MGYA00248361	ERZ724077	root:Engineered:Bioreactor	0.4122
MGYA00248362/ERZ724072_FASTA.fasta	MGYA00248362	ERZ724072	root:Engineered:Bioreactor	0.486
MGYA00248363/ERZ724074_FASTA.fasta	MGYA00248363	ERZ724074	root:Engineered:Bioreactor	0.296
MGYA00248364/ERZ724076_FASTA.fasta	MGYA00248364	ERZ724076	root:Engineered:Bioreactor	0.388
MGYA00248365/ERZ724098_FASTA.fasta	MGYA00248365	ERZ724098	root:Engineered:Bioreactor	0.7714
MGYA00248366/ERZ724100_FASTA.fasta	MGYA00248366	ERZ724100	root:Engineered:Bioreactor	0.8192
MGYA00248367/ERZ724099_FASTA.fasta	MGYA00248367	ERZ724099	root:Engineered:Bioreactor	0.811
MGYA00248368/ERZ724101_FASTA.fasta	MGYA00248368	ERZ724101	root:Engineered:Bioreactor	0.8032
MGYA00248370/ERZ747222_FASTA.fasta	MGYA00248370	ERZ747222	root:Engineered:Bioreactor	0.5592
MGYA00248371/ERZ761576_FASTA.fasta	MGYA00248371	ERZ761576	root:Environmental:Aquatic:Lentic:Brackish	0.3928
MGYA00248372/ERZ761617_FASTA.fasta	MGYA00248372	ERZ761617	root:Engineered:Food production	0.4264
MGYA00248373/ERZ761619_FASTA.fasta	MGYA00248373	ERZ761619	root:Engineered:Food production	0.4484
MGYA00248374/ERZ761621_FASTA.fasta	MGYA00248374	ERZ761621	root:Engineered:Food production	0.4504
MGYA00248375/ERZ761623_FASTA.fasta	MGYA00248375	ERZ761623	root:Engineered:Food production	0.0294
MGYA00248376/ERZ761625_FASTA.fasta	MGYA00248376	ERZ761625	root:Engineered:Food production	0.0342
MGYA00248377/ERZ761627_FASTA.fasta	MGYA00248377	ERZ761627	root:Engineered:Food production	0.0348
MGYA00248378/ERZ761629_FASTA.fasta	MGYA00248378	ERZ761629	root:Engineered:Food production	0.0464
MGYA00248379/ERZ761631_FASTA.fasta	MGYA00248379	ERZ761631	root:Engineered:Food production	0.3852
MGYA00248380/ERZ761616_FASTA.fasta	MGYA00248380	ERZ761616	root:Engineered:Food production	0.4162
MGYA00248381/ERZ761618_FASTA.fasta	MGYA00248381	ERZ761618	root:Engineered:Food production	0.045
MGYA00248382/ERZ761620_FASTA.fasta	MGYA00248382	ERZ761620	root:Engineered:Food production	0.0342
MGYA00248383/ERZ761622_FASTA.fasta	MGYA00248383	ERZ761622	root:Engineered:Food production	0.443
MGYA00248384/ERZ761624_FASTA.fasta	MGYA00248384	ERZ761624	root:Engineered:Food production	0.0294
MGYA00248385/ERZ761626_FASTA.fasta	MGYA00248385	ERZ761626	root:Engineered:Food production	0.4342
MGYA00248386/ERZ761628_FASTA.fasta	MGYA00248386	ERZ761628	root:Engineered:Food production	0.0342
MGYA00248387/ERZ761630_FASTA.fasta	MGYA00248387	ERZ761630	root:Engineered:Food production	0.4202
MGYA00248388/ERZ761636_FASTA.fasta	MGYA00248388	ERZ761636	root:Environmental:Aquatic:Lentic:Brackish	0.6692
MGYA00248389/ERZ764730_FASTA.fasta	MGYA00248389	ERZ764730	root:Engineered:Wastewater:Activated Sludge	0.8626
MGYA00248390/ERZ764753_FASTA.fasta	MGYA00248390	ERZ764753	root:Engineered:Wastewater:Activated Sludge	0.7988
MGYA00248391/ERZ764845_FASTA.fasta	MGYA00248391	ERZ764845	root:Engineered:Bioreactor	0.7302
MGYA00248392/ERZ767044_FASTA.fasta	MGYA00248392	ERZ767044	root:Environmental:Aquatic:Estuary	0.849
MGYA00248393/ERZ767088_FASTA.fasta	MGYA00248393	ERZ767088	root:Environmental:Aquatic:Estuary	0.824
MGYA00248394/ERZ767334_FASTA.fasta	MGYA00248394	ERZ767334	root:Environmental:Aquatic:Estuary	0.822
MGYA00248395/ERZ767383_FASTA.fasta	MGYA00248395	ERZ767383	root:Environmental:Aquatic:Estuary	0.8168
MGYA00248396/ERZ767408_FASTA.fasta	MGYA00248396	ERZ767408	root:Environmental:Aquatic:Estuary	0.8184
MGYA00248397/ERZ773284_FASTA.fasta	MGYA00248397	ERZ773284	root:Engineered:Wastewater:Activated Sludge	0.858
MGYA00248398/ERZ773287_FASTA.fasta	MGYA00248398	ERZ773287	root:Engineered:Bioreactor	0.5744
MGYA00248399/ERZ773290_FASTA.fasta	MGYA00248399	ERZ773290	root:Engineered:Bioreactor	0.5362
MGYA00248400/ERZ773357_FASTA.fasta	MGYA00248400	ERZ773357	root:Engineered:Biogas plant	0.826
MGYA00248401/ERZ773360_FASTA.fasta	MGYA00248401	ERZ773360	root:Engineered:Bioreactor	0.8708
MGYA00248402/ERZ650115_FASTA.fasta	MGYA00248402	ERZ650115	root:Host-associated:Mammals:Digestive system:Fecal	0.183
MGYA00248403/ERZ650117_FASTA.fasta	MGYA00248403	ERZ650117	root:Host-associated:Mammals:Digestive system:Fecal	0.1524
MGYA00248404/ERZ650119_FASTA.fasta	MGYA00248404	ERZ650119	root:Host-associated:Mammals:Digestive system:Fecal	0.14
MGYA00248405/ERZ650121_FASTA.fasta	MGYA00248405	ERZ650121	root:Host-associated:Mammals:Digestive system:Fecal	0.0948
MGYA00248406/ERZ650123_FASTA.fasta	MGYA00248406	ERZ650123	root:Host-associated:Mammals:Digestive system:Fecal	0.277
MGYA00248407/ERZ650125_FASTA.fasta	MGYA00248407	ERZ650125	root:Host-associated:Mammals:Digestive system:Fecal	0.2916
MGYA00248408/ERZ650127_FASTA.fasta	MGYA00248408	ERZ650127	root:Host-associated:Mammals:Digestive system:Fecal	0.1596
MGYA00248409/ERZ650129_FASTA.fasta	MGYA00248409	ERZ650129	root:Host-associated:Mammals:Digestive system:Fecal	0.291
MGYA00248410/ERZ650131_FASTA.fasta	MGYA00248410	ERZ650131	root:Host-associated:Mammals:Digestive system:Fecal	0.2366
MGYA00248411/ERZ650133_FASTA.fasta	MGYA00248411	ERZ650133	root:Host-associated:Mammals:Digestive system:Fecal	0.2352
MGYA00248412/ERZ650135_FASTA.fasta	MGYA00248412	ERZ650135	root:Host-associated:Mammals:Digestive system:Fecal	0.232
MGYA00248413/ERZ650137_FASTA.fasta	MGYA00248413	ERZ650137	root:Host-associated:Mammals:Digestive system:Fecal	0.0912
MGYA00248414/ERZ650139_FASTA.fasta	MGYA00248414	ERZ650139	root:Host-associated:Mammals:Digestive system:Fecal	0.248
MGYA00248415/ERZ650141_FASTA.fasta	MGYA00248415	ERZ650141	root:Host-associated:Mammals:Digestive system:Fecal	0.1974
MGYA00248416/ERZ650143_FASTA.fasta	MGYA00248416	ERZ650143	root:Host-associated:Mammals:Digestive system:Fecal	0.1854
MGYA00248417/ERZ650145_FASTA.fasta	MGYA00248417	ERZ650145	root:Host-associated:Mammals:Digestive system:Fecal	0.1374
MGYA00248418/ERZ650147_FASTA.fasta	MGYA00248418	ERZ650147	root:Host-associated:Mammals:Digestive system:Fecal	0.1188
MGYA00248419/ERZ650149_FASTA.fasta	MGYA00248419	ERZ650149	root:Host-associated:Mammals:Digestive system:Fecal	0.1898
MGYA00248420/ERZ650116_FASTA.fasta	MGYA00248420	ERZ650116	root:Host-associated:Mammals:Digestive system:Fecal	0.2486
MGYA00248421/ERZ650118_FASTA.fasta	MGYA00248421	ERZ650118	root:Host-associated:Mammals:Digestive system:Fecal	0.2346
MGYA00248422/ERZ650120_FASTA.fasta	MGYA00248422	ERZ650120	root:Host-associated:Mammals:Digestive system:Fecal	0.1152
MGYA00248423/ERZ650122_FASTA.fasta	MGYA00248423	ERZ650122	root:Host-associated:Mammals:Digestive system:Fecal	0.1666
MGYA00248424/ERZ650124_FASTA.fasta	MGYA00248424	ERZ650124	root:Host-associated:Mammals:Digestive system:Fecal	0.2132
MGYA00248425/ERZ650126_FASTA.fasta	MGYA00248425	ERZ650126	root:Host-associated:Mammals:Digestive system:Fecal	0.1024
MGYA00248426/ERZ650128_FASTA.fasta	MGYA00248426	ERZ650128	root:Host-associated:Mammals:Digestive system:Fecal	0.1994
MGYA00248427/ERZ650130_FASTA.fasta	MGYA00248427	ERZ650130	root:Host-associated:Mammals:Digestive system:Fecal	0.1526
MGYA00248428/ERZ650132_FASTA.fasta	MGYA00248428	ERZ650132	root:Host-associated:Mammals:Digestive system:Fecal	0.148
MGYA00248429/ERZ650134_FASTA.fasta	MGYA00248429	ERZ650134	root:Host-associated:Mammals:Digestive system:Fecal	0.1644
MGYA00248430/ERZ650136_FASTA.fasta	MGYA00248430	ERZ650136	root:Host-associated:Mammals:Digestive system:Fecal	0.171
MGYA00248431/ERZ650138_FASTA.fasta	MGYA00248431	ERZ650138	root:Host-associated:Mammals:Digestive system:Fecal	0.2398
MGYA00248432/ERZ650140_FASTA.fasta	MGYA00248432	ERZ650140	root:Host-associated:Mammals:Digestive system:Fecal	0.2332
MGYA00248433/ERZ650142_FASTA.fasta	MGYA00248433	ERZ650142	root:Host-associated:Mammals:Digestive system:Fecal	0.211
MGYA00248434/ERZ650144_FASTA.fasta	MGYA00248434	ERZ650144	root:Host-associated:Mammals:Digestive system:Fecal	0.247
MGYA00248435/ERZ650146_FASTA.fasta	MGYA00248435	ERZ650146	root:Host-associated:Mammals:Digestive system:Fecal	0.2158
MGYA00248436/ERZ650148_FASTA.fasta	MGYA00248436	ERZ650148	root:Host-associated:Mammals:Digestive system:Fecal	0.1898
MGYA00248437/ERZ650150_FASTA.fasta	MGYA00248437	ERZ650150	root:Host-associated:Mammals:Digestive system:Fecal	0.256
MGYA00248438/ERZ650151_FASTA.fasta	MGYA00248438	ERZ650151	root:Host-associated:Mammals:Digestive system:Fecal	0.2642
MGYA00248439/ERZ584659_FASTA.fasta	MGYA00248439	ERZ584659	root:Environmental:Aquatic:Marine	0.3884
MGYA00248440/ERZ584563_FASTA.fasta	MGYA00248440	ERZ584563	root:Environmental:Aquatic:Marine	0.2454
MGYA00248441/ERZ584565_FASTA.fasta	MGYA00248441	ERZ584565	root:Environmental:Aquatic:Marine	0.2402
MGYA00248442/ERZ584567_FASTA.fasta	MGYA00248442	ERZ584567	root:Environmental:Aquatic:Marine	0.2016
MGYA00248443/ERZ584569_FASTA.fasta	MGYA00248443	ERZ584569	root:Environmental:Aquatic:Marine	0.2668
MGYA00248444/ERZ584571_FASTA.fasta	MGYA00248444	ERZ584571	root:Environmental:Aquatic:Marine	0.1784
MGYA00248445/ERZ584573_FASTA.fasta	MGYA00248445	ERZ584573	root:Environmental:Aquatic:Marine	0.2398
MGYA00248446/ERZ584575_FASTA.fasta	MGYA00248446	ERZ584575	root:Environmental:Aquatic:Marine	0.1876
MGYA00248447/ERZ584577_FASTA.fasta	MGYA00248447	ERZ584577	root:Environmental:Aquatic:Marine	0.1594
MGYA00248448/ERZ584579_FASTA.fasta	MGYA00248448	ERZ584579	root:Environmental:Aquatic:Marine	0.2512
MGYA00248449/ERZ584581_FASTA.fasta	MGYA00248449	ERZ584581	root:Environmental:Aquatic:Marine	0.2658
MGYA00248450/ERZ584560_FASTA.fasta	MGYA00248450	ERZ584560	root:Environmental:Aquatic:Marine	0.258
MGYA00248451/ERZ584562_FASTA.fasta	MGYA00248451	ERZ584562	root:Environmental:Aquatic:Marine	0.073
MGYA00248452/ERZ584564_FASTA.fasta	MGYA00248452	ERZ584564	root:Environmental:Aquatic:Marine	0.1992
MGYA00248453/ERZ584566_FASTA.fasta	MGYA00248453	ERZ584566	root:Environmental:Aquatic:Marine	0.2374
MGYA00248454/ERZ584568_FASTA.fasta	MGYA00248454	ERZ584568	root:Environmental:Aquatic:Marine	0.262
MGYA00248455/ERZ584570_FASTA.fasta	MGYA00248455	ERZ584570	root:Environmental:Aquatic:Marine	0.2614
MGYA00248456/ERZ584572_FASTA.fasta	MGYA00248456	ERZ584572	root:Environmental:Aquatic:Marine	0.2538
MGYA00248457/ERZ584574_FASTA.fasta	MGYA00248457	ERZ584574	root:Environmental:Aquatic:Marine	0.0774
MGYA00248458/ERZ584576_FASTA.fasta	MGYA00248458	ERZ584576	root:Environmental:Aquatic:Marine	0.2554
MGYA00248459/ERZ584578_FASTA.fasta	MGYA00248459	ERZ584578	root:Environmental:Aquatic:Marine	0.2444
MGYA00248460/ERZ584580_FASTA.fasta	MGYA00248460	ERZ584580	root:Environmental:Aquatic:Marine	0.2496
MGYA00248461/ERZ584582_FASTA.fasta	MGYA00248461	ERZ584582	root:Environmental:Aquatic:Marine	0.1776
MGYA00248462/ERZ651162_FASTA.fasta	MGYA00248462	ERZ651162	root:Environmental:Aquatic:Marine	0.2004
MGYA00248463/ERZ548848_FASTA.fasta	MGYA00248463	ERZ548848	root:Host-associated:Human:Digestive system:Large int	0.4762
MGYA00248464/ERZ548849_FASTA.fasta	MGYA00248464	ERZ548849	root:Host-associated:Human:Digestive system:Large int	0.5054
MGYA00248465/ERZ534230_FASTA.fasta	MGYA00248465	ERZ534230	root:Environmental:Aquatic:Marine	0.6476
MGYA00248466/ERZ534189_FASTA.fasta	MGYA00248466	ERZ534189	root:Environmental:Aquatic:Marine	0.2454
MGYA00248467/ERZ534190_FASTA.fasta	MGYA00248467	ERZ534190	root:Environmental:Aquatic:Marine	0.088
MGYA00248468/ERZ534191_FASTA.fasta	MGYA00248468	ERZ534191	root:Environmental:Aquatic:Marine	0.2158
MGYA00248469/ERZ534192_FASTA.fasta	MGYA00248469	ERZ534192	root:Environmental:Aquatic:Marine	0.223
MGYA00248470/ERZ534193_FASTA.fasta	MGYA00248470	ERZ534193	root:Environmental:Aquatic:Marine	0.1712
MGYA00248471/ERZ534194_FASTA.fasta	MGYA00248471	ERZ534194	root:Environmental:Aquatic:Marine	0.1582
MGYA00248472/ERZ534195_FASTA.fasta	MGYA00248472	ERZ534195	root:Environmental:Aquatic:Marine	0.2454
MGYA00248473/ERZ534196_FASTA.fasta	MGYA00248473	ERZ534196	root:Environmental:Aquatic:Marine	0.2168
MGYA00248474/ERZ534197_FASTA.fasta	MGYA00248474	ERZ534197	root:Environmental:Aquatic:Marine	0.2454
MGYA00248475/ERZ534198_FASTA.fasta	MGYA00248475	ERZ534198	root:Environmental:Aquatic:Marine	0.1798
MGYA00248476/ERZ534199_FASTA.fasta	MGYA00248476	ERZ534199	root:Environmental:Aquatic:Marine	0.1062
MGYA00248477/ERZ534200_FASTA.fasta	MGYA00248477	ERZ534200	root:Environmental:Aquatic:Marine	0.2508
MGYA00248478/ERZ534201_FASTA.fasta	MGYA00248478	ERZ534201	root:Environmental:Aquatic:Marine	0.1504
MGYA00248479/ERZ534202_FASTA.fasta	MGYA00248479	ERZ534202	root:Environmental:Aquatic:Marine	0.2946
MGYA00248480/ERZ534203_FASTA.fasta	MGYA00248480	ERZ534203	root:Environmental:Aquatic:Marine	0.098
MGYA00248481/ERZ534204_FASTA.fasta	MGYA00248481	ERZ534204	root:Environmental:Aquatic:Marine	0.2348
MGYA00248482/ERZ534205_FASTA.fasta	MGYA00248482	ERZ534205	root:Environmental:Aquatic:Marine	0.2272
MGYA00248483/ERZ534206_FASTA.fasta	MGYA00248483	ERZ534206	root:Environmental:Aquatic:Marine	0.1884
MGYA00248484/ERZ534207_FASTA.fasta	MGYA00248484	ERZ534207	root:Environmental:Aquatic:Marine	0.2454
MGYA00248485/ERZ534208_FASTA.fasta	MGYA00248485	ERZ534208	root:Environmental:Aquatic:Marine	0.0464
MGYA00248486/ERZ534209_FASTA.fasta	MGYA00248486	ERZ534209	root:Environmental:Aquatic:Marine	0.1538
MGYA00248487/ERZ534210_FASTA.fasta	MGYA00248487	ERZ534210	root:Environmental:Aquatic:Marine	0.2534
MGYA00248488/ERZ534211_FASTA.fasta	MGYA00248488	ERZ534211	root:Environmental:Aquatic:Marine	0.2248
MGYA00248489/ERZ534212_FASTA.fasta	MGYA00248489	ERZ534212	root:Environmental:Aquatic:Marine	0.2276
MGYA00248490/ERZ534213_FASTA.fasta	MGYA00248490	ERZ534213	root:Environmental:Aquatic:Marine	0.2156
MGYA00248491/ERZ534214_FASTA.fasta	MGYA00248491	ERZ534214	root:Environmental:Aquatic:Marine	0.304
MGYA00248492/ERZ534215_FASTA.fasta	MGYA00248492	ERZ534215	root:Environmental:Aquatic:Marine	0.0822
MGYA00248493/ERZ534216_FASTA.fasta	MGYA00248493	ERZ534216	root:Environmental:Aquatic:Marine	0.1786
MGYA00248494/ERZ534217_FASTA.fasta	MGYA00248494	ERZ534217	root:Environmental:Aquatic:Marine	0.179
MGYA00248495/ERZ534218_FASTA.fasta	MGYA00248495	ERZ534218	root:Environmental:Aquatic:Marine	0.1704
MGYA00248496/ERZ534219_FASTA.fasta	MGYA00248496	ERZ534219	root:Environmental:Aquatic:Marine	0.2454
MGYA00248497/ERZ534220_FASTA.fasta	MGYA00248497	ERZ534220	root:Environmental:Aquatic:Marine	0.2078
MGYA00248498/ERZ534221_FASTA.fasta	MGYA00248498	ERZ534221	root:Environmental:Aquatic:Marine	0.224
MGYA00248499/ERZ534222_FASTA.fasta	MGYA00248499	ERZ534222	root:Environmental:Aquatic:Marine	0.231
MGYA00248500/ERZ534223_FASTA.fasta	MGYA00248500	ERZ534223	root:Environmental:Aquatic:Marine	0.2076
MGYA00248501/ERZ534224_FASTA.fasta	MGYA00248501	ERZ534224	root:Environmental:Aquatic:Marine	0.1426
MGYA00248502/ERZ534225_FASTA.fasta	MGYA00248502	ERZ534225	root:Environmental:Aquatic:Marine	0.2454
MGYA00248503/ERZ534226_FASTA.fasta	MGYA00248503	ERZ534226	root:Environmental:Aquatic:Marine	0.1236
MGYA00248504/ERZ534227_FASTA.fasta	MGYA00248504	ERZ534227	root:Environmental:Aquatic:Marine	0.2332
MGYA00248505/ERZ534228_FASTA.fasta	MGYA00248505	ERZ534228	root:Environmental:Aquatic:Marine	0.2496
MGYA00248506/ERZ534147_FASTA.fasta	MGYA00248506	ERZ534147	root:Environmental:Aquatic:Marine	0.0996
MGYA00248507/ERZ534149_FASTA.fasta	MGYA00248507	ERZ534149	root:Environmental:Aquatic:Marine	0.2466
MGYA00248508/ERZ534151_FASTA.fasta	MGYA00248508	ERZ534151	root:Environmental:Aquatic:Marine	0.174
MGYA00248509/ERZ534153_FASTA.fasta	MGYA00248509	ERZ534153	root:Environmental:Aquatic:Marine	0.2728
MGYA00248510/ERZ534155_FASTA.fasta	MGYA00248510	ERZ534155	root:Environmental:Aquatic:Marine	0.191
MGYA00248511/ERZ534157_FASTA.fasta	MGYA00248511	ERZ534157	root:Environmental:Aquatic:Marine	0.1706
MGYA00248512/ERZ534159_FASTA.fasta	MGYA00248512	ERZ534159	root:Environmental:Aquatic:Marine	0.0716
MGYA00248513/ERZ534161_FASTA.fasta	MGYA00248513	ERZ534161	root:Environmental:Aquatic:Marine	0.374
MGYA00248514/ERZ534163_FASTA.fasta	MGYA00248514	ERZ534163	root:Environmental:Aquatic:Marine	0.2294
MGYA00248515/ERZ534165_FASTA.fasta	MGYA00248515	ERZ534165	root:Environmental:Aquatic:Marine	0.0712
MGYA00248516/ERZ534167_FASTA.fasta	MGYA00248516	ERZ534167	root:Environmental:Aquatic:Marine	0.1656
MGYA00248517/ERZ534169_FASTA.fasta	MGYA00248517	ERZ534169	root:Environmental:Aquatic:Marine	0.2366
MGYA00248518/ERZ534171_FASTA.fasta	MGYA00248518	ERZ534171	root:Environmental:Aquatic:Marine	0.1062
MGYA00248519/ERZ534173_FASTA.fasta	MGYA00248519	ERZ534173	root:Environmental:Aquatic:Marine	0.095
MGYA00248520/ERZ534146_FASTA.fasta	MGYA00248520	ERZ534146	root:Environmental:Aquatic:Marine	0.1166
MGYA00248521/ERZ534148_FASTA.fasta	MGYA00248521	ERZ534148	root:Environmental:Aquatic:Marine	0.2126
MGYA00248522/ERZ534150_FASTA.fasta	MGYA00248522	ERZ534150	root:Environmental:Aquatic:Marine	0.0828
MGYA00248523/ERZ534152_FASTA.fasta	MGYA00248523	ERZ534152	root:Environmental:Aquatic:Marine	0.0866
MGYA00248524/ERZ534154_FASTA.fasta	MGYA00248524	ERZ534154	root:Environmental:Aquatic:Marine	0.083
MGYA00248525/ERZ534156_FASTA.fasta	MGYA00248525	ERZ534156	root:Environmental:Aquatic:Marine	0.2168
MGYA00248526/ERZ534158_FASTA.fasta	MGYA00248526	ERZ534158	root:Environmental:Aquatic:Marine	0.2082
MGYA00248527/ERZ534160_FASTA.fasta	MGYA00248527	ERZ534160	root:Environmental:Aquatic:Marine	0.2126
MGYA00248528/ERZ534162_FASTA.fasta	MGYA00248528	ERZ534162	root:Environmental:Aquatic:Marine	0.1918
MGYA00248529/ERZ534164_FASTA.fasta	MGYA00248529	ERZ534164	root:Environmental:Aquatic:Marine	0.0848
MGYA00248530/ERZ534166_FASTA.fasta	MGYA00248530	ERZ534166	root:Environmental:Aquatic:Marine	0.2404
MGYA00248531/ERZ534168_FASTA.fasta	MGYA00248531	ERZ534168	root:Environmental:Aquatic:Marine	0.2488
MGYA00248532/ERZ534170_FASTA.fasta	MGYA00248532	ERZ534170	root:Environmental:Aquatic:Marine	0.2384
MGYA00248533/ERZ534172_FASTA.fasta	MGYA00248533	ERZ534172	root:Environmental:Aquatic:Marine	0.2246
MGYA00248534/ERZ534174_FASTA.fasta	MGYA00248534	ERZ534174	root:Environmental:Aquatic:Marine	0.2456
MGYA00248535/ERZ534175_FASTA.fasta	MGYA00248535	ERZ534175	root:Environmental:Aquatic:Marine	0.2024
MGYA00248536/ERZ534176_FASTA.fasta	MGYA00248536	ERZ534176	root:Environmental:Aquatic:Marine	0.2578
MGYA00248537/ERZ534177_FASTA.fasta	MGYA00248537	ERZ534177	root:Environmental:Aquatic:Marine	0.2428
MGYA00248538/ERZ534178_FASTA.fasta	MGYA00248538	ERZ534178	root:Environmental:Aquatic:Marine	0.081
MGYA00248539/ERZ534179_FASTA.fasta	MGYA00248539	ERZ534179	root:Environmental:Aquatic:Marine	0.164
MGYA00248540/ERZ534180_FASTA.fasta	MGYA00248540	ERZ534180	root:Environmental:Aquatic:Marine	0.0564
MGYA00248541/ERZ534181_FASTA.fasta	MGYA00248541	ERZ534181	root:Environmental:Aquatic:Marine	0.1332
MGYA00248542/ERZ534182_FASTA.fasta	MGYA00248542	ERZ534182	root:Environmental:Aquatic:Marine	0.0408
MGYA00248543/ERZ534183_FASTA.fasta	MGYA00248543	ERZ534183	root:Environmental:Aquatic:Marine	0.22
MGYA00248544/ERZ534184_FASTA.fasta	MGYA00248544	ERZ534184	root:Environmental:Aquatic:Marine	0.1746
MGYA00248545/ERZ534185_FASTA.fasta	MGYA00248545	ERZ534185	root:Environmental:Aquatic:Marine	0.2496
MGYA00248546/ERZ534186_FASTA.fasta	MGYA00248546	ERZ534186	root:Environmental:Aquatic:Marine	0.1834
MGYA00248547/ERZ534187_FASTA.fasta	MGYA00248547	ERZ534187	root:Environmental:Aquatic:Marine	0.2542
MGYA00248548/ERZ534188_FASTA.fasta	MGYA00248548	ERZ534188	root:Environmental:Aquatic:Marine	0.2556
MGYA00248549/ERZ534008_FASTA.fasta	MGYA00248549	ERZ534008	root:Environmental:Aquatic:Freshwater:Pond:Sediment	0.7378
MGYA00248550/ERZ534010_FASTA.fasta	MGYA00248550	ERZ534010	root:Environmental:Aquatic:Freshwater:Pond:Sediment	0.8034
MGYA00248551/ERZ534009_FASTA.fasta	MGYA00248551	ERZ534009	root:Environmental:Aquatic:Freshwater:Pond:Sediment	0.8812
MGYA00248552/ERZ534011_FASTA.fasta	MGYA00248552	ERZ534011	root:Environmental:Aquatic:Freshwater:Pond:Sediment	0.8582
MGYA00248553/ERZ529321_FASTA.fasta	MGYA00248553	ERZ529321	root:Environmental:Aquatic:Marine	0.9136
MGYA00248554/ERZ529323_FASTA.fasta	MGYA00248554	ERZ529323	root:Environmental:Aquatic:Marine	0.9104
MGYA00248555/ERZ529325_FASTA.fasta	MGYA00248555	ERZ529325	root:Environmental:Aquatic:Marine	0.77
MGYA00248556/ERZ529322_FASTA.fasta	MGYA00248556	ERZ529322	root:Environmental:Aquatic:Marine	0.908
MGYA00248557/ERZ529324_FASTA.fasta	MGYA00248557	ERZ529324	root:Environmental:Aquatic:Marine	0.84
MGYA00248558/ERZ511489_FASTA.fasta	MGYA00248558	ERZ511489	root:Environmental:Aquatic:Marine	0.8086
MGYA00248559/ERZ511487_FASTA.fasta	MGYA00248559	ERZ511487	root:Environmental:Aquatic:Marine:Intertidal zone:Salt	0.552
MGYA00248560/ERZ511486_FASTA.fasta	MGYA00248560	ERZ511486	root:Environmental:Aquatic:Marine:Intertidal zone:Salt	0.7766
MGYA00248561/ERZ511485_FASTA.fasta	MGYA00248561	ERZ511485	root:Environmental:Aquatic:Marine:Pelagic	0.9432
MGYA00248562/ERZ511474_FASTA.fasta	MGYA00248562	ERZ511474	root:Environmental:Aquatic:Thermal springs:Sediment	0.8204
MGYA00248563/ERZ511470_FASTA.fasta	MGYA00248563	ERZ511470	root:Environmental:Aquatic:Marine	0.62
MGYA00248564/ERZ511469_FASTA.fasta	MGYA00248564	ERZ511469	root:Environmental:Aquatic:Marine:Sediment	0.8266
MGYA00248565/ERZ511460_FASTA.fasta	MGYA00248565	ERZ511460	root:Environmental:Aquatic:Marine:Intertidal zone:Estu	0.7952
MGYA00248566/ERZ511459_FASTA.fasta	MGYA00248566	ERZ511459	root:Environmental:Aquatic:Marine:Intertidal zone:Estu	0.8048
MGYA00248567/ERZ508656_FASTA.fasta	MGYA00248567	ERZ508656	root:Environmental:Aquatic:Marine:Intertidal zone:Estu	0.8824
MGYA00248568/ERZ508609_FASTA.fasta	MGYA00248568	ERZ508609	root:Environmental:Aquatic:Marine	0.0786
MGYA00248569/ERZ508611_FASTA.fasta	MGYA00248569	ERZ508611	root:Environmental:Aquatic:Marine	0.2346
MGYA00248570/ERZ508613_FASTA.fasta	MGYA00248570	ERZ508613	root:Environmental:Aquatic:Marine	0.225
MGYA00248571/ERZ508615_FASTA.fasta	MGYA00248571	ERZ508615	root:Environmental:Aquatic:Marine	0.2478
MGYA00248572/ERZ508617_FASTA.fasta	MGYA00248572	ERZ508617	root:Environmental:Aquatic:Marine	0.2496
MGYA00248573/ERZ508619_FASTA.fasta	MGYA00248573	ERZ508619	root:Environmental:Aquatic:Marine	0.1234
MGYA00248574/ERZ508621_FASTA.fasta	MGYA00248574	ERZ508621	root:Environmental:Aquatic:Marine	0.2574
MGYA00248575/ERZ508623_FASTA.fasta	MGYA00248575	ERZ508623	root:Environmental:Aquatic:Marine	0.237
MGYA00248576/ERZ508625_FASTA.fasta	MGYA00248576	ERZ508625	root:Environmental:Aquatic:Marine	0.1692
MGYA00248577/ERZ508627_FASTA.fasta	MGYA00248577	ERZ508627	root:Environmental:Aquatic:Marine	0.2904
MGYA00248578/ERZ508629_FASTA.fasta	MGYA00248578	ERZ508629	root:Environmental:Aquatic:Marine	0.2454
MGYA00248579/ERZ508631_FASTA.fasta	MGYA00248579	ERZ508631	root:Environmental:Aquatic:Marine	0.2306
MGYA00248580/ERZ508635_FASTA.fasta	MGYA00248580	ERZ508635	root:Environmental:Aquatic:Marine	0.1986
MGYA00248581/ERZ508636_FASTA.fasta	MGYA00248581	ERZ508636	root:Environmental:Aquatic:Marine	0.167
MGYA00248582/ERZ508638_FASTA.fasta	MGYA00248582	ERZ508638	root:Environmental:Aquatic:Marine	0.1522
MGYA00248583/ERZ508610_FASTA.fasta	MGYA00248583	ERZ508610	root:Environmental:Aquatic:Marine	0.0476
MGYA00248584/ERZ508612_FASTA.fasta	MGYA00248584	ERZ508612	root:Environmental:Aquatic:Marine	0.0866

MGYA00248585/ERZ508614_FASTA.fasta	MGYA00248585	ERZ508614	root:Environmental:Aquatic:Marine	0.1244
MGYA00248586/ERZ508616_FASTA.fasta	MGYA00248586	ERZ508616	root:Environmental:Aquatic:Marine	0.1844
MGYA00248587/ERZ508618_FASTA.fasta	MGYA00248587	ERZ508618	root:Environmental:Aquatic:Marine	0.0492
MGYA00248588/ERZ508620_FASTA.fasta	MGYA00248588	ERZ508620	root:Environmental:Aquatic:Marine	0.391
MGYA00248589/ERZ508622_FASTA.fasta	MGYA00248589	ERZ508622	root:Environmental:Aquatic:Marine	0.1528
MGYA00248590/ERZ508624_FASTA.fasta	MGYA00248590	ERZ508624	root:Environmental:Aquatic:Marine	0.1042
MGYA00248591/ERZ508626_FASTA.fasta	MGYA00248591	ERZ508626	root:Environmental:Aquatic:Marine	0.199
MGYA00248592/ERZ508628_FASTA.fasta	MGYA00248592	ERZ508628	root:Environmental:Aquatic:Marine	0.1534
MGYA00248593/ERZ508630_FASTA.fasta	MGYA00248593	ERZ508630	root:Environmental:Aquatic:Marine	0.2454
MGYA00248594/ERZ508632_FASTA.fasta	MGYA00248594	ERZ508632	root:Environmental:Aquatic:Marine	0.2604
MGYA00248595/ERZ508633_FASTA.fasta	MGYA00248595	ERZ508633	root:Environmental:Aquatic:Marine	0.2454
MGYA00248596/ERZ508637_FASTA.fasta	MGYA00248596	ERZ508637	root:Environmental:Aquatic:Marine	0.2442
MGYA00248597/ERZ508639_FASTA.fasta	MGYA00248597	ERZ508639	root:Environmental:Aquatic:Marine	0.3464
MGYA00248598/ERZ508640_FASTA.fasta	MGYA00248598	ERZ508640	root:Environmental:Aquatic:Marine	0.0474
MGYA00248599/ERZ508641_FASTA.fasta	MGYA00248599	ERZ508641	root:Environmental:Aquatic:Marine	0.1966
MGYA00248600/ERZ508642_FASTA.fasta	MGYA00248600	ERZ508642	root:Environmental:Aquatic:Marine	0.3532
MGYA00248601/ERZ508643_FASTA.fasta	MGYA00248601	ERZ508643	root:Environmental:Aquatic:Marine	0.0678
MGYA00248602/ERZ508644_FASTA.fasta	MGYA00248602	ERZ508644	root:Environmental:Aquatic:Marine	0.1154
MGYA00248603/ERZ508645_FASTA.fasta	MGYA00248603	ERZ508645	root:Environmental:Aquatic:Marine	0.2608
MGYA00248604/ERZ508646_FASTA.fasta	MGYA00248604	ERZ508646	root:Environmental:Aquatic:Marine	0.1862
MGYA00248605/ERZ508647_FASTA.fasta	MGYA00248605	ERZ508647	root:Environmental:Aquatic:Marine	0.165
MGYA00248606/ERZ508648_FASTA.fasta	MGYA00248606	ERZ508648	root:Environmental:Aquatic:Marine	0.2794
MGYA00248607/ERZ508649_FASTA.fasta	MGYA00248607	ERZ508649	root:Environmental:Aquatic:Marine	0.2414
MGYA00248608/ERZ508650_FASTA.fasta	MGYA00248608	ERZ508650	root:Environmental:Aquatic:Marine	0.1834
MGYA00248609/ERZ508651_FASTA.fasta	MGYA00248609	ERZ508651	root:Environmental:Aquatic:Marine	0.0414
MGYA00248610/ERZ508652_FASTA.fasta	MGYA00248610	ERZ508652	root:Environmental:Aquatic:Marine	0.0454
MGYA00248611/ERZ508654_FASTA.fasta	MGYA00248611	ERZ508654	root:Environmental:Aquatic:Marine	0.1756
MGYA00248612/ERZ508653_FASTA.fasta	MGYA00248612	ERZ508653	root:Environmental:Aquatic:Marine	0.1086
MGYA00248613/ERZ508655_FASTA.fasta	MGYA00248613	ERZ508655	root:Environmental:Aquatic:Marine	0.1764
MGYA00248614/ERZ508606_FASTA.fasta	MGYA00248614	ERZ508606	root:Environmental:Aquatic:Marine:Intertidal zone:Salt	0.4484
MGYA00248615/ERZ508603_FASTA.fasta	MGYA00248615	ERZ508603	root:Environmental:Aquatic:Marine	0.739
MGYA00248616/ERZ508602_FASTA.fasta	MGYA00248616	ERZ508602	root:Environmental:Aquatic:Marine	0.781
MGYA00248617/ERZ508598_FASTA.fasta	MGYA00248617	ERZ508598	root:Environmental:Aquatic:Marine:Intertidal zone:Salt	0.486
MGYA00248618/ERZ508597_FASTA.fasta	MGYA00248618	ERZ508597	root:Environmental:Aquatic:Marine:Intertidal zone:Salt	0.4522
MGYA00248619/ERZ508594_FASTA.fasta	MGYA00248619	ERZ508594	root:Environmental:Aquatic:Marine:Intertidal zone:Estu	0.743
MGYA00248620/ERZ508592_FASTA.fasta	MGYA00248620	ERZ508592	root:Environmental:Aquatic:Marine:Intertidal zone:Estu	0.6984
MGYA00248621/ERZ508587_FASTA.fasta	MGYA00248621	ERZ508587	root:Environmental:Aquatic:Marine:Intertidal zone:Estu	0.7164
MGYA00248622/ERZ508571_FASTA.fasta	MGYA00248622	ERZ508571	root:Environmental:Aquatic:Marine:Intertidal zone:Estu	0.819
MGYA00248623/ERZ508561_FASTA.fasta	MGYA00248623	ERZ508561	root:Environmental:Aquatic:Marine:Intertidal zone:Estu	0.8338
MGYA00248624/ERZ508555_FASTA.fasta	MGYA00248624	ERZ508555	root:Environmental:Aquatic:Marine:Intertidal zone:Estu	0.8356
MGYA00248625/ERZ508552_FASTA.fasta	MGYA00248625	ERZ508552	root:Environmental:Aquatic:Marine	0.8186
MGYA00248626/ERZ508543_FASTA.fasta	MGYA00248626	ERZ508543	root:Host-associated:Mollusca	0.5958
MGYA00248627/ERZ508539_FASTA.fasta	MGYA00248627	ERZ508539	root:Environmental:Aquatic:Marine:Hydrothermal vents:	0.7634
MGYA00248628/ERZ508525_FASTA.fasta	MGYA00248628	ERZ508525	root:Environmental:Aquatic:Marine	0.851
MGYA00248629/ERZ508524_FASTA.fasta	MGYA00248629	ERZ508524	root:Environmental:Aquatic:Marine	0.834
MGYA00248630/ERZ508518_FASTA.fasta	MGYA00248630	ERZ508518	root:Environmental:Aquatic:Marine	0.8592
MGYA00248631/ERZ508512_FASTA.fasta	MGYA00248631	ERZ508512	root:Environmental:Aquatic:Marine	0.807
MGYA00248632/ERZ508511_FASTA.fasta	MGYA00248632	ERZ508511	root:Environmental:Aquatic:Marine	0.8028
MGYA00248633/ERZ508508_FASTA.fasta	MGYA00248633	ERZ508508	root:Environmental:Aquatic:Marine:Intertidal zone:Salt	0.54
MGYA00248634/ERZ508507_FASTA.fasta	MGYA00248634	ERZ508507	root:Environmental:Aquatic:Marine:Intertidal zone:Estu	0.825
MGYA00248635/ERZ508505_FASTA.fasta	MGYA00248635	ERZ508505	root:Environmental:Aquatic:Marine	0.9286
MGYA00248636/ERZ505789_FASTA.fasta	MGYA00248636	ERZ505789	root:Host-associated:Human:Digestive system:Large in	0.2388
MGYA00248637/ERZ505827_FASTA.fasta	MGYA00248637	ERZ505827	root:Host-associated:Human:Digestive system:Large in	0.2352
MGYA00248638/ERZ507555_FASTA.fasta	MGYA00248638	ERZ507555	root:Host-associated:Human:Digestive system:Large in	0.2906
MGYA00248639/ERZ507557_FASTA.fasta	MGYA00248639	ERZ507557	root:Host-associated:Human:Digestive system:Large in	0.2766
MGYA00248640/ERZ507559_FASTA.fasta	MGYA00248640	ERZ507559	root:Host-associated:Human:Digestive system:Large in	0.2422
MGYA00248641/ERZ507561_FASTA.fasta	MGYA00248641	ERZ507561	root:Host-associated:Human:Digestive system:Large in	0.3598
MGYA00248642/ERZ507502_FASTA.fasta	MGYA00248642	ERZ507502	root:Host-associated:Human:Digestive system:Large in	0.2938
MGYA00248643/ERZ507504_FASTA.fasta	MGYA00248643	ERZ507504	root:Host-associated:Human:Digestive system:Large in	0.3934
MGYA00248644/ERZ507506_FASTA.fasta	MGYA00248644	ERZ507506	root:Host-associated:Human:Digestive system:Large in	0.209
MGYA00248645/ERZ507508_FASTA.fasta	MGYA00248645	ERZ507508	root:Host-associated:Human:Digestive system:Large in	0.0708
MGYA00248646/ERZ507510_FASTA.fasta	MGYA00248646	ERZ507510	root:Host-associated:Human:Digestive system:Large in	0.2786
MGYA00248647/ERZ507512_FASTA.fasta	MGYA00248647	ERZ507512	root:Host-associated:Human:Digestive system:Large in	0.3816
MGYA00248648/ERZ507514_FASTA.fasta	MGYA00248648	ERZ507514	root:Host-associated:Human:Digestive system:Large in	0.2512
MGYA00248649/ERZ507516_FASTA.fasta	MGYA00248649	ERZ507516	root:Host-associated:Human:Digestive system:Large in	0.3274
MGYA00248650/ERZ507518_FASTA.fasta	MGYA00248650	ERZ507518	root:Host-associated:Human:Digestive system:Large in	0.247
MGYA00248651/ERZ507520_FASTA.fasta	MGYA00248651	ERZ507520	root:Host-associated:Human:Digestive system:Large in	0.3706
MGYA00248652/ERZ507522_FASTA.fasta	MGYA00248652	ERZ507522	root:Host-associated:Human:Digestive system:Large in	0.2372
MGYA00248653/ERZ507524_FASTA.fasta	MGYA00248653	ERZ507524	root:Host-associated:Human:Digestive system:Large in	0.1984
MGYA00248654/ERZ507526_FASTA.fasta	MGYA00248654	ERZ507526	root:Host-associated:Human:Digestive system:Large in	0.3104
MGYA00248655/ERZ507528_FASTA.fasta	MGYA00248655	ERZ507528	root:Host-associated:Human:Digestive system:Large in	0.254
MGYA00248656/ERZ507530_FASTA.fasta	MGYA00248656	ERZ507530	root:Host-associated:Human:Digestive system:Large in	0.3022
MGYA00248657/ERZ507532_FASTA.fasta	MGYA00248657	ERZ507532	root:Host-associated:Human:Digestive system:Large in	0.4436
MGYA00248658/ERZ507534_FASTA.fasta	MGYA00248658	ERZ507534	root:Host-associated:Human:Digestive system:Large in	0.4394
MGYA00248659/ERZ507536_FASTA.fasta	MGYA00248659	ERZ507536	root:Host-associated:Human:Digestive system:Large in	0.2572
MGYA00248660/ERZ507538_FASTA.fasta	MGYA00248660	ERZ507538	root:Host-associated:Human:Digestive system:Large in	0.1842
MGYA00248661/ERZ507540_FASTA.fasta	MGYA00248661	ERZ507540	root:Host-associated:Human:Digestive system:Large in	0.2268
MGYA00248662/ERZ507542_FASTA.fasta	MGYA00248662	ERZ507542	root:Host-associated:Human:Digestive system:Large in	0.3602
MGYA00248663/ERZ507544_FASTA.fasta	MGYA00248663	ERZ507544	root:Host-associated:Human:Digestive system:Large in	0.2324
MGYA00248664/ERZ507546_FASTA.fasta	MGYA00248664	ERZ507546	root:Host-associated:Human:Digestive system:Large in	0.3162
MGYA00248665/ERZ507548_FASTA.fasta	MGYA00248665	ERZ507548	root:Host-associated:Human:Digestive system:Large in	0.3558
MGYA00248666/ERZ507550_FASTA.fasta	MGYA00248666	ERZ507550	root:Host-associated:Human:Digestive system:Large in	0.1284
MGYA00248667/ERZ507552_FASTA.fasta	MGYA00248667	ERZ507552	root:Host-associated:Human:Digestive system:Large in	0.2568
MGYA00248668/ERZ507554_FASTA.fasta	MGYA00248668	ERZ507554	root:Host-associated:Human:Digestive system:Large in	0.3458
MGYA00248669/ERZ507556_FASTA.fasta	MGYA00248669	ERZ507556	root:Host-associated:Human:Digestive system:Large in	0.1816
MGYA00248670/ERZ507558_FASTA.fasta	MGYA00248670	ERZ507558	root:Host-associated:Human:Digestive system:Large in	0.3274
MGYA00248671/ERZ507560_FASTA.fasta	MGYA00248671	ERZ507560	root:Host-associated:Human:Digestive system:Large in	0.302
MGYA00248672/ERZ507562_FASTA.fasta	MGYA00248672	ERZ507562	root:Host-associated:Human:Digestive system:Large in	0.2674
MGYA00248673/ERZ507500_FASTA.fasta	MGYA00248673	ERZ507500	root:Host-associated:Human:Digestive system:Large in	0.2582
MGYA00248674/ERZ507501_FASTA.fasta	MGYA00248674	ERZ507501	root:Host-associated:Human:Digestive system:Large in	0.268
MGYA00248675/ERZ507503_FASTA.fasta	MGYA00248675	ERZ507503	root:Host-associated:Human:Digestive system:Large in	0.39
MGYA00248676/ERZ507505_FASTA.fasta	MGYA00248676	ERZ507505	root:Host-associated:Human:Digestive system:Large in	0.2244
MGYA00248677/ERZ507507_FASTA.fasta	MGYA00248677	ERZ507507	root:Host-associated:Human:Digestive system:Large in	0.2926
MGYA00248678/ERZ507509_FASTA.fasta	MGYA00248678	ERZ507509	root:Host-associated:Human:Digestive system:Large in	0.296
MGYA00248679/ERZ507511_FASTA.fasta	MGYA00248679	ERZ507511	root:Host-associated:Human:Digestive system:Large in	0.28
MGYA00248680/ERZ507513_FASTA.fasta	MGYA00248680	ERZ507513	root:Host-associated:Human:Digestive system:Large in	0.3234
MGYA00248681/ERZ507515_FASTA.fasta	MGYA00248681	ERZ507515	root:Host-associated:Human:Digestive system:Large in	0.2102
MGYA00248682/ERZ507517_FASTA.fasta	MGYA00248682	ERZ507517	root:Host-associated:Human:Digestive system:Large in	0.2616
MGYA00248683/ERZ507519_FASTA.fasta	MGYA00248683	ERZ507519	root:Host-associated:Human:Digestive system:Large in	0.3274
MGYA00248684/ERZ507521_FASTA.fasta	MGYA00248684	ERZ507521	root:Host-associated:Human:Digestive system:Large in	0.2394
MGYA00248685/ERZ507523_FASTA.fasta	MGYA00248685	ERZ507523	root:Host-associated:Human:Digestive system:Large in	0.2402
MGYA00248686/ERZ507525_FASTA.fasta	MGYA00248686	ERZ507525	root:Host-associated:Human:Digestive system:Large in	0.261
MGYA00248687/ERZ507527_FASTA.fasta	MGYA00248687	ERZ507527	root:Host-associated:Human:Digestive system:Large in	0.3254
MGYA00248688/ERZ507529_FASTA.fasta	MGYA00248688	ERZ507529	root:Host-associated:Human:Digestive system:Large in	0.2798
MGYA00248689/ERZ507531_FASTA.fasta	MGYA00248689	ERZ507531	root:Host-associated:Human:Digestive system:Large in	0.2548
MGYA00248690/ERZ507533_FASTA.fasta	MGYA00248690	ERZ507533	root:Host-associated:Human:Digestive system:Large in	0.2716
MGYA00248691/ERZ507535_FASTA.fasta	MGYA00248691	ERZ507535	root:Host-associated:Human:Digestive system:Large in	0.4234
MGYA00248692/ERZ507537_FASTA.fasta	MGYA00248692	ERZ507537	root:Host-associated:Human:Digestive system:Large in	0.1138
MGYA00248693/ERZ507539_FASTA.fasta	MGYA00248693	ERZ507539	root:Host-associated:Human:Digestive system:Large in	0.2726
MGYA00248694/ERZ507541_FASTA.fasta	MGYA00248694	ERZ507541	root:Host-associated:Human:Digestive system:Large in	0.292
MGYA00248695/ERZ507543_FASTA.fasta	MGYA00248695	ERZ507543	root:Host-associated:Human:Digestive system:Large in	0.2686
MGYA00248696/ERZ507545_FASTA.fasta	MGYA00248696	ERZ507545	root:Host-associated:Human:Digestive system:Large in	0.221
MGYA00248697/ERZ507547_FASTA.fasta	MGYA00248697	ERZ507547	root:Host-associated:Human:Digestive system:Large in	0.245
MGYA00248698/ERZ507549_FASTA.fasta	MGYA00248698	ERZ507549	root:Host-associated:Human:Digestive system:Large in	0.2108
MGYA00248699/ERZ507551_FASTA.fasta	MGYA00248699	ERZ507551	root:Host-associated:Human:Digestive system:Large in	0.3498
MGYA00248700/ERZ507553_FASTA.fasta	MGYA00248700	ERZ507553	root:Host-associated:Human:Digestive system:Large in	0.297
MGYA00248701/ERZ505754_FASTA.fasta	MGYA00248701	ERZ505754	root:Host-associated:Human:Digestive system:Large in	0.5036
MGYA00248702/ERZ505755_FASTA.fasta	MGYA00248702	ERZ505755	root:Host-associated:Human:Digestive system:Large in	0.4526
MGYA00248703/ERZ505756_FASTA.fasta	MGYA00248703	ERZ505756	root:Host-associated:Human:Digestive system:Large in	0.2084
MGYA00248704/ERZ505748_FASTA.fasta	MGYA00248704	ERZ505748	root:Host-associated:Human:Digestive system:Large in	0.4704
MGYA00248705/ERZ505749_FASTA.fasta	MGYA00248705	ERZ505749	root:Host-associated:Human:Digestive system:Large in	0.4418
MGYA00248706/ERZ505750_FASTA.fasta	MGYA00248706	ERZ505750	root:Host-associated:Human:Digestive system:Large in	0.5394
MGYA00248707/ERZ505751_FASTA.fasta	MGYA00248707	ERZ505751	root:Host-associated:Human:Digestive system:Large in	0.457
MGYA00248708/ERZ505752_FASTA.fasta	MGYA00248708	ERZ505752	root:Host-associated:Human:Digestive system:Large in	0.5154
MGYA00248709/ERZ505753_FASTA.fasta	MGYA00248709	ERZ505753	root:Host-associated:Human:Digestive system:Large in	0.3482
MGYA00248710/ERZ505731_FASTA.fasta	MGYA00248710	ERZ505731	root:Host-associated:Human:Digestive system:Large in	0.5218
MGYA00248711/ERZ505732_FASTA.fasta	MGYA00248711	ERZ505732	root:Host-associated:Human:Digestive system:Large in	0.4852
MGYA00248712/ERZ505734_FASTA.fasta	MGYA00248712	ERZ505734	root:Host-associated:Human:Digestive system:Large in	0.445
MGYA00248713/ERZ505735_FASTA.fasta	MGYA00248713	ERZ505735	root:Host-associated:Human:Digestive system:Large in	0.4396
MGYA00248714/ERZ505736_FASTA.fasta	MGYA00248714	ERZ505736	root:Host-associated:Human:Digestive system:Large in	0.4988
MGYA00248715/ERZ505737_FASTA.fasta	MGYA00248715	ERZ505737	root:Host-associated:Human:Digestive system:Large in	0.5144
MGYA00248716/ERZ505738_FASTA.fasta	MGYA00248716	ERZ505738	root:Host-associated:Human:Digestive system:Large in	0.4806
MGYA00248717/ERZ505739_FASTA.fasta	MGYA00248717	ERZ505739	root:Host-associated:Human:Digestive system:Large in	0.5096
MGYA00248718/ERZ505740_FASTA.fasta	MGYA00248718	ERZ505740	root:Host-associated:Human:Digestive system:Large in	0.4934
MGYA00248719/ERZ505368_FASTA.fasta	MGYA00248719	ERZ505368	root:Environmental:Aquatic:Marine	0.6748
MGYA00248720/ERZ505370_FASTA.fasta	MGYA00248720	ERZ505370	root:Environmental:Aquatic:Marine	0.4176
MGYA00248721/ERZ505372_FASTA.fasta	MGYA00248721	ERZ505372	root:Environmental:Aquatic:Marine	0.3526
MGYA00248722/ERZ505374_FASTA.fasta	MGYA00248722	ERZ505374	root:Environmental:Aquatic:Marine	0.796
MGYA00248723/ERZ505376_FASTA.fasta	MGYA00248723	ERZ505376	root:Environmental:Aquatic:Marine	0.8728
MGYA00248724/ERZ505380_FASTA.fasta	MGYA00248724	ERZ505380	root:Environmental:Aquatic:Marine	0.3594
MGYA00248725/ERZ505367_FASTA.fasta	MGYA00248725	ERZ505367	root:Environmental:Aquatic:Marine	0.5902
MGYA00248726/ERZ505369_FASTA.fasta	MGYA00248726	ERZ505369	root:Environmental:Aquatic:Marine	0.6818
MGYA00248727/ERZ505371_FASTA.fasta	MGYA00248727	ERZ505371	root:Environmental:Aquatic:Marine	0.326
MGYA00248728/ERZ505373_FASTA.fasta	MGYA00248728	ERZ505373	root:Environmental:Aquatic:Marine	0.7344
MGYA00248729/ERZ505375_FASTA.fasta	MGYA00248729	ERZ505375	root:Environmental:Aquatic:Marine	0.8108
MGYA00248730/ERZ505377_FASTA.fasta	MGYA00248730	ERZ505377	root:Environmental:Aquatic:Marine	0.637
MGYA00248731/ERZ505379_FASTA.fasta	MGYA00248731	ERZ505379	root:Environmental:Aquatic:Marine	0.8822
MGYA00248732/ERZ505381_FASTA.fasta	MGYA00248732	ERZ505381	root:Environmental:Aquatic:Marine	0.6606
MGYA00248733/ERZ505383_FASTA.fasta	MGYA00248733	ERZ505383	root:Environmental:Aquatic:Marine	0.7796
MGYA00248734/ERZ505382_FASTA.fasta	MGYA00248734	ERZ505382	root:Environmental:Aquatic:Marine	0.4082
MGYA00248735/ERZ505355_FASTA.fasta	MGYA00248735	ERZ505355	root:Environmental:Aquatic:Thermal springs:Hot (42-90	0.6816
MGYA00248736/ERZ505331_FASTA.fasta	MGYA00248736	ERZ505331	root:Host-associated:Human:Digestive system:Large in	0.3166
MGYA00248737/ERZ505333_FASTA.fasta	MGYA00248737	ERZ505333	root:Host-associated:Human:Digestive system:Large in	0.532
MGYA00248738/ERZ505335_FASTA.fasta	MGYA00248738	ERZ505335	root:Host-associated:Human:Digestive system:Large in	0.4756
MGYA00248739/ERZ505327_FASTA.fasta	MGYA00248739	ERZ505327	root:Host-associated:Human:Digestive system:Large in	0.52
MGYA00248740/ERZ505329_FASTA.fasta	MGYA00248740	ERZ505329	root:Host-associated:Human:Digestive system:Large in	0.4778
MGYA00248741/ERZ505328_FASTA.fasta	MGYA00248741	ERZ505328	root:Host-associated:Human:Digestive system:Large in	0.47
MGYA00248742/ERZ505330_FASTA.fasta	MGYA00248742	ERZ505330	root:Host-associated:Human:Digestive system:Large in	0.4008
MGYA00248743/ERZ505332_FASTA.fasta	MGYA00248743	ERZ505332	root:Host-associated:Human:Digestive system:Large in	0.529
MGYA00248744/ERZ505334_FASTA.fasta	MGYA00248744	ERZ505334	root:Host-associated:Human:Digestive system:Large in	0.4198
MGYA00248745/ERZ505336_FASTA.fasta	MGYA00248745	ERZ505336	root:Host-associated:Human:Digestive system:Large in	0.534
MGYA00248746/ERZ505312_FASTA.fasta	MGYA00248746	ERZ505312	root:Host-associated:Human:Digestive system	0.0652
MGYA00248747/ERZ505314_FASTA.fasta	MGYA00248747	ERZ505314	root:Host-associated:Human:Digestive system	0.0738
MGYA00248748/ERZ505311_FASTA.fasta	MGYA00248748	ERZ505311	root:Host-associated:Human:Digestive system	0.0598
MGYA00248749/ERZ505313_FASTA.fasta	MGYA00248749	ERZ505313	root:Host-associated:Human:Digestive system	0.106
MGYA00248750/ERZ505293_FASTA.fasta	MGYA00248750	ERZ505293	root:Environmental:Aquatic:Marine	0.7922
MGYA00248751/ERZ505283_FASTA.fasta	MGYA00248751	ERZ505283	root:Environmental:Aquatic:Marine	0.7822
MGYA00248752/ERZ505273_FASTA.fasta	MGYA00248752	ERZ505273	root:Environmental:Aquatic:Marine	0.824
MGYA00248753/ERZ505268_FASTA.fasta	MGYA00248753	ERZ505268	root:Environmental:Aquatic:Marine	0.8338
MGYA00248754/ERZ505257_FASTA.fasta	MGYA00248754	ERZ505257	root:Environmental:Aquatic:Marine	0.7592
MGYA00248755/ERZ505250_FASTA.fasta	MGYA00248755	ERZ505250	root:Environmental:Aquatic:Marine	0.7982
MGYA00248756/ERZ505249_FASTA.fasta	MGYA00248756	ERZ505249	root:Environmental:Aquatic:Marine	0.8022
MGYA00248757/ERZ505241_FASTA.fasta	MGYA00248757	ERZ505241	root:Environmental:Aquatic:Marine	0.8624
MGYA00248758/ERZ505211_FASTA.fasta	MGYA00248758	ERZ505211	root:Environmental:Aquatic:Marine	0.1686
MGYA00248759/ERZ505200_FASTA.fasta	MGYA00248759	ERZ505200	root:Environmental:Aquatic:Freshwater:Lotic:Sediment	0.5422
MGYA00248760/ERZ505202_FASTA.fasta	MGYA00248760	ERZ505202	root:Environmental:Aquatic:Freshwater:Lotic:Sediment	0.3956
MGYA00248761/ERZ505204_FASTA.fasta	MGYA00248761	ERZ505204	root:Environmental:Aquatic:Freshwater:Lotic:Sediment	0.6194
MGYA00248762/ERZ505201_FASTA.fasta	MGYA00248762	ERZ505201	root:Environmental:Aquatic:Freshwater:Lotic:Sediment	0.5722
MGYA00248763/ERZ505203_FASTA.fasta	MGYA00248763	ERZ505203	root:Environmental:Aquatic:Freshwater:Lotic:Sediment	0.5572
MGYA00248764/ERZ505190_FASTA.fasta	MGYA00248764	ERZ505190	root:Host-associated:Human:Digestive system	0.5852
MGYA00248765/ERZ505192_FASTA.fasta	MGYA00248765	ERZ505192	root:Host-associated:Human:Digestive system	0.4234
MGYA00248766/ERZ505189_FASTA.fasta	MGYA00248766	ERZ505189	root:Host-associated:Human:Digestive system	0.3536
MGYA00248767/ERZ505191_FASTA.fasta	MGYA00248767	ERZ505191	root:Host-associated:Human:Digestive system	0.4944
MGYA00248769/ERZ505139_FASTA.fasta	MGYA00248769	ERZ505139	root:Environmental:Aquatic:Freshwater:Lotic:Sediment	0.4422
MGYA00248770/ERZ505138_FASTA.fasta	MGYA00248770	ERZ505138	root:Environmental:Aquatic:Freshwater:Lotic:Acidic	0.23
MGYA00248771/ERZ505113_FASTA.fasta	MGYA00248771	ERZ505113	root:Environmental:Aquatic:Marine:Intertidal zone:Cora	0.0462
MGYA00248772/ERZ505115_FASTA.fasta	MGYA00248772	ERZ505115	root:Environmental:Aquatic:Marine:Intertidal zone:Cora	0.037
MGYA00248773/ERZ505117_FASTA.fasta	MGYA00248773	ERZ505117	root:Environmental:Aquatic:Marine:Intertidal zone:Cora	0.0312
MGYA00248774/ERZ505119_FASTA.fasta	MGYA00248774	ERZ505119	root:Environmental:Aquatic:Marine:Intertidal zone:Cora	0.0974
MGYA00248775/ERZ505121_FASTA.fasta	MGYA00248775	ERZ505121	root:Environmental:Aquatic:Marine:Intertidal zone:Cora	0.115
MGYA00248776/ERZ505123_FASTA.fasta	MGYA00248776	ERZ505123	root:Environmental:Aquatic:Marine:Intertidal zone:Cora	0.2678
MGYA00248777/ERZ505125_FASTA.fasta	MGYA00248777	ERZ505125	root:Environmental:Aquatic:Marine:Intertidal zone:Cora	0.3058
MGYA00248778/ERZ505127_FASTA.fasta	MGYA00248778	ERZ505127	root:Environmental:Aquatic:Marine:Intertidal zone:Cora	0.1602
MGYA00248779/ERZ505129_FASTA.fasta	MGYA00248779	ERZ505129	root:Environmental:Aquatic:Marine:Intertidal zone:Cora	0.0946
MGYA00248780/ERZ505114_FASTA.fasta	MGYA00248780	ERZ505114	root:Environmental:Aquatic:Marine:Intertidal zone:Cora	0.0294
MGYA00248781/ERZ505116_FASTA.fasta	MGYA00248781	ERZ505116	root:Environmental:Aquatic:Marine:Intertidal zone:Cora	0.034
MGYA00248782/ERZ505118_FASTA.fasta	MGYA00248782	ERZ505118	root:Environmental:Aquatic:Marine:Intertidal zone:Cora	0.0938
MGYA00248783/ERZ505120_FASTA.fasta	MGYA00248783	ERZ505120	root:Environmental:Aquatic:Marine:Intertidal zone:Cora	0.056
MGYA00248784/ERZ505122_FASTA.fasta	MGYA00248784	ERZ505122	root:Environmental:Aquatic:Marine:Intertidal zone:Cora	0.139
MGYA00248785/ERZ505124_FASTA.fasta	MGYA00248785	ERZ505124	root:Environmental:Aquatic:Marine:Intertidal zone:Cora	0.4006
MGYA00248786/ERZ505126_FASTA.fasta	MGYA00248786	ERZ505126	root:Environmental:Aquatic:Marine:Intertidal zone:Cora	0.1628
MGYA00248787/ERZ505128_FASTA.fasta	MGYA00248787	ERZ505128	root:Environmental:Aquatic:Marine:Intertidal zone:Cora	0.0398
MGYA00248788/ERZ505131_FASTA.fasta	MGYA00248788	ERZ505131	root:Environmental:Aquatic:Marine:Intertidal zone:Cora	0.0578
MGYA00248789/ERZ505133_FASTA.fasta	MGYA00248789	ERZ505133	root:Environmental:Aquatic:Marine:Intertidal zone:Cora	0.058
MGYA00248790/ERZ505130_FASTA.fasta	MGYA00248790	ERZ505130	root:Environmental:Aquatic:Marine:Intertidal zone:Cora	0.0602
MGYA00248791/ERZ505132_FASTA.fasta	MGYA00248791	ERZ505132	root:Environmental:Aquatic:Marine:Intertidal zone:Cora	0.096
MGYA00248792/ERZ505134_FASTA.fasta	MGYA00248792	ERZ505134	root:Environmental:Aquatic:Marine:Intertidal zone:Cora	0.2992
MGYA00248793/ERZ505018_FASTA_1.fasta	MGYA00248793	ERZ505018	root:Host-associated:Human:Digestive system:Oral	0.6036
MGYA00248793/ERZ505018_FASTA_2.fasta	MGYA00248793	ERZ505018	root:Host-associated:Human:Digestive system:Oral	0.36
MGYA00248794/ERZ505019_FASTA.fasta	MGYA00248794	ERZ505019	root:Host-associated:Human:Digestive system:Oral	0.4602
MGYA00248795/ERZ505020_FASTA.fasta	MGYA00248795	ERZ505020	root:Host-associated:Human:Digestive system:Oral	0.5098
MGYA00248796/ERZ505025_FASTA.fasta	MGYA00248796	ERZ505025	root:Host-associated:Human:Digestive system:Oral	0.4928
MGYA00248797/ERZ505026_FASTA.fasta	MGYA00248797	ERZ505026	root:Host-associated:Human:Digestive system:Oral	0.2616
MGYA00248798/ERZ505027_FASTA.fasta	MGYA00248798	ERZ505027	root:Host-associated:Human:Digestive system:Oral	0.4366
MGYA00248799/ERZ505028_FASTA.fasta	MGYA00248799	ERZ505028	root:Host-associated:Human:Digestive system:Oral	0.5142
MGYA00248800/ERZ505029_FASTA.fasta	MGYA00248800	ERZ505029	root:Host-associated:Human:Digestive system:Oral	0.5636
MGYA00248801/ERZ505030_FASTA.fasta	MGYA00248801	ERZ505030	root:Host-associated:Human:Digestive system:Oral	0.3182
MGYA00248802/ERZ505031_FASTA.fasta	MGYA00248802	ERZ505031	root:Host-associated:Human:Digestive system:Oral	0.4608
MGYA00248803/ERZ505032_FASTA.fasta	MGYA00248803	ERZ505032	root:Host-associated:Human:Digestive system:Oral	0.4858
MGYA00248804/ERZ505033_FASTA.fasta	MGYA00248804	ERZ505033	root:Host-associated:Human:Digestive system:Oral	0.451
MGYA00248805/ERZ505034_FASTA.fasta	MGYA00248805	ERZ505034	root:Host-associated:Human:Digestive system:Oral	0.412
MGYA00248806/ERZ505035_FASTA.fasta	MGYA00248806	ERZ505035	root:Host-associated:Human:Digestive system:Oral	0.3036
MGYA00248807/ERZ505036_FASTA.fasta	MGYA00248807	ERZ505036	root:Host-associated:Human:Digestive system:Oral	0.5024
MGYA00248808/ERZ505037_FASTA.fasta	MGYA00248808	ERZ505037	root:Host-associated:Human:Digestive system:Oral	0.5596
MGYA00248809/ERZ505038_FASTA.fasta	MGYA00248809	ERZ505038	root:Host-associated:Human:Digestive system:Oral	0.2126
MGYA00248810/ERZ505039_FASTA.fasta	MGYA00248810	ERZ505039	root:Host-associated:Human:Digestive system:Oral	0.4928
MGYA00248811/ERZ505040_FASTA.fasta	MGYA00248811	ERZ505040	root:Host-associated:Human:Digestive system:Oral	0.3838
MGYA00248812/ERZ505041_FASTA.fasta	MGYA00248812	ERZ505041	root:Host-associated:Human:Digestive system:Oral	0.4746
MGYA00248813/ERZ505042_FASTA.fasta	MGYA00248813	ERZ505042	root:Host-associated:Human:Digestive system:Oral	0.53
MGYA00248814/ERZ505043_FASTA.fasta	MGYA00248814	ERZ505043	root:Host-associated:Human:Digestive system:Oral	0.5026
MGYA00248815/ERZ505044_FASTA.fasta	MGYA00248815	ERZ505044	root:Host-associated:Human:Digestive system:Oral	0.5768
MGYA00248816/ERZ505045_FASTA.fasta	MGYA00248816	ERZ505045	root:Host-associated:Human:Digestive system:Oral	0.4102
MGYA00248817/ERZ505017_FASTA_1.fasta	MGYA00248817	ERZ505017	root:Host-associated:Human:Digestive system:Oral	0.624
MGYA00248817/ERZ505017_FASTA_2.fasta	MGYA00248817	ERZ505017	root:Host-associated:Human:Digestive system:Oral	0.461
MGYA00248818/ERZ505021_FASTA.fasta	MGYA00248818	ERZ505021	root:Host-associated:Human:Digestive system:Oral	0.422
MGYA00248819/ERZ505022_FASTA.fasta	MGYA00248819	ERZ505022	root:Host-associated:Human:Digestive system:Oral	0.5568
MGYA00248820/ERZ505023_FASTA.fasta	MGYA00248820	ERZ505023	root:Host-associated:Human:Digestive system:Oral	0.1508
MGYA00248821/ERZ500987_FASTA.fasta	MGYA00248821	ERZ500987	root:Host-associated:Human:Skin	0.4014
MGYA00248822/ERZ500989_FASTA.fasta	MGYA00248822	ERZ500989	root:Host-associated:Human:Skin	0.3976
MGYA00248823/ERZ500991_FASTA.fasta	MGYA00248823	ERZ500991	root:Host-associated:Human:Skin	0.3426
MGYA00248824/ERZ500993_FASTA.fasta	MGYA00248824	ERZ500993	root:Host-associated:Human:Skin	0.1538
MGYA00248825/ERZ500995_FASTA.fasta	MGYA00248825	ERZ500995	root:Host-associated:Human:Skin	0.3522
MGYA00248826/ERZ500997_FASTA.fasta	MGYA00248826	ERZ500997	root:Host-associated:Human:Skin	0.2782
MGYA00248827/ERZ500999_FASTA.fasta	MGYA00248827	ERZ500999	root:Host-associated:Human:Skin	0.3156
MGYA00248828/ERZ501001_FASTA.fasta	MGYA00248828	ERZ501001	root:Host-associated:Human:Skin	0.2346
MGYA00248829/ERZ501003_FASTA.fasta	MGYA00248829	ERZ501003	root:Host-associated:Human:Skin	0.2718
MGYA00248830/ERZ501005_FASTA.fasta	MGYA00248830	ERZ501005	root:Host-associated:Human:Skin	0.4662
MGYA00248831/ERZ501007_FASTA.fasta	MGYA00248831	ERZ501007	root:Host-associated:Human:Skin	0.1254
MGYA00248832/ERZ501009_FASTA.fasta	MGYA00248832	ERZ501009	root:Host-associated:Human:Skin	0.2194
MGYA00248833/ERZ501011_FASTA.fasta	MGYA00248833	ERZ501011	root:Host-associated:Human:Skin	0.4044
MGYA00248834/ERZ501013_FASTA.fasta	MGYA00248834	ERZ501013	root:Host-associated:Human:Skin	0.475
MGYA00248835/ERZ501015_FASTA.fasta	MGYA00248835	ERZ501015	root:Host-associated:Human:Skin	0.1966
MGYA00248836/ERZ501017_FASTA.fasta	MGYA00248836	ERZ501017	root:Host-associated:Human:Skin	0.132
MGYA00248837/ERZ501019_FASTA.fasta	MGYA00248837	ERZ501019	root:Host-associated:Human:Skin	0.1602
MGYA00248838/ERZ501021_FASTA.fasta	MGYA00248838	ERZ501021	root:Host-associated:Human:Skin	0.3062
MGYA00248839/ERZ501023_FASTA.fasta	MGYA00248839	ERZ501023	root:Host-associated:Human:Skin	0.3898
MGYA00248840/ERZ501025_FASTA.fasta	MGYA00248840	ERZ501025	root:Host-associated:Human:Skin	0.1086
MGYA00248841/ERZ501027_FASTA.fasta	MGYA00248841	ERZ501027	root:Host-associated:Human:Skin	0.0862
MGYA00248842/ERZ501029_FASTA.fasta	MGYA00248842	ERZ501029	root:Host-associated:Human:Skin	0.172
MGYA00248843/ERZ501031_FASTA.fasta	MGYA00248843	ERZ501031	root:Host-associated:Human:Skin	0.468
MGYA00248844/ERZ501033_FASTA.fasta	MGYA00248844	ERZ501033	root:Host-associated:Human:Skin	0.3874
MGYA00248845/ERZ501035_FASTA.fasta	MGYA00248845	ERZ501035	root:Host-associated:Human:Skin	0.1308
MGYA00248846/ERZ501037_FASTA.fasta	MGYA00248846	ERZ501037	root:Host-associated:Human:Skin	0.1286
MGYA00248847/ERZ501039_FASTA.fasta	MGYA00248847	ERZ501039	root:Host-associated:Human:Skin	0.293
MGYA00248848/ERZ501041_FASTA.fasta	MGYA00248848	ERZ501041	root:Host-associated:Human:Skin	0.3982
MGYA00248849/ERZ501043_FASTA.fasta	MGYA00248849	ERZ501043	root:Host-associated:Human:Skin	0.165
MGYA00248850/ERZ501045_FASTA.fasta	MGYA00248850	ERZ501045	root:Host-associated:Human:Skin	0.1648
MGYA00248851/ERZ501047_FASTA.fasta	MGYA00248851	ERZ501047	root:Host-associated:Human:Skin	0.5546
MGYA00248852/ERZ501049_FASTA.fasta	MGYA00248852	ERZ501049	root:Host-associated:Human:Skin	0.332
MGYA00248853/ERZ501051_FASTA.fasta	MGYA00248853	ERZ501051	root:Host-associated:Human:Skin	0.1422
MGYA00248854/ERZ501053_FASTA.fasta	MGYA00248854	ERZ501053	root:Host-associated:Human:Skin	0.2668
MGYA00248855/ERZ501055_FASTA.fasta	MGYA00248855	ERZ501055	root:Host-associated:Human:Skin	0.3314
MGYA00248856/ERZ501057_FASTA.fasta	MGYA00248856	ERZ501057	root:Host-associated:Human:Skin	0.1466
MGYA00248857/ERZ501059_FASTA.fasta	MGYA00248857	ERZ501059	root:Host-associated:Human:Skin	0.2152
MGYA00248858/ERZ501061_FASTA.fasta	MGYA00248858	ERZ501061	root:Host-associated:Human:Skin	0.3466
MGYA00248859/ERZ501063_FASTA.fasta	MGYA00248859	ERZ501063	root:Host-associated:Human:Skin	0.2228
MGYA00248860/ERZ501065_FASTA.fasta	MGYA00248860	ERZ501065	root:Host-associated:Human:Skin	0.1974
MGYA00248861/ERZ501067_FASTA.fasta	MGYA00248861	ERZ501067	root:Host-associated:Human:Skin	0.3138
MGYA00248862/ERZ500975_FASTA.fasta	MGYA00248862	ERZ500975	root:Host-associated:Human:Skin	0.3192
MGYA00248863/ERZ500977_FASTA.fasta	MGYA00248863	ERZ500977	root:Host-associated:Human:Skin	0.3896
MGYA00248864/ERZ500979_FASTA.fasta	MGYA00248864	ERZ500979	root:Host-associated:Human:Skin	0.2004
MGYA00248865/ERZ500981_FASTA.fasta	MGYA00248865	ERZ500981	root:Host-associated:Human:Skin	0.1534
MGYA00248866/ERZ500983_FASTA.fasta	MGYA00248866	ERZ500983	root:Host-associated:Human:Skin	0.2732
MGYA00248867/ERZ500985_FASTA.fasta	MGYA00248867	ERZ500985	root:Host-associated:Human:Skin	0.2792
MGYA00248868/ERZ500974_FASTA.fasta	MGYA00248868	ERZ500974	root:Host-associated:Human:Skin	0.4766
MGYA00248869/ERZ500976_FASTA.fasta	MGYA00248869	ERZ500976	root:Host-associated:Human:Skin	0.4016
MGYA00248870/ERZ500978_FASTA.fasta	MGYA00248870	ERZ500978	root:Host-associated:Human:Skin	0.3112
MGYA00248871/ERZ500980_FASTA.fasta	MGYA00248871	ERZ500980	root:Host-associated:Human:Skin	0.1962
MGYA00248872/ERZ500982_FASTA.fasta	MGYA00248872	ERZ500982	root:Host-associated:Human:Skin	0.3198
MGYA00248873/ERZ500984_FASTA.fasta	MGYA00248873	ERZ500984	root:Host-associated:Human:Skin	0.1584
MGYA00248874/ERZ500986_FASTA.fasta	MGYA00248874	ERZ500986	root:Host-associated:Human:Skin	0.1214
MGYA00248875/ERZ500988_FASTA.fasta	MGYA00248875	ERZ500988	root:Host-associated:Human:Skin	0.1814
MGYA00248876/ERZ500990_FASTA.fasta	MGYA00248876	ERZ500990	root:Host-associated:Human:Skin	0.379
MGYA00248877/ERZ500992_FASTA.fasta	MGYA00248877	ERZ500992	root:Host-associated:Human:Skin	0.1798
MGYA00248878/ERZ500994_FASTA.fasta	MGYA00248878	ERZ500994	root:Host-associated:Human:Skin	0.5794
MGYA00248879/ERZ500996_FASTA.fasta	MGYA00248879	ERZ500996	root:Host-associated:Human:Skin	0.164
MGYA00248880/ERZ500998_FASTA.fasta	MGYA00248880	ERZ500998	root:Host-associated:Human:Skin	0.1442
MGYA00248881/ERZ501000_FASTA.fasta	MGYA00248881	ERZ501000	root:Host-associated:Human:Skin	0.387
MGYA00248882/ERZ501002_FASTA.fasta	MGYA00248882	ERZ501002	root:Host-associated:Human:Skin	0.2742
MGYA00248883/ERZ501004_FASTA.fasta	MGYA00248883	ERZ501004	root:Host-associated:Human:Skin	0.148
MGYA00248884/ERZ501006_FASTA.fasta	MGYA00248884	ERZ501006	root:Host-associated:Human:Skin	0.3038
MGYA00248885/ERZ501008_FASTA.fasta	MGYA00248885	ERZ501008	root:Host-associated:Human:Skin	0.1036
MGYA00248886/ERZ501010_FASTA.fasta	MGYA00248886	ERZ501010	root:Host-associated:Human:Skin	0.3214
MGYA00248887/ERZ501012_FASTA.fasta	MGYA00248887	ERZ501012	root:Host-associated:Human:Skin	0.298
MGYA00248888/ERZ501014_FASTA.fasta	MGYA00248888	ERZ501014	root:Host-associated:Human:Skin	0.3912
MGYA00248889/ERZ501016_FASTA.fasta	MGYA00248889	ERZ501016	root:Host-associated:Human:Skin	0.566
MGYA00248890/ERZ501018_FASTA.fasta	MGYA00248890	ERZ501018	root:Host-associated:Human:Skin	0.2366
MGYA00248891/ERZ501020_FASTA.fasta	MGYA00248891	ERZ501020	root:Host-associated:Human:Skin	0.2784
MGYA00248892/ERZ501022_FASTA.fasta	MGYA00248892	ERZ501022	root:Host-associated:Human:Skin	0.1668
MGYA00248893/ERZ501024_FASTA.fasta	MGYA00248893	ERZ501024	root:Host-associated:Human:Skin	0.4136
MGYA00248894/ERZ501026_FASTA.fasta	MGYA00248894	ERZ501026	root:Host-associated:Human:Skin	0.1956
MGYA00248895/ERZ501028_FASTA.fasta	MGYA00248895	ERZ501028	root:Host-associated:Human:Skin	0.4908
MGYA00248896/ERZ501030_FASTA.fasta	MGYA00248896	ERZ501030	root:Host-associated:Human:Skin	0.2158
MGYA00248897/ERZ501032_FASTA.fasta	MGYA00248897	ERZ501032	root:Host-associated:Human:Skin	0.3272
MGYA00248898/ERZ501034_FASTA.fasta	MGYA00248898	ERZ501034	root:Host-associated:Human:Skin	0.1746
MGYA00248899/ERZ501036_FASTA.fasta	MGYA00248899	ERZ501036	root:Host-associated:Human:Skin	0.2986
MGYA00248900/ERZ501038_FASTA.fasta	MGYA00248900	ERZ501038	root:Host-associated:Human:Skin	0.178
MGYA00248901/ERZ501040_FASTA.fasta	MGYA00248901	ERZ501040	root:Host-associated:Human:Skin	0.2978
MGYA00248902/ERZ501042_FASTA.fasta	MGYA00248902	ERZ501042	root:Host-associated:Human:Skin	0.1898
MGYA00248903/ERZ501044_FASTA.fasta	MGYA00248903	ERZ501044	root:Host-associated:Human:Skin	0.4098
MGYA00248904/ERZ501046_FASTA.fasta	MGYA00248904	ERZ501046	root:Host-associated:Human:Skin	0.3574
MGYA00248905/ERZ501048_FASTA.fasta	MGYA00248905	ERZ501048	root:Host-associated:Human:Skin	0.358
MGYA00248906/ERZ501050_FASTA.fasta	MGYA00248906	ERZ501050	root:Host-associated:Human:Skin	0.2878
MGYA00248907/ERZ501052_FASTA.fasta	MGYA00248907	ERZ501052	root:Host-associated:Human:Skin	0.4088
MGYA00248908/ERZ501054_FASTA.fasta	MGYA00248908	ERZ501054	root:Host-associated:Human:Skin	0.3288
MGYA00248909/ERZ501056_FASTA.fasta	MGYA00248909	ERZ501056	root:Host-associated:Human:Skin	0.1734
MGYA00248910/ERZ501058_FASTA.fasta	MGYA00248910	ERZ501058	root:Host-associated:Human:Skin	0.2786
MGYA00248911/ERZ501060_FASTA.fasta	MGYA00248911	ERZ501060	root:Host-associated:Human:Skin	0.3366
MGYA00248912/ERZ501062_FASTA.fasta	MGYA00248912	ERZ501062	root:Host-associated:Human:Skin	0.1322
MGYA00248913/ERZ501064_FASTA.fasta	MGYA00248913	ERZ501064	root:Host-associated:Human:Skin	0.4544
MGYA00248914/ERZ501066_FASTA.fasta	MGYA00248914	ERZ501066	root:Host-associated:Human:Skin	0.1492
MGYA00248915/ERZ493868_FASTA.fasta	MGYA00248915	ERZ493868	root:Environmental:Aquatic:Marine:Intertidal zone:Cora	0.1586
MGYA00248916/ERZ650638_FASTA.fasta	MGYA00248916	ERZ650638	root:Environmental:Aquatic:Marine:Oceanic:Photic zon	0.673
MGYA00248917/ERZ483439_FASTA.fasta	MGYA00248917	ERZ483439	root:Environmental:Aquatic:Thermal springs	0.3722
MGYA00248918/ERZ483259_FASTA.fasta	MGYA00248918	ERZ483259	root:Environmental:Aquatic:Thermal springs:Hot (42-90	0.2482
MGYA00248919/ERZ651126_FASTA.fasta	MGYA00248919	ERZ651126	root:Environmental:Aquatic:Lentic:Brackish	0.119
MGYA00248920/ERZ651128_FASTA.fasta	MGYA00248920	ERZ651128	root:Environmental:Aquatic:Lentic:Brackish	0.1336
MGYA00248921/ERZ651130_FASTA.fasta	MGYA00248921	ERZ651130	root:Environmental:Aquatic:Lentic:Brackish	0.2874
MGYA00248922/ERZ651132_FASTA.fasta	MGYA00248922	ERZ651132	root:Environmental:Aquatic:Lentic:Brackish	0.2454
MGYA00248923/ERZ651134_FASTA.fasta	MGYA00248923	ERZ651134	root:Environmental:Aquatic:Lentic:Brackish	0.0618
MGYA00248924/ERZ651136_FASTA.fasta	MGYA00248924	ERZ651136	root:Environmental:Aquatic:Lentic:Brackish	0.2484
MGYA00248925/ERZ651138_FASTA.fasta	MGYA00248925	ERZ651138	root:Environmental:Aquatic:Lentic:Brackish	0.0568
MGYA00248926/ERZ651140_FASTA.fasta	MGYA00248926	ERZ651140	root:Environmental:Aquatic:Lentic:Brackish	0.3064
MGYA00248927/ERZ651144_FASTA.fasta	MGYA00248927	ERZ651144	root:Environmental:Aquatic:Lentic:Brackish	0.2454
MGYA00248928/ERZ651146_FASTA.fasta	MGYA00248928	ERZ651146	root:Environmental:Aquatic:Lentic:Brackish	0.2506
MGYA00248929/ERZ651148_FASTA.fasta	MGYA00248929	ERZ651148	root:Environmental:Aquatic:Lentic:Brackish	0.2496
MGYA00248930/ERZ651150_FASTA.fasta	MGYA00248930	ERZ651150	root:Environmental:Aquatic:Lentic:Brackish	0.13
MGYA00248931/ERZ651152_FASTA.fasta	MGYA00248931	ERZ651152	root:Environmental:Aquatic:Lentic:Brackish	0.2454
MGYA00248932/ERZ651124_FASTA.fasta	MGYA00248932	ERZ651124	root:Environmental:Aquatic:Lentic:Brackish	0.1046
MGYA00248933/ERZ651123_FASTA.fasta	MGYA00248933	ERZ651123	root:Environmental:Aquatic:Lentic:Brackish	0.1186
MGYA00248934/ERZ651125_FASTA.fasta	MGYA00248934	ERZ651125	root:Environmental:Aquatic:Lentic:Brackish	0.2326
MGYA00248935/ERZ651127_FASTA.fasta	MGYA00248935	ERZ651127	root:Environmental:Aquatic:Lentic:Brackish	0.1058
MGYA00248936/ERZ651129_FASTA.fasta	MGYA00248936	ERZ651129	root:Environmental:Aquatic:Lentic:Brackish	0.2312
MGYA00248937/ERZ651131_FASTA.fasta	MGYA00248937	ERZ651131	root:Environmental:Aquatic:Lentic:Brackish	0.207
MGYA00248938/ERZ651133_FASTA.fasta	MGYA00248938	ERZ651133	root:Environmental:Aquatic:Lentic:Brackish	0.2332
MGYA00248939/ERZ651135_FASTA.fasta	MGYA00248939	ERZ651135	root:Environmental:Aquatic:Lentic:Brackish	0.1828
MGYA00248940/ERZ651137_FASTA.fasta	MGYA00248940	ERZ651137	root:Environmental:Aquatic:Lentic:Brackish	0.1488
MGYA00248941/ERZ651139_FASTA.fasta	MGYA00248941	ERZ651139	root:Environmental:Aquatic:Lentic:Brackish	0.2238
MGYA00248942/ERZ651141_FASTA.fasta	MGYA00248942	ERZ651141	root:Environmental:Aquatic:Lentic:Brackish	0.0436
MGYA00248943/ERZ651143_FASTA.fasta	MGYA00248943	ERZ651143	root:Environmental:Aquatic:Lentic:Brackish	0.0578
MGYA00248944/ERZ651145_FASTA.fasta	MGYA00248944	ERZ651145	root:Environmental:Aquatic:Lentic:Brackish	0.084
MGYA00248945/ERZ651147_FASTA.fasta	MGYA00248945	ERZ651147	root:Environmental:Aquatic:Lentic:Brackish	0.2322
MGYA00248946/ERZ651149_FASTA.fasta	MGYA00248946	ERZ651149	root:Environmental:Aquatic:Lentic:Brackish	0.086
MGYA00248947/ERZ651151_FASTA.fasta	MGYA00248947	ERZ651151	root:Environmental:Aquatic:Lentic:Brackish	0.0516
MGYA00248948/ERZ781173_FASTA.fasta	MGYA00248948	ERZ781173	root:Engineered:Bioreactor	0.1948
MGYA00248959/ERZ746984_FASTA.fasta	MGYA00248959	ERZ746984	root:Engineered:Solid waste:Composting	0.085
MGYA00248961/ERZ504957_FASTA.fasta	MGYA00248961	ERZ504957	root:Host-associated:Human:Digestive system:Oral	0.462
MGYA00248962/ERZ504959_FASTA.fasta	MGYA00248962	ERZ504959	root:Host-associated:Human:Digestive system:Oral	0.083
MGYA00248963/ERZ504961_FASTA.fasta	MGYA00248963	ERZ504961	root:Host-associated:Human:Digestive system:Oral	0.282
MGYA00248964/ERZ504962_FASTA.fasta	MGYA00248964	ERZ504962	root:Host-associated:Human:Digestive system:Oral	0.2404
MGYA00248965/ERZ504967_FASTA.fasta	MGYA00248965	ERZ504967	root:Host-associated:Human:Digestive system:Oral	0.2096
MGYA00248966/ERZ504969_FASTA.fasta	MGYA00248966	ERZ504969	root:Host-associated:Human:Digestive system:Oral	0.0698
MGYA00248967/ERZ504939_FASTA.fasta	MGYA00248967	ERZ504939	root:Host-associated:Human:Digestive system:Oral	0.2558
MGYA00248968/ERZ504941_FASTA.fasta	MGYA00248968	ERZ504941	root:Host-associated:Human:Digestive system:Oral	0.2442
MGYA00248969/ERZ504943_FASTA.fasta	MGYA00248969	ERZ504943	root:Host-associated:Human:Digestive system:Oral	0.25
MGYA00248970/ERZ504945_FASTA.fasta	MGYA00248970	ERZ504945	root:Host-associated:Human:Digestive system:Oral	0.4434
MGYA00248971/ERZ504947_FASTA.fasta	MGYA00248971	ERZ504947	root:Host-associated:Human:Digestive system:Oral	0.314
MGYA00248972/ERZ504949_FASTA.fasta	MGYA00248972	ERZ504949	root:Host-associated:Human:Digestive system:Oral	0.3404
MGYA00248973/ERZ504951_FASTA.fasta	MGYA00248973	ERZ504951	root:Host-associated:Human:Digestive system:Oral	0.3634
MGYA00248974/ERZ504955_FASTA.fasta	MGYA00248974	ERZ504955	root:Host-associated:Human:Digestive system:Oral	0.2414
MGYA00248975/ERZ504940_FASTA.fasta	MGYA00248975	ERZ504940	root:Host-associated:Human:Digestive system:Oral	0.2484
MGYA00248976/ERZ504942_FASTA.fasta	MGYA00248976	ERZ504942	root:Host-associated:Human:Digestive system:Oral	0.3892
MGYA00248977/ERZ504944_FASTA.fasta	MGYA00248977	ERZ504944	root:Host-associated:Human:Digestive system:Oral	0.1796
MGYA00248978/ERZ504946_FASTA.fasta	MGYA00248978	ERZ504946	root:Host-associated:Human:Digestive system:Oral	0.3096
MGYA00248979/ERZ504948_FASTA.fasta	MGYA00248979	ERZ504948	root:Host-associated:Human:Digestive system:Oral	0.2968
MGYA00248980/ERZ504950_FASTA.fasta	MGYA00248980	ERZ504950	root:Host-associated:Human:Digestive system:Oral	0.6452
MGYA00248981/ERZ504952_FASTA.fasta	MGYA00248981	ERZ504952	root:Host-associated:Human:Digestive system:Oral	0.2572
MGYA00248982/ERZ504954_FASTA.fasta	MGYA00248982	ERZ504954	root:Host-associated:Human:Digestive system:Oral	0.2874
MGYA00248983/ERZ504956_FASTA.fasta	MGYA00248983	ERZ504956	root:Host-associated:Human:Digestive system:Oral	0.2746
MGYA00248984/ERZ504958_FASTA.fasta	MGYA00248984	ERZ504958	root:Host-associated:Human:Digestive system:Oral	0.523
MGYA00248985/ERZ504964_FASTA.fasta	MGYA00248985	ERZ504964	root:Host-associated:Human:Digestive system:Oral	0.588
MGYA00248986/ERZ504966_FASTA.fasta	MGYA00248986	ERZ504966	root:Host-associated:Human:Digestive system:Oral	0.2646
MGYA00248987/ERZ504968_FASTA.fasta	MGYA00248987	ERZ504968	root:Host-associated:Human:Digestive system:Oral	0.223
MGYA00249072/ERZ650318_FASTA.fasta	MGYA00249072	ERZ650318	root:Environmental:Aquatic:Lentic:Brackish	0.2326
MGYA00249073/ERZ650320_FASTA.fasta	MGYA00249073	ERZ650320	root:Environmental:Aquatic:Lentic:Brackish	0.228
MGYA00249074/ERZ650322_FASTA.fasta	MGYA00249074	ERZ650322	root:Environmental:Aquatic:Lentic:Brackish	0.2496
MGYA00249075/ERZ650324_FASTA.fasta	MGYA00249075	ERZ650324	root:Environmental:Aquatic:Lentic:Brackish	0.2496
MGYA00249076/ERZ650326_FASTA.fasta	MGYA00249076	ERZ650326	root:Environmental:Aquatic:Lentic:Brackish	0.2454
MGYA00249077/ERZ650328_FASTA.fasta	MGYA00249077	ERZ650328	root:Environmental:Aquatic:Lentic:Brackish	0.107
MGYA00249078/ERZ650330_FASTA.fasta	MGYA00249078	ERZ650330	root:Environmental:Aquatic:Lentic:Brackish	0.215
MGYA00249079/ERZ650332_FASTA.fasta	MGYA00249079	ERZ650332	root:Environmental:Aquatic:Lentic:Brackish	0.2322
MGYA00249080/ERZ650334_FASTA.fasta	MGYA00249080	ERZ650334	root:Environmental:Aquatic:Lentic:Brackish	0.25
MGYA00249081/ERZ650336_FASTA.fasta	MGYA00249081	ERZ650336	root:Environmental:Aquatic:Lentic:Brackish	0.2654
MGYA00249082/ERZ650338_FASTA.fasta	MGYA00249082	ERZ650338	root:Environmental:Aquatic:Lentic:Brackish	0.197
MGYA00249083/ERZ650319_FASTA.fasta	MGYA00249083	ERZ650319	root:Environmental:Aquatic:Lentic:Brackish	0.1648
MGYA00249084/ERZ650321_FASTA.fasta	MGYA00249084	ERZ650321	root:Environmental:Aquatic:Lentic:Brackish	0.186
MGYA00249085/ERZ650323_FASTA.fasta	MGYA00249085	ERZ650323	root:Environmental:Aquatic:Lentic:Brackish	0.2454
MGYA00249086/ERZ650325_FASTA.fasta	MGYA00249086	ERZ650325	root:Environmental:Aquatic:Lentic:Brackish	0.2124
MGYA00249087/ERZ650327_FASTA.fasta	MGYA00249087	ERZ650327	root:Environmental:Aquatic:Lentic:Brackish	0.191
MGYA00249088/ERZ650329_FASTA.fasta	MGYA00249088	ERZ650329	root:Environmental:Aquatic:Lentic:Brackish	0.1082
MGYA00249089/ERZ650331_FASTA.fasta	MGYA00249089	ERZ650331	root:Environmental:Aquatic:Lentic:Brackish	0.1112
MGYA00249090/ERZ650333_FASTA.fasta	MGYA00249090	ERZ650333	root:Environmental:Aquatic:Lentic:Brackish	0.2122
MGYA00249091/ERZ650335_FASTA.fasta	MGYA00249091	ERZ650335	root:Environmental:Aquatic:Lentic:Brackish	0.1204
MGYA00249092/ERZ650337_FASTA.fasta	MGYA00249092	ERZ650337	root:Environmental:Aquatic:Lentic:Brackish	0.2238
MGYA00249093/ERZ650339_FASTA.fasta	MGYA00249093	ERZ650339	root:Environmental:Aquatic:Lentic:Brackish	0.2454
MGYA00249094/ERZ650345_FASTA.fasta	MGYA00249094	ERZ650345	root:Environmental:Aquatic:Marine:Brackish	0.8708
MGYA00249095/ERZ650666_FASTA.fasta	MGYA00249095	ERZ650666	root:Environmental:Aquatic:Marine:Brackish	0.9298
MGYA00249096/ERZ650668_FASTA.fasta	MGYA00249096	ERZ650668	root:Environmental:Aquatic:Marine:Brackish	0.979
MGYA00249097/ERZ651159_FASTA.fasta	MGYA00249097	ERZ651159	root:Environmental:Aquatic:Marine	0.808
MGYA00249098/ERZ651773_FASTA.fasta	MGYA00249098	ERZ651773	root:Environmental:Aquatic:Marine:Intertidal zone:Estu	0.8238
MGYA00249099/ERZ651774_FASTA.fasta	MGYA00249099	ERZ651774	root:Environmental:Aquatic:Marine:Intertidal zone:Estu	0.8216
MGYA00249100/ERZ651805_FASTA.fasta	MGYA00249100	ERZ651805	root:Engineered:Solid waste:Composting:Bioreactor	0.0574
MGYA00249101/ERZ652144_FASTA.fasta	MGYA00249101	ERZ652144	root:Host-associated:Human:Digestive system:Large in	0.3558
MGYA00249102/ERZ652146_FASTA.fasta	MGYA00249102	ERZ652146	root:Host-associated:Human:Digestive system:Large in	0.2322
MGYA00249103/ERZ652148_FASTA.fasta	MGYA00249103	ERZ652148	root:Host-associated:Human:Digestive system:Large in	0.3312
MGYA00249104/ERZ652150_FASTA.fasta	MGYA00249104	ERZ652150	root:Host-associated:Human:Digestive system:Large in	0.2642
MGYA00249105/ERZ652158_FASTA.fasta	MGYA00249105	ERZ652158	root:Host-associated:Human:Digestive system:Large in	0.1708
MGYA00249106/ERZ652160_FASTA.fasta	MGYA00249106	ERZ652160	root:Host-associated:Human:Digestive system:Large in	0.3284
MGYA00249107/ERZ652162_FASTA.fasta	MGYA00249107	ERZ652162	root:Host-associated:Human:Digestive system:Large in	0.232
MGYA00249108/ERZ652172_FASTA.fasta	MGYA00249108	ERZ652172	root:Host-associated:Human:Digestive system:Large in	0.1782
MGYA00249109/ERZ652174_FASTA.fasta	MGYA00249109	ERZ652174	root:Host-associated:Human:Digestive system:Large in	0.319
MGYA00249110/ERZ652176_FASTA.fasta	MGYA00249110	ERZ652176	root:Host-associated:Human:Digestive system:Large in	0.406
MGYA00249111/ERZ652178_FASTA.fasta	MGYA00249111	ERZ652178	root:Host-associated:Human:Digestive system:Large in	0.2312
MGYA00249112/ERZ652180_FASTA.fasta	MGYA00249112	ERZ652180	root:Host-associated:Human:Digestive system:Large in	0.356
MGYA00249113/ERZ652182_FASTA.fasta	MGYA00249113	ERZ652182	root:Host-associated:Human:Digestive system:Large in	0.453
MGYA00249114/ERZ652184_FASTA.fasta	MGYA00249114	ERZ652184	root:Host-associated:Human:Digestive system:Large in	0.3592
MGYA00249115/ERZ652186_FASTA.fasta	MGYA00249115	ERZ652186	root:Host-associated:Human:Digestive system:Large in	0.2852
MGYA00249116/ERZ652188_FASTA.fasta	MGYA00249116	ERZ652188	root:Host-associated:Human:Digestive system:Large in	0.2818
MGYA00249117/ERZ652190_FASTA.fasta	MGYA00249117	ERZ652190	root:Host-associated:Human:Digestive system:Large in	0.267
MGYA00249118/ERZ652192_FASTA.fasta	MGYA00249118	ERZ652192	root:Host-associated:Human:Digestive system:Large in	0.4112
MGYA00249119/ERZ652194_FASTA.fasta	MGYA00249119	ERZ652194	root:Host-associated:Human:Digestive system:Large in	0.3782
MGYA00249120/ERZ652196_FASTA.fasta	MGYA00249120	ERZ652196	root:Host-associated:Human:Digestive system:Large in	0.3608
MGYA00249121/ERZ652198_FASTA.fasta	MGYA00249121	ERZ652198	root:Host-associated:Human:Digestive system:Large in	0.2986
MGYA00249122/ERZ652200_FASTA.fasta	MGYA00249122	ERZ652200	root:Host-associated:Human:Digestive system:Large in	0.2932
MGYA00249123/ERZ652202_FASTA.fasta	MGYA00249123	ERZ652202	root:Host-associated:Human:Digestive system:Large in	0.295
MGYA00249124/ERZ652204_FASTA.fasta	MGYA00249124	ERZ652204	root:Host-associated:Human:Digestive system:Large in	0.1286
MGYA00249125/ERZ652206_FASTA.fasta	MGYA00249125	ERZ652206	root:Host-associated:Human:Digestive system:Large in	0.451
MGYA00249126/ERZ652208_FASTA.fasta	MGYA00249126	ERZ652208	root:Host-associated:Human:Digestive system:Large in	0.3424
MGYA00249127/ERZ652210_FASTA.fasta	MGYA00249127	ERZ652210	root:Host-associated:Human:Digestive system:Large in	0.3998
MGYA00249128/ERZ652212_FASTA.fasta	MGYA00249128	ERZ652212	root:Host-associated:Human:Digestive system:Large in	0.3362
MGYA00249129/ERZ652216_FASTA.fasta	MGYA00249129	ERZ652216	root:Host-associated:Human:Digestive system:Large in	0.319
MGYA00249130/ERZ651938_FASTA.fasta	MGYA00249130	ERZ651938	root:Host-associated:Human:Digestive system:Large in	0.3272
MGYA00249131/ERZ651940_FASTA.fasta	MGYA00249131	ERZ651940	root:Host-associated:Human:Digestive system:Large in	0.2306
MGYA00249132/ERZ651942_FASTA.fasta	MGYA00249132	ERZ651942	root:Host-associated:Human:Digestive system:Large in	0.428
MGYA00249133/ERZ651944_FASTA.fasta	MGYA00249133	ERZ651944	root:Host-associated:Human:Digestive system:Large in	0.2604
MGYA00249134/ERZ651946_FASTA.fasta	MGYA00249134	ERZ651946	root:Host-associated:Human:Digestive system:Large in	0.1684
MGYA00249135/ERZ651948_FASTA.fasta	MGYA00249135	ERZ651948	root:Host-associated:Human:Digestive system:Large in	0.267
MGYA00249136/ERZ651950_FASTA.fasta	MGYA00249136	ERZ651950	root:Host-associated:Human:Digestive system:Large in	0.4922
MGYA00249137/ERZ651952_FASTA.fasta	MGYA00249137	ERZ651952	root:Host-associated:Human:Digestive system:Large in	0.4028
MGYA00249138/ERZ651954_FASTA.fasta	MGYA00249138	ERZ651954	root:Host-associated:Human:Digestive system:Large in	0.2832
MGYA00249139/ERZ651956_FASTA.fasta	MGYA00249139	ERZ651956	root:Host-associated:Human:Digestive system:Large in	0.3728
MGYA00249140/ERZ651958_FASTA.fasta	MGYA00249140	ERZ651958	root:Host-associated:Human:Digestive system:Large in	0.4032
MGYA00249141/ERZ651960_FASTA.fasta	MGYA00249141	ERZ651960	root:Host-associated:Human:Digestive system:Large in	0.2776
MGYA00249142/ERZ651962_FASTA.fasta	MGYA00249142	ERZ651962	root:Host-associated:Human:Digestive system:Large in	0.3386
MGYA00249143/ERZ651964_FASTA.fasta	MGYA00249143	ERZ651964	root:Host-associated:Human:Digestive system:Large in	0.3086
MGYA00249144/ERZ651966_FASTA.fasta	MGYA00249144	ERZ651966	root:Host-associated:Human:Digestive system:Large in	0.434
MGYA00249145/ERZ651968_FASTA.fasta	MGYA00249145	ERZ651968	root:Host-associated:Human:Digestive system:Large in	0.3206
MGYA00249146/ERZ651970_FASTA.fasta	MGYA00249146	ERZ651970	root:Host-associated:Human:Digestive system:Large in	0.3772
MGYA00249147/ERZ652218_FASTA.fasta	MGYA00249147	ERZ652218	root:Host-associated:Human:Digestive system:Large in	0.3182
MGYA00249148/ERZ652220_FASTA.fasta	MGYA00249148	ERZ652220	root:Host-associated:Human:Digestive system:Large in	0.2734
MGYA00249149/ERZ652222_FASTA.fasta	MGYA00249149	ERZ652222	root:Host-associated:Human:Digestive system:Large in	0.2736
MGYA00249150/ERZ652224_FASTA.fasta	MGYA00249150	ERZ652224	root:Host-associated:Human:Digestive system:Large in	0.2928
MGYA00249151/ERZ652228_FASTA.fasta	MGYA00249151	ERZ652228	root:Host-associated:Human:Digestive system:Large in	0.363
MGYA00249152/ERZ652230_FASTA.fasta	MGYA00249152	ERZ652230	root:Host-associated:Human:Digestive system:Large in	0.2036
MGYA00249153/ERZ652232_FASTA.fasta	MGYA00249153	ERZ652232	root:Host-associated:Human:Digestive system:Large in	0.3098
MGYA00249154/ERZ652234_FASTA.fasta	MGYA00249154	ERZ652234	root:Host-associated:Human:Digestive system:Large in	0.3036
MGYA00249155/ERZ652236_FASTA.fasta	MGYA00249155	ERZ652236	root:Host-associated:Human:Digestive system:Large in	0.2354
MGYA00249156/ERZ652240_FASTA.fasta	MGYA00249156	ERZ652240	root:Host-associated:Human:Digestive system:Large in	0.2974
MGYA00249157/ERZ651989_FASTA.fasta	MGYA00249157	ERZ651989	root:Host-associated:Human:Digestive system:Large in	0.106
MGYA00249158/ERZ651991_FASTA.fasta	MGYA00249158	ERZ651991	root:Host-associated:Human:Digestive system:Large in	0.308
MGYA00249159/ERZ651993_FASTA.fasta	MGYA00249159	ERZ651993	root:Host-associated:Human:Digestive system:Large in	0.2804
MGYA00249160/ERZ651995_FASTA.fasta	MGYA00249160	ERZ651995	root:Host-associated:Human:Digestive system:Large in	0.323
MGYA00249161/ERZ651997_FASTA.fasta	MGYA00249161	ERZ651997	root:Host-associated:Human:Digestive system:Large in	0.247
MGYA00249162/ERZ651999_FASTA.fasta	MGYA00249162	ERZ651999	root:Host-associated:Human:Digestive system:Large in	0.2178
MGYA00249163/ERZ652001_FASTA.fasta	MGYA00249163	ERZ652001	root:Host-associated:Human:Digestive system:Large in	0.246
MGYA00249164/ERZ652003_FASTA.fasta	MGYA00249164	ERZ652003	root:Host-associated:Human:Digestive system:Large in	0.3478
MGYA00249165/ERZ652005_FASTA.fasta	MGYA00249165	ERZ652005	root:Host-associated:Human:Digestive system:Large in	0.4228
MGYA00249166/ERZ652007_FASTA.fasta	MGYA00249166	ERZ652007	root:Host-associated:Human:Digestive system:Large in	0.3722
MGYA00249167/ERZ652009_FASTA.fasta	MGYA00249167	ERZ652009	root:Host-associated:Human:Digestive system:Large in	0.3894
MGYA00249168/ERZ652011_FASTA.fasta	MGYA00249168	ERZ652011	root:Host-associated:Human:Digestive system:Large in	0.2538
MGYA00249169/ERZ652013_FASTA.fasta	MGYA00249169	ERZ652013	root:Host-associated:Human:Digestive system:Large in	0.2976
MGYA00249170/ERZ652015_FASTA.fasta	MGYA00249170	ERZ652015	root:Host-associated:Human:Digestive system:Large in	0.2388
MGYA00249171/ERZ652017_FASTA.fasta	MGYA00249171	ERZ652017	root:Host-associated:Human:Digestive system:Large in	0.3572
MGYA00249172/ERZ652019_FASTA.fasta	MGYA00249172	ERZ652019	root:Host-associated:Human:Digestive system:Large in	0.4084
MGYA00249173/ERZ652675_FASTA.fasta	MGYA00249173	ERZ652675	root:Host-associated:Human:Digestive system:Large in	0.272
MGYA00249174/ERZ652676_FASTA.fasta	MGYA00249174	ERZ652676	root:Host-associated:Human:Digestive system:Large in	0.0662
MGYA00249175/ERZ652678_FASTA.fasta	MGYA00249175	ERZ652678	root:Host-associated:Human:Digestive system:Large in	0.3496
MGYA00249176/ERZ652680_FASTA.fasta	MGYA00249176	ERZ652680	root:Host-associated:Human:Digestive system:Large in	0.357
MGYA00249177/ERZ652682_FASTA.fasta	MGYA00249177	ERZ652682	root:Host-associated:Human:Digestive system:Large in	0.2138
MGYA00249178/ERZ652684_FASTA.fasta	MGYA00249178	ERZ652684	root:Host-associated:Human:Digestive system:Large in	0.2402
MGYA00249179/ERZ652686_FASTA.fasta	MGYA00249179	ERZ652686	root:Host-associated:Human:Digestive system:Large in	0.0876
MGYA00249180/ERZ652688_FASTA.fasta	MGYA00249180	ERZ652688	root:Host-associated:Human:Digestive system:Large in	0.2826
MGYA00249181/ERZ652063_FASTA.fasta	MGYA00249181	ERZ652063	root:Host-associated:Human:Digestive system:Large in	0.4088
MGYA00249182/ERZ652065_FASTA.fasta	MGYA00249182	ERZ652065	root:Host-associated:Human:Digestive system:Large in	0.3824
MGYA00249183/ERZ652067_FASTA.fasta	MGYA00249183	ERZ652067	root:Host-associated:Human:Digestive system:Large in	0.3292
MGYA00249184/ERZ652069_FASTA.fasta	MGYA00249184	ERZ652069	root:Host-associated:Human:Digestive system:Large in	0.2672
MGYA00249185/ERZ652071_FASTA.fasta	MGYA00249185	ERZ652071	root:Host-associated:Human:Digestive system:Large in	0.3384
MGYA00249186/ERZ652073_FASTA.fasta	MGYA00249186	ERZ652073	root:Host-associated:Human:Digestive system:Large in	0.25
MGYA00249187/ERZ652075_FASTA.fasta	MGYA00249187	ERZ652075	root:Host-associated:Human:Digestive system:Large in	0.3814
MGYA00249188/ERZ652077_FASTA.fasta	MGYA00249188	ERZ652077	root:Host-associated:Human:Digestive system:Large in	0.3606
MGYA00249189/ERZ652081_FASTA.fasta	MGYA00249189	ERZ652081	root:Host-associated:Human:Digestive system:Large in	0.228
MGYA00249190/ERZ652083_FASTA.fasta	MGYA00249190	ERZ652083	root:Host-associated:Human:Digestive system:Large in	0.5154
MGYA00249191/ERZ652085_FASTA.fasta	MGYA00249191	ERZ652085	root:Host-associated:Human:Digestive system:Large in	0.243
MGYA00249192/ERZ652087_FASTA.fasta	MGYA00249192	ERZ652087	root:Host-associated:Human:Digestive system:Large in	0.3642
MGYA00249193/ERZ652089_FASTA.fasta	MGYA00249193	ERZ652089	root:Host-associated:Human:Digestive system:Large in	0.375
MGYA00249194/ERZ652091_FASTA.fasta	MGYA00249194	ERZ652091	root:Host-associated:Human:Digestive system:Large in	0.3236
MGYA00249195/ERZ652093_FASTA.fasta	MGYA00249195	ERZ652093	root:Host-associated:Human:Digestive system:Large in	0.221
MGYA00249196/ERZ652095_FASTA.fasta	MGYA00249196	ERZ652095	root:Host-associated:Human:Digestive system:Large in	0.4456
MGYA00249197/ERZ652097_FASTA.fasta	MGYA00249197	ERZ652097	root:Host-associated:Human:Digestive system:Large in	0.3014
MGYA00249198/ERZ652099_FASTA.fasta	MGYA00249198	ERZ652099	root:Host-associated:Human:Digestive system:Large in	0.3614
MGYA00249199/ERZ652101_FASTA.fasta	MGYA00249199	ERZ652101	root:Host-associated:Human:Digestive system:Large in	0.3552
MGYA00249200/ERZ651972_FASTA.fasta	MGYA00249200	ERZ651972	root:Host-associated:Human:Digestive system:Large in	0.2798
MGYA00249201/ERZ651974_FASTA.fasta	MGYA00249201	ERZ651974	root:Host-associated:Human:Digestive system:Large in	0.3256
MGYA00249202/ERZ651976_FASTA.fasta	MGYA00249202	ERZ651976	root:Host-associated:Human:Digestive system:Large in	0.5066
MGYA00249203/ERZ651978_FASTA.fasta	MGYA00249203	ERZ651978	root:Host-associated:Human:Digestive system:Large in	0.355
MGYA00249204/ERZ651980_FASTA.fasta	MGYA00249204	ERZ651980	root:Host-associated:Human:Digestive system:Large in	0.3084
MGYA00249205/ERZ651982_FASTA.fasta	MGYA00249205	ERZ651982	root:Host-associated:Human:Digestive system:Large in	0.2452
MGYA00249206/ERZ651984_FASTA.fasta	MGYA00249206	ERZ651984	root:Host-associated:Human:Digestive system:Large in	0.3332
MGYA00249207/ERZ651986_FASTA.fasta	MGYA00249207	ERZ651986	root:Host-associated:Human:Digestive system:Large in	0.2276
MGYA00249208/ERZ652286_FASTA.fasta	MGYA00249208	ERZ652286	root:Host-associated:Human:Digestive system:Large in	0.2184
MGYA00249209/ERZ652288_FASTA.fasta	MGYA00249209	ERZ652288	root:Host-associated:Human:Digestive system:Large in	0.272
MGYA00249210/ERZ652290_FASTA.fasta	MGYA00249210	ERZ652290	root:Host-associated:Human:Digestive system:Large in	0.3158
MGYA00249211/ERZ652292_FASTA.fasta	MGYA00249211	ERZ652292	root:Host-associated:Human:Digestive system:Large in	0.2076
MGYA00249212/ERZ652294_FASTA.fasta	MGYA00249212	ERZ652294	root:Host-associated:Human:Digestive system:Large in	0.2456
MGYA00249213/ERZ652296_FASTA.fasta	MGYA00249213	ERZ652296	root:Host-associated:Human:Digestive system:Large in	0.1172
MGYA00249214/ERZ652298_FASTA.fasta	MGYA00249214	ERZ652298	root:Host-associated:Human:Digestive system:Large in	0.2508
MGYA00249215/ERZ652300_FASTA.fasta	MGYA00249215	ERZ652300	root:Host-associated:Human:Digestive system:Large in	0.223
MGYA00249216/ERZ652302_FASTA.fasta	MGYA00249216	ERZ652302	root:Host-associated:Human:Digestive system:Large in	0.1448
MGYA00249217/ERZ652304_FASTA.fasta	MGYA00249217	ERZ652304	root:Host-associated:Human:Digestive system:Large in	0.097
MGYA00249218/ERZ652306_FASTA.fasta	MGYA00249218	ERZ652306	root:Host-associated:Human:Digestive system:Large in	0.4112
MGYA00249219/ERZ652308_FASTA.fasta	MGYA00249219	ERZ652308	root:Host-associated:Human:Digestive system:Large in	0.3838
MGYA00249220/ERZ652310_FASTA.fasta	MGYA00249220	ERZ652310	root:Host-associated:Human:Digestive system:Large in	0.2124
MGYA00249221/ERZ652313_FASTA.fasta	MGYA00249221	ERZ652313	root:Host-associated:Human:Digestive system:Large in	0.2254
MGYA00249222/ERZ652315_FASTA.fasta	MGYA00249222	ERZ652315	root:Host-associated:Human:Digestive system:Large in	0.4284
MGYA00249223/ERZ652317_FASTA.fasta	MGYA00249223	ERZ652317	root:Host-associated:Human:Digestive system:Large in	0.3228
MGYA00249224/ERZ652319_FASTA.fasta	MGYA00249224	ERZ652319	root:Host-associated:Human:Digestive system:Large in	0.3082
MGYA00249225/ERZ652321_FASTA.fasta	MGYA00249225	ERZ652321	root:Host-associated:Human:Digestive system:Large in	0.2766
MGYA00249226/ERZ652323_FASTA.fasta	MGYA00249226	ERZ652323	root:Host-associated:Human:Digestive system:Large in	0.3156
MGYA00249227/ERZ652021_FASTA.fasta	MGYA00249227	ERZ652021	root:Host-associated:Human:Digestive system:Large in	0.3914
MGYA00249228/ERZ652023_FASTA.fasta	MGYA00249228	ERZ652023	root:Host-associated:Human:Digestive system:Large in	0.1974
MGYA00249229/ERZ652025_FASTA.fasta	MGYA00249229	ERZ652025	root:Host-associated:Human:Digestive system:Large in	0.3614
MGYA00249230/ERZ652027_FASTA.fasta	MGYA00249230	ERZ652027	root:Host-associated:Human:Digestive system:Large in	0.2794
MGYA00249231/ERZ652029_FASTA.fasta	MGYA00249231	ERZ652029	root:Host-associated:Human:Digestive system:Large in	0.3162
MGYA00249232/ERZ652031_FASTA.fasta	MGYA00249232	ERZ652031	root:Host-associated:Human:Digestive system:Large in	0.3678
MGYA00249233/ERZ652033_FASTA.fasta	MGYA00249233	ERZ652033	root:Host-associated:Human:Digestive system:Large in	0.3436
MGYA00249234/ERZ652035_FASTA.fasta	MGYA00249234	ERZ652035	root:Host-associated:Human:Digestive system:Large in	0.2926
MGYA00249235/ERZ652037_FASTA.fasta	MGYA00249235	ERZ652037	root:Host-associated:Human:Digestive system:Large in	0.4716
MGYA00249236/ERZ652039_FASTA.fasta	MGYA00249236	ERZ652039	root:Host-associated:Human:Digestive system:Large in	0.2524
MGYA00249237/ERZ652041_FASTA.fasta	MGYA00249237	ERZ652041	root:Host-associated:Human:Digestive system:Large in	0.2232
MGYA00249238/ERZ652043_FASTA.fasta	MGYA00249238	ERZ652043	root:Host-associated:Human:Digestive system:Large in	0.3114
MGYA00249239/ERZ652045_FASTA.fasta	MGYA00249239	ERZ652045	root:Host-associated:Human:Digestive system:Large in	0.4328
MGYA00249240/ERZ652047_FASTA.fasta	MGYA00249240	ERZ652047	root:Host-associated:Human:Digestive system:Large in	0.4618
MGYA00249241/ERZ652049_FASTA.fasta	MGYA00249241	ERZ652049	root:Host-associated:Human:Digestive system:Large in	0.3352
MGYA00249242/ERZ652051_FASTA.fasta	MGYA00249242	ERZ652051	root:Host-associated:Human:Digestive system:Large in	0.1702

MGYA00249243/ERZ652053_FASTA.fasta	MGYA00249243	ERZ652053	root:Host-associated:Human:Digestive system:Large in/	0.3794
MGYA00249244/ERZ652055_FASTA.fasta	MGYA00249244	ERZ652055	root:Host-associated:Human:Digestive system:Large in/	0.3078
MGYA00249245/ERZ652057_FASTA.fasta	MGYA00249245	ERZ652057	root:Host-associated:Human:Digestive system:Large in/	0.3288
MGYA00249246/ERZ652059_FASTA.fasta	MGYA00249246	ERZ652059	root:Host-associated:Human:Digestive system:Large in/	0.298
MGYA00249247/ERZ652690_FASTA.fasta	MGYA00249247	ERZ652690	root:Host-associated:Human:Digestive system:Large in/	0.4444
MGYA00249248/ERZ652692_FASTA.fasta	MGYA00249248	ERZ652692	root:Host-associated:Human:Digestive system:Large in/	0.1832
MGYA00249249/ERZ652694_FASTA.fasta	MGYA00249249	ERZ652694	root:Host-associated:Human:Digestive system:Large in/	0.2314
MGYA00249250/ERZ652696_FASTA.fasta	MGYA00249250	ERZ652696	root:Host-associated:Human:Digestive system:Large in/	0.2682
MGYA00249251/ERZ652698_FASTA.fasta	MGYA00249251	ERZ652698	root:Host-associated:Human:Digestive system:Large in/	0.1282
MGYA00249252/ERZ652700_FASTA.fasta	MGYA00249252	ERZ652700	root:Host-associated:Human:Digestive system:Large in/	0.2276
MGYA00249253/ERZ652702_FASTA.fasta	MGYA00249253	ERZ652702	root:Host-associated:Human:Digestive system:Large in/	0.439
MGYA00249254/ERZ652103_FASTA.fasta	MGYA00249254	ERZ652103	root:Host-associated:Human:Digestive system:Large in/	0.3474
MGYA00249255/ERZ652105_FASTA.fasta	MGYA00249255	ERZ652105	root:Host-associated:Human:Digestive system:Large in/	0.398
MGYA00249256/ERZ652107_FASTA.fasta	MGYA00249256	ERZ652107	root:Host-associated:Human:Digestive system:Large in/	0.405
MGYA00249257/ERZ652109_FASTA.fasta	MGYA00249257	ERZ652109	root:Host-associated:Human:Digestive system:Large in/	0.3892
MGYA00249258/ERZ652111_FASTA.fasta	MGYA00249258	ERZ652111	root:Host-associated:Human:Digestive system:Large in/	0.3512
MGYA00249259/ERZ652113_FASTA.fasta	MGYA00249259	ERZ652113	root:Host-associated:Human:Digestive system:Large in/	0.3748
MGYA00249260/ERZ652115_FASTA.fasta	MGYA00249260	ERZ652115	root:Host-associated:Human:Digestive system:Large in/	0.433
MGYA00249261/ERZ652117_FASTA.fasta	MGYA00249261	ERZ652117	root:Host-associated:Human:Digestive system:Large in/	0.2374
MGYA00249262/ERZ652119_FASTA.fasta	MGYA00249262	ERZ652119	root:Host-associated:Human:Digestive system:Large in/	0.3898
MGYA00249263/ERZ652121_FASTA.fasta	MGYA00249263	ERZ652121	root:Host-associated:Human:Digestive system:Large in/	0.3132
MGYA00249264/ERZ652123_FASTA.fasta	MGYA00249264	ERZ652123	root:Host-associated:Human:Digestive system:Large in/	0.3272
MGYA00249265/ERZ652125_FASTA.fasta	MGYA00249265	ERZ652125	root:Host-associated:Human:Digestive system:Large in/	0.3154
MGYA00249266/ERZ652127_FASTA.fasta	MGYA00249266	ERZ652127	root:Host-associated:Human:Digestive system:Large in/	0.3622
MGYA00249267/ERZ652129_FASTA.fasta	MGYA00249267	ERZ652129	root:Host-associated:Human:Digestive system:Large in/	0.428
MGYA00249268/ERZ652131_FASTA.fasta	MGYA00249268	ERZ652131	root:Host-associated:Human:Digestive system:Large in/	0.2258
MGYA00249269/ERZ652133_FASTA.fasta	MGYA00249269	ERZ652133	root:Host-associated:Human:Digestive system:Large in/	0.2962
MGYA00249270/ERZ652135_FASTA.fasta	MGYA00249270	ERZ652135	root:Host-associated:Human:Digestive system:Large in/	0.322
MGYA00249271/ERZ652137_FASTA.fasta	MGYA00249271	ERZ652137	root:Host-associated:Human:Digestive system:Large in/	0.3372
MGYA00249272/ERZ652325_FASTA.fasta	MGYA00249272	ERZ652325	root:Host-associated:Human:Digestive system:Large in/	0.4844
MGYA00249273/ERZ652327_FASTA.fasta	MGYA00249273	ERZ652327	root:Host-associated:Human:Digestive system:Large in/	0.2742
MGYA00249274/ERZ652329_FASTA.fasta	MGYA00249274	ERZ652329	root:Host-associated:Human:Digestive system:Large in/	0.2582
MGYA00249275/ERZ652331_FASTA.fasta	MGYA00249275	ERZ652331	root:Host-associated:Human:Digestive system:Large in/	0.2872
MGYA00249276/ERZ652333_FASTA.fasta	MGYA00249276	ERZ652333	root:Host-associated:Human:Digestive system:Large in/	0.2652
MGYA00249277/ERZ652335_FASTA.fasta	MGYA00249277	ERZ652335	root:Host-associated:Human:Digestive system:Large in/	0.2684
MGYA00249278/ERZ652337_FASTA.fasta	MGYA00249278	ERZ652337	root:Host-associated:Human:Digestive system:Large in/	0.2684
MGYA00249279/ERZ652339_FASTA.fasta	MGYA00249279	ERZ652339	root:Host-associated:Human:Digestive system:Large in/	0.1846
MGYA00249280/ERZ652341_FASTA.fasta	MGYA00249280	ERZ652341	root:Host-associated:Human:Digestive system:Large in/	0.2654
MGYA00249281/ERZ652343_FASTA.fasta	MGYA00249281	ERZ652343	root:Host-associated:Human:Digestive system:Large in/	0.2988
MGYA00249282/ERZ652345_FASTA.fasta	MGYA00249282	ERZ652345	root:Host-associated:Human:Digestive system:Large in/	0.28
MGYA00249283/ERZ652347_FASTA.fasta	MGYA00249283	ERZ652347	root:Host-associated:Human:Digestive system:Large in/	0.3132
MGYA00249284/ERZ652351_FASTA.fasta	MGYA00249284	ERZ652351	root:Host-associated:Human:Digestive system:Large in/	0.359
MGYA00249285/ERZ652353_FASTA.fasta	MGYA00249285	ERZ652353	root:Host-associated:Human:Digestive system:Large in/	0.3562
MGYA00249286/ERZ652355_FASTA.fasta	MGYA00249286	ERZ652355	root:Host-associated:Human:Digestive system:Large in/	0.2886
MGYA00249287/ERZ652357_FASTA.fasta	MGYA00249287	ERZ652357	root:Host-associated:Human:Digestive system:Large in/	0.3514
MGYA00249288/ERZ652359_FASTA.fasta	MGYA00249288	ERZ652359	root:Host-associated:Human:Digestive system:Large in/	0.2862
MGYA00249289/ERZ652061_FASTA.fasta	MGYA00249289	ERZ652061	root:Host-associated:Human:Digestive system:Large in/	0.2632
MGYA00249290/ERZ652139_FASTA.fasta	MGYA00249290	ERZ652139	root:Host-associated:Human:Digestive system:Large in/	0.5198
MGYA00249291/ERZ652361_FASTA.fasta	MGYA00249291	ERZ652361	root:Host-associated:Human:Digestive system:Large in/	0.201
MGYA00249292/ERZ652365_FASTA.fasta	MGYA00249292	ERZ652365	root:Host-associated:Human:Digestive system:Large in/	0.336
MGYA00249293/ERZ652367_FASTA.fasta	MGYA00249293	ERZ652367	root:Host-associated:Human:Digestive system:Large in/	0.4658
MGYA00249294/ERZ652369_FASTA.fasta	MGYA00249294	ERZ652369	root:Host-associated:Human:Digestive system:Large in/	0.1696
MGYA00249295/ERZ652371_FASTA.fasta	MGYA00249295	ERZ652371	root:Host-associated:Human:Digestive system:Large in/	0.2946
MGYA00249296/ERZ652373_FASTA.fasta	MGYA00249296	ERZ652373	root:Host-associated:Human:Digestive system:Large in/	0.236
MGYA00249297/ERZ652375_FASTA.fasta	MGYA00249297	ERZ652375	root:Host-associated:Human:Digestive system:Large in/	0.2064
MGYA00249298/ERZ652377_FASTA.fasta	MGYA00249298	ERZ652377	root:Host-associated:Human:Digestive system:Large in/	0.2476
MGYA00249299/ERZ652379_FASTA.fasta	MGYA00249299	ERZ652379	root:Host-associated:Human:Digestive system:Large in/	0.312
MGYA00249300/ERZ652381_FASTA.fasta	MGYA00249300	ERZ652381	root:Host-associated:Human:Digestive system:Large in/	0.165
MGYA00249301/ERZ652383_FASTA.fasta	MGYA00249301	ERZ652383	root:Host-associated:Human:Digestive system:Large in/	0.2982
MGYA00249302/ERZ652385_FASTA.fasta	MGYA00249302	ERZ652385	root:Host-associated:Human:Digestive system:Large in/	0.2024
MGYA00249303/ERZ652387_FASTA.fasta	MGYA00249303	ERZ652387	root:Host-associated:Human:Digestive system:Large in/	0.3068
MGYA00249304/ERZ652389_FASTA.fasta	MGYA00249304	ERZ652389	root:Host-associated:Human:Digestive system:Large in/	0.2884
MGYA00249305/ERZ652391_FASTA.fasta	MGYA00249305	ERZ652391	root:Host-associated:Human:Digestive system:Large in/	0.4074
MGYA00249306/ERZ652393_FASTA.fasta	MGYA00249306	ERZ652393	root:Host-associated:Human:Digestive system:Large in/	0.1514
MGYA00249307/ERZ652395_FASTA.fasta	MGYA00249307	ERZ652395	root:Host-associated:Human:Digestive system:Large in/	0.2394
MGYA00249308/ERZ652397_FASTA.fasta	MGYA00249308	ERZ652397	root:Host-associated:Human:Digestive system:Large in/	0.3628
MGYA00249309/ERZ652399_FASTA.fasta	MGYA00249309	ERZ652399	root:Host-associated:Human:Digestive system:Large in/	0.2518
MGYA00249310/ERZ652401_FASTA.fasta	MGYA00249310	ERZ652401	root:Host-associated:Human:Digestive system:Large in/	0.2646
MGYA00249311/ERZ652270_FASTA.fasta	MGYA00249311	ERZ652270	root:Host-associated:Human:Digestive system:Large in/	0.2564
MGYA00249312/ERZ652274_FASTA.fasta	MGYA00249312	ERZ652274	root:Host-associated:Human:Digestive system:Large in/	0.303
MGYA00249313/ERZ652276_FASTA.fasta	MGYA00249313	ERZ652276	root:Host-associated:Human:Digestive system:Large in/	0.0728
MGYA00249314/ERZ652278_FASTA.fasta	MGYA00249314	ERZ652278	root:Host-associated:Human:Digestive system:Large in/	0.358
MGYA00249315/ERZ652280_FASTA.fasta	MGYA00249315	ERZ652280	root:Host-associated:Human:Digestive system:Large in/	0.326
MGYA00249316/ERZ652282_FASTA.fasta	MGYA00249316	ERZ652282	root:Host-associated:Human:Digestive system:Large in/	0.2436
MGYA00249317/ERZ652284_FASTA.fasta	MGYA00249317	ERZ652284	root:Host-associated:Human:Digestive system:Large in/	0.2868
MGYA00249318/ERZ652596_FASTA.fasta	MGYA00249318	ERZ652596	root:Host-associated:Human:Digestive system:Large in/	0.2428
MGYA00249319/ERZ652598_FASTA.fasta	MGYA00249319	ERZ652598	root:Host-associated:Human:Digestive system:Large in/	0.3468
MGYA00249320/ERZ652600_FASTA.fasta	MGYA00249320	ERZ652600	root:Host-associated:Human:Digestive system:Large in/	0.2552
MGYA00249321/ERZ652602_FASTA.fasta	MGYA00249321	ERZ652602	root:Host-associated:Human:Digestive system:Large in/	0.3894
MGYA00249322/ERZ652604_FASTA.fasta	MGYA00249322	ERZ652604	root:Host-associated:Human:Digestive system:Large in/	0.1722
MGYA00249323/ERZ652606_FASTA.fasta	MGYA00249323	ERZ652606	root:Host-associated:Human:Digestive system:Large in/	0.3184
MGYA00249324/ERZ652608_FASTA.fasta	MGYA00249324	ERZ652608	root:Host-associated:Human:Digestive system:Large in/	0.1968
MGYA00249325/ERZ652610_FASTA.fasta	MGYA00249325	ERZ652610	root:Host-associated:Human:Digestive system:Large in/	0.161
MGYA00249326/ERZ652612_FASTA.fasta	MGYA00249326	ERZ652612	root:Host-associated:Human:Digestive system:Large in/	0.4446
MGYA00249327/ERZ652614_FASTA.fasta	MGYA00249327	ERZ652614	root:Host-associated:Human:Digestive system:Large in/	0.2694
MGYA00249328/ERZ652616_FASTA.fasta	MGYA00249328	ERZ652616	root:Host-associated:Human:Digestive system:Large in/	0.259
MGYA00249329/ERZ652618_FASTA.fasta	MGYA00249329	ERZ652618	root:Host-associated:Human:Digestive system:Large in/	0.3274
MGYA00249330/ERZ652620_FASTA.fasta	MGYA00249330	ERZ652620	root:Host-associated:Human:Digestive system:Large in/	0.2924
MGYA00249331/ERZ652624_FASTA.fasta	MGYA00249331	ERZ652624	root:Host-associated:Human:Digestive system:Large in/	0.2052
MGYA00249332/ERZ652626_FASTA.fasta	MGYA00249332	ERZ652626	root:Host-associated:Human:Digestive system:Large in/	0.2906
MGYA00249333/ERZ652628_FASTA.fasta	MGYA00249333	ERZ652628	root:Host-associated:Human:Digestive system:Large in/	0.0678
MGYA00249334/ERZ652630_FASTA.fasta	MGYA00249334	ERZ652630	root:Host-associated:Human:Digestive system:Large in/	0.2998
MGYA00249335/ERZ652632_FASTA.fasta	MGYA00249335	ERZ652632	root:Host-associated:Human:Digestive system:Large in/	0.3436
MGYA00249336/ERZ652634_FASTA.fasta	MGYA00249336	ERZ652634	root:Host-associated:Human:Digestive system:Large in/	0.3026
MGYA00249337/ERZ652636_FASTA.fasta	MGYA00249337	ERZ652636	root:Host-associated:Human:Digestive system:Large in/	0.3044
MGYA00249338/ERZ652638_FASTA.fasta	MGYA00249338	ERZ652638	root:Host-associated:Human:Digestive system:Large in/	0.1708
MGYA00249339/ERZ652640_FASTA.fasta	MGYA00249339	ERZ652640	root:Host-associated:Human:Digestive system:Large in/	0.3314
MGYA00249340/ERZ652642_FASTA.fasta	MGYA00249340	ERZ652642	root:Host-associated:Human:Digestive system:Large in/	0.2332
MGYA00249341/ERZ652644_FASTA.fasta	MGYA00249341	ERZ652644	root:Host-associated:Human:Digestive system:Large in/	0.1518
MGYA00249342/ERZ652646_FASTA.fasta	MGYA00249342	ERZ652646	root:Host-associated:Human:Digestive system:Large in/	0.3606
MGYA00249343/ERZ652648_FASTA.fasta	MGYA00249343	ERZ652648	root:Host-associated:Human:Digestive system:Large in/	0.2886
MGYA00249344/ERZ652650_FASTA.fasta	MGYA00249344	ERZ652650	root:Host-associated:Human:Digestive system:Large in/	0.2618
MGYA00249345/ERZ652652_FASTA.fasta	MGYA00249345	ERZ652652	root:Host-associated:Human:Digestive system:Large in/	0.3452
MGYA00249346/ERZ652654_FASTA.fasta	MGYA00249346	ERZ652654	root:Host-associated:Human:Digestive system:Large in/	0.2222
MGYA00249347/ERZ652227_FASTA.fasta	MGYA00249347	ERZ652227	root:Host-associated:Human:Digestive system:Large in/	0.1904
MGYA00249348/ERZ652229_FASTA.fasta	MGYA00249348	ERZ652229	root:Host-associated:Human:Digestive system:Large in/	0.2708
MGYA00249349/ERZ652231_FASTA.fasta	MGYA00249349	ERZ652231	root:Host-associated:Human:Digestive system:Large in/	0.2832
MGYA00249350/ERZ652233_FASTA.fasta	MGYA00249350	ERZ652233	root:Host-associated:Human:Digestive system:Large in/	0.27
MGYA00249351/ERZ652235_FASTA.fasta	MGYA00249351	ERZ652235	root:Host-associated:Human:Digestive system:Large in/	0.4266
MGYA00249352/ERZ652237_FASTA.fasta	MGYA00249352	ERZ652237	root:Host-associated:Human:Digestive system:Large in/	0.5108
MGYA00249353/ERZ652239_FASTA.fasta	MGYA00249353	ERZ652239	root:Host-associated:Human:Digestive system:Large in/	0.3966
MGYA00249354/ERZ652241_FASTA.fasta	MGYA00249354	ERZ652241	root:Host-associated:Human:Digestive system:Large in/	0.3468
MGYA00249355/ERZ652243_FASTA.fasta	MGYA00249355	ERZ652243	root:Host-associated:Human:Digestive system:Large in/	0.464
MGYA00249356/ERZ652245_FASTA.fasta	MGYA00249356	ERZ652245	root:Host-associated:Human:Digestive system:Large in/	0.2976
MGYA00249357/ERZ652247_FASTA.fasta	MGYA00249357	ERZ652247	root:Host-associated:Human:Digestive system:Large in/	0.1862
MGYA00249358/ERZ652249_FASTA.fasta	MGYA00249358	ERZ652249	root:Host-associated:Human:Digestive system:Large in/	0.3076
MGYA00249359/ERZ652251_FASTA.fasta	MGYA00249359	ERZ652251	root:Host-associated:Human:Digestive system:Large in/	0.2338
MGYA00249360/ERZ652253_FASTA.fasta	MGYA00249360	ERZ652253	root:Host-associated:Human:Digestive system:Large in/	0.3248
MGYA00249361/ERZ652255_FASTA.fasta	MGYA00249361	ERZ652255	root:Host-associated:Human:Digestive system:Large in/	0.2916
MGYA00249362/ERZ652257_FASTA.fasta	MGYA00249362	ERZ652257	root:Host-associated:Human:Digestive system:Large in/	0.1564
MGYA00249363/ERZ652259_FASTA.fasta	MGYA00249363	ERZ652259	root:Host-associated:Human:Digestive system:Large in/	0.3098
MGYA00249364/ERZ652261_FASTA.fasta	MGYA00249364	ERZ652261	root:Host-associated:Human:Digestive system:Large in/	0.4266
MGYA00249365/ERZ652263_FASTA.fasta	MGYA00249365	ERZ652263	root:Host-associated:Human:Digestive system:Large in/	0.323
MGYA00249366/ERZ652265_FASTA.fasta	MGYA00249366	ERZ652265	root:Host-associated:Human:Digestive system:Large in/	0.3214
MGYA00249367/ERZ652697_FASTA.fasta	MGYA00249367	ERZ652697	root:Host-associated:Human:Digestive system:Large in/	0.2558
MGYA00249368/ERZ652699_FASTA.fasta	MGYA00249368	ERZ652699	root:Host-associated:Human:Digestive system:Large in/	0.2452
MGYA00249369/ERZ652701_FASTA.fasta	MGYA00249369	ERZ652701	root:Host-associated:Human:Digestive system:Large in/	0.3434
MGYA00249370/ERZ652605_FASTA.fasta	MGYA00249370	ERZ652605	root:Host-associated:Human:Digestive system:Large in/	0.2904
MGYA00249371/ERZ652607_FASTA.fasta	MGYA00249371	ERZ652607	root:Host-associated:Human:Digestive system:Large in/	0.269
MGYA00249372/ERZ652609_FASTA.fasta	MGYA00249372	ERZ652609	root:Host-associated:Human:Digestive system:Large in/	0.3256
MGYA00249373/ERZ652611_FASTA.fasta	MGYA00249373	ERZ652611	root:Host-associated:Human:Digestive system:Large in/	0.2554
MGYA00249374/ERZ652613_FASTA.fasta	MGYA00249374	ERZ652613	root:Host-associated:Human:Digestive system:Large in/	0.2382
MGYA00249375/ERZ652615_FASTA.fasta	MGYA00249375	ERZ652615	root:Host-associated:Human:Digestive system:Large in/	0.4552
MGYA00249376/ERZ652617_FASTA.fasta	MGYA00249376	ERZ652617	root:Host-associated:Human:Digestive system:Large in/	0.3474
MGYA00249377/ERZ652619_FASTA.fasta	MGYA00249377	ERZ652619	root:Host-associated:Human:Digestive system:Large in/	0.2672
MGYA00249378/ERZ652621_FASTA.fasta	MGYA00249378	ERZ652621	root:Host-associated:Human:Digestive system:Large in/	0.2322
MGYA00249379/ERZ652623_FASTA.fasta	MGYA00249379	ERZ652623	root:Host-associated:Human:Digestive system:Large in/	0.1704
MGYA00249380/ERZ652625_FASTA.fasta	MGYA00249380	ERZ652625	root:Host-associated:Human:Digestive system:Large in/	0.4178
MGYA00249381/ERZ652627_FASTA.fasta	MGYA00249381	ERZ652627	root:Host-associated:Human:Digestive system:Large in/	0.1286
MGYA00249382/ERZ652629_FASTA.fasta	MGYA00249382	ERZ652629	root:Host-associated:Human:Digestive system:Large in/	0.0998
MGYA00249383/ERZ652631_FASTA.fasta	MGYA00249383	ERZ652631	root:Host-associated:Human:Digestive system:Large in/	0.2754
MGYA00249384/ERZ652633_FASTA.fasta	MGYA00249384	ERZ652633	root:Host-associated:Human:Digestive system:Large in/	0.2552
MGYA00249385/ERZ652635_FASTA.fasta	MGYA00249385	ERZ652635	root:Host-associated:Human:Digestive system:Large in/	0.2114
MGYA00249386/ERZ652637_FASTA.fasta	MGYA00249386	ERZ652637	root:Host-associated:Human:Digestive system:Large in/	0.4008
MGYA00249387/ERZ652141_FASTA.fasta	MGYA00249387	ERZ652141	root:Host-associated:Human:Digestive system:Large in/	0.3632
MGYA00249388/ERZ652143_FASTA.fasta	MGYA00249388	ERZ652143	root:Host-associated:Human:Digestive system:Large in/	0.3616
MGYA00249389/ERZ652145_FASTA.fasta	MGYA00249389	ERZ652145	root:Host-associated:Human:Digestive system:Large in/	0.3552
MGYA00249390/ERZ652147_FASTA.fasta	MGYA00249390	ERZ652147	root:Host-associated:Human:Digestive system:Large in/	0.1186
MGYA00249391/ERZ652149_FASTA.fasta	MGYA00249391	ERZ652149	root:Host-associated:Human:Digestive system:Large in/	0.3984
MGYA00249392/ERZ652155_FASTA.fasta	MGYA00249392	ERZ652155	root:Host-associated:Human:Digestive system:Large in/	0.412
MGYA00249393/ERZ652157_FASTA.fasta	MGYA00249393	ERZ652157	root:Host-associated:Human:Digestive system:Large in/	0.1774
MGYA00249394/ERZ652656_FASTA.fasta	MGYA00249394	ERZ652656	root:Host-associated:Human:Digestive system:Large in/	0.3108
MGYA00249395/ERZ652658_FASTA.fasta	MGYA00249395	ERZ652658	root:Host-associated:Human:Digestive system:Large in/	0.0934
MGYA00249396/ERZ652660_FASTA.fasta	MGYA00249396	ERZ652660	root:Host-associated:Human:Digestive system:Large in/	0.2462
MGYA00249397/ERZ652662_FASTA.fasta	MGYA00249397	ERZ652662	root:Host-associated:Human:Digestive system:Large in/	0.3092
MGYA00249398/ERZ652664_FASTA.fasta	MGYA00249398	ERZ652664	root:Host-associated:Human:Digestive system:Large in/	0.2316
MGYA00249399/ERZ652666_FASTA.fasta	MGYA00249399	ERZ652666	root:Host-associated:Human:Digestive system:Large in/	0.3974
MGYA00249400/ERZ652668_FASTA.fasta	MGYA00249400	ERZ652668	root:Host-associated:Human:Digestive system:Large in/	0.39
MGYA00249401/ERZ652672_FASTA.fasta	MGYA00249401	ERZ652672	root:Host-associated:Human:Digestive system:Large in/	0.4078
MGYA00249402/ERZ652674_FASTA.fasta	MGYA00249402	ERZ652674	root:Host-associated:Human:Digestive system:Large in/	0.3202
MGYA00249403/ERZ652267_FASTA.fasta	MGYA00249403	ERZ652267	root:Host-associated:Human:Digestive system:Large in/	0.2464
MGYA00249404/ERZ652269_FASTA.fasta	MGYA00249404	ERZ652269	root:Host-associated:Human:Digestive system:Large in/	0.283
MGYA00249405/ERZ652271_FASTA.fasta	MGYA00249405	ERZ652271	root:Host-associated:Human:Digestive system:Large in/	0.2568
MGYA00249406/ERZ652273_FASTA.fasta	MGYA00249406	ERZ652273	root:Host-associated:Human:Digestive system:Large in/	0.2982
MGYA00249407/ERZ652275_FASTA.fasta	MGYA00249407	ERZ652275	root:Host-associated:Human:Digestive system:Large in/	0.2494
MGYA00249408/ERZ652277_FASTA.fasta	MGYA00249408	ERZ652277	root:Host-associated:Human:Digestive system:Large in/	0.272
MGYA00249409/ERZ652279_FASTA.fasta	MGYA00249409	ERZ652279	root:Host-associated:Human:Digestive system:Large in/	0.1456
MGYA00249410/ERZ652281_FASTA.fasta	MGYA00249410	ERZ652281	root:Host-associated:Human:Digestive system:Large in/	0.2532
MGYA00249411/ERZ652283_FASTA.fasta	MGYA00249411	ERZ652283	root:Host-associated:Human:Digestive system:Large in/	0.3348
MGYA00249412/ERZ652285_FASTA.fasta	MGYA00249412	ERZ652285	root:Host-associated:Human:Digestive system:Large in/	0.1614
MGYA00249413/ERZ652374_FASTA.fasta	MGYA00249413	ERZ652374	root:Host-associated:Human:Digestive system:Large in/	0.3758
MGYA00249414/ERZ652376_FASTA.fasta	MGYA00249414	ERZ652376	root:Host-associated:Human:Digestive system:Large in/	0.3304
MGYA00249415/ERZ652378_FASTA.fasta	MGYA00249415	ERZ652378	root:Host-associated:Human:Digestive system:Large in/	0.1806
MGYA00249416/ERZ652380_FASTA.fasta	MGYA00249416	ERZ652380	root:Host-associated:Human:Digestive system:Large in/	0.1992
MGYA00249417/ERZ651939_FASTA.fasta	MGYA00249417	ERZ651939	root:Host-associated:Human:Digestive system:Large in/	0.142
MGYA00249418/ERZ651943_FASTA.fasta	MGYA00249418	ERZ651943	root:Host-associated:Human:Digestive system:Large in/	0.2318
MGYA00249419/ERZ651945_FASTA.fasta	MGYA00249419	ERZ651945	root:Host-associated:Human:Digestive system:Large in/	0.232
MGYA00249420/ERZ651947_FASTA.fasta	MGYA00249420	ERZ651947	root:Host-associated:Human:Digestive system:Large in/	0.11
MGYA00249421/ERZ651949_FASTA.fasta	MGYA00249421	ERZ651949	root:Host-associated:Human:Digestive system:Large in/	0.3256
MGYA00249422/ERZ651951_FASTA.fasta	MGYA00249422	ERZ651951	root:Host-associated:Human:Digestive system:Large in/	0.2548
MGYA00249423/ERZ651953_FASTA.fasta	MGYA00249423	ERZ651953	root:Host-associated:Human:Digestive system:Large in/	0.2406
MGYA00249424/ERZ651955_FASTA.fasta	MGYA00249424	ERZ651955	root:Host-associated:Human:Digestive system:Large in/	0.2886
MGYA00249425/ERZ651957_FASTA.fasta	MGYA00249425	ERZ651957	root:Host-associated:Human:Digestive system:Large in/	0.2204
MGYA00249426/ERZ652159_FASTA.fasta	MGYA00249426	ERZ652159	root:Host-associated:Human:Digestive system:Large in/	0.291
MGYA00249427/ERZ652163_FASTA.fasta	MGYA00249427	ERZ652163	root:Host-associated:Human:Digestive system:Large in/	0.1672
MGYA00249428/ERZ652173_FASTA.fasta	MGYA00249428	ERZ652173	root:Host-associated:Human:Digestive system:Large in/	0.3562
MGYA00249429/ERZ652175_FASTA.fasta	MGYA00249429	ERZ652175	root:Host-associated:Human:Digestive system:Large in/	0.1706
MGYA00249430/ERZ652177_FASTA.fasta	MGYA00249430	ERZ652177	root:Host-associated:Human:Digestive system:Large in/	0.3082
MGYA00249431/ERZ652179_FASTA.fasta	MGYA00249431	ERZ652179	root:Host-associated:Human:Digestive system:Large in/	0.2498
MGYA00249432/ERZ652181_FASTA.fasta	MGYA00249432	ERZ652181	root:Host-associated:Human:Digestive system:Large in/	0.3692
MGYA00249433/ERZ652183_FASTA.fasta	MGYA00249433	ERZ652183	root:Host-associated:Human:Digestive system:Large in/	0.2388
MGYA00249434/ERZ652185_FASTA.fasta	MGYA00249434	ERZ652185	root:Host-associated:Human:Digestive system:Large in/	0.29
MGYA00249435/ERZ652187_FASTA.fasta	MGYA00249435	ERZ652187	root:Host-associated:Human:Digestive system:Large in/	0.3484
MGYA00249436/ERZ652189_FASTA.fasta	MGYA00249436	ERZ652189	root:Host-associated:Human:Digestive system:Large in/	0.3692
MGYA00249437/ERZ652191_FASTA.fasta	MGYA00249437	ERZ652191	root:Host-associated:Human:Digestive system:Large in/	0.3816
MGYA00249438/ERZ652193_FASTA.fasta	MGYA00249438	ERZ652193	root:Host-associated:Human:Digestive system:Large in/	0.3332
MGYA00249439/ERZ652195_FASTA.fasta	MGYA00249439	ERZ652195	root:Host-associated:Human:Digestive system:Large in/	0.2622
MGYA00249440/ERZ652197_FASTA.fasta	MGYA00249440	ERZ652197	root:Host-associated:Human:Digestive system:Large in/	0.3756
MGYA00249441/ERZ652382_FASTA.fasta	MGYA00249441	ERZ652382	root:Host-associated:Human:Digestive system:Large in/	0.3454
MGYA00249442/ERZ652384_FASTA.fasta	MGYA00249442	ERZ652384	root:Host-associated:Human:Digestive system:Large in/	0.2988
MGYA00249443/ERZ652386_FASTA.fasta	MGYA00249443	ERZ652386	root:Host-associated:Human:Digestive system:Large in/	0.219
MGYA00249444/ERZ652388_FASTA.fasta	MGYA00249444	ERZ652388	root:Host-associated:Human:Digestive system:Large in/	0.3156
MGYA00249445/ERZ652390_FASTA.fasta	MGYA00249445	ERZ652390	root:Host-associated:Human:Digestive system:Large in/	0.3232
MGYA00249446/ERZ652392_FASTA.fasta	MGYA00249446	ERZ652392	root:Host-associated:Human:Digestive system:Large in/	0.206
MGYA00249447/ERZ652394_FASTA.fasta	MGYA00249447	ERZ652394	root:Host-associated:Human:Digestive system:Large in/	0.2004
MGYA00249448/ERZ652396_FASTA.fasta	MGYA00249448	ERZ652396	root:Host-associated:Human:Digestive system:Large in/	0.1702
MGYA00249449/ERZ652398_FASTA.fasta	MGYA00249449	ERZ652398	root:Host-associated:Human:Digestive system:Large in/	0.0768
MGYA00249450/ERZ652400_FASTA.fasta	MGYA00249450	ERZ652400	root:Host-associated:Human:Digestive system:Large in/	0.2348
MGYA00249451/ERZ652402_FASTA.fasta	MGYA00249451	ERZ652402	root:Host-associated:Human:Digestive system:Large in/	0.311
MGYA00249452/ERZ652404_FASTA.fasta	MGYA00249452	ERZ652404	root:Host-associated:Human:Digestive system:Large in/	0.3152
MGYA00249453/ERZ652406_FASTA.fasta	MGYA00249453	ERZ652406	root:Host-associated:Human:Digestive system:Large in/	0.2306
MGYA00249454/ERZ652408_FASTA.fasta	MGYA00249454	ERZ652408	root:Host-associated:Human:Digestive system:Large in/	0.3352
MGYA00249455/ERZ652410_FASTA.fasta	MGYA00249455	ERZ652410	root:Host-associated:Human:Digestive system:Large in/	0.3316
MGYA00249456/ERZ652412_FASTA.fasta	MGYA00249456	ERZ652412	root:Host-associated:Human:Digestive system:Large in/	0.347
MGYA00249457/ERZ652414_FASTA.fasta	MGYA00249457	ERZ652414	root:Host-associated:Human:Digestive system:Large in/	0.4004
MGYA00249458/ERZ652416_FASTA.fasta	MGYA00249458	ERZ652416	root:Host-associated:Human:Digestive system:Large in/	0.255
MGYA00249459/ERZ651959_FASTA.fasta	MGYA00249459	ERZ651959	root:Host-associated:Human:Digestive system:Large in/	0.2486
MGYA00249460/ERZ651961_FASTA.fasta	MGYA00249460	ERZ651961	root:Host-associated:Human:Digestive system:Large in/	0.3
MGYA00249461/ERZ651963_FASTA.fasta	MGYA00249461	ERZ651963	root:Host-associated:Human:Digestive system:Large in/	0.3686
MGYA00249462/ERZ651965_FASTA.fasta	MGYA00249462	ERZ651965	root:Host-associated:Human:Digestive system:Large in/	0.2814
MGYA00249463/ERZ651967_FASTA.fasta	MGYA00249463	ERZ651967	root:Host-associated:Human:Digestive system:Large in/	0.2374
MGYA00249464/ERZ651969_FASTA.fasta	MGYA00249464	ERZ651969	root:Host-associated:Human:Digestive system:Large in/	0.4034
MGYA00249465/ERZ651971_FASTA.fasta	MGYA00249465	ERZ651971	root:Host-associated:Human:Digestive system:Large in/	0.3692
MGYA00249466/ERZ651973_FASTA.fasta	MGYA00249466	ERZ651973	root:Host-associated:Human:Digestive system:Large in/	0.484
MGYA00249467/ERZ651975_FASTA.fasta	MGYA00249467	ERZ651975	root:Host-associated:Human:Digestive system:Large in/	0.2226
MGYA00249468/ERZ651977_FASTA.fasta	MGYA00249468	ERZ651977	root:Host-associated:Human:Digestive system:Large in/	0.2852
MGYA00249469/ERZ651979_FASTA.fasta	MGYA00249469	ERZ651979	root:Host-associated:Human:Digestive system:Large in/	0.2678
MGYA00249470/ERZ651981_FASTA.fasta	MGYA00249470	ERZ651981	root:Host-associated:Human:Digestive system:Large in/	0.3962
MGYA00249471/ERZ651983_FASTA.fasta	MGYA00249471	ERZ651983	root:Host-associated:Human:Digestive system:Large in/	0.385
MGYA00249472/ERZ651985_FASTA.fasta	MGYA00249472	ERZ651985	root:Host-associated:Human:Digestive system:Large in/	0.2738
MGYA00249473/ERZ652639_FASTA.fasta	MGYA00249473	ERZ652639	root:Host-associated:Human:Digestive system:Large in/	0.2452
MGYA00249474/ERZ652199_FASTA.fasta	MGYA00249474	ERZ652199	root:Host-associated:Human:Digestive system:Large in/	0.5018
MGYA00249475/ERZ652201_FASTA.fasta	MGYA00249475	ERZ652201	root:Host-associated:Human:Digestive system:Large in/	0.2042
MGYA00249476/ERZ652203_FASTA.fasta	MGYA00249476	ERZ652203	root:Host-associated:Human:Digestive system:Large in/	0.3836
MGYA00249477/ERZ652205_FASTA.fasta	MGYA00249477	ERZ652205	root:Host-associated:Human:Digestive system:Large in/	0.2998
MGYA00249478/ERZ652207_FASTA.fasta	MGYA00249478	ERZ652207	root:Host-associated:Human:Digestive system:Large in/	0.2814
MGYA00249479/ERZ652209_FASTA.fasta	MGYA00249479	ERZ652209	root:Host-associated:Human:Digestive system:Large in/	0.216
MGYA00249480/ERZ652211_FASTA.fasta	MGYA00249480	ERZ652211	root:Host-associated:Human:Digestive system:Large in/	0.2718
MGYA00249481/ERZ652213_FASTA.fasta	MGYA00249481	ERZ652213	root:Host-associated:Human:Digestive system:Large in/	0.2388
MGYA00249482/ERZ652214_FASTA.fasta	MGYA00249482	ERZ652214	root:Host-associated:Human:Digestive system:Large in/	0.268
MGYA00249483/ERZ652215_FASTA.fasta	MGYA00249483	ERZ652215	root:Host-associated:Human:Digestive system:Large in/	0.1064
MGYA00249484/ERZ652217_FASTA.fasta	MGYA00249484	ERZ652217	root:Host-associated:Human:Digestive system:Large in/	0.3216
MGYA00249485/ERZ652219_FASTA.fasta	MGYA00249485	ERZ652219	root:Host-associated:Human:Digestive system:Large in/	0.3018
MGYA00249486/ERZ652221_FASTA.fasta	MGYA00249486	ERZ652221	root:Host-associated:Human:Digestive system:Large in/	0.2874
MGYA00249487/ERZ652225_FASTA.fasta	MGYA00249487	ERZ652225	root:Host-associated:Human:Digestive system:Large in/	0.2016
MGYA00249488/ERZ652503_FASTA.fasta	MGYA00249488	ERZ652503	root:Host-associated:Human:Digestive system:Large in/	0.4686
MGYA00249489/ERZ652505_FASTA.fasta	MGYA00249489	ERZ652505	root:Host-associated:Human:Digestive system:Large in/	0.2448
MGYA00249490/ERZ652507_FASTA.fasta	MGYA00249490	ERZ652507	root:Host-associated:Human:Digestive system:Large in/	0.2046
MGYA00249491/ERZ652509_FASTA.fasta	MGYA00249491	ERZ652509	root:Host-associated:Human:Digestive system:Large in/	0.4172
MGYA00249492/ERZ652511_FASTA.fasta	MGYA00249492	ERZ652511	root:Host-associated:Human:Digestive system:Large in/	0.3258
MGYA00249493/ERZ652418_FASTA.fasta	MGYA00249493	ERZ652418	root:Host-associated:Human:Digestive system:Large in/	0.3522
MGYA00249494/ERZ652420_FASTA.fasta	MGYA00249494	ERZ652420	root:Host-associated:Human:Digestive system:Large in/	0.3036
MGYA00249495/ERZ652422_FASTA.fasta	MGYA00249495	ERZ652422	root:Host-associated:Human:Digestive system:Large in/	0.3544
MGYA00249496/ERZ652424_FASTA.fasta	MGYA00249496	ERZ652424	root:Host-associated:Human:Digestive system:Large in/	0.3224
MGYA00249497/ERZ652426_FASTA.fasta	MGYA00249497	ERZ652426	root:Host-associated:Human:Digestive system:Large in/	0.2352
MGYA00249498/ERZ652428_FASTA.fasta	MGYA00249498	ERZ652428	root:Host-associated:Human:Digestive system:Large in/	0.341
MGYA00249499/ERZ652430_FASTA.fasta	MGYA00249499	ERZ652430	root:Host-associated:Human:Digestive system:Large in/	0.356
MGYA00249500/ERZ652432_FASTA.fasta	MGYA00249500	ERZ652432	root:Host-associated:Human:Digestive system:Large in/	0.4516
MGYA00249501/ERZ652434_FASTA.fasta	MGYA00249501	ERZ652434	root:Host-associated:Human:Digestive system:Large in/	0.2382
MGYA00249502/ERZ652436_FASTA.fasta	MGYA00249502	ERZ652436	root:Host-associated:Human:Digestive system:Large in/	0.3252
MGYA00249503/ERZ652438_FASTA.fasta	MGYA00249503	ERZ652438	root:Host-associated:Human:Digestive system:Large in/	0.3004
MGYA00249504/ERZ652441_FASTA.fasta	MGYA00249504	ERZ652441	root:Host-associated:Human:Digestive system:Large in/	0.3328
MGYA00249505/ERZ652442_FASTA.fasta	MGYA00249505	ERZ652442	root:Host-associated:Human:Digestive system:Large in/	0.2944
MGYA00249506/ERZ652444_FASTA.fasta	MGYA00249506	ERZ652444	root:Host-associated:Human:Digestive system:Large in/	0.0604
MGYA00249507/ERZ652446_FASTA.fasta	MGYA00249507	ERZ652446	root:Host-associated:Human:Digestive system:Large in/	0.332
MGYA00249508/ERZ652448_FASTA.fasta	MGYA00249508	ERZ652448	root:Host-associated:Human:Digestive system:Large in/	0.3082
MGYA00249509/ERZ652450_FASTA.fasta	MGYA00249509	ERZ652450	root:Host-associated:Human:Digestive system:Large in/	0.2428
MGYA00249510/ERZ652641_FASTA.fasta	MGYA00249510	ERZ652641	root:Host-associated:Human:Digestive system:Large in/	0.364
MGYA00249511/ERZ652643_FASTA.fasta	MGYA00249511	ERZ652643	root:Host-associated:Human:Digestive system:Large in/	0.3124
MGYA00249512/ERZ652645_FASTA.fasta	MGYA00249512	ERZ652645	root:Host-associated:Human:Digestive system:Large in/	0.3636
MGYA00249513/ERZ652647_FASTA.fasta	MGYA00249513	ERZ652647	root:Host-associated:Human:Digestive system:Large in/	0.1928
MGYA00249514/ERZ652649_FASTA.fasta	MGYA00249514	ERZ652649	root:Host-associated:Human:Digestive system:Large in/	0.2712
MGYA00249515/ERZ652651_FASTA.fasta	MGYA00249515	ERZ652651	root:Host-associated:Human:Digestive system:Large in/	0.1458
MGYA00249516/ERZ652653_FASTA.fasta	MGYA00249516	ERZ652653	root:Host-associated:Human:Digestive system:Large in/	0.2804
MGYA00249517/ERZ652655_FASTA.fasta	MGYA00249517	ERZ652655	root:Host-associated:Human:Digestive system:Large in/	0.2592
MGYA00249518/ERZ652657_FASTA.fasta	MGYA00249518	ERZ652657	root:Host-associated:Human:Digestive system:Large in/	0.3314
MGYA00249519/ERZ652659_FASTA.fasta	MGYA00249519	ERZ652659	root:Host-associated:Human:Digestive system:Large in/	0.375
MGYA00249520/ERZ652661_FASTA.fasta	MGYA00249520	ERZ652661	root:Host-associated:Human:Digestive system:Large in/	0.3906
MGYA00249521/ERZ652663_FASTA.fasta	MGYA00249521	ERZ652663	root:Host-associated:Human:Digestive system:Large in/	0.3244
MGYA00249522/ERZ652665_FASTA.fasta	MGYA00249522	ERZ652665	root:Host-associated:Human:Digestive system:Large in/	0.2748
MGYA00249523/ERZ652667_FASTA.fasta	MGYA00249523	ERZ652667	root:Host-associated:Human:Digestive system:Large in/	0.253
MGYA00249524/ERZ652669_FASTA.fasta	MGYA00249524	ERZ652669	root:Host-associated:Human:Digestive system:Large in/	0.2444
MGYA00249525/ERZ652671_FASTA.fasta	MGYA00249525	ERZ652671	root:Host-associated:Human:Digestive system:Large in/	0.3378
MGYA00249526/ERZ652673_FASTA.fasta	MGYA00249526	ERZ652673	root:Host-associated:Human:Digestive system:Large in/	0.2328
MGYA00249527/ERZ652513_FASTA.fasta	MGYA00249527	ERZ652513	root:Host-associated:Human:Digestive system:Large in/	0.3202
MGYA00249528/ERZ652515_FASTA.fasta	MGYA00249528	ERZ652515	root:Host-associated:Human:Digestive system:Large in/	0.3
MGYA00249529/ERZ652517_FASTA.fasta	MGYA00249529	ERZ652517	root:Host-associated:Human:Digestive system:Large in/	0.292
MGYA00249530/ERZ652519_FASTA.fasta	MGYA00249530	ERZ652519	root:Host-associated:Human:Digestive system:Large in/	0.0522
MGYA00249531/ERZ652521_FASTA.fasta	MGYA00249531	ERZ652521	root:Host-associated:Human:Digestive system:Large in/	0.347
MGYA00249532/ERZ652523_FASTA.fasta	MGYA00249532	ERZ652523	root:Host-associated:Human:Digestive system:Large in/	0.2802
MGYA00249533/ERZ652525_FASTA.fasta	MGYA00249533	ERZ652525	root:Host-associated:Human:Digestive system:Large in/	0.2868
MGYA00249534/ERZ652527_FASTA.fasta	MGYA00249534	ERZ652527	root:Host-associated:Human:Digestive system:Large in/	0.337
MGYA00249535/ERZ652529_FASTA.fasta	MGYA00249535	ERZ652529	root:Host-associated:Human:Digestive system:Large in/	0.2718
MGYA00249536/ERZ652531_FASTA.fasta	MGYA00249536	ERZ652531	root:Host-associated:Human:Digestive system:Large in/	0.3352
MGYA00249537/ERZ652533_FASTA.fasta	MGYA00249537	ERZ652533	root:Host-associated:Human:Digestive system:Large in/	0.2126
MGYA00249538/ERZ652535_FASTA.fasta	MGYA00249538	ERZ652535	root:Host-associated:Human:Digestive system:Large in/	0.3584
MGYA00249539/ERZ652537_FASTA.fasta	MGYA00249539	ERZ652537	root:Host-associated:Human:Digestive system:Large in/	0.357
MGYA00249540/ERZ652539_FASTA.fasta	MGYA00249540	ERZ652539	root:Host-associated:Human:Digestive system:Large in/	0.2244
MGYA00249541/ERZ652541_FASTA.fasta	MGYA00249541	ERZ652541	root:Host-associated:Human:Digestive system:Large in/	0.2336
MGYA00249542/ERZ652543_FASTA.fasta	MGYA00249542	ERZ652543	root:Host-associated:Human:Digestive system:Large in/	0.2352
MGYA00249543/ERZ652545_FASTA.fasta	MGYA00249543	ERZ652545	root:Host-associated:Human:Digestive system:Large in/	0.2874
MGYA00249544/ERZ652547_FASTA.fasta	MGYA00249544	ERZ652547	root:Host-associated:Human:Digestive system:Large in/	0.3612
MGYA00249545/ERZ652549_FASTA.fasta	MGYA00249545	ERZ652549	root:Host-associated:Human:Digestive system:Large in/	0.123
MGYA00249546/ERZ652551_FASTA.fasta	MGYA00249546	ERZ652551	root:Host-associated:Human:Digestive system:Large in/	0.3372
MGYA00249547/ERZ652452_FASTA.fasta	MGYA00249547	ERZ652452	root:Host-associated:Human:Digestive system:Large in/	0.3602
MGYA00249548/ERZ652454_FASTA.fasta	MGYA00249548	ERZ652454	root:Host-associated:Human:Digestive system:Large in/	0.2438
MGYA00249549/ERZ652456_FASTA.fasta	MGYA00249549	ERZ652456	root:Host-associated:Human:Digestive system:Large in/	0.3498
MGYA00249550/ERZ652458_FASTA.fasta	MGYA00249550	ERZ652458	root:Host-associated:Human:Digestive system:Large in/	0.2376
MGYA00249551/ERZ652460_FASTA.fasta	MGYA00249551	ERZ652460	root:Host-associated:Human:Digestive system:Large in/	0.2706
MGYA00249552/ERZ652462_FASTA.fasta	MGYA00249552	ERZ652462	root:Host-associated:Human:Digestive system:Large in/	0.1968
MGYA00249553/ERZ652464_FASTA.fasta	MGYA00249553	ERZ652464	root:Host-associated:Human:Digestive system:Large in/	0.2848
MGYA00249554/ERZ652466_FASTA.fasta	MGYA00249554	ERZ652466	root:Host-associated:Human:Digestive system:Large in/	0.327
MGYA00249555/ERZ652468_FASTA.fasta	MGYA00249555	ERZ652468	root:Host-associated:Human:Digestive system:Large in/	0.2812
MGYA00249556/ERZ652470_FASTA.fasta	MGYA00249556	ERZ652470	root:Host-associated:Human:Digestive system:Large in/	0.2656
MGYA00249557/ERZ652472_FASTA.fasta	MGYA00249557	ERZ652472	root:Host-associated:Human:Digestive system:Large in/	0.1734
MGYA00249558/ERZ652474_FASTA.fasta	MGYA00249558	ERZ652474	root:Host-associated:Human:Digestive system:Large in/	0.3308
MGYA00249559/ERZ652476_FASTA.fasta	MGYA00249559	ERZ652476	root:Host-associated:Human:Digestive system:Large in/	0.246
MGYA00249560/ERZ652478_FASTA.fasta	MGYA00249560	ERZ652478	root:Host-associated:Human:Digestive system:Large in/	0.3206
MGYA00249561/ERZ652480_FASTA.fasta	MGYA00249561	ERZ652480	root:Host-associated:Human:Digestive system:Large in/	0.2068
MGYA00249562/ERZ652482_FASTA.fasta	MGYA00249562	ERZ652482	root:Host-associated:Human:Digestive system:Large in/	0.2546
MGYA00249563/ERZ652484_FASTA.fasta	MGYA00249563	ERZ652484	root:Host-associated:Human:Digestive system:Large in/	0.2934
MGYA00249564/ERZ652486_FASTA.fasta	MGYA00249564	ERZ652486	root:Host-associated:Human:Digestive system:Large in/	0.3392
MGYA00249565/ERZ652488_FASTA.fasta	MGYA00249565	ERZ652488	root:Host-associated:Human:Digestive system:Large in/	0.2526
MGYA00249566/ERZ652490_FASTA.fasta	MGYA00249566	ERZ652490	root:Host-associated:Human:Digestive system:Large in/	0.089
MGYA00249567/ERZ652677_FASTA.fasta	MGYA00249567	ERZ652677	root:Host-associated:Human:Digestive system:Large in/	0.2942
MGYA00249568/ERZ652679_FASTA.fasta	MGYA00249568	ERZ652679	root:Host-associated:Human:Digestive system:Large in/	0.2704
MGYA00249569/ERZ652681_FASTA.fasta	MGYA00249569	ERZ652681	root:Host-associated:Human:Digestive system:Large in/	0.2086
MGYA00249570/ERZ652683_FASTA.fasta	MGYA00249570	ERZ652683	root:Host-associated:Human:Digestive system:Large in/	0.134
MGYA00249571/ERZ652685_FASTA.fasta	MGYA00249571	ERZ652685	root:Host-associated:Human:Digestive system:Large in/	0.3096
MGYA00249572/ERZ652687_FASTA.fasta	MGYA00249572	ERZ652687	root:Host-associated:Human:Digestive system:Large in/	0.3628
MGYA00249573/ERZ652689_FASTA.fasta	MGYA00249573	ERZ652689	root:Host-associated:Human:Digestive system:Large in/	0.2286
MGYA00249574/ERZ652691_FASTA.fasta	MGYA00249574	ERZ652691	root:Host-associated:Human:Digestive system:Large in/	0.3986
MGYA00249575/ERZ652693_FASTA.fasta	MGYA00249575	ERZ652693	root:Host-associated:Human:Digestive system:Large in/	0.225
MGYA00249576/ERZ652695_FASTA.fasta	MGYA00249576	ERZ652695	root:Host-associated:Human:Digestive system:Large in/	0.2944
MGYA00249577/ERZ652553_FASTA.fasta	MGYA00249577	ERZ652553	root:Host-associated:Human:Digestive system:Large in/	0.3256
MGYA00249578/ERZ652555_FASTA.fasta	MGYA00249578	ERZ652555	root:Host-associated:Human:Digestive system:Large in/	0.1526
MGYA00249579/ERZ652557_FASTA.fasta	MGYA00249579	ERZ652557	root:Host-associated:Human:Digestive system:Large in/	0.234
MGYA00249580/ERZ652559_FASTA.fasta	MGYA00249580	ERZ652559	root:Host-associated:Human:Digestive system:Large in/	0.199
MGYA00249581/ERZ652561_FASTA.fasta	MGYA00249581	ERZ652561	root:Host-associated:Human:Digestive system:Large in/	0.321
MGYA00249582/ERZ652563_FASTA.fasta	MGYA00249582	ERZ652563	root:Host-associated:Human:Digestive system:Large in/	0.2568
MGYA00249583/ERZ652565_FASTA.fasta	MGYA00249583	ERZ652565	root:Host-associated:Human:Digestive system:Large in/	0.078
MGYA00249584/ERZ652567_FASTA.fasta	MGYA00249584	ERZ652567	root:Host-associated:Human:Digestive system:Large in/	0.2678
MGYA00249585/ERZ652569_FASTA.fasta	MGYA00249585	ERZ652569	root:Host-associated:Human:Digestive system:Large in/	0.3296
MGYA00249586/ERZ652571_FASTA.fasta	MGYA00249586	ERZ652571	root:Host-associated:Human:Digestive system:Large in/	0.3124
MGYA00249587/ERZ652573_FASTA.fasta	MGYA00249587	ERZ652573	root:Host-associated:Human:Digestive system:Large in/	0.3948
MGYA00249588/ERZ652575_FASTA.fasta	MGYA00249588	ERZ652575	root:Host-associated:Human:Digestive system:Large in/	0.2744
MGYA00249589/ERZ652577_FASTA.fasta	MGYA00249589	ERZ652577	root:Host-associated:Human:Digestive system:Large in/	0.273
MGYA00249590/ERZ652579_FASTA.fasta	MGYA00249590	ERZ652579	root:Host-associated:Human:Digestive system:Large in/	0.3412
MGYA00249591/ERZ652581_FASTA.fasta	MGYA00249591	ERZ652581	root:Host-associated:Human:Digestive system:Large in/	0.314
MGYA00249592/ERZ652583_FASTA.fasta	MGYA00249592	ERZ652583	root:Host-associated:Human:Digestive system:Large in/	0.347
MGYA00249593/ERZ652585_FASTA.fasta	MGYA00249593	ERZ652585	root:Host-associated:Human:Digestive system:Large in/	0.3118
MGYA00249594/ERZ652587_FASTA.fasta	MGYA00249594	ERZ652587	root:Host-associated:Human:Digestive system:Large in/	0.4364
MGYA00249595/ERZ652589_FASTA.fasta	MGYA00249595	ERZ652589	root:Host-associated:Human:Digestive system:Large in/	0.3294
MGYA00249596/ERZ652591_FASTA.fasta	MGYA00249596	ERZ652591	root:Host-associated:Human:Digestive system:Large in/	0.3598
MGYA00249597/ERZ652492_FASTA.fasta	MGYA00249597	ERZ652492	root:Host-associated:Human:Digestive system:Large in/	0.2108
MGYA00249598/ERZ652494_FASTA.fasta	MGYA00249598	ERZ652494	root:Host-associated:Human:Digestive system:Large in/	0.2748
MGYA00249599/ERZ652496_FASTA.fasta	MGYA00249599	ERZ652496	root:Host-associated:Human:Digestive system:Large in/	0.248
MGYA00249600/ERZ652498_FASTA.fasta	MGYA00249600	ERZ652498	root:Host-associated:Human:Digestive system:Large in/	0.3448
MGYA00249601/ERZ652500_FASTA.fasta	MGYA00249601	ERZ652500	root:Host-associated:Human:Digestive system:Large in/	0.2518
MGYA00249602/ERZ652502_FASTA.fasta	MGYA00249602	ERZ652502	root:Host-associated:Human:Digestive system:Large in/	0.308
MGYA00249603/ERZ652504_FASTA.fasta	MGYA00249603	ERZ652504	root:Host-associated:Human:Digestive system:Large in/	0.2356
MGYA00249604/ERZ652506_FASTA.fasta	MGYA00249604	ERZ652506	root:Host-associated:Human:Digestive system:Large in/	0.253
MGYA00249605/ERZ652508_FASTA.fasta	MGYA00249605	ERZ652508	root:Host-associated:Human:Digestive system:Large in/	0.3454
MGYA00249606/ERZ652510_FASTA.fasta	MGYA00249606	ERZ652510	root:Host-associated:Human:Digestive system:Large in/	0.087
MGYA00249607/ERZ652512_FASTA.fasta	MGYA00249607	ERZ652512	root:Host-associated:Human:Digestive system:Large in/	0.2216
MGYA00249608/ERZ652514_FASTA.fasta	MGYA00249608	ERZ652514	root:Host-associated:Human:Digestive system:Large in/	0.3284
MGYA00249609/ERZ652516_FASTA.fasta	MGYA00249609	ERZ652516	root:Host-associated:Human:Digestive system:Large in/	0.3628
MGYA00249610/ERZ652518_FASTA.fasta	MGYA00249610	ERZ652518	root:Host-associated:Human:Digestive system:Large in/	0.0818
MGYA00249611/ERZ652520_FASTA.fasta	MGYA00249611	ERZ652520	root:Host-associated:Human:Digestive system:Large in/	0.1532
MGYA00249612/ERZ652522_FASTA.fasta	MGYA00249612	ERZ652522	root:Host-associated:Human:Digestive system:Large in/	0.3772
MGYA00249613/ERZ652524_FASTA.fasta	MGYA00249613	ERZ652524	root:Host-associated:Human:Digestive system:Large in/	0.2906
MGYA00249614/ERZ652526_FASTA.fasta	MGYA00249614	ERZ652526	root:Host-associated:Human:Digestive system:Large in/	0.3138
MGYA00249615/ERZ652528_FASTA.fasta	MGYA00249615	ERZ652528	root:Host-associated:Human:Digestive system:Large in/	0.2962
MGYA00249616/ERZ652530_FASTA.fasta	MGYA00249616	ERZ652530	root:Host-associated:Human:Digestive system:Large in/	0.1542
MGYA00249617/ERZ652403_FASTA.fasta	MGYA00249617	ERZ652403	root:Host-associated:Human:Digestive system:Large in/	0.332
MGYA00249618/ERZ652405_FASTA.fasta	MGYA00249618	ERZ652405	root:Host-associated:Human:Digestive system:Large in/	0.2884
MGYA00249619/ERZ652593_FASTA.fasta	MGYA00249619	ERZ652593	root:Host-associated:Human:Digestive system:Large in/	0.2748
MGYA00249620/ERZ652595_FASTA.fasta	MGYA00249620	ERZ652595	root:Host-associated:Human:Digestive system:Large in/	0.1742
MGYA00249621/ERZ652597_FASTA.fasta	MGYA00249621	ERZ652597	root:Host-associated:Human:Digestive system:Large in/	0.3624
MGYA00249622/ERZ652599_FASTA.fasta	MGYA00249622	ERZ652599	root:Host-associated:Human:Digestive system:Large in/	0.3682
MGYA00249623/ERZ652601_FASTA.fasta	MGYA00249623	ERZ652601	root:Host-associated:Human:Digestive system:Large in/	0.1794
MGYA00249624/ERZ652603_FASTA.fasta	MGYA00249624	ERZ652603	root:Host-associated:Human:Digestive system:Large in/	0.09
MGYA00249625/ERZ652407_FASTA.fasta	MGYA00249625	ERZ652407	root:Host-associated:Human:Digestive system:Large in/	0.338
MGYA00249626/ERZ652409_FASTA.fasta	MGYA00249626	ERZ652409	root:Host-associated:Human:Digestive system:Large in/	0.277
MGYA00249627/ERZ652411_FASTA.fasta	MGYA00249627	ERZ652411	root:Host-associated:Human:Digestive system:Large in/	0.206
MGYA00249628/ERZ652413_FASTA.fasta	MGYA00249628	ERZ652413	root:Host-associated:Human:Digestive system:Large in/	0.3464
MGYA00249629/ERZ652415_FASTA.fasta	MGYA00249629	ERZ652415	root:Host-associated:Human:Digestive system:Large in/	0.3512
MGYA00249630/ERZ652417_FASTA.fasta	MGYA00249630	ERZ652417	root:Host-associated:Human:Digestive system:Large in/	0.2168
MGYA00249631/ERZ652419_FASTA.fasta	MGYA00249631	ERZ652419	root:Host-associated:Human:Digestive system:Large in/	0.3516
MGYA00249632/ERZ652421_FASTA.fasta	MGYA00249632	ERZ652421	root:Host-associated:Human:Digestive system:Large in/	0.3388
MGYA00249633/ERZ652423_FASTA.fasta	MGYA00249633	ERZ652423	root:Host-associated:Human:Digestive system:Large in/	0.3964
MGYA00249634/ERZ652425_FASTA.fasta	MGYA00249634	ERZ652425	root:Host-associated:Human:Digestive system:Large in/	0.3216
MGYA00249635/ERZ651987_FASTA.fasta	MGYA00249635	ERZ651987	root:Host-associated:Human:Digestive system:Large in/	0.305
MGYA00249636/ERZ651988_FASTA.fasta	MGYA00249636	ERZ651988	root:Host-associated:Human:Digestive system:Large in/	0.397
MGYA00249637/ERZ651990_FASTA.fasta	MGYA00249637	ERZ651990	root:Host-associated:Human:Digestive system:Large in/	0.2616
MGYA00249638/ERZ651992_FASTA.fasta	MGYA00249638	ERZ651992	root:Host-associated:Human:Digestive system:Large in/	0.3216
MGYA00249639/ERZ651994_FASTA.fasta	MGYA00249639	ERZ651994	root:Host-associated:Human:Digestive system:Large in/	0.4216
MGYA00249640/ERZ651996_FASTA.fasta	MGYA00249640	ERZ651996	root:Host-associated:Human:Digestive system:Large in/	0.2358
MGYA00249641/ERZ652532_FASTA.fasta	MGYA00249641	ERZ652532	root:Host-associated:Human:Digestive system:Large in/	0.3988
MGYA00249642/ERZ652534_FASTA.fasta	MGYA00249642	ERZ652534	root:Host-associated:Human:Digestive system:Large in/	0.3616
MGYA00249643/ERZ652536_FASTA.fasta	MGYA00249643	ERZ652536	root:Host-associated:Human:Digestive system:Large in/	0.2568
MGYA00249644/ERZ652538_FASTA.fasta	MGYA00249644	ERZ652538	root:Host-associated:Human:Digestive system:Large in/	0.4102
MGYA00249645/ERZ652540_FASTA.fasta	MGYA00249645	ERZ652540	root:Host-associated:Human:Digestive system:Large in/	0.3514
MGYA00249646/ERZ652542_FASTA.fasta	MGYA00249646	ERZ652542	root:Host-associated:Human:Digestive system:Large in/	0.1318
MGYA00249647/ERZ652544_FASTA.fasta	MGYA00249647	ERZ652544	root:Host-associated:Human:Digestive system:Large in/	0.298
MGYA00249648/ERZ652546_FASTA.fasta	MGYA00249648	ERZ652546	root:Host-associated:Human:Digestive system:Large in/	0.2378
MGYA00249649/ERZ652548_FASTA.fasta	MGYA00249649	ERZ652548	root:Host-associated:Human:Digestive system:Large in/	0.2992
MGYA00249650/ERZ652550_FASTA.fasta	MGYA00249650	ERZ652550	root:Host-associated:Human:Digestive system:Large in/	0.3062
MGYA00249651/ERZ652427_FASTA.fasta	MGYA00249651	ERZ652427	root:Host-associated:Human:Digestive system:Large in/	0.3358
MGYA00249652/ERZ652429_FASTA.fasta	MGYA00249652	ERZ652429	root:Host-associated:Human:Digestive system:Large in/	0.3554
MGYA00249653/ERZ652431_FASTA.fasta	MGYA00249653	ERZ652431	root:Host-associated:Human:Digestive system:Large in/	0.2668
MGYA00249654/ERZ652433_FASTA.fasta	MGYA00249654	ERZ652433	root:Host-associated:Human:Digestive system:Large in/	0.3162
MGYA00249655/ERZ652435_FASTA.fasta	MGYA00249655	ERZ652435	root:Host-associated:Human:Digestive system:Large in/	0.3022
MGYA00249656/ERZ652437_FASTA.fasta	MGYA00249656	ERZ652437	root:Host-associated:Human:Digestive system:Large in/	0.3902
MGYA00249657/ERZ652439_FASTA.fasta	MGYA00249657	ERZ652439	root:Host-associated:Human:Digestive system:Large in/	0.2636
MGYA00249658/ERZ652445_FASTA.fasta	MGYA00249658	ERZ652445	root:Host-associated:Human:Digestive system:Large in/	0.4008
MGYA00249659/ERZ652447_FASTA.fasta	MGYA00249659	ERZ652447	root:Host-associated:Human:Digestive system:Large in/	0.316
MGYA00249660/ERZ652449_FASTA.fasta	MGYA00249660	ERZ652449	root:Host-associated:Human:Digestive system:Large in/	0.2946
MGYA00249661/ERZ652451_FASTA.fasta	MGYA00249661	ERZ652451	root:Host-associated:Human:Digestive system:Large in/	0.32
MGYA00249662/ERZ652453_FASTA.fasta	MGYA00249662	ERZ652453	root:Host-associated:Human:Digestive system:Large in/	0.261
MGYA00249663/ERZ652455_FASTA.fasta	MGYA00249663	ERZ652455	root:Host-associated:Human:Digestive system:Large in/	0.2712
MGYA00249664/ERZ652457_FASTA.fasta	MGYA00249664	ERZ652457	root:Host-associated:Human:Digestive system:Large in/	0.2476
MGYA00249665/ERZ652459_FASTA.fasta	MGYA00249665	ERZ652459	root:Host-associated:Human:Digestive system:Large in/	0.263
MGYA00249666/ERZ652461_FASTA.fasta	MGYA00249666	ERZ652461	root:Host-associated:Human:Digestive system:Large in/	0.1436
MGYA00249667/ERZ652463_FASTA.fasta	MGYA00249667	ERZ652463	root:Host-associated:Human:Digestive system:Large in/	0.3354
MGYA00249668/ERZ652465_FASTA.fasta	MGYA00249668	ERZ652465	root:Host-associated:Human:Digestive system:Large in/	0.2516
MGYA00249669/ERZ652467_FASTA.fasta	MGYA00249669	ERZ652467	root:Host-associated:Human:Digestive system:Large in/	0.2588
MGYA00249670/ERZ651998_FASTA.fasta	MGYA00249670	ERZ651998	root:Host-associated:Human:Digestive system:Large in/	0.2102
MGYA00249671/ERZ652000_FASTA.fasta	MGYA00249671	ERZ652000	root:Host-associated:Human:Digestive system:Large in/	0.3304
MGYA00249672/ERZ652002_FASTA.fasta	MGYA00249672	ERZ652002	root:Host-associated:Human:Digestive system:Large in/	0.3154
MGYA00249673/ERZ652004_FASTA.fasta	MGYA00249673	ERZ652004	root:Host-associated:Human:Digestive system:Large in/	0.3716
MGYA00249674/ERZ652006_FASTA.fasta	MGYA00249674	ERZ652006	root:Host-associated:Human:Digestive system:Large in/	0.2964
MGYA00249675/ERZ652008_FASTA.fasta	MGYA00249675	ERZ652008	root:Host-associated:Human:Digestive system:Large in/	0.242
MGYA00249676/ERZ652010_FASTA.fasta	MGYA00249676	ERZ652010	root:Host-associated:Human:Digestive system:Large in/	0.3386
MGYA00249677/ERZ652014_FASTA.fasta	MGYA00249677	ERZ652014	root:Host-associated:Human:Digestive system:Large in/	0.1474
MGYA00249678/ERZ652016_FASTA.fasta	MGYA00249678	ERZ652016	root:Host-associated:Human:Digestive system:Large in/	0.155
MGYA00249679/ERZ652018_FASTA.fasta	MGYA00249679	ERZ652018	root:Host-associated:Human:Digestive system:Large in/	0.3134
MGYA00249680/ERZ652020_FASTA.fasta	MGYA00249680	ERZ652020	root:Host-associated:Human:Digestive system:Large in/	0.3306
MGYA00249681/ERZ652022_FASTA.fasta	MGYA00249681	ERZ652022	root:Host-associated:Human:Digestive system:Large in/	0.397
MGYA00249682/ERZ652024_FASTA.fasta	MGYA00249682	ERZ652024	root:Host-associated:Human:Digestive system:Large in/	0.4024
MGYA00249683/ERZ652026_FASTA.fasta	MGYA00249683	ERZ652026	root:Host-associated:Human:Digestive system:Large in/	0.2172
MGYA00249684/ERZ652028_FASTA.fasta	MGYA00249684	ERZ652028	root:Host-associated:Human:Digestive system:Large in/	0.3494
MGYA00249685/ERZ652030_FASTA.fasta	MGYA00249685	ERZ652030	root:Host-associated:Human:Digestive system:Large in/	0.4012
MGYA00249686/ERZ652032_FASTA.fasta	MGYA00249686	ERZ652032	root:Host-associated:Human:Digestive system:Large in/	0.3068
MGYA00249687/ERZ652034_FASTA.fasta	MGYA00249687	ERZ652034	root:Host-associated:Human:Digestive system:Large in/	0.1778
MGYA00249688/ERZ652036_FASTA.fasta	MGYA00249688	ERZ652036	root:Host-associated:Human:Digestive system:Large in/	0.3226
MGYA00249689/ERZ652552_FASTA.fasta	MGYA00249689	ERZ652552	root:Host-associated:Human:Digestive system:Large in/	0.2358
MGYA00249690/ERZ652554_FASTA.fasta	MGYA00249690	ERZ652554	root:Host-associated:Human:Digestive system:Large in/	0.3148
MGYA00249691/ERZ652556_FASTA.fasta	MGYA00249691	ERZ652556	root:Host-associated:Human:Digestive system:Large in/	0.2116
MGYA00249692/ERZ652558_FASTA.fasta	MGYA00249692	ERZ652558	root:Host-associated:Human:Digestive system:Large in/	0.3612
MGYA00249693/ERZ652560_FASTA.fasta	MGYA00249693	ERZ652560	root:Host-associated:Human:Digestive system:Large in/	0.3194
MGYA00249694/ERZ652562_FASTA.fasta	MGYA00249694	ERZ652562	root:Host-associated:Human:Digestive system:Large in/	0.2592
MGYA00249695/ERZ652564_FASTA.fasta	MGYA00249695	ERZ652564	root:Host-associated:Human:Digestive system:Large in/	0.2868
MGYA00249696/ERZ652566_FASTA.fasta	MGYA00249696	ERZ652566	root:Host-associated:Human:Digestive system:Large in/	0.2548
MGYA00249697/ERZ652568_FASTA.fasta	MGYA00249697	ERZ652568	root:Host-associated:Human:Digestive system:Large in/	0.3102
MGYA00249698/ERZ652570_FASTA.fasta	MGYA00249698	ERZ652570	root:Host-associated:Human:Digestive system:Large in/	0.2648
MGYA00249699/ERZ652572_FASTA.fasta	MGYA00249699	ERZ652572	root:Host-associated:Human:Digestive system:Large in/	0.2646
MGYA00249700/ERZ652574_FASTA.fasta	MGYA00249700	ERZ652574	root:Host-associated:Human:Digestive system:Large in/	0.479
MGYA00249701/ERZ652576_FASTA.fasta	MGYA00249701	ERZ652576	root:Host-associated:Human:Digestive system:Large in/	0.3878
MGYA00249702/ERZ652578_FASTA.fasta	MGYA00249702	ERZ652578	root:Host-associated:Human:Digestive system:Large in/	0.2304
MGYA00249703/ERZ652580_FASTA.fasta	MGYA00249703	ERZ652580	root:Host-associated:Human:Digestive system:Large in/	0.3176
MGYA00249704/ERZ652582_FASTA.fasta	MGYA00249704	ERZ652582	root:Host-associated:Human:Digestive system:Large in/	0.1828
MGYA00249705/ERZ652584_FASTA.fasta	MGYA00249705	ERZ652584	root:Host-associated:Human:Digestive system:Large in/	0.4036
MGYA00249706/ERZ652586_FASTA.fasta	MGYA00249706	ERZ652586	root:Host-associated:Human:Digestive system:Large in/	0.2996
MGYA00249707/ERZ652588_FASTA.fasta	MGYA00249707	ERZ652588	root:Host-associated:Human:Digestive system:Large in/	0.2786
MGYA00249708/ERZ652590_FASTA.fasta	MGYA00249708	ERZ652590	root:Host-associated:Human:Digestive system:Large in/	0.1974
MGYA00249709/ERZ652469_FASTA.fasta	MGYA00249709	ERZ652469	root:Host-associated:Human:Digestive system:Large in/	0.2248
MGYA00249710/ERZ652471_FASTA.fasta	MGYA00249710	ERZ652471	root:Host-associated:Human:Digestive system:Large in/	0.2824
MGYA00249711/ERZ652473_FASTA.fasta	MGYA00249711	ERZ652473	root:Host-associated:Human:Digestive system:Large in/	0.2732
MGYA00249712/ERZ652475_FASTA.fasta	MGYA00249712	ERZ652475	root:Host-associated:Human:Digestive system:Large in/	0.2718
MGYA00249713/ERZ652477_FASTA.fasta	MGYA00249713	ERZ652477	root:Host-associated:Human:Digestive system:Large in/	0.3168
MGYA00249714/ERZ652479_FASTA.fasta	MGYA00249714	ERZ652479	root:Host-associated:Human:Digestive system:Large in/	0.3826
MGYA00249715/ERZ652481_FASTA.fasta	MGYA00249715	ERZ652481	root:Host-associated:Human:Digestive system:Large in/	0.3248
MGYA00249716/ERZ652483_FASTA.fasta	MGYA00249716	ERZ652483	root:Host-associated:Human:Digestive system:Large in/	0.4286
MGYA00249717/ERZ652038_FASTA.fasta	MGYA00249717	ERZ652038	root:Host-associated:Human:Digestive system:Large in/	0.2556
MGYA00249718/ERZ652040_FASTA.fasta	MGYA00249718	ERZ652040	root:Host-associated:Human:Digestive system:Large in/	0.374
MGYA00249719/ERZ652042_FASTA.fasta	MGYA00249719	ERZ652042	root:Host-associated:Human:Digestive system:Large in/	0.29
MGYA00249720/ERZ652044_FASTA.fasta	MGYA00249720	ERZ652044	root:Host-associated:Human:Digestive system:Large in/	0.3248
MGYA00249721/ERZ652046_FASTA.fasta	MGYA00249721	ERZ652046	root:Host-associated:Human:Digestive system:Large in/	0.4148
MGYA00249722/ERZ652048_FASTA.fasta	MGYA00249722	ERZ652048	root:Host-associated:Human:Digestive system:Large in/	0.3554
MGYA00249723/ERZ652050_FASTA.fasta	MGYA00249723	ERZ652050	root:Host-associated:Human:Digestive system:Large in/	0.293
MGYA00249724/ERZ652052_FASTA.fasta	MGYA00249724	ERZ652052	root:Host-associated:Human:Digestive system:Large in/	0.3374
MGYA00249725/ERZ652054_FASTA.fasta	MGYA00249725	ERZ652054	root:Host-associated:Human:Digestive system:Large in/	0.5044
MGYA00249726/ERZ652056_FASTA.fasta	MGYA00249726	ERZ652056	root:Host-associated:Human:Digestive system:Large in/	0.2894
MGYA00249727/ERZ652058_FASTA.fasta	MGYA00249727	ERZ652058	root:Host-associated:Human:Digestive system:Large in/	0.3544
MGYA00249728/ERZ652060_FASTA.fasta	MGYA00249728	ERZ652060	root:Host-associated:Human:Digestive system:Large in/	0.3582
MGYA00249729/ERZ652062_FASTA.fasta	MGYA00249729	ERZ652062	root:Host-associated:Human:Digestive system:Large in/	0.4184
MGYA00249730/ERZ652064_FASTA.fasta	MGYA00249730	ERZ652064	root:Host-associated:Human:Digestive system:Large in/	0.4002
MGYA00249731/ERZ652066_FASTA.fasta	MGYA00249731	ERZ652066	root:Host-associated:Human:Digestive system:Large in/	0.2784
MGYA00249732/ERZ652068_FASTA.fasta	MGYA00249732	ERZ652068	root:Host-associated:Human:Digestive system:Large in/	0.3076
MGYA00249733/ERZ652070_FASTA.fasta	MGYA00249733	ERZ652070	root:Host-associated:Human:Digestive system:Large in/	0.3764
MGYA00249734/ERZ652072_FASTA.fasta	MGYA00249734	ERZ652072	root:Host-associated:Human:Digestive system:Large in/	0.3592
MGYA00249735/ERZ652074_FASTA.fasta	MGYA00249735	ERZ652074	root:Host-associated:Human:Digestive system:Large in/	0.3176
MGYA00249736/ERZ652076_FASTA.fasta	MGYA00249736	ERZ652076	root:Host-associated:Human:Digestive system:Large in/	0.2388
MGYA00249737/ERZ652592_FASTA.fasta	MGYA00249737	ERZ652592	root:Host-associated:Human:Digestive system:Large in/	0.0856
MGYA00249738/ERZ652594_FASTA.fasta	MGYA00249738	ERZ652594	root:Host-associated:Human:Digestive system:Large in/	0.3266
MGYA00249739/ERZ652487_FASTA.fasta	MGYA00249739	ERZ652487	root:Host-associated:Human:Digestive system:Large in/	0.356
MGYA00249740/ERZ652489_FASTA.fasta	MGYA00249740	ERZ652489	root:Host-associated:Human:Digestive system:Large in/	0.2624
MGYA00249741/ERZ652491_FASTA.fasta	MGYA00249741	ERZ652491	root:Host-associated:Human:Digestive system:Large in/	0.2888
MGYA00249742/ERZ652493_FASTA.fasta	MGYA00249742	ERZ652493	root:Host-associated:Human:Digestive system:Large in/	0.4156
MGYA00249743/ERZ652495_FASTA.fasta	MGYA00249743	ERZ652495	root:Host-associated:Human:Digestive system:Large in/	0.333
MGYA00249744/ERZ652497_FASTA.fasta	MGYA00249744	ERZ652497	root:Host-associated:Human:Digestive system:Large in/	0.2582
MGYA00249745/ERZ652499_FASTA.fasta	MGYA00249745	ERZ652499	root:Host-associated:Human:Digestive system:Large in/	0.3322
MGYA00249746/ERZ652078_FASTA.fasta	MGYA00249746	ERZ652078	root:Host-associated:Human:Digestive system:Large in/	0.3522
MGYA00249747/ERZ652080_FASTA.fasta	MGYA00249747	ERZ652080	root:Host-associated:Human:Digestive system:Large in/	0.4636
MGYA00249748/ERZ652082_FASTA.fasta	MGYA00249748	ERZ652082	root:Host-associated:Human:Digestive system:Large in/	0.2654
MGYA00249749/ERZ652084_FASTA.fasta	MGYA00249749	ERZ652084	root:Host-associated:Human:Digestive system:Large in/	0.437
MGYA00249750/ERZ652086_FASTA.fasta	MGYA00249750	ERZ652086	root:Host-associated:Human:Digestive system:Large in/	0.3012
MGYA00249751/ERZ652088_FASTA.fasta	MGYA00249751	ERZ652088	root:Host-associated:Human:Digestive system:Large in/	0.2648
MGYA00249752/ERZ652090_FASTA.fasta	MGYA00249752	ERZ652090	root:Host-associated:Human:Digestive system:Large in/	0.3778
MGYA00249753/ERZ652092_FASTA.fasta	MGYA00249753	ERZ652092	root:Host-associated:Human:Digestive system:Large in/	0.439
MGYA00249754/ERZ652094_FASTA.fasta	MGYA00249754	ERZ652094	root:Host-associated:Human:Digestive system:Large in/	0.3428
MGYA00249755/ERZ652096_FASTA.fasta	MGYA00249755	ERZ652096	root:Host-associated:Human:Digestive system:Large in/	0.2384
MGYA00249756/ERZ652098_FASTA.fasta	MGYA00249756	ERZ652098	root:Host-associated:Human:Digestive system:Large in/	0.2346
MGYA00249757/ERZ652100_FASTA.fasta	MGYA00249757	ERZ652100	root:Host-associated:Human:Digestive system:Large in/	0.3592
MGYA00249758/ERZ652102_FASTA.fasta	MGYA00249758	ERZ652102	root:Host-associated:Human:Digestive system:Large in/	0.337
MGYA00249759/ERZ652104_FASTA.fasta	MGYA00249759	ERZ652104	root:Host-associated:Human:Digestive system:Large in/	0.4116
MGYA00249760/ERZ652106_FASTA.fasta	MGYA00249760	ERZ652106	root:Host-associated:Human:Digestive system:Large in/	0.403
MGYA00249761/ERZ652108_FASTA.fasta	MGYA00249761	ERZ652108	root:Host-associated:Human:Digestive system:Large in/	0.3626
MGYA00249762/ERZ652110_FASTA.fasta	MGYA00249762	ERZ652110	root:Host-associated:Human:Digestive system:Large in/	0.1858
MGYA00249763/ERZ652112_FASTA.fasta	MGYA00249763	ERZ652112	root:Host-associated:Human:Digestive system:Large in/	0.3092
MGYA00249764/ERZ652114_FASTA.fasta	MGYA00249764	ERZ652114	root:Host-associated:Human:Digestive system:Large in/	0.452
MGYA00249765/ERZ652116_FASTA.fasta	MGYA00249765	ERZ652116	root:Host-associated:Human:Digestive system:Large in/	0.2376
MGYA00249766/ERZ652501_FASTA.fasta	MGYA00249766	ERZ652501	root:Host-associated:Human:Digestive system:Large in/	0.1932
MGYA00249767/ERZ652118_FASTA.fasta	MGYA00249767	ERZ652118	root:Host-associated:Human:Digestive system:Large in/	0.2284
MGYA00249768/ERZ652120_FASTA.fasta	MGYA00249768	ERZ652120	root:Host-associated:Human:Digestive system:Large in/	0.2326
MGYA00249769/ERZ652122_FASTA.fasta	MGYA00249769	ERZ652122	root:Host-associated:Human:Digestive system:Large in/	0.4944
MGYA00249770/ERZ652124_FASTA.fasta	MGYA00249770	ERZ652124	root:Host-associated:Human:Digestive system:Large in/	0.3552
MGYA00249771/ERZ652126_FASTA.fasta	MGYA00249771	ERZ652126	root:Host-associated:Human:Digestive system:Large in/	0.3664
MGYA00249772/ERZ652128_FASTA.fasta	MGYA00249772	ERZ652128	root:Host-associated:Human:Digestive system:Large in/	0.3252
MGYA00249773/ERZ652130_FASTA.fasta	MGYA00249773	ERZ652130	root:Host-associated:Human:Digestive system:Large in/	0.4028
MGYA00249774/ERZ652132_FASTA.fasta	MGYA00249774	ERZ652132	root:Host-associated:Human:Digestive system:Large in/	0.2836
MGYA00249775/ERZ652134_FASTA.fasta	MGYA00249775	ERZ652134	root:Host-associated:Human:Digestive system:Large in/	0.477
MGYA00249776/ERZ652136_FASTA.fasta	MGYA00249776	ERZ652136	root:Host-associated:Human:Digestive system:Large in/	0.4032
MGYA00249777/ERZ652138_FASTA.fasta	MGYA00249777	ERZ652138	root:Host-associated:Human:Digestive system:Large in/	0.2996
MGYA00249778/ERZ652140_FASTA.fasta	MGYA00249778	ERZ652140	root:Host-associated:Human:Digestive system:Large in/	0.1922
MGYA00249779/ERZ652142_FASTA.fasta	MGYA00249779	ERZ652142	root:Host-associated:Human:Digestive system:Large in/	0.2902
MGYA00249780/ERZ652287_FASTA.fasta	MGYA00249780	ERZ652287	root:Host-associated:Human:Digestive system:Large in/	0.3518
MGYA00249781/ERZ652289_FASTA.fasta	MGYA00249781	ERZ652289	root:Host-associated:Human:Digestive system:Large in/	0.2744
MGYA00249782/ERZ652291_FASTA.fasta	MGYA00249782	ERZ652291	root:Host-associated:Human:Digestive system:Large in/	0.159
MGYA00249783/ERZ652293_FASTA.fasta	MGYA00249783	ERZ652293	root:Host-associated:Human:Digestive system:Large in/	0.151
MGYA00249784/ERZ652295_FASTA.fasta	MGYA00249784	ERZ652295	root:Host-associated:Human:Digestive system:Large in/	0.207
MGYA00249785/ERZ652297_FASTA.fasta	MGYA00249785	ERZ652297	root:Host-associated:Human:Digestive system:Large in/	0.2804
MGYA00249786/ERZ652299_FASTA.fasta	MGYA00249786	ERZ652299	root:Host-associated:Human:Digestive system:Large in/	0.4522
MGYA00249787/ERZ652301_FASTA.fasta	MGYA00249787	ERZ652301	root:Host-associated:Human:Digestive system:Large in/	0.2222
MGYA00249788/ERZ652303_FASTA.fasta	MGYA00249788	ERZ652303	root:Host-associated:Human:Digestive system:Large in/	0.1828
MGYA00249789/ERZ652305_FASTA.fasta	MGYA00249789	ERZ652305	root:Host-associated:Human:Digestive system:Large in/	0.1154
MGYA00249790/ERZ652307_FASTA.fasta	MGYA00249790	ERZ652307	root:Host-associated:Human:Digestive system:Large in/	0.276
MGYA00249791/ERZ652309_FASTA.fasta	MGYA00249791	ERZ652309	root:Host-associated:Human:Digestive system:Large in/	0.255
MGYA00249792/ERZ652311_FASTA.fasta	MGYA00249792	ERZ652311	root:Host-associated:Human:Digestive system:Large in/	0.3264
MGYA00249793/ERZ652314_FASTA.fasta	MGYA00249793	ERZ652314	root:Host-associated:Human:Digestive system:Large in/	0.2832
MGYA00249794/ERZ652316_FASTA.fasta	MGYA00249794	ERZ652316	root:Host-associated:Human:Digestive system:Large in/	0.238
MGYA00249795/ERZ652318_FASTA.fasta	MGYA00249795	ERZ652318	root:Host-associated:Human:Digestive system:Large in/	0.1364
MGYA00249796/ERZ652320_FASTA.fasta	MGYA00249796	ERZ652320	root:Host-associated:Human:Digestive system:Large in/	0.1482
MGYA00249797/ERZ652322_FASTA.fasta	MGYA00249797	ERZ652322	root:Host-associated:Human:Digestive system:Large in/	0.283
MGYA00249798/ERZ652324_FASTA.fasta	MGYA00249798	ERZ652324	root:Host-associated:Human:Digestive system:Large in/	0.3562
MGYA00249799/ERZ652326_FASTA.fasta	MGYA00249799	ERZ652326	root:Host-associated:Human:Digestive system:Large in/	0.2698
MGYA00249800/ERZ652328_FASTA.fasta	MGYA00249800	ERZ652328	root:Host-associated:Human:Digestive system:Large in/	0.3374
MGYA00249801/ERZ652330_FASTA.fasta	MGYA00249801	ERZ652330	root:Host-associated:Human:Digestive system:Large in/	0.279
MGYA00249802/ERZ652332_FASTA.fasta	MGYA00249802	ERZ652332	root:Host-associated:Human:Digestive system:Large in/	0.3244
MGYA00249803/ERZ652334_FASTA.fasta	MGYA00249803	ERZ652334	root:Host-associated:Human:Digestive system:Large in/	0.257
MGYA00249804/ERZ652336_FASTA.fasta	MGYA00249804	ERZ652336	root:Host-associated:Human:Digestive system:Large in/	0.2552
MGYA00249805/ERZ652338_FASTA.fasta	MGYA00249805	ERZ652338	root:Host-associated:Human:Digestive system:Large in/	0.3338
MGYA00249806/ERZ652340_FASTA.fasta	MGYA00249806	ERZ652340	root:Host-associated:Human:Digestive system:Large in/	0.3542

MGYA00249807/ERZ652342_FASTA.fasta	MGYA00249807	ERZ652342	root:Host-associated:Human:Digestive system:Large in	0.2214
MGYA00249808/ERZ652344_FASTA.fasta	MGYA00249808	ERZ652344	root:Host-associated:Human:Digestive system:Large in	0.2846
MGYA00249809/ERZ652346_FASTA.fasta	MGYA00249809	ERZ652346	root:Host-associated:Human:Digestive system:Large in	0.3074
MGYA00249810/ERZ652348_FASTA.fasta	MGYA00249810	ERZ652348	root:Host-associated:Human:Digestive system:Large in	0.277
MGYA00249811/ERZ652350_FASTA.fasta	MGYA00249811	ERZ652350	root:Host-associated:Human:Digestive system:Large in	0.2402
MGYA00249812/ERZ652352_FASTA.fasta	MGYA00249812	ERZ652352	root:Host-associated:Human:Digestive system:Large in	0.2924
MGYA00249813/ERZ652354_FASTA.fasta	MGYA00249813	ERZ652354	root:Host-associated:Human:Digestive system:Large in	0.2296
MGYA00249814/ERZ652356_FASTA.fasta	MGYA00249814	ERZ652356	root:Host-associated:Human:Digestive system:Large in	0.0942
MGYA00249815/ERZ652358_FASTA.fasta	MGYA00249815	ERZ652358	root:Host-associated:Human:Digestive system:Large in	0.2238
MGYA00249816/ERZ652360_FASTA.fasta	MGYA00249816	ERZ652360	root:Host-associated:Human:Digestive system:Large in	0.2218
MGYA00249817/ERZ652362_FASTA.fasta	MGYA00249817	ERZ652362	root:Host-associated:Human:Digestive system:Large in	0.25
MGYA00249818/ERZ652363_FASTA.fasta	MGYA00249818	ERZ652363	root:Host-associated:Human:Digestive system:Large in	0.282
MGYA00249819/ERZ652364_FASTA.fasta	MGYA00249819	ERZ652364	root:Host-associated:Human:Digestive system:Large in	0.353
MGYA00249820/ERZ652366_FASTA.fasta	MGYA00249820	ERZ652366	root:Host-associated:Human:Digestive system:Large in	0.2332
MGYA00249821/ERZ652368_FASTA.fasta	MGYA00249821	ERZ652368	root:Host-associated:Human:Digestive system:Large in	0.3
MGYA00249822/ERZ652370_FASTA.fasta	MGYA00249822	ERZ652370	root:Host-associated:Human:Digestive system:Large in	0.2822
MGYA00249823/ERZ652372_FASTA.fasta	MGYA00249823	ERZ652372	root:Host-associated:Human:Digestive system:Large in	0.3208
MGYA00249824/ERZ652242_FASTA.fasta	MGYA00249824	ERZ652242	root:Host-associated:Human:Digestive system:Large in	0.435
MGYA00249825/ERZ652244_FASTA.fasta	MGYA00249825	ERZ652244	root:Host-associated:Human:Digestive system:Large in	0.3558
MGYA00249826/ERZ652246_FASTA.fasta	MGYA00249826	ERZ652246	root:Host-associated:Human:Digestive system:Large in	0.2788
MGYA00249827/ERZ652248_FASTA.fasta	MGYA00249827	ERZ652248	root:Host-associated:Human:Digestive system:Large in	0.1498
MGYA00249828/ERZ652250_FASTA.fasta	MGYA00249828	ERZ652250	root:Host-associated:Human:Digestive system:Large in	0.3156
MGYA00249829/ERZ652252_FASTA.fasta	MGYA00249829	ERZ652252	root:Host-associated:Human:Digestive system:Large in	0.235
MGYA00249830/ERZ652254_FASTA.fasta	MGYA00249830	ERZ652254	root:Host-associated:Human:Digestive system:Large in	0.272
MGYA00249831/ERZ652256_FASTA.fasta	MGYA00249831	ERZ652256	root:Host-associated:Human:Digestive system:Large in	0.2896
MGYA00249832/ERZ652258_FASTA.fasta	MGYA00249832	ERZ652258	root:Host-associated:Human:Digestive system:Large in	0.2672
MGYA00249833/ERZ652260_FASTA.fasta	MGYA00249833	ERZ652260	root:Host-associated:Human:Digestive system:Large in	0.3262
MGYA00249834/ERZ652262_FASTA.fasta	MGYA00249834	ERZ652262	root:Host-associated:Human:Digestive system:Large in	0.3542
MGYA00249835/ERZ652264_FASTA.fasta	MGYA00249835	ERZ652264	root:Host-associated:Human:Digestive system:Large in	0.402
MGYA00249836/ERZ652266_FASTA.fasta	MGYA00249836	ERZ652266	root:Host-associated:Human:Digestive system:Large in	0.3154
MGYA00249837/ERZ652268_FASTA.fasta	MGYA00249837	ERZ652268	root:Host-associated:Human:Digestive system:Large in	0.1504
MGYA00249838/ERZ747220_FASTA.fasta	MGYA00249838	ERZ747220	root:Engineered:Biogas plant	0.7702
MGYA00249839/ERZ747224_FASTA.fasta	MGYA00249839	ERZ747224	root:Engineered:Wastewater:Activated Sludge	0.82
MGYA00249840/ERZ761561_FASTA.fasta	MGYA00249840	ERZ761561	root:Host-associated:Plants	0.1498
MGYA00249841/ERZ761562_FASTA.fasta	MGYA00249841	ERZ761562	root:Engineered:Wastewater:Activated Sludge	0.9092
MGYA00249842/ERZ761565_FASTA.fasta	MGYA00249842	ERZ761565	root:Engineered:Food production	0.0768
MGYA00249843/ERZ761567_FASTA.fasta	MGYA00249843	ERZ761567	root:Host-associated:Plants	0.048
MGYA00249844/ERZ761568_FASTA.fasta	MGYA00249844	ERZ761568	root:Host-associated:Plants	0.0502
MGYA00249845/ERZ761571_FASTA.fasta	MGYA00249845	ERZ761571	root:Host-associated:Plants	0.052
MGYA00249846/ERZ761613_FASTA.fasta	MGYA00249846	ERZ761613	root:Engineered:Bioreactor	0.7906
MGYA00249847/ERZ761614_FASTA.fasta	MGYA00249847	ERZ761614	root:Engineered:Bioreactor	0.8168
MGYA00249848/ERZ761632_FASTA.fasta	MGYA00249848	ERZ761632	root:Environmental:Aquatic:Lentic:Brackish	0.3822
MGYA00249849/ERZ761633_FASTA.fasta	MGYA00249849	ERZ761633	root:Environmental:Aquatic:Lentic:Brackish	0.3646
MGYA00249850/ERZ761637_FASTA.fasta	MGYA00249850	ERZ761637	root:Environmental:Aquatic:Lentic:Brackish	0.6638
MGYA00249851/ERZ761641_FASTA.fasta	MGYA00249851	ERZ761641	root:Environmental:Aquatic:Lentic:Brackish	0.3646
MGYA00249852/ERZ764866_FASTA.fasta	MGYA00249852	ERZ764866	root:Engineered:Bioreactor	0.8454
MGYA00249853/ERZ766980_FASTA.fasta	MGYA00249853	ERZ766980	root:Environmental:Aquatic:Marine	0.9178
MGYA00249854/ERZ766990_FASTA.fasta	MGYA00249854	ERZ766990	root:Environmental:Aquatic:Marine:Oceanic	0.7578
MGYA00249855/ERZ767000_FASTA.fasta	MGYA00249855	ERZ767000	root:Environmental:Aquatic:Marine	0.7274
MGYA00249856/ERZ767009_FASTA.fasta	MGYA00249856	ERZ767009	root:Environmental:Aquatic:Marine:Coastal:Sediment	0.858
MGYA00249857/ERZ767061_FASTA.fasta	MGYA00249857	ERZ767061	root:Environmental:Aquatic:Estuary	0.8758
MGYA00249858/ERZ767071_FASTA.fasta	MGYA00249858	ERZ767071	root:Environmental:Aquatic:Estuary	0.811
MGYA00249859/ERZ767158_FASTA.fasta	MGYA00249859	ERZ767158	root:Environmental:Aquatic:Estuary	0.8074
MGYA00249860/ERZ767235_FASTA.fasta	MGYA00249860	ERZ767235	root:Environmental:Aquatic:Estuary	0.7956
MGYA00249861/ERZ767478_FASTA.fasta	MGYA00249861	ERZ767478	root:Environmental:Aquatic:Estuary	0.817
MGYA00249862/ERZ772711_FASTA.fasta	MGYA00249862	ERZ772711	root:Engineered:Food production:Dairy products	0.0778
MGYA00249863/ERZ772713_FASTA.fasta	MGYA00249863	ERZ772713	root:Engineered:Food production:Dairy products	0.0778
MGYA00249864/ERZ772715_FASTA.fasta	MGYA00249864	ERZ772715	root:Engineered:Food production:Dairy products	0.0778
MGYA00249865/ERZ772717_FASTA.fasta	MGYA00249865	ERZ772717	root:Engineered:Food production:Dairy products	0.3922
MGYA00249866/ERZ772719_FASTA.fasta	MGYA00249866	ERZ772719	root:Engineered:Food production:Dairy products	0.37
MGYA00249867/ERZ772721_FASTA.fasta	MGYA00249867	ERZ772721	root:Engineered:Food production:Dairy products	0.3748
MGYA00249868/ERZ772723_FASTA.fasta	MGYA00249868	ERZ772723	root:Engineered:Food production:Dairy products	0.0768
MGYA00249869/ERZ772725_FASTA.fasta	MGYA00249869	ERZ772725	root:Engineered:Food production:Dairy products	0.3874
MGYA00249870/ERZ772727_FASTA.fasta	MGYA00249870	ERZ772727	root:Engineered:Food production:Dairy products	0.0778
MGYA00249871/ERZ772729_FASTA.fasta	MGYA00249871	ERZ772729	root:Engineered:Food production:Dairy products	0.3922
MGYA00249872/ERZ772731_FASTA.fasta	MGYA00249872	ERZ772731	root:Engineered:Food production:Dairy products	0.08
MGYA00249873/ERZ772733_FASTA.fasta	MGYA00249873	ERZ772733	root:Engineered:Food production:Dairy products	0.4258
MGYA00249874/ERZ772735_FASTA.fasta	MGYA00249874	ERZ772735	root:Engineered:Food production:Dairy products	0.081
MGYA00249875/ERZ772737_FASTA.fasta	MGYA00249875	ERZ772737	root:Engineered:Food production:Dairy products	0.415
MGYA00249876/ERZ772739_FASTA.fasta	MGYA00249876	ERZ772739	root:Engineered:Food production:Dairy products	0.3706
MGYA00249877/ERZ772712_FASTA.fasta	MGYA00249877	ERZ772712	root:Engineered:Food production:Dairy products	0.0734
MGYA00249878/ERZ772714_FASTA.fasta	MGYA00249878	ERZ772714	root:Engineered:Food production:Dairy products	0.4002
MGYA00249879/ERZ772716_FASTA.fasta	MGYA00249879	ERZ772716	root:Engineered:Food production:Dairy products	0.0768
MGYA00249880/ERZ772718_FASTA.fasta	MGYA00249880	ERZ772718	root:Engineered:Food production:Dairy products	0.4264
MGYA00249881/ERZ772720_FASTA.fasta	MGYA00249881	ERZ772720	root:Engineered:Food production:Dairy products	0.3706
MGYA00249882/ERZ772722_FASTA.fasta	MGYA00249882	ERZ772722	root:Engineered:Food production:Dairy products	0.0788
MGYA00249883/ERZ772724_FASTA.fasta	MGYA00249883	ERZ772724	root:Engineered:Food production:Dairy products	0.4028
MGYA00249884/ERZ772741_FASTA.fasta	MGYA00249884	ERZ772741	root:Engineered:Food production:Dairy products	0.4028
MGYA00249885/ERZ772743_FASTA.fasta	MGYA00249885	ERZ772743	root:Engineered:Food production:Dairy products	0.0734
MGYA00249886/ERZ772745_FASTA.fasta	MGYA00249886	ERZ772745	root:Engineered:Food production:Dairy products	0.0734
MGYA00249887/ERZ772726_FASTA.fasta	MGYA00249887	ERZ772726	root:Engineered:Food production:Dairy products	0.0778
MGYA00249888/ERZ772728_FASTA.fasta	MGYA00249888	ERZ772728	root:Engineered:Food production:Dairy products	0.3874
MGYA00249889/ERZ772730_FASTA.fasta	MGYA00249889	ERZ772730	root:Engineered:Food production:Dairy products	0.0778
MGYA00249890/ERZ772732_FASTA.fasta	MGYA00249890	ERZ772732	root:Engineered:Food production:Dairy products	0.0734
MGYA00249891/ERZ772734_FASTA.fasta	MGYA00249891	ERZ772734	root:Engineered:Food production:Dairy products	0.0734
MGYA00249892/ERZ772736_FASTA.fasta	MGYA00249892	ERZ772736	root:Engineered:Food production:Dairy products	0.3812
MGYA00249893/ERZ772738_FASTA.fasta	MGYA00249893	ERZ772738	root:Engineered:Food production:Dairy products	0.3856
MGYA00249894/ERZ772740_FASTA.fasta	MGYA00249894	ERZ772740	root:Engineered:Food production:Dairy products	0.0778
MGYA00249895/ERZ772742_FASTA.fasta	MGYA00249895	ERZ772742	root:Engineered:Food production:Dairy products	0.079
MGYA00249896/ERZ772744_FASTA.fasta	MGYA00249896	ERZ772744	root:Engineered:Food production:Dairy products	0.375
MGYA00249897/ERZ772746_FASTA.fasta	MGYA00249897	ERZ772746	root:Engineered:Food production:Dairy products	0.3772
MGYA00249898/ERZ772748_FASTA.fasta	MGYA00249898	ERZ772748	root:Engineered:Solid waste:Composting	0.5628
MGYA00249899/ERZ773282_FASTA.fasta	MGYA00249899	ERZ773282	root:Engineered:Wastewater:Activated Sludge	0.8052
MGYA00249900/ERZ773281_FASTA.fasta	MGYA00249900	ERZ773281	root:Engineered:Wastewater:Activated Sludge	0.9154
MGYA00249901/ERZ773285_FASTA.fasta	MGYA00249901	ERZ773285	root:Engineered:Bioreactor	0.8322
MGYA00249902/ERZ773289_FASTA.fasta	MGYA00249902	ERZ773289	root:Engineered:Bioreactor	0.551
MGYA00249903/ERZ773355_FASTA.fasta	MGYA00249903	ERZ773355	root:Engineered:Wastewater:Activated Sludge	0.852
MGYA00249904/ERZ773356_FASTA.fasta	MGYA00249904	ERZ773356	root:Engineered:Biogas plant	0.6304
MGYA00249905/ERZ773358_FASTA.fasta	MGYA00249905	ERZ773358	root:Engineered:Biogas plant:Wet fermentation	0.5844
MGYA00249906/ERZ773362_FASTA.fasta	MGYA00249906	ERZ773362	root:Engineered:Wastewater:Activated Sludge	0.636
MGYA00249907/ERZ773363_FASTA.fasta	MGYA00249907	ERZ773363	root:Engineered:Wastewater:Activated Sludge	0.649
MGYA00249908/ERZ773478_FASTA.fasta	MGYA00249908	ERZ773478	root:Host-associated:Human:Digestive system	0.5426
MGYA00249909/ERZ773480_FASTA.fasta	MGYA00249909	ERZ773480	root:Host-associated:Human:Digestive system	0.4604
MGYA00249910/ERZ773482_FASTA.fasta	MGYA00249910	ERZ773482	root:Host-associated:Human:Digestive system	0.356
MGYA00249911/ERZ773484_FASTA.fasta	MGYA00249911	ERZ773484	root:Host-associated:Human:Digestive system	0.434
MGYA00249912/ERZ773449_FASTA.fasta	MGYA00249912	ERZ773449	root:Host-associated:Human:Digestive system	0.4418
MGYA00249913/ERZ773451_FASTA.fasta	MGYA00249913	ERZ773451	root:Host-associated:Human:Digestive system	0.4732
MGYA00249914/ERZ773453_FASTA.fasta	MGYA00249914	ERZ773453	root:Host-associated:Human:Digestive system	0.387
MGYA00249915/ERZ773455_FASTA.fasta	MGYA00249915	ERZ773455	root:Host-associated:Human:Digestive system	0.549
MGYA00249916/ERZ773457_FASTA.fasta	MGYA00249916	ERZ773457	root:Host-associated:Human:Digestive system	0.5368
MGYA00249917/ERZ773459_FASTA.fasta	MGYA00249917	ERZ773459	root:Host-associated:Human:Digestive system	0.4528
MGYA00249918/ERZ773461_FASTA.fasta	MGYA00249918	ERZ773461	root:Host-associated:Human:Digestive system	0.5262
MGYA00249919/ERZ773463_FASTA.fasta	MGYA00249919	ERZ773463	root:Host-associated:Human:Digestive system	0.4792
MGYA00249920/ERZ773465_FASTA.fasta	MGYA00249920	ERZ773465	root:Host-associated:Human:Digestive system	0.4138
MGYA00249921/ERZ773467_FASTA.fasta	MGYA00249921	ERZ773467	root:Host-associated:Human:Digestive system	0.3432
MGYA00249922/ERZ773469_FASTA.fasta	MGYA00249922	ERZ773469	root:Host-associated:Human:Digestive system	0.4412
MGYA00249923/ERZ773471_FASTA.fasta	MGYA00249923	ERZ773471	root:Host-associated:Human:Digestive system	0.356
MGYA00249924/ERZ773473_FASTA.fasta	MGYA00249924	ERZ773473	root:Host-associated:Human:Digestive system	0.4714
MGYA00249925/ERZ773475_FASTA.fasta	MGYA00249925	ERZ773475	root:Host-associated:Human:Digestive system	0.4704
MGYA00249926/ERZ773477_FASTA.fasta	MGYA00249926	ERZ773477	root:Host-associated:Human:Digestive system	0.4178
MGYA00249927/ERZ773435_FASTA.fasta	MGYA00249927	ERZ773435	root:Host-associated:Human:Digestive system	0.5088
MGYA00249928/ERZ773437_FASTA.fasta	MGYA00249928	ERZ773437	root:Host-associated:Human:Digestive system	0.5422
MGYA00249929/ERZ773439_FASTA.fasta	MGYA00249929	ERZ773439	root:Host-associated:Human:Digestive system	0.6084
MGYA00249930/ERZ773441_FASTA.fasta	MGYA00249930	ERZ773441	root:Host-associated:Human:Digestive system	0.46
MGYA00249931/ERZ773443_FASTA.fasta	MGYA00249931	ERZ773443	root:Host-associated:Human:Digestive system	0.4632
MGYA00249932/ERZ773445_FASTA.fasta	MGYA00249932	ERZ773445	root:Host-associated:Human:Digestive system	0.4874
MGYA00249933/ERZ773447_FASTA.fasta	MGYA00249933	ERZ773447	root:Host-associated:Human:Digestive system	0.4966
MGYA00249934/ERZ773479_FASTA.fasta	MGYA00249934	ERZ773479	root:Host-associated:Human:Digestive system	0.4292
MGYA00249935/ERZ773481_FASTA.fasta	MGYA00249935	ERZ773481	root:Host-associated:Human:Digestive system	0.4616
MGYA00249936/ERZ773483_FASTA.fasta	MGYA00249936	ERZ773483	root:Host-associated:Human:Digestive system	0.3598
MGYA00249937/ERZ773486_FASTA.fasta	MGYA00249937	ERZ773486	root:Host-associated:Human:Digestive system	0.4814
MGYA00249938/ERZ773488_FASTA.fasta	MGYA00249938	ERZ773488	root:Host-associated:Human:Digestive system	0.3778
MGYA00249939/ERZ773490_FASTA.fasta	MGYA00249939	ERZ773490	root:Host-associated:Human:Digestive system	0.4304
MGYA00249940/ERZ773492_FASTA.fasta	MGYA00249940	ERZ773492	root:Host-associated:Human:Digestive system	0.5692
MGYA00249941/ERZ773494_FASTA.fasta	MGYA00249941	ERZ773494	root:Host-associated:Human:Digestive system	0.45
MGYA00249942/ERZ773496_FASTA.fasta	MGYA00249942	ERZ773496	root:Host-associated:Human:Digestive system	0.4334
MGYA00249943/ERZ773498_FASTA.fasta	MGYA00249943	ERZ773498	root:Host-associated:Human:Digestive system	0.393
MGYA00249944/ERZ773500_FASTA.fasta	MGYA00249944	ERZ773500	root:Host-associated:Human:Digestive system	0.5202
MGYA00249945/ERZ773502_FASTA.fasta	MGYA00249945	ERZ773502	root:Host-associated:Human:Digestive system	0.3856
MGYA00249946/ERZ773504_FASTA.fasta	MGYA00249946	ERZ773504	root:Host-associated:Human:Digestive system	0.4448
MGYA00249947/ERZ773506_FASTA.fasta	MGYA00249947	ERZ773506	root:Host-associated:Human:Digestive system	0.4172
MGYA00249948/ERZ773508_FASTA.fasta	MGYA00249948	ERZ773508	root:Host-associated:Human:Digestive system	0.4984
MGYA00249949/ERZ773464_FASTA.fasta	MGYA00249949	ERZ773464	root:Host-associated:Human:Digestive system	0.4542
MGYA00249950/ERZ773466_FASTA.fasta	MGYA00249950	ERZ773466	root:Host-associated:Human:Digestive system	0.5034
MGYA00249951/ERZ773468_FASTA.fasta	MGYA00249951	ERZ773468	root:Host-associated:Human:Digestive system	0.3902
MGYA00249952/ERZ773470_FASTA.fasta	MGYA00249952	ERZ773470	root:Host-associated:Human:Digestive system	0.545
MGYA00249953/ERZ773472_FASTA.fasta	MGYA00249953	ERZ773472	root:Host-associated:Human:Digestive system	0.4012
MGYA00249954/ERZ773474_FASTA.fasta	MGYA00249954	ERZ773474	root:Host-associated:Human:Digestive system	0.4718
MGYA00249955/ERZ773476_FASTA.fasta	MGYA00249955	ERZ773476	root:Host-associated:Human:Digestive system	0.4828
MGYA00249956/ERZ773533_FASTA.fasta	MGYA00249956	ERZ773533	root:Host-associated:Human:Digestive system	0.5074
MGYA00249957/ERZ773374_FASTA.fasta	MGYA00249957	ERZ773374	root:Host-associated:Human:Digestive system	0.4104
MGYA00249958/ERZ773376_FASTA.fasta	MGYA00249958	ERZ773376	root:Host-associated:Human:Digestive system	0.463
MGYA00249959/ERZ773450_FASTA.fasta	MGYA00249959	ERZ773450	root:Host-associated:Human:Digestive system	0.3792
MGYA00249960/ERZ773452_FASTA.fasta	MGYA00249960	ERZ773452	root:Host-associated:Human:Digestive system	0.4132
MGYA00249961/ERZ773454_FASTA.fasta	MGYA00249961	ERZ773454	root:Host-associated:Human:Digestive system	0.4344
MGYA00249962/ERZ773456_FASTA.fasta	MGYA00249962	ERZ773456	root:Host-associated:Human:Digestive system	0.467
MGYA00249963/ERZ773458_FASTA.fasta	MGYA00249963	ERZ773458	root:Host-associated:Human:Digestive system	0.5154
MGYA00249964/ERZ773460_FASTA.fasta	MGYA00249964	ERZ773460	root:Host-associated:Human:Digestive system	0.517
MGYA00249965/ERZ773462_FASTA.fasta	MGYA00249965	ERZ773462	root:Host-associated:Human:Digestive system	0.329
MGYA00249966/ERZ773507_FASTA.fasta	MGYA00249966	ERZ773507	root:Host-associated:Human:Digestive system	0.4552
MGYA00249967/ERZ773509_FASTA.fasta	MGYA00249967	ERZ773509	root:Host-associated:Human:Digestive system	0.4646
MGYA00249968/ERZ773511_FASTA.fasta	MGYA00249968	ERZ773511	root:Host-associated:Human:Digestive system	0.3322
MGYA00249969/ERZ773513_FASTA.fasta	MGYA00249969	ERZ773513	root:Host-associated:Human:Digestive system	0.5408
MGYA00249970/ERZ773515_FASTA.fasta	MGYA00249970	ERZ773515	root:Host-associated:Human:Digestive system	0.5134
MGYA00249971/ERZ773517_FASTA.fasta	MGYA00249971	ERZ773517	root:Host-associated:Human:Digestive system	0.5302
MGYA00249972/ERZ773519_FASTA.fasta	MGYA00249972	ERZ773519	root:Host-associated:Human:Digestive system	0.4794
MGYA00249973/ERZ773521_FASTA.fasta	MGYA00249973	ERZ773521	root:Host-associated:Human:Digestive system	0.5402
MGYA00249974/ERZ773523_FASTA.fasta	MGYA00249974	ERZ773523	root:Host-associated:Human:Digestive system	0.4948
MGYA00249975/ERZ773525_FASTA.fasta	MGYA00249975	ERZ773525	root:Host-associated:Human:Digestive system	0.2738
MGYA00249976/ERZ773529_FASTA.fasta	MGYA00249976	ERZ773529	root:Host-associated:Human:Digestive system	0.48
MGYA00249977/ERZ773531_FASTA.fasta	MGYA00249977	ERZ773531	root:Host-associated:Human:Digestive system	0.2676
MGYA00249978/ERZ773485_FASTA.fasta	MGYA00249978	ERZ773485	root:Host-associated:Human:Digestive system	0.3254
MGYA00249979/ERZ773487_FASTA.fasta	MGYA00249979	ERZ773487	root:Host-associated:Human:Digestive system	0.3654
MGYA00249980/ERZ773489_FASTA.fasta	MGYA00249980	ERZ773489	root:Host-associated:Human:Digestive system	0.4908
MGYA00249981/ERZ773491_FASTA.fasta	MGYA00249981	ERZ773491	root:Host-associated:Human:Digestive system	0.2994
MGYA00249982/ERZ773493_FASTA.fasta	MGYA00249982	ERZ773493	root:Host-associated:Human:Digestive system	0.5264
MGYA00249983/ERZ773495_FASTA.fasta	MGYA00249983	ERZ773495	root:Host-associated:Human:Digestive system	0.4538
MGYA00249984/ERZ773497_FASTA.fasta	MGYA00249984	ERZ773497	root:Host-associated:Human:Digestive system	0.5688
MGYA00249985/ERZ773499_FASTA.fasta	MGYA00249985	ERZ773499	root:Host-associated:Human:Digestive system	0.3326
MGYA00249986/ERZ773501_FASTA.fasta	MGYA00249986	ERZ773501	root:Host-associated:Human:Digestive system	0.3612
MGYA00249987/ERZ773503_FASTA.fasta	MGYA00249987	ERZ773503	root:Host-associated:Human:Digestive system	0.3234
MGYA00249988/ERZ773505_FASTA.fasta	MGYA00249988	ERZ773505	root:Host-associated:Human:Digestive system	0.5004
MGYA00249989/ERZ773378_FASTA.fasta	MGYA00249989	ERZ773378	root:Host-associated:Human:Digestive system	0.4494
MGYA00249990/ERZ773387_FASTA.fasta	MGYA00249990	ERZ773387	root:Host-associated:Human:Digestive system	0.4812
MGYA00249991/ERZ773389_FASTA.fasta	MGYA00249991	ERZ773389	root:Host-associated:Human:Digestive system	0.471
MGYA00249992/ERZ773391_FASTA.fasta	MGYA00249992	ERZ773391	root:Host-associated:Human:Digestive system	0.2836
MGYA00249993/ERZ773393_FASTA.fasta	MGYA00249993	ERZ773393	root:Host-associated:Human:Digestive system	0.4864
MGYA00249994/ERZ773395_FASTA.fasta	MGYA00249994	ERZ773395	root:Host-associated:Human:Digestive system	0.431
MGYA00249995/ERZ773397_FASTA.fasta	MGYA00249995	ERZ773397	root:Host-associated:Human:Digestive system	0.4948
MGYA00249996/ERZ773399_FASTA.fasta	MGYA00249996	ERZ773399	root:Host-associated:Human:Digestive system	0.3864
MGYA00249997/ERZ773401_FASTA.fasta	MGYA00249997	ERZ773401	root:Host-associated:Human:Digestive system	0.452
MGYA00249998/ERZ773403_FASTA.fasta	MGYA00249998	ERZ773403	root:Host-associated:Human:Digestive system	0.3588
MGYA00249999/ERZ773405_FASTA.fasta	MGYA00249999	ERZ773405	root:Host-associated:Human:Digestive system	0.507
MGYA00250000/ERZ773412_FASTA.fasta	MGYA00250000	ERZ773412	root:Host-associated:Human:Digestive system	0.3402
MGYA00250001/ERZ773414_FASTA.fasta	MGYA00250001	ERZ773414	root:Host-associated:Human:Digestive system	0.4526
MGYA00250002/ERZ773416_FASTA.fasta	MGYA00250002	ERZ773416	root:Host-associated:Human:Digestive system	0.5814
MGYA00250003/ERZ773418_FASTA.fasta	MGYA00250003	ERZ773418	root:Host-associated:Human:Digestive system	0.3574
MGYA00250004/ERZ773420_FASTA.fasta	MGYA00250004	ERZ773420	root:Host-associated:Human:Digestive system	0.4788
MGYA00250005/ERZ773422_FASTA.fasta	MGYA00250005	ERZ773422	root:Host-associated:Human:Digestive system	0.5086
MGYA00250006/ERZ773424_FASTA.fasta	MGYA00250006	ERZ773424	root:Host-associated:Human:Digestive system	0.5442
MGYA00250007/ERZ773426_FASTA.fasta	MGYA00250007	ERZ773426	root:Host-associated:Human:Digestive system	0.3528
MGYA00250008/ERZ773428_FASTA.fasta	MGYA00250008	ERZ773428	root:Host-associated:Human:Digestive system	0.5256
MGYA00250009/ERZ773430_FASTA.fasta	MGYA00250009	ERZ773430	root:Host-associated:Human:Digestive system	0.5446
MGYA00250010/ERZ773407_FASTA.fasta	MGYA00250010	ERZ773407	root:Host-associated:Human:Digestive system	0.4552
MGYA00250011/ERZ773409_FASTA.fasta	MGYA00250011	ERZ773409	root:Host-associated:Human:Digestive system	0.4438
MGYA00250012/ERZ773411_FASTA.fasta	MGYA00250012	ERZ773411	root:Host-associated:Human:Digestive system	0.3986
MGYA00250013/ERZ773413_FASTA.fasta	MGYA00250013	ERZ773413	root:Host-associated:Human:Digestive system	0.4094
MGYA00250014/ERZ773415_FASTA.fasta	MGYA00250014	ERZ773415	root:Host-associated:Human:Digestive system	0.4166
MGYA00250015/ERZ773417_FASTA.fasta	MGYA00250015	ERZ773417	root:Host-associated:Human:Digestive system	0.4146
MGYA00250016/ERZ773419_FASTA.fasta	MGYA00250016	ERZ773419	root:Host-associated:Human:Digestive system	0.4324
MGYA00250017/ERZ773421_FASTA.fasta	MGYA00250017	ERZ773421	root:Host-associated:Human:Digestive system	0.4436
MGYA00250018/ERZ773423_FASTA.fasta	MGYA00250018	ERZ773423	root:Host-associated:Human:Digestive system	0.4586
MGYA00250019/ERZ773425_FASTA.fasta	MGYA00250019	ERZ773425	root:Host-associated:Human:Digestive system	0.5204
MGYA00250020/ERZ773427_FASTA.fasta	MGYA00250020	ERZ773427	root:Host-associated:Human:Digestive system	0.2658
MGYA00250021/ERZ773429_FASTA.fasta	MGYA00250021	ERZ773429	root:Host-associated:Human:Digestive system	0.3914
MGYA00250022/ERZ773431_FASTA.fasta	MGYA00250022	ERZ773431	root:Host-associated:Human:Digestive system	0.3992
MGYA00250023/ERZ773433_FASTA.fasta	MGYA00250023	ERZ773433	root:Host-associated:Human:Digestive system	0.446
MGYA00250024/ERZ773432_FASTA.fasta	MGYA00250024	ERZ773432	root:Host-associated:Human:Digestive system	0.4422
MGYA00250025/ERZ773434_FASTA.fasta	MGYA00250025	ERZ773434	root:Host-associated:Human:Digestive system	0.4006
MGYA00250026/ERZ773436_FASTA.fasta	MGYA00250026	ERZ773436	root:Host-associated:Human:Digestive system	0.4316
MGYA00250027/ERZ773438_FASTA.fasta	MGYA00250027	ERZ773438	root:Host-associated:Human:Digestive system	0.4176
MGYA00250028/ERZ773440_FASTA.fasta	MGYA00250028	ERZ773440	root:Host-associated:Human:Digestive system	0.5396
MGYA00250029/ERZ773442_FASTA.fasta	MGYA00250029	ERZ773442	root:Host-associated:Human:Digestive system	0.429
MGYA00250030/ERZ773444_FASTA.fasta	MGYA00250030	ERZ773444	root:Host-associated:Human:Digestive system	0.4336
MGYA00250031/ERZ773446_FASTA.fasta	MGYA00250031	ERZ773446	root:Host-associated:Human:Digestive system	0.4952
MGYA00250032/ERZ773448_FASTA.fasta	MGYA00250032	ERZ773448	root:Host-associated:Human:Digestive system	0.5392
MGYA00250033/ERZ773364_FASTA.fasta	MGYA00250033	ERZ773364	root:Host-associated:Human:Digestive system	0.3972
MGYA00250034/ERZ773366_FASTA.fasta	MGYA00250034	ERZ773366	root:Host-associated:Human:Digestive system	0.4292
MGYA00250035/ERZ773368_FASTA.fasta	MGYA00250035	ERZ773368	root:Host-associated:Human:Digestive system	0.398
MGYA00250036/ERZ773370_FASTA.fasta	MGYA00250036	ERZ773370	root:Host-associated:Human:Digestive system	0.5094
MGYA00250037/ERZ773372_FASTA.fasta	MGYA00250037	ERZ773372	root:Host-associated:Human:Digestive system	0.3018
MGYA00250038/ERZ773381_FASTA.fasta	MGYA00250038	ERZ773381	root:Host-associated:Human:Digestive system	0.4188
MGYA00250039/ERZ773383_FASTA.fasta	MGYA00250039	ERZ773383	root:Host-associated:Human:Digestive system	0.4772
MGYA00250040/ERZ773385_FASTA.fasta	MGYA00250040	ERZ773385	root:Host-associated:Human:Digestive system	0.4492
MGYA00250041/ERZ773530_FASTA.fasta	MGYA00250041	ERZ773530	root:Host-associated:Human:Digestive system	0.3108
MGYA00250042/ERZ773532_FASTA.fasta	MGYA00250042	ERZ773532	root:Host-associated:Human:Digestive system	0.574
MGYA00250043/ERZ773534_FASTA.fasta	MGYA00250043	ERZ773534	root:Host-associated:Human:Digestive system	0.3208
MGYA00250044/ERZ773365_FASTA.fasta	MGYA00250044	ERZ773365	root:Host-associated:Human:Digestive system	0.5314
MGYA00250045/ERZ773367_FASTA.fasta	MGYA00250045	ERZ773367	root:Host-associated:Human:Digestive system	0.342
MGYA00250046/ERZ773369_FASTA.fasta	MGYA00250046	ERZ773369	root:Host-associated:Human:Digestive system	0.4426
MGYA00250047/ERZ773371_FASTA.fasta	MGYA00250047	ERZ773371	root:Host-associated:Human:Digestive system	0.5296
MGYA00250048/ERZ773373_FASTA.fasta	MGYA00250048	ERZ773373	root:Host-associated:Human:Digestive system	0.5976
MGYA00250049/ERZ773375_FASTA.fasta	MGYA00250049	ERZ773375	root:Host-associated:Human:Digestive system	0.3736
MGYA00250050/ERZ773377_FASTA.fasta	MGYA00250050	ERZ773377	root:Host-associated:Human:Digestive system	0.4444
MGYA00250051/ERZ773379_FASTA.fasta	MGYA00250051	ERZ773379	root:Host-associated:Human:Digestive system	0.4082
MGYA00250052/ERZ773380_FASTA.fasta	MGYA00250052	ERZ773380	root:Host-associated:Human:Digestive system	0.444
MGYA00250053/ERZ773382_FASTA.fasta	MGYA00250053	ERZ773382	root:Host-associated:Human:Digestive system	0.2936
MGYA00250054/ERZ773384_FASTA.fasta	MGYA00250054	ERZ773384	root:Host-associated:Human:Digestive system	0.408
MGYA00250055/ERZ773386_FASTA.fasta	MGYA00250055	ERZ773386	root:Host-associated:Human:Digestive system	0.4218
MGYA00250056/ERZ773388_FASTA.fasta	MGYA00250056	ERZ773388	root:Host-associated:Human:Digestive system	0.4128
MGYA00250057/ERZ773390_FASTA.fasta	MGYA00250057	ERZ773390	root:Host-associated:Human:Digestive system	0.378
MGYA00250058/ERZ773392_FASTA.fasta	MGYA00250058	ERZ773392	root:Host-associated:Human:Digestive system	0.3236
MGYA00250059/ERZ773394_FASTA.fasta	MGYA00250059	ERZ773394	root:Host-associated:Human:Digestive system	0.5426
MGYA00250060/ERZ773396_FASTA.fasta	MGYA00250060	ERZ773396	root:Host-associated:Human:Digestive system	0.367
MGYA00250061/ERZ773398_FASTA.fasta	MGYA00250061	ERZ773398	root:Host-associated:Human:Digestive system	0.4934
MGYA00250062/ERZ773400_FASTA.fasta	MGYA00250062	ERZ773400	root:Host-associated:Human:Digestive system	0.456
MGYA00250063/ERZ773402_FASTA.fasta	MGYA00250063	ERZ773402	root:Host-associated:Human:Digestive system	0.4732
MGYA00250064/ERZ773510_FASTA.fasta	MGYA00250064	ERZ773510	root:Host-associated:Human:Digestive system	0.3938
MGYA00250065/ERZ773512_FASTA.fasta	MGYA00250065	ERZ773512	root:Host-associated:Human:Digestive system	0.5166
MGYA00250066/ERZ773514_FASTA.fasta	MGYA00250066	ERZ773514	root:Host-associated:Human:Digestive system	0.5742
MGYA00250067/ERZ773516_FASTA.fasta	MGYA00250067	ERZ773516	root:Host-associated:Human:Digestive system	0.461
MGYA00250068/ERZ773518_FASTA.fasta	MGYA00250068	ERZ773518	root:Host-associated:Human:Digestive system	0.427
MGYA00250069/ERZ773520_FASTA.fasta	MGYA00250069	ERZ773520	root:Host-associated:Human:Digestive system	0.5752
MGYA00250070/ERZ773522_FASTA.fasta	MGYA00250070	ERZ773522	root:Host-associated:Human:Digestive system	0.4148
MGYA00250071/ERZ773524_FASTA.fasta	MGYA00250071	ERZ773524	root:Host-associated:Human:Digestive system	0.44
MGYA00250072/ERZ773404_FASTA.fasta	MGYA00250072	ERZ773404	root:Host-associated:Human:Digestive system	0.4418
MGYA00250073/ERZ773406_FASTA.fasta	MGYA00250073	ERZ773406	root:Host-associated:Human:Digestive system	0.5002
MGYA00250074/ERZ773408_FASTA.fasta	MGYA00250074	ERZ773408	root:Host-associated:Human:Digestive system	0.4204
MGYA00250075/ERZ773410_FASTA.fasta	MGYA00250075	ERZ773410	root:Host-associated:Human:Digestive system	0.474
MGYA00250076/ERZ650197_FASTA.fasta	MGYA00250076	ERZ650197	root:Environmental:Aquatic:Estuary:Sediment	0.8206
MGYA00250077/ERZ650199_FASTA.fasta	MGYA00250077	ERZ650199	root:Environmental:Aquatic:Estuary:Sediment	0.6532
MGYA00250078/ERZ650206_FASTA.fasta	MGYA00250078	ERZ650206	root:Environmental:Aquatic:Estuary:Sediment	0.5242
MGYA00250079/ERZ650211_FASTA.fasta	MGYA00250079	ERZ650211	root:Environmental:Aquatic:Estuary:Sediment	0.643
MGYA00250080/ERZ650213_FASTA.fasta	MGYA00250080	ERZ650213	root:Environmental:Aquatic:Estuary:Sediment	0.6244
MGYA00250081/ERZ650215_FASTA.fasta	MGYA00250081	ERZ650215	root:Environmental:Aquatic:Estuary:Sediment	0.4422
MGYA00250082/ERZ650217_FASTA.fasta	MGYA00250082	ERZ650217	root:Environmental:Aquatic:Estuary:Sediment	0.5826
MGYA00250083/ERZ650219_FASTA.fasta	MGYA00250083	ERZ650219	root:Environmental:Aquatic:Estuary:Sediment	0.7228
MGYA00250084/ERZ650200_FASTA.fasta	MGYA00250084	ERZ650200	root:Environmental:Aquatic:Estuary:Sediment	0.792
MGYA00250085/ERZ650202_FASTA.fasta	MGYA00250085	ERZ650202	root:Environmental:Aquatic:Estuary:Sediment	0.7448
MGYA00250086/ERZ650204_FASTA.fasta	MGYA00250086	ERZ650204	root:Environmental:Aquatic:Estuary:Sediment	0.687
MGYA00250087/ERZ650207_FASTA.fasta	MGYA00250087	ERZ650207	root:Environmental:Aquatic:Estuary:Sediment	0.7188
MGYA00250088/ERZ650209_FASTA.fasta	MGYA00250088	ERZ650209	root:Environmental:Aquatic:Estuary:Sediment	0.4714
MGYA00250089/ERZ650208_FASTA.fasta	MGYA00250089	ERZ650208	root:Environmental:Aquatic:Estuary:Sediment	0.7636
MGYA00250090/ERZ650210_FASTA.fasta	MGYA00250090	ERZ650210	root:Environmental:Aquatic:Estuary:Sediment	0.66
MGYA00250091/ERZ650201_FASTA.fasta	MGYA00250091	ERZ650201	root:Environmental:Aquatic:Estuary:Sediment	0.747
MGYA00250092/ERZ650203_FASTA.fasta	MGYA00250092	ERZ650203	root:Environmental:Aquatic:Estuary:Sediment	0.736
MGYA00250093/ERZ650205_FASTA.fasta	MGYA00250093	ERZ650205	root:Environmental:Aquatic:Estuary:Sediment	0.6568
MGYA00250094/ERZ650212_FASTA.fasta	MGYA00250094	ERZ650212	root:Environmental:Aquatic:Estuary:Sediment	0.6844
MGYA00250095/ERZ650214_FASTA.fasta	MGYA00250095	ERZ650214	root:Environmental:Aquatic:Estuary:Sediment	0.4966
MGYA00250096/ERZ650216_FASTA.fasta	MGYA00250096	ERZ650216	root:Environmental:Aquatic:Estuary:Sediment	0.7194
MGYA00250097/ERZ650218_FASTA.fasta	MGYA00250097	ERZ650218	root:Environmental:Aquatic:Estuary:Sediment	0.7206
MGYA00250098/ERZ650220_FASTA.fasta	MGYA00250098	ERZ650220	root:Environmental:Aquatic:Estuary:Sediment	0.6668
MGYA00250099/ERZ650198_FASTA.fasta	MGYA00250099	ERZ650198	root:Environmental:Aquatic:Estuary:Sediment	0.676
MGYA00250100/ERZ650196_FASTA.fasta	MGYA00250100	ERZ650196	root:Environmental:Aquatic:Estuary:Sediment	0.8322
MGYA00250101/ERZ650185_FASTA.fasta	MGYA00250101	ERZ650185	root:Environmental:Aquatic:Marine:Sediment	0.0316
MGYA00250102/ERZ650187_FASTA.fasta	MGYA00250102	ERZ650187	root:Environmental:Aquatic:Marine:Sediment	0.049
MGYA00250103/ERZ650189_FASTA.fasta	MGYA00250103	ERZ650189	root:Environmental:Aquatic:Marine:Sediment	0.3764
MGYA00250104/ERZ650191_FASTA.fasta	MGYA00250104	ERZ650191	root:Environmental:Aquatic:Marine:Sediment	0.1646
MGYA00250105/ERZ650193_FASTA.fasta	MGYA00250105	ERZ650193	root:Environmental:Aquatic:Marine:Sediment	0.038
MGYA00250106/ERZ650184_FASTA.fasta	MGYA00250106	ERZ650184	root:Environmental:Aquatic:Marine:Sediment	0.0316
MGYA00250107/ERZ650186_FASTA.fasta	MGYA00250107	ERZ650186	root:Environmental:Aquatic:Marine:Sediment	0.14
MGYA00250108/ERZ650188_FASTA.fasta	MGYA00250108	ERZ650188	root:Environmental:Aquatic:Marine:Sediment	0.0294
MGYA00250109/ERZ650190_FASTA.fasta	MGYA00250109	ERZ650190	root:Environmental:Aquatic:Marine:Sediment	0.2788
MGYA00250110/ERZ650192_FASTA.fasta	MGYA00250110	ERZ650192	root:Environmental:Aquatic:Marine:Sediment	0.1914
MGYA00250111/ERZ650178_FASTA.fasta	MGYA00250111	ERZ650178	root:Environmental:Aquatic:Marine	0.0342
MGYA00250112/ERZ650180_FASTA.fasta	MGYA00250112	ERZ650180	root:Environmental:Aquatic:Marine	0.0678
MGYA00250113/ERZ650182_FASTA.fasta	MGYA00250113	ERZ650182	root:Environmental:Aquatic:Marine	0.0362
MGYA00250114/ERZ650177_FASTA.fasta	MGYA00250114	ERZ650177	root:Environmental:Aquatic:Marine	0.0344
MGYA00250115/ERZ650179_FASTA.fasta	MGYA00250115	ERZ650179	root:Environmental:Aquatic:Marine	0.067
MGYA00250116/ERZ650181_FASTA.fasta	MGYA00250116	ERZ650181	root:Environmental:Aquatic:Marine	0.0348
MGYA00250117/ERZ650183_FASTA.fasta	MGYA00250117	ERZ650183	root:Environmental:Aquatic:Marine	0.0712
MGYA00250118/ERZ650176_FASTA.fasta	MGYA00250118	ERZ650176	root:Environmental:Aquatic:Marine	0.0464
MGYA00250119/ERZ724591_FASTA.fasta	MGYA00250119	ERZ724591	root:Environmental:Aquatic:Marine	0.0346
MGYA00345647/ERZ802819_FASTA.fasta	MGYA00345647	ERZ802819	root:Environmental:Aquatic:Marine	0.6826
MGYA00345648/ERZ802821_FASTA.fasta	MGYA00345648	ERZ802821	root:Environmental:Aquatic:Marine	0.6718
MGYA00345649/ERZ802822_FASTA.fasta	MGYA00345649	ERZ802822	root:Environmental:Aquatic:Marine	0.7122
MGYA00345650/ERZ802824_FASTA.fasta	MGYA00345650	ERZ802824	root:Environmental:Aquatic:Marine	0.7302
MGYA00345651/ERZ802826_FASTA.fasta	MGYA00345651	ERZ802826	root:Environmental:Aquatic:Marine	0.6416
MGYA00345652/ERZ802827_FASTA.fasta	MGYA00345652	ERZ802827	root:Environmental:Aquatic:Marine	0.6272
MGYA00345653/ERZ802829_FASTA.fasta	MGYA00345653	ERZ802829	root:Environmental:Aquatic:Marine	0.1858
MGYA00345654/ERZ802830_FASTA.fasta	MGYA00345654	ERZ802830	root:Environmental:Aquatic:Marine	0.7572
MGYA00345655/ERZ802831_FASTA.fasta	MGYA00345655	ERZ802831	root:Environmental:Aquatic:Marine	0.6904
MGYA00345656/ERZ802832_FASTA.fasta	MGYA00345656	ERZ802832	root:Environmental:Aquatic:Marine	0.4854
MGYA00345657/ERZ802834_FASTA.fasta	MGYA00345657	ERZ802834	root:Environmental:Aquatic:Marine	0.6028
MGYA00345658/ERZ802836_FASTA.fasta	MGYA00345658	ERZ802836	root:Environmental:Aquatic:Marine	0.4364
MGYA00345659/ERZ802838_FASTA.fasta	MGYA00345659	ERZ802838	root:Environmental:Aquatic:Marine	0.622
MGYA00345660/ERZ802840_FASTA.fasta	MGYA00345660	ERZ802840	root:Environmental:Aquatic:Marine	0.5972
MGYA00345661/ERZ802844_FASTA.fasta	MGYA00345661	ERZ802844	root:Environmental:Aquatic:Marine	0.7664
MGYA00345662/ERZ802846_FASTA.fasta	MGYA00345662	ERZ802846	root:Environmental:Aquatic:Marine	0.8182
MGYA00345663/ERZ802847_FASTA.fasta	MGYA00345663	ERZ802847	root:Environmental:Aquatic:Marine	0.7596
MGYA00345664/ERZ802849_FASTA.fasta	MGYA00345664	ERZ802849	root:Environmental:Aquatic:Marine	0.7894
MGYA00345665/ERZ802851_FASTA.fasta	MGYA00345665	ERZ802851	root:Environmental:Aquatic:Marine	0.6968
MGYA00345666/ERZ802853_FASTA.fasta	MGYA00345666	ERZ802853	root:Environmental:Aquatic:Marine	0.6782
MGYA00345667/ERZ802855_FASTA.fasta	MGYA00345667	ERZ802855	root:Environmental:Aquatic:Marine	0.652
MGYA00345668/ERZ802859_FASTA.fasta	MGYA00345668	ERZ802859	root:Environmental:Aquatic:Marine	0.319
MGYA00345669/ERZ802861_FASTA.fasta	MGYA00345669	ERZ802861	root:Environmental:Aquatic:Marine	0.6468
MGYA00345670/ERZ802864_FASTA.fasta	MGYA00345670	ERZ802864	root:Environmental:Aquatic:Marine	0.7312
MGYA00345671/ERZ802866_FASTA.fasta	MGYA00345671	ERZ802866	root:Environmental:Aquatic:Marine	0.7496
MGYA00345672/ERZ802870_FASTA.fasta	MGYA00345672	ERZ802870	root:Environmental:Aquatic:Marine	0.7324
MGYA00345673/ERZ802872_FASTA.fasta	MGYA00345673	ERZ802872	root:Environmental:Aquatic:Marine	0.4218
MGYA00345674/ERZ802873_FASTA.fasta	MGYA00345674	ERZ802873	root:Environmental:Aquatic:Marine	0.7298
MGYA00345675/ERZ806357_FASTA.fasta	MGYA00345675	ERZ806357	root:Environmental:Aquatic:Marine	0.7304
MGYA00345676/ERZ806358_FASTA.fasta	MGYA00345676	ERZ806358	root:Environmental:Aquatic:Marine	0.7192
MGYA00345677/ERZ806360_FASTA.fasta	MGYA00345677	ERZ806360	root:Environmental:Aquatic:Marine	0.7522
MGYA00345678/ERZ806361_FASTA.fasta	MGYA00345678	ERZ806361	root:Environmental:Aquatic:Marine	0.72
MGYA00345679/ERZ806362_FASTA.fasta	MGYA00345679	ERZ806362	root:Environmental:Aquatic:Marine	0.7346
MGYA00345680/ERZ806363_FASTA.fasta	MGYA00345680	ERZ806363	root:Environmental:Aquatic:Marine	0.7424
MGYA00345681/ERZ806364_FASTA.fasta	MGYA00345681	ERZ806364	root:Environmental:Aquatic:Marine	0.8026
MGYA00345682/ERZ806365_FASTA.fasta	MGYA00345682	ERZ806365	root:Environmental:Aquatic:Marine	0.6554
MGYA00345683/ERZ806366_FASTA.fasta	MGYA00345683	ERZ806366	root:Environmental:Aquatic:Marine	0.2254
MGYA00345684/ERZ806367_FASTA.fasta	MGYA00345684	ERZ806367	root:Environmental:Aquatic:Marine	0.6372
MGYA00345685/ERZ806368_FASTA.fasta	MGYA00345685	ERZ806368	root:Environmental:Aquatic:Marine	0.7324
MGYA00345686/ERZ806370_FASTA.fasta	MGYA00345686	ERZ806370	root:Environmental:Aquatic:Marine	0.7626
MGYA00345687/ERZ806371_FASTA.fasta	MGYA00345687	ERZ806371	root:Environmental:Aquatic:Marine	0.696
MGYA00345688/ERZ806372_FASTA.fasta	MGYA00345688	ERZ806372	root:Environmental:Aquatic:Marine	0.638
MGYA00345689/ERZ806373_FASTA.fasta	MGYA00345689	ERZ806373	root:Environmental:Aquatic:Marine	0.641
MGYA00345690/ERZ806374_FASTA.fasta	MGYA00345690	ERZ806374	root:Environmental:Aquatic:Marine	0.2922
MGYA00345691/ERZ806375_FASTA.fasta	MGYA00345691	ERZ806375	root:Environmental:Aquatic:Marine	0.747
MGYA00345692/ERZ806376_FASTA.fasta	MGYA00345692	ERZ806376	root:Environmental:Aquatic:Marine	0.6576
MGYA00345693/ERZ806377_FASTA.fasta	MGYA00345693	ERZ806377	root:Environmental:Aquatic:Marine	0.6472
MGYA00345694/ERZ806378_FASTA.fasta	MGYA00345694	ERZ806378	root:Environmental:Aquatic:Marine	0.6662
MGYA00345695/ERZ806379_FASTA.fasta	MGYA00345695	ERZ806379	root:Environmental:Aquatic:Marine	0.7596
MGYA00345696/ERZ806380_FASTA.fasta	MGYA00345696	ERZ806380	root:Environmental:Aquatic:Marine	0.7522
MGYA00354805/ERZ841119_FASTA.fasta	MGYA00354805	ERZ841119	root:Environmental:Aquatic:Freshwater:Sediment	0.7514
MGYA00354806/ERZ841129_FASTA.fasta	MGYA00354806	ERZ841129	root:Environmental:Aquatic:Freshwater:Sediment	0.743
MGYA00354807/ERZ841138_FASTA.fasta	MGYA00354807	ERZ841138	root:Environmental:Aquatic:Freshwater:Sediment	0.723
MGYA00354808/ERZ841144_FASTA.fasta	MGYA00354808	ERZ841144	root:Environmental:Aquatic:Freshwater:Sediment	0.7524
MGYA00354809/ERZ841149_FASTA.fasta	MGYA00354809	ERZ841149	root:Environmental:Aquatic:Freshwater:Sediment	0.7716
MGYA00354810/ERZ841155_FASTA.fasta	MGYA00354810	ERZ841155	root:Environmental:Aquatic:Freshwater:Sediment	0.7742
MGYA00354811/ERZ841162_FASTA.fasta	MGYA00354811	ERZ841162	root:Environmental:Aquatic:Freshwater:Sediment	0.7876
MGYA00354812/ERZ841172_FASTA.fasta	MGYA00354812	ERZ841172	root:Environmental:Aquatic:Freshwater:Sediment	0.7946
MGYA00354813/ERZ841185_FASTA.fasta	MGYA00354813	ERZ841185	root:Environmental:Aquatic:Freshwater:Sediment	0.7802
MGYA00367508/ERZ828930_FASTA.fasta	MGYA00367508	ERZ828930	root:Environmental:Aquatic:Marine	0.0736
MGYA00367509/ERZ828932_FASTA.fasta	MGYA00367509	ERZ828932	root:Environmental:Aquatic:Marine	0.1724
MGYA00367510/ERZ828934_FASTA.fasta	MGYA00367510	ERZ828934	root:Environmental:Aquatic:Marine	0.7482
MGYA00367511/ERZ828940_FASTA.fasta	MGYA00367511	ERZ828940	root:Environmental:Aquatic:Marine	0.7214
MGYA00367512/ERZ828944_FASTA.fasta	MGYA00367512	ERZ828944	root:Environmental:Aquatic:Marine	0.737
MGYA00367513/ERZ828946_FASTA.fasta	MGYA00367513	ERZ828946	root:Environmental:Aquatic:Marine	0.733
MGYA00367514/ERZ828948_FASTA.fasta	MGYA00367514	ERZ828948	root:Environmental:Aquatic:Marine	0.7024
MGYA00367515/ERZ828950_FASTA.fasta	MGYA00367515	ERZ828950	root:Environmental:Aquatic:Marine	0.7984
MGYA00367516/ERZ829053_FASTA.fasta	MGYA00367516	ERZ829053	root:Environmental:Aquatic:Marine	0.8076
MGYA00367517/ERZ829055_FASTA.fasta	MGYA00367517	ERZ829055	root:Environmental:Aquatic:Marine	0.781
MGYA00367518/ERZ829058_FASTA.fasta	MGYA00367518	ERZ829058	root:Environmental:Aquatic:Marine	0.3618
MGYA00367519/ERZ829061_FASTA.fasta	MGYA00367519	ERZ829061	root:Environmental:Aquatic:Marine	0.7826
MGYA00367520/ERZ829063_FASTA.fasta	MGYA00367520	ERZ829063	root:Environmental:Aquatic:Marine	0.413
MGYA00367521/ERZ829065_FASTA.fasta	MGYA00367521	ERZ829065	root:Environmental:Aquatic:Marine	0.7304
MGYA00367522/ERZ829075_FASTA.fasta	MGYA00367522	ERZ829075	root:Environmental:Aquatic:Marine	0.9794
MGYA00367523/ERZ829332_FASTA.fasta	MGYA00367523	ERZ829332	root:Environmental:Aquatic:Marine	0.7492
MGYA00367524/ERZ829407_FASTA.fasta	MGYA00367524	ERZ829407	root:Environmental:Aquatic:Marine	0.7656
MGYA00367525/ERZ829562_FASTA.fasta	MGYA00367525	ERZ829562	root:Environmental:Aquatic:Marine	0.9498
MGYA00367526/ERZ829801_FASTA.fasta	MGYA00367526	ERZ829801	root:Environmental:Aquatic:Marine	0.6458
MGYA00367527/ERZ829845_FASTA.fasta	MGYA00367527	ERZ829845	root:Environmental:Aquatic:Marine	0.64
MGYA00367528/ERZ829891_FASTA.fasta	MGYA00367528	ERZ829891	root:Environmental:Aquatic:Marine	0.6472
MGYA00367529/ERZ829933_FASTA.fasta	MGYA00367529	ERZ829933	root:Environmental:Aquatic:Marine	0.6232
MGYA00367530/ERZ829971_FASTA.fasta	MGYA00367530	ERZ829971	root:Environmental:Aquatic:Marine	0.7326
MGYA00367531/ERZ830098_FASTA.fasta	MGYA00367531	ERZ830098	root:Environmental:Aquatic:Marine	0.8626
MGYA00367532/ERZ830535_FASTA.fasta	MGYA00367532	ERZ830535	root:Environmental:Aquatic:Marine	0.7818
MGYA00367533/ERZ830869_FASTA.fasta	MGYA00367533	ERZ830869	root:Environmental:Aquatic:Marine	0.7556
MGYA00367534/ERZ831056_FASTA.fasta	MGYA00367534	ERZ831056	root:Environmental:Aquatic:Marine	0.6026
MGYA00367535/ERZ831144_FASTA.fasta	MGYA00367535	ERZ831144	root:Environmental:Aquatic:Marine	0.943
MGYA00367536/ERZ831412_FASTA.fasta	MGYA00367536	ERZ831412	root:Environmental:Aquatic:Marine	0.637
MGYA00367537/ERZ831446_FASTA.fasta	MGYA00367537	ERZ831446	root:Environmental:Aquatic:Marine	0.8158
MGYA00367538/ERZ831745_FASTA.fasta	MGYA00367538	ERZ831745	root:Environmental:Aquatic:Marine	0.924
MGYA00367539/ERZ832020_FASTA.fasta	MGYA00367539	ERZ832020	root:Environmental:Aquatic:Marine	0.5594
MGYA00367540/ERZ832104_FASTA.fasta	MGYA00367540	ERZ832104	root:Environmental:Aquatic:Marine	0.8326
MGYA00367541/ERZ832643_FASTA.fasta	MGYA00367541	ERZ832643	root:Environmental:Aquatic:Marine	0.8424
MGYA00367542/ERZ832854_FASTA.fasta	MGYA00367542	ERZ832854	root:Environmental:Aquatic:Marine	0.565
MGYA00367543/ERZ832923_FASTA.fasta	MGYA00367543	ERZ832923	root:Environmental:Aquatic:Marine	0.6028
MGYA00367544/ERZ833000_FASTA.fasta	MGYA00367544	ERZ833000	root:Environmental:Aquatic:Marine	0.9324
MGYA00367545/ERZ833197_FASTA.fasta	MGYA00367545	ERZ833197	root:Environmental:Aquatic:Marine	0.6652
MGYA00367546/ERZ833247_FASTA.fasta	MGYA00367546	ERZ833247	root:Environmental:Aquatic:Marine	0.602
MGYA00367547/ERZ833316_FASTA.fasta	MGYA00367547	ERZ833316	root:Environmental:Aquatic:Marine	0.6646
MGYA00367548/ERZ833381_FASTA.fasta	MGYA00367548	ERZ833381	root:Environmental:Aquatic:Marine	0.7928
MGYA00367549/ERZ833507_FASTA.fasta	MGYA00367549	ERZ833507	root:Environmental:Aquatic:Marine	0.6376
MGYA00367550/ERZ833580_FASTA.fasta	MGYA00367550	ERZ833580	root:Environmental:Aquatic:Marine	0.796
MGYA00367551/ERZ833786_FASTA.fasta	MGYA00367551	ERZ833786	root:Environmental:Aquatic:Marine	0.6306
MGYA00367552/ERZ833884_FASTA.fasta	MGYA00367552	ERZ833884	root:Environmental:Aquatic:Marine	0.721
MGYA00367553/ERZ833910_FASTA.fasta	MGYA00367553	ERZ833910	root:Environmental:Aquatic:Marine	0.639
MGYA00367554/ERZ834050_FASTA.fasta	MGYA00367554	ERZ834050	root:Environmental:Aquatic:Marine	0.8504
MGYA00367555/ERZ834397_FASTA.fasta	MGYA00367555	ERZ834397	root:Environmental:Aquatic:Marine	0.9378
MGYA00367556/ERZ834656_FASTA.fasta	MGYA00367556	ERZ834656	root:Environmental:Aquatic:Marine	0.9726
MGYA00367557/ERZ834839_FASTA.fasta	MGYA00367557	ERZ834839	root:Environmental:Aquatic:Marine	0.742
MGYA00367558/ERZ834897_FASTA.fasta	MGYA00367558	ERZ834897	root:Environmental:Aquatic:Marine	0.7898
MGYA00367559/ERZ835002_FASTA.fasta	MGYA00367559	ERZ835002	root:Environmental:Aquatic:Marine	0.7792
MGYA00367560/ERZ835323_FASTA.fasta	MGYA00367560	ERZ835323	root:Environmental:Aquatic:Marine	0.652
MGYA00367561/ERZ835454_FASTA.fasta	MGYA00367561	ERZ835454	root:Environmental:Aquatic:Marine	0.6442
MGYA00367562/ERZ835625_FASTA.fasta	MGYA00367562	ERZ835625	root:Environmental:Aquatic:Marine	0.7872
MGYA00367563/ERZ835863_FASTA.fasta	MGYA00367563	ERZ835863	root:Environmental:Aquatic:Marine	0.7192
MGYA00367564/ERZ836051_FASTA.fasta	MGYA00367564	ERZ836051	root:Environmental:Aquatic:Marine	0.8808
MGYA00367565/ERZ836228_FASTA.fasta	MGYA00367565	ERZ836228	root:Environmental:Aquatic:Marine	0.5862
MGYA00367566/ERZ836272_FASTA.fasta	MGYA00367566	ERZ836272	root:Environmental:Aquatic:Marine	0.7892
MGYA00367567/ERZ836337_FASTA.fasta	MGYA00367567	ERZ836337	root:Environmental:Aquatic:Marine	0.8322
MGYA00367568/ERZ836445_FASTA.fasta	MGYA00367568	ERZ836445	root:Environmental:Aquatic:Marine	0.9786
MGYA00367569/ERZ836686_FASTA.fasta	MGYA00367569	ERZ836686	root:Environmental:Aquatic:Marine	0.8116
MGYA00367570/ERZ836958_FASTA.fasta	MGYA00367570	ERZ836958	root:Environmental:Aquatic:Marine	0.7784
MGYA00367571/ERZ837174_FASTA.fasta	MGYA00367571	ERZ837174	root:Environmental:Aquatic:Marine	0.795
MGYA00367572/ERZ837273_FASTA.fasta	MGYA00367572	ERZ837273	root:Environmental:Aquatic:Marine	0.9516
MGYA00367573/ERZ837530_FASTA.fasta	MGYA00367573	ERZ837530	root:Environmental:Aquatic:Marine	0.7412
MGYA00367574/ERZ837885_FASTA.fasta	MGYA00367574	ERZ837885	root:Environmental:Aquatic:Marine	0.8466
MGYA00367575/ERZ838136_FASTA.fasta	MGYA00367575	ERZ838136	root:Environmental:Aquatic:Marine	0.913
MGYA00367576/ERZ838294_FASTA.fasta	MGYA00367576	ERZ838294	root:Environmental:Aquatic:Marine	0.8218
MGYA00367577/ERZ838379_FASTA.fasta	MGYA00367577	ERZ838379	root:Environmental:Aquatic:Marine	0.588
MGYA00367578/ERZ838418_FASTA.fasta	MGYA00367578	ERZ838418	root:Environmental:Aquatic:Marine	0.6866
MGYA00367579/ERZ838492_FASTA.fasta	MGYA00367579	ERZ838492	root:Environmental:Aquatic:Marine	0.6646
MGYA00367580/ERZ838699_FASTA.fasta	MGYA00367580	ERZ838699	root:Environmental:Aquatic:Marine	0.5866
MGYA00367581/ERZ838778_FASTA.fasta	MGYA00367581	ERZ838778	root:Environmental:Aquatic:Marine	0.8464
MGYA00367582/ERZ839027_FASTA.fasta	MGYA00367582	ERZ839027	root:Environmental:Aquatic:Marine	0.778
MGYA00367583/ERZ839095_FASTA.fasta	MGYA00367583	ERZ839095	root:Environmental:Aquatic:Marine	0.6928
MGYA00367584/ERZ839150_FASTA.fasta	MGYA00367584	ERZ839150	root:Environmental:Aquatic:Marine	0.6976
MGYA00367585/ERZ839268_FASTA.fasta	MGYA00367585	ERZ839268	root:Environmental:Aquatic:Marine	0.62
MGYA00367586/ERZ839331_FASTA.fasta	MGYA00367586	ERZ839331	root:Environmental:Aquatic:Marine	0.5778
MGYA00367587/ERZ839424_FASTA.fasta	MGYA00367587	ERZ839424	root:Environmental:Aquatic:Marine	0.8886
MGYA00367588/ERZ839637_FASTA.fasta	MGYA00367588	ERZ839637	root:Environmental:Aquatic:Marine	0.8274
MGYA00367589/ERZ839685_FASTA.fasta	MGYA00367589	ERZ839685	root:Environmental:Aquatic:Marine	0.5638
MGYA00367590/ERZ839733_FASTA.fasta	MGYA00367590	ERZ839733	root:Environmental:Aquatic:Marine	0.7948
MGYA00367591/ERZ839901_FASTA.fasta	MGYA00367591	ERZ839901	root:Environmental:Aquatic:Marine	0.79
MGYA00367592/ERZ840032_FASTA.fasta	MGYA00367592	ERZ840032	root:Environmental:Aquatic:Marine	0.6442
MGYA00367593/ERZ840062_FASTA.fasta	MGYA00367593	ERZ840062	root:Environmental:Aquatic:Marine	0.7694
MGYA00367594/ERZ840267_FASTA.fasta	MGYA00367594	ERZ840267	root:Environmental:Aquatic:Marine	0.7634
MGYA00367595/ERZ840327_FASTA.fasta	MGYA00367595	ERZ840327	root:Environmental:Aquatic:Marine	0.7134
MGYA00367596/ERZ840367_FASTA.fasta	MGYA00367596	ERZ840367	root:Environmental:Aquatic:Marine	0.888
MGYA00367597/ERZ840511_FASTA.fasta	MGYA00367597	ERZ840511	root:Environmental:Aquatic:Marine	0.798
MGYA00367598/ERZ840530_FASTA.fasta	MGYA00367598	ERZ840530	root:Environmental:Aquatic:Marine	0.745
MGYA00367599/ERZ840537_FASTA.fasta	MGYA00367599	ERZ840537	root:Environmental:Aquatic:Marine	0.9414
MGYA00367600/ERZ840547_FASTA.fasta	MGYA00367600	ERZ840547	root:Environmental:Aquatic:Marine	0.8436
MGYA00367601/ERZ840554_FASTA.fasta	MGYA00367601	ERZ840554	root:Environmental:Aquatic:Marine	0.8024
MGYA00367602/ERZ840559_FASTA.fasta	MGYA00367602	ERZ840559	root:Environmental:Aquatic:Marine	0.7628
MGYA00367603/ERZ840564_FASTA.fasta	MGYA00367603	ERZ840564	root:Environmental:Aquatic:Marine	0.8142
MGYA00367604/ERZ840571_FASTA.fasta	MGYA00367604	ERZ840571	root:Environmental:Aquatic:Marine	0.7676
MGYA00367605/ERZ840577_FASTA.fasta	MGYA00367605	ERZ840577	root:Environmental:Aquatic:Marine	0.8574
MGYA00367606/ERZ840584_FASTA.fasta	MGYA00367606	ERZ840584	root:Environmental:Aquatic:Marine	0.8222
MGYA00367607/ERZ840587_FASTA.fasta	MGYA00367607	ERZ840587	root:Environmental:Aquatic:Marine	0.7672
MGYA00367608/ERZ840589_FASTA.fasta	MGYA00367608	ERZ840589	root:Environmental:Aquatic:Marine	0.567
MGYA00367609/ERZ840591_FASTA.fasta	MGYA00367609	ERZ840591	root:Environmental:Aquatic:Marine	0.6112
MGYA00367610/ERZ840593_FASTA.fasta	MGYA00367610	ERZ840593	root:Environmental:Aquatic:Marine	0.8068
MGYA00367611/ERZ840605_FASTA.fasta	MGYA00367611	ERZ840605	root:Environmental:Aquatic:Marine	0.9648
MGYA00367612/ERZ840834_FASTA.fasta	MGYA00367612	ERZ840834	root:Environmental:Aquatic:Marine	0.6184
MGYA00367613/ERZ840836_FASTA.fasta	MGYA00367613	ERZ840836	root:Environmental:Aquatic:Marine	0.6132
MGYA00367614/ERZ840839_FASTA.fasta	MGYA00367614	ERZ840839	root:Environmental:Aquatic:Marine	0.9804
MGYA00367615/ERZ840850_FASTA.fasta	MGYA00367615	ERZ840850	root:Environmental:Aquatic:Marine	0.8808
MGYA00367616/ERZ840857_FASTA.fasta	MGYA00367616	ERZ840857	root:Environmental:Aquatic:Marine	0.8222
MGYA00367617/ERZ840860_FASTA.fasta	MGYA00367617	ERZ840860	root:Environmental:Aquatic:Marine	0.9234
MGYA00367618/ERZ840877_FASTA.fasta	MGYA00367618	ERZ840877	root:Environmental:Aquatic:Marine	0.7874
MGYA00367619/ERZ840881_FASTA.fasta	MGYA00367619	ERZ840881	root:Environmental:Aquatic:Marine	0.8772
MGYA00367620/ERZ840905_FASTA.fasta	MGYA00367620	ERZ840905	root:Environmental:Aquatic:Marine	0.81
MGYA00367621/ERZ840919_FASTA.fasta	MGYA00367621	ERZ840919	root:Environmental:Aquatic:Marine	0.739
MGYA00367622/ERZ840932_FASTA.fasta	MGYA00367622	ERZ840932	root:Environmental:Aquatic:Marine	0.7918
MGYA00367623/ERZ840934_FASTA.fasta	MGYA00367623	ERZ840934	root:Environmental:Aquatic:Marine	0.6384
MGYA00367624/ERZ840938_FASTA.fasta	MGYA00367624	ERZ840938	root:Environmental:Aquatic:Marine	0.6724
MGYA00367625/ERZ840950_FASTA.fasta	MGYA00367625	ERZ840950	root:Environmental:Aquatic:Marine	0.7672
MGYA00367626/ERZ840959_FASTA.fasta	MGYA00367626	ERZ840959	root:Environmental:Aquatic:Marine	0.8334
MGYA00367627/ERZ840991_FASTA.fasta	MGYA00367627	ERZ840991	root:Environmental:Aquatic:Marine	0.7974
MGYA00367628/ERZ840995_FASTA.fasta	MGYA00367628	ERZ840995	root:Environmental:Aquatic:Marine	0.7322
MGYA00367629/ERZ841001_FASTA.fasta	MGYA00367629	ERZ841001	root:Environmental:Aquatic:Marine	0.8014
MGYA00367630/ERZ841003_FASTA.fasta	MGYA00367630	ERZ841003	root:Environmental:Aquatic:Marine	0.6582
MGYA00367631/ERZ841010_FASTA.fasta	MGYA00367631	ERZ841010	root:Environmental:Aquatic:Marine	0.9458
MGYA00367632/ERZ841028_FASTA.fasta	MGYA00367632	ERZ841028	root:Environmental:Aquatic:Marine	0.8162
MGYA00367633/ERZ841040_FASTA.fasta	MGYA00367633	ERZ841040	root:Environmental:Aquatic:Marine	0.7698
MGYA00367634/ERZ841112_FASTA.fasta	MGYA00367634	ERZ841112	root:Environmental:Aquatic:Marine	0.8362
MGYA00367635/ERZ841115_FASTA.fasta	MGYA00367635	ERZ841115	root:Environmental:Aquatic:Marine	0.621
MGYA00367636/ERZ841130_FASTA.fasta	MGYA00367636	ERZ841130	root:Environmental:Aquatic:Marine	0.7952
MGYA00367637/ERZ841132_FASTA.fasta	MGYA00367637	ERZ841132	root:Environmental:Aquatic:Marine	0.7732
MGYA00367638/ERZ841139_FASTA.fasta	MGYA00367638	ERZ841139	root:Environmental:Aquatic:Marine	0.9804
MGYA00367639/ERZ841151_FASTA.fasta	MGYA00367639	ERZ841151	root:Environmental:Aquatic:Marine	0.6708
MGYA00367640/ERZ841154_FASTA.fasta	MGYA00367640	ERZ841154	root:Environmental:Aquatic:Marine	0.8232
MGYA00367641/ERZ841176_FASTA.fasta	MGYA00367641	ERZ841176	root:Environmental:Aquatic:Marine	0.8086
MGYA00367642/ERZ841189_FASTA.fasta	MGYA00367642	ERZ841189	root:Environmental:Aquatic:Marine	0.811
MGYA00367643/ERZ841196_FASTA.fasta	MGYA00367643	ERZ841196	root:Environmental:Aquatic:Marine	0.7724
MGYA00367644/ERZ841232_FASTA.fasta	MGYA00367644	ERZ841232	root:Environmental:Aquatic:Marine	0.8434
MGYA00367645/ERZ841263_FASTA.fasta	MGYA00367645	ERZ841263	root:Environmental:Aquatic:Marine	0.666
MGYA00367646/ERZ841276_FASTA.fasta	MGYA00367646	ERZ841276	root:Environmental:Aquatic:Marine	0.7522
MGYA00367647/ERZ841280_FASTA.fasta	MGYA00367647	ERZ841280	root:Environmental:Aquatic:Marine	0.8436
MGYA00367648/ERZ841312_FASTA.fasta	MGYA00367648	ERZ841312	root:Environmental:Aquatic:Marine	0.7584
MGYA00367649/ERZ841315_FASTA.fasta	MGYA00367649	ERZ841315	root:Environmental:Aquatic:Marine	0.8822
MGYA00367650/ERZ841319_FASTA.fasta	MGYA00367650	ERZ841319	root:Environmental:Aquatic:Marine	0.75
MGYA00367651/ERZ841322_FASTA.fasta	MGYA00367651	ERZ841322	root:Environmental:Aquatic:Marine	0.6902
MGYA00367652/ERZ841327_FASTA.fasta	MGYA00367652	ERZ841327	root:Environmental:Aquatic:Marine	0.8782
MGYA00367653/ERZ841349_FASTA.fasta	MGYA00367653	ERZ841349	root:Environmental:Aquatic:Marine	0.8444
MGYA00367654/ERZ841379_FASTA.fasta	MGYA00367654	ERZ841379	root:Environmental:Aquatic:Marine	0.622
MGYA00367655/ERZ841381_FASTA.fasta	MGYA00367655	ERZ841381	root:Environmental:Aquatic:Marine	0.8026
MGYA00367656/ERZ841384_FASTA.fasta	MGYA00367656	ERZ841384	root:Environmental:Aquatic:Marine	0.8766
MGYA00367657/ERZ841392_FASTA.fasta	MGYA00367657	ERZ841392	root:Environmental:Aquatic:Marine	0.6732
MGYA00367658/ERZ841394_FASTA.fasta	MGYA00367658	ERZ841394	root:Environmental:Aquatic:Marine	0.7192
MGYA00367659/ERZ841401_FASTA.fasta	MGYA00367659	ERZ841401	root:Environmental:Aquatic:Marine	0.619
MGYA00367660/ERZ841404_FASTA.fasta	MGYA00367660	ERZ841404	root:Environmental:Aquatic:Marine	0.7966
MGYA00367661/ERZ841410_FASTA.fasta	MGYA00367661	ERZ841410	root:Environmental:Aquatic:Marine	0.8584
MGYA00367662/ERZ841423_FASTA.fasta	MGYA00367662	ERZ841423	root:Environmental:Aquatic:Marine	0.819
MGYA00367663/ERZ841426_FASTA.fasta	MGYA00367663	ERZ841426	root:Environmental:Aquatic:Marine	0.7982
MGYA00367664/ERZ841434_FASTA.fasta	MGYA00367664	ERZ841434	root:Environmental:Aquatic:Marine	0.6298
MGYA00367665/ERZ841436_FASTA.fasta	MGYA00367665	ERZ841436	root:Environmental:Aquatic:Marine	0.7564
MGYA00367666/ERZ841439_FASTA.fasta	MGYA00367666	ERZ841439	root:Environmental:Aquatic:Marine	0.907
MGYA00367667/ERZ841449_FASTA.fasta	MGYA00367667	ERZ841449	root:Environmental:Aquatic:Marine	0.7956
MGYA00367668/ERZ841457_FASTA.fasta	MGYA00367668	ERZ841457	root:Environmental:Aquatic:Marine	0.6374
MGYA00367669/ERZ841459_FASTA.fasta	MGYA00367669	ERZ841459	root:Environmental:Aquatic:Marine	0.7182
MGYA00367670/ERZ841462_FASTA.fasta	MGYA00367670	ERZ841462	root:Environmental:Aquatic:Marine	0.7654
MGYA00367671/ERZ841464_FASTA.fasta	MGYA00367671	ERZ841464	root:Environmental:Aquatic:Marine	0.8094
MGYA00367672/ERZ841467_FASTA.fasta	MGYA00367672	ERZ841467	root:Environmental:Aquatic:Marine	0.7694
MGYA00367673/ERZ841475_FASTA.fasta	MGYA00367673	ERZ841475	root:Environmental:Aquatic:Marine	0.6386
MGYA00367674/ERZ841477_FASTA.fasta	MGYA00367674	ERZ841477	root:Environmental:Aquatic:Marine	0.7484
MGYA00367675/ERZ841480_FASTA.fasta	MGYA00367675	ERZ841480	root:Environmental:Aquatic:Marine	0.6604
MGYA00367676/ERZ841483_FASTA.fasta	MGYA00367676	ERZ841483	root:Environmental:Aquatic:Marine	0.7648
MGYA00367677/ERZ841489_FASTA.fasta	MGYA00367677	ERZ841489	root:Environmental:Aquatic:Marine	0.7992
MGYA00367678/ERZ841497_FASTA.fasta	MGYA00367678	ERZ841497	root:Environmental:Aquatic:Marine	0.9782
MGYA00367679/ERZ841558_FASTA.fasta	MGYA00367679	ERZ841558	root:Environmental:Aquatic:Marine	0.7984
MGYA00367680/ERZ841566_FASTA.fasta	MGYA00367680	ERZ841566	root:Environmental:Aquatic:Marine	0.759
MGYA00367681/ERZ841573_FASTA.fasta	MGYA00367681	ERZ841573	root:Environmental:Aquatic:Marine	0.8898
MGYA00367682/ERZ841618_FASTA.fasta	MGYA00367682	ERZ841618	root:Environmental:Aquatic:Marine	0.6442
MGYA00367683/ERZ841638_FASTA.fasta	MGYA00367683	ERZ841638	root:Environmental:Aquatic:Marine	0.738
MGYA00367684/ERZ841645_FASTA.fasta	MGYA00367684	ERZ841645	root:Environmental:Aquatic:Marine	0.824
MGYA00367685/ERZ841657_FASTA.fasta	MGYA00367685	ERZ841657	root:Environmental:Aquatic:Marine	0.7916
MGYA00367686/ERZ841659_FASTA.fasta	MGYA00367686	ERZ841659	root:Environmental:Aquatic:Marine	0.5788
MGYA00367687/ERZ841662_FASTA.fasta	MGYA00367687	ERZ841662	root:Environmental:Aquatic:Marine	0.8832
MGYA00367688/ERZ841695_FASTA.fasta	MGYA00367688	ERZ841695	root:Environmental:Aquatic:Marine	0.7496
MGYA00367689/ERZ841701_FASTA.fasta	MGYA00367689	ERZ841701	root:Environmental:Aquatic:Marine	0.8356
MGYA00367690/ERZ841705_FASTA.fasta	MGYA00367690	ERZ841705	root:Environmental:Aquatic:Marine	0.6946
MGYA00367691/ERZ841738_FASTA.fasta	MGYA00367691	ERZ841738	root:Environmental:Aquatic:Marine	0.774
MGYA00367692/ERZ841741_FASTA.fasta	MGYA00367692	ERZ841741	root:Environmental:Aquatic:Marine	0.8454
MGYA00367693/ERZ841744_FASTA.fasta	MGYA00367693	ERZ841744	root:Environmental:Aquatic:Marine	0.6632
MGYA00367694/ERZ841748_FASTA.fasta	MGYA00367694	ERZ841748	root:Environmental:Aquatic:Marine	0.7178
MGYA00367695/ERZ841795_FASTA.fasta	MGYA00367695	ERZ841795	root:Environmental:Aquatic:Marine	0.731
MGYA00367696/ERZ841799_FASTA.fasta	MGYA00367696	ERZ841799	root:Environmental:Aquatic:Marine	0.9274
MGYA00367697/ERZ841809_FASTA.fasta	MGYA00367697	ERZ841809	root:Environmental:Aquatic:Marine	0.9316
MGYA00367698/ERZ841818_FASTA.fasta	MGYA00367698	ERZ841818	root:Environmental:Aquatic:Marine	0.745
MGYA00367699/ERZ841820_FASTA.fasta	MGYA00367699	ERZ841820	root:Environmental:Aquatic:Marine	0.6606

MGYA00367700/ERZ841824_FASTA.fasta	MGYA00367700	ERZ841824	root:Environmental:Aquatic:Marine	0.873
MGYA00367701/ERZ841839_FASTA.fasta	MGYA00367701	ERZ841839	root:Environmental:Aquatic:Marine	0.7322
MGYA00367702/ERZ841842_FASTA.fasta	MGYA00367702	ERZ841842	root:Environmental:Aquatic:Marine	0.8024
MGYA00367703/ERZ841863_FASTA.fasta	MGYA00367703	ERZ841863	root:Environmental:Aquatic:Marine	0.8056
MGYA00367704/ERZ841878_FASTA.fasta	MGYA00367704	ERZ841878	root:Environmental:Aquatic:Marine	0.6302
MGYA00367705/ERZ841881_FASTA.fasta	MGYA00367705	ERZ841881	root:Environmental:Aquatic:Marine	0.6032
MGYA00367706/ERZ841884_FASTA.fasta	MGYA00367706	ERZ841884	root:Environmental:Aquatic:Marine	0.7428
MGYA00367707/ERZ841895_FASTA.fasta	MGYA00367707	ERZ841895	root:Environmental:Aquatic:Marine	0.6128
MGYA00367708/ERZ841907_FASTA.fasta	MGYA00367708	ERZ841907	root:Environmental:Aquatic:Marine	0.8724
MGYA00367709/ERZ841911_FASTA.fasta	MGYA00367709	ERZ841911	root:Environmental:Aquatic:Marine	0.8034
MGYA00367710/ERZ841929_FASTA.fasta	MGYA00367710	ERZ841929	root:Environmental:Aquatic:Marine	0.7586
MGYA00367711/ERZ841932_FASTA.fasta	MGYA00367711	ERZ841932	root:Environmental:Aquatic:Marine	0.7196
MGYA00367712/ERZ841934_FASTA.fasta	MGYA00367712	ERZ841934	root:Environmental:Aquatic:Marine	0.8146
MGYA00367713/ERZ841954_FASTA.fasta	MGYA00367713	ERZ841954	root:Environmental:Aquatic:Marine	0.7854
MGYA00367714/ERZ841958_FASTA.fasta	MGYA00367714	ERZ841958	root:Environmental:Aquatic:Marine	0.8158
MGYA00367715/ERZ841973_FASTA.fasta	MGYA00367715	ERZ841973	root:Environmental:Aquatic:Marine	0.7966
MGYA00367716/ERZ841976_FASTA.fasta	MGYA00367716	ERZ841976	root:Environmental:Aquatic:Marine	0.7826
MGYA00367717/ERZ841978_FASTA.fasta	MGYA00367717	ERZ841978	root:Environmental:Aquatic:Marine	0.6302
MGYA00367718/ERZ841980_FASTA.fasta	MGYA00367718	ERZ841980	root:Environmental:Aquatic:Marine	0.805
MGYA00367719/ERZ841982_FASTA.fasta	MGYA00367719	ERZ841982	root:Environmental:Aquatic:Marine	0.5566
MGYA00367720/ERZ841987_FASTA.fasta	MGYA00367720	ERZ841987	root:Environmental:Aquatic:Marine	0.9154
MGYA00367721/ERZ842008_FASTA.fasta	MGYA00367721	ERZ842008	root:Environmental:Aquatic:Marine	0.7762
MGYA00367722/ERZ842010_FASTA.fasta	MGYA00367722	ERZ842010	root:Environmental:Aquatic:Marine	0.6418
MGYA00367723/ERZ842013_FASTA.fasta	MGYA00367723	ERZ842013	root:Environmental:Aquatic:Marine	0.8956
MGYA00367724/ERZ842040_FASTA.fasta	MGYA00367724	ERZ842040	root:Environmental:Aquatic:Marine	0.8994
MGYA00367725/ERZ842061_FASTA.fasta	MGYA00367725	ERZ842061	root:Environmental:Aquatic:Marine	0.7608
MGYA00367726/ERZ842064_FASTA.fasta	MGYA00367726	ERZ842064	root:Environmental:Aquatic:Marine	0.8442
MGYA00367727/ERZ842083_FASTA.fasta	MGYA00367727	ERZ842083	root:Environmental:Aquatic:Marine	0.8084
MGYA00367728/ERZ842085_FASTA.fasta	MGYA00367728	ERZ842085	root:Environmental:Aquatic:Marine	0.8454
MGYA00367729/ERZ842087_FASTA.fasta	MGYA00367729	ERZ842087	root:Environmental:Aquatic:Marine	0.7072
MGYA00367730/ERZ842091_FASTA.fasta	MGYA00367730	ERZ842091	root:Environmental:Aquatic:Marine	0.977
MGYA00367731/ERZ842108_FASTA.fasta	MGYA00367731	ERZ842108	root:Environmental:Aquatic:Marine	0.7144
MGYA00367732/ERZ842113_FASTA.fasta	MGYA00367732	ERZ842113	root:Environmental:Aquatic:Marine	0.8016
MGYA00367733/ERZ842116_FASTA.fasta	MGYA00367733	ERZ842116	root:Environmental:Aquatic:Marine	0.7284
MGYA00367734/ERZ842120_FASTA.fasta	MGYA00367734	ERZ842120	root:Environmental:Aquatic:Marine	0.6166
MGYA00367735/ERZ842122_FASTA.fasta	MGYA00367735	ERZ842122	root:Environmental:Aquatic:Marine	0.753
MGYA00367736/ERZ842124_FASTA.fasta	MGYA00367736	ERZ842124	root:Environmental:Aquatic:Marine	0.706
MGYA00367737/ERZ842127_FASTA.fasta	MGYA00367737	ERZ842127	root:Environmental:Aquatic:Marine	0.722
MGYA00367738/ERZ842130_FASTA.fasta	MGYA00367738	ERZ842130	root:Environmental:Aquatic:Marine	0.7994
MGYA00367739/ERZ842135_FASTA.fasta	MGYA00367739	ERZ842135	root:Environmental:Aquatic:Marine	0.7534
MGYA00367740/ERZ842199_FASTA.fasta	MGYA00367740	ERZ842199	root:Environmental:Aquatic:Marine	0.8542
MGYA00367741/ERZ842390_FASTA.fasta	MGYA00367741	ERZ842390	root:Environmental:Aquatic:Marine	0.9464
MGYA00367742/ERZ842400_FASTA.fasta	MGYA00367742	ERZ842400	root:Environmental:Aquatic:Marine	0.8702
MGYA00367743/ERZ842402_FASTA.fasta	MGYA00367743	ERZ842402	root:Environmental:Aquatic:Marine	0.8436
MGYA00367744/ERZ842407_FASTA.fasta	MGYA00367744	ERZ842407	root:Environmental:Aquatic:Marine	0.9798
MGYA00367745/ERZ842408_FASTA.fasta	MGYA00367745	ERZ842408	root:Environmental:Aquatic:Marine	0.8236
MGYA00367746/ERZ842555_FASTA.fasta	MGYA00367746	ERZ842555	root:Environmental:Aquatic:Marine	0.9674
MGYA00367747/ERZ842953_FASTA.fasta	MGYA00367747	ERZ842953	root:Environmental:Aquatic:Marine	0.8256
MGYA00367748/ERZ843003_FASTA.fasta	MGYA00367748	ERZ843003	root:Environmental:Aquatic:Marine	0.8574
MGYA00367749/ERZ843394_FASTA.fasta	MGYA00367749	ERZ843394	root:Environmental:Aquatic:Marine	0.8022
MGYA00367750/ERZ843524_FASTA.fasta	MGYA00367750	ERZ843524	root:Environmental:Aquatic:Marine	0.972
MGYA00367751/ERZ843936_FASTA.fasta	MGYA00367751	ERZ843936	root:Environmental:Aquatic:Marine	0.7948
MGYA00367752/ERZ843989_FASTA.fasta	MGYA00367752	ERZ843989	root:Environmental:Aquatic:Marine	0.7264
MGYA00367753/ERZ844199_FASTA.fasta	MGYA00367753	ERZ844199	root:Environmental:Aquatic:Marine	0.8046
MGYA00367754/ERZ856576_FASTA.fasta	MGYA00367754	ERZ856576	root:Environmental:Aquatic:Marine	0.899
MGYA00367755/ERZ376968_FASTA_2.fasta	MGYA00367755	ERZ376968	root:Environmental:Aquatic:Marine	0.712
MGYA00374899/ERZ801025_FASTA.fasta	MGYA00374899	ERZ801025	root:Environmental:Aquatic:Estuary	0.8274
MGYA00374947/ERZ511473_FASTA.fasta	MGYA00374947	ERZ511473	root:Environmental:Aquatic:Thermal springs:Sediment	0.7428
MGYA00374948/ERZ746981_FASTA.fasta	MGYA00374948	ERZ746981	root:Engineered:Food production:Fermented beverage	0.4866
MGYA00374949/ERZ747221_FASTA.fasta	MGYA00374949	ERZ747221	root:Engineered:Biogas plant	0.7336
MGYA00374950/ERZ761574_FASTA.fasta	MGYA00374950	ERZ761574	root:Environmental:Aquatic:Lentic:Brackish	0.3366
MGYA00374951/ERZ764778_FASTA.fasta	MGYA00374951	ERZ764778	root:Engineered:Bioreactor	0.7132
MGYA00374952/ERZ767114_FASTA.fasta	MGYA00374952	ERZ767114	root:Environmental:Aquatic:Estuary	0.8502
MGYA00374953/ERZ767285_FASTA.fasta	MGYA00374953	ERZ767285	root:Environmental:Aquatic:Estuary	0.845
MGYA00374954/ERZ767310_FASTA.fasta	MGYA00374954	ERZ767310	root:Environmental:Aquatic:Estuary	0.8476
MGYA00374955/ERZ773359_FASTA.fasta	MGYA00374955	ERZ773359	root:Engineered:Biogas plant	0.585
MGYA00374956/ERZ777003_FASTA.fasta	MGYA00374956	ERZ777003	root:Engineered:Bioreactor	0.777
MGYA00374957/ERZ777004_FASTA.fasta	MGYA00374957	ERZ777004	root:Host-associated:Plants	0.03
MGYA00374958/ERZ777005_FASTA.fasta	MGYA00374958	ERZ777005	root:Host-associated:Plants	0.478
MGYA00374959/ERZ777006_FASTA.fasta	MGYA00374959	ERZ777006	root:Host-associated:Plants	0.423
MGYA00374960/ERZ777007_FASTA.fasta	MGYA00374960	ERZ777007	root:Host-associated:Plants:Phylloplane	0.0998
MGYA00374961/ERZ777008_FASTA.fasta	MGYA00374961	ERZ777008	root:Host-associated:Plants:Phylloplane	0.0486
MGYA00374962/ERZ777009_FASTA.fasta	MGYA00374962	ERZ777009	root:Host-associated:Plants	0.1164
MGYA00374963/ERZ777010_FASTA.fasta	MGYA00374963	ERZ777010	root:Engineered:Bioreactor	0.7518
MGYA00374964/ERZ777011_FASTA.fasta	MGYA00374964	ERZ777011	root:Host-associated:Mammals:Respiratory system	0.4524
MGYA00374965/ERZ777305_FASTA.fasta	MGYA00374965	ERZ777305	root:Host-associated:Mammals:Respiratory system	0.458
MGYA00374966/ERZ777306_FASTA.fasta	MGYA00374966	ERZ777306	root:Host-associated:Mammals:Respiratory system	0.5548
MGYA00374967/ERZ777307_FASTA.fasta	MGYA00374967	ERZ777307	root:Host-associated:Plants:Rhizosphere	0.482
MGYA00374968/ERZ777308_FASTA.fasta	MGYA00374968	ERZ777308	root:Host-associated:Plants:Rhizosphere	0.4926
MGYA00374969/ERZ777309_FASTA.fasta	MGYA00374969	ERZ777309	root:Host-associated:Plants:Rhizosphere	0.5356
MGYA00374970/ERZ777310_FASTA.fasta	MGYA00374970	ERZ777310	root:Host-associated:Plants:Phylloplane	0.1066
MGYA00374971/ERZ777311_FASTA.fasta	MGYA00374971	ERZ777311	root:Host-associated:Plants:Phylloplane	0.0548
MGYA00374972/ERZ777312_FASTA.fasta	MGYA00374972	ERZ777312	root:Host-associated:Plants:Phylloplane	0.1146
MGYA00374973/ERZ777313_FASTA.fasta	MGYA00374973	ERZ777313	root:Host-associated:Plants:Phylloplane	0.095
MGYA00374974/ERZ777316_FASTA.fasta	MGYA00374974	ERZ777316	root:Host-associated:Mammals:Respiratory system	0.418
MGYA00374975/ERZ777317_FASTA.fasta	MGYA00374975	ERZ777317	root:Host-associated:Plants:Rhizosphere	0.4768
MGYA00374976/ERZ778036_FASTA.fasta	MGYA00374976	ERZ778036	root:Engineered:Wastewater:Activated Sludge	0.83
MGYA00374977/ERZ778037_FASTA.fasta	MGYA00374977	ERZ778037	root:Engineered:Wastewater:Activated Sludge	0.8128
MGYA00374978/ERZ778038_FASTA.fasta	MGYA00374978	ERZ778038	root:Engineered:Wastewater:Activated Sludge	0.8446
MGYA00374979/ERZ778039_FASTA.fasta	MGYA00374979	ERZ778039	root:Engineered:Bioreactor	0.7192
MGYA00374980/ERZ778040_FASTA.fasta	MGYA00374980	ERZ778040	root:Engineered:Bioreactor	0.768
MGYA00374981/ERZ778489_FASTA.fasta	MGYA00374981	ERZ778489	root:Host-associated:Mammals:Digestive system:Fecal	0.5444
MGYA00374982/ERZ778524_FASTA.fasta	MGYA00374982	ERZ778524	root:Engineered:Biogas plant:Wet fermentation	0.6888
MGYA00374983/ERZ778602_FASTA.fasta	MGYA00374983	ERZ778602	root:Environmental:Aquatic:Estuary	0.8566
MGYA00374984/ERZ778604_FASTA.fasta	MGYA00374984	ERZ778604	root:Environmental:Aquatic:Marine:Intertidal zone:Oil-c	0.9394
MGYA00374985/ERZ778607_FASTA.fasta	MGYA00374985	ERZ778607	root:Engineered:Biogas plant:Wet fermentation	0.8264
MGYA00374986/ERZ779608_FASTA.fasta	MGYA00374986	ERZ779608	root:Host-associated:Mammals:Digestive system:Fecal	0.4572
MGYA00374987/ERZ780236_FASTA.fasta	MGYA00374987	ERZ780236	root:Engineered:Bioreactor	0.4854
MGYA00374988/ERZ780650_FASTA.fasta	MGYA00374988	ERZ780650	root:Host-associated:Plants:Rhizosphere	0.5556
MGYA00374989/ERZ780651_FASTA.fasta	MGYA00374989	ERZ780651	root:Host-associated:Plants:Rhizosphere	0.5202
MGYA00374990/ERZ780652_FASTA.fasta	MGYA00374990	ERZ780652	root:Host-associated:Plants:Rhizosphere	0.6072
MGYA00374991/ERZ780653_FASTA.fasta	MGYA00374991	ERZ780653	root:Host-associated:Plants:Rhizosphere	0.6616
MGYA00374992/ERZ780654_FASTA.fasta	MGYA00374992	ERZ780654	root:Host-associated:Plants:Rhizosphere	0.638
MGYA00374993/ERZ780655_FASTA.fasta	MGYA00374993	ERZ780655	root:Host-associated:Plants:Rhizosphere	0.5696
MGYA00374994/ERZ780656_FASTA.fasta	MGYA00374994	ERZ780656	root:Host-associated:Plants:Rhizosphere	0.4988
MGYA00374995/ERZ780657_FASTA.fasta	MGYA00374995	ERZ780657	root:Host-associated:Plants:Rhizosphere	0.6012
MGYA00374996/ERZ780658_FASTA.fasta	MGYA00374996	ERZ780658	root:Host-associated:Plants:Rhizosphere	0.6262
MGYA00374997/ERZ780659_FASTA.fasta	MGYA00374997	ERZ780659	root:Host-associated:Plants:Rhizosphere	0.7816
MGYA00374998/ERZ780660_FASTA.fasta	MGYA00374998	ERZ780660	root:Host-associated:Plants:Rhizosphere	0.5192
MGYA00374999/ERZ780661_FASTA.fasta	MGYA00374999	ERZ780661	root:Host-associated:Plants:Rhizosphere	0.589
MGYA00375000/ERZ780662_FASTA.fasta	MGYA00375000	ERZ780662	root:Host-associated:Plants:Rhizosphere	0.5528
MGYA00375001/ERZ780663_FASTA.fasta	MGYA00375001	ERZ780663	root:Host-associated:Plants:Rhizosphere	0.5202
MGYA00375002/ERZ780664_FASTA.fasta	MGYA00375002	ERZ780664	root:Host-associated:Plants:Rhizosphere	0.644
MGYA00375003/ERZ780665_FASTA.fasta	MGYA00375003	ERZ780665	root:Host-associated:Plants:Rhizosphere	0.538
MGYA00375004/ERZ780666_FASTA.fasta	MGYA00375004	ERZ780666	root:Host-associated:Plants:Rhizosphere	0.516
MGYA00375005/ERZ780667_FASTA.fasta	MGYA00375005	ERZ780667	root:Host-associated:Plants	0.1046
MGYA00375006/ERZ780668_FASTA.fasta	MGYA00375006	ERZ780668	root:Host-associated:Plants	0.0976
MGYA00375007/ERZ780669_FASTA.fasta	MGYA00375007	ERZ780669	root:Host-associated:Plants	0.03
MGYA00375008/ERZ780670_FASTA.fasta	MGYA00375008	ERZ780670	root:Host-associated:Plants	0.0308
MGYA00375009/ERZ780671_FASTA.fasta	MGYA00375009	ERZ780671	root:Host-associated:Plants	0.3146
MGYA00375010/ERZ780672_FASTA.fasta	MGYA00375010	ERZ780672	root:Host-associated:Plants	0.171
MGYA00375011/ERZ780673_FASTA.fasta	MGYA00375011	ERZ780673	root:Host-associated:Plants	0.0706
MGYA00375012/ERZ780674_FASTA.fasta	MGYA00375012	ERZ780674	root:Host-associated:Plants	0.0872
MGYA00375013/ERZ780675_FASTA.fasta	MGYA00375013	ERZ780675	root:Host-associated:Plants	0.099
MGYA00375014/ERZ780676_FASTA.fasta	MGYA00375014	ERZ780676	root:Host-associated:Plants	0.1082
MGYA00375015/ERZ780677_FASTA.fasta	MGYA00375015	ERZ780677	root:Host-associated:Plants	0.1372
MGYA00375016/ERZ780678_FASTA.fasta	MGYA00375016	ERZ780678	root:Host-associated:Plants	0.137
MGYA00375017/ERZ780679_FASTA.fasta	MGYA00375017	ERZ780679	root:Host-associated:Plants	0.1114
MGYA00375018/ERZ780680_FASTA.fasta	MGYA00375018	ERZ780680	root:Host-associated:Plants	0.1358
MGYA00375019/ERZ780681_FASTA.fasta	MGYA00375019	ERZ780681	root:Host-associated:Plants	0.0706
MGYA00375020/ERZ780682_FASTA.fasta	MGYA00375020	ERZ780682	root:Host-associated:Plants	0.0426
MGYA00375021/ERZ780683_FASTA.fasta	MGYA00375021	ERZ780683	root:Host-associated:Plants	0.1294
MGYA00375022/ERZ780684_FASTA.fasta	MGYA00375022	ERZ780684	root:Host-associated:Plants	0.111
MGYA00375023/ERZ780685_FASTA.fasta	MGYA00375023	ERZ780685	root:Host-associated:Plants	0.093
MGYA00375024/ERZ780686_FASTA.fasta	MGYA00375024	ERZ780686	root:Host-associated:Plants	0.0958
MGYA00375025/ERZ780687_FASTA.fasta	MGYA00375025	ERZ780687	root:Host-associated:Plants	0.1984
MGYA00375026/ERZ780688_FASTA.fasta	MGYA00375026	ERZ780688	root:Host-associated:Plants	0.07
MGYA00375027/ERZ780689_FASTA.fasta	MGYA00375027	ERZ780689	root:Host-associated:Plants	0.0554
MGYA00375028/ERZ780690_FASTA.fasta	MGYA00375028	ERZ780690	root:Host-associated:Plants	0.168
MGYA00375029/ERZ780691_FASTA.fasta	MGYA00375029	ERZ780691	root:Host-associated:Plants	0.0468
MGYA00375030/ERZ780692_FASTA.fasta	MGYA00375030	ERZ780692	root:Host-associated:Plants	0.0948
MGYA00375031/ERZ780693_FASTA.fasta	MGYA00375031	ERZ780693	root:Host-associated:Plants	0.1442
MGYA00375032/ERZ780694_FASTA.fasta	MGYA00375032	ERZ780694	root:Host-associated:Plants	0.1218
MGYA00375033/ERZ781209_FASTA.fasta	MGYA00375033	ERZ781209	root:Host-associated:Mammals:Digestive system:Fecal	0.4356
MGYA00375034/ERZ781224_FASTA.fasta	MGYA00375034	ERZ781224	root:Host-associated:Mammals:Digestive system:Large	0.4988
MGYA00375035/ERZ781225_FASTA.fasta	MGYA00375035	ERZ781225	root:Host-associated:Mammals:Digestive system:Large	0.5568
MGYA00375036/ERZ781226_FASTA.fasta	MGYA00375036	ERZ781226	root:Host-associated:Plants:Rhizosphere	0.6912
MGYA00375037/ERZ781227_FASTA.fasta	MGYA00375037	ERZ781227	root:Host-associated:Plants:Rhizosphere	0.7886
MGYA00375038/ERZ781228_FASTA.fasta	MGYA00375038	ERZ781228	root:Host-associated:Plants:Rhizosphere	0.9396
MGYA00375039/ERZ781229_FASTA.fasta	MGYA00375039	ERZ781229	root:Host-associated:Plants	0.4616
MGYA00375040/ERZ781230_FASTA.fasta	MGYA00375040	ERZ781230	root:Host-associated:Plants	0.1242
MGYA00375041/ERZ781231_FASTA.fasta	MGYA00375041	ERZ781231	root:Host-associated:Plants	0.073
MGYA00375042/ERZ781232_FASTA.fasta	MGYA00375042	ERZ781232	root:Host-associated:Plants	0.0662
MGYA00375043/ERZ781233_FASTA.fasta	MGYA00375043	ERZ781233	root:Host-associated:Plants	0.0894
MGYA00375044/ERZ781234_FASTA.fasta	MGYA00375044	ERZ781234	root:Host-associated:Plants	0.1256
MGYA00375045/ERZ781244_FASTA.fasta	MGYA00375045	ERZ781244	root:Host-associated:Plants	0.7944
MGYA00375046/ERZ781245_FASTA.fasta	MGYA00375046	ERZ781245	root:Engineered:Solid waste:Composting	0.5222
MGYA00375047/ERZ781355_FASTA.fasta	MGYA00375047	ERZ781355	root:Environmental:Aquatic:Marine	0.6522
MGYA00375048/ERZ781356_FASTA.fasta	MGYA00375048	ERZ781356	root:Environmental:Aquatic:Freshwater:Pond	0.9266
MGYA00375049/ERZ781357_FASTA.fasta	MGYA00375049	ERZ781357	root:Environmental:Aquatic:Freshwater:Pond	0.8938
MGYA00375050/ERZ781358_FASTA.fasta	MGYA00375050	ERZ781358	root:Environmental:Aquatic:Freshwater:Pond:Sediment	0.8892
MGYA00375051/ERZ781359_FASTA.fasta	MGYA00375051	ERZ781359	root:Environmental:Aquatic:Thermal springs:Hot (42-90	0.967
MGYA00375052/ERZ781360_FASTA.fasta	MGYA00375052	ERZ781360	root:Environmental:Aquatic:Freshwater:Lentic:Sedimen	0.5302
MGYA00375053/ERZ781361_FASTA.fasta	MGYA00375053	ERZ781361	root:Environmental:Aquatic:Freshwater:Lentic:Sedimen	0.5922
MGYA00375054/ERZ781362_FASTA.fasta	MGYA00375054	ERZ781362	root:Environmental:Aquatic:Marine	0.9104
MGYA00375055/ERZ781363_FASTA.fasta	MGYA00375055	ERZ781363	root:Environmental:Aquatic:Marine	0.9136
MGYA00375056/ERZ781364_FASTA.fasta	MGYA00375056	ERZ781364	root:Environmental:Aquatic:Marine	0.9138
MGYA00375057/ERZ781365_FASTA.fasta	MGYA00375057	ERZ781365	root:Environmental:Aquatic:Marine	0.796
MGYA00375058/ERZ781366_FASTA.fasta	MGYA00375058	ERZ781366	root:Environmental:Aquatic:Marine	0.848
MGYA00375059/ERZ781367_FASTA.fasta	MGYA00375059	ERZ781367	root:Environmental:Aquatic:Marine	0.8322
MGYA00375060/ERZ781368_FASTA.fasta	MGYA00375060	ERZ781368	root:Environmental:Aquatic:Marine	0.8696
MGYA00375061/ERZ781369_FASTA.fasta	MGYA00375061	ERZ781369	root:Environmental:Aquatic:Estuary	0.8022
MGYA00375062/ERZ781370_FASTA.fasta	MGYA00375062	ERZ781370	root:Environmental:Aquatic:Estuary	0.6776
MGYA00375063/ERZ781371_FASTA.fasta	MGYA00375063	ERZ781371	root:Environmental:Aquatic:Estuary	0.8066
MGYA00375064/ERZ781372_FASTA.fasta	MGYA00375064	ERZ781372	root:Environmental:Aquatic:Estuary	0.7878
MGYA00375065/ERZ781373_FASTA.fasta	MGYA00375065	ERZ781373	root:Environmental:Aquatic:Marine	0.861
MGYA00375066/ERZ781374_FASTA.fasta	MGYA00375066	ERZ781374	root:Environmental:Aquatic:Marine	0.7644
MGYA00375067/ERZ781375_FASTA.fasta	MGYA00375067	ERZ781375	root:Environmental:Aquatic:Marine	0.649
MGYA00375068/ERZ781376_FASTA.fasta	MGYA00375068	ERZ781376	root:Environmental:Aquatic:Marine	0.769
MGYA00375069/ERZ781377_FASTA.fasta	MGYA00375069	ERZ781377	root:Environmental:Aquatic:Marine	0.8126
MGYA00375070/ERZ781378_FASTA.fasta	MGYA00375070	ERZ781378	root:Environmental:Aquatic:Marine	0.8952
MGYA00375071/ERZ781379_FASTA.fasta	MGYA00375071	ERZ781379	root:Environmental:Aquatic:Marine	0.8528
MGYA00375072/ERZ781380_FASTA.fasta	MGYA00375072	ERZ781380	root:Environmental:Aquatic:Marine:Coastal	0.695
MGYA00375073/ERZ781381_FASTA.fasta	MGYA00375073	ERZ781381	root:Environmental:Aquatic:Estuary	0.8654
MGYA00375074/ERZ781382_FASTA.fasta	MGYA00375074	ERZ781382	root:Host-associated:Algae:Red algae	0.9276
MGYA00375075/ERZ781383_FASTA.fasta	MGYA00375075	ERZ781383	root:Environmental:Aquatic:Marine:Oceanic	0.8616
MGYA00375076/ERZ781384_FASTA.fasta	MGYA00375076	ERZ781384	root:Environmental:Aquatic:Marine:Oceanic	0.8474
MGYA00375077/ERZ781385_FASTA.fasta	MGYA00375077	ERZ781385	root:Environmental:Aquatic:Marine:Oceanic	0.8822
MGYA00375078/ERZ781386_FASTA.fasta	MGYA00375078	ERZ781386	root:Environmental:Aquatic:Marine:Oceanic	0.8784
MGYA00375079/ERZ781387_FASTA.fasta	MGYA00375079	ERZ781387	root:Environmental:Aquatic:Marine:Oceanic	0.8972
MGYA00375080/ERZ781388_FASTA.fasta	MGYA00375080	ERZ781388	root:Environmental:Aquatic:Marine:Oceanic	0.9006
MGYA00375081/ERZ781389_FASTA.fasta	MGYA00375081	ERZ781389	root:Environmental:Aquatic:Marine:Oceanic	0.8598
MGYA00375082/ERZ781390_FASTA.fasta	MGYA00375082	ERZ781390	root:Environmental:Aquatic:Marine:Oceanic	0.8776
MGYA00375083/ERZ781391_FASTA.fasta	MGYA00375083	ERZ781391	root:Environmental:Aquatic:Marine:Oceanic	0.8684
MGYA00375084/ERZ781392_FASTA.fasta	MGYA00375084	ERZ781392	root:Environmental:Aquatic:Marine:Oceanic	0.8804
MGYA00375085/ERZ781393_FASTA.fasta	MGYA00375085	ERZ781393	root:Environmental:Aquatic:Marine:Sediment	0.487
MGYA00375086/ERZ781394_FASTA.fasta	MGYA00375086	ERZ781394	root:Environmental:Aquatic:Marine:Sediment	0.8228
MGYA00375087/ERZ781395_FASTA.fasta	MGYA00375087	ERZ781395	root:Environmental:Aquatic:Marine:Coastal	0.9374
MGYA00375088/ERZ781396_FASTA.fasta	MGYA00375088	ERZ781396	root:Environmental:Aquatic:Marine:Coastal	0.9114
MGYA00375089/ERZ781397_FASTA.fasta	MGYA00375089	ERZ781397	root:Environmental:Aquatic:Freshwater:Lentic:Sedimen	0.7192
MGYA00375090/ERZ782917_FASTA.fasta	MGYA00375090	ERZ782917	root:Environmental:Aquatic:Marine	0.7278
MGYA00375091/ERZ782918_FASTA.fasta	MGYA00375091	ERZ782918	root:Environmental:Aquatic:Estuary	0.8152
MGYA00375092/ERZ782919_FASTA.fasta	MGYA00375092	ERZ782919	root:Environmental:Aquatic:Marine:Pelagic	0.9052
MGYA00375093/ERZ782921_FASTA.fasta	MGYA00375093	ERZ782921	root:Engineered:Solid waste:Landfill	0.7848
MGYA00375094/ERZ791559_FASTA.fasta	MGYA00375094	ERZ791559	root:Environmental:Aquatic:Estuary:Sediment	0.75
MGYA00375095/ERZ791560_FASTA.fasta	MGYA00375095	ERZ791560	root:Environmental:Aquatic:Thermal springs:Hot (42-90	0.6974
MGYA00375096/ERZ791561_FASTA.fasta	MGYA00375096	ERZ791561	root:Environmental:Aquatic:Marine:Oceanic	0.8586
MGYA00375097/ERZ791562_FASTA.fasta	MGYA00375097	ERZ791562	root:Environmental:Aquatic:Estuary:Sediment	0.8456
MGYA00375098/ERZ791563_FASTA.fasta	MGYA00375098	ERZ791563	root:Environmental:Aquatic:Marine:Oceanic	0.8128
MGYA00375099/ERZ791564_FASTA.fasta	MGYA00375099	ERZ791564	root:Environmental:Aquatic:Marine:Pelagic	0.8484
MGYA00375100/ERZ791565_FASTA.fasta	MGYA00375100	ERZ791565	root:Environmental:Aquatic:Marine:Pelagic	0.8696
MGYA00375101/ERZ791566_FASTA.fasta	MGYA00375101	ERZ791566	root:Environmental:Aquatic:Marine:Pelagic	0.8626
MGYA00375102/ERZ791567_FASTA.fasta	MGYA00375102	ERZ791567	root:Environmental:Aquatic:Marine:Pelagic	0.871
MGYA00375103/ERZ791568_FASTA.fasta	MGYA00375103	ERZ791568	root:Environmental:Aquatic:Freshwater:Lotic:Sediment	0.892
MGYA00375104/ERZ794994_FASTA.fasta	MGYA00375104	ERZ794994	root:Engineered:Bioreactor	0.7062
MGYA00375105/ERZ795013_FASTA.fasta	MGYA00375105	ERZ795013	root:Engineered:Bioreactor	0.578
MGYA00375106/ERZ795014_FASTA.fasta	MGYA00375106	ERZ795014	root:Engineered:Wastewater	0.7986
MGYA00375107/ERZ795015_FASTA.fasta	MGYA00375107	ERZ795015	root:Engineered:Wastewater:Industrial wastewater	0.7616
MGYA00375108/ERZ795016_FASTA.fasta	MGYA00375108	ERZ795016	root:Engineered:Wastewater:Industrial wastewater	0.7578
MGYA00375109/ERZ795017_FASTA.fasta	MGYA00375109	ERZ795017	root:Engineered:Wastewater:Industrial wastewater	0.659
MGYA00375110/ERZ795018_FASTA.fasta	MGYA00375110	ERZ795018	root:Engineered:Wastewater:Industrial wastewater	0.769
MGYA00375111/ERZ795019_FASTA.fasta	MGYA00375111	ERZ795019	root:Engineered:Wastewater:Industrial wastewater	0.7844
MGYA00375112/ERZ795020_FASTA.fasta	MGYA00375112	ERZ795020	root:Engineered:Wastewater	0.708
MGYA00375113/ERZ795021_FASTA.fasta	MGYA00375113	ERZ795021	root:Engineered:Wastewater	0.745
MGYA00375114/ERZ795022_FASTA.fasta	MGYA00375114	ERZ795022	root:Engineered:Wastewater	0.7548
MGYA00375115/ERZ795023_FASTA.fasta	MGYA00375115	ERZ795023	root:Engineered:Wastewater	0.5468
MGYA00375116/ERZ795024_FASTA.fasta	MGYA00375116	ERZ795024	root:Engineered:Wastewater	0.6476
MGYA00375117/ERZ795025_FASTA.fasta	MGYA00375117	ERZ795025	root:Engineered:Wastewater	0.8492
MGYA00375118/ERZ795026_FASTA.fasta	MGYA00375118	ERZ795026	root:Engineered:Wastewater:Activated Sludge	0.8446
MGYA00375119/ERZ795027_FASTA.fasta	MGYA00375119	ERZ795027	root:Engineered:Wastewater:Activated Sludge	0.8832
MGYA00375120/ERZ795028_FASTA.fasta	MGYA00375120	ERZ795028	root:Engineered:Wastewater:Activated Sludge	0.835
MGYA00375121/ERZ795029_FASTA.fasta	MGYA00375121	ERZ795029	root:Engineered:Wastewater:Activated Sludge	0.843
MGYA00375122/ERZ795030_FASTA.fasta	MGYA00375122	ERZ795030	root:Engineered:Wastewater:Activated Sludge	0.8324
MGYA00375123/ERZ795031_FASTA.fasta	MGYA00375123	ERZ795031	root:Engineered:Wastewater:Activated Sludge	0.8868
MGYA00375124/ERZ795032_FASTA.fasta	MGYA00375124	ERZ795032	root:Engineered:Wastewater:Activated Sludge	0.6286
MGYA00375125/ERZ795041_FASTA.fasta	MGYA00375125	ERZ795041	root:Engineered:Wastewater:Activated Sludge	0.7718
MGYA00375126/ERZ795042_FASTA.fasta	MGYA00375126	ERZ795042	root:Engineered:Solid waste:Composting	0.7408
MGYA00375127/ERZ795043_FASTA.fasta	MGYA00375127	ERZ795043	root:Engineered:Solid waste:Composting	0.745
MGYA00375128/ERZ795044_FASTA.fasta	MGYA00375128	ERZ795044	root:Engineered:Wastewater:Activated Sludge	0.8944
MGYA00375129/ERZ795045_FASTA.fasta	MGYA00375129	ERZ795045	root:Engineered:Wastewater:Activated Sludge	0.7602
MGYA00375130/ERZ795046_FASTA.fasta	MGYA00375130	ERZ795046	root:Engineered:Wastewater:Activated Sludge	0.7702
MGYA00375131/ERZ795047_FASTA.fasta	MGYA00375131	ERZ795047	root:Engineered:Wastewater:Activated Sludge	0.8048
MGYA00375132/ERZ795048_FASTA.fasta	MGYA00375132	ERZ795048	root:Engineered:Wastewater:Activated Sludge	0.8462
MGYA00375133/ERZ795049_FASTA.fasta	MGYA00375133	ERZ795049	root:Engineered:Wastewater:Activated Sludge	0.8166
MGYA00375134/ERZ795050_FASTA.fasta	MGYA00375134	ERZ795050	root:Engineered:Wastewater:Activated Sludge	0.8418
MGYA00375135/ERZ795051_FASTA.fasta	MGYA00375135	ERZ795051	root:Engineered:Wastewater:Activated Sludge	0.8348
MGYA00375136/ERZ795052_FASTA.fasta	MGYA00375136	ERZ795052	root:Engineered:Wastewater:Activated Sludge	0.8712
MGYA00375137/ERZ795053_FASTA.fasta	MGYA00375137	ERZ795053	root:Engineered:Wastewater:Activated Sludge	0.9102
MGYA00375138/ERZ795054_FASTA.fasta	MGYA00375138	ERZ795054	root:Engineered:Wastewater:Activated Sludge	0.8416
MGYA00375139/ERZ795055_FASTA.fasta	MGYA00375139	ERZ795055	root:Engineered:Wastewater:Activated Sludge	0.8712
MGYA00375140/ERZ795056_FASTA.fasta	MGYA00375140	ERZ795056	root:Engineered:Wastewater:Activated Sludge	0.9332
MGYA00375141/ERZ795057_FASTA.fasta	MGYA00375141	ERZ795057	root:Engineered:Wastewater:Activated Sludge	0.8372
MGYA00375142/ERZ795058_FASTA.fasta	MGYA00375142	ERZ795058	root:Engineered:Wastewater:Activated Sludge	0.8412
MGYA00375143/ERZ795059_FASTA.fasta	MGYA00375143	ERZ795059	root:Engineered:Wastewater:Activated Sludge	0.9056
MGYA00375144/ERZ795060_FASTA.fasta	MGYA00375144	ERZ795060	root:Engineered:Wastewater:Activated Sludge	0.8746
MGYA00375145/ERZ795061_FASTA.fasta	MGYA00375145	ERZ795061	root:Engineered:Wastewater:Activated Sludge	0.893
MGYA00375146/ERZ795062_FASTA.fasta	MGYA00375146	ERZ795062	root:Engineered:Wastewater:Activated Sludge	0.8994
MGYA00375147/ERZ795063_FASTA.fasta	MGYA00375147	ERZ795063	root:Engineered:Wastewater:Activated Sludge	0.871
MGYA00375148/ERZ795064_FASTA.fasta	MGYA00375148	ERZ795064	root:Engineered:Wastewater:Activated Sludge	0.8692
MGYA00375149/ERZ795065_FASTA.fasta	MGYA00375149	ERZ795065	root:Engineered:Bioreactor	0.8254
MGYA00375150/ERZ795066_FASTA.fasta	MGYA00375150	ERZ795066	root:Engineered:Wastewater:Activated Sludge	0.7316
MGYA00375151/ERZ795067_FASTA.fasta	MGYA00375151	ERZ795067	root:Engineered:Wastewater:Activated Sludge	0.754
MGYA00375152/ERZ795068_FASTA.fasta	MGYA00375152	ERZ795068	root:Engineered:Bioreactor	0.78
MGYA00375153/ERZ795069_FASTA.fasta	MGYA00375153	ERZ795069	root:Engineered:Bioreactor	0.7572
MGYA00375154/ERZ795070_FASTA.fasta	MGYA00375154	ERZ795070	root:Engineered:Bioreactor	0.7514
MGYA00375155/ERZ795071_FASTA.fasta	MGYA00375155	ERZ795071	root:Engineered:Bioreactor	0.7576
MGYA00375156/ERZ795072_FASTA.fasta	MGYA00375156	ERZ795072	root:Engineered:Bioreactor	0.7438
MGYA00375157/ERZ795073_FASTA.fasta	MGYA00375157	ERZ795073	root:Engineered:Bioreactor	0.692
MGYA00375158/ERZ795074_FASTA.fasta	MGYA00375158	ERZ795074	root:Engineered:Food production:Fermented beverage	0.621
MGYA00375159/ERZ795075_FASTA.fasta	MGYA00375159	ERZ795075	root:Engineered:Wastewater:Activated Sludge	0.912
MGYA00375160/ERZ795076_FASTA.fasta	MGYA00375160	ERZ795076	root:Engineered:Wastewater:Activated Sludge	0.8588
MGYA00375161/ERZ795077_FASTA.fasta	MGYA00375161	ERZ795077	root:Engineered:Bioreactor	0.814
MGYA00375162/ERZ795078_FASTA.fasta	MGYA00375162	ERZ795078	root:Engineered:Bioreactor	0.6002
MGYA00375163/ERZ795079_FASTA.fasta	MGYA00375163	ERZ795079	root:Engineered:Bioreactor	0.5912
MGYA00375164/ERZ795080_FASTA.fasta	MGYA00375164	ERZ795080	root:Engineered:Bioreactor	0.5538
MGYA00375165/ERZ795081_FASTA.fasta	MGYA00375165	ERZ795081	root:Engineered:Bioreactor	0.582
MGYA00375166/ERZ795082_FASTA.fasta	MGYA00375166	ERZ795082	root:Engineered:Bioreactor	0.5816
MGYA00375167/ERZ795083_FASTA.fasta	MGYA00375167	ERZ795083	root:Engineered:Bioreactor	0.5646
MGYA00375168/ERZ795084_FASTA.fasta	MGYA00375168	ERZ795084	root:Engineered:Bioreactor	0.5538
MGYA00375169/ERZ795085_FASTA.fasta	MGYA00375169	ERZ795085	root:Engineered:Bioreactor	0.5768
MGYA00375170/ERZ795086_FASTA.fasta	MGYA00375170	ERZ795086	root:Engineered:Bioreactor	0.5568
MGYA00375171/ERZ795087_FASTA.fasta	MGYA00375171	ERZ795087	root:Engineered:Bioreactor	0.5596
MGYA00375172/ERZ795088_FASTA.fasta	MGYA00375172	ERZ795088	root:Engineered:Bioreactor	0.5666
MGYA00375173/ERZ795089_FASTA.fasta	MGYA00375173	ERZ795089	root:Engineered:Bioreactor	0.5176
MGYA00375174/ERZ795090_FASTA.fasta	MGYA00375174	ERZ795090	root:Engineered:Bioreactor	0.5722
MGYA00375175/ERZ795091_FASTA.fasta	MGYA00375175	ERZ795091	root:Engineered:Bioreactor	0.5602
MGYA00375176/ERZ795092_FASTA.fasta	MGYA00375176	ERZ795092	root:Engineered:Bioreactor	0.5674
MGYA00375177/ERZ795093_FASTA.fasta	MGYA00375177	ERZ795093	root:Engineered:Bioreactor	0.5592
MGYA00375178/ERZ795094_FASTA.fasta	MGYA00375178	ERZ795094	root:Engineered:Bioreactor	0.6102
MGYA00375179/ERZ795095_FASTA.fasta	MGYA00375179	ERZ795095	root:Engineered:Bioreactor	0.5072
MGYA00375180/ERZ795096_FASTA.fasta	MGYA00375180	ERZ795096	root:Engineered:Bioreactor	0.512
MGYA00375181/ERZ795097_FASTA.fasta	MGYA00375181	ERZ795097	root:Engineered:Bioreactor	0.615
MGYA00375182/ERZ795098_FASTA.fasta	MGYA00375182	ERZ795098	root:Engineered:Bioreactor	0.6456
MGYA00375183/ERZ795099_FASTA.fasta	MGYA00375183	ERZ795099	root:Engineered:Bioreactor	0.5568
MGYA00375184/ERZ795100_FASTA.fasta	MGYA00375184	ERZ795100	root:Engineered:Bioreactor	0.5836
MGYA00375185/ERZ795101_FASTA.fasta	MGYA00375185	ERZ795101	root:Engineered:Bioreactor	0.5106
MGYA00375186/ERZ795102_FASTA.fasta	MGYA00375186	ERZ795102	root:Engineered:Bioreactor	0.4852
MGYA00375187/ERZ795103_FASTA.fasta	MGYA00375187	ERZ795103	root:Engineered:Solid waste:Composting	0.8346
MGYA00375188/ERZ795104_FASTA.fasta	MGYA00375188	ERZ795104	root:Engineered:Solid waste:Composting	0.0972
MGYA00375189/ERZ795105_FASTA.fasta	MGYA00375189	ERZ795105	root:Engineered:Bioreactor	0.852
MGYA00375190/ERZ795106_FASTA.fasta	MGYA00375190	ERZ795106	root:Host-associated:Algae	0.3226
MGYA00375191/ERZ795107_FASTA.fasta	MGYA00375191	ERZ795107	root:Host-associated:Algae	0.3498
MGYA00375192/ERZ795108_FASTA.fasta	MGYA00375192	ERZ795108	root:Engineered:Bioreactor	0.7564
MGYA00375193/ERZ795127_FASTA.fasta	MGYA00375193	ERZ795127	root:Environmental:Aquatic:Freshwater:Lotic	0.8626
MGYA00375194/ERZ795381_FASTA.fasta	MGYA00375194	ERZ795381	root:Engineered:Wastewater:Activated Sludge	0.7562
MGYA00375195/ERZ795388_FASTA.fasta	MGYA00375195	ERZ795388	root:Engineered:Solid waste:Composting	0.655
MGYA00375196/ERZ795389_FASTA.fasta	MGYA00375196	ERZ795389	root:Engineered:Wastewater:Activated Sludge	0.8532
MGYA00375197/ERZ795390_FASTA.fasta	MGYA00375197	ERZ795390	root:Engineered:Bioreactor	0.683
MGYA00375198/ERZ795391_FASTA.fasta	MGYA00375198	ERZ795391	root:Engineered:Bioreactor	0.9312
MGYA00375199/ERZ795392_FASTA.fasta	MGYA00375199	ERZ795392	root:Engineered:Bioreactor	0.5576
MGYA00375200/ERZ795397_FASTA.fasta	MGYA00375200	ERZ795397	root:Engineered:Wastewater:Activated Sludge	0.8962
MGYA00375201/ERZ795404_FASTA.fasta	MGYA00375201	ERZ795404	root:Engineered:Bioreactor	0.567
MGYA00375202/ERZ795405_FASTA.fasta	MGYA00375202	ERZ795405	root:Engineered:Bioreactor	0.5538
MGYA00375203/ERZ795387_FASTA.fasta	MGYA00375203	ERZ795387	root:Engineered:Solid waste:Composting	0.7786
MGYA00375561/ERZ746980_FASTA.fasta	MGYA00375561	ERZ746980	root:Engineered:Wastewater:Activated Sludge	0.8088
MGYA00375562/ERZ766972_FASTA.fasta	MGYA00375562	ERZ766972	root:Environmental:Aquatic:Marine	0.8818
MGYA00375563/ERZ781400_FASTA.fasta	MGYA00375563	ERZ781400	root:Engineered:Wastewater	0.7636
MGYA00375564/ERZ781401_FASTA.fasta	MGYA00375564	ERZ781401	root:Engineered:Wastewater	0.73
MGYA00375565/ERZ781402_FASTA.fasta	MGYA00375565	ERZ781402	root:Engineered:Wastewater	0.7306
MGYA00375566/ERZ781403_FASTA.fasta	MGYA00375566	ERZ781403	root:Engineered:Wastewater	0.7278
MGYA00375567/ERZ781407_FASTA.fasta	MGYA00375567	ERZ781407	root:Environmental:Aquatic:Thermal springs:Hot (42-90	0.3652
MGYA00375568/ERZ781408_FASTA.fasta	MGYA00375568	ERZ781408	root:Environmental:Aquatic:Thermal springs:Hot (42-90	0.3546
MGYA00375569/ERZ794910_FASTA.fasta	MGYA00375569	ERZ794910	root:Engineered:Solid waste:Composting	0.5424
MGYA00375570/ERZ794911_FASTA.fasta	MGYA00375570	ERZ794911	root:Engineered:Solid waste:Composting	0.5752
MGYA00375571/ERZ795007_FASTA.fasta	MGYA00375571	ERZ795007	root:Engineered:Food production	0.0294
MGYA00375572/ERZ795008_FASTA.fasta	MGYA00375572	ERZ795008	root:Engineered:Food production	0.0294
MGYA00375573/ERZ795009_FASTA.fasta	MGYA00375573	ERZ795009	root:Engineered:Wastewater:Activated Sludge	0.8296
MGYA00375574/ERZ795010_FASTA.fasta	MGYA00375574	ERZ795010	root:Engineered:Wastewater:Activated Sludge	0.8008
MGYA00375575/ERZ795011_FASTA.fasta	MGYA00375575	ERZ795011	root:Engineered:Bioreactor	0.572
MGYA00375576/ERZ795012_FASTA.fasta	MGYA00375576	ERZ795012	root:Engineered:Bioreactor	0.532
MGYA00375577/ERZ795125_FASTA.fasta	MGYA00375577	ERZ795125	root:Environmental:Aquatic:Freshwater:Lotic:Sediment	0.6598
MGYA00375578/ERZ795126_FASTA.fasta	MGYA00375578	ERZ795126	root:Environmental:Aquatic:Freshwater:Lotic:Sediment	0.744
MGYA00375579/ERZ795384_FASTA.fasta	MGYA00375579	ERZ795384	root:Engineered:Bioreactor	0.5012
MGYA00375580/ERZ795386_FASTA.fasta	MGYA00375580	ERZ795386	root:Engineered:Bioreactor	0.5086
MGYA00375581/ERZ795395_FASTA.fasta	MGYA00375581	ERZ795395	root:Engineered:Food production	0.438
MGYA00375582/ERZ795396_FASTA.fasta	MGYA00375582	ERZ795396	root:Engineered:Food production	0.5096
MGYA00375583/ERZ781404_FASTA.fasta	MGYA00375583	ERZ781404	root:Engineered:Wastewater:Activated Sludge	0.7616
MGYA00375584/ERZ781405_FASTA.fasta	MGYA00375584	ERZ781405	root:Engineered:Wastewater:Activated Sludge	0.7996
MGYA00375585/ERZ781406_FASTA.fasta	MGYA00375585	ERZ781406	root:Engineered:Wastewater:Activated Sludge	0.7816
MGYA00375586/ERZ795004_FASTA.fasta	MGYA00375586	ERZ795004	root:Engineered:Food production:Fermented vegetable	0.4796
MGYA00375587/ERZ795005_FASTA.fasta	MGYA00375587	ERZ795005	root:Engineered:Food production:Fermented vegetable	0.4988
MGYA00375588/ERZ795006_FASTA.fasta	MGYA00375588	ERZ795006	root:Engineered:Food production:Fermented vegetable	0.499
MGYA00375589/ERZ795038_FASTA.fasta	MGYA00375589	ERZ795038	root:Engineered:Bioreactor	0.8632
MGYA00375590/ERZ795039_FASTA.fasta	MGYA00375590	ERZ795039	root:Engineered:Bioreactor	0.8766
MGYA00375591/ERZ795040_FASTA.fasta	MGYA00375591	ERZ795040	root:Engineered:Bioreactor	0.869
MGYA00375592/ERZ795122_FASTA.fasta	MGYA00375592	ERZ795122	root:Environmental:Aquatic:Estuary:Sediment	0.7726
MGYA00375593/ERZ795123_FASTA.fasta	MGYA00375593	ERZ795123	root:Environmental:Aquatic:Estuary:Sediment	0.6614
MGYA00375594/ERZ795124_FASTA.fasta	MGYA00375594	ERZ795124	root:Environmental:Aquatic:Estuary:Sediment	0.4108
MGYA00375597/ERZ778520_FASTA.fasta	MGYA00375597	ERZ778520	root:Host-associated:Plants:Root	0.5484
MGYA00375598/ERZ778521_FASTA.fasta	MGYA00375598	ERZ778521	root:Host-associated:Plants:Root	0.5148
MGYA00375599/ERZ778522_FASTA.fasta	MGYA00375599	ERZ778522	root:Host-associated:Plants:Root	0.5858
MGYA00375600/ERZ778523_FASTA.fasta	MGYA00375600	ERZ778523	root:Host-associated:Plants:Root	0.5052
MGYA00375601/ERZ781409_FASTA.fasta	MGYA00375601	ERZ781409	root:Engineered:Wastewater	0.2834
MGYA00375602/ERZ781410_FASTA.fasta	MGYA00375602	ERZ781410	root:Engineered:Wastewater	0.48
MGYA00375603/ERZ781411_FASTA.fasta	MGYA00375603	ERZ781411	root:Engineered:Wastewater	0.2308
MGYA00375604/ERZ781412_FASTA.fasta	MGYA00375604	ERZ781412	root:Engineered:Wastewater	0.3292
MGYA00375605/ERZ795000_FASTA.fasta	MGYA00375605	ERZ795000	root:Engineered:Bioreactor:Continuous culture:Marine i	0.6774
MGYA00375606/ERZ795001_FASTA.fasta	MGYA00375606	ERZ795001	root:Engineered:Bioreactor:Continuous culture:Marine i	0.439
MGYA00375607/ERZ795002_FASTA.fasta	MGYA00375607	ERZ795002	root:Engineered:Bioreactor:Continuous culture:Marine i	0.632
MGYA00375608/ERZ795003_FASTA.fasta	MGYA00375608	ERZ795003	root:Engineered:Bioreactor:Continuous culture:Marine i	0.601
MGYA00375614/ERZ483444_FASTA.fasta	MGYA00375614	ERZ483444	root:Environmental:Terrestrial:Soil:Grasslands	0.525
MGYA00375619/ERZ776998_FASTA.fasta	MGYA00375619	ERZ776998	root:Engineered:Food production:Fermented vegetable	0.5602
MGYA00375620/ERZ776999_FASTA.fasta	MGYA00375620	ERZ776999	root:Engineered:Food production:Fermented vegetable	0.5328
MGYA00375621/ERZ777000_FASTA.fasta	MGYA00375621	ERZ777000	root:Engineered:Food production:Fermented vegetable	0.5202
MGYA00375622/ERZ777001_FASTA.fasta	MGYA00375622	ERZ777001	root:Engineered:Food production:Fermented vegetable	0.6042
MGYA00375623/ERZ777002_FASTA.fasta	MGYA00375623	ERZ777002	root:Engineered:Food production:Fermented vegetable	0.5672
MGYA00375624/ERZ794995_FASTA.fasta	MGYA00375624	ERZ794995	root:Engineered:Bioreactor	0.5132
MGYA00375625/ERZ794996_FASTA.fasta	MGYA00375625	ERZ794996	root:Engineered:Bioreactor	0.7014
MGYA00375626/ERZ794997_FASTA.fasta	MGYA00375626	ERZ794997	root:Engineered:Bioreactor	0.6196
MGYA00375627/ERZ794998_FASTA.fasta	MGYA00375627	ERZ794998	root:Engineered:Bioreactor	0.7792
MGYA00375628/ERZ794999_FASTA.fasta	MGYA00375628	ERZ794999	root:Engineered:Bioreactor	0.6922
MGYA00375629/ERZ795033_FASTA.fasta	MGYA00375629	ERZ795033	root:Engineered:Bioreactor	0.8312
MGYA00375630/ERZ795034_FASTA.fasta	MGYA00375630	ERZ795034	root:Engineered:Bioreactor	0.9052
MGYA00375631/ERZ795035_FASTA.fasta	MGYA00375631	ERZ795035	root:Engineered:Bioreactor	0.9044
MGYA00375632/ERZ795036_FASTA.fasta	MGYA00375632	ERZ795036	root:Engineered:Bioreactor	0.8672
MGYA00375633/ERZ795037_FASTA.fasta	MGYA00375633	ERZ795037	root:Engineered:Bioreactor	0.899
MGYA00375634/ERZ841351_FASTA.fasta	MGYA00375634	ERZ841351	root:Environmental:Aquatic:Marine:Oceanic:Photic zon	0.6768
MGYA00375635/ERZ842531_FASTA.fasta	MGYA00375635	ERZ842531	root:Environmental:Aquatic:Marine:Oceanic:Photic zon	0.702
MGYA00375636/ERZ842562_FASTA.fasta	MGYA00375636	ERZ842562	root:Environmental:Aquatic:Marine:Oceanic:Photic zon	0.6544
MGYA00375637/ERZ842656_FASTA.fasta	MGYA00375637	ERZ842656	root:Environmental:Aquatic:Marine:Oceanic:Photic zon	0.7006
MGYA00375638/ERZ842729_FASTA.fasta	MGYA00375638	ERZ842729	root:Environmental:Aquatic:Marine:Oceanic:Photic zon	0.6454
MGYA00375639/ERZ780644_FASTA.fasta	MGYA00375639	ERZ780644	root:Host-associated:Microbial:Bacteria	0.4802
MGYA00375640/ERZ780645_FASTA.fasta	MGYA00375640	ERZ780645	root:Host-associated:Microbial:Bacteria	0.3116
MGYA00375641/ERZ780646_FASTA.fasta	MGYA00375641	ERZ780646	root:Host-associated:Microbial:Bacteria	0.6146
MGYA00375642/ERZ780647_FASTA.fasta	MGYA00375642	ERZ780647	root:Host-associated:Microbial:Bacteria	0.5264
MGYA00375643/ERZ780648_FASTA.fasta	MGYA00375643	ERZ780648	root:Host-associated:Microbial:Bacteria	0.621
MGYA00375644/ERZ780649_FASTA.fasta	MGYA00375644	ERZ780649	root:Host-associated:Microbial:Bacteria	0.372
MGYA00375645/ERZ795398_FASTA.fasta	MGYA00375645	ERZ795398	root:Environmental:Aquatic:Freshwater:Groundwater	0.7992
MGYA00375646/ERZ795399_FASTA.fasta	MGYA00375646	ERZ795399	root:Environmental:Aquatic:Freshwater:Groundwater	0.788
MGYA00375647/ERZ795400_FASTA.fasta	MGYA00375647	ERZ795400	root:Environmental:Aquatic:Freshwater:Groundwater	0.7936
MGYA00375648/ERZ795401_FASTA.fasta	MGYA00375648	ERZ795401	root:Environmental:Aquatic:Freshwater:Groundwater	0.787
MGYA00375649/ERZ795402_FASTA.fasta	MGYA00375649	ERZ795402	root:Environmental:Aquatic:Freshwater:Groundwater	0.7872
MGYA00375650/ERZ795403_FASTA.fasta	MGYA00375650	ERZ795403	root:Environmental:Aquatic:Freshwater:Groundwater	0.7618
MGYA00375651/ERZ658656_FASTA.fasta	MGYA00375651	ERZ658656	root:Host-associated:Human:Digestive system:Large int	0.443
MGYA00375652/ERZ658686_FASTA.fasta	MGYA00375652	ERZ658686	root:Host-associated:Human:Digestive system:Large int	0.5968
MGYA00375653/ERZ658687_FASTA.fasta	MGYA00375653	ERZ658687	root:Host-associated:Human:Digestive system:Large int	0.3346
MGYA00375654/ERZ658688_FASTA.fasta	MGYA00375654	ERZ658688	root:Host-associated:Human:Digestive system:Large int	0.5228
MGYA00375655/ERZ658689_FASTA.fasta	MGYA00375655	ERZ658689	root:Host-associated:Human:Digestive system:Large int	0.4886
MGYA00375656/ERZ658706_FASTA.fasta	MGYA00375656	ERZ658706	root:Host-associated:Human:Digestive system:Large int	0.5782
MGYA00375657/ERZ658707_FASTA.fasta	MGYA00375657	ERZ658707	root:Host-associated:Human:Digestive system:Large int	0.3882
MGYA00375658/ERZ658716_FASTA.fasta	MGYA00375658	ERZ658716	root:Host-associated:Human:Digestive system:Large int	0.4502
MGYA00375659/ERZ658717_FASTA.fasta	MGYA00375659	ERZ658717	root:Host-associated:Human:Digestive system:Large int	0.5684
MGYA00375660/ERZ781235_FASTA.fasta	MGYA00375660	ERZ781235	root:Environmental:Aquatic:Freshwater:Lotic:Sediment	0.7974
MGYA00375661/ERZ781236_FASTA.fasta	MGYA00375661	ERZ781236	root:Environmental:Aquatic:Freshwater:Lotic:Sediment	0.7986
MGYA00375662/ERZ781237_FASTA.fasta	MGYA00375662	ERZ781237	root:Environmental:Aquatic:Freshwater:Lotic:Sediment	0.799
MGYA00375663/ERZ781238_FASTA.fasta	MGYA00375663	ERZ781238	root:Environmental:Aquatic:Freshwater:Lotic:Sediment	0.8158
MGYA00375664/ERZ781239_FASTA.fasta	MGYA00375664	ERZ781239	root:Environmental:Aquatic:Freshwater:Lotic:Sediment	0.7522
MGYA00375665/ERZ781240_FASTA.fasta	MGYA00375665	ERZ781240	root:Environmental:Aquatic:Freshwater:Lotic:Sediment	0.8014
MGYA00375666/ERZ781241_FASTA.fasta	MGYA00375666	ERZ781241	root:Environmental:Aquatic:Freshwater:Lotic:Sediment	0.7864
MGYA00375667/ERZ781242_FASTA.fasta	MGYA00375667	ERZ781242	root:Environmental:Aquatic:Freshwater:Lotic:Sediment	0.7786
MGYA00375668/ERZ781243_FASTA.fasta	MGYA00375668	ERZ781243	root:Environmental:Aquatic:Freshwater:Lotic:Sediment	0.772
MGYA00375669/ERZ782915_FASTA.fasta	MGYA00375669	ERZ782915	root:Engineered:Wastewater:Activated Sludge	0.7162
MGYA00375670/ERZ782916_FASTA.fasta	MGYA00375670	ERZ782916	root:Engineered:Wastewater:Activated Sludge	0.7324
MGYA00375671/ERZ782923_FASTA.fasta	MGYA00375671	ERZ782923	root:Engineered:Wastewater:Activated Sludge	0.391
MGYA00375672/ERZ782924_FASTA.fasta	MGYA00375672	ERZ782924	root:Engineered:Wastewater:Activated Sludge	0.383
MGYA00375673/ERZ782925_FASTA.fasta	MGYA00375673	ERZ782925	root:Engineered:Wastewater:Activated Sludge	0.4346
MGYA00375674/ERZ782926_FASTA.fasta	MGYA00375674	ERZ782926	root:Engineered:Wastewater:Activated Sludge	0.377
MGYA00375675/ERZ782927_FASTA.fasta	MGYA00375675	ERZ782927	root:Engineered:Wastewater:Activated Sludge	0.3822
MGYA00375676/ERZ782928_FASTA.fasta	MGYA00375676	ERZ782928	root:Engineered:Wastewater:Activated Sludge	0.4024
MGYA00375677/ERZ782929_FASTA.fasta	MGYA00375677	ERZ782929	root:Engineered:Wastewater:Activated Sludge	0.404
MGYA00375678/ERZ782930_FASTA.fasta	MGYA00375678	ERZ782930	root:Engineered:Wastewater:Activated Sludge	0.3806
MGYA00375684/ERZ794970_FASTA.fasta	MGYA00375684	ERZ794970	root:Engineered:Solid waste:Composting	0.6342
MGYA00375685/ERZ794971_FASTA.fasta	MGYA00375685	ERZ794971	root:Engineered:Solid waste:Composting	0.816
MGYA00375686/ERZ794972_FASTA.fasta	MGYA00375686	ERZ794972	root:Engineered:Solid waste:Composting	0.849
MGYA00375687/ERZ794973_FASTA.fasta	MGYA00375687	ERZ794973	root:Engineered:Solid waste:Composting	0.8352
MGYA00375688/ERZ794974_FASTA.fasta	MGYA00375688	ERZ794974	root:Engineered:Solid waste:Composting	0.6704
MGYA00375689/ERZ794975_FASTA.fasta	MGYA00375689	ERZ794975	root:Engineered:Solid waste:Composting	0.832
MGYA00375690/ERZ794976_FASTA.fasta	MGYA00375690	ERZ794976	root:Engineered:Solid waste:Composting	0.8328
MGYA00375691/ERZ794977_FASTA.fasta	MGYA00375691	ERZ794977	root:Engineered:Solid waste:Composting	0.7774
MGYA00375692/ERZ794978_FASTA.fasta	MGYA00375692	ERZ794978	root:Engineered:Solid waste:Composting	0.8226
MGYA00375693/ERZ794979_FASTA.fasta	MGYA00375693	ERZ794979	root:Engineered:Solid waste:Composting	0.8248
MGYA00375694/ERZ794980_FASTA.fasta	MGYA00375694	ERZ794980	root:Engineered:Solid waste:Composting	0.8208
MGYA00375695/ERZ794981_FASTA.fasta	MGYA00375695	ERZ794981	root:Engineered:Solid waste:Composting	0.6874
MGYA00375696/ERZ795109_FASTA.fasta	MGYA00375696	ERZ795109	root:Environmental:Aquatic:Freshwater	0.7078
MGYA00375697/ERZ795110_FASTA.fasta	MGYA00375697	ERZ795110	root:Environmental:Aquatic:Freshwater	0.6586
MGYA00375698/ERZ795111_FASTA.fasta	MGYA00375698	ERZ795111	root:Environmental:Aquatic:Freshwater	0.6494
MGYA00375699/ERZ795112_FASTA.fasta	MGYA00375699	ERZ795112	root:Environmental:Aquatic:Freshwater	0.7502
MGYA00375700/ERZ795113_FASTA.fasta	MGYA00375700	ERZ795113	root:Environmental:Aquatic:Freshwater	0.71
MGYA00375701/ERZ795114_FASTA.fasta	MGYA00375701	ERZ795114	root:Environmental:Aquatic:Freshwater	0.624
MGYA00375702/ERZ795115_FASTA.fasta	MGYA00375702	ERZ795115	root:Environmental:Aquatic:Freshwater	0.8294
MGYA00375703/ERZ795116_FASTA.fasta	MGYA00375703	ERZ795116	root:Environmental:Aquatic:Freshwater	0.7608
MGYA00375704/ERZ795117_FASTA.fasta	MGYA00375704	ERZ795117	root:Environmental:Aquatic:Freshwater	0.6672
MGYA00375705/ERZ795118_FASTA.fasta	MGYA00375705	ERZ795118	root:Environmental:Aquatic:Freshwater	0.7514
MGYA00375706/ERZ795119_FASTA.fasta	MGYA00375706	ERZ795119	root:Environmental:Aquatic:Freshwater	0.7692
MGYA00375707/ERZ795120_FASTA.fasta	MGYA00375707	ERZ795120	root:Environmental:Aquatic:Freshwater	0.6118
MGYA00375708/ERZ795121_FASTA.fasta	MGYA00375708	ERZ795121	root:Environmental:Aquatic:Freshwater	0.7734
MGYA00375709/ERZ781210_FASTA.fasta	MGYA00375709	ERZ781210	root:Host-associated:Mammals:Digestive system	0.4668
MGYA00375710/ERZ781211_FASTA.fasta	MGYA00375710	ERZ781211	root:Host-associated:Mammals:Digestive system	0.5038
MGYA00375711/ERZ781212_FASTA.fasta	MGYA00375711	ERZ781212	root:Host-associated:Mammals:Digestive system	0.4344
MGYA00375712/ERZ781213_FASTA.fasta	MGYA00375712	ERZ781213	root:Host-associated:Mammals:Digestive system	0.4596
MGYA00375713/ERZ781214_FASTA.fasta	MGYA00375713	ERZ781214	root:Host-associated:Mammals:Digestive system	0.4628
MGYA00375714/ERZ781215_FASTA.fasta	MGYA00375714	ERZ781215	root:Host-associated:Mammals:Digestive system	0.4056
MGYA00375715/ERZ781216_FASTA.fasta	MGYA00375715	ERZ781216	root:Host-associated:Mammals:Digestive system	0.4344
MGYA00375716/ERZ781217_FASTA.fasta	MGYA00375716	ERZ781217	root:Host-associated:Mammals:Digestive system	0.4544
MGYA00375717/ERZ781218_FASTA.fasta	MGYA00375717	ERZ781218	root:Host-associated:Mammals:Digestive system	0.4304
MGYA00375718/ERZ781219_FASTA.fasta	MGYA00375718	ERZ781219	root:Host-associated:Mammals:Digestive system	0.4596
MGYA00375719/ERZ781220_FASTA.fasta	MGYA00375719	ERZ781220	root:Host-associated:Mammals:Digestive system	0.4474
MGYA00375720/ERZ781221_FASTA.fasta	MGYA00375720	ERZ781221	root:Host-associated:Mammals:Digestive system	0.4444
MGYA00375721/ERZ781222_FASTA.fasta	MGYA00375721	ERZ781222	root:Host-associated:Mammals:Digestive system	0.4684
MGYA00375722/ERZ781223_FASTA.fasta	MGYA00375722	ERZ781223	root:Host-associated:Mammals:Digestive system	0.4578
MGYA00375730/ERZ650672_FASTA.fasta	MGYA00375730	ERZ650672	root:Host-associated:Human:Digestive system:Large int	0.4446
MGYA00375731/ERZ794954_FASTA.fasta	MGYA00375731	ERZ794954	root:Engineered:Food production	0.2996
MGYA00375732/ERZ794955_FASTA.fasta	MGYA00375732	ERZ794955	root:Engineered:Food production	0.238
MGYA00375733/ERZ794956_FASTA.fasta	MGYA00375733	ERZ794956	root:Engineered:Food production	0.1674
MGYA00375734/ERZ794957_FASTA.fasta	MGYA00375734	ERZ794957	root:Engineered:Food production	0.2174
MGYA00375735/ERZ794958_FASTA.fasta	MGYA00375735	ERZ794958	root:Engineered:Food production	0.0522
MGYA00375736/ERZ794959_FASTA.fasta	MGYA00375736	ERZ794959	root:Engineered:Food production	0.133
MGYA00375737/ERZ794960_FASTA.fasta	MGYA00375737	ERZ794960	root:Engineered:Food production	0.2434
MGYA00375738/ERZ794961_FASTA.fasta	MGYA00375738	ERZ794961	root:Engineered:Food production	0.034
MGYA00375739/ERZ794962_FASTA.fasta	MGYA00375739	ERZ794962	root:Engineered:Food production	0.1568
MGYA00375740/ERZ794963_FASTA.fasta	MGYA00375740	ERZ794963	root:Engineered:Food production	0.2304
MGYA00375741/ERZ794964_FASTA.fasta	MGYA00375741	ERZ794964	root:Engineered:Food production	0.0988
MGYA00375742/ERZ794965_FASTA.fasta	MGYA00375742	ERZ794965	root:Engineered:Food production	0.0298
MGYA00375743/ERZ794966_FASTA.fasta	MGYA00375743	ERZ794966	root:Engineered:Food production	0.2524
MGYA00375744/ERZ794967_FASTA.fasta	MGYA00375744	ERZ794967	root:Engineered:Food production	0.0406
MGYA00375745/ERZ794968_FASTA.fasta	MGYA00375745	ERZ794968	root:Engineered:Food production	0.1904
MGYA00375746/ERZ794969_FASTA.fasta	MGYA00375746	ERZ794969	root:Engineered:Food production	0.2114
MGYA00375747/ERZ778017_FASTA.fasta	MGYA00375747	ERZ778017	root:Engineered:Bioreactor	0.8466
MGYA00375748/ERZ778018_FASTA.fasta	MGYA00375748	ERZ778018	root:Engineered:Bioreactor	0.826
MGYA00375750/ERZ778020_FASTA.fasta	MGYA00375750	ERZ778020	root:Engineered:Bioreactor	0.7636
MGYA00375751/ERZ778021_FASTA.fasta	MGYA00375751	ERZ778021	root:Engineered:Bioreactor	0.8798
MGYA00375752/ERZ778022_FASTA.fasta	MGYA00375752	ERZ778022	root:Engineered:Bioreactor	0.8658
MGYA00375753/ERZ778023_FASTA.fasta	MGYA00375753	ERZ778023	root:Engineered:Bioreactor	0.79
MGYA00375754/ERZ778024_FASTA.fasta	MGYA00375754	ERZ778024	root:Engineered:Bioreactor	0.8692
MGYA00375755/ERZ778025_FASTA.fasta	MGYA00375755	ERZ778025	root:Engineered:Bioreactor	0.8932
MGYA00375756/ERZ778026_FASTA.fasta	MGYA00375756	ERZ778026	root:Engineered:Bioreactor	0.765
MGYA00375757/ERZ778027_FASTA.fasta	MGYA00375757	ERZ778027	root:Engineered:Bioreactor	0.8062
MGYA00375758/ERZ778028_FASTA.fasta	MGYA00375758	ERZ778028	root:Engineered:Bioreactor	0.9086
MGYA00375759/ERZ778029_FASTA.fasta	MGYA00375759	ERZ778029	root:Engineered:Bioreactor	0.7472
MGYA00375760/ERZ778030_FASTA.fasta	MGYA00375760	ERZ778030	root:Engineered:Bioreactor	0.8654
MGYA00375761/ERZ778031_FASTA.fasta	MGYA00375761	ERZ778031	root:Engineered:Bioreactor	0.8578
MGYA00375762/ERZ778032_FASTA.fasta	MGYA00375762	ERZ778032	root:Engineered:Bioreactor	0.8824
MGYA00375763/ERZ778033_FASTA.fasta	MGYA00375763	ERZ778033	root:Engineered:Bioreactor	0.8138
MGYA00375764/ERZ778034_FASTA.fasta	MGYA00375764	ERZ778034	root:Engineered:Bioreactor	0.7928
MGYA00375765/ERZ778035_FASTA.fasta	MGYA00375765	ERZ778035	root:Engineered:Bioreactor	0.8696
MGYA00375769/ERZ500960_FASTA.fasta	MGYA00375769	ERZ500960	root:Host-associated:Human:Circulatory system	0.4446
MGYA00375770/ERZ500961_FASTA.fasta	MGYA00375770	ERZ500961	root:Host-associated:Human:Circulatory system	0.326
MGYA00375771/ERZ500963_FASTA.fasta	MGYA00375771	ERZ500963	root:Host-associated:Human:Circulatory system	0.3494
MGYA00375772/ERZ500966_FASTA.fasta	MGYA00375772	ERZ500966	root:Host-associated:Human:Circulatory system	0.2412
MGYA00375773/ERZ500968_FASTA.fasta	MGYA00375773	ERZ500968	root:Host-associated:Human:Circulatory system	0.0862
MGYA00375774/ERZ500969_FASTA.fasta	MGYA00375774	ERZ500969	root:Host-associated:Human:Circulatory system	0.1094
MGYA00375775/ERZ500971_FASTA.fasta	MGYA00375775	ERZ500971	root:Host-associated:Human:Circulatory system	0.3026
MGYA00375776/ERZ500972_FASTA.fasta	MGYA00375776	ERZ500972	root:Host-associated:Human:Circulatory system	0.3422
MGYA00375779/ERZ794912_FASTA.fasta	MGYA00375779	ERZ794912	root:Engineered:Wastewater:Activated Sludge	0.8492
MGYA00375780/ERZ794913_FASTA.fasta	MGYA00375780	ERZ794913	root:Engineered:Wastewater:Activated Sludge	0.8204
MGYA00375781/ERZ794914_FASTA.fasta	MGYA00375781	ERZ794914	root:Engineered:Wastewater:Activated Sludge	0.8314
MGYA00375782/ERZ794915_FASTA.fasta	MGYA00375782	ERZ794915	root:Engineered:Wastewater:Activated Sludge	0.7578
MGYA00375783/ERZ794916_FASTA.fasta	MGYA00375783	ERZ794916	root:Engineered:Wastewater:Activated Sludge	0.9058
MGYA00375784/ERZ794917_FASTA.fasta	MGYA00375784	ERZ794917	root:Engineered:Wastewater:Activated Sludge	0.8
MGYA00375785/ERZ794918_FASTA.fasta	MGYA00375785	ERZ794918	root:Engineered:Wastewater:Activated Sludge	0.7908
MGYA00375786/ERZ794919_FASTA.fasta	MGYA00375786	ERZ794919	root:Engineered:Wastewater:Activated Sludge	0.7538
MGYA00375787/ERZ794920_FASTA.fasta	MGYA00375787	ERZ794920	root:Engineered:Wastewater:Activated Sludge	0.8928
MGYA00375788/ERZ794921_FASTA.fasta	MGYA00375788	ERZ794921	root:Engineered:Wastewater:Activated Sludge	0.9036
MGYA00375789/ERZ794928_FASTA.fasta	MGYA00375789	ERZ794928	root:Engineered:Wastewater:Activated Sludge	0.8082
MGYA00375790/ERZ794929_FASTA.fasta	MGYA00375790	ERZ794929	root:Engineered:Wastewater:Activated Sludge	0.8936
MGYA00375791/ERZ794930_FASTA.fasta	MGYA00375791	ERZ794930	root:Engineered:Wastewater:Activated Sludge	0.8422
MGYA00375792/ERZ794931_FASTA.fasta	MGYA00375792	ERZ794931	root:Engineered:Wastewater:Activated Sludge	0.864
MGYA00375793/ERZ794932_FASTA.fasta	MGYA00375793	ERZ794932	root:Engineered:Wastewater:Activated Sludge	0.8032
MGYA00375794/ERZ794933_FASTA.fasta	MGYA00375794	ERZ794933	root:Engineered:Wastewater:Activated Sludge	0.7784
MGYA00375795/ERZ842394_FASTA.fasta	MGYA00375795	ERZ842394	root:Engineered:Wastewater:Activated Sludge	0.8394
MGYA00375796/ERZ842397_FASTA.fasta	MGYA00375796	ERZ842397	root:Engineered:Wastewater:Activated Sludge	0.8452
MGYA00375813/ERZ817765_FASTA.fasta	MGYA00375813	ERZ817765	root:Host-associated:Human:Digestive system	0.206
MGYA00375814/ERZ818680_FASTA.fasta	MGYA00375814	ERZ818680	root:Host-associated:Human:Digestive system	0.0412
MGYA00375815/ERZ819601_FASTA.fasta	MGYA00375815	ERZ819601	root:Host-associated:Human:Digestive system	0.0992
MGYA00375816/ERZ819620_FASTA.fasta	MGYA00375816	ERZ819620	root:Host-associated:Human:Digestive system	0.1002
MGYA00375817/ERZ819624_FASTA.fasta	MGYA00375817	ERZ819624	root:Host-associated:Human:Digestive system	0.0986
MGYA00375818/ERZ819632_FASTA.fasta	MGYA00375818	ERZ819632	root:Host-associated:Human:Digestive system	0.0644
MGYA00375819/ERZ819634_FASTA.fasta	MGYA00375819	ERZ819634	root:Host-associated:Human:Digestive system	0.0626
MGYA00375820/ERZ819636_FASTA.fasta	MGYA00375820	ERZ819636	root:Host-associated:Human:Digestive system	0.0926
MGYA00375821/ERZ819648_FASTA.fasta	MGYA00375821	ERZ819648	root:Host-associated:Human:Digestive system	0.0902
MGYA00375822/ERZ819661_FASTA.fasta	MGYA00375822	ERZ819661	root:Host-associated:Human:Digestive system	0.082
MGYA00375823/ERZ819663_FASTA.fasta	MGYA00375823	ERZ819663	root:Host-associated:Human:Digestive system	0.07
MGYA00375824/ERZ781246_FASTA.fasta	MGYA00375824	ERZ781246	root:Host-associated:Human:Digestive system	0.4754
MGYA00375825/ERZ781247_FASTA.fasta	MGYA00375825	ERZ781247	root:Host-associated:Human:Digestive system	0.473
MGYA00375826/ERZ781248_FASTA.fasta	MGYA00375826	ERZ781248	root:Host-associated:Human:Digestive system	0.3492
MGYA00375827/ERZ781249_FASTA.fasta	MGYA00375827	ERZ781249	root:Host-associated:Human:Digestive system	0.458
MGYA00375828/ERZ781250_FASTA.fasta	MGYA00375828	ERZ781250	root:Host-associated:Human:Digestive system	0.3282
MGYA00375829/ERZ781251_FASTA.fasta	MGYA00375829	ERZ781251	root:Host-associated:Human:Digestive system	0.415
MGYA00375830/ERZ781252_FASTA.fasta	MGYA00375830	ERZ781252	root:Host-associated:Human:Digestive system	0.3482
MGYA00375831/ERZ781253_FASTA.fasta	MGYA00375831	ERZ781253	root:Host-associated:Human:Digestive system	0.528
MGYA00375832/ERZ781254_FASTA.fasta	MGYA00375832	ERZ781254	root:Host-associated:Human:Digestive system	0.4674
MGYA00375833/ERZ781255_FASTA.fasta	MGYA00375833	ERZ781255	root:Host-associated:Human:Digestive system	0.3358
MGYA00375834/ERZ781256_FASTA.fasta	MGYA00375834	ERZ781256	root:Host-associated:Human:Digestive system	0.489
MGYA00375835/ERZ781257_FASTA.fasta	MGYA00375835	ERZ781257	root:Host-associated:Human:Digestive system	0.3404
MGYA00375836/ERZ781258_FASTA.fasta	MGYA00375836	ERZ781258	root:Host-associated:Human:Digestive system	0.458
MGYA00375837/ERZ781259_FASTA.fasta	MGYA00375837	ERZ781259	root:Host-associated:Human:Digestive system	0.3416
MGYA00375838/ERZ781260_FASTA.fasta	MGYA00375838	ERZ781260	root:Host-associated:Human:Digestive system	0.5222
MGYA00375839/ERZ781261_FASTA.fasta	MGYA00375839	ERZ781261	root:Host-associated:Human:Digestive system	0.3414
MGYA00375840/ERZ781262_FASTA.fasta	MGYA00375840	ERZ781262	root:Host-associated:Human:Digestive system	0.5376
MGYA00375841/ERZ781263_FASTA.fasta	MGYA00375841	ERZ781263	root:Host-associated:Human:Digestive system	0.4578
MGYA00375842/ERZ781264_FASTA.fasta	MGYA00375842	ERZ781264	root:Host-associated:Human:Digestive system	0.3804
MGYA00375843/ERZ781265_FASTA.fasta	MGYA00375843	ERZ781265	root:Host-associated:Human:Digestive system	0.4578
MGYA00375844/ERZ781266_FASTA.fasta	MGYA00375844	ERZ781266	root:Host-associated:Human:Digestive system	0.3098
MGYA00375845/ERZ781267_FASTA.fasta	MGYA00375845	ERZ781267	root:Host-associated:Human:Digestive system	0.45
MGYA00375846/ERZ781268_FASTA.fasta	MGYA00375846	ERZ781268	root:Host-associated:Human:Digestive system	0.3098
MGYA00375847/ERZ781269_FASTA.fasta	MGYA00375847	ERZ781269	root:Host-associated:Human:Digestive system	0.375
MGYA00375848/ERZ781270_FASTA.fasta	MGYA00375848	ERZ781270	root:Host-associated:Human:Digestive system	0.3542
MGYA00375849/ERZ781271_FASTA.fasta	MGYA00375849	ERZ781271	root:Host-associated:Human:Digestive system	0.3736
MGYA00375850/ERZ781272_FASTA.fasta	MGYA00375850	ERZ781272	root:Host-associated:Human:Digestive system	0.5376
MGYA00375851/ERZ781273_FASTA.fasta	MGYA00375851	ERZ781273	root:Host-associated:Human:Digestive system	0.4138
MGYA00375852/ERZ781274_FASTA.fasta	MGYA00375852	ERZ781274	root:Host-associated:Human:Digestive system	0.3968
MGYA00375853/ERZ781275_FASTA.fasta	MGYA00375853	ERZ781275	root:Host-associated:Human:Digestive system	0.513
MGYA00375854/ERZ724560_FASTA.fasta	MGYA00375854	ERZ724560	root:Environmental:Aquatic:Marine:Sediment	0.4346
MGYA00375855/ERZ724561_FASTA.fasta	MGYA00375855	ERZ724561	root:Environmental:Aquatic:Marine:Sediment	0.52

MGYA00375856/ERZ724562_FASTA.fasta	MGYA00375856	ERZ724562	root:Environmental:Aquatic:Marine:Sediment	0.416
MGYA00375857/ERZ724563_FASTA.fasta	MGYA00375857	ERZ724563	root:Environmental:Aquatic:Marine:Sediment	0.768
MGYA00375858/ERZ724564_FASTA.fasta	MGYA00375858	ERZ724564	root:Environmental:Aquatic:Marine:Sediment	0.352
MGYA00375859/ERZ724565_FASTA.fasta	MGYA00375859	ERZ724565	root:Environmental:Aquatic:Marine:Sediment	0.1894
MGYA00375860/ERZ724566_FASTA.fasta	MGYA00375860	ERZ724566	root:Environmental:Aquatic:Marine:Sediment	0.3546
MGYA00375861/ERZ724567_FASTA.fasta	MGYA00375861	ERZ724567	root:Environmental:Aquatic:Marine:Sediment	0.1664
MGYA00375862/ERZ724568_FASTA.fasta	MGYA00375862	ERZ724568	root:Environmental:Aquatic:Marine:Sediment	0.6226
MGYA00375863/ERZ724569_FASTA.fasta	MGYA00375863	ERZ724569	root:Environmental:Aquatic:Marine:Sediment	0.7482
MGYA00375864/ERZ724570_FASTA.fasta	MGYA00375864	ERZ724570	root:Environmental:Aquatic:Marine:Sediment	0.3702
MGYA00375865/ERZ724571_FASTA.fasta	MGYA00375865	ERZ724571	root:Environmental:Aquatic:Marine:Sediment	0.6892
MGYA00375866/ERZ724572_FASTA.fasta	MGYA00375866	ERZ724572	root:Environmental:Aquatic:Marine:Sediment	0.5496
MGYA00375867/ERZ724573_FASTA.fasta	MGYA00375867	ERZ724573	root:Environmental:Aquatic:Marine:Sediment	0.2562
MGYA00375868/ERZ724574_FASTA.fasta	MGYA00375868	ERZ724574	root:Environmental:Aquatic:Marine:Sediment	0.4906
MGYA00375869/ERZ724575_FASTA.fasta	MGYA00375869	ERZ724575	root:Environmental:Aquatic:Marine:Sediment	0.735
MGYA00375870/ERZ724576_FASTA.fasta	MGYA00375870	ERZ724576	root:Environmental:Aquatic:Marine:Sediment	0.1788
MGYA00375871/ERZ724577_FASTA.fasta	MGYA00375871	ERZ724577	root:Environmental:Aquatic:Marine:Sediment	0.525
MGYA00375872/ERZ724578_FASTA.fasta	MGYA00375872	ERZ724578	root:Environmental:Aquatic:Marine:Sediment	0.4662
MGYA00375873/ERZ724579_FASTA.fasta	MGYA00375873	ERZ724579	root:Environmental:Aquatic:Marine:Sediment	0.232
MGYA00375874/ERZ724580_FASTA.fasta	MGYA00375874	ERZ724580	root:Environmental:Aquatic:Marine:Sediment	0.7766
MGYA00375875/ERZ724581_FASTA.fasta	MGYA00375875	ERZ724581	root:Environmental:Aquatic:Marine:Sediment	0.484
MGYA00375876/ERZ724582_FASTA.fasta	MGYA00375876	ERZ724582	root:Environmental:Aquatic:Marine:Sediment	0.1696
MGYA00375877/ERZ724583_FASTA.fasta	MGYA00375877	ERZ724583	root:Environmental:Aquatic:Marine:Sediment	0.8054
MGYA00375878/ERZ724584_FASTA.fasta	MGYA00375878	ERZ724584	root:Environmental:Aquatic:Marine:Sediment	0.8034
MGYA00375879/ERZ724585_FASTA.fasta	MGYA00375879	ERZ724585	root:Environmental:Aquatic:Marine:Sediment	0.7814
MGYA00375880/ERZ724586_FASTA.fasta	MGYA00375880	ERZ724586	root:Environmental:Aquatic:Marine:Sediment	0.768
MGYA00375881/ERZ724587_FASTA.fasta	MGYA00375881	ERZ724587	root:Environmental:Aquatic:Marine:Sediment	0.4546
MGYA00375882/ERZ724588_FASTA.fasta	MGYA00375882	ERZ724588	root:Environmental:Aquatic:Marine:Sediment	0.3634
MGYA00375883/ERZ724589_FASTA.fasta	MGYA00375883	ERZ724589	root:Environmental:Aquatic:Marine:Sediment	0.2822
MGYA00375884/ERZ724590_FASTA.fasta	MGYA00375884	ERZ724590	root:Environmental:Aquatic:Marine:Sediment	0.449
MGYA00375885/ERZ776967_FASTA.fasta	MGYA00375885	ERZ776967	root:Host-associated:Mammals:Digestive system	0.1196
MGYA00375886/ERZ776968_FASTA.fasta	MGYA00375886	ERZ776968	root:Host-associated:Mammals:Digestive system	0.1292
MGYA00375887/ERZ776969_FASTA.fasta	MGYA00375887	ERZ776969	root:Host-associated:Mammals:Digestive system	0.231
MGYA00375888/ERZ776970_FASTA.fasta	MGYA00375888	ERZ776970	root:Host-associated:Mammals:Digestive system	0.1748
MGYA00375889/ERZ776971_FASTA.fasta	MGYA00375889	ERZ776971	root:Host-associated:Mammals:Digestive system	0.0806
MGYA00375890/ERZ776972_FASTA.fasta	MGYA00375890	ERZ776972	root:Host-associated:Mammals:Digestive system	0.1512
MGYA00375891/ERZ776973_FASTA.fasta	MGYA00375891	ERZ776973	root:Host-associated:Mammals:Digestive system	0.134
MGYA00375892/ERZ776974_FASTA.fasta	MGYA00375892	ERZ776974	root:Host-associated:Mammals:Digestive system	0.2662
MGYA00375893/ERZ776975_FASTA.fasta	MGYA00375893	ERZ776975	root:Host-associated:Mammals:Digestive system	0.2766
MGYA00375894/ERZ776976_FASTA.fasta	MGYA00375894	ERZ776976	root:Host-associated:Mammals:Digestive system	0.13
MGYA00375895/ERZ776977_FASTA.fasta	MGYA00375895	ERZ776977	root:Host-associated:Mammals:Digestive system	0.1498
MGYA00375896/ERZ776978_FASTA.fasta	MGYA00375896	ERZ776978	root:Host-associated:Mammals:Digestive system	0.129
MGYA00375897/ERZ776979_FASTA.fasta	MGYA00375897	ERZ776979	root:Host-associated:Mammals:Digestive system	0.1966
MGYA00375898/ERZ776980_FASTA.fasta	MGYA00375898	ERZ776980	root:Host-associated:Mammals:Digestive system	0.307
MGYA00375899/ERZ776981_FASTA.fasta	MGYA00375899	ERZ776981	root:Host-associated:Mammals:Digestive system	0.1038
MGYA00375900/ERZ776982_FASTA.fasta	MGYA00375900	ERZ776982	root:Host-associated:Mammals:Digestive system	0.2922
MGYA00375901/ERZ776983_FASTA.fasta	MGYA00375901	ERZ776983	root:Host-associated:Mammals:Digestive system	0.3094
MGYA00375902/ERZ776984_FASTA.fasta	MGYA00375902	ERZ776984	root:Host-associated:Mammals:Digestive system	0.318
MGYA00375903/ERZ776985_FASTA.fasta	MGYA00375903	ERZ776985	root:Host-associated:Mammals:Digestive system	0.1614
MGYA00375904/ERZ776986_FASTA.fasta	MGYA00375904	ERZ776986	root:Host-associated:Mammals:Digestive system	0.1
MGYA00375905/ERZ776987_FASTA.fasta	MGYA00375905	ERZ776987	root:Host-associated:Mammals:Digestive system	0.2906
MGYA00375906/ERZ776988_FASTA.fasta	MGYA00375906	ERZ776988	root:Host-associated:Mammals:Digestive system	0.1804
MGYA00375907/ERZ776989_FASTA.fasta	MGYA00375907	ERZ776989	root:Host-associated:Mammals:Digestive system	0.274
MGYA00375908/ERZ776990_FASTA.fasta	MGYA00375908	ERZ776990	root:Host-associated:Mammals:Digestive system	0.274
MGYA00375909/ERZ776991_FASTA.fasta	MGYA00375909	ERZ776991	root:Host-associated:Mammals:Digestive system	0.1072
MGYA00375910/ERZ776992_FASTA.fasta	MGYA00375910	ERZ776992	root:Host-associated:Mammals:Digestive system	0.258
MGYA00375911/ERZ776993_FASTA.fasta	MGYA00375911	ERZ776993	root:Host-associated:Mammals:Digestive system	0.1486
MGYA00375912/ERZ776994_FASTA.fasta	MGYA00375912	ERZ776994	root:Host-associated:Mammals:Digestive system	0.283
MGYA00375913/ERZ776995_FASTA.fasta	MGYA00375913	ERZ776995	root:Host-associated:Mammals:Digestive system	0.068
MGYA00375914/ERZ776996_FASTA.fasta	MGYA00375914	ERZ776996	root:Host-associated:Mammals:Digestive system	0.1096
MGYA00375915/ERZ776997_FASTA.fasta	MGYA00375915	ERZ776997	root:Host-associated:Mammals:Digestive system	0.1688
MGYA00375916/ERZ782882_FASTA.fasta	MGYA00375916	ERZ782882	root:Host-associated:Human:Skin	0.219
MGYA00375917/ERZ782883_FASTA.fasta	MGYA00375917	ERZ782883	root:Host-associated:Human:Skin	0.3352
MGYA00375918/ERZ782884_FASTA.fasta	MGYA00375918	ERZ782884	root:Host-associated:Human:Skin	0.1058
MGYA00375919/ERZ782885_FASTA.fasta	MGYA00375919	ERZ782885	root:Host-associated:Human:Skin	0.1988
MGYA00375920/ERZ782886_FASTA.fasta	MGYA00375920	ERZ782886	root:Host-associated:Human:Skin	0.3008
MGYA00375921/ERZ782887_FASTA.fasta	MGYA00375921	ERZ782887	root:Host-associated:Human:Skin	0.1898
MGYA00375922/ERZ782888_FASTA.fasta	MGYA00375922	ERZ782888	root:Host-associated:Human:Skin	0.3228
MGYA00375923/ERZ782889_FASTA.fasta	MGYA00375923	ERZ782889	root:Host-associated:Human:Skin	0.2066
MGYA00375924/ERZ782890_FASTA.fasta	MGYA00375924	ERZ782890	root:Host-associated:Human:Skin	0.4906
MGYA00375925/ERZ782891_FASTA.fasta	MGYA00375925	ERZ782891	root:Host-associated:Human:Skin	0.233
MGYA00375926/ERZ782892_FASTA.fasta	MGYA00375926	ERZ782892	root:Host-associated:Human:Skin	0.1892
MGYA00375927/ERZ782893_FASTA.fasta	MGYA00375927	ERZ782893	root:Host-associated:Human:Skin	0.2244
MGYA00375928/ERZ782894_FASTA.fasta	MGYA00375928	ERZ782894	root:Host-associated:Human:Skin	0.4296
MGYA00375929/ERZ782895_FASTA.fasta	MGYA00375929	ERZ782895	root:Host-associated:Human:Skin	0.231
MGYA00375930/ERZ782896_FASTA.fasta	MGYA00375930	ERZ782896	root:Host-associated:Human:Skin	0.2712
MGYA00375931/ERZ782897_FASTA.fasta	MGYA00375931	ERZ782897	root:Host-associated:Human:Skin	0.2304
MGYA00375932/ERZ782898_FASTA.fasta	MGYA00375932	ERZ782898	root:Host-associated:Human:Skin	0.216
MGYA00375933/ERZ782899_FASTA.fasta	MGYA00375933	ERZ782899	root:Host-associated:Human:Skin	0.2356
MGYA00375934/ERZ782900_FASTA.fasta	MGYA00375934	ERZ782900	root:Host-associated:Human:Skin	0.2354
MGYA00375935/ERZ782901_FASTA.fasta	MGYA00375935	ERZ782901	root:Host-associated:Human:Skin	0.2126
MGYA00375936/ERZ782902_FASTA.fasta	MGYA00375936	ERZ782902	root:Host-associated:Human:Skin	0.2022
MGYA00375937/ERZ782903_FASTA.fasta	MGYA00375937	ERZ782903	root:Host-associated:Human:Skin	0.2162
MGYA00375938/ERZ782904_FASTA.fasta	MGYA00375938	ERZ782904	root:Host-associated:Human:Skin	0.2352
MGYA00375939/ERZ782905_FASTA.fasta	MGYA00375939	ERZ782905	root:Host-associated:Human:Skin	0.2908
MGYA00375940/ERZ782906_FASTA.fasta	MGYA00375940	ERZ782906	root:Host-associated:Human:Skin	0.2212
MGYA00375941/ERZ782907_FASTA.fasta	MGYA00375941	ERZ782907	root:Host-associated:Human:Skin	0.1556
MGYA00375942/ERZ782908_FASTA.fasta	MGYA00375942	ERZ782908	root:Host-associated:Human:Skin	0.219
MGYA00375943/ERZ782909_FASTA.fasta	MGYA00375943	ERZ782909	root:Host-associated:Human:Skin	0.4324
MGYA00375944/ERZ782910_FASTA.fasta	MGYA00375944	ERZ782910	root:Host-associated:Human:Skin	0.1944
MGYA00375945/ERZ782911_FASTA.fasta	MGYA00375945	ERZ782911	root:Host-associated:Human:Skin	0.4384
MGYA00375946/ERZ782912_FASTA.fasta	MGYA00375946	ERZ782912	root:Host-associated:Human:Skin	0.2936
MGYA00375947/ERZ782913_FASTA.fasta	MGYA00375947	ERZ782913	root:Host-associated:Human:Skin	0.1986
MGYA00375948/ERZ782914_FASTA.fasta	MGYA00375948	ERZ782914	root:Host-associated:Human:Skin	0.4122
MGYA00375964/ERZ650948_FASTA.fasta	MGYA00375964	ERZ650948	root:Host-associated:Human:Digestive system:Large in	0.3986
MGYA00375965/ERZ650949_FASTA.fasta	MGYA00375965	ERZ650949	root:Host-associated:Human:Digestive system:Large in	0.4252
MGYA00375966/ERZ650951_FASTA.fasta	MGYA00375966	ERZ650951	root:Host-associated:Human:Digestive system:Large in	0.4466
MGYA00375967/ERZ650952_FASTA.fasta	MGYA00375967	ERZ650952	root:Host-associated:Human:Digestive system:Large in	0.2944
MGYA00375968/ERZ650954_FASTA.fasta	MGYA00375968	ERZ650954	root:Host-associated:Human:Digestive system:Large in	0.464
MGYA00375969/ERZ650956_FASTA.fasta	MGYA00375969	ERZ650956	root:Host-associated:Human:Digestive system:Large in	0.3458
MGYA00375970/ERZ650957_FASTA.fasta	MGYA00375970	ERZ650957	root:Host-associated:Human:Digestive system:Large in	0.4222
MGYA00375971/ERZ650959_FASTA.fasta	MGYA00375971	ERZ650959	root:Host-associated:Human:Digestive system:Large in	0.3276
MGYA00375972/ERZ650960_FASTA.fasta	MGYA00375972	ERZ650960	root:Host-associated:Human:Digestive system:Large in	0.532
MGYA00375973/ERZ650962_FASTA.fasta	MGYA00375973	ERZ650962	root:Host-associated:Human:Digestive system:Large in	0.0508
MGYA00375974/ERZ650964_FASTA.fasta	MGYA00375974	ERZ650964	root:Host-associated:Human:Digestive system:Large in	0.3986
MGYA00375975/ERZ650965_FASTA.fasta	MGYA00375975	ERZ650965	root:Host-associated:Human:Digestive system:Large in	0.4592
MGYA00375976/ERZ650967_FASTA.fasta	MGYA00375976	ERZ650967	root:Host-associated:Human:Digestive system:Large in	0.3792
MGYA00375977/ERZ650968_FASTA.fasta	MGYA00375977	ERZ650968	root:Host-associated:Human:Digestive system:Large in	0.4052
MGYA00375978/ERZ650970_FASTA.fasta	MGYA00375978	ERZ650970	root:Host-associated:Human:Digestive system:Large in	0.4908
MGYA00375979/ERZ650971_FASTA.fasta	MGYA00375979	ERZ650971	root:Host-associated:Human:Digestive system:Large in	0.403
MGYA00375980/ERZ650975_FASTA.fasta	MGYA00375980	ERZ650975	root:Host-associated:Human:Digestive system:Large in	0.2896
MGYA00375981/ERZ650976_FASTA.fasta	MGYA00375981	ERZ650976	root:Host-associated:Human:Digestive system:Large in	0.4146
MGYA00375982/ERZ650977_FASTA.fasta	MGYA00375982	ERZ650977	root:Host-associated:Human:Digestive system:Large in	0.3824
MGYA00375983/ERZ650979_FASTA.fasta	MGYA00375983	ERZ650979	root:Host-associated:Human:Digestive system:Large in	0.2974
MGYA00375984/ERZ650982_FASTA.fasta	MGYA00375984	ERZ650982	root:Host-associated:Human:Digestive system:Large in	0.3584
MGYA00375985/ERZ650983_FASTA.fasta	MGYA00375985	ERZ650983	root:Host-associated:Human:Digestive system:Large in	0.3902
MGYA00375986/ERZ650987_FASTA.fasta	MGYA00375986	ERZ650987	root:Host-associated:Human:Digestive system:Large in	0.4104
MGYA00375987/ERZ650988_FASTA.fasta	MGYA00375987	ERZ650988	root:Host-associated:Human:Digestive system:Large in	0.3968
MGYA00375988/ERZ650989_FASTA.fasta	MGYA00375988	ERZ650989	root:Host-associated:Human:Digestive system:Large in	0.301
MGYA00375989/ERZ650993_FASTA.fasta	MGYA00375989	ERZ650993	root:Host-associated:Human:Digestive system:Large in	0.2882
MGYA00375990/ERZ650995_FASTA.fasta	MGYA00375990	ERZ650995	root:Host-associated:Human:Digestive system:Large in	0.3248
MGYA00375991/ERZ650997_FASTA.fasta	MGYA00375991	ERZ650997	root:Host-associated:Human:Digestive system:Large in	0.4876
MGYA00375994/ERZ483179_FASTA.fasta	MGYA00375994	ERZ483179	root:Environmental:Aquatic:Marine:Hydrothermal vents	0.3134
MGYA00375995/ERZ483182_FASTA.fasta	MGYA00375995	ERZ483182	root:Environmental:Aquatic:Marine:Hydrothermal vents	0.2454
MGYA00375996/ERZ483184_FASTA.fasta	MGYA00375996	ERZ483184	root:Environmental:Aquatic:Marine:Hydrothermal vents	0.32
MGYA00375997/ERZ483185_FASTA.fasta	MGYA00375997	ERZ483185	root:Environmental:Aquatic:Marine:Hydrothermal vents	0.4602
MGYA00375998/ERZ483188_FASTA.fasta	MGYA00375998	ERZ483188	root:Environmental:Aquatic:Marine:Hydrothermal vents	0.2908
MGYA00375999/ERZ483191_FASTA.fasta	MGYA00375999	ERZ483191	root:Environmental:Aquatic:Marine:Hydrothermal vents	0.1804
MGYA00376000/ERZ483194_FASTA.fasta	MGYA00376000	ERZ483194	root:Environmental:Aquatic:Marine:Hydrothermal vents	0.291
MGYA00376001/ERZ483196_FASTA.fasta	MGYA00376001	ERZ483196	root:Environmental:Aquatic:Marine:Hydrothermal vents	0.2036
MGYA00376002/ERZ483197_FASTA.fasta	MGYA00376002	ERZ483197	root:Environmental:Aquatic:Marine:Hydrothermal vents	0.2816
MGYA00376003/ERZ483200_FASTA.fasta	MGYA00376003	ERZ483200	root:Environmental:Aquatic:Marine:Hydrothermal vents	0.5342
MGYA00376004/ERZ483201_FASTA.fasta	MGYA00376004	ERZ483201	root:Environmental:Aquatic:Marine:Hydrothermal vents	0.5142
MGYA00376005/ERZ483202_FASTA.fasta	MGYA00376005	ERZ483202	root:Environmental:Aquatic:Marine:Hydrothermal vents	0.3746
MGYA00376006/ERZ483203_FASTA.fasta	MGYA00376006	ERZ483203	root:Environmental:Aquatic:Marine:Hydrothermal vents	0.4228
MGYA00376007/ERZ483205_FASTA.fasta	MGYA00376007	ERZ483205	root:Environmental:Aquatic:Marine:Hydrothermal vents	0.5388
MGYA00376008/ERZ483206_FASTA.fasta	MGYA00376008	ERZ483206	root:Environmental:Aquatic:Marine:Hydrothermal vents	0.4556
MGYA00376009/ERZ483208_FASTA.fasta	MGYA00376009	ERZ483208	root:Environmental:Aquatic:Marine:Hydrothermal vents	0.4854
MGYA00376010/ERZ483211_FASTA.fasta	MGYA00376010	ERZ483211	root:Environmental:Aquatic:Marine:Hydrothermal vents	0.5126
MGYA00376011/ERZ483215_FASTA.fasta	MGYA00376011	ERZ483215	root:Environmental:Aquatic:Marine:Hydrothermal vents	0.1462
MGYA00376012/ERZ483218_FASTA.fasta	MGYA00376012	ERZ483218	root:Environmental:Aquatic:Marine:Hydrothermal vents	0.1462
MGYA00376013/ERZ483222_FASTA.fasta	MGYA00376013	ERZ483222	root:Environmental:Aquatic:Marine:Hydrothermal vents	0.35
MGYA00376014/ERZ483223_FASTA.fasta	MGYA00376014	ERZ483223	root:Environmental:Aquatic:Marine:Hydrothermal vents	0.4502
MGYA00376015/ERZ483224_FASTA.fasta	MGYA00376015	ERZ483224	root:Environmental:Aquatic:Marine:Hydrothermal vents	0.4304
MGYA00376016/ERZ483225_FASTA.fasta	MGYA00376016	ERZ483225	root:Environmental:Aquatic:Marine:Hydrothermal vents	0.4376
MGYA00376033/ERZ841514_FASTA.fasta	MGYA00376033	ERZ841514	root:Host-associated:Human:Digestive system:Large in	0.185
MGYA00376034/ERZ841621_FASTA.fasta	MGYA00376034	ERZ841621	root:Host-associated:Human:Digestive system:Large in	0.288
MGYA00376035/ERZ724502_FASTA.fasta	MGYA00376035	ERZ724502	root:Engineered:Wastewater	0.8798
MGYA00376036/ERZ724503_FASTA.fasta	MGYA00376036	ERZ724503	root:Engineered:Wastewater	0.8586
MGYA00376037/ERZ724504_FASTA.fasta	MGYA00376037	ERZ724504	root:Engineered:Wastewater	0.8912
MGYA00376038/ERZ724505_FASTA.fasta	MGYA00376038	ERZ724505	root:Engineered:Wastewater	0.7286
MGYA00376039/ERZ724506_FASTA.fasta	MGYA00376039	ERZ724506	root:Engineered:Wastewater	0.8376
MGYA00376040/ERZ724507_FASTA.fasta	MGYA00376040	ERZ724507	root:Engineered:Wastewater	0.8434
MGYA00376041/ERZ724508_FASTA.fasta	MGYA00376041	ERZ724508	root:Engineered:Wastewater	0.866
MGYA00376042/ERZ724509_FASTA.fasta	MGYA00376042	ERZ724509	root:Engineered:Wastewater	0.7808
MGYA00376043/ERZ724510_FASTA.fasta	MGYA00376043	ERZ724510	root:Engineered:Wastewater	0.8802
MGYA00376044/ERZ724511_FASTA.fasta	MGYA00376044	ERZ724511	root:Engineered:Wastewater	0.8152
MGYA00376045/ERZ724512_FASTA.fasta	MGYA00376045	ERZ724512	root:Engineered:Wastewater	0.8146
MGYA00376046/ERZ724513_FASTA.fasta	MGYA00376046	ERZ724513	root:Engineered:Wastewater	0.845
MGYA00376047/ERZ724514_FASTA.fasta	MGYA00376047	ERZ724514	root:Engineered:Wastewater	0.8408
MGYA00376048/ERZ724515_FASTA.fasta	MGYA00376048	ERZ724515	root:Engineered:Wastewater	0.8634
MGYA00376049/ERZ724516_FASTA.fasta	MGYA00376049	ERZ724516	root:Engineered:Wastewater	0.936
MGYA00376050/ERZ724517_FASTA.fasta	MGYA00376050	ERZ724517	root:Engineered:Wastewater	0.9138
MGYA00376051/ERZ724518_FASTA.fasta	MGYA00376051	ERZ724518	root:Engineered:Wastewater	0.8648
MGYA00376052/ERZ724519_FASTA.fasta	MGYA00376052	ERZ724519	root:Engineered:Wastewater	0.892
MGYA00376053/ERZ724520_FASTA.fasta	MGYA00376053	ERZ724520	root:Engineered:Wastewater	0.8972
MGYA00376054/ERZ724521_FASTA.fasta	MGYA00376054	ERZ724521	root:Engineered:Wastewater	0.8684
MGYA00376055/ERZ724522_FASTA.fasta	MGYA00376055	ERZ724522	root:Engineered:Wastewater	0.5174
MGYA00376056/ERZ724523_FASTA.fasta	MGYA00376056	ERZ724523	root:Engineered:Wastewater	0.889
MGYA00376057/ERZ724524_FASTA.fasta	MGYA00376057	ERZ724524	root:Engineered:Wastewater	0.9008
MGYA00376058/ERZ724525_FASTA.fasta	MGYA00376058	ERZ724525	root:Engineered:Wastewater	0.8718
MGYA00376059/ERZ724526_FASTA.fasta	MGYA00376059	ERZ724526	root:Engineered:Wastewater	0.8872
MGYA00376060/ERZ724527_FASTA.fasta	MGYA00376060	ERZ724527	root:Engineered:Wastewater	0.9102
MGYA00376061/ERZ724528_FASTA.fasta	MGYA00376061	ERZ724528	root:Engineered:Wastewater	0.7596
MGYA00376062/ERZ724529_FASTA.fasta	MGYA00376062	ERZ724529	root:Engineered:Wastewater	0.9044
MGYA00376063/ERZ724530_FASTA.fasta	MGYA00376063	ERZ724530	root:Engineered:Wastewater	0.8738
MGYA00376064/ERZ724531_FASTA.fasta	MGYA00376064	ERZ724531	root:Engineered:Wastewater	0.856
MGYA00376065/ERZ724532_FASTA.fasta	MGYA00376065	ERZ724532	root:Engineered:Wastewater	0.7204
MGYA00376066/ERZ724533_FASTA.fasta	MGYA00376066	ERZ724533	root:Engineered:Wastewater	0.8944
MGYA00376067/ERZ724534_FASTA.fasta	MGYA00376067	ERZ724534	root:Engineered:Wastewater	0.8608
MGYA00376068/ERZ724535_FASTA.fasta	MGYA00376068	ERZ724535	root:Engineered:Wastewater	0.818
MGYA00376069/ERZ724536_FASTA.fasta	MGYA00376069	ERZ724536	root:Engineered:Wastewater	0.8836
MGYA00376070/ERZ724537_FASTA.fasta	MGYA00376070	ERZ724537	root:Engineered:Wastewater	0.8834
MGYA00376071/ERZ724538_FASTA.fasta	MGYA00376071	ERZ724538	root:Engineered:Wastewater	0.6718
MGYA00376072/ERZ724539_FASTA.fasta	MGYA00376072	ERZ724539	root:Engineered:Wastewater	0.8664
MGYA00376073/ERZ724540_FASTA.fasta	MGYA00376073	ERZ724540	root:Engineered:Wastewater	0.8544
MGYA00376074/ERZ724541_FASTA.fasta	MGYA00376074	ERZ724541	root:Engineered:Wastewater	0.8624
MGYA00376075/ERZ724542_FASTA.fasta	MGYA00376075	ERZ724542	root:Engineered:Wastewater	0.8792
MGYA00376076/ERZ724543_FASTA.fasta	MGYA00376076	ERZ724543	root:Engineered:Wastewater	0.6772
MGYA00376077/ERZ724544_FASTA.fasta	MGYA00376077	ERZ724544	root:Engineered:Wastewater	0.7816
MGYA00376078/ERZ724545_FASTA.fasta	MGYA00376078	ERZ724545	root:Engineered:Wastewater	0.8774
MGYA00376079/ERZ724546_FASTA.fasta	MGYA00376079	ERZ724546	root:Engineered:Wastewater	0.874
MGYA00376080/ERZ724547_FASTA.fasta	MGYA00376080	ERZ724547	root:Engineered:Wastewater	0.7754
MGYA00376081/ERZ724548_FASTA.fasta	MGYA00376081	ERZ724548	root:Engineered:Wastewater	0.8578
MGYA00376082/ERZ724549_FASTA.fasta	MGYA00376082	ERZ724549	root:Engineered:Wastewater	0.7026
MGYA00376083/ERZ724550_FASTA.fasta	MGYA00376083	ERZ724550	root:Engineered:Wastewater	0.8942
MGYA00376084/ERZ724551_FASTA.fasta	MGYA00376084	ERZ724551	root:Engineered:Wastewater	0.8888
MGYA00376085/ERZ724552_FASTA.fasta	MGYA00376085	ERZ724552	root:Engineered:Wastewater	0.6238
MGYA00376086/ERZ724553_FASTA.fasta	MGYA00376086	ERZ724553	root:Engineered:Wastewater	0.8794
MGYA00376087/ERZ724554_FASTA.fasta	MGYA00376087	ERZ724554	root:Engineered:Wastewater	0.8748
MGYA00376088/ERZ724555_FASTA.fasta	MGYA00376088	ERZ724555	root:Engineered:Wastewater	0.8474
MGYA00376089/ERZ724556_FASTA.fasta	MGYA00376089	ERZ724556	root:Engineered:Wastewater	0.898
MGYA00376090/ERZ724557_FASTA.fasta	MGYA00376090	ERZ724557	root:Engineered:Wastewater	0.8488
MGYA00376091/ERZ724558_FASTA.fasta	MGYA00376091	ERZ724558	root:Engineered:Wastewater	0.8634
MGYA00376092/ERZ724559_FASTA.fasta	MGYA00376092	ERZ724559	root:Engineered:Wastewater	0.9176
MGYA00376154/ERZ493297_FASTA.fasta	MGYA00376154	ERZ493297	root:Host-associated:Human:Digestive system	0.0536
MGYA00376155/ERZ493298_FASTA.fasta	MGYA00376155	ERZ493298	root:Host-associated:Human:Digestive system	0.0778
MGYA00376156/ERZ493299_FASTA.fasta	MGYA00376156	ERZ493299	root:Host-associated:Human:Digestive system	0.0682
MGYA00376157/ERZ650221_FASTA.fasta	MGYA00376157	ERZ650221	root:Engineered:Bioremediation:Terephthalate:Wastew	0.8464
MGYA00376158/ERZ650222_FASTA.fasta	MGYA00376158	ERZ650222	root:Engineered:Bioremediation:Terephthalate:Wastew	0.8402
MGYA00376159/ERZ650223_FASTA.fasta	MGYA00376159	ERZ650223	root:Engineered:Bioremediation:Terephthalate:Wastew	0.8586
MGYA00376160/ERZ650224_FASTA.fasta	MGYA00376160	ERZ650224	root:Engineered:Bioremediation:Terephthalate:Wastew	0.7594
MGYA00376161/ERZ650225_FASTA.fasta	MGYA00376161	ERZ650225	root:Engineered:Bioremediation:Terephthalate:Wastew	0.7632
MGYA00376162/ERZ650226_FASTA.fasta	MGYA00376162	ERZ650226	root:Engineered:Bioremediation:Terephthalate:Wastew	0.769
MGYA00376163/ERZ650227_FASTA.fasta	MGYA00376163	ERZ650227	root:Engineered:Bioremediation:Terephthalate:Wastew	0.8078
MGYA00376164/ERZ650228_FASTA.fasta	MGYA00376164	ERZ650228	root:Engineered:Bioremediation:Terephthalate:Wastew	0.7994
MGYA00376165/ERZ650229_FASTA.fasta	MGYA00376165	ERZ650229	root:Engineered:Bioremediation:Terephthalate:Wastew	0.8246
MGYA00376166/ERZ650230_FASTA.fasta	MGYA00376166	ERZ650230	root:Engineered:Bioremediation:Terephthalate:Wastew	0.8154
MGYA00376167/ERZ650231_FASTA.fasta	MGYA00376167	ERZ650231	root:Engineered:Bioremediation:Terephthalate:Wastew	0.8428
MGYA00376168/ERZ650232_FASTA.fasta	MGYA00376168	ERZ650232	root:Engineered:Bioremediation:Terephthalate:Wastew	0.7864
MGYA00376169/ERZ650233_FASTA.fasta	MGYA00376169	ERZ650233	root:Engineered:Bioremediation:Terephthalate:Wastew	0.8668
MGYA00376170/ERZ650234_FASTA.fasta	MGYA00376170	ERZ650234	root:Engineered:Bioremediation:Terephthalate:Wastew	0.8082
MGYA00376171/ERZ650235_FASTA.fasta	MGYA00376171	ERZ650235	root:Engineered:Bioremediation:Terephthalate:Wastew	0.6686
MGYA00376172/ERZ650236_FASTA.fasta	MGYA00376172	ERZ650236	root:Engineered:Bioremediation:Terephthalate:Wastew	0.8198
MGYA00376173/ERZ650237_FASTA.fasta	MGYA00376173	ERZ650237	root:Engineered:Bioremediation:Terephthalate:Wastew	0.6326
MGYA00376174/ERZ650238_FASTA.fasta	MGYA00376174	ERZ650238	root:Engineered:Bioremediation:Terephthalate:Wastew	0.7124
MGYA00376175/ERZ650239_FASTA.fasta	MGYA00376175	ERZ650239	root:Engineered:Bioremediation:Terephthalate:Wastew	0.7888
MGYA00376176/ERZ650240_FASTA.fasta	MGYA00376176	ERZ650240	root:Engineered:Bioremediation:Terephthalate:Wastew	0.647
MGYA00376177/ERZ650241_FASTA.fasta	MGYA00376177	ERZ650241	root:Engineered:Bioremediation:Terephthalate:Wastew	0.785
MGYA00376178/ERZ650242_FASTA.fasta	MGYA00376178	ERZ650242	root:Engineered:Bioremediation:Terephthalate:Wastew	0.8702
MGYA00376179/ERZ650243_FASTA.fasta	MGYA00376179	ERZ650243	root:Engineered:Bioremediation:Terephthalate:Wastew	0.8712
MGYA00376180/ERZ650244_FASTA.fasta	MGYA00376180	ERZ650244	root:Engineered:Bioremediation:Terephthalate:Wastew	0.5824
MGYA00376181/ERZ650245_FASTA.fasta	MGYA00376181	ERZ650245	root:Engineered:Bioremediation:Terephthalate:Wastew	0.7802
MGYA00376182/ERZ650246_FASTA.fasta	MGYA00376182	ERZ650246	root:Engineered:Bioremediation:Terephthalate:Wastew	0.558
MGYA00376183/ERZ650247_FASTA.fasta	MGYA00376183	ERZ650247	root:Engineered:Bioremediation:Terephthalate:Wastew	0.8198
MGYA00376184/ERZ650248_FASTA.fasta	MGYA00376184	ERZ650248	root:Engineered:Bioremediation:Terephthalate:Wastew	0.8192
MGYA00376185/ERZ650249_FASTA.fasta	MGYA00376185	ERZ650249	root:Engineered:Bioremediation:Terephthalate:Wastew	0.7638
MGYA00376186/ERZ650250_FASTA.fasta	MGYA00376186	ERZ650250	root:Engineered:Bioremediation:Terephthalate:Wastew	0.807
MGYA00376187/ERZ650251_FASTA.fasta	MGYA00376187	ERZ650251	root:Engineered:Bioremediation:Terephthalate:Wastew	0.6692
MGYA00376188/ERZ650252_FASTA.fasta	MGYA00376188	ERZ650252	root:Engineered:Bioremediation:Terephthalate:Wastew	0.7468
MGYA00376189/ERZ650253_FASTA.fasta	MGYA00376189	ERZ650253	root:Engineered:Bioremediation:Terephthalate:Wastew	0.8278
MGYA00376190/ERZ650254_FASTA.fasta	MGYA00376190	ERZ650254	root:Engineered:Bioremediation:Terephthalate:Wastew	0.8208
MGYA00376191/ERZ650255_FASTA.fasta	MGYA00376191	ERZ650255	root:Engineered:Bioremediation:Terephthalate:Wastew	0.8448
MGYA00376192/ERZ650256_FASTA.fasta	MGYA00376192	ERZ650256	root:Engineered:Bioremediation:Terephthalate:Wastew	0.84
MGYA00376193/ERZ650257_FASTA.fasta	MGYA00376193	ERZ650257	root:Engineered:Bioremediation:Terephthalate:Wastew	0.8532
MGYA00376194/ERZ650258_FASTA.fasta	MGYA00376194	ERZ650258	root:Engineered:Bioremediation:Terephthalate:Wastew	0.8868
MGYA00376195/ERZ650259_FASTA.fasta	MGYA00376195	ERZ650259	root:Engineered:Bioremediation:Terephthalate:Wastew	0.8576
MGYA00376196/ERZ650260_FASTA.fasta	MGYA00376196	ERZ650260	root:Engineered:Bioremediation:Terephthalate:Wastew	0.8354
MGYA00376197/ERZ650261_FASTA.fasta	MGYA00376197	ERZ650261	root:Engineered:Bioremediation:Terephthalate:Wastew	0.7596
MGYA00376198/ERZ650262_FASTA.fasta	MGYA00376198	ERZ650262	root:Engineered:Bioremediation:Terephthalate:Wastew	0.7892
MGYA00376199/ERZ650263_FASTA.fasta	MGYA00376199	ERZ650263	root:Engineered:Bioremediation:Terephthalate:Wastew	0.807
MGYA00376200/ERZ650264_FASTA.fasta	MGYA00376200	ERZ650264	root:Engineered:Bioremediation:Terephthalate:Wastew	0.8188
MGYA00376201/ERZ650265_FASTA.fasta	MGYA00376201	ERZ650265	root:Engineered:Bioremediation:Terephthalate:Wastew	0.8376
MGYA00376202/ERZ650266_FASTA.fasta	MGYA00376202	ERZ650266	root:Engineered:Bioremediation:Terephthalate:Wastew	0.6638
MGYA00376203/ERZ650267_FASTA.fasta	MGYA00376203	ERZ650267	root:Engineered:Bioremediation:Terephthalate:Wastew	0.6936
MGYA00376204/ERZ650268_FASTA.fasta	MGYA00376204	ERZ650268	root:Engineered:Bioremediation:Terephthalate:Wastew	0.819
MGYA00376205/ERZ650269_FASTA.fasta	MGYA00376205	ERZ650269	root:Engineered:Bioremediation:Terephthalate:Wastew	0.7236
MGYA00376206/ERZ650270_FASTA.fasta	MGYA00376206	ERZ650270	root:Engineered:Bioremediation:Terephthalate:Wastew	0.7524
MGYA00376207/ERZ650271_FASTA.fasta	MGYA00376207	ERZ650271	root:Engineered:Bioremediation:Terephthalate:Wastew	0.7858
MGYA00376208/ERZ650272_FASTA.fasta	MGYA00376208	ERZ650272	root:Engineered:Bioremediation:Terephthalate:Wastew	0.8286
MGYA00376209/ERZ650273_FASTA.fasta	MGYA00376209	ERZ650273	root:Engineered:Bioremediation:Terephthalate:Wastew	0.7846
MGYA00376210/ERZ650274_FASTA.fasta	MGYA00376210	ERZ650274	root:Engineered:Bioremediation:Terephthalate:Wastew	0.8702
MGYA00376211/ERZ650275_FASTA.fasta	MGYA00376211	ERZ650275	root:Engineered:Bioremediation:Terephthalate:Wastew	0.6412
MGYA00376212/ERZ650276_FASTA.fasta	MGYA00376212	ERZ650276	root:Engineered:Bioremediation:Terephthalate:Wastew	0.6722
MGYA00376213/ERZ650277_FASTA.fasta	MGYA00376213	ERZ650277	root:Engineered:Bioremediation:Terephthalate:Wastew	0.8434
MGYA00376214/ERZ650278_FASTA.fasta	MGYA00376214	ERZ650278	root:Engineered:Bioremediation:Terephthalate:Wastew	0.779
MGYA00376215/ERZ650279_FASTA.fasta	MGYA00376215	ERZ650279	root:Engineered:Bioremediation:Terephthalate:Wastew	0.834
MGYA00376216/ERZ650280_FASTA.fasta	MGYA00376216	ERZ650280	root:Engineered:Bioremediation:Terephthalate:Wastew	0.748
MGYA00376217/ERZ650281_FASTA.fasta	MGYA00376217	ERZ650281	root:Engineered:Bioremediation:Terephthalate:Wastew	0.7732
MGYA00376218/ERZ650282_FASTA.fasta	MGYA00376218	ERZ650282	root:Engineered:Bioremediation:Terephthalate:Wastew	0.8656
MGYA00376219/ERZ650283_FASTA.fasta	MGYA00376219	ERZ650283	root:Engineered:Bioremediation:Terephthalate:Wastew	0.833
MGYA00376220/ERZ650284_FASTA.fasta	MGYA00376220	ERZ650284	root:Engineered:Bioremediation:Terephthalate:Wastew	0.7006
MGYA00376221/ERZ650285_FASTA.fasta	MGYA00376221	ERZ650285	root:Engineered:Bioremediation:Terephthalate:Wastew	0.6964
MGYA00376222/ERZ650286_FASTA.fasta	MGYA00376222	ERZ650286	root:Engineered:Bioremediation:Terephthalate:Wastew	0.8126
MGYA00376223/ERZ650287_FASTA.fasta	MGYA00376223	ERZ650287	root:Engineered:Bioremediation:Terephthalate:Wastew	0.6636
MGYA00376224/ERZ650288_FASTA.fasta	MGYA00376224	ERZ650288	root:Engineered:Bioremediation:Terephthalate:Wastew	0.8864
MGYA00376225/ERZ650289_FASTA.fasta	MGYA00376225	ERZ650289	root:Engineered:Bioremediation:Terephthalate:Wastew	0.8426
MGYA00376226/ERZ650290_FASTA.fasta	MGYA00376226	ERZ650290	root:Engineered:Bioremediation:Terephthalate:Wastew	0.8308
MGYA00376227/ERZ650291_FASTA.fasta	MGYA00376227	ERZ650291	root:Engineered:Bioremediation:Terephthalate:Wastew	0.6986
MGYA00376228/ERZ650292_FASTA.fasta	MGYA00376228	ERZ650292	root:Engineered:Bioremediation:Terephthalate:Wastew	0.7888
MGYA00376229/ERZ650293_FASTA.fasta	MGYA00376229	ERZ650293	root:Engineered:Bioremediation:Terephthalate:Wastew	0.7718
MGYA00376230/ERZ650294_FASTA.fasta	MGYA00376230	ERZ650294	root:Engineered:Bioremediation:Terephthalate:Wastew	0.8528
MGYA00376231/ERZ650295_FASTA.fasta	MGYA00376231	ERZ650295	root:Engineered:Bioremediation:Terephthalate:Wastew	0.7954
MGYA00376232/ERZ650296_FASTA.fasta	MGYA00376232	ERZ650296	root:Engineered:Bioremediation:Terephthalate:Wastew	0.7592
MGYA00376233/ERZ650297_FASTA.fasta	MGYA00376233	ERZ650297	root:Engineered:Bioremediation:Terephthalate:Wastew	0.87
MGYA00376234/ERZ650298_FASTA.fasta	MGYA00376234	ERZ650298	root:Engineered:Bioremediation:Terephthalate:Wastew	0.8302
MGYA00376235/ERZ650299_FASTA.fasta	MGYA00376235	ERZ650299	root:Engineered:Bioremediation:Terephthalate:Wastew	0.7684
MGYA00376236/ERZ650300_FASTA.fasta	MGYA00376236	ERZ650300	root:Engineered:Bioremediation:Terephthalate:Wastew	0.8626
MGYA00376237/ERZ650301_FASTA.fasta	MGYA00376237	ERZ650301	root:Engineered:Bioremediation:Terephthalate:Wastew	0.67
MGYA00376238/ERZ650302_FASTA.fasta	MGYA00376238	ERZ650302	root:Engineered:Bioremediation:Terephthalate:Wastew	0.8542
MGYA00376239/ERZ650303_FASTA.fasta	MGYA00376239	ERZ650303	root:Engineered:Bioremediation:Terephthalate:Wastew	0.7752
MGYA00376240/ERZ650304_FASTA.fasta	MGYA00376240	ERZ650304	root:Engineered:Bioremediation:Terephthalate:Wastew	0.724
MGYA00376241/ERZ650305_FASTA.fasta	MGYA00376241	ERZ650305	root:Engineered:Bioremediation:Terephthalate:Wastew	0.8608
MGYA00376242/ERZ650306_FASTA.fasta	MGYA00376242	ERZ650306	root:Engineered:Bioremediation:Terephthalate:Wastew	0.8118
MGYA00376243/ERZ650307_FASTA.fasta	MGYA00376243	ERZ650307	root:Engineered:Bioremediation:Terephthalate:Wastew	0.8538
MGYA00376244/ERZ650308_FASTA.fasta	MGYA00376244	ERZ650308	root:Engineered:Bioremediation:Terephthalate:Wastew	0.7436
MGYA00376245/ERZ650309_FASTA.fasta	MGYA00376245	ERZ650309	root:Engineered:Bioremediation:Terephthalate:Wastew	0.8664
MGYA00376246/ERZ650310_FASTA.fasta	MGYA00376246	ERZ650310	root:Engineered:Bioremediation:Terephthalate:Wastew	0.7064
MGYA00376247/ERZ650311_FASTA.fasta	MGYA00376247	ERZ650311	root:Engineered:Bioremediation:Terephthalate:Wastew	0.6346
MGYA00376248/ERZ650312_FASTA.fasta	MGYA00376248	ERZ650312	root:Engineered:Bioremediation:Terephthalate:Wastew	0.8328
MGYA00376249/ERZ812616_FASTA.fasta	MGYA00376249	ERZ812616	root:Host-associated:Human:Digestive system:Large in	0.508
MGYA00377259/ERZ485761_FASTA.fasta	MGYA00377259	ERZ485761	root:Host-associated:Human:Digestive system:Large in	0.5606
MGYA00377260/ERZ485764_FASTA.fasta	MGYA00377260	ERZ485764	root:Host-associated:Human:Digestive system:Large in	0.4584
MGYA00377261/ERZ485768_FASTA.fasta	MGYA00377261	ERZ485768	root:Host-associated:Human:Digestive system:Large in	0.4826
MGYA00377262/ERZ485772_FASTA.fasta	MGYA00377262	ERZ485772	root:Host-associated:Human:Digestive system:Large in	0.4328
MGYA00377263/ERZ485776_FASTA.fasta	MGYA00377263	ERZ485776	root:Host-associated:Human:Digestive system:Large in	0.5246
MGYA00377264/ERZ485777_FASTA.fasta	MGYA00377264	ERZ485777	root:Host-associated:Human:Digestive system:Large in	0.5546
MGYA00377265/ERZ485778_FASTA.fasta	MGYA00377265	ERZ485778	root:Host-associated:Human:Digestive system:Large in	0.4232
MGYA00377266/ERZ485779_FASTA.fasta	MGYA00377266	ERZ485779	root:Host-associated:Human:Digestive system:Large in	0.5748
MGYA00377267/ERZ485781_FASTA.fasta	MGYA00377267	ERZ485781	root:Host-associated:Human:Digestive system:Large in	0.424
MGYA00377268/ERZ485784_FASTA.fasta	MGYA00377268	ERZ485784	root:Host-associated:Human:Digestive system:Large in	0.5864
MGYA00377269/ERZ485785_FASTA.fasta	MGYA00377269	ERZ485785	root:Host-associated:Human:Digestive system:Large in	0.3736
MGYA00377270/ERZ485786_FASTA.fasta	MGYA00377270	ERZ485786	root:Host-associated:Human:Digestive system:Large in	0.4048
MGYA00377271/ERZ485791_FASTA.fasta	MGYA00377271	ERZ485791	root:Host-associated:Human:Digestive system:Large in	0.4522
MGYA00377272/ERZ485793_FASTA.fasta	MGYA00377272	ERZ485793	root:Host-associated:Human:Digestive system:Large in	0.5174
MGYA00377273/ERZ485794_FASTA.fasta	MGYA00377273	ERZ485794	root:Host-associated:Human:Digestive system:Large in	0.4702
MGYA00377274/ERZ485797_FASTA.fasta	MGYA00377274	ERZ485797	root:Host-associated:Human:Digestive system:Large in	0.4078
MGYA00377275/ERZ485799_FASTA.fasta	MGYA00377275	ERZ485799	root:Host-associated:Human:Digestive system:Large in	0.4992
MGYA00377276/ERZ485801_FASTA.fasta	MGYA00377276	ERZ485801	root:Host-associated:Human:Digestive system:Large in	0.4946
MGYA00377277/ERZ485804_FASTA.fasta	MGYA00377277	ERZ485804	root:Host-associated:Human:Digestive system:Large in	0.4798
MGYA00377278/ERZ485805_FASTA.fasta	MGYA00377278	ERZ485805	root:Host-associated:Human:Digestive system:Large in	0.46
MGYA00377279/ERZ485808_FASTA.fasta	MGYA00377279	ERZ485808	root:Host-associated:Human:Digestive system:Large in	0.8502
MGYA00377280/ERZ485811_FASTA.fasta	MGYA00377280	ERZ485811	root:Host-associated:Human:Digestive system:Large in	0.3882
MGYA00377281/ERZ485813_FASTA.fasta	MGYA00377281	ERZ485813	root:Host-associated:Human:Digestive system:Large in	0.4384
MGYA00377282/ERZ485815_FASTA.fasta	MGYA00377282	ERZ485815	root:Host-associated:Human:Digestive system:Large in	0.414
MGYA00377283/ERZ485817_FASTA.fasta	MGYA00377283	ERZ485817	root:Host-associated:Human:Digestive system:Large in	0.4594
MGYA00377284/ERZ485819_FASTA.fasta	MGYA00377284	ERZ485819	root:Host-associated:Human:Digestive system:Large in	0.4128
MGYA00377285/ERZ485820_FASTA.fasta	MGYA00377285	ERZ485820	root:Host-associated:Human:Digestive system:Large in	0.4482
MGYA00377286/ERZ485821_FASTA.fasta	MGYA00377286	ERZ485821	root:Host-associated:Human:Digestive system:Large in	0.499
MGYA00377287/ERZ485824_FASTA.fasta	MGYA00377287	ERZ485824	root:Host-associated:Human:Digestive system:Large in	0.5332
MGYA00377288/ERZ485829_FASTA.fasta	MGYA00377288	ERZ485829	root:Host-associated:Human:Digestive system:Large in	0.4144
MGYA00377289/ERZ485833_FASTA.fasta	MGYA00377289	ERZ485833	root:Host-associated:Human:Digestive system:Large in	0.4376
MGYA00377290/ERZ485834_FASTA.fasta	MGYA00377290	ERZ485834	root:Host-associated:Human:Digestive system:Large in	0.3732
MGYA00377291/ERZ485835_FASTA.fasta	MGYA00377291	ERZ485835	root:Host-associated:Human:Digestive system:Large in	0.4058
MGYA00377292/ERZ485836_FASTA.fasta	MGYA00377292	ERZ485836	root:Host-associated:Human:Digestive system:Large in	0.5178
MGYA00377293/ERZ485837_FASTA.fasta	MGYA00377293	ERZ485837	root:Host-associated:Human:Digestive system:Large in	0.5896
MGYA00377294/ERZ485841_FASTA.fasta	MGYA00377294	ERZ485841	root:Host-associated:Human:Digestive system:Large in	0.385
MGYA00377295/ERZ485846_FASTA.fasta	MGYA00377295	ERZ485846	root:Host-associated:Human:Digestive system:Large in	0.4382
MGYA00377296/ERZ485847_FASTA.fasta	MGYA00377296	ERZ485847	root:Host-associated:Human:Digestive system:Large in	0.5048
MGYA00377297/ERZ485849_FASTA.fasta	MGYA00377297	ERZ485849	root:Host-associated:Human:Digestive system:Large in	0.572
MGYA00377298/ERZ485851_FASTA.fasta	MGYA00377298	ERZ485851	root:Host-associated:Human:Digestive system:Large in	0.3666
MGYA00377299/ERZ485852_FASTA.fasta	MGYA00377299	ERZ485852	root:Host-associated:Human:Digestive system:Large in	0.4306
MGYA00377300/ERZ485854_FASTA.fasta	MGYA00377300	ERZ485854	root:Host-associated:Human:Digestive system:Large in	0.4406
MGYA00377301/ERZ485858_FASTA.fasta	MGYA00377301	ERZ485858	root:Host-associated:Human:Digestive system:Large in	0.4566
MGYA00377302/ERZ485860_FASTA.fasta	MGYA00377302	ERZ485860	root:Host-associated:Human:Digestive system:Large in	0.512
