# Supplementary material for: Structural color in the bacterial domain: The ecogenomics of a 2-dimensional optical phenotype
Source: Proc Natl Acad Sci U S A. 2024 Jul 11;121(29):e2309757121. doi: 10.1073/pnas.2309757121 (PMC11260094; doi:10.1073/pnas.2309757121)
Supplement: Supplementary file 11 — Dataset S05 (PDF) [file pnas.2309757121.sd05.pdf]

Assembly	Analysis accession	Analysis assembly accessio	Sample biome lineage	SC score	Depth	Contigs >= 500 bp	Predicted proteins
MGYA00237873/ERZ529320_FASTA.fasta	MGYA00237873	ERZ529320	root:Environmental:Aquatic:Marine	0.0294	No depth information	1072	1041
MGYA00237874/ERZ529319_FASTA.fasta	MGYA00237874	ERZ529319	root:Environmental:Aquatic:Marine	0.0294	No depth information	5513	5134
MGYA00238080/ERZ650175_FASTA.fasta	MGYA00238080	ERZ650175	root:Environmental:Aquatic:Marine	0.0294	No depth information	1396	1216
MGYA00153470/OLKC01_FASTA.fasta	MGYA00153470	ERZ484799	root:Environmental:Aquatic:Marine	0.031	Surface	3029	6238
MGYA00153469/OKRO01_FASTA.fasta	MGYA00153469	ERZ484800	root:Environmental:Aquatic:Marine	0.0316	5m	523	1410
MGYA00238028/ERZ584644_FASTA.fasta	MGYA00238028	ERZ584644	root:Environmental:Aquatic:Marine	0.0328	No depth information	801	1229
MGYA00250111/ERZ650178_FASTA.fasta	MGYA00250111	ERZ650178	root:Environmental:Aquatic:Marine	0.0342	No depth information	620	943
MGYA00250114/ERZ650177_FASTA.fasta	MGYA00250114	ERZ650177	root:Environmental:Aquatic:Marine	0.0344	No depth information	650	972
MGYA00250119/ERZ724591_FASTA.fasta	MGYA00250119	ERZ724591	root:Environmental:Aquatic:Marine	0.0346	No depth information	633	882
MGYA00250116/ERZ650181_FASTA.fasta	MGYA00250116	ERZ650181	root:Environmental:Aquatic:Marine	0.0348	No depth information	702	1044
MGYA00250113/ERZ650182_FASTA.fasta	MGYA00250113	ERZ650182	root:Environmental:Aquatic:Marine	0.0362	No depth information	547	778
MGYA00248542/ERZ534182_FASTA.fasta	MGYA00248542	ERZ534182	root:Environmental:Aquatic:Marine	0.0408	No depth information	697	1065
MGYA00248609/ERZ508651_FASTA.fasta	MGYA00248609	ERZ508651	root:Environmental:Aquatic:Marine	0.0414	No depth information	996	1522
MGYA00238059/ERZ584645_FASTA.fasta	MGYA00238059	ERZ584645	root:Environmental:Aquatic:Marine	0.043	No depth information	1488	2221
MGYA00248610/ERZ508652_FASTA.fasta	MGYA00248610	ERZ508652	root:Environmental:Aquatic:Marine	0.0454	No depth information	1077	1591
MGYA00248485/ERZ534208_FASTA.fasta	MGYA00248485	ERZ534208	root:Environmental:Aquatic:Marine	0.0464	No depth information	724	1051
MGYA00250118/ERZ650176_FASTA.fasta	MGYA00250118	ERZ650176	root:Environmental:Aquatic:Marine	0.0464	No depth information	748	1146
MGYA00248598/ERZ508640_FASTA.fasta	MGYA00248598	ERZ508640	root:Environmental:Aquatic:Marine	0.0474	No depth information	792	1153
MGYA00248583/ERZ508610_FASTA.fasta	MGYA00248583	ERZ508610	root:Environmental:Aquatic:Marine	0.0476	No depth information	513	812
MGYA00238037/ERZ584620_FASTA.fasta	MGYA00238037	ERZ584620	root:Environmental:Aquatic:Marine	0.049	No depth information	715	1056
MGYA00248587/ERZ508618_FASTA.fasta	MGYA00248587	ERZ508618	root:Environmental:Aquatic:Marine	0.0492	No depth information	1417	2153
MGYA00238032/ERZ584652_FASTA.fasta	MGYA00238032	ERZ584652	root:Environmental:Aquatic:Marine	0.0534	No depth information	531	817
MGYA00238044/ERZ584634_FASTA.fasta	MGYA00238044	ERZ584634	root:Environmental:Aquatic:Marine	0.0548	No depth information	512	812
MGYA00238054/ERZ584633_FASTA.fasta	MGYA00238054	ERZ584633	root:Environmental:Aquatic:Marine	0.0548	No depth information	680	985
MGYA00248540/ERZ534180_FASTA.fasta	MGYA00248540	ERZ534180	root:Environmental:Aquatic:Marine	0.0564	No depth information	791	1164
MGYA00238053/ERZ584631_FASTA.fasta	MGYA00238053	ERZ584631	root:Environmental:Aquatic:Marine	0.0566	No depth information	621	914
MGYA00238057/ERZ584641_FASTA.fasta	MGYA00238057	ERZ584641	root:Environmental:Aquatic:Marine	0.065	No depth information	594	924
MGYA00238062/ERZ584651_FASTA.fasta	MGYA00238062	ERZ584651	root:Environmental:Aquatic:Marine	0.0662	No depth information	1078	1653
MGYA00250115/ERZ650179_FASTA.fasta	MGYA00250115	ERZ650179	root:Environmental:Aquatic:Marine	0.067	No depth information	1313	1723
MGYA00248601/ERZ508643_FASTA.fasta	MGYA00248601	ERZ508643	root:Environmental:Aquatic:Marine	0.0678	No depth information	1596	2431
MGYA00250112/ERZ650180_FASTA.fasta	MGYA00250112	ERZ650180	root:Environmental:Aquatic:Marine	0.0678	No depth information	1163	1519
MGYA00238026/ERZ584640_FASTA.fasta	MGYA00238026	ERZ584640	root:Environmental:Aquatic:Marine	0.0686	No depth information	808	1185
MGYA00238049/ERZ584623_FASTA.fasta	MGYA00238049	ERZ584623	root:Environmental:Aquatic:Marine	0.0694	No depth information	1026	1574
MGYA00248515/ERZ534165_FASTA.fasta	MGYA00248515	ERZ534165	root:Environmental:Aquatic:Marine	0.0712	No depth information	1160	1794
MGYA00250117/ERZ650183_FASTA.fasta	MGYA00250117	ERZ650183	root:Environmental:Aquatic:Marine	0.0712	No depth information	1319	1738
MGYA00248512/ERZ534159_FASTA.fasta	MGYA00248512	ERZ534159	root:Environmental:Aquatic:Marine	0.0716	No depth information	1414	2173
MGYA00248451/ERZ584562_FASTA.fasta	MGYA00248451	ERZ584562	root:Environmental:Aquatic:Marine	0.073	No depth information	1343	2066
MGYA00367508/ERZ828930_FASTA.fasta	MGYA00367508	ERZ828930	root:Environmental:Aquatic:Marine	0.0736	5m	5699	15291
MGYA00248457/ERZ584574_FASTA.fasta	MGYA00248457	ERZ584574	root:Environmental:Aquatic:Marine	0.0774	No depth information	832	1271
MGYA00248568/ERZ508609_FASTA.fasta	MGYA00248568	ERZ508609	root:Environmental:Aquatic:Marine	0.0786	No depth information	1461	2249
MGYA00238060/ERZ584647_FASTA.fasta	MGYA00238060	ERZ584647	root:Environmental:Aquatic:Marine	0.0792	No depth information	581	941
MGYA00248538/ERZ534178_FASTA.fasta	MGYA00248538	ERZ534178	root:Environmental:Aquatic:Marine	0.081	No depth information	500	744
MGYA00238048/ERZ584621_FASTA.fasta	MGYA00238048	ERZ584621	root:Environmental:Aquatic:Marine	0.0814	No depth information	1609	2495
MGYA00248492/ERZ534215_FASTA.fasta	MGYA00248492	ERZ534215	root:Environmental:Aquatic:Marine	0.0822	No depth information	1039	1525
MGYA00248522/ERZ534150_FASTA.fasta	MGYA00248522	ERZ534150	root:Environmental:Aquatic:Marine	0.0828	No depth information	1119	1654
MGYA00248524/ERZ534154_FASTA.fasta	MGYA00248524	ERZ534154	root:Environmental:Aquatic:Marine	0.083	No depth information	706	1006
MGYA00248529/ERZ534164_FASTA.fasta	MGYA00248529	ERZ534164	root:Environmental:Aquatic:Marine	0.0848	No depth information	1130	1797
MGYA00248523/ERZ534152_FASTA.fasta	MGYA00248523	ERZ534152	root:Environmental:Aquatic:Marine	0.0866	No depth information	2216	3482
MGYA00248584/ERZ508612_FASTA.fasta	MGYA00248584	ERZ508612	root:Environmental:Aquatic:Marine	0.0866	No depth information	1165	1726
MGYA00248467/ERZ534190_FASTA.fasta	MGYA00248467	ERZ534190	root:Environmental:Aquatic:Marine	0.088	No depth information	1444	2179
MGYA00238000/ERZ584587_FASTA.fasta	MGYA00238000	ERZ584587	root:Environmental:Aquatic:Marine	0.0888	No depth information	2838	4971
MGYA00237982/ERZ584540_FASTA.fasta	MGYA00237982	ERZ584540	root:Environmental:Aquatic:Marine	0.0932	No depth information	2716	4785
MGYA00248519/ERZ534173_FASTA.fasta	MGYA00248519	ERZ534173	root:Environmental:Aquatic:Marine	0.095	No depth information	1737	2644
MGYA00248480/ERZ534203_FASTA.fasta	MGYA00248480	ERZ534203	root:Environmental:Aquatic:Marine	0.098	No depth information	1285	1920
MGYA00248506/ERZ534147_FASTA.fasta	MGYA00248506	ERZ534147	root:Environmental:Aquatic:Marine	0.0996	No depth information	1584	2443
MGYA00248590/ERZ508624_FASTA.fasta	MGYA00248590	ERZ508624	root:Environmental:Aquatic:Marine	0.1042	No depth information	2367	3812
MGYA00248476/ERZ534199_FASTA.fasta	MGYA00248476	ERZ534199	root:Environmental:Aquatic:Marine	0.1062	No depth information	1942	2936
MGYA00248518/ERZ534171_FASTA.fasta	MGYA00248518	ERZ534171	root:Environmental:Aquatic:Marine	0.1062	No depth information	1602	2452
MGYA00248612/ERZ508653_FASTA.fasta	MGYA00248612	ERZ508653	root:Environmental:Aquatic:Marine	0.1086	No depth information	2450	4057
MGYA00136613/ERZ475168_FASTA.fasta	MGYA00136613	ERZ475168	root:Environmental:Aquatic:Marine	0.11	1m	1138	9189
MGYA00238046/ERZ584617_FASTA.fasta	MGYA00238046	ERZ584617	root:Environmental:Aquatic:Marine	0.1136	No depth information	1513	2334
MGYA00248602/ERZ508644_FASTA.fasta	MGYA00248602	ERZ508644	root:Environmental:Aquatic:Marine	0.1154	No depth information	2803	5210
MGYA00248520/ERZ534146_FASTA.fasta	MGYA00248520	ERZ534146	root:Environmental:Aquatic:Marine	0.1166	No depth information	2026	3217
MGYA00238061/ERZ584649_FASTA.fasta	MGYA00238061	ERZ584649	root:Environmental:Aquatic:Marine	0.1204	No depth information	1984	3093
MGYA00248573/ERZ508619_FASTA.fasta	MGYA00248573	ERZ508619	root:Environmental:Aquatic:Marine	0.1234	No depth information	2762	4864
MGYA00248503/ERZ534226_FASTA.fasta	MGYA00248503	ERZ534226	root:Environmental:Aquatic:Marine	0.1236	No depth information	2547	4072
MGYA00248585/ERZ508614_FASTA.fasta	MGYA00248585	ERZ508614	root:Environmental:Aquatic:Marine	0.1244	No depth information	1072	1731
MGYA00238030/ERZ584648_FASTA.fasta	MGYA00238030	ERZ584648	root:Environmental:Aquatic:Marine	0.1272	No depth information	1694	2646
MGYA00238024/ERZ584637_FASTA.fasta	MGYA00238024	ERZ584637	root:Environmental:Aquatic:Marine	0.1278	No depth information	2165	3442
MGYA00248541/ERZ534181_FASTA.fasta	MGYA00248541	ERZ534181	root:Environmental:Aquatic:Marine	0.1332	No depth information	1943	3105
MGYA00238003/ERZ584593_FASTA.fasta	MGYA00238003	ERZ584593	root:Environmental:Aquatic:Marine	0.1388	No depth information	2694	4440
MGYA00238038/ERZ584622_FASTA.fasta	MGYA00238038	ERZ584622	root:Environmental:Aquatic:Marine	0.139	No depth information	1861	2881
MGYA00237966/ERZ584545_FASTA.fasta	MGYA00237966	ERZ584545	root:Environmental:Aquatic:Marine	0.1412	No depth information	2625	4363
MGYA00238051/ERZ584627_FASTA.fasta	MGYA00238051	ERZ584627	root:Environmental:Aquatic:Marine	0.142	No depth information	2650	4493
MGYA00248501/ERZ534224_FASTA.fasta	MGYA00248501	ERZ534224	root:Environmental:Aquatic:Marine	0.1426	No depth information	1698	2542
MGYA00238050/ERZ584625_FASTA.fasta	MGYA00238050	ERZ584625	root:Environmental:Aquatic:Marine	0.1464	No depth information	2516	4143
MGYA00238029/ERZ584646_FASTA.fasta	MGYA00238029	ERZ584646	root:Environmental:Aquatic:Marine	0.1494	No depth information	2662	4629
MGYA00248478/ERZ534201_FASTA.fasta	MGYA00248478	ERZ534201	root:Environmental:Aquatic:Marine	0.1504	No depth information	2625	4483
MGYA00238039/ERZ584624_FASTA.fasta	MGYA00238039	ERZ584624	root:Environmental:Aquatic:Marine	0.1512	No depth information	621	1037
MGYA00248582/ERZ508638_FASTA.fasta	MGYA00248582	ERZ508638	root:Environmental:Aquatic:Marine	0.1522	No depth information	2386	3892
MGYA00238020/ERZ584608_FASTA.fasta	MGYA00238020	ERZ584608	root:Environmental:Aquatic:Marine	0.1524	No depth information	2313	3769
MGYA00248589/ERZ508622_FASTA.fasta	MGYA00248589	ERZ508622	root:Environmental:Aquatic:Marine	0.1528	No depth information	2923	5643
MGYA00248592/ERZ508628_FASTA.fasta	MGYA00248592	ERZ508628	root:Environmental:Aquatic:Marine	0.1534	No depth information	2728	5151
MGYA00238040/ERZ584626_FASTA.fasta	MGYA00238040	ERZ584626	root:Environmental:Aquatic:Marine	0.1536	No depth information	2331	3755
MGYA00248486/ERZ534209_FASTA.fasta	MGYA00248486	ERZ534209	root:Environmental:Aquatic:Marine	0.1538	No depth information	2753	4640
MGYA00238036/ERZ584618_FASTA.fasta	MGYA00238036	ERZ584618	root:Environmental:Aquatic:Marine	0.154	No depth information	1759	2673
MGYA00238007/ERZ584601_FASTA.fasta	MGYA00238007	ERZ584601	root:Environmental:Aquatic:Marine	0.1548	No depth information	2755	4694
MGYA00237978/ERZ584532_FASTA.fasta	MGYA00237978	ERZ584532	root:Environmental:Aquatic:Marine	0.1552	No depth information	2698	5521
MGYA00238052/ERZ584629_FASTA.fasta	MGYA00238052	ERZ584629	root:Environmental:Aquatic:Marine	0.156	No depth information	2237	3591
MGYA00238034/ERZ584657_FASTA.fasta	MGYA00238034	ERZ584657	root:Environmental:Aquatic:Marine	0.1564	No depth information	2482	5615
MGYA00248471/ERZ534194_FASTA.fasta	MGYA00248471	ERZ534194	root:Environmental:Aquatic:Marine	0.1582	No depth information	2869	5177
MGYA00248447/ERZ584577_FASTA.fasta	MGYA00248447	ERZ584577	root:Environmental:Aquatic:Marine	0.1594	No depth information	2841	5127
MGYA00248539/ERZ534179_FASTA.fasta	MGYA00248539	ERZ534179	root:Environmental:Aquatic:Marine	0.164	No depth information	2561	4277
MGYA00248605/ERZ508647_FASTA.fasta	MGYA00248605	ERZ508647	root:Environmental:Aquatic:Marine	0.165	No depth information	2850	5620
MGYA00248516/ERZ534167_FASTA.fasta	MGYA00248516	ERZ534167	root:Environmental:Aquatic:Marine	0.1656	No depth information	2916	5411
MGYA00248581/ERZ508636_FASTA.fasta	MGYA00248581	ERZ508636	root:Environmental:Aquatic:Marine	0.167	No depth information	2396	3862
MGYA00248758/ERZ505211_FASTA.fasta	MGYA00248758	ERZ505211	root:Environmental:Aquatic:Marine	0.1686	No depth information	33760	38316
MGYA00248576/ERZ508625_FASTA.fasta	MGYA00248576	ERZ508625	root:Environmental:Aquatic:Marine	0.1692	No depth information	2828	5305
MGYA00237995/ERZ584609_FASTA.fasta	MGYA00237995	ERZ584609	root:Environmental:Aquatic:Marine	0.1696	No depth information	2570	4347
MGYA00248495/ERZ534218_FASTA.fasta	MGYA00248495	ERZ534218	root:Environmental:Aquatic:Marine	0.1704	No depth information	1281	1954
MGYA00248511/ERZ534157_FASTA.fasta	MGYA00248511	ERZ534157	root:Environmental:Aquatic:Marine	0.1706	No depth information	2839	5470
MGYA00248470/ERZ534193_FASTA.fasta	MGYA00248470	ERZ534193	root:Environmental:Aquatic:Marine	0.1712	No depth information	2790	5557
MGYA00367509/ERZ828932_FASTA.fasta	MGYA00367509	ERZ828932	root:Environmental:Aquatic:Marine	0.1724	5m	3670	8964
MGYA00238047/ERZ584619_FASTA.fasta	MGYA00238047	ERZ584619	root:Environmental:Aquatic:Marine	0.1736	No depth information	1931	3020
MGYA00248508/ERZ534151_FASTA.fasta	MGYA00248508	ERZ534151	root:Environmental:Aquatic:Marine	0.174	No depth information	2598	4437
MGYA00248544/ERZ534184_FASTA.fasta	MGYA00248544	ERZ534184	root:Environmental:Aquatic:Marine	0.1746	No depth information	2844	5238
MGYA00238016/ERZ584600_FASTA.fasta	MGYA00238016	ERZ584600	root:Environmental:Aquatic:Marine	0.1752	No depth information	2778	4783
MGYA00248611/ERZ508654_FASTA.fasta	MGYA00248611	ERZ508654	root:Environmental:Aquatic:Marine	0.1756	No depth information	2844	5087
MGYA00238019/ERZ584606_FASTA.fasta	MGYA00238019	ERZ584606	root:Environmental:Aquatic:Marine	0.1758	No depth information	2521	5794
MGYA00248613/ERZ508655_FASTA.fasta	MGYA00248613	ERZ508655	root:Environmental:Aquatic:Marine	0.1764	No depth information	2762	4878
MGYA00248461/ERZ584582_FASTA.fasta	MGYA00248461	ERZ584582	root:Environmental:Aquatic:Marine	0.1776	No depth information	2848	5290
MGYA00248444/ERZ584571_FASTA.fasta	MGYA00248444	ERZ584571	root:Environmental:Aquatic:Marine	0.1784	No depth information	2894	5215
MGYA00248493/ERZ534216_FASTA.fasta	MGYA00248493	ERZ534216	root:Environmental:Aquatic:Marine	0.1786	No depth information	2398	3855
MGYA00248494/ERZ534217_FASTA.fasta	MGYA00248494	ERZ534217	root:Environmental:Aquatic:Marine	0.179	No depth information	1903	5390
MGYA00248475/ERZ534198_FASTA.fasta	MGYA00248475	ERZ534198	root:Environmental:Aquatic:Marine	0.1798	No depth information	2768	5378
MGYA00237955/ERZ584523_FASTA.fasta	MGYA00237955	ERZ584523	root:Environmental:Aquatic:Marine	0.1816	No depth information	2733	5691
MGYA00248546/ERZ534186_FASTA.fasta	MGYA00248546	ERZ534186	root:Environmental:Aquatic:Marine	0.1834	No depth information	2853	5477
MGYA00248608/ERZ508650_FASTA.fasta	MGYA00248608	ERZ508650	root:Environmental:Aquatic:Marine	0.1834	No depth information	2850	5391
MGYA00238023/ERZ584614_FASTA.fasta	MGYA00238023	ERZ584614	root:Environmental:Aquatic:Marine	0.1836	No depth information	2702	4651
MGYA00248586/ERZ508616_FASTA.fasta	MGYA00248586	ERZ508616	root:Environmental:Aquatic:Marine	0.1844	No depth information	2408	3956
MGYA00238008/ERZ584584_FASTA.fasta	MGYA00238008	ERZ584584	root:Environmental:Aquatic:Marine	0.1852	No depth information	2907	5683
MGYA00345653/ERZ802829_FASTA.fasta	MGYA00345653	ERZ802829	root:Environmental:Aquatic:Marine	0.1858	3	243695	223859
MGYA00248604/ERZ508646_FASTA.fasta	MGYA00248604	ERZ508646	root:Environmental:Aquatic:Marine	0.1862	No depth information	2268	3664
MGYA00237965/ERZ584543_FASTA.fasta	MGYA00237965	ERZ584543	root:Environmental:Aquatic:Marine	0.187	No depth information	2657	5580
MGYA00248446/ERZ584575_FASTA.fasta	MGYA00248446	ERZ584575	root:Environmental:Aquatic:Marine	0.1876	No depth information	2861	5133
MGYA00237956/ERZ584525_FASTA.fasta	MGYA00237956	ERZ584525	root:Environmental:Aquatic:Marine	0.188	No depth information	3169	6257
MGYA00248483/ERZ534206_FASTA.fasta	MGYA00248483	ERZ534206	root:Environmental:Aquatic:Marine	0.1884	No depth information	2929	5331
MGYA00248510/ERZ534155_FASTA.fasta	MGYA00248510	ERZ534155	root:Environmental:Aquatic:Marine	0.191	No depth information	2639	5750
MGYA00248528/ERZ534162_FASTA.fasta	MGYA00248528	ERZ534162	root:Environmental:Aquatic:Marine	0.1918	No depth information	2474	5717
MGYA00237958/ERZ584529_FASTA.fasta	MGYA00237958	ERZ584529	root:Environmental:Aquatic:Marine	0.1918	No depth information	2672	5729
MGYA00238031/ERZ584650_FASTA.fasta	MGYA00238031	ERZ584650	root:Environmental:Aquatic:Marine	0.1932	No depth information	2730	5057
MGYA00238013/ERZ584594_FASTA.fasta	MGYA00238013	ERZ584594	root:Environmental:Aquatic:Marine	0.195	No depth information	2426	5869
MGYA00248599/ERZ508641_FASTA.fasta	MGYA00248599	ERZ508641	root:Environmental:Aquatic:Marine	0.1966	No depth information	2632	4494
MGYA00238033/ERZ584654_FASTA.fasta	MGYA00238033	ERZ584654	root:Environmental:Aquatic:Marine	0.1966	No depth information	2588	4379
MGYA00237999/ERZ584585_FASTA.fasta	MGYA00237999	ERZ584585	root:Environmental:Aquatic:Marine	0.1968	No depth information	1665	5417
MGYA00238025/ERZ584638_FASTA.fasta	MGYA00238025	ERZ584638	root:Environmental:Aquatic:Marine	0.1968	No depth information	2458	4085
MGYA00237967/ERZ584547_FASTA.fasta	MGYA00237967	ERZ584547	root:Environmental:Aquatic:Marine	0.1972	No depth information	2895	5389
MGYA00248580/ERZ508635_FASTA.fasta	MGYA00248580	ERZ508635	root:Environmental:Aquatic:Marine	0.1986	No depth information	2454	4135
MGYA00248591/ERZ508626_FASTA.fasta	MGYA00248591	ERZ508626	root:Environmental:Aquatic:Marine	0.199	No depth information	2711	4677
MGYA00248452/ERZ584564_FASTA.fasta	MGYA00248452	ERZ584564	root:Environmental:Aquatic:Marine	0.1992	No depth information	2791	5223
MGYA00237997/ERZ584613_FASTA.fasta	MGYA00237997	ERZ584613	root:Environmental:Aquatic:Marine	0.1998	No depth information	2357	5766
MGYA00248462/ERZ651162_FASTA.fasta	MGYA00248462	ERZ651162	root:Environmental:Aquatic:Marine	0.2004	No depth information	2550	5507
MGYA00237988/ERZ584552_FASTA.fasta	MGYA00237988	ERZ584552	root:Environmental:Aquatic:Marine	0.2008	No depth information	2481	5614
MGYA00238058/ERZ584643_FASTA.fasta	MGYA00238058	ERZ584643	root:Environmental:Aquatic:Marine	0.2008	No depth information	2536	5799
MGYA00248442/ERZ584567_FASTA.fasta	MGYA00248442	ERZ584567	root:Environmental:Aquatic:Marine	0.2016	No depth information	1914	5470
MGYA00238056/ERZ584639_FASTA.fasta	MGYA00238056	ERZ584639	root:Environmental:Aquatic:Marine	0.202	No depth information	766	1323
MGYA00248535/ERZ534175_FASTA.fasta	MGYA00248535	ERZ534175	root:Environmental:Aquatic:Marine	0.2024	No depth information	2408	5768
MGYA00237973/ERZ584559_FASTA.fasta	MGYA00237973	ERZ584559	root:Environmental:Aquatic:Marine	0.2026	No depth information	1653	5444
MGYA00237963/ERZ584539_FASTA.fasta	MGYA00237963	ERZ584539	root:Environmental:Aquatic:Marine	0.2036	No depth information	1658	5401
MGYA00248500/ERZ534223_FASTA.fasta	MGYA00248500	ERZ534223	root:Environmental:Aquatic:Marine	0.2076	No depth information	2450	5300
MGYA00248497/ERZ534220_FASTA.fasta	MGYA00248497	ERZ534220	root:Environmental:Aquatic:Marine	0.2078	No depth information	2534	5585
MGYA00248526/ERZ534158_FASTA.fasta	MGYA00248526	ERZ534158	root:Environmental:Aquatic:Marine	0.2082	No depth information	1847	5669
MGYA00238022/ERZ584612_FASTA.fasta	MGYA00238022	ERZ584612	root:Environmental:Aquatic:Marine	0.2096	No depth information	2908	5653
MGYA00238017/ERZ584602_FASTA.fasta	MGYA00238017	ERZ584602	root:Environmental:Aquatic:Marine	0.2106	No depth information	2711	5655
MGYA00238001/ERZ584589_FASTA.fasta	MGYA00238001	ERZ584589	root:Environmental:Aquatic:Marine	0.211	No depth information	2447	5626
MGYA00248521/ERZ534148_FASTA.fasta	MGYA00248521	ERZ534148	root:Environmental:Aquatic:Marine	0.2126	No depth information	804	4867
MGYA00248527/ERZ534160_FASTA.fasta	MGYA00248527	ERZ534160	root:Environmental:Aquatic:Marine	0.2126	No depth information	1026	5047
MGYA00237979/ERZ584534_FASTA.fasta	MGYA00237979	ERZ584534	root:Environmental:Aquatic:Marine	0.2128	No depth information	1412	5321
MGYA00248490/ERZ534213_FASTA.fasta	MGYA00248490	ERZ534213	root:Environmental:Aquatic:Marine	0.2156	No depth information	2558	4224
MGYA00238014/ERZ584596_FASTA.fasta	MGYA00238014	ERZ584596	root:Environmental:Aquatic:Marine	0.2156	No depth information	1501	5257
MGYA00248468/ERZ534191_FASTA.fasta	MGYA00248468	ERZ534191	root:Environmental:Aquatic:Marine	0.2158	No depth information	825	4822
MGYA00248473/ERZ534196_FASTA.fasta	MGYA00248473	ERZ534196	root:Environmental:Aquatic:Marine	0.2168	No depth information	522	4584
MGYA00248525/ERZ534156_FASTA.fasta	MGYA00248525	ERZ534156	root:Environmental:Aquatic:Marine	0.2168	No depth information	872	4899
MGYA00237994/ERZ584607_FASTA.fasta	MGYA00237994	ERZ584607	root:Environmental:Aquatic:Marine	0.2168	No depth information	541	4699
MGYA00238064/ERZ584655_FASTA.fasta	MGYA00238064	ERZ584655	root:Environmental:Aquatic:Marine	0.2168	No depth information	2516	5647
MGYA00238035/ERZ584616_FASTA.fasta	MGYA00238035	ERZ584616	root:Environmental:Aquatic:Marine	0.2176	No depth information	2217	5649
MGYA00248543/ERZ534183_FASTA.fasta	MGYA00248543	ERZ534183	root:Environmental:Aquatic:Marine	0.22	No depth information	864	4869
MGYA00237998/ERZ584615_FASTA.fasta	MGYA00237998	ERZ584615	root:Environmental:Aquatic:Marine	0.22	No depth information	840	4972
MGYA00238004/ERZ584595_FASTA.fasta	MGYA00238004	ERZ584595	root:Environmental:Aquatic:Marine	0.22	No depth information	1330	5309
MGYA00248469/ERZ534192_FASTA.fasta	MGYA00248469	ERZ534192	root:Environmental:Aquatic:Marine	0.223	No depth information	2088	5382
MGYA00248498/ERZ534221_FASTA.fasta	MGYA00248498	ERZ534221	root:Environmental:Aquatic:Marine	0.224	No depth information	2683	5571
MGYA00237970/ERZ584553_FASTA.fasta	MGYA00237970	ERZ584553	root:Environmental:Aquatic:Marine	0.2242	No depth information	1180	5109
MGYA00248533/ERZ534172_FASTA.fasta	MGYA00248533	ERZ534172	root:Environmental:Aquatic:Marine	0.2246	No depth information	2758	5678
MGYA00248488/ERZ534211_FASTA.fasta	MGYA00248488	ERZ534211	root:Environmental:Aquatic:Marine	0.2248	No depth information	2373	5543
MGYA00237957/ERZ584527_FASTA.fasta	MGYA00237957	ERZ584527	root:Environmental:Aquatic:Marine	0.2248	No depth information	1761	5465
MGYA00248570/ERZ508613_FASTA.fasta	MGYA00248570	ERZ508613	root:Environmental:Aquatic:Marine	0.225	No depth information	2333	5812
MGYA00345683/ERZ806366_FASTA.fasta	MGYA00345683	ERZ806366	root:Environmental:Aquatic:Marine	0.2254	3	689124	630920
MGYA00248482/ERZ534205_FASTA.fasta	MGYA00248482	ERZ534205	root:Environmental:Aquatic:Marine	0.2272	No depth information	1707	5297
MGYA00237977/ERZ584530_FASTA.fasta	MGYA00237977	ERZ584530	root:Environmental:Aquatic:Marine	0.2272	No depth information	2745	6154
MGYA00237986/ERZ584548_FASTA.fasta	MGYA00237986	ERZ584548	root:Environmental:Aquatic:Marine	0.2274	No depth information	1916	5636
MGYA00248489/ERZ534212_FASTA.fasta	MGYA00248489	ERZ534212	root:Environmental:Aquatic:Marine	0.2276	No depth information	1172	4997
MGYA00237968/ERZ584549_FASTA.fasta	MGYA00237968	ERZ584549	root:Environmental:Aquatic:Marine	0.2276	No depth information	1634	5333
MGYA00238018/ERZ584604_FASTA.fasta	MGYA00238018	ERZ584604	root:Environmental:Aquatic:Marine	0.2286	No depth information	948	5052
MGYA00238042/ERZ584630_FASTA.fasta	MGYA00238042	ERZ584630	root:Environmental:Aquatic:Marine	0.2288	No depth information	2688	4756
MGYA00248514/ERZ534163_FASTA.fasta	MGYA00248514	ERZ534163	root:Environmental:Aquatic:Marine	0.2294	No depth information	1394	5288
MGYA00237996/ERZ584611_FASTA.fasta	MGYA00237996	ERZ584611	root:Environmental:Aquatic:Marine	0.2294	No depth information	2031	5756
MGYA00248579/ERZ508631_FASTA.fasta	MGYA00248579	ERZ508631	root:Environmental:Aquatic:Marine	0.2306	No depth information	2761	5712
MGYA00248499/ERZ534222_FASTA.fasta	MGYA00248499	ERZ534222	root:Environmental:Aquatic:Marine	0.231	No depth information	998	4925
MGYA00238045/ERZ584635_FASTA.fasta	MGYA00238045	ERZ584635	root:Environmental:Aquatic:Marine	0.233	No depth information	2763	5465
MGYA00248504/ERZ534227_FASTA.fasta	MGYA00248504	ERZ534227	root:Environmental:Aquatic:Marine	0.2332	No depth information	630	4721
MGYA00136637/ERZ475128_FASTA.fasta	MGYA00136637	ERZ475128	root:Environmental:Aquatic:Marine	0.2334	2m	19536	105425
MGYA00238063/ERZ584653_FASTA.fasta	MGYA00238063	ERZ584653	root:Environmental:Aquatic:Marine	0.234	No depth information	2254	3832
MGYA00248569/ERZ508611_FASTA.fasta	MGYA00248569	ERZ508611	root:Environmental:Aquatic:Marine	0.2346	No depth information	2591	5795
MGYA00248481/ERZ534204_FASTA.fasta	MGYA00248481	ERZ534204	root:Environmental:Aquatic:Marine	0.2348	No depth information	2417	5551
MGYA00248517/ERZ534169_FASTA.fasta	MGYA00248517	ERZ534169	root:Environmental:Aquatic:Marine	0.2366	No depth information	2742	4562
MGYA00237969/ERZ584551_FASTA.fasta	MGYA00237969	ERZ584551	root:Environmental:Aquatic:Marine	0.2368	No depth information	2412	5734
MGYA00248575/ERZ508623_FASTA.fasta	MGYA00248575	ERZ508623	root:Environmental:Aquatic:Marine	0.237	No depth information	1653	5545
MGYA00248453/ERZ584566_FASTA.fasta	MGYA00248453	ERZ584566	root:Environmental:Aquatic:Marine	0.2374	No depth information	1361	5234
MGYA00248532/ERZ534170_FASTA.fasta	MGYA00248532	ERZ534170	root:Environmental:Aquatic:Marine	0.2384	No depth information	991	5035
MGYA00237971/ERZ584555_FASTA.fasta	MGYA00237971	ERZ584555	root:Environmental:Aquatic:Marine	0.2384	No depth information	1511	5484
MGYA00248445/ERZ584573_FASTA.fasta	MGYA00248445	ERZ584573	root:Environmental:Aquatic:Marine	0.2398	No depth information	1987	5546
MGYA00237993/ERZ584605_FASTA.fasta	MGYA00237993	ERZ584605	root:Environmental:Aquatic:Marine	0.2398	No depth information	1805	5587
MGYA00248441/ERZ584565_FASTA.fasta	MGYA00248441	ERZ584565	root:Environmental:Aquatic:Marine	0.2402	No depth information	2654	5580
MGYA00248530/ERZ534166_FASTA.fasta	MGYA00248530	ERZ534166	root:Environmental:Aquatic:Marine	0.2404	No depth information	660	4800
MGYA00237976/ERZ584528_FASTA.fasta	MGYA00237976	ERZ584528	root:Environmental:Aquatic:Marine	0.241	No depth information	2631	5717
MGYA00248607/ERZ508649_FASTA.fasta	MGYA00248607	ERZ508649	root:Environmental:Aquatic:Marine	0.2414	No depth information	2465	5781
MGYA00248537/ERZ534177_FASTA.fasta	MGYA00248537	ERZ534177	root:Environmental:Aquatic:Marine	0.2428	No depth information	2681	4501
MGYA00248596/ERZ508637_FASTA.fasta	MGYA00248596	ERZ508637	root:Environmental:Aquatic:Marine	0.2442	No depth information	1995	5542
MGYA00248459/ERZ584578_FASTA.fasta	MGYA00248459	ERZ584578	root:Environmental:Aquatic:Marine	0.2444	No depth information	1428	5229
MGYA00237989/ERZ584554_FASTA.fasta	MGYA00237989	ERZ584554	root:Environmental:Aquatic:Marine	0.245	No depth information	2024	5591
MGYA00248440/ERZ584563_FASTA.fasta	MGYA00248440	ERZ584563	root:Environmental:Aquatic:Marine	0.2454	No depth information	535	4604
MGYA00248466/ERZ534189_FASTA.fasta	MGYA00248466	ERZ534189	root:Environmental:Aquatic:Marine	0.2454	No depth information	1158	5083
MGYA00248472/ERZ534195_FASTA.fasta	MGYA00248472	ERZ534195	root:Environmental:Aquatic:Marine	0.2454	No depth information	1466	5156
MGYA00248474/ERZ534197_FASTA.fasta	MGYA00248474	ERZ534197	root:Environmental:Aquatic:Marine	0.2454	No depth information	725	4768
MGYA00248484/ERZ534207_FASTA.fasta	MGYA00248484	ERZ534207	root:Environmental:Aquatic:Marine	0.2454	No depth information	991	4910
MGYA00248496/ERZ534219_FASTA.fasta	MGYA00248496	ERZ534219	root:Environmental:Aquatic:Marine	0.2454	No depth information	593	4592
MGYA00248502/ERZ534225_FASTA.fasta	MGYA00248502	ERZ534225	root:Environmental:Aquatic:Marine	0.2454	No depth information	692	4750
MGYA00248578/ERZ508629_FASTA.fasta	MGYA00248578	ERZ508629	root:Environmental:Aquatic:Marine	0.2454	No depth information	1552	5357
MGYA00248593/ERZ508630_FASTA.fasta	MGYA00248593	ERZ508630	root:Environmental:Aquatic:Marine	0.2454	No depth information	736	4857
MGYA00248595/ERZ508633_FASTA.fasta	MGYA00248595	ERZ508633	root:Environmental:Aquatic:Marine	0.2454	No depth information	890	4933
MGYA00237964/ERZ584541_FASTA.fasta	MGYA00237964	ERZ584541	root:Environmental:Aquatic:Marine	0.2454	No depth information	544	4725
MGYA00237972/ERZ584557_FASTA.fasta	MGYA00237972	ERZ584557	root:Environmental:Aquatic:Marine	0.2454	No depth information	599	4690
MGYA00237984/ERZ584544_FASTA.fasta	MGYA00237984	ERZ584544	root:Environmental:Aquatic:Marine	0.2454	No depth information	599	4689
MGYA00237987/ERZ584550_FASTA.fasta	MGYA00237987	ERZ584550	root:Environmental:Aquatic:Marine	0.2454	No depth information	755	4848
MGYA00238021/ERZ584610_FASTA.fasta	MGYA00238021	ERZ584610	root:Environmental:Aquatic:Marine	0.2454	No depth information	1692	5463
MGYA00248534/ERZ534174_FASTA.fasta	MGYA00248534	ERZ534174	root:Environmental:Aquatic:Marine	0.2456	No depth information	1489	6005
MGYA00238015/ERZ584598_FASTA.fasta	MGYA00238015	ERZ584598	root:Environmental:Aquatic:Marine	0.2462	No depth information	587	4832
MGYA00248507/ERZ534149_FASTA.fasta	MGYA00248507	ERZ534149	root:Environmental:Aquatic:Marine	0.2466	No depth information	1211	5177
MGYA00237974/ERZ584522_FASTA.fasta	MGYA00237974	ERZ584522	root:Environmental:Aquatic:Marine	0.2466	No depth information	707	4777
MGYA00238041/ERZ584628_FASTA.fasta	MGYA00238041	ERZ584628	root:Environmental:Aquatic:Marine	0.2476	No depth information	1463	5268
MGYA00248571/ERZ508615_FASTA.fasta	MGYA00248571	ERZ508615	root:Environmental:Aquatic:Marine	0.2478	No depth information	1074	5185
MGYA00238012/ERZ584592_FASTA.fasta	MGYA00238012	ERZ584592	root:Environmental:Aquatic:Marine	0.2486	No depth information	2910	5489
MGYA00248531/ERZ534168_FASTA.fasta	MGYA00248531	ERZ534168	root:Environmental:Aquatic:Marine	0.2488	No depth information	993	5664
MGYA00238011/ERZ584590_FASTA.fasta	MGYA00238011	ERZ584590	root:Environmental:Aquatic:Marine	0.2492	No depth information	2562	5822
MGYA00248460/ERZ584580_FASTA.fasta	MGYA00248460	ERZ584580	root:Environmental:Aquatic:Marine	0.2496	No depth information	906	4907
MGYA00248505/ERZ534228_FASTA.fasta	MGYA00248505	ERZ534228	root:Environmental:Aquatic:Marine	0.2496	No depth information	1285	5061
MGYA00248545/ERZ534185_FASTA.fasta	MGYA00248545	ERZ534185	root:Environmental:Aquatic:Marine	0.2496	No depth information	531	4671
MGYA00248572/ERZ508617_FASTA.fasta	MGYA00248572	ERZ508617	root:Environmental:Aquatic:Marine	0.2496	No depth information	1915	5628
MGYA00237961/ERZ584535_FASTA.fasta	MGYA00237961	ERZ584535	root:Environmental:Aquatic:Marine	0.2496	No depth information	927	5077
MGYA00237980/ERZ584536_FASTA.fasta	MGYA00237980	ERZ584536	root:Environmental:Aquatic:Marine	0.2496	No depth information	1111	5062
MGYA00237990/ERZ584556_FASTA.fasta	MGYA00237990	ERZ584556	root:Environmental:Aquatic:Marine	0.2496	No depth information	1448	5327
MGYA00237992/ERZ584603_FASTA.fasta	MGYA00237992	ERZ584603	root:Environmental:Aquatic:Marine	0.2496	No depth information	877	5024
MGYA00238002/ERZ584591_FASTA.fasta	MGYA00238002	ERZ584591	root:Environmental:Aquatic:Marine	0.2496	No depth information	1794	5452
MGYA00238006/ERZ584599_FASTA.fasta	MGYA00238006	ERZ584599	root:Environmental:Aquatic:Marine	0.2496	No depth information	936	4972
MGYA00237983/ERZ584542_FASTA.fasta	MGYA00237983	ERZ584542	root:Environmental:Aquatic:Marine	0.25	No depth information	935	4955
MGYA00248477/ERZ534200_FASTA.fasta	MGYA00248477	ERZ534200	root:Environmental:Aquatic:Marine	0.2508	No depth information	2033	5403
MGYA00238005/ERZ584597_FASTA.fasta	MGYA00238005	ERZ584597	root:Environmental:Aquatic:Marine	0.2508	No depth information	1370	5204
MGYA00248448/ERZ584579_FASTA.fasta	MGYA00248448	ERZ584579	root:Environmental:Aquatic:Marine	0.2512	No depth information	693	4821
MGYA00237960/ERZ584533_FASTA.fasta	MGYA00237960	ERZ584533	root:Environmental:Aquatic:Marine	0.2512	No depth information	628	4867
MGYA00248487/ERZ534210_FASTA.fasta	MGYA00248487	ERZ534210	root:Environmental:Aquatic:Marine	0.2534	No depth information	2309	5535
MGYA00248456/ERZ584572_FASTA.fasta	MGYA00248456	ERZ584572	root:Environmental:Aquatic:Marine	0.2538	No depth information	1761	5389
MGYA00248547/ERZ534187_FASTA.fasta	MGYA00248547	ERZ534187	root:Environmental:Aquatic:Marine	0.2542	No depth information	1209	5099
MGYA00237991/ERZ584558_FASTA.fasta	MGYA00237991	ERZ584558	root:Environmental:Aquatic:Marine	0.2544	No depth information	843	5325
MGYA00248458/ERZ584576_FASTA.fasta	MGYA00248458	ERZ584576	root:Environmental:Aquatic:Marine	0.2554	No depth information	918	4894
MGYA00248548/ERZ534188_FASTA.fasta	MGYA00248548	ERZ534188	root:Environmental:Aquatic:Marine	0.2556	No depth information	1363	5394
MGYA00248574/ERZ508621_FASTA.fasta	MGYA00248574	ERZ508621	root:Environmental:Aquatic:Marine	0.2574	No depth information	2323	6477
MGYA00248536/ERZ534176_FASTA.fasta	MGYA00248536	ERZ534176	root:Environmental:Aquatic:Marine	0.2578	No depth information	995	5506
MGYA00237959/ERZ584531_FASTA.fasta	MGYA00237959	ERZ584531	root:Environmental:Aquatic:Marine	0.2578	No depth information	529	4924
MGYA00248450/ERZ584560_FASTA.fasta	MGYA00248450	ERZ584560	root:Environmental:Aquatic:Marine	0.258	No depth information	852	4931
MGYA00248594/ERZ508632_FASTA.fasta	MGYA00248594	ERZ508632	root:Environmental:Aquatic:Marine	0.2604	No depth information	706	5205
MGYA00248603/ERZ508645_FASTA.fasta	MGYA00248603	ERZ508645	root:Environmental:Aquatic:Marine	0.2608	No depth information	1065	5020
MGYA00238027/ERZ584642_FASTA.fasta	MGYA00238027	ERZ584642	root:Environmental:Aquatic:Marine	0.261	No depth information	660	4793
MGYA00248455/ERZ584570_FASTA.fasta	MGYA00248455	ERZ584570	root:Environmental:Aquatic:Marine	0.2614	No depth information	638	4714
MGYA00248454/ERZ584568_FASTA.fasta	MGYA00248454	ERZ584568	root:Environmental:Aquatic:Marine	0.262	No depth information	2273	5537
MGYA00136674/ERZ475224_FASTA.fasta	MGYA00136674	ERZ475224	root:Environmental:Aquatic:Marine	0.2624	0m	12083	87421
MGYA00237985/ERZ584546_FASTA.fasta	MGYA00237985	ERZ584546	root:Environmental:Aquatic:Marine	0.2628	No depth information	1531	6474
MGYA00237981/ERZ584538_FASTA.fasta	MGYA00237981	ERZ584538	root:Environmental:Aquatic:Marine	0.2648	No depth information	515	5025
MGYA00237975/ERZ584526_FASTA.fasta	MGYA00237975	ERZ584526	root:Environmental:Aquatic:Marine	0.265	No depth information	571	5626
MGYA00248449/ERZ584581_FASTA.fasta	MGYA00248449	ERZ584581	root:Environmental:Aquatic:Marine	0.2658	No depth information	558	4631
MGYA00238043/ERZ584632_FASTA.fasta	MGYA00238043	ERZ584632	root:Environmental:Aquatic:Marine	0.266	No depth information	622	4918
MGYA00248443/ERZ584569_FASTA.fasta	MGYA00248443	ERZ584569	root:Environmental:Aquatic:Marine	0.2668	No depth information	2057	5494
MGYA00238010/ERZ584588_FASTA.fasta	MGYA00238010	ERZ584588	root:Environmental:Aquatic:Marine	0.2682	No depth information	894	4968
MGYA00238009/ERZ584586_FASTA.fasta	MGYA00238009	ERZ584586	root:Environmental:Aquatic:Marine	0.2692	No depth information	2132	5685
MGYA00136727/ERZ475251_FASTA.fasta	MGYA00136727	ERZ475251	root:Environmental:Aquatic:Marine	0.2726	0m	2695	37447
MGYA00248509/ERZ534153_FASTA.fasta	MGYA00248509	ERZ534153	root:Environmental:Aquatic:Marine	0.2728	No depth information	1686	6885
MGYA00237962/ERZ584537_FASTA.fasta	MGYA00237962	ERZ584537	root:Environmental:Aquatic:Marine	0.2766	No depth information	1630	5410
MGYA00248606/ERZ508648_FASTA.fasta	MGYA00248606	ERZ508648	root:Environmental:Aquatic:Marine	0.2794	No depth information	2339	5609
MGYA00238055/ERZ584636_FASTA.fasta	MGYA00238055	ERZ584636	root:Environmental:Aquatic:Marine	0.2852	No depth information	939	4952
MGYA00136661/ERZ475197_FASTA.fasta	MGYA00136661	ERZ475197	root:Environmental:Aquatic:Marine	0.2858	3m	10777	53358
MGYA00248577/ERZ508627_FASTA.fasta	MGYA00248577	ERZ508627	root:Environmental:Aquatic:Marine	0.2904	No depth information	968	5528
MGYA00345690/ERZ806374_FASTA.fasta	MGYA00345690	ERZ806374	root:Environmental:Aquatic:Marine	0.2922	3	362202	379253
MGYA00248479/ERZ534202_FASTA.fasta	MGYA00248479	ERZ534202	root:Environmental:Aquatic:Marine	0.2946	No depth information	1360	5066
MGYA00136686/ERZ475123_FASTA.fasta	MGYA00136686	ERZ475123	root:Environmental:Aquatic:Marine	0.2994	0.5m	15207	106989
MGYA00248491/ERZ534214_FASTA.fasta	MGYA00248491	ERZ534214	root:Environmental:Aquatic:Marine	0.304	No depth information	2462	5487
MGYA00136713/ERZ475245_FASTA.fasta	MGYA00136713	ERZ475245	root:Environmental:Aquatic:Marine	0.3166	50m	13915	61669
MGYA00345668/ERZ802859_FASTA.fasta	MGYA00345668	ERZ802859	root:Environmental:Aquatic:Marine	0.319	3	137033	132842
MGYA00136698/ERZ475243_FASTA.fasta	MGYA00136698	ERZ475243	root:Environmental:Aquatic:Marine	0.3234	0m	9039	68819
MGYA00136738/ERZ475252_FASTA.fasta	MGYA00136738	ERZ475252	root:Environmental:Aquatic:Marine	0.3234	15m	9647	57682
MGYA00136749/ERZ475124_FASTA.fasta	MGYA00136749	ERZ475124	root:Environmental:Aquatic:Marine	0.3254	0m	19557	129400
MGYA00248727/ERZ505371_FASTA.fasta	MGYA00248727	ERZ505371	root:Environmental:Aquatic:Marine	0.326	No depth information	39839	78832
MGYA00136702/ERZ475188_FASTA.fasta	MGYA00136702	ERZ475188	root:Environmental:Aquatic:Marine	0.3286	2.4m	6827	49566
MGYA00136730/ERZ475191_FASTA.fasta	MGYA00136730	ERZ475191	root:Environmental:Aquatic:Marine	0.3348	3m	16395	70207
MGYA00136737/ERZ475165_FASTA.fasta	MGYA00136737	ERZ475165	root:Environmental:Aquatic:Marine	0.3354	0m	32902	150320
MGYA00136668/ERZ475133_FASTA.fasta	MGYA00136668	ERZ475133	root:Environmental:Aquatic:Marine	0.3412	0m	13049	77784
MGYA00136624/ERZ475248_FASTA.fasta	MGYA00136624	ERZ475248	root:Environmental:Aquatic:Marine	0.3462	0m	9433	77998
MGYA00248597/ERZ508639_FASTA.fasta	MGYA00248597	ERZ508639	root:Environmental:Aquatic:Marine	0.3464	No depth information	3147	9347
MGYA00136638/ERZ475255_FASTA.fasta	MGYA00136638	ERZ475255	root:Environmental:Aquatic:Marine	0.3496	0m	14368	76470
MGYA00248721/ERZ505372_FASTA.fasta	MGYA00248721	ERZ505372	root:Environmental:Aquatic:Marine	0.3526	No depth information	68582	132420
MGYA00136619/ERZ475225_FASTA.fasta	MGYA00136619	ERZ475225	root:Environmental:Aquatic:Marine	0.3532	1m	21041	121590
MGYA00248600/ERZ508642_FASTA.fasta	MGYA00248600	ERZ508642	root:Environmental:Aquatic:Marine	0.3532	No depth information	3746	10063
MGYA00136731/ERZ475226_FASTA.fasta	MGYA00136731	ERZ475226	root:Environmental:Aquatic:Marine	0.3538	1m	18913	103892
MGYA00004358/ERR700148_FASTA_nt_reads.fas	MGYA00004358	ERR700148	root:Environmental:Aquatic:Marine	0.3572	No depth information	7551	422306
MGYA00248724/ERZ505380_FASTA.fasta	MGYA00248724	ERZ505380	root:Environmental:Aquatic:Marine	0.3594	No depth information	78538	116838
MGYA00367518/ERZ829058_FASTA.fasta	MGYA00367518	ERZ829058	root:Environmental:Aquatic:Marine	0.3618	70m	16741	29143
MGYA00136707/ERZ475215_FASTA.fasta	MGYA00136707	ERZ475215	root:Environmental:Aquatic:Marine	0.3634	0m	3925	16871
MGYA00136717/ERZ475239_FASTA.fasta	MGYA00136717	ERZ475239	root:Environmental:Aquatic:Marine	0.366	4m	9520	84230
MGYA00136658/ERZ475249_FASTA.fasta	MGYA00136658	ERZ475249	root:Environmental:Aquatic:Marine	0.3704	0m	3556	39302
MGYA00136691/ERZ475250_FASTA.fasta	MGYA00136691	ERZ475250	root:Environmental:Aquatic:Marine	0.372	0m	7050	62095
MGYA00136712/ERZ475237_FASTA.fasta	MGYA00136712	ERZ475237	root:Environmental:Aquatic:Marine	0.372	0m	13681	73079
MGYA00248513/ERZ534161_FASTA.fasta	MGYA00248513	ERZ534161	root:Environmental:Aquatic:Marine	0.374	No depth information	2866	8760
MGYA00136721/ERZ475160_FASTA.fasta	MGYA00136721	ERZ475160	root:Environmental:Aquatic:Marine	0.3786	75m	40140	226459
MGYA00004363/ERR700153_FASTA_nt_reads.fas	MGYA00004363	ERR700153	root:Environmental:Aquatic:Marine	0.3812	No depth information	8355	219593
MGYA00136682/ERZ475142_FASTA.fasta	MGYA00136682	ERZ475142	root:Environmental:Aquatic:Marine	0.3874	0.1m	12225	81238
MGYA00248439/ERZ584659_FASTA.fasta	MGYA00248439	ERZ584659	root:Environmental:Aquatic:Marine	0.3884	No depth information	255988	664378
MGYA00248588/ERZ508620_FASTA.fasta	MGYA00248588	ERZ508620	root:Environmental:Aquatic:Marine	0.391	No depth information	3625	10698
MGYA00136701/ERZ475216_FASTA.fasta	MGYA00136701	ERZ475216	root:Environmental:Aquatic:Marine	0.394	0m	31223	116545
MGYA00136659/ERZ475195_FASTA.fasta	MGYA00136659	ERZ475195	root:Environmental:Aquatic:Marine	0.396	3m	17059	79062
MGYA00136639/ERZ475253_FASTA.fasta	MGYA00136639	ERZ475253	root:Environmental:Aquatic:Marine	0.3996	0m	8249	54003
MGYA00136742/ERZ475140_FASTA.fasta	MGYA00136742	ERZ475140	root:Environmental:Aquatic:Marine	0.3996	0m	42309	213540
MGYA00136724/ERZ475241_FASTA.fasta	MGYA00136724	ERZ475241	root:Environmental:Aquatic:Marine	0.3998	0.2m	16983	75874
MGYA00136741/ERZ475219_FASTA.fasta	MGYA00136741	ERZ475219	root:Environmental:Aquatic:Marine	0.4036	0m	33301	138069
MGYA00136680/ERZ475135_FASTA.fasta	MGYA00136680	ERZ475135	root:Environmental:Aquatic:Marine	0.408	0.5m	27521	124206
MGYA00248734/ERZ505382_FASTA.fasta	MGYA00248734	ERZ505382	root:Environmental:Aquatic:Marine	0.4082	No depth information	67371	113609
MGYA00136651/ERZ475238_FASTA.fasta	MGYA00136651	ERZ475238	root:Environmental:Aquatic:Marine	0.4104	5m	15219	82343
MGYA00367520/ERZ829063_FASTA.fasta	MGYA00367520	ERZ829063	root:Environmental:Aquatic:Marine	0.413	70m	16647	29132
MGYA00248720/ERZ505370_FASTA.fasta	MGYA00248720	ERZ505370	root:Environmental:Aquatic:Marine	0.4176	No depth information	38204	74069
MGYA00136626/ERZ475161_FASTA.fasta	MGYA00136626	ERZ475161	root:Environmental:Aquatic:Marine	0.4184	1m	39180	186232
MGYA00345673/ERZ802872_FASTA.fasta	MGYA00345673	ERZ802872	root:Environmental:Aquatic:Marine	0.4218	3	225342	256445
MGYA00136640/ERZ475221_FASTA.fasta	MGYA00136640	ERZ475221	root:Environmental:Aquatic:Marine	0.4294	0m	19993	121012
MGYA00136700/ERZ475242_FASTA.fasta	MGYA00136700	ERZ475242	root:Environmental:Aquatic:Marine	0.43	0m	13633	73783
MGYA00345658/ERZ802836_FASTA.fasta	MGYA00345658	ERZ802836	root:Environmental:Aquatic:Marine	0.4364	3	81600	99166
MGYA00136663/ERZ475138_FASTA.fasta	MGYA00136663	ERZ475138	root:Environmental:Aquatic:Marine	0.4392	1m	26300	108649
MGYA00136652/ERZ475240_FASTA.fasta	MGYA00136652	ERZ475240	root:Environmental:Aquatic:Marine	0.444	0m	18037	85686
MGYA00136714/ERZ475134_FASTA.fasta	MGYA00136714	ERZ475134	root:Environmental:Aquatic:Marine	0.4468	0.5m	19370	132756
MGYA00237870/ERZ529317_FASTA.fasta	MGYA00237870	ERZ529317	root:Environmental:Aquatic:Marine	0.4518	275.0-287.0	15099	48017
MGYA00136708/ERZ475259_FASTA.fasta	MGYA00136708	ERZ475259	root:Environmental:Aquatic:Marine	0.4532	1m	50298	220671
MGYA00136697/ERZ475198_FASTA.fasta	MGYA00136697	ERZ475198	root:Environmental:Aquatic:Marine	0.4544	3m	16840	74897
MGYA00136664/ERZ475258_FASTA.fasta	MGYA00136664	ERZ475258	root:Environmental:Aquatic:Marine	0.4644	1m	22157	118644
MGYA00136675/ERZ475208_FASTA.fasta	MGYA00136675	ERZ475208	root:Environmental:Aquatic:Marine	0.47	2m	33689	139998
MGYA00136728/ERZ475213_FASTA.fasta	MGYA00136728	ERZ475213	root:Environmental:Aquatic:Marine	0.4738	5m	19823	120501
MGYA00136632/ERZ475229_FASTA.fasta	MGYA00136632	ERZ475229	root:Environmental:Aquatic:Marine	0.4756	0m	23574	123053
MGYA00237871/ERZ529316_FASTA.fasta	MGYA00237871	ERZ529316	root:Environmental:Aquatic:Marine	0.4758	275.0-287.0	43054	95098
MGYA00136703/ERZ475125_FASTA.fasta	MGYA00136703	ERZ475125	root:Environmental:Aquatic:Marine	0.4762	0m	20440	112502
MGYA00136644/ERZ475194_FASTA.fasta	MGYA00136644	ERZ475194	root:Environmental:Aquatic:Marine	0.478	3m	18437	80711
MGYA00136617/ERZ475247_FASTA.fasta	MGYA00136617	ERZ475247	root:Environmental:Aquatic:Marine	0.4784	0m	26821	102812
MGYA00136614/ERZ475162_FASTA.fasta	MGYA00136614	ERZ475162	root:Environmental:Aquatic:Marine	0.4802	2m	41777	211044
MGYA00136656/ERZ475206_FASTA.fasta	MGYA00136656	ERZ475206	root:Environmental:Aquatic:Marine	0.484	2m	36431	159514
MGYA00136615/ERZ475227_FASTA.fasta	MGYA00136615	ERZ475227	root:Environmental:Aquatic:Marine	0.4852	5m	19875	110061
MGYA00345656/ERZ802832_FASTA.fasta	MGYA00345656	ERZ802832	root:Environmental:Aquatic:Marine	0.4854	3	144420	193961
MGYA00136710/ERZ475193_FASTA.fasta	MGYA00136710	ERZ475193	root:Environmental:Aquatic:Marine	0.487	75m	21527	151916
MGYA00136679/ERZ475256_FASTA.fasta	MGYA00136679	ERZ475256	root:Environmental:Aquatic:Marine	0.4892	0m	12854	60308
MGYA00136631/ERZ475131_FASTA.fasta	MGYA00136631	ERZ475131	root:Environmental:Aquatic:Marine	0.4904	0m	27948	131361
MGYA00136699/ERZ475144_FASTA.fasta	MGYA00136699	ERZ475144	root:Environmental:Aquatic:Marine	0.4906	0.1m	56657	272103
MGYA00136725/ERZ475217_FASTA.fasta	MGYA00136725	ERZ475217	root:Environmental:Aquatic:Marine	0.4938	0m	56253	230647
MGYA00136716/ERZ475130_FASTA.fasta	MGYA00136716	ERZ475130	root:Environmental:Aquatic:Marine	0.497	0m	45329	236216
MGYA00136678/ERZ475177_FASTA.fasta	MGYA00136678	ERZ475177	root:Environmental:Aquatic:Marine	0.4998	0.2m	61627	307819
MGYA00136670/ERZ475189_FASTA.fasta	MGYA00136670	ERZ475189	root:Environmental:Aquatic:Marine	0.5004	3m	28769	118583
MGYA00237877/ERZ529326_FASTA.fasta	MGYA00237877	ERZ529326	root:Environmental:Aquatic:Marine	0.5012	No depth information	4840	13079
MGYA00136746/ERZ475246_FASTA.fasta	MGYA00136746	ERZ475246	root:Environmental:Aquatic:Marine	0.5034	39m	19289	78302
MGYA00136623/ERZ475158_FASTA.fasta	MGYA00136623	ERZ475158	root:Environmental:Aquatic:Marine	0.5052	2m	55762	252136
MGYA00136740/ERZ475207_FASTA.fasta	MGYA00136740	ERZ475207	root:Environmental:Aquatic:Marine	0.5052	1m	49159	182349
MGYA00136706/ERZ475244_FASTA.fasta	MGYA00136706	ERZ475244	root:Environmental:Aquatic:Marine	0.5128	0m	11660	55213
MGYA00136723/ERZ475220_FASTA.fasta	MGYA00136723	ERZ475220	root:Environmental:Aquatic:Marine	0.5152	0m	22462	135511
MGYA00237872/ERZ529318_FASTA.fasta	MGYA00237872	ERZ529318	root:Environmental:Aquatic:Marine	0.5152	275.0-287.0	19214	55385
MGYA00136657/ERZ475146_FASTA.fasta	MGYA00136657	ERZ475146	root:Environmental:Aquatic:Marine	0.5164	0m	25369	90123
MGYA00136688/ERZ475232_FASTA.fasta	MGYA00136688	ERZ475232	root:Environmental:Aquatic:Marine	0.5192	1m	28002	115323
MGYA00136636/ERZ473095_FASTA.fasta	MGYA00136636	ERZ473095	root:Environmental:Aquatic:Marine	0.5262	1m	29430	46539
MGYA00136676/ERZ475196_FASTA.fasta	MGYA00136676	ERZ475196	root:Environmental:Aquatic:Marine	0.5314	3m	34410	143215
MGYA00136693/ERZ475155_FASTA.fasta	MGYA00136693	ERZ475155	root:Environmental:Aquatic:Marine	0.5322	1m	51400	232219
MGYA00136660/ERZ475143_FASTA.fasta	MGYA00136660	ERZ475143	root:Environmental:Aquatic:Marine	0.533	0m	42609	208216
MGYA00237644/ERZ505207_FASTA.fasta	MGYA00237644	ERZ505207	root:Environmental:Aquatic:Marine	0.534	No depth information	55621	100469
MGYA00136654/ERZ475136_FASTA.fasta	MGYA00136654	ERZ475136	root:Environmental:Aquatic:Marine	0.5374	0.5m	28884	122814
MGYA00136685/ERZ475257_FASTA.fasta	MGYA00136685	ERZ475257	root:Environmental:Aquatic:Marine	0.5408	0m	21058	100091
MGYA00136633/ERZ475200_FASTA.fasta	MGYA00136633	ERZ475200	root:Environmental:Aquatic:Marine	0.5444	3m	32727	137661
MGYA00136683/ERZ475218_FASTA.fasta	MGYA00136683	ERZ475218	root:Environmental:Aquatic:Marine	0.5458	50m	58326	262979
MGYA00136729/ERZ475183_FASTA.fasta	MGYA00136729	ERZ475183	root:Environmental:Aquatic:Marine	0.5472	0m	39806	160282
MGYA00136690/ERZ475231_FASTA.fasta	MGYA00136690	ERZ475231	root:Environmental:Aquatic:Marine	0.5526	1m	37680	120884
MGYA00136622/ERZ475181_FASTA.fasta	MGYA00136622	ERZ475181	root:Environmental:Aquatic:Marine	0.5532	0m	54572	248924
MGYA00136627/ERZ475149_FASTA.fasta	MGYA00136627	ERZ475149	root:Environmental:Aquatic:Marine	0.5542	0.1m	39248	213596
MGYA00136726/ERZ475154_FASTA.fasta	MGYA00136726	ERZ475154	root:Environmental:Aquatic:Marine	0.5544	1m	51820	230355
MGYA00136719/ERZ475167_FASTA.fasta	MGYA00136719	ERZ475167	root:Environmental:Aquatic:Marine	0.5564	0m	75874	347109
MGYA00367719/ERZ841982_FASTA.fasta	MGYA00367719	ERZ841982	root:Environmental:Aquatic:Marine	0.5566	5m	83975	158093
MGYA00367539/ERZ832020_FASTA.fasta	MGYA00367539	ERZ832020	root:Environmental:Aquatic:Marine	0.5594	5m	211524	413814
MGYA00136641/ERZ475205_FASTA.fasta	MGYA00136641	ERZ475205	root:Environmental:Aquatic:Marine	0.5616	2m	30014	111753
MGYA00367589/ERZ839685_FASTA.fasta	MGYA00367589	ERZ839685	root:Environmental:Aquatic:Marine	0.5638	5m	120626	199824
MGYA00136667/ERZ475172_FASTA.fasta	MGYA00136667	ERZ475172	root:Environmental:Aquatic:Marine	0.5642	1m	57674	282145
MGYA00136630/ERZ475254_FASTA.fasta	MGYA00136630	ERZ475254	root:Environmental:Aquatic:Marine	0.5644	4.12m	15761	65371
MGYA00367542/ERZ832854_FASTA.fasta	MGYA00367542	ERZ832854	root:Environmental:Aquatic:Marine	0.565	5m	139325	246267
MGYA00367608/ERZ840589_FASTA.fasta	MGYA00367608	ERZ840589	root:Environmental:Aquatic:Marine	0.567	5m	202001	373776
MGYA00136692/ERZ475180_FASTA.fasta	MGYA00136692	ERZ475180	root:Environmental:Aquatic:Marine	0.5678	0m	73449	308522
MGYA00136677/ERZ475260_FASTA.fasta	MGYA00136677	ERZ475260	root:Environmental:Aquatic:Marine	0.5682	0m	43883	182877
MGYA00136645/ERZ475199_FASTA.fasta	MGYA00136645	ERZ475199	root:Environmental:Aquatic:Marine	0.573	3m	34259	140086
MGYA00136620/ERZ475211_FASTA.fasta	MGYA00136620	ERZ475211	root:Environmental:Aquatic:Marine	0.5742	1m	48644	193831
MGYA00136752/ERZ475171_FASTA.fasta	MGYA00136752	ERZ475171	root:Environmental:Aquatic:Marine	0.575	0m	80933	353769
MGYA00136625/ERZ475222_FASTA.fasta	MGYA00136625	ERZ475222	root:Environmental:Aquatic:Marine	0.5754	5m	33600	131154
MGYA00136642/ERZ475152_FASTA.fasta	MGYA00136642	ERZ475152	root:Environmental:Aquatic:Marine	0.5772	0m	57272	281865
MGYA00367586/ERZ839331_FASTA.fasta	MGYA00367586	ERZ839331	root:Environmental:Aquatic:Marine	0.5778	5m	136739	255894
MGYA00367686/ERZ841659_FASTA.fasta	MGYA00367686	ERZ841659	root:Environmental:Aquatic:Marine	0.5788	5m	203423	367206
MGYA00136635/ERZ475212_FASTA.fasta	MGYA00136635	ERZ475212	root:Environmental:Aquatic:Marine	0.583	1m	52958	245781
MGYA00367565/ERZ836228_FASTA.fasta	MGYA00367565	ERZ836228	root:Environmental:Aquatic:Marine	0.5862	5m	139951	255399
MGYA00367580/ERZ838699_FASTA.fasta	MGYA00367580	ERZ838699	root:Environmental:Aquatic:Marine	0.5866	80m	146786	299122
MGYA00136747/ERZ475137_FASTA.fasta	MGYA00136747	ERZ475137	root:Environmental:Aquatic:Marine	0.587	1m	28373	123585
MGYA00136744/ERZ475141_FASTA.fasta	MGYA00136744	ERZ475141	root:Environmental:Aquatic:Marine	0.5878	0m	39720	192791
MGYA00367577/ERZ838379_FASTA.fasta	MGYA00367577	ERZ838379	root:Environmental:Aquatic:Marine	0.588	5m	76986	123940
MGYA00248725/ERZ505367_FASTA.fasta	MGYA00248725	ERZ505367	root:Environmental:Aquatic:Marine	0.5902	No depth information	104681	194956
MGYA00136750/ERZ475139_FASTA.fasta	MGYA00136750	ERZ475139	root:Environmental:Aquatic:Marine	0.5926	0.8m	51675	210808
MGYA00136629/ERZ475182_FASTA.fasta	MGYA00136629	ERZ475182	root:Environmental:Aquatic:Marine	0.594	1m	54188	239650
MGYA00136681/ERZ475203_FASTA.fasta	MGYA00136681	ERZ475203	root:Environmental:Aquatic:Marine	0.5952	0.1m	37963	150131
MGYA00136655/ERZ475186_FASTA.fasta	MGYA00136655	ERZ475186	root:Environmental:Aquatic:Marine	0.5956	0m	50226	215184
MGYA00345660/ERZ802840_FASTA.fasta	MGYA00345660	ERZ802840	root:Environmental:Aquatic:Marine	0.5972	3	639778	733567
MGYA00136687/ERZ475202_FASTA.fasta	MGYA00136687	ERZ475202	root:Environmental:Aquatic:Marine	0.5974	3m	57521	235067
MGYA00237657/ERZ505235_FASTA.fasta	MGYA00237657	ERZ505235	root:Environmental:Aquatic:Marine	0.5988	No depth information	125857	235332
MGYA00367546/ERZ833247_FASTA.fasta	MGYA00367546	ERZ833247	root:Environmental:Aquatic:Marine	0.602	5m	229497	406380
MGYA00367534/ERZ831056_FASTA.fasta	MGYA00367534	ERZ831056	root:Environmental:Aquatic:Marine	0.6026	5m	201462	391305
MGYA00345657/ERZ802834_FASTA.fasta	MGYA00345657	ERZ802834	root:Environmental:Aquatic:Marine	0.6028	3	126520	161344
MGYA00367543/ERZ832923_FASTA.fasta	MGYA00367543	ERZ832923	root:Environmental:Aquatic:Marine	0.6028	5m	203403	356387
MGYA00367705/ERZ841881_FASTA.fasta	MGYA00367705	ERZ841881	root:Environmental:Aquatic:Marine	0.6032	5m	164043	303393
MGYA00237658/ERZ505237_FASTA.fasta	MGYA00237658	ERZ505237	root:Environmental:Aquatic:Marine	0.6042	No depth information	103079	234429
MGYA00237846/ERZ508604_FASTA.fasta	MGYA00237846	ERZ508604	root:Environmental:Aquatic:Marine	0.6076	No depth information	22546	77451
MGYA00367609/ERZ840591_FASTA.fasta	MGYA00367609	ERZ840591	root:Environmental:Aquatic:Marine	0.6112	188m	216842	381327
MGYA00136715/ERZ475173_FASTA.fasta	MGYA00136715	ERZ475173	root:Environmental:Aquatic:Marine	0.6114	0.5m	53816	321417
MGYA00136736/ERZ475201_FASTA.fasta	MGYA00136736	ERZ475201	root:Environmental:Aquatic:Marine	0.612	3m	54177	246845
MGYA00367707/ERZ841895_FASTA.fasta	MGYA00367707	ERZ841895	root:Environmental:Aquatic:Marine	0.6128	5m	378820	717948
MGYA00136720/ERZ475187_FASTA.fasta	MGYA00136720	ERZ475187	root:Environmental:Aquatic:Marine	0.6128	2m	37191	148696
MGYA00367613/ERZ840836_FASTA.fasta	MGYA00367613	ERZ840836	root:Environmental:Aquatic:Marine	0.6132	120m	137546	220894
MGYA00139971/ERZ477649_FASTA.fasta	MGYA00139971	ERZ477649	root:Environmental:Aquatic:Marine	0.6138	No depth information	163217	1208030
MGYA00136643/ERZ475261_FASTA.fasta	MGYA00136643	ERZ475261	root:Environmental:Aquatic:Marine	0.6154	2m	36409	136864
MGYA00136653/ERZ475150_FASTA.fasta	MGYA00136653	ERZ475150	root:Environmental:Aquatic:Marine	0.6162	0m	62544	232989
MGYA00367734/ERZ842120_FASTA.fasta	MGYA00367734	ERZ842120	root:Environmental:Aquatic:Marine	0.6166	5m	190247	329186
MGYA00367612/ERZ840834_FASTA.fasta	MGYA00367612	ERZ840834	root:Environmental:Aquatic:Marine	0.6184	5m	150699	271071
MGYA00136669/ERZ475163_FASTA.fasta	MGYA00136669	ERZ475163	root:Environmental:Aquatic:Marine	0.6188	0m	71150	320379
MGYA00367659/ERZ841401_FASTA.fasta	MGYA00367659	ERZ841401	root:Environmental:Aquatic:Marine	0.619	5m	355528	647670
MGYA00248563/ERZ511470_FASTA.fasta	MGYA00248563	ERZ511470	root:Environmental:Aquatic:Marine	0.62	No depth information	287668	629672
MGYA00367585/ERZ839268_FASTA.fasta	MGYA00367585	ERZ839268	root:Environmental:Aquatic:Marine	0.62	30m	97257	165417
MGYA00136666/ERZ475148_FASTA.fasta	MGYA00136666	ERZ475148	root:Environmental:Aquatic:Marine	0.6202	0.5m	54467	207435
MGYA00367635/ERZ841115_FASTA.fasta	MGYA00367635	ERZ841115	root:Environmental:Aquatic:Marine	0.621	5m	254231	435945
MGYA00345659/ERZ802838_FASTA.fasta	MGYA00345659	ERZ802838	root:Environmental:Aquatic:Marine	0.622	3	642019	837335
MGYA00367654/ERZ841379_FASTA.fasta	MGYA00367654	ERZ841379	root:Environmental:Aquatic:Marine	0.622	5m	201017	352780
MGYA00367529/ERZ829933_FASTA.fasta	MGYA00367529	ERZ829933	root:Environmental:Aquatic:Marine	0.6232	30m	112257	202578
MGYA00136732/ERZ475151_FASTA.fasta	MGYA00136732	ERZ475151	root:Environmental:Aquatic:Marine	0.6258	1m	40514	224715
MGYA00136612/ERZ475228_FASTA.fasta	MGYA00136612	ERZ475228	root:Environmental:Aquatic:Marine	0.626	2m	47766	213711
MGYA00345652/ERZ802827_FASTA.fasta	MGYA00345652	ERZ802827	root:Environmental:Aquatic:Marine	0.6272	3	266500	476606
MGYA00136694/ERZ475223_FASTA.fasta	MGYA00136694	ERZ475223	root:Environmental:Aquatic:Marine	0.6276	0m	38344	171430
MGYA00367664/ERZ841434_FASTA.fasta	MGYA00367664	ERZ841434	root:Environmental:Aquatic:Marine	0.6298	5m	141005	263651
MGYA00367704/ERZ841878_FASTA.fasta	MGYA00367704	ERZ841878	root:Environmental:Aquatic:Marine	0.6302	5m	166471	304498
MGYA00367717/ERZ841978_FASTA.fasta	MGYA00367717	ERZ841978	root:Environmental:Aquatic:Marine	0.6302	50m	176339	329727
MGYA00136751/ERZ475169_FASTA.fasta	MGYA00136751	ERZ475169	root:Environmental:Aquatic:Marine	0.6304	0m	32052	134980
MGYA00367551/ERZ833786_FASTA.fasta	MGYA00367551	ERZ833786	root:Environmental:Aquatic:Marine	0.6306	5m	187012	339904
MGYA00136646/ERZ475230_FASTA.fasta	MGYA00136646	ERZ475230	root:Environmental:Aquatic:Marine	0.6318	0.5m	26254	132291
MGYA00136735/ERZ475175_FASTA.fasta	MGYA00136735	ERZ475175	root:Environmental:Aquatic:Marine	0.6336	2m	60357	259928
MGYA00139096/ERZ477576_FASTA.fasta	MGYA00139096	ERZ477576	root:Environmental:Aquatic:Marine	0.6366	5m	148992	1276504
MGYA00248730/ERZ505377_FASTA.fasta	MGYA00248730	ERZ505377	root:Environmental:Aquatic:Marine	0.637	No depth information	44009	71651
MGYA00367536/ERZ831412_FASTA.fasta	MGYA00367536	ERZ831412	root:Environmental:Aquatic:Marine	0.637	40m	174146	310758
MGYA00345684/ERZ806367_FASTA.fasta	MGYA00345684	ERZ806367	root:Environmental:Aquatic:Marine	0.6372	3	728905	712079
MGYA00367668/ERZ841457_FASTA.fasta	MGYA00367668	ERZ841457	root:Environmental:Aquatic:Marine	0.6374	5m	400952	766502
MGYA00136650/ERZ475176_FASTA.fasta	MGYA00136650	ERZ475176	root:Environmental:Aquatic:Marine	0.6376	0m	48059	215737
MGYA00367549/ERZ833507_FASTA.fasta	MGYA00367549	ERZ833507	root:Environmental:Aquatic:Marine	0.6376	60m	205051	401446
MGYA00345688/ERZ806372_FASTA.fasta	MGYA00345688	ERZ806372	root:Environmental:Aquatic:Marine	0.638	3	334547	526811
MGYA00367623/ERZ840934_FASTA.fasta	MGYA00367623	ERZ840934	root:Environmental:Aquatic:Marine	0.6384	50m	177483	306673
MGYA00367673/ERZ841475_FASTA.fasta	MGYA00367673	ERZ841475	root:Environmental:Aquatic:Marine	0.6386	30m	227893	398577
MGYA00367553/ERZ833910_FASTA.fasta	MGYA00367553	ERZ833910	root:Environmental:Aquatic:Marine	0.639	30m	231243	407083
MGYA00367527/ERZ829845_FASTA.fasta	MGYA00367527	ERZ829845	root:Environmental:Aquatic:Marine	0.64	40m	203049	370123
MGYA00345689/ERZ806373_FASTA.fasta	MGYA00345689	ERZ806373	root:Environmental:Aquatic:Marine	0.641	3	274207	425579
MGYA00345651/ERZ802826_FASTA.fasta	MGYA00345651	ERZ802826	root:Environmental:Aquatic:Marine	0.6416	3	205093	346146
MGYA00367722/ERZ842010_FASTA.fasta	MGYA00367722	ERZ842010	root:Environmental:Aquatic:Marine	0.6418	5m	206594	383532
MGYA00367561/ERZ835454_FASTA.fasta	MGYA00367561	ERZ835454	root:Environmental:Aquatic:Marine	0.6442	5m	337747	670641
MGYA00367592/ERZ840032_FASTA.fasta	MGYA00367592	ERZ840032	root:Environmental:Aquatic:Marine	0.6442	65m	203216	345876
MGYA00367682/ERZ841618_FASTA.fasta	MGYA00367682	ERZ841618	root:Environmental:Aquatic:Marine	0.6442	5m	471167	865374
MGYA00367526/ERZ829801_FASTA.fasta	MGYA00367526	ERZ829801	root:Environmental:Aquatic:Marine	0.6458	25m	178185	339073
MGYA00345669/ERZ802861_FASTA.fasta	MGYA00345669	ERZ802861	root:Environmental:Aquatic:Marine	0.6468	3	477709	614322
MGYA00345693/ERZ806377_FASTA.fasta	MGYA00345693	ERZ806377	root:Environmental:Aquatic:Marine	0.6472	3	476066	701504
MGYA00367528/ERZ829891_FASTA.fasta	MGYA00367528	ERZ829891	root:Environmental:Aquatic:Marine	0.6472	5m	187585	378256
MGYA00136722/ERZ475126_FASTA.fasta	MGYA00136722	ERZ475126	root:Environmental:Aquatic:Marine	0.6472	2m	21677	111468
MGYA00248465/ERZ534230_FASTA.fasta	MGYA00248465	ERZ534230	root:Environmental:Aquatic:Marine	0.6476	No depth information	323249	689526
MGYA00136689/ERZ475204_FASTA.fasta	MGYA00136689	ERZ475204	root:Environmental:Aquatic:Marine	0.6478	0.1m	30206	152041
MGYA00375067/ERZ781375_FASTA.fasta	MGYA00375067	ERZ781375	root:Environmental:Aquatic:Marine	0.649	No depth information	401156	699663
MGYA00345667/ERZ802855_FASTA.fasta	MGYA00345667	ERZ802855	root:Environmental:Aquatic:Marine	0.652	3	773433	929130
MGYA00367560/ERZ835323_FASTA.fasta	MGYA00367560	ERZ835323	root:Environmental:Aquatic:Marine	0.652	30m	209094	370507
MGYA00375047/ERZ781355_FASTA.fasta	MGYA00375047	ERZ781355	root:Environmental:Aquatic:Marine	0.6522	No depth information	227121	373728
MGYA00136748/ERZ475170_FASTA.fasta	MGYA00136748	ERZ475170	root:Environmental:Aquatic:Marine	0.655	0.1m	69658	320537
MGYA00345682/ERZ806365_FASTA.fasta	MGYA00345682	ERZ806365	root:Environmental:Aquatic:Marine	0.6554	3	397276	677426
MGYA00345692/ERZ806376_FASTA.fasta	MGYA00345692	ERZ806376	root:Environmental:Aquatic:Marine	0.6576	3	484915	681339
MGYA00136711/ERZ475156_FASTA.fasta	MGYA00136711	ERZ475156	root:Environmental:Aquatic:Marine	0.6576	1m	60845	245366
MGYA00367630/ERZ841003_FASTA.fasta	MGYA00367630	ERZ841003	root:Environmental:Aquatic:Marine	0.6582	5m	205614	374175
MGYA00136621/ERZ475178_FASTA.fasta	MGYA00136621	ERZ475178	root:Environmental:Aquatic:Marine	0.6602	0.2m	45707	254259
MGYA00367675/ERZ841480_FASTA.fasta	MGYA00367675	ERZ841480	root:Environmental:Aquatic:Marine	0.6604	340m	170169	328003
MGYA00248732/ERZ505381_FASTA.fasta	MGYA00248732	ERZ505381	root:Environmental:Aquatic:Marine	0.6606	No depth information	74533	113065
MGYA00367699/ERZ841820_FASTA.fasta	MGYA00367699	ERZ841820	root:Environmental:Aquatic:Marine	0.6606	5m	149368	233871
MGYA00136709/ERZ475145_FASTA.fasta	MGYA00136709	ERZ475145	root:Environmental:Aquatic:Marine	0.662	1m	85701	301490
MGYA00367693/ERZ841744_FASTA.fasta	MGYA00367693	ERZ841744	root:Environmental:Aquatic:Marine	0.6632	5m	185691	320362
MGYA00367547/ERZ833316_FASTA.fasta	MGYA00367547	ERZ833316	root:Environmental:Aquatic:Marine	0.6646	17m	186742	357137
MGYA00367579/ERZ838492_FASTA.fasta	MGYA00367579	ERZ838492	root:Environmental:Aquatic:Marine	0.6646	5m	661127	1161239
MGYA00367545/ERZ833197_FASTA.fasta	MGYA00367545	ERZ833197	root:Environmental:Aquatic:Marine	0.6652	65m	209240	356734
MGYA00367645/ERZ841263_FASTA.fasta	MGYA00367645	ERZ841263	root:Environmental:Aquatic:Marine	0.666	188m	461069	823793
MGYA00345694/ERZ806378_FASTA.fasta	MGYA00345694	ERZ806378	root:Environmental:Aquatic:Marine	0.6662	3	977115	1007296
MGYA00136733/ERZ475236_FASTA.fasta	MGYA00136733	ERZ475236	root:Environmental:Aquatic:Marine	0.6678	10m	38891	130938
MGYA00136648/ERZ475190_FASTA.fasta	MGYA00136648	ERZ475190	root:Environmental:Aquatic:Marine	0.6698	3m	53178	218369
MGYA00367639/ERZ841151_FASTA.fasta	MGYA00367639	ERZ841151	root:Environmental:Aquatic:Marine	0.6708	60m	221277	415045
MGYA00345648/ERZ802821_FASTA.fasta	MGYA00345648	ERZ802821	root:Environmental:Aquatic:Marine	0.6718	3	289283	523842
MGYA00136745/ERZ475157_FASTA.fasta	MGYA00136745	ERZ475157	root:Environmental:Aquatic:Marine	0.6718	1m	93404	375846
MGYA00367624/ERZ840938_FASTA.fasta	MGYA00367624	ERZ840938	root:Environmental:Aquatic:Marine	0.6724	5m	700568	1180983
MGYA00136705/ERZ475159_FASTA.fasta	MGYA00136705	ERZ475159	root:Environmental:Aquatic:Marine	0.6728	0m	65214	242026
MGYA00367657/ERZ841392_FASTA.fasta	MGYA00367657	ERZ841392	root:Environmental:Aquatic:Marine	0.6732	5m	133237	267749
MGYA00136704/ERZ475184_FASTA.fasta	MGYA00136704	ERZ475184	root:Environmental:Aquatic:Marine	0.6736	0m	42861	175352
MGYA00248719/ERZ505368_FASTA.fasta	MGYA00248719	ERZ505368	root:Environmental:Aquatic:Marine	0.6748	No depth information	139965	265044
MGYA00136628/ERZ475127_FASTA.fasta	MGYA00136628	ERZ475127	root:Environmental:Aquatic:Marine	0.6782	2m	28281	130597
MGYA00345666/ERZ802853_FASTA.fasta	MGYA00345666	ERZ802853	root:Environmental:Aquatic:Marine	0.6782	3	264302	459129
MGYA00136718/ERZ475129_FASTA.fasta	MGYA00136718	ERZ475129	root:Environmental:Aquatic:Marine	0.6808	2m	38890	158643
MGYA00248726/ERZ505369_FASTA.fasta	MGYA00248726	ERZ505369	root:Environmental:Aquatic:Marine	0.6818	No depth information	161860	299575
MGYA00345647/ERZ802819_FASTA.fasta	MGYA00345647	ERZ802819	root:Environmental:Aquatic:Marine	0.6826	3	327051	602419
MGYA00237794/ERZ508530_FASTA.fasta	MGYA00237794	ERZ508530	root:Environmental:Aquatic:Marine	0.6836	No depth information	66063	135456
MGYA00237800/ERZ508537_FASTA.fasta	MGYA00237800	ERZ508537	root:Environmental:Aquatic:Marine	0.6842	No depth information	188918	380979
MGYA00367578/ERZ838418_FASTA.fasta	MGYA00367578	ERZ838418	root:Environmental:Aquatic:Marine	0.6866	5m	158033	280982
MGYA00367651/ERZ841322_FASTA.fasta	MGYA00367651	ERZ841322	root:Environmental:Aquatic:Marine	0.6902	488m	173648	277621
MGYA00345655/ERZ802831_FASTA.fasta	MGYA00345655	ERZ802831	root:Environmental:Aquatic:Marine	0.6904	3	274731	507518
MGYA00367583/ERZ839095_FASTA.fasta	MGYA00367583	ERZ839095	root:Environmental:Aquatic:Marine	0.6928	75m	187262	335448
MGYA00136616/ERZ475214_FASTA.fasta	MGYA00136616	ERZ475214	root:Environmental:Aquatic:Marine	0.6934	1m	27013	107071
MGYA00367690/ERZ841705_FASTA.fasta	MGYA00367690	ERZ841705	root:Environmental:Aquatic:Marine	0.6946	5m	542837	974255
MGYA00345687/ERZ806371_FASTA.fasta	MGYA00345687	ERZ806371	root:Environmental:Aquatic:Marine	0.696	3	312322	501552
MGYA00345665/ERZ802851_FASTA.fasta	MGYA00345665	ERZ802851	root:Environmental:Aquatic:Marine	0.6968	3	260714	436058
MGYA00136647/ERZ475192_FASTA.fasta	MGYA00136647	ERZ475192	root:Environmental:Aquatic:Marine	0.697	3m	63208	235088
MGYA00367584/ERZ839150_FASTA.fasta	MGYA00367584	ERZ839150	root:Environmental:Aquatic:Marine	0.6976	50m	247512	453462
MGYA00136739/ERZ475233_FASTA.fasta	MGYA00136739	ERZ475233	root:Environmental:Aquatic:Marine	0.698	0m	41744	150507
MGYA00136673/ERZ475132_FASTA.fasta	MGYA00136673	ERZ475132	root:Environmental:Aquatic:Marine	0.699	2m	18085	88959
MGYA00367514/ERZ828948_FASTA.fasta	MGYA00367514	ERZ828948	root:Environmental:Aquatic:Marine	0.7024	5m	269129	502579
MGYA00367736/ERZ842124_FASTA.fasta	MGYA00367736	ERZ842124	root:Environmental:Aquatic:Marine	0.706	5m	362067	664615
MGYA00367729/ERZ842087_FASTA.fasta	MGYA00367729	ERZ842087	root:Environmental:Aquatic:Marine	0.7072	5m	328475	528368
MGYA00136695/ERZ475209_FASTA.fasta	MGYA00136695	ERZ475209	root:Environmental:Aquatic:Marine	0.711	1m	55896	236014
MGYA00096440/ERZ376968_FASTA_2.fasta	MGYA00096440	ERZ376968	root:Environmental:Aquatic:Marine	0.7118	No depth information	690219	3264949
MGYA00367755/ERZ376968_FASTA_2.fasta	MGYA00367755	ERZ376968	root:Environmental:Aquatic:Marine	0.712	No depth information	703058	3366815
MGYA00345649/ERZ802822_FASTA.fasta	MGYA00345649	ERZ802822	root:Environmental:Aquatic:Marine	0.7122	3	245745	493715
MGYA00367595/ERZ840327_FASTA.fasta	MGYA00367595	ERZ840327	root:Environmental:Aquatic:Marine	0.7134	17m	667078	1258968
MGYA00367731/ERZ842108_FASTA.fasta	MGYA00367731	ERZ842108	root:Environmental:Aquatic:Marine	0.7144	340m	374185	702558
MGYA00237793/ERZ508529_FASTA.fasta	MGYA00237793	ERZ508529	root:Environmental:Aquatic:Marine	0.7176	No depth information	52391	93785
MGYA00367694/ERZ841748_FASTA.fasta	MGYA00367694	ERZ841748	root:Environmental:Aquatic:Marine	0.7178	80m	701083	1376006
MGYA00367669/ERZ841459_FASTA.fasta	MGYA00367669	ERZ841459	root:Environmental:Aquatic:Marine	0.7182	50m	180867	314099
MGYA00345676/ERZ806358_FASTA.fasta	MGYA00345676	ERZ806358	root:Environmental:Aquatic:Marine	0.7192	3	527527	949659
MGYA00367563/ERZ835863_FASTA.fasta	MGYA00367563	ERZ835863	root:Environmental:Aquatic:Marine	0.7192	5m	818014	1613317
MGYA00367658/ERZ841394_FASTA.fasta	MGYA00367658	ERZ841394	root:Environmental:Aquatic:Marine	0.7192	60m	668603	1547420
MGYA00367711/ERZ841932_FASTA.fasta	MGYA00367711	ERZ841932	root:Environmental:Aquatic:Marine	0.7196	40m	252615	492890
MGYA00345678/ERZ806361_FASTA.fasta	MGYA00345678	ERZ806361	root:Environmental:Aquatic:Marine	0.72	3	533765	968670
MGYA00136743/ERZ475153_FASTA.fasta	MGYA00136743	ERZ475153	root:Environmental:Aquatic:Marine	0.7202	0m	74084	283045
MGYA00136618/ERZ475185_FASTA.fasta	MGYA00136618	ERZ475185	root:Environmental:Aquatic:Marine	0.7204	20m	44383	170895
MGYA00096440/ERZ376968_FASTA_1.fasta	MGYA00096440	ERZ376968	root:Environmental:Aquatic:Marine	0.7208	No depth information	946810	2736667
MGYA00367552/ERZ833884_FASTA.fasta	MGYA00367552	ERZ833884	root:Environmental:Aquatic:Marine	0.721	177m	160601	312630
MGYA00367511/ERZ828940_FASTA.fasta	MGYA00367511	ERZ828940	root:Environmental:Aquatic:Marine	0.7214	5m	264850	495905
MGYA00367737/ERZ842127_FASTA.fasta	MGYA00367737	ERZ842127	root:Environmental:Aquatic:Marine	0.722	5m	280217	495416
MGYA00367752/ERZ843989_FASTA.fasta	MGYA00367752	ERZ843989	root:Environmental:Aquatic:Marine	0.7264	5m	1041041	1892052
MGYA00249855/ERZ767000_FASTA.fasta	MGYA00249855	ERZ767000	root:Environmental:Aquatic:Marine	0.7274	No depth information	180976	317284
MGYA00375090/ERZ782917_FASTA.fasta	MGYA00375090	ERZ782917	root:Environmental:Aquatic:Marine	0.7278	No depth information	183711	320047
MGYA00367733/ERZ842116_FASTA.fasta	MGYA00367733	ERZ842116	root:Environmental:Aquatic:Marine	0.7284	5m	249776	489924
MGYA00136672/ERZ475147_FASTA.fasta	MGYA00136672	ERZ475147	root:Environmental:Aquatic:Marine	0.729	0.2m	62402	224254
MGYA00237670/ERZ505269_FASTA.fasta	MGYA00237670	ERZ505269	root:Environmental:Aquatic:Marine	0.729	No depth information	81646	125865
MGYA00345674/ERZ802873_FASTA.fasta	MGYA00345674	ERZ802873	root:Environmental:Aquatic:Marine	0.7298	3	356253	684167
MGYA00345650/ERZ802824_FASTA.fasta	MGYA00345650	ERZ802824	root:Environmental:Aquatic:Marine	0.7302	3	278588	514662
MGYA00345675/ERZ806357_FASTA.fasta	MGYA00345675	ERZ806357	root:Environmental:Aquatic:Marine	0.7304	3	445824	779533
MGYA00367521/ERZ829065_FASTA.fasta	MGYA00367521	ERZ829065	root:Environmental:Aquatic:Marine	0.7304	5m	239308	441961

MGYA00367695/ERZ841795_FASTA.fasta	MGYA00367695	ERZ841795	root:Environmental:Aquatic:Marine	0.731	700m		144531	250044
MGYA00345670/ERZ802864_FASTA.fasta	MGYA00345670	ERZ802864	root:Environmental:Aquatic:Marine	0.7312		3	463753	919374
MGYA00367628/ERZ840995_FASTA.fasta	MGYA00367628	ERZ840995	root:Environmental:Aquatic:Marine	0.7322	80m		236470	425024
MGYA00367701/ERZ841839_FASTA.fasta	MGYA00367701	ERZ841839	root:Environmental:Aquatic:Marine	0.7322	100m		248698	450837
MGYA00345672/ERZ802870_FASTA.fasta	MGYA00345672	ERZ802870	root:Environmental:Aquatic:Marine	0.7324		3	460026	836140
MGYA00345685/ERZ806368_FASTA.fasta	MGYA00345685	ERZ806368	root:Environmental:Aquatic:Marine	0.7324		3	602393	1164783
MGYA00136684/ERZ475235_FASTA.fasta	MGYA00136684	ERZ475235	root:Environmental:Aquatic:Marine	0.7326	1m		36573	111543
MGYA00367530/ERZ829971_FASTA.fasta	MGYA00367530	ERZ829971	root:Environmental:Aquatic:Marine	0.7326	177m		245028	502070
MGYA00367513/ERZ828946_FASTA.fasta	MGYA00367513	ERZ828946	root:Environmental:Aquatic:Marine	0.733	55m		261256	509982
MGYA00237785/ERZ508519_FASTA.fasta	MGYA00237785	ERZ508519	root:Environmental:Aquatic:Marine	0.7332	No depth information		17047	71104
MGYA00248728/ERZ505373_FASTA.fasta	MGYA00248728	ERZ505373	root:Environmental:Aquatic:Marine	0.7344	No depth information		43748	78927
MGYA00345679/ERZ806362_FASTA.fasta	MGYA00345679	ERZ806362	root:Environmental:Aquatic:Marine	0.7346		3	493987	879833
MGYA00367512/ERZ828944_FASTA.fasta	MGYA00367512	ERZ828944	root:Environmental:Aquatic:Marine	0.737	55m		264628	517504
MGYA00237878/ERZ529328_FASTA.fasta	MGYA00237878	ERZ529328	root:Environmental:Aquatic:Marine	0.7372	No depth information		5263	19504
MGYA00367683/ERZ841638_FASTA.fasta	MGYA00367683	ERZ841638	root:Environmental:Aquatic:Marine	0.738	150m		368011	642952
MGYA00248615/ERZ508603_FASTA.fasta	MGYA00248615	ERZ508603	root:Environmental:Aquatic:Marine	0.739	No depth information		20126	86604
MGYA00367621/ERZ840919_FASTA.fasta	MGYA00367621	ERZ840919	root:Environmental:Aquatic:Marine	0.739	5m		648917	1158311
MGYA00136671/ERZ475234_FASTA.fasta	MGYA00136671	ERZ475234	root:Environmental:Aquatic:Marine	0.7402	10m		29270	109568
MGYA00367573/ERZ837530_FASTA.fasta	MGYA00367573	ERZ837530	root:Environmental:Aquatic:Marine	0.7412	5m		592668	1162240
MGYA00367557/ERZ834839_FASTA.fasta	MGYA00367557	ERZ834839	root:Environmental:Aquatic:Marine	0.742	40m		249022	489859
MGYA00345680/ERZ806363_FASTA.fasta	MGYA00345680	ERZ806363	root:Environmental:Aquatic:Marine	0.7424		3	453545	809974
MGYA00367706/ERZ841884_FASTA.fasta	MGYA00367706	ERZ841884	root:Environmental:Aquatic:Marine	0.7428	5m		750520	1356038
MGYA00237890/ERZ534239_FASTA.fasta	MGYA00237890	ERZ534239	root:Environmental:Aquatic:Marine	0.7444	No depth information		351884	818334
MGYA00367598/ERZ840530_FASTA.fasta	MGYA00367598	ERZ840530	root:Environmental:Aquatic:Marine	0.745	600m		169117	314374
MGYA00367698/ERZ841818_FASTA.fasta	MGYA00367698	ERZ841818	root:Environmental:Aquatic:Marine	0.745	5m		202901	391894
MGYA00345691/ERZ806375_FASTA.fasta	MGYA00345691	ERZ806375	root:Environmental:Aquatic:Marine	0.747		3	645421	1270185
MGYA00367510/ERZ828934_FASTA.fasta	MGYA00367510	ERZ828934	root:Environmental:Aquatic:Marine	0.7482	25m		556953	911143
MGYA00367674/ERZ841477_FASTA.fasta	MGYA00367674	ERZ841477	root:Environmental:Aquatic:Marine	0.7484	30m		217946	397488
MGYA00367523/ERZ829332_FASTA.fasta	MGYA00367523	ERZ829332	root:Environmental:Aquatic:Marine	0.7492	5m		143323	248161
MGYA00345671/ERZ802866_FASTA.fasta	MGYA00345671	ERZ802866	root:Environmental:Aquatic:Marine	0.7496		3	448516	891500
MGYA00367688/ERZ841695_FASTA.fasta	MGYA00367688	ERZ841695	root:Environmental:Aquatic:Marine	0.7496	800m		125808	220945
MGYA00367650/ERZ841319_FASTA.fasta	MGYA00367650	ERZ841319	root:Environmental:Aquatic:Marine	0.75	120m		345649	634273
MGYA00345677/ERZ806360_FASTA.fasta	MGYA00345677	ERZ806360	root:Environmental:Aquatic:Marine	0.7522		3	525884	930084
MGYA00345696/ERZ806380_FASTA.fasta	MGYA00345696	ERZ806380	root:Environmental:Aquatic:Marine	0.7522		3	690888	1109653
MGYA00367646/ERZ841276_FASTA.fasta	MGYA00367646	ERZ841276	root:Environmental:Aquatic:Marine	0.7522	5m		251689	453967
MGYA00237797/ERZ508534_FASTA.fasta	MGYA00237797	ERZ508534	root:Environmental:Aquatic:Marine	0.7528	No depth information		77520	151149
MGYA00367735/ERZ842122_FASTA.fasta	MGYA00367735	ERZ842122	root:Environmental:Aquatic:Marine	0.753	270m		200591	385183
MGYA00367739/ERZ842135_FASTA.fasta	MGYA00367739	ERZ842135	root:Environmental:Aquatic:Marine	0.7534	5m		128760	221848
MGYA00367533/ERZ830869_FASTA.fasta	MGYA00367533	ERZ830869	root:Environmental:Aquatic:Marine	0.7556	150m		378354	662429
MGYA00367665/ERZ841436_FASTA.fasta	MGYA00367665	ERZ841436	root:Environmental:Aquatic:Marine	0.7564	40m		252592	497008
MGYA00345654/ERZ802830_FASTA.fasta	MGYA00345654	ERZ802830	root:Environmental:Aquatic:Marine	0.7572		3	534732	1065267
MGYA00367648/ERZ841312_FASTA.fasta	MGYA00367648	ERZ841312	root:Environmental:Aquatic:Marine	0.7584	25m		605804	1192613
MGYA00367710/ERZ841929_FASTA.fasta	MGYA00367710	ERZ841929	root:Environmental:Aquatic:Marine	0.7586	5m		395053	719063
MGYA00367680/ERZ841566_FASTA.fasta	MGYA00367680	ERZ841566	root:Environmental:Aquatic:Marine	0.759	5m		233751	443370
MGYA00248754/ERZ505257_FASTA.fasta	MGYA00248754	ERZ505257	root:Environmental:Aquatic:Marine	0.7592	No depth information		70333	134884
MGYA00345663/ERZ802847_FASTA.fasta	MGYA00345663	ERZ802847	root:Environmental:Aquatic:Marine	0.7596		3	452529	899066
MGYA00345695/ERZ806379_FASTA.fasta	MGYA00345695	ERZ806379	root:Environmental:Aquatic:Marine	0.7596		3	791838	1482820
MGYA00367725/ERZ842061_FASTA.fasta	MGYA00367725	ERZ842061	root:Environmental:Aquatic:Marine	0.7608	120m		339099	618583
MGYA00345686/ERZ806370_FASTA.fasta	MGYA00345686	ERZ806370	root:Environmental:Aquatic:Marine	0.7626		3	627539	1211110
MGYA00367602/ERZ840559_FASTA.fasta	MGYA00367602	ERZ840559	root:Environmental:Aquatic:Marine	0.7628	5m		241108	451202
MGYA00367594/ERZ840267_FASTA.fasta	MGYA00367594	ERZ840267	root:Environmental:Aquatic:Marine	0.7634	40m		181045	350354
MGYA00375066/ERZ781374_FASTA.fasta	MGYA00375066	ERZ781374	root:Environmental:Aquatic:Marine	0.7644	No depth information		462492	811838
MGYA00367676/ERZ841483_FASTA.fasta	MGYA00367676	ERZ841483	root:Environmental:Aquatic:Marine	0.7648	30m		433432	769342
MGYA00367670/ERZ841462_FASTA.fasta	MGYA00367670	ERZ841462	root:Environmental:Aquatic:Marine	0.7654	90m		169234	279269
MGYA00367524/ERZ829407_FASTA.fasta	MGYA00367524	ERZ829407	root:Environmental:Aquatic:Marine	0.7656	177m		466348	953855
MGYA00237876/ERZ529329_FASTA.fasta	MGYA00237876	ERZ529329	root:Environmental:Aquatic:Marine	0.7658	No depth information		5580	26600
MGYA00345661/ERZ802844_FASTA.fasta	MGYA00345661	ERZ802844	root:Environmental:Aquatic:Marine	0.7664		3	500823	951696
MGYA00367607/ERZ840587_FASTA.fasta	MGYA00367607	ERZ840587	root:Environmental:Aquatic:Marine	0.7672	150m		120054	202902
MGYA00367625/ERZ840950_FASTA.fasta	MGYA00367625	ERZ840950	root:Environmental:Aquatic:Marine	0.7672	5m		648650	1151448
MGYA00367604/ERZ840571_FASTA.fasta	MGYA00367604	ERZ840571	root:Environmental:Aquatic:Marine	0.7676	270m		419537	813462
MGYA00375068/ERZ781376_FASTA.fasta	MGYA00375068	ERZ781376	root:Environmental:Aquatic:Marine	0.769	No depth information		287877	493665
MGYA00367593/ERZ840062_FASTA.fasta	MGYA00367593	ERZ840062	root:Environmental:Aquatic:Marine	0.7694	66m		833596	1578150
MGYA00367672/ERZ841467_FASTA.fasta	MGYA00367672	ERZ841467	root:Environmental:Aquatic:Marine	0.7694	5m		608783	1290587
MGYA00367633/ERZ841040_FASTA.fasta	MGYA00367633	ERZ841040	root:Environmental:Aquatic:Marine	0.7698	5m		244148	524025
MGYA00248555/ERZ529325_FASTA.fasta	MGYA00248555	ERZ529325	root:Environmental:Aquatic:Marine	0.77		50	37336	89909
MGYA00237653/ERZ505226_FASTA.fasta	MGYA00237653	ERZ505226	root:Environmental:Aquatic:Marine	0.7712	No depth information		95392	172282
MGYA00136696/ERZ475210_FASTA.fasta	MGYA00136696	ERZ475210	root:Environmental:Aquatic:Marine	0.7716	1m		73148	302615
MGYA00237669/ERZ505265_FASTA.fasta	MGYA00237669	ERZ505265	root:Environmental:Aquatic:Marine	0.7718	No depth information		103805	186256
MGYA00367643/ERZ841196_FASTA.fasta	MGYA00367643	ERZ841196	root:Environmental:Aquatic:Marine	0.7724	5m		835909	1553247
MGYA00367637/ERZ841132_FASTA.fasta	MGYA00367637	ERZ841132	root:Environmental:Aquatic:Marine	0.7732	40m		251711	495246
MGYA00237791/ERZ508527_FASTA.fasta	MGYA00237791	ERZ508527	root:Environmental:Aquatic:Marine	0.7736	No depth information		96314	171579
MGYA00367691/ERZ841738_FASTA.fasta	MGYA00367691	ERZ841738	root:Environmental:Aquatic:Marine	0.774	375m		253715	467596
MGYA00237805/ERZ508546_FASTA.fasta	MGYA00237805	ERZ508546	root:Environmental:Aquatic:Marine	0.7748	No depth information		120127	286174
MGYA00237875/ERZ529327_FASTA.fasta	MGYA00237875	ERZ529327	root:Environmental:Aquatic:Marine	0.7748	No depth information		3591	22253
MGYA00136662/ERZ475166_FASTA.fasta	MGYA00136662	ERZ475166	root:Environmental:Aquatic:Marine	0.7752	0m		54227	253922
MGYA00367721/ERZ842008_FASTA.fasta	MGYA00367721	ERZ842008	root:Environmental:Aquatic:Marine	0.7762	150m		364275	634873
MGYA00367582/ERZ839027_FASTA.fasta	MGYA00367582	ERZ839027	root:Environmental:Aquatic:Marine	0.778	375m		251527	463771
MGYA00240995/ERZ534231_FASTA.fasta	MGYA00240995	ERZ534231	root:Environmental:Aquatic:Marine	0.7782	No depth information		301178	644676
MGYA00367570/ERZ836958_FASTA.fasta	MGYA00367570	ERZ836958	root:Environmental:Aquatic:Marine	0.7784	75m		880848	1587528
MGYA00367559/ERZ835002_FASTA.fasta	MGYA00367559	ERZ835002	root:Environmental:Aquatic:Marine	0.7792	5m		864045	1531295
MGYA00132146/ERZ312764_FASTA.fasta	MGYA00132146	ERZ312764	root:Environmental:Aquatic:Marine	0.7794		74	4254	164405
MGYA00248733/ERZ505383_FASTA.fasta	MGYA00248733	ERZ505383	root:Environmental:Aquatic:Marine	0.7796	No depth information		58728	95784
MGYA00237798/ERZ508535_FASTA.fasta	MGYA00237798	ERZ508535	root:Environmental:Aquatic:Marine	0.7796	No depth information		143780	290504
MGYA00248616/ERZ508602_FASTA.fasta	MGYA00248616	ERZ508602	root:Environmental:Aquatic:Marine	0.781	No depth information		15081	51290
MGYA00367517/ERZ829055_FASTA.fasta	MGYA00367517	ERZ829055	root:Environmental:Aquatic:Marine	0.781	70m		470254	964186
MGYA00367532/ERZ830535_FASTA.fasta	MGYA00367532	ERZ830535	root:Environmental:Aquatic:Marine	0.7818	5m		769985	1449637
MGYA00248751/ERZ505283_FASTA.fasta	MGYA00248751	ERZ505283	root:Environmental:Aquatic:Marine	0.7822	No depth information		101192	168856
MGYA00367519/ERZ829061_FASTA.fasta	MGYA00367519	ERZ829061	root:Environmental:Aquatic:Marine	0.7826	70m		425937	1001828
MGYA00367716/ERZ841976_FASTA.fasta	MGYA00367716	ERZ841976	root:Environmental:Aquatic:Marine	0.7826	375m		253102	462678
MGYA00240993/ERZ508538_FASTA.fasta	MGYA00240993	ERZ508538	root:Environmental:Aquatic:Marine	0.7852	No depth information		187875	366571
MGYA00367713/ERZ841954_FASTA.fasta	MGYA00367713	ERZ841954	root:Environmental:Aquatic:Marine	0.7854	800m		123617	231272
MGYA00240992/ERZ505281_FASTA.fasta	MGYA00240992	ERZ505281	root:Environmental:Aquatic:Marine	0.786	No depth information		43266	80316
MGYA00367562/ERZ835625_FASTA.fasta	MGYA00367562	ERZ835625	root:Environmental:Aquatic:Marine	0.7872	5m		712373	1275987
MGYA00237789/ERZ508523_FASTA.fasta	MGYA00237789	ERZ508523	root:Environmental:Aquatic:Marine	0.7872	No depth information		89969	163093
MGYA00367618/ERZ840877_FASTA.fasta	MGYA00367618	ERZ840877	root:Environmental:Aquatic:Marine	0.7874	450m		241191	449656
MGYA00237787/ERZ508521_FASTA.fasta	MGYA00237787	ERZ508521	root:Environmental:Aquatic:Marine	0.7886	No depth information		404192	885579
MGYA00367566/ERZ836272_FASTA.fasta	MGYA00367566	ERZ836272	root:Environmental:Aquatic:Marine	0.7892	150m		369388	645679
MGYA00345664/ERZ802849_FASTA.fasta	MGYA00345664	ERZ802849	root:Environmental:Aquatic:Marine	0.7894		3	359114	628618
MGYA00367558/ERZ834897_FASTA.fasta	MGYA00367558	ERZ834897	root:Environmental:Aquatic:Marine	0.7898	120m		345022	631861
MGYA00367591/ERZ839901_FASTA.fasta	MGYA00367591	ERZ839901	root:Environmental:Aquatic:Marine	0.79	5m		773585	1404630
MGYA00237879/ERZ529330_FASTA.fasta	MGYA00237879	ERZ529330	root:Environmental:Aquatic:Marine	0.791	No depth information		3131	30592
MGYA00237672/ERZ505275_FASTA.fasta	MGYA00237672	ERZ505275	root:Environmental:Aquatic:Marine	0.7912	No depth information		106755	213429
MGYA00367685/ERZ841657_FASTA.fasta	MGYA00367685	ERZ841657	root:Environmental:Aquatic:Marine	0.7916	5m		237795	448481
MGYA00367622/ERZ840932_FASTA.fasta	MGYA00367622	ERZ840932	root:Environmental:Aquatic:Marine	0.7918	480m		111800	204957
MGYA00248750/ERZ505293_FASTA.fasta	MGYA00248750	ERZ505293	root:Environmental:Aquatic:Marine	0.7922	No depth information		120488	220955
MGYA00367548/ERZ833381_FASTA.fasta	MGYA00367548	ERZ833381	root:Environmental:Aquatic:Marine	0.7928	60m		259202	463486
MGYA00237651/ERZ505222_FASTA.fasta	MGYA00237651	ERZ505222	root:Environmental:Aquatic:Marine	0.7934	No depth information		106233	216898
MGYA00237847/ERZ508605_FASTA.fasta	MGYA00237847	ERZ508605	root:Environmental:Aquatic:Marine	0.7936	No depth information		29224	82718
MGYA00367590/ERZ839733_FASTA.fasta	MGYA00367590	ERZ839733	root:Environmental:Aquatic:Marine	0.7948	65m		492013	845772
MGYA00367751/ERZ843936_FASTA.fasta	MGYA00367751	ERZ843936	root:Environmental:Aquatic:Marine	0.7948	5m		1078988	1850060
MGYA00367571/ERZ837174_FASTA.fasta	MGYA00367571	ERZ837174	root:Environmental:Aquatic:Marine	0.795	450m		237048	442909
MGYA00367636/ERZ841130_FASTA.fasta	MGYA00367636	ERZ841130	root:Environmental:Aquatic:Marine	0.7952	5m		257606	466294
MGYA00367667/ERZ841449_FASTA.fasta	MGYA00367667	ERZ841449	root:Environmental:Aquatic:Marine	0.7956	120m		893235	1538071
MGYA00248722/ERZ505374_FASTA.fasta	MGYA00248722	ERZ505374	root:Environmental:Aquatic:Marine	0.796	No depth information		66952	118185
MGYA00367550/ERZ833580_FASTA.fasta	MGYA00367550	ERZ833580	root:Environmental:Aquatic:Marine	0.796	5m		722303	1363604
MGYA00375057/ERZ781365_FASTA.fasta	MGYA00375057	ERZ781365	root:Environmental:Aquatic:Marine	0.796	No depth information		404173	717253
MGYA00237663/ERZ505246_FASTA.fasta	MGYA00237663	ERZ505246	root:Environmental:Aquatic:Marine	0.7964	No depth information		108977	217426
MGYA00367660/ERZ841404_FASTA.fasta	MGYA00367660	ERZ841404	root:Environmental:Aquatic:Marine	0.7966	50m		697131	1572723
MGYA00367715/ERZ841973_FASTA.fasta	MGYA00367715	ERZ841973	root:Environmental:Aquatic:Marine	0.7966	5m		248191	447104
MGYA00367627/ERZ840991_FASTA.fasta	MGYA00367627	ERZ840991	root:Environmental:Aquatic:Marine	0.7974	450m		241637	451186
MGYA00367597/ERZ840511_FASTA.fasta	MGYA00367597	ERZ840511	root:Environmental:Aquatic:Marine	0.798	5m		843058	1487389
MGYA00248755/ERZ505250_FASTA.fasta	MGYA00248755	ERZ505250	root:Environmental:Aquatic:Marine	0.7982	No depth information		138993	299654
MGYA00367663/ERZ841426_FASTA.fasta	MGYA00367663	ERZ841426	root:Environmental:Aquatic:Marine	0.7982	80m		701001	1377807
MGYA00367515/ERZ828950_FASTA.fasta	MGYA00367515	ERZ828950	root:Environmental:Aquatic:Marine	0.7984	5m		343922	927101
MGYA00367679/ERZ841558_FASTA.fasta	MGYA00367679	ERZ841558	root:Environmental:Aquatic:Marine	0.7984	50m		187343	326228
MGYA00367677/ERZ841489_FASTA.fasta	MGYA00367677	ERZ841489	root:Environmental:Aquatic:Marine	0.7992	60m		953635	1937356
MGYA00237659/ERZ505238_FASTA.fasta	MGYA00237659	ERZ505238	root:Environmental:Aquatic:Marine	0.7992	No depth information		77995	158909
MGYA00367738/ERZ842130_FASTA.fasta	MGYA00367738	ERZ842130	root:Environmental:Aquatic:Marine	0.7994	450m		276925	496273
MGYA00367629/ERZ841001_FASTA.fasta	MGYA00367629	ERZ841001	root:Environmental:Aquatic:Marine	0.8014	600m		787013	1493738
MGYA00367732/ERZ842113_FASTA.fasta	MGYA00367732	ERZ842113	root:Environmental:Aquatic:Marine	0.8016	40m		431230	800269
MGYA00248756/ERZ505249_FASTA.fasta	MGYA00248756	ERZ505249	root:Environmental:Aquatic:Marine	0.8022	No depth information		101451	179008
MGYA00367749/ERZ843394_FASTA.fasta	MGYA00367749	ERZ843394	root:Environmental:Aquatic:Marine	0.8022	488m		1134784	1863570
MGYA00367601/ERZ840554_FASTA.fasta	MGYA00367601	ERZ840554	root:Environmental:Aquatic:Marine	0.8024	850m		203275	361260
MGYA00367702/ERZ841842_FASTA.fasta	MGYA00367702	ERZ841842	root:Environmental:Aquatic:Marine	0.8024	5m		516783	1179264
MGYA00345681/ERZ806364_FASTA.fasta	MGYA00345681	ERZ806364	root:Environmental:Aquatic:Marine	0.8026		3	741307	1350128
MGYA00367655/ERZ841381_FASTA.fasta	MGYA00367655	ERZ841381	root:Environmental:Aquatic:Marine	0.8026	120m		344710	630938
MGYA00248632/ERZ508511_FASTA.fasta	MGYA00248632	ERZ508511	root:Environmental:Aquatic:Marine	0.8028	No depth information		36193	114276
MGYA00367709/ERZ841911_FASTA.fasta	MGYA00367709	ERZ841911	root:Environmental:Aquatic:Marine	0.8034	25m		709823	1336030
MGYA00367753/ERZ844199_FASTA.fasta	MGYA00367753	ERZ844199	root:Environmental:Aquatic:Marine	0.8046	5m		1046220	1790677
MGYA00237655/ERZ505230_FASTA.fasta	MGYA00237655	ERZ505230	root:Environmental:Aquatic:Marine	0.8046	No depth information		83551	185953
MGYA00237799/ERZ508536_FASTA.fasta	MGYA00237799	ERZ508536	root:Environmental:Aquatic:Marine	0.8048	No depth information		264187	502256
MGYA00367718/ERZ841980_FASTA.fasta	MGYA00367718	ERZ841980	root:Environmental:Aquatic:Marine	0.805	800m		160281	280044
MGYA00367703/ERZ841863_FASTA.fasta	MGYA00367703	ERZ841863	root:Environmental:Aquatic:Marine	0.8056	5m		667808	1232286
MGYA00367610/ERZ840593_FASTA.fasta	MGYA00367610	ERZ840593	root:Environmental:Aquatic:Marine	0.8068	5m		711696	1449726
MGYA00248631/ERZ508512_FASTA.fasta	MGYA00248631	ERZ508512	root:Environmental:Aquatic:Marine	0.807	No depth information		36883	108714
MGYA00237676/ERZ505291_FASTA.fasta	MGYA00237676	ERZ505291	root:Environmental:Aquatic:Marine	0.8072	No depth information		136963	301942
MGYA00367516/ERZ829053_FASTA.fasta	MGYA00367516	ERZ829053	root:Environmental:Aquatic:Marine	0.8076	5m		376716	748456
MGYA00249097/ERZ651159_FASTA.fasta	MGYA00249097	ERZ651159	root:Environmental:Aquatic:Marine	0.808	No depth information		87772	172476
MGYA00237660/ERZ505240_FASTA.fasta	MGYA00237660	ERZ505240	root:Environmental:Aquatic:Marine	0.8082	No depth information		102664	201755
MGYA00367727/ERZ842083_FASTA.fasta	MGYA00367727	ERZ842083	root:Environmental:Aquatic:Marine	0.8084	5m		489334	987439
MGYA00248558/ERZ511489_FASTA.fasta	MGYA00248558	ERZ511489	root:Environmental:Aquatic:Marine	0.8086	No depth information		19224	92633
MGYA00367641/ERZ841176_FASTA.fasta	MGYA00367641	ERZ841176	root:Environmental:Aquatic:Marine	0.8086	5m		531494	1176937
MGYA00367671/ERZ841464_FASTA.fasta	MGYA00367671	ERZ841464	root:Environmental:Aquatic:Marine	0.8094	5m		157343	277291
MGYA00237666/ERZ505259_FASTA.fasta	MGYA00237666	ERZ505259	root:Environmental:Aquatic:Marine	0.8096	No depth information		111186	196205
MGYA00237845/ERZ508601_FASTA.fasta	MGYA00237845	ERZ508601	root:Environmental:Aquatic:Marine	0.8096	No depth information		42447	111072
MGYA00367620/ERZ840905_FASTA.fasta	MGYA00367620	ERZ840905	root:Environmental:Aquatic:Marine	0.81	60m		681428	1632201
MGYA00248729/ERZ505375_FASTA.fasta	MGYA00248729	ERZ505375	root:Environmental:Aquatic:Marine	0.8108	No depth information		97895	150283
MGYA00367642/ERZ841189_FASTA.fasta	MGYA00367642	ERZ841189	root:Environmental:Aquatic:Marine	0.811	375m		252949	466239
MGYA00367569/ERZ836686_FASTA.fasta	MGYA00367569	ERZ836686	root:Environmental:Aquatic:Marine	0.8116	5-160m		819006	1723752
MGYA00237804/ERZ508545_FASTA.fasta	MGYA00237804	ERZ508545	root:Environmental:Aquatic:Marine	0.8118	No depth information		239146	564230
MGYA00237645/ERZ505209_FASTA.fasta	MGYA00237645	ERZ505209	root:Environmental:Aquatic:Marine	0.812	No depth information		109086	187872
MGYA00375069/ERZ781377_FASTA.fasta	MGYA00375069	ERZ781377	root:Environmental:Aquatic:Marine	0.8126	No depth information		355891	657554
MGYA00367603/ERZ840564_FASTA.fasta	MGYA00367603	ERZ840564	root:Environmental:Aquatic:Marine	0.8142	5m		251581	453041
MGYA00367712/ERZ841934_FASTA.fasta	MGYA00367712	ERZ841934	root:Environmental:Aquatic:Marine	0.8146	150m		827999	1428588
MGYA00237661/ERZ505243_FASTA.fasta	MGYA00237661	ERZ505243	root:Environmental:Aquatic:Marine	0.8154	No depth information		90483	181320
MGYA00367537/ERZ831446_FASTA.fasta	MGYA00367537	ERZ831446	root:Environmental:Aquatic:Marine	0.8158	5m		954365	1856329
MGYA00367714/ERZ841958_FASTA.fasta	MGYA00367714	ERZ841958	root:Environmental:Aquatic:Marine	0.8158	5m		882783	1509913
MGYA00237667/ERZ505261_FASTA.fasta	MGYA00237667	ERZ505261	root:Environmental:Aquatic:Marine	0.8158	No depth information		123586	206856
MGYA00367632/ERZ841028_FASTA.fasta	MGYA00367632	ERZ841028	root:Environmental:Aquatic:Marine	0.8162	30m		462700	977999
MGYA00237556/ERZ505227_FASTA.fasta	MGYA00237556	ERZ505227	root:Environmental:Aquatic:Marine	0.8174	No depth information		109152	226772
MGYA00345662/ERZ802846_FASTA.fasta	MGYA00345662	ERZ802846	root:Environmental:Aquatic:Marine	0.8182		3	482029	987652
MGYA00248625/ERZ508552_FASTA.fasta	MGYA00248625	ERZ508552	root:Environmental:Aquatic:Marine	0.8186	No depth information		253659	501792
MGYA00367662/ERZ841423_FASTA.fasta	MGYA00367662	ERZ841423	root:Environmental:Aquatic:Marine	0.819	600m		246267	440507
MGYA00367576/ERZ838294_FASTA.fasta	MGYA00367576	ERZ838294	root:Environmental:Aquatic:Marine	0.8218	5m		336776	636006
MGYA00367606/ERZ840584_FASTA.fasta	MGYA00367606	ERZ840584	root:Environmental:Aquatic:Marine	0.8222	5m		871862	1505861
MGYA00367616/ERZ840857_FASTA.fasta	MGYA00367616	ERZ840857	root:Environmental:Aquatic:Marine	0.8222	5m		345135	688361
MGYA00367640/ERZ841154_FASTA.fasta	MGYA00367640	ERZ841154	root:Environmental:Aquatic:Marine	0.8232	5m		764796	1392696
MGYA00367745/ERZ842408_FASTA.fasta	MGYA00367745	ERZ842408	root:Environmental:Aquatic:Marine	0.8236	115m		1341467	2354269
MGYA00248752/ERZ505273_FASTA.fasta	MGYA00248752	ERZ505273	root:Environmental:Aquatic:Marine	0.824	No depth information		133975	242291
MGYA00367684/ERZ841645_FASTA.fasta	MGYA00367684	ERZ841645	root:Environmental:Aquatic:Marine	0.824	5m		799494	1435751
MGYA00237652/ERZ505224_FASTA.fasta	MGYA00237652	ERZ505224	root:Environmental:Aquatic:Marine	0.8242	No depth information		128232	269662
MGYA00238135/ERZ651158_FASTA.fasta	MGYA00238135	ERZ651158	root:Environmental:Aquatic:Marine	0.8242	No depth information		151178	335565
MGYA00237784/ERZ508517_FASTA.fasta	MGYA00237784	ERZ508517	root:Environmental:Aquatic:Marine	0.825	No depth information		39333	126251
MGYA00367747/ERZ842953_FASTA.fasta	MGYA00367747	ERZ842953	root:Environmental:Aquatic:Marine	0.8256	90m		1131858	2038984
MGYA00136634/ERZ475164_FASTA.fasta	MGYA00136634	ERZ475164	root:Environmental:Aquatic:Marine	0.8264	0m		66591	288644
MGYA00237780/ERZ508510_FASTA.fasta	MGYA00237780	ERZ508510	root:Environmental:Aquatic:Marine	0.8268	No depth information		23233	68134
MGYA00367588/ERZ839637_FASTA.fasta	MGYA00367588	ERZ839637	root:Environmental:Aquatic:Marine	0.8274	1000m		186767	322200
MGYA00237649/ERZ505219_FASTA.fasta	MGYA00237649	ERZ505219	root:Environmental:Aquatic:Marine	0.8274	No depth information		160388	323697
MGYA00237678/ERZ505297_FASTA.fasta	MGYA00237678	ERZ505297	root:Environmental:Aquatic:Marine	0.8274	No depth information		133651	285791
MGYA00237675/ERZ505285_FASTA.fasta	MGYA00237675	ERZ505285	root:Environmental:Aquatic:Marine	0.8288	No depth information		138111	291119
MGYA00367567/ERZ836337_FASTA.fasta	MGYA00367567	ERZ836337	root:Environmental:Aquatic:Marine	0.8322	600m		249732	446728
MGYA00375059/ERZ781367_FASTA.fasta	MGYA00375059	ERZ781367	root:Environmental:Aquatic:Marine	0.8322	No depth information		341411	601712
MGYA00367540/ERZ832104_FASTA.fasta	MGYA00367540	ERZ832104	root:Environmental:Aquatic:Marine	0.8326	5m		667225	1220694
MGYA00238065/ERZ584658_FASTA.fasta	MGYA00238065	ERZ584658	root:Environmental:Aquatic:Marine	0.833	No depth information		216618	609939
MGYA00367626/ERZ840959_FASTA.fasta	MGYA00367626	ERZ840959	root:Environmental:Aquatic:Marine	0.8334	5m		553143	1098076
MGYA00248753/ERZ505268_FASTA.fasta	MGYA00248753	ERZ505268	root:Environmental:Aquatic:Marine	0.8338	No depth information		164488	332870
MGYA00248629/ERZ508524_FASTA.fasta	MGYA00248629	ERZ508524	root:Environmental:Aquatic:Marine	0.834	No depth information		160776	317939
MGYA00237792/ERZ508528_FASTA.fasta	MGYA00237792	ERZ508528	root:Environmental:Aquatic:Marine	0.8342	No depth information		85275	170330
MGYA00367689/ERZ841701_FASTA.fasta	MGYA00367689	ERZ841701	root:Environmental:Aquatic:Marine	0.8356	790m		209290	376643
MGYA00367634/ERZ841112_FASTA.fasta	MGYA00367634	ERZ841112	root:Environmental:Aquatic:Marine	0.8362	380m		170942	308914
MGYA00248557/ERZ529324_FASTA.fasta	MGYA00248557	ERZ529324	root:Environmental:Aquatic:Marine	0.84		50	36387	92021
MGYA00237654/ERZ505229_FASTA.fasta	MGYA00237654	ERZ505229	root:Environmental:Aquatic:Marine	0.842	No depth information		123474	272817
MGYA00367541/ERZ832643_FASTA.fasta	MGYA00367541	ERZ832643	root:Environmental:Aquatic:Marine	0.8424	5m		796665	1707677
MGYA00237673/ERZ505277_FASTA.fasta	MGYA00237673	ERZ505277	root:Environmental:Aquatic:Marine	0.8424	No depth information		132698	279780
MGYA00367644/ERZ841232_FASTA.fasta	MGYA00367644	ERZ841232	root:Environmental:Aquatic:Marine	0.8434	5m		844235	1510973
MGYA00367600/ERZ840547_FASTA.fasta	MGYA00367600	ERZ840547	root:Environmental:Aquatic:Marine	0.8436	600m		250548	447576
MGYA00367647/ERZ841280_FASTA.fasta	MGYA00367647	ERZ841280	root:Environmental:Aquatic:Marine	0.8436	50m		844574	1566185
MGYA00367743/ERZ842402_FASTA.fasta	MGYA00367743	ERZ842402	root:Environmental:Aquatic:Marine	0.8436	115m		1002666	1980655
MGYA00237848/ERZ508608_FASTA.fasta	MGYA00237848	ERZ508608	root:Environmental:Aquatic:Marine	0.8438	No depth information		41166	117065
MGYA00367726/ERZ842064_FASTA.fasta	MGYA00367726	ERZ842064	root:Environmental:Aquatic:Marine	0.8442	5m		947882	1768667
MGYA00367653/ERZ841349_FASTA.fasta	MGYA00367653	ERZ841349	root:Environmental:Aquatic:Marine	0.8444	60m		952299	1689031
MGYA00237677/ERZ505295_FASTA.fasta	MGYA00237677	ERZ505295	root:Environmental:Aquatic:Marine	0.8452	No depth information		102394	218480
MGYA00367692/ERZ841741_FASTA.fasta	MGYA00367692	ERZ841741	root:Environmental:Aquatic:Marine	0.8454	480m		202056	380066
MGYA00367728/ERZ842085_FASTA.fasta	MGYA00367728	ERZ842085	root:Environmental:Aquatic:Marine	0.8454	1000m		293637	506154
MGYA00237790/ERZ508526_FASTA.fasta	MGYA00237790	ERZ508526	root:Environmental:Aquatic:Marine	0.8456	No depth information		137162	236231
MGYA00367581/ERZ838778_FASTA.fasta	MGYA00367581	ERZ838778	root:Environmental:Aquatic:Marine	0.8464	50m		893317	1877797
MGYA00367574/ERZ837885_FASTA.fasta	MGYA00367574	ERZ837885	root:Environmental:Aquatic:Marine	0.8466	40m		795024	1489879
MGYA00237668/ERZ505262_FASTA.fasta	MGYA00237668	ERZ505262	root:Environmental:Aquatic:Marine	0.8468	No depth information		130134	274800
MGYA00237810/ERZ508553_FASTA.fasta	MGYA00237810	ERZ508553	root:Environmental:Aquatic:Marine	0.8468	No depth information		199938	443756
MGYA00375058/ERZ781366_FASTA.fasta	MGYA00375058	ERZ781366	root:Environmental:Aquatic:Marine	0.848	No depth information		374551	669639
MGYA00237778/ERZ508506_FASTA.fasta	MGYA00237778	ERZ508506	root:Environmental:Aquatic:Marine	0.8502	No depth information		249787	560428
MGYA00367554/ERZ834050_FASTA.fasta	MGYA00367554	ERZ834050	root:Environmental:Aquatic:Marine	0.8504	5m		912979	1804019
MGYA00237665/ERZ505255_FASTA.fasta	MGYA00237665	ERZ505255	root:Environmental:Aquatic:Marine	0.8504	No depth information		106420	237472
MGYA00248628/ERZ508525_FASTA.fasta	MGYA00248628	ERZ508525	root:Environmental:Aquatic:Marine	0.851	No depth information		178552	372802
MGYA00237811/ERZ508554_FASTA.fasta	MGYA00237811	ERZ508554	root:Environmental:Aquatic:Marine	0.8512	No depth information		312731	646298
MGYA00375071/ERZ781379_FASTA.fasta	MGYA00375071	ERZ781379	root:Environmental:Aquatic:Marine	0.8528	No depth information		395072	693157
MGYA00237796/ERZ508532_FASTA.fasta	MGYA00237796	ERZ508532	root:Environmental:Aquatic:Marine	0.853	No depth information		107258	245463
MGYA00367740/ERZ842199_FASTA.fasta	MGYA00367740	ERZ842199	root:Environmental:Aquatic:Marine	0.8542	45m		1054517	2024225
MGYA00367605/ERZ840577_FASTA.fasta	MGYA00367605	ERZ840577	root:Environmental:Aquatic:Marine	0.8574	5m		737170	1270543
MGYA00367748/ERZ843003_FASTA.fasta	MGYA00367748	ERZ843003	root:Environmental:Aquatic:Marine	0.8574	40m		1116744	2012996
MGYA00238138/ERZ651775_FASTA.fasta	MGYA00238138	ERZ651775	root:Environmental:Aquatic:Marine	0.8576	No depth information		367941	806439
MGYA00367661/ERZ841410_FASTA.fasta	MGYA00367661	ERZ841410	root:Environmental:Aquatic:Marine	0.8584	40m		742768	1672713
MGYA00248630/ERZ508518_FASTA.fasta	MGYA00248630	ERZ508518	root:Environmental:Aquatic:Marine	0.8592	No depth information		50484	128232
MGYA00237662/ERZ505245_FASTA.fasta	MGYA00237662	ERZ505245	root:Environmental:Aquatic:Marine	0.8598	No depth information		171836	368992
MGYA00375065/ERZ781373_FASTA.fasta	MGYA00375065	ERZ781373	root:Environmental:Aquatic:Marine	0.861	No depth information		402461	751020
MGYA00237664/ERZ505252_FASTA.fasta	MGYA00237664	ERZ505252	root:Environmental:Aquatic:Marine	0.8616	No depth information		136604	305904
MGYA00248757/ERZ505241_FASTA.fasta	MGYA00248757	ERZ505241	root:Environmental:Aquatic:Marine	0.8624	No depth information		110963	248856
MGYA00367531/ERZ830098_FASTA.fasta	MGYA00367531	ERZ830098	root:Environmental:Aquatic:Marine	0.8626	5m		998974	1707912
MGYA00237656/ERZ505234_FASTA.fasta	MGYA00237656	ERZ505234	root:Environmental:Aquatic:Marine	0.8668	No depth information		181835	394624
MGYA00237795/ERZ508531_FASTA.fasta	MGYA00237795	ERZ508531	root:Environmental:Aquatic:Marine	0.867	No depth information		144043	295770
MGYA00375060/ERZ781368_FASTA.fasta	MGYA00375060	ERZ781368	root:Environmental:Aquatic:Marine	0.8696	No depth information		342382	654980
MGYA00367742/ERZ842400_FASTA.fasta	MGYA00367742	ERZ842400	root:Environmental:Aquatic:Marine	0.8702	80m		1207037	2112608
MGYA00367708/ERZ841907_FASTA.fasta	MGYA00367708	ERZ841907	root:Environmental:Aquatic:Marine	0.8724	5m		329653	614808
MGYA00248723/ERZ505376_FASTA.fasta	MGYA00248723	ERZ505376	root:Environmental:Aquatic:Marine	0.8728	No depth information		258943	431362
MGYA00367700/ERZ841824_FASTA.fasta	MGYA00367700	ERZ841824	root:Environmental:Aquatic:Marine	0.873	5m		685220	1523353
MGYA00237809/ERZ508551_FASTA.fasta	MGYA00237809	ERZ508551	root:Environmental:Aquatic:Marine	0.8756	No depth information		299873	607579
MGYA00237788/ERZ508522_FASTA.fasta	MGYA00237788	ERZ508522	root:Environmental:Aquatic:Marine	0.8758	No depth information		147825	303250
MGYA00367656/ERZ841384_FASTA.fasta	MGYA00367656	ERZ841384	root:Environmental:Aquatic:Marine	0.8766	5m		405139	717383
MGYA00367619/ERZ840881_FASTA.fasta	MGYA00367619	ERZ840881	root:Environmental:Aquatic:Marine	0.8772	5m		946481	2186496
MGYA00367652/ERZ841327_FASTA.fasta	MGYA00367652	ERZ841327	root:Environmental:Aquatic:Marine	0.8782	350m		891736	1532361
MGYA00136665/ERZ475174_FASTA.fasta	MGYA00136665	ERZ475174	root:Environmental:Aquatic:Marine	0.8786	0m		56662	263841
MGYA00238165/ERZ766961_FASTA.fasta	MGYA00238165	ERZ766961	root:Environmental:Aquatic:Marine	0.8796		1	712998	1445514
MGYA00367564/ERZ836051_FASTA.fasta	MGYA00367564	ERZ836051	root:Environmental:Aquatic:Marine	0.8808	5m		318994	599076
MGYA00367615/ERZ840850_FASTA.fasta	MGYA00367615	ERZ840850	root:Environmental:Aquatic:Marine	0.8808	800m		201213	351593
MGYA00375562/ERZ766972_FASTA.fasta	MGYA00375562	ERZ766972	root:Environmental:Aquatic:Marine	0.8818		1	1003543	1947016
MGYA00248731/ERZ505379_FASTA.fasta	MGYA00248731	ERZ505379	root:Environmental:Aquatic:Marine	0.8822	No depth information		238125	400514
MGYA00367649/ERZ841315_FASTA.fasta	MGYA00367649	ERZ841315	root:Environmental:Aquatic:Marine	0.8822	600m		255369	457097
MGYA00237647/ERZ505214_FASTA.fasta	MGYA00237647	ERZ505214	root:Environmental:Aquatic:Marine	0.883	No depth information		240054	489736
MGYA00367687/ERZ841662_FASTA.fasta	MGYA00367687	ERZ841662	root:Environmental:Aquatic:Marine	0.8832	90m		376896	839104
MGYA00237650/ERZ505221_FASTA.fasta	MGYA00237650	ERZ505221	root:Environmental:Aquatic:Marine	0.8838	No depth information		89618	196219
MGYA00237674/ERZ505278_FASTA.fasta	MGYA00237674	ERZ505278	root:Environmental:Aquatic:Marine	0.8844	No depth information		136089	286806
MGYA00367596/ERZ840367_FASTA.fasta	MGYA00367596	ERZ840367	root:Environmental:Aquatic:Marine	0.888	5m		671329	1575981
MGYA00237648/ERZ505217_FASTA.fasta	MGYA00237648	ERZ505217	root:Environmental:Aquatic:Marine	0.8884	No depth information		145604	331625
MGYA00367587/ERZ839424_FASTA.fasta	MGYA00367587	ERZ839424	root:Environmental:Aquatic:Marine	0.8886	380m		988867	1725979
MGYA00237779/ERZ508509_FASTA.fasta	MGYA00237779	ERZ508509	root:Environmental:Aquatic:Marine	0.8892	No depth information		57773	182607
MGYA00367681/ERZ841573_FASTA.fasta	MGYA00367681	ERZ841573	root:Environmental:Aquatic:Marine	0.8898	650m		832019	1437252
MGYA00237786/ERZ508520_FASTA.fasta	MGYA00237786	ERZ508520	root:Environmental:Aquatic:Marine	0.892	No depth information		56398	181080
MGYA00237646/ERZ505213_FASTA.fasta	MGYA00237646	ERZ505213	root:Environmental:Aquatic:Marine	0.8932	No depth information		154222	316094
MGYA00375070/ERZ781378_FASTA.fasta	MGYA00375070	ERZ781378	root:Environmental:Aquatic:Marine	0.8952	No depth information		488896	860630
MGYA00367723/ERZ842013_FASTA.fasta	MGYA00367723	ERZ842013	root:Environmental:Aquatic:Marine	0.8956	800m		788859	1365912
MGYA00237776/ERZ505498_FASTA.fasta	MGYA00237776	ERZ505498	root:Environmental:Aquatic:Marine	0.8976	No depth information		285631	705762
MGYA00367754/ERZ856576_FASTA.fasta	MGYA00367754	ERZ856576	root:Environmental:Aquatic:Marine	0.899	40m		547577	1061282
MGYA00367724/ERZ842040_FASTA.fasta	MGYA00367724	ERZ842040	root:Environmental:Aquatic:Marine	0.8994	5m		775011	1637247
MGYA00367666/ERZ841439_FASTA.fasta	MGYA00367666	ERZ841439	root:Environmental:Aquatic:Marine	0.907	125m		671324	1490886
MGYA00248556/ERZ529322_FASTA.fasta	MGYA00248556	ERZ529322	root:Environmental:Aquatic:Marine	0.908		50	37382	95858
MGYA00136649/ERZ475179_FASTA.fasta	MGYA00136649	ERZ475179	root:Environmental:Aquatic:Marine	0.9084	0m		57923	378144
MGYA00248554/ERZ529323_FASTA.fasta	MGYA00248554	ERZ529323	root:Environmental:Aquatic:Marine	0.9104		50	25751	77287
MGYA00375054/ERZ781362_FASTA.fasta	MGYA00375054	ERZ781362	root:Environmental:Aquatic:Marine	0.9104	No depth information		437541	981087
MGYA00237671/ERZ505271_FASTA.fasta	MGYA00237671	ERZ505271	root:Environmental:Aquatic:Marine	0.9112	No depth information		141595	301389
MGYA00367575/ERZ838136_FASTA.fasta	MGYA00367575	ERZ838136	root:Environmental:Aquatic:Marine	0.913	790m		714773	1443294
MGYA00248553/ERZ529321_FASTA.fasta	MGYA00248553	ERZ529321	root:Environmental:Aquatic:Marine	0.9136		50	41093	112709
MGYA00375055/ERZ781363_FASTA.fasta	MGYA00375055	ERZ781363	root:Environmental:Aquatic:Marine	0.9136	No depth information		338209	765371
MGYA00375056/ERZ781364_FASTA.fasta	MGYA00375056	ERZ781364	root:Environmental:Aquatic:Marine	0.9138	No depth information		247650	578383
MGYA00367720/ERZ841987_FASTA.fasta	MGYA00367720	ERZ841987	root:Environmental:Aquatic:Marine	0.9154	140m		903729	2131495
MGYA00249853/ERZ766980_FASTA.fasta	MGYA00249853	ERZ766980	root:Environmental:Aquatic:Marine	0.9178		1	574199	1177490
MGYA00367617/ERZ840860_FASTA.fasta	MGYA00367617	ERZ840860	root:Environmental:Aquatic:Marine	0.9234	5m		632349	1616752
MGYA00367538/ERZ831745_FASTA.fasta	MGYA00367538	ERZ831745	root:Environmental:Aquatic:Marine	0.924	35m		558312	1296988
MGYA00367696/ERZ841799_FASTA.fasta	MGYA00367696	ERZ841799	root:Environmental:Aquatic:Marine	0.9274	480m		424411	921204
MGYA00248635/ERZ508505_FASTA.fasta	MGYA00248635	ERZ508505	root:Environmental:Aquatic:Marine	0.9286	No depth information		261376	646133
MGYA00367697/ERZ841809_FASTA.fasta	MGYA00367697	ERZ841809	root:Environmental:Aquatic:Marine	0.9316	100m		695418	1239680
MGYA00367544/ERZ833000_FASTA.fasta	MGYA00367544	ERZ833000	root:Environmental:Aquatic:Marine	0.9324	380m		567852	1011213
MGYA00367555/ERZ834397_FASTA.fasta	MGYA00367555	ERZ834397	root:Environmental:Aquatic:Marine	0.9378	5m		854050	1535991
MGYA00367599/ERZ840537_FASTA.fasta	MGYA00367599	ERZ840537	root:Environmental:Aquatic:Marine	0.9414	40m		666781	1428589
MGYA00367535/ERZ831144_FASTA.fasta	MGYA00367535	ERZ831144	root:Environmental:Aquatic:Marine	0.943	850m		802939	1346818
MGYA00367631/ERZ841010_FASTA.fasta	MGYA00367631	ERZ841010	root:Environmental:Aquatic:Marine	0.9458	5m		624756	1250823
MGYA00367741/ERZ842390_FASTA.fasta	MGYA00367741	ERZ842390	root:Environmental:Aquatic:Marine	0.9464	550m		1086978	1795964
MGYA00367525/ERZ829562_FASTA.fasta	MGYA00367525	ERZ829562	root:Environmental:Aquatic:Marine	0.9498	700m		675430	1147967
MGYA00367572/ERZ837273_FASTA.fasta	MGYA00367572	ERZ837273	root:Environmental:Aquatic:Marine	0.9516	800m		412065	732604
MGYA00367611/ERZ840605_FASTA.fasta	MGYA00367611	ERZ840605	root:Environmental:Aquatic:Marine	0.9648	800m		413318	789973
MGYA00367746/ERZ842555_FASTA.fasta	MGYA00367746	ERZ842555	root:Environmental:Aquatic:Marine	0.9674	250m		1104581	1974960
MGYA00367750/ERZ843524_FASTA.fasta	MGYA00367750	ERZ843524	root:Environmental:Aquatic:Marine	0.972	740m		1017506	1682405
MGYA00367556/ERZ834656_FASTA.fasta	MGYA00367556	ERZ834656	root:Environmental:Aquatic:Marine	0.9726	640m		730448	1479253
MGYA00367730/ERZ842091_FASTA.fasta	MGYA00367730	ERZ842091	root:Environmental:Aquatic:Marine	0.977	590m		930041	1639589
MGYA00367678/ERZ841497_FASTA.fasta	MGYA00367678	ERZ841497	root:Environmental:Aquatic:Marine	0.9782	1000m		729344	1328407
MGYA00367568/ERZ836445_FASTA.fasta	MGYA00367568	ERZ836445	root:Environmental:Aquatic:Marine	0.9786	800m		894267	1554923
MGYA00367522/ERZ829075_FASTA.fasta	MGYA00367522	ERZ829075	root:Environmental:Aquatic:Marine	0.9794	800m		980223	1667371
MGYA00367744/ERZ842407_FASTA.fasta	MGYA00367744	ERZ842407	root:Environmental:Aquatic:Marine	0.9798	155m		1064942	1866060
MGYA00367614/ERZ840839_FASTA.fasta	MGYA00367614	ERZ840839	root:Environmental:Aquatic:Marine	0.9804	640m		812177	1570697
MGYA00367638/ERZ841139_FASTA.fasta	MGYA00367638	ERZ841139	root:Environmental:Aquatic:Marine	0.9804	696m		896013	1753607
