# Supplementary material for: Structural color in the bacterial domain: The ecogenomics of a 2-dimensional optical phenotype
Source: Proc Natl Acad Sci U S A. 2024 Jul 11;121(29):e2309757121. doi: 10.1073/pnas.2309757121 (PMC11260094; doi:10.1073/pnas.2309757121)
Supplement: Supplementary file 12 — Dataset S06 (PDF) [file pnas.2309757121.sd06.pdf]

Run	Date	Depth	Contigs >= 500 bp	Predicted proteins	SC score
SRR7632678	2014-03-21	4000m	79935	228124	0.735
SRR7632646	2014-04-02	4000m	80552	240718	0.7384
SRR7632643	2014-04-13	4000m	88235	281832	0.7984
SRR7632644	2014-04-25	4000m	130902	415970	0.8706
SRR7632674	2014-05-07	4000m	88559	195304	0.6886
SRR7632675	2014-05-18	4000m	168091	474928	0.8424
SRR7632679	2014-05-30	4000m	80854	249414	0.8682
SRR7632639	2014-06-11	4000m	152664	405834	0.8596
SRR7632653	2014-06-22	4000m	158549	437505	0.7766
SRR7632680	2014-07-04	4000m	145099	365709	0.7488
SRR7632641	2014-07-16	4000m	70329	226198	0.7982
SRR7632672	2014-07-27	4000m	108592	322194	0.888
SRR7632640	2014-08-08	4000m	147236	460736	0.9152
SRR7632671	2014-08-20	4000m	144518	445977	0.8374
SRR7632648	2014-08-31	4000m	156774	477360	0.8688
SRR7632676	2014-09-12	4000m	75989	205890	0.6488
SRR7632677	2014-09-24	4000m	116633	366968	0.8314
SRR7632642	2014-10-05	4000m	110299	348376	0.807
SRR7632673	2014-10-17	4000m	180643	474549	0.8036
SRR7632645	2014-10-29	4000m	145314	377431	0.7696
SRR7632647	2014-11-09	4000m	344000	1114706	0.8828
SRR7648292	2015-04-15	4000m	191489	595703	0.8912
SRR7648298	2015-04-25	4000m	221267	599699	0.9688
SRR7648339	2015-05-05	4000m	134832	414714	0.8134
SRR7648287	2015-05-16	4000m	165641	509240	0.875
SRR7648335	2015-05-26	4000m	181802	496805	0.8454
SRR7648348	2015-06-05	4000m	168274	516222	0.844
SRR7648286	2015-06-15	4000m	190125	568420	0.8192
SRR7648343	2015-06-25	4000m	244013	720903	0.8648
SRR7648291	2015-07-06	4000m	202525	625121	0.8646
SRR7648349	2015-07-16	4000m	221997	662707	0.8822
SRR7648296	2015-07-26	4000m	212732	433774	0.643
SRR7648290	2015-08-05	4000m	183913	475641	0.7912
SRR7648341	2015-08-15	4000m	232439	705453	0.8958
SRR7648288	2015-08-26	4000m	233759	713080	0.9418
SRR7648273	2015-09-05	4000m	228933	680566	0.9204
SRR7648289	2015-09-15	4000m	140991	438557	0.9388
SRR7648332	2015-09-25	4000m	273800	740439	0.9288
SRR7648326	2015-10-05	4000m	229090	647861	0.9028
SRR7648350	2015-10-16	4000m	262359	810241	0.9224
SRR7648270	2015-10-26	4000m	258942	557972	0.772
SRR7648321	2015-11-05	4000m	263127	609664	0.849
SRR7648274	2016-06-05	4000m	561925	955137	0.6824
SRR7648285	2016-06-13	4000m	130077	337387	0.792
SRR7648346	2016-06-21	4000m	190295	471489	0.8636
SRR7648297	2016-06-30	4000m	170066	562992	0.8032
SRR7648333	2016-07-08	4000m	203949	562663	0.8676
SRR7648345	2016-07-16	4000m	216692	460475	0.8786
SRR7648347	2016-07-24	4000m	131621	385499	0.8126

SRR7648327	2016-08-01	4000m	177830	564784	0.9002
SRR7648294	2016-08-10	4000m	138911	435641	0.9068
SRR7648336	2016-08-26	4000m	313962	760612	0.8678
SRR7648295	2016-09-03	4000m	124722	404270	0.898
SRR7648320	2016-09-11	4000m	25331	78510	0.7138
SRR7648331	2016-09-20	4000m	274142	840356	0.7708
SRR7648299	2016-09-28	4000m	109493	350051	0.7776
SRR7648284	2016-10-06	4000m	119901	326089	0.7494
SRR7648337	2016-10-14	4000m	36887	108472	0.6756
SRR7648334	2016-10-22	4000m	112883	384275	0.8308
SRR7648310	2016-10-31	4000m	119847	262324	0.6828
SRR7648293	2016-11-08	4000m	510779	802105	0.29
SRR7648338	2016-11-16	4000m	124116	374232	0.8652
