# Supplementary material for: Structural color in the bacterial domain: The ecogenomics of a 2-dimensional optical phenotype
Source: Proc Natl Acad Sci U S A. 2024 Jul 11;121(29):e2309757121. doi: 10.1073/pnas.2309757121 (PMC11260094; doi:10.1073/pnas.2309757121)
Supplement: Supplementary file 7 — Dataset S01 (PDF) [file pnas.2309757121.sd01.pdf]

							STRING	Selected by	pairwise	pairwise
							Flavobacterium johnsoniae UW101			
							Muricauda ruestringensis			
							Cellulophaga lytica H1			
							Marinobacter algicola HM-33			
							Final clustering			
							RandomForest Gini importance		0.047783	0.059525
							Function		annotation	annotation
								Order in clustering	1	2
species	Short name	SC	SC_reteste	RF score	order_in_t	Phylum	Strain	number of genes	group_2538	group_268
Virgibacillus dokdonensis	HM-38	1	1	0.18350464	1	Firmicutes	Virgibacillus dokdonensis HM-38	61	0	0
Leptothrix ochracea	L12	0	0	0.11461661	2	Proteobacteria	Leptothrix ochracea L12	17	0	0
Enterobacter cloacae	HM-20	0	0	0.3488463	3	Proteobacteria	Enterobacter cloacae HM-20	59	0	0
Algicola bacteriolytica	HM-41	1	1	0.388364	4	Proteobacteria	Algicola bacteriolytica HM-41	69	0	0
Alteromonas mediterranea	HM-65	1	1	0.52042629	5	Proteobacteria	Alteromonas mediterranea HM-65	78	0	0
Pseudomonas aeruginosa	PAO1	0	1	0.64950884	6	Proteobacteria	Pseudomonas aeruginosa PAO1	69	1	1
Zooshikella ganghwensis	HM-19	0	0	0.209375	7	Proteobacteria	Zooshikella ganghwensis HM-19	57	0	0
Zooshikella ganghwensis	HM-66	0	0	0.20998415	8	Proteobacteria	Zooshikella ganghwensis HM-66	58	0	0
Marinobacter santoriniensis	HM-31	1	1	0.77571283	9	Proteobacteria	Marinobacter santoriniensis HM-31	65	0	0
Marinobacter salinus	HM-67	1	1	0.85937107	10	Proteobacteria	Marinobacter salinus HM-67	68	0	0
Marinobacter salinus	HM-69	1	1	0.84788093	11	Proteobacteria	Marinobacter salinus HM-69	75	0	0
Marinobacter salarius	HM-43	1	1	0.70449949	12	Proteobacteria	Marinobacter salarius HM-43	68	0	1
Marinobacter salarius	HM-39	1	1	0.75435496	13	Proteobacteria	Marinobacter salarius HM-39	62	0	0
Marinobacter salarius	HM-34	1	1	0.8997991	14	Proteobacteria	Marinobacter salarius HM-34	66	0	0
Marinobacter salarius	HM-35	1	1	0.89982337	15	Proteobacteria	Marinobacter salarius HM-35	66	0	0
Marinobacter salarius	HM-64	1	1	0.90739339	16	Proteobacteria	Marinobacter salarius HM-64	72	0	0
Marinobacter algicola	HM-30	1	1	0.78371628	17	Proteobacteria	Marinobacter algicola HM-30	142	1	1
Marinobacter adhaerens	HM-42	1	1	0.74022699	18	Proteobacteria	Marinobacter adhaerens HM-42	66	1	0
Marinobacter adhaerens	HM-06	1	1	0.83164557	19	Proteobacteria	Marinobacter adhaerens HM-06	67	0	0
Marinobacter adhaerens	HM-44	1	1	0.86643234	20	Proteobacteria	Marinobacter adhaerens HM-44	72	0	0
Marinobacter algicola	HM-28	1	1	0.9223203	21	Proteobacteria	Marinobacter algicola HM-28	72	0	0
Marinobacter algicola	HM-32	1	1	0.9223423	22	Proteobacteria	Marinobacter algicola HM-32	72	0	0
Marinobacter algicola	HM-33	1	1	0.92303861	23	Proteobacteria	Marinobacter algicola HM-33	72	0	0
Marinobacter algicola	HM-36	1	1	0.92173252	24	Proteobacteria	Marinobacter algicola HM-36	72	0	0
Cyclobacterium marinum	HM-11	0	1	0.59792166	25	Bacteroidota	Cyclobacterium marinum HM-11	114	0	0
Porphyromonas gingivalis	KCOM3131	0	0	0.05490196	26	Bacteroidota	Porphyromonas gingivalis KCOM3131	35	0	0
Polaribacter dokdonensis	DSW5	1	1	0.65775536	27	Bacteroidota	Polaribacter dokdonensis DSW5	127	0	0
Tenacibaculum soleae	LL0412.1.7	1	1	0.76509386	28	Bacteroidota	Tenacibaculum soleae LL0412.1.7	116	1	1
Tenacibaculum ovolyticum	daSA-8	1	1	0.83785841	29	Bacteroidota	Tenacibaculum ovolyticum daSA-8	139	1	1
Tenacibaculum ovolyticum	DSM18103	1	1	0.89295632	30	Bacteroidota	Tenacibaculum ovolyticum DSM18103	139	1	1
Tenacibaculum lutimaris	DSM16505T	1	1	0.69711299	31	Bacteroidota	Tenacibaculum lutimaris DSM16505T	115	1	1
Tenacibaculum mesophilum	GCA_001027945.1	1	1	0.90352412	32	Bacteroidota	Tenacibaculum mesophilum GCA_001027945.1	125	1	1
Tenacibaculum discolor	DSM18842	1	1	0.86651584	33	Bacteroidota	Tenacibaculum discolor DSM18842	125	1	1
Tenacibaculum gallaicum	DSM18841	1	1	0.94392286	34	Bacteroidota	Tenacibaculum gallaicum DSM18841	138	1	1
Tenacibaculum gallaicum	HM-45	1	1	0.92403798	35	Bacteroidota	Tenacibaculum gallaicum HM-45	137	1	1
Tenacibaculum gallaicum	HM-46	1	1	0.92684747	36	Bacteroidota	Tenacibaculum gallaicum HM-46	137	1	1
Tenacibaculum gallaicum	HM-47	1	1	0.92579505	37	Bacteroidota	Tenacibaculum gallaicum HM-47	137	1	1
Flavobacterium phocarum	HM-10	0	0	0.08624801	38	Bacteroidota	Flavobacterium phocarum HM-10	64	0	0
Flavobacterium gelidilacus	DSM15343	0	0	0.08639053	39	Bacteroidota	Flavobacterium gelidilacus DSM15343	80	0	0
Flavobacterium degerlachei	DSM15718	0	0	0.20221169	40	Bacteroidota	Flavobacterium degerlachei DSM15718	108	0	0
Flavobacterium succinicans	DD5b	0	0	0.11181602	41	Bacteroidota	Flavobacterium succinicans DD5b	82	0	0
Flavobacterium psychrophilum	V4-33	0	0	0.08076923	42	Bacteroidota	Flavobacterium psychrophilum V4-33	57	0	0
Flavobacterium aquidurense	DSM18293	1	1	0.66133536	43	Bacteroidota	Flavobacterium aquidurense DSM18293	128	0	0
Flavobacterium piscis	IR1	1	1	0.68860759	44	Bacteroidota	Flavobacterium piscis IR1	124	0	0
Flavobacterium anhuiense	FS2	1	1	0.72921914	45	Bacteroidota	Flavobacterium anhuiense FS2	133	0	0
Flavobacterium johnsoniae	DSM2064	1	1	0.82296409	46	Bacteroidota	Flavobacterium johnsoniae DSM2064	136	1	1
Flavobacterium johnsoniae	UW101	1	1	0.823143	47	Bacteroidota	Flavobacterium johnsoniae UW101	136	1	1
Olleya marilimosa	CAM030	0	0	0.27004388	48	Bacteroidota	Olleya marilimosa CAM030	97	0	0
Winogradskyella eximia	CECT7946	0	0	0.38570865	49	Bacteroidota	Winogradskyella eximia CECT7946	108	0	0
Psychroserpens burtonensis	DSM12212	0	0	0.26317845	50	Bacteroidota	Psychroserpens burtonensis DSM12212	89	0	0
Algibacter lectus	HM-75	1	1	0.67915514	51	Bacteroidota	Algibacter lectus HM-75	132	0	0
Algibacter lectus	HM-02	1	1	0.63231968	52	Bacteroidota	Algibacter lectus HM-02	139	0	0
Algibacter lectus	DSM15365	1	1	0.61873583	53	Bacteroidota	Algibacter lectus DSM15365	128	0	0
Algibacter lectus	HM-73	1	1	0.4885477	54	Bacteroidota	Algibacter lectus HM-73	123	0	0
Aquimarina latercula	DSM2041	1	1	0.75262631	55	Bacteroidota	Aquimarina latercula DSM2041	149	1	1
Dokdonia donghaensis	DSW1	0	0	0.14308114	56	Bacteroidota	Dokdonia donghaensis DSW1	94	0	0
Nonlabens dokdonensis	DSW6	0	0	0.13618369	57	Bacteroidota	Nonlabens dokdonensis DSW6	69	0	0
Zunongwangia profunda	HM-12	0	0	0.37210216	58	Bacteroidota	Zunongwangia profunda HM-12	114	0	0
Salegentibacter mishustinae	DSM23404	0	0	0.06201248	59	Bacteroidota	Salegentibacter mishustinae DSM23404	84	0	0
Salegentibacter salegens	ACAM48	0	0	0.30799373	60	Bacteroidota	Salegentibacter salegens ACAM48	98	0	0
Salegentibacter salarius	KCTC12974	0	0	0.02949273	61	Bacteroidota	Salegentibacter salarius KCTC12974	81	0	0
Mesonia algae	DSM15361	0	0	0.09967453	62	Bacteroidota	Mesonia algae DSM15361	65	0	0
Gramella echinicola	HM-13	0	0	0.07260329	63	Bacteroidota	Gramella echinicola HM-13	96	1	1
Gramella portivictoriae	HM-14	0	0	0.19565217	64	Bacteroidota	Gramella portivictoriae HM-14	92	0	0
Ulvibacter antarcticus	DSM23424	0	0	0.17538341	65	Bacteroidota	Ulvibacter antarcticus DSM23424	86	1	1
Gillisia marina	HM-09	0	0	0.0553224	66	Bacteroidota	Gillisia marina HM-09	70	0	0
Cellulophaga tyrosinoxydans	DSM21164	1	1	0.5673101	67	Bacteroidota	Cellulophaga tyrosinoxydans DSM21164	128	0	0
Cellulophaga baltica	18	1	1	0.77527515	68	Bacteroidota	Cellulophaga baltica 18	154	0	0
Cellulophaga baltica	NN016038	1	1	0.81503911	69	Bacteroidota	Cellulophaga baltica NN016038	150	0	0
Cellulophaga pacifica	HM-15	1	1	0.85197124	70	Bacteroidota	Cellulophaga pacifica HM-15	135	0	0
Cellulophaga pacifica	HM-48	1	1	0.83404255	71	Bacteroidota	Cellulophaga pacifica HM-48	140	0	0
Arenibacter algicola	HM-07	1	1	0.54467657	72	Bacteroidota	Arenibacter algicola HM-07	138	1	1
Arenibacter nanhaiticus	CGMCC1.8863	1	1	0.70500127	73	Bacteroidota	Arenibacter nanhaiticus CGMCC1.8863	131	1	1
Maribacter forsetii	HM-61	0	0	0.47910095	74	Bacteroidota	Maribacter forsetii HM-61	133	0	0
Maribacter dokdonensis	HM-08	1	1	0.81300403	75	Bacteroidota	Maribacter dokdonensis HM-08	139	0	0
Maribacter dokdonensis	DSW8	1	1	0.79083816	76	Bacteroidota	Maribacter dokdonensis DSW8	143	0	0
Maribacter dokdonensis	HM-62	1	1	0.7819511	77	Bacteroidota	Maribacter dokdonensis HM-62	146	0	0
Maribacter dokdonensis	MAR_2009_71	1	1	0.81924346	78	Bacteroidota	Maribacter dokdonensis MAR_2009_71	149	0	0
Zobellia galactanivorans	DSM1208	0	1	0.60724017	79	Bacteroidota	Zobellia galactanivorans DSM1208	135	0	0
Zobellia uliginosa	DSM2061	0	1	0.58966327	80	Bacteroidota	Zobellia uliginosa DSM2061	133	0	0
Kriegella aquimaris	DSM19866	0	1	0.68048973	81	Bacteroidota	Kriegella aquimaris DSM19866	149	1	1
Zobellia spp	HM-68	1	1	0.53460861	82	Bacteroidota	Zobellia spp HM-68	155	1	1
Muricauda flavescens	HM-29	1	1	0.64609982	83	Bacteroidota	Muricauda flavescens HM-29	125	0	0
Muricauda ruestringensis	HM-25	1	1	0.76571573	84	Bacteroidota	Muricauda ruestringensis HM-25	139	1	1
Muricauda ruestringensis	HM-40	1	1	0.73240506	85	Bacteroidota	Muricauda ruestringensis HM-40	129	0	0
Muricauda ruestringensis	HM-21	1	1	0.92844545	86	Bacteroidota	Muricauda ruestringensis HM-21	139	1	1
Muricauda ruestringensis	HM-22	1	1	0.91506572	87	Bacteroidota	Muricauda ruestringensis HM-22	138	1	1
Muricauda ruestringensis	HM-23	1	1	0.91493671	88	Bacteroidota	Muricauda ruestringensis HM-23	138	1	1
Muricauda ruestringensis	HM-24	1	1	0.9140665	89	Bacteroidota	Muricauda ruestringensis HM-24	138	1	1
Muricauda ruestringensis	HM-37	1	1	0.92788217	90	Bacteroidota	Muricauda ruestringensis HM-37	139	1	1
Cellulophaga spp	HM-74	1	1	0.74305205	91	Bacteroidota	Cellulophaga spp HM-74	140	0	0
Cellulophaga fucicola	DSM24786	1	1	0.90718858	92	Bacteroidota	Cellulophaga fucicola DSM24786	157	1	1
Cellulophaga spp	HM-04	1	1	0.96499622	93	Bacteroidota	Cellulophaga spp HM-04	159	0	0
Cellulophaga spp	HM-26	1	1	0.9833795	94	Bacteroidota	Cellulophaga spp HM-26	163	0	0
Cellulophaga spp	HM-01	1	1	0.97721519	95	Bacteroidota	Cellulophaga spp HM-01	162	0	0
Cellulophaga spp	HM-03	1	1	0.97750562	96	Bacteroidota	Cellulophaga spp HM-03	162	0	0
Cellulophaga spp	HM-05	1	1	0.97767745	97	Bacteroidota	Cellulophaga spp HM-05	158	0	0
Cellulophaga spp	HM-27	1	1	0.97551224	98	Bacteroidota	Cellulophaga spp HM-27	159	0	0
Cellulophaga omnivescoria	WSC	1	1	0.98503298	99	Bacteroidota	Cellulophaga omnivescoria WSC	155	0	0
Cellulophaga lytica	HM-59		1	0.99108734	100	Bacteroidota	Cellulophaga lytica HM-59	160	0	0
Cellulophaga lytica	HM-49	1	1	0.9982247	101	Bacteroidota	Cellulophaga lytica HM-49	159	0	0
Cellulophaga lytica	HM-50	1	1	0.99823988	102	Bacteroidota	Cellulophaga lytica HM-50	159	0	0
Cellulophaga lytica	HM-56	1	1	0.98150925	103	Bacteroidota	Cellulophaga lytica HM-56	159	0	0
Cellulophaga lytica	DAU203	1	1	0.97183807	104	Bacteroidota	Cellulophaga lytica DAU203	160	0	0
Cellulophaga lytica	CL8139	1	1	0.98994975	105	Bacteroidota	Cellulophaga lytica CL8139	162	0	0
Cellulophaga lytica	DSM7489	1	1	0.98920141	106	Bacteroidota	Cellulophaga lytica DSM7489	156	0	0
Cellulophaga lytica	HI1	1	1	0.99086526	107	Bacteroidota	Cellulophaga lytica HI1	160	0	0
Cellulophaga lytica	HM-51	1	1	0.99949495	108	Bacteroidota	Cellulophaga lytica HM-51	161	0	0
Cellulophaga lytica	HM-52	1	1	0.99745353	109	Bacteroidota	Cellulophaga lytica HM-52	162	0	0
Cellulophaga lytica	HM-53	1	1	0.99899649	110	Bacteroidota	Cellulophaga lytica HM-53	159	0	0
Cellulophaga lytica	HM-54	1	1	0.99570599	111	Bacteroidota	Cellulophaga lytica HM-54	156	0	0
Cellulophaga lytica	HM-55	1	1	0.99949303	112	Bacteroidota	Cellulophaga lytica HM-55	161	0	0
Cellulophaga lytica	HM-57	1	1	0.9816629	113	Bacteroidota	Cellulophaga lytica HM-57	160	0	0
Cellulophaga lytica	HM-58	1	1	0.98150025	114	Bacteroidota	Cellulophaga lytica HM-58	160	0	0
Cellulophaga lytica	HM-60	1	1	0.98845382	115	Bacteroidota	Cellulophaga lytica HM-60	159	0	0
Cellulophaga lytica	HM-63	1	1	0.98175456	116	Bacteroidota	Cellulophaga lytica HM-63	162	0	0
Cellulophaga lytica	KL-A	1	1	0.99577745	117	Bacteroidota	Cellulophaga lytica KL-A	162	0	0

pairwise	pairwise,pe	fisherexact	pairwise	permutatio	pairwise	pairwise	pairwise,pe	pairwise	permutatio	pairwise,pe	permutatio	permutatio	pairwise	mutant	pairwise,pe	permutatio	permutatio	fisherexact	permutatio	permutatio	permutatio	permutatio
																			carbohydra	carbohydra	carbohydra	gliding
																gliding						
															gliding	gliding	gliding		carbohydra	carbohydra	carbohydrate2	
															gliding	gliding	gliding		carbohydra	carbohydra	carbohydra	gliding
0.03503	0.032678	0.2907187	0.114607	0.059989	0.040745	0.099484	0.121186	0.06201311	0.029677	0.074725	0.045673	0.046329	0.040702	0.109878	0.054885	0.024178	0.051888	0.386711	0.055411	0.057332	0.03007	0.027094
Transcriptio	KEGG: fbc:F	COGs: COG17	KEGG: cat:C	InterPro IPR	Cytosine-sp	Major facilit	InterPro IPR	COGs: COG294	KEGG: fbc:F	COGs: COG	COGs: COG	annotation	KEGG: bth:	annotation	COGs: COG	KEGG: fbc:F	KEGG: fbc:F	KEGG: rbi:R	Beta-N-acet	COGs: COG	Alpha-1,2-m	KEGG: rbi:R
76	77	78	79	80	81	82	83	84	85	86	87	88	89	90	91	92	93	94	95	96	97	98
**yojI**	**group_167**	**group_2532**	**group_245**	**oprO**	**bspRIM**	**mdtG**	**merT**	**group_1652**	**group_163**	**group_168**	**group_189**	**group_222**	**group_170**	**hsdR**	**dacB**	**group_209**	**group_217**	**group_256**	**exoI**	**nagB**	**group_198**	**group_184**

pairwise	pairwise	pairwise	mutant,pai	pairwise,pe	pairwise,pe	pairwise	pairwise,pe	pairwise,pe	mutant	permutatio	permutatio	permutatio	permutatio	permutatio	pairwise	permutatio	pairwise,pe	pairwise,pe	permutatio	pairwise	permutatio	pairwise,pe
			gliding	carbohydra	carbohydrate2		carbohydra	gliding									gliding	gliding			gliding	
carbohydra	carbohydrate1		gliding	gliding	gliding	gliding		gliding				gliding	gliding		pterin		gliding	gliding	gliding	carbohydrate1		
gliding	gliding	carbohydra	gliding		gliding			gliding				gliding			pterin		gliding	gliding	gliding	carbohydrate1		
carbohydra	carbohydra	carbohydra	gliding	carbohydra	gliding	gliding	carbohydra	gliding				gliding	gliding		pterin		gliding	gliding	gliding	carbohydra	gliding	
0.034898	0.033318	0.071647	0.034098	0.0289	0.027607	0.0426	0.029526	0.022098	0.026941	0.045652	0.096863	0.039535	0.023188	0.031366	0.12485	0.218983	0.127462	0.175968	0.046437	0.02609	0.027215	0.045764
Uncharacte	Xylose isom	Polyphosph	Galactokin	KEGG: fbc:F	KEGG: fbc:F	PHP domai	KEGG: fbc:F	Aldose 1-ep	Major facili	Tetratricop	KEGG: rbi:R	KEGG: gfo:G	KEGG: fbc:F	KEGG: fbc:F	MOSC dom	Carboxyma	KEGG: rbi:R	Putative m	KEGG: fbc:F	COGs: COG	KEGG: fbc:F	Pentapepti
99	100	101	102	103	104	105	106	107	108	109	110	111	112	113	114	115	116	117	118	119	120	121
**group_102**	**iolE**	**ppgK**	**galK**	**group_169**	**group_167**	**ywqE**	**group_168**	**lacX**	**group_156**	**group_177**	**group_179**	**group_124**	**group_156**	**group_120**	**yiiM**	**group_195**	**group_156**	**group_566**	**group_107**	**group_108**	**group_126**	**mfpA**

permutatio	pairwise	pairwise,pe	permutatio	pairwise,pe	permutatio	pairwise,pe	pairwise,pe	permutatio	mutant	permutatio	permutatio	permutatio	mutant	pairwise,pe	mutant	mutant	mutant	mutant	mutant	mutant	mutant	mutant
				porphyrin		carbohydrate2																
				porphyrin																		
				porphyrin	pterin	gliding																
				porphyrin	pterin	carbohydrate																
0.054795	0.118841	0.086941	0.031419	0.068487	0.019187	0.05221	0.02245	0.052021	0.060008	0.02903	0.034035	0.043248	0.055066	0.027602	0.118374	0.029795	0.035968	0.114596	0.022541	0.043458	0.051752	0.039008
COGs: COG	KEGG: fbc:F	KEGG: fbc:F	HAMAP: m	COGs: COG	KEGG: fbc:F	KEGG: fbc:F	KEGG: cat:C	COGs: COG	KEGG: rbi:R	annotation	KEGG: fbc:F	Ferric uptak	Peptidase M	annotation	COGs: COG	Transcriptio	Uncharacte	GH3 auxin-r	KEGG: fbc:F	Putative m	Amidophos	RagB/SusD
122	123	124	125	126	127	128	129	130	131	132	133	134	135	136	137	138	139	140	141	142	143	144
**fepA**	**group_162**	**bag**	**yflT**	**cysG**	**group_160**	**xynA1**	**group_197**	**group_155**	**group_431**	**group_165**	**group_159**	**perR**	**lpqL**	**group_311**	**hmgA**	**kstR2**	**murK**	**group_184**	**group_123**	**group_44**	**purF**	**susD_1**

pairwise,pe	permutatio	permutatio	fisherexact	fisherexact	fisherexact,	pairwise,pe	pairwise,pe	mutant,per	permutatio	permutatio	pairwise,pe	pairwise	pairwise,pe	pairwise,pe	permutatio	permutatio	fisherexact	fisherexact	fisherexact	fisherexact	fisherexact,	fisherexact,
											acetolactate			acetolactate			methionine	pterin	pterin	pterin	pterin	pterin
gliding	gliding																	pterin	pterin	pterin	pterin	pterin
		gliding									acetolactate			acetolactate			methionine	pterin	pterin	pterin	pterin	pterin
											acetolactate						methionine	pterin	pterin	pterin	pterin	pterin
gliding	gliding	gliding									acetolactate			acetolactate			methionine	pterin	pterin	pterin	pterin	pterin
0.040788	0.060837	0.112106	0.181121	0.349269	0.436774	0.049378	0.065096	0.056675	0.171969	0.053596	0.095646	0.044361	0.061093	0.246702	0.13283	0.085663	0.345002	0.559166	0.612646	0.550768	0.383919	0.702979
Glycosyl tra	COGs: COG	KEGG: fbc:F	COGs: COG	ThiJ/PfpI do	COGs: COG	Uncharacte	Uncharacte	Transcriptio	InterPro IPR	Uncharacte	COGs: COG	COGs: COG	Putative m	COGs: COG	HAMAP: Rib	Peptidase S	S-methyltr	Molybdopt	COGs: COG	Molybdenu	Isoquinolin	Nucleotide
145	146	147	148	149	150	151	152	153	154	155	156	157	158	159	160	161	162	163	164	165	166	167
**arnT**	**group_116**	**group_173**	**ydjA**	**inhA**	**quiP**	**group_746**	**group_747**	**rhaR_1**	**group_534**	**secE2**	**gpx1**	**mtfA**	**yidD**	**ygeA**	**rpmH**	**umuD**	**metE**	**moaE**	**moaC2**	**moeA**	**iorA_2**	**nboR**

iorB	pucA	ilvD	ilvB	ilvC	ilvH	argE	argG	argH	ggt	glnII	xylB	cysH	sir	sumT	moaA	nlhH	glpQ_2	metH_1	guaB_2	degA_2	cirA_1	guaA	fabF_1	bicA_2	relA	inlJ_2	trmD	maeB	fabG_1	acuA	wbpA
fisherexact,	fisherexact,	permutatio	permutatio	permutatio	permutatio	permutation	permutatio	permutatio	permutation	fisherexact	fisherexact	permutatio	fisherexact,	fisherexact,	fisherexact	permutatio	permutatio	permutatio	permutatio	mutant	mutant	mutant	mutant	mutant	mutant	mutant	mutant	mutant	mutant	mutant	mutant
pterin	pterin	acetolactat	acetolactat	acetolactat	acetolactat	acetolactate	acetolactat	acetolactat	acetolactate	acetolactate		porphyrin	porphyrin	porphyrin	pterin	carbohydrate2		methionine													
pterin	pterin	acetolactat	acetolactat	acetolactat	acetolactat	acetolactate	acetolactat	acetolactat	acetolactate	acetolactat	carbohydra	porphyrin	porphyrin	porphyrin	pterin		carbohydra	methionine													
pterin	pterin	acetolactat	acetolactat	acetolactat	acetolactat	acetolactate	acetolactat	acetolactat	acetolactate	acetolactat	carbohydra	porphyrin	porphyrin	porphyrin	pterin		carbohydra	methionine													
pterin	pterin	acetolactat	acetolactat	acetolactat	acetolactat	acetolactate	acetolactat	acetolactat	acetolactate	acetolactat	carbohydra	porphyrin	porphyrin	porphyrin	pterin		carbohydra	methionine													
pterin	pterin	acetolactat	acetolactat	acetolactat	acetolactat	acetolactate	acetolactat	acetolactat	acetolactate	acetolactat	carbohydra	porphyrin	porphyrin	porphyrin	pterin	carbohydra	carbohydra	methionine													
0.162663	0.222827	0.009583	0.011277	0.012192	0.004222	0.036291647	0.029787	0.02308	0.04739948	0.226433	0.205111	0.01187	0.128091	0.084127	0.276241	0.040009	0.019372	0.010527	0.060211	0.008189	0.011128	0.00148	0.000443	0.000969	0.001227	0.040388	0	0	0	0	0
Aldehyde de	KshC-Cox1-	COGs: COG	Acetolactat	Ketol-acid r	COGs: COG	COGs: COG0624	COGs: COG	COGs: COG	COGs: COG040	COGs: COG	COGs: COG	COGs: COG	COGs: COG	Uroporphyr	Molybdenu	KEGG: gfo:	COGs: COG	COGs: COG	Putative sig	Transcriptio	TonB-depen	Glutamine	COGs: COG	COGs: COG	[P]ppGpp s	GTP cyclohy	tRNA (guan	Malic prote	3-oxoacyl-(	Acetate/Co	Nucleotide sugar dehydrogenase; COGs: COG[illegible]
168	169	170	171	172	173	174	175	176	177	178	179	180	181	182	183	184	185	186	187	188	189	190	191	192	193	194	195	196	197	198	199
0	0	1	1	1	1	0	1	1	1	0	1	1	1	1	1	1	1	1	1	1	0	1	1	1	1	0	1	1	1	1	1
0	0	0	1	1	1	0	1	1	0	1	0	1	0	0	0	1	0	0	0	0	0	0	0	0	0	1	1	1	1	1	1
0	0	1	1	1	1	1	1	1	1	1	1	1	1	1	1	1	1	1	0	1	1	1	1	1	1	1	1	1	1	1	1
1	1	1	1	1	1	1	1	1	1	1	0	1	1	1	1	0	1	1	1	1	1	1	1	1	1	1	1	1	1	1	1
1	1	1	1	1	1	1	1	1	1	1	1	1	1	1	1	1	1	1	1	1	1	1	1	1	1	1	1	1	1	1	1
1	1	1	1	1	1	1	1	1	1	1	1	1	1	1	1	1	1	1	0	1	1	1	1	1	1	0	1	1	1	1	1
1	1	1	1	1	1	1	1	1	1	1	1	1	1	1	1	0	1	1	1	1	1	1	1	1	1	1	1	1	1	1	1
1	1	1	1	1	1	1	1	1	1	1	1	1	1	1	1	0	1	1	1	1	1	1	1	1	1	1	1	1	1	1	1
1	1	1	1	1	1	1	1	1	1	1	1	1	1	1	1	1	1	1	1	0	1	1	1	1	1	0	1	1	1	1	1
1	1	1	1	1	1	1	1	1	1	1	1	1	1	1	1	1	1	1	1	1	1	1	1	1	1	0	1	1	1	1	1
1	1	1	1	1	1	1	1	1	1	1	1	1	1	1	1	1	1	1	1	1	1	1	1	1	1	1	1	1	1	1	1
1	1	1	1	1	1	1	1	1	1	1	1	1	1	1	1	1	1	1	1	1	1	1	1	1	1	1	1	1	1	1	1
1	1	1	1	1	1	1	1	1	1	1	1	1	1	1	1	1	1	1	1	1	1	1	1	1	1	1	1	1	1	1	1
1	1	1	1	1	1	1	1	1	1	1	1	1	1	1	1	1	1	1	1	1	1	1	1	1	1	1	1	1	1	1	1
1	1	1	1	1	1	1	1	1	1	1	1	1	1	1	1	1	1	1	1	1	1	1	1	1	1	1	1	1	1	1	1
1	1	1	1	1	1	1	1	1	1	1	1	1	1	1	1	1	1	1	1	1	1	1	1	1	1	1	1	1	1	1	1
1	1	1	1	1	1	1	1	1	1	1	1	1	1	1	1	1	1	1	1	1	1	1	1	1	1	1	1	1	1	1	1
1	1	1	1	1	1	1	1	1	1	1	1	1	1	1	1	1	1	1	1	0	1	1	1	1	1	1	1	1	1	1	1
1	1	1	1	1	1	1	1	1	1	1	1	1	1	1	1	1	1	1	1	1	1	1	1	1	1	1	1	1	1	1	1
1	1	1	1	1	1	1	1	1	1	1	1	1	1	1	1	1	1	1	1	1	1	1	1	1	1	0	1	1	1	1	1
1	1	1	1	1	1	1	1	1	1	1	1	1	1	1	1	1	1	1	1	1	1	1	1	1	1	1	1	1	1	1	1
1	1	1	1	1	1	1	1	1	1	1	1	1	1	1	1	1	1	1	1	1	1	1	1	1	1	1	1	1	1	1	1
1	1	1	1	1	1	1	1	1	1	1	1	1	1	1	1	1	1	1	1	1	1	1	1	1	1	1	1	1	1	1	1
1	1	1	1	1	1	1	1	1	1	1	1	1	1	1	1	1	1	1	1	1	1	1	1	1	1	1	1	1	1	1	1
1	1	1	1	1	1	1	1	1	1	1	1	1	1	1	1	1	1	1	1	1	1	1	1	1	1	1	1	1	1	1	1
0	0	0	0	0	0	0	0	0	0	0	0	0	0	1	0	0	0	0	0	0	1	1	1	1	1	1	1	1	1	1	1
1	1	1	1	1	1	1	1	1	0	1	1	1	1	1	1	1	1	1	1	1	1	1	1	1	1	1	1	1	1	1	1
[illegible]	[illegible]	[illegible]	[illegible]	[illegible]	[illegible]	[illegible]	[illegible]	[illegible]	[illegible]	[illegible]	[illegible]	[illegible]	[illegible]	[illegible]	[illegible]	[illegible]	[illegible]	[illegible]	[illegible]	[illegible]	[illegible]	[illegible]	[illegible]	[illegible]	[illegible]	[illegible]	[illegible]	[illegible]	[illegible]	[illegible]	[illegible]

Species	queryItem	orthologname	stringId	identity
Cellulophaga lytica DSM 7489	HI1_00780	argH	867900.Celly_0829	100
Cellulophaga lytica DSM 7489	HI1_00313	group_1441	867900.Celly_0376	98.6
Cellulophaga lytica DSM 7489	HI1_00315	ygeA	867900.Celly_0378	99.6
Cellulophaga lytica DSM 7489	HI1_00773	argG	867900.Celly_0822	99.7
Cellulophaga lytica DSM 7489	HI1_00779	argE	867900.Celly_0828	99.4
Cellulophaga lytica DSM 7489	HI1_01711	ggt	867900.Celly_1759	99.3
Cellulophaga lytica DSM 7489	HI1_02368	glnII	867900.Celly_2396	100
Cellulophaga lytica DSM 7489	HI1_02734	ilvB	867900.Celly_2747	100
Cellulophaga lytica DSM 7489	HI1_02735	ilvH	867900.Celly_2748	100
Cellulophaga lytica DSM 7489	HI1_03165	gpx1	867900.Celly_3169	100
Cellulophaga lytica DSM 7489	HI1_02736	ilvC	867900.Celly_2749	100

Cellulophaga lytica DSM 7489	HI1_02733	ilvD	867900.Celly_2746	100
Cellulophaga lytica DSM 7489	HI1_00495	glpQ_2	867900.Celly_0551	99.7
Cellulophaga lytica DSM 7489	HI1_00740	xylB	867900.Celly_0790	99.4
Cellulophaga lytica DSM 7489	HI1_00788	group_1082	867900.Celly_0840	100
Cellulophaga lytica DSM 7489	HI1_00790	glpQ_2	867900.Celly_0842	98.3
Cellulophaga lytica DSM 7489	HI1_01427	glpQ_2	867900.Celly_1462	99.3
Cellulophaga lytica DSM 7489	HI1_02241	ppgK	867900.Celly_2272	100
Cellulophaga lytica DSM 7489	HI1_03090	xylB	867900.Celly_3094	100
Cellulophaga lytica DSM 7489	HI1_00741	xylB	867900.Celly_0791	100
Cellulophaga lytica DSM 7489	HI1_03364	exoI	867900.Celly_0065	100
Cellulophaga lytica DSM 7489	HI1_02006	nagB	867900.Celly_2044	99.7
Cellulophaga lytica DSM 7489	HI1_02306	xynA1	867900.Celly_2335	98.6

Cellulophaga lytica DSM 7489	HI1_02308	exoI	867900.Celly_2337	99.7
Cellulophaga lytica DSM 7489	HI1_02309	group_198	867900.Celly_2338	99.6
Cellulophaga lytica DSM 7489	HI1_02312	group_198	867900.Celly_2341	98.6
Cellulophaga lytica DSM 7489	HI1_03322	group_2095	867900.Celly_0023	100
Cellulophaga lytica DSM 7489	HI1_00129	group_1242	867900.Celly_0197	98.8
Cellulophaga lytica DSM 7489	HI1_00326	group_1739	867900.Celly_0389	99.8
Cellulophaga lytica DSM 7489	HI1_01284	group_566	867900.Celly_1321	100
Cellulophaga lytica DSM 7489	HI1_01285	group_1561	867900.Celly_1322	99.6
Cellulophaga lytica DSM 7489	HI1_01500	pepN_1	867900.Celly_1556	100
Cellulophaga lytica DSM 7489	HI1_01603	group_197	867900.Celly_1656	100
Cellulophaga lytica DSM 7489	HI1_01608	group_102	867900.Celly_1661	99.6
Cellulophaga lytica DSM 7489	HI1_01609	group_102	867900.Celly_1662	99.6
Cellulophaga lytica DSM 7489	HI1_01610	iolE	867900.Celly_1663	100
Cellulophaga lytica DSM 7489	HI1_01799	xynA1	867900.Celly_1842	99.9
Cellulophaga lytica DSM 7489	HI1_01808	group_2382	867900.Celly_1850	100
Cellulophaga lytica DSM 7489	HI1_01870	group_1242	867900.Celly_1912	99.4
Cellulophaga lytica DSM 7489	HI1_01895	group_2227	867900.Celly_1935	100
Cellulophaga lytica DSM 7489	HI1_02092	group_1672	867900.Celly_2130	100
Cellulophaga lytica DSM 7489	HI1_02145	group_1075	867900.Celly_2184	98.8

Cellulophaga lytica DSM 7489	HI1_02167	lacX	867900.Celly_2206	100
Cellulophaga lytica DSM 7489	HI1_02330	galK	867900.Celly_2359	99.7
Cellulophaga lytica DSM 7489	HI1_02487	dacB	867900.Celly_2506	99.8
Cellulophaga lytica DSM 7489	HI1_02571	group_2173	867900.Celly_2589	100
Cellulophaga lytica DSM 7489	HI1_02664	group_1961	867900.Celly_2677	100
Cellulophaga lytica DSM 7489	HI1_02676	group_2132	867900.Celly_2689	100
Cellulophaga lytica DSM 7489	HI1_02759	group_1698	867900.Celly_2772	100
Cellulophaga lytica DSM 7489	HI1_02816	ply	867900.Celly_2830	100
Cellulophaga lytica DSM 7489	HI1_02855	group_1441	867900.Celly_2869	100
Cellulophaga lytica DSM 7489	HI1_03113	group_566	867900.Celly_3118	100
Cellulophaga lytica DSM 7489	HI1_01936	metH_1	867900.Celly_1976	100
Cellulophaga lytica DSM 7489	HI1_01937	metH_1	867900.Celly_1977	99.9
Cellulophaga lytica DSM 7489	HI1_01872	metE	867900.Celly_1914	97
Cellulophaga lytica DSM 7489	HI1_00689	sumT	867900.Celly_0739	99.6
Cellulophaga lytica DSM 7489	HI1_00692	group_1833	867900.Celly_0742	99.5

Cellulophaga lytica DSM 7489	HI1_01929	cysH	867900.Celly_1969	100
Cellulophaga lytica DSM 7489	HI1_01932	sir	867900.Celly_1972	100
Cellulophaga lytica DSM 7489	HI1_01933	sumT	867900.Celly_1973	100
Cellulophaga lytica DSM 7489	HI1_01934	cysG	867900.Celly_1974	100
Cellulophaga lytica DSM 7489	HI1_00439	yiiM	867900.Celly_0499	100
Cellulophaga lytica DSM 7489	HI1_01521	moaC2	867900.Celly_1576	99.3
Cellulophaga lytica DSM 7489	HI1_01522	moaE	867900.Celly_1577	98.6
Cellulophaga lytica DSM 7489	HI1_01753	iorA_2	867900.Celly_1797	100
Cellulophaga lytica DSM 7489	HI1_01754	iorB	867900.Celly_1798	99.4
Cellulophaga lytica DSM 7489	HI1_02660	group_1607	867900.Celly_2673	100
Cellulophaga lytica DSM 7489	HI1_02786	iorA_2	867900.Celly_2799	100

Cellulophaga lytica DSM 7489	HI1_02787	iorB	867900.Celly_2800	99.6
Cellulophaga lytica DSM 7489	HI1_02789	moaA	867900.Celly_2802	100
Cellulophaga lytica DSM 7489	HI1_02792	moeA	867900.Celly_2805	100
Cellulophaga lytica DSM 7489	HI1_02793	pucA	867900.Celly_2806	100
Cellulophaga lytica DSM 7489	HI1_02794	nboR	867900.Celly_2807	100
Cellulophaga lytica DSM 7489	HI1_03326	group_1208	867900.Celly_0027	100
Cellulophaga lytica DSM 7489	HI1_03339	group_2453	867900.Celly_0040	99.1
Cellulophaga lytica DSM 7489	HI1_00010	group_2955	867900.Celly_0080	100
Cellulophaga lytica DSM 7489	HI1_03381	group_2955	867900.Celly_0080	97
Cellulophaga lytica DSM 7489	HI1_00067	yfiT	867900.Celly_0135	100
Cellulophaga lytica DSM 7489	HI1_00076	group_1267	867900.Celly_0144	99.5
Cellulophaga lytica DSM 7489	HI1_00185	group_1634	867900.Celly_0253	100
Cellulophaga lytica DSM 7489	HI1_00201	celZ	867900.Celly_0269	100
Cellulophaga lytica DSM 7489	HI1_00258	group_1164	867900.Celly_0321	100
Cellulophaga lytica DSM 7489	HI1_00282	group_1688	867900.Celly_0345	100
Cellulophaga lytica DSM 7489	HI1_00284	mdtG	867900.Celly_0347	100
Cellulophaga lytica DSM 7489	HI1_00285	mdtG	867900.Celly_0347	100
Cellulophaga lytica DSM 7489	HI1_00286	inhA	867900.Celly_0348	100

Cellulophaga lytica DSM 7489	HI1_00301	rppH	867900.Celly_0365	93.6
Cellulophaga lytica DSM 7489	HI1_00629	group_197	867900.Celly_0681	99.4
Cellulophaga lytica DSM 7489	HI1_00674	group_1685	867900.Celly_0723	98.3
Cellulophaga lytica DSM 7489	HI1_00677	mtfA	867900.Celly_0727	99.6
Cellulophaga lytica DSM 7489	HI1_00699	group_1896	867900.Celly_0749	99.6
Cellulophaga lytica DSM 7489	HI1_00798	ydjA	867900.Celly_0850	100
Cellulophaga lytica DSM 7489	HI1_00815	glmU	867900.Celly_0866	100
Cellulophaga lytica DSM 7489	HI1_00825	quiP	867900.Celly_0876	100
Cellulophaga lytica DSM 7489	HI1_00880	group_1689	867900.Celly_0935	99.2
Cellulophaga lytica DSM 7489	HI1_00882	perR	867900.Celly_0937	94.4
Cellulophaga lytica DSM 7489	HI1_00919	celZ	867900.Celly_0965	100
Cellulophaga lytica DSM 7489	HI1_00928	ymfA	867900.Celly_0975	97.9
Cellulophaga lytica DSM 7489	HI1_00937	yjoA	867900.Celly_0984	100
Cellulophaga lytica DSM 7489	HI1_00939	group_2080	867900.Celly_0986	100
Cellulophaga lytica DSM 7489	HI1_00950	bspRIM	867900.Celly_0997	100
Cellulophaga lytica DSM 7489	HI1_00977	merT	867900.Celly_1023	98.6
Cellulophaga lytica DSM 7489	HI1_00985	group_534	867900.Celly_1031	99.3

Cellulophaga lytica DSM 7489	HI1_00997	umuD	867900.Celly_1043	99.3
Cellulophaga lytica DSM 7489	HI1_01023	group_1625	867900.Celly_1063	99.3
Cellulophaga lytica DSM 7489	HI1_01066	group_1961	867900.Celly_1106	100
Cellulophaga lytica DSM 7489	HI1_01079	group_1285	867900.Celly_1116	98.4
Cellulophaga lytica DSM 7489	HI1_01153	group_1954	867900.Celly_1191	100
Cellulophaga lytica DSM 7489	HI1_01246	nlhH	867900.Celly_1283	99.7
Cellulophaga lytica DSM 7489	HI1_01300	group_2038	867900.Celly_1335	99.1
Cellulophaga lytica DSM 7489	HI1_01303	group_1599	867900.Celly_1338	99.2
Cellulophaga lytica DSM 7489	HI1_01341	yqjI	867900.Celly_1377	99.1
Cellulophaga lytica DSM 7489	HI1_01346	group_1678	867900.Celly_1381	91.8
Cellulophaga lytica DSM 7489	HI1_01366	group_2300	867900.Celly_1401	99.3
Cellulophaga lytica DSM 7489	HI1_01383	group_1562	867900.Celly_1418	99.3
Cellulophaga lytica DSM 7489	HI1_01405	group_197	867900.Celly_1440	99.5
Cellulophaga lytica DSM 7489	HI1_01429	rhaR_1	867900.Celly_1464	99.2
Cellulophaga lytica DSM 7489	HI1_01473	guaB_2	867900.Celly_1524	100
Cellulophaga lytica DSM 7489	HI1_01475	mfpA	867900.Celly_1526	90.5
Cellulophaga lytica DSM 7489	HI1_01496	hpcD	867900.Celly_1552	99.1
Cellulophaga lytica DSM 7489	HI1_01571	bag	867900.Celly_1625	99.6
Cellulophaga lytica DSM 7489	HI1_01577	bspRIM	867900.Celly_1631	99.8
Cellulophaga lytica DSM 7489	HI1_01599	oprO	867900.Celly_1652	99.8

Cellulophaga lytica DSM 7489	HI1_01643	group_2917	867900.Celly_1696	99.8
Cellulophaga lytica DSM 7489	HI1_01736	group_1705	867900.Celly_1783	100
Cellulophaga lytica DSM 7489	HI1_01855	secE2	867900.Celly_1897	100
Cellulophaga lytica DSM 7489	HI1_01868	group_1652	867900.Celly_1910	99.5
Cellulophaga lytica DSM 7489	HI1_01876	mdtG	867900.Celly_1918	99.7
Cellulophaga lytica DSM 7489	HI1_02028	group_2532	867900.Celly_2066	100
Cellulophaga lytica DSM 7489	HI1_02091	group_1694	867900.Celly_2129	100
Cellulophaga lytica DSM 7489	HI1_02141	mtfA	867900.Celly_2180	99.6
Cellulophaga lytica DSM 7489	HI1_02155	group_2163	867900.Celly_2194	100
Cellulophaga lytica DSM 7489	HI1_02211	group_1705	867900.Celly_2243	100
Cellulophaga lytica DSM 7489	HI1_02214	group_1843	867900.Celly_2246	99.7
Cellulophaga lytica DSM 7489	HI1_02269	yidD	867900.Celly_2300	100
Cellulophaga lytica DSM 7489	HI1_02316	xynA1	867900.Celly_2345	99.6
Cellulophaga lytica DSM 7489	HI1_02366	group_1848	867900.Celly_2394	100
Cellulophaga lytica DSM 7489	HI1_02473	group_2433	867900.Celly_2493	100
Cellulophaga lytica DSM 7489	HI1_02502	group_1791	867900.Celly_2521	99.6
Cellulophaga lytica DSM 7489	HI1_02504	group_1772	867900.Celly_2523	100
Cellulophaga lytica DSM 7489	HI1_02535	group_2568	867900.Celly_2554	100
Cellulophaga lytica DSM 7489	HI1_02632	ywqE	867900.Celly_2645	99.2
Cellulophaga lytica DSM 7489	HI1_02701	group_197	867900.Celly_2714	100
Cellulophaga lytica DSM 7489	HI1_02754	arnT	867900.Celly_2767	99.4

Cellulophaga lytica DSM 7489	HI1_02865	cotH	867900.Celly_2879	98.8
Cellulophaga lytica DSM 7489	HI1_02873	mtrR_2	867900.Celly_2887	100
Cellulophaga lytica DSM 7489	HI1_02874	group_2135	867900.Celly_2888	100
Cellulophaga lytica DSM 7489	HI1_02875	group_1559	867900.Celly_2889	99.8
Cellulophaga lytica DSM 7489	HI1_02889	umuD	867900.Celly_2903	98
Cellulophaga lytica DSM 7489	HI1_02907	group_2162	867900.Celly_2919	100
Cellulophaga lytica DSM 7489	HI1_02908	group_2162	867900.Celly_2920	100
Cellulophaga lytica DSM 7489	HI1_03004	quiP	867900.Celly_3016	99.5
Cellulophaga lytica DSM 7489	HI1_03032	group_1748	867900.Celly_3044	96.9
Cellulophaga lytica DSM 7489	HI1_03033	perR	867900.Celly_3045	100
Cellulophaga lytica DSM 7489	HI1_03057	group_2097	867900.Celly_3060	95.3
Cellulophaga lytica DSM 7489	HI1_03058	group_2822	867900.Celly_3061	98.7
Cellulophaga lytica DSM 7489	HI1_03121	fepA	867900.Celly_3126	100
Cellulophaga lytica DSM 7489	HI1_03156	bag	867900.Celly_3160	100
Cellulophaga lytica DSM 7489	HI1_01443	perR	867900.Celly_3220	47.1
Cellulophaga lytica DSM 7489	HI1_03214	perR	867900.Celly_3220	100
Cellulophaga lytica DSM 7489	HI1_03262	group_1728	867900.Celly_3266	98.8
Cellulophaga lytica DSM 7489	HI1_03263	group_2063	867900.Celly_3267	99
Cellulophaga lytica DSM 7489	HI1_03264	group_2200	867900.Celly_3268	99.1
Cellulophaga lytica DSM 7489	HI1_03269	group_746	867900.Celly_3273	100
Cellulophaga lytica DSM 7489	HI1_03270	group_747	867900.Celly_3274	100
Cellulophaga lytica DSM 7489	HI1_00097	rpmH	867900.Celly_0165	100
Flavobacterium johnsoniae UW101	IR1_03726	ggt	376686.Fjoh_4506	80.5

Flavobacterium johnsoniae UW101	IR1_03462	ilvD	376686.Fjoh_2863	93.2
Flavobacterium johnsoniae UW101	IR1_03368	ygeA	376686.Fjoh_2497	55.9
Flavobacterium johnsoniae UW101	IR1_02404	ygeA	376686.Fjoh_2497	85.2
Flavobacterium johnsoniae UW101	IR1_04000	ygeA	376686.Fjoh_2497	45.7
Flavobacterium johnsoniae UW101	IR1_02054	argH	376686.Fjoh_3427	95.1
Flavobacterium johnsoniae UW101	IR1_03464	ilvH	376686.Fjoh_2861	93.6
Flavobacterium johnsoniae UW101	IR1_03463	ilvB	376686.Fjoh_2862	91
Flavobacterium johnsoniae UW101	IR1_01069	ilvB	376686.Fjoh_3836	29
Flavobacterium johnsoniae UW101	IR1_02055	argE	376686.Fjoh_3428	92.1
Flavobacterium johnsoniae UW101	IR1_01846	gpx1	376686.Fjoh_2944	82.6
Flavobacterium johnsoniae UW101	IR1_02545	glnII	376686.Fjoh_2344	96.2
Flavobacterium johnsoniae UW101	IR1_02063	argG	376686.Fjoh_3435	95.2
Flavobacterium johnsoniae UW101	IR1_03465	ilvC	376686.Fjoh_2860	97.5
Flavobacterium johnsoniae UW101	IR1_01333	xynA1	376686.Fjoh_2035	81.4
Flavobacterium johnsoniae UW101	IR1_01327	nagB	376686.Fjoh_2029	93.1
Flavobacterium johnsoniae UW101	IR1_02514	nagB	376686.Fjoh_2381	93
Flavobacterium johnsoniae UW101	IR1_01337	exoI	376686.Fjoh_2039	78.1
Flavobacterium johnsoniae UW101	IR1_01330	group_198	376686.Fjoh_2032	84.2
Flavobacterium johnsoniae UW101	IR1_02540	group_198	376686.Fjoh_2358	91.4
Flavobacterium johnsoniae UW101	IR1_03288	group_198	376686.Fjoh_3125	33.7
Flavobacterium johnsoniae UW101	IR1_01339	group_198	376686.Fjoh_2041	83.6
Flavobacterium johnsoniae UW101	IR1_01531	group_198	376686.Fjoh_2715	83.3
Flavobacterium johnsoniae UW101	IR1_03841	group_198	376686.Fjoh_3124	86.1
Flavobacterium johnsoniae UW101	IR1_01530	group_198	376686.Fjoh_2714	84
Flavobacterium johnsoniae UW101	IR1_03784	nagB	376686.Fjoh_4557	94.9
Flavobacterium johnsoniae UW101	IR1_03783	exoI	376686.Fjoh_4556	84
Flavobacterium johnsoniae UW101	IR1_00109	xynA1	376686.Fjoh_1765	77.8
Flavobacterium johnsoniae UW101	IR1_02410	rhaR_1	376686.Fjoh_2491	72.3
Flavobacterium johnsoniae UW101	IR1_01155	rhaR_1	376686.Fjoh_2491	46.2
Flavobacterium johnsoniae UW101	IR1_04415	group_1694	376686.Fjoh_2215	47.3
Flavobacterium johnsoniae UW101	IR1_04414	group_1672	376686.Fjoh_2216	69.8

Flavobacterium johnsoniae UW101	IR1_02836	nlhH	376686.Fjoh_0134	88.2
Flavobacterium johnsoniae UW101	IR1_03167	group_1843	376686.Fjoh_0682	56.5
Flavobacterium johnsoniae UW101	IR1_00573	group_2366	376686.Fjoh_1294	69.9
Flavobacterium johnsoniae UW101	IR1_01567	group_1688	376686.Fjoh_4945	80.5
Flavobacterium johnsoniae UW101	IR1_01566	group_1688	376686.Fjoh_4945	70.8
Flavobacterium johnsoniae UW101	IR1_00723	galK	376686.Fjoh_1114	85.2
Flavobacterium johnsoniae UW101	IR1_04775	group_566	376686.Fjoh_0980	85.5
Flavobacterium johnsoniae UW101	IR1_04774	group_1561	376686.Fjoh_0981	57.1
Flavobacterium johnsoniae UW101	IR1_02066	lacX	376686.Fjoh_3438	72.8
Flavobacterium johnsoniae UW101	IR1_03097	group_1267	376686.Fjoh_0424	82.9
Flavobacterium johnsoniae UW101	IR1_01874	group_1848	376686.Fjoh_2767	91.1
Flavobacterium johnsoniae UW101	IR1_00358	metH_1	376686.Fjoh_1510	92.1
Flavobacterium johnsoniae UW101	IR1_03365	metE	376686.Fjoh_3839	93
Flavobacterium johnsoniae UW101	IR1_00359	metH_1	376686.Fjoh_1508	94.3
Flavobacterium johnsoniae UW101	IR1_00345	metE	376686.Fjoh_1519	89.7
Flavobacterium johnsoniae UW101	IR1_00362	sumT	376686.Fjoh_1505	87.5
Flavobacterium johnsoniae UW101	IR1_04220	sumT	376686.Fjoh_4641	83.2
Flavobacterium johnsoniae UW101	IR1_00361	cysG	376686.Fjoh_1506	92.2
Flavobacterium johnsoniae UW101	IR1_00366	cysH	376686.Fjoh_1501	85.6
Flavobacterium johnsoniae UW101	IR1_00363	sir	376686.Fjoh_1504	86.6
Flavobacterium johnsoniae UW101	IR1_02672	iorB	376686.Fjoh_4200	83.7
Flavobacterium johnsoniae UW101	IR1_04217	moaE	376686.Fjoh_4638	95.1
Flavobacterium johnsoniae UW101	IR1_00465	glpQ_2	376686.Fjoh_1397	75.2
Flavobacterium johnsoniae UW101	IR1_02670	iorA_2	376686.Fjoh_4202	87.1
Flavobacterium johnsoniae UW101	IR1_01472	xylB	376686.Fjoh_4885	82.1
Flavobacterium johnsoniae UW101	IR1_04222	moeA	376686.Fjoh_4644	73.5
Flavobacterium johnsoniae UW101	IR1_04216	moaC2	376686.Fjoh_4637	88.8
Flavobacterium johnsoniae UW101	IR1_04215	moaA	376686.Fjoh_4636	82.2
Flavobacterium johnsoniae UW101	IR1_02139	glpQ_2	376686.Fjoh_3520	71
Flavobacterium johnsoniae UW101	IR1_00853	pucA	376686.Fjoh_4205	30.7
Flavobacterium johnsoniae UW101	IR1_02667	pucA	376686.Fjoh_4205	71.8
Flavobacterium johnsoniae UW101	IR1_02668	nboR	376686.Fjoh_4204	58.3
Flavobacterium johnsoniae UW101	IR1_03149	rhaR_1	376686.Fjoh_0538	42

Flavobacterium johnsoniae UW101	IR1_00577	rhaR_1	376686.Fjoh_1291	69.1
Flavobacterium johnsoniae UW101	IR1_02619	yidD	376686.Fjoh_2267	92.4
Flavobacterium johnsoniae UW101	IR1_03689	group_1075	376686.Fjoh_0159	67.6
Flavobacterium johnsoniae UW101	IR1_01364	group_1748	376686.Fjoh_3287	69
Flavobacterium johnsoniae UW101	IR1_01420	bag	376686.Fjoh_4930	77.6
Flavobacterium johnsoniae UW101	IR1_01615	bag	376686.Fjoh_4995	69.2
Flavobacterium johnsoniae UW101	IR1_04375	group_2366	376686.Fjoh_4797	44.8
Flavobacterium johnsoniae UW101	IR1_02759	group_1839	376686.Fjoh_0055	63.3
Flavobacterium johnsoniae UW101	IR1_02798	rpmH	376686.Fjoh_0100	100
Flavobacterium johnsoniae UW101	IR1_00369	group_1705	376686.Fjoh_1497	53.7
Flavobacterium johnsoniae UW101	IR1_00033	group_2135	376686.Fjoh_1822	76
Flavobacterium johnsoniae UW101	IR1_01475	group_746	376686.Fjoh_4882	89.8
Flavobacterium johnsoniae UW101	IR1_01474	group_747	376686.Fjoh_4883	82.4
Flavobacterium johnsoniae UW101	IR1_03104	group_1652	376686.Fjoh_0430	79
Flavobacterium johnsoniae UW101	IR1_04501	group_1896	376686.Fjoh_2122	83.4
Flavobacterium johnsoniae UW101	IR1_03407	group_1082	376686.Fjoh_2933	80.6
Flavobacterium johnsoniae UW101	IR1_02252	group_2080	376686.Fjoh_2964	70.2
Flavobacterium johnsoniae UW101	IR1_01864	group_2080	376686.Fjoh_2964	94.1
Flavobacterium johnsoniae UW101	IR1_01613	ywqE	376686.Fjoh_4993	65.7
Flavobacterium johnsoniae UW101	IR1_01262	quiP	376686.Fjoh_0502	87.3
Flavobacterium johnsoniae UW101	IR1_00614	group_1728	376686.Fjoh_1252	85.6
Flavobacterium johnsoniae UW101	IR1_02865	mfpA	376686.Fjoh_0182	92.6
Flavobacterium johnsoniae UW101	IR1_02413	mfpA	376686.Fjoh_2486	92.1
Flavobacterium johnsoniae UW101	IR1_02031	mfpA	376686.Fjoh_3404	73.4
Flavobacterium johnsoniae UW101	IR1_04110	group_2917	376686.Fjoh_0745	91.1
Flavobacterium johnsoniae UW101	IR1_01871	mdtG	376686.Fjoh_2773	89.3
Flavobacterium johnsoniae UW101	IR1_00808	merT	376686.Fjoh_3089	59.9
Flavobacterium johnsoniae UW101	IR1_01569	celZ	376686.Fjoh_4946	78.9
Flavobacterium johnsoniae UW101	IR1_01635	paiA_2	376686.Fjoh_3300	58.1
Flavobacterium johnsoniae UW101	IR1_02032	paiA_2	376686.Fjoh_3407	64.2
Flavobacterium johnsoniae UW101	IR1_01938	xylB	376686.Fjoh_2000	87.6
Flavobacterium johnsoniae UW101	IR1_04073	xylB	376686.Fjoh_2000	43.9
Flavobacterium johnsoniae UW101	IR1_04428	nlhH	376686.Fjoh_2198	84.4
Flavobacterium johnsoniae UW101	IR1_01051	prc_1	376686.Fjoh_1277	49.3
Flavobacterium johnsoniae UW101	IR1_01829	prc_1	376686.Fjoh_1277	44.7
Flavobacterium johnsoniae UW101	IR1_00558	prc_1	376686.Fjoh_1277	48.7
Flavobacterium johnsoniae UW101	IR1_04446	nap	376686.Fjoh_2171	77.4
Flavobacterium johnsoniae UW101	IR1_03231	lgrD_1	376686.Fjoh_2093	31.1
Flavobacterium johnsoniae UW101	IR1_03231	lgrD_1	376686.Fjoh_2093	35.1

Flavobacterium johnsoniae UW101	IR1_00328	merT	376686.Fjoh_1541	73.6
Flavobacterium johnsoniae UW101	IR1_04570	nlhH	376686.Fjoh_2023	91.7
Flavobacterium johnsoniae UW101	IR1_04115	group_1625	376686.Fjoh_0739	69.5
Flavobacterium johnsoniae UW101	IR1_03914	perR	376686.Fjoh_0931	85.2
Flavobacterium johnsoniae UW101	IR1_04769	group_1599	376686.Fjoh_0986	85
Flavobacterium johnsoniae UW101	IR1_00613	group_2063	376686.Fjoh_1253	80.8
Flavobacterium johnsoniae UW101	IR1_00612	group_2200	376686.Fjoh_1254	85.3
Flavobacterium johnsoniae UW101	IR1_00972	group_1653	376686.Fjoh_1929	90.7
Flavobacterium johnsoniae UW101	IR1_03347	pepN_1	376686.Fjoh_1979	28.4
Flavobacterium johnsoniae UW101	IR1_02485	norB	376686.Fjoh_2413	91.5
Flavobacterium johnsoniae UW101	IR1_02395	group_1208	376686.Fjoh_2507	62.8
Flavobacterium johnsoniae UW101	IR1_03459	group_1607	376686.Fjoh_2866	91.1
Flavobacterium johnsoniae UW101	IR1_02134	group_2300	376686.Fjoh_3514	69.1
Flavobacterium johnsoniae UW101	IR1_03324	group_1739	376686.Fjoh_3759	87.1
Flavobacterium johnsoniae UW101	IR1_00989	group_197	376686.Fjoh_3800	38
Flavobacterium johnsoniae UW101	IR1_01535	group_1559	376686.Fjoh_3959	88.8
Flavobacterium johnsoniae UW101	IR1_03540	nlhH	376686.Fjoh_4253	70.1
Flavobacterium johnsoniae UW101	IR1_03553	nlhH	376686.Fjoh_4266	78.1
Flavobacterium johnsoniae UW101	IR1_02020	nlhH	376686.Fjoh_4266	31.8
Flavobacterium johnsoniae UW101	IR1_04207	group_1833	376686.Fjoh_4626	90.1
Marinobacter algicola DG893	HM-33_00508	argG	443152.MDG893_01075	99
Marinobacter algicola DG893	HM-33_03120	argH	443152.MDG893_14735	98.1
Marinobacter algicola DG893	HM-33_03936	argE	443152.MDG893_10361	97.1
Marinobacter algicola DG893	HM-33_00719	ilvD	443152.MDG893_00030	97.7
Marinobacter algicola DG893	HM-33_03858	ilvH	443152.MDG893_12844	100
Marinobacter algicola DG893	HM-33_03859	ilvB	443152.MDG893_12849	98.4
Marinobacter algicola DG893	HM-33_00799	ggt	443152.MDG893_08955	89.4
Marinobacter algicola DG893	HM-33_02788	glnII	443152.MDG893_16197	96.7
Marinobacter algicola DG893	HM-33_03541	glnII	443152.MDG893_13564	98.7
Marinobacter algicola DG893	HM-33_01677	argE	443152.MDG893_20369	97.2
Marinobacter algicola DG893	HM-33_01455	gpx1	443152.MDG893_05544	97.5
Marinobacter algicola DG893	HM-33_01908	ilvB	443152.MDG893_04272	95.9
Marinobacter algicola DG893	HM-33_02816	gpx1	443152.MDG893_16322	91.1
Marinobacter algicola DG893	HM-33_01123	ilvB	443152.MDG893_07170	89.1
Marinobacter algicola DG893	HM-33_02462	ilvB	443152.MDG893_10856	88.7

Marinobacter algicola DG893	HM-33_03857	ilvC	443152.MDG893_12839	98.8
Marinobacter algicola DG893	HM-33_01622	ilvD	443152.MDG893_20104	97.1
Marinobacter algicola DG893	HM-33_00727	xylB	443152.MDG893_05159	98
Marinobacter algicola DG893	HM-33_01517	glpQ1	443152.MDG893_19429	93
Marinobacter algicola DG893	HM-33_02715	xylB	443152.MDG893_11889	95.5
Marinobacter algicola DG893	HM-33_03101	gnl	443152.MDG893_14640	92.6
Marinobacter algicola DG893	HM-33_00687	glpQ_2	443152.MDG893_00180	85.5
Marinobacter algicola DG893	HM-33_00635	glpQ_2	443152.MDG893_00432	94
Marinobacter algicola DG893	HM-33_01039	xylB	443152.MDG893_07635	80.2
Marinobacter algicola DG893	HM-33_00725	xylB	443152.MDG893_07635	34.6
Marinobacter algicola DG893	HM-33_01038	xylB	443152.MDG893_07640	92.7
Marinobacter algicola DG893	HM-33_01209	metH_1	443152.MDG893_06745	96.1
Marinobacter algicola DG893	HM-33_03980	metE	443152.MDG893_13334	93.7
Marinobacter algicola DG893	HM-33_01231	sumT	443152.MDG893_06630	97.1
Marinobacter algicola DG893	HM-33_01268	cysH	443152.MDG893_06439	98.8
Marinobacter algicola DG893	HM-33_01212	sir	443152.MDG893_06730	97.1
Marinobacter algicola DG893	HM-33_03749	iorB	443152.MDG893_09466	88.3
Marinobacter algicola DG893	HM-33_03748	iorA_2	443152.MDG893_09471	88.2
Marinobacter algicola DG893	HM-33_00649	nboR	443152.MDG893_00365	78.8
Marinobacter algicola DG893	HM-33_03130	moaC2	443152.MDG893_14785	92.1
Marinobacter algicola DG893	HM-33_03131	moeA	443152.MDG893_14790	85.4
Marinobacter algicola DG893	HM-33_03133	moaE	443152.MDG893_14800	94.6
Marinobacter algicola DG893	HM-33_03195	moeA	443152.MDG893_15527	92.4
Marinobacter algicola DG893	HM-33_01081	pucA	443152.MDG893_07390	75.9
Marinobacter algicola DG893	HM-33_03367	moaA	443152.MDG893_03465	91.8
Marinobacter algicola DG893	HM-33_03465	moaC2	443152.MDG893_12630	96.9
Marinobacter algicola DG893	HM-33_01886	moaA	443152.MDG893_04347	94
Marinobacter algicola DG893	HM-33_03671	guaB_2	443152.MDG893_17227	98.1
Marinobacter algicola DG893	HM-33_03448	ilvD	443152.MDG893_12420	97.1
Marinobacter algicola DG893	HM-33_02279	nlhH	443152.MDG893_17857	81.8
Marinobacter algicola DG893	HM-33_00770	inhA	443152.MDG893_18679	28
Marinobacter algicola DG893	HM-33_03408	group_2135	443152.MDG893_12223	89.7
Marinobacter algicola DG893	HM-33_02747	group_2038	443152.MDG893_16042	93.5
Marinobacter algicola DG893	HM-33_02758	mhqP	443152.MDG893_16102	93.1
Marinobacter algicola DG893	HM-33_00306	nlhH	443152.MDG893_02030	92.6

Marinobacter algicola DG893	HM-33_00267	secE2	443152.MDG893_02235	97.2
Marinobacter algicola DG893	HM-33_00847	ygeA	443152.MDG893_08716	87.4
Marinobacter algicola DG893	HM-33_01656	mtrR_2	443152.MDG893_20279	94
Marinobacter algicola DG893	HM-33_01705	group_1833	443152.MDG893_20504	88.7
Marinobacter algicola DG893	HM-33_00756	group_1739	443152.MDG893_05314	94.8
Marinobacter algicola DG893	HM-33_03263	group_1685	443152.MDG893_03810	97.9
Marinobacter algicola DG893	HM-33_02216	yidD	443152.MDG893_18182	92.6
Marinobacter algicola DG893	HM-33_00125	rhaR_1	443152.MDG893_02795	88.5
Marinobacter algicola DG893	HM-33_01310	group_534	443152.MDG893_06224	96.1
Marinobacter algicola DG893	HM-33_01193	mtrR_2	443152.MDG893_06810	97.1
Marinobacter algicola DG893	HM-33_00904	ydjA	443152.MDG893_08270	82.3
Marinobacter algicola DG893	HM-33_02901	merT	443152.MDG893_09936	73
Marinobacter algicola DG893	HM-33_02385	quiP	443152.MDG893_10496	94.3
Marinobacter algicola DG893	HM-33_02394	rimK_3	443152.MDG893_10536	92.7
Marinobacter algicola DG893	HM-33_02488	group_2038	443152.MDG893_10976	94.1
Marinobacter algicola DG893	HM-33_02551	mtfA	443152.MDG893_11196	82.5
Marinobacter algicola DG893	HM-33_00550	umuD	443152.MDG893_00867	96.5
Marinobacter algicola DG893	HM-33_02902	merT	443152.MDG893_09931	60
Muricauda ruestringensis B1	HM-21_01276	argH	886377.Murru_0934	90.8
Muricauda ruestringensis B1	HM-21_01766	ilvD	886377.Murru_3252	93
Muricauda ruestringensis B1	HM-21_02570	glnII	886377.Murru_0414	85.7
Muricauda ruestringensis B1	HM-21_01284	argG	886377.Murru_0926	92.1

Muricauda ruestringensis B1	HM-21_01277	argE	886377.Murru_0933	84.8
Muricauda ruestringensis B1	HM-21_01806	ggt	886377.Murru_3163	83.2
Muricauda ruestringensis B1	HM-21_01770	ilvC	886377.Murru_3249	95.7
Muricauda ruestringensis B1	HM-21_01769	ilvH	886377.Murru_3250	88.4
Muricauda ruestringensis B1	HM-21_01767	ilvB	886377.Murru_3251	87.2
Muricauda ruestringensis B1	HM-21_01267	xylB	886377.Murru_0946	89.4
Muricauda ruestringensis B1	HM-21_01967	group_102	886377.Murru_0591	60.4
Muricauda ruestringensis B1	HM-21_01268	group_1082	886377.Murru_0945	63.6
Muricauda ruestringensis B1	HM-21_01264	glpQ_2	886377.Murru_0949	72.3
Muricauda ruestringensis B1	HM-21_01044	xylB	886377.Murru_1160	81.8
Muricauda ruestringensis B1	HM-21_00646	group_102	886377.Murru_1413	68.6

Muricauda ruestringensis B1	HM-21_00611	iolE	886377.Murru_1526	94
Muricauda ruestringensis B1	HM-21_00610	group_102	886377.Murru_1527	72.4
Muricauda ruestringensis B1	HM-21_00609	group_102	886377.Murru_1528	84.2
Muricauda ruestringensis B1	HM-21_00536	xylB	886377.Murru_1605	77.6
Muricauda ruestringensis B1	HM-21_00912	group_102	886377.Murru_2765	73.6
Muricauda ruestringensis B1	HM-21_01932	group_102	886377.Murru_3307	77.7
Muricauda ruestringensis B1	HM-21_01933	group_102	886377.Murru_3308	76.8
Muricauda ruestringensis B1	HM-21_01605	group_1164	886377.Murru_0035	71.3
Muricauda ruestringensis B1	HM-21_01502	group_566	886377.Murru_0132	70.9
Muricauda ruestringensis B1	HM-21_02726	group_2095	886377.Murru_0294	78.8
Muricauda ruestringensis B1	HM-21_02690	arnT	886377.Murru_0325	80.1
Muricauda ruestringensis B1	HM-21_01227	qdoI	886377.Murru_0461	52.5
Muricauda ruestringensis B1	HM-21_02543	galK	886377.Murru_0564	52.3
Muricauda ruestringensis B1	HM-21_02399	lacX	886377.Murru_0616	67.5
Muricauda ruestringensis B1	HM-21_02420	group_1075	886377.Murru_0636	52.5
Muricauda ruestringensis B1	HM-21_02481	group_1672	886377.Murru_0694	71.5

Muricauda ruestringensis B1	HM-21_02482	group_1694	886377.Murru_0695	56.9
Muricauda ruestringensis B1	HM-21_00824	group_566	886377.Murru_1321	75.9
Muricauda ruestringensis B1	HM-21_00823	group_1561	886377.Murru_1322	67.5
Muricauda ruestringensis B1	HM-21_00733	group_1562	886377.Murru_1430	65
Muricauda ruestringensis B1	HM-21_00574	group_2580	886377.Murru_1561	61.4
Muricauda ruestringensis B1	HM-21_02394	xynA1	886377.Murru_1821	60
Muricauda ruestringensis B1	HM-21_00130	group_2227	886377.Murru_1914	65.5
Muricauda ruestringensis B1	HM-21_00026	group_1961	886377.Murru_2003	60
Muricauda ruestringensis B1	HM-21_02192	ywqE	886377.Murru_2333	56.9
Muricauda ruestringensis B1	HM-21_03257	group_1242	886377.Murru_2387	58.5
Muricauda ruestringensis B1	HM-21_02110	group_2132	886377.Murru_2974	92
Muricauda ruestringensis B1	HM-21_01681	group_566	886377.Murru_3439	58.1
Muricauda ruestringensis B1	HM-21_00068	metH_1	886377.Murru_1966	88.4
Muricauda ruestringensis B1	HM-21_03457	metH_1	886377.Murru_1966	55
Muricauda ruestringensis B1	HM-21_00066	metH_1	886377.Murru_1967	84.7
Muricauda ruestringensis B1	HM-21_00375	cysG	886377.Murru_1681	85.3

Muricauda ruestringensis B1	HM-21_00076	cysH	886377.Murru_1958	92.8
Muricauda ruestringensis B1	HM-21_00073	sir	886377.Murru_1961	87.8
Muricauda ruestringensis B1	HM-21_00072	sumT	886377.Murru_1962	81.8
Muricauda ruestringensis B1	HM-21_02956	iorB	886377.Murru_0166	62.2
Muricauda ruestringensis B1	HM-21_01929	iorB	886377.Murru_0166	48.3
Muricauda ruestringensis B1	HM-21_01467	iorB	886377.Murru_0166	82.2
Muricauda ruestringensis B1	HM-21_02957	iorB	886377.Murru_0166	60.3
Muricauda ruestringensis B1	HM-21_01930	iorA_2	886377.Murru_0167	70.9
Muricauda ruestringensis B1	HM-21_01466	iorA_2	886377.Murru_0167	86.8

Muricauda ruestringensis B1	HM-21_02955	iorA_2	886377.Murru_0167	77.5
Muricauda ruestringensis B1	HM-21_00314	umuD	886377.Murru_1723	83.9
Muricauda ruestringensis B1	HM-21_03321	yiiM	886377.Murru_2186	67
Muricauda ruestringensis B1	HM-21_01923	moeA	886377.Murru_3377	28.3
Muricauda ruestringensis B1	HM-21_01927	moaA	886377.Murru_3378	33.1
Muricauda ruestringensis B1	HM-21_00492	moaC2	886377.Murru_3379	69.3
Muricauda ruestringensis B1	HM-21_00493	moaE	886377.Murru_3380	71.1
Muricauda ruestringensis B1	HM-21_01921	nboR	886377.Murru_3383	37.2
Muricauda ruestringensis B1	HM-21_01922	pucA	886377.Murru_3384	33
Muricauda ruestringensis B1	HM-21_01827	inhA	886377.Murru_0044	71.3
Muricauda ruestringensis B1	HM-21_01595	group_1267	886377.Murru_0047	69.8
Muricauda ruestringensis B1	HM-21_01518	group_1772	886377.Murru_0119	70.3
Muricauda ruestringensis B1	HM-21_01516	group_1791	886377.Murru_0121	87

Muricauda ruestringensis B1	HM-21_01493	dacB	886377.Murru_0139	65.1
Muricauda ruestringensis B1	HM-21_01475	group_2532	886377.Murru_0161	68.6
Muricauda ruestringensis B1	HM-21_00544	group_2689	886377.Murru_0169	76.1
Muricauda ruestringensis B1	HM-21_03125	inhA	886377.Murru_0180	29.9
Muricauda ruestringensis B1	HM-21_00332	nagB	886377.Murru_0236	56.9
Muricauda ruestringensis B1	HM-21_02722	group_1208	886377.Murru_0298	64.8
Muricauda ruestringensis B1	HM-21_02682	exoI	886377.Murru_0331	74.5
Muricauda ruestringensis B1	HM-21_00360	exoI	886377.Murru_0331	33.1
Muricauda ruestringensis B1	HM-21_02597	group_1688	886377.Murru_0392	82.9
Muricauda ruestringensis B1	HM-21_02577	group_1739	886377.Murru_0406	81
Muricauda ruestringensis B1	HM-21_02567	group_1848	886377.Murru_0418	80.9
Muricauda ruestringensis B1	HM-21_02358	ppgK	886377.Murru_0513	88.1
Muricauda ruestringensis B1	HM-21_02364	group_2200	886377.Murru_0521	42.7
Muricauda ruestringensis B1	HM-21_02365	group_2063	886377.Murru_0522	75.8
Muricauda ruestringensis B1	HM-21_02366	group_1728	886377.Murru_0523	77.9
Muricauda ruestringensis B1	HM-21_02940	exoI	886377.Murru_0547	33.2

Muricauda ruestringensis B1	HM-21_02961	rhaR_1	886377.Murru_0555	43.9
Muricauda ruestringensis B1	HM-21_02531	umuD	886377.Murru_0739	81
Muricauda ruestringensis B1	HM-21_01882	umuD	886377.Murru_0739	53.8
Muricauda ruestringensis B1	HM-21_01443	group_197	886377.Murru_0786	85.6
Muricauda ruestringensis B1	HM-21_00859	umuD	886377.Murru_0798	47.6
Muricauda ruestringensis B1	HM-21_01428	umuD	886377.Murru_0798	54.8
Muricauda ruestringensis B1	HM-21_01887	umuD	886377.Murru_0798	57.1
Muricauda ruestringensis B1	HM-21_01430	umuD	886377.Murru_0798	75
Muricauda ruestringensis B1	HM-21_01859	xynA1	886377.Murru_0937	28.7
Muricauda ruestringensis B1	HM-21_02265	xynA1	886377.Murru_0937	27.7
Muricauda ruestringensis B1	HM-21_00398	xynA1	886377.Murru_0937	28.8
Muricauda ruestringensis B1	HM-21_03146	xynA1	886377.Murru_0937	32.3
Muricauda ruestringensis B1	HM-21_01252	bag	886377.Murru_0959	63.5
Muricauda ruestringensis B1	HM-21_00884	rhaR_1	886377.Murru_0966	25.6
Muricauda ruestringensis B1	HM-21_01246	quiP	886377.Murru_0969	82.4
Muricauda ruestringensis B1	HM-21_02972	group_1559	886377.Murru_1017	75.2
Muricauda ruestringensis B1	HM-21_01153	group_534	886377.Murru_1061	87.5
Muricauda ruestringensis B1	HM-21_01069	group_1625	886377.Murru_1130	58.4

Muricauda ruestringensis B1	HM-21_00967	group_1954	886377.Murru_1237	86
Muricauda ruestringensis B1	HM-21_00963	group_1718	886377.Murru_1240	86.6
Muricauda ruestringensis B1	HM-21_02866	group_1718	886377.Murru_1240	66.9
Muricauda ruestringensis B1	HM-21_00836	bspRIM	886377.Murru_1309	80.3
Muricauda ruestringensis B1	HM-21_00812	group_1599	886377.Murru_1338	65.6
Muricauda ruestringensis B1	HM-21_00723	paiA_2	886377.Murru_1420	67.1
Muricauda ruestringensis B1	HM-21_00603	oprO	886377.Murru_1535	74.7
Muricauda ruestringensis B1	HM-21_00514	group_747	886377.Murru_1587	85.4
Muricauda ruestringensis B1	HM-21_00515	group_746	886377.Murru_1588	90.2
Muricauda ruestringensis B1	HM-21_00484	guaB_2	886377.Murru_1622	91
Muricauda ruestringensis B1	HM-21_00482	mfpA	886377.Murru_1624	73.2
Muricauda ruestringensis B1	HM-21_00308	ydjA	886377.Murru_1729	89.1
Muricauda ruestringensis B1	HM-21_00305	fepA	886377.Murru_1732	83.4
Muricauda ruestringensis B1	HM-21_02385	ppgK	886377.Murru_1789	26.5
Muricauda ruestringensis B1	HM-21_00230	xynA1	886377.Murru_1821	78.1
Muricauda ruestringensis B1	HM-21_00161	secE2	886377.Murru_1889	90.9
Muricauda ruestringensis B1	HM-21_00341	group_2173	886377.Murru_1903	71.3
Muricauda ruestringensis B1	HM-21_00036	group_1698	886377.Murru_1994	73.8
Muricauda ruestringensis B1	HM-21_01871	group_1718	886377.Murru_2028	45.3
Muricauda ruestringensis B1	HM-21_03154	group_1718	886377.Murru_2028	63.6
Muricauda ruestringensis B1	HM-21_03033	group_1653	886377.Murru_2149	94.7

Muricauda ruestringensis B1	HM-21_03172	xynA1	886377.Murru_2182	53.1
Muricauda ruestringensis B1	HM-21_03316	xynA1	886377.Murru_2182	79
Muricauda ruestringensis B1	HM-21_00388	perR	886377.Murru_2340	73.4
Muricauda ruestringensis B1	HM-21_00283	perR	886377.Murru_2461	73.2
Muricauda ruestringensis B1	HM-21_00054	group_2538	886377.Murru_2768	46.5
Muricauda ruestringensis B1	HM-21_00057	group_2681	886377.Murru_2772	67.1
Muricauda ruestringensis B1	HM-21_01962	group_1689	886377.Murru_2888	77.3
Muricauda ruestringensis B1	HM-21_02081	group_197	886377.Murru_2996	80.5
Muricauda ruestringensis B1	HM-21_02630	yfiT	886377.Murru_3046	73.7
Muricauda ruestringensis B1	HM-21_02631	paiA_2	886377.Murru_3047	60.7
Muricauda ruestringensis B1	HM-21_02005	rhaR_1	886377.Murru_3095	57.2
Muricauda ruestringensis B1	HM-21_01872	group_2080	886377.Murru_3214	85.5
Muricauda ruestringensis B1	HM-21_01758	quiP	886377.Murru_3261	77.1
Muricauda ruestringensis B1	HM-21_01571	rpmH	886377.Murru_0060	96.2

bitscore	preferredName
837.8	argH
710.3	Celly_0376
484.6	Celly_0378
787.7	Celly_0822
703.7	Celly_0828
1105.9	Celly_1759
716.5	Celly_2396
1144	Celly_2747
338.6	Celly_2748
370.2	Celly_3169
976.9	ilvC

1114.8	ilvD
627.5	Celly_0551
1031.6	Celly_0790
348.6	Celly_0840
442.2	Celly_0842
575.9	Celly_1462
518.1	Celly_2272
998.4	Celly_3094
1007.7	glpK
1087.4	Celly_0065
1292.3	Celly_2044
735.7	Celly_2335

1543.1	Celly_2337
1503	Celly_2338
1574.3	Celly_2341
422.2	Celly_0023
333.2	Celly_0197
843.6	Celly_0389
1327	Celly_1321
1451.8	Celly_1322
2346.2	Celly_1556
1493.4	Celly_1656
558.1	Celly_1661
520.8	Celly_1662
744.2	Celly_1663
1780.8	Celly_1842
788.1	Celly_1850
344.4	Celly_1912
384.8	Celly_1935
951	Celly_2130
1135.9	Celly_2184

528.9	Celly_2206
747.3	Celly_2359
848.2	Celly_2506
487.6	Celly_2589
307.4	Celly_2677
382.1	Celly_2689
411.4	Celly_2772
1239.9	Celly_2830
742.3	Celly_2869
1243	Celly_3118
736.9	Celly_1976
1783.1	Celly_1977
1499.6	metE
492.7	Celly_0739
385.6	Celly_0742

432.6	Celly_1969
1380.5	Celly_1972
493.8	Celly_1973
392.1	Celly_1974
449.5	Celly_0499
272.3	Celly_1576
293.1	Celly_1577
322	Celly_1797
1430.6	Celly_1798
1014.2	Celly_2673
313.9	Celly_2799

1431.4	Celly_2800
646	Celly_2802
785.4	Celly_2805
664.5	Celly_2806
370.2	Celly_2807
199.1	Celly_0027
218.4	Celly_0040
563.9	Celly_0080
271.2	Celly_0080
382.9	Celly_0135
375.2	Celly_0144
207.6	Celly_0253
647.1	Celly_0269
282.3	Celly_0321
637.1	Celly_0345
588.2	Celly_0347
169.5	Celly_0347
505	Celly_0348

334.7	Celly_0365
1618.2	Celly_0681
335.9	Celly_0723
511.1	Celly_0727
1090.5	Celly_0749
386.3	Celly_0850
589.3	Celly_0866
1500	Celly_0876
248.1	Celly_0935
242.3	Celly_0937
2011.1	Celly_0965
456.8	Celly_0975
358.2	Celly_0984
280	Celly_0986
852.4	Celly_0997
256.1	Celly_1023
595.9	Celly_1031

290	Celly_1043
299.7	Celly_1063
257.3	Celly_1106
260.8	Celly_1116
409.1	Celly_1191
618.2	Celly_1283
240.7	Celly_1335
254.2	Celly_1338
218.8	Celly_1377
389.8	Celly_1381
581.6	Celly_1401
289.3	Celly_1418
1715.7	Celly_1440
693.7	Celly_1464
305.8	Celly_1524
118.2	Celly_1526
231.1	Celly_1552
568.9	Celly_1625
828.9	Celly_1631
816.6	Celly_1652

981.9	Celly_1696
471.9	Celly_1783
124	Celly_1897
413.3	Celly_1910
711.1	Celly_1918
501.9	Celly_2066
370.2	Celly_2129
526.6	Celly_2180
258.1	Celly_2194
372.1	Celly_2243
563.9	Celly_2246
157.1	Celly_2300
1602.8	Celly_2345
98.2	Celly_2394
382.1	Celly_2493
435.6	Celly_2521
842.4	Celly_2523
225.7	Celly_2554
493.8	Celly_2645
1585.1	Celly_2714
938.7	Celly_2767

968.4	Celly_2879
417.9	Celly_2887
295	Celly_2888
889	Celly_2889
390.6	Celly_2903
189.5	Celly_2919
180.6	Celly_2920
1614.4	Celly_3016
325.1	Celly_3044
260.8	Celly_3045
121.7	Celly_3060
303.9	Celly_3061
1486.9	Celly_3126
557.8	Celly_3160
149.4	Celly_3220
275	Celly_3220
1324.7	Celly_3266
194.1	Celly_3267
183.3	Celly_3268
365.5	Celly_3273
270	Celly_3274
98.2	rpmH
911.8	Fjoh_4506

1047.3	Fjoh_2863
289.7	Fjoh_2497
409.8	Fjoh_2497
216.5	Fjoh_2497
805.1	argH
311.6	Fjoh_2861
1038.5	Fjoh_2862
243.8	Fjoh_3836
664.5	Fjoh_3428
336.3	Fjoh_2944
698.4	Fjoh_2344
773.1	Fjoh_3435
965.3	ilvC
627.5	Fjoh_2035
1209.9	Fjoh_2029
1227.6	Fjoh_2381
1281.2	Fjoh_2039
1332.4	Fjoh_2032
1474.1	Fjoh_2358
417.9	Fjoh_3125
1302.3	Fjoh_2041
1337	Fjoh_2715
1392.5	Fjoh_3124
1335.9	Fjoh_2714
1252.7	Fjoh_4557
1203	Fjoh_4556
1318.5	Fjoh_1765
524.2	Fjoh_2491
325.5	Fjoh_2491
176.8	Fjoh_2215
679.9	Fjoh_2216

558.5	Fjoh_0134
286.2	Fjoh_0682
676.8	Fjoh_1294
582	Fjoh_4945
518.1	Fjoh_4945
682.9	Fjoh_1114
659.8	sprD
538.5	sprC
432.2	Fjoh_3438
310.5	Fjoh_0424
86.7	Fjoh_2767
1655.6	Fjoh_1510
657.5	Fjoh_3839
643.7	Fjoh_1508
1415.2	metE
442.2	Fjoh_1505
432.6	Fjoh_4641
354.8	Fjoh_1506
429.1	Fjoh_1501
1232.6	Fjoh_1504
1233	Fjoh_4200
281.6	Fjoh_4638
357.5	Fjoh_1397
381.3	Fjoh_4202
872.1	Fjoh_4885
573.5	Fjoh_4644
539.7	Fjoh_4637
551.6	Fjoh_4636
367.5	Fjoh_3520
158.3	Fjoh_4205
524.2	Fjoh_4205
233.4	Fjoh_4204
252.3	Fjoh_0538

734.6	Fjoh_1291
164.5	Fjoh_2267
449.1	Fjoh_0159
230.7	Fjoh_3287
466.1	Fjoh_4930
471.1	Fjoh_4995
446.8	Fjoh_4797
62.8	Fjoh_0055
65.1	rpmH
169.5	Fjoh_1497
241.1	Fjoh_1822
355.1	Fjoh_4882
263.8	Fjoh_4883
353.2	Fjoh_0430
936.4	Fjoh_2122
288.5	Fjoh_2933
203.8	Fjoh_2964
267.3	Fjoh_2964
339.3	Fjoh_4993
1431.4	Fjoh_0502
908.7	Fjoh_1252
116.3	Fjoh_0182
144.4	Fjoh_2486
88.2	Fjoh_3404
915.2	Fjoh_0745
708.4	Fjoh_2773
246.1	Fjoh_3089
541.6	Fjoh_4946
209.9	Fjoh_3300
221.5	Fjoh_3407
900.6	Fjoh_2000
400.6	Fjoh_2000
482.3	Fjoh_2198
516.2	Fjoh_1277
465.7	Fjoh_1277
506.5	Fjoh_1277
491.1	Fjoh_2171
368.2	Fjoh_2093
121.3	Fjoh_2093

171.4	Fjoh_1541
673.7	Fjoh_2023
227.6	Fjoh_0739
226.1	Fjoh_0931
218.4	Fjoh_0986
158.3	Fjoh_1253
167.5	Fjoh_1254
137.5	Fjoh_1929
378.6	Fjoh_1979
1403.7	Fjoh_2413
118.2	Fjoh_2507
919.1	Fjoh_2866
419.1	Fjoh_3514
698.4	Fjoh_3759
519.6	Fjoh_3800
809.3	Fjoh_3959
452.2	Fjoh_4253
487.3	Fjoh_4266
127.9	Fjoh_4266
354.4	Fjoh_4626
810.4	argG
898.7	argH
762.7	dapE
1194.1	EDM45818.1
297.4	EDM46422.1
1149.8	EDM46423.1
1014.6	EDM46789.1
901.4	EDM46999.1
951	EDM47472.1
776.2	EDM47712.1
332	EDM47845.1
1074.7	EDM48374.1
327.4	EDM48598.1
1041.6	EDM49158.1
1026.2	EDM49695.1

669.1	ilvC
1067	ilvD
989.6	EDM45841.1
870.9	EDM46611.1
964.1	EDM47089.1
713.4	EDM47215.1
1008.8	EDM47432.1
517.7	EDM47554.1
873.2	EDM49251.1
183.3	EDM49251.1
979.9	EDM49252.1
2375.9	EDM49073.1
1458.4	metE
549.3	EDM46759.1
499.6	EDM49051.1
1100.1	EDM49070.1
1345.5	EDM46720.1
295.4	EDM46721.1
287.7	EDM47469.1
345.1	EDM47865.1
672.9	EDM47866.1
290	EDM47868.1
783.1	EDM48224.1
500	EDM49202.1
616.7	moaA
298.9	moaC
771.5	pqqE
218	EDM45789.1
1158.3	EDM45878.1
500	EDM46026.1
69.7	EDM46371.1
315.8	EDM46564.1
237.3	EDM46968.1
259.2	EDM46980.1
570.9	EDM47026.1

142.9	EDM47067.1
396.7	EDM47154.1
438.3	EDM47694.1
365.9	EDM47739.1
748	EDM47799.1
270.8	EDM48169.1
193.4	EDM48497.1
601.7	EDM48852.1
572.4	EDM49008.1
413.7	EDM49086.1
306.2	EDM49378.1
176.4	EDM49511.1
1705.6	EDM49623.1
581.6	EDM49631.1
229.6	EDM49719.1
470.3	EDM49763.1
385.6	lexA
107.8	merP
773.9	argH
1042.3	ilvD
634	Murru_0414
733	Murru_0926

611.7	Murru_0933
951.4	Murru_3163
941	Murru_3249
304.7	Murru_3250
1032.3	Murru_3251
918.3	glpK
310.1	Murru_0591
226.9	Murru_0945
341.3	Murru_0949
927.9	Murru_1160
387.5	Murru_1413

708.8	Murru_1526
346.7	Murru_1527
442.6	Murru_1528
797.7	Murru_1605
699.9	Murru_2765
531.6	Murru_3307
327.4	Murru_3308
222.2	Murru_0035
825.1	Murru_0132
338.6	Murru_0294
906	Murru_0325
192.6	Murru_0461
396.4	Murru_0564
372.9	Murru_0616
605.5	Murru_0636
727.6	Murru_0694

200.3	Murru_0695
1034.2	Murru_1321
1055	Murru_1322
200.7	Murru_1430
170.2	Murru_1561
1084.3	Murru_1821
253.1	Murru_1914
199.5	Murru_2003
293.1	Murru_2333
214.5	Murru_2387
355.1	Murru_2974
492.7	Murru_3439
592.8	Murru_1966
377.1	Murru_1966
1536.5	Murru_1967
303.9	Murru_1681

405.2	Murru_1958
1241.9	Murru_1961
411	Murru_1962
963.4	Murru_0166
686.8	Murru_0166
1245.7	Murru_0166
915.6	Murru_0166
241.9	Murru_0167
288.1	Murru_0167

255.4	Murru_0167
250.8	Murru_1723
315.1	Murru_2186
166.4	Murru_3377
218.4	Murru_3378
193	Murru_3379
215.7	Murru_3380
110.2	Murru_3383
171	Murru_3384
358.2	Murru_0044
283.9	Murru_0047
604.4	Murru_0119
396.7	Murru_0121

577	Murru_0139
363.2	Murru_0161
533.1	Murru_0169
148.3	Murru_0180
755	Murru_0236
125.6	Murru_0298
817	Murru_0331
285.8	Murru_0331
586.6	Murru_0392
708.8	Murru_0406
82	Murru_0418
461.8	Murru_0513
74.3	Murru_0521
155.2	Murru_0522
829.7	Murru_0523
350.1	Murru_0547

482.3	Murru_0555
132.5	Murru_0739
63.2	Murru_0739
1469.9	Murru_0786
67	Murru_0798
63.5	Murru_0798
70.1	Murru_0798
62.4	Murru_0798
262.7	Murru_0937
256.1	Murru_0937
241.1	Murru_0937
406	Murru_0937
388.7	Murru_0959
64.7	Murru_0966
1260.4	Murru_0969
716.8	Murru_1017
556.6	Murru_1061
196.1	Murru_1130

356.7	Murru_1237
291.2	Murru_1240
198.4	Murru_1240
690.3	Murru_1309
178.7	Murru_1338
247.7	Murru_1420
637.5	Murru_1535
245.7	Murru_1587
340.5	Murru_1588
288.9	Murru_1622
82	Murru_1624
356.3	Murru_1729
1287.3	Murru_1732
100.1	Murru_1789
1471.4	Murru_1821
114.8	Murru_1889
355.9	Murru_1903
132.9	Murru_1994
121.3	Murru_2028
189.1	Murru_2028
142.1	Murru_2149

815.5	Murru_2182
1252.7	Murru_2182
199.1	Murru_2340
229.9	Murru_2461
189.1	Murru_2768
117.9	Murru_2772
196.4	Murru_2888
1330.5	Murru_2996
298.9	Murru_3046
208.4	Murru_3047
190.7	Murru_3095
249.2	Murru_3214
1327.8	Murru_3261
93.6	rpmH

annotation
COGs: COG0165 Argininosuccinate lyase; HAMAP: Argininosuccinate lyase; InterPro IPR009049: IPR022761; KEGG: fbc:FB2170_08244 argininosuccinate lyase; PFAM: Lyase 1, N-terminal; PRIAM: Argininosuccinate lyase; SPTR: Argininosuccinate lyase; TIGRFAM: Argininosuccinate lyase; PFAM: Lyase; TIGRFAM: argininosuccinate lyase
Major royal jelly protein; InterPro IPR003534; KEGG: gbm:Gbem_1195 major royal jelly-like protein; PFAM: Major royal jelly-related; SPTR: Putative uncharacterized protein; PFAM: Major royal jelly protein
COGs: COG1794 Aspartate racemase; InterPro IPR015942: IPR004380; KEGG: rmr:Rmar_2418 aspartate racemase; PFAM: Asp/Glu/hydantoin racemase; SPTR: Aspartate racemase; TIGRFAM: Aspartate racemase; PFAM: Asp/Glu/Hydantoin racemase; TIGRFAM: aspartate racemase
COGs: COG0137 Argininosuccinate synthase; InterPro IPR001518; KEGG: fbc:FB2170_08294 argininosuccinate synthase; PFAM: Argininosuccinate synthase; PRIAM: Argininosuccinate synthase; SPTR: Argininosuccinate synthase; PFAM: Arginosuccinate synthase; TIGRFAM: argininosuccinate synthase
COGs: COG0624 Acetylornithine deacetylase/Succinyl-diaminopimelate desuccinylase and related deacylase; InterPro IPR002933: IPR011650; KEGG: fbc:FB2170_08249 acetylornithine deacetylase; PFAM: Peptidase M20; Peptidase M20, dimerisation; PRIAM: Succinyl-diaminopimelate desuccinylase; SPTR: Acetylornithine deacetylase; PFAM: Peptidase family M20/M25/M40; Peptidase dimerisation domain
COGs: COG0405 Gamma-glutamyltransferase; InterPro IPR000101; KEGG: fbc:FB2170_15563 putative gamma-glutamyltranspeptidase; PFAM: Gamma-glutamyltranspeptidase; SPTR: Putative gamma-glutamyltranspeptidase; TIGRFAM: Gamma-glutamyltranspeptidase; PFAM: Gamma-glutamyltranspeptidase; TIGRFAM: gamma-glutamyltranspeptidase
COGs: COG0174 Glutamine synthetase; InterPro IPR008147: IPR008146; KEGG: fbc:FB2170_01327 glutamine synthetase II; PFAM: Glutamine synthetase, catalytic domain; Glutamine synthetase, beta-Grasp; PRIAM: Glutamate--ammonia ligase; SPTR: Glutamine synthetase; PFAM: Glutamine synthetase, catalytic domain; Glutamine synthetase, beta-Grasp domain
Acetolactate synthase; COGs: COG0028 Thiamine pyrophosphate-requiring protein; InterPro IPR012001: IPR012000: IPR011766: IPR012846; KEGG: fbc:FB2170_12296 acetolactate synthase large subunit; PFAM: Thiamine pyrophosphate enzyme, N-terminal TPP-binding domain; Thiamine pyrophosphate enzyme, central domain; Thiamine pyrophosphate enzyme, C-terminal TPP-binding; PRIAM: Acetolactate synthase; SPTR: Acetolactate synthase large subunit; TIGRFAM: Acetolactate synthase, large subunit, biosynthetic; PFAM: Thiamine pyrophosphate enzyme, central domain; Thiamine pyrophosphate enzyme, N-terminal T [...]
COGs: COG0440 Acetolactate synthase small (regulatory) subunit; InterPro IPR002912: IPR019455: IPR004789; KEGG: fbc:FB2170_12291 putative acetohydroxyacid synthase small subunit; PFAM: Acetolactate synthase, small subunit, C-terminal; Amino acid-binding ACT; SPTR: Putative acetohydroxyacid synthase small subunit; TIGRFAM: Acetolactate synthase, small subunit; PFAM: ACT domain; Small subunit of acetolactate synthase; TIGRFAM: acetolactate synthase, small subunit
COGs: COG0386 Glutathione peroxidase; InterPro IPR000889; KEGG: nve:NEMVE_v1g225874 hypothetical protein; PFAM: Glutathione peroxidase; PRIAM: Peroxiredoxin; SPTR: Glutathione peroxidase; PFAM: Glutathione peroxidase
Ketol-acid reductoisomerase (NADP(+)); Involved in the biosynthesis of branched-chain amino acids (BCAA). Catalyzes an alkyl-migration followed by a ketol- acid reduction of (S)-2-acetolactate (S2AL) to yield (R)-2,3-dihydroxy-isovalerate. In the isomerase reaction, S2AL is rearranged via a Mg-dependent methyl migration to produce 3- hydroxy-3-methyl-2-ketobutyrate (HMKB). In the reductase reaction, this 2-ketoacid undergoes a metal-dependent reduction by NADPH to yield (R)-2,3-dihydroxy-isovalerate; Belongs to the ketol-acid reductoisomerase family

COGs: COG0129 Dihydroxyacid dehydratase/phosphogluconate dehydratase; HAMAP: Dihydroxy-acid dehydratase; InterPro IPR000581: IPR004404; KEGG: fbc:FB2170_12301 dihydroxy-acid dehydratase; PFAM: Dihydroxy-acid/6-phosphogluconate dehydratase; PRIAM: Dihydroxy-acid dehydratase; SPTR: Dihydroxy-acid dehydratase; TIGRFAM: Dihydroxy-acid dehydratase; PFAM: Dehydratase family; TIGRFAM: dihydroxy-acid dehydratase; Belongs to the IlvD/Edd family
COGs: COG0584 Glycerophosphoryl diester phosphodiesterase; InterPro IPR004129; KEGG: zpr:ZPR_0250 glycerophosphoryl diester phosphodiesterase; PFAM: Glycerophosphoryl diester phosphodiesterase; SPTR: Glycerophosphoryl diester phosphodiesterase; PFAM: Glycerophosphoryl diester phosphodiesterase family
COGs: COG0578 Glycerol-3-phosphate dehydrogenase; InterPro IPR006076; KEGG: zpr:ZPR_0766 FAD dependent oxidoreductase; PFAM: FAD dependent oxidoreductase; PRIAM: Glycerol-3-phosphate dehydrogenase; SPTR: FAD dependent oxidoreductase; PFAM: FAD dependent oxidoreductase
COGs: COG3797 conserved hypothetical protein; InterPro IPR012545; KEGG: fbc:FB2170_08199 hypothetical protein; PFAM: Protein of unknown function DUF1697; SPTR: Putative uncharacterized protein; PFAM: Protein of unknown function (DUF1697)
COGs: COG0584 Glycerophosphoryl diester phosphodiesterase; InterPro IPR004129; KEGG: fbc:FB2170_08189 glcerophosphoryl diester phosphodiesterase; PFAM: Glycerophosphoryl diester phosphodiesterase; SPTR: Glcerophosphoryl diester phosphodiesterase; PFAM: Glycerophosphoryl diester phosphodiesterase family
COGs: COG0584 Glycerophosphoryl diester phosphodiesterase; InterPro IPR004129; KEGG: coc:Coch_1204 glycerophosphoryl diester phosphodiesterase; PFAM: Glycerophosphoryl diester phosphodiesterase; SPTR: Glycerophosphoryl diester phosphodiesterase family protein; PFAM: Glycerophosphoryl diester phosphodiesterase family
Polyphosphate--glucose phosphotransferase; COGs: COG1940 Transcriptional regulator/sugar kinase; InterPro IPR000600; KEGG: rbi:RB2501_10397 ROK family protein; PFAM: ROK; PRIAM: Polyphosphate--glucose phosphotransferase; SPTR: ROK family protein; PFAM: ROK family
Xylulokinase; COGs: COG1070 Sugar (pentulose and hexulose) kinase; InterPro IPR018484: IPR018485; KEGG: fjo:Fjoh_2000 carbohydrate kinase, FGGY; PFAM: Carbohydrate kinase, FGGY, N-terminal; Carbohydrate kinase, FGGY, C-terminal; PRIAM: Xylulokinase; SPTR: Carbohydrate kinase, FGGY; PFAM: FGGY family of carbohydrate kinases, N-terminal domain; FGGY family of carbohydrate kinases, C-terminal domain
Glycerol kinase; Key enzyme in the regulation of glycerol uptake and metabolism. Catalyzes the phosphorylation of glycerol to yield sn- glycerol 3-phosphate; Belongs to the FGGY kinase family
Beta-N-acetylhexosaminidase; COGs: COG3525 N-acetyl-beta-hexosaminidase; InterPro IPR015882: IPR015883; KEGG: fbc:FB2170_00805 beta-N-acetylhexosaminidase; PFAM: Glycoside hydrolase, family 20, catalytic core; Acetylhexosaminidase, subunit a/b; PRIAM: Beta-N-acetylhexosaminidase; SPTR: Beta-N-acetylhexosaminidase; PFAM: Glycosyl hydrolase family 20, catalytic domain; Glycosyl hydrolase family 20, domain 2
COGs: COG0363 6-phosphogluconolactonase/Glucosamine-6-phosphate isomerase/deaminase; InterPro IPR004547: IPR006148: IPR003737; KEGG: fjo:Fjoh_2381 glucosamine-6-phosphate deaminase-like protein; PFAM: Glucosamine/galactosamine-6-phosphate isomerase; N-acetylglucosaminyl phosphatidylinositol deacetylase; PRIAM: Glucosamine-6-phosphate deaminase; SPTR: Glucosamine-6-phosphate isomerase; TIGRFAM: Glucosamine-6-phosphate isomerase; PFAM: Glucosamine-6-phosphate isomerases/6-phosphogluconolactonase; GlcNAc-PI de-N-acetylase; TIGRFAM: glucosamine-6-phosphate isomerase
KEGG: zpr:ZPR_4181 carbohydrate-binding family 9-like protein; SPTR: Carbohydrate-binding family 9-like protein; PFAM: Domain of unknown function (DUF1083)

Beta-N-acetylhexosaminidase; COGs: COG3525 N-acetyl-beta-hexosaminidase; InterPro IPR015882: IPR015883: IPR000421; KEGG: zpr:ZPR_4178 beta-N-acetylhexosaminidase; PFAM: Glycoside hydrolase, family 20, catalytic core; Acetylhexosaminidase, subunit a/b; Coagulation factor 5/8 type, C-terminal; PRIAM: Beta-N-acetylhexosaminidase; SPTR: Beta-N-acetylhexosaminidase; PFAM: F5/8 type C domain; Glycosyl hydrolase family 20, catalytic domain; Glycosyl hydrolase family 20, domain 2
Alpha-1,2-mannosidase; COGs: COG3537 Putative alpha-1 2-mannosidase; InterPro IPR012939: IPR005887; KEGG: zpr:ZPR_4177 glycosyl hydrolase family 92; PFAM: Glycosyl hydrolase 92; SPTR: Glycosyl hydrolase family 92; TIGRFAM: Alpha-1,2-mannosidase, putative; PFAM: Glycosyl hydrolase family 92; TIGRFAM: alpha-1,2-mannosidase, putative
Alpha-1,2-mannosidase; COGs: COG3537 Putative alpha-1 2-mannosidase; InterPro IPR012939: IPR005887; KEGG: pat:Patl_0120 putative alpha-1,2-mannosidase; PFAM: Glycosyl hydrolase 92; SPTR: Putative alpha-1,2-mannosidase; TIGRFAM: Alpha-1,2-mannosidase, putative; PFAM: Glycosyl hydrolase family 92; TIGRFAM: alpha-1,2-mannosidase, putative
KEGG: fbc:FB2170_09166 hypothetical protein; SPTR: Putative uncharacterized protein
KEGG: gfo:GFO_1871 secreted protein; SPTR: Putative uncharacterized protein
KEGG: fbc:FB2170_01292 hypothetical protein; SPTR: Putative uncharacterized protein; PFAM: Protein of unknown function (DUF3570)
Putative membrane protein; InterPro IPR019861; KEGG: fbc:FB2170_05935 hypothetical protein; SPTR: Putative uncharacterized protein; TIGRFAM: Conserved hypothetical protein CHP03519, membrane, Bacteroidetes; PFAM: Protein of unknown function (DUF3308); TIGRFAM: Bacteroidetes-specific putative membrane protein
KEGG: rbi:RB2501_06860 hypothetical protein; SPTR: Putative uncharacterized protein
Peptidase M1 membrane alanine aminopeptidase; COGs: COG0308 Aminopeptidase N; InterPro IPR014782; KEGG: sli:Slin_4874 hypothetical protein; PFAM: Peptidase M1, membrane alanine aminopeptidase, N-terminal; SPTR: Putative uncharacterized protein; PFAM: Peptidase family M1
KEGG: cat:CA2559_11328 hypothetical protein; SPTR: Putative uncharacterized protein
Uncharacterized protein; InterPro IPR010496; KEGG: rbi:RB2501_05690 putative multi-domain protein; PFAM: Protein of unknown function DUF1080; SPTR: Putative multi-domain protein; PFAM: Domain of Unknown Function (DUF1080)
Uncharacterized protein; InterPro IPR010496; KEGG: rbi:RB2501_05695 probable secreted glycosyl hydrolase; PFAM: Protein of unknown function DUF1080; SPTR: Probable secreted glycosyl hydrolase; PFAM: Domain of Unknown Function (DUF1080)
Xylose isomerase domain-containing protein TIM barrel; COGs: COG1082 Sugar phosphate isomerase/epimerase; InterPro IPR012307: IPR011418; KEGG: rbi:RB2501_05700 hypothetical protein; PFAM: Xylose isomerase, TIM barrel domain; AP endonuclease, family 2, C-terminal; SPTR: Putative uncharacterized protein; PFAM: AP endonuclease family 2 C terminus; Xylose isomerase-like TIM barrel
KEGG: fbc:FB2170_03165 putative membrane associated hydrolase; SPTR: Putative membrane associated hydrolase; PFAM: Domain of unknown function (DUF1083)
Sodium/hydrogen exchanger; COGs: COG0025 NhaP-type Na+/H+ and K+/H+ antiporter; InterPro IPR006153; KEGG: fbc:FB2170_03115 hypothetical protein; PFAM: Cation/H+ exchanger; SPTR: Putative uncharacterized protein; PFAM: Sodium/hydrogen exchanger family
KEGG: fbc:FB2170_15458 hypothetical protein; SPTR: Putative uncharacterized protein
KEGG: fbc:FB2170_02790 hypothetical protein; SPTR: Putative uncharacterized protein
KEGG: fbc:FB2170_02590 hypothetical protein; SPTR: Putative uncharacterized protein
KEGG: fbc:FB2170_02315 hypothetical protein; SPTR: Putative uncharacterized protein

Aldose 1-epimerase; InterPro IPR008183; KEGG: fbc:FB2170_02195 hypothetical protein; PFAM: Aldose 1-epimerase; SPTR: Putative uncharacterized protein; PFAM: Aldose 1-epimerase
Galactokinase; COGs: COG0153 Galactokinase; InterPro IPR019539: IPR006204: IPR013750: IPR000705; KEGG: nve:NEMVE_v1g6609 hypothetical protein; PFAM: Galactokinase galactose-binding domain; GHMP kinase; GHMP kinase, C-terminal; PRIAM: Galactokinase; SPTR: Galactokinase; TIGRFAM: Galactokinase; PFAM: Galactokinase galactose-binding signature; GHMP kinases C terminal; GHMP kinases N terminal domain; TIGRFAM: galactokinase
COGs: COG2027 D-alanyl-D-alanine carboxypeptidase (penicillin-binding protein 4); InterPro IPR000667; KEGG: fbc:FB2170_14348 D-alanyl-D-alanine carboxypeptidase/D-alanyl-D-alanine-endopeptidase; PFAM: Peptidase S13, D-Ala-D-Ala carboxypeptidase C; SPTR: D-alanyl-D-alanine carboxypeptidase/D-alanyl-D-alanine-endopeptidase; PFAM: D-Ala-D-Ala carboxypeptidase 3 (S13) family
KEGG: fbc:FB2170_14763 Tat (twin-arginine translocation) pathway signal sequence domain protein; SPTR: Putative uncharacterized protein
KEGG: nve:NEMVE_v1g198360 hypothetical protein; SPTR: Putative uncharacterized protein
KEGG: fbc:FB2170_12891 hypothetical protein; SPTR: Putative uncharacterized protein
KEGG: fjo:Fjoh_0630 hypothetical protein; SPTR: Putative uncharacterized protein
Thiol-activated cytolysin; InterPro IPR001869; KEGG: fbc:FB2170_11951 listeriolysin O; PFAM: Thiol-activated cytolysin; SPTR: Listeriolysin O; PFAM: Thiol-activated cytolysin
Major royal jelly protein; InterPro IPR003534; KEGG: avn:Avin_35790 hypothetical protein; PFAM: Major royal jelly-related; SPTR: Putative uncharacterized protein; PFAM: Major royal jelly protein
Putative membrane protein; InterPro IPR019861: IPR007730; KEGG: fbc:FB2170_15033 hypothetical protein; PFAM: Sporulation-related domain; SPTR: Putative uncharacterized protein; TIGRFAM: Conserved hypothetical protein CHP03519, membrane, Bacteroidetes; PFAM: Protein of unknown function (DUF3308); Sporulation related domain; TIGRFAM: Bacteroidetes-specific putative membrane protein
COGs: COG0646 Methionine synthase I (cobalamin-dependent) methyltransferase domain; InterPro IPR003726; KEGG: fbc:FB2170_17141 5-methyltetrahydrofolate--homocysteine methyltransferase; PFAM: Homocysteine S-methyltransferase; PRIAM: Methionine synthase; SPTR: 5-methyltetrahydrofolate--homocysteine methyltransferase; PFAM: Homocysteine S-methyltransferase
COGs: COG1410 Methionine synthase I cobalamin-binding domain; InterProIPR011822: IPR000489: IPR003759: IPR006158: IPR 004223; KEGG: fbc:FB2170_17131 5-methyltetrahydrofolate--homocysteine methyltransferase; PFAM: Pterin-binding; Methionine synthase, cobalamin (vitamin B12)-binding module, cap; Cobalamin (vitamin B12)-binding; Vitamin B12-dependent methionine synthase, activation domain; PRIAM: Methionine synthase; SPTR: 5-methyltetrahydrofolate--homocysteine methyltransferase; TIGRFAM: 5-methyltetrahydrofolate--homocysteine methyltransferase; PFAM: Pterin binding enzyme; Vitamin B12 de [...]
5-methyltetrahydropteroyltriglutamate--homocysteine methyltransferase; Catalyzes the transfer of a methyl group from 5- methyltetrahydrofolate to homocysteine resulting in methionine formation
Uroporphyrin-III C-methyltransferase; COGs: COG0007 Uroporphyrinogen-III methylase; InterPro IPR006366: IPR000878; KEGG: fjo:Fjoh_1505 uroporphyrin-III C-methyltransferase; PFAM: Tetrapyrrole methylase; PRIAM: Uroporphyrinogen-III C-methyltransferase; SPTR: Uroporphyrinogen-III C-methyltransferase; TIGRFAM: Uroporphyrin-III C-methyltransferase, C-terminal; PFAM: Tetrapyrrole (Corrin/Porphyrin) Methylases; TIGRFAM: uroporphyrin-III C-methyltransferase; Belongs to the precorrin methyltransferase family
KEGG: fjo:Fjoh_4626 hypothetical protein; SPTR: Putative uncharacterized protein

COGs: COG0175 3'-phosphoadenosine 5'-phosphosulfate sulfotransferase (PAPS reductase)/FAD synthetase; InterPro IPR002500; KEGG: fbc:FB2170_17176 possible 3'-phosphoadenosine 5'-phosphosulfate sulfotransferase; PFAM: Phosphoadenosine phosphosulphate reductase; SPTR: Possible 3'-phosphoadenosine 5'-phosphosulfate sulfotransferase; PFAM: Phosphoadenosine phosphosulfate reductase family; TIGRFAM: phosophoadenylyl-sulfate reductase (thioredoxin)
COGs: COG0155 Sulfite reductase beta subunit (hemoprotein); InterPro IPR005117: IPR006067: IPR007842; KEGG: fbc:FB2170_17161 probable sulfite reductase; PFAM: Nitrite/sulphite reductase 4Fe-4S domain; Nitrite/sulphite reductase, hemoprotein beta-component, ferrodoxin-like; HEPN; PRIAM: Ferredoxin--nitrite reductase; SPTR: Probable sulfite reductase; PFAM: HEPN domain; Nitrite and sulphite reductase 4Fe-4S domain; Nitrite/Sulfite reductase ferredoxin-like half domain
Uroporphyrin-III C-methyltransferase; COGs: COG0007 Uroporphyrinogen-III methylase; InterPro IPR006366: IPR000878; KEGG: fbc:FB2170_17156 uroporphyrin-III C-methyltransferase; PFAM: Tetrapyrrole methylase; PRIAM: Uroporphyrinogen-III C-methyltransferase; SPTR: Uroporphyrin-III C-methyltransferase; TIGRFAM: Uroporphyrin-III C-methyltransferase, C-terminal; PFAM: Tetrapyrrole (Corrin/Porphyrin) Methylases; TIGRFAM: uroporphyrin-III C-methyltransferase; Belongs to the precorrin methyltransferase family
COGs: COG1648 Siroheme synthase (precorrin-2 oxidase/ferrochelatase domain); InterPro IPR006367; KEGG: fbc:FB2170_17151 uroporphyrin-III C-methyltransferase; SPTR: Uroporphyrin-III C-methyltransferase; TIGRFAM: Sirohaem synthase, N-terminal; TIGRFAM: siroheme synthase, N-terminal domain
MOSC domain containing protein; COGs: COG2258 conserved hypothetical protein; InterPro IPR005302; KEGG: fbc:FB2170_16046 hypothetical protein; PFAM: Molybdenum cofactor sulfurase, C-terminal; SPTR: Putative uncharacterized protein; PFAM: MOSC domain
COGs: COG0315 Molybdenum cofactor biosynthesis enzyme; InterPro IPR023045: IPR002820; KEGG: dfe:Dfer_3066 bifunctional molybdenum cofactor biosynthesis protein MoaC/MogA; PFAM: Molybdopterin cofactor biosynthesis C (MoaC) domain; SPTR: Molybdenum cofactor synthesis domain protein; TIGRFAM: Molybdenum cofactor biosynthesis C; PFAM: MoaC family; TIGRFAM: molybdenum cofactor biosynthesis protein MoaC
Molybdopterin biosynthesis MoaE protein; COGs: COG0314 Molybdopterin converting factor large subunit; InterPro IPR003448; KEGG: rbi:RB2501_05280 molybdopterinconverting factor, subunit; PFAM: Molybdopterin biosynthesis MoaE; SPTR: Molybdopterinconverting factor, subunit; PFAM: MoaE protein
Isoquinoline 1-oxidoreductase; COGs: COG2080 Aerobic-type carbon monoxide dehydrogenase small subunit CoxS/CutS homologs; InterPro IPR001041: IPR002888; KEGG: sli:Slin_5426 (2Fe-2S)-binding domain protein; PFAM: [2Fe-2S]-binding; Ferredoxin; PRIAM: Isoquinoline 1-oxidoreductase; SPTR: Probable oxidoreductase; PFAM: 2Fe-2S iron-sulfur cluster binding domain; [2Fe-2S] binding domain
Aldehyde dehydrogenase (Pyrroloquinoline-quinone); COGs: COG1529 Aerobic-type carbon monoxide dehydrogenase large subunit CoxL/CutL homologs; InterPro IPR006311: IPR008274; KEGG: bid:Bind_2919 aldehyde oxidase and xanthine dehydrogenase molybdopterin binding; PFAM: Aldehyde oxidase/xanthine dehydrogenase, molybdopterin binding; PRIAM: Aldehyde dehydrogenase (pyrroloquinoline-quinone); SPTR: Twin-arginine translocation pathway signal; PFAM: Molybdopterin-binding domain of aldehyde dehydrogenase; TIGRFAM: Tat (twin-arginine translocation) pathway signal sequence
KEGG: fbc:FB2170_13136 hypothetical protein; SPTR: Putative uncharacterized protein
Isoquinoline 1-oxidoreductase; COGs: COG2080 Aerobic-type carbon monoxide dehydrogenase small subunit CoxS/CutS homologs; InterPro IPR001041: IPR002888; KEGG: fbc:FB2170_09341 ferredoxin; PFAM: [2Fe-2S]-binding; Ferredoxin; PRIAM: Isoquinoline 1-oxidoreductase; SPTR: (2Fe-2S)-binding protein; PFAM: 2Fe-2S iron-sulfur cluster binding domain; [2Fe-2S] binding domain

Aldehyde dehydrogenase (Pyrroloquinoline-quinone); COGs: COG1529 Aerobic-type carbon monoxide dehydrogenase large subunit CoxL/CutL homologs; InterPro IPR008274: IPR006311; KEGG: fbc:FB2170_09346 isoquinoline 1-oxidoreductase, beta subunit; PFAM: Aldehyde oxidase/xanthine dehydrogenase, molybdopterin binding; PRIAM: Aldehyde dehydrogenase (pyrroloquinoline-quinone); SPTR: Putative uncharacterized protein; PFAM: Molybdopterin-binding domain of aldehyde dehydrogenase
Molybdenum cofactor biosynthesis protein A; Catalyzes the cyclization of GTP to (8S)-3',8-cyclo-7,8-dihydroguanosine 5'-triphosphate
Molybdenum cofactor synthesis domain protein; COGs: COG0303 Molybdopterin biosynthesis enzyme; InterPro IPR001453: IPR005110: IPR005111: IPR020817; KEGG: fbc:FB2170_09391 molybdopterin biosynthesis; PFAM: MoeA, N-terminal and linker domain; Molybdopterin binding; MoeA, C-terminal, domain IV; SMART: Molybdopterin binding; SPTR: Molybdopterin biosynthesis; TIGRFAM: Molybdenum cofactor synthesis; PFAM: Probable molybdopterin binding domain; MoeA N-terminal region (domain I and II); MoeA C-terminal region (domain IV); TIGRFAM: molybdenum cofactor synthesis domain
XshC-Cox1-family protein; COGs: COG1975 Xanthine and CO dehydrogenase maturation factor XdhC/CoxF family; InterPro IPR003777; KEGG: fbc:FB2170_11426 hypothetical protein; PFAM: XdhC- CoxI; SPTR: Putative uncharacterized protein; PFAM: XdhC and CoxI family
Nucleotide-diphospho-sugar transferase; COGs: COG2068 Uncharacterized MobA-related protein; KEGG: zpr:ZPR_2972 nucleotide-diphospho-sugar transferase; SPTR: Nucleotide-diphospho-sugar transferase; PFAM: Uncharacterized protein family UPF0007
KEGG: fbc:FB2170_09146 hypothetical protein; SPTR: Putative uncharacterized protein; PFAM: Protein of unknown function (DUF2752)
KEGG: cat:CA2559_02190 hypothetical protein; SPTR: Putative uncharacterized protein
KEGG: rbi:RB2501_11462 Zn-dependent alcohol dehydrogenase, class III; SPTR: Putative uncharacterized protein; PFAM: Glycosyl transferase family 2
KEGG: rbi:RB2501_11462 Zn-dependent alcohol dehydrogenase, class III; SPTR: Putative uncharacterized protein; PFAM: Glycosyl transferase family 2
HAMAP: metal-dependent hydrolase; KEGG: fbc:FB2170_01070 hypothetical protein; SPTR: Putative uncharacterized protein
KEGG: fbc:FB2170_13733 adenylosuccinate lyase; SPTR: Adenylosuccinate lyase
KEGG: fbc:FB2170_09601 hypothetical protein; SPTR: Putative uncharacterized protein
Cellulase; InterPro IPR001547; KEGG: chu:CHU_2103 endoglucanase; PFAM: Glycoside hydrolase, family 5; PRIAM: Cellulase; SPTR: Endoglucanase, glycoside hydrolase family 5 protein; PFAM: Cellulase (glycosyl hydrolase family 5); Belongs to the glycosyl hydrolase 5 (cellulase A) family
COGs: COG2020 Putative protein-S-isoprenylcysteine methyltransferase; KEGG: fbc:FB2170_13673 hypothetical protein; SPTR: Putative uncharacterized protein; PFAM: Isoprenylcysteine carboxyl methyltransferase (ICMT) family
KEGG: fbc:FB2170_01197 hypothetical protein; SPTR: Putative uncharacterized protein
Major facilitator superfamily MFS_1; COGs: COG2814 Arabinose efflux permease; InterPro IPR011701; KEGG: fbc:FB2170_01207 putative integral membrane transport protein; PFAM: Major facilitator superfamily MFS-1; SPTR: Putative integral membrane transport protein; PFAM: Major Facilitator Superfamily
Major facilitator superfamily MFS_1; COGs: COG2814 Arabinose efflux permease; InterPro IPR011701; KEGG: fbc:FB2170_01207 putative integral membrane transport protein; PFAM: Major facilitator superfamily MFS-1; SPTR: Putative integral membrane transport protein; PFAM: Major Facilitator Superfamily
ThiJ/PfpI domain-containing protein; COGs: COG4977 Transcriptional regulator containing an amidase domain and an AraC-type DNA-binding HTH domain; InterPro IPR002818; KEGG: fbc:FB2170_01212 hypothetical protein; PFAM: ThiJ/PfpI; SPTR: Putative uncharacterized protein; PFAM: DJ-1/PfpI family

NUDIX hydrolase; InterPro IPR000086; KEGG: vsp:VS_II0417 MutT/NUDIX protein; PFAM: NUDIX hydrolase domain; SPTR: Phosphohydrolase (MutT/nudix family protein); PFAM: NUDIX domain
KEGG: cat:CA2559_05500 hypothetical protein; SPTR: Putative uncharacterized protein
COGs: COG3193 Uncharacterized protein possibly involved in utilization of glycolate and propanediol; InterPro IPR005624; KEGG: fba:FIC_02561 protein containing DUF336; PFAM: Domain of unknown function DUF336; SPTR: Putative uncharacterized protein; PFAM: Domain of unknown function (DUF336)
COGs: COG3228 conserved hypothetical protein; InterPro IPR010384; KEGG: zpr:ZPR_0756 hypothetical protein; PFAM: Protein of unknown function DUF980; SPTR: Putative uncharacterized protein; PFAM: Phosphoenolpyruvate:glucose-phosphotransferase regulator; Belongs to the MtfA family
COGs: COG1649 conserved hypothetical protein; InterPro IPR003790; KEGG: phe:Phep_1349 protein of unknown function DUF187; PFAM: Protein of unknown function DUF187; SPTR: Putative uncharacterized protein; PFAM: Uncharacterised BCR, COG1649
COGs: COG0778 Nitroreductase; InterPro IPR000415; KEGG: rbi:RB2501_04095 hypothetical protein; PFAM: Nitroreductase-like; SPTR: Putative uncharacterized protein; PFAM: Nitroreductase family
KEGG: rbi:RB2501_04205 hypothetical protein; SPTR: Putative uncharacterized protein; PFAM: Nucleotidyl transferase
COGs: COG2366 Protein related to penicillin acylase; InterPro IPR002692; KEGG: fbc:FB2170_07894 penicillin amidase family protein; PFAM: Peptidase S45, penicillin amidase; PRIAM: Glutaryl-7-aminocephalosporanic-acid acylase; SPTR: Penicillin amidase family protein; PFAM: Penicillin amidase
COGs: COG4898 conserved hypothetical protein; InterPro IPR014580; KEGG: fbc:FB2170_15433 hypothetical protein; PFAM: Uncharacterised conserved protein UCP033199; SPTR: Putative uncharacterized protein; PFAM: Uncharacterized protein conserved in bacteria (DUF2200)
Ferric uptake regulator, Fur family; COGs: COG0735 Fe2+/Zn2+ uptake regulation protein; InterPro IPR002481; KEGG: lby:Lbys_0800 transcription regulator; PFAM: Ferric-uptake regulator; SPTR: Putative transcription regulator; PFAM: Ferric uptake regulator family; Belongs to the Fur family
Cellulase; InterPro IPR001547: IPR000601: IPR006626; KEGG: sde:Sde_3237 cellulase; PFAM: Glycoside hydrolase, family 5; PKD domain; PRIAM: Cellulase; SPTR: Putative endoglucanase; PFAM: Cellulase (glycosyl hydrolase family 5); TIGRFAM: parallel beta-helix repeat (two copies)
KEGG: fjo:Fjoh_4554 hypothetical protein; SPTR: Putative uncharacterized protein; PFAM: Protein of unknown function (DUF3592)
DinB family protein; InterPro IPR007837; KEGG: cpi:Cpin_4454 DinB family protein; PFAM: DinB; SPTR: Putative uncharacterized protein; PFAM: DinB family
COGs: COG3558 conserved hypothetical protein; InterPro IPR009783; KEGG: cpi:Cpin_7146 protein of unknown function DUF1348; PFAM: Protein of unknown function DUF1348; SPTR: Putative uncharacterized protein; PFAM: Protein of unknown function (DUF1348)
Cytosine-specific methyltransferase; COGs: COG0270 Site-specific DNA methylase; InterPro IPR000551: IPR001525; KEGG: wch:wcw_1690 C-5 cytosine-specific DNA methylase; PFAM: C-5 cytosine methyltransferase; HTH transcriptional regulator, MerR; PRIAM: DNA (cytosine-5-)-methyltransferase; SPTR: Cytosine-specific methyltransferase; TIGRFAM: C-5 cytosine methyltransferase; PFAM: C-5 cytosine-specific DNA methylase; TIGRFAM: DNA-methyltransferase (dcm); DNA binding domain, excisionase family
InterPro IPR006121; KEGG: fjo:Fjoh_1541 heavy metal transport/detoxification protein; PFAM: Heavy metal transport/detoxification protein; SPTR: Putative uncharacterized protein; PFAM: Heavy-metal-associated domain
InterPro IPR003347; KEGG: rbi:RB2501_08045 hypothetical protein; SMART: Transcription factor jumonji/aspartyl beta-hydroxylase; SPTR: Putative uncharacterized protein; PFAM: Cupin superfamily protein

Peptidase S24/S26A/S26B, conserved region; COGs: COG1974 SOS-response transcriptional repressors (RecA-mediated autopeptidase); InterPro IPR019759; KEGG: fjo:Fjoh_1967 putative prophage repressor; PFAM: Peptidase S24/S26A/S26B, conserved region; SPTR: Putative UmuD/RumA DNA repair protein; PFAM: Peptidase S24-like
KEGG: fbc:FB2170_07015 hypothetical protein; SPTR: Putative uncharacterized protein
KEGG: cow:Calow_0789 major facilitator superfamily mfs_1; SPTR: Putative uncharacterized protein
COGs: COG4922 conserved hypothetical protein; InterPro IPR009959; KEGG: cat:CA2559_10133 hypothetical protein; PFAM: Protein of unknown function DUF1486; SPTR: Putative uncharacterized protein; PFAM: SnoaL-like polyketide cyclase
Carboxymuconolactone decarboxylase; InterPro IPR003779; KEGG: cat:CA2559_02815 probable fusion protein; PFAM: Carboxymuconolactone decarboxylase; SPTR: Carboxymuconolactone decarboxylase; PFAM: Carboxymuconolactone decarboxylase family; TIGRFAM: uncharacterized peroxidase-related enzyme; alkylhydroperoxidase AhpD family core domain
KEGG: gfo:GFO_0370 secreted lipase/esterase; SPTR: Esterase; PFAM: Phospholipase/Carboxylesterase
YCII-related protein; COGs: COG3795 conserved hypothetical protein; InterPro IPR005545; KEGG: chu:CHU_2475 hypothetical protein; PFAM: YCII-related; SPTR: DGPFAETKE domain protein; PFAM: YCII-related domain
KEGG: fbc:FB2170_05880 hypothetical protein; SPTR: Putative uncharacterized protein
Transcriptional regulator, PadR-like family; COGs: COG1695 transcriptional regulator protein; InterPro IPR005149; KEGG: fbc:FB2170_05680 hypothetical protein; PFAM: Transcription regulator PadR N-terminal-like; SPTR: Putative uncharacterized protein; PFAM: Transcriptional regulator PadR-like family
KEGG: fbc:FB2170_05670 hypothetical protein; SPTR: Putative uncharacterized protein
KEGG: coc:Coch_1883 hypothetical protein; SPTR: Putative uncharacterized protein
KEGG: fbc:FB2170_05510 hypothetical protein; SPTR: Putative uncharacterized protein
KEGG: fjo:Fjoh_4670 hypothetical protein; SPTR: Putative uncharacterized protein
Transcriptional regulator, AraC family; COGs: COG2207 AraC-type DNA-binding domain-containing protein; InterPro IPR000005: IPR018060; KEGG: sli:Slin_4331 transcriptional regulator, AraC family; PFAM: HTH transcriptional regulator, AraC; SMART: Helix-turn-helix, AraC type, DNA binding domain; SPTR: Transcriptional regulator araC family protein; PFAM: Bacterial regulatory helix-turn-helix proteins, AraC family
Putative signal transduction protein with CBS domains; COGs: COG2524 transcriptional regulator protein; InterPro IPR000644; KEGG: fbc:FB2170_04405 CBS domain protein; PFAM: Cystathionine beta-synthase, core; SMART: Cystathionine beta-synthase, core; SPTR: CBS domain protein; PFAM: CBS domain
Pentapeptide repeat protein; InterPro IPR001646; KEGG: fbc:FB2170_04390 hypothetical protein; PFAM: Pentapeptide repeat; SPTR: Putative uncharacterized protein; PFAM: Pentapeptide repeats (8 copies)
4-oxalocrotonate tautomerase; COGs: COG3232 5-carboxymethyl-2-hydroxymuconate isomerase; InterPro IPR004370; KEGG: fbc:FB2170_00320 putative 5-carboxymethyl-2-hydroxymuconate delta-isomerase; PFAM: 4-oxalocrotonate tautomerase; SPTR: Putative 5-carboxymethyl-2-hydroxymuconate delta-isomerase; PFAM: 5-carboxymethyl-2-hydroxymuconate isomerase
KEGG: fbc:FB2170_08119 hypothetical protein; SPTR: Putative uncharacterized protein
DNA-cytosine methyltransferase; COGs: COG0270 Site-specific DNA methylase; InterPro IPR001525; KEGG: mhr:MHR_0071 cytosine-specific methyltransferase; PFAM: C-5 cytosine methyltransferase; SPTR: Cytosine-specific methyltransferase; TIGRFAM: C-5 cytosine methyltransferase; PFAM: C-5 cytosine-specific DNA methylase; TIGRFAM: DNA-methyltransferase (dcm); DNA binding domain, excisionase family; Belongs to the class I-like SAM-binding methyltransferase superfamily. C5-methyltransferase family
InterPro IPR010870; KEGG: fbc:FB2170_04855 phosphate-selective porin O and P; PFAM: Phosphate-selective porin O/P; SPTR: Phosphate-selective porin O and P; PFAM: Phosphate-selective porin O and P

COGs: COG1488 Nicotinic acid phosphoribosyltransferase; InterPro IPR015977; KEGG: fjo:Fjoh_0745 putative nicotinate phosphoribosyltransferase; PFAM: Nicotinate phosphoribosyltransferase-like; PRIAM: Nicotinamide phosphoribosyltransferase; SPTR: Nicotinamide phosphoribosyltransferase; PFAM: Nicotinate phosphoribosyltransferase (NAPRTase) family
KEGG: bth:BT_4352 hypothetical protein; SPTR: Putative uncharacterized protein
Uncharacterized protein; InterPro IPR009923; KEGG: fbc:FB2170_02910 hypothetical protein; PFAM: Dodecin flavoprotein; SPTR: Putative uncharacterized protein; PFAM: Protein of unknown function (DUF1458)
COGs: COG2949 Uncharacterized membrane protein; InterPro IPR003848; KEGG: fbc:FB2170_02845 putative transport-related membrane protein; PFAM: Domain of unknown function DUF218; SPTR: Putative transport-related membrane protein; PFAM: DUF218 domain
Major facilitator superfamily MFS_1; COGs: COG2814 Arabinose efflux permease; InterPro IPR011701; KEGG: ebi:EbC_03990 permease, MFS family; PFAM: Major facilitator superfamily MFS-1; SPTR: Putative uncharacterized protein; PFAM: Major Facilitator Superfamily
COGs: COG1723 conserved hypothetical protein; InterPro IPR003734; KEGG: cat:CA2559_00505 hypothetical protein; PFAM: Protein of unknown function DUF155; SPTR: Putative uncharacterized protein; PFAM: Uncharacterised ACR, YagE family COG1723
KEGG: fbc:FB2170_02595 hypothetical protein; SPTR: Putative uncharacterized protein
COGs: COG3228 conserved hypothetical protein; InterPro IPR010384; KEGG: cat:CA2559_12488 hypothetical protein; PFAM: Protein of unknown function DUF980; SPTR: Putative uncharacterized protein; PFAM: Phosphoenolpyruvate:glucose-phosphotransferase regulator; Belongs to the MtfA family
InterPro IPR013096; KEGG: fba:FIC_00886 hypothetical protein; PFAM: Cupin 2, conserved barrel; SPTR: Putative uncharacterized protein; PFAM: Cupin domain
RDD domain containing protein; InterPro IPR010432; KEGG: amr:AM1_G0015 hypothetical protein; PFAM: RDD; SPTR: Putative uncharacterized protein; PFAM: RDD family
LytTr DNA-binding region; InterPro IPR007492; KEGG: fjo:Fjoh_0682 response regulator receiver protein; PFAM: LytTr, DNA-binding domain; SMART: LytTr, DNA-binding domain; SPTR: Putative uncharacterized protein; PFAM: LytTr DNA-binding domain
Putative membrane protein insertion efficiency factor; Could be involved in insertion of integral membrane proteins into the membrane
KEGG: fba:FIC_01564 hypothetical protein; SPTR: Putative uncharacterized protein; PFAM: Domain of unknown function (DUF1083)
KEGG: rbi:RB2501_10997 hypothetical protein; SPTR: Putative uncharacterized protein
KEGG: zpr:ZPR_2090 hypothetical protein; SPTR: Putative uncharacterized protein
KEGG: rbi:RB2501_00631 hypothetical protein; SPTR: Putative uncharacterized protein
Tetratricopeptide TPR_1 repeat-containing protein; InterPro IPR001440: IPR019734; KEGG: rbi:RB2501_00621 hypothetical protein; PFAM: Tetratricopeptide TPR-1; SPTR: Putative uncharacterized protein
KEGG: rbi:RB2501_05175 hypothetical protein; SPTR: Putative uncharacterized protein
PHP domain protein; COGs: COG4464 Capsular polysaccharide biosynthesis protein; InterPro IPR004013; KEGG: fbc:FB2170_10121 capsular polysaccharide biosynthesis protein, putative; PFAM: PHP, C-terminal; SPTR: Capsular polysaccharide biosynthesis protein, putative
KEGG: fbc:FB2170_12751 hypothetical protein; SPTR: Putative uncharacterized protein
Glycosyl transferase family 39; COGs: COG1807 4-amino-4-deoxy-L-arabinose transferase and related glycosyltransferase of PMT family; InterPro IPR003342; KEGG: fba:FIC_02407 hypothetical protein; PFAM: Glycosyl transferase, family 39; SPTR: Putative uncharacterized protein; PFAM: Dolichyl-phosphate-mannose-protein mannosyltransferase

COGs: COG5337 Spore coat assembly protein; InterPro IPR014867; KEGG: sfr:Sfri_0739 spore coat assembly protein-like protein; PFAM: Spore coat protein CotH; SPTR: Spore coat protein CotH; PFAM: CotH protein
Regulatory protein TetR; InterPro IPR001647; KEGG: rbi:RB2501_04325 putative transcriptional regulator; PFAM: Transcription regulator, TetR-like, DNA-binding, bacterial/archaeal; SPTR: Transcriptional regulator, TetR family protein; PFAM: Bacterial regulatory proteins, tetR family
Uncharacterized protein; InterPro IPR009305; KEGG: fjo:Fjoh_1822 hypothetical protein; SPTR: Putative uncharacterized protein; PFAM: Protein of unknown function (DUF962)
COGs: COG1538 Outer membrane protein; InterPro IPR003423; KEGG: cat:CA2559_06470 hypothetical protein; PFAM: Outer membrane efflux protein; SPTR: Putative uncharacterized protein; PFAM: Outer membrane efflux protein
Phage repressor like transcriptional regulator, XRE family; Represses a number of genes involved in the response to DNA damage (SOS response)
Uncharacterized protein; KEGG: pkn:PKH_122370 DNA-directed RNA polymerase 3 largest subunit; SPTR: Putative uncharacterized protein
KEGG: sbi:SORBI_03g004950 hypothetical protein; SPTR: Putative uncharacterized protein
COGs: COG2366 Protein related to penicillin acylase; InterPro IPR002692; KEGG: fbc:FB2170_12451 penicillin amidase; PFAM: Peptidase S45, penicillin amidase; PRIAM: Acyl-homoserine-lactone acylase; SPTR: Penicillin amidase; PFAM: Penicillin amidase
KEGG: fjo:Fjoh_3287 hypothetical protein; SPTR: Putative uncharacterized protein
KEGG: zpr:ZPR_3182 putative transcription regulator; SPTR: Putative uncharacterized protein; PFAM: Ferric uptake regulator family
KEGG: chu:CHU_0965 hypothetical protein; SPTR: Putative uncharacterized protein
KEGG: fbc:FB2170_14538 hypothetical protein; SPTR: Putative uncharacterized protein
COGs: COG1629 Outer membrane receptor protein mostly Fe transport; InterPro IPR012910: IPR000531; KEGG: cat:CA2559_04680 TonB-dependent receptor; PFAM: TonB-dependent receptor, beta-barrel; TonB-dependent receptor, plug; SPTR: TonB-dependent receptor; PFAM: TonB-dependent Receptor Plug Domain
KEGG: fbc:FB2170_10439 hypothetical protein; SPTR: Putative uncharacterized protein
COGs: COG0735 Fe2+/Zn2+ uptake regulation protein; KEGG: gfo:GFO_0027 Fur family transcriptional regulator protein; SPTR: Ferric uptake regulation protein; PFAM: Ferric uptake regulator family; Belongs to the Fur family
COGs: COG0735 Fe2+/Zn2+ uptake regulation protein; KEGG: gfo:GFO_0027 Fur family transcriptional regulator protein; SPTR: Ferric uptake regulation protein; PFAM: Ferric uptake regulator family; Belongs to the Fur family
PepSY-associated TM helix domain protein; COGs: COG3182 Uncharacterized iron-regulated membrane protein; InterPro IPR005625; KEGG: fjo:Fjoh_1252 PepSY-associated TM helix domain-containing protein; PFAM: PepSY-associated TM helix; SPTR: PepSY-associated TM helix domain protein
KEGG: fjo:Fjoh_1253 hypothetical protein; SPTR: Putative uncharacterized protein
KEGG: fjo:Fjoh_1254 hypothetical protein; SPTR: Putative uncharacterized protein
Uncharacterized protein; InterPro IPR007272; KEGG: gfo:GFO_0978 YeeE/YedE domain-containing protein; PFAM: Domain of unknown function DUF395, YeeE/YedE; SPTR: Membrane protein containing YeeE/YedE domain; PFAM: YeeE/YedE family (DUF395)
Uncharacterized protein; InterPro IPR007272; KEGG: rbi:RB2501_07280 hypothetical protein; PFAM: Domain of unknown function DUF395, YeeE/YedE; SPTR: Putative uncharacterized protein; PFAM: YeeE/YedE family (DUF395)
HAMAP: Ribosomal protein L34; InterPro IPR000271; KEGG: cat:CA2559_12828 hypothetical protein; PFAM: Ribosomal protein L34; SPTR: 50S ribosomal protein L34; TIGRFAM: Ribosomal protein L34; PFAM: Ribosomal protein L34; TIGRFAM: ribosomal protein L34, bacterial type
TIGRFAM: gamma-glutamyltransferase; PFAM: gamma-glutamyltranspeptidase

TIGRFAM: dihydroxy-acid dehydratase; PFAM: dihydroxy-acid and 6-phosphogluconate dehydratase; Belongs to the IlvD/Edd family
TIGRFAM: aspartate racemase; PFAM: Asp/Glu racemase; Belongs to the aspartate/glutamate racemases family
TIGRFAM: aspartate racemase; PFAM: Asp/Glu racemase; Belongs to the aspartate/glutamate racemases family
TIGRFAM: aspartate racemase; PFAM: Asp/Glu racemase; Belongs to the aspartate/glutamate racemases family
TIGRFAM: argininosuccinate lyase; PFAM: fumarate lyase
TIGRFAM: acetolactate synthase, small subunit; PFAM: amino acid-binding ACT domain protein
TIGRFAM: acetolactate synthase, large subunit, biosynthetic type; PFAM: thiamine pyrophosphate enzyme domain protein TPP-binding; thiamine pyrophosphate enzyme, central region; thiamine pyrophosphate enzyme TPP binding domain protein
PFAM: thiamine pyrophosphate enzyme domain protein TPP-binding; thiamine pyrophosphate enzyme, central region; thiamine pyrophosphate enzyme TPP binding domain protein; Belongs to the TPP enzyme family
PFAM: peptidase M20; peptidase dimerisation domain protein
PFAM: glutathione peroxidase; Belongs to the glutathione peroxidase family
PFAM: glutamine synthetase, catalytic region; glutamine synthetase, beta-Grasp
PFAM: argininosuccinate synthase; ExsB family protein
Ketol-acid reductoisomerase (NADP(+)); Involved in the biosynthesis of branched-chain amino acids (BCAA). Catalyzes an alkyl-migration followed by a ketol- acid reduction of (S)-2-acetolactate (S2AL) to yield (R)-2,3-dihydroxy-isovalerate. In the isomerase reaction, S2AL is rearranged via a Mg-dependent methyl migration to produce 3- hydroxy-3-methyl-2-ketobutyrate (HMKB). In the reductase reaction, this 2-ketoacid undergoes a metal-dependent reduction by NADPH to yield (R)-2,3-dihydroxy-isovalerate; Belongs to the ketol-acid reductoisomerase family
Uncharacterized protein; N-terminus exhibits some similarity to carbohydrate binding module family 9 (CBM9) domain members
TIGRFAM: glucosamine-6-phosphate isomerase; PFAM: LmbE family protein; glucosamine/galactosamine-6-phosphate isomerase
TIGRFAM: glucosamine-6-phosphate isomerase; PFAM: LmbE family protein; glucosamine/galactosamine-6-phosphate isomerase
PFAM: glycoside hydrolase, family 20
Glycoside hydrolase family 92; Distantly related to alpha-mannosidases; predicted lipoprotein
Glycoside hydrolase family 92; Distantly related to alpha-mannosidases; predicted lipoprotein
Glycoside hydrolase family 92; Distantly related to alpha-mannosidases; predicted lipoprotein
Glycoside hydrolase family 92; Distantly related to alpha-mannosidases
Glycoside hydrolase family 92; Distantly related to alpha-mannosidases
Glycoside hydrolase family 92; Distantly related to alpha-mannosidases
Glycoside hydrolase family 2; Distantly related to alpha-mannosidases
Glucosamine-6-phosphate deaminase; Catalyzes the reversible isomerization-deamination of glucosamine 6-phosphate (GlcN6P) to form fructose 6-phosphate (Fru6P) and ammonium ion
Beta-N-acetylglucosaminidase-like protein Glycoside hydrolase family 20; Related to Beta-N-acetylglucosaminidase
Uncharacterized protein; N-terminus exhibits some similarity to carbohydrate binding module family 9 (CBM9) domain members
Transcriptional regulator, AraC family; PFAM: helix-turn-helix- domain containing protein, AraC type
Transcriptional regulator, AraC family; PFAM: helix-turn-helix- domain containing protein, AraC type
Predicted lipoprotein
Predicted lipoprotein

annotation not available
annotation not available
annotation not available
annotation not available
annotation not available
TIGRFAM: galactokinase; PFAM: GHMP kinase; GHMP kinase, C terminal domain protein; Belongs to the GHMP kinase family. GalK subfamily
SprD; Protein involved ingliding motility
SprC; Protein involved in gliding motility
PFAM: Aldose 1-epimerase
annotation not available
annotation not available
TIGRFAM: methionine synthase; PFAM: dihydropteroate synthase, DHPS; Methionine synthase, B12-binding module, cap domain protein; Vitamin B12 dependent methionine synthase, activation region; cobalamin B12-binding domain protein
PFAM: Methionine synthase, vitamin-B12 independent; Cobalamin-independent synthase MetE, N-terminal domain protein
PFAM: homocysteine S-methyltransferase
5-methyltetrahydropteroyltriglutamate--homocysteine methyltransferase; Catalyzes the transfer of a methyl group from 5- methyltetrahydrofolate to homocysteine resulting in methionine formation; Belongs to the vitamin-B12 independent methionine synthase family
TIGRFAM: uroporphyrin-III C-methyltransferase; PFAM: Uroporphyrin-III C/tetrapyrrole (Corrin/Porphyrin) methyltransferase
TIGRFAM: uroporphyrin-III C-methyltransferase; PFAM: Uroporphyrin-III C/tetrapyrrole (Corrin/Porphyrin) methyltransferase
TIGRFAM: siroheme synthase
Phosphoadenylylsulfate reductase (Thioredoxin); Reduction of activated sulfate into sulfite
PFAM: nitrite/sulfite reductase, hemoprotein beta-component, ferrodoxin domain protein; nitrite and sulphite reductase 4Fe-4S region; HEPN domain protein
Xanthine dehydrogenase, molybdenum binding subunit apoprotein; PFAM: aldehyde oxidase and xanthine dehydrogenase, a/b hammerhead; aldehyde oxidase and xanthine dehydrogenase, molybdopterin binding
PFAM: molybdopterin biosynthesis MoaE protein
PFAM: glycerophosphoryl diester phosphodiesterase
PFAM: ferredoxin; [2Fe-2S]-binding domain protein
PFAM: FAD dependent oxidoreductase
Molybdopterin molybdochelatase; TIGRFAM: molybdenum cofactor synthesis domain; PFAM: molybdopterin binding domain; MoeA domain protein, domain I and II; MoeA domain protein, domain IV
GTP cyclohydrolase subunit MoaC; TIGRFAM: molybdenum cofactor synthesis domain; PFAM: molybdopterin binding domain; molybdopterin cofactor biosynthesis MoaC region
GTP cyclohydrolase subunit MoaA; Catalyzes the cyclization of GTP to (8S)-3',8-cyclo-7,8- dihydroguanosine 5'-triphosphate
Glycerophosphoryl diester phosphodiesterase; Predicted lipoprotein
Alanine dehydrogenase; PFAM: protein of unknown function DUF182
Alanine dehydrogenase; PFAM: protein of unknown function DUF182
annotation not available
Transcriptional regulator, AraC family; PFAM: helix-turn-helix- domain containing protein, AraC type

Response regulator receiver protein; PFAM: helix-turn-helix- domain containing protein, AraC type; TPR repeat-containing protein; SMART: Tetratricopeptide domain protein
Putative membrane protein insertion efficiency factor; Could be involved in insertion of integral membrane proteins into the membrane
Predicted lipoprotein
Predicted lipoprotein
Predicted lipoprotein
Predicted lipoprotein
PFAM: Vitamin K epoxide reductase
PFAM: TPR repeat-containing protein; Tetratricopeptide TPR_2 repeat protein; SMART: Tetratricopeptide domain protein
PFAM: ribosomal protein L34; Belongs to the bacterial ribosomal protein bL34 family
PFAM: RDD domain containing protein
PFAM: protein of unknown function DUF962
PFAM: protein of unknown function DUF395, YeeE/YedE
PFAM: protein of unknown function DUF395, YeeE/YedE
PFAM: protein of unknown function DUF218
PFAM: protein of unknown function DUF187
PFAM: protein of unknown function DUF1697
PFAM: protein of unknown function DUF1348
PFAM: protein of unknown function DUF1348
PFAM: PHP C-terminal domain protein
PFAM: peptidase S45, penicillin amidase
PFAM: PepSY-associated TM helix domain protein
PFAM: pentapeptide repeat protein
PFAM: pentapeptide repeat protein
PFAM: pentapeptide repeat protein
PFAM: Nicotinate phosphoribosyltransferase and related
PFAM: major facilitator superfamily MFS_1
PFAM: Heavy metal transport/detoxification protein
PFAM: glycoside hydrolase, family 5; Belongs to the glycosyl hydrolase 5 (cellulase A) family
PFAM: GCN5-related N-acetyltransferase
PFAM: GCN5-related N-acetyltransferase
PFAM: carbohydrate kinase, FGGY
PFAM: carbohydrate kinase, FGGY
Peptidase family S9-like protein; Classified as 'non-peptidase homologue' by MEROPS; may not have peptidase activity; alpha/beta hydrolase fold-3 domain
Peptidase family S41-like protein; Classified as 'non-peptidase homologue' by MEROPS; may not have peptidase activity
Peptidase family S41-like protein; Classified as 'non-peptidase homologue' by MEROPS; may not have peptidase activity
Peptidase family S41-like protein; Classified as 'non-peptidase homologue' by MEROPS; may not have peptidase activity
Peptidase family S33; Predicted lipoprotein; alpha/beta hydrolase fold protein
Nonribosomal peptide synthesis
Nonribosomal peptide synthesis

Heavy metal transport/detoxification protein; Predicted lipoprotein
Glycoside hydrolase family 43; Distantly related to beta-D/alpha-L glycosidases
annotation not available
annotation not available
annotation not available
annotation not available
annotation not available
annotation not available
annotation not available
annotation not available
annotation not available
annotation not available
annotation not available
annotation not available
annotation not available
annotation not available
annotation not available
annotation not available
annotation not available
annotation not available
COG0137 Argininosuccinate synthase; Belongs to the argininosuccinate synthase family. Type 1 subfamily
COG0165 Argininosuccinate lyase
Succinyl-diaminopimelate desuccinylase; Catalyzes the hydrolysis of N-succinyl-L,L- diaminopimelic acid (SDAP), forming succinate and LL-2,6- diaminoheptanedioate (DAP), an intermediate involved in the bacterial biosynthesis of lysine and meso-diaminopimelic acid, an essential component of bacterial cell walls; Belongs to the peptidase M20A family. DapE subfamily
COG0129 Dihydroxyacid dehydratase/phosphogluconate dehydratase; Belongs to the IlvD/Edd family
COG0440 Acetolactate synthase, small (regulatory) subunit
Acetolactate synthase; COG0028 Thiamine pyrophosphate-requiring enzymes [acetolactate synthase, pyruvate dehydrogenase (cytochrome), glyoxylate carboligase, phosphonopyruvate decarboxylase]
COG0405 Gamma-glutamyltransferase
COG0174 Glutamine synthetase
COG0174 Glutamine synthetase
COG0624 Acetylornithine deacetylase/Succinyl-diaminopimelate desuccinylase and related deacylases; Belongs to the peptidase M20A family. ArgE subfamily
COG0386 Glutathione peroxidase; Belongs to the glutathione peroxidase family
COG0028 Thiamine pyrophosphate-requiring enzymes [acetolactate synthase, pyruvate dehydrogenase (cytochrome), glyoxylate carboligase, phosphonopyruvate decarboxylase]; Belongs to the TPP enzyme family
COG0386 Glutathione peroxidase; Belongs to the glutathione peroxidase family
COG0028 Thiamine pyrophosphate-requiring enzymes [acetolactate synthase, pyruvate dehydrogenase (cytochrome), glyoxylate carboligase, phosphonopyruvate decarboxylase]; Belongs to the TPP enzyme family
Acetolactate synthase; COG0028 Thiamine pyrophosphate-requiring enzymes [acetolactate synthase, pyruvate dehydrogenase (cytochrome), glyoxylate carboligase, phosphonopyruvate decarboxylase]

Ketol-acid reductoisomerase (NADP(+)); Involved in the biosynthesis of branched-chain amino acids (BCAA). Catalyzes an alkyl-migration followed by a ketol- acid reduction of (S)-2-acetolactate (S2AL) to yield (R)-2,3-dihydroxy-isovalerate. In the isomerase reaction, S2AL is rearranged via a Mg-dependent methyl migration to produce 3- hydroxy-3-methyl-2-ketobutyrate (HMKB). In the reductase reaction, this 2-ketoacid undergoes a metal-dependent reduction by NADPH to yield (R)-2,3-dihydroxy-isovalerate; Belongs to the ketol-acid reductoisomerase family
COG0129 Dihydroxyacid dehydratase/phosphogluconate dehydratase; Belongs to the IlvD/Edd family
Glycerol kinase; Key enzyme in the regulation of glycerol uptake and metabolism. Catalyzes the phosphorylation of glycerol to yield sn- glycerol 3-phosphate; Belongs to the FGGY kinase family
COG0584 Glycerophosphoryl diester phosphodiesterase
Glycerol kinase; Key enzyme in the regulation of glycerol uptake and metabolism. Catalyzes the phosphorylation of glycerol to yield sn- glycerol 3-phosphate; Belongs to the FGGY kinase family
Putative enzyme; COG3386 Gluconolactonase
COG0584 Glycerophosphoryl diester phosphodiesterase
COG0584 Glycerophosphoryl diester phosphodiesterase
FAD dependent oxidoreductase; COG0578 Glycerol-3-phosphate dehydrogenase
FAD dependent oxidoreductase; COG0578 Glycerol-3-phosphate dehydrogenase
COG1070 Sugar (pentulose and hexulose) kinases
COG0646 Methionine synthase I (cobalamin-dependent), methyltransferase domain
5-methyltetrahydropteroyltriglutamate--homocysteine methyltransferase; Catalyzes the transfer of a methyl group from 5- methyltetrahydrofolate to homocysteine resulting in methionine formation
COG0007 Uroporphyrinogen-III methylase; Belongs to the precorrin methyltransferase family
Phosphoadenosine phosphosulfate reductase; Reduction of activated sulfate into sulfite
COG0155 Sulfite reductase, beta subunit (hemoprotein)
COG1529 Aerobic-type carbon monoxide dehydrogenase, large subunit CoxL/CutL homologs
Isoquinoline 1-oxidoreductase, alpha subunit; COG2080 Aerobic-type carbon monoxide dehydrogenase, small subunit CoxS/CutS homologs
Purine catabolism protein pucB; COG2068 Uncharacterized MobA-related protein
Molybdenum cofactor biosynthesis protein B; May be involved in the biosynthesis of molybdopterin
COG0303 Molybdopterin biosynthesis enzyme
COG0314 Molybdopterin converting factor, large subunit
COG0303 Molybdopterin biosynthesis enzyme
Uncharacterized protein; COG1975 Xanthine and CO dehydrogenases maturation factor, XdhC/CoxF family
GTP 3',8-cyclase; Catalyzes the cyclization of GTP to (8S)-3',8-cyclo-7,8- dihydroguanosine 5'-triphosphate; Belongs to the radical SAM superfamily. MoaA family
Cyclic pyranopterin monophosphate synthase; Catalyzes the conversion of (8S)-3',8-cyclo-7,8- dihydroguanosine 5'-triphosphate to cyclic pyranopterin monophosphate (cPMP); Belongs to the MoaC family
Coenzyme PQQ synthesis protein E; COG0535 Predicted Fe-S oxidoreductases
COG0517 FOG: CBS domain
COG0129 Dihydroxyacid dehydratase/phosphogluconate dehydratase; Belongs to the IlvD/Edd family
Probable lipase/esterase; COG0657 Esterase/lipase
COG4977 Transcriptional regulator containing an amidase domain and an AraC-type DNA-binding HTH domain
Putative PRS2 protein; COG4539 Predicted membrane protein
COG3795 Uncharacterized protein conserved in bacteria
DoxD-like family protein; COG2259 Predicted membrane protein
COG0657 Esterase/lipase

Uncharacterized protein
COG1794 Aspartate racemase; Belongs to the aspartate/glutamate racemases family
COG1309 Transcriptional regulator
annotation not available
annotation not available
Putative glcG protein; COG3193 Uncharacterized protein, possibly involved in utilization of glycolate and propanediol
Putative membrane protein insertion efficiency factor; Could be involved in insertion of integral membrane proteins into the membrane
Transcriptional regulatory protein; COG0477 Permeases of the major facilitator superfamily
annotation not available
COG1309 Transcriptional regulator
COG0778 Nitroreductase
annotation not available
Peptidase S45, penicillin amidase; COG2366 Protein related to penicillin acylase
Alpha-L-glutamate ligase-related protein; COG0189 Glutathione synthase/Ribosomal protein S6 modification enzyme (glutaminyl transferase)
COG3795 Uncharacterized protein conserved in bacteria
COG3228 Uncharacterized protein conserved in bacteria; Belongs to the MtfA family
LexA repressor; Represses a number of genes involved in the response to DNA damage (SOS response), including recA and lexA. In the presence of single-stranded DNA, RecA interacts with LexA causing an autocatalytic cleavage which disrupts the DNA-binding part of LexA, leading to derepression of the SOS regulon and eventually DNA repair
Periplasmic mercury ion-binding protein; Mercury scavenger that specifically binds to one mercury ion and which passes it to the mercuric reductase (MerA) via the MerT protein
PFAM: Lyase; TIGRFAM: argininosuccinate lyase; COGs: COG0165 Argininosuccinate lyase; HAMAP: Argininosuccinate lyase; InterPro IPR022761:IPR009049; KEGG: fbc:FB2170_08244 argininosuccinate lyase; PFAM: Lyase 1, N-terminal; PRIAM: Argininosuccinate lyase; SPTR: Argininosuccinate lyase; TIGRFAM: Argininosuccinate lyase
PFAM: Dehydratase family; TIGRFAM: dihydroxy-acid dehydratase; COGs: COG0129 Dihydroxyacid dehydratase/phosphogluconate dehydratase; HAMAP: Dihydroxy-acid dehydratase; InterPro IPR004404:IPR000581; KEGG: rbi:RB2501_14414 dihydroxy-acid dehydratase; PFAM: Dihydroxy-acid/6-phosphogluconate dehydratase; PRIAM: Dihydroxy-acid dehydratase; SPTR: Dihydroxy-acid dehydratase; TIGRFAM: Dihydroxy-acid dehydratase; Belongs to the IlvD/Edd family
PFAM: Glutamine synthetase, catalytic domain; Glutamine synthetase, beta-Grasp domain; COGs: COG0174 Glutamine synthetase; InterPro IPR008147:IPR008146; KEGG: fbc:FB2170_01327 glutamine synthetase II; PFAM: Glutamine synthetase, catalytic domain; Glutamine synthetase, beta-Grasp; PRIAM: Glutamate--ammonia ligase; SPTR: Glutamine synthetase
PFAM: Arginosuccinate synthase; TIGRFAM: argininosuccinate synthase; COGs: COG0137 Argininosuccinate synthase; InterPro IPR001518; KEGG: fbc:FB2170_08294 argininosuccinate synthase; PFAM: Argininosuccinate synthase; PRIAM: Argininosuccinate synthase; SPTR: Argininosuccinate synthase; Belongs to the argininosuccinate synthase family. Type 1 subfamily

Succinyl-diaminopimelate desuccinylase; PFAM: Peptidase family M20/M25/M40; Peptidase dimerisation domain; COGs: COG0624 Acetylornithine deacetylase/Succinyl-diaminopimelate desuccinylase and related deacylase; InterPro IPR002933:IPR011650; KEGG: fbc:FB2170_08249 acetylornithine deacetylase; PFAM: Peptidase M20; Peptidase M20, dimerisation; PRIAM: Succinyl-diaminopimelate desuccinylase; SPTR: Acetylornithine deacetylase
Gamma-glutamyltransferase; PFAM: Gamma-glutamyltranspeptidase; TIGRFAM: gamma-glutamyltranspeptidase; COGs: COG0405 Gamma-glutamyltransferase; InterPro IPR000101; KEGG: rbi:RB2501_14579 gamma-glutamyltranspeptidase; PFAM: Gamma-glutamyltranspeptidase; PRIAM: Gamma-glutamyltransferase; SPTR: Gamma-glutamyltranspeptidase; TIGRFAM: Gamma-glutamyltranspeptidase
Ketol-acid reductoisomerase (NADP(+)); Involved in the biosynthesis of branched-chain amino acids (BCAA). Catalyzes an alkyl-migration followed by a ketol- acid reduction of (S)-2-acetolactate (S2AL) to yield (R)-2,3-dihydroxy-isovalerate. In the isomerase reaction, S2AL is rearranged via a Mg-dependent methyl migration to produce 3- hydroxy-3-methyl-2-ketobutyrate (HMKB). In the reductase reaction, this 2-ketoacid undergoes a metal-dependent reduction by NADPH to yield (R)-2,3-dihydroxy-isovalerate
PFAM: ACT domain; Small subunit of acetolactate synthase; TIGRFAM: acetolactate synthase, small subunit; COGs: COG0440 Acetolactate synthase small (regulatory) subunit; InterPro IPR004789:IPR002912:IPR019455; KEGG: rbi:RB2501_14424 putative acetohydroxyacid synthase small subunit; PFAM: Acetolactate synthase, small subunit, C-terminal; Amino acid-binding ACT; SPTR: Putative acetohydroxyacid synthase small subunit; TIGRFAM: Acetolactate synthase, small subunit
Acetolactate synthase; PFAM: Thiamine pyrophosphate enzyme, central domain; Thiamine pyrophosphate enzyme, N-terminal TPP binding domain; Thiamine pyrophosphate enzyme, C-terminal TPP binding domain; TIGRFAM: acetolactate synthase, large subunit, biosynthetic type; COGs: COG0028 Thiamine pyrophosphate-requiring protein; InterPro IPR012846:IPR012001:IPR012000:IPR011766; KEGG: fbc:FB2170_12296 acetolactate synthase large subunit; PFAM: Thiamine pyrophosphate enzyme, N-terminal TPP-binding domain; Thiamine pyrophosphate enzyme, central domain; Thiamine pyrophosphate enzyme, C-terminal TPP [...]
Glycerol kinase; Key enzyme in the regulation of glycerol uptake and metabolism. Catalyzes the phosphorylation of glycerol to yield sn- glycerol 3-phosphate; Belongs to the FGGY kinase family
Uncharacterized protein; PFAM: Domain of Unknown Function (DUF1080); InterPro IPR010496; KEGG: dfe:Dfer_3433 protein of unknown function DUF1080; PFAM: Protein of unknown function DUF1080; SPTR: Putative uncharacterized protein
PFAM: Protein of unknown function (DUF1697); COGs: COG3797 conserved hypothetical protein; InterPro IPR012545; KEGG: fbc:FB2170_08199 hypothetical protein; PFAM: Protein of unknown function DUF1697; SPTR: Putative uncharacterized protein
PFAM: Glycerophosphoryl diester phosphodiesterase family; COGs: COG0584 Glycerophosphoryl diester phosphodiesterase; InterPro IPR004129; KEGG: fbc:FB2170_08189 glcerophosphoryl diester phosphodiesterase; PFAM: Glycerophosphoryl diester phosphodiesterase; SPTR: Glcerophosphoryl diester phosphodiesterase
Glycerol-3-phosphate dehydrogenase; PFAM: FAD dependent oxidoreductase; COGs: COG0578 Glycerol-3-phosphate dehydrogenase; InterPro IPR006076; KEGG: fbc:FB2170_06880 GlpD; PFAM: FAD dependent oxidoreductase; PRIAM: Glycerol-3-phosphate dehydrogenase; SPTR: GlpD; Belongs to the FAD-dependent glycerol-3-phosphate dehydrogenase family
Uncharacterized protein; PFAM: Domain of Unknown Function (DUF1080); InterPro IPR010496; KEGG: rbi:RB2501_06280 putative secreted glycosyl hydrolase; PFAM: Protein of unknown function DUF1080; SPTR: Probable secreted glycosyl hydrolase

PFAM: AP endonuclease family 2 C terminus; Xylose isomerase-like TIM barrel; COGs: COG1082 Sugar phosphate isomerase/epimerase; InterPro IPR012307:IPR011418; KEGG: fbc:FB2170_04950 hypothetical protein; PFAM: Xylose isomerase, TIM barrel domain; AP endonuclease, family 2, C-terminal; SPTR: Putative uncharacterized protein
Uncharacterized protein; PFAM: Domain of Unknown Function (DUF1080); InterPro IPR010496; KEGG: rbi:RB2501_05695 putative secreted glycosyl hydrolase; PFAM: Protein of unknown function DUF1080; SPTR: Probable secreted glycosyl hydrolase
Uncharacterized protein; PFAM: Domain of Unknown Function (DUF1080); InterPro IPR010496; KEGG: rbi:RB2501_05690 hypothetical protein; PFAM: Protein of unknown function DUF1080; SPTR: Putative multi-domain protein
Xylulokinase; PFAM: FGGY family of carbohydrate kinases, N-terminal domain; FGGY family of carbohydrate kinases, C-terminal domain; COGs: COG1070 Sugar (pentulose and hexulose) kinase; InterPro IPR018484:IPR018485; KEGG: rbi:RB2501_04535 putative xylulose kinase; PFAM: Carbohydrate kinase, FGGY, N-terminal; Carbohydrate kinase, FGGY, C-terminal; PRIAM: Xylulokinase; SPTR: Putative xylulose kinase
Uncharacterized protein; PFAM: Domain of Unknown Function (DUF1080); InterPro IPR010496; KEGG: fbc:FB2170_00670 hypothetical protein; PFAM: Protein of unknown function DUF1080; SPTR: Putative uncharacterized protein
Uncharacterized protein; PFAM: Domain of Unknown Function (DUF1080); InterPro IPR010496; KEGG: fbc:FB2170_13101 putative glycosyl hydrolase; PFAM: Protein of unknown function DUF1080; SPTR: Probable large multifunctional protein-putative glycosyl hydrolase
Uncharacterized protein; PFAM: Domain of Unknown Function (DUF1080); InterPro IPR010496; KEGG: fbc:FB2170_07170 putative secreted glycosyl hydrolase; PFAM: Protein of unknown function DUF1080; SPTR: Probable secreted glycosyl hydrolase
Uncharacterized protein; PFAM: Isoprenylcysteine carboxyl methyltransferase (ICMT) family; COGs: COG2020 Putative protein-S-isoprenylcysteine methyltransferase; KEGG: rbi:RB2501_00116 putative protein-S-isoprenylcysteine methyltransferase; SPTR: Predicted protein-S-isoprenylcysteine methyltransferase
Putative membrane protein; PFAM: Protein of unknown function (DUF3308); TIGRFAM: Bacteroidetes-specific putative membrane protein; InterPro IPR019861; KEGG: rbi:RB2501_06865 hypothetical protein; SPTR: Putative uncharacterized protein; TIGRFAM: Conserved hypothetical protein CHP03519, membrane, Bacteroidetes
KEGG: fbc:FB2170_09166 hypothetical protein; SPTR: Putative uncharacterized protein
Glycosyl transferase family 39; COGs: COG1807 4-amino-4-deoxy-L-arabinose transferase and related glycosyltransferase of PMT family; InterPro IPR003342; KEGG: fbc:FB2170_00720 dolichyl-phosphate-mannose-protein mannosyltransferase family protein; PFAM: Glycosyl transferase, family 39; SPTR: Dolichyl-phosphate-mannose-protein mannosyltransferase-family protein
PFAM: Cupin domain; InterPro IPR013096; KEGG: rbi:RB2501_03435 polyketide synthesis domain-containing protein; PFAM: Cupin 2, conserved barrel; SPTR: Polyketide synthesis domain protein
PFAM: Galactokinase galactose-binding signature; GHMP kinases C terminal; GHMP kinases N terminal domain; TIGRFAM: galactokinase; COGs: COG0153 Galactokinase; InterPro IPR000705:IPR019539:IPR006204:IPR013750; KEGG: fbc:FB2170_14568 galactokinase; PFAM: GHMP kinase; Galactokinase galactose-binding domain; GHMP kinase, C-terminal; PRIAM: Galactokinase; SPTR: Galactokinase; TIGRFAM: Galactokinase; Belongs to the GHMP kinase family. GalK subfamily
PFAM: Aldose 1-epimerase; InterPro IPR008183; KEGG: fbc:FB2170_02195 hypothetical protein; PFAM: Aldose 1-epimerase; SPTR: Putative uncharacterized protein
KEGG: rbi:RB2501_10070 hypothetical protein; SPTR: Putative uncharacterized protein
KEGG: fbc:FB2170_02590 hypothetical protein; SPTR: Putative uncharacterized protein

KEGG: fbc:FB2170_02595 hypothetical protein; SPTR: Putative uncharacterized protein
Putative membrane protein; PFAM: Protein of unknown function (DUF3308); Sporulation related domain; TIGRFAM: Bacteroidetes-specific putative membrane protein; InterPro IPR007730:IPR019861; KEGG: rbi:RB2501_06865 hypothetical protein; PFAM: Sporulation-related domain; SPTR: Putative uncharacterized protein; TIGRFAM: Conserved hypothetical protein CHP03519, membrane, Bacteroidetes
KEGG: rbi:RB2501_06860 hypothetical protein; SPTR: Putative uncharacterized protein
KEGG: fbc:FB2170_05510 hypothetical protein; SPTR: Putative uncharacterized protein
KEGG: fbc:FB2170_10636 hypothetical protein; SPTR: Putative uncharacterized protein
Uncharacterized protein; PFAM: Domain of unknown function (DUF1083); KEGG: fbc:FB2170_03170 hypothetical protein; SPTR: Putative uncharacterized protein
KEGG: fbc:FB2170_02790 hypothetical protein; SPTR: Putative uncharacterized protein
Uncharacterized protein
COGs: COG4464 Capsular polysaccharide biosynthesis protein; KEGG: fbc:FB2170_10121 capsular polysaccharide biosynthesis protein; SPTR: Capsular polysaccharide biosynthesis protein, putative
KEGG: fbc:FB2170_15458 hypothetical protein; SPTR: Putative uncharacterized protein
Sporulation domain-containing protein; PFAM: Sporulation related domain; InterPro IPR007730; KEGG: fbc:FB2170_12891 hypothetical protein; PFAM: Sporulation-related domain; SPTR: Putative uncharacterized protein
Putative membrane protein; PFAM: Protein of unknown function (DUF3308); TIGRFAM: Bacteroidetes-specific putative membrane protein; InterPro IPR019861; KEGG: rbi:RB2501_15909 hypothetical protein; SPTR: Putative uncharacterized protein; TIGRFAM: Conserved hypothetical protein CHP03519, membrane, Bacteroidetes
Methionine synthase; PFAM: Homocysteine S-methyltransferase; TIGRFAM: 5-methyltetrahydrofolate--homocysteine methyltransferase; COGs: COG0646 Methionine synthase I (cobalamin-dependent) methyltransferase domain; InterPro IPR003726; KEGG: fbc:FB2170_17141 5-methyltetrahydrofolate--homocysteine methyltransferase; PFAM: Homocysteine S-methyltransferase; PRIAM: Methionine synthase; SPTR: 5-methyltetrahydrofolate--homocysteine methyltransferase
Methionine synthase; PFAM: Homocysteine S-methyltransferase; TIGRFAM: 5-methyltetrahydrofolate--homocysteine methyltransferase; COGs: COG0646 Methionine synthase I (cobalamin-dependent) methyltransferase domain; InterPro IPR003726; KEGG: fbc:FB2170_17141 5-methyltetrahydrofolate--homocysteine methyltransferase; PFAM: Homocysteine S-methyltransferase; PRIAM: Methionine synthase; SPTR: 5-methyltetrahydrofolate--homocysteine methyltransferase
Methionine synthase; PFAM: Pterin binding enzyme; Vitamin B12 dependent methionine synthase, activation domain; B12 binding domain; TIGRFAM: 5-methyltetrahydrofolate--homocysteine methyltransferase; COGs: COG1410 Methionine synthase I cobalamin-binding domain; InterProIPR011822:IPR000489:IPR003759:IPR006158:IPR 004223; KEGG: fbc:FB2170_17131 5-methyltetrahydrofolate--homocysteine methyltransferase; PFAM: Vitamin B12-dependent methionine synthase, activation domain; Pterin-binding; Methionine synthase, cobalamin (vitamin B12)-binding module, cap; Cobalamin (vitamin B12)-binding; PRIAM: [...]
TIGRFAM: siroheme synthase, N-terminal domain; COGs: COG1648 Siroheme synthase (precorrin-2 oxidase/ferrochelatase domain); InterPro IPR006367; KEGG: gfo:GFO_0329 siroheme synthase; SPTR: Siroheme synthase (Precorrin-2 oxidase); TIGRFAM: Sirohaem synthase, N-terminal

PFAM: Phosphoadenosine phosphosulfate reductase family; TIGRFAM: phosophoadenylyl-sulfate reductase (thioredoxin); COGs: COG0175 3'-phosphoadenosine 5'-phosphosulfate sulfotransferase (PAPS reductase)/FAD synthetase; InterPro IPR002500; KEGG: fbc:FB2170_17176 putative 3'-phosphoadenosine 5'-phosphosulfate sulfotransferase; PFAM: Phosphoadenosine phosphosulphate reductase; SPTR: Possible 3'-phosphoadenosine 5'-phosphosulfate sulfotransferase
PFAM: HEPN domain; Nitrite and sulphite reductase 4Fe-4S domain; Nitrite/Sulfite reductase ferredoxin-like half domain; COGs: COG0155 Sulfite reductase beta subunit (hemoprotein); InterPro IPR005117:IPR006067:IPR007842; KEGG: fbc:FB2170_17161 putative sulfite reductase; PFAM: Nitrite/sulphite reductase 4Fe-4S domain; Nitrite/sulphite reductase, hemoprotein beta-component, ferrodoxin-like; HEPN; PRIAM: Ferredoxin--nitrite reductase; SPTR: Probable sulfite reductase
Uroporphyrin-III C-methyltransferase; PFAM: Tetrapyrrole (Corrin/Porphyrin) Methylases; TIGRFAM: uroporphyrin-III C-methyltransferase; COGs: COG0007 Uroporphyrinogen-III methylase; InterPro IPR006366:IPR000878; KEGG: fbc:FB2170_17156 uroporphyrin-III C-methyltransferase; PFAM: Tetrapyrrole methylase; PRIAM: Uroporphyrinogen-III C-methyltransferase; SPTR: Uroporphyrin-III C-methyltransferase; TIGRFAM: Uroporphyrin-III C-methyltransferase, C-terminal; Belongs to the precorrin methyltransferase family
Isoquinoline 1-oxidoreductase; PFAM: Molybdopterin-binding domain of aldehyde dehydrogenase; COGs: COG1529 Aerobic-type carbon monoxide dehydrogenase large subunit CoxL/CutL homologs; InterPro IPR006311:IPR008274; KEGG: rbi:RB2501_02795 isoquinoline 1-oxidoreductase subunit beta; PFAM: Aldehyde oxidase/xanthine dehydrogenase, molybdopterin binding; PRIAM: Isoquinoline 1-oxidoreductase; SPTR: Isoquinoline 1-oxidoreductase, beta subunit
Isoquinoline 1-oxidoreductase; PFAM: Molybdopterin-binding domain of aldehyde dehydrogenase; COGs: COG1529 Aerobic-type carbon monoxide dehydrogenase large subunit CoxL/CutL homologs; InterPro IPR006311:IPR008274; KEGG: rbi:RB2501_02795 isoquinoline 1-oxidoreductase subunit beta; PFAM: Aldehyde oxidase/xanthine dehydrogenase, molybdopterin binding; PRIAM: Isoquinoline 1-oxidoreductase; SPTR: Isoquinoline 1-oxidoreductase, beta subunit
Isoquinoline 1-oxidoreductase; PFAM: Molybdopterin-binding domain of aldehyde dehydrogenase; COGs: COG1529 Aerobic-type carbon monoxide dehydrogenase large subunit CoxL/CutL homologs; InterPro IPR006311:IPR008274; KEGG: rbi:RB2501_02795 isoquinoline 1-oxidoreductase subunit beta; PFAM: Aldehyde oxidase/xanthine dehydrogenase, molybdopterin binding; PRIAM: Isoquinoline 1-oxidoreductase; SPTR: Isoquinoline 1-oxidoreductase, beta subunit
Isoquinoline 1-oxidoreductase; PFAM: Molybdopterin-binding domain of aldehyde dehydrogenase; COGs: COG1529 Aerobic-type carbon monoxide dehydrogenase large subunit CoxL/CutL homologs; InterPro IPR006311:IPR008274; KEGG: rbi:RB2501_02795 isoquinoline 1-oxidoreductase subunit beta; PFAM: Aldehyde oxidase/xanthine dehydrogenase, molybdopterin binding; PRIAM: Isoquinoline 1-oxidoreductase; SPTR: Isoquinoline 1-oxidoreductase, beta subunit
Isoquinoline 1-oxidoreductase; PFAM: 2Fe-2S iron-sulfur cluster binding domain; [2Fe-2S] binding domain; COGs: COG2080 Aerobic-type carbon monoxide dehydrogenase small subunit CoxS/CutS homologs; InterPro IPR001041:IPR002888; KEGG: rbi:RB2501_01016 isoquinoline 1-oxidoreductase subunit alpha; PFAM: [2Fe-2S]-binding; Ferredoxin; PRIAM: Isoquinoline 1-oxidoreductase; SPTR: Isoquinoline 1-oxidoreductase, alpha subunit
Isoquinoline 1-oxidoreductase; PFAM: 2Fe-2S iron-sulfur cluster binding domain; [2Fe-2S] binding domain; COGs: COG2080 Aerobic-type carbon monoxide dehydrogenase small subunit CoxS/CutS homologs; InterPro IPR001041:IPR002888; KEGG: rbi:RB2501_01016 isoquinoline 1-oxidoreductase subunit alpha; PFAM: [2Fe-2S]-binding; Ferredoxin; PRIAM: Isoquinoline 1-oxidoreductase; SPTR: Isoquinoline 1-oxidoreductase, alpha subunit

Isoquinoline 1-oxidoreductase; PFAM: 2Fe-2S iron-sulfur cluster binding domain; [2Fe-2S] binding domain; COGs: COG2080 Aerobic-type carbon monoxide dehydrogenase small subunit CoxS/CutS homologs; InterPro IPR001041:IPR002888; KEGG: rbi:RB2501_01016 isoquinoline 1-oxidoreductase subunit alpha; PFAM: [2Fe-2S]-binding; Ferredoxin; PRIAM: Isoquinoline 1-oxidoreductase; SPTR: Isoquinoline 1-oxidoreductase, alpha subunit
Peptidase S24/S26A/S26B, conserved region; PFAM: Peptidase S24-like; COGs: COG1974 SOS-response transcriptional repressors (RecA-mediated autopeptidase); InterPro IPR019759; KEGG: fjo:Fjoh_1967 putative prophage repressor; PFAM: Peptidase S24/S26A/S26B, conserved region; SPTR: Putative UmuD/RumA DNA repair protein
PFAM: MOSC domain; COGs: COG2258 conserved hypothetical protein; InterPro IPR005302; KEGG: fbc:FB2170_16046 hypothetical protein; PFAM: Molybdenum cofactor sulfurase, C-terminal; SPTR: Putative uncharacterized protein
Molybdenum cofactor synthesis domain protein; PFAM: Probable molybdopterin binding domain; MoeA N-terminal region (domain I and II); MoeA C-terminal region (domain IV); TIGRFAM: molybdenum cofactor synthesis domain; COGs: COG0303 Molybdopterin biosynthesis enzyme; InterPro IPR005110:IPR001453:IPR005111:IPR020817; KEGG: gfo:GFO_0455 molybdopterin biosynthesis protein MoeA; PFAM: MoeA, N-terminal and linker domain; Molybdopterin binding; MoeA, C-terminal, domain IV; SMART: Molybdopterin binding; SPTR: Molybdopterin biosynthesis protein moeA; TIGRFAM: Molybdenum cofactor synthesis
Molybdenum cofactor biosynthesis protein A; Catalyzes the cyclization of GTP to (8S)-3',8-cyclo-7,8-dihydroguanosine 5'-triphosphate
PFAM: MoaC family; Probable molybdopterin binding domain; TIGRFAM: molybdenum cofactor biosynthesis protein MoaC; molybdenum cofactor synthesis domain; COGs: COG0315 Molybdenum cofactor biosynthesis enzyme; InterPro IPR002820:IPR001453:IPR020817; KEGG: gfo:GFO_0453 bifunctional molybdenum cofactor biosynthesis protein MoaC/MogA; PFAM: Molybdopterin cofactor biosynthesis C (MoaC) domain; Molybdopterin binding; SMART: Molybdopterin binding; SPTR: Molybdenum cofactor biosynthesis bifunctional protein; TIGRFAM: Molybdenum cofactor synthesis
PFAM: MoaE protein; COGs: COG0314 Molybdopterin converting factor large subunit; InterPro IPR003448; KEGG: gfo:GFO_0452 molybdopterin converting factor subunit 2; PFAM: Molybdopterin biosynthesis MoaE; SPTR: Molybdopterin converting factor subunit 2
4-diphosphocytidyl-2C-methyl-D-erythritol synthase; PFAM: Uncharacterized protein family UPF0007; COGs: COG2068 Uncharacterized MobA-related protein; InterPro IPR001228; KEGG: gfo:GFO_0449 MobA-like molybdenum cofactor biosynthesis protein; PFAM: 4-diphosphocytidyl-2C-methyl-D-erythritol synthase; SPTR: MobA-like molybdenum cofactor biosynthesis protein
Xanthine dehydrogenase; PFAM: XdhC and CoxI family; COGs: COG1975 Xanthine and CO dehydrogenase maturation factor XdhC/CoxF family; InterPro IPR003777; KEGG: gfo:GFO_0448 XdhC/CoxI type molybdenum-dependent oxidoreductase accessory protein; PFAM: XdhC- CoxI; PRIAM: Xanthine dehydrogenase; SPTR: XdhC/CoxI type molybdenum-dependent oxidoreductase accessory protein
ThiJ/PfpI domain-containing protein; PFAM: DJ-1/PfpI family; COGs: COG4977 Transcriptional regulator containing an amidase domain and an AraC-type DNA-binding HTH domain; InterPro IPR002818; KEGG: gfo:GFO_3027 hypothetical protein; PFAM: ThiJ/PfpI; SPTR: Putative uncharacterized protein
KEGG: fbc:FB2170_13733 adenylosuccinate lyase; SPTR: Adenylosuccinate lyase
Uncharacterized protein; InterPro IPR019734; KEGG: rbi:RB2501_00621 hypothetical protein; SPTR: Putative uncharacterized protein
KEGG: rbi:RB2501_00631 hypothetical protein; SPTR: Putative uncharacterized protein

PFAM: D-Ala-D-Ala carboxypeptidase 3 (S13) family; COGs: COG2027 D-alanyl-D-alanine carboxypeptidase (penicillin-binding protein 4); InterPro IPR000667; KEGG: fbc:FB2170_14348 D-alanyl-D-alanine carboxypeptidase/D-alanyl-D-alanine-endopeptidase; PFAM: Peptidase S13, D-Ala-D-Ala carboxypeptidase C; SPTR: D-alanyl-D-alanine carboxypeptidase/D-alanyl-D-alanine-endopeptidase
Uncharacterized protein; PFAM: Uncharacterised ACR, YagE family COG1723; COGs: COG1723 conserved hypothetical protein; InterPro IPR003734; KEGG: cat:CA2559_00505 hypothetical protein; PFAM: Protein of unknown function DUF155; SPTR: Putative uncharacterized protein
KEGG: mtt:Ftrac_3621 hypothetical protein; SPTR: Putative outer membrane insertion C-signal
PFAM: DJ-1/PfpI family; Bacterial regulatory helix-turn-helix proteins, AraC family; COGs: COG4977 Transcriptional regulator containing an amidase domain and an AraC-type DNA-binding HTH domain; InterPro IPR018060:IPR002818:IPR000005; KEGG: chu:CHU_3787 transcriptional regulator; PFAM: HTH transcriptional regulator, AraC; ThiJ/PfpI; SMART: Helix-turn-helix, AraC type, DNA binding domain; SPTR: Transcriptional regulator, AraC family with amidase-like domain
PFAM: Glucosamine-6-phosphate isomerases/6-phosphogluconolactonase; GlcNAc-PI de-N-acetylase; TIGRFAM: glucosamine-6-phosphate isomerase; COGs: COG0363 6-phosphogluconolactonase/Glucosamine-6-phosphate isomerase/deaminase; InterPro IPR004547:IPR006148:IPR003737; KEGG: rbi:RB2501_01555 glucosamine-6-phosphate deaminase-like protein; PFAM: Glucosamine/galactosamine-6-phosphate isomerase; N-acetylglucosaminyl phosphatidylinositol deacetylase; PRIAM: Glucosamine-6-phosphate deaminase; SPTR: Glucosamine-6-phosphate deaminase; TIGRFAM: Glucosamine-6-phosphate isomerase
PFAM: Protein of unknown function (DUF2752); KEGG: fbc:FB2170_09146 hypothetical protein; SPTR: Putative uncharacterized protein
Beta-N-acetylhexosaminidase; PFAM: Glycosyl hydrolase family 20, catalytic domain; Glycosyl hydrolase family 20, domain 2; COGs: COG3525 N-acetyl-beta-hexosaminidase; InterPro IPR015882:IPR015883; KEGG: fbc:FB2170_00805 beta-N-acetylhexosaminidase; PFAM: Glycoside hydrolase, family 20, catalytic core; Acetylhexosaminidase, subunit a/b; PRIAM: Beta-N-acetylhexosaminidase; SPTR: Beta-N-acetylhexosaminidase
Beta-N-acetylhexosaminidase; PFAM: Glycosyl hydrolase family 20, catalytic domain; Glycosyl hydrolase family 20, domain 2; COGs: COG3525 N-acetyl-beta-hexosaminidase; InterPro IPR015882:IPR015883; KEGG: fbc:FB2170_00805 beta-N-acetylhexosaminidase; PFAM: Glycoside hydrolase, family 20, catalytic core; Acetylhexosaminidase, subunit a/b; PRIAM: Beta-N-acetylhexosaminidase; SPTR: Beta-N-acetylhexosaminidase
KEGG: rbi:RB2501_11127 hypothetical protein; SPTR: Putative uncharacterized protein
PFAM: Protein of unknown function (DUF3570); KEGG: fbc:FB2170_01292 hypothetical protein; SPTR: Putative uncharacterized protein
KEGG: fbc:FB2170_01362 adenine phosphoribosyltransferase; SPTR: Putative uncharacterized protein
Polyphosphate--glucose phosphotransferase; PFAM: ROK family; COGs: COG1940 Transcriptional regulator/sugar kinase; InterPro IPR000600; KEGG: rbi:RB2501_10397 ROK family protein; PFAM: ROK; PRIAM: Polyphosphate--glucose phosphotransferase; SPTR: ROK family protein
KEGG: fjo:Fjoh_1254 hypothetical protein; SPTR: Putative uncharacterized protein
KEGG: fjo:Fjoh_1253 hypothetical protein; SPTR: Putative uncharacterized protein
PepSY-associated TM helix domain protein; COGs: COG3182 Uncharacterized iron-regulated membrane protein; InterPro IPR005625; KEGG: fjo:Fjoh_1252 PepSY-associated TM helix domain-containing protein; PFAM: PepSY-associated TM helix; SPTR: Membrane protein, putative
PFAM: Glycosyl hydrolase family 20, catalytic domain; Glycosyl hydrolase family 20, domain 2; COGs: COG3525 N-acetyl-beta-hexosaminidase; InterPro IPR015882:IPR015883; KEGG: amu:Amuc_1815 beta-N-acetylhexosaminidase; PFAM: Glycoside hydrolase, family 20, catalytic core; Acetylhexosaminidase, subunit a/b; SPTR: Beta-N-acetylhexosaminidase

PFAM: Bacterial regulatory helix-turn-helix proteins, AraC family; InterPro IPR019734:IPR018060:IPR001440:IPR000005; KEGG: fba:FIC_01251 transcriptional regulator; PFAM: HTH transcriptional regulator, AraC; Tetratricopeptide TPR-1; SMART: Helix-turn-helix, AraC type, DNA binding domain; Tetratricopeptide repeat; SPTR: TPR-repeat-containing protein
Helix-turn-helix domain protein; PFAM: Helix-turn-helix; InterPro IPR001387; KEGG: chu:CHU_2935 hypothetical protein; PFAM: Helix-turn-helix type 3; SMART: Helix-turn-helix type 3; SPTR: Putative uncharacterized protein
Helix-turn-helix domain protein; PFAM: Helix-turn-helix; InterPro IPR001387; KEGG: chu:CHU_2935 hypothetical protein; PFAM: Helix-turn-helix type 3; SMART: Helix-turn-helix type 3; SPTR: Putative uncharacterized protein
KEGG: cat:CA2559_05500 hypothetical protein; SPTR: Putative uncharacterized protein
Helix-turn-helix domain protein; PFAM: Helix-turn-helix; InterPro IPR001387; KEGG: cpi:Cpin_2560 transcriptional regulator, XRE family; PFAM: Helix-turn-helix type 3; SMART: Helix-turn-helix type 3; SPTR: Transcriptional regulator, XRE family
Helix-turn-helix domain protein; PFAM: Helix-turn-helix; InterPro IPR001387; KEGG: cpi:Cpin_2560 transcriptional regulator, XRE family; PFAM: Helix-turn-helix type 3; SMART: Helix-turn-helix type 3; SPTR: Transcriptional regulator, XRE family
Helix-turn-helix domain protein; PFAM: Helix-turn-helix; InterPro IPR001387; KEGG: cpi:Cpin_2560 transcriptional regulator, XRE family; PFAM: Helix-turn-helix type 3; SMART: Helix-turn-helix type 3; SPTR: Transcriptional regulator, XRE family
Helix-turn-helix domain protein; PFAM: Helix-turn-helix; InterPro IPR001387; KEGG: cpi:Cpin_2560 transcriptional regulator, XRE family; PFAM: Helix-turn-helix type 3; SMART: Helix-turn-helix type 3; SPTR: Transcriptional regulator, XRE family
Putative membrane associated hydrolase; PFAM: Domain of unknown function (DUF1083); KEGG: fbc:FB2170_11461 putative membrane associated hydrolase; SPTR: Putative membrane associated hydrolase
Putative membrane associated hydrolase; PFAM: Domain of unknown function (DUF1083); KEGG: fbc:FB2170_11461 putative membrane associated hydrolase; SPTR: Putative membrane associated hydrolase
Putative membrane associated hydrolase; PFAM: Domain of unknown function (DUF1083); KEGG: fbc:FB2170_11461 putative membrane associated hydrolase; SPTR: Putative membrane associated hydrolase
Putative membrane associated hydrolase; PFAM: Domain of unknown function (DUF1083); KEGG: fbc:FB2170_11461 putative membrane associated hydrolase; SPTR: Putative membrane associated hydrolase
KEGG: fbc:FB2170_08119 hypothetical protein; SPTR: Putative uncharacterized protein
PFAM: Bacterial regulatory helix-turn-helix proteins, AraC family; COGs: COG2207 AraC-type DNA-binding domain-containing protein; InterPro IPR000005:IPR018060; KEGG: rbi:RB2501_08520 DNA binding protein; PFAM: HTH transcriptional regulator, AraC; SMART: Helix-turn-helix, AraC type, DNA binding domain; SPTR: DNA binding protein
PFAM: Penicillin amidase; COGs: COG2366 Protein related to penicillin acylase; InterPro IPR002692; KEGG: fbc:FB2170_07894 penicillin amidase family protein; PFAM: Peptidase S45, penicillin amidase; PRIAM: Glutaryl-7-aminocephalosporanic-acid acylase; SPTR: Penicillin amidase family protein
PFAM: Outer membrane efflux protein; COGs: COG1538 Outer membrane protein; KEGG: rbi:RB2501_08275 outer membrane efflux protein; SPTR: Putative uncharacterized protein
InterPro IPR003347; KEGG: fbc:FB2170_07444 hypothetical protein; SMART: Transcription factor jumonji/aspartyl beta-hydroxylase; SPTR: JmjC domain protein
KEGG: fbc:FB2170_07015 hypothetical protein; SPTR: Putative uncharacterized protein

PFAM: Carboxymuconolactone decarboxylase family; TIGRFAM: uncharacterized peroxidase-related enzyme; alkylhydroperoxidase AhpD family core domain; InterPro IPR003779; KEGG: cat:CA2559_02815 probable fusion protein; PFAM: Carboxymuconolactone decarboxylase; SPTR: Carboxymuconolactone decarboxylase
KEGG: vsp:VS_II1421 hypothetical protein; SPTR: Putative uncharacterized protein
KEGG: vsp:VS_II1421 hypothetical protein; SPTR: Putative uncharacterized protein
PFAM: C-5 cytosine-specific DNA methylase; TIGRFAM: DNA-methyltransferase (dcm); COGs: COG0270 Site-specific DNA methylase; InterPro IPR001525; KEGG: fbc:FB2170_06040 DNA (cytosine-5-)-methyltransferase; PFAM: C-5 cytosine methyltransferase; SPTR: Cytosine-specific methyltransferase; TIGRFAM: C-5 cytosine methyltransferase
KEGG: fbc:FB2170_05880 hypothetical protein; SPTR: Putative uncharacterized protein
GCN5-related N-acetyltransferase; PFAM: Acetyltransferase (GNAT) family; InterPro IPR000182; KEGG: fbc:FB2170_05445 transcriptional regulator; PFAM: GCN5-related N-acetyltransferase (GNAT) domain; SPTR: Transcriptional regulator
PFAM: Phosphate-selective porin O and P; InterPro IPR010870; KEGG: fbc:FB2170_04855 phosphate-selective porin O and P; PFAM: Phosphate-selective porin O/P; SPTR: Phosphate-selective porin O and P
Uncharacterized protein; PFAM: YeeE/YedE family (DUF395); InterPro IPR007272; KEGG: zpr:ZPR_2396 YeeE/YedE domain-containing protein; PFAM: Domain of unknown function DUF395, YeeE/YedE; SPTR: YeeE/YedE domain-containing protein
Uncharacterized protein; PFAM: YeeE/YedE family (DUF395); InterPro IPR007272; KEGG: fbc:FB2170_01597 hypothetical protein; PFAM: Domain of unknown function DUF395, YeeE/YedE; SPTR: Putative uncharacterized protein
Putative signal transduction protein with CBS domains; PFAM: CBS domain; COGs: COG2524 transcriptional regulator protein; InterPro IPR000644; KEGG: rbi:RB2501_05205 CBS domain-containing protein; PFAM: Cystathionine beta-synthase, core; SMART: Cystathionine beta-synthase, core; SPTR: CBS domain protein
PFAM: Pentapeptide repeats (8 copies); InterPro IPR001646; KEGG: fbc:FB2170_04390 hypothetical protein; PFAM: Pentapeptide repeat; SPTR: Putative uncharacterized protein
PFAM: Nitroreductase family; COGs: COG0778 Nitroreductase; InterPro IPR000415; KEGG: rbi:RB2501_04095 hypothetical protein; PFAM: Nitroreductase-like; SPTR: Putative uncharacterized protein
PFAM: TonB-dependent Receptor Plug Domain; COGs: COG1629 Outer membrane receptor protein mostly Fe transport; InterPro IPR012910:IPR000531; KEGG: cat:CA2559_04680 TonB-dependent receptor; PFAM: TonB-dependent receptor, beta-barrel; TonB-dependent receptor, plug; SPTR: TonB-dependent receptor
Glucokinase; PFAM: ROK family; TIGRFAM: ROK family protein (putative glucokinase); COGs: COG1940 Transcriptional regulator/sugar kinase; InterPro IPR000600; KEGG: fbc:FB2170_07529 putative ROK family transcriptional repressor; PFAM: ROK; PRIAM: Glucokinase; SPTR: Putative ROK family transcriptional repressor
Uncharacterized protein; PFAM: Domain of unknown function (DUF1083); KEGG: fbc:FB2170_03170 hypothetical protein; SPTR: Putative uncharacterized protein
PFAM: Protein of unknown function (DUF1458); InterPro IPR009923; KEGG: fbc:FB2170_02910 hypothetical protein; PFAM: Dodecin flavoprotein; SPTR: Putative uncharacterized protein
KEGG: rbi:RB2501_09525 Tat (twin-arginine translocation) pathway signal sequence domain-containing protein; SPTR: Tat (Twin-arginine translocation) pathway signal sequence domain protein
KEGG: fbc:FB2170_17021 hypothetical protein; SPTR: Putative uncharacterized protein
KEGG: cat:CA2559_01845 hypothetical protein; SPTR: Putative uncharacterized protein
KEGG: cat:CA2559_01845 hypothetical protein; SPTR: Putative uncharacterized protein
Uncharacterized protein; PFAM: PspC domain; KEGG: rbi:RB2501_02160 hypothetical protein; SPTR: Putative uncharacterized protein

Putative membrane associated hydrolase; PFAM: Domain of unknown function (DUF1083); KEGG: fbc:FB2170_16081 putative membrane associated hydrolase; SPTR: Putative membrane associated hydrolase
Putative membrane associated hydrolase; PFAM: Domain of unknown function (DUF1083); KEGG: fbc:FB2170_16081 putative membrane associated hydrolase; SPTR: Putative membrane associated hydrolase
PFAM: Ferric uptake regulator family; COGs: COG0735 Fe2+/Zn2+ uptake regulation protein; KEGG: lby:Lbys_0800 transcription regulator; SPTR: Probable transcription regulator
Uncharacterized protein; PFAM: Ferric uptake regulator family; COGs: COG0735 Fe2+/Zn2+ uptake regulation protein; KEGG: rbi:RB2501_04910 hypothetical protein; SPTR: Putative uncharacterized protein; Belongs to the Fur family
Regulatory protein MarR; PFAM: MarR family; InterPro IPR000835; KEGG: fbc:FB2170_11026 transcriptional regulator; PFAM: HTH transcriptional regulator, MarR; SMART: HTH transcriptional regulator, MarR; SPTR: Transcriptional regulator
Thioredoxin; KEGG: fbc:FB2170_11036 thioredoxin; SPTR: Thioredoxin
PFAM: Uncharacterized protein conserved in bacteria (DUF2200); COGs: COG4898 conserved hypothetical protein; InterPro IPR014580; KEGG: cpi:Cpin_6024 hypothetical protein; PFAM: Uncharacterised conserved protein UCP033199; SPTR: Putative uncharacterized protein
KEGG: fbc:FB2170_12751 hypothetical protein; SPTR: Putative uncharacterized protein
Metal-dependent hydrolase; PFAM: Mycothiol maleylpyruvate isomerase N-terminal domain; HAMAP: metal-dependent hydrolase; KEGG: rbi:RB2501_11247 metal-dependent hydrolase; SPTR: Putative uncharacterized protein
GCN5-related N-acetyltransferase; PFAM: Acetyltransferase (GNAT) family; InterPro IPR000182; KEGG: rbi:RB2501_11252 acetyltransferase (GNAT) family protein; PFAM: GCN5-related N-acetyltransferase (GNAT) domain; SPTR: Acetyltransferase (GNAT) family protein
PFAM: Bacterial regulatory helix-turn-helix proteins, AraC family; COGs: COG2207 AraC-type DNA-binding domain-containing protein; InterPro IPR000005:IPR018060; KEGG: fba:FIC_01251 transcriptional regulator; PFAM: HTH transcriptional regulator, AraC; SMART: Helix-turn-helix, AraC type, DNA binding domain; SPTR: DNA-binding response regulator, AraC family protein
PFAM: Protein of unknown function (DUF1348); COGs: COG3558 conserved hypothetical protein; InterPro IPR009783; KEGG: cpi:Cpin_7146 protein of unknown function DUF1348; PFAM: Protein of unknown function DUF1348; SPTR: Putative uncharacterized protein
Acyl-homoserine-lactone acylase; PFAM: Penicillin amidase; COGs: COG2366 Protein related to penicillin acylase; InterPro IPR002692; KEGG: fbc:FB2170_12451 penicillin amidase; PFAM: Peptidase S45, penicillin amidase; PRIAM: Acyl-homoserine-lactone acylase; SPTR: Penicillin amidase
PFAM: Ribosomal protein L34; TIGRFAM: ribosomal protein L34, bacterial type; HAMAP: Ribosomal protein L34; InterPro IPR000271; KEGG: coc:Coch_0279 ribosomal protein L34; PFAM: Ribosomal protein L34; SPTR: 50S ribosomal protein L34; TIGRFAM: Ribosomal protein L34; Belongs to the bacterial ribosomal protein bL34 family

cluster
acetolactate
acetolactate
acetolactate
acetolactate
acetolactate
acetolactate
acetolactate
acetolactate
acetolactate
acetolactate
acetolactate

acetolactate
carbohydrate1
carbohydrate1
carbohydrate1
carbohydrate1
carbohydrate1
carbohydrate1
carbohydrate1
carbohydrate1
carbohydrate2
carbohydrate2
carbohydrate2

carbohydrate2
carbohydrate2
carbohydrate2
gliding
gliding
gliding
gliding
gliding
gliding
gliding
gliding
gliding
gliding
gliding
gliding
gliding
gliding
gliding
gliding

gliding
gliding
gliding
gliding
gliding
gliding
gliding
gliding
gliding
gliding
methionine
methionine
methionine
porphyrin
porphyrin

porphyrin
porphyrin
porphyrin
porphyrin
pterin
pterin
pterin
pterin
pterin
pterin
pterin

pterin
pterin
pterin
pterin
pterin

acetolactate

acetolactate
acetolactate
acetolactate
acetolactate
acetolactate
acetolactate
acetolactate
acetolactate
acetolactate
acetolactate
acetolactate
acetolactate
acetolactate
carbohydrate1
carbohydrate1
carbohydrate1
carbohydrate1
carbohydrate1
carbohydrate1
carbohydrate1
carbohydrate1
carbohydrate1
carbohydrate1
carbohydrate1
carbohydrate1
carbohydrate1
carbohydrate2
carbohydrate2
carbohydrate2
carbohydrate2
carbohydrate2

carbohydrate2
carbohydrate2
carbohydrate2
carbohydrate2
carbohydrate2
gliding
gliding
gliding
gliding
gliding
gliding
methionine
methionine
methionine
methionine
porphyrin
porphyrin
porphyrin
porphyrin
porphyrin
pterin
pterin
pterin
pterin
pterin
pterin
pterin
pterin
pterin
pterin
pterin
pterin

acetolactate
acetolactate
acetolactate
acetolactate
acetolactate
acetolactate
acetolactate
acetolactate
acetolactate
acetolactate
acetolactate
acetolactate
acetolactate
acetolactate
acetolactate

acetolactate
acetolactate
carbohydrate1
carbohydrate1
carbohydrate1
carbohydrate1
carbohydrate1
carbohydrate1
carbohydrate1
carbohydrate1
carbohydrate1
methionine
methionine
porphyrin
porphyrin
porphyrin
pterin
pterin
pterin
pterin
pterin
pterin
pterin
pterin
pterin
pterin
pterin

acetolactate
acetolactate
acetolactate
acetolactate

acetolactate
acetolactate
acetolactate
acetolactate
acetolactate
carbohydrate1
carbohydrate1
carbohydrate1
carbohydrate1
carbohydrate1
carbohydrate1

carbohydrate1
carbohydrate1
carbohydrate1
carbohydrate1
carbohydrate1
carbohydrate1
carbohydrate1
gliding
gliding
gliding
gliding
gliding
gliding
gliding
gliding
gliding

gliding
gliding
gliding
gliding
gliding
gliding
gliding
gliding
gliding
gliding
gliding
gliding
methionine
methionine
methionine
porphyrin

porphyrin
porphyrin
porphyrin
pterin
pterin
pterin
pterin
pterin
pterin

pterin
pterin
pterin
pterin
pterin
pterin
pterin
pterin
pterin

zraR_2	18_00001	zraR_2
zraR_2	18_01989	zraR_2
zraR_2	18_00448	zraR_2
zraR_2	18_04018	zraR_2
zraR_2	18_03981	zraR_2
zraR_2	18_04017	zraR_2
zraR_2	18_04020	zraR_2
zraR_2	18_03680	zraR_2
zraR_2	18_04016	zraR_2
zraR_2	18_03805	zraR_2
zraR_2	18_04015	zraR_2
zraR_2	18_04019	zraR_2
zraR_2	18_02726	zraR_2
zraR_2	18_01796	zraR_2
zraR_2	18_03465	zraR_2
zraR_2	18_00648	zraR_2
zraR_2	18_00595	zraR_2
zraR_2	18_03575	zraR_2
zraR_2	18_03574	zraR_2
zraR_2	18_03464	zraR_2
zraR_2	18_00913	zraR_2
zraR_2	18_03453	zraR_2
zraR_2	18_02393	zraR_2
zraR_2	18_00596	zraR_2
zraR_2	18_00123	zraR_2
zraR_2	18_02394	zraR_2
zraR_2	18_00972	zraR_2
zraR_2	18_00100	zraR_2
zraR_2	18_01410	zraR_2
zraR_2	18_01432	zraR_2
zraR_2	18_03226	zraR_2
zraR_2	18_02412	zraR_2
zraR_2	18_01411	zraR_2
zraR_2	18_03558	zraR_2
zraR_2	18_03559	zraR_2
zraR_2	18_01813	zraR_2
zraR_2	18_00122	zraR_2
zraR_2	18_01317	zraR_2
zraR_2	18_03452	zraR_2
zraR_2	18_01208	zraR_2
zraR_2	18_02199	zraR_2
zraR_2	18_00574	zraR_2
zraR_2	18_03896	zraR_2
zraR_2	18_01812	zraR_2
zraR_2	18_00023	zraR_2
zraR_2	18_03948	zraR_2
zraR_2	18_02380	zraR_2
zraR_2	18_01894	zraR_2

zraR_2	18_01497	zraR_2
zraR_2	18_00086	zraR_2
zraR_2	18_03816	zraR_2
zraR_2	18_00746	zraR_2
zraR_2	18_03831	zraR_2
zraR_2	18_00744	zraR_2
zraR_2	18_01826	zraR_2
zraR_2	18_00162	zraR_2
zraR_2	18_03225	zraR_2
zraR_2	18_02423	zraR_2
zraR_2	18_02920	zraR_2
zraR_2	18_03560	zraR_2
zraR_2	18_01435	zraR_2
zraR_2	18_02421	zraR_2
zraR_2	18_00575	zraR_2
zraR_2	18_03943	zraR_2
zraR_2	18_00327	zraR_2
zraR_2	18_01692	zraR_2
zraR_2	18_01723	zraR_2
zraR_2	18_01315	zraR_2
zraR_2	18_00474	zraR_2
zraR_2	18_02537	zraR_2
zraR_2	18_00649	zraR_2
zraR_2	18_00638	zraR_2
zraR_2	18_03492	zraR_2
zraR_2	18_00862	zraR_2
zraR_2	18_01457	zraR_2
zraR_2	18_03254	zraR_2
zraR_2	18_00931	zraR_2
zraR_2	18_01190	zraR_2
zraR_2	18_01409	zraR_2
zraR_2	18_02643	zraR_2
zraR_2	18_01437	zraR_2
zraR_2	ACAM48_02839	zraR_2
zraR_2	ACAM48_02563	zraR_2
zraR_2	ACAM48_02442	zraR_2
zraR_2	ACAM48_02659	zraR_2
zraR_2	ACAM48_03411	zraR_2
zraR_2	ACAM48_01772	zraR_2
zraR_2	ACAM48_03125	zraR_2
zraR_2	ACAM48_02043	zraR_2
zraR_2	ACAM48_01388	zraR_2
zraR_2	ACAM48_01389	zraR_2
zraR_2	ACAM48_02044	zraR_2
zraR_2	ACAM48_02014	zraR_2
zraR_2	ACAM48_01551	zraR_2
zraR_2	ACAM48_00442	zraR_2
zraR_2	ACAM48_02015	zraR_2

zraR_2	ACAM48_00682	zraR_2
zraR_2	ACAM48_01542	zraR_2
zraR_2	ACAM48_01550	zraR_2
zraR_2	ACAM48_02513	zraR_2
zraR_2	ACAM48_03351	zraR_2
zraR_2	ACAM48_00140	zraR_2
zraR_2	ACAM48_00108	zraR_2
zraR_2	ACAM48_00680	zraR_2
zraR_2	ACAM48_02544	zraR_2
zraR_2	ACAM48_00482	zraR_2
zraR_2	ACAM48_02178	zraR_2
zraR_2	ACAM48_00749	zraR_2
zraR_2	ACAM48_01283	zraR_2
zraR_2	ACAM48_00662	zraR_2
zraR_2	ACAM48_00139	zraR_2
zraR_2	ACAM48_00663	zraR_2
zraR_2	ACAM48_01182	zraR_2
zraR_2	ACAM48_01855	zraR_2
zraR_2	ACAM48_00443	zraR_2
zraR_2	ACAM48_03196	zraR_2
zraR_2	ACAM48_01946	zraR_2
zraR_2	ACAM48_03533	zraR_2
zraR_2	ACAM48_00354	zraR_2
zraR_2	ACAM48_02781	zraR_2
zraR_2	ACAM48_03197	zraR_2
zraR_2	ACAM48_02658	zraR_2
zraR_2	ACAM48_01945	zraR_2
zraR_2	ACAM48_00816	zraR_2
zraR_2	ACAM48_02667	zraR_2
zraR_2	ACAM48_01541	zraR_2
zraR_2	ACAM48_00109	zraR_2
zraR_2	ACAM48_00133	zraR_2
zraR_2	ACAM48_02506	zraR_2
zraR_2	ACAM48_02094	zraR_2
zraR_2	ACAM48_02770	zraR_2
zraR_2	ACAM48_03210	zraR_2
zraR_2	ACAM48_01580	zraR_2
zraR_2	ACAM48_00032	zraR_2
zraR_2	ACAM48_02426	zraR_2
zraR_2	ACAM48_02427	zraR_2
zraR_2	CAM030_01158	zraR_2
zraR_2	CAM030_01231	zraR_2
zraR_2	CAM030_02669	zraR_2
zraR_2	CAM030_02135	zraR_2
zraR_2	CAM030_01975	zraR_2
zraR_2	CAM030_00402	zraR_2
zraR_2	CAM030_00360	zraR_2
zraR_2	CAM030_01884	zraR_2

zraR_2	CAM030_00359	zraR_2
zraR_2	CAM030_02134	zraR_2
zraR_2	CAM030_01885	zraR_2
zraR_2	CAM030_00403	zraR_2
zraR_2	CAM030_01976	zraR_2
zraR_2	CAM030_02192	zraR_2
zraR_2	CAM030_00874	zraR_2
zraR_2	CAM030_02209	zraR_2
zraR_2	CAM030_01894	zraR_2
zraR_2	CAM030_02756	zraR_2
zraR_2	CAM030_02194	zraR_2
zraR_2	CAM030_00022	zraR_2
zraR_2	CAM030_00923	zraR_2
zraR_2	CAM030_02035	zraR_2
zraR_2	CAM030_02036	zraR_2
zraR_2	CAM030_01385	zraR_2
zraR_2	CAM030_02200	zraR_2
zraR_2	CAM030_00027	zraR_2
zraR_2	CAM030_00651	zraR_2
zraR_2	CAM030_00776	zraR_2
zraR_2	CAM030_00926	zraR_2
zraR_2	CAM030_00662	zraR_2
zraR_2	CAM030_01883	zraR_2
zraR_2	CAM030_02440	zraR_2
zraR_2	CAM030_01886	zraR_2
zraR_2	CAM030_01895	zraR_2
zraR_2	CAM030_00574	zraR_2
zraR_2	CAM030_01441	zraR_2
zraR_2	CAM030_01967	zraR_2
zraR_2	CAM030_02409	zraR_2
zraR_2	CAM030_02103	zraR_2
zraR_2	CAM030_01298	zraR_2
zraR_2	CAM030_01910	zraR_2
zraR_2	CAM030_02441	zraR_2
zraR_2	CAM030_00931	zraR_2
zraR_2	CAM030_02526	zraR_2
zraR_2	CAM030_00435	zraR_2
zraR_2	CAM030_03063	zraR_2
zraR_2	CECT7946_00713	zraR_2
zraR_2	CECT7946_03571	zraR_2
zraR_2	CECT7946_00954	zraR_2
zraR_2	CECT7946_00450	zraR_2
zraR_2	CECT7946_00578	zraR_2
zraR_2	CECT7946_02492	zraR_2
zraR_2	CECT7946_00947	zraR_2
zraR_2	CECT7946_02006	zraR_2
zraR_2	CECT7946_00449	zraR_2
zraR_2	CECT7946_02493	zraR_2

zraR_2	CECT7946_03465	zraR_2
zraR_2	CECT7946_02359	zraR_2
zraR_2	CECT7946_02650	zraR_2
zraR_2	CECT7946_02189	zraR_2
zraR_2	CECT7946_03466	zraR_2
zraR_2	CECT7946_02007	zraR_2
zraR_2	CECT7946_03282	zraR_2
zraR_2	CECT7946_02651	zraR_2
zraR_2	CECT7946_00760	zraR_2
zraR_2	CECT7946_00501	zraR_2
zraR_2	CECT7946_02474	zraR_2
zraR_2	CECT7946_02358	zraR_2
zraR_2	CECT7946_00517	zraR_2
zraR_2	CECT7946_01268	zraR_2
zraR_2	CECT7946_00813	zraR_2
zraR_2	CECT7946_00276	zraR_2
zraR_2	CECT7946_00362	zraR_2
zraR_2	CECT7946_00865	zraR_2
zraR_2	CECT7946_02404	zraR_2
zraR_2	CECT7946_00503	zraR_2
zraR_2	CECT7946_01021	zraR_2
zraR_2	CECT7946_01006	zraR_2
zraR_2	CECT7946_01778	zraR_2
zraR_2	CECT7946_00252	zraR_2
zraR_2	CECT7946_00908	zraR_2
zraR_2	CECT7946_02733	zraR_2
zraR_2	CECT7946_01022	zraR_2
zraR_2	CECT7946_02367	zraR_2
zraR_2	CECT7946_00986	zraR_2
zraR_2	CECT7946_00814	zraR_2
zraR_2	CECT7946_02137	zraR_2
zraR_2	CECT7946_01007	zraR_2
zraR_2	CECT7946_00403	zraR_2
zraR_2	CECT7946_01364	zraR_2
zraR_2	CECT7946_01197	zraR_2
zraR_2	CECT7946_00717	zraR_2
zraR_2	CECT7946_02804	zraR_2
zraR_2	CECT7946_03260	zraR_2
zraR_2	CECT7946_03130	zraR_2
zraR_2	CECT7946_00242	zraR_2
zraR_2	CECT7946_01604	zraR_2
zraR_2	CECT7946_01581	zraR_2
zraR_2	CECT7946_02758	zraR_2
zraR_2	CGMCC1.8863_00655	zraR_2
zraR_2	CGMCC1.8863_02892	zraR_2
zraR_2	CGMCC1.8863_00145	zraR_2
zraR_2	CGMCC1.8863_01655	zraR_2
zraR_2	CGMCC1.8863_01964	zraR_2

zraR_2	CGMCC1.8863_01925	zraR_2
zraR_2	CGMCC1.8863_02828	zraR_2
zraR_2	CGMCC1.8863_00339	zraR_2
zraR_2	CGMCC1.8863_01620	zraR_2
zraR_2	CGMCC1.8863_00570	zraR_2
zraR_2	CGMCC1.8863_02452	zraR_2
zraR_2	CGMCC1.8863_03277	zraR_2
zraR_2	CGMCC1.8863_01249	zraR_2
zraR_2	CGMCC1.8863_01609	zraR_2
zraR_2	CGMCC1.8863_02934	zraR_2
zraR_2	CGMCC1.8863_00239	zraR_2
zraR_2	CGMCC1.8863_01669	zraR_2
zraR_2	CGMCC1.8863_01250	zraR_2
zraR_2	CGMCC1.8863_02935	zraR_2
zraR_2	CGMCC1.8863_00158	zraR_2
zraR_2	CGMCC1.8863_00240	zraR_2
zraR_2	CGMCC1.8863_01914	zraR_2
zraR_2	CGMCC1.8863_01668	zraR_2
zraR_2	CGMCC1.8863_01915	zraR_2
zraR_2	CGMCC1.8863_00929	zraR_2
zraR_2	CGMCC1.8863_02700	zraR_2
zraR_2	CGMCC1.8863_01840	zraR_2
zraR_2	CGMCC1.8863_02629	zraR_2
zraR_2	CGMCC1.8863_03279	zraR_2
zraR_2	CGMCC1.8863_00411	zraR_2
zraR_2	CGMCC1.8863_00410	zraR_2
zraR_2	CGMCC1.8863_01985	zraR_2
zraR_2	CGMCC1.8863_00743	zraR_2
zraR_2	CGMCC1.8863_02254	zraR_2
zraR_2	CGMCC1.8863_02208	zraR_2
zraR_2	CGMCC1.8863_03206	zraR_2
zraR_2	CGMCC1.8863_01579	zraR_2
zraR_2	CGMCC1.8863_00077	zraR_2
zraR_2	CGMCC1.8863_03202	zraR_2
zraR_2	CGMCC1.8863_00258	zraR_2
zraR_2	CGMCC1.8863_03199	zraR_2
zraR_2	CGMCC1.8863_03562	zraR_2
zraR_2	CGMCC1.8863_02675	zraR_2
zraR_2	CGMCC1.8863_00385	zraR_2
zraR_2	CGMCC1.8863_02676	zraR_2
zraR_2	CGMCC1.8863_02726	zraR_2
zraR_2	CGMCC1.8863_01324	zraR_2
zraR_2	CGMCC1.8863_02512	zraR_2
zraR_2	CGMCC1.8863_03458	zraR_2
zraR_2	CGMCC1.8863_00157	zraR_2
zraR_2	CGMCC1.8863_00165	zraR_2
zraR_2	CGMCC1.8863_00483	zraR_2
zraR_2	CGMCC1.8863_00915	zraR_2

zraR_2	CGMCC1.8863_01861	zraR_2
zraR_2	CGMCC1.8863_03507	zraR_2
zraR_2	CGMCC1.8863_03563	zraR_2
zraR_2	CGMCC1.8863_01999	zraR_2
zraR_2	CL8139_00518	zraR_2
zraR_2	CL8139_03185	zraR_2
zraR_2	CL8139_03113	zraR_2
zraR_2	CL8139_03135	zraR_2
zraR_2	CL8139_02933	zraR_2
zraR_2	CL8139_02183	zraR_2
zraR_2	CL8139_02395	zraR_2
zraR_2	CL8139_02550	zraR_2
zraR_2	CL8139_01285	zraR_2
zraR_2	CL8139_02394	zraR_2
zraR_2	CL8139_02184	zraR_2
zraR_2	CL8139_02549	zraR_2
zraR_2	CL8139_01284	zraR_2
zraR_2	CL8139_03106	zraR_2
zraR_2	CL8139_03175	zraR_2
zraR_2	CL8139_01663	zraR_2
zraR_2	CL8139_03176	zraR_2
zraR_2	CL8139_00952	zraR_2
zraR_2	CL8139_03105	zraR_2
zraR_2	CL8139_01664	zraR_2
zraR_2	CL8139_02115	zraR_2
zraR_2	CL8139_01732	zraR_2
zraR_2	CL8139_00116	zraR_2
zraR_2	CL8139_02258	zraR_2
zraR_2	CL8139_00058	zraR_2
zraR_2	CL8139_02250	zraR_2
zraR_2	CL8139_01722	zraR_2
zraR_2	CL8139_02820	zraR_2
zraR_2	CL8139_03056	zraR_2
zraR_2	CL8139_01595	zraR_2
zraR_2	CL8139_00093	zraR_2
zraR_2	CL8139_01204	zraR_2
zraR_2	CL8139_02270	zraR_2
zraR_2	CL8139_02268	zraR_2
zraR_2	CL8139_02383	zraR_2
zraR_2	CL8139_02571	zraR_2
zraR_2	CL8139_01681	zraR_2
zraR_2	CL8139_00079	zraR_2
zraR_2	CL8139_03327	zraR_2
zraR_2	CL8139_02606	zraR_2
zraR_2	CL8139_01736	zraR_2
zraR_2	CL8139_02414	zraR_2
zraR_2	CL8139_01680	zraR_2
zraR_2	CL8139_00537	zraR_2

zraR_2	CL8139_00484	zraR_2
zraR_2	CL8139_01376	zraR_2
zraR_2	CL8139_00266	zraR_2
zraR_2	CL8139_03353	zraR_2
zraR_2	CL8139_01550	zraR_2
zraR_2	CL8139_00875	zraR_2
zraR_2	CL8139_00349	zraR_2
zraR_2	CL8139_02932	zraR_2
zraR_2	DAU203_00445	zraR_2
zraR_2	DAU203_02476	zraR_2
zraR_2	DAU203_02485	zraR_2
zraR_2	DAU203_02621	zraR_2
zraR_2	DAU203_02600	zraR_2
zraR_2	DAU203_00616	zraR_2
zraR_2	DAU203_00969	zraR_2
zraR_2	DAU203_03166	zraR_2
zraR_2	DAU203_01446	zraR_2
zraR_2	DAU203_03466	zraR_2
zraR_2	DAU203_03165	zraR_2
zraR_2	DAU203_00970	zraR_2
zraR_2	DAU203_01445	zraR_2
zraR_2	DAU203_03467	zraR_2
zraR_2	DAU203_03355	zraR_2
zraR_2	DAU203_02466	zraR_2
zraR_2	DAU203_01269	zraR_2
zraR_2	DAU203_02467	zraR_2
zraR_2	DAU203_01729	zraR_2
zraR_2	DAU203_03356	zraR_2
zraR_2	DAU203_01270	zraR_2
zraR_2	DAU203_01042	zraR_2
zraR_2	DAU203_02647	zraR_2
zraR_2	DAU203_01927	zraR_2
zraR_2	DAU203_01338	zraR_2
zraR_2	DAU203_00523	zraR_2
zraR_2	DAU203_01330	zraR_2
zraR_2	DAU203_02657	zraR_2
zraR_2	DAU203_00068	zraR_2
zraR_2	DAU203_03405	zraR_2
zraR_2	DAU203_01203	zraR_2
zraR_2	DAU203_01904	zraR_2
zraR_2	DAU203_00221	zraR_2
zraR_2	DAU203_01350	zraR_2
zraR_2	DAU203_01348	zraR_2
zraR_2	DAU203_03154	zraR_2
zraR_2	DAU203_03186	zraR_2
zraR_2	DAU203_03109	zraR_2
zraR_2	DAU203_01287	zraR_2
zraR_2	DAU203_03331	zraR_2

zraR_2	DAU203_02125	zraR_2
zraR_2	DAU203_02353	zraR_2
zraR_2	DAU203_02643	zraR_2
zraR_2	DAU203_00847	zraR_2
zraR_2	DAU203_01286	zraR_2
zraR_2	DAU203_00348	zraR_2
zraR_2	DAU203_00477	zraR_2
zraR_2	DAU203_01098	zraR_2
zraR_2	DAU203_03110	zraR_2
zraR_2	DAU203_00820	zraR_2
zraR_2	DAU203_02151	zraR_2
zraR_2	DAU203_02169	zraR_2
zraR_2	DAU203_01651	zraR_2
zraR_2	DAU203_01571	zraR_2
zraR_2	DD5b_01716	zraR_2
zraR_2	DD5b_00886	zraR_2
zraR_2	DD5b_01008	zraR_2
zraR_2	DD5b_00470	zraR_2
zraR_2	DD5b_01868	zraR_2
zraR_2	DD5b_00480	zraR_2
zraR_2	DD5b_01869	zraR_2
zraR_2	DD5b_01438	zraR_2
zraR_2	DD5b_01689	zraR_2
zraR_2	DD5b_01688	zraR_2
zraR_2	DD5b_00390	zraR_2
zraR_2	DD5b_01437	zraR_2
zraR_2	DD5b_01618	zraR_2
zraR_2	DD5b_00902	zraR_2
zraR_2	DD5b_01040	zraR_2
zraR_2	DD5b_02723	zraR_2
zraR_2	DD5b_00942	zraR_2
zraR_2	DD5b_00774	zraR_2
zraR_2	DD5b_01389	zraR_2
zraR_2	DD5b_00584	zraR_2
zraR_2	DD5b_02067	zraR_2
zraR_2	DD5b_01629	zraR_2
zraR_2	DD5b_00698	zraR_2
zraR_2	DD5b_00079	zraR_2
zraR_2	DD5b_00518	zraR_2
zraR_2	DD5b_02741	zraR_2
zraR_2	DD5b_00893	zraR_2
zraR_2	DD5b_01718	zraR_2
zraR_2	DD5b_01630	zraR_2
zraR_2	DD5b_01819	zraR_2
zraR_2	DD5b_02225	zraR_2
zraR_2	DD5b_00055	zraR_2
zraR_2	DD5b_02498	zraR_2
zraR_2	DD5b_02696	zraR_2

zraR_2	DD5b_01336	zraR_2
zraR_2	DD5b_02064	zraR_2
zraR_2	DD5b_00391	zraR_2
zraR_2	DD5b_01715	zraR_2
zraR_2	DD5b_00339	zraR_2
zraR_2	DSM1208_03297	zraR_2
zraR_2	DSM1208_03486	zraR_2
zraR_2	DSM1208_04504	zraR_2
zraR_2	DSM1208_03655	zraR_2
zraR_2	DSM1208_00334	zraR_2
zraR_2	DSM1208_00166	zraR_2
zraR_2	DSM1208_00235	zraR_2
zraR_2	DSM1208_02134	zraR_2
zraR_2	DSM1208_00998	zraR_2
zraR_2	DSM1208_04435	zraR_2
zraR_2	DSM1208_02216	zraR_2
zraR_2	DSM1208_00258	zraR_2
zraR_2	DSM1208_00209	zraR_2
zraR_2	DSM1208_03367	zraR_2
zraR_2	DSM1208_00177	zraR_2
zraR_2	DSM1208_01501	zraR_2
zraR_2	DSM1208_02068	zraR_2
zraR_2	DSM1208_00068	zraR_2
zraR_2	DSM1208_00687	zraR_2
zraR_2	DSM1208_03975	zraR_2
zraR_2	DSM1208_03976	zraR_2
zraR_2	DSM1208_04569	zraR_2
zraR_2	DSM1208_01254	zraR_2
zraR_2	DSM1208_01253	zraR_2
zraR_2	DSM1208_03694	zraR_2
zraR_2	DSM1208_01300	zraR_2
zraR_2	DSM1208_04570	zraR_2
zraR_2	DSM1208_03693	zraR_2
zraR_2	DSM1208_03429	zraR_2
zraR_2	DSM1208_04474	zraR_2
zraR_2	DSM1208_01234	zraR_2
zraR_2	DSM1208_04169	zraR_2
zraR_2	DSM1208_02543	zraR_2
zraR_2	DSM1208_01050	zraR_2
zraR_2	DSM1208_02882	zraR_2
zraR_2	DSM1208_03993	zraR_2
zraR_2	DSM1208_04134	zraR_2
zraR_2	DSM1208_03844	zraR_2
zraR_2	DSM1208_03747	zraR_2
zraR_2	DSM1208_01885	zraR_2
zraR_2	DSM1208_02732	zraR_2
zraR_2	DSM1208_03319	zraR_2
zraR_2	DSM1208_00308	zraR_2

zraR_2	DSM1208_03050	zraR_2
zraR_2	DSM1208_00893	zraR_2
zraR_2	DSM1208_00190	zraR_2
zraR_2	DSM1208_00590	zraR_2
zraR_2	DSM1208_00218	zraR_2
zraR_2	DSM1208_04488	zraR_2
zraR_2	DSM1208_03850	zraR_2
zraR_2	DSM1208_03400	zraR_2
zraR_2	DSM1208_03401	zraR_2
zraR_2	DSM1208_02663	zraR_2
zraR_2	DSM1208_02921	zraR_2
zraR_2	DSM1208_03435	zraR_2
zraR_2	DSM1208_00658	zraR_2
zraR_2	DSM1208_04009	zraR_2
zraR_2	DSM1208_04049	zraR_2
zraR_2	DSM1208_04007	zraR_2
zraR_2	DSM1208_01382	zraR_2
zraR_2	DSM1208_02484	zraR_2
zraR_2	DSM1208_02805	zraR_2
zraR_2	DSM1208_02870	zraR_2
zraR_2	DSM1208_01505	zraR_2
zraR_2	DSM1208_02872	zraR_2
zraR_2	DSM1208_04351	zraR_2
zraR_2	DSM1208_03437	zraR_2
zraR_2	DSM1208_04446	zraR_2
zraR_2	DSM1208_03749	zraR_2
zraR_2	DSM1208_02952	zraR_2
zraR_2	DSM1208_00787	zraR_2
zraR_2	DSM1208_01052	zraR_2
zraR_2	DSM1208_01116	zraR_2
zraR_2	DSM1208_03438	zraR_2
zraR_2	DSM1208_01301	zraR_2
zraR_2	DSM1208_01809	zraR_2
zraR_2	DSM1208_01292	zraR_2
zraR_2	DSM1208_02542	zraR_2
zraR_2	DSM1208_01585	zraR_2
zraR_2	DSM1208_00754	zraR_2
zraR_2	DSM1208_02174	zraR_2
zraR_2	DSM1208_01798	zraR_2
zraR_2	DSM1208_02630	zraR_2
zraR_2	DSM1208_00348	zraR_2
zraR_2	DSM1208_04501	zraR_2
zraR_2	DSM1208_04269	zraR_2
zraR_2	DSM1208_03992	zraR_2
zraR_2	DSM1208_02580	zraR_2
zraR_2	DSM1208_03109	zraR_2
zraR_2	DSM1208_04477	zraR_2
zraR_2	DSM12212_02278	zraR_2

zraR_2	DSM12212_00966	zraR_2
zraR_2	DSM12212_00440	zraR_2
zraR_2	DSM12212_00439	zraR_2
zraR_2	DSM12212_02341	zraR_2
zraR_2	DSM12212_01066	zraR_2
zraR_2	DSM12212_00442	zraR_2
zraR_2	DSM12212_01455	zraR_2
zraR_2	DSM12212_02901	zraR_2
zraR_2	DSM12212_01364	zraR_2
zraR_2	DSM12212_01448	zraR_2
zraR_2	DSM12212_02348	zraR_2
zraR_2	DSM12212_01363	zraR_2
zraR_2	DSM12212_01891	zraR_2
zraR_2	DSM12212_01892	zraR_2
zraR_2	DSM12212_01619	zraR_2
zraR_2	DSM12212_02349	zraR_2
zraR_2	DSM12212_00443	zraR_2
zraR_2	DSM12212_01135	zraR_2
zraR_2	DSM12212_00600	zraR_2
zraR_2	DSM12212_03310	zraR_2
zraR_2	DSM12212_01406	zraR_2
zraR_2	DSM12212_03311	zraR_2
zraR_2	DSM12212_00438	zraR_2
zraR_2	DSM12212_00218	zraR_2
zraR_2	DSM12212_00809	zraR_2
zraR_2	DSM12212_00429	zraR_2
zraR_2	DSM12212_01281	zraR_2
zraR_2	DSM12212_00635	zraR_2
zraR_2	DSM12212_01680	zraR_2
zraR_2	DSM12212_00437	zraR_2
zraR_2	DSM12212_01618	zraR_2
zraR_2	DSM12212_02791	zraR_2
zraR_2	DSM12212_01578	zraR_2
zraR_2	DSM12212_01523	zraR_2
zraR_2	DSM12212_01952	zraR_2
zraR_2	DSM12212_00188	zraR_2
zraR_2	DSM12212_02808	zraR_2
zraR_2	DSM12212_02942	zraR_2
zraR_2	DSM12212_02879	zraR_2
zraR_2	DSM12212_00445	zraR_2
zraR_2	DSM12212_00441	zraR_2
zraR_2	DSM12212_00444	zraR_2
zraR_2	DSM12212_03142	zraR_2
zraR_2	DSM12212_00728	zraR_2
zraR_2	DSM12212_00579	zraR_2
zraR_2	DSM12212_01156	zraR_2
zraR_2	DSM12212_00423	zraR_2
zraR_2	DSM12212_01326	zraR_2

zraR_2	DSM12212_02020	zraR_2
zraR_2	DSM12212_00171	zraR_2
zraR_2	DSM12212_02803	zraR_2
zraR_2	DSM12212_02802	zraR_2
zraR_2	DSM12212_01825	zraR_2
zraR_2	DSM12212_02970	zraR_2
zraR_2	DSM12212_02809	zraR_2
zraR_2	DSM15343_00175	zraR_2
zraR_2	DSM15343_02504	zraR_2
zraR_2	DSM15343_01784	zraR_2
zraR_2	DSM15343_00212	zraR_2
zraR_2	DSM15343_00404	zraR_2
zraR_2	DSM15343_00782	zraR_2
zraR_2	DSM15343_00783	zraR_2
zraR_2	DSM15343_02274	zraR_2
zraR_2	DSM15343_00230	zraR_2
zraR_2	DSM15343_02275	zraR_2
zraR_2	DSM15343_01169	zraR_2
zraR_2	DSM15343_00853	zraR_2
zraR_2	DSM15343_00336	zraR_2
zraR_2	DSM15343_00231	zraR_2
zraR_2	DSM15343_00274	zraR_2
zraR_2	DSM15343_02245	zraR_2
zraR_2	DSM15343_01110	zraR_2
zraR_2	DSM15343_00722	zraR_2
zraR_2	DSM15343_00724	zraR_2
zraR_2	DSM15343_00852	zraR_2
zraR_2	DSM15343_01385	zraR_2
zraR_2	DSM15343_00334	zraR_2
zraR_2	DSM15343_00873	zraR_2
zraR_2	DSM15343_00173	zraR_2
zraR_2	DSM15343_00199	zraR_2
zraR_2	DSM15343_00273	zraR_2
zraR_2	DSM15343_00309	zraR_2
zraR_2	DSM15343_00776	zraR_2
zraR_2	DSM15343_01829	zraR_2
zraR_2	DSM15343_01858	zraR_2
zraR_2	DSM15343_01851	zraR_2
zraR_2	DSM15343_02851	zraR_2
zraR_2	DSM15343_02099	zraR_2
zraR_2	DSM15343_00775	zraR_2
zraR_2	DSM15343_02577	zraR_2
zraR_2	DSM15343_02794	zraR_2
zraR_2	DSM15343_00072	zraR_2
zraR_2	DSM15343_02025	zraR_2
zraR_2	DSM15343_01355	zraR_2
zraR_2	DSM15343_00869	zraR_2
zraR_2	DSM15343_02853	zraR_2

zraR_2	DSM15343_01411	zraR_2
zraR_2	DSM15343_01292	zraR_2
zraR_2	DSM15343_01962	zraR_2
zraR_2	DSM15343_01726	zraR_2
zraR_2	DSM15361_02137	zraR_2
zraR_2	DSM15361_00250	zraR_2
zraR_2	DSM15361_02650	zraR_2
zraR_2	DSM15361_00149	zraR_2
zraR_2	DSM15361_00990	zraR_2
zraR_2	DSM15361_01721	zraR_2
zraR_2	DSM15361_01720	zraR_2
zraR_2	DSM15361_00150	zraR_2
zraR_2	DSM15361_00393	zraR_2
zraR_2	DSM15361_00979	zraR_2
zraR_2	DSM15361_02055	zraR_2
zraR_2	DSM15361_00183	zraR_2
zraR_2	DSM15361_02690	zraR_2
zraR_2	DSM15361_02222	zraR_2
zraR_2	DSM15361_00679	zraR_2
zraR_2	DSM15361_00242	zraR_2
zraR_2	DSM15361_02712	zraR_2
zraR_2	DSM15361_02040	zraR_2
zraR_2	DSM15361_02113	zraR_2
zraR_2	DSM15361_02056	zraR_2
zraR_2	DSM15361_01951	zraR_2
zraR_2	DSM15361_01950	zraR_2
zraR_2	DSM15361_00695	zraR_2
zraR_2	DSM15361_01980	zraR_2
zraR_2	DSM15361_00980	zraR_2
zraR_2	DSM15361_00797	zraR_2
zraR_2	DSM15361_01799	zraR_2
zraR_2	DSM15361_01321	zraR_2
zraR_2	DSM15361_00251	zraR_2
zraR_2	DSM15361_01037	zraR_2
zraR_2	DSM15361_00079	zraR_2
zraR_2	DSM15361_01772	zraR_2
zraR_2	DSM15361_00397	zraR_2
zraR_2	DSM15361_01304	zraR_2
zraR_2	DSM15361_01809	zraR_2
zraR_2	DSM15361_01774	zraR_2
zraR_2	DSM15361_01064	zraR_2
zraR_2	DSM15365_00407	zraR_2
zraR_2	DSM15365_02418	zraR_2
zraR_2	DSM15365_02820	zraR_2
zraR_2	DSM15365_02949	zraR_2
zraR_2	DSM15365_00399	zraR_2
zraR_2	DSM15365_02677	zraR_2
zraR_2	DSM15365_02463	zraR_2

zraR_2	DSM15365_02395	zraR_2
zraR_2	DSM15365_02772	zraR_2
zraR_2	DSM15365_01968	zraR_2
zraR_2	DSM15365_03083	zraR_2
zraR_2	DSM15365_03309	zraR_2
zraR_2	DSM15365_01805	zraR_2
zraR_2	DSM15365_01082	zraR_2
zraR_2	DSM15365_03807	zraR_2
zraR_2	DSM15365_03273	zraR_2
zraR_2	DSM15365_00833	zraR_2
zraR_2	DSM15365_03795	zraR_2
zraR_2	DSM15365_00834	zraR_2
zraR_2	DSM15365_00138	zraR_2
zraR_2	DSM15365_00137	zraR_2
zraR_2	DSM15365_00720	zraR_2
zraR_2	DSM15365_02075	zraR_2
zraR_2	DSM15365_01679	zraR_2
zraR_2	DSM15365_00909	zraR_2
zraR_2	DSM15365_01723	zraR_2
zraR_2	DSM15365_00721	zraR_2
zraR_2	DSM15365_01724	zraR_2
zraR_2	DSM15365_03184	zraR_2
zraR_2	DSM15365_03892	zraR_2
zraR_2	DSM15365_02047	zraR_2
zraR_2	DSM15365_02438	zraR_2
zraR_2	DSM15365_02045	zraR_2
zraR_2	DSM15365_02077	zraR_2
zraR_2	DSM15365_00024	zraR_2
zraR_2	DSM15365_00026	zraR_2
zraR_2	DSM15365_00873	zraR_2
zraR_2	DSM15365_00953	zraR_2
zraR_2	DSM15365_00959	zraR_2
zraR_2	DSM15365_02834	zraR_2
zraR_2	DSM15365_02507	zraR_2
zraR_2	DSM15365_00752	zraR_2
zraR_2	DSM15365_02512	zraR_2
zraR_2	DSM15365_02835	zraR_2
zraR_2	DSM15365_02511	zraR_2
zraR_2	DSM15365_00078	zraR_2
zraR_2	DSM15365_02464	zraR_2
zraR_2	DSM15365_03687	zraR_2
zraR_2	DSM15365_01070	zraR_2
zraR_2	DSM15365_02102	zraR_2
zraR_2	DSM15365_00809	zraR_2
zraR_2	DSM15365_01440	zraR_2
zraR_2	DSM15365_01766	zraR_2
zraR_2	DSM15365_02146	zraR_2
zraR_2	DSM15365_01166	zraR_2

zraR_2	DSM15365_02033	zraR_2
zraR_2	DSM15365_02034	zraR_2
zraR_2	DSM15365_01849	zraR_2
zraR_2	DSM15365_03885	zraR_2
zraR_2	DSM15365_02053	zraR_2
zraR_2	DSM15365_02325	zraR_2
zraR_2	DSM15365_03378	zraR_2
zraR_2	DSM15365_00962	zraR_2
zraR_2	DSM15365_02442	zraR_2
zraR_2	DSM15365_00420	zraR_2
zraR_2	DSM15365_00027	zraR_2
zraR_2	DSM15365_00281	zraR_2
zraR_2	DSM15365_03082	zraR_2
zraR_2	DSM15365_01294	zraR_2
zraR_2	DSM15365_02934	zraR_2
zraR_2	DSM15365_01314	zraR_2
zraR_2	DSM15365_02020	zraR_2
zraR_2	DSM15365_00718	zraR_2
zraR_2	DSM15365_00320	zraR_2
zraR_2	DSM15365_02219	zraR_2
zraR_2	DSM15365_01366	zraR_2
zraR_2	DSM15365_02658	zraR_2
zraR_2	DSM15365_02143	zraR_2
zraR_2	DSM15365_00757	zraR_2
zraR_2	DSM15365_02626	zraR_2
zraR_2	DSM15365_02839	zraR_2
zraR_2	DSM15365_01581	zraR_2
zraR_2	DSM15365_01224	zraR_2
zraR_2	DSM15365_01604	zraR_2
zraR_2	DSM15365_02076	zraR_2
zraR_2	DSM15718_01927	zraR_2
zraR_2	DSM15718_01820	zraR_2
zraR_2	DSM15718_01406	zraR_2
zraR_2	DSM15718_01569	zraR_2
zraR_2	DSM15718_02130	zraR_2
zraR_2	DSM15718_01694	zraR_2
zraR_2	DSM15718_01696	zraR_2
zraR_2	DSM15718_01937	zraR_2
zraR_2	DSM15718_02057	zraR_2
zraR_2	DSM15718_00154	zraR_2
zraR_2	DSM15718_01487	zraR_2
zraR_2	DSM15718_00123	zraR_2
zraR_2	DSM15718_00780	zraR_2
zraR_2	DSM15718_00969	zraR_2
zraR_2	DSM15718_00817	zraR_2
zraR_2	DSM15718_02233	zraR_2
zraR_2	DSM15718_03011	zraR_2
zraR_2	DSM15718_00227	zraR_2

zraR_2	DSM15718_00226	zraR_2
zraR_2	DSM15718_03012	zraR_2
zraR_2	DSM15718_02234	zraR_2
zraR_2	DSM15718_01936	zraR_2
zraR_2	DSM15718_01355	zraR_2
zraR_2	DSM15718_01469	zraR_2
zraR_2	DSM15718_00954	zraR_2
zraR_2	DSM15718_01941	zraR_2
zraR_2	DSM15718_02023	zraR_2
zraR_2	DSM15718_01354	zraR_2
zraR_2	DSM15718_01433	zraR_2
zraR_2	DSM15718_01403	zraR_2
zraR_2	DSM15718_02526	zraR_2
zraR_2	DSM15718_03109	zraR_2
zraR_2	DSM15718_01940	zraR_2
zraR_2	DSM15718_02014	zraR_2
zraR_2	DSM15718_01335	zraR_2
zraR_2	DSM15718_00397	zraR_2
zraR_2	DSM15718_00191	zraR_2
zraR_2	DSM15718_00272	zraR_2
zraR_2	DSM15718_01956	zraR_2
zraR_2	DSM15718_00389	zraR_2
zraR_2	DSM15718_02283	zraR_2
zraR_2	DSM15718_02285	zraR_2
zraR_2	DSM15718_03108	zraR_2
zraR_2	DSM15718_02094	zraR_2
zraR_2	DSM15718_01477	zraR_2
zraR_2	DSM15718_00888	zraR_2
zraR_2	DSM15718_01126	zraR_2
zraR_2	DSM15718_01912	zraR_2
zraR_2	DSM15718_03380	zraR_2
zraR_2	DSM15718_00422	zraR_2
zraR_2	DSM15718_00887	zraR_2
zraR_2	DSM15718_01278	zraR_2
zraR_2	DSM15718_01393	zraR_2
zraR_2	DSM15718_00153	zraR_2
zraR_2	DSM15718_01574	zraR_2
zraR_2	DSM15718_00466	zraR_2
zraR_2	DSM15718_00349	zraR_2
zraR_2	DSM15718_02489	zraR_2
zraR_2	DSM15718_02522	zraR_2
zraR_2	DSM15718_02342	zraR_2
zraR_2	DSM15718_03216	zraR_2
zraR_2	DSM15718_02508	zraR_2
zraR_2	DSM15718_01305	zraR_2
zraR_2	DSM15718_01919	zraR_2
zraR_2	DSM15718_01695	zraR_2
zraR_2	DSM15718_01405	zraR_2

zraR_2	DSM15718_01404	zraR_2
zraR_2	DSM15718_03193	zraR_2
zraR_2	DSM16505T_01183	zraR_2
zraR_2	DSM16505T_01318	zraR_2
zraR_2	DSM16505T_02023	zraR_2
zraR_2	DSM16505T_00127	zraR_2
zraR_2	DSM16505T_00538	zraR_2
zraR_2	DSM16505T_02294	zraR_2
zraR_2	DSM16505T_02293	zraR_2
zraR_2	DSM16505T_00537	zraR_2
zraR_2	DSM16505T_00986	zraR_2
zraR_2	DSM16505T_01078	zraR_2
zraR_2	DSM16505T_02568	zraR_2
zraR_2	DSM16505T_01738	zraR_2
zraR_2	DSM16505T_02535	zraR_2
zraR_2	DSM16505T_00985	zraR_2
zraR_2	DSM16505T_00601	zraR_2
zraR_2	DSM16505T_00817	zraR_2
zraR_2	DSM16505T_02365	zraR_2
zraR_2	DSM16505T_02575	zraR_2
zraR_2	DSM16505T_00844	zraR_2
zraR_2	DSM16505T_01109	zraR_2
zraR_2	DSM16505T_01852	zraR_2
zraR_2	DSM16505T_01028	zraR_2
zraR_2	DSM16505T_02567	zraR_2
zraR_2	DSM16505T_00126	zraR_2
zraR_2	DSM16505T_00141	zraR_2
zraR_2	DSM16505T_01041	zraR_2
zraR_2	DSM16505T_01520	zraR_2
zraR_2	DSM16505T_02481	zraR_2
zraR_2	DSM16505T_01904	zraR_2
zraR_2	DSM16505T_01306	zraR_2
zraR_2	DSM18103_01429	zraR_2
zraR_2	DSM18103_02613	zraR_2
zraR_2	DSM18103_01475	zraR_2
zraR_2	DSM18103_00953	zraR_2
zraR_2	DSM18103_02709	zraR_2
zraR_2	DSM18103_02710	zraR_2
zraR_2	DSM18103_00952	zraR_2
zraR_2	DSM18103_00255	zraR_2
zraR_2	DSM18103_02612	zraR_2
zraR_2	DSM18103_00256	zraR_2
zraR_2	DSM18103_02344	zraR_2
zraR_2	DSM18103_02345	zraR_2
zraR_2	DSM18103_01443	zraR_2
zraR_2	DSM18103_01244	zraR_2
zraR_2	DSM18103_03693	zraR_2
zraR_2	DSM18103_02552	zraR_2

zraR_2	DSM18103_01341	zraR_2
zraR_2	DSM18103_01474	zraR_2
zraR_2	DSM18103_00240	zraR_2
zraR_2	DSM18103_00370	zraR_2
zraR_2	DSM18103_00779	zraR_2
zraR_2	DSM18103_02647	zraR_2
zraR_2	DSM18103_00384	zraR_2
zraR_2	DSM18103_01927	zraR_2
zraR_2	DSM18103_00382	zraR_2
zraR_2	DSM18103_03682	zraR_2
zraR_2	DSM18103_02067	zraR_2
zraR_2	DSM18103_01015	zraR_2
zraR_2	DSM18103_03152	zraR_2
zraR_2	DSM18103_02280	zraR_2
zraR_2	DSM18103_02265	zraR_2
zraR_2	DSM18103_00119	zraR_2
zraR_2	DSM18103_03434	zraR_2
zraR_2	DSM18103_02621	zraR_2
zraR_2	DSM18103_02052	zraR_2
zraR_2	DSM18103_02526	zraR_2
zraR_2	DSM18103_00239	zraR_2
zraR_2	DSM18103_01428	zraR_2
zraR_2	DSM18103_02928	zraR_2
zraR_2	DSM18103_02312	zraR_2
zraR_2	DSM18103_01982	zraR_2
zraR_2	DSM18103_00251	zraR_2
zraR_2	DSM18103_03248	zraR_2
zraR_2	DSM18103_00483	zraR_2
zraR_2	DSM18103_03121	zraR_2
zraR_2	DSM18103_02211	zraR_2
zraR_2	DSM18103_02341	zraR_2
zraR_2	DSM18103_03301	zraR_2
zraR_2	DSM18103_03692	zraR_2
zraR_2	DSM18293_04607	zraR_2
zraR_2	DSM18293_02906	zraR_2
zraR_2	DSM18293_03355	zraR_2
zraR_2	DSM18293_03338	zraR_2
zraR_2	DSM18293_01310	zraR_2
zraR_2	DSM18293_01215	zraR_2
zraR_2	DSM18293_04245	zraR_2
zraR_2	DSM18293_02576	zraR_2
zraR_2	DSM18293_02849	zraR_2
zraR_2	DSM18293_00868	zraR_2
zraR_2	DSM18293_00932	zraR_2
zraR_2	DSM18293_01042	zraR_2
zraR_2	DSM18293_03291	zraR_2
zraR_2	DSM18293_04142	zraR_2
zraR_2	DSM18293_04143	zraR_2

zraR_2	DSM18293_01511	zraR_2
zraR_2	DSM18293_00931	zraR_2
zraR_2	DSM18293_04198	zraR_2
zraR_2	DSM18293_00708	zraR_2
zraR_2	DSM18293_02246	zraR_2
zraR_2	DSM18293_01299	zraR_2
zraR_2	DSM18293_02848	zraR_2
zraR_2	DSM18293_01764	zraR_2
zraR_2	DSM18293_00707	zraR_2
zraR_2	DSM18293_03367	zraR_2
zraR_2	DSM18293_01309	zraR_2
zraR_2	DSM18293_01340	zraR_2
zraR_2	DSM18293_04244	zraR_2
zraR_2	DSM18293_02263	zraR_2
zraR_2	DSM18293_04239	zraR_2
zraR_2	DSM18293_04314	zraR_2
zraR_2	DSM18293_02843	zraR_2
zraR_2	DSM18293_04246	zraR_2
zraR_2	DSM18293_01510	zraR_2
zraR_2	DSM18293_02809	zraR_2
zraR_2	DSM18293_00249	zraR_2
zraR_2	DSM18293_03764	zraR_2
zraR_2	DSM18293_02654	zraR_2
zraR_2	DSM18293_00234	zraR_2
zraR_2	DSM18293_02691	zraR_2
zraR_2	DSM18293_01487	zraR_2
zraR_2	DSM18293_01035	zraR_2
zraR_2	DSM18293_00427	zraR_2
zraR_2	DSM18293_04435	zraR_2
zraR_2	DSM18293_02851	zraR_2
zraR_2	DSM18293_01765	zraR_2
zraR_2	DSM18293_03743	zraR_2
zraR_2	DSM18293_04389	zraR_2
zraR_2	DSM18293_03945	zraR_2
zraR_2	DSM18293_04199	zraR_2
zraR_2	DSM18293_02856	zraR_2
zraR_2	DSM18293_04387	zraR_2
zraR_2	DSM18293_03156	zraR_2
zraR_2	DSM18293_03889	zraR_2
zraR_2	DSM18293_04388	zraR_2
zraR_2	DSM18293_02271	zraR_2
zraR_2	DSM18293_01104	zraR_2
zraR_2	DSM18293_02319	zraR_2
zraR_2	DSM18293_03292	zraR_2
zraR_2	DSM18293_00414	zraR_2
zraR_2	DSM18293_01597	zraR_2
zraR_2	DSM18293_02781	zraR_2
zraR_2	DSM18293_01168	zraR_2

zraR_2	DSM18293_01689	zraR_2
zraR_2	DSM18293_02844	zraR_2
zraR_2	DSM18293_00093	zraR_2
zraR_2	DSM18293_02134	zraR_2
zraR_2	DSM18293_02273	zraR_2
zraR_2	DSM18293_04585	zraR_2
zraR_2	DSM18293_00867	zraR_2
zraR_2	DSM18293_00413	zraR_2
zraR_2	DSM18293_04590	zraR_2
zraR_2	DSM18293_02318	zraR_2
zraR_2	DSM18293_00435	zraR_2
zraR_2	DSM18293_00677	zraR_2
zraR_2	DSM18293_03368	zraR_2
zraR_2	DSM18293_01738	zraR_2
zraR_2	DSM18293_00192	zraR_2
zraR_2	DSM18293_02352	zraR_2
zraR_2	DSM18293_01115	zraR_2
zraR_2	DSM18293_01703	zraR_2
zraR_2	DSM18293_02084	zraR_2
zraR_2	DSM18293_03976	zraR_2
zraR_2	DSM18293_03153	zraR_2
zraR_2	DSM18293_00037	zraR_2
zraR_2	DSM18293_04338	zraR_2
zraR_2	DSM18293_03493	zraR_2
zraR_2	DSM18293_04241	zraR_2
zraR_2	DSM18293_00668	zraR_2
zraR_2	DSM18293_02875	zraR_2
zraR_2	DSM18293_04247	zraR_2
zraR_2	DSM18293_04248	zraR_2
zraR_2	DSM18293_03414	zraR_2
zraR_2	DSM18293_01672	zraR_2
zraR_2	DSM18293_03492	zraR_2
zraR_2	DSM18293_00720	zraR_2
zraR_2	DSM18841_02317	zraR_2
zraR_2	DSM18841_03012	zraR_2
zraR_2	DSM18841_02714	zraR_2
zraR_2	DSM18841_00398	zraR_2
zraR_2	DSM18841_00861	zraR_2
zraR_2	DSM18841_01331	zraR_2
zraR_2	DSM18841_01332	zraR_2
zraR_2	DSM18841_00862	zraR_2
zraR_2	DSM18841_00397	zraR_2
zraR_2	DSM18841_02797	zraR_2
zraR_2	DSM18841_00887	zraR_2
zraR_2	DSM18841_01840	zraR_2
zraR_2	DSM18841_02975	zraR_2
zraR_2	DSM18841_02977	zraR_2
zraR_2	DSM18841_01720	zraR_2

zraR_2	DSM18841_00455	zraR_2
zraR_2	DSM18841_02931	zraR_2
zraR_2	DSM18841_03074	zraR_2
zraR_2	DSM18841_02790	zraR_2
zraR_2	DSM18841_00711	zraR_2
zraR_2	DSM18841_01588	zraR_2
zraR_2	DSM18841_00908	zraR_2
zraR_2	DSM18841_02798	zraR_2
zraR_2	DSM18841_02715	zraR_2
zraR_2	DSM18841_01073	zraR_2
zraR_2	DSM18841_00964	zraR_2
zraR_2	DSM18841_00921	zraR_2
zraR_2	DSM18841_02144	zraR_2
zraR_2	DSM18841_01790	zraR_2
zraR_2	DSM18841_02861	zraR_2
zraR_2	DSM18841_02004	zraR_2
zraR_2	DSM18841_02804	zraR_2
zraR_2	DSM18841_00486	zraR_2
zraR_2	DSM18841_02302	zraR_2
zraR_2	DSM18841_01657	zraR_2
zraR_2	DSM18841_01288	zraR_2
zraR_2	DSM18842_01228	zraR_2
zraR_2	DSM18842_02532	zraR_2
zraR_2	DSM18842_00834	zraR_2
zraR_2	DSM18842_01677	zraR_2
zraR_2	DSM18842_01678	zraR_2
zraR_2	DSM18842_00835	zraR_2
zraR_2	DSM18842_02733	zraR_2
zraR_2	DSM18842_02018	zraR_2
zraR_2	DSM18842_00355	zraR_2
zraR_2	DSM18842_02770	zraR_2
zraR_2	DSM18842_02016	zraR_2
zraR_2	DSM18842_02764	zraR_2
zraR_2	DSM18842_02133	zraR_2
zraR_2	DSM18842_00494	zraR_2
zraR_2	DSM18842_02772	zraR_2
zraR_2	DSM18842_01487	zraR_2
zraR_2	DSM18842_00772	zraR_2
zraR_2	DSM18842_03054	zraR_2
zraR_2	DSM18842_00030	zraR_2
zraR_2	DSM18842_02727	zraR_2
zraR_2	DSM18842_00610	zraR_2
zraR_2	DSM18842_00311	zraR_2
zraR_2	DSM18842_02272	zraR_2
zraR_2	DSM18842_00399	zraR_2
zraR_2	DSM18842_02734	zraR_2
zraR_2	DSM18842_02531	zraR_2
zraR_2	DSM18842_00387	zraR_2

zraR_2	DSM18842_00814	zraR_2
zraR_2	DSM18842_02085	zraR_2
zraR_2	DSM18842_02841	zraR_2
zraR_2	DSM18842_02317	zraR_2
zraR_2	DSM18842_00742	zraR_2
zraR_2	DSM18842_01215	zraR_2
zraR_2	DSM18842_01745	zraR_2
zraR_2	DSM18842_01455	zraR_2
zraR_2	DSM18842_03164	zraR_2
zraR_2	DSM19866_01161	zraR_2
zraR_2	DSM19866_02563	zraR_2
zraR_2	DSM19866_02077	zraR_2
zraR_2	DSM19866_02571	zraR_2
zraR_2	DSM19866_02389	zraR_2
zraR_2	DSM19866_04926	zraR_2
zraR_2	DSM19866_04839	zraR_2
zraR_2	DSM19866_04870	zraR_2
zraR_2	DSM19866_02019	zraR_2
zraR_2	DSM19866_02213	zraR_2
zraR_2	DSM19866_04884	zraR_2
zraR_2	DSM19866_01163	zraR_2
zraR_2	DSM19866_00538	zraR_2
zraR_2	DSM19866_04782	zraR_2
zraR_2	DSM19866_04130	zraR_2
zraR_2	DSM19866_04129	zraR_2
zraR_2	DSM19866_01282	zraR_2
zraR_2	DSM19866_04406	zraR_2
zraR_2	DSM19866_01656	zraR_2
zraR_2	DSM19866_04680	zraR_2
zraR_2	DSM19866_00037	zraR_2
zraR_2	DSM19866_04407	zraR_2
zraR_2	DSM19866_02495	zraR_2
zraR_2	DSM19866_02494	zraR_2
zraR_2	DSM19866_02237	zraR_2
zraR_2	DSM19866_02708	zraR_2
zraR_2	DSM19866_02238	zraR_2
zraR_2	DSM19866_03666	zraR_2
zraR_2	DSM19866_03156	zraR_2
zraR_2	DSM19866_04681	zraR_2
zraR_2	DSM19866_02239	zraR_2
zraR_2	DSM19866_03619	zraR_2
zraR_2	DSM19866_03667	zraR_2
zraR_2	DSM19866_02815	zraR_2
zraR_2	DSM19866_01437	zraR_2
zraR_2	DSM19866_00159	zraR_2
zraR_2	DSM19866_01262	zraR_2
zraR_2	DSM19866_01260	zraR_2
zraR_2	DSM19866_02248	zraR_2

zraR_2	DSM19866_02814	zraR_2
zraR_2	DSM19866_00070	zraR_2
zraR_2	DSM19866_03845	zraR_2
zraR_2	DSM19866_02056	zraR_2
zraR_2	DSM19866_02236	zraR_2
zraR_2	DSM19866_04233	zraR_2
zraR_2	DSM19866_04114	zraR_2
zraR_2	DSM19866_02864	zraR_2
zraR_2	DSM19866_01792	zraR_2
zraR_2	DSM19866_00327	zraR_2
zraR_2	DSM19866_02564	zraR_2
zraR_2	DSM19866_02447	zraR_2
zraR_2	DSM19866_04487	zraR_2
zraR_2	DSM19866_01237	zraR_2
zraR_2	DSM19866_00862	zraR_2
zraR_2	DSM19866_03503	zraR_2
zraR_2	DSM19866_03869	zraR_2
zraR_2	DSM19866_04852	zraR_2
zraR_2	DSM19866_02480	zraR_2
zraR_2	DSM19866_02879	zraR_2
zraR_2	DSM19866_04851	zraR_2
zraR_2	DSM19866_04423	zraR_2
zraR_2	DSM19866_03372	zraR_2
zraR_2	DSM19866_02474	zraR_2
zraR_2	DSM19866_04102	zraR_2
zraR_2	DSM19866_00570	zraR_2
zraR_2	DSM19866_03546	zraR_2
zraR_2	DSM19866_03241	zraR_2
zraR_2	DSM19866_04800	zraR_2
zraR_2	DSM19866_04898	zraR_2
zraR_2	DSM19866_04168	zraR_2
zraR_2	DSM19866_03357	zraR_2
zraR_2	DSM19866_02416	zraR_2
zraR_2	DSM19866_00245	zraR_2
zraR_2	DSM19866_01575	zraR_2
zraR_2	DSM19866_02627	zraR_2
zraR_2	DSM19866_00445	zraR_2
zraR_2	DSM19866_02256	zraR_2
zraR_2	DSM19866_02054	zraR_2
zraR_2	DSM19866_00808	zraR_2
zraR_2	DSM19866_00160	zraR_2
zraR_2	DSM19866_00153	zraR_2
zraR_2	DSM19866_03643	zraR_2
zraR_2	DSM19866_03807	zraR_2
zraR_2	DSM19866_01791	zraR_2
zraR_2	DSM19866_04887	zraR_2
zraR_2	DSM19866_03358	zraR_2
zraR_2	DSM19866_03618	zraR_2

zraR_2	DSM19866_02865	zraR_2
zraR_2	DSM19866_04051	zraR_2
zraR_2	DSM19866_03711	zraR_2
zraR_2	DSM19866_04808	zraR_2
zraR_2	DSM19866_04263	zraR_2
zraR_2	DSM19866_02039	zraR_2
zraR_2	DSM19866_03981	zraR_2
zraR_2	DSM19866_02693	zraR_2
zraR_2	DSM19866_04115	zraR_2
zraR_2	DSM19866_00868	zraR_2
zraR_2	DSM19866_02117	zraR_2
zraR_2	DSM19866_01671	zraR_2
zraR_2	DSM2041_03757	zraR_2
zraR_2	DSM2041_00375	zraR_2
zraR_2	DSM2041_04575	zraR_2
zraR_2	DSM2041_00319	zraR_2
zraR_2	DSM2041_05160	zraR_2
zraR_2	DSM2041_01459	zraR_2
zraR_2	DSM2041_04510	zraR_2
zraR_2	DSM2041_00857	zraR_2
zraR_2	DSM2041_00600	zraR_2
zraR_2	DSM2041_02813	zraR_2
zraR_2	DSM2041_04461	zraR_2
zraR_2	DSM2041_04462	zraR_2
zraR_2	DSM2041_04010	zraR_2
zraR_2	DSM2041_04101	zraR_2
zraR_2	DSM2041_02503	zraR_2
zraR_2	DSM2041_00664	zraR_2
zraR_2	DSM2041_05250	zraR_2
zraR_2	DSM2041_04062	zraR_2
zraR_2	DSM2041_02047	zraR_2
zraR_2	DSM2041_02504	zraR_2
zraR_2	DSM2041_04835	zraR_2
zraR_2	DSM2041_01438	zraR_2
zraR_2	DSM2041_04836	zraR_2
zraR_2	DSM2041_00663	zraR_2
zraR_2	DSM2041_04212	zraR_2
zraR_2	DSM2041_01381	zraR_2
zraR_2	DSM2041_03614	zraR_2
zraR_2	DSM2041_01914	zraR_2
zraR_2	DSM2041_05358	zraR_2
zraR_2	DSM2041_03613	zraR_2
zraR_2	DSM2041_05249	zraR_2
zraR_2	DSM2041_02941	zraR_2
zraR_2	DSM2041_00764	zraR_2
zraR_2	DSM2041_04213	zraR_2
zraR_2	DSM2041_00716	zraR_2
zraR_2	DSM2041_02855	zraR_2

zraR_2	DSM2041_01420	zraR_2
zraR_2	DSM2041_05445	zraR_2
zraR_2	DSM2041_00132	zraR_2
zraR_2	DSM2041_00415	zraR_2
zraR_2	DSM2041_01380	zraR_2
zraR_2	DSM2041_02347	zraR_2
zraR_2	DSM2041_00715	zraR_2
zraR_2	DSM2041_04768	zraR_2
zraR_2	DSM2041_03716	zraR_2
zraR_2	DSM2041_04894	zraR_2
zraR_2	DSM2041_01116	zraR_2
zraR_2	DSM2041_03782	zraR_2
zraR_2	DSM2041_04149	zraR_2
zraR_2	DSM2041_04063	zraR_2
zraR_2	DSM2041_00343	zraR_2
zraR_2	DSM2041_04256	zraR_2
zraR_2	DSM2041_00824	zraR_2
zraR_2	DSM2041_00826	zraR_2
zraR_2	DSM2041_01847	zraR_2
zraR_2	DSM2041_02075	zraR_2
zraR_2	DSM2041_00828	zraR_2
zraR_2	DSM2041_04769	zraR_2
zraR_2	DSM2041_03928	zraR_2
zraR_2	DSM2041_05384	zraR_2
zraR_2	DSM2041_01988	zraR_2
zraR_2	DSM2041_00599	zraR_2
zraR_2	DSM2041_03213	zraR_2
zraR_2	DSM2041_03929	zraR_2
zraR_2	DSM2041_03230	zraR_2
zraR_2	DSM2041_03857	zraR_2
zraR_2	DSM2041_00456	zraR_2
zraR_2	DSM2041_04603	zraR_2
zraR_2	DSM2041_03536	zraR_2
zraR_2	DSM2041_05110	zraR_2
zraR_2	DSM2041_05478	zraR_2
zraR_2	DSM2041_05035	zraR_2
zraR_2	DSM2041_02732	zraR_2
zraR_2	DSM2041_00397	zraR_2
zraR_2	DSM2041_00615	zraR_2
zraR_2	DSM2041_02094	zraR_2
zraR_2	DSM2041_00400	zraR_2
zraR_2	DSM2041_00396	zraR_2
zraR_2	DSM2041_00806	zraR_2
zraR_2	DSM2041_02269	zraR_2
zraR_2	DSM2041_02299	zraR_2
zraR_2	DSM2041_04962	zraR_2
zraR_2	DSM2041_02268	zraR_2
zraR_2	DSM2041_01897	zraR_2

zraR_2	DSM2041_04255	zraR_2
zraR_2	DSM2041_01292	zraR_2
zraR_2	DSM2041_03384	zraR_2
zraR_2	DSM2041_04283	zraR_2
zraR_2	DSM2041_03165	zraR_2
zraR_2	DSM2041_00113	zraR_2
zraR_2	DSM2041_00117	zraR_2
zraR_2	DSM2041_01166	zraR_2
zraR_2	DSM2041_04458	zraR_2
zraR_2	DSM2041_04864	zraR_2
zraR_2	DSM2041_01492	zraR_2
zraR_2	DSM2041_04282	zraR_2
zraR_2	DSM2041_00730	zraR_2
zraR_2	DSM2041_04527	zraR_2
zraR_2	DSM2041_01330	zraR_2
zraR_2	DSM2041_05040	zraR_2
zraR_2	DSM2061_01752	zraR_2
zraR_2	DSM2061_00646	zraR_2
zraR_2	DSM2061_01403	zraR_2
zraR_2	DSM2061_01599	zraR_2
zraR_2	DSM2061_01872	zraR_2
zraR_2	DSM2061_00354	zraR_2
zraR_2	DSM2061_03685	zraR_2
zraR_2	DSM2061_00721	zraR_2
zraR_2	DSM2061_00221	zraR_2
zraR_2	DSM2061_00127	zraR_2
zraR_2	DSM2061_00290	zraR_2
zraR_2	DSM2061_01712	zraR_2
zraR_2	DSM2061_00344	zraR_2
zraR_2	DSM2061_00479	zraR_2
zraR_2	DSM2061_00481	zraR_2
zraR_2	DSM2061_00484	zraR_2
zraR_2	DSM2061_02181	zraR_2
zraR_2	DSM2061_00830	zraR_2
zraR_2	DSM2061_00831	zraR_2
zraR_2	DSM2061_02755	zraR_2
zraR_2	DSM2061_03587	zraR_2
zraR_2	DSM2061_00585	zraR_2
zraR_2	DSM2061_02756	zraR_2
zraR_2	DSM2061_00584	zraR_2
zraR_2	DSM2061_00677	zraR_2
zraR_2	DSM2061_00965	zraR_2
zraR_2	DSM2061_01187	zraR_2
zraR_2	DSM2061_02782	zraR_2
zraR_2	DSM2061_02567	zraR_2
zraR_2	DSM2061_00848	zraR_2
zraR_2	DSM2061_00959	zraR_2
zraR_2	DSM2061_01337	zraR_2

zraR_2	DSM2061_02521	zraR_2
zraR_2	DSM2061_04367	zraR_2
zraR_2	DSM2061_04030	zraR_2
zraR_2	DSM2061_00339	zraR_2
zraR_2	DSM2061_03442	zraR_2
zraR_2	DSM2061_01730	zraR_2
zraR_2	DSM2061_00365	zraR_2
zraR_2	DSM2061_00070	zraR_2
zraR_2	DSM2061_02394	zraR_2
zraR_2	DSM2061_01652	zraR_2
zraR_2	DSM2061_02085	zraR_2
zraR_2	DSM2061_00662	zraR_2
zraR_2	DSM2061_00281	zraR_2
zraR_2	DSM2061_01182	zraR_2
zraR_2	DSM2061_04148	zraR_2
zraR_2	DSM2061_01653	zraR_2
zraR_2	DSM2061_02152	zraR_2
zraR_2	DSM2061_03998	zraR_2
zraR_2	DSM2061_00863	zraR_2
zraR_2	DSM2061_02903	zraR_2
zraR_2	DSM2061_00861	zraR_2
zraR_2	DSM2061_03997	zraR_2
zraR_2	DSM2061_01279	zraR_2
zraR_2	DSM2061_00980	zraR_2
zraR_2	DSM2061_01275	zraR_2
zraR_2	DSM2061_04354	zraR_2
zraR_2	DSM2061_01306	zraR_2
zraR_2	DSM2061_03034	zraR_2
zraR_2	DSM2061_04356	zraR_2
zraR_2	DSM2061_00908	zraR_2
zraR_2	DSM2061_00785	zraR_2
zraR_2	DSM2061_01648	zraR_2
zraR_2	DSM2061_04029	zraR_2
zraR_2	DSM2061_04220	zraR_2
zraR_2	DSM2061_00710	zraR_2
zraR_2	DSM2061_01336	zraR_2
zraR_2	DSM2061_02285	zraR_2
zraR_2	DSM2061_03337	zraR_2
zraR_2	DSM2061_02569	zraR_2
zraR_2	DSM2061_01807	zraR_2
zraR_2	DSM2061_02633	zraR_2
zraR_2	DSM2061_01647	zraR_2
zraR_2	DSM2061_02761	zraR_2
zraR_2	DSM2061_03586	zraR_2
zraR_2	DSM2061_00649	zraR_2
zraR_2	DSM2061_03595	zraR_2
zraR_2	DSM2061_02253	zraR_2
zraR_2	DSM2061_03112	zraR_2

zraR_2	DSM2061_03719	zraR_2
zraR_2	DSM2061_03327	zraR_2
zraR_2	DSM2061_01244	zraR_2
zraR_2	DSM2061_04407	zraR_2
zraR_2	DSM2061_02760	zraR_2
zraR_2	DSM2061_01272	zraR_2
zraR_2	DSM2061_04094	zraR_2
zraR_2	DSM2061_00847	zraR_2
zraR_2	DSM2061_03419	zraR_2
zraR_2	DSM2061_03420	zraR_2
zraR_2	DSM2061_02052	zraR_2
zraR_2	DSM2064_01113	zraR_2
zraR_2	DSM2064_01453	zraR_2
zraR_2	DSM2064_01442	zraR_2
zraR_2	DSM2064_04500	zraR_2
zraR_2	DSM2064_04563	zraR_2
zraR_2	DSM2064_00549	zraR_2
zraR_2	DSM2064_01386	zraR_2
zraR_2	DSM2064_01063	zraR_2
zraR_2	DSM2064_03976	zraR_2
zraR_2	DSM2064_00784	zraR_2
zraR_2	DSM2064_04855	zraR_2
zraR_2	DSM2064_05134	zraR_2
zraR_2	DSM2064_04121	zraR_2
zraR_2	DSM2064_01617	zraR_2
zraR_2	DSM2064_00897	zraR_2
zraR_2	DSM2064_00896	zraR_2
zraR_2	DSM2064_03703	zraR_2
zraR_2	DSM2064_04176	zraR_2
zraR_2	DSM2064_05135	zraR_2
zraR_2	DSM2064_00834	zraR_2
zraR_2	DSM2064_04418	zraR_2
zraR_2	DSM2064_00785	zraR_2
zraR_2	DSM2064_05244	zraR_2
zraR_2	DSM2064_01394	zraR_2
zraR_2	DSM2064_02453	zraR_2
zraR_2	DSM2064_01387	zraR_2
zraR_2	DSM2064_02632	zraR_2
zraR_2	DSM2064_00300	zraR_2
zraR_2	DSM2064_01299	zraR_2
zraR_2	DSM2064_01393	zraR_2
zraR_2	DSM2064_02989	zraR_2
zraR_2	DSM2064_02633	zraR_2
zraR_2	DSM2064_03004	zraR_2
zraR_2	DSM2064_04177	zraR_2
zraR_2	DSM2064_03704	zraR_2
zraR_2	DSM2064_03838	zraR_2
zraR_2	DSM2064_00790	zraR_2

zraR_2	DSM2064_03349	zraR_2
zraR_2	DSM2064_03722	zraR_2
zraR_2	DSM2064_02360	zraR_2
zraR_2	DSM2064_01577	zraR_2
zraR_2	DSM2064_04966	zraR_2
zraR_2	DSM2064_03740	zraR_2
zraR_2	DSM2064_00562	zraR_2
zraR_2	DSM2064_01493	zraR_2
zraR_2	DSM2064_01496	zraR_2
zraR_2	DSM2064_01528	zraR_2
zraR_2	DSM2064_00632	zraR_2
zraR_2	DSM2064_02350	zraR_2
zraR_2	DSM2064_04217	zraR_2
zraR_2	DSM2064_01573	zraR_2
zraR_2	DSM2064_01375	zraR_2
zraR_2	DSM2064_00299	zraR_2
zraR_2	DSM2064_03185	zraR_2
zraR_2	DSM2064_03373	zraR_2
zraR_2	DSM2064_00833	zraR_2
zraR_2	DSM2064_01930	zraR_2
zraR_2	DSM2064_00783	zraR_2
zraR_2	DSM2064_00476	zraR_2
zraR_2	DSM2064_03011	zraR_2
zraR_2	DSM2064_04064	zraR_2
zraR_2	DSM2064_00327	zraR_2
zraR_2	DSM2064_04417	zraR_2
zraR_2	DSM2064_04611	zraR_2
zraR_2	DSM2064_02339	zraR_2
zraR_2	DSM2064_03625	zraR_2
zraR_2	DSM2064_03953	zraR_2
zraR_2	DSM2064_04027	zraR_2
zraR_2	DSM2064_05058	zraR_2
zraR_2	DSM2064_04467	zraR_2
zraR_2	DSM2064_00398	zraR_2
zraR_2	DSM2064_05150	zraR_2
zraR_2	DSM2064_04387	zraR_2
zraR_2	DSM2064_02874	zraR_2
zraR_2	DSM2064_01618	zraR_2
zraR_2	DSM2064_02338	zraR_2
zraR_2	DSM2064_04610	zraR_2
zraR_2	DSM2064_01417	zraR_2
zraR_2	DSM2064_02604	zraR_2
zraR_2	DSM2064_05243	zraR_2
zraR_2	DSM2064_04060	zraR_2
zraR_2	DSM2064_00366	zraR_2
zraR_2	DSM2064_02813	zraR_2
zraR_2	DSM2064_03152	zraR_2
zraR_2	DSM2064_04250	zraR_2

zraR_2	DSM2064_01934	zraR_2
zraR_2	DSM2064_04438	zraR_2
zraR_2	DSM2064_00746	zraR_2
zraR_2	DSM2064_03857	zraR_2
zraR_2	DSM2064_00242	zraR_2
zraR_2	DSM2064_04471	zraR_2
zraR_2	DSM2064_01357	zraR_2
zraR_2	DSM2064_00788	zraR_2
zraR_2	DSM2064_02595	zraR_2
zraR_2	DSM2064_01257	zraR_2
zraR_2	DSM2064_01419	zraR_2
zraR_2	DSM2064_00782	zraR_2
zraR_2	DSM2064_00781	zraR_2
zraR_2	DSM2064_04914	zraR_2
zraR_2	DSM2064_01086	zraR_2
zraR_2	DSM2064_04287	zraR_2
zraR_2	DSM2064_01568	zraR_2
zraR_2	DSM2064_01310	zraR_2
zraR_2	DSM2064_04933	zraR_2
zraR_2	DSM2064_01201	zraR_2
zraR_2	DSM21164_03181	zraR_2
zraR_2	DSM21164_02176	zraR_2
zraR_2	DSM21164_02631	zraR_2
zraR_2	DSM21164_00084	zraR_2
zraR_2	DSM21164_02123	zraR_2
zraR_2	DSM21164_01910	zraR_2
zraR_2	DSM21164_02789	zraR_2
zraR_2	DSM21164_02105	zraR_2
zraR_2	DSM21164_02104	zraR_2
zraR_2	DSM21164_02790	zraR_2
zraR_2	DSM21164_01223	zraR_2
zraR_2	DSM21164_01222	zraR_2
zraR_2	DSM21164_00881	zraR_2
zraR_2	DSM21164_00936	zraR_2
zraR_2	DSM21164_01171	zraR_2
zraR_2	DSM21164_01909	zraR_2
zraR_2	DSM21164_00490	zraR_2
zraR_2	DSM21164_01790	zraR_2
zraR_2	DSM21164_00069	zraR_2
zraR_2	DSM21164_02741	zraR_2
zraR_2	DSM21164_00572	zraR_2
zraR_2	DSM21164_00070	zraR_2
zraR_2	DSM21164_02561	zraR_2
zraR_2	DSM21164_00659	zraR_2
zraR_2	DSM21164_01255	zraR_2
zraR_2	DSM21164_02009	zraR_2
zraR_2	DSM21164_02850	zraR_2
zraR_2	DSM21164_02738	zraR_2

zraR_2	DSM21164_01101	zraR_2
zraR_2	DSM21164_01103	zraR_2
zraR_2	DSM21164_02175	zraR_2
zraR_2	DSM21164_03190	zraR_2
zraR_2	DSM21164_00055	zraR_2
zraR_2	DSM21164_01780	zraR_2
zraR_2	DSM21164_01778	zraR_2
zraR_2	DSM21164_00440	zraR_2
zraR_2	DSM21164_02630	zraR_2
zraR_2	DSM21164_00929	zraR_2
zraR_2	DSM21164_02297	zraR_2
zraR_2	DSM21164_01394	zraR_2
zraR_2	DSM21164_02546	zraR_2
zraR_2	DSM21164_00179	zraR_2
zraR_2	DSM21164_00150	zraR_2
zraR_2	DSM21164_00575	zraR_2
zraR_2	DSM21164_01352	zraR_2
zraR_2	DSM21164_01254	zraR_2
zraR_2	DSM21164_01170	zraR_2
zraR_2	DSM21164_01180	zraR_2
zraR_2	DSM21164_00438	zraR_2
zraR_2	DSM21164_02669	zraR_2
zraR_2	DSM21164_00923	zraR_2
zraR_2	DSM21164_00420	zraR_2
zraR_2	DSM21164_00674	zraR_2
zraR_2	DSM21164_02685	zraR_2
zraR_2	DSM23404_00152	zraR_2
zraR_2	DSM23404_02568	zraR_2
zraR_2	DSM23404_02077	zraR_2
zraR_2	DSM23404_02295	zraR_2
zraR_2	DSM23404_00753	zraR_2
zraR_2	DSM23404_00246	zraR_2
zraR_2	DSM23404_01845	zraR_2
zraR_2	DSM23404_01676	zraR_2
zraR_2	DSM23404_01677	zraR_2
zraR_2	DSM23404_01846	zraR_2
zraR_2	DSM23404_01817	zraR_2
zraR_2	DSM23404_01818	zraR_2
zraR_2	DSM23404_00141	zraR_2
zraR_2	DSM23404_01133	zraR_2
zraR_2	DSM23404_02640	zraR_2
zraR_2	DSM23404_01317	zraR_2
zraR_2	DSM23404_02007	zraR_2
zraR_2	DSM23404_00142	zraR_2
zraR_2	DSM23404_01386	zraR_2
zraR_2	DSM23404_01352	zraR_2
zraR_2	DSM23404_01353	zraR_2
zraR_2	DSM23404_02642	zraR_2

zraR_2	DSM23404_02101	zraR_2
zraR_2	DSM23404_01098	zraR_2
zraR_2	DSM23404_01973	zraR_2
zraR_2	DSM23404_02089	zraR_2
zraR_2	DSM23404_02659	zraR_2
zraR_2	DSM23404_01318	zraR_2
zraR_2	DSM23404_02658	zraR_2
zraR_2	DSM23404_02717	zraR_2
zraR_2	DSM23404_00166	zraR_2
zraR_2	DSM23404_01225	zraR_2
zraR_2	DSM23404_02566	zraR_2
zraR_2	DSM23404_01132	zraR_2
zraR_2	DSM23404_02296	zraR_2
zraR_2	DSM23404_00140	zraR_2
zraR_2	DSM23404_01500	zraR_2
zraR_2	DSM23404_00436	zraR_2
zraR_2	DSM23404_02715	zraR_2
zraR_2	DSM23404_02159	zraR_2
zraR_2	DSM23404_00139	zraR_2
zraR_2	DSM23404_00807	zraR_2
zraR_2	DSM23404_02567	zraR_2
zraR_2	DSM23404_02289	zraR_2
zraR_2	DSM23404_01385	zraR_2
zraR_2	DSM23404_01360	zraR_2
zraR_2	DSM23404_02014	zraR_2
zraR_2	DSM23404_01895	zraR_2
zraR_2	DSM23404_02190	zraR_2
zraR_2	DSM23404_02710	zraR_2
zraR_2	DSM23404_00095	zraR_2
zraR_2	DSM23404_00911	zraR_2
zraR_2	DSM23404_00671	zraR_2
zraR_2	DSM23404_00590	zraR_2
zraR_2	DSM23404_02090	zraR_2
zraR_2	DSM23404_01449	zraR_2
zraR_2	DSM23404_00165	zraR_2
zraR_2	DSM23404_03298	zraR_2
zraR_2	DSM23404_00091	zraR_2
zraR_2	DSM23404_03382	zraR_2
zraR_2	DSM23424_01015	zraR_2
zraR_2	DSM23424_02273	zraR_2
zraR_2	DSM23424_02966	zraR_2
zraR_2	DSM23424_02440	zraR_2
zraR_2	DSM23424_02534	zraR_2
zraR_2	DSM23424_02295	zraR_2
zraR_2	DSM23424_00145	zraR_2
zraR_2	DSM23424_00664	zraR_2
zraR_2	DSM23424_00701	zraR_2
zraR_2	DSM23424_03180	zraR_2

zraR_2	DSM23424_01701	zraR_2
zraR_2	DSM23424_00700	zraR_2
zraR_2	DSM23424_03386	zraR_2
zraR_2	DSM23424_00526	zraR_2
zraR_2	DSM23424_02002	zraR_2
zraR_2	DSM23424_02001	zraR_2
zraR_2	DSM23424_00741	zraR_2
zraR_2	DSM23424_01702	zraR_2
zraR_2	DSM23424_02268	zraR_2
zraR_2	DSM23424_02037	zraR_2
zraR_2	DSM23424_02634	zraR_2
zraR_2	DSM23424_03096	zraR_2
zraR_2	DSM23424_00202	zraR_2
zraR_2	DSM23424_01607	zraR_2
zraR_2	DSM23424_00544	zraR_2
zraR_2	DSM23424_01306	zraR_2
zraR_2	DSM23424_00763	zraR_2
zraR_2	DSM23424_01307	zraR_2
zraR_2	DSM23424_00615	zraR_2
zraR_2	DSM23424_03093	zraR_2
zraR_2	DSM23424_01259	zraR_2
zraR_2	DSM23424_00740	zraR_2
zraR_2	DSM23424_00241	zraR_2
zraR_2	DSM23424_01185	zraR_2
zraR_2	DSM23424_03095	zraR_2
zraR_2	DSM23424_02400	zraR_2
zraR_2	DSM23424_03043	zraR_2
zraR_2	DSM23424_02512	zraR_2
zraR_2	DSM23424_02180	zraR_2
zraR_2	DSM23424_02146	zraR_2
zraR_2	DSM23424_00879	zraR_2
zraR_2	DSM23424_02036	zraR_2
zraR_2	DSM23424_01184	zraR_2
zraR_2	DSM23424_00663	zraR_2
zraR_2	DSM23424_03094	zraR_2
zraR_2	DSM23424_00670	zraR_2
zraR_2	DSM23424_02781	zraR_2
zraR_2	DSM23424_00126	zraR_2
zraR_2	DSM23424_01368	zraR_2
zraR_2	DSM23424_00246	zraR_2
zraR_2	DSM23424_00307	zraR_2
zraR_2	DSM23424_02979	zraR_2
zraR_2	DSM23424_00553	zraR_2
zraR_2	DSM23424_01364	zraR_2
zraR_2	DSM24786_01532	zraR_2
zraR_2	DSM24786_02517	zraR_2
zraR_2	DSM24786_03115	zraR_2
zraR_2	DSM24786_00116	zraR_2

zraR_2	DSM24786_01032	zraR_2
zraR_2	DSM24786_00573	zraR_2
zraR_2	DSM24786_01149	zraR_2
zraR_2	DSM24786_03135	zraR_2
zraR_2	DSM24786_00617	zraR_2
zraR_2	DSM24786_02934	zraR_2
zraR_2	DSM24786_00974	zraR_2
zraR_2	DSM24786_00616	zraR_2
zraR_2	DSM24786_02933	zraR_2
zraR_2	DSM24786_03136	zraR_2
zraR_2	DSM24786_00975	zraR_2
zraR_2	DSM24786_01542	zraR_2
zraR_2	DSM24786_01541	zraR_2
zraR_2	DSM24786_00832	zraR_2
zraR_2	DSM24786_00833	zraR_2
zraR_2	DSM24786_00935	zraR_2
zraR_2	DSM24786_00936	zraR_2
zraR_2	DSM24786_02252	zraR_2
zraR_2	DSM24786_02780	zraR_2
zraR_2	DSM24786_02712	zraR_2
zraR_2	DSM24786_01165	zraR_2
zraR_2	DSM24786_00834	zraR_2
zraR_2	DSM24786_02713	zraR_2
zraR_2	DSM24786_02122	zraR_2
zraR_2	DSM24786_02600	zraR_2
zraR_2	DSM24786_02772	zraR_2
zraR_2	DSM24786_00770	zraR_2
zraR_2	DSM24786_00578	zraR_2
zraR_2	DSM24786_01588	zraR_2
zraR_2	DSM24786_01251	zraR_2
zraR_2	DSM24786_00888	zraR_2
zraR_2	DSM24786_02674	zraR_2
zraR_2	DSM24786_02143	zraR_2
zraR_2	DSM24786_02790	zraR_2
zraR_2	DSM24786_02792	zraR_2
zraR_2	DSM24786_00605	zraR_2
zraR_2	DSM24786_00637	zraR_2
zraR_2	DSM24786_02729	zraR_2
zraR_2	DSM24786_01368	zraR_2
zraR_2	DSM24786_00806	zraR_2
zraR_2	DSM24786_01920	zraR_2
zraR_2	DSM24786_02293	zraR_2
zraR_2	DSM24786_01339	zraR_2
zraR_2	DSM24786_02728	zraR_2
zraR_2	DSM24786_02422	zraR_2
zraR_2	DSM24786_03212	zraR_2
zraR_2	DSM24786_03324	zraR_2
zraR_2	DSM24786_01894	zraR_2

zraR_2	DSM24786_01876	zraR_2
zraR_2	DSM24786_02347	zraR_2
zraR_2	DSM24786_01669	zraR_2
zraR_2	DSM24786_03058	zraR_2
zraR_2	DSM7489_00429	zraR_2
zraR_2	DSM7489_02391	zraR_2
zraR_2	DSM7489_02510	zraR_2
zraR_2	DSM7489_02487	zraR_2
zraR_2	DSM7489_00602	zraR_2
zraR_2	DSM7489_00903	zraR_2
zraR_2	DSM7489_02998	zraR_2
zraR_2	DSM7489_01358	zraR_2
zraR_2	DSM7489_03333	zraR_2
zraR_2	DSM7489_02997	zraR_2
zraR_2	DSM7489_00904	zraR_2
zraR_2	DSM7489_01357	zraR_2
zraR_2	DSM7489_03334	zraR_2
zraR_2	DSM7489_03191	zraR_2
zraR_2	DSM7489_02381	zraR_2
zraR_2	DSM7489_01178	zraR_2
zraR_2	DSM7489_02382	zraR_2
zraR_2	DSM7489_01648	zraR_2
zraR_2	DSM7489_03192	zraR_2
zraR_2	DSM7489_01179	zraR_2
zraR_2	DSM7489_02522	zraR_2
zraR_2	DSM7489_01838	zraR_2
zraR_2	DSM7489_01248	zraR_2
zraR_2	DSM7489_00510	zraR_2
zraR_2	DSM7489_01240	zraR_2
zraR_2	DSM7489_03281	zraR_2
zraR_2	DSM7489_02532	zraR_2
zraR_2	DSM7489_00068	zraR_2
zraR_2	DSM7489_03244	zraR_2
zraR_2	DSM7489_01110	zraR_2
zraR_2	DSM7489_01815	zraR_2
zraR_2	DSM7489_00214	zraR_2
zraR_2	DSM7489_01260	zraR_2
zraR_2	DSM7489_01258	zraR_2
zraR_2	DSM7489_02986	zraR_2
zraR_2	DSM7489_03018	zraR_2
zraR_2	DSM7489_01196	zraR_2
zraR_2	DSM7489_03167	zraR_2
zraR_2	DSM7489_02037	zraR_2
zraR_2	DSM7489_02269	zraR_2
zraR_2	DSM7489_02518	zraR_2
zraR_2	DSM7489_00780	zraR_2
zraR_2	DSM7489_01195	zraR_2
zraR_2	DSM7489_00333	zraR_2

zraR_2	DSM7489_00461	zraR_2
zraR_2	DSM7489_01017	zraR_2
zraR_2	DSM7489_00753	zraR_2
zraR_2	DSM7489_02063	zraR_2
zraR_2	DSM7489_02081	zraR_2
zraR_2	DSM7489_01570	zraR_2
zraR_2	DSM7489_01474	zraR_2
zraR_2	DSW1_02148	zraR_2
zraR_2	DSW1_00957	zraR_2
zraR_2	DSW1_00379	zraR_2
zraR_2	DSW1_00532	zraR_2
zraR_2	DSW1_02885	zraR_2
zraR_2	DSW1_00439	zraR_2
zraR_2	DSW1_02739	zraR_2
zraR_2	DSW1_02469	zraR_2
zraR_2	DSW1_00956	zraR_2
zraR_2	DSW1_01715	zraR_2
zraR_2	DSW1_02740	zraR_2
zraR_2	DSW1_00229	zraR_2
zraR_2	DSW1_00228	zraR_2
zraR_2	DSW1_01714	zraR_2
zraR_2	DSW1_01344	zraR_2
zraR_2	DSW1_00573	zraR_2
zraR_2	DSW1_00366	zraR_2
zraR_2	DSW1_00563	zraR_2
zraR_2	DSW1_00385	zraR_2
zraR_2	DSW1_00842	zraR_2
zraR_2	DSW1_00364	zraR_2
zraR_2	DSW1_02661	zraR_2
zraR_2	DSW1_00450	zraR_2
zraR_2	DSW1_02078	zraR_2
zraR_2	DSW1_01853	zraR_2
zraR_2	DSW1_01851	zraR_2
zraR_2	DSW1_02680	zraR_2
zraR_2	DSW1_00512	zraR_2
zraR_2	DSW1_02681	zraR_2
zraR_2	DSW1_02230	zraR_2
zraR_2	DSW1_00178	zraR_2
zraR_2	DSW1_01310	zraR_2
zraR_2	DSW1_02676	zraR_2
zraR_2	DSW1_00026	zraR_2
zraR_2	DSW1_01487	zraR_2
zraR_2	DSW1_00793	zraR_2
zraR_2	DSW1_01978	zraR_2
zraR_2	DSW1_02290	zraR_2
zraR_2	DSW1_00518	zraR_2
zraR_2	DSW1_01343	zraR_2
zraR_2	DSW1_01345	zraR_2

zraR_2	DSW1_00409	zraR_2
zraR_2	DSW1_01802	zraR_2
zraR_2	DSW5_02540	zraR_2
zraR_2	DSW5_02541	zraR_2
zraR_2	DSW5_02656	zraR_2
zraR_2	DSW5_02538	zraR_2
zraR_2	DSW5_01913	zraR_2
zraR_2	DSW5_01912	zraR_2
zraR_2	DSW5_00835	zraR_2
zraR_2	DSW5_02539	zraR_2
zraR_2	DSW5_02514	zraR_2
zraR_2	DSW5_00622	zraR_2
zraR_2	DSW5_00623	zraR_2
zraR_2	DSW5_00836	zraR_2
zraR_2	DSW5_02276	zraR_2
zraR_2	DSW5_02671	zraR_2
zraR_2	DSW5_02441	zraR_2
zraR_2	DSW5_02742	zraR_2
zraR_2	DSW5_02429	zraR_2
zraR_2	DSW5_02439	zraR_2
zraR_2	DSW5_01070	zraR_2
zraR_2	DSW5_00562	zraR_2
zraR_2	DSW5_02655	zraR_2
zraR_2	DSW5_01764	zraR_2
zraR_2	DSW5_02300	zraR_2
zraR_2	DSW5_02653	zraR_2
zraR_2	DSW5_01683	zraR_2
zraR_2	DSW5_02274	zraR_2
zraR_2	DSW5_01073	zraR_2
zraR_2	DSW5_00147	zraR_2
zraR_2	DSW5_01462	zraR_2
zraR_2	DSW5_00804	zraR_2
zraR_2	DSW5_00683	zraR_2
zraR_2	DSW5_00598	zraR_2
zraR_2	DSW5_01688	zraR_2
zraR_2	DSW5_00727	zraR_2
zraR_2	DSW5_01163	zraR_2
zraR_2	DSW5_00133	zraR_2
zraR_2	DSW5_00962	zraR_2
zraR_2	DSW5_02262	zraR_2
zraR_2	DSW5_02453	zraR_2
zraR_2	DSW5_02649	zraR_2
zraR_2	DSW5_00345	zraR_2
zraR_2	DSW5_01368	zraR_2
zraR_2	DSW5_00774	zraR_2
zraR_2	DSW5_01801	zraR_2
zraR_2	DSW5_01889	zraR_2
zraR_2	DSW6_00320	zraR_2

zraR_2	DSW6_00564	zraR_2
zraR_2	DSW6_03149	zraR_2
zraR_2	DSW6_01869	zraR_2
zraR_2	DSW6_01837	zraR_2
zraR_2	DSW6_01021	zraR_2
zraR_2	DSW6_01022	zraR_2
zraR_2	DSW6_02478	zraR_2
zraR_2	DSW6_03475	zraR_2
zraR_2	DSW6_01453	zraR_2
zraR_2	DSW6_03476	zraR_2
zraR_2	DSW6_00458	zraR_2
zraR_2	DSW6_01836	zraR_2
zraR_2	DSW6_01454	zraR_2
zraR_2	DSW6_00722	zraR_2
zraR_2	DSW6_00459	zraR_2
zraR_2	DSW6_00927	zraR_2
zraR_2	DSW6_01585	zraR_2
zraR_2	DSW6_02271	zraR_2
zraR_2	DSW6_02176	zraR_2
zraR_2	DSW6_01084	zraR_2
zraR_2	DSW6_00024	zraR_2
zraR_2	DSW6_03111	zraR_2
zraR_2	DSW6_00022	zraR_2
zraR_2	DSW6_02957	zraR_2
zraR_2	DSW6_00186	zraR_2
zraR_2	DSW6_01820	zraR_2
zraR_2	DSW6_00600	zraR_2
zraR_2	DSW6_00786	zraR_2
zraR_2	DSW6_02923	zraR_2
zraR_2	DSW6_02354	zraR_2
zraR_2	DSW6_02485	zraR_2
zraR_2	DSW6_02573	zraR_2
zraR_2	DSW6_03073	zraR_2
zraR_2	DSW6_00838	zraR_2
zraR_2	DSW6_00723	zraR_2
zraR_2	DSW6_02463	zraR_2
zraR_2	DSW6_01587	zraR_2
zraR_2	DSW6_02464	zraR_2
zraR_2	DSW6_01745	zraR_2
zraR_2	DSW6_02791	zraR_2
zraR_2	DSW6_00187	zraR_2
zraR_2	DSW6_01664	zraR_2
zraR_2	DSW6_02483	zraR_2
zraR_2	DSW8_03111	zraR_2
zraR_2	DSW8_03154	zraR_2
zraR_2	DSW8_01272	zraR_2
zraR_2	DSW8_03558	zraR_2
zraR_2	DSW8_01937	zraR_2

zraR_2	DSW8_02105	zraR_2
zraR_2	DSW8_03371	zraR_2
zraR_2	DSW8_02679	zraR_2
zraR_2	DSW8_03692	zraR_2
zraR_2	DSW8_00207	zraR_2
zraR_2	DSW8_00220	zraR_2
zraR_2	DSW8_02627	zraR_2
zraR_2	DSW8_00540	zraR_2
zraR_2	DSW8_00208	zraR_2
zraR_2	DSW8_02678	zraR_2
zraR_2	DSW8_00541	zraR_2
zraR_2	DSW8_03634	zraR_2
zraR_2	DSW8_03633	zraR_2
zraR_2	DSW8_00108	zraR_2
zraR_2	DSW8_02309	zraR_2
zraR_2	DSW8_02869	zraR_2
zraR_2	DSW8_01369	zraR_2
zraR_2	DSW8_02873	zraR_2
zraR_2	DSW8_01904	zraR_2
zraR_2	DSW8_00327	zraR_2
zraR_2	DSW8_02331	zraR_2
zraR_2	DSW8_00757	zraR_2
zraR_2	DSW8_00221	zraR_2
zraR_2	DSW8_00107	zraR_2
zraR_2	DSW8_02104	zraR_2
zraR_2	DSW8_01054	zraR_2
zraR_2	DSW8_01076	zraR_2
zraR_2	DSW8_03153	zraR_2
zraR_2	DSW8_03388	zraR_2
zraR_2	DSW8_03416	zraR_2
zraR_2	DSW8_03192	zraR_2
zraR_2	DSW8_02516	zraR_2
zraR_2	DSW8_01093	zraR_2
zraR_2	DSW8_01080	zraR_2
zraR_2	DSW8_03720	zraR_2
zraR_2	DSW8_01960	zraR_2
zraR_2	DSW8_03721	zraR_2
zraR_2	DSW8_01584	zraR_2
zraR_2	DSW8_02555	zraR_2
zraR_2	DSW8_01483	zraR_2
zraR_2	DSW8_03470	zraR_2
zraR_2	DSW8_00996	zraR_2
zraR_2	DSW8_01086	zraR_2
zraR_2	DSW8_03370	zraR_2
zraR_2	DSW8_00159	zraR_2
zraR_2	DSW8_01084	zraR_2
zraR_2	DSW8_00037	zraR_2
zraR_2	DSW8_03372	zraR_2

zraR_2	DSW8_03373	zraR_2
zraR_2	DSW8_02585	zraR_2
zraR_2	DSW8_02350	zraR_2
zraR_2	DSW8_03243	zraR_2
zraR_2	DSW8_03086	zraR_2
zraR_2	DSW8_01426	zraR_2
zraR_2	DSW8_02368	zraR_2
zraR_2	DSW8_02875	zraR_2
zraR_2	DSW8_02634	zraR_2
zraR_2	DSW8_03186	zraR_2
zraR_2	DSW8_02819	zraR_2
zraR_2	DSW8_01055	zraR_2
zraR_2	DSW8_02626	zraR_2
zraR_2	DSW8_01229	zraR_2
zraR_2	DSW8_02684	zraR_2
zraR_2	DSW8_01330	zraR_2
zraR_2	DSW8_02683	zraR_2
zraR_2	DSW8_03211	zraR_2
zraR_2	DSW8_02344	zraR_2
zraR_2	DSW8_03315	zraR_2
zraR_2	DSW8_02118	zraR_2
zraR_2	DSW8_02369	zraR_2
zraR_2	DSW8_01401	zraR_2
zraR_2	DSW8_01031	zraR_2
zraR_2	DSW8_02332	zraR_2
zraR_2	DSW8_02959	zraR_2
zraR_2	F52_03534	zraR_2
zraR_2	F52_01790	zraR_2
zraR_2	F52_03556	zraR_2
zraR_2	F52_00905	zraR_2
zraR_2	F52_04598	zraR_2
zraR_2	F52_00928	zraR_2
zraR_2	F52_04135	zraR_2
zraR_2	F52_01662	zraR_2
zraR_2	F52_00861	zraR_2
zraR_2	F52_02007	zraR_2
zraR_2	F52_04275	zraR_2
zraR_2	F52_03731	zraR_2
zraR_2	F52_02073	zraR_2
zraR_2	F52_00766	zraR_2
zraR_2	F52_00767	zraR_2
zraR_2	F52_03853	zraR_2
zraR_2	F52_02008	zraR_2
zraR_2	F52_00823	zraR_2
zraR_2	F52_01377	zraR_2
zraR_2	F52_00929	zraR_2
zraR_2	F52_03036	zraR_2
zraR_2	F52_04404	zraR_2

zraR_2	F52_00860	zraR_2
zraR_2	F52_03706	zraR_2
zraR_2	F52_03037	zraR_2
zraR_2	F52_03077	zraR_2
zraR_2	F52_00855	zraR_2
zraR_2	F52_03854	zraR_2
zraR_2	F52_02341	zraR_2
zraR_2	F52_03730	zraR_2
zraR_2	F52_02512	zraR_2
zraR_2	F52_00315	zraR_2
zraR_2	F52_04496	zraR_2
zraR_2	F52_03870	zraR_2
zraR_2	F52_02757	zraR_2
zraR_2	F52_01827	zraR_2
zraR_2	F52_03888	zraR_2
zraR_2	F52_03090	zraR_2
zraR_2	F52_04563	zraR_2
zraR_2	F52_04433	zraR_2
zraR_2	F52_04043	zraR_2
zraR_2	F52_03984	zraR_2
zraR_2	F52_01378	zraR_2
zraR_2	F52_00290	zraR_2
zraR_2	F52_00477	zraR_2
zraR_2	F52_00824	zraR_2
zraR_2	F52_04038	zraR_2
zraR_2	F52_00862	zraR_2
zraR_2	F52_01212	zraR_2
zraR_2	F52_03070	zraR_2
zraR_2	F52_04222	zraR_2
zraR_2	F52_01353	zraR_2
zraR_2	F52_00938	zraR_2
zraR_2	F52_02735	zraR_2
zraR_2	F52_03780	zraR_2
zraR_2	F52_01310	zraR_2
zraR_2	F52_01915	zraR_2
zraR_2	F52_00004	zraR_2
zraR_2	F52_04187	zraR_2
zraR_2	F52_02023	zraR_2
zraR_2	F52_03638	zraR_2
zraR_2	F52_03231	zraR_2
zraR_2	F52_01028	zraR_2
zraR_2	F52_00651	zraR_2
zraR_2	F52_02734	zraR_2
zraR_2	F52_02074	zraR_2
zraR_2	F52_00937	zraR_2
zraR_2	F52_01101	zraR_2
zraR_2	F52_01318	zraR_2
zraR_2	F52_03009	zraR_2

zraR_2	F52_01093	zraR_2
zraR_2	F52_04405	zraR_2
zraR_2	F52_04216	zraR_2
zraR_2	F52_03277	zraR_2
zraR_2	F52_00507	zraR_2
zraR_2	F52_02344	zraR_2
zraR_2	F52_03595	zraR_2
zraR_2	F52_00857	zraR_2
zraR_2	F52_03000	zraR_2
zraR_2	F52_04441	zraR_2
zraR_2	F52_00008	zraR_2
zraR_2	F52_03483	zraR_2
zraR_2	F52_00683	zraR_2
zraR_2	F52_01047	zraR_2
zraR_2	F52_00863	zraR_2
zraR_2	F52_00864	zraR_2
zraR_2	F52_03566	zraR_2
zraR_2	F52_04478	zraR_2
zraR_2	F52_03184	zraR_2
zraR_2	F52_03043	zraR_2
zraR_2	GCA_001027945.1_01687	zraR_2
zraR_2	GCA_001027945.1_00215	zraR_2
zraR_2	GCA_001027945.1_02397	zraR_2
zraR_2	GCA_001027945.1_00216	zraR_2
zraR_2	GCA_001027945.1_02396	zraR_2
zraR_2	GCA_001027945.1_00761	zraR_2
zraR_2	GCA_001027945.1_02655	zraR_2
zraR_2	GCA_001027945.1_00602	zraR_2
zraR_2	GCA_001027945.1_02055	zraR_2
zraR_2	GCA_001027945.1_02044	zraR_2
zraR_2	GCA_001027945.1_01613	zraR_2
zraR_2	GCA_001027945.1_01088	zraR_2
zraR_2	GCA_001027945.1_02354	zraR_2
zraR_2	GCA_001027945.1_02057	zraR_2
zraR_2	GCA_001027945.1_00150	zraR_2
zraR_2	GCA_001027945.1_01039	zraR_2
zraR_2	GCA_001027945.1_00768	zraR_2
zraR_2	GCA_001027945.1_03137	zraR_2
zraR_2	GCA_001027945.1_02351	zraR_2
zraR_2	GCA_001027945.1_02870	zraR_2
zraR_2	GCA_001027945.1_01516	zraR_2
zraR_2	GCA_001027945.1_00760	zraR_2
zraR_2	GCA_001027945.1_02012	zraR_2
zraR_2	GCA_001027945.1_01463	zraR_2
zraR_2	GCA_001027945.1_01797	zraR_2
zraR_2	GCA_001027945.1_01169	zraR_2
zraR_2	GCA_001027945.1_00660	zraR_2
zraR_2	GCA_001027945.1_02827	zraR_2

zraR_2	GCA_001027945.1_00739	zraR_2
zraR_2	GCA_001027945.1_00119	zraR_2
zraR_2	GCA_001027945.1_01085	zraR_2
zraR_2	GCA_001027945.1_00193	zraR_2
zraR_2	GCA_001027945.1_00754	zraR_2
zraR_2	GCA_001027945.1_02447	zraR_2
zraR_2	GCA_001027945.1_02448	zraR_2
zraR_2	HI1_00361	zraR_2
zraR_2	HI1_02324	zraR_2
zraR_2	HI1_02442	zraR_2
zraR_2	HI1_02419	zraR_2
zraR_2	HI1_00534	zraR_2
zraR_2	HI1_00835	zraR_2
zraR_2	HI1_02939	zraR_2
zraR_2	HI1_01299	zraR_2
zraR_2	HI1_03279	zraR_2
zraR_2	HI1_02938	zraR_2
zraR_2	HI1_00836	zraR_2
zraR_2	HI1_01298	zraR_2
zraR_2	HI1_03280	zraR_2
zraR_2	HI1_03138	zraR_2
zraR_2	HI1_02314	zraR_2
zraR_2	HI1_01120	zraR_2
zraR_2	HI1_02315	zraR_2
zraR_2	HI1_01569	zraR_2
zraR_2	HI1_03139	zraR_2
zraR_2	HI1_01121	zraR_2
zraR_2	HI1_00896	zraR_2
zraR_2	HI1_02454	zraR_2
zraR_2	HI1_01766	zraR_2
zraR_2	HI1_01190	zraR_2
zraR_2	HI1_00442	zraR_2
zraR_2	HI1_01182	zraR_2
zraR_2	HI1_03224	zraR_2
zraR_2	HI1_02464	zraR_2
zraR_2	HI1_03367	zraR_2
zraR_2	HI1_03188	zraR_2
zraR_2	HI1_01050	zraR_2
zraR_2	HI1_01743	zraR_2
zraR_2	HI1_00146	zraR_2
zraR_2	HI1_01202	zraR_2
zraR_2	HI1_01200	zraR_2
zraR_2	HI1_02927	zraR_2
zraR_2	HI1_02959	zraR_2
zraR_2	HI1_01138	zraR_2
zraR_2	HI1_03114	zraR_2
zraR_2	HI1_01964	zraR_2
zraR_2	HI1_02196	zraR_2

zraR_2	HI1_02450	zraR_2
zraR_2	HI1_00715	zraR_2
zraR_2	HI1_01137	zraR_2
zraR_2	HI1_00265	zraR_2
zraR_2	HI1_00393	zraR_2
zraR_2	HI1_00955	zraR_2
zraR_2	HI1_00688	zraR_2
zraR_2	HI1_01990	zraR_2
zraR_2	HI1_02008	zraR_2
zraR_2	HI1_01490	zraR_2
zraR_2	HI1_01415	zraR_2
zraR_2	IR1_04527	zraR_2
zraR_2	IR1_01951	zraR_2
zraR_2	IR1_01376	zraR_2
zraR_2	IR1_04575	zraR_2
zraR_2	IR1_03293	zraR_2
zraR_2	IR1_03845	zraR_2
zraR_2	IR1_04267	zraR_2
zraR_2	IR1_03204	zraR_2
zraR_2	IR1_01791	zraR_2
zraR_2	IR1_02915	zraR_2
zraR_2	IR1_01219	zraR_2
zraR_2	IR1_03798	zraR_2
zraR_2	IR1_01147	zraR_2
zraR_2	IR1_03227	zraR_2
zraR_2	IR1_02083	zraR_2
zraR_2	IR1_02082	zraR_2
zraR_2	IR1_01218	zraR_2
zraR_2	IR1_03742	zraR_2
zraR_2	IR1_01592	zraR_2
zraR_2	IR1_03631	zraR_2
zraR_2	IR1_03881	zraR_2
zraR_2	IR1_02030	zraR_2
zraR_2	IR1_01703	zraR_2
zraR_2	IR1_01128	zraR_2
zraR_2	IR1_01792	zraR_2
zraR_2	IR1_02928	zraR_2
zraR_2	IR1_00932	zraR_2
zraR_2	IR1_01966	zraR_2
zraR_2	IR1_01591	zraR_2
zraR_2	IR1_01509	zraR_2
zraR_2	IR1_01516	zraR_2
zraR_2	IR1_03741	zraR_2
zraR_2	IR1_03630	zraR_2
zraR_2	IR1_01435	zraR_2
zraR_2	IR1_02235	zraR_2
zraR_2	IR1_01796	zraR_2
zraR_2	IR1_03909	zraR_2

zraR_2	IR1_00278	zraR_2
zraR_2	IR1_01790	zraR_2
zraR_2	IR1_02549	zraR_2
zraR_2	IR1_01574	zraR_2
zraR_2	IR1_02826	zraR_2
zraR_2	IR1_01414	zraR_2
zraR_2	IR1_01908	zraR_2
zraR_2	IR1_04595	zraR_2
zraR_2	IR1_02576	zraR_2
zraR_2	IR1_03301	zraR_2
zraR_2	IR1_03299	zraR_2
zraR_2	IR1_02400	zraR_2
zraR_2	IR1_03202	zraR_2
zraR_2	IR1_01129	zraR_2
zraR_2	IR1_04406	zraR_2
zraR_2	IR1_02029	zraR_2
zraR_2	IR1_03228	zraR_2
zraR_2	IR1_03300	zraR_2
zraR_2	IR1_03185	zraR_2
zraR_2	IR1_04169	zraR_2
zraR_2	IR1_00925	zraR_2
zraR_2	IR1_01146	zraR_2
zraR_2	IR1_03431	zraR_2
zraR_2	IR1_03496	zraR_2
zraR_2	IR1_00526	zraR_2
zraR_2	IR1_04209	zraR_2
zraR_2	IR1_01664	zraR_2
zraR_2	IR1_04238	zraR_2
zraR_2	IR1_02726	zraR_2
zraR_2	IR1_00017	zraR_2
zraR_2	IR1_00527	zraR_2
zraR_2	IR1_03495	zraR_2
zraR_2	IR1_00504	zraR_2
zraR_2	IR1_03384	zraR_2
zraR_2	IR1_01967	zraR_2
zraR_2	IR1_01434	zraR_2
zraR_2	IR1_03134	zraR_2
zraR_2	IR1_02682	zraR_2
zraR_2	IR1_03401	zraR_2
zraR_2	IR1_00081	zraR_2
zraR_2	IR1_01744	zraR_2
zraR_2	IR1_02376	zraR_2
zraR_2	IR1_00287	zraR_2
zraR_2	IR1_03905	zraR_2
zraR_2	IR1_02256	zraR_2
zraR_2	IR1_03557	zraR_2
zraR_2	IR1_01760	zraR_2
zraR_2	IR1_02685	zraR_2

zraR_2	IR1_03585	zraR_2
zraR_2	IR1_01711	zraR_2
zraR_2	IR1_01794	zraR_2
zraR_2	IR1_01789	zraR_2
zraR_2	IR1_01788	zraR_2
zraR_2	IR1_00056	zraR_2
zraR_2	IR1_02666	zraR_2
zraR_2	IR1_00833	zraR_2
zraR_2	IR1_01390	zraR_2
zraR_2	IR1_00847	zraR_2
zraR_2	IR1_01710	zraR_2
zraR_2	IR1_04797	zraR_2
zraR_2	IR1_02686	zraR_2
zraR_2	IR1_01391	zraR_2
zraR_2	IR1_01389	zraR_2
zraR_2	IR1_01102	zraR_2
zraR_2	KCOM3131_00634	zraR_2
zraR_2	KCOM3131_01605	zraR_2
zraR_2	KCOM3131_01838	zraR_2
zraR_2	KCOM3131_00955	zraR_2
zraR_2	KCOM3131_00135	zraR_2
zraR_2	KCOM3131_00114	zraR_2
zraR_2	KCOM3131_00136	zraR_2
zraR_2	KCOM3131_01606	zraR_2
zraR_2	KCOM3131_01139	zraR_2
zraR_2	KCOM3131_00113	zraR_2
zraR_2	KCOM3131_00954	zraR_2
zraR_2	KCOM3131_00849	zraR_2
zraR_2	KCOM3131_01941	zraR_2
zraR_2	KCOM3131_00417	zraR_2
zraR_2	KCOM3131_01372	zraR_2
zraR_2	KCOM3131_01016	zraR_2
zraR_2	KCOM3131_01748	zraR_2
zraR_2	KCTC12974_00884	zraR_2
zraR_2	KCTC12974_02918	zraR_2
zraR_2	KCTC12974_01288	zraR_2
zraR_2	KCTC12974_01013	zraR_2
zraR_2	KCTC12974_00622	zraR_2
zraR_2	KCTC12974_00818	zraR_2
zraR_2	KCTC12974_00939	zraR_2
zraR_2	KCTC12974_00940	zraR_2
zraR_2	KCTC12974_00819	zraR_2
zraR_2	KCTC12974_00791	zraR_2
zraR_2	KCTC12974_02300	zraR_2
zraR_2	KCTC12974_00792	zraR_2
zraR_2	KCTC12974_00895	zraR_2
zraR_2	KCTC12974_02979	zraR_2
zraR_2	KCTC12974_02846	zraR_2

zraR_2	KCTC12974_00894	zraR_2
zraR_2	KCTC12974_00084	zraR_2
zraR_2	KCTC12974_00049	zraR_2
zraR_2	KCTC12974_00896	zraR_2
zraR_2	KCTC12974_02976	zraR_2
zraR_2	KCTC12974_02336	zraR_2
zraR_2	KCTC12974_02660	zraR_2
zraR_2	KCTC12974_00431	zraR_2
zraR_2	KCTC12974_02928	zraR_2
zraR_2	KCTC12974_02939	zraR_2
zraR_2	KCTC12974_02959	zraR_2
zraR_2	KCTC12974_02960	zraR_2
zraR_2	KCTC12974_00050	zraR_2
zraR_2	KCTC12974_02621	zraR_2
zraR_2	KCTC12974_02822	zraR_2
zraR_2	KCTC12974_02301	zraR_2
zraR_2	KCTC12974_01289	zraR_2
zraR_2	KCTC12974_00205	zraR_2
zraR_2	KCTC12974_00555	zraR_2
zraR_2	KCTC12974_01152	zraR_2
zraR_2	KCTC12974_00897	zraR_2
zraR_2	KCTC12974_00345	zraR_2
zraR_2	KCTC12974_01282	zraR_2
zraR_2	KCTC12974_00083	zraR_2
zraR_2	KCTC12974_00057	zraR_2
zraR_2	KCTC12974_02854	zraR_2
zraR_2	KCTC12974_01181	zraR_2
zraR_2	KCTC12974_02847	zraR_2
zraR_2	KCTC12974_02929	zraR_2
zraR_2	KCTC12974_00262	zraR_2
zraR_2	KCTC12974_02934	zraR_2
zraR_2	KCTC12974_00156	zraR_2
zraR_2	KCTC12974_01453	zraR_2
zraR_2	KL-A_01777	zraR_2
zraR_2	KL-A_01671	zraR_2
zraR_2	KL-A_01046	zraR_2
zraR_2	KL-A_01068	zraR_2
zraR_2	KL-A_02561	zraR_2
zraR_2	KL-A_02368	zraR_2
zraR_2	KL-A_00488	zraR_2
zraR_2	KL-A_00871	zraR_2
zraR_2	KL-A_01859	zraR_2
zraR_2	KL-A_00489	zraR_2
zraR_2	KL-A_02367	zraR_2
zraR_2	KL-A_00872	zraR_2
zraR_2	KL-A_01858	zraR_2
zraR_2	KL-A_01981	zraR_2
zraR_2	KL-A_01661	zraR_2

zraR_2	KL-A_03006	zraR_2
zraR_2	KL-A_01662	zraR_2
zraR_2	KL-A_01450	zraR_2
zraR_2	KL-A_01980	zraR_2
zraR_2	KL-A_03007	zraR_2
zraR_2	KL-A_02199	zraR_2
zraR_2	KL-A_01034	zraR_2
zraR_2	KL-A_00433	zraR_2
zraR_2	KL-A_02137	zraR_2
zraR_2	KL-A_01696	zraR_2
zraR_2	KL-A_02129	zraR_2
zraR_2	KL-A_01912	zraR_2
zraR_2	KL-A_01024	zraR_2
zraR_2	KL-A_03280	zraR_2
zraR_2	KL-A_01931	zraR_2
zraR_2	KL-A_02056	zraR_2
zraR_2	KL-A_00456	zraR_2
zraR_2	KL-A_01210	zraR_2
zraR_2	KL-A_02149	zraR_2
zraR_2	KL-A_02147	zraR_2
zraR_2	KL-A_00500	zraR_2
zraR_2	KL-A_00468	zraR_2
zraR_2	KL-A_00545	zraR_2
zraR_2	KL-A_03024	zraR_2
zraR_2	KL-A_02005	zraR_2
zraR_2	KL-A_00233	zraR_2
zraR_2	KL-A_00004	zraR_2
zraR_2	KL-A_01038	zraR_2
zraR_2	KL-A_02446	zraR_2
zraR_2	KL-A_03023	zraR_2
zraR_2	KL-A_02660	zraR_2
zraR_2	KL-A_01745	zraR_2
zraR_2	KL-A_02260	zraR_2
zraR_2	KL-A_00544	zraR_2
zraR_2	KL-A_02473	zraR_2
zraR_2	KL-A_00207	zraR_2
zraR_2	KL-A_00189	zraR_2
zraR_2	KL-A_02861	zraR_2
zraR_2	KL-A_00752	zraR_2
zraR_2	L12_00338	zraR_2
zraR_2	L12_00339	zraR_2
zraR_2	L12_00301	zraR_2
zraR_2	L12_00346	zraR_2
zraR_2	LL0412.1.7_00495	zraR_2
zraR_2	LL0412.1.7_01662	zraR_2
zraR_2	LL0412.1.7_00494	zraR_2
zraR_2	LL0412.1.7_01661	zraR_2
zraR_2	LL0412.1.7_01104	zraR_2

zraR_2	LL0412.1.7_00785	zraR_2
zraR_2	LL0412.1.7_02632	zraR_2
zraR_2	LL0412.1.7_02466	zraR_2
zraR_2	LL0412.1.7_02048	zraR_2
zraR_2	LL0412.1.7_00960	zraR_2
zraR_2	LL0412.1.7_01640	zraR_2
zraR_2	LL0412.1.7_02190	zraR_2
zraR_2	LL0412.1.7_00348	zraR_2
zraR_2	LL0412.1.7_01811	zraR_2
zraR_2	LL0412.1.7_00841	zraR_2
zraR_2	LL0412.1.7_01861	zraR_2
zraR_2	LL0412.1.7_02471	zraR_2
zraR_2	LL0412.1.7_01057	zraR_2
zraR_2	LL0412.1.7_02633	zraR_2
zraR_2	LL0412.1.7_02467	zraR_2
zraR_2	LL0412.1.7_02630	zraR_2
zraR_2	LL0412.1.7_02449	zraR_2
zraR_2	LL0412.1.7_01755	zraR_2
zraR_2	LL0412.1.7_01996	zraR_2
zraR_2	LL0412.1.7_02206	zraR_2
zraR_2	LL0412.1.7_02593	zraR_2
zraR_2	LL0412.1.7_01349	zraR_2
zraR_2	LL0412.1.7_01711	zraR_2
zraR_2	MAR_2009_71_03802	zraR_2
zraR_2	MAR_2009_71_03760	zraR_2
zraR_2	MAR_2009_71_02715	zraR_2
zraR_2	MAR_2009_71_01762	zraR_2
zraR_2	MAR_2009_71_01444	zraR_2
zraR_2	MAR_2009_71_01325	zraR_2
zraR_2	MAR_2009_71_03558	zraR_2
zraR_2	MAR_2009_71_00774	zraR_2
zraR_2	MAR_2009_71_00264	zraR_2
zraR_2	MAR_2009_71_03005	zraR_2
zraR_2	MAR_2009_71_00827	zraR_2
zraR_2	MAR_2009_71_03018	zraR_2
zraR_2	MAR_2009_71_03338	zraR_2
zraR_2	MAR_2009_71_03006	zraR_2
zraR_2	MAR_2009_71_00775	zraR_2
zraR_2	MAR_2009_71_03339	zraR_2
zraR_2	MAR_2009_71_02784	zraR_2
zraR_2	MAR_2009_71_02783	zraR_2
zraR_2	MAR_2009_71_02909	zraR_2
zraR_2	MAR_2009_71_03840	zraR_2
zraR_2	MAR_2009_71_00588	zraR_2
zraR_2	MAR_2009_71_03874	zraR_2
zraR_2	MAR_2009_71_02082	zraR_2
zraR_2	MAR_2009_71_00011	zraR_2
zraR_2	MAR_2009_71_00584	zraR_2

zraR_2	MAR_2009_71_01478	zraR_2
zraR_2	MAR_2009_71_01129	zraR_2
zraR_2	MAR_2009_71_01108	zraR_2
zraR_2	MAR_2009_71_02667	zraR_2
zraR_2	MAR_2009_71_03019	zraR_2
zraR_2	MAR_2009_71_02908	zraR_2
zraR_2	MAR_2009_71_02387	zraR_2
zraR_2	MAR_2009_71_02369	zraR_2
zraR_2	MAR_2009_71_03848	zraR_2
zraR_2	MAR_2009_71_03761	zraR_2
zraR_2	MAR_2009_71_03542	zraR_2
zraR_2	MAR_2009_71_00167	zraR_2
zraR_2	MAR_2009_71_03723	zraR_2
zraR_2	MAR_2009_71_00936	zraR_2
zraR_2	MAR_2009_71_02352	zraR_2
zraR_2	MAR_2009_71_02365	zraR_2
zraR_2	MAR_2009_71_00236	zraR_2
zraR_2	MAR_2009_71_01421	zraR_2
zraR_2	MAR_2009_71_00235	zraR_2
zraR_2	MAR_2009_71_01841	zraR_2
zraR_2	MAR_2009_71_00897	zraR_2
zraR_2	MAR_2009_71_01942	zraR_2
zraR_2	MAR_2009_71_00116	zraR_2
zraR_2	MAR_2009_71_02445	zraR_2
zraR_2	MAR_2009_71_03937	zraR_2
zraR_2	MAR_2009_71_02359	zraR_2
zraR_2	MAR_2009_71_03559	zraR_2
zraR_2	MAR_2009_71_02960	zraR_2
zraR_2	MAR_2009_71_02361	zraR_2
zraR_2	MAR_2009_71_03557	zraR_2
zraR_2	MAR_2009_71_01326	zraR_2
zraR_2	MAR_2009_71_02835	zraR_2
zraR_2	MAR_2009_71_03847	zraR_2
zraR_2	MAR_2009_71_00869	zraR_2
zraR_2	MAR_2009_71_01089	zraR_2
zraR_2	MAR_2009_71_03676	zraR_2
zraR_2	MAR_2009_71_00372	zraR_2
zraR_2	MAR_2009_71_02002	zraR_2
zraR_2	MAR_2009_71_01071	zraR_2
zraR_2	MAR_2009_71_00582	zraR_2
zraR_2	MAR_2009_71_00820	zraR_2
zraR_2	MAR_2009_71_03729	zraR_2
zraR_2	MAR_2009_71_00635	zraR_2
zraR_2	MAR_2009_71_02386	zraR_2
zraR_2	MAR_2009_71_00828	zraR_2
zraR_2	MAR_2009_71_02208	zraR_2
zraR_2	MAR_2009_71_00769	zraR_2
zraR_2	MAR_2009_71_02121	zraR_2

zraR_2	MAR_2009_71_03556	zraR_2
zraR_2	MAR_2009_71_00770	zraR_2
zraR_2	MAR_2009_71_01095	zraR_2
zraR_2	MAR_2009_71_03607	zraR_2
zraR_2	MAR_2009_71_01312	zraR_2
zraR_2	MAR_2009_71_01070	zraR_2
zraR_2	MAR_2009_71_03955	zraR_2
zraR_2	MAR_2009_71_02052	zraR_2
zraR_2	MAR_2009_71_02410	zraR_2
zraR_2	MAR_2009_71_03969	zraR_2
zraR_2	MAR_2009_71_01107	zraR_2
zraR_2	MAR_2009_71_00498	zraR_2
zraR_2	MAR_2009_71_01445	zraR_2
zraR_2	NN016038_00001	zraR_2
zraR_2	NN016038_02005	zraR_2
zraR_2	NN016038_03857	zraR_2
zraR_2	NN016038_03721	zraR_2
zraR_2	NN016038_02773	zraR_2
zraR_2	NN016038_01817	zraR_2
zraR_2	NN016038_03501	zraR_2
zraR_2	NN016038_00663	zraR_2
zraR_2	NN016038_00609	zraR_2
zraR_2	NN016038_03615	zraR_2
zraR_2	NN016038_03614	zraR_2
zraR_2	NN016038_03500	zraR_2
zraR_2	NN016038_00923	zraR_2
zraR_2	NN016038_03489	zraR_2
zraR_2	NN016038_02404	zraR_2
zraR_2	NN016038_00610	zraR_2
zraR_2	NN016038_00126	zraR_2
zraR_2	NN016038_02405	zraR_2
zraR_2	NN016038_00991	zraR_2
zraR_2	NN016038_00106	zraR_2
zraR_2	NN016038_01440	zraR_2
zraR_2	NN016038_01419	zraR_2
zraR_2	NN016038_02423	zraR_2
zraR_2	NN016038_01420	zraR_2
zraR_2	NN016038_03272	zraR_2
zraR_2	NN016038_03598	zraR_2
zraR_2	NN016038_01834	zraR_2
zraR_2	NN016038_00125	zraR_2
zraR_2	NN016038_01328	zraR_2
zraR_2	NN016038_02214	zraR_2
zraR_2	NN016038_03488	zraR_2
zraR_2	NN016038_01229	zraR_2
zraR_2	NN016038_03599	zraR_2
zraR_2	NN016038_02686	zraR_2
zraR_2	NN016038_00584	zraR_2

zraR_2	NN016038_03949	zraR_2
zraR_2	NN016038_01833	zraR_2
zraR_2	NN016038_00024	zraR_2
zraR_2	NN016038_04004	zraR_2
zraR_2	NN016038_02391	zraR_2
zraR_2	NN016038_01920	zraR_2
zraR_2	NN016038_01505	zraR_2
zraR_2	NN016038_00092	zraR_2
zraR_2	NN016038_03868	zraR_2
zraR_2	NN016038_00766	zraR_2
zraR_2	NN016038_03884	zraR_2
zraR_2	NN016038_00764	zraR_2
zraR_2	NN016038_00169	zraR_2
zraR_2	NN016038_01847	zraR_2
zraR_2	NN016038_03270	zraR_2
zraR_2	NN016038_02968	zraR_2
zraR_2	NN016038_02434	zraR_2
zraR_2	NN016038_01443	zraR_2
zraR_2	NN016038_03600	zraR_2
zraR_2	NN016038_02432	zraR_2
zraR_2	NN016038_00585	zraR_2
zraR_2	NN016038_00331	zraR_2
zraR_2	NN016038_01699	zraR_2
zraR_2	NN016038_01731	zraR_2
zraR_2	NN016038_01744	zraR_2
zraR_2	NN016038_01325	zraR_2
zraR_2	NN016038_00483	zraR_2
zraR_2	NN016038_02560	zraR_2
zraR_2	NN016038_00664	zraR_2
zraR_2	NN016038_00653	zraR_2
zraR_2	NN016038_03529	zraR_2
zraR_2	NN016038_01465	zraR_2
zraR_2	NN016038_03296	zraR_2
zraR_2	NN016038_00941	zraR_2
zraR_2	NN016038_01211	zraR_2
zraR_2	NN016038_02718	zraR_2
zraR_2	NN016038_04028	zraR_2
zraR_2	NN016038_01228	zraR_2
zraR_2	NN016038_01445	zraR_2
zraR_2	NN016038_03271	zraR_2
zraR_2	PAO1_04103	zraR_2
zraR_2	PAO1_01655	zraR_2
zraR_2	PAO1_04074	zraR_2
zraR_2	PAO1_03150	zraR_2
zraR_2	PAO1_05151	zraR_2
zraR_2	PAO1_00959	zraR_2
zraR_2	PAO1_03584	zraR_2
zraR_2	PAO1_05667	zraR_2

zraR_2	PAO1_02660	zraR_2
zraR_2	PAO1_05023	zraR_2
zraR_2	PAO1_04523	zraR_2
zraR_2	PAO1_02925	zraR_2
zraR_2	PAO1_04243	zraR_2
zraR_2	PAO1_00960	zraR_2
zraR_2	PAO1_05537	zraR_2
zraR_2	PAO1_01281	zraR_2
zraR_2	PAO1_03302	zraR_2
zraR_2	PAO1_02247	zraR_2
zraR_2	PAO1_00474	zraR_2
zraR_2	PAO1_02767	zraR_2
zraR_2	PAO1_01852	zraR_2
zraR_2	PAO1_01216	zraR_2
zraR_2	PAO1_04939	zraR_2
zraR_2	PAO1_02908	zraR_2
zraR_2	PAO1_04231	zraR_2
zraR_2	PAO1_01194	zraR_2
zraR_2	PAO1_01681	zraR_2
zraR_2	PAO1_00420	zraR_2
zraR_2	PAO1_02909	zraR_2
zraR_2	PAO1_01477	zraR_2
zraR_2	PAO1_01496	zraR_2
zraR_2	PAO1_05052	zraR_2
zraR_2	PAO1_03315	zraR_2
zraR_2	PAO1_05375	zraR_2
zraR_2	PAO1_03182	zraR_2
zraR_2	PAO1_01478	zraR_2
zraR_2	PAO1_00780	zraR_2
zraR_2	PAO1_00184	zraR_2
zraR_2	PAO1_04164	zraR_2
zraR_2	PAO1_02594	zraR_2
zraR_2	PAO1_05538	zraR_2
zraR_2	PAO1_00419	zraR_2
zraR_2	PAO1_03464	zraR_2
zraR_2	PAO1_03827	zraR_2
zraR_2	PAO1_02897	zraR_2
zraR_2	PAO1_00424	zraR_2
zraR_2	PAO1_02593	zraR_2
zraR_2	PAO1_05541	zraR_2
zraR_2	PAO1_04160	zraR_2
zraR_2	PAO1_04938	zraR_2
zraR_2	PAO1_01195	zraR_2
zraR_2	PAO1_04248	zraR_2
zraR_2	PAO1_04886	zraR_2
zraR_2	PAO1_01135	zraR_2
zraR_2	PAO1_02984	zraR_2
zraR_2	PAO1_02410	zraR_2

zraR_2	PAO1_01299	zraR_2
zraR_2	PAO1_04026	zraR_2
zraR_2	PAO1_02037	zraR_2
zraR_2	PAO1_02054	zraR_2
zraR_2	PAO1_03386	zraR_2
zraR_2	PAO1_00035	zraR_2
zraR_2	PAO1_05153	zraR_2
zraR_2	PAO1_04076	zraR_2
zraR_2	PAO1_04004	zraR_2
zraR_2	PAO1_02648	zraR_2
zraR_2	PAO1_02736	zraR_2
zraR_2	PAO1_01374	zraR_2
zraR_2	PAO1_04695	zraR_2
zraR_2	PAO1_03826	zraR_2
zraR_2	PAO1_02768	zraR_2
zraR_2	PAO1_03317	zraR_2
zraR_2	PAO1_01436	zraR_2
zraR_2	PAO1_00475	zraR_2
zraR_2	PAO1_05294	zraR_2
zraR_2	PAO1_00781	zraR_2
zraR_2	PAO1_05053	zraR_2
zraR_2	PAO1_00961	zraR_2
zraR_2	PAO1_01851	zraR_2
zraR_2	PAO1_05374	zraR_2
zraR_2	PAO1_04694	zraR_2
zraR_2	PAO1_03301	zraR_2
zraR_2	PAO1_01375	zraR_2
zraR_2	PAO1_04232	zraR_2
zraR_2	PAO1_02647	zraR_2
zraR_2	PAO1_04328	zraR_2
zraR_2	PAO1_00616	zraR_2
zraR_2	PAO1_02664	zraR_2
zraR_2	PAO1_01499	zraR_2
zraR_2	PAO1_04887	zraR_2
zraR_2	PAO1_02042	zraR_2
zraR_2	PAO1_01437	zraR_2
zraR_2	PAO1_03151	zraR_2
zraR_2	PAO1_03727	zraR_2
zraR_2	PAO1_00546	zraR_2
zraR_2	PAO1_04223	zraR_2
zraR_2	PAO1_02427	zraR_2
zraR_2	PAO1_02432	zraR_2
zraR_2	PAO1_01133	zraR_2
zraR_2	PAO1_02745	zraR_2
zraR_2	PAO1_02006	zraR_2
zraR_2	PAO1_05295	zraR_2
zraR_2	PAO1_05340	zraR_2
zraR_2	PAO1_05666	zraR_2

zraR_2	PAO1_05694	zraR_2
zraR_2	PAO1_02067	zraR_2
zraR_2	PAO1_01233	zraR_2
zraR_2	PAO1_02521	zraR_2
zraR_2	PAO1_01708	zraR_2
zraR_2	PAO1_04278	zraR_2
zraR_2	PAO1_04149	zraR_2
zraR_2	PAO1_00900	zraR_2
zraR_2	PAO1_04060	zraR_2
zraR_2	PAO1_04729	zraR_2
zraR_2	PAO1_00920	zraR_2
zraR_2	PAO1_02777	zraR_2
zraR_2	PAO1_03331	zraR_2
zraR_2	PAO1_05339	zraR_2
zraR_2	PAO1_04432	zraR_2
zraR_2	PAO1_04541	zraR_2
zraR_2	PAO1_01134	zraR_2
zraR_2	PAO1_02983	zraR_2
zraR_2	PAO1_02549	zraR_2
zraR_2	PAO1_04539	zraR_2
zraR_2	PAO1_04637	zraR_2
zraR_2	PAO1_04435	zraR_2
zraR_2	PAO1_04327	zraR_2
zraR_2	PAO1_01759	zraR_2
zraR_2	PAO1_04209	zraR_2
zraR_2	PAO1_02448	zraR_2
zraR_2	PAO1_02039	zraR_2
zraR_2	PAO1_03837	zraR_2
zraR_2	PAO1_01585	zraR_2
zraR_2	PAO1_00666	zraR_2
zraR_2	PAO1_00539	zraR_2
zraR_2	PAO1_04358	zraR_2
zraR_2	PAO1_03384	zraR_2
zraR_2	PAO1_02353	zraR_2
zraR_2	PAO1_02735	zraR_2
zraR_2	PAO1_03019	zraR_2
zraR_2	PAO1_02110	zraR_2
zraR_2	PAO1_01663	zraR_2
zraR_2	PAO1_00281	zraR_2
zraR_2	PAO1_00805	zraR_2
zraR_2	PAO1_03694	zraR_2
zraR_2	PAO1_00772	zraR_2
zraR_2	PAO1_05561	zraR_2
zraR_2	PAO1_03001	zraR_2
zraR_2	PAO1_01420	zraR_2
zraR_2	PAO1_04579	zraR_2
zraR_2	PAO1_04199	zraR_2
zraR_2	PAO1_00577	zraR_2

zraR_2	PAO1_05519	zraR_2
zraR_2	PAO1_02348	zraR_2
zraR_2	PAO1_03466	zraR_2
zraR_2	PAO1_00423	zraR_2
zraR_2	PAO1_00180	zraR_2
zraR_2	PAO1_03829	zraR_2
zraR_2	PAO1_00425	zraR_2
zraR_2	PAO1_04522	zraR_2
zraR_2	PAO1_04315	zraR_2
zraR_2	PAO1_04427	zraR_2
zraR_2	PAO1_01217	zraR_2
zraR_2	PAO1_03955	zraR_2
zraR_2	PAO1_03183	zraR_2
zraR_2	PAO1_01525	zraR_2
zraR_2	PAO1_01643	zraR_2
zraR_2	PAO1_02669	zraR_2
zraR_2	PAO1_02550	zraR_2
zraR_2	PAO1_02559	zraR_2
zraR_2	PAO1_03541	zraR_2
zraR_2	PAO1_02405	zraR_2
zraR_2	PAO1_04055	zraR_2
zraR_2	PAO1_02558	zraR_2
zraR_2	PAO1_00817	zraR_2
zraR_2	PAO1_01266	zraR_2
zraR_2	PAO1_00168	zraR_2
zraR_2	PAO1_01145	zraR_2
zraR_2	PAO1_00891	zraR_2
zraR_2	PAO1_04864	zraR_2
zraR_2	PAO1_01807	zraR_2
zraR_2	PAO1_03894	zraR_2
zraR_2	PAO1_01806	zraR_2
zraR_2	PAO1_04049	zraR_2
zraR_2	PAO1_02589	zraR_2
zraR_2	PAO1_03722	zraR_2
zraR_2	PAO1_00034	zraR_2
zraR_2	PAO1_04970	zraR_2
zraR_2	PAO1_04203	zraR_2
zraR_2	PAO1_02581	zraR_2
zraR_2	PAO1_02884	zraR_2
zraR_2	UW101_04366	zraR_2
zraR_2	UW101_04026	zraR_2
zraR_2	UW101_04037	zraR_2
zraR_2	UW101_02113	zraR_2
zraR_2	UW101_02176	zraR_2
zraR_2	UW101_03260	zraR_2
zraR_2	UW101_04093	zraR_2
zraR_2	UW101_04416	zraR_2
zraR_2	UW101_04911	zraR_2

zraR_2	UW101_03495	zraR_2
zraR_2	UW101_00247	zraR_2
zraR_2	UW101_00574	zraR_2
zraR_2	UW101_04765	zraR_2
zraR_2	UW101_00671	zraR_2
zraR_2	UW101_03608	zraR_2
zraR_2	UW101_03607	zraR_2
zraR_2	UW101_05183	zraR_2
zraR_2	UW101_04710	zraR_2
zraR_2	UW101_00575	zraR_2
zraR_2	UW101_03545	zraR_2
zraR_2	UW101_04468	zraR_2
zraR_2	UW101_03496	zraR_2
zraR_2	UW101_02853	zraR_2
zraR_2	UW101_04085	zraR_2
zraR_2	UW101_01507	zraR_2
zraR_2	UW101_04092	zraR_2
zraR_2	UW101_01686	zraR_2
zraR_2	UW101_03011	zraR_2
zraR_2	UW101_04180	zraR_2
zraR_2	UW101_04086	zraR_2
zraR_2	UW101_02043	zraR_2
zraR_2	UW101_01687	zraR_2
zraR_2	UW101_02058	zraR_2
zraR_2	UW101_04709	zraR_2
zraR_2	UW101_05182	zraR_2
zraR_2	UW101_05048	zraR_2
zraR_2	UW101_03501	zraR_2
zraR_2	UW101_02451	zraR_2
zraR_2	UW101_05164	zraR_2
zraR_2	UW101_01414	zraR_2
zraR_2	UW101_03902	zraR_2
zraR_2	UW101_00136	zraR_2
zraR_2	UW101_05146	zraR_2
zraR_2	UW101_03273	zraR_2
zraR_2	UW101_03986	zraR_2
zraR_2	UW101_03983	zraR_2
zraR_2	UW101_03951	zraR_2
zraR_2	UW101_03343	zraR_2
zraR_2	UW101_01404	zraR_2
zraR_2	UW101_04668	zraR_2
zraR_2	UW101_03906	zraR_2
zraR_2	UW101_04104	zraR_2
zraR_2	UW101_03010	zraR_2
zraR_2	UW101_02615	zraR_2
zraR_2	UW101_02427	zraR_2
zraR_2	UW101_03544	zraR_2
zraR_2	UW101_00985	zraR_2

zraR_2	UW101_03494	zraR_2
zraR_2	UW101_03187	zraR_2
zraR_2	UW101_02065	zraR_2
zraR_2	UW101_04823	zraR_2
zraR_2	UW101_03038	zraR_2
zraR_2	UW101_04469	zraR_2
zraR_2	UW101_00491	zraR_2
zraR_2	UW101_01393	zraR_2
zraR_2	UW101_05261	zraR_2
zraR_2	UW101_04934	zraR_2
zraR_2	UW101_04860	zraR_2
zraR_2	UW101_00044	zraR_2
zraR_2	UW101_02080	zraR_2
zraR_2	UW101_03109	zraR_2
zraR_2	UW101_00590	zraR_2
zraR_2	UW101_04499	zraR_2
zraR_2	UW101_01928	zraR_2
zraR_2	UW101_00672	zraR_2
zraR_2	UW101_01392	zraR_2
zraR_2	UW101_00492	zraR_2
zraR_2	UW101_04062	zraR_2
zraR_2	UW101_01658	zraR_2
zraR_2	UW101_02852	zraR_2
zraR_2	UW101_04827	zraR_2
zraR_2	UW101_03077	zraR_2
zraR_2	UW101_01867	zraR_2
zraR_2	UW101_02648	zraR_2
zraR_2	UW101_04635	zraR_2
zraR_2	UW101_00989	zraR_2
zraR_2	UW101_04448	zraR_2
zraR_2	UW101_03457	zraR_2
zraR_2	UW101_05029	zraR_2
zraR_2	UW101_02953	zraR_2
zraR_2	UW101_02084	zraR_2
zraR_2	UW101_04122	zraR_2
zraR_2	UW101_03499	zraR_2
zraR_2	UW101_01649	zraR_2
zraR_2	UW101_04222	zraR_2
zraR_2	UW101_04060	zraR_2
zraR_2	UW101_03493	zraR_2
zraR_2	UW101_03492	zraR_2
zraR_2	UW101_00188	zraR_2
zraR_2	UW101_04393	zraR_2
zraR_2	UW101_04599	zraR_2
zraR_2	UW101_03911	zraR_2
zraR_2	UW101_04169	zraR_2
zraR_2	UW101_00169	zraR_2
zraR_2	UW101_04278	zraR_2

zraR_2	V4-33_00336	zraR_2
zraR_2	V4-33_01379	zraR_2
zraR_2	V4-33_00335	zraR_2
zraR_2	V4-33_01737	zraR_2
zraR_2	V4-33_01310	zraR_2
zraR_2	V4-33_01738	zraR_2
zraR_2	V4-33_01428	zraR_2
zraR_2	V4-33_01593	zraR_2
zraR_2	V4-33_01309	zraR_2
zraR_2	V4-33_00929	zraR_2
zraR_2	V4-33_00927	zraR_2
zraR_2	V4-33_00901	zraR_2
zraR_2	V4-33_01600	zraR_2
zraR_2	V4-33_00034	zraR_2
zraR_2	V4-33_00639	zraR_2
zraR_2	V4-33_00688	zraR_2
zraR_2	V4-33_01895	zraR_2
zraR_2	V4-33_02270	zraR_2
zraR_2	V4-33_00976	zraR_2
zraR_2	V4-33_00676	zraR_2
zraR_2	V4-33_01986	zraR_2
zraR_2	V4-33_01062	zraR_2
zraR_2	V4-33_00031	zraR_2
zraR_2	V4-33_01450	zraR_2
zraR_2	V4-33_00765	zraR_2
zraR_2	V4-33_00230	zraR_2
zraR_2	V4-33_00915	zraR_2
zraR_2	V4-33_01665	zraR_2
zraR_2	W5C_03170	zraR_2
zraR_2	W5C_02711	zraR_2
zraR_2	W5C_02718	zraR_2
zraR_2	W5C_01015	zraR_2
zraR_2	W5C_00994	zraR_2
zraR_2	W5C_01240	zraR_2
zraR_2	W5C_01546	zraR_2
zraR_2	W5C_02207	zraR_2
zraR_2	W5C_00891	zraR_2
zraR_2	W5C_01877	zraR_2
zraR_2	W5C_02206	zraR_2
zraR_2	W5C_01547	zraR_2
zraR_2	W5C_01878	zraR_2
zraR_2	W5C_00890	zraR_2
zraR_2	W5C_01741	zraR_2
zraR_2	W5C_02701	zraR_2
zraR_2	W5C_02702	zraR_2
zraR_2	W5C_00342	zraR_2
zraR_2	W5C_01742	zraR_2
zraR_2	W5C_02019	zraR_2

zraR_2	W5C_01027	zraR_2
zraR_2	W5C_00524	zraR_2
zraR_2	W5C_02788	zraR_2
zraR_2	W5C_01616	zraR_2
zraR_2	W5C_01037	zraR_2
zraR_2	W5C_00803	zraR_2
zraR_2	W5C_01791	zraR_2
zraR_2	W5C_01957	zraR_2
zraR_2	W5C_00123	zraR_2
zraR_2	W5C_00501	zraR_2
zraR_2	W5C_01636	zraR_2
zraR_2	W5C_03320	zraR_2
zraR_2	W5C_01634	zraR_2
zraR_2	W5C_02196	zraR_2
zraR_2	W5C_01624	zraR_2
zraR_2	W5C_02227	zraR_2
zraR_2	W5C_02195	zraR_2
zraR_2	W5C_02020	zraR_2
zraR_2	W5C_02037	zraR_2
zraR_2	W5C_01380	zraR_2
zraR_2	W5C_02364	zraR_2
zraR_2	W5C_03021	zraR_2
zraR_2	W5C_01023	zraR_2
zraR_2	W5C_01406	zraR_2
zraR_2	W5C_02036	zraR_2
zraR_2	W5C_02770	zraR_2
zraR_2	W5C_03141	zraR_2
zraR_2	W5C_03060	zraR_2
zraR_2	W5C_01386	zraR_2
zraR_2	W5C_02995	zraR_2
zraR_2	W5C_03321	zraR_2
zraR_2	W5C_02978	zraR_2
zraR_2	W5C_00259	zraR_2
zraR_2	W5C_03304	zraR_2
zraR_2	W5C_02600	zraR_2
zraR_2	ZONMW-01_01881	zraR_2
zraR_2	ZONMW-01_02828	zraR_2
zraR_2	ZONMW-01_02837	zraR_2
zraR_2	ZONMW-01_01527	zraR_2
zraR_2	ZONMW-01_02798	zraR_2
zraR_2	ZONMW-01_02540	zraR_2
zraR_2	ZONMW-01_00773	zraR_2
zraR_2	ZONMW-01_03135	zraR_2
zraR_2	ZONMW-01_00774	zraR_2
zraR_2	ZONMW-01_02541	zraR_2
zraR_2	ZONMW-01_03399	zraR_2
zraR_2	ZONMW-01_03134	zraR_2
zraR_2	ZONMW-01_03400	zraR_2

zraR_2	ZONMW-01_00594	zraR_2
zraR_2	ZONMW-01_01199	zraR_2
zraR_2	ZONMW-01_01198	zraR_2
zraR_2	ZONMW-01_00095	zraR_2
zraR_2	ZONMW-01_01088	zraR_2
zraR_2	ZONMW-01_01575	zraR_2
zraR_2	ZONMW-01_01087	zraR_2
zraR_2	ZONMW-01_01026	zraR_2
zraR_2	ZONMW-01_00292	zraR_2
zraR_2	ZONMW-01_01963	zraR_2
zraR_2	ZONMW-01_01034	zraR_2
zraR_2	ZONMW-01_01585	zraR_2
zraR_2	ZONMW-01_01234	zraR_2
zraR_2	ZONMW-01_03047	zraR_2
zraR_2	ZONMW-01_00542	zraR_2
zraR_2	ZONMW-01_01156	zraR_2
zraR_2	ZONMW-01_00269	zraR_2
zraR_2	ZONMW-01_01016	zraR_2
zraR_2	ZONMW-01_01014	zraR_2
zraR_2	ZONMW-01_02098	zraR_2
zraR_2	ZONMW-01_00593	zraR_2
zraR_2	ZONMW-01_00785	zraR_2
zraR_2	ZONMW-01_00754	zraR_2
zraR_2	ZONMW-01_00786	zraR_2
zraR_2	ZONMW-01_01071	zraR_2
zraR_2	ZONMW-01_02651	zraR_2
zraR_2	ZONMW-01_00618	zraR_2
zraR_2	ZONMW-01_00487	zraR_2
zraR_2	ZONMW-01_02922	zraR_2
zraR_2	ZONMW-01_02673	zraR_2
zraR_2	ZONMW-01_01072	zraR_2
zraR_2	ZONMW-01_01777	zraR_2
zraR_2	ZONMW-01_01917	zraR_2
zraR_2	ZONMW-01_00512	zraR_2
zraR_2	ZONMW-01_01505	zraR_2
zraR_2	ZONMW-01_00008	zraR_2
zraR_2	ZONMW-01_01317	zraR_2
zraR_2	ZONMW-01_01571	zraR_2
zraR_2	ZONMW-01_02472	zraR_2
zraR_2	ZONMW-02_02997	zraR_2
zraR_2	ZONMW-02_00543	zraR_2
zraR_2	ZONMW-02_02297	zraR_2
zraR_2	ZONMW-02_01204	zraR_2
zraR_2	ZONMW-02_00493	zraR_2
zraR_2	ZONMW-02_02989	zraR_2
zraR_2	ZONMW-02_02282	zraR_2
zraR_2	ZONMW-02_02534	zraR_2
zraR_2	ZONMW-02_02637	zraR_2

zraR_2	ZONMW-02_00247	zraR_2
zraR_2	ZONMW-02_03390	zraR_2
zraR_2	ZONMW-02_03053	zraR_2
zraR_2	ZONMW-02_03598	zraR_2
zraR_2	ZONMW-02_02857	zraR_2
zraR_2	ZONMW-02_00206	zraR_2
zraR_2	ZONMW-02_02858	zraR_2
zraR_2	ZONMW-02_00780	zraR_2
zraR_2	ZONMW-02_03041	zraR_2
zraR_2	ZONMW-02_00781	zraR_2
zraR_2	ZONMW-02_01163	zraR_2
zraR_2	ZONMW-02_02197	zraR_2
zraR_2	ZONMW-02_01626	zraR_2
zraR_2	ZONMW-02_03447	zraR_2
zraR_2	ZONMW-02_03786	zraR_2
zraR_2	ZONMW-02_02131	zraR_2
zraR_2	ZONMW-02_01164	zraR_2
zraR_2	ZONMW-02_02087	zraR_2
zraR_2	ZONMW-02_00565	zraR_2
zraR_2	ZONMW-02_03474	zraR_2
zraR_2	ZONMW-02_00093	zraR_2
zraR_2	ZONMW-02_03445	zraR_2
zraR_2	ZONMW-02_02718	zraR_2
zraR_2	ZONMW-02_01291	zraR_2
zraR_2	ZONMW-02_01293	zraR_2
zraR_2	ZONMW-02_00517	zraR_2
zraR_2	ZONMW-02_01677	zraR_2
zraR_2	ZONMW-02_03634	zraR_2
zraR_2	ZONMW-02_01795	zraR_2
zraR_2	ZONMW-02_01219	zraR_2
zraR_2	ZONMW-02_00913	zraR_2
zraR_2	ZONMW-02_00583	zraR_2
zraR_2	ZONMW-02_01896	zraR_2
zraR_2	ZONMW-02_01509	zraR_2
zraR_2	ZONMW-02_02624	zraR_2
zraR_2	ZONMW-02_03421	zraR_2
zraR_2	ZONMW-02_02836	zraR_2
zraR_2	ZONMW-02_01893	zraR_2
zraR_2	ZONMW-02_03476	zraR_2
zraR_2	ZONMW-02_01562	zraR_2
zraR_2	ZONMW-02_01670	zraR_2
zraR_2	ZONMW-02_03488	zraR_2
zraR_2	ZONMW-02_03487	zraR_2
zraR_2	ZONMW-02_03129	zraR_2
zraR_2	ZONMW-02_03348	zraR_2
zraR_2	ZONMW-02_01588	zraR_2
zraR_2	ZONMW-02_03468	zraR_2
zraR_2	ZONMW-02_02348	zraR_2

zraR_2	ZONMW-02_00424	zraR_2
zraR_2	ZONMW-02_00310	zraR_2
zraR_2	ZONMW-02_01680	zraR_2
zraR_2	ZONMW-02_00569	zraR_2
zraR_2	ZONMW-02_03655	zraR_2
zraR_2	ZONMW-02_01294	zraR_2
zraR_2	ZONMW-02_03231	zraR_2
zraR_2	ZONMW-02_03597	zraR_2
zraR_2	ZONMW-02_03752	zraR_2
zraR_2	ZONMW-02_02912	zraR_2
zraR_2	ZONMW-02_03159	zraR_2
zraR_2	ZONMW-02_00869	zraR_2
zraR_2	ZONMW-02_03503	zraR_2
zraR_2	ZONMW-02_01161	zraR_2
zraR_2	ZONMW-02_02453	zraR_2
zraR_2	ZONMW-02_02038	zraR_2
zraR_2	ZONMW-02_02768	zraR_2
zraR_2	ZONMW-02_01443	zraR_2
zraR_2	ZONMW-02_03446	zraR_2
zraR_2	ZONMW-03_02865	zraR_2
zraR_2	ZONMW-03_01591	zraR_2
zraR_2	ZONMW-03_01600	zraR_2
zraR_2	ZONMW-03_00680	zraR_2
zraR_2	ZONMW-03_03108	zraR_2
zraR_2	ZONMW-03_01821	zraR_2
zraR_2	ZONMW-03_00151	zraR_2
zraR_2	ZONMW-03_02370	zraR_2
zraR_2	ZONMW-03_00152	zraR_2
zraR_2	ZONMW-03_01822	zraR_2
zraR_2	ZONMW-03_03432	zraR_2
zraR_2	ZONMW-03_02369	zraR_2
zraR_2	ZONMW-03_03431	zraR_2
zraR_2	ZONMW-03_02488	zraR_2
zraR_2	ZONMW-03_01581	zraR_2
zraR_2	ZONMW-03_01582	zraR_2
zraR_2	ZONMW-03_00821	zraR_2
zraR_2	ZONMW-03_02157	zraR_2
zraR_2	ZONMW-03_00632	zraR_2
zraR_2	ZONMW-03_02156	zraR_2
zraR_2	ZONMW-03_02095	zraR_2
zraR_2	ZONMW-03_01018	zraR_2
zraR_2	ZONMW-03_02947	zraR_2
zraR_2	ZONMW-03_02103	zraR_2
zraR_2	ZONMW-03_00622	zraR_2
zraR_2	ZONMW-03_01546	zraR_2
zraR_2	ZONMW-03_02282	zraR_2
zraR_2	ZONMW-03_02436	zraR_2
zraR_2	ZONMW-03_02225	zraR_2

zraR_2	ZONMW-03_00995	zraR_2
zraR_2	ZONMW-03_02085	zraR_2
zraR_2	ZONMW-03_02083	zraR_2
zraR_2	ZONMW-03_02612	zraR_2
zraR_2	ZONMW-03_02487	zraR_2
zraR_2	ZONMW-03_00163	zraR_2
zraR_2	ZONMW-03_00132	zraR_2
zraR_2	ZONMW-03_00164	zraR_2
zraR_2	ZONMW-03_02140	zraR_2
zraR_2	ZONMW-03_01677	zraR_2
zraR_2	ZONMW-03_02512	zraR_2
zraR_2	ZONMW-03_01213	zraR_2
zraR_2	ZONMW-03_01934	zraR_2
zraR_2	ZONMW-03_01699	zraR_2
zraR_2	ZONMW-03_02141	zraR_2
zraR_2	ZONMW-03_02762	zraR_2
zraR_2	ZONMW-03_02901	zraR_2
zraR_2	ZONMW-03_01238	zraR_2
zraR_2	ZONMW-03_01275	zraR_2
zraR_2	ZONMW-03_00733	zraR_2
zraR_2	ZONMW-03_01463	zraR_2
zraR_2	ZONMW-03_00636	zraR_2
zraR_2	ZONMW-03_03075	zraR_2
zraR_2	ZONMW-04_02585	zraR_2
zraR_2	ZONMW-04_00091	zraR_2
zraR_2	ZONMW-04_00082	zraR_2
zraR_2	ZONMW-04_02667	zraR_2
zraR_2	ZONMW-04_02808	zraR_2
zraR_2	ZONMW-04_01198	zraR_2
zraR_2	ZONMW-04_03026	zraR_2
zraR_2	ZONMW-04_01155	zraR_2
zraR_2	ZONMW-04_00834	zraR_2
zraR_2	ZONMW-04_01156	zraR_2
zraR_2	ZONMW-04_03027	zraR_2
zraR_2	ZONMW-04_03368	zraR_2
zraR_2	ZONMW-04_00833	zraR_2
zraR_2	ZONMW-04_03367	zraR_2
zraR_2	ZONMW-04_00969	zraR_2
zraR_2	ZONMW-04_00101	zraR_2
zraR_2	ZONMW-04_02125	zraR_2
zraR_2	ZONMW-04_01661	zraR_2
zraR_2	ZONMW-04_00100	zraR_2
zraR_2	ZONMW-04_02618	zraR_2
zraR_2	ZONMW-04_01662	zraR_2
zraR_2	ZONMW-04_01724	zraR_2
zraR_2	ZONMW-04_00654	zraR_2
zraR_2	ZONMW-04_02500	zraR_2
zraR_2	ZONMW-04_01716	zraR_2

zraR_2	ZONMW-04_02608	zraR_2
zraR_2	ZONMW-04_00747	zraR_2
zraR_2	ZONMW-04_00917	zraR_2
zraR_2	ZONMW-04_01591	zraR_2
zraR_2	ZONMW-04_00677	zraR_2
zraR_2	ZONMW-04_01734	zraR_2
zraR_2	ZONMW-04_01736	zraR_2
zraR_2	ZONMW-04_01943	zraR_2
zraR_2	ZONMW-04_00968	zraR_2
zraR_2	ZONMW-04_01167	zraR_2
zraR_2	ZONMW-04_01136	zraR_2
zraR_2	ZONMW-04_01168	zraR_2
zraR_2	ZONMW-04_01678	zraR_2
zraR_2	ZONMW-04_02315	zraR_2
zraR_2	ZONMW-04_00993	zraR_2
zraR_2	ZONMW-04_00459	zraR_2
zraR_2	ZONMW-04_02930	zraR_2
zraR_2	ZONMW-04_02293	zraR_2
zraR_2	ZONMW-04_01677	zraR_2
zraR_2	ZONMW-04_02761	zraR_2
zraR_2	ZONMW-04_02548	zraR_2
zraR_2	ZONMW-04_00433	zraR_2
zraR_2	ZONMW-04_00415	zraR_2
zraR_2	ZONMW-04_02211	zraR_2
zraR_2	ZONMW-04_00232	zraR_2
zraR_2	ZONMW-04_02622	zraR_2
zraR_2	ZONMW-05_02214	zraR_2
zraR_2	ZONMW-05_01739	zraR_2
zraR_2	ZONMW-05_01748	zraR_2
zraR_2	ZONMW-05_02907	zraR_2
zraR_2	ZONMW-05_03065	zraR_2
zraR_2	ZONMW-05_03270	zraR_2
zraR_2	ZONMW-05_00406	zraR_2
zraR_2	ZONMW-05_00104	zraR_2
zraR_2	ZONMW-05_00407	zraR_2
zraR_2	ZONMW-05_03271	zraR_2
zraR_2	ZONMW-05_03361	zraR_2
zraR_2	ZONMW-05_00103	zraR_2
zraR_2	ZONMW-05_03360	zraR_2
zraR_2	ZONMW-05_00221	zraR_2
zraR_2	ZONMW-05_01729	zraR_2
zraR_2	ZONMW-05_01010	zraR_2
zraR_2	ZONMW-05_01940	zraR_2
zraR_2	ZONMW-05_01730	zraR_2
zraR_2	ZONMW-05_00894	zraR_2
zraR_2	ZONMW-05_01941	zraR_2
zraR_2	ZONMW-05_02003	zraR_2
zraR_2	ZONMW-05_00419	zraR_2

zraR_2	ZONMW-05_01171	zraR_2
zraR_2	ZONMW-05_02131	zraR_2
zraR_2	ZONMW-05_01995	zraR_2
zraR_2	ZONMW-05_00884	zraR_2
zraR_2	ZONMW-05_01684	zraR_2
zraR_2	ZONMW-05_00016	zraR_2
zraR_2	ZONMW-05_00169	zraR_2
zraR_2	ZONMW-05_01871	zraR_2
zraR_2	ZONMW-05_01148	zraR_2
zraR_2	ZONMW-05_02013	zraR_2
zraR_2	ZONMW-05_02015	zraR_2
zraR_2	ZONMW-05_02426	zraR_2
zraR_2	ZONMW-05_00220	zraR_2
zraR_2	ZONMW-05_00418	zraR_2
zraR_2	ZONMW-05_00387	zraR_2
zraR_2	ZONMW-05_01957	zraR_2
zraR_2	ZONMW-05_02831	zraR_2
zraR_2	ZONMW-05_00245	zraR_2
zraR_2	ZONMW-05_01366	zraR_2
zraR_2	ZONMW-05_02995	zraR_2
zraR_2	ZONMW-05_02809	zraR_2
zraR_2	ZONMW-05_01956	zraR_2
zraR_2	ZONMW-05_02311	zraR_2
zraR_2	ZONMW-05_02177	zraR_2
zraR_2	ZONMW-05_01392	zraR_2
zraR_2	ZONMW-05_01410	zraR_2
zraR_2	ZONMW-05_00926	zraR_2
zraR_2	ZONMW-05_01592	zraR_2
zraR_2	ZONMW-05_00898	zraR_2
zraR_2	ZONMW-05_02655	zraR_2
zraR_2	ZONMW-06_01378	zraR_2
zraR_2	ZONMW-06_02356	zraR_2
zraR_2	ZONMW-06_01220	zraR_2
zraR_2	ZONMW-06_01870	zraR_2
zraR_2	ZONMW-06_03814	zraR_2
zraR_2	ZONMW-06_02871	zraR_2
zraR_2	ZONMW-06_01307	zraR_2
zraR_2	ZONMW-06_00815	zraR_2
zraR_2	ZONMW-06_00052	zraR_2
zraR_2	ZONMW-06_03169	zraR_2
zraR_2	ZONMW-06_01010	zraR_2
zraR_2	ZONMW-06_00427	zraR_2
zraR_2	ZONMW-06_03815	zraR_2
zraR_2	ZONMW-06_01527	zraR_2
zraR_2	ZONMW-06_03764	zraR_2
zraR_2	ZONMW-06_00051	zraR_2
zraR_2	ZONMW-06_01511	zraR_2
zraR_2	ZONMW-06_03379	zraR_2

zraR_2	ZONMW-06_03734	zraR_2
zraR_2	ZONMW-06_02912	zraR_2
zraR_2	ZONMW-06_03545	zraR_2
zraR_2	ZONMW-06_00257	zraR_2
zraR_2	ZONMW-06_03763	zraR_2
zraR_2	ZONMW-06_03463	zraR_2
zraR_2	ZONMW-06_01221	zraR_2
zraR_2	ZONMW-06_01534	zraR_2
zraR_2	ZONMW-06_03818	zraR_2
zraR_2	ZONMW-06_00441	zraR_2
zraR_2	ZONMW-06_03735	zraR_2
zraR_2	ZONMW-06_00219	zraR_2
zraR_2	ZONMW-06_03405	zraR_2
zraR_2	ZONMW-06_03926	zraR_2
zraR_2	ZONMW-06_01093	zraR_2
zraR_2	ZONMW-06_02911	zraR_2
zraR_2	ZONMW-06_01950	zraR_2
zraR_2	ZONMW-06_00675	zraR_2
zraR_2	ZONMW-06_02058	zraR_2
zraR_2	ZONMW-06_00816	zraR_2
zraR_2	ZONMW-06_01512	zraR_2
zraR_2	ZONMW-06_01740	zraR_2
zraR_2	ZONMW-06_03397	zraR_2
zraR_2	ZONMW-06_03799	zraR_2
zraR_2	ZONMW-06_01225	zraR_2
zraR_2	ZONMW-06_01380	zraR_2
zraR_2	ZONMW-06_01224	zraR_2
zraR_2	ZONMW-06_00475	zraR_2
zraR_2	ZONMW-06_02057	zraR_2
zraR_2	ZONMW-06_03314	zraR_2
zraR_2	ZONMW-06_03975	zraR_2
zraR_2	ZONMW-06_03677	zraR_2
zraR_2	ZONMW-06_00050	zraR_2
zraR_2	ZONMW-06_01741	zraR_2
zraR_2	ZONMW-06_00428	zraR_2
zraR_2	ZONMW-06_00262	zraR_2
zraR_2	ZONMW-06_02933	zraR_2
zraR_2	ZONMW-06_03974	zraR_2
zraR_2	ZONMW-06_03678	zraR_2
zraR_2	ZONMW-06_01528	zraR_2
zraR_2	ZONMW-06_03447	zraR_2
zraR_2	ZONMW-06_02408	zraR_2
zraR_2	ZONMW-06_03376	zraR_2
zraR_2	ZONMW-06_00577	zraR_2
zraR_2	ZONMW-06_03817	zraR_2
zraR_2	ZONMW-06_02848	zraR_2
zraR_2	ZONMW-06_03446	zraR_2
zraR_2	ZONMW-06_00137	zraR_2

zraR_2	ZONMW-06_01949	zraR_2
zraR_2	ZONMW-06_03407	zraR_2
zraR_2	ZONMW-06_02647	zraR_2
zraR_2	ZONMW-06_02932	zraR_2
zraR_2	ZONMW-06_00744	zraR_2
zraR_2	ZONMW-06_03780	zraR_2
zraR_2	ZONMW-06_01364	zraR_2
zraR_2	ZONMW-06_00834	zraR_2
zraR_2	ZONMW-06_03240	zraR_2
zraR_2	ZONMW-06_03287	zraR_2
zraR_2	ZONMW-06_03406	zraR_2
zraR_2	ZONMW-06_01226	zraR_2
zraR_2	ZONMW-06_03462	zraR_2
zraR_2	ZONMW-06_00264	zraR_2
zraR_2	ZONMW-06_01547	zraR_2
zraR_2	ZONMW-06_02296	zraR_2
zraR_2	ZONMW-06_00476	zraR_2
zraR_2	ZONMW-06_03547	zraR_2
zraR_2	ZONMW-06_01069	zraR_2
zraR_2	ZONMW-06_00136	zraR_2
zraR_2	ZONMW-06_00220	zraR_2
zraR_2	ZONMW-06_02967	zraR_2
zraR_2	ZONMW-06_01707	zraR_2
zraR_2	ZONMW-06_01235	zraR_2
zraR_2	ZONMW-06_02327	zraR_2
zraR_2	ZONMW-06_00646	zraR_2
zraR_2	ZONMW-06_02745	zraR_2
zraR_2	ZONMW-06_00263	zraR_2
zraR_2	ZONMW-06_01004	zraR_2
zraR_2	ZONMW-06_01760	zraR_2
zraR_2	ZONMW-06_00238	zraR_2
zraR_2	ZONMW-06_01533	zraR_2
zraR_2	ZONMW-06_03798	zraR_2
zraR_2	ZONMW-06_01058	zraR_2
zraR_2	ZONMW-06_02428	zraR_2
zraR_2	ZONMW-06_01092	zraR_2
zraR_2	ZONMW-06_01094	zraR_2
zraR_2	ZONMW-06_00449	zraR_2
zraR_2	ZONMW-06_01766	zraR_2
zraR_2	ZONMW-06_00143	zraR_2
zraR_2	ZONMW-06_02094	zraR_2
zraR_2	ZONMW-07_02096	zraR_2
zraR_2	ZONMW-07_00881	zraR_2
zraR_2	ZONMW-07_01097	zraR_2
zraR_2	ZONMW-07_01625	zraR_2
zraR_2	ZONMW-07_01361	zraR_2
zraR_2	ZONMW-07_01283	zraR_2
zraR_2	ZONMW-07_04016	zraR_2

zraR_2	ZONMW-07_03047	zraR_2
zraR_2	ZONMW-07_00432	zraR_2
zraR_2	ZONMW-07_00417	zraR_2
zraR_2	ZONMW-07_02965	zraR_2
zraR_2	ZONMW-07_03000	zraR_2
zraR_2	ZONMW-07_03445	zraR_2
zraR_2	ZONMW-07_00099	zraR_2
zraR_2	ZONMW-07_02784	zraR_2
zraR_2	ZONMW-07_02814	zraR_2
zraR_2	ZONMW-07_00573	zraR_2
zraR_2	ZONMW-07_00574	zraR_2
zraR_2	ZONMW-07_02936	zraR_2
zraR_2	ZONMW-07_02924	zraR_2
zraR_2	ZONMW-07_00644	zraR_2
zraR_2	ZONMW-07_04044	zraR_2
zraR_2	ZONMW-07_03374	zraR_2
zraR_2	ZONMW-07_01025	zraR_2
zraR_2	ZONMW-07_02935	zraR_2
zraR_2	ZONMW-07_03375	zraR_2
zraR_2	ZONMW-07_02923	zraR_2
zraR_2	ZONMW-07_02585	zraR_2
zraR_2	ZONMW-07_00442	zraR_2
zraR_2	ZONMW-07_00783	zraR_2
zraR_2	ZONMW-07_02590	zraR_2
zraR_2	ZONMW-07_03691	zraR_2
zraR_2	ZONMW-07_01379	zraR_2
zraR_2	ZONMW-07_02420	zraR_2
zraR_2	ZONMW-07_03194	zraR_2
zraR_2	ZONMW-07_03634	zraR_2
zraR_2	ZONMW-07_01306	zraR_2
zraR_2	ZONMW-07_03989	zraR_2
zraR_2	ZONMW-07_02523	zraR_2
zraR_2	ZONMW-07_01841	zraR_2
zraR_2	ZONMW-07_01100	zraR_2
zraR_2	ZONMW-07_04070	zraR_2
zraR_2	ZONMW-07_01961	zraR_2
zraR_2	ZONMW-07_01969	zraR_2
zraR_2	ZONMW-07_03883	zraR_2
zraR_2	ZONMW-07_00978	zraR_2
zraR_2	ZONMW-07_00894	zraR_2
zraR_2	ZONMW-07_02191	zraR_2
zraR_2	ZONMW-07_00977	zraR_2
zraR_2	ZONMW-07_00059	zraR_2
zraR_2	ZONMW-07_03761	zraR_2
zraR_2	ZONMW-07_01613	zraR_2
zraR_2	ZONMW-07_03726	zraR_2
zraR_2	ZONMW-07_00202	zraR_2
zraR_2	ZONMW-07_03096	zraR_2

zraR_2	ZONMW-07_02827	zraR_2
zraR_2	ZONMW-07_03279	zraR_2
zraR_2	ZONMW-07_03730	zraR_2
zraR_2	ZONMW-07_02271	zraR_2
zraR_2	ZONMW-07_03733	zraR_2
zraR_2	ZONMW-07_00437	zraR_2
zraR_2	ZONMW-07_00645	zraR_2
zraR_2	ZONMW-07_02533	zraR_2
zraR_2	ZONMW-07_03195	zraR_2
zraR_2	ZONMW-07_01026	zraR_2
zraR_2	ZONMW-07_04063	zraR_2
zraR_2	ZONMW-07_03282	zraR_2
zraR_2	ZONMW-07_02358	zraR_2
zraR_2	ZONMW-07_01163	zraR_2
zraR_2	ZONMW-07_00635	zraR_2
zraR_2	ZONMW-07_00480	zraR_2
zraR_2	ZONMW-07_01796	zraR_2
zraR_2	ZONMW-07_02567	zraR_2
zraR_2	ZONMW-07_04034	zraR_2
zraR_2	ZONMW-07_03288	zraR_2
zraR_2	ZONMW-07_00256	zraR_2
zraR_2	ZONMW-07_01396	zraR_2
zraR_2	ZONMW-07_04155	zraR_2
zraR_2	ZONMW-07_03782	zraR_2
zraR_2	ZONMW-07_03061	zraR_2
zraR_2	ZONMW-07_00780	zraR_2
zraR_2	ZONMW-07_00624	zraR_2
zraR_2	ZONMW-07_03916	zraR_2
zraR_2	ZONMW-07_00459	zraR_2
zraR_2	ZONMW-07_01271	zraR_2
zraR_2	ZONMW-07_02272	zraR_2
zraR_2	ZONMW-07_03457	zraR_2
zraR_2	ZONMW-07_01310	zraR_2
zraR_2	ZONMW-08_03140	zraR_2
zraR_2	ZONMW-08_03183	zraR_2
zraR_2	ZONMW-08_01080	zraR_2
zraR_2	ZONMW-08_01926	zraR_2
zraR_2	ZONMW-08_01462	zraR_2
zraR_2	ZONMW-08_01869	zraR_2
zraR_2	ZONMW-08_02366	zraR_2
zraR_2	ZONMW-08_00292	zraR_2
zraR_2	ZONMW-08_03340	zraR_2
zraR_2	ZONMW-08_02205	zraR_2
zraR_2	ZONMW-08_00345	zraR_2
zraR_2	ZONMW-08_02218	zraR_2
zraR_2	ZONMW-08_02852	zraR_2
zraR_2	ZONMW-08_02206	zraR_2
zraR_2	ZONMW-08_00293	zraR_2

zraR_2	ZONMW-08_02853	zraR_2
zraR_2	ZONMW-08_02001	zraR_2
zraR_2	ZONMW-08_02000	zraR_2
zraR_2	ZONMW-08_02108	zraR_2
zraR_2	ZONMW-08_00104	zraR_2
zraR_2	ZONMW-08_03880	zraR_2
zraR_2	ZONMW-08_00100	zraR_2
zraR_2	ZONMW-08_01176	zraR_2
zraR_2	ZONMW-08_00651	zraR_2
zraR_2	ZONMW-08_00626	zraR_2
zraR_2	ZONMW-08_03671	zraR_2
zraR_2	ZONMW-08_02219	zraR_2
zraR_2	ZONMW-08_02107	zraR_2
zraR_2	ZONMW-08_00633	zraR_2
zraR_2	ZONMW-08_00854	zraR_2
zraR_2	ZONMW-08_00877	zraR_2
zraR_2	ZONMW-08_03182	zraR_2
zraR_2	ZONMW-08_02382	zraR_2
zraR_2	ZONMW-08_02883	zraR_2
zraR_2	ZONMW-08_03220	zraR_2
zraR_2	ZONMW-08_00456	zraR_2
zraR_2	ZONMW-08_00894	zraR_2
zraR_2	ZONMW-08_00881	zraR_2
zraR_2	ZONMW-08_03368	zraR_2
zraR_2	ZONMW-08_01772	zraR_2
zraR_2	ZONMW-08_03369	zraR_2
zraR_2	ZONMW-08_01383	zraR_2
zraR_2	ZONMW-08_00417	zraR_2
zraR_2	ZONMW-08_01283	zraR_2
zraR_2	ZONMW-08_02935	zraR_2
zraR_2	ZONMW-08_03444	zraR_2
zraR_2	ZONMW-08_03121	zraR_2
zraR_2	ZONMW-08_03071	zraR_2
zraR_2	ZONMW-08_00887	zraR_2
zraR_2	ZONMW-08_01730	zraR_2
zraR_2	ZONMW-08_02365	zraR_2
zraR_2	ZONMW-08_02159	zraR_2
zraR_2	ZONMW-08_00885	zraR_2
zraR_2	ZONMW-08_02367	zraR_2
zraR_2	ZONMW-08_01868	zraR_2
zraR_2	ZONMW-08_02068	zraR_2
zraR_2	ZONMW-08_02368	zraR_2
zraR_2	ZONMW-08_03122	zraR_2
zraR_2	ZONMW-08_00388	zraR_2
zraR_2	ZONMW-08_00607	zraR_2
zraR_2	ZONMW-08_03795	zraR_2
zraR_2	ZONMW-08_03690	zraR_2
zraR_2	ZONMW-08_01226	zraR_2

zraR_2	ZONMW-08_00589	zraR_2
zraR_2	ZONMW-08_00098	zraR_2
zraR_2	ZONMW-08_00338	zraR_2
zraR_2	ZONMW-08_03214	zraR_2
zraR_2	ZONMW-08_00151	zraR_2
zraR_2	ZONMW-08_00855	zraR_2
zraR_2	ZONMW-08_00346	zraR_2
zraR_2	ZONMW-08_01037	zraR_2
zraR_2	ZONMW-08_00287	zraR_2
zraR_2	ZONMW-08_01138	zraR_2
zraR_2	ZONMW-08_00288	zraR_2
zraR_2	ZONMW-08_03241	zraR_2
zraR_2	ZONMW-08_00613	zraR_2
zraR_2	ZONMW-08_02308	zraR_2
zraR_2	ZONMW-08_01882	zraR_2
zraR_2	ZONMW-08_00588	zraR_2
zraR_2	ZONMW-08_03049	zraR_2
zraR_2	ZONMW-08_01202	zraR_2
zraR_2	ZONMW-08_00827	zraR_2
zraR_2	ZONMW-08_00625	zraR_2
zraR_2	ZONMW-08_00015	zraR_2
zraR_2	ZONMW-08_01729	zraR_2
zraR_2	ZONMW-09_01676	zraR_2
zraR_2	ZONMW-09_02497	zraR_2
zraR_2	ZONMW-09_00576	zraR_2
zraR_2	ZONMW-09_01258	zraR_2
zraR_2	ZONMW-09_00082	zraR_2
zraR_2	ZONMW-09_01880	zraR_2
zraR_2	ZONMW-09_02009	zraR_2
zraR_2	ZONMW-09_01138	zraR_2
zraR_2	ZONMW-09_01568	zraR_2
zraR_2	ZONMW-09_02162	zraR_2
zraR_2	ZONMW-09_00083	zraR_2
zraR_2	ZONMW-09_01567	zraR_2
zraR_2	ZONMW-09_00027	zraR_2
zraR_2	ZONMW-09_02163	zraR_2
zraR_2	ZONMW-09_01180	zraR_2
zraR_2	ZONMW-09_00480	zraR_2
zraR_2	ZONMW-09_02499	zraR_2
zraR_2	ZONMW-09_00267	zraR_2
zraR_2	ZONMW-09_00645	zraR_2
zraR_2	ZONMW-09_02270	zraR_2
zraR_2	ZONMW-09_00778	zraR_2
zraR_2	ZONMW-09_02418	zraR_2
zraR_2	ZONMW-09_00777	zraR_2
zraR_2	ZONMW-09_02486	zraR_2
zraR_2	ZONMW-09_01137	zraR_2
zraR_2	ZONMW-09_01000	zraR_2

zraR_2	ZONMW-09_00582	zraR_2
zraR_2	ZONMW-09_02639	zraR_2
zraR_2	ZONMW-09_01217	zraR_2
zraR_2	ZONMW-09_01768	zraR_2
zraR_2	ZONMW-09_01656	zraR_2
zraR_2	ZONMW-09_00575	zraR_2
zraR_2	ZONMW-09_02323	zraR_2
zraR_2	ZONMW-09_00475	zraR_2
zraR_2	ZONMW-09_00644	zraR_2
zraR_2	ZONMW-09_02304	zraR_2
zraR_2	ZONMW-09_02684	zraR_2
zraR_2	ZONMW-09_01675	zraR_2
zraR_2	ZONMW-10_01649	zraR_2
zraR_2	ZONMW-10_02065	zraR_2
zraR_2	ZONMW-10_01487	zraR_2
zraR_2	ZONMW-10_00096	zraR_2
zraR_2	ZONMW-10_00188	zraR_2
zraR_2	ZONMW-10_00097	zraR_2
zraR_2	ZONMW-10_00938	zraR_2
zraR_2	ZONMW-10_00937	zraR_2
zraR_2	ZONMW-10_01796	zraR_2
zraR_2	ZONMW-10_01733	zraR_2
zraR_2	ZONMW-10_02493	zraR_2
zraR_2	ZONMW-10_00633	zraR_2
zraR_2	ZONMW-10_01528	zraR_2
zraR_2	ZONMW-10_01751	zraR_2
zraR_2	ZONMW-10_00881	zraR_2
zraR_2	ZONMW-10_01782	zraR_2
zraR_2	ZONMW-10_01058	zraR_2
zraR_2	ZONMW-10_02308	zraR_2
zraR_2	ZONMW-10_01734	zraR_2
zraR_2	ZONMW-10_00429	zraR_2
zraR_2	ZONMW-10_02107	zraR_2
zraR_2	ZONMW-10_00839	zraR_2
zraR_2	ZONMW-10_01140	zraR_2
zraR_2	ZONMW-10_00190	zraR_2
zraR_2	ZONMW-10_02494	zraR_2
zraR_2	ZONMW-10_01621	zraR_2
zraR_2	ZONMW-10_00872	zraR_2
zraR_2	ZONMW-10_01092	zraR_2
zraR_2	ZONMW-10_01431	zraR_2
zraR_2	ZONMW-10_01030	zraR_2
zraR_2	ZONMW-10_00432	zraR_2
zraR_2	ZONMW-10_02324	zraR_2
zraR_2	ZONMW-11_00069	zraR_2
zraR_2	ZONMW-11_01230	zraR_2
zraR_2	ZONMW-11_00990	zraR_2
zraR_2	ZONMW-11_04732	zraR_2

zraR_2	ZONMW-11_01027	zraR_2
zraR_2	ZONMW-11_02050	zraR_2
zraR_2	ZONMW-11_00980	zraR_2
zraR_2	ZONMW-11_00567	zraR_2
zraR_2	ZONMW-11_01051	zraR_2
zraR_2	ZONMW-11_00878	zraR_2
zraR_2	ZONMW-11_02243	zraR_2
zraR_2	ZONMW-11_03852	zraR_2
zraR_2	ZONMW-11_03853	zraR_2
zraR_2	ZONMW-11_00566	zraR_2
zraR_2	ZONMW-11_03649	zraR_2
zraR_2	ZONMW-11_01052	zraR_2
zraR_2	ZONMW-11_00885	zraR_2
zraR_2	ZONMW-11_03175	zraR_2
zraR_2	ZONMW-11_04373	zraR_2
zraR_2	ZONMW-11_02246	zraR_2
zraR_2	ZONMW-11_03413	zraR_2
zraR_2	ZONMW-11_03650	zraR_2
zraR_2	ZONMW-11_01689	zraR_2
zraR_2	ZONMW-11_00809	zraR_2
zraR_2	ZONMW-11_01412	zraR_2
zraR_2	ZONMW-11_03575	zraR_2
zraR_2	ZONMW-11_00459	zraR_2
zraR_2	ZONMW-11_03936	zraR_2
zraR_2	ZONMW-11_04487	zraR_2
zraR_2	ZONMW-11_04527	zraR_2
zraR_2	ZONMW-11_02973	zraR_2
zraR_2	ZONMW-11_00569	zraR_2
zraR_2	ZONMW-11_00291	zraR_2
zraR_2	ZONMW-11_04291	zraR_2
zraR_2	ZONMW-11_03214	zraR_2
zraR_2	ZONMW-11_01582	zraR_2
zraR_2	ZONMW-11_02729	zraR_2
zraR_2	ZONMW-11_00986	zraR_2
zraR_2	ZONMW-11_04233	zraR_2
zraR_2	ZONMW-11_00244	zraR_2
zraR_2	ZONMW-11_01907	zraR_2
zraR_2	ZONMW-11_02978	zraR_2
zraR_2	ZONMW-11_03503	zraR_2
zraR_2	ZONMW-11_03491	zraR_2
zraR_2	ZONMW-11_00886	zraR_2
zraR_2	ZONMW-11_00758	zraR_2
zraR_2	ZONMW-11_04624	zraR_2
zraR_2	ZONMW-11_03014	zraR_2
zraR_2	ZONMW-11_01167	zraR_2
zraR_2	ZONMW-11_03528	zraR_2
zraR_2	ZONMW-11_03799	zraR_2
zraR_2	ZONMW-11_04223	zraR_2

zraR_2	ZONMW-11_04592	zraR_2
zraR_2	ZONMW-11_00056	zraR_2
zraR_2	ZONMW-11_01012	zraR_2
zraR_2	ZONMW-11_03529	zraR_2
zraR_2	ZONMW-11_00057	zraR_2
zraR_2	ZONMW-11_02049	zraR_2
zraR_2	ZONMW-11_00243	zraR_2
zraR_2	ZONMW-11_02168	zraR_2
zraR_2	ZONMW-11_00240	zraR_2
zraR_2	ZONMW-11_00237	zraR_2
zraR_2	ZONMW-11_01688	zraR_2
zraR_2	ZONMW-11_00460	zraR_2
zraR_2	ZONMW-11_04764	zraR_2
zraR_2	ZONMW-11_01778	zraR_2
zraR_2	ZONMW-11_04667	zraR_2
zraR_2	ZONMW-11_03492	zraR_2
zraR_2	ZONMW-11_00756	zraR_2
zraR_2	ZONMW-11_03182	zraR_2
zraR_2	ZONMW-11_00001	zraR_2
zraR_2	ZONMW-11_00324	zraR_2
zraR_2	ZONMW-11_01480	zraR_2
zraR_2	ZONMW-11_02345	zraR_2
zraR_2	ZONMW-12_01473	zraR_2
zraR_2	ZONMW-12_04240	zraR_2
zraR_2	ZONMW-12_02015	zraR_2
zraR_2	ZONMW-12_00312	zraR_2
zraR_2	ZONMW-12_03798	zraR_2
zraR_2	ZONMW-12_03622	zraR_2
zraR_2	ZONMW-12_03078	zraR_2
zraR_2	ZONMW-12_00911	zraR_2
zraR_2	ZONMW-12_03164	zraR_2
zraR_2	ZONMW-12_01297	zraR_2
zraR_2	ZONMW-12_02781	zraR_2
zraR_2	ZONMW-12_01634	zraR_2
zraR_2	ZONMW-12_00621	zraR_2
zraR_2	ZONMW-12_03425	zraR_2
zraR_2	ZONMW-12_02212	zraR_2
zraR_2	ZONMW-12_00938	zraR_2
zraR_2	ZONMW-12_00620	zraR_2
zraR_2	ZONMW-12_03225	zraR_2
zraR_2	ZONMW-12_03224	zraR_2
zraR_2	ZONMW-12_00939	zraR_2
zraR_2	ZONMW-12_02359	zraR_2
zraR_2	ZONMW-12_02929	zraR_2
zraR_2	ZONMW-12_01620	zraR_2
zraR_2	ZONMW-12_03079	zraR_2
zraR_2	ZONMW-12_03657	zraR_2
zraR_2	ZONMW-12_02320	zraR_2

zraR_2	ZONMW-12_03086	zraR_2
zraR_2	ZONMW-12_01636	zraR_2
zraR_2	ZONMW-12_00810	zraR_2
zraR_2	ZONMW-12_03902	zraR_2
zraR_2	ZONMW-12_04076	zraR_2
zraR_2	ZONMW-12_02862	zraR_2
zraR_2	ZONMW-12_00765	zraR_2
zraR_2	ZONMW-12_01456	zraR_2
zraR_2	ZONMW-12_02378	zraR_2
zraR_2	ZONMW-12_02593	zraR_2
zraR_2	ZONMW-12_02086	zraR_2
zraR_2	ZONMW-12_03474	zraR_2
zraR_2	ZONMW-12_01480	zraR_2
zraR_2	ZONMW-12_03659	zraR_2
zraR_2	ZONMW-12_02930	zraR_2
zraR_2	ZONMW-12_03857	zraR_2
zraR_2	ZONMW-12_00183	zraR_2
zraR_2	ZONMW-12_01619	zraR_2
zraR_2	ZONMW-12_02462	zraR_2
zraR_2	ZONMW-12_00048	zraR_2
zraR_2	ZONMW-12_00975	zraR_2
zraR_2	ZONMW-12_04511	zraR_2
zraR_2	ZONMW-12_02791	zraR_2
zraR_2	ZONMW-12_00809	zraR_2
zraR_2	ZONMW-12_03163	zraR_2
zraR_2	ZONMW-12_03547	zraR_2
zraR_2	ZONMW-12_04269	zraR_2
zraR_2	ZONMW-12_02360	zraR_2
zraR_2	ZONMW-12_02211	zraR_2
zraR_2	ZONMW-12_00513	zraR_2
zraR_2	ZONMW-12_03335	zraR_2
zraR_2	ZONMW-12_01369	zraR_2
zraR_2	ZONMW-12_00805	zraR_2
zraR_2	ZONMW-12_04015	zraR_2
zraR_2	ZONMW-12_03936	zraR_2
zraR_2	ZONMW-12_01238	zraR_2
zraR_2	ZONMW-12_00333	zraR_2
zraR_2	ZONMW-12_00846	zraR_2
zraR_2	ZONMW-12_00334	zraR_2
zraR_2	ZONMW-12_03172	zraR_2
zraR_2	ZONMW-12_04042	zraR_2
zraR_2	ZONMW-12_03087	zraR_2
zraR_2	ZONMW-12_04041	zraR_2
zraR_2	ZONMW-12_02574	zraR_2
zraR_2	ZONMW-12_01639	zraR_2
zraR_2	ZONMW-12_01602	zraR_2
zraR_2	ZONMW-12_00188	zraR_2
zraR_2	ZONMW-12_01203	zraR_2

zraR_2	ZONMW-12_00833	zraR_2
zraR_2	ZONMW-12_00834	zraR_2
zraR_2	ZONMW-12_00655	zraR_2
zraR_2	ZONMW-12_04018	zraR_2
zraR_2	ZONMW-12_02988	zraR_2
zraR_2	ZONMW-12_01479	zraR_2
zraR_2	ZONMW-12_02861	zraR_2
zraR_2	ZONMW-12_04278	zraR_2
zraR_2	ZONMW-12_01812	zraR_2
zraR_2	ZONMW-12_03592	zraR_2
zraR_2	ZONMW-12_02764	zraR_2
zraR_2	ZONMW-12_02137	zraR_2
zraR_2	ZONMW-12_02604	zraR_2
zraR_2	ZONMW-12_00960	zraR_2
zraR_2	ZONMW-12_02321	zraR_2
zraR_2	ZONMW-12_04223	zraR_2
zraR_2	ZONMW-12_04017	zraR_2
zraR_2	ZONMW-12_04014	zraR_2
zraR_2	ZONMW-12_04016	zraR_2
zraR_2	ZONMW-12_04510	zraR_2
zraR_2	ZONMW-13_00603	zraR_2
zraR_2	ZONMW-13_02413	zraR_2
zraR_2	ZONMW-13_00070	zraR_2
zraR_2	ZONMW-13_00155	zraR_2
zraR_2	ZONMW-13_01693	zraR_2
zraR_2	ZONMW-13_02958	zraR_2
zraR_2	ZONMW-13_00939	zraR_2
zraR_2	ZONMW-13_00637	zraR_2
zraR_2	ZONMW-13_01510	zraR_2
zraR_2	ZONMW-13_02080	zraR_2
zraR_2	ZONMW-13_02380	zraR_2
zraR_2	ZONMW-13_02379	zraR_2
zraR_2	ZONMW-13_02861	zraR_2
zraR_2	ZONMW-13_00086	zraR_2
zraR_2	ZONMW-13_00938	zraR_2
zraR_2	ZONMW-13_02145	zraR_2
zraR_2	ZONMW-13_00087	zraR_2
zraR_2	ZONMW-13_02959	zraR_2
zraR_2	ZONMW-13_02860	zraR_2
zraR_2	ZONMW-13_02908	zraR_2
zraR_2	ZONMW-13_01910	zraR_2
zraR_2	ZONMW-13_02989	zraR_2
zraR_2	ZONMW-13_00149	zraR_2
zraR_2	ZONMW-13_03093	zraR_2
zraR_2	ZONMW-13_00993	zraR_2
zraR_2	ZONMW-13_00256	zraR_2
zraR_2	ZONMW-13_03051	zraR_2
zraR_2	ZONMW-13_02805	zraR_2

zraR_2	ZONMW-13_02804	zraR_2
zraR_2	ZONMW-13_00290	zraR_2
zraR_2	ZONMW-13_01911	zraR_2
zraR_2	ZONMW-13_02138	zraR_2
zraR_2	ZONMW-13_00122	zraR_2
zraR_2	ZONMW-13_02992	zraR_2
zraR_2	ZONMW-13_01297	zraR_2
zraR_2	ZONMW-13_02705	zraR_2
zraR_2	ZONMW-13_00156	zraR_2
zraR_2	ZONMW-13_03126	zraR_2
zraR_2	ZONMW-13_02526	zraR_2
zraR_2	ZONMW-13_02787	zraR_2
zraR_2	ZONMW-13_00589	zraR_2
zraR_2	ZONMW-13_01768	zraR_2
zraR_2	ZONMW-13_02811	zraR_2
zraR_2	ZONMW-13_00754	zraR_2
zraR_2	ZONMW-13_02527	zraR_2
zraR_2	ZONMW-13_03127	zraR_2
zraR_2	ZONMW-13_01308	zraR_2
zraR_2	ZONMW-13_00295	zraR_2
zraR_2	ZONMW-13_00761	zraR_2
zraR_2	ZONMW-13_02843	zraR_2
zraR_2	ZONMW-13_02572	zraR_2
zraR_2	ZONMW-13_01250	zraR_2
zraR_2	ZONMW-13_02079	zraR_2
zraR_2	ZONMW-13_02960	zraR_2
zraR_2	ZONMW-13_02028	zraR_2
zraR_2	ZONMW-13_00093	zraR_2
zraR_2	ZONMW-14_00181	zraR_2
zraR_2	ZONMW-14_02462	zraR_2
zraR_2	ZONMW-14_02697	zraR_2
zraR_2	ZONMW-14_02488	zraR_2
zraR_2	ZONMW-14_00936	zraR_2
zraR_2	ZONMW-14_01165	zraR_2
zraR_2	ZONMW-14_02600	zraR_2
zraR_2	ZONMW-14_02277	zraR_2
zraR_2	ZONMW-14_02755	zraR_2
zraR_2	ZONMW-14_01166	zraR_2
zraR_2	ZONMW-14_00165	zraR_2
zraR_2	ZONMW-14_00166	zraR_2
zraR_2	ZONMW-14_02601	zraR_2
zraR_2	ZONMW-14_00614	zraR_2
zraR_2	ZONMW-14_02754	zraR_2
zraR_2	ZONMW-14_02671	zraR_2
zraR_2	ZONMW-14_01692	zraR_2
zraR_2	ZONMW-14_00024	zraR_2
zraR_2	ZONMW-14_00764	zraR_2
zraR_2	ZONMW-14_00332	zraR_2

zraR_2	ZONMW-14_02793	zraR_2
zraR_2	ZONMW-14_00147	zraR_2
zraR_2	ZONMW-14_00452	zraR_2
zraR_2	ZONMW-14_00671	zraR_2
zraR_2	ZONMW-14_00782	zraR_2
zraR_2	ZONMW-14_02887	zraR_2
zraR_2	ZONMW-14_01986	zraR_2
zraR_2	ZONMW-14_02052	zraR_2
zraR_2	ZONMW-14_02051	zraR_2
zraR_2	ZONMW-14_00296	zraR_2
zraR_2	ZONMW-14_00781	zraR_2
zraR_2	ZONMW-14_02885	zraR_2
zraR_2	ZONMW-14_00525	zraR_2
zraR_2	ZONMW-14_01861	zraR_2
zraR_2	ZONMW-14_02066	zraR_2
zraR_2	ZONMW-14_01008	zraR_2
zraR_2	ZONMW-14_02044	zraR_2
zraR_2	ZONMW-14_02691	zraR_2
zraR_2	ZONMW-14_01292	zraR_2
zraR_2	ZONMW-14_00867	zraR_2
zraR_2	ZONMW-14_02698	zraR_2
zraR_2	ZONMW-14_00866	zraR_2
zraR_2	ZONMW-14_00672	zraR_2
zraR_2	ZONMW-14_00291	zraR_2
zraR_2	ZONMW-14_01284	zraR_2
zraR_2	ZONMW-14_02002	zraR_2
zraR_2	ZONMW-14_02901	zraR_2
zraR_2	ZONMW-14_00615	zraR_2
zraR_2	ZONMW-14_01912	zraR_2
zraR_2	ZONMW-14_02747	zraR_2
zraR_2	ZONMW-15_01492	zraR_2
zraR_2	ZONMW-15_01454	zraR_2
zraR_2	ZONMW-15_01731	zraR_2
zraR_2	ZONMW-15_02080	zraR_2
zraR_2	ZONMW-15_01202	zraR_2
zraR_2	ZONMW-15_01687	zraR_2
zraR_2	ZONMW-15_03358	zraR_2
zraR_2	ZONMW-15_00891	zraR_2
zraR_2	ZONMW-15_03011	zraR_2
zraR_2	ZONMW-15_00940	zraR_2
zraR_2	ZONMW-15_00699	zraR_2
zraR_2	ZONMW-15_00939	zraR_2
zraR_2	ZONMW-15_03012	zraR_2
zraR_2	ZONMW-15_00698	zraR_2
zraR_2	ZONMW-15_00717	zraR_2
zraR_2	ZONMW-15_00284	zraR_2
zraR_2	ZONMW-15_02949	zraR_2
zraR_2	ZONMW-15_01629	zraR_2

zraR_2	ZONMW-15_01488	zraR_2
zraR_2	ZONMW-15_01866	zraR_2
zraR_2	ZONMW-15_02119	zraR_2
zraR_2	ZONMW-15_02948	zraR_2
zraR_2	ZONMW-15_01478	zraR_2
zraR_2	ZONMW-15_00535	zraR_2
zraR_2	ZONMW-15_02195	zraR_2
zraR_2	ZONMW-15_02146	zraR_2
zraR_2	ZONMW-15_03034	zraR_2
zraR_2	ZONMW-15_03315	zraR_2
zraR_2	ZONMW-15_01887	zraR_2
zraR_2	ZONMW-15_03346	zraR_2
zraR_2	ZONMW-15_03317	zraR_2
zraR_2	ZONMW-15_01141	zraR_2
zraR_2	ZONMW-15_00732	zraR_2
zraR_2	ZONMW-15_00172	zraR_2
zraR_2	ZONMW-15_00306	zraR_2
zraR_2	ZONMW-15_01476	zraR_2
zraR_2	ZONMW-15_01919	zraR_2
zraR_2	ZONMW-15_02468	zraR_2
zraR_2	ZONMW-15_02127	zraR_2
zraR_2	ZONMW-15_03359	zraR_2
zraR_2	ZONMW-15_00890	zraR_2
zraR_2	ZONMW-15_01676	zraR_2
zraR_2	ZONMW-15_02775	zraR_2
zraR_2	ZONMW-15_01294	zraR_2
zraR_2	ZONMW-15_01269	zraR_2
zraR_2	ZONMW-15_00170	zraR_2
zraR_2	ZONMW-15_01056	zraR_2
zraR_2	ZONMW-15_02128	zraR_2
zraR_2	ZONMW-15_00637	zraR_2
zraR_2	ZONMW-15_00055	zraR_2
zraR_2	ZONMW-15_00900	zraR_2
zraR_2	ZONMW-15_02250	zraR_2
zraR_2	ZONMW-15_02453	zraR_2
zraR_2	ZONMW-15_01670	zraR_2
zraR_2	ZONMW-15_00322	zraR_2
zraR_2	ZONMW-15_00046	zraR_2
zraR_2	ZONMW-15_01610	zraR_2
zraR_2	ZONMW-15_02117	zraR_2
zraR_2	ZONMW-15_00308	zraR_2
zraR_2	ZONMW-15_03035	zraR_2
zraR_2	ZONMW-19_03053	zraR_2
zraR_2	ZONMW-19_04362	zraR_2
zraR_2	ZONMW-19_00842	zraR_2
zraR_2	ZONMW-19_03504	zraR_2
zraR_2	ZONMW-19_00369	zraR_2
zraR_2	ZONMW-19_00388	zraR_2

zraR_2	ZONMW-19_00173	zraR_2
zraR_2	ZONMW-19_02781	zraR_2
zraR_2	ZONMW-19_01268	zraR_2
zraR_2	ZONMW-19_00919	zraR_2
zraR_2	ZONMW-19_01115	zraR_2
zraR_2	ZONMW-19_01114	zraR_2
zraR_2	ZONMW-19_00559	zraR_2
zraR_2	ZONMW-19_03737	zraR_2
zraR_2	ZONMW-19_02753	zraR_2
zraR_2	ZONMW-19_03607	zraR_2
zraR_2	ZONMW-19_00296	zraR_2
zraR_2	ZONMW-19_03436	zraR_2
zraR_2	ZONMW-19_04898	zraR_2
zraR_2	ZONMW-19_00174	zraR_2
zraR_2	ZONMW-19_00046	zraR_2
zraR_2	ZONMW-19_03606	zraR_2
zraR_2	ZONMW-19_02752	zraR_2
zraR_2	ZONMW-19_00047	zraR_2
zraR_2	ZONMW-19_02017	zraR_2
zraR_2	ZONMW-19_02229	zraR_2
zraR_2	ZONMW-19_00915	zraR_2
zraR_2	ZONMW-19_03663	zraR_2
zraR_2	ZONMW-19_03646	zraR_2
zraR_2	ZONMW-19_02253	zraR_2
zraR_2	ZONMW-19_02873	zraR_2
zraR_2	ZONMW-19_03678	zraR_2
zraR_2	ZONMW-19_03188	zraR_2
zraR_2	ZONMW-19_02783	zraR_2
zraR_2	ZONMW-19_00920	zraR_2
zraR_2	ZONMW-19_03994	zraR_2
zraR_2	ZONMW-19_03736	zraR_2
zraR_2	ZONMW-19_01516	zraR_2
zraR_2	ZONMW-19_04491	zraR_2
zraR_2	ZONMW-19_00394	zraR_2
zraR_2	ZONMW-19_03664	zraR_2
zraR_2	ZONMW-19_00128	zraR_2
zraR_2	ZONMW-19_00600	zraR_2
zraR_2	ZONMW-19_01508	zraR_2
zraR_2	ZONMW-19_01345	zraR_2
zraR_2	ZONMW-19_04303	zraR_2
zraR_2	ZONMW-19_00641	zraR_2
zraR_2	ZONMW-19_03821	zraR_2
zraR_2	ZONMW-19_00573	zraR_2
zraR_2	ZONMW-19_01304	zraR_2
zraR_2	ZONMW-19_00985	zraR_2
zraR_2	ZONMW-19_02782	zraR_2
zraR_2	ZONMW-19_02621	zraR_2
zraR_2	ZONMW-19_02409	zraR_2

zraR_2	ZONMW-19_03437	zraR_2
zraR_2	ZONMW-19_03786	zraR_2
zraR_2	ZONMW-19_00956	zraR_2
zraR_2	ZONMW-19_00560	zraR_2
zraR_2	ZONMW-19_03175	zraR_2
zraR_2	ZONMW-19_03187	zraR_2
zraR_2	ZONMW-19_00642	zraR_2
zraR_2	ZONMW-19_02901	zraR_2
zraR_2	ZONMW-19_02408	zraR_2
zraR_2	ZONMW-19_01779	zraR_2
zraR_2	ZONMW-19_02846	zraR_2
zraR_2	ZONMW-19_01498	zraR_2
zraR_2	ZONMW-19_01518	zraR_2
zraR_2	ZONMW-19_00652	zraR_2
zraR_2	ZONMW-19_00955	zraR_2
zraR_2	ZONMW-19_03787	zraR_2
zraR_2	ZONMW-19_00501	zraR_2
zraR_2	ZONMW-19_03805	zraR_2
zraR_2	ZONMW-19_04005	zraR_2
zraR_2	ZONMW-19_03238	zraR_2
zraR_2	ZONMW-19_02098	zraR_2
zraR_2	ZONMW-19_03906	zraR_2
zraR_2	ZONMW-19_01283	zraR_2
zraR_2	ZONMW-19_01517	zraR_2
zraR_2	ZONMW-19_02252	zraR_2
zraR_2	ZONMW-19_00500	zraR_2
zraR_2	ZONMW-19_03174	zraR_2
zraR_2	ZONMW-19_02895	zraR_2
zraR_2	ZONMW-19_02899	zraR_2
zraR_2	ZONMW-19_03680	zraR_2
zraR_2	ZONMW-19_00151	zraR_2
zraR_2	ZONMW-19_01305	zraR_2
zraR_2	ZONMW-19_01928	zraR_2
zraR_2	ZONMW-19_04808	zraR_2
zraR_2	ZONMW-19_03406	zraR_2
zraR_2	ZONMW-19_03986	zraR_2
zraR_2	ZONMW-19_01193	zraR_2
zraR_2	ZONMW-19_00191	zraR_2
zraR_2	ZONMW-19_00916	zraR_2
zraR_2	ZONMW-19_03153	zraR_2
zraR_2	ZONMW-19_00914	zraR_2
zraR_2	ZONMW-19_03645	zraR_2
zraR_2	ZONMW-19_03173	zraR_2
zraR_2	ZONMW-19_01098	zraR_2
zraR_2	ZONMW-19_01344	zraR_2
zraR_2	ZONMW-19_02894	zraR_2
zraR_2	ZONMW-19_01259	zraR_2
zraR_2	ZONMW-19_01784	zraR_2

zraR_2	ZONMW-19_02572	zraR_2
zraR_2	ZONMW-19_03701	zraR_2
zraR_2	ZONMW-19_04658	zraR_2
zraR_2	ZONMW-19_00938	zraR_2
zraR_2	ZONMW-19_03828	zraR_2
zraR_2	ZONMW-20_02841	zraR_2
zraR_2	ZONMW-20_02083	zraR_2
zraR_2	ZONMW-20_00292	zraR_2
zraR_2	ZONMW-20_00490	zraR_2
zraR_2	ZONMW-20_02437	zraR_2
zraR_2	ZONMW-20_02436	zraR_2
zraR_2	ZONMW-20_01382	zraR_2
zraR_2	ZONMW-20_03036	zraR_2
zraR_2	ZONMW-20_04083	zraR_2
zraR_2	ZONMW-20_04082	zraR_2
zraR_2	ZONMW-20_02069	zraR_2
zraR_2	ZONMW-20_01381	zraR_2
zraR_2	ZONMW-20_03385	zraR_2
zraR_2	ZONMW-20_03037	zraR_2
zraR_2	ZONMW-20_02657	zraR_2
zraR_2	ZONMW-20_02346	zraR_2
zraR_2	ZONMW-20_01874	zraR_2
zraR_2	ZONMW-20_01718	zraR_2
zraR_2	ZONMW-20_02869	zraR_2
zraR_2	ZONMW-20_03605	zraR_2
zraR_2	ZONMW-20_03740	zraR_2
zraR_2	ZONMW-20_01057	zraR_2
zraR_2	ZONMW-20_00079	zraR_2
zraR_2	ZONMW-20_03657	zraR_2
zraR_2	ZONMW-20_02985	zraR_2
zraR_2	ZONMW-20_02983	zraR_2
zraR_2	ZONMW-20_01520	zraR_2
zraR_2	ZONMW-20_00109	zraR_2
zraR_2	ZONMW-20_03544	zraR_2
zraR_2	ZONMW-20_03733	zraR_2
zraR_2	ZONMW-20_01516	zraR_2
zraR_2	ZONMW-20_03656	zraR_2
zraR_2	ZONMW-20_01719	zraR_2
zraR_2	ZONMW-20_00491	zraR_2
zraR_2	ZONMW-20_03606	zraR_2
zraR_2	ZONMW-20_01147	zraR_2
zraR_2	ZONMW-20_02070	zraR_2
zraR_2	ZONMW-20_00711	zraR_2
zraR_2	ZONMW-20_01923	zraR_2
zraR_2	ZONMW-20_03555	zraR_2
zraR_2	ZONMW-20_01241	zraR_2
zraR_2	ZONMW-20_01146	zraR_2
zraR_2	ZONMW-20_04285	zraR_2

zraR_2	ZONMW-20_03478	zraR_2
zraR_2	ZONMW-20_02581	zraR_2
zraR_2	ZONMW-20_03459	zraR_2
zraR_2	ZONMW-20_03893	zraR_2
zraR_2	ZONMW-20_03032	zraR_2
zraR_2	ZONMW-20_00451	zraR_2
zraR_2	ZONMW-20_02278	zraR_2
zraR_2	ZONMW-20_03717	zraR_2
zraR_2	ZONMW-20_01059	zraR_2
zraR_2	ZONMW-20_02868	zraR_2
zraR_2	ZONMW-20_03386	zraR_2
zraR_2	ZONMW-20_00078	zraR_2
zraR_2	ZONMW-20_02084	zraR_2
zraR_2	ZONMW-20_00108	zraR_2
zraR_2	ZONMW-20_01507	zraR_2
zraR_2	ZONMW-20_03417	zraR_2
zraR_2	ZONMW-20_00772	zraR_2
zraR_2	ZONMW-20_01125	zraR_2
zraR_2	ZONMW-20_01140	zraR_2
zraR_2	ZONMW-20_03967	zraR_2
zraR_2	ZONMW-20_00114	zraR_2
zraR_2	ZONMW-20_02201	zraR_2
zraR_2	ZONMW-20_00304	zraR_2
zraR_2	ZONMW-20_00071	zraR_2
zraR_2	ZONMW-20_00087	zraR_2
zraR_2	ZONMW-20_03282	zraR_2
zraR_2	ZONMW-20_03188	zraR_2
zraR_2	ZONMW-20_01148	zraR_2
zraR_2	ZONMW-20_01876	zraR_2
zraR_2	ZONMW-20_02659	zraR_2
zraR_2	ZONMW-20_00469	zraR_2
zraR_2	ZONMW-20_04007	zraR_2
zraR_2	ZONMW-20_02607	zraR_2
zraR_2	ZONMW-20_02085	zraR_2
zraR_2	ZONMW-20_03110	zraR_2
zraR_2	ZONMW-20_01700	zraR_2
zraR_2	ZONMW-20_03373	zraR_2
zraR_2	ZONMW-20_00740	zraR_2
zraR_2	ZONMW-20_01095	zraR_2
zraR_2	ZONMW-20_01096	zraR_2
zraR_2	ZONMW-20_00739	zraR_2
zraR_2	ZONMW-20_00745	zraR_2
zraR_2	ZONMW-20_03928	zraR_2
zraR_2	ZONMW-21_03470	zraR_2
zraR_2	ZONMW-21_02915	zraR_2
zraR_2	ZONMW-21_02887	zraR_2
zraR_2	ZONMW-21_01499	zraR_2
zraR_2	ZONMW-21_03081	zraR_2

zraR_2	ZONMW-21_01186	zraR_2
zraR_2	ZONMW-21_01234	zraR_2
zraR_2	ZONMW-21_01667	zraR_2
zraR_2	ZONMW-21_02874	zraR_2
zraR_2	ZONMW-21_01666	zraR_2
zraR_2	ZONMW-21_01233	zraR_2
zraR_2	ZONMW-21_02875	zraR_2
zraR_2	ZONMW-21_02190	zraR_2
zraR_2	ZONMW-21_02122	zraR_2
zraR_2	ZONMW-21_00643	zraR_2
zraR_2	ZONMW-21_02191	zraR_2
zraR_2	ZONMW-21_02267	zraR_2
zraR_2	ZONMW-21_02613	zraR_2
zraR_2	ZONMW-21_00343	zraR_2
zraR_2	ZONMW-21_03319	zraR_2
zraR_2	ZONMW-21_03093	zraR_2
zraR_2	ZONMW-21_02390	zraR_2
zraR_2	ZONMW-21_01047	zraR_2
zraR_2	ZONMW-21_03056	zraR_2
zraR_2	ZONMW-21_02933	zraR_2
zraR_2	ZONMW-21_00280	zraR_2
zraR_2	ZONMW-21_02188	zraR_2
zraR_2	ZONMW-21_01716	zraR_2
zraR_2	ZONMW-21_00030	zraR_2
zraR_2	ZONMW-21_00506	zraR_2
zraR_2	ZONMW-21_01374	zraR_2
zraR_2	ZONMW-21_02187	zraR_2
zraR_2	ZONMW-21_01496	zraR_2
zraR_2	ZONMW-21_00942	zraR_2
zraR_2	ZONMW-21_01185	zraR_2
zraR_2	ZONMW-21_01194	zraR_2
zraR_2	ZONMW-21_00943	zraR_2
zraR_2	ZONMW-21_00718	zraR_2
zraR_2	ZONMW-21_01999	zraR_2
zraR_2	ZONMW-21_01729	zraR_2
zraR_2	ZONMW-21_00277	zraR_2
zraR_2	ZONMW-21_00664	zraR_2
zraR_2	ZONMW-21_01436	zraR_2
zraR_2	ZONMW-21_00765	zraR_2
zraR_2	ZONMW-21_03422	zraR_2
zraR_2	ZONMW-21_01952	zraR_2
zraR_2	ZONMW-21_01857	zraR_2
zraR_2	ZONMW-21_01449	zraR_2
zraR_2	ZONMW-21_00060	zraR_2
zraR_2	ZONMW-21_00442	zraR_2
zraR_2	ZONMW-22_03483	zraR_2
zraR_2	ZONMW-22_02909	zraR_2
zraR_2	ZONMW-22_02937	zraR_2

zraR_2	ZONMW-22_02224	zraR_2
zraR_2	ZONMW-22_03106	zraR_2
zraR_2	ZONMW-22_01188	zraR_2
zraR_2	ZONMW-22_01236	zraR_2
zraR_2	ZONMW-22_02057	zraR_2
zraR_2	ZONMW-22_02950	zraR_2
zraR_2	ZONMW-22_02058	zraR_2
zraR_2	ZONMW-22_01235	zraR_2
zraR_2	ZONMW-22_02949	zraR_2
zraR_2	ZONMW-22_01535	zraR_2
zraR_2	ZONMW-22_01603	zraR_2
zraR_2	ZONMW-22_00644	zraR_2
zraR_2	ZONMW-22_01534	zraR_2
zraR_2	ZONMW-22_03441	zraR_2
zraR_2	ZONMW-22_02496	zraR_2
zraR_2	ZONMW-22_00343	zraR_2
zraR_2	ZONMW-22_03406	zraR_2
zraR_2	ZONMW-22_03118	zraR_2
zraR_2	ZONMW-22_02718	zraR_2
zraR_2	ZONMW-22_01048	zraR_2
zraR_2	ZONMW-22_03081	zraR_2
zraR_2	ZONMW-22_02891	zraR_2
zraR_2	ZONMW-22_00280	zraR_2
zraR_2	ZONMW-22_01537	zraR_2
zraR_2	ZONMW-22_02008	zraR_2
zraR_2	ZONMW-22_00030	zraR_2
zraR_2	ZONMW-22_00506	zraR_2
zraR_2	ZONMW-22_01376	zraR_2
zraR_2	ZONMW-22_01538	zraR_2
zraR_2	ZONMW-22_02227	zraR_2
zraR_2	ZONMW-22_00943	zraR_2
zraR_2	ZONMW-22_01187	zraR_2
zraR_2	ZONMW-22_01196	zraR_2
zraR_2	ZONMW-22_00944	zraR_2
zraR_2	ZONMW-22_00719	zraR_2
zraR_2	ZONMW-22_01727	zraR_2
zraR_2	ZONMW-22_01995	zraR_2
zraR_2	ZONMW-22_00277	zraR_2
zraR_2	ZONMW-22_00665	zraR_2
zraR_2	ZONMW-22_01438	zraR_2
zraR_2	ZONMW-22_00766	zraR_2
zraR_2	ZONMW-22_02275	zraR_2
zraR_2	ZONMW-22_01774	zraR_2
zraR_2	ZONMW-22_01869	zraR_2
zraR_2	ZONMW-22_01451	zraR_2
zraR_2	ZONMW-22_00060	zraR_2
zraR_2	ZONMW-22_00442	zraR_2
zraR_2	ZONMW-23_03477	zraR_2

zraR_2	ZONMW-23_02961	zraR_2
zraR_2	ZONMW-23_02933	zraR_2
zraR_2	ZONMW-23_00327	zraR_2
zraR_2	ZONMW-23_03127	zraR_2
zraR_2	ZONMW-23_02300	zraR_2
zraR_2	ZONMW-23_02348	zraR_2
zraR_2	ZONMW-23_00494	zraR_2
zraR_2	ZONMW-23_02920	zraR_2
zraR_2	ZONMW-23_00493	zraR_2
zraR_2	ZONMW-23_02347	zraR_2
zraR_2	ZONMW-23_02921	zraR_2
zraR_2	ZONMW-23_01016	zraR_2
zraR_2	ZONMW-23_00948	zraR_2
zraR_2	ZONMW-23_01758	zraR_2
zraR_2	ZONMW-23_01017	zraR_2
zraR_2	ZONMW-23_01093	zraR_2
zraR_2	ZONMW-23_00054	zraR_2
zraR_2	ZONMW-23_01458	zraR_2
zraR_2	ZONMW-23_03365	zraR_2
zraR_2	ZONMW-23_03139	zraR_2
zraR_2	ZONMW-23_02742	zraR_2
zraR_2	ZONMW-23_02161	zraR_2
zraR_2	ZONMW-23_03102	zraR_2
zraR_2	ZONMW-23_02979	zraR_2
zraR_2	ZONMW-23_01395	zraR_2
zraR_2	ZONMW-23_01014	zraR_2
zraR_2	ZONMW-23_00543	zraR_2
zraR_2	ZONMW-23_01145	zraR_2
zraR_2	ZONMW-23_01621	zraR_2
zraR_2	ZONMW-23_02489	zraR_2
zraR_2	ZONMW-23_01013	zraR_2
zraR_2	ZONMW-23_00324	zraR_2
zraR_2	ZONMW-23_02056	zraR_2
zraR_2	ZONMW-23_02299	zraR_2
zraR_2	ZONMW-23_02308	zraR_2
zraR_2	ZONMW-23_02057	zraR_2
zraR_2	ZONMW-23_01833	zraR_2
zraR_2	ZONMW-23_00825	zraR_2
zraR_2	ZONMW-23_00556	zraR_2
zraR_2	ZONMW-23_01392	zraR_2
zraR_2	ZONMW-23_01779	zraR_2
zraR_2	ZONMW-23_02551	zraR_2
zraR_2	ZONMW-23_01880	zraR_2
zraR_2	ZONMW-23_00276	zraR_2
zraR_2	ZONMW-23_00778	zraR_2
zraR_2	ZONMW-23_00683	zraR_2
zraR_2	ZONMW-23_02564	zraR_2
zraR_2	ZONMW-23_01175	zraR_2

zraR_2	ZONMW-23_01557	zraR_2
zraR_2	ZONMW-24_03476	zraR_2
zraR_2	ZONMW-24_02932	zraR_2
zraR_2	ZONMW-24_02960	zraR_2
zraR_2	ZONMW-24_01988	zraR_2
zraR_2	ZONMW-24_03128	zraR_2
zraR_2	ZONMW-24_00273	zraR_2
zraR_2	ZONMW-24_00225	zraR_2
zraR_2	ZONMW-24_02682	zraR_2
zraR_2	ZONMW-24_02973	zraR_2
zraR_2	ZONMW-24_02683	zraR_2
zraR_2	ZONMW-24_00226	zraR_2
zraR_2	ZONMW-24_02972	zraR_2
zraR_2	ZONMW-24_01559	zraR_2
zraR_2	ZONMW-24_01627	zraR_2
zraR_2	ZONMW-24_00817	zraR_2
zraR_2	ZONMW-24_01558	zraR_2
zraR_2	ZONMW-24_01482	zraR_2
zraR_2	ZONMW-24_02261	zraR_2
zraR_2	ZONMW-24_01116	zraR_2
zraR_2	ZONMW-24_03367	zraR_2
zraR_2	ZONMW-24_03140	zraR_2
zraR_2	ZONMW-24_02414	zraR_2
zraR_2	ZONMW-24_00413	zraR_2
zraR_2	ZONMW-24_03103	zraR_2
zraR_2	ZONMW-24_02914	zraR_2
zraR_2	ZONMW-24_01179	zraR_2
zraR_2	ZONMW-24_01561	zraR_2
zraR_2	ZONMW-24_02633	zraR_2
zraR_2	ZONMW-24_01430	zraR_2
zraR_2	ZONMW-24_00953	zraR_2
zraR_2	ZONMW-24_00085	zraR_2
zraR_2	ZONMW-24_01562	zraR_2
zraR_2	ZONMW-24_01991	zraR_2
zraR_2	ZONMW-24_00518	zraR_2
zraR_2	ZONMW-24_00274	zraR_2
zraR_2	ZONMW-24_00265	zraR_2
zraR_2	ZONMW-24_00517	zraR_2
zraR_2	ZONMW-24_00742	zraR_2
zraR_2	ZONMW-24_01750	zraR_2
zraR_2	ZONMW-24_02620	zraR_2
zraR_2	ZONMW-24_01182	zraR_2
zraR_2	ZONMW-24_00796	zraR_2
zraR_2	ZONMW-24_00023	zraR_2
zraR_2	ZONMW-24_00695	zraR_2
zraR_2	ZONMW-24_02039	zraR_2
zraR_2	ZONMW-24_01797	zraR_2
zraR_2	ZONMW-24_01892	zraR_2

zraR_2	ZONMW-24_00010	zraR_2
zraR_2	ZONMW-24_01400	zraR_2
zraR_2	ZONMW-24_01017	zraR_2
zraR_2	ZONMW-25_02162	zraR_2
zraR_2	ZONMW-25_03170	zraR_2
zraR_2	ZONMW-25_01862	zraR_2
zraR_2	ZONMW-25_01931	zraR_2
zraR_2	ZONMW-25_02483	zraR_2
zraR_2	ZONMW-25_02484	zraR_2
zraR_2	ZONMW-25_01457	zraR_2
zraR_2	ZONMW-25_00166	zraR_2
zraR_2	ZONMW-25_01557	zraR_2
zraR_2	ZONMW-25_01558	zraR_2
zraR_2	ZONMW-25_01456	zraR_2
zraR_2	ZONMW-25_00873	zraR_2
zraR_2	ZONMW-25_00415	zraR_2
zraR_2	ZONMW-25_00341	zraR_2
zraR_2	ZONMW-25_00340	zraR_2
zraR_2	ZONMW-25_02588	zraR_2
zraR_2	ZONMW-25_00165	zraR_2
zraR_2	ZONMW-25_00591	zraR_2
zraR_2	ZONMW-25_02268	zraR_2
zraR_2	ZONMW-25_02589	zraR_2
zraR_2	ZONMW-25_01598	zraR_2
zraR_2	ZONMW-25_02974	zraR_2
zraR_2	ZONMW-25_03009	zraR_2
zraR_2	ZONMW-25_03439	zraR_2
zraR_2	ZONMW-25_00205	zraR_2
zraR_2	ZONMW-25_02564	zraR_2
zraR_2	ZONMW-25_02055	zraR_2
zraR_2	ZONMW-25_01981	zraR_2
zraR_2	ZONMW-25_02389	zraR_2
zraR_2	ZONMW-25_00146	zraR_2
zraR_2	ZONMW-25_01834	zraR_2
zraR_2	ZONMW-25_02906	zraR_2
zraR_2	ZONMW-25_03010	zraR_2
zraR_2	ZONMW-25_01505	zraR_2
zraR_2	ZONMW-25_01954	zraR_2
zraR_2	ZONMW-25_02839	zraR_2
zraR_2	ZONMW-25_00755	zraR_2
zraR_2	ZONMW-25_03016	zraR_2
zraR_2	ZONMW-25_01023	zraR_2
zraR_2	ZONMW-25_00590	zraR_2
zraR_2	ZONMW-25_03015	zraR_2
zraR_2	ZONMW-25_02909	zraR_2
zraR_2	ZONMW-25_01615	zraR_2
zraR_2	ZONMW-25_01170	zraR_2
zraR_2	ZONMW-25_03171	zraR_2

zraR_2	ZONMW-25_00987	zraR_2
zraR_2	ZONMW-25_03341	zraR_2
zraR_2	ZONMW-25_00064	zraR_2
zraR_2	ZONMW-26_02882	zraR_2
zraR_2	ZONMW-26_01554	zraR_2
zraR_2	ZONMW-26_01545	zraR_2
zraR_2	ZONMW-26_00017	zraR_2
zraR_2	ZONMW-26_03132	zraR_2
zraR_2	ZONMW-26_02028	zraR_2
zraR_2	ZONMW-26_00559	zraR_2
zraR_2	ZONMW-26_00856	zraR_2
zraR_2	ZONMW-26_00558	zraR_2
zraR_2	ZONMW-26_02027	zraR_2
zraR_2	ZONMW-26_03320	zraR_2
zraR_2	ZONMW-26_00857	zraR_2
zraR_2	ZONMW-26_03321	zraR_2
zraR_2	ZONMW-26_00736	zraR_2
zraR_2	ZONMW-26_01564	zraR_2
zraR_2	ZONMW-26_01054	zraR_2
zraR_2	ZONMW-26_02457	zraR_2
zraR_2	ZONMW-26_01563	zraR_2
zraR_2	ZONMW-26_00066	zraR_2
zraR_2	ZONMW-26_02456	zraR_2
zraR_2	ZONMW-26_02397	zraR_2
zraR_2	ZONMW-26_00546	zraR_2
zraR_2	ZONMW-26_01225	zraR_2
zraR_2	ZONMW-26_02800	zraR_2
zraR_2	ZONMW-26_02405	zraR_2
zraR_2	ZONMW-26_00076	zraR_2
zraR_2	ZONMW-26_00944	zraR_2
zraR_2	ZONMW-26_00788	zraR_2
zraR_2	ZONMW-26_02524	zraR_2
zraR_2	ZONMW-26_01199	zraR_2
zraR_2	ZONMW-26_02387	zraR_2
zraR_2	ZONMW-26_02385	zraR_2
zraR_2	ZONMW-26_02690	zraR_2
zraR_2	ZONMW-26_00737	zraR_2
zraR_2	ZONMW-26_00547	zraR_2
zraR_2	ZONMW-26_00578	zraR_2
zraR_2	ZONMW-26_02441	zraR_2
zraR_2	ZONMW-26_02171	zraR_2
zraR_2	ZONMW-26_00712	zraR_2
zraR_2	ZONMW-26_01419	zraR_2
zraR_2	ZONMW-26_01921	zraR_2
zraR_2	ZONMW-26_02149	zraR_2
zraR_2	ZONMW-26_02442	zraR_2
zraR_2	ZONMW-26_02976	zraR_2
zraR_2	ZONMW-26_02846	zraR_2

zraR_2	ZONMW-26_01674	zraR_2
zraR_2	ZONMW-26_01445	zraR_2
zraR_2	ZONMW-26_01859	zraR_2
zraR_2	ZONMW-26_00969	zraR_2
zraR_2	ZONMW-26_00062	zraR_2
zraR_2	ZONMW-26_03093	zraR_2
zraR_2	ZONMW-27_02459	zraR_2
zraR_2	ZONMW-27_00088	zraR_2
zraR_2	ZONMW-27_00079	zraR_2
zraR_2	ZONMW-27_03124	zraR_2
zraR_2	ZONMW-27_03154	zraR_2
zraR_2	ZONMW-27_01276	zraR_2
zraR_2	ZONMW-27_02901	zraR_2
zraR_2	ZONMW-27_01232	zraR_2
zraR_2	ZONMW-27_00931	zraR_2
zraR_2	ZONMW-27_01233	zraR_2
zraR_2	ZONMW-27_02902	zraR_2
zraR_2	ZONMW-27_03371	zraR_2
zraR_2	ZONMW-27_00930	zraR_2
zraR_2	ZONMW-27_03372	zraR_2
zraR_2	ZONMW-27_01044	zraR_2
zraR_2	ZONMW-27_00098	zraR_2
zraR_2	ZONMW-27_00818	zraR_2
zraR_2	ZONMW-27_01637	zraR_2
zraR_2	ZONMW-27_00097	zraR_2
zraR_2	ZONMW-27_03081	zraR_2
zraR_2	ZONMW-27_01636	zraR_2
zraR_2	ZONMW-27_01577	zraR_2
zraR_2	ZONMW-27_00648	zraR_2
zraR_2	ZONMW-27_02541	zraR_2
zraR_2	ZONMW-27_01585	zraR_2
zraR_2	ZONMW-27_03071	zraR_2
zraR_2	ZONMW-27_00843	zraR_2
zraR_2	ZONMW-27_00992	zraR_2
zraR_2	ZONMW-27_01705	zraR_2
zraR_2	ZONMW-27_00671	zraR_2
zraR_2	ZONMW-27_01567	zraR_2
zraR_2	ZONMW-27_01565	zraR_2
zraR_2	ZONMW-27_02114	zraR_2
zraR_2	ZONMW-27_01043	zraR_2
zraR_2	ZONMW-27_01244	zraR_2
zraR_2	ZONMW-27_01213	zraR_2
zraR_2	ZONMW-27_01245	zraR_2
zraR_2	ZONMW-27_01621	zraR_2
zraR_2	ZONMW-27_02294	zraR_2
zraR_2	ZONMW-27_01068	zraR_2
zraR_2	ZONMW-27_00453	zraR_2
zraR_2	ZONMW-27_03016	zraR_2

zraR_2	ZONMW-27_02272	zraR_2
zraR_2	ZONMW-27_01622	zraR_2
zraR_2	ZONMW-27_03253	zraR_2
zraR_2	ZONMW-27_02495	zraR_2
zraR_2	ZONMW-27_00427	zraR_2
zraR_2	ZONMW-27_00409	zraR_2
zraR_2	ZONMW-27_02629	zraR_2
zraR_2	ZONMW-27_00227	zraR_2
zraR_2	ZONMW-27_03085	zraR_2
zraR_2	ZONMW-27_02812	zraR_2
zraR_2	ZONMW-28_01669	zraR_2
zraR_2	ZONMW-28_00274	zraR_2
zraR_2	ZONMW-28_02712	zraR_2
zraR_2	ZONMW-28_02036	zraR_2
zraR_2	ZONMW-28_00687	zraR_2
zraR_2	ZONMW-28_03494	zraR_2
zraR_2	ZONMW-28_01141	zraR_2
zraR_2	ZONMW-28_01232	zraR_2
zraR_2	ZONMW-28_01233	zraR_2
zraR_2	ZONMW-28_02979	zraR_2
zraR_2	ZONMW-28_01014	zraR_2
zraR_2	ZONMW-28_01955	zraR_2
zraR_2	ZONMW-28_03191	zraR_2
zraR_2	ZONMW-28_01995	zraR_2
zraR_2	ZONMW-28_00325	zraR_2
zraR_2	ZONMW-28_01143	zraR_2
zraR_2	ZONMW-28_01895	zraR_2
zraR_2	ZONMW-28_00655	zraR_2
zraR_2	ZONMW-28_04017	zraR_2
zraR_2	ZONMW-28_02096	zraR_2
zraR_2	ZONMW-28_01142	zraR_2
zraR_2	ZONMW-28_01954	zraR_2
zraR_2	ZONMW-28_04018	zraR_2
zraR_2	ZONMW-28_03869	zraR_2
zraR_2	ZONMW-28_02181	zraR_2
zraR_2	ZONMW-28_01186	zraR_2
zraR_2	ZONMW-28_02283	zraR_2
zraR_2	ZONMW-28_01001	zraR_2
zraR_2	ZONMW-28_01996	zraR_2
zraR_2	ZONMW-28_01499	zraR_2
zraR_2	ZONMW-28_01896	zraR_2
zraR_2	ZONMW-28_03165	zraR_2
zraR_2	ZONMW-28_00968	zraR_2
zraR_2	ZONMW-28_02632	zraR_2
zraR_2	ZONMW-28_01146	zraR_2
zraR_2	ZONMW-28_03173	zraR_2
zraR_2	ZONMW-28_02527	zraR_2
zraR_2	ZONMW-28_03418	zraR_2

zraR_2	ZONMW-28_01811	zraR_2
zraR_2	ZONMW-28_01453	zraR_2
zraR_2	ZONMW-28_01332	zraR_2
zraR_2	ZONMW-28_00346	zraR_2
zraR_2	ZONMW-28_03737	zraR_2
zraR_2	ZONMW-28_00686	zraR_2
zraR_2	ZONMW-28_01130	zraR_2
zraR_2	ZONMW-28_01891	zraR_2
zraR_2	ZONMW-28_02529	zraR_2
zraR_2	ZONMW-28_00345	zraR_2
zraR_2	ZONMW-28_00525	zraR_2
zraR_2	ZONMW-28_03257	zraR_2
zraR_2	ZONMW-28_01667	zraR_2
zraR_2	ZONMW-28_00916	zraR_2
zraR_2	ZONMW-28_01231	zraR_2
zraR_2	ZONMW-28_00780	zraR_2
zraR_2	ZONMW-28_03420	zraR_2
zraR_2	ZONMW-28_03649	zraR_2
zraR_2	ZONMW-28_03740	zraR_2
zraR_2	ZONMW-28_00656	zraR_2
zraR_2	ZONMW-28_03944	zraR_2
zraR_2	ZONMW-28_03164	zraR_2
zraR_2	ZONMW-28_03650	zraR_2
zraR_2	ZONMW-28_01129	zraR_2
zraR_2	ZONMW-28_00216	zraR_2
zraR_2	ZONMW-28_03194	zraR_2
zraR_2	ZONMW-28_02190	zraR_2
zraR_2	ZONMW-28_01465	zraR_2
zraR_2	ZONMW-28_01145	zraR_2
zraR_2	ZONMW-28_02061	zraR_2
zraR_2	ZONMW-28_02633	zraR_2
zraR_2	ZONMW-28_03163	zraR_2
zraR_2	ZONMW-28_03739	zraR_2
zraR_2	ZONMW-28_01972	zraR_2
zraR_2	ZONMW-28_03943	zraR_2
zraR_2	ZONMW-28_01682	zraR_2
zraR_2	ZONMW-28_03071	zraR_2
zraR_2	ZONMW-28_00777	zraR_2
zraR_2	ZONMW-28_03121	zraR_2
zraR_2	ZONMW-28_00085	zraR_2
zraR_2	ZONMW-28_00917	zraR_2
zraR_2	ZONMW-28_01013	zraR_2
zraR_2	ZONMW-28_00524	zraR_2
zraR_2	ZONMW-28_00311	zraR_2
zraR_2	ZONMW-28_02589	zraR_2
zraR_2	ZONMW-28_02282	zraR_2
zraR_2	ZONMW-28_00967	zraR_2
zraR_2	ZONMW-28_01185	zraR_2

zraR_2	ZONMW-28_03557	zraR_2
zraR_2	ZONMW-28_01454	zraR_2
zraR_2	ZONMW-28_01331	zraR_2
zraR_2	ZONMW-28_03282	zraR_2
zraR_2	ZONMW-28_01495	zraR_2
zraR_2	ZONMW-28_02528	zraR_2
zraR_2	ZONMW-28_01843	zraR_2
zraR_2	ZONMW-28_00293	zraR_2
zraR_2	ZONMW-28_00768	zraR_2
zraR_2	ZONMW-28_02188	zraR_2
zraR_2	ZONMW-28_01908	zraR_2
zraR_2	ZONMW-28_03872	zraR_2
zraR_2	ZONMW-28_02200	zraR_2
zraR_2	ZONMW-28_00812	zraR_2
zraR_2	ZONMW-28_01496	zraR_2
zraR_2	ZONMW-28_02543	zraR_2
zraR_2	ZONMW-28_02413	zraR_2
zraR_2	ZONMW-28_01814	zraR_2
zraR_2	ZONMW-28_02532	zraR_2
zraR_2	ZONMW-28_02095	zraR_2
zraR_2	ZONMW-28_00324	zraR_2
zraR_2	ZONMW-28_03529	zraR_2
zraR_2	ZONMW-28_01497	zraR_2
zraR_2	ZONMW-28_00671	zraR_2
zraR_2	ZONMW-28_00203	zraR_2
zraR_2	ZONMW-28_01892	zraR_2
zraR_2	ZONMW-28_02186	zraR_2
zraR_2	ZONMW-28_01890	zraR_2
zraR_2	ZONMW-28_03488	zraR_2
zraR_2	ZONMW-28_00073	zraR_2
zraR_2	ZONMW-28_01775	zraR_2
zraR_2	ZONMW-28_00070	zraR_2
zraR_2	ZONMW-28_02345	zraR_2
zraR_2	ZONMW-28_00993	zraR_2
zraR_2	ZONMW-28_03011	zraR_2
zraR_2	ZONMW-28_00490	zraR_2
zraR_2	ZONMW-29_02242	zraR_2
zraR_2	ZONMW-29_01502	zraR_2
zraR_2	ZONMW-29_02604	zraR_2
zraR_2	ZONMW-29_03124	zraR_2
zraR_2	ZONMW-29_02152	zraR_2
zraR_2	ZONMW-29_00819	zraR_2
zraR_2	ZONMW-29_00820	zraR_2
zraR_2	ZONMW-29_02151	zraR_2
zraR_2	ZONMW-29_03125	zraR_2
zraR_2	ZONMW-29_02060	zraR_2
zraR_2	ZONMW-29_00770	zraR_2
zraR_2	ZONMW-29_02693	zraR_2

zraR_2	ZONMW-29_01000	zraR_2
zraR_2	ZONMW-29_01327	zraR_2
zraR_2	ZONMW-29_01395	zraR_2
zraR_2	ZONMW-29_02059	zraR_2
zraR_2	ZONMW-29_02694	zraR_2
zraR_2	ZONMW-29_01394	zraR_2
zraR_2	ZONMW-29_00999	zraR_2
zraR_2	ZONMW-29_01591	zraR_2
zraR_2	ZONMW-29_01779	zraR_2
zraR_2	ZONMW-29_00286	zraR_2
zraR_2	ZONMW-29_01392	zraR_2
zraR_2	ZONMW-29_02171	zraR_2
zraR_2	ZONMW-29_02844	zraR_2
zraR_2	ZONMW-29_02629	zraR_2
zraR_2	ZONMW-29_00157	zraR_2
zraR_2	ZONMW-29_02924	zraR_2
zraR_2	ZONMW-29_02261	zraR_2
zraR_2	ZONMW-29_00769	zraR_2
zraR_2	ZONMW-29_00526	zraR_2
zraR_2	ZONMW-29_01812	zraR_2
zraR_2	ZONMW-29_01470	zraR_2
zraR_2	ZONMW-29_00175	zraR_2
zraR_2	ZONMW-29_02510	zraR_2
zraR_2	ZONMW-29_00921	zraR_2
zraR_2	ZONMW-29_01391	zraR_2
zraR_2	ZONMW-29_00525	zraR_2
zraR_2	ZONMW-29_00778	zraR_2
zraR_2	ZONMW-29_01271	zraR_2
zraR_2	ZONMW-29_01963	zraR_2
zraR_2	ZONMW-29_02077	zraR_2
zraR_2	ZONMW-29_02927	zraR_2
zraR_2	ZONMW-29_02359	zraR_2
zraR_2	ZONMW-29_00318	zraR_2
zraR_2	ZONMW-29_02314	zraR_2
zraR_2	ZONMW-29_02259	zraR_2
zraR_2	ZONMW-29_01229	zraR_2
zraR_2	ZONMW-29_02231	zraR_2
zraR_2	ZONMW-29_00996	zraR_2
zraR_2	ZONMW-29_02049	zraR_2
zraR_2	ZONMW-30_01624	zraR_2
zraR_2	ZONMW-30_00972	zraR_2
zraR_2	ZONMW-30_00902	zraR_2
zraR_2	ZONMW-30_00503	zraR_2
zraR_2	ZONMW-30_00502	zraR_2
zraR_2	ZONMW-30_02205	zraR_2
zraR_2	ZONMW-30_02620	zraR_2
zraR_2	ZONMW-30_01104	zraR_2
zraR_2	ZONMW-30_01105	zraR_2

zraR_2	ZONMW-30_02204	zraR_2
zraR_2	ZONMW-30_02713	zraR_2
zraR_2	ZONMW-30_01189	zraR_2
zraR_2	ZONMW-30_03284	zraR_2
zraR_2	ZONMW-30_03283	zraR_2
zraR_2	ZONMW-30_00166	zraR_2
zraR_2	ZONMW-30_02619	zraR_2
zraR_2	ZONMW-30_00732	zraR_2
zraR_2	ZONMW-30_00167	zraR_2
zraR_2	ZONMW-30_02400	zraR_2
zraR_2	ZONMW-30_00596	zraR_2
zraR_2	ZONMW-30_01870	zraR_2
zraR_2	ZONMW-30_03238	zraR_2
zraR_2	ZONMW-30_00147	zraR_2
zraR_2	ZONMW-30_01745	zraR_2
zraR_2	ZONMW-30_01818	zraR_2
zraR_2	ZONMW-30_00044	zraR_2
zraR_2	ZONMW-30_02600	zraR_2
zraR_2	ZONMW-30_01000	zraR_2
zraR_2	ZONMW-30_01491	zraR_2
zraR_2	ZONMW-30_01869	zraR_2
zraR_2	ZONMW-30_01961	zraR_2
zraR_2	ZONMW-30_01051	zraR_2
zraR_2	ZONMW-30_00879	zraR_2
zraR_2	ZONMW-30_00654	zraR_2
zraR_2	ZONMW-30_00356	zraR_2
zraR_2	ZONMW-30_01863	zraR_2
zraR_2	ZONMW-30_00461	zraR_2
zraR_2	ZONMW-30_01341	zraR_2
zraR_2	ZONMW-30_01864	zraR_2
zraR_2	ZONMW-30_01488	zraR_2
zraR_2	ZONMW-30_02383	zraR_2
zraR_2	ZONMW-30_03179	zraR_2
zraR_2	ZONMW-30_01308	zraR_2
zraR_2	ZONMW-30_03318	zraR_2
zraR_2	ZONMW-31_03423	zraR_2
zraR_2	ZONMW-31_03289	zraR_2
zraR_2	ZONMW-31_01979	zraR_2
zraR_2	ZONMW-31_02979	zraR_2
zraR_2	ZONMW-31_01636	zraR_2
zraR_2	ZONMW-31_01266	zraR_2
zraR_2	ZONMW-31_01084	zraR_2
zraR_2	ZONMW-31_00385	zraR_2
zraR_2	ZONMW-31_00384	zraR_2
zraR_2	ZONMW-31_00990	zraR_2
zraR_2	ZONMW-31_00274	zraR_2
zraR_2	ZONMW-31_02245	zraR_2
zraR_2	ZONMW-31_03019	zraR_2

zraR_2	ZONMW-31_00243	zraR_2
zraR_2	ZONMW-31_02451	zraR_2
zraR_2	ZONMW-31_00273	zraR_2
zraR_2	ZONMW-31_03718	zraR_2
zraR_2	ZONMW-31_02231	zraR_2
zraR_2	ZONMW-31_00803	zraR_2
zraR_2	ZONMW-31_03018	zraR_2
zraR_2	ZONMW-31_02244	zraR_2
zraR_2	ZONMW-31_02550	zraR_2
zraR_2	ZONMW-31_01809	zraR_2
zraR_2	ZONMW-31_00244	zraR_2
zraR_2	ZONMW-31_00548	zraR_2
zraR_2	ZONMW-31_02339	zraR_2
zraR_2	ZONMW-31_01131	zraR_2
zraR_2	ZONMW-31_01903	zraR_2
zraR_2	ZONMW-31_00236	zraR_2
zraR_2	ZONMW-31_02881	zraR_2
zraR_2	ZONMW-31_02535	zraR_2
zraR_2	ZONMW-31_03229	zraR_2
zraR_2	ZONMW-31_01476	zraR_2
zraR_2	ZONMW-31_00574	zraR_2
zraR_2	ZONMW-31_01085	zraR_2
zraR_2	ZONMW-31_03720	zraR_2
zraR_2	ZONMW-31_00989	zraR_2
zraR_2	ZONMW-31_03425	zraR_2
zraR_2	ZONMW-31_00300	zraR_2
zraR_2	ZONMW-31_01746	zraR_2
zraR_2	ZONMW-31_01477	zraR_2
zraR_2	ZONMW-31_03129	zraR_2
zraR_2	ZONMW-31_03040	zraR_2
zraR_2	ZONMW-31_03228	zraR_2
zraR_2	ZONMW-31_00774	zraR_2
zraR_2	ZONMW-31_03306	zraR_2
zraR_2	ZONMW-31_03041	zraR_2
zraR_2	ZONMW-31_00299	zraR_2
zraR_2	ZONMW-31_03130	zraR_2
zraR_2	ZONMW-31_03697	zraR_2
zraR_2	ZONMW-31_02882	zraR_2
zraR_2	ZONMW-31_02957	zraR_2
zraR_2	ZONMW-31_02467	zraR_2
zraR_2	ZONMW-31_03723	zraR_2
zraR_2	ZONMW-31_01904	zraR_2
zraR_2	ZONMW-31_02489	zraR_2
zraR_2	ZONMW-31_02194	zraR_2
zraR_2	ZONMW-31_01811	zraR_2
zraR_2	ZONMW-31_03409	zraR_2
zraR_2	ZONMW-31_01720	zraR_2
zraR_2	ZONMW-31_02466	zraR_2

zraR_2	ZONMW-31_01130	zraR_2
zraR_2	ZONMW-31_03722	zraR_2
zraR_2	ZONMW-31_02219	zraR_2
zraR_2	ZONMW-31_02452	zraR_2
zraR_2	ZONMW-31_02232	zraR_2
zraR_2	ZONMW-31_02337	zraR_2
zraR_2	ZONMW-31_01875	zraR_2
zraR_2	ZONMW-31_01038	zraR_2
zraR_2	ZONMW-31_00549	zraR_2
zraR_2	ZONMW-31_03523	zraR_2
zraR_2	ZONMW-31_02885	zraR_2
zraR_2	ZONMW-31_00773	zraR_2
zraR_2	ZONMW-31_01595	zraR_2
zraR_2	ZONMW-31_02838	zraR_2
zraR_2	ZONMW-31_03307	zraR_2
zraR_2	ZONMW-31_01260	zraR_2
zraR_2	ZONMW-31_03407	zraR_2
zraR_2	ZONMW-31_00934	zraR_2
zraR_2	ZONMW-31_01008	zraR_2
zraR_2	ZONMW-31_00240	zraR_2
zraR_2	ZONMW-31_03659	zraR_2
zraR_2	ZONMW-31_00235	zraR_2
zraR_2	ZONMW-31_02738	zraR_2
zraR_2	ZONMW-31_03308	zraR_2
zraR_2	ZONMW-31_00811	zraR_2
zraR_2	ZONMW-31_03305	zraR_2
zraR_2	ZONMW-31_01444	zraR_2
zraR_2	ZONMW-31_03304	zraR_2
zraR_2	ZONMW-31_00471	zraR_2
zraR_2	ZONMW-31_03749	zraR_2
zraR_2	ZONMW-32_02303	zraR_2
zraR_2	ZONMW-32_01065	zraR_2
zraR_2	ZONMW-32_03164	zraR_2
zraR_2	ZONMW-32_02441	zraR_2
zraR_2	ZONMW-32_00404	zraR_2
zraR_2	ZONMW-32_00579	zraR_2
zraR_2	ZONMW-32_01549	zraR_2
zraR_2	ZONMW-32_01640	zraR_2
zraR_2	ZONMW-32_01641	zraR_2
zraR_2	ZONMW-32_01463	zraR_2
zraR_2	ZONMW-32_00077	zraR_2
zraR_2	ZONMW-32_02017	zraR_2
zraR_2	ZONMW-32_00716	zraR_2
zraR_2	ZONMW-32_02401	zraR_2
zraR_2	ZONMW-32_01116	zraR_2
zraR_2	ZONMW-32_01551	zraR_2
zraR_2	ZONMW-32_02077	zraR_2
zraR_2	ZONMW-32_00436	zraR_2

zraR_2	ZONMW-32_03874	zraR_2
zraR_2	ZONMW-32_02501	zraR_2
zraR_2	ZONMW-32_01550	zraR_2
zraR_2	ZONMW-32_02018	zraR_2
zraR_2	ZONMW-32_03875	zraR_2
zraR_2	ZONMW-32_03807	zraR_2
zraR_2	ZONMW-32_02586	zraR_2
zraR_2	ZONMW-32_01594	zraR_2
zraR_2	ZONMW-32_02688	zraR_2
zraR_2	ZONMW-32_00090	zraR_2
zraR_2	ZONMW-32_02402	zraR_2
zraR_2	ZONMW-32_01906	zraR_2
zraR_2	ZONMW-32_02076	zraR_2
zraR_2	ZONMW-32_00690	zraR_2
zraR_2	ZONMW-32_00123	zraR_2
zraR_2	ZONMW-32_03244	zraR_2
zraR_2	ZONMW-32_01554	zraR_2
zraR_2	ZONMW-32_00698	zraR_2
zraR_2	ZONMW-32_02823	zraR_2
zraR_2	ZONMW-32_03563	zraR_2
zraR_2	ZONMW-32_02161	zraR_2
zraR_2	ZONMW-32_01860	zraR_2
zraR_2	ZONMW-32_01740	zraR_2
zraR_2	ZONMW-32_01137	zraR_2
zraR_2	ZONMW-32_03928	zraR_2
zraR_2	ZONMW-32_00405	zraR_2
zraR_2	ZONMW-32_01538	zraR_2
zraR_2	ZONMW-32_02081	zraR_2
zraR_2	ZONMW-32_02821	zraR_2
zraR_2	ZONMW-32_01136	zraR_2
zraR_2	ZONMW-32_01316	zraR_2
zraR_2	ZONMW-32_00782	zraR_2
zraR_2	ZONMW-32_02305	zraR_2
zraR_2	ZONMW-32_00175	zraR_2
zraR_2	ZONMW-32_01639	zraR_2
zraR_2	ZONMW-32_00311	zraR_2
zraR_2	ZONMW-32_03561	zraR_2
zraR_2	ZONMW-32_03677	zraR_2
zraR_2	ZONMW-32_03925	zraR_2
zraR_2	ZONMW-32_00435	zraR_2
zraR_2	ZONMW-32_03942	zraR_2
zraR_2	ZONMW-32_00689	zraR_2
zraR_2	ZONMW-32_03678	zraR_2
zraR_2	ZONMW-32_01537	zraR_2
zraR_2	ZONMW-32_01007	zraR_2
zraR_2	ZONMW-32_00719	zraR_2
zraR_2	ZONMW-32_02595	zraR_2
zraR_2	ZONMW-32_01872	zraR_2

zraR_2	ZONMW-32_01553	zraR_2
zraR_2	ZONMW-32_02466	zraR_2
zraR_2	ZONMW-32_03243	zraR_2
zraR_2	ZONMW-32_00688	zraR_2
zraR_2	ZONMW-32_03926	zraR_2
zraR_2	ZONMW-32_02000	zraR_2
zraR_2	ZONMW-32_03943	zraR_2
zraR_2	ZONMW-32_02290	zraR_2
zraR_2	ZONMW-32_03338	zraR_2
zraR_2	ZONMW-32_00314	zraR_2
zraR_2	ZONMW-32_03288	zraR_2
zraR_2	ZONMW-32_00876	zraR_2
zraR_2	ZONMW-32_00174	zraR_2
zraR_2	ZONMW-32_00078	zraR_2
zraR_2	ZONMW-32_01315	zraR_2
zraR_2	ZONMW-32_01102	zraR_2
zraR_2	ZONMW-32_02761	zraR_2
zraR_2	ZONMW-32_02687	zraR_2
zraR_2	ZONMW-32_00124	zraR_2
zraR_2	ZONMW-32_01593	zraR_2
zraR_2	ZONMW-32_00642	zraR_2
zraR_2	ZONMW-32_01861	zraR_2
zraR_2	ZONMW-32_01739	zraR_2
zraR_2	ZONMW-32_03407	zraR_2
zraR_2	ZONMW-32_01902	zraR_2
zraR_2	ZONMW-32_02822	zraR_2
zraR_2	ZONMW-32_02129	zraR_2
zraR_2	ZONMW-32_01084	zraR_2
zraR_2	ZONMW-32_00323	zraR_2
zraR_2	ZONMW-32_02593	zraR_2
zraR_2	ZONMW-32_02064	zraR_2
zraR_2	ZONMW-32_03810	zraR_2
zraR_2	ZONMW-32_02605	zraR_2
zraR_2	ZONMW-32_00279	zraR_2
zraR_2	ZONMW-32_01903	zraR_2
zraR_2	ZONMW-32_02807	zraR_2
zraR_2	ZONMW-32_02937	zraR_2
zraR_2	ZONMW-32_02158	zraR_2
zraR_2	ZONMW-32_02818	zraR_2
zraR_2	ZONMW-32_02500	zraR_2
zraR_2	ZONMW-32_01115	zraR_2
zraR_2	ZONMW-32_00614	zraR_2
zraR_2	ZONMW-32_01904	zraR_2
zraR_2	ZONMW-32_00420	zraR_2
zraR_2	ZONMW-32_00994	zraR_2
zraR_2	ZONMW-32_02080	zraR_2
zraR_2	ZONMW-32_02591	zraR_2
zraR_2	ZONMW-32_02082	zraR_2

zraR_2	ZONMW-32_00573	zraR_2
zraR_2	ZONMW-32_00864	zraR_2
zraR_2	ZONMW-32_02197	zraR_2
zraR_2	ZONMW-32_00861	zraR_2
zraR_2	ZONMW-32_03005	zraR_2
zraR_2	ZONMW-32_00098	zraR_2
zraR_2	ZONMW-32_01491	zraR_2
zraR_2	ZONMW-32_01281	zraR_2
zraR_2	ZONMW-33_02294	zraR_2
zraR_2	ZONMW-33_01054	zraR_2
zraR_2	ZONMW-33_03110	zraR_2
zraR_2	ZONMW-33_02677	zraR_2
zraR_2	ZONMW-33_00271	zraR_2
zraR_2	ZONMW-33_00447	zraR_2
zraR_2	ZONMW-33_01539	zraR_2
zraR_2	ZONMW-33_01630	zraR_2
zraR_2	ZONMW-33_01631	zraR_2
zraR_2	ZONMW-33_01453	zraR_2
zraR_2	ZONMW-33_03338	zraR_2
zraR_2	ZONMW-33_02008	zraR_2
zraR_2	ZONMW-33_00584	zraR_2
zraR_2	ZONMW-33_02717	zraR_2
zraR_2	ZONMW-33_01105	zraR_2
zraR_2	ZONMW-33_01541	zraR_2
zraR_2	ZONMW-33_02068	zraR_2
zraR_2	ZONMW-33_00303	zraR_2
zraR_2	ZONMW-33_03630	zraR_2
zraR_2	ZONMW-33_02617	zraR_2
zraR_2	ZONMW-33_01540	zraR_2
zraR_2	ZONMW-33_02009	zraR_2
zraR_2	ZONMW-33_03629	zraR_2
zraR_2	ZONMW-33_03740	zraR_2
zraR_2	ZONMW-33_02532	zraR_2
zraR_2	ZONMW-33_01584	zraR_2
zraR_2	ZONMW-33_02430	zraR_2
zraR_2	ZONMW-33_03351	zraR_2
zraR_2	ZONMW-33_02716	zraR_2
zraR_2	ZONMW-33_01896	zraR_2
zraR_2	ZONMW-33_02067	zraR_2
zraR_2	ZONMW-33_00558	zraR_2
zraR_2	ZONMW-33_03384	zraR_2
zraR_2	ZONMW-33_03030	zraR_2
zraR_2	ZONMW-33_01544	zraR_2
zraR_2	ZONMW-33_00566	zraR_2
zraR_2	ZONMW-33_02925	zraR_2
zraR_2	ZONMW-33_03913	zraR_2
zraR_2	ZONMW-33_02152	zraR_2
zraR_2	ZONMW-33_01850	zraR_2

zraR_2	ZONMW-33_01730	zraR_2
zraR_2	ZONMW-33_01126	zraR_2
zraR_2	ZONMW-33_03733	zraR_2
zraR_2	ZONMW-33_00272	zraR_2
zraR_2	ZONMW-33_01528	zraR_2
zraR_2	ZONMW-33_02072	zraR_2
zraR_2	ZONMW-33_02927	zraR_2
zraR_2	ZONMW-33_01125	zraR_2
zraR_2	ZONMW-33_01305	zraR_2
zraR_2	ZONMW-33_00650	zraR_2
zraR_2	ZONMW-33_02296	zraR_2
zraR_2	ZONMW-33_00042	zraR_2
zraR_2	ZONMW-33_01629	zraR_2
zraR_2	ZONMW-33_00178	zraR_2
zraR_2	ZONMW-33_03911	zraR_2
zraR_2	ZONMW-33_03544	zraR_2
zraR_2	ZONMW-33_03730	zraR_2
zraR_2	ZONMW-33_00302	zraR_2
zraR_2	ZONMW-33_03866	zraR_2
zraR_2	ZONMW-33_00557	zraR_2
zraR_2	ZONMW-33_03543	zraR_2
zraR_2	ZONMW-33_01527	zraR_2
zraR_2	ZONMW-33_00996	zraR_2
zraR_2	ZONMW-33_00587	zraR_2
zraR_2	ZONMW-33_02523	zraR_2
zraR_2	ZONMW-33_01862	zraR_2
zraR_2	ZONMW-33_01543	zraR_2
zraR_2	ZONMW-33_02652	zraR_2
zraR_2	ZONMW-33_03031	zraR_2
zraR_2	ZONMW-33_00556	zraR_2
zraR_2	ZONMW-33_03731	zraR_2
zraR_2	ZONMW-33_01991	zraR_2
zraR_2	ZONMW-33_03867	zraR_2
zraR_2	ZONMW-33_02281	zraR_2
zraR_2	ZONMW-33_00728	zraR_2
zraR_2	ZONMW-33_00181	zraR_2
zraR_2	ZONMW-33_00678	zraR_2
zraR_2	ZONMW-33_00866	zraR_2
zraR_2	ZONMW-33_00041	zraR_2
zraR_2	ZONMW-33_03339	zraR_2
zraR_2	ZONMW-33_01304	zraR_2
zraR_2	ZONMW-33_01091	zraR_2
zraR_2	ZONMW-33_02987	zraR_2
zraR_2	ZONMW-33_02431	zraR_2
zraR_2	ZONMW-33_03385	zraR_2
zraR_2	ZONMW-33_01583	zraR_2
zraR_2	ZONMW-33_00510	zraR_2
zraR_2	ZONMW-33_01851	zraR_2

zraR_2	ZONMW-33_01729	zraR_2
zraR_2	ZONMW-33_03410	zraR_2
zraR_2	ZONMW-33_01892	zraR_2
zraR_2	ZONMW-33_02926	zraR_2
zraR_2	ZONMW-33_02120	zraR_2
zraR_2	ZONMW-33_01073	zraR_2
zraR_2	ZONMW-33_00190	zraR_2
zraR_2	ZONMW-33_02525	zraR_2
zraR_2	ZONMW-33_02055	zraR_2
zraR_2	ZONMW-33_03737	zraR_2
zraR_2	ZONMW-33_02513	zraR_2
zraR_2	ZONMW-33_00146	zraR_2
zraR_2	ZONMW-33_01893	zraR_2
zraR_2	ZONMW-33_02941	zraR_2
zraR_2	ZONMW-33_02811	zraR_2
zraR_2	ZONMW-33_02149	zraR_2
zraR_2	ZONMW-33_02930	zraR_2
zraR_2	ZONMW-33_02618	zraR_2
zraR_2	ZONMW-33_01104	zraR_2
zraR_2	ZONMW-33_00482	zraR_2
zraR_2	ZONMW-33_01894	zraR_2
zraR_2	ZONMW-33_00287	zraR_2
zraR_2	ZONMW-33_00983	zraR_2
zraR_2	ZONMW-33_02071	zraR_2
zraR_2	ZONMW-33_02527	zraR_2
zraR_2	ZONMW-33_02073	zraR_2
zraR_2	ZONMW-33_00441	zraR_2
zraR_2	ZONMW-33_00854	zraR_2
zraR_2	ZONMW-33_02188	zraR_2
zraR_2	ZONMW-33_00851	zraR_2
zraR_2	ZONMW-33_02743	zraR_2
zraR_2	ZONMW-33_03359	zraR_2
zraR_2	ZONMW-33_01481	zraR_2
zraR_2	ZONMW-33_01270	zraR_2
zraR_2	ZONMW-34_01256	zraR_2
zraR_2	ZONMW-34_03172	zraR_2
zraR_2	ZONMW-34_02485	zraR_2
zraR_2	ZONMW-34_02546	zraR_2
zraR_2	ZONMW-34_01970	zraR_2
zraR_2	ZONMW-34_00648	zraR_2
zraR_2	ZONMW-34_01641	zraR_2
zraR_2	ZONMW-34_02919	zraR_2
zraR_2	ZONMW-34_00824	zraR_2
zraR_2	ZONMW-34_00016	zraR_2
zraR_2	ZONMW-34_00017	zraR_2
zraR_2	ZONMW-34_02458	zraR_2
zraR_2	ZONMW-34_00380	zraR_2
zraR_2	ZONMW-34_02138	zraR_2

zraR_2	ZONMW-34_03013	zraR_2
zraR_2	ZONMW-34_02918	zraR_2
zraR_2	ZONMW-34_00681	zraR_2
zraR_2	ZONMW-34_02011	zraR_2
zraR_2	ZONMW-34_02186	zraR_2
zraR_2	ZONMW-34_02139	zraR_2
zraR_2	ZONMW-34_00948	zraR_2
zraR_2	ZONMW-34_02869	zraR_2
zraR_2	ZONMW-34_03638	zraR_2
zraR_2	ZONMW-34_02840	zraR_2
zraR_2	ZONMW-34_03014	zraR_2
zraR_2	ZONMW-34_01750	zraR_2
zraR_2	ZONMW-34_00922	zraR_2
zraR_2	ZONMW-34_02010	zraR_2
zraR_2	ZONMW-34_02185	zraR_2
zraR_2	ZONMW-34_02917	zraR_2
zraR_2	ZONMW-34_02623	zraR_2
zraR_2	ZONMW-34_02914	zraR_2
zraR_2	ZONMW-34_00649	zraR_2
zraR_2	ZONMW-34_00930	zraR_2
zraR_2	ZONMW-34_03425	zraR_2
zraR_2	ZONMW-34_03423	zraR_2
zraR_2	ZONMW-34_00572	zraR_2
zraR_2	ZONMW-34_01317	zraR_2
zraR_2	ZONMW-34_00199	zraR_2
zraR_2	ZONMW-34_00113	zraR_2
zraR_2	ZONMW-34_00235	zraR_2
zraR_2	ZONMW-34_02033	zraR_2
zraR_2	ZONMW-34_00231	zraR_2
zraR_2	ZONMW-34_02190	zraR_2
zraR_2	ZONMW-34_03362	zraR_2
zraR_2	ZONMW-34_02929	zraR_2
zraR_2	ZONMW-34_01316	zraR_2
zraR_2	ZONMW-34_01014	zraR_2
zraR_2	ZONMW-34_01497	zraR_2
zraR_2	ZONMW-34_00015	zraR_2
zraR_2	ZONMW-34_03436	zraR_2
zraR_2	ZONMW-34_03170	zraR_2
zraR_2	ZONMW-34_03546	zraR_2
zraR_2	ZONMW-34_02036	zraR_2
zraR_2	ZONMW-34_03843	zraR_2
zraR_2	ZONMW-34_02930	zraR_2
zraR_2	ZONMW-34_03547	zraR_2
zraR_2	ZONMW-34_00680	zraR_2
zraR_2	ZONMW-34_01199	zraR_2
zraR_2	ZONMW-34_00951	zraR_2
zraR_2	ZONMW-34_00211	zraR_2
zraR_2	ZONMW-34_01767	zraR_2

zraR_2	ZONMW-34_01946	zraR_2
zraR_2	ZONMW-34_02622	zraR_2
zraR_2	ZONMW-34_00920	zraR_2
zraR_2	ZONMW-34_03065	zraR_2
zraR_2	ZONMW-34_02035	zraR_2
zraR_2	ZONMW-34_02121	zraR_2
zraR_2	ZONMW-34_00552	zraR_2
zraR_2	ZONMW-34_03842	zraR_2
zraR_2	ZONMW-34_03184	zraR_2
zraR_2	ZONMW-34_01094	zraR_2
zraR_2	ZONMW-34_01041	zraR_2
zraR_2	ZONMW-34_02770	zraR_2
zraR_2	ZONMW-34_01766	zraR_2
zraR_2	ZONMW-34_00921	zraR_2
zraR_2	ZONMW-34_01496	zraR_2
zraR_2	ZONMW-34_01751	zraR_2
zraR_2	ZONMW-34_00381	zraR_2
zraR_2	ZONMW-34_02841	zraR_2
zraR_2	ZONMW-34_00874	zraR_2
zraR_2	ZONMW-34_01052	zraR_2
zraR_2	ZONMW-34_03040	zraR_2
zraR_2	ZONMW-34_00495	zraR_2
zraR_2	ZONMW-34_00200	zraR_2
zraR_2	ZONMW-34_00112	zraR_2
zraR_2	ZONMW-34_02253	zraR_2
zraR_2	ZONMW-34_03435	zraR_2
zraR_2	ZONMW-34_02750	zraR_2
zraR_2	ZONMW-34_02173	zraR_2
zraR_2	ZONMW-34_03364	zraR_2
zraR_2	ZONMW-34_03635	zraR_2
zraR_2	ZONMW-34_02756	zraR_2
zraR_2	ZONMW-34_01830	zraR_2
zraR_2	ZONMW-34_00232	zraR_2
zraR_2	ZONMW-34_00818	zraR_2
zraR_2	ZONMW-34_02870	zraR_2
zraR_2	ZONMW-34_03374	zraR_2
zraR_2	ZONMW-34_03252	zraR_2
zraR_2	ZONMW-34_03570	zraR_2
zraR_2	ZONMW-34_02915	zraR_2
zraR_2	ZONMW-34_00863	zraR_2
zraR_2	ZONMW-34_00233	zraR_2
zraR_2	ZONMW-34_01188	zraR_2
zraR_2	ZONMW-34_02189	zraR_2
zraR_2	ZONMW-34_02191	zraR_2
zraR_2	ZONMW-34_02342	zraR_2
zraR_2	ZONMW-34_03626	zraR_2
zraR_2	ZONMW-34_01462	zraR_2
zraR_2	ZONMW-34_01673	zraR_2

zraR_2	ZONMW-35_00329	zraR_2
zraR_2	ZONMW-35_03065	zraR_2
zraR_2	ZONMW-35_02449	zraR_2
zraR_2	ZONMW-35_02510	zraR_2
zraR_2	ZONMW-35_01662	zraR_2
zraR_2	ZONMW-35_00741	zraR_2
zraR_2	ZONMW-35_03605	zraR_2
zraR_2	ZONMW-35_02886	zraR_2
zraR_2	ZONMW-35_02639	zraR_2
zraR_2	ZONMW-35_01374	zraR_2
zraR_2	ZONMW-35_01373	zraR_2
zraR_2	ZONMW-35_02422	zraR_2
zraR_2	ZONMW-35_01010	zraR_2
zraR_2	ZONMW-35_02111	zraR_2
zraR_2	ZONMW-35_02979	zraR_2
zraR_2	ZONMW-35_02885	zraR_2
zraR_2	ZONMW-35_00708	zraR_2
zraR_2	ZONMW-35_01703	zraR_2
zraR_2	ZONMW-35_02063	zraR_2
zraR_2	ZONMW-35_02110	zraR_2
zraR_2	ZONMW-35_02295	zraR_2
zraR_2	ZONMW-35_02836	zraR_2
zraR_2	ZONMW-35_03854	zraR_2
zraR_2	ZONMW-35_02807	zraR_2
zraR_2	ZONMW-35_02978	zraR_2
zraR_2	ZONMW-35_01441	zraR_2
zraR_2	ZONMW-35_02321	zraR_2
zraR_2	ZONMW-35_01702	zraR_2
zraR_2	ZONMW-35_02064	zraR_2
zraR_2	ZONMW-35_02884	zraR_2
zraR_2	ZONMW-35_02587	zraR_2
zraR_2	ZONMW-35_02881	zraR_2
zraR_2	ZONMW-35_00740	zraR_2
zraR_2	ZONMW-35_02313	zraR_2
zraR_2	ZONMW-35_03390	zraR_2
zraR_2	ZONMW-35_03392	zraR_2
zraR_2	ZONMW-35_00817	zraR_2
zraR_2	ZONMW-35_00390	zraR_2
zraR_2	ZONMW-35_01191	zraR_2
zraR_2	ZONMW-35_01278	zraR_2
zraR_2	ZONMW-35_01155	zraR_2
zraR_2	ZONMW-35_01725	zraR_2
zraR_2	ZONMW-35_01159	zraR_2
zraR_2	ZONMW-35_02059	zraR_2
zraR_2	ZONMW-35_03272	zraR_2
zraR_2	ZONMW-35_02896	zraR_2
zraR_2	ZONMW-35_00389	zraR_2
zraR_2	ZONMW-35_02229	zraR_2

zraR_2	ZONMW-35_00570	zraR_2
zraR_2	ZONMW-35_01375	zraR_2
zraR_2	ZONMW-35_03379	zraR_2
zraR_2	ZONMW-35_03067	zraR_2
zraR_2	ZONMW-35_03507	zraR_2
zraR_2	ZONMW-35_01728	zraR_2
zraR_2	ZONMW-35_03783	zraR_2
zraR_2	ZONMW-35_02897	zraR_2
zraR_2	ZONMW-35_03508	zraR_2
zraR_2	ZONMW-35_00709	zraR_2
zraR_2	ZONMW-35_00272	zraR_2
zraR_2	ZONMW-35_02292	zraR_2
zraR_2	ZONMW-35_01179	zraR_2
zraR_2	ZONMW-35_01458	zraR_2
zraR_2	ZONMW-35_01638	zraR_2
zraR_2	ZONMW-35_02586	zraR_2
zraR_2	ZONMW-35_02323	zraR_2
zraR_2	ZONMW-35_02927	zraR_2
zraR_2	ZONMW-35_01727	zraR_2
zraR_2	ZONMW-35_02128	zraR_2
zraR_2	ZONMW-35_00837	zraR_2
zraR_2	ZONMW-35_03784	zraR_2
zraR_2	ZONMW-35_03053	zraR_2
zraR_2	ZONMW-35_02149	zraR_2
zraR_2	ZONMW-35_02202	zraR_2
zraR_2	ZONMW-35_00147	zraR_2
zraR_2	ZONMW-35_01457	zraR_2
zraR_2	ZONMW-35_02322	zraR_2
zraR_2	ZONMW-35_00569	zraR_2
zraR_2	ZONMW-35_01442	zraR_2
zraR_2	ZONMW-35_01009	zraR_2
zraR_2	ZONMW-35_02808	zraR_2
zraR_2	ZONMW-35_02369	zraR_2
zraR_2	ZONMW-35_02191	zraR_2
zraR_2	ZONMW-35_02952	zraR_2
zraR_2	ZONMW-35_00894	zraR_2
zraR_2	ZONMW-35_01190	zraR_2
zraR_2	ZONMW-35_01279	zraR_2
zraR_2	ZONMW-35_01996	zraR_2
zraR_2	ZONMW-35_03380	zraR_2
zraR_2	ZONMW-35_00127	zraR_2
zraR_2	ZONMW-35_02076	zraR_2
zraR_2	ZONMW-35_03270	zraR_2
zraR_2	ZONMW-35_03857	zraR_2
zraR_2	ZONMW-35_00133	zraR_2
zraR_2	ZONMW-35_01521	zraR_2
zraR_2	ZONMW-35_01158	zraR_2
zraR_2	ZONMW-35_02645	zraR_2

zraR_2	ZONMW-35_02837	zraR_2
zraR_2	ZONMW-35_03260	zraR_2
zraR_2	ZONMW-35_03213	zraR_2
zraR_2	ZONMW-35_03594	zraR_2
zraR_2	ZONMW-35_02882	zraR_2
zraR_2	ZONMW-35_02380	zraR_2
zraR_2	ZONMW-35_01157	zraR_2
zraR_2	ZONMW-35_00261	zraR_2
zraR_2	ZONMW-35_02060	zraR_2
zraR_2	ZONMW-35_02058	zraR_2
zraR_2	ZONMW-35_01907	zraR_2
zraR_2	ZONMW-35_03538	zraR_2
zraR_2	ZONMW-35_00535	zraR_2
zraR_2	ZONMW-35_03637	zraR_2
zraR_2	ZONMW-36_01942	zraR_2
zraR_2	ZONMW-36_00274	zraR_2
zraR_2	ZONMW-36_02892	zraR_2
zraR_2	ZONMW-36_02575	zraR_2
zraR_2	ZONMW-36_01098	zraR_2
zraR_2	ZONMW-36_00924	zraR_2
zraR_2	ZONMW-36_01817	zraR_2
zraR_2	ZONMW-36_01726	zraR_2
zraR_2	ZONMW-36_01725	zraR_2
zraR_2	ZONMW-36_00672	zraR_2
zraR_2	ZONMW-36_03075	zraR_2
zraR_2	ZONMW-36_02228	zraR_2
zraR_2	ZONMW-36_00787	zraR_2
zraR_2	ZONMW-36_02535	zraR_2
zraR_2	ZONMW-36_00325	zraR_2
zraR_2	ZONMW-36_01815	zraR_2
zraR_2	ZONMW-36_02168	zraR_2
zraR_2	ZONMW-36_01066	zraR_2
zraR_2	ZONMW-36_03625	zraR_2
zraR_2	ZONMW-36_02635	zraR_2
zraR_2	ZONMW-36_01816	zraR_2
zraR_2	ZONMW-36_02227	zraR_2
zraR_2	ZONMW-36_03626	zraR_2
zraR_2	ZONMW-36_03689	zraR_2
zraR_2	ZONMW-36_02720	zraR_2
zraR_2	ZONMW-36_01772	zraR_2
zraR_2	ZONMW-36_02822	zraR_2
zraR_2	ZONMW-36_03088	zraR_2
zraR_2	ZONMW-36_02536	zraR_2
zraR_2	ZONMW-36_01459	zraR_2
zraR_2	ZONMW-36_02169	zraR_2
zraR_2	ZONMW-36_00813	zraR_2
zraR_2	ZONMW-36_03121	zraR_2
zraR_2	ZONMW-36_02972	zraR_2

zraR_2	ZONMW-36_01812	zraR_2
zraR_2	ZONMW-36_00805	zraR_2
zraR_2	ZONMW-36_02437	zraR_2
zraR_2	ZONMW-36_03862	zraR_2
zraR_2	ZONMW-36_02084	zraR_2
zraR_2	ZONMW-36_01505	zraR_2
zraR_2	ZONMW-36_01626	zraR_2
zraR_2	ZONMW-36_00346	zraR_2
zraR_2	ZONMW-36_03682	zraR_2
zraR_2	ZONMW-36_01097	zraR_2
zraR_2	ZONMW-36_01828	zraR_2
zraR_2	ZONMW-36_02164	zraR_2
zraR_2	ZONMW-36_02439	zraR_2
zraR_2	ZONMW-36_00345	zraR_2
zraR_2	ZONMW-36_00525	zraR_2
zraR_2	ZONMW-36_00721	zraR_2
zraR_2	ZONMW-36_01940	zraR_2
zraR_2	ZONMW-36_01327	zraR_2
zraR_2	ZONMW-36_01727	zraR_2
zraR_2	ZONMW-36_01191	zraR_2
zraR_2	ZONMW-36_03860	zraR_2
zraR_2	ZONMW-36_03434	zraR_2
zraR_2	ZONMW-36_03679	zraR_2
zraR_2	ZONMW-36_01067	zraR_2
zraR_2	ZONMW-36_03939	zraR_2
zraR_2	ZONMW-36_00814	zraR_2
zraR_2	ZONMW-36_03435	zraR_2
zraR_2	ZONMW-36_01829	zraR_2
zraR_2	ZONMW-36_00216	zraR_2
zraR_2	ZONMW-36_00784	zraR_2
zraR_2	ZONMW-36_02729	zraR_2
zraR_2	ZONMW-36_01493	zraR_2
zraR_2	ZONMW-36_01813	zraR_2
zraR_2	ZONMW-36_02600	zraR_2
zraR_2	ZONMW-36_02971	zraR_2
zraR_2	ZONMW-36_00815	zraR_2
zraR_2	ZONMW-36_03680	zraR_2
zraR_2	ZONMW-36_02245	zraR_2
zraR_2	ZONMW-36_03938	zraR_2
zraR_2	ZONMW-36_01955	zraR_2
zraR_2	ZONMW-36_03184	zraR_2
zraR_2	ZONMW-36_01188	zraR_2
zraR_2	ZONMW-36_03234	zraR_2
zraR_2	ZONMW-36_00085	zraR_2
zraR_2	ZONMW-36_01328	zraR_2
zraR_2	ZONMW-36_03076	zraR_2
zraR_2	ZONMW-36_00524	zraR_2
zraR_2	ZONMW-36_00311	zraR_2

zraR_2	ZONMW-36_02499	zraR_2
zraR_2	ZONMW-36_02821	zraR_2
zraR_2	ZONMW-36_03122	zraR_2
zraR_2	ZONMW-36_01773	zraR_2
zraR_2	ZONMW-36_00861	zraR_2
zraR_2	ZONMW-36_01504	zraR_2
zraR_2	ZONMW-36_01627	zraR_2
zraR_2	ZONMW-36_03267	zraR_2
zraR_2	ZONMW-36_01463	zraR_2
zraR_2	ZONMW-36_02438	zraR_2
zraR_2	ZONMW-36_02116	zraR_2
zraR_2	ZONMW-36_00293	zraR_2
zraR_2	ZONMW-36_01179	zraR_2
zraR_2	ZONMW-36_02727	zraR_2
zraR_2	ZONMW-36_02181	zraR_2
zraR_2	ZONMW-36_03686	zraR_2
zraR_2	ZONMW-36_02739	zraR_2
zraR_2	ZONMW-36_01223	zraR_2
zraR_2	ZONMW-36_01462	zraR_2
zraR_2	ZONMW-36_02453	zraR_2
zraR_2	ZONMW-36_02323	zraR_2
zraR_2	ZONMW-36_02087	zraR_2
zraR_2	ZONMW-36_02442	zraR_2
zraR_2	ZONMW-36_02634	zraR_2
zraR_2	ZONMW-36_00324	zraR_2
zraR_2	ZONMW-36_00889	zraR_2
zraR_2	ZONMW-36_01461	zraR_2
zraR_2	ZONMW-36_01082	zraR_2
zraR_2	ZONMW-36_00203	zraR_2
zraR_2	ZONMW-36_02165	zraR_2
zraR_2	ZONMW-36_02725	zraR_2
zraR_2	ZONMW-36_02163	zraR_2
zraR_2	ZONMW-36_00930	zraR_2
zraR_2	ZONMW-36_00073	zraR_2
zraR_2	ZONMW-36_02048	zraR_2
zraR_2	ZONMW-36_00070	zraR_2
zraR_2	ZONMW-36_02255	zraR_2
zraR_2	ZONMW-36_03096	zraR_2
zraR_2	ZONMW-36_00704	zraR_2
zraR_2	ZONMW-36_00490	zraR_2
zraR_2	ZONMW-37_03462	zraR_2
zraR_2	ZONMW-37_02919	zraR_2
zraR_2	ZONMW-37_02947	zraR_2
zraR_2	ZONMW-37_02554	zraR_2
zraR_2	ZONMW-37_03115	zraR_2
zraR_2	ZONMW-37_01176	zraR_2
zraR_2	ZONMW-37_01128	zraR_2
zraR_2	ZONMW-37_02045	zraR_2

zraR_2	ZONMW-37_02960	zraR_2
zraR_2	ZONMW-37_02044	zraR_2
zraR_2	ZONMW-37_01129	zraR_2
zraR_2	ZONMW-37_02959	zraR_2
zraR_2	ZONMW-37_01558	zraR_2
zraR_2	ZONMW-37_01626	zraR_2
zraR_2	ZONMW-37_00643	zraR_2
zraR_2	ZONMW-37_01557	zraR_2
zraR_2	ZONMW-37_01481	zraR_2
zraR_2	ZONMW-37_02280	zraR_2
zraR_2	ZONMW-37_00343	zraR_2
zraR_2	ZONMW-37_03415	zraR_2
zraR_2	ZONMW-37_03127	zraR_2
zraR_2	ZONMW-37_02660	zraR_2
zraR_2	ZONMW-37_01315	zraR_2
zraR_2	ZONMW-37_03090	zraR_2
zraR_2	ZONMW-37_02901	zraR_2
zraR_2	ZONMW-37_00280	zraR_2
zraR_2	ZONMW-37_01560	zraR_2
zraR_2	ZONMW-37_02094	zraR_2
zraR_2	ZONMW-37_00029	zraR_2
zraR_2	ZONMW-37_00506	zraR_2
zraR_2	ZONMW-37_00988	zraR_2
zraR_2	ZONMW-37_01561	zraR_2
zraR_2	ZONMW-37_02551	zraR_2
zraR_2	ZONMW-37_01420	zraR_2
zraR_2	ZONMW-37_01177	zraR_2
zraR_2	ZONMW-37_01168	zraR_2
zraR_2	ZONMW-37_01419	zraR_2
zraR_2	ZONMW-37_00718	zraR_2
zraR_2	ZONMW-37_01750	zraR_2
zraR_2	ZONMW-37_02107	zraR_2
zraR_2	ZONMW-37_00277	zraR_2
zraR_2	ZONMW-37_00664	zraR_2
zraR_2	ZONMW-37_00926	zraR_2
zraR_2	ZONMW-37_00765	zraR_2
zraR_2	ZONMW-37_02503	zraR_2
zraR_2	ZONMW-37_01797	zraR_2
zraR_2	ZONMW-37_03479	zraR_2
zraR_2	ZONMW-37_00913	zraR_2
zraR_2	ZONMW-37_00059	zraR_2
zraR_2	ZONMW-37_00442	zraR_2
zraR_2	ZONMW-38_00530	zraR_2
zraR_2	ZONMW-38_02415	zraR_2
zraR_2	ZONMW-38_02807	zraR_2
zraR_2	ZONMW-38_01804	zraR_2
zraR_2	ZONMW-38_00228	zraR_2
zraR_2	ZONMW-38_02786	zraR_2

zraR_2	ZONMW-38_02414	zraR_2
zraR_2	ZONMW-38_00042	zraR_2
zraR_2	ZONMW-38_02806	zraR_2
zraR_2	ZONMW-38_02545	zraR_2
zraR_2	ZONMW-38_01805	zraR_2
zraR_2	ZONMW-38_02877	zraR_2
zraR_2	ZONMW-38_00229	zraR_2
zraR_2	ZONMW-38_00043	zraR_2
zraR_2	ZONMW-38_02546	zraR_2
zraR_2	ZONMW-38_03329	zraR_2
zraR_2	ZONMW-38_02876	zraR_2
zraR_2	ZONMW-38_01061	zraR_2
zraR_2	ZONMW-38_02582	zraR_2
zraR_2	ZONMW-38_02076	zraR_2
zraR_2	ZONMW-38_02785	zraR_2
zraR_2	ZONMW-38_01002	zraR_2
zraR_2	ZONMW-38_00845	zraR_2
zraR_2	ZONMW-38_00594	zraR_2
zraR_2	ZONMW-38_01235	zraR_2
zraR_2	ZONMW-38_00412	zraR_2
zraR_2	ZONMW-38_01236	zraR_2
zraR_2	ZONMW-38_02135	zraR_2
zraR_2	ZONMW-38_03328	zraR_2
zraR_2	ZONMW-38_02583	zraR_2
zraR_2	ZONMW-38_00595	zraR_2
zraR_2	ZONMW-38_02476	zraR_2
zraR_2	ZONMW-38_00836	zraR_2
zraR_2	ZONMW-38_02132	zraR_2
zraR_2	ZONMW-38_01972	zraR_2
zraR_2	ZONMW-38_01086	zraR_2
zraR_2	ZONMW-38_00558	zraR_2
zraR_2	ZONMW-38_01722	zraR_2
zraR_2	ZONMW-38_02288	zraR_2
zraR_2	ZONMW-38_02045	zraR_2
zraR_2	ZONMW-38_01060	zraR_2
zraR_2	ZONMW-38_02075	zraR_2
zraR_2	ZONMW-38_03950	zraR_2
zraR_2	ZONMW-38_03156	zraR_2
zraR_2	ZONMW-38_02569	zraR_2
zraR_2	ZONMW-38_00902	zraR_2
zraR_2	ZONMW-38_00326	zraR_2
zraR_2	ZONMW-38_03668	zraR_2
zraR_2	ZONMW-38_02017	zraR_2
zraR_2	ZONMW-38_02473	zraR_2
zraR_2	ZONMW-38_03070	zraR_2
zraR_2	ZONMW-38_00694	zraR_2
zraR_2	ZONMW-38_02201	zraR_2
zraR_2	ZONMW-38_01111	zraR_2

zraR_2	ZONMW-38_02693	zraR_2
zraR_2	ZONMW-38_00527	zraR_2
zraR_2	ZONMW-38_01853	zraR_2
zraR_2	ZONMW-38_03614	zraR_2
zraR_2	ZONMW-38_03026	zraR_2
zraR_2	ZONMW-38_00557	zraR_2
zraR_2	ZONMW-38_03854	zraR_2
zraR_2	ZONMW-38_00947	zraR_2
zraR_2	ZONMW-38_04135	zraR_2
zraR_2	ZONMW-38_02200	zraR_2
zraR_2	ZONMW-38_02448	zraR_2
zraR_2	ZONMW-38_03106	zraR_2
zraR_2	ZONMW-38_00936	zraR_2
zraR_2	ZONMW-38_01434	zraR_2
zraR_2	ZONMW-38_01703	zraR_2
zraR_2	ZONMW-38_00180	zraR_2
zraR_2	ZONMW-38_01090	zraR_2
zraR_2	ZONMW-39_00746	zraR_2
zraR_2	ZONMW-39_02018	zraR_2
zraR_2	ZONMW-39_01737	zraR_2
zraR_2	ZONMW-39_02088	zraR_2
zraR_2	ZONMW-39_01577	zraR_2
zraR_2	ZONMW-39_02222	zraR_2
zraR_2	ZONMW-39_00058	zraR_2
zraR_2	ZONMW-39_01111	zraR_2
zraR_2	ZONMW-39_01288	zraR_2
zraR_2	ZONMW-39_02597	zraR_2
zraR_2	ZONMW-39_02518	zraR_2
zraR_2	ZONMW-39_01990	zraR_2
zraR_2	ZONMW-39_02517	zraR_2
zraR_2	ZONMW-39_02446	zraR_2
zraR_2	ZONMW-39_03083	zraR_2
zraR_2	ZONMW-39_01411	zraR_2
zraR_2	ZONMW-39_02595	zraR_2
zraR_2	ZONMW-39_01144	zraR_2
zraR_2	ZONMW-39_02745	zraR_2
zraR_2	ZONMW-39_02181	zraR_2
zraR_2	ZONMW-39_01466	zraR_2
zraR_2	ZONMW-39_01412	zraR_2
zraR_2	ZONMW-39_02819	zraR_2
zraR_2	ZONMW-39_02596	zraR_2
zraR_2	ZONMW-39_04094	zraR_2
zraR_2	ZONMW-39_01578	zraR_2
zraR_2	ZONMW-39_02182	zraR_2
zraR_2	ZONMW-39_03733	zraR_2
zraR_2	ZONMW-39_01385	zraR_2
zraR_2	ZONMW-39_01465	zraR_2
zraR_2	ZONMW-39_02744	zraR_2

zraR_2	ZONMW-39_03956	zraR_2
zraR_2	ZONMW-39_02592	zraR_2
zraR_2	ZONMW-39_01112	zraR_2
zraR_2	ZONMW-39_04212	zraR_2
zraR_2	ZONMW-39_04091	zraR_2
zraR_2	ZONMW-39_02256	zraR_2
zraR_2	ZONMW-39_03245	zraR_2
zraR_2	ZONMW-39_03251	zraR_2
zraR_2	ZONMW-39_00718	zraR_2
zraR_2	ZONMW-39_01036	zraR_2
zraR_2	ZONMW-39_00383	zraR_2
zraR_2	ZONMW-39_03357	zraR_2
zraR_2	ZONMW-39_03329	zraR_2
zraR_2	ZONMW-39_02414	zraR_2
zraR_2	ZONMW-39_02445	zraR_2
zraR_2	ZONMW-39_03253	zraR_2
zraR_2	ZONMW-39_03769	zraR_2
zraR_2	ZONMW-39_02158	zraR_2
zraR_2	ZONMW-39_02608	zraR_2
zraR_2	ZONMW-39_01470	zraR_2
zraR_2	ZONMW-39_03217	zraR_2
zraR_2	ZONMW-39_00384	zraR_2
zraR_2	ZONMW-39_02885	zraR_2
zraR_2	ZONMW-39_00201	zraR_2
zraR_2	ZONMW-39_02519	zraR_2
zraR_2	ZONMW-39_01739	zraR_2
zraR_2	ZONMW-39_03832	zraR_2
zraR_2	ZONMW-39_02155	zraR_2
zraR_2	ZONMW-39_04055	zraR_2
zraR_2	ZONMW-39_03831	zraR_2
zraR_2	ZONMW-39_02609	zraR_2
zraR_2	ZONMW-39_00792	zraR_2
zraR_2	ZONMW-39_02822	zraR_2
zraR_2	ZONMW-39_03345	zraR_2
zraR_2	ZONMW-39_03716	zraR_2
zraR_2	ZONMW-39_03770	zraR_2
zraR_2	ZONMW-39_02246	zraR_2
zraR_2	ZONMW-39_02593	zraR_2
zraR_2	ZONMW-39_03955	zraR_2
zraR_2	ZONMW-39_01383	zraR_2
zraR_2	ZONMW-39_02695	zraR_2
zraR_2	ZONMW-39_02156	zraR_2
zraR_2	ZONMW-39_01394	zraR_2
zraR_2	ZONMW-39_04056	zraR_2
zraR_2	ZONMW-39_01017	zraR_2
zraR_2	ZONMW-39_01725	zraR_2
zraR_2	ZONMW-39_03983	zraR_2
zraR_2	ZONMW-39_02961	zraR_2

zraR_2	ZONMW-39_02654	zraR_2
zraR_2	ZONMW-39_02912	zraR_2
zraR_2	ZONMW-39_03717	zraR_2
zraR_2	ZONMW-39_01143	zraR_2
zraR_2	ZONMW-39_01384	zraR_2
zraR_2	ZONMW-39_00202	zraR_2
zraR_2	ZONMW-39_03732	zraR_2
zraR_2	ZONMW-39_02724	zraR_2
zraR_2	ZONMW-39_01337	zraR_2
zraR_2	ZONMW-39_00964	zraR_2
zraR_2	ZONMW-39_03356	zraR_2
zraR_2	ZONMW-39_02415	zraR_2
zraR_2	ZONMW-39_03084	zraR_2
zraR_2	ZONMW-39_03246	zraR_2
zraR_2	ZONMW-39_01453	zraR_2
zraR_2	ZONMW-39_01005	zraR_2
zraR_2	ZONMW-39_03219	zraR_2
zraR_2	ZONMW-39_00405	zraR_2
zraR_2	ZONMW-39_02354	zraR_2
zraR_2	ZONMW-39_03229	zraR_2
zraR_2	ZONMW-39_01861	zraR_2
zraR_2	ZONMW-39_03624	zraR_2
zraR_2	ZONMW-39_01325	zraR_2
zraR_2	ZONMW-39_00817	zraR_2
zraR_2	ZONMW-39_01469	zraR_2
zraR_2	ZONMW-39_01471	zraR_2
zraR_2	ZONMW-39_02562	zraR_2
zraR_2	ZONMW-39_03417	zraR_2
zraR_2	ZONMW-39_02701	zraR_2
zraR_2	ZONMW-39_01617	zraR_2
zraR_2	ZONMW-39_02677	zraR_2
zraR_2	ZONMW-39_01282	zraR_2
zraR_2	ZONMW-39_00237	zraR_2
zraR_2	ZONMW-39_00026	zraR_2
zraR_2	ZONMW-39_01991	zraR_2
zraR_2	ZONMW-40_01903	zraR_2
zraR_2	ZONMW-40_01977	zraR_2
zraR_2	ZONMW-40_01595	zraR_2
zraR_2	ZONMW-40_00828	zraR_2
zraR_2	ZONMW-40_01097	zraR_2
zraR_2	ZONMW-40_01018	zraR_2
zraR_2	ZONMW-40_00094	zraR_2
zraR_2	ZONMW-40_01859	zraR_2
zraR_2	ZONMW-40_00047	zraR_2
zraR_2	ZONMW-40_00048	zraR_2
zraR_2	ZONMW-40_02426	zraR_2
zraR_2	ZONMW-40_00740	zraR_2
zraR_2	ZONMW-40_00581	zraR_2

zraR_2	ZONMW-40_01858	zraR_2
zraR_2	ZONMW-40_02427	zraR_2
zraR_2	ZONMW-40_03305	zraR_2
zraR_2	ZONMW-40_00741	zraR_2
zraR_2	ZONMW-40_00608	zraR_2
zraR_2	ZONMW-40_00607	zraR_2
zraR_2	ZONMW-40_03333	zraR_2
zraR_2	ZONMW-40_03399	zraR_2
zraR_2	ZONMW-40_00824	zraR_2
zraR_2	ZONMW-40_01189	zraR_2
zraR_2	ZONMW-40_02231	zraR_2
zraR_2	ZONMW-40_02023	zraR_2
zraR_2	ZONMW-40_00205	zraR_2
zraR_2	ZONMW-40_00851	zraR_2
zraR_2	ZONMW-40_01593	zraR_2
zraR_2	ZONMW-40_02130	zraR_2
zraR_2	ZONMW-40_01240	zraR_2
zraR_2	ZONMW-40_01125	zraR_2
zraR_2	ZONMW-40_00704	zraR_2
zraR_2	ZONMW-40_02695	zraR_2
zraR_2	ZONMW-40_01323	zraR_2
zraR_2	ZONMW-40_00346	zraR_2
zraR_2	ZONMW-40_00095	zraR_2
zraR_2	ZONMW-40_00340	zraR_2
zraR_2	ZONMW-40_02379	zraR_2
zraR_2	ZONMW-40_00995	zraR_2
zraR_2	ZONMW-40_02742	zraR_2
zraR_2	ZONMW-40_00345	zraR_2
zraR_2	ZONMW-40_00339	zraR_2
zraR_2	ZONMW-40_01791	zraR_2
zraR_2	ZONMW-40_00086	zraR_2
zraR_2	ZONMW-40_03196	zraR_2
zraR_2	ZONMW-40_02692	zraR_2
zraR_2	ZONMW-40_01307	zraR_2
zraR_2	ZONMW-40_03365	zraR_2
zraR_2	ZONMW-40_03063	zraR_2
zraR_2	ZONMW-40_03068	zraR_2
zraR_2	ZONMW-40_02947	zraR_2
zraR_2	ZONMW-40_03450	zraR_2
zraR_2	ZONMW-40_00965	zraR_2
zraR_2	ZONMW-41_01224	zraR_2
zraR_2	ZONMW-41_00154	zraR_2
zraR_2	ZONMW-41_01429	zraR_2
zraR_2	ZONMW-41_00831	zraR_2
zraR_2	ZONMW-41_00622	zraR_2
zraR_2	ZONMW-41_00990	zraR_2
zraR_2	ZONMW-41_01449	zraR_2
zraR_2	ZONMW-41_01085	zraR_2

zraR_2	ZONMW-41_02342	zraR_2
zraR_2	ZONMW-41_00220	zraR_2
zraR_2	ZONMW-41_01975	zraR_2
zraR_2	ZONMW-41_02087	zraR_2
zraR_2	ZONMW-41_01529	zraR_2
zraR_2	ZONMW-41_01647	zraR_2
zraR_2	ZONMW-41_01651	zraR_2
zraR_2	ZONMW-41_00650	zraR_2
zraR_2	ZONMW-41_02086	zraR_2
zraR_2	ZONMW-41_00097	zraR_2
zraR_2	ZONMW-41_03086	zraR_2
zraR_2	ZONMW-41_00026	zraR_2
zraR_2	ZONMW-41_01408	zraR_2
zraR_2	ZONMW-41_01553	zraR_2
zraR_2	ZONMW-41_00221	zraR_2
zraR_2	ZONMW-41_03066	zraR_2
zraR_2	ZONMW-41_03019	zraR_2
zraR_2	ZONMW-41_00096	zraR_2
zraR_2	ZONMW-41_01347	zraR_2
zraR_2	ZONMW-41_01829	zraR_2
zraR_2	ZONMW-41_00183	zraR_2
zraR_2	ZONMW-41_02051	zraR_2
zraR_2	ZONMW-41_00025	zraR_2
zraR_2	ZONMW-41_01411	zraR_2
zraR_2	ZONMW-41_00368	zraR_2
zraR_2	ZONMW-41_01968	zraR_2
zraR_2	ZONMW-41_00185	zraR_2
zraR_2	ZONMW-41_02709	zraR_2
zraR_2	ZONMW-41_02432	zraR_2
zraR_2	ZONMW-41_01138	zraR_2
zraR_2	ZONMW-41_00087	zraR_2
zraR_2	ZONMW-41_01993	zraR_2
zraR_2	ZONMW-41_01554	zraR_2
zraR_2	ZONMW-41_01257	zraR_2
zraR_2	ZONMW-41_01137	zraR_2
zraR_2	ZONMW-41_01318	zraR_2
zraR_2	ZONMW-41_01525	zraR_2
zraR_2	ZONMW-41_00788	zraR_2
zraR_2	ZONMW-41_03065	zraR_2
zraR_2	ZONMW-41_00525	zraR_2
zraR_2	ZONMW-41_02984	zraR_2
zraR_2	ZONMW-41_01457	zraR_2
zraR_2	ZONMW-41_01994	zraR_2
zraR_2	ZONMW-41_00850	zraR_2
zraR_2	ZONMW-41_00219	zraR_2
zraR_2	ZONMW-41_00603	zraR_2
zraR_2	ZONMW-41_00793	zraR_2
zraR_2	ZONMW-41_00789	zraR_2

zraR_2	ZONMW-41_00630	zraR_2
zraR_2	ZONMW-41_00526	zraR_2
zraR_2	ZONMW-41_01346	zraR_2
zraR_2	ZONMW-41_00581	zraR_2
zraR_2	ZONMW-41_01524	zraR_2
zraR_2	ZONMW-41_01828	zraR_2
zraR_2	ZONMW-41_01578	zraR_2
zraR_2	ZONMW-41_02774	zraR_2
zraR_2	ZONMW-41_02050	zraR_2
zraR_2	ZONMW-41_01971	zraR_2
zraR_2	ZONMW-41_03018	zraR_2
zraR_2	ZONMW-41_00582	zraR_2
zraR_2	ZONMW-41_01650	zraR_2
zraR_2	ZONMW-41_00991	zraR_2
zraR_2	ZONMW-41_01815	zraR_2
zraR_2	ZONMW-41_01534	zraR_2
zraR_2	ZONMW-41_01363	zraR_2
zraR_2	ZONMW-41_00527	zraR_2
zraR_2	ZONMW-41_00100	zraR_2
zraR_2	ZONMW-41_00849	zraR_2
zraR_2	ZONMW-41_03123	zraR_2
zraR_2	ZONMW-41_01817	zraR_2
zraR_2	ZONMW-41_03022	zraR_2
zraR_2	ZONMW-41_00659	zraR_2
zraR_2	ZONMW-41_02279	zraR_2
zraR_2	ZONMW-42_03871	zraR_2
zraR_2	ZONMW-42_03515	zraR_2
zraR_2	ZONMW-42_02530	zraR_2
zraR_2	ZONMW-42_01129	zraR_2
zraR_2	ZONMW-42_00227	zraR_2
zraR_2	ZONMW-42_03044	zraR_2
zraR_2	ZONMW-42_00449	zraR_2
zraR_2	ZONMW-42_03261	zraR_2
zraR_2	ZONMW-42_01601	zraR_2
zraR_2	ZONMW-42_00226	zraR_2
zraR_2	ZONMW-42_00130	zraR_2
zraR_2	ZONMW-42_03031	zraR_2
zraR_2	ZONMW-42_02391	zraR_2
zraR_2	ZONMW-42_01722	zraR_2
zraR_2	ZONMW-42_01367	zraR_2
zraR_2	ZONMW-42_00131	zraR_2
zraR_2	ZONMW-42_00265	zraR_2
zraR_2	ZONMW-42_00938	zraR_2
zraR_2	ZONMW-42_03476	zraR_2
zraR_2	ZONMW-42_00294	zraR_2
zraR_2	ZONMW-42_00407	zraR_2
zraR_2	ZONMW-42_01363	zraR_2
zraR_2	ZONMW-42_00697	zraR_2

zraR_2	ZONMW-42_00266	zraR_2
zraR_2	ZONMW-42_03759	zraR_2
zraR_2	ZONMW-42_03657	zraR_2
zraR_2	ZONMW-42_03160	zraR_2
zraR_2	ZONMW-42_00293	zraR_2
zraR_2	ZONMW-42_00964	zraR_2
zraR_2	ZONMW-42_01635	zraR_2
zraR_2	ZONMW-42_02390	zraR_2
zraR_2	ZONMW-42_00223	zraR_2
zraR_2	ZONMW-42_00298	zraR_2
zraR_2	ZONMW-42_00408	zraR_2
zraR_2	ZONMW-42_03337	zraR_2
zraR_2	ZONMW-42_04078	zraR_2
zraR_2	ZONMW-42_03382	zraR_2
zraR_2	ZONMW-42_00045	zraR_2
zraR_2	ZONMW-42_02369	zraR_2
zraR_2	ZONMW-42_01919	zraR_2
zraR_2	ZONMW-42_01602	zraR_2
zraR_2	ZONMW-42_01558	zraR_2
zraR_2	ZONMW-42_00956	zraR_2
zraR_2	ZONMW-42_00241	zraR_2
zraR_2	ZONMW-42_02528	zraR_2
zraR_2	ZONMW-42_01920	zraR_2
zraR_2	ZONMW-42_03124	zraR_2
zraR_2	ZONMW-42_00873	zraR_2
zraR_2	ZONMW-42_03655	zraR_2
zraR_2	ZONMW-42_02271	zraR_2
zraR_2	ZONMW-42_02368	zraR_2
zraR_2	ZONMW-42_00132	zraR_2
zraR_2	ZONMW-42_01634	zraR_2
zraR_2	ZONMW-42_01366	zraR_2
zraR_2	ZONMW-42_02272	zraR_2
zraR_2	ZONMW-42_03125	zraR_2
zraR_2	ZONMW-42_00965	zraR_2
zraR_2	ZONMW-42_04019	zraR_2
zraR_2	ZONMW-42_00935	zraR_2
zraR_2	ZONMW-42_03366	zraR_2
zraR_2	ZONMW-42_00224	zraR_2
zraR_2	ZONMW-42_00472	zraR_2
zraR_2	ZONMW-42_00666	zraR_2
zraR_2	ZONMW-42_02911	zraR_2
zraR_2	ZONMW-42_03764	zraR_2
zraR_2	ZONMW-42_04079	zraR_2
zraR_2	ZONMW-42_00966	zraR_2
zraR_2	ZONMW-42_01311	zraR_2
zraR_2	ZONMW-42_00642	zraR_2
zraR_2	ZONMW-42_02544	zraR_2
zraR_2	ZONMW-42_00010	zraR_2

zraR_2	ZONMW-42_00800	zraR_2
zraR_2	ZONMW-42_00846	zraR_2
zraR_2	ZONMW-42_00667	zraR_2
zraR_2	ZONMW-42_01352	zraR_2
zraR_2	ZONMW-42_00696	zraR_2
zraR_2	ZONMW-42_03766	zraR_2
zraR_2	ZONMW-42_02914	zraR_2
zraR_2	ZONMW-42_03336	zraR_2
zraR_2	ZONMW-42_00242	zraR_2
zraR_2	ZONMW-42_01008	zraR_2
zraR_2	ZONMW-42_03381	zraR_2
zraR_2	ZONMW-42_00046	zraR_2
zraR_2	ZONMW-42_04157	zraR_2
zraR_2	ZONMW-42_02962	zraR_2
zraR_2	ZONMW-42_00561	zraR_2
zraR_2	ZONMW-42_00683	zraR_2
zraR_2	ZONMW-42_03765	zraR_2
zraR_2	ZONMW-42_03025	zraR_2
zraR_2	ZONMW-42_01364	zraR_2
zraR_2	ZONMW-42_01028	zraR_2
zraR_2	ZONMW-42_02779	zraR_2
zraR_2	ZONMW-42_03670	zraR_2
zraR_2	ZONMW-42_00297	zraR_2
zraR_2	ZONMW-42_03472	zraR_2
zraR_2	ZONMW-42_00299	zraR_2
zraR_2	ZONMW-42_01885	zraR_2
zraR_2	ZONMW-42_03867	zraR_2
zraR_2	ZONMW-43_03057	zraR_2
zraR_2	ZONMW-43_03496	zraR_2
zraR_2	ZONMW-43_01096	zraR_2
zraR_2	ZONMW-43_01173	zraR_2
zraR_2	ZONMW-43_01933	zraR_2
zraR_2	ZONMW-43_01474	zraR_2
zraR_2	ZONMW-43_00188	zraR_2
zraR_2	ZONMW-43_01627	zraR_2
zraR_2	ZONMW-43_03995	zraR_2
zraR_2	ZONMW-43_00363	zraR_2
zraR_2	ZONMW-43_00932	zraR_2
zraR_2	ZONMW-43_00931	zraR_2
zraR_2	ZONMW-43_01069	zraR_2
zraR_2	ZONMW-43_02342	zraR_2
zraR_2	ZONMW-43_03211	zraR_2
zraR_2	ZONMW-43_03872	zraR_2
zraR_2	ZONMW-43_00221	zraR_2
zraR_2	ZONMW-43_01515	zraR_2
zraR_2	ZONMW-43_03260	zraR_2
zraR_2	ZONMW-43_02701	zraR_2
zraR_2	ZONMW-43_03997	zraR_2

zraR_2	ZONMW-43_03212	zraR_2
zraR_2	ZONMW-43_00481	zraR_2
zraR_2	ZONMW-43_01919	zraR_2
zraR_2	ZONMW-43_03996	zraR_2
zraR_2	ZONMW-43_01932	zraR_2
zraR_2	ZONMW-43_02603	zraR_2
zraR_2	ZONMW-43_03873	zraR_2
zraR_2	ZONMW-43_03366	zraR_2
zraR_2	ZONMW-43_00455	zraR_2
zraR_2	ZONMW-43_01514	zraR_2
zraR_2	ZONMW-43_03259	zraR_2
zraR_2	ZONMW-43_01250	zraR_2
zraR_2	ZONMW-43_04001	zraR_2
zraR_2	ZONMW-43_00189	zraR_2
zraR_2	ZONMW-43_00463	zraR_2
zraR_2	ZONMW-43_02648	zraR_2
zraR_2	ZONMW-43_02646	zraR_2
zraR_2	ZONMW-43_03932	zraR_2
zraR_2	ZONMW-43_00112	zraR_2
zraR_2	ZONMW-43_02903	zraR_2
zraR_2	ZONMW-43_00749	zraR_2
zraR_2	ZONMW-43_00836	zraR_2
zraR_2	ZONMW-43_00714	zraR_2
zraR_2	ZONMW-43_01537	zraR_2
zraR_2	ZONMW-43_03264	zraR_2
zraR_2	ZONMW-43_02682	zraR_2
zraR_2	ZONMW-43_03984	zraR_2
zraR_2	ZONMW-43_02902	zraR_2
zraR_2	ZONMW-43_00548	zraR_2
zraR_2	ZONMW-43_01757	zraR_2
zraR_2	ZONMW-43_00933	zraR_2
zraR_2	ZONMW-43_03494	zraR_2
zraR_2	ZONMW-43_03654	zraR_2
zraR_2	ZONMW-43_00103	zraR_2
zraR_2	ZONMW-43_00718	zraR_2
zraR_2	ZONMW-43_01540	zraR_2
zraR_2	ZONMW-43_03796	zraR_2
zraR_2	ZONMW-43_03653	zraR_2
zraR_2	ZONMW-43_03983	zraR_2
zraR_2	ZONMW-43_00220	zraR_2
zraR_2	ZONMW-43_03102	zraR_2
zraR_2	ZONMW-43_00484	zraR_2
zraR_2	ZONMW-43_00737	zraR_2
zraR_2	ZONMW-43_04000	zraR_2
zraR_2	ZONMW-43_03383	zraR_2
zraR_2	ZONMW-43_01450	zraR_2
zraR_2	ZONMW-43_01920	zraR_2
zraR_2	ZONMW-43_01249	zraR_2

zraR_2	ZONMW-43_00453	zraR_2
zraR_2	ZONMW-43_04081	zraR_2
zraR_2	ZONMW-43_01539	zraR_2
zraR_2	ZONMW-43_03194	zraR_2
zraR_2	ZONMW-43_03795	zraR_2
zraR_2	ZONMW-43_00097	zraR_2
zraR_2	ZONMW-43_03508	zraR_2
zraR_2	ZONMW-43_04138	zraR_2
zraR_2	ZONMW-43_03591	zraR_2
zraR_2	ZONMW-43_00575	zraR_2
zraR_2	ZONMW-43_03412	zraR_2
zraR_2	ZONMW-43_03382	zraR_2
zraR_2	ZONMW-43_00454	zraR_2
zraR_2	ZONMW-43_01758	zraR_2
zraR_2	ZONMW-43_03367	zraR_2
zraR_2	ZONMW-43_02343	zraR_2
zraR_2	ZONMW-43_02684	zraR_2
zraR_2	ZONMW-43_00407	zraR_2
zraR_2	ZONMW-43_00586	zraR_2
zraR_2	ZONMW-43_04106	zraR_2
zraR_2	ZONMW-43_00040	zraR_2
zraR_2	ZONMW-43_00748	zraR_2
zraR_2	ZONMW-43_00837	zraR_2
zraR_2	ZONMW-43_02683	zraR_2
zraR_2	ZONMW-43_01979	zraR_2
zraR_2	ZONMW-43_02434	zraR_2
zraR_2	ZONMW-43_03247	zraR_2
zraR_2	ZONMW-43_02702	zraR_2
zraR_2	ZONMW-43_02680	zraR_2
zraR_2	ZONMW-43_02606	zraR_2
zraR_2	ZONMW-43_03898	zraR_2
zraR_2	ZONMW-43_01340	zraR_2
zraR_2	ZONMW-43_00717	zraR_2
zraR_2	ZONMW-43_00357	zraR_2
zraR_2	ZONMW-43_02670	zraR_2
zraR_2	ZONMW-43_04058	zraR_2
zraR_2	ZONMW-43_03519	zraR_2
zraR_2	ZONMW-43_00396	zraR_2
zraR_2	ZONMW-43_00716	zraR_2
zraR_2	ZONMW-43_03113	zraR_2
zraR_2	ZONMW-43_03263	zraR_2
zraR_2	ZONMW-43_03265	zraR_2
zraR_2	ZONMW-43_01889	zraR_2
zraR_2	ZONMW-43_03606	zraR_2
zraR_2	ZONMW-43_04478	zraR_2
zraR_2	ZONMW-43_01792	zraR_2
zraR_2	ZONMW-43_01595	zraR_2
zraR_2	ZONMW-43_03959	zraR_2

zraR_2	ZONMW-44_02340	zraR_2
zraR_2	ZONMW-44_01122	zraR_2
zraR_2	ZONMW-44_02507	zraR_2
zraR_2	ZONMW-44_00150	zraR_2
zraR_2	ZONMW-44_02996	zraR_2
zraR_2	ZONMW-44_00848	zraR_2
zraR_2	ZONMW-44_01412	zraR_2
zraR_2	ZONMW-44_01994	zraR_2
zraR_2	ZONMW-44_02842	zraR_2
zraR_2	ZONMW-44_02208	zraR_2
zraR_2	ZONMW-44_03627	zraR_2
zraR_2	ZONMW-44_00561	zraR_2
zraR_2	ZONMW-44_02995	zraR_2
zraR_2	ZONMW-44_03046	zraR_2
zraR_2	ZONMW-44_01995	zraR_2
zraR_2	ZONMW-44_00579	zraR_2
zraR_2	ZONMW-44_03695	zraR_2
zraR_2	ZONMW-44_03075	zraR_2
zraR_2	ZONMW-44_00890	zraR_2
zraR_2	ZONMW-44_02565	zraR_2
zraR_2	ZONMW-44_01791	zraR_2
zraR_2	ZONMW-44_03047	zraR_2
zraR_2	ZONMW-44_02647	zraR_2
zraR_2	ZONMW-44_02506	zraR_2
zraR_2	ZONMW-44_00554	zraR_2
zraR_2	ZONMW-44_02992	zraR_2
zraR_2	ZONMW-44_03642	zraR_2
zraR_2	ZONMW-44_03074	zraR_2
zraR_2	ZONMW-44_01827	zraR_2
zraR_2	ZONMW-44_03669	zraR_2
zraR_2	ZONMW-44_03800	zraR_2
zraR_2	ZONMW-44_03079	zraR_2
zraR_2	ZONMW-44_00889	zraR_2
zraR_2	ZONMW-44_02068	zraR_2
zraR_2	ZONMW-44_00029	zraR_2
zraR_2	ZONMW-44_01531	zraR_2
zraR_2	ZONMW-44_01385	zraR_2
zraR_2	ZONMW-44_03930	zraR_2
zraR_2	ZONMW-44_01411	zraR_2
zraR_2	ZONMW-44_01909	zraR_2
zraR_2	ZONMW-44_00578	zraR_2
zraR_2	ZONMW-44_03128	zraR_2
zraR_2	ZONMW-44_00331	zraR_2
zraR_2	ZONMW-44_03677	zraR_2
zraR_2	ZONMW-44_03011	zraR_2
zraR_2	ZONMW-44_02502	zraR_2
zraR_2	ZONMW-44_02338	zraR_2
zraR_2	ZONMW-44_02503	zraR_2

zraR_2	ZONMW-44_03611	zraR_2
zraR_2	ZONMW-44_01729	zraR_2
zraR_2	ZONMW-44_01386	zraR_2
zraR_2	ZONMW-44_03760	zraR_2
zraR_2	ZONMW-44_00323	zraR_2
zraR_2	ZONMW-44_03129	zraR_2
zraR_2	ZONMW-44_03223	zraR_2
zraR_2	ZONMW-44_01996	zraR_2
zraR_2	ZONMW-44_03628	zraR_2
zraR_2	ZONMW-44_01786	zraR_2
zraR_2	ZONMW-44_00910	zraR_2
zraR_2	ZONMW-44_02067	zraR_2
zraR_2	ZONMW-44_03222	zraR_2
zraR_2	ZONMW-44_00560	zraR_2
zraR_2	ZONMW-44_03610	zraR_2
zraR_2	ZONMW-44_02663	zraR_2
zraR_2	ZONMW-44_01076	zraR_2
zraR_2	ZONMW-44_03698	zraR_2
zraR_2	ZONMW-44_02993	zraR_2
zraR_2	ZONMW-44_00825	zraR_2
zraR_2	ZONMW-44_02664	zraR_2
zraR_2	ZONMW-44_00030	zraR_2
zraR_2	ZONMW-44_03667	zraR_2
zraR_2	ZONMW-44_00501	zraR_2
zraR_2	ZONMW-44_02689	zraR_2
zraR_2	ZONMW-44_00909	zraR_2
zraR_2	ZONMW-44_00216	zraR_2
zraR_2	ZONMW-44_03030	zraR_2
zraR_2	ZONMW-44_02355	zraR_2
zraR_2	ZONMW-44_02912	zraR_2
zraR_2	ZONMW-44_00245	zraR_2
zraR_2	ZONMW-44_02398	zraR_2
zraR_2	ZONMW-44_03787	zraR_2
zraR_2	ZONMW-44_03668	zraR_2
zraR_2	ZONMW-44_02501	zraR_2
zraR_2	ZONMW-44_02648	zraR_2
zraR_2	ZONMW-44_01784	zraR_2
zraR_2	ZONMW-44_00541	zraR_2
zraR_2	ZONMW-44_01728	zraR_2
zraR_2	ZONMW-44_02563	zraR_2
zraR_2	ZONMW-44_02258	zraR_2
zraR_2	ZONMW-44_01910	zraR_2
zraR_2	ZONMW-44_02394	zraR_2
zraR_2	ZONMW-44_01826	zraR_2
zraR_2	ZONMW-44_00953	zraR_2
zraR_2	ZONMW-44_00324	zraR_2
zraR_2	ZONMW-44_02492	zraR_2
zraR_2	ZONMW-44_01151	zraR_2

zraR_2	ZONMW-44_00733	zraR_2
zraR_2	ZONMW-44_01785	zraR_2
zraR_2	ZONMW-44_02202	zraR_2
zraR_2	ZONMW-44_00346	zraR_2
zraR_2	ZONMW-44_01808	zraR_2
zraR_2	ZONMW-44_00555	zraR_2
zraR_2	ZONMW-44_00334	zraR_2
zraR_2	ZONMW-44_03012	zraR_2
zraR_2	ZONMW-44_02247	zraR_2
zraR_2	ZONMW-44_03304	zraR_2
zraR_2	ZONMW-44_03078	zraR_2
zraR_2	ZONMW-44_03080	zraR_2
zraR_2	ZONMW-44_03928	zraR_2
zraR_2	ZONMW-44_01756	zraR_2
zraR_2	ZONMW-44_00257	zraR_2
zraR_2	ZONMW-44_01903	zraR_2
zraR_2	ZONMW-44_01349	zraR_2
zraR_2	ZONMW-44_04102	zraR_2
zraR_2	ZONMW-45_03173	zraR_2
zraR_2	ZONMW-45_02421	zraR_2
zraR_2	ZONMW-45_02824	zraR_2
zraR_2	ZONMW-45_03095	zraR_2
zraR_2	ZONMW-45_02350	zraR_2
zraR_2	ZONMW-45_01391	zraR_2
zraR_2	ZONMW-45_02349	zraR_2
zraR_2	ZONMW-45_03094	zraR_2
zraR_2	ZONMW-45_01392	zraR_2
zraR_2	ZONMW-45_01232	zraR_2
zraR_2	ZONMW-45_01233	zraR_2
zraR_2	ZONMW-45_02914	zraR_2
zraR_2	ZONMW-45_01439	zraR_2
zraR_2	ZONMW-45_02250	zraR_2
zraR_2	ZONMW-45_02252	zraR_2
zraR_2	ZONMW-45_00212	zraR_2
zraR_2	ZONMW-45_02771	zraR_2
zraR_2	ZONMW-45_00595	zraR_2
zraR_2	ZONMW-45_02511	zraR_2
zraR_2	ZONMW-45_01489	zraR_2
zraR_2	ZONMW-45_02863	zraR_2
zraR_2	ZONMW-45_02907	zraR_2
zraR_2	ZONMW-45_01069	zraR_2
zraR_2	ZONMW-45_00870	zraR_2
zraR_2	ZONMW-45_01459	zraR_2
zraR_2	ZONMW-45_02915	zraR_2
zraR_2	ZONMW-45_02823	zraR_2
zraR_2	ZONMW-45_02203	zraR_2
zraR_2	ZONMW-45_02958	zraR_2
zraR_2	ZONMW-45_01472	zraR_2

zraR_2	ZONMW-45_01577	zraR_2
zraR_2	ZONMW-45_00262	zraR_2
zraR_2	ZONMW-45_03131	zraR_2
zraR_2	ZONMW-45_00065	zraR_2
zraR_2	ZONMW-45_00625	zraR_2
zraR_2	ZONMW-45_03188	zraR_2
zraR_2	ZONMW-45_00497	zraR_2
zraR_2	ZONMW-45_00798	zraR_2
zraR_2	ZONMW-45_03077	zraR_2
zraR_2	ZONMW-45_01163	zraR_2
zraR_2	ZONMW-45_03114	zraR_2
zraR_2	ZONMW-46_02927	zraR_2
zraR_2	ZONMW-46_00187	zraR_2
zraR_2	ZONMW-46_02390	zraR_2
zraR_2	ZONMW-46_01287	zraR_2
zraR_2	ZONMW-46_01212	zraR_2
zraR_2	ZONMW-46_01646	zraR_2
zraR_2	ZONMW-46_01211	zraR_2
zraR_2	ZONMW-46_01288	zraR_2
zraR_2	ZONMW-46_01647	zraR_2
zraR_2	ZONMW-46_02616	zraR_2
zraR_2	ZONMW-46_02617	zraR_2
zraR_2	ZONMW-46_03027	zraR_2
zraR_2	ZONMW-46_01694	zraR_2
zraR_2	ZONMW-46_03165	zraR_2
zraR_2	ZONMW-46_03163	zraR_2
zraR_2	ZONMW-46_00427	zraR_2
zraR_2	ZONMW-46_01140	zraR_2
zraR_2	ZONMW-46_00864	zraR_2
zraR_2	ZONMW-46_02733	zraR_2
zraR_2	ZONMW-46_01744	zraR_2
zraR_2	ZONMW-46_02860	zraR_2
zraR_2	ZONMW-46_03034	zraR_2
zraR_2	ZONMW-46_00598	zraR_2
zraR_2	ZONMW-46_01004	zraR_2
zraR_2	ZONMW-46_01714	zraR_2
zraR_2	ZONMW-46_03026	zraR_2
zraR_2	ZONMW-46_02391	zraR_2
zraR_2	ZONMW-46_01879	zraR_2
zraR_2	ZONMW-46_01768	zraR_2
zraR_2	ZONMW-46_01727	zraR_2
zraR_2	ZONMW-46_02088	zraR_2
zraR_2	ZONMW-46_00477	zraR_2
zraR_2	ZONMW-46_02805	zraR_2
zraR_2	ZONMW-46_00280	zraR_2
zraR_2	ZONMW-46_00834	zraR_2
zraR_2	ZONMW-46_02912	zraR_2
zraR_2	ZONMW-46_01342	zraR_2

zraR_2	ZONMW-46_01076	zraR_2
zraR_2	ZONMW-46_00158	zraR_2
zraR_2	ZONMW-46_02547	zraR_2
zraR_2	ZONMW-46_01268	zraR_2
zraR_2	ZONMW-47_02946	zraR_2
zraR_2	ZONMW-47_00318	zraR_2
zraR_2	ZONMW-47_02380	zraR_2
zraR_2	ZONMW-47_01565	zraR_2
zraR_2	ZONMW-47_00961	zraR_2
zraR_2	ZONMW-47_01843	zraR_2
zraR_2	ZONMW-47_00962	zraR_2
zraR_2	ZONMW-47_01564	zraR_2
zraR_2	ZONMW-47_01842	zraR_2
zraR_2	ZONMW-47_02606	zraR_2
zraR_2	ZONMW-47_02607	zraR_2
zraR_2	ZONMW-47_03018	zraR_2
zraR_2	ZONMW-47_01795	zraR_2
zraR_2	ZONMW-47_03157	zraR_2
zraR_2	ZONMW-47_03155	zraR_2
zraR_2	ZONMW-47_00078	zraR_2
zraR_2	ZONMW-47_01033	zraR_2
zraR_2	ZONMW-47_00864	zraR_2
zraR_2	ZONMW-47_02724	zraR_2
zraR_2	ZONMW-47_01745	zraR_2
zraR_2	ZONMW-47_02851	zraR_2
zraR_2	ZONMW-47_03025	zraR_2
zraR_2	ZONMW-47_00598	zraR_2
zraR_2	ZONMW-47_01169	zraR_2
zraR_2	ZONMW-47_01775	zraR_2
zraR_2	ZONMW-47_03017	zraR_2
zraR_2	ZONMW-47_02381	zraR_2
zraR_2	ZONMW-47_01610	zraR_2
zraR_2	ZONMW-47_01721	zraR_2
zraR_2	ZONMW-47_01762	zraR_2
zraR_2	ZONMW-47_01973	zraR_2
zraR_2	ZONMW-47_00028	zraR_2
zraR_2	ZONMW-47_02796	zraR_2
zraR_2	ZONMW-47_00225	zraR_2
zraR_2	ZONMW-47_00834	zraR_2
zraR_2	ZONMW-47_02961	zraR_2
zraR_2	ZONMW-47_01510	zraR_2
zraR_2	ZONMW-47_01097	zraR_2
zraR_2	ZONMW-47_00347	zraR_2
zraR_2	ZONMW-47_02537	zraR_2
zraR_2	ZONMW-47_01584	zraR_2
zraR_2	ZONMW-48_02716	zraR_2
zraR_2	ZONMW-48_01865	zraR_2
zraR_2	ZONMW-48_01829	zraR_2

zraR_2	ZONMW-48_00118	zraR_2
zraR_2	ZONMW-48_02738	zraR_2
zraR_2	ZONMW-48_02491	zraR_2
zraR_2	ZONMW-48_00073	zraR_2
zraR_2	ZONMW-48_01402	zraR_2
zraR_2	ZONMW-48_00304	zraR_2
zraR_2	ZONMW-48_03130	zraR_2
zraR_2	ZONMW-48_00354	zraR_2
zraR_2	ZONMW-48_02103	zraR_2
zraR_2	ZONMW-48_00353	zraR_2
zraR_2	ZONMW-48_03131	zraR_2
zraR_2	ZONMW-48_02104	zraR_2
zraR_2	ZONMW-48_02085	zraR_2
zraR_2	ZONMW-48_00933	zraR_2
zraR_2	ZONMW-48_01644	zraR_2
zraR_2	ZONMW-48_00019	zraR_2
zraR_2	ZONMW-48_01861	zraR_2
zraR_2	ZONMW-48_01450	zraR_2
zraR_2	ZONMW-48_01745	zraR_2
zraR_2	ZONMW-48_02687	zraR_2
zraR_2	ZONMW-48_01645	zraR_2
zraR_2	ZONMW-48_01851	zraR_2
zraR_2	ZONMW-48_02266	zraR_2
zraR_2	ZONMW-48_02612	zraR_2
zraR_2	ZONMW-48_02660	zraR_2
zraR_2	ZONMW-48_00232	zraR_2
zraR_2	ZONMW-48_02964	zraR_2
zraR_2	ZONMW-48_01472	zraR_2
zraR_2	ZONMW-48_00230	zraR_2
zraR_2	ZONMW-48_02552	zraR_2
zraR_2	ZONMW-48_02070	zraR_2
zraR_2	ZONMW-48_00818	zraR_2
zraR_2	ZONMW-48_00943	zraR_2
zraR_2	ZONMW-48_01506	zraR_2
zraR_2	ZONMW-48_01849	zraR_2
zraR_2	ZONMW-48_03019	zraR_2
zraR_2	ZONMW-48_02680	zraR_2
zraR_2	ZONMW-48_01403	zraR_2
zraR_2	ZONMW-48_00303	zraR_2
zraR_2	ZONMW-48_00062	zraR_2
zraR_2	ZONMW-48_03253	zraR_2
zraR_2	ZONMW-48_02402	zraR_2
zraR_2	ZONMW-48_02427	zraR_2
zraR_2	ZONMW-48_00816	zraR_2
zraR_2	ZONMW-48_00472	zraR_2
zraR_2	ZONMW-48_02679	zraR_2
zraR_2	ZONMW-48_02164	zraR_2
zraR_2	ZONMW-48_00716	zraR_2

zraR_2	ZONMW-48_00314	zraR_2
zraR_2	ZONMW-48_01746	zraR_2
zraR_2	ZONMW-48_01360	zraR_2
zraR_2	ZONMW-48_03034	zraR_2
zraR_2	ZONMW-48_00056	zraR_2
zraR_2	ZONMW-48_00959	zraR_2
zraR_2	ZONMW-48_00707	zraR_2
zraR_2	ZONMW-48_01981	zraR_2
zraR_2	ZONMW-48_02689	zraR_2
zraR_2	ZONMW-48_00945	zraR_2
zraR_2	ZONMW-49_01954	zraR_2
zraR_2	ZONMW-49_00518	zraR_2
zraR_2	ZONMW-49_00060	zraR_2
zraR_2	ZONMW-49_00038	zraR_2
zraR_2	ZONMW-49_02737	zraR_2
zraR_2	ZONMW-49_02097	zraR_2
zraR_2	ZONMW-49_01680	zraR_2
zraR_2	ZONMW-49_01244	zraR_2
zraR_2	ZONMW-49_03033	zraR_2
zraR_2	ZONMW-49_01679	zraR_2
zraR_2	ZONMW-49_02098	zraR_2
zraR_2	ZONMW-49_01245	zraR_2
zraR_2	ZONMW-49_03034	zraR_2
zraR_2	ZONMW-49_03013	zraR_2
zraR_2	ZONMW-49_00528	zraR_2
zraR_2	ZONMW-49_03105	zraR_2
zraR_2	ZONMW-49_00527	zraR_2
zraR_2	ZONMW-49_00954	zraR_2
zraR_2	ZONMW-49_03012	zraR_2
zraR_2	ZONMW-49_03106	zraR_2
zraR_2	ZONMW-49_02154	zraR_2
zraR_2	ZONMW-49_00072	zraR_2
zraR_2	ZONMW-49_01379	zraR_2
zraR_2	ZONMW-49_02689	zraR_2
zraR_2	ZONMW-49_02031	zraR_2
zraR_2	ZONMW-49_02697	zraR_2
zraR_2	ZONMW-49_00082	zraR_2
zraR_2	ZONMW-49_03287	zraR_2
zraR_2	ZONMW-49_02963	zraR_2
zraR_2	ZONMW-49_02858	zraR_2
zraR_2	ZONMW-49_01355	zraR_2
zraR_2	ZONMW-49_02463	zraR_2
zraR_2	ZONMW-49_02677	zraR_2
zraR_2	ZONMW-49_02679	zraR_2
zraR_2	ZONMW-49_01668	zraR_2
zraR_2	ZONMW-49_01700	zraR_2
zraR_2	ZONMW-49_01622	zraR_2
zraR_2	ZONMW-49_03123	zraR_2

zraR_2	ZONMW-49_03261	zraR_2
zraR_2	ZONMW-49_00877	zraR_2
zraR_2	ZONMW-49_00642	zraR_2
zraR_2	ZONMW-49_00068	zraR_2
zraR_2	ZONMW-49_02319	zraR_2
zraR_2	ZONMW-49_03122	zraR_2
zraR_2	ZONMW-49_01862	zraR_2
zraR_2	ZONMW-49_01985	zraR_2
zraR_2	ZONMW-49_02207	zraR_2
zraR_2	ZONMW-49_01623	zraR_2
zraR_2	ZONMW-49_02346	zraR_2
zraR_2	ZONMW-49_00851	zraR_2
zraR_2	ZONMW-49_00833	zraR_2
zraR_2	ZONMW-49_01037	zraR_2
zraR_2	ZONMW-49_01122	zraR_2
zraR_2	ZONMW-50_02550	zraR_2
zraR_2	ZONMW-50_00519	zraR_2
zraR_2	ZONMW-50_00426	zraR_2
zraR_2	ZONMW-50_00448	zraR_2
zraR_2	ZONMW-50_02663	zraR_2
zraR_2	ZONMW-50_01891	zraR_2
zraR_2	ZONMW-50_01734	zraR_2
zraR_2	ZONMW-50_01245	zraR_2
zraR_2	ZONMW-50_02999	zraR_2
zraR_2	ZONMW-50_01735	zraR_2
zraR_2	ZONMW-50_01892	zraR_2
zraR_2	ZONMW-50_01246	zraR_2
zraR_2	ZONMW-50_02998	zraR_2
zraR_2	ZONMW-50_02842	zraR_2
zraR_2	ZONMW-50_00529	zraR_2
zraR_2	ZONMW-50_03101	zraR_2
zraR_2	ZONMW-50_00528	zraR_2
zraR_2	ZONMW-50_00955	zraR_2
zraR_2	ZONMW-50_02843	zraR_2
zraR_2	ZONMW-50_03102	zraR_2
zraR_2	ZONMW-50_01948	zraR_2
zraR_2	ZONMW-50_00414	zraR_2
zraR_2	ZONMW-50_01381	zraR_2
zraR_2	ZONMW-50_02483	zraR_2
zraR_2	ZONMW-50_02627	zraR_2
zraR_2	ZONMW-50_02491	zraR_2
zraR_2	ZONMW-50_00404	zraR_2
zraR_2	ZONMW-50_03295	zraR_2
zraR_2	ZONMW-50_02892	zraR_2
zraR_2	ZONMW-50_02798	zraR_2
zraR_2	ZONMW-50_01357	zraR_2
zraR_2	ZONMW-50_02257	zraR_2
zraR_2	ZONMW-50_02471	zraR_2

zraR_2	ZONMW-50_02473	zraR_2
zraR_2	ZONMW-50_01746	zraR_2
zraR_2	ZONMW-50_01714	zraR_2
zraR_2	ZONMW-50_01792	zraR_2
zraR_2	ZONMW-50_03119	zraR_2
zraR_2	ZONMW-50_03248	zraR_2
zraR_2	ZONMW-50_00878	zraR_2
zraR_2	ZONMW-50_00643	zraR_2
zraR_2	ZONMW-50_00418	zraR_2
zraR_2	ZONMW-50_02141	zraR_2
zraR_2	ZONMW-50_03118	zraR_2
zraR_2	ZONMW-50_03043	zraR_2
zraR_2	ZONMW-50_02581	zraR_2
zraR_2	ZONMW-50_02001	zraR_2
zraR_2	ZONMW-50_01791	zraR_2
zraR_2	ZONMW-50_02114	zraR_2
zraR_2	ZONMW-50_00852	zraR_2
zraR_2	ZONMW-50_00834	zraR_2
zraR_2	ZONMW-50_01038	zraR_2
zraR_2	ZONMW-50_01123	zraR_2
zraR_2	ZONMW-51_02260	zraR_2
zraR_2	ZONMW-51_01187	zraR_2
zraR_2	ZONMW-51_00057	zraR_2
zraR_2	ZONMW-51_00035	zraR_2
zraR_2	ZONMW-51_03038	zraR_2
zraR_2	ZONMW-51_01755	zraR_2
zraR_2	ZONMW-51_00578	zraR_2
zraR_2	ZONMW-51_02854	zraR_2
zraR_2	ZONMW-51_01927	zraR_2
zraR_2	ZONMW-51_00577	zraR_2
zraR_2	ZONMW-51_01756	zraR_2
zraR_2	ZONMW-51_02855	zraR_2
zraR_2	ZONMW-51_01926	zraR_2
zraR_2	ZONMW-51_02032	zraR_2
zraR_2	ZONMW-51_01177	zraR_2
zraR_2	ZONMW-51_03161	zraR_2
zraR_2	ZONMW-51_01178	zraR_2
zraR_2	ZONMW-51_01310	zraR_2
zraR_2	ZONMW-51_01817	zraR_2
zraR_2	ZONMW-51_02031	zraR_2
zraR_2	ZONMW-51_03162	zraR_2
zraR_2	ZONMW-51_00069	zraR_2
zraR_2	ZONMW-51_01496	zraR_2
zraR_2	ZONMW-51_02589	zraR_2
zraR_2	ZONMW-51_02337	zraR_2
zraR_2	ZONMW-51_02581	zraR_2
zraR_2	ZONMW-51_00079	zraR_2
zraR_2	ZONMW-51_01839	zraR_2

zraR_2	ZONMW-51_01982	zraR_2
zraR_2	ZONMW-51_02916	zraR_2
zraR_2	ZONMW-51_01473	zraR_2
zraR_2	ZONMW-51_02440	zraR_2
zraR_2	ZONMW-51_02601	zraR_2
zraR_2	ZONMW-51_02599	zraR_2
zraR_2	ZONMW-51_00566	zraR_2
zraR_2	ZONMW-51_00598	zraR_2
zraR_2	ZONMW-51_00521	zraR_2
zraR_2	ZONMW-51_03179	zraR_2
zraR_2	ZONMW-51_02056	zraR_2
zraR_2	ZONMW-51_00834	zraR_2
zraR_2	ZONMW-51_01062	zraR_2
zraR_2	ZONMW-51_00065	zraR_2
zraR_2	ZONMW-51_01637	zraR_2
zraR_2	ZONMW-51_03178	zraR_2
zraR_2	ZONMW-51_02145	zraR_2
zraR_2	ZONMW-51_02291	zraR_2
zraR_2	ZONMW-51_03110	zraR_2
zraR_2	ZONMW-51_00522	zraR_2
zraR_2	ZONMW-51_01610	zraR_2
zraR_2	ZONMW-51_00860	zraR_2
zraR_2	ZONMW-51_00878	zraR_2
zraR_2	ZONMW-51_01230	zraR_2
zraR_2	ZONMW-51_02729	zraR_2
zraR_2	ZONMW-51_01816	zraR_2
zraR_2	ZONMW-52_02672	zraR_2
zraR_2	ZONMW-52_02880	zraR_2
zraR_2	ZONMW-52_00030	zraR_2
zraR_2	ZONMW-52_00008	zraR_2
zraR_2	ZONMW-52_03036	zraR_2
zraR_2	ZONMW-52_01898	zraR_2
zraR_2	ZONMW-52_00535	zraR_2
zraR_2	ZONMW-52_02191	zraR_2
zraR_2	ZONMW-52_00846	zraR_2
zraR_2	ZONMW-52_00534	zraR_2
zraR_2	ZONMW-52_01899	zraR_2
zraR_2	ZONMW-52_02190	zraR_2
zraR_2	ZONMW-52_00847	zraR_2
zraR_2	ZONMW-52_00740	zraR_2
zraR_2	ZONMW-52_02870	zraR_2
zraR_2	ZONMW-52_03120	zraR_2
zraR_2	ZONMW-52_02871	zraR_2
zraR_2	ZONMW-52_01037	zraR_2
zraR_2	ZONMW-52_01960	zraR_2
zraR_2	ZONMW-52_00741	zraR_2
zraR_2	ZONMW-52_03121	zraR_2
zraR_2	ZONMW-52_00042	zraR_2

zraR_2	ZONMW-52_01236	zraR_2
zraR_2	ZONMW-52_02082	zraR_2
zraR_2	ZONMW-52_02752	zraR_2
zraR_2	ZONMW-52_02074	zraR_2
zraR_2	ZONMW-52_00052	zraR_2
zraR_2	ZONMW-52_00934	zraR_2
zraR_2	ZONMW-52_00790	zraR_2
zraR_2	ZONMW-52_02957	zraR_2
zraR_2	ZONMW-52_01213	zraR_2
zraR_2	ZONMW-52_02423	zraR_2
zraR_2	ZONMW-52_02094	zraR_2
zraR_2	ZONMW-52_02092	zraR_2
zraR_2	ZONMW-52_00523	zraR_2
zraR_2	ZONMW-52_00555	zraR_2
zraR_2	ZONMW-52_03138	zraR_2
zraR_2	ZONMW-52_00716	zraR_2
zraR_2	ZONMW-52_01434	zraR_2
zraR_2	ZONMW-52_01663	zraR_2
zraR_2	ZONMW-52_00038	zraR_2
zraR_2	ZONMW-52_01780	zraR_2
zraR_2	ZONMW-52_03137	zraR_2
zraR_2	ZONMW-52_02574	zraR_2
zraR_2	ZONMW-52_02703	zraR_2
zraR_2	ZONMW-52_02009	zraR_2
zraR_2	ZONMW-52_01753	zraR_2
zraR_2	ZONMW-52_01460	zraR_2
zraR_2	ZONMW-52_01478	zraR_2
zraR_2	ZONMW-52_00959	zraR_2
zraR_2	ZONMW-52_02317	zraR_2
zraR_2	ZONMW-52_01959	zraR_2
zraR_2	ZONMW-53_02425	zraR_2
zraR_2	ZONMW-53_00035	zraR_2
zraR_2	ZONMW-53_02625	zraR_2
zraR_2	ZONMW-53_02648	zraR_2
zraR_2	ZONMW-53_03100	zraR_2
zraR_2	ZONMW-53_01858	zraR_2
zraR_2	ZONMW-53_01290	zraR_2
zraR_2	ZONMW-53_02785	zraR_2
zraR_2	ZONMW-53_01589	zraR_2
zraR_2	ZONMW-53_01289	zraR_2
zraR_2	ZONMW-53_01857	zraR_2
zraR_2	ZONMW-53_02784	zraR_2
zraR_2	ZONMW-53_01590	zraR_2
zraR_2	ZONMW-53_01474	zraR_2
zraR_2	ZONMW-53_00045	zraR_2
zraR_2	ZONMW-53_03167	zraR_2
zraR_2	ZONMW-53_00044	zraR_2
zraR_2	ZONMW-53_00797	zraR_2

zraR_2	ZONMW-53_01792	zraR_2
zraR_2	ZONMW-53_01475	zraR_2
zraR_2	ZONMW-53_03166	zraR_2
zraR_2	ZONMW-53_02613	zraR_2
zraR_2	ZONMW-53_00589	zraR_2
zraR_2	ZONMW-53_02757	zraR_2
zraR_2	ZONMW-53_02502	zraR_2
zraR_2	ZONMW-53_02765	zraR_2
zraR_2	ZONMW-53_02603	zraR_2
zraR_2	ZONMW-53_01677	zraR_2
zraR_2	ZONMW-53_01524	zraR_2
zraR_2	ZONMW-53_02964	zraR_2
zraR_2	ZONMW-53_00612	zraR_2
zraR_2	ZONMW-53_02210	zraR_2
zraR_2	ZONMW-53_02745	zraR_2
zraR_2	ZONMW-53_02747	zraR_2
zraR_2	ZONMW-53_01278	zraR_2
zraR_2	ZONMW-53_01310	zraR_2
zraR_2	ZONMW-53_01233	zraR_2
zraR_2	ZONMW-53_03149	zraR_2
zraR_2	ZONMW-53_01448	zraR_2
zraR_2	ZONMW-53_00393	zraR_2
zraR_2	ZONMW-53_00162	zraR_2
zraR_2	ZONMW-53_02617	zraR_2
zraR_2	ZONMW-53_01976	zraR_2
zraR_2	ZONMW-53_03150	zraR_2
zraR_2	ZONMW-53_02326	zraR_2
zraR_2	ZONMW-53_02456	zraR_2
zraR_2	ZONMW-53_01739	zraR_2
zraR_2	ZONMW-53_01234	zraR_2
zraR_2	ZONMW-53_02003	zraR_2
zraR_2	ZONMW-53_00367	zraR_2
zraR_2	ZONMW-53_00349	zraR_2
zraR_2	ZONMW-53_00877	zraR_2
zraR_2	ZONMW-53_02906	zraR_2
zraR_2	ZONMW-54_02485	zraR_2
zraR_2	ZONMW-54_00993	zraR_2
zraR_2	ZONMW-54_02467	zraR_2
zraR_2	ZONMW-54_02194	zraR_2
zraR_2	ZONMW-54_01947	zraR_2
zraR_2	ZONMW-54_01968	zraR_2
zraR_2	ZONMW-54_01002	zraR_2
zraR_2	ZONMW-54_03102	zraR_2
zraR_2	ZONMW-54_02213	zraR_2
zraR_2	ZONMW-54_01427	zraR_2
zraR_2	ZONMW-54_00007	zraR_2
zraR_2	ZONMW-54_01136	zraR_2
zraR_2	ZONMW-54_01428	zraR_2

zraR_2	ZONMW-54_02214	zraR_2
zraR_2	ZONMW-54_00006	zraR_2
zraR_2	ZONMW-54_01135	zraR_2
zraR_2	ZONMW-54_01249	zraR_2
zraR_2	ZONMW-54_00983	zraR_2
zraR_2	ZONMW-54_03131	zraR_2
zraR_2	ZONMW-54_00984	zraR_2
zraR_2	ZONMW-54_00272	zraR_2
zraR_2	ZONMW-54_01935	zraR_2
zraR_2	ZONMW-54_01248	zraR_2
zraR_2	ZONMW-54_03132	zraR_2
zraR_2	ZONMW-54_00446	zraR_2
zraR_2	ZONMW-54_02900	zraR_2
zraR_2	ZONMW-54_02562	zraR_2
zraR_2	ZONMW-54_02908	zraR_2
zraR_2	ZONMW-54_01925	zraR_2
zraR_2	ZONMW-54_01048	zraR_2
zraR_2	ZONMW-54_01196	zraR_2
zraR_2	ZONMW-54_02968	zraR_2
zraR_2	ZONMW-54_00423	zraR_2
zraR_2	ZONMW-54_02700	zraR_2
zraR_2	ZONMW-54_02888	zraR_2
zraR_2	ZONMW-54_02890	zraR_2
zraR_2	ZONMW-54_01439	zraR_2
zraR_2	ZONMW-54_01407	zraR_2
zraR_2	ZONMW-54_01484	zraR_2
zraR_2	ZONMW-54_03149	zraR_2
zraR_2	ZONMW-54_01273	zraR_2
zraR_2	ZONMW-54_00641	zraR_2
zraR_2	ZONMW-54_01939	zraR_2
zraR_2	ZONMW-54_02078	zraR_2
zraR_2	ZONMW-54_03148	zraR_2
zraR_2	ZONMW-54_02368	zraR_2
zraR_2	ZONMW-54_02516	zraR_2
zraR_2	ZONMW-54_02296	zraR_2
zraR_2	ZONMW-54_01483	zraR_2
zraR_2	ZONMW-54_02051	zraR_2
zraR_2	ZONMW-54_00667	zraR_2
zraR_2	ZONMW-54_00685	zraR_2
zraR_2	ZONMW-54_00188	zraR_2
zraR_2	ZONMW-54_00866	zraR_2
zraR_2	ZONMW-54_00122	zraR_2
zraR_2	ZONMW-55_01728	zraR_2
zraR_2	ZONMW-55_00033	zraR_2
zraR_2	ZONMW-55_01481	zraR_2
zraR_2	ZONMW-55_01503	zraR_2
zraR_2	ZONMW-55_01558	zraR_2
zraR_2	ZONMW-55_02077	zraR_2

zraR_2	ZONMW-55_00946	zraR_2
zraR_2	ZONMW-55_02787	zraR_2
zraR_2	ZONMW-55_02249	zraR_2
zraR_2	ZONMW-55_00947	zraR_2
zraR_2	ZONMW-55_02078	zraR_2
zraR_2	ZONMW-55_02786	zraR_2
zraR_2	ZONMW-55_02248	zraR_2
zraR_2	ZONMW-55_02354	zraR_2
zraR_2	ZONMW-55_00043	zraR_2
zraR_2	ZONMW-55_03165	zraR_2
zraR_2	ZONMW-55_00042	zraR_2
zraR_2	ZONMW-55_00766	zraR_2
zraR_2	ZONMW-55_02139	zraR_2
zraR_2	ZONMW-55_02353	zraR_2
zraR_2	ZONMW-55_03164	zraR_2
zraR_2	ZONMW-55_01469	zraR_2
zraR_2	ZONMW-55_00584	zraR_2
zraR_2	ZONMW-55_02757	zraR_2
zraR_2	ZONMW-55_01651	zraR_2
zraR_2	ZONMW-55_02765	zraR_2
zraR_2	ZONMW-55_01459	zraR_2
zraR_2	ZONMW-55_02161	zraR_2
zraR_2	ZONMW-55_02304	zraR_2
zraR_2	ZONMW-55_02972	zraR_2
zraR_2	ZONMW-55_00607	zraR_2
zraR_2	ZONMW-55_02531	zraR_2
zraR_2	ZONMW-55_02745	zraR_2
zraR_2	ZONMW-55_02747	zraR_2
zraR_2	ZONMW-55_00958	zraR_2
zraR_2	ZONMW-55_00926	zraR_2
zraR_2	ZONMW-55_01003	zraR_2
zraR_2	ZONMW-55_03147	zraR_2
zraR_2	ZONMW-55_02378	zraR_2
zraR_2	ZONMW-55_00386	zraR_2
zraR_2	ZONMW-55_00158	zraR_2
zraR_2	ZONMW-55_01473	zraR_2
zraR_2	ZONMW-55_01959	zraR_2
zraR_2	ZONMW-55_03148	zraR_2
zraR_2	ZONMW-55_01827	zraR_2
zraR_2	ZONMW-55_01697	zraR_2
zraR_2	ZONMW-55_03077	zraR_2
zraR_2	ZONMW-55_01002	zraR_2
zraR_2	ZONMW-55_01932	zraR_2
zraR_2	ZONMW-55_00360	zraR_2
zraR_2	ZONMW-55_00342	zraR_2
zraR_2	ZONMW-55_00843	zraR_2
zraR_2	ZONMW-55_02912	zraR_2
zraR_2	ZONMW-55_02138	zraR_2

zraR_2	ZONMW-56_01882	zraR_2
zraR_2	ZONMW-56_02774	zraR_2
zraR_2	ZONMW-56_00707	zraR_2
zraR_2	ZONMW-56_00729	zraR_2
zraR_2	ZONMW-56_02987	zraR_2
zraR_2	ZONMW-56_01357	zraR_2
zraR_2	ZONMW-56_00182	zraR_2
zraR_2	ZONMW-56_02551	zraR_2
zraR_2	ZONMW-56_02119	zraR_2
zraR_2	ZONMW-56_00183	zraR_2
zraR_2	ZONMW-56_01356	zraR_2
zraR_2	ZONMW-56_02550	zraR_2
zraR_2	ZONMW-56_02120	zraR_2
zraR_2	ZONMW-56_02002	zraR_2
zraR_2	ZONMW-56_02764	zraR_2
zraR_2	ZONMW-56_03125	zraR_2
zraR_2	ZONMW-56_02765	zraR_2
zraR_2	ZONMW-56_02313	zraR_2
zraR_2	ZONMW-56_01301	zraR_2
zraR_2	ZONMW-56_02003	zraR_2
zraR_2	ZONMW-56_03124	zraR_2
zraR_2	ZONMW-56_00695	zraR_2
zraR_2	ZONMW-56_03042	zraR_2
zraR_2	ZONMW-56_02438	zraR_2
zraR_2	ZONMW-56_01801	zraR_2
zraR_2	ZONMW-56_02430	zraR_2
zraR_2	ZONMW-56_00685	zraR_2
zraR_2	ZONMW-56_02207	zraR_2
zraR_2	ZONMW-56_02052	zraR_2
zraR_2	ZONMW-56_02852	zraR_2
zraR_2	ZONMW-56_03065	zraR_2
zraR_2	ZONMW-56_01698	zraR_2
zraR_2	ZONMW-56_02450	zraR_2
zraR_2	ZONMW-56_02448	zraR_2
zraR_2	ZONMW-56_00194	zraR_2
zraR_2	ZONMW-56_00162	zraR_2
zraR_2	ZONMW-56_00239	zraR_2
zraR_2	ZONMW-56_03107	zraR_2
zraR_2	ZONMW-56_00023	zraR_2
zraR_2	ZONMW-56_00929	zraR_2
zraR_2	ZONMW-56_01162	zraR_2
zraR_2	ZONMW-56_00699	zraR_2
zraR_2	ZONMW-56_01477	zraR_2
zraR_2	ZONMW-56_03108	zraR_2
zraR_2	ZONMW-56_01975	zraR_2
zraR_2	ZONMW-56_01850	zraR_2
zraR_2	ZONMW-56_01252	zraR_2
zraR_2	ZONMW-56_00238	zraR_2

zraR_2	ZONMW-56_01504	zraR_2
zraR_2	ZONMW-56_00955	zraR_2
zraR_2	ZONMW-56_00973	zraR_2
zraR_2	ZONMW-56_02229	zraR_2
zraR_2	ZONMW-56_02673	zraR_2
zraR_2	ZONMW-56_01302	zraR_2
zraR_2	ZONMW-57_02256	zraR_2
zraR_2	ZONMW-57_01580	zraR_2
zraR_2	ZONMW-57_00914	zraR_2
zraR_2	ZONMW-57_00936	zraR_2
zraR_2	ZONMW-57_03061	zraR_2
zraR_2	ZONMW-57_01867	zraR_2
zraR_2	ZONMW-57_00395	zraR_2
zraR_2	ZONMW-57_02692	zraR_2
zraR_2	ZONMW-57_00101	zraR_2
zraR_2	ZONMW-57_00396	zraR_2
zraR_2	ZONMW-57_01868	zraR_2
zraR_2	ZONMW-57_02691	zraR_2
zraR_2	ZONMW-57_00100	zraR_2
zraR_2	ZONMW-57_00214	zraR_2
zraR_2	ZONMW-57_01570	zraR_2
zraR_2	ZONMW-57_03144	zraR_2
zraR_2	ZONMW-57_01571	zraR_2
zraR_2	ZONMW-57_02441	zraR_2
zraR_2	ZONMW-57_00213	zraR_2
zraR_2	ZONMW-57_03145	zraR_2
zraR_2	ZONMW-57_03033	zraR_2
zraR_2	ZONMW-57_00902	zraR_2
zraR_2	ZONMW-57_01030	zraR_2
zraR_2	ZONMW-57_02661	zraR_2
zraR_2	ZONMW-57_02339	zraR_2
zraR_2	ZONMW-57_02669	zraR_2
zraR_2	ZONMW-57_00892	zraR_2
zraR_2	ZONMW-57_00013	zraR_2
zraR_2	ZONMW-57_00164	zraR_2
zraR_2	ZONMW-57_02890	zraR_2
zraR_2	ZONMW-57_01007	zraR_2
zraR_2	ZONMW-57_02045	zraR_2
zraR_2	ZONMW-57_02649	zraR_2
zraR_2	ZONMW-57_02651	zraR_2
zraR_2	ZONMW-57_00407	zraR_2
zraR_2	ZONMW-57_00375	zraR_2
zraR_2	ZONMW-57_00452	zraR_2
zraR_2	ZONMW-57_03162	zraR_2
zraR_2	ZONMW-57_00238	zraR_2
zraR_2	ZONMW-57_01229	zraR_2
zraR_2	ZONMW-57_01459	zraR_2
zraR_2	ZONMW-57_00906	zraR_2

zraR_2	ZONMW-57_01749	zraR_2
zraR_2	ZONMW-57_03161	zraR_2
zraR_2	ZONMW-57_02159	zraR_2
zraR_2	ZONMW-57_02290	zraR_2
zraR_2	ZONMW-57_02981	zraR_2
zraR_2	ZONMW-57_00451	zraR_2
zraR_2	ZONMW-57_01722	zraR_2
zraR_2	ZONMW-57_01255	zraR_2
zraR_2	ZONMW-57_01273	zraR_2
zraR_2	ZONMW-57_02360	zraR_2
zraR_2	ZONMW-57_02818	zraR_2
zraR_2	ZONMW-58_02280	zraR_2
zraR_2	ZONMW-58_01004	zraR_2
zraR_2	ZONMW-58_00057	zraR_2
zraR_2	ZONMW-58_00035	zraR_2
zraR_2	ZONMW-58_03082	zraR_2
zraR_2	ZONMW-58_01891	zraR_2
zraR_2	ZONMW-58_00576	zraR_2
zraR_2	ZONMW-58_02716	zraR_2
zraR_2	ZONMW-58_00865	zraR_2
zraR_2	ZONMW-58_00575	zraR_2
zraR_2	ZONMW-58_01892	zraR_2
zraR_2	ZONMW-58_02715	zraR_2
zraR_2	ZONMW-58_00866	zraR_2
zraR_2	ZONMW-58_00757	zraR_2
zraR_2	ZONMW-58_01014	zraR_2
zraR_2	ZONMW-58_03203	zraR_2
zraR_2	ZONMW-58_01013	zraR_2
zraR_2	ZONMW-58_02466	zraR_2
zraR_2	ZONMW-58_00758	zraR_2
zraR_2	ZONMW-58_03202	zraR_2
zraR_2	ZONMW-58_02960	zraR_2
zraR_2	ZONMW-58_00069	zraR_2
zraR_2	ZONMW-58_01554	zraR_2
zraR_2	ZONMW-58_02686	zraR_2
zraR_2	ZONMW-58_02363	zraR_2
zraR_2	ZONMW-58_02694	zraR_2
zraR_2	ZONMW-58_00079	zraR_2
zraR_2	ZONMW-58_00953	zraR_2
zraR_2	ZONMW-58_00807	zraR_2
zraR_2	ZONMW-58_02914	zraR_2
zraR_2	ZONMW-58_01577	zraR_2
zraR_2	ZONMW-58_02033	zraR_2
zraR_2	ZONMW-58_02674	zraR_2
zraR_2	ZONMW-58_02676	zraR_2
zraR_2	ZONMW-58_00564	zraR_2
zraR_2	ZONMW-58_00596	zraR_2
zraR_2	ZONMW-58_00519	zraR_2

zraR_2	ZONMW-58_03185	zraR_2
zraR_2	ZONMW-58_00733	zraR_2
zraR_2	ZONMW-58_01355	zraR_2
zraR_2	ZONMW-58_01125	zraR_2
zraR_2	ZONMW-58_00065	zraR_2
zraR_2	ZONMW-58_01773	zraR_2
zraR_2	ZONMW-58_03186	zraR_2
zraR_2	ZONMW-58_02183	zraR_2
zraR_2	ZONMW-58_02314	zraR_2
zraR_2	ZONMW-58_03012	zraR_2
zraR_2	ZONMW-58_00520	zraR_2
zraR_2	ZONMW-58_01746	zraR_2
zraR_2	ZONMW-58_01329	zraR_2
zraR_2	ZONMW-58_01311	zraR_2
zraR_2	ZONMW-58_02385	zraR_2
zraR_2	ZONMW-58_02842	zraR_2
zraR_2	ZONMW-59_02496	zraR_2
zraR_2	ZONMW-59_02752	zraR_2
zraR_2	ZONMW-59_00947	zraR_2
zraR_2	ZONMW-59_00970	zraR_2
zraR_2	ZONMW-59_03137	zraR_2
zraR_2	ZONMW-59_01704	zraR_2
zraR_2	ZONMW-59_00411	zraR_2
zraR_2	ZONMW-59_03010	zraR_2
zraR_2	ZONMW-59_00101	zraR_2
zraR_2	ZONMW-59_00412	zraR_2
zraR_2	ZONMW-59_01705	zraR_2
zraR_2	ZONMW-59_03011	zraR_2
zraR_2	ZONMW-59_00100	zraR_2
zraR_2	ZONMW-59_00207	zraR_2
zraR_2	ZONMW-59_02762	zraR_2
zraR_2	ZONMW-59_03260	zraR_2
zraR_2	ZONMW-59_02761	zraR_2
zraR_2	ZONMW-59_02025	zraR_2
zraR_2	ZONMW-59_00206	zraR_2
zraR_2	ZONMW-59_03259	zraR_2
zraR_2	ZONMW-59_01766	zraR_2
zraR_2	ZONMW-59_00897	zraR_2
zraR_2	ZONMW-59_01032	zraR_2
zraR_2	ZONMW-59_02694	zraR_2
zraR_2	ZONMW-59_02577	zraR_2
zraR_2	ZONMW-59_02702	zraR_2
zraR_2	ZONMW-59_00886	zraR_2
zraR_2	ZONMW-59_00013	zraR_2
zraR_2	ZONMW-59_00157	zraR_2
zraR_2	ZONMW-59_03059	zraR_2
zraR_2	ZONMW-59_01009	zraR_2
zraR_2	ZONMW-59_02252	zraR_2

zraR_2	ZONMW-59_02682	zraR_2
zraR_2	ZONMW-59_02684	zraR_2
zraR_2	ZONMW-59_00423	zraR_2
zraR_2	ZONMW-59_00391	zraR_2
zraR_2	ZONMW-59_03242	zraR_2
zraR_2	ZONMW-59_00231	zraR_2
zraR_2	ZONMW-59_01230	zraR_2
zraR_2	ZONMW-59_01464	zraR_2
zraR_2	ZONMW-59_00901	zraR_2
zraR_2	ZONMW-59_01585	zraR_2
zraR_2	ZONMW-59_03243	zraR_2
zraR_2	ZONMW-59_02402	zraR_2
zraR_2	ZONMW-59_02528	zraR_2
zraR_2	ZONMW-59_01820	zraR_2
zraR_2	ZONMW-59_01558	zraR_2
zraR_2	ZONMW-59_01256	zraR_2
zraR_2	ZONMW-59_01274	zraR_2
zraR_2	ZONMW-59_02108	zraR_2
zraR_2	ZONMW-59_02887	zraR_2
zraR_2	ZONMW-60_02365	zraR_2
zraR_2	ZONMW-60_00978	zraR_2
zraR_2	ZONMW-60_02347	zraR_2
zraR_2	ZONMW-60_02068	zraR_2
zraR_2	ZONMW-60_00967	zraR_2
zraR_2	ZONMW-60_00913	zraR_2
zraR_2	ZONMW-60_00934	zraR_2
zraR_2	ZONMW-60_03073	zraR_2
zraR_2	ZONMW-60_00969	zraR_2
zraR_2	ZONMW-60_02087	zraR_2
zraR_2	ZONMW-60_00422	zraR_2
zraR_2	ZONMW-60_02820	zraR_2
zraR_2	ZONMW-60_00102	zraR_2
zraR_2	ZONMW-60_00423	zraR_2
zraR_2	ZONMW-60_00968	zraR_2
zraR_2	ZONMW-60_02088	zraR_2
zraR_2	ZONMW-60_02819	zraR_2
zraR_2	ZONMW-60_00101	zraR_2
zraR_2	ZONMW-60_00240	zraR_2
zraR_2	ZONMW-60_00988	zraR_2
zraR_2	ZONMW-60_03155	zraR_2
zraR_2	ZONMW-60_00987	zraR_2
zraR_2	ZONMW-60_01708	zraR_2
zraR_2	ZONMW-60_00239	zraR_2
zraR_2	ZONMW-60_03156	zraR_2
zraR_2	ZONMW-60_00901	zraR_2
zraR_2	ZONMW-60_02700	zraR_2
zraR_2	ZONMW-60_02440	zraR_2
zraR_2	ZONMW-60_02692	zraR_2

zraR_2	ZONMW-60_00891	zraR_2
zraR_2	ZONMW-60_00014	zraR_2
zraR_2	ZONMW-60_00190	zraR_2
zraR_2	ZONMW-60_03007	zraR_2
zraR_2	ZONMW-60_01549	zraR_2
zraR_2	ZONMW-60_02548	zraR_2
zraR_2	ZONMW-60_02712	zraR_2
zraR_2	ZONMW-60_02710	zraR_2
zraR_2	ZONMW-60_00434	zraR_2
zraR_2	ZONMW-60_00402	zraR_2
zraR_2	ZONMW-60_03173	zraR_2
zraR_2	ZONMW-60_00263	zraR_2
zraR_2	ZONMW-60_01340	zraR_2
zraR_2	ZONMW-60_00905	zraR_2
zraR_2	ZONMW-60_01966	zraR_2
zraR_2	ZONMW-60_03172	zraR_2
zraR_2	ZONMW-60_02248	zraR_2
zraR_2	ZONMW-60_02396	zraR_2
zraR_2	ZONMW-60_02174	zraR_2
zraR_2	ZONMW-60_01939	zraR_2
zraR_2	ZONMW-60_01314	zraR_2
zraR_2	ZONMW-60_01296	zraR_2
zraR_2	ZONMW-60_01787	zraR_2
zraR_2	ZONMW-60_03357	zraR_2
zraR_2	ZONMW-60_01116	zraR_2
zraR_2	ZONMW-60_02935	zraR_2
zraR_2	ZONMW-61_01067	zraR_2
zraR_2	ZONMW-61_02330	zraR_2
zraR_2	ZONMW-61_02924	zraR_2
zraR_2	ZONMW-61_00466	zraR_2
zraR_2	ZONMW-61_03229	zraR_2
zraR_2	ZONMW-61_01672	zraR_2
zraR_2	ZONMW-61_03217	zraR_2
zraR_2	ZONMW-61_02020	zraR_2
zraR_2	ZONMW-61_03218	zraR_2
zraR_2	ZONMW-61_01673	zraR_2
zraR_2	ZONMW-61_02021	zraR_2
zraR_2	ZONMW-61_02398	zraR_2
zraR_2	ZONMW-61_01730	zraR_2
zraR_2	ZONMW-61_02222	zraR_2
zraR_2	ZONMW-61_02399	zraR_2
zraR_2	ZONMW-61_01866	zraR_2
zraR_2	ZONMW-61_01473	zraR_2
zraR_2	ZONMW-61_00233	zraR_2
zraR_2	ZONMW-61_02302	zraR_2
zraR_2	ZONMW-61_02469	zraR_2
zraR_2	ZONMW-61_00596	zraR_2
zraR_2	ZONMW-61_01192	zraR_2

zraR_2	ZONMW-61_02468	zraR_2
zraR_2	ZONMW-61_01844	zraR_2
zraR_2	ZONMW-61_00829	zraR_2
zraR_2	ZONMW-61_03230	zraR_2
zraR_2	ZONMW-61_04042	zraR_2
zraR_2	ZONMW-61_01037	zraR_2
zraR_2	ZONMW-61_02821	zraR_2
zraR_2	ZONMW-61_00027	zraR_2
zraR_2	ZONMW-61_00846	zraR_2
zraR_2	ZONMW-61_00833	zraR_2
zraR_2	ZONMW-61_03720	zraR_2
zraR_2	ZONMW-61_00559	zraR_2
zraR_2	ZONMW-61_03721	zraR_2
zraR_2	ZONMW-61_03003	zraR_2
zraR_2	ZONMW-61_01801	zraR_2
zraR_2	ZONMW-61_00926	zraR_2
zraR_2	ZONMW-61_02767	zraR_2
zraR_2	ZONMW-61_01477	zraR_2
zraR_2	ZONMW-61_03636	zraR_2
zraR_2	ZONMW-61_00839	zraR_2
zraR_2	ZONMW-61_00837	zraR_2
zraR_2	ZONMW-61_04060	zraR_2
zraR_2	ZONMW-61_00801	zraR_2
zraR_2	ZONMW-61_02238	zraR_2
zraR_2	ZONMW-61_02520	zraR_2
zraR_2	ZONMW-61_00467	zraR_2
zraR_2	ZONMW-61_02237	zraR_2
zraR_2	ZONMW-61_01771	zraR_2
zraR_2	ZONMW-61_00185	zraR_2
zraR_2	ZONMW-61_00992	zraR_2
zraR_2	ZONMW-61_03917	zraR_2
zraR_2	ZONMW-61_03418	zraR_2
zraR_2	ZONMW-61_00210	zraR_2
zraR_2	ZONMW-61_00164	zraR_2
zraR_2	ZONMW-61_01471	zraR_2
zraR_2	ZONMW-61_01723	zraR_2
zraR_2	ZONMW-61_01526	zraR_2
zraR_2	ZONMW-61_00802	zraR_2
zraR_2	ZONMW-61_01667	zraR_2
zraR_2	ZONMW-61_02626	zraR_2
zraR_2	ZONMW-61_01731	zraR_2
zraR_2	ZONMW-61_01154	zraR_2
zraR_2	ZONMW-61_02236	zraR_2
zraR_2	ZONMW-61_01668	zraR_2
zraR_2	ZONMW-61_02846	zraR_2
zraR_2	ZONMW-61_02845	zraR_2
zraR_2	ZONMW-61_03692	zraR_2
zraR_2	ZONMW-61_02578	zraR_2

zraR_2	ZONMW-61_00191	zraR_2
zraR_2	ZONMW-61_03185	zraR_2
zraR_2	ZONMW-61_00455	zraR_2
zraR_2	ZONMW-61_04003	zraR_2
zraR_2	ZONMW-61_03065	zraR_2
zraR_2	ZONMW-61_03847	zraR_2
zraR_2	ZONMW-61_03061	zraR_2
zraR_2	ZONMW-62_03038	zraR_2
zraR_2	ZONMW-62_03080	zraR_2
zraR_2	ZONMW-62_03853	zraR_2
zraR_2	ZONMW-62_02849	zraR_2
zraR_2	ZONMW-62_00835	zraR_2
zraR_2	ZONMW-62_01984	zraR_2
zraR_2	ZONMW-62_01993	zraR_2
zraR_2	ZONMW-62_03292	zraR_2
zraR_2	ZONMW-62_01418	zraR_2
zraR_2	ZONMW-62_03737	zraR_2
zraR_2	ZONMW-62_00560	zraR_2
zraR_2	ZONMW-62_00547	zraR_2
zraR_2	ZONMW-62_01471	zraR_2
zraR_2	ZONMW-62_00222	zraR_2
zraR_2	ZONMW-62_00559	zraR_2
zraR_2	ZONMW-62_01419	zraR_2
zraR_2	ZONMW-62_00221	zraR_2
zraR_2	ZONMW-62_00759	zraR_2
zraR_2	ZONMW-62_00760	zraR_2
zraR_2	ZONMW-62_00657	zraR_2
zraR_2	ZONMW-62_01229	zraR_2
zraR_2	ZONMW-62_02945	zraR_2
zraR_2	ZONMW-62_03351	zraR_2
zraR_2	ZONMW-62_01225	zraR_2
zraR_2	ZONMW-62_02542	zraR_2
zraR_2	ZONMW-62_01788	zraR_2
zraR_2	ZONMW-62_01755	zraR_2
zraR_2	ZONMW-62_00874	zraR_2
zraR_2	ZONMW-62_00546	zraR_2
zraR_2	ZONMW-62_00658	zraR_2
zraR_2	ZONMW-62_01994	zraR_2
zraR_2	ZONMW-62_02622	zraR_2
zraR_2	ZONMW-62_03029	zraR_2
zraR_2	ZONMW-62_02645	zraR_2
zraR_2	ZONMW-62_03079	zraR_2
zraR_2	ZONMW-62_00012	zraR_2
zraR_2	ZONMW-62_03506	zraR_2
zraR_2	ZONMW-62_03118	zraR_2
zraR_2	ZONMW-62_01580	zraR_2
zraR_2	ZONMW-62_02663	zraR_2
zraR_2	ZONMW-62_02649	zraR_2

zraR_2	ZONMW-62_03765	zraR_2
zraR_2	ZONMW-62_02584	zraR_2
zraR_2	ZONMW-62_03766	zraR_2
zraR_2	ZONMW-62_02231	zraR_2
zraR_2	ZONMW-62_01541	zraR_2
zraR_2	ZONMW-62_02132	zraR_2
zraR_2	ZONMW-62_03455	zraR_2
zraR_2	ZONMW-62_01093	zraR_2
zraR_2	ZONMW-62_02655	zraR_2
zraR_2	ZONMW-62_03291	zraR_2
zraR_2	ZONMW-62_00607	zraR_2
zraR_2	ZONMW-62_02653	zraR_2
zraR_2	ZONMW-62_00697	zraR_2
zraR_2	ZONMW-62_03293	zraR_2
zraR_2	ZONMW-62_03030	zraR_2
zraR_2	ZONMW-62_01511	zraR_2
zraR_2	ZONMW-62_01736	zraR_2
zraR_2	ZONMW-62_03165	zraR_2
zraR_2	ZONMW-62_03624	zraR_2
zraR_2	ZONMW-62_02993	zraR_2
zraR_2	ZONMW-62_01771	zraR_2
zraR_2	ZONMW-62_01718	zraR_2
zraR_2	ZONMW-62_01223	zraR_2
zraR_2	ZONMW-62_01464	zraR_2
zraR_2	ZONMW-62_03112	zraR_2
zraR_2	ZONMW-62_01278	zraR_2
zraR_2	ZONMW-62_02623	zraR_2
zraR_2	ZONMW-62_01472	zraR_2
zraR_2	ZONMW-62_02806	zraR_2
zraR_2	ZONMW-62_01413	zraR_2
zraR_2	ZONMW-62_02905	zraR_2
zraR_2	ZONMW-62_03294	zraR_2
zraR_2	ZONMW-62_01414	zraR_2
zraR_2	ZONMW-62_01742	zraR_2
zraR_2	ZONMW-62_03240	zraR_2
zraR_2	ZONMW-62_01973	zraR_2
zraR_2	ZONMW-62_01717	zraR_2
zraR_2	ZONMW-62_03308	zraR_2
zraR_2	ZONMW-62_02974	zraR_2
zraR_2	ZONMW-62_02599	zraR_2
zraR_2	ZONMW-62_01754	zraR_2
zraR_2	ZONMW-62_01129	zraR_2
zraR_2	ZONMW-62_03836	zraR_2
zraR_2	ZONMW-63_02427	zraR_2
zraR_2	ZONMW-63_00977	zraR_2
zraR_2	ZONMW-63_02048	zraR_2
zraR_2	ZONMW-63_02070	zraR_2
zraR_2	ZONMW-63_02953	zraR_2

zraR_2	ZONMW-63_02724	zraR_2
zraR_2	ZONMW-63_00063	zraR_2
zraR_2	ZONMW-63_02382	zraR_2
zraR_2	ZONMW-63_01865	zraR_2
zraR_2	ZONMW-63_00064	zraR_2
zraR_2	ZONMW-63_02723	zraR_2
zraR_2	ZONMW-63_02383	zraR_2
zraR_2	ZONMW-63_01864	zraR_2
zraR_2	ZONMW-63_02612	zraR_2
zraR_2	ZONMW-63_00987	zraR_2
zraR_2	ZONMW-63_03177	zraR_2
zraR_2	ZONMW-63_00986	zraR_2
zraR_2	ZONMW-63_00608	zraR_2
zraR_2	ZONMW-63_02611	zraR_2
zraR_2	ZONMW-63_03176	zraR_2
zraR_2	ZONMW-63_02666	zraR_2
zraR_2	ZONMW-63_02036	zraR_2
zraR_2	ZONMW-63_00786	zraR_2
zraR_2	ZONMW-63_02207	zraR_2
zraR_2	ZONMW-63_02508	zraR_2
zraR_2	ZONMW-63_02215	zraR_2
zraR_2	ZONMW-63_02026	zraR_2
zraR_2	ZONMW-63_01777	zraR_2
zraR_2	ZONMW-63_02561	zraR_2
zraR_2	ZONMW-63_02820	zraR_2
zraR_2	ZONMW-63_00763	zraR_2
zraR_2	ZONMW-63_01638	zraR_2
zraR_2	ZONMW-63_02195	zraR_2
zraR_2	ZONMW-63_02197	zraR_2
zraR_2	ZONMW-63_00076	zraR_2
zraR_2	ZONMW-63_00043	zraR_2
zraR_2	ZONMW-63_00122	zraR_2
zraR_2	ZONMW-63_03159	zraR_2
zraR_2	ZONMW-63_02638	zraR_2
zraR_2	ZONMW-63_01336	zraR_2
zraR_2	ZONMW-63_01103	zraR_2
zraR_2	ZONMW-63_02040	zraR_2
zraR_2	ZONMW-63_01485	zraR_2
zraR_2	ZONMW-63_03160	zraR_2
zraR_2	ZONMW-63_03106	zraR_2
zraR_2	ZONMW-63_02459	zraR_2
zraR_2	ZONMW-63_03006	zraR_2
zraR_2	ZONMW-63_00121	zraR_2
zraR_2	ZONMW-63_01458	zraR_2
zraR_2	ZONMW-63_01310	zraR_2
zraR_2	ZONMW-63_01292	zraR_2
zraR_2	ZONMW-63_00523	zraR_2
zraR_2	ZONMW-63_03309	zraR_2

zraR_2	ZONMW-63_02256	zraR_2
zraR_2	ZONMW-64_02219	zraR_2
zraR_2	ZONMW-64_01698	zraR_2
zraR_2	ZONMW-64_03523	zraR_2
zraR_2	ZONMW-64_02051	zraR_2
zraR_2	ZONMW-64_00204	zraR_2
zraR_2	ZONMW-64_03067	zraR_2
zraR_2	ZONMW-64_01289	zraR_2
zraR_2	ZONMW-64_00380	zraR_2
zraR_2	ZONMW-64_01217	zraR_2
zraR_2	ZONMW-64_01216	zraR_2
zraR_2	ZONMW-64_02507	zraR_2
zraR_2	ZONMW-64_00741	zraR_2
zraR_2	ZONMW-64_01340	zraR_2
zraR_2	ZONMW-64_03810	zraR_2
zraR_2	ZONMW-64_00237	zraR_2
zraR_2	ZONMW-64_02092	zraR_2
zraR_2	ZONMW-64_01388	zraR_2
zraR_2	ZONMW-64_01286	zraR_2
zraR_2	ZONMW-64_01947	zraR_2
zraR_2	ZONMW-64_01341	zraR_2
zraR_2	ZONMW-64_00499	zraR_2
zraR_2	ZONMW-64_01288	zraR_2
zraR_2	ZONMW-64_03888	zraR_2
zraR_2	ZONMW-64_01504	zraR_2
zraR_2	ZONMW-64_03784	zraR_2
zraR_2	ZONMW-64_03809	zraR_2
zraR_2	ZONMW-64_01842	zraR_2
zraR_2	ZONMW-64_00473	zraR_2
zraR_2	ZONMW-64_01579	zraR_2
zraR_2	ZONMW-64_02091	zraR_2
zraR_2	ZONMW-64_01387	zraR_2
zraR_2	ZONMW-64_04182	zraR_2
zraR_2	ZONMW-64_01283	zraR_2
zraR_2	ZONMW-64_00205	zraR_2
zraR_2	ZONMW-64_00481	zraR_2
zraR_2	ZONMW-64_03265	zraR_2
zraR_2	ZONMW-64_03267	zraR_2
zraR_2	ZONMW-64_02196	zraR_2
zraR_2	ZONMW-64_00129	zraR_2
zraR_2	ZONMW-64_03441	zraR_2
zraR_2	ZONMW-64_01034	zraR_2
zraR_2	ZONMW-64_01121	zraR_2
zraR_2	ZONMW-64_00998	zraR_2
zraR_2	ZONMW-64_02114	zraR_2
zraR_2	ZONMW-64_01392	zraR_2
zraR_2	ZONMW-64_03197	zraR_2
zraR_2	ZONMW-64_01300	zraR_2

zraR_2	ZONMW-64_03442	zraR_2
zraR_2	ZONMW-64_00566	zraR_2
zraR_2	ZONMW-64_02841	zraR_2
zraR_2	ZONMW-64_01218	zraR_2
zraR_2	ZONMW-64_03255	zraR_2
zraR_2	ZONMW-64_01700	zraR_2
zraR_2	ZONMW-64_03996	zraR_2
zraR_2	ZONMW-64_00120	zraR_2
zraR_2	ZONMW-64_01002	zraR_2
zraR_2	ZONMW-64_02117	zraR_2
zraR_2	ZONMW-64_04116	zraR_2
zraR_2	ZONMW-64_03997	zraR_2
zraR_2	ZONMW-64_01301	zraR_2
zraR_2	ZONMW-64_00236	zraR_2
zraR_2	ZONMW-64_02272	zraR_2
zraR_2	ZONMW-64_00502	zraR_2
zraR_2	ZONMW-64_01022	zraR_2
zraR_2	ZONMW-64_01284	zraR_2
zraR_2	ZONMW-64_01859	zraR_2
zraR_2	ZONMW-64_02027	zraR_2
zraR_2	ZONMW-64_01503	zraR_2
zraR_2	ZONMW-64_04181	zraR_2
zraR_2	ZONMW-64_00471	zraR_2
zraR_2	ZONMW-64_02760	zraR_2
zraR_2	ZONMW-64_02116	zraR_2
zraR_2	ZONMW-64_01323	zraR_2
zraR_2	ZONMW-64_04115	zraR_2
zraR_2	ZONMW-64_00114	zraR_2
zraR_2	ZONMW-64_01685	zraR_2
zraR_2	ZONMW-64_00646	zraR_2
zraR_2	ZONMW-64_03902	zraR_2
zraR_2	ZONMW-64_02725	zraR_2
zraR_2	ZONMW-64_00593	zraR_2
zraR_2	ZONMW-64_01858	zraR_2
zraR_2	ZONMW-64_00472	zraR_2
zraR_2	ZONMW-64_02840	zraR_2
zraR_2	ZONMW-64_01843	zraR_2
zraR_2	ZONMW-64_00740	zraR_2
zraR_2	ZONMW-64_00425	zraR_2
zraR_2	ZONMW-64_00604	zraR_2
zraR_2	ZONMW-64_02785	zraR_2
zraR_2	ZONMW-64_01033	zraR_2
zraR_2	ZONMW-64_01122	zraR_2
zraR_2	ZONMW-64_03906	zraR_2
zraR_2	ZONMW-64_00702	zraR_2
zraR_2	ZONMW-64_01455	zraR_2
zraR_2	ZONMW-64_03256	zraR_2
zraR_2	ZONMW-64_02411	zraR_2

zraR_2	ZONMW-64_01375	zraR_2
zraR_2	ZONMW-64_01946	zraR_2
zraR_2	ZONMW-64_03199	zraR_2
zraR_2	ZONMW-64_03787	zraR_2
zraR_2	ZONMW-64_00726	zraR_2
zraR_2	ZONMW-64_02161	zraR_2
zraR_2	ZONMW-64_01923	zraR_2
zraR_2	ZONMW-64_01001	zraR_2
zraR_2	ZONMW-64_00374	zraR_2
zraR_2	ZONMW-64_03209	zraR_2
zraR_2	ZONMW-64_02607	zraR_2
zraR_2	ZONMW-64_02653	zraR_2
zraR_2	ZONMW-64_00414	zraR_2
zraR_2	ZONMW-64_01000	zraR_2
zraR_2	ZONMW-64_02283	zraR_2
zraR_2	ZONMW-64_01391	zraR_2
zraR_2	ZONMW-64_01393	zraR_2
zraR_2	ZONMW-64_01571	zraR_2
zraR_2	ZONMW-64_01526	zraR_2
zraR_2	ZONMW-64_02740	zraR_2
zraR_2	ZONMW-64_02805	zraR_2
zraR_2	ZONMW-64_03099	zraR_2
zraR_2	ZONMW-64_04069	zraR_2
zraR_2	ZONMW-65_02115	zraR_2
zraR_2	ZONMW-65_02543	zraR_2
zraR_2	ZONMW-65_01303	zraR_2
zraR_2	ZONMW-65_02951	zraR_2
zraR_2	ZONMW-65_01296	zraR_2
zraR_2	ZONMW-65_00089	zraR_2
zraR_2	ZONMW-65_02913	zraR_2
zraR_2	ZONMW-65_02065	zraR_2
zraR_2	ZONMW-65_02915	zraR_2
zraR_2	ZONMW-65_02618	zraR_2
zraR_2	ZONMW-65_01190	zraR_2
zraR_2	ZONMW-65_03076	zraR_2
zraR_2	ZONMW-65_00142	zraR_2
zraR_2	ZONMW-65_00346	zraR_2
zraR_2	ZONMW-65_01468	zraR_2
zraR_2	ZONMW-65_01429	zraR_2
zraR_2	ZONMW-65_02204	zraR_2
zraR_2	ZONMW-65_02549	zraR_2
zraR_2	ZONMW-65_01177	zraR_2
zraR_2	ZONMW-65_01295	zraR_2
zraR_2	ZONMW-65_00061	zraR_2
zraR_2	ZONMW-65_01006	zraR_2
zraR_2	ZONMW-65_03451	zraR_2
zraR_2	ZONMW-65_00345	zraR_2
zraR_2	ZONMW-65_03142	zraR_2

zraR_2	ZONMW-65_03415	zraR_2
zraR_2	ZONMW-65_00980	zraR_2
zraR_2	ZONMW-65_03625	zraR_2
zraR_2	ZONMW-65_03249	zraR_2
zraR_2	ZONMW-65_01934	zraR_2
zraR_2	ZONMW-65_01428	zraR_2
zraR_2	ZONMW-65_00062	zraR_2
zraR_2	ZONMW-65_02616	zraR_2
zraR_2	ZONMW-65_03590	zraR_2
zraR_2	ZONMW-65_00979	zraR_2
zraR_2	ZONMW-65_00134	zraR_2
zraR_2	ZONMW-65_02548	zraR_2
zraR_2	ZONMW-65_02914	zraR_2
zraR_2	ZONMW-65_01176	zraR_2
zraR_2	ZONMW-65_03462	zraR_2
zraR_2	ZONMW-65_02856	zraR_2
zraR_2	ZONMW-65_02205	zraR_2
zraR_2	ZONMW-65_00347	zraR_2
zraR_2	ZONMW-65_00030	zraR_2
zraR_2	ZONMW-65_00610	zraR_2
zraR_2	ZONMW-65_03616	zraR_2
zraR_2	ZONMW-65_00978	zraR_2
zraR_2	ZONMW-65_00029	zraR_2
zraR_2	ZONMW-65_00135	zraR_2
zraR_2	ZONMW-65_02614	zraR_2
zraR_2	ZONMW-65_01037	zraR_2
zraR_2	ZONMW-65_01042	zraR_2
zraR_2	ZONMW-65_03784	zraR_2
zraR_2	ZONMW-65_01933	zraR_2
zraR_2	ZONMW-65_03452	zraR_2
zraR_2	ZONMW-65_00283	zraR_2
zraR_2	ZONMW-65_02517	zraR_2
zraR_2	ZONMW-65_03618	zraR_2
zraR_2	ZONMW-65_03143	zraR_2
zraR_2	ZONMW-65_03420	zraR_2
zraR_2	ZONMW-65_00554	zraR_2
zraR_2	ZONMW-65_03255	zraR_2
zraR_2	ZONMW-65_00630	zraR_2
zraR_2	ZONMW-65_02855	zraR_2
zraR_2	ZONMW-65_03342	zraR_2
zraR_2	ZONMW-65_00227	zraR_2
zraR_2	ZONMW-65_01570	zraR_2
zraR_2	ZONMW-65_01997	zraR_2
zraR_2	ZONMW-65_00946	zraR_2
zraR_2	ZONMW-65_00402	zraR_2
zraR_2	ZONMW-65_00059	zraR_2
zraR_2	ZONMW-65_01192	zraR_2
zraR_2	ZONMW-65_01590	zraR_2

zraR_2	ZONMW-65_02906	zraR_2
zraR_2	ZONMW-65_00937	zraR_2
zraR_2	ZONMW-65_03622	zraR_2
zraR_2	ZONMW-65_00141	zraR_2
zraR_2	ZONMW-65_01078	zraR_2
zraR_2	ZONMW-66_02736	zraR_2
zraR_2	ZONMW-66_00895	zraR_2
zraR_2	ZONMW-66_01586	zraR_2
zraR_2	ZONMW-66_01389	zraR_2
zraR_2	ZONMW-66_00142	zraR_2
zraR_2	ZONMW-66_04890	zraR_2
zraR_2	ZONMW-66_00123	zraR_2
zraR_2	ZONMW-66_00737	zraR_2
zraR_2	ZONMW-66_03828	zraR_2
zraR_2	ZONMW-66_00337	zraR_2
zraR_2	ZONMW-66_04743	zraR_2
zraR_2	ZONMW-66_00242	zraR_2
zraR_2	ZONMW-66_01225	zraR_2
zraR_2	ZONMW-66_01224	zraR_2
zraR_2	ZONMW-66_01627	zraR_2
zraR_2	ZONMW-66_03851	zraR_2
zraR_2	ZONMW-66_03641	zraR_2
zraR_2	ZONMW-66_03493	zraR_2
zraR_2	ZONMW-66_02152	zraR_2
zraR_2	ZONMW-66_03759	zraR_2
zraR_2	ZONMW-66_04655	zraR_2
zraR_2	ZONMW-66_00736	zraR_2
zraR_2	ZONMW-66_01252	zraR_2
zraR_2	ZONMW-66_03494	zraR_2
zraR_2	ZONMW-66_03642	zraR_2
zraR_2	ZONMW-66_01251	zraR_2
zraR_2	ZONMW-66_02786	zraR_2
zraR_2	ZONMW-66_01898	zraR_2
zraR_2	ZONMW-66_00246	zraR_2
zraR_2	ZONMW-66_02935	zraR_2
zraR_2	ZONMW-66_02969	zraR_2
zraR_2	ZONMW-66_03634	zraR_2
zraR_2	ZONMW-66_04396	zraR_2
zraR_2	ZONMW-66_02311	zraR_2
zraR_2	ZONMW-66_02649	zraR_2
zraR_2	ZONMW-66_03830	zraR_2
zraR_2	ZONMW-66_00241	zraR_2
zraR_2	ZONMW-66_01835	zraR_2
zraR_2	ZONMW-66_03850	zraR_2
zraR_2	ZONMW-66_01704	zraR_2
zraR_2	ZONMW-66_03156	zraR_2
zraR_2	ZONMW-66_00117	zraR_2
zraR_2	ZONMW-66_03950	zraR_2

zraR_2	ZONMW-66_00782	zraR_2
zraR_2	ZONMW-66_02183	zraR_2
zraR_2	ZONMW-66_01696	zraR_2
zraR_2	ZONMW-66_04000	zraR_2
zraR_2	ZONMW-66_04066	zraR_2
zraR_2	ZONMW-66_02751	zraR_2
zraR_2	ZONMW-66_03746	zraR_2
zraR_2	ZONMW-66_02545	zraR_2
zraR_2	ZONMW-66_01520	zraR_2
zraR_2	ZONMW-66_02955	zraR_2
zraR_2	ZONMW-66_03829	zraR_2
zraR_2	ZONMW-66_00010	zraR_2
zraR_2	ZONMW-66_00301	zraR_2
zraR_2	ZONMW-66_03758	zraR_2
zraR_2	ZONMW-66_04196	zraR_2
zraR_2	ZONMW-66_04240	zraR_2
zraR_2	ZONMW-66_01628	zraR_2
zraR_2	ZONMW-66_02326	zraR_2
zraR_2	ZONMW-66_02312	zraR_2
zraR_2	ZONMW-66_02752	zraR_2
zraR_2	ZONMW-66_02470	zraR_2
zraR_2	ZONMW-66_00300	zraR_2
zraR_2	ZONMW-66_01937	zraR_2
zraR_2	ZONMW-66_04479	zraR_2
zraR_2	ZONMW-66_01687	zraR_2
zraR_2	ZONMW-66_01706	zraR_2
zraR_2	ZONMW-66_00489	zraR_2
zraR_2	ZONMW-66_04241	zraR_2
zraR_2	ZONMW-66_04197	zraR_2
zraR_2	ZONMW-66_00099	zraR_2
zraR_2	ZONMW-66_02048	zraR_2
zraR_2	ZONMW-66_04456	zraR_2
zraR_2	ZONMW-66_03562	zraR_2
zraR_2	ZONMW-66_02424	zraR_2
zraR_2	ZONMW-66_04737	zraR_2
zraR_2	ZONMW-66_00352	zraR_2
zraR_2	ZONMW-66_01705	zraR_2
zraR_2	ZONMW-66_02968	zraR_2
zraR_2	ZONMW-66_00098	zraR_2
zraR_2	ZONMW-66_02327	zraR_2
zraR_2	ZONMW-66_03094	zraR_2
zraR_2	ZONMW-66_02472	zraR_2
zraR_2	ZONMW-66_05000	zraR_2
zraR_2	ZONMW-66_02651	zraR_2
zraR_2	ZONMW-66_00759	zraR_2
zraR_2	ZONMW-66_01521	zraR_2
zraR_2	ZONMW-66_03556	zraR_2
zraR_2	ZONMW-66_04854	zraR_2

zraR_2	ZONMW-66_04569	zraR_2
zraR_2	ZONMW-66_01667	zraR_2
zraR_2	ZONMW-66_04361	zraR_2
zraR_2	ZONMW-66_02867	zraR_2
zraR_2	ZONMW-66_00719	zraR_2
zraR_2	ZONMW-66_00245	zraR_2
zraR_2	ZONMW-66_04034	zraR_2
zraR_2	ZONMW-66_00247	zraR_2
zraR_2	ZONMW-66_02934	zraR_2
zraR_2	ZONMW-66_02328	zraR_2
zraR_2	ZONMW-66_01208	zraR_2
zraR_2	ZONMW-66_03999	zraR_2
zraR_2	ZONMW-66_03093	zraR_2
zraR_2	ZONMW-66_02279	zraR_2
zraR_2	ZONMW-66_01932	zraR_2
zraR_2	ZONMW-66_04895	zraR_2
zraR_2	ZONMW-66_02821	zraR_2
zraR_2	ZONMW-66_00373	zraR_2
zraR_2	ZONMW-66_01713	zraR_2
zraR_2	ZONMW-66_00222	zraR_2
zraR_2	ZONMW-67_02059	zraR_2
zraR_2	ZONMW-67_00050	zraR_2
zraR_2	ZONMW-67_03373	zraR_2
zraR_2	ZONMW-67_00175	zraR_2
zraR_2	ZONMW-67_02369	zraR_2
zraR_2	ZONMW-67_01575	zraR_2
zraR_2	ZONMW-67_00868	zraR_2
zraR_2	ZONMW-67_00495	zraR_2
zraR_2	ZONMW-67_01046	zraR_2
zraR_2	ZONMW-67_00429	zraR_2
zraR_2	ZONMW-67_00431	zraR_2
zraR_2	ZONMW-67_00365	zraR_2
zraR_2	ZONMW-67_02962	zraR_2
zraR_2	ZONMW-67_00494	zraR_2
zraR_2	ZONMW-67_01164	zraR_2
zraR_2	ZONMW-67_02411	zraR_2
zraR_2	ZONMW-67_00335	zraR_2
zraR_2	ZONMW-67_00430	zraR_2
zraR_2	ZONMW-67_00364	zraR_2
zraR_2	ZONMW-67_02976	zraR_2
zraR_2	ZONMW-67_03239	zraR_2
zraR_2	ZONMW-67_01720	zraR_2
zraR_2	ZONMW-67_03301	zraR_2
zraR_2	ZONMW-67_01146	zraR_2
zraR_2	ZONMW-67_00434	zraR_2
zraR_2	ZONMW-67_00174	zraR_2
zraR_2	ZONMW-67_03134	zraR_2
zraR_2	ZONMW-67_01138	zraR_2

zraR_2	ZONMW-67_00336	zraR_2
zraR_2	ZONMW-67_02808	zraR_2
zraR_2	ZONMW-67_03052	zraR_2
zraR_2	ZONMW-67_00331	zraR_2
zraR_2	ZONMW-67_00369	zraR_2
zraR_2	ZONMW-67_00590	zraR_2
zraR_2	ZONMW-67_02963	zraR_2
zraR_2	ZONMW-67_01445	zraR_2
zraR_2	ZONMW-67_03087	zraR_2
zraR_2	ZONMW-67_03086	zraR_2
zraR_2	ZONMW-67_00417	zraR_2
zraR_2	ZONMW-67_01259	zraR_2
zraR_2	ZONMW-67_01446	zraR_2
zraR_2	ZONMW-67_00048	zraR_2
zraR_2	ZONMW-67_03132	zraR_2
zraR_2	ZONMW-67_02431	zraR_2
zraR_2	ZONMW-67_03522	zraR_2
zraR_2	ZONMW-67_02288	zraR_2
zraR_2	ZONMW-67_02410	zraR_2
zraR_2	ZONMW-67_00869	zraR_2
zraR_2	ZONMW-67_02646	zraR_2
zraR_2	ZONMW-67_02432	zraR_2
zraR_2	ZONMW-67_03523	zraR_2
zraR_2	ZONMW-67_03620	zraR_2
zraR_2	ZONMW-67_00416	zraR_2
zraR_2	ZONMW-67_02009	zraR_2
zraR_2	ZONMW-67_01167	zraR_2
zraR_2	ZONMW-67_02346	zraR_2
zraR_2	ZONMW-67_02108	zraR_2
zraR_2	ZONMW-67_00493	zraR_2
zraR_2	ZONMW-67_03302	zraR_2
zraR_2	ZONMW-67_01136	zraR_2
zraR_2	ZONMW-67_03619	zraR_2
zraR_2	ZONMW-67_00395	zraR_2
zraR_2	ZONMW-67_00065	zraR_2
zraR_2	ZONMW-67_01643	zraR_2
zraR_2	ZONMW-67_02580	zraR_2
zraR_2	ZONMW-67_01691	zraR_2
zraR_2	ZONMW-67_02230	zraR_2
zraR_2	ZONMW-67_01137	zraR_2
zraR_2	ZONMW-67_03240	zraR_2
zraR_2	ZONMW-67_01097	zraR_2
zraR_2	ZONMW-67_02986	zraR_2
zraR_2	ZONMW-67_02648	zraR_2
zraR_2	ZONMW-67_00433	zraR_2
zraR_2	ZONMW-67_00589	zraR_2
zraR_2	ZONMW-67_02467	zraR_2
zraR_2	ZONMW-67_01260	zraR_2

zraR_2	ZONMW-67_00798	zraR_2
zraR_2	ZONMW-67_00106	zraR_2
zraR_2	ZONMW-67_01040	zraR_2
zraR_2	ZONMW-67_00694	zraR_2
zraR_2	ZONMW-67_02289	zraR_2
zraR_2	ZONMW-67_02975	zraR_2
zraR_2	ZONMW-67_01086	zraR_2
zraR_2	ZONMW-67_02260	zraR_2
zraR_2	ZONMW-67_01991	zraR_2
zraR_2	ZONMW-67_00332	zraR_2
zraR_2	ZONMW-67_00330	zraR_2
zraR_2	ZONMW-67_02647	zraR_2
zraR_2	ZONMW-67_00821	zraR_2
zraR_2	ZONMW-67_02568	zraR_2
zraR_2	ZONMW-67_02816	zraR_2
zraR_2	ZONMW-67_02155	zraR_2
zraR_2	ZONMW-67_03011	zraR_2
zraR_2	ZONMW-67_01411	zraR_2
zraR_2	ZONMW-67_02154	zraR_2
zraR_2	ZONMW-68_02288	zraR_2
zraR_2	ZONMW-68_02389	zraR_2
zraR_2	ZONMW-68_00220	zraR_2
zraR_2	ZONMW-68_02632	zraR_2
zraR_2	ZONMW-68_03342	zraR_2
zraR_2	ZONMW-68_03509	zraR_2
zraR_2	ZONMW-68_03790	zraR_2
zraR_2	ZONMW-68_01429	zraR_2
zraR_2	ZONMW-68_03059	zraR_2
zraR_2	ZONMW-68_03463	zraR_2
zraR_2	ZONMW-68_00142	zraR_2
zraR_2	ZONMW-68_02287	zraR_2
zraR_2	ZONMW-68_00965	zraR_2
zraR_2	ZONMW-68_03606	zraR_2
zraR_2	ZONMW-68_03714	zraR_2
zraR_2	ZONMW-68_02902	zraR_2
zraR_2	ZONMW-68_02332	zraR_2
zraR_2	ZONMW-68_03713	zraR_2
zraR_2	ZONMW-68_00742	zraR_2
zraR_2	ZONMW-68_01071	zraR_2
zraR_2	ZONMW-68_04170	zraR_2
zraR_2	ZONMW-68_00788	zraR_2
zraR_2	ZONMW-68_02331	zraR_2
zraR_2	ZONMW-68_01587	zraR_2
zraR_2	ZONMW-68_04010	zraR_2
zraR_2	ZONMW-68_01070	zraR_2
zraR_2	ZONMW-68_03160	zraR_2
zraR_2	ZONMW-68_00789	zraR_2
zraR_2	ZONMW-68_01586	zraR_2

zraR_2	ZONMW-68_03268	zraR_2
zraR_2	ZONMW-68_02903	zraR_2
zraR_2	ZONMW-68_04302	zraR_2
zraR_2	ZONMW-68_03267	zraR_2
zraR_2	ZONMW-68_02598	zraR_2
zraR_2	ZONMW-68_00199	zraR_2
zraR_2	ZONMW-68_02904	zraR_2
zraR_2	ZONMW-68_03424	zraR_2
zraR_2	ZONMW-68_01588	zraR_2
zraR_2	ZONMW-68_02969	zraR_2
zraR_2	ZONMW-68_02659	zraR_2
zraR_2	ZONMW-68_00289	zraR_2
zraR_2	ZONMW-68_03697	zraR_2
zraR_2	ZONMW-68_02388	zraR_2
zraR_2	ZONMW-68_01436	zraR_2
zraR_2	ZONMW-68_03111	zraR_2
zraR_2	ZONMW-68_01368	zraR_2
zraR_2	ZONMW-68_01265	zraR_2
zraR_2	ZONMW-68_02257	zraR_2
zraR_2	ZONMW-68_02322	zraR_2
zraR_2	ZONMW-68_01770	zraR_2
zraR_2	ZONMW-68_03477	zraR_2
zraR_2	ZONMW-68_00624	zraR_2
zraR_2	ZONMW-68_00303	zraR_2
zraR_2	ZONMW-68_03476	zraR_2
zraR_2	ZONMW-68_02316	zraR_2
zraR_2	ZONMW-68_03684	zraR_2
zraR_2	ZONMW-68_03573	zraR_2
zraR_2	ZONMW-68_02105	zraR_2
zraR_2	ZONMW-68_00665	zraR_2
zraR_2	ZONMW-68_02906	zraR_2
zraR_2	ZONMW-68_02412	zraR_2
zraR_2	ZONMW-68_02376	zraR_2
zraR_2	ZONMW-68_02378	zraR_2
zraR_2	ZONMW-68_02901	zraR_2
zraR_2	ZONMW-68_04376	zraR_2
zraR_2	ZONMW-68_02895	zraR_2
zraR_2	ZONMW-68_04766	zraR_2
zraR_2	ZONMW-68_00290	zraR_2
zraR_2	ZONMW-68_01399	zraR_2
zraR_2	ZONMW-68_04841	zraR_2
zraR_2	ZONMW-68_01963	zraR_2
zraR_2	ZONMW-68_03025	zraR_2
zraR_2	ZONMW-68_00197	zraR_2
zraR_2	ZONMW-68_01573	zraR_2
zraR_2	ZONMW-68_02203	zraR_2
zraR_2	ZONMW-68_02596	zraR_2
zraR_2	ZONMW-68_02604	zraR_2

zraR_2	ZONMW-68_02774	zraR_2
zraR_2	ZONMW-68_00765	zraR_2
zraR_2	ZONMW-68_00924	zraR_2
zraR_2	ZONMW-68_02773	zraR_2
zraR_2	ZONMW-68_04284	zraR_2
zraR_2	ZONMW-68_00741	zraR_2
zraR_2	ZONMW-68_02544	zraR_2
zraR_2	ZONMW-68_03432	zraR_2
zraR_2	ZONMW-68_03005	zraR_2
zraR_2	ZONMW-68_00180	zraR_2
zraR_2	ZONMW-68_03150	zraR_2
zraR_2	ZONMW-68_02493	zraR_2
zraR_2	ZONMW-68_00087	zraR_2
zraR_2	ZONMW-68_03630	zraR_2
zraR_2	ZONMW-68_00832	zraR_2
zraR_2	ZONMW-68_01612	zraR_2
zraR_2	ZONMW-68_03698	zraR_2
zraR_2	ZONMW-68_04285	zraR_2
zraR_2	ZONMW-68_00258	zraR_2
zraR_2	ZONMW-68_00431	zraR_2
zraR_2	ZONMW-68_04156	zraR_2
zraR_2	ZONMW-68_04972	zraR_2
zraR_2	ZONMW-69_01884	zraR_2
zraR_2	ZONMW-69_02659	zraR_2
zraR_2	ZONMW-69_03374	zraR_2
zraR_2	ZONMW-69_02784	zraR_2
zraR_2	ZONMW-69_02417	zraR_2
zraR_2	ZONMW-69_01426	zraR_2
zraR_2	ZONMW-69_00915	zraR_2
zraR_2	ZONMW-69_00242	zraR_2
zraR_2	ZONMW-69_01093	zraR_2
zraR_2	ZONMW-69_00176	zraR_2
zraR_2	ZONMW-69_00178	zraR_2
zraR_2	ZONMW-69_00112	zraR_2
zraR_2	ZONMW-69_02992	zraR_2
zraR_2	ZONMW-69_00241	zraR_2
zraR_2	ZONMW-69_01210	zraR_2
zraR_2	ZONMW-69_02459	zraR_2
zraR_2	ZONMW-69_00082	zraR_2
zraR_2	ZONMW-69_00177	zraR_2
zraR_2	ZONMW-69_00111	zraR_2
zraR_2	ZONMW-69_02978	zraR_2
zraR_2	ZONMW-69_03240	zraR_2
zraR_2	ZONMW-69_02223	zraR_2
zraR_2	ZONMW-69_03302	zraR_2
zraR_2	ZONMW-69_01192	zraR_2
zraR_2	ZONMW-69_00181	zraR_2
zraR_2	ZONMW-69_02783	zraR_2

zraR_2	ZONMW-69_03135	zraR_2
zraR_2	ZONMW-69_01184	zraR_2
zraR_2	ZONMW-69_00083	zraR_2
zraR_2	ZONMW-69_00605	zraR_2
zraR_2	ZONMW-69_02901	zraR_2
zraR_2	ZONMW-69_00078	zraR_2
zraR_2	ZONMW-69_00116	zraR_2
zraR_2	ZONMW-69_00337	zraR_2
zraR_2	ZONMW-69_02991	zraR_2
zraR_2	ZONMW-69_01555	zraR_2
zraR_2	ZONMW-69_02866	zraR_2
zraR_2	ZONMW-69_02867	zraR_2
zraR_2	ZONMW-69_00164	zraR_2
zraR_2	ZONMW-69_01742	zraR_2
zraR_2	ZONMW-69_01283	zraR_2
zraR_2	ZONMW-69_01554	zraR_2
zraR_2	ZONMW-69_02657	zraR_2
zraR_2	ZONMW-69_03133	zraR_2
zraR_2	ZONMW-69_02479	zraR_2
zraR_2	ZONMW-69_03524	zraR_2
zraR_2	ZONMW-69_02336	zraR_2
zraR_2	ZONMW-69_02458	zraR_2
zraR_2	ZONMW-69_00916	zraR_2
zraR_2	ZONMW-69_00443	zraR_2
zraR_2	ZONMW-69_02480	zraR_2
zraR_2	ZONMW-69_03525	zraR_2
zraR_2	ZONMW-69_03622	zraR_2
zraR_2	ZONMW-69_00163	zraR_2
zraR_2	ZONMW-69_01934	zraR_2
zraR_2	ZONMW-69_01213	zraR_2
zraR_2	ZONMW-69_02394	zraR_2
zraR_2	ZONMW-69_01835	zraR_2
zraR_2	ZONMW-69_00240	zraR_2
zraR_2	ZONMW-69_03303	zraR_2
zraR_2	ZONMW-69_01182	zraR_2
zraR_2	ZONMW-69_03621	zraR_2
zraR_2	ZONMW-69_00142	zraR_2
zraR_2	ZONMW-69_02674	zraR_2
zraR_2	ZONMW-69_01358	zraR_2
zraR_2	ZONMW-69_00377	zraR_2
zraR_2	ZONMW-69_01310	zraR_2
zraR_2	ZONMW-69_02278	zraR_2
zraR_2	ZONMW-69_01183	zraR_2
zraR_2	ZONMW-69_03241	zraR_2
zraR_2	ZONMW-69_01144	zraR_2
zraR_2	ZONMW-69_02968	zraR_2
zraR_2	ZONMW-69_00445	zraR_2
zraR_2	ZONMW-69_00180	zraR_2

zraR_2	ZONMW-69_00336	zraR_2
zraR_2	ZONMW-69_02515	zraR_2
zraR_2	ZONMW-69_01741	zraR_2
zraR_2	ZONMW-69_00845	zraR_2
zraR_2	ZONMW-69_02715	zraR_2
zraR_2	ZONMW-69_01087	zraR_2
zraR_2	ZONMW-69_00741	zraR_2
zraR_2	ZONMW-69_02337	zraR_2
zraR_2	ZONMW-69_02979	zraR_2
zraR_2	ZONMW-69_01133	zraR_2
zraR_2	ZONMW-69_02308	zraR_2
zraR_2	ZONMW-69_01952	zraR_2
zraR_2	ZONMW-69_00079	zraR_2
zraR_2	ZONMW-69_00077	zraR_2
zraR_2	ZONMW-69_00444	zraR_2
zraR_2	ZONMW-69_00868	zraR_2
zraR_2	ZONMW-69_00365	zraR_2
zraR_2	ZONMW-69_00613	zraR_2
zraR_2	ZONMW-69_01788	zraR_2
zraR_2	ZONMW-69_02943	zraR_2
zraR_2	ZONMW-69_01589	zraR_2
zraR_2	ZONMW-69_01789	zraR_2
zraR_2	ZONMW-72_03508	zraR_2
zraR_2	ZONMW-72_01047	zraR_2
zraR_2	ZONMW-72_00969	zraR_2
zraR_2	ZONMW-72_04227	zraR_2
zraR_2	ZONMW-72_01691	zraR_2
zraR_2	ZONMW-72_00698	zraR_2
zraR_2	ZONMW-72_01844	zraR_2
zraR_2	ZONMW-72_01779	zraR_2
zraR_2	ZONMW-72_04213	zraR_2
zraR_2	ZONMW-72_01482	zraR_2
zraR_2	ZONMW-72_01565	zraR_2
zraR_2	ZONMW-72_00745	zraR_2
zraR_2	ZONMW-72_01637	zraR_2
zraR_2	ZONMW-72_01756	zraR_2
zraR_2	ZONMW-72_03244	zraR_2
zraR_2	ZONMW-72_03243	zraR_2
zraR_2	ZONMW-72_01566	zraR_2
zraR_2	ZONMW-72_00801	zraR_2
zraR_2	ZONMW-72_02089	zraR_2
zraR_2	ZONMW-72_03420	zraR_2
zraR_2	ZONMW-72_03191	zraR_2
zraR_2	ZONMW-72_01656	zraR_2
zraR_2	ZONMW-72_04214	zraR_2
zraR_2	ZONMW-72_01495	zraR_2
zraR_2	ZONMW-72_02345	zraR_2
zraR_2	ZONMW-72_04004	zraR_2

zraR_2	ZONMW-72_02088	zraR_2
zraR_2	ZONMW-72_02015	zraR_2
zraR_2	ZONMW-72_02022	zraR_2
zraR_2	ZONMW-72_00802	zraR_2
zraR_2	ZONMW-72_03421	zraR_2
zraR_2	ZONMW-72_01941	zraR_2
zraR_2	ZONMW-72_02797	zraR_2
zraR_2	ZONMW-72_04218	zraR_2
zraR_2	ZONMW-72_02223	zraR_2
zraR_2	ZONMW-72_00406	zraR_2
zraR_2	ZONMW-72_04212	zraR_2
zraR_2	ZONMW-72_02497	zraR_2
zraR_2	ZONMW-72_02071	zraR_2
zraR_2	ZONMW-72_01393	zraR_2
zraR_2	ZONMW-72_01920	zraR_2
zraR_2	ZONMW-72_01004	zraR_2
zraR_2	ZONMW-72_04247	zraR_2
zraR_2	ZONMW-72_02470	zraR_2
zraR_2	ZONMW-72_01683	zraR_2
zraR_2	ZONMW-72_01685	zraR_2
zraR_2	ZONMW-72_02933	zraR_2
zraR_2	ZONMW-72_01781	zraR_2
zraR_2	ZONMW-72_01655	zraR_2
zraR_2	ZONMW-72_03190	zraR_2
zraR_2	ZONMW-72_01755	zraR_2
zraR_2	ZONMW-72_01684	zraR_2
zraR_2	ZONMW-72_02338	zraR_2
zraR_2	ZONMW-72_01638	zraR_2
zraR_2	ZONMW-72_04187	zraR_2
zraR_2	ZONMW-72_03569	zraR_2
zraR_2	ZONMW-72_00178	zraR_2
zraR_2	ZONMW-72_01902	zraR_2
zraR_2	ZONMW-72_02161	zraR_2
zraR_2	ZONMW-72_01873	zraR_2
zraR_2	ZONMW-72_01293	zraR_2
zraR_2	ZONMW-72_00665	zraR_2
zraR_2	ZONMW-72_00179	zraR_2
zraR_2	ZONMW-72_03570	zraR_2
zraR_2	ZONMW-72_00156	zraR_2
zraR_2	ZONMW-72_03463	zraR_2
zraR_2	ZONMW-72_04005	zraR_2
zraR_2	ZONMW-72_01940	zraR_2
zraR_2	ZONMW-72_01086	zraR_2
zraR_2	ZONMW-72_00992	zraR_2
zraR_2	ZONMW-72_03480	zraR_2
zraR_2	ZONMW-72_00601	zraR_2
zraR_2	ZONMW-72_03639	zraR_2
zraR_2	ZONMW-72_02909	zraR_2

zraR_2	ZONMW-72_00397	zraR_2
zraR_2	ZONMW-72_02219	zraR_2
zraR_2	ZONMW-72_02818	zraR_2
zraR_2	ZONMW-72_04124	zraR_2
zraR_2	ZONMW-72_03623	zraR_2
zraR_2	ZONMW-72_00995	zraR_2
zraR_2	ZONMW-72_04042	zraR_2
zraR_2	ZONMW-72_04414	zraR_2
zraR_2	ZONMW-72_04216	zraR_2
zraR_2	ZONMW-72_04211	zraR_2
zraR_2	ZONMW-72_04210	zraR_2
zraR_2	ZONMW-72_00626	zraR_2
zraR_2	ZONMW-72_04372	zraR_2
zraR_2	ZONMW-72_02618	zraR_2
zraR_2	ZONMW-72_00983	zraR_2
zraR_2	ZONMW-72_02604	zraR_2
zraR_2	ZONMW-72_04413	zraR_2
zraR_2	ZONMW-72_00996	zraR_2
zraR_2	ZONMW-72_00984	zraR_2
zraR_2	ZONMW-72_00982	zraR_2
zraR_2	ZONMW-73_00694	zraR_2
zraR_2	ZONMW-73_02720	zraR_2
zraR_2	ZONMW-73_00688	zraR_2
zraR_2	ZONMW-73_01154	zraR_2
zraR_2	ZONMW-73_00439	zraR_2
zraR_2	ZONMW-73_00412	zraR_2
zraR_2	ZONMW-73_01448	zraR_2
zraR_2	ZONMW-73_02281	zraR_2
zraR_2	ZONMW-73_01991	zraR_2
zraR_2	ZONMW-73_02170	zraR_2
zraR_2	ZONMW-73_01230	zraR_2
zraR_2	ZONMW-73_00229	zraR_2
zraR_2	ZONMW-73_03031	zraR_2
zraR_2	ZONMW-73_02447	zraR_2
zraR_2	ZONMW-73_02483	zraR_2
zraR_2	ZONMW-73_03347	zraR_2
zraR_2	ZONMW-73_03032	zraR_2
zraR_2	ZONMW-73_03346	zraR_2
zraR_2	ZONMW-73_00727	zraR_2
zraR_2	ZONMW-73_03247	zraR_2
zraR_2	ZONMW-73_03538	zraR_2
zraR_2	ZONMW-73_03566	zraR_2
zraR_2	ZONMW-73_03537	zraR_2
zraR_2	ZONMW-73_00854	zraR_2
zraR_2	ZONMW-73_00726	zraR_2
zraR_2	ZONMW-73_02613	zraR_2
zraR_2	ZONMW-73_01472	zraR_2
zraR_2	ZONMW-73_01085	zraR_2

zraR_2	ZONMW-73_02703	zraR_2
zraR_2	ZONMW-73_02705	zraR_2
zraR_2	ZONMW-73_02593	zraR_2
zraR_2	ZONMW-73_01369	zraR_2
zraR_2	ZONMW-73_00856	zraR_2
zraR_2	ZONMW-73_00855	zraR_2
zraR_2	ZONMW-73_03270	zraR_2
zraR_2	ZONMW-73_00197	zraR_2
zraR_2	ZONMW-73_00527	zraR_2
zraR_2	ZONMW-73_01975	zraR_2
zraR_2	ZONMW-73_01970	zraR_2
zraR_2	ZONMW-73_00070	zraR_2
zraR_2	ZONMW-73_01971	zraR_2
zraR_2	ZONMW-73_00750	zraR_2
zraR_2	ZONMW-73_02690	zraR_2
zraR_2	ZONMW-73_01486	zraR_2
zraR_2	ZONMW-73_03010	zraR_2
zraR_2	ZONMW-73_03489	zraR_2
zraR_2	ZONMW-73_02449	zraR_2
zraR_2	ZONMW-73_03565	zraR_2
zraR_2	ZONMW-73_01782	zraR_2
zraR_2	ZONMW-73_02697	zraR_2
zraR_2	ZONMW-73_03792	zraR_2
zraR_2	ZONMW-73_03643	zraR_2
zraR_2	ZONMW-73_01273	zraR_2
zraR_2	ZONMW-73_01532	zraR_2
zraR_2	ZONMW-73_02612	zraR_2
zraR_2	ZONMW-73_01088	zraR_2
zraR_2	ZONMW-73_00199	zraR_2
zraR_2	ZONMW-73_00613	zraR_2
zraR_2	ZONMW-73_01370	zraR_2
zraR_2	ZONMW-73_02918	zraR_2
zraR_2	ZONMW-73_02169	zraR_2
zraR_2	ZONMW-73_00189	zraR_2
zraR_2	ZONMW-73_00807	zraR_2
zraR_2	ZONMW-73_03811	zraR_2
zraR_2	ZONMW-73_00729	zraR_2
zraR_2	ZONMW-73_02240	zraR_2
zraR_2	ZONMW-73_02635	zraR_2
zraR_2	ZONMW-73_03854	zraR_2
zraR_2	ZONMW-73_02649	zraR_2
zraR_2	ZONMW-73_03584	zraR_2
zraR_2	ZONMW-73_03126	zraR_2
zraR_2	ZONMW-73_03470	zraR_2
zraR_2	ZONMW-73_01471	zraR_2
zraR_2	ZONMW-73_03794	zraR_2
zraR_2	ZONMW-74_02847	zraR_2
zraR_2	ZONMW-74_00525	zraR_2

zraR_2	ZONMW-74_01628	zraR_2
zraR_2	ZONMW-74_02506	zraR_2
zraR_2	ZONMW-74_02872	zraR_2
zraR_2	ZONMW-74_02873	zraR_2
zraR_2	ZONMW-74_00557	zraR_2
zraR_2	ZONMW-74_01549	zraR_2
zraR_2	ZONMW-74_01672	zraR_2
zraR_2	ZONMW-74_00558	zraR_2
zraR_2	ZONMW-74_00208	zraR_2
zraR_2	ZONMW-74_00281	zraR_2
zraR_2	ZONMW-74_03282	zraR_2
zraR_2	ZONMW-74_00444	zraR_2
zraR_2	ZONMW-74_00520	zraR_2
zraR_2	ZONMW-74_01245	zraR_2
zraR_2	ZONMW-74_02213	zraR_2
zraR_2	ZONMW-74_02212	zraR_2
zraR_2	ZONMW-74_00947	zraR_2
zraR_2	ZONMW-74_00793	zraR_2
zraR_2	ZONMW-74_03010	zraR_2
zraR_2	ZONMW-74_00797	zraR_2
zraR_2	ZONMW-74_02407	zraR_2
zraR_2	ZONMW-74_00767	zraR_2
zraR_2	ZONMW-74_02363	zraR_2
zraR_2	ZONMW-74_00352	zraR_2
zraR_2	ZONMW-74_02759	zraR_2
zraR_2	ZONMW-74_02163	zraR_2
zraR_2	ZONMW-74_02148	zraR_2
zraR_2	ZONMW-74_01623	zraR_2
zraR_2	ZONMW-74_00121	zraR_2
zraR_2	ZONMW-74_01683	zraR_2
zraR_2	ZONMW-74_00243	zraR_2
zraR_2	ZONMW-74_02234	zraR_2
zraR_2	ZONMW-74_00426	zraR_2
zraR_2	ZONMW-74_01168	zraR_2
zraR_2	ZONMW-74_01362	zraR_2
zraR_2	ZONMW-74_02605	zraR_2
zraR_2	ZONMW-74_02785	zraR_2
zraR_2	ZONMW-74_01285	zraR_2
zraR_2	ZONMW-74_02233	zraR_2
zraR_2	ZONMW-74_02295	zraR_2
zraR_2	ZONMW-74_02067	zraR_2
zraR_2	ZONMW-74_01124	zraR_2
zraR_2	ZONMW-74_01412	zraR_2
zraR_2	ZONMW-74_03280	zraR_2
zraR_2	ZONMW-74_00896	zraR_2
zraR_2	ZONMW-74_01275	zraR_2
zraR_2	ZONMW-74_01319	zraR_2
zraR_2	ZONMW-74_00207	zraR_2

zraR_2	ZONMW-74_01267	zraR_2
zraR_2	ZONMW-74_01256	zraR_2
zraR_2	ZONMW-75_03168	zraR_2
zraR_2	ZONMW-75_02741	zraR_2
zraR_2	ZONMW-75_02681	zraR_2
zraR_2	ZONMW-75_00068	zraR_2
zraR_2	ZONMW-75_01886	zraR_2
zraR_2	ZONMW-75_03174	zraR_2
zraR_2	ZONMW-75_01756	zraR_2
zraR_2	ZONMW-75_03260	zraR_2
zraR_2	ZONMW-75_02099	zraR_2
zraR_2	ZONMW-75_02105	zraR_2
zraR_2	ZONMW-75_01701	zraR_2
zraR_2	ZONMW-75_02365	zraR_2
zraR_2	ZONMW-75_03520	zraR_2
zraR_2	ZONMW-75_01114	zraR_2
zraR_2	ZONMW-75_03438	zraR_2
zraR_2	ZONMW-75_00345	zraR_2
zraR_2	ZONMW-75_03340	zraR_2
zraR_2	ZONMW-75_01074	zraR_2
zraR_2	ZONMW-75_01842	zraR_2
zraR_2	ZONMW-75_00346	zraR_2
zraR_2	ZONMW-75_01444	zraR_2
zraR_2	ZONMW-75_01443	zraR_2
zraR_2	ZONMW-75_03431	zraR_2
zraR_2	ZONMW-75_01631	zraR_2
zraR_2	ZONMW-75_02453	zraR_2
zraR_2	ZONMW-75_02177	zraR_2
zraR_2	ZONMW-75_02176	zraR_2
zraR_2	ZONMW-75_03607	zraR_2
zraR_2	ZONMW-75_02990	zraR_2
zraR_2	ZONMW-75_03802	zraR_2
zraR_2	ZONMW-75_00981	zraR_2
zraR_2	ZONMW-75_00422	zraR_2
zraR_2	ZONMW-75_03605	zraR_2
zraR_2	ZONMW-75_01632	zraR_2
zraR_2	ZONMW-75_01868	zraR_2
zraR_2	ZONMW-75_03591	zraR_2
zraR_2	ZONMW-75_03045	zraR_2
zraR_2	ZONMW-75_03599	zraR_2
zraR_2	ZONMW-75_00884	zraR_2
zraR_2	ZONMW-75_01935	zraR_2
zraR_2	ZONMW-75_00475	zraR_2
zraR_2	ZONMW-75_02740	zraR_2
zraR_2	ZONMW-75_03388	zraR_2
zraR_2	ZONMW-75_02727	zraR_2
zraR_2	ZONMW-75_01500	zraR_2
zraR_2	ZONMW-75_03003	zraR_2

zraR_2	ZONMW-75_00776	zraR_2
zraR_2	ZONMW-75_01825	zraR_2
zraR_2	ZONMW-75_03007	zraR_2
zraR_2	ZONMW-75_02511	zraR_2
zraR_2	ZONMW-75_02138	zraR_2
zraR_2	ZONMW-75_00323	zraR_2
zraR_2	ZONMW-75_02558	zraR_2
zraR_2	ZONMW-75_02907	zraR_2
zraR_2	ZONMW-75_02169	zraR_2
zraR_2	ZONMW-75_00469	zraR_2
zraR_2	ZONMW-75_02058	zraR_2
zraR_2	ZONMW-75_03592	zraR_2
zraR_2	ZONMW-75_02455	zraR_2
zraR_2	ZONMW-75_02426	zraR_2
zraR_2	ZONMW-75_03514	zraR_2
zraR_2	ZONMW-75_00120	zraR_2
zraR_2	ZONMW-75_01166	zraR_2
zraR_2	ZONMW-75_03226	zraR_2
zraR_2	ZONMW-75_00478	zraR_2
zraR_2	ZONMW-75_03155	zraR_2
zraR_2	ZONMW-75_03044	zraR_2
zraR_2	ZONMW-75_01675	zraR_2
zraR_2	ZONMW-75_02364	zraR_2
zraR_2	ZONMW-75_02829	zraR_2
zraR_2	ZONMW-75_02726	zraR_2
zraR_2	ZONMW-75_00387	zraR_2
zraR_2	ZONMW-75_00923	zraR_2
zraR_2	ZONMW-75_03578	zraR_2
zraR_2	ZONMW-75_01629	zraR_2
zraR_2	ZONMW-75_00245	zraR_2
zraR_2	ZONMW-75_01865	zraR_2
zraR_2	ZONMW-75_02593	zraR_2
zraR_2	ZONMW-75_01420	zraR_2
zraR_2	ZONMW-75_00519	zraR_2
zraR_2	ZONMW-75_02857	zraR_2
zraR_2	ZONMW-75_02454	zraR_2
zraR_2	da5A-8_00978	zraR_2
zraR_2	da5A-8_00380	zraR_2
zraR_2	da5A-8_01667	zraR_2
zraR_2	da5A-8_01282	zraR_2
zraR_2	da5A-8_01135	zraR_2
zraR_2	da5A-8_01134	zraR_2
zraR_2	da5A-8_02707	zraR_2
zraR_2	da5A-8_01283	zraR_2
zraR_2	da5A-8_02708	zraR_2
zraR_2	da5A-8_03019	zraR_2
zraR_2	da5A-8_00381	zraR_2
zraR_2	da5A-8_03020	zraR_2

zraR_2	da5A-8_02280	zraR_2
zraR_2	da5A-8_01470	zraR_2
zraR_2	da5A-8_00096	zraR_2
zraR_2	da5A-8_01061	zraR_2
zraR_2	da5A-8_01666	zraR_2
zraR_2	da5A-8_00198	zraR_2
zraR_2	da5A-8_01441	zraR_2
zraR_2	da5A-8_00346	zraR_2
zraR_2	da5A-8_03217	zraR_2
zraR_2	da5A-8_03565	zraR_2
zraR_2	da5A-8_00833	zraR_2
zraR_2	da5A-8_02057	zraR_2
zraR_2	da5A-8_03595	zraR_2
zraR_2	da5A-8_03592	zraR_2
zraR_2	da5A-8_01219	zraR_2
zraR_2	da5A-8_01779	zraR_2
zraR_2	da5A-8_00128	zraR_2
zraR_2	da5A-8_02689	zraR_2
zraR_2	da5A-8_01677	zraR_2
zraR_2	da5A-8_00310	zraR_2
zraR_2	da5A-8_00372	zraR_2
zraR_2	da5A-8_02245	zraR_2
zraR_2	da5A-8_00074	zraR_2
zraR_2	da5A-8_03786	zraR_2
zraR_2	da5A-8_00979	zraR_2
zraR_2	da5A-8_02609	zraR_2
zraR_2	da5A-8_02991	zraR_2
zraR_2	da5A-8_02876	zraR_2
zraR_2	da5A-8_02703	zraR_2
zraR_2	da5A-8_00607	zraR_2
zraR_2	da5A-8_02182	zraR_2
zraR_2	da5A-8_01817	zraR_2
zraR_2	da5A-8_03416	zraR_2
zraR_2	da5A-8_03016	zraR_2
zraR_2	da5A-8_03370	zraR_2
zraR_2	da5A-8_03611	zraR_2
zraR_2	da5A-8_00318	zraR_2
pyrH	18_01530	pyrH
pyrH	ACAM48_02063	pyrH
pyrH	CAM030_01324	pyrH
pyrH	CECT7946_01762	pyrH
pyrH	CGMCC1.8863_01063	pyrH
pyrH	CL8139_00144	pyrH
pyrH	DAU203_01955	pyrH
pyrH	DD5b_01894	pyrH
pyrH	DSM1208_02255	pyrH
pyrH	DSM12212_01698	pyrH
pyrH	DSM15343_00813	pyrH

pyrH	DSM15361_01405	pyrH
pyrH	DSM15365_03400	pyrH
pyrH	DSM15718_02989	pyrH
pyrH	DSM16505T_00863	pyrH
pyrH	DSM18103_03170	pyrH
pyrH	DSM18293_00907	pyrH
pyrH	DSM18841_00731	pyrH
pyrH	DSM18842_00589	pyrH
pyrH	DSM19866_00648	pyrH
pyrH	DSM2041_04631	pyrH
pyrH	DSM2061_03785	pyrH
pyrH	DSM2064_05165	pyrH
pyrH	DSM21164_00347	pyrH
pyrH	DSM23404_01865	pyrH
pyrH	DSM23424_00632	pyrH
pyrH	DSM24786_02093	pyrH
pyrH	DSM7489_01866	pyrH
pyrH	DSW1_00165	pyrH
pyrH	DSW5_00768	pyrH
pyrH	DSW6_00990	pyrH
pyrH	DSW8_01691	pyrH
pyrH	F52_02036	pyrH
pyrH	GCA_001027945.1_03157	pyrH
pyrH	HI1_01794	pyrH
pyrH	IR1_01190	pyrH
pyrH	KCOM3131_00707	pyrH
pyrH	KCTC12974_02069	pyrH
pyrH	KL-A_00405	pyrH
pyrH	L12_00273	pyrH
pyrH	LL0412.1.7_00864	pyrH
pyrH	MAR_2009_71_01726	pyrH
pyrH	NN016038_01537	pyrH
pyrH	PAO1_03777	pyrH
pyrH	UW101_00605	pyrH
pyrH	V4-33_00415	pyrH
pyrH	W5C_00552	pyrH
pyrH	ZONMW-01_00320	pyrH
pyrH	ZONMW-02_00326	pyrH
pyrH	ZONMW-03_01046	pyrH
pyrH	ZONMW-04_00626	pyrH
pyrH	ZONMW-05_01199	pyrH
pyrH	ZONMW-06_03530	pyrH
pyrH	ZONMW-07_00331	pyrH
pyrH	ZONMW-08_01508	pyrH
pyrH	ZONMW-09_00100	pyrH
pyrH	ZONMW-10_00557	pyrH
pyrH	ZONMW-11_03048	pyrH
pyrH	ZONMW-12_00427	pyrH

pyrH	ZONMW-13_00921	pyrH
pyrH	ZONMW-14_01183	pyrH
pyrH	ZONMW-15_00386	pyrH
pyrH	ZONMW-19_00794	pyrH
pyrH	ZONMW-20_04123	pyrH
pyrH	ZONMW-21_00235	pyrH
pyrH	ZONMW-22_00235	pyrH
pyrH	ZONMW-23_01350	pyrH
pyrH	ZONMW-24_01224	pyrH
pyrH	ZONMW-25_03062	pyrH
pyrH	ZONMW-26_01253	pyrH
pyrH	ZONMW-27_00620	pyrH
pyrH	ZONMW-28_03403	pyrH
pyrH	ZONMW-29_02969	pyrH
pyrH	ZONMW-30_01442	pyrH
pyrH	ZONMW-31_02385	pyrH
pyrH	ZONMW-32_03578	pyrH
pyrH	ZONMW-33_03928	pyrH
pyrH	ZONMW-34_03408	pyrH
pyrH	ZONMW-35_03407	pyrH
pyrH	ZONMW-36_03877	pyrH
pyrH	ZONMW-37_00235	pyrH
pyrH	ZONMW-38_00857	pyrH
pyrH	ZONMW-39_03268	pyrH
pyrH	ZONMW-40_02648	pyrH
pyrH	ZONMW-41_02496	pyrH
pyrH	ZONMW-42_02872	pyrH
pyrH	ZONMW-43_02631	pyrH
pyrH	ZONMW-44_02580	pyrH
pyrH	ZONMW-45_01089	pyrH
pyrH	ZONMW-46_00578	pyrH
pyrH	ZONMW-47_00578	pyrH
pyrH	ZONMW-48_01023	pyrH
pyrH	ZONMW-49_01408	pyrH
pyrH	ZONMW-50_01410	pyrH
pyrH	ZONMW-51_01524	pyrH
pyrH	ZONMW-52_01264	pyrH
pyrH	ZONMW-53_00561	pyrH
pyrH	ZONMW-54_00474	pyrH
pyrH	ZONMW-55_00556	pyrH
pyrH	ZONMW-56_03014	pyrH
pyrH	ZONMW-57_01058	pyrH
pyrH	ZONMW-58_01526	pyrH
pyrH	ZONMW-59_01060	pyrH
pyrH	ZONMW-60_01507	pyrH
pyrH	ZONMW-61_02887	pyrH
pyrH	ZONMW-62_02340	pyrH
pyrH	ZONMW-63_00814	pyrH

pyrH	ZONMW-64_03282	pyrH
pyrH	ZONMW-65_00906	pyrH
pyrH	ZONMW-66_00181	pyrH
pyrH	ZONMW-67_03171	pyrH
pyrH	ZONMW-68_03907	pyrH
pyrH	ZONMW-69_03172	pyrH
pyrH	ZONMW-72_01594	pyrH
pyrH	ZONMW-73_03189	pyrH
pyrH	ZONMW-74_01836	pyrH
pyrH	ZONMW-75_01182	pyrH
pyrH	da5A-8_01748	pyrH
dnaA	18_03604	dnaA
dnaA	ACAM48_01417	dnaA
dnaA	CAM030_00333	dnaA
dnaA	CECT7946_03491	dnaA
dnaA	CGMCC1.8863_02961	dnaA
dnaA	CL8139_01264	dnaA
dnaA	DAU203_00001	dnaA
dnaA	DD5b_00682	dnaA
dnaA	DSM1208_00001	dnaA
dnaA	DSM12212_01921	dnaA
dnaA	DSM15343_02783	dnaA
dnaA	DSM15361_01693	dnaA
dnaA	DSM15365_00113	dnaA
dnaA	DSM15718_01113	dnaA
dnaA	DSM16505T_02211	dnaA
dnaA	DSM18103_00444	dnaA
dnaA	DSM18293_01611	dnaA
dnaA	DSM18841_01956	dnaA
dnaA	DSM18842_01809	dnaA
dnaA	DSM19866_04713	dnaA
dnaA	DSM2041_04808	dnaA
dnaA	DSM2061_00559	dnaA
dnaA	DSM2064_03610	dnaA
dnaA	DSM21164_02818	dnaA
dnaA	DSM23404_01702	dnaA
dnaA	DSM23424_01723	dnaA
dnaA	DSM24786_00995	dnaA
dnaA	DSM7489_00001	dnaA
dnaA	DSW1_01583	dnaA
dnaA	DSW5_00279	dnaA
dnaA	DSW6_00001	dnaA
dnaA	DSW8_00573	dnaA
dnaA	F52_03766	dnaA
dnaA	GCA_001027945.1_02306	dnaA
dnaA	HI1_03300	dnaA
dnaA	IR1_01678	dnaA
dnaA	KCOM3131_00942	dnaA

dnaA	KCTC12974_00967	dnaA
dnaA	KL-A_01838	dnaA
dnaA	L12_00499	dnaA
dnaA	LL0412.1.7_01508	dnaA
dnaA	MAR_2009_71_03370	dnaA
dnaA	NN016038_03644	dnaA
dnaA	PAO1_00001	dnaA
dnaA	PAO1_00979	dnaA
dnaA	UW101_00005	dnaA
dnaA	V4-33_01117	dnaA
dnaA	W5C_00870	dnaA
dnaA	ZONMW-01_03114	dnaA
dnaA	ZONMW-02_00805	dnaA
dnaA	ZONMW-03_02349	dnaA
dnaA	ZONMW-04_00813	dnaA
dnaA	ZONMW-05_00083	dnaA
dnaA	ZONMW-06_01266	dnaA
dnaA	ZONMW-06_03873	dnaA
dnaA	ZONMW-07_03406	dnaA
dnaA	ZONMW-08_02557	dnaA
dnaA	ZONMW-09_02188	dnaA
dnaA	ZONMW-10_01609	dnaA
dnaA	ZONMW-11_03862	dnaA
dnaA	ZONMW-12_03642	dnaA
dnaA	ZONMW-13_02354	dnaA
dnaA	ZONMW-14_02627	dnaA
dnaA	ZONMW-15_02982	dnaA
dnaA	ZONMW-19_03329	dnaA
dnaA	ZONMW-19_00020	dnaA
dnaA	ZONMW-20_00677	dnaA
dnaA	ZONMW-20_03050	dnaA
dnaA	ZONMW-21_01641	dnaA
dnaA	ZONMW-22_02083	dnaA
dnaA	ZONMW-23_00468	dnaA
dnaA	ZONMW-24_02708	dnaA
dnaA	ZONMW-25_01433	dnaA
dnaA	ZONMW-26_00877	dnaA
dnaA	ZONMW-27_00910	dnaA
dnaA	ZONMW-28_01744	dnaA
dnaA	ZONMW-28_03816	dnaA
dnaA	ZONMW-29_03150	dnaA
dnaA	ZONMW-30_02181	dnaA
dnaA	ZONMW-31_00045	dnaA
dnaA	ZONMW-31_02602	dnaA
dnaA	ZONMW-32_02228	dnaA
dnaA	ZONMW-32_03754	dnaA
dnaA	ZONMW-33_02219	dnaA
dnaA	ZONMW-33_03793	dnaA

dnaA	ZONMW-34_02372	dnaA
dnaA	ZONMW-34_03691	dnaA
dnaA	ZONMW-35_01877	dnaA
dnaA	ZONMW-35_00016	dnaA
dnaA	ZONMW-36_02017	dnaA
dnaA	ZONMW-36_03742	dnaA
dnaA	ZONMW-37_02019	dnaA
dnaA	ZONMW-38_02739	dnaA
dnaA	ZONMW-39_01664	dnaA
dnaA	ZONMW-39_03549	dnaA
dnaA	ZONMW-40_02451	dnaA
dnaA	ZONMW-41_00397	dnaA
dnaA	ZONMW-41_03047	dnaA
dnaA	ZONMW-42_02755	dnaA
dnaA	ZONMW-42_03213	dnaA
dnaA	ZONMW-43_01860	dnaA
dnaA	ZONMW-43_02551	dnaA
dnaA	ZONMW-44_02463	dnaA
dnaA	ZONMW-44_03853	dnaA
dnaA	ZONMW-45_00103	dnaA
dnaA	ZONMW-46_00318	dnaA
dnaA	ZONMW-47_00187	dnaA
dnaA	ZONMW-48_03101	dnaA
dnaA	ZONMW-49_03054	dnaA
dnaA	ZONMW-50_02978	dnaA
dnaA	ZONMW-51_01906	dnaA
dnaA	ZONMW-52_00867	dnaA
dnaA	ZONMW-53_01610	dnaA
dnaA	ZONMW-54_01115	dnaA
dnaA	ZONMW-55_02228	dnaA
dnaA	ZONMW-56_02140	dnaA
dnaA	ZONMW-57_00080	dnaA
dnaA	ZONMW-58_00886	dnaA
dnaA	ZONMW-59_00080	dnaA
dnaA	ZONMW-60_00081	dnaA
dnaA	ZONMW-61_02057	dnaA
dnaA	ZONMW-62_00189	dnaA
dnaA	ZONMW-63_01844	dnaA
dnaA	ZONMW-64_01619	dnaA
dnaA	ZONMW-64_03732	dnaA
dnaA	ZONMW-65_02369	dnaA
dnaA	ZONMW-65_00563	dnaA
dnaA	ZONMW-66_02366	dnaA
dnaA	ZONMW-66_01279	dnaA
dnaA	ZONMW-67_00145	dnaA
dnaA	ZONMW-67_01773	dnaA
dnaA	ZONMW-68_01040	dnaA
dnaA	ZONMW-69_02754	dnaA

dnaA	ZONMW-69_02170	dnaA
dnaA	ZONMW-72_02175	dnaA
dnaA	ZONMW-73_00782	dnaA
dnaA	ZONMW-74_00583	dnaA
dnaA	ZONMW-75_01467	dnaA
dnaA	da5A-8_03276	dnaA
rpsT	18_00068	rpsT
rpsT	ACAM48_03382	rpsT
rpsT	CAM030_01506	rpsT
rpsT	CECT7946_01084	rpsT
rpsT	CGMCC1.8863_00661	rpsT
rpsT	CL8139_02044	rpsT
rpsT	DAU203_02431	rpsT
rpsT	DD5b_01391	rpsT
rpsT	DSM1208_02673	rpsT
rpsT	DSM12212_02059	rpsT
rpsT	DSM15343_01252	rpsT
rpsT	DSM15361_02435	rpsT
rpsT	DSM15365_00567	rpsT
rpsT	DSM15718_03147	rpsT
rpsT	DSM16505T_00067	rpsT
rpsT	DSM18103_01385	rpsT
rpsT	DSM18293_01489	rpsT
rpsT	DSM18841_02569	rpsT
rpsT	DSM18842_02469	rpsT
rpsT	DSM19866_00466	rpsT
rpsT	DSM2041_02819	rpsT
rpsT	DSM2061_04159	rpsT
rpsT	DSM2064_03738	rpsT
rpsT	DSM21164_02090	rpsT
rpsT	DSM23404_00783	rpsT
rpsT	DSM23424_03238	rpsT
rpsT	DSM24786_01578	rpsT
rpsT	DSM7489_02346	rpsT
rpsT	DSW1_01630	rpsT
rpsT	DSW5_00745	rpsT
rpsT	DSW6_02061	rpsT
rpsT	DSW8_01319	rpsT
rpsT	F52_03886	rpsT
rpsT	GCA_001027945.1_00970	rpsT
rpsT	HI1_02279	rpsT
rpsT	IR1_01556	rpsT
rpsT	KCOM3131_01279	rpsT
rpsT	KCTC12974_01043	rpsT
rpsT	KL-A_01626	rpsT
rpsT	L12_00495	rpsT
rpsT	LL0412.1.7_00066	rpsT
rpsT	MAR_2009_71_02132	rpsT

rpsT	NN016038_00073	rpsT
rpsT	PAO1_04711	rpsT
rpsT	UW101_05148	rpsT
rpsT	V4-33_01300	rpsT
rpsT	W5C_02680	rpsT
rpsT	ZONMW-01_01223	rpsT
rpsT	ZONMW-02_01001	rpsT
rpsT	ZONMW-03_01557	rpsT
rpsT	ZONMW-04_00135	rpsT
rpsT	ZONMW-05_01695	rpsT
rpsT	ZONMW-06_03953	rpsT
rpsT	ZONMW-07_02189	rpsT
rpsT	ZONMW-08_01127	rpsT
rpsT	ZONMW-09_01284	rpsT
rpsT	ZONMW-10_01060	rpsT
rpsT	ZONMW-11_02472	rpsT
rpsT	ZONMW-12_01325	rpsT
rpsT	ZONMW-13_01719	rpsT
rpsT	ZONMW-14_00962	rpsT
rpsT	ZONMW-15_00682	rpsT
rpsT	ZONMW-19_00610	rpsT
rpsT	ZONMW-20_01041	rpsT
rpsT	ZONMW-21_02330	rpsT
rpsT	ZONMW-22_02779	rpsT
rpsT	ZONMW-23_02801	rpsT
rpsT	ZONMW-24_02354	rpsT
rpsT	ZONMW-25_02664	rpsT
rpsT	ZONMW-26_01588	rpsT
rpsT	ZONMW-27_00132	rpsT
rpsT	ZONMW-28_03929	rpsT
rpsT	ZONMW-29_01651	rpsT
rpsT	ZONMW-30_00281	rpsT
rpsT	ZONMW-31_03213	rpsT
rpsT	ZONMW-32_03957	rpsT
rpsT	ZONMW-33_03881	rpsT
rpsT	ZONMW-34_03827	rpsT
rpsT	ZONMW-35_03798	rpsT
rpsT	ZONMW-36_03924	rpsT
rpsT	ZONMW-37_02600	rpsT
rpsT	ZONMW-38_01377	rpsT
rpsT	ZONMW-39_04071	rpsT
rpsT	ZONMW-40_03140	rpsT
rpsT	ZONMW-41_03146	rpsT
rpsT	ZONMW-42_02354	rpsT
rpsT	ZONMW-43_03780	rpsT
rpsT	ZONMW-44_03143	rpsT
rpsT	ZONMW-45_02468	rpsT
rpsT	ZONMW-46_02461	rpsT

rpsT	ZONMW-47_02451	rpsT
rpsT	ZONMW-48_02119	rpsT
rpsT	ZONMW-49_00563	rpsT
rpsT	ZONMW-50_00564	rpsT
rpsT	ZONMW-51_01142	rpsT
rpsT	ZONMW-52_02835	rpsT
rpsT	ZONMW-53_00080	rpsT
rpsT	ZONMW-54_00947	rpsT
rpsT	ZONMW-55_00078	rpsT
rpsT	ZONMW-56_02729	rpsT
rpsT	ZONMW-57_01535	rpsT
rpsT	ZONMW-58_01049	rpsT
rpsT	ZONMW-59_02797	rpsT
rpsT	ZONMW-60_01023	rpsT
rpsT	ZONMW-61_01144	rpsT
rpsT	ZONMW-62_02896	rpsT
rpsT	ZONMW-63_01022	rpsT
rpsT	ZONMW-64_04100	rpsT
rpsT	ZONMW-65_01734	rpsT
rpsT	ZONMW-66_03414	rpsT
rpsT	ZONMW-67_03604	rpsT
rpsT	ZONMW-68_03367	rpsT
rpsT	ZONMW-69_03606	rpsT
rpsT	ZONMW-72_02054	rpsT
rpsT	ZONMW-73_03721	rpsT
rpsT	ZONMW-74_00841	rpsT
rpsT	ZONMW-75_00672	rpsT
rpsT	da5A-8_01021	rpsT
nqrF	18_01107	nqrF
nqrF	18_01758	nqrF
nqrF	ACAM48_02455	nqrF
nqrF	ACAM48_01646	nqrF
nqrF	CAM030_00990	nqrF
nqrF	CAM030_01453	nqrF
nqrF	CECT7946_00200	nqrF
nqrF	CECT7946_02723	nqrF
nqrF	CECT7946_00897	nqrF
nqrF	CECT7946_02512	nqrF
nqrF	CGMCC1.8863_00832	nqrF
nqrF	CGMCC1.8863_00305	nqrF
nqrF	CGMCC1.8863_00612	nqrF
nqrF	CGMCC1.8863_02035	nqrF
nqrF	CL8139_01854	nqrF
nqrF	CL8139_03016	nqrF
nqrF	CL8139_03040	nqrF
nqrF	DAU203_01494	nqrF
nqrF	DAU203_00652	nqrF
nqrF	DAU203_03421	nqrF

nqrF	DD5b_02119	nqrF
nqrF	DSM1208_01709	nqrF
nqrF	DSM1208_00719	nqrF
nqrF	DSM12212_00107	nqrF
nqrF	DSM12212_01568	nqrF
nqrF	DSM12212_02739	nqrF
nqrF	DSM15343_01023	nqrF
nqrF	DSM15343_02139	nqrF
nqrF	DSM15343_01991	nqrF
nqrF	DSM15343_00010	nqrF
nqrF	DSM15361_00562	nqrF
nqrF	DSM15361_01194	nqrF
nqrF	DSM15361_00913	nqrF
nqrF	DSM15361_02427	nqrF
nqrF	DSM15365_03018	nqrF
nqrF	DSM15365_01442	nqrF
nqrF	DSM15718_02606	nqrF
nqrF	DSM15718_00221	nqrF
nqrF	DSM16505T_00401	nqrF
nqrF	DSM16505T_00247	nqrF
nqrF	DSM18103_02999	nqrF
nqrF	DSM18103_01711	nqrF
nqrF	DSM18293_03050	nqrF
nqrF	DSM18293_02459	nqrF
nqrF	DSM18293_01338	nqrF
nqrF	DSM18841_00230	nqrF
nqrF	DSM18841_00047	nqrF
nqrF	DSM18842_00989	nqrF
nqrF	DSM18842_01162	nqrF
nqrF	DSM19866_01941	nqrF
nqrF	DSM19866_02168	nqrF
nqrF	DSM2041_00179	nqrF
nqrF	DSM2041_01204	nqrF
nqrF	DSM2041_05319	nqrF
nqrF	DSM2041_03540	nqrF
nqrF	DSM2061_03242	nqrF
nqrF	DSM2061_02219	nqrF
nqrF	DSM2064_02045	nqrF
nqrF	DSM21164_00762	nqrF
nqrF	DSM21164_00120	nqrF
nqrF	DSM23404_02065	nqrF
nqrF	DSM23404_00972	nqrF
nqrF	DSM23404_01140	nqrF
nqrF	DSM23424_00301	nqrF
nqrF	DSM23424_02357	nqrF
nqrF	DSM23424_02475	nqrF
nqrF	DSM24786_02985	nqrF
nqrF	DSM24786_03460	nqrF

nqrF	DSM7489_01400	nqrF
nqrF	DSM7489_00638	nqrF
nqrF	DSM7489_03289	nqrF
nqrF	DSW1_00666	nqrF
nqrF	DSW1_01103	nqrF
nqrF	DSW1_00201	nqrF
nqrF	DSW5_00890	nqrF
nqrF	DSW5_00633	nqrF
nqrF	DSW6_01318	nqrF
nqrF	DSW6_02119	nqrF
nqrF	DSW6_01818	nqrF
nqrF	DSW8_02231	nqrF
nqrF	DSW8_03822	nqrF
nqrF	F52_02445	nqrF
nqrF	GCA_001027945.1_00380	nqrF
nqrF	GCA_001027945.1_02023	nqrF
nqrF	GCA_001027945.1_00582	nqrF
nqrF	HI1_01340	nqrF
nqrF	HI1_00570	nqrF
nqrF	HI1_03235	nqrF
nqrF	IR1_04695	nqrF
nqrF	IR1_00918	nqrF
nqrF	KCOM3131_01000	nqrF
nqrF	KCTC12974_02904	nqrF
nqrF	KCTC12974_02056	nqrF
nqrF	KCTC12974_02292	nqrF
nqrF	KL-A_00826	nqrF
nqrF	KL-A_03084	nqrF
nqrF	KL-A_01904	nqrF
nqrF	L12_00494	nqrF
nqrF	LL0412.1.7_00575	nqrF
nqrF	LL0412.1.7_00214	nqrF
nqrF	MAR_2009_71_01208	nqrF
nqrF	MAR_2009_71_00306	nqrF
nqrF	NN016038_01121	nqrF
nqrF	NN016038_01779	nqrF
nqrF	PAO1_05593	nqrF
nqrF	PAO1_05056	nqrF
nqrF	PAO1_02744	nqrF
nqrF	PAO1_02586	nqrF
nqrF	PAO1_05072	nqrF
nqrF	PAO1_03620	nqrF
nqrF	PAO1_02584	nqrF
nqrF	PAO1_03099	nqrF
nqrF	PAO1_00558	nqrF
nqrF	PAO1_04765	nqrF
nqrF	PAO1_03516	nqrF
nqrF	PAO1_04471	nqrF

nqrF	PAO1_05412	nqrF
nqrF	UW101_01100	nqrF
nqrF	V4-33_02390	nqrF
nqrF	V4-33_01767	nqrF
nqrF	W5C_01100	nqrF
nqrF	W5C_01202	nqrF
nqrF	ZONMW-01_02396	nqrF
nqrF	ZONMW-01_03171	nqrF
nqrF	ZONMW-02_01825	nqrF
nqrF	ZONMW-02_01898	nqrF
nqrF	ZONMW-03_02999	nqrF
nqrF	ZONMW-03_03347	nqrF
nqrF	ZONMW-04_02441	nqrF
nqrF	ZONMW-04_03189	nqrF
nqrF	ZONMW-05_02582	nqrF
nqrF	ZONMW-05_03320	nqrF
nqrF	ZONMW-06_00732	nqrF
nqrF	ZONMW-06_00464	nqrF
nqrF	ZONMW-06_03281	nqrF
nqrF	ZONMW-06_03331	nqrF
nqrF	ZONMW-06_00557	nqrF
nqrF	ZONMW-06_01853	nqrF
nqrF	ZONMW-07_01474	nqrF
nqrF	ZONMW-07_01971	nqrF
nqrF	ZONMW-07_03872	nqrF
nqrF	ZONMW-07_01013	nqrF
nqrF	ZONMW-08_00729	nqrF
nqrF	ZONMW-08_03298	nqrF
nqrF	ZONMW-09_00348	nqrF
nqrF	ZONMW-09_01915	nqrF
nqrF	ZONMW-10_00396	nqrF
nqrF	ZONMW-10_01185	nqrF
nqrF	ZONMW-11_03354	nqrF
nqrF	ZONMW-11_00803	nqrF
nqrF	ZONMW-11_02583	nqrF
nqrF	ZONMW-11_02075	nqrF
nqrF	ZONMW-12_00249	nqrF
nqrF	ZONMW-12_02196	nqrF
nqrF	ZONMW-13_00347	nqrF
nqrF	ZONMW-13_02614	nqrF
nqrF	ZONMW-13_00639	nqrF
nqrF	ZONMW-14_00243	nqrF
nqrF	ZONMW-14_01623	nqrF
nqrF	ZONMW-14_00009	nqrF
nqrF	ZONMW-15_02564	nqrF
nqrF	ZONMW-15_01238	nqrF
nqrF	ZONMW-15_02070	nqrF
nqrF	ZONMW-19_03807	nqrF

nqrF	ZONMW-19_00263	nqrF
nqrF	ZONMW-19_01590	nqrF
nqrF	ZONMW-19_04040	nqrF
nqrF	ZONMW-19_01897	nqrF
nqrF	ZONMW-19_01135	nqrF
nqrF	ZONMW-20_00382	nqrF
nqrF	ZONMW-20_02405	nqrF
nqrF	ZONMW-20_03589	nqrF
nqrF	ZONMW-20_02987	nqrF
nqrF	ZONMW-20_01430	nqrF
nqrF	ZONMW-20_00893	nqrF
nqrF	ZONMW-21_00767	nqrF
nqrF	ZONMW-21_03114	nqrF
nqrF	ZONMW-21_02299	nqrF
nqrF	ZONMW-22_00768	nqrF
nqrF	ZONMW-22_03139	nqrF
nqrF	ZONMW-22_02810	nqrF
nqrF	ZONMW-23_01882	nqrF
nqrF	ZONMW-23_03160	nqrF
nqrF	ZONMW-23_02832	nqrF
nqrF	ZONMW-24_00693	nqrF
nqrF	ZONMW-24_03161	nqrF
nqrF	ZONMW-24_02324	nqrF
nqrF	ZONMW-25_01695	nqrF
nqrF	ZONMW-25_01897	nqrF
nqrF	ZONMW-25_00215	nqrF
nqrF	ZONMW-26_03019	nqrF
nqrF	ZONMW-26_03273	nqrF
nqrF	ZONMW-27_02739	nqrF
nqrF	ZONMW-27_03343	nqrF
nqrF	ZONMW-28_00978	nqrF
nqrF	ZONMW-28_03238	nqrF
nqrF	ZONMW-28_03115	nqrF
nqrF	ZONMW-28_01028	nqrF
nqrF	ZONMW-28_02729	nqrF
nqrF	ZONMW-29_00407	nqrF
nqrF	ZONMW-29_02568	nqrF
nqrF	ZONMW-30_02299	nqrF
nqrF	ZONMW-30_00936	nqrF
nqrF	ZONMW-31_03583	nqrF
nqrF	ZONMW-31_00826	nqrF
nqrF	ZONMW-31_02188	nqrF
nqrF	ZONMW-31_01770	nqrF
nqrF	ZONMW-31_00917	nqrF
nqrF	ZONMW-31_01711	nqrF
nqrF	ZONMW-31_01994	nqrF
nqrF	ZONMW-31_00285	nqrF
nqrF	ZONMW-32_00113	nqrF

nqrF	ZONMW-32_00763	nqrF
nqrF	ZONMW-32_03294	nqrF
nqrF	ZONMW-32_00063	nqrF
nqrF	ZONMW-32_03147	nqrF
nqrF	ZONMW-33_03374	nqrF
nqrF	ZONMW-33_00631	nqrF
nqrF	ZONMW-33_00684	nqrF
nqrF	ZONMW-33_03324	nqrF
nqrF	ZONMW-33_03127	nqrF
nqrF	ZONMW-34_00391	nqrF
nqrF	ZONMW-34_03071	nqrF
nqrF	ZONMW-34_00995	nqrF
nqrF	ZONMW-34_03886	nqrF
nqrF	ZONMW-34_01047	nqrF
nqrF	ZONMW-34_02529	nqrF
nqrF	ZONMW-35_00999	nqrF
nqrF	ZONMW-35_02921	nqrF
nqrF	ZONMW-35_02248	nqrF
nqrF	ZONMW-35_03880	nqrF
nqrF	ZONMW-35_02196	nqrF
nqrF	ZONMW-35_02493	nqrF
nqrF	ZONMW-36_03111	nqrF
nqrF	ZONMW-36_00740	nqrF
nqrF	ZONMW-36_03228	nqrF
nqrF	ZONMW-36_03061	nqrF
nqrF	ZONMW-36_02875	nqrF
nqrF	ZONMW-37_00767	nqrF
nqrF	ZONMW-37_03148	nqrF
nqrF	ZONMW-37_02570	nqrF
nqrF	ZONMW-38_02099	nqrF
nqrF	ZONMW-39_03636	nqrF
nqrF	ZONMW-39_03094	nqrF
nqrF	ZONMW-39_02635	nqrF
nqrF	ZONMW-39_02689	nqrF
nqrF	ZONMW-39_01633	nqrF
nqrF	ZONMW-39_02866	nqrF
nqrF	ZONMW-39_02918	nqrF
nqrF	ZONMW-39_02071	nqrF
nqrF	ZONMW-40_00438	nqrF
nqrF	ZONMW-40_01613	nqrF
nqrF	ZONMW-40_01059	nqrF
nqrF	ZONMW-41_00964	nqrF
nqrF	ZONMW-41_01215	nqrF
nqrF	ZONMW-41_00165	nqrF
nqrF	ZONMW-41_00164	nqrF
nqrF	ZONMW-41_02459	nqrF
nqrF	ZONMW-42_01520	nqrF
nqrF	ZONMW-42_03099	nqrF

nqrF	ZONMW-42_01305	nqrF
nqrF	ZONMW-42_00839	nqrF
nqrF	ZONMW-42_00890	nqrF
nqrF	ZONMW-42_01146	nqrF
nqrF	ZONMW-43_02817	nqrF
nqrF	ZONMW-43_02353	nqrF
nqrF	ZONMW-43_04466	nqrF
nqrF	ZONMW-43_03578	nqrF
nqrF	ZONMW-43_04075	nqrF
nqrF	ZONMW-43_00528	nqrF
nqrF	ZONMW-43_00581	nqrF
nqrF	ZONMW-43_01156	nqrF
nqrF	ZONMW-44_01492	nqrF
nqrF	ZONMW-44_01740	nqrF
nqrF	ZONMW-44_00232	nqrF
nqrF	ZONMW-44_00495	nqrF
nqrF	ZONMW-44_03793	nqrF
nqrF	ZONMW-44_03743	nqrF
nqrF	ZONMW-44_01634	nqrF
nqrF	ZONMW-44_00167	nqrF
nqrF	ZONMW-45_00380	nqrF
nqrF	ZONMW-45_02056	nqrF
nqrF	ZONMW-46_01459	nqrF
nqrF	ZONMW-46_02226	nqrF
nqrF	ZONMW-47_01393	nqrF
nqrF	ZONMW-47_02306	nqrF
nqrF	ZONMW-48_00617	nqrF
nqrF	ZONMW-48_02458	nqrF
nqrF	ZONMW-48_02748	nqrF
nqrF	ZONMW-49_01197	nqrF
nqrF	ZONMW-49_03184	nqrF
nqrF	ZONMW-50_01198	nqrF
nqrF	ZONMW-50_03180	nqrF
nqrF	ZONMW-51_02803	nqrF
nqrF	ZONMW-51_03241	nqrF
nqrF	ZONMW-52_02242	nqrF
nqrF	ZONMW-52_03182	nqrF
nqrF	ZONMW-53_02831	nqrF
nqrF	ZONMW-53_03210	nqrF
nqrF	ZONMW-54_00047	nqrF
nqrF	ZONMW-54_03207	nqrF
nqrF	ZONMW-55_02838	nqrF
nqrF	ZONMW-55_03208	nqrF
nqrF	ZONMW-56_02598	nqrF
nqrF	ZONMW-56_03202	nqrF
nqrF	ZONMW-57_02743	nqrF
nqrF	ZONMW-57_03224	nqrF
nqrF	ZONMW-58_02767	nqrF

nqrF	ZONMW-58_03227	nqrF
nqrF	ZONMW-59_02963	nqrF
nqrF	ZONMW-59_03284	nqrF
nqrF	ZONMW-60_02860	nqrF
nqrF	ZONMW-60_03273	nqrF
nqrF	ZONMW-61_00320	nqrF
nqrF	ZONMW-61_03893	nqrF
nqrF	ZONMW-62_01870	nqrF
nqrF	ZONMW-62_03695	nqrF
nqrF	ZONMW-63_02331	nqrF
nqrF	ZONMW-63_03201	nqrF
nqrF	ZONMW-64_00730	nqrF
nqrF	ZONMW-64_02712	nqrF
nqrF	ZONMW-64_02754	nqrF
nqrF	ZONMW-64_00546	nqrF
nqrF	ZONMW-64_00599	nqrF
nqrF	ZONMW-64_03506	nqrF
nqrF	ZONMW-65_00333	nqrF
nqrF	ZONMW-65_03208	nqrF
nqrF	ZONMW-65_00416	nqrF
nqrF	ZONMW-65_02939	nqrF
nqrF	ZONMW-66_04938	nqrF
nqrF	ZONMW-66_00927	nqrF
nqrF	ZONMW-66_00421	nqrF
nqrF	ZONMW-66_00022	nqrF
nqrF	ZONMW-66_00636	nqrF
nqrF	ZONMW-66_04121	nqrF
nqrF	ZONMW-66_00549	nqrF
nqrF	ZONMW-67_02832	nqrF
nqrF	ZONMW-67_03031	nqrF
nqrF	ZONMW-67_01682	nqrF
nqrF	ZONMW-67_01212	nqrF
nqrF	ZONMW-67_00685	nqrF
nqrF	ZONMW-67_03390	nqrF
nqrF	ZONMW-68_00073	nqrF
nqrF	ZONMW-68_01654	nqrF
nqrF	ZONMW-69_00629	nqrF
nqrF	ZONMW-69_02923	nqrF
nqrF	ZONMW-69_01319	nqrF
nqrF	ZONMW-69_01258	nqrF
nqrF	ZONMW-69_00732	nqrF
nqrF	ZONMW-69_03391	nqrF
nqrF	ZONMW-72_03316	nqrF
nqrF	ZONMW-73_00047	nqrF
nqrF	ZONMW-73_03089	nqrF
nqrF	ZONMW-74_01502	nqrF
nqrF	ZONMW-74_03432	nqrF
nqrF	ZONMW-75_02303	nqrF

nqrF	ZONMW-75_00774	nqrF
nqrF	da5A-8_01969	nqrF
nqrF	da5A-8_00777	nqrF
rpsl	18_01534	rpsl
rpsl	ACAM48_02029	rpsl
rpsl	CAM030_01327	rpsl
rpsl	CECT7946_01759	rpsl
rpsl	CGMCC1.8863_01069	rpsl
rpsl	CL8139_00147	rpsl
rpsl	DAU203_01958	rpsl
rpsl	DD5b_01830	rpsl
rpsl	DSM1208_02259	rpsl
rpsl	DSM12212_01710	rpsl
rpsl	DSM15343_02587	rpsl
rpsl	DSM15361_01401	rpsl
rpsl	DSM15365_03403	rpsl
rpsl	DSM15718_01287	rpsl
rpsl	DSM16505T_00867	rpsl
rpsl	DSM18103_03173	rpsl
rpsl	DSM18293_00700	rpsl
rpsl	DSM18841_00734	rpsl
rpsl	DSM18842_00586	rpsl
rpsl	DSM19866_00643	rpsl
rpsl	DSM2041_04634	rpsl
rpsl	DSM2061_03789	rpsl
rpsl	DSM2064_02623	rpsl
rpsl	DSM21164_00343	rpsl
rpsl	DSM23404_01830	rpsl
rpsl	DSM23424_00635	rpsl
rpsl	DSM24786_02090	rpsl
rpsl	DSM7489_01869	rpsl
rpsl	DSW1_00161	rpsl
rpsl	DSW5_00906	rpsl
rpsl	DSW6_01014	rpsl
rpsl	DSW8_01687	rpsl
rpsl	F52_03029	rpsl
rpsl	GCA_001027945.1_03160	rpsl
rpsl	HI1_01797	rpsl
rpsl	IR1_00258	rpsl
rpsl	KCOM3131_00438	rpsl
rpsl	KCTC12974_00804	rpsl
rpsl	KL-A_00402	rpsl
rpsl	L12_00485	rpsl
rpsl	LL0412.1.7_00867	rpsl
rpsl	MAR_2009_71_01730	rpsl
rpsl	NN016038_01541	rpsl
rpsl	PAO1_04575	rpsl
rpsl	UW101_01677	rpsl

rpsl	V4-33_00435	rpsl
rpsl	W5C_00555	rpsl
rpsl	ZONMW-01_00323	rpsl
rpsl	ZONMW-02_00329	rpsl
rpsl	ZONMW-03_01049	rpsl
rpsl	ZONMW-04_00623	rpsl
rpsl	ZONMW-05_01202	rpsl
rpsl	ZONMW-06_03054	rpsl
rpsl	ZONMW-07_00335	rpsl
rpsl	ZONMW-08_01504	rpsl
rpsl	ZONMW-09_00038	rpsl
rpsl	ZONMW-10_00642	rpsl
rpsl	ZONMW-11_01524	rpsl
rpsl	ZONMW-12_00636	rpsl
rpsl	ZONMW-13_00955	rpsl
rpsl	ZONMW-14_01151	rpsl
rpsl	ZONMW-15_00390	rpsl
rpsl	ZONMW-19_00466	rpsl
rpsl	ZONMW-20_02819	rpsl
rpsl	ZONMW-21_00232	rpsl
rpsl	ZONMW-22_00232	rpsl
rpsl	ZONMW-23_01347	rpsl
rpsl	ZONMW-24_01227	rpsl
rpsl	ZONMW-25_03065	rpsl
rpsl	ZONMW-26_01256	rpsl
rpsl	ZONMW-27_00617	rpsl
rpsl	ZONMW-28_03368	rpsl
rpsl	ZONMW-29_02972	rpsl
rpsl	ZONMW-30_01439	rpsl
rpsl	ZONMW-31_03637	rpsl
rpsl	ZONMW-32_03493	rpsl
rpsl	ZONMW-33_03496	rpsl
rpsl	ZONMW-34_03709	rpsl
rpsl	ZONMW-35_03672	rpsl
rpsl	ZONMW-36_03353	rpsl
rpsl	ZONMW-37_00232	rpsl
rpsl	ZONMW-38_01881	rpsl
rpsl	ZONMW-39_03573	rpsl
rpsl	ZONMW-40_02645	rpsl
rpsl	ZONMW-41_01194	rpsl
rpsl	ZONMW-42_04226	rpsl
rpsl	ZONMW-43_02763	rpsl
rpsl	ZONMW-44_01040	rpsl
rpsl	ZONMW-45_01093	rpsl
rpsl	ZONMW-46_00574	rpsl
rpsl	ZONMW-47_00574	rpsl
rpsl	ZONMW-48_01027	rpsl
rpsl	ZONMW-49_01411	rpsl

rpsl	ZONMW-50_01413	rpsl
rpsl	ZONMW-51_01527	rpsl
rpsl	ZONMW-52_01267	rpsl
rpsl	ZONMW-53_00558	rpsl
rpsl	ZONMW-54_00477	rpsl
rpsl	ZONMW-55_00553	rpsl
rpsl	ZONMW-56_03011	rpsl
rpsl	ZONMW-57_01061	rpsl
rpsl	ZONMW-58_01523	rpsl
rpsl	ZONMW-59_01063	rpsl
rpsl	ZONMW-60_01504	rpsl
rpsl	ZONMW-61_02891	rpsl
rpsl	ZONMW-62_02336	rpsl
rpsl	ZONMW-63_00817	rpsl
rpsl	ZONMW-64_03688	rpsl
rpsl	ZONMW-65_00292	rpsl
rpsl	ZONMW-66_00064	rpsl
rpsl	ZONMW-67_02555	rpsl
rpsl	ZONMW-68_03902	rpsl
rpsl	ZONMW-69_02603	rpsl
rpsl	ZONMW-72_00426	rpsl
rpsl	ZONMW-73_03184	rpsl
rpsl	ZONMW-74_01833	rpsl
rpsl	ZONMW-75_01187	rpsl
rpsl	da5A-8_01745	rpsl
wbpA	18_02494	wbpA
wbpA	18_02496	wbpA
wbpA	ACAM48_00066	wbpA
wbpA	ACAM48_00601	wbpA
wbpA	ACAM48_00067	wbpA
wbpA	CAM030_02148	wbpA
wbpA	CAM030_01007	wbpA
wbpA	CECT7946_03590	wbpA
wbpA	CECT7946_01371	wbpA
wbpA	CGMCC1.8863_01515	wbpA
wbpA	CGMCC1.8863_01514	wbpA
wbpA	CL8139_02969	wbpA
wbpA	CL8139_02962	wbpA
wbpA	CL8139_02970	wbpA
wbpA	CL8139_02132	wbpA
wbpA	DAU203_02816	wbpA
wbpA	DAU203_01469	wbpA
wbpA	DAU203_01467	wbpA
wbpA	DD5b_02377	wbpA
wbpA	DD5b_02345	wbpA
wbpA	DD5b_02378	wbpA
wbpA	DSM1208_03623	wbpA
wbpA	DSM1208_01908	wbpA

wbpA	DSM1208_03631	wbpA
wbpA	DSM12212_00334	wbpA
wbpA	DSM12212_00096	wbpA
wbpA	DSM15343_01609	wbpA
wbpA	DSM15343_01608	wbpA
wbpA	DSM15361_02165	wbpA
wbpA	DSM15361_00112	wbpA
wbpA	DSM15361_00111	wbpA
wbpA	DSM15365_01868	wbpA
wbpA	DSM15365_01867	wbpA
wbpA	DSM15365_00356	wbpA
wbpA	DSM15365_03034	wbpA
wbpA	DSM15718_00710	wbpA
wbpA	DSM15718_00690	wbpA
wbpA	DSM15718_00688	wbpA
wbpA	DSM16505T_02628	wbpA
wbpA	DSM16505T_02626	wbpA
wbpA	DSM18103_03377	wbpA
wbpA	DSM18103_03382	wbpA
wbpA	DSM18103_03379	wbpA
wbpA	DSM18293_02454	wbpA
wbpA	DSM18293_02452	wbpA
wbpA	DSM18841_02737	wbpA
wbpA	DSM18841_02730	wbpA
wbpA	DSM18841_02970	wbpA
wbpA	DSM18841_02731	wbpA
wbpA	DSM18841_02733	wbpA
wbpA	DSM18842_02672	wbpA
wbpA	DSM18842_02666	wbpA
wbpA	DSM19866_04500	wbpA
wbpA	DSM19866_04499	wbpA
wbpA	DSM2041_01959	wbpA
wbpA	DSM2041_00226	wbpA
wbpA	DSM2041_00192	wbpA
wbpA	DSM2061_01431	wbpA
wbpA	DSM2061_01428	wbpA
wbpA	DSM2064_04724	wbpA
wbpA	DSM2064_04722	wbpA
wbpA	DSM21164_01719	wbpA
wbpA	DSM21164_01726	wbpA
wbpA	DSM21164_01717	wbpA
wbpA	DSM23404_01425	wbpA
wbpA	DSM23404_01018	wbpA
wbpA	DSM23404_01424	wbpA
wbpA	DSM23424_02681	wbpA
wbpA	DSM23424_00319	wbpA
wbpA	DSM24786_00274	wbpA
wbpA	DSM24786_02953	wbpA

wbpA	DSM7489_02686	wbpA
wbpA	DSM7489_02678	wbpA
wbpA	DSM7489_01379	wbpA
wbpA	DSM7489_02687	wbpA
wbpA	DSM7489_01377	wbpA
wbpA	DSW1_02196	wbpA
wbpA	DSW1_02189	wbpA
wbpA	DSW5_00245	wbpA
wbpA	DSW5_00243	wbpA
wbpA	DSW6_03448	wbpA
wbpA	DSW6_03447	wbpA
wbpA	DSW8_00878	wbpA
wbpA	DSW8_00875	wbpA
wbpA	F52_01545	wbpA
wbpA	F52_01543	wbpA
wbpA	GCA_001027945.1_00814	wbpA
wbpA	GCA_001027945.1_00820	wbpA
wbpA	HI1_02626	wbpA
wbpA	HI1_01318	wbpA
wbpA	IR1_03033	wbpA
wbpA	IR1_03035	wbpA
wbpA	IR1_00915	wbpA
wbpA	KCOM3131_01335	wbpA
wbpA	KCOM3131_01105	wbpA
wbpA	KCOM3131_01783	wbpA
wbpA	KCTC12974_00126	wbpA
wbpA	KCTC12974_00123	wbpA
wbpA	KL-A_01371	wbpA
wbpA	KL-A_00850	wbpA
wbpA	KL-A_00852	wbpA
wbpA	L12_00465	wbpA
wbpA	L12_00085	wbpA
wbpA	LL0412.1.7_01910	wbpA
wbpA	LL0412.1.7_01907	wbpA
wbpA	MAR_2009_71_02566	wbpA
wbpA	MAR_2009_71_02569	wbpA
wbpA	NN016038_02527	wbpA
wbpA	NN016038_02529	wbpA
wbpA	PAO1_03269	wbpA
wbpA	PAO1_02085	wbpA
wbpA	PAO1_03662	wbpA
wbpA	PAO1_03681	wbpA
wbpA	UW101_00378	wbpA
wbpA	UW101_00380	wbpA
wbpA	V4-33_01205	wbpA
wbpA	V4-33_01206	wbpA
wbpA	V4-33_01204	wbpA
wbpA	W5C_00739	wbpA

wbpA	W5C_00732	wbpA
wbpA	W5C_00740	wbpA
wbpA	W5C_01858	wbpA
wbpA	ZONMW-01_01731	wbpA
wbpA	ZONMW-01_02372	wbpA
wbpA	ZONMW-01_02370	wbpA
wbpA	ZONMW-02_03330	wbpA
wbpA	ZONMW-02_03331	wbpA
wbpA	ZONMW-02_02946	wbpA
wbpA	ZONMW-03_00476	wbpA
wbpA	ZONMW-03_02975	wbpA
wbpA	ZONMW-03_02973	wbpA
wbpA	ZONMW-04_01415	wbpA
wbpA	ZONMW-04_02474	wbpA
wbpA	ZONMW-05_00745	wbpA
wbpA	ZONMW-05_00744	wbpA
wbpA	ZONMW-05_02559	wbpA
wbpA	ZONMW-06_01055	wbpA
wbpA	ZONMW-06_03123	wbpA
wbpA	ZONMW-07_02482	wbpA
wbpA	ZONMW-07_02298	wbpA
wbpA	ZONMW-07_01495	wbpA
wbpA	ZONMW-07_02481	wbpA
wbpA	ZONMW-08_03564	wbpA
wbpA	ZONMW-08_03569	wbpA
wbpA	ZONMW-09_02545	wbpA
wbpA	ZONMW-09_02547	wbpA
wbpA	ZONMW-10_02435	wbpA
wbpA	ZONMW-10_01423	wbpA
wbpA	ZONMW-11_01043	wbpA
wbpA	ZONMW-11_04702	wbpA
wbpA	ZONMW-11_03873	wbpA
wbpA	ZONMW-12_01597	wbpA
wbpA	ZONMW-12_00666	wbpA
wbpA	ZONMW-13_01222	wbpA
wbpA	ZONMW-13_00976	wbpA
wbpA	ZONMW-13_00975	wbpA
wbpA	ZONMW-13_01221	wbpA
wbpA	ZONMW-14_01931	wbpA
wbpA	ZONMW-14_02775	wbpA
wbpA	ZONMW-14_01932	wbpA
wbpA	ZONMW-15_01403	wbpA
wbpA	ZONMW-15_02534	wbpA
wbpA	ZONMW-15_01402	wbpA
wbpA	ZONMW-19_02922	wbpA
wbpA	ZONMW-19_02923	wbpA
wbpA	ZONMW-20_00845	wbpA
wbpA	ZONMW-20_03912	wbpA

wbpA	ZONMW-21_00794	wbpA
wbpA	ZONMW-21_02205	wbpA
wbpA	ZONMW-22_00795	wbpA
wbpA	ZONMW-22_01520	wbpA
wbpA	ZONMW-23_01909	wbpA
wbpA	ZONMW-23_01031	wbpA
wbpA	ZONMW-24_00666	wbpA
wbpA	ZONMW-24_01544	wbpA
wbpA	ZONMW-25_00326	wbpA
wbpA	ZONMW-25_00333	wbpA
wbpA	ZONMW-26_00230	wbpA
wbpA	ZONMW-26_03297	wbpA
wbpA	ZONMW-26_03299	wbpA
wbpA	ZONMW-27_01883	wbpA
wbpA	ZONMW-27_01884	wbpA
wbpA	ZONMW-27_02716	wbpA
wbpA	ZONMW-28_03526	wbpA
wbpA	ZONMW-28_02932	wbpA
wbpA	ZONMW-28_02847	wbpA
wbpA	ZONMW-29_01410	wbpA
wbpA	ZONMW-29_01403	wbpA
wbpA	ZONMW-30_03331	wbpA
wbpA	ZONMW-30_03276	wbpA
wbpA	ZONMW-31_01327	wbpA
wbpA	ZONMW-31_02117	wbpA
wbpA	ZONMW-31_01576	wbpA
wbpA	ZONMW-31_01303	wbpA
wbpA	ZONMW-32_00611	wbpA
wbpA	ZONMW-32_01416	wbpA
wbpA	ZONMW-32_03028	wbpA
wbpA	ZONMW-33_00479	wbpA
wbpA	ZONMW-33_01406	wbpA
wbpA	ZONMW-33_03245	wbpA
wbpA	ZONMW-34_00860	wbpA
wbpA	ZONMW-34_03223	wbpA
wbpA	ZONMW-34_01594	wbpA
wbpA	ZONMW-34_00855	wbpA
wbpA	ZONMW-35_02383	wbpA
wbpA	ZONMW-35_03184	wbpA
wbpA	ZONMW-35_00665	wbpA
wbpA	ZONMW-35_02388	wbpA
wbpA	ZONMW-36_00892	wbpA
wbpA	ZONMW-36_00625	wbpA
wbpA	ZONMW-36_03546	wbpA
wbpA	ZONMW-37_00794	wbpA
wbpA	ZONMW-37_01543	wbpA
wbpA	ZONMW-38_00914	wbpA
wbpA	ZONMW-38_02631	wbpA

wbpA	ZONMW-38_01654	wbpA
wbpA	ZONMW-38_01514	wbpA
wbpA	ZONMW-38_00915	wbpA
wbpA	ZONMW-39_01322	wbpA
wbpA	ZONMW-39_01917	wbpA
wbpA	ZONMW-39_00106	wbpA
wbpA	ZONMW-40_00623	wbpA
wbpA	ZONMW-40_00616	wbpA
wbpA	ZONMW-41_02584	wbpA
wbpA	ZONMW-41_01691	wbpA
wbpA	ZONMW-42_01037	wbpA
wbpA	ZONMW-42_03945	wbpA
wbpA	ZONMW-42_03943	wbpA
wbpA	ZONMW-42_03306	wbpA
wbpA	ZONMW-43_00393	wbpA
wbpA	ZONMW-43_00990	wbpA
wbpA	ZONMW-43_01675	wbpA
wbpA	ZONMW-44_03948	wbpA
wbpA	ZONMW-44_02244	wbpA
wbpA	ZONMW-44_02796	wbpA
wbpA	ZONMW-45_02695	wbpA
wbpA	ZONMW-45_02699	wbpA
wbpA	ZONMW-45_02245	wbpA
wbpA	ZONMW-45_02691	wbpA
wbpA	ZONMW-46_03094	wbpA
wbpA	ZONMW-46_03090	wbpA
wbpA	ZONMW-46_03170	wbpA
wbpA	ZONMW-46_03097	wbpA
wbpA	ZONMW-47_03085	wbpA
wbpA	ZONMW-47_03081	wbpA
wbpA	ZONMW-47_03162	wbpA
wbpA	ZONMW-47_03089	wbpA
wbpA	ZONMW-48_01764	wbpA
wbpA	ZONMW-48_00647	wbpA
wbpA	ZONMW-48_01762	wbpA
wbpA	ZONMW-49_00241	wbpA
wbpA	ZONMW-49_01225	wbpA
wbpA	ZONMW-50_00244	wbpA
wbpA	ZONMW-50_01226	wbpA
wbpA	ZONMW-51_00229	wbpA
wbpA	ZONMW-51_02835	wbpA
wbpA	ZONMW-51_02837	wbpA
wbpA	ZONMW-52_00213	wbpA
wbpA	ZONMW-52_02210	wbpA
wbpA	ZONMW-52_02208	wbpA
wbpA	ZONMW-53_00949	wbpA
wbpA	ZONMW-53_00942	wbpA
wbpA	ZONMW-53_02806	wbpA

wbpA	ZONMW-53_00950	wbpA
wbpA	ZONMW-53_02804	wbpA
wbpA	ZONMW-54_01777	wbpA
wbpA	ZONMW-54_00026	wbpA
wbpA	ZONMW-55_01297	wbpA
wbpA	ZONMW-55_02806	wbpA
wbpA	ZONMW-55_02804	wbpA
wbpA	ZONMW-56_00526	wbpA
wbpA	ZONMW-56_02570	wbpA
wbpA	ZONMW-57_00740	wbpA
wbpA	ZONMW-57_00747	wbpA
wbpA	ZONMW-57_02711	wbpA
wbpA	ZONMW-57_00739	wbpA
wbpA	ZONMW-57_02709	wbpA
wbpA	ZONMW-58_00231	wbpA
wbpA	ZONMW-58_00224	wbpA
wbpA	ZONMW-58_02735	wbpA
wbpA	ZONMW-58_00232	wbpA
wbpA	ZONMW-58_02733	wbpA
wbpA	ZONMW-59_00728	wbpA
wbpA	ZONMW-59_02991	wbpA
wbpA	ZONMW-60_00741	wbpA
wbpA	ZONMW-60_02839	wbpA
wbpA	ZONMW-61_03817	wbpA
wbpA	ZONMW-61_03818	wbpA
wbpA	ZONMW-62_00974	wbpA
wbpA	ZONMW-62_00975	wbpA
wbpA	ZONMW-63_00413	wbpA
wbpA	ZONMW-63_00421	wbpA
wbpA	ZONMW-63_02363	wbpA
wbpA	ZONMW-63_00412	wbpA
wbpA	ZONMW-63_02365	wbpA
wbpA	ZONMW-64_00411	wbpA
wbpA	ZONMW-64_02586	wbpA
wbpA	ZONMW-64_03019	wbpA
wbpA	ZONMW-65_01643	wbpA
wbpA	ZONMW-65_02975	wbpA
wbpA	ZONMW-65_00865	wbpA
wbpA	ZONMW-66_00865	wbpA
wbpA	ZONMW-66_00864	wbpA
wbpA	ZONMW-67_01083	wbpA
wbpA	ZONMW-67_01071	wbpA
wbpA	ZONMW-67_01531	wbpA
wbpA	ZONMW-68_01252	wbpA
wbpA	ZONMW-68_01253	wbpA
wbpA	ZONMW-69_01130	wbpA
wbpA	ZONMW-69_01118	wbpA
wbpA	ZONMW-69_01470	wbpA

wbpA	ZONMW-72_01187	wbpA
wbpA	ZONMW-72_01185	wbpA
wbpA	ZONMW-73_02499	wbpA
wbpA	ZONMW-73_00034	wbpA
wbpA	ZONMW-74_03086	wbpA
wbpA	ZONMW-74_03087	wbpA
wbpA	ZONMW-74_01529	wbpA
wbpA	ZONMW-75_02041	wbpA
wbpA	ZONMW-75_00012	wbpA
wbpA	ZONMW-75_02042	wbpA
wbpA	da5A-8_00254	wbpA
wbpA	da5A-8_00250	wbpA
fur	18_02211	fur
fur	ACAM48_01339	fur
fur	CAM030_01589	fur
fur	CECT7946_03195	fur
fur	CGMCC1.8863_01132	fur
fur	CL8139_01140	fur
fur	DAU203_00284	fur
fur	DD5b_00721	fur
fur	DSM1208_03525	fur
fur	DSM12212_02448	fur
fur	DSM15343_01910	fur
fur	DSM15361_01546	fur
fur	DSM15365_00074	fur
fur	DSM15718_01153	fur
fur	DSM16505T_01382	fur
fur	DSM18103_00916	fur
fur	DSM18293_03664	fur
fur	DSM18841_02395	fur
fur	DSM18842_01309	fur
fur	DSM19866_04617	fur
fur	DSM2041_05495	fur
fur	DSM2061_01538	fur
fur	DSM2064_03468	fur
fur	DSM21164_03099	fur
fur	DSM23404_01637	fur
fur	DSM23424_01879	fur
fur	DSM24786_01085	fur
fur	DSM7489_00278	fur
fur	DSW1_02495	fur
fur	DSW5_02187	fur
fur	DSW6_03294	fur
fur	DSW8_00982	fur
fur	F52_00206	fur
fur	GCA_001027945.1_01970	fur
fur	HI1_00210	fur
fur	IR1_04408	fur

fur	KCOM3131_00357	fur
fur	KCTC12974_01830	fur
fur	KL-A_01274	fur
fur	L12_00049	fur
fur	LL0412.1.7_02030	fur
fur	MAR_2009_71_02459	fur
fur	NN016038_02227	fur
fur	PAO1_04926	fur
fur	UW101_02332	fur
fur	V4-33_01814	fur
fur	W5C_00187	fur
fur	ZONMW-01_02162	fur
fur	ZONMW-02_00916	fur
fur	ZONMW-03_02548	fur
fur	ZONMW-04_02007	fur
fur	ZONMW-05_02362	fur
fur	ZONMW-06_00299	fur
fur	ZONMW-07_01748	fur
fur	ZONMW-08_03458	fur
fur	ZONMW-09_02069	fur
fur	ZONMW-10_01014	fur
fur	ZONMW-11_02024	fur
fur	ZONMW-11_00289	fur
fur	ZONMW-11_01478	fur
fur	ZONMW-12_01772	fur
fur	ZONMW-13_02475	fur
fur	ZONMW-14_02528	fur
fur	ZONMW-15_02675	fur
fur	ZONMW-19_00689	fur
fur	ZONMW-20_02338	fur
fur	ZONMW-21_02742	fur
fur	ZONMW-22_02367	fur
fur	ZONMW-23_00183	fur
fur	ZONMW-24_02132	fur
fur	ZONMW-25_00658	fur
fur	ZONMW-26_02754	fur
fur	ZONMW-27_02178	fur
fur	ZONMW-28_01528	fur
fur	ZONMW-29_01906	fur
fur	ZONMW-30_02856	fur
fur	ZONMW-31_00612	fur
fur	ZONMW-32_01935	fur
fur	ZONMW-33_01926	fur
fur	ZONMW-34_00261	fur
fur	ZONMW-35_01129	fur
fur	ZONMW-36_01430	fur
fur	ZONMW-37_02409	fur
fur	ZONMW-38_00590	fur

fur	ZONMW-38_03363	fur
fur	ZONMW-38_03098	fur
fur	ZONMW-39_04036	fur
fur	ZONMW-40_01695	fur
fur	ZONMW-41_01496	fur
fur	ZONMW-42_03791	fur
fur	ZONMW-43_00691	fur
fur	ZONMW-44_03489	fur
fur	ZONMW-45_01808	fur
fur	ZONMW-46_01908	fur
fur	ZONMW-47_02153	fur
fur	ZONMW-48_02850	fur
fur	ZONMW-49_03224	fur
fur	ZONMW-50_03210	fur
fur	ZONMW-51_02375	fur
fur	ZONMW-52_02359	fur
fur	ZONMW-53_02274	fur
fur	ZONMW-54_02764	fur
fur	ZONMW-55_02466	fur
fur	ZONMW-56_01762	fur
fur	ZONMW-57_02109	fur
fur	ZONMW-58_01969	fur
fur	ZONMW-59_02188	fur
fur	ZONMW-60_02485	fur
fur	ZONMW-61_03625	fur
fur	ZONMW-62_01079	fur
fur	ZONMW-63_01574	fur
fur	ZONMW-64_00953	fur
fur	ZONMW-65_00494	fur
fur	ZONMW-66_00452	fur
fur	ZONMW-67_02673	fur
fur	ZONMW-68_01135	fur
fur	ZONMW-69_00470	fur
fur	ZONMW-72_03183	fur
fur	ZONMW-73_00747	fur
fur	ZONMW-74_02544	fur
fur	ZONMW-75_01503	fur
fur	da5A-8_01369	fur
rplM	18_01535	rplM
rplM	ACAM48_02028	rplM
rplM	CAM030_01328	rplM
rplM	CECT7946_01758	rplM
rplM	CGMCC1.8863_01070	rplM
rplM	CL8139_00148	rplM
rplM	DAU203_01959	rplM
rplM	DD5b_01831	rplM
rplM	DSM1208_02260	rplM
rplM	DSM12212_01711	rplM

rplM	DSM15343_02588	rplM
rplM	DSM15361_01400	rplM
rplM	DSM15365_03404	rplM
rplM	DSM15718_01288	rplM
rplM	DSM16505T_00868	rplM
rplM	DSM18103_03174	rplM
rplM	DSM18293_00701	rplM
rplM	DSM18841_00735	rplM
rplM	DSM18842_00585	rplM
rplM	DSM19866_00642	rplM
rplM	DSM2041_04635	rplM
rplM	DSM2061_03790	rplM
rplM	DSM2064_02624	rplM
rplM	DSM21164_00342	rplM
rplM	DSM23404_01829	rplM
rplM	DSM23424_00636	rplM
rplM	DSM24786_02089	rplM
rplM	DSM7489_01870	rplM
rplM	DSW1_00160	rplM
rplM	DSW5_00905	rplM
rplM	DSW6_01013	rplM
rplM	DSW8_01686	rplM
rplM	F52_03030	rplM
rplM	GCA_001027945.1_03161	rplM
rplM	HI1_01798	rplM
rplM	IR1_00257	rplM
rplM	KCOM3131_00439	rplM
rplM	KCTC12974_00803	rplM
rplM	KL-A_00401	rplM
rplM	L12_00484	rplM
rplM	LL0412.1.7_00868	rplM
rplM	MAR_2009_71_01731	rplM
rplM	NN016038_01542	rplM
rplM	PAO1_04576	rplM
rplM	UW101_01678	rplM
rplM	V4-33_00436	rplM
rplM	W5C_00556	rplM
rplM	ZONMW-01_00324	rplM
rplM	ZONMW-02_00330	rplM
rplM	ZONMW-03_01050	rplM
rplM	ZONMW-04_00622	rplM
rplM	ZONMW-05_01203	rplM
rplM	ZONMW-06_03055	rplM
rplM	ZONMW-07_00336	rplM
rplM	ZONMW-08_01503	rplM
rplM	ZONMW-09_00037	rplM
rplM	ZONMW-10_00643	rplM
rplM	ZONMW-11_01525	rplM

rplM	ZONMW-12_00637	rplM
rplM	ZONMW-13_00956	rplM
rplM	ZONMW-14_01150	rplM
rplM	ZONMW-15_00391	rplM
rplM	ZONMW-19_00467	rplM
rplM	ZONMW-20_02818	rplM
rplM	ZONMW-21_00231	rplM
rplM	ZONMW-22_00231	rplM
rplM	ZONMW-23_01346	rplM
rplM	ZONMW-24_01228	rplM
rplM	ZONMW-25_03066	rplM
rplM	ZONMW-26_01257	rplM
rplM	ZONMW-27_00616	rplM
rplM	ZONMW-28_03369	rplM
rplM	ZONMW-29_02973	rplM
rplM	ZONMW-30_01438	rplM
rplM	ZONMW-31_03638	rplM
rplM	ZONMW-32_03494	rplM
rplM	ZONMW-33_03497	rplM
rplM	ZONMW-34_03708	rplM
rplM	ZONMW-35_03671	rplM
rplM	ZONMW-36_03354	rplM
rplM	ZONMW-37_00231	rplM
rplM	ZONMW-38_01882	rplM
rplM	ZONMW-39_03572	rplM
rplM	ZONMW-40_02644	rplM
rplM	ZONMW-41_01193	rplM
rplM	ZONMW-42_04225	rplM
rplM	ZONMW-43_02762	rplM
rplM	ZONMW-44_01041	rplM
rplM	ZONMW-45_01094	rplM
rplM	ZONMW-46_00573	rplM
rplM	ZONMW-47_00573	rplM
rplM	ZONMW-48_01028	rplM
rplM	ZONMW-49_01412	rplM
rplM	ZONMW-50_01414	rplM
rplM	ZONMW-51_01528	rplM
rplM	ZONMW-52_01268	rplM
rplM	ZONMW-53_00557	rplM
rplM	ZONMW-54_00478	rplM
rplM	ZONMW-55_00552	rplM
rplM	ZONMW-56_03010	rplM
rplM	ZONMW-57_01062	rplM
rplM	ZONMW-58_01522	rplM
rplM	ZONMW-59_01064	rplM
rplM	ZONMW-60_01503	rplM
rplM	ZONMW-61_02892	rplM
rplM	ZONMW-62_02335	rplM

rplM	ZONMW-63_00818	rplM
rplM	ZONMW-64_03689	rplM
rplM	ZONMW-65_00293	rplM
rplM	ZONMW-66_00065	rplM
rplM	ZONMW-67_02556	rplM
rplM	ZONMW-68_03901	rplM
rplM	ZONMW-69_02604	rplM
rplM	ZONMW-72_00427	rplM
rplM	ZONMW-73_03183	rplM
rplM	ZONMW-74_01832	rplM
rplM	ZONMW-75_01188	rplM
rplM	da5A-8_01744	rplM
rplS	18_03615	rplS
rplS	ACAM48_01428	rplS
rplS	CAM030_01803	rplS
rplS	CECT7946_03504	rplS
rplS	CGMCC1.8863_02972	rplS
rplS	CL8139_01251	rplS
rplS	DAU203_00014	rplS
rplS	DD5b_00140	rplS
rplS	DSM1208_00013	rplS
rplS	DSM12212_01940	rplS
rplS	DSM15343_00344	rplS
rplS	DSM15361_01873	rplS
rplS	DSM15365_00100	rplS
rplS	DSM15718_03044	rplS
rplS	DSM16505T_01919	rplS
rplS	DSM18103_00553	rplS
rplS	DSM18293_03621	rplS
rplS	DSM18841_02017	rplS
rplS	DSM18842_02330	rplS
rplS	DSM19866_04725	rplS
rplS	DSM2041_04797	rplS
rplS	DSM2061_00541	rplS
rplS	DSM2064_03508	rplS
rplS	DSM21164_02829	rplS
rplS	DSM23404_01713	rplS
rplS	DSM23424_01739	rplS
rplS	DSM24786_01008	rplS
rplS	DSM7489_00014	rplS
rplS	DSW1_01629	rplS
rplS	DSW5_02749	rplS
rplS	DSW6_00122	rplS
rplS	DSW8_00585	rplS
rplS	F52_00168	rplS
rplS	GCA_001027945.1_02852	rplS
rplS	HI1_03313	rplS
rplS	IR1_04440	rplS

rplS	KCOM3131_00979	rplS
rplS	KCTC12974_02237	rplS
rplS	KL-A_01825	rplS
rplS	L12_00509	rplS
rplS	LL0412.1.7_01979	rplS
rplS	MAR_2009_71_03382	rplS
rplS	NN016038_03655	rplS
rplS	PAO1_03865	rplS
rplS	UW101_02292	rplS
rplS	V4-33_01783	rplS
rplS	W5C_00857	rplS
rplS	ZONMW-01_03101	rplS
rplS	ZONMW-02_00892	rplS
rplS	ZONMW-03_02336	rplS
rplS	ZONMW-04_00800	rplS
rplS	ZONMW-05_00070	rplS
rplS	ZONMW-06_02897	rplS
rplS	ZONMW-07_03417	rplS
rplS	ZONMW-08_02545	rplS
rplS	ZONMW-09_02199	rplS
rplS	ZONMW-10_01850	rplS
rplS	ZONMW-11_00382	rplS
rplS	ZONMW-12_03630	rplS
rplS	ZONMW-13_02343	rplS
rplS	ZONMW-14_02638	rplS
rplS	ZONMW-15_02970	rplS
rplS	ZONMW-19_00409	rplS
rplS	ZONMW-20_02724	rplS
rplS	ZONMW-21_01628	rplS
rplS	ZONMW-22_02096	rplS
rplS	ZONMW-23_00455	rplS
rplS	ZONMW-24_02721	rplS
rplS	ZONMW-25_01422	rplS
rplS	ZONMW-26_00890	rplS
rplS	ZONMW-27_00897	rplS
rplS	ZONMW-28_02009	rplS
rplS	ZONMW-29_03161	rplS
rplS	ZONMW-30_02170	rplS
rplS	ZONMW-31_03005	rplS
rplS	ZONMW-32_02415	rplS
rplS	ZONMW-33_02703	rplS
rplS	ZONMW-34_01997	rplS
rplS	ZONMW-35_01689	rplS
rplS	ZONMW-36_02549	rplS
rplS	ZONMW-37_02006	rplS
rplS	ZONMW-38_00806	rplS
rplS	ZONMW-39_02195	rplS
rplS	ZONMW-40_02462	rplS

rplS	ZONMW-41_01310	rplS
rplS	ZONMW-42_00422	rplS
rplS	ZONMW-43_01501	rplS
rplS	ZONMW-44_00875	rplS
rplS	ZONMW-45_00052	rplS
rplS	ZONMW-46_00267	rplS
rplS	ZONMW-47_00238	rplS
rplS	ZONMW-48_03089	rplS
rplS	ZONMW-49_03067	rplS
rplS	ZONMW-50_02965	rplS
rplS	ZONMW-51_01893	rplS
rplS	ZONMW-52_00880	rplS
rplS	ZONMW-53_01623	rplS
rplS	ZONMW-54_01102	rplS
rplS	ZONMW-55_02215	rplS
rplS	ZONMW-56_02153	rplS
rplS	ZONMW-57_00067	rplS
rplS	ZONMW-58_00899	rplS
rplS	ZONMW-59_00067	rplS
rplS	ZONMW-60_00068	rplS
rplS	ZONMW-61_02069	rplS
rplS	ZONMW-62_00177	rplS
rplS	ZONMW-63_01831	rplS
rplS	ZONMW-64_02078	rplS
rplS	ZONMW-65_01173	rplS
rplS	ZONMW-66_02236	rplS
rplS	ZONMW-67_02396	rplS
rplS	ZONMW-68_01028	rplS
rplS	ZONMW-69_02444	rplS
rplS	ZONMW-72_03151	rplS
rplS	ZONMW-73_00771	rplS
rplS	ZONMW-74_00595	rplS
rplS	ZONMW-75_01479	rplS
rplS	da5A-8_02200	rplS
mnmE	18_00401	mnmE
mnmE	18_02604	mnmE
mnmE	18_00404	mnmE
mnmE	ACAM48_02950	mnmE
mnmE	ACAM48_01293	mnmE
mnmE	ACAM48_02949	mnmE
mnmE	CAM030_02715	mnmE
mnmE	CAM030_02718	mnmE
mnmE	CAM030_01881	mnmE
mnmE	CECT7946_01097	mnmE
mnmE	CECT7946_01086	mnmE
mnmE	CECT7946_03027	mnmE
mnmE	CGMCC1.8863_02568	mnmE
mnmE	CGMCC1.8863_02287	mnmE

mnmE	CGMCC1.8863_02566	mnmE
mnmE	CL8139_02747	mnmE
mnmE	CL8139_03044	mnmE
mnmE	CL8139_02750	mnmE
mnmE	DAU203_00734	mnmE
mnmE	DAU203_03417	mnmE
mnmE	DAU203_00737	mnmE
mnmE	DD5b_00235	mnmE
mnmE	DD5b_00290	mnmE
mnmE	DD5b_00238	mnmE
mnmE	DSM1208_00956	mnmE
mnmE	DSM1208_02072	mnmE
mnmE	DSM1208_00958	mnmE
mnmE	DSM12212_03190	mnmE
mnmE	DSM12212_03188	mnmE
mnmE	DSM12212_00906	mnmE
mnmE	DSM15343_02125	mnmE
mnmE	DSM15343_00120	mnmE
mnmE	DSM15343_02121	mnmE
mnmE	DSM15361_01207	mnmE
mnmE	DSM15361_01209	mnmE
mnmE	DSM15361_02049	mnmE
mnmE	DSM15365_03499	mnmE
mnmE	DSM15365_03952	mnmE
mnmE	DSM15365_03497	mnmE
mnmE	DSM15718_01002	mnmE
mnmE	DSM15718_00206	mnmE
mnmE	DSM15718_01000	mnmE
mnmE	DSM16505T_00917	mnmE
mnmE	DSM16505T_02688	mnmE
mnmE	DSM16505T_00919	mnmE
mnmE	DSM18103_01483	mnmE
mnmE	DSM18103_02461	mnmE
mnmE	DSM18103_01481	mnmE
mnmE	DSM18293_02167	mnmE
mnmE	DSM18293_03391	mnmE
mnmE	DSM18293_02166	mnmE
mnmE	DSM18841_00788	mnmE
mnmE	DSM18841_02944	mnmE
mnmE	DSM18841_00790	mnmE
mnmE	DSM18842_00525	mnmE
mnmE	DSM18842_02052	mnmE
mnmE	DSM18842_00523	mnmE
mnmE	DSM19866_00923	mnmE
mnmE	DSM19866_04463	mnmE
mnmE	DSM19866_00924	mnmE
mnmE	DSM2041_01187	mnmE
mnmE	DSM2041_02240	mnmE

mnmE	DSM2041_01184	mnmE
mnmE	DSM2061_02461	mnmE
mnmE	DSM2061_01350	mnmE
mnmE	DSM2061_02463	mnmE
mnmE	DSM2064_02908	mnmE
mnmE	DSM2064_05216	mnmE
mnmE	DSM2064_02907	mnmE
mnmE	DSM21164_01380	mnmE
mnmE	DSM21164_01690	mnmE
mnmE	DSM21164_01377	mnmE
mnmE	DSM23404_01194	mnmE
mnmE	DSM23404_01589	mnmE
mnmE	DSM23404_01195	mnmE
mnmE	DSM23424_03059	mnmE
mnmE	DSM23424_02291	mnmE
mnmE	DSM23424_03062	mnmE
mnmE	DSM24786_01409	mnmE
mnmE	DSM24786_01406	mnmE
mnmE	DSM24786_00900	mnmE
mnmE	DSM7489_00721	mnmE
mnmE	DSM7489_03256	mnmE
mnmE	DSM7489_00725	mnmE
mnmE	DSW1_00288	mnmE
mnmE	DSW1_02165	mnmE
mnmE	DSW1_02913	mnmE
mnmE	DSW1_00002	mnmE
mnmE	DSW1_00003	mnmE
mnmE	DSW1_00004	mnmE
mnmE	DSW5_01497	mnmE
mnmE	DSW5_02427	mnmE
mnmE	DSW5_01499	mnmE
mnmE	DSW6_01897	mnmE
mnmE	DSW6_02481	mnmE
mnmE	DSW6_00475	mnmE
mnmE	DSW8_02935	mnmE
mnmE	DSW8_00860	mnmE
mnmE	DSW8_02933	mnmE
mnmE	F52_03174	mnmE
mnmE	F52_03175	mnmE
mnmE	F52_03418	mnmE
mnmE	GCA_001027945.1_01317	mnmE
mnmE	GCA_001027945.1_02017	mnmE
mnmE	GCA_001027945.1_01319	mnmE
mnmE	HI1_00652	mnmE
mnmE	HI1_03200	mnmE
mnmE	HI1_00655	mnmE
mnmE	IR1_01022	mnmE
mnmE	IR1_01986	mnmE

mnmE	IR1_01024	mnmE
mnmE	KCOM3131_01031	mnmE
mnmE	KCOM3131_01986	mnmE
mnmE	KCOM3131_01032	mnmE
mnmE	KCTC12974_02225	mnmE
mnmE	KCTC12974_01791	mnmE
mnmE	KCTC12974_02226	mnmE
mnmE	KL-A_02764	mnmE
mnmE	KL-A_01919	mnmE
mnmE	KL-A_02761	mnmE
mnmE	L12_00289	mnmE
mnmE	LL0412.1.7_00930	mnmE
mnmE	LL0412.1.7_02150	mnmE
mnmE	LL0412.1.7_00932	mnmE
mnmE	MAR_2009_71_00524	mnmE
mnmE	MAR_2009_71_02587	mnmE
mnmE	MAR_2009_71_00526	mnmE
mnmE	NN016038_00403	mnmE
mnmE	NN016038_02654	mnmE
mnmE	NN016038_00406	mnmE
mnmE	PAO1_03923	mnmE
mnmE	PAO1_05750	mnmE
mnmE	PAO1_00796	mnmE
mnmE	UW101_01962	mnmE
mnmE	UW101_02825	mnmE
mnmE	UW101_01961	mnmE
mnmE	V4-33_01872	mnmE
mnmE	V4-33_00602	mnmE
mnmE	V4-33_01873	mnmE
mnmE	W5C_01358	mnmE
mnmE	W5C_01803	mnmE
mnmE	W5C_01361	mnmE
mnmE	ZONMW-01_03244	mnmE
mnmE	ZONMW-01_00530	mnmE
mnmE	ZONMW-01_03241	mnmE
mnmE	ZONMW-02_00438	mnmE
mnmE	ZONMW-02_01419	mnmE
mnmE	ZONMW-02_00436	mnmE
mnmE	ZONMW-03_03206	mnmE
mnmE	ZONMW-03_02424	mnmE
mnmE	ZONMW-03_03203	mnmE
mnmE	ZONMW-04_03163	mnmE
mnmE	ZONMW-04_00905	mnmE
mnmE	ZONMW-04_03166	mnmE
mnmE	ZONMW-05_03171	mnmE
mnmE	ZONMW-05_00157	mnmE
mnmE	ZONMW-05_03168	mnmE
mnmE	ZONMW-06_02477	mnmE

mnmE	ZONMW-06_01261	mnmE
mnmE	ZONMW-06_02874	mnmE
mnmE	ZONMW-07_03557	mnmE
mnmE	ZONMW-07_02657	mnmE
mnmE	ZONMW-07_03561	mnmE
mnmE	ZONMW-08_00040	mnmE
mnmE	ZONMW-08_03590	mnmE
mnmE	ZONMW-08_00042	mnmE
mnmE	ZONMW-09_02508	mnmE
mnmE	ZONMW-09_01522	mnmE
mnmE	ZONMW-09_01679	mnmE
mnmE	ZONMW-10_01172	mnmE
mnmE	ZONMW-10_02509	mnmE
mnmE	ZONMW-10_01171	mnmE
mnmE	ZONMW-11_01328	mnmE
mnmE	ZONMW-11_03582	mnmE
mnmE	ZONMW-11_01327	mnmE
mnmE	ZONMW-12_01990	mnmE
mnmE	ZONMW-12_01992	mnmE
mnmE	ZONMW-12_03735	mnmE
mnmE	ZONMW-13_00058	mnmE
mnmE	ZONMW-13_00059	mnmE
mnmE	ZONMW-13_03115	mnmE
mnmE	ZONMW-14_02453	mnmE
mnmE	ZONMW-14_01957	mnmE
mnmE	ZONMW-14_02454	mnmE
mnmE	ZONMW-15_03163	mnmE
mnmE	ZONMW-15_02906	mnmE
mnmE	ZONMW-15_03162	mnmE
mnmE	ZONMW-19_02448	mnmE
mnmE	ZONMW-19_03333	mnmE
mnmE	ZONMW-19_00373	mnmE
mnmE	ZONMW-20_03023	mnmE
mnmE	ZONMW-20_00674	mnmE
mnmE	ZONMW-20_02971	mnmE
mnmE	ZONMW-21_01407	mnmE
mnmE	ZONMW-21_02169	mnmE
mnmE	ZONMW-21_01405	mnmE
mnmE	ZONMW-22_01409	mnmE
mnmE	ZONMW-22_01556	mnmE
mnmE	ZONMW-22_01407	mnmE
mnmE	ZONMW-23_02522	mnmE
mnmE	ZONMW-23_00995	mnmE
mnmE	ZONMW-23_02520	mnmE
mnmE	ZONMW-24_00052	mnmE
mnmE	ZONMW-24_01580	mnmE
mnmE	ZONMW-24_00054	mnmE
mnmE	ZONMW-25_03114	mnmE

mnmE	ZONMW-25_00364	mnmE
mnmE	ZONMW-25_03112	mnmE
mnmE	ZONMW-26_02240	mnmE
mnmE	ZONMW-26_00800	mnmE
mnmE	ZONMW-26_02236	mnmE
mnmE	ZONMW-27_02342	mnmE
mnmE	ZONMW-27_00980	mnmE
mnmE	ZONMW-27_02339	mnmE
mnmE	ZONMW-28_00154	mnmE
mnmE	ZONMW-28_01749	mnmE
mnmE	ZONMW-28_02033	mnmE
mnmE	ZONMW-29_00961	mnmE
mnmE	ZONMW-29_01379	mnmE
mnmE	ZONMW-29_00959	mnmE
mnmE	ZONMW-30_00684	mnmE
mnmE	ZONMW-30_03299	mnmE
mnmE	ZONMW-30_00682	mnmE
mnmE	ZONMW-31_02800	mnmE
mnmE	ZONMW-31_00050	mnmE
mnmE	ZONMW-31_02982	mnmE
mnmE	ZONMW-32_00945	mnmE
mnmE	ZONMW-32_02223	mnmE
mnmE	ZONMW-32_02438	mnmE
mnmE	ZONMW-33_00935	mnmE
mnmE	ZONMW-33_02214	mnmE
mnmE	ZONMW-33_02680	mnmE
mnmE	ZONMW-34_01142	mnmE
mnmE	ZONMW-34_02367	mnmE
mnmE	ZONMW-34_01973	mnmE
mnmE	ZONMW-35_00215	mnmE
mnmE	ZONMW-35_01882	mnmE
mnmE	ZONMW-35_01665	mnmE
mnmE	ZONMW-36_00154	mnmE
mnmE	ZONMW-36_02022	mnmE
mnmE	ZONMW-36_02572	mnmE
mnmE	ZONMW-37_00955	mnmE
mnmE	ZONMW-37_01579	mnmE
mnmE	ZONMW-37_00957	mnmE
mnmE	ZONMW-38_02436	mnmE
mnmE	ZONMW-38_01352	mnmE
mnmE	ZONMW-38_02744	mnmE
mnmE	ZONMW-39_00863	mnmE
mnmE	ZONMW-39_01659	mnmE
mnmE	ZONMW-39_02219	mnmE
mnmE	ZONMW-40_02777	mnmE
mnmE	ZONMW-40_00511	mnmE
mnmE	ZONMW-40_02775	mnmE
mnmE	ZONMW-41_01385	mnmE

mnmE	ZONMW-41_00392	mnmE
mnmE	ZONMW-41_00472	mnmE
mnmE	ZONMW-42_02700	mnmE
mnmE	ZONMW-42_02750	mnmE
mnmE	ZONMW-42_00446	mnmE
mnmE	ZONMW-43_03159	mnmE
mnmE	ZONMW-43_01865	mnmE
mnmE	ZONMW-43_01477	mnmE
mnmE	ZONMW-44_03353	mnmE
mnmE	ZONMW-44_02468	mnmE
mnmE	ZONMW-44_00851	mnmE
mnmE	ZONMW-45_01145	mnmE
mnmE	ZONMW-45_03236	mnmE
mnmE	ZONMW-45_01147	mnmE
mnmE	ZONMW-46_00522	mnmE
mnmE	ZONMW-46_03251	mnmE
mnmE	ZONMW-46_00520	mnmE
mnmE	ZONMW-47_00522	mnmE
mnmE	ZONMW-47_03243	mnmE
mnmE	ZONMW-47_00520	mnmE
mnmE	ZONMW-48_00520	mnmE
mnmE	ZONMW-48_01688	mnmE
mnmE	ZONMW-48_00516	mnmE
mnmE	ZONMW-49_02379	mnmE
mnmE	ZONMW-49_02951	mnmE
mnmE	ZONMW-49_02376	mnmE
mnmE	ZONMW-50_02081	mnmE
mnmE	ZONMW-50_02904	mnmE
mnmE	ZONMW-50_02084	mnmE
mnmE	ZONMW-51_01575	mnmE
mnmE	ZONMW-51_01970	mnmE
mnmE	ZONMW-51_01578	mnmE
mnmE	ZONMW-52_01718	mnmE
mnmE	ZONMW-52_00802	mnmE
mnmE	ZONMW-52_01721	mnmE
mnmE	ZONMW-53_02040	mnmE
mnmE	ZONMW-53_01536	mnmE
mnmE	ZONMW-53_02037	mnmE
mnmE	ZONMW-54_02019	mnmE
mnmE	ZONMW-54_01184	mnmE
mnmE	ZONMW-54_02022	mnmE
mnmE	ZONMW-55_01897	mnmE
mnmE	ZONMW-55_02292	mnmE
mnmE	ZONMW-55_01900	mnmE
mnmE	ZONMW-56_01538	mnmE
mnmE	ZONMW-56_02064	mnmE
mnmE	ZONMW-56_01535	mnmE
mnmE	ZONMW-57_01662	mnmE

mnmE	ZONMW-57_00152	mnmE
mnmE	ZONMW-57_01665	mnmE
mnmE	ZONMW-58_01686	mnmE
mnmE	ZONMW-58_00819	mnmE
mnmE	ZONMW-58_01689	mnmE
mnmE	ZONMW-59_01525	mnmE
mnmE	ZONMW-59_00145	mnmE
mnmE	ZONMW-59_01528	mnmE
mnmE	ZONMW-60_01877	mnmE
mnmE	ZONMW-60_00178	mnmE
mnmE	ZONMW-60_01880	mnmE
mnmE	ZONMW-61_01352	mnmE
mnmE	ZONMW-61_03806	mnmE
mnmE	ZONMW-61_01353	mnmE
mnmE	ZONMW-62_01154	mnmE
mnmE	ZONMW-62_00954	mnmE
mnmE	ZONMW-62_01157	mnmE
mnmE	ZONMW-63_01398	mnmE
mnmE	ZONMW-63_02549	mnmE
mnmE	ZONMW-63_01401	mnmE
mnmE	ZONMW-64_02329	mnmE
mnmE	ZONMW-64_01614	mnmE
mnmE	ZONMW-64_02054	mnmE
mnmE	ZONMW-65_01698	mnmE
mnmE	ZONMW-65_02365	mnmE
mnmE	ZONMW-65_01767	mnmE
mnmE	ZONMW-66_00682	mnmE
mnmE	ZONMW-66_02370	mnmE
mnmE	ZONMW-66_00138	mnmE
mnmE	ZONMW-67_01943	mnmE
mnmE	ZONMW-67_00148	mnmE
mnmE	ZONMW-67_02372	mnmE
mnmE	ZONMW-68_04086	mnmE
mnmE	ZONMW-68_01288	mnmE
mnmE	ZONMW-68_04084	mnmE
mnmE	ZONMW-69_02000	mnmE
mnmE	ZONMW-69_02757	mnmE
mnmE	ZONMW-69_02420	mnmE
mnmE	ZONMW-72_02435	mnmE
mnmE	ZONMW-72_04024	mnmE
mnmE	ZONMW-72_02437	mnmE
mnmE	ZONMW-73_01321	mnmE
mnmE	ZONMW-73_03417	mnmE
mnmE	ZONMW-73_01323	mnmE
mnmE	ZONMW-74_03181	mnmE
mnmE	ZONMW-74_03179	mnmE
mnmE	ZONMW-74_00492	mnmE
mnmE	ZONMW-75_01348	mnmE

mnmE	ZONMW-75_01346	mnmE
mnmE	ZONMW-75_03021	mnmE
mnmE	da5A-8_01684	mnmE
mnmE	da5A-8_03094	mnmE
mnmE	da5A-8_01682	mnmE
bshA_2	18_00698	bshA_2
bshA_2	18_02468	bshA_2
bshA_2	18_03291	bshA_2
bshA_2	ACAM48_03063	bshA_2
bshA_2	ACAM48_03062	bshA_2
bshA_2	ACAM48_02809	bshA_2
bshA_2	ACAM48_00813	bshA_2
bshA_2	ACAM48_00092	bshA_2
bshA_2	ACAM48_03056	bshA_2
bshA_2	ACAM48_03055	bshA_2
bshA_2	ACAM48_00094	bshA_2
bshA_2	ACAM48_03033	bshA_2
bshA_2	ACAM48_03462	bshA_2
bshA_2	ACAM48_03060	bshA_2
bshA_2	ACAM48_00091	bshA_2
bshA_2	ACAM48_03041	bshA_2
bshA_2	ACAM48_00927	bshA_2
bshA_2	ACAM48_00929	bshA_2
bshA_2	ACAM48_01761	bshA_2
bshA_2	CAM030_01711	bshA_2
bshA_2	CAM030_00717	bshA_2
bshA_2	CAM030_01709	bshA_2
bshA_2	CAM030_02153	bshA_2
bshA_2	CAM030_01703	bshA_2
bshA_2	CAM030_01694	bshA_2
bshA_2	CAM030_01708	bshA_2
bshA_2	CECT7946_01381	bshA_2
bshA_2	CECT7946_03620	bshA_2
bshA_2	CECT7946_02293	bshA_2
bshA_2	CECT7946_03610	bshA_2
bshA_2	CECT7946_00837	bshA_2
bshA_2	CECT7946_03613	bshA_2
bshA_2	CECT7946_01376	bshA_2
bshA_2	CECT7946_01382	bshA_2
bshA_2	CECT7946_03653	bshA_2
bshA_2	CECT7946_00847	bshA_2
bshA_2	CECT7946_01384	bshA_2
bshA_2	CECT7946_00158	bshA_2
bshA_2	CECT7946_03616	bshA_2
bshA_2	CECT7946_03617	bshA_2
bshA_2	CECT7946_00845	bshA_2
bshA_2	CECT7946_01752	bshA_2
bshA_2	CGMCC1.8863_03557	bshA_2

bshA_2	CGMCC1.8863_00668	bshA_2
bshA_2	CGMCC1.8863_03551	bshA_2
bshA_2	CGMCC1.8863_01520	bshA_2
bshA_2	CGMCC1.8863_01523	bshA_2
bshA_2	CGMCC1.8863_01517	bshA_2
bshA_2	CGMCC1.8863_01521	bshA_2
bshA_2	CGMCC1.8863_03552	bshA_2
bshA_2	CGMCC1.8863_00801	bshA_2
bshA_2	CGMCC1.8863_03556	bshA_2
bshA_2	CL8139_02798	bshA_2
bshA_2	CL8139_01838	bshA_2
bshA_2	CL8139_02876	bshA_2
bshA_2	DAU203_02784	bshA_2
bshA_2	DAU203_02807	bshA_2
bshA_2	DAU203_00080	bshA_2
bshA_2	DAU203_02810	bshA_2
bshA_2	DAU203_02811	bshA_2
bshA_2	DAU203_02804	bshA_2
bshA_2	DAU203_02813	bshA_2
bshA_2	DD5b_00189	bshA_2
bshA_2	DD5b_01004	bshA_2
bshA_2	DD5b_00029	bshA_2
bshA_2	DD5b_00197	bshA_2
bshA_2	DD5b_00191	bshA_2
bshA_2	DD5b_02135	bshA_2
bshA_2	DD5b_00733	bshA_2
bshA_2	DD5b_01711	bshA_2
bshA_2	DD5b_01345	bshA_2
bshA_2	DD5b_01706	bshA_2
bshA_2	DD5b_00192	bshA_2
bshA_2	DD5b_01707	bshA_2
bshA_2	DD5b_02341	bshA_2
bshA_2	DSM1208_03598	bshA_2
bshA_2	DSM1208_03618	bshA_2
bshA_2	DSM1208_03612	bshA_2
bshA_2	DSM1208_01912	bshA_2
bshA_2	DSM1208_04187	bshA_2
bshA_2	DSM1208_01909	bshA_2
bshA_2	DSM1208_03611	bshA_2
bshA_2	DSM1208_03614	bshA_2
bshA_2	DSM1208_01078	bshA_2
bshA_2	DSM1208_01079	bshA_2
bshA_2	DSM1208_01072	bshA_2
bshA_2	DSM12212_00355	bshA_2
bshA_2	DSM12212_00365	bshA_2
bshA_2	DSM12212_00572	bshA_2
bshA_2	DSM12212_00359	bshA_2
bshA_2	DSM12212_00361	bshA_2

bshA_2	DSM12212_00744	bshA_2
bshA_2	DSM12212_02689	bshA_2
bshA_2	DSM12212_01006	bshA_2
bshA_2	DSM12212_00095	bshA_2
bshA_2	DSM12212_00343	bshA_2
bshA_2	DSM12212_00742	bshA_2
bshA_2	DSM12212_00353	bshA_2
bshA_2	DSM12212_00743	bshA_2
bshA_2	DSM12212_00349	bshA_2
bshA_2	DSM12212_00348	bshA_2
bshA_2	DSM12212_00356	bshA_2
bshA_2	DSM12212_00740	bshA_2
bshA_2	DSM12212_00362	bshA_2
bshA_2	DSM12212_00358	bshA_2
bshA_2	DSM15343_01653	bshA_2
bshA_2	DSM15343_00152	bshA_2
bshA_2	DSM15343_01646	bshA_2
bshA_2	DSM15343_01041	bshA_2
bshA_2	DSM15343_01620	bshA_2
bshA_2	DSM15343_02884	bshA_2
bshA_2	DSM15343_01888	bshA_2
bshA_2	DSM15343_01651	bshA_2
bshA_2	DSM15343_02598	bshA_2
bshA_2	DSM15343_01650	bshA_2
bshA_2	DSM15361_02180	bshA_2
bshA_2	DSM15361_02186	bshA_2
bshA_2	DSM15361_00095	bshA_2
bshA_2	DSM15361_02176	bshA_2
bshA_2	DSM15361_00842	bshA_2
bshA_2	DSM15361_00544	bshA_2
bshA_2	DSM15361_00092	bshA_2
bshA_2	DSM15361_00096	bshA_2
bshA_2	DSM15361_02191	bshA_2
bshA_2	DSM15361_00867	bshA_2
bshA_2	DSM15361_02184	bshA_2
bshA_2	DSM15361_02190	bshA_2
bshA_2	DSM15361_02193	bshA_2
bshA_2	DSM15361_00105	bshA_2
bshA_2	DSM15361_02178	bshA_2
bshA_2	DSM15365_01952	bshA_2
bshA_2	DSM15365_00659	bshA_2
bshA_2	DSM15365_03591	bshA_2
bshA_2	DSM15365_01883	bshA_2
bshA_2	DSM15365_01322	bshA_2
bshA_2	DSM15365_01885	bshA_2
bshA_2	DSM15365_03111	bshA_2
bshA_2	DSM15365_01320	bshA_2
bshA_2	DSM15365_01321	bshA_2

bshA_2	DSM15365_00687	bshA_2
bshA_2	DSM15365_00669	bshA_2
bshA_2	DSM15365_03592	bshA_2
bshA_2	DSM15365_00663	bshA_2
bshA_2	DSM15365_01876	bshA_2
bshA_2	DSM15365_00714	bshA_2
bshA_2	DSM15365_00346	bshA_2
bshA_2	DSM15365_00664	bshA_2
bshA_2	DSM15365_00348	bshA_2
bshA_2	DSM15365_00347	bshA_2
bshA_2	DSM15365_03035	bshA_2
bshA_2	DSM15718_00976	bshA_2
bshA_2	DSM15718_02593	bshA_2
bshA_2	DSM15718_00755	bshA_2
bshA_2	DSM15718_00361	bshA_2
bshA_2	DSM15718_00720	bshA_2
bshA_2	DSM15718_00764	bshA_2
bshA_2	DSM15718_00702	bshA_2
bshA_2	DSM15718_01162	bshA_2
bshA_2	DSM15718_00825	bshA_2
bshA_2	DSM15718_00759	bshA_2
bshA_2	DSM16505T_01400	bshA_2
bshA_2	DSM16505T_02615	bshA_2
bshA_2	DSM18103_00898	bshA_2
bshA_2	DSM18103_03655	bshA_2
bshA_2	DSM18103_01159	bshA_2
bshA_2	DSM18103_02036	bshA_2
bshA_2	DSM18103_02038	bshA_2
bshA_2	DSM18293_01048	bshA_2
bshA_2	DSM18293_02476	bshA_2
bshA_2	DSM18293_02496	bshA_2
bshA_2	DSM18293_02492	bshA_2
bshA_2	DSM18293_02510	bshA_2
bshA_2	DSM18293_02507	bshA_2
bshA_2	DSM18293_02470	bshA_2
bshA_2	DSM18293_02515	bshA_2
bshA_2	DSM18293_02587	bshA_2
bshA_2	DSM18293_03684	bshA_2
bshA_2	DSM18293_03063	bshA_2
bshA_2	DSM18293_01542	bshA_2
bshA_2	DSM18293_03686	bshA_2
bshA_2	DSM18293_02514	bshA_2
bshA_2	DSM18841_02414	bshA_2
bshA_2	DSM18841_02746	bshA_2
bshA_2	DSM18841_02747	bshA_2
bshA_2	DSM18841_00581	bshA_2
bshA_2	DSM18842_02675	bshA_2
bshA_2	DSM18842_01329	bshA_2

bshA_2	DSM18842_02676	bshA_2
bshA_2	DSM18842_02679	bshA_2
bshA_2	DSM18842_00647	bshA_2
bshA_2	DSM19866_04535	bshA_2
bshA_2	DSM19866_04526	bshA_2
bshA_2	DSM19866_04501	bshA_2
bshA_2	DSM19866_04506	bshA_2
bshA_2	DSM19866_01388	bshA_2
bshA_2	DSM19866_04508	bshA_2
bshA_2	DSM19866_04511	bshA_2
bshA_2	DSM19866_04509	bshA_2
bshA_2	DSM19866_04520	bshA_2
bshA_2	DSM19866_01386	bshA_2
bshA_2	DSM2041_01502	bshA_2
bshA_2	DSM2041_05003	bshA_2
bshA_2	DSM2041_02633	bshA_2
bshA_2	DSM2041_01949	bshA_2
bshA_2	DSM2041_03740	bshA_2
bshA_2	DSM2041_00197	bshA_2
bshA_2	DSM2041_01953	bshA_2
bshA_2	DSM2041_01944	bshA_2
bshA_2	DSM2041_00194	bshA_2
bshA_2	DSM2041_00202	bshA_2
bshA_2	DSM2041_01954	bshA_2
bshA_2	DSM2041_02405	bshA_2
bshA_2	DSM2041_02406	bshA_2
bshA_2	DSM2041_02629	bshA_2
bshA_2	DSM2061_01462	bshA_2
bshA_2	DSM2061_01438	bshA_2
bshA_2	DSM2061_01442	bshA_2
bshA_2	DSM2061_03463	bshA_2
bshA_2	DSM2061_03464	bshA_2
bshA_2	DSM2061_01448	bshA_2
bshA_2	DSM2061_01449	bshA_2
bshA_2	DSM2061_04284	bshA_2
bshA_2	DSM2061_02592	bshA_2
bshA_2	DSM2061_02594	bshA_2
bshA_2	DSM2064_04115	bshA_2
bshA_2	DSM2064_04781	bshA_2
bshA_2	DSM2064_02032	bshA_2
bshA_2	DSM2064_04788	bshA_2
bshA_2	DSM2064_03174	bshA_2
bshA_2	DSM2064_04868	bshA_2
bshA_2	DSM2064_03447	bshA_2
bshA_2	DSM2064_04739	bshA_2
bshA_2	DSM2064_04772	bshA_2
bshA_2	DSM2064_02033	bshA_2
bshA_2	DSM2064_04787	bshA_2

bshA_2	DSM2064_04754	bshA_2
bshA_2	DSM2064_03170	bshA_2
bshA_2	DSM2064_04740	bshA_2
bshA_2	DSM21164_02143	bshA_2
bshA_2	DSM21164_01747	bshA_2
bshA_2	DSM21164_02139	bshA_2
bshA_2	DSM21164_02138	bshA_2
bshA_2	DSM23404_00310	bshA_2
bshA_2	DSM23404_00309	bshA_2
bshA_2	DSM23404_00314	bshA_2
bshA_2	DSM23404_02509	bshA_2
bshA_2	DSM23404_01405	bshA_2
bshA_2	DSM23404_00339	bshA_2
bshA_2	DSM23404_01403	bshA_2
bshA_2	DSM23404_01406	bshA_2
bshA_2	DSM23404_00192	bshA_2
bshA_2	DSM23404_00688	bshA_2
bshA_2	DSM23404_03169	bshA_2
bshA_2	DSM23404_01395	bshA_2
bshA_2	DSM23404_00312	bshA_2
bshA_2	DSM23404_02713	bshA_2
bshA_2	DSM23404_01415	bshA_2
bshA_2	DSM23404_01011	bshA_2
bshA_2	DSM23404_00329	bshA_2
bshA_2	DSM23424_02717	bshA_2
bshA_2	DSM23424_01038	bshA_2
bshA_2	DSM23424_02711	bshA_2
bshA_2	DSM23424_01085	bshA_2
bshA_2	DSM23424_02258	bshA_2
bshA_2	DSM23424_02715	bshA_2
bshA_2	DSM23424_03153	bshA_2
bshA_2	DSM23424_00320	bshA_2
bshA_2	DSM23424_02714	bshA_2
bshA_2	DSM23424_01857	bshA_2
bshA_2	DSM24786_00242	bshA_2
bshA_2	DSM24786_00261	bshA_2
bshA_2	DSM24786_00269	bshA_2
bshA_2	DSM24786_00270	bshA_2
bshA_2	DSM7489_02656	bshA_2
bshA_2	DSW1_02438	bshA_2
bshA_2	DSW1_01564	bshA_2
bshA_2	DSW1_01521	bshA_2
bshA_2	DSW1_01529	bshA_2
bshA_2	DSW1_00647	bshA_2
bshA_2	DSW1_01534	bshA_2
bshA_2	DSW1_01536	bshA_2
bshA_2	DSW1_01533	bshA_2
bshA_2	DSW1_01674	bshA_2

bshA_2	DSW1_02185	bshA_2
bshA_2	DSW5_02199	bshA_2
bshA_2	DSW5_01434	bshA_2
bshA_2	DSW5_01773	bshA_2
bshA_2	DSW5_00843	bshA_2
bshA_2	DSW5_00227	bshA_2
bshA_2	DSW6_00087	bshA_2
bshA_2	DSW6_02993	bshA_2
bshA_2	DSW6_02247	bshA_2
bshA_2	DSW6_01406	bshA_2
bshA_2	DSW6_02242	bshA_2
bshA_2	DSW6_02246	bshA_2
bshA_2	DSW6_00372	bshA_2
bshA_2	DSW8_00901	bshA_2
bshA_2	DSW8_02250	bshA_2
bshA_2	DSW8_00888	bshA_2
bshA_2	DSW8_02251	bshA_2
bshA_2	DSW8_01810	bshA_2
bshA_2	DSW8_01816	bshA_2
bshA_2	DSW8_02253	bshA_2
bshA_2	DSW8_01815	bshA_2
bshA_2	F52_04269	bshA_2
bshA_2	F52_00488	bshA_2
bshA_2	F52_01675	bshA_2
bshA_2	F52_01594	bshA_2
bshA_2	F52_01599	bshA_2
bshA_2	F52_01600	bshA_2
bshA_2	F52_00224	bshA_2
bshA_2	F52_00492	bshA_2
bshA_2	F52_01561	bshA_2
bshA_2	F52_02430	bshA_2
bshA_2	GCA_001027945.1_01940	bshA_2
bshA_2	GCA_001027945.1_00808	bshA_2
bshA_2	HI1_02590	bshA_2
bshA_2	HI1_01323	bshA_2
bshA_2	HI1_02616	bshA_2
bshA_2	HI1_02619	bshA_2
bshA_2	HI1_01321	bshA_2
bshA_2	HI1_02610	bshA_2
bshA_2	IR1_03804	bshA_2
bshA_2	IR1_02994	bshA_2
bshA_2	IR1_02991	bshA_2
bshA_2	IR1_02986	bshA_2
bshA_2	IR1_02900	bshA_2
bshA_2	IR1_04707	bshA_2
bshA_2	IR1_02635	bshA_2
bshA_2	IR1_02987	bshA_2
bshA_2	KCOM3131_01682	bshA_2

bshA_2	KCOM3131_01779	bshA_2
bshA_2	KCOM3131_01437	bshA_2
bshA_2	KCOM3131_01121	bshA_2
bshA_2	KCOM3131_01786	bshA_2
bshA_2	KCOM3131_01683	bshA_2
bshA_2	KCTC12974_02523	bshA_2
bshA_2	KCTC12974_02522	bshA_2
bshA_2	KCTC12974_01732	bshA_2
bshA_2	KCTC12974_02529	bshA_2
bshA_2	KCTC12974_02407	bshA_2
bshA_2	KCTC12974_00103	bshA_2
bshA_2	KCTC12974_00104	bshA_2
bshA_2	KCTC12974_01433	bshA_2
bshA_2	KCTC12974_00842	bshA_2
bshA_2	KCTC12974_02530	bshA_2
bshA_2	KCTC12974_02409	bshA_2
bshA_2	KCTC12974_00093	bshA_2
bshA_2	KCTC12974_02525	bshA_2
bshA_2	KCTC12974_00106	bshA_2
bshA_2	KCTC12974_00857	bshA_2
bshA_2	KCTC12974_02543	bshA_2
bshA_2	KCTC12974_02411	bshA_2
bshA_2	KL-A_01405	bshA_2
bshA_2	KL-A_01378	bshA_2
bshA_2	KL-A_01382	bshA_2
bshA_2	KL-A_01385	bshA_2
bshA_2	KL-A_01377	bshA_2
bshA_2	KL-A_01374	bshA_2
bshA_2	KL-A_00843	bshA_2
bshA_2	KL-A_01373	bshA_2
bshA_2	L12_00475	bshA_2
bshA_2	L12_00479	bshA_2
bshA_2	L12_00476	bshA_2
bshA_2	LL0412.1.7_02047	bshA_2
bshA_2	LL0412.1.7_00233	bshA_2
bshA_2	LL0412.1.7_00942	bshA_2
bshA_2	LL0412.1.7_00944	bshA_2
bshA_2	MAR_2009_71_02541	bshA_2
bshA_2	MAR_2009_71_01189	bshA_2
bshA_2	MAR_2009_71_02554	bshA_2
bshA_2	MAR_2009_71_01188	bshA_2
bshA_2	MAR_2009_71_01611	bshA_2
bshA_2	MAR_2009_71_01609	bshA_2
bshA_2	MAR_2009_71_01186	bshA_2
bshA_2	MAR_2009_71_01607	bshA_2
bshA_2	MAR_2009_71_01608	bshA_2
bshA_2	MAR_2009_71_01613	bshA_2
bshA_2	NN016038_02503	bshA_2

bshA_2	NN016038_00720	bshA_2
bshA_2	NN016038_03334	bshA_2
bshA_2	NN016038_02522	bshA_2
bshA_2	NN016038_00726	bshA_2
bshA_2	NN016038_02518	bshA_2
bshA_2	NN016038_00725	bshA_2
bshA_2	PAO1_05174	bshA_2
bshA_2	PAO1_01431	bshA_2
bshA_2	PAO1_00868	bshA_2
bshA_2	PAO1_01425	bshA_2
bshA_2	PAO1_03164	bshA_2
bshA_2	PAO1_05632	bshA_2
bshA_2	PAO1_05631	bshA_2
bshA_2	PAO1_04450	bshA_2
bshA_2	PAO1_02310	bshA_2
bshA_2	PAO1_05633	bshA_2
bshA_2	PAO1_01429	bshA_2
bshA_2	PAO1_03329	bshA_2
bshA_2	PAO1_05180	bshA_2
bshA_2	UW101_04771	bshA_2
bshA_2	UW101_00321	bshA_2
bshA_2	UW101_01087	bshA_2
bshA_2	UW101_00314	bshA_2
bshA_2	UW101_02626	bshA_2
bshA_2	UW101_00234	bshA_2
bshA_2	UW101_02353	bshA_2
bshA_2	UW101_00363	bshA_2
bshA_2	UW101_00330	bshA_2
bshA_2	UW101_01088	bshA_2
bshA_2	UW101_00315	bshA_2
bshA_2	UW101_00348	bshA_2
bshA_2	UW101_02630	bshA_2
bshA_2	UW101_00362	bshA_2
bshA_2	V4-33_00736	bshA_2
bshA_2	V4-33_01161	bshA_2
bshA_2	V4-33_01171	bshA_2
bshA_2	V4-33_01155	bshA_2
bshA_2	V4-33_01538	bshA_2
bshA_2	V4-33_01913	bshA_2
bshA_2	V4-33_01156	bshA_2
bshA_2	V4-33_01774	bshA_2
bshA_2	V4-33_01546	bshA_2
bshA_2	W5C_00782	bshA_2
bshA_2	W5C_00414	bshA_2
bshA_2	W5C_01082	bshA_2
bshA_2	ZONMW-01_01709	bshA_2
bshA_2	ZONMW-01_01726	bshA_2
bshA_2	ZONMW-01_01717	bshA_2

bshA_2	ZONMW-01_03040	bshA_2
bshA_2	ZONMW-01_01725	bshA_2
bshA_2	ZONMW-01_02376	bshA_2
bshA_2	ZONMW-02_02547	bshA_2
bshA_2	ZONMW-02_01103	bshA_2
bshA_2	ZONMW-02_03190	bshA_2
bshA_2	ZONMW-02_02619	bshA_2
bshA_2	ZONMW-02_00022	bshA_2
bshA_2	ZONMW-02_03191	bshA_2
bshA_2	ZONMW-02_03323	bshA_2
bshA_2	ZONMW-02_01113	bshA_2
bshA_2	ZONMW-02_01107	bshA_2
bshA_2	ZONMW-02_01157	bshA_2
bshA_2	ZONMW-02_01837	bshA_2
bshA_2	ZONMW-02_01108	bshA_2
bshA_2	ZONMW-02_02935	bshA_2
bshA_2	ZONMW-03_00498	bshA_2
bshA_2	ZONMW-03_00481	bshA_2
bshA_2	ZONMW-03_00490	bshA_2
bshA_2	ZONMW-03_02275	bshA_2
bshA_2	ZONMW-03_00482	bshA_2
bshA_2	ZONMW-03_02979	bshA_2
bshA_2	ZONMW-04_01437	bshA_2
bshA_2	ZONMW-04_01420	bshA_2
bshA_2	ZONMW-04_01429	bshA_2
bshA_2	ZONMW-04_01424	bshA_2
bshA_2	ZONMW-04_01419	bshA_2
bshA_2	ZONMW-04_02458	bshA_2
bshA_2	ZONMW-04_02459	bshA_2
bshA_2	ZONMW-05_00761	bshA_2
bshA_2	ZONMW-05_00754	bshA_2
bshA_2	ZONMW-05_00009	bshA_2
bshA_2	ZONMW-05_00738	bshA_2
bshA_2	ZONMW-05_00753	bshA_2
bshA_2	ZONMW-06_02039	bshA_2
bshA_2	ZONMW-06_02025	bshA_2
bshA_2	ZONMW-06_00513	bshA_2
bshA_2	ZONMW-06_03692	bshA_2
bshA_2	ZONMW-06_03700	bshA_2
bshA_2	ZONMW-06_02036	bshA_2
bshA_2	ZONMW-06_03699	bshA_2
bshA_2	ZONMW-06_02919	bshA_2
bshA_2	ZONMW-06_01042	bshA_2
bshA_2	ZONMW-06_02001	bshA_2
bshA_2	ZONMW-07_02506	bshA_2
bshA_2	ZONMW-07_02489	bshA_2
bshA_2	ZONMW-07_02488	bshA_2
bshA_2	ZONMW-07_02486	bshA_2

bshA_2	ZONMW-07_02485	bshA_2
bshA_2	ZONMW-07_01498	bshA_2
bshA_2	ZONMW-07_01231	bshA_2
bshA_2	ZONMW-07_01232	bshA_2
bshA_2	ZONMW-07_01236	bshA_2
bshA_2	ZONMW-08_03540	bshA_2
bshA_2	ZONMW-08_03562	bshA_2
bshA_2	ZONMW-08_03556	bshA_2
bshA_2	ZONMW-08_01660	bshA_2
bshA_2	ZONMW-08_03555	bshA_2
bshA_2	ZONMW-08_03557	bshA_2
bshA_2	ZONMW-08_00845	bshA_2
bshA_2	ZONMW-08_01626	bshA_2
bshA_2	ZONMW-08_01629	bshA_2
bshA_2	ZONMW-08_01657	bshA_2
bshA_2	ZONMW-08_01628	bshA_2
bshA_2	ZONMW-09_02474	bshA_2
bshA_2	ZONMW-09_02654	bshA_2
bshA_2	ZONMW-09_01141	bshA_2
bshA_2	ZONMW-09_02280	bshA_2
bshA_2	ZONMW-09_02282	bshA_2
bshA_2	ZONMW-09_01188	bshA_2
bshA_2	ZONMW-09_02294	bshA_2
bshA_2	ZONMW-09_02477	bshA_2
bshA_2	ZONMW-09_02475	bshA_2
bshA_2	ZONMW-09_02089	bshA_2
bshA_2	ZONMW-09_02274	bshA_2
bshA_2	ZONMW-09_02554	bshA_2
bshA_2	ZONMW-09_02478	bshA_2
bshA_2	ZONMW-10_02211	bshA_2
bshA_2	ZONMW-10_02456	bshA_2
bshA_2	ZONMW-10_00381	bshA_2
bshA_2	ZONMW-10_01557	bshA_2
bshA_2	ZONMW-10_01555	bshA_2
bshA_2	ZONMW-10_01560	bshA_2
bshA_2	ZONMW-10_01558	bshA_2
bshA_2	ZONMW-10_01001	bshA_2
bshA_2	ZONMW-10_02458	bshA_2
bshA_2	ZONMW-10_01043	bshA_2
bshA_2	ZONMW-10_01568	bshA_2
bshA_2	ZONMW-11_03634	bshA_2
bshA_2	ZONMW-11_04443	bshA_2
bshA_2	ZONMW-11_01746	bshA_2
bshA_2	ZONMW-11_04041	bshA_2
bshA_2	ZONMW-11_04040	bshA_2
bshA_2	ZONMW-11_03291	bshA_2
bshA_2	ZONMW-11_01140	bshA_2
bshA_2	ZONMW-11_00705	bshA_2

bshA_2	ZONMW-11_04299	bshA_2
bshA_2	ZONMW-11_04619	bshA_2
bshA_2	ZONMW-11_01116	bshA_2
bshA_2	ZONMW-11_01142	bshA_2
bshA_2	ZONMW-11_04305	bshA_2
bshA_2	ZONMW-11_00703	bshA_2
bshA_2	ZONMW-12_03498	bshA_2
bshA_2	ZONMW-12_03497	bshA_2
bshA_2	ZONMW-12_03500	bshA_2
bshA_2	ZONMW-12_02278	bshA_2
bshA_2	ZONMW-12_03506	bshA_2
bshA_2	ZONMW-12_03934	bshA_2
bshA_2	ZONMW-12_01133	bshA_2
bshA_2	ZONMW-12_03501	bshA_2
bshA_2	ZONMW-12_01130	bshA_2
bshA_2	ZONMW-12_01132	bshA_2
bshA_2	ZONMW-13_03161	bshA_2
bshA_2	ZONMW-13_03162	bshA_2
bshA_2	ZONMW-13_02083	bshA_2
bshA_2	ZONMW-13_02764	bshA_2
bshA_2	ZONMW-13_03170	bshA_2
bshA_2	ZONMW-13_02767	bshA_2
bshA_2	ZONMW-13_02763	bshA_2
bshA_2	ZONMW-13_02766	bshA_2
bshA_2	ZONMW-13_02945	bshA_2
bshA_2	ZONMW-13_02778	bshA_2
bshA_2	ZONMW-13_02772	bshA_2
bshA_2	ZONMW-13_03164	bshA_2
bshA_2	ZONMW-13_01450	bshA_2
bshA_2	ZONMW-13_00982	bshA_2
bshA_2	ZONMW-13_02124	bshA_2
bshA_2	ZONMW-13_02841	bshA_2
bshA_2	ZONMW-13_03165	bshA_2
bshA_2	ZONMW-13_02950	bshA_2
bshA_2	ZONMW-14_01757	bshA_2
bshA_2	ZONMW-14_01756	bshA_2
bshA_2	ZONMW-14_00611	bshA_2
bshA_2	ZONMW-14_02090	bshA_2
bshA_2	ZONMW-14_02079	bshA_2
bshA_2	ZONMW-14_01722	bshA_2
bshA_2	ZONMW-14_02097	bshA_2
bshA_2	ZONMW-14_01751	bshA_2
bshA_2	ZONMW-14_01754	bshA_2
bshA_2	ZONMW-14_02779	bshA_2
bshA_2	ZONMW-14_02337	bshA_2
bshA_2	ZONMW-14_02075	bshA_2
bshA_2	ZONMW-14_02338	bshA_2
bshA_2	ZONMW-14_02000	bshA_2

bshA_2	ZONMW-14_02092	bshA_2
bshA_2	ZONMW-14_01753	bshA_2
bshA_2	ZONMW-14_02783	bshA_2
bshA_2	ZONMW-14_02782	bshA_2
bshA_2	ZONMW-15_01409	bshA_2
bshA_2	ZONMW-15_01422	bshA_2
bshA_2	ZONMW-15_03341	bshA_2
bshA_2	ZONMW-15_02467	bshA_2
bshA_2	ZONMW-15_01414	bshA_2
bshA_2	ZONMW-19_02293	bshA_2
bshA_2	ZONMW-19_02292	bshA_2
bshA_2	ZONMW-19_01984	bshA_2
bshA_2	ZONMW-19_02290	bshA_2
bshA_2	ZONMW-19_01793	bshA_2
bshA_2	ZONMW-19_02822	bshA_2
bshA_2	ZONMW-19_02787	bshA_2
bshA_2	ZONMW-19_01989	bshA_2
bshA_2	ZONMW-19_01792	bshA_2
bshA_2	ZONMW-19_00476	bshA_2
bshA_2	ZONMW-20_00826	bshA_2
bshA_2	ZONMW-20_00814	bshA_2
bshA_2	ZONMW-20_04052	bshA_2
bshA_2	ZONMW-20_00813	bshA_2
bshA_2	ZONMW-20_04065	bshA_2
bshA_2	ZONMW-21_02220	bshA_2
bshA_2	ZONMW-21_02209	bshA_2
bshA_2	ZONMW-21_02213	bshA_2
bshA_2	ZONMW-21_02211	bshA_2
bshA_2	ZONMW-21_02200	bshA_2
bshA_2	ZONMW-22_01505	bshA_2
bshA_2	ZONMW-22_01516	bshA_2
bshA_2	ZONMW-22_01512	bshA_2
bshA_2	ZONMW-22_01514	bshA_2
bshA_2	ZONMW-22_01525	bshA_2
bshA_2	ZONMW-23_01046	bshA_2
bshA_2	ZONMW-23_01035	bshA_2
bshA_2	ZONMW-23_01039	bshA_2
bshA_2	ZONMW-23_01037	bshA_2
bshA_2	ZONMW-23_01026	bshA_2
bshA_2	ZONMW-24_01529	bshA_2
bshA_2	ZONMW-24_01540	bshA_2
bshA_2	ZONMW-24_01536	bshA_2
bshA_2	ZONMW-24_01538	bshA_2
bshA_2	ZONMW-24_01549	bshA_2
bshA_2	ZONMW-25_01714	bshA_2
bshA_2	ZONMW-25_00302	bshA_2
bshA_2	ZONMW-26_00198	bshA_2
bshA_2	ZONMW-26_00220	bshA_2

bshA_2	ZONMW-26_00218	bshA_2
bshA_2	ZONMW-26_00951	bshA_2
bshA_2	ZONMW-26_00225	bshA_2
bshA_2	ZONMW-26_00224	bshA_2
bshA_2	ZONMW-26_00227	bshA_2
bshA_2	ZONMW-26_03291	bshA_2
bshA_2	ZONMW-27_01867	bshA_2
bshA_2	ZONMW-27_01874	bshA_2
bshA_2	ZONMW-27_00836	bshA_2
bshA_2	ZONMW-27_01890	bshA_2
bshA_2	ZONMW-27_01875	bshA_2
bshA_2	ZONMW-28_03517	bshA_2
bshA_2	ZONMW-28_02869	bshA_2
bshA_2	ZONMW-28_00925	bshA_2
bshA_2	ZONMW-28_00551	bshA_2
bshA_2	ZONMW-28_00545	bshA_2
bshA_2	ZONMW-28_03678	bshA_2
bshA_2	ZONMW-28_03673	bshA_2
bshA_2	ZONMW-28_03519	bshA_2
bshA_2	ZONMW-28_00552	bshA_2
bshA_2	ZONMW-28_02453	bshA_2
bshA_2	ZONMW-28_03679	bshA_2
bshA_2	ZONMW-28_01988	bshA_2
bshA_2	ZONMW-28_02896	bshA_2
bshA_2	ZONMW-29_01434	bshA_2
bshA_2	ZONMW-30_03074	bshA_2
bshA_2	ZONMW-31_01513	bshA_2
bshA_2	ZONMW-31_01310	bshA_2
bshA_2	ZONMW-31_01497	bshA_2
bshA_2	ZONMW-31_00866	bshA_2
bshA_2	ZONMW-31_03156	bshA_2
bshA_2	ZONMW-31_03155	bshA_2
bshA_2	ZONMW-31_03026	bshA_2
bshA_2	ZONMW-31_01298	bshA_2
bshA_2	ZONMW-31_01297	bshA_2
bshA_2	ZONMW-31_01537	bshA_2
bshA_2	ZONMW-31_01500	bshA_2
bshA_2	ZONMW-32_00602	bshA_2
bshA_2	ZONMW-32_01354	bshA_2
bshA_2	ZONMW-32_00166	bshA_2
bshA_2	ZONMW-32_01342	bshA_2
bshA_2	ZONMW-32_01336	bshA_2
bshA_2	ZONMW-32_03706	bshA_2
bshA_2	ZONMW-32_03701	bshA_2
bshA_2	ZONMW-32_00604	bshA_2
bshA_2	ZONMW-32_01343	bshA_2
bshA_2	ZONMW-32_02897	bshA_2
bshA_2	ZONMW-32_03707	bshA_2

bshA_2	ZONMW-32_02394	bshA_2
bshA_2	ZONMW-32_01381	bshA_2
bshA_2	ZONMW-33_00470	bshA_2
bshA_2	ZONMW-33_01343	bshA_2
bshA_2	ZONMW-33_00033	bshA_2
bshA_2	ZONMW-33_01331	bshA_2
bshA_2	ZONMW-33_01325	bshA_2
bshA_2	ZONMW-33_03515	bshA_2
bshA_2	ZONMW-33_03520	bshA_2
bshA_2	ZONMW-33_00472	bshA_2
bshA_2	ZONMW-33_01332	bshA_2
bshA_2	ZONMW-33_02851	bshA_2
bshA_2	ZONMW-33_03514	bshA_2
bshA_2	ZONMW-33_02724	bshA_2
bshA_2	ZONMW-33_01370	bshA_2
bshA_2	ZONMW-34_01528	bshA_2
bshA_2	ZONMW-34_01515	bshA_2
bshA_2	ZONMW-34_00436	bshA_2
bshA_2	ZONMW-34_03869	bshA_2
bshA_2	ZONMW-34_03864	bshA_2
bshA_2	ZONMW-34_03863	bshA_2
bshA_2	ZONMW-34_03613	bshA_2
bshA_2	ZONMW-34_02018	bshA_2
bshA_2	ZONMW-34_01556	bshA_2
bshA_2	ZONMW-34_01517	bshA_2
bshA_2	ZONMW-35_00601	bshA_2
bshA_2	ZONMW-35_00588	bshA_2
bshA_2	ZONMW-35_00954	bshA_2
bshA_2	ZONMW-35_03862	bshA_2
bshA_2	ZONMW-35_03867	bshA_2
bshA_2	ZONMW-35_03868	bshA_2
bshA_2	ZONMW-35_03551	bshA_2
bshA_2	ZONMW-35_01710	bshA_2
bshA_2	ZONMW-35_00629	bshA_2
bshA_2	ZONMW-35_00590	bshA_2
bshA_2	ZONMW-36_00901	bshA_2
bshA_2	ZONMW-36_00563	bshA_2
bshA_2	ZONMW-36_01336	bshA_2
bshA_2	ZONMW-36_00551	bshA_2
bshA_2	ZONMW-36_00545	bshA_2
bshA_2	ZONMW-36_03463	bshA_2
bshA_2	ZONMW-36_03458	bshA_2
bshA_2	ZONMW-36_00899	bshA_2
bshA_2	ZONMW-36_00552	bshA_2
bshA_2	ZONMW-36_02363	bshA_2
bshA_2	ZONMW-36_03464	bshA_2
bshA_2	ZONMW-36_02528	bshA_2
bshA_2	ZONMW-36_00590	bshA_2

bshA_2	ZONMW-37_01528	bshA_2
bshA_2	ZONMW-37_01539	bshA_2
bshA_2	ZONMW-37_01535	bshA_2
bshA_2	ZONMW-37_01537	bshA_2
bshA_2	ZONMW-37_01548	bshA_2
bshA_2	ZONMW-38_03429	bshA_2
bshA_2	ZONMW-38_00909	bshA_2
bshA_2	ZONMW-38_00910	bshA_2
bshA_2	ZONMW-38_00907	bshA_2
bshA_2	ZONMW-38_00908	bshA_2
bshA_2	ZONMW-39_01314	bshA_2
bshA_2	ZONMW-39_00170	bshA_2
bshA_2	ZONMW-39_03140	bshA_2
bshA_2	ZONMW-39_00183	bshA_2
bshA_2	ZONMW-39_03808	bshA_2
bshA_2	ZONMW-39_03803	bshA_2
bshA_2	ZONMW-39_03802	bshA_2
bshA_2	ZONMW-39_02174	bshA_2
bshA_2	ZONMW-39_00141	bshA_2
bshA_2	ZONMW-39_00181	bshA_2
bshA_2	ZONMW-40_00653	bshA_2
bshA_2	ZONMW-40_00419	bshA_2
bshA_2	ZONMW-41_00833	bshA_2
bshA_2	ZONMW-41_01877	bshA_2
bshA_2	ZONMW-41_01685	bshA_2
bshA_2	ZONMW-41_01690	bshA_2
bshA_2	ZONMW-42_04050	bshA_2
bshA_2	ZONMW-42_04048	bshA_2
bshA_2	ZONMW-42_04133	bshA_2
bshA_2	ZONMW-42_02296	bshA_2
bshA_2	ZONMW-42_02295	bshA_2
bshA_2	ZONMW-42_04109	bshA_2
bshA_2	ZONMW-42_01047	bshA_2
bshA_2	ZONMW-42_00400	bshA_2
bshA_2	ZONMW-42_01044	bshA_2
bshA_2	ZONMW-42_03346	bshA_2
bshA_2	ZONMW-43_01741	bshA_2
bshA_2	ZONMW-43_02400	bshA_2
bshA_2	ZONMW-43_00385	bshA_2
bshA_2	ZONMW-43_01740	bshA_2
bshA_2	ZONMW-43_03629	bshA_2
bshA_2	ZONMW-43_03624	bshA_2
bshA_2	ZONMW-43_03623	bshA_2
bshA_2	ZONMW-43_03562	bshA_2
bshA_2	ZONMW-43_01522	bshA_2
bshA_2	ZONMW-43_01713	bshA_2
bshA_2	ZONMW-44_02734	bshA_2
bshA_2	ZONMW-44_03954	bshA_2

bshA_2	ZONMW-44_01691	bshA_2
bshA_2	ZONMW-44_03208	bshA_2
bshA_2	ZONMW-44_02727	bshA_2
bshA_2	ZONMW-44_03200	bshA_2
bshA_2	ZONMW-44_03949	bshA_2
bshA_2	ZONMW-44_01403	bshA_2
bshA_2	ZONMW-44_01401	bshA_2
bshA_2	ZONMW-44_03201	bshA_2
bshA_2	ZONMW-44_00897	bshA_2
bshA_2	ZONMW-44_02236	bshA_2
bshA_2	ZONMW-44_03968	bshA_2
bshA_2	ZONMW-44_02759	bshA_2
bshA_2	ZONMW-44_03955	bshA_2
bshA_2	ZONMW-45_01826	bshA_2
bshA_2	ZONMW-45_02709	bshA_2
bshA_2	ZONMW-45_00729	bshA_2
bshA_2	ZONMW-46_01926	bshA_2
bshA_2	ZONMW-46_03080	bshA_2
bshA_2	ZONMW-46_00730	bshA_2
bshA_2	ZONMW-47_02135	bshA_2
bshA_2	ZONMW-47_03071	bshA_2
bshA_2	ZONMW-47_00730	bshA_2
bshA_2	ZONMW-48_01797	bshA_2
bshA_2	ZONMW-48_02957	bshA_2
bshA_2	ZONMW-48_01773	bshA_2
bshA_2	ZONMW-48_02958	bshA_2
bshA_2	ZONMW-48_00635	bshA_2
bshA_2	ZONMW-48_01770	bshA_2
bshA_2	ZONMW-48_01779	bshA_2
bshA_2	ZONMW-48_03020	bshA_2
bshA_2	ZONMW-49_00208	bshA_2
bshA_2	ZONMW-49_00236	bshA_2
bshA_2	ZONMW-49_00228	bshA_2
bshA_2	ZONMW-49_00237	bshA_2
bshA_2	ZONMW-50_00277	bshA_2
bshA_2	ZONMW-50_00249	bshA_2
bshA_2	ZONMW-50_00257	bshA_2
bshA_2	ZONMW-50_00248	bshA_2
bshA_2	ZONMW-51_00206	bshA_2
bshA_2	ZONMW-51_00220	bshA_2
bshA_2	ZONMW-51_00224	bshA_2
bshA_2	ZONMW-51_02819	bshA_2
bshA_2	ZONMW-51_00225	bshA_2
bshA_2	ZONMW-51_00216	bshA_2
bshA_2	ZONMW-52_00177	bshA_2
bshA_2	ZONMW-52_00203	bshA_2
bshA_2	ZONMW-52_02226	bshA_2
bshA_2	ZONMW-52_00206	bshA_2

bshA_2	ZONMW-52_00197	bshA_2
bshA_2	ZONMW-53_00924	bshA_2
bshA_2	ZONMW-53_02814	bshA_2
bshA_2	ZONMW-54_01799	bshA_2
bshA_2	ZONMW-54_00031	bshA_2
bshA_2	ZONMW-54_01789	bshA_2
bshA_2	ZONMW-54_01782	bshA_2
bshA_2	ZONMW-54_01781	bshA_2
bshA_2	ZONMW-54_00029	bshA_2
bshA_2	ZONMW-55_01331	bshA_2
bshA_2	ZONMW-55_01302	bshA_2
bshA_2	ZONMW-55_02822	bshA_2
bshA_2	ZONMW-55_01306	bshA_2
bshA_2	ZONMW-55_01301	bshA_2
bshA_2	ZONMW-55_01311	bshA_2
bshA_2	ZONMW-56_00558	bshA_2
bshA_2	ZONMW-56_00536	bshA_2
bshA_2	ZONMW-56_00531	bshA_2
bshA_2	ZONMW-56_00538	bshA_2
bshA_2	ZONMW-56_00529	bshA_2
bshA_2	ZONMW-56_00532	bshA_2
bshA_2	ZONMW-57_00765	bshA_2
bshA_2	ZONMW-57_02727	bshA_2
bshA_2	ZONMW-58_00206	bshA_2
bshA_2	ZONMW-58_02751	bshA_2
bshA_2	ZONMW-59_00761	bshA_2
bshA_2	ZONMW-59_00734	bshA_2
bshA_2	ZONMW-59_00741	bshA_2
bshA_2	ZONMW-59_00733	bshA_2
bshA_2	ZONMW-60_00764	bshA_2
bshA_2	ZONMW-60_02844	bshA_2
bshA_2	ZONMW-60_00754	bshA_2
bshA_2	ZONMW-60_00747	bshA_2
bshA_2	ZONMW-60_00746	bshA_2
bshA_2	ZONMW-60_02842	bshA_2
bshA_2	ZONMW-61_03547	bshA_2
bshA_2	ZONMW-61_00686	bshA_2
bshA_2	ZONMW-61_03538	bshA_2
bshA_2	ZONMW-61_00715	bshA_2
bshA_2	ZONMW-61_03537	bshA_2
bshA_2	ZONMW-61_00683	bshA_2
bshA_2	ZONMW-61_00719	bshA_2
bshA_2	ZONMW-61_00716	bshA_2
bshA_2	ZONMW-62_00997	bshA_2
bshA_2	ZONMW-62_01846	bshA_2
bshA_2	ZONMW-62_02457	bshA_2
bshA_2	ZONMW-62_00989	bshA_2
bshA_2	ZONMW-62_02456	bshA_2

bshA_2	ZONMW-62_00988	bshA_2
bshA_2	ZONMW-62_02454	bshA_2
bshA_2	ZONMW-63_00440	bshA_2
bshA_2	ZONMW-63_02347	bshA_2
bshA_2	ZONMW-64_02877	bshA_2
bshA_2	ZONMW-64_00402	bshA_2
bshA_2	ZONMW-64_00672	bshA_2
bshA_2	ZONMW-64_02860	bshA_2
bshA_2	ZONMW-64_04021	bshA_2
bshA_2	ZONMW-64_02866	bshA_2
bshA_2	ZONMW-64_04231	bshA_2
bshA_2	ZONMW-64_02867	bshA_2
bshA_2	ZONMW-64_04230	bshA_2
bshA_2	ZONMW-64_02696	bshA_2
bshA_2	ZONMW-64_00401	bshA_2
bshA_2	ZONMW-64_00403	bshA_2
bshA_2	ZONMW-64_02099	bshA_2
bshA_2	ZONMW-64_02905	bshA_2
bshA_2	ZONMW-65_01634	bshA_2
bshA_2	ZONMW-65_01618	bshA_2
bshA_2	ZONMW-65_01635	bshA_2
bshA_2	ZONMW-65_02981	bshA_2
bshA_2	ZONMW-66_01994	bshA_2
bshA_2	ZONMW-66_04625	bshA_2
bshA_2	ZONMW-66_01995	bshA_2
bshA_2	ZONMW-66_02929	bshA_2
bshA_2	ZONMW-66_01997	bshA_2
bshA_2	ZONMW-66_01923	bshA_2
bshA_2	ZONMW-66_02021	bshA_2
bshA_2	ZONMW-66_03099	bshA_2
bshA_2	ZONMW-66_02924	bshA_2
bshA_2	ZONMW-66_01924	bshA_2
bshA_2	ZONMW-66_00074	bshA_2
bshA_2	ZONMW-67_01068	bshA_2
bshA_2	ZONMW-67_01464	bshA_2
bshA_2	ZONMW-67_01475	bshA_2
bshA_2	ZONMW-67_03547	bshA_2
bshA_2	ZONMW-67_00624	bshA_2
bshA_2	ZONMW-67_03537	bshA_2
bshA_2	ZONMW-67_01067	bshA_2
bshA_2	ZONMW-67_02168	bshA_2
bshA_2	ZONMW-67_03546	bshA_2
bshA_2	ZONMW-67_01064	bshA_2
bshA_2	ZONMW-67_02418	bshA_2
bshA_2	ZONMW-67_01466	bshA_2
bshA_2	ZONMW-67_01496	bshA_2
bshA_2	ZONMW-68_01218	bshA_2
bshA_2	ZONMW-68_01227	bshA_2

bshA_2	ZONMW-68_04808	bshA_2
bshA_2	ZONMW-68_01251	bshA_2
bshA_2	ZONMW-68_01246	bshA_2
bshA_2	ZONMW-68_01244	bshA_2
bshA_2	ZONMW-68_01243	bshA_2
bshA_2	ZONMW-68_01242	bshA_2
bshA_2	ZONMW-68_04266	bshA_2
bshA_2	ZONMW-68_01233	bshA_2
bshA_2	ZONMW-69_01115	bshA_2
bshA_2	ZONMW-69_01536	bshA_2
bshA_2	ZONMW-69_01525	bshA_2
bshA_2	ZONMW-69_03549	bshA_2
bshA_2	ZONMW-69_00671	bshA_2
bshA_2	ZONMW-69_03539	bshA_2
bshA_2	ZONMW-69_01114	bshA_2
bshA_2	ZONMW-69_01775	bshA_2
bshA_2	ZONMW-69_03548	bshA_2
bshA_2	ZONMW-69_01111	bshA_2
bshA_2	ZONMW-69_02466	bshA_2
bshA_2	ZONMW-69_01534	bshA_2
bshA_2	ZONMW-69_01504	bshA_2
bshA_2	ZONMW-72_00739	bshA_2
bshA_2	ZONMW-72_01226	bshA_2
bshA_2	ZONMW-72_01229	bshA_2
bshA_2	ZONMW-72_01234	bshA_2
bshA_2	ZONMW-72_01467	bshA_2
bshA_2	ZONMW-72_03304	bshA_2
bshA_2	ZONMW-72_03383	bshA_2
bshA_2	ZONMW-72_01233	bshA_2
bshA_2	ZONMW-73_03690	bshA_2
bshA_2	ZONMW-73_00902	bshA_2
bshA_2	ZONMW-73_02198	bshA_2
bshA_2	ZONMW-73_00733	bshA_2
bshA_2	ZONMW-73_03688	bshA_2
bshA_2	ZONMW-73_03726	bshA_2
bshA_2	ZONMW-73_03727	bshA_2
bshA_2	ZONMW-73_03681	bshA_2
bshA_2	ZONMW-73_00032	bshA_2
bshA_2	ZONMW-73_00028	bshA_2
bshA_2	ZONMW-73_03687	bshA_2
bshA_2	ZONMW-73_00658	bshA_2
bshA_2	ZONMW-73_00664	bshA_2
bshA_2	ZONMW-74_03053	bshA_2
bshA_2	ZONMW-74_03074	bshA_2
bshA_2	ZONMW-74_03081	bshA_2
bshA_2	ZONMW-74_03082	bshA_2
bshA_2	ZONMW-75_01565	bshA_2
bshA_2	ZONMW-75_01959	bshA_2

bshA_2	ZONMW-75_00915	bshA_2
bshA_2	ZONMW-75_02037	bshA_2
bshA_2	ZONMW-75_02031	bshA_2
bshA_2	ZONMW-75_02394	bshA_2
bshA_2	ZONMW-75_00916	bshA_2
bshA_2	ZONMW-75_02026	bshA_2
bshA_2	ZONMW-75_03644	bshA_2
bshA_2	ZONMW-75_02034	bshA_2
bshA_2	ZONMW-75_01592	bshA_2
bshA_2	ZONMW-75_02317	bshA_2
bshA_2	ZONMW-75_01576	bshA_2
bshA_2	ZONMW-75_01569	bshA_2
bshA_2	ZONMW-75_02039	bshA_2
bshA_2	ZONMW-75_01625	bshA_2
bshA_2	ZONMW-75_00009	bshA_2
bshA_2	ZONMW-75_01570	bshA_2
bshA_2	ZONMW-75_01586	bshA_2
bshA_2	ZONMW-75_00005	bshA_2
bshA_2	ZONMW-75_00917	bshA_2
bshA_2	ZONMW-75_00010	bshA_2
bshA_2	ZONMW-75_00007	bshA_2
bshA_2	da5A-8_00977	bshA_2
bshA_2	da5A-8_01352	bshA_2
bshA_2	da5A-8_01553	bshA_2
bshA_2	da5A-8_00264	bshA_2
bshA_2	da5A-8_00258	bshA_2
rplT	18_02056	rplT
rplT	ACAM48_01442	rplT
rplT	CAM030_01792	rplT
rplT	CECT7946_02775	rplT
rplT	CGMCC1.8863_02815	rplT
rplT	CL8139_03218	rplT
rplT	DAU203_02553	rplT
rplT	DD5b_01121	rplT
rplT	DSM1208_02926	rplT
rplT	DSM12212_03455	rplT
rplT	DSM15343_02863	rplT
rplT	DSM15361_02235	rplT
rplT	DSM15365_01301	rplT
rplT	DSM15718_03370	rplT
rplT	DSM16505T_00003	rplT
rplT	DSM18103_01549	rplT
rplT	DSM18293_02790	rplT
rplT	DSM18841_02475	rplT
rplT	DSM18842_02382	rplT
rplT	DSM19866_02696	rplT
rplT	DSM2041_05499	rplT
rplT	DSM2061_04410	rplT

rplT	DSM2064_05069	rplT
rplT	DSM21164_03002	rplT
rplT	DSM23404_00004	rplT
rplT	DSM23424_03343	rplT
rplT	DSM24786_01473	rplT
rplT	DSM7489_02444	rplT
rplT	DSW1_00064	rplT
rplT	DSW5_01775	rplT
rplT	DSW6_00219	rplT
rplT	DSW8_01027	rplT
rplT	F52_01924	rplT
rplT	GCA_001027945.1_01092	rplT
rplT	HI1_02376	rplT
rplT	IR1_02716	rplT
rplT	IR1_04803	rplT
rplT	KCOM3131_01690	rplT
rplT	KCTC12974_00686	rplT
rplT	KL-A_03248	rplT
rplT	L12_00028	rplT
rplT	LL0412.1.7_00004	rplT
rplT	MAR_2009_71_02414	rplT
rplT	NN016038_02074	rplT
rplT	PAO1_02824	rplT
rplT	UW101_00033	rplT
rplT	V4-33_00807	rplT
rplT	W5C_02096	rplT
rplT	ZONMW-01_03269	rplT
rplT	ZONMW-02_00856	rplT
rplT	ZONMW-03_03399	rplT
rplT	ZONMW-04_00004	rplT
rplT	ZONMW-05_01824	rplT
rplT	ZONMW-06_03485	rplT
rplT	ZONMW-07_03778	rplT
rplT	ZONMW-08_00823	rplT
rplT	ZONMW-09_01791	rplT
rplT	ZONMW-10_01435	rplT
rplT	ZONMW-11_00948	rplT
rplT	ZONMW-12_04459	rplT
rplT	ZONMW-13_03143	rplT
rplT	ZONMW-14_01775	rplT
rplT	ZONMW-15_02039	rplT
rplT	ZONMW-19_02930	rplT
rplT	ZONMW-20_01637	rplT
rplT	ZONMW-21_03390	rplT
rplT	ZONMW-22_03335	rplT
rplT	ZONMW-23_03436	rplT
rplT	ZONMW-24_03438	rplT
rplT	ZONMW-25_03132	rplT

rplT	ZONMW-26_01463	rplT
rplT	ZONMW-27_00004	rplT
rplT	ZONMW-28_02305	rplT
rplT	ZONMW-29_02902	rplT
rplT	ZONMW-30_02871	rplT
rplT	ZONMW-31_02429	rplT
rplT	ZONMW-32_02711	rplT
rplT	ZONMW-33_02407	rplT
rplT	ZONMW-34_01729	rplT
rplT	ZONMW-35_01420	rplT
rplT	ZONMW-36_03838	rplT
rplT	ZONMW-37_03344	rplT
rplT	ZONMW-38_00213	rplT
rplT	ZONMW-39_03755	rplT
rplT	ZONMW-40_03468	rplT
rplT	ZONMW-41_01493	rplT
rplT	ZONMW-42_00718	rplT
rplT	ZONMW-43_03345	rplT
rplT	ZONMW-44_02625	rplT
rplT	ZONMW-45_02680	rplT
rplT	ZONMW-46_02543	rplT
rplT	ZONMW-47_02533	rplT
rplT	ZONMW-48_03342	rplT
rplT	ZONMW-49_00488	rplT
rplT	ZONMW-50_00489	rplT
rplT	ZONMW-51_01216	rplT
rplT	ZONMW-52_02910	rplT
rplT	ZONMW-53_00004	rplT
rplT	ZONMW-54_01032	rplT
rplT	ZONMW-55_00004	rplT
rplT	ZONMW-56_02806	rplT
rplT	ZONMW-57_01614	rplT
rplT	ZONMW-58_00970	rplT
rplT	ZONMW-59_02722	rplT
rplT	ZONMW-60_03254	rplT
rplT	ZONMW-61_04007	rplT
rplT	ZONMW-62_02595	rplT
rplT	ZONMW-63_03287	rplT
rplT	ZONMW-64_01821	rplT
rplT	ZONMW-65_03083	rplT
rplT	ZONMW-66_00857	rplT
rplT	ZONMW-67_03218	rplT
rplT	ZONMW-68_02496	rplT
rplT	ZONMW-69_03219	rplT
rplT	ZONMW-72_01283	rplT
rplT	ZONMW-73_02288	rplT
rplT	ZONMW-74_00695	rplT
rplT	ZONMW-75_00935	rplT

rplT	da5A-8_00210	rplT
rpmA	18_00664	rpmA
rpmA	ACAM48_02647	rpmA
rpmA	CAM030_02088	rpmA
rpmA	CECT7946_01937	rpmA
rpmA	CGMCC1.8863_00138	rpmA
rpmA	CL8139_01393	rpmA
rpmA	DAU203_01115	rpmA
rpmA	DD5b_00368	rpmA
rpmA	DSM1208_01315	rpmA
rpmA	DSM12212_02104	rpmA
rpmA	DSM15343_02565	rpmA
rpmA	DSM15361_00260	rpmA
rpmA	DSM15365_03340	rpmA
rpmA	DSM15718_01264	rpmA
rpmA	DSM16505T_00591	rpmA
rpmA	DSM18103_01005	rpmA
rpmA	DSM18293_00628	rpmA
rpmA	DSM18841_00447	rpmA
rpmA	DSM18842_00782	rpmA
rpmA	DSM19866_03606	rpmA
rpmA	DSM2041_00586	rpmA
rpmA	DSM2061_03574	rpmA
rpmA	DSM2064_02558	rpmA
rpmA	DSM21164_01157	rpmA
rpmA	DSM23404_02307	rpmA
rpmA	DSM23424_00094	rpmA
rpmA	DSM24786_03342	rpmA
rpmA	DSM7489_01034	rpmA
rpmA	DSW1_02909	rpmA
rpmA	DSW5_01048	rpmA
rpmA	DSW6_01566	rpmA
rpmA	DSW8_02611	rpmA
rpmA	F52_02955	rpmA
rpmA	GCA_001027945.1_00160	rpmA
rpmA	HI1_00972	rpmA
rpmA	IR1_00323	rpmA
rpmA	KCOM3131_00490	rpmA
rpmA	KCTC12974_02018	rpmA
rpmA	KL-A_02277	rpmA
rpmA	L12_00184	rpmA
rpmA	LL0412.1.7_00554	rpmA
rpmA	MAR_2009_71_00842	rpmA
rpmA	NN016038_00679	rpmA
rpmA	PAO1_04715	rpmA
rpmA	UW101_01612	rpmA
rpmA	V4-33_02138	rpmA
rpmA	W5C_03077	rpmA

rpmA	ZONMW-01_02906	rpmA
rpmA	ZONMW-02_00273	rpmA
rpmA	ZONMW-03_01950	rpmA
rpmA	ZONMW-04_02914	rpmA
rpmA	ZONMW-05_03011	rpmA
rpmA	ZONMW-06_03950	rpmA
rpmA	ZONMW-07_00656	rpmA
rpmA	ZONMW-08_00360	rpmA
rpmA	ZONMW-09_00565	rpmA
rpmA	ZONMW-10_00612	rpmA
rpmA	ZONMW-11_02698	rpmA
rpmA	ZONMW-12_00477	rpmA
rpmA	ZONMW-13_00750	rpmA
rpmA	ZONMW-14_01296	rpmA
rpmA	ZONMW-15_00875	rpmA
rpmA	ZONMW-19_00607	rpmA
rpmA	ZONMW-20_02865	rpmA
rpmA	ZONMW-21_01167	rpmA
rpmA	ZONMW-22_01169	rpmA
rpmA	ZONMW-23_02281	rpmA
rpmA	ZONMW-24_00292	rpmA
rpmA	ZONMW-25_03308	rpmA
rpmA	ZONMW-26_01905	rpmA
rpmA	ZONMW-27_03032	rpmA
rpmA	ZONMW-28_03926	rpmA
rpmA	ZONMW-29_00758	rpmA
rpmA	ZONMW-30_00444	rpmA
rpmA	ZONMW-31_03210	rpmA
rpmA	ZONMW-32_03960	rpmA
rpmA	ZONMW-33_03884	rpmA
rpmA	ZONMW-34_03824	rpmA
rpmA	ZONMW-35_03801	rpmA
rpmA	ZONMW-36_03921	rpmA
rpmA	ZONMW-37_01195	rpmA
rpmA	ZONMW-38_02310	rpmA
rpmA	ZONMW-39_04074	rpmA
rpmA	ZONMW-40_00107	rpmA
rpmA	ZONMW-41_02673	rpmA
rpmA	ZONMW-42_02351	rpmA
rpmA	ZONMW-43_03777	rpmA
rpmA	ZONMW-44_03146	rpmA
rpmA	ZONMW-45_00586	rpmA
rpmA	ZONMW-46_00873	rpmA
rpmA	ZONMW-47_00873	rpmA
rpmA	ZONMW-48_00288	rpmA
rpmA	ZONMW-49_02224	rpmA
rpmA	ZONMW-50_02018	rpmA
rpmA	ZONMW-51_03093	rpmA

rpmA	ZONMW-52_02026	rpmA
rpmA	ZONMW-53_01722	rpmA
rpmA	ZONMW-54_02313	rpmA
rpmA	ZONMW-55_03060	rpmA
rpmA	ZONMW-56_01235	rpmA
rpmA	ZONMW-57_02964	rpmA
rpmA	ZONMW-58_03029	rpmA
rpmA	ZONMW-59_01837	rpmA
rpmA	ZONMW-60_02191	rpmA
rpmA	ZONMW-61_01745	rpmA
rpmA	ZONMW-62_01485	rpmA
rpmA	ZONMW-63_03023	rpmA
rpmA	ZONMW-64_04097	rpmA
rpmA	ZONMW-65_01220	rpmA
rpmA	ZONMW-66_03411	rpmA
rpmA	ZONMW-67_03601	rpmA
rpmA	ZONMW-68_00729	rpmA
rpmA	ZONMW-69_03603	rpmA
rpmA	ZONMW-72_03788	rpmA
rpmA	ZONMW-73_01748	rpmA
rpmA	ZONMW-74_02052	rpmA
rpmA	ZONMW-75_01136	rpmA
rpmA	da5A-8_01229	rpmA
surA_1	18_00502	surA_1
surA_1	18_01925	surA_1
surA_1	ACAM48_02887	surA_1
surA_1	ACAM48_01472	surA_1
surA_1	CAM030_02259	surA_1
surA_1	CAM030_01757	surA_1
surA_1	CAM030_01755	surA_1
surA_1	CECT7946_00729	surA_1
surA_1	CECT7946_03541	surA_1
surA_1	CECT7946_03543	surA_1
surA_1	CGMCC1.8863_02480	surA_1
surA_1	CGMCC1.8863_02784	surA_1
surA_1	CL8139_02446	surA_1
surA_1	CL8139_00469	surA_1
surA_1	DAU203_00878	surA_1
surA_1	DAU203_00492	surA_1
surA_1	DD5b_01738	surA_1
surA_1	DD5b_02808	surA_1
surA_1	DSM1208_01151	surA_1
surA_1	DSM1208_02858	surA_1
surA_1	DSM12212_02637	surA_1
surA_1	DSM12212_02198	surA_1
surA_1	DSM12212_02200	surA_1
surA_1	DSM15343_00673	surA_1
surA_1	DSM15343_02797	surA_1

surA_1	DSM15343_02799	surA_1
surA_1	DSM15361_00199	surA_1
surA_1	DSM15361_02125	surA_1
surA_1	DSM15361_02127	surA_1
surA_1	DSM15365_03757	surA_1
surA_1	DSM15365_00046	surA_1
surA_1	DSM15365_00044	surA_1
surA_1	DSM15718_02210	surA_1
surA_1	DSM15718_01074	surA_1
surA_1	DSM16505T_01058	surA_1
surA_1	DSM16505T_01803	surA_1
surA_1	DSM16505T_01801	surA_1
surA_1	DSM18103_02496	surA_1
surA_1	DSM18103_00792	surA_1
surA_1	DSM18103_00794	surA_1
surA_1	DSM18293_00504	surA_1
surA_1	DSM18293_02237	surA_1
surA_1	DSM18841_00944	surA_1
surA_1	DSM18841_01535	surA_1
surA_1	DSM18842_00370	surA_1
surA_1	DSM18842_02220	surA_1
surA_1	DSM18842_02222	surA_1
surA_1	DSM19866_03768	surA_1
surA_1	DSM19866_00077	surA_1
surA_1	DSM2041_00779	surA_1
surA_1	DSM2041_03361	surA_1
surA_1	DSM2041_03363	surA_1
surA_1	DSM2061_02665	surA_1
surA_1	DSM2061_04342	surA_1
surA_1	DSM2064_02426	surA_1
surA_1	DSM2064_02982	surA_1
surA_1	DSM21164_01322	surA_1
surA_1	DSM21164_02893	surA_1
surA_1	DSM23404_01772	surA_1
surA_1	DSM23404_00033	surA_1
surA_1	DSM23424_00830	surA_1
surA_1	DSM23424_02772	surA_1
surA_1	DSM23424_02774	surA_1
surA_1	DSM24786_01312	surA_1
surA_1	DSM24786_02569	surA_1
surA_1	DSM7489_00811	surA_1
surA_1	DSM7489_00476	surA_1
surA_1	DSW1_00096	surA_1
surA_1	DSW1_02296	surA_1
surA_1	DSW1_02298	surA_1
surA_1	DSW5_00686	surA_1
surA_1	DSW5_01835	surA_1
surA_1	DSW5_01833	surA_1

surA_1	DSW6_01941	surA_1
surA_1	DSW6_02318	surA_1
surA_1	DSW6_02316	surA_1
surA_1	DSW8_02787	surA_1
surA_1	DSW8_01103	surA_1
surA_1	F52_02833	surA_1
surA_1	F52_03097	surA_1
surA_1	GCA_001027945.1_01479	surA_1
surA_1	GCA_001027945.1_02118	surA_1
surA_1	HI1_00746	surA_1
surA_1	HI1_00408	surA_1
surA_1	IR1_00447	surA_1
surA_1	IR1_00949	surA_1
surA_1	KCOM3131_00363	surA_1
surA_1	KCOM3131_00403	surA_1
surA_1	KCTC12974_01356	surA_1
surA_1	KCTC12974_00715	surA_1
surA_1	KL-A_02415	surA_1
surA_1	KL-A_01730	surA_1
surA_1	L12_00359	surA_1
surA_1	LL0412.1.7_01086	surA_1
surA_1	LL0412.1.7_02260	surA_1
surA_1	MAR_2009_71_00667	surA_1
surA_1	MAR_2009_71_02342	surA_1
surA_1	NN016038_00511	surA_1
surA_1	NN016038_01954	surA_1
surA_1	PAO1_04307	surA_1
surA_1	PAO1_02058	surA_1
surA_1	PAO1_00609	surA_1
surA_1	PAO1_03996	surA_1
surA_1	PAO1_01859	surA_1
surA_1	PAO1_00718	surA_1
surA_1	UW101_01480	surA_1
surA_1	UW101_02036	surA_1
surA_1	V4-33_02351	surA_1
surA_1	V4-33_01078	surA_1
surA_1	V4-33_01079	surA_1
surA_1	W5C_01438	surA_1
surA_1	W5C_03126	surA_1
surA_1	ZONMW-01_02704	surA_1
surA_1	ZONMW-01_01932	surA_1
surA_1	ZONMW-02_03009	surA_1
surA_1	ZONMW-02_01312	surA_1
surA_1	ZONMW-02_01310	surA_1
surA_1	ZONMW-03_01730	surA_1
surA_1	ZONMW-03_02916	surA_1
surA_1	ZONMW-04_02262	surA_1
surA_1	ZONMW-04_02531	surA_1

surA_1	ZONMW-05_02778	surA_1
surA_1	ZONMW-05_02162	surA_1
surA_1	ZONMW-06_00800	surA_1
surA_1	ZONMW-06_00125	surA_1
surA_1	ZONMW-06_03256	surA_1
surA_1	ZONMW-06_00448	surA_1
surA_1	ZONMW-07_00513	surA_1
surA_1	ZONMW-07_03809	surA_1
surA_1	ZONMW-08_00183	surA_1
surA_1	ZONMW-08_00904	surA_1
surA_1	ZONMW-09_00653	surA_1
surA_1	ZONMW-09_01818	surA_1
surA_1	ZONMW-10_00032	surA_1
surA_1	ZONMW-10_01593	surA_1
surA_1	ZONMW-10_01595	surA_1
surA_1	ZONMW-11_02216	surA_1
surA_1	ZONMW-11_01201	surA_1
surA_1	ZONMW-11_01199	surA_1
surA_1	ZONMW-12_01046	surA_1
surA_1	ZONMW-12_03853	surA_1
surA_1	ZONMW-13_00049	surA_1
surA_1	ZONMW-13_01583	surA_1
surA_1	ZONMW-14_02443	surA_1
surA_1	ZONMW-14_00811	surA_1
surA_1	ZONMW-15_01025	surA_1
surA_1	ZONMW-15_02164	surA_1
surA_1	ZONMW-19_02152	surA_1
surA_1	ZONMW-19_00517	surA_1
surA_1	ZONMW-19_02806	surA_1
surA_1	ZONMW-20_00836	surA_1
surA_1	ZONMW-20_01015	surA_1
surA_1	ZONMW-20_01324	surA_1
surA_1	ZONMW-21_01340	surA_1
surA_1	ZONMW-21_03350	surA_1
surA_1	ZONMW-22_01342	surA_1
surA_1	ZONMW-22_03375	surA_1
surA_1	ZONMW-23_02455	surA_1
surA_1	ZONMW-23_03396	surA_1
surA_1	ZONMW-24_00119	surA_1
surA_1	ZONMW-24_03398	surA_1
surA_1	ZONMW-25_02878	surA_1
surA_1	ZONMW-25_02805	surA_1
surA_1	ZONMW-26_02118	surA_1
surA_1	ZONMW-26_02831	surA_1
surA_1	ZONMW-27_02241	surA_1
surA_1	ZONMW-27_02510	surA_1
surA_1	ZONMW-28_00702	surA_1
surA_1	ZONMW-28_01320	surA_1

surA_1	ZONMW-28_03090	surA_1
surA_1	ZONMW-28_02385	surA_1
surA_1	ZONMW-28_00994	surA_1
surA_1	ZONMW-29_00880	surA_1
surA_1	ZONMW-29_02211	surA_1
surA_1	ZONMW-30_00620	surA_1
surA_1	ZONMW-30_02930	surA_1
surA_1	ZONMW-31_00458	surA_1
surA_1	ZONMW-31_01069	surA_1
surA_1	ZONMW-31_01685	surA_1
surA_1	ZONMW-31_00810	surA_1
surA_1	ZONMW-32_00389	surA_1
surA_1	ZONMW-32_01728	surA_1
surA_1	ZONMW-32_03319	surA_1
surA_1	ZONMW-32_02965	surA_1
surA_1	ZONMW-32_00097	surA_1
surA_1	ZONMW-33_00256	surA_1
surA_1	ZONMW-33_01718	surA_1
surA_1	ZONMW-33_00709	surA_1
surA_1	ZONMW-33_02783	surA_1
surA_1	ZONMW-33_03358	surA_1
surA_1	ZONMW-34_00101	surA_1
surA_1	ZONMW-34_00631	surA_1
surA_1	ZONMW-34_01075	surA_1
surA_1	ZONMW-35_01290	surA_1
surA_1	ZONMW-35_00758	surA_1
surA_1	ZONMW-35_02168	surA_1
surA_1	ZONMW-36_01113	surA_1
surA_1	ZONMW-36_01638	surA_1
surA_1	ZONMW-36_03203	surA_1
surA_1	ZONMW-36_02295	surA_1
surA_1	ZONMW-36_03095	surA_1
surA_1	ZONMW-37_01022	surA_1
surA_1	ZONMW-37_03384	surA_1
surA_1	ZONMW-38_01747	surA_1
surA_1	ZONMW-38_02983	surA_1
surA_1	ZONMW-39_02426	surA_1
surA_1	ZONMW-39_01095	surA_1
surA_1	ZONMW-39_02943	surA_1
surA_1	ZONMW-40_02708	surA_1
surA_1	ZONMW-40_01227	surA_1
surA_1	ZONMW-41_00504	surA_1
surA_1	ZONMW-41_01733	surA_1
surA_1	ZONMW-41_01400	surA_1
surA_1	ZONMW-41_02661	surA_1
surA_1	ZONMW-42_00057	surA_1
surA_1	ZONMW-42_01587	surA_1
surA_1	ZONMW-42_00816	surA_1

surA_1	ZONMW-43_00848	surA_1
surA_1	ZONMW-43_00173	surA_1
surA_1	ZONMW-43_00609	surA_1
surA_1	ZONMW-44_01426	surA_1
surA_1	ZONMW-44_01921	surA_1
surA_1	ZONMW-44_04154	surA_1
surA_1	ZONMW-44_03649	surA_1
surA_1	ZONMW-45_01498	surA_1
surA_1	ZONMW-45_00920	surA_1
surA_1	ZONMW-46_01752	surA_1
surA_1	ZONMW-46_00954	surA_1
surA_1	ZONMW-47_01737	surA_1
surA_1	ZONMW-47_01219	surA_1
surA_1	ZONMW-48_00441	surA_1
surA_1	ZONMW-48_02643	surA_1
surA_1	ZONMW-49_02288	surA_1
surA_1	ZONMW-49_02000	surA_1
surA_1	ZONMW-50_02172	surA_1
surA_1	ZONMW-50_02596	surA_1
surA_1	ZONMW-51_01668	surA_1
surA_1	ZONMW-51_02306	surA_1
surA_1	ZONMW-52_01811	surA_1
surA_1	ZONMW-52_02718	surA_1
surA_1	ZONMW-53_01945	surA_1
surA_1	ZONMW-53_02471	surA_1
surA_1	ZONMW-54_02109	surA_1
surA_1	ZONMW-54_02531	surA_1
surA_1	ZONMW-55_01990	surA_1
surA_1	ZONMW-55_01682	surA_1
surA_1	ZONMW-56_01446	surA_1
surA_1	ZONMW-56_01835	surA_1
surA_1	ZONMW-57_01780	surA_1
surA_1	ZONMW-57_02305	surA_1
surA_1	ZONMW-58_01804	surA_1
surA_1	ZONMW-58_02329	surA_1
surA_1	ZONMW-59_01616	surA_1
surA_1	ZONMW-59_02543	surA_1
surA_1	ZONMW-60_01998	surA_1
surA_1	ZONMW-60_02412	surA_1
surA_1	ZONMW-61_01559	surA_1
surA_1	ZONMW-61_00857	surA_1
surA_1	ZONMW-62_01310	surA_1
surA_1	ZONMW-62_02673	surA_1
surA_1	ZONMW-63_01516	surA_1
surA_1	ZONMW-63_02474	surA_1
surA_1	ZONMW-64_01133	surA_1
surA_1	ZONMW-64_00188	surA_1
surA_1	ZONMW-64_00627	surA_1

surA_1	ZONMW-64_01572	surA_1
surA_1	ZONMW-65_01725	surA_1
surA_1	ZONMW-65_00313	surA_1
surA_1	ZONMW-66_01655	surA_1
surA_1	ZONMW-66_02696	surA_1
surA_1	ZONMW-67_00853	surA_1
surA_1	ZONMW-67_00576	surA_1
surA_1	ZONMW-67_01657	surA_1
surA_1	ZONMW-67_02815	surA_1
surA_1	ZONMW-68_00885	surA_1
surA_1	ZONMW-68_02650	surA_1
surA_1	ZONMW-69_00900	surA_1
surA_1	ZONMW-69_00323	surA_1
surA_1	ZONMW-69_01344	surA_1
surA_1	ZONMW-69_00612	surA_1
surA_1	ZONMW-72_00099	surA_1
surA_1	ZONMW-72_02362	surA_1
surA_1	ZONMW-73_03263	surA_1
surA_1	ZONMW-73_01570	surA_1
surA_1	ZONMW-73_01568	surA_1
surA_1	ZONMW-74_03288	surA_1
surA_1	ZONMW-74_02375	surA_1
surA_1	ZONMW-75_03399	surA_1
surA_1	ZONMW-75_00898	surA_1
surA_1	ZONMW-75_00896	surA_1
surA_1	da5A-8_00044	surA_1
surA_1	da5A-8_03150	surA_1
surA_1	da5A-8_01454	surA_1
trxB	18_01664	trxB
trxB	18_02176	trxB
trxB	18_02521	trxB
trxB	ACAM48_00507	trxB
trxB	ACAM48_02036	trxB
trxB	ACAM48_01898	trxB
trxB	CAM030_02143	trxB
trxB	CAM030_00145	trxB
trxB	CECT7946_00458	trxB
trxB	CECT7946_02680	trxB
trxB	CECT7946_02977	trxB
trxB	CGMCC1.8863_01853	trxB
trxB	CGMCC1.8863_01294	trxB
trxB	CGMCC1.8863_01243	trxB
trxB	CGMCC1.8863_01680	trxB
trxB	CL8139_03298	trxB
trxB	CL8139_02176	trxB
trxB	CL8139_01710	trxB
trxB	DAU203_02096	trxB
trxB	DAU203_00962	trxB

trxB	DAU203_02669	trxB
trxB	DD5b_01809	trxB
trxB	DD5b_00521	trxB
trxB	DSM1208_00813	trxB
trxB	DSM1208_01247	trxB
trxB	DSM1208_01416	trxB
trxB	DSM1208_00150	trxB
trxB	DSM12212_01371	trxB
trxB	DSM12212_02812	trxB
trxB	DSM15343_00766	trxB
trxB	DSM15343_01816	trxB
trxB	DSM15361_00142	trxB
trxB	DSM15361_02357	trxB
trxB	DSM15365_00620	trxB
trxB	DSM15365_02929	trxB
trxB	DSM15365_00025	trxB
trxB	DSM15718_02148	trxB
trxB	DSM15718_02887	trxB
trxB	DSM16505T_00618	trxB
trxB	DSM16505T_01652	trxB
trxB	DSM16505T_02558	trxB
trxB	DSM18103_01029	trxB
trxB	DSM18103_02911	trxB
trxB	DSM18103_02663	trxB
trxB	DSM18293_00594	trxB
trxB	DSM18293_02608	trxB
trxB	DSM18841_00472	trxB
trxB	DSM18841_03133	trxB
trxB	DSM18841_02822	trxB
trxB	DSM18842_00755	trxB
trxB	DSM18842_03151	trxB
trxB	DSM18842_02742	trxB
trxB	DSM19866_02288	trxB
trxB	DSM19866_03673	trxB
trxB	DSM19866_04017	trxB
trxB	DSM19866_01816	trxB
trxB	DSM2041_04960	trxB
trxB	DSM2041_04248	trxB
trxB	DSM2041_02591	trxB
trxB	DSM2061_02314	trxB
trxB	DSM2061_02771	trxB
trxB	DSM2061_02936	trxB
trxB	DSM2061_00385	trxB
trxB	DSM2064_02520	trxB
trxB	DSM2064_04886	trxB
trxB	DSM21164_00207	trxB
trxB	DSM21164_01229	trxB
trxB	DSM21164_02766	trxB

trxB	DSM23404_01076	trxB
trxB	DSM23404_01838	trxB
trxB	DSM23404_01267	trxB
trxB	DSM23424_00708	trxB
trxB	DSM23424_01558	trxB
trxB	DSM24786_01949	trxB
trxB	DSM24786_03128	trxB
trxB	DSM24786_00129	trxB
trxB	DSM7489_02008	trxB
trxB	DSM7489_00896	trxB
trxB	DSM7489_02544	trxB
trxB	DSW1_00964	trxB
trxB	DSW1_02777	trxB
trxB	DSW5_01089	trxB
trxB	DSW5_01719	trxB
trxB	DSW5_00955	trxB
trxB	DSW5_00159	trxB
trxB	DSW6_02177	trxB
trxB	DSW6_01765	trxB
trxB	DSW6_00011	trxB
trxB	DSW8_03053	trxB
trxB	DSW8_02701	trxB
trxB	DSW8_00341	trxB
trxB	DSW8_03336	trxB
trxB	F52_02923	trxB
trxB	F52_01764	trxB
trxB	GCA_001027945.1_02677	trxB
trxB	GCA_001027945.1_00133	trxB
trxB	GCA_001027945.1_00726	trxB
trxB	HI1_01935	trxB
trxB	HI1_00828	trxB
trxB	HI1_02476	trxB
trxB	IR1_00360	trxB
trxB	IR1_02884	trxB
trxB	KCOM3131_01794	trxB
trxB	KCOM3131_00226	trxB
trxB	KCTC12974_02358	trxB
trxB	KCTC12974_00811	trxB
trxB	KCTC12974_01775	trxB
trxB	KL-A_00262	trxB
trxB	KL-A_02375	trxB
trxB	KL-A_01012	trxB
trxB	L12_00375	trxB
trxB	LL0412.1.7_02681	trxB
trxB	LL0412.1.7_00332	trxB
trxB	LL0412.1.7_01821	trxB
trxB	MAR_2009_71_00401	trxB
trxB	MAR_2009_71_00752	trxB

trxB	MAR_2009_71_03139	trxB
trxB	MAR_2009_71_03593	trxB
trxB	NN016038_01671	trxB
trxB	NN016038_02194	trxB
trxB	NN016038_02544	trxB
trxB	PAO1_00875	trxB
trxB	PAO1_02695	trxB
trxB	PAO1_00146	trxB
trxB	UW101_01574	trxB
trxB	UW101_00216	trxB
trxB	V4-33_01577	trxB
trxB	V4-33_00591	trxB
trxB	W5C_00696	trxB
trxB	W5C_01539	trxB
trxB	W5C_01049	trxB
trxB	ZONMW-01_00460	trxB
trxB	ZONMW-01_02533	trxB
trxB	ZONMW-01_01598	trxB
trxB	ZONMW-02_01061	trxB
trxB	ZONMW-02_03165	trxB
trxB	ZONMW-02_01292	trxB
trxB	ZONMW-03_01186	trxB
trxB	ZONMW-03_01814	trxB
trxB	ZONMW-03_00609	trxB
trxB	ZONMW-04_00488	trxB
trxB	ZONMW-04_03019	trxB
trxB	ZONMW-05_01337	trxB
trxB	ZONMW-05_03263	trxB
trxB	ZONMW-05_00871	trxB
trxB	ZONMW-06_01190	trxB
trxB	ZONMW-06_03153	trxB
trxB	ZONMW-07_01538	trxB
trxB	ZONMW-07_00616	trxB
trxB	ZONMW-07_02979	trxB
trxB	ZONMW-08_03722	trxB
trxB	ZONMW-08_00269	trxB
trxB	ZONMW-08_02655	trxB
trxB	ZONMW-08_02331	trxB
trxB	ZONMW-09_00075	trxB
trxB	ZONMW-09_00883	trxB
trxB	ZONMW-10_00087	trxB
trxB	ZONMW-10_02094	trxB
trxB	ZONMW-11_00876	trxB
trxB	ZONMW-11_00941	trxB
trxB	ZONMW-11_03160	trxB
trxB	ZONMW-11_01396	trxB
trxB	ZONMW-12_02064	trxB
trxB	ZONMW-12_00628	trxB

trxB	ZONMW-12_01555	trxB
trxB	ZONMW-13_01158	trxB
trxB	ZONMW-13_00946	trxB
trxB	ZONMW-13_02304	trxB
trxB	ZONMW-14_01435	trxB
trxB	ZONMW-14_01158	trxB
trxB	ZONMW-14_02225	trxB
trxB	ZONMW-15_01328	trxB
trxB	ZONMW-15_02489	trxB
trxB	ZONMW-19_00474	trxB
trxB	ZONMW-20_02390	trxB
trxB	ZONMW-20_02272	trxB
trxB	ZONMW-21_00071	trxB
trxB	ZONMW-21_01242	trxB
trxB	ZONMW-21_01477	trxB
trxB	ZONMW-22_00071	trxB
trxB	ZONMW-22_01244	trxB
trxB	ZONMW-22_02246	trxB
trxB	ZONMW-23_01186	trxB
trxB	ZONMW-23_02356	trxB
trxB	ZONMW-23_00305	trxB
trxB	ZONMW-24_01389	trxB
trxB	ZONMW-24_00217	trxB
trxB	ZONMW-24_02010	trxB
trxB	ZONMW-25_03184	trxB
trxB	ZONMW-25_02475	trxB
trxB	ZONMW-25_00494	trxB
trxB	ZONMW-26_01391	trxB
trxB	ZONMW-26_02035	trxB
trxB	ZONMW-26_00089	trxB
trxB	ZONMW-27_00482	trxB
trxB	ZONMW-27_02894	trxB
trxB	ZONMW-27_01753	trxB
trxB	ZONMW-28_02962	trxB
trxB	ZONMW-28_01791	trxB
trxB	ZONMW-29_02477	trxB
trxB	ZONMW-29_00828	trxB
trxB	ZONMW-29_01524	trxB
trxB	ZONMW-30_00844	trxB
trxB	ZONMW-30_00511	trxB
trxB	ZONMW-30_02006	trxB
trxB	ZONMW-31_01621	trxB
trxB	ZONMW-31_00149	trxB
trxB	ZONMW-32_01446	trxB
trxB	ZONMW-32_02181	trxB
trxB	ZONMW-33_01436	trxB
trxB	ZONMW-33_02172	trxB
trxB	ZONMW-34_01624	trxB

trxB	ZONMW-34_02321	trxB
trxB	ZONMW-35_00695	trxB
trxB	ZONMW-35_01928	trxB
trxB	ZONMW-36_00655	trxB
trxB	ZONMW-36_02064	trxB
trxB	ZONMW-37_00070	trxB
trxB	ZONMW-37_01120	trxB
trxB	ZONMW-37_02532	trxB
trxB	ZONMW-38_03404	trxB
trxB	ZONMW-38_00494	trxB
trxB	ZONMW-38_01286	trxB
trxB	ZONMW-38_01136	trxB
trxB	ZONMW-38_01202	trxB
trxB	ZONMW-39_00075	trxB
trxB	ZONMW-39_01588	trxB
trxB	ZONMW-40_00957	trxB
trxB	ZONMW-40_00039	trxB
trxB	ZONMW-40_01575	trxB
trxB	ZONMW-41_01002	trxB
trxB	ZONMW-41_02887	trxB
trxB	ZONMW-42_03721	trxB
trxB	ZONMW-42_03277	trxB
trxB	ZONMW-43_01644	trxB
trxB	ZONMW-43_01918	trxB
trxB	ZONMW-44_04028	trxB
trxB	ZONMW-44_02826	trxB
trxB	ZONMW-45_00611	trxB
trxB	ZONMW-45_02284	trxB
trxB	ZONMW-45_02930	trxB
trxB	ZONMW-46_00848	trxB
trxB	ZONMW-46_03131	trxB
trxB	ZONMW-46_03011	trxB
trxB	ZONMW-47_00848	trxB
trxB	ZONMW-47_03123	trxB
trxB	ZONMW-47_03002	trxB
trxB	ZONMW-48_02368	trxB
trxB	ZONMW-48_02998	trxB
trxB	ZONMW-49_01550	trxB
trxB	ZONMW-49_02090	trxB
trxB	ZONMW-49_00094	trxB
trxB	ZONMW-50_01552	trxB
trxB	ZONMW-50_01884	trxB
trxB	ZONMW-50_00392	trxB
trxB	ZONMW-51_00805	trxB
trxB	ZONMW-51_01748	trxB
trxB	ZONMW-51_00091	trxB
trxB	ZONMW-52_01405	trxB
trxB	ZONMW-52_01891	trxB

trxB	ZONMW-52_00064	trxB
trxB	ZONMW-53_00420	trxB
trxB	ZONMW-53_01865	trxB
trxB	ZONMW-53_02591	trxB
trxB	ZONMW-54_00614	trxB
trxB	ZONMW-54_02206	trxB
trxB	ZONMW-54_01913	trxB
trxB	ZONMW-55_00415	trxB
trxB	ZONMW-55_02070	trxB
trxB	ZONMW-55_01447	trxB
trxB	ZONMW-56_00900	trxB
trxB	ZONMW-56_01364	trxB
trxB	ZONMW-56_00673	trxB
trxB	ZONMW-57_01200	trxB
trxB	ZONMW-57_01860	trxB
trxB	ZONMW-57_00880	trxB
trxB	ZONMW-58_01384	trxB
trxB	ZONMW-58_01884	trxB
trxB	ZONMW-58_00091	trxB
trxB	ZONMW-59_01201	trxB
trxB	ZONMW-59_01697	trxB
trxB	ZONMW-59_00874	trxB
trxB	ZONMW-60_01367	trxB
trxB	ZONMW-60_02080	trxB
trxB	ZONMW-60_00879	trxB
trxB	ZONMW-61_03451	trxB
trxB	ZONMW-61_01653	trxB
trxB	ZONMW-61_03358	trxB
trxB	ZONMW-61_03205	trxB
trxB	ZONMW-61_02597	trxB
trxB	ZONMW-62_03595	trxB
trxB	ZONMW-62_01396	trxB
trxB	ZONMW-62_00428	trxB
trxB	ZONMW-62_03257	trxB
trxB	ZONMW-63_03344	trxB
trxB	ZONMW-63_02731	trxB
trxB	ZONMW-63_02014	trxB
trxB	ZONMW-64_03050	trxB
trxB	ZONMW-64_01505	trxB
trxB	ZONMW-65_02479	trxB
trxB	ZONMW-65_02528	trxB
trxB	ZONMW-66_00072	trxB
trxB	ZONMW-67_01559	trxB
trxB	ZONMW-67_00246	trxB
trxB	ZONMW-68_01542	trxB
trxB	ZONMW-68_00796	trxB
trxB	ZONMW-68_02797	trxB
trxB	ZONMW-68_00631	trxB

trxB	ZONMW-69_01442	trxB
trxB	ZONMW-69_02855	trxB
trxB	ZONMW-72_00012	trxB
trxB	ZONMW-72_01451	trxB
trxB	ZONMW-73_02313	trxB
trxB	ZONMW-73_00319	trxB
trxB	ZONMW-73_02594	trxB
trxB	ZONMW-74_01210	trxB
trxB	ZONMW-74_02865	trxB
trxB	ZONMW-74_03001	trxB
trxB	ZONMW-75_00614	trxB
trxB	ZONMW-75_03378	trxB
trxB	ZONMW-75_00885	trxB
trxB	da5A-8_01202	trxB
trxB	da5A-8_02593	trxB
trxB	da5A-8_00330	trxB
oxyR	18_01469	oxyR
oxyR	18_02442	oxyR
oxyR	18_02194	oxyR
oxyR	18_03470	oxyR
oxyR	18_02370	oxyR
oxyR	ACAM48_01024	oxyR
oxyR	CAM030_00427	oxyR
oxyR	CAM030_00157	oxyR
oxyR	CAM030_03040	oxyR
oxyR	CAM030_03043	oxyR
oxyR	CECT7946_02968	oxyR
oxyR	CECT7946_03150	oxyR
oxyR	CGMCC1.8863_00496	oxyR
oxyR	CGMCC1.8863_00424	oxyR
oxyR	CGMCC1.8863_03497	oxyR
oxyR	CGMCC1.8863_00881	oxyR
oxyR	CGMCC1.8863_01695	oxyR
oxyR	CL8139_02148	oxyR
oxyR	CL8139_02684	oxyR
oxyR	CL8139_00366	oxyR
oxyR	DAU203_00933	oxyR
oxyR	DAU203_02682	oxyR
oxyR	DAU203_01553	oxyR
oxyR	DD5b_01174	oxyR
oxyR	DD5b_00424	oxyR
oxyR	DD5b_02279	oxyR
oxyR	DSM1208_04078	oxyR
oxyR	DSM1208_00129	oxyR
oxyR	DSM1208_00294	oxyR
oxyR	DSM1208_01737	oxyR
oxyR	DSM1208_03147	oxyR
oxyR	DSM12212_02822	oxyR

oxyR	DSM12212_02671	oxyR
oxyR	DSM15343_02329	oxyR
oxyR	DSM15343_01693	oxyR
oxyR	DSM15343_02542	oxyR
oxyR	DSM15361_00748	oxyR
oxyR	DSM15361_02793	oxyR
oxyR	DSM15361_02289	oxyR
oxyR	DSM15361_00061	oxyR
oxyR	DSM15365_01368	oxyR
oxyR	DSM15365_00373	oxyR
oxyR	DSM15718_01364	oxyR
oxyR	DSM15718_01893	oxyR
oxyR	DSM15718_02720	oxyR
oxyR	DSM15718_00870	oxyR
oxyR	DSM16505T_02530	oxyR
oxyR	DSM16505T_01857	oxyR
oxyR	DSM18103_01281	oxyR
oxyR	DSM18103_02205	oxyR
oxyR	DSM18103_03517	oxyR
oxyR	DSM18103_02870	oxyR
oxyR	DSM18103_00788	oxyR
oxyR	DSM18103_00345	oxyR
oxyR	DSM18103_01735	oxyR
oxyR	DSM18293_01097	oxyR
oxyR	DSM18293_01263	oxyR
oxyR	DSM18293_00368	oxyR
oxyR	DSM18293_03198	oxyR
oxyR	DSM18293_01759	oxyR
oxyR	DSM18293_04293	oxyR
oxyR	DSM18293_00784	oxyR
oxyR	DSM18841_02926	oxyR
oxyR	DSM18841_01592	oxyR
oxyR	DSM18841_01152	oxyR
oxyR	DSM18842_02781	oxyR
oxyR	DSM18842_02276	oxyR
oxyR	DSM19866_01780	oxyR
oxyR	DSM2041_01445	oxyR
oxyR	DSM2041_02579	oxyR
oxyR	DSM2041_01797	oxyR
oxyR	DSM2041_05423	oxyR
oxyR	DSM2061_00910	oxyR
oxyR	DSM2061_00404	oxyR
oxyR	DSM2061_03268	oxyR
oxyR	DSM2064_04071	oxyR
oxyR	DSM2064_00729	oxyR
oxyR	DSM2064_01860	oxyR
oxyR	DSM2064_03920	oxyR
oxyR	DSM2064_02286	oxyR

oxyR	DSM2064_04483	oxyR
oxyR	DSM2064_00305	oxyR
oxyR	DSM2064_01282	oxyR
oxyR	DSM2064_01743	oxyR
oxyR	DSM21164_00410	oxyR
oxyR	DSM21164_01771	oxyR
oxyR	DSM21164_02752	oxyR
oxyR	DSM21164_00914	oxyR
oxyR	DSM23404_02855	oxyR
oxyR	DSM23424_01547	oxyR
oxyR	DSM24786_03094	oxyR
oxyR	DSM24786_00141	oxyR
oxyR	DSM24786_03043	oxyR
oxyR	DSM24786_03041	oxyR
oxyR	DSM7489_00868	oxyR
oxyR	DSM7489_02556	oxyR
oxyR	DSM7489_01457	oxyR
oxyR	DSW1_01789	oxyR
oxyR	DSW1_00489	oxyR
oxyR	DSW5_00694	oxyR
oxyR	DSW5_02132	oxyR
oxyR	DSW5_02584	oxyR
oxyR	DSW6_03403	oxyR
oxyR	DSW6_00754	oxyR
oxyR	DSW8_01758	oxyR
oxyR	DSW8_00422	oxyR
oxyR	DSW8_02760	oxyR
oxyR	DSW8_03354	oxyR
oxyR	DSW8_00252	oxyR
oxyR	F52_04229	oxyR
oxyR	F52_00941	oxyR
oxyR	F52_04091	oxyR
oxyR	F52_02678	oxyR
oxyR	F52_02294	oxyR
oxyR	F52_00968	oxyR
oxyR	F52_01372	oxyR
oxyR	F52_02198	oxyR
oxyR	GCA_001027945.1_00611	oxyR
oxyR	GCA_001027945.1_02867	oxyR
oxyR	GCA_001027945.1_02129	oxyR
oxyR	GCA_001027945.1_03003	oxyR
oxyR	HI1_00800	oxyR
oxyR	HI1_02489	oxyR
oxyR	HI1_01398	oxyR
oxyR	IR1_04202	oxyR
oxyR	IR1_03950	oxyR
oxyR	IR1_04308	oxyR
oxyR	IR1_00591	oxyR

oxyR	IR1_03445	oxyR
oxyR	IR1_03206	oxyR
oxyR	IR1_04098	oxyR
oxyR	KCOM3131_01249	oxyR
oxyR	KCTC12974_02135	oxyR
oxyR	KL-A_02403	oxyR
oxyR	KL-A_03234	oxyR
oxyR	KL-A_03445	oxyR
oxyR	KL-A_00999	oxyR
oxyR	KL-A_00769	oxyR
oxyR	L12_00395	oxyR
oxyR	L12_00442	oxyR
oxyR	LL0412.1.7_00177	oxyR
oxyR	LL0412.1.7_02627	oxyR
oxyR	MAR_2009_71_01662	oxyR
oxyR	MAR_2009_71_03223	oxyR
oxyR	MAR_2009_71_03575	oxyR
oxyR	MAR_2009_71_00693	oxyR
oxyR	MAR_2009_71_03051	oxyR
oxyR	MAR_2009_71_00694	oxyR
oxyR	NN016038_01477	oxyR
oxyR	NN016038_02477	oxyR
oxyR	NN016038_02209	oxyR
oxyR	NN016038_03506	oxyR
oxyR	NN016038_02381	oxyR
oxyR	PAO1_03717	oxyR
oxyR	PAO1_05199	oxyR
oxyR	PAO1_02580	oxyR
oxyR	PAO1_01880	oxyR
oxyR	PAO1_05393	oxyR
oxyR	PAO1_03899	oxyR
oxyR	PAO1_00540	oxyR
oxyR	PAO1_03554	oxyR
oxyR	PAO1_02117	oxyR
oxyR	PAO1_02060	oxyR
oxyR	PAO1_01348	oxyR
oxyR	PAO1_05619	oxyR
oxyR	PAO1_00904	oxyR
oxyR	PAO1_00224	oxyR
oxyR	PAO1_02179	oxyR
oxyR	PAO1_00503	oxyR
oxyR	PAO1_00158	oxyR
oxyR	PAO1_05521	oxyR
oxyR	PAO1_04334	oxyR
oxyR	PAO1_03230	oxyR
oxyR	PAO1_05364	oxyR
oxyR	PAO1_03710	oxyR
oxyR	PAO1_00196	oxyR

oxyR	PAO1_02278	oxyR
oxyR	PAO1_03517	oxyR
oxyR	PAO1_00239	oxyR
oxyR	PAO1_00842	oxyR
oxyR	PAO1_05255	oxyR
oxyR	PAO1_00763	oxyR
oxyR	PAO1_05081	oxyR
oxyR	PAO1_01453	oxyR
oxyR	PAO1_02140	oxyR
oxyR	PAO1_01801	oxyR
oxyR	PAO1_02539	oxyR
oxyR	PAO1_05610	oxyR
oxyR	PAO1_01785	oxyR
oxyR	PAO1_03901	oxyR
oxyR	PAO1_00032	oxyR
oxyR	PAO1_03753	oxyR
oxyR	PAO1_01613	oxyR
oxyR	PAO1_02679	oxyR
oxyR	PAO1_05563	oxyR
oxyR	PAO1_05353	oxyR
oxyR	PAO1_02407	oxyR
oxyR	PAO1_02567	oxyR
oxyR	PAO1_00728	oxyR
oxyR	PAO1_01180	oxyR
oxyR	PAO1_01367	oxyR
oxyR	PAO1_03023	oxyR
oxyR	PAO1_02981	oxyR
oxyR	PAO1_00139	oxyR
oxyR	PAO1_01462	oxyR
oxyR	PAO1_01916	oxyR
oxyR	PAO1_02504	oxyR
oxyR	PAO1_02519	oxyR
oxyR	PAO1_02188	oxyR
oxyR	PAO1_02947	oxyR
oxyR	PAO1_04123	oxyR
oxyR	PAO1_02979	oxyR
oxyR	PAO1_01167	oxyR
oxyR	PAO1_00809	oxyR
oxyR	PAO1_00458	oxyR
oxyR	PAO1_04305	oxyR
oxyR	PAO1_04240	oxyR
oxyR	PAO1_02605	oxyR
oxyR	PAO1_02388	oxyR
oxyR	PAO1_03232	oxyR
oxyR	PAO1_05470	oxyR
oxyR	PAO1_02939	oxyR
oxyR	PAO1_00903	oxyR
oxyR	PAO1_02119	oxyR

oxyR	PAO1_01909	oxyR
oxyR	PAO1_04023	oxyR
oxyR	PAO1_00841	oxyR
oxyR	PAO1_02186	oxyR
oxyR	PAO1_05159	oxyR
oxyR	PAO1_02488	oxyR
oxyR	PAO1_02022	oxyR
oxyR	PAO1_03247	oxyR
oxyR	PAO1_05069	oxyR
oxyR	PAO1_03336	oxyR
oxyR	PAO1_03439	oxyR
oxyR	PAO1_00295	oxyR
oxyR	PAO1_01103	oxyR
oxyR	PAO1_00223	oxyR
oxyR	PAO1_01238	oxyR
oxyR	PAO1_00038	oxyR
oxyR	PAO1_03688	oxyR
oxyR	PAO1_02850	oxyR
oxyR	PAO1_00062	oxyR
oxyR	PAO1_00165	oxyR
oxyR	PAO1_01184	oxyR
oxyR	PAO1_00213	oxyR
oxyR	PAO1_02618	oxyR
oxyR	PAO1_03834	oxyR
oxyR	PAO1_01177	oxyR
oxyR	PAO1_03032	oxyR
oxyR	PAO1_02623	oxyR
oxyR	PAO1_01351	oxyR
oxyR	PAO1_02330	oxyR
oxyR	PAO1_01439	oxyR
oxyR	PAO1_00129	oxyR
oxyR	PAO1_01221	oxyR
oxyR	PAO1_02292	oxyR
oxyR	PAO1_02761	oxyR
oxyR	PAO1_00186	oxyR
oxyR	PAO1_00491	oxyR
oxyR	PAO1_04276	oxyR
oxyR	PAO1_03970	oxyR
oxyR	PAO1_02455	oxyR
oxyR	PAO1_01302	oxyR
oxyR	PAO1_02949	oxyR
oxyR	PAO1_02339	oxyR
oxyR	PAO1_00278	oxyR
oxyR	PAO1_00489	oxyR
oxyR	PAO1_01260	oxyR
oxyR	PAO1_02562	oxyR
oxyR	PAO1_02935	oxyR
oxyR	PAO1_01035	oxyR

oxyR	UW101_04816	oxyR
oxyR	UW101_03440	oxyR
oxyR	UW101_00914	oxyR
oxyR	UW101_04967	oxyR
oxyR	UW101_01340	oxyR
oxyR	UW101_02096	oxyR
oxyR	UW101_03016	oxyR
oxyR	UW101_04197	oxyR
oxyR	UW101_00797	oxyR
oxyR	V4-33_01646	oxyR
oxyR	V4-33_00737	oxyR
oxyR	W5C_01511	oxyR
oxyR	W5C_03325	oxyR
oxyR	W5C_00928	oxyR
oxyR	W5C_01059	oxyR
oxyR	W5C_01154	oxyR
oxyR	ZONMW-01_02504	oxyR
oxyR	ZONMW-01_01606	oxyR
oxyR	ZONMW-01_02453	oxyR
oxyR	ZONMW-02_01273	oxyR
oxyR	ZONMW-03_01785	oxyR
oxyR	ZONMW-03_00601	oxyR
oxyR	ZONMW-03_03056	oxyR
oxyR	ZONMW-04_02990	oxyR
oxyR	ZONMW-04_01539	oxyR
oxyR	ZONMW-04_02388	oxyR
oxyR	ZONMW-05_03234	oxyR
oxyR	ZONMW-05_00863	oxyR
oxyR	ZONMW-05_02636	oxyR
oxyR	ZONMW-06_01645	oxyR
oxyR	ZONMW-06_01925	oxyR
oxyR	ZONMW-06_02113	oxyR
oxyR	ZONMW-06_02095	oxyR
oxyR	ZONMW-06_01987	oxyR
oxyR	ZONMW-06_00776	oxyR
oxyR	ZONMW-06_01825	oxyR
oxyR	ZONMW-06_03710	oxyR
oxyR	ZONMW-06_01692	oxyR
oxyR	ZONMW-06_01724	oxyR
oxyR	ZONMW-06_01720	oxyR
oxyR	ZONMW-06_01783	oxyR
oxyR	ZONMW-06_00664	oxyR
oxyR	ZONMW-06_02703	oxyR
oxyR	ZONMW-06_00755	oxyR
oxyR	ZONMW-06_00637	oxyR
oxyR	ZONMW-06_01770	oxyR
oxyR	ZONMW-06_03282	oxyR
oxyR	ZONMW-06_00655	oxyR

oxyR	ZONMW-06_02285	oxyR
oxyR	ZONMW-06_02349	oxyR
oxyR	ZONMW-06_01156	oxyR
oxyR	ZONMW-06_02288	oxyR
oxyR	ZONMW-06_01716	oxyR
oxyR	ZONMW-06_02718	oxyR
oxyR	ZONMW-06_01785	oxyR
oxyR	ZONMW-06_01789	oxyR
oxyR	ZONMW-06_00794	oxyR
oxyR	ZONMW-06_00669	oxyR
oxyR	ZONMW-06_02840	oxyR
oxyR	ZONMW-06_03167	oxyR
oxyR	ZONMW-06_03946	oxyR
oxyR	ZONMW-06_02533	oxyR
oxyR	ZONMW-06_01757	oxyR
oxyR	ZONMW-06_01285	oxyR
oxyR	ZONMW-06_02425	oxyR
oxyR	ZONMW-06_02329	oxyR
oxyR	ZONMW-06_02541	oxyR
oxyR	ZONMW-06_02307	oxyR
oxyR	ZONMW-06_01140	oxyR
oxyR	ZONMW-06_01696	oxyR
oxyR	ZONMW-06_01780	oxyR
oxyR	ZONMW-07_00268	oxyR
oxyR	ZONMW-07_03491	oxyR
oxyR	ZONMW-08_01571	oxyR
oxyR	ZONMW-08_02732	oxyR
oxyR	ZONMW-08_00210	oxyR
oxyR	ZONMW-08_02349	oxyR
oxyR	ZONMW-08_03830	oxyR
oxyR	ZONMW-09_00873	oxyR
oxyR	ZONMW-10_02294	oxyR
oxyR	ZONMW-10_02014	oxyR
oxyR	ZONMW-11_04108	oxyR
oxyR	ZONMW-12_03961	oxyR
oxyR	ZONMW-12_01543	oxyR
oxyR	ZONMW-12_04289	oxyR
oxyR	ZONMW-12_03806	oxyR
oxyR	ZONMW-13_02294	oxyR
oxyR	ZONMW-14_02215	oxyR
oxyR	ZONMW-15_00331	oxyR
oxyR	ZONMW-15_02472	oxyR
oxyR	ZONMW-15_03274	oxyR
oxyR	ZONMW-19_03438	oxyR
oxyR	ZONMW-19_01125	oxyR
oxyR	ZONMW-19_03834	oxyR
oxyR	ZONMW-19_02383	oxyR
oxyR	ZONMW-19_02780	oxyR

oxyR	ZONMW-19_03905	oxyR
oxyR	ZONMW-19_00981	oxyR
oxyR	ZONMW-19_00222	oxyR
oxyR	ZONMW-19_02207	oxyR
oxyR	ZONMW-19_00834	oxyR
oxyR	ZONMW-19_04369	oxyR
oxyR	ZONMW-19_04591	oxyR
oxyR	ZONMW-19_01379	oxyR
oxyR	ZONMW-19_02040	oxyR
oxyR	ZONMW-19_03032	oxyR
oxyR	ZONMW-19_03728	oxyR
oxyR	ZONMW-19_04636	oxyR
oxyR	ZONMW-19_02495	oxyR
oxyR	ZONMW-19_03829	oxyR
oxyR	ZONMW-19_03998	oxyR
oxyR	ZONMW-20_01477	oxyR
oxyR	ZONMW-20_00513	oxyR
oxyR	ZONMW-20_00515	oxyR
oxyR	ZONMW-20_02216	oxyR
oxyR	ZONMW-20_03564	oxyR
oxyR	ZONMW-20_03957	oxyR
oxyR	ZONMW-20_03105	oxyR
oxyR	ZONMW-20_01502	oxyR
oxyR	ZONMW-20_00436	oxyR
oxyR	ZONMW-20_03945	oxyR
oxyR	ZONMW-20_03430	oxyR
oxyR	ZONMW-20_03722	oxyR
oxyR	ZONMW-20_03502	oxyR
oxyR	ZONMW-20_03856	oxyR
oxyR	ZONMW-20_03848	oxyR
oxyR	ZONMW-20_00475	oxyR
oxyR	ZONMW-20_01466	oxyR
oxyR	ZONMW-20_01566	oxyR
oxyR	ZONMW-20_02924	oxyR
oxyR	ZONMW-20_00243	oxyR
oxyR	ZONMW-20_03946	oxyR
oxyR	ZONMW-20_02123	oxyR
oxyR	ZONMW-20_00701	oxyR
oxyR	ZONMW-20_01458	oxyR
oxyR	ZONMW-20_00276	oxyR
oxyR	ZONMW-20_00325	oxyR
oxyR	ZONMW-20_00879	oxyR
oxyR	ZONMW-20_02042	oxyR
oxyR	ZONMW-20_00586	oxyR
oxyR	ZONMW-20_00538	oxyR
oxyR	ZONMW-20_00405	oxyR
oxyR	ZONMW-20_00229	oxyR
oxyR	ZONMW-20_03133	oxyR

oxyR	ZONMW-20_03280	oxyR
oxyR	ZONMW-20_02805	oxyR
oxyR	ZONMW-20_01915	oxyR
oxyR	ZONMW-20_01065	oxyR
oxyR	ZONMW-20_01042	oxyR
oxyR	ZONMW-20_03924	oxyR
oxyR	ZONMW-20_04016	oxyR
oxyR	ZONMW-20_02635	oxyR
oxyR	ZONMW-20_00681	oxyR
oxyR	ZONMW-20_00993	oxyR
oxyR	ZONMW-20_02932	oxyR
oxyR	ZONMW-20_00716	oxyR
oxyR	ZONMW-20_00540	oxyR
oxyR	ZONMW-20_00439	oxyR
oxyR	ZONMW-20_02627	oxyR
oxyR	ZONMW-20_03961	oxyR
oxyR	ZONMW-20_03299	oxyR
oxyR	ZONMW-20_02297	oxyR
oxyR	ZONMW-20_02270	oxyR
oxyR	ZONMW-20_04151	oxyR
oxyR	ZONMW-20_00514	oxyR
oxyR	ZONMW-20_00672	oxyR
oxyR	ZONMW-20_00500	oxyR
oxyR	ZONMW-21_00309	oxyR
oxyR	ZONMW-21_02540	oxyR
oxyR	ZONMW-21_01495	oxyR
oxyR	ZONMW-22_00309	oxyR
oxyR	ZONMW-22_02569	oxyR
oxyR	ZONMW-22_02228	oxyR
oxyR	ZONMW-23_01424	oxyR
oxyR	ZONMW-23_02593	oxyR
oxyR	ZONMW-23_00323	oxyR
oxyR	ZONMW-24_01150	oxyR
oxyR	ZONMW-24_02564	oxyR
oxyR	ZONMW-24_01992	oxyR
oxyR	ZONMW-25_02879	oxyR
oxyR	ZONMW-25_00478	oxyR
oxyR	ZONMW-25_02161	oxyR
oxyR	ZONMW-26_02064	oxyR
oxyR	ZONMW-26_00097	oxyR
oxyR	ZONMW-26_03073	oxyR
oxyR	ZONMW-27_02856	oxyR
oxyR	ZONMW-27_01765	oxyR
oxyR	ZONMW-27_02793	oxyR
oxyR	ZONMW-28_02658	oxyR
oxyR	ZONMW-28_03880	oxyR
oxyR	ZONMW-28_00489	oxyR
oxyR	ZONMW-28_00471	oxyR

oxyR	ZONMW-28_01782	oxyR
oxyR	ZONMW-28_02759	oxyR
oxyR	ZONMW-28_00350	oxyR
oxyR	ZONMW-28_02133	oxyR
oxyR	ZONMW-28_00708	oxyR
oxyR	ZONMW-28_02823	oxyR
oxyR	ZONMW-28_02819	oxyR
oxyR	ZONMW-28_02170	oxyR
oxyR	ZONMW-28_03425	oxyR
oxyR	ZONMW-28_01850	oxyR
oxyR	ZONMW-28_00873	oxyR
oxyR	ZONMW-28_01920	oxyR
oxyR	ZONMW-28_03116	oxyR
oxyR	ZONMW-28_02787	oxyR
oxyR	ZONMW-28_00824	oxyR
oxyR	ZONMW-28_00315	oxyR
oxyR	ZONMW-28_02069	oxyR
oxyR	ZONMW-28_03922	oxyR
oxyR	ZONMW-28_02977	oxyR
oxyR	ZONMW-28_02359	oxyR
oxyR	ZONMW-28_01787	oxyR
oxyR	ZONMW-28_00101	oxyR
oxyR	ZONMW-28_02375	oxyR
oxyR	ZONMW-28_01864	oxyR
oxyR	ZONMW-28_01925	oxyR
oxyR	ZONMW-28_00290	oxyR
oxyR	ZONMW-28_00081	oxyR
oxyR	ZONMW-29_00013	oxyR
oxyR	ZONMW-29_01506	oxyR
oxyR	ZONMW-29_00168	oxyR
oxyR	ZONMW-30_01518	oxyR
oxyR	ZONMW-30_02023	oxyR
oxyR	ZONMW-30_01623	oxyR
oxyR	ZONMW-31_01925	oxyR
oxyR	ZONMW-31_03166	oxyR
oxyR	ZONMW-31_01443	oxyR
oxyR	ZONMW-31_01425	oxyR
oxyR	ZONMW-31_02023	oxyR
oxyR	ZONMW-31_01046	oxyR
oxyR	ZONMW-31_00076	oxyR
oxyR	ZONMW-31_00155	oxyR
oxyR	ZONMW-31_02944	oxyR
oxyR	ZONMW-31_03296	oxyR
oxyR	ZONMW-31_01635	oxyR
oxyR	ZONMW-31_01063	oxyR
oxyR	ZONMW-31_00972	oxyR
oxyR	ZONMW-31_01712	oxyR
oxyR	ZONMW-31_00191	oxyR

oxyR	ZONMW-31_00122	oxyR
oxyR	ZONMW-31_02940	oxyR
oxyR	ZONMW-31_03206	oxyR
oxyR	ZONMW-31_00978	oxyR
oxyR	ZONMW-31_03662	oxyR
oxyR	ZONMW-31_00210	oxyR
oxyR	ZONMW-32_03218	oxyR
oxyR	ZONMW-32_03989	oxyR
oxyR	ZONMW-32_01280	oxyR
oxyR	ZONMW-32_01262	oxyR
oxyR	ZONMW-32_02190	oxyR
oxyR	ZONMW-32_03116	oxyR
oxyR	ZONMW-32_01141	oxyR
oxyR	ZONMW-32_02538	oxyR
oxyR	ZONMW-32_00383	oxyR
oxyR	ZONMW-32_03052	oxyR
oxyR	ZONMW-32_03056	oxyR
oxyR	ZONMW-32_02575	oxyR
oxyR	ZONMW-32_03556	oxyR
oxyR	ZONMW-32_02122	oxyR
oxyR	ZONMW-32_00218	oxyR
oxyR	ZONMW-32_02052	oxyR
oxyR	ZONMW-32_03293	oxyR
oxyR	ZONMW-32_03088	oxyR
oxyR	ZONMW-32_00267	oxyR
oxyR	ZONMW-32_01106	oxyR
oxyR	ZONMW-32_02474	oxyR
oxyR	ZONMW-32_03964	oxyR
oxyR	ZONMW-32_01461	oxyR
oxyR	ZONMW-32_02991	oxyR
oxyR	ZONMW-32_00892	oxyR
oxyR	ZONMW-32_02185	oxyR
oxyR	ZONMW-32_02975	oxyR
oxyR	ZONMW-32_02108	oxyR
oxyR	ZONMW-32_02047	oxyR
oxyR	ZONMW-32_01081	oxyR
oxyR	ZONMW-32_00872	oxyR
oxyR	ZONMW-33_03056	oxyR
oxyR	ZONMW-33_03979	oxyR
oxyR	ZONMW-33_01269	oxyR
oxyR	ZONMW-33_01251	oxyR
oxyR	ZONMW-33_02181	oxyR
oxyR	ZONMW-33_03157	oxyR
oxyR	ZONMW-33_01130	oxyR
oxyR	ZONMW-33_02580	oxyR
oxyR	ZONMW-33_00250	oxyR
oxyR	ZONMW-33_03221	oxyR
oxyR	ZONMW-33_03217	oxyR

oxyR	ZONMW-33_02543	oxyR
oxyR	ZONMW-33_02113	oxyR
oxyR	ZONMW-33_00085	oxyR
oxyR	ZONMW-33_02043	oxyR
oxyR	ZONMW-33_00683	oxyR
oxyR	ZONMW-33_03185	oxyR
oxyR	ZONMW-33_03906	oxyR
oxyR	ZONMW-33_00134	oxyR
oxyR	ZONMW-33_01095	oxyR
oxyR	ZONMW-33_02644	oxyR
oxyR	ZONMW-33_03888	oxyR
oxyR	ZONMW-33_01451	oxyR
oxyR	ZONMW-33_02757	oxyR
oxyR	ZONMW-33_02176	oxyR
oxyR	ZONMW-33_00882	oxyR
oxyR	ZONMW-33_02773	oxyR
oxyR	ZONMW-33_02099	oxyR
oxyR	ZONMW-33_02038	oxyR
oxyR	ZONMW-33_01070	oxyR
oxyR	ZONMW-33_00862	oxyR
oxyR	ZONMW-34_03768	oxyR
oxyR	ZONMW-34_03778	oxyR
oxyR	ZONMW-34_02598	oxyR
oxyR	ZONMW-34_01443	oxyR
oxyR	ZONMW-34_01461	oxyR
oxyR	ZONMW-34_02334	oxyR
oxyR	ZONMW-34_02499	oxyR
oxyR	ZONMW-34_02424	oxyR
oxyR	ZONMW-34_01880	oxyR
oxyR	ZONMW-34_00625	oxyR
oxyR	ZONMW-34_03621	oxyR
oxyR	ZONMW-34_01847	oxyR
oxyR	ZONMW-34_01046	oxyR
oxyR	ZONMW-34_01289	oxyR
oxyR	ZONMW-34_02236	oxyR
oxyR	ZONMW-34_01321	oxyR
oxyR	ZONMW-34_03263	oxyR
oxyR	ZONMW-34_01267	oxyR
oxyR	ZONMW-34_01295	oxyR
oxyR	ZONMW-34_01939	oxyR
oxyR	ZONMW-34_03820	oxyR
oxyR	ZONMW-34_01639	oxyR
oxyR	ZONMW-34_02333	oxyR
oxyR	ZONMW-34_01676	oxyR
oxyR	ZONMW-34_03272	oxyR
oxyR	ZONMW-34_02156	oxyR
oxyR	ZONMW-34_01294	oxyR
oxyR	ZONMW-34_02217	oxyR

oxyR	ZONMW-34_01290	oxyR
oxyR	ZONMW-34_02758	oxyR
oxyR	ZONMW-34_01299	oxyR
oxyR	ZONMW-35_03731	oxyR
oxyR	ZONMW-35_03762	oxyR
oxyR	ZONMW-35_02562	oxyR
oxyR	ZONMW-35_00516	oxyR
oxyR	ZONMW-35_00534	oxyR
oxyR	ZONMW-35_01915	oxyR
oxyR	ZONMW-35_02463	oxyR
oxyR	ZONMW-35_03154	oxyR
oxyR	ZONMW-35_01571	oxyR
oxyR	ZONMW-35_00764	oxyR
oxyR	ZONMW-35_03543	oxyR
oxyR	ZONMW-35_01538	oxyR
oxyR	ZONMW-35_02197	oxyR
oxyR	ZONMW-35_00362	oxyR
oxyR	ZONMW-35_02013	oxyR
oxyR	ZONMW-35_00394	oxyR
oxyR	ZONMW-35_03224	oxyR
oxyR	ZONMW-35_00340	oxyR
oxyR	ZONMW-35_00368	oxyR
oxyR	ZONMW-35_01631	oxyR
oxyR	ZONMW-35_03805	oxyR
oxyR	ZONMW-35_03603	oxyR
oxyR	ZONMW-35_01916	oxyR
oxyR	ZONMW-35_03640	oxyR
oxyR	ZONMW-35_03233	oxyR
oxyR	ZONMW-35_02093	oxyR
oxyR	ZONMW-35_00367	oxyR
oxyR	ZONMW-35_02032	oxyR
oxyR	ZONMW-35_00363	oxyR
oxyR	ZONMW-35_00135	oxyR
oxyR	ZONMW-35_00372	oxyR
oxyR	ZONMW-36_02946	oxyR
oxyR	ZONMW-36_03980	oxyR
oxyR	ZONMW-36_00489	oxyR
oxyR	ZONMW-36_00471	oxyR
oxyR	ZONMW-36_02055	oxyR
oxyR	ZONMW-36_02844	oxyR
oxyR	ZONMW-36_00350	oxyR
oxyR	ZONMW-36_02672	oxyR
oxyR	ZONMW-36_01119	oxyR
oxyR	ZONMW-36_03522	oxyR
oxyR	ZONMW-36_03518	oxyR
oxyR	ZONMW-36_02709	oxyR
oxyR	ZONMW-36_02123	oxyR
oxyR	ZONMW-36_01284	oxyR

oxyR	ZONMW-36_02193	oxyR
oxyR	ZONMW-36_03229	oxyR
oxyR	ZONMW-36_03486	oxyR
oxyR	ZONMW-36_03855	oxyR
oxyR	ZONMW-36_01235	oxyR
oxyR	ZONMW-36_00315	oxyR
oxyR	ZONMW-36_02608	oxyR
oxyR	ZONMW-36_03917	oxyR
oxyR	ZONMW-36_00670	oxyR
oxyR	ZONMW-36_02269	oxyR
oxyR	ZONMW-36_02060	oxyR
oxyR	ZONMW-36_00101	oxyR
oxyR	ZONMW-36_02285	oxyR
oxyR	ZONMW-36_02137	oxyR
oxyR	ZONMW-36_02198	oxyR
oxyR	ZONMW-36_00290	oxyR
oxyR	ZONMW-36_00081	oxyR
oxyR	ZONMW-37_00309	oxyR
oxyR	ZONMW-37_02810	oxyR
oxyR	ZONMW-37_02550	oxyR
oxyR	ZONMW-38_03334	oxyR
oxyR	ZONMW-38_00797	oxyR
oxyR	ZONMW-38_01171	oxyR
oxyR	ZONMW-38_02012	oxyR
oxyR	ZONMW-38_02767	oxyR
oxyR	ZONMW-38_00050	oxyR
oxyR	ZONMW-38_01265	oxyR
oxyR	ZONMW-38_03119	oxyR
oxyR	ZONMW-38_00772	oxyR
oxyR	ZONMW-38_00699	oxyR
oxyR	ZONMW-38_02198	oxyR
oxyR	ZONMW-38_01640	oxyR
oxyR	ZONMW-39_02641	oxyR
oxyR	ZONMW-39_00704	oxyR
oxyR	ZONMW-39_03923	oxyR
oxyR	ZONMW-39_00256	oxyR
oxyR	ZONMW-39_00238	oxyR
oxyR	ZONMW-39_04135	oxyR
oxyR	ZONMW-39_01600	oxyR
oxyR	ZONMW-39_00379	oxyR
oxyR	ZONMW-39_02040	oxyR
oxyR	ZONMW-39_02311	oxyR
oxyR	ZONMW-39_01947	oxyR
oxyR	ZONMW-39_00416	oxyR
oxyR	ZONMW-39_01089	oxyR
oxyR	ZONMW-39_01959	oxyR
oxyR	ZONMW-39_02989	oxyR
oxyR	ZONMW-39_02917	oxyR

oxyR	ZONMW-39_01551	oxyR
oxyR	ZONMW-39_01523	oxyR
oxyR	ZONMW-39_01441	oxyR
oxyR	ZONMW-39_00412	oxyR
oxyR	ZONMW-39_01878	oxyR
oxyR	ZONMW-39_01886	oxyR
oxyR	ZONMW-39_00407	oxyR
oxyR	ZONMW-39_02674	oxyR
oxyR	ZONMW-39_02264	oxyR
oxyR	ZONMW-39_04078	oxyR
oxyR	ZONMW-39_00060	oxyR
oxyR	ZONMW-39_01599	oxyR
oxyR	ZONMW-39_01435	oxyR
oxyR	ZONMW-39_01503	oxyR
oxyR	ZONMW-39_00408	oxyR
oxyR	ZONMW-39_00730	oxyR
oxyR	ZONMW-39_03443	oxyR
oxyR	ZONMW-39_00400	oxyR
oxyR	ZONMW-40_01538	oxyR
oxyR	ZONMW-40_03510	oxyR
oxyR	ZONMW-41_00860	oxyR
oxyR	ZONMW-41_02764	oxyR
oxyR	ZONMW-41_02137	oxyR
oxyR	ZONMW-41_02889	oxyR
oxyR	ZONMW-41_01503	oxyR
oxyR	ZONMW-41_02759	oxyR
oxyR	ZONMW-41_02325	oxyR
oxyR	ZONMW-41_00335	oxyR
oxyR	ZONMW-41_02928	oxyR
oxyR	ZONMW-41_01031	oxyR
oxyR	ZONMW-41_00455	oxyR
oxyR	ZONMW-41_00130	oxyR
oxyR	ZONMW-41_02309	oxyR
oxyR	ZONMW-41_00878	oxyR
oxyR	ZONMW-41_00995	oxyR
oxyR	ZONMW-41_00249	oxyR
oxyR	ZONMW-41_02502	oxyR
oxyR	ZONMW-41_02009	oxyR
oxyR	ZONMW-41_02789	oxyR
oxyR	ZONMW-41_00159	oxyR
oxyR	ZONMW-41_00987	oxyR
oxyR	ZONMW-41_01775	oxyR
oxyR	ZONMW-41_00106	oxyR
oxyR	ZONMW-41_01821	oxyR
oxyR	ZONMW-41_01267	oxyR
oxyR	ZONMW-42_01232	oxyR
oxyR	ZONMW-42_01074	oxyR
oxyR	ZONMW-42_01867	oxyR

oxyR	ZONMW-42_01884	oxyR
oxyR	ZONMW-42_01566	oxyR
oxyR	ZONMW-42_02306	oxyR
oxyR	ZONMW-42_03508	oxyR
oxyR	ZONMW-42_01482	oxyR
oxyR	ZONMW-42_01541	oxyR
oxyR	ZONMW-42_01463	oxyR
oxyR	ZONMW-42_00841	oxyR
oxyR	ZONMW-42_00681	oxyR
oxyR	ZONMW-42_00346	oxyR
oxyR	ZONMW-42_02957	oxyR
oxyR	ZONMW-42_01581	oxyR
oxyR	ZONMW-42_00480	oxyR
oxyR	ZONMW-42_02347	oxyR
oxyR	ZONMW-42_03263	oxyR
oxyR	ZONMW-42_01183	oxyR
oxyR	ZONMW-42_02772	oxyR
oxyR	ZONMW-42_03667	oxyR
oxyR	ZONMW-42_00325	oxyR
oxyR	ZONMW-43_03584	oxyR
oxyR	ZONMW-43_03414	oxyR
oxyR	ZONMW-43_03731	oxyR
oxyR	ZONMW-43_01225	oxyR
oxyR	ZONMW-43_03040	oxyR
oxyR	ZONMW-43_01793	oxyR
oxyR	ZONMW-43_01906	oxyR
oxyR	ZONMW-43_03909	oxyR
oxyR	ZONMW-43_01126	oxyR
oxyR	ZONMW-43_01020	oxyR
oxyR	ZONMW-43_01391	oxyR
oxyR	ZONMW-43_00167	oxyR
oxyR	ZONMW-43_03601	oxyR
oxyR	ZONMW-43_01038	oxyR
oxyR	ZONMW-43_01357	oxyR
oxyR	ZONMW-43_00641	oxyR
oxyR	ZONMW-43_00580	oxyR
oxyR	ZONMW-43_03911	oxyR
oxyR	ZONMW-43_03316	oxyR
oxyR	ZONMW-43_00024	oxyR
oxyR	ZONMW-43_03905	oxyR
oxyR	ZONMW-43_02907	oxyR
oxyR	ZONMW-43_00974	oxyR
oxyR	ZONMW-43_04042	oxyR
oxyR	ZONMW-43_03892	oxyR
oxyR	ZONMW-43_03919	oxyR
oxyR	ZONMW-43_03046	oxyR
oxyR	ZONMW-43_03900	oxyR
oxyR	ZONMW-43_01443	oxyR

oxyR	ZONMW-43_03773	oxyR
oxyR	ZONMW-43_01629	oxyR
oxyR	ZONMW-43_01907	oxyR
oxyR	ZONMW-43_01105	oxyR
oxyR	ZONMW-43_04033	oxyR
oxyR	ZONMW-43_03230	oxyR
oxyR	ZONMW-43_03901	oxyR
oxyR	ZONMW-43_03297	oxyR
oxyR	ZONMW-43_01023	oxyR
oxyR	ZONMW-43_02428	oxyR
oxyR	ZONMW-44_00238	oxyR
oxyR	ZONMW-44_00406	oxyR
oxyR	ZONMW-44_00422	oxyR
oxyR	ZONMW-44_00051	oxyR
oxyR	ZONMW-44_01330	oxyR
oxyR	ZONMW-44_01348	oxyR
oxyR	ZONMW-44_01450	oxyR
oxyR	ZONMW-44_00195	oxyR
oxyR	ZONMW-44_03190	oxyR
oxyR	ZONMW-44_00367	oxyR
oxyR	ZONMW-44_01542	oxyR
oxyR	ZONMW-44_00689	oxyR
oxyR	ZONMW-44_01471	oxyR
oxyR	ZONMW-44_01564	oxyR
oxyR	ZONMW-44_03792	oxyR
oxyR	ZONMW-44_01551	oxyR
oxyR	ZONMW-44_01187	oxyR
oxyR	ZONMW-44_01175	oxyR
oxyR	ZONMW-44_04062	oxyR
oxyR	ZONMW-44_01129	oxyR
oxyR	ZONMW-44_01184	oxyR
oxyR	ZONMW-44_00704	oxyR
oxyR	ZONMW-44_01432	oxyR
oxyR	ZONMW-44_01537	oxyR
oxyR	ZONMW-44_00817	oxyR
oxyR	ZONMW-44_02840	oxyR
oxyR	ZONMW-44_03150	oxyR
oxyR	ZONMW-44_03410	oxyR
oxyR	ZONMW-44_03301	oxyR
oxyR	ZONMW-44_01149	oxyR
oxyR	ZONMW-44_01171	oxyR
oxyR	ZONMW-44_04077	oxyR
oxyR	ZONMW-45_02516	oxyR
oxyR	ZONMW-45_00866	oxyR
oxyR	ZONMW-45_01779	oxyR
oxyR	ZONMW-46_02738	oxyR
oxyR	ZONMW-46_01008	oxyR
oxyR	ZONMW-46_00018	oxyR

oxyR	ZONMW-47_02729	oxyR
oxyR	ZONMW-47_01165	oxyR
oxyR	ZONMW-47_00487	oxyR
oxyR	ZONMW-48_00968	oxyR
oxyR	ZONMW-48_03015	oxyR
oxyR	ZONMW-48_03384	oxyR
oxyR	ZONMW-49_02062	oxyR
oxyR	ZONMW-49_00107	oxyR
oxyR	ZONMW-49_01139	oxyR
oxyR	ZONMW-50_01856	oxyR
oxyR	ZONMW-50_00379	oxyR
oxyR	ZONMW-50_01140	oxyR
oxyR	ZONMW-51_01720	oxyR
oxyR	ZONMW-51_00104	oxyR
oxyR	ZONMW-51_02746	oxyR
oxyR	ZONMW-52_01863	oxyR
oxyR	ZONMW-52_00076	oxyR
oxyR	ZONMW-52_02300	oxyR
oxyR	ZONMW-53_01893	oxyR
oxyR	ZONMW-53_02578	oxyR
oxyR	ZONMW-53_02889	oxyR
oxyR	ZONMW-54_02164	oxyR
oxyR	ZONMW-54_01014	oxyR
oxyR	ZONMW-54_01901	oxyR
oxyR	ZONMW-54_00105	oxyR
oxyR	ZONMW-55_02042	oxyR
oxyR	ZONMW-55_01434	oxyR
oxyR	ZONMW-55_02895	oxyR
oxyR	ZONMW-56_01392	oxyR
oxyR	ZONMW-56_00660	oxyR
oxyR	ZONMW-56_02656	oxyR
oxyR	ZONMW-57_01832	oxyR
oxyR	ZONMW-57_00866	oxyR
oxyR	ZONMW-57_02800	oxyR
oxyR	ZONMW-58_01856	oxyR
oxyR	ZONMW-58_00105	oxyR
oxyR	ZONMW-58_02824	oxyR
oxyR	ZONMW-59_01669	oxyR
oxyR	ZONMW-59_00861	oxyR
oxyR	ZONMW-59_02904	oxyR
oxyR	ZONMW-60_02047	oxyR
oxyR	ZONMW-60_03363	oxyR
oxyR	ZONMW-60_00959	oxyR
oxyR	ZONMW-60_00865	oxyR
oxyR	ZONMW-60_02918	oxyR
oxyR	ZONMW-61_00762	oxyR
oxyR	ZONMW-61_04020	oxyR
oxyR	ZONMW-61_03269	oxyR

oxyR	ZONMW-61_01714	oxyR
oxyR	ZONMW-62_02407	oxyR
oxyR	ZONMW-62_00340	oxyR
oxyR	ZONMW-62_01337	oxyR
oxyR	ZONMW-62_03275	oxyR
oxyR	ZONMW-62_00515	oxyR
oxyR	ZONMW-63_02759	oxyR
oxyR	ZONMW-63_03303	oxyR
oxyR	ZONMW-63_02000	oxyR
oxyR	ZONMW-63_02273	oxyR
oxyR	ZONMW-64_02718	oxyR
oxyR	ZONMW-64_04157	oxyR
oxyR	ZONMW-64_03575	oxyR
oxyR	ZONMW-64_03310	oxyR
oxyR	ZONMW-64_02804	oxyR
oxyR	ZONMW-64_01518	oxyR
oxyR	ZONMW-64_02173	oxyR
oxyR	ZONMW-64_03476	oxyR
oxyR	ZONMW-64_02556	oxyR
oxyR	ZONMW-64_01973	oxyR
oxyR	ZONMW-64_00182	oxyR
oxyR	ZONMW-64_02735	oxyR
oxyR	ZONMW-64_02538	oxyR
oxyR	ZONMW-64_00903	oxyR
oxyR	ZONMW-64_02149	oxyR
oxyR	ZONMW-64_00598	oxyR
oxyR	ZONMW-64_02175	oxyR
oxyR	ZONMW-64_01444	oxyR
oxyR	ZONMW-64_01258	oxyR
oxyR	ZONMW-64_00044	oxyR
oxyR	ZONMW-64_02168	oxyR
oxyR	ZONMW-64_03437	oxyR
oxyR	ZONMW-64_02623	oxyR
oxyR	ZONMW-64_02155	oxyR
oxyR	ZONMW-64_02183	oxyR
oxyR	ZONMW-64_02208	oxyR
oxyR	ZONMW-64_02163	oxyR
oxyR	ZONMW-64_02020	oxyR
oxyR	ZONMW-64_04093	oxyR
oxyR	ZONMW-64_03065	oxyR
oxyR	ZONMW-64_01517	oxyR
oxyR	ZONMW-64_02632	oxyR
oxyR	ZONMW-64_01358	oxyR
oxyR	ZONMW-64_02164	oxyR
oxyR	ZONMW-64_01425	oxyR
oxyR	ZONMW-64_02553	oxyR
oxyR	ZONMW-64_02405	oxyR
oxyR	ZONMW-65_00724	oxyR

oxyR	ZONMW-65_01019	oxyR
oxyR	ZONMW-65_02560	oxyR
oxyR	ZONMW-65_02282	oxyR
oxyR	ZONMW-65_02606	oxyR
oxyR	ZONMW-65_01401	oxyR
oxyR	ZONMW-65_03523	oxyR
oxyR	ZONMW-65_00721	oxyR
oxyR	ZONMW-65_03021	oxyR
oxyR	ZONMW-65_01572	oxyR
oxyR	ZONMW-65_02613	oxyR
oxyR	ZONMW-65_01407	oxyR
oxyR	ZONMW-65_00164	oxyR
oxyR	ZONMW-65_01989	oxyR
oxyR	ZONMW-65_02994	oxyR
oxyR	ZONMW-65_01649	oxyR
oxyR	ZONMW-65_01441	oxyR
oxyR	ZONMW-65_01116	oxyR
oxyR	ZONMW-66_04305	oxyR
oxyR	ZONMW-66_01235	oxyR
oxyR	ZONMW-66_02943	oxyR
oxyR	ZONMW-66_02064	oxyR
oxyR	ZONMW-66_03827	oxyR
oxyR	ZONMW-66_04799	oxyR
oxyR	ZONMW-66_02951	oxyR
oxyR	ZONMW-66_03212	oxyR
oxyR	ZONMW-66_01398	oxyR
oxyR	ZONMW-66_01594	oxyR
oxyR	ZONMW-66_00318	oxyR
oxyR	ZONMW-66_04628	oxyR
oxyR	ZONMW-66_01336	oxyR
oxyR	ZONMW-66_03109	oxyR
oxyR	ZONMW-66_04388	oxyR
oxyR	ZONMW-66_04805	oxyR
oxyR	ZONMW-66_03956	oxyR
oxyR	ZONMW-66_00377	oxyR
oxyR	ZONMW-66_02939	oxyR
oxyR	ZONMW-66_02948	oxyR
oxyR	ZONMW-66_04439	oxyR
oxyR	ZONMW-67_03025	oxyR
oxyR	ZONMW-67_03320	oxyR
oxyR	ZONMW-67_03557	oxyR
oxyR	ZONMW-67_01410	oxyR
oxyR	ZONMW-67_00776	oxyR
oxyR	ZONMW-67_02135	oxyR
oxyR	ZONMW-67_03418	oxyR
oxyR	ZONMW-67_01392	oxyR
oxyR	ZONMW-67_00795	oxyR
oxyR	ZONMW-67_02066	oxyR

oxyR	ZONMW-67_01573	oxyR
oxyR	ZONMW-67_00831	oxyR
oxyR	ZONMW-67_02149	oxyR
oxyR	ZONMW-67_01683	oxyR
oxyR	ZONMW-67_00221	oxyR
oxyR	ZONMW-67_02089	oxyR
oxyR	ZONMW-67_03154	oxyR
oxyR	ZONMW-67_02141	oxyR
oxyR	ZONMW-67_00753	oxyR
oxyR	ZONMW-67_00282	oxyR
oxyR	ZONMW-67_00659	oxyR
oxyR	ZONMW-67_03138	oxyR
oxyR	ZONMW-67_02143	oxyR
oxyR	ZONMW-67_03597	oxyR
oxyR	ZONMW-67_00724	oxyR
oxyR	ZONMW-67_00742	oxyR
oxyR	ZONMW-67_02093	oxyR
oxyR	ZONMW-67_00847	oxyR
oxyR	ZONMW-67_02107	oxyR
oxyR	ZONMW-67_02337	oxyR
oxyR	ZONMW-67_00202	oxyR
oxyR	ZONMW-67_02092	oxyR
oxyR	ZONMW-67_01994	oxyR
oxyR	ZONMW-67_01829	oxyR
oxyR	ZONMW-67_03045	oxyR
oxyR	ZONMW-67_00299	oxyR
oxyR	ZONMW-68_02761	oxyR
oxyR	ZONMW-69_02929	oxyR
oxyR	ZONMW-69_03321	oxyR
oxyR	ZONMW-69_03559	oxyR
oxyR	ZONMW-69_01590	oxyR
oxyR	ZONMW-69_00823	oxyR
oxyR	ZONMW-69_01808	oxyR
oxyR	ZONMW-69_03419	oxyR
oxyR	ZONMW-69_01608	oxyR
oxyR	ZONMW-69_00842	oxyR
oxyR	ZONMW-69_01877	oxyR
oxyR	ZONMW-69_01428	oxyR
oxyR	ZONMW-69_00878	oxyR
oxyR	ZONMW-69_01794	oxyR
oxyR	ZONMW-69_01318	oxyR
oxyR	ZONMW-69_02830	oxyR
oxyR	ZONMW-69_01854	oxyR
oxyR	ZONMW-69_03155	oxyR
oxyR	ZONMW-69_01802	oxyR
oxyR	ZONMW-69_00800	oxyR
oxyR	ZONMW-69_00029	oxyR
oxyR	ZONMW-69_00706	oxyR

oxyR	ZONMW-69_03139	oxyR
oxyR	ZONMW-69_01800	oxyR
oxyR	ZONMW-69_03599	oxyR
oxyR	ZONMW-69_00771	oxyR
oxyR	ZONMW-69_00789	oxyR
oxyR	ZONMW-69_01850	oxyR
oxyR	ZONMW-69_00894	oxyR
oxyR	ZONMW-69_01836	oxyR
oxyR	ZONMW-69_02385	oxyR
oxyR	ZONMW-69_02811	oxyR
oxyR	ZONMW-69_01851	oxyR
oxyR	ZONMW-69_01949	oxyR
oxyR	ZONMW-69_02114	oxyR
oxyR	ZONMW-69_02908	oxyR
oxyR	ZONMW-69_00046	oxyR
oxyR	ZONMW-72_01909	oxyR
oxyR	ZONMW-72_02264	oxyR
oxyR	ZONMW-72_01803	oxyR
oxyR	ZONMW-72_00243	oxyR
oxyR	ZONMW-72_04173	oxyR
oxyR	ZONMW-72_01777	oxyR
oxyR	ZONMW-72_02753	oxyR
oxyR	ZONMW-73_02608	oxyR
oxyR	ZONMW-73_02633	oxyR
oxyR	ZONMW-73_00409	oxyR
oxyR	ZONMW-73_01576	oxyR
oxyR	ZONMW-74_03347	oxyR
oxyR	ZONMW-74_02982	oxyR
oxyR	ZONMW-74_01284	oxyR
oxyR	ZONMW-75_00841	oxyR
oxyR	ZONMW-75_00420	oxyR
oxyR	ZONMW-75_01278	oxyR
oxyR	ZONMW-75_03276	oxyR
oxyR	da5A-8_02327	oxyR
oxyR	da5A-8_03422	oxyR
oxyR	da5A-8_03633	oxyR
oxyR	da5A-8_01450	oxyR
oxyR	da5A-8_03743	oxyR
rpmI	18_02057	rpmI
rpmI	ACAM48_01441	rpmI
rpmI	CAM030_01793	rpmI
rpmI	CECT7946_02774	rpmI
rpmI	CGMCC1.8863_02816	rpmI
rpmI	CL8139_03219	rpmI
rpmI	DAU203_02554	rpmI
rpmI	DD5b_01122	rpmI
rpmI	DSM1208_02928	rpmI
rpmI	DSM12212_03454	rpmI

rpml	DSM15343_02864	rpml
rpml	DSM15361_02236	rpml
rpml	DSM15365_01300	rpml
rpml	DSM15718_03369	rpml
rpml	DSM16505T_00002	rpml
rpml	DSM18103_01548	rpml
rpml	DSM18293_02791	rpml
rpml	DSM18841_02474	rpml
rpml	DSM18842_02381	rpml
rpml	DSM19866_02697	rpml
rpml	DSM2041_05500	rpml
rpml	DSM2061_04411	rpml
rpml	DSM2064_05070	rpml
rpml	DSM21164_03003	rpml
rpml	DSM23404_00003	rpml
rpml	DSM23424_03342	rpml
rpml	DSM24786_01472	rpml
rpml	DSM7489_02445	rpml
rpml	DSW1_00063	rpml
rpml	DSW5_01776	rpml
rpml	DSW6_00220	rpml
rpml	DSW8_01026	rpml
rpml	F52_01925	rpml
rpml	GCA_001027945.1_01091	rpml
rpml	HI1_02377	rpml
rpml	IR1_02715	rpml
rpml	KCOM3131_01691	rpml
rpml	KCTC12974_00685	rpml
rpml	KL-A_03249	rpml
rpml	L12_00027	rpml
rpml	LL0412.1.7_00003	rpml
rpml	MAR_2009_71_02415	rpml
rpml	NN016038_02075	rpml
rpml	PAO1_02825	rpml
rpml	UW101_00032	rpml
rpml	V4-33_00808	rpml
rpml	W5C_02095	rpml
rpml	ZONMW-01_03268	rpml
rpml	ZONMW-02_00855	rpml
rpml	ZONMW-03_03398	rpml
rpml	ZONMW-04_00003	rpml
rpml	ZONMW-05_01825	rpml
rpml	ZONMW-06_03484	rpml
rpml	ZONMW-07_03777	rpml
rpml	ZONMW-08_00822	rpml
rpml	ZONMW-09_01790	rpml
rpml	ZONMW-10_01434	rpml
rpml	ZONMW-11_00949	rpml

rpml	ZONMW-12_04458	rpml
rpml	ZONMW-13_03142	rpml
rpml	ZONMW-14_01776	rpml
rpml	ZONMW-15_02038	rpml
rpml	ZONMW-19_02929	rpml
rpml	ZONMW-20_01638	rpml
rpml	ZONMW-21_03391	rpml
rpml	ZONMW-22_03334	rpml
rpml	ZONMW-23_03437	rpml
rpml	ZONMW-24_03439	rpml
rpml	ZONMW-25_03131	rpml
rpml	ZONMW-26_01462	rpml
rpml	ZONMW-27_00003	rpml
rpml	ZONMW-28_02304	rpml
rpml	ZONMW-29_02903	rpml
rpml	ZONMW-30_02870	rpml
rpml	ZONMW-31_02430	rpml
rpml	ZONMW-32_02710	rpml
rpml	ZONMW-33_02408	rpml
rpml	ZONMW-34_01730	rpml
rpml	ZONMW-35_01421	rpml
rpml	ZONMW-36_03839	rpml
rpml	ZONMW-37_03343	rpml
rpml	ZONMW-38_00214	rpml
rpml	ZONMW-39_03754	rpml
rpml	ZONMW-40_03467	rpml
rpml	ZONMW-41_01492	rpml
rpml	ZONMW-42_00717	rpml
rpml	ZONMW-43_03346	rpml
rpml	ZONMW-44_02626	rpml
rpml	ZONMW-45_02681	rpml
rpml	ZONMW-46_02544	rpml
rpml	ZONMW-47_02534	rpml
rpml	ZONMW-48_03341	rpml
rpml	ZONMW-49_00487	rpml
rpml	ZONMW-50_00488	rpml
rpml	ZONMW-51_01217	rpml
rpml	ZONMW-52_02911	rpml
rpml	ZONMW-53_00003	rpml
rpml	ZONMW-54_01033	rpml
rpml	ZONMW-55_00003	rpml
rpml	ZONMW-56_02807	rpml
rpml	ZONMW-57_01615	rpml
rpml	ZONMW-58_00969	rpml
rpml	ZONMW-59_02721	rpml
rpml	ZONMW-60_03255	rpml
rpml	ZONMW-61_04008	rpml
rpml	ZONMW-62_02594	rpml

rpmI	ZONMW-63_03288	rpmI
rpmI	ZONMW-64_01822	rpmI
rpmI	ZONMW-65_03082	rpmI
rpmI	ZONMW-66_00858	rpmI
rpmI	ZONMW-67_03219	rpmI
rpmI	ZONMW-68_02497	rpmI
rpmI	ZONMW-69_03220	rpmI
rpmI	ZONMW-72_01282	rpmI
rpmI	ZONMW-73_02289	rpmI
rpmI	ZONMW-74_00694	rpmI
rpmI	ZONMW-75_00936	rpmI
rpmI	da5A-8_00209	rpmI
rpsB	18_00665	rpsB
rpsB	18_01533	rpsB
rpsB	18_00793	rpsB
rpsB	ACAM48_02646	rpsB
rpsB	ACAM48_02030	rpsB
rpsB	CAM030_02087	rpsB
rpsB	CAM030_02892	rpsB
rpsB	CAM030_01326	rpsB
rpsB	CECT7946_01936	rpsB
rpsB	CECT7946_01906	rpsB
rpsB	CECT7946_01760	rpsB
rpsB	CGMCC1.8863_00139	rpsB
rpsB	CGMCC1.8863_02162	rpsB
rpsB	CGMCC1.8863_01068	rpsB
rpsB	CL8139_01394	rpsB
rpsB	CL8139_00146	rpsB
rpsB	CL8139_01634	rpsB
rpsB	DAU203_01116	rpsB
rpsB	DAU203_01957	rpsB
rpsB	DAU203_01241	rpsB
rpsB	DD5b_00369	rpsB
rpsB	DD5b_01829	rpsB
rpsB	DSM1208_01316	rpsB
rpsB	DSM1208_02258	rpsB
rpsB	DSM1208_01439	rpsB
rpsB	DSM12212_02105	rpsB
rpsB	DSM12212_01709	rpsB
rpsB	DSM15343_02564	rpsB
rpsB	DSM15343_00688	rpsB
rpsB	DSM15343_02586	rpsB
rpsB	DSM15361_00261	rpsB
rpsB	DSM15361_01402	rpsB
rpsB	DSM15365_03341	rpsB
rpsB	DSM15365_03252	rpsB
rpsB	DSM15365_03402	rpsB
rpsB	DSM15718_01263	rpsB

rpsB	DSM15718_01286	rpsB
rpsB	DSM16505T_00590	rpsB
rpsB	DSM16505T_00480	rpsB
rpsB	DSM16505T_00866	rpsB
rpsB	DSM18103_01004	rpsB
rpsB	DSM18103_02814	rpsB
rpsB	DSM18103_03172	rpsB
rpsB	DSM18293_00627	rpsB
rpsB	DSM18293_01854	rpsB
rpsB	DSM18293_00699	rpsB
rpsB	DSM18841_00446	rpsB
rpsB	DSM18841_00323	rpsB
rpsB	DSM18841_00733	rpsB
rpsB	DSM18842_00783	rpsB
rpsB	DSM18842_00906	rpsB
rpsB	DSM18842_00587	rpsB
rpsB	DSM19866_03605	rpsB
rpsB	DSM19866_00644	rpsB
rpsB	DSM19866_03485	rpsB
rpsB	DSM2041_00585	rpsB
rpsB	DSM2041_04633	rpsB
rpsB	DSM2061_03573	rpsB
rpsB	DSM2061_03788	rpsB
rpsB	DSM2061_02961	rpsB
rpsB	DSM2064_02557	rpsB
rpsB	DSM2064_00203	rpsB
rpsB	DSM2064_02622	rpsB
rpsB	DSM21164_01156	rpsB
rpsB	DSM21164_00344	rpsB
rpsB	DSM21164_01048	rpsB
rpsB	DSM23404_02308	rpsB
rpsB	DSM23404_01831	rpsB
rpsB	DSM23424_00095	rpsB
rpsB	DSM23424_00466	rpsB
rpsB	DSM23424_00634	rpsB
rpsB	DSM23424_01988	rpsB
rpsB	DSM24786_03343	rpsB
rpsB	DSM24786_02091	rpsB
rpsB	DSM24786_02889	rpsB
rpsB	DSM7489_01035	rpsB
rpsB	DSM7489_01868	rpsB
rpsB	DSM7489_01150	rpsB
rpsB	DSW1_02910	rpsB
rpsB	DSW1_00728	rpsB
rpsB	DSW1_00162	rpsB
rpsB	DSW5_01049	rpsB
rpsB	DSW5_00907	rpsB
rpsB	DSW5_00185	rpsB

rpsB	DSW6_01565	rpsB
rpsB	DSW6_01015	rpsB
rpsB	DSW8_02610	rpsB
rpsB	DSW8_01688	rpsB
rpsB	DSW8_03651	rpsB
rpsB	DSW8_02502	rpsB
rpsB	F52_02954	rpsB
rpsB	F52_04376	rpsB
rpsB	F52_03028	rpsB
rpsB	GCA_001027945.1_00161	rpsB
rpsB	GCA_001027945.1_03159	rpsB
rpsB	HI1_00973	rpsB
rpsB	HI1_01796	rpsB
rpsB	HI1_01092	rpsB
rpsB	IR1_00324	rpsB
rpsB	IR1_01835	rpsB
rpsB	IR1_00259	rpsB
rpsB	KCOM3131_00491	rpsB
rpsB	KCOM3131_00437	rpsB
rpsB	KCTC12974_02017	rpsB
rpsB	KCTC12974_00805	rpsB
rpsB	KL-A_02278	rpsB
rpsB	KL-A_00403	rpsB
rpsB	KL-A_02095	rpsB
rpsB	L12_00270	rpsB
rpsB	L12_00271	rpsB
rpsB	LL0412.1.7_00553	rpsB
rpsB	LL0412.1.7_00866	rpsB
rpsB	MAR_2009_71_00843	rpsB
rpsB	MAR_2009_71_01729	rpsB
rpsB	MAR_2009_71_02803	rpsB
rpsB	MAR_2009_71_00951	rpsB
rpsB	NN016038_00680	rpsB
rpsB	NN016038_01540	rpsB
rpsB	NN016038_00813	rpsB
rpsB	PAO1_04716	rpsB
rpsB	PAO1_03779	rpsB
rpsB	UW101_01611	rpsB
rpsB	UW101_02914	rpsB
rpsB	UW101_01676	rpsB
rpsB	V4-33_02139	rpsB
rpsB	V4-33_00434	rpsB
rpsB	W5C_03078	rpsB
rpsB	W5C_00554	rpsB
rpsB	W5C_01993	rpsB
rpsB	ZONMW-01_02905	rpsB
rpsB	ZONMW-01_00322	rpsB
rpsB	ZONMW-01_01115	rpsB

rpsB	ZONMW-02_00274	rpsB
rpsB	ZONMW-02_00185	rpsB
rpsB	ZONMW-02_00328	rpsB
rpsB	ZONMW-03_01951	rpsB
rpsB	ZONMW-03_01048	rpsB
rpsB	ZONMW-03_02184	rpsB
rpsB	ZONMW-04_02913	rpsB
rpsB	ZONMW-04_00624	rpsB
rpsB	ZONMW-04_01634	rpsB
rpsB	ZONMW-05_03012	rpsB
rpsB	ZONMW-05_01201	rpsB
rpsB	ZONMW-05_01912	rpsB
rpsB	ZONMW-06_03949	rpsB
rpsB	ZONMW-06_03528	rpsB
rpsB	ZONMW-07_00657	rpsB
rpsB	ZONMW-07_00334	rpsB
rpsB	ZONMW-07_00725	rpsB
rpsB	ZONMW-08_00361	rpsB
rpsB	ZONMW-08_01505	rpsB
rpsB	ZONMW-08_02020	rpsB
rpsB	ZONMW-08_00470	rpsB
rpsB	ZONMW-09_00564	rpsB
rpsB	ZONMW-09_00039	rpsB
rpsB	ZONMW-10_00611	rpsB
rpsB	ZONMW-10_00641	rpsB
rpsB	ZONMW-11_02697	rpsB
rpsB	ZONMW-11_01523	rpsB
rpsB	ZONMW-12_00476	rpsB
rpsB	ZONMW-12_00635	rpsB
rpsB	ZONMW-13_00749	rpsB
rpsB	ZONMW-13_00954	rpsB
rpsB	ZONMW-14_01297	rpsB
rpsB	ZONMW-14_01152	rpsB
rpsB	ZONMW-15_00874	rpsB
rpsB	ZONMW-15_00389	rpsB
rpsB	ZONMW-15_01789	rpsB
rpsB	ZONMW-19_00606	rpsB
rpsB	ZONMW-19_00796	rpsB
rpsB	ZONMW-20_02864	rpsB
rpsB	ZONMW-20_04125	rpsB
rpsB	ZONMW-21_01166	rpsB
rpsB	ZONMW-21_00233	rpsB
rpsB	ZONMW-21_01036	rpsB
rpsB	ZONMW-22_01168	rpsB
rpsB	ZONMW-22_00233	rpsB
rpsB	ZONMW-22_01037	rpsB
rpsB	ZONMW-23_02280	rpsB
rpsB	ZONMW-23_01348	rpsB

rpsB	ZONMW-23_02150	rpsB
rpsB	ZONMW-24_00293	rpsB
rpsB	ZONMW-24_01226	rpsB
rpsB	ZONMW-24_00424	rpsB
rpsB	ZONMW-25_03307	rpsB
rpsB	ZONMW-25_03064	rpsB
rpsB	ZONMW-25_02041	rpsB
rpsB	ZONMW-26_01904	rpsB
rpsB	ZONMW-26_01255	rpsB
rpsB	ZONMW-26_02484	rpsB
rpsB	ZONMW-27_03033	rpsB
rpsB	ZONMW-27_00618	rpsB
rpsB	ZONMW-27_01663	rpsB
rpsB	ZONMW-28_03925	rpsB
rpsB	ZONMW-28_03401	rpsB
rpsB	ZONMW-29_00757	rpsB
rpsB	ZONMW-29_02971	rpsB
rpsB	ZONMW-29_00607	rpsB
rpsB	ZONMW-30_00443	rpsB
rpsB	ZONMW-30_01440	rpsB
rpsB	ZONMW-30_01756	rpsB
rpsB	ZONMW-31_03209	rpsB
rpsB	ZONMW-31_02387	rpsB
rpsB	ZONMW-32_03961	rpsB
rpsB	ZONMW-32_03580	rpsB
rpsB	ZONMW-33_03885	rpsB
rpsB	ZONMW-33_03930	rpsB
rpsB	ZONMW-34_03823	rpsB
rpsB	ZONMW-34_03406	rpsB
rpsB	ZONMW-35_03802	rpsB
rpsB	ZONMW-35_03409	rpsB
rpsB	ZONMW-36_03920	rpsB
rpsB	ZONMW-36_03879	rpsB
rpsB	ZONMW-37_01196	rpsB
rpsB	ZONMW-37_00233	rpsB
rpsB	ZONMW-37_01326	rpsB
rpsB	ZONMW-38_02308	rpsB
rpsB	ZONMW-38_00855	rpsB
rpsB	ZONMW-39_04075	rpsB
rpsB	ZONMW-39_03270	rpsB
rpsB	ZONMW-40_00108	rpsB
rpsB	ZONMW-40_02646	rpsB
rpsB	ZONMW-40_03306	rpsB
rpsB	ZONMW-40_00222	rpsB
rpsB	ZONMW-41_02672	rpsB
rpsB	ZONMW-41_02498	rpsB
rpsB	ZONMW-42_02350	rpsB
rpsB	ZONMW-42_02874	rpsB

rpsB	ZONMW-43_03776	rpsB
rpsB	ZONMW-43_02629	rpsB
rpsB	ZONMW-44_03147	rpsB
rpsB	ZONMW-44_02582	rpsB
rpsB	ZONMW-45_00585	rpsB
rpsB	ZONMW-45_00477	rpsB
rpsB	ZONMW-45_01092	rpsB
rpsB	ZONMW-46_00874	rpsB
rpsB	ZONMW-46_01362	rpsB
rpsB	ZONMW-46_00575	rpsB
rpsB	ZONMW-47_00874	rpsB
rpsB	ZONMW-47_01490	rpsB
rpsB	ZONMW-47_00575	rpsB
rpsB	ZONMW-48_00287	rpsB
rpsB	ZONMW-48_01026	rpsB
rpsB	ZONMW-48_00176	rpsB
rpsB	ZONMW-49_02225	rpsB
rpsB	ZONMW-49_01410	rpsB
rpsB	ZONMW-49_02896	rpsB
rpsB	ZONMW-50_02019	rpsB
rpsB	ZONMW-50_01412	rpsB
rpsB	ZONMW-50_02760	rpsB
rpsB	ZONMW-51_03092	rpsB
rpsB	ZONMW-51_01526	rpsB
rpsB	ZONMW-51_02878	rpsB
rpsB	ZONMW-52_02027	rpsB
rpsB	ZONMW-52_01266	rpsB
rpsB	ZONMW-52_02996	rpsB
rpsB	ZONMW-53_01721	rpsB
rpsB	ZONMW-53_00559	rpsB
rpsB	ZONMW-53_03003	rpsB
rpsB	ZONMW-54_02314	rpsB
rpsB	ZONMW-54_00476	rpsB
rpsB	ZONMW-54_03004	rpsB
rpsB	ZONMW-55_03059	rpsB
rpsB	ZONMW-55_00554	rpsB
rpsB	ZONMW-55_03010	rpsB
rpsB	ZONMW-56_01234	rpsB
rpsB	ZONMW-56_03012	rpsB
rpsB	ZONMW-56_02889	rpsB
rpsB	ZONMW-57_02963	rpsB
rpsB	ZONMW-57_01060	rpsB
rpsB	ZONMW-57_02852	rpsB
rpsB	ZONMW-58_03030	rpsB
rpsB	ZONMW-58_01524	rpsB
rpsB	ZONMW-58_02876	rpsB
rpsB	ZONMW-59_01838	rpsB
rpsB	ZONMW-59_01062	rpsB

rpsB	ZONMW-59_03098	rpsB
rpsB	ZONMW-60_02192	rpsB
rpsB	ZONMW-60_01505	rpsB
rpsB	ZONMW-60_02970	rpsB
rpsB	ZONMW-61_01746	rpsB
rpsB	ZONMW-61_02890	rpsB
rpsB	ZONMW-61_00041	rpsB
rpsB	ZONMW-61_02414	rpsB
rpsB	ZONMW-62_01486	rpsB
rpsB	ZONMW-62_02337	rpsB
rpsB	ZONMW-62_00740	rpsB
rpsB	ZONMW-62_01595	rpsB
rpsB	ZONMW-63_03024	rpsB
rpsB	ZONMW-63_00816	rpsB
rpsB	ZONMW-63_02860	rpsB
rpsB	ZONMW-64_04096	rpsB
rpsB	ZONMW-64_03284	rpsB
rpsB	ZONMW-65_01221	rpsB
rpsB	ZONMW-65_00904	rpsB
rpsB	ZONMW-66_03410	rpsB
rpsB	ZONMW-66_00179	rpsB
rpsB	ZONMW-67_03600	rpsB
rpsB	ZONMW-67_03173	rpsB
rpsB	ZONMW-68_00728	rpsB
rpsB	ZONMW-68_03903	rpsB
rpsB	ZONMW-68_00605	rpsB
rpsB	ZONMW-69_03602	rpsB
rpsB	ZONMW-69_03174	rpsB
rpsB	ZONMW-72_03787	rpsB
rpsB	ZONMW-72_03065	rpsB
rpsB	ZONMW-72_00425	rpsB
rpsB	ZONMW-73_01749	rpsB
rpsB	ZONMW-73_03185	rpsB
rpsB	ZONMW-74_02051	rpsB
rpsB	ZONMW-74_01834	rpsB
rpsB	ZONMW-74_02178	rpsB
rpsB	ZONMW-75_01137	rpsB
rpsB	ZONMW-75_01051	rpsB
rpsB	ZONMW-75_01186	rpsB
rpsB	da5A-8_01230	rpsB
rpsB	da5A-8_01872	rpsB
rpsB	da5A-8_01746	rpsB
clpC	18_02358	clpC
clpC	18_03036	clpC
clpC	18_03085	clpC
clpC	ACAM48_01972	clpC
clpC	ACAM48_03488	clpC
clpC	CAM030_00755	clpC

clpC	CAM030_00383	clpC
clpC	CECT7946_01483	clpC
clpC	CECT7946_01510	clpC
clpC	CGMCC1.8863_03218	clpC
clpC	CGMCC1.8863_02921	clpC
clpC	CL8139_00420	clpC
clpC	CL8139_02023	clpC
clpC	DAU203_02567	clpC
clpC	DAU203_02829	clpC
clpC	DAU203_00758	clpC
clpC	DD5b_00260	clpC
clpC	DSM1208_00450	clpC
clpC	DSM1208_04556	clpC
clpC	DSM1208_00390	clpC
clpC	DSM12212_00537	clpC
clpC	DSM12212_01862	clpC
clpC	DSM15343_00320	clpC
clpC	DSM15343_02243	clpC
clpC	DSM15343_00605	clpC
clpC	DSM15361_00971	clpC
clpC	DSM15361_01257	clpC
clpC	DSM15361_01674	clpC
clpC	DSM15365_02088	clpC
clpC	DSM15365_02477	clpC
clpC	DSM15365_02802	clpC
clpC	DSM15718_01812	clpC
clpC	DSM15718_01568	clpC
clpC	DSM16505T_02032	clpC
clpC	DSM16505T_01493	clpC
clpC	DSM18103_02015	clpC
clpC	DSM18103_00095	clpC
clpC	DSM18103_01961	clpC
clpC	DSM18293_04021	clpC
clpC	DSM18293_04181	clpC
clpC	DSM18841_01278	clpC
clpC	DSM18841_02115	clpC
clpC	DSM18841_02556	clpC
clpC	DSM18842_02913	clpC
clpC	DSM18842_01458	clpC
clpC	DSM18842_02456	clpC
clpC	DSM19866_03253	clpC
clpC	DSM19866_04666	clpC
clpC	DSM2041_04075	clpC
clpC	DSM2041_04859	clpC
clpC	DSM2041_04066	clpC
clpC	DSM2061_01939	clpC
clpC	DSM2061_00598	clpC
clpC	DSM2061_00305	clpC

clpC	DSM2064_03102	clpC
clpC	DSM2064_00857	clpC
clpC	DSM2064_00712	clpC
clpC	DSM21164_01801	clpC
clpC	DSM21164_01457	clpC
clpC	DSM23404_01306	clpC
clpC	DSM23404_00667	clpC
clpC	DSM23424_01009	clpC
clpC	DSM23424_01676	clpC
clpC	DSM24786_00035	clpC
clpC	DSM24786_00287	clpC
clpC	DSM7489_02458	clpC
clpC	DSM7489_02700	clpC
clpC	DSW1_02087	clpC
clpC	DSW1_02053	clpC
clpC	DSW5_00051	clpC
clpC	DSW5_02162	clpC
clpC	DSW6_02522	clpC
clpC	DSW6_02934	clpC
clpC	DSW8_03102	clpC
clpC	DSW8_00527	clpC
clpC	F52_00561	clpC
clpC	F52_00805	clpC
clpC	F52_00458	clpC
clpC	GCA_001027945.1_02917	clpC
clpC	GCA_001027945.1_01858	clpC
clpC	GCA_001027945.1_00983	clpC
clpC	GCA_001027945.1_01488	clpC
clpC	HI1_02390	clpC
clpC	HI1_02639	clpC
clpC	IR1_02315	clpC
clpC	IR1_02048	clpC
clpC	IR1_00728	clpC
clpC	KCOM3131_00950	clpC
clpC	KCOM3131_01815	clpC
clpC	KCTC12974_00007	clpC
clpC	KCTC12974_01458	clpC
clpC	KL-A_03105	clpC
clpC	KL-A_01358	clpC
clpC	KL-A_03332	clpC
clpC	L12_00309	clpC
clpC	LL0412.1.7_01336	clpC
clpC	LL0412.1.7_02167	clpC
clpC	MAR_2009_71_00032	clpC
clpC	MAR_2009_71_03325	clpC
clpC	NN016038_02369	clpC
clpC	NN016038_03068	clpC
clpC	NN016038_03122	clpC

clpC	PAO1_04689	clpC
clpC	PAO1_00470	clpC
clpC	PAO1_02699	clpC
clpC	PAO1_02444	clpC
clpC	PAO1_00096	clpC
clpC	PAO1_01707	clpC
clpC	PAO1_03483	clpC
clpC	PAO1_02806	clpC
clpC	UW101_02698	clpC
clpC	UW101_03568	clpC
clpC	UW101_03423	clpC
clpC	V4-33_00721	clpC
clpC	V4-33_01673	clpC
clpC	W5C_00969	clpC
clpC	W5C_00753	clpC
clpC	ZONMW-01_03351	clpC
clpC	ZONMW-01_01744	clpC
clpC	ZONMW-01_03370	clpC
clpC	ZONMW-02_03434	clpC
clpC	ZONMW-02_00597	clpC
clpC	ZONMW-03_00720	clpC
clpC	ZONMW-03_00463	clpC
clpC	ZONMW-03_03372	clpC
clpC	ZONMW-04_03300	clpC
clpC	ZONMW-04_01402	clpC
clpC	ZONMW-05_02867	clpC
clpC	ZONMW-05_00726	clpC
clpC	ZONMW-06_03969	clpC
clpC	ZONMW-06_03157	clpC
clpC	ZONMW-06_03779	clpC
clpC	ZONMW-06_01213	clpC
clpC	ZONMW-07_03627	clpC
clpC	ZONMW-07_03360	clpC
clpC	ZONMW-08_03022	clpC
clpC	ZONMW-08_02839	clpC
clpC	ZONMW-09_00733	clpC
clpC	ZONMW-09_01462	clpC
clpC	ZONMW-10_00818	clpC
clpC	ZONMW-10_00755	clpC
clpC	ZONMW-11_01508	clpC
clpC	ZONMW-11_01755	clpC
clpC	ZONMW-12_03121	clpC
clpC	ZONMW-12_03987	clpC
clpC	ZONMW-13_01956	clpC
clpC	ZONMW-13_01433	clpC
clpC	ZONMW-14_00737	clpC
clpC	ZONMW-14_02353	clpC
clpC	ZONMW-15_01506	clpC

clpC	ZONMW-15_02025	clpC
clpC	ZONMW-19_00495	clpC
clpC	ZONMW-19_00241	clpC
clpC	ZONMW-19_00653	clpC
clpC	ZONMW-20_02778	clpC
clpC	ZONMW-20_02395	clpC
clpC	ZONMW-20_03448	clpC
clpC	ZONMW-20_01758	clpC
clpC	ZONMW-21_03199	clpC
clpC	ZONMW-21_01683	clpC
clpC	ZONMW-22_03317	clpC
clpC	ZONMW-22_02041	clpC
clpC	ZONMW-23_03338	clpC
clpC	ZONMW-23_00510	clpC
clpC	ZONMW-24_03340	clpC
clpC	ZONMW-24_02666	clpC
clpC	ZONMW-25_01227	clpC
clpC	ZONMW-25_01475	clpC
clpC	ZONMW-26_03236	clpC
clpC	ZONMW-26_00243	clpC
clpC	ZONMW-26_03382	clpC
clpC	ZONMW-27_03285	clpC
clpC	ZONMW-27_01902	clpC
clpC	ZONMW-28_03949	clpC
clpC	ZONMW-28_02967	clpC
clpC	ZONMW-28_01971	clpC
clpC	ZONMW-29_02271	clpC
clpC	ZONMW-29_03108	clpC
clpC	ZONMW-29_02360	clpC
clpC	ZONMW-29_00672	clpC
clpC	ZONMW-30_02526	clpC
clpC	ZONMW-30_02223	clpC
clpC	ZONMW-31_03234	clpC
clpC	ZONMW-31_01625	clpC
clpC	ZONMW-32_03937	clpC
clpC	ZONMW-32_01451	clpC
clpC	ZONMW-32_02001	clpC
clpC	ZONMW-33_03861	clpC
clpC	ZONMW-33_01441	clpC
clpC	ZONMW-33_01992	clpC
clpC	ZONMW-34_03848	clpC
clpC	ZONMW-34_01629	clpC
clpC	ZONMW-34_02122	clpC
clpC	ZONMW-35_03778	clpC
clpC	ZONMW-35_00700	clpC
clpC	ZONMW-35_02127	clpC
clpC	ZONMW-36_03944	clpC
clpC	ZONMW-36_00660	clpC

clpC	ZONMW-36_02244	clpC
clpC	ZONMW-37_03326	clpC
clpC	ZONMW-37_02061	clpC
clpC	ZONMW-38_01942	clpC
clpC	ZONMW-38_00622	clpC
clpC	ZONMW-39_04050	clpC
clpC	ZONMW-39_00070	clpC
clpC	ZONMW-39_01395	clpC
clpC	ZONMW-39_01570	clpC
clpC	ZONMW-40_02905	clpC
clpC	ZONMW-40_02408	clpC
clpC	ZONMW-41_02425	clpC
clpC	ZONMW-41_01006	clpC
clpC	ZONMW-42_02374	clpC
clpC	ZONMW-42_03273	clpC
clpC	ZONMW-43_03801	clpC
clpC	ZONMW-43_01639	clpC
clpC	ZONMW-43_03195	clpC
clpC	ZONMW-43_01940	clpC
clpC	ZONMW-44_03123	clpC
clpC	ZONMW-44_02830	clpC
clpC	ZONMW-44_03031	clpC
clpC	ZONMW-44_02514	clpC
clpC	ZONMW-45_03087	clpC
clpC	ZONMW-45_01548	clpC
clpC	ZONMW-45_02481	clpC
clpC	ZONMW-46_00148	clpC
clpC	ZONMW-46_02059	clpC
clpC	ZONMW-46_02474	clpC
clpC	ZONMW-47_00357	clpC
clpC	ZONMW-47_02002	clpC
clpC	ZONMW-47_02464	clpC
clpC	ZONMW-48_01879	clpC
clpC	ZONMW-48_01613	clpC
clpC	ZONMW-49_00007	clpC
clpC	ZONMW-49_00254	clpC
clpC	ZONMW-50_00479	clpC
clpC	ZONMW-50_00231	clpC
clpC	ZONMW-51_00006	clpC
clpC	ZONMW-51_00242	clpC
clpC	ZONMW-52_03239	clpC
clpC	ZONMW-52_00226	clpC
clpC	ZONMW-53_03253	clpC
clpC	ZONMW-53_00963	clpC
clpC	ZONMW-54_03228	clpC
clpC	ZONMW-54_01764	clpC
clpC	ZONMW-55_01532	clpC
clpC	ZONMW-55_01284	clpC

clpC	ZONMW-56_00758	clpC
clpC	ZONMW-56_00513	clpC
clpC	ZONMW-57_03245	clpC
clpC	ZONMW-57_00726	clpC
clpC	ZONMW-58_00006	clpC
clpC	ZONMW-58_00245	clpC
clpC	ZONMW-59_00999	clpC
clpC	ZONMW-59_00715	clpC
clpC	ZONMW-60_03312	clpC
clpC	ZONMW-60_00728	clpC
clpC	ZONMW-61_02685	clpC
clpC	ZONMW-61_02007	clpC
clpC	ZONMW-62_03372	clpC
clpC	ZONMW-62_00235	clpC
clpC	ZONMW-63_02099	clpC
clpC	ZONMW-63_00399	clpC
clpC	ZONMW-64_04121	clpC
clpC	ZONMW-64_03055	clpC
clpC	ZONMW-64_01324	clpC
clpC	ZONMW-65_01812	clpC
clpC	ZONMW-65_02483	clpC
clpC	ZONMW-66_00093	clpC
clpC	ZONMW-66_00950	clpC
clpC	ZONMW-66_00488	clpC
clpC	ZONMW-67_03625	clpC
clpC	ZONMW-67_01563	clpC
clpC	ZONMW-67_00394	clpC
clpC	ZONMW-67_00182	clpC
clpC	ZONMW-68_03841	clpC
clpC	ZONMW-68_01087	clpC
clpC	ZONMW-69_03627	clpC
clpC	ZONMW-69_01438	clpC
clpC	ZONMW-69_00141	clpC
clpC	ZONMW-69_02791	clpC
clpC	ZONMW-72_02877	clpC
clpC	ZONMW-72_03209	clpC
clpC	ZONMW-72_04464	clpC
clpC	ZONMW-73_01116	clpC
clpC	ZONMW-73_00510	clpC
clpC	ZONMW-73_03896	clpC
clpC	ZONMW-74_03224	clpC
clpC	ZONMW-74_03102	clpC
clpC	ZONMW-75_02498	clpC
clpC	ZONMW-75_01823	clpC
clpC	da5A-8_00534	clpC
clpC	da5A-8_02667	clpC
clpC	da5A-8_02900	clpC
alkH	18_00791	alkH

alkH	18_02005	alkH
alkH	18_03952	alkH
alkH	18_03317	alkH
alkH	18_03314	alkH
alkH	18_03031	alkH
alkH	18_03024	alkH
alkH	18_01750	alkH
alkH	18_03555	alkH
alkH	18_02943	alkH
alkH	ACAM48_02270	alkH
alkH	ACAM48_03482	alkH
alkH	ACAM48_00483	alkH
alkH	ACAM48_01124	alkH
alkH	ACAM48_00791	alkH
alkH	ACAM48_02856	alkH
alkH	ACAM48_01656	alkH
alkH	ACAM48_01460	alkH
alkH	CAM030_02890	alkH
alkH	CAM030_02681	alkH
alkH	CAM030_01968	alkH
alkH	CAM030_00671	alkH
alkH	CAM030_00137	alkH
alkH	CAM030_01445	alkH
alkH	CAM030_00456	alkH
alkH	CECT7946_01908	alkH
alkH	CECT7946_00942	alkH
alkH	CECT7946_02985	alkH
alkH	CECT7946_02366	alkH
alkH	CECT7946_00718	alkH
alkH	CECT7946_00671	alkH
alkH	CECT7946_00889	alkH
alkH	CECT7946_02712	alkH
alkH	CECT7946_01730	alkH
alkH	CGMCC1.8863_02163	alkH
alkH	CGMCC1.8863_02465	alkH
alkH	CGMCC1.8863_02453	alkH
alkH	CGMCC1.8863_00621	alkH
alkH	CGMCC1.8863_03367	alkH
alkH	CL8139_01632	alkH
alkH	CL8139_00529	alkH
alkH	CL8139_00350	alkH
alkH	CL8139_00912	alkH
alkH	CL8139_01728	alkH
alkH	CL8139_00112	alkH
alkH	CL8139_02015	alkH
alkH	CL8139_03008	alkH
alkH	CL8139_00924	alkH
alkH	CL8139_00348	alkH

alkH	DAU203_01239	alkH
alkH	DAU203_00434	alkH
alkH	DAU203_01570	alkH
alkH	DAU203_01688	alkH
alkH	DAU203_02651	alkH
alkH	DAU203_01923	alkH
alkH	DAU203_02928	alkH
alkH	DAU203_00660	alkH
alkH	DAU203_01700	alkH
alkH	DAU203_01572	alkH
alkH	DD5b_02236	alkH
alkH	DD5b_00595	alkH
alkH	DD5b_00586	alkH
alkH	DD5b_00600	alkH
alkH	DD5b_02669	alkH
alkH	DD5b_00889	alkH
alkH	DD5b_00599	alkH
alkH	DD5b_00598	alkH
alkH	DSM1208_01436	alkH
alkH	DSM1208_04469	alkH
alkH	DSM1208_04305	alkH
alkH	DSM1208_02662	alkH
alkH	DSM1208_03046	alkH
alkH	DSM1208_03987	alkH
alkH	DSM1208_04385	alkH
alkH	DSM1208_00728	alkH
alkH	DSM1208_03852	alkH
alkH	DSM12212_03387	alkH
alkH	DSM12212_03344	alkH
alkH	DSM12212_02798	alkH
alkH	DSM12212_01509	alkH
alkH	DSM12212_02728	alkH
alkH	DSM12212_01734	alkH
alkH	DSM12212_01556	alkH
alkH	DSM15343_02479	alkH
alkH	DSM15343_00070	alkH
alkH	DSM15343_02841	alkH
alkH	DSM15343_01982	alkH
alkH	DSM15343_02273	alkH
alkH	DSM15343_02148	alkH
alkH	DSM15361_00344	alkH
alkH	DSM15361_01913	alkH
alkH	DSM15361_00727	alkH
alkH	DSM15361_01773	alkH
alkH	DSM15361_00729	alkH
alkH	DSM15361_02308	alkH
alkH	DSM15361_02417	alkH
alkH	DSM15361_01183	alkH

alkH	DSM15365_03250	alkH
alkH	DSM15365_02905	alkH
alkH	DSM15365_01295	alkH
alkH	DSM15365_00758	alkH
alkH	DSM15365_02743	alkH
alkH	DSM15365_02427	alkH
alkH	DSM15365_02428	alkH
alkH	DSM15365_01587	alkH
alkH	DSM15365_00576	alkH
alkH	DSM15365_01907	alkH
alkH	DSM15365_02505	alkH
alkH	DSM15718_02695	alkH
alkH	DSM15718_01304	alkH
alkH	DSM15718_01576	alkH
alkH	DSM15718_00151	alkH
alkH	DSM15718_00846	alkH
alkH	DSM15718_01720	alkH
alkH	DSM15718_00498	alkH
alkH	DSM15718_01484	alkH
alkH	DSM16505T_00483	alkH
alkH	DSM16505T_02260	alkH
alkH	DSM16505T_01851	alkH
alkH	DSM16505T_00260	alkH
alkH	DSM16505T_00120	alkH
alkH	DSM16505T_00933	alkH
alkH	DSM18103_02817	alkH
alkH	DSM18103_02142	alkH
alkH	DSM18103_01591	alkH
alkH	DSM18103_01423	alkH
alkH	DSM18103_01723	alkH
alkH	DSM18103_02649	alkH
alkH	DSM18293_00317	alkH
alkH	DSM18293_01701	alkH
alkH	DSM18293_01294	alkH
alkH	DSM18293_01414	alkH
alkH	DSM18293_02043	alkH
alkH	DSM18293_01213	alkH
alkH	DSM18841_00325	alkH
alkH	DSM18841_01689	alkH
alkH	DSM18841_01587	alkH
alkH	DSM18841_00060	alkH
alkH	DSM18841_02606	alkH
alkH	DSM18841_00822	alkH
alkH	DSM18842_00904	alkH
alkH	DSM18842_01716	alkH
alkH	DSM18842_02271	alkH
alkH	DSM18842_01149	alkH
alkH	DSM18842_02524	alkH

alkH	DSM18842_00496	alkH
alkH	DSM19866_03488	alkH
alkH	DSM19866_01299	alkH
alkH	DSM19866_00176	alkH
alkH	DSM19866_00106	alkH
alkH	DSM19866_03347	alkH
alkH	DSM19866_04118	alkH
alkH	DSM19866_01472	alkH
alkH	DSM19866_01481	alkH
alkH	DSM19866_02177	alkH
alkH	DSM19866_04230	alkH
alkH	DSM2041_00141	alkH
alkH	DSM2041_01127	alkH
alkH	DSM2041_00419	alkH
alkH	DSM2041_03714	alkH
alkH	DSM2041_03551	alkH
alkH	DSM2041_04965	alkH
alkH	DSM2041_01215	alkH
alkH	DSM2041_04147	alkH
alkH	DSM2041_01761	alkH
alkH	DSM2061_02958	alkH
alkH	DSM2061_00683	alkH
alkH	DSM2061_04147	alkH
alkH	DSM2061_00066	alkH
alkH	DSM2061_00929	alkH
alkH	DSM2061_00842	alkH
alkH	DSM2061_00755	alkH
alkH	DSM2061_02228	alkH
alkH	DSM2061_01180	alkH
alkH	DSM2061_00215	alkH
alkH	DSM2064_02229	alkH
alkH	DSM2064_00392	alkH
alkH	DSM2064_01062	alkH
alkH	DSM2064_03808	alkH
alkH	DSM2064_02772	alkH
alkH	DSM2064_03978	alkH
alkH	DSM21164_01050	alkH
alkH	DSM21164_01460	alkH
alkH	DSM21164_01465	alkH
alkH	DSM21164_00128	alkH
alkH	DSM21164_02735	alkH
alkH	DSM23404_02990	alkH
alkH	DSM23404_00672	alkH
alkH	DSM23404_01097	alkH
alkH	DSM23404_02531	alkH
alkH	DSM23404_00982	alkH
alkH	DSM23404_00022	alkH
alkH	DSM23424_00464	alkH

alkH	DSM23424_02517	alkH
alkH	DSM23424_02288	alkH
alkH	DSM23424_01093	alkH
alkH	DSM23424_01237	alkH
alkH	DSM23424_03031	alkH
alkH	DSM23424_02463	alkH
alkH	DSM23424_01923	alkH
alkH	DSM23424_02370	alkH
alkH	DSM24786_02887	alkH
alkH	DSM24786_02506	alkH
alkH	DSM24786_01589	alkH
alkH	DSM24786_02300	alkH
alkH	DSM24786_03057	alkH
alkH	DSM24786_02320	alkH
alkH	DSM24786_00881	alkH
alkH	DSM24786_02126	alkH
alkH	DSM24786_00389	alkH
alkH	DSM24786_02287	alkH
alkH	DSM24786_03468	alkH
alkH	DSM24786_00580	alkH
alkH	DSM7489_01148	alkH
alkH	DSM7489_00418	alkH
alkH	DSM7489_01473	alkH
alkH	DSM7489_01607	alkH
alkH	DSM7489_02526	alkH
alkH	DSM7489_01834	alkH
alkH	DSM7489_02798	alkH
alkH	DSM7489_00646	alkH
alkH	DSM7489_01619	alkH
alkH	DSM7489_03283	alkH
alkH	DSM7489_01475	alkH
alkH	DSW1_00731	alkH
alkH	DSW1_01376	alkH
alkH	DSW1_01904	alkH
alkH	DSW1_02154	alkH
alkH	DSW1_00212	alkH
alkH	DSW1_01114	alkH
alkH	DSW1_02655	alkH
alkH	DSW1_00361	alkH
alkH	DSW5_01311	alkH
alkH	DSW5_00416	alkH
alkH	DSW5_02771	alkH
alkH	DSW5_00200	alkH
alkH	DSW5_00925	alkH
alkH	DSW5_01754	alkH
alkH	DSW5_00560	alkH
alkH	DSW6_01112	alkH
alkH	DSW6_01996	alkH

alkH	DSW6_03200	alkH
alkH	DSW6_03074	alkH
alkH	DSW6_02884	alkH
alkH	DSW6_01809	alkH
alkH	DSW6_02139	alkH
alkH	DSW6_02828	alkH
alkH	DSW8_02504	alkH
alkH	DSW8_03207	alkH
alkH	DSW8_01331	alkH
alkH	DSW8_00333	alkH
alkH	DSW8_02357	alkH
alkH	DSW8_03595	alkH
alkH	DSW8_03813	alkH
alkH	DSW8_01091	alkH
alkH	F52_02626	alkH
alkH	F52_04506	alkH
alkH	F52_01316	alkH
alkH	F52_03044	alkH
alkH	F52_03955	alkH
alkH	F52_03313	alkH
alkH	F52_01760	alkH
alkH	F52_04137	alkH
alkH	F52_04494	alkH
alkH	GCA_001027945.1_00273	alkH
alkH	GCA_001027945.1_02479	alkH
alkH	GCA_001027945.1_02871	alkH
alkH	GCA_001027945.1_00565	alkH
alkH	GCA_001027945.1_00917	alkH
alkH	GCA_001027945.1_01615	alkH
alkH	HI1_01090	alkH
alkH	HI1_00350	alkH
alkH	HI1_01414	alkH
alkH	HI1_01528	alkH
alkH	HI1_02458	alkH
alkH	HI1_01762	alkH
alkH	HI1_02738	alkH
alkH	HI1_00578	alkH
alkH	HI1_01540	alkH
alkH	HI1_03226	alkH
alkH	HI1_01416	alkH
alkH	IR1_00640	alkH
alkH	IR1_03399	alkH
alkH	IR1_03664	alkH
alkH	IR1_01491	alkH
alkH	IR1_00122	alkH
alkH	IR1_04265	alkH
alkH	IR1_02694	alkH
alkH	KCOM3131_01340	alkH

alkH	KCTC12974_01856	alkH
alkH	KCTC12974_01452	alkH
alkH	KCTC12974_02683	alkH
alkH	KCTC12974_02337	alkH
alkH	KCTC12974_01710	alkH
alkH	KCTC12974_02066	alkH
alkH	KCTC12974_00704	alkH
alkH	KL-A_02093	alkH
alkH	KL-A_01788	alkH
alkH	KL-A_00753	alkH
alkH	KL-A_02823	alkH
alkH	KL-A_01030	alkH
alkH	KL-A_00437	alkH
alkH	KL-A_00717	alkH
alkH	KL-A_03076	alkH
alkH	KL-A_02811	alkH
alkH	KL-A_01910	alkH
alkH	KL-A_00751	alkH
alkH	L12_00518	alkH
alkH	LL0412.1.7_00442	alkH
alkH	LL0412.1.7_02550	alkH
alkH	LL0412.1.7_00100	alkH
alkH	LL0412.1.7_00227	alkH
alkH	LL0412.1.7_00958	alkH
alkH	MAR_2009_71_00948	alkH
alkH	MAR_2009_71_03706	alkH
alkH	MAR_2009_71_02120	alkH
alkH	MAR_2009_71_03131	alkH
alkH	MAR_2009_71_01082	alkH
alkH	MAR_2009_71_02752	alkH
alkH	MAR_2009_71_00315	alkH
alkH	MAR_2009_71_02354	alkH
alkH	NN016038_00811	alkH
alkH	NN016038_02022	alkH
alkH	NN016038_04008	alkH
alkH	NN016038_03357	alkH
alkH	NN016038_03354	alkH
alkH	NN016038_03063	alkH
alkH	NN016038_03056	alkH
alkH	NN016038_01771	alkH
alkH	NN016038_03595	alkH
alkH	PAO1_01062	alkH
alkH	PAO1_05554	alkH
alkH	PAO1_00271	alkH
alkH	PAO1_05066	alkH
alkH	PAO1_05489	alkH
alkH	PAO1_00136	alkH
alkH	PAO1_03693	alkH

alkH	PAO1_00807	alkH
alkH	PAO1_00771	alkH
alkH	PAO1_04254	alkH
alkH	PAO1_02046	alkH
alkH	PAO1_04150	alkH
alkH	PAO1_03626	alkH
alkH	PAO1_04202	alkH
alkH	PAO1_00925	alkH
alkH	PAO1_04320	alkH
alkH	PAO1_00225	alkH
alkH	PAO1_02190	alkH
alkH	PAO1_02396	alkH
alkH	PAO1_00375	alkH
alkH	PAO1_02289	alkH
alkH	PAO1_01291	alkH
alkH	UW101_01283	alkH
alkH	UW101_03103	alkH
alkH	UW101_04417	alkH
alkH	UW101_05078	alkH
alkH	UW101_01826	alkH
alkH	UW101_04909	alkH
alkH	V4-33_02246	alkH
alkH	V4-33_00394	alkH
alkH	V4-33_00803	alkH
alkH	W5C_01991	alkH
alkH	W5C_03323	alkH
alkH	W5C_03182	alkH
alkH	W5C_00917	alkH
alkH	W5C_00295	alkH
alkH	W5C_01031	alkH
alkH	W5C_00520	alkH
alkH	W5C_02482	alkH
alkH	W5C_01194	alkH
alkH	W5C_00307	alkH
alkH	ZONMW-01_01117	alkH
alkH	ZONMW-01_01870	alkH
alkH	ZONMW-01_01235	alkH
alkH	ZONMW-01_00053	alkH
alkH	ZONMW-01_02471	alkH
alkH	ZONMW-01_00034	alkH
alkH	ZONMW-01_00288	alkH
alkH	ZONMW-01_01579	alkH
alkH	ZONMW-01_02282	alkH
alkH	ZONMW-01_03179	alkH
alkH	ZONMW-01_00065	alkH
alkH	ZONMW-01_02473	alkH
alkH	ZONMW-02_00183	alkH
alkH	ZONMW-02_03610	alkH

alkH	ZONMW-02_00619	alkH
alkH	ZONMW-02_03753	alkH
alkH	ZONMW-02_00553	alkH
alkH	ZONMW-02_00529	alkH
alkH	ZONMW-02_00554	alkH
alkH	ZONMW-02_02045	alkH
alkH	ZONMW-02_01017	alkH
alkH	ZONMW-02_02597	alkH
alkH	ZONMW-03_02186	alkH
alkH	ZONMW-03_02854	alkH
alkH	ZONMW-03_01545	alkH
alkH	ZONMW-03_00778	alkH
alkH	ZONMW-03_03074	alkH
alkH	ZONMW-03_00759	alkH
alkH	ZONMW-03_01014	alkH
alkH	ZONMW-03_00628	alkH
alkH	ZONMW-03_00366	alkH
alkH	ZONMW-03_03339	alkH
alkH	ZONMW-03_00790	alkH
alkH	ZONMW-03_03076	alkH
alkH	ZONMW-04_01632	alkH
alkH	ZONMW-04_02596	alkH
alkH	ZONMW-04_02183	alkH
alkH	ZONMW-04_00658	alkH
alkH	ZONMW-04_02164	alkH
alkH	ZONMW-04_02614	alkH
alkH	ZONMW-04_01301	alkH
alkH	ZONMW-04_02370	alkH
alkH	ZONMW-04_03197	alkH
alkH	ZONMW-04_02152	alkH
alkH	ZONMW-05_01910	alkH
alkH	ZONMW-05_02225	alkH
alkH	ZONMW-05_01683	alkH
alkH	ZONMW-05_00971	alkH
alkH	ZONMW-05_02654	alkH
alkH	ZONMW-05_00952	alkH
alkH	ZONMW-05_01167	alkH
alkH	ZONMW-05_00890	alkH
alkH	ZONMW-05_00625	alkH
alkH	ZONMW-05_03328	alkH
alkH	ZONMW-05_00983	alkH
alkH	ZONMW-05_02656	alkH
alkH	ZONMW-06_01196	alkH
alkH	ZONMW-06_01646	alkH
alkH	ZONMW-06_00343	alkH
alkH	ZONMW-06_01732	alkH
alkH	ZONMW-06_02744	alkH
alkH	ZONMW-06_01509	alkH

alkH	ZONMW-06_00247	alkH
alkH	ZONMW-06_03841	alkH
alkH	ZONMW-06_02711	alkH
alkH	ZONMW-06_00775	alkH
alkH	ZONMW-06_00747	alkH
alkH	ZONMW-06_03229	alkH
alkH	ZONMW-06_00076	alkH
alkH	ZONMW-07_00723	alkH
alkH	ZONMW-07_00194	alkH
alkH	ZONMW-07_00189	alkH
alkH	ZONMW-07_00225	alkH
alkH	ZONMW-07_00622	alkH
alkH	ZONMW-07_00793	alkH
alkH	ZONMW-07_03001	alkH
alkH	ZONMW-07_00835	alkH
alkH	ZONMW-07_01111	alkH
alkH	ZONMW-07_02193	alkH
alkH	ZONMW-07_02992	alkH
alkH	ZONMW-08_00468	alkH
alkH	ZONMW-08_03237	alkH
alkH	ZONMW-08_01139	alkH
alkH	ZONMW-08_02647	alkH
alkH	ZONMW-08_00600	alkH
alkH	ZONMW-08_01963	alkH
alkH	ZONMW-08_03289	alkH
alkH	ZONMW-08_00892	alkH
alkH	ZONMW-09_00461	alkH
alkH	ZONMW-09_00903	alkH
alkH	ZONMW-09_00723	alkH
alkH	ZONMW-09_01924	alkH
alkH	ZONMW-10_00309	alkH
alkH	ZONMW-10_02507	alkH
alkH	ZONMW-10_01304	alkH
alkH	ZONMW-10_01194	alkH
alkH	ZONMW-10_01679	alkH
alkH	ZONMW-11_03395	alkH
alkH	ZONMW-11_03183	alkH
alkH	ZONMW-11_04511	alkH
alkH	ZONMW-11_03391	alkH
alkH	ZONMW-11_02166	alkH
alkH	ZONMW-11_01071	alkH
alkH	ZONMW-11_00532	alkH
alkH	ZONMW-12_00016	alkH
alkH	ZONMW-12_02610	alkH
alkH	ZONMW-12_03131	alkH
alkH	ZONMW-12_02085	alkH
alkH	ZONMW-12_02186	alkH
alkH	ZONMW-12_02986	alkH

alkH	ZONMW-12_02376	alkH
alkH	ZONMW-13_00476	alkH
alkH	ZONMW-13_01967	alkH
alkH	ZONMW-13_03094	alkH
alkH	ZONMW-13_02333	alkH
alkH	ZONMW-13_02624	alkH
alkH	ZONMW-13_02324	alkH
alkH	ZONMW-14_00084	alkH
alkH	ZONMW-14_02250	alkH
alkH	ZONMW-14_02900	alkH
alkH	ZONMW-14_00727	alkH
alkH	ZONMW-14_01613	alkH
alkH	ZONMW-14_02242	alkH
alkH	ZONMW-15_01791	alkH
alkH	ZONMW-15_01909	alkH
alkH	ZONMW-15_02454	alkH
alkH	ZONMW-15_02461	alkH
alkH	ZONMW-15_01246	alkH
alkH	ZONMW-15_02144	alkH
alkH	ZONMW-19_02042	alkH
alkH	ZONMW-19_04001	alkH
alkH	ZONMW-19_00393	alkH
alkH	ZONMW-19_02097	alkH
alkH	ZONMW-19_01276	alkH
alkH	ZONMW-19_04567	alkH
alkH	ZONMW-19_03629	alkH
alkH	ZONMW-19_03631	alkH
alkH	ZONMW-19_02539	alkH
alkH	ZONMW-19_03350	alkH
alkH	ZONMW-19_01783	alkH
alkH	ZONMW-19_03160	alkH
alkH	ZONMW-19_04363	alkH
alkH	ZONMW-20_02228	alkH
alkH	ZONMW-20_02809	alkH
alkH	ZONMW-20_00160	alkH
alkH	ZONMW-20_00417	alkH
alkH	ZONMW-20_00373	alkH
alkH	ZONMW-20_00476	alkH
alkH	ZONMW-20_03875	alkH
alkH	ZONMW-20_00605	alkH
alkH	ZONMW-20_04028	alkH
alkH	ZONMW-20_01124	alkH
alkH	ZONMW-20_01109	alkH
alkH	ZONMW-20_01667	alkH
alkH	ZONMW-21_01037	alkH
alkH	ZONMW-21_01772	alkH
alkH	ZONMW-21_03123	alkH
alkH	ZONMW-21_01910	alkH

alkH	ZONMW-21_02388	alkH
alkH	ZONMW-22_01038	alkH
alkH	ZONMW-22_01952	alkH
alkH	ZONMW-22_03148	alkH
alkH	ZONMW-22_01816	alkH
alkH	ZONMW-22_02720	alkH
alkH	ZONMW-23_02151	alkH
alkH	ZONMW-23_00599	alkH
alkH	ZONMW-23_03169	alkH
alkH	ZONMW-23_00736	alkH
alkH	ZONMW-23_02744	alkH
alkH	ZONMW-24_00423	alkH
alkH	ZONMW-24_01975	alkH
alkH	ZONMW-24_03170	alkH
alkH	ZONMW-24_01839	alkH
alkH	ZONMW-24_02412	alkH
alkH	ZONMW-25_02042	alkH
alkH	ZONMW-25_00838	alkH
alkH	ZONMW-25_01906	alkH
alkH	ZONMW-25_00885	alkH
alkH	ZONMW-25_02566	alkH
alkH	ZONMW-26_02486	alkH
alkH	ZONMW-26_02893	alkH
alkH	ZONMW-26_03092	alkH
alkH	ZONMW-26_01221	alkH
alkH	ZONMW-26_00995	alkH
alkH	ZONMW-26_01014	alkH
alkH	ZONMW-26_00070	alkH
alkH	ZONMW-26_00341	alkH
alkH	ZONMW-26_03265	alkH
alkH	ZONMW-26_01026	alkH
alkH	ZONMW-26_03094	alkH
alkH	ZONMW-27_01665	alkH
alkH	ZONMW-27_02448	alkH
alkH	ZONMW-27_02811	alkH
alkH	ZONMW-27_02656	alkH
alkH	ZONMW-27_02675	alkH
alkH	ZONMW-27_03077	alkH
alkH	ZONMW-27_00652	alkH
alkH	ZONMW-27_01449	alkH
alkH	ZONMW-27_03335	alkH
alkH	ZONMW-27_02687	alkH
alkH	ZONMW-27_02813	alkH
alkH	ZONMW-28_01793	alkH
alkH	ZONMW-28_01577	alkH
alkH	ZONMW-28_03120	alkH
alkH	ZONMW-28_04014	alkH
alkH	ZONMW-28_02412	alkH

alkH	ZONMW-28_01484	alkH
alkH	ZONMW-28_02389	alkH
alkH	ZONMW-28_01167	alkH
alkH	ZONMW-28_00749	alkH
alkH	ZONMW-28_03061	alkH
alkH	ZONMW-28_01257	alkH
alkH	ZONMW-29_00609	alkH
alkH	ZONMW-29_00046	alkH
alkH	ZONMW-29_01053	alkH
alkH	ZONMW-29_02559	alkH
alkH	ZONMW-29_01131	alkH
alkH	ZONMW-29_02846	alkH
alkH	ZONMW-30_01755	alkH
alkH	ZONMW-30_01151	alkH
alkH	ZONMW-30_00927	alkH
alkH	ZONMW-30_01201	alkH
alkH	ZONMW-30_00149	alkH
alkH	ZONMW-31_00143	alkH
alkH	ZONMW-31_01833	alkH
alkH	ZONMW-31_00166	alkH
alkH	ZONMW-31_00659	alkH
alkH	ZONMW-31_02837	alkH
alkH	ZONMW-31_02105	alkH
alkH	ZONMW-31_01045	alkH
alkH	ZONMW-31_02249	alkH
alkH	ZONMW-31_01719	alkH
alkH	ZONMW-31_02941	alkH
alkH	ZONMW-31_00563	alkH
alkH	ZONMW-31_02875	alkH
alkH	ZONMW-31_00332	alkH
alkH	ZONMW-31_02185	alkH
alkH	ZONMW-31_01022	alkH
alkH	ZONMW-31_01006	alkH
alkH	ZONMW-31_00409	alkH
alkH	ZONMW-32_02179	alkH
alkH	ZONMW-32_01984	alkH
alkH	ZONMW-32_03289	alkH
alkH	ZONMW-32_03871	alkH
alkH	ZONMW-32_02938	alkH
alkH	ZONMW-32_01891	alkH
alkH	ZONMW-32_02961	alkH
alkH	ZONMW-32_01575	alkH
alkH	ZONMW-32_00342	alkH
alkH	ZONMW-32_03348	alkH
alkH	ZONMW-32_01665	alkH
alkH	ZONMW-33_02170	alkH
alkH	ZONMW-33_01975	alkH
alkH	ZONMW-33_00679	alkH

alkH	ZONMW-33_03633	alkH
alkH	ZONMW-33_02810	alkH
alkH	ZONMW-33_01881	alkH
alkH	ZONMW-33_02787	alkH
alkH	ZONMW-33_01565	alkH
alkH	ZONMW-33_00209	alkH
alkH	ZONMW-33_00738	alkH
alkH	ZONMW-33_01655	alkH
alkH	ZONMW-34_02319	alkH
alkH	ZONMW-34_00310	alkH
alkH	ZONMW-34_03010	alkH
alkH	ZONMW-34_01042	alkH
alkH	ZONMW-34_02335	alkH
alkH	ZONMW-34_03074	alkH
alkH	ZONMW-34_00220	alkH
alkH	ZONMW-34_03276	alkH
alkH	ZONMW-34_03894	alkH
alkH	ZONMW-34_02895	alkH
alkH	ZONMW-34_00588	alkH
alkH	ZONMW-34_03092	alkH
alkH	ZONMW-34_03292	alkH
alkH	ZONMW-34_00041	alkH
alkH	ZONMW-35_01930	alkH
alkH	ZONMW-35_01080	alkH
alkH	ZONMW-35_02982	alkH
alkH	ZONMW-35_02201	alkH
alkH	ZONMW-35_01914	alkH
alkH	ZONMW-35_02918	alkH
alkH	ZONMW-35_01170	alkH
alkH	ZONMW-35_03237	alkH
alkH	ZONMW-35_01821	alkH
alkH	ZONMW-35_02862	alkH
alkH	ZONMW-35_00801	alkH
alkH	ZONMW-35_03145	alkH
alkH	ZONMW-35_03253	alkH
alkH	ZONMW-35_01349	alkH
alkH	ZONMW-36_02066	alkH
alkH	ZONMW-36_01381	alkH
alkH	ZONMW-36_03233	alkH
alkH	ZONMW-36_03622	alkH
alkH	ZONMW-36_02322	alkH
alkH	ZONMW-36_01474	alkH
alkH	ZONMW-36_02299	alkH
alkH	ZONMW-36_01791	alkH
alkH	ZONMW-36_01160	alkH
alkH	ZONMW-36_03174	alkH
alkH	ZONMW-36_01701	alkH
alkH	ZONMW-37_01325	alkH

alkH	ZONMW-37_02150	alkH
alkH	ZONMW-37_03157	alkH
alkH	ZONMW-37_01839	alkH
alkH	ZONMW-37_02658	alkH
alkH	ZONMW-38_00647	alkH
alkH	ZONMW-38_03386	alkH
alkH	ZONMW-38_00315	alkH
alkH	ZONMW-38_02764	alkH
alkH	ZONMW-38_01062	alkH
alkH	ZONMW-38_02033	alkH
alkH	ZONMW-38_02695	alkH
alkH	ZONMW-38_00104	alkH
alkH	ZONMW-38_02021	alkH
alkH	ZONMW-38_00643	alkH
alkH	ZONMW-38_01076	alkH
alkH	ZONMW-38_02993	alkH
alkH	ZONMW-38_00684	alkH
alkH	ZONMW-38_03373	alkH
alkH	ZONMW-39_01586	alkH
alkH	ZONMW-39_03763	alkH
alkH	ZONMW-39_02667	alkH
alkH	ZONMW-39_02995	alkH
alkH	ZONMW-39_02749	alkH
alkH	ZONMW-39_01601	alkH
alkH	ZONMW-39_01862	alkH
alkH	ZONMW-39_00766	alkH
alkH	ZONMW-39_02648	alkH
alkH	ZONMW-39_02913	alkH
alkH	ZONMW-39_03336	alkH
alkH	ZONMW-39_02686	alkH
alkH	ZONMW-39_01631	alkH
alkH	ZONMW-39_02573	alkH
alkH	ZONMW-39_03622	alkH
alkH	ZONMW-39_01052	alkH
alkH	ZONMW-39_01817	alkH
alkH	ZONMW-39_02493	alkH
alkH	ZONMW-40_00221	alkH
alkH	ZONMW-40_00565	alkH
alkH	ZONMW-40_01050	alkH
alkH	ZONMW-40_02985	alkH
alkH	ZONMW-40_02233	alkH
alkH	ZONMW-41_00528	alkH
alkH	ZONMW-41_01627	alkH
alkH	ZONMW-41_00127	alkH
alkH	ZONMW-41_00433	alkH
alkH	ZONMW-41_01348	alkH
alkH	ZONMW-41_01866	alkH
alkH	ZONMW-42_03729	alkH

alkH	ZONMW-42_01231	alkH
alkH	ZONMW-42_03574	alkH
alkH	ZONMW-42_00562	alkH
alkH	ZONMW-42_01302	alkH
alkH	ZONMW-42_03348	alkH
alkH	ZONMW-42_02393	alkH
alkH	ZONMW-42_00198	alkH
alkH	ZONMW-42_00594	alkH
alkH	ZONMW-42_01565	alkH
alkH	ZONMW-42_01533	alkH
alkH	ZONMW-42_00791	alkH
alkH	ZONMW-42_00106	alkH
alkH	ZONMW-43_01923	alkH
alkH	ZONMW-43_03595	alkH
alkH	ZONMW-43_03413	alkH
alkH	ZONMW-43_03077	alkH
alkH	ZONMW-43_00635	alkH
alkH	ZONMW-43_03869	alkH
alkH	ZONMW-43_00576	alkH
alkH	ZONMW-43_01905	alkH
alkH	ZONMW-43_00728	alkH
alkH	ZONMW-43_04470	alkH
alkH	ZONMW-43_04029	alkH
alkH	ZONMW-43_00980	alkH
alkH	ZONMW-43_04072	alkH
alkH	ZONMW-43_00128	alkH
alkH	ZONMW-43_03418	alkH
alkH	ZONMW-43_03962	alkH
alkH	ZONMW-43_04013	alkH
alkH	ZONMW-43_00907	alkH
alkH	ZONMW-44_04022	alkH
alkH	ZONMW-44_00421	alkH
alkH	ZONMW-44_00249	alkH
alkH	ZONMW-44_03534	alkH
alkH	ZONMW-44_00732	alkH
alkH	ZONMW-44_00492	alkH
alkH	ZONMW-44_00581	alkH
alkH	ZONMW-44_01801	alkH
alkH	ZONMW-44_02395	alkH
alkH	ZONMW-44_02969	alkH
alkH	ZONMW-44_00697	alkH
alkH	ZONMW-44_01451	alkH
alkH	ZONMW-44_01479	alkH
alkH	ZONMW-44_02902	alkH
alkH	ZONMW-44_01970	alkH
alkH	ZONMW-45_00479	alkH
alkH	ZONMW-45_00769	alkH
alkH	ZONMW-45_00871	alkH

alkH	ZONMW-45_02069	alkH
alkH	ZONMW-45_02817	alkH
alkH	ZONMW-45_01352	alkH
alkH	ZONMW-46_01360	alkH
alkH	ZONMW-46_01105	alkH
alkH	ZONMW-46_01003	alkH
alkH	ZONMW-46_02213	alkH
alkH	ZONMW-46_02397	alkH
alkH	ZONMW-46_01607	alkH
alkH	ZONMW-47_01492	alkH
alkH	ZONMW-47_01068	alkH
alkH	ZONMW-47_01170	alkH
alkH	ZONMW-47_02319	alkH
alkH	ZONMW-47_02387	alkH
alkH	ZONMW-47_01882	alkH
alkH	ZONMW-48_00178	alkH
alkH	ZONMW-48_01496	alkH
alkH	ZONMW-48_03033	alkH
alkH	ZONMW-48_03026	alkH
alkH	ZONMW-48_02450	alkH
alkH	ZONMW-48_02662	alkH
alkH	ZONMW-49_02894	alkH
alkH	ZONMW-49_01943	alkH
alkH	ZONMW-49_01123	alkH
alkH	ZONMW-49_01000	alkH
alkH	ZONMW-49_00076	alkH
alkH	ZONMW-49_01375	alkH
alkH	ZONMW-49_00357	alkH
alkH	ZONMW-49_03176	alkH
alkH	ZONMW-49_00988	alkH
alkH	ZONMW-49_01121	alkH
alkH	ZONMW-50_02762	alkH
alkH	ZONMW-50_02539	alkH
alkH	ZONMW-50_01124	alkH
alkH	ZONMW-50_01001	alkH
alkH	ZONMW-50_00410	alkH
alkH	ZONMW-50_01377	alkH
alkH	ZONMW-50_00128	alkH
alkH	ZONMW-50_03172	alkH
alkH	ZONMW-50_00989	alkH
alkH	ZONMW-50_01122	alkH
alkH	ZONMW-51_02880	alkH
alkH	ZONMW-51_02249	alkH
alkH	ZONMW-51_02730	alkH
alkH	ZONMW-51_01267	alkH
alkH	ZONMW-51_00073	alkH
alkH	ZONMW-51_01492	alkH
alkH	ZONMW-51_00344	alkH

alkH	ZONMW-51_03233	alkH
alkH	ZONMW-51_01279	alkH
alkH	ZONMW-51_02728	alkH
alkH	ZONMW-52_02994	alkH
alkH	ZONMW-52_02661	alkH
alkH	ZONMW-52_02316	alkH
alkH	ZONMW-52_00996	alkH
alkH	ZONMW-52_00046	alkH
alkH	ZONMW-52_01232	alkH
alkH	ZONMW-52_00329	alkH
alkH	ZONMW-52_03190	alkH
alkH	ZONMW-52_01008	alkH
alkH	ZONMW-52_02318	alkH
alkH	ZONMW-53_03001	alkH
alkH	ZONMW-53_02414	alkH
alkH	ZONMW-53_02905	alkH
alkH	ZONMW-53_00840	alkH
alkH	ZONMW-53_02609	alkH
alkH	ZONMW-53_00593	alkH
alkH	ZONMW-53_01062	alkH
alkH	ZONMW-53_03202	alkH
alkH	ZONMW-53_00828	alkH
alkH	ZONMW-53_02907	alkH
alkH	ZONMW-54_03002	alkH
alkH	ZONMW-54_02474	alkH
alkH	ZONMW-54_00121	alkH
alkH	ZONMW-54_00224	alkH
alkH	ZONMW-54_01931	alkH
alkH	ZONMW-54_00442	alkH
alkH	ZONMW-54_01661	alkH
alkH	ZONMW-54_03199	alkH
alkH	ZONMW-54_00236	alkH
alkH	ZONMW-54_00123	alkH
alkH	ZONMW-55_03008	alkH
alkH	ZONMW-55_01739	alkH
alkH	ZONMW-55_02911	alkH
alkH	ZONMW-55_00806	alkH
alkH	ZONMW-55_01465	alkH
alkH	ZONMW-55_00588	alkH
alkH	ZONMW-55_01182	alkH
alkH	ZONMW-55_03200	alkH
alkH	ZONMW-55_00794	alkH
alkH	ZONMW-55_02913	alkH
alkH	ZONMW-56_02887	alkH
alkH	ZONMW-56_01893	alkH
alkH	ZONMW-56_02672	alkH
alkH	ZONMW-56_02266	alkH
alkH	ZONMW-56_00691	alkH

alkH	ZONMW-56_03046	alkH
alkH	ZONMW-56_00410	alkH
alkH	ZONMW-56_03210	alkH
alkH	ZONMW-56_02278	alkH
alkH	ZONMW-56_02674	alkH
alkH	ZONMW-57_02854	alkH
alkH	ZONMW-57_02245	alkH
alkH	ZONMW-57_02817	alkH
alkH	ZONMW-57_02397	alkH
alkH	ZONMW-57_00898	alkH
alkH	ZONMW-57_01026	alkH
alkH	ZONMW-57_00623	alkH
alkH	ZONMW-57_03216	alkH
alkH	ZONMW-57_02409	alkH
alkH	ZONMW-57_02819	alkH
alkH	ZONMW-58_02878	alkH
alkH	ZONMW-58_02269	alkH
alkH	ZONMW-58_02841	alkH
alkH	ZONMW-58_02422	alkH
alkH	ZONMW-58_00073	alkH
alkH	ZONMW-58_01558	alkH
alkH	ZONMW-58_00348	alkH
alkH	ZONMW-58_03235	alkH
alkH	ZONMW-58_02434	alkH
alkH	ZONMW-58_02843	alkH
alkH	ZONMW-59_03096	alkH
alkH	ZONMW-59_02485	alkH
alkH	ZONMW-59_02888	alkH
alkH	ZONMW-59_02071	alkH
alkH	ZONMW-59_00892	alkH
alkH	ZONMW-59_01028	alkH
alkH	ZONMW-59_00611	alkH
alkH	ZONMW-59_03292	alkH
alkH	ZONMW-59_02059	alkH
alkH	ZONMW-59_02886	alkH
alkH	ZONMW-60_02972	alkH
alkH	ZONMW-60_03361	alkH
alkH	ZONMW-60_02354	alkH
alkH	ZONMW-60_00948	alkH
alkH	ZONMW-60_01750	alkH
alkH	ZONMW-60_02934	alkH
alkH	ZONMW-60_00897	alkH
alkH	ZONMW-60_00626	alkH
alkH	ZONMW-60_03281	alkH
alkH	ZONMW-60_01738	alkH
alkH	ZONMW-60_02936	alkH
alkH	ZONMW-61_00039	alkH
alkH	ZONMW-61_01049	alkH

alkH	ZONMW-61_01155	alkH
alkH	ZONMW-61_03342	alkH
alkH	ZONMW-61_00176	alkH
alkH	ZONMW-61_02369	alkH
alkH	ZONMW-61_03884	alkH
alkH	ZONMW-61_00844	alkH
alkH	ZONMW-62_01592	alkH
alkH	ZONMW-62_03135	alkH
alkH	ZONMW-62_02906	alkH
alkH	ZONMW-62_00436	alkH
alkH	ZONMW-62_01729	alkH
alkH	ZONMW-62_00798	alkH
alkH	ZONMW-62_03686	alkH
alkH	ZONMW-62_02660	alkH
alkH	ZONMW-63_02858	alkH
alkH	ZONMW-63_03305	alkH
alkH	ZONMW-63_02416	alkH
alkH	ZONMW-63_00950	alkH
alkH	ZONMW-63_02257	alkH
alkH	ZONMW-63_00560	alkH
alkH	ZONMW-63_02032	alkH
alkH	ZONMW-63_00782	alkH
alkH	ZONMW-63_00296	alkH
alkH	ZONMW-63_03209	alkH
alkH	ZONMW-63_00572	alkH
alkH	ZONMW-63_02255	alkH
alkH	ZONMW-64_01500	alkH
alkH	ZONMW-64_02729	alkH
alkH	ZONMW-64_02239	alkH
alkH	ZONMW-64_00897	alkH
alkH	ZONMW-64_03813	alkH
alkH	ZONMW-64_00594	alkH
alkH	ZONMW-64_01519	alkH
alkH	ZONMW-64_02652	alkH
alkH	ZONMW-64_01013	alkH
alkH	ZONMW-64_02636	alkH
alkH	ZONMW-64_03905	alkH
alkH	ZONMW-64_02751	alkH
alkH	ZONMW-64_01264	alkH
alkH	ZONMW-64_00145	alkH
alkH	ZONMW-64_01776	alkH
alkH	ZONMW-64_01192	alkH
alkH	ZONMW-64_01536	alkH
alkH	ZONMW-65_02338	alkH
alkH	ZONMW-65_03041	alkH
alkH	ZONMW-65_00752	alkH
alkH	ZONMW-65_01206	alkH
alkH	ZONMW-65_00163	alkH

alkH	ZONMW-65_01464	alkH
alkH	ZONMW-65_03091	alkH
alkH	ZONMW-65_01865	alkH
alkH	ZONMW-65_01285	alkH
alkH	ZONMW-66_03111	alkH
alkH	ZONMW-66_02432	alkH
alkH	ZONMW-66_04424	alkH
alkH	ZONMW-66_00118	alkH
alkH	ZONMW-66_02423	alkH
alkH	ZONMW-66_00345	alkH
alkH	ZONMW-66_00876	alkH
alkH	ZONMW-66_00874	alkH
alkH	ZONMW-66_01076	alkH
alkH	ZONMW-66_02854	alkH
alkH	ZONMW-66_01933	alkH
alkH	ZONMW-66_04027	alkH
alkH	ZONMW-66_02053	alkH
alkH	ZONMW-67_00238	alkH
alkH	ZONMW-67_03078	alkH
alkH	ZONMW-67_02958	alkH
alkH	ZONMW-67_00830	alkH
alkH	ZONMW-67_02719	alkH
alkH	ZONMW-67_02229	alkH
alkH	ZONMW-67_02637	alkH
alkH	ZONMW-67_00454	alkH
alkH	ZONMW-67_00787	alkH
alkH	ZONMW-67_00519	alkH
alkH	ZONMW-68_00608	alkH
alkH	ZONMW-68_03775	alkH
alkH	ZONMW-68_02577	alkH
alkH	ZONMW-68_04577	alkH
alkH	ZONMW-68_03701	alkH
alkH	ZONMW-68_04296	alkH
alkH	ZONMW-68_01849	alkH
alkH	ZONMW-68_01857	alkH
alkH	ZONMW-68_01646	alkH
alkH	ZONMW-68_02966	alkH
alkH	ZONMW-69_02847	alkH
alkH	ZONMW-69_02875	alkH
alkH	ZONMW-69_02996	alkH
alkH	ZONMW-69_00877	alkH
alkH	ZONMW-69_00516	alkH
alkH	ZONMW-69_02277	alkH
alkH	ZONMW-69_00434	alkH
alkH	ZONMW-69_00201	alkH
alkH	ZONMW-69_00834	alkH
alkH	ZONMW-69_00266	alkH
alkH	ZONMW-72_00292	alkH

alkH	ZONMW-72_03478	alkH
alkH	ZONMW-72_00878	alkH
alkH	ZONMW-72_01997	alkH
alkH	ZONMW-72_00561	alkH
alkH	ZONMW-72_01846	alkH
alkH	ZONMW-73_00646	alkH
alkH	ZONMW-73_02611	alkH
alkH	ZONMW-73_01075	alkH
alkH	ZONMW-73_01902	alkH
alkH	ZONMW-73_03517	alkH
alkH	ZONMW-73_00943	alkH
alkH	ZONMW-73_01978	alkH
alkH	ZONMW-74_02177	alkH
alkH	ZONMW-74_00381	alkH
alkH	ZONMW-74_01372	alkH
alkH	ZONMW-74_02604	alkH
alkH	ZONMW-74_00043	alkH
alkH	ZONMW-74_01357	alkH
alkH	ZONMW-74_03424	alkH
alkH	ZONMW-74_02365	alkH
alkH	ZONMW-74_02730	alkH
alkH	ZONMW-75_01049	alkH
alkH	ZONMW-75_02717	alkH
alkH	ZONMW-75_02828	alkH
alkH	ZONMW-75_01748	alkH
alkH	ZONMW-75_01421	alkH
alkH	ZONMW-75_03371	alkH
alkH	ZONMW-75_01924	alkH
alkH	ZONMW-75_01878	alkH
alkH	ZONMW-75_01879	alkH
alkH	ZONMW-75_00514	alkH
alkH	ZONMW-75_00658	alkH
alkH	ZONMW-75_02004	alkH
alkH	da5A-8_01869	alkH
alkH	da5A-8_02796	alkH
alkH	da5A-8_00650	alkH
alkH	da5A-8_00983	alkH
alkH	da5A-8_00789	alkH
alkH	da5A-8_00344	alkH
gtaB	18_01132	gtaB
gtaB	18_01082	gtaB
gtaB	ACAM48_00059	gtaB
gtaB	ACAM48_02470	gtaB
gtaB	CAM030_01025	gtaB
gtaB	CAM030_00968	gtaB
gtaB	CECT7946_00146	gtaB
gtaB	CECT7946_00179	gtaB
gtaB	CECT7946_02164	gtaB

gtaB	CGMCC1.8863_00791	gtaB
gtaB	CL8139_02135	gtaB
gtaB	CL8139_01875	gtaB
gtaB	DAU203_01464	gtaB
gtaB	DAU203_01517	gtaB
gtaB	DD5b_01115	gtaB
gtaB	DD5b_02099	gtaB
gtaB	DSM1208_01677	gtaB
gtaB	DSM12212_00072	gtaB
gtaB	DSM12212_00127	gtaB
gtaB	DSM15343_01049	gtaB
gtaB	DSM15343_00995	gtaB
gtaB	DSM15361_00533	gtaB
gtaB	DSM15361_00582	gtaB
gtaB	DSM15365_03050	gtaB
gtaB	DSM15365_03009	gtaB
gtaB	DSM15365_03042	gtaB
gtaB	DSM15718_00728	gtaB
gtaB	DSM15718_02628	gtaB
gtaB	DSM16505T_02610	gtaB
gtaB	DSM16505T_00530	gtaB
gtaB	DSM18103_03398	gtaB
gtaB	DSM18103_02891	gtaB
gtaB	DSM18293_02483	gtaB
gtaB	DSM18293_03028	gtaB
gtaB	DSM18841_02740	gtaB
gtaB	DSM18841_00388	gtaB
gtaB	DSM18842_02688	gtaB
gtaB	DSM18842_00846	gtaB
gtaB	DSM19866_01901	gtaB
gtaB	DSM19866_01912	gtaB
gtaB	DSM2041_00231	gtaB
gtaB	DSM2041_00222	gtaB
gtaB	DSM2041_00159	gtaB
gtaB	DSM2061_03212	gtaB
gtaB	DSM2064_04749	gtaB
gtaB	DSM2064_02061	gtaB
gtaB	DSM2064_04731	gtaB
gtaB	DSM2064_04733	gtaB
gtaB	DSM21164_00736	gtaB
gtaB	DSM21164_00787	gtaB
gtaB	DSM23404_01430	gtaB
gtaB	DSM23404_02050	gtaB
gtaB	DSM23424_00338	gtaB
gtaB	DSM23424_00286	gtaB
gtaB	DSM24786_02950	gtaB
gtaB	DSM24786_03006	gtaB
gtaB	DSM7489_01374	gtaB

gtaB	DSM7489_01421	gtaB
gtaB	DSW1_02194	gtaB
gtaB	DSW1_00685	gtaB
gtaB	DSW5_00224	gtaB
gtaB	DSW5_00851	gtaB
gtaB	DSW5_00234	gtaB
gtaB	DSW5_00831	gtaB
gtaB	DSW6_03450	gtaB
gtaB	DSW6_03441	gtaB
gtaB	DSW6_01500	gtaB
gtaB	DSW8_02257	gtaB
gtaB	F52_01571	gtaB
gtaB	F52_02461	gtaB
gtaB	GCA_001027945.1_00803	gtaB
gtaB	GCA_001027945.1_00225	gtaB
gtaB	HI1_01315	gtaB
gtaB	HI1_01362	gtaB
gtaB	IR1_03013	gtaB
gtaB	IR1_03184	gtaB
gtaB	IR1_04679	gtaB
gtaB	KCOM3131_01549	gtaB
gtaB	KCTC12974_00132	gtaB
gtaB	KCTC12974_02889	gtaB
gtaB	KL-A_00855	gtaB
gtaB	KL-A_00805	gtaB
gtaB	L12_00031	gtaB
gtaB	LL0412.1.7_01894	gtaB
gtaB	LL0412.1.7_00487	gtaB
gtaB	MAR_2009_71_01182	gtaB
gtaB	NN016038_01146	gtaB
gtaB	NN016038_01099	gtaB
gtaB	PAO1_05337	gtaB
gtaB	PAO1_02086	gtaB
gtaB	PAO1_00612	gtaB
gtaB	UW101_00353	gtaB
gtaB	UW101_01116	gtaB
gtaB	UW101_00371	gtaB
gtaB	UW101_00369	gtaB
gtaB	V4-33_01202	gtaB
gtaB	V4-33_02370	gtaB
gtaB	W5C_01861	gtaB
gtaB	W5C_01120	gtaB
gtaB	ZONMW-01_02367	gtaB
gtaB	ZONMW-01_02418	gtaB
gtaB	ZONMW-02_03568	gtaB
gtaB	ZONMW-02_01816	gtaB
gtaB	ZONMW-03_02970	gtaB
gtaB	ZONMW-03_03021	gtaB

gtaB	ZONMW-04_02477	gtaB
gtaB	ZONMW-04_02420	gtaB
gtaB	ZONMW-05_02556	gtaB
gtaB	ZONMW-05_02603	gtaB
gtaB	ZONMW-06_01028	gtaB
gtaB	ZONMW-06_00122	gtaB
gtaB	ZONMW-06_02005	gtaB
gtaB	ZONMW-07_01491	gtaB
gtaB	ZONMW-08_00704	gtaB
gtaB	ZONMW-09_02543	gtaB
gtaB	ZONMW-09_00332	gtaB
gtaB	ZONMW-10_02439	gtaB
gtaB	ZONMW-10_00411	gtaB
gtaB	ZONMW-11_01993	gtaB
gtaB	ZONMW-11_00339	gtaB
gtaB	ZONMW-12_00728	gtaB
gtaB	ZONMW-12_00230	gtaB
gtaB	ZONMW-13_01219	gtaB
gtaB	ZONMW-13_00331	gtaB
gtaB	ZONMW-13_01207	gtaB
gtaB	ZONMW-14_01129	gtaB
gtaB	ZONMW-14_00259	gtaB
gtaB	ZONMW-15_02530	gtaB
gtaB	ZONMW-15_02581	gtaB
gtaB	ZONMW-19_00349	gtaB
gtaB	ZONMW-19_01768	gtaB
gtaB	ZONMW-19_02155	gtaB
gtaB	ZONMW-20_04047	gtaB
gtaB	ZONMW-20_03543	gtaB
gtaB	ZONMW-20_04050	gtaB
gtaB	ZONMW-21_00798	gtaB
gtaB	ZONMW-21_00752	gtaB
gtaB	ZONMW-22_00799	gtaB
gtaB	ZONMW-22_00753	gtaB
gtaB	ZONMW-23_01913	gtaB
gtaB	ZONMW-23_01867	gtaB
gtaB	ZONMW-24_00662	gtaB
gtaB	ZONMW-24_00708	gtaB
gtaB	ZONMW-25_01724	gtaB
gtaB	ZONMW-25_01680	gtaB
gtaB	ZONMW-26_03302	gtaB
gtaB	ZONMW-26_03040	gtaB
gtaB	ZONMW-27_02713	gtaB
gtaB	ZONMW-27_02760	gtaB
gtaB	ZONMW-28_01317	gtaB
gtaB	ZONMW-28_02892	gtaB
gtaB	ZONMW-29_00438	gtaB
gtaB	ZONMW-29_00392	gtaB

gtaB	ZONMW-30_02274	gtaB
gtaB	ZONMW-30_02314	gtaB
gtaB	ZONMW-31_01285	gtaB
gtaB	ZONMW-31_01533	gtaB
gtaB	ZONMW-31_00455	gtaB
gtaB	ZONMW-32_01725	gtaB
gtaB	ZONMW-32_01377	gtaB
gtaB	ZONMW-33_01715	gtaB
gtaB	ZONMW-33_01366	gtaB
gtaB	ZONMW-34_00098	gtaB
gtaB	ZONMW-34_01552	gtaB
gtaB	ZONMW-35_01293	gtaB
gtaB	ZONMW-35_00625	gtaB
gtaB	ZONMW-36_01641	gtaB
gtaB	ZONMW-36_00586	gtaB
gtaB	ZONMW-37_00798	gtaB
gtaB	ZONMW-37_00752	gtaB
gtaB	ZONMW-38_00904	gtaB
gtaB	ZONMW-39_02429	gtaB
gtaB	ZONMW-39_00145	gtaB
gtaB	ZONMW-40_00408	gtaB
gtaB	ZONMW-40_00453	gtaB
gtaB	ZONMW-41_02587	gtaB
gtaB	ZONMW-41_02585	gtaB
gtaB	ZONMW-41_00348	gtaB
gtaB	ZONMW-42_03976	gtaB
gtaB	ZONMW-42_00060	gtaB
gtaB	ZONMW-42_04180	gtaB
gtaB	ZONMW-43_00851	gtaB
gtaB	ZONMW-43_01717	gtaB
gtaB	ZONMW-44_01924	gtaB
gtaB	ZONMW-44_02755	gtaB
gtaB	ZONMW-44_03962	gtaB
gtaB	ZONMW-44_02232	gtaB
gtaB	ZONMW-45_02702	gtaB
gtaB	ZONMW-45_00541	gtaB
gtaB	ZONMW-46_03087	gtaB
gtaB	ZONMW-46_01298	gtaB
gtaB	ZONMW-47_03078	gtaB
gtaB	ZONMW-47_01554	gtaB
gtaB	ZONMW-48_00651	gtaB
gtaB	ZONMW-48_00643	gtaB
gtaB	ZONMW-48_00600	gtaB
gtaB	ZONMW-49_01228	gtaB
gtaB	ZONMW-49_01176	gtaB
gtaB	ZONMW-50_01229	gtaB
gtaB	ZONMW-50_01177	gtaB
gtaB	ZONMW-51_02831	gtaB

gtaB	ZONMW-51_02782	gtaB
gtaB	ZONMW-52_02214	gtaB
gtaB	ZONMW-52_02264	gtaB
gtaB	ZONMW-53_02801	gtaB
gtaB	ZONMW-53_02853	gtaB
gtaB	ZONMW-54_00023	gtaB
gtaB	ZONMW-54_00069	gtaB
gtaB	ZONMW-55_02810	gtaB
gtaB	ZONMW-55_02859	gtaB
gtaB	ZONMW-56_02567	gtaB
gtaB	ZONMW-56_02619	gtaB
gtaB	ZONMW-57_02715	gtaB
gtaB	ZONMW-57_02763	gtaB
gtaB	ZONMW-58_02739	gtaB
gtaB	ZONMW-58_02787	gtaB
gtaB	ZONMW-59_02994	gtaB
gtaB	ZONMW-59_02941	gtaB
gtaB	ZONMW-60_02836	gtaB
gtaB	ZONMW-60_02882	gtaB
gtaB	ZONMW-61_00287	gtaB
gtaB	ZONMW-62_01840	gtaB
gtaB	ZONMW-63_02359	gtaB
gtaB	ZONMW-63_02310	gtaB
gtaB	ZONMW-64_01136	gtaB
gtaB	ZONMW-64_02901	gtaB
gtaB	ZONMW-65_02989	gtaB
gtaB	ZONMW-65_00866	gtaB
gtaB	ZONMW-66_01016	gtaB
gtaB	ZONMW-66_01948	gtaB
gtaB	ZONMW-66_01658	gtaB
gtaB	ZONMW-66_03938	gtaB
gtaB	ZONMW-66_02462	gtaB
gtaB	ZONMW-66_03103	gtaB
gtaB	ZONMW-67_01492	gtaB
gtaB	ZONMW-67_00573	gtaB
gtaB	ZONMW-68_00035	gtaB
gtaB	ZONMW-68_00045	gtaB
gtaB	ZONMW-69_01508	gtaB
gtaB	ZONMW-69_00320	gtaB
gtaB	ZONMW-72_01207	gtaB
gtaB	ZONMW-72_03332	gtaB
gtaB	ZONMW-73_00018	gtaB
gtaB	ZONMW-73_00056	gtaB
gtaB	ZONMW-74_01532	gtaB
gtaB	ZONMW-75_02329	gtaB
gtaB	ZONMW-75_02294	gtaB
gtaB	da5A-8_00274	gtaB
gtaB	da5A-8_00012	gtaB

dapA	18_03585	dapA
dapA	ACAM48_01401	dapA
dapA	ACAM48_03257	dapA
dapA	CAM030_00348	dapA
dapA	CECT7946_03477	dapA
dapA	CGMCC1.8863_02945	dapA
dapA	CGMCC1.8863_02743	dapA
dapA	CGMCC1.8863_02730	dapA
dapA	CL8139_01274	dapA
dapA	DAU203_03477	dapA
dapA	DD5b_02253	dapA
dapA	DSM1208_04580	dapA
dapA	DSM1208_03851	dapA
dapA	DSM1208_01006	dapA
dapA	DSM1208_01014	dapA
dapA	DSM1208_01008	dapA
dapA	DSM12212_01904	dapA
dapA	DSM15343_02289	dapA
dapA	DSM15361_01708	dapA
dapA	DSM15365_00127	dapA
dapA	DSM15365_02506	dapA
dapA	DSM15718_00237	dapA
dapA	DSM16505T_02188	dapA
dapA	DSM16505T_00934	dapA
dapA	DSM18103_00424	dapA
dapA	DSM18103_02648	dapA
dapA	DSM18103_02672	dapA
dapA	DSM18293_04133	dapA
dapA	DSM18841_01934	dapA
dapA	DSM18841_00823	dapA
dapA	DSM18842_01826	dapA
dapA	DSM18842_00495	dapA
dapA	DSM19866_04694	dapA
dapA	DSM19866_03870	dapA
dapA	DSM19866_04186	dapA
dapA	DSM19866_01182	dapA
dapA	DSM19866_02880	dapA
dapA	DSM19866_00911	dapA
dapA	DSM19866_01425	dapA
dapA	DSM19866_01180	dapA
dapA	DSM2041_04824	dapA
dapA	DSM2041_04148	dapA
dapA	DSM2061_00574	dapA
dapA	DSM2061_01181	dapA
dapA	DSM2061_00282	dapA
dapA	DSM2061_00277	dapA
dapA	DSM2061_00254	dapA
dapA	DSM2064_00906	dapA

dapA	DSM21164_02800	dapA
dapA	DSM21164_02737	dapA
dapA	DSM23404_01687	dapA
dapA	DSM23424_01713	dapA
dapA	DSM24786_00985	dapA
dapA	DSM24786_00579	dapA
dapA	DSM7489_03344	dapA
dapA	DSM7489_03282	dapA
dapA	DSW1_02750	dapA
dapA	DSW1_00363	dapA
dapA	DSW5_00312	dapA
dapA	DSW5_00561	dapA
dapA	DSW6_03488	dapA
dapA	DSW8_00551	dapA
dapA	DSW8_01092	dapA
dapA	F52_00756	dapA
dapA	F52_04495	dapA
dapA	GCA_001027945.1_02290	dapA
dapA	GCA_001027945.1_01614	dapA
dapA	HI1_03290	dapA
dapA	HI1_03225	dapA
dapA	IR1_02093	dapA
dapA	KCOM3131_00838	dapA
dapA	KCTC12974_00951	dapA
dapA	KL-A_01848	dapA
dapA	KL-A_01911	dapA
dapA	L12_00317	dapA
dapA	LL0412.1.7_01493	dapA
dapA	LL0412.1.7_00959	dapA
dapA	MAR_2009_71_03349	dapA
dapA	MAR_2009_71_02353	dapA
dapA	NN016038_03625	dapA
dapA	PAO1_01042	dapA
dapA	PAO1_00229	dapA
dapA	PAO1_01292	dapA
dapA	PAO1_04319	dapA
dapA	UW101_03617	dapA
dapA	V4-33_01747	dapA
dapA	W5C_00880	dapA
dapA	ZONMW-01_03124	dapA
dapA	ZONMW-02_00791	dapA
dapA	ZONMW-03_02359	dapA
dapA	ZONMW-04_00823	dapA
dapA	ZONMW-05_00093	dapA
dapA	ZONMW-06_03102	dapA
dapA	ZONMW-07_03385	dapA
dapA	ZONMW-07_02078	dapA
dapA	ZONMW-07_03882	dapA

dapA	ZONMW-07_02192	dapA
dapA	ZONMW-07_03657	dapA
dapA	ZONMW-07_01968	dapA
dapA	ZONMW-07_03902	dapA
dapA	ZONMW-07_02077	dapA
dapA	ZONMW-08_02863	dapA
dapA	ZONMW-08_00893	dapA
dapA	ZONMW-09_02173	dapA
dapA	ZONMW-10_00947	dapA
dapA	ZONMW-11_02554	dapA
dapA	ZONMW-11_03323	dapA
dapA	ZONMW-11_00812	dapA
dapA	ZONMW-11_01384	dapA
dapA	ZONMW-11_04161	dapA
dapA	ZONMW-12_00949	dapA
dapA	ZONMW-12_02377	dapA
dapA	ZONMW-13_02369	dapA
dapA	ZONMW-14_02611	dapA
dapA	ZONMW-15_03001	dapA
dapA	ZONMW-15_02145	dapA
dapA	ZONMW-19_00067	dapA
dapA	ZONMW-19_04366	dapA
dapA	ZONMW-20_03060	dapA
dapA	ZONMW-20_03246	dapA
dapA	ZONMW-20_02823	dapA
dapA	ZONMW-21_01655	dapA
dapA	ZONMW-21_02389	dapA
dapA	ZONMW-21_02934	dapA
dapA	ZONMW-22_02069	dapA
dapA	ZONMW-22_02719	dapA
dapA	ZONMW-22_02890	dapA
dapA	ZONMW-23_00482	dapA
dapA	ZONMW-23_02743	dapA
dapA	ZONMW-23_02980	dapA
dapA	ZONMW-24_02694	dapA
dapA	ZONMW-24_02413	dapA
dapA	ZONMW-24_02913	dapA
dapA	ZONMW-25_01446	dapA
dapA	ZONMW-25_02565	dapA
dapA	ZONMW-26_00867	dapA
dapA	ZONMW-27_00920	dapA
dapA	ZONMW-28_02910	dapA
dapA	ZONMW-29_03135	dapA
dapA	ZONMW-29_02389	dapA
dapA	ZONMW-29_02845	dapA
dapA	ZONMW-30_02194	dapA
dapA	ZONMW-30_00148	dapA
dapA	ZONMW-30_03154	dapA

dapA	ZONMW-31_01555	dapA
dapA	ZONMW-32_01395	dapA
dapA	ZONMW-33_01384	dapA
dapA	ZONMW-34_01570	dapA
dapA	ZONMW-35_00643	dapA
dapA	ZONMW-36_00604	dapA
dapA	ZONMW-37_02033	dapA
dapA	ZONMW-37_02659	dapA
dapA	ZONMW-37_02900	dapA
dapA	ZONMW-38_00070	dapA
dapA	ZONMW-38_00881	dapA
dapA	ZONMW-38_02762	dapA
dapA	ZONMW-39_00127	dapA
dapA	ZONMW-40_02437	dapA
dapA	ZONMW-40_02232	dapA
dapA	ZONMW-40_01913	dapA
dapA	ZONMW-40_01914	dapA
dapA	ZONMW-41_03111	dapA
dapA	ZONMW-42_03326	dapA
dapA	ZONMW-43_01697	dapA
dapA	ZONMW-43_01412	dapA
dapA	ZONMW-44_02773	dapA
dapA	ZONMW-45_00121	dapA
dapA	ZONMW-45_01353	dapA
dapA	ZONMW-46_00336	dapA
dapA	ZONMW-46_01608	dapA
dapA	ZONMW-47_00169	dapA
dapA	ZONMW-47_01881	dapA
dapA	ZONMW-48_03120	dapA
dapA	ZONMW-48_02661	dapA
dapA	ZONMW-49_03044	dapA
dapA	ZONMW-50_02988	dapA
dapA	ZONMW-51_01916	dapA
dapA	ZONMW-52_00857	dapA
dapA	ZONMW-53_01600	dapA
dapA	ZONMW-54_01125	dapA
dapA	ZONMW-55_02238	dapA
dapA	ZONMW-56_02130	dapA
dapA	ZONMW-57_00090	dapA
dapA	ZONMW-58_00876	dapA
dapA	ZONMW-59_00090	dapA
dapA	ZONMW-60_00091	dapA
dapA	ZONMW-61_02031	dapA
dapA	ZONMW-61_00845	dapA
dapA	ZONMW-62_00211	dapA
dapA	ZONMW-62_02661	dapA
dapA	ZONMW-63_01854	dapA
dapA	ZONMW-64_02997	dapA

dapA	ZONMW-65_00575	dapA
dapA	ZONMW-66_00565	dapA
dapA	ZONMW-66_02056	dapA
dapA	ZONMW-67_01510	dapA
dapA	ZONMW-68_01058	dapA
dapA	ZONMW-68_01769	dapA
dapA	ZONMW-68_02920	dapA
dapA	ZONMW-68_00304	dapA
dapA	ZONMW-68_04064	dapA
dapA	ZONMW-68_02279	dapA
dapA	ZONMW-68_03954	dapA
dapA	ZONMW-68_01805	dapA
dapA	ZONMW-68_04099	dapA
dapA	ZONMW-69_01490	dapA
dapA	ZONMW-72_03254	dapA
dapA	ZONMW-73_03336	dapA
dapA	ZONMW-73_03477	dapA
dapA	ZONMW-73_01977	dapA
dapA	ZONMW-73_01976	dapA
dapA	ZONMW-74_00568	dapA
dapA	ZONMW-74_02364	dapA
dapA	ZONMW-74_03247	dapA
dapA	ZONMW-75_01454	dapA
dapA	da5A-8_03257	dapA
dapA	da5A-8_00345	dapA
dapA	da5A-8_02845	dapA
hemN_1	18_01446	hemN_1
hemN_1	18_01477	hemN_1
hemN_1	ACAM48_01823	hemN_1
hemN_1	ACAM48_03005	hemN_1
hemN_1	CAM030_01926	hemN_1
hemN_1	CAM030_02184	hemN_1
hemN_1	CECT7946_03140	hemN_1
hemN_1	CECT7946_00494	hemN_1
hemN_1	CGMCC1.8863_00471	hemN_1
hemN_1	CGMCC1.8863_00506	hemN_1
hemN_1	CL8139_01763	hemN_1
hemN_1	CL8139_00119	hemN_1
hemN_1	DAU203_02330	hemN_1
hemN_1	DAU203_01930	hemN_1
hemN_1	DD5b_01327	hemN_1
hemN_1	DD5b_01700	hemN_1
hemN_1	DSM1208_02145	hemN_1
hemN_1	DSM1208_02187	hemN_1
hemN_1	DSM12212_00398	hemN_1
hemN_1	DSM12212_01399	hemN_1
hemN_1	DSM15343_01364	hemN_1
hemN_1	DSM15343_00731	hemN_1

hemN_1	DSM15361_01495	hemN_1
hemN_1	DSM15361_00017	hemN_1
hemN_1	DSM15365_00731	hemN_1
hemN_1	DSM15365_02183	hemN_1
hemN_1	DSM15718_00339	hemN_1
hemN_1	DSM15718_02291	hemN_1
hemN_1	DSM16505T_02476	hemN_1
hemN_1	DSM16505T_01010	hemN_1
hemN_1	DSM18103_03243	hemN_1
hemN_1	DSM18103_02547	hemN_1
hemN_1	DSM18293_03985	hemN_1
hemN_1	DSM18293_00464	hemN_1
hemN_1	DSM18841_02856	hemN_1
hemN_1	DSM18841_00891	hemN_1
hemN_1	DSM18842_02845	hemN_1
hemN_1	DSM18842_00416	hemN_1
hemN_1	DSM19866_04837	hemN_1
hemN_1	DSM19866_01324	hemN_1
hemN_1	DSM2041_03616	hemN_1
hemN_1	DSM2041_04156	hemN_1
hemN_1	DSM2061_03698	hemN_1
hemN_1	DSM2061_03732	hemN_1
hemN_1	DSM2064_03143	hemN_1
hemN_1	DSM2064_02385	hemN_1
hemN_1	DSM21164_00431	hemN_1
hemN_1	DSM21164_00401	hemN_1
hemN_1	DSM23404_02420	hemN_1
hemN_1	DSM23404_00361	hemN_1
hemN_1	DSM23424_02026	hemN_1
hemN_1	DSM23424_00752	hemN_1
hemN_1	DSM24786_01701	hemN_1
hemN_1	DSM24786_02119	hemN_1
hemN_1	DSM7489_02246	hemN_1
hemN_1	DSM7489_01841	hemN_1
hemN_1	DSW1_00876	hemN_1
hemN_1	DSW5_02451	hemN_1
hemN_1	DSW5_00569	hemN_1
hemN_1	DSW6_00200	hemN_1
hemN_1	DSW6_01462	hemN_1
hemN_1	DSW8_03303	hemN_1
hemN_1	DSW8_01749	hemN_1
hemN_1	F52_00516	hemN_1
hemN_1	F52_02793	hemN_1
hemN_1	GCA_001027945.1_00665	hemN_1
hemN_1	GCA_001027945.1_01534	hemN_1
hemN_1	HI1_02172	hemN_1
hemN_1	HI1_01769	hemN_1
hemN_1	IR1_02367	hemN_1

hemN_1	IR1_03179	hemN_1
hemN_1	IR1_00482	hemN_1
hemN_1	KCOM3131_00350	hemN_1
hemN_1	KCTC12974_02795	hemN_1
hemN_1	KCTC12974_02571	hemN_1
hemN_1	KL-A_00027	hemN_1
hemN_1	KL-A_00430	hemN_1
hemN_1	L12_00302	hemN_1
hemN_1	LL0412.1.7_01751	hemN_1
hemN_1	LL0412.1.7_01038	hemN_1
hemN_1	MAR_2009_71_03619	hemN_1
hemN_1	MAR_2009_71_01671	hemN_1
hemN_1	NN016038_01454	hemN_1
hemN_1	NN016038_01484	hemN_1
hemN_1	PAO1_01587	hemN_1
hemN_1	PAO1_00396	hemN_1
hemN_1	UW101_02657	hemN_1
hemN_1	UW101_01439	hemN_1
hemN_1	V4-33_01073	hemN_1
hemN_1	V4-33_00184	hemN_1
hemN_1	W5C_02816	hemN_1
hemN_1	W5C_00527	hemN_1
hemN_1	ZONMW-01_01343	hemN_1
hemN_1	ZONMW-01_00295	hemN_1
hemN_1	ZONMW-02_01174	hemN_1
hemN_1	ZONMW-02_02791	hemN_1
hemN_1	ZONMW-03_01437	hemN_1
hemN_1	ZONMW-03_01021	hemN_1
hemN_1	ZONMW-04_00254	hemN_1
hemN_1	ZONMW-04_00651	hemN_1
hemN_1	ZONMW-05_01571	hemN_1
hemN_1	ZONMW-05_01174	hemN_1
hemN_1	ZONMW-06_00435	hemN_1
hemN_1	ZONMW-06_01897	hemN_1
hemN_1	ZONMW-07_00243	hemN_1
hemN_1	ZONMW-07_00276	hemN_1
hemN_1	ZONMW-08_02296	hemN_1
hemN_1	ZONMW-08_01563	hemN_1
hemN_1	ZONMW-09_00042	hemN_1
hemN_1	ZONMW-10_01021	hemN_1
hemN_1	ZONMW-10_00066	hemN_1
hemN_1	ZONMW-11_03930	hemN_1
hemN_1	ZONMW-11_00530	hemN_1
hemN_1	ZONMW-12_02927	hemN_1
hemN_1	ZONMW-12_02447	hemN_1
hemN_1	ZONMW-13_00094	hemN_1
hemN_1	ZONMW-13_00893	hemN_1
hemN_1	ZONMW-14_00171	hemN_1

hemN_1	ZONMW-14_00170	hemN_1
hemN_1	ZONMW-14_01209	hemN_1
hemN_1	ZONMW-15_00311	hemN_1
hemN_1	ZONMW-15_00339	hemN_1
hemN_1	ZONMW-19_00150	hemN_1
hemN_1	ZONMW-19_00935	hemN_1
hemN_1	ZONMW-20_03658	hemN_1
hemN_1	ZONMW-20_00131	hemN_1
hemN_1	ZONMW-21_00266	hemN_1
hemN_1	ZONMW-21_00300	hemN_1
hemN_1	ZONMW-22_00266	hemN_1
hemN_1	ZONMW-22_00300	hemN_1
hemN_1	ZONMW-23_01381	hemN_1
hemN_1	ZONMW-23_01415	hemN_1
hemN_1	ZONMW-24_01193	hemN_1
hemN_1	ZONMW-24_01159	hemN_1
hemN_1	ZONMW-25_02920	hemN_1
hemN_1	ZONMW-25_02890	hemN_1
hemN_1	ZONMW-26_01699	hemN_1
hemN_1	ZONMW-26_01228	hemN_1
hemN_1	ZONMW-27_00248	hemN_1
hemN_1	ZONMW-27_00645	hemN_1
hemN_1	ZONMW-28_01006	hemN_1
hemN_1	ZONMW-28_02685	hemN_1
hemN_1	ZONMW-29_02939	hemN_1
hemN_1	ZONMW-29_00005	hemN_1
hemN_1	ZONMW-30_01477	hemN_1
hemN_1	ZONMW-30_01507	hemN_1
hemN_1	ZONMW-31_00791	hemN_1
hemN_1	ZONMW-31_01952	hemN_1
hemN_1	ZONMW-32_00085	hemN_1
hemN_1	ZONMW-32_03191	hemN_1
hemN_1	ZONMW-33_03346	hemN_1
hemN_1	ZONMW-33_03083	hemN_1
hemN_1	ZONMW-34_02571	hemN_1
hemN_1	ZONMW-35_02535	hemN_1
hemN_1	ZONMW-36_03083	hemN_1
hemN_1	ZONMW-36_02919	hemN_1
hemN_1	ZONMW-37_00266	hemN_1
hemN_1	ZONMW-37_00300	hemN_1
hemN_1	ZONMW-38_01372	hemN_1
hemN_1	ZONMW-39_02114	hemN_1
hemN_1	ZONMW-40_02681	hemN_1
hemN_1	ZONMW-40_01548	hemN_1
hemN_1	ZONMW-41_00181	hemN_1
hemN_1	ZONMW-41_02121	hemN_1
hemN_1	ZONMW-42_03103	hemN_1
hemN_1	ZONMW-42_01101	hemN_1

hemN_1	ZONMW-43_01198	hemN_1
hemN_1	ZONMW-44_03635	hemN_1
hemN_1	ZONMW-44_00123	hemN_1
hemN_1	ZONMW-45_03136	hemN_1
hemN_1	ZONMW-45_01443	hemN_1
hemN_1	ZONMW-46_02810	hemN_1
hemN_1	ZONMW-46_01698	hemN_1
hemN_1	ZONMW-47_02801	hemN_1
hemN_1	ZONMW-47_01791	hemN_1
hemN_1	ZONMW-48_00948	hemN_1
hemN_1	ZONMW-48_00977	hemN_1
hemN_1	ZONMW-49_00666	hemN_1
hemN_1	ZONMW-49_01383	hemN_1
hemN_1	ZONMW-50_00667	hemN_1
hemN_1	ZONMW-50_01385	hemN_1
hemN_1	ZONMW-51_01041	hemN_1
hemN_1	ZONMW-51_01499	hemN_1
hemN_1	ZONMW-52_01641	hemN_1
hemN_1	ZONMW-52_01239	hemN_1
hemN_1	ZONMW-53_00184	hemN_1
hemN_1	ZONMW-53_00586	hemN_1
hemN_1	ZONMW-54_00846	hemN_1
hemN_1	ZONMW-54_00449	hemN_1
hemN_1	ZONMW-55_00179	hemN_1
hemN_1	ZONMW-55_00581	hemN_1
hemN_1	ZONMW-56_01139	hemN_1
hemN_1	ZONMW-56_03039	hemN_1
hemN_1	ZONMW-57_01438	hemN_1
hemN_1	ZONMW-57_01033	hemN_1
hemN_1	ZONMW-58_01146	hemN_1
hemN_1	ZONMW-58_01551	hemN_1
hemN_1	ZONMW-59_01440	hemN_1
hemN_1	ZONMW-59_01035	hemN_1
hemN_1	ZONMW-60_01135	hemN_1
hemN_1	ZONMW-60_01532	hemN_1
hemN_1	ZONMW-61_03173	hemN_1
hemN_1	ZONMW-61_00771	hemN_1
hemN_1	ZONMW-62_03228	hemN_1
hemN_1	ZONMW-62_02398	hemN_1
hemN_1	ZONMW-63_01127	hemN_1
hemN_1	ZONMW-63_00789	hemN_1
hemN_1	ZONMW-64_01583	hemN_1
hemN_1	ZONMW-64_03548	hemN_1
hemN_1	ZONMW-65_02576	hemN_1
hemN_1	ZONMW-65_00832	hemN_1
hemN_1	ZONMW-66_00760	hemN_1
hemN_1	ZONMW-66_00226	hemN_1
hemN_1	ZONMW-67_02804	hemN_1

hemN_1	ZONMW-67_03347	hemN_1
hemN_1	ZONMW-68_03459	hemN_1
hemN_1	ZONMW-68_03743	hemN_1
hemN_1	ZONMW-69_00601	hemN_1
hemN_1	ZONMW-69_03348	hemN_1
hemN_1	ZONMW-72_04340	hemN_1
hemN_1	ZONMW-72_00134	hemN_1
hemN_1	ZONMW-73_00716	hemN_1
hemN_1	ZONMW-73_02940	hemN_1
hemN_1	ZONMW-74_00918	hemN_1
hemN_1	ZONMW-74_01862	hemN_1
hemN_1	ZONMW-75_01643	hemN_1
hemN_1	ZONMW-75_00279	hemN_1
hemN_1	da5A-8_00602	hemN_1
hemN_1	da5A-8_00092	hemN_1
sdsA	18_01265	sdsA
sdsA	18_01685	sdsA
sdsA	ACAM48_02083	sdsA
sdsA	ACAM48_00531	sdsA
sdsA	CAM030_01291	sdsA
sdsA	CAM030_01431	sdsA
sdsA	CECT7946_01803	sdsA
sdsA	CECT7946_02701	sdsA
sdsA	CGMCC1.8863_00988	sdsA
sdsA	CGMCC1.8863_01321	sdsA
sdsA	CL8139_01012	sdsA
sdsA	CL8139_03320	sdsA
sdsA	DAU203_01789	sdsA
sdsA	DAU203_02118	sdsA
sdsA	DD5b_01548	sdsA
sdsA	DD5b_02690	sdsA
sdsA	DSM1208_01880	sdsA
sdsA	DSM1208_00791	sdsA
sdsA	DSM12212_01669	sdsA
sdsA	DSM12212_01584	sdsA
sdsA	DSM15343_00846	sdsA
sdsA	DSM15343_02020	sdsA
sdsA	DSM15361_01428	sdsA
sdsA	DSM15361_01090	sdsA
sdsA	DSM15365_03366	sdsA
sdsA	DSM15365_00593	sdsA
sdsA	DSM15718_02389	sdsA
sdsA	DSM15718_00471	sdsA
sdsA	DSM16505T_00826	sdsA
sdsA	DSM16505T_01018	sdsA
sdsA	DSM18103_03127	sdsA
sdsA	DSM18103_02535	sdsA
sdsA	DSM18293_00349	sdsA

sdsA	DSM18293_02079	sdsA
sdsA	DSM18841_00692	sdsA
sdsA	DSM18841_00900	sdsA
sdsA	DSM18842_00635	sdsA
sdsA	DSM18842_00406	sdsA
sdsA	DSM19866_02134	sdsA
sdsA	DSM19866_02263	sdsA
sdsA	DSM2041_05201	sdsA
sdsA	DSM2041_04275	sdsA
sdsA	DSM2061_03434	sdsA
sdsA	DSM2061_02290	sdsA
sdsA	DSM2064_02267	sdsA
sdsA	DSM2064_02806	sdsA
sdsA	DSM21164_00604	sdsA
sdsA	DSM21164_00186	sdsA
sdsA	DSM23404_01885	sdsA
sdsA	DSM23404_01054	sdsA
sdsA	DSM23424_00611	sdsA
sdsA	DSM23424_02394	sdsA
sdsA	DSM24786_02193	sdsA
sdsA	DSM24786_01927	sdsA
sdsA	DSM7489_01708	sdsA
sdsA	DSM7489_02030	sdsA
sdsA	DSW1_00280	sdsA
sdsA	DSW1_02813	sdsA
sdsA	DSW5_01257	sdsA
sdsA	DSW5_01546	sdsA
sdsA	DSW6_01739	sdsA
sdsA	DSW6_01896	sdsA
sdsA	DSW8_02041	sdsA
sdsA	DSW8_03080	sdsA
sdsA	F52_02657	sdsA
sdsA	F52_03282	sdsA
sdsA	GCA_001027945.1_03116	sdsA
sdsA	GCA_001027945.1_01524	sdsA
sdsA	HI1_01629	sdsA
sdsA	HI1_01957	sdsA
sdsA	IR1_00602	sdsA
sdsA	IR1_00086	sdsA
sdsA	IR1_00907	sdsA
sdsA	KCOM3131_00080	sdsA
sdsA	KCOM3131_00790	sdsA
sdsA	KCTC12974_02089	sdsA
sdsA	KCTC12974_02385	sdsA
sdsA	KL-A_01510	sdsA
sdsA	KL-A_00240	sdsA
sdsA	L12_00181	sdsA
sdsA	LL0412.1.7_00825	sdsA

sdsA	LL0412.1.7_01049	sdsA
sdsA	MAR_2009_71_01390	sdsA
sdsA	MAR_2009_71_00378	sdsA
sdsA	NN016038_01289	sdsA
sdsA	NN016038_01692	sdsA
sdsA	PAO1_04717	sdsA
sdsA	PAO1_04171	sdsA
sdsA	UW101_01321	sdsA
sdsA	UW101_01860	sdsA
sdsA	V4-33_02326	sdsA
sdsA	V4-33_01993	sdsA
sdsA	W5C_00404	sdsA
sdsA	W5C_00718	sdsA
sdsA	ZONMW-01_00155	sdsA
sdsA	ZONMW-01_00480	sdsA
sdsA	ZONMW-02_00298	sdsA
sdsA	ZONMW-02_01034	sdsA
sdsA	ZONMW-03_00881	sdsA
sdsA	ZONMW-03_01206	sdsA
sdsA	ZONMW-04_02065	sdsA
sdsA	ZONMW-04_00466	sdsA
sdsA	ZONMW-05_01070	sdsA
sdsA	ZONMW-05_01359	sdsA
sdsA	ZONMW-06_03948	sdsA
sdsA	ZONMW-06_03019	sdsA
sdsA	ZONMW-07_01326	sdsA
sdsA	ZONMW-07_01169	sdsA
sdsA	ZONMW-08_01804	sdsA
sdsA	ZONMW-08_03696	sdsA
sdsA	ZONMW-09_00121	sdsA
sdsA	ZONMW-09_01763	sdsA
sdsA	ZONMW-10_00140	sdsA
sdsA	ZONMW-10_01087	sdsA
sdsA	ZONMW-11_02701	sdsA
sdsA	ZONMW-11_01968	sdsA
sdsA	ZONMW-12_00406	sdsA
sdsA	ZONMW-12_02647	sdsA
sdsA	ZONMW-13_00179	sdsA
sdsA	ZONMW-13_01181	sdsA
sdsA	ZONMW-14_00415	sdsA
sdsA	ZONMW-14_01413	sdsA
sdsA	ZONMW-15_00115	sdsA
sdsA	ZONMW-15_01303	sdsA
sdsA	ZONMW-19_00605	sdsA
sdsA	ZONMW-19_01949	sdsA
sdsA	ZONMW-20_02863	sdsA
sdsA	ZONMW-20_01357	sdsA
sdsA	ZONMW-21_00585	sdsA

sdsA	ZONMW-21_00037	sdsA
sdsA	ZONMW-22_00585	sdsA
sdsA	ZONMW-22_00037	sdsA
sdsA	ZONMW-23_01700	sdsA
sdsA	ZONMW-23_01152	sdsA
sdsA	ZONMW-24_00875	sdsA
sdsA	ZONMW-24_01423	sdsA
sdsA	ZONMW-25_02235	sdsA
sdsA	ZONMW-25_01964	sdsA
sdsA	ZONMW-26_01114	sdsA
sdsA	ZONMW-26_01412	sdsA
sdsA	ZONMW-27_00758	sdsA
sdsA	ZONMW-27_00460	sdsA
sdsA	ZONMW-28_03924	sdsA
sdsA	ZONMW-28_03334	sdsA
sdsA	ZONMW-29_00250	sdsA
sdsA	ZONMW-29_02499	sdsA
sdsA	ZONMW-30_01693	sdsA
sdsA	ZONMW-30_00869	sdsA
sdsA	ZONMW-31_03208	sdsA
sdsA	ZONMW-31_03603	sdsA
sdsA	ZONMW-32_03962	sdsA
sdsA	ZONMW-32_03459	sdsA
sdsA	ZONMW-33_03886	sdsA
sdsA	ZONMW-33_03462	sdsA
sdsA	ZONMW-34_03822	sdsA
sdsA	ZONMW-34_03743	sdsA
sdsA	ZONMW-35_03803	sdsA
sdsA	ZONMW-35_03706	sdsA
sdsA	ZONMW-36_03919	sdsA
sdsA	ZONMW-36_03319	sdsA
sdsA	ZONMW-37_00585	sdsA
sdsA	ZONMW-37_00036	sdsA
sdsA	ZONMW-38_02446	sdsA
sdsA	ZONMW-38_02482	sdsA
sdsA	ZONMW-39_04076	sdsA
sdsA	ZONMW-39_03608	sdsA
sdsA	ZONMW-40_01375	sdsA
sdsA	ZONMW-40_00986	sdsA
sdsA	ZONMW-41_02881	sdsA
sdsA	ZONMW-41_02671	sdsA
sdsA	ZONMW-42_02349	sdsA
sdsA	ZONMW-42_03810	sdsA
sdsA	ZONMW-42_01760	sdsA
sdsA	ZONMW-43_03775	sdsA
sdsA	ZONMW-43_02797	sdsA
sdsA	ZONMW-44_03148	sdsA
sdsA	ZONMW-44_01005	sdsA

sdsA	ZONMW-45_01047	sdsA
sdsA	ZONMW-45_01453	sdsA
sdsA	ZONMW-46_00620	sdsA
sdsA	ZONMW-46_01708	sdsA
sdsA	ZONMW-47_00620	sdsA
sdsA	ZONMW-47_01781	sdsA
sdsA	ZONMW-48_00776	sdsA
sdsA	ZONMW-48_02393	sdsA
sdsA	ZONMW-49_01255	sdsA
sdsA	ZONMW-49_01572	sdsA
sdsA	ZONMW-50_01257	sdsA
sdsA	ZONMW-50_01574	sdsA
sdsA	ZONMW-51_01370	sdsA
sdsA	ZONMW-51_00827	sdsA
sdsA	ZONMW-52_01112	sdsA
sdsA	ZONMW-52_01427	sdsA
sdsA	ZONMW-53_00737	sdsA
sdsA	ZONMW-53_00400	sdsA
sdsA	ZONMW-54_00332	sdsA
sdsA	ZONMW-54_00634	sdsA
sdsA	ZONMW-55_00706	sdsA
sdsA	ZONMW-55_00393	sdsA
sdsA	ZONMW-56_02373	sdsA
sdsA	ZONMW-56_00922	sdsA
sdsA	ZONMW-57_02502	sdsA
sdsA	ZONMW-57_01222	sdsA
sdsA	ZONMW-58_02527	sdsA
sdsA	ZONMW-58_01362	sdsA
sdsA	ZONMW-59_01965	sdsA
sdsA	ZONMW-59_01223	sdsA
sdsA	ZONMW-60_01648	sdsA
sdsA	ZONMW-60_01347	sdsA
sdsA	ZONMW-61_00526	sdsA
sdsA	ZONMW-61_03424	sdsA
sdsA	ZONMW-62_02058	sdsA
sdsA	ZONMW-62_03618	sdsA
sdsA	ZONMW-63_00668	sdsA
sdsA	ZONMW-63_01343	sdsA
sdsA	ZONMW-64_04095	sdsA
sdsA	ZONMW-64_03654	sdsA
sdsA	ZONMW-65_01222	sdsA
sdsA	ZONMW-65_01797	sdsA
sdsA	ZONMW-66_03409	sdsA
sdsA	ZONMW-66_01507	sdsA
sdsA	ZONMW-67_03599	sdsA
sdsA	ZONMW-67_02521	sdsA
sdsA	ZONMW-68_00274	sdsA
sdsA	ZONMW-68_01566	sdsA

sdsA	ZONMW-69_03601	sdsA
sdsA	ZONMW-69_02569	sdsA
sdsA	ZONMW-72_00254	sdsA
sdsA	ZONMW-72_00596	sdsA
sdsA	ZONMW-73_02799	sdsA
sdsA	ZONMW-73_02340	sdsA
sdsA	ZONMW-74_01958	sdsA
sdsA	ZONMW-74_01175	sdsA
sdsA	ZONMW-75_01161	sdsA
sdsA	ZONMW-75_00641	sdsA
sdsA	da5A-8_01805	sdsA
sdsA	da5A-8_00081	sdsA
recQ_1	18_03778	recQ_1
recQ_1	18_02147	recQ_1
recQ_1	18_03428	recQ_1
recQ_1	18_02713	recQ_1
recQ_1	18_03397	recQ_1
recQ_1	18_03647	recQ_1
recQ_1	18_03426	recQ_1
recQ_1	18_03396	recQ_1
recQ_1	18_03440	recQ_1
recQ_1	ACAM48_03071	recQ_1
recQ_1	ACAM48_01011	recQ_1
recQ_1	ACAM48_00650	recQ_1
recQ_1	ACAM48_01776	recQ_1
recQ_1	ACAM48_00901	recQ_1
recQ_1	ACAM48_03493	recQ_1
recQ_1	ACAM48_02115	recQ_1
recQ_1	CAM030_01721	recQ_1
recQ_1	CAM030_00296	recQ_1
recQ_1	CAM030_00616	recQ_1
recQ_1	CAM030_02627	recQ_1
recQ_1	CAM030_00678	recQ_1
recQ_1	CAM030_00266	recQ_1
recQ_1	CAM030_00617	recQ_1
recQ_1	CAM030_02730	recQ_1
recQ_1	CAM030_00438	recQ_1
recQ_1	CAM030_01592	recQ_1
recQ_1	CAM030_01552	recQ_1
recQ_1	CECT7946_01116	recQ_1
recQ_1	CECT7946_03630	recQ_1
recQ_1	CECT7946_02858	recQ_1
recQ_1	CECT7946_01213	recQ_1
recQ_1	CECT7946_01207	recQ_1
recQ_1	CECT7946_01329	recQ_1
recQ_1	CECT7946_02918	recQ_1
recQ_1	CECT7946_01565	recQ_1
recQ_1	CECT7946_03424	recQ_1

recQ_1	CECT7946_03219	recQ_1
recQ_1	CECT7946_01074	recQ_1
recQ_1	CGMCC1.8863_00639	recQ_1
recQ_1	CGMCC1.8863_03375	recQ_1
recQ_1	CGMCC1.8863_00321	recQ_1
recQ_1	CGMCC1.8863_02602	recQ_1
recQ_1	CGMCC1.8863_00310	recQ_1
recQ_1	CGMCC1.8863_01793	recQ_1
recQ_1	CGMCC1.8863_01706	recQ_1
recQ_1	CGMCC1.8863_02711	recQ_1
recQ_1	CGMCC1.8863_02692	recQ_1
recQ_1	CGMCC1.8863_00340	recQ_1
recQ_1	CL8139_02858	recQ_1
recQ_1	CL8139_03070	recQ_1
recQ_1	CL8139_00677	recQ_1
recQ_1	CL8139_02825	recQ_1
recQ_1	CL8139_00676	recQ_1
recQ_1	CL8139_01802	recQ_1
recQ_1	CL8139_00657	recQ_1
recQ_1	CL8139_02374	recQ_1
recQ_1	CL8139_02171	recQ_1
recQ_1	DAU203_00030	recQ_1
recQ_1	DAU203_03391	recQ_1
recQ_1	DAU203_03011	recQ_1
recQ_1	DAU203_00063	recQ_1
recQ_1	DAU203_03010	recQ_1
recQ_1	DAU203_00344	recQ_1
recQ_1	DAU203_02990	recQ_1
recQ_1	DAU203_03145	recQ_1
recQ_1	DAU203_00957	recQ_1
recQ_1	DD5b_01273	recQ_1
recQ_1	DD5b_00179	recQ_1
recQ_1	DD5b_00571	recQ_1
recQ_1	DD5b_01307	recQ_1
recQ_1	DD5b_01009	recQ_1
recQ_1	DD5b_00827	recQ_1
recQ_1	DD5b_00699	recQ_1
recQ_1	DD5b_02206	recQ_1
recQ_1	DSM1208_04125	recQ_1
recQ_1	DSM1208_02848	recQ_1
recQ_1	DSM1208_01971	recQ_1
recQ_1	DSM1208_03804	recQ_1
recQ_1	DSM1208_04124	recQ_1
recQ_1	DSM1208_00039	recQ_1
recQ_1	DSM1208_00123	recQ_1
recQ_1	DSM1208_01029	recQ_1
recQ_1	DSM1208_00102	recQ_1
recQ_1	DSM1208_04130	recQ_1

recQ_1	DSM12212_00377	recQ_1
recQ_1	DSM12212_03038	recQ_1
recQ_1	DSM12212_00598	recQ_1
recQ_1	DSM12212_02303	recQ_1
recQ_1	DSM12212_00574	recQ_1
recQ_1	DSM12212_01967	recQ_1
recQ_1	DSM12212_00599	recQ_1
recQ_1	DSM12212_03297	recQ_1
recQ_1	DSM12212_01813	recQ_1
recQ_1	DSM12212_03515	recQ_1
recQ_1	DSM15343_01319	recQ_1
recQ_1	DSM15343_01328	recQ_1
recQ_1	DSM15343_01663	recQ_1
recQ_1	DSM15343_02766	recQ_1
recQ_1	DSM15343_00103	recQ_1
recQ_1	DSM15343_01275	recQ_1
recQ_1	DSM15343_00402	recQ_1
recQ_1	DSM15343_01830	recQ_1
recQ_1	DSM15343_02917	recQ_1
recQ_1	DSM15361_00136	recQ_1
recQ_1	DSM15361_02089	recQ_1
recQ_1	DSM15361_00869	recQ_1
recQ_1	DSM15361_02028	recQ_1
recQ_1	DSM15361_00888	recQ_1
recQ_1	DSM15361_00889	recQ_1
recQ_1	DSM15361_02322	recQ_1
recQ_1	DSM15361_00868	recQ_1
recQ_1	DSM15361_00878	recQ_1
recQ_1	DSM15361_00895	recQ_1
recQ_1	DSM15365_00282	recQ_1
recQ_1	DSM15365_00648	recQ_1
recQ_1	DSM15365_00313	recQ_1
recQ_1	DSM15365_02489	recQ_1
recQ_1	DSM15365_01719	recQ_1
recQ_1	DSM15365_00324	recQ_1
recQ_1	DSM15365_01462	recQ_1
recQ_1	DSM15365_02069	recQ_1
recQ_1	DSM15365_01720	recQ_1
recQ_1	DSM15365_03779	recQ_1
recQ_1	DSM15365_01242	recQ_1
recQ_1	DSM15365_00528	recQ_1
recQ_1	DSM15718_02024	recQ_1
recQ_1	DSM15718_00776	recQ_1
recQ_1	DSM15718_00896	recQ_1
recQ_1	DSM15718_02845	recQ_1
recQ_1	DSM15718_01309	recQ_1
recQ_1	DSM15718_01490	recQ_1
recQ_1	DSM15718_02060	recQ_1

recQ_1	DSM15718_00320	recQ_1
recQ_1	DSM15718_00967	recQ_1
recQ_1	DSM15718_01127	recQ_1
recQ_1	DSM15718_02561	recQ_1
recQ_1	DSM16505T_01438	recQ_1
recQ_1	DSM16505T_00442	recQ_1
recQ_1	DSM16505T_01526	recQ_1
recQ_1	DSM16505T_01124	recQ_1
recQ_1	DSM16505T_01806	recQ_1
recQ_1	DSM16505T_01315	recQ_1
recQ_1	DSM16505T_01290	recQ_1
recQ_1	DSM16505T_00265	recQ_1
recQ_1	DSM16505T_00245	recQ_1
recQ_1	DSM18103_00263	recQ_1
recQ_1	DSM18103_01304	recQ_1
recQ_1	DSM18103_00797	recQ_1
recQ_1	DSM18103_03348	recQ_1
recQ_1	DSM18103_00036	recQ_1
recQ_1	DSM18103_00107	recQ_1
recQ_1	DSM18103_03047	recQ_1
recQ_1	DSM18103_00843	recQ_1
recQ_1	DSM18103_01731	recQ_1
recQ_1	DSM18103_01707	recQ_1
recQ_1	DSM18103_00129	recQ_1
recQ_1	DSM18103_01217	recQ_1
recQ_1	DSM18293_03888	recQ_1
recQ_1	DSM18293_01041	recQ_1
recQ_1	DSM18293_02527	recQ_1
recQ_1	DSM18293_01835	recQ_1
recQ_1	DSM18293_02678	recQ_1
recQ_1	DSM18293_01792	recQ_1
recQ_1	DSM18293_03845	recQ_1
recQ_1	DSM18293_04049	recQ_1
recQ_1	DSM18293_01596	recQ_1
recQ_1	DSM18293_03102	recQ_1
recQ_1	DSM18841_00273	recQ_1
recQ_1	DSM18841_02151	recQ_1
recQ_1	DSM18841_01019	recQ_1
recQ_1	DSM18841_01539	recQ_1
recQ_1	DSM18841_02314	recQ_1
recQ_1	DSM18841_02456	recQ_1
recQ_1	DSM18841_02286	recQ_1
recQ_1	DSM18841_00064	recQ_1
recQ_1	DSM18841_00045	recQ_1
recQ_1	DSM18842_01493	recQ_1
recQ_1	DSM18842_00948	recQ_1
recQ_1	DSM18842_00299	recQ_1
recQ_1	DSM18842_02225	recQ_1

recQ_1	DSM18842_01225	recQ_1
recQ_1	DSM18842_01368	recQ_1
recQ_1	DSM18842_01197	recQ_1
recQ_1	DSM18842_01142	recQ_1
recQ_1	DSM18842_01164	recQ_1
recQ_1	DSM19866_01741	recQ_1
recQ_1	DSM19866_04000	recQ_1
recQ_1	DSM19866_00068	recQ_1
recQ_1	DSM19866_04294	recQ_1
recQ_1	DSM19866_04001	recQ_1
recQ_1	DSM19866_04750	recQ_1
recQ_1	DSM19866_03996	recQ_1
recQ_1	DSM19866_03676	recQ_1
recQ_1	DSM2041_05326	recQ_1
recQ_1	DSM2041_02709	recQ_1
recQ_1	DSM2041_01622	recQ_1
recQ_1	DSM2041_01410	recQ_1
recQ_1	DSM2041_00556	recQ_1
recQ_1	DSM2041_02080	recQ_1
recQ_1	DSM2041_04067	recQ_1
recQ_1	DSM2041_04892	recQ_1
recQ_1	DSM2041_01419	recQ_1
recQ_1	DSM2041_01733	recQ_1
recQ_1	DSM2061_00944	recQ_1
recQ_1	DSM2061_04333	recQ_1
recQ_1	DSM2061_03532	recQ_1
recQ_1	DSM2061_01226	recQ_1
recQ_1	DSM2061_01364	recQ_1
recQ_1	DSM2061_00943	recQ_1
recQ_1	DSM2061_00411	recQ_1
recQ_1	DSM2061_00514	recQ_1
recQ_1	DSM2061_01316	recQ_1
recQ_1	DSM2061_00948	recQ_1
recQ_1	DSM2064_03071	recQ_1
recQ_1	DSM2064_03235	recQ_1
recQ_1	DSM2064_04800	recQ_1
recQ_1	DSM2064_00223	recQ_1
recQ_1	DSM2064_04953	recQ_1
recQ_1	DSM2064_00267	recQ_1
recQ_1	DSM2064_03285	recQ_1
recQ_1	DSM2064_04122	recQ_1
recQ_1	DSM2064_03626	recQ_1
recQ_1	DSM2064_01994	recQ_1
recQ_1	DSM21164_02356	recQ_1
recQ_1	DSM21164_03087	recQ_1
recQ_1	DSM21164_02458	recQ_1
recQ_1	DSM21164_02605	recQ_1
recQ_1	DSM21164_01648	recQ_1

recQ_1	DSM21164_01942	recQ_1
recQ_1	DSM21164_02603	recQ_1
recQ_1	DSM21164_01943	recQ_1
recQ_1	DSM21164_02618	recQ_1
recQ_1	DSM23404_00301	recQ_1
recQ_1	DSM23404_02868	recQ_1
recQ_1	DSM23404_02671	recQ_1
recQ_1	DSM23404_03191	recQ_1
recQ_1	DSM23404_02464	recQ_1
recQ_1	DSM23404_01538	recQ_1
recQ_1	DSM23404_01913	recQ_1
recQ_1	DSM23424_01806	recQ_1
recQ_1	DSM23424_00507	recQ_1
recQ_1	DSM23424_02726	recQ_1
recQ_1	DSM23424_01390	recQ_1
recQ_1	DSM23424_01156	recQ_1
recQ_1	DSM23424_01208	recQ_1
recQ_1	DSM23424_02838	recQ_1
recQ_1	DSM23424_01218	recQ_1
recQ_1	DSM23424_01414	recQ_1
recQ_1	DSM23424_01209	recQ_1
recQ_1	DSM23424_01141	recQ_1
recQ_1	DSM24786_01214	recQ_1
recQ_1	DSM24786_00869	recQ_1
recQ_1	DSM24786_00476	recQ_1
recQ_1	DSM24786_02418	recQ_1
recQ_1	DSM24786_00475	recQ_1
recQ_1	DSM24786_01246	recQ_1
recQ_1	DSM24786_00455	recQ_1
recQ_1	DSM24786_00597	recQ_1
recQ_1	DSM24786_03122	recQ_1
recQ_1	DSM7489_00030	recQ_1
recQ_1	DSM7489_03227	recQ_1
recQ_1	DSM7489_02881	recQ_1
recQ_1	DSM7489_00063	recQ_1
recQ_1	DSM7489_02880	recQ_1
recQ_1	DSM7489_00329	recQ_1
recQ_1	DSM7489_02860	recQ_1
recQ_1	DSM7489_02977	recQ_1
recQ_1	DSM7489_00891	recQ_1
recQ_1	DSW1_01676	recQ_1
recQ_1	DSW1_02704	recQ_1
recQ_1	DSW1_00381	recQ_1
recQ_1	DSW1_01641	recQ_1
recQ_1	DSW1_02030	recQ_1
recQ_1	DSW1_02143	recQ_1
recQ_1	DSW1_02044	recQ_1
recQ_1	DSW1_01669	recQ_1

recQ_1	DSW5_02294	recQ_1
recQ_1	DSW5_01830	recQ_1
recQ_1	DSW5_01510	recQ_1
recQ_1	DSW5_02371	recQ_1
recQ_1	DSW5_02419	recQ_1
recQ_1	DSW5_02129	recQ_1
recQ_1	DSW5_01357	recQ_1
recQ_1	DSW5_00355	recQ_1
recQ_1	DSW5_01449	recQ_1
recQ_1	DSW5_01395	recQ_1
recQ_1	DSW5_01521	recQ_1
recQ_1	DSW6_02074	recQ_1
recQ_1	DSW6_02251	recQ_1
recQ_1	DSW6_01593	recQ_1
recQ_1	DSW6_02763	recQ_1
recQ_1	DSW6_02491	recQ_1
recQ_1	DSW6_02952	recQ_1
recQ_1	DSW6_02418	recQ_1
recQ_1	DSW6_00224	recQ_1
recQ_1	DSW8_01109	recQ_1
recQ_1	DSW8_01897	recQ_1
recQ_1	DSW8_00081	recQ_1
recQ_1	DSW8_03376	recQ_1
recQ_1	DSW8_00362	recQ_1
recQ_1	DSW8_00615	recQ_1
recQ_1	DSW8_00301	recQ_1
recQ_1	DSW8_01138	recQ_1
recQ_1	DSW8_00354	recQ_1
recQ_1	F52_00596	recQ_1
recQ_1	F52_00394	recQ_1
recQ_1	F52_01612	recQ_1
recQ_1	F52_04393	recQ_1
recQ_1	F52_01813	recQ_1
recQ_1	F52_03177	recQ_1
recQ_1	F52_01404	recQ_1
recQ_1	F52_04276	recQ_1
recQ_1	F52_00436	recQ_1
recQ_1	F52_03781	recQ_1
recQ_1	F52_02397	recQ_1
recQ_1	GCA_001027945.1_00341	recQ_1
recQ_1	GCA_001027945.1_01791	recQ_1
recQ_1	GCA_001027945.1_01409	recQ_1
recQ_1	GCA_001027945.1_02122	recQ_1
recQ_1	GCA_001027945.1_02682	recQ_1
recQ_1	GCA_001027945.1_01911	recQ_1
recQ_1	GCA_001027945.1_00562	recQ_1
recQ_1	GCA_001027945.1_00526	recQ_1
recQ_1	HI1_03329	recQ_1

recQ_1	HI1_03174	recQ_1
recQ_1	HI1_02820	recQ_1
recQ_1	HI1_03362	recQ_1
recQ_1	HI1_02819	recQ_1
recQ_1	HI1_00261	recQ_1
recQ_1	HI1_02800	recQ_1
recQ_1	HI1_02918	recQ_1
recQ_1	HI1_00823	recQ_1
recQ_1	IR1_03797	recQ_1
recQ_1	IR1_02289	recQ_1
recQ_1	IR1_02975	recQ_1
recQ_1	IR1_01816	recQ_1
recQ_1	IR1_02838	recQ_1
recQ_1	IR1_03473	recQ_1
recQ_1	IR1_02495	recQ_1
recQ_1	IR1_02439	recQ_1
recQ_1	IR1_01663	recQ_1
recQ_1	IR1_04761	recQ_1
recQ_1	KCOM3131_00402	recQ_1
recQ_1	KCOM3131_00605	recQ_1
recQ_1	KCOM3131_01073	recQ_1
recQ_1	KCOM3131_00878	recQ_1
recQ_1	KCOM3131_00834	recQ_1
recQ_1	KCOM3131_00859	recQ_1
recQ_1	KCTC12974_02514	recQ_1
recQ_1	KCTC12974_02122	recQ_1
recQ_1	KCTC12974_02947	recQ_1
recQ_1	KCTC12974_00464	recQ_1
recQ_1	KCTC12974_02747	recQ_1
recQ_1	KCTC12974_00243	recQ_1
recQ_1	KCTC12974_00372	recQ_1
recQ_1	KL-A_01809	recQ_1
recQ_1	KL-A_01945	recQ_1
recQ_1	KL-A_00635	recQ_1
recQ_1	KL-A_03285	recQ_1
recQ_1	KL-A_00636	recQ_1
recQ_1	KL-A_02664	recQ_1
recQ_1	KL-A_00655	recQ_1
recQ_1	KL-A_00509	recQ_1
recQ_1	KL-A_02380	recQ_1
recQ_1	L12_00324	recQ_1
recQ_1	LL0412.1.7_02573	recQ_1
recQ_1	LL0412.1.7_00717	recQ_1
recQ_1	LL0412.1.7_02444	recQ_1
recQ_1	LL0412.1.7_00187	recQ_1
recQ_1	LL0412.1.7_02256	recQ_1
recQ_1	LL0412.1.7_02463	recQ_1
recQ_1	LL0412.1.7_02214	recQ_1

recQ_1	LL0412.1.7_02191	recQ_1
recQ_1	LL0412.1.7_00405	recQ_1
recQ_1	LL0412.1.7_00213	recQ_1
recQ_1	LL0412.1.7_02473	recQ_1
recQ_1	LL0412.1.7_00765	recQ_1
recQ_1	MAR_2009_71_02336	recQ_1
recQ_1	MAR_2009_71_01481	recQ_1
recQ_1	MAR_2009_71_02882	recQ_1
recQ_1	MAR_2009_71_03554	recQ_1
recQ_1	MAR_2009_71_03161	recQ_1
recQ_1	MAR_2009_71_03412	recQ_1
recQ_1	MAR_2009_71_03105	recQ_1
recQ_1	MAR_2009_71_02306	recQ_1
recQ_1	MAR_2009_71_03153	recQ_1
recQ_1	NN016038_03830	recQ_1
recQ_1	NN016038_02164	recQ_1
recQ_1	NN016038_03464	recQ_1
recQ_1	NN016038_02762	recQ_1
recQ_1	NN016038_03432	recQ_1
recQ_1	NN016038_03687	recQ_1
recQ_1	NN016038_03462	recQ_1
recQ_1	NN016038_03431	recQ_1
recQ_1	NN016038_03476	recQ_1
recQ_1	PAO1_04078	recQ_1
recQ_1	PAO1_03462	recQ_1
recQ_1	PAO1_00465	recQ_1
recQ_1	PAO1_00439	recQ_1
recQ_1	PAO1_03986	recQ_1
recQ_1	PAO1_02941	recQ_1
recQ_1	PAO1_03588	recQ_1
recQ_1	UW101_02729	recQ_1
recQ_1	UW101_02565	recQ_1
recQ_1	UW101_00302	recQ_1
recQ_1	UW101_02934	recQ_1
recQ_1	UW101_00149	recQ_1
recQ_1	UW101_02978	recQ_1
recQ_1	UW101_02515	recQ_1
recQ_1	UW101_04764	recQ_1
recQ_1	UW101_05260	recQ_1
recQ_1	UW101_01049	recQ_1
recQ_1	V4-33_01002	recQ_1
recQ_1	V4-33_01142	recQ_1
recQ_1	V4-33_01925	recQ_1
recQ_1	V4-33_00633	recQ_1
recQ_1	V4-33_00857	recQ_1
recQ_1	V4-33_01727	recQ_1
recQ_1	V4-33_01607	recQ_1
recQ_1	V4-33_00638	recQ_1

recQ_1	V4-33_02430	recQ_1
recQ_1	W5C_00841	recQ_1
recQ_1	W5C_01777	recQ_1
recQ_1	W5C_02561	recQ_1
recQ_1	W5C_00808	recQ_1
recQ_1	W5C_02562	recQ_1
recQ_1	W5C_02774	recQ_1
recQ_1	W5C_02542	recQ_1
recQ_1	W5C_02182	recQ_1
recQ_1	W5C_01534	recQ_1
recQ_1	W5C_00932	recQ_1
recQ_1	ZONMW-01_03085	recQ_1
recQ_1	ZONMW-01_00558	recQ_1
recQ_1	ZONMW-01_00908	recQ_1
recQ_1	ZONMW-01_00909	recQ_1
recQ_1	ZONMW-01_01773	recQ_1
recQ_1	ZONMW-01_03052	recQ_1
recQ_1	ZONMW-01_02342	recQ_1
recQ_1	ZONMW-01_00798	recQ_1
recQ_1	ZONMW-01_02528	recQ_1
recQ_1	ZONMW-02_03228	recQ_1
recQ_1	ZONMW-02_01091	recQ_1
recQ_1	ZONMW-02_02905	recQ_1
recQ_1	ZONMW-02_00609	recQ_1
recQ_1	ZONMW-02_02138	recQ_1
recQ_1	ZONMW-02_02916	recQ_1
recQ_1	ZONMW-02_01919	recQ_1
recQ_1	ZONMW-02_03453	recQ_1
recQ_1	ZONMW-02_02137	recQ_1
recQ_1	ZONMW-02_03025	recQ_1
recQ_1	ZONMW-02_00841	recQ_1
recQ_1	ZONMW-03_02320	recQ_1
recQ_1	ZONMW-03_02452	recQ_1
recQ_1	ZONMW-03_00286	recQ_1
recQ_1	ZONMW-03_00287	recQ_1
recQ_1	ZONMW-03_02758	recQ_1
recQ_1	ZONMW-03_02287	recQ_1
recQ_1	ZONMW-03_00306	recQ_1
recQ_1	ZONMW-03_00176	recQ_1
recQ_1	ZONMW-03_01809	recQ_1
recQ_1	ZONMW-04_00784	recQ_1
recQ_1	ZONMW-04_00933	recQ_1
recQ_1	ZONMW-04_03055	recQ_1
recQ_1	ZONMW-04_03054	recQ_1
recQ_1	ZONMW-04_02765	recQ_1
recQ_1	ZONMW-04_00752	recQ_1
recQ_1	ZONMW-04_01240	recQ_1
recQ_1	ZONMW-04_01180	recQ_1

recQ_1	ZONMW-04_03014	recQ_1
recQ_1	ZONMW-05_00054	recQ_1
recQ_1	ZONMW-05_00185	recQ_1
recQ_1	ZONMW-05_00544	recQ_1
recQ_1	ZONMW-05_00545	recQ_1
recQ_1	ZONMW-05_02315	recQ_1
recQ_1	ZONMW-05_00021	recQ_1
recQ_1	ZONMW-05_00565	recQ_1
recQ_1	ZONMW-05_00431	recQ_1
recQ_1	ZONMW-05_03258	recQ_1
recQ_1	ZONMW-06_02229	recQ_1
recQ_1	ZONMW-06_03843	recQ_1
recQ_1	ZONMW-06_01178	recQ_1
recQ_1	ZONMW-06_03012	recQ_1
recQ_1	ZONMW-06_03175	recQ_1
recQ_1	ZONMW-07_02249	recQ_1
recQ_1	ZONMW-07_03247	recQ_1
recQ_1	ZONMW-07_03969	recQ_1
recQ_1	ZONMW-07_01882	recQ_1
recQ_1	ZONMW-07_01839	recQ_1
recQ_1	ZONMW-07_01131	recQ_1
recQ_1	ZONMW-07_03210	recQ_1
recQ_1	ZONMW-07_02576	recQ_1
recQ_1	ZONMW-08_00910	recQ_1
recQ_1	ZONMW-08_01726	recQ_1
recQ_1	ZONMW-08_02081	recQ_1
recQ_1	ZONMW-08_02370	recQ_1
recQ_1	ZONMW-08_02676	recQ_1
recQ_1	ZONMW-08_02515	recQ_1
recQ_1	ZONMW-08_02620	recQ_1
recQ_1	ZONMW-08_00939	recQ_1
recQ_1	ZONMW-08_02668	recQ_1
recQ_1	ZONMW-09_02110	recQ_1
recQ_1	ZONMW-09_02222	recQ_1
recQ_1	ZONMW-09_02466	recQ_1
recQ_1	ZONMW-09_01173	recQ_1
recQ_1	ZONMW-09_00790	recQ_1
recQ_1	ZONMW-09_01466	recQ_1
recQ_1	ZONMW-09_01083	recQ_1
recQ_1	ZONMW-09_01068	recQ_1
recQ_1	ZONMW-09_01238	recQ_1
recQ_1	ZONMW-09_00713	recQ_1
recQ_1	ZONMW-09_02432	recQ_1
recQ_1	ZONMW-10_01809	recQ_1
recQ_1	ZONMW-10_01973	recQ_1
recQ_1	ZONMW-10_01546	recQ_1
recQ_1	ZONMW-10_01629	recQ_1
recQ_1	ZONMW-10_00842	recQ_1

recQ_1	ZONMW-10_01886	recQ_1
recQ_1	ZONMW-10_02064	recQ_1
recQ_1	ZONMW-10_02108	recQ_1
recQ_1	ZONMW-10_00347	recQ_1
recQ_1	ZONMW-11_02082	recQ_1
recQ_1	ZONMW-11_00536	recQ_1
recQ_1	ZONMW-11_03714	recQ_1
recQ_1	ZONMW-11_00829	recQ_1
recQ_1	ZONMW-11_03639	recQ_1
recQ_1	ZONMW-11_04658	recQ_1
recQ_1	ZONMW-11_01926	recQ_1
recQ_1	ZONMW-11_03420	recQ_1
recQ_1	ZONMW-11_02614	recQ_1
recQ_1	ZONMW-11_03009	recQ_1
recQ_1	ZONMW-11_02647	recQ_1
recQ_1	ZONMW-12_01897	recQ_1
recQ_1	ZONMW-12_03219	recQ_1
recQ_1	ZONMW-12_03489	recQ_1
recQ_1	ZONMW-12_03782	recQ_1
recQ_1	ZONMW-12_03707	recQ_1
recQ_1	ZONMW-12_00738	recQ_1
recQ_1	ZONMW-13_01646	recQ_1
recQ_1	ZONMW-13_02193	recQ_1
recQ_1	ZONMW-13_01337	recQ_1
recQ_1	ZONMW-13_03153	recQ_1
recQ_1	ZONMW-13_02738	recQ_1
recQ_1	ZONMW-13_01898	recQ_1
recQ_1	ZONMW-13_02341	recQ_1
recQ_1	ZONMW-14_01823	recQ_1
recQ_1	ZONMW-14_00558	recQ_1
recQ_1	ZONMW-14_01765	recQ_1
recQ_1	ZONMW-14_00794	recQ_1
recQ_1	ZONMW-14_02114	recQ_1
recQ_1	ZONMW-14_02640	recQ_1
recQ_1	ZONMW-15_01870	recQ_1
recQ_1	ZONMW-15_03032	recQ_1
recQ_1	ZONMW-15_02653	recQ_1
recQ_1	ZONMW-15_01845	recQ_1
recQ_1	ZONMW-15_02922	recQ_1
recQ_1	ZONMW-15_01869	recQ_1
recQ_1	ZONMW-15_02424	recQ_1
recQ_1	ZONMW-15_01847	recQ_1
recQ_1	ZONMW-15_01831	recQ_1
recQ_1	ZONMW-19_03384	recQ_1
recQ_1	ZONMW-19_04015	recQ_1
recQ_1	ZONMW-19_04534	recQ_1
recQ_1	ZONMW-19_00632	recQ_1
recQ_1	ZONMW-20_00873	recQ_1

recQ_1	ZONMW-20_02890	recQ_1
recQ_1	ZONMW-20_02961	recQ_1
recQ_1	ZONMW-20_02521	recQ_1
recQ_1	ZONMW-20_00839	recQ_1
recQ_1	ZONMW-20_00619	recQ_1
recQ_1	ZONMW-21_02718	recQ_1
recQ_1	ZONMW-21_02149	recQ_1
recQ_1	ZONMW-21_01603	recQ_1
recQ_1	ZONMW-22_02391	recQ_1
recQ_1	ZONMW-22_01576	recQ_1
recQ_1	ZONMW-22_02121	recQ_1
recQ_1	ZONMW-23_00159	recQ_1
recQ_1	ZONMW-23_00975	recQ_1
recQ_1	ZONMW-23_00430	recQ_1
recQ_1	ZONMW-24_02156	recQ_1
recQ_1	ZONMW-24_01600	recQ_1
recQ_1	ZONMW-24_02746	recQ_1
recQ_1	ZONMW-25_00681	recQ_1
recQ_1	ZONMW-25_02460	recQ_1
recQ_1	ZONMW-25_00389	recQ_1
recQ_1	ZONMW-25_01395	recQ_1
recQ_1	ZONMW-26_00906	recQ_1
recQ_1	ZONMW-26_00772	recQ_1
recQ_1	ZONMW-26_00423	recQ_1
recQ_1	ZONMW-26_00422	recQ_1
recQ_1	ZONMW-26_02980	recQ_1
recQ_1	ZONMW-26_00939	recQ_1
recQ_1	ZONMW-26_00402	recQ_1
recQ_1	ZONMW-26_00534	recQ_1
recQ_1	ZONMW-26_02040	recQ_1
recQ_1	ZONMW-27_00881	recQ_1
recQ_1	ZONMW-27_01008	recQ_1
recQ_1	ZONMW-27_02332	recQ_1
recQ_1	ZONMW-27_01368	recQ_1
recQ_1	ZONMW-27_01369	recQ_1
recQ_1	ZONMW-27_03249	recQ_1
recQ_1	ZONMW-27_00848	recQ_1
recQ_1	ZONMW-27_01389	recQ_1
recQ_1	ZONMW-27_01257	recQ_1
recQ_1	ZONMW-27_02889	recQ_1
recQ_1	ZONMW-28_00359	recQ_1
recQ_1	ZONMW-28_03327	recQ_1
recQ_1	ZONMW-28_01820	recQ_1
recQ_1	ZONMW-28_03792	recQ_1
recQ_1	ZONMW-28_02986	recQ_1
recQ_1	ZONMW-29_01884	recQ_1
recQ_1	ZONMW-29_00841	recQ_1
recQ_1	ZONMW-29_01351	recQ_1

recQ_1	ZONMW-29_02300	recQ_1
recQ_1	ZONMW-29_02397	recQ_1
recQ_1	ZONMW-29_03189	recQ_1
recQ_1	ZONMW-30_03011	recQ_1
recQ_1	ZONMW-30_00526	recQ_1
recQ_1	ZONMW-30_02688	recQ_1
recQ_1	ZONMW-30_02144	recQ_1
recQ_1	ZONMW-31_03357	recQ_1
recQ_1	ZONMW-31_03595	recQ_1
recQ_1	ZONMW-31_02638	recQ_1
recQ_1	ZONMW-31_00174	recQ_1
recQ_1	ZONMW-31_01642	recQ_1
recQ_1	ZONMW-32_01150	recQ_1
recQ_1	ZONMW-32_03452	recQ_1
recQ_1	ZONMW-32_02152	recQ_1
recQ_1	ZONMW-32_03731	recQ_1
recQ_1	ZONMW-32_01470	recQ_1
recQ_1	ZONMW-33_01139	recQ_1
recQ_1	ZONMW-33_03455	recQ_1
recQ_1	ZONMW-33_02143	recQ_1
recQ_1	ZONMW-33_04045	recQ_1
recQ_1	ZONMW-33_01460	recQ_1
recQ_1	ZONMW-34_01330	recQ_1
recQ_1	ZONMW-34_03750	recQ_1
recQ_1	ZONMW-34_02277	recQ_1
recQ_1	ZONMW-34_02661	recQ_1
recQ_1	ZONMW-34_01648	recQ_1
recQ_1	ZONMW-35_00403	recQ_1
recQ_1	ZONMW-35_03713	recQ_1
recQ_1	ZONMW-35_01972	recQ_1
recQ_1	ZONMW-35_00038	recQ_1
recQ_1	ZONMW-35_03612	recQ_1
recQ_1	ZONMW-36_00359	recQ_1
recQ_1	ZONMW-36_03312	recQ_1
recQ_1	ZONMW-36_02093	recQ_1
recQ_1	ZONMW-36_04046	recQ_1
recQ_1	ZONMW-36_00679	recQ_1
recQ_1	ZONMW-37_02385	recQ_1
recQ_1	ZONMW-37_01599	recQ_1
recQ_1	ZONMW-37_01981	recQ_1
recQ_1	ZONMW-38_01340	recQ_1
recQ_1	ZONMW-38_01152	recQ_1
recQ_1	ZONMW-38_02420	recQ_1
recQ_1	ZONMW-38_02894	recQ_1
recQ_1	ZONMW-38_02419	recQ_1
recQ_1	ZONMW-39_00370	recQ_1
recQ_1	ZONMW-39_03615	recQ_1
recQ_1	ZONMW-39_01552	recQ_1

recQ_1	ZONMW-39_03527	recQ_1
recQ_1	ZONMW-39_00051	recQ_1
recQ_1	ZONMW-40_01719	recQ_1
recQ_1	ZONMW-40_00486	recQ_1
recQ_1	ZONMW-40_02489	recQ_1
recQ_1	ZONMW-41_02590	recQ_1
recQ_1	ZONMW-41_00301	recQ_1
recQ_1	ZONMW-41_02713	recQ_1
recQ_1	ZONMW-41_00820	recQ_1
recQ_1	ZONMW-41_00823	recQ_1
recQ_1	ZONMW-41_01116	recQ_1
recQ_1	ZONMW-41_02012	recQ_1
recQ_1	ZONMW-41_01094	recQ_1
recQ_1	ZONMW-41_00724	recQ_1
recQ_1	ZONMW-41_02220	recQ_1
recQ_1	ZONMW-42_01738	recQ_1
recQ_1	ZONMW-42_03237	recQ_1
recQ_1	ZONMW-42_03709	recQ_1
recQ_1	ZONMW-42_03803	recQ_1
recQ_1	ZONMW-42_00735	recQ_1
recQ_1	ZONMW-43_02916	recQ_1
recQ_1	ZONMW-43_02804	recQ_1
recQ_1	ZONMW-43_01957	recQ_1
recQ_1	ZONMW-43_02528	recQ_1
recQ_1	ZONMW-43_01620	recQ_1
recQ_1	ZONMW-44_01204	recQ_1
recQ_1	ZONMW-44_03880	recQ_1
recQ_1	ZONMW-44_04040	recQ_1
recQ_1	ZONMW-44_00998	recQ_1
recQ_1	ZONMW-44_02848	recQ_1
recQ_1	ZONMW-45_00423	recQ_1
recQ_1	ZONMW-45_01584	recQ_1
recQ_1	ZONMW-45_02152	recQ_1
recQ_1	ZONMW-45_00916	recQ_1
recQ_1	ZONMW-45_03176	recQ_1
recQ_1	ZONMW-45_01868	recQ_1
recQ_1	ZONMW-45_03165	recQ_1
recQ_1	ZONMW-45_02074	recQ_1
recQ_1	ZONMW-45_02054	recQ_1
recQ_1	ZONMW-46_01416	recQ_1
recQ_1	ZONMW-46_02095	recQ_1
recQ_1	ZONMW-46_01828	recQ_1
recQ_1	ZONMW-46_00958	recQ_1
recQ_1	ZONMW-46_02924	recQ_1
recQ_1	ZONMW-46_01968	recQ_1
recQ_1	ZONMW-46_02897	recQ_1
recQ_1	ZONMW-46_02208	recQ_1
recQ_1	ZONMW-46_02228	recQ_1

recQ_1	ZONMW-47_01436	recQ_1
recQ_1	ZONMW-47_01966	recQ_1
recQ_1	ZONMW-47_01661	recQ_1
recQ_1	ZONMW-47_01215	recQ_1
recQ_1	ZONMW-47_02949	recQ_1
recQ_1	ZONMW-47_02093	recQ_1
recQ_1	ZONMW-47_02976	recQ_1
recQ_1	ZONMW-47_02324	recQ_1
recQ_1	ZONMW-47_02304	recQ_1
recQ_1	ZONMW-48_01454	recQ_1
recQ_1	ZONMW-48_03151	recQ_1
recQ_1	ZONMW-48_02872	recQ_1
recQ_1	ZONMW-48_02873	recQ_1
recQ_1	ZONMW-48_01429	recQ_1
recQ_1	ZONMW-48_01672	recQ_1
recQ_1	ZONMW-48_01453	recQ_1
recQ_1	ZONMW-48_03063	recQ_1
recQ_1	ZONMW-48_01431	recQ_1
recQ_1	ZONMW-48_01415	recQ_1
recQ_1	ZONMW-49_03083	recQ_1
recQ_1	ZONMW-49_02977	recQ_1
recQ_1	ZONMW-49_00438	recQ_1
recQ_1	ZONMW-49_03292	recQ_1
recQ_1	ZONMW-49_00437	recQ_1
recQ_1	ZONMW-49_01858	recQ_1
recQ_1	ZONMW-49_00418	recQ_1
recQ_1	ZONMW-49_01659	recQ_1
recQ_1	ZONMW-49_02085	recQ_1
recQ_1	ZONMW-50_02949	recQ_1
recQ_1	ZONMW-50_02878	recQ_1
recQ_1	ZONMW-50_00047	recQ_1
recQ_1	ZONMW-50_03290	recQ_1
recQ_1	ZONMW-50_00048	recQ_1
recQ_1	ZONMW-50_03039	recQ_1
recQ_1	ZONMW-50_00067	recQ_1
recQ_1	ZONMW-50_01755	recQ_1
recQ_1	ZONMW-50_01879	recQ_1
recQ_1	ZONMW-51_01877	recQ_1
recQ_1	ZONMW-51_01996	recQ_1
recQ_1	ZONMW-51_00427	recQ_1
recQ_1	ZONMW-51_01844	recQ_1
recQ_1	ZONMW-51_00426	recQ_1
recQ_1	ZONMW-51_02141	recQ_1
recQ_1	ZONMW-51_00406	recQ_1
recQ_1	ZONMW-51_00557	recQ_1
recQ_1	ZONMW-51_01743	recQ_1
recQ_1	ZONMW-52_00896	recQ_1
recQ_1	ZONMW-52_00776	recQ_1

recQ_1	ZONMW-52_00411	recQ_1
recQ_1	ZONMW-52_00929	recQ_1
recQ_1	ZONMW-52_00410	recQ_1
recQ_1	ZONMW-52_02570	recQ_1
recQ_1	ZONMW-52_00391	recQ_1
recQ_1	ZONMW-52_00514	recQ_1
recQ_1	ZONMW-52_01886	recQ_1
recQ_1	ZONMW-53_01639	recQ_1
recQ_1	ZONMW-53_01510	recQ_1
recQ_1	ZONMW-53_01143	recQ_1
recQ_1	ZONMW-53_01672	recQ_1
recQ_1	ZONMW-53_01142	recQ_1
recQ_1	ZONMW-53_02322	recQ_1
recQ_1	ZONMW-53_01123	recQ_1
recQ_1	ZONMW-53_01269	recQ_1
recQ_1	ZONMW-53_01870	recQ_1
recQ_1	ZONMW-54_01086	recQ_1
recQ_1	ZONMW-54_01213	recQ_1
recQ_1	ZONMW-54_01578	recQ_1
recQ_1	ZONMW-54_01053	recQ_1
recQ_1	ZONMW-54_01579	recQ_1
recQ_1	ZONMW-54_02364	recQ_1
recQ_1	ZONMW-54_01599	recQ_1
recQ_1	ZONMW-54_01448	recQ_1
recQ_1	ZONMW-54_02201	recQ_1
recQ_1	ZONMW-55_02199	recQ_1
recQ_1	ZONMW-55_02318	recQ_1
recQ_1	ZONMW-55_01099	recQ_1
recQ_1	ZONMW-55_02166	recQ_1
recQ_1	ZONMW-55_01100	recQ_1
recQ_1	ZONMW-55_01831	recQ_1
recQ_1	ZONMW-55_01120	recQ_1
recQ_1	ZONMW-55_00967	recQ_1
recQ_1	ZONMW-55_02065	recQ_1
recQ_1	ZONMW-56_02169	recQ_1
recQ_1	ZONMW-56_02038	recQ_1
recQ_1	ZONMW-56_00329	recQ_1
recQ_1	ZONMW-56_02202	recQ_1
recQ_1	ZONMW-56_00330	recQ_1
recQ_1	ZONMW-56_01979	recQ_1
recQ_1	ZONMW-56_00349	recQ_1
recQ_1	ZONMW-56_00203	recQ_1
recQ_1	ZONMW-56_01369	recQ_1
recQ_1	ZONMW-57_00051	recQ_1
recQ_1	ZONMW-57_00178	recQ_1
recQ_1	ZONMW-57_00541	recQ_1
recQ_1	ZONMW-57_00018	recQ_1
recQ_1	ZONMW-57_00542	recQ_1

recQ_1	ZONMW-57_02155	recQ_1
recQ_1	ZONMW-57_00562	recQ_1
recQ_1	ZONMW-57_00416	recQ_1
recQ_1	ZONMW-57_01855	recQ_1
recQ_1	ZONMW-58_00915	recQ_1
recQ_1	ZONMW-58_00793	recQ_1
recQ_1	ZONMW-58_00430	recQ_1
recQ_1	ZONMW-58_00948	recQ_1
recQ_1	ZONMW-58_00429	recQ_1
recQ_1	ZONMW-58_02179	recQ_1
recQ_1	ZONMW-58_00409	recQ_1
recQ_1	ZONMW-58_00555	recQ_1
recQ_1	ZONMW-58_01879	recQ_1
recQ_1	ZONMW-59_00051	recQ_1
recQ_1	ZONMW-59_00171	recQ_1
recQ_1	ZONMW-59_00529	recQ_1
recQ_1	ZONMW-59_00018	recQ_1
recQ_1	ZONMW-59_00530	recQ_1
recQ_1	ZONMW-59_02398	recQ_1
recQ_1	ZONMW-59_00549	recQ_1
recQ_1	ZONMW-59_00432	recQ_1
recQ_1	ZONMW-59_01692	recQ_1
recQ_1	ZONMW-60_00052	recQ_1
recQ_1	ZONMW-60_00204	recQ_1
recQ_1	ZONMW-60_00544	recQ_1
recQ_1	ZONMW-60_00019	recQ_1
recQ_1	ZONMW-60_00545	recQ_1
recQ_1	ZONMW-60_02244	recQ_1
recQ_1	ZONMW-60_00564	recQ_1
recQ_1	ZONMW-60_00443	recQ_1
recQ_1	ZONMW-60_02075	recQ_1
recQ_1	ZONMW-61_00864	recQ_1
recQ_1	ZONMW-61_00599	recQ_1
recQ_1	ZONMW-61_02440	recQ_1
recQ_1	ZONMW-61_02234	recQ_1
recQ_1	ZONMW-61_01842	recQ_1
recQ_1	ZONMW-61_03313	recQ_1
recQ_1	ZONMW-61_02099	recQ_1
recQ_1	ZONMW-61_00895	recQ_1
recQ_1	ZONMW-61_03374	recQ_1
recQ_1	ZONMW-62_02679	recQ_1
recQ_1	ZONMW-62_02539	recQ_1
recQ_1	ZONMW-62_00684	recQ_1
recQ_1	ZONMW-62_03297	recQ_1
recQ_1	ZONMW-62_00147	recQ_1
recQ_1	ZONMW-62_00463	recQ_1
recQ_1	ZONMW-62_02708	recQ_1
recQ_1	ZONMW-62_00414	recQ_1

recQ_1	ZONMW-63_01815	recQ_1
recQ_1	ZONMW-63_02576	recQ_1
recQ_1	ZONMW-63_00215	recQ_1
recQ_1	ZONMW-63_01782	recQ_1
recQ_1	ZONMW-63_00216	recQ_1
recQ_1	ZONMW-63_03110	recQ_1
recQ_1	ZONMW-63_00235	recQ_1
recQ_1	ZONMW-63_00085	recQ_1
recQ_1	ZONMW-63_02736	recQ_1
recQ_1	ZONMW-64_03428	recQ_1
recQ_1	ZONMW-64_03647	recQ_1
recQ_1	ZONMW-64_01476	recQ_1
recQ_1	ZONMW-64_03711	recQ_1
recQ_1	ZONMW-64_03074	recQ_1
recQ_1	ZONMW-65_02001	recQ_1
recQ_1	ZONMW-65_00201	recQ_1
recQ_1	ZONMW-65_00834	recQ_1
recQ_1	ZONMW-65_00413	recQ_1
recQ_1	ZONMW-65_01835	recQ_1
recQ_1	ZONMW-65_00727	recQ_1
recQ_1	ZONMW-65_01307	recQ_1
recQ_1	ZONMW-66_04405	recQ_1
recQ_1	ZONMW-66_04060	recQ_1
recQ_1	ZONMW-66_04170	recQ_1
recQ_1	ZONMW-66_02742	recQ_1
recQ_1	ZONMW-67_01280	recQ_1
recQ_1	ZONMW-67_01798	recQ_1
recQ_1	ZONMW-67_00259	recQ_1
recQ_1	ZONMW-67_02513	recQ_1
recQ_1	ZONMW-67_01582	recQ_1
recQ_1	ZONMW-68_02722	recQ_1
recQ_1	ZONMW-68_04559	recQ_1
recQ_1	ZONMW-68_02661	recQ_1
recQ_1	ZONMW-68_04888	recQ_1
recQ_1	ZONMW-68_04558	recQ_1
recQ_1	ZONMW-68_01003	recQ_1
recQ_1	ZONMW-68_04563	recQ_1
recQ_1	ZONMW-68_00800	recQ_1
recQ_1	ZONMW-69_01721	recQ_1
recQ_1	ZONMW-69_03637	recQ_1
recQ_1	ZONMW-69_02145	recQ_1
recQ_1	ZONMW-69_00006	recQ_1
recQ_1	ZONMW-69_02561	recQ_1
recQ_1	ZONMW-69_01419	recQ_1
recQ_1	ZONMW-72_00746	recQ_1
recQ_1	ZONMW-72_02851	recQ_1
recQ_1	ZONMW-72_01245	recQ_1
recQ_1	ZONMW-72_03084	recQ_1

recQ_1	ZONMW-72_01405	recQ_1
recQ_1	ZONMW-72_03592	recQ_1
recQ_1	ZONMW-72_02551	recQ_1
recQ_1	ZONMW-72_02972	recQ_1
recQ_1	ZONMW-72_02160	recQ_1
recQ_1	ZONMW-72_04153	recQ_1
recQ_1	ZONMW-73_01698	recQ_1
recQ_1	ZONMW-73_00597	recQ_1
recQ_1	ZONMW-73_03701	recQ_1
recQ_1	ZONMW-73_02885	recQ_1
recQ_1	ZONMW-73_00500	recQ_1
recQ_1	ZONMW-73_00860	recQ_1
recQ_1	ZONMW-73_01499	recQ_1
recQ_1	ZONMW-73_02596	recQ_1
recQ_1	ZONMW-73_00859	recQ_1
recQ_1	ZONMW-73_03231	recQ_1
recQ_1	ZONMW-73_03523	recQ_1
recQ_1	ZONMW-74_00095	recQ_1
recQ_1	ZONMW-74_00615	recQ_1
recQ_1	ZONMW-74_00471	recQ_1
recQ_1	ZONMW-74_00094	recQ_1
recQ_1	ZONMW-74_00649	recQ_1
recQ_1	ZONMW-74_02292	recQ_1
recQ_1	ZONMW-74_00177	recQ_1
recQ_1	ZONMW-74_00198	recQ_1
recQ_1	ZONMW-74_02861	recQ_1
recQ_1	ZONMW-75_01673	recQ_1
recQ_1	ZONMW-75_02199	recQ_1
recQ_1	ZONMW-75_01550	recQ_1
recQ_1	ZONMW-75_03242	recQ_1
recQ_1	ZONMW-75_01811	recQ_1
recQ_1	ZONMW-75_00754	recQ_1
recQ_1	ZONMW-75_03221	recQ_1
recQ_1	ZONMW-75_02441	recQ_1
recQ_1	ZONMW-75_02198	recQ_1
recQ_1	ZONMW-75_03415	recQ_1
recQ_1	ZONMW-75_03113	recQ_1
recQ_1	da5A-8_01296	recQ_1
recQ_1	da5A-8_02715	recQ_1
recQ_1	da5A-8_01099	recQ_1
recQ_1	da5A-8_03153	recQ_1
recQ_1	da5A-8_01659	recQ_1
recQ_1	da5A-8_03533	recQ_1
recQ_1	da5A-8_02677	recQ_1
recQ_1	da5A-8_01920	recQ_1
recQ_1	da5A-8_00797	recQ_1
recQ_1	da5A-8_00771	recQ_1
recQ_1	da5A-8_03396	recQ_1

recQ_1	da5A-8_01492	recQ_1
ftsH	18_00413	ftsH
ftsH	18_01180	ftsH
ftsH	18_01818	ftsH
ftsH	ACAM48_03426	ftsH
ftsH	CAM030_02779	ftsH
ftsH	CAM030_00634	ftsH
ftsH	CECT7946_03295	ftsH
ftsH	CGMCC1.8863_01587	ftsH
ftsH	CL8139_02915	ftsH
ftsH	DAU203_00599	ftsH
ftsH	DD5b_00820	ftsH
ftsH	DD5b_01847	ftsH
ftsH	DSM1208_03708	ftsH
ftsH	DSM1208_00666	ftsH
ftsH	DSM12212_01123	ftsH
ftsH	DSM15343_01344	ftsH
ftsH	DSM15361_01500	ftsH
ftsH	DSM15361_02061	ftsH
ftsH	DSM15365_03854	ftsH
ftsH	DSM15365_03825	ftsH
ftsH	DSM15718_00327	ftsH
ftsH	DSM16505T_02251	ftsH
ftsH	DSM16505T_02370	ftsH
ftsH	DSM18103_02167	ftsH
ftsH	DSM18103_01626	ftsH
ftsH	DSM18103_02676	ftsH
ftsH	DSM18293_04037	ftsH
ftsH	DSM18841_01674	ftsH
ftsH	DSM18841_02666	ftsH
ftsH	DSM18841_03069	ftsH
ftsH	DSM18842_01731	ftsH
ftsH	DSM18842_00035	ftsH
ftsH	DSM18842_02576	ftsH
ftsH	DSM19866_04393	ftsH
ftsH	DSM19866_00562	ftsH
ftsH	DSM19866_01487	ftsH
ftsH	DSM2041_02798	ftsH
ftsH	DSM2041_05119	ftsH
ftsH	DSM2041_01148	ftsH
ftsH	DSM2061_02160	ftsH
ftsH	DSM2064_03085	ftsH
ftsH	DSM2064_03043	ftsH
ftsH	DSM2064_00486	ftsH
ftsH	DSM2064_02990	ftsH
ftsH	DSM2064_01163	ftsH
ftsH	DSM2064_00597	ftsH
ftsH	DSM21164_00683	ftsH

ftsH	DSM21164_00063	ftsH
ftsH	DSM21164_00992	ftsH
ftsH	DSM23404_00738	ftsH
ftsH	DSM23404_03287	ftsH
ftsH	DSM23424_02573	ftsH
ftsH	DSM23424_02159	ftsH
ftsH	DSM24786_03417	ftsH
ftsH	DSM24786_01396	ftsH
ftsH	DSM24786_01660	ftsH
ftsH	DSM7489_00585	ftsH
ftsH	DSW1_02880	ftsH
ftsH	DSW5_00379	ftsH
ftsH	DSW6_02071	ftsH
ftsH	DSW6_00547	ftsH
ftsH	DSW8_03712	ftsH
ftsH	F52_00579	ftsH
ftsH	F52_01188	ftsH
ftsH	F52_03987	ftsH
ftsH	F52_04529	ftsH
ftsH	F52_01055	ftsH
ftsH	GCA_001027945.1_02464	ftsH
ftsH	GCA_001027945.1_01044	ftsH
ftsH	GCA_001027945.1_00860	ftsH
ftsH	HI1_00517	ftsH
ftsH	IR1_02300	ftsH
ftsH	IR1_01749	ftsH
ftsH	IR1_00895	ftsH
ftsH	KCOM3131_00984	ftsH
ftsH	KCTC12974_01335	ftsH
ftsH	KCTC12974_00998	ftsH
ftsH	KL-A_02544	ftsH
ftsH	L12_00012	ftsH
ftsH	LL0412.1.7_02565	ftsH
ftsH	LL0412.1.7_01684	ftsH
ftsH	MAR_2009_71_00244	ftsH
ftsH	NN016038_01201	ftsH
ftsH	NN016038_01839	ftsH
ftsH	NN016038_00418	ftsH
ftsH	PAO1_04913	ftsH
ftsH	PAO1_00671	ftsH
ftsH	UW101_02715	ftsH
ftsH	UW101_02757	ftsH
ftsH	UW101_03197	ftsH
ftsH	UW101_02044	ftsH
ftsH	UW101_04316	ftsH
ftsH	UW101_03308	ftsH
ftsH	V4-33_00579	ftsH
ftsH	W5C_01257	ftsH

ftsH	ZONMW-01_02781	ftsH
ftsH	ZONMW-02_03100	ftsH
ftsH	ZONMW-02_03072	ftsH
ftsH	ZONMW-03_03125	ftsH
ftsH	ZONMW-04_02825	ftsH
ftsH	ZONMW-05_03082	ftsH
ftsH	ZONMW-06_00309	ftsH
ftsH	ZONMW-06_02617	ftsH
ftsH	ZONMW-07_00162	ftsH
ftsH	ZONMW-07_02819	ftsH
ftsH	ZONMW-08_03360	ftsH
ftsH	ZONMW-09_01579	ftsH
ftsH	ZONMW-09_01864	ftsH
ftsH	ZONMW-10_01980	ftsH
ftsH	ZONMW-10_00595	ftsH
ftsH	ZONMW-10_02357	ftsH
ftsH	ZONMW-11_00062	ftsH
ftsH	ZONMW-11_01929	ftsH
ftsH	ZONMW-12_01965	ftsH
ftsH	ZONMW-12_04106	ftsH
ftsH	ZONMW-12_00524	ftsH
ftsH	ZONMW-13_01677	ftsH
ftsH	ZONMW-14_00921	ftsH
ftsH	ZONMW-15_01585	ftsH
ftsH	ZONMW-15_01149	ftsH
ftsH	ZONMW-19_00678	ftsH
ftsH	ZONMW-19_04057	ftsH
ftsH	ZONMW-19_03225	ftsH
ftsH	ZONMW-20_02874	ftsH
ftsH	ZONMW-20_03602	ftsH
ftsH	ZONMW-21_03064	ftsH
ftsH	ZONMW-22_03089	ftsH
ftsH	ZONMW-23_03110	ftsH
ftsH	ZONMW-24_03111	ftsH
ftsH	ZONMW-25_01530	ftsH
ftsH	ZONMW-25_01842	ftsH
ftsH	ZONMW-25_01181	ftsH
ftsH	ZONMW-26_03149	ftsH
ftsH	ZONMW-27_03171	ftsH
ftsH	ZONMW-28_01539	ftsH
ftsH	ZONMW-28_00382	ftsH
ftsH	ZONMW-29_02346	ftsH
ftsH	ZONMW-29_02621	ftsH
ftsH	ZONMW-29_00034	ftsH
ftsH	ZONMW-30_01076	ftsH
ftsH	ZONMW-30_00992	ftsH
ftsH	ZONMW-31_00623	ftsH
ftsH	ZONMW-31_02684	ftsH

ftsH	ZONMW-32_01946	ftsH
ftsH	ZONMW-32_01173	ftsH
ftsH	ZONMW-33_01937	ftsH
ftsH	ZONMW-33_01162	ftsH
ftsH	ZONMW-34_00272	ftsH
ftsH	ZONMW-35_01118	ftsH
ftsH	ZONMW-36_01419	ftsH
ftsH	ZONMW-36_00382	ftsH
ftsH	ZONMW-37_03098	ftsH
ftsH	ZONMW-38_02980	ftsH
ftsH	ZONMW-39_04025	ftsH
ftsH	ZONMW-40_01117	ftsH
ftsH	ZONMW-41_00616	ftsH
ftsH	ZONMW-42_03609	ftsH
ftsH	ZONMW-42_02566	ftsH
ftsH	ZONMW-43_00680	ftsH
ftsH	ZONMW-44_03499	ftsH
ftsH	ZONMW-44_03908	ftsH
ftsH	ZONMW-45_00784	ftsH
ftsH	ZONMW-45_02858	ftsH
ftsH	ZONMW-45_01982	ftsH
ftsH	ZONMW-46_01090	ftsH
ftsH	ZONMW-46_02855	ftsH
ftsH	ZONMW-46_02331	ftsH
ftsH	ZONMW-47_01083	ftsH
ftsH	ZONMW-47_02846	ftsH
ftsH	ZONMW-47_02201	ftsH
ftsH	ZONMW-48_02544	ftsH
ftsH	ZONMW-49_02754	ftsH
ftsH	ZONMW-50_02680	ftsH
ftsH	ZONMW-51_03021	ftsH
ftsH	ZONMW-52_03053	ftsH
ftsH	ZONMW-53_03083	ftsH
ftsH	ZONMW-54_03085	ftsH
ftsH	ZONMW-55_01575	ftsH
ftsH	ZONMW-56_02970	ftsH
ftsH	ZONMW-57_03078	ftsH
ftsH	ZONMW-58_03099	ftsH
ftsH	ZONMW-59_03154	ftsH
ftsH	ZONMW-60_03090	ftsH
ftsH	ZONMW-61_03712	ftsH
ftsH	ZONMW-62_03757	ftsH
ftsH	ZONMW-63_02936	ftsH
ftsH	ZONMW-64_00942	ftsH
ftsH	ZONMW-65_02426	ftsH
ftsH	ZONMW-66_00463	ftsH
ftsH	ZONMW-66_04228	ftsH
ftsH	ZONMW-66_01671	ftsH

ftsH	ZONMW-67_02683	ftsH
ftsH	ZONMW-67_01842	ftsH
ftsH	ZONMW-68_03581	ftsH
ftsH	ZONMW-68_01863	ftsH
ftsH	ZONMW-69_00480	ftsH
ftsH	ZONMW-69_02101	ftsH
ftsH	ZONMW-72_02862	ftsH
ftsH	ZONMW-72_03634	ftsH
ftsH	ZONMW-72_04352	ftsH
ftsH	ZONMW-73_01177	ftsH
ftsH	ZONMW-73_01209	ftsH
ftsH	ZONMW-73_02744	ftsH
ftsH	ZONMW-73_03475	ftsH
ftsH	ZONMW-74_02490	ftsH
ftsH	ZONMW-74_03128	ftsH
ftsH	ZONMW-74_01307	ftsH
ftsH	ZONMW-75_03484	ftsH
ftsH	ZONMW-75_03456	ftsH
ftsH	da5A-8_02768	ftsH
ftsH	da5A-8_00689	ftsH
ftsH	da5A-8_02841	ftsH
pglF	18_00693	pglF
pglF	18_01133	pglF
pglF	18_00694	pglF
pglF	18_01120	pglF
pglF	18_02495	pglF
pglF	18_01509	pglF
pglF	18_02486	pglF
pglF	18_02498	pglF
pglF	ACAM48_00068	pglF
pglF	ACAM48_02333	pglF
pglF	ACAM48_00058	pglF
pglF	ACAM48_00064	pglF
pglF	ACAM48_01804	pglF
pglF	ACAM48_00103	pglF
pglF	ACAM48_00076	pglF
pglF	CAM030_01021	pglF
pglF	CAM030_01026	pglF
pglF	CAM030_01690	pglF
pglF	CAM030_01006	pglF
pglF	CAM030_01019	pglF
pglF	CAM030_02149	pglF
pglF	CAM030_01666	pglF
pglF	CAM030_02370	pglF
pglF	CAM030_01008	pglF
pglF	CECT7946_00149	pglF
pglF	CECT7946_00145	pglF
pglF	CECT7946_01370	pglF

pglF	CECT7946_00151	pglF
pglF	CECT7946_00820	pglF
pglF	CECT7946_03591	pglF
pglF	CECT7946_02640	pglF
pglF	CECT7946_01388	pglF
pglF	CECT7946_02165	pglF
pglF	CGMCC1.8863_03545	pglF
pglF	CGMCC1.8863_00804	pglF
pglF	CGMCC1.8863_00790	pglF
pglF	CGMCC1.8863_01513	pglF
pglF	CGMCC1.8863_03547	pglF
pglF	CGMCC1.8863_01512	pglF
pglF	CGMCC1.8863_01045	pglF
pglF	CGMCC1.8863_01505	pglF
pglF	CL8139_01841	pglF
pglF	CL8139_00248	pglF
pglF	CL8139_02971	pglF
pglF	CL8139_02136	pglF
pglF	CL8139_00950	pglF
pglF	CL8139_03189	pglF
pglF	CL8139_02964	pglF
pglF	CL8139_02973	pglF
pglF	DAU203_01463	pglF
pglF	DAU203_01481	pglF
pglF	DAU203_02817	pglF
pglF	DAU203_01727	pglF
pglF	DAU203_02480	pglF
pglF	DAU203_02819	pglF
pglF	DD5b_02344	pglF
pglF	DD5b_02376	pglF
pglF	DD5b_02379	pglF
pglF	DD5b_02134	pglF
pglF	DD5b_02348	pglF
pglF	DD5b_02644	pglF
pglF	DD5b_01517	pglF
pglF	DD5b_01114	pglF
pglF	DD5b_02337	pglF
pglF	DD5b_02369	pglF
pglF	DSM1208_03064	pglF
pglF	DSM1208_01676	pglF
pglF	DSM1208_03624	pglF
pglF	DSM1208_03632	pglF
pglF	DSM1208_01920	pglF
pglF	DSM1208_03063	pglF
pglF	DSM1208_03630	pglF
pglF	DSM1208_02236	pglF
pglF	DSM1208_03634	pglF
pglF	DSM12212_00071	pglF

pglF	DSM12212_00097	pglF
pglF	DSM12212_00335	pglF
pglF	DSM12212_03446	pglF
pglF	DSM12212_01643	pglF
pglF	DSM12212_00080	pglF
pglF	DSM12212_00751	pglF
pglF	DSM15343_01034	pglF
pglF	DSM15343_01036	pglF
pglF	DSM15343_01607	pglF
pglF	DSM15343_01610	pglF
pglF	DSM15343_01957	pglF
pglF	DSM15343_01627	pglF
pglF	DSM15361_02179	pglF
pglF	DSM15361_00549	pglF
pglF	DSM15361_00532	pglF
pglF	DSM15361_02166	pglF
pglF	DSM15361_00960	pglF
pglF	DSM15361_00103	pglF
pglF	DSM15361_00122	pglF
pglF	DSM15365_00358	pglF
pglF	DSM15365_03033	pglF
pglF	DSM15365_03051	pglF
pglF	DSM15365_00357	pglF
pglF	DSM15365_00343	pglF
pglF	DSM15365_01866	pglF
pglF	DSM15365_02251	pglF
pglF	DSM15365_01864	pglF
pglF	DSM15365_01859	pglF
pglF	DSM15365_03047	pglF
pglF	DSM15718_00694	pglF
pglF	DSM15718_00727	pglF
pglF	DSM15718_00687	pglF
pglF	DSM15718_00693	pglF
pglF	DSM15718_02595	pglF
pglF	DSM15718_00709	pglF
pglF	DSM15718_00514	pglF
pglF	DSM15718_00732	pglF
pglF	DSM16505T_02607	pglF
pglF	DSM16505T_02625	pglF
pglF	DSM16505T_02611	pglF
pglF	DSM16505T_01542	pglF
pglF	DSM18103_03400	pglF
pglF	DSM18103_03383	pglF
pglF	DSM18103_03401	pglF
pglF	DSM18103_03384	pglF
pglF	DSM18103_03410	pglF
pglF	DSM18293_02461	pglF
pglF	DSM18293_02456	pglF

pglF	DSM18293_02451	pglF
pglF	DSM18293_03062	pglF
pglF	DSM18293_02462	pglF
pglF	DSM18293_02466	pglF
pglF	DSM18293_02015	pglF
pglF	DSM18293_02484	pglF
pglF	DSM18293_04451	pglF
pglF	DSM18841_02739	pglF
pglF	DSM18841_02734	pglF
pglF	DSM18841_02763	pglF
pglF	DSM18842_02690	pglF
pglF	DSM18842_02667	pglF
pglF	DSM18842_02671	pglF
pglF	DSM18842_02687	pglF
pglF	DSM18842_02702	pglF
pglF	DSM19866_04522	pglF
pglF	DSM19866_01923	pglF
pglF	DSM19866_01900	pglF
pglF	DSM19866_04497	pglF
pglF	DSM19866_04496	pglF
pglF	DSM19866_04523	pglF
pglF	DSM19866_00669	pglF
pglF	DSM19866_04491	pglF
pglF	DSM19866_01907	pglF
pglF	DSM19866_02453	pglF
pglF	DSM2041_00196	pglF
pglF	DSM2041_00195	pglF
pglF	DSM2041_00224	pglF
pglF	DSM2041_01960	pglF
pglF	DSM2041_00232	pglF
pglF	DSM2041_00191	pglF
pglF	DSM2041_03930	pglF
pglF	DSM2041_03750	pglF
pglF	DSM2041_00780	pglF
pglF	DSM2041_01930	pglF
pglF	DSM2061_00079	pglF
pglF	DSM2061_03211	pglF
pglF	DSM2061_01430	pglF
pglF	DSM2061_03474	pglF
pglF	DSM2061_00078	pglF
pglF	DSM2061_01427	pglF
pglF	DSM2061_01429	pglF
pglF	DSM2061_03766	pglF
pglF	DSM2061_01425	pglF
pglF	DSM2064_04750	pglF
pglF	DSM2064_04726	pglF
pglF	DSM2064_04721	pglF
pglF	DSM2064_04751	pglF

pglF	DSM2064_02034	pglF
pglF	DSM2064_02747	pglF
pglF	DSM2064_04752	pglF
pglF	DSM21164_02146	pglF
pglF	DSM21164_00735	pglF
pglF	DSM21164_01720	pglF
pglF	DSM21164_02145	pglF
pglF	DSM21164_01718	pglF
pglF	DSM21164_00371	pglF
pglF	DSM21164_01725	pglF
pglF	DSM21164_01715	pglF
pglF	DSM23404_01020	pglF
pglF	DSM23404_01423	pglF
pglF	DSM23404_03048	pglF
pglF	DSM23404_01431	pglF
pglF	DSM23404_01019	pglF
pglF	DSM23404_01426	pglF
pglF	DSM23404_02439	pglF
pglF	DSM23404_01391	pglF
pglF	DSM23424_00318	pglF
pglF	DSM23424_02683	pglF
pglF	DSM23424_00339	pglF
pglF	DSM23424_02648	pglF
pglF	DSM23424_00575	pglF
pglF	DSM23424_02328	pglF
pglF	DSM23424_00573	pglF
pglF	DSM23424_02262	pglF
pglF	DSM23424_00334	pglF
pglF	DSM24786_02972	pglF
pglF	DSM24786_00275	pglF
pglF	DSM24786_02949	pglF
pglF	DSM24786_02254	pglF
pglF	DSM24786_01528	pglF
pglF	DSM24786_02954	pglF
pglF	DSM24786_00277	pglF
pglF	DSM7489_02667	pglF
pglF	DSM7489_01387	pglF
pglF	DSM7489_02668	pglF
pglF	DSM7489_02688	pglF
pglF	DSM7489_01373	pglF
pglF	DSM7489_01646	pglF
pglF	DSM7489_02396	pglF
pglF	DSM7489_02681	pglF
pglF	DSM7489_02690	pglF
pglF	DSW1_01407	pglF
pglF	DSW1_00641	pglF
pglF	DSW1_02195	pglF
pglF	DSW1_02197	pglF

pglF	DSW1_00650	pglF
pglF	DSW1_00642	pglF
pglF	DSW1_02190	pglF
pglF	DSW1_01179	pglF
pglF	DSW1_02184	pglF
pglF	DSW1_02176	pglF
pglF	DSW5_00241	pglF
pglF	DSW5_00221	pglF
pglF	DSW5_00242	pglF
pglF	DSW5_01641	pglF
pglF	DSW5_00852	pglF
pglF	DSW5_00217	pglF
pglF	DSW5_00240	pglF
pglF	DSW5_00225	pglF
pglF	DSW6_01400	pglF
pglF	DSW6_03451	pglF
pglF	DSW6_03449	pglF
pglF	DSW6_01325	pglF
pglF	DSW6_01401	pglF
pglF	DSW6_01890	pglF
pglF	DSW6_03420	pglF
pglF	DSW6_03427	pglF
pglF	DSW6_03432	pglF
pglF	DSW6_03446	pglF
pglF	DSW8_01834	pglF
pglF	DSW8_02258	pglF
pglF	DSW8_01835	pglF
pglF	DSW8_02247	pglF
pglF	DSW8_00877	pglF
pglF	DSW8_00876	pglF
pglF	DSW8_01712	pglF
pglF	DSW8_00873	pglF
pglF	F52_01565	pglF
pglF	F52_01552	pglF
pglF	F52_01547	pglF
pglF	F52_01542	pglF
pglF	F52_01551	pglF
pglF	F52_02433	pglF
pglF	F52_03335	pglF
pglF	F52_01572	pglF
pglF	GCA_001027945.1_00801	pglF
pglF	HI1_01326	pglF
pglF	HI1_02627	pglF
pglF	HI1_01314	pglF
pglF	HI1_01567	pglF
pglF	HI1_02328	pglF
pglF	HI1_02629	pglF
pglF	IR1_03031	pglF

pglF	IR1_04706	pglF
pglF	IR1_03036	pglF
pglF	IR1_00148	pglF
pglF	IR1_03012	pglF
pglF	KCOM3131_01326	pglF
pglF	KCOM3131_01325	pglF
pglF	KCOM3131_01546	pglF
pglF	KCOM3131_00461	pglF
pglF	KCOM3131_00921	pglF
pglF	KCTC12974_00136	pglF
pglF	KCTC12974_01644	pglF
pglF	KCTC12974_00127	pglF
pglF	KCTC12974_02775	pglF
pglF	KCTC12974_00089	pglF
pglF	KCTC12974_00121	pglF
pglF	KL-A_00839	pglF
pglF	KL-A_01370	pglF
pglF	KL-A_00856	pglF
pglF	KL-A_01448	pglF
pglF	KL-A_01675	pglF
pglF	KL-A_01368	pglF
pglF	L12_00382	pglF
pglF	L12_00466	pglF
pglF	LL0412.1.7_01892	pglF
pglF	LL0412.1.7_01906	pglF
pglF	LL0412.1.7_01895	pglF
pglF	LL0412.1.7_00107	pglF
pglF	MAR_2009_71_01587	pglF
pglF	MAR_2009_71_01181	pglF
pglF	MAR_2009_71_01586	pglF
pglF	MAR_2009_71_01192	pglF
pglF	MAR_2009_71_02567	pglF
pglF	MAR_2009_71_02568	pglF
pglF	MAR_2009_71_01705	pglF
pglF	MAR_2009_71_02571	pglF
pglF	NN016038_00713	pglF
pglF	NN016038_01147	pglF
pglF	NN016038_00714	pglF
pglF	NN016038_01134	pglF
pglF	NN016038_02528	pglF
pglF	NN016038_01516	pglF
pglF	NN016038_02531	pglF
pglF	PAO1_01424	pglF
pglF	PAO1_05637	pglF
pglF	PAO1_05638	pglF
pglF	PAO1_05335	pglF
pglF	PAO1_04197	pglF
pglF	PAO1_03455	pglF

pglF	PAO1_03254	pglF
pglF	UW101_00352	pglF
pglF	UW101_00376	pglF
pglF	UW101_00381	pglF
pglF	UW101_00351	pglF
pglF	UW101_01089	pglF
pglF	UW101_01801	pglF
pglF	UW101_00350	pglF
pglF	V4-33_01547	pglF
pglF	V4-33_01548	pglF
pglF	V4-33_01207	pglF
pglF	V4-33_01203	pglF
pglF	V4-33_02400	pglF
pglF	V4-33_00384	pglF
pglF	V4-33_01185	pglF
pglF	V4-33_01197	pglF
pglF	W5C_01857	pglF
pglF	W5C_01087	pglF
pglF	W5C_01856	pglF
pglF	W5C_00741	pglF
pglF	W5C_01862	pglF
pglF	W5C_00340	pglF
pglF	W5C_02713	pglF
pglF	W5C_00734	pglF
pglF	W5C_00743	pglF
pglF	ZONMW-01_02366	pglF
pglF	ZONMW-01_02382	pglF
pglF	ZONMW-01_01732	pglF
pglF	ZONMW-01_00093	pglF
pglF	ZONMW-01_02832	pglF
pglF	ZONMW-01_01734	pglF
pglF	ZONMW-02_02948	pglF
pglF	ZONMW-02_03569	pglF
pglF	ZONMW-02_01836	pglF
pglF	ZONMW-02_02947	pglF
pglF	ZONMW-02_03332	pglF
pglF	ZONMW-02_03677	pglF
pglF	ZONMW-02_02420	pglF
pglF	ZONMW-02_03334	pglF
pglF	ZONMW-02_03339	pglF
pglF	ZONMW-03_02969	pglF
pglF	ZONMW-03_02985	pglF
pglF	ZONMW-03_00475	pglF
pglF	ZONMW-03_00819	pglF
pglF	ZONMW-03_01595	pglF
pglF	ZONMW-03_00473	pglF
pglF	ZONMW-04_02457	pglF
pglF	ZONMW-04_02456	pglF

pglF	ZONMW-04_02454	pglF
pglF	ZONMW-04_01414	pglF
pglF	ZONMW-04_02478	pglF
pglF	ZONMW-04_02127	pglF
pglF	ZONMW-04_00087	pglF
pglF	ZONMW-04_02473	pglF
pglF	ZONMW-04_01412	pglF
pglF	ZONMW-05_02555	pglF
pglF	ZONMW-05_02569	pglF
pglF	ZONMW-05_00743	pglF
pglF	ZONMW-05_00742	pglF
pglF	ZONMW-05_01008	pglF
pglF	ZONMW-05_01743	pglF
pglF	ZONMW-05_00748	pglF
pglF	ZONMW-05_00736	pglF
pglF	ZONMW-06_01026	pglF
pglF	ZONMW-06_03098	pglF
pglF	ZONMW-06_03124	pglF
pglF	ZONMW-06_01048	pglF
pglF	ZONMW-06_01047	pglF
pglF	ZONMW-07_01245	pglF
pglF	ZONMW-07_01505	pglF
pglF	ZONMW-07_01489	pglF
pglF	ZONMW-07_02480	pglF
pglF	ZONMW-07_01244	pglF
pglF	ZONMW-07_02476	pglF
pglF	ZONMW-07_00311	pglF
pglF	ZONMW-07_02468	pglF
pglF	ZONMW-08_01645	pglF
pglF	ZONMW-08_01625	pglF
pglF	ZONMW-08_00703	pglF
pglF	ZONMW-08_03570	pglF
pglF	ZONMW-08_03568	pglF
pglF	ZONMW-08_01646	pglF
pglF	ZONMW-08_03565	pglF
pglF	ZONMW-08_01529	pglF
pglF	ZONMW-08_03572	pglF
pglF	ZONMW-09_00356	pglF
pglF	ZONMW-09_02541	pglF
pglF	ZONMW-09_02544	pglF
pglF	ZONMW-09_02540	pglF
pglF	ZONMW-09_00715	pglF
pglF	ZONMW-09_02298	pglF
pglF	ZONMW-10_02437	pglF
pglF	ZONMW-10_00385	pglF
pglF	ZONMW-10_01422	pglF
pglF	ZONMW-10_01294	pglF
pglF	ZONMW-10_01584	pglF

pglF	ZONMW-10_02440	pglF
pglF	ZONMW-11_03460	pglF
pglF	ZONMW-11_00699	pglF
pglF	ZONMW-11_03756	pglF
pglF	ZONMW-11_03288	pglF
pglF	ZONMW-11_03459	pglF
pglF	ZONMW-11_01044	pglF
pglF	ZONMW-11_01045	pglF
pglF	ZONMW-11_01513	pglF
pglF	ZONMW-11_04314	pglF
pglF	ZONMW-11_04313	pglF
pglF	ZONMW-11_00340	pglF
pglF	ZONMW-11_02109	pglF
pglF	ZONMW-12_01596	pglF
pglF	ZONMW-12_00668	pglF
pglF	ZONMW-12_00727	pglF
pglF	ZONMW-12_00257	pglF
pglF	ZONMW-12_00667	pglF
pglF	ZONMW-12_01126	pglF
pglF	ZONMW-12_03535	pglF
pglF	ZONMW-12_00958	pglF
pglF	ZONMW-12_03139	pglF
pglF	ZONMW-12_01600	pglF
pglF	ZONMW-12_04046	pglF
pglF	ZONMW-13_02947	pglF
pglF	ZONMW-13_00355	pglF
pglF	ZONMW-13_02948	pglF
pglF	ZONMW-13_02744	pglF
pglF	ZONMW-13_01223	pglF
pglF	ZONMW-13_01220	pglF
pglF	ZONMW-13_01974	pglF
pglF	ZONMW-13_00599	pglF
pglF	ZONMW-13_02782	pglF
pglF	ZONMW-13_00983	pglF
pglF	ZONMW-13_01212	pglF
pglF	ZONMW-14_02772	pglF
pglF	ZONMW-14_00235	pglF
pglF	ZONMW-14_01131	pglF
pglF	ZONMW-14_01128	pglF
pglF	ZONMW-14_02774	pglF
pglF	ZONMW-14_01930	pglF
pglF	ZONMW-14_00720	pglF
pglF	ZONMW-14_02071	pglF
pglF	ZONMW-15_01405	pglF
pglF	ZONMW-15_02529	pglF
pglF	ZONMW-15_01406	pglF
pglF	ZONMW-15_02549	pglF
pglF	ZONMW-15_00366	pglF

pglF	ZONMW-15_01400	pglF
pglF	ZONMW-19_00348	pglF
pglF	ZONMW-19_02924	pglF
pglF	ZONMW-19_02908	pglF
pglF	ZONMW-19_04202	pglF
pglF	ZONMW-20_04061	pglF
pglF	ZONMW-20_04049	pglF
pglF	ZONMW-20_04060	pglF
pglF	ZONMW-20_03913	pglF
pglF	ZONMW-20_02560	pglF
pglF	ZONMW-20_00822	pglF
pglF	ZONMW-21_00799	pglF
pglF	ZONMW-21_00779	pglF
pglF	ZONMW-21_02197	pglF
pglF	ZONMW-21_00257	pglF
pglF	ZONMW-21_02202	pglF
pglF	ZONMW-22_00800	pglF
pglF	ZONMW-22_00780	pglF
pglF	ZONMW-22_01528	pglF
pglF	ZONMW-22_00257	pglF
pglF	ZONMW-22_01523	pglF
pglF	ZONMW-23_01914	pglF
pglF	ZONMW-23_01894	pglF
pglF	ZONMW-23_01023	pglF
pglF	ZONMW-23_01372	pglF
pglF	ZONMW-23_01028	pglF
pglF	ZONMW-24_00661	pglF
pglF	ZONMW-24_00681	pglF
pglF	ZONMW-24_01552	pglF
pglF	ZONMW-24_01202	pglF
pglF	ZONMW-24_01547	pglF
pglF	ZONMW-25_00317	pglF
pglF	ZONMW-25_01706	pglF
pglF	ZONMW-25_01725	pglF
pglF	ZONMW-25_00318	pglF
pglF	ZONMW-25_00334	pglF
pglF	ZONMW-25_02936	pglF
pglF	ZONMW-25_00328	pglF
pglF	ZONMW-25_00336	pglF
pglF	ZONMW-26_03006	pglF
pglF	ZONMW-26_00231	pglF
pglF	ZONMW-26_03303	pglF
pglF	ZONMW-26_01052	pglF
pglF	ZONMW-26_01550	pglF
pglF	ZONMW-26_00233	pglF
pglF	ZONMW-27_02712	pglF
pglF	ZONMW-27_02726	pglF
pglF	ZONMW-27_01885	pglF

pglF	ZONMW-27_01886	pglF
pglF	ZONMW-27_00820	pglF
pglF	ZONMW-27_00084	pglF
pglF	ZONMW-27_01880	pglF
pglF	ZONMW-27_01892	pglF
pglF	ZONMW-28_03513	pglF
pglF	ZONMW-28_02906	pglF
pglF	ZONMW-28_02933	pglF
pglF	ZONMW-28_03543	pglF
pglF	ZONMW-28_03534	pglF
pglF	ZONMW-28_03522	pglF
pglF	ZONMW-29_01400	pglF
pglF	ZONMW-29_01420	pglF
pglF	ZONMW-29_00421	pglF
pglF	ZONMW-29_00439	pglF
pglF	ZONMW-29_01419	pglF
pglF	ZONMW-29_02238	pglF
pglF	ZONMW-29_01402	pglF
pglF	ZONMW-29_02950	pglF
pglF	ZONMW-29_01408	pglF
pglF	ZONMW-30_02271	pglF
pglF	ZONMW-30_03344	pglF
pglF	ZONMW-30_02288	pglF
pglF	ZONMW-30_02273	pglF
pglF	ZONMW-30_02272	pglF
pglF	ZONMW-30_03343	pglF
pglF	ZONMW-30_03277	pglF
pglF	ZONMW-30_01461	pglF
pglF	ZONMW-30_03271	pglF
pglF	ZONMW-30_03279	pglF
pglF	ZONMW-31_01301	pglF
pglF	ZONMW-31_01284	pglF
pglF	ZONMW-31_01302	pglF
pglF	ZONMW-31_01551	pglF
pglF	ZONMW-31_01577	pglF
pglF	ZONMW-31_01318	pglF
pglF	ZONMW-32_00598	pglF
pglF	ZONMW-32_01391	pglF
pglF	ZONMW-32_01417	pglF
pglF	ZONMW-32_00628	pglF
pglF	ZONMW-32_00619	pglF
pglF	ZONMW-32_00607	pglF
pglF	ZONMW-33_00466	pglF
pglF	ZONMW-33_01380	pglF
pglF	ZONMW-33_01407	pglF
pglF	ZONMW-33_00496	pglF
pglF	ZONMW-33_00487	pglF
pglF	ZONMW-33_00475	pglF

pglF	ZONMW-34_00853	pglF
pglF	ZONMW-34_01566	pglF
pglF	ZONMW-34_01595	pglF
pglF	ZONMW-34_00843	pglF
pglF	ZONMW-34_00857	pglF
pglF	ZONMW-35_02390	pglF
pglF	ZONMW-35_00639	pglF
pglF	ZONMW-35_00666	pglF
pglF	ZONMW-35_02620	pglF
pglF	ZONMW-35_02386	pglF
pglF	ZONMW-36_00905	pglF
pglF	ZONMW-36_00600	pglF
pglF	ZONMW-36_00626	pglF
pglF	ZONMW-36_00875	pglF
pglF	ZONMW-36_00884	pglF
pglF	ZONMW-36_00896	pglF
pglF	ZONMW-37_00799	pglF
pglF	ZONMW-37_00779	pglF
pglF	ZONMW-37_01551	pglF
pglF	ZONMW-37_00257	pglF
pglF	ZONMW-37_01546	pglF
pglF	ZONMW-38_00913	pglF
pglF	ZONMW-38_02629	pglF
pglF	ZONMW-38_01509	pglF
pglF	ZONMW-38_00410	pglF
pglF	ZONMW-38_00903	pglF
pglF	ZONMW-39_00131	pglF
pglF	ZONMW-39_00104	pglF
pglF	ZONMW-39_01305	pglF
pglF	ZONMW-39_01318	pglF
pglF	ZONMW-39_00105	pglF
pglF	ZONMW-40_00632	pglF
pglF	ZONMW-40_00427	pglF
pglF	ZONMW-40_00407	pglF
pglF	ZONMW-40_00631	pglF
pglF	ZONMW-40_00615	pglF
pglF	ZONMW-40_02667	pglF
pglF	ZONMW-40_00621	pglF
pglF	ZONMW-40_00612	pglF
pglF	ZONMW-40_01596	pglF
pglF	ZONMW-41_01682	pglF
pglF	ZONMW-41_02586	pglF
pglF	ZONMW-41_01681	pglF
pglF	ZONMW-41_02583	pglF
pglF	ZONMW-41_02582	pglF
pglF	ZONMW-41_02215	pglF
pglF	ZONMW-42_01046	pglF
pglF	ZONMW-42_01045	pglF

pglF	ZONMW-42_03975	pglF
pglF	ZONMW-42_03330	pglF
pglF	ZONMW-42_03305	pglF
pglF	ZONMW-42_03050	pglF
pglF	ZONMW-42_03946	pglF
pglF	ZONMW-42_03947	pglF
pglF	ZONMW-43_01701	pglF
pglF	ZONMW-43_01674	pglF
pglF	ZONMW-43_00389	pglF
pglF	ZONMW-44_03950	pglF
pglF	ZONMW-44_02769	pglF
pglF	ZONMW-44_02797	pglF
pglF	ZONMW-44_02237	pglF
pglF	ZONMW-44_02227	pglF
pglF	ZONMW-44_03967	pglF
pglF	ZONMW-45_02701	pglF
pglF	ZONMW-45_02721	pglF
pglF	ZONMW-45_02730	pglF
pglF	ZONMW-45_01921	pglF
pglF	ZONMW-46_03088	pglF
pglF	ZONMW-46_03068	pglF
pglF	ZONMW-46_03059	pglF
pglF	ZONMW-46_02270	pglF
pglF	ZONMW-47_03079	pglF
pglF	ZONMW-47_03059	pglF
pglF	ZONMW-47_03050	pglF
pglF	ZONMW-47_02262	pglF
pglF	ZONMW-48_00652	pglF
pglF	ZONMW-48_00632	pglF
pglF	ZONMW-48_01763	pglF
pglF	ZONMW-48_00644	pglF
pglF	ZONMW-48_01003	pglF
pglF	ZONMW-48_01760	pglF
pglF	ZONMW-49_01210	pglF
pglF	ZONMW-49_00242	pglF
pglF	ZONMW-49_01229	pglF
pglF	ZONMW-49_00956	pglF
pglF	ZONMW-49_00514	pglF
pglF	ZONMW-49_00244	pglF
pglF	ZONMW-50_01211	pglF
pglF	ZONMW-50_00243	pglF
pglF	ZONMW-50_01230	pglF
pglF	ZONMW-50_00957	pglF
pglF	ZONMW-50_00515	pglF
pglF	ZONMW-50_00241	pglF
pglF	ZONMW-51_02816	pglF
pglF	ZONMW-51_00230	pglF
pglF	ZONMW-51_02832	pglF

pglF	ZONMW-51_01308	pglF
pglF	ZONMW-51_02829	pglF
pglF	ZONMW-51_01191	pglF
pglF	ZONMW-51_00232	pglF
pglF	ZONMW-52_02229	pglF
pglF	ZONMW-52_00214	pglF
pglF	ZONMW-52_02213	pglF
pglF	ZONMW-52_01035	pglF
pglF	ZONMW-52_02216	pglF
pglF	ZONMW-52_02885	pglF
pglF	ZONMW-52_00216	pglF
pglF	ZONMW-53_02818	pglF
pglF	ZONMW-53_00951	pglF
pglF	ZONMW-53_02800	pglF
pglF	ZONMW-53_00799	pglF
pglF	ZONMW-53_00031	pglF
pglF	ZONMW-53_00944	pglF
pglF	ZONMW-53_00953	pglF
pglF	ZONMW-53_00934	pglF
pglF	ZONMW-54_00034	pglF
pglF	ZONMW-54_01776	pglF
pglF	ZONMW-54_00022	pglF
pglF	ZONMW-54_00270	pglF
pglF	ZONMW-54_00997	pglF
pglF	ZONMW-54_01774	pglF
pglF	ZONMW-55_02825	pglF
pglF	ZONMW-55_01296	pglF
pglF	ZONMW-55_02809	pglF
pglF	ZONMW-55_00768	pglF
pglF	ZONMW-55_02812	pglF
pglF	ZONMW-55_00029	pglF
pglF	ZONMW-55_01294	pglF
pglF	ZONMW-56_02585	pglF
pglF	ZONMW-56_00525	pglF
pglF	ZONMW-56_02566	pglF
pglF	ZONMW-56_02311	pglF
pglF	ZONMW-56_02779	pglF
pglF	ZONMW-56_00523	pglF
pglF	ZONMW-57_02730	pglF
pglF	ZONMW-57_00738	pglF
pglF	ZONMW-57_02714	pglF
pglF	ZONMW-57_02439	pglF
pglF	ZONMW-57_02717	pglF
pglF	ZONMW-57_01584	pglF
pglF	ZONMW-57_00745	pglF
pglF	ZONMW-57_00736	pglF
pglF	ZONMW-57_00755	pglF
pglF	ZONMW-58_02754	pglF

pglF	ZONMW-58_00233	pglF
pglF	ZONMW-58_02738	pglF
pglF	ZONMW-58_02464	pglF
pglF	ZONMW-58_02741	pglF
pglF	ZONMW-58_01000	pglF
pglF	ZONMW-58_00226	pglF
pglF	ZONMW-58_00235	pglF
pglF	ZONMW-58_00216	pglF
pglF	ZONMW-59_02976	pglF
pglF	ZONMW-59_00727	pglF
pglF	ZONMW-59_02995	pglF
pglF	ZONMW-59_02027	pglF
pglF	ZONMW-59_02748	pglF
pglF	ZONMW-59_00725	pglF
pglF	ZONMW-60_02847	pglF
pglF	ZONMW-60_00740	pglF
pglF	ZONMW-60_02835	pglF
pglF	ZONMW-60_01710	pglF
pglF	ZONMW-60_00974	pglF
pglF	ZONMW-60_00738	pglF
pglF	ZONMW-61_00694	pglF
pglF	ZONMW-61_03529	pglF
pglF	ZONMW-61_00726	pglF
pglF	ZONMW-61_00286	pglF
pglF	ZONMW-61_00693	pglF
pglF	ZONMW-61_00303	pglF
pglF	ZONMW-61_03816	pglF
pglF	ZONMW-61_02866	pglF
pglF	ZONMW-61_03814	pglF
pglF	ZONMW-62_02477	pglF
pglF	ZONMW-62_00980	pglF
pglF	ZONMW-62_01839	pglF
pglF	ZONMW-62_01854	pglF
pglF	ZONMW-62_00973	pglF
pglF	ZONMW-62_02478	pglF
pglF	ZONMW-62_02362	pglF
pglF	ZONMW-62_00971	pglF
pglF	ZONMW-63_00430	pglF
pglF	ZONMW-63_02344	pglF
pglF	ZONMW-63_00429	pglF
pglF	ZONMW-63_00411	pglF
pglF	ZONMW-63_02360	pglF
pglF	ZONMW-63_00606	pglF
pglF	ZONMW-63_02357	pglF
pglF	ZONMW-63_00973	pglF
pglF	ZONMW-63_00418	pglF
pglF	ZONMW-63_00409	pglF
pglF	ZONMW-64_02993	pglF

pglF	ZONMW-64_03020	pglF
pglF	ZONMW-64_00407	pglF
pglF	ZONMW-65_02993	pglF
pglF	ZONMW-65_01644	pglF
pglF	ZONMW-65_00854	pglF
pglF	ZONMW-65_00966	pglF
pglF	ZONMW-66_01017	pglF
pglF	ZONMW-66_00863	pglF
pglF	ZONMW-66_02464	pglF
pglF	ZONMW-66_02461	pglF
pglF	ZONMW-66_00687	pglF
pglF	ZONMW-66_00693	pglF
pglF	ZONMW-66_03933	pglF
pglF	ZONMW-66_04085	pglF
pglF	ZONMW-67_02167	pglF
pglF	ZONMW-67_01506	pglF
pglF	ZONMW-67_01061	pglF
pglF	ZONMW-67_01532	pglF
pglF	ZONMW-67_01082	pglF
pglF	ZONMW-68_01231	pglF
pglF	ZONMW-68_00056	pglF
pglF	ZONMW-68_00034	pglF
pglF	ZONMW-68_01256	pglF
pglF	ZONMW-68_01230	pglF
pglF	ZONMW-68_01255	pglF
pglF	ZONMW-68_03928	pglF
pglF	ZONMW-68_01261	pglF
pglF	ZONMW-68_00040	pglF
pglF	ZONMW-69_01776	pglF
pglF	ZONMW-69_01494	pglF
pglF	ZONMW-69_01108	pglF
pglF	ZONMW-69_01469	pglF
pglF	ZONMW-69_01129	pglF
pglF	ZONMW-72_01189	pglF
pglF	ZONMW-72_03305	pglF
pglF	ZONMW-72_01184	pglF
pglF	ZONMW-72_00535	pglF
pglF	ZONMW-72_01208	pglF
pglF	ZONMW-73_00017	pglF
pglF	ZONMW-73_00035	pglF
pglF	ZONMW-73_02500	pglF
pglF	ZONMW-73_00331	pglF
pglF	ZONMW-73_02504	pglF
pglF	ZONMW-74_01534	pglF
pglF	ZONMW-74_01515	pglF
pglF	ZONMW-74_03088	pglF
pglF	ZONMW-74_03089	pglF
pglF	ZONMW-74_01251	pglF

pglF	ZONMW-74_03092	pglF
pglF	ZONMW-75_03617	pglF
pglF	ZONMW-75_02330	pglF
pglF	ZONMW-75_02314	pglF
pglF	ZONMW-75_01588	pglF
pglF	ZONMW-75_02043	pglF
pglF	ZONMW-75_03616	pglF
pglF	ZONMW-75_00196	pglF
pglF	ZONMW-75_02045	pglF
pglF	ZONMW-75_02050	pglF
pglF	da5A-8_00276	pglF
pglF	da5A-8_00253	pglF
pglF	da5A-8_00277	pglF
pglF	da5A-8_00261	pglF
pglF	da5A-8_00286	pglF
atpA	18_00719	atpA
atpA	18_01090	atpA
atpA	ACAM48_02614	atpA
atpA	ACAM48_02465	atpA
atpA	CAM030_02964	atpA
atpA	CAM030_00974	atpA
atpA	CECT7946_00343	atpA
atpA	CECT7946_00185	atpA
atpA	CGMCC1.8863_02228	atpA
atpA	CGMCC1.8863_00848	atpA
atpA	CL8139_01420	atpA
atpA	CL8139_01870	atpA
atpA	DAU203_01143	atpA
atpA	DAU203_01512	atpA
atpA	DD5b_02307	atpA
atpA	DD5b_02105	atpA
atpA	DSM1208_01359	atpA
atpA	DSM1208_03719	atpA
atpA	DSM1208_03726	atpA
atpA	DSM1208_01723	atpA
atpA	DSM12212_00279	atpA
atpA	DSM12212_00122	atpA
atpA	DSM15343_00917	atpA
atpA	DSM15343_01008	atpA
atpA	DSM15361_00293	atpA
atpA	DSM15361_00578	atpA
atpA	DSM15365_00980	atpA
atpA	DSM15365_03005	atpA
atpA	DSM15718_02441	atpA
atpA	DSM15718_02621	atpA
atpA	DSM16505T_00716	atpA
atpA	DSM16505T_00412	atpA
atpA	DSM18103_01180	atpA

atpA	DSM18103_03008	atpA
atpA	DSM18293_03257	atpA
atpA	DSM18293_03039	atpA
atpA	DSM18841_00596	atpA
atpA	DSM18841_00240	atpA
atpA	DSM18842_02953	atpA
atpA	DSM18842_00982	atpA
atpA	DSM19866_03576	atpA
atpA	DSM19866_04381	atpA
atpA	DSM19866_01957	atpA
atpA	DSM2041_00549	atpA
atpA	DSM2041_00166	atpA
atpA	DSM2061_02881	atpA
atpA	DSM2061_03253	atpA
atpA	DSM2064_01806	atpA
atpA	DSM2064_02056	atpA
atpA	DSM21164_01129	atpA
atpA	DSM21164_00778	atpA
atpA	DSM23404_02341	atpA
atpA	DSM23404_02055	atpA
atpA	DSM23424_00115	atpA
atpA	DSM23424_00292	atpA
atpA	DSM24786_03376	atpA
atpA	DSM24786_03001	atpA
atpA	DSM7489_01060	atpA
atpA	DSM7489_01416	atpA
atpA	DSW1_00401	atpA
atpA	DSW1_00677	atpA
atpA	DSW5_00810	atpA
atpA	DSW5_01305	atpA
atpA	DSW6_01172	atpA
atpA	DSW6_01305	atpA
atpA	DSW8_02577	atpA
atpA	DSW8_02217	atpA
atpA	F52_02242	atpA
atpA	F52_02456	atpA
atpA	GCA_001027945.1_01259	atpA
atpA	GCA_001027945.1_00370	atpA
atpA	HI1_00999	atpA
atpA	HI1_01357	atpA
atpA	IR1_04025	atpA
atpA	IR1_04684	atpA
atpA	KCOM3131_00630	atpA
atpA	KCOM3131_00629	atpA
atpA	KCTC12974_01984	atpA
atpA	KCTC12974_02894	atpA
atpA	KL-A_02303	atpA
atpA	KL-A_00810	atpA

atpA	L12_00326	atpA
atpA	LL0412.1.7_00731	atpA
atpA	LL0412.1.7_00565	atpA
atpA	MAR_2009_71_00875	atpA
atpA	MAR_2009_71_01223	atpA
atpA	NN016038_00738	atpA
atpA	NN016038_01107	atpA
atpA	PAO1_01743	atpA
atpA	PAO1_01140	atpA
atpA	PAO1_05737	atpA
atpA	PAO1_05739	atpA
atpA	UW101_00860	atpA
atpA	UW101_01111	atpA
atpA	V4-33_00099	atpA
atpA	V4-33_02380	atpA
atpA	W5C_03103	atpA
atpA	W5C_01115	atpA
atpA	ZONMW-01_02878	atpA
atpA	ZONMW-01_02413	atpA
atpA	ZONMW-02_01699	atpA
atpA	ZONMW-02_01812	atpA
atpA	ZONMW-03_01978	atpA
atpA	ZONMW-03_03016	atpA
atpA	ZONMW-04_02884	atpA
atpA	ZONMW-04_02425	atpA
atpA	ZONMW-05_03039	atpA
atpA	ZONMW-05_02598	atpA
atpA	ZONMW-06_03400	atpA
atpA	ZONMW-06_01240	atpA
atpA	ZONMW-06_01554	atpA
atpA	ZONMW-06_01242	atpA
atpA	ZONMW-06_01561	atpA
atpA	ZONMW-07_03125	atpA
atpA	ZONMW-07_01463	atpA
atpA	ZONMW-08_00395	atpA
atpA	ZONMW-08_00743	atpA
atpA	ZONMW-09_00528	atpA
atpA	ZONMW-09_00337	atpA
atpA	ZONMW-10_00528	atpA
atpA	ZONMW-10_00407	atpA
atpA	ZONMW-11_02357	atpA
atpA	ZONMW-11_00509	atpA
atpA	ZONMW-12_00444	atpA
atpA	ZONMW-12_00235	atpA
atpA	ZONMW-13_00713	atpA
atpA	ZONMW-13_00336	atpA
atpA	ZONMW-14_01333	atpA
atpA	ZONMW-14_00254	atpA

atpA	ZONMW-15_00842	atpA
atpA	ZONMW-15_02576	atpA
atpA	ZONMW-19_01511	atpA
atpA	ZONMW-19_01056	atpA
atpA	ZONMW-19_01058	atpA
atpA	ZONMW-20_02664	atpA
atpA	ZONMW-20_00655	atpA
atpA	ZONMW-20_01943	atpA
atpA	ZONMW-20_00653	atpA
atpA	ZONMW-21_01134	atpA
atpA	ZONMW-21_00756	atpA
atpA	ZONMW-22_01136	atpA
atpA	ZONMW-22_00757	atpA
atpA	ZONMW-23_02248	atpA
atpA	ZONMW-23_01871	atpA
atpA	ZONMW-24_00325	atpA
atpA	ZONMW-24_00704	atpA
atpA	ZONMW-25_03485	atpA
atpA	ZONMW-25_00600	atpA
atpA	ZONMW-25_01684	atpA
atpA	ZONMW-25_00599	atpA
atpA	ZONMW-26_01877	atpA
atpA	ZONMW-26_03035	atpA
atpA	ZONMW-27_03060	atpA
atpA	ZONMW-27_02755	atpA
atpA	ZONMW-28_03170	atpA
atpA	ZONMW-28_01769	atpA
atpA	ZONMW-28_01767	atpA
atpA	ZONMW-29_00729	atpA
atpA	ZONMW-29_00396	atpA
atpA	ZONMW-30_00420	atpA
atpA	ZONMW-30_02310	atpA
atpA	ZONMW-31_00068	atpA
atpA	ZONMW-31_02632	atpA
atpA	ZONMW-31_03536	atpA
atpA	ZONMW-31_02625	atpA
atpA	ZONMW-31_00066	atpA
atpA	ZONMW-31_03542	atpA
atpA	ZONMW-32_00695	atpA
atpA	ZONMW-32_02203	atpA
atpA	ZONMW-32_02205	atpA
atpA	ZONMW-33_00563	atpA
atpA	ZONMW-33_02194	atpA
atpA	ZONMW-33_02196	atpA
atpA	ZONMW-34_00927	atpA
atpA	ZONMW-34_02347	atpA
atpA	ZONMW-34_02349	atpA
atpA	ZONMW-35_02316	atpA

atpA	ZONMW-35_01902	atpA
atpA	ZONMW-35_01900	atpA
atpA	ZONMW-36_00808	atpA
atpA	ZONMW-36_02042	atpA
atpA	ZONMW-36_02040	atpA
atpA	ZONMW-37_01228	atpA
atpA	ZONMW-37_00756	atpA
atpA	ZONMW-38_00826	atpA
atpA	ZONMW-38_03836	atpA
atpA	ZONMW-38_03838	atpA
atpA	ZONMW-39_01639	atpA
atpA	ZONMW-39_01641	atpA
atpA	ZONMW-40_00134	atpA
atpA	ZONMW-40_00449	atpA
atpA	ZONMW-41_03071	atpA
atpA	ZONMW-41_00379	atpA
atpA	ZONMW-41_00381	atpA
atpA	ZONMW-42_00959	atpA
atpA	ZONMW-42_02731	atpA
atpA	ZONMW-42_01343	atpA
atpA	ZONMW-42_02733	atpA
atpA	ZONMW-42_01336	atpA
atpA	ZONMW-43_00460	atpA
atpA	ZONMW-43_01884	atpA
atpA	ZONMW-43_01882	atpA
atpA	ZONMW-44_03674	atpA
atpA	ZONMW-44_02487	atpA
atpA	ZONMW-44_00534	atpA
atpA	ZONMW-44_02485	atpA
atpA	ZONMW-44_00527	atpA
atpA	ZONMW-45_00746	atpA
atpA	ZONMW-45_00390	atpA
atpA	ZONMW-46_00714	atpA
atpA	ZONMW-46_01449	atpA
atpA	ZONMW-47_00714	atpA
atpA	ZONMW-47_01403	atpA
atpA	ZONMW-48_00256	atpA
atpA	ZONMW-48_00605	atpA
atpA	ZONMW-49_02250	atpA
atpA	ZONMW-49_01181	atpA
atpA	ZONMW-50_02044	atpA
atpA	ZONMW-50_01182	atpA
atpA	ZONMW-51_03064	atpA
atpA	ZONMW-51_02787	atpA
atpA	ZONMW-52_02055	atpA
atpA	ZONMW-52_02259	atpA
atpA	ZONMW-53_01693	atpA
atpA	ZONMW-53_02848	atpA

atpA	ZONMW-54_02339	atpA
atpA	ZONMW-54_00064	atpA
atpA	ZONMW-55_03031	atpA
atpA	ZONMW-55_02854	atpA
atpA	ZONMW-56_01206	atpA
atpA	ZONMW-56_02614	atpA
atpA	ZONMW-57_02935	atpA
atpA	ZONMW-57_02758	atpA
atpA	ZONMW-58_03058	atpA
atpA	ZONMW-58_02782	atpA
atpA	ZONMW-59_01866	atpA
atpA	ZONMW-59_02946	atpA
atpA	ZONMW-60_02219	atpA
atpA	ZONMW-60_02877	atpA
atpA	ZONMW-61_01778	atpA
atpA	ZONMW-61_00332	atpA
atpA	ZONMW-62_01518	atpA
atpA	ZONMW-62_01884	atpA
atpA	ZONMW-63_03051	atpA
atpA	ZONMW-63_02315	atpA
atpA	ZONMW-64_00478	atpA
atpA	ZONMW-64_01595	atpA
atpA	ZONMW-64_01597	atpA
atpA	ZONMW-65_00985	atpA
atpA	ZONMW-65_02349	atpA
atpA	ZONMW-65_02351	atpA
atpA	ZONMW-66_01699	atpA
atpA	ZONMW-66_01757	atpA
atpA	ZONMW-66_01755	atpA
atpA	ZONMW-67_01143	atpA
atpA	ZONMW-67_00166	atpA
atpA	ZONMW-67_00164	atpA
atpA	ZONMW-68_00697	atpA
atpA	ZONMW-68_00096	atpA
atpA	ZONMW-69_01189	atpA
atpA	ZONMW-69_02775	atpA
atpA	ZONMW-69_02773	atpA
atpA	ZONMW-72_04390	atpA
atpA	ZONMW-72_03327	atpA
atpA	ZONMW-73_00235	atpA
atpA	ZONMW-73_00140	atpA
atpA	ZONMW-74_02105	atpA
atpA	ZONMW-74_01480	atpA
atpA	ZONMW-75_00496	atpA
atpA	ZONMW-75_02289	atpA
atpA	da5A-8_01532	atpA
atpA	da5A-8_01959	atpA
bepE_1	18_00133	bepE_1

bepE_1	18_00817	bepE_1
bepE_1	18_02868	bepE_1
bepE_1	18_02865	bepE_1
bepE_1	18_01261	bepE_1
bepE_1	18_00457	bepE_1
bepE_1	ACAM48_02276	bepE_1
bepE_1	ACAM48_01524	bepE_1
bepE_1	ACAM48_00188	bepE_1
bepE_1	ACAM48_03287	bepE_1
bepE_1	ACAM48_01520	bepE_1
bepE_1	ACAM48_00425	bepE_1
bepE_1	ACAM48_01740	bepE_1
bepE_1	ACAM48_02794	bepE_1
bepE_1	ACAM48_02087	bepE_1
bepE_1	ACAM48_02910	bepE_1
bepE_1	CAM030_01133	bepE_1
bepE_1	CAM030_02916	bepE_1
bepE_1	CAM030_01946	bepE_1
bepE_1	CAM030_00125	bepE_1
bepE_1	CAM030_02443	bepE_1
bepE_1	CAM030_00811	bepE_1
bepE_1	CAM030_02431	bepE_1
bepE_1	CAM030_01287	bepE_1
bepE_1	CAM030_02422	bepE_1
bepE_1	CAM030_02449	bepE_1
bepE_1	CECT7946_01878	bepE_1
bepE_1	CECT7946_02440	bepE_1
bepE_1	CECT7946_00429	bepE_1
bepE_1	CECT7946_01807	bepE_1
bepE_1	CECT7946_01001	bepE_1
bepE_1	CGMCC1.8863_02141	bepE_1
bepE_1	CGMCC1.8863_01019	bepE_1
bepE_1	CGMCC1.8863_02993	bepE_1
bepE_1	CGMCC1.8863_00728	bepE_1
bepE_1	CGMCC1.8863_00194	bepE_1
bepE_1	CGMCC1.8863_00984	bepE_1
bepE_1	CGMCC1.8863_02066	bepE_1
bepE_1	CL8139_00946	bepE_1
bepE_1	CL8139_03180	bepE_1
bepE_1	CL8139_01008	bepE_1
bepE_1	DAU203_01723	bepE_1
bepE_1	DAU203_02471	bepE_1
bepE_1	DAU203_03112	bepE_1
bepE_1	DAU203_01785	bepE_1
bepE_1	DAU203_03118	bepE_1
bepE_1	DD5b_02032	bepE_1
bepE_1	DD5b_01543	bepE_1
bepE_1	DD5b_01204	bepE_1

bepE_1	DD5b_00394	bepE_1
bepE_1	DSM1208_02815	bepE_1
bepE_1	DSM1208_01467	bepE_1
bepE_1	DSM1208_03292	bepE_1
bepE_1	DSM1208_01101	bepE_1
bepE_1	DSM1208_03933	bepE_1
bepE_1	DSM1208_01874	bepE_1
bepE_1	DSM1208_00758	bepE_1
bepE_1	DSM12212_02605	bepE_1
bepE_1	DSM12212_02387	bepE_1
bepE_1	DSM12212_03172	bepE_1
bepE_1	DSM12212_01665	bepE_1
bepE_1	DSM12212_02956	bepE_1
bepE_1	DSM12212_02517	bepE_1
bepE_1	DSM12212_02926	bepE_1
bepE_1	DSM15343_02049	bepE_1
bepE_1	DSM15343_02456	bepE_1
bepE_1	DSM15343_00421	bepE_1
bepE_1	DSM15343_01757	bepE_1
bepE_1	DSM15343_00271	bepE_1
bepE_1	DSM15343_00850	bepE_1
bepE_1	DSM15343_02656	bepE_1
bepE_1	DSM15361_00349	bepE_1
bepE_1	DSM15361_00934	bepE_1
bepE_1	DSM15361_01432	bepE_1
bepE_1	DSM15361_01309	bepE_1
bepE_1	DSM15361_01813	bepE_1
bepE_1	DSM15361_01835	bepE_1
bepE_1	DSM15361_00760	bepE_1
bepE_1	DSM15361_01821	bepE_1
bepE_1	DSM15365_02245	bepE_1
bepE_1	DSM15365_03263	bepE_1
bepE_1	DSM15365_03362	bepE_1
bepE_1	DSM15365_03769	bepE_1
bepE_1	DSM15365_00433	bepE_1
bepE_1	DSM15718_01797	bepE_1
bepE_1	DSM15718_01383	bepE_1
bepE_1	DSM15718_01430	bepE_1
bepE_1	DSM15718_02395	bepE_1
bepE_1	DSM15718_01689	bepE_1
bepE_1	DSM15718_01353	bepE_1
bepE_1	DSM16505T_01830	bepE_1
bepE_1	DSM16505T_00144	bepE_1
bepE_1	DSM16505T_00091	bepE_1
bepE_1	DSM16505T_02425	bepE_1
bepE_1	DSM16505T_01293	bepE_1
bepE_1	DSM16505T_00830	bepE_1
bepE_1	DSM16505T_01165	bepE_1

bepE_1	DSM18103_01213	bepE_1
bepE_1	DSM18103_00030	bepE_1
bepE_1	DSM18103_03131	bepE_1
bepE_1	DSM18103_03579	bepE_1
bepE_1	DSM18103_00682	bepE_1
bepE_1	DSM18103_02451	bepE_1
bepE_1	DSM18293_01405	bepE_1
bepE_1	DSM18293_04279	bepE_1
bepE_1	DSM18293_00077	bepE_1
bepE_1	DSM18293_03169	bepE_1
bepE_1	DSM18293_01706	bepE_1
bepE_1	DSM18293_00354	bepE_1
bepE_1	DSM18293_03372	bepE_1
bepE_1	DSM18293_00050	bepE_1
bepE_1	DSM18293_04252	bepE_1
bepE_1	DSM18293_04253	bepE_1
bepE_1	DSM18841_01556	bepE_1
bepE_1	DSM18841_02697	bepE_1
bepE_1	DSM18841_02309	bepE_1
bepE_1	DSM18841_00697	bepE_1
bepE_1	DSM18841_01066	bepE_1
bepE_1	DSM18842_02246	bepE_1
bepE_1	DSM18842_02547	bepE_1
bepE_1	DSM18842_02495	bepE_1
bepE_1	DSM18842_01200	bepE_1
bepE_1	DSM18842_00631	bepE_1
bepE_1	DSM18842_00256	bepE_1
bepE_1	DSM19866_03459	bepE_1
bepE_1	DSM19866_03462	bepE_1
bepE_1	DSM19866_04375	bepE_1
bepE_1	DSM19866_02536	bepE_1
bepE_1	DSM19866_00372	bepE_1
bepE_1	DSM19866_03823	bepE_1
bepE_1	DSM19866_02128	bepE_1
bepE_1	DSM19866_03406	bepE_1
bepE_1	DSM19866_02352	bepE_1
bepE_1	DSM2041_01081	bepE_1
bepE_1	DSM2041_03906	bepE_1
bepE_1	DSM2041_05205	bepE_1
bepE_1	DSM2041_00820	bepE_1
bepE_1	DSM2041_03896	bepE_1
bepE_1	DSM2041_00790	bepE_1
bepE_1	DSM2041_01058	bepE_1
bepE_1	DSM2041_01817	bepE_1
bepE_1	DSM2061_04299	bepE_1
bepE_1	DSM2061_02990	bepE_1
bepE_1	DSM2061_02258	bepE_1
bepE_1	DSM2061_02619	bepE_1

bepE_1	DSM2061_03428	bepE_1
bepE_1	DSM2061_03755	bepE_1
bepE_1	DSM2061_03139	bepE_1
bepE_1	DSM2064_04291	bepE_1
bepE_1	DSM2064_03815	bepE_1
bepE_1	DSM2064_00669	bepE_1
bepE_1	DSM2064_01176	bepE_1
bepE_1	DSM2064_04408	bepE_1
bepE_1	DSM2064_00657	bepE_1
bepE_1	DSM2064_01896	bepE_1
bepE_1	DSM2064_00363	bepE_1
bepE_1	DSM2064_02272	bepE_1
bepE_1	DSM2064_05240	bepE_1
bepE_1	DSM2064_00777	bepE_1
bepE_1	DSM2064_04424	bepE_1
bepE_1	DSM2064_04235	bepE_1
bepE_1	DSM2064_04414	bepE_1
bepE_1	DSM2064_04215	bepE_1
bepE_1	DSM2064_01378	bepE_1
bepE_1	DSM2064_00497	bepE_1
bepE_1	DSM2064_00498	bepE_1
bepE_1	DSM2064_04260	bepE_1
bepE_1	DSM2064_00776	bepE_1
bepE_1	DSM21164_02776	bepE_1
bepE_1	DSM21164_01507	bepE_1
bepE_1	DSM21164_01511	bepE_1
bepE_1	DSM21164_01369	bepE_1
bepE_1	DSM21164_00608	bepE_1
bepE_1	DSM23404_02996	bepE_1
bepE_1	DSM23404_03268	bepE_1
bepE_1	DSM23404_00869	bepE_1
bepE_1	DSM23404_00157	bepE_1
bepE_1	DSM23404_01319	bepE_1
bepE_1	DSM23404_01889	bepE_1
bepE_1	DSM23404_02142	bepE_1
bepE_1	DSM23404_02145	bepE_1
bepE_1	DSM23404_03241	bepE_1
bepE_1	DSM23404_01746	bepE_1
bepE_1	DSM23404_02146	bepE_1
bepE_1	DSM23424_00455	bepE_1
bepE_1	DSM23424_01578	bepE_1
bepE_1	DSM23424_02076	bepE_1
bepE_1	DSM23424_03089	bepE_1
bepE_1	DSM23424_00607	bepE_1
bepE_1	DSM23424_00868	bepE_1
bepE_1	DSM24786_02259	bepE_1
bepE_1	DSM24786_01537	bepE_1
bepE_1	DSM24786_00426	bepE_1

bepE_1	DSM24786_02197	bepE_1
bepE_1	DSM24786_01861	bepE_1
bepE_1	DSM7489_01641	bepE_1
bepE_1	DSM7489_02386	bepE_1
bepE_1	DSM7489_03268	bepE_1
bepE_1	DSM7489_01704	bepE_1
bepE_1	DSW1_00276	bepE_1
bepE_1	DSW1_00012	bepE_1
bepE_1	DSW1_02921	bepE_1
bepE_1	DSW5_01253	bepE_1
bepE_1	DSW5_00865	bepE_1
bepE_1	DSW5_01811	bepE_1
bepE_1	DSW6_00711	bepE_1
bepE_1	DSW6_01735	bepE_1
bepE_1	DSW6_00699	bepE_1
bepE_1	DSW6_00825	bepE_1
bepE_1	DSW8_02468	bepE_1
bepE_1	DSW8_02022	bepE_1
bepE_1	DSW8_02476	bepE_1
bepE_1	DSW8_03222	bepE_1
bepE_1	DSW8_03257	bepE_1
bepE_1	DSW8_03648	bepE_1
bepE_1	DSW8_02053	bepE_1
bepE_1	DSW8_02835	bepE_1
bepE_1	DSW8_00437	bepE_1
bepE_1	F52_03962	bepE_1
bepE_1	F52_00947	bepE_1
bepE_1	F52_03501	bepE_1
bepE_1	F52_03619	bepE_1
bepE_1	F52_02327	bepE_1
bepE_1	F52_01322	bepE_1
bepE_1	F52_02662	bepE_1
bepE_1	F52_04408	bepE_1
bepE_1	F52_03609	bepE_1
bepE_1	F52_00868	bepE_1
bepE_1	F52_01201	bepE_1
bepE_1	F52_01755	bepE_1
bepE_1	F52_01177	bepE_1
bepE_1	F52_01743	bepE_1
bepE_1	F52_01176	bepE_1
bepE_1	F52_00869	bepE_1
bepE_1	GCA_001027945.1_02523	bepE_1
bepE_1	GCA_001027945.1_01642	bepE_1
bepE_1	GCA_001027945.1_01340	bepE_1
bepE_1	GCA_001027945.1_00948	bepE_1
bepE_1	GCA_001027945.1_01078	bepE_1
bepE_1	GCA_001027945.1_03120	bepE_1
bepE_1	GCA_001027945.1_01364	bepE_1

bepE_1	HI1_01562	bepE_1
bepE_1	HI1_02319	bepE_1
bepE_1	HI1_03232	bepE_1
bepE_1	HI1_03212	bepE_1
bepE_1	HI1_01625	bepE_1
bepE_1	HI1_03037	bepE_1
bepE_1	IR1_01506	bepE_1
bepE_1	IR1_01925	bepE_1
bepE_1	IR1_01321	bepE_1
bepE_1	IR1_03572	bepE_1
bepE_1	IR1_03260	bepE_1
bepE_1	IR1_03404	bepE_1
bepE_1	IR1_00597	bepE_1
bepE_1	IR1_01970	bepE_1
bepE_1	IR1_03566	bepE_1
bepE_1	IR1_03371	bepE_1
bepE_1	IR1_03251	bepE_1
bepE_1	IR1_03855	bepE_1
bepE_1	IR1_02192	bepE_1
bepE_1	IR1_00787	bepE_1
bepE_1	IR1_00786	bepE_1
bepE_1	KCOM3131_00290	bepE_1
bepE_1	KCOM3131_00861	bepE_1
bepE_1	KCOM3131_00390	bepE_1
bepE_1	KCOM3131_00392	bepE_1
bepE_1	KCTC12974_01849	bepE_1
bepE_1	KCTC12974_00294	bepE_1
bepE_1	KCTC12974_00879	bepE_1
bepE_1	KCTC12974_00018	bepE_1
bepE_1	KCTC12974_01139	bepE_1
bepE_1	KCTC12974_02093	bepE_1
bepE_1	KCTC12974_01380	bepE_1
bepE_1	KL-A_01443	bepE_1
bepE_1	KL-A_01666	bepE_1
bepE_1	KL-A_00542	bepE_1
bepE_1	KL-A_01506	bepE_1
bepE_1	KL-A_02698	bepE_1
bepE_1	KL-A_00536	bepE_1
bepE_1	L12_00413	bepE_1
bepE_1	L12_00412	bepE_1
bepE_1	LL0412.1.7_01422	bepE_1
bepE_1	LL0412.1.7_00762	bepE_1
bepE_1	LL0412.1.7_01833	bepE_1
bepE_1	LL0412.1.7_02748	bepE_1
bepE_1	LL0412.1.7_00829	bepE_1
bepE_1	LL0412.1.7_01825	bepE_1
bepE_1	MAR_2009_71_00985	bepE_1
bepE_1	MAR_2009_71_00977	bepE_1

bepE_1	MAR_2009_71_03697	bepE_1
bepE_1	MAR_2009_71_02034	bepE_1
bepE_1	MAR_2009_71_02800	bepE_1
bepE_1	MAR_2009_71_01378	bepE_1
bepE_1	MAR_2009_71_00619	bepE_1
bepE_1	MAR_2009_71_03236	bepE_1
bepE_1	NN016038_00136	bepE_1
bepE_1	NN016038_00837	bepE_1
bepE_1	NN016038_02920	bepE_1
bepE_1	NN016038_02457	bepE_1
bepE_1	NN016038_02917	bepE_1
bepE_1	NN016038_01285	bepE_1
bepE_1	NN016038_00466	bepE_1
bepE_1	PAO1_00437	bepE_1
bepE_1	PAO1_02564	bepE_1
bepE_1	PAO1_04748	bepE_1
bepE_1	PAO1_03644	bepE_1
bepE_1	PAO1_02081	bepE_1
bepE_1	PAO1_04338	bepE_1
bepE_1	PAO1_04517	bepE_1
bepE_1	PAO1_02598	bepE_1
bepE_1	PAO1_01476	bepE_1
bepE_1	PAO1_02597	bepE_1
bepE_1	PAO1_02590	bepE_1
bepE_1	PAO1_03799	bepE_1
bepE_1	PAO1_00164	bepE_1
bepE_1	UW101_04595	bepE_1
bepE_1	UW101_05071	bepE_1
bepE_1	UW101_03380	bepE_1
bepE_1	UW101_04303	bepE_1
bepE_1	UW101_04478	bepE_1
bepE_1	UW101_03368	bepE_1
bepE_1	UW101_00950	bepE_1
bepE_1	UW101_03074	bepE_1
bepE_1	UW101_01326	bepE_1
bepE_1	UW101_02849	bepE_1
bepE_1	UW101_03488	bepE_1
bepE_1	UW101_04462	bepE_1
bepE_1	UW101_04650	bepE_1
bepE_1	UW101_04472	bepE_1
bepE_1	UW101_04670	bepE_1
bepE_1	UW101_04101	bepE_1
bepE_1	UW101_03208	bepE_1
bepE_1	UW101_03209	bepE_1
bepE_1	UW101_04625	bepE_1
bepE_1	UW101_03487	bepE_1
bepE_1	V4-33_00844	bepE_1
bepE_1	V4-33_00795	bepE_1

bepE_1	V4-33_01717	bepE_1
bepE_1	V4-33_02322	bepE_1
bepE_1	W5C_00335	bepE_1
bepE_1	W5C_02706	bepE_1
bepE_1	W5C_00400	bepE_1
bepE_1	W5C_02966	bepE_1
bepE_1	ZONMW-01_00089	bepE_1
bepE_1	ZONMW-01_02823	bepE_1
bepE_1	ZONMW-01_00151	bepE_1
bepE_1	ZONMW-02_00196	bepE_1
bepE_1	ZONMW-02_00741	bepE_1
bepE_1	ZONMW-02_02070	bepE_1
bepE_1	ZONMW-02_00294	bepE_1
bepE_1	ZONMW-02_03668	bepE_1
bepE_1	ZONMW-03_00814	bepE_1
bepE_1	ZONMW-03_01586	bepE_1
bepE_1	ZONMW-03_00877	bepE_1
bepE_1	ZONMW-04_02131	bepE_1
bepE_1	ZONMW-04_00096	bepE_1
bepE_1	ZONMW-04_00893	bepE_1
bepE_1	ZONMW-04_02069	bepE_1
bepE_1	ZONMW-05_01004	bepE_1
bepE_1	ZONMW-05_01734	bepE_1
bepE_1	ZONMW-05_01066	bepE_1
bepE_1	ZONMW-06_02721	bepE_1
bepE_1	ZONMW-06_00144	bepE_1
bepE_1	ZONMW-06_01254	bepE_1
bepE_1	ZONMW-06_01447	bepE_1
bepE_1	ZONMW-06_01564	bepE_1
bepE_1	ZONMW-06_03429	bepE_1
bepE_1	ZONMW-06_01568	bepE_1
bepE_1	ZONMW-06_00347	bepE_1
bepE_1	ZONMW-07_00749	bepE_1
bepE_1	ZONMW-07_01107	bepE_1
bepE_1	ZONMW-07_00465	bepE_1
bepE_1	ZONMW-07_01330	bepE_1
bepE_1	ZONMW-07_02990	bepE_1
bepE_1	ZONMW-07_02430	bepE_1
bepE_1	ZONMW-08_00504	bepE_1
bepE_1	ZONMW-08_00496	bepE_1
bepE_1	ZONMW-08_03816	bepE_1
bepE_1	ZONMW-08_02246	bepE_1
bepE_1	ZONMW-08_02017	bepE_1
bepE_1	ZONMW-08_01816	bepE_1
bepE_1	ZONMW-08_00135	bepE_1
bepE_1	ZONMW-08_02751	bepE_1
bepE_1	ZONMW-09_02057	bepE_1
bepE_1	ZONMW-09_00803	bepE_1

bepE_1	ZONMW-09_00125	bepE_1
bepE_1	ZONMW-09_01668	bepE_1
bepE_1	ZONMW-09_00677	bepE_1
bepE_1	ZONMW-10_00245	bepE_1
bepE_1	ZONMW-10_02190	bepE_1
bepE_1	ZONMW-10_00144	bepE_1
bepE_1	ZONMW-10_02497	bepE_1
bepE_1	ZONMW-10_01845	bepE_1
bepE_1	ZONMW-10_00770	bepE_1
bepE_1	ZONMW-11_04569	bepE_1
bepE_1	ZONMW-11_00927	bepE_1
bepE_1	ZONMW-11_02863	bepE_1
bepE_1	ZONMW-11_03203	bepE_1
bepE_1	ZONMW-11_00661	bepE_1
bepE_1	ZONMW-11_00332	bepE_1
bepE_1	ZONMW-11_04384	bepE_1
bepE_1	ZONMW-11_03794	bepE_1
bepE_1	ZONMW-11_00471	bepE_1
bepE_1	ZONMW-11_00673	bepE_1
bepE_1	ZONMW-11_00887	bepE_1
bepE_1	ZONMW-11_04198	bepE_1
bepE_1	ZONMW-11_01664	bepE_1
bepE_1	ZONMW-11_02465	bepE_1
bepE_1	ZONMW-11_04126	bepE_1
bepE_1	ZONMW-12_03926	bepE_1
bepE_1	ZONMW-12_00022	bepE_1
bepE_1	ZONMW-12_04238	bepE_1
bepE_1	ZONMW-12_02871	bepE_1
bepE_1	ZONMW-12_02432	bepE_1
bepE_1	ZONMW-12_02482	bepE_1
bepE_1	ZONMW-12_01445	bepE_1
bepE_1	ZONMW-12_00401	bepE_1
bepE_1	ZONMW-12_01255	bepE_1
bepE_1	ZONMW-12_01250	bepE_1
bepE_1	ZONMW-12_02938	bepE_1
bepE_1	ZONMW-12_00558	bepE_1
bepE_1	ZONMW-12_02500	bepE_1
bepE_1	ZONMW-12_00993	bepE_1
bepE_1	ZONMW-12_02411	bepE_1
bepE_1	ZONMW-12_01023	bepE_1
bepE_1	ZONMW-12_03851	bepE_1
bepE_1	ZONMW-12_01448	bepE_1
bepE_1	ZONMW-13_00471	bepE_1
bepE_1	ZONMW-13_00865	bepE_1
bepE_1	ZONMW-13_03005	bepE_1
bepE_1	ZONMW-13_02845	bepE_1
bepE_1	ZONMW-13_00183	bepE_1
bepE_1	ZONMW-13_00106	bepE_1

bepE_1	ZONMW-13_00025	bepE_1
bepE_1	ZONMW-14_00090	bepE_1
bepE_1	ZONMW-14_01231	bepE_1
bepE_1	ZONMW-14_01388	bepE_1
bepE_1	ZONMW-14_00411	bepE_1
bepE_1	ZONMW-14_00656	bepE_1
bepE_1	ZONMW-14_02656	bepE_1
bepE_1	ZONMW-14_02418	bepE_1
bepE_1	ZONMW-15_01170	bepE_1
bepE_1	ZONMW-15_01763	bepE_1
bepE_1	ZONMW-15_00111	bepE_1
bepE_1	ZONMW-15_01073	bepE_1
bepE_1	ZONMW-19_03451	bepE_1
bepE_1	ZONMW-19_03643	bepE_1
bepE_1	ZONMW-19_03612	bepE_1
bepE_1	ZONMW-19_01685	bepE_1
bepE_1	ZONMW-19_03695	bepE_1
bepE_1	ZONMW-19_03369	bepE_1
bepE_1	ZONMW-19_03376	bepE_1
bepE_1	ZONMW-19_00637	bepE_1
bepE_1	ZONMW-19_02694	bepE_1
bepE_1	ZONMW-19_03739	bepE_1
bepE_1	ZONMW-19_03588	bepE_1
bepE_1	ZONMW-20_02208	bepE_1
bepE_1	ZONMW-20_03069	bepE_1
bepE_1	ZONMW-20_01291	bepE_1
bepE_1	ZONMW-20_02780	bepE_1
bepE_1	ZONMW-20_00731	bepE_1
bepE_1	ZONMW-20_03034	bepE_1
bepE_1	ZONMW-20_04079	bepE_1
bepE_1	ZONMW-20_04080	bepE_1
bepE_1	ZONMW-20_02290	bepE_1
bepE_1	ZONMW-20_03938	bepE_1
bepE_1	ZONMW-21_01012	bepE_1
bepE_1	ZONMW-21_03361	bepE_1
bepE_1	ZONMW-21_00575	bepE_1
bepE_1	ZONMW-21_00591	bepE_1
bepE_1	ZONMW-21_01362	bepE_1
bepE_1	ZONMW-21_02217	bepE_1
bepE_1	ZONMW-22_01013	bepE_1
bepE_1	ZONMW-22_03364	bepE_1
bepE_1	ZONMW-22_00575	bepE_1
bepE_1	ZONMW-22_00591	bepE_1
bepE_1	ZONMW-22_01364	bepE_1
bepE_1	ZONMW-22_01508	bepE_1
bepE_1	ZONMW-23_02126	bepE_1
bepE_1	ZONMW-23_03407	bepE_1
bepE_1	ZONMW-23_01690	bepE_1

bepE_1	ZONMW-23_01706	bepE_1
bepE_1	ZONMW-23_02477	bepE_1
bepE_1	ZONMW-23_01043	bepE_1
bepE_1	ZONMW-24_00448	bepE_1
bepE_1	ZONMW-24_03409	bepE_1
bepE_1	ZONMW-24_00884	bepE_1
bepE_1	ZONMW-24_00869	bepE_1
bepE_1	ZONMW-24_00097	bepE_1
bepE_1	ZONMW-24_01532	bepE_1
bepE_1	ZONMW-25_01360	bepE_1
bepE_1	ZONMW-25_02016	bepE_1
bepE_1	ZONMW-25_02176	bepE_1
bepE_1	ZONMW-25_00157	bepE_1
bepE_1	ZONMW-25_02228	bepE_1
bepE_1	ZONMW-25_01204	bepE_1
bepE_1	ZONMW-25_03100	bepE_1
bepE_1	ZONMW-25_02241	bepE_1
bepE_1	ZONMW-25_02851	bepE_1
bepE_1	ZONMW-25_01216	bepE_1
bepE_1	ZONMW-26_01048	bepE_1
bepE_1	ZONMW-26_01559	bepE_1
bepE_1	ZONMW-26_01110	bepE_1
bepE_1	ZONMW-27_00825	bepE_1
bepE_1	ZONMW-27_00093	bepE_1
bepE_1	ZONMW-27_00762	bepE_1
bepE_1	ZONMW-28_02397	bepE_1
bepE_1	ZONMW-28_01910	bepE_1
bepE_1	ZONMW-28_01756	bepE_1
bepE_1	ZONMW-28_01336	bepE_1
bepE_1	ZONMW-28_02538	bepE_1
bepE_1	ZONMW-28_02478	bepE_1
bepE_1	ZONMW-28_02533	bepE_1
bepE_1	ZONMW-28_02253	bepE_1
bepE_1	ZONMW-28_01476	bepE_1
bepE_1	ZONMW-28_01581	bepE_1
bepE_1	ZONMW-28_02534	bepE_1
bepE_1	ZONMW-29_00584	bepE_1
bepE_1	ZONMW-29_02863	bepE_1
bepE_1	ZONMW-29_02286	bepE_1
bepE_1	ZONMW-29_00188	bepE_1
bepE_1	ZONMW-29_00241	bepE_1
bepE_1	ZONMW-29_00257	bepE_1
bepE_1	ZONMW-29_00909	bepE_1
bepE_1	ZONMW-30_02109	bepE_1
bepE_1	ZONMW-30_01781	bepE_1
bepE_1	ZONMW-30_01638	bepE_1
bepE_1	ZONMW-30_02611	bepE_1
bepE_1	ZONMW-30_01686	bepE_1

bepE_1	ZONMW-30_02969	bepE_1
bepE_1	ZONMW-30_00670	bepE_1
bepE_1	ZONMW-30_01700	bepE_1
bepE_1	ZONMW-30_00642	bepE_1
bepE_1	ZONMW-30_02980	bepE_1
bepE_1	ZONMW-31_02536	bepE_1
bepE_1	ZONMW-31_00472	bepE_1
bepE_1	ZONMW-31_00056	bepE_1
bepE_1	ZONMW-31_02213	bepE_1
bepE_1	ZONMW-31_02481	bepE_1
bepE_1	ZONMW-31_02210	bepE_1
bepE_1	ZONMW-31_00795	bepE_1
bepE_1	ZONMW-31_00663	bepE_1
bepE_1	ZONMW-32_02953	bepE_1
bepE_1	ZONMW-32_02062	bepE_1
bepE_1	ZONMW-32_02216	bepE_1
bepE_1	ZONMW-32_01744	bepE_1
bepE_1	ZONMW-32_02812	bepE_1
bepE_1	ZONMW-32_02872	bepE_1
bepE_1	ZONMW-32_02817	bepE_1
bepE_1	ZONMW-32_02658	bepE_1
bepE_1	ZONMW-32_01883	bepE_1
bepE_1	ZONMW-32_01988	bepE_1
bepE_1	ZONMW-32_02816	bepE_1
bepE_1	ZONMW-33_02795	bepE_1
bepE_1	ZONMW-33_02053	bepE_1
bepE_1	ZONMW-33_02207	bepE_1
bepE_1	ZONMW-33_01734	bepE_1
bepE_1	ZONMW-33_02936	bepE_1
bepE_1	ZONMW-33_02876	bepE_1
bepE_1	ZONMW-33_02931	bepE_1
bepE_1	ZONMW-33_02460	bepE_1
bepE_1	ZONMW-33_01873	bepE_1
bepE_1	ZONMW-33_01979	bepE_1
bepE_1	ZONMW-33_02932	bepE_1
bepE_1	ZONMW-34_02171	bepE_1
bepE_1	ZONMW-34_00117	bepE_1
bepE_1	ZONMW-34_02359	bepE_1
bepE_1	ZONMW-34_03454	bepE_1
bepE_1	ZONMW-34_03369	bepE_1
bepE_1	ZONMW-34_03365	bepE_1
bepE_1	ZONMW-34_00218	bepE_1
bepE_1	ZONMW-34_01783	bepE_1
bepE_1	ZONMW-34_00314	bepE_1
bepE_1	ZONMW-35_02078	bepE_1
bepE_1	ZONMW-35_01274	bepE_1
bepE_1	ZONMW-35_01890	bepE_1
bepE_1	ZONMW-35_03361	bepE_1

bepE_1	ZONMW-35_03265	bepE_1
bepE_1	ZONMW-35_03269	bepE_1
bepE_1	ZONMW-35_01172	bepE_1
bepE_1	ZONMW-35_01474	bepE_1
bepE_1	ZONMW-35_01076	bepE_1
bepE_1	ZONMW-36_02307	bepE_1
bepE_1	ZONMW-36_02183	bepE_1
bepE_1	ZONMW-36_02029	bepE_1
bepE_1	ZONMW-36_01622	bepE_1
bepE_1	ZONMW-36_02448	bepE_1
bepE_1	ZONMW-36_02388	bepE_1
bepE_1	ZONMW-36_02443	bepE_1
bepE_1	ZONMW-36_02792	bepE_1
bepE_1	ZONMW-36_01482	bepE_1
bepE_1	ZONMW-36_01377	bepE_1
bepE_1	ZONMW-36_02444	bepE_1
bepE_1	ZONMW-37_01350	bepE_1
bepE_1	ZONMW-37_03373	bepE_1
bepE_1	ZONMW-37_00575	bepE_1
bepE_1	ZONMW-37_00591	bepE_1
bepE_1	ZONMW-37_01000	bepE_1
bepE_1	ZONMW-37_01531	bepE_1
bepE_1	ZONMW-38_02935	bepE_1
bepE_1	ZONMW-39_01451	bepE_1
bepE_1	ZONMW-39_02410	bepE_1
bepE_1	ZONMW-39_01652	bepE_1
bepE_1	ZONMW-39_03224	bepE_1
bepE_1	ZONMW-39_03220	bepE_1
bepE_1	ZONMW-39_00427	bepE_1
bepE_1	ZONMW-39_00689	bepE_1
bepE_1	ZONMW-39_03699	bepE_1
bepE_1	ZONMW-39_03338	bepE_1
bepE_1	ZONMW-39_02999	bepE_1
bepE_1	ZONMW-40_03520	bepE_1
bepE_1	ZONMW-40_00246	bepE_1
bepE_1	ZONMW-40_03050	bepE_1
bepE_1	ZONMW-40_02259	bepE_1
bepE_1	ZONMW-40_01417	bepE_1
bepE_1	ZONMW-40_00749	bepE_1
bepE_1	ZONMW-40_01383	bepE_1
bepE_1	ZONMW-40_02758	bepE_1
bepE_1	ZONMW-40_01369	bepE_1
bepE_1	ZONMW-40_01933	bepE_1
bepE_1	ZONMW-40_02730	bepE_1
bepE_1	ZONMW-41_02949	bepE_1
bepE_1	ZONMW-41_03160	bepE_1
bepE_1	ZONMW-41_01709	bepE_1
bepE_1	ZONMW-41_00577	bepE_1

bepE_1	ZONMW-41_00239	bepE_1
bepE_1	ZONMW-41_00537	bepE_1
bepE_1	ZONMW-41_01539	bepE_1
bepE_1	ZONMW-41_00173	bepE_1
bepE_1	ZONMW-41_00993	bepE_1
bepE_1	ZONMW-42_00587	bepE_1
bepE_1	ZONMW-42_00041	bepE_1
bepE_1	ZONMW-42_02745	bepE_1
bepE_1	ZONMW-42_02453	bepE_1
bepE_1	ZONMW-42_00680	bepE_1
bepE_1	ZONMW-42_01333	bepE_1
bepE_1	ZONMW-42_00651	bepE_1
bepE_1	ZONMW-42_03726	bepE_1
bepE_1	ZONMW-42_02934	bepE_1
bepE_1	ZONMW-42_01329	bepE_1
bepE_1	ZONMW-42_03570	bepE_1
bepE_1	ZONMW-43_04384	bepE_1
bepE_1	ZONMW-43_03245	bepE_1
bepE_1	ZONMW-43_00832	bepE_1
bepE_1	ZONMW-43_01872	bepE_1
bepE_1	ZONMW-43_02675	bepE_1
bepE_1	ZONMW-43_02679	bepE_1
bepE_1	ZONMW-43_00730	bepE_1
bepE_1	ZONMW-43_01294	bepE_1
bepE_1	ZONMW-43_04376	bepE_1
bepE_1	ZONMW-43_00631	bepE_1
bepE_1	ZONMW-44_00707	bepE_1
bepE_1	ZONMW-44_00082	bepE_1
bepE_1	ZONMW-44_01902	bepE_1
bepE_1	ZONMW-44_02473	bepE_1
bepE_1	ZONMW-44_00341	bepE_1
bepE_1	ZONMW-44_00272	bepE_1
bepE_1	ZONMW-44_00642	bepE_1
bepE_1	ZONMW-44_00335	bepE_1
bepE_1	ZONMW-44_00524	bepE_1
bepE_1	ZONMW-44_02681	bepE_1
bepE_1	ZONMW-44_00520	bepE_1
bepE_1	ZONMW-44_00074	bepE_1
bepE_1	ZONMW-44_03538	bepE_1
bepE_1	ZONMW-45_00898	bepE_1
bepE_1	ZONMW-45_03197	bepE_1
bepE_1	ZONMW-45_02448	bepE_1
bepE_1	ZONMW-45_03181	bepE_1
bepE_1	ZONMW-45_01052	bepE_1
bepE_1	ZONMW-45_02196	bepE_1
bepE_1	ZONMW-46_00976	bepE_1
bepE_1	ZONMW-46_02373	bepE_1
bepE_1	ZONMW-46_02441	bepE_1

bepE_1	ZONMW-46_02919	bepE_1
bepE_1	ZONMW-46_00615	bepE_1
bepE_1	ZONMW-46_01872	bepE_1
bepE_1	ZONMW-47_01197	bepE_1
bepE_1	ZONMW-47_03236	bepE_1
bepE_1	ZONMW-47_02431	bepE_1
bepE_1	ZONMW-47_02954	bepE_1
bepE_1	ZONMW-47_00615	bepE_1
bepE_1	ZONMW-47_01617	bepE_1
bepE_1	ZONMW-48_02523	bepE_1
bepE_1	ZONMW-48_00150	bepE_1
bepE_1	ZONMW-48_00772	bepE_1
bepE_1	ZONMW-48_00489	bepE_1
bepE_1	ZONMW-49_00960	bepE_1
bepE_1	ZONMW-49_00523	bepE_1
bepE_1	ZONMW-49_01625	bepE_1
bepE_1	ZONMW-49_01251	bepE_1
bepE_1	ZONMW-49_01778	bepE_1
bepE_1	ZONMW-49_01631	bepE_1
bepE_1	ZONMW-50_00961	bepE_1
bepE_1	ZONMW-50_00524	bepE_1
bepE_1	ZONMW-50_01789	bepE_1
bepE_1	ZONMW-50_01253	bepE_1
bepE_1	ZONMW-50_01636	bepE_1
bepE_1	ZONMW-50_01783	bepE_1
bepE_1	ZONMW-51_01303	bepE_1
bepE_1	ZONMW-51_01182	bepE_1
bepE_1	ZONMW-51_00524	bepE_1
bepE_1	ZONMW-51_01366	bepE_1
bepE_1	ZONMW-51_00530	bepE_1
bepE_1	ZONMW-52_01030	bepE_1
bepE_1	ZONMW-52_02875	bepE_1
bepE_1	ZONMW-52_01108	bepE_1
bepE_1	ZONMW-52_00639	bepE_1
bepE_1	ZONMW-53_00804	bepE_1
bepE_1	ZONMW-53_00040	bepE_1
bepE_1	ZONMW-53_01236	bepE_1
bepE_1	ZONMW-53_00741	bepE_1
bepE_1	ZONMW-53_01242	bepE_1
bepE_1	ZONMW-54_00265	bepE_1
bepE_1	ZONMW-54_00988	bepE_1
bepE_1	ZONMW-54_01481	bepE_1
bepE_1	ZONMW-54_00328	bepE_1
bepE_1	ZONMW-54_01475	bepE_1
bepE_1	ZONMW-55_00772	bepE_1
bepE_1	ZONMW-55_00038	bepE_1
bepE_1	ZONMW-55_01000	bepE_1
bepE_1	ZONMW-55_00710	bepE_1

bepE_1	ZONMW-55_00994	bepE_1
bepE_1	ZONMW-56_02307	bepE_1
bepE_1	ZONMW-56_02769	bepE_1
bepE_1	ZONMW-56_00236	bepE_1
bepE_1	ZONMW-56_02369	bepE_1
bepE_1	ZONMW-56_00230	bepE_1
bepE_1	ZONMW-57_02434	bepE_1
bepE_1	ZONMW-57_01575	bepE_1
bepE_1	ZONMW-57_00449	bepE_1
bepE_1	ZONMW-57_02498	bepE_1
bepE_1	ZONMW-57_00443	bepE_1
bepE_1	ZONMW-58_02459	bepE_1
bepE_1	ZONMW-58_01009	bepE_1
bepE_1	ZONMW-58_00522	bepE_1
bepE_1	ZONMW-58_02523	bepE_1
bepE_1	ZONMW-58_00528	bepE_1
bepE_1	ZONMW-59_02031	bepE_1
bepE_1	ZONMW-59_02757	bepE_1
bepE_1	ZONMW-59_00915	bepE_1
bepE_1	ZONMW-59_01969	bepE_1
bepE_1	ZONMW-59_00308	bepE_1
bepE_1	ZONMW-60_01715	bepE_1
bepE_1	ZONMW-60_00983	bepE_1
bepE_1	ZONMW-60_00166	bepE_1
bepE_1	ZONMW-60_01652	bepE_1
bepE_1	ZONMW-60_00160	bepE_1
bepE_1	ZONMW-61_00074	bepE_1
bepE_1	ZONMW-61_00067	bepE_1
bepE_1	ZONMW-61_02615	bepE_1
bepE_1	ZONMW-61_03143	bepE_1
bepE_1	ZONMW-61_03938	bepE_1
bepE_1	ZONMW-61_01412	bepE_1
bepE_1	ZONMW-61_00516	bepE_1
bepE_1	ZONMW-61_01510	bepE_1
bepE_1	ZONMW-61_01357	bepE_1
bepE_1	ZONMW-61_03510	bepE_1
bepE_1	ZONMW-62_01629	bepE_1
bepE_1	ZONMW-62_01621	bepE_1
bepE_1	ZONMW-62_03144	bepE_1
bepE_1	ZONMW-62_03180	bepE_1
bepE_1	ZONMW-62_00743	bepE_1
bepE_1	ZONMW-62_02046	bepE_1
bepE_1	ZONMW-62_01262	bepE_1
bepE_1	ZONMW-62_00323	bepE_1
bepE_1	ZONMW-62_03539	bepE_1
bepE_1	ZONMW-63_00601	bepE_1
bepE_1	ZONMW-63_00982	bepE_1
bepE_1	ZONMW-63_00119	bepE_1

bepE_1	ZONMW-63_00664	bepE_1
bepE_1	ZONMW-63_00113	bepE_1
bepE_1	ZONMW-64_01373	bepE_1
bepE_1	ZONMW-64_01117	bepE_1
bepE_1	ZONMW-64_01607	bepE_1
bepE_1	ZONMW-64_03236	bepE_1
bepE_1	ZONMW-64_03204	bepE_1
bepE_1	ZONMW-64_03200	bepE_1
bepE_1	ZONMW-64_01015	bepE_1
bepE_1	ZONMW-64_01875	bepE_1
bepE_1	ZONMW-64_00893	bepE_1
bepE_1	ZONMW-65_01578	bepE_1
bepE_1	ZONMW-65_02013	bepE_1
bepE_1	ZONMW-65_00367	bepE_1
bepE_1	ZONMW-65_01167	bepE_1
bepE_1	ZONMW-65_02055	bepE_1
bepE_1	ZONMW-65_00486	bepE_1
bepE_1	ZONMW-65_00417	bepE_1
bepE_1	ZONMW-65_00505	bepE_1
bepE_1	ZONMW-65_00236	bepE_1
bepE_1	ZONMW-65_03066	bepE_1
bepE_1	ZONMW-65_03580	bepE_1
bepE_1	ZONMW-65_03629	bepE_1
bepE_1	ZONMW-65_00485	bepE_1
bepE_1	ZONMW-65_00542	bepE_1
bepE_1	ZONMW-65_03510	bepE_1
bepE_1	ZONMW-65_03585	bepE_1
bepE_1	ZONMW-65_03514	bepE_1
bepE_1	ZONMW-65_01470	bepE_1
bepE_1	ZONMW-66_00106	bepE_1
bepE_1	ZONMW-66_02932	bepE_1
bepE_1	ZONMW-66_03488	bepE_1
bepE_1	ZONMW-66_03617	bepE_1
bepE_1	ZONMW-66_03877	bepE_1
bepE_1	ZONMW-66_00499	bepE_1
bepE_1	ZONMW-66_00506	bepE_1
bepE_1	ZONMW-66_02747	bepE_1
bepE_1	ZONMW-66_04434	bepE_1
bepE_1	ZONMW-66_03853	bepE_1
bepE_1	ZONMW-66_03810	bepE_1
bepE_1	ZONMW-67_02204	bepE_1
bepE_1	ZONMW-67_00594	bepE_1
bepE_1	ZONMW-67_00152	bepE_1
bepE_1	ZONMW-67_02881	bepE_1
bepE_1	ZONMW-67_03270	bepE_1
bepE_1	ZONMW-67_02991	bepE_1
bepE_1	ZONMW-67_02723	bepE_1
bepE_1	ZONMW-68_04928	bepE_1

bepE_1	ZONMW-68_00583	bepE_1
bepE_1	ZONMW-68_00580	bepE_1
bepE_1	ZONMW-68_02101	bepE_1
bepE_1	ZONMW-68_03057	bepE_1
bepE_1	ZONMW-68_02367	bepE_1
bepE_1	ZONMW-68_00940	bepE_1
bepE_1	ZONMW-68_03648	bepE_1
bepE_1	ZONMW-68_00268	bepE_1
bepE_1	ZONMW-68_04586	bepE_1
bepE_1	ZONMW-68_01473	bepE_1
bepE_1	ZONMW-68_02041	bepE_1
bepE_1	ZONMW-69_02252	bepE_1
bepE_1	ZONMW-69_00341	bepE_1
bepE_1	ZONMW-69_02761	bepE_1
bepE_1	ZONMW-69_03073	bepE_1
bepE_1	ZONMW-69_03271	bepE_1
bepE_1	ZONMW-69_02963	bepE_1
bepE_1	ZONMW-69_00520	bepE_1
bepE_1	ZONMW-72_02012	bepE_1
bepE_1	ZONMW-72_01021	bepE_1
bepE_1	ZONMW-72_00914	bepE_1
bepE_1	ZONMW-72_04055	bepE_1
bepE_1	ZONMW-72_01723	bepE_1
bepE_1	ZONMW-72_03483	bepE_1
bepE_1	ZONMW-72_00249	bepE_1
bepE_1	ZONMW-72_04008	bepE_1
bepE_1	ZONMW-72_04115	bepE_1
bepE_1	ZONMW-72_03450	bepE_1
bepE_1	ZONMW-72_01732	bepE_1
bepE_1	ZONMW-72_00688	bepE_1
bepE_1	ZONMW-72_03685	bepE_1
bepE_1	ZONMW-72_02664	bepE_1
bepE_1	ZONMW-72_02665	bepE_1
bepE_1	ZONMW-73_03629	bepE_1
bepE_1	ZONMW-73_01810	bepE_1
bepE_1	ZONMW-73_03384	bepE_1
bepE_1	ZONMW-73_00267	bepE_1
bepE_1	ZONMW-73_02795	bepE_1
bepE_1	ZONMW-73_00260	bepE_1
bepE_1	ZONMW-73_00630	bepE_1
bepE_1	ZONMW-74_00805	bepE_1
bepE_1	ZONMW-74_01279	bepE_1
bepE_1	ZONMW-74_00791	bepE_1
bepE_1	ZONMW-74_00080	bepE_1
bepE_1	ZONMW-74_02770	bepE_1
bepE_1	ZONMW-74_01962	bepE_1
bepE_1	ZONMW-74_01101	bepE_1
bepE_1	ZONMW-75_00202	bepE_1

bepE_1	ZONMW-75_01064	bepE_1
bepE_1	ZONMW-75_01231	bepE_1
bepE_1	ZONMW-75_01833	bepE_1
bepE_1	ZONMW-75_01157	bepE_1
bepE_1	ZONMW-75_02120	bepE_1
bepE_1	ZONMW-75_01220	bepE_1
bepE_1	ZONMW-75_03140	bepE_1
bepE_1	da5A-8_01499	bepE_1
bepE_1	da5A-8_03539	bepE_1
bepE_1	da5A-8_01801	bepE_1
bepE_1	da5A-8_01156	bepE_1
bepE_1	da5A-8_02048	bepE_1
dck	18_01611	dck
dck	18_01648	dck
dck	ACAM48_00405	dck
dck	ACAM48_00477	dck
dck	CAM030_02360	dck
dck	CAM030_02390	dck
dck	CECT7946_02571	dck
dck	CECT7946_02648	dck
dck	CGMCC1.8863_02247	dck
dck	CGMCC1.8863_01281	dck
dck	CL8139_03253	dck
dck	CL8139_03283	dck
dck	DAU203_02051	dck
dck	DAU203_02081	dck
dck	DD5b_02631	dck
dck	DD5b_01788	dck
dck	DSM1208_02354	dck
dck	DSM1208_00833	dck
dck	DSM12212_03534	dck
dck	DSM12212_01621	dck
dck	DSM15343_01945	dck
dck	DSM15343_00592	dck
dck	DSM15361_01152	dck
dck	DSM15361_01115	dck
dck	DSM15365_03462	dck
dck	DSM15365_03491	dck
dck	DSM15718_00568	dck
dck	DSM15718_02167	dck
dck	DSM16505T_00959	dck
dck	DSM16505T_00966	dck
dck	DSM18103_02623	dck
dck	DSM18103_02614	dck
dck	DSM18293_02003	dck
dck	DSM18293_00568	dck
dck	DSM18841_00851	dck
dck	DSM18841_00860	dck

dck	DSM18842_00468	dck
dck	DSM18842_00459	dck
dck	DSM19866_01012	dck
dck	DSM19866_02308	dck
dck	DSM2041_04728	dck
dck	DSM2041_04210	dck
dck	DSM2061_03867	dck
dck	DSM2061_02335	dck
dck	DSM2064_02736	dck
dck	DSM2064_02493	dck
dck	DSM21164_00257	dck
dck	DSM21164_00222	dck
dck	DSM23404_01172	dck
dck	DSM23404_01100	dck
dck	DSM23424_03269	dck
dck	DSM23424_02347	dck
dck	DSM24786_01994	dck
dck	DSM24786_01964	dck
dck	DSM7489_01962	dck
dck	DSM7489_01993	dck
dck	DSW1_00282	dck
dck	DSW1_00232	dck
dck	DSW5_00759	dck
dck	DSW5_00624	dck
dck	DSW6_01118	dck
dck	DSW6_01451	dck
dck	DSW8_01614	dck
dck	DSW8_03032	dck
dck	F52_03348	dck
dck	F52_02898	dck
dck	GCA_001027945.1_01586	dck
dck	GCA_001027945.1_01577	dck
dck	HI1_01890	dck
dck	HI1_01920	dck
dck	IR1_00160	dck
dck	IR1_00391	dck
dck	KCOM3131_01595	dck
dck	KCTC12974_02271	dck
dck	KCTC12974_02334	dck
dck	KL-A_00309	dck
dck	KL-A_00279	dck
dck	L12_00502	dck
dck	LL0412.1.7_00982	dck
dck	LL0412.1.7_00989	dck
dck	MAR_2009_71_01811	dck
dck	MAR_2009_71_00422	dck
dck	NN016038_01619	dck
dck	NN016038_01656	dck

dck	PAO1_04889	dck
dck	PAO1_00598	dck
dck	UW101_01790	dck
dck	UW101_01547	dck
dck	V4-33_00376	dck
dck	V4-33_00239	dck
dck	W5C_00650	dck
dck	W5C_00680	dck
dck	ZONMW-01_00414	dck
dck	ZONMW-01_00444	dck
dck	ZONMW-02_00386	dck
dck	ZONMW-02_00429	dck
dck	ZONMW-03_01140	dck
dck	ZONMW-03_01170	dck
dck	ZONMW-04_00533	dck
dck	ZONMW-04_00503	dck
dck	ZONMW-05_01291	dck
dck	ZONMW-05_01321	dck
dck	ZONMW-06_01943	dck
dck	ZONMW-06_01947	dck
dck	ZONMW-07_01213	dck
dck	ZONMW-07_01524	dck
dck	ZONMW-08_01413	dck
dck	ZONMW-08_03744	dck
dck	ZONMW-09_01685	dck
dck	ZONMW-09_01737	dck
dck	ZONMW-10_01282	dck
dck	ZONMW-10_02416	dck
dck	ZONMW-11_00506	dck
dck	ZONMW-11_00249	dck
dck	ZONMW-12_01285	dck
dck	ZONMW-12_02088	dck
dck	ZONMW-13_01083	dck
dck	ZONMW-13_01134	dck
dck	ZONMW-14_01508	dck
dck	ZONMW-14_01460	dck
dck	ZONMW-15_01378	dck
dck	ZONMW-15_01343	dck
dck	ZONMW-19_02406	dck
dck	ZONMW-20_00928	dck
dck	ZONMW-21_00140	dck
dck	ZONMW-21_00084	dck
dck	ZONMW-22_00140	dck
dck	ZONMW-22_00084	dck
dck	ZONMW-23_01255	dck
dck	ZONMW-23_01199	dck
dck	ZONMW-24_01320	dck
dck	ZONMW-24_01376	dck

dck	ZONMW-25_02322	dck
dck	ZONMW-25_03367	dck
dck	ZONMW-26_01346	dck
dck	ZONMW-26_01376	dck
dck	ZONMW-27_00527	dck
dck	ZONMW-27_00497	dck
dck	ZONMW-28_02635	dck
dck	ZONMW-28_02639	dck
dck	ZONMW-28_01937	dck
dck	ZONMW-29_02413	dck
dck	ZONMW-29_02464	dck
dck	ZONMW-30_00786	dck
dck	ZONMW-30_00830	dck
dck	ZONMW-31_01910	dck
dck	ZONMW-31_01906	dck
dck	ZONMW-32_03241	dck
dck	ZONMW-32_03237	dck
dck	ZONMW-32_02035	dck
dck	ZONMW-33_03033	dck
dck	ZONMW-33_03037	dck
dck	ZONMW-33_02026	dck
dck	ZONMW-34_02616	dck
dck	ZONMW-34_02620	dck
dck	ZONMW-35_02580	dck
dck	ZONMW-35_02584	dck
dck	ZONMW-36_02969	dck
dck	ZONMW-36_02965	dck
dck	ZONMW-36_02210	dck
dck	ZONMW-37_00139	dck
dck	ZONMW-37_00083	dck
dck	ZONMW-38_02717	dck
dck	ZONMW-38_02987	dck
dck	ZONMW-38_02718	dck
dck	ZONMW-39_03949	dck
dck	ZONMW-39_03953	dck
dck	ZONMW-40_00904	dck
dck	ZONMW-40_00948	dck
dck	ZONMW-41_01837	dck
dck	ZONMW-42_01060	dck
dck	ZONMW-42_04082	dck
dck	ZONMW-43_01243	dck
dck	ZONMW-43_01247	dck
dck	ZONMW-44_00036	dck
dck	ZONMW-44_00032	dck
dck	ZONMW-45_01381	dck
dck	ZONMW-45_01390	dck
dck	ZONMW-46_01636	dck
dck	ZONMW-46_01645	dck

dck	ZONMW-47_01853	dck
dck	ZONMW-47_01844	dck
dck	ZONMW-48_02318	dck
dck	ZONMW-48_02353	dck
dck	ZONMW-49_01505	dck
dck	ZONMW-49_01535	dck
dck	ZONMW-50_01507	dck
dck	ZONMW-50_01537	dck
dck	ZONMW-51_00760	dck
dck	ZONMW-51_00790	dck
dck	ZONMW-52_01360	dck
dck	ZONMW-52_01390	dck
dck	ZONMW-53_00465	dck
dck	ZONMW-53_00435	dck
dck	ZONMW-54_00569	dck
dck	ZONMW-54_00599	dck
dck	ZONMW-55_00460	dck
dck	ZONMW-55_00430	dck
dck	ZONMW-56_00855	dck
dck	ZONMW-56_00885	dck
dck	ZONMW-57_01155	dck
dck	ZONMW-57_01185	dck
dck	ZONMW-58_01429	dck
dck	ZONMW-58_01399	dck
dck	ZONMW-59_01156	dck
dck	ZONMW-59_01186	dck
dck	ZONMW-60_01412	dck
dck	ZONMW-60_01382	dck
dck	ZONMW-61_02974	dck
dck	ZONMW-61_03473	dck
dck	ZONMW-62_02261	dck
dck	ZONMW-62_03575	dck
dck	ZONMW-63_00910	dck
dck	ZONMW-63_03359	dck
dck	ZONMW-64_04175	dck
dck	ZONMW-64_04179	dck
dck	ZONMW-65_02867	dck
dck	ZONMW-65_00355	dck
dck	ZONMW-66_00298	dck
dck	ZONMW-67_03308	dck
dck	ZONMW-67_03304	dck
dck	ZONMW-68_03283	dck
dck	ZONMW-68_01517	dck
dck	ZONMW-69_03309	dck
dck	ZONMW-69_03305	dck
dck	ZONMW-72_00523	dck
dck	ZONMW-72_00043	dck
dck	ZONMW-73_02104	dck

dck	ZONMW-73_01328	dck
dck	ZONMW-74_01738	dck
dck	ZONMW-74_01699	dck
dck	ZONMW-75_01308	dck
dck	ZONMW-75_01339	dck
dck	da5A-8_00370	dck
dck	da5A-8_00379	dck
yhgF	18_01363	yhgF
yhgF	18_02319	yhgF
yhgF	ACAM48_01096	yhgF
yhgF	ACAM48_01038	yhgF
yhgF	CAM030_01221	yhgF
yhgF	CAM030_02063	yhgF
yhgF	CECT7946_00301	yhgF
yhgF	CECT7946_03526	yhgF
yhgF	CGMCC1.8863_03187	yhgF
yhgF	CGMCC1.8863_03322	yhgF
yhgF	CL8139_01054	yhgF
yhgF	CL8139_01241	yhgF
yhgF	DAU203_01832	yhgF
yhgF	DAU203_00184	yhgF
yhgF	DD5b_02156	yhgF
yhgF	DD5b_00075	yhgF
yhgF	DSM1208_02016	yhgF
yhgF	DSM1208_00487	yhgF
yhgF	DSM12212_00234	yhgF
yhgF	DSM12212_00784	yhgF
yhgF	DSM15343_01056	yhgF
yhgF	DSM15343_00375	yhgF
yhgF	DSM15361_01103	yhgF
yhgF	DSM15361_01492	yhgF
yhgF	DSM15365_03259	yhgF
yhgF	DSM15365_03917	yhgF
yhgF	DSM15718_02582	yhgF
yhgF	DSM15718_01581	yhgF
yhgF	DSM16505T_00301	yhgF
yhgF	DSM16505T_01469	yhgF
yhgF	DSM18103_01776	yhgF
yhgF	DSM18103_00190	yhgF
yhgF	DSM18293_03075	yhgF
yhgF	DSM18293_01913	yhgF
yhgF	DSM18841_00104	yhgF
yhgF	DSM18841_02069	yhgF
yhgF	DSM18842_01106	yhgF
yhgF	DSM18842_01401	yhgF
yhgF	DSM19866_02968	yhgF
yhgF	DSM19866_03211	yhgF
yhgF	DSM2041_00239	yhgF

yhgF	DSM2041_01479	yhgF
yhgF	DSM2061_02833	yhgF
yhgF	DSM2061_01977	yhgF
yhgF	DSM2064_02018	yhgF
yhgF	DSM2064_00175	yhgF
yhgF	DSM21164_00531	yhgF
yhgF	DSM21164_01843	yhgF
yhgF	DSM23404_02792	yhgF
yhgF	DSM23404_02843	yhgF
yhgF	DSM23424_00512	yhgF
yhgF	DSM23424_00969	yhgF
yhgF	DSM24786_03275	yhgF
yhgF	DSM24786_00061	yhgF
yhgF	DSM7489_01753	yhgF
yhgF	DSM7489_00178	yhgF
yhgF	DSW1_00481	yhgF
yhgF	DSW1_02602	yhgF
yhgF	DSW5_01787	yhgF
yhgF	DSW6_03208	yhgF
yhgF	DSW8_01894	yhgF
yhgF	DSW8_03509	yhgF
yhgF	F52_02419	yhgF
yhgF	F52_04329	yhgF
yhgF	GCA_001027945.1_00492	yhgF
yhgF	GCA_001027945.1_01885	yhgF
yhgF	HI1_01679	yhgF
yhgF	HI1_00110	yhgF
yhgF	IR1_04736	yhgF
yhgF	IR1_01879	yhgF
yhgF	KCOM3131_01318	yhgF
yhgF	KCTC12974_01577	yhgF
yhgF	KL-A_02875	yhgF
yhgF	KL-A_01174	yhgF
yhgF	L12_00214	yhgF
yhgF	LL0412.1.7_00680	yhgF
yhgF	LL0412.1.7_02508	yhgF
yhgF	MAR_2009_71_01486	yhgF
yhgF	MAR_2009_71_00077	yhgF
yhgF	NN016038_01375	yhgF
yhgF	NN016038_02330	yhgF
yhgF	PAO1_05376	yhgF
yhgF	PAO1_03272	yhgF
yhgF	PAO1_03253	yhgF
yhgF	UW101_01073	yhgF
yhgF	UW101_02886	yhgF
yhgF	V4-33_02408	yhgF
yhgF	V4-33_01701	yhgF
yhgF	W5C_00449	yhgF

yhgF	W5C_00087	yhgF
yhgF	ZONMW-01_00191	yhgF
yhgF	ZONMW-01_02065	yhgF
yhgF	ZONMW-02_00192	yhgF
yhgF	ZONMW-02_01383	yhgF
yhgF	ZONMW-03_00917	yhgF
yhgF	ZONMW-03_02646	yhgF
yhgF	ZONMW-04_00730	yhgF
yhgF	ZONMW-04_01908	yhgF
yhgF	ZONMW-05_01104	yhgF
yhgF	ZONMW-05_02462	yhgF
yhgF	ZONMW-06_01835	yhgF
yhgF	ZONMW-06_02607	yhgF
yhgF	ZONMW-06_00523	yhgF
yhgF	ZONMW-07_00043	yhgF
yhgF	ZONMW-07_03582	yhgF
yhgF	ZONMW-08_01721	yhgF
yhgF	ZONMW-08_02974	yhgF
yhgF	ZONMW-09_00855	yhgF
yhgF	ZONMW-09_02268	yhgF
yhgF	ZONMW-10_00364	yhgF
yhgF	ZONMW-10_01881	yhgF
yhgF	ZONMW-11_00709	yhgF
yhgF	ZONMW-11_01187	yhgF
yhgF	ZONMW-12_01504	yhgF
yhgF	ZONMW-12_03471	yhgF
yhgF	ZONMW-13_03054	yhgF
yhgF	ZONMW-14_00450	yhgF
yhgF	ZONMW-15_00238	yhgF
yhgF	ZONMW-15_01540	yhgF
yhgF	ZONMW-19_01172	yhgF
yhgF	ZONMW-19_00113	yhgF
yhgF	ZONMW-19_02919	yhgF
yhgF	ZONMW-20_03383	yhgF
yhgF	ZONMW-20_03781	yhgF
yhgF	ZONMW-20_01323	yhgF
yhgF	ZONMW-21_00468	yhgF
yhgF	ZONMW-21_03231	yhgF
yhgF	ZONMW-22_00468	yhgF
yhgF	ZONMW-22_03285	yhgF
yhgF	ZONMW-23_01583	yhgF
yhgF	ZONMW-23_03306	yhgF
yhgF	ZONMW-24_00991	yhgF
yhgF	ZONMW-24_03308	yhgF
yhgF	ZONMW-25_02132	yhgF
yhgF	ZONMW-25_01264	yhgF
yhgF	ZONMW-26_01150	yhgF
yhgF	ZONMW-26_02654	yhgF

yhgF	ZONMW-27_00721	yhgF
yhgF	ZONMW-27_02079	yhgF
yhgF	ZONMW-28_02747	yhgF
yhgF	ZONMW-28_00026	yhgF
yhgF	ZONMW-28_00905	yhgF
yhgF	ZONMW-29_00129	yhgF
yhgF	ZONMW-29_01757	yhgF
yhgF	ZONMW-30_01594	yhgF
yhgF	ZONMW-30_02489	yhgF
yhgF	ZONMW-31_02011	yhgF
yhgF	ZONMW-31_02694	yhgF
yhgF	ZONMW-31_00874	yhgF
yhgF	ZONMW-32_03128	yhgF
yhgF	ZONMW-32_00817	yhgF
yhgF	ZONMW-32_00186	yhgF
yhgF	ZONMW-33_03145	yhgF
yhgF	ZONMW-33_00807	yhgF
yhgF	ZONMW-33_00053	yhgF
yhgF	ZONMW-34_02511	yhgF
yhgF	ZONMW-34_02708	yhgF
yhgF	ZONMW-34_00449	yhgF
yhgF	ZONMW-35_02475	yhgF
yhgF	ZONMW-35_00085	yhgF
yhgF	ZONMW-35_00941	yhgF
yhgF	ZONMW-36_02856	yhgF
yhgF	ZONMW-36_00026	yhgF
yhgF	ZONMW-36_01316	yhgF
yhgF	ZONMW-37_00468	yhgF
yhgF	ZONMW-37_03294	yhgF
yhgF	ZONMW-38_04078	yhgF
yhgF	ZONMW-38_02973	yhgF
yhgF	ZONMW-38_00479	yhgF
yhgF	ZONMW-38_02433	yhgF
yhgF	ZONMW-38_01382	yhgF
yhgF	ZONMW-39_02052	yhgF
yhgF	ZONMW-39_03488	yhgF
yhgF	ZONMW-39_04181	yhgF
yhgF	ZONMW-40_01459	yhgF
yhgF	ZONMW-40_02869	yhgF
yhgF	ZONMW-41_00132	yhgF
yhgF	ZONMW-41_01612	yhgF
yhgF	ZONMW-41_00456	yhgF
yhgF	ZONMW-42_01164	yhgF
yhgF	ZONMW-42_02576	yhgF
yhgF	ZONMW-42_04165	yhgF
yhgF	ZONMW-43_01138	yhgF
yhgF	ZONMW-43_02476	yhgF
yhgF	ZONMW-43_04351	yhgF

yhgF	ZONMW-44_00185	yhgF
yhgF	ZONMW-44_03918	yhgF
yhgF	ZONMW-44_01681	yhgF
yhgF	ZONMW-45_02111	yhgF
yhgF	ZONMW-45_01898	yhgF
yhgF	ZONMW-46_02171	yhgF
yhgF	ZONMW-46_01998	yhgF
yhgF	ZONMW-47_02361	yhgF
yhgF	ZONMW-47_02063	yhgF
yhgF	ZONMW-48_00884	yhgF
yhgF	ZONMW-48_01913	yhgF
yhgF	ZONMW-49_01297	yhgF
yhgF	ZONMW-49_02499	yhgF
yhgF	ZONMW-50_01299	yhgF
yhgF	ZONMW-50_02293	yhgF
yhgF	ZONMW-51_01412	yhgF
yhgF	ZONMW-51_02476	yhgF
yhgF	ZONMW-52_01154	yhgF
yhgF	ZONMW-52_02459	yhgF
yhgF	ZONMW-53_00697	yhgF
yhgF	ZONMW-53_02174	yhgF
yhgF	ZONMW-54_00364	yhgF
yhgF	ZONMW-54_02664	yhgF
yhgF	ZONMW-55_00668	yhgF
yhgF	ZONMW-55_02567	yhgF
yhgF	ZONMW-56_03183	yhgF
yhgF	ZONMW-56_01662	yhgF
yhgF	ZONMW-57_00947	yhgF
yhgF	ZONMW-57_02009	yhgF
yhgF	ZONMW-58_01637	yhgF
yhgF	ZONMW-58_02069	yhgF
yhgF	ZONMW-59_01924	yhgF
yhgF	ZONMW-59_02288	yhgF
yhgF	ZONMW-60_01611	yhgF
yhgF	ZONMW-60_02584	yhgF
yhgF	ZONMW-61_00602	yhgF
yhgF	ZONMW-61_02729	yhgF
yhgF	ZONMW-62_02534	yhgF
yhgF	ZONMW-62_03416	yhgF
yhgF	ZONMW-63_00706	yhgF
yhgF	ZONMW-63_01674	yhgF
yhgF	ZONMW-64_03488	yhgF
yhgF	ZONMW-64_02453	yhgF
yhgF	ZONMW-64_00014	yhgF
yhgF	ZONMW-65_02242	yhgF
yhgF	ZONMW-65_00587	yhgF
yhgF	ZONMW-66_00512	yhgF
yhgF	ZONMW-66_00611	yhgF

yhgF	ZONMW-66_00868	yhgF
yhgF	ZONMW-67_03408	yhgF
yhgF	ZONMW-67_01852	yhgF
yhgF	ZONMW-67_00634	yhgF
yhgF	ZONMW-68_00398	yhgF
yhgF	ZONMW-68_02134	yhgF
yhgF	ZONMW-69_03409	yhgF
yhgF	ZONMW-69_02091	yhgF
yhgF	ZONMW-69_00681	yhgF
yhgF	ZONMW-72_03275	yhgF
yhgF	ZONMW-72_03021	yhgF
yhgF	ZONMW-73_01806	yhgF
yhgF	ZONMW-73_02388	yhgF
yhgF	ZONMW-74_01928	yhgF
yhgF	ZONMW-74_02662	yhgF
yhgF	ZONMW-75_01058	yhgF
yhgF	ZONMW-75_03065	yhgF
yhgF	da5A-8_02404	yhgF
yhgF	da5A-8_03090	yhgF
ychF	18_00256	ychF
ychF	18_03515	ychF
ychF	ACAM48_00280	ychF
ychF	ACAM48_01009	ychF
ychF	CAM030_02637	ychF
ychF	CAM030_00554	ychF
ychF	CECT7946_03379	ychF
ychF	CECT7946_01405	ychF
ychF	CGMCC1.8863_01390	ychF
ychF	CGMCC1.8863_02322	ychF
ychF	CL8139_01482	ychF
ychF	CL8139_01890	ychF
ychF	DAU203_02237	ychF
ychF	DAU203_03201	ychF
ychF	DD5b_02448	ychF
ychF	DD5b_01256	ychF
ychF	DSM1208_02393	ychF
ychF	DSM1208_03964	ychF
ychF	DSM12212_01052	ychF
ychF	DSM12212_01322	ychF
ychF	DSM15343_01540	ychF
ychF	DSM15343_01463	ychF
ychF	DSM15361_02616	ychF
ychF	DSM15361_00991	ychF
ychF	DSM15365_02292	ychF
ychF	DSM15365_01927	ychF
ychF	DSM15718_00621	ychF
ychF	DSM15718_01999	ychF
ychF	DSM16505T_00009	ychF

ychF	DSM16505T_01434	ychF
ychF	DSM18103_01554	ychF
ychF	DSM18103_00853	ychF
ychF	DSM18293_02392	ychF
ychF	DSM18293_03906	ychF
ychF	DSM18841_02481	ychF
ychF	DSM18841_02452	ychF
ychF	DSM18842_02388	ychF
ychF	DSM18842_01364	ychF
ychF	DSM19866_01050	ychF
ychF	DSM19866_04196	ychF
ychF	DSM2041_03034	ychF
ychF	DSM2041_02049	ychF
ychF	DSM2061_03904	ychF
ychF	DSM2061_01076	ychF
ychF	DSM2064_04663	ychF
ychF	DSM2064_03220	ychF
ychF	DSM21164_02255	ychF
ychF	DSM21164_02692	ychF
ychF	DSM23404_00511	ychF
ychF	DSM23404_02870	ychF
ychF	DSM23424_02912	ychF
ychF	DSM23424_01282	ychF
ychF	DSM24786_01795	ychF
ychF	DSM24786_00652	ychF
ychF	DSM7489_02150	ychF
ychF	DSM7489_03033	ychF
ychF	DSW1_01221	ychF
ychF	DSW1_01811	ychF
ychF	DSW5_00546	ychF
ychF	DSW5_02100	ychF
ychF	DSW6_01971	ychF
ychF	DSW6_02616	ychF
ychF	DSW8_01578	ychF
ychF	DSW8_00137	ychF
ychF	F52_01485	ychF
ychF	F52_00449	ychF
ychF	GCA_001027945.1_01098	ychF
ychF	GCA_001027945.1_01915	ychF
ychF	HI1_02076	ychF
ychF	HI1_02974	ychF
ychF	IR1_03091	ychF
ychF	IR1_02426	ychF
ychF	KCOM3131_00075	ychF
ychF	KCOM3131_00985	ychF
ychF	KCTC12974_01098	ychF
ychF	KCTC12974_02120	ychF
ychF	KL-A_00121	ychF

ychF	KL-A_02993	ychF
ychF	L12_00185	ychF
ychF	LL0412.1.7_00009	ychF
ychF	LL0412.1.7_02733	ychF
ychF	MAR_2009_71_01847	ychF
ychF	MAR_2009_71_02938	ychF
ychF	NN016038_00266	ychF
ychF	NN016038_03555	ychF
ychF	PAO1_04714	ychF
ychF	PAO1_04827	ychF
ychF	UW101_00439	ychF
ychF	UW101_02580	ychF
ychF	V4-33_01265	ychF
ychF	V4-33_00616	ychF
ychF	W5C_02909	ychF
ychF	W5C_02242	ychF
ychF	ZONMW-01_01436	ychF
ychF	ZONMW-01_00739	ychF
ychF	ZONMW-02_02380	ychF
ychF	ZONMW-02_02578	ychF
ychF	ZONMW-03_01344	ychF
ychF	ZONMW-03_00117	ychF
ychF	ZONMW-04_00349	ychF
ychF	ZONMW-04_01121	ychF
ychF	ZONMW-05_01476	ychF
ychF	ZONMW-05_00372	ychF
ychF	ZONMW-06_03951	ychF
ychF	ZONMW-06_02953	ychF
ychF	ZONMW-07_02016	ychF
ychF	ZONMW-07_02390	ychF
ychF	ZONMW-08_01377	ychF
ychF	ZONMW-08_02137	ychF
ychF	ZONMW-09_01376	ychF
ychF	ZONMW-09_00755	ychF
ychF	ZONMW-10_01360	ychF
ychF	ZONMW-10_02285	ychF
ychF	ZONMW-11_02174	ychF
ychF	ZONMW-11_00098	ychF
ychF	ZONMW-12_02695	ychF
ychF	ZONMW-12_02269	ychF
ychF	ZONMW-13_01852	ychF
ychF	ZONMW-13_01932	ychF
ychF	ZONMW-14_01084	ychF
ychF	ZONMW-14_00760	ychF
ychF	ZONMW-15_00812	ychF
ychF	ZONMW-15_02275	ychF
ychF	ZONMW-19_00608	ychF
ychF	ZONMW-19_01925	ychF

ychF	ZONMW-20_02867	ychF
ychF	ZONMW-20_01746	ychF
ychF	ZONMW-21_02499	ychF
ychF	ZONMW-21_02021	ychF
ychF	ZONMW-22_02610	ychF
ychF	ZONMW-22_01705	ychF
ychF	ZONMW-23_02634	ychF
ychF	ZONMW-23_00847	ychF
ychF	ZONMW-24_02523	ychF
ychF	ZONMW-24_01728	ychF
ychF	ZONMW-25_02395	ychF
ychF	ZONMW-25_01043	ychF
ychF	ZONMW-26_01793	ychF
ychF	ZONMW-26_00593	ychF
ychF	ZONMW-27_00343	ychF
ychF	ZONMW-27_01198	ychF
ychF	ZONMW-28_03927	ychF
ychF	ZONMW-28_03762	ychF
ychF	ZONMW-29_02742	ychF
ychF	ZONMW-29_01289	ychF
ychF	ZONMW-30_00050	ychF
ychF	ZONMW-30_01359	ychF
ychF	ZONMW-31_03211	ychF
ychF	ZONMW-31_03059	ychF
ychF	ZONMW-32_03959	ychF
ychF	ZONMW-32_03903	ychF
ychF	ZONMW-33_03883	ychF
ychF	ZONMW-33_03708	ychF
ychF	ZONMW-34_03825	ychF
ychF	ZONMW-34_02057	ychF
ychF	ZONMW-35_03800	ychF
ychF	ZONMW-35_01749	ychF
ychF	ZONMW-36_03922	ychF
ychF	ZONMW-36_03657	ychF
ychF	ZONMW-37_02769	ychF
ychF	ZONMW-37_01728	ychF
ychF	ZONMW-38_02312	ychF
ychF	ZONMW-38_02795	ychF
ychF	ZONMW-39_04073	ychF
ychF	ZONMW-39_02134	ychF
ychF	ZONMW-40_02135	ychF
ychF	ZONMW-40_03016	ychF
ychF	ZONMW-41_02674	ychF
ychF	ZONMW-41_02890	ychF
ychF	ZONMW-42_02352	ychF
ychF	ZONMW-42_00370	ychF
ychF	ZONMW-43_03778	ychF
ychF	ZONMW-43_01561	ychF

ychF	ZONMW-44_03145	ychF
ychF	ZONMW-44_00930	ychF
ychF	ZONMW-45_02674	ychF
ychF	ZONMW-45_01864	ychF
ychF	ZONMW-46_02537	ychF
ychF	ZONMW-46_01964	ychF
ychF	ZONMW-47_02527	ychF
ychF	ZONMW-47_02097	ychF
ychF	ZONMW-48_01993	ychF
ychF	ZONMW-48_01334	ychF
ychF	ZONMW-49_00762	ychF
ychF	ZONMW-49_01715	ychF
ychF	ZONMW-50_00763	ychF
ychF	ZONMW-50_01699	ychF
ychF	ZONMW-51_00946	ychF
ychF	ZONMW-51_00613	ychF
ychF	ZONMW-52_01546	ychF
ychF	ZONMW-52_00570	ychF
ychF	ZONMW-53_00281	ychF
ychF	ZONMW-53_01325	ychF
ychF	ZONMW-54_00750	ychF
ychF	ZONMW-54_01392	ychF
ychF	ZONMW-55_00274	ychF
ychF	ZONMW-55_00911	ychF
ychF	ZONMW-56_01043	ychF
ychF	ZONMW-56_00147	ychF
ychF	ZONMW-57_01343	ychF
ychF	ZONMW-57_00360	ychF
ychF	ZONMW-58_01241	ychF
ychF	ZONMW-58_00611	ychF
ychF	ZONMW-59_01344	ychF
ychF	ZONMW-59_00376	ychF
ychF	ZONMW-60_01231	ychF
ychF	ZONMW-60_00387	ychF
ychF	ZONMW-61_03008	ychF
ychF	ZONMW-61_02499	ychF
ychF	ZONMW-62_02226	ychF
ychF	ZONMW-62_00628	ychF
ychF	ZONMW-63_01222	ychF
ychF	ZONMW-63_00028	ychF
ychF	ZONMW-64_04098	ychF
ychF	ZONMW-64_02138	ychF
ychF	ZONMW-65_01219	ychF
ychF	ZONMW-65_03002	ychF
ychF	ZONMW-66_03412	ychF
ychF	ZONMW-66_03553	ychF
ychF	ZONMW-67_03602	ychF
ychF	ZONMW-67_02452	ychF

ychF	ZONMW-68_04654	ychF
ychF	ZONMW-68_02930	ychF
ychF	ZONMW-69_03604	ychF
ychF	ZONMW-69_02500	ychF
ychF	ZONMW-72_01129	ychF
ychF	ZONMW-72_02959	ychF
ychF	ZONMW-73_03046	ychF
ychF	ZONMW-73_00956	ychF
ychF	ZONMW-74_01049	ychF
ychF	ZONMW-74_00259	ychF
ychF	ZONMW-75_00154	ychF
ychF	ZONMW-75_01985	ychF
ychF	da5A-8_00215	ychF
ychF	da5A-8_01303	ychF
parE	18_00088	parE
parE	18_03513	parE
parE	ACAM48_00389	parE
parE	ACAM48_00684	parE
parE	CAM030_02872	parE
parE	CAM030_00556	parE
parE	CECT7946_03255	parE
parE	CECT7946_01403	parE
parE	CGMCC1.8863_02844	parE
parE	CGMCC1.8863_02321	parE
parE	CL8139_02063	parE
parE	CL8139_01889	parE
parE	DAU203_02412	parE
parE	DAU203_03200	parE
parE	DD5b_00743	parE
parE	DD5b_01195	parE
parE	DSM1208_02651	parE
parE	DSM1208_04367	parE
parE	DSM12212_01159	parE
parE	DSM12212_01323	parE
parE	DSM15343_02896	parE
parE	DSM15343_01465	parE
parE	DSM15361_02538	parE
parE	DSM15361_00989	parE
parE	DSM15365_00181	parE
parE	DSM15365_01925	parE
parE	DSM15718_01175	parE
parE	DSM15718_01997	parE
parE	DSM16505T_01108	parE
parE	DSM16505T_01722	parE
parE	DSM18103_01326	parE
parE	DSM18103_02367	parE
parE	DSM18293_03697	parE
parE	DSM18293_03913	parE

parE	DSM18841_01005	parE
parE	DSM18841_01812	parE
parE	DSM18842_00313	parE
parE	DSM18842_02107	parE
parE	DSM19866_00449	parE
parE	DSM19866_04192	parE
parE	DSM2041_04980	parE
parE	DSM2041_02038	parE
parE	DSM2041_05584	parE
parE	DSM2061_04133	parE
parE	DSM2061_00768	parE
parE	DSM2064_03436	parE
parE	DSM2064_03213	parE
parE	DSM21164_02113	parE
parE	DSM21164_02690	parE
parE	DSM23404_00404	parE
parE	DSM23404_02638	parE
parE	DSM23424_02822	parE
parE	DSM23424_01280	parE
parE	DSM24786_01599	parE
parE	DSM24786_00651	parE
parE	DSM7489_02327	parE
parE	DSM7489_03032	parE
parE	DSW1_01390	parE
parE	DSW1_01952	parE
parE	DSW5_00540	parE
parE	DSW5_02111	parE
parE	DSW6_00405	parE
parE	DSW6_02888	parE
parE	DSW8_01343	parE
parE	DSW8_00141	parE
parE	F52_00236	parE
parE	F52_00456	parE
parE	GCA_001027945.1_01421	parE
parE	GCA_001027945.1_02146	parE
parE	HI1_02260	parE
parE	HI1_02973	parE
parE	IR1_02624	parE
parE	IR1_02420	parE
parE	KCOM3131_01295	parE
parE	KCOM3131_00443	parE
parE	KCTC12974_00588	parE
parE	KCTC12974_02981	parE
parE	KL-A_01607	parE
parE	KL-A_02994	parE
parE	L12_00323	parE
parE	LL0412.1.7_01127	parE
parE	LL0412.1.7_02339	parE

parE	MAR_2009_71_02108	parE
parE	MAR_2009_71_02942	parE
parE	NN016038_00094	parE
parE	NN016038_03553	parE
parE	PAO1_00004	parE
parE	PAO1_05136	parE
parE	UW101_02364	parE
parE	UW101_02587	parE
parE	V4-33_00505	parE
parE	V4-33_00618	parE
parE	W5C_02661	parE
parE	W5C_02241	parE
parE	ZONMW-01_01245	parE
parE	ZONMW-01_00740	parE
parE	ZONMW-02_03731	parE
parE	ZONMW-02_02580	parE
parE	ZONMW-03_01535	parE
parE	ZONMW-03_00118	parE
parE	ZONMW-04_00155	parE
parE	ZONMW-04_01122	parE
parE	ZONMW-05_01673	parE
parE	ZONMW-05_00373	parE
parE	ZONMW-06_01270	parE
parE	ZONMW-06_00901	parE
parE	ZONMW-07_02172	parE
parE	ZONMW-07_02348	parE
parE	ZONMW-08_01150	parE
parE	ZONMW-08_02141	parE
parE	ZONMW-09_01298	parE
parE	ZONMW-09_00754	parE
parE	ZONMW-10_00992	parE
parE	ZONMW-10_02286	parE
parE	ZONMW-11_02390	parE
parE	ZONMW-11_00955	parE
parE	ZONMW-12_01339	parE
parE	ZONMW-12_02268	parE
parE	ZONMW-13_01734	parE
parE	ZONMW-13_01933	parE
parE	ZONMW-14_00978	parE
parE	ZONMW-14_00759	parE
parE	ZONMW-15_00704	parE
parE	ZONMW-15_02273	parE
parE	ZONMW-19_03326	parE
parE	ZONMW-19_00335	parE
parE	ZONMW-20_00680	parE
parE	ZONMW-20_00064	parE
parE	ZONMW-21_02348	parE
parE	ZONMW-21_02019	parE

parE	ZONMW-22_02760	parE
parE	ZONMW-22_01707	parE
parE	ZONMW-23_02784	parE
parE	ZONMW-23_00845	parE
parE	ZONMW-24_02372	parE
parE	ZONMW-24_01730	parE
parE	ZONMW-25_02644	parE
parE	ZONMW-25_01041	parE
parE	ZONMW-26_01608	parE
parE	ZONMW-26_00592	parE
parE	ZONMW-27_00152	parE
parE	ZONMW-27_01199	parE
parE	ZONMW-28_01740	parE
parE	ZONMW-28_00622	parE
parE	ZONMW-29_03267	parE
parE	ZONMW-29_01288	parE
parE	ZONMW-30_00261	parE
parE	ZONMW-30_01357	parE
parE	ZONMW-31_00041	parE
parE	ZONMW-31_01162	parE
parE	ZONMW-32_02232	parE
parE	ZONMW-32_00469	parE
parE	ZONMW-33_02223	parE
parE	ZONMW-33_00336	parE
parE	ZONMW-34_02376	parE
parE	ZONMW-34_00714	parE
parE	ZONMW-35_01873	parE
parE	ZONMW-35_02748	parE
parE	ZONMW-36_02013	parE
parE	ZONMW-36_01033	parE
parE	ZONMW-37_02618	parE
parE	ZONMW-37_01730	parE
parE	ZONMW-38_02734	parE
parE	ZONMW-38_02148	parE
parE	ZONMW-39_01668	parE
parE	ZONMW-39_01176	parE
parE	ZONMW-40_03121	parE
parE	ZONMW-40_03014	parE
parE	ZONMW-41_00400	parE
parE	ZONMW-41_02119	parE
parE	ZONMW-42_02759	parE
parE	ZONMW-42_01665	parE
parE	ZONMW-43_01856	parE
parE	ZONMW-43_00254	parE
parE	ZONMW-43_02192	parE
parE	ZONMW-44_02459	parE
parE	ZONMW-44_02098	parE
parE	ZONMW-45_02138	parE

parE	ZONMW-45_00240	parE
parE	ZONMW-46_01814	parE
parE	ZONMW-46_00455	parE
parE	ZONMW-47_01675	parE
parE	ZONMW-47_00050	parE
parE	ZONMW-48_02098	parE
parE	ZONMW-48_01336	parE
parE	ZONMW-49_00583	parE
parE	ZONMW-49_01714	parE
parE	ZONMW-50_00584	parE
parE	ZONMW-50_01700	parE
parE	ZONMW-51_01122	parE
parE	ZONMW-51_00612	parE
parE	ZONMW-52_02815	parE
parE	ZONMW-52_00569	parE
parE	ZONMW-53_00100	parE
parE	ZONMW-53_01324	parE
parE	ZONMW-54_00927	parE
parE	ZONMW-54_01393	parE
parE	ZONMW-55_00098	parE
parE	ZONMW-55_00912	parE
parE	ZONMW-56_02709	parE
parE	ZONMW-56_00148	parE
parE	ZONMW-57_01515	parE
parE	ZONMW-57_00361	parE
parE	ZONMW-58_01069	parE
parE	ZONMW-58_00610	parE
parE	ZONMW-59_02817	parE
parE	ZONMW-59_00377	parE
parE	ZONMW-60_01043	parE
parE	ZONMW-60_00388	parE
parE	ZONMW-61_01166	parE
parE	ZONMW-61_02502	parE
parE	ZONMW-62_02918	parE
parE	ZONMW-62_00625	parE
parE	ZONMW-63_01043	parE
parE	ZONMW-63_00029	parE
parE	ZONMW-64_01623	parE
parE	ZONMW-64_00270	parE
parE	ZONMW-65_02372	parE
parE	ZONMW-65_00404	parE
parE	ZONMW-66_02363	parE
parE	ZONMW-66_01030	parE
parE	ZONMW-67_00141	parE
parE	ZONMW-67_00936	parE
parE	ZONMW-68_03351	parE
parE	ZONMW-68_02926	parE
parE	ZONMW-69_02750	parE

parE	ZONMW-69_00983	parE
parE	ZONMW-72_03372	parE
parE	ZONMW-72_02953	parE
parE	ZONMW-73_02924	parE
parE	ZONMW-73_00954	parE
parE	ZONMW-74_00860	parE
parE	ZONMW-74_00257	parE
parE	ZONMW-75_02820	parE
parE	ZONMW-75_01987	parE
parE	da5A-8_01077	parE
parE	da5A-8_02558	parE
pyrB	18_00542	pyrB
pyrB	18_02321	pyrB
pyrB	ACAM48_03545	pyrB
pyrB	ACAM48_01002	pyrB
pyrB	CAM030_01103	pyrB
pyrB	CAM030_02066	pyrB
pyrB	CECT7946_00665	pyrB
pyrB	CECT7946_03524	pyrB
pyrB	CGMCC1.8863_01206	pyrB
pyrB	CGMCC1.8863_03320	pyrB
pyrB	CL8139_02476	pyrB
pyrB	CL8139_01243	pyrB
pyrB	DAU203_00908	pyrB
pyrB	DAU203_00182	pyrB
pyrB	DD5b_01027	pyrB
pyrB	DSM1208_01193	pyrB
pyrB	DSM1208_00485	pyrB
pyrB	DSM12212_00782	pyrB
pyrB	DSM15343_01483	pyrB
pyrB	DSM15361_01641	pyrB
pyrB	DSM15365_01566	pyrB
pyrB	DSM15365_00152	pyrB
pyrB	DSM15718_01560	pyrB
pyrB	DSM15718_01546	pyrB
pyrB	DSM16505T_01622	pyrB
pyrB	DSM16505T_01860	pyrB
pyrB	DSM18103_00933	pyrB
pyrB	DSM18103_03708	pyrB
pyrB	DSM18293_04173	pyrB
pyrB	DSM18293_01009	pyrB
pyrB	DSM18841_02990	pyrB
pyrB	DSM18841_01595	pyrB
pyrB	DSM18842_02003	pyrB
pyrB	DSM18842_02279	pyrB
pyrB	DSM19866_03724	pyrB
pyrB	DSM19866_03213	pyrB
pyrB	DSM2041_02373	pyrB

pyrB	DSM2041_01482	pyrB
pyrB	DSM2061_02706	pyrB
pyrB	DSM2061_01975	pyrB
pyrB	DSM2064_00865	pyrB
pyrB	DSM2064_04152	pyrB
pyrB	DSM21164_01288	pyrB
pyrB	DSM21164_01841	pyrB
pyrB	DSM23404_01488	pyrB
pyrB	DSM23404_02879	pyrB
pyrB	DSM23424_03025	pyrB
pyrB	DSM23424_00971	pyrB
pyrB	DSM24786_01282	pyrB
pyrB	DSM24786_00063	pyrB
pyrB	DSM7489_00842	pyrB
pyrB	DSM7489_00176	pyrB
pyrB	DSW1_01160	pyrB
pyrB	DSW1_02268	pyrB
pyrB	DSW5_02336	pyrB
pyrB	DSW5_00341	pyrB
pyrB	DSW6_03204	pyrB
pyrB	DSW8_02746	pyrB
pyrB	DSW8_03517	pyrB
pyrB	F52_00797	pyrB
pyrB	F52_04300	pyrB
pyrB	GCA_001027945.1_02072	pyrB
pyrB	GCA_001027945.1_02863	pyrB
pyrB	HI1_00777	pyrB
pyrB	HI1_00108	pyrB
pyrB	IR1_02057	pyrB
pyrB	IR1_03771	pyrB
pyrB	KCOM3131_00453	pyrB
pyrB	KCTC12974_00193	pyrB
pyrB	KCTC12974_02108	pyrB
pyrB	KL-A_03215	pyrB
pyrB	KL-A_01172	pyrB
pyrB	L12_00098	pyrB
pyrB	LL0412.1.7_02012	pyrB
pyrB	LL0412.1.7_02624	pyrB
pyrB	MAR_2009_71_00707	pyrB
pyrB	MAR_2009_71_00069	pyrB
pyrB	NN016038_00551	pyrB
pyrB	NN016038_02332	pyrB
pyrB	PAO1_03659	pyrB
pyrB	PAO1_05346	pyrB
pyrB	PAO1_00413	pyrB
pyrB	UW101_03576	pyrB
pyrB	UW101_04734	pyrB
pyrB	V4-33_01703	pyrB

pyrB	W5C_01468	pyrB
pyrB	W5C_00085	pyrB
pyrB	ZONMW-01_03418	pyrB
pyrB	ZONMW-01_02063	pyrB
pyrB	ZONMW-02_02025	pyrB
pyrB	ZONMW-02_00766	pyrB
pyrB	ZONMW-03_01761	pyrB
pyrB	ZONMW-03_02648	pyrB
pyrB	ZONMW-04_02231	pyrB
pyrB	ZONMW-04_01906	pyrB
pyrB	ZONMW-05_02748	pyrB
pyrB	ZONMW-05_02464	pyrB
pyrB	ZONMW-06_01552	pyrB
pyrB	ZONMW-06_02908	pyrB
pyrB	ZONMW-06_03741	pyrB
pyrB	ZONMW-07_00561	pyrB
pyrB	ZONMW-07_03584	pyrB
pyrB	ZONMW-08_00223	pyrB
pyrB	ZONMW-08_02982	pyrB
pyrB	ZONMW-09_02632	pyrB
pyrB	ZONMW-09_02669	pyrB
pyrB	ZONMW-10_00696	pyrB
pyrB	ZONMW-11_02959	pyrB
pyrB	ZONMW-11_02053	pyrB
pyrB	ZONMW-12_03323	pyrB
pyrB	ZONMW-12_02918	pyrB
pyrB	ZONMW-13_01285	pyrB
pyrB	ZONMW-13_03047	pyrB
pyrB	ZONMW-14_01873	pyrB
pyrB	ZONMW-14_00455	pyrB
pyrB	ZONMW-15_00992	pyrB
pyrB	ZONMW-15_01538	pyrB
pyrB	ZONMW-19_01272	pyrB
pyrB	ZONMW-19_02615	pyrB
pyrB	ZONMW-20_03253	pyrB
pyrB	ZONMW-20_03242	pyrB
pyrB	ZONMW-20_03239	pyrB
pyrB	ZONMW-21_01280	pyrB
pyrB	ZONMW-21_03229	pyrB
pyrB	ZONMW-22_01282	pyrB
pyrB	ZONMW-22_03287	pyrB
pyrB	ZONMW-23_02394	pyrB
pyrB	ZONMW-23_03308	pyrB
pyrB	ZONMW-24_00179	pyrB
pyrB	ZONMW-24_03310	pyrB
pyrB	ZONMW-25_02420	pyrB
pyrB	ZONMW-25_01262	pyrB
pyrB	ZONMW-26_02088	pyrB

pyrB	ZONMW-26_02652	pyrB
pyrB	ZONMW-27_02210	pyrB
pyrB	ZONMW-27_02077	pyrB
pyrB	ZONMW-28_01999	pyrB
pyrB	ZONMW-28_02368	pyrB
pyrB	ZONMW-28_01902	pyrB
pyrB	ZONMW-29_00657	pyrB
pyrB	ZONMW-29_01759	pyrB
pyrB	ZONMW-30_00566	pyrB
pyrB	ZONMW-30_02491	pyrB
pyrB	ZONMW-31_03015	pyrB
pyrB	ZONMW-31_00250	pyrB
pyrB	ZONMW-31_00983	pyrB
pyrB	ZONMW-32_02405	pyrB
pyrB	ZONMW-32_02982	pyrB
pyrB	ZONMW-32_02070	pyrB
pyrB	ZONMW-33_02713	pyrB
pyrB	ZONMW-33_02766	pyrB
pyrB	ZONMW-33_02061	pyrB
pyrB	ZONMW-34_02007	pyrB
pyrB	ZONMW-34_02179	pyrB
pyrB	ZONMW-35_01699	pyrB
pyrB	ZONMW-35_02070	pyrB
pyrB	ZONMW-36_02539	pyrB
pyrB	ZONMW-36_02278	pyrB
pyrB	ZONMW-36_02175	pyrB
pyrB	ZONMW-37_01082	pyrB
pyrB	ZONMW-37_03296	pyrB
pyrB	ZONMW-38_00994	pyrB
pyrB	ZONMW-38_03483	pyrB
pyrB	ZONMW-38_01631	pyrB
pyrB	ZONMW-38_03732	pyrB
pyrB	ZONMW-39_02185	pyrB
pyrB	ZONMW-39_01459	pyrB
pyrB	ZONMW-40_03426	pyrB
pyrB	ZONMW-40_02871	pyrB
pyrB	ZONMW-41_02604	pyrB
pyrB	ZONMW-41_02808	pyrB
pyrB	ZONMW-42_00411	pyrB
pyrB	ZONMW-42_01345	pyrB
pyrB	ZONMW-42_00287	pyrB
pyrB	ZONMW-43_01511	pyrB
pyrB	ZONMW-43_03253	pyrB
pyrB	ZONMW-44_00536	pyrB
pyrB	ZONMW-44_00886	pyrB
pyrB	ZONMW-44_03068	pyrB
pyrB	ZONMW-45_02265	pyrB
pyrB	ZONMW-45_00863	pyrB

pyrB	ZONMW-46_03150	pyrB
pyrB	ZONMW-46_01011	pyrB
pyrB	ZONMW-47_03142	pyrB
pyrB	ZONMW-47_01162	pyrB
pyrB	ZONMW-48_00408	pyrB
pyrB	ZONMW-48_01911	pyrB
pyrB	ZONMW-49_02258	pyrB
pyrB	ZONMW-49_02501	pyrB
pyrB	ZONMW-50_02202	pyrB
pyrB	ZONMW-50_02295	pyrB
pyrB	ZONMW-51_01698	pyrB
pyrB	ZONMW-51_02478	pyrB
pyrB	ZONMW-52_01841	pyrB
pyrB	ZONMW-52_02461	pyrB
pyrB	ZONMW-53_01915	pyrB
pyrB	ZONMW-53_02172	pyrB
pyrB	ZONMW-54_02139	pyrB
pyrB	ZONMW-54_02662	pyrB
pyrB	ZONMW-55_02020	pyrB
pyrB	ZONMW-55_02569	pyrB
pyrB	ZONMW-56_01416	pyrB
pyrB	ZONMW-56_01660	pyrB
pyrB	ZONMW-57_01811	pyrB
pyrB	ZONMW-57_02007	pyrB
pyrB	ZONMW-58_01835	pyrB
pyrB	ZONMW-58_02071	pyrB
pyrB	ZONMW-59_01647	pyrB
pyrB	ZONMW-59_02290	pyrB
pyrB	ZONMW-60_02028	pyrB
pyrB	ZONMW-60_02586	pyrB
pyrB	ZONMW-61_01610	pyrB
pyrB	ZONMW-61_02721	pyrB
pyrB	ZONMW-62_01350	pyrB
pyrB	ZONMW-62_03408	pyrB
pyrB	ZONMW-63_01546	pyrB
pyrB	ZONMW-63_01676	pyrB
pyrB	ZONMW-64_02088	pyrB
pyrB	ZONMW-64_01381	pyrB
pyrB	ZONMW-65_01434	pyrB
pyrB	ZONMW-65_01473	pyrB
pyrB	ZONMW-65_00649	pyrB
pyrB	ZONMW-66_00341	pyrB
pyrB	ZONMW-66_00004	pyrB
pyrB	ZONMW-67_02407	pyrB
pyrB	ZONMW-67_00342	pyrB
pyrB	ZONMW-68_00846	pyrB
pyrB	ZONMW-68_02132	pyrB
pyrB	ZONMW-69_02455	pyrB

pyrB	ZONMW-69_00089	pyrB
pyrB	ZONMW-72_03218	pyrB
pyrB	ZONMW-72_00772	pyrB
pyrB	ZONMW-73_03328	pyrB
pyrB	ZONMW-73_03366	pyrB
pyrB	ZONMW-74_03319	pyrB
pyrB	ZONMW-74_02664	pyrB
pyrB	ZONMW-75_00531	pyrB
pyrB	ZONMW-75_01415	pyrB
pyrB	da5A-8_01386	pyrB
pyrB	da5A-8_03636	pyrB
tqsA	18_02450	tqsA
tqsA	ACAM48_00796	tqsA
tqsA	ACAM48_01825	tqsA
tqsA	CAM030_00744	tqsA
tqsA	CAM030_01784	tqsA
tqsA	CECT7946_01472	tqsA
tqsA	CECT7946_02781	tqsA
tqsA	CGMCC1.8863_00684	tqsA
tqsA	CGMCC1.8863_02025	tqsA
tqsA	CGMCC1.8863_01370	tqsA
tqsA	CL8139_02782	tqsA
tqsA	DAU203_02768	tqsA
tqsA	DD5b_00034	tqsA
tqsA	DSM1208_03582	tqsA
tqsA	DSM1208_01041	tqsA
tqsA	DSM12212_00555	tqsA
tqsA	DSM12212_03460	tqsA
tqsA	DSM15343_00370	tqsA
tqsA	DSM15343_01120	tqsA
tqsA	DSM15361_00827	tqsA
tqsA	DSM15361_01793	tqsA
tqsA	DSM15365_01983	tqsA
tqsA	DSM15718_00995	tqsA
tqsA	DSM15718_00089	tqsA
tqsA	DSM15718_00273	tqsA
tqsA	DSM16505T_00526	tqsA
tqsA	DSM18103_02887	tqsA
tqsA	DSM18293_01070	tqsA
tqsA	DSM18293_01882	tqsA
tqsA	DSM18841_00384	tqsA
tqsA	DSM18842_00850	tqsA
tqsA	DSM19866_04556	tqsA
tqsA	DSM19866_02340	tqsA
tqsA	DSM2041_03853	tqsA
tqsA	DSM2061_01478	tqsA
tqsA	DSM2061_02557	tqsA
tqsA	DSM2064_04097	tqsA

tqsA	DSM2064_00381	tqsA
tqsA	DSM21164_01763	tqsA
tqsA	DSM23404_02526	tqsA
tqsA	DSM23404_02419	tqsA
tqsA	DSM23424_01021	tqsA
tqsA	DSM24786_00226	tqsA
tqsA	DSM7489_02640	tqsA
tqsA	DSW1_02211	tqsA
tqsA	DSW5_00032	tqsA
tqsA	DSW6_02393	tqsA
tqsA	DSW8_00918	tqsA
tqsA	F52_04251	tqsA
tqsA	F52_04355	tqsA
tqsA	GCA_001027945.1_00229	tqsA
tqsA	HI1_02574	tqsA
tqsA	IR1_03824	tqsA
tqsA	IR1_01857	tqsA
tqsA	KCOM3131_00755	tqsA
tqsA	KCTC12974_01715	tqsA
tqsA	KCTC12974_02796	tqsA
tqsA	KL-A_01421	tqsA
tqsA	L12_00498	tqsA
tqsA	LL0412.1.7_00483	tqsA
tqsA	MAR_2009_71_02524	tqsA
tqsA	NN016038_02485	tqsA
tqsA	PAO1_01039	tqsA
tqsA	UW101_04789	tqsA
tqsA	UW101_03092	tqsA
tqsA	V4-33_01527	tqsA
tqsA	W5C_00766	tqsA
tqsA	ZONMW-01_01693	tqsA
tqsA	ZONMW-02_02519	tqsA
tqsA	ZONMW-03_00514	tqsA
tqsA	ZONMW-04_01453	tqsA
tqsA	ZONMW-05_00777	tqsA
tqsA	ZONMW-06_03104	tqsA
tqsA	ZONMW-06_02734	tqsA
tqsA	ZONMW-07_00166	tqsA
tqsA	ZONMW-07_01682	tqsA
tqsA	ZONMW-07_01997	tqsA
tqsA	ZONMW-08_03523	tqsA
tqsA	ZONMW-09_01157	tqsA
tqsA	ZONMW-10_02227	tqsA
tqsA	ZONMW-10_00922	tqsA
tqsA	ZONMW-11_00099	tqsA
tqsA	ZONMW-11_03812	tqsA
tqsA	ZONMW-11_03483	tqsA
tqsA	ZONMW-11_00539	tqsA

tqsA	ZONMW-12_02297	tqsA
tqsA	ZONMW-13_02099	tqsA
tqsA	ZONMW-13_02036	tqsA
tqsA	ZONMW-14_00595	tqsA
tqsA	ZONMW-14_00675	tqsA
tqsA	ZONMW-15_01439	tqsA
tqsA	ZONMW-19_00065	tqsA
tqsA	ZONMW-19_00019	tqsA
tqsA	ZONMW-20_03057	tqsA
tqsA	ZONMW-20_01484	tqsA
tqsA	ZONMW-20_03325	tqsA
tqsA	ZONMW-21_02238	tqsA
tqsA	ZONMW-22_01487	tqsA
tqsA	ZONMW-23_01064	tqsA
tqsA	ZONMW-24_01511	tqsA
tqsA	ZONMW-25_00286	tqsA
tqsA	ZONMW-26_00182	tqsA
tqsA	ZONMW-27_01851	tqsA
tqsA	ZONMW-28_02912	tqsA
tqsA	ZONMW-28_02245	tqsA
tqsA	ZONMW-28_02161	tqsA
tqsA	ZONMW-29_01450	tqsA
tqsA	ZONMW-30_03090	tqsA
tqsA	ZONMW-31_02548	tqsA
tqsA	ZONMW-31_01557	tqsA
tqsA	ZONMW-32_01397	tqsA
tqsA	ZONMW-32_02650	tqsA
tqsA	ZONMW-32_02566	tqsA
tqsA	ZONMW-33_01386	tqsA
tqsA	ZONMW-33_02468	tqsA
tqsA	ZONMW-33_02552	tqsA
tqsA	ZONMW-34_01572	tqsA
tqsA	ZONMW-34_01790	tqsA
tqsA	ZONMW-34_01854	tqsA
tqsA	ZONMW-35_00645	tqsA
tqsA	ZONMW-35_01481	tqsA
tqsA	ZONMW-35_01545	tqsA
tqsA	ZONMW-36_00606	tqsA
tqsA	ZONMW-36_02784	tqsA
tqsA	ZONMW-36_02700	tqsA
tqsA	ZONMW-37_01510	tqsA
tqsA	ZONMW-38_02346	tqsA
tqsA	ZONMW-38_01158	tqsA
tqsA	ZONMW-38_00234	tqsA
tqsA	ZONMW-38_01586	tqsA
tqsA	ZONMW-39_00125	tqsA
tqsA	ZONMW-39_02396	tqsA
tqsA	ZONMW-39_02335	tqsA

tqsA	ZONMW-40_00669	tqsA
tqsA	ZONMW-41_03114	tqsA
tqsA	ZONMW-41_02191	tqsA
tqsA	ZONMW-42_03324	tqsA
tqsA	ZONMW-42_00572	tqsA
tqsA	ZONMW-43_01695	tqsA
tqsA	ZONMW-43_01300	tqsA
tqsA	ZONMW-43_01364	tqsA
tqsA	ZONMW-44_02775	tqsA
tqsA	ZONMW-44_00721	tqsA
tqsA	ZONMW-45_00537	tqsA
tqsA	ZONMW-46_01302	tqsA
tqsA	ZONMW-47_01550	tqsA
tqsA	ZONMW-48_01814	tqsA
tqsA	ZONMW-49_00192	tqsA
tqsA	ZONMW-50_00293	tqsA
tqsA	ZONMW-51_00190	tqsA
tqsA	ZONMW-52_00161	tqsA
tqsA	ZONMW-53_00908	tqsA
tqsA	ZONMW-54_01815	tqsA
tqsA	ZONMW-55_01347	tqsA
tqsA	ZONMW-56_00574	tqsA
tqsA	ZONMW-57_00781	tqsA
tqsA	ZONMW-58_00190	tqsA
tqsA	ZONMW-59_00777	tqsA
tqsA	ZONMW-60_00780	tqsA
tqsA	ZONMW-61_03564	tqsA
tqsA	ZONMW-62_01014	tqsA
tqsA	ZONMW-63_00456	tqsA
tqsA	ZONMW-64_02999	tqsA
tqsA	ZONMW-64_01881	tqsA
tqsA	ZONMW-64_01944	tqsA
tqsA	ZONMW-65_00570	tqsA
tqsA	ZONMW-65_03453	tqsA
tqsA	ZONMW-65_03402	tqsA
tqsA	ZONMW-66_00563	tqsA
tqsA	ZONMW-66_01280	tqsA
tqsA	ZONMW-67_01512	tqsA
tqsA	ZONMW-67_02217	tqsA
tqsA	ZONMW-67_00763	tqsA
tqsA	ZONMW-68_01196	tqsA
tqsA	ZONMW-68_01484	tqsA
tqsA	ZONMW-69_01488	tqsA
tqsA	ZONMW-69_02265	tqsA
tqsA	ZONMW-69_00810	tqsA
tqsA	ZONMW-72_00719	tqsA
tqsA	ZONMW-72_03043	tqsA
tqsA	ZONMW-73_01417	tqsA

tqsA	ZONMW-74_03034	tqsA
tqsA	ZONMW-75_03535	tqsA
tqsA	da5A-8_00008	tqsA
yceA	18_00144	yceA
yceA	18_00314	yceA
yceA	18_03544	yceA
yceA	18_01851	yceA
yceA	18_00517	yceA
yceA	18_01067	yceA
yceA	18_02677	yceA
yceA	ACAM48_01909	yceA
yceA	ACAM48_03282	yceA
yceA	ACAM48_03450	yceA
yceA	ACAM48_02485	yceA
yceA	ACAM48_03283	yceA
yceA	CAM030_01931	yceA
yceA	CAM030_01938	yceA
yceA	CAM030_02608	yceA
yceA	CAM030_02609	yceA
yceA	CAM030_02235	yceA
yceA	CAM030_00955	yceA
yceA	CAM030_02698	yceA
yceA	CECT7946_01043	yceA
yceA	CECT7946_02462	yceA
yceA	CECT7946_01980	yceA
yceA	CECT7946_01979	yceA
yceA	CECT7946_00618	yceA
yceA	CECT7946_00210	yceA
yceA	CECT7946_02725	yceA
yceA	CECT7946_00211	yceA
yceA	CGMCC1.8863_03125	yceA
yceA	CGMCC1.8863_01652	yceA
yceA	CGMCC1.8863_01385	yceA
yceA	CGMCC1.8863_01559	yceA
yceA	CGMCC1.8863_01188	yceA
yceA	CGMCC1.8863_00865	yceA
yceA	CL8139_01766	yceA
yceA	CL8139_01297	yceA
yceA	CL8139_01508	yceA
yceA	CL8139_02454	yceA
yceA	CL8139_02883	yceA
yceA	CL8139_00384	yceA
yceA	DAU203_02327	yceA
yceA	DAU203_03454	yceA
yceA	DAU203_02211	yceA
yceA	DAU203_00886	yceA
yceA	DAU203_00566	yceA
yceA	DAU203_01536	yceA

yceA	DD5b_00506	yceA
yceA	DD5b_02034	yceA
yceA	DD5b_00507	yceA
yceA	DD5b_02035	yceA
yceA	DD5b_02665	yceA
yceA	DD5b_02216	yceA
yceA	DSM1208_02511	yceA
yceA	DSM1208_00285	yceA
yceA	DSM1208_04259	yceA
yceA	DSM1208_02386	yceA
yceA	DSM1208_00284	yceA
yceA	DSM1208_00637	yceA
yceA	DSM1208_01161	yceA
yceA	DSM1208_01742	yceA
yceA	DSM12212_02366	yceA
yceA	DSM12212_02036	yceA
yceA	DSM12212_02367	yceA
yceA	DSM12212_02326	yceA
yceA	DSM12212_02325	yceA
yceA	DSM12212_01477	yceA
yceA	DSM12212_00141	yceA
yceA	DSM12212_01571	yceA
yceA	DSM15343_00084	yceA
yceA	DSM15343_01366	yceA
yceA	DSM15343_01354	yceA
yceA	DSM15343_00981	yceA
yceA	DSM15343_02137	yceA
yceA	DSM15343_01728	yceA
yceA	DSM15361_02667	yceA
yceA	DSM15361_01939	yceA
yceA	DSM15361_01937	yceA
yceA	DSM15361_00243	yceA
yceA	DSM15361_00432	yceA
yceA	DSM15361_01938	yceA
yceA	DSM15365_00545	yceA
yceA	DSM15365_02962	yceA
yceA	DSM15365_03875	yceA
yceA	DSM15365_01206	yceA
yceA	DSM15365_03142	yceA
yceA	DSM15365_03874	yceA
yceA	DSM15365_03522	yceA
yceA	DSM15718_01713	yceA
yceA	DSM15718_00944	yceA
yceA	DSM15718_02322	yceA
yceA	DSM15718_00827	yceA
yceA	DSM15718_01789	yceA
yceA	DSM15718_01719	yceA
yceA	DSM15718_02499	yceA

yceA	DSM15718_02323	yceA
yceA	DSM15718_00503	yceA
yceA	DSM16505T_00216	yceA
yceA	DSM16505T_00215	yceA
yceA	DSM16505T_00877	yceA
yceA	DSM16505T_00597	yceA
yceA	DSM16505T_00596	yceA
yceA	DSM18103_01680	yceA
yceA	DSM18103_01679	yceA
yceA	DSM18103_01531	yceA
yceA	DSM18103_03458	yceA
yceA	DSM18103_01011	yceA
yceA	DSM18103_01010	yceA
yceA	DSM18293_01422	yceA
yceA	DSM18293_01415	yceA
yceA	DSM18293_03172	yceA
yceA	DSM18293_03174	yceA
yceA	DSM18293_02030	yceA
yceA	DSM18841_00018	yceA
yceA	DSM18841_00017	yceA
yceA	DSM18841_00745	yceA
yceA	DSM18841_00140	yceA
yceA	DSM18841_00451	yceA
yceA	DSM18842_02650	yceA
yceA	DSM18842_02649	yceA
yceA	DSM18842_00574	yceA
yceA	DSM18842_01066	yceA
yceA	DSM18842_00776	yceA
yceA	DSM18842_00777	yceA
yceA	DSM19866_04344	yceA
yceA	DSM19866_04354	yceA
yceA	DSM19866_04345	yceA
yceA	DSM19866_04348	yceA
yceA	DSM19866_00600	yceA
yceA	DSM19866_03753	yceA
yceA	DSM19866_01978	yceA
yceA	DSM2041_03693	yceA
yceA	DSM2041_03589	yceA
yceA	DSM2041_03162	yceA
yceA	DSM2041_03692	yceA
yceA	DSM2041_03161	yceA
yceA	DSM2041_00741	yceA
yceA	DSM2041_00052	yceA
yceA	DSM2061_04023	yceA
yceA	DSM2061_03897	yceA
yceA	DSM2061_02133	yceA
yceA	DSM2061_02674	yceA
yceA	DSM2061_03273	yceA

yceA	DSM2064_03796	yceA
yceA	DSM2064_03807	yceA
yceA	DSM2064_01893	yceA
yceA	DSM2064_01891	yceA
yceA	DSM2064_02763	yceA
yceA	DSM21164_02156	yceA
yceA	DSM21164_02722	yceA
yceA	DSM21164_02286	yceA
yceA	DSM21164_01313	yceA
yceA	DSM21164_00032	yceA
yceA	DSM21164_00804	yceA
yceA	DSM23404_01277	yceA
yceA	DSM23404_00873	yceA
yceA	DSM23404_00701	yceA
yceA	DSM23404_02035	yceA
yceA	DSM23404_00872	yceA
yceA	DSM23404_01262	yceA
yceA	DSM23424_01976	yceA
yceA	DSM23424_03213	yceA
yceA	DSM23424_03214	yceA
yceA	DSM23424_00818	yceA
yceA	DSM23424_00271	yceA
yceA	DSM23424_02354	yceA
yceA	DSM24786_01704	yceA
yceA	DSM24786_00960	yceA
yceA	DSM24786_01826	yceA
yceA	DSM24786_01304	yceA
yceA	DSM24786_03386	yceA
yceA	DSM24786_03025	yceA
yceA	DSM7489_02243	yceA
yceA	DSM7489_03321	yceA
yceA	DSM7489_02124	yceA
yceA	DSM7489_00820	yceA
yceA	DSM7489_00553	yceA
yceA	DSM7489_01440	yceA
yceA	DSW1_01353	yceA
yceA	DSW1_01451	yceA
yceA	DSW1_01351	yceA
yceA	DSW1_01450	yceA
yceA	DSW1_01352	yceA
yceA	DSW1_00112	yceA
yceA	DSW1_00546	yceA
yceA	DSW1_01099	yceA
yceA	DSW5_00913	yceA
yceA	DSW5_00912	yceA
yceA	DSW5_01576	yceA
yceA	DSW5_00888	yceA
yceA	DSW5_01461	yceA

yceA	DSW6_00290	yceA
yceA	DSW6_00747	yceA
yceA	DSW6_00292	yceA
yceA	DSW6_00614	yceA
yceA	DSW6_00291	yceA
yceA	DSW6_00892	yceA
yceA	DSW6_01438	yceA
yceA	DSW8_01461	yceA
yceA	DSW8_03299	yceA
yceA	DSW8_01586	yceA
yceA	DSW8_03744	yceA
yceA	DSW8_02778	yceA
yceA	DSW8_02194	yceA
yceA	F52_03945	yceA
yceA	F52_01729	yceA
yceA	F52_01728	yceA
yceA	F52_03954	yceA
yceA	F52_02324	yceA
yceA	F52_02322	yceA
yceA	F52_03320	yceA
yceA	GCA_001027945.1_00554	yceA
yceA	GCA_001027945.1_00555	yceA
yceA	GCA_001027945.1_03173	yceA
yceA	GCA_001027945.1_00456	yceA
yceA	GCA_001027945.1_00154	yceA
yceA	GCA_001027945.1_00155	yceA
yceA	HI1_02169	yceA
yceA	HI1_03267	yceA
yceA	HI1_02050	yceA
yceA	HI1_00755	yceA
yceA	HI1_00485	yceA
yceA	HI1_01381	yceA
yceA	IR1_01476	yceA
yceA	IR1_01490	yceA
yceA	IR1_03923	yceA
yceA	IR1_03924	yceA
yceA	IR1_00135	yceA
yceA	KCOM3131_01556	yceA
yceA	KCOM3131_01311	yceA
yceA	KCOM3131_01555	yceA
yceA	KCOM3131_01287	yceA
yceA	KCOM3131_01594	yceA
yceA	KCOM3131_00108	yceA
yceA	KCTC12974_01765	yceA
yceA	KCTC12974_01419	yceA
yceA	KCTC12974_00290	yceA
yceA	KCTC12974_02874	yceA
yceA	KCTC12974_00291	yceA

yceA	KL-A_00030	yceA
yceA	KL-A_01871	yceA
yceA	KL-A_00147	yceA
yceA	KL-A_03193	yceA
yceA	KL-A_02512	yceA
yceA	KL-A_00786	yceA
yceA	L12_00211	yceA
yceA	LL0412.1.7_00168	yceA
yceA	LL0412.1.7_00167	yceA
yceA	LL0412.1.7_00648	yceA
yceA	LL0412.1.7_00352	yceA
yceA	LL0412.1.7_00879	yceA
yceA	MAR_2009_71_01967	yceA
yceA	MAR_2009_71_03623	yceA
yceA	MAR_2009_71_01839	yceA
yceA	MAR_2009_71_00212	yceA
yceA	MAR_2009_71_00676	yceA
yceA	MAR_2009_71_01246	yceA
yceA	NN016038_00146	yceA
yceA	NN016038_03585	yceA
yceA	NN016038_00318	yceA
yceA	NN016038_01874	yceA
yceA	NN016038_00527	yceA
yceA	NN016038_01083	yceA
yceA	PAO1_03017	yceA
yceA	PAO1_00884	yceA
yceA	PAO1_00604	yceA
yceA	PAO1_00858	yceA
yceA	PAO1_05623	yceA
yceA	PAO1_04041	yceA
yceA	PAO1_02681	yceA
yceA	UW101_05090	yceA
yceA	UW101_05079	yceA
yceA	UW101_00947	yceA
yceA	UW101_00945	yceA
yceA	UW101_01817	yceA
yceA	V4-33_01724	yceA
yceA	V4-33_01723	yceA
yceA	V4-33_00495	yceA
yceA	V4-33_00008	yceA
yceA	V4-33_00938	yceA
yceA	W5C_02818	yceA
yceA	W5C_00903	yceA
yceA	W5C_02932	yceA
yceA	W5C_03254	yceA
yceA	W5C_01446	yceA
yceA	W5C_01139	yceA
yceA	ZONMW-01_01346	yceA

yceA	ZONMW-01_03147	yceA
yceA	ZONMW-01_01463	yceA
yceA	ZONMW-01_00551	yceA
yceA	ZONMW-01_02750	yceA
yceA	ZONMW-01_02713	yceA
yceA	ZONMW-01_02437	yceA
yceA	ZONMW-02_00974	yceA
yceA	ZONMW-02_00728	yceA
yceA	ZONMW-02_03119	yceA
yceA	ZONMW-02_00720	yceA
yceA	ZONMW-02_03118	yceA
yceA	ZONMW-02_02749	yceA
yceA	ZONMW-02_00048	yceA
yceA	ZONMW-02_00461	yceA
yceA	ZONMW-02_00049	yceA
yceA	ZONMW-03_01434	yceA
yceA	ZONMW-03_02382	yceA
yceA	ZONMW-03_01317	yceA
yceA	ZONMW-03_02445	yceA
yceA	ZONMW-03_03156	yceA
yceA	ZONMW-03_01739	yceA
yceA	ZONMW-03_03040	yceA
yceA	ZONMW-04_00257	yceA
yceA	ZONMW-04_00846	yceA
yceA	ZONMW-04_00373	yceA
yceA	ZONMW-04_00926	yceA
yceA	ZONMW-04_02856	yceA
yceA	ZONMW-04_02253	yceA
yceA	ZONMW-04_02403	yceA
yceA	ZONMW-05_01568	yceA
yceA	ZONMW-05_00116	yceA
yceA	ZONMW-05_01452	yceA
yceA	ZONMW-05_00178	yceA
yceA	ZONMW-05_03113	yceA
yceA	ZONMW-05_02770	yceA
yceA	ZONMW-05_02621	yceA
yceA	ZONMW-06_01543	yceA
yceA	ZONMW-06_01302	yceA
yceA	ZONMW-06_02143	yceA
yceA	ZONMW-06_01599	yceA
yceA	ZONMW-06_00510	yceA
yceA	ZONMW-06_00381	yceA
yceA	ZONMW-06_00605	yceA
yceA	ZONMW-06_00397	yceA
yceA	ZONMW-06_00437	yceA
yceA	ZONMW-07_01702	yceA
yceA	ZONMW-07_00403	yceA
yceA	ZONMW-07_02011	yceA

yceA	ZONMW-07_02072	yceA
yceA	ZONMW-07_02849	yceA
yceA	ZONMW-07_01446	yceA
yceA	ZONMW-08_01261	yceA
yceA	ZONMW-08_02292	yceA
yceA	ZONMW-08_01385	yceA
yceA	ZONMW-08_03391	yceA
yceA	ZONMW-08_00192	yceA
yceA	ZONMW-08_00766	yceA
yceA	ZONMW-09_00891	yceA
yceA	ZONMW-09_00900	yceA
yceA	ZONMW-09_01836	yceA
yceA	ZONMW-09_00641	yceA
yceA	ZONMW-09_00318	yceA
yceA	ZONMW-09_01835	yceA
yceA	ZONMW-10_01695	yceA
yceA	ZONMW-10_01694	yceA
yceA	ZONMW-10_01656	yceA
yceA	ZONMW-10_01962	yceA
yceA	ZONMW-10_00459	yceA
yceA	ZONMW-10_02468	yceA
yceA	ZONMW-11_00631	yceA
yceA	ZONMW-11_00657	yceA
yceA	ZONMW-11_01067	yceA
yceA	ZONMW-11_02808	yceA
yceA	ZONMW-11_03514	yceA
yceA	ZONMW-11_02238	yceA
yceA	ZONMW-11_00041	yceA
yceA	ZONMW-11_02239	yceA
yceA	ZONMW-12_02810	yceA
yceA	ZONMW-12_03206	yceA
yceA	ZONMW-12_01936	yceA
yceA	ZONMW-12_00211	yceA
yceA	ZONMW-12_00171	yceA
yceA	ZONMW-12_03881	yceA
yceA	ZONMW-12_00213	yceA
yceA	ZONMW-13_02827	yceA
yceA	ZONMW-13_02319	yceA
yceA	ZONMW-13_02956	yceA
yceA	ZONMW-13_02826	yceA
yceA	ZONMW-13_00765	yceA
yceA	ZONMW-13_01650	yceA
yceA	ZONMW-13_00318	yceA
yceA	ZONMW-14_02010	yceA
yceA	ZONMW-14_02237	yceA
yceA	ZONMW-14_02722	yceA
yceA	ZONMW-14_00894	yceA
yceA	ZONMW-14_02012	yceA

yceA	ZONMW-14_01280	yceA
yceA	ZONMW-14_00274	yceA
yceA	ZONMW-15_01179	yceA
yceA	ZONMW-15_02782	yceA
yceA	ZONMW-15_01121	yceA
yceA	ZONMW-15_01016	yceA
yceA	ZONMW-15_02594	yceA
yceA	ZONMW-19_03881	yceA
yceA	ZONMW-19_02147	yceA
yceA	ZONMW-19_02262	yceA
yceA	ZONMW-19_00871	yceA
yceA	ZONMW-19_02062	yceA
yceA	ZONMW-19_00860	yceA
yceA	ZONMW-20_03994	yceA
yceA	ZONMW-20_00831	yceA
yceA	ZONMW-20_00454	yceA
yceA	ZONMW-20_04084	yceA
yceA	ZONMW-20_02894	yceA
yceA	ZONMW-20_03370	yceA
yceA	ZONMW-20_01453	yceA
yceA	ZONMW-21_00522	yceA
yceA	ZONMW-21_00521	yceA
yceA	ZONMW-21_03035	yceA
yceA	ZONMW-21_01298	yceA
yceA	ZONMW-21_00731	yceA
yceA	ZONMW-22_00522	yceA
yceA	ZONMW-22_00521	yceA
yceA	ZONMW-22_03060	yceA
yceA	ZONMW-22_01300	yceA
yceA	ZONMW-22_00732	yceA
yceA	ZONMW-23_01637	yceA
yceA	ZONMW-23_01636	yceA
yceA	ZONMW-23_03081	yceA
yceA	ZONMW-23_02412	yceA
yceA	ZONMW-23_01846	yceA
yceA	ZONMW-24_00937	yceA
yceA	ZONMW-24_00938	yceA
yceA	ZONMW-24_03082	yceA
yceA	ZONMW-24_00161	yceA
yceA	ZONMW-24_00729	yceA
yceA	ZONMW-25_02190	yceA
yceA	ZONMW-25_00177	yceA
yceA	ZONMW-25_02188	yceA
yceA	ZONMW-25_02189	yceA
yceA	ZONMW-25_01812	yceA
yceA	ZONMW-25_01663	yceA
yceA	ZONMW-26_01702	yceA
yceA	ZONMW-26_00844	yceA

yceA	ZONMW-26_01817	yceA
yceA	ZONMW-26_00779	yceA
yceA	ZONMW-26_03180	yceA
yceA	ZONMW-26_02110	yceA
yceA	ZONMW-26_03058	yceA
yceA	ZONMW-27_00251	yceA
yceA	ZONMW-27_00943	yceA
yceA	ZONMW-27_00367	yceA
yceA	ZONMW-27_01001	yceA
yceA	ZONMW-27_03202	yceA
yceA	ZONMW-27_02232	yceA
yceA	ZONMW-27_02778	yceA
yceA	ZONMW-28_02594	yceA
yceA	ZONMW-28_00441	yceA
yceA	ZONMW-28_00928	yceA
yceA	ZONMW-28_01066	yceA
yceA	ZONMW-28_01005	yceA
yceA	ZONMW-29_00197	yceA
yceA	ZONMW-29_00196	yceA
yceA	ZONMW-29_02651	yceA
yceA	ZONMW-29_00374	yceA
yceA	ZONMW-30_01648	yceA
yceA	ZONMW-30_02631	yceA
yceA	ZONMW-30_01646	yceA
yceA	ZONMW-30_01647	yceA
yceA	ZONMW-30_01022	yceA
yceA	ZONMW-30_02333	yceA
yceA	ZONMW-31_03247	yceA
yceA	ZONMW-31_02223	yceA
yceA	ZONMW-31_03707	yceA
yceA	ZONMW-31_00026	yceA
yceA	ZONMW-31_00863	yceA
yceA	ZONMW-31_00718	yceA
yceA	ZONMW-31_03243	yceA
yceA	ZONMW-31_00732	yceA
yceA	ZONMW-31_00792	yceA
yceA	ZONMW-31_02508	yceA
yceA	ZONMW-32_02756	yceA
yceA	ZONMW-32_01232	yceA
yceA	ZONMW-32_00163	yceA
yceA	ZONMW-32_00025	yceA
yceA	ZONMW-32_00086	yceA
yceA	ZONMW-33_02992	yceA
yceA	ZONMW-33_01221	yceA
yceA	ZONMW-33_00030	yceA
yceA	ZONMW-33_03286	yceA
yceA	ZONMW-33_03347	yceA
yceA	ZONMW-34_02871	yceA

yceA	ZONMW-34_02868	yceA
yceA	ZONMW-34_00433	yceA
yceA	ZONMW-34_02883	yceA
yceA	ZONMW-34_00347	yceA
yceA	ZONMW-34_01300	yceA
yceA	ZONMW-34_01413	yceA
yceA	ZONMW-35_02838	yceA
yceA	ZONMW-35_02835	yceA
yceA	ZONMW-35_00957	yceA
yceA	ZONMW-35_02850	yceA
yceA	ZONMW-35_01043	yceA
yceA	ZONMW-35_00373	yceA
yceA	ZONMW-35_00486	yceA
yceA	ZONMW-36_02504	yceA
yceA	ZONMW-36_00441	yceA
yceA	ZONMW-36_01339	yceA
yceA	ZONMW-36_03023	yceA
yceA	ZONMW-36_03084	yceA
yceA	ZONMW-37_00522	yceA
yceA	ZONMW-37_00521	yceA
yceA	ZONMW-37_03069	yceA
yceA	ZONMW-37_01064	yceA
yceA	ZONMW-37_00731	yceA
yceA	ZONMW-38_03158	yceA
yceA	ZONMW-38_02815	yceA
yceA	ZONMW-38_00669	yceA
yceA	ZONMW-38_01468	yceA
yceA	ZONMW-38_00678	yceA
yceA	ZONMW-38_02504	yceA
yceA	ZONMW-38_03151	yceA
yceA	ZONMW-38_03754	yceA
yceA	ZONMW-38_00675	yceA
yceA	ZONMW-38_02519	yceA
yceA	ZONMW-38_00522	yceA
yceA	ZONMW-38_00523	yceA
yceA	ZONMW-39_00287	yceA
yceA	ZONMW-39_03137	yceA
yceA	ZONMW-39_03036	yceA
yceA	ZONMW-39_00399	yceA
yceA	ZONMW-40_01412	yceA
yceA	ZONMW-40_01147	yceA
yceA	ZONMW-40_00469	yceA
yceA	ZONMW-41_00243	yceA
yceA	ZONMW-41_02921	yceA
yceA	ZONMW-41_01495	yceA
yceA	ZONMW-41_02924	yceA
yceA	ZONMW-41_00625	yceA
yceA	ZONMW-41_00155	yceA

yceA	ZONMW-41_01910	yceA
yceA	ZONMW-41_00369	yceA
yceA	ZONMW-41_02690	yceA
yceA	ZONMW-41_02552	yceA
yceA	ZONMW-41_00989	yceA
yceA	ZONMW-42_01356	yceA
yceA	ZONMW-42_04212	yceA
yceA	ZONMW-42_04246	yceA
yceA	ZONMW-42_02790	yceA
yceA	ZONMW-42_02929	yceA
yceA	ZONMW-42_01837	yceA
yceA	ZONMW-42_01280	yceA
yceA	ZONMW-42_04237	yceA
yceA	ZONMW-42_04136	yceA
yceA	ZONMW-42_01400	yceA
yceA	ZONMW-42_03439	yceA
yceA	ZONMW-42_03461	yceA
yceA	ZONMW-43_02694	yceA
yceA	ZONMW-43_02397	yceA
yceA	ZONMW-43_02309	yceA
yceA	ZONMW-43_03891	yceA
yceA	ZONMW-43_03010	yceA
yceA	ZONMW-44_00545	yceA
yceA	ZONMW-44_02432	yceA
yceA	ZONMW-44_01300	yceA
yceA	ZONMW-44_00468	yceA
yceA	ZONMW-44_01694	yceA
yceA	ZONMW-44_03571	yceA
yceA	ZONMW-44_01594	yceA
yceA	ZONMW-44_03587	yceA
yceA	ZONMW-44_03637	yceA
yceA	ZONMW-45_02027	yceA
yceA	ZONMW-45_02026	yceA
yceA	ZONMW-45_03057	yceA
yceA	ZONMW-45_01105	yceA
yceA	ZONMW-45_00591	yceA
yceA	ZONMW-46_02255	yceA
yceA	ZONMW-46_02256	yceA
yceA	ZONMW-46_01563	yceA
yceA	ZONMW-46_00562	yceA
yceA	ZONMW-46_00868	yceA
yceA	ZONMW-47_02277	yceA
yceA	ZONMW-47_02276	yceA
yceA	ZONMW-47_01289	yceA
yceA	ZONMW-47_00562	yceA
yceA	ZONMW-47_00868	yceA
yceA	ZONMW-48_02514	yceA
yceA	ZONMW-48_03246	yceA

yceA	ZONMW-48_02572	yceA
yceA	ZONMW-48_00432	yceA
yceA	ZONMW-48_00587	yceA
yceA	ZONMW-49_00669	yceA
yceA	ZONMW-49_03021	yceA
yceA	ZONMW-49_00791	yceA
yceA	ZONMW-49_02280	yceA
yceA	ZONMW-49_02786	yceA
yceA	ZONMW-49_01157	yceA
yceA	ZONMW-50_00670	yceA
yceA	ZONMW-50_03011	yceA
yceA	ZONMW-50_00792	yceA
yceA	ZONMW-50_02180	yceA
yceA	ZONMW-50_02712	yceA
yceA	ZONMW-50_01158	yceA
yceA	ZONMW-51_01038	yceA
yceA	ZONMW-51_01939	yceA
yceA	ZONMW-51_00920	yceA
yceA	ZONMW-51_01676	yceA
yceA	ZONMW-51_02989	yceA
yceA	ZONMW-51_02763	yceA
yceA	ZONMW-52_01638	yceA
yceA	ZONMW-52_00834	yceA
yceA	ZONMW-52_01520	yceA
yceA	ZONMW-52_01819	yceA
yceA	ZONMW-52_03085	yceA
yceA	ZONMW-52_02283	yceA
yceA	ZONMW-53_00187	yceA
yceA	ZONMW-53_01577	yceA
yceA	ZONMW-53_00307	yceA
yceA	ZONMW-53_01937	yceA
yceA	ZONMW-53_03051	yceA
yceA	ZONMW-53_02872	yceA
yceA	ZONMW-54_00843	yceA
yceA	ZONMW-54_01148	yceA
yceA	ZONMW-54_00727	yceA
yceA	ZONMW-54_02117	yceA
yceA	ZONMW-54_03053	yceA
yceA	ZONMW-54_00088	yceA
yceA	ZONMW-55_00182	yceA
yceA	ZONMW-55_02261	yceA
yceA	ZONMW-55_00300	yceA
yceA	ZONMW-55_01998	yceA
yceA	ZONMW-55_01607	yceA
yceA	ZONMW-55_02878	yceA
yceA	ZONMW-56_01136	yceA
yceA	ZONMW-56_02107	yceA
yceA	ZONMW-56_01015	yceA

yceA	ZONMW-56_01438	yceA
yceA	ZONMW-56_02937	yceA
yceA	ZONMW-56_02638	yceA
yceA	ZONMW-57_01435	yceA
yceA	ZONMW-57_00113	yceA
yceA	ZONMW-57_01315	yceA
yceA	ZONMW-57_01788	yceA
yceA	ZONMW-57_03110	yceA
yceA	ZONMW-57_02782	yceA
yceA	ZONMW-58_01149	yceA
yceA	ZONMW-58_00853	yceA
yceA	ZONMW-58_01269	yceA
yceA	ZONMW-58_01812	yceA
yceA	ZONMW-58_03131	yceA
yceA	ZONMW-58_02806	yceA
yceA	ZONMW-59_01437	yceA
yceA	ZONMW-59_00113	yceA
yceA	ZONMW-59_01316	yceA
yceA	ZONMW-59_01624	yceA
yceA	ZONMW-59_03186	yceA
yceA	ZONMW-59_02922	yceA
yceA	ZONMW-60_01138	yceA
yceA	ZONMW-60_00114	yceA
yceA	ZONMW-60_01254	yceA
yceA	ZONMW-60_02006	yceA
yceA	ZONMW-60_03122	yceA
yceA	ZONMW-60_02901	yceA
yceA	ZONMW-61_03168	yceA
yceA	ZONMW-61_01254	yceA
yceA	ZONMW-61_03169	yceA
yceA	ZONMW-61_03154	yceA
yceA	ZONMW-61_03001	yceA
yceA	ZONMW-61_03167	yceA
yceA	ZONMW-61_01568	yceA
yceA	ZONMW-61_03744	yceA
yceA	ZONMW-61_00360	yceA
yceA	ZONMW-62_02106	yceA
yceA	ZONMW-62_03224	yceA
yceA	ZONMW-62_02233	yceA
yceA	ZONMW-62_03788	yceA
yceA	ZONMW-62_01319	yceA
yceA	ZONMW-62_01907	yceA
yceA	ZONMW-63_01130	yceA
yceA	ZONMW-63_01877	yceA
yceA	ZONMW-63_01250	yceA
yceA	ZONMW-63_01524	yceA
yceA	ZONMW-63_02904	yceA
yceA	ZONMW-63_02291	yceA

yceA	ZONMW-64_03245	yceA
yceA	ZONMW-64_03406	yceA
yceA	ZONMW-64_00675	yceA
yceA	ZONMW-64_00774	yceA
yceA	ZONMW-64_01582	yceA
yceA	ZONMW-64_02154	yceA
yceA	ZONMW-64_03341	yceA
yceA	ZONMW-65_03751	yceA
yceA	ZONMW-65_03712	yceA
yceA	ZONMW-65_00097	yceA
yceA	ZONMW-65_00729	yceA
yceA	ZONMW-65_02154	yceA
yceA	ZONMW-65_02612	yceA
yceA	ZONMW-65_01384	yceA
yceA	ZONMW-65_03370	yceA
yceA	ZONMW-66_04422	yceA
yceA	ZONMW-66_01650	yceA
yceA	ZONMW-66_02978	yceA
yceA	ZONMW-66_02309	yceA
yceA	ZONMW-66_02606	yceA
yceA	ZONMW-66_01568	yceA
yceA	ZONMW-67_02982	yceA
yceA	ZONMW-67_01361	yceA
yceA	ZONMW-67_00621	yceA
yceA	ZONMW-67_02759	yceA
yceA	ZONMW-67_02776	yceA
yceA	ZONMW-67_02805	yceA
yceA	ZONMW-67_00712	yceA
yceA	ZONMW-68_00561	yceA
yceA	ZONMW-68_02019	yceA
yceA	ZONMW-68_01330	yceA
yceA	ZONMW-68_01328	yceA
yceA	ZONMW-68_04648	yceA
yceA	ZONMW-68_03540	yceA
yceA	ZONMW-68_00875	yceA
yceA	ZONMW-68_00121	yceA
yceA	ZONMW-69_02972	yceA
yceA	ZONMW-69_01639	yceA
yceA	ZONMW-69_00668	yceA
yceA	ZONMW-69_00556	yceA
yceA	ZONMW-69_00573	yceA
yceA	ZONMW-69_00602	yceA
yceA	ZONMW-69_00759	yceA
yceA	ZONMW-72_01982	yceA
yceA	ZONMW-72_01996	yceA
yceA	ZONMW-72_02237	yceA
yceA	ZONMW-72_02238	yceA
yceA	ZONMW-72_00548	yceA

yceA	ZONMW-73_01905	yceA
yceA	ZONMW-73_00915	yceA
yceA	ZONMW-73_01156	yceA
yceA	ZONMW-73_01157	yceA
yceA	ZONMW-73_03144	yceA
yceA	ZONMW-73_00119	yceA
yceA	ZONMW-73_01298	yceA
yceA	ZONMW-73_03575	yceA
yceA	ZONMW-73_00118	yceA
yceA	ZONMW-74_00953	yceA
yceA	ZONMW-74_00785	yceA
yceA	ZONMW-74_01058	yceA
yceA	ZONMW-74_00786	yceA
yceA	ZONMW-74_02457	yceA
yceA	ZONMW-74_01446	yceA
yceA	ZONMW-75_00694	yceA
yceA	ZONMW-75_03504	yceA
yceA	ZONMW-75_02768	yceA
yceA	ZONMW-75_03503	yceA
yceA	ZONMW-75_02874	yceA
yceA	ZONMW-75_00941	yceA
yceA	ZONMW-75_01371	yceA
yceA	da5A-8_00744	yceA
yceA	da5A-8_00743	yceA
yceA	da5A-8_01731	yceA
yceA	da5A-8_02439	yceA
yceA	da5A-8_01223	yceA
yceA	da5A-8_01224	yceA
sigA_1	18_01729	sigA_1
sigA_1	18_03445	sigA_1
sigA_1	18_03274	sigA_1
sigA_1	ACAM48_01673	sigA_1
sigA_1	CAM030_01376	sigA_1
sigA_1	CAM030_02357	sigA_1
sigA_1	CECT7946_00860	sigA_1
sigA_1	CECT7946_02568	sigA_1
sigA_1	CGMCC1.8863_00635	sigA_1
sigA_1	CGMCC1.8863_00346	sigA_1
sigA_1	CL8139_02710	sigA_1
sigA_1	CL8139_02380	sigA_1
sigA_1	DAU203_00684	sigA_1
sigA_1	DAU203_03151	sigA_1
sigA_1	DD5b_02720	sigA_1
sigA_1	DSM1208_00746	sigA_1
sigA_1	DSM1208_04000	sigA_1
sigA_1	DSM12212_02699	sigA_1
sigA_1	DSM12212_03537	sigA_1
sigA_1	DSM15343_02063	sigA_1

sigA_1	DSM15361_02392	sigA_1
sigA_1	DSM15361_01155	sigA_1
sigA_1	DSM15361_00948	sigA_1
sigA_1	DSM15365_00772	sigA_1
sigA_1	DSM15365_01118	sigA_1
sigA_1	DSM15718_00441	sigA_1
sigA_1	DSM16505T_00079	sigA_1
sigA_1	DSM16505T_00925	sigA_1
sigA_1	DSM18103_01398	sigA_1
sigA_1	DSM18293_00522	sigA_1
sigA_1	DSM18841_02581	sigA_1
sigA_1	DSM18841_00814	sigA_1
sigA_1	DSM18842_02481	sigA_1
sigA_1	DSM18842_00505	sigA_1
sigA_1	DSM19866_02200	sigA_1
sigA_1	DSM19866_04107	sigA_1
sigA_1	DSM19866_03990	sigA_1
sigA_1	DSM2041_03580	sigA_1
sigA_1	DSM2061_02245	sigA_1
sigA_1	DSM2061_00856	sigA_1
sigA_1	DSM2061_00958	sigA_1
sigA_1	DSM2064_02444	sigA_1
sigA_1	DSM21164_00144	sigA_1
sigA_1	DSM21164_02623	sigA_1
sigA_1	DSM23404_00999	sigA_1
sigA_1	DSM23424_02435	sigA_1
sigA_1	DSM23424_01227	sigA_1
sigA_1	DSM24786_03493	sigA_1
sigA_1	DSM24786_00602	sigA_1
sigA_1	DSM24786_00532	sigA_1
sigA_1	DSM7489_00670	sigA_1
sigA_1	DSM7489_02983	sigA_1
sigA_1	DSW1_01368	sigA_1
sigA_1	DSW1_02110	sigA_1
sigA_1	DSW5_00625	sigA_1
sigA_1	DSW5_01018	sigA_1
sigA_1	DSW6_03088	sigA_1
sigA_1	DSW8_03797	sigA_1
sigA_1	DSW8_00228	sigA_1
sigA_1	DSW8_02867	sigA_1
sigA_1	F52_02851	sigA_1
sigA_1	GCA_001027945.1_00958	sigA_1
sigA_1	GCA_001027945.1_01627	sigA_1
sigA_1	HI1_00602	sigA_1
sigA_1	HI1_02924	sigA_1
sigA_1	IR1_00429	sigA_1
sigA_1	KCOM3131_00247	sigA_1
sigA_1	KCTC12974_02426	sigA_1

sigA_1	KL-A_03052	sigA_1
sigA_1	KL-A_00503	sigA_1
sigA_1	L12_00336	sigA_1
sigA_1	L12_00335	sigA_1
sigA_1	LL0412.1.7_00078	sigA_1
sigA_1	MAR_2009_71_00331	sigA_1
sigA_1	MAR_2009_71_03026	sigA_1
sigA_1	MAR_2009_71_00589	sigA_1
sigA_1	NN016038_01750	sigA_1
sigA_1	NN016038_03481	sigA_1
sigA_1	NN016038_03317	sigA_1
sigA_1	PAO1_00591	sigA_1
sigA_1	PAO1_03745	sigA_1
sigA_1	PAO1_00386	sigA_1
sigA_1	PAO1_01495	sigA_1
sigA_1	UW101_01498	sigA_1
sigA_1	V4-33_01963	sigA_1
sigA_1	W5C_01171	sigA_1
sigA_1	W5C_02188	sigA_1
sigA_1	ZONMW-01_03202	sigA_1
sigA_1	ZONMW-01_00793	sigA_1
sigA_1	ZONMW-01_00846	sigA_1
sigA_1	ZONMW-02_02293	sigA_1
sigA_1	ZONMW-02_02672	sigA_1
sigA_1	ZONMW-03_03316	sigA_1
sigA_1	ZONMW-03_00171	sigA_1
sigA_1	ZONMW-03_00224	sigA_1
sigA_1	ZONMW-04_03220	sigA_1
sigA_1	ZONMW-04_01175	sigA_1
sigA_1	ZONMW-04_03118	sigA_1
sigA_1	ZONMW-05_03351	sigA_1
sigA_1	ZONMW-05_00426	sigA_1
sigA_1	ZONMW-05_00482	sigA_1
sigA_1	ZONMW-06_01937	sigA_1
sigA_1	ZONMW-06_03892	sigA_1
sigA_1	ZONMW-06_01427	sigA_1
sigA_1	ZONMW-06_03760	sigA_1
sigA_1	ZONMW-06_03380	sigA_1
sigA_1	ZONMW-07_01128	sigA_1
sigA_1	ZONMW-07_02571	sigA_1
sigA_1	ZONMW-07_02628	sigA_1
sigA_1	ZONMW-08_03273	sigA_1
sigA_1	ZONMW-08_02226	sigA_1
sigA_1	ZONMW-08_00105	sigA_1
sigA_1	ZONMW-09_01945	sigA_1
sigA_1	ZONMW-10_01120	sigA_1
sigA_1	ZONMW-11_00942	sigA_1
sigA_1	ZONMW-12_02171	sigA_1

sigA_1	ZONMW-12_01579	sigA_1
sigA_1	ZONMW-13_02641	sigA_1
sigA_1	ZONMW-14_01595	sigA_1
sigA_1	ZONMW-15_01263	sigA_1
sigA_1	ZONMW-15_01911	sigA_1
sigA_1	ZONMW-15_03366	sigA_1
sigA_1	ZONMW-19_00766	sigA_1
sigA_1	ZONMW-19_02139	sigA_1
sigA_1	ZONMW-19_03168	sigA_1
sigA_1	ZONMW-19_02018	sigA_1
sigA_1	ZONMW-20_00028	sigA_1
sigA_1	ZONMW-20_00322	sigA_1
sigA_1	ZONMW-20_03340	sigA_1
sigA_1	ZONMW-20_01932	sigA_1
sigA_1	ZONMW-20_02696	sigA_1
sigA_1	ZONMW-21_03142	sigA_1
sigA_1	ZONMW-21_01948	sigA_1
sigA_1	ZONMW-21_00406	sigA_1
sigA_1	ZONMW-22_03167	sigA_1
sigA_1	ZONMW-22_01778	sigA_1
sigA_1	ZONMW-22_00406	sigA_1
sigA_1	ZONMW-23_03188	sigA_1
sigA_1	ZONMW-23_00774	sigA_1
sigA_1	ZONMW-23_01521	sigA_1
sigA_1	ZONMW-24_03189	sigA_1
sigA_1	ZONMW-24_01801	sigA_1
sigA_1	ZONMW-24_01053	sigA_1
sigA_1	ZONMW-25_01922	sigA_1
sigA_1	ZONMW-25_00983	sigA_1
sigA_1	ZONMW-25_00880	sigA_1
sigA_1	ZONMW-25_00353	sigA_1
sigA_1	ZONMW-26_03242	sigA_1
sigA_1	ZONMW-26_00539	sigA_1
sigA_1	ZONMW-26_00486	sigA_1
sigA_1	ZONMW-27_03312	sigA_1
sigA_1	ZONMW-27_01252	sigA_1
sigA_1	ZONMW-27_01306	sigA_1
sigA_1	ZONMW-28_02645	sigA_1
sigA_1	ZONMW-28_03835	sigA_1
sigA_1	ZONMW-28_01617	sigA_1
sigA_1	ZONMW-28_01951	sigA_1
sigA_1	ZONMW-28_03190	sigA_1
sigA_1	ZONMW-29_02543	sigA_1
sigA_1	ZONMW-29_01226	sigA_1
sigA_1	ZONMW-29_01382	sigA_1
sigA_1	ZONMW-30_00911	sigA_1
sigA_1	ZONMW-30_01304	sigA_1
sigA_1	ZONMW-30_01196	sigA_1

sigA_1	ZONMW-30_03296	sigA_1
sigA_1	ZONMW-31_01916	sigA_1
sigA_1	ZONMW-31_02583	sigA_1
sigA_1	ZONMW-31_03478	sigA_1
sigA_1	ZONMW-31_00269	sigA_1
sigA_1	ZONMW-32_03231	sigA_1
sigA_1	ZONMW-32_03773	sigA_1
sigA_1	ZONMW-32_02354	sigA_1
sigA_1	ZONMW-32_02021	sigA_1
sigA_1	ZONMW-32_00715	sigA_1
sigA_1	ZONMW-33_03043	sigA_1
sigA_1	ZONMW-33_03774	sigA_1
sigA_1	ZONMW-33_02346	sigA_1
sigA_1	ZONMW-33_02012	sigA_1
sigA_1	ZONMW-33_00583	sigA_1
sigA_1	ZONMW-34_02610	sigA_1
sigA_1	ZONMW-34_03672	sigA_1
sigA_1	ZONMW-34_03109	sigA_1
sigA_1	ZONMW-34_02142	sigA_1
sigA_1	ZONMW-34_00947	sigA_1
sigA_1	ZONMW-35_02574	sigA_1
sigA_1	ZONMW-35_03821	sigA_1
sigA_1	ZONMW-35_03128	sigA_1
sigA_1	ZONMW-35_02107	sigA_1
sigA_1	ZONMW-35_02296	sigA_1
sigA_1	ZONMW-36_02959	sigA_1
sigA_1	ZONMW-36_03723	sigA_1
sigA_1	ZONMW-36_01890	sigA_1
sigA_1	ZONMW-36_02224	sigA_1
sigA_1	ZONMW-36_00788	sigA_1
sigA_1	ZONMW-37_03176	sigA_1
sigA_1	ZONMW-37_01801	sigA_1
sigA_1	ZONMW-37_00406	sigA_1
sigA_1	ZONMW-38_01344	sigA_1
sigA_1	ZONMW-38_00850	sigA_1
sigA_1	ZONMW-38_01618	sigA_1
sigA_1	ZONMW-38_03354	sigA_1
sigA_1	ZONMW-38_01402	sigA_1
sigA_1	ZONMW-38_02911	sigA_1
sigA_1	ZONMW-38_01619	sigA_1
sigA_1	ZONMW-38_01932	sigA_1
sigA_1	ZONMW-39_03943	sigA_1
sigA_1	ZONMW-39_04127	sigA_1
sigA_1	ZONMW-39_01799	sigA_1
sigA_1	ZONMW-39_01416	sigA_1
sigA_1	ZONMW-39_02818	sigA_1
sigA_1	ZONMW-40_01031	sigA_1
sigA_1	ZONMW-40_02951	sigA_1

sigA_1	ZONMW-40_00598	sigA_1
sigA_1	ZONMW-41_01369	sigA_1
sigA_1	ZONMW-41_01249	sigA_1
sigA_1	ZONMW-41_01915	sigA_1
sigA_1	ZONMW-41_03085	sigA_1
sigA_1	ZONMW-42_01066	sigA_1
sigA_1	ZONMW-42_03194	sigA_1
sigA_1	ZONMW-42_02472	sigA_1
sigA_1	ZONMW-42_00269	sigA_1
sigA_1	ZONMW-42_00939	sigA_1
sigA_1	ZONMW-43_01237	sigA_1
sigA_1	ZONMW-43_02570	sigA_1
sigA_1	ZONMW-43_03435	sigA_1
sigA_1	ZONMW-43_03215	sigA_1
sigA_1	ZONMW-43_00480	sigA_1
sigA_1	ZONMW-44_00042	sigA_1
sigA_1	ZONMW-44_03834	sigA_1
sigA_1	ZONMW-44_00662	sigA_1
sigA_1	ZONMW-44_03050	sigA_1
sigA_1	ZONMW-44_03694	sigA_1
sigA_1	ZONMW-45_02457	sigA_1
sigA_1	ZONMW-45_01344	sigA_1
sigA_1	ZONMW-46_02450	sigA_1
sigA_1	ZONMW-46_01599	sigA_1
sigA_1	ZONMW-47_02440	sigA_1
sigA_1	ZONMW-47_01890	sigA_1
sigA_1	ZONMW-48_02433	sigA_1
sigA_1	ZONMW-48_01410	sigA_1
sigA_1	ZONMW-48_01498	sigA_1
sigA_1	ZONMW-49_03152	sigA_1
sigA_1	ZONMW-49_01665	sigA_1
sigA_1	ZONMW-50_03148	sigA_1
sigA_1	ZONMW-50_01749	sigA_1
sigA_1	ZONMW-51_03209	sigA_1
sigA_1	ZONMW-51_00563	sigA_1
sigA_1	ZONMW-52_03214	sigA_1
sigA_1	ZONMW-52_00520	sigA_1
sigA_1	ZONMW-53_03178	sigA_1
sigA_1	ZONMW-53_01275	sigA_1
sigA_1	ZONMW-54_03175	sigA_1
sigA_1	ZONMW-54_01442	sigA_1
sigA_1	ZONMW-55_03176	sigA_1
sigA_1	ZONMW-55_00961	sigA_1
sigA_1	ZONMW-56_03234	sigA_1
sigA_1	ZONMW-56_00197	sigA_1
sigA_1	ZONMW-57_03192	sigA_1
sigA_1	ZONMW-57_00410	sigA_1
sigA_1	ZONMW-58_03259	sigA_1

sigA_1	ZONMW-58_00561	sigA_1
sigA_1	ZONMW-59_03316	sigA_1
sigA_1	ZONMW-59_00426	sigA_1
sigA_1	ZONMW-60_03305	sigA_1
sigA_1	ZONMW-60_00437	sigA_1
sigA_1	ZONMW-61_03868	sigA_1
sigA_1	ZONMW-61_03237	sigA_1
sigA_1	ZONMW-61_01845	sigA_1
sigA_1	ZONMW-61_01478	sigA_1
sigA_1	ZONMW-62_03670	sigA_1
sigA_1	ZONMW-62_00539	sigA_1
sigA_1	ZONMW-62_01231	sigA_1
sigA_1	ZONMW-63_03233	sigA_1
sigA_1	ZONMW-63_00079	sigA_1
sigA_1	ZONMW-64_04169	sigA_1
sigA_1	ZONMW-64_03751	sigA_1
sigA_1	ZONMW-64_01759	sigA_1
sigA_1	ZONMW-64_01344	sigA_1
sigA_1	ZONMW-64_00498	sigA_1
sigA_1	ZONMW-65_00363	sigA_1
sigA_1	ZONMW-65_01216	sigA_1
sigA_1	ZONMW-65_02882	sigA_1
sigA_1	ZONMW-65_01005	sigA_1
sigA_1	ZONMW-66_00209	sigA_1
sigA_1	ZONMW-66_01642	sigA_1
sigA_1	ZONMW-66_01961	sigA_1
sigA_1	ZONMW-66_02787	sigA_1
sigA_1	ZONMW-66_01716	sigA_1
sigA_1	ZONMW-67_03314	sigA_1
sigA_1	ZONMW-67_01754	sigA_1
sigA_1	ZONMW-67_02863	sigA_1
sigA_1	ZONMW-67_00361	sigA_1
sigA_1	ZONMW-67_01163	sigA_1
sigA_1	ZONMW-68_01625	sigA_1
sigA_1	ZONMW-68_03690	sigA_1
sigA_1	ZONMW-68_04565	sigA_1
sigA_1	ZONMW-69_03315	sigA_1
sigA_1	ZONMW-69_02189	sigA_1
sigA_1	ZONMW-69_03091	sigA_1
sigA_1	ZONMW-69_00108	sigA_1
sigA_1	ZONMW-69_01209	sigA_1
sigA_1	ZONMW-72_00081	sigA_1
sigA_1	ZONMW-73_03627	sigA_1
sigA_1	ZONMW-73_02022	sigA_1
sigA_1	ZONMW-74_03401	sigA_1
sigA_1	ZONMW-74_00203	sigA_1
sigA_1	ZONMW-75_03781	sigA_1
sigA_1	ZONMW-75_02954	sigA_1

sigA_1	da5A-8_01008	sigA_1
maeB	18_01563	maeB
maeB	18_02404	maeB
maeB	ACAM48_02697	maeB
maeB	ACAM48_00961	maeB
maeB	CAM030_02307	maeB
maeB	CECT7946_01696	maeB
maeB	CECT7946_02684	maeB
maeB	CGMCC1.8863_01096	maeB
maeB	CGMCC1.8863_00222	maeB
maeB	CL8139_00183	maeB
maeB	DAU203_01994	maeB
maeB	DD5b_02580	maeB
maeB	DD5b_01134	maeB
maeB	DSM1208_02288	maeB
maeB	DSM12212_01760	maeB
maeB	DSM12212_02977	maeB
maeB	DSM15343_02627	maeB
maeB	DSM15361_01372	maeB
maeB	DSM15365_00807	maeB
maeB	DSM15365_00609	maeB
maeB	DSM15718_03311	maeB
maeB	DSM15718_00285	maeB
maeB	DSM16505T_00655	maeB
maeB	DSM18103_01078	maeB
maeB	DSM18103_03363	maeB
maeB	DSM18293_01938	maeB
maeB	DSM18293_04072	maeB
maeB	DSM18841_00526	maeB
maeB	DSM18842_00700	maeB
maeB	DSM18842_02654	maeB
maeB	DSM19866_02774	maeB
maeB	DSM19866_02505	maeB
maeB	DSM2041_04667	maeB
maeB	DSM2041_05306	maeB
maeB	DSM2061_03817	maeB
maeB	DSM2064_02667	maeB
maeB	DSM2064_00967	maeB
maeB	DSM21164_00311	maeB
maeB	DSM21164_01792	maeB
maeB	DSM23404_02259	maeB
maeB	DSM23404_03319	maeB
maeB	DSM23424_00030	maeB
maeB	DSM24786_02056	maeB
maeB	DSM7489_01905	maeB
maeB	DSW1_01058	maeB
maeB	DSW5_01611	maeB
maeB	DSW5_01894	maeB

maeB	DSW6_01045	maeB
maeB	DSW6_00132	maeB
maeB	DSW8_01656	maeB
maeB	DSW8_03225	maeB
maeB	F52_03411	maeB
maeB	F52_00695	maeB
maeB	GCA_001027945.1_00080	maeB
maeB	GCA_001027945.1_00829	maeB
maeB	HI1_01833	maeB
maeB	IR1_00224	maeB
maeB	IR1_03645	maeB
maeB	IR1_02146	maeB
maeB	IR1_04789	maeB
maeB	KCOM3131_00523	maeB
maeB	KCOM3131_01845	maeB
maeB	KCTC12974_01252	maeB
maeB	KCTC12974_00532	maeB
maeB	KL-A_00366	maeB
maeB	L12_00060	maeB
maeB	LL0412.1.7_00286	maeB
maeB	LL0412.1.7_01924	maeB
maeB	MAR_2009_71_01769	maeB
maeB	MAR_2009_71_03694	maeB
maeB	NN016038_01570	maeB
maeB	NN016038_02415	maeB
maeB	PAO1_05216	maeB
maeB	PAO1_00861	maeB
maeB	PAO1_03593	maeB
maeB	UW101_01721	maeB
maeB	UW101_03678	maeB
maeB	V4-33_02040	maeB
maeB	W5C_00591	maeB
maeB	ZONMW-01_00358	maeB
maeB	ZONMW-02_02834	maeB
maeB	ZONMW-02_01050	maeB
maeB	ZONMW-03_01084	maeB
maeB	ZONMW-04_00588	maeB
maeB	ZONMW-05_01237	maeB
maeB	ZONMW-06_03724	maeB
maeB	ZONMW-06_02476	maeB
maeB	ZONMW-07_00364	maeB
maeB	ZONMW-07_02950	maeB
maeB	ZONMW-08_01455	maeB
maeB	ZONMW-08_03813	maeB
maeB	ZONMW-09_00005	maeB
maeB	ZONMW-10_00670	maeB
maeB	ZONMW-11_01097	maeB
maeB	ZONMW-12_01101	maeB

maeB	ZONMW-12_04069	maeB
maeB	ZONMW-13_01018	maeB
maeB	ZONMW-14_01573	maeB
maeB	ZONMW-15_00417	maeB
maeB	ZONMW-15_00218	maeB
maeB	ZONMW-19_01031	maeB
maeB	ZONMW-19_00483	maeB
maeB	ZONMW-20_03083	maeB
maeB	ZONMW-20_02034	maeB
maeB	ZONMW-20_00545	maeB
maeB	ZONMW-21_00186	maeB
maeB	ZONMW-22_00186	maeB
maeB	ZONMW-23_01301	maeB
maeB	ZONMW-24_01274	maeB
maeB	ZONMW-25_02273	maeB
maeB	ZONMW-26_01291	maeB
maeB	ZONMW-27_00582	maeB
maeB	ZONMW-28_03895	maeB
maeB	ZONMW-28_00155	maeB
maeB	ZONMW-28_01927	maeB
maeB	ZONMW-29_03024	maeB
maeB	ZONMW-30_00737	maeB
maeB	ZONMW-31_03180	maeB
maeB	ZONMW-31_02801	maeB
maeB	ZONMW-32_04004	maeB
maeB	ZONMW-32_00946	maeB
maeB	ZONMW-32_02045	maeB
maeB	ZONMW-33_03963	maeB
maeB	ZONMW-33_00936	maeB
maeB	ZONMW-33_02036	maeB
maeB	ZONMW-34_03792	maeB
maeB	ZONMW-35_03748	maeB
maeB	ZONMW-36_03965	maeB
maeB	ZONMW-36_00155	maeB
maeB	ZONMW-36_02200	maeB
maeB	ZONMW-37_00185	maeB
maeB	ZONMW-38_00241	maeB
maeB	ZONMW-38_02264	maeB
maeB	ZONMW-38_02472	maeB
maeB	ZONMW-38_01262	maeB
maeB	ZONMW-39_04149	maeB
maeB	ZONMW-40_02593	maeB
maeB	ZONMW-41_01891	maeB
maeB	ZONMW-41_02397	maeB
maeB	ZONMW-42_02320	maeB
maeB	ZONMW-42_02701	maeB
maeB	ZONMW-43_03745	maeB
maeB	ZONMW-44_03177	maeB

maeB	ZONMW-44_03352	maeB
maeB	ZONMW-45_00667	maeB
maeB	ZONMW-46_00792	maeB
maeB	ZONMW-47_00792	maeB
maeB	ZONMW-48_01054	maeB
maeB	ZONMW-48_00864	maeB
maeB	ZONMW-49_01447	maeB
maeB	ZONMW-50_01449	maeB
maeB	ZONMW-51_00703	maeB
maeB	ZONMW-52_01303	maeB
maeB	ZONMW-53_00522	maeB
maeB	ZONMW-54_00513	maeB
maeB	ZONMW-55_00517	maeB
maeB	ZONMW-56_00796	maeB
maeB	ZONMW-57_01097	maeB
maeB	ZONMW-58_01487	maeB
maeB	ZONMW-59_01099	maeB
maeB	ZONMW-60_01468	maeB
maeB	ZONMW-61_02931	maeB
maeB	ZONMW-61_03942	maeB
maeB	ZONMW-62_02303	maeB
maeB	ZONMW-62_03147	maeB
maeB	ZONMW-63_00853	maeB
maeB	ZONMW-64_04143	maeB
maeB	ZONMW-65_02894	maeB
maeB	ZONMW-65_01445	maeB
maeB	ZONMW-66_01116	maeB
maeB	ZONMW-66_00081	maeB
maeB	ZONMW-67_03570	maeB
maeB	ZONMW-67_01944	maeB
maeB	ZONMW-68_03226	maeB
maeB	ZONMW-68_02339	maeB
maeB	ZONMW-69_03572	maeB
maeB	ZONMW-69_01999	maeB
maeB	ZONMW-72_00459	maeB
maeB	ZONMW-72_03406	maeB
maeB	ZONMW-72_03999	maeB
maeB	ZONMW-73_03009	maeB
maeB	ZONMW-73_02325	maeB
maeB	ZONMW-74_01800	maeB
maeB	ZONMW-75_00322	maeB
maeB	ZONMW-75_00625	maeB
maeB	da5A-8_01610	maeB
maeB	da5A-8_00236	maeB
ftsE	18_00059	ftsE
ftsE	18_03695	ftsE
ftsE	18_00588	ftsE
ftsE	18_03881	ftsE

ftsE	18_03355	ftsE
ftsE	18_01582	ftsE
ftsE	18_00102	ftsE
ftsE	18_02845	ftsE
ftsE	18_00928	ftsE
ftsE	18_03229	ftsE
ftsE	18_00889	ftsE
ftsE	18_02408	ftsE
ftsE	18_00628	ftsE
ftsE	18_00333	ftsE
ftsE	18_02143	ftsE
ftsE	18_02062	ftsE
ftsE	18_00927	ftsE
ftsE	18_01175	ftsE
ftsE	18_01158	ftsE
ftsE	18_03853	ftsE
ftsE	18_00081	ftsE
ftsE	18_01560	ftsE
ftsE	18_03348	ftsE
ftsE	18_02140	ftsE
ftsE	18_00462	ftsE
ftsE	18_03209	ftsE
ftsE	18_02792	ftsE
ftsE	18_00094	ftsE
ftsE	18_03053	ftsE
ftsE	18_01101	ftsE
ftsE	18_03899	ftsE
ftsE	18_01194	ftsE
ftsE	18_03668	ftsE
ftsE	ACAM48_01623	ftsE
ftsE	ACAM48_01067	ftsE
ftsE	ACAM48_01490	ftsE
ftsE	ACAM48_00410	ftsE
ftsE	ACAM48_00086	ftsE
ftsE	ACAM48_02712	ftsE
ftsE	ACAM48_03299	ftsE
ftsE	ACAM48_03020	ftsE
ftsE	ACAM48_01943	ftsE
ftsE	ACAM48_03067	ftsE
ftsE	ACAM48_01315	ftsE
ftsE	ACAM48_00665	ftsE
ftsE	ACAM48_02454	ftsE
ftsE	ACAM48_00519	ftsE
ftsE	ACAM48_01596	ftsE
ftsE	ACAM48_01597	ftsE
ftsE	ACAM48_00918	ftsE
ftsE	ACAM48_00137	ftsE
ftsE	ACAM48_01502	ftsE

ftsE	ACAM48_01938	ftsE
ftsE	ACAM48_03208	ftsE
ftsE	ACAM48_00265	ftsE
ftsE	ACAM48_00177	ftsE
ftsE	ACAM48_03455	ftsE
ftsE	ACAM48_03029	ftsE
ftsE	ACAM48_03347	ftsE
ftsE	ACAM48_03023	ftsE
ftsE	ACAM48_03316	ftsE
ftsE	ACAM48_00411	ftsE
ftsE	ACAM48_02792	ftsE
ftsE	ACAM48_03165	ftsE
ftsE	ACAM48_01253	ftsE
ftsE	ACAM48_02457	ftsE
ftsE	ACAM48_02143	ftsE
ftsE	CAM030_01511	ftsE
ftsE	CAM030_00219	ftsE
ftsE	CAM030_01728	ftsE
ftsE	CAM030_00863	ftsE
ftsE	CAM030_02324	ftsE
ftsE	CAM030_00686	ftsE
ftsE	CAM030_00772	ftsE
ftsE	CAM030_01717	ftsE
ftsE	CAM030_02574	ftsE
ftsE	CAM030_00948	ftsE
ftsE	CAM030_02771	ftsE
ftsE	CAM030_01686	ftsE
ftsE	CAM030_00855	ftsE
ftsE	CAM030_00018	ftsE
ftsE	CAM030_01415	ftsE
ftsE	CAM030_00240	ftsE
ftsE	CAM030_02585	ftsE
ftsE	CAM030_02420	ftsE
ftsE	CAM030_00912	ftsE
ftsE	CAM030_01682	ftsE
ftsE	CAM030_01131	ftsE
ftsE	CAM030_01836	ftsE
ftsE	CAM030_00039	ftsE
ftsE	CAM030_00488	ftsE
ftsE	CAM030_00259	ftsE
ftsE	CAM030_00983	ftsE
ftsE	CECT7946_01029	ftsE
ftsE	CECT7946_02961	ftsE
ftsE	CECT7946_02283	ftsE
ftsE	CECT7946_01650	ftsE
ftsE	CECT7946_01967	ftsE
ftsE	CECT7946_01261	ftsE
ftsE	CECT7946_01680	ftsE

ftsE	CECT7946_03584	ftsE
ftsE	CECT7946_01421	ftsE
ftsE	CECT7946_03368	ftsE
ftsE	CECT7946_01209	ftsE
ftsE	CECT7946_01009	ftsE
ftsE	CECT7946_00220	ftsE
ftsE	CECT7946_02012	ftsE
ftsE	CECT7946_02604	ftsE
ftsE	CECT7946_03627	ftsE
ftsE	CECT7946_02685	ftsE
ftsE	CECT7946_03649	ftsE
ftsE	CECT7946_03400	ftsE
ftsE	CECT7946_02020	ftsE
ftsE	CECT7946_02926	ftsE
ftsE	CECT7946_02452	ftsE
ftsE	CECT7946_02453	ftsE
ftsE	CECT7946_03597	ftsE
ftsE	CECT7946_01015	ftsE
ftsE	CECT7946_01957	ftsE
ftsE	CECT7946_03586	ftsE
ftsE	CECT7946_01601	ftsE
ftsE	CECT7946_00264	ftsE
ftsE	CECT7946_00049	ftsE
ftsE	CECT7946_01493	ftsE
ftsE	CECT7946_02159	ftsE
ftsE	CECT7946_00999	ftsE
ftsE	CECT7946_01743	ftsE
ftsE	CECT7946_01450	ftsE
ftsE	CECT7946_01793	ftsE
ftsE	CECT7946_03214	ftsE
ftsE	CECT7946_02270	ftsE
ftsE	CECT7946_00803	ftsE
ftsE	CECT7946_00194	ftsE
ftsE	CECT7946_02134	ftsE
ftsE	CECT7946_01061	ftsE
ftsE	CGMCC1.8863_03586	ftsE
ftsE	CGMCC1.8863_01748	ftsE
ftsE	CGMCC1.8863_01241	ftsE
ftsE	CGMCC1.8863_03445	ftsE
ftsE	CGMCC1.8863_03019	ftsE
ftsE	CGMCC1.8863_00216	ftsE
ftsE	CGMCC1.8863_01110	ftsE
ftsE	CGMCC1.8863_02864	ftsE
ftsE	CGMCC1.8863_00968	ftsE
ftsE	CGMCC1.8863_02337	ftsE
ftsE	CGMCC1.8863_01308	ftsE
ftsE	CGMCC1.8863_01309	ftsE
ftsE	CGMCC1.8863_03379	ftsE

ftsE	CGMCC1.8863_00207	ftsE
ftsE	CGMCC1.8863_02094	ftsE
ftsE	CGMCC1.8863_01375	ftsE
ftsE	CGMCC1.8863_00892	ftsE
ftsE	CGMCC1.8863_01770	ftsE
ftsE	CGMCC1.8863_00994	ftsE
ftsE	CGMCC1.8863_03420	ftsE
ftsE	CGMCC1.8863_02839	ftsE
ftsE	CGMCC1.8863_03382	ftsE
ftsE	CGMCC1.8863_00714	ftsE
ftsE	CGMCC1.8863_02522	ftsE
ftsE	CGMCC1.8863_02413	ftsE
ftsE	CGMCC1.8863_02852	ftsE
ftsE	CGMCC1.8863_02641	ftsE
ftsE	CGMCC1.8863_00834	ftsE
ftsE	CGMCC1.8863_03462	ftsE
ftsE	CGMCC1.8863_00006	ftsE
ftsE	CGMCC1.8863_00839	ftsE
ftsE	CGMCC1.8863_00007	ftsE
ftsE	CL8139_03149	ftsE
ftsE	CL8139_02632	ftsE
ftsE	CL8139_02174	ftsE
ftsE	CL8139_02338	ftsE
ftsE	CL8139_00198	ftsE
ftsE	CL8139_01910	ftsE
ftsE	CL8139_02069	ftsE
ftsE	CL8139_00269	ftsE
ftsE	CL8139_00725	ftsE
ftsE	CL8139_03305	ftsE
ftsE	CL8139_02854	ftsE
ftsE	CL8139_01677	ftsE
ftsE	CL8139_00337	ftsE
ftsE	CL8139_01669	ftsE
ftsE	CL8139_02158	ftsE
ftsE	CL8139_01518	ftsE
ftsE	CL8139_01683	ftsE
ftsE	CL8139_00270	ftsE
ftsE	CL8139_00797	ftsE
ftsE	CL8139_00918	ftsE
ftsE	CL8139_01018	ftsE
ftsE	CL8139_02058	ftsE
ftsE	CL8139_02851	ftsE
ftsE	CL8139_02402	ftsE
ftsE	CL8139_01746	ftsE
ftsE	CL8139_02896	ftsE
ftsE	CL8139_02533	ftsE
ftsE	CL8139_02497	ftsE
ftsE	CL8139_01861	ftsE

ftsE	CL8139_00541	ftsE
ftsE	CL8139_00886	ftsE
ftsE	CL8139_02312	ftsE
ftsE	CL8139_00809	ftsE
ftsE	DAU203_02440	ftsE
ftsE	DAU203_02734	ftsE
ftsE	DAU203_00960	ftsE
ftsE	DAU203_00132	ftsE
ftsE	DAU203_02009	ftsE
ftsE	DAU203_03221	ftsE
ftsE	DAU203_02406	ftsE
ftsE	DAU203_00817	ftsE
ftsE	DAU203_03065	ftsE
ftsE	DAU203_02103	ftsE
ftsE	DAU203_00034	ftsE
ftsE	DAU203_01283	ftsE
ftsE	DAU203_03073	ftsE
ftsE	DAU203_01275	ftsE
ftsE	DAU203_00944	ftsE
ftsE	DAU203_02201	ftsE
ftsE	DAU203_01289	ftsE
ftsE	DAU203_00816	ftsE
ftsE	DAU203_01624	ftsE
ftsE	DAU203_01694	ftsE
ftsE	DAU203_01795	ftsE
ftsE	DAU203_02417	ftsE
ftsE	DAU203_00037	ftsE
ftsE	DAU203_00835	ftsE
ftsE	DAU203_00097	ftsE
ftsE	DAU203_00579	ftsE
ftsE	DAU203_02880	ftsE
ftsE	DAU203_02844	ftsE
ftsE	DAU203_01503	ftsE
ftsE	DAU203_00352	ftsE
ftsE	DAU203_03100	ftsE
ftsE	DAU203_01662	ftsE
ftsE	DAU203_00159	ftsE
ftsE	DAU203_01612	ftsE
ftsE	DD5b_00610	ftsE
ftsE	DD5b_00155	ftsE
ftsE	DD5b_00538	ftsE
ftsE	DD5b_02596	ftsE
ftsE	DD5b_01471	ftsE
ftsE	DD5b_02680	ftsE
ftsE	DD5b_01270	ftsE
ftsE	DD5b_00435	ftsE
ftsE	DD5b_00735	ftsE
ftsE	DD5b_01632	ftsE

ftsE	DD5b_00329	ftsE
ftsE	DD5b_00183	ftsE
ftsE	DD5b_02180	ftsE
ftsE	DD5b_02481	ftsE
ftsE	DD5b_00696	ftsE
ftsE	DD5b_01382	ftsE
ftsE	DD5b_01468	ftsE
ftsE	DD5b_02832	ftsE
ftsE	DD5b_00616	ftsE
ftsE	DD5b_02751	ftsE
ftsE	DD5b_01025	ftsE
ftsE	DD5b_02114	ftsE
ftsE	DD5b_00220	ftsE
ftsE	DD5b_01960	ftsE
ftsE	DSM1208_02680	ftsE
ftsE	DSM1208_00085	ftsE
ftsE	DSM1208_01244	ftsE
ftsE	DSM1208_02745	ftsE
ftsE	DSM1208_04118	ftsE
ftsE	DSM1208_02305	ftsE
ftsE	DSM1208_04115	ftsE
ftsE	DSM1208_01706	ftsE
ftsE	DSM1208_00211	ftsE
ftsE	DSM1208_03945	ftsE
ftsE	DSM1208_02640	ftsE
ftsE	DSM1208_01582	ftsE
ftsE	DSM1208_03347	ftsE
ftsE	DSM1208_01991	ftsE
ftsE	DSM1208_02845	ftsE
ftsE	DSM1208_04178	ftsE
ftsE	DSM1208_00855	ftsE
ftsE	DSM1208_04101	ftsE
ftsE	DSM1208_04177	ftsE
ftsE	DSM1208_00805	ftsE
ftsE	DSM1208_03730	ftsE
ftsE	DSM1208_01476	ftsE
ftsE	DSM1208_00216	ftsE
ftsE	DSM1208_02728	ftsE
ftsE	DSM1208_01774	ftsE
ftsE	DSM1208_01581	ftsE
ftsE	DSM1208_01104	ftsE
ftsE	DSM1208_02655	ftsE
ftsE	DSM1208_02842	ftsE
ftsE	DSM1208_04165	ftsE
ftsE	DSM1208_03876	ftsE
ftsE	DSM1208_04540	ftsE
ftsE	DSM1208_02154	ftsE
ftsE	DSM1208_02782	ftsE

ftsE	DSM1208_04194	ftsE
ftsE	DSM1208_04206	ftsE
ftsE	DSM1208_01713	ftsE
ftsE	DSM1208_00060	ftsE
ftsE	DSM12212_02052	ftsE
ftsE	DSM12212_00466	ftsE
ftsE	DSM12212_00856	ftsE
ftsE	DSM12212_02245	ftsE
ftsE	DSM12212_03462	ftsE
ftsE	DSM12212_01303	ftsE
ftsE	DSM12212_01777	ftsE
ftsE	DSM12212_00328	ftsE
ftsE	DSM12212_00604	ftsE
ftsE	DSM12212_02358	ftsE
ftsE	DSM12212_00373	ftsE
ftsE	DSM12212_01010	ftsE
ftsE	DSM12212_02159	ftsE
ftsE	DSM12212_00153	ftsE
ftsE	DSM12212_01600	ftsE
ftsE	DSM12212_00672	ftsE
ftsE	DSM12212_01032	ftsE
ftsE	DSM12212_00762	ftsE
ftsE	DSM12212_00673	ftsE
ftsE	DSM12212_03328	ftsE
ftsE	DSM12212_00504	ftsE
ftsE	DSM12212_02404	ftsE
ftsE	DSM12212_02875	ftsE
ftsE	DSM12212_00409	ftsE
ftsE	DSM12212_02087	ftsE
ftsE	DSM12212_02269	ftsE
ftsE	DSM12212_00330	ftsE
ftsE	DSM12212_00200	ftsE
ftsE	DSM12212_02954	ftsE
ftsE	DSM12212_01842	ftsE
ftsE	DSM12212_01712	ftsE
ftsE	DSM12212_01223	ftsE
ftsE	DSM12212_00843	ftsE
ftsE	DSM12212_00113	ftsE
ftsE	DSM12212_00337	ftsE
ftsE	DSM15343_00452	ftsE
ftsE	DSM15343_02741	ftsE
ftsE	DSM15343_01259	ftsE
ftsE	DSM15343_00050	ftsE
ftsE	DSM15343_00754	ftsE
ftsE	DSM15343_03052	ftsE
ftsE	DSM15343_02000	ftsE
ftsE	DSM15343_00249	ftsE
ftsE	DSM15343_01115	ftsE

ftsE	DSM15343_02891	ftsE
ftsE	DSM15343_01209	ftsE
ftsE	DSM15343_01659	ftsE
ftsE	DSM15343_01448	ftsE
ftsE	DSM15343_00098	ftsE
ftsE	DSM15343_01516	ftsE
ftsE	DSM15343_01546	ftsE
ftsE	DSM15343_00773	ftsE
ftsE	DSM15343_00096	ftsE
ftsE	DSM15343_00577	ftsE
ftsE	DSM15343_02932	ftsE
ftsE	DSM15343_01826	ftsE
ftsE	DSM15343_01744	ftsE
ftsE	DSM15343_01202	ftsE
ftsE	DSM15343_01743	ftsE
ftsE	DSM15343_01632	ftsE
ftsE	DSM15343_01719	ftsE
ftsE	DSM15343_01212	ftsE
ftsE	DSM15343_01192	ftsE
ftsE	DSM15343_02315	ftsE
ftsE	DSM15343_01834	ftsE
ftsE	DSM15343_02110	ftsE
ftsE	DSM15343_00476	ftsE
ftsE	DSM15343_00405	ftsE
ftsE	DSM15343_02814	ftsE
ftsE	DSM15343_01017	ftsE
ftsE	DSM15361_02440	ftsE
ftsE	DSM15361_01867	ftsE
ftsE	DSM15361_02099	ftsE
ftsE	DSM15361_01438	ftsE
ftsE	DSM15361_00929	ftsE
ftsE	DSM15361_01356	ftsE
ftsE	DSM15361_02160	ftsE
ftsE	DSM15361_02043	ftsE
ftsE	DSM15361_00421	ftsE
ftsE	DSM15361_02085	ftsE
ftsE	DSM15361_02750	ftsE
ftsE	DSM15361_02471	ftsE
ftsE	DSM15361_00115	ftsE
ftsE	DSM15361_01100	ftsE
ftsE	DSM15361_02168	ftsE
ftsE	DSM15361_01581	ftsE
ftsE	DSM15361_00379	ftsE
ftsE	DSM15361_00029	ftsE
ftsE	DSM15361_01445	ftsE
ftsE	DSM15361_02162	ftsE
ftsE	DSM15361_01311	ftsE
ftsE	DSM15361_02225	ftsE

ftsE	DSM15361_01684	ftsE
ftsE	DSM15361_00568	ftsE
ftsE	DSM15361_02687	ftsE
ftsE	DSM15361_01965	ftsE
ftsE	DSM15365_00558	ftsE
ftsE	DSM15365_01505	ftsE
ftsE	DSM15365_00179	ftsE
ftsE	DSM15365_01543	ftsE
ftsE	DSM15365_00630	ftsE
ftsE	DSM15365_01738	ftsE
ftsE	DSM15365_02261	ftsE
ftsE	DSM15365_03221	ftsE
ftsE	DSM15365_00652	ftsE
ftsE	DSM15365_02141	ftsE
ftsE	DSM15365_01793	ftsE
ftsE	DSM15365_02017	ftsE
ftsE	DSM15365_02201	ftsE
ftsE	DSM15365_00586	ftsE
ftsE	DSM15365_00608	ftsE
ftsE	DSM15365_01514	ftsE
ftsE	DSM15365_03597	ftsE
ftsE	DSM15365_01749	ftsE
ftsE	DSM15365_00690	ftsE
ftsE	DSM15365_02016	ftsE
ftsE	DSM15365_01973	ftsE
ftsE	DSM15365_00927	ftsE
ftsE	DSM15365_00277	ftsE
ftsE	DSM15365_00694	ftsE
ftsE	DSM15365_00041	ftsE
ftsE	DSM15365_03176	ftsE
ftsE	DSM15365_00431	ftsE
ftsE	DSM15365_03406	ftsE
ftsE	DSM15365_01263	ftsE
ftsE	DSM15365_02162	ftsE
ftsE	DSM15365_01484	ftsE
ftsE	DSM15365_03013	ftsE
ftsE	DSM15365_02598	ftsE
ftsE	DSM15365_00506	ftsE
ftsE	DSM15718_01916	ftsE
ftsE	DSM15718_02829	ftsE
ftsE	DSM15718_00890	ftsE
ftsE	DSM15718_03059	ftsE
ftsE	DSM15718_03295	ftsE
ftsE	DSM15718_03085	ftsE
ftsE	DSM15718_03020	ftsE
ftsE	DSM15718_00141	ftsE
ftsE	DSM15718_00490	ftsE
ftsE	DSM15718_00950	ftsE

ftsE	DSM15718_02509	ftsE
ftsE	DSM15718_00801	ftsE
ftsE	DSM15718_01166	ftsE
ftsE	DSM15718_02012	ftsE
ftsE	DSM15718_00772	ftsE
ftsE	DSM15718_00885	ftsE
ftsE	DSM15718_00605	ftsE
ftsE	DSM15718_02678	ftsE
ftsE	DSM15718_02143	ftsE
ftsE	DSM15718_01961	ftsE
ftsE	DSM15718_01700	ftsE
ftsE	DSM15718_01699	ftsE
ftsE	DSM15718_01124	ftsE
ftsE	DSM15718_00736	ftsE
ftsE	DSM15718_00391	ftsE
ftsE	DSM15718_00195	ftsE
ftsE	DSM15718_03094	ftsE
ftsE	DSM15718_02781	ftsE
ftsE	DSM15718_01402	ftsE
ftsE	DSM15718_01549	ftsE
ftsE	DSM15718_02822	ftsE
ftsE	DSM15718_00407	ftsE
ftsE	DSM15718_02612	ftsE
ftsE	DSM16505T_02580	ftsE
ftsE	DSM16505T_01540	ftsE
ftsE	DSM16505T_02286	ftsE
ftsE	DSM16505T_02512	ftsE
ftsE	DSM16505T_02303	ftsE
ftsE	DSM16505T_01641	ftsE
ftsE	DSM16505T_02451	ftsE
ftsE	DSM16505T_00531	ftsE
ftsE	DSM16505T_01567	ftsE
ftsE	DSM16505T_00181	ftsE
ftsE	DSM16505T_01105	ftsE
ftsE	DSM16505T_01530	ftsE
ftsE	DSM16505T_00763	ftsE
ftsE	DSM16505T_00471	ftsE
ftsE	DSM16505T_00819	ftsE
ftsE	DSM16505T_01066	ftsE
ftsE	DSM16505T_01369	ftsE
ftsE	DSM16505T_00533	ftsE
ftsE	DSM16505T_02296	ftsE
ftsE	DSM16505T_00421	ftsE
ftsE	DSM16505T_02271	ftsE
ftsE	DSM16505T_01038	ftsE
ftsE	DSM16505T_01317	ftsE
ftsE	DSM16505T_02060	ftsE
ftsE	DSM16505T_02397	ftsE

ftsE	DSM16505T_02538	ftsE
ftsE	DSM18103_03429	ftsE
ftsE	DSM18103_00279	ftsE
ftsE	DSM18103_03532	ftsE
ftsE	DSM18103_02717	ftsE
ftsE	DSM18103_02467	ftsE
ftsE	DSM18103_00237	ftsE
ftsE	DSM18103_02698	ftsE
ftsE	DSM18103_02037	ftsE
ftsE	DSM18103_02158	ftsE
ftsE	DSM18103_02892	ftsE
ftsE	DSM18103_00751	ftsE
ftsE	DSM18103_03316	ftsE
ftsE	DSM18103_00818	ftsE
ftsE	DSM18103_01456	ftsE
ftsE	DSM18103_01322	ftsE
ftsE	DSM18103_03609	ftsE
ftsE	DSM18103_00267	ftsE
ftsE	DSM18103_00322	ftsE
ftsE	DSM18103_02802	ftsE
ftsE	DSM18103_00145	ftsE
ftsE	DSM18103_00355	ftsE
ftsE	DSM18103_01644	ftsE
ftsE	DSM18103_01221	ftsE
ftsE	DSM18103_01993	ftsE
ftsE	DSM18103_03017	ftsE
ftsE	DSM18103_00944	ftsE
ftsE	DSM18103_01169	ftsE
ftsE	DSM18103_02708	ftsE
ftsE	DSM18103_02752	ftsE
ftsE	DSM18103_02515	ftsE
ftsE	DSM18103_00038	ftsE
ftsE	DSM18103_00711	ftsE
ftsE	DSM18103_01345	ftsE
ftsE	DSM18103_01276	ftsE
ftsE	DSM18103_00141	ftsE
ftsE	DSM18103_00135	ftsE
ftsE	DSM18103_01968	ftsE
ftsE	DSM18293_01173	ftsE
ftsE	DSM18293_02699	ftsE
ftsE	DSM18293_01828	ftsE
ftsE	DSM18293_03640	ftsE
ftsE	DSM18293_03847	ftsE
ftsE	DSM18293_00382	ftsE
ftsE	DSM18293_02179	ftsE
ftsE	DSM18293_04296	ftsE
ftsE	DSM18293_01954	ftsE
ftsE	DSM18293_00088	ftsE

ftsE	DSM18293_01558	ftsE
ftsE	DSM18293_02548	ftsE
ftsE	DSM18293_03688	ftsE
ftsE	DSM18293_03895	ftsE
ftsE	DSM18293_02523	ftsE
ftsE	DSM18293_02364	ftsE
ftsE	DSM18293_02317	ftsE
ftsE	DSM18293_00411	ftsE
ftsE	DSM18293_00602	ftsE
ftsE	DSM18293_00285	ftsE
ftsE	DSM18293_01111	ftsE
ftsE	DSM18293_02993	ftsE
ftsE	DSM18293_01481	ftsE
ftsE	DSM18293_04058	ftsE
ftsE	DSM18293_01551	ftsE
ftsE	DSM18293_01728	ftsE
ftsE	DSM18293_02705	ftsE
ftsE	DSM18293_02146	ftsE
ftsE	DSM18293_01016	ftsE
ftsE	DSM18293_03579	ftsE
ftsE	DSM18293_01599	ftsE
ftsE	DSM18293_03048	ftsE
ftsE	DSM18293_03033	ftsE
ftsE	DSM18293_02729	ftsE
ftsE	DSM18293_02486	ftsE
ftsE	DSM18841_02785	ftsE
ftsE	DSM18841_02166	ftsE
ftsE	DSM18841_01339	ftsE
ftsE	DSM18841_02898	ftsE
ftsE	DSM18841_02626	ftsE
ftsE	DSM18841_01321	ftsE
ftsE	DSM18841_01681	ftsE
ftsE	DSM18841_01238	ftsE
ftsE	DSM18841_02188	ftsE
ftsE	DSM18841_01305	ftsE
ftsE	DSM18841_00389	ftsE
ftsE	DSM18841_02649	ftsE
ftsE	DSM18841_01002	ftsE
ftsE	DSM18841_02155	ftsE
ftsE	DSM18841_00952	ftsE
ftsE	DSM18841_00314	ftsE
ftsE	DSM18841_00640	ftsE
ftsE	DSM18841_00249	ftsE
ftsE	DSM18841_02378	ftsE
ftsE	DSM18841_00394	ftsE
ftsE	DSM18841_01330	ftsE
ftsE	DSM18841_03007	ftsE
ftsE	DSM18841_00919	ftsE

ftsE	DSM18841_02316	ftsE
ftsE	DSM18841_01412	ftsE
ftsE	DSM18841_02934	ftsE
ftsE	DSM18841_02099	ftsE
ftsE	DSM18841_01885	ftsE
ftsE	DSM18841_02041	ftsE
ftsE	DSM18842_02722	ftsE
ftsE	DSM18842_01508	ftsE
ftsE	DSM18842_01685	ftsE
ftsE	DSM18842_02805	ftsE
ftsE	DSM18842_03170	ftsE
ftsE	DSM18842_01762	ftsE
ftsE	DSM18842_01668	ftsE
ftsE	DSM18842_03175	ftsE
ftsE	DSM18842_01724	ftsE
ftsE	DSM18842_02872	ftsE
ftsE	DSM18842_01532	ftsE
ftsE	DSM18842_00845	ftsE
ftsE	DSM18842_02593	ftsE
ftsE	DSM18842_00317	ftsE
ftsE	DSM18842_02999	ftsE
ftsE	DSM18842_01497	ftsE
ftsE	DSM18842_00918	ftsE
ftsE	DSM18842_00973	ftsE
ftsE	DSM18842_03056	ftsE
ftsE	DSM18842_01285	ftsE
ftsE	DSM18842_00841	ftsE
ftsE	DSM18842_01676	ftsE
ftsE	DSM18842_01703	ftsE
ftsE	DSM18842_00389	ftsE
ftsE	DSM18842_01227	ftsE
ftsE	DSM18842_01957	ftsE
ftsE	DSM18842_02761	ftsE
ftsE	DSM18842_01438	ftsE
ftsE	DSM18842_02350	ftsE
ftsE	DSM19866_00475	ftsE
ftsE	DSM19866_01685	ftsE
ftsE	DSM19866_03675	ftsE
ftsE	DSM19866_00262	ftsE
ftsE	DSM19866_03394	ftsE
ftsE	DSM19866_02788	ftsE
ftsE	DSM19866_04040	ftsE
ftsE	DSM19866_00430	ftsE
ftsE	DSM19866_01932	ftsE
ftsE	DSM19866_04212	ftsE
ftsE	DSM19866_00052	ftsE
ftsE	DSM19866_04411	ftsE
ftsE	DSM19866_03375	ftsE

ftsE	DSM19866_00065	ftsE
ftsE	DSM19866_04362	ftsE
ftsE	DSM19866_03373	ftsE
ftsE	DSM19866_02277	ftsE
ftsE	DSM19866_02949	ftsE
ftsE	DSM19866_03361	ftsE
ftsE	DSM19866_04361	ftsE
ftsE	DSM19866_04844	ftsE
ftsE	DSM19866_02648	ftsE
ftsE	DSM19866_04775	ftsE
ftsE	DSM19866_02012	ftsE
ftsE	DSM19866_01276	ftsE
ftsE	DSM19866_02335	ftsE
ftsE	DSM19866_00241	ftsE
ftsE	DSM19866_02669	ftsE
ftsE	DSM19866_00453	ftsE
ftsE	DSM19866_00062	ftsE
ftsE	DSM19866_00292	ftsE
ftsE	DSM19866_03820	ftsE
ftsE	DSM19866_04650	ftsE
ftsE	DSM19866_01632	ftsE
ftsE	DSM19866_04829	ftsE
ftsE	DSM19866_01948	ftsE
ftsE	DSM19866_00214	ftsE
ftsE	DSM2041_02925	ftsE
ftsE	DSM2041_02435	ftsE
ftsE	DSM2041_04454	ftsE
ftsE	DSM2041_02688	ftsE
ftsE	DSM2041_04888	ftsE
ftsE	DSM2041_05360	ftsE
ftsE	DSM2041_04448	ftsE
ftsE	DSM2041_04683	ftsE
ftsE	DSM2041_02723	ftsE
ftsE	DSM2041_00133	ftsE
ftsE	DSM2041_03063	ftsE
ftsE	DSM2041_02070	ftsE
ftsE	DSM2041_02271	ftsE
ftsE	DSM2041_02713	ftsE
ftsE	DSM2041_01304	ftsE
ftsE	DSM2041_02404	ftsE
ftsE	DSM2041_01789	ftsE
ftsE	DSM2041_03112	ftsE
ftsE	DSM2041_00009	ftsE
ftsE	DSM2041_03079	ftsE
ftsE	DSM2041_04258	ftsE
ftsE	DSM2041_03879	ftsE
ftsE	DSM2041_05389	ftsE
ftsE	DSM2041_00295	ftsE

ftsE	DSM2041_05095	ftsE
ftsE	DSM2041_05009	ftsE
ftsE	DSM2041_05390	ftsE
ftsE	DSM2041_00438	ftsE
ftsE	DSM2041_02281	ftsE
ftsE	DSM2041_01858	ftsE
ftsE	DSM2041_00100	ftsE
ftsE	DSM2041_00817	ftsE
ftsE	DSM2041_03286	ftsE
ftsE	DSM2041_02721	ftsE
ftsE	DSM2041_02642	ftsE
ftsE	DSM2041_05321	ftsE
ftsE	DSM2041_03595	ftsE
ftsE	DSM2041_02496	ftsE
ftsE	DSM2041_01665	ftsE
ftsE	DSM2041_01863	ftsE
ftsE	DSM2041_03226	ftsE
ftsE	DSM2041_00175	ftsE
ftsE	DSM2041_01253	ftsE
ftsE	DSM2041_02262	ftsE
ftsE	DSM2041_02258	ftsE
ftsE	DSM2041_02782	ftsE
ftsE	DSM2041_05192	ftsE
ftsE	DSM2061_04168	ftsE
ftsE	DSM2061_00465	ftsE
ftsE	DSM2061_02774	ftsE
ftsE	DSM2061_04233	ftsE
ftsE	DSM2061_03402	ftsE
ftsE	DSM2061_03834	ftsE
ftsE	DSM2061_00937	ftsE
ftsE	DSM2061_01553	ftsE
ftsE	DSM2061_04091	ftsE
ftsE	DSM2061_03239	ftsE
ftsE	DSM2061_03109	ftsE
ftsE	DSM2061_01094	ftsE
ftsE	DSM2061_03553	ftsE
ftsE	DSM2061_04122	ftsE
ftsE	DSM2061_03638	ftsE
ftsE	DSM2061_04330	ftsE
ftsE	DSM2061_02304	ftsE
ftsE	DSM2061_02764	ftsE
ftsE	DSM2061_02357	ftsE
ftsE	DSM2061_04216	ftsE
ftsE	DSM2061_03302	ftsE
ftsE	DSM2061_04271	ftsE
ftsE	DSM2061_03108	ftsE
ftsE	DSM2061_02622	ftsE
ftsE	DSM2061_04141	ftsE

ftsE	DSM2061_04327	ftsE
ftsE	DSM2061_00971	ftsE
ftsE	DSM2061_02999	ftsE
ftsE	DSM2061_02768	ftsE
ftsE	DSM2061_01156	ftsE
ftsE	DSM2061_03752	ftsE
ftsE	DSM2061_00613	ftsE
ftsE	DSM2061_03244	ftsE
ftsE	DSM2061_00492	ftsE
ftsE	DSM2064_04023	ftsE
ftsE	DSM2064_04974	ftsE
ftsE	DSM2064_00230	ftsE
ftsE	DSM2064_03490	ftsE
ftsE	DSM2064_02167	ftsE
ftsE	DSM2064_03283	ftsE
ftsE	DSM2064_02303	ftsE
ftsE	DSM2064_03890	ftsE
ftsE	DSM2064_02925	ftsE
ftsE	DSM2064_01324	ftsE
ftsE	DSM2064_02683	ftsE
ftsE	DSM2064_03671	ftsE
ftsE	DSM2064_04826	ftsE
ftsE	DSM2064_04393	ftsE
ftsE	DSM2064_03445	ftsE
ftsE	DSM2064_01350	ftsE
ftsE	DSM2064_04588	ftsE
ftsE	DSM2064_04796	ftsE
ftsE	DSM2064_02529	ftsE
ftsE	DSM2064_04631	ftsE
ftsE	DSM2064_04609	ftsE
ftsE	DSM2064_02336	ftsE
ftsE	DSM2064_02186	ftsE
ftsE	DSM2064_02095	ftsE
ftsE	DSM2064_00760	ftsE
ftsE	DSM2064_04755	ftsE
ftsE	DSM2064_03623	ftsE
ftsE	DSM2064_03744	ftsE
ftsE	DSM2064_03062	ftsE
ftsE	DSM2064_03676	ftsE
ftsE	DSM2064_03233	ftsE
ftsE	DSM2064_04146	ftsE
ftsE	DSM2064_04983	ftsE
ftsE	DSM2064_02886	ftsE
ftsE	DSM2064_00337	ftsE
ftsE	DSM2064_03548	ftsE
ftsE	DSM2064_05009	ftsE
ftsE	DSM2064_03014	ftsE
ftsE	DSM2064_02047	ftsE

ftsE	DSM21164_02081	ftsE
ftsE	DSM21164_02407	ftsE
ftsE	DSM21164_01231	ftsE
ftsE	DSM21164_01994	ftsE
ftsE	DSM21164_00294	ftsE
ftsE	DSM21164_01189	ftsE
ftsE	DSM21164_02124	ftsE
ftsE	DSM21164_01532	ftsE
ftsE	DSM21164_01898	ftsE
ftsE	DSM21164_02564	ftsE
ftsE	DSM21164_00926	ftsE
ftsE	DSM21164_02303	ftsE
ftsE	DSM21164_03083	ftsE
ftsE	DSM21164_00707	ftsE
ftsE	DSM21164_00958	ftsE
ftsE	DSM21164_00927	ftsE
ftsE	DSM21164_01966	ftsE
ftsE	DSM21164_02103	ftsE
ftsE	DSM21164_00314	ftsE
ftsE	DSM21164_03079	ftsE
ftsE	DSM21164_01583	ftsE
ftsE	DSM21164_01364	ftsE
ftsE	DSM21164_02117	ftsE
ftsE	DSM21164_01441	ftsE
ftsE	DSM21164_01697	ftsE
ftsE	DSM21164_02013	ftsE
ftsE	DSM21164_00688	ftsE
ftsE	DSM21164_02377	ftsE
ftsE	DSM21164_00769	ftsE
ftsE	DSM23404_00954	ftsE
ftsE	DSM23404_02814	ftsE
ftsE	DSM23404_00052	ftsE
ftsE	DSM23404_01168	ftsE
ftsE	DSM23404_01413	ftsE
ftsE	DSM23404_02244	ftsE
ftsE	DSM23404_00349	ftsE
ftsE	DSM23404_01613	ftsE
ftsE	DSM23404_00861	ftsE
ftsE	DSM23404_00305	ftsE
ftsE	DSM23404_02656	ftsE
ftsE	DSM23404_01790	ftsE
ftsE	DSM23404_01066	ftsE
ftsE	DSM23404_02066	ftsE
ftsE	DSM23404_00891	ftsE
ftsE	DSM23404_00526	ftsE
ftsE	DSM23404_00064	ftsE
ftsE	DSM23404_01355	ftsE
ftsE	DSM23404_03174	ftsE

ftsE	DSM23404_00332	ftsE
ftsE	DSM23404_02063	ftsE
ftsE	DSM23404_00346	ftsE
ftsE	DSM23404_00695	ftsE
ftsE	DSM23404_02148	ftsE
ftsE	DSM23404_00207	ftsE
ftsE	DSM23404_00811	ftsE
ftsE	DSM23404_00654	ftsE
ftsE	DSM23404_00913	ftsE
ftsE	DSM23404_01940	ftsE
ftsE	DSM23404_00841	ftsE
ftsE	DSM23424_02560	ftsE
ftsE	DSM23424_01458	ftsE
ftsE	DSM23424_02752	ftsE
ftsE	DSM23424_01221	ftsE
ftsE	DSM23424_01129	ftsE
ftsE	DSM23424_02675	ftsE
ftsE	DSM23424_01303	ftsE
ftsE	DSM23424_00012	ftsE
ftsE	DSM23424_00265	ftsE
ftsE	DSM23424_01117	ftsE
ftsE	DSM23424_01182	ftsE
ftsE	DSM23424_03147	ftsE
ftsE	DSM23424_02722	ftsE
ftsE	DSM23424_03002	ftsE
ftsE	DSM23424_00071	ftsE
ftsE	DSM23424_02928	ftsE
ftsE	DSM23424_01136	ftsE
ftsE	DSM23424_01432	ftsE
ftsE	DSM23424_02083	ftsE
ftsE	DSM23424_00403	ftsE
ftsE	DSM23424_02082	ftsE
ftsE	DSM23424_00168	ftsE
ftsE	DSM23424_01848	ftsE
ftsE	DSM23424_00232	ftsE
ftsE	DSM23424_02677	ftsE
ftsE	DSM23424_02010	ftsE
ftsE	DSM23424_02630	ftsE
ftsE	DSM23424_00891	ftsE
ftsE	DSM23424_01962	ftsE
ftsE	DSM23424_01912	ftsE
ftsE	DSM23424_01481	ftsE
ftsE	DSM23424_02685	ftsE
ftsE	DSM24786_01569	ftsE
ftsE	DSM24786_00195	ftsE
ftsE	DSM24786_03126	ftsE
ftsE	DSM24786_00111	ftsE
ftsE	DSM24786_00546	ftsE

ftsE	DSM24786_02041	ftsE
ftsE	DSM24786_01605	ftsE
ftsE	DSM24786_00542	ftsE
ftsE	DSM24786_00536	ftsE
ftsE	DSM24786_01942	ftsE
ftsE	DSM24786_01218	ftsE
ftsE	DSM24786_02725	ftsE
ftsE	DSM24786_00524	ftsE
ftsE	DSM24786_03104	ftsE
ftsE	DSM24786_02731	ftsE
ftsE	DSM24786_01836	ftsE
ftsE	DSM24786_02368	ftsE
ftsE	DSM24786_02294	ftsE
ftsE	DSM24786_02717	ftsE
ftsE	DSM24786_02380	ftsE
ftsE	DSM24786_00666	ftsE
ftsE	DSM24786_02187	ftsE
ftsE	DSM24786_01112	ftsE
ftsE	DSM24786_01594	ftsE
ftsE	DSM24786_01221	ftsE
ftsE	DSM24786_01351	ftsE
ftsE	DSM24786_03399	ftsE
ftsE	DSM24786_00356	ftsE
ftsE	DSM24786_00321	ftsE
ftsE	DSM24786_02337	ftsE
ftsE	DSM24786_02992	ftsE
ftsE	DSM24786_00085	ftsE
ftsE	DSM24786_02426	ftsE
ftsE	DSM7489_02355	ftsE
ftsE	DSM7489_02608	ftsE
ftsE	DSM7489_00894	ftsE
ftsE	DSM7489_00130	ftsE
ftsE	DSM7489_01920	ftsE
ftsE	DSM7489_03053	ftsE
ftsE	DSM7489_02321	ftsE
ftsE	DSM7489_00750	ftsE
ftsE	DSM7489_02930	ftsE
ftsE	DSM7489_02015	ftsE
ftsE	DSM7489_00034	ftsE
ftsE	DSM7489_01192	ftsE
ftsE	DSM7489_02938	ftsE
ftsE	DSM7489_01184	ftsE
ftsE	DSM7489_00878	ftsE
ftsE	DSM7489_02114	ftsE
ftsE	DSM7489_01198	ftsE
ftsE	DSM7489_00749	ftsE
ftsE	DSM7489_01613	ftsE
ftsE	DSM7489_01714	ftsE

ftsE	DSM7489_02332	ftsE
ftsE	DSM7489_00037	ftsE
ftsE	DSM7489_00768	ftsE
ftsE	DSM7489_00095	ftsE
ftsE	DSM7489_00566	ftsE
ftsE	DSM7489_02751	ftsE
ftsE	DSM7489_02715	ftsE
ftsE	DSM7489_01407	ftsE
ftsE	DSM7489_00337	ftsE
ftsE	DSM7489_01581	ftsE
ftsE	DSM7489_00156	ftsE
ftsE	DSM7489_01538	ftsE
ftsE	DSW1_02514	ftsE
ftsE	DSW1_02420	ftsE
ftsE	DSW1_02008	ftsE
ftsE	DSW1_02113	ftsE
ftsE	DSW1_00583	ftsE
ftsE	DSW1_01080	ftsE
ftsE	DSW1_00539	ftsE
ftsE	DSW1_01506	ftsE
ftsE	DSW1_01201	ftsE
ftsE	DSW1_02080	ftsE
ftsE	DSW1_01568	ftsE
ftsE	DSW1_02708	ftsE
ftsE	DSW1_00067	ftsE
ftsE	DSW1_02833	ftsE
ftsE	DSW1_02370	ftsE
ftsE	DSW1_01512	ftsE
ftsE	DSW1_00979	ftsE
ftsE	DSW1_00072	ftsE
ftsE	DSW1_01667	ftsE
ftsE	DSW1_01030	ftsE
ftsE	DSW1_01483	ftsE
ftsE	DSW1_00855	ftsE
ftsE	DSW1_01508	ftsE
ftsE	DSW1_01650	ftsE
ftsE	DSW1_00014	ftsE
ftsE	DSW1_02923	ftsE
ftsE	DSW1_02339	ftsE
ftsE	DSW1_02589	ftsE
ftsE	DSW1_00668	ftsE
ftsE	DSW1_00663	ftsE
ftsE	DSW5_00678	ftsE
ftsE	DSW5_02620	ftsE
ftsE	DSW5_00322	ftsE
ftsE	DSW5_01906	ftsE
ftsE	DSW5_02506	ftsE
ftsE	DSW5_02379	ftsE

ftsE	DSW5_00438	ftsE
ftsE	DSW5_02298	ftsE
ftsE	DSW5_00520	ftsE
ftsE	DSW5_00616	ftsE
ftsE	DSW5_02361	ftsE
ftsE	DSW5_01245	ftsE
ftsE	DSW5_02674	ftsE
ftsE	DSW5_01378	ftsE
ftsE	DSW5_01900	ftsE
ftsE	DSW5_01296	ftsE
ftsE	DSW5_00832	ftsE
ftsE	DSW5_01920	ftsE
ftsE	DSW5_00717	ftsE
ftsE	DSW5_02131	ftsE
ftsE	DSW5_02766	ftsE
ftsE	DSW5_00316	ftsE
ftsE	DSW5_00151	ftsE
ftsE	DSW6_00444	ftsE
ftsE	DSW6_03147	ftsE
ftsE	DSW6_00917	ftsE
ftsE	DSW6_00391	ftsE
ftsE	DSW6_02639	ftsE
ftsE	DSW6_01421	ftsE
ftsE	DSW6_01548	ftsE
ftsE	DSW6_02359	ftsE
ftsE	DSW6_00261	ftsE
ftsE	DSW6_02461	ftsE
ftsE	DSW6_00043	ftsE
ftsE	DSW6_03370	ftsE
ftsE	DSW6_01214	ftsE
ftsE	DSW6_00146	ftsE
ftsE	DSW6_02959	ftsE
ftsE	DSW6_00076	ftsE
ftsE	DSW6_00426	ftsE
ftsE	DSW6_01850	ftsE
ftsE	DSW6_00144	ftsE
ftsE	DSW6_01276	ftsE
ftsE	DSW6_02267	ftsE
ftsE	DSW6_02454	ftsE
ftsE	DSW6_01289	ftsE
ftsE	DSW6_01616	ftsE
ftsE	DSW6_02204	ftsE
ftsE	DSW6_02589	ftsE
ftsE	DSW6_00827	ftsE
ftsE	DSW6_02703	ftsE
ftsE	DSW6_02395	ftsE
ftsE	DSW6_00110	ftsE
ftsE	DSW6_02861	ftsE

ftsE	DSW6_01316	ftsE
ftsE	DSW6_00479	ftsE
ftsE	DSW6_02214	ftsE
ftsE	DSW8_01310	ftsE
ftsE	DSW8_00662	ftsE
ftsE	DSW8_02704	ftsE
ftsE	DSW8_01214	ftsE
ftsE	DSW8_00310	ftsE
ftsE	DSW8_01639	ftsE
ftsE	DSW8_02641	ftsE
ftsE	DSW8_00120	ftsE
ftsE	DSW8_01357	ftsE
ftsE	DSW8_02347	ftsE
ftsE	DSW8_01142	ftsE
ftsE	DSW8_02697	ftsE
ftsE	DSW8_03009	ftsE
ftsE	DSW8_01113	ftsE
ftsE	DSW8_00638	ftsE
ftsE	DSW8_02687	ftsE
ftsE	DSW8_02391	ftsE
ftsE	DSW8_02161	ftsE
ftsE	DSW8_02348	ftsE
ftsE	DSW8_01336	ftsE
ftsE	DSW8_01126	ftsE
ftsE	DSW8_01184	ftsE
ftsE	DSW8_00826	ftsE
ftsE	DSW8_00005	ftsE
ftsE	DSW8_02831	ftsE
ftsE	DSW8_00513	ftsE
ftsE	DSW8_01233	ftsE
ftsE	DSW8_02226	ftsE
ftsE	F52_04183	ftsE
ftsE	F52_01837	ftsE
ftsE	F52_04399	ftsE
ftsE	F52_00184	ftsE
ftsE	F52_02589	ftsE
ftsE	F52_02700	ftsE
ftsE	F52_04643	ftsE
ftsE	F52_02100	ftsE
ftsE	F52_01997	ftsE
ftsE	F52_00375	ftsE
ftsE	F52_03163	ftsE
ftsE	F52_04012	ftsE
ftsE	F52_03395	ftsE
ftsE	F52_03819	ftsE
ftsE	F52_03633	ftsE
ftsE	F52_01635	ftsE
ftsE	F52_00228	ftsE

ftsE	F52_04006	ftsE
ftsE	F52_01608	ftsE
ftsE	F52_00936	ftsE
ftsE	F52_04444	ftsE
ftsE	F52_02732	ftsE
ftsE	F52_02931	ftsE
ftsE	F52_02606	ftsE
ftsE	F52_01458	ftsE
ftsE	F52_02493	ftsE
ftsE	F52_03892	ftsE
ftsE	F52_03778	ftsE
ftsE	F52_01574	ftsE
ftsE	F52_00606	ftsE
ftsE	F52_03824	ftsE
ftsE	F52_01869	ftsE
ftsE	F52_00438	ftsE
ftsE	F52_04295	ftsE
ftsE	F52_01844	ftsE
ftsE	F52_03219	ftsE
ftsE	F52_01346	ftsE
ftsE	F52_01424	ftsE
ftsE	F52_02447	ftsE
ftsE	F52_03068	ftsE
ftsE	F52_04641	ftsE
ftsE	GCA_001027945.1_00773	ftsE
ftsE	GCA_001027945.1_01777	ftsE
ftsE	GCA_001027945.1_02389	ftsE
ftsE	GCA_001027945.1_00741	ftsE
ftsE	GCA_001027945.1_00628	ftsE
ftsE	GCA_001027945.1_00893	ftsE
ftsE	GCA_001027945.1_01225	ftsE
ftsE	GCA_001027945.1_02471	ftsE
ftsE	GCA_001027945.1_00224	ftsE
ftsE	GCA_001027945.1_00690	ftsE
ftsE	GCA_001027945.1_01757	ftsE
ftsE	GCA_001027945.1_00877	ftsE
ftsE	GCA_001027945.1_01425	ftsE
ftsE	GCA_001027945.1_01787	ftsE
ftsE	GCA_001027945.1_03061	ftsE
ftsE	GCA_001027945.1_00010	ftsE
ftsE	GCA_001027945.1_00304	ftsE
ftsE	GCA_001027945.1_00361	ftsE
ftsE	GCA_001027945.1_00731	ftsE
ftsE	GCA_001027945.1_00220	ftsE
ftsE	GCA_001027945.1_02399	ftsE
ftsE	GCA_001027945.1_02499	ftsE
ftsE	GCA_001027945.1_01506	ftsE
ftsE	GCA_001027945.1_02684	ftsE

ftsE	GCA_001027945.1_02189	ftsE
ftsE	GCA_001027945.1_02749	ftsE
ftsE	GCA_001027945.1_00599	ftsE
ftsE	GCA_001027945.1_02605	ftsE
ftsE	HI1_02288	ftsE
ftsE	HI1_02541	ftsE
ftsE	HI1_00826	ftsE
ftsE	HI1_00062	ftsE
ftsE	HI1_01848	ftsE
ftsE	HI1_02994	ftsE
ftsE	HI1_02254	ftsE
ftsE	HI1_00685	ftsE
ftsE	HI1_02869	ftsE
ftsE	HI1_01942	ftsE
ftsE	HI1_03333	ftsE
ftsE	HI1_01134	ftsE
ftsE	HI1_02877	ftsE
ftsE	HI1_01126	ftsE
ftsE	HI1_00810	ftsE
ftsE	HI1_02040	ftsE
ftsE	HI1_01140	ftsE
ftsE	HI1_00684	ftsE
ftsE	HI1_01534	ftsE
ftsE	HI1_01635	ftsE
ftsE	HI1_02265	ftsE
ftsE	HI1_03336	ftsE
ftsE	HI1_00703	ftsE
ftsE	HI1_00028	ftsE
ftsE	HI1_00498	ftsE
ftsE	HI1_02690	ftsE
ftsE	HI1_02654	ftsE
ftsE	HI1_01348	ftsE
ftsE	HI1_00269	ftsE
ftsE	HI1_02903	ftsE
ftsE	HI1_01501	ftsE
ftsE	HI1_00088	ftsE
ftsE	HI1_01463	ftsE
ftsE	IR1_04242	ftsE
ftsE	IR1_02817	ftsE
ftsE	IR1_01809	ftsE
ftsE	IR1_04426	ftsE
ftsE	IR1_00686	ftsE
ftsE	IR1_02493	ftsE
ftsE	IR1_00568	ftsE
ftsE	IR1_04373	ftsE
ftsE	IR1_01075	ftsE
ftsE	IR1_01012	ftsE
ftsE	IR1_03656	ftsE

ftsE	IR1_01323	ftsE
ftsE	IR1_00208	ftsE
ftsE	IR1_03580	ftsE
ftsE	IR1_01630	ftsE
ftsE	IR1_02949	ftsE
ftsE	IR1_03241	ftsE
ftsE	IR1_02631	ftsE
ftsE	IR1_03850	ftsE
ftsE	IR1_02979	ftsE
ftsE	IR1_01533	ftsE
ftsE	IR1_00529	ftsE
ftsE	IR1_03122	ftsE
ftsE	IR1_00347	ftsE
ftsE	IR1_04641	ftsE
ftsE	IR1_03494	ftsE
ftsE	IR1_00669	ftsE
ftsE	IR1_03010	ftsE
ftsE	IR1_01418	ftsE
ftsE	IR1_01666	ftsE
ftsE	IR1_01315	ftsE
ftsE	IR1_02279	ftsE
ftsE	IR1_01625	ftsE
ftsE	IR1_02437	ftsE
ftsE	IR1_03731	ftsE
ftsE	IR1_03423	ftsE
ftsE	IR1_02804	ftsE
ftsE	IR1_00007	ftsE
ftsE	IR1_03776	ftsE
ftsE	IR1_02784	ftsE
ftsE	IR1_03348	ftsE
ftsE	IR1_04693	ftsE
ftsE	IR1_01701	ftsE
ftsE	IR1_04606	ftsE
ftsE	IR1_03879	ftsE
ftsE	IR1_00923	ftsE
ftsE	KCOM3131_01283	ftsE
ftsE	KCOM3131_01422	ftsE
ftsE	KCOM3131_01421	ftsE
ftsE	KCOM3131_02042	ftsE
ftsE	KCOM3131_00006	ftsE
ftsE	KCOM3131_01426	ftsE
ftsE	KCOM3131_00168	ftsE
ftsE	KCOM3131_01461	ftsE
ftsE	KCOM3131_00520	ftsE
ftsE	KCOM3131_01655	ftsE
ftsE	KCOM3131_00179	ftsE
ftsE	KCOM3131_00988	ftsE
ftsE	KCOM3131_01925	ftsE

ftsE	KCOM3131_00217	ftsE
ftsE	KCOM3131_01242	ftsE
ftsE	KCOM3131_01730	ftsE
ftsE	KCOM3131_01380	ftsE
ftsE	KCOM3131_00914	ftsE
ftsE	KCOM3131_00920	ftsE
ftsE	KCOM3131_00536	ftsE
ftsE	KCOM3131_00201	ftsE
ftsE	KCOM3131_01440	ftsE
ftsE	KCOM3131_01712	ftsE
ftsE	KCTC12974_02038	ftsE
ftsE	KCTC12974_01547	ftsE
ftsE	KCTC12974_00734	ftsE
ftsE	KCTC12974_02275	ftsE
ftsE	KCTC12974_02028	ftsE
ftsE	KCTC12974_01237	ftsE
ftsE	KCTC12974_02556	ftsE
ftsE	KCTC12974_01805	ftsE
ftsE	KCTC12974_02518	ftsE
ftsE	KCTC12974_00302	ftsE
ftsE	KCTC12974_02962	ftsE
ftsE	KCTC12974_02905	ftsE
ftsE	KCTC12974_02371	ftsE
ftsE	KCTC12974_02245	ftsE
ftsE	KCTC12974_01113	ftsE
ftsE	KCTC12974_00746	ftsE
ftsE	KCTC12974_00052	ftsE
ftsE	KCTC12974_02548	ftsE
ftsE	KCTC12974_00483	ftsE
ftsE	KCTC12974_02902	ftsE
ftsE	KCTC12974_02553	ftsE
ftsE	KCTC12974_01425	ftsE
ftsE	KCTC12974_01141	ftsE
ftsE	KCTC12974_00673	ftsE
ftsE	KCTC12974_00341	ftsE
ftsE	KCTC12974_01517	ftsE
ftsE	KCTC12974_00260	ftsE
ftsE	KCTC12974_00399	ftsE
ftsE	KCTC12974_00313	ftsE
ftsE	KL-A_01635	ftsE
ftsE	KL-A_00947	ftsE
ftsE	KL-A_02377	ftsE
ftsE	KL-A_01125	ftsE
ftsE	KL-A_02672	ftsE
ftsE	KL-A_00351	ftsE
ftsE	KL-A_02973	ftsE
ftsE	KL-A_01601	ftsE
ftsE	KL-A_02476	ftsE

ftsE	KL-A_00587	ftsE
ftsE	KL-A_00255	ftsE
ftsE	KL-A_01805	ftsE
ftsE	KL-A_03020	ftsE
ftsE	KL-A_00579	ftsE
ftsE	KL-A_03012	ftsE
ftsE	KL-A_02393	ftsE
ftsE	KL-A_00157	ftsE
ftsE	KL-A_02477	ftsE
ftsE	KL-A_03026	ftsE
ftsE	KL-A_02817	ftsE
ftsE	KL-A_01516	ftsE
ftsE	KL-A_01612	ftsE
ftsE	KL-A_01802	ftsE
ftsE	KL-A_02458	ftsE
ftsE	KL-A_01090	ftsE
ftsE	KL-A_02525	ftsE
ftsE	KL-A_01307	ftsE
ftsE	KL-A_01343	ftsE
ftsE	KL-A_00819	ftsE
ftsE	KL-A_02656	ftsE
ftsE	KL-A_00554	ftsE
ftsE	KL-A_02850	ftsE
ftsE	KL-A_01152	ftsE
ftsE	KL-A_03263	ftsE
ftsE	L12_00217	ftsE
ftsE	L12_00513	ftsE
ftsE	L12_00432	ftsE
ftsE	L12_00166	ftsE
ftsE	LL0412.1.7_01866	ftsE
ftsE	LL0412.1.7_02428	ftsE
ftsE	LL0412.1.7_02639	ftsE
ftsE	LL0412.1.7_01654	ftsE
ftsE	LL0412.1.7_01784	ftsE
ftsE	LL0412.1.7_00943	ftsE
ftsE	LL0412.1.7_02469	ftsE
ftsE	LL0412.1.7_00488	ftsE
ftsE	LL0412.1.7_01672	ftsE
ftsE	LL0412.1.7_02334	ftsE
ftsE	LL0412.1.7_01720	ftsE
ftsE	LL0412.1.7_02406	ftsE
ftsE	LL0412.1.7_02558	ftsE
ftsE	LL0412.1.7_00138	ftsE
ftsE	LL0412.1.7_02440	ftsE
ftsE	LL0412.1.7_01130	ftsE
ftsE	LL0412.1.7_00433	ftsE
ftsE	LL0412.1.7_02312	ftsE
ftsE	LL0412.1.7_00768	ftsE

ftsE	LL0412.1.7_01092	ftsE
ftsE	LL0412.1.7_01272	ftsE
ftsE	LL0412.1.7_01160	ftsE
ftsE	LL0412.1.7_00490	ftsE
ftsE	LL0412.1.7_00724	ftsE
ftsE	LL0412.1.7_01663	ftsE
ftsE	LL0412.1.7_02216	ftsE
ftsE	LL0412.1.7_01614	ftsE
ftsE	LL0412.1.7_01068	ftsE
ftsE	LL0412.1.7_01539	ftsE
ftsE	LL0412.1.7_02134	ftsE
ftsE	LL0412.1.7_01814	ftsE
ftsE	LL0412.1.7_02228	ftsE
ftsE	LL0412.1.7_00556	ftsE
ftsE	MAR_2009_71_02141	ftsE
ftsE	MAR_2009_71_03459	ftsE
ftsE	MAR_2009_71_00750	ftsE
ftsE	MAR_2009_71_02223	ftsE
ftsE	MAR_2009_71_03113	ftsE
ftsE	MAR_2009_71_01786	ftsE
ftsE	MAR_2009_71_00813	ftsE
ftsE	MAR_2009_71_02921	ftsE
ftsE	MAR_2009_71_02094	ftsE
ftsE	MAR_2009_71_01092	ftsE
ftsE	MAR_2009_71_02303	ftsE
ftsE	MAR_2009_71_00756	ftsE
ftsE	MAR_2009_71_00445	ftsE
ftsE	MAR_2009_71_02332	ftsE
ftsE	MAR_2009_71_03435	ftsE
ftsE	MAR_2009_71_00766	ftsE
ftsE	MAR_2009_71_01048	ftsE
ftsE	MAR_2009_71_01279	ftsE
ftsE	MAR_2009_71_01091	ftsE
ftsE	MAR_2009_71_02115	ftsE
ftsE	MAR_2009_71_02318	ftsE
ftsE	MAR_2009_71_02260	ftsE
ftsE	MAR_2009_71_02620	ftsE
ftsE	MAR_2009_71_02816	ftsE
ftsE	MAR_2009_71_00623	ftsE
ftsE	MAR_2009_71_03311	ftsE
ftsE	MAR_2009_71_02204	ftsE
ftsE	MAR_2009_71_01212	ftsE
ftsE	MAR_2009_71_00747	ftsE
ftsE	MAR_2009_71_03831	ftsE
ftsE	NN016038_00060	ftsE
ftsE	NN016038_03736	ftsE
ftsE	NN016038_00602	ftsE
ftsE	NN016038_03934	ftsE

ftsE	NN016038_01159	ftsE
ftsE	NN016038_03400	ftsE
ftsE	NN016038_01589	ftsE
ftsE	NN016038_00108	ftsE
ftsE	NN016038_02896	ftsE
ftsE	NN016038_00938	ftsE
ftsE	NN016038_03275	ftsE
ftsE	NN016038_00898	ftsE
ftsE	NN016038_02419	ftsE
ftsE	NN016038_00643	ftsE
ftsE	NN016038_00337	ftsE
ftsE	NN016038_02160	ftsE
ftsE	NN016038_02080	ftsE
ftsE	NN016038_00937	ftsE
ftsE	NN016038_01196	ftsE
ftsE	NN016038_01179	ftsE
ftsE	NN016038_03906	ftsE
ftsE	NN016038_01567	ftsE
ftsE	NN016038_03255	ftsE
ftsE	NN016038_00087	ftsE
ftsE	NN016038_03393	ftsE
ftsE	NN016038_02157	ftsE
ftsE	NN016038_00471	ftsE
ftsE	NN016038_02839	ftsE
ftsE	NN016038_00100	ftsE
ftsE	NN016038_03085	ftsE
ftsE	NN016038_01119	ftsE
ftsE	NN016038_03952	ftsE
ftsE	NN016038_01214	ftsE
ftsE	NN016038_03708	ftsE
ftsE	PAO1_05167	ftsE
ftsE	PAO1_01151	ftsE
ftsE	PAO1_03339	ftsE
ftsE	PAO1_04274	ftsE
ftsE	PAO1_00886	ftsE
ftsE	PAO1_04354	ftsE
ftsE	PAO1_01284	ftsE
ftsE	PAO1_04353	ftsE
ftsE	PAO1_03526	ftsE
ftsE	PAO1_01934	ftsE
ftsE	PAO1_02423	ftsE
ftsE	PAO1_01378	ftsE
ftsE	PAO1_05686	ftsE
ftsE	PAO1_04650	ftsE
ftsE	PAO1_02469	ftsE
ftsE	PAO1_05244	ftsE
ftsE	PAO1_04323	ftsE
ftsE	PAO1_03365	ftsE

ftsE	PAO1_00384	ftsE
ftsE	PAO1_02414	ftsE
ftsE	PAO1_01294	ftsE
ftsE	PAO1_05264	ftsE
ftsE	PAO1_03963	ftsE
ftsE	PAO1_03091	ftsE
ftsE	PAO1_00189	ftsE
ftsE	PAO1_02462	ftsE
ftsE	PAO1_00308	ftsE
ftsE	PAO1_03636	ftsE
ftsE	PAO1_03564	ftsE
ftsE	PAO1_00334	ftsE
ftsE	PAO1_05029	ftsE
ftsE	PAO1_04018	ftsE
ftsE	PAO1_01861	ftsE
ftsE	PAO1_05325	ftsE
ftsE	PAO1_00286	ftsE
ftsE	PAO1_05543	ftsE
ftsE	PAO1_02958	ftsE
ftsE	PAO1_03730	ftsE
ftsE	PAO1_02124	ftsE
ftsE	PAO1_01106	ftsE
ftsE	PAO1_00919	ftsE
ftsE	PAO1_04065	ftsE
ftsE	PAO1_04743	ftsE
ftsE	PAO1_05634	ftsE
ftsE	PAO1_01918	ftsE
ftsE	PAO1_05076	ftsE
ftsE	PAO1_00618	ftsE
ftsE	PAO1_00212	ftsE
ftsE	PAO1_05407	ftsE
ftsE	PAO1_03297	ftsE
ftsE	PAO1_03014	ftsE
ftsE	PAO1_02911	ftsE
ftsE	PAO1_00142	ftsE
ftsE	PAO1_03503	ftsE
ftsE	PAO1_02402	ftsE
ftsE	PAO1_04866	ftsE
ftsE	PAO1_04649	ftsE
ftsE	PAO1_01107	ftsE
ftsE	PAO1_03569	ftsE
ftsE	PAO1_04599	ftsE
ftsE	PAO1_03028	ftsE
ftsE	PAO1_03795	ftsE
ftsE	PAO1_02366	ftsE
ftsE	PAO1_02025	ftsE
ftsE	PAO1_04605	ftsE
ftsE	PAO1_03323	ftsE

ftsE	PAO1_04744	ftsE
ftsE	PAO1_04165	ftsE
ftsE	PAO1_02008	ftsE
ftsE	PAO1_03660	ftsE
ftsE	PAO1_05428	ftsE
ftsE	PAO1_03124	ftsE
ftsE	PAO1_01466	ftsE
ftsE	PAO1_05683	ftsE
ftsE	PAO1_02380	ftsE
ftsE	PAO1_05557	ftsE
ftsE	PAO1_03494	ftsE
ftsE	PAO1_04192	ftsE
ftsE	PAO1_03432	ftsE
ftsE	PAO1_00079	ftsE
ftsE	PAO1_03495	ftsE
ftsE	PAO1_05077	ftsE
ftsE	PAO1_03513	ftsE
ftsE	PAO1_02479	ftsE
ftsE	PAO1_05028	ftsE
ftsE	PAO1_01515	ftsE
ftsE	PAO1_04289	ftsE
ftsE	PAO1_01426	ftsE
ftsE	UW101_04864	ftsE
ftsE	UW101_00128	ftsE
ftsE	UW101_02941	ftsE
ftsE	UW101_02310	ftsE
ftsE	UW101_01221	ftsE
ftsE	UW101_02517	ftsE
ftsE	UW101_01357	ftsE
ftsE	UW101_04997	ftsE
ftsE	UW101_01979	ftsE
ftsE	UW101_04155	ftsE
ftsE	UW101_01737	ftsE
ftsE	UW101_05215	ftsE
ftsE	UW101_00276	ftsE
ftsE	UW101_04493	ftsE
ftsE	UW101_02355	ftsE
ftsE	UW101_04129	ftsE
ftsE	UW101_02201	ftsE
ftsE	UW101_00306	ftsE
ftsE	UW101_01583	ftsE
ftsE	UW101_00471	ftsE
ftsE	UW101_00493	ftsE
ftsE	UW101_01390	ftsE
ftsE	UW101_01240	ftsE
ftsE	UW101_01149	ftsE
ftsE	UW101_03471	ftsE
ftsE	UW101_00347	ftsE

ftsE	UW101_05263	ftsE
ftsE	UW101_05142	ftsE
ftsE	UW101_02738	ftsE
ftsE	UW101_05210	ftsE
ftsE	UW101_02567	ftsE
ftsE	UW101_04740	ftsE
ftsE	UW101_00119	ftsE
ftsE	UW101_01940	ftsE
ftsE	UW101_03048	ftsE
ftsE	UW101_02252	ftsE
ftsE	UW101_00093	ftsE
ftsE	UW101_02068	ftsE
ftsE	UW101_01102	ftsE
ftsE	V4-33_01330	ftsE
ftsE	V4-33_01049	ftsE
ftsE	V4-33_00748	ftsE
ftsE	V4-33_00510	ftsE
ftsE	V4-33_01787	ftsE
ftsE	V4-33_01861	ftsE
ftsE	V4-33_00402	ftsE
ftsE	V4-33_00497	ftsE
ftsE	V4-33_00561	ftsE
ftsE	V4-33_00604	ftsE
ftsE	V4-33_00341	ftsE
ftsE	V4-33_01146	ftsE
ftsE	V4-33_01413	ftsE
ftsE	V4-33_00229	ftsE
ftsE	V4-33_02313	ftsE
ftsE	V4-33_00833	ftsE
ftsE	V4-33_02193	ftsE
ftsE	V4-33_01287	ftsE
ftsE	V4-33_01177	ftsE
ftsE	V4-33_00565	ftsE
ftsE	V4-33_01566	ftsE
ftsE	V4-33_01910	ftsE
ftsE	V4-33_00535	ftsE
ftsE	V4-33_01878	ftsE
ftsE	V4-33_00953	ftsE
ftsE	V4-33_01672	ftsE
ftsE	V4-33_00641	ftsE
ftsE	V4-33_02005	ftsE
ftsE	W5C_02689	ftsE
ftsE	W5C_01280	ftsE
ftsE	W5C_01537	ftsE
ftsE	W5C_00039	ftsE
ftsE	W5C_01498	ftsE
ftsE	W5C_02144	ftsE
ftsE	W5C_00606	ftsE

ftsE	W5C_02653	ftsE
ftsE	W5C_02262	ftsE
ftsE	W5C_01383	ftsE
ftsE	W5C_00703	ftsE
ftsE	W5C_00837	ftsE
ftsE	W5C_02033	ftsE
ftsE	W5C_02131	ftsE
ftsE	W5C_01521	ftsE
ftsE	W5C_00231	ftsE
ftsE	W5C_02139	ftsE
ftsE	W5C_02025	ftsE
ftsE	W5C_02039	ftsE
ftsE	W5C_01382	ftsE
ftsE	W5C_02942	ftsE
ftsE	W5C_00410	ftsE
ftsE	W5C_00007	ftsE
ftsE	W5C_02766	ftsE
ftsE	W5C_02666	ftsE
ftsE	W5C_00834	ftsE
ftsE	W5C_01394	ftsE
ftsE	W5C_03241	ftsE
ftsE	W5C_02430	ftsE
ftsE	W5C_02394	ftsE
ftsE	W5C_01106	ftsE
ftsE	W5C_00269	ftsE
ftsE	W5C_00301	ftsE
ftsE	W5C_00065	ftsE
ftsE	W5C_00220	ftsE
ftsE	ZONMW-01_01214	ftsE
ftsE	ZONMW-01_01658	ftsE
ftsE	ZONMW-01_02531	ftsE
ftsE	ZONMW-01_02016	ftsE
ftsE	ZONMW-01_00373	ftsE
ftsE	ZONMW-01_00719	ftsE
ftsE	ZONMW-01_01251	ftsE
ftsE	ZONMW-01_00859	ftsE
ftsE	ZONMW-01_00467	ftsE
ftsE	ZONMW-01_03081	ftsE
ftsE	ZONMW-01_01075	ftsE
ftsE	ZONMW-01_00851	ftsE
ftsE	ZONMW-01_01473	ftsE
ftsE	ZONMW-01_02515	ftsE
ftsE	ZONMW-01_01082	ftsE
ftsE	ZONMW-01_01069	ftsE
ftsE	ZONMW-01_00059	ftsE
ftsE	ZONMW-01_03013	ftsE
ftsE	ZONMW-01_00161	ftsE
ftsE	ZONMW-01_01781	ftsE

ftsE	ZONMW-01_01984	ftsE
ftsE	ZONMW-01_01240	ftsE
ftsE	ZONMW-01_03078	ftsE
ftsE	ZONMW-01_01510	ftsE
ftsE	ZONMW-01_02661	ftsE
ftsE	ZONMW-01_02763	ftsE
ftsE	ZONMW-01_02235	ftsE
ftsE	ZONMW-01_02200	ftsE
ftsE	ZONMW-01_02404	ftsE
ftsE	ZONMW-01_00018	ftsE
ftsE	ZONMW-01_03002	ftsE
ftsE	ZONMW-01_02043	ftsE
ftsE	ZONMW-02_00992	ftsE
ftsE	ZONMW-02_01965	ftsE
ftsE	ZONMW-02_03729	ftsE
ftsE	ZONMW-02_02006	ftsE
ftsE	ZONMW-02_01071	ftsE
ftsE	ZONMW-02_02116	ftsE
ftsE	ZONMW-02_02410	ftsE
ftsE	ZONMW-02_01095	ftsE
ftsE	ZONMW-02_01565	ftsE
ftsE	ZONMW-02_00151	ftsE
ftsE	ZONMW-02_02056	ftsE
ftsE	ZONMW-02_03506	ftsE
ftsE	ZONMW-02_01049	ftsE
ftsE	ZONMW-02_01976	ftsE
ftsE	ZONMW-02_01027	ftsE
ftsE	ZONMW-02_02470	ftsE
ftsE	ZONMW-02_01944	ftsE
ftsE	ZONMW-02_03196	ftsE
ftsE	ZONMW-02_02077	ftsE
ftsE	ZONMW-02_02107	ftsE
ftsE	ZONMW-02_02078	ftsE
ftsE	ZONMW-02_01133	ftsE
ftsE	ZONMW-02_01644	ftsE
ftsE	ZONMW-02_03507	ftsE
ftsE	ZONMW-02_01137	ftsE
ftsE	ZONMW-02_03235	ftsE
ftsE	ZONMW-02_01307	ftsE
ftsE	ZONMW-02_02529	ftsE
ftsE	ZONMW-02_00085	ftsE
ftsE	ZONMW-02_03666	ftsE
ftsE	ZONMW-02_00332	ftsE
ftsE	ZONMW-02_00821	ftsE
ftsE	ZONMW-02_01548	ftsE
ftsE	ZONMW-02_02061	ftsE
ftsE	ZONMW-02_01820	ftsE
ftsE	ZONMW-02_00953	ftsE

ftsE	ZONMW-03_01566	ftsE
ftsE	ZONMW-03_00549	ftsE
ftsE	ZONMW-03_01812	ftsE
ftsE	ZONMW-03_02695	ftsE
ftsE	ZONMW-03_01099	ftsE
ftsE	ZONMW-03_00097	ftsE
ftsE	ZONMW-03_01529	ftsE
ftsE	ZONMW-03_00237	ftsE
ftsE	ZONMW-03_01193	ftsE
ftsE	ZONMW-03_02316	ftsE
ftsE	ZONMW-03_02144	ftsE
ftsE	ZONMW-03_00229	ftsE
ftsE	ZONMW-03_01307	ftsE
ftsE	ZONMW-03_01796	ftsE
ftsE	ZONMW-03_02151	ftsE
ftsE	ZONMW-03_02138	ftsE
ftsE	ZONMW-03_00784	ftsE
ftsE	ZONMW-03_03297	ftsE
ftsE	ZONMW-03_00887	ftsE
ftsE	ZONMW-03_02766	ftsE
ftsE	ZONMW-03_02727	ftsE
ftsE	ZONMW-03_01540	ftsE
ftsE	ZONMW-03_02313	ftsE
ftsE	ZONMW-03_01270	ftsE
ftsE	ZONMW-03_01687	ftsE
ftsE	ZONMW-03_03143	ftsE
ftsE	ZONMW-03_00413	ftsE
ftsE	ZONMW-03_00448	ftsE
ftsE	ZONMW-03_03007	ftsE
ftsE	ZONMW-03_00743	ftsE
ftsE	ZONMW-03_03286	ftsE
ftsE	ZONMW-03_02668	ftsE
ftsE	ZONMW-04_00126	ftsE
ftsE	ZONMW-04_01488	ftsE
ftsE	ZONMW-04_03017	ftsE
ftsE	ZONMW-04_01859	ftsE
ftsE	ZONMW-04_00573	ftsE
ftsE	ZONMW-04_01107	ftsE
ftsE	ZONMW-04_00161	ftsE
ftsE	ZONMW-04_03105	ftsE
ftsE	ZONMW-04_00481	ftsE
ftsE	ZONMW-04_00780	ftsE
ftsE	ZONMW-04_01674	ftsE
ftsE	ZONMW-04_03113	ftsE
ftsE	ZONMW-04_00383	ftsE
ftsE	ZONMW-04_03001	ftsE
ftsE	ZONMW-04_01667	ftsE
ftsE	ZONMW-04_01680	ftsE

ftsE	ZONMW-04_02158	ftsE
ftsE	ZONMW-04_02059	ftsE
ftsE	ZONMW-04_02757	ftsE
ftsE	ZONMW-04_01828	ftsE
ftsE	ZONMW-04_00150	ftsE
ftsE	ZONMW-04_00777	ftsE
ftsE	ZONMW-04_02305	ftsE
ftsE	ZONMW-04_02843	ftsE
ftsE	ZONMW-04_01352	ftsE
ftsE	ZONMW-04_01387	ftsE
ftsE	ZONMW-04_02434	ftsE
ftsE	ZONMW-04_02200	ftsE
ftsE	ZONMW-04_03242	ftsE
ftsE	ZONMW-04_01886	ftsE
ftsE	ZONMW-05_01704	ftsE
ftsE	ZONMW-05_00812	ftsE
ftsE	ZONMW-05_03261	ftsE
ftsE	ZONMW-05_02510	ftsE
ftsE	ZONMW-05_00917	ftsE
ftsE	ZONMW-05_01252	ftsE
ftsE	ZONMW-05_00358	ftsE
ftsE	ZONMW-05_01667	ftsE
ftsE	ZONMW-05_00495	ftsE
ftsE	ZONMW-05_01344	ftsE
ftsE	ZONMW-05_00050	ftsE
ftsE	ZONMW-05_01953	ftsE
ftsE	ZONMW-05_00487	ftsE
ftsE	ZONMW-05_01442	ftsE
ftsE	ZONMW-05_03245	ftsE
ftsE	ZONMW-05_01946	ftsE
ftsE	ZONMW-05_01959	ftsE
ftsE	ZONMW-05_00977	ftsE
ftsE	ZONMW-05_01076	ftsE
ftsE	ZONMW-05_02307	ftsE
ftsE	ZONMW-05_02541	ftsE
ftsE	ZONMW-05_01678	ftsE
ftsE	ZONMW-05_00047	ftsE
ftsE	ZONMW-05_02821	ftsE
ftsE	ZONMW-05_03100	ftsE
ftsE	ZONMW-05_00676	ftsE
ftsE	ZONMW-05_00711	ftsE
ftsE	ZONMW-05_02589	ftsE
ftsE	ZONMW-05_00936	ftsE
ftsE	ZONMW-05_02720	ftsE
ftsE	ZONMW-05_02484	ftsE
ftsE	ZONMW-06_03140	ftsE
ftsE	ZONMW-06_02807	ftsE
ftsE	ZONMW-06_01273	ftsE

ftsE	ZONMW-06_01274	ftsE
ftsE	ZONMW-06_03809	ftsE
ftsE	ZONMW-06_02200	ftsE
ftsE	ZONMW-06_02038	ftsE
ftsE	ZONMW-06_01472	ftsE
ftsE	ZONMW-06_03272	ftsE
ftsE	ZONMW-06_02665	ftsE
ftsE	ZONMW-06_02550	ftsE
ftsE	ZONMW-06_00489	ftsE
ftsE	ZONMW-06_00107	ftsE
ftsE	ZONMW-06_02525	ftsE
ftsE	ZONMW-06_00325	ftsE
ftsE	ZONMW-06_02138	ftsE
ftsE	ZONMW-06_00352	ftsE
ftsE	ZONMW-06_02909	ftsE
ftsE	ZONMW-06_03205	ftsE
ftsE	ZONMW-06_02644	ftsE
ftsE	ZONMW-06_03758	ftsE
ftsE	ZONMW-06_01215	ftsE
ftsE	ZONMW-06_01824	ftsE
ftsE	ZONMW-06_00433	ftsE
ftsE	ZONMW-06_01604	ftsE
ftsE	ZONMW-06_00225	ftsE
ftsE	ZONMW-06_00099	ftsE
ftsE	ZONMW-06_02715	ftsE
ftsE	ZONMW-06_00021	ftsE
ftsE	ZONMW-06_01823	ftsE
ftsE	ZONMW-06_02625	ftsE
ftsE	ZONMW-06_03146	ftsE
ftsE	ZONMW-06_00395	ftsE
ftsE	ZONMW-06_02199	ftsE
ftsE	ZONMW-06_02214	ftsE
ftsE	ZONMW-06_00729	ftsE
ftsE	ZONMW-06_02587	ftsE
ftsE	ZONMW-06_00988	ftsE
ftsE	ZONMW-06_02555	ftsE
ftsE	ZONMW-06_01609	ftsE
ftsE	ZONMW-06_01413	ftsE
ftsE	ZONMW-06_00326	ftsE
ftsE	ZONMW-06_01569	ftsE
ftsE	ZONMW-06_00730	ftsE
ftsE	ZONMW-06_02514	ftsE
ftsE	ZONMW-06_03238	ftsE
ftsE	ZONMW-06_02622	ftsE
ftsE	ZONMW-06_01216	ftsE
ftsE	ZONMW-06_01519	ftsE
ftsE	ZONMW-06_01640	ftsE
ftsE	ZONMW-06_01734	ftsE

ftsE	ZONMW-06_01681	ftsE
ftsE	ZONMW-06_01636	ftsE
ftsE	ZONMW-06_02727	ftsE
ftsE	ZONMW-06_01735	ftsE
ftsE	ZONMW-06_02728	ftsE
ftsE	ZONMW-06_02699	ftsE
ftsE	ZONMW-06_00983	ftsE
ftsE	ZONMW-06_00455	ftsE
ftsE	ZONMW-06_00231	ftsE
ftsE	ZONMW-06_00011	ftsE
ftsE	ZONMW-06_00801	ftsE
ftsE	ZONMW-06_01097	ftsE
ftsE	ZONMW-06_00530	ftsE
ftsE	ZONMW-06_01844	ftsE
ftsE	ZONMW-06_03934	ftsE
ftsE	ZONMW-06_02465	ftsE
ftsE	ZONMW-06_00529	ftsE
ftsE	ZONMW-06_02219	ftsE
ftsE	ZONMW-06_02403	ftsE
ftsE	ZONMW-06_00196	ftsE
ftsE	ZONMW-06_00223	ftsE
ftsE	ZONMW-06_03239	ftsE
ftsE	ZONMW-06_02808	ftsE
ftsE	ZONMW-06_03365	ftsE
ftsE	ZONMW-07_00801	ftsE
ftsE	ZONMW-07_03321	ftsE
ftsE	ZONMW-07_00618	ftsE
ftsE	ZONMW-07_01808	ftsE
ftsE	ZONMW-07_01920	ftsE
ftsE	ZONMW-07_00148	ftsE
ftsE	ZONMW-07_00378	ftsE
ftsE	ZONMW-07_00160	ftsE
ftsE	ZONMW-07_01054	ftsE
ftsE	ZONMW-07_03285	ftsE
ftsE	ZONMW-07_03303	ftsE
ftsE	ZONMW-07_02375	ftsE
ftsE	ZONMW-07_01183	ftsE
ftsE	ZONMW-07_02033	ftsE
ftsE	ZONMW-07_02245	ftsE
ftsE	ZONMW-07_01182	ftsE
ftsE	ZONMW-07_02001	ftsE
ftsE	ZONMW-07_02691	ftsE
ftsE	ZONMW-07_02116	ftsE
ftsE	ZONMW-07_03284	ftsE
ftsE	ZONMW-07_03080	ftsE
ftsE	ZONMW-07_01792	ftsE
ftsE	ZONMW-07_02177	ftsE
ftsE	ZONMW-07_02242	ftsE

ftsE	ZONMW-07_04211	ftsE
ftsE	ZONMW-07_02167	ftsE
ftsE	ZONMW-07_00469	ftsE
ftsE	ZONMW-07_03344	ftsE
ftsE	ZONMW-07_01421	ftsE
ftsE	ZONMW-07_01859	ftsE
ftsE	ZONMW-07_01472	ftsE
ftsE	ZONMW-08_01118	ftsE
ftsE	ZONMW-08_02468	ftsE
ftsE	ZONMW-08_00266	ftsE
ftsE	ZONMW-08_01022	ftsE
ftsE	ZONMW-08_02629	ftsE
ftsE	ZONMW-08_01438	ftsE
ftsE	ZONMW-08_00331	ftsE
ftsE	ZONMW-08_02120	ftsE
ftsE	ZONMW-08_01164	ftsE
ftsE	ZONMW-08_00610	ftsE
ftsE	ZONMW-08_00942	ftsE
ftsE	ZONMW-08_00273	ftsE
ftsE	ZONMW-08_03767	ftsE
ftsE	ZONMW-08_00914	ftsE
ftsE	ZONMW-08_02492	ftsE
ftsE	ZONMW-08_00284	ftsE
ftsE	ZONMW-08_00566	ftsE
ftsE	ZONMW-08_00798	ftsE
ftsE	ZONMW-08_00609	ftsE
ftsE	ZONMW-08_01144	ftsE
ftsE	ZONMW-08_00927	ftsE
ftsE	ZONMW-08_00985	ftsE
ftsE	ZONMW-08_03624	ftsE
ftsE	ZONMW-08_02037	ftsE
ftsE	ZONMW-08_00139	ftsE
ftsE	ZONMW-08_02825	ftsE
ftsE	ZONMW-08_01041	ftsE
ftsE	ZONMW-08_00733	ftsE
ftsE	ZONMW-08_00263	ftsE
ftsE	ZONMW-09_01776	ftsE
ftsE	ZONMW-09_00836	ftsE
ftsE	ZONMW-09_02459	ftsE
ftsE	ZONMW-09_01687	ftsE
ftsE	ZONMW-09_02272	ftsE
ftsE	ZONMW-09_02437	ftsE
ftsE	ZONMW-09_01197	ftsE
ftsE	ZONMW-09_00183	ftsE
ftsE	ZONMW-09_00910	ftsE
ftsE	ZONMW-09_00775	ftsE
ftsE	ZONMW-09_01553	ftsE
ftsE	ZONMW-09_02036	ftsE

ftsE	ZONMW-09_02470	ftsE
ftsE	ZONMW-09_00420	ftsE
ftsE	ZONMW-09_01752	ftsE
ftsE	ZONMW-09_01954	ftsE
ftsE	ZONMW-09_01390	ftsE
ftsE	ZONMW-09_00816	ftsE
ftsE	ZONMW-09_01547	ftsE
ftsE	ZONMW-09_02694	ftsE
ftsE	ZONMW-09_01195	ftsE
ftsE	ZONMW-09_02096	ftsE
ftsE	ZONMW-09_00496	ftsE
ftsE	ZONMW-09_01666	ftsE
ftsE	ZONMW-09_01470	ftsE
ftsE	ZONMW-09_01501	ftsE
ftsE	ZONMW-09_00346	ftsE
ftsE	ZONMW-09_01221	ftsE
ftsE	ZONMW-09_01468	ftsE
ftsE	ZONMW-10_02032	ftsE
ftsE	ZONMW-10_01517	ftsE
ftsE	ZONMW-10_00845	ftsE
ftsE	ZONMW-10_01902	ftsE
ftsE	ZONMW-10_01740	ftsE
ftsE	ZONMW-10_02126	ftsE
ftsE	ZONMW-10_02039	ftsE
ftsE	ZONMW-10_00924	ftsE
ftsE	ZONMW-10_00463	ftsE
ftsE	ZONMW-10_02270	ftsE
ftsE	ZONMW-10_00996	ftsE
ftsE	ZONMW-10_00445	ftsE
ftsE	ZONMW-10_01343	ftsE
ftsE	ZONMW-10_01550	ftsE
ftsE	ZONMW-10_02405	ftsE
ftsE	ZONMW-10_00398	ftsE
ftsE	ZONMW-10_01573	ftsE
ftsE	ZONMW-10_01484	ftsE
ftsE	ZONMW-10_01054	ftsE
ftsE	ZONMW-10_02129	ftsE
ftsE	ZONMW-10_01756	ftsE
ftsE	ZONMW-10_00331	ftsE
ftsE	ZONMW-10_02110	ftsE
ftsE	ZONMW-10_01150	ftsE
ftsE	ZONMW-10_02066	ftsE
ftsE	ZONMW-10_00699	ftsE
ftsE	ZONMW-10_00210	ftsE
ftsE	ZONMW-10_02105	ftsE
ftsE	ZONMW-11_02441	ftsE
ftsE	ZONMW-11_02448	ftsE
ftsE	ZONMW-11_02230	ftsE

ftsE	ZONMW-11_02367	ftsE
ftsE	ZONMW-11_01924	ftsE
ftsE	ZONMW-11_01656	ftsE
ftsE	ZONMW-11_00694	ftsE
ftsE	ZONMW-11_00419	ftsE
ftsE	ZONMW-11_03787	ftsE
ftsE	ZONMW-11_01362	ftsE
ftsE	ZONMW-11_01783	ftsE
ftsE	ZONMW-11_00160	ftsE
ftsE	ZONMW-11_01890	ftsE
ftsE	ZONMW-11_03609	ftsE
ftsE	ZONMW-11_01000	ftsE
ftsE	ZONMW-11_03708	ftsE
ftsE	ZONMW-11_03610	ftsE
ftsE	ZONMW-11_01162	ftsE
ftsE	ZONMW-11_00767	ftsE
ftsE	ZONMW-11_03532	ftsE
ftsE	ZONMW-11_03007	ftsE
ftsE	ZONMW-11_03854	ftsE
ftsE	ZONMW-11_03807	ftsE
ftsE	ZONMW-11_02170	ftsE
ftsE	ZONMW-11_04063	ftsE
ftsE	ZONMW-11_03445	ftsE
ftsE	ZONMW-11_02189	ftsE
ftsE	ZONMW-11_03539	ftsE
ftsE	ZONMW-11_01650	ftsE
ftsE	ZONMW-11_04285	ftsE
ftsE	ZONMW-11_00872	ftsE
ftsE	ZONMW-11_01869	ftsE
ftsE	ZONMW-11_00232	ftsE
ftsE	ZONMW-11_00095	ftsE
ftsE	ZONMW-11_03690	ftsE
ftsE	ZONMW-11_02475	ftsE
ftsE	ZONMW-11_03512	ftsE
ftsE	ZONMW-11_04651	ftsE
ftsE	ZONMW-11_04109	ftsE
ftsE	ZONMW-11_01699	ftsE
ftsE	ZONMW-12_02217	ftsE
ftsE	ZONMW-12_03238	ftsE
ftsE	ZONMW-12_03867	ftsE
ftsE	ZONMW-12_02892	ftsE
ftsE	ZONMW-12_02229	ftsE
ftsE	ZONMW-12_04252	ftsE
ftsE	ZONMW-12_01086	ftsE
ftsE	ZONMW-12_02944	ftsE
ftsE	ZONMW-12_03523	ftsE
ftsE	ZONMW-12_04078	ftsE
ftsE	ZONMW-12_00331	ftsE

ftsE	ZONMW-12_03493	ftsE
ftsE	ZONMW-12_02055	ftsE
ftsE	ZONMW-12_02834	ftsE
ftsE	ZONMW-12_00489	ftsE
ftsE	ZONMW-12_02712	ftsE
ftsE	ZONMW-12_00347	ftsE
ftsE	ZONMW-12_03880	ftsE
ftsE	ZONMW-12_00320	ftsE
ftsE	ZONMW-12_03516	ftsE
ftsE	ZONMW-12_00005	ftsE
ftsE	ZONMW-12_02512	ftsE
ftsE	ZONMW-12_02148	ftsE
ftsE	ZONMW-12_03521	ftsE
ftsE	ZONMW-12_03897	ftsE
ftsE	ZONMW-12_01248	ftsE
ftsE	ZONMW-12_03016	ftsE
ftsE	ZONMW-12_02575	ftsE
ftsE	ZONMW-12_00095	ftsE
ftsE	ZONMW-12_00690	ftsE
ftsE	ZONMW-12_00244	ftsE
ftsE	ZONMW-12_00788	ftsE
ftsE	ZONMW-12_03906	ftsE
ftsE	ZONMW-12_04153	ftsE
ftsE	ZONMW-12_00867	ftsE
ftsE	ZONMW-12_01282	ftsE
ftsE	ZONMW-12_02893	ftsE
ftsE	ZONMW-13_02595	ftsE
ftsE	ZONMW-13_02232	ftsE
ftsE	ZONMW-13_01603	ftsE
ftsE	ZONMW-13_02154	ftsE
ftsE	ZONMW-13_01091	ftsE
ftsE	ZONMW-13_02759	ftsE
ftsE	ZONMW-13_01035	ftsE
ftsE	ZONMW-13_02931	ftsE
ftsE	ZONMW-13_02813	ftsE
ftsE	ZONMW-13_03129	ftsE
ftsE	ZONMW-13_03157	ftsE
ftsE	ZONMW-13_01913	ftsE
ftsE	ZONMW-13_00429	ftsE
ftsE	ZONMW-13_01167	ftsE
ftsE	ZONMW-13_01866	ftsE
ftsE	ZONMW-13_03137	ftsE
ftsE	ZONMW-13_01615	ftsE
ftsE	ZONMW-13_02176	ftsE
ftsE	ZONMW-13_02942	ftsE
ftsE	ZONMW-13_02210	ftsE
ftsE	ZONMW-13_00216	ftsE
ftsE	ZONMW-13_02934	ftsE

ftsE	ZONMW-13_01457	ftsE
ftsE	ZONMW-13_00108	ftsE
ftsE	ZONMW-13_01422	ftsE
ftsE	ZONMW-13_01465	ftsE
ftsE	ZONMW-13_00345	ftsE
ftsE	ZONMW-13_02904	ftsE
ftsE	ZONMW-14_01642	ftsE
ftsE	ZONMW-14_02152	ftsE
ftsE	ZONMW-14_00832	ftsE
ftsE	ZONMW-14_01506	ftsE
ftsE	ZONMW-14_01938	ftsE
ftsE	ZONMW-14_00660	ftsE
ftsE	ZONMW-14_01557	ftsE
ftsE	ZONMW-14_01712	ftsE
ftsE	ZONMW-14_02019	ftsE
ftsE	ZONMW-14_02042	ftsE
ftsE	ZONMW-14_00779	ftsE
ftsE	ZONMW-14_01427	ftsE
ftsE	ZONMW-14_01761	ftsE
ftsE	ZONMW-14_01098	ftsE
ftsE	ZONMW-14_00846	ftsE
ftsE	ZONMW-14_02130	ftsE
ftsE	ZONMW-14_00135	ftsE
ftsE	ZONMW-14_01731	ftsE
ftsE	ZONMW-14_02330	ftsE
ftsE	ZONMW-14_01715	ftsE
ftsE	ZONMW-14_00377	ftsE
ftsE	ZONMW-14_02658	ftsE
ftsE	ZONMW-14_02364	ftsE
ftsE	ZONMW-14_02320	ftsE
ftsE	ZONMW-14_02872	ftsE
ftsE	ZONMW-14_00245	ftsE
ftsE	ZONMW-14_01688	ftsE
ftsE	ZONMW-14_00005	ftsE
ftsE	ZONMW-15_00673	ftsE
ftsE	ZONMW-15_02845	ftsE
ftsE	ZONMW-15_00947	ftsE
ftsE	ZONMW-15_00523	ftsE
ftsE	ZONMW-15_02903	ftsE
ftsE	ZONMW-15_00435	ftsE
ftsE	ZONMW-15_00718	ftsE
ftsE	ZONMW-15_01673	ftsE
ftsE	ZONMW-15_01927	ftsE
ftsE	ZONMW-15_02291	ftsE
ftsE	ZONMW-15_00908	ftsE
ftsE	ZONMW-15_02649	ftsE
ftsE	ZONMW-15_02769	ftsE
ftsE	ZONMW-15_01674	ftsE

ftsE	ZONMW-15_00014	ftsE
ftsE	ZONMW-15_01708	ftsE
ftsE	ZONMW-15_00497	ftsE
ftsE	ZONMW-15_00696	ftsE
ftsE	ZONMW-15_02643	ftsE
ftsE	ZONMW-15_00646	ftsE
ftsE	ZONMW-15_00415	ftsE
ftsE	ZONMW-15_01923	ftsE
ftsE	ZONMW-15_00711	ftsE
ftsE	ZONMW-15_01068	ftsE
ftsE	ZONMW-15_02392	ftsE
ftsE	ZONMW-15_02349	ftsE
ftsE	ZONMW-15_02567	ftsE
ftsE	ZONMW-15_00538	ftsE
ftsE	ZONMW-15_00033	ftsE
ftsE	ZONMW-15_02444	ftsE
ftsE	ZONMW-19_00346	ftsE
ftsE	ZONMW-19_03084	ftsE
ftsE	ZONMW-19_03082	ftsE
ftsE	ZONMW-19_04578	ftsE
ftsE	ZONMW-19_02596	ftsE
ftsE	ZONMW-19_02595	ftsE
ftsE	ZONMW-19_01866	ftsE
ftsE	ZONMW-19_00480	ftsE
ftsE	ZONMW-19_02852	ftsE
ftsE	ZONMW-19_00866	ftsE
ftsE	ZONMW-19_01284	ftsE
ftsE	ZONMW-19_02877	ftsE
ftsE	ZONMW-19_04349	ftsE
ftsE	ZONMW-19_00004	ftsE
ftsE	ZONMW-19_02082	ftsE
ftsE	ZONMW-19_03166	ftsE
ftsE	ZONMW-19_03699	ftsE
ftsE	ZONMW-19_01582	ftsE
ftsE	ZONMW-19_01674	ftsE
ftsE	ZONMW-19_03864	ftsE
ftsE	ZONMW-19_01903	ftsE
ftsE	ZONMW-19_01310	ftsE
ftsE	ZONMW-19_03858	ftsE
ftsE	ZONMW-19_03475	ftsE
ftsE	ZONMW-19_01794	ftsE
ftsE	ZONMW-19_01904	ftsE
ftsE	ZONMW-19_03129	ftsE
ftsE	ZONMW-19_02314	ftsE
ftsE	ZONMW-19_02415	ftsE
ftsE	ZONMW-19_02046	ftsE
ftsE	ZONMW-19_03008	ftsE
ftsE	ZONMW-19_01360	ftsE

ftsE	ZONMW-19_04339	ftsE
ftsE	ZONMW-19_01395	ftsE
ftsE	ZONMW-19_01402	ftsE
ftsE	ZONMW-19_03479	ftsE
ftsE	ZONMW-19_00023	ftsE
ftsE	ZONMW-19_01257	ftsE
ftsE	ZONMW-19_01759	ftsE
ftsE	ZONMW-19_01160	ftsE
ftsE	ZONMW-19_00728	ftsE
ftsE	ZONMW-19_00897	ftsE
ftsE	ZONMW-19_03228	ftsE
ftsE	ZONMW-19_03360	ftsE
ftsE	ZONMW-19_02557	ftsE
ftsE	ZONMW-19_00991	ftsE
ftsE	ZONMW-19_01967	ftsE
ftsE	ZONMW-20_03784	ftsE
ftsE	ZONMW-20_01315	ftsE
ftsE	ZONMW-20_02491	ftsE
ftsE	ZONMW-20_01316	ftsE
ftsE	ZONMW-20_02392	ftsE
ftsE	ZONMW-20_00423	ftsE
ftsE	ZONMW-20_02391	ftsE
ftsE	ZONMW-20_02639	ftsE
ftsE	ZONMW-20_00754	ftsE
ftsE	ZONMW-20_02312	ftsE
ftsE	ZONMW-20_02508	ftsE
ftsE	ZONMW-20_01582	ftsE
ftsE	ZONMW-20_02149	ftsE
ftsE	ZONMW-20_02703	ftsE
ftsE	ZONMW-20_02615	ftsE
ftsE	ZONMW-20_00488	ftsE
ftsE	ZONMW-20_01473	ftsE
ftsE	ZONMW-20_01927	ftsE
ftsE	ZONMW-20_01588	ftsE
ftsE	ZONMW-20_02442	ftsE
ftsE	ZONMW-20_01004	ftsE
ftsE	ZONMW-20_04095	ftsE
ftsE	ZONMW-20_02398	ftsE
ftsE	ZONMW-20_03803	ftsE
ftsE	ZONMW-20_04184	ftsE
ftsE	ZONMW-20_00536	ftsE
ftsE	ZONMW-20_02431	ftsE
ftsE	ZONMW-20_03348	ftsE
ftsE	ZONMW-20_03483	ftsE
ftsE	ZONMW-20_03533	ftsE
ftsE	ZONMW-20_02090	ftsE
ftsE	ZONMW-20_03690	ftsE
ftsE	ZONMW-20_02488	ftsE

ftsE	ZONMW-20_03338	ftsE
ftsE	ZONMW-20_02105	ftsE
ftsE	ZONMW-20_00666	ftsE
ftsE	ZONMW-20_03093	ftsE
ftsE	ZONMW-20_02470	ftsE
ftsE	ZONMW-20_04086	ftsE
ftsE	ZONMW-20_00915	ftsE
ftsE	ZONMW-20_00577	ftsE
ftsE	ZONMW-20_03464	ftsE
ftsE	ZONMW-20_01446	ftsE
ftsE	ZONMW-20_04191	ftsE
ftsE	ZONMW-20_00464	ftsE
ftsE	ZONMW-20_01266	ftsE
ftsE	ZONMW-20_01447	ftsE
ftsE	ZONMW-20_03352	ftsE
ftsE	ZONMW-20_01341	ftsE
ftsE	ZONMW-20_01627	ftsE
ftsE	ZONMW-20_01896	ftsE
ftsE	ZONMW-20_03204	ftsE
ftsE	ZONMW-20_00004	ftsE
ftsE	ZONMW-20_01261	ftsE
ftsE	ZONMW-20_01913	ftsE
ftsE	ZONMW-20_00942	ftsE
ftsE	ZONMW-20_02019	ftsE
ftsE	ZONMW-20_00237	ftsE
ftsE	ZONMW-20_01723	ftsE
ftsE	ZONMW-20_04087	ftsE
ftsE	ZONMW-20_03739	ftsE
ftsE	ZONMW-20_01597	ftsE
ftsE	ZONMW-20_02221	ftsE
ftsE	ZONMW-20_02258	ftsE
ftsE	ZONMW-20_01410	ftsE
ftsE	ZONMW-20_03616	ftsE
ftsE	ZONMW-20_03835	ftsE
ftsE	ZONMW-20_03754	ftsE
ftsE	ZONMW-20_02559	ftsE
ftsE	ZONMW-20_02849	ftsE
ftsE	ZONMW-20_03744	ftsE
ftsE	ZONMW-20_01852	ftsE
ftsE	ZONMW-20_03347	ftsE
ftsE	ZONMW-20_00066	ftsE
ftsE	ZONMW-20_00638	ftsE
ftsE	ZONMW-20_03762	ftsE
ftsE	ZONMW-20_03895	ftsE
ftsE	ZONMW-20_00463	ftsE
ftsE	ZONMW-20_02132	ftsE
ftsE	ZONMW-20_02482	ftsE
ftsE	ZONMW-20_03812	ftsE

ftsE	ZONMW-20_02524	ftsE
ftsE	ZONMW-20_03431	ftsE
ftsE	ZONMW-20_01064	ftsE
ftsE	ZONMW-20_00546	ftsE
ftsE	ZONMW-20_00894	ftsE
ftsE	ZONMW-20_02234	ftsE
ftsE	ZONMW-20_02591	ftsE
ftsE	ZONMW-20_03534	ftsE
ftsE	ZONMW-20_00914	ftsE
ftsE	ZONMW-20_02242	ftsE
ftsE	ZONMW-20_00535	ftsE
ftsE	ZONMW-20_03482	ftsE
ftsE	ZONMW-20_03753	ftsE
ftsE	ZONMW-20_02855	ftsE
ftsE	ZONMW-20_02222	ftsE
ftsE	ZONMW-20_01674	ftsE
ftsE	ZONMW-20_04141	ftsE
ftsE	ZONMW-20_02539	ftsE
ftsE	ZONMW-20_01912	ftsE
ftsE	ZONMW-20_02554	ftsE
ftsE	ZONMW-20_01631	ftsE
ftsE	ZONMW-21_02323	ftsE
ftsE	ZONMW-21_01554	ftsE
ftsE	ZONMW-21_02636	ftsE
ftsE	ZONMW-21_03395	ftsE
ftsE	ZONMW-21_00169	ftsE
ftsE	ZONMW-21_02970	ftsE
ftsE	ZONMW-21_02357	ftsE
ftsE	ZONMW-21_02035	ftsE
ftsE	ZONMW-21_00052	ftsE
ftsE	ZONMW-21_02714	ftsE
ftsE	ZONMW-21_00939	ftsE
ftsE	ZONMW-21_02512	ftsE
ftsE	ZONMW-21_00932	ftsE
ftsE	ZONMW-21_01583	ftsE
ftsE	ZONMW-21_00395	ftsE
ftsE	ZONMW-21_00950	ftsE
ftsE	ZONMW-21_02609	ftsE
ftsE	ZONMW-21_00691	ftsE
ftsE	ZONMW-21_01386	ftsE
ftsE	ZONMW-21_03403	ftsE
ftsE	ZONMW-21_02666	ftsE
ftsE	ZONMW-21_02343	ftsE
ftsE	ZONMW-21_02710	ftsE
ftsE	ZONMW-21_01699	ftsE
ftsE	ZONMW-21_02091	ftsE
ftsE	ZONMW-21_02578	ftsE
ftsE	ZONMW-22_02786	ftsE

ftsE	ZONMW-22_02169	ftsE
ftsE	ZONMW-22_02473	ftsE
ftsE	ZONMW-22_02301	ftsE
ftsE	ZONMW-22_00169	ftsE
ftsE	ZONMW-22_02854	ftsE
ftsE	ZONMW-22_02751	ftsE
ftsE	ZONMW-22_01691	ftsE
ftsE	ZONMW-22_00052	ftsE
ftsE	ZONMW-22_02395	ftsE
ftsE	ZONMW-22_00940	ftsE
ftsE	ZONMW-22_02597	ftsE
ftsE	ZONMW-22_00933	ftsE
ftsE	ZONMW-22_02141	ftsE
ftsE	ZONMW-22_00395	ftsE
ftsE	ZONMW-22_00951	ftsE
ftsE	ZONMW-22_02500	ftsE
ftsE	ZONMW-22_00692	ftsE
ftsE	ZONMW-22_01388	ftsE
ftsE	ZONMW-22_02294	ftsE
ftsE	ZONMW-22_02443	ftsE
ftsE	ZONMW-22_02765	ftsE
ftsE	ZONMW-22_02399	ftsE
ftsE	ZONMW-22_02025	ftsE
ftsE	ZONMW-22_01634	ftsE
ftsE	ZONMW-22_02531	ftsE
ftsE	ZONMW-23_02808	ftsE
ftsE	ZONMW-23_00382	ftsE
ftsE	ZONMW-23_00077	ftsE
ftsE	ZONMW-23_00249	ftsE
ftsE	ZONMW-23_01284	ftsE
ftsE	ZONMW-23_03016	ftsE
ftsE	ZONMW-23_02775	ftsE
ftsE	ZONMW-23_00861	ftsE
ftsE	ZONMW-23_01167	ftsE
ftsE	ZONMW-23_00155	ftsE
ftsE	ZONMW-23_02053	ftsE
ftsE	ZONMW-23_02621	ftsE
ftsE	ZONMW-23_02046	ftsE
ftsE	ZONMW-23_00410	ftsE
ftsE	ZONMW-23_01510	ftsE
ftsE	ZONMW-23_02064	ftsE
ftsE	ZONMW-23_00050	ftsE
ftsE	ZONMW-23_01806	ftsE
ftsE	ZONMW-23_02501	ftsE
ftsE	ZONMW-23_00257	ftsE
ftsE	ZONMW-23_00107	ftsE
ftsE	ZONMW-23_02789	ftsE
ftsE	ZONMW-23_00151	ftsE

ftsE	ZONMW-23_00526	ftsE
ftsE	ZONMW-23_00917	ftsE
ftsE	ZONMW-23_00019	ftsE
ftsE	ZONMW-24_02347	ftsE
ftsE	ZONMW-24_02794	ftsE
ftsE	ZONMW-24_02238	ftsE
ftsE	ZONMW-24_02066	ftsE
ftsE	ZONMW-24_01291	ftsE
ftsE	ZONMW-24_02877	ftsE
ftsE	ZONMW-24_02381	ftsE
ftsE	ZONMW-24_01714	ftsE
ftsE	ZONMW-24_01408	ftsE
ftsE	ZONMW-24_02160	ftsE
ftsE	ZONMW-24_00521	ftsE
ftsE	ZONMW-24_02536	ftsE
ftsE	ZONMW-24_00528	ftsE
ftsE	ZONMW-24_02766	ftsE
ftsE	ZONMW-24_01064	ftsE
ftsE	ZONMW-24_00510	ftsE
ftsE	ZONMW-24_02265	ftsE
ftsE	ZONMW-24_00769	ftsE
ftsE	ZONMW-24_00073	ftsE
ftsE	ZONMW-24_02058	ftsE
ftsE	ZONMW-24_02208	ftsE
ftsE	ZONMW-24_02367	ftsE
ftsE	ZONMW-24_02164	ftsE
ftsE	ZONMW-24_02650	ftsE
ftsE	ZONMW-24_01658	ftsE
ftsE	ZONMW-24_02296	ftsE
ftsE	ZONMW-25_02673	ftsE
ftsE	ZONMW-25_02749	ftsE
ftsE	ZONMW-25_02473	ftsE
ftsE	ZONMW-25_00922	ftsE
ftsE	ZONMW-25_02290	ftsE
ftsE	ZONMW-25_02637	ftsE
ftsE	ZONMW-25_00020	ftsE
ftsE	ZONMW-25_01562	ftsE
ftsE	ZONMW-25_00153	ftsE
ftsE	ZONMW-25_01058	ftsE
ftsE	ZONMW-25_03215	ftsE
ftsE	ZONMW-25_03192	ftsE
ftsE	ZONMW-25_00685	ftsE
ftsE	ZONMW-25_03019	ftsE
ftsE	ZONMW-25_03026	ftsE
ftsE	ZONMW-25_02377	ftsE
ftsE	ZONMW-25_01375	ftsE
ftsE	ZONMW-25_03039	ftsE
ftsE	ZONMW-25_01643	ftsE

ftsE	ZONMW-25_00726	ftsE
ftsE	ZONMW-25_01555	ftsE
ftsE	ZONMW-25_00256	ftsE
ftsE	ZONMW-25_02650	ftsE
ftsE	ZONMW-25_00688	ftsE
ftsE	ZONMW-25_00351	ftsE
ftsE	ZONMW-25_03097	ftsE
ftsE	ZONMW-25_01693	ftsE
ftsE	ZONMW-25_01491	ftsE
ftsE	ZONMW-25_00440	ftsE
ftsE	ZONMW-25_00759	ftsE
ftsE	ZONMW-25_00093	ftsE
ftsE	ZONMW-25_00786	ftsE
ftsE	ZONMW-26_01579	ftsE
ftsE	ZONMW-26_00148	ftsE
ftsE	ZONMW-26_02037	ftsE
ftsE	ZONMW-26_02604	ftsE
ftsE	ZONMW-26_01306	ftsE
ftsE	ZONMW-26_00607	ftsE
ftsE	ZONMW-26_01614	ftsE
ftsE	ZONMW-26_00473	ftsE
ftsE	ZONMW-26_01399	ftsE
ftsE	ZONMW-26_00910	ftsE
ftsE	ZONMW-26_02445	ftsE
ftsE	ZONMW-26_00481	ftsE
ftsE	ZONMW-26_01827	ftsE
ftsE	ZONMW-26_02053	ftsE
ftsE	ZONMW-26_02452	ftsE
ftsE	ZONMW-26_02439	ftsE
ftsE	ZONMW-26_01020	ftsE
ftsE	ZONMW-26_03353	ftsE
ftsE	ZONMW-26_01120	ftsE
ftsE	ZONMW-26_02972	ftsE
ftsE	ZONMW-26_02572	ftsE
ftsE	ZONMW-26_01603	ftsE
ftsE	ZONMW-26_00913	ftsE
ftsE	ZONMW-26_02161	ftsE
ftsE	ZONMW-26_03167	ftsE
ftsE	ZONMW-26_00293	ftsE
ftsE	ZONMW-26_00258	ftsE
ftsE	ZONMW-26_03026	ftsE
ftsE	ZONMW-26_00979	ftsE
ftsE	ZONMW-26_03342	ftsE
ftsE	ZONMW-26_02632	ftsE
ftsE	ZONMW-27_00123	ftsE
ftsE	ZONMW-27_01816	ftsE
ftsE	ZONMW-27_02892	ftsE
ftsE	ZONMW-27_02030	ftsE

ftsE	ZONMW-27_02620	ftsE
ftsE	ZONMW-27_00567	ftsE
ftsE	ZONMW-27_01184	ftsE
ftsE	ZONMW-27_00158	ftsE
ftsE	ZONMW-27_01319	ftsE
ftsE	ZONMW-27_00475	ftsE
ftsE	ZONMW-27_00877	ftsE
ftsE	ZONMW-27_01625	ftsE
ftsE	ZONMW-27_01311	ftsE
ftsE	ZONMW-27_00377	ftsE
ftsE	ZONMW-27_02876	ftsE
ftsE	ZONMW-27_01632	ftsE
ftsE	ZONMW-27_01619	ftsE
ftsE	ZONMW-27_02681	ftsE
ftsE	ZONMW-27_02605	ftsE
ftsE	ZONMW-27_00752	ftsE
ftsE	ZONMW-27_03257	ftsE
ftsE	ZONMW-27_01999	ftsE
ftsE	ZONMW-27_00147	ftsE
ftsE	ZONMW-27_00874	ftsE
ftsE	ZONMW-27_02284	ftsE
ftsE	ZONMW-27_03189	ftsE
ftsE	ZONMW-27_01951	ftsE
ftsE	ZONMW-27_01916	ftsE
ftsE	ZONMW-27_02746	ftsE
ftsE	ZONMW-27_02639	ftsE
ftsE	ZONMW-27_02594	ftsE
ftsE	ZONMW-27_02057	ftsE
ftsE	ZONMW-28_02950	ftsE
ftsE	ZONMW-28_01735	ftsE
ftsE	ZONMW-28_02089	ftsE
ftsE	ZONMW-28_01734	ftsE
ftsE	ZONMW-28_01139	ftsE
ftsE	ZONMW-28_00387	ftsE
ftsE	ZONMW-28_03970	ftsE
ftsE	ZONMW-28_03106	ftsE
ftsE	ZONMW-28_00949	ftsE
ftsE	ZONMW-28_00093	ftsE
ftsE	ZONMW-28_01629	ftsE
ftsE	ZONMW-28_00446	ftsE
ftsE	ZONMW-28_00107	ftsE
ftsE	ZONMW-28_01300	ftsE
ftsE	ZONMW-28_01556	ftsE
ftsE	ZONMW-28_01051	ftsE
ftsE	ZONMW-28_01362	ftsE
ftsE	ZONMW-28_02440	ftsE
ftsE	ZONMW-28_02815	ftsE
ftsE	ZONMW-28_01586	ftsE

ftsE	ZONMW-28_01949	ftsE
ftsE	ZONMW-28_01461	ftsE
ftsE	ZONMW-28_03775	ftsE
ftsE	ZONMW-28_01998	ftsE
ftsE	ZONMW-28_01008	ftsE
ftsE	ZONMW-28_02760	ftsE
ftsE	ZONMW-28_02761	ftsE
ftsE	ZONMW-28_02393	ftsE
ftsE	ZONMW-28_01217	ftsE
ftsE	ZONMW-28_01290	ftsE
ftsE	ZONMW-28_03069	ftsE
ftsE	ZONMW-28_01195	ftsE
ftsE	ZONMW-28_02586	ftsE
ftsE	ZONMW-28_01375	ftsE
ftsE	ZONMW-28_00388	ftsE
ftsE	ZONMW-28_00086	ftsE
ftsE	ZONMW-28_01598	ftsE
ftsE	ZONMW-28_03268	ftsE
ftsE	ZONMW-28_03764	ftsE
ftsE	ZONMW-28_03997	ftsE
ftsE	ZONMW-28_00798	ftsE
ftsE	ZONMW-28_02403	ftsE
ftsE	ZONMW-28_03054	ftsE
ftsE	ZONMW-28_00374	ftsE
ftsE	ZONMW-28_01019	ftsE
ftsE	ZONMW-28_04056	ftsE
ftsE	ZONMW-28_01557	ftsE
ftsE	ZONMW-28_02955	ftsE
ftsE	ZONMW-28_03998	ftsE
ftsE	ZONMW-28_00118	ftsE
ftsE	ZONMW-28_02792	ftsE
ftsE	ZONMW-28_02404	ftsE
ftsE	ZONMW-28_00046	ftsE
ftsE	ZONMW-28_04051	ftsE
ftsE	ZONMW-28_03473	ftsE
ftsE	ZONMW-28_00988	ftsE
ftsE	ZONMW-28_00166	ftsE
ftsE	ZONMW-28_01887	ftsE
ftsE	ZONMW-28_00896	ftsE
ftsE	ZONMW-28_02174	ftsE
ftsE	ZONMW-28_02738	ftsE
ftsE	ZONMW-28_01430	ftsE
ftsE	ZONMW-28_00701	ftsE
ftsE	ZONMW-28_03910	ftsE
ftsE	ZONMW-28_00799	ftsE
ftsE	ZONMW-28_01471	ftsE
ftsE	ZONMW-28_02088	ftsE
ftsE	ZONMW-28_00897	ftsE

ftsE	ZONMW-28_00369	ftsE
ftsE	ZONMW-28_03269	ftsE
ftsE	ZONMW-28_01457	ftsE
ftsE	ZONMW-28_00224	ftsE
ftsE	ZONMW-28_03070	ftsE
ftsE	ZONMW-28_01632	ftsE
ftsE	ZONMW-28_03203	ftsE
ftsE	ZONMW-29_01643	ftsE
ftsE	ZONMW-29_01498	ftsE
ftsE	ZONMW-29_03241	ftsE
ftsE	ZONMW-29_02719	ftsE
ftsE	ZONMW-29_03041	ftsE
ftsE	ZONMW-29_03276	ftsE
ftsE	ZONMW-29_02154	ftsE
ftsE	ZONMW-29_00785	ftsE
ftsE	ZONMW-29_01164	ftsE
ftsE	ZONMW-29_01783	ftsE
ftsE	ZONMW-29_01880	ftsE
ftsE	ZONMW-29_02485	ftsE
ftsE	ZONMW-29_00522	ftsE
ftsE	ZONMW-29_02716	ftsE
ftsE	ZONMW-29_00530	ftsE
ftsE	ZONMW-29_00516	ftsE
ftsE	ZONMW-29_02043	ftsE
ftsE	ZONMW-29_00344	ftsE
ftsE	ZONMW-29_02149	ftsE
ftsE	ZONMW-29_01837	ftsE
ftsE	ZONMW-29_00933	ftsE
ftsE	ZONMW-29_03262	ftsE
ftsE	ZONMW-29_01877	ftsE
ftsE	ZONMW-29_03212	ftsE
ftsE	ZONMW-29_01484	ftsE
ftsE	ZONMW-29_01217	ftsE
ftsE	ZONMW-29_02102	ftsE
ftsE	ZONMW-29_00405	ftsE
ftsE	ZONMW-29_01808	ftsE
ftsE	ZONMW-30_00290	ftsE
ftsE	ZONMW-30_03398	ftsE
ftsE	ZONMW-30_00513	ftsE
ftsE	ZONMW-30_01240	ftsE
ftsE	ZONMW-30_00754	ftsE
ftsE	ZONMW-30_00254	ftsE
ftsE	ZONMW-30_01109	ftsE
ftsE	ZONMW-30_02607	ftsE
ftsE	ZONMW-30_01373	ftsE
ftsE	ZONMW-30_00468	ftsE
ftsE	ZONMW-30_00852	ftsE
ftsE	ZONMW-30_03015	ftsE

ftsE	ZONMW-30_01860	ftsE
ftsE	ZONMW-30_01855	ftsE
ftsE	ZONMW-30_00031	ftsE
ftsE	ZONMW-30_01932	ftsE
ftsE	ZONMW-30_02124	ftsE
ftsE	ZONMW-30_01933	ftsE
ftsE	ZONMW-30_01842	ftsE
ftsE	ZONMW-30_02354	ftsE
ftsE	ZONMW-30_03056	ftsE
ftsE	ZONMW-30_01102	ftsE
ftsE	ZONMW-30_03410	ftsE
ftsE	ZONMW-30_00267	ftsE
ftsE	ZONMW-30_03018	ftsE
ftsE	ZONMW-30_03294	ftsE
ftsE	ZONMW-30_00667	ftsE
ftsE	ZONMW-30_02301	ftsE
ftsE	ZONMW-30_02239	ftsE
ftsE	ZONMW-30_02738	ftsE
ftsE	ZONMW-30_00352	ftsE
ftsE	ZONMW-30_03121	ftsE
ftsE	ZONMW-30_03381	ftsE
ftsE	ZONMW-30_00325	ftsE
ftsE	ZONMW-31_01610	ftsE
ftsE	ZONMW-31_02918	ftsE
ftsE	ZONMW-31_00038	ftsE
ftsE	ZONMW-31_03264	ftsE
ftsE	ZONMW-31_00037	ftsE
ftsE	ZONMW-31_00309	ftsE
ftsE	ZONMW-31_01498	ftsE
ftsE	ZONMW-31_01331	ftsE
ftsE	ZONMW-31_03512	ftsE
ftsE	ZONMW-31_00112	ftsE
ftsE	ZONMW-31_00075	ftsE
ftsE	ZONMW-31_00844	ftsE
ftsE	ZONMW-31_00162	ftsE
ftsE	ZONMW-31_03016	ftsE
ftsE	ZONMW-31_02946	ftsE
ftsE	ZONMW-31_01706	ftsE
ftsE	ZONMW-31_01402	ftsE
ftsE	ZONMW-31_02765	ftsE
ftsE	ZONMW-31_00668	ftsE
ftsE	ZONMW-31_00440	ftsE
ftsE	ZONMW-31_01332	ftsE
ftsE	ZONMW-31_03704	ftsE
ftsE	ZONMW-31_01671	ftsE
ftsE	ZONMW-31_00267	ftsE
ftsE	ZONMW-31_00639	ftsE
ftsE	ZONMW-31_02486	ftsE

ftsE	ZONMW-31_00730	ftsE
ftsE	ZONMW-31_03586	ftsE
ftsE	ZONMW-31_02161	ftsE
ftsE	ZONMW-31_02024	ftsE
ftsE	ZONMW-31_01615	ftsE
ftsE	ZONMW-31_03742	ftsE
ftsE	ZONMW-31_00433	ftsE
ftsE	ZONMW-31_02107	ftsE
ftsE	ZONMW-31_02775	ftsE
ftsE	ZONMW-31_00554	ftsE
ftsE	ZONMW-31_02156	ftsE
ftsE	ZONMW-31_03743	ftsE
ftsE	ZONMW-31_02714	ftsE
ftsE	ZONMW-31_01244	ftsE
ftsE	ZONMW-31_00356	ftsE
ftsE	ZONMW-31_03369	ftsE
ftsE	ZONMW-31_00525	ftsE
ftsE	ZONMW-31_02209	ftsE
ftsE	ZONMW-31_02099	ftsE
ftsE	ZONMW-31_01239	ftsE
ftsE	ZONMW-31_03585	ftsE
ftsE	ZONMW-31_02041	ftsE
ftsE	ZONMW-31_00640	ftsE
ftsE	ZONMW-31_02108	ftsE
ftsE	ZONMW-31_02235	ftsE
ftsE	ZONMW-31_02543	ftsE
ftsE	ZONMW-31_00196	ftsE
ftsE	ZONMW-31_00197	ftsE
ftsE	ZONMW-31_02058	ftsE
ftsE	ZONMW-31_00817	ftsE
ftsE	ZONMW-31_02542	ftsE
ftsE	ZONMW-31_01070	ftsE
ftsE	ZONMW-31_02042	ftsE
ftsE	ZONMW-31_00232	ftsE
ftsE	ZONMW-31_00882	ftsE
ftsE	ZONMW-31_02003	ftsE
ftsE	ZONMW-31_03194	ftsE
ftsE	ZONMW-31_02812	ftsE
ftsE	ZONMW-31_00881	ftsE
ftsE	ZONMW-31_02025	ftsE
ftsE	ZONMW-31_03702	ftsE
ftsE	ZONMW-31_03365	ftsE
ftsE	ZONMW-31_00552	ftsE
ftsE	ZONMW-31_02103	ftsE
ftsE	ZONMW-31_02919	ftsE
ftsE	ZONMW-31_02853	ftsE
ftsE	ZONMW-31_01802	ftsE
ftsE	ZONMW-31_00287	ftsE

ftsE	ZONMW-31_00293	ftsE
ftsE	ZONMW-32_01434	ftsE
ftsE	ZONMW-32_02237	ftsE
ftsE	ZONMW-32_02494	ftsE
ftsE	ZONMW-32_02238	ftsE
ftsE	ZONMW-32_01547	ftsE
ftsE	ZONMW-32_01178	ftsE
ftsE	ZONMW-32_03827	ftsE
ftsE	ZONMW-32_03303	ftsE
ftsE	ZONMW-32_00142	ftsE
ftsE	ZONMW-32_00884	ftsE
ftsE	ZONMW-32_02343	ftsE
ftsE	ZONMW-32_01237	ftsE
ftsE	ZONMW-32_00898	ftsE
ftsE	ZONMW-32_01708	ftsE
ftsE	ZONMW-32_01963	ftsE
ftsE	ZONMW-32_00040	ftsE
ftsE	ZONMW-32_01769	ftsE
ftsE	ZONMW-32_02910	ftsE
ftsE	ZONMW-32_03060	ftsE
ftsE	ZONMW-32_01993	ftsE
ftsE	ZONMW-32_02023	ftsE
ftsE	ZONMW-32_01868	ftsE
ftsE	ZONMW-32_03890	ftsE
ftsE	ZONMW-32_02404	ftsE
ftsE	ZONMW-32_00083	ftsE
ftsE	ZONMW-32_03115	ftsE
ftsE	ZONMW-32_03114	ftsE
ftsE	ZONMW-32_02957	ftsE
ftsE	ZONMW-32_01625	ftsE
ftsE	ZONMW-32_01698	ftsE
ftsE	ZONMW-32_03340	ftsE
ftsE	ZONMW-32_01603	ftsE
ftsE	ZONMW-32_02764	ftsE
ftsE	ZONMW-32_01782	ftsE
ftsE	ZONMW-32_01179	ftsE
ftsE	ZONMW-32_00877	ftsE
ftsE	ZONMW-32_02373	ftsE
ftsE	ZONMW-32_03393	ftsE
ftsE	ZONMW-32_03901	ftsE
ftsE	ZONMW-32_03854	ftsE
ftsE	ZONMW-32_00293	ftsE
ftsE	ZONMW-32_02947	ftsE
ftsE	ZONMW-32_03355	ftsE
ftsE	ZONMW-32_01165	ftsE
ftsE	ZONMW-32_00072	ftsE
ftsE	ZONMW-32_00557	ftsE
ftsE	ZONMW-32_01964	ftsE

ftsE	ZONMW-32_01439	ftsE
ftsE	ZONMW-32_03855	ftsE
ftsE	ZONMW-32_00909	ftsE
ftsE	ZONMW-32_03083	ftsE
ftsE	ZONMW-32_02946	ftsE
ftsE	ZONMW-32_00837	ftsE
ftsE	ZONMW-32_00552	ftsE
ftsE	ZONMW-32_03508	ftsE
ftsE	ZONMW-32_00103	ftsE
ftsE	ZONMW-32_00957	ftsE
ftsE	ZONMW-32_02085	ftsE
ftsE	ZONMW-32_00195	ftsE
ftsE	ZONMW-32_02579	ftsE
ftsE	ZONMW-32_03137	ftsE
ftsE	ZONMW-32_01837	ftsE
ftsE	ZONMW-32_00390	ftsE
ftsE	ZONMW-32_03976	ftsE
ftsE	ZONMW-32_00292	ftsE
ftsE	ZONMW-32_01878	ftsE
ftsE	ZONMW-32_02493	ftsE
ftsE	ZONMW-32_00194	ftsE
ftsE	ZONMW-32_01160	ftsE
ftsE	ZONMW-32_03394	ftsE
ftsE	ZONMW-32_01864	ftsE
ftsE	ZONMW-32_01015	ftsE
ftsE	ZONMW-32_03339	ftsE
ftsE	ZONMW-32_02340	ftsE
ftsE	ZONMW-32_00728	ftsE
ftsE	ZONMW-33_01424	ftsE
ftsE	ZONMW-33_02228	ftsE
ftsE	ZONMW-33_02624	ftsE
ftsE	ZONMW-33_02229	ftsE
ftsE	ZONMW-33_01537	ftsE
ftsE	ZONMW-33_01167	ftsE
ftsE	ZONMW-33_03677	ftsE
ftsE	ZONMW-33_00693	ftsE
ftsE	ZONMW-33_00009	ftsE
ftsE	ZONMW-33_00874	ftsE
ftsE	ZONMW-33_02334	ftsE
ftsE	ZONMW-33_01226	ftsE
ftsE	ZONMW-33_00888	ftsE
ftsE	ZONMW-33_01698	ftsE
ftsE	ZONMW-33_01954	ftsE
ftsE	ZONMW-33_03301	ftsE
ftsE	ZONMW-33_01759	ftsE
ftsE	ZONMW-33_02838	ftsE
ftsE	ZONMW-33_03213	ftsE
ftsE	ZONMW-33_01984	ftsE

ftsE	ZONMW-33_02014	ftsE
ftsE	ZONMW-33_01858	ftsE
ftsE	ZONMW-33_03695	ftsE
ftsE	ZONMW-33_02714	ftsE
ftsE	ZONMW-33_03344	ftsE
ftsE	ZONMW-33_03158	ftsE
ftsE	ZONMW-33_03159	ftsE
ftsE	ZONMW-33_02791	ftsE
ftsE	ZONMW-33_01615	ftsE
ftsE	ZONMW-33_01688	ftsE
ftsE	ZONMW-33_00730	ftsE
ftsE	ZONMW-33_01593	ftsE
ftsE	ZONMW-33_02984	ftsE
ftsE	ZONMW-33_01772	ftsE
ftsE	ZONMW-33_01168	ftsE
ftsE	ZONMW-33_00867	ftsE
ftsE	ZONMW-33_02365	ftsE
ftsE	ZONMW-33_03396	ftsE
ftsE	ZONMW-33_03706	ftsE
ftsE	ZONMW-33_03650	ftsE
ftsE	ZONMW-33_00160	ftsE
ftsE	ZONMW-33_02801	ftsE
ftsE	ZONMW-33_00745	ftsE
ftsE	ZONMW-33_01154	ftsE
ftsE	ZONMW-33_03333	ftsE
ftsE	ZONMW-33_00425	ftsE
ftsE	ZONMW-33_01955	ftsE
ftsE	ZONMW-33_01429	ftsE
ftsE	ZONMW-33_03649	ftsE
ftsE	ZONMW-33_00899	ftsE
ftsE	ZONMW-33_03190	ftsE
ftsE	ZONMW-33_02802	ftsE
ftsE	ZONMW-33_00827	ftsE
ftsE	ZONMW-33_00420	ftsE
ftsE	ZONMW-33_03808	ftsE
ftsE	ZONMW-33_03364	ftsE
ftsE	ZONMW-33_00947	ftsE
ftsE	ZONMW-33_02076	ftsE
ftsE	ZONMW-33_00062	ftsE
ftsE	ZONMW-33_02539	ftsE
ftsE	ZONMW-33_03136	ftsE
ftsE	ZONMW-33_01827	ftsE
ftsE	ZONMW-33_00257	ftsE
ftsE	ZONMW-33_03900	ftsE
ftsE	ZONMW-33_00159	ftsE
ftsE	ZONMW-33_01868	ftsE
ftsE	ZONMW-33_02625	ftsE
ftsE	ZONMW-33_00061	ftsE

ftsE	ZONMW-33_01149	ftsE
ftsE	ZONMW-33_03397	ftsE
ftsE	ZONMW-33_01854	ftsE
ftsE	ZONMW-33_01004	ftsE
ftsE	ZONMW-33_00729	ftsE
ftsE	ZONMW-33_02331	ftsE
ftsE	ZONMW-33_00596	ftsE
ftsE	ZONMW-34_01612	ftsE
ftsE	ZONMW-34_02381	ftsE
ftsE	ZONMW-34_01919	ftsE
ftsE	ZONMW-34_02382	ftsE
ftsE	ZONMW-34_02921	ftsE
ftsE	ZONMW-34_01361	ftsE
ftsE	ZONMW-34_01059	ftsE
ftsE	ZONMW-34_02965	ftsE
ftsE	ZONMW-34_01876	ftsE
ftsE	ZONMW-34_00412	ftsE
ftsE	ZONMW-34_00521	ftsE
ftsE	ZONMW-34_01418	ftsE
ftsE	ZONMW-34_03127	ftsE
ftsE	ZONMW-34_02791	ftsE
ftsE	ZONMW-34_00289	ftsE
ftsE	ZONMW-34_00362	ftsE
ftsE	ZONMW-34_00081	ftsE
ftsE	ZONMW-34_00319	ftsE
ftsE	ZONMW-34_02144	ftsE
ftsE	ZONMW-34_02008	ftsE
ftsE	ZONMW-34_03600	ftsE
ftsE	ZONMW-34_00207	ftsE
ftsE	ZONMW-34_00386	ftsE
ftsE	ZONMW-34_02497	ftsE
ftsE	ZONMW-34_02498	ftsE
ftsE	ZONMW-34_01096	ftsE
ftsE	ZONMW-34_03043	ftsE
ftsE	ZONMW-34_02994	ftsE
ftsE	ZONMW-34_02830	ftsE
ftsE	ZONMW-34_02771	ftsE
ftsE	ZONMW-34_02064	ftsE
ftsE	ZONMW-34_02995	ftsE
ftsE	ZONMW-34_01110	ftsE
ftsE	ZONMW-34_03085	ftsE
ftsE	ZONMW-34_01345	ftsE
ftsE	ZONMW-34_01617	ftsE
ftsE	ZONMW-34_00290	ftsE
ftsE	ZONMW-34_02802	ftsE
ftsE	ZONMW-34_00071	ftsE
ftsE	ZONMW-34_00802	ftsE
ftsE	ZONMW-34_03196	ftsE

ftsE	ZONMW-34_02065	ftsE
ftsE	ZONMW-34_02728	ftsE
ftsE	ZONMW-34_03299	ftsE
ftsE	ZONMW-34_02231	ftsE
ftsE	ZONMW-34_03284	ftsE
ftsE	ZONMW-34_03883	ftsE
ftsE	ZONMW-34_00454	ftsE
ftsE	ZONMW-34_01362	ftsE
ftsE	ZONMW-34_03131	ftsE
ftsE	ZONMW-34_00797	ftsE
ftsE	ZONMW-34_03882	ftsE
ftsE	ZONMW-34_03200	ftsE
ftsE	ZONMW-34_02481	ftsE
ftsE	ZONMW-34_01843	ftsE
ftsE	ZONMW-34_02194	ftsE
ftsE	ZONMW-34_01920	ftsE
ftsE	ZONMW-34_02520	ftsE
ftsE	ZONMW-34_00464	ftsE
ftsE	ZONMW-34_00176	ftsE
ftsE	ZONMW-34_00632	ftsE
ftsE	ZONMW-34_01152	ftsE
ftsE	ZONMW-34_03807	ftsE
ftsE	ZONMW-34_02232	ftsE
ftsE	ZONMW-34_03283	ftsE
ftsE	ZONMW-34_00453	ftsE
ftsE	ZONMW-34_01204	ftsE
ftsE	ZONMW-34_00203	ftsE
ftsE	ZONMW-34_01340	ftsE
ftsE	ZONMW-34_01095	ftsE
ftsE	ZONMW-34_03466	ftsE
ftsE	ZONMW-34_00960	ftsE
ftsE	ZONMW-34_00463	ftsE
ftsE	ZONMW-35_00683	ftsE
ftsE	ZONMW-35_01868	ftsE
ftsE	ZONMW-35_01611	ftsE
ftsE	ZONMW-35_01867	ftsE
ftsE	ZONMW-35_02888	ftsE
ftsE	ZONMW-35_00434	ftsE
ftsE	ZONMW-35_02184	ftsE
ftsE	ZONMW-35_03027	ftsE
ftsE	ZONMW-35_01567	ftsE
ftsE	ZONMW-35_00978	ftsE
ftsE	ZONMW-35_00868	ftsE
ftsE	ZONMW-35_00491	ftsE
ftsE	ZONMW-35_03110	ftsE
ftsE	ZONMW-35_00168	ftsE
ftsE	ZONMW-35_01101	ftsE
ftsE	ZONMW-35_01028	ftsE

ftsE	ZONMW-35_01309	ftsE
ftsE	ZONMW-35_01071	ftsE
ftsE	ZONMW-35_02105	ftsE
ftsE	ZONMW-35_01700	ftsE
ftsE	ZONMW-35_03564	ftsE
ftsE	ZONMW-35_01183	ftsE
ftsE	ZONMW-35_01004	ftsE
ftsE	ZONMW-35_02461	ftsE
ftsE	ZONMW-35_02462	ftsE
ftsE	ZONMW-35_02147	ftsE
ftsE	ZONMW-35_02949	ftsE
ftsE	ZONMW-35_02998	ftsE
ftsE	ZONMW-35_02797	ftsE
ftsE	ZONMW-35_00148	ftsE
ftsE	ZONMW-35_01756	ftsE
ftsE	ZONMW-35_02997	ftsE
ftsE	ZONMW-35_02133	ftsE
ftsE	ZONMW-35_03901	ftsE
ftsE	ZONMW-35_00418	ftsE
ftsE	ZONMW-35_00688	ftsE
ftsE	ZONMW-35_01100	ftsE
ftsE	ZONMW-35_00179	ftsE
ftsE	ZONMW-35_01319	ftsE
ftsE	ZONMW-35_02661	ftsE
ftsE	ZONMW-35_03157	ftsE
ftsE	ZONMW-35_01757	ftsE
ftsE	ZONMW-35_00105	ftsE
ftsE	ZONMW-35_03335	ftsE
ftsE	ZONMW-35_02018	ftsE
ftsE	ZONMW-35_03245	ftsE
ftsE	ZONMW-35_03883	ftsE
ftsE	ZONMW-35_00936	ftsE
ftsE	ZONMW-35_00435	ftsE
ftsE	ZONMW-35_03106	ftsE
ftsE	ZONMW-35_02666	ftsE
ftsE	ZONMW-35_03884	ftsE
ftsE	ZONMW-35_03161	ftsE
ftsE	ZONMW-35_02445	ftsE
ftsE	ZONMW-35_01534	ftsE
ftsE	ZONMW-35_02055	ftsE
ftsE	ZONMW-35_01612	ftsE
ftsE	ZONMW-35_02484	ftsE
ftsE	ZONMW-35_00926	ftsE
ftsE	ZONMW-35_01215	ftsE
ftsE	ZONMW-35_00757	ftsE
ftsE	ZONMW-35_00225	ftsE
ftsE	ZONMW-35_03732	ftsE
ftsE	ZONMW-35_02017	ftsE

ftsE	ZONMW-35_03244	ftsE
ftsE	ZONMW-35_00937	ftsE
ftsE	ZONMW-35_00277	ftsE
ftsE	ZONMW-35_01187	ftsE
ftsE	ZONMW-35_00413	ftsE
ftsE	ZONMW-35_02148	ftsE
ftsE	ZONMW-35_03349	ftsE
ftsE	ZONMW-35_02283	ftsE
ftsE	ZONMW-35_00927	ftsE
ftsE	ZONMW-36_00643	ftsE
ftsE	ZONMW-36_02008	ftsE
ftsE	ZONMW-36_02628	ftsE
ftsE	ZONMW-36_02007	ftsE
ftsE	ZONMW-36_01819	ftsE
ftsE	ZONMW-36_00387	ftsE
ftsE	ZONMW-36_03578	ftsE
ftsE	ZONMW-36_03219	ftsE
ftsE	ZONMW-36_01360	ftsE
ftsE	ZONMW-36_00093	ftsE
ftsE	ZONMW-36_01902	ftsE
ftsE	ZONMW-36_00446	ftsE
ftsE	ZONMW-36_00107	ftsE
ftsE	ZONMW-36_01658	ftsE
ftsE	ZONMW-36_01402	ftsE
ftsE	ZONMW-36_03038	ftsE
ftsE	ZONMW-36_01596	ftsE
ftsE	ZONMW-36_02350	ftsE
ftsE	ZONMW-36_03514	ftsE
ftsE	ZONMW-36_01372	ftsE
ftsE	ZONMW-36_02222	ftsE
ftsE	ZONMW-36_01497	ftsE
ftsE	ZONMW-36_03644	ftsE
ftsE	ZONMW-36_02538	ftsE
ftsE	ZONMW-36_03081	ftsE
ftsE	ZONMW-36_02843	ftsE
ftsE	ZONMW-36_02842	ftsE
ftsE	ZONMW-36_02303	ftsE
ftsE	ZONMW-36_01741	ftsE
ftsE	ZONMW-36_01668	ftsE
ftsE	ZONMW-36_03182	ftsE
ftsE	ZONMW-36_01763	ftsE
ftsE	ZONMW-36_02496	ftsE
ftsE	ZONMW-36_01583	ftsE
ftsE	ZONMW-36_00388	ftsE
ftsE	ZONMW-36_00086	ftsE
ftsE	ZONMW-36_01871	ftsE
ftsE	ZONMW-36_03253	ftsE
ftsE	ZONMW-36_03655	ftsE

ftsE	ZONMW-36_03605	ftsE
ftsE	ZONMW-36_01209	ftsE
ftsE	ZONMW-36_02313	ftsE
ftsE	ZONMW-36_03167	ftsE
ftsE	ZONMW-36_00374	ftsE
ftsE	ZONMW-36_03070	ftsE
ftsE	ZONMW-36_00946	ftsE
ftsE	ZONMW-36_01401	ftsE
ftsE	ZONMW-36_00648	ftsE
ftsE	ZONMW-36_03606	ftsE
ftsE	ZONMW-36_00118	ftsE
ftsE	ZONMW-36_03491	ftsE
ftsE	ZONMW-36_02314	ftsE
ftsE	ZONMW-36_00046	ftsE
ftsE	ZONMW-36_00951	ftsE
ftsE	ZONMW-36_03798	ftsE
ftsE	ZONMW-36_03101	ftsE
ftsE	ZONMW-36_00166	ftsE
ftsE	ZONMW-36_02160	ftsE
ftsE	ZONMW-36_01307	ftsE
ftsE	ZONMW-36_02713	ftsE
ftsE	ZONMW-36_02865	ftsE
ftsE	ZONMW-36_01528	ftsE
ftsE	ZONMW-36_01112	ftsE
ftsE	ZONMW-36_03950	ftsE
ftsE	ZONMW-36_01210	ftsE
ftsE	ZONMW-36_01487	ftsE
ftsE	ZONMW-36_02627	ftsE
ftsE	ZONMW-36_01308	ftsE
ftsE	ZONMW-36_00369	ftsE
ftsE	ZONMW-36_03254	ftsE
ftsE	ZONMW-36_01501	ftsE
ftsE	ZONMW-36_00224	ftsE
ftsE	ZONMW-36_03183	ftsE
ftsE	ZONMW-36_01905	ftsE
ftsE	ZONMW-36_00775	ftsE
ftsE	ZONMW-37_02593	ftsE
ftsE	ZONMW-37_01932	ftsE
ftsE	ZONMW-37_02303	ftsE
ftsE	ZONMW-37_02475	ftsE
ftsE	ZONMW-37_00168	ftsE
ftsE	ZONMW-37_02864	ftsE
ftsE	ZONMW-37_02627	ftsE
ftsE	ZONMW-37_01714	ftsE
ftsE	ZONMW-37_00051	ftsE
ftsE	ZONMW-37_02381	ftsE
ftsE	ZONMW-37_01423	ftsE
ftsE	ZONMW-37_02782	ftsE

ftsE	ZONMW-37_01430	ftsE
ftsE	ZONMW-37_01961	ftsE
ftsE	ZONMW-37_00395	ftsE
ftsE	ZONMW-37_01412	ftsE
ftsE	ZONMW-37_02276	ftsE
ftsE	ZONMW-37_00691	ftsE
ftsE	ZONMW-37_00976	ftsE
ftsE	ZONMW-37_02484	ftsE
ftsE	ZONMW-37_02333	ftsE
ftsE	ZONMW-37_02613	ftsE
ftsE	ZONMW-37_02377	ftsE
ftsE	ZONMW-37_02077	ftsE
ftsE	ZONMW-37_01657	ftsE
ftsE	ZONMW-37_02245	ftsE
ftsE	ZONMW-38_02361	ftsE
ftsE	ZONMW-38_00600	ftsE
ftsE	ZONMW-38_02362	ftsE
ftsE	ZONMW-38_00564	ftsE
ftsE	ZONMW-38_02813	ftsE
ftsE	ZONMW-38_03829	ftsE
ftsE	ZONMW-38_01026	ftsE
ftsE	ZONMW-38_00563	ftsE
ftsE	ZONMW-38_03830	ftsE
ftsE	ZONMW-38_01035	ftsE
ftsE	ZONMW-38_00359	ftsE
ftsE	ZONMW-38_01034	ftsE
ftsE	ZONMW-38_00360	ftsE
ftsE	ZONMW-38_02145	ftsE
ftsE	ZONMW-38_03494	ftsE
ftsE	ZONMW-38_02725	ftsE
ftsE	ZONMW-38_02234	ftsE
ftsE	ZONMW-38_03889	ftsE
ftsE	ZONMW-38_04041	ftsE
ftsE	ZONMW-38_02584	ftsE
ftsE	ZONMW-38_03087	ftsE
ftsE	ZONMW-38_01886	ftsE
ftsE	ZONMW-38_01742	ftsE
ftsE	ZONMW-38_02550	ftsE
ftsE	ZONMW-38_03147	ftsE
ftsE	ZONMW-38_02636	ftsE
ftsE	ZONMW-38_02883	ftsE
ftsE	ZONMW-38_02834	ftsE
ftsE	ZONMW-38_02595	ftsE
ftsE	ZONMW-38_00717	ftsE
ftsE	ZONMW-38_01885	ftsE
ftsE	ZONMW-38_00977	ftsE
ftsE	ZONMW-38_01237	ftsE
ftsE	ZONMW-38_01813	ftsE

ftsE	ZONMW-38_01656	ftsE
ftsE	ZONMW-38_04010	ftsE
ftsE	ZONMW-38_02651	ftsE
ftsE	ZONMW-38_02667	ftsE
ftsE	ZONMW-38_00091	ftsE
ftsE	ZONMW-38_02551	ftsE
ftsE	ZONMW-38_00596	ftsE
ftsE	ZONMW-38_02869	ftsE
ftsE	ZONMW-38_01439	ftsE
ftsE	ZONMW-38_01115	ftsE
ftsE	ZONMW-38_00926	ftsE
ftsE	ZONMW-38_01802	ftsE
ftsE	ZONMW-38_00454	ftsE
ftsE	ZONMW-38_02668	ftsE
ftsE	ZONMW-38_02922	ftsE
ftsE	ZONMW-38_00978	ftsE
ftsE	ZONMW-38_01028	ftsE
ftsE	ZONMW-38_03236	ftsE
ftsE	ZONMW-38_00426	ftsE
ftsE	ZONMW-38_02235	ftsE
ftsE	ZONMW-38_01754	ftsE
ftsE	ZONMW-38_02081	ftsE
ftsE	ZONMW-38_01552	ftsE
ftsE	ZONMW-38_02919	ftsE
ftsE	ZONMW-38_01291	ftsE
ftsE	ZONMW-38_03096	ftsE
ftsE	ZONMW-38_04167	ftsE
ftsE	ZONMW-38_01731	ftsE
ftsE	ZONMW-38_01976	ftsE
ftsE	ZONMW-38_02160	ftsE
ftsE	ZONMW-38_02850	ftsE
ftsE	ZONMW-38_03953	ftsE
ftsE	ZONMW-38_03083	ftsE
ftsE	ZONMW-38_02241	ftsE
ftsE	ZONMW-38_00425	ftsE
ftsE	ZONMW-38_01761	ftsE
ftsE	ZONMW-38_01495	ftsE
ftsE	ZONMW-38_02080	ftsE
ftsE	ZONMW-38_00887	ftsE
ftsE	ZONMW-38_00718	ftsE
ftsE	ZONMW-38_01279	ftsE
ftsE	ZONMW-38_01247	ftsE
ftsE	ZONMW-38_01672	ftsE
ftsE	ZONMW-38_02676	ftsE
ftsE	ZONMW-38_01851	ftsE
ftsE	ZONMW-38_02046	ftsE
ftsE	ZONMW-38_02661	ftsE
ftsE	ZONMW-38_00206	ftsE

ftsE	ZONMW-38_00614	ftsE
ftsE	ZONMW-38_03317	ftsE
ftsE	ZONMW-38_00338	ftsE
ftsE	ZONMW-38_00662	ftsE
ftsE	ZONMW-38_03886	ftsE
ftsE	ZONMW-38_03574	ftsE
ftsE	ZONMW-38_03946	ftsE
ftsE	ZONMW-38_01542	ftsE
ftsE	ZONMW-38_01958	ftsE
ftsE	ZONMW-38_01013	ftsE
ftsE	ZONMW-38_00090	ftsE
ftsE	ZONMW-38_01285	ftsE
ftsE	ZONMW-38_01242	ftsE
ftsE	ZONMW-38_02238	ftsE
ftsE	ZONMW-38_01271	ftsE
ftsE	ZONMW-38_00120	ftsE
ftsE	ZONMW-38_03884	ftsE
ftsE	ZONMW-38_04228	ftsE
ftsE	ZONMW-38_00176	ftsE
ftsE	ZONMW-38_03024	ftsE
ftsE	ZONMW-38_02886	ftsE
ftsE	ZONMW-38_03627	ftsE
ftsE	ZONMW-38_04011	ftsE
ftsE	ZONMW-38_02849	ftsE
ftsE	ZONMW-38_00175	ftsE
ftsE	ZONMW-38_00617	ftsE
ftsE	ZONMW-38_01000	ftsE
ftsE	ZONMW-39_00087	ftsE
ftsE	ZONMW-39_01673	ftsE
ftsE	ZONMW-39_02283	ftsE
ftsE	ZONMW-39_01674	ftsE
ftsE	ZONMW-39_02655	ftsE
ftsE	ZONMW-39_03419	ftsE
ftsE	ZONMW-39_02599	ftsE
ftsE	ZONMW-39_00340	ftsE
ftsE	ZONMW-39_04189	ftsE
ftsE	ZONMW-39_02927	ftsE
ftsE	ZONMW-39_02794	ftsE
ftsE	ZONMW-39_02315	ftsE
ftsE	ZONMW-39_03117	ftsE
ftsE	ZONMW-39_02262	ftsE
ftsE	ZONMW-39_01784	ftsE
ftsE	ZONMW-39_00282	ftsE
ftsE	ZONMW-39_00910	ftsE
ftsE	ZONMW-39_00989	ftsE
ftsE	ZONMW-39_04008	ftsE
ftsE	ZONMW-39_03051	ftsE
ftsE	ZONMW-39_02453	ftsE

ftsE	ZONMW-39_03004	ftsE
ftsE	ZONMW-39_04170	ftsE
ftsE	ZONMW-39_01418	ftsE
ftsE	ZONMW-39_01572	ftsE
ftsE	ZONMW-39_01963	ftsE
ftsE	ZONMW-39_02184	ftsE
ftsE	ZONMW-39_00714	ftsE
ftsE	ZONMW-39_01835	ftsE
ftsE	ZONMW-39_03089	ftsE
ftsE	ZONMW-39_02721	ftsE
ftsE	ZONMW-39_02662	ftsE
ftsE	ZONMW-39_00768	ftsE
ftsE	ZONMW-39_02038	ftsE
ftsE	ZONMW-39_03349	ftsE
ftsE	ZONMW-39_02963	ftsE
ftsE	ZONMW-39_02039	ftsE
ftsE	ZONMW-39_02764	ftsE
ftsE	ZONMW-39_02548	ftsE
ftsE	ZONMW-39_04194	ftsE
ftsE	ZONMW-39_04165	ftsE
ftsE	ZONMW-39_03440	ftsE
ftsE	ZONMW-39_03629	ftsE
ftsE	ZONMW-39_02132	ftsE
ftsE	ZONMW-39_02763	ftsE
ftsE	ZONMW-39_02463	ftsE
ftsE	ZONMW-39_02978	ftsE
ftsE	ZONMW-39_00355	ftsE
ftsE	ZONMW-39_04007	ftsE
ftsE	ZONMW-39_00082	ftsE
ftsE	ZONMW-39_04164	ftsE
ftsE	ZONMW-39_03154	ftsE
ftsE	ZONMW-39_00339	ftsE
ftsE	ZONMW-39_00899	ftsE
ftsE	ZONMW-39_01266	ftsE
ftsE	ZONMW-39_01944	ftsE
ftsE	ZONMW-39_03468	ftsE
ftsE	ZONMW-39_03630	ftsE
ftsE	ZONMW-39_01518	ftsE
ftsE	ZONMW-39_01870	ftsE
ftsE	ZONMW-39_04176	ftsE
ftsE	ZONMW-39_00769	ftsE
ftsE	ZONMW-39_01779	ftsE
ftsE	ZONMW-39_01261	ftsE
ftsE	ZONMW-39_01573	ftsE
ftsE	ZONMW-39_03067	ftsE
ftsE	ZONMW-39_01474	ftsE
ftsE	ZONMW-39_02661	ftsE
ftsE	ZONMW-39_02282	ftsE

ftsE	ZONMW-39_02061	ftsE
ftsE	ZONMW-39_01096	ftsE
ftsE	ZONMW-39_00854	ftsE
ftsE	ZONMW-39_04209	ftsE
ftsE	ZONMW-39_01519	ftsE
ftsE	ZONMW-39_01871	ftsE
ftsE	ZONMW-39_02441	ftsE
ftsE	ZONMW-39_01940	ftsE
ftsE	ZONMW-39_04177	ftsE
ftsE	ZONMW-39_03381	ftsE
ftsE	ZONMW-39_03353	ftsE
ftsE	ZONMW-39_00787	ftsE
ftsE	ZONMW-39_00360	ftsE
ftsE	ZONMW-39_02962	ftsE
ftsE	ZONMW-39_02014	ftsE
ftsE	ZONMW-39_02831	ftsE
ftsE	ZONMW-40_03148	ftsE
ftsE	ZONMW-40_03255	ftsE
ftsE	ZONMW-40_01934	ftsE
ftsE	ZONMW-40_00036	ftsE
ftsE	ZONMW-40_00588	ftsE
ftsE	ZONMW-40_02576	ftsE
ftsE	ZONMW-40_03029	ftsE
ftsE	ZONMW-40_00080	ftsE
ftsE	ZONMW-40_03112	ftsE
ftsE	ZONMW-40_00753	ftsE
ftsE	ZONMW-40_01723	ftsE
ftsE	ZONMW-40_01944	ftsE
ftsE	ZONMW-40_00313	ftsE
ftsE	ZONMW-40_00969	ftsE
ftsE	ZONMW-40_00336	ftsE
ftsE	ZONMW-40_02117	ftsE
ftsE	ZONMW-40_00328	ftsE
ftsE	ZONMW-40_01281	ftsE
ftsE	ZONMW-40_01765	ftsE
ftsE	ZONMW-40_00600	ftsE
ftsE	ZONMW-40_03127	ftsE
ftsE	ZONMW-40_01727	ftsE
ftsE	ZONMW-40_02755	ftsE
ftsE	ZONMW-40_02522	ftsE
ftsE	ZONMW-40_02394	ftsE
ftsE	ZONMW-40_03211	ftsE
ftsE	ZONMW-40_01795	ftsE
ftsE	ZONMW-40_00440	ftsE
ftsE	ZONMW-41_03034	ftsE
ftsE	ZONMW-41_02338	ftsE
ftsE	ZONMW-41_01985	ftsE
ftsE	ZONMW-41_02138	ftsE

ftsE	ZONMW-41_02748	ftsE
ftsE	ZONMW-41_00798	ftsE
ftsE	ZONMW-41_01997	ftsE
ftsE	ZONMW-41_02080	ftsE
ftsE	ZONMW-41_02566	ftsE
ftsE	ZONMW-41_02571	ftsE
ftsE	ZONMW-41_00978	ftsE
ftsE	ZONMW-41_01780	ftsE
ftsE	ZONMW-41_02349	ftsE
ftsE	ZONMW-41_03028	ftsE
ftsE	ZONMW-41_02625	ftsE
ftsE	ZONMW-41_01934	ftsE
ftsE	ZONMW-41_01917	ftsE
ftsE	ZONMW-41_02044	ftsE
ftsE	ZONMW-41_00488	ftsE
ftsE	ZONMW-41_00523	ftsE
ftsE	ZONMW-41_02797	ftsE
ftsE	ZONMW-41_00211	ftsE
ftsE	ZONMW-41_00425	ftsE
ftsE	ZONMW-41_01176	ftsE
ftsE	ZONMW-41_03014	ftsE
ftsE	ZONMW-41_00177	ftsE
ftsE	ZONMW-41_00017	ftsE
ftsE	ZONMW-41_02419	ftsE
ftsE	ZONMW-41_00725	ftsE
ftsE	ZONMW-41_00763	ftsE
ftsE	ZONMW-41_00608	ftsE
ftsE	ZONMW-41_02608	ftsE
ftsE	ZONMW-41_01182	ftsE
ftsE	ZONMW-41_00678	ftsE
ftsE	ZONMW-41_01986	ftsE
ftsE	ZONMW-41_00797	ftsE
ftsE	ZONMW-41_02040	ftsE
ftsE	ZONMW-41_00609	ftsE
ftsE	ZONMW-41_01293	ftsE
ftsE	ZONMW-42_03290	ftsE
ftsE	ZONMW-42_00501	ftsE
ftsE	ZONMW-42_02762	ftsE
ftsE	ZONMW-42_02763	ftsE
ftsE	ZONMW-42_02939	ftsE
ftsE	ZONMW-42_00232	ftsE
ftsE	ZONMW-42_01780	ftsE
ftsE	ZONMW-42_03052	ftsE
ftsE	ZONMW-42_02424	ftsE
ftsE	ZONMW-42_00832	ftsE
ftsE	ZONMW-42_00622	ftsE
ftsE	ZONMW-42_02633	ftsE
ftsE	ZONMW-42_03076	ftsE

ftsE	ZONMW-42_00075	ftsE
ftsE	ZONMW-42_02652	ftsE
ftsE	ZONMW-42_00766	ftsE
ftsE	ZONMW-42_04012	ftsE
ftsE	ZONMW-42_03593	ftsE
ftsE	ZONMW-42_03565	ftsE
ftsE	ZONMW-42_00410	ftsE
ftsE	ZONMW-42_00645	ftsE
ftsE	ZONMW-42_00194	ftsE
ftsE	ZONMW-42_00271	ftsE
ftsE	ZONMW-42_01842	ftsE
ftsE	ZONMW-42_01273	ftsE
ftsE	ZONMW-42_03459	ftsE
ftsE	ZONMW-42_00590	ftsE
ftsE	ZONMW-42_02628	ftsE
ftsE	ZONMW-42_00173	ftsE
ftsE	ZONMW-42_01752	ftsE
ftsE	ZONMW-42_03446	ftsE
ftsE	ZONMW-42_04111	ftsE
ftsE	ZONMW-42_03284	ftsE
ftsE	ZONMW-42_01517	ftsE
ftsE	ZONMW-42_03009	ftsE
ftsE	ZONMW-42_01781	ftsE
ftsE	ZONMW-42_02596	ftsE
ftsE	ZONMW-42_02563	ftsE
ftsE	ZONMW-42_01268	ftsE
ftsE	ZONMW-42_02496	ftsE
ftsE	ZONMW-42_03592	ftsE
ftsE	ZONMW-42_02663	ftsE
ftsE	ZONMW-42_02383	ftsE
ftsE	ZONMW-42_01518	ftsE
ftsE	ZONMW-42_00083	ftsE
ftsE	ZONMW-42_02448	ftsE
ftsE	ZONMW-42_01236	ftsE
ftsE	ZONMW-42_00578	ftsE
ftsE	ZONMW-42_01328	ftsE
ftsE	ZONMW-42_01240	ftsE
ftsE	ZONMW-42_01198	ftsE
ftsE	ZONMW-42_01474	ftsE
ftsE	ZONMW-42_00579	ftsE
ftsE	ZONMW-42_00798	ftsE
ftsE	ZONMW-42_03360	ftsE
ftsE	ZONMW-42_02966	ftsE
ftsE	ZONMW-42_02941	ftsE
ftsE	ZONMW-42_01588	ftsE
ftsE	ZONMW-42_00302	ftsE
ftsE	ZONMW-42_03004	ftsE
ftsE	ZONMW-42_01380	ftsE

ftsE	ZONMW-42_01155	ftsE
ftsE	ZONMW-42_02335	ftsE
ftsE	ZONMW-42_03707	ftsE
ftsE	ZONMW-42_01379	ftsE
ftsE	ZONMW-42_01747	ftsE
ftsE	ZONMW-42_04014	ftsE
ftsE	ZONMW-42_00500	ftsE
ftsE	ZONMW-42_03405	ftsE
ftsE	ZONMW-42_00799	ftsE
ftsE	ZONMW-42_03378	ftsE
ftsE	ZONMW-42_01187	ftsE
ftsE	ZONMW-42_02950	ftsE
ftsE	ZONMW-42_00924	ftsE
ftsE	ZONMW-43_01656	ftsE
ftsE	ZONMW-43_01851	ftsE
ftsE	ZONMW-43_01423	ftsE
ftsE	ZONMW-43_01850	ftsE
ftsE	ZONMW-43_03993	ftsE
ftsE	ZONMW-43_02944	ftsE
ftsE	ZONMW-43_04362	ftsE
ftsE	ZONMW-43_00593	ftsE
ftsE	ZONMW-43_03824	ftsE
ftsE	ZONMW-43_01387	ftsE
ftsE	ZONMW-43_02380	ftsE
ftsE	ZONMW-43_03542	ftsE
ftsE	ZONMW-43_00066	ftsE
ftsE	ZONMW-43_03015	ftsE
ftsE	ZONMW-43_03451	ftsE
ftsE	ZONMW-43_04495	ftsE
ftsE	ZONMW-43_00663	ftsE
ftsE	ZONMW-43_02324	ftsE
ftsE	ZONMW-43_01042	ftsE
ftsE	ZONMW-43_00867	ftsE
ftsE	ZONMW-43_01032	ftsE
ftsE	ZONMW-43_00626	ftsE
ftsE	ZONMW-43_02890	ftsE
ftsE	ZONMW-43_03217	ftsE
ftsE	ZONMW-43_01938	ftsE
ftsE	ZONMW-43_01512	ftsE
ftsE	ZONMW-43_03549	ftsE
ftsE	ZONMW-43_00741	ftsE
ftsE	ZONMW-43_03079	ftsE
ftsE	ZONMW-43_03928	ftsE
ftsE	ZONMW-43_02348	ftsE
ftsE	ZONMW-43_03600	ftsE
ftsE	ZONMW-43_01124	ftsE
ftsE	ZONMW-43_01125	ftsE
ftsE	ZONMW-43_04136	ftsE

ftsE	ZONMW-43_04103	ftsE
ftsE	ZONMW-43_02406	ftsE
ftsE	ZONMW-43_03853	ftsE
ftsE	ZONMW-43_00963	ftsE
ftsE	ZONMW-43_02810	ftsE
ftsE	ZONMW-43_01563	ftsE
ftsE	ZONMW-43_02884	ftsE
ftsE	ZONMW-43_02424	ftsE
ftsE	ZONMW-43_03854	ftsE
ftsE	ZONMW-43_04122	ftsE
ftsE	ZONMW-43_04448	ftsE
ftsE	ZONMW-43_02931	ftsE
ftsE	ZONMW-43_02811	ftsE
ftsE	ZONMW-43_00662	ftsE
ftsE	ZONMW-43_01651	ftsE
ftsE	ZONMW-43_04484	ftsE
ftsE	ZONMW-43_00877	ftsE
ftsE	ZONMW-43_00341	ftsE
ftsE	ZONMW-43_01017	ftsE
ftsE	ZONMW-43_02883	ftsE
ftsE	ZONMW-43_02456	ftsE
ftsE	ZONMW-43_03311	ftsE
ftsE	ZONMW-43_04021	ftsE
ftsE	ZONMW-43_02748	ftsE
ftsE	ZONMW-43_04346	ftsE
ftsE	ZONMW-43_02945	ftsE
ftsE	ZONMW-43_03455	ftsE
ftsE	ZONMW-43_00336	ftsE
ftsE	ZONMW-43_01013	ftsE
ftsE	ZONMW-43_01092	ftsE
ftsE	ZONMW-43_01937	ftsE
ftsE	ZONMW-43_01353	ftsE
ftsE	ZONMW-43_03080	ftsE
ftsE	ZONMW-43_03761	ftsE
ftsE	ZONMW-43_03268	ftsE
ftsE	ZONMW-43_03599	ftsE
ftsE	ZONMW-43_01424	ftsE
ftsE	ZONMW-43_01147	ftsE
ftsE	ZONMW-43_02407	ftsE
ftsE	ZONMW-43_00772	ftsE
ftsE	ZONMW-43_00174	ftsE
ftsE	ZONMW-43_03149	ftsE
ftsE	ZONMW-43_03312	ftsE
ftsE	ZONMW-43_04022	ftsE
ftsE	ZONMW-43_04347	ftsE
ftsE	ZONMW-43_03097	ftsE
ftsE	ZONMW-43_00745	ftsE
ftsE	ZONMW-43_02926	ftsE

ftsE	ZONMW-43_04137	ftsE
ftsE	ZONMW-43_01028	ftsE
ftsE	ZONMW-43_00493	ftsE
ftsE	ZONMW-44_02813	ftsE
ftsE	ZONMW-44_00795	ftsE
ftsE	ZONMW-44_02456	ftsE
ftsE	ZONMW-44_02455	ftsE
ftsE	ZONMW-44_03001	ftsE
ftsE	ZONMW-44_01402	ftsE
ftsE	ZONMW-44_03970	ftsE
ftsE	ZONMW-44_01231	ftsE
ftsE	ZONMW-44_00618	ftsE
ftsE	ZONMW-44_04138	ftsE
ftsE	ZONMW-44_02706	ftsE
ftsE	ZONMW-44_03426	ftsE
ftsE	ZONMW-44_01715	ftsE
ftsE	ZONMW-44_01939	ftsE
ftsE	ZONMW-44_03401	ftsE
ftsE	ZONMW-44_03515	ftsE
ftsE	ZONMW-44_01305	ftsE
ftsE	ZONMW-44_03543	ftsE
ftsE	ZONMW-44_00254	ftsE
ftsE	ZONMW-44_00887	ftsE
ftsE	ZONMW-44_02878	ftsE
ftsE	ZONMW-44_02686	ftsE
ftsE	ZONMW-44_02965	ftsE
ftsE	ZONMW-44_03052	ftsE
ftsE	ZONMW-44_02512	ftsE
ftsE	ZONMW-44_00196	ftsE
ftsE	ZONMW-44_03633	ftsE
ftsE	ZONMW-44_01821	ftsE
ftsE	ZONMW-44_01947	ftsE
ftsE	ZONMW-44_00463	ftsE
ftsE	ZONMW-44_00701	ftsE
ftsE	ZONMW-44_02945	ftsE
ftsE	ZONMW-44_00197	ftsE
ftsE	ZONMW-44_03900	ftsE
ftsE	ZONMW-44_02819	ftsE
ftsE	ZONMW-44_03585	ftsE
ftsE	ZONMW-44_01232	ftsE
ftsE	ZONMW-44_01219	ftsE
ftsE	ZONMW-44_01495	ftsE
ftsE	ZONMW-44_03464	ftsE
ftsE	ZONMW-44_02185	ftsE
ftsE	ZONMW-44_03431	ftsE
ftsE	ZONMW-44_00458	ftsE
ftsE	ZONMW-44_00059	ftsE
ftsE	ZONMW-44_02305	ftsE

ftsE	ZONMW-44_03516	ftsE
ftsE	ZONMW-44_00519	ftsE
ftsE	ZONMW-44_01494	ftsE
ftsE	ZONMW-44_03390	ftsE
ftsE	ZONMW-44_02910	ftsE
ftsE	ZONMW-44_03903	ftsE
ftsE	ZONMW-44_02511	ftsE
ftsE	ZONMW-44_00571	ftsE
ftsE	ZONMW-44_00427	ftsE
ftsE	ZONMW-44_00378	ftsE
ftsE	ZONMW-44_00431	ftsE
ftsE	ZONMW-44_00714	ftsE
ftsE	ZONMW-44_00715	ftsE
ftsE	ZONMW-44_00685	ftsE
ftsE	ZONMW-44_02180	ftsE
ftsE	ZONMW-44_01750	ftsE
ftsE	ZONMW-44_01815	ftsE
ftsE	ZONMW-44_02955	ftsE
ftsE	ZONMW-44_01425	ftsE
ftsE	ZONMW-44_03083	ftsE
ftsE	ZONMW-44_01674	ftsE
ftsE	ZONMW-44_00253	ftsE
ftsE	ZONMW-44_00176	ftsE
ftsE	ZONMW-44_03162	ftsE
ftsE	ZONMW-44_03341	ftsE
ftsE	ZONMW-44_01675	ftsE
ftsE	ZONMW-44_01214	ftsE
ftsE	ZONMW-44_01081	ftsE
ftsE	ZONMW-44_01850	ftsE
ftsE	ZONMW-44_01823	ftsE
ftsE	ZONMW-44_02911	ftsE
ftsE	ZONMW-44_00796	ftsE
ftsE	ZONMW-44_03709	ftsE
ftsE	ZONMW-45_02902	ftsE
ftsE	ZONMW-45_01602	ftsE
ftsE	ZONMW-45_02342	ftsE
ftsE	ZONMW-45_02539	ftsE
ftsE	ZONMW-45_02359	ftsE
ftsE	ZONMW-45_00777	ftsE
ftsE	ZONMW-45_01693	ftsE
ftsE	ZONMW-45_01621	ftsE
ftsE	ZONMW-45_02409	ftsE
ftsE	ZONMW-45_00542	ftsE
ftsE	ZONMW-45_01965	ftsE
ftsE	ZONMW-45_02134	ftsE
ftsE	ZONMW-45_01588	ftsE
ftsE	ZONMW-45_00467	ftsE
ftsE	ZONMW-45_00992	ftsE

ftsE	ZONMW-45_00399	ftsE
ftsE	ZONMW-45_02582	ftsE
ftsE	ZONMW-45_00547	ftsE
ftsE	ZONMW-45_02351	ftsE
ftsE	ZONMW-45_02426	ftsE
ftsE	ZONMW-45_01470	ftsE
ftsE	ZONMW-45_03174	ftsE
ftsE	ZONMW-45_01258	ftsE
ftsE	ZONMW-45_00554	ftsE
ftsE	ZONMW-45_02508	ftsE
ftsE	ZONMW-45_01530	ftsE
ftsE	ZONMW-45_00167	ftsE
ftsE	ZONMW-45_00030	ftsE
ftsE	ZONMW-45_00557	ftsE
ftsE	ZONMW-46_03039	ftsE
ftsE	ZONMW-46_02113	ftsE
ftsE	ZONMW-46_01204	ftsE
ftsE	ZONMW-46_02761	ftsE
ftsE	ZONMW-46_01221	ftsE
ftsE	ZONMW-46_01097	ftsE
ftsE	ZONMW-46_00104	ftsE
ftsE	ZONMW-46_02132	ftsE
ftsE	ZONMW-46_00175	ftsE
ftsE	ZONMW-46_01297	ftsE
ftsE	ZONMW-46_02314	ftsE
ftsE	ZONMW-46_01810	ftsE
ftsE	ZONMW-46_02099	ftsE
ftsE	ZONMW-46_01372	ftsE
ftsE	ZONMW-46_00675	ftsE
ftsE	ZONMW-46_01440	ftsE
ftsE	ZONMW-46_02976	ftsE
ftsE	ZONMW-46_01292	ftsE
ftsE	ZONMW-46_01213	ftsE
ftsE	ZONMW-46_00192	ftsE
ftsE	ZONMW-46_01725	ftsE
ftsE	ZONMW-46_02926	ftsE
ftsE	ZONMW-46_02642	ftsE
ftsE	ZONMW-46_00905	ftsE
ftsE	ZONMW-46_02730	ftsE
ftsE	ZONMW-46_02041	ftsE
ftsE	ZONMW-46_00382	ftsE
ftsE	ZONMW-46_00245	ftsE
ftsE	ZONMW-46_00902	ftsE
ftsE	ZONMW-47_03030	ftsE
ftsE	ZONMW-47_01948	ftsE
ftsE	ZONMW-47_00969	ftsE
ftsE	ZONMW-47_02752	ftsE
ftsE	ZONMW-47_00952	ftsE

ftsE	ZONMW-47_01076	ftsE
ftsE	ZONMW-47_00401	ftsE
ftsE	ZONMW-47_01929	ftsE
ftsE	ZONMW-47_00330	ftsE
ftsE	ZONMW-47_01555	ftsE
ftsE	ZONMW-47_02218	ftsE
ftsE	ZONMW-47_01679	ftsE
ftsE	ZONMW-47_01962	ftsE
ftsE	ZONMW-47_01480	ftsE
ftsE	ZONMW-47_00675	ftsE
ftsE	ZONMW-47_01412	ftsE
ftsE	ZONMW-47_02897	ftsE
ftsE	ZONMW-47_01560	ftsE
ftsE	ZONMW-47_00960	ftsE
ftsE	ZONMW-47_00313	ftsE
ftsE	ZONMW-47_01764	ftsE
ftsE	ZONMW-47_02947	ftsE
ftsE	ZONMW-47_02632	ftsE
ftsE	ZONMW-47_00905	ftsE
ftsE	ZONMW-47_02721	ftsE
ftsE	ZONMW-47_02020	ftsE
ftsE	ZONMW-47_00123	ftsE
ftsE	ZONMW-47_00260	ftsE
ftsE	ZONMW-47_00902	ftsE
ftsE	ZONMW-48_02128	ftsE
ftsE	ZONMW-48_01195	ftsE
ftsE	ZONMW-48_00361	ftsE
ftsE	ZONMW-48_02278	ftsE
ftsE	ZONMW-48_01072	ftsE
ftsE	ZONMW-48_02084	ftsE
ftsE	ZONMW-48_00059	ftsE
ftsE	ZONMW-48_01514	ftsE
ftsE	ZONMW-48_01318	ftsE
ftsE	ZONMW-48_00322	ftsE
ftsE	ZONMW-48_02877	ftsE
ftsE	ZONMW-48_03259	ftsE
ftsE	ZONMW-48_00060	ftsE
ftsE	ZONMW-48_00675	ftsE
ftsE	ZONMW-48_00094	ftsE
ftsE	ZONMW-48_02304	ftsE
ftsE	ZONMW-48_02106	ftsE
ftsE	ZONMW-48_02883	ftsE
ftsE	ZONMW-48_02155	ftsE
ftsE	ZONMW-48_01052	ftsE
ftsE	ZONMW-48_01510	ftsE
ftsE	ZONMW-48_02091	ftsE
ftsE	ZONMW-48_00484	ftsE
ftsE	ZONMW-48_01220	ftsE

ftsE	ZONMW-48_01262	ftsE
ftsE	ZONMW-48_00614	ftsE
ftsE	ZONMW-48_02263	ftsE
ftsE	ZONMW-48_00694	ftsE
ftsE	ZONMW-48_01704	ftsE
ftsE	ZONMW-48_03043	ftsE
ftsE	ZONMW-48_01128	ftsE
ftsE	ZONMW-49_00554	ftsE
ftsE	ZONMW-49_00159	ftsE
ftsE	ZONMW-49_02088	ftsE
ftsE	ZONMW-49_02548	ftsE
ftsE	ZONMW-49_01462	ftsE
ftsE	ZONMW-49_01735	ftsE
ftsE	ZONMW-49_00589	ftsE
ftsE	ZONMW-49_02349	ftsE
ftsE	ZONMW-49_01579	ftsE
ftsE	ZONMW-49_01557	ftsE
ftsE	ZONMW-49_03087	ftsE
ftsE	ZONMW-49_03119	ftsE
ftsE	ZONMW-49_01587	ftsE
ftsE	ZONMW-49_03111	ftsE
ftsE	ZONMW-49_02072	ftsE
ftsE	ZONMW-49_00801	ftsE
ftsE	ZONMW-49_03125	ftsE
ftsE	ZONMW-49_02350	ftsE
ftsE	ZONMW-49_01058	ftsE
ftsE	ZONMW-49_00994	ftsE
ftsE	ZONMW-49_01261	ftsE
ftsE	ZONMW-49_00578	ftsE
ftsE	ZONMW-49_03090	ftsE
ftsE	ZONMW-49_02331	ftsE
ftsE	ZONMW-49_02582	ftsE
ftsE	ZONMW-49_02773	ftsE
ftsE	ZONMW-49_00305	ftsE
ftsE	ZONMW-49_00269	ftsE
ftsE	ZONMW-49_01190	ftsE
ftsE	ZONMW-49_01866	ftsE
ftsE	ZONMW-49_01026	ftsE
ftsE	ZONMW-49_02521	ftsE
ftsE	ZONMW-49_01070	ftsE
ftsE	ZONMW-50_00555	ftsE
ftsE	ZONMW-50_00326	ftsE
ftsE	ZONMW-50_01882	ftsE
ftsE	ZONMW-50_02342	ftsE
ftsE	ZONMW-50_01464	ftsE
ftsE	ZONMW-50_01679	ftsE
ftsE	ZONMW-50_00590	ftsE
ftsE	ZONMW-50_02111	ftsE

ftsE	ZONMW-50_01835	ftsE
ftsE	ZONMW-50_01559	ftsE
ftsE	ZONMW-50_02945	ftsE
ftsE	ZONMW-50_03115	ftsE
ftsE	ZONMW-50_01827	ftsE
ftsE	ZONMW-50_03107	ftsE
ftsE	ZONMW-50_01866	ftsE
ftsE	ZONMW-50_00802	ftsE
ftsE	ZONMW-50_03121	ftsE
ftsE	ZONMW-50_02110	ftsE
ftsE	ZONMW-50_01059	ftsE
ftsE	ZONMW-50_00995	ftsE
ftsE	ZONMW-50_01263	ftsE
ftsE	ZONMW-50_00579	ftsE
ftsE	ZONMW-50_02942	ftsE
ftsE	ZONMW-50_02129	ftsE
ftsE	ZONMW-50_02376	ftsE
ftsE	ZONMW-50_02699	ftsE
ftsE	ZONMW-50_00180	ftsE
ftsE	ZONMW-50_00216	ftsE
ftsE	ZONMW-50_01191	ftsE
ftsE	ZONMW-50_03047	ftsE
ftsE	ZONMW-50_01027	ftsE
ftsE	ZONMW-50_02315	ftsE
ftsE	ZONMW-50_01071	ftsE
ftsE	ZONMW-51_01151	ftsE
ftsE	ZONMW-51_00156	ftsE
ftsE	ZONMW-51_01746	ftsE
ftsE	ZONMW-51_02525	ftsE
ftsE	ZONMW-51_00718	ftsE
ftsE	ZONMW-51_00633	ftsE
ftsE	ZONMW-51_01116	ftsE
ftsE	ZONMW-51_01607	ftsE
ftsE	ZONMW-51_00476	ftsE
ftsE	ZONMW-51_00812	ftsE
ftsE	ZONMW-51_01873	ftsE
ftsE	ZONMW-51_03175	ftsE
ftsE	ZONMW-51_00484	ftsE
ftsE	ZONMW-51_03167	ftsE
ftsE	ZONMW-51_01730	ftsE
ftsE	ZONMW-51_00910	ftsE
ftsE	ZONMW-51_03181	ftsE
ftsE	ZONMW-51_01606	ftsE
ftsE	ZONMW-51_01273	ftsE
ftsE	ZONMW-51_01376	ftsE
ftsE	ZONMW-51_01127	ftsE
ftsE	ZONMW-51_01870	ftsE
ftsE	ZONMW-51_01625	ftsE

ftsE	ZONMW-51_02559	ftsE
ftsE	ZONMW-51_03002	ftsE
ftsE	ZONMW-51_00293	ftsE
ftsE	ZONMW-51_00257	ftsE
ftsE	ZONMW-51_02796	ftsE
ftsE	ZONMW-51_02149	ftsE
ftsE	ZONMW-51_00512	ftsE
ftsE	ZONMW-51_01241	ftsE
ftsE	ZONMW-51_02498	ftsE
ftsE	ZONMW-51_03274	ftsE
ftsE	ZONMW-52_02844	ftsE
ftsE	ZONMW-52_00128	ftsE
ftsE	ZONMW-52_01889	ftsE
ftsE	ZONMW-52_02508	ftsE
ftsE	ZONMW-52_01318	ftsE
ftsE	ZONMW-52_00590	ftsE
ftsE	ZONMW-52_02809	ftsE
ftsE	ZONMW-52_01750	ftsE
ftsE	ZONMW-52_00461	ftsE
ftsE	ZONMW-52_01412	ftsE
ftsE	ZONMW-52_00900	ftsE
ftsE	ZONMW-52_03134	ftsE
ftsE	ZONMW-52_00469	ftsE
ftsE	ZONMW-52_03126	ftsE
ftsE	ZONMW-52_01873	ftsE
ftsE	ZONMW-52_01510	ftsE
ftsE	ZONMW-52_03140	ftsE
ftsE	ZONMW-52_01749	ftsE
ftsE	ZONMW-52_01002	ftsE
ftsE	ZONMW-52_01118	ftsE
ftsE	ZONMW-52_02820	ftsE
ftsE	ZONMW-52_00903	ftsE
ftsE	ZONMW-52_01768	ftsE
ftsE	ZONMW-52_02542	ftsE
ftsE	ZONMW-52_03072	ftsE
ftsE	ZONMW-52_00277	ftsE
ftsE	ZONMW-52_00241	ftsE
ftsE	ZONMW-52_02250	ftsE
ftsE	ZONMW-52_02578	ftsE
ftsE	ZONMW-52_00970	ftsE
ftsE	ZONMW-52_02481	ftsE
ftsE	ZONMW-52_03259	ftsE
ftsE	ZONMW-53_00071	ftsE
ftsE	ZONMW-53_02526	ftsE
ftsE	ZONMW-53_01867	ftsE
ftsE	ZONMW-53_02125	ftsE
ftsE	ZONMW-53_00507	ftsE
ftsE	ZONMW-53_01345	ftsE

ftsE	ZONMW-53_00106	ftsE
ftsE	ZONMW-53_02006	ftsE
ftsE	ZONMW-53_01192	ftsE
ftsE	ZONMW-53_00413	ftsE
ftsE	ZONMW-53_01643	ftsE
ftsE	ZONMW-53_03153	ftsE
ftsE	ZONMW-53_01200	ftsE
ftsE	ZONMW-53_03161	ftsE
ftsE	ZONMW-53_01883	ftsE
ftsE	ZONMW-53_00317	ftsE
ftsE	ZONMW-53_03147	ftsE
ftsE	ZONMW-53_02007	ftsE
ftsE	ZONMW-53_00834	ftsE
ftsE	ZONMW-53_00731	ftsE
ftsE	ZONMW-53_00095	ftsE
ftsE	ZONMW-53_01646	ftsE
ftsE	ZONMW-53_01988	ftsE
ftsE	ZONMW-53_02090	ftsE
ftsE	ZONMW-53_03064	ftsE
ftsE	ZONMW-53_01014	ftsE
ftsE	ZONMW-53_00978	ftsE
ftsE	ZONMW-53_02839	ftsE
ftsE	ZONMW-53_02330	ftsE
ftsE	ZONMW-53_00866	ftsE
ftsE	ZONMW-53_02152	ftsE
ftsE	ZONMW-53_03283	ftsE
ftsE	ZONMW-54_00956	ftsE
ftsE	ZONMW-54_01848	ftsE
ftsE	ZONMW-54_02204	ftsE
ftsE	ZONMW-54_02615	ftsE
ftsE	ZONMW-54_00528	ftsE
ftsE	ZONMW-54_01372	ftsE
ftsE	ZONMW-54_00921	ftsE
ftsE	ZONMW-54_02048	ftsE
ftsE	ZONMW-54_01528	ftsE
ftsE	ZONMW-54_00621	ftsE
ftsE	ZONMW-54_01082	ftsE
ftsE	ZONMW-54_03145	ftsE
ftsE	ZONMW-54_01520	ftsE
ftsE	ZONMW-54_03137	ftsE
ftsE	ZONMW-54_02183	ftsE
ftsE	ZONMW-54_00717	ftsE
ftsE	ZONMW-54_02047	ftsE
ftsE	ZONMW-54_03151	ftsE
ftsE	ZONMW-54_00165	ftsE
ftsE	ZONMW-54_00230	ftsE
ftsE	ZONMW-54_00338	ftsE
ftsE	ZONMW-54_00932	ftsE

ftsE	ZONMW-54_01079	ftsE
ftsE	ZONMW-54_02066	ftsE
ftsE	ZONMW-54_02581	ftsE
ftsE	ZONMW-54_03066	ftsE
ftsE	ZONMW-54_01713	ftsE
ftsE	ZONMW-54_01749	ftsE
ftsE	ZONMW-54_00055	ftsE
ftsE	ZONMW-54_02372	ftsE
ftsE	ZONMW-54_01493	ftsE
ftsE	ZONMW-54_00198	ftsE
ftsE	ZONMW-54_00153	ftsE
ftsE	ZONMW-54_02642	ftsE
ftsE	ZONMW-55_00069	ftsE
ftsE	ZONMW-55_01381	ftsE
ftsE	ZONMW-55_02068	ftsE
ftsE	ZONMW-55_02616	ftsE
ftsE	ZONMW-55_00502	ftsE
ftsE	ZONMW-55_00891	ftsE
ftsE	ZONMW-55_00104	ftsE
ftsE	ZONMW-55_01929	ftsE
ftsE	ZONMW-55_01050	ftsE
ftsE	ZONMW-55_00408	ftsE
ftsE	ZONMW-55_02195	ftsE
ftsE	ZONMW-55_03151	ftsE
ftsE	ZONMW-55_01042	ftsE
ftsE	ZONMW-55_03159	ftsE
ftsE	ZONMW-55_02052	ftsE
ftsE	ZONMW-55_00310	ftsE
ftsE	ZONMW-55_03145	ftsE
ftsE	ZONMW-55_01928	ftsE
ftsE	ZONMW-55_00800	ftsE
ftsE	ZONMW-55_00700	ftsE
ftsE	ZONMW-55_00093	ftsE
ftsE	ZONMW-55_02192	ftsE
ftsE	ZONMW-55_01947	ftsE
ftsE	ZONMW-55_02650	ftsE
ftsE	ZONMW-55_01594	ftsE
ftsE	ZONMW-55_01233	ftsE
ftsE	ZONMW-55_01269	ftsE
ftsE	ZONMW-55_02845	ftsE
ftsE	ZONMW-55_01823	ftsE
ftsE	ZONMW-55_01012	ftsE
ftsE	ZONMW-55_00832	ftsE
ftsE	ZONMW-55_02589	ftsE
ftsE	ZONMW-55_03241	ftsE
ftsE	ZONMW-56_02738	ftsE
ftsE	ZONMW-56_00607	ftsE
ftsE	ZONMW-56_01366	ftsE

ftsE	ZONMW-56_01613	ftsE
ftsE	ZONMW-56_00811	ftsE
ftsE	ZONMW-56_00127	ftsE
ftsE	ZONMW-56_02703	ftsE
ftsE	ZONMW-56_01507	ftsE
ftsE	ZONMW-56_00278	ftsE
ftsE	ZONMW-56_00907	ftsE
ftsE	ZONMW-56_02173	ftsE
ftsE	ZONMW-56_03111	ftsE
ftsE	ZONMW-56_00270	ftsE
ftsE	ZONMW-56_03119	ftsE
ftsE	ZONMW-56_01382	ftsE
ftsE	ZONMW-56_01005	ftsE
ftsE	ZONMW-56_03105	ftsE
ftsE	ZONMW-56_01508	ftsE
ftsE	ZONMW-56_02272	ftsE
ftsE	ZONMW-56_02379	ftsE
ftsE	ZONMW-56_02714	ftsE
ftsE	ZONMW-56_02176	ftsE
ftsE	ZONMW-56_01489	ftsE
ftsE	ZONMW-56_01579	ftsE
ftsE	ZONMW-56_02950	ftsE
ftsE	ZONMW-56_00462	ftsE
ftsE	ZONMW-56_00498	ftsE
ftsE	ZONMW-56_02605	ftsE
ftsE	ZONMW-56_01971	ftsE
ftsE	ZONMW-56_02240	ftsE
ftsE	ZONMW-56_01640	ftsE
ftsE	ZONMW-56_03251	ftsE
ftsE	ZONMW-57_01544	ftsE
ftsE	ZONMW-57_00814	ftsE
ftsE	ZONMW-57_01858	ftsE
ftsE	ZONMW-57_01960	ftsE
ftsE	ZONMW-57_01112	ftsE
ftsE	ZONMW-57_00340	ftsE
ftsE	ZONMW-57_01509	ftsE
ftsE	ZONMW-57_01719	ftsE
ftsE	ZONMW-57_00491	ftsE
ftsE	ZONMW-57_01207	ftsE
ftsE	ZONMW-57_00047	ftsE
ftsE	ZONMW-57_03158	ftsE
ftsE	ZONMW-57_00483	ftsE
ftsE	ZONMW-57_03150	ftsE
ftsE	ZONMW-57_01842	ftsE
ftsE	ZONMW-57_01305	ftsE
ftsE	ZONMW-57_03164	ftsE
ftsE	ZONMW-57_01718	ftsE
ftsE	ZONMW-57_02403	ftsE

ftsE	ZONMW-57_02508	ftsE
ftsE	ZONMW-57_01520	ftsE
ftsE	ZONMW-57_00044	ftsE
ftsE	ZONMW-57_01737	ftsE
ftsE	ZONMW-57_01926	ftsE
ftsE	ZONMW-57_03097	ftsE
ftsE	ZONMW-57_00674	ftsE
ftsE	ZONMW-57_00711	ftsE
ftsE	ZONMW-57_02749	ftsE
ftsE	ZONMW-57_02163	ftsE
ftsE	ZONMW-57_02371	ftsE
ftsE	ZONMW-57_01987	ftsE
ftsE	ZONMW-57_03293	ftsE
ftsE	ZONMW-58_01040	ftsE
ftsE	ZONMW-58_00157	ftsE
ftsE	ZONMW-58_01882	ftsE
ftsE	ZONMW-58_02118	ftsE
ftsE	ZONMW-58_01472	ftsE
ftsE	ZONMW-58_00631	ftsE
ftsE	ZONMW-58_01075	ftsE
ftsE	ZONMW-58_01743	ftsE
ftsE	ZONMW-58_00480	ftsE
ftsE	ZONMW-58_01377	ftsE
ftsE	ZONMW-58_00919	ftsE
ftsE	ZONMW-58_03189	ftsE
ftsE	ZONMW-58_00488	ftsE
ftsE	ZONMW-58_03197	ftsE
ftsE	ZONMW-58_01866	ftsE
ftsE	ZONMW-58_01279	ftsE
ftsE	ZONMW-58_03183	ftsE
ftsE	ZONMW-58_01742	ftsE
ftsE	ZONMW-58_02428	ftsE
ftsE	ZONMW-58_02533	ftsE
ftsE	ZONMW-58_01064	ftsE
ftsE	ZONMW-58_00922	ftsE
ftsE	ZONMW-58_01761	ftsE
ftsE	ZONMW-58_02152	ftsE
ftsE	ZONMW-58_03118	ftsE
ftsE	ZONMW-58_00297	ftsE
ftsE	ZONMW-58_00260	ftsE
ftsE	ZONMW-58_02773	ftsE
ftsE	ZONMW-58_02187	ftsE
ftsE	ZONMW-58_02396	ftsE
ftsE	ZONMW-58_02091	ftsE
ftsE	ZONMW-58_03284	ftsE
ftsE	ZONMW-59_02788	ftsE
ftsE	ZONMW-59_00809	ftsE
ftsE	ZONMW-59_01695	ftsE

ftsE	ZONMW-59_02337	ftsE
ftsE	ZONMW-59_01114	ftsE
ftsE	ZONMW-59_00356	ftsE
ftsE	ZONMW-59_02823	ftsE
ftsE	ZONMW-59_01555	ftsE
ftsE	ZONMW-59_00481	ftsE
ftsE	ZONMW-59_01208	ftsE
ftsE	ZONMW-59_00047	ftsE
ftsE	ZONMW-59_03246	ftsE
ftsE	ZONMW-59_00473	ftsE
ftsE	ZONMW-59_03254	ftsE
ftsE	ZONMW-59_01679	ftsE
ftsE	ZONMW-59_01306	ftsE
ftsE	ZONMW-59_01554	ftsE
ftsE	ZONMW-59_03240	ftsE
ftsE	ZONMW-59_02065	ftsE
ftsE	ZONMW-59_01959	ftsE
ftsE	ZONMW-59_02812	ftsE
ftsE	ZONMW-59_00044	ftsE
ftsE	ZONMW-59_01573	ftsE
ftsE	ZONMW-59_02371	ftsE
ftsE	ZONMW-59_03173	ftsE
ftsE	ZONMW-59_00663	ftsE
ftsE	ZONMW-59_00700	ftsE
ftsE	ZONMW-59_02955	ftsE
ftsE	ZONMW-59_02406	ftsE
ftsE	ZONMW-59_02097	ftsE
ftsE	ZONMW-59_02310	ftsE
ftsE	ZONMW-59_02137	ftsE
ftsE	ZONMW-60_01014	ftsE
ftsE	ZONMW-60_00813	ftsE
ftsE	ZONMW-60_02078	ftsE
ftsE	ZONMW-60_02635	ftsE
ftsE	ZONMW-60_01453	ftsE
ftsE	ZONMW-60_00367	ftsE
ftsE	ZONMW-60_01049	ftsE
ftsE	ZONMW-60_01936	ftsE
ftsE	ZONMW-60_01360	ftsE
ftsE	ZONMW-60_00493	ftsE
ftsE	ZONMW-60_00048	ftsE
ftsE	ZONMW-60_03169	ftsE
ftsE	ZONMW-60_00485	ftsE
ftsE	ZONMW-60_03161	ftsE
ftsE	ZONMW-60_02057	ftsE
ftsE	ZONMW-60_01264	ftsE
ftsE	ZONMW-60_03175	ftsE
ftsE	ZONMW-60_01935	ftsE
ftsE	ZONMW-60_01812	ftsE

ftsE	ZONMW-60_01744	ftsE
ftsE	ZONMW-60_01642	ftsE
ftsE	ZONMW-60_01038	ftsE
ftsE	ZONMW-60_00045	ftsE
ftsE	ZONMW-60_01954	ftsE
ftsE	ZONMW-60_02669	ftsE
ftsE	ZONMW-60_03109	ftsE
ftsE	ZONMW-60_00677	ftsE
ftsE	ZONMW-60_00713	ftsE
ftsE	ZONMW-60_02868	ftsE
ftsE	ZONMW-60_02252	ftsE
ftsE	ZONMW-60_00458	ftsE
ftsE	ZONMW-60_01776	ftsE
ftsE	ZONMW-60_01824	ftsE
ftsE	ZONMW-60_02609	ftsE
ftsE	ZONMW-61_01135	ftsE
ftsE	ZONMW-61_02145	ftsE
ftsE	ZONMW-61_01651	ftsE
ftsE	ZONMW-61_00975	ftsE
ftsE	ZONMW-61_03321	ftsE
ftsE	ZONMW-61_02949	ftsE
ftsE	ZONMW-61_02481	ftsE
ftsE	ZONMW-61_01711	ftsE
ftsE	ZONMW-61_00188	ftsE
ftsE	ZONMW-61_00899	ftsE
ftsE	ZONMW-61_03106	ftsE
ftsE	ZONMW-61_01178	ftsE
ftsE	ZONMW-61_00868	ftsE
ftsE	ZONMW-61_01657	ftsE
ftsE	ZONMW-61_03497	ftsE
ftsE	ZONMW-61_00141	ftsE
ftsE	ZONMW-61_01664	ftsE
ftsE	ZONMW-61_00392	ftsE
ftsE	ZONMW-61_02121	ftsE
ftsE	ZONMW-61_00996	ftsE
ftsE	ZONMW-61_00187	ftsE
ftsE	ZONMW-61_01160	ftsE
ftsE	ZONMW-61_00881	ftsE
ftsE	ZONMW-61_00943	ftsE
ftsE	ZONMW-61_02245	ftsE
ftsE	ZONMW-61_02427	ftsE
ftsE	ZONMW-61_01514	ftsE
ftsE	ZONMW-61_01993	ftsE
ftsE	ZONMW-61_00322	ftsE
ftsE	ZONMW-62_02887	ftsE
ftsE	ZONMW-62_00100	ftsE
ftsE	ZONMW-62_01393	ftsE
ftsE	ZONMW-62_02791	ftsE

ftsE	ZONMW-62_00455	ftsE
ftsE	ZONMW-62_02286	ftsE
ftsE	ZONMW-62_01457	ftsE
ftsE	ZONMW-62_00645	ftsE
ftsE	ZONMW-62_03925	ftsE
ftsE	ZONMW-62_02932	ftsE
ftsE	ZONMW-62_01739	ftsE
ftsE	ZONMW-62_02712	ftsE
ftsE	ZONMW-62_01400	ftsE
ftsE	ZONMW-62_03552	ftsE
ftsE	ZONMW-62_02683	ftsE
ftsE	ZONMW-62_00124	ftsE
ftsE	ZONMW-62_01410	ftsE
ftsE	ZONMW-62_01695	ftsE
ftsE	ZONMW-62_01940	ftsE
ftsE	ZONMW-62_01738	ftsE
ftsE	ZONMW-62_02911	ftsE
ftsE	ZONMW-62_02696	ftsE
ftsE	ZONMW-62_02755	ftsE
ftsE	ZONMW-62_00387	ftsE
ftsE	ZONMW-62_00921	ftsE
ftsE	ZONMW-62_00729	ftsE
ftsE	ZONMW-62_01266	ftsE
ftsE	ZONMW-62_00249	ftsE
ftsE	ZONMW-62_02810	ftsE
ftsE	ZONMW-62_01874	ftsE
ftsE	ZONMW-62_01390	ftsE
ftsE	ZONMW-63_01013	ftsE
ftsE	ZONMW-63_00489	ftsE
ftsE	ZONMW-63_02733	ftsE
ftsE	ZONMW-63_01723	ftsE
ftsE	ZONMW-63_00868	ftsE
ftsE	ZONMW-63_00008	ftsE
ftsE	ZONMW-63_01049	ftsE
ftsE	ZONMW-63_01455	ftsE
ftsE	ZONMW-63_00164	ftsE
ftsE	ZONMW-63_03337	ftsE
ftsE	ZONMW-63_01811	ftsE
ftsE	ZONMW-63_03163	ftsE
ftsE	ZONMW-63_00156	ftsE
ftsE	ZONMW-63_03171	ftsE
ftsE	ZONMW-63_02749	ftsE
ftsE	ZONMW-63_01260	ftsE
ftsE	ZONMW-63_01454	ftsE
ftsE	ZONMW-63_03157	ftsE
ftsE	ZONMW-63_00566	ftsE
ftsE	ZONMW-63_00674	ftsE
ftsE	ZONMW-63_01037	ftsE

ftsE	ZONMW-63_01808	ftsE
ftsE	ZONMW-63_01473	ftsE
ftsE	ZONMW-63_01758	ftsE
ftsE	ZONMW-63_02917	ftsE
ftsE	ZONMW-63_00348	ftsE
ftsE	ZONMW-63_00384	ftsE
ftsE	ZONMW-63_02324	ftsE
ftsE	ZONMW-63_03102	ftsE
ftsE	ZONMW-63_00534	ftsE
ftsE	ZONMW-63_01696	ftsE
ftsE	ZONMW-63_03375	ftsE
ftsE	ZONMW-64_03038	ftsE
ftsE	ZONMW-64_01628	ftsE
ftsE	ZONMW-64_02000	ftsE
ftsE	ZONMW-64_01629	ftsE
ftsE	ZONMW-64_01291	ftsE
ftsE	ZONMW-64_03399	ftsE
ftsE	ZONMW-64_00003	ftsE
ftsE	ZONMW-64_00611	ftsE
ftsE	ZONMW-64_03858	ftsE
ftsE	ZONMW-64_01969	ftsE
ftsE	ZONMW-64_00692	ftsE
ftsE	ZONMW-64_02676	ftsE
ftsE	ZONMW-64_00082	ftsE
ftsE	ZONMW-64_03336	ftsE
ftsE	ZONMW-64_01743	ftsE
ftsE	ZONMW-64_02376	ftsE
ftsE	ZONMW-64_00925	ftsE
ftsE	ZONMW-64_00759	ftsE
ftsE	ZONMW-64_02534	ftsE
ftsE	ZONMW-64_01152	ftsE
ftsE	ZONMW-64_02544	ftsE
ftsE	ZONMW-64_00888	ftsE
ftsE	ZONMW-64_03584	ftsE
ftsE	ZONMW-64_01346	ftsE
ftsE	ZONMW-64_02089	ftsE
ftsE	ZONMW-64_02683	ftsE
ftsE	ZONMW-64_02241	ftsE
ftsE	ZONMW-64_02192	ftsE
ftsE	ZONMW-64_00735	ftsE
ftsE	ZONMW-64_02734	ftsE
ftsE	ZONMW-64_03474	ftsE
ftsE	ZONMW-64_03475	ftsE
ftsE	ZONMW-64_01026	ftsE
ftsE	ZONMW-64_00648	ftsE
ftsE	ZONMW-64_02782	ftsE
ftsE	ZONMW-64_03829	ftsE
ftsE	ZONMW-64_01248	ftsE

ftsE	ZONMW-64_02140	ftsE
ftsE	ZONMW-64_03590	ftsE
ftsE	ZONMW-64_02401	ftsE
ftsE	ZONMW-64_03828	ftsE
ftsE	ZONMW-64_00664	ftsE
ftsE	ZONMW-64_01783	ftsE
ftsE	ZONMW-64_03413	ftsE
ftsE	ZONMW-64_00924	ftsE
ftsE	ZONMW-64_03043	ftsE
ftsE	ZONMW-64_04072	ftsE
ftsE	ZONMW-64_02365	ftsE
ftsE	ZONMW-64_01162	ftsE
ftsE	ZONMW-64_00358	ftsE
ftsE	ZONMW-64_02644	ftsE
ftsE	ZONMW-64_02559	ftsE
ftsE	ZONMW-64_03591	ftsE
ftsE	ZONMW-64_02433	ftsE
ftsE	ZONMW-64_03129	ftsE
ftsE	ZONMW-64_03398	ftsE
ftsE	ZONMW-64_01439	ftsE
ftsE	ZONMW-64_00019	ftsE
ftsE	ZONMW-64_01739	ftsE
ftsE	ZONMW-64_04071	ftsE
ftsE	ZONMW-64_01566	ftsE
ftsE	ZONMW-64_00353	ftsE
ftsE	ZONMW-64_02563	ftsE
ftsE	ZONMW-64_02484	ftsE
ftsE	ZONMW-64_02242	ftsE
ftsE	ZONMW-64_01396	ftsE
ftsE	ZONMW-64_02733	ftsE
ftsE	ZONMW-64_02001	ftsE
ftsE	ZONMW-64_03497	ftsE
ftsE	ZONMW-64_01058	ftsE
ftsE	ZONMW-64_00189	ftsE
ftsE	ZONMW-64_02319	ftsE
ftsE	ZONMW-64_04128	ftsE
ftsE	ZONMW-64_01440	ftsE
ftsE	ZONMW-64_02643	ftsE
ftsE	ZONMW-64_00018	ftsE
ftsE	ZONMW-64_02267	ftsE
ftsE	ZONMW-64_01030	ftsE
ftsE	ZONMW-64_03418	ftsE
ftsE	ZONMW-64_00647	ftsE
ftsE	ZONMW-64_03229	ftsE
ftsE	ZONMW-64_02548	ftsE
ftsE	ZONMW-64_00511	ftsE
ftsE	ZONMW-64_00028	ftsE
ftsE	ZONMW-65_00806	ftsE

ftsE	ZONMW-65_01169	ftsE
ftsE	ZONMW-65_03555	ftsE
ftsE	ZONMW-65_01830	ftsE
ftsE	ZONMW-65_00935	ftsE
ftsE	ZONMW-65_02907	ftsE
ftsE	ZONMW-65_03466	ftsE
ftsE	ZONMW-65_03767	ftsE
ftsE	ZONMW-65_03527	ftsE
ftsE	ZONMW-65_00194	ftsE
ftsE	ZONMW-65_01709	ftsE
ftsE	ZONMW-65_02878	ftsE
ftsE	ZONMW-65_01032	ftsE
ftsE	ZONMW-65_03648	ftsE
ftsE	ZONMW-65_02840	ftsE
ftsE	ZONMW-65_00880	ftsE
ftsE	ZONMW-65_00275	ftsE
ftsE	ZONMW-65_02860	ftsE
ftsE	ZONMW-65_02273	ftsE
ftsE	ZONMW-65_02121	ftsE
ftsE	ZONMW-65_01655	ftsE
ftsE	ZONMW-65_01684	ftsE
ftsE	ZONMW-65_02029	ftsE
ftsE	ZONMW-65_02552	ftsE
ftsE	ZONMW-65_00686	ftsE
ftsE	ZONMW-65_03492	ftsE
ftsE	ZONMW-65_01776	ftsE
ftsE	ZONMW-65_02234	ftsE
ftsE	ZONMW-65_00269	ftsE
ftsE	ZONMW-65_01033	ftsE
ftsE	ZONMW-65_01395	ftsE
ftsE	ZONMW-65_00858	ftsE
ftsE	ZONMW-65_02995	ftsE
ftsE	ZONMW-65_02577	ftsE
ftsE	ZONMW-65_03559	ftsE
ftsE	ZONMW-65_00876	ftsE
ftsE	ZONMW-66_01019	ftsE
ftsE	ZONMW-66_04291	ftsE
ftsE	ZONMW-66_04293	ftsE
ftsE	ZONMW-66_03637	ftsE
ftsE	ZONMW-66_02906	ftsE
ftsE	ZONMW-66_02905	ftsE
ftsE	ZONMW-66_03336	ftsE
ftsE	ZONMW-66_00078	ftsE
ftsE	ZONMW-66_02574	ftsE
ftsE	ZONMW-66_01562	ftsE
ftsE	ZONMW-66_00353	ftsE
ftsE	ZONMW-66_03320	ftsE
ftsE	ZONMW-66_00910	ftsE

ftsE	ZONMW-66_01295	ftsE
ftsE	ZONMW-66_02408	ftsE
ftsE	ZONMW-66_01959	ftsE
ftsE	ZONMW-66_00371	ftsE
ftsE	ZONMW-66_00413	ftsE
ftsE	ZONMW-66_03125	ftsE
ftsE	ZONMW-66_03720	ftsE
ftsE	ZONMW-66_03289	ftsE
ftsE	ZONMW-66_01526	ftsE
ftsE	ZONMW-66_00702	ftsE
ftsE	ZONMW-66_01922	ftsE
ftsE	ZONMW-66_03288	ftsE
ftsE	ZONMW-66_00890	ftsE
ftsE	ZONMW-66_02587	ftsE
ftsE	ZONMW-66_00307	ftsE
ftsE	ZONMW-66_03115	ftsE
ftsE	ZONMW-66_02456	ftsE
ftsE	ZONMW-66_02485	ftsE
ftsE	ZONMW-66_04428	ftsE
ftsE	ZONMW-66_01351	ftsE
ftsE	ZONMW-66_01358	ftsE
ftsE	ZONMW-66_00706	ftsE
ftsE	ZONMW-66_01276	ftsE
ftsE	ZONMW-66_02277	ftsE
ftsE	ZONMW-66_00263	ftsE
ftsE	ZONMW-66_00524	ftsE
ftsE	ZONMW-66_00987	ftsE
ftsE	ZONMW-66_02222	ftsE
ftsE	ZONMW-66_03787	ftsE
ftsE	ZONMW-66_02709	ftsE
ftsE	ZONMW-66_02767	ftsE
ftsE	ZONMW-66_02961	ftsE
ftsE	ZONMW-66_03033	ftsE
ftsE	ZONMW-67_01547	ftsE
ftsE	ZONMW-67_02315	ftsE
ftsE	ZONMW-67_00138	ftsE
ftsE	ZONMW-67_00137	ftsE
ftsE	ZONMW-67_00426	ftsE
ftsE	ZONMW-67_02566	ftsE
ftsE	ZONMW-67_01305	ftsE
ftsE	ZONMW-67_02911	ftsE
ftsE	ZONMW-67_00212	ftsE
ftsE	ZONMW-67_01674	ftsE
ftsE	ZONMW-67_02849	ftsE
ftsE	ZONMW-67_00556	ftsE
ftsE	ZONMW-67_01894	ftsE
ftsE	ZONMW-67_00016	ftsE
ftsE	ZONMW-67_01366	ftsE

ftsE	ZONMW-67_03014	ftsE
ftsE	ZONMW-67_02408	ftsE
ftsE	ZONMW-67_02728	ftsE
ftsE	ZONMW-67_02699	ftsE
ftsE	ZONMW-67_01611	ftsE
ftsE	ZONMW-67_00359	ftsE
ftsE	ZONMW-67_00180	ftsE
ftsE	ZONMW-67_02773	ftsE
ftsE	ZONMW-67_03054	ftsE
ftsE	ZONMW-67_01552	ftsE
ftsE	ZONMW-67_03419	ftsE
ftsE	ZONMW-67_01306	ftsE
ftsE	ZONMW-67_03420	ftsE
ftsE	ZONMW-67_00466	ftsE
ftsE	ZONMW-67_02970	ftsE
ftsE	ZONMW-67_01905	ftsE
ftsE	ZONMW-67_02296	ftsE
ftsE	ZONMW-67_01872	ftsE
ftsE	ZONMW-67_01024	ftsE
ftsE	ZONMW-67_01295	ftsE
ftsE	ZONMW-67_02221	ftsE
ftsE	ZONMW-67_02887	ftsE
ftsE	ZONMW-67_00548	ftsE
ftsE	ZONMW-67_00179	ftsE
ftsE	ZONMW-67_02700	ftsE
ftsE	ZONMW-67_03080	ftsE
ftsE	ZONMW-67_02210	ftsE
ftsE	ZONMW-67_02822	ftsE
ftsE	ZONMW-67_03431	ftsE
ftsE	ZONMW-67_02605	ftsE
ftsE	ZONMW-67_00327	ftsE
ftsE	ZONMW-67_02992	ftsE
ftsE	ZONMW-67_01019	ftsE
ftsE	ZONMW-67_03059	ftsE
ftsE	ZONMW-67_00641	ftsE
ftsE	ZONMW-67_03585	ftsE
ftsE	ZONMW-67_00854	ftsE
ftsE	ZONMW-67_03081	ftsE
ftsE	ZONMW-67_03399	ftsE
ftsE	ZONMW-67_01955	ftsE
ftsE	ZONMW-67_02626	ftsE
ftsE	ZONMW-67_02316	ftsE
ftsE	ZONMW-67_00640	ftsE
ftsE	ZONMW-67_01290	ftsE
ftsE	ZONMW-67_02211	ftsE
ftsE	ZONMW-67_02014	ftsE
ftsE	ZONMW-67_03015	ftsE
ftsE	ZONMW-67_01641	ftsE

ftsE	ZONMW-67_01640	ftsE
ftsE	ZONMW-67_01178	ftsE
ftsE	ZONMW-67_02245	ftsE
ftsE	ZONMW-68_03376	ftsE
ftsE	ZONMW-68_04142	ftsE
ftsE	ZONMW-68_00799	ftsE
ftsE	ZONMW-68_04061	ftsE
ftsE	ZONMW-68_04599	ftsE
ftsE	ZONMW-68_04529	ftsE
ftsE	ZONMW-68_03240	ftsE
ftsE	ZONMW-68_03332	ftsE
ftsE	ZONMW-68_00065	ftsE
ftsE	ZONMW-68_02945	ftsE
ftsE	ZONMW-68_04982	ftsE
ftsE	ZONMW-68_04622	ftsE
ftsE	ZONMW-68_02664	ftsE
ftsE	ZONMW-68_00522	ftsE
ftsE	ZONMW-68_04624	ftsE
ftsE	ZONMW-68_00380	ftsE
ftsE	ZONMW-68_01552	ftsE
ftsE	ZONMW-68_04281	ftsE
ftsE	ZONMW-68_00972	ftsE
ftsE	ZONMW-68_03470	ftsE
ftsE	ZONMW-68_00157	ftsE
ftsE	ZONMW-68_01489	ftsE
ftsE	ZONMW-68_04837	ftsE
ftsE	ZONMW-68_03355	ftsE
ftsE	ZONMW-68_02667	ftsE
ftsE	ZONMW-68_04028	ftsE
ftsE	ZONMW-68_00937	ftsE
ftsE	ZONMW-68_01103	ftsE
ftsE	ZONMW-68_04193	ftsE
ftsE	ZONMW-68_03451	ftsE
ftsE	ZONMW-68_00081	ftsE
ftsE	ZONMW-68_03809	ftsE
ftsE	ZONMW-68_02466	ftsE
ftsE	ZONMW-68_02457	ftsE
ftsE	ZONMW-68_04939	ftsE
ftsE	ZONMW-69_01454	ftsE
ftsE	ZONMW-69_02363	ftsE
ftsE	ZONMW-69_02747	ftsE
ftsE	ZONMW-69_02746	ftsE
ftsE	ZONMW-69_00173	ftsE
ftsE	ZONMW-69_00363	ftsE
ftsE	ZONMW-69_01696	ftsE
ftsE	ZONMW-69_03043	ftsE
ftsE	ZONMW-69_02821	ftsE
ftsE	ZONMW-69_01327	ftsE

ftsE	ZONMW-69_00646	ftsE
ftsE	ZONMW-69_00303	ftsE
ftsE	ZONMW-69_02049	ftsE
ftsE	ZONMW-69_02625	ftsE
ftsE	ZONMW-69_01634	ftsE
ftsE	ZONMW-69_02940	ftsE
ftsE	ZONMW-69_02456	ftsE
ftsE	ZONMW-69_00525	ftsE
ftsE	ZONMW-69_00496	ftsE
ftsE	ZONMW-69_01390	ftsE
ftsE	ZONMW-69_00106	ftsE
ftsE	ZONMW-69_02789	ftsE
ftsE	ZONMW-69_00570	ftsE
ftsE	ZONMW-69_02899	ftsE
ftsE	ZONMW-69_01449	ftsE
ftsE	ZONMW-69_03420	ftsE
ftsE	ZONMW-69_01695	ftsE
ftsE	ZONMW-69_03421	ftsE
ftsE	ZONMW-69_00213	ftsE
ftsE	ZONMW-69_02984	ftsE
ftsE	ZONMW-69_02038	ftsE
ftsE	ZONMW-69_02344	ftsE
ftsE	ZONMW-69_02071	ftsE
ftsE	ZONMW-69_01071	ftsE
ftsE	ZONMW-69_01706	ftsE
ftsE	ZONMW-69_02269	ftsE
ftsE	ZONMW-69_03067	ftsE
ftsE	ZONMW-69_00295	ftsE
ftsE	ZONMW-69_02788	ftsE
ftsE	ZONMW-69_00497	ftsE
ftsE	ZONMW-69_02873	ftsE
ftsE	ZONMW-69_02258	ftsE
ftsE	ZONMW-69_00619	ftsE
ftsE	ZONMW-69_03432	ftsE
ftsE	ZONMW-69_00402	ftsE
ftsE	ZONMW-69_00074	ftsE
ftsE	ZONMW-69_02962	ftsE
ftsE	ZONMW-69_01066	ftsE
ftsE	ZONMW-69_02894	ftsE
ftsE	ZONMW-69_00688	ftsE
ftsE	ZONMW-69_03587	ftsE
ftsE	ZONMW-69_00901	ftsE
ftsE	ZONMW-69_02872	ftsE
ftsE	ZONMW-69_03400	ftsE
ftsE	ZONMW-69_01988	ftsE
ftsE	ZONMW-69_00423	ftsE
ftsE	ZONMW-69_02364	ftsE
ftsE	ZONMW-69_00687	ftsE

ftsE	ZONMW-69_01711	ftsE
ftsE	ZONMW-69_02259	ftsE
ftsE	ZONMW-69_01929	ftsE
ftsE	ZONMW-69_02939	ftsE
ftsE	ZONMW-69_01360	ftsE
ftsE	ZONMW-69_01361	ftsE
ftsE	ZONMW-69_01224	ftsE
ftsE	ZONMW-69_02293	ftsE
ftsE	ZONMW-72_01869	ftsE
ftsE	ZONMW-72_01384	ftsE
ftsE	ZONMW-72_03091	ftsE
ftsE	ZONMW-72_03165	ftsE
ftsE	ZONMW-72_00338	ftsE
ftsE	ZONMW-72_02553	ftsE
ftsE	ZONMW-72_00220	ftsE
ftsE	ZONMW-72_04074	ftsE
ftsE	ZONMW-72_03874	ftsE
ftsE	ZONMW-72_02425	ftsE
ftsE	ZONMW-72_00886	ftsE
ftsE	ZONMW-72_00916	ftsE
ftsE	ZONMW-72_00475	ftsE
ftsE	ZONMW-72_04047	ftsE
ftsE	ZONMW-72_02127	ftsE
ftsE	ZONMW-72_01270	ftsE
ftsE	ZONMW-72_01742	ftsE
ftsE	ZONMW-72_03379	ftsE
ftsE	ZONMW-72_00693	ftsE
ftsE	ZONMW-72_01241	ftsE
ftsE	ZONMW-72_02039	ftsE
ftsE	ZONMW-72_00181	ftsE
ftsE	ZONMW-72_01098	ftsE
ftsE	ZONMW-72_03764	ftsE
ftsE	ZONMW-72_03833	ftsE
ftsE	ZONMW-72_03571	ftsE
ftsE	ZONMW-72_00321	ftsE
ftsE	ZONMW-72_01210	ftsE
ftsE	ZONMW-72_01924	ftsE
ftsE	ZONMW-72_02163	ftsE
ftsE	ZONMW-72_00908	ftsE
ftsE	ZONMW-72_02841	ftsE
ftsE	ZONMW-72_02122	ftsE
ftsE	ZONMW-72_02970	ftsE
ftsE	ZONMW-72_00812	ftsE
ftsE	ZONMW-72_03502	ftsE
ftsE	ZONMW-72_01371	ftsE
ftsE	ZONMW-72_00675	ftsE
ftsE	ZONMW-72_00767	ftsE
ftsE	ZONMW-72_01351	ftsE

ftsE	ZONMW-72_03742	ftsE
ftsE	ZONMW-72_03318	ftsE
ftsE	ZONMW-73_03596	ftsE
ftsE	ZONMW-73_01630	ftsE
ftsE	ZONMW-73_02928	ftsE
ftsE	ZONMW-73_00593	ftsE
ftsE	ZONMW-73_03753	ftsE
ftsE	ZONMW-73_03658	ftsE
ftsE	ZONMW-73_03305	ftsE
ftsE	ZONMW-73_03654	ftsE
ftsE	ZONMW-73_03697	ftsE
ftsE	ZONMW-73_02212	ftsE
ftsE	ZONMW-73_03500	ftsE
ftsE	ZONMW-73_03814	ftsE
ftsE	ZONMW-73_01621	ftsE
ftsE	ZONMW-73_01646	ftsE
ftsE	ZONMW-73_00911	ftsE
ftsE	ZONMW-73_02223	ftsE
ftsE	ZONMW-73_03729	ftsE
ftsE	ZONMW-73_03787	ftsE
ftsE	ZONMW-73_02347	ftsE
ftsE	ZONMW-73_00928	ftsE
ftsE	ZONMW-73_00927	ftsE
ftsE	ZONMW-73_03815	ftsE
ftsE	ZONMW-73_02326	ftsE
ftsE	ZONMW-73_01847	ftsE
ftsE	ZONMW-73_01426	ftsE
ftsE	ZONMW-73_01004	ftsE
ftsE	ZONMW-73_03783	ftsE
ftsE	ZONMW-73_02914	ftsE
ftsE	ZONMW-73_01565	ftsE
ftsE	ZONMW-73_00624	ftsE
ftsE	ZONMW-73_03178	ftsE
ftsE	ZONMW-73_02366	ftsE
ftsE	ZONMW-73_03400	ftsE
ftsE	ZONMW-73_00050	ftsE
ftsE	ZONMW-73_03851	ftsE
ftsE	ZONMW-74_00834	ftsE
ftsE	ZONMW-74_02927	ftsE
ftsE	ZONMW-74_02863	ftsE
ftsE	ZONMW-74_02711	ftsE
ftsE	ZONMW-74_00496	ftsE
ftsE	ZONMW-74_01785	ftsE
ftsE	ZONMW-74_00135	ftsE
ftsE	ZONMW-74_00866	ftsE
ftsE	ZONMW-74_00272	ftsE
ftsE	ZONMW-74_01189	ftsE
ftsE	ZONMW-74_02686	ftsE

ftsE	ZONMW-74_00370	ftsE
ftsE	ZONMW-74_02217	ftsE
ftsE	ZONMW-74_00366	ftsE
ftsE	ZONMW-74_00619	ftsE
ftsE	ZONMW-74_02230	ftsE
ftsE	ZONMW-74_01068	ftsE
ftsE	ZONMW-74_00681	ftsE
ftsE	ZONMW-74_01367	ftsE
ftsE	ZONMW-74_00855	ftsE
ftsE	ZONMW-74_00622	ftsE
ftsE	ZONMW-74_01954	ftsE
ftsE	ZONMW-74_02773	ftsE
ftsE	ZONMW-74_01392	ftsE
ftsE	ZONMW-74_02468	ftsE
ftsE	ZONMW-74_03394	ftsE
ftsE	ZONMW-74_03358	ftsE
ftsE	ZONMW-74_01498	ftsE
ftsE	ZONMW-74_02299	ftsE
ftsE	ZONMW-74_01422	ftsE
ftsE	ZONMW-74_02236	ftsE
ftsE	ZONMW-74_02333	ftsE
ftsE	ZONMW-75_00681	ftsE
ftsE	ZONMW-75_00585	ftsE
ftsE	ZONMW-75_02822	ftsE
ftsE	ZONMW-75_00548	ftsE
ftsE	ZONMW-75_02157	ftsE
ftsE	ZONMW-75_00186	ftsE
ftsE	ZONMW-75_02555	ftsE
ftsE	ZONMW-75_01554	ftsE
ftsE	ZONMW-75_02124	ftsE
ftsE	ZONMW-75_01021	ftsE
ftsE	ZONMW-75_03575	ftsE
ftsE	ZONMW-75_00730	ftsE
ftsE	ZONMW-75_02148	ftsE
ftsE	ZONMW-75_00262	ftsE
ftsE	ZONMW-75_03647	ftsE
ftsE	ZONMW-75_00574	ftsE
ftsE	ZONMW-75_00648	ftsE
ftsE	ZONMW-75_01595	ftsE
ftsE	ZONMW-75_03574	ftsE
ftsE	ZONMW-75_00626	ftsE
ftsE	ZONMW-75_00440	ftsE
ftsE	ZONMW-75_01679	ftsE
ftsE	ZONMW-75_01599	ftsE
ftsE	ZONMW-75_03525	ftsE
ftsE	ZONMW-75_00893	ftsE
ftsE	ZONMW-75_00973	ftsE
ftsE	ZONMW-75_02132	ftsE

ftsE	ZONMW-75_03142	ftsE
ftsE	ZONMW-75_01190	ftsE
ftsE	ZONMW-75_03093	ftsE
ftsE	ZONMW-75_02582	ftsE
ftsE	ZONMW-75_02298	ftsE
ftsE	ZONMW-75_00857	ftsE
ftsE	da5A-8_00305	ftsE
ftsE	da5A-8_02731	ftsE
ftsE	da5A-8_00165	ftsE
ftsE	da5A-8_01127	ftsE
ftsE	da5A-8_01764	ftsE
ftsE	da5A-8_03046	ftsE
ftsE	da5A-8_00324	ftsE
ftsE	da5A-8_03788	ftsE
ftsE	da5A-8_02816	ftsE
ftsE	da5A-8_02778	ftsE
ftsE	da5A-8_00013	ftsE
ftsE	da5A-8_03432	ftsE
ftsE	da5A-8_01413	ftsE
ftsE	da5A-8_03176	ftsE
ftsE	da5A-8_02267	ftsE
ftsE	da5A-8_01081	ftsE
ftsE	da5A-8_02719	ftsE
ftsE	da5A-8_03694	ftsE
ftsE	da5A-8_03700	ftsE
ftsE	da5A-8_02237	ftsE
ftsE	da5A-8_01885	ftsE
ftsE	da5A-8_03383	ftsE
ftsE	da5A-8_02367	ftsE
ftsE	da5A-8_00708	ftsE
ftsE	da5A-8_01488	ftsE
ftsE	da5A-8_02865	ftsE
ftsE	da5A-8_01950	ftsE
ftsE	da5A-8_00021	ftsE
ftsE	da5A-8_01543	ftsE
ftsE	da5A-8_01136	ftsE
ftsE	da5A-8_02498	ftsE
ftsE	da5A-8_00064	ftsE
ftsE	da5A-8_03772	ftsE
ftsE	da5A-8_02952	ftsE
ftsE	da5A-8_01058	ftsE
ftsE	da5A-8_02322	ftsE
ftsE	da5A-8_03387	ftsE
ftsE	da5A-8_02893	ftsE
yjjV	18_01517	yjjV
yjjV	ACAM48_02079	yjjV
yjjV	CAM030_01306	yjjV
yjjV	CECT7946_01776	yjjV

yjjV	CGMCC1.8863_01050	yjjV
yjjV	CL8139_00130	yjjV
yjjV	DAU203_01941	yjjV
yjjV	DD5b_01555	yjjV
yjjV	DD5b_00090	yjjV
yjjV	DSM1208_02242	yjjV
yjjV	DSM12212_01682	yjjV
yjjV	DSM15343_00833	yjjV
yjjV	DSM15343_02339	yjjV
yjjV	DSM15361_01424	yjjV
yjjV	DSM15365_03386	yjjV
yjjV	DSM15718_02382	yjjV
yjjV	DSM15718_00078	yjjV
yjjV	DSM16505T_00273	yjjV
yjjV	DSM16505T_00971	yjjV
yjjV	DSM18103_01744	yjjV
yjjV	DSM18103_02604	yjjV
yjjV	DSM18293_00364	yjjV
yjjV	DSM18293_01898	yjjV
yjjV	DSM18293_01241	yjjV
yjjV	DSM18841_00076	yjjV
yjjV	DSM18842_01134	yjjV
yjjV	DSM19866_00661	yjjV
yjjV	DSM2041_04591	yjjV
yjjV	DSM2061_03772	yjjV
yjjV	DSM2064_02282	yjjV
yjjV	DSM2064_03946	yjjV
yjjV	DSM2064_00189	yjjV
yjjV	DSM21164_00361	yjjV
yjjV	DSM23404_01881	yjjV
yjjV	DSM23424_00618	yjjV
yjjV	DSM24786_02107	yjjV
yjjV	DSM7489_01852	yjjV
yjjV	DSW1_02903	yjjV
yjjV	DSW5_01448	yjjV
yjjV	DSW6_01879	yjjV
yjjV	DSW8_01704	yjjV
yjjV	F52_02674	yjjV
yjjV	F52_04111	yjjV
yjjV	F52_04341	yjjV
yjjV	GCA_001027945.1_00522	yjjV
yjjV	HI1_01780	yjjV
yjjV	IR1_00595	yjjV
yjjV	IR1_04290	yjjV
yjjV	IR1_01869	yjjV
yjjV	KCOM3131_00081	yjjV
yjjV	KCOM3131_01185	yjjV
yjjV	KCTC12974_02085	yjjV

yjjV	KL-A_00419	yjjV
yjjV	L12_00445	yjjV
yjjV	LL0412.1.7_00709	yjjV
yjjV	LL0412.1.7_00994	yjjV
yjjV	MAR_2009_71_01713	yjjV
yjjV	NN016038_01524	yjjV
yjjV	PAO1_03686	yjjV
yjjV	PAO1_03063	yjjV
yjjV	PAO1_02968	yjjV
yjjV	UW101_01336	yjjV
yjjV	UW101_04941	yjjV
yjjV	UW101_02900	yjjV
yjjV	V4-33_02210	yjjV
yjjV	V4-33_01654	yjjV
yjjV	W5C_00538	yjjV
yjjV	ZONMW-01_00306	yjjV
yjjV	ZONMW-02_00312	yjjV
yjjV	ZONMW-03_01032	yjjV
yjjV	ZONMW-04_00640	yjjV
yjjV	ZONMW-05_01185	yjjV
yjjV	ZONMW-06_03825	yjjV
yjjV	ZONMW-06_03056	yjjV
yjjV	ZONMW-07_00318	yjjV
yjjV	ZONMW-08_01521	yjjV
yjjV	ZONMW-09_00116	yjjV
yjjV	ZONMW-10_00560	yjjV
yjjV	ZONMW-10_00749	yjjV
yjjV	ZONMW-11_00858	yjjV
yjjV	ZONMW-12_00410	yjjV
yjjV	ZONMW-13_00905	yjjV
yjjV	ZONMW-14_01199	yjjV
yjjV	ZONMW-15_00373	yjjV
yjjV	ZONMW-19_02487	yjjV
yjjV	ZONMW-19_03564	yjjV
yjjV	ZONMW-19_03614	yjjV
yjjV	ZONMW-20_04167	yjjV
yjjV	ZONMW-20_00890	yjjV
yjjV	ZONMW-20_01078	yjjV
yjjV	ZONMW-21_00250	yjjV
yjjV	ZONMW-22_00250	yjjV
yjjV	ZONMW-23_01365	yjjV
yjjV	ZONMW-24_01209	yjjV
yjjV	ZONMW-25_03049	yjjV
yjjV	ZONMW-26_01239	yjjV
yjjV	ZONMW-27_00634	yjjV
yjjV	ZONMW-28_01152	yjjV
yjjV	ZONMW-28_03370	yjjV
yjjV	ZONMW-29_02956	yjjV

yjjV	ZONMW-30_01455	yjjV
yjjV	ZONMW-31_00316	yjjV
yjjV	ZONMW-31_03639	yjjV
yjjV	ZONMW-32_01560	yjjV
yjjV	ZONMW-32_03495	yjjV
yjjV	ZONMW-33_01550	yjjV
yjjV	ZONMW-33_03498	yjjV
yjjV	ZONMW-34_02909	yjjV
yjjV	ZONMW-34_03707	yjjV
yjjV	ZONMW-35_02876	yjjV
yjjV	ZONMW-35_03670	yjjV
yjjV	ZONMW-36_01806	yjjV
yjjV	ZONMW-36_03355	yjjV
yjjV	ZONMW-37_00250	yjjV
yjjV	ZONMW-38_02124	yjjV
yjjV	ZONMW-38_02594	yjjV
yjjV	ZONMW-39_02587	yjjV
yjjV	ZONMW-39_03571	yjjV
yjjV	ZONMW-40_02661	yjjV
yjjV	ZONMW-41_01793	yjjV
yjjV	ZONMW-41_02196	yjjV
yjjV	ZONMW-42_00215	yjjV
yjjV	ZONMW-42_04224	yjjV
yjjV	ZONMW-43_04006	yjjV
yjjV	ZONMW-43_02761	yjjV
yjjV	ZONMW-44_02985	yjjV
yjjV	ZONMW-44_01042	yjjV
yjjV	ZONMW-45_02083	yjjV
yjjV	ZONMW-46_02199	yjjV
yjjV	ZONMW-47_02333	yjjV
yjjV	ZONMW-48_01010	yjjV
yjjV	ZONMW-49_01394	yjjV
yjjV	ZONMW-50_01396	yjjV
yjjV	ZONMW-51_01510	yjjV
yjjV	ZONMW-52_01250	yjjV
yjjV	ZONMW-53_00575	yjjV
yjjV	ZONMW-54_00460	yjjV
yjjV	ZONMW-55_00570	yjjV
yjjV	ZONMW-56_03028	yjjV
yjjV	ZONMW-57_01044	yjjV
yjjV	ZONMW-58_01540	yjjV
yjjV	ZONMW-59_01046	yjjV
yjjV	ZONMW-60_01521	yjjV
yjjV	ZONMW-61_02874	yjjV
yjjV	ZONMW-62_02353	yjjV
yjjV	ZONMW-63_00800	yjjV
yjjV	ZONMW-64_01278	yjjV
yjjV	ZONMW-64_03690	yjjV

yjjV	ZONMW-65_00458	yjjV
yjjV	ZONMW-65_01769	yjjV
yjjV	ZONMW-65_01311	yjjV
yjjV	ZONMW-66_00385	yjjV
yjjV	ZONMW-66_03921	yjjV
yjjV	ZONMW-66_03486	yjjV
yjjV	ZONMW-67_00439	yjjV
yjjV	ZONMW-67_02557	yjjV
yjjV	ZONMW-68_03920	yjjV
yjjV	ZONMW-69_00186	yjjV
yjjV	ZONMW-69_02605	yjjV
yjjV	ZONMW-72_00247	yjjV
yjjV	ZONMW-72_01821	yjjV
yjjV	ZONMW-72_03031	yjjV
yjjV	ZONMW-73_03204	yjjV
yjjV	ZONMW-73_03436	yjjV
yjjV	ZONMW-74_01849	yjjV
yjjV	ZONMW-75_01168	yjjV
yjjV	da5A-8_00809	yjjV
yjjV	da5A-8_00389	yjjV
narB	18_00528	narB
narB	18_02377	narB
narB	18_00918	narB
narB	18_02298	narB
narB	18_00934	narB
narB	18_02893	narB
narB	18_01413	narB
narB	18_03001	narB
narB	18_01373	narB
narB	ACAM48_02765	narB
narB	ACAM48_01964	narB
narB	ACAM48_01123	narB
narB	ACAM48_01692	narB
narB	ACAM48_01958	narB
narB	ACAM48_02125	narB
narB	ACAM48_00190	narB
narB	ACAM48_02217	narB
narB	ACAM48_01051	narB
narB	CAM030_02224	narB
narB	CAM030_02041	narB
narB	CAM030_00436	narB
narB	CAM030_00118	narB
narB	CECT7946_00608	narB
narB	CECT7946_02395	narB
narB	CECT7946_01575	narB
narB	CGMCC1.8863_01195	narB
narB	CGMCC1.8863_03332	narB
narB	CGMCC1.8863_02951	narB

narB	CGMCC1.8863_00283	narB
narB	CL8139_02465	narB
narB	CL8139_01719	narB
narB	CL8139_00273	narB
narB	CL8139_01226	narB
narB	CL8139_00264	narB
narB	CL8139_01928	narB
narB	DAU203_00897	narB
narB	DAU203_02660	narB
narB	DAU203_00813	narB
narB	DAU203_00199	narB
narB	DAU203_00822	narB
narB	DAU203_03238	narB
narB	DD5b_01148	narB
narB	DSM1208_01179	narB
narB	DSM1208_04485	narB
narB	DSM1208_01577	narB
narB	DSM1208_00502	narB
narB	DSM1208_03100	narB
narB	DSM1208_01588	narB
narB	DSM1208_03906	narB
narB	DSM1208_04423	narB
narB	DSM1208_02026	narB
narB	DSM12212_01468	narB
narB	DSM12212_00801	narB
narB	DSM12212_01823	narB
narB	DSM15343_00453	narB
narB	DSM15343_00515	narB
narB	DSM15361_01993	narB
narB	DSM15361_02287	narB
narB	DSM15361_01562	narB
narB	DSM15361_00213	narB
narB	DSM15365_01186	narB
narB	DSM15365_02842	narB
narB	DSM15365_02051	narB
narB	DSM15365_02097	narB
narB	DSM15365_02057	narB
narB	DSM15365_02058	narB
narB	DSM15365_02138	narB
narB	DSM15718_01783	narB
narB	DSM15718_01917	narB
narB	DSM15718_01508	narB
narB	DSM16505T_00666	narB
narB	DSM16505T_02672	narB
narB	DSM16505T_01060	narB
narB	DSM16505T_02058	narB
narB	DSM18103_01091	narB
narB	DSM18103_00675	narB

narB	DSM18103_00364	narB
narB	DSM18103_00215	narB
narB	DSM18103_01462	narB
narB	DSM18293_01116	narB
narB	DSM18293_01100	narB
narB	DSM18293_01175	narB
narB	DSM18293_03567	narB
narB	DSM18841_00539	narB
narB	DSM18841_03110	narB
narB	DSM18841_02965	narB
narB	DSM18841_00947	narB
narB	DSM18841_01408	narB
narB	DSM18842_00688	narB
narB	DSM18842_02024	narB
narB	DSM18842_00368	narB
narB	DSM18842_01961	narB
narB	DSM19866_03737	narB
narB	DSM19866_02477	narB
narB	DSM19866_02422	narB
narB	DSM19866_02411	narB
narB	DSM19866_02406	narB
narB	DSM19866_00760	narB
narB	DSM19866_02994	narB
narB	DSM19866_01543	narB
narB	DSM2041_00718	narB
narB	DSM2041_01117	narB
narB	DSM2041_05393	narB
narB	DSM2041_05382	narB
narB	DSM2041_01602	narB
narB	DSM2041_01802	narB
narB	DSM2041_01115	narB
narB	DSM2041_04879	narB
narB	DSM2041_01080	narB
narB	DSM2041_02908	narB
narB	DSM2061_02691	narB
narB	DSM2061_00665	narB
narB	DSM2061_03104	narB
narB	DSM2061_01993	narB
narB	DSM2061_03115	narB
narB	DSM2061_00234	narB
narB	DSM2061_01126	narB
narB	DSM2061_00733	narB
narB	DSM2061_02822	narB
narB	DSM2064_04059	narB
narB	DSM2064_04068	narB
narB	DSM2064_04015	narB
narB	DSM2064_03559	narB
narB	DSM21164_01301	narB

narB	DSM21164_00931	narB
narB	DSM21164_01857	narB
narB	DSM21164_00920	narB
narB	DSM21164_00482	narB
narB	DSM21164_01481	narB
narB	DSM23404_02195	narB
narB	DSM23404_03365	narB
narB	DSM23404_03362	narB
narB	DSM23404_02764	narB
narB	DSM23404_01920	narB
narB	DSM23404_02944	narB
narB	DSM23404_02133	narB
narB	DSM23404_02830	narB
narB	DSM23424_00806	narB
narB	DSM23424_00954	narB
narB	DSM23424_02119	narB
narB	DSM23424_00522	narB
narB	DSM24786_01293	narB
narB	DSM24786_00767	narB
narB	DSM24786_01369	narB
narB	DSM24786_01364	narB
narB	DSM24786_00046	narB
narB	DSM24786_00683	narB
narB	DSM7489_00831	narB
narB	DSM7489_02535	narB
narB	DSM7489_00746	narB
narB	DSM7489_00193	narB
narB	DSM7489_00755	narB
narB	DSM7489_03070	narB
narB	DSW1_00132	narB
narB	DSW1_02630	narB
narB	DSW1_00499	narB
narB	DSW1_01660	narB
narB	DSW5_01559	narB
narB	DSW5_02589	narB
narB	DSW5_00036	narB
narB	DSW5_00973	narB
narB	DSW5_01403	narB
narB	DSW6_01788	narB
narB	DSW6_03457	narB
narB	DSW6_00883	narB
narB	DSW6_00652	narB
narB	DSW6_02109	narB
narB	DSW8_02769	narB
narB	DSW8_03195	narB
narB	DSW8_02351	narB
narB	DSW8_03495	narB
narB	DSW8_02341	narB

narB	DSW8_01772	narB
narB	DSW8_01880	narB
narB	DSW8_03576	narB
narB	F52_04215	narB
narB	F52_04226	narB
narB	F52_04176	narB
narB	F52_03142	narB
narB	F52_00103	narB
narB	F52_04609	narB
narB	GCA_001027945.1_00067	narB
narB	GCA_001027945.1_02058	narB
narB	GCA_001027945.1_01477	narB
narB	GCA_001027945.1_02186	narB
narB	HI1_00766	narB
narB	HI1_02467	narB
narB	HI1_00681	narB
narB	HI1_00125	narB
narB	HI1_00690	narB
narB	HI1_03011	narB
narB	IR1_04212	narB
narB	IR1_04205	narB
narB	IR1_04244	narB
narB	IR1_04226	narB
narB	IR1_04484	narB
narB	IR1_04192	narB
narB	IR1_00912	narB
narB	KCOM3131_00062	narB
narB	KCOM3131_00927	narB
narB	KCTC12974_01186	narB
narB	KCTC12974_02684	narB
narB	KCTC12974_00381	narB
narB	KCTC12974_01904	narB
narB	KCTC12974_01564	narB
narB	KL-A_03204	narB
narB	KL-A_01021	narB
narB	KL-A_02480	narB
narB	KL-A_01189	narB
narB	KL-A_02471	narB
narB	KL-A_02956	narB
narB	L12_00073	narB
narB	LL0412.1.7_00273	narB
narB	LL0412.1.7_02123	narB
narB	LL0412.1.7_02488	narB
narB	LL0412.1.7_01088	narB
narB	LL0412.1.7_01536	narB
narB	MAR_2009_71_00684	narB
narB	MAR_2009_71_03720	narB
narB	MAR_2009_71_01088	narB

narB	MAR_2009_71_00091	narB
narB	MAR_2009_71_01098	narB
narB	MAR_2009_71_01648	narB
narB	MAR_2009_71_01500	narB
narB	MAR_2009_71_02733	narB
narB	NN016038_00538	narB
narB	NN016038_02388	narB
narB	NN016038_00928	narB
narB	NN016038_02309	narB
narB	NN016038_00944	narB
narB	NN016038_02944	narB
narB	NN016038_01422	narB
narB	NN016038_03036	narB
narB	NN016038_01385	narB
narB	PAO1_01828	narB
narB	PAO1_05526	narB
narB	PAO1_01211	narB
narB	PAO1_03656	narB
narB	PAO1_02795	narB
narB	PAO1_05355	narB
narB	PAO1_04976	narB
narB	PAO1_04977	narB
narB	PAO1_02088	narB
narB	PAO1_02322	narB
narB	PAO1_04995	narB
narB	PAO1_01631	narB
narB	PAO1_03096	narB
narB	UW101_04828	narB
narB	UW101_04819	narB
narB	UW101_04872	narB
narB	UW101_02241	narB
narB	V4-33_00679	narB
narB	V4-33_01643	narB
narB	W5C_01457	narB
narB	W5C_01040	narB
narB	W5C_01379	narB
narB	W5C_00102	narB
narB	W5C_01388	narB
narB	W5C_02277	narB
narB	ZONMW-01_03407	narB
narB	ZONMW-01_01588	narB
narB	ZONMW-01_02646	narB
narB	ZONMW-01_02655	narB
narB	ZONMW-01_02078	narB
narB	ZONMW-01_00702	narB
narB	ZONMW-02_02732	narB
narB	ZONMW-02_01222	narB
narB	ZONMW-02_03470	narB

narB	ZONMW-02_03464	narB
narB	ZONMW-02_03425	narB
narB	ZONMW-02_03463	narB
narB	ZONMW-02_01568	narB
narB	ZONMW-03_01750	narB
narB	ZONMW-03_00619	narB
narB	ZONMW-03_01672	narB
narB	ZONMW-03_01681	narB
narB	ZONMW-03_02632	narB
narB	ZONMW-03_00080	narB
narB	ZONMW-04_02242	narB
narB	ZONMW-04_02605	narB
narB	ZONMW-04_02320	narB
narB	ZONMW-04_02311	narB
narB	ZONMW-04_01922	narB
narB	ZONMW-04_01088	narB
narB	ZONMW-05_02759	narB
narB	ZONMW-05_00881	narB
narB	ZONMW-05_02836	narB
narB	ZONMW-05_02827	narB
narB	ZONMW-05_02448	narB
narB	ZONMW-05_00341	narB
narB	ZONMW-06_00057	narB
narB	ZONMW-06_01450	narB
narB	ZONMW-06_02398	narB
narB	ZONMW-06_01667	narB
narB	ZONMW-06_01203	narB
narB	ZONMW-06_01929	narB
narB	ZONMW-06_02313	narB
narB	ZONMW-06_02357	narB
narB	ZONMW-06_02449	narB
narB	ZONMW-06_01420	narB
narB	ZONMW-06_01792	narB
narB	ZONMW-06_03328	narB
narB	ZONMW-06_00150	narB
narB	ZONMW-07_00572	narB
narB	ZONMW-07_00897	narB
narB	ZONMW-07_03281	narB
narB	ZONMW-07_03291	narB
narB	ZONMW-07_03391	narB
narB	ZONMW-07_02291	narB
narB	ZONMW-07_03034	narB
narB	ZONMW-08_00201	narB
narB	ZONMW-08_03223	narB
narB	ZONMW-08_00606	narB
narB	ZONMW-08_02960	narB
narB	ZONMW-08_00616	narB
narB	ZONMW-08_01585	narB

narB	ZONMW-08_01706	narB
narB	ZONMW-08_01944	narB
narB	ZONMW-09_00633	narB
narB	ZONMW-09_02113	narB
narB	ZONMW-09_02255	narB
narB	ZONMW-09_00152	narB
narB	ZONMW-09_00218	narB
narB	ZONMW-10_02033	narB
narB	ZONMW-10_02424	narB
narB	ZONMW-10_00723	narB
narB	ZONMW-11_03215	narB
narB	ZONMW-11_03224	narB
narB	ZONMW-11_03227	narB
narB	ZONMW-11_00896	narB
narB	ZONMW-11_01741	narB
narB	ZONMW-11_01226	narB
narB	ZONMW-11_00627	narB
narB	ZONMW-11_03275	narB
narB	ZONMW-12_01195	narB
narB	ZONMW-12_03457	narB
narB	ZONMW-12_00360	narB
narB	ZONMW-12_03566	narB
narB	ZONMW-12_01890	narB
narB	ZONMW-12_01268	narB
narB	ZONMW-12_00550	narB
narB	ZONMW-12_01056	narB
narB	ZONMW-13_00772	narB
narB	ZONMW-13_01342	narB
narB	ZONMW-13_03067	narB
narB	ZONMW-13_00235	narB
narB	ZONMW-14_01273	narB
narB	ZONMW-14_01819	narB
narB	ZONMW-14_00436	narB
narB	ZONMW-14_01975	narB
narB	ZONMW-14_00356	narB
narB	ZONMW-15_01005	narB
narB	ZONMW-15_01682	narB
narB	ZONMW-15_01667	narB
narB	ZONMW-15_00235	narB
narB	ZONMW-15_01555	narB
narB	ZONMW-15_03087	narB
narB	ZONMW-15_00250	narB
narB	ZONMW-19_01119	narB
narB	ZONMW-19_00543	narB
narB	ZONMW-19_00599	narB
narB	ZONMW-19_01701	narB
narB	ZONMW-19_01587	narB
narB	ZONMW-19_02710	narB

narB	ZONMW-19_04232	narB
narB	ZONMW-20_02579	narB
narB	ZONMW-20_00154	narB
narB	ZONMW-20_03410	narB
narB	ZONMW-20_01605	narB
narB	ZONMW-20_03557	narB
narB	ZONMW-20_03731	narB
narB	ZONMW-20_00555	narB
narB	ZONMW-20_03634	narB
narB	ZONMW-20_03563	narB
narB	ZONMW-20_03633	narB
narB	ZONMW-20_00556	narB
narB	ZONMW-20_03732	narB
narB	ZONMW-20_00954	narB
narB	ZONMW-20_03288	narB
narB	ZONMW-20_00904	narB
narB	ZONMW-21_01290	narB
narB	ZONMW-21_00317	narB
narB	ZONMW-21_01752	narB
narB	ZONMW-21_03252	narB
narB	ZONMW-22_01292	narB
narB	ZONMW-22_00317	narB
narB	ZONMW-22_01972	narB
narB	ZONMW-22_03264	narB
narB	ZONMW-23_02404	narB
narB	ZONMW-23_01432	narB
narB	ZONMW-23_00579	narB
narB	ZONMW-23_03286	narB
narB	ZONMW-24_00169	narB
narB	ZONMW-24_01142	narB
narB	ZONMW-24_02597	narB
narB	ZONMW-24_03287	narB
narB	ZONMW-25_02992	narB
narB	ZONMW-25_01283	narB
narB	ZONMW-25_00818	narB
narB	ZONMW-25_02549	narB
narB	ZONMW-25_03157	narB
narB	ZONMW-26_02099	narB
narB	ZONMW-26_00079	narB
narB	ZONMW-26_02176	narB
narB	ZONMW-26_02167	narB
narB	ZONMW-26_02668	narB
narB	ZONMW-26_00626	narB
narB	ZONMW-27_02221	narB
narB	ZONMW-27_03068	narB
narB	ZONMW-27_02299	narB
narB	ZONMW-27_02290	narB
narB	ZONMW-27_02092	narB

narB	ZONMW-27_01163	narB
narB	ZONMW-28_01599	narB
narB	ZONMW-28_01238	narB
narB	ZONMW-28_02505	narB
narB	ZONMW-28_01593	narB
narB	ZONMW-28_00234	narB
narB	ZONMW-28_02806	narB
narB	ZONMW-28_01600	narB
narB	ZONMW-28_02654	narB
narB	ZONMW-28_00273	narB
narB	ZONMW-28_01624	narB
narB	ZONMW-28_00182	narB
narB	ZONMW-28_03241	narB
narB	ZONMW-28_01342	narB
narB	ZONMW-29_00669	narB
narB	ZONMW-29_01738	narB
narB	ZONMW-29_01029	narB
narB	ZONMW-30_00577	narB
narB	ZONMW-30_02470	narB
narB	ZONMW-30_01130	narB
narB	ZONMW-30_01916	narB
narB	ZONMW-30_01526	narB
narB	ZONMW-31_00390	narB
narB	ZONMW-31_03269	narB
narB	ZONMW-31_02072	narB
narB	ZONMW-31_01921	narB
narB	ZONMW-31_00335	narB
narB	ZONMW-31_03288	narB
narB	ZONMW-31_03471	narB
narB	ZONMW-31_03645	narB
narB	ZONMW-31_01767	narB
narB	ZONMW-31_00478	narB
narB	ZONMW-32_02372	narB
narB	ZONMW-32_01646	narB
narB	ZONMW-32_02845	narB
narB	ZONMW-32_02378	narB
narB	ZONMW-32_01025	narB
narB	ZONMW-32_03069	narB
narB	ZONMW-32_02371	narB
narB	ZONMW-32_03222	narB
narB	ZONMW-32_01064	narB
narB	ZONMW-32_02347	narB
narB	ZONMW-32_00973	narB
narB	ZONMW-32_00766	narB
narB	ZONMW-32_01750	narB
narB	ZONMW-33_02364	narB
narB	ZONMW-33_01636	narB
narB	ZONMW-33_02903	narB

narB	ZONMW-33_02370	narB
narB	ZONMW-33_01014	narB
narB	ZONMW-33_03204	narB
narB	ZONMW-33_02363	narB
narB	ZONMW-33_03052	narB
narB	ZONMW-33_01053	narB
narB	ZONMW-33_02339	narB
narB	ZONMW-33_00962	narB
narB	ZONMW-33_00634	narB
narB	ZONMW-33_01740	narB
narB	ZONMW-34_03086	narB
narB	ZONMW-34_00022	narB
narB	ZONMW-34_03080	narB
narB	ZONMW-34_01222	narB
narB	ZONMW-34_02467	narB
narB	ZONMW-34_03087	narB
narB	ZONMW-34_02602	narB
narB	ZONMW-34_01255	narB
narB	ZONMW-34_03116	narB
narB	ZONMW-34_01167	narB
narB	ZONMW-34_00998	narB
narB	ZONMW-34_03468	narB
narB	ZONMW-34_00123	narB
narB	ZONMW-35_03902	narB
narB	ZONMW-35_01368	narB
narB	ZONMW-35_03896	narB
narB	ZONMW-35_00295	narB
narB	ZONMW-35_02431	narB
narB	ZONMW-35_03903	narB
narB	ZONMW-35_02566	narB
narB	ZONMW-35_00328	narB
narB	ZONMW-35_03121	narB
narB	ZONMW-35_00240	narB
narB	ZONMW-35_02245	narB
narB	ZONMW-35_03347	narB
narB	ZONMW-35_01268	narB
narB	ZONMW-36_01872	narB
narB	ZONMW-36_01720	narB
narB	ZONMW-36_02415	narB
narB	ZONMW-36_01866	narB
narB	ZONMW-36_00234	narB
narB	ZONMW-36_03505	narB
narB	ZONMW-36_01873	narB
narB	ZONMW-36_02950	narB
narB	ZONMW-36_00273	narB
narB	ZONMW-36_01897	narB
narB	ZONMW-36_00182	narB
narB	ZONMW-36_00737	narB

narB	ZONMW-36_01616	narB
narB	ZONMW-37_01072	narB
narB	ZONMW-37_00317	narB
narB	ZONMW-37_02130	narB
narB	ZONMW-37_03273	narB
narB	ZONMW-38_00655	narB
narB	ZONMW-38_02862	narB
narB	ZONMW-38_03463	narB
narB	ZONMW-38_03617	narB
narB	ZONMW-38_02351	narB
narB	ZONMW-38_01260	narB
narB	ZONMW-38_02469	narB
narB	ZONMW-38_00211	narB
narB	ZONMW-39_04193	narB
narB	ZONMW-39_02512	narB
narB	ZONMW-39_04199	narB
narB	ZONMW-39_02000	narB
narB	ZONMW-39_04192	narB
narB	ZONMW-39_03927	narB
narB	ZONMW-39_03771	narB
narB	ZONMW-39_00747	narB
narB	ZONMW-39_01792	narB
narB	ZONMW-39_00838	narB
narB	ZONMW-39_02869	narB
narB	ZONMW-39_02404	narB
narB	ZONMW-40_03416	narB
narB	ZONMW-40_02047	narB
narB	ZONMW-40_02052	narB
narB	ZONMW-40_03228	narB
narB	ZONMW-40_01530	narB
narB	ZONMW-40_02850	narB
narB	ZONMW-41_00230	narB
narB	ZONMW-41_00315	narB
narB	ZONMW-41_00192	narB
narB	ZONMW-41_00009	narB
narB	ZONMW-41_01230	narB
narB	ZONMW-41_02353	narB
narB	ZONMW-42_02450	narB
narB	ZONMW-42_02643	narB
narB	ZONMW-42_00125	narB
narB	ZONMW-42_02451	narB
narB	ZONMW-42_02445	narB
narB	ZONMW-42_03907	narB
narB	ZONMW-42_01212	narB
narB	ZONMW-42_03733	narB
narB	ZONMW-42_01070	narB
narB	ZONMW-42_03516	narB
narB	ZONMW-42_02479	narB

narB	ZONMW-42_03691	narB
narB	ZONMW-42_00887	narB
narB	ZONMW-42_00035	narB
narB	ZONMW-43_04449	narB
narB	ZONMW-43_02253	narB
narB	ZONMW-43_04367	narB
narB	ZONMW-43_00926	narB
narB	ZONMW-43_04443	narB
narB	ZONMW-43_01078	narB
narB	ZONMW-43_04450	narB
narB	ZONMW-43_01229	narB
narB	ZONMW-43_03058	narB
narB	ZONMW-43_02291	narB
narB	ZONMW-43_03442	narB
narB	ZONMW-43_03134	narB
narB	ZONMW-43_00531	narB
narB	ZONMW-43_00826	narB
narB	ZONMW-44_01989	narB
narB	ZONMW-44_00301	narB
narB	ZONMW-44_00640	narB
narB	ZONMW-44_01086	narB
narB	ZONMW-44_00392	narB
narB	ZONMW-44_04015	narB
narB	ZONMW-44_00047	narB
narB	ZONMW-44_01165	narB
narB	ZONMW-44_01121	narB
narB	ZONMW-44_03325	narB
narB	ZONMW-44_02282	narB
narB	ZONMW-44_03746	narB
narB	ZONMW-44_01896	narB
narB	ZONMW-45_00680	narB
narB	ZONMW-45_02306	narB
narB	ZONMW-45_02240	narB
narB	ZONMW-45_01500	narB
narB	ZONMW-45_01253	narB
narB	ZONMW-46_00779	narB
narB	ZONMW-46_03109	narB
narB	ZONMW-46_03175	narB
narB	ZONMW-46_01754	narB
narB	ZONMW-46_02637	narB
narB	ZONMW-47_00779	narB
narB	ZONMW-47_03101	narB
narB	ZONMW-47_03167	narB
narB	ZONMW-47_01735	narB
narB	ZONMW-47_02627	narB
narB	ZONMW-48_00421	narB
narB	ZONMW-48_00068	narB
narB	ZONMW-48_00053	narB

narB	ZONMW-48_00881	narB
narB	ZONMW-48_01928	narB
narB	ZONMW-48_03444	narB
narB	ZONMW-48_00896	narB
narB	ZONMW-49_02269	narB
narB	ZONMW-49_00085	narB
narB	ZONMW-49_02353	narB
narB	ZONMW-49_02484	narB
narB	ZONMW-49_02344	narB
narB	ZONMW-49_01752	narB
narB	ZONMW-50_02191	narB
narB	ZONMW-50_00401	narB
narB	ZONMW-50_02107	narB
narB	ZONMW-50_02278	narB
narB	ZONMW-50_02116	narB
narB	ZONMW-50_01662	narB
narB	ZONMW-51_01687	narB
narB	ZONMW-51_00082	narB
narB	ZONMW-51_01603	narB
narB	ZONMW-51_02461	narB
narB	ZONMW-51_01612	narB
narB	ZONMW-51_00648	narB
narB	ZONMW-52_01830	narB
narB	ZONMW-52_00055	narB
narB	ZONMW-52_01746	narB
narB	ZONMW-52_02444	narB
narB	ZONMW-52_01755	narB
narB	ZONMW-52_00607	narB
narB	ZONMW-53_01926	narB
narB	ZONMW-53_02600	narB
narB	ZONMW-53_02010	narB
narB	ZONMW-53_02189	narB
narB	ZONMW-53_02001	narB
narB	ZONMW-53_01362	narB
narB	ZONMW-54_02128	narB
narB	ZONMW-54_01922	narB
narB	ZONMW-54_02044	narB
narB	ZONMW-54_02679	narB
narB	ZONMW-54_02053	narB
narB	ZONMW-54_01355	narB
narB	ZONMW-55_02009	narB
narB	ZONMW-55_01456	narB
narB	ZONMW-55_01925	narB
narB	ZONMW-55_02552	narB
narB	ZONMW-55_01934	narB
narB	ZONMW-55_00876	narB
narB	ZONMW-56_01427	narB
narB	ZONMW-56_00682	narB

narB	ZONMW-56_01511	narB
narB	ZONMW-56_01677	narB
narB	ZONMW-56_01502	narB
narB	ZONMW-56_00112	narB
narB	ZONMW-57_01799	narB
narB	ZONMW-57_00889	narB
narB	ZONMW-57_01715	narB
narB	ZONMW-57_02024	narB
narB	ZONMW-57_01724	narB
narB	ZONMW-57_00323	narB
narB	ZONMW-58_01823	narB
narB	ZONMW-58_00082	narB
narB	ZONMW-58_01739	narB
narB	ZONMW-58_02054	narB
narB	ZONMW-58_01748	narB
narB	ZONMW-58_00648	narB
narB	ZONMW-59_01635	narB
narB	ZONMW-59_00883	narB
narB	ZONMW-59_01551	narB
narB	ZONMW-59_02273	narB
narB	ZONMW-59_01560	narB
narB	ZONMW-59_00339	narB
narB	ZONMW-60_02017	narB
narB	ZONMW-60_00888	narB
narB	ZONMW-60_01932	narB
narB	ZONMW-60_02569	narB
narB	ZONMW-60_01941	narB
narB	ZONMW-60_00350	narB
narB	ZONMW-61_01577	narB
narB	ZONMW-61_01040	narB
narB	ZONMW-61_00182	narB
narB	ZONMW-61_00194	narB
narB	ZONMW-61_02743	narB
narB	ZONMW-61_00745	narB
narB	ZONMW-61_02350	narB
narB	ZONMW-61_00616	narB
narB	ZONMW-62_01328	narB
narB	ZONMW-62_03121	narB
narB	ZONMW-62_01735	narB
narB	ZONMW-62_03430	narB
narB	ZONMW-62_01745	narB
narB	ZONMW-62_02421	narB
narB	ZONMW-62_02520	narB
narB	ZONMW-62_00817	narB
narB	ZONMW-63_01535	narB
narB	ZONMW-63_02023	narB
narB	ZONMW-63_01451	narB
narB	ZONMW-63_01659	narB

narB	ZONMW-63_01460	narB
narB	ZONMW-63_01962	narB
narB	ZONMW-64_01782	narB
narB	ZONMW-64_00830	narB
narB	ZONMW-64_01211	narB
narB	ZONMW-64_01788	narB
narB	ZONMW-64_02498	narB
narB	ZONMW-64_01781	narB
narB	ZONMW-64_03579	narB
narB	ZONMW-64_02220	narB
narB	ZONMW-64_00792	narB
narB	ZONMW-64_01752	narB
narB	ZONMW-64_02304	narB
narB	ZONMW-64_03223	narB
narB	ZONMW-64_00549	narB
narB	ZONMW-64_01111	narB
narB	ZONMW-65_03635	narB
narB	ZONMW-65_02922	narB
narB	ZONMW-65_01551	narB
narB	ZONMW-65_01391	narB
narB	ZONMW-65_03158	narB
narB	ZONMW-66_01229	narB
narB	ZONMW-66_01611	narB
narB	ZONMW-66_02182	narB
narB	ZONMW-66_02394	narB
narB	ZONMW-66_00418	narB
narB	ZONMW-66_03150	narB
narB	ZONMW-66_01680	narB
narB	ZONMW-67_02885	narB
narB	ZONMW-67_00500	narB
narB	ZONMW-67_02884	narB
narB	ZONMW-67_02890	narB
narB	ZONMW-67_02019	narB
narB	ZONMW-67_03445	narB
narB	ZONMW-67_00214	narB
narB	ZONMW-67_03316	narB
narB	ZONMW-67_01215	narB
narB	ZONMW-67_02123	narB
narB	ZONMW-67_02058	narB
narB	ZONMW-67_00007	narB
narB	ZONMW-67_01970	narB
narB	ZONMW-67_00600	narB
narB	ZONMW-68_00860	narB
narB	ZONMW-68_02319	narB
narB	ZONMW-68_01391	narB
narB	ZONMW-68_02154	narB
narB	ZONMW-68_01404	narB
narB	ZONMW-68_01409	narB

narB	ZONMW-68_02435	narB
narB	ZONMW-68_02432	narB
narB	ZONMW-68_00416	narB
narB	ZONMW-68_02079	narB
narB	ZONMW-68_01928	narB
narB	ZONMW-69_03069	narB
narB	ZONMW-69_00247	narB
narB	ZONMW-69_03070	narB
narB	ZONMW-69_03064	narB
narB	ZONMW-69_01924	narB
narB	ZONMW-69_03446	narB
narB	ZONMW-69_02823	narB
narB	ZONMW-69_03317	narB
narB	ZONMW-69_01261	narB
narB	ZONMW-69_01820	narB
narB	ZONMW-69_01885	narB
narB	ZONMW-69_02616	narB
narB	ZONMW-69_01973	narB
narB	ZONMW-69_00347	narB
narB	ZONMW-72_01899	narB
narB	ZONMW-72_01906	narB
narB	ZONMW-72_01867	narB
narB	ZONMW-72_01885	narB
narB	ZONMW-72_03107	narB
narB	ZONMW-73_03161	narB
narB	ZONMW-73_02699	narB
narB	ZONMW-73_01491	narB
narB	ZONMW-73_01507	narB
narB	ZONMW-73_01505	narB
narB	ZONMW-73_03308	narB
narB	ZONMW-74_03305	narB
narB	ZONMW-74_01612	narB
narB	ZONMW-74_02650	narB
narB	ZONMW-74_01617	narB
narB	ZONMW-74_00572	narB
narB	ZONMW-74_00296	narB
narB	ZONMW-75_02893	narB
narB	ZONMW-75_02737	narB
narB	ZONMW-75_03611	narB
narB	ZONMW-75_02507	narB
narB	ZONMW-75_02430	narB
narB	ZONMW-75_02431	narB
narB	ZONMW-75_02552	narB
narB	da5A-8_01597	narB
narB	da5A-8_02039	narB
narB	da5A-8_02063	narB
narB	da5A-8_02086	narB
narB	da5A-8_02261	narB

exoA	18_00733	exoA
exoA	18_02400	exoA
exoA	ACAM48_02600	exoA
exoA	CAM030_00807	exoA
exoA	CECT7946_00432	exoA
exoA	CECT7946_02507	exoA
exoA	CGMCC1.8863_02216	exoA
exoA	CGMCC1.8863_02724	exoA
exoA	CL8139_01603	exoA
exoA	DAU203_01211	exoA
exoA	DAU203_00299	exoA
exoA	DD5b_01993	exoA
exoA	DSM1208_01372	exoA
exoA	DSM1208_01765	exoA
exoA	DSM12212_03175	exoA
exoA	DSM15343_00932	exoA
exoA	DSM15361_00237	exoA
exoA	DSM15365_00893	exoA
exoA	DSM15718_02456	exoA
exoA	DSM16505T_00425	exoA
exoA	DSM18103_03022	exoA
exoA	DSM18293_03244	exoA
exoA	DSM18293_02256	exoA
exoA	DSM18841_00254	exoA
exoA	DSM18842_00968	exoA
exoA	DSM19866_03555	exoA
exoA	DSM19866_02498	exoA
exoA	DSM2041_00530	exoA
exoA	DSM2061_02893	exoA
exoA	DSM2064_01819	exoA
exoA	DSM2064_02998	exoA
exoA	DSM21164_01116	exoA
exoA	DSM21164_01903	exoA
exoA	DSM23404_02353	exoA
exoA	DSM23424_00134	exoA
exoA	DSM24786_02682	exoA
exoA	DSM24786_01100	exoA
exoA	DSM7489_01118	exoA
exoA	DSM7489_00293	exoA
exoA	DSW1_00413	exoA
exoA	DSW5_00881	exoA
exoA	DSW6_01481	exoA
exoA	DSW8_02564	exoA
exoA	DSW8_02172	exoA
exoA	F52_02257	exoA
exoA	F52_03083	exoA
exoA	GCA_001027945.1_00357	exoA
exoA	HI1_01058	exoA

exoA	HI1_00225	exoA
exoA	IR1_04006	exoA
exoA	KCOM3131_01248	exoA
exoA	KCTC12974_01971	exoA
exoA	KL-A_02064	exoA
exoA	KL-A_03179	exoA
exoA	L12_00240	exoA
exoA	LL0412.1.7_00358	exoA
exoA	MAR_2009_71_00888	exoA
exoA	MAR_2009_71_01268	exoA
exoA	NN016038_00753	exoA
exoA	NN016038_02411	exoA
exoA	PAO1_05509	exoA
exoA	PAO1_02616	exoA
exoA	PAO1_04303	exoA
exoA	UW101_00873	exoA
exoA	UW101_02052	exoA
exoA	V4-33_00048	exoA
exoA	W5C_01965	exoA
exoA	W5C_00202	exoA
exoA	ZONMW-01_01148	exoA
exoA	ZONMW-01_02177	exoA
exoA	ZONMW-02_01608	exoA
exoA	ZONMW-03_02217	exoA
exoA	ZONMW-03_02533	exoA
exoA	ZONMW-04_01599	exoA
exoA	ZONMW-04_02022	exoA
exoA	ZONMW-05_01879	exoA
exoA	ZONMW-05_02347	exoA
exoA	ZONMW-06_01916	exoA
exoA	ZONMW-06_02173	exoA
exoA	ZONMW-07_03136	exoA
exoA	ZONMW-07_02944	exoA
exoA	ZONMW-08_00408	exoA
exoA	ZONMW-08_00788	exoA
exoA	ZONMW-09_00517	exoA
exoA	ZONMW-09_01541	exoA
exoA	ZONMW-10_00511	exoA
exoA	ZONMW-11_01448	exoA
exoA	ZONMW-12_00430	exoA
exoA	ZONMW-13_00698	exoA
exoA	ZONMW-14_01345	exoA
exoA	ZONMW-15_00828	exoA
exoA	ZONMW-19_00882	exoA
exoA	ZONMW-19_02496	exoA
exoA	ZONMW-20_01670	exoA
exoA	ZONMW-21_01122	exoA
exoA	ZONMW-22_01124	exoA

exoA	ZONMW-23_02236	exoA
exoA	ZONMW-24_00337	exoA
exoA	ZONMW-25_02113	exoA
exoA	ZONMW-26_02516	exoA
exoA	ZONMW-26_02769	exoA
exoA	ZONMW-27_01697	exoA
exoA	ZONMW-27_02193	exoA
exoA	ZONMW-28_02666	exoA
exoA	ZONMW-28_00412	exoA
exoA	ZONMW-29_00718	exoA
exoA	ZONMW-29_02124	exoA
exoA	ZONMW-30_00408	exoA
exoA	ZONMW-31_01934	exoA
exoA	ZONMW-31_01361	exoA
exoA	ZONMW-32_03210	exoA
exoA	ZONMW-32_01203	exoA
exoA	ZONMW-33_03064	exoA
exoA	ZONMW-33_01192	exoA
exoA	ZONMW-34_02590	exoA
exoA	ZONMW-34_01386	exoA
exoA	ZONMW-35_02554	exoA
exoA	ZONMW-35_00459	exoA
exoA	ZONMW-36_02938	exoA
exoA	ZONMW-36_00412	exoA
exoA	ZONMW-37_01240	exoA
exoA	ZONMW-38_01493	exoA
exoA	ZONMW-39_03915	exoA
exoA	ZONMW-39_00314	exoA
exoA	ZONMW-40_00146	exoA
exoA	ZONMW-41_00727	exoA
exoA	ZONMW-42_01083	exoA
exoA	ZONMW-42_01807	exoA
exoA	ZONMW-43_01217	exoA
exoA	ZONMW-43_02969	exoA
exoA	ZONMW-44_00104	exoA
exoA	ZONMW-44_01258	exoA
exoA	ZONMW-45_00404	exoA
exoA	ZONMW-46_01435	exoA
exoA	ZONMW-47_01417	exoA
exoA	ZONMW-48_00242	exoA
exoA	ZONMW-49_02866	exoA
exoA	ZONMW-49_03209	exoA
exoA	ZONMW-50_02790	exoA
exoA	ZONMW-50_03225	exoA
exoA	ZONMW-51_02908	exoA
exoA	ZONMW-51_02360	exoA
exoA	ZONMW-52_02965	exoA
exoA	ZONMW-52_02344	exoA

exoA	ZONMW-53_02972	exoA
exoA	ZONMW-53_02289	exoA
exoA	ZONMW-54_02976	exoA
exoA	ZONMW-54_02779	exoA
exoA	ZONMW-55_02980	exoA
exoA	ZONMW-55_02451	exoA
exoA	ZONMW-56_02860	exoA
exoA	ZONMW-56_01777	exoA
exoA	ZONMW-57_02882	exoA
exoA	ZONMW-57_02124	exoA
exoA	ZONMW-58_02906	exoA
exoA	ZONMW-58_01954	exoA
exoA	ZONMW-59_03067	exoA
exoA	ZONMW-59_02173	exoA
exoA	ZONMW-60_02999	exoA
exoA	ZONMW-60_02470	exoA
exoA	ZONMW-61_01792	exoA
exoA	ZONMW-61_00383	exoA
exoA	ZONMW-62_01532	exoA
exoA	ZONMW-62_01929	exoA
exoA	ZONMW-63_02828	exoA
exoA	ZONMW-63_01559	exoA
exoA	ZONMW-64_03567	exoA
exoA	ZONMW-64_03374	exoA
exoA	ZONMW-65_03114	exoA
exoA	ZONMW-66_02298	exoA
exoA	ZONMW-66_00376	exoA
exoA	ZONMW-67_03328	exoA
exoA	ZONMW-67_01332	exoA
exoA	ZONMW-68_00676	exoA
exoA	ZONMW-68_02335	exoA
exoA	ZONMW-69_03329	exoA
exoA	ZONMW-69_01669	exoA
exoA	ZONMW-72_02320	exoA
exoA	ZONMW-73_01832	exoA
exoA	ZONMW-74_02155	exoA
exoA	ZONMW-75_00405	exoA
exoA	da5A-8_01945	exoA
frdA	18_01142	frdA
frdA	ACAM48_02130	frdA
frdA	ACAM48_01950	frdA
frdA	CAM030_00832	frdA
frdA	CECT7946_01941	frdA
frdA	CGMCC1.8863_00877	frdA
frdA	CGMCC1.8863_00778	frdA
frdA	CL8139_01885	frdA
frdA	DAU203_01527	frdA
frdA	DD5b_01945	frdA

frdA	DD5b_02766	frdA
frdA	DSM1208_01759	frdA
frdA	DSM12212_02101	frdA
frdA	DSM15343_02425	frdA
frdA	DSM15361_01459	frdA
frdA	DSM15365_03277	frdA
frdA	DSM15365_03308	frdA
frdA	DSM15718_02925	frdA
frdA	DSM16505T_00456	frdA
frdA	DSM18103_03074	frdA
frdA	DSM18103_01207	frdA
frdA	DSM18293_00843	frdA
frdA	DSM18293_04265	frdA
frdA	DSM18841_00297	frdA
frdA	DSM18841_03113	frdA
frdA	DSM18842_00935	frdA
frdA	DSM19866_01993	frdA
frdA	DSM19866_02618	frdA
frdA	DSM2041_04475	frdA
frdA	DSM2061_03288	frdA
frdA	DSM2064_01658	frdA
frdA	DSM2064_00755	frdA
frdA	DSM2064_01254	frdA
frdA	DSM21164_00727	frdA
frdA	DSM23404_01925	frdA
frdA	DSM23424_00147	frdA
frdA	DSM24786_03016	frdA
frdA	DSM7489_01431	frdA
frdA	DSW1_01003	frdA
frdA	DSW5_01352	frdA
frdA	DSW6_01492	frdA
frdA	DSW8_02181	frdA
frdA	F52_02117	frdA
frdA	F52_00886	frdA
frdA	F52_04453	frdA
frdA	GCA_001027945.1_00314	frdA
frdA	HI1_01372	frdA
frdA	IR1_01113	frdA
frdA	IR1_01909	frdA
frdA	KCOM3131_01507	frdA
frdA	KCOM3131_01562	frdA
frdA	KCTC12974_00386	frdA
frdA	KL-A_00795	frdA
frdA	L12_00457	frdA
frdA	LL0412.1.7_00414	frdA
frdA	MAR_2009_71_01259	frdA
frdA	NN016038_01156	frdA
frdA	PAO1_01627	frdA

frdA	PAO1_00786	frdA
frdA	PAO1_02370	frdA
frdA	UW101_00712	frdA
frdA	UW101_03466	frdA
frdA	UW101_04225	frdA
frdA	V4-33_00079	frdA
frdA	W5C_01130	frdA
frdA	W5C_00756	frdA
frdA	ZONMW-01_02428	frdA
frdA	ZONMW-02_00210	frdA
frdA	ZONMW-02_00246	frdA
frdA	ZONMW-03_03031	frdA
frdA	ZONMW-04_02411	frdA
frdA	ZONMW-05_02613	frdA
frdA	ZONMW-06_02453	frdA
frdA	ZONMW-06_02885	frdA
frdA	ZONMW-07_01433	frdA
frdA	ZONMW-08_00779	frdA
frdA	ZONMW-09_00508	frdA
frdA	ZONMW-10_00220	frdA
frdA	ZONMW-11_00255	frdA
frdA	ZONMW-11_01989	frdA
frdA	ZONMW-12_00077	frdA
frdA	ZONMW-12_02352	frdA
frdA	ZONMW-13_00230	frdA
frdA	ZONMW-14_00361	frdA
frdA	ZONMW-15_02521	frdA
frdA	ZONMW-19_02706	frdA
frdA	ZONMW-19_00383	frdA
frdA	ZONMW-20_03137	frdA
frdA	ZONMW-20_02361	frdA
frdA	ZONMW-20_02963	frdA
frdA	ZONMW-21_00714	frdA
frdA	ZONMW-22_00715	frdA
frdA	ZONMW-23_01829	frdA
frdA	ZONMW-24_00746	frdA
frdA	ZONMW-25_01629	frdA
frdA	ZONMW-25_00604	frdA
frdA	ZONMW-26_03050	frdA
frdA	ZONMW-27_02770	frdA
frdA	ZONMW-28_00178	frdA
frdA	ZONMW-28_02021	frdA
frdA	ZONMW-28_01021	frdA
frdA	ZONMW-29_00362	frdA
frdA	ZONMW-30_02369	frdA
frdA	ZONMW-30_02777	frdA
frdA	ZONMW-31_02829	frdA
frdA	ZONMW-31_02993	frdA

frdA	ZONMW-32_00969	frdA
frdA	ZONMW-32_02427	frdA
frdA	ZONMW-32_00070	frdA
frdA	ZONMW-33_00958	frdA
frdA	ZONMW-33_02691	frdA
frdA	ZONMW-33_03331	frdA
frdA	ZONMW-34_01163	frdA
frdA	ZONMW-34_01984	frdA
frdA	ZONMW-35_00236	frdA
frdA	ZONMW-35_01676	frdA
frdA	ZONMW-36_00178	frdA
frdA	ZONMW-36_02561	frdA
frdA	ZONMW-36_03068	frdA
frdA	ZONMW-37_00714	frdA
frdA	ZONMW-38_00183	frdA
frdA	ZONMW-38_02538	frdA
frdA	ZONMW-39_00842	frdA
frdA	ZONMW-39_02208	frdA
frdA	ZONMW-40_01260	frdA
frdA	ZONMW-41_00897	frdA
frdA	ZONMW-41_00464	frdA
frdA	ZONMW-42_03695	frdA
frdA	ZONMW-42_00435	frdA
frdA	ZONMW-43_03138	frdA
frdA	ZONMW-43_01488	frdA
frdA	ZONMW-44_03329	frdA
frdA	ZONMW-44_00862	frdA
frdA	ZONMW-45_00450	frdA
frdA	ZONMW-45_02303	frdA
frdA	ZONMW-46_01389	frdA
frdA	ZONMW-46_03112	frdA
frdA	ZONMW-47_01463	frdA
frdA	ZONMW-47_03104	frdA
frdA	ZONMW-48_00660	frdA
frdA	ZONMW-49_01166	frdA
frdA	ZONMW-50_01167	frdA
frdA	ZONMW-51_02772	frdA
frdA	ZONMW-52_02274	frdA
frdA	ZONMW-53_02863	frdA
frdA	ZONMW-54_00079	frdA
frdA	ZONMW-55_02869	frdA
frdA	ZONMW-56_02629	frdA
frdA	ZONMW-57_02773	frdA
frdA	ZONMW-58_02797	frdA
frdA	ZONMW-59_02931	frdA
frdA	ZONMW-60_02892	frdA
frdA	ZONMW-61_00374	frdA
frdA	ZONMW-62_01920	frdA

frdA	ZONMW-63_02300	frdA
frdA	ZONMW-64_02308	frdA
frdA	ZONMW-64_02065	frdA
frdA	ZONMW-65_00789	frdA
frdA	ZONMW-65_01759	frdA
frdA	ZONMW-66_03146	frdA
frdA	ZONMW-66_00128	frdA
frdA	ZONMW-67_01966	frdA
frdA	ZONMW-67_02383	frdA
frdA	ZONMW-68_00135	frdA
frdA	ZONMW-68_04748	frdA
frdA	ZONMW-69_01977	frdA
frdA	ZONMW-69_02431	frdA
frdA	ZONMW-72_01671	frdA
frdA	ZONMW-72_01005	frdA
frdA	ZONMW-73_01835	frdA
frdA	ZONMW-73_01724	frdA
frdA	ZONMW-74_01460	frdA
frdA	ZONMW-75_01078	frdA
frdA	ZONMW-75_01113	frdA
frdA	da5A-8_01908	frdA
frdA	da5A-8_01505	frdA
infB	18_00106	infB
infB	18_00966	infB
infB	18_00209	infB
infB	18_01864	infB
infB	18_01875	infB
infB	18_01545	infB
infB	18_01661	infB
infB	ACAM48_01504	infB
infB	ACAM48_02374	infB
infB	ACAM48_00302	infB
infB	ACAM48_03094	infB
infB	ACAM48_03107	infB
infB	ACAM48_02683	infB
infB	ACAM48_00073	infB
infB	ACAM48_00063	infB
infB	CAM030_02583	infB
infB	CAM030_01072	infB
infB	CAM030_02817	infB
infB	CAM030_02545	infB
infB	CAM030_02534	infB
infB	CAM030_02288	infB
infB	CAM030_01412	infB
infB	CECT7946_03664	infB
infB	CECT7946_00092	infB
infB	CECT7946_03335	infB
infB	CECT7946_01154	infB

infB	CECT7946_01166	infB
infB	CECT7946_01193	infB
infB	CECT7946_01715	infB
infB	CECT7946_00832	infB
infB	CGMCC1.8863_02870	infB
infB	CGMCC1.8863_03521	infB
infB	CGMCC1.8863_01411	infB
infB	CGMCC1.8863_01543	infB
infB	CGMCC1.8863_01532	infB
infB	CGMCC1.8863_01082	infB
infB	CGMCC1.8863_01291	infB
infB	CL8139_02084	infB
infB	CL8139_00766	infB
infB	CL8139_01452	infB
infB	CL8139_00311	infB
infB	CL8139_00322	infB
infB	CL8139_00161	infB
infB	CL8139_03294	infB
infB	DAU203_02389	infB
infB	DAU203_01405	infB
infB	DAU203_02264	infB
infB	DAU203_00553	infB
infB	DAU203_00542	infB
infB	DAU203_01972	infB
infB	DAU203_02092	infB
infB	DD5b_01388	infB
infB	DD5b_01970	infB
infB	DD5b_02797	infB
infB	DD5b_02422	infB
infB	DD5b_02807	infB
infB	DD5b_00386	infB
infB	DD5b_01805	infB
infB	DSM1208_02634	infB
infB	DSM1208_01617	infB
infB	DSM1208_02421	infB
infB	DSM1208_00624	infB
infB	DSM1208_00613	infB
infB	DSM1208_02273	infB
infB	DSM1208_00816	infB
infB	DSM12212_00989	infB
infB	DSM12212_00023	infB
infB	DSM12212_00007	infB
infB	DSM12212_01081	infB
infB	DSM12212_03222	infB
infB	DSM12212_03234	infB
infB	DSM12212_01746	infB
infB	DSM12212_00079	infB
infB	DSM15343_01253	infB

infB	DSM15343_02412	infB
infB	DSM15343_02189	infB
infB	DSM15343_01561	infB
infB	DSM15343_02203	infB
infB	DSM15343_02613	infB
infB	DSM15361_02462	infB
infB	DSM15361_00487	infB
infB	DSM15361_02596	infB
infB	DSM15361_02498	infB
infB	DSM15361_02509	infB
infB	DSM15361_01386	infB
infB	DSM15361_00539	infB
infB	DSM15365_03583	infB
infB	DSM15365_03117	infB
infB	DSM15365_01132	infB
infB	DSM15365_03620	infB
infB	DSM15365_00303	infB
infB	DSM15365_02228	infB
infB	DSM15365_00624	infB
infB	DSM15718_00396	infB
infB	DSM15718_02909	infB
infB	DSM15718_01060	infB
infB	DSM15718_00646	infB
infB	DSM15718_03342	infB
infB	DSM15718_01072	infB
infB	DSM15718_02152	infB
infB	DSM16505T_01405	infB
infB	DSM16505T_00850	infB
infB	DSM16505T_01229	infB
infB	DSM16505T_02492	infB
infB	DSM16505T_02119	infB
infB	DSM16505T_00704	infB
infB	DSM16505T_01657	infB
infB	DSM18103_00894	infB
infB	DSM18103_03156	infB
infB	DSM18103_02434	infB
infB	DSM18103_01889	infB
infB	DSM18103_03259	infB
infB	DSM18103_00620	infB
infB	DSM18103_01163	infB
infB	DSM18103_02906	infB
infB	DSM18293_01486	infB
infB	DSM18293_00760	infB
infB	DSM18293_02225	infB
infB	DSM18293_02412	infB
infB	DSM18293_02236	infB
infB	DSM18293_00719	infB
infB	DSM18293_00590	infB

infB	DSM18841_02419	infB
infB	DSM18841_00718	infB
infB	DSM18841_01162	infB
infB	DSM18841_02872	infB
infB	DSM18841_01483	infB
infB	DSM18841_00585	infB
infB	DSM18841_03127	infB
infB	DSM18842_01334	infB
infB	DSM18842_00603	infB
infB	DSM18842_02363	infB
infB	DSM18842_00142	infB
infB	DSM18842_02830	infB
infB	DSM18842_01898	infB
infB	DSM18842_02941	infB
infB	DSM18842_03146	infB
infB	DSM19866_00425	infB
infB	DSM19866_05021	infB
infB	DSM19866_01075	infB
infB	DSM19866_00614	infB
infB	DSM19866_00625	infB
infB	DSM19866_02751	infB
infB	DSM19866_02292	infB
infB	DSM2041_04997	infB
infB	DSM2041_00484	infB
infB	DSM2041_02980	infB
infB	DSM2041_05057	infB
infB	DSM2041_05069	infB
infB	DSM2041_04649	infB
infB	DSM2041_04244	infB
infB	DSM2061_04116	infB
infB	DSM2061_03155	infB
infB	DSM2061_03932	infB
infB	DSM2061_02120	infB
infB	DSM2061_02109	infB
infB	DSM2061_03802	infB
infB	DSM2061_02317	infB
infB	DSM2064_03741	infB
infB	DSM2064_01773	infB
infB	DSM2064_02968	infB
infB	DSM2064_04682	infB
infB	DSM2064_02980	infB
infB	DSM2064_02645	infB
infB	DSM2064_02516	infB
infB	DSM21164_02128	infB
infB	DSM21164_00884	infB
infB	DSM21164_02223	infB
infB	DSM21164_00018	infB
infB	DSM21164_00007	infB

infB	DSM21164_00330	infB
infB	DSM21164_00210	infB
infB	DSM23404_00066	infB
infB	DSM23404_03089	infB
infB	DSM23404_00488	infB
infB	DSM23404_00277	infB
infB	DSM23404_00264	infB
infB	DSM23404_02273	infB
infB	DSM23404_01418	infB
infB	DSM23404_01427	infB
infB	DSM23424_03162	infB
infB	DSM23424_00382	infB
infB	DSM23424_02889	infB
infB	DSM23424_03117	infB
infB	DSM23424_03103	infB
infB	DSM23424_00047	infB
infB	DSM23424_01081	infB
infB	DSM23424_02245	infB
infB	DSM24786_01624	infB
infB	DSM24786_02862	infB
infB	DSM24786_01767	infB
infB	DSM24786_01458	infB
infB	DSM24786_01469	infB
infB	DSM24786_02076	infB
infB	DSM24786_01953	infB
infB	DSM7489_02306	infB
infB	DSM7489_01317	infB
infB	DSM7489_02180	infB
infB	DSM7489_00540	infB
infB	DSM7489_00529	infB
infB	DSM7489_01883	infB
infB	DSM7489_02004	infB
infB	DSW1_01558	infB
infB	DSW1_00151	infB
infB	DSW1_01261	infB
infB	DSW1_02692	infB
infB	DSW1_01751	infB
infB	DSW1_01041	infB
infB	DSW1_01733	infB
infB	DSW5_02354	infB
infB	DSW5_00791	infB
infB	DSW5_00477	infB
infB	DSW5_00062	infB
infB	DSW5_02535	infB
infB	DSW5_02790	infB
infB	DSW5_01715	infB
infB	DSW6_00273	infB
infB	DSW6_01261	infB

infB	DSW6_02023	infB
infB	DSW6_00522	infB
infB	DSW6_02038	infB
infB	DSW6_01705	infB
infB	DSW6_02209	infB
infB	DSW8_01391	infB
infB	DSW8_02311	infB
infB	DSW8_01532	infB
infB	DSW8_03758	infB
infB	DSW8_03769	infB
infB	DSW8_01671	infB
infB	DSW8_03050	infB
infB	F52_03889	infB
infB	F52_02218	infB
infB	F52_03111	infB
infB	F52_01503	infB
infB	F52_03099	infB
infB	F52_03048	infB
infB	F52_02919	infB
infB	GCA_001027945.1_01928	infB
infB	GCA_001027945.1_03144	infB
infB	GCA_001027945.1_02736	infB
infB	GCA_001027945.1_02993	infB
infB	GCA_001027945.1_00649	infB
infB	GCA_001027945.1_02247	infB
infB	GCA_001027945.1_01280	infB
infB	GCA_001027945.1_02671	infB
infB	HI1_02239	infB
infB	HI1_01257	infB
infB	HI1_02106	infB
infB	HI1_00472	infB
infB	HI1_00461	infB
infB	HI1_01811	infB
infB	HI1_01931	infB
infB	IR1_01415	infB
infB	IR1_04070	infB
infB	IR1_00962	infB
infB	IR1_03075	infB
infB	IR1_00951	infB
infB	IR1_00242	infB
infB	IR1_00364	infB
infB	KCOM3131_00230	infB
infB	KCOM3131_01367	infB
infB	KCOM3131_00744	infB
infB	KCOM3131_01191	infB
infB	KCOM3131_00427	infB
infB	KCOM3131_01239	infB
infB	KCOM3131_01936	infB

infB	KCTC12974_00748	infB
infB	KCTC12974_01602	infB
infB	KCTC12974_01078	infB
infB	KCTC12974_02490	infB
infB	KCTC12974_00281	infB
infB	KCTC12974_02477	infB
infB	KCTC12974_01266	infB
infB	KCTC12974_00128	infB
infB	KCTC12974_00282	infB
infB	KL-A_01586	infB
infB	KL-A_00916	infB
infB	KL-A_00093	infB
infB	KL-A_02498	infB
infB	KL-A_03425	infB
infB	KL-A_00388	infB
infB	KL-A_00266	infB
infB	L12_00373	infB
infB	LL0412.1.7_02705	infB
infB	LL0412.1.7_00845	infB
infB	LL0412.1.7_02529	infB
infB	LL0412.1.7_01225	infB
infB	LL0412.1.7_01766	infB
infB	LL0412.1.7_01602	infB
infB	LL0412.1.7_00228	infB
infB	LL0412.1.7_02675	infB
infB	MAR_2009_71_02061	infB
infB	MAR_2009_71_01127	infB
infB	MAR_2009_71_01893	infB
infB	MAR_2009_71_00198	infB
infB	MAR_2009_71_00187	infB
infB	MAR_2009_71_01749	infB
infB	MAR_2009_71_00404	infB
infB	NN016038_00112	infB
infB	NN016038_00985	infB
infB	NN016038_00216	infB
infB	NN016038_01887	infB
infB	NN016038_01898	infB
infB	NN016038_01552	infB
infB	NN016038_01668	infB
infB	PAO1_05287	infB
infB	PAO1_00792	infB
infB	PAO1_04397	infB
infB	PAO1_02134	infB
infB	PAO1_04396	infB
infB	PAO1_04409	infB
infB	PAO1_04905	infB
infB	PAO1_04031	infB
infB	PAO1_04971	infB

infB	UW101_05145	infB
infB	UW101_00827	infB
infB	UW101_02022	infB
infB	UW101_00420	infB
infB	UW101_02034	infB
infB	UW101_01699	infB
infB	UW101_01570	infB
infB	V4-33_00451	infB
infB	V4-33_02163	infB
infB	V4-33_01249	infB
infB	V4-33_01093	infB
infB	V4-33_01082	infB
infB	V4-33_00450	infB
infB	W5C_02636	infB
infB	W5C_01698	infB
infB	W5C_02881	infB
infB	W5C_03268	infB
infB	W5C_03279	infB
infB	W5C_00569	infB
infB	W5C_00692	infB
infB	ZONMW-01_01269	infB
infB	ZONMW-01_00950	infB
infB	ZONMW-01_01409	infB
infB	ZONMW-01_02736	infB
infB	ZONMW-01_02725	infB
infB	ZONMW-01_00337	infB
infB	ZONMW-01_00456	infB
infB	ZONMW-02_03183	infB
infB	ZONMW-02_00026	infB
infB	ZONMW-02_02684	infB
infB	ZONMW-02_03770	infB
infB	ZONMW-02_02895	infB
infB	ZONMW-02_02444	infB
infB	ZONMW-02_01065	infB
infB	ZONMW-02_03559	infB
infB	ZONMW-03_01511	infB
infB	ZONMW-03_02019	infB
infB	ZONMW-03_01371	infB
infB	ZONMW-03_03170	infB
infB	ZONMW-03_03181	infB
infB	ZONMW-03_01063	infB
infB	ZONMW-03_01182	infB
infB	ZONMW-04_00179	infB
infB	ZONMW-04_01804	infB
infB	ZONMW-04_00321	infB
infB	ZONMW-04_02870	infB
infB	ZONMW-04_02881	infB
infB	ZONMW-04_00609	infB

infB	ZONMW-04_00492	infB
infB	ZONMW-05_01648	infB
infB	ZONMW-05_02079	infB
infB	ZONMW-05_01504	infB
infB	ZONMW-05_03127	infB
infB	ZONMW-05_03138	infB
infB	ZONMW-05_01216	infB
infB	ZONMW-05_01333	infB
infB	ZONMW-06_03681	infB
infB	ZONMW-06_02878	infB
infB	ZONMW-06_03616	infB
infB	ZONMW-06_03629	infB
infB	ZONMW-06_03628	infB
infB	ZONMW-06_03057	infB
infB	ZONMW-06_00317	infB
infB	ZONMW-06_00962	infB
infB	ZONMW-07_01061	infB
infB	ZONMW-07_02131	infB
infB	ZONMW-07_01564	infB
infB	ZONMW-07_02862	infB
infB	ZONMW-07_02873	infB
infB	ZONMW-07_00348	infB
infB	ZONMW-07_01535	infB
infB	ZONMW-08_01194	infB
infB	ZONMW-08_00649	infB
infB	ZONMW-08_01333	infB
infB	ZONMW-08_03405	infB
infB	ZONMW-08_03416	infB
infB	ZONMW-08_01476	infB
infB	ZONMW-08_03725	infB
infB	ZONMW-09_01953	infB
infB	ZONMW-09_00396	infB
infB	ZONMW-09_01352	infB
infB	ZONMW-09_01981	infB
infB	ZONMW-09_01992	infB
infB	ZONMW-09_01091	infB
infB	ZONMW-09_00018	infB
infB	ZONMW-10_01057	infB
infB	ZONMW-10_00208	infB
infB	ZONMW-10_01229	infB
infB	ZONMW-10_01380	infB
infB	ZONMW-10_01592	infB
infB	ZONMW-10_00657	infB
infB	ZONMW-11_01693	infB
infB	ZONMW-11_02039	infB
infB	ZONMW-11_03053	infB
infB	ZONMW-11_00199	infB
infB	ZONMW-11_03066	infB

infB	ZONMW-11_03614	infB
infB	ZONMW-11_03871	infB
infB	ZONMW-12_02054	infB
infB	ZONMW-12_00296	infB
infB	ZONMW-12_01421	infB
infB	ZONMW-12_03395	infB
infB	ZONMW-12_03409	infB
infB	ZONMW-12_01114	infB
infB	ZONMW-12_00664	infB
infB	ZONMW-13_01620	infB
infB	ZONMW-13_00393	infB
infB	ZONMW-13_01825	infB
infB	ZONMW-13_01541	infB
infB	ZONMW-13_01528	infB
infB	ZONMW-13_01002	infB
infB	ZONMW-13_01154	infB
infB	ZONMW-14_00851	infB
infB	ZONMW-14_00196	infB
infB	ZONMW-14_01061	infB
infB	ZONMW-14_02852	infB
infB	ZONMW-14_02260	infB
infB	ZONMW-14_02802	infB
infB	ZONMW-14_01439	infB
infB	ZONMW-14_02102	infB
infB	ZONMW-14_02771	infB
infB	ZONMW-15_01156	infB
infB	ZONMW-15_01633	infB
infB	ZONMW-15_00774	infB
infB	ZONMW-15_01107	infB
infB	ZONMW-15_01096	infB
infB	ZONMW-15_00400	infB
infB	ZONMW-15_01331	infB
infB	ZONMW-19_01004	infB
infB	ZONMW-19_00377	infB
infB	ZONMW-19_01628	infB
infB	ZONMW-19_01616	infB
infB	ZONMW-19_01629	infB
infB	ZONMW-19_03573	infB
infB	ZONMW-19_00669	infB
infB	ZONMW-19_01381	infB
infB	ZONMW-20_03654	infB
infB	ZONMW-20_02968	infB
infB	ZONMW-20_00570	infB
infB	ZONMW-20_03444	infB
infB	ZONMW-20_01082	infB
infB	ZONMW-20_00312	infB
infB	ZONMW-20_02883	infB
infB	ZONMW-20_03873	infB

infB	ZONMW-21_02359	infB
infB	ZONMW-21_00851	infB
infB	ZONMW-21_02469	infB
infB	ZONMW-21_03021	infB
infB	ZONMW-21_03008	infB
infB	ZONMW-21_00201	infB
infB	ZONMW-21_00074	infB
infB	ZONMW-22_02749	infB
infB	ZONMW-22_00852	infB
infB	ZONMW-22_02640	infB
infB	ZONMW-22_03046	infB
infB	ZONMW-22_03033	infB
infB	ZONMW-22_00201	infB
infB	ZONMW-22_00074	infB
infB	ZONMW-23_02773	infB
infB	ZONMW-23_01966	infB
infB	ZONMW-23_02664	infB
infB	ZONMW-23_03067	infB
infB	ZONMW-23_03054	infB
infB	ZONMW-23_01316	infB
infB	ZONMW-23_01189	infB
infB	ZONMW-24_02383	infB
infB	ZONMW-24_00609	infB
infB	ZONMW-24_02493	infB
infB	ZONMW-24_03068	infB
infB	ZONMW-24_03055	infB
infB	ZONMW-24_01259	infB
infB	ZONMW-24_01386	infB
infB	ZONMW-25_02635	infB
infB	ZONMW-25_01780	infB
infB	ZONMW-25_03457	infB
infB	ZONMW-25_01798	infB
infB	ZONMW-25_01786	infB
infB	ZONMW-25_03493	infB
infB	ZONMW-25_01718	infB
infB	ZONMW-26_01632	infB
infB	ZONMW-26_02318	infB
infB	ZONMW-26_01766	infB
infB	ZONMW-26_03194	infB
infB	ZONMW-26_03205	infB
infB	ZONMW-26_01270	infB
infB	ZONMW-26_01387	infB
infB	ZONMW-27_00175	infB
infB	ZONMW-27_01493	infB
infB	ZONMW-27_00315	infB
infB	ZONMW-27_03216	infB
infB	ZONMW-27_03227	infB
infB	ZONMW-27_00603	infB

infB	ZONMW-27_00486	infB
infB	ZONMW-28_03653	infB
infB	ZONMW-28_02028	infB
infB	ZONMW-28_03599	infB
infB	ZONMW-28_03588	infB
infB	ZONMW-28_03600	infB
infB	ZONMW-28_03371	infB
infB	ZONMW-28_01547	infB
infB	ZONMW-28_00562	infB
infB	ZONMW-29_03278	infB
infB	ZONMW-29_00495	infB
infB	ZONMW-29_02771	infB
infB	ZONMW-29_02664	infB
infB	ZONMW-29_02676	infB
infB	ZONMW-29_03002	infB
infB	ZONMW-29_02474	infB
infB	ZONMW-30_00252	infB
infB	ZONMW-30_01895	infB
infB	ZONMW-30_00076	infB
infB	ZONMW-30_01036	infB
infB	ZONMW-30_01048	infB
infB	ZONMW-30_00716	infB
infB	ZONMW-30_00841	infB
infB	ZONMW-31_03133	infB
infB	ZONMW-31_02986	infB
infB	ZONMW-31_03068	infB
infB	ZONMW-31_03081	infB
infB	ZONMW-31_03080	infB
infB	ZONMW-31_03640	infB
infB	ZONMW-31_00631	infB
infB	ZONMW-31_01321	infB
infB	ZONMW-31_00116	infB
infB	ZONMW-31_01217	infB
infB	ZONMW-32_03681	infB
infB	ZONMW-32_02434	infB
infB	ZONMW-32_03627	infB
infB	ZONMW-32_03616	infB
infB	ZONMW-32_03628	infB
infB	ZONMW-32_03496	infB
infB	ZONMW-32_01954	infB
infB	ZONMW-32_00530	infB
infB	ZONMW-33_03540	infB
infB	ZONMW-33_02684	infB
infB	ZONMW-33_03594	infB
infB	ZONMW-33_03593	infB
infB	ZONMW-33_03605	infB
infB	ZONMW-33_03499	infB
infB	ZONMW-33_01945	infB

infB	ZONMW-33_00398	infB
infB	ZONMW-34_03550	infB
infB	ZONMW-34_01977	infB
infB	ZONMW-34_03497	infB
infB	ZONMW-34_03485	infB
infB	ZONMW-34_03498	infB
infB	ZONMW-34_03706	infB
infB	ZONMW-34_00280	infB
infB	ZONMW-34_00775	infB
infB	ZONMW-35_03511	infB
infB	ZONMW-35_01669	infB
infB	ZONMW-35_03458	infB
infB	ZONMW-35_03446	infB
infB	ZONMW-35_03459	infB
infB	ZONMW-35_03669	infB
infB	ZONMW-35_01110	infB
infB	ZONMW-35_02688	infB
infB	ZONMW-36_03438	infB
infB	ZONMW-36_02568	infB
infB	ZONMW-36_03384	infB
infB	ZONMW-36_03373	infB
infB	ZONMW-36_03385	infB
infB	ZONMW-36_03356	infB
infB	ZONMW-36_01411	infB
infB	ZONMW-36_00973	infB
infB	ZONMW-37_02629	infB
infB	ZONMW-37_00851	infB
infB	ZONMW-37_02739	infB
infB	ZONMW-37_03055	infB
infB	ZONMW-37_03042	infB
infB	ZONMW-37_00200	infB
infB	ZONMW-37_00073	infB
infB	ZONMW-38_01950	infB
infB	ZONMW-38_01373	infB
infB	ZONMW-38_01918	infB
infB	ZONMW-38_01917	infB
infB	ZONMW-38_00867	infB
infB	ZONMW-39_03828	infB
infB	ZONMW-39_02215	infB
infB	ZONMW-39_03882	infB
infB	ZONMW-39_03881	infB
infB	ZONMW-39_03894	infB
infB	ZONMW-39_03570	infB
infB	ZONMW-39_04017	infB
infB	ZONMW-39_01239	infB
infB	ZONMW-40_03110	infB
infB	ZONMW-40_00354	infB
infB	ZONMW-40_02164	infB

infB	ZONMW-40_01161	infB
infB	ZONMW-40_01174	infB
infB	ZONMW-40_02613	infB
infB	ZONMW-40_00414	infB
infB	ZONMW-41_01842	infB
infB	ZONMW-41_00469	infB
infB	ZONMW-41_01942	infB
infB	ZONMW-41_01941	infB
infB	ZONMW-41_01953	infB
infB	ZONMW-41_01936	infB
infB	ZONMW-41_02885	infB
infB	ZONMW-41_02533	infB
infB	ZONMW-41_03192	infB
infB	ZONMW-42_02276	infB
infB	ZONMW-42_00442	infB
infB	ZONMW-42_02210	infB
infB	ZONMW-42_02223	infB
infB	ZONMW-42_02222	infB
infB	ZONMW-42_04223	infB
infB	ZONMW-42_03601	infB
infB	ZONMW-42_02983	infB
infB	ZONMW-43_03650	infB
infB	ZONMW-43_01481	infB
infB	ZONMW-43_03703	infB
infB	ZONMW-43_03702	infB
infB	ZONMW-43_03715	infB
infB	ZONMW-43_02760	infB
infB	ZONMW-43_00672	infB
infB	ZONMW-43_00314	infB
infB	ZONMW-44_03219	infB
infB	ZONMW-44_00855	infB
infB	ZONMW-44_03271	infB
infB	ZONMW-44_03284	infB
infB	ZONMW-44_03272	infB
infB	ZONMW-44_01043	infB
infB	ZONMW-44_03507	infB
infB	ZONMW-44_02159	infB
infB	ZONMW-45_01831	infB
infB	ZONMW-45_01076	infB
infB	ZONMW-45_00016	infB
infB	ZONMW-45_01769	infB
infB	ZONMW-45_02565	infB
infB	ZONMW-45_01327	infB
infB	ZONMW-45_00733	infB
infB	ZONMW-45_02289	infB
infB	ZONMW-46_01931	infB
infB	ZONMW-46_00591	infB
infB	ZONMW-46_00231	infB

infB	ZONMW-46_00028	infB
infB	ZONMW-46_02787	infB
infB	ZONMW-46_02711	infB
infB	ZONMW-46_00726	infB
infB	ZONMW-46_03126	infB
infB	ZONMW-47_02130	infB
infB	ZONMW-47_00591	infB
infB	ZONMW-47_00274	infB
infB	ZONMW-47_00477	infB
infB	ZONMW-47_02778	infB
infB	ZONMW-47_02701	infB
infB	ZONMW-47_00726	infB
infB	ZONMW-47_03118	infB
infB	ZONMW-48_02537	infB
infB	ZONMW-48_00023	infB
infB	ZONMW-48_02028	infB
infB	ZONMW-48_02586	infB
infB	ZONMW-48_02597	infB
infB	ZONMW-48_01037	infB
infB	ZONMW-48_02365	infB
infB	ZONMW-49_00604	infB
infB	ZONMW-49_02622	infB
infB	ZONMW-49_00732	infB
infB	ZONMW-49_02800	infB
infB	ZONMW-49_02811	infB
infB	ZONMW-49_01425	infB
infB	ZONMW-49_01546	infB
infB	ZONMW-50_00605	infB
infB	ZONMW-50_02416	infB
infB	ZONMW-50_00733	infB
infB	ZONMW-50_02726	infB
infB	ZONMW-50_02737	infB
infB	ZONMW-50_01427	infB
infB	ZONMW-50_01548	infB
infB	ZONMW-51_01101	infB
infB	ZONMW-51_02678	infB
infB	ZONMW-51_00975	infB
infB	ZONMW-51_02975	infB
infB	ZONMW-51_02964	infB
infB	ZONMW-51_00681	infB
infB	ZONMW-51_00801	infB
infB	ZONMW-52_02794	infB
infB	ZONMW-52_02149	infB
infB	ZONMW-52_01575	infB
infB	ZONMW-52_03099	infB
infB	ZONMW-52_03110	infB
infB	ZONMW-52_01281	infB
infB	ZONMW-52_01401	infB

infB	ZONMW-53_00121	infB
infB	ZONMW-53_02690	infB
infB	ZONMW-53_00250	infB
infB	ZONMW-53_03037	infB
infB	ZONMW-53_03026	infB
infB	ZONMW-53_00544	infB
infB	ZONMW-53_00424	infB
infB	ZONMW-54_00906	infB
infB	ZONMW-54_02829	infB
infB	ZONMW-54_00780	infB
infB	ZONMW-54_03039	infB
infB	ZONMW-54_03028	infB
infB	ZONMW-54_00491	infB
infB	ZONMW-54_00610	infB
infB	ZONMW-55_00119	infB
infB	ZONMW-55_02690	infB
infB	ZONMW-55_00245	infB
infB	ZONMW-55_01621	infB
infB	ZONMW-55_01632	infB
infB	ZONMW-55_00539	infB
infB	ZONMW-55_00419	infB
infB	ZONMW-56_01200	infB
infB	ZONMW-56_02511	infB
infB	ZONMW-56_01073	infB
infB	ZONMW-56_02923	infB
infB	ZONMW-56_02912	infB
infB	ZONMW-56_00774	infB
infB	ZONMW-56_00896	infB
infB	ZONMW-57_01494	infB
infB	ZONMW-57_02579	infB
infB	ZONMW-57_01372	infB
infB	ZONMW-57_03124	infB
infB	ZONMW-57_03135	infB
infB	ZONMW-57_01075	infB
infB	ZONMW-57_01196	infB
infB	ZONMW-58_01090	infB
infB	ZONMW-58_02604	infB
infB	ZONMW-58_01212	infB
infB	ZONMW-58_03145	infB
infB	ZONMW-58_03156	infB
infB	ZONMW-58_01509	infB
infB	ZONMW-58_01388	infB
infB	ZONMW-59_02838	infB
infB	ZONMW-59_02627	infB
infB	ZONMW-59_01374	infB
infB	ZONMW-59_03199	infB
infB	ZONMW-59_03210	infB
infB	ZONMW-59_01077	infB

infB	ZONMW-59_01197	infB
infB	ZONMW-60_01068	infB
infB	ZONMW-60_02779	infB
infB	ZONMW-60_01201	infB
infB	ZONMW-60_03135	infB
infB	ZONMW-60_03146	infB
infB	ZONMW-60_01490	infB
infB	ZONMW-60_01371	infB
infB	ZONMW-61_01216	infB
infB	ZONMW-61_00227	infB
infB	ZONMW-61_01322	infB
infB	ZONMW-61_03758	infB
infB	ZONMW-61_03769	infB
infB	ZONMW-61_02911	infB
infB	ZONMW-61_03454	infB
infB	ZONMW-62_02966	infB
infB	ZONMW-62_01786	infB
infB	ZONMW-62_02181	infB
infB	ZONMW-62_03802	infB
infB	ZONMW-62_03813	infB
infB	ZONMW-62_02318	infB
infB	ZONMW-62_03592	infB
infB	ZONMW-63_01064	infB
infB	ZONMW-63_02140	infB
infB	ZONMW-63_01193	infB
infB	ZONMW-63_02890	infB
infB	ZONMW-63_02879	infB
infB	ZONMW-63_00831	infB
infB	ZONMW-63_03348	infB
infB	ZONMW-64_04000	infB
infB	ZONMW-64_02058	infB
infB	ZONMW-64_04209	infB
infB	ZONMW-64_03691	infB
infB	ZONMW-64_00934	infB
infB	ZONMW-64_00331	infB
infB	ZONMW-64_03948	infB
infB	ZONMW-64_04221	infB
infB	ZONMW-65_00131	infB
infB	ZONMW-65_01764	infB
infB	ZONMW-65_01816	infB
infB	ZONMW-65_03025	infB
infB	ZONMW-65_02539	infB
infB	ZONMW-65_01071	infB
infB	ZONMW-65_01128	infB
infB	ZONMW-66_04270	infB
infB	ZONMW-66_00134	infB
infB	ZONMW-66_01157	infB
infB	ZONMW-66_01145	infB

infB	ZONMW-66_01158	infB
infB	ZONMW-66_04812	infB
infB	ZONMW-66_00472	infB
infB	ZONMW-66_01338	infB
infB	ZONMW-67_03526	infB
infB	ZONMW-67_02376	infB
infB	ZONMW-67_03473	infB
infB	ZONMW-67_03461	infB
infB	ZONMW-67_03474	infB
infB	ZONMW-67_02558	infB
infB	ZONMW-67_02691	infB
infB	ZONMW-67_01079	infB
infB	ZONMW-67_00998	infB
infB	ZONMW-68_03327	infB
infB	ZONMW-68_04704	infB
infB	ZONMW-68_04464	infB
infB	ZONMW-68_03526	infB
infB	ZONMW-68_03515	infB
infB	ZONMW-68_03203	infB
infB	ZONMW-68_01538	infB
infB	ZONMW-69_03528	infB
infB	ZONMW-69_02424	infB
infB	ZONMW-69_03475	infB
infB	ZONMW-69_03463	infB
infB	ZONMW-69_03476	infB
infB	ZONMW-69_02606	infB
infB	ZONMW-69_00488	infB
infB	ZONMW-69_01126	infB
infB	ZONMW-69_01045	infB
infB	ZONMW-72_01921	infB
infB	ZONMW-72_02781	infB
infB	ZONMW-72_02375	infB
infB	ZONMW-72_01145	infB
infB	ZONMW-72_02364	infB
infB	ZONMW-72_00441	infB
infB	ZONMW-72_00016	infB
infB	ZONMW-73_03863	infB
infB	ZONMW-73_02199	infB
infB	ZONMW-73_02119	infB
infB	ZONMW-73_01881	infB
infB	ZONMW-73_03121	infB
infB	ZONMW-73_01341	infB
infB	ZONMW-73_02309	infB
infB	ZONMW-74_00871	infB
infB	ZONMW-74_01588	infB
infB	ZONMW-74_01025	infB
infB	ZONMW-74_02443	infB
infB	ZONMW-74_02432	infB

infB	ZONMW-74_01820	infB
infB	ZONMW-74_01214	infB
infB	ZONMW-75_03639	infB
infB	ZONMW-75_02400	infB
infB	ZONMW-75_02942	infB
infB	ZONMW-75_03797	infB
infB	ZONMW-75_03785	infB
infB	ZONMW-75_00236	infB
infB	ZONMW-75_00610	infB
infB	da5A-8_01348	infB
infB	da5A-8_01762	infB
infB	da5A-8_00436	infB
infB	da5A-8_00878	infB
infB	da5A-8_00618	infB
infB	da5A-8_03570	infB
infB	da5A-8_01549	infB
infB	da5A-8_02588	infB
acsA	18_00120	acsA
acsA	18_02625	acsA
acsA	18_03704	acsA
acsA	ACAM48_00972	acsA
acsA	ACAM48_01077	acsA
acsA	CAM030_01979	acsA
acsA	CAM030_01978	acsA
acsA	CAM030_00200	acsA
acsA	CECT7946_02355	acsA
acsA	CECT7946_02356	acsA
acsA	CECT7946_00298	acsA
acsA	CECT7946_03112	acsA
acsA	CECT7946_03554	acsA
acsA	CGMCC1.8863_01176	acsA
acsA	CGMCC1.8863_01736	acsA
acsA	CL8139_03229	acsA
acsA	CL8139_02645	acsA
acsA	DAU203_02027	acsA
acsA	DAU203_02721	acsA
acsA	DD5b_01687	acsA
acsA	DD5b_01373	acsA
acsA	DSM1208_03567	acsA
acsA	DSM1208_00070	acsA
acsA	DSM1208_00093	acsA
acsA	DSM12212_00860	acsA
acsA	DSM12212_00451	acsA
acsA	DSM15343_00233	acsA
acsA	DSM15343_00232	acsA
acsA	DSM15343_00221	acsA
acsA	DSM15343_02225	acsA
acsA	DSM15343_01168	acsA

acsA	DSM15361_02355	acsA
acsA	DSM15361_01858	acsA
acsA	DSM15365_01297	acsA
acsA	DSM15365_03690	acsA
acsA	DSM15365_01534	acsA
acsA	DSM15718_02236	acsA
acsA	DSM15718_00860	acsA
acsA	DSM15718_02235	acsA
acsA	DSM15718_02225	acsA
acsA	DSM15718_01478	acsA
acsA	DSM15718_01872	acsA
acsA	DSM16505T_01849	acsA
acsA	DSM16505T_00748	acsA
acsA	DSM16505T_01365	acsA
acsA	DSM16505T_01366	acsA
acsA	DSM18103_01473	acsA
acsA	DSM18103_03525	acsA
acsA	DSM18103_02969	acsA
acsA	DSM18103_03610	acsA
acsA	DSM18103_00063	acsA
acsA	DSM18103_00064	acsA
acsA	DSM18103_00257	acsA
acsA	DSM18103_02967	acsA
acsA	DSM18293_01784	acsA
acsA	DSM18293_01228	acsA
acsA	DSM18841_01585	acsA
acsA	DSM18841_01292	acsA
acsA	DSM18841_02374	acsA
acsA	DSM18841_02375	acsA
acsA	DSM18841_01299	acsA
acsA	DSM18841_01306	acsA
acsA	DSM18841_01301	acsA
acsA	DSM18841_01294	acsA
acsA	DSM18841_01295	acsA
acsA	DSM18842_02269	acsA
acsA	DSM18842_02984	acsA
acsA	DSM18842_01281	acsA
acsA	DSM18842_01282	acsA
acsA	DSM19866_04572	acsA
acsA	DSM19866_01002	acsA
acsA	DSM19866_04074	acsA
acsA	DSM19866_01694	acsA
acsA	DSM19866_04077	acsA
acsA	DSM19866_04080	acsA
acsA	DSM19866_04079	acsA
acsA	DSM2041_05248	acsA
acsA	DSM2041_01496	acsA
acsA	DSM2041_03123	acsA

acsA	DSM2041_03124	acsA
acsA	DSM2041_03119	acsA
acsA	DSM2041_03117	acsA
acsA	DSM2041_02444	acsA
acsA	DSM2041_03115	acsA
acsA	DSM2041_03122	acsA
acsA	DSM2041_01940	acsA
acsA	DSM2041_03120	acsA
acsA	DSM2061_01497	acsA
acsA	DSM2061_01415	acsA
acsA	DSM2061_00482	acsA
acsA	DSM2061_01413	acsA
acsA	DSM2061_01417	acsA
acsA	DSM2061_00457	acsA
acsA	DSM2064_00279	acsA
acsA	DSM2064_04582	acsA
acsA	DSM2064_04580	acsA
acsA	DSM2064_04576	acsA
acsA	DSM2064_03962	acsA
acsA	DSM2064_04589	acsA
acsA	DSM2064_04584	acsA
acsA	DSM2064_04575	acsA
acsA	DSM2064_00606	acsA
acsA	DSM2064_04577	acsA
acsA	DSM2064_04579	acsA
acsA	DSM21164_01938	acsA
acsA	DSM21164_02417	acsA
acsA	DSM23404_02907	acsA
acsA	DSM23404_02805	acsA
acsA	DSM23404_01026	acsA
acsA	DSM23424_01559	acsA
acsA	DSM23424_01151	acsA
acsA	DSM23424_01466	acsA
acsA	DSM24786_02023	acsA
acsA	DSM24786_00180	acsA
acsA	DSM7489_01938	acsA
acsA	DSM7489_02595	acsA
acsA	DSW1_00378	acsA
acsA	DSW1_02098	acsA
acsA	DSW5_00323	acsA
acsA	DSW5_01890	acsA
acsA	DSW5_01891	acsA
acsA	DSW6_00461	acsA
acsA	DSW6_00460	acsA
acsA	DSW6_00162	acsA
acsA	DSW6_00160	acsA
acsA	DSW6_00161	acsA
acsA	DSW6_02932	acsA

acsA	DSW6_00152	acsA
acsA	DSW6_00154	acsA
acsA	DSW6_00155	acsA
acsA	DSW6_00151	acsA
acsA	DSW6_00157	acsA
acsA	DSW6_00153	acsA
acsA	DSW6_00156	acsA
acsA	DSW8_00938	acsA
acsA	DSW8_00649	acsA
acsA	DSW8_00674	acsA
acsA	DSW8_01832	acsA
acsA	F52_01396	acsA
acsA	F52_04125	acsA
acsA	GCA_001027945.1_02873	acsA
acsA	GCA_001027945.1_00014	acsA
acsA	GCA_001027945.1_00013	acsA
acsA	HI1_01866	acsA
acsA	HI1_02528	acsA
acsA	IR1_03466	acsA
acsA	IR1_03967	acsA
acsA	IR1_03230	acsA
acsA	IR1_03234	acsA
acsA	IR1_04277	acsA
acsA	IR1_03242	acsA
acsA	IR1_03237	acsA
acsA	IR1_03233	acsA
acsA	KCOM3131_00007	acsA
acsA	KCOM3131_01781	acsA
acsA	KCOM3131_00606	acsA
acsA	KCTC12974_02152	acsA
acsA	KCTC12974_02399	acsA
acsA	KCTC12974_01538	acsA
acsA	KCTC12974_02398	acsA
acsA	KL-A_00333	acsA
acsA	KL-A_00960	acsA
acsA	L12_00227	acsA
acsA	LL0412.1.7_02631	acsA
acsA	LL0412.1.7_02308	acsA
acsA	LL0412.1.7_02307	acsA
acsA	MAR_2009_71_02503	acsA
acsA	MAR_2009_71_03446	acsA
acsA	MAR_2009_71_03470	acsA
acsA	MAR_2009_71_01589	acsA
acsA	NN016038_00124	acsA
acsA	NN016038_02675	acsA
acsA	NN016038_03745	acsA
acsA	PAO1_04894	acsA
acsA	PAO1_00914	acsA

acsA	PAO1_03691	acsA
acsA	PAO1_02627	acsA
acsA	PAO1_02059	acsA
acsA	PAO1_01028	acsA
acsA	PAO1_03985	acsA
acsA	PAO1_04329	acsA
acsA	PAO1_03415	acsA
acsA	PAO1_02629	acsA
acsA	PAO1_03414	acsA
acsA	PAO1_04359	acsA
acsA	PAO1_04052	acsA
acsA	PAO1_02495	acsA
acsA	PAO1_02377	acsA
acsA	PAO1_02473	acsA
acsA	PAO1_02472	acsA
acsA	PAO1_01661	acsA
acsA	PAO1_02471	acsA
acsA	PAO1_04356	acsA
acsA	PAO1_04207	acsA
acsA	PAO1_03445	acsA
acsA	PAO1_01252	acsA
acsA	PAO1_04357	acsA
acsA	PAO1_01258	acsA
acsA	PAO1_02995	acsA
acsA	PAO1_03447	acsA
acsA	UW101_02990	acsA
acsA	UW101_02195	acsA
acsA	UW101_02193	acsA
acsA	UW101_02189	acsA
acsA	UW101_04925	acsA
acsA	UW101_02202	acsA
acsA	UW101_02197	acsA
acsA	UW101_02188	acsA
acsA	UW101_03317	acsA
acsA	UW101_02190	acsA
acsA	UW101_02192	acsA
acsA	V4-33_00854	acsA
acsA	V4-33_01628	acsA
acsA	W5C_00625	acsA
acsA	W5C_01293	acsA
acsA	ZONMW-01_00391	acsA
acsA	ZONMW-01_01644	acsA
acsA	ZONMW-02_03755	acsA
acsA	ZONMW-02_01506	acsA
acsA	ZONMW-02_01997	acsA
acsA	ZONMW-03_01117	acsA
acsA	ZONMW-03_00563	acsA
acsA	ZONMW-04_00555	acsA

acsA	ZONMW-04_01501	acsA
acsA	ZONMW-05_01270	acsA
acsA	ZONMW-05_00825	acsA
acsA	ZONMW-06_02849	acsA
acsA	ZONMW-06_03129	acsA
acsA	ZONMW-06_02731	acsA
acsA	ZONMW-06_01369	acsA
acsA	ZONMW-06_01691	acsA
acsA	ZONMW-06_02085	acsA
acsA	ZONMW-06_03448	acsA
acsA	ZONMW-06_02659	acsA
acsA	ZONMW-06_01493	acsA
acsA	ZONMW-06_00836	acsA
acsA	ZONMW-06_00887	acsA
acsA	ZONMW-06_02049	acsA
acsA	ZONMW-06_01641	acsA
acsA	ZONMW-06_02522	acsA
acsA	ZONMW-06_00792	acsA
acsA	ZONMW-06_01539	acsA
acsA	ZONMW-07_02736	acsA
acsA	ZONMW-07_03174	acsA
acsA	ZONMW-08_03502	acsA
acsA	ZONMW-08_02481	acsA
acsA	ZONMW-08_02457	acsA
acsA	ZONMW-08_01643	acsA
acsA	ZONMW-09_00146	acsA
acsA	ZONMW-09_00844	acsA
acsA	ZONMW-10_00710	acsA
acsA	ZONMW-10_00711	acsA
acsA	ZONMW-10_02003	acsA
acsA	ZONMW-10_02463	acsA
acsA	ZONMW-11_02683	acsA
acsA	ZONMW-11_03731	acsA
acsA	ZONMW-12_03744	acsA
acsA	ZONMW-12_01486	acsA
acsA	ZONMW-13_03106	acsA
acsA	ZONMW-13_00963	acsA
acsA	ZONMW-13_02241	acsA
acsA	ZONMW-14_01948	acsA
acsA	ZONMW-14_01145	acsA
acsA	ZONMW-14_02159	acsA
acsA	ZONMW-15_03233	acsA
acsA	ZONMW-15_03222	acsA
acsA	ZONMW-15_02853	acsA
acsA	ZONMW-19_01730	acsA
acsA	ZONMW-19_01189	acsA
acsA	ZONMW-19_02488	acsA
acsA	ZONMW-19_01478	acsA

acsA	ZONMW-19_02564	acsA
acsA	ZONMW-19_02447	acsA
acsA	ZONMW-19_00487	acsA
acsA	ZONMW-19_01558	acsA
acsA	ZONMW-19_03398	acsA
acsA	ZONMW-19_02562	acsA
acsA	ZONMW-19_00900	acsA
acsA	ZONMW-19_00969	acsA
acsA	ZONMW-19_02890	acsA
acsA	ZONMW-19_01109	acsA
acsA	ZONMW-19_00968	acsA
acsA	ZONMW-20_03727	acsA
acsA	ZONMW-20_01802	acsA
acsA	ZONMW-20_00251	acsA
acsA	ZONMW-20_02248	acsA
acsA	ZONMW-20_02241	acsA
acsA	ZONMW-21_02158	acsA
acsA	ZONMW-21_01543	acsA
acsA	ZONMW-22_01567	acsA
acsA	ZONMW-22_02180	acsA
acsA	ZONMW-23_00984	acsA
acsA	ZONMW-23_00371	acsA
acsA	ZONMW-24_01591	acsA
acsA	ZONMW-24_02805	acsA
acsA	ZONMW-25_00377	acsA
acsA	ZONMW-25_02735	acsA
acsA	ZONMW-26_01324	acsA
acsA	ZONMW-26_00135	acsA
acsA	ZONMW-27_00549	acsA
acsA	ZONMW-27_01803	acsA
acsA	ZONMW-28_02060	acsA
acsA	ZONMW-28_02938	acsA
acsA	ZONMW-28_02407	acsA
acsA	ZONMW-28_01677	acsA
acsA	ZONMW-28_00499	acsA
acsA	ZONMW-28_02856	acsA
acsA	ZONMW-28_03994	acsA
acsA	ZONMW-28_02834	acsA
acsA	ZONMW-28_00864	acsA
acsA	ZONMW-28_00682	acsA
acsA	ZONMW-28_00110	acsA
acsA	ZONMW-28_00710	acsA
acsA	ZONMW-28_01188	acsA
acsA	ZONMW-28_00533	acsA
acsA	ZONMW-28_03773	acsA
acsA	ZONMW-28_03772	acsA
acsA	ZONMW-28_00637	acsA
acsA	ZONMW-29_01365	acsA

acsA	ZONMW-29_01557	acsA
acsA	ZONMW-30_02675	acsA
acsA	ZONMW-30_02073	acsA
acsA	ZONMW-31_02958	acsA
acsA	ZONMW-31_01599	acsA
acsA	ZONMW-31_02545	acsA
acsA	ZONMW-31_03414	acsA
acsA	ZONMW-31_00091	acsA
acsA	ZONMW-31_01453	acsA
acsA	ZONMW-31_03732	acsA
acsA	ZONMW-31_02500	acsA
acsA	ZONMW-31_02465	acsA
acsA	ZONMW-31_02112	acsA
acsA	ZONMW-31_03739	acsA
acsA	ZONMW-31_01092	acsA
acsA	ZONMW-31_02768	acsA
acsA	ZONMW-31_02046	acsA
acsA	ZONMW-31_02098	acsA
acsA	ZONMW-31_01061	acsA
acsA	ZONMW-31_02226	acsA
acsA	ZONMW-31_01485	acsA
acsA	ZONMW-31_01148	acsA
acsA	ZONMW-31_02509	acsA
acsA	ZONMW-32_02465	acsA
acsA	ZONMW-32_01422	acsA
acsA	ZONMW-32_02943	acsA
acsA	ZONMW-32_02295	acsA
acsA	ZONMW-32_01290	acsA
acsA	ZONMW-32_03019	acsA
acsA	ZONMW-32_03851	acsA
acsA	ZONMW-32_03041	acsA
acsA	ZONMW-32_00227	acsA
acsA	ZONMW-32_00409	acsA
acsA	ZONMW-32_00901	acsA
acsA	ZONMW-32_00381	acsA
acsA	ZONMW-32_01596	acsA
acsA	ZONMW-32_01324	acsA
acsA	ZONMW-32_03892	acsA
acsA	ZONMW-32_03893	acsA
acsA	ZONMW-32_00454	acsA
acsA	ZONMW-33_02653	acsA
acsA	ZONMW-33_01412	acsA
acsA	ZONMW-33_02805	acsA
acsA	ZONMW-33_02286	acsA
acsA	ZONMW-33_01279	acsA
acsA	ZONMW-33_03254	acsA
acsA	ZONMW-33_03653	acsA
acsA	ZONMW-33_03232	acsA

acsA	ZONMW-33_00094	acsA
acsA	ZONMW-33_00276	acsA
acsA	ZONMW-33_00891	acsA
acsA	ZONMW-33_00248	acsA
acsA	ZONMW-33_01586	acsA
acsA	ZONMW-33_01313	acsA
acsA	ZONMW-33_03697	acsA
acsA	ZONMW-33_03698	acsA
acsA	ZONMW-33_00321	acsA
acsA	ZONMW-34_01947	acsA
acsA	ZONMW-34_01600	acsA
acsA	ZONMW-34_03287	acsA
acsA	ZONMW-34_03179	acsA
acsA	ZONMW-34_01471	acsA
acsA	ZONMW-34_03233	acsA
acsA	ZONMW-34_02991	acsA
acsA	ZONMW-34_02984	acsA
acsA	ZONMW-34_01765	acsA
acsA	ZONMW-34_02429	acsA
acsA	ZONMW-34_03876	acsA
acsA	ZONMW-34_03210	acsA
acsA	ZONMW-34_03887	acsA
acsA	ZONMW-34_03890	acsA
acsA	ZONMW-34_00654	acsA
acsA	ZONMW-34_03235	acsA
acsA	ZONMW-34_03195	acsA
acsA	ZONMW-34_02794	acsA
acsA	ZONMW-34_00623	acsA
acsA	ZONMW-34_02838	acsA
acsA	ZONMW-34_01505	acsA
acsA	ZONMW-34_00699	acsA
acsA	ZONMW-34_02837	acsA
acsA	ZONMW-35_01639	acsA
acsA	ZONMW-35_00671	acsA
acsA	ZONMW-35_03248	acsA
acsA	ZONMW-35_03058	acsA
acsA	ZONMW-35_00544	acsA
acsA	ZONMW-35_03194	acsA
acsA	ZONMW-35_03001	acsA
acsA	ZONMW-35_03008	acsA
acsA	ZONMW-35_01456	acsA
acsA	ZONMW-35_03890	acsA
acsA	ZONMW-35_03171	acsA
acsA	ZONMW-35_03879	acsA
acsA	ZONMW-35_01825	acsA
acsA	ZONMW-35_00735	acsA
acsA	ZONMW-35_02393	acsA
acsA	ZONMW-35_03196	acsA

acsA	ZONMW-35_03156	acsA
acsA	ZONMW-35_00171	acsA
acsA	ZONMW-35_00766	acsA
acsA	ZONMW-35_02805	acsA
acsA	ZONMW-35_00578	acsA
acsA	ZONMW-35_02763	acsA
acsA	ZONMW-35_02804	acsA
acsA	ZONMW-36_02599	acsA
acsA	ZONMW-36_00631	acsA
acsA	ZONMW-36_02317	acsA
acsA	ZONMW-36_01950	acsA
acsA	ZONMW-36_00499	acsA
acsA	ZONMW-36_03555	acsA
acsA	ZONMW-36_03602	acsA
acsA	ZONMW-36_03533	acsA
acsA	ZONMW-36_01275	acsA
acsA	ZONMW-36_01093	acsA
acsA	ZONMW-36_00110	acsA
acsA	ZONMW-36_01121	acsA
acsA	ZONMW-36_01770	acsA
acsA	ZONMW-36_00533	acsA
acsA	ZONMW-36_03646	acsA
acsA	ZONMW-36_03647	acsA
acsA	ZONMW-36_01048	acsA
acsA	ZONMW-37_01590	acsA
acsA	ZONMW-37_01921	acsA
acsA	ZONMW-38_02094	acsA
acsA	ZONMW-38_02526	acsA
acsA	ZONMW-38_00623	acsA
acsA	ZONMW-38_00192	acsA
acsA	ZONMW-38_00060	acsA
acsA	ZONMW-38_01228	acsA
acsA	ZONMW-38_01710	acsA
acsA	ZONMW-38_02011	acsA
acsA	ZONMW-38_00059	acsA
acsA	ZONMW-38_02152	acsA
acsA	ZONMW-38_00464	acsA
acsA	ZONMW-38_01482	acsA
acsA	ZONMW-38_00010	acsA
acsA	ZONMW-38_00973	acsA
acsA	ZONMW-38_02231	acsA
acsA	ZONMW-38_00011	acsA
acsA	ZONMW-39_02245	acsA
acsA	ZONMW-39_00099	acsA
acsA	ZONMW-39_01867	acsA
acsA	ZONMW-39_01730	acsA
acsA	ZONMW-39_01611	acsA
acsA	ZONMW-39_00227	acsA

acsA	ZONMW-39_01908	acsA
acsA	ZONMW-39_03718	acsA
acsA	ZONMW-39_02775	acsA
acsA	ZONMW-39_02767	acsA
acsA	ZONMW-39_01952	acsA
acsA	ZONMW-39_00773	acsA
acsA	ZONMW-39_01930	acsA
acsA	ZONMW-39_01117	acsA
acsA	ZONMW-39_01161	acsA
acsA	ZONMW-39_03762	acsA
acsA	ZONMW-39_01945	acsA
acsA	ZONMW-39_00907	acsA
acsA	ZONMW-39_01087	acsA
acsA	ZONMW-39_04172	acsA
acsA	ZONMW-39_00193	acsA
acsA	ZONMW-39_02124	acsA
acsA	ZONMW-40_00499	acsA
acsA	ZONMW-40_03260	acsA
acsA	ZONMW-41_01972	acsA
acsA	ZONMW-41_01562	acsA
acsA	ZONMW-41_02514	acsA
acsA	ZONMW-41_02162	acsA
acsA	ZONMW-41_02455	acsA
acsA	ZONMW-42_00471	acsA
acsA	ZONMW-42_03300	acsA
acsA	ZONMW-42_00575	acsA
acsA	ZONMW-42_02539	acsA
acsA	ZONMW-42_01894	acsA
acsA	ZONMW-42_00668	acsA
acsA	ZONMW-42_00630	acsA
acsA	ZONMW-42_02404	acsA
acsA	ZONMW-42_01607	acsA
acsA	ZONMW-42_01235	acsA
acsA	ZONMW-42_02655	acsA
acsA	ZONMW-42_01579	acsA
acsA	ZONMW-42_01361	acsA
acsA	ZONMW-42_01651	acsA
acsA	ZONMW-43_01451	acsA
acsA	ZONMW-43_01668	acsA
acsA	ZONMW-43_04018	acsA
acsA	ZONMW-43_03503	acsA
acsA	ZONMW-43_00614	acsA
acsA	ZONMW-43_01895	acsA
acsA	ZONMW-43_01783	acsA
acsA	ZONMW-43_04068	acsA
acsA	ZONMW-43_03850	acsA
acsA	ZONMW-43_03843	acsA
acsA	ZONMW-43_03381	acsA

acsA	ZONMW-43_03084	acsA
acsA	ZONMW-43_01003	acsA
acsA	ZONMW-43_00194	acsA
acsA	ZONMW-43_02270	acsA
acsA	ZONMW-43_03964	acsA
acsA	ZONMW-43_04436	acsA
acsA	ZONMW-43_01018	acsA
acsA	ZONMW-43_04498	acsA
acsA	ZONMW-43_00165	acsA
acsA	ZONMW-43_02892	acsA
acsA	ZONMW-43_00239	acsA
acsA	ZONMW-43_01571	acsA
acsA	ZONMW-44_00826	acsA
acsA	ZONMW-44_02802	acsA
acsA	ZONMW-44_00718	acsA
acsA	ZONMW-44_02350	acsA
acsA	ZONMW-44_00368	acsA
acsA	ZONMW-44_01358	acsA
acsA	ZONMW-44_02662	acsA
acsA	ZONMW-44_02701	acsA
acsA	ZONMW-44_00597	acsA
acsA	ZONMW-44_01394	acsA
acsA	ZONMW-44_02084	acsA
acsA	ZONMW-44_00426	acsA
acsA	ZONMW-44_03398	acsA
acsA	ZONMW-44_01434	acsA
acsA	ZONMW-44_00549	acsA
acsA	ZONMW-44_01405	acsA
acsA	ZONMW-45_00873	acsA
acsA	ZONMW-45_03073	acsA
acsA	ZONMW-45_02586	acsA
acsA	ZONMW-45_02585	acsA
acsA	ZONMW-45_02410	acsA
acsA	ZONMW-45_02405	acsA
acsA	ZONMW-45_02403	acsA
acsA	ZONMW-45_02399	acsA
acsA	ZONMW-45_03071	acsA
acsA	ZONMW-46_01001	acsA
acsA	ZONMW-46_00162	acsA
acsA	ZONMW-46_02972	acsA
acsA	ZONMW-46_02973	acsA
acsA	ZONMW-46_00176	acsA
acsA	ZONMW-46_00171	acsA
acsA	ZONMW-46_00169	acsA
acsA	ZONMW-46_00165	acsA
acsA	ZONMW-46_00164	acsA
acsA	ZONMW-47_01172	acsA
acsA	ZONMW-47_00343	acsA

acsA	ZONMW-47_02901	acsA
acsA	ZONMW-47_02900	acsA
acsA	ZONMW-47_00329	acsA
acsA	ZONMW-47_00334	acsA
acsA	ZONMW-47_00336	acsA
acsA	ZONMW-47_00340	acsA
acsA	ZONMW-47_00341	acsA
acsA	ZONMW-48_03157	acsA
acsA	ZONMW-48_01187	acsA
acsA	ZONMW-49_01480	acsA
acsA	ZONMW-49_00146	acsA
acsA	ZONMW-50_01482	acsA
acsA	ZONMW-50_00340	acsA
acsA	ZONMW-51_00736	acsA
acsA	ZONMW-51_00143	acsA
acsA	ZONMW-52_01336	acsA
acsA	ZONMW-52_00115	acsA
acsA	ZONMW-53_00489	acsA
acsA	ZONMW-53_02539	acsA
acsA	ZONMW-54_00546	acsA
acsA	ZONMW-54_01861	acsA
acsA	ZONMW-55_00484	acsA
acsA	ZONMW-55_01395	acsA
acsA	ZONMW-56_00829	acsA
acsA	ZONMW-56_00621	acsA
acsA	ZONMW-57_01130	acsA
acsA	ZONMW-57_00827	acsA
acsA	ZONMW-58_01454	acsA
acsA	ZONMW-58_00144	acsA
acsA	ZONMW-59_01132	acsA
acsA	ZONMW-59_00822	acsA
acsA	ZONMW-60_01435	acsA
acsA	ZONMW-60_00826	acsA
acsA	ZONMW-61_03582	acsA
acsA	ZONMW-61_02132	acsA
acsA	ZONMW-61_02156	acsA
acsA	ZONMW-61_02595	acsA
acsA	ZONMW-61_00697	acsA
acsA	ZONMW-62_01035	acsA
acsA	ZONMW-62_00113	acsA
acsA	ZONMW-62_00089	acsA
acsA	ZONMW-62_02475	acsA
acsA	ZONMW-63_00886	acsA
acsA	ZONMW-63_00502	acsA
acsA	ZONMW-64_02028	acsA
acsA	ZONMW-64_03026	acsA
acsA	ZONMW-64_02647	acsA
acsA	ZONMW-64_01690	acsA

acsA	ZONMW-64_00876	acsA
acsA	ZONMW-64_02815	acsA
acsA	ZONMW-64_02597	acsA
acsA	ZONMW-64_03832	acsA
acsA	ZONMW-64_03839	acsA
acsA	ZONMW-64_01857	acsA
acsA	ZONMW-64_02246	acsA
acsA	ZONMW-64_02573	acsA
acsA	ZONMW-64_04067	acsA
acsA	ZONMW-64_00210	acsA
acsA	ZONMW-64_00813	acsA
acsA	ZONMW-64_02558	acsA
acsA	ZONMW-64_02373	acsA
acsA	ZONMW-64_00180	acsA
acsA	ZONMW-64_03582	acsA
acsA	ZONMW-64_02849	acsA
acsA	ZONMW-64_01534	acsA
acsA	ZONMW-64_00255	acsA
acsA	ZONMW-64_02148	acsA
acsA	ZONMW-65_03256	acsA
acsA	ZONMW-65_03409	acsA
acsA	ZONMW-65_00331	acsA
acsA	ZONMW-65_03304	acsA
acsA	ZONMW-65_03640	acsA
acsA	ZONMW-65_02593	acsA
acsA	ZONMW-65_03638	acsA
acsA	ZONMW-65_03637	acsA
acsA	ZONMW-65_02843	acsA
acsA	ZONMW-65_03643	acsA
acsA	ZONMW-66_00292	acsA
acsA	ZONMW-66_03701	acsA
acsA	ZONMW-66_00384	acsA
acsA	ZONMW-66_03389	acsA
acsA	ZONMW-66_01308	acsA
acsA	ZONMW-66_02760	acsA
acsA	ZONMW-66_00085	acsA
acsA	ZONMW-66_02206	acsA
acsA	ZONMW-66_04234	acsA
acsA	ZONMW-66_02762	acsA
acsA	ZONMW-66_02219	acsA
acsA	ZONMW-66_03768	acsA
acsA	ZONMW-66_03089	acsA
acsA	ZONMW-66_02355	acsA
acsA	ZONMW-66_01219	acsA
acsA	ZONMW-67_02347	acsA
acsA	ZONMW-67_01537	acsA
acsA	ZONMW-67_02214	acsA
acsA	ZONMW-67_00060	acsA

acsA	ZONMW-67_03146	acsA
acsA	ZONMW-67_01420	acsA
acsA	ZONMW-67_02190	acsA
acsA	ZONMW-67_03252	acsA
acsA	ZONMW-67_02929	acsA
acsA	ZONMW-67_02936	acsA
acsA	ZONMW-67_00874	acsA
acsA	ZONMW-67_01456	acsA
acsA	ZONMW-67_00925	acsA
acsA	ZONMW-67_01897	acsA
acsA	ZONMW-67_00845	acsA
acsA	ZONMW-67_02978	acsA
acsA	ZONMW-68_01180	acsA
acsA	ZONMW-68_03657	acsA
acsA	ZONMW-68_03655	acsA
acsA	ZONMW-68_04133	acsA
acsA	ZONMW-68_04957	acsA
acsA	ZONMW-68_03659	acsA
acsA	ZONMW-68_03660	acsA
acsA	ZONMW-69_02395	acsA
acsA	ZONMW-69_01464	acsA
acsA	ZONMW-69_02262	acsA
acsA	ZONMW-69_02669	acsA
acsA	ZONMW-69_03147	acsA
acsA	ZONMW-69_01580	acsA
acsA	ZONMW-69_02237	acsA
acsA	ZONMW-69_03253	acsA
acsA	ZONMW-69_03025	acsA
acsA	ZONMW-69_03018	acsA
acsA	ZONMW-69_00921	acsA
acsA	ZONMW-69_01544	acsA
acsA	ZONMW-69_00972	acsA
acsA	ZONMW-69_02046	acsA
acsA	ZONMW-69_00892	acsA
acsA	ZONMW-69_02976	acsA
acsA	ZONMW-72_03599	acsA
acsA	ZONMW-72_02281	acsA
acsA	ZONMW-72_01753	acsA
acsA	ZONMW-72_01749	acsA
acsA	ZONMW-72_01834	acsA
acsA	ZONMW-72_01741	acsA
acsA	ZONMW-72_01746	acsA
acsA	ZONMW-72_01750	acsA
acsA	ZONMW-73_02934	acsA
acsA	ZONMW-73_01599	acsA
acsA	ZONMW-74_01765	acsA
acsA	ZONMW-74_03071	acsA
acsA	ZONMW-74_02938	acsA

acsA	ZONMW-75_02826	acsA
acsA	ZONMW-75_03010	acsA
acsA	ZONMW-75_03622	acsA
acsA	ZONMW-75_03618	acsA
acsA	ZONMW-75_00558	acsA
acsA	ZONMW-75_03620	acsA
acsA	da5A-8_01665	acsA
acsA	da5A-8_03625	acsA
acsA	da5A-8_02635	acsA
acsA	da5A-8_02636	acsA
acsA	da5A-8_02709	acsA
plsC	18_01072	plsC
plsC	ACAM48_02480	plsC
plsC	CAM030_00960	plsC
plsC	CECT7946_00203	plsC
plsC	CGMCC1.8863_00860	plsC
plsC	CL8139_00378	plsC
plsC	DAU203_01541	plsC
plsC	DD5b_02094	plsC
plsC	DSM1208_01735	plsC
plsC	DSM12212_00136	plsC
plsC	DSM15343_00986	plsC
plsC	DSM15361_00437	plsC
plsC	DSM15365_03134	plsC
plsC	DSM15718_02546	plsC
plsC	DSM15718_01235	plsC
plsC	DSM16505T_00643	plsC
plsC	DSM18103_01060	plsC
plsC	DSM18293_03109	plsC
plsC	DSM18841_00513	plsC
plsC	DSM18842_00713	plsC
plsC	DSM19866_01970	plsC
plsC	DSM2041_00057	plsC
plsC	DSM2061_03266	plsC
plsC	DSM2064_01983	plsC
plsC	DSM21164_00798	plsC
plsC	DSM23404_02040	plsC
plsC	DSM23424_00275	plsC
plsC	DSM24786_03031	plsC
plsC	DSM7489_01445	plsC
plsC	DSW1_00550	plsC
plsC	DSW5_00876	plsC
plsC	DSW5_00181	plsC
plsC	DSW6_01447	plsC
plsC	DSW8_02199	plsC
plsC	F52_02390	plsC
plsC	GCA_001027945.1_00093	plsC
plsC	HI1_01386	plsC

plsC	IR1_04766	plsC
plsC	KCOM3131_01361	plsC
plsC	KCTC12974_02879	plsC
plsC	KL-A_00781	plsC
plsC	L12_00113	plsC
plsC	LL0412.1.7_00304	plsC
plsC	MAR_2009_71_01241	plsC
plsC	NN016038_01089	plsC
plsC	PAO1_03382	plsC
plsC	PAO1_02608	plsC
plsC	UW101_01038	plsC
plsC	V4-33_02167	plsC
plsC	W5C_01144	plsC
plsC	ZONMW-01_02443	plsC
plsC	ZONMW-02_00043	plsC
plsC	ZONMW-03_03046	plsC
plsC	ZONMW-04_02398	plsC
plsC	ZONMW-05_02626	plsC
plsC	ZONMW-06_00035	plsC
plsC	ZONMW-06_00272	plsC
plsC	ZONMW-06_03802	plsC
plsC	ZONMW-06_01252	plsC
plsC	ZONMW-07_01451	plsC
plsC	ZONMW-08_00761	plsC
plsC	ZONMW-09_00323	plsC
plsC	ZONMW-10_00422	plsC
plsC	ZONMW-11_00157	plsC
plsC	ZONMW-12_00220	plsC
plsC	ZONMW-13_00323	plsC
plsC	ZONMW-14_00269	plsC
plsC	ZONMW-15_02589	plsC
plsC	ZONMW-19_01956	plsC
plsC	ZONMW-20_00082	plsC
plsC	ZONMW-20_00252	plsC
plsC	ZONMW-21_00738	plsC
plsC	ZONMW-22_00739	plsC
plsC	ZONMW-23_01853	plsC
plsC	ZONMW-24_00722	plsC
plsC	ZONMW-25_01669	plsC
plsC	ZONMW-26_03063	plsC
plsC	ZONMW-27_02783	plsC
plsC	ZONMW-28_01133	plsC
plsC	ZONMW-28_03387	plsC
plsC	ZONMW-29_00380	plsC
plsC	ZONMW-30_02327	plsC
plsC	ZONMW-31_02902	plsC
plsC	ZONMW-31_00370	plsC
plsC	ZONMW-31_00303	plsC

plsC	ZONMW-31_00583	plsC
plsC	ZONMW-32_01541	plsC
plsC	ZONMW-32_03594	plsC
plsC	ZONMW-33_01531	plsC
plsC	ZONMW-33_03944	plsC
plsC	ZONMW-34_02816	plsC
plsC	ZONMW-34_02926	plsC
plsC	ZONMW-34_03391	plsC
plsC	ZONMW-35_02783	plsC
plsC	ZONMW-35_02893	plsC
plsC	ZONMW-35_03424	plsC
plsC	ZONMW-36_01825	plsC
plsC	ZONMW-36_03893	plsC
plsC	ZONMW-37_00738	plsC
plsC	ZONMW-38_00638	plsC
plsC	ZONMW-38_02769	plsC
plsC	ZONMW-39_02534	plsC
plsC	ZONMW-39_03284	plsC
plsC	ZONMW-39_02605	plsC
plsC	ZONMW-40_00463	plsC
plsC	ZONMW-41_02594	plsC
plsC	ZONMW-42_00159	plsC
plsC	ZONMW-42_00238	plsC
plsC	ZONMW-42_03774	plsC
plsC	ZONMW-42_02743	plsC
plsC	ZONMW-43_00948	plsC
plsC	ZONMW-43_03987	plsC
plsC	ZONMW-43_02613	plsC
plsC	ZONMW-44_02931	plsC
plsC	ZONMW-44_01774	plsC
plsC	ZONMW-44_03008	plsC
plsC	ZONMW-44_02475	plsC
plsC	ZONMW-45_00654	plsC
plsC	ZONMW-46_00805	plsC
plsC	ZONMW-47_00805	plsC
plsC	ZONMW-48_00592	plsC
plsC	ZONMW-49_01151	plsC
plsC	ZONMW-50_01152	plsC
plsC	ZONMW-51_02758	plsC
plsC	ZONMW-52_02288	plsC
plsC	ZONMW-53_02877	plsC
plsC	ZONMW-54_00093	plsC
plsC	ZONMW-55_02883	plsC
plsC	ZONMW-56_02644	plsC
plsC	ZONMW-57_02788	plsC
plsC	ZONMW-58_02812	plsC
plsC	ZONMW-59_02916	plsC
plsC	ZONMW-60_02906	plsC

plsC	ZONMW-61_00354	plsC
plsC	ZONMW-62_01902	plsC
plsC	ZONMW-63_02285	plsC
plsC	ZONMW-64_01233	plsC
plsC	ZONMW-64_01297	plsC
plsC	ZONMW-64_03299	plsC
plsC	ZONMW-65_00137	plsC
plsC	ZONMW-66_01514	plsC
plsC	ZONMW-67_00154	plsC
plsC	ZONMW-68_00110	plsC
plsC	ZONMW-69_02763	plsC
plsC	ZONMW-72_04148	plsC
plsC	ZONMW-73_00126	plsC
plsC	ZONMW-74_01441	plsC
plsC	ZONMW-75_02417	plsC
plsC	da5A-8_01621	plsC
fabG_1	18_00025	fabG_1
fabG_1	18_03484	fabG_1
fabG_1	18_01313	fabG_1
fabG_1	18_02169	fabG_1
fabG_1	18_00922	fabG_1
fabG_1	18_02007	fabG_1
fabG_1	18_03533	fabG_1
fabG_1	18_03532	fabG_1
fabG_1	18_03669	fabG_1
fabG_1	18_03233	fabG_1
fabG_1	18_02656	fabG_1
fabG_1	18_00028	fabG_1
fabG_1	18_04004	fabG_1
fabG_1	18_02911	fabG_1
fabG_1	18_02932	fabG_1
fabG_1	18_01997	fabG_1
fabG_1	18_02927	fabG_1
fabG_1	18_03050	fabG_1
fabG_1	18_03316	fabG_1
fabG_1	18_03997	fabG_1
fabG_1	18_02720	fabG_1
fabG_1	18_00232	fabG_1
fabG_1	18_02734	fabG_1
fabG_1	18_00799	fabG_1
fabG_1	18_00842	fabG_1
fabG_1	18_00337	fabG_1
fabG_1	18_02048	fabG_1
fabG_1	18_03303	fabG_1
fabG_1	18_02783	fabG_1
fabG_1	18_03152	fabG_1
fabG_1	ACAM48_00441	fabG_1
fabG_1	ACAM48_00166	fabG_1

fabG_1	ACAM48_01844	fabG_1
fabG_1	ACAM48_02863	fabG_1
fabG_1	ACAM48_03398	fabG_1
fabG_1	ACAM48_02746	fabG_1
fabG_1	ACAM48_00752	fabG_1
fabG_1	ACAM48_01795	fabG_1
fabG_1	ACAM48_01256	fabG_1
fabG_1	ACAM48_01381	fabG_1
fabG_1	ACAM48_00580	fabG_1
fabG_1	ACAM48_01799	fabG_1
fabG_1	ACAM48_00552	fabG_1
fabG_1	ACAM48_00261	fabG_1
fabG_1	ACAM48_00978	fabG_1
fabG_1	ACAM48_02263	fabG_1
fabG_1	ACAM48_00768	fabG_1
fabG_1	ACAM48_01461	fabG_1
fabG_1	ACAM48_00842	fabG_1
fabG_1	ACAM48_00767	fabG_1
fabG_1	ACAM48_00080	fabG_1
fabG_1	CAM030_00584	fabG_1
fabG_1	CAM030_00629	fabG_1
fabG_1	CAM030_02852	fabG_1
fabG_1	CAM030_00396	fabG_1
fabG_1	CAM030_02683	fabG_1
fabG_1	CAM030_02894	fabG_1
fabG_1	CAM030_00307	fabG_1
fabG_1	CAM030_02752	fabG_1
fabG_1	CAM030_00475	fabG_1
fabG_1	CECT7946_01354	fabG_1
fabG_1	CECT7946_02614	fabG_1
fabG_1	CECT7946_02995	fabG_1
fabG_1	CECT7946_02037	fabG_1
fabG_1	CECT7946_03560	fabG_1
fabG_1	CECT7946_00293	fabG_1
fabG_1	CECT7946_00557	fabG_1
fabG_1	CECT7946_01626	fabG_1
fabG_1	CECT7946_03192	fabG_1
fabG_1	CECT7946_01496	fabG_1
fabG_1	CECT7946_00940	fabG_1
fabG_1	CECT7946_00529	fabG_1
fabG_1	CECT7946_01902	fabG_1
fabG_1	CECT7946_02847	fabG_1
fabG_1	CECT7946_03404	fabG_1
fabG_1	CECT7946_02080	fabG_1
fabG_1	CECT7946_02052	fabG_1
fabG_1	CGMCC1.8863_00408	fabG_1
fabG_1	CGMCC1.8863_00377	fabG_1
fabG_1	CGMCC1.8863_00658	fabG_1

fabG_1	CGMCC1.8863_00395	fabG_1
fabG_1	CGMCC1.8863_02638	fabG_1
fabG_1	CGMCC1.8863_01988	fabG_1
fabG_1	CGMCC1.8863_02615	fabG_1
fabG_1	CGMCC1.8863_02122	fabG_1
fabG_1	CGMCC1.8863_00456	fabG_1
fabG_1	CGMCC1.8863_02156	fabG_1
fabG_1	CGMCC1.8863_01369	fabG_1
fabG_1	CGMCC1.8863_01016	fabG_1
fabG_1	CGMCC1.8863_01832	fabG_1
fabG_1	CGMCC1.8863_01833	fabG_1
fabG_1	CL8139_02580	fabG_1
fabG_1	CL8139_00531	fabG_1
fabG_1	CL8139_00372	fabG_1
fabG_1	CL8139_00361	fabG_1
fabG_1	CL8139_02704	fabG_1
fabG_1	CL8139_03187	fabG_1
fabG_1	CL8139_01330	fabG_1
fabG_1	CL8139_00523	fabG_1
fabG_1	CL8139_02127	fabG_1
fabG_1	CL8139_02494	fabG_1
fabG_1	CL8139_01326	fabG_1
fabG_1	CL8139_00282	fabG_1
fabG_1	CL8139_01522	fabG_1
fabG_1	CL8139_00370	fabG_1
fabG_1	CL8139_01640	fabG_1
fabG_1	CL8139_03080	fabG_1
fabG_1	CL8139_01038	fabG_1
fabG_1	CL8139_00715	fabG_1
fabG_1	DAU203_03177	fabG_1
fabG_1	DAU203_00432	fabG_1
fabG_1	DAU203_01547	fabG_1
fabG_1	DAU203_01559	fabG_1
fabG_1	DAU203_00314	fabG_1
fabG_1	DAU203_02478	fabG_1
fabG_1	DAU203_00440	fabG_1
fabG_1	DAU203_02841	fabG_1
fabG_1	DAU203_01247	fabG_1
fabG_1	DAU203_00804	fabG_1
fabG_1	DAU203_02197	fabG_1
fabG_1	DAU203_01549	fabG_1
fabG_1	DAU203_03381	fabG_1
fabG_1	DAU203_03055	fabG_1
fabG_1	DAU203_01815	fabG_1
fabG_1	DD5b_00956	fabG_1
fabG_1	DD5b_00946	fabG_1
fabG_1	DD5b_01751	fabG_1
fabG_1	DD5b_02361	fabG_1

fabG_1	DD5b_01014	fabG_1
fabG_1	DD5b_02259	fabG_1
fabG_1	DD5b_00647	fabG_1
fabG_1	DD5b_02509	fabG_1
fabG_1	DD5b_02356	fabG_1
fabG_1	DD5b_02619	fabG_1
fabG_1	DD5b_00018	fabG_1
fabG_1	DD5b_00233	fabG_1
fabG_1	DSM1208_03328	fabG_1
fabG_1	DSM1208_04256	fabG_1
fabG_1	DSM1208_01013	fabG_1
fabG_1	DSM1208_04343	fabG_1
fabG_1	DSM1208_02535	fabG_1
fabG_1	DSM1208_02528	fabG_1
fabG_1	DSM1208_01062	fabG_1
fabG_1	DSM1208_04471	fabG_1
fabG_1	DSM1208_00185	fabG_1
fabG_1	DSM1208_00268	fabG_1
fabG_1	DSM1208_03316	fabG_1
fabG_1	DSM1208_04509	fabG_1
fabG_1	DSM1208_01170	fabG_1
fabG_1	DSM1208_03330	fabG_1
fabG_1	DSM1208_01063	fabG_1
fabG_1	DSM1208_02397	fabG_1
fabG_1	DSM1208_02001	fabG_1
fabG_1	DSM1208_01034	fabG_1
fabG_1	DSM1208_04543	fabG_1
fabG_1	DSM1208_04494	fabG_1
fabG_1	DSM1208_03657	fabG_1
fabG_1	DSM1208_00304	fabG_1
fabG_1	DSM1208_00243	fabG_1
fabG_1	DSM1208_04087	fabG_1
fabG_1	DSM1208_03015	fabG_1
fabG_1	DSM1208_02508	fabG_1
fabG_1	DSM1208_03146	fabG_1
fabG_1	DSM1208_02961	fabG_1
fabG_1	DSM1208_01532	fabG_1
fabG_1	DSM1208_04252	fabG_1
fabG_1	DSM1208_03090	fabG_1
fabG_1	DSM1208_01448	fabG_1
fabG_1	DSM1208_00370	fabG_1
fabG_1	DSM1208_00859	fabG_1
fabG_1	DSM1208_00314	fabG_1
fabG_1	DSM1208_03813	fabG_1
fabG_1	DSM1208_04218	fabG_1
fabG_1	DSM1208_03053	fabG_1
fabG_1	DSM1208_03869	fabG_1
fabG_1	DSM1208_00264	fabG_1

fabG_1	DSM1208_00265	fabG_1
fabG_1	DSM1208_00371	fabG_1
fabG_1	DSM12212_00718	fabG_1
fabG_1	DSM12212_01845	fabG_1
fabG_1	DSM12212_02780	fabG_1
fabG_1	DSM12212_03381	fabG_1
fabG_1	DSM12212_03345	fabG_1
fabG_1	DSM12212_03288	fabG_1
fabG_1	DSM12212_03028	fabG_1
fabG_1	DSM12212_02165	fabG_1
fabG_1	DSM12212_01204	fabG_1
fabG_1	DSM15343_00284	fabG_1
fabG_1	DSM15343_02361	fabG_1
fabG_1	DSM15343_00341	fabG_1
fabG_1	DSM15343_00479	fabG_1
fabG_1	DSM15343_01513	fabG_1
fabG_1	DSM15343_00129	fabG_1
fabG_1	DSM15343_01265	fabG_1
fabG_1	DSM15361_01760	fabG_1
fabG_1	DSM15361_00705	fabG_1
fabG_1	DSM15361_01802	fabG_1
fabG_1	DSM15361_00806	fabG_1
fabG_1	DSM15361_01681	fabG_1
fabG_1	DSM15361_00214	fabG_1
fabG_1	DSM15361_00950	fabG_1
fabG_1	DSM15361_01657	fabG_1
fabG_1	DSM15361_00335	fabG_1
fabG_1	DSM15361_00033	fabG_1
fabG_1	DSM15361_00751	fabG_1
fabG_1	DSM15365_00477	fabG_1
fabG_1	DSM15365_01840	fabG_1
fabG_1	DSM15365_03800	fabG_1
fabG_1	DSM15365_00786	fabG_1
fabG_1	DSM15365_02903	fabG_1
fabG_1	DSM15365_02832	fabG_1
fabG_1	DSM15365_01994	fabG_1
fabG_1	DSM15365_03157	fabG_1
fabG_1	DSM15365_00403	fabG_1
fabG_1	DSM15365_00474	fabG_1
fabG_1	DSM15365_02815	fabG_1
fabG_1	DSM15365_02400	fabG_1
fabG_1	DSM15365_00210	fabG_1
fabG_1	DSM15365_03509	fabG_1
fabG_1	DSM15365_01261	fabG_1
fabG_1	DSM15365_02006	fabG_1
fabG_1	DSM15365_03233	fabG_1
fabG_1	DSM15365_02971	fabG_1
fabG_1	DSM15365_02333	fabG_1

fabG_1	DSM15365_02629	fabG_1
fabG_1	DSM15365_00015	fabG_1
fabG_1	DSM15365_03571	fabG_1
fabG_1	DSM15365_02738	fabG_1
fabG_1	DSM15365_00164	fabG_1
fabG_1	DSM15718_01943	fabG_1
fabG_1	DSM15718_01366	fabG_1
fabG_1	DSM15718_01106	fabG_1
fabG_1	DSM15718_02219	fabG_1
fabG_1	DSM15718_01134	fabG_1
fabG_1	DSM15718_00962	fabG_1
fabG_1	DSM15718_02701	fabG_1
fabG_1	DSM15718_00603	fabG_1
fabG_1	DSM15718_02793	fabG_1
fabG_1	DSM15718_00218	fabG_1
fabG_1	DSM15718_01004	fabG_1
fabG_1	DSM16505T_01512	fabG_1
fabG_1	DSM16505T_00753	fabG_1
fabG_1	DSM16505T_02258	fabG_1
fabG_1	DSM16505T_02324	fabG_1
fabG_1	DSM16505T_00891	fabG_1
fabG_1	DSM16505T_00476	fabG_1
fabG_1	DSM16505T_00292	fabG_1
fabG_1	DSM16505T_02406	fabG_1
fabG_1	DSM16505T_01039	fabG_1
fabG_1	DSM16505T_01555	fabG_1
fabG_1	DSM16505T_00993	fabG_1
fabG_1	DSM18103_00242	fabG_1
fabG_1	DSM18103_01605	fabG_1
fabG_1	DSM18103_00847	fabG_1
fabG_1	DSM18103_01841	fabG_1
fabG_1	DSM18103_01804	fabG_1
fabG_1	DSM18103_01516	fabG_1
fabG_1	DSM18103_02145	fabG_1
fabG_1	DSM18103_00346	fabG_1
fabG_1	DSM18103_02807	fabG_1
fabG_1	DSM18103_03389	fabG_1
fabG_1	DSM18103_01815	fabG_1
fabG_1	DSM18103_01763	fabG_1
fabG_1	DSM18103_02376	fabG_1
fabG_1	DSM18103_02581	fabG_1
fabG_1	DSM18293_02734	fabG_1
fabG_1	DSM18293_01353	fabG_1
fabG_1	DSM18293_01616	fabG_1
fabG_1	DSM18293_01475	fabG_1
fabG_1	DSM18293_02909	fabG_1
fabG_1	DSM18293_01755	fabG_1
fabG_1	DSM18293_04547	fabG_1

fabG_1	DSM18293_02806	fabG_1
fabG_1	DSM18293_04421	fabG_1
fabG_1	DSM18293_02828	fabG_1
fabG_1	DSM18293_00045	fabG_1
fabG_1	DSM18293_04609	fabG_1
fabG_1	DSM18293_02983	fabG_1
fabG_1	DSM18293_00005	fabG_1
fabG_1	DSM18293_01588	fabG_1
fabG_1	DSM18293_04243	fabG_1
fabG_1	DSM18293_01036	fabG_1
fabG_1	DSM18293_00029	fabG_1
fabG_1	DSM18293_04350	fabG_1
fabG_1	DSM18293_00003	fabG_1
fabG_1	DSM18293_00816	fabG_1
fabG_1	DSM18293_04319	fabG_1
fabG_1	DSM18293_04423	fabG_1
fabG_1	DSM18293_02361	fabG_1
fabG_1	DSM18293_04365	fabG_1
fabG_1	DSM18293_01821	fabG_1
fabG_1	DSM18293_01082	fabG_1
fabG_1	DSM18293_00044	fabG_1
fabG_1	DSM18841_02135	fabG_1
fabG_1	DSM18841_02829	fabG_1
fabG_1	DSM18841_01687	fabG_1
fabG_1	DSM18841_01389	fabG_1
fabG_1	DSM18841_03031	fabG_1
fabG_1	DSM18841_00319	fabG_1
fabG_1	DSM18841_01382	fabG_1
fabG_1	DSM18841_02177	fabG_1
fabG_1	DSM18841_03148	fabG_1
fabG_1	DSM18841_00096	fabG_1
fabG_1	DSM18841_00097	fabG_1
fabG_1	DSM18842_01478	fabG_1
fabG_1	DSM18842_02747	fabG_1
fabG_1	DSM18842_02989	fabG_1
fabG_1	DSM18842_01718	fabG_1
fabG_1	DSM18842_01986	fabG_1
fabG_1	DSM18842_01645	fabG_1
fabG_1	DSM18842_00341	fabG_1
fabG_1	DSM18842_00913	fabG_1
fabG_1	DSM18842_00560	fabG_1
fabG_1	DSM18842_02360	fabG_1
fabG_1	DSM18842_01113	fabG_1
fabG_1	DSM18842_01519	fabG_1
fabG_1	DSM18842_00339	fabG_1
fabG_1	DSM18842_00436	fabG_1
fabG_1	DSM19866_00745	fabG_1
fabG_1	DSM19866_02378	fabG_1

fabG_1	DSM19866_04160	fabG_1
fabG_1	DSM19866_01297	fabG_1
fabG_1	DSM19866_03723	fabG_1
fabG_1	DSM19866_02959	fabG_1
fabG_1	DSM19866_00162	fabG_1
fabG_1	DSM19866_01056	fabG_1
fabG_1	DSM19866_02418	fabG_1
fabG_1	DSM19866_04221	fabG_1
fabG_1	DSM19866_04304	fabG_1
fabG_1	DSM19866_02403	fabG_1
fabG_1	DSM19866_05004	fabG_1
fabG_1	DSM19866_00676	fabG_1
fabG_1	DSM19866_04058	fabG_1
fabG_1	DSM19866_02547	fabG_1
fabG_1	DSM19866_04958	fabG_1
fabG_1	DSM19866_02846	fabG_1
fabG_1	DSM19866_01538	fabG_1
fabG_1	DSM19866_02460	fabG_1
fabG_1	DSM19866_03479	fabG_1
fabG_1	DSM19866_04023	fabG_1
fabG_1	DSM19866_02735	fabG_1
fabG_1	DSM19866_04653	fabG_1
fabG_1	DSM19866_03420	fabG_1
fabG_1	DSM19866_02341	fabG_1
fabG_1	DSM19866_04281	fabG_1
fabG_1	DSM19866_01640	fabG_1
fabG_1	DSM19866_03951	fabG_1
fabG_1	DSM19866_03320	fabG_1
fabG_1	DSM19866_02722	fabG_1
fabG_1	DSM19866_04012	fabG_1
fabG_1	DSM19866_03149	fabG_1
fabG_1	DSM2041_01425	fabG_1
fabG_1	DSM2041_01456	fabG_1
fabG_1	DSM2041_03462	fabG_1
fabG_1	DSM2041_01430	fabG_1
fabG_1	DSM2041_03074	fabG_1
fabG_1	DSM2041_03888	fabG_1
fabG_1	DSM2041_01434	fabG_1
fabG_1	DSM2041_01633	fabG_1
fabG_1	DSM2041_02389	fabG_1
fabG_1	DSM2041_01126	fabG_1
fabG_1	DSM2041_02294	fabG_1
fabG_1	DSM2041_04570	fabG_1
fabG_1	DSM2041_04573	fabG_1
fabG_1	DSM2041_01449	fabG_1
fabG_1	DSM2041_01757	fabG_1
fabG_1	DSM2041_00084	fabG_1
fabG_1	DSM2041_05324	fabG_1

fabG_1	DSM2041_03095	fabG_1
fabG_1	DSM2041_01758	fabG_1
fabG_1	DSM2041_02298	fabG_1
fabG_1	DSM2041_02475	fabG_1
fabG_1	DSM2041_03321	fabG_1
fabG_1	DSM2041_01756	fabG_1
fabG_1	DSM2041_03658	fabG_1
fabG_1	DSM2041_01762	fabG_1
fabG_1	DSM2041_01764	fabG_1
fabG_1	DSM2061_01721	fabG_1
fabG_1	DSM2061_00793	fabG_1
fabG_1	DSM2061_00680	fabG_1
fabG_1	DSM2061_02707	fabG_1
fabG_1	DSM2061_02579	fabG_1
fabG_1	DSM2061_02749	fabG_1
fabG_1	DSM2061_04046	fabG_1
fabG_1	DSM2061_01719	fabG_1
fabG_1	DSM2061_01733	fabG_1
fabG_1	DSM2061_02580	fabG_1
fabG_1	DSM2061_01575	fabG_1
fabG_1	DSM2061_03563	fabG_1
fabG_1	DSM2061_02682	fabG_1
fabG_1	DSM2061_03908	fabG_1
fabG_1	DSM2061_00642	fabG_1
fabG_1	DSM2061_01264	fabG_1
fabG_1	DSM2061_01330	fabG_1
fabG_1	DSM2061_01401	fabG_1
fabG_1	DSM2061_00610	fabG_1
fabG_1	DSM2061_01852	fabG_1
fabG_1	DSM2061_00369	fabG_1
fabG_1	DSM2061_01855	fabG_1
fabG_1	DSM2061_02548	fabG_1
fabG_1	DSM2061_00656	fabG_1
fabG_1	DSM2061_01830	fabG_1
fabG_1	DSM2061_00827	fabG_1
fabG_1	DSM2061_01572	fabG_1
fabG_1	DSM2061_00035	fabG_1
fabG_1	DSM2061_00915	fabG_1
fabG_1	DSM2061_04020	fabG_1
fabG_1	DSM2061_01777	fabG_1
fabG_1	DSM2061_02970	fabG_1
fabG_1	DSM2061_03063	fabG_1
fabG_1	DSM2061_02361	fabG_1
fabG_1	DSM2061_01784	fabG_1
fabG_1	DSM2061_01217	fabG_1
fabG_1	DSM2061_01001	fabG_1
fabG_1	DSM2061_04372	fabG_1
fabG_1	DSM2061_01163	fabG_1

fabG_1	DSM2061_01836	fabG_1
fabG_1	DSM2061_01770	fabG_1
fabG_1	DSM2061_01263	fabG_1
fabG_1	DSM2064_01186	fabG_1
fabG_1	DSM2064_04474	fabG_1
fabG_1	DSM2064_05015	fabG_1
fabG_1	DSM2064_03605	fabG_1
fabG_1	DSM2064_04253	fabG_1
fabG_1	DSM2064_03750	fabG_1
fabG_1	DSM2064_01116	fabG_1
fabG_1	DSM2064_04429	fabG_1
fabG_1	DSM2064_00309	fabG_1
fabG_1	DSM2064_01303	fabG_1
fabG_1	DSM2064_01468	fabG_1
fabG_1	DSM2064_01570	fabG_1
fabG_1	DSM2064_00786	fabG_1
fabG_1	DSM2064_01189	fabG_1
fabG_1	DSM2064_01466	fabG_1
fabG_1	DSM2064_02105	fabG_1
fabG_1	DSM2064_04445	fabG_1
fabG_1	DSM2064_04255	fabG_1
fabG_1	DSM2064_03635	fabG_1
fabG_1	DSM2064_03041	fabG_1
fabG_1	DSM2064_04127	fabG_1
fabG_1	DSM2064_01316	fabG_1
fabG_1	DSM2064_00643	fabG_1
fabG_1	DSM2064_02866	fabG_1
fabG_1	DSM2064_01091	fabG_1
fabG_1	DSM2064_04601	fabG_1
fabG_1	DSM2064_02316	fabG_1
fabG_1	DSM2064_00722	fabG_1
fabG_1	DSM2064_01698	fabG_1
fabG_1	DSM2064_01574	fabG_1
fabG_1	DSM2064_01535	fabG_1
fabG_1	DSM2064_04628	fabG_1
fabG_1	DSM2064_01490	fabG_1
fabG_1	DSM2064_03028	fabG_1
fabG_1	DSM2064_00621	fabG_1
fabG_1	DSM2064_01311	fabG_1
fabG_1	DSM2064_01522	fabG_1
fabG_1	DSM2064_04606	fabG_1
fabG_1	DSM2064_01425	fabG_1
fabG_1	DSM2064_01488	fabG_1
fabG_1	DSM2064_01569	fabG_1
fabG_1	DSM2064_04578	fabG_1
fabG_1	DSM2064_04430	fabG_1
fabG_1	DSM2064_01420	fabG_1
fabG_1	DSM2064_04288	fabG_1

fabG_1	DSM21164_02661	fabG_1
fabG_1	DSM21164_02709	fabG_1
fabG_1	DSM21164_02710	fabG_1
fabG_1	DSM21164_02907	fabG_1
fabG_1	DSM21164_01677	fabG_1
fabG_1	DSM21164_02910	fabG_1
fabG_1	DSM21164_01444	fabG_1
fabG_1	DSM21164_02242	fabG_1
fabG_1	DSM21164_01000	fabG_1
fabG_1	DSM21164_01040	fabG_1
fabG_1	DSM21164_01636	fabG_1
fabG_1	DSM21164_02307	fabG_1
fabG_1	DSM21164_01592	fabG_1
fabG_1	DSM21164_02980	fabG_1
fabG_1	DSM23404_01134	fabG_1
fabG_1	DSM23404_01216	fabG_1
fabG_1	DSM23404_01327	fabG_1
fabG_1	DSM23404_03222	fabG_1
fabG_1	DSM23404_00768	fabG_1
fabG_1	DSM23404_02556	fabG_1
fabG_1	DSM23404_02448	fabG_1
fabG_1	DSM23404_00723	fabG_1
fabG_1	DSM23404_02444	fabG_1
fabG_1	DSM23404_00657	fabG_1
fabG_1	DSM23404_02985	fabG_1
fabG_1	DSM23404_02901	fabG_1
fabG_1	DSM23404_00530	fabG_1
fabG_1	DSM23404_02486	fabG_1
fabG_1	DSM23404_00023	fabG_1
fabG_1	DSM23424_03197	fabG_1
fabG_1	DSM23424_01647	fabG_1
fabG_1	DSM23424_01627	fabG_1
fabG_1	DSM23424_00081	fabG_1
fabG_1	DSM23424_01146	fabG_1
fabG_1	DSM23424_01249	fabG_1
fabG_1	DSM23424_01620	fabG_1
fabG_1	DSM23424_01908	fabG_1
fabG_1	DSM23424_02516	fabG_1
fabG_1	DSM23424_01230	fabG_1
fabG_1	DSM23424_00472	fabG_1
fabG_1	DSM23424_01382	fabG_1
fabG_1	DSM23424_02933	fabG_1
fabG_1	DSM23424_01065	fabG_1
fabG_1	DSM23424_02745	fabG_1
fabG_1	DSM23424_00330	fabG_1
fabG_1	DSM24786_02504	fabG_1
fabG_1	DSM24786_00628	fabG_1
fabG_1	DSM24786_02461	fabG_1

fabG_1	DSM24786_03037	fabG_1
fabG_1	DSM24786_00026	fabG_1
fabG_1	DSM24786_03048	fabG_1
fabG_1	DSM24786_02512	fabG_1
fabG_1	DSM24786_01530	fabG_1
fabG_1	DSM24786_00318	fabG_1
fabG_1	DSM24786_03183	fabG_1
fabG_1	DSM24786_02691	fabG_1
fabG_1	DSM24786_01378	fabG_1
fabG_1	DSM24786_01840	fabG_1
fabG_1	DSM24786_00859	fabG_1
fabG_1	DSM7489_03009	fabG_1
fabG_1	DSM7489_00416	fabG_1
fabG_1	DSM7489_01451	fabG_1
fabG_1	DSM7489_01462	fabG_1
fabG_1	DSM7489_00302	fabG_1
fabG_1	DSM7489_02394	fabG_1
fabG_1	DSM7489_00424	fabG_1
fabG_1	DSM7489_02712	fabG_1
fabG_1	DSM7489_01156	fabG_1
fabG_1	DSM7489_00733	fabG_1
fabG_1	DSM7489_02110	fabG_1
fabG_1	DSM7489_01453	fabG_1
fabG_1	DSM7489_03217	fabG_1
fabG_1	DSM7489_01734	fabG_1
fabG_1	DSM7489_02920	fabG_1
fabG_1	DSW1_00895	fabG_1
fabG_1	DSW1_01988	fabG_1
fabG_1	DSW1_01375	fabG_1
fabG_1	DSW1_02342	fabG_1
fabG_1	DSW1_00790	fabG_1
fabG_1	DSW1_01655	fabG_1
fabG_1	DSW1_02161	fabG_1
fabG_1	DSW1_01197	fabG_1
fabG_1	DSW1_01946	fabG_1
fabG_1	DSW1_01724	fabG_1
fabG_1	DSW1_01836	fabG_1
fabG_1	DSW5_01475	fabG_1
fabG_1	DSW5_02254	fabG_1
fabG_1	DSW5_01478	fabG_1
fabG_1	DSW5_00988	fabG_1
fabG_1	DSW5_00998	fabG_1
fabG_1	DSW5_02616	fabG_1
fabG_1	DSW5_02770	fabG_1
fabG_1	DSW5_00414	fabG_1
fabG_1	DSW5_01308	fabG_1
fabG_1	DSW5_00172	fabG_1
fabG_1	DSW5_01146	fabG_1

fabG_1	DSW5_01238	fabG_1
fabG_1	DSW5_00433	fabG_1
fabG_1	DSW5_00359	fabG_1
fabG_1	DSW5_01978	fabG_1
fabG_1	DSW6_02800	fabG_1
fabG_1	DSW6_00312	fabG_1
fabG_1	DSW6_01527	fabG_1
fabG_1	DSW6_00322	fabG_1
fabG_1	DSW6_02005	fabG_1
fabG_1	DSW6_00325	fabG_1
fabG_1	DSW6_02434	fabG_1
fabG_1	DSW6_02850	fabG_1
fabG_1	DSW6_01100	fabG_1
fabG_1	DSW6_02632	fabG_1
fabG_1	DSW6_00463	fabG_1
fabG_1	DSW6_01967	fabG_1
fabG_1	DSW6_02421	fabG_1
fabG_1	DSW6_03056	fabG_1
fabG_1	DSW8_03151	fabG_1
fabG_1	DSW8_00167	fabG_1
fabG_1	DSW8_02878	fabG_1
fabG_1	DSW8_00492	fabG_1
fabG_1	DSW8_00254	fabG_1
fabG_1	DSW8_02903	fabG_1
fabG_1	DSW8_03206	fabG_1
fabG_1	DSW8_01292	fabG_1
fabG_1	DSW8_03148	fabG_1
fabG_1	DSW8_03163	fabG_1
fabG_1	DSW8_01555	fabG_1
fabG_1	DSW8_03143	fabG_1
fabG_1	DSW8_00515	fabG_1
fabG_1	DSW8_00334	fabG_1
fabG_1	DSW8_00594	fabG_1
fabG_1	DSW8_01285	fabG_1
fabG_1	DSW8_02494	fabG_1
fabG_1	DSW8_02451	fabG_1
fabG_1	DSW8_01288	fabG_1
fabG_1	DSW8_00090	fabG_1
fabG_1	DSW8_00453	fabG_1
fabG_1	DSW8_03005	fabG_1
fabG_1	DSW8_01390	fabG_1
fabG_1	DSW8_00837	fabG_1
fabG_1	DSW8_00346	fabG_1
fabG_1	DSW8_02446	fabG_1
fabG_1	DSW8_02144	fabG_1
fabG_1	F52_02510	fabG_1
fabG_1	F52_00011	fabG_1
fabG_1	F52_01874	fabG_1

fabG_1	F52_03761	fabG_1
fabG_1	F52_01370	fabG_1
fabG_1	F52_03531	fabG_1
fabG_1	F52_03604	fabG_1
fabG_1	F52_03183	fabG_1
fabG_1	F52_00959	fabG_1
fabG_1	F52_02513	fabG_1
fabG_1	F52_02993	fabG_1
fabG_1	F52_02503	fabG_1
fabG_1	F52_01104	fabG_1
fabG_1	F52_00984	fabG_1
fabG_1	F52_03490	fabG_1
fabG_1	F52_03788	fabG_1
fabG_1	F52_00859	fabG_1
fabG_1	F52_01098	fabG_1
fabG_1	F52_03587	fabG_1
fabG_1	F52_04281	fabG_1
fabG_1	F52_02154	fabG_1
fabG_1	F52_03562	fabG_1
fabG_1	F52_00671	fabG_1
fabG_1	F52_02712	fabG_1
fabG_1	F52_03210	fabG_1
fabG_1	F52_03238	fabG_1
fabG_1	F52_04553	fabG_1
fabG_1	F52_04039	fabG_1
fabG_1	F52_04575	fabG_1
fabG_1	F52_01455	fabG_1
fabG_1	F52_04463	fabG_1
fabG_1	F52_01754	fabG_1
fabG_1	F52_00077	fabG_1
fabG_1	F52_03690	fabG_1
fabG_1	F52_01739	fabG_1
fabG_1	F52_01095	fabG_1
fabG_1	F52_01434	fabG_1
fabG_1	F52_01733	fabG_1
fabG_1	F52_01048	fabG_1
fabG_1	F52_01761	fabG_1
fabG_1	F52_00660	fabG_1
fabG_1	F52_03128	fabG_1
fabG_1	F52_00653	fabG_1
fabG_1	F52_00668	fabG_1
fabG_1	F52_03603	fabG_1
fabG_1	F52_00684	fabG_1
fabG_1	F52_03689	fabG_1
fabG_1	GCA_001027945.1_01805	fabG_1
fabG_1	GCA_001027945.1_02164	fabG_1
fabG_1	GCA_001027945.1_02477	fabG_1
fabG_1	GCA_001027945.1_01202	fabG_1

fabG_1	GCA_001027945.1_03195	fabG_1
fabG_1	GCA_001027945.1_02128	fabG_1
fabG_1	GCA_001027945.1_02739	fabG_1
fabG_1	GCA_001027945.1_01843	fabG_1
fabG_1	GCA_001027945.1_00499	fabG_1
fabG_1	GCA_001027945.1_01767	fabG_1
fabG_1	GCA_001027945.1_00310	fabG_1
fabG_1	GCA_001027945.1_00309	fabG_1
fabG_1	GCA_001027945.1_01556	fabG_1
fabG_1	GCA_001027945.1_01337	fabG_1
fabG_1	GCA_001027945.1_01555	fabG_1
fabG_1	HI1_02950	fabG_1
fabG_1	HI1_00348	fabG_1
fabG_1	HI1_01392	fabG_1
fabG_1	HI1_01403	fabG_1
fabG_1	HI1_00234	fabG_1
fabG_1	HI1_02326	fabG_1
fabG_1	HI1_00356	fabG_1
fabG_1	HI1_00909	fabG_1
fabG_1	HI1_02651	fabG_1
fabG_1	HI1_00672	fabG_1
fabG_1	HI1_02036	fabG_1
fabG_1	HI1_01394	fabG_1
fabG_1	HI1_01098	fabG_1
fabG_1	HI1_01672	fabG_1
fabG_1	HI1_03164	fabG_1
fabG_1	HI1_01658	fabG_1
fabG_1	HI1_02859	fabG_1
fabG_1	IR1_02780	fabG_1
fabG_1	IR1_01684	fabG_1
fabG_1	IR1_01932	fabG_1
fabG_1	IR1_03442	fabG_1
fabG_1	IR1_03307	fabG_1
fabG_1	IR1_03975	fabG_1
fabG_1	IR1_03562	fabG_1
fabG_1	IR1_03683	fabG_1
fabG_1	IR1_01294	fabG_1
fabG_1	IR1_00054	fabG_1
fabG_1	IR1_04586	fabG_1
fabG_1	IR1_01656	fabG_1
fabG_1	IR1_04630	fabG_1
fabG_1	IR1_04580	fabG_1
fabG_1	IR1_03791	fabG_1
fabG_1	IR1_01775	fabG_1
fabG_1	IR1_03550	fabG_1
fabG_1	IR1_03169	fabG_1
fabG_1	IR1_03222	fabG_1
fabG_1	IR1_03663	fabG_1

fabG_1	IR1_04141	fabG_1
fabG_1	IR1_00979	fabG_1
fabG_1	IR1_03125	fabG_1
fabG_1	IR1_04556	fabG_1
fabG_1	IR1_03679	fabG_1
fabG_1	IR1_03217	fabG_1
fabG_1	IR1_03561	fabG_1
fabG_1	IR1_03680	fabG_1
fabG_1	KCOM3131_01370	fabG_1
fabG_1	KCOM3131_00902	fabG_1
fabG_1	KCOM3131_00176	fabG_1
fabG_1	KCOM3131_01384	fabG_1
fabG_1	KCTC12974_00003	fabG_1
fabG_1	KCTC12974_02812	fabG_1
fabG_1	KCTC12974_00026	fabG_1
fabG_1	KCTC12974_02299	fabG_1
fabG_1	KCTC12974_01028	fabG_1
fabG_1	KCTC12974_01687	fabG_1
fabG_1	KCTC12974_02766	fabG_1
fabG_1	KCTC12974_00983	fabG_1
fabG_1	KCTC12974_01520	fabG_1
fabG_1	KCTC12974_01861	fabG_1
fabG_1	KCTC12974_01117	fabG_1
fabG_1	KCTC12974_02770	fabG_1
fabG_1	KCTC12974_02158	fabG_1
fabG_1	KCTC12974_02733	fabG_1
fabG_1	KCTC12974_00705	fabG_1
fabG_1	KCTC12974_02298	fabG_1
fabG_1	KL-A_00477	fabG_1
fabG_1	KL-A_01790	fabG_1
fabG_1	KL-A_00775	fabG_1
fabG_1	KL-A_00764	fabG_1
fabG_1	KL-A_03170	fabG_1
fabG_1	KL-A_01673	fabG_1
fabG_1	KL-A_01782	fabG_1
fabG_1	KL-A_01346	fabG_1
fabG_1	KL-A_02101	fabG_1
fabG_1	KL-A_03403	fabG_1
fabG_1	KL-A_00161	fabG_1
fabG_1	KL-A_00773	fabG_1
fabG_1	KL-A_01955	fabG_1
fabG_1	KL-A_01540	fabG_1
fabG_1	KL-A_00597	fabG_1
fabG_1	L12_00223	fabG_1
fabG_1	LL0412.1.7_02458	fabG_1
fabG_1	LL0412.1.7_00374	fabG_1
fabG_1	LL0412.1.7_01270	fabG_1
fabG_1	LL0412.1.7_01698	fabG_1

fabG_1	LL0412.1.7_02552	fabG_1
fabG_1	LL0412.1.7_00899	fabG_1
fabG_1	LL0412.1.7_00438	fabG_1
fabG_1	LL0412.1.7_02532	fabG_1
fabG_1	LL0412.1.7_00687	fabG_1
fabG_1	LL0412.1.7_02416	fabG_1
fabG_1	MAR_2009_71_03763	fabG_1
fabG_1	MAR_2009_71_02968	fabG_1
fabG_1	MAR_2009_71_00579	fabG_1
fabG_1	MAR_2009_71_03059	fabG_1
fabG_1	MAR_2009_71_00554	fabG_1
fabG_1	MAR_2009_71_03707	fabG_1
fabG_1	MAR_2009_71_02159	fabG_1
fabG_1	MAR_2009_71_03766	fabG_1
fabG_1	MAR_2009_71_03751	fabG_1
fabG_1	MAR_2009_71_01870	fabG_1
fabG_1	MAR_2009_71_03771	fabG_1
fabG_1	MAR_2009_71_03313	fabG_1
fabG_1	MAR_2009_71_03653	fabG_1
fabG_1	MAR_2009_71_03132	fabG_1
fabG_1	MAR_2009_71_03391	fabG_1
fabG_1	MAR_2009_71_03854	fabG_1
fabG_1	MAR_2009_71_00959	fabG_1
fabG_1	MAR_2009_71_01002	fabG_1
fabG_1	MAR_2009_71_02163	fabG_1
fabG_1	MAR_2009_71_03859	fabG_1
fabG_1	MAR_2009_71_02891	fabG_1
fabG_1	MAR_2009_71_03251	fabG_1
fabG_1	MAR_2009_71_02793	fabG_1
fabG_1	MAR_2009_71_00449	fabG_1
fabG_1	MAR_2009_71_02063	fabG_1
fabG_1	MAR_2009_71_02610	fabG_1
fabG_1	MAR_2009_71_03145	fabG_1
fabG_1	MAR_2009_71_01007	fabG_1
fabG_1	NN016038_00026	fabG_1
fabG_1	NN016038_03521	fabG_1
fabG_1	NN016038_03709	fabG_1
fabG_1	NN016038_01323	fabG_1
fabG_1	NN016038_02187	fabG_1
fabG_1	NN016038_00932	fabG_1
fabG_1	NN016038_02024	fabG_1
fabG_1	NN016038_03573	fabG_1
fabG_1	NN016038_03572	fabG_1
fabG_1	NN016038_03240	fabG_1
fabG_1	NN016038_02961	fabG_1
fabG_1	NN016038_02733	fabG_1
fabG_1	NN016038_00029	fabG_1
fabG_1	NN016038_04047	fabG_1

fabG_1	NN016038_02981	fabG_1
fabG_1	NN016038_02014	fabG_1
fabG_1	NN016038_02976	fabG_1
fabG_1	NN016038_03082	fabG_1
fabG_1	NN016038_03356	fabG_1
fabG_1	NN016038_04040	fabG_1
fabG_1	NN016038_00241	fabG_1
fabG_1	NN016038_02781	fabG_1
fabG_1	NN016038_02576	fabG_1
fabG_1	NN016038_00819	fabG_1
fabG_1	NN016038_00862	fabG_1
fabG_1	NN016038_00341	fabG_1
fabG_1	NN016038_02066	fabG_1
fabG_1	NN016038_03218	fabG_1
fabG_1	NN016038_02830	fabG_1
fabG_1	NN016038_03189	fabG_1
fabG_1	NN016038_03347	fabG_1
fabG_1	NN016038_03346	fabG_1
fabG_1	PAO1_01694	fabG_1
fabG_1	PAO1_01882	fabG_1
fabG_1	PAO1_03506	fabG_1
fabG_1	PAO1_02208	fabG_1
fabG_1	PAO1_04227	fabG_1
fabG_1	PAO1_02065	fabG_1
fabG_1	PAO1_03236	fabG_1
fabG_1	PAO1_05704	fabG_1
fabG_1	PAO1_03633	fabG_1
fabG_1	PAO1_04279	fabG_1
fabG_1	PAO1_04293	fabG_1
fabG_1	PAO1_00187	fabG_1
fabG_1	PAO1_04532	fabG_1
fabG_1	PAO1_03071	fabG_1
fabG_1	PAO1_04218	fabG_1
fabG_1	PAO1_02989	fabG_1
fabG_1	PAO1_05707	fabG_1
fabG_1	PAO1_01578	fabG_1
fabG_1	PAO1_01383	fabG_1
fabG_1	PAO1_01881	fabG_1
fabG_1	PAO1_01058	fabG_1
fabG_1	PAO1_03214	fabG_1
fabG_1	PAO1_02585	fabG_1
fabG_1	PAO1_03548	fabG_1
fabG_1	PAO1_05323	fabG_1
fabG_1	PAO1_03629	fabG_1
fabG_1	PAO1_02626	fabG_1
fabG_1	PAO1_04085	fabG_1
fabG_1	PAO1_01510	fabG_1
fabG_1	PAO1_02994	fabG_1

fabG_1	PAO1_01419	fabG_1
fabG_1	PAO1_02163	fabG_1
fabG_1	PAO1_03392	fabG_1
fabG_1	PAO1_03442	fabG_1
fabG_1	PAO1_00672	fabG_1
fabG_1	PAO1_03448	fabG_1
fabG_1	PAO1_00123	fabG_1
fabG_1	PAO1_04998	fabG_1
fabG_1	PAO1_04208	fabG_1
fabG_1	PAO1_04008	fabG_1
fabG_1	PAO1_04949	fabG_1
fabG_1	PAO1_05074	fabG_1
fabG_1	PAO1_03283	fabG_1
fabG_1	PAO1_01369	fabG_1
fabG_1	PAO1_03020	fabG_1
fabG_1	PAO1_03558	fabG_1
fabG_1	PAO1_05201	fabG_1
fabG_1	UW101_04293	fabG_1
fabG_1	UW101_02087	fabG_1
fabG_1	UW101_00087	fabG_1
fabG_1	UW101_00010	fabG_1
fabG_1	UW101_04632	fabG_1
fabG_1	UW101_05136	fabG_1
fabG_1	UW101_04363	fabG_1
fabG_1	UW101_04457	fabG_1
fabG_1	UW101_03020	fabG_1
fabG_1	UW101_04176	fabG_1
fabG_1	UW101_04011	fabG_1
fabG_1	UW101_03909	fabG_1
fabG_1	UW101_03497	fabG_1
fabG_1	UW101_04290	fabG_1
fabG_1	UW101_04013	fabG_1
fabG_1	UW101_01159	fabG_1
fabG_1	UW101_04441	fabG_1
fabG_1	UW101_04630	fabG_1
fabG_1	UW101_05251	fabG_1
fabG_1	UW101_02759	fabG_1
fabG_1	UW101_04759	fabG_1
fabG_1	UW101_04163	fabG_1
fabG_1	UW101_03354	fabG_1
fabG_1	UW101_01920	fabG_1
fabG_1	UW101_04388	fabG_1
fabG_1	UW101_00501	fabG_1
fabG_1	UW101_01370	fabG_1
fabG_1	UW101_03433	fabG_1
fabG_1	UW101_00752	fabG_1
fabG_1	UW101_03905	fabG_1
fabG_1	UW101_03944	fabG_1

fabG_1	UW101_00474	fabG_1
fabG_1	UW101_03989	fabG_1
fabG_1	UW101_02772	fabG_1
fabG_1	UW101_03332	fabG_1
fabG_1	UW101_04168	fabG_1
fabG_1	UW101_03957	fabG_1
fabG_1	UW101_00496	fabG_1
fabG_1	UW101_04054	fabG_1
fabG_1	UW101_03991	fabG_1
fabG_1	UW101_03910	fabG_1
fabG_1	UW101_02191	fabG_1
fabG_1	UW101_04456	fabG_1
fabG_1	UW101_04059	fabG_1
fabG_1	UW101_04598	fabG_1
fabG_1	V4-33_00876	fabG_1
fabG_1	V4-33_01042	fabG_1
fabG_1	V4-33_02206	fabG_1
fabG_1	V4-33_00959	fabG_1
fabG_1	V4-33_00923	fabG_1
fabG_1	V4-33_01291	fabG_1
fabG_1	V4-33_01188	fabG_1
fabG_1	V4-33_00990	fabG_1
fabG_1	V4-33_01075	fabG_1
fabG_1	W5C_03184	fabG_1
fabG_1	W5C_02218	fabG_1
fabG_1	W5C_00209	fabG_1
fabG_1	W5C_03228	fabG_1
fabG_1	W5C_01159	fabG_1
fabG_1	W5C_01902	fabG_1
fabG_1	W5C_01897	fabG_1
fabG_1	W5C_03175	fabG_1
fabG_1	W5C_02391	fabG_1
fabG_1	W5C_01370	fabG_1
fabG_1	W5C_01151	fabG_1
fabG_1	W5C_02946	fabG_1
fabG_1	W5C_01999	fabG_1
fabG_1	W5C_01767	fabG_1
fabG_1	W5C_02122	fabG_1
fabG_1	W5C_00437	fabG_1
fabG_1	ZONMW-01_00762	fabG_1
fabG_1	ZONMW-01_01868	fabG_1
fabG_1	ZONMW-01_01823	fabG_1
fabG_1	ZONMW-01_02830	fabG_1
fabG_1	ZONMW-01_02650	fabG_1
fabG_1	ZONMW-01_02458	fabG_1
fabG_1	ZONMW-01_02193	fabG_1
fabG_1	ZONMW-01_01876	fabG_1
fabG_1	ZONMW-01_02197	fabG_1

fabG_1	ZONMW-01_01109	fabG_1
fabG_1	ZONMW-01_02637	fabG_1
fabG_1	ZONMW-01_02449	fabG_1
fabG_1	ZONMW-01_01477	fabG_1
fabG_1	ZONMW-01_00568	fabG_1
fabG_1	ZONMW-01_00869	fabG_1
fabG_1	ZONMW-01_00181	fabG_1
fabG_1	ZONMW-02_01334	fabG_1
fabG_1	ZONMW-02_01972	fabG_1
fabG_1	ZONMW-02_03357	fabG_1
fabG_1	ZONMW-02_03046	fabG_1
fabG_1	ZONMW-02_02812	fabG_1
fabG_1	ZONMW-02_03608	fabG_1
fabG_1	ZONMW-02_00064	fabG_1
fabG_1	ZONMW-02_02509	fabG_1
fabG_1	ZONMW-02_02993	fabG_1
fabG_1	ZONMW-02_01337	fabG_1
fabG_1	ZONMW-02_01217	fabG_1
fabG_1	ZONMW-02_01199	fabG_1
fabG_1	ZONMW-02_03294	fabG_1
fabG_1	ZONMW-02_00823	fabG_1
fabG_1	ZONMW-02_02498	fabG_1
fabG_1	ZONMW-02_00448	fabG_1
fabG_1	ZONMW-02_00166	fabG_1
fabG_1	ZONMW-02_02340	fabG_1
fabG_1	ZONMW-02_02161	fabG_1
fabG_1	ZONMW-02_03838	fabG_1
fabG_1	ZONMW-02_03171	fabG_1
fabG_1	ZONMW-02_00624	fabG_1
fabG_1	ZONMW-03_00140	fabG_1
fabG_1	ZONMW-03_02852	fabG_1
fabG_1	ZONMW-03_02808	fabG_1
fabG_1	ZONMW-03_01593	fabG_1
fabG_1	ZONMW-03_01676	fabG_1
fabG_1	ZONMW-03_03061	fabG_1
fabG_1	ZONMW-03_02517	fabG_1
fabG_1	ZONMW-03_02860	fabG_1
fabG_1	ZONMW-03_00451	fabG_1
fabG_1	ZONMW-03_02178	fabG_1
fabG_1	ZONMW-03_01663	fabG_1
fabG_1	ZONMW-03_03052	fabG_1
fabG_1	ZONMW-03_01303	fabG_1
fabG_1	ZONMW-03_02462	fabG_1
fabG_1	ZONMW-03_00247	fabG_1
fabG_1	ZONMW-03_00907	fabG_1
fabG_1	ZONMW-04_01144	fabG_1
fabG_1	ZONMW-04_02598	fabG_1
fabG_1	ZONMW-04_02719	fabG_1

fabG_1	ZONMW-04_00089	fabG_1
fabG_1	ZONMW-04_02316	fabG_1
fabG_1	ZONMW-04_02383	fabG_1
fabG_1	ZONMW-04_02032	fabG_1
fabG_1	ZONMW-04_02590	fabG_1
fabG_1	ZONMW-04_01193	fabG_1
fabG_1	ZONMW-04_01390	fabG_1
fabG_1	ZONMW-04_01640	fabG_1
fabG_1	ZONMW-04_01192	fabG_1
fabG_1	ZONMW-04_02329	fabG_1
fabG_1	ZONMW-04_02392	fabG_1
fabG_1	ZONMW-04_00387	fabG_1
fabG_1	ZONMW-04_00943	fabG_1
fabG_1	ZONMW-04_03095	fabG_1
fabG_1	ZONMW-04_02039	fabG_1
fabG_1	ZONMW-05_00395	fabG_1
fabG_1	ZONMW-05_02227	fabG_1
fabG_1	ZONMW-05_02271	fabG_1
fabG_1	ZONMW-05_01741	fabG_1
fabG_1	ZONMW-05_02832	fabG_1
fabG_1	ZONMW-05_02641	fabG_1
fabG_1	ZONMW-05_02337	fabG_1
fabG_1	ZONMW-05_02219	fabG_1
fabG_1	ZONMW-05_00714	fabG_1
fabG_1	ZONMW-05_01918	fabG_1
fabG_1	ZONMW-05_00444	fabG_1
fabG_1	ZONMW-05_02845	fabG_1
fabG_1	ZONMW-05_02632	fabG_1
fabG_1	ZONMW-05_01438	fabG_1
fabG_1	ZONMW-05_00195	fabG_1
fabG_1	ZONMW-05_00505	fabG_1
fabG_1	ZONMW-05_01096	fabG_1
fabG_1	ZONMW-06_02239	fabG_1
fabG_1	ZONMW-06_00692	fabG_1
fabG_1	ZONMW-06_02768	fabG_1
fabG_1	ZONMW-06_02835	fabG_1
fabG_1	ZONMW-06_03293	fabG_1
fabG_1	ZONMW-06_01368	fabG_1
fabG_1	ZONMW-06_00555	fabG_1
fabG_1	ZONMW-06_01491	fabG_1
fabG_1	ZONMW-06_01073	fabG_1
fabG_1	ZONMW-06_00893	fabG_1
fabG_1	ZONMW-06_02651	fabG_1
fabG_1	ZONMW-06_00629	fabG_1
fabG_1	ZONMW-06_02838	fabG_1
fabG_1	ZONMW-06_01690	fabG_1
fabG_1	ZONMW-06_01439	fabG_1
fabG_1	ZONMW-06_00385	fabG_1

fabG_1	ZONMW-06_00888	fabG_1
fabG_1	ZONMW-06_03423	fabG_1
fabG_1	ZONMW-06_03574	fabG_1
fabG_1	ZONMW-06_00688	fabG_1
fabG_1	ZONMW-06_00281	fabG_1
fabG_1	ZONMW-06_00580	fabG_1
fabG_1	ZONMW-06_02798	fabG_1
fabG_1	ZONMW-06_02769	fabG_1
fabG_1	ZONMW-06_02382	fabG_1
fabG_1	ZONMW-06_03585	fabG_1
fabG_1	ZONMW-06_02548	fabG_1
fabG_1	ZONMW-06_00610	fabG_1
fabG_1	ZONMW-06_01280	fabG_1
fabG_1	ZONMW-06_02179	fabG_1
fabG_1	ZONMW-06_01540	fabG_1
fabG_1	ZONMW-06_01284	fabG_1
fabG_1	ZONMW-07_03636	fabG_1
fabG_1	ZONMW-07_02541	fabG_1
fabG_1	ZONMW-07_00774	fabG_1
fabG_1	ZONMW-07_00775	fabG_1
fabG_1	ZONMW-07_03647	fabG_1
fabG_1	ZONMW-07_01046	fabG_1
fabG_1	ZONMW-07_02311	fabG_1
fabG_1	ZONMW-07_00303	fabG_1
fabG_1	ZONMW-07_02929	fabG_1
fabG_1	ZONMW-07_03347	fabG_1
fabG_1	ZONMW-07_02020	fabG_1
fabG_1	ZONMW-07_02158	fabG_1
fabG_1	ZONMW-07_00891	fabG_1
fabG_1	ZONMW-07_00215	fabG_1
fabG_1	ZONMW-07_03443	fabG_1
fabG_1	ZONMW-07_00056	fabG_1
fabG_1	ZONMW-07_03760	fabG_1
fabG_1	ZONMW-07_00731	fabG_1
fabG_1	ZONMW-07_03078	fabG_1
fabG_1	ZONMW-07_01060	fabG_1
fabG_1	ZONMW-07_01995	fabG_1
fabG_1	ZONMW-07_02641	fabG_1
fabG_1	ZONMW-07_02621	fabG_1
fabG_1	ZONMW-07_01004	fabG_1
fabG_1	ZONMW-07_01037	fabG_1
fabG_1	ZONMW-07_02155	fabG_1
fabG_1	ZONMW-08_03180	fabG_1
fabG_1	ZONMW-08_03549	fabG_1
fabG_1	ZONMW-08_02167	fabG_1
fabG_1	ZONMW-08_00095	fabG_1
fabG_1	ZONMW-08_03548	fabG_1
fabG_1	ZONMW-08_00071	fabG_1

fabG_1	ZONMW-08_03236	fabG_1
fabG_1	ZONMW-08_03822	fabG_1
fabG_1	ZONMW-08_01100	fabG_1
fabG_1	ZONMW-08_03177	fabG_1
fabG_1	ZONMW-08_03192	fabG_1
fabG_1	ZONMW-08_03553	fabG_1
fabG_1	ZONMW-08_03172	fabG_1
fabG_1	ZONMW-08_01356	fabG_1
fabG_1	ZONMW-08_02827	fabG_1
fabG_1	ZONMW-08_02261	fabG_1
fabG_1	ZONMW-08_02648	fabG_1
fabG_1	ZONMW-08_02536	fabG_1
fabG_1	ZONMW-08_03115	fabG_1
fabG_1	ZONMW-08_01093	fabG_1
fabG_1	ZONMW-08_00478	fabG_1
fabG_1	ZONMW-08_00521	fabG_1
fabG_1	ZONMW-08_01096	fabG_1
fabG_1	ZONMW-08_03110	fabG_1
fabG_1	ZONMW-08_02766	fabG_1
fabG_1	ZONMW-08_02090	fabG_1
fabG_1	ZONMW-08_02010	fabG_1
fabG_1	ZONMW-08_03771	fabG_1
fabG_1	ZONMW-08_01193	fabG_1
fabG_1	ZONMW-08_03614	fabG_1
fabG_1	ZONMW-08_02660	fabG_1
fabG_1	ZONMW-08_00526	fabG_1
fabG_1	ZONMW-09_01009	fabG_1
fabG_1	ZONMW-09_00922	fabG_1
fabG_1	ZONMW-09_00608	fabG_1
fabG_1	ZONMW-09_01092	fabG_1
fabG_1	ZONMW-09_01560	fabG_1
fabG_1	ZONMW-09_00957	fabG_1
fabG_1	ZONMW-09_00708	fabG_1
fabG_1	ZONMW-09_01504	fabG_1
fabG_1	ZONMW-09_01394	fabG_1
fabG_1	ZONMW-09_01118	fabG_1
fabG_1	ZONMW-09_00305	fabG_1
fabG_1	ZONMW-09_01069	fabG_1
fabG_1	ZONMW-10_00826	fabG_1
fabG_1	ZONMW-10_01670	fabG_1
fabG_1	ZONMW-10_01589	fabG_1
fabG_1	ZONMW-10_00384	fabG_1
fabG_1	ZONMW-10_00702	fabG_1
fabG_1	ZONMW-10_02047	fabG_1
fabG_1	ZONMW-10_02466	fabG_1
fabG_1	ZONMW-10_00296	fabG_1
fabG_1	ZONMW-10_01340	fabG_1
fabG_1	ZONMW-10_02465	fabG_1

fabG_1	ZONMW-11_01387	fabG_1
fabG_1	ZONMW-11_02438	fabG_1
fabG_1	ZONMW-11_02796	fabG_1
fabG_1	ZONMW-11_03121	fabG_1
fabG_1	ZONMW-11_00212	fabG_1
fabG_1	ZONMW-11_03325	fabG_1
fabG_1	ZONMW-11_02249	fabG_1
fabG_1	ZONMW-11_01901	fabG_1
fabG_1	ZONMW-11_00897	fabG_1
fabG_1	ZONMW-11_00424	fabG_1
fabG_1	ZONMW-11_00594	fabG_1
fabG_1	ZONMW-11_01359	fabG_1
fabG_1	ZONMW-11_00738	fabG_1
fabG_1	ZONMW-11_00874	fabG_1
fabG_1	ZONMW-11_04169	fabG_1
fabG_1	ZONMW-11_00182	fabG_1
fabG_1	ZONMW-11_02399	fabG_1
fabG_1	ZONMW-11_02278	fabG_1
fabG_1	ZONMW-11_02676	fabG_1
fabG_1	ZONMW-11_01469	fabG_1
fabG_1	ZONMW-11_03669	fabG_1
fabG_1	ZONMW-11_01076	fabG_1
fabG_1	ZONMW-11_04168	fabG_1
fabG_1	ZONMW-11_01165	fabG_1
fabG_1	ZONMW-11_01212	fabG_1
fabG_1	ZONMW-11_02087	fabG_1
fabG_1	ZONMW-11_02657	fabG_1
fabG_1	ZONMW-11_03132	fabG_1
fabG_1	ZONMW-11_01543	fabG_1
fabG_1	ZONMW-11_01539	fabG_1
fabG_1	ZONMW-11_03131	fabG_1
fabG_1	ZONMW-11_00790	fabG_1
fabG_1	ZONMW-11_04677	fabG_1
fabG_1	ZONMW-11_00006	fabG_1
fabG_1	ZONMW-11_02005	fabG_1
fabG_1	ZONMW-12_02865	fabG_1
fabG_1	ZONMW-12_03302	fabG_1
fabG_1	ZONMW-12_01310	fabG_1
fabG_1	ZONMW-12_02559	fabG_1
fabG_1	ZONMW-12_02850	fabG_1
fabG_1	ZONMW-12_04212	fabG_1
fabG_1	ZONMW-12_01176	fabG_1
fabG_1	ZONMW-12_04257	fabG_1
fabG_1	ZONMW-12_00080	fabG_1
fabG_1	ZONMW-12_03609	fabG_1
fabG_1	ZONMW-12_04497	fabG_1
fabG_1	ZONMW-12_04363	fabG_1
fabG_1	ZONMW-12_02314	fabG_1

fabG_1	ZONMW-12_00361	fabG_1
fabG_1	ZONMW-12_01748	fabG_1
fabG_1	ZONMW-12_03166	fabG_1
fabG_1	ZONMW-12_02857	fabG_1
fabG_1	ZONMW-12_02476	fabG_1
fabG_1	ZONMW-12_03977	fabG_1
fabG_1	ZONMW-12_04366	fabG_1
fabG_1	ZONMW-12_01655	fabG_1
fabG_1	ZONMW-12_03379	fabG_1
fabG_1	ZONMW-12_04213	fabG_1
fabG_1	ZONMW-12_04281	fabG_1
fabG_1	ZONMW-12_01482	fabG_1
fabG_1	ZONMW-12_02534	fabG_1
fabG_1	ZONMW-12_00796	fabG_1
fabG_1	ZONMW-12_02355	fabG_1
fabG_1	ZONMW-12_02341	fabG_1
fabG_1	ZONMW-12_04277	fabG_1
fabG_1	ZONMW-12_02715	fabG_1
fabG_1	ZONMW-12_04272	fabG_1
fabG_1	ZONMW-12_02522	fabG_1
fabG_1	ZONMW-12_03957	fabG_1
fabG_1	ZONMW-12_04298	fabG_1
fabG_1	ZONMW-12_04297	fabG_1
fabG_1	ZONMW-12_01871	fabG_1
fabG_1	ZONMW-12_02868	fabG_1
fabG_1	ZONMW-12_04276	fabG_1
fabG_1	ZONMW-12_04324	fabG_1
fabG_1	ZONMW-13_02717	fabG_1
fabG_1	ZONMW-13_02505	fabG_1
fabG_1	ZONMW-13_00556	fabG_1
fabG_1	ZONMW-13_01144	fabG_1
fabG_1	ZONMW-13_00968	fabG_1
fabG_1	ZONMW-13_02116	fabG_1
fabG_1	ZONMW-13_00534	fabG_1
fabG_1	ZONMW-13_01706	fabG_1
fabG_1	ZONMW-13_00627	fabG_1
fabG_1	ZONMW-13_01425	fabG_1
fabG_1	ZONMW-13_00876	fabG_1
fabG_1	ZONMW-13_01979	fabG_1
fabG_1	ZONMW-13_00841	fabG_1
fabG_1	ZONMW-13_00118	fabG_1
fabG_1	ZONMW-13_02661	fabG_1
fabG_1	ZONMW-13_00483	fabG_1
fabG_1	ZONMW-13_01978	fabG_1
fabG_1	ZONMW-13_02057	fabG_1
fabG_1	ZONMW-13_02325	fabG_1
fabG_1	ZONMW-13_01204	fabG_1
fabG_1	ZONMW-13_01870	fabG_1

fabG_1	ZONMW-13_00625	fabG_1
fabG_1	ZONMW-13_00626	fabG_1
fabG_1	ZONMW-14_00536	fabG_1
fabG_1	ZONMW-14_02497	fabG_1
fabG_1	ZONMW-14_00578	fabG_1
fabG_1	ZONMW-14_00949	fabG_1
fabG_1	ZONMW-14_01450	fabG_1
fabG_1	ZONMW-14_01140	fabG_1
fabG_1	ZONMW-14_02361	fabG_1
fabG_1	ZONMW-14_00715	fabG_1
fabG_1	ZONMW-14_00477	fabG_1
fabG_1	ZONMW-14_01239	fabG_1
fabG_1	ZONMW-14_02668	fabG_1
fabG_1	ZONMW-14_00716	fabG_1
fabG_1	ZONMW-14_00078	fabG_1
fabG_1	ZONMW-14_01103	fabG_1
fabG_1	ZONMW-14_00635	fabG_1
fabG_1	ZONMW-14_00386	fabG_1
fabG_1	ZONMW-15_00602	fabG_1
fabG_1	ZONMW-15_02242	fabG_1
fabG_1	ZONMW-15_03022	fabG_1
fabG_1	ZONMW-15_03393	fabG_1
fabG_1	ZONMW-15_01678	fabG_1
fabG_1	ZONMW-15_00168	fabG_1
fabG_1	ZONMW-15_03052	fabG_1
fabG_1	ZONMW-15_03048	fabG_1
fabG_1	ZONMW-15_03394	fabG_1
fabG_1	ZONMW-15_00599	fabG_1
fabG_1	ZONMW-15_00799	fabG_1
fabG_1	ZONMW-15_02395	fabG_1
fabG_1	ZONMW-15_02663	fabG_1
fabG_1	ZONMW-15_02934	fabG_1
fabG_1	ZONMW-15_01782	fabG_1
fabG_1	ZONMW-15_01740	fabG_1
fabG_1	ZONMW-15_00459	fabG_1
fabG_1	ZONMW-15_02765	fabG_1
fabG_1	ZONMW-15_01836	fabG_1
fabG_1	ZONMW-15_02357	fabG_1
fabG_1	ZONMW-15_02827	fabG_1
fabG_1	ZONMW-15_01917	fabG_1
fabG_1	ZONMW-19_01605	fabG_1
fabG_1	ZONMW-19_02994	fabG_1
fabG_1	ZONMW-19_04533	fabG_1
fabG_1	ZONMW-19_02479	fabG_1
fabG_1	ZONMW-19_02633	fabG_1
fabG_1	ZONMW-19_02554	fabG_1
fabG_1	ZONMW-19_03846	fabG_1
fabG_1	ZONMW-19_04038	fabG_1

fabG_1	ZONMW-19_03073	fabG_1
fabG_1	ZONMW-19_00102	fabG_1
fabG_1	ZONMW-19_00422	fabG_1
fabG_1	ZONMW-19_01322	fabG_1
fabG_1	ZONMW-19_02312	fabG_1
fabG_1	ZONMW-19_02468	fabG_1
fabG_1	ZONMW-19_01217	fabG_1
fabG_1	ZONMW-19_03818	fabG_1
fabG_1	ZONMW-20_00537	fabG_1
fabG_1	ZONMW-20_02219	fabG_1
fabG_1	ZONMW-20_00184	fabG_1
fabG_1	ZONMW-20_02140	fabG_1
fabG_1	ZONMW-20_00151	fabG_1
fabG_1	ZONMW-20_04160	fabG_1
fabG_1	ZONMW-20_02250	fabG_1
fabG_1	ZONMW-20_03843	fabG_1
fabG_1	ZONMW-20_00158	fabG_1
fabG_1	ZONMW-20_03310	fabG_1
fabG_1	ZONMW-20_03281	fabG_1
fabG_1	ZONMW-20_00212	fabG_1
fabG_1	ZONMW-20_01649	fabG_1
fabG_1	ZONMW-20_00234	fabG_1
fabG_1	ZONMW-20_03298	fabG_1
fabG_1	ZONMW-20_00510	fabG_1
fabG_1	ZONMW-20_04324	fabG_1
fabG_1	ZONMW-20_02645	fabG_1
fabG_1	ZONMW-20_02585	fabG_1
fabG_1	ZONMW-20_03506	fabG_1
fabG_1	ZONMW-20_00217	fabG_1
fabG_1	ZONMW-20_01263	fabG_1
fabG_1	ZONMW-20_00352	fabG_1
fabG_1	ZONMW-20_00396	fabG_1
fabG_1	ZONMW-20_01514	fabG_1
fabG_1	ZONMW-21_00347	fabG_1
fabG_1	ZONMW-21_01991	fabG_1
fabG_1	ZONMW-21_00357	fabG_1
fabG_1	ZONMW-21_00481	fabG_1
fabG_1	ZONMW-21_01279	fabG_1
fabG_1	ZONMW-21_00069	fabG_1
fabG_1	ZONMW-21_00571	fabG_1
fabG_1	ZONMW-21_02492	fabG_1
fabG_1	ZONMW-21_00704	fabG_1
fabG_1	ZONMW-21_01696	fabG_1
fabG_1	ZONMW-21_01030	fabG_1
fabG_1	ZONMW-21_03460	fabG_1
fabG_1	ZONMW-21_00918	fabG_1
fabG_1	ZONMW-21_02517	fabG_1
fabG_1	ZONMW-21_00987	fabG_1

fabG_1	ZONMW-21_00221	fabG_1
fabG_1	ZONMW-22_00347	fabG_1
fabG_1	ZONMW-22_01735	fabG_1
fabG_1	ZONMW-22_00357	fabG_1
fabG_1	ZONMW-22_00481	fabG_1
fabG_1	ZONMW-22_01281	fabG_1
fabG_1	ZONMW-22_00069	fabG_1
fabG_1	ZONMW-22_00571	fabG_1
fabG_1	ZONMW-22_02617	fabG_1
fabG_1	ZONMW-22_00705	fabG_1
fabG_1	ZONMW-22_02028	fabG_1
fabG_1	ZONMW-22_01031	fabG_1
fabG_1	ZONMW-22_03473	fabG_1
fabG_1	ZONMW-22_00919	fabG_1
fabG_1	ZONMW-22_02592	fabG_1
fabG_1	ZONMW-22_00988	fabG_1
fabG_1	ZONMW-22_00221	fabG_1
fabG_1	ZONMW-23_01462	fabG_1
fabG_1	ZONMW-23_00817	fabG_1
fabG_1	ZONMW-23_01472	fabG_1
fabG_1	ZONMW-23_01596	fabG_1
fabG_1	ZONMW-23_02393	fabG_1
fabG_1	ZONMW-23_01184	fabG_1
fabG_1	ZONMW-23_01686	fabG_1
fabG_1	ZONMW-23_02641	fabG_1
fabG_1	ZONMW-23_01819	fabG_1
fabG_1	ZONMW-23_00523	fabG_1
fabG_1	ZONMW-23_02144	fabG_1
fabG_1	ZONMW-23_03467	fabG_1
fabG_1	ZONMW-23_02032	fabG_1
fabG_1	ZONMW-23_02616	fabG_1
fabG_1	ZONMW-23_02101	fabG_1
fabG_1	ZONMW-23_01336	fabG_1
fabG_1	ZONMW-24_01112	fabG_1
fabG_1	ZONMW-24_01758	fabG_1
fabG_1	ZONMW-24_01102	fabG_1
fabG_1	ZONMW-24_00978	fabG_1
fabG_1	ZONMW-24_00180	fabG_1
fabG_1	ZONMW-24_01391	fabG_1
fabG_1	ZONMW-24_00888	fabG_1
fabG_1	ZONMW-24_02516	fabG_1
fabG_1	ZONMW-24_00756	fabG_1
fabG_1	ZONMW-24_02653	fabG_1
fabG_1	ZONMW-24_00430	fabG_1
fabG_1	ZONMW-24_03469	fabG_1
fabG_1	ZONMW-24_00542	fabG_1
fabG_1	ZONMW-24_02541	fabG_1
fabG_1	ZONMW-24_00473	fabG_1

fabG_1	ZONMW-24_01239	fabG_1
fabG_1	ZONMW-25_00220	fabG_1
fabG_1	ZONMW-25_00128	fabG_1
fabG_1	ZONMW-25_01015	fabG_1
fabG_1	ZONMW-25_02421	fabG_1
fabG_1	ZONMW-25_00204	fabG_1
fabG_1	ZONMW-25_01540	fabG_1
fabG_1	ZONMW-25_02401	fabG_1
fabG_1	ZONMW-25_02223	fabG_1
fabG_1	ZONMW-25_00535	fabG_1
fabG_1	ZONMW-25_01487	fabG_1
fabG_1	ZONMW-25_02035	fabG_1
fabG_1	ZONMW-25_01987	fabG_1
fabG_1	ZONMW-25_00398	fabG_1
fabG_1	ZONMW-25_02430	fabG_1
fabG_1	ZONMW-25_02373	fabG_1
fabG_1	ZONMW-25_00430	fabG_1
fabG_1	ZONMW-25_01125	fabG_1
fabG_1	ZONMW-26_02895	fabG_1
fabG_1	ZONMW-26_00570	fabG_1
fabG_1	ZONMW-26_02936	fabG_1
fabG_1	ZONMW-26_01552	fabG_1
fabG_1	ZONMW-26_02172	fabG_1
fabG_1	ZONMW-26_03078	fabG_1
fabG_1	ZONMW-26_02785	fabG_1
fabG_1	ZONMW-26_02887	fabG_1
fabG_1	ZONMW-26_00255	fabG_1
fabG_1	ZONMW-26_02478	fabG_1
fabG_1	ZONMW-26_00522	fabG_1
fabG_1	ZONMW-26_02185	fabG_1
fabG_1	ZONMW-26_03069	fabG_1
fabG_1	ZONMW-26_01831	fabG_1
fabG_1	ZONMW-26_00762	fabG_1
fabG_1	ZONMW-26_00463	fabG_1
fabG_1	ZONMW-26_01140	fabG_1
fabG_1	ZONMW-27_01221	fabG_1
fabG_1	ZONMW-27_02446	fabG_1
fabG_1	ZONMW-27_02400	fabG_1
fabG_1	ZONMW-27_00086	fabG_1
fabG_1	ZONMW-27_02295	fabG_1
fabG_1	ZONMW-27_02798	fabG_1
fabG_1	ZONMW-27_02203	fabG_1
fabG_1	ZONMW-27_02454	fabG_1
fabG_1	ZONMW-27_01269	fabG_1
fabG_1	ZONMW-27_01913	fabG_1
fabG_1	ZONMW-27_01657	fabG_1
fabG_1	ZONMW-27_01268	fabG_1
fabG_1	ZONMW-27_02308	fabG_1

fabG_1	ZONMW-27_02789	fabG_1
fabG_1	ZONMW-27_00381	fabG_1
fabG_1	ZONMW-27_01018	fabG_1
fabG_1	ZONMW-27_01329	fabG_1
fabG_1	ZONMW-27_00732	fabG_1
fabG_1	ZONMW-28_03989	fabG_1
fabG_1	ZONMW-28_02074	fabG_1
fabG_1	ZONMW-28_03127	fabG_1
fabG_1	ZONMW-28_01678	fabG_1
fabG_1	ZONMW-28_02071	fabG_1
fabG_1	ZONMW-28_03992	fabG_1
fabG_1	ZONMW-28_03561	fabG_1
fabG_1	ZONMW-28_00630	fabG_1
fabG_1	ZONMW-28_00014	fabG_1
fabG_1	ZONMW-28_01834	fabG_1
fabG_1	ZONMW-28_00827	fabG_1
fabG_1	ZONMW-28_01607	fabG_1
fabG_1	ZONMW-28_01061	fabG_1
fabG_1	ZONMW-28_02833	fabG_1
fabG_1	ZONMW-28_03248	fabG_1
fabG_1	ZONMW-28_00872	fabG_1
fabG_1	ZONMW-28_00636	fabG_1
fabG_1	ZONMW-28_03435	fabG_1
fabG_1	ZONMW-28_00102	fabG_1
fabG_1	ZONMW-28_01293	fabG_1
fabG_1	ZONMW-28_03140	fabG_1
fabG_1	ZONMW-28_03429	fabG_1
fabG_1	ZONMW-28_03445	fabG_1
fabG_1	ZONMW-28_02344	fabG_1
fabG_1	ZONMW-28_02169	fabG_1
fabG_1	ZONMW-28_00248	fabG_1
fabG_1	ZONMW-28_00089	fabG_1
fabG_1	ZONMW-28_00406	fabG_1
fabG_1	ZONMW-28_01189	fabG_1
fabG_1	ZONMW-28_00816	fabG_1
fabG_1	ZONMW-29_01601	fabG_1
fabG_1	ZONMW-29_00214	fabG_1
fabG_1	ZONMW-29_01263	fabG_1
fabG_1	ZONMW-29_02748	fabG_1
fabG_1	ZONMW-29_02131	fabG_1
fabG_1	ZONMW-29_01656	fabG_1
fabG_1	ZONMW-29_01487	fabG_1
fabG_1	ZONMW-29_00237	fabG_1
fabG_1	ZONMW-29_00601	fabG_1
fabG_1	ZONMW-29_02712	fabG_1
fabG_1	ZONMW-29_01342	fabG_1
fabG_1	ZONMW-29_00556	fabG_1
fabG_1	ZONMW-29_01561	fabG_1

fabG_1	ZONMW-29_02113	fabG_1
fabG_1	ZONMW-30_02582	fabG_1
fabG_1	ZONMW-30_01333	fabG_1
fabG_1	ZONMW-30_00565	fabG_1
fabG_1	ZONMW-30_00056	fabG_1
fabG_1	ZONMW-30_01086	fabG_1
fabG_1	ZONMW-30_01681	fabG_1
fabG_1	ZONMW-30_01966	fabG_1
fabG_1	ZONMW-30_02235	fabG_1
fabG_1	ZONMW-30_01762	fabG_1
fabG_1	ZONMW-30_01811	fabG_1
fabG_1	ZONMW-30_02698	fabG_1
fabG_1	ZONMW-30_00554	fabG_1
fabG_1	ZONMW-30_00027	fabG_1
fabG_1	ZONMW-30_02728	fabG_1
fabG_1	ZONMW-30_03198	fabG_1
fabG_1	ZONMW-31_03348	fabG_1
fabG_1	ZONMW-31_02857	fabG_1
fabG_1	ZONMW-31_02935	fabG_1
fabG_1	ZONMW-31_03733	fabG_1
fabG_1	ZONMW-31_01726	fabG_1
fabG_1	ZONMW-31_03413	fabG_1
fabG_1	ZONMW-31_01871	fabG_1
fabG_1	ZONMW-31_03736	fabG_1
fabG_1	ZONMW-31_00923	fabG_1
fabG_1	ZONMW-31_02938	fabG_1
fabG_1	ZONMW-31_01153	fabG_1
fabG_1	ZONMW-31_00090	fabG_1
fabG_1	ZONMW-31_02492	fabG_1
fabG_1	ZONMW-31_00722	fabG_1
fabG_1	ZONMW-31_03488	fabG_1
fabG_1	ZONMW-31_01853	fabG_1
fabG_1	ZONMW-31_01755	fabG_1
fabG_1	ZONMW-31_02116	fabG_1
fabG_1	ZONMW-31_02302	fabG_1
fabG_1	ZONMW-31_01355	fabG_1
fabG_1	ZONMW-31_02858	fabG_1
fabG_1	ZONMW-31_00604	fabG_1
fabG_1	ZONMW-31_02342	fabG_1
fabG_1	ZONMW-31_02293	fabG_1
fabG_1	ZONMW-31_00032	fabG_1
fabG_1	ZONMW-31_02225	fabG_1
fabG_1	ZONMW-32_03846	fabG_1
fabG_1	ZONMW-32_02479	fabG_1
fabG_1	ZONMW-32_03282	fabG_1
fabG_1	ZONMW-32_02294	fabG_1
fabG_1	ZONMW-32_02476	fabG_1
fabG_1	ZONMW-32_03849	fabG_1

fabG_1	ZONMW-32_00646	fabG_1
fabG_1	ZONMW-32_00461	fabG_1
fabG_1	ZONMW-32_00805	fabG_1
fabG_1	ZONMW-32_02138	fabG_1
fabG_1	ZONMW-32_00264	fabG_1
fabG_1	ZONMW-32_02364	fabG_1
fabG_1	ZONMW-32_00030	fabG_1
fabG_1	ZONMW-32_03042	fabG_1
fabG_1	ZONMW-32_00773	fabG_1
fabG_1	ZONMW-32_00219	fabG_1
fabG_1	ZONMW-32_00455	fabG_1
fabG_1	ZONMW-32_03546	fabG_1
fabG_1	ZONMW-32_00893	fabG_1
fabG_1	ZONMW-32_01701	fabG_1
fabG_1	ZONMW-32_00665	fabG_1
fabG_1	ZONMW-32_03552	fabG_1
fabG_1	ZONMW-32_03536	fabG_1
fabG_1	ZONMW-32_03006	fabG_1
fabG_1	ZONMW-32_02574	fabG_1
fabG_1	ZONMW-32_01039	fabG_1
fabG_1	ZONMW-32_00880	fabG_1
fabG_1	ZONMW-32_01197	fabG_1
fabG_1	ZONMW-32_01597	fabG_1
fabG_1	ZONMW-32_00275	fabG_1
fabG_1	ZONMW-33_03658	fabG_1
fabG_1	ZONMW-33_02639	fabG_1
fabG_1	ZONMW-33_00672	fabG_1
fabG_1	ZONMW-33_02285	fabG_1
fabG_1	ZONMW-33_02642	fabG_1
fabG_1	ZONMW-33_03655	fabG_1
fabG_1	ZONMW-33_00514	fabG_1
fabG_1	ZONMW-33_00328	fabG_1
fabG_1	ZONMW-33_00795	fabG_1
fabG_1	ZONMW-33_02129	fabG_1
fabG_1	ZONMW-33_00131	fabG_1
fabG_1	ZONMW-33_02356	fabG_1
fabG_1	ZONMW-33_03291	fabG_1
fabG_1	ZONMW-33_03231	fabG_1
fabG_1	ZONMW-33_00641	fabG_1
fabG_1	ZONMW-33_00086	fabG_1
fabG_1	ZONMW-33_00322	fabG_1
fabG_1	ZONMW-33_03846	fabG_1
fabG_1	ZONMW-33_00883	fabG_1
fabG_1	ZONMW-33_01691	fabG_1
fabG_1	ZONMW-33_00533	fabG_1
fabG_1	ZONMW-33_03852	fabG_1
fabG_1	ZONMW-33_03836	fabG_1
fabG_1	ZONMW-33_02742	fabG_1

fabG_1	ZONMW-33_02544	fabG_1
fabG_1	ZONMW-33_01028	fabG_1
fabG_1	ZONMW-33_00870	fabG_1
fabG_1	ZONMW-33_01186	fabG_1
fabG_1	ZONMW-33_01587	fabG_1
fabG_1	ZONMW-33_00142	fabG_1
fabG_1	ZONMW-34_02985	fabG_1
fabG_1	ZONMW-34_01933	fabG_1
fabG_1	ZONMW-34_00475	fabG_1
fabG_1	ZONMW-34_01035	fabG_1
fabG_1	ZONMW-34_02988	fabG_1
fabG_1	ZONMW-34_03180	fabG_1
fabG_1	ZONMW-34_00878	fabG_1
fabG_1	ZONMW-34_01936	fabG_1
fabG_1	ZONMW-34_00706	fabG_1
fabG_1	ZONMW-34_02262	fabG_1
fabG_1	ZONMW-34_03225	fabG_1
fabG_1	ZONMW-34_03100	fabG_1
fabG_1	ZONMW-34_01005	fabG_1
fabG_1	ZONMW-34_00700	fabG_1
fabG_1	ZONMW-34_00352	fabG_1
fabG_1	ZONMW-34_03337	fabG_1
fabG_1	ZONMW-34_03056	fabG_1
fabG_1	ZONMW-34_00499	fabG_1
fabG_1	ZONMW-34_00074	fabG_1
fabG_1	ZONMW-34_00897	fabG_1
fabG_1	ZONMW-34_01848	fabG_1
fabG_1	ZONMW-34_03327	fabG_1
fabG_1	ZONMW-34_01230	fabG_1
fabG_1	ZONMW-34_02773	fabG_1
fabG_1	ZONMW-34_01282	fabG_1
fabG_1	ZONMW-34_01380	fabG_1
fabG_1	ZONMW-34_02836	fabG_1
fabG_1	ZONMW-35_03007	fabG_1
fabG_1	ZONMW-35_01625	fabG_1
fabG_1	ZONMW-35_00915	fabG_1
fabG_1	ZONMW-35_02208	fabG_1
fabG_1	ZONMW-35_03004	fabG_1
fabG_1	ZONMW-35_03057	fabG_1
fabG_1	ZONMW-35_02365	fabG_1
fabG_1	ZONMW-35_01628	fabG_1
fabG_1	ZONMW-35_02756	fabG_1
fabG_1	ZONMW-35_01987	fabG_1
fabG_1	ZONMW-35_03186	fabG_1
fabG_1	ZONMW-35_03137	fabG_1
fabG_1	ZONMW-35_02238	fabG_1
fabG_1	ZONMW-35_02762	fabG_1
fabG_1	ZONMW-35_01038	fabG_1

fabG_1	ZONMW-35_03297	fabG_1
fabG_1	ZONMW-35_02936	fabG_1
fabG_1	ZONMW-35_00890	fabG_1
fabG_1	ZONMW-35_01316	fabG_1
fabG_1	ZONMW-35_02346	fabG_1
fabG_1	ZONMW-35_01539	fabG_1
fabG_1	ZONMW-35_03307	fabG_1
fabG_1	ZONMW-35_00303	fabG_1
fabG_1	ZONMW-35_00150	fabG_1
fabG_1	ZONMW-35_00355	fabG_1
fabG_1	ZONMW-35_00453	fabG_1
fabG_1	ZONMW-35_02803	fabG_1
fabG_1	ZONMW-36_03597	fabG_1
fabG_1	ZONMW-36_02613	fabG_1
fabG_1	ZONMW-36_03240	fabG_1
fabG_1	ZONMW-36_01951	fabG_1
fabG_1	ZONMW-36_02610	fabG_1
fabG_1	ZONMW-36_03600	fabG_1
fabG_1	ZONMW-36_00857	fabG_1
fabG_1	ZONMW-36_01041	fabG_1
fabG_1	ZONMW-36_00014	fabG_1
fabG_1	ZONMW-36_02107	fabG_1
fabG_1	ZONMW-36_01238	fabG_1
fabG_1	ZONMW-36_01880	fabG_1
fabG_1	ZONMW-36_03028	fabG_1
fabG_1	ZONMW-36_03532	fabG_1
fabG_1	ZONMW-36_00730	fabG_1
fabG_1	ZONMW-36_01283	fabG_1
fabG_1	ZONMW-36_01047	fabG_1
fabG_1	ZONMW-36_03760	fabG_1
fabG_1	ZONMW-36_00102	fabG_1
fabG_1	ZONMW-36_01665	fabG_1
fabG_1	ZONMW-36_00838	fabG_1
fabG_1	ZONMW-36_03754	fabG_1
fabG_1	ZONMW-36_03770	fabG_1
fabG_1	ZONMW-36_02254	fabG_1
fabG_1	ZONMW-36_02708	fabG_1
fabG_1	ZONMW-36_00248	fabG_1
fabG_1	ZONMW-36_00089	fabG_1
fabG_1	ZONMW-36_00406	fabG_1
fabG_1	ZONMW-36_01769	fabG_1
fabG_1	ZONMW-36_01227	fabG_1
fabG_1	ZONMW-37_00347	fabG_1
fabG_1	ZONMW-37_01758	fabG_1
fabG_1	ZONMW-37_00357	fabG_1
fabG_1	ZONMW-37_00481	fabG_1
fabG_1	ZONMW-37_01083	fabG_1
fabG_1	ZONMW-37_00068	fabG_1

fabG_1	ZONMW-37_00571	fabG_1
fabG_1	ZONMW-37_02762	fabG_1
fabG_1	ZONMW-37_00704	fabG_1
fabG_1	ZONMW-37_02074	fabG_1
fabG_1	ZONMW-37_01332	fabG_1
fabG_1	ZONMW-37_03428	fabG_1
fabG_1	ZONMW-37_01444	fabG_1
fabG_1	ZONMW-37_02787	fabG_1
fabG_1	ZONMW-37_01375	fabG_1
fabG_1	ZONMW-37_00220	fabG_1
fabG_1	ZONMW-38_02005	fabG_1
fabG_1	ZONMW-38_03247	fabG_1
fabG_1	ZONMW-38_00341	fabG_1
fabG_1	ZONMW-38_01172	fabG_1
fabG_1	ZONMW-38_00960	fabG_1
fabG_1	ZONMW-38_00448	fabG_1
fabG_1	ZONMW-38_02831	fabG_1
fabG_1	ZONMW-38_01510	fabG_1
fabG_1	ZONMW-38_02240	fabG_1
fabG_1	ZONMW-38_00055	fabG_1
fabG_1	ZONMW-38_03165	fabG_1
fabG_1	ZONMW-38_00892	fabG_1
fabG_1	ZONMW-38_01873	fabG_1
fabG_1	ZONMW-38_02232	fabG_1
fabG_1	ZONMW-38_03369	fabG_1
fabG_1	ZONMW-38_01778	fabG_1
fabG_1	ZONMW-38_03507	fabG_1
fabG_1	ZONMW-39_02774	fabG_1
fabG_1	ZONMW-39_02269	fabG_1
fabG_1	ZONMW-39_00945	fabG_1
fabG_1	ZONMW-39_02906	fabG_1
fabG_1	ZONMW-39_02771	fabG_1
fabG_1	ZONMW-39_03765	fabG_1
fabG_1	ZONMW-39_02266	fabG_1
fabG_1	ZONMW-39_01729	fabG_1
fabG_1	ZONMW-39_01341	fabG_1
fabG_1	ZONMW-39_02305	fabG_1
fabG_1	ZONMW-39_01168	fabG_1
fabG_1	ZONMW-39_02634	fabG_1
fabG_1	ZONMW-39_01916	fabG_1
fabG_1	ZONMW-39_01538	fabG_1
fabG_1	ZONMW-39_00777	fabG_1
fabG_1	ZONMW-39_01612	fabG_1
fabG_1	ZONMW-39_02876	fabG_1
fabG_1	ZONMW-39_01809	fabG_1
fabG_1	ZONMW-39_01162	fabG_1
fabG_1	ZONMW-39_03041	fabG_1
fabG_1	ZONMW-39_03192	fabG_1

fabG_1	ZONMW-39_02707	fabG_1
fabG_1	ZONMW-39_02460	fabG_1
fabG_1	ZONMW-39_00968	fabG_1
fabG_1	ZONMW-39_01360	fabG_1
fabG_1	ZONMW-39_01783	fabG_1
fabG_1	ZONMW-39_00320	fabG_1
fabG_1	ZONMW-39_02341	fabG_1
fabG_1	ZONMW-39_00414	fabG_1
fabG_1	ZONMW-39_03182	fabG_1
fabG_1	ZONMW-39_03438	fabG_1
fabG_1	ZONMW-39_00703	fabG_1
fabG_1	ZONMW-39_02564	fabG_1
fabG_1	ZONMW-39_00778	fabG_1
fabG_1	ZONMW-40_00785	fabG_1
fabG_1	ZONMW-40_02017	fabG_1
fabG_1	ZONMW-40_03188	fabG_1
fabG_1	ZONMW-40_02279	fabG_1
fabG_1	ZONMW-40_03427	fabG_1
fabG_1	ZONMW-40_01868	fabG_1
fabG_1	ZONMW-40_02141	fabG_1
fabG_1	ZONMW-40_00854	fabG_1
fabG_1	ZONMW-40_02269	fabG_1
fabG_1	ZONMW-40_02397	fabG_1
fabG_1	ZONMW-40_01387	fabG_1
fabG_1	ZONMW-40_00228	fabG_1
fabG_1	ZONMW-40_03065	fabG_1
fabG_1	ZONMW-40_00271	fabG_1
fabG_1	ZONMW-40_00861	fabG_1
fabG_1	ZONMW-40_03348	fabG_1
fabG_1	ZONMW-40_01957	fabG_1
fabG_1	ZONMW-40_01248	fabG_1
fabG_1	ZONMW-40_01968	fabG_1
fabG_1	ZONMW-40_03057	fabG_1
fabG_1	ZONMW-40_02111	fabG_1
fabG_1	ZONMW-40_02093	fabG_1
fabG_1	ZONMW-40_03069	fabG_1
fabG_1	ZONMW-40_02299	fabG_1
fabG_1	ZONMW-40_00289	fabG_1
fabG_1	ZONMW-40_03053	fabG_1
fabG_1	ZONMW-40_03061	fabG_1
fabG_1	ZONMW-41_00485	fabG_1
fabG_1	ZONMW-41_01349	fabG_1
fabG_1	ZONMW-41_00946	fabG_1
fabG_1	ZONMW-41_00561	fabG_1
fabG_1	ZONMW-41_00275	fabG_1
fabG_1	ZONMW-41_01484	fabG_1
fabG_1	ZONMW-41_01489	fabG_1
fabG_1	ZONMW-41_02556	fabG_1

fabG_1	ZONMW-41_01665	fabG_1
fabG_1	ZONMW-41_01032	fabG_1
fabG_1	ZONMW-41_00879	fabG_1
fabG_1	ZONMW-42_01728	fabG_1
fabG_1	ZONMW-42_00485	fabG_1
fabG_1	ZONMW-42_00852	fabG_1
fabG_1	ZONMW-42_00540	fabG_1
fabG_1	ZONMW-42_00156	fabG_1
fabG_1	ZONMW-42_02540	fabG_1
fabG_1	ZONMW-42_01657	fabG_1
fabG_1	ZONMW-42_01002	fabG_1
fabG_1	ZONMW-42_01456	fabG_1
fabG_1	ZONMW-42_00482	fabG_1
fabG_1	ZONMW-42_00638	fabG_1
fabG_1	ZONMW-42_02461	fabG_1
fabG_1	ZONMW-42_01652	fabG_1
fabG_1	ZONMW-42_03449	fabG_1
fabG_1	ZONMW-42_03641	fabG_1
fabG_1	ZONMW-42_02491	fabG_1
fabG_1	ZONMW-42_00982	fabG_1
fabG_1	ZONMW-42_01419	fabG_1
fabG_1	ZONMW-42_00511	fabG_1
fabG_1	ZONMW-42_00539	fabG_1
fabG_1	ZONMW-42_03630	fabG_1
fabG_1	ZONMW-42_03549	fabG_1
fabG_1	ZONMW-42_02635	fabG_1
fabG_1	ZONMW-42_01801	fabG_1
fabG_1	ZONMW-42_01360	fabG_1
fabG_1	ZONMW-42_02771	fabG_1
fabG_1	ZONMW-42_02767	fabG_1
fabG_1	ZONMW-43_03408	fabG_1
fabG_1	ZONMW-43_02233	fabG_1
fabG_1	ZONMW-43_03844	fabG_1
fabG_1	ZONMW-43_01438	fabG_1
fabG_1	ZONMW-43_00569	fabG_1
fabG_1	ZONMW-43_00012	fabG_1
fabG_1	ZONMW-43_02217	fabG_1
fabG_1	ZONMW-43_03847	fabG_1
fabG_1	ZONMW-43_02224	fabG_1
fabG_1	ZONMW-43_03504	fabG_1
fabG_1	ZONMW-43_02286	fabG_1
fabG_1	ZONMW-43_01441	fabG_1
fabG_1	ZONMW-43_02258	fabG_1
fabG_1	ZONMW-43_00411	fabG_1
fabG_1	ZONMW-43_00246	fabG_1
fabG_1	ZONMW-43_03577	fabG_1
fabG_1	ZONMW-43_01894	fabG_1
fabG_1	ZONMW-43_00988	fabG_1

fabG_1	ZONMW-43_02269	fabG_1
fabG_1	ZONMW-43_04467	fabG_1
fabG_1	ZONMW-43_01970	fabG_1
fabG_1	ZONMW-43_02243	fabG_1
fabG_1	ZONMW-43_03088	fabG_1
fabG_1	ZONMW-43_03426	fabG_1
fabG_1	ZONMW-43_00538	fabG_1
fabG_1	ZONMW-43_02314	fabG_1
fabG_1	ZONMW-43_00240	fabG_1
fabG_1	ZONMW-43_02264	fabG_1
fabG_1	ZONMW-43_02226	fabG_1
fabG_1	ZONMW-43_02710	fabG_1
fabG_1	ZONMW-43_04090	fabG_1
fabG_1	ZONMW-43_00044	fabG_1
fabG_1	ZONMW-43_00874	fabG_1
fabG_1	ZONMW-43_00430	fabG_1
fabG_1	ZONMW-43_01358	fabG_1
fabG_1	ZONMW-43_03907	fabG_1
fabG_1	ZONMW-43_02720	fabG_1
fabG_1	ZONMW-43_02422	fabG_1
fabG_1	ZONMW-43_03918	fabG_1
fabG_1	ZONMW-43_02230	fabG_1
fabG_1	ZONMW-43_02963	fabG_1
fabG_1	ZONMW-43_02293	fabG_1
fabG_1	ZONMW-43_03089	fabG_1
fabG_1	ZONMW-44_01194	fabG_1
fabG_1	ZONMW-44_01512	fabG_1
fabG_1	ZONMW-44_00756	fabG_1
fabG_1	ZONMW-44_00812	fabG_1
fabG_1	ZONMW-44_03781	fabG_1
fabG_1	ZONMW-44_02351	fabG_1
fabG_1	ZONMW-44_02015	fabG_1
fabG_1	ZONMW-44_01641	fabG_1
fabG_1	ZONMW-44_00599	fabG_1
fabG_1	ZONMW-44_02262	fabG_1
fabG_1	ZONMW-44_02090	fabG_1
fabG_1	ZONMW-44_02693	fabG_1
fabG_1	ZONMW-44_01572	fabG_1
fabG_1	ZONMW-44_00815	fabG_1
fabG_1	ZONMW-44_00369	fabG_1
fabG_1	ZONMW-44_00231	fabG_1
fabG_1	ZONMW-44_04108	fabG_1
fabG_1	ZONMW-44_00650	fabG_1
fabG_1	ZONMW-44_03575	fabG_1
fabG_1	ZONMW-44_02085	fabG_1
fabG_1	ZONMW-44_03651	fabG_1
fabG_1	ZONMW-44_02300	fabG_1
fabG_1	ZONMW-44_01518	fabG_1

fabG_1	ZONMW-44_02549	fabG_1
fabG_1	ZONMW-44_01765	fabG_1
fabG_1	ZONMW-44_01620	fabG_1
fabG_1	ZONMW-44_00786	fabG_1
fabG_1	ZONMW-44_00757	fabG_1
fabG_1	ZONMW-44_01097	fabG_1
fabG_1	ZONMW-44_02538	fabG_1
fabG_1	ZONMW-44_03424	fabG_1
fabG_1	ZONMW-44_01589	fabG_1
fabG_1	ZONMW-44_02449	fabG_1
fabG_1	ZONMW-44_01252	fabG_1
fabG_1	ZONMW-44_00548	fabG_1
fabG_1	ZONMW-44_02445	fabG_1
fabG_1	ZONMW-45_01568	fabG_1
fabG_1	ZONMW-45_02938	fabG_1
fabG_1	ZONMW-45_00771	fabG_1
fabG_1	ZONMW-45_02383	fabG_1
fabG_1	ZONMW-45_01229	fabG_1
fabG_1	ZONMW-45_00472	fabG_1
fabG_1	ZONMW-45_02104	fabG_1
fabG_1	ZONMW-45_00020	fabG_1
fabG_1	ZONMW-45_01666	fabG_1
fabG_1	ZONMW-45_01611	fabG_1
fabG_1	ZONMW-45_01665	fabG_1
fabG_1	ZONMW-45_01415	fabG_1
fabG_1	ZONMW-46_02079	fabG_1
fabG_1	ZONMW-46_03003	fabG_1
fabG_1	ZONMW-46_01103	fabG_1
fabG_1	ZONMW-46_01245	fabG_1
fabG_1	ZONMW-46_02613	fabG_1
fabG_1	ZONMW-46_01367	fabG_1
fabG_1	ZONMW-46_02178	fabG_1
fabG_1	ZONMW-46_00235	fabG_1
fabG_1	ZONMW-46_00131	fabG_1
fabG_1	ZONMW-46_02122	fabG_1
fabG_1	ZONMW-46_00132	fabG_1
fabG_1	ZONMW-46_01670	fabG_1
fabG_1	ZONMW-47_01982	fabG_1
fabG_1	ZONMW-47_02994	fabG_1
fabG_1	ZONMW-47_01070	fabG_1
fabG_1	ZONMW-47_00928	fabG_1
fabG_1	ZONMW-47_02603	fabG_1
fabG_1	ZONMW-47_01485	fabG_1
fabG_1	ZONMW-47_02354	fabG_1
fabG_1	ZONMW-47_00270	fabG_1
fabG_1	ZONMW-47_00374	fabG_1
fabG_1	ZONMW-47_01939	fabG_1
fabG_1	ZONMW-47_00373	fabG_1

fabG_1	ZONMW-47_01819	fabG_1
fabG_1	ZONMW-48_02199	fabG_1
fabG_1	ZONMW-48_01368	fabG_1
fabG_1	ZONMW-48_03395	fabG_1
fabG_1	ZONMW-48_03141	fabG_1
fabG_1	ZONMW-48_01625	fabG_1
fabG_1	ZONMW-48_00064	fabG_1
fabG_1	ZONMW-48_00814	fabG_1
fabG_1	ZONMW-48_03407	fabG_1
fabG_1	ZONMW-48_03403	fabG_1
fabG_1	ZONMW-48_01624	fabG_1
fabG_1	ZONMW-48_02202	fabG_1
fabG_1	ZONMW-48_02006	fabG_1
fabG_1	ZONMW-48_01217	fabG_1
fabG_1	ZONMW-48_02862	fabG_1
fabG_1	ZONMW-48_01660	fabG_1
fabG_1	ZONMW-48_00169	fabG_1
fabG_1	ZONMW-48_03393	fabG_1
fabG_1	ZONMW-48_00127	fabG_1
fabG_1	ZONMW-48_01096	fabG_1
fabG_1	ZONMW-48_02715	fabG_1
fabG_1	ZONMW-48_01137	fabG_1
fabG_1	ZONMW-48_03263	fabG_1
fabG_1	ZONMW-48_01420	fabG_1
fabG_1	ZONMW-48_01254	fabG_1
fabG_1	ZONMW-48_01504	fabG_1
fabG_1	ZONMW-49_01691	fabG_1
fabG_1	ZONMW-49_01941	fabG_1
fabG_1	ZONMW-49_01145	fabG_1
fabG_1	ZONMW-49_01134	fabG_1
fabG_1	ZONMW-49_03200	fabG_1
fabG_1	ZONMW-49_00516	fabG_1
fabG_1	ZONMW-49_01949	fabG_1
fabG_1	ZONMW-49_00266	fabG_1
fabG_1	ZONMW-49_02362	fabG_1
fabG_1	ZONMW-49_00805	fabG_1
fabG_1	ZONMW-49_01143	fabG_1
fabG_1	ZONMW-49_02902	fabG_1
fabG_1	ZONMW-49_02987	fabG_1
fabG_1	ZONMW-49_01281	fabG_1
fabG_1	ZONMW-49_00478	fabG_1
fabG_1	ZONMW-50_01723	fabG_1
fabG_1	ZONMW-50_02537	fabG_1
fabG_1	ZONMW-50_01146	fabG_1
fabG_1	ZONMW-50_01135	fabG_1
fabG_1	ZONMW-50_03234	fabG_1
fabG_1	ZONMW-50_00517	fabG_1
fabG_1	ZONMW-50_02545	fabG_1

fabG_1	ZONMW-50_00219	fabG_1
fabG_1	ZONMW-50_02098	fabG_1
fabG_1	ZONMW-50_00806	fabG_1
fabG_1	ZONMW-50_01144	fabG_1
fabG_1	ZONMW-50_02754	fabG_1
fabG_1	ZONMW-50_02868	fabG_1
fabG_1	ZONMW-50_01283	fabG_1
fabG_1	ZONMW-50_00007	fabG_1
fabG_1	ZONMW-51_00589	fabG_1
fabG_1	ZONMW-51_02247	fabG_1
fabG_1	ZONMW-51_02752	fabG_1
fabG_1	ZONMW-51_02741	fabG_1
fabG_1	ZONMW-51_02351	fabG_1
fabG_1	ZONMW-51_01189	fabG_1
fabG_1	ZONMW-51_02255	fabG_1
fabG_1	ZONMW-51_00254	fabG_1
fabG_1	ZONMW-51_01594	fabG_1
fabG_1	ZONMW-51_00906	fabG_1
fabG_1	ZONMW-51_02750	fabG_1
fabG_1	ZONMW-51_02872	fabG_1
fabG_1	ZONMW-51_02006	fabG_1
fabG_1	ZONMW-51_01396	fabG_1
fabG_1	ZONMW-51_00466	fabG_1
fabG_1	ZONMW-52_00546	fabG_1
fabG_1	ZONMW-52_02659	fabG_1
fabG_1	ZONMW-52_02294	fabG_1
fabG_1	ZONMW-52_02305	fabG_1
fabG_1	ZONMW-52_02335	fabG_1
fabG_1	ZONMW-52_02667	fabG_1
fabG_1	ZONMW-52_00238	fabG_1
fabG_1	ZONMW-52_01737	fabG_1
fabG_1	ZONMW-52_01506	fabG_1
fabG_1	ZONMW-52_02296	fabG_1
fabG_1	ZONMW-52_03002	fabG_1
fabG_1	ZONMW-52_02883	fabG_1
fabG_1	ZONMW-52_00766	fabG_1
fabG_1	ZONMW-52_01138	fabG_1
fabG_1	ZONMW-52_00451	fabG_1
fabG_1	ZONMW-52_02882	fabG_1
fabG_1	ZONMW-53_01301	fabG_1
fabG_1	ZONMW-53_02412	fabG_1
fabG_1	ZONMW-53_02883	fabG_1
fabG_1	ZONMW-53_02894	fabG_1
fabG_1	ZONMW-53_02298	fabG_1
fabG_1	ZONMW-53_00033	fabG_1
fabG_1	ZONMW-53_02420	fabG_1
fabG_1	ZONMW-53_00975	fabG_1
fabG_1	ZONMW-53_02023	fabG_1

fabG_1	ZONMW-53_00321	fabG_1
fabG_1	ZONMW-53_02885	fabG_1
fabG_1	ZONMW-53_03009	fabG_1
fabG_1	ZONMW-53_01500	fabG_1
fabG_1	ZONMW-53_00709	fabG_1
fabG_1	ZONMW-53_01182	fabG_1
fabG_1	ZONMW-54_02472	fabG_1
fabG_1	ZONMW-54_01416	fabG_1
fabG_1	ZONMW-54_00099	fabG_1
fabG_1	ZONMW-54_00110	fabG_1
fabG_1	ZONMW-54_02794	fabG_1
fabG_1	ZONMW-54_00995	fabG_1
fabG_1	ZONMW-54_02480	fabG_1
fabG_1	ZONMW-54_01752	fabG_1
fabG_1	ZONMW-54_02035	fabG_1
fabG_1	ZONMW-54_00713	fabG_1
fabG_1	ZONMW-54_03010	fabG_1
fabG_1	ZONMW-54_00101	fabG_1
fabG_1	ZONMW-54_01223	fabG_1
fabG_1	ZONMW-54_01538	fabG_1
fabG_1	ZONMW-54_00358	fabG_1
fabG_1	ZONMW-55_00935	fabG_1
fabG_1	ZONMW-55_01741	fabG_1
fabG_1	ZONMW-55_02889	fabG_1
fabG_1	ZONMW-55_02900	fabG_1
fabG_1	ZONMW-55_02442	fabG_1
fabG_1	ZONMW-55_00031	fabG_1
fabG_1	ZONMW-55_01733	fabG_1
fabG_1	ZONMW-55_01272	fabG_1
fabG_1	ZONMW-55_01916	fabG_1
fabG_1	ZONMW-55_00314	fabG_1
fabG_1	ZONMW-55_02891	fabG_1
fabG_1	ZONMW-55_03016	fabG_1
fabG_1	ZONMW-55_02328	fabG_1
fabG_1	ZONMW-55_00680	fabG_1
fabG_1	ZONMW-55_01060	fabG_1
fabG_1	ZONMW-56_00171	fabG_1
fabG_1	ZONMW-56_01895	fabG_1
fabG_1	ZONMW-56_02650	fabG_1
fabG_1	ZONMW-56_02661	fabG_1
fabG_1	ZONMW-56_01786	fabG_1
fabG_1	ZONMW-56_01887	fabG_1
fabG_1	ZONMW-56_00501	fabG_1
fabG_1	ZONMW-56_01520	fabG_1
fabG_1	ZONMW-56_01001	fabG_1
fabG_1	ZONMW-56_02652	fabG_1
fabG_1	ZONMW-56_02895	fabG_1
fabG_1	ZONMW-56_02028	fabG_1

fabG_1	ZONMW-56_02776	fabG_1
fabG_1	ZONMW-56_02777	fabG_1
fabG_1	ZONMW-56_02399	fabG_1
fabG_1	ZONMW-56_00288	fabG_1
fabG_1	ZONMW-57_00384	fabG_1
fabG_1	ZONMW-57_02243	fabG_1
fabG_1	ZONMW-57_02794	fabG_1
fabG_1	ZONMW-57_02805	fabG_1
fabG_1	ZONMW-57_02133	fabG_1
fabG_1	ZONMW-57_01582	fabG_1
fabG_1	ZONMW-57_02251	fabG_1
fabG_1	ZONMW-57_00714	fabG_1
fabG_1	ZONMW-57_01702	fabG_1
fabG_1	ZONMW-57_01301	fabG_1
fabG_1	ZONMW-57_02796	fabG_1
fabG_1	ZONMW-57_02846	fabG_1
fabG_1	ZONMW-57_00188	fabG_1
fabG_1	ZONMW-57_02528	fabG_1
fabG_1	ZONMW-57_00501	fabG_1
fabG_1	ZONMW-58_00587	fabG_1
fabG_1	ZONMW-58_02267	fabG_1
fabG_1	ZONMW-58_02818	fabG_1
fabG_1	ZONMW-58_02829	fabG_1
fabG_1	ZONMW-58_01945	fabG_1
fabG_1	ZONMW-58_01002	fabG_1
fabG_1	ZONMW-58_02275	fabG_1
fabG_1	ZONMW-58_00257	fabG_1
fabG_1	ZONMW-58_01726	fabG_1
fabG_1	ZONMW-58_01283	fabG_1
fabG_1	ZONMW-58_02820	fabG_1
fabG_1	ZONMW-58_02870	fabG_1
fabG_1	ZONMW-58_00783	fabG_1
fabG_1	ZONMW-58_02553	fabG_1
fabG_1	ZONMW-58_00470	fabG_1
fabG_1	ZONMW-59_00400	fabG_1
fabG_1	ZONMW-59_02483	fabG_1
fabG_1	ZONMW-59_02910	fabG_1
fabG_1	ZONMW-59_02899	fabG_1
fabG_1	ZONMW-59_02164	fabG_1
fabG_1	ZONMW-59_02750	fabG_1
fabG_1	ZONMW-59_02491	fabG_1
fabG_1	ZONMW-59_00703	fabG_1
fabG_1	ZONMW-59_01542	fabG_1
fabG_1	ZONMW-59_01302	fabG_1
fabG_1	ZONMW-59_02908	fabG_1
fabG_1	ZONMW-59_03104	fabG_1
fabG_1	ZONMW-59_00181	fabG_1
fabG_1	ZONMW-59_01939	fabG_1

fabG_1	ZONMW-59_00491	fabG_1
fabG_1	ZONMW-60_02352	fabG_1
fabG_1	ZONMW-60_00411	fabG_1
fabG_1	ZONMW-60_02912	fabG_1
fabG_1	ZONMW-60_02923	fabG_1
fabG_1	ZONMW-60_02455	fabG_1
fabG_1	ZONMW-60_00976	fabG_1
fabG_1	ZONMW-60_02360	fabG_1
fabG_1	ZONMW-60_00716	fabG_1
fabG_1	ZONMW-60_01923	fabG_1
fabG_1	ZONMW-60_01268	fabG_1
fabG_1	ZONMW-60_02964	fabG_1
fabG_1	ZONMW-60_02914	fabG_1
fabG_1	ZONMW-60_00214	fabG_1
fabG_1	ZONMW-60_00503	fabG_1
fabG_1	ZONMW-60_01622	fabG_1
fabG_1	ZONMW-61_02593	fabG_1
fabG_1	ZONMW-61_02585	fabG_1
fabG_1	ZONMW-61_02528	fabG_1
fabG_1	ZONMW-61_02599	fabG_1
fabG_1	ZONMW-61_01468	fabG_1
fabG_1	ZONMW-61_02589	fabG_1
fabG_1	ZONMW-61_01047	fabG_1
fabG_1	ZONMW-61_03271	fabG_1
fabG_1	ZONMW-61_01444	fabG_1
fabG_1	ZONMW-61_02404	fabG_1
fabG_1	ZONMW-61_01118	fabG_1
fabG_1	ZONMW-61_03343	fabG_1
fabG_1	ZONMW-61_01079	fabG_1
fabG_1	ZONMW-61_01062	fabG_1
fabG_1	ZONMW-61_03119	fabG_1
fabG_1	ZONMW-61_02078	fabG_1
fabG_1	ZONMW-61_01995	fabG_1
fabG_1	ZONMW-61_01348	fabG_1
fabG_1	ZONMW-61_02636	fabG_1
fabG_1	ZONMW-61_02409	fabG_1
fabG_1	ZONMW-61_01114	fabG_1
fabG_1	ZONMW-61_01591	fabG_1
fabG_1	ZONMW-61_00091	fabG_1
fabG_1	ZONMW-61_00049	fabG_1
fabG_1	ZONMW-61_01818	fabG_1
fabG_1	ZONMW-61_02629	fabG_1
fabG_1	ZONMW-61_01929	fabG_1
fabG_1	ZONMW-61_02450	fabG_1
fabG_1	ZONMW-61_01205	fabG_1
fabG_1	ZONMW-61_03501	fabG_1
fabG_1	ZONMW-61_01817	fabG_1
fabG_1	ZONMW-61_02674	fabG_1

fabG_1	ZONMW-61_01215	fabG_1
fabG_1	ZONMW-61_03783	fabG_1
fabG_1	ZONMW-61_03364	fabG_1
fabG_1	ZONMW-61_00098	fabG_1
fabG_1	ZONMW-62_03077	fabG_1
fabG_1	ZONMW-62_00599	fabG_1
fabG_1	ZONMW-62_01220	fabG_1
fabG_1	ZONMW-62_03827	fabG_1
fabG_1	ZONMW-62_01188	fabG_1
fabG_1	ZONMW-62_03134	fabG_1
fabG_1	ZONMW-62_00507	fabG_1
fabG_1	ZONMW-62_03641	fabG_1
fabG_1	ZONMW-62_02869	fabG_1
fabG_1	ZONMW-62_03074	fabG_1
fabG_1	ZONMW-62_03089	fabG_1
fabG_1	ZONMW-62_03849	fabG_1
fabG_1	ZONMW-62_02204	fabG_1
fabG_1	ZONMW-62_03852	fabG_1
fabG_1	ZONMW-62_03069	fabG_1
fabG_1	ZONMW-62_03194	fabG_1
fabG_1	ZONMW-62_00247	fabG_1
fabG_1	ZONMW-62_00435	fabG_1
fabG_1	ZONMW-62_00168	fabG_1
fabG_1	ZONMW-62_03897	fabG_1
fabG_1	ZONMW-62_02862	fabG_1
fabG_1	ZONMW-62_01646	fabG_1
fabG_1	ZONMW-62_01603	fabG_1
fabG_1	ZONMW-62_02865	fabG_1
fabG_1	ZONMW-62_03903	fabG_1
fabG_1	ZONMW-62_00675	fabG_1
fabG_1	ZONMW-62_00307	fabG_1
fabG_1	ZONMW-62_00750	fabG_1
fabG_1	ZONMW-62_03548	fabG_1
fabG_1	ZONMW-62_02964	fabG_1
fabG_1	ZONMW-62_03841	fabG_1
fabG_1	ZONMW-62_00931	fabG_1
fabG_1	ZONMW-62_00422	fabG_1
fabG_1	ZONMW-62_01651	fabG_1
fabG_1	ZONMW-62_03898	fabG_1
fabG_1	ZONMW-63_00052	fabG_1
fabG_1	ZONMW-63_02414	fabG_1
fabG_1	ZONMW-63_02279	fabG_1
fabG_1	ZONMW-63_02268	fabG_1
fabG_1	ZONMW-63_01550	fabG_1
fabG_1	ZONMW-63_00975	fabG_1
fabG_1	ZONMW-63_02422	fabG_1
fabG_1	ZONMW-63_00387	fabG_1
fabG_1	ZONMW-63_01438	fabG_1

fabG_1	ZONMW-63_01264	fabG_1
fabG_1	ZONMW-63_02277	fabG_1
fabG_1	ZONMW-63_02866	fabG_1
fabG_1	ZONMW-63_02586	fabG_1
fabG_1	ZONMW-63_00694	fabG_1
fabG_1	ZONMW-63_00174	fabG_1
fabG_1	ZONMW-64_00851	fabG_1
fabG_1	ZONMW-64_03838	fabG_1
fabG_1	ZONMW-64_02015	fabG_1
fabG_1	ZONMW-64_00587	fabG_1
fabG_1	ZONMW-64_00036	fabG_1
fabG_1	ZONMW-64_00867	fabG_1
fabG_1	ZONMW-64_03835	fabG_1
fabG_1	ZONMW-64_00860	fabG_1
fabG_1	ZONMW-64_01689	fabG_1
fabG_1	ZONMW-64_00797	fabG_1
fabG_1	ZONMW-64_02018	fabG_1
fabG_1	ZONMW-64_00825	fabG_1
fabG_1	ZONMW-64_00429	fabG_1
fabG_1	ZONMW-64_00262	fabG_1
fabG_1	ZONMW-64_02711	fabG_1
fabG_1	ZONMW-64_02589	fabG_1
fabG_1	ZONMW-64_00814	fabG_1
fabG_1	ZONMW-64_01464	fabG_1
fabG_1	ZONMW-64_00840	fabG_1
fabG_1	ZONMW-64_02250	fabG_1
fabG_1	ZONMW-64_01768	fabG_1
fabG_1	ZONMW-64_00556	fabG_1
fabG_1	ZONMW-64_00769	fabG_1
fabG_1	ZONMW-64_00256	fabG_1
fabG_1	ZONMW-64_00819	fabG_1
fabG_1	ZONMW-64_04065	fabG_1
fabG_1	ZONMW-64_00858	fabG_1
fabG_1	ZONMW-64_03167	fabG_1
fabG_1	ZONMW-64_02769	fabG_1
fabG_1	ZONMW-64_01159	fabG_1
fabG_1	ZONMW-64_00448	fabG_1
fabG_1	ZONMW-64_01936	fabG_1
fabG_1	ZONMW-64_03157	fabG_1
fabG_1	ZONMW-64_02171	fabG_1
fabG_1	ZONMW-64_02399	fabG_1
fabG_1	ZONMW-64_02182	fabG_1
fabG_1	ZONMW-64_03380	fabG_1
fabG_1	ZONMW-64_00854	fabG_1
fabG_1	ZONMW-64_00790	fabG_1
fabG_1	ZONMW-64_02251	fabG_1
fabG_1	ZONMW-65_00511	fabG_1
fabG_1	ZONMW-65_00373	fabG_1

fabG_1	ZONMW-65_00512	fabG_1
fabG_1	ZONMW-65_00451	fabG_1
fabG_1	ZONMW-65_01495	fabG_1
fabG_1	ZONMW-65_02568	fabG_1
fabG_1	ZONMW-65_03425	fabG_1
fabG_1	ZONMW-65_02314	fabG_1
fabG_1	ZONMW-65_02429	fabG_1
fabG_1	ZONMW-65_03078	fabG_1
fabG_1	ZONMW-65_00765	fabG_1
fabG_1	ZONMW-65_01804	fabG_1
fabG_1	ZONMW-65_02410	fabG_1
fabG_1	ZONMW-65_01533	fabG_1
fabG_1	ZONMW-65_00058	fabG_1
fabG_1	ZONMW-65_00802	fabG_1
fabG_1	ZONMW-65_01591	fabG_1
fabG_1	ZONMW-65_01847	fabG_1
fabG_1	ZONMW-65_02061	fabG_1
fabG_1	ZONMW-66_00436	fabG_1
fabG_1	ZONMW-66_02477	fabG_1
fabG_1	ZONMW-66_02622	fabG_1
fabG_1	ZONMW-66_00393	fabG_1
fabG_1	ZONMW-66_02883	fabG_1
fabG_1	ZONMW-66_02770	fabG_1
fabG_1	ZONMW-66_01885	fabG_1
fabG_1	ZONMW-66_00020	fabG_1
fabG_1	ZONMW-66_02381	fabG_1
fabG_1	ZONMW-66_00600	fabG_1
fabG_1	ZONMW-66_02249	fabG_1
fabG_1	ZONMW-66_02937	fabG_1
fabG_1	ZONMW-66_01538	fabG_1
fabG_1	ZONMW-66_02589	fabG_1
fabG_1	ZONMW-66_00662	fabG_1
fabG_1	ZONMW-66_04022	fabG_1
fabG_1	ZONMW-66_03941	fabG_1
fabG_1	ZONMW-66_02938	fabG_1
fabG_1	ZONMW-66_03749	fabG_1
fabG_1	ZONMW-67_02930	fabG_1
fabG_1	ZONMW-67_02332	fabG_1
fabG_1	ZONMW-67_01697	fabG_1
fabG_1	ZONMW-67_00061	fabG_1
fabG_1	ZONMW-67_01101	fabG_1
fabG_1	ZONMW-67_02933	fabG_1
fabG_1	ZONMW-67_00671	fabG_1
fabG_1	ZONMW-67_02335	fabG_1
fabG_1	ZONMW-67_00266	fabG_1
fabG_1	ZONMW-67_00738	fabG_1
fabG_1	ZONMW-67_00931	fabG_1
fabG_1	ZONMW-67_01838	fabG_1

fabG_1	ZONMW-67_03032	fabG_1
fabG_1	ZONMW-67_03147	fabG_1
fabG_1	ZONMW-67_02763	fabG_1
fabG_1	ZONMW-67_00926	fabG_1
fabG_1	ZONMW-67_02182	fabG_1
fabG_1	ZONMW-67_02875	fabG_1
fabG_1	ZONMW-67_03120	fabG_1
fabG_1	ZONMW-67_02253	fabG_1
fabG_1	ZONMW-67_01120	fabG_1
fabG_1	ZONMW-67_00722	fabG_1
fabG_1	ZONMW-67_02281	fabG_1
fabG_1	ZONMW-67_03110	fabG_1
fabG_1	ZONMW-67_02034	fabG_1
fabG_1	ZONMW-67_00034	fabG_1
fabG_1	ZONMW-67_01326	fabG_1
fabG_1	ZONMW-67_00017	fabG_1
fabG_1	ZONMW-67_02979	fabG_1
fabG_1	ZONMW-67_00131	fabG_1
fabG_1	ZONMW-68_01441	fabG_1
fabG_1	ZONMW-68_03792	fabG_1
fabG_1	ZONMW-68_02887	fabG_1
fabG_1	ZONMW-68_03777	fabG_1
fabG_1	ZONMW-68_00532	fabG_1
fabG_1	ZONMW-68_00523	fabG_1
fabG_1	ZONMW-68_02594	fabG_1
fabG_1	ZONMW-68_02956	fabG_1
fabG_1	ZONMW-68_01395	fabG_1
fabG_1	ZONMW-68_00390	fabG_1
fabG_1	ZONMW-68_00845	fabG_1
fabG_1	ZONMW-68_04768	fabG_1
fabG_1	ZONMW-68_01412	fabG_1
fabG_1	ZONMW-68_03934	fabG_1
fabG_1	ZONMW-68_04578	fabG_1
fabG_1	ZONMW-68_04660	fabG_1
fabG_1	ZONMW-68_01917	fabG_1
fabG_1	ZONMW-68_04689	fabG_1
fabG_1	ZONMW-68_03636	fabG_1
fabG_1	ZONMW-68_02308	fabG_1
fabG_1	ZONMW-68_04862	fabG_1
fabG_1	ZONMW-68_04368	fabG_1
fabG_1	ZONMW-68_01355	fabG_1
fabG_1	ZONMW-68_04541	fabG_1
fabG_1	ZONMW-68_00599	fabG_1
fabG_1	ZONMW-68_01100	fabG_1
fabG_1	ZONMW-68_01483	fabG_1
fabG_1	ZONMW-68_04901	fabG_1
fabG_1	ZONMW-68_04877	fabG_1
fabG_1	ZONMW-68_00538	fabG_1

fabG_1	ZONMW-68_01686	fabG_1
fabG_1	ZONMW-68_04325	fabG_1
fabG_1	ZONMW-68_04185	fabG_1
fabG_1	ZONMW-68_03173	fabG_1
fabG_1	ZONMW-68_04909	fabG_1
fabG_1	ZONMW-68_04551	fabG_1
fabG_1	ZONMW-68_01321	fabG_1
fabG_1	ZONMW-68_04900	fabG_1
fabG_1	ZONMW-69_03024	fabG_1
fabG_1	ZONMW-69_02380	fabG_1
fabG_1	ZONMW-69_01304	fabG_1
fabG_1	ZONMW-69_02670	fabG_1
fabG_1	ZONMW-69_01148	fabG_1
fabG_1	ZONMW-69_03021	fabG_1
fabG_1	ZONMW-69_00718	fabG_1
fabG_1	ZONMW-69_02383	fabG_1
fabG_1	ZONMW-69_00013	fabG_1
fabG_1	ZONMW-69_00785	fabG_1
fabG_1	ZONMW-69_00978	fabG_1
fabG_1	ZONMW-69_02105	fabG_1
fabG_1	ZONMW-69_02922	fabG_1
fabG_1	ZONMW-69_03148	fabG_1
fabG_1	ZONMW-69_00560	fabG_1
fabG_1	ZONMW-69_00973	fabG_1
fabG_1	ZONMW-69_02229	fabG_1
fabG_1	ZONMW-69_03079	fabG_1
fabG_1	ZONMW-69_03121	fabG_1
fabG_1	ZONMW-69_02301	fabG_1
fabG_1	ZONMW-69_01166	fabG_1
fabG_1	ZONMW-69_00769	fabG_1
fabG_1	ZONMW-69_02329	fabG_1
fabG_1	ZONMW-69_03111	fabG_1
fabG_1	ZONMW-69_01909	fabG_1
fabG_1	ZONMW-69_02643	fabG_1
fabG_1	ZONMW-69_01675	fabG_1
fabG_1	ZONMW-69_02626	fabG_1
fabG_1	ZONMW-69_02975	fabG_1
fabG_1	ZONMW-69_02740	fabG_1
fabG_1	ZONMW-72_01347	fabG_1
fabG_1	ZONMW-72_02181	fabG_1
fabG_1	ZONMW-72_01028	fabG_1
fabG_1	ZONMW-72_04176	fabG_1
fabG_1	ZONMW-72_04477	fabG_1
fabG_1	ZONMW-72_02289	fabG_1
fabG_1	ZONMW-72_04119	fabG_1
fabG_1	ZONMW-72_00859	fabG_1
fabG_1	ZONMW-72_04520	fabG_1
fabG_1	ZONMW-72_00628	fabG_1

fabG_1	ZONMW-72_04238	fabG_1
fabG_1	ZONMW-72_02153	fabG_1
fabG_1	ZONMW-72_03822	fabG_1
fabG_1	ZONMW-72_04232	fabG_1
fabG_1	ZONMW-72_00752	fabG_1
fabG_1	ZONMW-72_04197	fabG_1
fabG_1	ZONMW-72_04131	fabG_1
fabG_1	ZONMW-72_01051	fabG_1
fabG_1	ZONMW-72_01761	fabG_1
fabG_1	ZONMW-72_00879	fabG_1
fabG_1	ZONMW-72_02710	fabG_1
fabG_1	ZONMW-72_02392	fabG_1
fabG_1	ZONMW-72_01095	fabG_1
fabG_1	ZONMW-72_03536	fabG_1
fabG_1	ZONMW-72_00863	fabG_1
fabG_1	ZONMW-72_01766	fabG_1
fabG_1	ZONMW-72_04120	fabG_1
fabG_1	ZONMW-72_00862	fabG_1
fabG_1	ZONMW-73_02583	fabG_1
fabG_1	ZONMW-73_02458	fabG_1
fabG_1	ZONMW-73_02777	fabG_1
fabG_1	ZONMW-73_01405	fabG_1
fabG_1	ZONMW-73_02280	fabG_1
fabG_1	ZONMW-73_02529	fabG_1
fabG_1	ZONMW-73_00690	fabG_1
fabG_1	ZONMW-73_02586	fabG_1
fabG_1	ZONMW-73_01687	fabG_1
fabG_1	ZONMW-73_01100	fabG_1
fabG_1	ZONMW-73_02364	fabG_1
fabG_1	ZONMW-73_00093	fabG_1
fabG_1	ZONMW-73_01311	fabG_1
fabG_1	ZONMW-73_03572	fabG_1
fabG_1	ZONMW-73_01264	fabG_1
fabG_1	ZONMW-73_01137	fabG_1
fabG_1	ZONMW-73_00403	fabG_1
fabG_1	ZONMW-74_00944	fabG_1
fabG_1	ZONMW-74_00233	fabG_1
fabG_1	ZONMW-74_00867	fabG_1
fabG_1	ZONMW-74_01265	fabG_1
fabG_1	ZONMW-74_00921	fabG_1
fabG_1	ZONMW-74_00931	fabG_1
fabG_1	ZONMW-74_03398	fabG_1
fabG_1	ZONMW-74_01666	fabG_1
fabG_1	ZONMW-74_01318	fabG_1
fabG_1	ZONMW-74_01073	fabG_1
fabG_1	ZONMW-74_00718	fabG_1
fabG_1	ZONMW-74_01297	fabG_1
fabG_1	ZONMW-74_02186	fabG_1

fabG_1	ZONMW-74_01273	fabG_1
fabG_1	ZONMW-74_00388	fabG_1
fabG_1	ZONMW-74_01264	fabG_1
fabG_1	ZONMW-74_02747	fabG_1
fabG_1	ZONMW-74_01276	fabG_1
fabG_1	ZONMW-75_00877	fabG_1
fabG_1	ZONMW-75_00578	fabG_1
fabG_1	ZONMW-75_02067	fabG_1
fabG_1	ZONMW-75_00955	fabG_1
fabG_1	ZONMW-75_00299	fabG_1
fabG_1	ZONMW-75_02715	fabG_1
fabG_1	ZONMW-75_02209	fabG_1
fabG_1	ZONMW-75_03547	fabG_1
fabG_1	ZONMW-75_00705	fabG_1
fabG_1	ZONMW-75_03172	fabG_1
fabG_1	ZONMW-75_00880	fabG_1
fabG_1	ZONMW-75_00715	fabG_1
fabG_1	ZONMW-75_03722	fabG_1
fabG_1	ZONMW-75_03095	fabG_1
fabG_1	ZONMW-75_01358	fabG_1
fabG_1	ZONMW-75_02647	fabG_1
fabG_1	ZONMW-75_01531	fabG_1
fabG_1	ZONMW-75_01034	fabG_1
fabG_1	ZONMW-75_00112	fabG_1
fabG_1	ZONMW-75_03383	fabG_1
fabG_1	ZONMW-75_03625	fabG_1
fabG_1	ZONMW-75_00065	fabG_1
fabG_1	ZONMW-75_01754	fabG_1
fabG_1	ZONMW-75_02182	fabG_1
fabG_1	da5A-8_02694	fabG_1
fabG_1	da5A-8_00665	fabG_1
fabG_1	da5A-8_01300	fabG_1
fabG_1	da5A-8_00923	fabG_1
fabG_1	da5A-8_00959	fabG_1
fabG_1	da5A-8_01717	fabG_1
fabG_1	da5A-8_02799	fabG_1
fabG_1	da5A-8_03745	fabG_1
fabG_1	da5A-8_01880	fabG_1
fabG_1	da5A-8_02890	fabG_1
fabG_1	da5A-8_00948	fabG_1
fabG_1	da5A-8_02392	fabG_1
fabG_1	da5A-8_02549	fabG_1
fabG_1	da5A-8_00413	fabG_1
ppiB_1	18_00892	ppiB_1
ppiB_1	18_03891	ppiB_1
ppiB_1	18_02229	ppiB_1
ppiB_1	18_02031	ppiB_1
ppiB_1	18_02719	ppiB_1

ppiB_1	18_02283	ppiB_1
ppiB_1	18_03417	ppiB_1
ppiB_1	ACAM48_01497	ppiB_1
ppiB_1	ACAM48_01331	ppiB_1
ppiB_1	ACAM48_01452	ppiB_1
ppiB_1	ACAM48_01015	ppiB_1
ppiB_1	ACAM48_00701	ppiB_1
ppiB_1	CAM030_01723	ppiB_1
ppiB_1	CAM030_00064	ppiB_1
ppiB_1	CAM030_01775	ppiB_1
ppiB_1	CAM030_02024	ppiB_1
ppiB_1	CAM030_00167	ppiB_1
ppiB_1	CAM030_02562	ppiB_1
ppiB_1	CECT7946_03632	ppiB_1
ppiB_1	CECT7946_02790	ppiB_1
ppiB_1	CECT7946_03174	ppiB_1
ppiB_1	CECT7946_01663	ppiB_1
ppiB_1	CECT7946_01982	ppiB_1
ppiB_1	CGMCC1.8863_00637	ppiB_1
ppiB_1	CGMCC1.8863_03454	ppiB_1
ppiB_1	CGMCC1.8863_01143	ppiB_1
ppiB_1	CGMCC1.8863_02802	ppiB_1
ppiB_1	CGMCC1.8863_03343	ppiB_1
ppiB_1	CGMCC1.8863_02703	ppiB_1
ppiB_1	CL8139_02655	ppiB_1
ppiB_1	CL8139_00376	ppiB_1
ppiB_1	CL8139_02332	ppiB_1
ppiB_1	CL8139_01150	ppiB_1
ppiB_1	CL8139_00613	ppiB_1
ppiB_1	CL8139_01214	ppiB_1
ppiB_1	CL8139_02072	ppiB_1
ppiB_1	DAU203_02711	ppiB_1
ppiB_1	DAU203_01543	ppiB_1
ppiB_1	DAU203_00138	ppiB_1
ppiB_1	DAU203_00274	ppiB_1
ppiB_1	DAU203_00422	ppiB_1
ppiB_1	DAU203_00211	ppiB_1
ppiB_1	DAU203_02403	ppiB_1
ppiB_1	DD5b_01469	ppiB_1
ppiB_1	DD5b_00151	ppiB_1
ppiB_1	DD5b_00791	ppiB_1
ppiB_1	DD5b_00503	ppiB_1
ppiB_1	DD5b_00790	ppiB_1
ppiB_1	DSM1208_00056	ppiB_1
ppiB_1	DSM1208_02736	ppiB_1
ppiB_1	DSM1208_03553	ppiB_1
ppiB_1	DSM1208_02906	ppiB_1
ppiB_1	DSM1208_00515	ppiB_1

ppiB_1	DSM1208_01259	ppiB_1
ppiB_1	DSM12212_00379	ppiB_1
ppiB_1	DSM12212_02462	ppiB_1
ppiB_1	DSM12212_02183	ppiB_1
ppiB_1	DSM12212_02853	ppiB_1
ppiB_1	DSM12212_03053	ppiB_1
ppiB_1	DSM12212_02215	ppiB_1
ppiB_1	DSM15343_00340	ppiB_1
ppiB_1	DSM15343_01185	ppiB_1
ppiB_1	DSM15343_01186	ppiB_1
ppiB_1	DSM15343_02838	ppiB_1
ppiB_1	DSM15361_02090	ppiB_1
ppiB_1	DSM15361_01740	ppiB_1
ppiB_1	DSM15361_01487	ppiB_1
ppiB_1	DSM15361_02783	ppiB_1
ppiB_1	DSM15361_00947	ppiB_1
ppiB_1	DSM15361_00006	ppiB_1
ppiB_1	DSM15365_00226	ppiB_1
ppiB_1	DSM15365_03496	ppiB_1
ppiB_1	DSM15365_01552	ppiB_1
ppiB_1	DSM15365_03833	ppiB_1
ppiB_1	DSM15365_01387	ppiB_1
ppiB_1	DSM15365_00310	ppiB_1
ppiB_1	DSM15365_02147	ppiB_1
ppiB_1	DSM15365_02110	ppiB_1
ppiB_1	DSM15718_02661	ppiB_1
ppiB_1	DSM15718_03049	ppiB_1
ppiB_1	DSM15718_02090	ppiB_1
ppiB_1	DSM15718_02091	ppiB_1
ppiB_1	DSM15718_02313	ppiB_1
ppiB_1	DSM15718_00836	ppiB_1
ppiB_1	DSM16505T_02343	ppiB_1
ppiB_1	DSM16505T_02121	ppiB_1
ppiB_1	DSM16505T_02342	ppiB_1
ppiB_1	DSM16505T_01502	ppiB_1
ppiB_1	DSM18103_01314	ppiB_1
ppiB_1	DSM18103_03203	ppiB_1
ppiB_1	DSM18103_00622	ppiB_1
ppiB_1	DSM18103_03202	ppiB_1
ppiB_1	DSM18103_00086	ppiB_1
ppiB_1	DSM18293_01553	ppiB_1
ppiB_1	DSM18293_03631	ppiB_1
ppiB_1	DSM18293_03791	ppiB_1
ppiB_1	DSM18293_02767	ppiB_1
ppiB_1	DSM18293_03790	ppiB_1
ppiB_1	DSM18841_03097	ppiB_1
ppiB_1	DSM18841_01485	ppiB_1
ppiB_1	DSM18841_03098	ppiB_1

ppiB_1	DSM18841_02124	ppiB_1
ppiB_1	DSM18842_00289	ppiB_1
ppiB_1	DSM18842_00008	ppiB_1
ppiB_1	DSM18842_01896	ppiB_1
ppiB_1	DSM18842_00007	ppiB_1
ppiB_1	DSM18842_01467	ppiB_1
ppiB_1	DSM19866_04772	ppiB_1
ppiB_1	DSM19866_01967	ppiB_1
ppiB_1	DSM19866_00249	ppiB_1
ppiB_1	DSM19866_04599	ppiB_1
ppiB_1	DSM19866_02678	ppiB_1
ppiB_1	DSM19866_00777	ppiB_1
ppiB_1	DSM19866_04053	ppiB_1
ppiB_1	DSM2041_03421	ppiB_1
ppiB_1	DSM2041_02697	ppiB_1
ppiB_1	DSM2041_03403	ppiB_1
ppiB_1	DSM2041_02568	ppiB_1
ppiB_1	DSM2061_00496	ppiB_1
ppiB_1	DSM2061_04224	ppiB_1
ppiB_1	DSM2061_01510	ppiB_1
ppiB_1	DSM2061_04391	ppiB_1
ppiB_1	DSM2061_02006	ppiB_1
ppiB_1	DSM2061_03630	ppiB_1
ppiB_1	DSM2064_03675	ppiB_1
ppiB_1	DSM2064_03499	ppiB_1
ppiB_1	DSM2064_03320	ppiB_1
ppiB_1	DSM2064_01401	ppiB_1
ppiB_1	DSM2064_05045	ppiB_1
ppiB_1	DSM2064_03321	ppiB_1
ppiB_1	DSM21164_00955	ppiB_1
ppiB_1	DSM21164_02005	ppiB_1
ppiB_1	DSM21164_02991	ppiB_1
ppiB_1	DSM21164_03117	ppiB_1
ppiB_1	DSM21164_01871	ppiB_1
ppiB_1	DSM21164_01642	ppiB_1
ppiB_1	DSM23404_00059	ppiB_1
ppiB_1	DSM23404_01629	ppiB_1
ppiB_1	DSM23404_00014	ppiB_1
ppiB_1	DSM23404_02864	ppiB_1
ppiB_1	DSM23404_02621	ppiB_1
ppiB_1	DSM23424_02727	ppiB_1
ppiB_1	DSM23424_03354	ppiB_1
ppiB_1	DSM23424_01900	ppiB_1
ppiB_1	DSM23424_01536	ppiB_1
ppiB_1	DSM24786_00169	ppiB_1
ppiB_1	DSM24786_03033	ppiB_1
ppiB_1	DSM24786_00106	ppiB_1
ppiB_1	DSM24786_01075	ppiB_1

ppiB_1	DSM24786_02494	ppiB_1
ppiB_1	DSM24786_01155	ppiB_1
ppiB_1	DSM24786_01609	ppiB_1
ppiB_1	DSM7489_02585	ppiB_1
ppiB_1	DSM7489_01447	ppiB_1
ppiB_1	DSM7489_00136	ppiB_1
ppiB_1	DSM7489_00268	ppiB_1
ppiB_1	DSM7489_00406	ppiB_1
ppiB_1	DSM7489_00204	ppiB_1
ppiB_1	DSM7489_02318	ppiB_1
ppiB_1	DSW1_02511	ppiB_1
ppiB_1	DSW1_01554	ppiB_1
ppiB_1	DSW1_01418	ppiB_1
ppiB_1	DSW1_01777	ppiB_1
ppiB_1	DSW5_02537	ppiB_1
ppiB_1	DSW5_02071	ppiB_1
ppiB_1	DSW5_02070	ppiB_1
ppiB_1	DSW5_02020	ppiB_1
ppiB_1	DSW5_02533	ppiB_1
ppiB_1	DSW5_01674	ppiB_1
ppiB_1	DSW6_00053	ppiB_1
ppiB_1	DSW6_02345	ppiB_1
ppiB_1	DSW6_03173	ppiB_1
ppiB_1	DSW6_02398	ppiB_1
ppiB_1	DSW6_00279	ppiB_1
ppiB_1	DSW6_03001	ppiB_1
ppiB_1	DSW8_00439	ppiB_1
ppiB_1	DSW8_01225	ppiB_1
ppiB_1	DSW8_00964	ppiB_1
ppiB_1	DSW8_01042	ppiB_1
ppiB_1	DSW8_03481	ppiB_1
ppiB_1	DSW8_00442	ppiB_1
ppiB_1	F52_03822	ppiB_1
ppiB_1	F52_00175	ppiB_1
ppiB_1	F52_00338	ppiB_1
ppiB_1	F52_01902	ppiB_1
ppiB_1	F52_00337	ppiB_1
ppiB_1	GCA_001027945.1_01013	ppiB_1
ppiB_1	GCA_001027945.1_02249	ppiB_1
ppiB_1	GCA_001027945.1_01012	ppiB_1
ppiB_1	GCA_001027945.1_01866	ppiB_1
ppiB_1	HI1_02518	ppiB_1
ppiB_1	HI1_01388	ppiB_1
ppiB_1	HI1_00068	ppiB_1
ppiB_1	HI1_00200	ppiB_1
ppiB_1	HI1_00338	ppiB_1
ppiB_1	HI1_00136	ppiB_1
ppiB_1	HI1_02251	ppiB_1

ppiB_1	IR1_01626	ppiB_1
ppiB_1	IR1_04435	ppiB_1
ppiB_1	IR1_02533	ppiB_1
ppiB_1	IR1_02742	ppiB_1
ppiB_1	IR1_02534	ppiB_1
ppiB_1	KCOM3131_00146	ppiB_1
ppiB_1	KCOM3131_00145	ppiB_1
ppiB_1	KCOM3131_00147	ppiB_1
ppiB_1	KCOM3131_01379	ppiB_1
ppiB_1	KCOM3131_01302	ppiB_1
ppiB_1	KCOM3131_01013	ppiB_1
ppiB_1	KCTC12974_00741	ppiB_1
ppiB_1	KCTC12974_00696	ppiB_1
ppiB_1	KCTC12974_01822	ppiB_1
ppiB_1	KCTC12974_02126	ppiB_1
ppiB_1	KCTC12974_02998	ppiB_1
ppiB_1	KL-A_00970	ppiB_1
ppiB_1	KL-A_00779	ppiB_1
ppiB_1	KL-A_01131	ppiB_1
ppiB_1	KL-A_01264	ppiB_1
ppiB_1	KL-A_02586	ppiB_1
ppiB_1	KL-A_01200	ppiB_1
ppiB_1	KL-A_01598	ppiB_1
ppiB_1	L12_00146	ppiB_1
ppiB_1	L12_00311	ppiB_1
ppiB_1	LL0412.1.7_01136	ppiB_1
ppiB_1	LL0412.1.7_01314	ppiB_1
ppiB_1	LL0412.1.7_01604	ppiB_1
ppiB_1	LL0412.1.7_01315	ppiB_1
ppiB_1	LL0412.1.7_02159	ppiB_1
ppiB_1	MAR_2009_71_03238	ppiB_1
ppiB_1	MAR_2009_71_02212	ppiB_1
ppiB_1	MAR_2009_71_02477	ppiB_1
ppiB_1	MAR_2009_71_02399	ppiB_1
ppiB_1	MAR_2009_71_00105	ppiB_1
ppiB_1	MAR_2009_71_03241	ppiB_1
ppiB_1	NN016038_00901	ppiB_1
ppiB_1	NN016038_03944	ppiB_1
ppiB_1	NN016038_02245	ppiB_1
ppiB_1	NN016038_02049	ppiB_1
ppiB_1	NN016038_02768	ppiB_1
ppiB_1	NN016038_02294	ppiB_1
ppiB_1	NN016038_03453	ppiB_1
ppiB_1	PAO1_03375	ppiB_1
ppiB_1	PAO1_04720	ppiB_1
ppiB_1	PAO1_03840	ppiB_1
ppiB_1	PAO1_01840	ppiB_1
ppiB_1	PAO1_03338	ppiB_1

ppiB_1	PAO1_05431	ppiB_1
ppiB_1	PAO1_04706	ppiB_1
ppiB_1	PAO1_00863	ppiB_1
ppiB_1	UW101_05211	ppiB_1
ppiB_1	UW101_02301	ppiB_1
ppiB_1	UW101_02480	ppiB_1
ppiB_1	UW101_04078	ppiB_1
ppiB_1	UW101_00057	ppiB_1
ppiB_1	UW101_02479	ppiB_1
ppiB_1	V4-33_01801	ppiB_1
ppiB_1	V4-33_01819	ppiB_1
ppiB_1	V4-33_01802	ppiB_1
ppiB_1	V4-33_01346	ppiB_1
ppiB_1	W5C_01304	ppiB_1
ppiB_1	W5C_01146	ppiB_1
ppiB_1	W5C_00045	ppiB_1
ppiB_1	W5C_00177	ppiB_1
ppiB_1	W5C_03194	ppiB_1
ppiB_1	W5C_00113	ppiB_1
ppiB_1	W5C_02650	ppiB_1
ppiB_1	ZONMW-01_01634	ppiB_1
ppiB_1	ZONMW-01_02445	ppiB_1
ppiB_1	ZONMW-01_02022	ppiB_1
ppiB_1	ZONMW-01_02152	ppiB_1
ppiB_1	ZONMW-01_01858	ppiB_1
ppiB_1	ZONMW-01_02088	ppiB_1
ppiB_1	ZONMW-01_01254	ppiB_1
ppiB_1	ZONMW-02_03278	ppiB_1
ppiB_1	ZONMW-02_00435	ppiB_1
ppiB_1	ZONMW-02_02015	ppiB_1
ppiB_1	ZONMW-02_03080	ppiB_1
ppiB_1	ZONMW-02_01253	ppiB_1
ppiB_1	ZONMW-02_02902	ppiB_1
ppiB_1	ZONMW-02_01561	ppiB_1
ppiB_1	ZONMW-02_03413	ppiB_1
ppiB_1	ZONMW-03_00573	ppiB_1
ppiB_1	ZONMW-03_03048	ppiB_1
ppiB_1	ZONMW-03_02689	ppiB_1
ppiB_1	ZONMW-03_02558	ppiB_1
ppiB_1	ZONMW-03_02843	ppiB_1
ppiB_1	ZONMW-03_02622	ppiB_1
ppiB_1	ZONMW-03_01526	ppiB_1
ppiB_1	ZONMW-04_01511	ppiB_1
ppiB_1	ZONMW-04_02396	ppiB_1
ppiB_1	ZONMW-04_01865	ppiB_1
ppiB_1	ZONMW-04_01997	ppiB_1
ppiB_1	ZONMW-04_02687	ppiB_1
ppiB_1	ZONMW-04_01933	ppiB_1

ppiB_1	ZONMW-04_00164	ppiB_1
ppiB_1	ZONMW-05_00835	ppiB_1
ppiB_1	ZONMW-05_02628	ppiB_1
ppiB_1	ZONMW-05_02504	ppiB_1
ppiB_1	ZONMW-05_02372	ppiB_1
ppiB_1	ZONMW-05_02236	ppiB_1
ppiB_1	ZONMW-05_02436	ppiB_1
ppiB_1	ZONMW-05_01664	ppiB_1
ppiB_1	ZONMW-06_01841	ppiB_1
ppiB_1	ZONMW-06_03270	ppiB_1
ppiB_1	ZONMW-06_02434	ppiB_1
ppiB_1	ZONMW-06_02190	ppiB_1
ppiB_1	ZONMW-06_03958	ppiB_1
ppiB_1	ZONMW-06_02915	ppiB_1
ppiB_1	ZONMW-06_00589	ppiB_1
ppiB_1	ZONMW-07_01390	ppiB_1
ppiB_1	ZONMW-07_01800	ppiB_1
ppiB_1	ZONMW-07_01758	ppiB_1
ppiB_1	ZONMW-07_03794	ppiB_1
ppiB_1	ZONMW-07_02282	ppiB_1
ppiB_1	ZONMW-07_03224	ppiB_1
ppiB_1	ZONMW-08_02753	ppiB_1
ppiB_1	ZONMW-08_01033	ppiB_1
ppiB_1	ZONMW-08_03476	ppiB_1
ppiB_1	ZONMW-08_00838	ppiB_1
ppiB_1	ZONMW-08_02946	ppiB_1
ppiB_1	ZONMW-08_02756	ppiB_1
ppiB_1	ZONMW-09_02141	ppiB_1
ppiB_1	ZONMW-09_02465	ppiB_1
ppiB_1	ZONMW-09_00943	ppiB_1
ppiB_1	ZONMW-09_01801	ppiB_1
ppiB_1	ZONMW-09_00871	ppiB_1
ppiB_1	ZONMW-09_01031	ppiB_1
ppiB_1	ZONMW-10_01908	ppiB_1
ppiB_1	ZONMW-10_01451	ppiB_1
ppiB_1	ZONMW-10_01926	ppiB_1
ppiB_1	ZONMW-10_01925	ppiB_1
ppiB_1	ZONMW-11_02785	ppiB_1
ppiB_1	ZONMW-11_02784	ppiB_1
ppiB_1	ZONMW-11_02783	ppiB_1
ppiB_1	ZONMW-11_00747	ppiB_1
ppiB_1	ZONMW-11_02456	ppiB_1
ppiB_1	ZONMW-11_00058	ppiB_1
ppiB_1	ZONMW-11_01161	ppiB_1
ppiB_1	ZONMW-11_02436	ppiB_1
ppiB_1	ZONMW-12_03230	ppiB_1
ppiB_1	ZONMW-12_03875	ppiB_1
ppiB_1	ZONMW-12_01782	ppiB_1

ppiB_1	ZONMW-12_03065	ppiB_1
ppiB_1	ZONMW-12_01534	ppiB_1
ppiB_1	ZONMW-12_03100	ppiB_1
ppiB_1	ZONMW-13_01610	ppiB_1
ppiB_1	ZONMW-13_02483	ppiB_1
ppiB_1	ZONMW-13_01565	ppiB_1
ppiB_1	ZONMW-13_02285	ppiB_1
ppiB_1	ZONMW-13_01996	ppiB_1
ppiB_1	ZONMW-14_00841	ppiB_1
ppiB_1	ZONMW-14_02520	ppiB_1
ppiB_1	ZONMW-14_02826	ppiB_1
ppiB_1	ZONMW-14_02206	ppiB_1
ppiB_1	ZONMW-14_00698	ppiB_1
ppiB_1	ZONMW-15_01705	ppiB_1
ppiB_1	ZONMW-15_00531	ppiB_1
ppiB_1	ZONMW-15_02694	ppiB_1
ppiB_1	ZONMW-15_02051	ppiB_1
ppiB_1	ZONMW-15_01570	ppiB_1
ppiB_1	ZONMW-15_01850	ppiB_1
ppiB_1	ZONMW-19_01164	ppiB_1
ppiB_1	ZONMW-19_00164	ppiB_1
ppiB_1	ZONMW-19_03576	ppiB_1
ppiB_1	ZONMW-19_03847	ppiB_1
ppiB_1	ZONMW-19_00615	ppiB_1
ppiB_1	ZONMW-20_03437	ppiB_1
ppiB_1	ZONMW-20_03183	ppiB_1
ppiB_1	ZONMW-20_03412	ppiB_1
ppiB_1	ZONMW-20_01254	ppiB_1
ppiB_1	ZONMW-20_01037	ppiB_1
ppiB_1	ZONMW-20_03435	ppiB_1
ppiB_1	ZONMW-21_02626	ppiB_1
ppiB_1	ZONMW-21_03376	ppiB_1
ppiB_1	ZONMW-21_02750	ppiB_1
ppiB_1	ZONMW-21_03265	ppiB_1
ppiB_1	ZONMW-21_01880	ppiB_1
ppiB_1	ZONMW-22_02483	ppiB_1
ppiB_1	ZONMW-22_03349	ppiB_1
ppiB_1	ZONMW-22_02359	ppiB_1
ppiB_1	ZONMW-22_03251	ppiB_1
ppiB_1	ZONMW-22_01846	ppiB_1
ppiB_1	ZONMW-23_00067	ppiB_1
ppiB_1	ZONMW-23_03422	ppiB_1
ppiB_1	ZONMW-23_00191	ppiB_1
ppiB_1	ZONMW-23_03273	ppiB_1
ppiB_1	ZONMW-23_00706	ppiB_1
ppiB_1	ZONMW-24_02248	ppiB_1
ppiB_1	ZONMW-24_03424	ppiB_1
ppiB_1	ZONMW-24_02124	ppiB_1

ppiB_1	ZONMW-24_03274	ppiB_1
ppiB_1	ZONMW-24_01869	ppiB_1
ppiB_1	ZONMW-25_00650	ppiB_1
ppiB_1	ZONMW-25_00929	ppiB_1
ppiB_1	ZONMW-25_03145	ppiB_1
ppiB_1	ZONMW-25_01296	ppiB_1
ppiB_1	ZONMW-25_00877	ppiB_1
ppiB_1	ZONMW-26_00125	ppiB_1
ppiB_1	ZONMW-26_03065	ppiB_1
ppiB_1	ZONMW-26_02610	ppiB_1
ppiB_1	ZONMW-26_02744	ppiB_1
ppiB_1	ZONMW-26_02904	ppiB_1
ppiB_1	ZONMW-26_02680	ppiB_1
ppiB_1	ZONMW-26_01617	ppiB_1
ppiB_1	ZONMW-27_01793	ppiB_1
ppiB_1	ZONMW-27_02785	ppiB_1
ppiB_1	ZONMW-27_02036	ppiB_1
ppiB_1	ZONMW-27_02168	ppiB_1
ppiB_1	ZONMW-27_02435	ppiB_1
ppiB_1	ZONMW-27_02104	ppiB_1
ppiB_1	ZONMW-27_00161	ppiB_1
ppiB_1	ZONMW-28_02741	ppiB_1
ppiB_1	ZONMW-28_03104	ppiB_1
ppiB_1	ZONMW-28_00197	ppiB_1
ppiB_1	ZONMW-28_00396	ppiB_1
ppiB_1	ZONMW-28_03934	ppiB_1
ppiB_1	ZONMW-28_01992	ppiB_1
ppiB_1	ZONMW-28_00853	ppiB_1
ppiB_1	ZONMW-29_01913	ppiB_1
ppiB_1	ZONMW-29_03234	ppiB_1
ppiB_1	ZONMW-29_02890	ppiB_1
ppiB_1	ZONMW-29_01725	ppiB_1
ppiB_1	ZONMW-29_01566	ppiB_1
ppiB_1	ZONMW-29_01136	ppiB_1
ppiB_1	ZONMW-30_02848	ppiB_1
ppiB_1	ZONMW-30_01247	ppiB_1
ppiB_1	ZONMW-30_02884	ppiB_1
ppiB_1	ZONMW-30_02457	ppiB_1
ppiB_1	ZONMW-30_01193	ppiB_1
ppiB_1	ZONMW-31_02006	ppiB_1
ppiB_1	ZONMW-31_03658	ppiB_1
ppiB_1	ZONMW-31_01699	ppiB_1
ppiB_1	ZONMW-31_01344	ppiB_1
ppiB_1	ZONMW-31_03022	ppiB_1
ppiB_1	ZONMW-31_03218	ppiB_1
ppiB_1	ZONMW-31_00930	ppiB_1
ppiB_1	ZONMW-32_03134	ppiB_1
ppiB_1	ZONMW-32_03305	ppiB_1

ppiB_1	ZONMW-32_00988	ppiB_1
ppiB_1	ZONMW-32_01187	ppiB_1
ppiB_1	ZONMW-32_03952	ppiB_1
ppiB_1	ZONMW-32_02398	ppiB_1
ppiB_1	ZONMW-32_00238	ppiB_1
ppiB_1	ZONMW-33_03139	ppiB_1
ppiB_1	ZONMW-33_00695	ppiB_1
ppiB_1	ZONMW-33_00977	ppiB_1
ppiB_1	ZONMW-33_01176	ppiB_1
ppiB_1	ZONMW-33_03876	ppiB_1
ppiB_1	ZONMW-33_02720	ppiB_1
ppiB_1	ZONMW-33_00105	ppiB_1
ppiB_1	ZONMW-34_02517	ppiB_1
ppiB_1	ZONMW-34_01061	ppiB_1
ppiB_1	ZONMW-34_01182	ppiB_1
ppiB_1	ZONMW-34_01370	ppiB_1
ppiB_1	ZONMW-34_03832	ppiB_1
ppiB_1	ZONMW-34_02014	ppiB_1
ppiB_1	ZONMW-34_00496	ppiB_1
ppiB_1	ZONMW-35_02481	ppiB_1
ppiB_1	ZONMW-35_02182	ppiB_1
ppiB_1	ZONMW-35_00255	ppiB_1
ppiB_1	ZONMW-35_00443	ppiB_1
ppiB_1	ZONMW-35_03793	ppiB_1
ppiB_1	ZONMW-35_01706	ppiB_1
ppiB_1	ZONMW-35_00893	ppiB_1
ppiB_1	ZONMW-36_02862	ppiB_1
ppiB_1	ZONMW-36_03217	ppiB_1
ppiB_1	ZONMW-36_00197	ppiB_1
ppiB_1	ZONMW-36_00396	ppiB_1
ppiB_1	ZONMW-36_03929	ppiB_1
ppiB_1	ZONMW-36_02532	ppiB_1
ppiB_1	ZONMW-36_01264	ppiB_1
ppiB_1	ZONMW-37_02293	ppiB_1
ppiB_1	ZONMW-37_03358	ppiB_1
ppiB_1	ZONMW-37_02417	ppiB_1
ppiB_1	ZONMW-37_03260	ppiB_1
ppiB_1	ZONMW-37_01869	ppiB_1
ppiB_1	ZONMW-38_03348	ppiB_1
ppiB_1	ZONMW-39_02058	ppiB_1
ppiB_1	ZONMW-39_02929	ppiB_1
ppiB_1	ZONMW-39_00823	ppiB_1
ppiB_1	ZONMW-39_00331	ppiB_1
ppiB_1	ZONMW-39_04066	ppiB_1
ppiB_1	ZONMW-39_02178	ppiB_1
ppiB_1	ZONMW-39_00965	ppiB_1
ppiB_1	ZONMW-40_01687	ppiB_1
ppiB_1	ZONMW-40_00595	ppiB_1

ppiB_1	ZONMW-40_03481	ppiB_1
ppiB_1	ZONMW-40_02838	ppiB_1
ppiB_1	ZONMW-40_03301	ppiB_1
ppiB_1	ZONMW-41_00013	ppiB_1
ppiB_1	ZONMW-41_01412	ppiB_1
ppiB_1	ZONMW-41_02557	ppiB_1
ppiB_1	ZONMW-41_01203	ppiB_1
ppiB_1	ZONMW-41_01861	ppiB_1
ppiB_1	ZONMW-41_02054	ppiB_1
ppiB_1	ZONMW-41_03151	ppiB_1
ppiB_1	ZONMW-42_01158	ppiB_1
ppiB_1	ZONMW-42_00830	ppiB_1
ppiB_1	ZONMW-42_03676	ppiB_1
ppiB_1	ZONMW-42_01790	ppiB_1
ppiB_1	ZONMW-42_02359	ppiB_1
ppiB_1	ZONMW-42_00404	ppiB_1
ppiB_1	ZONMW-42_01427	ppiB_1
ppiB_1	ZONMW-43_01144	ppiB_1
ppiB_1	ZONMW-43_00595	ppiB_1
ppiB_1	ZONMW-43_03119	ppiB_1
ppiB_1	ZONMW-43_02953	ppiB_1
ppiB_1	ZONMW-43_03785	ppiB_1
ppiB_1	ZONMW-43_01518	ppiB_1
ppiB_1	ZONMW-43_00041	ppiB_1
ppiB_1	ZONMW-44_00179	ppiB_1
ppiB_1	ZONMW-44_04140	ppiB_1
ppiB_1	ZONMW-44_03310	ppiB_1
ppiB_1	ZONMW-44_01241	ppiB_1
ppiB_1	ZONMW-44_03138	ppiB_1
ppiB_1	ZONMW-44_00893	ppiB_1
ppiB_1	ZONMW-44_01609	ppiB_1
ppiB_1	ZONMW-45_02160	ppiB_1
ppiB_1	ZONMW-45_02884	ppiB_1
ppiB_1	ZONMW-45_01329	ppiB_1
ppiB_1	ZONMW-45_02885	ppiB_1
ppiB_1	ZONMW-45_01557	ppiB_1
ppiB_1	ZONMW-46_01836	ppiB_1
ppiB_1	ZONMW-46_02881	ppiB_1
ppiB_1	ZONMW-46_02713	ppiB_1
ppiB_1	ZONMW-46_02882	ppiB_1
ppiB_1	ZONMW-46_02068	ppiB_1
ppiB_1	ZONMW-47_01653	ppiB_1
ppiB_1	ZONMW-47_02872	ppiB_1
ppiB_1	ZONMW-47_02703	ppiB_1
ppiB_1	ZONMW-47_02873	ppiB_1
ppiB_1	ZONMW-47_01993	ppiB_1
ppiB_1	ZONMW-48_00091	ppiB_1
ppiB_1	ZONMW-48_02270	ppiB_1

ppiB_1	ZONMW-48_02831	ppiB_1
ppiB_1	ZONMW-48_03354	ppiB_1
ppiB_1	ZONMW-48_01943	ppiB_1
ppiB_1	ZONMW-48_01434	ppiB_1
ppiB_1	ZONMW-49_00136	ppiB_1
ppiB_1	ZONMW-49_01149	ppiB_1
ppiB_1	ZONMW-49_02542	ppiB_1
ppiB_1	ZONMW-49_01932	ppiB_1
ppiB_1	ZONMW-49_03234	ppiB_1
ppiB_1	ZONMW-49_02473	ppiB_1
ppiB_1	ZONMW-49_00592	ppiB_1
ppiB_1	ZONMW-50_00350	ppiB_1
ppiB_1	ZONMW-50_01150	ppiB_1
ppiB_1	ZONMW-50_02336	ppiB_1
ppiB_1	ZONMW-50_02528	ppiB_1
ppiB_1	ZONMW-50_03200	ppiB_1
ppiB_1	ZONMW-50_02267	ppiB_1
ppiB_1	ZONMW-50_00593	ppiB_1
ppiB_1	ZONMW-51_00133	ppiB_1
ppiB_1	ZONMW-51_02756	ppiB_1
ppiB_1	ZONMW-51_02519	ppiB_1
ppiB_1	ZONMW-51_02237	ppiB_1
ppiB_1	ZONMW-51_02386	ppiB_1
ppiB_1	ZONMW-51_02450	ppiB_1
ppiB_1	ZONMW-51_01113	ppiB_1
ppiB_1	ZONMW-52_00105	ppiB_1
ppiB_1	ZONMW-52_02290	ppiB_1
ppiB_1	ZONMW-52_02502	ppiB_1
ppiB_1	ZONMW-52_02649	ppiB_1
ppiB_1	ZONMW-52_02369	ppiB_1
ppiB_1	ZONMW-52_02433	ppiB_1
ppiB_1	ZONMW-52_02806	ppiB_1
ppiB_1	ZONMW-53_02549	ppiB_1
ppiB_1	ZONMW-53_02879	ppiB_1
ppiB_1	ZONMW-53_02131	ppiB_1
ppiB_1	ZONMW-53_02264	ppiB_1
ppiB_1	ZONMW-53_02402	ppiB_1
ppiB_1	ZONMW-53_02200	ppiB_1
ppiB_1	ZONMW-53_00109	ppiB_1
ppiB_1	ZONMW-54_01872	ppiB_1
ppiB_1	ZONMW-54_00095	ppiB_1
ppiB_1	ZONMW-54_02621	ppiB_1
ppiB_1	ZONMW-54_02444	ppiB_1
ppiB_1	ZONMW-54_02754	ppiB_1
ppiB_1	ZONMW-54_02690	ppiB_1
ppiB_1	ZONMW-54_00918	ppiB_1
ppiB_1	ZONMW-55_01405	ppiB_1
ppiB_1	ZONMW-55_02885	ppiB_1

ppiB_1	ZONMW-55_02610	ppiB_1
ppiB_1	ZONMW-55_01751	ppiB_1
ppiB_1	ZONMW-55_02477	ppiB_1
ppiB_1	ZONMW-55_02541	ppiB_1
ppiB_1	ZONMW-55_00107	ppiB_1
ppiB_1	ZONMW-56_00631	ppiB_1
ppiB_1	ZONMW-56_02646	ppiB_1
ppiB_1	ZONMW-56_01619	ppiB_1
ppiB_1	ZONMW-56_01905	ppiB_1
ppiB_1	ZONMW-56_01752	ppiB_1
ppiB_1	ZONMW-56_01688	ppiB_1
ppiB_1	ZONMW-56_02700	ppiB_1
ppiB_1	ZONMW-57_00837	ppiB_1
ppiB_1	ZONMW-57_02790	ppiB_1
ppiB_1	ZONMW-57_01966	ppiB_1
ppiB_1	ZONMW-57_02233	ppiB_1
ppiB_1	ZONMW-57_02099	ppiB_1
ppiB_1	ZONMW-57_02035	ppiB_1
ppiB_1	ZONMW-57_01506	ppiB_1
ppiB_1	ZONMW-58_00134	ppiB_1
ppiB_1	ZONMW-58_02814	ppiB_1
ppiB_1	ZONMW-58_02112	ppiB_1
ppiB_1	ZONMW-58_02257	ppiB_1
ppiB_1	ZONMW-58_01979	ppiB_1
ppiB_1	ZONMW-58_02043	ppiB_1
ppiB_1	ZONMW-58_01078	ppiB_1
ppiB_1	ZONMW-59_00832	ppiB_1
ppiB_1	ZONMW-59_02914	ppiB_1
ppiB_1	ZONMW-59_02331	ppiB_1
ppiB_1	ZONMW-59_02472	ppiB_1
ppiB_1	ZONMW-59_02198	ppiB_1
ppiB_1	ZONMW-59_02262	ppiB_1
ppiB_1	ZONMW-59_02826	ppiB_1
ppiB_1	ZONMW-60_00836	ppiB_1
ppiB_1	ZONMW-60_02908	ppiB_1
ppiB_1	ZONMW-60_02629	ppiB_1
ppiB_1	ZONMW-60_02325	ppiB_1
ppiB_1	ZONMW-60_02495	ppiB_1
ppiB_1	ZONMW-60_02558	ppiB_1
ppiB_1	ZONMW-60_01052	ppiB_1
ppiB_1	ZONMW-61_01916	ppiB_1
ppiB_1	ZONMW-61_00988	ppiB_1
ppiB_1	ZONMW-61_03993	ppiB_1
ppiB_1	ZONMW-61_03607	ppiB_1
ppiB_1	ZONMW-61_02221	ppiB_1
ppiB_1	ZONMW-61_02756	ppiB_1
ppiB_1	ZONMW-61_01919	ppiB_1
ppiB_1	ZONMW-61_00144	ppiB_1

ppiB_1	ZONMW-62_00321	ppiB_1
ppiB_1	ZONMW-62_02802	ppiB_1
ppiB_1	ZONMW-62_01061	ppiB_1
ppiB_1	ZONMW-62_02610	ppiB_1
ppiB_1	ZONMW-62_03444	ppiB_1
ppiB_1	ZONMW-62_00318	ppiB_1
ppiB_1	ZONMW-63_00512	ppiB_1
ppiB_1	ZONMW-63_02283	ppiB_1
ppiB_1	ZONMW-63_01716	ppiB_1
ppiB_1	ZONMW-63_02405	ppiB_1
ppiB_1	ZONMW-63_01584	ppiB_1
ppiB_1	ZONMW-63_01648	ppiB_1
ppiB_1	ZONMW-63_01052	ppiB_1
ppiB_1	ZONMW-64_03494	ppiB_1
ppiB_1	ZONMW-64_00613	ppiB_1
ppiB_1	ZONMW-64_02289	ppiB_1
ppiB_1	ZONMW-64_03390	ppiB_1
ppiB_1	ZONMW-64_04105	ppiB_1
ppiB_1	ZONMW-64_02095	ppiB_1
ppiB_1	ZONMW-64_00059	ppiB_1
ppiB_1	ZONMW-65_03218	ppiB_1
ppiB_1	ZONMW-65_02238	ppiB_1
ppiB_1	ZONMW-65_03365	ppiB_1
ppiB_1	ZONMW-65_00390	ppiB_1
ppiB_1	ZONMW-65_01754	ppiB_1
ppiB_1	ZONMW-65_01740	ppiB_1
ppiB_1	ZONMW-65_03001	ppiB_1
ppiB_1	ZONMW-66_00520	ppiB_1
ppiB_1	ZONMW-66_00746	ppiB_1
ppiB_1	ZONMW-66_03900	ppiB_1
ppiB_1	ZONMW-66_01884	ppiB_1
ppiB_1	ZONMW-66_03419	ppiB_1
ppiB_1	ZONMW-67_03402	ppiB_1
ppiB_1	ZONMW-67_01672	ppiB_1
ppiB_1	ZONMW-67_01985	ppiB_1
ppiB_1	ZONMW-67_03609	ppiB_1
ppiB_1	ZONMW-67_02414	ppiB_1
ppiB_1	ZONMW-67_01315	ppiB_1
ppiB_1	ZONMW-67_00676	ppiB_1
ppiB_1	ZONMW-68_00975	ppiB_1
ppiB_1	ZONMW-68_00107	ppiB_1
ppiB_1	ZONMW-68_04845	ppiB_1
ppiB_1	ZONMW-68_01154	ppiB_1
ppiB_1	ZONMW-68_02479	ppiB_1
ppiB_1	ZONMW-68_02169	ppiB_1
ppiB_1	ZONMW-68_03632	ppiB_1
ppiB_1	ZONMW-69_03403	ppiB_1
ppiB_1	ZONMW-69_01329	ppiB_1

ppiB_1	ZONMW-69_01958	ppiB_1
ppiB_1	ZONMW-69_03611	ppiB_1
ppiB_1	ZONMW-69_02462	ppiB_1
ppiB_1	ZONMW-69_01686	ppiB_1
ppiB_1	ZONMW-69_00723	ppiB_1
ppiB_1	ZONMW-72_02123	ppiB_1
ppiB_1	ZONMW-72_03156	ppiB_1
ppiB_1	ZONMW-72_02513	ppiB_1
ppiB_1	ZONMW-72_01309	ppiB_1
ppiB_1	ZONMW-72_02512	ppiB_1
ppiB_1	ZONMW-73_01538	ppiB_1
ppiB_1	ZONMW-73_01324	ppiB_1
ppiB_1	ZONMW-73_03702	ppiB_1
ppiB_1	ZONMW-73_01198	ppiB_1
ppiB_1	ZONMW-73_03105	ppiB_1
ppiB_1	ZONMW-73_02882	ppiB_1
ppiB_1	ZONMW-73_03403	ppiB_1
ppiB_1	ZONMW-73_01124	ppiB_1
ppiB_1	ZONMW-74_02947	ppiB_1
ppiB_1	ZONMW-74_02705	ppiB_1
ppiB_1	ZONMW-74_02346	ppiB_1
ppiB_1	ZONMW-74_02555	ppiB_1
ppiB_1	ZONMW-74_02637	ppiB_1
ppiB_1	ZONMW-74_02323	ppiB_1
ppiB_1	ZONMW-75_03738	ppiB_1
ppiB_1	ZONMW-75_01345	ppiB_1
ppiB_1	ZONMW-75_00540	ppiB_1
ppiB_1	ZONMW-75_03464	ppiB_1
ppiB_1	ZONMW-75_02519	ppiB_1
ppiB_1	ZONMW-75_00821	ppiB_1
ppiB_1	ZONMW-75_03245	ppiB_1
ppiB_1	ZONMW-75_02568	ppiB_1
ppiB_1	da5A-8_01089	ppiB_1
ppiB_1	da5A-8_00564	ppiB_1
ppiB_1	da5A-8_03572	ppiB_1
ppiB_1	da5A-8_00563	ppiB_1
ppiB_1	da5A-8_02658	ppiB_1
mepM_1	18_00395	mepM_1
mepM_1	18_00754	mepM_1
mepM_1	18_01137	mepM_1
mepM_1	18_02373	mepM_1
mepM_1	18_02091	mepM_1
mepM_1	18_02127	mepM_1
mepM_1	ACAM48_02956	mepM_1
mepM_1	ACAM48_02167	mepM_1
mepM_1	ACAM48_02338	mepM_1
mepM_1	ACAM48_03367	mepM_1
mepM_1	ACAM48_02603	mepM_1

mepM_1	ACAM48_01061	mepM_1
mepM_1	CAM030_02702	mepM_1
mepM_1	CAM030_01202	mepM_1
mepM_1	CAM030_01031	mepM_1
mepM_1	CAM030_02031	mepM_1
mepM_1	CAM030_01672	mepM_1
mepM_1	CECT7946_01103	mepM_1
mepM_1	CECT7946_00374	mepM_1
mepM_1	CECT7946_00141	mepM_1
mepM_1	CECT7946_01334	mepM_1
mepM_1	CECT7946_02410	mepM_1
mepM_1	CECT7946_03458	mepM_1
mepM_1	CECT7946_02244	mepM_1
mepM_1	CGMCC1.8863_02574	mepM_1
mepM_1	CGMCC1.8863_02595	mepM_1
mepM_1	CGMCC1.8863_02199	mepM_1
mepM_1	CGMCC1.8863_00786	mepM_1
mepM_1	CGMCC1.8863_00111	mepM_1
mepM_1	CL8139_02736	mepM_1
mepM_1	CL8139_01585	mepM_1
mepM_1	CL8139_02140	mepM_1
mepM_1	CL8139_02866	mepM_1
mepM_1	CL8139_02839	mepM_1
mepM_1	DAU203_00723	mepM_1
mepM_1	DAU203_01184	mepM_1
mepM_1	DAU203_01459	mepM_1
mepM_1	DAU203_00022	mepM_1
mepM_1	DAU203_00049	mepM_1
mepM_1	DD5b_00227	mepM_1
mepM_1	DD5b_02004	mepM_1
mepM_1	DD5b_00606	mepM_1
mepM_1	DD5b_00932	mepM_1
mepM_1	DD5b_02143	mepM_1
mepM_1	DSM1208_00949	mepM_1
mepM_1	DSM1208_02989	mepM_1
mepM_1	DSM1208_01393	mepM_1
mepM_1	DSM1208_01672	mepM_1
mepM_1	DSM1208_03032	mepM_1
mepM_1	DSM12212_03198	mepM_1
mepM_1	DSM12212_00311	mepM_1
mepM_1	DSM12212_00067	mepM_1
mepM_1	DSM12212_00587	mepM_1
mepM_1	DSM12212_00814	mepM_1
mepM_1	DSM12212_03450	mepM_1
mepM_1	DSM12212_01881	mepM_1
mepM_1	DSM12212_01882	mepM_1
mepM_1	DSM15343_02132	mepM_1
mepM_1	DSM15343_02463	mepM_1

mepM_1	DSM15343_01959	mepM_1
mepM_1	DSM15343_02748	mepM_1
mepM_1	DSM15343_01054	mepM_1
mepM_1	DSM15361_01200	mepM_1
mepM_1	DSM15361_00634	mepM_1
mepM_1	DSM15361_00528	mepM_1
mepM_1	DSM15361_00239	mepM_1
mepM_1	DSM15361_01574	mepM_1
mepM_1	DSM15361_02705	mepM_1
mepM_1	DSM15361_01666	mepM_1
mepM_1	DSM15365_03505	mepM_1
mepM_1	DSM15365_00950	mepM_1
mepM_1	DSM15365_03055	mepM_1
mepM_1	DSM15365_01809	mepM_1
mepM_1	DSM15365_02106	mepM_1
mepM_1	DSM15365_00255	mepM_1
mepM_1	DSM15365_00756	mepM_1
mepM_1	DSM15718_01010	mepM_1
mepM_1	DSM15718_02746	mepM_1
mepM_1	DSM15718_02833	mepM_1
mepM_1	DSM15718_02585	mepM_1
mepM_1	DSM15718_00157	mepM_1
mepM_1	DSM16505T_00897	mepM_1
mepM_1	DSM16505T_00625	mepM_1
mepM_1	DSM16505T_00776	mepM_1
mepM_1	DSM16505T_01934	mepM_1
mepM_1	DSM16505T_01696	mepM_1
mepM_1	DSM16505T_01697	mepM_1
mepM_1	DSM16505T_02225	mepM_1
mepM_1	DSM18103_00626	mepM_1
mepM_1	DSM18103_01510	mepM_1
mepM_1	DSM18103_01044	mepM_1
mepM_1	DSM18103_00632	mepM_1
mepM_1	DSM18103_01241	mepM_1
mepM_1	DSM18293_02176	mepM_1
mepM_1	DSM18293_00797	mepM_1
mepM_1	DSM18293_02695	mepM_1
mepM_1	DSM18293_03072	mepM_1
mepM_1	DSM18293_01843	mepM_1
mepM_1	DSM18841_00763	mepM_1
mepM_1	DSM18841_00498	mepM_1
mepM_1	DSM18841_00654	mepM_1
mepM_1	DSM18841_02033	mepM_1
mepM_1	DSM18841_01646	mepM_1
mepM_1	DSM18841_01788	mepM_1
mepM_1	DSM18842_00554	mepM_1
mepM_1	DSM18842_01442	mepM_1
mepM_1	DSM18842_00732	mepM_1

mepM_1	DSM18842_03013	mepM_1
mepM_1	DSM18842_02344	mepM_1
mepM_1	DSM18842_02083	mepM_1
mepM_1	DSM18842_01757	mepM_1
mepM_1	DSM18842_01756	mepM_1
mepM_1	DSM19866_00916	mepM_1
mepM_1	DSM19866_03536	mepM_1
mepM_1	DSM19866_01896	mepM_1
mepM_1	DSM19866_01836	mepM_1
mepM_1	DSM19866_00005	mepM_1
mepM_1	DSM2041_01191	mepM_1
mepM_1	DSM2041_03394	mepM_1
mepM_1	DSM2041_00497	mepM_1
mepM_1	DSM2041_00236	mepM_1
mepM_1	DSM2041_05014	mepM_1
mepM_1	DSM2041_04082	mepM_1
mepM_1	DSM2041_01915	mepM_1
mepM_1	DSM2041_04842	mepM_1
mepM_1	DSM2041_03265	mepM_1
mepM_1	DSM2041_03264	mepM_1
mepM_1	DSM2041_03164	mepM_1
mepM_1	DSM2041_04549	mepM_1
mepM_1	DSM2061_02455	mepM_1
mepM_1	DSM2061_02913	mepM_1
mepM_1	DSM2061_03207	mepM_1
mepM_1	DSM2061_00052	mepM_1
mepM_1	DSM2064_02923	mepM_1
mepM_1	DSM2064_01717	mepM_1
mepM_1	DSM2064_04970	mepM_1
mepM_1	DSM2064_02021	mepM_1
mepM_1	DSM2064_00215	mepM_1
mepM_1	DSM21164_01388	mepM_1
mepM_1	DSM21164_01093	mepM_1
mepM_1	DSM21164_00731	mepM_1
mepM_1	DSM21164_03071	mepM_1
mepM_1	DSM21164_03026	mepM_1
mepM_1	DSM21164_03162	mepM_1
mepM_1	DSM23404_01188	mepM_1
mepM_1	DSM23404_01962	mepM_1
mepM_1	DSM23404_03053	mepM_1
mepM_1	DSM23404_00795	mepM_1
mepM_1	DSM23404_02350	mepM_1
mepM_1	DSM23404_02820	mepM_1
mepM_1	DSM23404_00902	mepM_1
mepM_1	DSM23424_03051	mepM_1
mepM_1	DSM23424_00186	mepM_1
mepM_1	DSM23424_00342	mepM_1
mepM_1	DSM23424_00944	mepM_1

mepM_1	DSM23424_00129	mepM_1
mepM_1	DSM23424_02342	mepM_1
mepM_1	DSM23424_00779	mepM_1
mepM_1	DSM23424_03339	mepM_1
mepM_1	DSM23424_01476	mepM_1
mepM_1	DSM24786_01420	mepM_1
mepM_1	DSM24786_02664	mepM_1
mepM_1	DSM24786_02945	mepM_1
mepM_1	DSM24786_01205	mepM_1
mepM_1	DSM24786_01232	mepM_1
mepM_1	DSM24786_00919	mepM_1
mepM_1	DSM7489_00710	mepM_1
mepM_1	DSM7489_01100	mepM_1
mepM_1	DSM7489_01369	mepM_1
mepM_1	DSM7489_00022	mepM_1
mepM_1	DSM7489_00049	mepM_1
mepM_1	DSW1_01096	mepM_1
mepM_1	DSW1_00348	mepM_1
mepM_1	DSW1_00637	mepM_1
mepM_1	DSW1_01624	mepM_1
mepM_1	DSW1_01500	mepM_1
mepM_1	DSW5_01472	mepM_1
mepM_1	DSW5_01084	mepM_1
mepM_1	DSW5_01101	mepM_1
mepM_1	DSW5_02314	mepM_1
mepM_1	DSW5_00362	mepM_1
mepM_1	DSW5_00554	mepM_1
mepM_1	DSW6_00091	mepM_1
mepM_1	DSW6_00877	mepM_1
mepM_1	DSW6_01393	mepM_1
mepM_1	DSW6_02094	mepM_1
mepM_1	DSW6_01324	mepM_1
mepM_1	DSW8_02940	mepM_1
mepM_1	DSW8_02546	mepM_1
mepM_1	DSW8_03361	mepM_1
mepM_1	DSW8_02262	mepM_1
mepM_1	DSW8_01000	mepM_1
mepM_1	DSW8_01145	mepM_1
mepM_1	F52_03165	mepM_1
mepM_1	F52_02175	mepM_1
mepM_1	F52_01831	mepM_1
mepM_1	F52_00048	mepM_1
mepM_1	F52_02421	mepM_1
mepM_1	F52_04385	mepM_1
mepM_1	GCA_001027945.1_03202	mepM_1
mepM_1	GCA_001027945.1_00109	mepM_1
mepM_1	GCA_001027945.1_03074	mepM_1
mepM_1	GCA_001027945.1_02756	mepM_1

mepM_1	GCA_001027945.1_02416	mepM_1
mepM_1	GCA_001027945.1_01171	mepM_1
mepM_1	HI1_00641	mepM_1
mepM_1	HI1_01040	mepM_1
mepM_1	HI1_01310	mepM_1
mepM_1	HI1_03321	mepM_1
mepM_1	HI1_03348	mepM_1
mepM_1	IR1_01014	mepM_1
mepM_1	IR1_04122	mepM_1
mepM_1	IR1_02822	mepM_1
mepM_1	IR1_04716	mepM_1
mepM_1	IR1_01824	mepM_1
mepM_1	KCOM3131_00986	mepM_1
mepM_1	KCTC12974_02219	mepM_1
mepM_1	KCTC12974_00420	mepM_1
mepM_1	KCTC12974_01639	mepM_1
mepM_1	KCTC12974_01974	mepM_1
mepM_1	KCTC12974_00357	mepM_1
mepM_1	KCTC12974_01553	mepM_1
mepM_1	KL-A_02775	mepM_1
mepM_1	KL-A_02046	mepM_1
mepM_1	KL-A_00860	mepM_1
mepM_1	KL-A_01817	mepM_1
mepM_1	KL-A_03299	mepM_1
mepM_1	L12_00490	mepM_1
mepM_1	L12_00354	mepM_1
mepM_1	LL0412.1.7_00906	mepM_1
mepM_1	LL0412.1.7_00318	mepM_1
mepM_1	LL0412.1.7_02248	mepM_1
mepM_1	LL0412.1.7_00782	mepM_1
mepM_1	LL0412.1.7_02358	mepM_1
mepM_1	MAR_2009_71_00519	mepM_1
mepM_1	MAR_2009_71_00906	mepM_1
mepM_1	MAR_2009_71_03568	mepM_1
mepM_1	MAR_2009_71_01177	mepM_1
mepM_1	MAR_2009_71_02441	mepM_1
mepM_1	MAR_2009_71_02300	mepM_1
mepM_1	NN016038_00397	mepM_1
mepM_1	NN016038_00774	mepM_1
mepM_1	NN016038_01151	mepM_1
mepM_1	NN016038_02384	mepM_1
mepM_1	NN016038_02109	mepM_1
mepM_1	NN016038_02145	mepM_1
mepM_1	PAO1_00681	mepM_1
mepM_1	PAO1_03911	mepM_1
mepM_1	PAO1_04547	mepM_1
mepM_1	PAO1_05540	mepM_1
mepM_1	PAO1_05303	mepM_1

mepM_1	PAO1_03746	mepM_1
mepM_1	PAO1_05091	mepM_1
mepM_1	PAO1_05734	mepM_1
mepM_1	UW101_01977	mepM_1
mepM_1	UW101_00771	mepM_1
mepM_1	UW101_00132	mepM_1
mepM_1	UW101_01076	mepM_1
mepM_1	UW101_02926	mepM_1
mepM_1	V4-33_01853	mepM_1
mepM_1	V4-33_01053	mepM_1
mepM_1	V4-33_00205	mepM_1
mepM_1	V4-33_02406	mepM_1
mepM_1	W5C_01347	mepM_1
mepM_1	W5C_01947	mepM_1
mepM_1	W5C_01866	mepM_1
mepM_1	W5C_00849	mepM_1
mepM_1	W5C_00822	mepM_1
mepM_1	W5C_02625	mepM_1
mepM_1	ZONMW-01_03255	mepM_1
mepM_1	ZONMW-01_01164	mepM_1
mepM_1	ZONMW-01_02362	mepM_1
mepM_1	ZONMW-01_03093	mepM_1
mepM_1	ZONMW-01_03066	mepM_1
mepM_1	ZONMW-01_01280	mepM_1
mepM_1	ZONMW-01_02601	mepM_1
mepM_1	ZONMW-02_00444	mepM_1
mepM_1	ZONMW-02_01667	mepM_1
mepM_1	ZONMW-02_03573	mepM_1
mepM_1	ZONMW-02_03386	mepM_1
mepM_1	ZONMW-02_03417	mepM_1
mepM_1	ZONMW-02_03258	mepM_1
mepM_1	ZONMW-03_03217	mepM_1
mepM_1	ZONMW-03_02233	mepM_1
mepM_1	ZONMW-03_02965	mepM_1
mepM_1	ZONMW-03_02328	mepM_1
mepM_1	ZONMW-03_02301	mepM_1
mepM_1	ZONMW-03_01500	mepM_1
mepM_1	ZONMW-03_01882	mepM_1
mepM_1	ZONMW-04_03152	mepM_1
mepM_1	ZONMW-04_01580	mepM_1
mepM_1	ZONMW-04_02482	mepM_1
mepM_1	ZONMW-04_00792	mepM_1
mepM_1	ZONMW-04_00766	mepM_1
mepM_1	ZONMW-04_00191	mepM_1
mepM_1	ZONMW-04_03309	mepM_1
mepM_1	ZONMW-05_03182	mepM_1
mepM_1	ZONMW-05_01861	mepM_1
mepM_1	ZONMW-05_02551	mepM_1

mepM_1	ZONMW-05_00062	mepM_1
mepM_1	ZONMW-05_00035	mepM_1
mepM_1	ZONMW-05_01636	mepM_1
mepM_1	ZONMW-05_02955	mepM_1
mepM_1	ZONMW-06_03026	mepM_1
mepM_1	ZONMW-06_01976	mepM_1
mepM_1	ZONMW-06_03893	mepM_1
mepM_1	ZONMW-06_00379	mepM_1
mepM_1	ZONMW-07_03903	mepM_1
mepM_1	ZONMW-07_03551	mepM_1
mepM_1	ZONMW-07_03978	mepM_1
mepM_1	ZONMW-07_00679	mepM_1
mepM_1	ZONMW-07_01485	mepM_1
mepM_1	ZONMW-07_01735	mepM_1
mepM_1	ZONMW-08_00035	mepM_1
mepM_1	ZONMW-08_00426	mepM_1
mepM_1	ZONMW-08_02356	mepM_1
mepM_1	ZONMW-08_00699	mepM_1
mepM_1	ZONMW-08_03440	mepM_1
mepM_1	ZONMW-08_00945	mepM_1
mepM_1	ZONMW-09_01517	mepM_1
mepM_1	ZONMW-09_00254	mepM_1
mepM_1	ZONMW-09_00361	mepM_1
mepM_1	ZONMW-09_01203	mepM_1
mepM_1	ZONMW-09_02244	mepM_1
mepM_1	ZONMW-10_01179	mepM_1
mepM_1	ZONMW-10_00255	mepM_1
mepM_1	ZONMW-10_00366	mepM_1
mepM_1	ZONMW-10_01521	mepM_1
mepM_1	ZONMW-11_03699	mepM_1
mepM_1	ZONMW-11_00337	mepM_1
mepM_1	ZONMW-11_00940	mepM_1
mepM_1	ZONMW-11_03703	mepM_1
mepM_1	ZONMW-11_00451	mepM_1
mepM_1	ZONMW-11_02196	mepM_1
mepM_1	ZONMW-11_00517	mepM_1
mepM_1	ZONMW-12_01984	mepM_1
mepM_1	ZONMW-12_00115	mepM_1
mepM_1	ZONMW-12_00260	mepM_1
mepM_1	ZONMW-12_00435	mepM_1
mepM_1	ZONMW-12_03447	mepM_1
mepM_1	ZONMW-12_03528	mepM_1
mepM_1	ZONMW-13_01068	mepM_1
mepM_1	ZONMW-13_00244	mepM_1
mepM_1	ZONMW-13_00360	mepM_1
mepM_1	ZONMW-13_03077	mepM_1
mepM_1	ZONMW-13_02921	mepM_1
mepM_1	ZONMW-13_00702	mepM_1

mepM_1	ZONMW-14_01524	mepM_1
mepM_1	ZONMW-14_00344	mepM_1
mepM_1	ZONMW-14_00230	mepM_1
mepM_1	ZONMW-14_01705	mepM_1
mepM_1	ZONMW-14_02916	mepM_1
mepM_1	ZONMW-14_01342	mepM_1
mepM_1	ZONMW-15_03170	mepM_1
mepM_1	ZONMW-15_03325	mepM_1
mepM_1	ZONMW-15_02525	mepM_1
mepM_1	ZONMW-15_03116	mepM_1
mepM_1	ZONMW-15_03155	mepM_1
mepM_1	ZONMW-19_00439	mepM_1
mepM_1	ZONMW-19_03213	mepM_1
mepM_1	ZONMW-19_00200	mepM_1
mepM_1	ZONMW-19_00767	mepM_1
mepM_1	ZONMW-19_02264	mepM_1
mepM_1	ZONMW-20_01850	mepM_1
mepM_1	ZONMW-20_00202	mepM_1
mepM_1	ZONMW-20_00829	mepM_1
mepM_1	ZONMW-20_00321	mepM_1
mepM_1	ZONMW-21_01415	mepM_1
mepM_1	ZONMW-21_01087	mepM_1
mepM_1	ZONMW-21_00804	mepM_1
mepM_1	ZONMW-21_02728	mepM_1
mepM_1	ZONMW-22_01417	mepM_1
mepM_1	ZONMW-22_01088	mepM_1
mepM_1	ZONMW-22_00805	mepM_1
mepM_1	ZONMW-22_02381	mepM_1
mepM_1	ZONMW-23_02530	mepM_1
mepM_1	ZONMW-23_02201	mepM_1
mepM_1	ZONMW-23_01919	mepM_1
mepM_1	ZONMW-23_00169	mepM_1
mepM_1	ZONMW-24_00044	mepM_1
mepM_1	ZONMW-24_00373	mepM_1
mepM_1	ZONMW-24_00656	mepM_1
mepM_1	ZONMW-24_02146	mepM_1
mepM_1	ZONMW-25_03122	mepM_1
mepM_1	ZONMW-25_02087	mepM_1
mepM_1	ZONMW-25_01730	mepM_1
mepM_1	ZONMW-25_00673	mepM_1
mepM_1	ZONMW-25_00004	mepM_1
mepM_1	ZONMW-25_01350	mepM_1
mepM_1	ZONMW-26_02251	mepM_1
mepM_1	ZONMW-26_02532	mepM_1
mepM_1	ZONMW-26_03307	mepM_1
mepM_1	ZONMW-26_00898	mepM_1
mepM_1	ZONMW-26_00925	mepM_1
mepM_1	ZONMW-26_01643	mepM_1

mepM_1	ZONMW-26_01969	mepM_1
mepM_1	ZONMW-27_02353	mepM_1
mepM_1	ZONMW-27_01715	mepM_1
mepM_1	ZONMW-27_02708	mepM_1
mepM_1	ZONMW-27_00889	mepM_1
mepM_1	ZONMW-27_00862	mepM_1
mepM_1	ZONMW-27_00186	mepM_1
mepM_1	ZONMW-27_02968	mepM_1
mepM_1	ZONMW-28_03341	mepM_1
mepM_1	ZONMW-28_02607	mepM_1
mepM_1	ZONMW-28_03836	mepM_1
mepM_1	ZONMW-28_01068	mepM_1
mepM_1	ZONMW-29_00969	mepM_1
mepM_1	ZONMW-29_00702	mepM_1
mepM_1	ZONMW-29_00444	mepM_1
mepM_1	ZONMW-29_01892	mepM_1
mepM_1	ZONMW-30_00693	mepM_1
mepM_1	ZONMW-30_00384	mepM_1
mepM_1	ZONMW-30_02266	mepM_1
mepM_1	ZONMW-30_03003	mepM_1
mepM_1	ZONMW-30_02099	mepM_1
mepM_1	ZONMW-31_03610	mepM_1
mepM_1	ZONMW-31_02077	mepM_1
mepM_1	ZONMW-31_01879	mepM_1
mepM_1	ZONMW-31_02582	mepM_1
mepM_1	ZONMW-31_00716	mepM_1
mepM_1	ZONMW-32_03466	mepM_1
mepM_1	ZONMW-32_03269	mepM_1
mepM_1	ZONMW-32_03774	mepM_1
mepM_1	ZONMW-32_00023	mepM_1
mepM_1	ZONMW-33_03469	mepM_1
mepM_1	ZONMW-33_03005	mepM_1
mepM_1	ZONMW-33_03773	mepM_1
mepM_1	ZONMW-33_03284	mepM_1
mepM_1	ZONMW-34_03736	mepM_1
mepM_1	ZONMW-34_02647	mepM_1
mepM_1	ZONMW-34_03671	mepM_1
mepM_1	ZONMW-34_00345	mepM_1
mepM_1	ZONMW-35_03699	mepM_1
mepM_1	ZONMW-35_02611	mepM_1
mepM_1	ZONMW-35_03822	mepM_1
mepM_1	ZONMW-35_01045	mepM_1
mepM_1	ZONMW-36_03326	mepM_1
mepM_1	ZONMW-36_02997	mepM_1
mepM_1	ZONMW-36_03722	mepM_1
mepM_1	ZONMW-36_03021	mepM_1
mepM_1	ZONMW-37_00947	mepM_1
mepM_1	ZONMW-37_01275	mepM_1

mepM_1	ZONMW-37_00804	mepM_1
mepM_1	ZONMW-37_02395	mepM_1
mepM_1	ZONMW-38_01117	mepM_1
mepM_1	ZONMW-38_04193	mepM_1
mepM_1	ZONMW-38_00508	mepM_1
mepM_1	ZONMW-38_02924	mepM_1
mepM_1	ZONMW-39_03601	mepM_1
mepM_1	ZONMW-39_03980	mepM_1
mepM_1	ZONMW-39_04126	mepM_1
mepM_1	ZONMW-39_03034	mepM_1
mepM_1	ZONMW-40_02787	mepM_1
mepM_1	ZONMW-40_00174	mepM_1
mepM_1	ZONMW-40_00402	mepM_1
mepM_1	ZONMW-40_01711	mepM_1
mepM_1	ZONMW-40_02544	mepM_1
mepM_1	ZONMW-41_01386	mepM_1
mepM_1	ZONMW-41_00652	mepM_1
mepM_1	ZONMW-41_02390	mepM_1
mepM_1	ZONMW-41_01248	mepM_1
mepM_1	ZONMW-41_01912	mepM_1
mepM_1	ZONMW-42_03817	mepM_1
mepM_1	ZONMW-42_04057	mepM_1
mepM_1	ZONMW-42_03193	mepM_1
mepM_1	ZONMW-42_03437	mepM_1
mepM_1	ZONMW-43_02790	mepM_1
mepM_1	ZONMW-43_02154	mepM_1
mepM_1	ZONMW-43_02141	mepM_1
mepM_1	ZONMW-43_01275	mepM_1
mepM_1	ZONMW-43_02571	mepM_1
mepM_1	ZONMW-43_02307	mepM_1
mepM_1	ZONMW-44_01012	mepM_1
mepM_1	ZONMW-44_00003	mepM_1
mepM_1	ZONMW-44_03833	mepM_1
mepM_1	ZONMW-44_03569	mepM_1
mepM_1	ZONMW-45_01124	mepM_1
mepM_1	ZONMW-45_01534	mepM_1
mepM_1	ZONMW-45_00636	mepM_1
mepM_1	ZONMW-45_01006	mepM_1
mepM_1	ZONMW-45_00038	mepM_1
mepM_1	ZONMW-45_00809	mepM_1
mepM_1	ZONMW-45_00264	mepM_1
mepM_1	ZONMW-46_00543	mepM_1
mepM_1	ZONMW-46_02045	mepM_1
mepM_1	ZONMW-46_00823	mepM_1
mepM_1	ZONMW-46_00661	mepM_1
mepM_1	ZONMW-46_00253	mepM_1
mepM_1	ZONMW-46_01065	mepM_1
mepM_1	ZONMW-46_00479	mepM_1

mepM_1	ZONMW-47_00543	mepM_1
mepM_1	ZONMW-47_02016	mepM_1
mepM_1	ZONMW-47_00823	mepM_1
mepM_1	ZONMW-47_00661	mepM_1
mepM_1	ZONMW-47_00252	mepM_1
mepM_1	ZONMW-47_01108	mepM_1
mepM_1	ZONMW-47_00026	mepM_1
mepM_1	ZONMW-48_00527	mepM_1
mepM_1	ZONMW-48_00220	mepM_1
mepM_1	ZONMW-48_00656	mepM_1
mepM_1	ZONMW-48_02933	mepM_1
mepM_1	ZONMW-48_02894	mepM_1
mepM_1	ZONMW-49_02390	mepM_1
mepM_1	ZONMW-49_02848	mepM_1
mepM_1	ZONMW-49_01233	mepM_1
mepM_1	ZONMW-49_03075	mepM_1
mepM_1	ZONMW-49_03306	mepM_1
mepM_1	ZONMW-50_02070	mepM_1
mepM_1	ZONMW-50_02808	mepM_1
mepM_1	ZONMW-50_01234	mepM_1
mepM_1	ZONMW-50_02957	mepM_1
mepM_1	ZONMW-50_03276	mepM_1
mepM_1	ZONMW-51_01564	mepM_1
mepM_1	ZONMW-51_02926	mepM_1
mepM_1	ZONMW-51_02843	mepM_1
mepM_1	ZONMW-51_01885	mepM_1
mepM_1	ZONMW-51_01858	mepM_1
mepM_1	ZONMW-52_01707	mepM_1
mepM_1	ZONMW-52_02947	mepM_1
mepM_1	ZONMW-52_02202	mepM_1
mepM_1	ZONMW-52_00888	mepM_1
mepM_1	ZONMW-52_00915	mepM_1
mepM_1	ZONMW-53_02051	mepM_1
mepM_1	ZONMW-53_02954	mepM_1
mepM_1	ZONMW-53_02796	mepM_1
mepM_1	ZONMW-53_01631	mepM_1
mepM_1	ZONMW-53_01658	mepM_1
mepM_1	ZONMW-54_02008	mepM_1
mepM_1	ZONMW-54_02958	mepM_1
mepM_1	ZONMW-54_00018	mepM_1
mepM_1	ZONMW-54_01094	mepM_1
mepM_1	ZONMW-54_01067	mepM_1
mepM_1	ZONMW-54_00895	mepM_1
mepM_1	ZONMW-55_01886	mepM_1
mepM_1	ZONMW-55_02962	mepM_1
mepM_1	ZONMW-55_02798	mepM_1
mepM_1	ZONMW-55_02207	mepM_1
mepM_1	ZONMW-55_02180	mepM_1

mepM_1	ZONMW-56_01549	mepM_1
mepM_1	ZONMW-56_02842	mepM_1
mepM_1	ZONMW-56_02562	mepM_1
mepM_1	ZONMW-56_02161	mepM_1
mepM_1	ZONMW-56_02188	mepM_1
mepM_1	ZONMW-57_01651	mepM_1
mepM_1	ZONMW-57_02900	mepM_1
mepM_1	ZONMW-57_02703	mepM_1
mepM_1	ZONMW-57_00059	mepM_1
mepM_1	ZONMW-57_00032	mepM_1
mepM_1	ZONMW-58_01675	mepM_1
mepM_1	ZONMW-58_02924	mepM_1
mepM_1	ZONMW-58_02727	mepM_1
mepM_1	ZONMW-58_00907	mepM_1
mepM_1	ZONMW-58_00934	mepM_1
mepM_1	ZONMW-59_01514	mepM_1
mepM_1	ZONMW-59_03049	mepM_1
mepM_1	ZONMW-59_02999	mepM_1
mepM_1	ZONMW-59_00059	mepM_1
mepM_1	ZONMW-59_00032	mepM_1
mepM_1	ZONMW-60_01866	mepM_1
mepM_1	ZONMW-60_03017	mepM_1
mepM_1	ZONMW-60_02831	mepM_1
mepM_1	ZONMW-60_00060	mepM_1
mepM_1	ZONMW-60_00033	mepM_1
mepM_1	ZONMW-60_01079	mepM_1
mepM_1	ZONMW-61_03827	mepM_1
mepM_1	ZONMW-61_03952	mepM_1
mepM_1	ZONMW-61_00282	mepM_1
mepM_1	ZONMW-61_03641	mepM_1
mepM_1	ZONMW-61_00903	mepM_1
mepM_1	ZONMW-62_01149	mepM_1
mepM_1	ZONMW-62_01550	mepM_1
mepM_1	ZONMW-62_03282	mepM_1
mepM_1	ZONMW-62_01835	mepM_1
mepM_1	ZONMW-62_01097	mepM_1
mepM_1	ZONMW-62_02715	mepM_1
mepM_1	ZONMW-63_01387	mepM_1
mepM_1	ZONMW-63_02810	mepM_1
mepM_1	ZONMW-63_02371	mepM_1
mepM_1	ZONMW-63_01823	mepM_1
mepM_1	ZONMW-63_01796	mepM_1
mepM_1	ZONMW-64_03661	mepM_1
mepM_1	ZONMW-64_04207	mepM_1
mepM_1	ZONMW-64_03752	mepM_1
mepM_1	ZONMW-64_00776	mepM_1
mepM_1	ZONMW-65_00004	mepM_1
mepM_1	ZONMW-65_00105	mepM_1

mepM_1	ZONMW-65_00742	mepM_1
mepM_1	ZONMW-65_01382	mepM_1
mepM_1	ZONMW-65_02182	mepM_1
mepM_1	ZONMW-65_01215	mepM_1
mepM_1	ZONMW-66_00037	mepM_1
mepM_1	ZONMW-66_03837	mepM_1
mepM_1	ZONMW-66_00710	mepM_1
mepM_1	ZONMW-66_00208	mepM_1
mepM_1	ZONMW-66_02980	mepM_1
mepM_1	ZONMW-67_02528	mepM_1
mepM_1	ZONMW-67_03276	mepM_1
mepM_1	ZONMW-67_01753	mepM_1
mepM_1	ZONMW-67_02757	mepM_1
mepM_1	ZONMW-68_04094	mepM_1
mepM_1	ZONMW-68_03882	mepM_1
mepM_1	ZONMW-68_04472	mepM_1
mepM_1	ZONMW-68_00655	mepM_1
mepM_1	ZONMW-68_00030	mepM_1
mepM_1	ZONMW-68_03802	mepM_1
mepM_1	ZONMW-68_02822	mepM_1
mepM_1	ZONMW-68_03978	mepM_1
mepM_1	ZONMW-69_02576	mepM_1
mepM_1	ZONMW-69_03277	mepM_1
mepM_1	ZONMW-69_02190	mepM_1
mepM_1	ZONMW-69_00554	mepM_1
mepM_1	ZONMW-72_02427	mepM_1
mepM_1	ZONMW-72_02729	mepM_1
mepM_1	ZONMW-72_01389	mepM_1
mepM_1	ZONMW-72_03295	mepM_1
mepM_1	ZONMW-72_03076	mepM_1
mepM_1	ZONMW-73_01315	mepM_1
mepM_1	ZONMW-73_01866	mepM_1
mepM_1	ZONMW-73_00013	mepM_1
mepM_1	ZONMW-73_00894	mepM_1
mepM_1	ZONMW-73_02887	mepM_1
mepM_1	ZONMW-73_01120	mepM_1
mepM_1	ZONMW-74_03187	mepM_1
mepM_1	ZONMW-74_02140	mepM_1
mepM_1	ZONMW-74_01538	mepM_1
mepM_1	ZONMW-74_00603	mepM_1
mepM_1	ZONMW-74_00630	mepM_1
mepM_1	ZONMW-75_01354	mepM_1
mepM_1	ZONMW-75_00466	mepM_1
mepM_1	ZONMW-75_02335	mepM_1
mepM_1	ZONMW-75_02515	mepM_1
mepM_1	ZONMW-75_03760	mepM_1
mepM_1	da5A-8_01997	mepM_1
mepM_1	da5A-8_01711	mepM_1

mepM_1	da5A-8_03038	mepM_1
mepM_1	da5A-8_01639	mepM_1
mepM_1	da5A-8_02003	mepM_1
mepM_1	da5A-8_01473	mepM_1
alr1	18_00883	alr1
alr1	ACAM48_02318	alr1
alr1	CAM030_01141	alr1
alr1	CECT7946_00041	alr1
alr1	CGMCC1.8863_02099	alr1
alr1	CL8139_01688	alr1
alr1	DAU203_01293	alr1
alr1	DD5b_01570	alr1
alr1	DSM1208_01480	alr1
alr1	DSM12212_02151	alr1
alr1	DSM15343_02384	alr1
alr1	DSM15343_00506	alr1
alr1	DSM15361_00463	alr1
alr1	DSM15365_03127	alr1
alr1	DSM15718_02376	alr1
alr1	DSM15718_01498	alr1
alr1	DSM16505T_00695	alr1
alr1	DSM16505T_00929	alr1
alr1	DSM18103_01156	alr1
alr1	DSM18293_00403	alr1
alr1	DSM18841_00578	alr1
alr1	DSM18841_00817	alr1
alr1	DSM18842_00650	alr1
alr1	DSM18842_00501	alr1
alr1	DSM19866_03379	alr1
alr1	DSM2041_00444	alr1
alr1	DSM2061_03003	alr1
alr1	DSM2064_02324	alr1
alr1	DSM21164_00963	alr1
alr1	DSM23404_03036	alr1
alr1	DSM23424_00409	alr1
alr1	DSM24786_02735	alr1
alr1	DSM7489_01203	alr1
alr1	DSW1_00766	alr1
alr1	DSW5_01431	alr1
alr1	DSW6_01294	alr1
alr1	DSW8_02395	alr1
alr1	F52_02721	alr1
alr1	GCA_001027945.1_01287	alr1
alr1	GCA_001027945.1_01623	alr1
alr1	HI1_01145	alr1
alr1	IR1_00538	alr1
alr1	KCOM3131_01832	alr1
alr1	KCTC12974_01656	alr1

alr1	KL-A_03031	alr1
alr1	L12_00112	alr1
alr1	LL0412.1.7_00236	alr1
alr1	MAR_2009_71_01044	alr1
alr1	NN016038_00893	alr1
alr1	PAO1_05479	alr1
alr1	PAO1_05097	alr1
alr1	UW101_01378	alr1
alr1	V4-33_00974	alr1
alr1	W5C_02043	alr1
alr1	ZONMW-01_01064	alr1
alr1	ZONMW-02_00036	alr1
alr1	ZONMW-03_02133	alr1
alr1	ZONMW-04_01684	alr1
alr1	ZONMW-05_01964	alr1
alr1	ZONMW-06_02970	alr1
alr1	ZONMW-07_02029	alr1
alr1	ZONMW-08_00562	alr1
alr1	ZONMW-09_00425	alr1
alr1	ZONMW-10_00181	alr1
alr1	ZONMW-10_00498	alr1
alr1	ZONMW-11_02760	alr1
alr1	ZONMW-11_02198	alr1
alr1	ZONMW-11_02952	alr1
alr1	ZONMW-12_00351	alr1
alr1	ZONMW-13_00433	alr1
alr1	ZONMW-14_00130	alr1
alr1	ZONMW-15_01714	alr1
alr1	ZONMW-19_03628	alr1
alr1	ZONMW-20_03707	alr1
alr1	ZONMW-20_01789	alr1
alr1	ZONMW-21_00954	alr1
alr1	ZONMW-22_00955	alr1
alr1	ZONMW-23_02068	alr1
alr1	ZONMW-24_00506	alr1
alr1	ZONMW-25_03044	alr1
alr1	ZONMW-26_02435	alr1
alr1	ZONMW-27_01615	alr1
alr1	ZONMW-28_03285	alr1
alr1	ZONMW-29_00534	alr1
alr1	ZONMW-30_01838	alr1
alr1	ZONMW-31_03526	alr1
alr1	ZONMW-32_03410	alr1
alr1	ZONMW-33_03413	alr1
alr1	ZONMW-34_02079	alr1
alr1	ZONMW-35_01771	alr1
alr1	ZONMW-36_03270	alr1
alr1	ZONMW-37_01408	alr1

alr1	ZONMW-38_02902	alr1
alr1	ZONMW-39_03688	alr1
alr1	ZONMW-40_00309	alr1
alr1	ZONMW-41_01158	alr1
alr1	ZONMW-41_02294	alr1
alr1	ZONMW-42_04154	alr1
alr1	ZONMW-43_02869	alr1
alr1	ZONMW-44_00956	alr1
alr1	ZONMW-45_00726	alr1
alr1	ZONMW-45_01347	alr1
alr1	ZONMW-46_00733	alr1
alr1	ZONMW-46_01602	alr1
alr1	ZONMW-47_00733	alr1
alr1	ZONMW-47_01887	alr1
alr1	ZONMW-48_00100	alr1
alr1	ZONMW-49_03130	alr1
alr1	ZONMW-50_03126	alr1
alr1	ZONMW-51_03186	alr1
alr1	ZONMW-52_03145	alr1
alr1	ZONMW-53_03142	alr1
alr1	ZONMW-54_03155	alr1
alr1	ZONMW-55_03140	alr1
alr1	ZONMW-56_03100	alr1
alr1	ZONMW-57_03169	alr1
alr1	ZONMW-58_03178	alr1
alr1	ZONMW-59_03235	alr1
alr1	ZONMW-60_03180	alr1
alr1	ZONMW-61_00137	alr1
alr1	ZONMW-62_01691	alr1
alr1	ZONMW-63_03152	alr1
alr1	ZONMW-64_03605	alr1
alr1	ZONMW-65_01270	alr1
alr1	ZONMW-66_00877	alr1
alr1	ZONMW-67_02469	alr1
alr1	ZONMW-68_04618	alr1
alr1	ZONMW-69_02517	alr1
alr1	ZONMW-72_00190	alr1
alr1	ZONMW-73_02207	alr1
alr1	ZONMW-74_02240	alr1
alr1	ZONMW-75_02411	alr1
alr1	da5A-8_01556	alr1
tal	18_00438	tal
tal	18_03051	tal
tal	ACAM48_02818	tal
tal	ACAM48_01255	tal
tal	CAM030_00397	tal
tal	CECT7946_01495	tal
tal	CGMCC1.8863_02639	tal

tal	CL8139_02495	tal
tal	DAU203_02842	tal
tal	DD5b_01016	tal
tal	DSM1208_00169	tal
tal	DSM1208_04542	tal
tal	DSM12212_01844	tal
tal	DSM15343_00478	tal
tal	DSM15361_01682	tal
tal	DSM15365_02951	tal
tal	DSM15365_03433	tal
tal	DSM15718_00960	tal
tal	DSM16505T_02323	tal
tal	DSM18103_01803	tal
tal	DSM18293_01344	tal
tal	DSM18293_01034	tal
tal	DSM18841_03030	tal
tal	DSM18842_01646	tal
tal	DSM19866_04652	tal
tal	DSM2041_05323	tal
tal	DSM2041_03976	tal
tal	DSM2061_00352	tal
tal	DSM2061_00163	tal
tal	DSM2061_00611	tal
tal	DSM2064_04482	tal
tal	DSM2064_04129	tal
tal	DSM21164_01443	tal
tal	DSM23404_00656	tal
tal	DSM23424_01909	tal
tal	DSM24786_00319	tal
tal	DSM7489_02713	tal
tal	DSW1_02341	tal
tal	DSW5_02614	tal
tal	DSW6_02701	tal
tal	DSW8_03319	tal
tal	F52_00019	tal
tal	F52_04282	tal
tal	GCA_001027945.1_01203	tal
tal	HI1_02652	tal
tal	IR1_01940	tal
tal	IR1_03790	tal
tal	KCOM3131_01215	tal
tal	KCTC12974_01519	tal
tal	KL-A_01345	tal
tal	L12_00171	tal
tal	LL0412.1.7_01697	tal
tal	MAR_2009_71_03603	tal
tal	NN016038_03083	tal
tal	PAO1_02895	tal

tal	UW101_02095	tal
tal	UW101_04757	tal
tal	V4-33_00958	tal
tal	W5C_02392	tal
tal	ZONMW-01_02198	tal
tal	ZONMW-02_00359	tal
tal	ZONMW-02_00399	tal
tal	ZONMW-03_00450	tal
tal	ZONMW-04_01389	tal
tal	ZONMW-05_00713	tal
tal	ZONMW-06_03324	tal
tal	ZONMW-07_03346	tal
tal	ZONMW-08_02312	tal
tal	ZONMW-09_01503	tal
tal	ZONMW-10_00701	tal
tal	ZONMW-11_01888	tal
tal	ZONMW-12_03976	tal
tal	ZONMW-13_01424	tal
tal	ZONMW-14_02362	tal
tal	ZONMW-15_02394	tal
tal	ZONMW-19_02733	tal
tal	ZONMW-19_03992	tal
tal	ZONMW-20_02474	tal
tal	ZONMW-20_01049	tal
tal	ZONMW-20_03082	tal
tal	ZONMW-21_01697	tal
tal	ZONMW-22_02027	tal
tal	ZONMW-23_00524	tal
tal	ZONMW-24_02652	tal
tal	ZONMW-25_01488	tal
tal	ZONMW-26_00256	tal
tal	ZONMW-27_01914	tal
tal	ZONMW-28_03245	tal
tal	ZONMW-29_01486	tal
tal	ZONMW-30_02236	tal
tal	ZONMW-31_01763	tal
tal	ZONMW-31_01590	tal
tal	ZONMW-32_00770	tal
tal	ZONMW-33_00638	tal
tal	ZONMW-34_01002	tal
tal	ZONMW-35_02241	tal
tal	ZONMW-36_00733	tal
tal	ZONMW-37_02075	tal
tal	ZONMW-38_02290	tal
tal	ZONMW-38_00966	tal
tal	ZONMW-39_02873	tal
tal	ZONMW-40_02396	tal
tal	ZONMW-40_01608	tal

tal	ZONMW-41_02697	tal
tal	ZONMW-42_00883	tal
tal	ZONMW-43_00535	tal
tal	ZONMW-44_03750	tal
tal	ZONMW-45_02382	tal
tal	ZONMW-46_01244	tal
tal	ZONMW-47_00929	tal
tal	ZONMW-48_01218	tal
tal	ZONMW-49_00267	tal
tal	ZONMW-50_00218	tal
tal	ZONMW-51_00255	tal
tal	ZONMW-52_00239	tal
tal	ZONMW-53_00976	tal
tal	ZONMW-54_01751	tal
tal	ZONMW-55_01271	tal
tal	ZONMW-56_00500	tal
tal	ZONMW-57_00713	tal
tal	ZONMW-58_00258	tal
tal	ZONMW-59_00702	tal
tal	ZONMW-60_00715	tal
tal	ZONMW-61_03189	tal
tal	ZONMW-61_03193	tal
tal	ZONMW-62_03244	tal
tal	ZONMW-63_00386	tal
tal	ZONMW-64_00553	tal
tal	ZONMW-65_00048	tal
tal	ZONMW-66_04209	tal
tal	ZONMW-66_01837	tal
tal	ZONMW-67_01219	tal
tal	ZONMW-68_01101	tal
tal	ZONMW-69_01265	tal
tal	ZONMW-72_01036	tal
tal	ZONMW-72_00753	tal
tal	ZONMW-73_00310	tal
tal	ZONMW-74_03397	tal
tal	ZONMW-75_01280	tal
tal	da5A-8_00960	tal
hemL	18_00386	hemL
hemL	18_02207	hemL
hemL	18_02081	hemL
hemL	18_00539	hemL
hemL	18_00683	hemL
hemL	ACAM48_02970	hemL
hemL	ACAM48_01130	hemL
hemL	ACAM48_01344	hemL
hemL	ACAM48_00953	hemL
hemL	ACAM48_03544	hemL
hemL	ACAM48_02629	hemL

hemL	CAM030_01488	hemL
hemL	CAM030_01586	hemL
hemL	CAM030_02020	hemL
hemL	CAM030_01102	hemL
hemL	CECT7946_02746	hemL
hemL	CECT7946_03199	hemL
hemL	CECT7946_02419	hemL
hemL	CECT7946_00666	hemL
hemL	CGMCC1.8863_02584	hemL
hemL	CGMCC1.8863_01129	hemL
hemL	CGMCC1.8863_01204	hemL
hemL	CGMCC1.8863_00103	hemL
hemL	CGMCC1.8863_00418	hemL
hemL	CGMCC1.8863_00121	hemL
hemL	CL8139_02727	hemL
hemL	CL8139_01137	hemL
hemL	CL8139_02475	hemL
hemL	CL8139_01188	hemL
hemL	CL8139_01411	hemL
hemL	DAU203_00714	hemL
hemL	DAU203_00287	hemL
hemL	DAU203_00907	hemL
hemL	DAU203_00237	hemL
hemL	DAU203_01132	hemL
hemL	DD5b_02764	hemL
hemL	DD5b_00589	hemL
hemL	DD5b_00593	hemL
hemL	DD5b_00585	hemL
hemL	DD5b_00812	hemL
hemL	DD5b_01355	hemL
hemL	DD5b_02319	hemL
hemL	DSM1208_00935	hemL
hemL	DSM1208_03522	hemL
hemL	DSM1208_01190	hemL
hemL	DSM1208_03023	hemL
hemL	DSM1208_01341	hemL
hemL	DSM1208_01522	hemL
hemL	DSM12212_01542	hemL
hemL	DSM12212_00086	hemL
hemL	DSM12212_02445	hemL
hemL	DSM12212_00825	hemL
hemL	DSM12212_00267	hemL
hemL	DSM12212_01717	hemL
hemL	DSM12212_01721	hemL
hemL	DSM12212_01723	hemL
hemL	DSM15343_02164	hemL
hemL	DSM15343_01907	hemL
hemL	DSM15343_00472	hemL

hemL	DSM15361_01161	hemL
hemL	DSM15361_02366	hemL
hemL	DSM15361_01543	hemL
hemL	DSM15361_00277	hemL
hemL	DSM15365_03530	hemL
hemL	DSM15365_00065	hemL
hemL	DSM15365_02116	hemL
hemL	DSM15365_01569	hemL
hemL	DSM15718_01030	hemL
hemL	DSM15718_00335	hemL
hemL	DSM15718_01734	hemL
hemL	DSM15718_01556	hemL
hemL	DSM16505T_01898	hemL
hemL	DSM16505T_01377	hemL
hemL	DSM16505T_01781	hemL
hemL	DSM16505T_01621	hemL
hemL	DSM16505T_00744	hemL
hemL	DSM18103_00478	hemL
hemL	DSM18103_00921	hemL
hemL	DSM18103_02970	hemL
hemL	DSM18103_01197	hemL
hemL	DSM18103_02304	hemL
hemL	DSM18103_00932	hemL
hemL	DSM18293_02195	hemL
hemL	DSM18293_01159	hemL
hemL	DSM18293_04029	hemL
hemL	DSM18293_04169	hemL
hemL	DSM18293_03266	hemL
hemL	DSM18841_01999	hemL
hemL	DSM18841_02387	hemL
hemL	DSM18841_02989	hemL
hemL	DSM18841_01513	hemL
hemL	DSM18841_00622	hemL
hemL	DSM18842_02313	hemL
hemL	DSM18842_01300	hemL
hemL	DSM18842_02201	hemL
hemL	DSM18842_02004	hemL
hemL	DSM18842_02978	hemL
hemL	DSM19866_00905	hemL
hemL	DSM19866_01240	hemL
hemL	DSM19866_01664	hemL
hemL	DSM19866_01845	hemL
hemL	DSM19866_03727	hemL
hemL	DSM19866_03411	hemL
hemL	DSM19866_03901	hemL
hemL	DSM19866_03584	hemL
hemL	DSM19866_00174	hemL
hemL	DSM2041_01311	hemL

hemL	DSM2041_02596	hemL
hemL	DSM2041_02376	hemL
hemL	DSM2041_04091	hemL
hemL	DSM2041_00563	hemL
hemL	DSM2041_01300	hemL
hemL	DSM2061_02442	hemL
hemL	DSM2061_01541	hemL
hemL	DSM2061_00043	hemL
hemL	DSM2061_02703	hemL
hemL	DSM2061_02859	hemL
hemL	DSM2061_03053	hemL
hemL	DSM2064_02943	hemL
hemL	DSM2064_04034	hemL
hemL	DSM2064_03094	hemL
hemL	DSM2064_01797	hemL
hemL	DSM2064_00868	hemL
hemL	DSM2064_04581	hemL
hemL	DSM21164_01402	hemL
hemL	DSM21164_03095	hemL
hemL	DSM21164_03015	hemL
hemL	DSM21164_01291	hemL
hemL	DSM21164_01137	hemL
hemL	DSM23404_01177	hemL
hemL	DSM23404_01642	hemL
hemL	DSM23404_03145	hemL
hemL	DSM23404_01489	hemL
hemL	DSM23404_02325	hemL
hemL	DSM23424_02391	hemL
hemL	DSM23424_02282	hemL
hemL	DSM23424_01876	hemL
hemL	DSM23424_00938	hemL
hemL	DSM23424_03026	hemL
hemL	DSM23424_00761	hemL
hemL	DSM24786_01429	hemL
hemL	DSM24786_01088	hemL
hemL	DSM24786_01283	hemL
hemL	DSM24786_01181	hemL
hemL	DSM24786_03360	hemL
hemL	DSM7489_00701	hemL
hemL	DSM7489_00281	hemL
hemL	DSM7489_00841	hemL
hemL	DSM7489_00230	hemL
hemL	DSM7489_01051	hemL
hemL	DSW1_01127	hemL
hemL	DSW1_02378	hemL
hemL	DSW1_01617	hemL
hemL	DSW1_00393	hemL
hemL	DSW1_01159	hemL

hemL	DSW5_02645	hemL
hemL	DSW5_02184	hemL
hemL	DSW5_01953	hemL
hemL	DSW5_02335	hemL
hemL	DSW6_02158	hemL
hemL	DSW6_03415	hemL
hemL	DSW6_00355	hemL
hemL	DSW6_01161	hemL
hemL	DSW8_02950	hemL
hemL	DSW8_00990	hemL
hemL	DSW8_02590	hemL
hemL	DSW8_01009	hemL
hemL	DSW8_02749	hemL
hemL	DSW8_01440	hemL
hemL	F52_03146	hemL
hemL	F52_04194	hemL
hemL	F52_00570	hemL
hemL	F52_00792	hemL
hemL	F52_04503	hemL
hemL	GCA_001027945.1_02833	hemL
hemL	GCA_001027945.1_01978	hemL
hemL	GCA_001027945.1_02097	hemL
hemL	GCA_001027945.1_02071	hemL
hemL	GCA_001027945.1_01245	hemL
hemL	HI1_00632	hemL
hemL	HI1_00213	hemL
hemL	HI1_00776	hemL
hemL	HI1_00162	hemL
hemL	HI1_00990	hemL
hemL	IR1_00990	hemL
hemL	IR1_04231	hemL
hemL	IR1_02308	hemL
hemL	IR1_04033	hemL
hemL	IR1_02061	hemL
hemL	KCOM3131_01338	hemL
hemL	KCOM3131_01089	hemL
hemL	KCTC12974_02266	hemL
hemL	KCTC12974_02677	hemL
hemL	KCTC12974_00905	hemL
hemL	KCTC12974_00508	hemL
hemL	KCTC12974_00194	hemL
hemL	KCTC12974_02000	hemL
hemL	KL-A_02784	hemL
hemL	KL-A_03191	hemL
hemL	KL-A_03214	hemL
hemL	KL-A_01226	hemL
hemL	KL-A_02294	hemL
hemL	L12_00097	hemL

hemL	LL0412.1.7_02000	hemL
hemL	LL0412.1.7_02025	hemL
hemL	LL0412.1.7_02275	hemL
hemL	LL0412.1.7_00750	hemL
hemL	LL0412.1.7_02013	hemL
hemL	MAR_2009_71_00508	hemL
hemL	MAR_2009_71_02451	hemL
hemL	MAR_2009_71_00864	hemL
hemL	MAR_2009_71_02432	hemL
hemL	MAR_2009_71_00704	hemL
hemL	MAR_2009_71_01988	hemL
hemL	NN016038_00388	hemL
hemL	NN016038_02222	hemL
hemL	NN016038_02099	hemL
hemL	NN016038_00548	hemL
hemL	NN016038_00698	hemL
hemL	PAO1_04106	hemL
hemL	PAO1_00272	hemL
hemL	PAO1_04217	hemL
hemL	PAO1_05706	hemL
hemL	PAO1_00543	hemL
hemL	PAO1_00922	hemL
hemL	PAO1_00138	hemL
hemL	PAO1_02484	hemL
hemL	PAO1_05490	hemL
hemL	PAO1_00305	hemL
hemL	PAO1_00227	hemL
hemL	PAO1_00431	hemL
hemL	PAO1_04969	hemL
hemL	UW101_01997	hemL
hemL	UW101_04853	hemL
hemL	UW101_02706	hemL
hemL	UW101_00851	hemL
hemL	UW101_03579	hemL
hemL	UW101_02194	hemL
hemL	V4-33_01113	hemL
hemL	V4-33_01679	hemL
hemL	V4-33_00713	hemL
hemL	V4-33_00780	hemL
hemL	V4-33_02249	hemL
hemL	W5C_01338	hemL
hemL	W5C_00190	hemL
hemL	W5C_01467	hemL
hemL	W5C_00139	hemL
hemL	W5C_02383	hemL
hemL	W5C_03094	hemL
hemL	ZONMW-01_03264	hemL
hemL	ZONMW-01_02165	hemL

hemL	ZONMW-01_03417	hemL
hemL	ZONMW-01_02114	hemL
hemL	ZONMW-01_02889	hemL
hemL	ZONMW-02_00468	hemL
hemL	ZONMW-02_00925	hemL
hemL	ZONMW-02_03407	hemL
hemL	ZONMW-02_02028	hemL
hemL	ZONMW-03_03226	hemL
hemL	ZONMW-03_02545	hemL
hemL	ZONMW-03_01760	hemL
hemL	ZONMW-03_02596	hemL
hemL	ZONMW-03_01967	hemL
hemL	ZONMW-04_03142	hemL
hemL	ZONMW-04_02010	hemL
hemL	ZONMW-04_02232	hemL
hemL	ZONMW-04_01959	hemL
hemL	ZONMW-04_02897	hemL
hemL	ZONMW-05_03192	hemL
hemL	ZONMW-05_02359	hemL
hemL	ZONMW-05_02749	hemL
hemL	ZONMW-05_02410	hemL
hemL	ZONMW-05_03028	hemL
hemL	ZONMW-06_01966	hemL
hemL	ZONMW-06_00340	hemL
hemL	ZONMW-06_01272	hemL
hemL	ZONMW-06_00487	hemL
hemL	ZONMW-06_01425	hemL
hemL	ZONMW-07_03540	hemL
hemL	ZONMW-07_01745	hemL
hemL	ZONMW-07_00563	hemL
hemL	ZONMW-07_01725	hemL
hemL	ZONMW-07_00197	hemL
hemL	ZONMW-07_00672	hemL
hemL	ZONMW-07_02051	hemL
hemL	ZONMW-08_00024	hemL
hemL	ZONMW-08_03450	hemL
hemL	ZONMW-08_00383	hemL
hemL	ZONMW-08_03431	hemL
hemL	ZONMW-08_00220	hemL
hemL	ZONMW-08_01240	hemL
hemL	ZONMW-09_01507	hemL
hemL	ZONMW-09_02072	hemL
hemL	ZONMW-09_02218	hemL
hemL	ZONMW-09_02633	hemL
hemL	ZONMW-09_00544	hemL
hemL	ZONMW-10_01210	hemL
hemL	ZONMW-10_01017	hemL
hemL	ZONMW-10_02289	hemL

hemL	ZONMW-10_00538	hemL
hemL	ZONMW-11_00145	hemL
hemL	ZONMW-11_00106	hemL
hemL	ZONMW-11_01075	hemL
hemL	ZONMW-11_03264	hemL
hemL	ZONMW-11_04242	hemL
hemL	ZONMW-11_02958	hemL
hemL	ZONMW-11_04360	hemL
hemL	ZONMW-12_01974	hemL
hemL	ZONMW-12_01767	hemL
hemL	ZONMW-12_03440	hemL
hemL	ZONMW-12_03324	hemL
hemL	ZONMW-12_00458	hemL
hemL	ZONMW-13_01078	hemL
hemL	ZONMW-13_02470	hemL
hemL	ZONMW-13_01286	hemL
hemL	ZONMW-13_03085	hemL
hemL	ZONMW-13_00728	hemL
hemL	ZONMW-14_01514	hemL
hemL	ZONMW-14_02533	hemL
hemL	ZONMW-14_02908	hemL
hemL	ZONMW-14_01872	hemL
hemL	ZONMW-14_01317	hemL
hemL	ZONMW-15_03179	hemL
hemL	ZONMW-15_02672	hemL
hemL	ZONMW-15_03106	hemL
hemL	ZONMW-15_00995	hemL
hemL	ZONMW-15_00856	hemL
hemL	ZONMW-19_01253	hemL
hemL	ZONMW-19_03367	hemL
hemL	ZONMW-19_03279	hemL
hemL	ZONMW-19_01281	hemL
hemL	ZONMW-19_04217	hemL
hemL	ZONMW-19_03811	hemL
hemL	ZONMW-19_04570	hemL
hemL	ZONMW-19_04569	hemL
hemL	ZONMW-20_04138	hemL
hemL	ZONMW-20_03341	hemL
hemL	ZONMW-20_02164	hemL
hemL	ZONMW-20_00020	hemL
hemL	ZONMW-20_01669	hemL
hemL	ZONMW-20_03415	hemL
hemL	ZONMW-20_02544	hemL
hemL	ZONMW-21_01425	hemL
hemL	ZONMW-21_02738	hemL
hemL	ZONMW-21_03298	hemL
hemL	ZONMW-21_01281	hemL
hemL	ZONMW-21_01150	hemL

hemL	ZONMW-21_00527	hemL
hemL	ZONMW-22_01427	hemL
hemL	ZONMW-22_02371	hemL
hemL	ZONMW-22_03218	hemL
hemL	ZONMW-22_01283	hemL
hemL	ZONMW-22_01152	hemL
hemL	ZONMW-22_00527	hemL
hemL	ZONMW-23_02540	hemL
hemL	ZONMW-23_00179	hemL
hemL	ZONMW-23_03240	hemL
hemL	ZONMW-23_02395	hemL
hemL	ZONMW-23_02264	hemL
hemL	ZONMW-23_01642	hemL
hemL	ZONMW-24_00034	hemL
hemL	ZONMW-24_02136	hemL
hemL	ZONMW-24_03241	hemL
hemL	ZONMW-24_00178	hemL
hemL	ZONMW-24_00309	hemL
hemL	ZONMW-24_00932	hemL
hemL	ZONMW-25_03360	hemL
hemL	ZONMW-25_00663	hemL
hemL	ZONMW-25_01330	hemL
hemL	ZONMW-25_02419	hemL
hemL	ZONMW-25_02195	hemL
hemL	ZONMW-26_02260	hemL
hemL	ZONMW-26_02757	hemL
hemL	ZONMW-26_02089	hemL
hemL	ZONMW-26_02706	hemL
hemL	ZONMW-26_01888	hemL
hemL	ZONMW-27_02362	hemL
hemL	ZONMW-27_02181	hemL
hemL	ZONMW-27_02211	hemL
hemL	ZONMW-27_02130	hemL
hemL	ZONMW-27_03049	hemL
hemL	ZONMW-28_02617	hemL
hemL	ZONMW-28_01574	hemL
hemL	ZONMW-28_01736	hemL
hemL	ZONMW-28_01619	hemL
hemL	ZONMW-28_00951	hemL
hemL	ZONMW-29_00979	hemL
hemL	ZONMW-29_01903	hemL
hemL	ZONMW-29_01687	hemL
hemL	ZONMW-29_00658	hemL
hemL	ZONMW-29_00737	hemL
hemL	ZONMW-30_00705	hemL
hemL	ZONMW-30_02861	hemL
hemL	ZONMW-30_00567	hemL
hemL	ZONMW-30_02427	hemL

hemL	ZONMW-30_01653	hemL
hemL	ZONMW-31_01889	hemL
hemL	ZONMW-31_00165	hemL
hemL	ZONMW-31_00656	hemL
hemL	ZONMW-31_00039	hemL
hemL	ZONMW-31_00156	hemL
hemL	ZONMW-31_00842	hemL
hemL	ZONMW-31_03476	hemL
hemL	ZONMW-32_03259	hemL
hemL	ZONMW-32_01981	hemL
hemL	ZONMW-32_02236	hemL
hemL	ZONMW-32_02352	hemL
hemL	ZONMW-32_00140	hemL
hemL	ZONMW-33_03015	hemL
hemL	ZONMW-33_01972	hemL
hemL	ZONMW-33_02227	hemL
hemL	ZONMW-33_02344	hemL
hemL	ZONMW-33_00007	hemL
hemL	ZONMW-34_02637	hemL
hemL	ZONMW-34_00307	hemL
hemL	ZONMW-34_02380	hemL
hemL	ZONMW-34_02768	hemL
hemL	ZONMW-34_03111	hemL
hemL	ZONMW-34_02306	hemL
hemL	ZONMW-34_00410	hemL
hemL	ZONMW-35_02601	hemL
hemL	ZONMW-35_01083	hemL
hemL	ZONMW-35_01869	hemL
hemL	ZONMW-35_00145	hemL
hemL	ZONMW-35_03126	hemL
hemL	ZONMW-35_01943	hemL
hemL	ZONMW-35_00980	hemL
hemL	ZONMW-36_02987	hemL
hemL	ZONMW-36_01384	hemL
hemL	ZONMW-36_02009	hemL
hemL	ZONMW-36_01892	hemL
hemL	ZONMW-36_01362	hemL
hemL	ZONMW-37_00937	hemL
hemL	ZONMW-37_02405	hemL
hemL	ZONMW-37_03227	hemL
hemL	ZONMW-37_01081	hemL
hemL	ZONMW-37_01212	hemL
hemL	ZONMW-37_00527	hemL
hemL	ZONMW-38_03188	hemL
hemL	ZONMW-38_00598	hemL
hemL	ZONMW-38_00949	hemL
hemL	ZONMW-38_01964	hemL
hemL	ZONMW-38_03041	hemL

hemL	ZONMW-38_00049	hemL
hemL	ZONMW-38_01412	hemL
hemL	ZONMW-38_02213	hemL
hemL	ZONMW-39_03970	hemL
hemL	ZONMW-39_02992	hemL
hemL	ZONMW-39_01672	hemL
hemL	ZONMW-39_01797	hemL
hemL	ZONMW-39_03114	hemL
hemL	ZONMW-40_02798	hemL
hemL	ZONMW-40_01699	hemL
hemL	ZONMW-40_02811	hemL
hemL	ZONMW-40_03425	hemL
hemL	ZONMW-40_01432	hemL
hemL	ZONMW-40_00124	hemL
hemL	ZONMW-41_02807	hemL
hemL	ZONMW-41_00435	hemL
hemL	ZONMW-41_00832	hemL
hemL	ZONMW-41_00750	hemL
hemL	ZONMW-42_04067	hemL
hemL	ZONMW-42_03577	hemL
hemL	ZONMW-42_02761	hemL
hemL	ZONMW-42_03080	hemL
hemL	ZONMW-42_02474	hemL
hemL	ZONMW-43_01265	hemL
hemL	ZONMW-43_00638	hemL
hemL	ZONMW-43_01852	hemL
hemL	ZONMW-43_03437	hemL
hemL	ZONMW-43_02377	hemL
hemL	ZONMW-44_00013	hemL
hemL	ZONMW-44_03531	hemL
hemL	ZONMW-44_02457	hemL
hemL	ZONMW-44_01717	hemL
hemL	ZONMW-44_00664	hemL
hemL	ZONMW-45_00070	hemL
hemL	ZONMW-45_01800	hemL
hemL	ZONMW-45_00940	hemL
hemL	ZONMW-45_02264	hemL
hemL	ZONMW-45_00978	hemL
hemL	ZONMW-46_00285	hemL
hemL	ZONMW-46_01900	hemL
hemL	ZONMW-46_00934	hemL
hemL	ZONMW-46_03151	hemL
hemL	ZONMW-46_00689	hemL
hemL	ZONMW-47_00220	hemL
hemL	ZONMW-47_02161	hemL
hemL	ZONMW-47_01239	hemL
hemL	ZONMW-47_03143	hemL
hemL	ZONMW-47_00689	hemL

hemL	ZONMW-48_00537	hemL
hemL	ZONMW-48_02853	hemL
hemL	ZONMW-48_02943	hemL
hemL	ZONMW-48_00411	hemL
hemL	ZONMW-48_00269	hemL
hemL	ZONMW-49_02399	hemL
hemL	ZONMW-49_03221	hemL
hemL	ZONMW-49_02259	hemL
hemL	ZONMW-49_02447	hemL
hemL	ZONMW-49_02241	hemL
hemL	ZONMW-50_02061	hemL
hemL	ZONMW-50_03213	hemL
hemL	ZONMW-50_02201	hemL
hemL	ZONMW-50_02241	hemL
hemL	ZONMW-50_02035	hemL
hemL	ZONMW-51_01555	hemL
hemL	ZONMW-51_02372	hemL
hemL	ZONMW-51_01697	hemL
hemL	ZONMW-51_02424	hemL
hemL	ZONMW-51_03075	hemL
hemL	ZONMW-52_01698	hemL
hemL	ZONMW-52_02356	hemL
hemL	ZONMW-52_01840	hemL
hemL	ZONMW-52_02407	hemL
hemL	ZONMW-52_02044	hemL
hemL	ZONMW-53_02060	hemL
hemL	ZONMW-53_02277	hemL
hemL	ZONMW-53_01916	hemL
hemL	ZONMW-53_02226	hemL
hemL	ZONMW-53_01704	hemL
hemL	ZONMW-54_01999	hemL
hemL	ZONMW-54_02767	hemL
hemL	ZONMW-54_02138	hemL
hemL	ZONMW-54_02716	hemL
hemL	ZONMW-54_02330	hemL
hemL	ZONMW-55_01877	hemL
hemL	ZONMW-55_02463	hemL
hemL	ZONMW-55_02019	hemL
hemL	ZONMW-55_02515	hemL
hemL	ZONMW-55_03042	hemL
hemL	ZONMW-56_01558	hemL
hemL	ZONMW-56_01765	hemL
hemL	ZONMW-56_01417	hemL
hemL	ZONMW-56_01714	hemL
hemL	ZONMW-56_01217	hemL
hemL	ZONMW-57_01642	hemL
hemL	ZONMW-57_02112	hemL
hemL	ZONMW-57_01810	hemL

hemL	ZONMW-57_02061	hemL
hemL	ZONMW-57_02946	hemL
hemL	ZONMW-58_01666	hemL
hemL	ZONMW-58_01966	hemL
hemL	ZONMW-58_01834	hemL
hemL	ZONMW-58_02017	hemL
hemL	ZONMW-58_03047	hemL
hemL	ZONMW-59_01505	hemL
hemL	ZONMW-59_02185	hemL
hemL	ZONMW-59_01646	hemL
hemL	ZONMW-59_02236	hemL
hemL	ZONMW-59_01855	hemL
hemL	ZONMW-60_01857	hemL
hemL	ZONMW-60_02482	hemL
hemL	ZONMW-60_02027	hemL
hemL	ZONMW-60_02532	hemL
hemL	ZONMW-60_03369	hemL
hemL	ZONMW-60_02208	hemL
hemL	ZONMW-61_03838	hemL
hemL	ZONMW-61_03630	hemL
hemL	ZONMW-61_03650	hemL
hemL	ZONMW-61_01766	hemL
hemL	ZONMW-61_01607	hemL
hemL	ZONMW-62_01138	hemL
hemL	ZONMW-62_01087	hemL
hemL	ZONMW-62_01506	hemL
hemL	ZONMW-62_01106	hemL
hemL	ZONMW-62_01347	hemL
hemL	ZONMW-62_02085	hemL
hemL	ZONMW-63_01378	hemL
hemL	ZONMW-63_01571	hemL
hemL	ZONMW-63_01545	hemL
hemL	ZONMW-63_01622	hemL
hemL	ZONMW-63_03297	hemL
hemL	ZONMW-63_03040	hemL
hemL	ZONMW-64_04197	hemL
hemL	ZONMW-64_00900	hemL
hemL	ZONMW-64_01627	hemL
hemL	ZONMW-64_01757	hemL
hemL	ZONMW-64_00705	hemL
hemL	ZONMW-65_01474	hemL
hemL	ZONMW-65_00162	hemL
hemL	ZONMW-65_01462	hemL
hemL	ZONMW-65_03567	hemL
hemL	ZONMW-66_02273	hemL
hemL	ZONMW-66_02716	hemL
hemL	ZONMW-66_01304	hemL
hemL	ZONMW-66_00350	hemL

hemL	ZONMW-66_03200	hemL
hemL	ZONMW-66_04126	hemL
hemL	ZONMW-66_04915	hemL
hemL	ZONMW-67_03286	hemL
hemL	ZONMW-67_02716	hemL
hemL	ZONMW-67_00139	hemL
hemL	ZONMW-67_00002	hemL
hemL	ZONMW-67_02848	hemL
hemL	ZONMW-68_04105	hemL
hemL	ZONMW-68_04163	hemL
hemL	ZONMW-68_04369	hemL
hemL	ZONMW-68_02831	hemL
hemL	ZONMW-68_04353	hemL
hemL	ZONMW-68_00849	hemL
hemL	ZONMW-68_04580	hemL
hemL	ZONMW-68_01735	hemL
hemL	ZONMW-68_00705	hemL
hemL	ZONMW-68_02579	hemL
hemL	ZONMW-69_03287	hemL
hemL	ZONMW-69_00513	hemL
hemL	ZONMW-69_02748	hemL
hemL	ZONMW-69_02611	hemL
hemL	ZONMW-69_00645	hemL
hemL	ZONMW-72_02403	hemL
hemL	ZONMW-72_01880	hemL
hemL	ZONMW-72_02870	hemL
hemL	ZONMW-72_04382	hemL
hemL	ZONMW-72_03222	hemL
hemL	ZONMW-73_01291	hemL
hemL	ZONMW-73_00741	hemL
hemL	ZONMW-73_02696	hemL
hemL	ZONMW-73_03325	hemL
hemL	ZONMW-74_03196	hemL
hemL	ZONMW-74_02541	hemL
hemL	ZONMW-74_03318	hemL
hemL	ZONMW-74_02612	hemL
hemL	ZONMW-75_01378	hemL
hemL	ZONMW-75_01512	hemL
hemL	ZONMW-75_02525	hemL
hemL	ZONMW-75_00528	hemL
hemL	da5A-8_02177	hemL
hemL	da5A-8_01374	hemL
hemL	da5A-8_01515	hemL
hemL	da5A-8_02983	hemL
hemL	da5A-8_01385	hemL
cca	18_03840	cca
cca	ACAM48_03460	cca
cca	CAM030_00044	cca

cca	CECT7946_02163	cca
cca	CGMCC1.8863_03415	cca
cca	CL8139_02809	cca
cca	DAU203_00083	cca
cca	DD5b_01405	cca
cca	DSM1208_02793	cca
cca	DSM12212_02264	cca
cca	DSM15343_01880	cca
cca	DSM15361_01586	cca
cca	DSM15365_01326	cca
cca	DSM15718_00364	cca
cca	DSM16505T_01541	cca
cca	DSM18103_00280	cca
cca	DSM18293_01452	cca
cca	DSM18841_02167	cca
cca	DSM18842_01509	cca
cca	DSM19866_00049	cca
cca	DSM2041_02635	cca
cca	DSM2061_04281	cca
cca	DSM2064_03771	cca
cca	DSM21164_03199	cca
cca	DSM23404_00690	cca
cca	DSM23424_01852	cca
cca	DSM24786_01262	cca
cca	DSM7489_00084	cca
cca	DSW1_01672	cca
cca	DSW5_02621	cca
cca	DSW6_00374	cca
cca	DSW8_01178	cca
cca	F52_03919	cca
cca	GCA_001027945.1_01776	cca
cca	HI1_00014	cca
cca	IR1_01444	cca
cca	KCOM3131_00064	cca
cca	KCTC12974_01430	cca
cca	KL-A_03353	cca
cca	L12_00501	cca
cca	LL0412.1.7_02427	cca
cca	MAR_2009_71_02267	cca
cca	NN016038_03893	cca
cca	PAO1_00599	cca
cca	PAO1_04888	cca
cca	UW101_05115	cca
cca	V4-33_01551	cca
cca	W5C_00792	cca
cca	ZONMW-01_03037	cca
cca	ZONMW-02_00881	cca
cca	ZONMW-03_02272	cca

cca	ZONMW-04_00736	cca
cca	ZONMW-05_00006	cca
cca	ZONMW-06_01948	cca
cca	ZONMW-06_00386	cca
cca	ZONMW-07_02696	cca
cca	ZONMW-08_00978	cca
cca	ZONMW-09_02091	cca
cca	ZONMW-10_01048	cca
cca	ZONMW-11_01033	cca
cca	ZONMW-12_03891	cca
cca	ZONMW-13_01452	cca
cca	ZONMW-14_02335	cca
cca	ZONMW-15_03336	cca
cca	ZONMW-19_02407	cca
cca	ZONMW-19_02146	cca
cca	ZONMW-20_00044	cca
cca	ZONMW-20_00927	cca
cca	ZONMW-21_02671	cca
cca	ZONMW-22_02438	cca
cca	ZONMW-23_00112	cca
cca	ZONMW-24_02203	cca
cca	ZONMW-25_00721	cca
cca	ZONMW-26_00954	cca
cca	ZONMW-27_00833	cca
cca	ZONMW-28_01060	cca
cca	ZONMW-28_02634	cca
cca	ZONMW-29_01842	cca
cca	ZONMW-30_03051	cca
cca	ZONMW-31_01905	cca
cca	ZONMW-31_00723	cca
cca	ZONMW-32_00031	cca
cca	ZONMW-32_03242	cca
cca	ZONMW-33_03292	cca
cca	ZONMW-33_03032	cca
cca	ZONMW-34_00353	cca
cca	ZONMW-34_02621	cca
cca	ZONMW-35_01037	cca
cca	ZONMW-35_02585	cca
cca	ZONMW-36_03029	cca
cca	ZONMW-36_02970	cca
cca	ZONMW-37_02338	cca
cca	ZONMW-38_03428	cca
cca	ZONMW-39_03042	cca
cca	ZONMW-39_03954	cca
cca	ZONMW-40_01760	cca
cca	ZONMW-41_01377	cca
cca	ZONMW-41_01836	cca
cca	ZONMW-42_04081	cca

cca	ZONMW-42_03450	cca
cca	ZONMW-43_02315	cca
cca	ZONMW-43_01248	cca
cca	ZONMW-44_00031	cca
cca	ZONMW-44_03576	cca
cca	ZONMW-45_01603	cca
cca	ZONMW-46_02114	cca
cca	ZONMW-47_01947	cca
cca	ZONMW-48_02954	cca
cca	ZONMW-49_03276	cca
cca	ZONMW-50_03306	cca
cca	ZONMW-51_01828	cca
cca	ZONMW-52_00945	cca
cca	ZONMW-53_01688	cca
cca	ZONMW-54_01037	cca
cca	ZONMW-55_02150	cca
cca	ZONMW-56_02218	cca
cca	ZONMW-57_00002	cca
cca	ZONMW-58_00964	cca
cca	ZONMW-59_00002	cca
cca	ZONMW-60_00003	cca
cca	ZONMW-61_00936	cca
cca	ZONMW-62_02748	cca
cca	ZONMW-63_01766	cca
cca	ZONMW-64_00768	cca
cca	ZONMW-64_04180	cca
cca	ZONMW-65_02868	cca
cca	ZONMW-65_00835	cca
cca	ZONMW-66_00299	cca
cca	ZONMW-66_01649	cca
cca	ZONMW-67_03303	cca
cca	ZONMW-67_02764	cca
cca	ZONMW-68_04022	cca
cca	ZONMW-69_03304	cca
cca	ZONMW-69_00561	cca
cca	ZONMW-72_01950	cca
cca	ZONMW-73_00795	cca
cca	ZONMW-74_00667	cca
cca	ZONMW-75_00911	cca
cca	da5A-8_02732	cca
pncC	18_01912	pncC
pncC	ACAM48_01240	pncC
pncC	CAM030_01633	pncC
pncC	CECT7946_02193	pncC
pncC	CGMCC1.8863_02772	pncC
pncC	CL8139_00455	pncC
pncC	DAU203_00506	pncC
pncC	DD5b_01461	pncC

pncC	DSM1208_00572	pncC
pncC	DSM12212_03088	pncC
pncC	DSM15343_01220	pncC
pncC	DSM15361_02254	pncC
pncC	DSM15365_00247	pncC
pncC	DSM15718_03102	pncC
pncC	DSM16505T_02317	pncC
pncC	DSM18103_01796	pncC
pncC	DSM18293_01527	pncC
pncC	DSM18841_03024	pncC
pncC	DSM18842_01652	pncC
pncC	DSM19866_00835	pncC
pncC	DSM2041_03295	pncC
pncC	DSM2061_02065	pncC
pncC	DSM2064_03688	pncC
pncC	DSM2064_01144	pncC
pncC	DSM21164_02880	pncC
pncC	DSM23404_00641	pncC
pncC	DSM23424_01786	pncC
pncC	DSM24786_02583	pncC
pncC	DSM7489_00490	pncC
pncC	DSW1_02722	pncC
pncC	DSW5_02607	pncC
pncC	DSW6_02192	pncC
pncC	DSW8_03434	pncC
pncC	F52_03837	pncC
pncC	F52_01084	pncC
pncC	F52_03511	pncC
pncC	GCA_001027945.1_01209	pncC
pncC	HI1_00422	pncC
pncC	IR1_01606	pncC
pncC	IR1_04786	pncC
pncC	KCOM3131_01275	pncC
pncC	KCTC12974_01500	pncC
pncC	KL-A_01716	pncC
pncC	L12_00342	pncC
pncC	L12_00063	pncC
pncC	LL0412.1.7_01691	pncC
pncC	MAR_2009_71_00150	pncC
pncC	NN016038_01941	pncC
pncC	PAO1_03741	pncC
pncC	PAO1_02207	pncC
pncC	UW101_05198	pncC
pncC	UW101_04335	pncC
pncC	V4-33_01341	pncC
pncC	W5C_03113	pncC
pncC	ZONMW-01_01946	pncC
pncC	ZONMW-02_03266	pncC

pncC	ZONMW-03_02930	pncC
pncC	ZONMW-04_02517	pncC
pncC	ZONMW-05_02148	pncC
pncC	ZONMW-06_03456	pncC
pncC	ZONMW-07_03822	pncC
pncC	ZONMW-08_02900	pncC
pncC	ZONMW-09_02023	pncC
pncC	ZONMW-10_02138	pncC
pncC	ZONMW-10_02332	pncC
pncC	ZONMW-10_01587	pncC
pncC	ZONMW-11_00992	pncC
pncC	ZONMW-12_03006	pncC
pncC	ZONMW-13_01413	pncC
pncC	ZONMW-14_02375	pncC
pncC	ZONMW-15_02177	pncC
pncC	ZONMW-19_00763	pncC
pncC	ZONMW-20_02072	pncC
pncC	ZONMW-20_02594	pncC
pncC	ZONMW-20_00216	pncC
pncC	ZONMW-21_03337	pncC
pncC	ZONMW-22_03388	pncC
pncC	ZONMW-23_03383	pncC
pncC	ZONMW-24_03385	pncC
pncC	ZONMW-25_02822	pncC
pncC	ZONMW-26_02817	pncC
pncC	ZONMW-27_02524	pncC
pncC	ZONMW-28_02276	pncC
pncC	ZONMW-29_02193	pncC
pncC	ZONMW-30_03217	pncC
pncC	ZONMW-31_02457	pncC
pncC	ZONMW-32_02681	pncC
pncC	ZONMW-33_02437	pncC
pncC	ZONMW-34_01757	pncC
pncC	ZONMW-35_01448	pncC
pncC	ZONMW-36_02815	pncC
pncC	ZONMW-37_03397	pncC
pncC	ZONMW-38_00897	pncC
pncC	ZONMW-39_03726	pncC
pncC	ZONMW-40_01210	pncC
pncC	ZONMW-41_01252	pncC
pncC	ZONMW-42_00690	pncC
pncC	ZONMW-43_03373	pncC
pncC	ZONMW-44_02654	pncC
pncC	ZONMW-45_02375	pncC
pncC	ZONMW-46_01237	pncC
pncC	ZONMW-47_00936	pncC
pncC	ZONMW-48_02630	pncC
pncC	ZONMW-49_02014	pncC

pncC	ZONMW-50_02610	pncC
pncC	ZONMW-51_02320	pncC
pncC	ZONMW-52_02732	pncC
pncC	ZONMW-53_02485	pncC
pncC	ZONMW-54_02545	pncC
pncC	ZONMW-55_01668	pncC
pncC	ZONMW-56_01821	pncC
pncC	ZONMW-57_02319	pncC
pncC	ZONMW-58_02343	pncC
pncC	ZONMW-59_02557	pncC
pncC	ZONMW-60_02426	pncC
pncC	ZONMW-61_02802	pncC
pncC	ZONMW-62_03489	pncC
pncC	ZONMW-63_02488	pncC
pncC	ZONMW-64_01849	pncC
pncC	ZONMW-65_00848	pncC
pncC	ZONMW-66_00212	pncC
pncC	ZONMW-67_03245	pncC
pncC	ZONMW-68_02231	pncC
pncC	ZONMW-69_03246	pncC
pncC	ZONMW-72_02103	pncC
pncC	ZONMW-73_01555	pncC
pncC	ZONMW-74_02391	pncC
pncC	ZONMW-75_03752	pncC
pncC	da5A-8_00967	pncC
rnr	18_01029	rnr
rnr	ACAM48_02361	rnr
rnr	CAM030_01060	rnr
rnr	CECT7946_00111	rnr
rnr	CGMCC1.8863_02282	rnr
rnr	CL8139_01836	rnr
rnr	DAU203_01423	rnr
rnr	DD5b_02189	rnr
rnr	DSM1208_01645	rnr
rnr	DSM12212_00041	rnr
rnr	DSM15343_01063	rnr
rnr	DSM15361_00502	rnr
rnr	DSM15365_03087	rnr
rnr	DSM15718_02578	rnr
rnr	DSM16505T_00503	rnr
rnr	DSM18103_02851	rnr
rnr	DSM18293_03080	rnr
rnr	DSM18841_00351	rnr
rnr	DSM18842_00880	rnr
rnr	DSM19866_04990	rnr
rnr	DSM2041_00267	rnr
rnr	DSM2061_03178	rnr
rnr	DSM2064_02014	rnr

rnr	DSM21164_00849	rnr
rnr	DSM23404_03077	rnr
rnr	DSM23424_00364	rnr
rnr	DSM24786_02907	rnr
rnr	DSM7489_01335	rnr
rnr	DSW1_00612	rnr
rnr	DSW5_01206	rnr
rnr	DSW6_01364	rnr
rnr	DSW8_02294	rnr
rnr	F52_02414	rnr
rnr	GCA_001027945.1_00260	rnr
rnr	HI1_01274	rnr
rnr	IR1_04742	rnr
rnr	KCOM3131_01281	rnr
rnr	KCTC12974_01615	rnr
rnr	KL-A_00897	rnr
rnr	L12_00277	rnr
rnr	LL0412.1.7_00461	rnr
rnr	MAR_2009_71_01144	rnr
rnr	NN016038_01045	rnr
rnr	PAO1_05104	rnr
rnr	UW101_01069	rnr
rnr	V4-33_02413	rnr
rnr	W5C_01715	rnr
rnr	ZONMW-01_00930	rnr
rnr	ZONMW-02_03604	rnr
rnr	ZONMW-03_01999	rnr
rnr	ZONMW-04_03345	rnr
rnr	ZONMW-05_02099	rnr
rnr	ZONMW-06_02977	rnr
rnr	ZONMW-06_01128	rnr
rnr	ZONMW-07_03829	rnr
rnr	ZONMW-08_00666	rnr
rnr	ZONMW-09_00385	rnr
rnr	ZONMW-10_00361	rnr
rnr	ZONMW-11_02702	rnr
rnr	ZONMW-12_00284	rnr
rnr	ZONMW-13_00381	rnr
rnr	ZONMW-14_00208	rnr
rnr	ZONMW-15_03245	rnr
rnr	ZONMW-19_00306	rnr
rnr	ZONMW-19_01858	rnr
rnr	ZONMW-20_03160	rnr
rnr	ZONMW-20_03488	rnr
rnr	ZONMW-21_00831	rnr
rnr	ZONMW-22_00832	rnr
rnr	ZONMW-23_01946	rnr
rnr	ZONMW-24_00629	rnr

rnr	ZONMW-25_01764	rnr
rnr	ZONMW-26_02298	rnr
rnr	ZONMW-27_01473	rnr
rnr	ZONMW-28_03292	rnr
rnr	ZONMW-28_01869	rnr
rnr	ZONMW-29_00473	rnr
rnr	ZONMW-30_03270	rnr
rnr	ZONMW-31_03533	rnr
rnr	ZONMW-31_00215	rnr
rnr	ZONMW-32_03417	rnr
rnr	ZONMW-32_02103	rnr
rnr	ZONMW-33_03420	rnr
rnr	ZONMW-33_02094	rnr
rnr	ZONMW-34_02086	rnr
rnr	ZONMW-34_02212	rnr
rnr	ZONMW-35_01778	rnr
rnr	ZONMW-35_02037	rnr
rnr	ZONMW-36_03277	rnr
rnr	ZONMW-36_02142	rnr
rnr	ZONMW-37_00831	rnr
rnr	ZONMW-38_02589	rnr
rnr	ZONMW-39_03681	rnr
rnr	ZONMW-39_01492	rnr
rnr	ZONMW-40_00369	rnr
rnr	ZONMW-41_01859	rnr
rnr	ZONMW-42_04147	rnr
rnr	ZONMW-42_00319	rnr
rnr	ZONMW-43_02862	rnr
rnr	ZONMW-43_03286	rnr
rnr	ZONMW-44_00963	rnr
rnr	ZONMW-44_03114	rnr
rnr	ZONMW-45_00505	rnr
rnr	ZONMW-46_01334	rnr
rnr	ZONMW-47_01518	rnr
rnr	ZONMW-48_03306	rnr
rnr	ZONMW-49_02605	rnr
rnr	ZONMW-50_02399	rnr
rnr	ZONMW-51_02695	rnr
rnr	ZONMW-52_02168	rnr
rnr	ZONMW-53_02673	rnr
rnr	ZONMW-54_02812	rnr
rnr	ZONMW-55_02673	rnr
rnr	ZONMW-56_02528	rnr
rnr	ZONMW-57_02561	rnr
rnr	ZONMW-58_02586	rnr
rnr	ZONMW-59_02609	rnr
rnr	ZONMW-60_02796	rnr
rnr	ZONMW-61_00248	rnr

rnr	ZONMW-62_01803	rnr
rnr	ZONMW-63_02122	rnr
rnr	ZONMW-64_03612	rnr
rnr	ZONMW-64_01414	rnr
rnr	ZONMW-65_02946	rnr
rnr	ZONMW-65_00076	rnr
rnr	ZONMW-66_02162	rnr
rnr	ZONMW-66_02130	rnr
rnr	ZONMW-67_02476	rnr
rnr	ZONMW-67_00304	rnr
rnr	ZONMW-68_00001	rnr
rnr	ZONMW-69_02524	rnr
rnr	ZONMW-69_00051	rnr
rnr	ZONMW-72_03269	rnr
rnr	ZONMW-73_02174	rnr
rnr	ZONMW-74_01570	rnr
rnr	ZONMW-75_02371	rnr
rnr	da5A-8_03598	rnr
def	18_03833	def
def	ACAM48_03475	def
def	CAM030_00061	def
def	CAM030_01386	def
def	CECT7946_02174	def
def	CGMCC1.8863_03406	def
def	CL8139_02818	def
def	DAU203_00076	def
def	DD5b_01339	def
def	DSM1208_02801	def
def	DSM1208_01173	def
def	DSM12212_02252	def
def	DSM15343_01898	def
def	DSM15361_01598	def
def	DSM15365_01316	def
def	DSM15365_02278	def
def	DSM15718_00353	def
def	DSM16505T_01552	def
def	DSM18103_00287	def
def	DSM18293_03973	def
def	DSM18841_02174	def
def	DSM18842_01516	def
def	DSM19866_00041	def
def	DSM2041_02619	def
def	DSM2061_04289	def
def	DSM2061_02685	def
def	DSM2064_03155	def
def	DSM21164_03192	def
def	DSM21164_01145	def
def	DSM23404_00679	def

def	DSM23424_01867	def
def	DSM24786_01253	def
def	DSM7489_00075	def
def	DSW1_02596	def
def	DSW5_02627	def
def	DSW6_00362	def
def	DSW8_01169	def
def	F52_00504	def
def	GCA_001027945.1_01770	def
def	HI1_00005	def
def	HI1_03374	def
def	IR1_02379	def
def	KCOM3131_00916	def
def	KCTC12974_01443	def
def	KL-A_03362	def
def	L12_00409	def
def	LL0412.1.7_02419	def
def	MAR_2009_71_02276	def
def	NN016038_03886	def
def	PAO1_00019	def
def	PAO1_01160	def
def	UW101_02645	def
def	V4-33_01661	def
def	W5C_00801	def
def	ZONMW-01_03045	def
def	ZONMW-02_00871	def
def	ZONMW-02_02394	def
def	ZONMW-03_02280	def
def	ZONMW-04_00745	def
def	ZONMW-05_00014	def
def	ZONMW-06_01316	def
def	ZONMW-07_02708	def
def	ZONMW-08_00969	def
def	ZONMW-09_02081	def
def	ZONMW-10_01033	def
def	ZONMW-11_03299	def
def	ZONMW-12_03883	def
def	ZONMW-13_01441	def
def	ZONMW-14_02345	def
def	ZONMW-15_03344	def
def	ZONMW-19_02663	def
def	ZONMW-19_01100	def
def	ZONMW-20_04244	def
def	ZONMW-21_02680	def
def	ZONMW-22_02429	def
def	ZONMW-23_00121	def
def	ZONMW-24_02194	def
def	ZONMW-25_00713	def

def	ZONMW-26_00946	def
def	ZONMW-27_00841	def
def	ZONMW-28_01726	def
def	ZONMW-29_01852	def
def	ZONMW-30_03043	def
def	ZONMW-31_00013	def
def	ZONMW-32_02246	def
def	ZONMW-33_02237	def
def	ZONMW-34_02391	def
def	ZONMW-35_01858	def
def	ZONMW-36_01999	def
def	ZONMW-37_02347	def
def	ZONMW-38_02356	def
def	ZONMW-39_01683	def
def	ZONMW-40_01752	def
def	ZONMW-41_00414	def
def	ZONMW-42_02809	def
def	ZONMW-43_01841	def
def	ZONMW-44_02420	def
def	ZONMW-45_01608	def
def	ZONMW-46_02119	def
def	ZONMW-47_01942	def
def	ZONMW-48_02962	def
def	ZONMW-49_03285	def
def	ZONMW-50_03297	def
def	ZONMW-51_01837	def
def	ZONMW-52_00936	def
def	ZONMW-53_01679	def
def	ZONMW-54_01046	def
def	ZONMW-55_02159	def
def	ZONMW-56_02209	def
def	ZONMW-57_00011	def
def	ZONMW-58_00955	def
def	ZONMW-59_00011	def
def	ZONMW-60_00012	def
def	ZONMW-61_00928	def
def	ZONMW-62_02739	def
def	ZONMW-63_01775	def
def	ZONMW-64_01638	def
def	ZONMW-65_02390	def
def	ZONMW-66_03069	def
def	ZONMW-66_01210	def
def	ZONMW-67_00117	def
def	ZONMW-68_04014	def
def	ZONMW-69_02726	def
def	ZONMW-72_02912	def
def	ZONMW-73_00805	def
def	ZONMW-73_02092	def

def	ZONMW-74_00656	def
def	ZONMW-75_00921	def
def	ZONMW-75_00168	def
def	da5A-8_03724	def
serA	18_01385	serA
serA	18_01386	serA
serA	18_02382	serA
serA	18_03641	serA
serA	18_03523	serA
serA	ACAM48_02579	serA
serA	ACAM48_00676	serA
serA	ACAM48_01324	serA
serA	ACAM48_00494	serA
serA	ACAM48_01902	serA
serA	CAM030_01242	serA
serA	CAM030_01244	serA
serA	CAM030_00548	serA
serA	CECT7946_01860	serA
serA	CECT7946_01857	serA
serA	CECT7946_01413	serA
serA	CGMCC1.8863_00563	serA
serA	CGMCC1.8863_00560	serA
serA	CGMCC1.8863_01799	serA
serA	CGMCC1.8863_00255	serA
serA	CGMCC1.8863_02327	serA
serA	CGMCC1.8863_00344	serA
serA	CGMCC1.8863_03289	serA
serA	CL8139_01086	serA
serA	CL8139_01088	serA
serA	CL8139_01806	serA
serA	CL8139_01895	serA
serA	DAU203_01852	serA
serA	DAU203_01854	serA
serA	DAU203_00340	serA
serA	DAU203_03206	serA
serA	DD5b_02016	serA
serA	DD5b_01263	serA
serA	DSM1208_03095	serA
serA	DSM1208_02044	serA
serA	DSM1208_03717	serA
serA	DSM1208_00033	serA
serA	DSM1208_04012	serA
serA	DSM1208_03957	serA
serA	DSM1208_04002	serA
serA	DSM12212_02529	serA
serA	DSM12212_02532	serA
serA	DSM12212_02409	serA
serA	DSM12212_01315	serA

serA	DSM15343_00937	serA
serA	DSM15343_01456	serA
serA	DSM15361_00612	serA
serA	DSM15361_00610	serA
serA	DSM15361_02358	serA
serA	DSM15361_00996	serA
serA	DSM15361_01110	serA
serA	DSM15365_03289	serA
serA	DSM15365_03287	serA
serA	DSM15365_00702	serA
serA	DSM15365_01935	serA
serA	DSM15718_02459	serA
serA	DSM15718_01040	serA
serA	DSM15718_01946	serA
serA	DSM15718_02006	serA
serA	DSM16505T_00730	serA
serA	DSM16505T_00728	serA
serA	DSM16505T_02266	serA
serA	DSM16505T_00822	serA
serA	DSM16505T_01429	serA
serA	DSM18103_01191	serA
serA	DSM18103_01189	serA
serA	DSM18103_02133	serA
serA	DSM18103_00861	serA
serA	DSM18103_02374	serA
serA	DSM18293_02323	serA
serA	DSM18293_03238	serA
serA	DSM18293_02199	serA
serA	DSM18293_02505	serA
serA	DSM18293_03900	serA
serA	DSM18293_02190	serA
serA	DSM18841_00609	serA
serA	DSM18841_00607	serA
serA	DSM18841_01704	serA
serA	DSM18841_02447	serA
serA	DSM18842_02967	serA
serA	DSM18842_02964	serA
serA	DSM18842_01708	serA
serA	DSM18842_03059	serA
serA	DSM18842_01359	serA
serA	DSM19866_03006	serA
serA	DSM19866_03008	serA
serA	DSM19866_01443	serA
serA	DSM19866_04384	serA
serA	DSM19866_04098	serA
serA	DSM19866_04745	serA
serA	DSM19866_04201	serA
serA	DSM2041_04524	serA

serA	DSM2041_04529	serA
serA	DSM2041_01028	serA
serA	DSM2041_03685	serA
serA	DSM2041_02062	serA
serA	DSM2041_02286	serA
serA	DSM2061_02807	serA
serA	DSM2061_02804	serA
serA	DSM2061_00866	serA
serA	DSM2061_00229	serA
serA	DSM2061_00520	serA
serA	DSM2061_01083	serA
serA	DSM2061_00858	serA
serA	DSM2064_00239	serA
serA	DSM2064_01826	serA
serA	DSM2064_02946	serA
serA	DSM2064_03226	serA
serA	DSM2064_02939	serA
serA	DSM21164_00511	serA
serA	DSM21164_00509	serA
serA	DSM21164_02351	serA
serA	DSM21164_02700	serA
serA	DSM23404_02375	serA
serA	DSM23404_02376	serA
serA	DSM23404_03329	serA
serA	DSM23404_02646	serA
serA	DSM23404_01622	serA
serA	DSM23404_01089	serA
serA	DSM23404_01271	serA
serA	DSM23424_00550	serA
serA	DSM23424_00554	serA
serA	DSM23424_01294	serA
serA	DSM24786_03249	serA
serA	DSM24786_03247	serA
serA	DSM24786_02414	serA
serA	DSM24786_00657	serA
serA	DSM7489_01781	serA
serA	DSM7489_01783	serA
serA	DSM7489_00325	serA
serA	DSM7489_03038	serA
serA	DSW1_00454	serA
serA	DSW1_00456	serA
serA	DSW1_02318	serA
serA	DSW5_01192	serA
serA	DSW5_01190	serA
serA	DSW5_00422	serA
serA	DSW5_02095	serA
serA	DSW6_01755	serA
serA	DSW6_01756	serA

serA	DSW6_03261	serA
serA	DSW8_01865	serA
serA	DSW8_01863	serA
serA	DSW8_01909	serA
serA	DSW8_00608	serA
serA	DSW8_00130	serA
serA	DSW8_00249	serA
serA	DSW8_00230	serA
serA	F52_01431	serA
serA	F52_02263	serA
serA	F52_03137	serA
serA	F52_00443	serA
serA	GCA_001027945.1_01277	serA
serA	GCA_001027945.1_01275	serA
serA	GCA_001027945.1_02494	serA
serA	GCA_001027945.1_01920	serA
serA	HI1_01707	serA
serA	HI1_01709	serA
serA	HI1_00257	serA
serA	HI1_02979	serA
serA	IR1_04001	serA
serA	IR1_00986	serA
serA	IR1_02432	serA
serA	IR1_00995	serA
serA	KCOM3131_01333	serA
serA	KCOM3131_01410	serA
serA	KCOM3131_01006	serA
serA	KCOM3131_01385	serA
serA	KCTC12974_01950	serA
serA	KCTC12974_01949	serA
serA	KCTC12974_00656	serA
serA	KCTC12974_00524	serA
serA	KCTC12974_02972	serA
serA	KCTC12974_01814	serA
serA	KCTC12974_02345	serA
serA	KCTC12974_01771	serA
serA	KL-A_02906	serA
serA	KL-A_02908	serA
serA	KL-A_02668	serA
serA	KL-A_02988	serA
serA	L12_00234	serA
serA	LL0412.1.7_00742	serA
serA	LL0412.1.7_00740	serA
serA	LL0412.1.7_02543	serA
serA	LL0412.1.7_02728	serA
serA	MAR_2009_71_01514	serA
serA	MAR_2009_71_01516	serA
serA	MAR_2009_71_01473	serA

serA	MAR_2009_71_03405	serA
serA	MAR_2009_71_02931	serA
serA	MAR_2009_71_03048	serA
serA	MAR_2009_71_03028	serA
serA	NN016038_01397	serA
serA	NN016038_01398	serA
serA	NN016038_02393	serA
serA	NN016038_03681	serA
serA	NN016038_03563	serA
serA	PAO1_00958	serA
serA	PAO1_00324	serA
serA	PAO1_04024	serA
serA	PAO1_02335	serA
serA	PAO1_04777	serA
serA	PAO1_01415	serA
serA	PAO1_01335	serA
serA	UW101_02950	serA
serA	UW101_00880	serA
serA	UW101_02000	serA
serA	UW101_02574	serA
serA	UW101_01993	serA
serA	V4-33_00050	serA
serA	V4-33_00611	serA
serA	W5C_00469	serA
serA	W5C_00471	serA
serA	W5C_02778	serA
serA	W5C_02247	serA
serA	ZONMW-01_00218	serA
serA	ZONMW-01_00220	serA
serA	ZONMW-01_01769	serA
serA	ZONMW-01_00734	serA
serA	ZONMW-02_00222	serA
serA	ZONMW-02_00220	serA
serA	ZONMW-02_01145	serA
serA	ZONMW-02_02567	serA
serA	ZONMW-03_00944	serA
serA	ZONMW-03_00946	serA
serA	ZONMW-03_02754	serA
serA	ZONMW-03_00112	serA
serA	ZONMW-04_00701	serA
serA	ZONMW-04_00699	serA
serA	ZONMW-04_02769	serA
serA	ZONMW-04_01116	serA
serA	ZONMW-05_01123	serA
serA	ZONMW-05_01125	serA
serA	ZONMW-05_02319	serA
serA	ZONMW-05_00367	serA
serA	ZONMW-06_00877	serA

serA	ZONMW-06_00481	serA
serA	ZONMW-06_02702	serA
serA	ZONMW-06_03174	serA
serA	ZONMW-06_01504	serA
serA	ZONMW-06_02847	serA
serA	ZONMW-07_00091	serA
serA	ZONMW-07_00089	serA
serA	ZONMW-07_03299	serA
serA	ZONMW-07_02906	serA
serA	ZONMW-07_03166	serA
serA	ZONMW-07_01888	serA
serA	ZONMW-07_02385	serA
serA	ZONMW-08_01691	serA
serA	ZONMW-08_01689	serA
serA	ZONMW-08_01735	serA
serA	ZONMW-08_02522	serA
serA	ZONMW-08_02130	serA
serA	ZONMW-08_03833	serA
serA	ZONMW-08_02228	serA
serA	ZONMW-09_00165	serA
serA	ZONMW-09_02430	serA
serA	ZONMW-09_00166	serA
serA	ZONMW-09_01524	serA
serA	ZONMW-09_00766	serA
serA	ZONMW-09_01744	serA
serA	ZONMW-10_00885	serA
serA	ZONMW-10_00508	serA
serA	ZONMW-10_02280	serA
serA	ZONMW-11_03627	serA
serA	ZONMW-11_01439	serA
serA	ZONMW-11_00996	serA
serA	ZONMW-11_00618	serA
serA	ZONMW-11_02484	serA
serA	ZONMW-11_02884	serA
serA	ZONMW-12_00582	serA
serA	ZONMW-12_00583	serA
serA	ZONMW-12_00581	serA
serA	ZONMW-12_00290	serA
serA	ZONMW-12_03834	serA
serA	ZONMW-12_01869	serA
serA	ZONMW-12_00761	serA
serA	ZONMW-12_02076	serA
serA	ZONMW-12_01559	serA
serA	ZONMW-13_00678	serA
serA	ZONMW-13_00677	serA
serA	ZONMW-13_02434	serA
serA	ZONMW-13_01922	serA
serA	ZONMW-13_01142	serA

serA	ZONMW-13_02315	serA
serA	ZONMW-14_01363	serA
serA	ZONMW-14_01364	serA
serA	ZONMW-14_02559	serA
serA	ZONMW-14_00768	serA
serA	ZONMW-14_01452	serA
serA	ZONMW-14_02233	serA
serA	ZONMW-15_00262	serA
serA	ZONMW-15_00264	serA
serA	ZONMW-15_02419	serA
serA	ZONMW-15_02283	serA
serA	ZONMW-19_04141	serA
serA	ZONMW-19_00304	serA
serA	ZONMW-19_03552	serA
serA	ZONMW-20_00368	serA
serA	ZONMW-20_00174	serA
serA	ZONMW-20_00898	serA
serA	ZONMW-20_04014	serA
serA	ZONMW-20_02005	serA
serA	ZONMW-21_00445	serA
serA	ZONMW-21_00441	serA
serA	ZONMW-21_01606	serA
serA	ZONMW-21_02026	serA
serA	ZONMW-22_00445	serA
serA	ZONMW-22_00441	serA
serA	ZONMW-22_02118	serA
serA	ZONMW-22_01700	serA
serA	ZONMW-23_01560	serA
serA	ZONMW-23_01556	serA
serA	ZONMW-23_00433	serA
serA	ZONMW-23_00852	serA
serA	ZONMW-24_01014	serA
serA	ZONMW-24_01018	serA
serA	ZONMW-24_02743	serA
serA	ZONMW-24_01723	serA
serA	ZONMW-25_02495	serA
serA	ZONMW-25_02497	serA
serA	ZONMW-25_00175	serA
serA	ZONMW-25_01399	serA
serA	ZONMW-25_01049	serA
serA	ZONMW-26_01175	serA
serA	ZONMW-26_01177	serA
serA	ZONMW-26_02984	serA
serA	ZONMW-26_00598	serA
serA	ZONMW-27_00695	serA
serA	ZONMW-27_00693	serA
serA	ZONMW-27_03245	serA
serA	ZONMW-27_01193	serA

serA	ZONMW-28_00648	serA
serA	ZONMW-28_00961	serA
serA	ZONMW-28_02985	serA
serA	ZONMW-28_02171	serA
serA	ZONMW-28_04008	serA
serA	ZONMW-28_02062	serA
serA	ZONMW-29_00106	serA
serA	ZONMW-29_00104	serA
serA	ZONMW-29_03186	serA
serA	ZONMW-29_02067	serA
serA	ZONMW-29_01294	serA
serA	ZONMW-29_01572	serA
serA	ZONMW-30_01573	serA
serA	ZONMW-30_01570	serA
serA	ZONMW-30_02629	serA
serA	ZONMW-30_02147	serA
serA	ZONMW-30_01363	serA
serA	ZONMW-31_01138	serA
serA	ZONMW-31_00837	serA
serA	ZONMW-31_02255	serA
serA	ZONMW-31_01641	serA
serA	ZONMW-31_02956	serA
serA	ZONMW-32_00443	serA
serA	ZONMW-32_00130	serA
serA	ZONMW-32_01469	serA
serA	ZONMW-32_02576	serA
serA	ZONMW-32_03865	serA
serA	ZONMW-32_02467	serA
serA	ZONMW-33_00310	serA
serA	ZONMW-33_03391	serA
serA	ZONMW-33_01459	serA
serA	ZONMW-33_02542	serA
serA	ZONMW-33_03639	serA
serA	ZONMW-33_02651	serA
serA	ZONMW-34_00689	serA
serA	ZONMW-34_00403	serA
serA	ZONMW-34_01647	serA
serA	ZONMW-34_01846	serA
serA	ZONMW-34_03005	serA
serA	ZONMW-34_01945	serA
serA	ZONMW-35_02773	serA
serA	ZONMW-35_00987	serA
serA	ZONMW-35_03611	serA
serA	ZONMW-35_01537	serA
serA	ZONMW-35_02987	serA
serA	ZONMW-35_01637	serA
serA	ZONMW-36_01059	serA
serA	ZONMW-36_03128	serA

serA	ZONMW-36_00678	serA
serA	ZONMW-36_02710	serA
serA	ZONMW-36_03616	serA
serA	ZONMW-36_02601	serA
serA	ZONMW-37_00445	serA
serA	ZONMW-37_00441	serA
serA	ZONMW-37_01984	serA
serA	ZONMW-37_01723	serA
serA	ZONMW-38_00125	serA
serA	ZONMW-38_00499	serA
serA	ZONMW-38_00561	serA
serA	ZONMW-38_00559	serA
serA	ZONMW-38_00346	serA
serA	ZONMW-38_03127	serA
serA	ZONMW-39_01151	serA
serA	ZONMW-39_03106	serA
serA	ZONMW-39_00052	serA
serA	ZONMW-39_02754	serA
serA	ZONMW-39_02247	serA
serA	ZONMW-40_01478	serA
serA	ZONMW-40_01875	serA
serA	ZONMW-40_01480	serA
serA	ZONMW-40_00732	serA
serA	ZONMW-40_02485	serA
serA	ZONMW-40_03020	serA
serA	ZONMW-41_02872	serA
serA	ZONMW-41_00778	serA
serA	ZONMW-41_01850	serA
serA	ZONMW-41_02640	serA
serA	ZONMW-41_03040	serA
serA	ZONMW-42_01641	serA
serA	ZONMW-42_03086	serA
serA	ZONMW-42_02398	serA
serA	ZONMW-42_00734	serA
serA	ZONMW-42_00473	serA
serA	ZONMW-43_00229	serA
serA	ZONMW-43_02366	serA
serA	ZONMW-43_01621	serA
serA	ZONMW-43_01356	serA
serA	ZONMW-43_03864	serA
serA	ZONMW-43_01449	serA
serA	ZONMW-44_02074	serA
serA	ZONMW-44_01723	serA
serA	ZONMW-44_00688	serA
serA	ZONMW-44_02847	serA
serA	ZONMW-44_00586	serA
serA	ZONMW-44_00824	serA
serA	ZONMW-45_00967	serA

serA	ZONMW-45_00965	serA
serA	ZONMW-45_00758	serA
serA	ZONMW-45_01859	serA
serA	ZONMW-46_00700	serA
serA	ZONMW-46_00702	serA
serA	ZONMW-46_01116	serA
serA	ZONMW-46_01959	serA
serA	ZONMW-47_00700	serA
serA	ZONMW-47_00702	serA
serA	ZONMW-47_01057	serA
serA	ZONMW-47_02102	serA
serA	ZONMW-48_00908	serA
serA	ZONMW-48_00910	serA
serA	ZONMW-48_03068	serA
serA	ZONMW-48_01326	serA
serA	ZONMW-49_01325	serA
serA	ZONMW-49_01327	serA
serA	ZONMW-49_01854	serA
serA	ZONMW-49_01720	serA
serA	ZONMW-50_01327	serA
serA	ZONMW-50_01329	serA
serA	ZONMW-50_03035	serA
serA	ZONMW-50_01694	serA
serA	ZONMW-51_01439	serA
serA	ZONMW-51_01441	serA
serA	ZONMW-51_02137	serA
serA	ZONMW-51_00618	serA
serA	ZONMW-52_01181	serA
serA	ZONMW-52_01183	serA
serA	ZONMW-52_02566	serA
serA	ZONMW-52_00575	serA
serA	ZONMW-53_00669	serA
serA	ZONMW-53_00667	serA
serA	ZONMW-53_02318	serA
serA	ZONMW-53_01330	serA
serA	ZONMW-54_00392	serA
serA	ZONMW-54_00394	serA
serA	ZONMW-54_02360	serA
serA	ZONMW-54_01387	serA
serA	ZONMW-55_00641	serA
serA	ZONMW-55_00639	serA
serA	ZONMW-55_01835	serA
serA	ZONMW-55_00906	serA
serA	ZONMW-56_03154	serA
serA	ZONMW-56_03152	serA
serA	ZONMW-56_01983	serA
serA	ZONMW-56_00142	serA
serA	ZONMW-57_00974	serA

serA	ZONMW-57_00976	serA
serA	ZONMW-57_02151	serA
serA	ZONMW-57_00355	serA
serA	ZONMW-58_01610	serA
serA	ZONMW-58_01608	serA
serA	ZONMW-58_02175	serA
serA	ZONMW-58_00616	serA
serA	ZONMW-59_01897	serA
serA	ZONMW-59_01895	serA
serA	ZONMW-59_02394	serA
serA	ZONMW-59_00371	serA
serA	ZONMW-60_01580	serA
serA	ZONMW-60_01578	serA
serA	ZONMW-60_02240	serA
serA	ZONMW-60_00382	serA
serA	ZONMW-61_00631	serA
serA	ZONMW-61_00633	serA
serA	ZONMW-61_03266	serA
serA	ZONMW-61_02092	serA
serA	ZONMW-61_02492	serA
serA	ZONMW-61_03239	serA
serA	ZONMW-62_02506	serA
serA	ZONMW-62_02504	serA
serA	ZONMW-62_02547	serA
serA	ZONMW-62_00154	serA
serA	ZONMW-62_00635	serA
serA	ZONMW-62_00518	serA
serA	ZONMW-62_00537	serA
serA	ZONMW-63_00732	serA
serA	ZONMW-63_00734	serA
serA	ZONMW-63_03114	serA
serA	ZONMW-63_00023	serA
serA	ZONMW-64_00245	serA
serA	ZONMW-64_00716	serA
serA	ZONMW-64_03073	serA
serA	ZONMW-64_03818	serA
serA	ZONMW-64_02026	serA
serA	ZONMW-65_02633	serA
serA	ZONMW-65_01557	serA
serA	ZONMW-65_00756	serA
serA	ZONMW-65_02294	serA
serA	ZONMW-65_03344	serA
serA	ZONMW-65_00116	serA
serA	ZONMW-66_03570	serA
serA	ZONMW-66_02160	serA
serA	ZONMW-66_00330	serA
serA	ZONMW-67_00914	serA
serA	ZONMW-67_02841	serA

serA	ZONMW-67_01581	serA
serA	ZONMW-67_02954	serA
serA	ZONMW-67_02344	serA
serA	ZONMW-68_00428	serA
serA	ZONMW-68_00432	serA
serA	ZONMW-68_01816	serA
serA	ZONMW-68_02057	serA
serA	ZONMW-68_03678	serA
serA	ZONMW-68_03803	serA
serA	ZONMW-68_01008	serA
serA	ZONMW-68_02935	serA
serA	ZONMW-69_00961	serA
serA	ZONMW-69_00638	serA
serA	ZONMW-69_01420	serA
serA	ZONMW-69_03000	serA
serA	ZONMW-69_02392	serA
serA	ZONMW-72_02315	serA
serA	ZONMW-72_02399	serA
serA	ZONMW-72_02965	serA
serA	ZONMW-72_02408	serA
serA	ZONMW-73_01798	serA
serA	ZONMW-73_01800	serA
serA	ZONMW-73_03775	serA
serA	ZONMW-73_00966	serA
serA	ZONMW-73_03208	serA
serA	ZONMW-74_01905	serA
serA	ZONMW-74_01903	serA
serA	ZONMW-74_02289	serA
serA	ZONMW-74_00263	serA
serA	ZONMW-75_01089	serA
serA	ZONMW-75_01087	serA
serA	ZONMW-75_01613	serA
serA	ZONMW-75_01975	serA
serA	da5A-8_01521	serA
serA	da5A-8_01523	serA
serA	da5A-8_02787	serA
serA	da5A-8_01312	serA
serA	da5A-8_02551	serA
pgi	18_00753	pgi
pgi	ACAM48_02166	pgi
pgi	CAM030_01203	pgi
pgi	CECT7946_00375	pgi
pgi	CGMCC1.8863_02200	pgi
pgi	CL8139_01586	pgi
pgi	DAU203_01185	pgi
pgi	DD5b_02003	pgi
pgi	DSM1208_01392	pgi
pgi	DSM12212_00312	pgi

pgi	DSM15343_02462	pgi
pgi	DSM15361_02302	pgi
pgi	DSM15365_00949	pgi
pgi	DSM15718_02747	pgi
pgi	DSM16505T_00626	pgi
pgi	DSM18103_01045	pgi
pgi	DSM18293_00794	pgi
pgi	DSM18841_00499	pgi
pgi	DSM18842_00731	pgi
pgi	DSM19866_03537	pgi
pgi	DSM2041_00496	pgi
pgi	DSM2061_02912	pgi
pgi	DSM2064_01720	pgi
pgi	DSM21164_01094	pgi
pgi	DSM23404_01961	pgi
pgi	DSM23424_02142	pgi
pgi	DSM24786_02665	pgi
pgi	DSM7489_01101	pgi
pgi	DSW1_00347	pgi
pgi	DSW5_01083	pgi
pgi	DSW6_00878	pgi
pgi	DSW8_02547	pgi
pgi	F52_02178	pgi
pgi	GCA_001027945.1_00108	pgi
pgi	HI1_01041	pgi
pgi	IR1_04119	pgi
pgi	KCOM3131_01663	pgi
pgi	KCTC12974_00419	pgi
pgi	KL-A_02047	pgi
pgi	L12_00170	pgi
pgi	LL0412.1.7_00317	pgi
pgi	MAR_2009_71_00905	pgi
pgi	NN016038_00773	pgi
pgi	PAO1_04893	pgi
pgi	UW101_00774	pgi
pgi	V4-33_00127	pgi
pgi	W5C_01948	pgi
pgi	ZONMW-01_01163	pgi
pgi	ZONMW-02_01666	pgi
pgi	ZONMW-03_02232	pgi
pgi	ZONMW-04_01581	pgi
pgi	ZONMW-05_01862	pgi
pgi	ZONMW-06_01944	pgi
pgi	ZONMW-07_00678	pgi
pgi	ZONMW-08_00425	pgi
pgi	ZONMW-09_00253	pgi
pgi	ZONMW-10_00254	pgi
pgi	ZONMW-11_00807	pgi

pgi	ZONMW-12_00114	pgi
pgi	ZONMW-13_00243	pgi
pgi	ZONMW-14_00345	pgi
pgi	ZONMW-15_03324	pgi
pgi	ZONMW-19_02402	pgi
pgi	ZONMW-20_03681	pgi
pgi	ZONMW-21_01088	pgi
pgi	ZONMW-22_01089	pgi
pgi	ZONMW-23_02202	pgi
pgi	ZONMW-24_00372	pgi
pgi	ZONMW-25_02088	pgi
pgi	ZONMW-26_02531	pgi
pgi	ZONMW-27_01714	pgi
pgi	ZONMW-28_02638	pgi
pgi	ZONMW-29_00701	pgi
pgi	ZONMW-30_00385	pgi
pgi	ZONMW-31_01909	pgi
pgi	ZONMW-32_03238	pgi
pgi	ZONMW-33_03036	pgi
pgi	ZONMW-34_02617	pgi
pgi	ZONMW-35_02581	pgi
pgi	ZONMW-36_02966	pgi
pgi	ZONMW-37_01274	pgi
pgi	ZONMW-38_00481	pgi
pgi	ZONMW-39_03950	pgi
pgi	ZONMW-39_00712	pgi
pgi	ZONMW-40_00173	pgi
pgi	ZONMW-41_02696	pgi
pgi	ZONMW-42_01059	pgi
pgi	ZONMW-43_01244	pgi
pgi	ZONMW-43_03926	pgi
pgi	ZONMW-44_00035	pgi
pgi	ZONMW-45_00637	pgi
pgi	ZONMW-46_00822	pgi
pgi	ZONMW-47_00822	pgi
pgi	ZONMW-48_00221	pgi
pgi	ZONMW-49_02849	pgi
pgi	ZONMW-50_02807	pgi
pgi	ZONMW-51_02925	pgi
pgi	ZONMW-52_02948	pgi
pgi	ZONMW-53_02955	pgi
pgi	ZONMW-54_02959	pgi
pgi	ZONMW-55_02963	pgi
pgi	ZONMW-56_02843	pgi
pgi	ZONMW-57_02899	pgi
pgi	ZONMW-58_02923	pgi
pgi	ZONMW-59_03050	pgi
pgi	ZONMW-60_03016	pgi

pgi	ZONMW-61_03953	pgi
pgi	ZONMW-62_01549	pgi
pgi	ZONMW-63_02811	pgi
pgi	ZONMW-64_04176	pgi
pgi	ZONMW-64_02190	pgi
pgi	ZONMW-65_00049	pgi
pgi	ZONMW-66_00294	pgi
pgi	ZONMW-67_03307	pgi
pgi	ZONMW-68_00656	pgi
pgi	ZONMW-69_03308	pgi
pgi	ZONMW-72_02732	pgi
pgi	ZONMW-73_01865	pgi
pgi	ZONMW-74_02141	pgi
pgi	ZONMW-75_00465	pgi
pgi	da5A-8_01638	pgi
uvrC	18_00142	uvrC
uvrC	18_03569	uvrC
uvrC	18_02962	uvrC
uvrC	18_00759	uvrC
uvrC	18_01908	uvrC
uvrC	18_02613	uvrC
uvrC	ACAM48_03213	uvrC
uvrC	ACAM48_01173	uvrC
uvrC	ACAM48_01244	uvrC
uvrC	ACAM48_02173	uvrC
uvrC	ACAM48_00971	uvrC
uvrC	CAM030_00287	uvrC
uvrC	CAM030_00365	uvrC
uvrC	CAM030_01196	uvrC
uvrC	CAM030_01630	uvrC
uvrC	CAM030_00176	uvrC
uvrC	CECT7946_01215	uvrC
uvrC	CECT7946_03461	uvrC
uvrC	CECT7946_02190	uvrC
uvrC	CECT7946_00367	uvrC
uvrC	CECT7946_02349	uvrC
uvrC	CGMCC1.8863_03123	uvrC
uvrC	CGMCC1.8863_02931	uvrC
uvrC	CGMCC1.8863_02769	uvrC
uvrC	CGMCC1.8863_02194	uvrC
uvrC	CGMCC1.8863_02296	uvrC
uvrC	CL8139_03058	uvrC
uvrC	CL8139_01288	uvrC
uvrC	CL8139_00451	uvrC
uvrC	CL8139_01580	uvrC
uvrC	CL8139_01536	uvrC
uvrC	DAU203_03403	uvrC
uvrC	DAU203_03463	uvrC

uvrC	DAU203_00510	uvrC
uvrC	DAU203_01179	uvrC
uvrC	DAU203_02183	uvrC
uvrC	DD5b_01464	uvrC
uvrC	DD5b_02010	uvrC
uvrC	DD5b_02785	uvrC
uvrC	DSM1208_02517	uvrC
uvrC	DSM1208_04565	uvrC
uvrC	DSM1208_00575	uvrC
uvrC	DSM1208_01399	uvrC
uvrC	DSM1208_03685	uvrC
uvrC	DSM12212_00592	uvrC
uvrC	DSM12212_01886	uvrC
uvrC	DSM12212_00301	uvrC
uvrC	DSM12212_03085	uvrC
uvrC	DSM15343_02265	uvrC
uvrC	DSM15343_01217	uvrC
uvrC	DSM15343_02469	uvrC
uvrC	DSM15343_00244	uvrC
uvrC	DSM15361_01724	uvrC
uvrC	DSM15361_02251	uvrC
uvrC	DSM15361_00629	uvrC
uvrC	DSM15365_03694	uvrC
uvrC	DSM15365_01917	uvrC
uvrC	DSM15365_00244	uvrC
uvrC	DSM15365_01000	uvrC
uvrC	DSM15365_00176	uvrC
uvrC	DSM15718_01475	uvrC
uvrC	DSM15718_00056	uvrC
uvrC	DSM15718_03099	uvrC
uvrC	DSM15718_02740	uvrC
uvrC	DSM15718_02227	uvrC
uvrC	DSM15718_00851	uvrC
uvrC	DSM15718_01047	uvrC
uvrC	DSM16505T_01426	uvrC
uvrC	DSM16505T_02163	uvrC
uvrC	DSM16505T_00577	uvrC
uvrC	DSM18103_00865	uvrC
uvrC	DSM18103_02214	uvrC
uvrC	DSM18103_00986	uvrC
uvrC	DSM18293_02257	uvrC
uvrC	DSM18293_04150	uvrC
uvrC	DSM18293_01530	uvrC
uvrC	DSM18293_02213	uvrC
uvrC	DSM18293_00804	uvrC
uvrC	DSM18293_03927	uvrC
uvrC	DSM18293_01786	uvrC
uvrC	DSM18841_02444	uvrC

uvrC	DSM18841_01905	uvrC
uvrC	DSM18841_00427	uvrC
uvrC	DSM18842_01356	uvrC
uvrC	DSM18842_01853	uvrC
uvrC	DSM18842_00800	uvrC
uvrC	DSM19866_00319	uvrC
uvrC	DSM19866_04676	uvrC
uvrC	DSM19866_00839	uvrC
uvrC	DSM19866_03525	uvrC
uvrC	DSM19866_04453	uvrC
uvrC	DSM2041_03054	uvrC
uvrC	DSM2041_04839	uvrC
uvrC	DSM2041_03289	uvrC
uvrC	DSM2041_00503	uvrC
uvrC	DSM2041_05215	uvrC
uvrC	DSM2061_04033	uvrC
uvrC	DSM2061_00589	uvrC
uvrC	DSM2061_01921	uvrC
uvrC	DSM2061_01282	uvrC
uvrC	DSM2061_01920	uvrC
uvrC	DSM2061_02068	uvrC
uvrC	DSM2061_02919	uvrC
uvrC	DSM2061_01341	uvrC
uvrC	DSM2064_02999	uvrC
uvrC	DSM2064_00887	uvrC
uvrC	DSM2064_03685	uvrC
uvrC	DSM2064_01711	uvrC
uvrC	DSM2064_03207	uvrC
uvrC	DSM2064_00277	uvrC
uvrC	DSM21164_02154	uvrC
uvrC	DSM21164_02784	uvrC
uvrC	DSM21164_01088	uvrC
uvrC	DSM21164_02876	uvrC
uvrC	DSM21164_01681	uvrC
uvrC	DSM23404_00908	uvrC
uvrC	DSM23404_02726	uvrC
uvrC	DSM23404_00645	uvrC
uvrC	DSM23404_01968	uvrC
uvrC	DSM23404_02908	uvrC
uvrC	DSM23424_02089	uvrC
uvrC	DSM23424_01698	uvrC
uvrC	DSM23424_01783	uvrC
uvrC	DSM23424_00192	uvrC
uvrC	DSM24786_00886	uvrC
uvrC	DSM24786_00971	uvrC
uvrC	DSM24786_02587	uvrC
uvrC	DSM24786_02659	uvrC
uvrC	DSM24786_01855	uvrC

uvrC	DSM7489_03242	uvrC
uvrC	DSM7489_03330	uvrC
uvrC	DSM7489_00494	uvrC
uvrC	DSM7489_01095	uvrC
uvrC	DSM7489_02095	uvrC
uvrC	DSW1_02123	uvrC
uvrC	DSW1_02736	uvrC
uvrC	DSW1_02725	uvrC
uvrC	DSW1_00568	uvrC
uvrC	DSW1_00423	uvrC
uvrC	DSW5_00627	uvrC
uvrC	DSW5_02092	uvrC
uvrC	DSW5_00108	uvrC
uvrC	DSW5_01058	uvrC
uvrC	DSW6_02452	uvrC
uvrC	DSW6_02931	uvrC
uvrC	DSW6_02297	uvrC
uvrC	DSW6_00870	uvrC
uvrC	DSW8_01460	uvrC
uvrC	DSW8_00536	uvrC
uvrC	DSW8_00035	uvrC
uvrC	DSW8_03430	uvrC
uvrC	DSW8_02542	uvrC
uvrC	DSW8_00853	uvrC
uvrC	F52_03082	uvrC
uvrC	F52_00774	uvrC
uvrC	F52_02169	uvrC
uvrC	F52_03834	uvrC
uvrC	F52_00462	uvrC
uvrC	F52_01398	uvrC
uvrC	F52_03123	uvrC
uvrC	GCA_001027945.1_01923	uvrC
uvrC	GCA_001027945.1_02787	uvrC
uvrC	GCA_001027945.1_00178	uvrC
uvrC	HI1_03186	uvrC
uvrC	HI1_03276	uvrC
uvrC	HI1_00426	uvrC
uvrC	HI1_01035	uvrC
uvrC	HI1_02022	uvrC
uvrC	IR1_02075	uvrC
uvrC	IR1_00936	uvrC
uvrC	IR1_01609	uvrC
uvrC	IR1_04128	uvrC
uvrC	IR1_02418	uvrC
uvrC	IR1_03467	uvrC
uvrC	IR1_00974	uvrC
uvrC	KCOM3131_00785	uvrC
uvrC	KCOM3131_00660	uvrC

uvrC	KCOM3131_01210	uvrC
uvrC	KCTC12974_00265	uvrC
uvrC	KCTC12974_02630	uvrC
uvrC	KCTC12974_01505	uvrC
uvrC	KCTC12974_00426	uvrC
uvrC	KCTC12974_02151	uvrC
uvrC	KL-A_01933	uvrC
uvrC	KL-A_01862	uvrC
uvrC	KL-A_01712	uvrC
uvrC	KL-A_02041	uvrC
uvrC	KL-A_00175	uvrC
uvrC	L12_00248	uvrC
uvrC	LL0412.1.7_02725	uvrC
uvrC	LL0412.1.7_02597	uvrC
uvrC	LL0412.1.7_00534	uvrC
uvrC	MAR_2009_71_01968	uvrC
uvrC	MAR_2009_71_02833	uvrC
uvrC	MAR_2009_71_03334	uvrC
uvrC	MAR_2009_71_00154	uvrC
uvrC	MAR_2009_71_00910	uvrC
uvrC	MAR_2009_71_02594	uvrC
uvrC	NN016038_00144	uvrC
uvrC	NN016038_03609	uvrC
uvrC	NN016038_03000	uvrC
uvrC	NN016038_00779	uvrC
uvrC	NN016038_01937	uvrC
uvrC	NN016038_02663	uvrC
uvrC	PAO1_02663	uvrC
uvrC	PAO1_01870	uvrC
uvrC	PAO1_03343	uvrC
uvrC	UW101_02053	uvrC
uvrC	UW101_03598	uvrC
uvrC	UW101_05201	uvrC
uvrC	UW101_00765	uvrC
uvrC	UW101_02593	uvrC
uvrC	UW101_02988	uvrC
uvrC	V4-33_01826	uvrC
uvrC	V4-33_01345	uvrC
uvrC	V4-33_00198	uvrC
uvrC	V4-33_01104	uvrC
uvrC	V4-33_00855	uvrC
uvrC	W5C_01789	uvrC
uvrC	W5C_00894	uvrC
uvrC	W5C_03109	uvrC
uvrC	W5C_01942	uvrC
uvrC	W5C_02960	uvrC
uvrC	ZONMW-01_00544	uvrC
uvrC	ZONMW-01_03138	uvrC

uvrC	ZONMW-01_01169	uvrC
uvrC	ZONMW-01_01491	uvrC
uvrC	ZONMW-02_01502	uvrC
uvrC	ZONMW-02_02587	uvrC
uvrC	ZONMW-02_03269	uvrC
uvrC	ZONMW-02_01719	uvrC
uvrC	ZONMW-02_03726	uvrC
uvrC	ZONMW-03_02438	uvrC
uvrC	ZONMW-03_02373	uvrC
uvrC	ZONMW-03_02238	uvrC
uvrC	ZONMW-03_01289	uvrC
uvrC	ZONMW-04_00919	uvrC
uvrC	ZONMW-04_00837	uvrC
uvrC	ZONMW-04_02513	uvrC
uvrC	ZONMW-04_01575	uvrC
uvrC	ZONMW-04_00401	uvrC
uvrC	ZONMW-05_00171	uvrC
uvrC	ZONMW-05_00107	uvrC
uvrC	ZONMW-05_02144	uvrC
uvrC	ZONMW-05_01856	uvrC
uvrC	ZONMW-05_01424	uvrC
uvrC	ZONMW-06_00339	uvrC
uvrC	ZONMW-06_02407	uvrC
uvrC	ZONMW-06_02146	uvrC
uvrC	ZONMW-06_03922	uvrC
uvrC	ZONMW-06_01514	uvrC
uvrC	ZONMW-07_01704	uvrC
uvrC	ZONMW-07_01029	uvrC
uvrC	ZONMW-07_03370	uvrC
uvrC	ZONMW-07_03825	uvrC
uvrC	ZONMW-07_00684	uvrC
uvrC	ZONMW-07_02668	uvrC
uvrC	ZONMW-08_01260	uvrC
uvrC	ZONMW-08_02848	uvrC
uvrC	ZONMW-08_02066	uvrC
uvrC	ZONMW-08_02896	uvrC
uvrC	ZONMW-08_00430	uvrC
uvrC	ZONMW-08_03597	uvrC
uvrC	ZONMW-09_01537	uvrC
uvrC	ZONMW-09_02158	uvrC
uvrC	ZONMW-09_02049	uvrC
uvrC	ZONMW-09_02020	uvrC
uvrC	ZONMW-09_00262	uvrC
uvrC	ZONMW-10_00753	uvrC
uvrC	ZONMW-10_00934	uvrC
uvrC	ZONMW-10_00265	uvrC
uvrC	ZONMW-10_02135	uvrC
uvrC	ZONMW-10_02207	uvrC

uvrC	ZONMW-11_03734	uvrC
uvrC	ZONMW-11_00520	uvrC
uvrC	ZONMW-11_00754	uvrC
uvrC	ZONMW-11_02685	uvrC
uvrC	ZONMW-12_01587	uvrC
uvrC	ZONMW-12_00934	uvrC
uvrC	ZONMW-12_03010	uvrC
uvrC	ZONMW-12_00123	uvrC
uvrC	ZONMW-12_03745	uvrC
uvrC	ZONMW-13_01645	uvrC
uvrC	ZONMW-13_02385	uvrC
uvrC	ZONMW-13_01416	uvrC
uvrC	ZONMW-13_00250	uvrC
uvrC	ZONMW-13_03103	uvrC
uvrC	ZONMW-14_00890	uvrC
uvrC	ZONMW-14_02595	uvrC
uvrC	ZONMW-14_02370	uvrC
uvrC	ZONMW-14_00337	uvrC
uvrC	ZONMW-14_01944	uvrC
uvrC	ZONMW-15_01177	uvrC
uvrC	ZONMW-15_03017	uvrC
uvrC	ZONMW-15_02181	uvrC
uvrC	ZONMW-15_03330	uvrC
uvrC	ZONMW-15_02914	uvrC
uvrC	ZONMW-19_00857	uvrC
uvrC	ZONMW-19_01778	uvrC
uvrC	ZONMW-20_01662	uvrC
uvrC	ZONMW-20_01450	uvrC
uvrC	ZONMW-20_01922	uvrC
uvrC	ZONMW-20_01840	uvrC
uvrC	ZONMW-21_02398	uvrC
uvrC	ZONMW-21_01672	uvrC
uvrC	ZONMW-21_03334	uvrC
uvrC	ZONMW-21_01082	uvrC
uvrC	ZONMW-21_02157	uvrC
uvrC	ZONMW-22_02710	uvrC
uvrC	ZONMW-22_02052	uvrC
uvrC	ZONMW-22_03391	uvrC
uvrC	ZONMW-22_01083	uvrC
uvrC	ZONMW-22_01568	uvrC
uvrC	ZONMW-23_02734	uvrC
uvrC	ZONMW-23_00499	uvrC
uvrC	ZONMW-23_03380	uvrC
uvrC	ZONMW-23_02196	uvrC
uvrC	ZONMW-23_00983	uvrC
uvrC	ZONMW-24_02422	uvrC
uvrC	ZONMW-24_02677	uvrC
uvrC	ZONMW-24_03382	uvrC

uvrC	ZONMW-24_00378	uvrC
uvrC	ZONMW-24_01592	uvrC
uvrC	ZONMW-25_02563	uvrC
uvrC	ZONMW-25_01461	uvrC
uvrC	ZONMW-25_02083	uvrC
uvrC	ZONMW-25_02825	uvrC
uvrC	ZONMW-25_00378	uvrC
uvrC	ZONMW-26_00786	uvrC
uvrC	ZONMW-26_00853	uvrC
uvrC	ZONMW-26_02813	uvrC
uvrC	ZONMW-26_02537	uvrC
uvrC	ZONMW-26_01845	uvrC
uvrC	ZONMW-27_00994	uvrC
uvrC	ZONMW-27_00934	uvrC
uvrC	ZONMW-27_02528	uvrC
uvrC	ZONMW-27_01720	uvrC
uvrC	ZONMW-27_00395	uvrC
uvrC	ZONMW-28_01573	uvrC
uvrC	ZONMW-28_00217	uvrC
uvrC	ZONMW-28_00438	uvrC
uvrC	ZONMW-28_03865	uvrC
uvrC	ZONMW-28_04020	uvrC
uvrC	ZONMW-29_02841	uvrC
uvrC	ZONMW-29_03119	uvrC
uvrC	ZONMW-29_02190	uvrC
uvrC	ZONMW-29_00706	uvrC
uvrC	ZONMW-29_01364	uvrC
uvrC	ZONMW-30_00146	uvrC
uvrC	ZONMW-30_02209	uvrC
uvrC	ZONMW-30_00380	uvrC
uvrC	ZONMW-30_03220	uvrC
uvrC	ZONMW-30_02676	uvrC
uvrC	ZONMW-31_00655	uvrC
uvrC	ZONMW-31_03698	uvrC
uvrC	ZONMW-31_01394	uvrC
uvrC	ZONMW-31_02554	uvrC
uvrC	ZONMW-31_02242	uvrC
uvrC	ZONMW-32_01980	uvrC
uvrC	ZONMW-32_01008	uvrC
uvrC	ZONMW-32_01229	uvrC
uvrC	ZONMW-32_03803	uvrC
uvrC	ZONMW-32_03877	uvrC
uvrC	ZONMW-33_01971	uvrC
uvrC	ZONMW-33_00997	uvrC
uvrC	ZONMW-33_01218	uvrC
uvrC	ZONMW-33_03744	uvrC
uvrC	ZONMW-33_03627	uvrC
uvrC	ZONMW-34_00306	uvrC

uvrC	ZONMW-34_01410	uvrC
uvrC	ZONMW-34_01200	uvrC
uvrC	ZONMW-34_03642	uvrC
uvrC	ZONMW-34_03016	uvrC
uvrC	ZONMW-35_01084	uvrC
uvrC	ZONMW-35_00483	uvrC
uvrC	ZONMW-35_00273	uvrC
uvrC	ZONMW-35_03850	uvrC
uvrC	ZONMW-35_02976	uvrC
uvrC	ZONMW-36_01385	uvrC
uvrC	ZONMW-36_00217	uvrC
uvrC	ZONMW-36_00438	uvrC
uvrC	ZONMW-36_03693	uvrC
uvrC	ZONMW-36_03628	uvrC
uvrC	ZONMW-37_02668	uvrC
uvrC	ZONMW-37_02050	uvrC
uvrC	ZONMW-37_03400	uvrC
uvrC	ZONMW-37_01280	uvrC
uvrC	ZONMW-37_01591	uvrC
uvrC	ZONMW-38_03426	uvrC
uvrC	ZONMW-38_00862	uvrC
uvrC	ZONMW-38_00129	uvrC
uvrC	ZONMW-38_00186	uvrC
uvrC	ZONMW-39_02991	uvrC
uvrC	ZONMW-39_00290	uvrC
uvrC	ZONMW-39_00791	uvrC
uvrC	ZONMW-39_04098	uvrC
uvrC	ZONMW-39_02742	uvrC
uvrC	ZONMW-40_02230	uvrC
uvrC	ZONMW-40_02422	uvrC
uvrC	ZONMW-40_00178	uvrC
uvrC	ZONMW-40_01207	uvrC
uvrC	ZONMW-40_00498	uvrC
uvrC	ZONMW-41_03177	uvrC
uvrC	ZONMW-41_00787	uvrC
uvrC	ZONMW-41_01967	uvrC
uvrC	ZONMW-42_03578	uvrC
uvrC	ZONMW-42_04018	uvrC
uvrC	ZONMW-42_01834	uvrC
uvrC	ZONMW-42_03164	uvrC
uvrC	ZONMW-42_02388	uvrC
uvrC	ZONMW-43_00639	uvrC
uvrC	ZONMW-43_03007	uvrC
uvrC	ZONMW-43_03101	uvrC
uvrC	ZONMW-43_02599	uvrC
uvrC	ZONMW-43_03875	uvrC
uvrC	ZONMW-44_03530	uvrC
uvrC	ZONMW-44_01077	uvrC

uvrC	ZONMW-44_01297	uvrC
uvrC	ZONMW-44_03804	uvrC
uvrC	ZONMW-44_00576	uvrC
uvrC	ZONMW-45_01856	uvrC
uvrC	ZONMW-45_00147	uvrC
uvrC	ZONMW-45_00567	uvrC
uvrC	ZONMW-46_01956	uvrC
uvrC	ZONMW-46_00362	uvrC
uvrC	ZONMW-46_00892	uvrC
uvrC	ZONMW-47_02105	uvrC
uvrC	ZONMW-47_00143	uvrC
uvrC	ZONMW-47_00892	uvrC
uvrC	ZONMW-48_02516	uvrC
uvrC	ZONMW-48_03136	uvrC
uvrC	ZONMW-48_03383	uvrC
uvrC	ZONMW-48_02626	uvrC
uvrC	ZONMW-48_00215	uvrC
uvrC	ZONMW-48_01680	uvrC
uvrC	ZONMW-49_02965	uvrC
uvrC	ZONMW-49_03030	uvrC
uvrC	ZONMW-49_02018	uvrC
uvrC	ZONMW-49_02843	uvrC
uvrC	ZONMW-49_00819	uvrC
uvrC	ZONMW-50_02890	uvrC
uvrC	ZONMW-50_03002	uvrC
uvrC	ZONMW-50_02614	uvrC
uvrC	ZONMW-50_02813	uvrC
uvrC	ZONMW-50_00820	uvrC
uvrC	ZONMW-51_01984	uvrC
uvrC	ZONMW-51_01930	uvrC
uvrC	ZONMW-51_02324	uvrC
uvrC	ZONMW-51_02931	uvrC
uvrC	ZONMW-51_00892	uvrC
uvrC	ZONMW-52_00788	uvrC
uvrC	ZONMW-52_00843	uvrC
uvrC	ZONMW-52_02736	uvrC
uvrC	ZONMW-52_02942	uvrC
uvrC	ZONMW-52_01492	uvrC
uvrC	ZONMW-53_01522	uvrC
uvrC	ZONMW-53_01586	uvrC
uvrC	ZONMW-53_02489	uvrC
uvrC	ZONMW-53_02949	uvrC
uvrC	ZONMW-53_00335	uvrC
uvrC	ZONMW-54_01198	uvrC
uvrC	ZONMW-54_01139	uvrC
uvrC	ZONMW-54_02549	uvrC
uvrC	ZONMW-54_02953	uvrC
uvrC	ZONMW-54_00699	uvrC

uvrC	ZONMW-55_02306	uvrC
uvrC	ZONMW-55_02252	uvrC
uvrC	ZONMW-55_01664	uvrC
uvrC	ZONMW-55_02957	uvrC
uvrC	ZONMW-55_00328	uvrC
uvrC	ZONMW-56_02050	uvrC
uvrC	ZONMW-56_02116	uvrC
uvrC	ZONMW-56_01817	uvrC
uvrC	ZONMW-56_02837	uvrC
uvrC	ZONMW-56_00987	uvrC
uvrC	ZONMW-57_00166	uvrC
uvrC	ZONMW-57_00104	uvrC
uvrC	ZONMW-57_02323	uvrC
uvrC	ZONMW-57_02905	uvrC
uvrC	ZONMW-57_01287	uvrC
uvrC	ZONMW-58_00805	uvrC
uvrC	ZONMW-58_00862	uvrC
uvrC	ZONMW-58_02347	uvrC
uvrC	ZONMW-58_02929	uvrC
uvrC	ZONMW-58_01297	uvrC
uvrC	ZONMW-59_00159	uvrC
uvrC	ZONMW-59_00104	uvrC
uvrC	ZONMW-59_02561	uvrC
uvrC	ZONMW-59_03044	uvrC
uvrC	ZONMW-59_01288	uvrC
uvrC	ZONMW-60_00192	uvrC
uvrC	ZONMW-60_00105	uvrC
uvrC	ZONMW-60_02430	uvrC
uvrC	ZONMW-60_03022	uvrC
uvrC	ZONMW-60_01282	uvrC
uvrC	ZONMW-61_01253	uvrC
uvrC	ZONMW-61_01419	uvrC
uvrC	ZONMW-61_02016	uvrC
uvrC	ZONMW-61_02806	uvrC
uvrC	ZONMW-61_03948	uvrC
uvrC	ZONMW-61_03799	uvrC
uvrC	ZONMW-62_02105	uvrC
uvrC	ZONMW-62_00226	uvrC
uvrC	ZONMW-62_00699	uvrC
uvrC	ZONMW-62_03493	uvrC
uvrC	ZONMW-62_01554	uvrC
uvrC	ZONMW-62_00947	uvrC
uvrC	ZONMW-63_02563	uvrC
uvrC	ZONMW-63_01868	uvrC
uvrC	ZONMW-63_02492	uvrC
uvrC	ZONMW-63_02805	uvrC
uvrC	ZONMW-63_01278	uvrC
uvrC	ZONMW-64_00901	uvrC

uvrC	ZONMW-64_03344	uvrC
uvrC	ZONMW-64_02271	uvrC
uvrC	ZONMW-64_03780	uvrC
uvrC	ZONMW-64_03807	uvrC
uvrC	ZONMW-65_02189	uvrC
uvrC	ZONMW-65_00611	uvrC
uvrC	ZONMW-65_03763	uvrC
uvrC	ZONMW-65_03245	uvrC
uvrC	ZONMW-66_01571	uvrC
uvrC	ZONMW-66_01938	uvrC
uvrC	ZONMW-67_02715	uvrC
uvrC	ZONMW-67_02010	uvrC
uvrC	ZONMW-67_01358	uvrC
uvrC	ZONMW-67_01724	uvrC
uvrC	ZONMW-67_02965	uvrC
uvrC	ZONMW-68_03126	uvrC
uvrC	ZONMW-68_04479	uvrC
uvrC	ZONMW-68_01075	uvrC
uvrC	ZONMW-68_02235	uvrC
uvrC	ZONMW-68_00641	uvrC
uvrC	ZONMW-68_01298	uvrC
uvrC	ZONMW-69_00512	uvrC
uvrC	ZONMW-69_01933	uvrC
uvrC	ZONMW-69_01642	uvrC
uvrC	ZONMW-69_02219	uvrC
uvrC	ZONMW-69_02989	uvrC
uvrC	ZONMW-72_03236	uvrC
uvrC	ZONMW-72_02349	uvrC
uvrC	ZONMW-72_02106	uvrC
uvrC	ZONMW-72_02723	uvrC
uvrC	ZONMW-72_02951	uvrC
uvrC	ZONMW-72_03598	uvrC
uvrC	ZONMW-72_02387	uvrC
uvrC	ZONMW-73_03547	uvrC
uvrC	ZONMW-73_00948	uvrC
uvrC	ZONMW-73_01552	uvrC
uvrC	ZONMW-73_00081	uvrC
uvrC	ZONMW-73_02931	uvrC
uvrC	ZONMW-74_00430	uvrC
uvrC	ZONMW-74_00554	uvrC
uvrC	ZONMW-74_02395	uvrC
uvrC	ZONMW-74_02135	uvrC
uvrC	ZONMW-74_01094	uvrC
uvrC	ZONMW-75_03013	uvrC
uvrC	ZONMW-75_01993	uvrC
uvrC	ZONMW-75_03749	uvrC
uvrC	ZONMW-75_02219	uvrC
uvrC	ZONMW-75_02825	uvrC

uvrC	da5A-8_01316	uvrC
uvrC	da5A-8_03413	uvrC
uvrC	da5A-8_01248	uvrC
htpG	18_01900	htpG
htpG	18_02550	htpG
htpG	ACAM48_03126	htpG
htpG	CAM030_01617	htpG
htpG	CECT7946_03208	htpG
htpG	CGMCC1.8863_02764	htpG
htpG	CL8139_00051	htpG
htpG	DAU203_00516	htpG
htpG	DD5b_00432	htpG
htpG	DSM1208_00583	htpG
htpG	DSM12212_03069	htpG
htpG	DSM15343_01196	htpG
htpG	DSM15361_02240	htpG
htpG	DSM15365_00227	htpG
htpG	DSM15718_00798	htpG
htpG	DSM16505T_01873	htpG
htpG	DSM18103_03663	htpG
htpG	DSM18293_02545	htpG
htpG	DSM18293_04012	htpG
htpG	DSM18841_01603	htpG
htpG	DSM18842_02288	htpG
htpG	DSM19866_00851	htpG
htpG	DSM2041_02731	htpG
htpG	DSM2061_02077	htpG
htpG	DSM2064_04823	htpG
htpG	DSM2064_03113	htpG
htpG	DSM21164_02858	htpG
htpG	DSM23404_00245	htpG
htpG	DSM23424_01773	htpG
htpG	DSM24786_02593	htpG
htpG	DSM7489_00500	htpG
htpG	DSW1_02520	htpG
htpG	DSW5_00349	htpG
htpG	DSW6_02430	htpG
htpG	DSW8_03422	htpG
htpG	F52_01632	htpG
htpG	GCA_001027945.1_02855	htpG
htpG	HI1_00432	htpG
htpG	IR1_02952	htpG
htpG	KCOM3131_00982	htpG
htpG	KCTC12974_00623	htpG
htpG	KL-A_01706	htpG
htpG	KL-A_03320	htpG
htpG	L12_00263	htpG
htpG	LL0412.1.7_02616	htpG

htpG	MAR_2009_71_00162	htpG
htpG	NN016038_01929	htpG
htpG	PAO1_01640	htpG
htpG	PAO1_01104	htpG
htpG	UW101_00279	htpG
htpG	UW101_02687	htpG
htpG	V4-33_01418	htpG
htpG	W5C_02781	htpG
htpG	ZONMW-01_01956	htpG
htpG	ZONMW-02_03277	htpG
htpG	ZONMW-03_02940	htpG
htpG	ZONMW-04_02507	htpG
htpG	ZONMW-05_02138	htpG
htpG	ZONMW-06_02578	htpG
htpG	ZONMW-07_04066	htpG
htpG	ZONMW-08_02888	htpG
htpG	ZONMW-09_02011	htpG
htpG	ZONMW-10_01249	htpG
htpG	ZONMW-11_03659	htpG
htpG	ZONMW-12_03427	htpG
htpG	ZONMW-12_02742	htpG
htpG	ZONMW-13_01508	htpG
htpG	ZONMW-14_02279	htpG
htpG	ZONMW-15_02190	htpG
htpG	ZONMW-19_04376	htpG
htpG	ZONMW-20_01280	htpG
htpG	ZONMW-21_03326	htpG
htpG	ZONMW-22_03399	htpG
htpG	ZONMW-23_03372	htpG
htpG	ZONMW-24_03374	htpG
htpG	ZONMW-25_03447	htpG
htpG	ZONMW-25_00882	htpG
htpG	ZONMW-26_02807	htpG
htpG	ZONMW-27_02534	htpG
htpG	ZONMW-28_00055	htpG
htpG	ZONMW-29_02179	htpG
htpG	ZONMW-29_01978	htpG
htpG	ZONMW-30_03230	htpG
htpG	ZONMW-31_02723	htpG
htpG	ZONMW-32_00846	htpG
htpG	ZONMW-33_00836	htpG
htpG	ZONMW-34_02737	htpG
htpG	ZONMW-35_00114	htpG
htpG	ZONMW-36_00055	htpG
htpG	ZONMW-37_03408	htpG
htpG	ZONMW-38_00117	htpG
htpG	ZONMW-39_03459	htpG
htpG	ZONMW-40_01197	htpG

htpG	ZONMW-41_03143	htpG
htpG	ZONMW-42_02605	htpG
htpG	ZONMW-43_02447	htpG
htpG	ZONMW-44_03455	htpG
htpG	ZONMW-45_00854	htpG
htpG	ZONMW-46_01020	htpG
htpG	ZONMW-47_01153	htpG
htpG	ZONMW-48_02617	htpG
htpG	ZONMW-49_02024	htpG
htpG	ZONMW-50_02620	htpG
htpG	ZONMW-51_02330	htpG
htpG	ZONMW-52_02742	htpG
htpG	ZONMW-53_02495	htpG
htpG	ZONMW-53_03295	htpG
htpG	ZONMW-54_02555	htpG
htpG	ZONMW-55_01658	htpG
htpG	ZONMW-56_01811	htpG
htpG	ZONMW-57_02329	htpG
htpG	ZONMW-58_02353	htpG
htpG	ZONMW-59_02567	htpG
htpG	ZONMW-60_02436	htpG
htpG	ZONMW-61_02815	htpG
htpG	ZONMW-61_01381	htpG
htpG	ZONMW-62_03501	htpG
htpG	ZONMW-63_02498	htpG
htpG	ZONMW-64_02424	htpG
htpG	ZONMW-65_03279	htpG
htpG	ZONMW-66_03294	htpG
htpG	ZONMW-67_01881	htpG
htpG	ZONMW-68_02244	htpG
htpG	ZONMW-69_02062	htpG
htpG	ZONMW-72_01267	htpG
htpG	ZONMW-73_01540	htpG
htpG	ZONMW-74_02401	htpG
htpG	ZONMW-75_03740	htpG
htpG	da5A-8_03646	htpG
hisS	18_03628	hisS
hisS	ACAM48_00886	hisS
hisS	CAM030_00281	hisS
hisS	CECT7946_02894	hisS
hisS	CGMCC1.8863_01811	hisS
hisS	CL8139_01820	hisS
hisS	DAU203_00323	hisS
hisS	DD5b_00693	hisS
hisS	DSM1208_00021	hisS
hisS	DSM12212_01950	hisS
hisS	DSM15343_02775	hisS
hisS	DSM15361_02019	hisS

hisS	DSM15365_00093	hisS
hisS	DSM15718_01121	hisS
hisS	DSM16505T_02382	hisS
hisS	DSM18103_00311	hisS
hisS	DSM18293_01603	hisS
hisS	DSM18841_03055	hisS
hisS	DSM18842_00046	hisS
hisS	DSM19866_04733	hisS
hisS	DSM2041_01402	hisS
hisS	DSM2061_00532	hisS
hisS	DSM2064_03619	hisS
hisS	DSM21164_02338	hisS
hisS	DSM23404_03205	hisS
hisS	DSM23424_01398	hisS
hisS	DSM24786_02396	hisS
hisS	DSM7489_00311	hisS
hisS	DSW1_01922	hisS
hisS	DSW5_01928	hisS
hisS	DSW6_02846	hisS
hisS	DSW8_00595	hisS
hisS	F52_03774	hisS
hisS	GCA_001027945.1_01061	hisS
hisS	HI1_00243	hisS
hisS	IR1_01670	hisS
hisS	KCOM3131_00909	hisS
hisS	KCTC12974_00448	hisS
hisS	KL-A_02686	hisS
hisS	L12_00293	hisS
hisS	LL0412.1.7_01282	hisS
hisS	MAR_2009_71_03392	hisS
hisS	NN016038_03668	hisS
hisS	PAO1_03926	hisS
hisS	UW101_05267	hisS
hisS	V4-33_01936	hisS
hisS	W5C_01597	hisS
hisS	ZONMW-01_01755	hisS
hisS	ZONMW-02_00898	hisS
hisS	ZONMW-03_02740	hisS
hisS	ZONMW-04_02783	hisS
hisS	ZONMW-05_02333	hisS
hisS	ZONMW-06_02480	hisS
hisS	ZONMW-07_04101	hisS
hisS	ZONMW-08_02535	hisS
hisS	ZONMW-09_02236	hisS
hisS	ZONMW-10_01613	hisS
hisS	ZONMW-11_01127	hisS
hisS	ZONMW-12_00752	hisS
hisS	ZONMW-13_02179	hisS

hisS	ZONMW-14_00806	hisS
hisS	ZONMW-15_02406	hisS
hisS	ZONMW-19_02451	hisS
hisS	ZONMW-20_03020	hisS
hisS	ZONMW-21_01618	hisS
hisS	ZONMW-22_02106	hisS
hisS	ZONMW-23_00445	hisS
hisS	ZONMW-24_02731	hisS
hisS	ZONMW-25_01413	hisS
hisS	ZONMW-26_02999	hisS
hisS	ZONMW-27_03231	hisS
hisS	ZONMW-28_00151	hisS
hisS	ZONMW-29_03170	hisS
hisS	ZONMW-30_02161	hisS
hisS	ZONMW-31_02797	hisS
hisS	ZONMW-32_00942	hisS
hisS	ZONMW-33_00932	hisS
hisS	ZONMW-34_01139	hisS
hisS	ZONMW-35_00212	hisS
hisS	ZONMW-36_00151	hisS
hisS	ZONMW-37_01996	hisS
hisS	ZONMW-38_02336	hisS
hisS	ZONMW-39_00866	hisS
hisS	ZONMW-40_02471	hisS
hisS	ZONMW-41_01382	hisS
hisS	ZONMW-42_02697	hisS
hisS	ZONMW-43_03162	hisS
hisS	ZONMW-44_03356	hisS
hisS	ZONMW-45_02844	hisS
hisS	ZONMW-46_02841	hisS
hisS	ZONMW-47_02832	hisS
hisS	ZONMW-48_03081	hisS
hisS	ZONMW-49_01840	hisS
hisS	ZONMW-50_03021	hisS
hisS	ZONMW-51_02123	hisS
hisS	ZONMW-52_02552	hisS
hisS	ZONMW-53_02302	hisS
hisS	ZONMW-54_02345	hisS
hisS	ZONMW-55_01849	hisS
hisS	ZONMW-56_01997	hisS
hisS	ZONMW-57_02137	hisS
hisS	ZONMW-58_02161	hisS
hisS	ZONMW-59_02380	hisS
hisS	ZONMW-60_02225	hisS
hisS	ZONMW-61_02079	hisS
hisS	ZONMW-62_00167	hisS
hisS	ZONMW-63_03128	hisS
hisS	ZONMW-64_02332	hisS

hisS	ZONMW-65_01695	hisS
hisS	ZONMW-66_00679	hisS
hisS	ZONMW-66_04643	hisS
hisS	ZONMW-67_01940	hisS
hisS	ZONMW-68_01020	hisS
hisS	ZONMW-69_02003	hisS
hisS	ZONMW-72_02167	hisS
hisS	ZONMW-73_00765	hisS
hisS	ZONMW-74_02277	hisS
hisS	ZONMW-75_01485	hisS
hisS	da5A-8_02227	hisS
surE	18_00493	surE
surE	ACAM48_02897	surE
surE	CAM030_02269	surE
surE	CECT7946_00740	surE
surE	CGMCC1.8863_02490	surE
surE	CL8139_02434	surE
surE	DAU203_00866	surE
surE	DD5b_01753	surE
surE	DSM1208_01142	surE
surE	DSM12212_02649	surE
surE	DSM15343_00660	surE
surE	DSM15361_00188	surE
surE	DSM15365_02348	surE
surE	DSM15718_02198	surE
surE	DSM16505T_00021	surE
surE	DSM18103_01566	surE
surE	DSM18293_00518	surE
surE	DSM18841_02492	surE
surE	DSM18842_02399	surE
surE	DSM19866_03777	surE
surE	DSM2041_05179	surE
surE	DSM2061_02656	surE
surE	DSM2064_02440	surE
surE	DSM21164_01331	surE
surE	DSM23404_01760	surE
surE	DSM23424_00843	surE
surE	DSM24786_01320	surE
surE	DSM7489_00799	surE
surE	DSW1_00086	surE
surE	DSW5_01691	surE
surE	DSW6_01980	surE
surE	DSW8_02796	surE
surE	DSW8_01375	surE
surE	F52_02847	surE
surE	GCA_001027945.1_01110	surE
surE	HI1_00734	surE
surE	IR1_00433	surE

surE	KCOM3131_01014	surE
surE	KCTC12974_01366	surE
surE	KCTC12974_01735	surE
surE	KL-A_02427	surE
surE	L12_00352	surE
surE	LL0412.1.7_00022	surE
surE	MAR_2009_71_00658	surE
surE	NN016038_00502	surE
surE	PAO1_03748	surE
surE	UW101_01494	surE
surE	V4-33_02253	surE
surE	W5C_01425	surE
surE	ZONMW-01_02692	surE
surE	ZONMW-02_02325	surE
surE	ZONMW-03_01718	surE
surE	ZONMW-04_02274	surE
surE	ZONMW-05_02790	surE
surE	ZONMW-06_03896	surE
surE	ZONMW-07_00503	surE
surE	ZONMW-08_00174	surE
surE	ZONMW-09_00664	surE
surE	ZONMW-10_00018	surE
surE	ZONMW-11_00032	surE
surE	ZONMW-12_01036	surE
surE	ZONMW-13_00039	surE
surE	ZONMW-14_02432	surE
surE	ZONMW-15_01034	surE
surE	ZONMW-19_00770	surE
surE	ZONMW-20_00319	surE
surE	ZONMW-21_01351	surE
surE	ZONMW-22_01353	surE
surE	ZONMW-23_02466	surE
surE	ZONMW-24_00108	surE
surE	ZONMW-25_02862	surE
surE	ZONMW-26_02130	surE
surE	ZONMW-27_02253	surE
surE	ZONMW-28_03839	surE
surE	ZONMW-29_00898	surE
surE	ZONMW-30_00631	surE
surE	ZONMW-31_02579	surE
surE	ZONMW-31_03738	surE
surE	ZONMW-32_03777	surE
surE	ZONMW-33_03770	surE
surE	ZONMW-34_03668	surE
surE	ZONMW-34_02990	surE
surE	ZONMW-35_03825	surE
surE	ZONMW-35_03002	surE
surE	ZONMW-36_03719	surE

surE	ZONMW-37_01011	surE
surE	ZONMW-38_01816	surE
surE	ZONMW-39_04123	surE
surE	ZONMW-39_02768	surE
surE	ZONMW-40_02718	surE
surE	ZONMW-41_01245	surE
surE	ZONMW-42_03190	surE
surE	ZONMW-43_02574	surE
surE	ZONMW-43_03849	surE
surE	ZONMW-44_03830	surE
surE	ZONMW-45_02663	surE
surE	ZONMW-46_02526	surE
surE	ZONMW-47_02516	surE
surE	ZONMW-48_00450	surE
surE	ZONMW-49_02300	surE
surE	ZONMW-50_02160	surE
surE	ZONMW-51_01656	surE
surE	ZONMW-52_01799	surE
surE	ZONMW-53_01957	surE
surE	ZONMW-54_02097	surE
surE	ZONMW-55_01978	surE
surE	ZONMW-56_01458	surE
surE	ZONMW-57_01768	surE
surE	ZONMW-58_01792	surE
surE	ZONMW-59_01604	surE
surE	ZONMW-60_01986	surE
surE	ZONMW-61_01550	surE
surE	ZONMW-62_01301	surE
surE	ZONMW-63_01504	surE
surE	ZONMW-64_03755	surE
surE	ZONMW-64_03833	surE
surE	ZONMW-65_01212	surE
surE	ZONMW-66_00205	surE
surE	ZONMW-67_01750	surE
surE	ZONMW-67_02935	surE
surE	ZONMW-68_00894	surE
surE	ZONMW-69_02193	surE
surE	ZONMW-69_03019	surE
surE	ZONMW-72_00085	surE
surE	ZONMW-73_01253	surE
surE	ZONMW-74_02803	surE
surE	ZONMW-75_00097	surE
surE	da5A-8_00226	surE
ribE	18_00906	ribE
ribE	ACAM48_02444	ribE
ribE	CAM030_01118	ribE
ribE	CECT7946_00060	ribE
ribE	CGMCC1.8863_02084	ribE

ribE	CL8139_00744	ribE
ribE	DAU203_01383	ribE
ribE	DD5b_01935	ribE
ribE	DSM1208_01566	ribE
ribE	DSM12212_03422	ribE
ribE	DSM15343_02405	ribE
ribE	DSM15361_00475	ribE
ribE	DSM15365_03230	ribE
ribE	DSM15718_02932	ribE
ribE	DSM16505T_00806	ribE
ribE	DSM18103_03623	ribE
ribE	DSM18293_00854	ribE
ribE	DSM18841_00683	ribE
ribE	DSM18842_03041	ribE
ribE	DSM19866_03328	ribE
ribE	DSM2041_00472	ribE
ribE	DSM2061_03093	ribE
ribE	DSM2064_01630	ribE
ribE	DSM21164_00943	ribE
ribE	DSM23404_02076	ribE
ribE	DSM23424_00390	ribE
ribE	DSM24786_02826	ribE
ribE	DSM7489_01293	ribE
ribE	DSW1_00142	ribE
ribE	DSW5_01120	ribE
ribE	DSW6_01191	ribE
ribE	DSW8_02379	ribE
ribE	F52_02086	ribE
ribE	GCA_001027945.1_03108	ribE
ribE	HI1_01235	ribE
ribE	IR1_01132	ribE
ribE	KCOM3131_00124	ribE
ribE	KCTC12974_02916	ribE
ribE	KL-A_02182	ribE
ribE	L12_00527	ribE
ribE	LL0412.1.7_00817	ribE
ribE	MAR_2009_71_01060	ribE
ribE	NN016038_00915	ribE
ribE	PAO1_04183	ribE
ribE	UW101_00684	ribE
ribE	V4-33_02304	ribE
ribE	W5C_01670	ribE
ribE	ZONMW-01_00980	ribE
ribE	ZONMW-02_00162	ribE
ribE	ZONMW-03_02049	ribE
ribE	ZONMW-04_01770	ribE
ribE	ZONMW-05_02049	ribE
ribE	ZONMW-06_03345	ribE

ribE	ZONMW-07_02043	ribE
ribE	ZONMW-08_00578	ribE
ribE	ZONMW-09_00410	ribE
ribE	ZONMW-10_00198	ribE
ribE	ZONMW-11_04543	ribE
ribE	ZONMW-12_00336	ribE
ribE	ZONMW-13_00419	ribE
ribE	ZONMW-14_00145	ribE
ribE	ZONMW-15_01693	ribE
ribE	ZONMW-19_01937	ribE
ribE	ZONMW-19_04718	ribE
ribE	ZONMW-20_01569	ribE
ribE	ZONMW-21_00924	ribE
ribE	ZONMW-22_00925	ribE
ribE	ZONMW-23_02038	ribE
ribE	ZONMW-24_00536	ribE
ribE	ZONMW-25_03034	ribE
ribE	ZONMW-26_02351	ribE
ribE	ZONMW-27_01531	ribE
ribE	ZONMW-28_03224	ribE
ribE	ZONMW-29_00508	ribE
ribE	ZONMW-30_01847	ribE
ribE	ZONMW-31_01783	ribE
ribE	ZONMW-32_00749	ribE
ribE	ZONMW-33_00617	ribE
ribE	ZONMW-34_00981	ribE
ribE	ZONMW-35_02262	ribE
ribE	ZONMW-36_00754	ribE
ribE	ZONMW-37_01438	ribE
ribE	ZONMW-38_00666	ribE
ribE	ZONMW-39_02852	ribE
ribE	ZONMW-40_00320	ribE
ribE	ZONMW-41_02414	ribE
ribE	ZONMW-41_01487	ribE
ribE	ZONMW-42_00904	ribE
ribE	ZONMW-43_00514	ribE
ribE	ZONMW-44_03729	ribE
ribE	ZONMW-45_01038	ribE
ribE	ZONMW-46_00629	ribE
ribE	ZONMW-47_00629	ribE
ribE	ZONMW-48_00079	ribE
ribE	ZONMW-49_02644	ribE
ribE	ZONMW-50_02438	ribE
ribE	ZONMW-51_02634	ribE
ribE	ZONMW-52_02127	ribE
ribE	ZONMW-53_02712	ribE
ribE	ZONMW-54_02855	ribE
ribE	ZONMW-55_02712	ribE

ribE	ZONMW-56_02483	ribE
ribE	ZONMW-57_02616	ribE
ribE	ZONMW-58_02641	ribE
ribE	ZONMW-59_02649	ribE
ribE	ZONMW-60_02746	ribE
ribE	ZONMW-61_00154	ribE
ribE	ZONMW-62_01707	ribE
ribE	ZONMW-63_02162	ribE
ribE	ZONMW-64_00532	ribE
ribE	ZONMW-65_01781	ribE
ribE	ZONMW-66_01495	ribE
ribE	ZONMW-66_04741	ribE
ribE	ZONMW-67_01198	ribE
ribE	ZONMW-68_04316	ribE
ribE	ZONMW-69_01244	ribE
ribE	ZONMW-72_01652	ribE
ribE	ZONMW-73_02268	ribE
ribE	ZONMW-74_01637	ribE
ribE	ZONMW-75_01030	ribE
ribE	da5A-8_01835	ribE
dapB	18_02251	dapB
dapB	ACAM48_00162	dapB
dapB	CAM030_00084	dapB
dapB	CECT7946_03000	dapB
dapB	CGMCC1.8863_01162	dapB
dapB	CL8139_01168	dapB
dapB	DAU203_00257	dapB
dapB	DD5b_00490	dapB
dapB	DSM1208_03560	dapB
dapB	DSM12212_02489	dapB
dapB	DSM15343_01801	dapB
dapB	DSM15361_01756	dapB
dapB	DSM15365_03856	dapB
dapB	DSM15718_02892	dapB
dapB	DSM16505T_02141	dapB
dapB	DSM18103_02238	dapB
dapB	DSM18293_02600	dapB
dapB	DSM18293_04261	dapB
dapB	DSM18841_01503	dapB
dapB	DSM18842_01875	dapB
dapB	DSM19866_04582	dapB
dapB	DSM2041_03448	dapB
dapB	DSM2061_01504	dapB
dapB	DSM2064_04879	dapB
dapB	DSM2064_00757	dapB
dapB	DSM21164_03135	dapB
dapB	DSM23404_01332	dapB
dapB	DSM23424_01651	dapB

dapB	DSM24786_01057	dapB
dapB	DSM7489_00250	dapB
dapB	DSW1_02784	dapB
dapB	DSW5_00088	dapB
dapB	DSW6_02772	dapB
dapB	DSW8_00946	dapB
dapB	F52_01686	dapB
dapB	F52_00884	dapB
dapB	GCA_001027945.1_02807	dapB
dapB	HI1_00182	dapB
dapB	IR1_02889	dapB
dapB	IR1_03484	dapB
dapB	KCOM3131_00794	dapB
dapB	KCTC12974_00031	dapB
dapB	KL-A_01246	dapB
dapB	L12_00051	dapB
dapB	LL0412.1.7_01453	dapB
dapB	MAR_2009_71_02495	dapB
dapB	NN016038_02263	dapB
dapB	PAO1_04921	dapB
dapB	UW101_00223	dapB
dapB	UW101_03468	dapB
dapB	V4-33_01038	dapB
dapB	W5C_00159	dapB
dapB	ZONMW-01_02134	dapB
dapB	ZONMW-02_03102	dapB
dapB	ZONMW-03_02576	dapB
dapB	ZONMW-04_01979	dapB
dapB	ZONMW-05_02390	dapB
dapB	ZONMW-06_00304	dapB
dapB	ZONMW-07_02217	dapB
dapB	ZONMW-08_03494	dapB
dapB	ZONMW-09_00926	dapB
dapB	ZONMW-10_01666	dapB
dapB	ZONMW-11_02516	dapB
dapB	ZONMW-12_03306	dapB
dapB	ZONMW-13_02501	dapB
dapB	ZONMW-14_02502	dapB
dapB	ZONMW-15_02711	dapB
dapB	ZONMW-19_00684	dapB
dapB	ZONMW-20_01027	dapB
dapB	ZONMW-21_02768	dapB
dapB	ZONMW-21_03237	dapB
dapB	ZONMW-22_02341	dapB
dapB	ZONMW-22_03279	dapB
dapB	ZONMW-23_00209	dapB
dapB	ZONMW-23_03301	dapB
dapB	ZONMW-24_02106	dapB

dapB	ZONMW-24_03302	dapB
dapB	ZONMW-25_00632	dapB
dapB	ZONMW-26_02726	dapB
dapB	ZONMW-27_02150	dapB
dapB	ZONMW-28_01533	dapB
dapB	ZONMW-29_01931	dapB
dapB	ZONMW-30_02830	dapB
dapB	ZONMW-31_00617	dapB
dapB	ZONMW-32_01940	dapB
dapB	ZONMW-33_01931	dapB
dapB	ZONMW-34_00266	dapB
dapB	ZONMW-35_01124	dapB
dapB	ZONMW-36_01425	dapB
dapB	ZONMW-37_02435	dapB
dapB	ZONMW-37_03288	dapB
dapB	ZONMW-38_03431	dapB
dapB	ZONMW-39_04031	dapB
dapB	ZONMW-40_01669	dapB
dapB	ZONMW-41_00698	dapB
dapB	ZONMW-42_03796	dapB
dapB	ZONMW-43_00686	dapB
dapB	ZONMW-44_03494	dapB
dapB	ZONMW-45_00950	dapB
dapB	ZONMW-46_00924	dapB
dapB	ZONMW-47_01249	dapB
dapB	ZONMW-48_02814	dapB
dapB	ZONMW-49_02427	dapB
dapB	ZONMW-50_02221	dapB
dapB	ZONMW-51_02404	dapB
dapB	ZONMW-52_02387	dapB
dapB	ZONMW-53_02246	dapB
dapB	ZONMW-54_02736	dapB
dapB	ZONMW-55_02495	dapB
dapB	ZONMW-56_01734	dapB
dapB	ZONMW-57_02081	dapB
dapB	ZONMW-58_01997	dapB
dapB	ZONMW-59_02216	dapB
dapB	ZONMW-60_02512	dapB
dapB	ZONMW-61_03590	dapB
dapB	ZONMW-62_01043	dapB
dapB	ZONMW-63_01602	dapB
dapB	ZONMW-64_00948	dapB
dapB	ZONMW-65_03407	dapB
dapB	ZONMW-66_00457	dapB
dapB	ZONMW-67_02678	dapB
dapB	ZONMW-68_01171	dapB
dapB	ZONMW-69_00475	dapB
dapB	ZONMW-72_01456	dapB

dapB	ZONMW-72_03581	dapB
dapB	ZONMW-73_01175	dapB
dapB	ZONMW-74_02573	dapB
dapB	ZONMW-75_03486	dapB
dapB	da5A-8_02916	dapB
rpoE_2	18_00166	rpoE_2
rpoE_2	18_00849	rpoE_2
rpoE_2	18_02970	rpoE_2
rpoE_2	18_00361	rpoE_2
rpoE_2	18_01087	rpoE_2
rpoE_2	18_03696	rpoE_2
rpoE_2	18_02825	rpoE_2
rpoE_2	18_00315	rpoE_2
rpoE_2	18_02313	rpoE_2
rpoE_2	18_00322	rpoE_2
rpoE_2	18_02555	rpoE_2
rpoE_2	18_02823	rpoE_2
rpoE_2	18_01802	rpoE_2
rpoE_2	18_03757	rpoE_2
rpoE_2	18_00844	rpoE_2
rpoE_2	18_00959	rpoE_2
rpoE_2	18_03516	rpoE_2
rpoE_2	18_01070	rpoE_2
rpoE_2	18_01686	rpoE_2
rpoE_2	18_02123	rpoE_2
rpoE_2	18_01799	rpoE_2
rpoE_2	18_02287	rpoE_2
rpoE_2	18_03232	rpoE_2
rpoE_2	18_03660	rpoE_2
rpoE_2	ACAM48_01576	rpoE_2
rpoE_2	ACAM48_01069	rpoE_2
rpoE_2	ACAM48_00274	rpoE_2
rpoE_2	ACAM48_01276	rpoE_2
rpoE_2	ACAM48_01866	rpoE_2
rpoE_2	ACAM48_01041	rpoE_2
rpoE_2	ACAM48_02482	rpoE_2
rpoE_2	ACAM48_00350	rpoE_2
rpoE_2	ACAM48_03364	rpoE_2
rpoE_2	ACAM48_01114	rpoE_2
rpoE_2	ACAM48_03414	rpoE_2
rpoE_2	ACAM48_01121	rpoE_2
rpoE_2	ACAM48_00912	rpoE_2
rpoE_2	CAM030_02855	rpoE_2
rpoE_2	CAM030_02762	rpoE_2
rpoE_2	CAM030_00205	rpoE_2
rpoE_2	CAM030_00532	rpoE_2
rpoE_2	CAM030_00218	rpoE_2
rpoE_2	CAM030_02646	rpoE_2

rpoE_2	CAM030_01551	rpoE_2
rpoE_2	CAM030_02661	rpoE_2
rpoE_2	CAM030_01669	rpoE_2
rpoE_2	CAM030_01432	rpoE_2
rpoE_2	CAM030_00958	rpoE_2
rpoE_2	CAM030_02056	rpoE_2
rpoE_2	CAM030_02666	rpoE_2
rpoE_2	CAM030_00188	rpoE_2
rpoE_2	CAM030_00247	rpoE_2
rpoE_2	CAM030_00172	rpoE_2
rpoE_2	CECT7946_03274	rpoE_2
rpoE_2	CECT7946_02965	rpoE_2
rpoE_2	CECT7946_00064	rpoE_2
rpoE_2	CECT7946_03105	rpoE_2
rpoE_2	CECT7946_02934	rpoE_2
rpoE_2	CECT7946_02324	rpoE_2
rpoE_2	CECT7946_03390	rpoE_2
rpoE_2	CECT7946_01073	rpoE_2
rpoE_2	CECT7946_01568	rpoE_2
rpoE_2	CECT7946_00957	rpoE_2
rpoE_2	CECT7946_02421	rpoE_2
rpoE_2	CECT7946_01303	rpoE_2
rpoE_2	CECT7946_00797	rpoE_2
rpoE_2	CECT7946_02703	rpoE_2
rpoE_2	CECT7946_01533	rpoE_2
rpoE_2	CECT7946_00205	rpoE_2
rpoE_2	CECT7946_01550	rpoE_2
rpoE_2	CECT7946_03222	rpoE_2
rpoE_2	CECT7946_03532	rpoE_2
rpoE_2	CECT7946_02241	rpoE_2
rpoE_2	CECT7946_00962	rpoE_2
rpoE_2	CECT7946_01659	rpoE_2
rpoE_2	CECT7946_03087	rpoE_2
rpoE_2	CECT7946_03143	rpoE_2
rpoE_2	CGMCC1.8863_01453	rpoE_2
rpoE_2	CGMCC1.8863_02541	rpoE_2
rpoE_2	CGMCC1.8863_03149	rpoE_2
rpoE_2	CGMCC1.8863_01029	rpoE_2
rpoE_2	CGMCC1.8863_01747	rpoE_2
rpoE_2	CGMCC1.8863_01977	rpoE_2
rpoE_2	CGMCC1.8863_01384	rpoE_2
rpoE_2	CGMCC1.8863_03276	rpoE_2
rpoE_2	CGMCC1.8863_01603	rpoE_2
rpoE_2	CGMCC1.8863_02355	rpoE_2
rpoE_2	CGMCC1.8863_01606	rpoE_2
rpoE_2	CGMCC1.8863_02356	rpoE_2
rpoE_2	CGMCC1.8863_00694	rpoE_2
rpoE_2	CGMCC1.8863_00061	rpoE_2

rpoE_2	CGMCC1.8863_03101	rpoE_2
rpoE_2	CGMCC1.8863_02017	rpoE_2
rpoE_2	CGMCC1.8863_01866	rpoE_2
rpoE_2	CGMCC1.8863_01892	rpoE_2
rpoE_2	CGMCC1.8863_00420	rpoE_2
rpoE_2	CGMCC1.8863_00862	rpoE_2
rpoE_2	CGMCC1.8863_01778	rpoE_2
rpoE_2	CGMCC1.8863_03339	rpoE_2
rpoE_2	CGMCC1.8863_00323	rpoE_2
rpoE_2	CL8139_01791	rpoE_2
rpoE_2	CL8139_02633	rpoE_2
rpoE_2	CL8139_01899	rpoE_2
rpoE_2	CL8139_03131	rpoE_2
rpoE_2	CL8139_01509	rpoE_2
rpoE_2	CL8139_00839	rpoE_2
rpoE_2	CL8139_02926	rpoE_2
rpoE_2	CL8139_02929	rpoE_2
rpoE_2	CL8139_02666	rpoE_2
rpoE_2	CL8139_02835	rpoE_2
rpoE_2	CL8139_03116	rpoE_2
rpoE_2	CL8139_00381	rpoE_2
rpoE_2	CL8139_03321	rpoE_2
rpoE_2	CL8139_01237	rpoE_2
rpoE_2	CL8139_00053	rpoE_2
rpoE_2	CL8139_01218	rpoE_2
rpoE_2	CL8139_00652	rpoE_2
rpoE_2	CL8139_02317	rpoE_2
rpoE_2	DAU203_02302	rpoE_2
rpoE_2	DAU203_02733	rpoE_2
rpoE_2	DAU203_00305	rpoE_2
rpoE_2	DAU203_03210	rpoE_2
rpoE_2	DAU203_02591	rpoE_2
rpoE_2	DAU203_01329	rpoE_2
rpoE_2	DAU203_02604	rpoE_2
rpoE_2	DAU203_02210	rpoE_2
rpoE_2	DAU203_00518	rpoE_2
rpoE_2	DAU203_02908	rpoE_2
rpoE_2	DAU203_00610	rpoE_2
rpoE_2	DAU203_00613	rpoE_2
rpoE_2	DAU203_02700	rpoE_2
rpoE_2	DAU203_00053	rpoE_2
rpoE_2	DAU203_02618	rpoE_2
rpoE_2	DAU203_01539	rpoE_2
rpoE_2	DAU203_02119	rpoE_2
rpoE_2	DAU203_00188	rpoE_2
rpoE_2	DAU203_00207	rpoE_2
rpoE_2	DAU203_02985	rpoE_2
rpoE_2	DAU203_00153	rpoE_2

rpoE_2	DD5b_01286	rpoE_2
rpoE_2	DD5b_00364	rpoE_2
rpoE_2	DD5b_00070	rpoE_2
rpoE_2	DD5b_00474	rpoE_2
rpoE_2	DD5b_01034	rpoE_2
rpoE_2	DD5b_02691	rpoE_2
rpoE_2	DD5b_02257	rpoE_2
rpoE_2	DD5b_00131	rpoE_2
rpoE_2	DD5b_01885	rpoE_2
rpoE_2	DD5b_00707	rpoE_2
rpoE_2	DD5b_01181	rpoE_2
rpoE_2	DD5b_02316	rpoE_2
rpoE_2	DD5b_01320	rpoE_2
rpoE_2	DD5b_02845	rpoE_2
rpoE_2	DSM1208_02480	rpoE_2
rpoE_2	DSM1208_02946	rpoE_2
rpoE_2	DSM1208_01023	rpoE_2
rpoE_2	DSM1208_02896	rpoE_2
rpoE_2	DSM1208_02229	rpoE_2
rpoE_2	DSM1208_00965	rpoE_2
rpoE_2	DSM1208_00345	rpoE_2
rpoE_2	DSM1208_00317	rpoE_2
rpoE_2	DSM1208_03385	rpoE_2
rpoE_2	DSM1208_00302	rpoE_2
rpoE_2	DSM1208_02208	rpoE_2
rpoE_2	DSM1208_03909	rpoE_2
rpoE_2	DSM1208_03789	rpoE_2
rpoE_2	DSM1208_00086	rpoE_2
rpoE_2	DSM1208_00890	rpoE_2
rpoE_2	DSM1208_00978	rpoE_2
rpoE_2	DSM1208_02385	rpoE_2
rpoE_2	DSM1208_03830	rpoE_2
rpoE_2	DSM1208_03912	rpoE_2
rpoE_2	DSM1208_00491	rpoE_2
rpoE_2	DSM1208_00869	rpoE_2
rpoE_2	DSM1208_00439	rpoE_2
rpoE_2	DSM1208_00681	rpoE_2
rpoE_2	DSM1208_03826	rpoE_2
rpoE_2	DSM1208_03774	rpoE_2
rpoE_2	DSM1208_03963	rpoE_2
rpoE_2	DSM1208_01739	rpoE_2
rpoE_2	DSM1208_04324	rpoE_2
rpoE_2	DSM1208_00051	rpoE_2
rpoE_2	DSM1208_00684	rpoE_2
rpoE_2	DSM1208_00511	rpoE_2
rpoE_2	DSM1208_01879	rpoE_2
rpoE_2	DSM12212_01143	rpoE_2
rpoE_2	DSM12212_02825	rpoE_2

rpoE_2	DSM12212_00495	rpoE_2
rpoE_2	DSM12212_02300	rpoE_2
rpoE_2	DSM12212_00455	rpoE_2
rpoE_2	DSM12212_00465	rpoE_2
rpoE_2	DSM12212_01265	rpoE_2
rpoE_2	DSM12212_01042	rpoE_2
rpoE_2	DSM12212_00827	rpoE_2
rpoE_2	DSM12212_02905	rpoE_2
rpoE_2	DSM12212_02909	rpoE_2
rpoE_2	DSM12212_01582	rpoE_2
rpoE_2	DSM12212_01655	rpoE_2
rpoE_2	DSM12212_00138	rpoE_2
rpoE_2	DSM12212_03292	rpoE_2
rpoE_2	DSM12212_02705	rpoE_2
rpoE_2	DSM12212_00790	rpoE_2
rpoE_2	DSM12212_00396	rpoE_2
rpoE_2	DSM12212_02704	rpoE_2
rpoE_2	DSM12212_00511	rpoE_2
rpoE_2	DSM12212_02857	rpoE_2
rpoE_2	DSM15343_01764	rpoE_2
rpoE_2	DSM15343_00285	rpoE_2
rpoE_2	DSM15343_02365	rpoE_2
rpoE_2	DSM15343_01306	rpoE_2
rpoE_2	DSM15343_01787	rpoE_2
rpoE_2	DSM15343_00984	rpoE_2
rpoE_2	DSM15343_01268	rpoE_2
rpoE_2	DSM15343_02021	rpoE_2
rpoE_2	DSM15343_01843	rpoE_2
rpoE_2	DSM15343_02823	rpoE_2
rpoE_2	DSM15343_01084	rpoE_2
rpoE_2	DSM15361_02552	rpoE_2
rpoE_2	DSM15361_01866	rpoE_2
rpoE_2	DSM15361_01007	rpoE_2
rpoE_2	DSM15361_00019	rpoE_2
rpoE_2	DSM15361_00903	rpoE_2
rpoE_2	DSM15361_00897	rpoE_2
rpoE_2	DSM15361_02765	rpoE_2
rpoE_2	DSM15361_01089	rpoE_2
rpoE_2	DSM15361_00435	rpoE_2
rpoE_2	DSM15361_02702	rpoE_2
rpoE_2	DSM15361_00123	rpoE_2
rpoE_2	DSM15361_02775	rpoE_2
rpoE_2	DSM15361_02647	rpoE_2
rpoE_2	DSM15361_02742	rpoE_2
rpoE_2	DSM15365_00186	rpoE_2
rpoE_2	DSM15365_00445	rpoE_2
rpoE_2	DSM15365_02532	rpoE_2
rpoE_2	DSM15365_01528	rpoE_2

rpoE_2	DSM15365_03428	rpoE_2
rpoE_2	DSM15365_02124	rpoE_2
rpoE_2	DSM15365_01506	rpoE_2
rpoE_2	DSM15365_00072	rpoE_2
rpoE_2	DSM15365_03556	rpoE_2
rpoE_2	DSM15365_03455	rpoE_2
rpoE_2	DSM15365_03086	rpoE_2
rpoE_2	DSM15365_02590	rpoE_2
rpoE_2	DSM15365_03815	rpoE_2
rpoE_2	DSM15365_02137	rpoE_2
rpoE_2	DSM15365_01638	rpoE_2
rpoE_2	DSM15365_03810	rpoE_2
rpoE_2	DSM15365_02650	rpoE_2
rpoE_2	DSM15365_00733	rpoE_2
rpoE_2	DSM15365_00447	rpoE_2
rpoE_2	DSM15365_01175	rpoE_2
rpoE_2	DSM15365_03136	rpoE_2
rpoE_2	DSM15365_00225	rpoE_2
rpoE_2	DSM15365_01476	rpoE_2
rpoE_2	DSM15718_00174	rpoE_2
rpoE_2	DSM15718_01081	rpoE_2
rpoE_2	DSM15718_01903	rpoE_2
rpoE_2	DSM15718_02298	rpoE_2
rpoE_2	DSM15718_02036	rpoE_2
rpoE_2	DSM15718_01777	rpoE_2
rpoE_2	DSM15718_02544	rpoE_2
rpoE_2	DSM15718_00821	rpoE_2
rpoE_2	DSM15718_03173	rpoE_2
rpoE_2	DSM15718_03127	rpoE_2
rpoE_2	DSM15718_00470	rpoE_2
rpoE_2	DSM15718_00101	rpoE_2
rpoE_2	DSM15718_02066	rpoE_2
rpoE_2	DSM15718_01038	rpoE_2
rpoE_2	DSM15718_02773	rpoE_2
rpoE_2	DSM16505T_02663	rpoE_2
rpoE_2	DSM16505T_00736	rpoE_2
rpoE_2	DSM16505T_00723	rpoE_2
rpoE_2	DSM16505T_01877	rpoE_2
rpoE_2	DSM16505T_01097	rpoE_2
rpoE_2	DSM16505T_01981	rpoE_2
rpoE_2	DSM16505T_00078	rpoE_2
rpoE_2	DSM16505T_01964	rpoE_2
rpoE_2	DSM16505T_02106	rpoE_2
rpoE_2	DSM16505T_00100	rpoE_2
rpoE_2	DSM16505T_00486	rpoE_2
rpoE_2	DSM16505T_01386	rpoE_2
rpoE_2	DSM16505T_01746	rpoE_2
rpoE_2	DSM16505T_01881	rpoE_2

rpoE_2	DSM16505T_02172	rpoE_2
rpoE_2	DSM16505T_01758	rpoE_2
rpoE_2	DSM16505T_01585	rpoE_2
rpoE_2	DSM18103_03668	rpoE_2
rpoE_2	DSM18103_03104	rpoE_2
rpoE_2	DSM18103_02569	rpoE_2
rpoE_2	DSM18103_01185	rpoE_2
rpoE_2	DSM18103_00465	rpoE_2
rpoE_2	DSM18103_03548	rpoE_2
rpoE_2	DSM18103_01312	rpoE_2
rpoE_2	DSM18103_03680	rpoE_2
rpoE_2	DSM18103_01397	rpoE_2
rpoE_2	DSM18103_00762	rpoE_2
rpoE_2	DSM18103_00609	rpoE_2
rpoE_2	DSM18103_01237	rpoE_2
rpoE_2	DSM18103_00854	rpoE_2
rpoE_2	DSM18103_02820	rpoE_2
rpoE_2	DSM18103_00913	rpoE_2
rpoE_2	DSM18103_00829	rpoE_2
rpoE_2	DSM18103_01396	rpoE_2
rpoE_2	DSM18103_01300	rpoE_2
rpoE_2	DSM18103_00406	rpoE_2
rpoE_2	DSM18103_00783	rpoE_2
rpoE_2	DSM18103_02801	rpoE_2
rpoE_2	DSM18103_03290	rpoE_2
rpoE_2	DSM18293_00308	rpoE_2
rpoE_2	DSM18293_04453	rpoE_2
rpoE_2	DSM18293_01269	rpoE_2
rpoE_2	DSM18293_01305	rpoE_2
rpoE_2	DSM18293_03874	rpoE_2
rpoE_2	DSM18293_04531	rpoE_2
rpoE_2	DSM18293_02956	rpoE_2
rpoE_2	DSM18293_01921	rpoE_2
rpoE_2	DSM18293_02582	rpoE_2
rpoE_2	DSM18293_02738	rpoE_2
rpoE_2	DSM18293_01323	rpoE_2
rpoE_2	DSM18293_02917	rpoE_2
rpoE_2	DSM18293_02080	rpoE_2
rpoE_2	DSM18293_03419	rpoE_2
rpoE_2	DSM18293_01749	rpoE_2
rpoE_2	DSM18293_01584	rpoE_2
rpoE_2	DSM18293_01868	rpoE_2
rpoE_2	DSM18293_03263	rpoE_2
rpoE_2	DSM18293_03838	rpoE_2
rpoE_2	DSM18293_02716	rpoE_2
rpoE_2	DSM18841_02249	rpoE_2
rpoE_2	DSM18841_00615	rpoE_2
rpoE_2	DSM18841_00603	rpoE_2

rpoE_2	DSM18841_01606	rpoE_2
rpoE_2	DSM18841_01266	rpoE_2
rpoE_2	DSM18841_00993	rpoE_2
rpoE_2	DSM18841_02084	rpoE_2
rpoE_2	DSM18841_01718	rpoE_2
rpoE_2	DSM18841_02397	rpoE_2
rpoE_2	DSM18841_02580	rpoE_2
rpoE_2	DSM18841_01470	rpoE_2
rpoE_2	DSM18841_00328	rpoE_2
rpoE_2	DSM18841_01213	rpoE_2
rpoE_2	DSM18841_01849	rpoE_2
rpoE_2	DSM18841_01981	rpoE_2
rpoE_2	DSM18841_01917	rpoE_2
rpoE_2	DSM18841_01022	rpoE_2
rpoE_2	DSM18841_01860	rpoE_2
rpoE_2	DSM18841_02206	rpoE_2
rpoE_2	DSM18842_01604	rpoE_2
rpoE_2	DSM18842_01104	rpoE_2
rpoE_2	DSM18842_01911	rpoE_2
rpoE_2	DSM18842_02960	rpoE_2
rpoE_2	DSM18842_02293	rpoE_2
rpoE_2	DSM18842_00324	rpoE_2
rpoE_2	DSM18842_02230	rpoE_2
rpoE_2	DSM18842_00901	rpoE_2
rpoE_2	DSM18842_03114	rpoE_2
rpoE_2	DSM18842_01312	rpoE_2
rpoE_2	DSM18842_00919	rpoE_2
rpoE_2	DSM18842_00096	rpoE_2
rpoE_2	DSM18842_02141	rpoE_2
rpoE_2	DSM18842_02297	rpoE_2
rpoE_2	DSM18842_02480	rpoE_2
rpoE_2	DSM18842_01841	rpoE_2
rpoE_2	DSM18842_00296	rpoE_2
rpoE_2	DSM18842_02152	rpoE_2
rpoE_2	DSM18842_01549	rpoE_2
rpoE_2	DSM19866_01122	rpoE_2
rpoE_2	DSM19866_02819	rpoE_2
rpoE_2	DSM19866_03116	rpoE_2
rpoE_2	DSM19866_04944	rpoE_2
rpoE_2	DSM19866_04477	rpoE_2
rpoE_2	DSM19866_01686	rpoE_2
rpoE_2	DSM19866_02600	rpoE_2
rpoE_2	DSM19866_03044	rpoE_2
rpoE_2	DSM19866_03413	rpoE_2
rpoE_2	DSM19866_02440	rpoE_2
rpoE_2	DSM19866_04943	rpoE_2
rpoE_2	DSM19866_01591	rpoE_2
rpoE_2	DSM19866_01044	rpoE_2

rpoE_2	DSM19866_00010	rpoE_2
rpoE_2	DSM19866_00483	rpoE_2
rpoE_2	DSM19866_00857	rpoE_2
rpoE_2	DSM19866_04242	rpoE_2
rpoE_2	DSM19866_01589	rpoE_2
rpoE_2	DSM19866_00544	rpoE_2
rpoE_2	DSM19866_01974	rpoE_2
rpoE_2	DSM19866_01715	rpoE_2
rpoE_2	DSM19866_01363	rpoE_2
rpoE_2	DSM19866_01802	rpoE_2
rpoE_2	DSM19866_04261	rpoE_2
rpoE_2	DSM19866_00988	rpoE_2
rpoE_2	DSM19866_02825	rpoE_2
rpoE_2	DSM19866_04140	rpoE_2
rpoE_2	DSM19866_00699	rpoE_2
rpoE_2	DSM19866_01727	rpoE_2
rpoE_2	DSM19866_02747	rpoE_2
rpoE_2	DSM19866_02262	rpoE_2
rpoE_2	DSM19866_00747	rpoE_2
rpoE_2	DSM19866_00541	rpoE_2
rpoE_2	DSM19866_04552	rpoE_2
rpoE_2	DSM19866_03974	rpoE_2
rpoE_2	DSM19866_00877	rpoE_2
rpoE_2	DSM19866_01793	rpoE_2
rpoE_2	DSM19866_00773	rpoE_2
rpoE_2	DSM19866_04762	rpoE_2
rpoE_2	DSM19866_04439	rpoE_2
rpoE_2	DSM19866_02133	rpoE_2
rpoE_2	DSM2041_03428	rpoE_2
rpoE_2	DSM2041_02937	rpoE_2
rpoE_2	DSM2041_02910	rpoE_2
rpoE_2	DSM2041_01225	rpoE_2
rpoE_2	DSM2041_01830	rpoE_2
rpoE_2	DSM2041_00514	rpoE_2
rpoE_2	DSM2041_03391	rpoE_2
rpoE_2	DSM2041_01159	rpoE_2
rpoE_2	DSM2041_02436	rpoE_2
rpoE_2	DSM2041_03660	rpoE_2
rpoE_2	DSM2041_03048	rpoE_2
rpoE_2	DSM2041_03261	rpoE_2
rpoE_2	DSM2041_04795	rpoE_2
rpoE_2	DSM2041_02107	rpoE_2
rpoE_2	DSM2041_03639	rpoE_2
rpoE_2	DSM2041_02533	rpoE_2
rpoE_2	DSM2041_02859	rpoE_2
rpoE_2	DSM2041_03521	rpoE_2
rpoE_2	DSM2041_00881	rpoE_2
rpoE_2	DSM2041_00883	rpoE_2

rpoE_2	DSM2041_02857	rpoE_2
rpoE_2	DSM2041_02218	rpoE_2
rpoE_2	DSM2041_02809	rpoE_2
rpoE_2	DSM2041_03350	rpoE_2
rpoE_2	DSM2041_00055	rpoE_2
rpoE_2	DSM2041_04277	rpoE_2
rpoE_2	DSM2041_01923	rpoE_2
rpoE_2	DSM2041_03255	rpoE_2
rpoE_2	DSM2041_01850	rpoE_2
rpoE_2	DSM2041_00020	rpoE_2
rpoE_2	DSM2041_02544	rpoE_2
rpoE_2	DSM2041_01912	rpoE_2
rpoE_2	DSM2041_05436	rpoE_2
rpoE_2	DSM2041_05084	rpoE_2
rpoE_2	DSM2041_05018	rpoE_2
rpoE_2	DSM2061_03993	rpoE_2
rpoE_2	DSM2061_00108	rpoE_2
rpoE_2	DSM2061_02512	rpoE_2
rpoE_2	DSM2061_00122	rpoE_2
rpoE_2	DSM2061_03760	rpoE_2
rpoE_2	DSM2061_03142	rpoE_2
rpoE_2	DSM2061_00464	rpoE_2
rpoE_2	DSM2061_01123	rpoE_2
rpoE_2	DSM2061_01816	rpoE_2
rpoE_2	DSM2061_01755	rpoE_2
rpoE_2	DSM2061_01317	rpoE_2
rpoE_2	DSM2061_03896	rpoE_2
rpoE_2	DSM2061_00895	rpoE_2
rpoE_2	DSM2061_01196	rpoE_2
rpoE_2	DSM2061_01121	rpoE_2
rpoE_2	DSM2061_01982	rpoE_2
rpoE_2	DSM2061_02175	rpoE_2
rpoE_2	DSM2061_01256	rpoE_2
rpoE_2	DSM2061_01200	rpoE_2
rpoE_2	DSM2061_00370	rpoE_2
rpoE_2	DSM2061_02391	rpoE_2
rpoE_2	DSM2061_00115	rpoE_2
rpoE_2	DSM2061_01077	rpoE_2
rpoE_2	DSM2061_03270	rpoE_2
rpoE_2	DSM2061_00815	rpoE_2
rpoE_2	DSM2061_02178	rpoE_2
rpoE_2	DSM2061_02002	rpoE_2
rpoE_2	DSM2061_02371	rpoE_2
rpoE_2	DSM2061_00429	rpoE_2
rpoE_2	DSM2061_00501	rpoE_2
rpoE_2	DSM2061_03433	rpoE_2
rpoE_2	DSM2064_04929	rpoE_2
rpoE_2	DSM2064_01512	rpoE_2

rpoE_2	DSM2064_00765	rpoE_2
rpoE_2	DSM2064_03893	rpoE_2
rpoE_2	DSM2064_02219	rpoE_2
rpoE_2	DSM2064_03911	rpoE_2
rpoE_2	DSM2064_03252	rpoE_2
rpoE_2	DSM2064_04942	rpoE_2
rpoE_2	DSM2064_04486	rpoE_2
rpoE_2	DSM2064_02132	rpoE_2
rpoE_2	DSM2064_04271	rpoE_2
rpoE_2	DSM2064_00165	rpoE_2
rpoE_2	DSM2064_00517	rpoE_2
rpoE_2	DSM2064_04862	rpoE_2
rpoE_2	DSM2064_05019	rpoE_2
rpoE_2	DSM2064_00683	rpoE_2
rpoE_2	DSM2064_01135	rpoE_2
rpoE_2	DSM2064_01456	rpoE_2
rpoE_2	DSM2064_02809	rpoE_2
rpoE_2	DSM2064_00317	rpoE_2
rpoE_2	DSM2064_05154	rpoE_2
rpoE_2	DSM2064_01364	rpoE_2
rpoE_2	DSM2064_03639	rpoE_2
rpoE_2	DSM2064_00369	rpoE_2
rpoE_2	DSM2064_03292	rpoE_2
rpoE_2	DSM2064_04995	rpoE_2
rpoE_2	DSM21164_02180	rpoE_2
rpoE_2	DSM21164_00487	rpoE_2
rpoE_2	DSM21164_00781	rpoE_2
rpoE_2	DSM21164_02287	rpoE_2
rpoE_2	DSM21164_02855	rpoE_2
rpoE_2	DSM21164_01847	rpoE_2
rpoE_2	DSM21164_00078	rpoE_2
rpoE_2	DSM21164_01553	rpoE_2
rpoE_2	DSM21164_03066	rpoE_2
rpoE_2	DSM21164_00081	rpoE_2
rpoE_2	DSM21164_02409	rpoE_2
rpoE_2	DSM21164_02693	rpoE_2
rpoE_2	DSM21164_00800	rpoE_2
rpoE_2	DSM21164_00185	rpoE_2
rpoE_2	DSM21164_02371	rpoE_2
rpoE_2	DSM21164_02439	rpoE_2
rpoE_2	DSM21164_01867	rpoE_2
rpoE_2	DSM23404_00884	rpoE_2
rpoE_2	DSM23404_00517	rpoE_2
rpoE_2	DSM23404_02812	rpoE_2
rpoE_2	DSM23404_00798	rpoE_2
rpoE_2	DSM23404_02120	rpoE_2
rpoE_2	DSM23404_01234	rpoE_2
rpoE_2	DSM23404_02840	rpoE_2

rpoE_2	DSM23404_02038	rpoE_2
rpoE_2	DSM23404_00440	rpoE_2
rpoE_2	DSM23404_01586	rpoE_2
rpoE_2	DSM23404_02773	rpoE_2
rpoE_2	DSM23404_02766	rpoE_2
rpoE_2	DSM23404_00750	rpoE_2
rpoE_2	DSM23404_03180	rpoE_2
rpoE_2	DSM23424_02846	rpoE_2
rpoE_2	DSM23424_01459	rpoE_2
rpoE_2	DSM23424_01320	rpoE_2
rpoE_2	DSM23424_02842	rpoE_2
rpoE_2	DSM23424_02919	rpoE_2
rpoE_2	DSM23424_02665	rpoE_2
rpoE_2	DSM23424_00964	rpoE_2
rpoE_2	DSM23424_01897	rpoE_2
rpoE_2	DSM23424_02396	rpoE_2
rpoE_2	DSM23424_02537	rpoE_2
rpoE_2	DSM23424_01494	rpoE_2
rpoE_2	DSM23424_00273	rpoE_2
rpoE_2	DSM23424_02437	rpoE_2
rpoE_2	DSM23424_02661	rpoE_2
rpoE_2	DSM23424_01532	rpoE_2
rpoE_2	DSM23424_01426	rpoE_2
rpoE_2	DSM24786_01729	rpoE_2
rpoE_2	DSM24786_00194	rpoE_2
rpoE_2	DSM24786_01106	rpoE_2
rpoE_2	DSM24786_01028	rpoE_2
rpoE_2	DSM24786_01037	rpoE_2
rpoE_2	DSM24786_01827	rpoE_2
rpoE_2	DSM24786_02595	rpoE_2
rpoE_2	DSM24786_01015	rpoE_2
rpoE_2	DSM24786_00365	rpoE_2
rpoE_2	DSM24786_01143	rpoE_2
rpoE_2	DSM24786_01146	rpoE_2
rpoE_2	DSM24786_00159	rpoE_2
rpoE_2	DSM24786_01236	rpoE_2
rpoE_2	DSM24786_03028	rpoE_2
rpoE_2	DSM24786_01926	rpoE_2
rpoE_2	DSM24786_00057	rpoE_2
rpoE_2	DSM24786_00714	rpoE_2
rpoE_2	DSM24786_02151	rpoE_2
rpoE_2	DSM24786_00039	rpoE_2
rpoE_2	DSM24786_00450	rpoE_2
rpoE_2	DSM24786_00091	rpoE_2
rpoE_2	DSM7489_02218	rpoE_2
rpoE_2	DSM7489_02607	rpoE_2
rpoE_2	DSM7489_03042	rpoE_2
rpoE_2	DSM7489_01239	rpoE_2

rpoE_2	DSM7489_02491	rpoE_2
rpoE_2	DSM7489_02123	rpoE_2
rpoE_2	DSM7489_02779	rpoE_2
rpoE_2	DSM7489_00596	rpoE_2
rpoE_2	DSM7489_00599	rpoE_2
rpoE_2	DSM7489_02574	rpoE_2
rpoE_2	DSM7489_00053	rpoE_2
rpoE_2	DSM7489_02507	rpoE_2
rpoE_2	DSM7489_01443	rpoE_2
rpoE_2	DSM7489_00503	rpoE_2
rpoE_2	DSM7489_02031	rpoE_2
rpoE_2	DSM7489_00182	rpoE_2
rpoE_2	DSM7489_00505	rpoE_2
rpoE_2	DSM7489_00200	rpoE_2
rpoE_2	DSM7489_02855	rpoE_2
rpoE_2	DSM7489_00151	rpoE_2
rpoE_2	DSW1_01879	rpoE_2
rpoE_2	DSW1_02109	rpoE_2
rpoE_2	DSW1_01210	rpoE_2
rpoE_2	DSW1_02067	rpoE_2
rpoE_2	DSW1_02870	rpoE_2
rpoE_2	DSW1_01360	rpoE_2
rpoE_2	DSW1_01687	rpoE_2
rpoE_2	DSW1_00548	rpoE_2
rpoE_2	DSW1_01239	rpoE_2
rpoE_2	DSW1_01304	rpoE_2
rpoE_2	DSW1_02615	rpoE_2
rpoE_2	DSW1_01497	rpoE_2
rpoE_2	DSW1_02866	rpoE_2
rpoE_2	DSW1_01781	rpoE_2
rpoE_2	DSW5_02573	rpoE_2
rpoE_2	DSW5_02520	rpoE_2
rpoE_2	DSW5_02152	rpoE_2
rpoE_2	DSW5_01186	rpoE_2
rpoE_2	DSW5_02285	rpoE_2
rpoE_2	DSW5_02412	rpoE_2
rpoE_2	DSW5_02685	rpoE_2
rpoE_2	DSW5_01930	rpoE_2
rpoE_2	DSW5_02576	rpoE_2
rpoE_2	DSW5_02635	rpoE_2
rpoE_2	DSW5_02345	rpoE_2
rpoE_2	DSW5_02089	rpoE_2
rpoE_2	DSW5_02388	rpoE_2
rpoE_2	DSW6_02412	rpoE_2
rpoE_2	DSW6_03380	rpoE_2
rpoE_2	DSW6_00477	rpoE_2
rpoE_2	DSW6_00080	rpoE_2
rpoE_2	DSW6_01300	rpoE_2

rpoE_2	DSW6_00107	rpoE_2
rpoE_2	DSW6_01445	rpoE_2
rpoE_2	DSW6_02553	rpoE_2
rpoE_2	DSW6_02065	rpoE_2
rpoE_2	DSW6_02942	rpoE_2
rpoE_2	DSW6_00908	rpoE_2
rpoE_2	DSW6_02612	rpoE_2
rpoE_2	DSW6_03460	rpoE_2
rpoE_2	DSW6_03214	rpoE_2
rpoE_2	DSW8_01488	rpoE_2
rpoE_2	DSW8_00374	rpoE_2
rpoE_2	DSW8_00044	rpoE_2
rpoE_2	DSW8_00665	rpoE_2
rpoE_2	DSW8_01452	rpoE_2
rpoE_2	DSW8_00794	rpoE_2
rpoE_2	DSW8_01587	rpoE_2
rpoE_2	DSW8_03393	rpoE_2
rpoE_2	DSW8_02213	rpoE_2
rpoE_2	DSW8_00792	rpoE_2
rpoE_2	DSW8_01150	rpoE_2
rpoE_2	DSW8_03698	rpoE_2
rpoE_2	DSW8_03695	rpoE_2
rpoE_2	DSW8_00719	rpoE_2
rpoE_2	DSW8_03505	rpoE_2
rpoE_2	DSW8_00191	rpoE_2
rpoE_2	DSW8_00136	rpoE_2
rpoE_2	DSW8_00014	rpoE_2
rpoE_2	DSW8_02197	rpoE_2
rpoE_2	DSW8_03081	rpoE_2
rpoE_2	DSW8_03677	rpoE_2
rpoE_2	DSW8_00270	rpoE_2
rpoE_2	DSW8_03485	rpoE_2
rpoE_2	DSW8_00631	rpoE_2
rpoE_2	DSW8_02048	rpoE_2
rpoE_2	F52_01113	rpoE_2
rpoE_2	F52_04059	rpoE_2
rpoE_2	F52_04084	rpoE_2
rpoE_2	F52_00881	rpoE_2
rpoE_2	F52_00421	rpoE_2
rpoE_2	F52_01804	rpoE_2
rpoE_2	F52_00053	rpoE_2
rpoE_2	F52_03993	rpoE_2
rpoE_2	F52_02556	rpoE_2
rpoE_2	F52_04323	rpoE_2
rpoE_2	F52_01157	rpoE_2
rpoE_2	F52_01670	rpoE_2
rpoE_2	F52_01878	rpoE_2
rpoE_2	F52_03519	rpoE_2

rpoE_2	F52_03281	rpoE_2
rpoE_2	F52_02027	rpoE_2
rpoE_2	F52_01363	rpoE_2
rpoE_2	F52_03792	rpoE_2
rpoE_2	F52_04364	rpoE_2
rpoE_2	F52_00390	rpoE_2
rpoE_2	F52_01858	rpoE_2
rpoE_2	GCA_001027945.1_02086	rpoE_2
rpoE_2	GCA_001027945.1_01253	rpoE_2
rpoE_2	GCA_001027945.1_00837	rpoE_2
rpoE_2	GCA_001027945.1_02234	rpoE_2
rpoE_2	GCA_001027945.1_01271	rpoE_2
rpoE_2	GCA_001027945.1_01433	rpoE_2
rpoE_2	GCA_001027945.1_00276	rpoE_2
rpoE_2	GCA_001027945.1_02330	rpoE_2
rpoE_2	GCA_001027945.1_00959	rpoE_2
rpoE_2	GCA_001027945.1_01968	rpoE_2
rpoE_2	GCA_001027945.1_01311	rpoE_2
rpoE_2	GCA_001027945.1_02933	rpoE_2
rpoE_2	GCA_001027945.1_02646	rpoE_2
rpoE_2	GCA_001027945.1_02846	rpoE_2
rpoE_2	GCA_001027945.1_02775	rpoE_2
rpoE_2	GCA_001027945.1_01406	rpoE_2
rpoE_2	GCA_001027945.1_02636	rpoE_2
rpoE_2	GCA_001027945.1_01739	rpoE_2
rpoE_2	GCA_001027945.1_00303	rpoE_2
rpoE_2	HI1_02144	rpoE_2
rpoE_2	HI1_02540	rpoE_2
rpoE_2	HI1_02983	rpoE_2
rpoE_2	HI1_01181	rpoE_2
rpoE_2	HI1_02423	rpoE_2
rpoE_2	HI1_02049	rpoE_2
rpoE_2	HI1_02718	rpoE_2
rpoE_2	HI1_00528	rpoE_2
rpoE_2	HI1_00531	rpoE_2
rpoE_2	HI1_02507	rpoE_2
rpoE_2	HI1_03352	rpoE_2
rpoE_2	HI1_02439	rpoE_2
rpoE_2	HI1_01384	rpoE_2
rpoE_2	HI1_00435	rpoE_2
rpoE_2	HI1_01958	rpoE_2
rpoE_2	HI1_00114	rpoE_2
rpoE_2	HI1_00437	rpoE_2
rpoE_2	HI1_00132	rpoE_2
rpoE_2	HI1_02795	rpoE_2
rpoE_2	HI1_00083	rpoE_2
rpoE_2	IR1_03690	rpoE_2
rpoE_2	IR1_01311	rpoE_2

rpoE_2	IR1_02453	rpoE_2
rpoE_2	IR1_04315	rpoE_2
rpoE_2	IR1_04593	rpoE_2
rpoE_2	IR1_00709	rpoE_2
rpoE_2	IR1_01887	rpoE_2
rpoE_2	IR1_02776	rpoE_2
rpoE_2	IR1_02906	rpoE_2
rpoE_2	IR1_01199	rpoE_2
rpoE_2	IR1_03268	rpoE_2
rpoE_2	IR1_00085	rpoE_2
rpoE_2	IR1_01847	rpoE_2
rpoE_2	IR1_04030	rpoE_2
rpoE_2	IR1_02501	rpoE_2
rpoE_2	IR1_02233	rpoE_2
rpoE_2	IR1_02791	rpoE_2
rpoE_2	KCOM3131_01199	rpoE_2
rpoE_2	KCOM3131_01153	rpoE_2
rpoE_2	KCOM3131_01463	rpoE_2
rpoE_2	KCOM3131_00608	rpoE_2
rpoE_2	KCOM3131_01299	rpoE_2
rpoE_2	KCOM3131_01894	rpoE_2
rpoE_2	KCTC12974_01104	rpoE_2
rpoE_2	KCTC12974_01545	rpoE_2
rpoE_2	KCTC12974_00354	rpoE_2
rpoE_2	KCTC12974_02176	rpoE_2
rpoE_2	KCTC12974_01574	rpoE_2
rpoE_2	KCTC12974_02877	rpoE_2
rpoE_2	KCTC12974_02693	rpoE_2
rpoE_2	KCTC12974_00551	rpoE_2
rpoE_2	KCTC12974_02686	rpoE_2
rpoE_2	KCTC12974_01010	rpoE_2
rpoE_2	KCTC12974_00475	rpoE_2
rpoE_2	KL-A_00055	rpoE_2
rpoE_2	KL-A_00948	rpoE_2
rpoE_2	KL-A_02984	rpoE_2
rpoE_2	KL-A_01081	rpoE_2
rpoE_2	KL-A_02128	rpoE_2
rpoE_2	KL-A_01064	rpoE_2
rpoE_2	KL-A_00148	rpoE_2
rpoE_2	KL-A_01279	rpoE_2
rpoE_2	KL-A_02555	rpoE_2
rpoE_2	KL-A_02558	rpoE_2
rpoE_2	KL-A_00981	rpoE_2
rpoE_2	KL-A_03295	rpoE_2
rpoE_2	KL-A_01049	rpoE_2
rpoE_2	KL-A_00783	rpoE_2
rpoE_2	KL-A_01703	rpoE_2
rpoE_2	KL-A_00239	rpoE_2

rpoE_2	KL-A_01178	rpoE_2
rpoE_2	KL-A_01701	rpoE_2
rpoE_2	KL-A_01196	rpoE_2
rpoE_2	KL-A_00660	rpoE_2
rpoE_2	KL-A_01146	rpoE_2
rpoE_2	L12_00460	rpoE_2
rpoE_2	LL0412.1.7_02756	rpoE_2
rpoE_2	LL0412.1.7_00736	rpoE_2
rpoE_2	LL0412.1.7_02060	rpoE_2
rpoE_2	LL0412.1.7_01137	rpoE_2
rpoE_2	LL0412.1.7_02057	rpoE_2
rpoE_2	LL0412.1.7_01433	rpoE_2
rpoE_2	LL0412.1.7_02066	rpoE_2
rpoE_2	LL0412.1.7_02032	rpoE_2
rpoE_2	LL0412.1.7_01590	rpoE_2
rpoE_2	LL0412.1.7_02324	rpoE_2
rpoE_2	LL0412.1.7_00077	rpoE_2
rpoE_2	LL0412.1.7_01478	rpoE_2
rpoE_2	LL0412.1.7_00185	rpoE_2
rpoE_2	LL0412.1.7_02075	rpoE_2
rpoE_2	LL0412.1.7_02390	rpoE_2
rpoE_2	MAR_2009_71_01937	rpoE_2
rpoE_2	MAR_2009_71_03173	rpoE_2
rpoE_2	MAR_2009_71_02842	rpoE_2
rpoE_2	MAR_2009_71_03461	rpoE_2
rpoE_2	MAR_2009_71_03862	rpoE_2
rpoE_2	MAR_2009_71_02580	rpoE_2
rpoE_2	MAR_2009_71_01975	rpoE_2
rpoE_2	MAR_2009_71_02652	rpoE_2
rpoE_2	MAR_2009_71_01838	rpoE_2
rpoE_2	MAR_2009_71_03537	rpoE_2
rpoE_2	MAR_2009_71_02654	rpoE_2
rpoE_2	MAR_2009_71_02295	rpoE_2
rpoE_2	MAR_2009_71_01227	rpoE_2
rpoE_2	MAR_2009_71_00258	rpoE_2
rpoE_2	MAR_2009_71_00261	rpoE_2
rpoE_2	MAR_2009_71_00081	rpoE_2
rpoE_2	MAR_2009_71_02990	rpoE_2
rpoE_2	MAR_2009_71_03931	rpoE_2
rpoE_2	MAR_2009_71_02937	rpoE_2
rpoE_2	MAR_2009_71_01243	rpoE_2
rpoE_2	MAR_2009_71_00377	rpoE_2
rpoE_2	MAR_2009_71_00279	rpoE_2
rpoE_2	MAR_2009_71_03074	rpoE_2
rpoE_2	MAR_2009_71_00101	rpoE_2
rpoE_2	MAR_2009_71_03514	rpoE_2
rpoE_2	MAR_2009_71_03428	rpoE_2
rpoE_2	MAR_2009_71_01383	rpoE_2

rpoE_2	MAR_2009_71_02825	rpoE_2
rpoE_2	NN016038_00173	rpoE_2
rpoE_2	NN016038_00869	rpoE_2
rpoE_2	NN016038_03009	rpoE_2
rpoE_2	NN016038_00365	rpoE_2
rpoE_2	NN016038_03737	rpoE_2
rpoE_2	NN016038_02874	rpoE_2
rpoE_2	NN016038_00319	rpoE_2
rpoE_2	NN016038_02324	rpoE_2
rpoE_2	NN016038_00326	rpoE_2
rpoE_2	NN016038_02604	rpoE_2
rpoE_2	NN016038_02872	rpoE_2
rpoE_2	NN016038_01823	rpoE_2
rpoE_2	NN016038_01104	rpoE_2
rpoE_2	NN016038_03807	rpoE_2
rpoE_2	NN016038_00864	rpoE_2
rpoE_2	NN016038_03556	rpoE_2
rpoE_2	NN016038_01086	rpoE_2
rpoE_2	NN016038_01693	rpoE_2
rpoE_2	NN016038_02141	rpoE_2
rpoE_2	NN016038_01820	rpoE_2
rpoE_2	NN016038_01925	rpoE_2
rpoE_2	NN016038_02298	rpoE_2
rpoE_2	NN016038_03700	rpoE_2
rpoE_2	PAO1_00155	rpoE_2
rpoE_2	PAO1_02459	rpoE_2
rpoE_2	PAO1_01972	rpoE_2
rpoE_2	PAO1_01339	rpoE_2
rpoE_2	PAO1_03530	rpoE_2
rpoE_2	PAO1_04027	rpoE_2
rpoE_2	PAO1_00787	rpoE_2
rpoE_2	PAO1_03401	rpoE_2
rpoE_2	PAO1_02497	rpoE_2
rpoE_2	PAO1_02998	rpoE_2
rpoE_2	PAO1_01402	rpoE_2
rpoE_2	PAO1_00484	rpoE_2
rpoE_2	PAO1_01823	rpoE_2
rpoE_2	PAO1_02157	rpoE_2
rpoE_2	PAO1_02538	rpoE_2
rpoE_2	PAO1_00693	rpoE_2
rpoE_2	PAO1_02113	rpoE_2
rpoE_2	PAO1_05063	rpoE_2
rpoE_2	UW101_00173	rpoE_2
rpoE_2	UW101_03967	rpoE_2
rpoE_2	UW101_03476	rpoE_2
rpoE_2	UW101_04994	rpoE_2
rpoE_2	UW101_01273	rpoE_2
rpoE_2	UW101_04976	rpoE_2

rpoE_2	UW101_02548	rpoE_2
rpoE_2	UW101_00160	rpoE_2
rpoE_2	UW101_02099	rpoE_2
rpoE_2	UW101_01186	rpoE_2
rpoE_2	UW101_04614	rpoE_2
rpoE_2	UW101_02876	rpoE_2
rpoE_2	UW101_03228	rpoE_2
rpoE_2	UW101_00240	rpoE_2
rpoE_2	UW101_00083	rpoE_2
rpoE_2	UW101_03394	rpoE_2
rpoE_2	UW101_04344	rpoE_2
rpoE_2	UW101_04023	rpoE_2
rpoE_2	UW101_01863	rpoE_2
rpoE_2	UW101_03028	rpoE_2
rpoE_2	UW101_00594	rpoE_2
rpoE_2	UW101_04115	rpoE_2
rpoE_2	UW101_05247	rpoE_2
rpoE_2	UW101_03080	rpoE_2
rpoE_2	UW101_02508	rpoE_2
rpoE_2	UW101_00107	rpoE_2
rpoE_2	V4-33_01017	rpoE_2
rpoE_2	V4-33_01374	rpoE_2
rpoE_2	V4-33_00600	rpoE_2
rpoE_2	V4-33_01992	rpoE_2
rpoE_2	V4-33_00526	rpoE_2
rpoE_2	V4-33_01843	rpoE_2
rpoE_2	V4-33_01363	rpoE_2
rpoE_2	W5C_02843	rpoE_2
rpoE_2	W5C_01281	rpoE_2
rpoE_2	W5C_02251	rpoE_2
rpoE_2	W5C_03313	rpoE_2
rpoE_2	W5C_03312	rpoE_2
rpoE_2	W5C_00998	rpoE_2
rpoE_2	W5C_02933	rpoE_2
rpoE_2	W5C_02458	rpoE_2
rpoE_2	W5C_01246	rpoE_2
rpoE_2	W5C_01243	rpoE_2
rpoE_2	W5C_01315	rpoE_2
rpoE_2	W5C_00818	rpoE_2
rpoE_2	W5C_03329	rpoE_2
rpoE_2	W5C_01012	rpoE_2
rpoE_2	W5C_01142	rpoE_2
rpoE_2	W5C_00719	rpoE_2
rpoE_2	W5C_02784	rpoE_2
rpoE_2	W5C_00091	rpoE_2
rpoE_2	W5C_00109	rpoE_2
rpoE_2	W5C_02537	rpoE_2
rpoE_2	W5C_00060	rpoE_2

rpoE_2	ZONMW-01_01371	rpoE_2
rpoE_2	ZONMW-01_02183	rpoE_2
rpoE_2	ZONMW-01_01657	rpoE_2
rpoE_2	ZONMW-01_00730	rpoE_2
rpoE_2	ZONMW-01_03313	rpoE_2
rpoE_2	ZONMW-01_01531	rpoE_2
rpoE_2	ZONMW-01_01289	rpoE_2
rpoE_2	ZONMW-01_01464	rpoE_2
rpoE_2	ZONMW-01_02263	rpoE_2
rpoE_2	ZONMW-01_02792	rpoE_2
rpoE_2	ZONMW-01_02795	rpoE_2
rpoE_2	ZONMW-01_03062	rpoE_2
rpoE_2	ZONMW-01_01623	rpoE_2
rpoE_2	ZONMW-01_01547	rpoE_2
rpoE_2	ZONMW-01_03342	rpoE_2
rpoE_2	ZONMW-01_02440	rpoE_2
rpoE_2	ZONMW-01_00481	rpoE_2
rpoE_2	ZONMW-01_01959	rpoE_2
rpoE_2	ZONMW-01_02069	rpoE_2
rpoE_2	ZONMW-01_02084	rpoE_2
rpoE_2	ZONMW-01_02037	rpoE_2
rpoE_2	ZONMW-01_02337	rpoE_2
rpoE_2	ZONMW-02_01990	rpoE_2
rpoE_2	ZONMW-02_01584	rpoE_2
rpoE_2	ZONMW-02_00918	rpoE_2
rpoE_2	ZONMW-02_01966	rpoE_2
rpoE_2	ZONMW-02_00379	rpoE_2
rpoE_2	ZONMW-02_03603	rpoE_2
rpoE_2	ZONMW-02_03061	rpoE_2
rpoE_2	ZONMW-02_01569	rpoE_2
rpoE_2	ZONMW-02_02237	rpoE_2
rpoE_2	ZONMW-02_03056	rpoE_2
rpoE_2	ZONMW-02_01176	rpoE_2
rpoE_2	ZONMW-02_00045	rpoE_2
rpoE_2	ZONMW-02_03279	rpoE_2
rpoE_2	ZONMW-02_01934	rpoE_2
rpoE_2	ZONMW-03_01409	rpoE_2
rpoE_2	ZONMW-03_02527	rpoE_2
rpoE_2	ZONMW-03_00550	rpoE_2
rpoE_2	ZONMW-03_00108	rpoE_2
rpoE_2	ZONMW-03_00693	rpoE_2
rpoE_2	ZONMW-03_00676	rpoE_2
rpoE_2	ZONMW-03_01491	rpoE_2
rpoE_2	ZONMW-03_01316	rpoE_2
rpoE_2	ZONMW-03_00385	rpoE_2
rpoE_2	ZONMW-03_03114	rpoE_2
rpoE_2	ZONMW-03_03111	rpoE_2
rpoE_2	ZONMW-03_02297	rpoE_2

rpoE_2	ZONMW-03_00584	rpoE_2
rpoE_2	ZONMW-03_00660	rpoE_2
rpoE_2	ZONMW-03_00711	rpoE_2
rpoE_2	ZONMW-03_03043	rpoE_2
rpoE_2	ZONMW-03_01207	rpoE_2
rpoE_2	ZONMW-03_02943	rpoE_2
rpoE_2	ZONMW-03_02642	rpoE_2
rpoE_2	ZONMW-03_02626	rpoE_2
rpoE_2	ZONMW-03_02674	rpoE_2
rpoE_2	ZONMW-03_00311	rpoE_2
rpoE_2	ZONMW-04_00283	rpoE_2
rpoE_2	ZONMW-04_01489	rpoE_2
rpoE_2	ZONMW-04_02663	rpoE_2
rpoE_2	ZONMW-04_00374	rpoE_2
rpoE_2	ZONMW-04_01324	rpoE_2
rpoE_2	ZONMW-04_02814	rpoE_2
rpoE_2	ZONMW-04_02811	rpoE_2
rpoE_2	ZONMW-04_00762	rpoE_2
rpoE_2	ZONMW-04_01522	rpoE_2
rpoE_2	ZONMW-04_02646	rpoE_2
rpoE_2	ZONMW-04_03291	rpoE_2
rpoE_2	ZONMW-04_02400	rpoE_2
rpoE_2	ZONMW-04_00465	rpoE_2
rpoE_2	ZONMW-04_02504	rpoE_2
rpoE_2	ZONMW-04_01912	rpoE_2
rpoE_2	ZONMW-04_01929	rpoE_2
rpoE_2	ZONMW-04_01245	rpoE_2
rpoE_2	ZONMW-04_01880	rpoE_2
rpoE_2	ZONMW-05_01542	rpoE_2
rpoE_2	ZONMW-05_00813	rpoE_2
rpoE_2	ZONMW-05_02894	rpoE_2
rpoE_2	ZONMW-05_02911	rpoE_2
rpoE_2	ZONMW-05_01451	rpoE_2
rpoE_2	ZONMW-05_00648	rpoE_2
rpoE_2	ZONMW-05_03071	rpoE_2
rpoE_2	ZONMW-05_03068	rpoE_2
rpoE_2	ZONMW-05_00031	rpoE_2
rpoE_2	ZONMW-05_00846	rpoE_2
rpoE_2	ZONMW-05_02928	rpoE_2
rpoE_2	ZONMW-05_02876	rpoE_2
rpoE_2	ZONMW-05_02624	rpoE_2
rpoE_2	ZONMW-05_01360	rpoE_2
rpoE_2	ZONMW-05_02135	rpoE_2
rpoE_2	ZONMW-05_02458	rpoE_2
rpoE_2	ZONMW-05_02440	rpoE_2
rpoE_2	ZONMW-05_00570	rpoE_2
rpoE_2	ZONMW-05_02489	rpoE_2
rpoE_2	ZONMW-06_02884	rpoE_2

rpoE_2	ZONMW-06_01961	rpoE_2
rpoE_2	ZONMW-06_02704	rpoE_2
rpoE_2	ZONMW-06_02316	rpoE_2
rpoE_2	ZONMW-06_01112	rpoE_2
rpoE_2	ZONMW-06_01469	rpoE_2
rpoE_2	ZONMW-06_01887	rpoE_2
rpoE_2	ZONMW-06_01419	rpoE_2
rpoE_2	ZONMW-07_01609	rpoE_2
rpoE_2	ZONMW-07_00409	rpoE_2
rpoE_2	ZONMW-07_03273	rpoE_2
rpoE_2	ZONMW-07_03921	rpoE_2
rpoE_2	ZONMW-07_01305	rpoE_2
rpoE_2	ZONMW-07_02895	rpoE_2
rpoE_2	ZONMW-07_00863	rpoE_2
rpoE_2	ZONMW-07_00984	rpoE_2
rpoE_2	ZONMW-07_03184	rpoE_2
rpoE_2	ZONMW-07_00424	rpoE_2
rpoE_2	ZONMW-07_02458	rpoE_2
rpoE_2	ZONMW-07_00851	rpoE_2
rpoE_2	ZONMW-07_03424	rpoE_2
rpoE_2	ZONMW-07_03664	rpoE_2
rpoE_2	ZONMW-07_02010	rpoE_2
rpoE_2	ZONMW-07_02728	rpoE_2
rpoE_2	ZONMW-07_03481	rpoE_2
rpoE_2	ZONMW-07_02402	rpoE_2
rpoE_2	ZONMW-07_02791	rpoE_2
rpoE_2	ZONMW-07_02399	rpoE_2
rpoE_2	ZONMW-07_02440	rpoE_2
rpoE_2	ZONMW-07_01449	rpoE_2
rpoE_2	ZONMW-07_03153	rpoE_2
rpoE_2	ZONMW-07_00109	rpoE_2
rpoE_2	ZONMW-07_01977	rpoE_2
rpoE_2	ZONMW-07_00951	rpoE_2
rpoE_2	ZONMW-07_01084	rpoE_2
rpoE_2	ZONMW-07_00286	rpoE_2
rpoE_2	ZONMW-07_02094	rpoE_2
rpoE_2	ZONMW-07_01816	rpoE_2
rpoE_2	ZONMW-07_01168	rpoE_2
rpoE_2	ZONMW-07_03578	rpoE_2
rpoE_2	ZONMW-07_02788	rpoE_2
rpoE_2	ZONMW-07_01866	rpoE_2
rpoE_2	ZONMW-07_02286	rpoE_2
rpoE_2	ZONMW-07_02449	rpoE_2
rpoE_2	ZONMW-08_01288	rpoE_2
rpoE_2	ZONMW-08_02687	rpoE_2
rpoE_2	ZONMW-08_02466	rpoE_2
rpoE_2	ZONMW-08_03107	rpoE_2
rpoE_2	ZONMW-08_03583	rpoE_2

rpoE_2	ZONMW-08_03131	rpoE_2
rpoE_2	ZONMW-08_01252	rpoE_2
rpoE_2	ZONMW-08_03656	rpoE_2
rpoE_2	ZONMW-08_01386	rpoE_2
rpoE_2	ZONMW-08_02387	rpoE_2
rpoE_2	ZONMW-08_00747	rpoE_2
rpoE_2	ZONMW-08_03658	rpoE_2
rpoE_2	ZONMW-08_00950	rpoE_2
rpoE_2	ZONMW-08_03346	rpoE_2
rpoE_2	ZONMW-08_03343	rpoE_2
rpoE_2	ZONMW-08_02412	rpoE_2
rpoE_2	ZONMW-08_02970	rpoE_2
rpoE_2	ZONMW-08_02190	rpoE_2
rpoE_2	ZONMW-08_03077	rpoE_2
rpoE_2	ZONMW-08_02136	rpoE_2
rpoE_2	ZONMW-08_02045	rpoE_2
rpoE_2	ZONMW-08_00763	rpoE_2
rpoE_2	ZONMW-08_03695	rpoE_2
rpoE_2	ZONMW-08_03325	rpoE_2
rpoE_2	ZONMW-08_02589	rpoE_2
rpoE_2	ZONMW-08_02950	rpoE_2
rpoE_2	ZONMW-08_02499	rpoE_2
rpoE_2	ZONMW-08_01811	rpoE_2
rpoE_2	ZONMW-09_00837	rpoE_2
rpoE_2	ZONMW-09_01381	rpoE_2
rpoE_2	ZONMW-09_02265	rpoE_2
rpoE_2	ZONMW-09_00991	rpoE_2
rpoE_2	ZONMW-09_01764	rpoE_2
rpoE_2	ZONMW-09_01877	rpoE_2
rpoE_2	ZONMW-09_01093	rpoE_2
rpoE_2	ZONMW-09_00811	rpoE_2
rpoE_2	ZONMW-09_00321	rpoE_2
rpoE_2	ZONMW-09_01206	rpoE_2
rpoE_2	ZONMW-09_00860	rpoE_2
rpoE_2	ZONMW-09_00867	rpoE_2
rpoE_2	ZONMW-10_00446	rpoE_2
rpoE_2	ZONMW-10_01825	rpoE_2
rpoE_2	ZONMW-10_01652	rpoE_2
rpoE_2	ZONMW-10_01088	rpoE_2
rpoE_2	ZONMW-10_01770	rpoE_2
rpoE_2	ZONMW-10_00535	rpoE_2
rpoE_2	ZONMW-10_02147	rpoE_2
rpoE_2	ZONMW-10_00911	rpoE_2
rpoE_2	ZONMW-11_01314	rpoE_2
rpoE_2	ZONMW-11_04712	rpoE_2
rpoE_2	ZONMW-11_01916	rpoE_2
rpoE_2	ZONMW-11_00617	rpoE_2
rpoE_2	ZONMW-11_00129	rpoE_2

rpoE_2	ZONMW-11_04139	rpoE_2
rpoE_2	ZONMW-11_03251	rpoE_2
rpoE_2	ZONMW-11_04073	rpoE_2
rpoE_2	ZONMW-11_04155	rpoE_2
rpoE_2	ZONMW-11_03403	rpoE_2
rpoE_2	ZONMW-11_02798	rpoE_2
rpoE_2	ZONMW-11_03942	rpoE_2
rpoE_2	ZONMW-11_03962	rpoE_2
rpoE_2	ZONMW-11_01154	rpoE_2
rpoE_2	ZONMW-11_00895	rpoE_2
rpoE_2	ZONMW-11_00666	rpoE_2
rpoE_2	ZONMW-11_00300	rpoE_2
rpoE_2	ZONMW-11_03349	rpoE_2
rpoE_2	ZONMW-11_03312	rpoE_2
rpoE_2	ZONMW-11_03557	rpoE_2
rpoE_2	ZONMW-11_04564	rpoE_2
rpoE_2	ZONMW-11_04427	rpoE_2
rpoE_2	ZONMW-11_03826	rpoE_2
rpoE_2	ZONMW-11_02561	rpoE_2
rpoE_2	ZONMW-11_04535	rpoE_2
rpoE_2	ZONMW-11_04012	rpoE_2
rpoE_2	ZONMW-11_02492	rpoE_2
rpoE_2	ZONMW-11_03887	rpoE_2
rpoE_2	ZONMW-11_03238	rpoE_2
rpoE_2	ZONMW-11_01292	rpoE_2
rpoE_2	ZONMW-11_02301	rpoE_2
rpoE_2	ZONMW-11_04485	rpoE_2
rpoE_2	ZONMW-11_04006	rpoE_2
rpoE_2	ZONMW-11_04572	rpoE_2
rpoE_2	ZONMW-11_00603	rpoE_2
rpoE_2	ZONMW-11_02184	rpoE_2
rpoE_2	ZONMW-11_01331	rpoE_2
rpoE_2	ZONMW-11_01111	rpoE_2
rpoE_2	ZONMW-11_04637	rpoE_2
rpoE_2	ZONMW-11_03896	rpoE_2
rpoE_2	ZONMW-12_03734	rpoE_2
rpoE_2	ZONMW-12_00479	rpoE_2
rpoE_2	ZONMW-12_03691	rpoE_2
rpoE_2	ZONMW-12_00820	rpoE_2
rpoE_2	ZONMW-12_03237	rpoE_2
rpoE_2	ZONMW-12_03608	rpoE_2
rpoE_2	ZONMW-12_00083	rpoE_2
rpoE_2	ZONMW-12_03540	rpoE_2
rpoE_2	ZONMW-12_03598	rpoE_2
rpoE_2	ZONMW-12_04236	rpoE_2
rpoE_2	ZONMW-12_01726	rpoE_2
rpoE_2	ZONMW-12_01694	rpoE_2
rpoE_2	ZONMW-12_02701	rpoE_2

rpoE_2	ZONMW-12_03981	rpoE_2
rpoE_2	ZONMW-12_01749	rpoE_2
rpoE_2	ZONMW-12_00914	rpoE_2
rpoE_2	ZONMW-12_01521	rpoE_2
rpoE_2	ZONMW-12_03467	rpoE_2
rpoE_2	ZONMW-12_04133	rpoE_2
rpoE_2	ZONMW-12_00055	rpoE_2
rpoE_2	ZONMW-12_00218	rpoE_2
rpoE_2	ZONMW-12_01373	rpoE_2
rpoE_2	ZONMW-12_03531	rpoE_2
rpoE_2	ZONMW-12_01294	rpoE_2
rpoE_2	ZONMW-12_04083	rpoE_2
rpoE_2	ZONMW-12_01530	rpoE_2
rpoE_2	ZONMW-12_04491	rpoE_2
rpoE_2	ZONMW-12_00696	rpoE_2
rpoE_2	ZONMW-13_02234	rpoE_2
rpoE_2	ZONMW-13_01857	rpoE_2
rpoE_2	ZONMW-13_02924	rpoE_2
rpoE_2	ZONMW-13_03057	rpoE_2
rpoE_2	ZONMW-13_02696	rpoE_2
rpoE_2	ZONMW-13_02274	rpoE_2
rpoE_2	ZONMW-13_00321	rpoE_2
rpoE_2	ZONMW-13_01772	rpoE_2
rpoE_2	ZONMW-13_02918	rpoE_2
rpoE_2	ZONMW-13_01690	rpoE_2
rpoE_2	ZONMW-13_02204	rpoE_2
rpoE_2	ZONMW-13_02281	rpoE_2
rpoE_2	ZONMW-13_01016	rpoE_2
rpoE_2	ZONMW-14_02153	rpoE_2
rpoE_2	ZONMW-14_01089	rpoE_2
rpoE_2	ZONMW-14_00515	rpoE_2
rpoE_2	ZONMW-14_02193	rpoE_2
rpoE_2	ZONMW-14_00271	rpoE_2
rpoE_2	ZONMW-14_01012	rpoE_2
rpoE_2	ZONMW-14_01702	rpoE_2
rpoE_2	ZONMW-14_00446	rpoE_2
rpoE_2	ZONMW-14_00933	rpoE_2
rpoE_2	ZONMW-14_02124	rpoE_2
rpoE_2	ZONMW-14_02202	rpoE_2
rpoE_2	ZONMW-15_00287	rpoE_2
rpoE_2	ZONMW-15_00464	rpoE_2
rpoE_2	ZONMW-15_02846	rpoE_2
rpoE_2	ZONMW-15_02781	rpoE_2
rpoE_2	ZONMW-15_02864	rpoE_2
rpoE_2	ZONMW-15_01544	rpoE_2
rpoE_2	ZONMW-15_02320	rpoE_2
rpoE_2	ZONMW-15_01196	rpoE_2
rpoE_2	ZONMW-15_01199	rpoE_2

rpoE_2	ZONMW-15_02871	rpoE_2
rpoE_2	ZONMW-15_01302	rpoE_2
rpoE_2	ZONMW-15_03151	rpoE_2
rpoE_2	ZONMW-15_00649	rpoE_2
rpoE_2	ZONMW-15_02493	rpoE_2
rpoE_2	ZONMW-15_02223	rpoE_2
rpoE_2	ZONMW-15_02276	rpoE_2
rpoE_2	ZONMW-15_02591	rpoE_2
rpoE_2	ZONMW-15_02438	rpoE_2
rpoE_2	ZONMW-15_01566	rpoE_2
rpoE_2	ZONMW-19_02686	rpoE_2
rpoE_2	ZONMW-19_00382	rpoE_2
rpoE_2	ZONMW-20_00520	rpoE_2
rpoE_2	ZONMW-20_02964	rpoE_2
rpoE_2	ZONMW-21_02421	rpoE_2
rpoE_2	ZONMW-21_02287	rpoE_2
rpoE_2	ZONMW-21_03000	rpoE_2
rpoE_2	ZONMW-21_01553	rpoE_2
rpoE_2	ZONMW-21_03160	rpoE_2
rpoE_2	ZONMW-21_00561	rpoE_2
rpoE_2	ZONMW-21_02504	rpoE_2
rpoE_2	ZONMW-21_00381	rpoE_2
rpoE_2	ZONMW-21_02300	rpoE_2
rpoE_2	ZONMW-21_03240	rpoE_2
rpoE_2	ZONMW-21_03075	rpoE_2
rpoE_2	ZONMW-21_02306	rpoE_2
rpoE_2	ZONMW-21_02057	rpoE_2
rpoE_2	ZONMW-21_02695	rpoE_2
rpoE_2	ZONMW-21_00586	rpoE_2
rpoE_2	ZONMW-21_03435	rpoE_2
rpoE_2	ZONMW-21_03177	rpoE_2
rpoE_2	ZONMW-21_03432	rpoE_2
rpoE_2	ZONMW-21_00734	rpoE_2
rpoE_2	ZONMW-21_03323	rpoE_2
rpoE_2	ZONMW-21_02799	rpoE_2
rpoE_2	ZONMW-21_03078	rpoE_2
rpoE_2	ZONMW-21_01737	rpoE_2
rpoE_2	ZONMW-21_03261	rpoE_2
rpoE_2	ZONMW-21_03090	rpoE_2
rpoE_2	ZONMW-21_01523	rpoE_2
rpoE_2	ZONMW-21_01589	rpoE_2
rpoE_2	ZONMW-21_03475	rpoE_2
rpoE_2	ZONMW-22_02687	rpoE_2
rpoE_2	ZONMW-22_03421	rpoE_2
rpoE_2	ZONMW-22_02824	rpoE_2
rpoE_2	ZONMW-22_02170	rpoE_2
rpoE_2	ZONMW-22_03185	rpoE_2
rpoE_2	ZONMW-22_00561	rpoE_2

rpoE_2	ZONMW-22_02605	rpoE_2
rpoE_2	ZONMW-22_00381	rpoE_2
rpoE_2	ZONMW-22_02809	rpoE_2
rpoE_2	ZONMW-22_03276	rpoE_2
rpoE_2	ZONMW-22_03100	rpoE_2
rpoE_2	ZONMW-22_02803	rpoE_2
rpoE_2	ZONMW-22_01669	rpoE_2
rpoE_2	ZONMW-22_02414	rpoE_2
rpoE_2	ZONMW-22_00586	rpoE_2
rpoE_2	ZONMW-22_03448	rpoE_2
rpoE_2	ZONMW-22_03202	rpoE_2
rpoE_2	ZONMW-22_02265	rpoE_2
rpoE_2	ZONMW-22_00735	rpoE_2
rpoE_2	ZONMW-22_03402	rpoE_2
rpoE_2	ZONMW-22_02310	rpoE_2
rpoE_2	ZONMW-22_03103	rpoE_2
rpoE_2	ZONMW-22_01987	rpoE_2
rpoE_2	ZONMW-22_03255	rpoE_2
rpoE_2	ZONMW-22_03115	rpoE_2
rpoE_2	ZONMW-22_02200	rpoE_2
rpoE_2	ZONMW-22_02135	rpoE_2
rpoE_2	ZONMW-22_03488	rpoE_2
rpoE_2	ZONMW-23_02711	rpoE_2
rpoE_2	ZONMW-23_01113	rpoE_2
rpoE_2	ZONMW-23_03046	rpoE_2
rpoE_2	ZONMW-23_00381	rpoE_2
rpoE_2	ZONMW-23_03206	rpoE_2
rpoE_2	ZONMW-23_01676	rpoE_2
rpoE_2	ZONMW-23_02629	rpoE_2
rpoE_2	ZONMW-23_01496	rpoE_2
rpoE_2	ZONMW-23_02831	rpoE_2
rpoE_2	ZONMW-23_03298	rpoE_2
rpoE_2	ZONMW-23_03121	rpoE_2
rpoE_2	ZONMW-23_02825	rpoE_2
rpoE_2	ZONMW-23_00883	rpoE_2
rpoE_2	ZONMW-23_00136	rpoE_2
rpoE_2	ZONMW-23_01701	rpoE_2
rpoE_2	ZONMW-23_03442	rpoE_2
rpoE_2	ZONMW-23_03223	rpoE_2
rpoE_2	ZONMW-23_00286	rpoE_2
rpoE_2	ZONMW-23_01849	rpoE_2
rpoE_2	ZONMW-23_03369	rpoE_2
rpoE_2	ZONMW-23_00240	rpoE_2
rpoE_2	ZONMW-23_03124	rpoE_2
rpoE_2	ZONMW-23_00564	rpoE_2
rpoE_2	ZONMW-23_03277	rpoE_2
rpoE_2	ZONMW-23_03136	rpoE_2
rpoE_2	ZONMW-23_00351	rpoE_2

rpoE_2	ZONMW-23_00416	rpoE_2
rpoE_2	ZONMW-23_03482	rpoE_2
rpoE_2	ZONMW-24_02445	rpoE_2
rpoE_2	ZONMW-24_01462	rpoE_2
rpoE_2	ZONMW-24_02847	rpoE_2
rpoE_2	ZONMW-24_02795	rpoE_2
rpoE_2	ZONMW-24_03207	rpoE_2
rpoE_2	ZONMW-24_00898	rpoE_2
rpoE_2	ZONMW-24_02528	rpoE_2
rpoE_2	ZONMW-24_01078	rpoE_2
rpoE_2	ZONMW-24_02325	rpoE_2
rpoE_2	ZONMW-24_03299	rpoE_2
rpoE_2	ZONMW-24_03122	rpoE_2
rpoE_2	ZONMW-24_02330	rpoE_2
rpoE_2	ZONMW-24_01692	rpoE_2
rpoE_2	ZONMW-24_02179	rpoE_2
rpoE_2	ZONMW-24_00874	rpoE_2
rpoE_2	ZONMW-24_03444	rpoE_2
rpoE_2	ZONMW-24_03224	rpoE_2
rpoE_2	ZONMW-24_02029	rpoE_2
rpoE_2	ZONMW-24_00726	rpoE_2
rpoE_2	ZONMW-24_03371	rpoE_2
rpoE_2	ZONMW-24_02075	rpoE_2
rpoE_2	ZONMW-24_03125	rpoE_2
rpoE_2	ZONMW-24_02612	rpoE_2
rpoE_2	ZONMW-24_03278	rpoE_2
rpoE_2	ZONMW-24_03137	rpoE_2
rpoE_2	ZONMW-24_02825	rpoE_2
rpoE_2	ZONMW-24_02760	rpoE_2
rpoE_2	ZONMW-24_03471	rpoE_2
rpoE_2	ZONMW-25_03274	rpoE_2
rpoE_2	ZONMW-25_00083	rpoE_2
rpoE_2	ZONMW-25_02748	rpoE_2
rpoE_2	ZONMW-25_02236	rpoE_2
rpoE_2	ZONMW-25_02216	rpoE_2
rpoE_2	ZONMW-25_02387	rpoE_2
rpoE_2	ZONMW-25_03164	rpoE_2
rpoE_2	ZONMW-25_01080	rpoE_2
rpoE_2	ZONMW-25_01856	rpoE_2
rpoE_2	ZONMW-25_01272	rpoE_2
rpoE_2	ZONMW-25_02811	rpoE_2
rpoE_2	ZONMW-25_03419	rpoE_2
rpoE_2	ZONMW-25_02709	rpoE_2
rpoE_2	ZONMW-25_01872	rpoE_2
rpoE_2	ZONMW-25_03412	rpoE_2
rpoE_2	ZONMW-25_01666	rpoE_2
rpoE_2	ZONMW-25_00700	rpoE_2
rpoE_2	ZONMW-25_01963	rpoE_2

rpoE_2	ZONMW-25_01859	rpoE_2
rpoE_2	ZONMW-25_03443	rpoE_2
rpoE_2	ZONMW-25_01292	rpoE_2
rpoE_2	ZONMW-25_01380	rpoE_2
rpoE_2	ZONMW-26_01728	rpoE_2
rpoE_2	ZONMW-26_02775	rpoE_2
rpoE_2	ZONMW-26_00147	rpoE_2
rpoE_2	ZONMW-26_00004	rpoE_2
rpoE_2	ZONMW-26_00021	rpoE_2
rpoE_2	ZONMW-26_01818	rpoE_2
rpoE_2	ZONMW-26_00321	rpoE_2
rpoE_2	ZONMW-26_03138	rpoE_2
rpoE_2	ZONMW-26_03135	rpoE_2
rpoE_2	ZONMW-26_00929	rpoE_2
rpoE_2	ZONMW-26_00114	rpoE_2
rpoE_2	ZONMW-26_00038	rpoE_2
rpoE_2	ZONMW-26_03227	rpoE_2
rpoE_2	ZONMW-26_03061	rpoE_2
rpoE_2	ZONMW-26_01413	rpoE_2
rpoE_2	ZONMW-26_02804	rpoE_2
rpoE_2	ZONMW-26_02658	rpoE_2
rpoE_2	ZONMW-26_02676	rpoE_2
rpoE_2	ZONMW-26_00397	rpoE_2
rpoE_2	ZONMW-26_02625	rpoE_2
rpoE_2	ZONMW-27_00277	rpoE_2
rpoE_2	ZONMW-27_01815	rpoE_2
rpoE_2	ZONMW-27_03133	rpoE_2
rpoE_2	ZONMW-27_03120	rpoE_2
rpoE_2	ZONMW-27_00368	rpoE_2
rpoE_2	ZONMW-27_01979	rpoE_2
rpoE_2	ZONMW-27_03160	rpoE_2
rpoE_2	ZONMW-27_03157	rpoE_2
rpoE_2	ZONMW-27_00858	rpoE_2
rpoE_2	ZONMW-27_01782	rpoE_2
rpoE_2	ZONMW-27_03294	rpoE_2
rpoE_2	ZONMW-27_02781	rpoE_2
rpoE_2	ZONMW-27_00459	rpoE_2
rpoE_2	ZONMW-27_02537	rpoE_2
rpoE_2	ZONMW-27_02083	rpoE_2
rpoE_2	ZONMW-27_02100	rpoE_2
rpoE_2	ZONMW-27_01394	rpoE_2
rpoE_2	ZONMW-27_02052	rpoE_2
rpoE_2	ZONMW-28_02022	rpoE_2
rpoE_2	ZONMW-28_03776	rpoE_2
rpoE_2	ZONMW-28_02622	rpoE_2
rpoE_2	ZONMW-28_03967	rpoE_2
rpoE_2	ZONMW-28_00758	rpoE_2
rpoE_2	ZONMW-28_02695	rpoE_2

rpoE_2	ZONMW-29_02821	rpoE_2
rpoE_2	ZONMW-29_00083	rpoE_2
rpoE_2	ZONMW-29_01618	rpoE_2
rpoE_2	ZONMW-29_00230	rpoE_2
rpoE_2	ZONMW-29_02737	rpoE_2
rpoE_2	ZONMW-29_01865	rpoE_2
rpoE_2	ZONMW-29_02610	rpoE_2
rpoE_2	ZONMW-29_01752	rpoE_2
rpoE_2	ZONMW-29_01189	rpoE_2
rpoE_2	ZONMW-29_02175	rpoE_2
rpoE_2	ZONMW-29_00251	rpoE_2
rpoE_2	ZONMW-29_01590	rpoE_2
rpoE_2	ZONMW-29_01243	rpoE_2
rpoE_2	ZONMW-29_00377	rpoE_2
rpoE_2	ZONMW-29_02500	rpoE_2
rpoE_2	ZONMW-29_02204	rpoE_2
rpoE_2	ZONMW-29_02607	rpoE_2
rpoE_2	ZONMW-29_03207	rpoE_2
rpoE_2	ZONMW-29_01729	rpoE_2
rpoE_2	ZONMW-29_02594	rpoE_2
rpoE_2	ZONMW-30_00123	rpoE_2
rpoE_2	ZONMW-30_03131	rpoE_2
rpoE_2	ZONMW-30_02086	rpoE_2
rpoE_2	ZONMW-30_01694	rpoE_2
rpoE_2	ZONMW-30_01674	rpoE_2
rpoE_2	ZONMW-30_00042	rpoE_2
rpoE_2	ZONMW-30_01396	rpoE_2
rpoE_2	ZONMW-30_00978	rpoE_2
rpoE_2	ZONMW-30_02481	rpoE_2
rpoE_2	ZONMW-30_02936	rpoE_2
rpoE_2	ZONMW-30_02048	rpoE_2
rpoE_2	ZONMW-30_00962	rpoE_2
rpoE_2	ZONMW-30_02330	rpoE_2
rpoE_2	ZONMW-30_03030	rpoE_2
rpoE_2	ZONMW-30_00870	rpoE_2
rpoE_2	ZONMW-30_00210	rpoE_2
rpoE_2	ZONMW-30_00975	rpoE_2
rpoE_2	ZONMW-30_03234	rpoE_2
rpoE_2	ZONMW-30_02461	rpoE_2
rpoE_2	ZONMW-30_02129	rpoE_2
rpoE_2	ZONMW-31_02992	rpoE_2
rpoE_2	ZONMW-31_03510	rpoE_2
rpoE_2	ZONMW-31_03470	rpoE_2
rpoE_2	ZONMW-31_02519	rpoE_2
rpoE_2	ZONMW-31_02262	rpoE_2
rpoE_2	ZONMW-31_01962	rpoE_2
rpoE_2	ZONMW-31_01893	rpoE_2
rpoE_2	ZONMW-31_00140	rpoE_2

rpoE_2	ZONMW-32_02428	rpoE_2
rpoE_2	ZONMW-32_03889	rpoE_2
rpoE_2	ZONMW-32_03254	rpoE_2
rpoE_2	ZONMW-32_03824	rpoE_2
rpoE_2	ZONMW-32_00333	rpoE_2
rpoE_2	ZONMW-32_03181	rpoE_2
rpoE_2	ZONMW-33_02690	rpoE_2
rpoE_2	ZONMW-33_03694	rpoE_2
rpoE_2	ZONMW-33_03020	rpoE_2
rpoE_2	ZONMW-33_03680	rpoE_2
rpoE_2	ZONMW-33_00200	rpoE_2
rpoE_2	ZONMW-33_03093	rpoE_2
rpoE_2	ZONMW-34_01983	rpoE_2
rpoE_2	ZONMW-34_03117	rpoE_2
rpoE_2	ZONMW-34_02632	rpoE_2
rpoE_2	ZONMW-34_02281	rpoE_2
rpoE_2	ZONMW-34_02962	rpoE_2
rpoE_2	ZONMW-34_00582	rpoE_2
rpoE_2	ZONMW-34_02561	rpoE_2
rpoE_2	ZONMW-34_02328	rpoE_2
rpoE_2	ZONMW-35_01675	rpoE_2
rpoE_2	ZONMW-35_03120	rpoE_2
rpoE_2	ZONMW-35_02596	rpoE_2
rpoE_2	ZONMW-35_01968	rpoE_2
rpoE_2	ZONMW-35_03030	rpoE_2
rpoE_2	ZONMW-35_00807	rpoE_2
rpoE_2	ZONMW-35_02525	rpoE_2
rpoE_2	ZONMW-35_01921	rpoE_2
rpoE_2	ZONMW-36_02562	rpoE_2
rpoE_2	ZONMW-36_03643	rpoE_2
rpoE_2	ZONMW-36_02982	rpoE_2
rpoE_2	ZONMW-36_03575	rpoE_2
rpoE_2	ZONMW-36_01169	rpoE_2
rpoE_2	ZONMW-36_02909	rpoE_2
rpoE_2	ZONMW-37_02691	rpoE_2
rpoE_2	ZONMW-37_01461	rpoE_2
rpoE_2	ZONMW-37_02834	rpoE_2
rpoE_2	ZONMW-37_01931	rpoE_2
rpoE_2	ZONMW-37_03194	rpoE_2
rpoE_2	ZONMW-37_00561	rpoE_2
rpoE_2	ZONMW-37_02774	rpoE_2
rpoE_2	ZONMW-37_00381	rpoE_2
rpoE_2	ZONMW-37_02571	rpoE_2
rpoE_2	ZONMW-37_03285	rpoE_2
rpoE_2	ZONMW-37_03109	rpoE_2
rpoE_2	ZONMW-37_02576	rpoE_2
rpoE_2	ZONMW-37_01692	rpoE_2
rpoE_2	ZONMW-37_02362	rpoE_2

rpoE_2	ZONMW-37_00586	rpoE_2
rpoE_2	ZONMW-37_03453	rpoE_2
rpoE_2	ZONMW-37_03211	rpoE_2
rpoE_2	ZONMW-37_02513	rpoE_2
rpoE_2	ZONMW-37_00734	rpoE_2
rpoE_2	ZONMW-37_03411	rpoE_2
rpoE_2	ZONMW-37_02466	rpoE_2
rpoE_2	ZONMW-37_03112	rpoE_2
rpoE_2	ZONMW-37_02115	rpoE_2
rpoE_2	ZONMW-37_03264	rpoE_2
rpoE_2	ZONMW-37_03124	rpoE_2
rpoE_2	ZONMW-37_01901	rpoE_2
rpoE_2	ZONMW-37_01967	rpoE_2
rpoE_2	ZONMW-37_03457	rpoE_2
rpoE_2	ZONMW-38_01810	rpoE_2
rpoE_2	ZONMW-38_01177	rpoE_2
rpoE_2	ZONMW-39_04169	rpoE_2
rpoE_2	ZONMW-39_02209	rpoE_2
rpoE_2	ZONMW-39_01791	rpoE_2
rpoE_2	ZONMW-39_03965	rpoE_2
rpoE_2	ZONMW-39_01556	rpoE_2
rpoE_2	ZONMW-39_02797	rpoE_2
rpoE_2	ZONMW-39_01046	rpoE_2
rpoE_2	ZONMW-39_02104	rpoE_2
rpoE_2	ZONMW-39_01594	rpoE_2
rpoE_2	ZONMW-40_02208	rpoE_2
rpoE_2	ZONMW-40_03080	rpoE_2
rpoE_2	ZONMW-40_01856	rpoE_2
rpoE_2	ZONMW-40_01394	rpoE_2
rpoE_2	ZONMW-40_00834	rpoE_2
rpoE_2	ZONMW-40_02127	rpoE_2
rpoE_2	ZONMW-40_02941	rpoE_2
rpoE_2	ZONMW-40_01103	rpoE_2
rpoE_2	ZONMW-40_02863	rpoE_2
rpoE_2	ZONMW-40_03281	rpoE_2
rpoE_2	ZONMW-40_01220	rpoE_2
rpoE_2	ZONMW-40_01880	rpoE_2
rpoE_2	ZONMW-40_01738	rpoE_2
rpoE_2	ZONMW-40_02314	rpoE_2
rpoE_2	ZONMW-40_02288	rpoE_2
rpoE_2	ZONMW-40_00466	rpoE_2
rpoE_2	ZONMW-40_00987	rpoE_2
rpoE_2	ZONMW-40_01100	rpoE_2
rpoE_2	ZONMW-40_01193	rpoE_2
rpoE_2	ZONMW-40_01961	rpoE_2
rpoE_2	ZONMW-40_02842	rpoE_2
rpoE_2	ZONMW-40_02034	rpoE_2
rpoE_2	ZONMW-40_01886	rpoE_2

rpoE_2	ZONMW-40_02516	rpoE_2
rpoE_2	ZONMW-40_01374	rpoE_2
rpoE_2	ZONMW-41_01759	rpoE_2
rpoE_2	ZONMW-41_01154	rpoE_2
rpoE_2	ZONMW-41_01416	rpoE_2
rpoE_2	ZONMW-41_01771	rpoE_2
rpoE_2	ZONMW-41_00465	rpoE_2
rpoE_2	ZONMW-41_02328	rpoE_2
rpoE_2	ZONMW-41_01670	rpoE_2
rpoE_2	ZONMW-41_00332	rpoE_2
rpoE_2	ZONMW-42_00436	rpoE_2
rpoE_2	ZONMW-42_02948	rpoE_2
rpoE_2	ZONMW-42_02480	rpoE_2
rpoE_2	ZONMW-42_00602	rpoE_2
rpoE_2	ZONMW-42_00181	rpoE_2
rpoE_2	ZONMW-42_02427	rpoE_2
rpoE_2	ZONMW-42_01111	rpoE_2
rpoE_2	ZONMW-42_02954	rpoE_2
rpoE_2	ZONMW-43_01487	rpoE_2
rpoE_2	ZONMW-43_02889	rpoE_2
rpoE_2	ZONMW-43_03443	rpoE_2
rpoE_2	ZONMW-43_01260	rpoE_2
rpoE_2	ZONMW-43_01953	rpoE_2
rpoE_2	ZONMW-43_03821	rpoE_2
rpoE_2	ZONMW-43_00122	rpoE_2
rpoE_2	ZONMW-43_01188	rpoE_2
rpoE_2	ZONMW-43_01912	rpoE_2
rpoE_2	ZONMW-44_00861	rpoE_2
rpoE_2	ZONMW-44_00018	rpoE_2
rpoE_2	ZONMW-44_01162	rpoE_2
rpoE_2	ZONMW-44_00690	rpoE_2
rpoE_2	ZONMW-44_02283	rpoE_2
rpoE_2	ZONMW-44_03098	rpoE_2
rpoE_2	ZONMW-44_00621	rpoE_2
rpoE_2	ZONMW-44_00133	rpoE_2
rpoE_2	ZONMW-44_04085	rpoE_2
rpoE_2	ZONMW-45_01196	rpoE_2
rpoE_2	ZONMW-45_00753	rpoE_2
rpoE_2	ZONMW-45_00848	rpoE_2
rpoE_2	ZONMW-45_02127	rpoE_2
rpoE_2	ZONMW-45_01511	rpoE_2
rpoE_2	ZONMW-45_02773	rpoE_2
rpoE_2	ZONMW-45_02458	rpoE_2
rpoE_2	ZONMW-45_01314	rpoE_2
rpoE_2	ZONMW-45_00482	rpoE_2
rpoE_2	ZONMW-45_01810	rpoE_2
rpoE_2	ZONMW-45_01717	rpoE_2
rpoE_2	ZONMW-45_00203	rpoE_2

rpoE_2	ZONMW-45_02947	rpoE_2
rpoE_2	ZONMW-45_00086	rpoE_2
rpoE_2	ZONMW-45_00137	rpoE_2
rpoE_2	ZONMW-45_02155	rpoE_2
rpoE_2	ZONMW-45_00192	rpoE_2
rpoE_2	ZONMW-45_01638	rpoE_2
rpoE_2	ZONMW-45_00466	rpoE_2
rpoE_2	ZONMW-46_02580	rpoE_2
rpoE_2	ZONMW-46_00707	rpoE_2
rpoE_2	ZONMW-46_01026	rpoE_2
rpoE_2	ZONMW-46_01803	rpoE_2
rpoE_2	ZONMW-46_02022	rpoE_2
rpoE_2	ZONMW-46_01138	rpoE_2
rpoE_2	ZONMW-46_02451	rpoE_2
rpoE_2	ZONMW-46_02698	rpoE_2
rpoE_2	ZONMW-46_01357	rpoE_2
rpoE_2	ZONMW-46_01910	rpoE_2
rpoE_2	ZONMW-46_00080	rpoE_2
rpoE_2	ZONMW-46_00418	rpoE_2
rpoE_2	ZONMW-46_02994	rpoE_2
rpoE_2	ZONMW-46_00301	rpoE_2
rpoE_2	ZONMW-46_00352	rpoE_2
rpoE_2	ZONMW-46_01831	rpoE_2
rpoE_2	ZONMW-46_00407	rpoE_2
rpoE_2	ZONMW-46_02149	rpoE_2
rpoE_2	ZONMW-46_01373	rpoE_2
rpoE_2	ZONMW-47_02570	rpoE_2
rpoE_2	ZONMW-47_00707	rpoE_2
rpoE_2	ZONMW-47_01147	rpoE_2
rpoE_2	ZONMW-47_01686	rpoE_2
rpoE_2	ZONMW-47_02039	rpoE_2
rpoE_2	ZONMW-47_01035	rpoE_2
rpoE_2	ZONMW-47_02441	rpoE_2
rpoE_2	ZONMW-47_02688	rpoE_2
rpoE_2	ZONMW-47_01495	rpoE_2
rpoE_2	ZONMW-47_02151	rpoE_2
rpoE_2	ZONMW-47_00425	rpoE_2
rpoE_2	ZONMW-47_00087	rpoE_2
rpoE_2	ZONMW-47_02985	rpoE_2
rpoE_2	ZONMW-47_00204	rpoE_2
rpoE_2	ZONMW-47_00153	rpoE_2
rpoE_2	ZONMW-47_01658	rpoE_2
rpoE_2	ZONMW-47_00098	rpoE_2
rpoE_2	ZONMW-47_01912	rpoE_2
rpoE_2	ZONMW-47_01479	rpoE_2
rpoE_2	ZONMW-48_00936	rpoE_2
rpoE_2	ZONMW-48_01101	rpoE_2
rpoE_2	ZONMW-48_01194	rpoE_2

rpoE_2	ZONMW-48_03247	rpoE_2
rpoE_2	ZONMW-48_01917	rpoE_2
rpoE_2	ZONMW-48_01176	rpoE_2
rpoE_2	ZONMW-48_01291	rpoE_2
rpoE_2	ZONMW-48_02497	rpoE_2
rpoE_2	ZONMW-48_02494	rpoE_2
rpoE_2	ZONMW-48_01710	rpoE_2
rpoE_2	ZONMW-48_01169	rpoE_2
rpoE_2	ZONMW-48_02394	rpoE_2
rpoE_2	ZONMW-48_02898	rpoE_2
rpoE_2	ZONMW-48_02152	rpoE_2
rpoE_2	ZONMW-48_02994	rpoE_2
rpoE_2	ZONMW-48_01387	rpoE_2
rpoE_2	ZONMW-48_01333	rpoE_2
rpoE_2	ZONMW-48_00590	rpoE_2
rpoE_2	ZONMW-48_03049	rpoE_2
rpoE_2	ZONMW-48_03200	rpoE_2
rpoE_2	ZONMW-48_01939	rpoE_2
rpoE_2	ZONMW-49_00694	rpoE_2
rpoE_2	ZONMW-49_00158	rpoE_2
rpoE_2	ZONMW-49_01724	rpoE_2
rpoE_2	ZONMW-49_02698	rpoE_2
rpoE_2	ZONMW-49_00042	rpoE_2
rpoE_2	ZONMW-49_00792	rpoE_2
rpoE_2	ZONMW-49_00333	rpoE_2
rpoE_2	ZONMW-49_02743	rpoE_2
rpoE_2	ZONMW-49_02740	rpoE_2
rpoE_2	ZONMW-49_00125	rpoE_2
rpoE_2	ZONMW-49_03302	rpoE_2
rpoE_2	ZONMW-49_00057	rpoE_2
rpoE_2	ZONMW-49_01154	rpoE_2
rpoE_2	ZONMW-49_01573	rpoE_2
rpoE_2	ZONMW-49_02495	rpoE_2
rpoE_2	ZONMW-49_02026	rpoE_2
rpoE_2	ZONMW-49_02477	rpoE_2
rpoE_2	ZONMW-49_00413	rpoE_2
rpoE_2	ZONMW-49_02527	rpoE_2
rpoE_2	ZONMW-50_00695	rpoE_2
rpoE_2	ZONMW-50_00327	rpoE_2
rpoE_2	ZONMW-50_01690	rpoE_2
rpoE_2	ZONMW-50_02492	rpoE_2
rpoE_2	ZONMW-50_00444	rpoE_2
rpoE_2	ZONMW-50_00793	rpoE_2
rpoE_2	ZONMW-50_00152	rpoE_2
rpoE_2	ZONMW-50_02669	rpoE_2
rpoE_2	ZONMW-50_02666	rpoE_2
rpoE_2	ZONMW-50_00361	rpoE_2
rpoE_2	ZONMW-50_03280	rpoE_2

rpoE_2	ZONMW-50_00429	rpoE_2
rpoE_2	ZONMW-50_01155	rpoE_2
rpoE_2	ZONMW-50_01575	rpoE_2
rpoE_2	ZONMW-50_02289	rpoE_2
rpoE_2	ZONMW-50_02622	rpoE_2
rpoE_2	ZONMW-50_02271	rpoE_2
rpoE_2	ZONMW-50_00072	rpoE_2
rpoE_2	ZONMW-50_02321	rpoE_2
rpoE_2	ZONMW-51_01013	rpoE_2
rpoE_2	ZONMW-51_00155	rpoE_2
rpoE_2	ZONMW-51_00622	rpoE_2
rpoE_2	ZONMW-51_02580	rpoE_2
rpoE_2	ZONMW-51_00039	rpoE_2
rpoE_2	ZONMW-51_00919	rpoE_2
rpoE_2	ZONMW-51_00321	rpoE_2
rpoE_2	ZONMW-51_03032	rpoE_2
rpoE_2	ZONMW-51_03035	rpoE_2
rpoE_2	ZONMW-51_00122	rpoE_2
rpoE_2	ZONMW-51_02195	rpoE_2
rpoE_2	ZONMW-51_01854	rpoE_2
rpoE_2	ZONMW-51_00054	rpoE_2
rpoE_2	ZONMW-51_02760	rpoE_2
rpoE_2	ZONMW-51_00828	rpoE_2
rpoE_2	ZONMW-51_02472	rpoE_2
rpoE_2	ZONMW-51_02332	rpoE_2
rpoE_2	ZONMW-51_02454	rpoE_2
rpoE_2	ZONMW-51_00401	rpoE_2
rpoE_2	ZONMW-51_02504	rpoE_2
rpoE_2	ZONMW-52_01613	rpoE_2
rpoE_2	ZONMW-52_00127	rpoE_2
rpoE_2	ZONMW-52_00579	rpoE_2
rpoE_2	ZONMW-52_02073	rpoE_2
rpoE_2	ZONMW-52_00012	rpoE_2
rpoE_2	ZONMW-52_01519	rpoE_2
rpoE_2	ZONMW-52_00305	rpoE_2
rpoE_2	ZONMW-52_03042	rpoE_2
rpoE_2	ZONMW-52_03039	rpoE_2
rpoE_2	ZONMW-52_00094	rpoE_2
rpoE_2	ZONMW-52_02608	rpoE_2
rpoE_2	ZONMW-52_00919	rpoE_2
rpoE_2	ZONMW-52_00027	rpoE_2
rpoE_2	ZONMW-52_02286	rpoE_2
rpoE_2	ZONMW-52_02745	rpoE_2
rpoE_2	ZONMW-52_01428	rpoE_2
rpoE_2	ZONMW-52_02455	rpoE_2
rpoE_2	ZONMW-52_02747	rpoE_2
rpoE_2	ZONMW-52_02437	rpoE_2
rpoE_2	ZONMW-52_00386	rpoE_2

rpoE_2	ZONMW-52_02487	rpoE_2
rpoE_2	ZONMW-53_00212	rpoE_2
rpoE_2	ZONMW-53_02527	rpoE_2
rpoE_2	ZONMW-53_01334	rpoE_2
rpoE_2	ZONMW-53_02644	rpoE_2
rpoE_2	ZONMW-53_00308	rpoE_2
rpoE_2	ZONMW-53_01042	rpoE_2
rpoE_2	ZONMW-53_03094	rpoE_2
rpoE_2	ZONMW-53_03097	rpoE_2
rpoE_2	ZONMW-53_02560	rpoE_2
rpoE_2	ZONMW-53_01662	rpoE_2
rpoE_2	ZONMW-53_02628	rpoE_2
rpoE_2	ZONMW-53_02875	rpoE_2
rpoE_2	ZONMW-53_00399	rpoE_2
rpoE_2	ZONMW-53_02178	rpoE_2
rpoE_2	ZONMW-53_02497	rpoE_2
rpoE_2	ZONMW-53_02196	rpoE_2
rpoE_2	ZONMW-53_01118	rpoE_2
rpoE_2	ZONMW-53_02146	rpoE_2
rpoE_2	ZONMW-54_00818	rpoE_2
rpoE_2	ZONMW-54_01849	rpoE_2
rpoE_2	ZONMW-54_02785	rpoE_2
rpoE_2	ZONMW-54_01383	rpoE_2
rpoE_2	ZONMW-54_02909	rpoE_2
rpoE_2	ZONMW-54_01964	rpoE_2
rpoE_2	ZONMW-54_00726	rpoE_2
rpoE_2	ZONMW-54_01685	rpoE_2
rpoE_2	ZONMW-54_03096	rpoE_2
rpoE_2	ZONMW-54_03099	rpoE_2
rpoE_2	ZONMW-54_01883	rpoE_2
rpoE_2	ZONMW-54_01950	rpoE_2
rpoE_2	ZONMW-54_00091	rpoE_2
rpoE_2	ZONMW-54_02558	rpoE_2
rpoE_2	ZONMW-54_00635	rpoE_2
rpoE_2	ZONMW-54_01063	rpoE_2
rpoE_2	ZONMW-54_02668	rpoE_2
rpoE_2	ZONMW-54_02686	rpoE_2
rpoE_2	ZONMW-54_01604	rpoE_2
rpoE_2	ZONMW-54_02636	rpoE_2
rpoE_2	ZONMW-55_00207	rpoE_2
rpoE_2	ZONMW-55_01382	rpoE_2
rpoE_2	ZONMW-55_00902	rpoE_2
rpoE_2	ZONMW-55_02766	rpoE_2
rpoE_2	ZONMW-55_01499	rpoE_2
rpoE_2	ZONMW-55_00301	rpoE_2
rpoE_2	ZONMW-55_01205	rpoE_2
rpoE_2	ZONMW-55_01564	rpoE_2
rpoE_2	ZONMW-55_01561	rpoE_2

rpoE_2	ZONMW-55_01416	rpoE_2
rpoE_2	ZONMW-55_01793	rpoE_2
rpoE_2	ZONMW-55_02176	rpoE_2
rpoE_2	ZONMW-55_01484	rpoE_2
rpoE_2	ZONMW-55_02881	rpoE_2
rpoE_2	ZONMW-55_00392	rpoE_2
rpoE_2	ZONMW-55_02563	rpoE_2
rpoE_2	ZONMW-55_01656	rpoE_2
rpoE_2	ZONMW-55_02545	rpoE_2
rpoE_2	ZONMW-55_01125	rpoE_2
rpoE_2	ZONMW-55_02595	rpoE_2
rpoE_2	ZONMW-56_01111	rpoE_2
rpoE_2	ZONMW-56_00608	rpoE_2
rpoE_2	ZONMW-56_00138	rpoE_2
rpoE_2	ZONMW-56_02429	rpoE_2
rpoE_2	ZONMW-56_00725	rpoE_2
rpoE_2	ZONMW-56_01014	rpoE_2
rpoE_2	ZONMW-56_00434	rpoE_2
rpoE_2	ZONMW-56_02981	rpoE_2
rpoE_2	ZONMW-56_02984	rpoE_2
rpoE_2	ZONMW-56_00642	rpoE_2
rpoE_2	ZONMW-56_02192	rpoE_2
rpoE_2	ZONMW-56_00710	rpoE_2
rpoE_2	ZONMW-56_02641	rpoE_2
rpoE_2	ZONMW-56_01808	rpoE_2
rpoE_2	ZONMW-56_00923	rpoE_2
rpoE_2	ZONMW-56_01666	rpoE_2
rpoE_2	ZONMW-56_01806	rpoE_2
rpoE_2	ZONMW-56_01684	rpoE_2
rpoE_2	ZONMW-56_00354	rpoE_2
rpoE_2	ZONMW-56_01634	rpoE_2
rpoE_2	ZONMW-57_01410	rpoE_2
rpoE_2	ZONMW-57_00815	rpoE_2
rpoE_2	ZONMW-57_00351	rpoE_2
rpoE_2	ZONMW-57_02670	rpoE_2
rpoE_2	ZONMW-57_00932	rpoE_2
rpoE_2	ZONMW-57_01314	rpoE_2
rpoE_2	ZONMW-57_02334	rpoE_2
rpoE_2	ZONMW-57_00646	rpoE_2
rpoE_2	ZONMW-57_03067	rpoE_2
rpoE_2	ZONMW-57_03064	rpoE_2
rpoE_2	ZONMW-57_00848	rpoE_2
rpoE_2	ZONMW-57_00028	rpoE_2
rpoE_2	ZONMW-57_00917	rpoE_2
rpoE_2	ZONMW-57_02785	rpoE_2
rpoE_2	ZONMW-57_02332	rpoE_2
rpoE_2	ZONMW-57_01223	rpoE_2
rpoE_2	ZONMW-57_02013	rpoE_2

rpoE_2	ZONMW-57_02031	rpoE_2
rpoE_2	ZONMW-57_00567	rpoE_2
rpoE_2	ZONMW-57_01981	rpoE_2
rpoE_2	ZONMW-58_01174	rpoE_2
rpoE_2	ZONMW-58_00156	rpoE_2
rpoE_2	ZONMW-58_00620	rpoE_2
rpoE_2	ZONMW-58_02695	rpoE_2
rpoE_2	ZONMW-58_00039	rpoE_2
rpoE_2	ZONMW-58_01270	rpoE_2
rpoE_2	ZONMW-58_02358	rpoE_2
rpoE_2	ZONMW-58_00325	rpoE_2
rpoE_2	ZONMW-58_03088	rpoE_2
rpoE_2	ZONMW-58_03085	rpoE_2
rpoE_2	ZONMW-58_00123	rpoE_2
rpoE_2	ZONMW-58_00938	rpoE_2
rpoE_2	ZONMW-58_00054	rpoE_2
rpoE_2	ZONMW-58_02809	rpoE_2
rpoE_2	ZONMW-58_02356	rpoE_2
rpoE_2	ZONMW-58_01361	rpoE_2
rpoE_2	ZONMW-58_02065	rpoE_2
rpoE_2	ZONMW-58_02047	rpoE_2
rpoE_2	ZONMW-58_00404	rpoE_2
rpoE_2	ZONMW-58_02097	rpoE_2
rpoE_2	ZONMW-59_01412	rpoE_2
rpoE_2	ZONMW-59_00810	rpoE_2
rpoE_2	ZONMW-59_00367	rpoE_2
rpoE_2	ZONMW-59_02703	rpoE_2
rpoE_2	ZONMW-59_00966	rpoE_2
rpoE_2	ZONMW-59_01315	rpoE_2
rpoE_2	ZONMW-59_00635	rpoE_2
rpoE_2	ZONMW-59_03143	rpoE_2
rpoE_2	ZONMW-59_03140	rpoE_2
rpoE_2	ZONMW-59_00843	rpoE_2
rpoE_2	ZONMW-59_00028	rpoE_2
rpoE_2	ZONMW-59_00950	rpoE_2
rpoE_2	ZONMW-59_02919	rpoE_2
rpoE_2	ZONMW-59_02570	rpoE_2
rpoE_2	ZONMW-59_01224	rpoE_2
rpoE_2	ZONMW-59_02284	rpoE_2
rpoE_2	ZONMW-59_02572	rpoE_2
rpoE_2	ZONMW-59_02266	rpoE_2
rpoE_2	ZONMW-59_00554	rpoE_2
rpoE_2	ZONMW-59_02316	rpoE_2
rpoE_2	ZONMW-60_01163	rpoE_2
rpoE_2	ZONMW-60_00814	rpoE_2
rpoE_2	ZONMW-60_02464	rpoE_2
rpoE_2	ZONMW-60_00378	rpoE_2
rpoE_2	ZONMW-60_02691	rpoE_2

rpoE_2	ZONMW-60_00930	rpoE_2
rpoE_2	ZONMW-60_01255	rpoE_2
rpoE_2	ZONMW-60_00649	rpoE_2
rpoE_2	ZONMW-60_03079	rpoE_2
rpoE_2	ZONMW-60_03076	rpoE_2
rpoE_2	ZONMW-60_00847	rpoE_2
rpoE_2	ZONMW-60_00029	rpoE_2
rpoE_2	ZONMW-60_03367	rpoE_2
rpoE_2	ZONMW-60_00916	rpoE_2
rpoE_2	ZONMW-60_02904	rpoE_2
rpoE_2	ZONMW-60_01346	rpoE_2
rpoE_2	ZONMW-60_02580	rpoE_2
rpoE_2	ZONMW-60_02562	rpoE_2
rpoE_2	ZONMW-60_00569	rpoE_2
rpoE_2	ZONMW-60_02614	rpoE_2
rpoE_2	ZONMW-61_01861	rpoE_2
rpoE_2	ZONMW-61_02147	rpoE_2
rpoE_2	ZONMW-61_00337	rpoE_2
rpoE_2	ZONMW-61_02277	rpoE_2
rpoE_2	ZONMW-61_03000	rpoE_2
rpoE_2	ZONMW-61_02733	rpoE_2
rpoE_2	ZONMW-61_00108	rpoE_2
rpoE_2	ZONMW-61_02279	rpoE_2
rpoE_2	ZONMW-61_03080	rpoE_2
rpoE_2	ZONMW-61_00908	rpoE_2
rpoE_2	ZONMW-61_03698	rpoE_2
rpoE_2	ZONMW-61_02498	rpoE_2
rpoE_2	ZONMW-61_00357	rpoE_2
rpoE_2	ZONMW-61_03423	rpoE_2
rpoE_2	ZONMW-61_03695	rpoE_2
rpoE_2	ZONMW-61_03675	rpoE_2
rpoE_2	ZONMW-61_03286	rpoE_2
rpoE_2	ZONMW-61_02752	rpoE_2
rpoE_2	ZONMW-61_02199	rpoE_2
rpoE_2	ZONMW-61_02115	rpoE_2
rpoE_2	ZONMW-62_02137	rpoE_2
rpoE_2	ZONMW-62_00393	rpoE_2
rpoE_2	ZONMW-62_00098	rpoE_2
rpoE_2	ZONMW-62_03906	rpoE_2
rpoE_2	ZONMW-62_00961	rpoE_2
rpoE_2	ZONMW-62_02097	rpoE_2
rpoE_2	ZONMW-62_00889	rpoE_2
rpoE_2	ZONMW-62_02234	rpoE_2
rpoE_2	ZONMW-62_00017	rpoE_2
rpoE_2	ZONMW-62_01888	rpoE_2
rpoE_2	ZONMW-62_00887	rpoE_2
rpoE_2	ZONMW-62_02720	rpoE_2
rpoE_2	ZONMW-62_03743	rpoE_2

rpoE_2	ZONMW-62_03740	rpoE_2
rpoE_2	ZONMW-62_00040	rpoE_2
rpoE_2	ZONMW-62_03420	rpoE_2
rpoE_2	ZONMW-62_00576	rpoE_2
rpoE_2	ZONMW-62_00629	rpoE_2
rpoE_2	ZONMW-62_00720	rpoE_2
rpoE_2	ZONMW-62_01904	rpoE_2
rpoE_2	ZONMW-62_03619	rpoE_2
rpoE_2	ZONMW-62_03722	rpoE_2
rpoE_2	ZONMW-62_00492	rpoE_2
rpoE_2	ZONMW-62_03440	rpoE_2
rpoE_2	ZONMW-62_00131	rpoE_2
rpoE_2	ZONMW-62_02051	rpoE_2
rpoE_2	ZONMW-63_01155	rpoE_2
rpoE_2	ZONMW-63_00490	rpoE_2
rpoE_2	ZONMW-63_00019	rpoE_2
rpoE_2	ZONMW-63_02216	rpoE_2
rpoE_2	ZONMW-63_02066	rpoE_2
rpoE_2	ZONMW-63_01251	rpoE_2
rpoE_2	ZONMW-63_00320	rpoE_2
rpoE_2	ZONMW-63_02947	rpoE_2
rpoE_2	ZONMW-63_02950	rpoE_2
rpoE_2	ZONMW-63_01982	rpoE_2
rpoE_2	ZONMW-63_01792	rpoE_2
rpoE_2	ZONMW-63_03299	rpoE_2
rpoE_2	ZONMW-63_02051	rpoE_2
rpoE_2	ZONMW-63_02288	rpoE_2
rpoE_2	ZONMW-63_02501	rpoE_2
rpoE_2	ZONMW-63_01342	rpoE_2
rpoE_2	ZONMW-63_01670	rpoE_2
rpoE_2	ZONMW-63_02503	rpoE_2
rpoE_2	ZONMW-63_01652	rpoE_2
rpoE_2	ZONMW-63_00240	rpoE_2
rpoE_2	ZONMW-63_01701	rpoE_2
rpoE_2	ZONMW-64_02064	rpoE_2
rpoE_2	ZONMW-64_03585	rpoE_2
rpoE_2	ZONMW-64_01751	rpoE_2
rpoE_2	ZONMW-64_04192	rpoE_2
rpoE_2	ZONMW-64_01480	rpoE_2
rpoE_2	ZONMW-64_03455	rpoE_2
rpoE_2	ZONMW-64_03861	rpoE_2
rpoE_2	ZONMW-64_00139	rpoE_2
rpoE_2	ZONMW-64_03538	rpoE_2
rpoE_2	ZONMW-64_01512	rpoE_2
rpoE_2	ZONMW-65_01894	rpoE_2
rpoE_2	ZONMW-65_01092	rpoE_2
rpoE_2	ZONMW-65_01134	rpoE_2
rpoE_2	ZONMW-65_01760	rpoE_2

rpoE_2	ZONMW-65_03522	rpoE_2
rpoE_2	ZONMW-65_00204	rpoE_2
rpoE_2	ZONMW-65_02291	rpoE_2
rpoE_2	ZONMW-65_01807	rpoE_2
rpoE_2	ZONMW-66_02671	rpoE_2
rpoE_2	ZONMW-66_00129	rpoE_2
rpoE_2	ZONMW-67_02382	rpoE_2
rpoE_2	ZONMW-67_03291	rpoE_2
rpoE_2	ZONMW-67_00008	rpoE_2
rpoE_2	ZONMW-67_02120	rpoE_2
rpoE_2	ZONMW-67_03356	rpoE_2
rpoE_2	ZONMW-67_02908	rpoE_2
rpoE_2	ZONMW-68_04417	rpoE_2
rpoE_2	ZONMW-68_02510	rpoE_2
rpoE_2	ZONMW-68_02532	rpoE_2
rpoE_2	ZONMW-68_03119	rpoE_2
rpoE_2	ZONMW-68_04141	rpoE_2
rpoE_2	ZONMW-68_00533	rpoE_2
rpoE_2	ZONMW-68_00481	rpoE_2
rpoE_2	ZONMW-68_02410	rpoE_2
rpoE_2	ZONMW-68_03497	rpoE_2
rpoE_2	ZONMW-68_04231	rpoE_2
rpoE_2	ZONMW-68_00111	rpoE_2
rpoE_2	ZONMW-68_02979	rpoE_2
rpoE_2	ZONMW-68_01980	rpoE_2
rpoE_2	ZONMW-68_02533	rpoE_2
rpoE_2	ZONMW-68_03003	rpoE_2
rpoE_2	ZONMW-68_04647	rpoE_2
rpoE_2	ZONMW-68_02425	rpoE_2
rpoE_2	ZONMW-68_03384	rpoE_2
rpoE_2	ZONMW-68_02252	rpoE_2
rpoE_2	ZONMW-68_01978	rpoE_2
rpoE_2	ZONMW-68_03600	rpoE_2
rpoE_2	ZONMW-68_02141	rpoE_2
rpoE_2	ZONMW-68_03983	rpoE_2
rpoE_2	ZONMW-68_02691	rpoE_2
rpoE_2	ZONMW-68_04241	rpoE_2
rpoE_2	ZONMW-68_02783	rpoE_2
rpoE_2	ZONMW-68_04069	rpoE_2
rpoE_2	ZONMW-68_03959	rpoE_2
rpoE_2	ZONMW-68_02867	rpoE_2
rpoE_2	ZONMW-68_01373	rpoE_2
rpoE_2	ZONMW-68_01200	rpoE_2
rpoE_2	ZONMW-68_02704	rpoE_2
rpoE_2	ZONMW-68_02987	rpoE_2
rpoE_2	ZONMW-68_03199	rpoE_2
rpoE_2	ZONMW-68_00118	rpoE_2
rpoE_2	ZONMW-68_01567	rpoE_2

rpoE_2	ZONMW-68_03603	rpoE_2
rpoE_2	ZONMW-68_02775	rpoE_2
rpoE_2	ZONMW-68_02165	rpoE_2
rpoE_2	ZONMW-68_01319	rpoE_2
rpoE_2	ZONMW-68_00992	rpoE_2
rpoE_2	ZONMW-68_00273	rpoE_2
rpoE_2	ZONMW-69_02430	rpoE_2
rpoE_2	ZONMW-69_03292	rpoE_2
rpoE_2	ZONMW-69_02617	rpoE_2
rpoE_2	ZONMW-69_01823	rpoE_2
rpoE_2	ZONMW-69_03357	rpoE_2
rpoE_2	ZONMW-69_03046	rpoE_2
rpoE_2	ZONMW-72_00852	rpoE_2
rpoE_2	ZONMW-72_00904	rpoE_2
rpoE_2	ZONMW-72_02986	rpoE_2
rpoE_2	ZONMW-72_01796	rpoE_2
rpoE_2	ZONMW-72_04245	rpoE_2
rpoE_2	ZONMW-72_00361	rpoE_2
rpoE_2	ZONMW-72_03013	rpoE_2
rpoE_2	ZONMW-72_01343	rpoE_2
rpoE_2	ZONMW-72_01473	rpoE_2
rpoE_2	ZONMW-72_01585	rpoE_2
rpoE_2	ZONMW-72_01715	rpoE_2
rpoE_2	ZONMW-72_00597	rpoE_2
rpoE_2	ZONMW-72_03053	rpoE_2
rpoE_2	ZONMW-72_04385	rpoE_2
rpoE_2	ZONMW-72_02545	rpoE_2
rpoE_2	ZONMW-72_02795	rpoE_2
rpoE_2	ZONMW-72_01358	rpoE_2
rpoE_2	ZONMW-73_03824	rpoE_2
rpoE_2	ZONMW-73_02922	rpoE_2
rpoE_2	ZONMW-73_00385	rpoE_2
rpoE_2	ZONMW-73_00489	rpoE_2
rpoE_2	ZONMW-73_00641	rpoE_2
rpoE_2	ZONMW-73_01606	rpoE_2
rpoE_2	ZONMW-73_02679	rpoE_2
rpoE_2	ZONMW-73_00557	rpoE_2
rpoE_2	ZONMW-73_01952	rpoE_2
rpoE_2	ZONMW-73_01629	rpoE_2
rpoE_2	ZONMW-73_00533	rpoE_2
rpoE_2	ZONMW-73_03047	rpoE_2
rpoE_2	ZONMW-73_02172	rpoE_2
rpoE_2	ZONMW-73_03309	rpoE_2
rpoE_2	ZONMW-73_01223	rpoE_2
rpoE_2	ZONMW-73_00714	rpoE_2
rpoE_2	ZONMW-73_00695	rpoE_2
rpoE_2	ZONMW-73_01345	rpoE_2
rpoE_2	ZONMW-73_02078	rpoE_2

rpoE_2	ZONMW-73_00124	rpoE_2
rpoE_2	ZONMW-73_01227	rpoE_2
rpoE_2	ZONMW-73_01537	rpoE_2
rpoE_2	ZONMW-73_01037	rpoE_2
rpoE_2	ZONMW-73_01662	rpoE_2
rpoE_2	ZONMW-74_00984	rpoE_2
rpoE_2	ZONMW-74_02587	rpoE_2
rpoE_2	ZONMW-74_00908	rpoE_2
rpoE_2	ZONMW-74_00361	rpoE_2
rpoE_2	ZONMW-74_01884	rpoE_2
rpoE_2	ZONMW-74_03441	rpoE_2
rpoE_2	ZONMW-74_01937	rpoE_2
rpoE_2	ZONMW-74_02928	rpoE_2
rpoE_2	ZONMW-74_01059	rpoE_2
rpoE_2	ZONMW-74_00021	rpoE_2
rpoE_2	ZONMW-74_02500	rpoE_2
rpoE_2	ZONMW-74_00636	rpoE_2
rpoE_2	ZONMW-74_02959	rpoE_2
rpoE_2	ZONMW-74_01705	rpoE_2
rpoE_2	ZONMW-74_03270	rpoE_2
rpoE_2	ZONMW-74_01091	rpoE_2
rpoE_2	ZONMW-74_01443	rpoE_2
rpoE_2	ZONMW-74_01174	rpoE_2
rpoE_2	ZONMW-74_02658	rpoE_2
rpoE_2	ZONMW-74_02403	rpoE_2
rpoE_2	ZONMW-74_02503	rpoE_2
rpoE_2	ZONMW-74_03285	rpoE_2
rpoE_2	ZONMW-74_00183	rpoE_2
rpoE_2	ZONMW-74_02641	rpoE_2
rpoE_2	ZONMW-74_02691	rpoE_2
rpoE_2	ZONMW-75_02815	rpoE_2
rpoE_2	ZONMW-75_02485	rpoE_2
rpoE_2	ZONMW-75_00563	rpoE_2
rpoE_2	ZONMW-75_02531	rpoE_2
rpoE_2	ZONMW-75_00584	rpoE_2
rpoE_2	ZONMW-75_01215	rpoE_2
rpoE_2	ZONMW-75_01505	rpoE_2
rpoE_2	ZONMW-75_01300	rpoE_2
rpoE_2	ZONMW-75_02369	rpoE_2
rpoE_2	ZONMW-75_02551	rpoE_2
rpoE_2	ZONMW-75_03304	rpoE_2
rpoE_2	ZONMW-75_02419	rpoE_2
rpoE_2	ZONMW-75_01645	rpoE_2
rpoE_2	ZONMW-75_03446	rpoE_2
rpoE_2	ZONMW-75_02633	rpoE_2
rpoE_2	ZONMW-75_02451	rpoE_2
rpoE_2	ZONMW-75_03441	rpoE_2
rpoE_2	ZONMW-75_03737	rpoE_2

rpoE_2	ZONMW-75_00738	rpoE_2
rpoE_2	da5A-8_01146	rpoE_2
rpoE_2	da5A-8_01831	rpoE_2
rpoE_2	da5A-8_00113	rpoE_2
rpoE_2	da5A-8_01527	rpoE_2
rpoE_2	da5A-8_02165	rpoE_2
rpoE_2	da5A-8_00181	rpoE_2
rpoE_2	da5A-8_01091	rpoE_2
rpoE_2	da5A-8_00200	rpoE_2
rpoE_2	da5A-8_01424	rpoE_2
rpoE_2	da5A-8_01009	rpoE_2
rpoE_2	da5A-8_02113	rpoE_2
rpoE_2	da5A-8_01304	rpoE_2
rpoE_2	da5A-8_01866	rpoE_2
rpoE_2	da5A-8_01366	rpoE_2
rpoE_2	da5A-8_03187	rpoE_2
rpoE_2	da5A-8_01010	rpoE_2
rpoE_2	da5A-8_02347	rpoE_2
rpoE_2	da5A-8_03239	rpoE_2
rpoE_2	da5A-8_01445	rpoE_2
rpoE_2	da5A-8_01886	rpoE_2
rpoE_2	da5A-8_03359	rpoE_2
rimM	18_01424	rimM
rimM	ACAM48_01995	rimM
rimM	CAM030_02160	rimM
rimM	CECT7946_00464	rimM
rimM	CGMCC1.8863_00574	rimM
rimM	CL8139_01029	rimM
rimM	DAU203_01806	rimM
rimM	DD5b_01644	rimM
rimM	DSM1208_02093	rimM
rimM	DSM12212_01375	rimM
rimM	DSM15343_00750	rimM
rimM	DSM15361_00134	rimM
rimM	DSM15365_02234	rimM
rimM	DSM15718_02350	rimM
rimM	DSM16505T_00176	rimM
rimM	DSM18103_01621	rimM
rimM	DSM18293_00385	rimM
rimM	DSM18841_02672	rimM
rimM	DSM18842_02570	rimM
rimM	DSM19866_03070	rimM
rimM	DSM2041_00693	rimM
rimM	DSM2061_03653	rimM
rimM	DSM2064_02307	rimM
rimM	DSM21164_00477	rimM
rimM	DSM23404_01801	rimM
rimM	DSM23424_00718	rimM

rimM	DSM24786_02174	rimM
rimM	DSM7489_01725	rimM
rimM	DSW1_00318	rimM
rimM	DSW5_00612	rimM
rimM	DSW6_00859	rimM
rimM	DSW8_01792	rimM
rimM	F52_02703	rimM
rimM	GCA_001027945.1_00854	rimM
rimM	HI1_01646	rimM
rimM	IR1_00556	rimM
rimM	KCOM3131_01666	rimM
rimM	KCTC12974_00771	rimM
rimM	KL-A_01531	rimM
rimM	L12_00507	rimM
rimM	LL0412.1.7_00132	rimM
rimM	MAR_2009_71_01628	rimM
rimM	NN016038_01433	rimM
rimM	PAO1_03867	rimM
rimM	UW101_01361	rimM
rimM	V4-33_00322	rimM
rimM	W5C_00427	rimM
rimM	ZONMW-01_00172	rimM
rimM	ZONMW-02_02437	rimM
rimM	ZONMW-03_00898	rimM
rimM	ZONMW-04_02048	rimM
rimM	ZONMW-05_01087	rimM
rimM	ZONMW-06_02899	rimM
rimM	ZONMW-07_00113	rimM
rimM	ZONMW-08_01605	rimM
rimM	ZONMW-09_00061	rimM
rimM	ZONMW-10_00080	rimM
rimM	ZONMW-11_00384	rimM
rimM	ZONMW-12_03186	rimM
rimM	ZONMW-13_00135	rimM
rimM	ZONMW-14_02678	rimM
rimM	ZONMW-15_00226	rimM
rimM	ZONMW-19_00411	rimM
rimM	ZONMW-20_02722	rimM
rimM	ZONMW-21_00322	rimM
rimM	ZONMW-22_00322	rimM
rimM	ZONMW-23_01437	rimM
rimM	ZONMW-24_01137	rimM
rimM	ZONMW-25_02545	rimM
rimM	ZONMW-26_01131	rimM
rimM	ZONMW-27_00741	rimM
rimM	ZONMW-28_02007	rimM
rimM	ZONMW-29_00025	rimM
rimM	ZONMW-30_01530	rimM

rimM	ZONMW-31_03007	rimM
rimM	ZONMW-32_02413	rimM
rimM	ZONMW-33_02705	rimM
rimM	ZONMW-34_01999	rimM
rimM	ZONMW-35_01691	rimM
rimM	ZONMW-36_02547	rimM
rimM	ZONMW-37_00322	rimM
rimM	ZONMW-38_00804	rimM
rimM	ZONMW-39_02193	rimM
rimM	ZONMW-40_01527	rimM
rimM	ZONMW-41_01308	rimM
rimM	ZONMW-42_00420	rimM
rimM	ZONMW-43_01503	rimM
rimM	ZONMW-44_00877	rimM
rimM	ZONMW-45_01988	rimM
rimM	ZONMW-46_02337	rimM
rimM	ZONMW-47_02195	rimM
rimM	ZONMW-48_00872	rimM
rimM	ZONMW-49_01272	rimM
rimM	ZONMW-50_01274	rimM
rimM	ZONMW-51_01387	rimM
rimM	ZONMW-52_01129	rimM
rimM	ZONMW-53_00718	rimM
rimM	ZONMW-54_00349	rimM
rimM	ZONMW-55_00689	rimM
rimM	ZONMW-56_02390	rimM
rimM	ZONMW-57_02519	rimM
rimM	ZONMW-58_02544	rimM
rimM	ZONMW-59_01948	rimM
rimM	ZONMW-60_01631	rimM
rimM	ZONMW-61_00737	rimM
rimM	ZONMW-62_02440	rimM
rimM	ZONMW-63_00685	rimM
rimM	ZONMW-64_02080	rimM
rimM	ZONMW-65_01171	rimM
rimM	ZONMW-66_02238	rimM
rimM	ZONMW-67_02398	rimM
rimM	ZONMW-68_00505	rimM
rimM	ZONMW-69_02446	rimM
rimM	ZONMW-72_00208	rimM
rimM	ZONMW-73_01335	rimM
rimM	ZONMW-74_01944	rimM
rimM	ZONMW-75_00230	rimM
rimM	da5A-8_00684	rimM
msrB_2	18_02126	msrB_2
msrB_2	18_02872	msrB_2
msrB_2	18_02873	msrB_2
msrB_2	18_02299	msrB_2

msrB_2	18_02871	msrB_2
msrB_2	ACAM48_03405	msrB_2
msrB_2	ACAM48_00667	msrB_2
msrB_2	ACAM48_01048	msrB_2
msrB_2	ACAM48_01049	msrB_2
msrB_2	CAM030_02673	msrB_2
msrB_2	CAM030_00770	msrB_2
msrB_2	CAM030_02045	msrB_2
msrB_2	CAM030_02044	msrB_2
msrB_2	CECT7946_00950	msrB_2
msrB_2	CECT7946_02479	msrB_2
msrB_2	CECT7946_02476	msrB_2
msrB_2	CECT7946_02393	msrB_2
msrB_2	CECT7946_02481	msrB_2
msrB_2	CGMCC1.8863_03389	msrB_2
msrB_2	CGMCC1.8863_02494	msrB_2
msrB_2	CGMCC1.8863_01692	msrB_2
msrB_2	CGMCC1.8863_03331	msrB_2
msrB_2	CGMCC1.8863_02495	msrB_2
msrB_2	CL8139_02838	msrB_2
msrB_2	CL8139_00241	msrB_2
msrB_2	CL8139_01230	msrB_2
msrB_2	CL8139_01229	msrB_2
msrB_2	CL8139_00240	msrB_2
msrB_2	DAU203_00050	msrB_2
msrB_2	DAU203_03341	msrB_2
msrB_2	DAU203_00195	msrB_2
msrB_2	DAU203_00196	msrB_2
msrB_2	DAU203_03340	msrB_2
msrB_2	DD5b_01269	msrB_2
msrB_2	DD5b_00174	msrB_2
msrB_2	DD5b_00173	msrB_2
msrB_2	DSM1208_02829	msrB_2
msrB_2	DSM1208_01137	msrB_2
msrB_2	DSM1208_01139	msrB_2
msrB_2	DSM1208_00500	msrB_2
msrB_2	DSM1208_01136	msrB_2
msrB_2	DSM12212_02897	msrB_2
msrB_2	DSM12212_02331	msrB_2
msrB_2	DSM12212_01442	msrB_2
msrB_2	DSM12212_00798	msrB_2
msrB_2	DSM12212_02332	msrB_2
msrB_2	DSM15343_01450	msrB_2
msrB_2	DSM15343_01078	msrB_2
msrB_2	DSM15343_00041	msrB_2
msrB_2	DSM15361_02047	msrB_2
msrB_2	DSM15361_02044	msrB_2
msrB_2	DSM15361_00882	msrB_2

msrB_2	DSM15361_01565	msrB_2
msrB_2	DSM15361_01564	msrB_2
msrB_2	DSM15365_03803	msrB_2
msrB_2	DSM15365_03441	msrB_2
msrB_2	DSM15365_03444	msrB_2
msrB_2	DSM15365_02094	msrB_2
msrB_2	DSM15365_03440	msrB_2
msrB_2	DSM15718_01772	msrB_2
msrB_2	DSM15718_02011	msrB_2
msrB_2	DSM15718_00847	msrB_2
msrB_2	DSM15718_01773	msrB_2
msrB_2	DSM15718_01595	msrB_2
msrB_2	DSM16505T_01298	msrB_2
msrB_2	DSM16505T_01666	msrB_2
msrB_2	DSM16505T_01360	msrB_2
msrB_2	DSM16505T_01667	msrB_2
msrB_2	DSM18103_03361	msrB_2
msrB_2	DSM18103_01488	msrB_2
msrB_2	DSM18103_00057	msrB_2
msrB_2	DSM18103_01487	msrB_2
msrB_2	DSM18293_02534	msrB_2
msrB_2	DSM18293_01849	msrB_2
msrB_2	DSM18293_04397	msrB_2
msrB_2	DSM18293_02535	msrB_2
msrB_2	DSM18293_03991	msrB_2
msrB_2	DSM18841_02292	msrB_2
msrB_2	DSM18841_03118	msrB_2
msrB_2	DSM18841_02369	msrB_2
msrB_2	DSM18841_03117	msrB_2
msrB_2	DSM18842_01205	msrB_2
msrB_2	DSM18842_03137	msrB_2
msrB_2	DSM18842_01276	msrB_2
msrB_2	DSM18842_03136	msrB_2
msrB_2	DSM19866_00006	msrB_2
msrB_2	DSM19866_03784	msrB_2
msrB_2	DSM19866_03780	msrB_2
msrB_2	DSM19866_00758	msrB_2
msrB_2	DSM19866_03785	msrB_2
msrB_2	DSM2041_02930	msrB_2
msrB_2	DSM2041_02068	msrB_2
msrB_2	DSM2041_02836	msrB_2
msrB_2	DSM2041_01605	msrB_2
msrB_2	DSM2041_01604	msrB_2
msrB_2	DSM2061_04313	msrB_2
msrB_2	DSM2061_02653	msrB_2
msrB_2	DSM2061_02654	msrB_2
msrB_2	DSM2061_01991	msrB_2
msrB_2	DSM2061_02652	msrB_2

msrB_2	DSM2064_04810	msrB_2
msrB_2	DSM2064_00208	msrB_2
msrB_2	DSM2064_04811	msrB_2
msrB_2	DSM2064_03133	msrB_2
msrB_2	DSM21164_03069	msrB_2
msrB_2	DSM21164_01499	msrB_2
msrB_2	DSM21164_01498	msrB_2
msrB_2	DSM21164_01856	msrB_2
msrB_2	DSM21164_01500	msrB_2
msrB_2	DSM23404_00759	msrB_2
msrB_2	DSM23404_02654	msrB_2
msrB_2	DSM23404_02467	msrB_2
msrB_2	DSM23404_02833	msrB_2
msrB_2	DSM23404_02832	msrB_2
msrB_2	DSM23424_02530	msrB_2
msrB_2	DSM23424_01201	msrB_2
msrB_2	DSM23424_01196	msrB_2
msrB_2	DSM23424_00956	msrB_2
msrB_2	DSM23424_01202	msrB_2
msrB_2	DSM24786_01233	msrB_2
msrB_2	DSM24786_00816	msrB_2
msrB_2	DSM24786_00821	msrB_2
msrB_2	DSM24786_00050	msrB_2
msrB_2	DSM24786_00049	msrB_2
msrB_2	DSM24786_00815	msrB_2
msrB_2	DSM7489_00050	msrB_2
msrB_2	DSM7489_03177	msrB_2
msrB_2	DSM7489_00189	msrB_2
msrB_2	DSM7489_00190	msrB_2
msrB_2	DSM7489_03176	msrB_2
msrB_2	DSW1_02889	msrB_2
msrB_2	DSW1_00812	msrB_2
msrB_2	DSW1_02628	msrB_2
msrB_2	DSW1_02629	msrB_2
msrB_2	DSW5_02416	msrB_2
msrB_2	DSW5_01493	msrB_2
msrB_2	DSW5_01490	msrB_2
msrB_2	DSW5_02029	msrB_2
msrB_2	DSW5_01494	msrB_2
msrB_2	DSW6_00568	msrB_2
msrB_2	DSW6_02961	msrB_2
msrB_2	DSW6_02687	msrB_2
msrB_2	DSW6_02415	msrB_2
msrB_2	DSW6_02416	msrB_2
msrB_2	DSW8_01146	msrB_2
msrB_2	DSW8_02799	msrB_2
msrB_2	DSW8_03350	msrB_2
msrB_2	DSW8_03496	msrB_2

msrB_2	DSW8_02800	msrB_2
msrB_2	F52_01620	msrB_2
msrB_2	F52_04380	msrB_2
msrB_2	F52_01621	msrB_2
msrB_2	F52_00522	msrB_2
msrB_2	GCA_001027945.1_01152	msrB_2
msrB_2	GCA_001027945.1_02662	msrB_2
msrB_2	GCA_001027945.1_00019	msrB_2
msrB_2	GCA_001027945.1_02661	msrB_2
msrB_2	HI1_03349	msrB_2
msrB_2	HI1_03124	msrB_2
msrB_2	HI1_00121	msrB_2
msrB_2	HI1_00122	msrB_2
msrB_2	HI1_03123	msrB_2
msrB_2	IR1_02969	msrB_2
msrB_2	IR1_01831	msrB_2
msrB_2	IR1_02968	msrB_2
msrB_2	IR1_02361	msrB_2
msrB_2	KCOM3131_01082	msrB_2
msrB_2	KCTC12974_01019	msrB_2
msrB_2	KCTC12974_02964	msrB_2
msrB_2	KCTC12974_02743	msrB_2
msrB_2	KCTC12974_01567	msrB_2
msrB_2	KCTC12974_01566	msrB_2
msrB_2	KL-A_03298	msrB_2
msrB_2	KL-A_01995	msrB_2
msrB_2	KL-A_01185	msrB_2
msrB_2	KL-A_01186	msrB_2
msrB_2	KL-A_01996	msrB_2
msrB_2	L12_00356	msrB_2
msrB_2	LL0412.1.7_02199	msrB_2
msrB_2	LL0412.1.7_00925	msrB_2
msrB_2	LL0412.1.7_02302	msrB_2
msrB_2	LL0412.1.7_00926	msrB_2
msrB_2	MAR_2009_71_02299	msrB_2
msrB_2	MAR_2009_71_00655	msrB_2
msrB_2	MAR_2009_71_03579	msrB_2
msrB_2	MAR_2009_71_00090	msrB_2
msrB_2	MAR_2009_71_00654	msrB_2
msrB_2	NN016038_02144	msrB_2
msrB_2	NN016038_02924	msrB_2
msrB_2	NN016038_02925	msrB_2
msrB_2	NN016038_02310	msrB_2
msrB_2	NN016038_02923	msrB_2
msrB_2	PAO1_05188	msrB_2
msrB_2	PAO1_02928	msrB_2
msrB_2	UW101_00292	msrB_2
msrB_2	UW101_02919	msrB_2

msrB_2	UW101_00291	msrB_2
msrB_2	UW101_02667	msrB_2
msrB_2	V4-33_00605	msrB_2
msrB_2	V4-33_01626	msrB_2
msrB_2	V4-33_01627	msrB_2
msrB_2	W5C_00821	msrB_2
msrB_2	W5C_02374	msrB_2
msrB_2	W5C_00098	msrB_2
msrB_2	W5C_00099	msrB_2
msrB_2	W5C_02373	msrB_2
msrB_2	ZONMW-01_03065	msrB_2
msrB_2	ZONMW-01_00608	msrB_2
msrB_2	ZONMW-01_02076	msrB_2
msrB_2	ZONMW-01_00609	msrB_2
msrB_2	ZONMW-02_03049	msrB_2
msrB_2	ZONMW-02_00365	msrB_2
msrB_2	ZONMW-02_00368	msrB_2
msrB_2	ZONMW-02_03428	msrB_2
msrB_2	ZONMW-02_00364	msrB_2
msrB_2	ZONMW-03_02300	msrB_2
msrB_2	ZONMW-03_02502	msrB_2
msrB_2	ZONMW-03_02635	msrB_2
msrB_2	ZONMW-03_02634	msrB_2
msrB_2	ZONMW-03_02503	msrB_2
msrB_2	ZONMW-04_00765	msrB_2
msrB_2	ZONMW-04_00983	msrB_2
msrB_2	ZONMW-04_01919	msrB_2
msrB_2	ZONMW-04_01920	msrB_2
msrB_2	ZONMW-04_00984	msrB_2
msrB_2	ZONMW-05_00034	msrB_2
msrB_2	ZONMW-05_00235	msrB_2
msrB_2	ZONMW-05_02451	msrB_2
msrB_2	ZONMW-05_02450	msrB_2
msrB_2	ZONMW-05_00236	msrB_2
msrB_2	ZONMW-06_00270	msrB_2
msrB_2	ZONMW-06_01513	msrB_2
msrB_2	ZONMW-06_00271	msrB_2
msrB_2	ZONMW-07_02733	msrB_2
msrB_2	ZONMW-07_00498	msrB_2
msrB_2	ZONMW-07_03495	msrB_2
msrB_2	ZONMW-07_02292	msrB_2
msrB_2	ZONMW-07_00497	msrB_2
msrB_2	ZONMW-08_00946	msrB_2
msrB_2	ZONMW-08_00171	msrB_2
msrB_2	ZONMW-08_02345	msrB_2
msrB_2	ZONMW-08_02961	msrB_2
msrB_2	ZONMW-08_00170	msrB_2
msrB_2	ZONMW-09_01885	msrB_2

msrB_2	ZONMW-09_00773	msrB_2
msrB_2	ZONMW-09_01090	msrB_2
msrB_2	ZONMW-09_02258	msrB_2
msrB_2	ZONMW-09_02257	msrB_2
msrB_2	ZONMW-10_02271	msrB_2
msrB_2	ZONMW-10_00900	msrB_2
msrB_2	ZONMW-10_01792	msrB_2
msrB_2	ZONMW-10_01793	msrB_2
msrB_2	ZONMW-11_04021	msrB_2
msrB_2	ZONMW-11_01411	msrB_2
msrB_2	ZONMW-11_01462	msrB_2
msrB_2	ZONMW-11_01463	msrB_2
msrB_2	ZONMW-12_01302	msrB_2
msrB_2	ZONMW-12_04079	msrB_2
msrB_2	ZONMW-12_03460	msrB_2
msrB_2	ZONMW-12_03459	msrB_2
msrB_2	ZONMW-13_01698	msrB_2
msrB_2	ZONMW-13_01915	msrB_2
msrB_2	ZONMW-13_02040	msrB_2
msrB_2	ZONMW-13_03064	msrB_2
msrB_2	ZONMW-13_03065	msrB_2
msrB_2	ZONMW-14_00941	msrB_2
msrB_2	ZONMW-14_00777	msrB_2
msrB_2	ZONMW-14_00665	msrB_2
msrB_2	ZONMW-14_00439	msrB_2
msrB_2	ZONMW-14_00438	msrB_2
msrB_2	ZONMW-15_03154	msrB_2
msrB_2	ZONMW-15_01447	msrB_2
msrB_2	ZONMW-15_02475	msrB_2
msrB_2	ZONMW-15_01553	msrB_2
msrB_2	ZONMW-15_01554	msrB_2
msrB_2	ZONMW-15_01446	msrB_2
msrB_2	ZONMW-19_02916	msrB_2
msrB_2	ZONMW-19_01266	msrB_2
msrB_2	ZONMW-20_03198	msrB_2
msrB_2	ZONMW-20_01689	msrB_2
msrB_2	ZONMW-21_02698	msrB_2
msrB_2	ZONMW-21_02034	msrB_2
msrB_2	ZONMW-21_03249	msrB_2
msrB_2	ZONMW-21_03250	msrB_2
msrB_2	ZONMW-22_02411	msrB_2
msrB_2	ZONMW-22_01692	msrB_2
msrB_2	ZONMW-22_03267	msrB_2
msrB_2	ZONMW-22_03266	msrB_2
msrB_2	ZONMW-23_00139	msrB_2
msrB_2	ZONMW-23_00860	msrB_2
msrB_2	ZONMW-23_03289	msrB_2
msrB_2	ZONMW-23_03288	msrB_2

msrB_2	ZONMW-24_02176	msrB_2
msrB_2	ZONMW-24_01715	msrB_2
msrB_2	ZONMW-24_03290	msrB_2
msrB_2	ZONMW-24_03289	msrB_2
msrB_2	ZONMW-25_00697	msrB_2
msrB_2	ZONMW-25_01057	msrB_2
msrB_2	ZONMW-25_01281	msrB_2
msrB_2	ZONMW-25_01282	msrB_2
msrB_2	ZONMW-26_00926	msrB_2
msrB_2	ZONMW-26_00722	msrB_2
msrB_2	ZONMW-26_02665	msrB_2
msrB_2	ZONMW-26_02666	msrB_2
msrB_2	ZONMW-26_00721	msrB_2
msrB_2	ZONMW-27_00861	msrB_2
msrB_2	ZONMW-27_01058	msrB_2
msrB_2	ZONMW-27_02090	msrB_2
msrB_2	ZONMW-27_01059	msrB_2
msrB_2	ZONMW-28_01505	msrB_2
msrB_2	ZONMW-28_04019	msrB_2
msrB_2	ZONMW-28_01506	msrB_2
msrB_2	ZONMW-29_01868	msrB_2
msrB_2	ZONMW-29_01163	msrB_2
msrB_2	ZONMW-29_01742	msrB_2
msrB_2	ZONMW-29_01741	msrB_2
msrB_2	ZONMW-30_03027	msrB_2
msrB_2	ZONMW-30_01372	msrB_2
msrB_2	ZONMW-30_02472	msrB_2
msrB_2	ZONMW-30_02471	msrB_2
msrB_2	ZONMW-31_00579	msrB_2
msrB_2	ZONMW-31_02243	msrB_2
msrB_2	ZONMW-31_00580	msrB_2
msrB_2	ZONMW-32_01912	msrB_2
msrB_2	ZONMW-32_03876	msrB_2
msrB_2	ZONMW-32_01913	msrB_2
msrB_2	ZONMW-33_01902	msrB_2
msrB_2	ZONMW-33_03628	msrB_2
msrB_2	ZONMW-33_01903	msrB_2
msrB_2	ZONMW-34_00241	msrB_2
msrB_2	ZONMW-34_03015	msrB_2
msrB_2	ZONMW-34_00242	msrB_2
msrB_2	ZONMW-35_01149	msrB_2
msrB_2	ZONMW-35_02977	msrB_2
msrB_2	ZONMW-35_01148	msrB_2
msrB_2	ZONMW-36_01453	msrB_2
msrB_2	ZONMW-36_03627	msrB_2
msrB_2	ZONMW-36_01452	msrB_2
msrB_2	ZONMW-37_02365	msrB_2
msrB_2	ZONMW-37_01715	msrB_2

msrB_2	ZONMW-37_03276	msrB_2
msrB_2	ZONMW-37_03275	msrB_2
msrB_2	ZONMW-38_00736	msrB_2
msrB_2	ZONMW-38_02178	msrB_2
msrB_2	ZONMW-39_03323	msrB_2
msrB_2	ZONMW-39_02743	msrB_2
msrB_2	ZONMW-39_03322	msrB_2
msrB_2	ZONMW-40_01735	msrB_2
msrB_2	ZONMW-40_03028	msrB_2
msrB_2	ZONMW-40_02853	msrB_2
msrB_2	ZONMW-40_02852	msrB_2
msrB_2	ZONMW-41_01432	msrB_2
msrB_2	ZONMW-41_02057	msrB_2
msrB_2	ZONMW-41_01707	msrB_2
msrB_2	ZONMW-41_01726	msrB_2
msrB_2	ZONMW-42_03772	msrB_2
msrB_2	ZONMW-42_02389	msrB_2
msrB_2	ZONMW-42_03773	msrB_2
msrB_2	ZONMW-43_00708	msrB_2
msrB_2	ZONMW-43_03874	msrB_2
msrB_2	ZONMW-43_00707	msrB_2
msrB_2	ZONMW-44_01778	msrB_2
msrB_2	ZONMW-44_00577	msrB_2
msrB_2	ZONMW-44_01777	msrB_2
msrB_2	ZONMW-45_03159	msrB_2
msrB_2	ZONMW-45_02298	msrB_2
msrB_2	ZONMW-45_02591	msrB_2
msrB_2	ZONMW-45_02299	msrB_2
msrB_2	ZONMW-46_02903	msrB_2
msrB_2	ZONMW-46_03117	msrB_2
msrB_2	ZONMW-46_02967	msrB_2
msrB_2	ZONMW-46_03116	msrB_2
msrB_2	ZONMW-47_02970	msrB_2
msrB_2	ZONMW-47_03109	msrB_2
msrB_2	ZONMW-47_02906	msrB_2
msrB_2	ZONMW-47_03108	msrB_2
msrB_2	ZONMW-48_02895	msrB_2
msrB_2	ZONMW-48_01822	msrB_2
msrB_2	ZONMW-48_03012	msrB_2
msrB_2	ZONMW-48_01926	msrB_2
msrB_2	ZONMW-48_01927	msrB_2
msrB_2	ZONMW-48_01821	msrB_2
msrB_2	ZONMW-49_03305	msrB_2
msrB_2	ZONMW-49_03251	msrB_2
msrB_2	ZONMW-49_02488	msrB_2
msrB_2	ZONMW-49_02487	msrB_2
msrB_2	ZONMW-49_03252	msrB_2
msrB_2	ZONMW-50_03277	msrB_2

msrB_2	ZONMW-50_03258	msrB_2
msrB_2	ZONMW-50_02282	msrB_2
msrB_2	ZONMW-50_02281	msrB_2
msrB_2	ZONMW-50_03257	msrB_2
msrB_2	ZONMW-51_01857	msrB_2
msrB_2	ZONMW-51_02046	msrB_2
msrB_2	ZONMW-51_02465	msrB_2
msrB_2	ZONMW-51_02464	msrB_2
msrB_2	ZONMW-51_02047	msrB_2
msrB_2	ZONMW-52_00916	msrB_2
msrB_2	ZONMW-52_00726	msrB_2
msrB_2	ZONMW-52_02448	msrB_2
msrB_2	ZONMW-52_02447	msrB_2
msrB_2	ZONMW-52_00725	msrB_2
msrB_2	ZONMW-53_01659	msrB_2
msrB_2	ZONMW-53_01458	msrB_2
msrB_2	ZONMW-53_02185	msrB_2
msrB_2	ZONMW-53_02186	msrB_2
msrB_2	ZONMW-53_01457	msrB_2
msrB_2	ZONMW-54_01066	msrB_2
msrB_2	ZONMW-54_01263	msrB_2
msrB_2	ZONMW-54_02675	msrB_2
msrB_2	ZONMW-54_02676	msrB_2
msrB_2	ZONMW-54_01264	msrB_2
msrB_2	ZONMW-55_02179	msrB_2
msrB_2	ZONMW-55_02368	msrB_2
msrB_2	ZONMW-55_02556	msrB_2
msrB_2	ZONMW-55_02555	msrB_2
msrB_2	ZONMW-55_02369	msrB_2
msrB_2	ZONMW-56_02189	msrB_2
msrB_2	ZONMW-56_00013	msrB_2
msrB_2	ZONMW-56_01673	msrB_2
msrB_2	ZONMW-56_01674	msrB_2
msrB_2	ZONMW-56_00014	msrB_2
msrB_2	ZONMW-57_00031	msrB_2
msrB_2	ZONMW-57_00228	msrB_2
msrB_2	ZONMW-57_02020	msrB_2
msrB_2	ZONMW-57_02021	msrB_2
msrB_2	ZONMW-57_00229	msrB_2
msrB_2	ZONMW-58_00935	msrB_2
msrB_2	ZONMW-58_00743	msrB_2
msrB_2	ZONMW-58_02058	msrB_2
msrB_2	ZONMW-58_02057	msrB_2
msrB_2	ZONMW-58_00742	msrB_2
msrB_2	ZONMW-59_00031	msrB_2
msrB_2	ZONMW-59_00221	msrB_2
msrB_2	ZONMW-59_02277	msrB_2
msrB_2	ZONMW-59_02276	msrB_2

msrB_2	ZONMW-59_00222	msrB_2
msrB_2	ZONMW-60_00032	msrB_2
msrB_2	ZONMW-60_00253	msrB_2
msrB_2	ZONMW-60_02573	msrB_2
msrB_2	ZONMW-60_02572	msrB_2
msrB_2	ZONMW-60_00254	msrB_2
msrB_2	ZONMW-61_00904	msrB_2
msrB_2	ZONMW-61_01547	msrB_2
msrB_2	ZONMW-61_04016	msrB_2
msrB_2	ZONMW-61_02742	msrB_2
msrB_2	ZONMW-61_01546	msrB_2
msrB_2	ZONMW-62_02716	msrB_2
msrB_2	ZONMW-62_01298	msrB_2
msrB_2	ZONMW-62_03271	msrB_2
msrB_2	ZONMW-62_03429	msrB_2
msrB_2	ZONMW-62_01297	msrB_2
msrB_2	ZONMW-63_01795	msrB_2
msrB_2	ZONMW-63_02627	msrB_2
msrB_2	ZONMW-63_01663	msrB_2
msrB_2	ZONMW-63_01662	msrB_2
msrB_2	ZONMW-63_02628	msrB_2
msrB_2	ZONMW-64_00992	msrB_2
msrB_2	ZONMW-64_03808	msrB_2
msrB_2	ZONMW-64_00991	msrB_2
msrB_2	ZONMW-65_03186	msrB_2
msrB_2	ZONMW-65_02460	msrB_2
msrB_2	ZONMW-66_04559	msrB_2
msrB_2	ZONMW-66_00335	msrB_2
msrB_2	ZONMW-67_02964	msrB_2
msrB_2	ZONMW-67_02653	msrB_2
msrB_2	ZONMW-67_02654	msrB_2
msrB_2	ZONMW-68_03979	msrB_2
msrB_2	ZONMW-68_00901	msrB_2
msrB_2	ZONMW-68_00897	msrB_2
msrB_2	ZONMW-68_02152	msrB_2
msrB_2	ZONMW-68_00902	msrB_2
msrB_2	ZONMW-69_02990	msrB_2
msrB_2	ZONMW-69_00450	msrB_2
msrB_2	ZONMW-69_00451	msrB_2
msrB_2	ZONMW-72_01251	msrB_2
msrB_2	ZONMW-72_03069	msrB_2
msrB_2	ZONMW-72_01252	msrB_2
msrB_2	ZONMW-72_04334	msrB_2
msrB_2	ZONMW-73_03623	msrB_2
msrB_2	ZONMW-73_00318	msrB_2
msrB_2	ZONMW-73_00323	msrB_2
msrB_2	ZONMW-73_01494	msrB_2
msrB_2	ZONMW-73_00317	msrB_2

msrB_2	ZONMW-74_00631	msrB_2
msrB_2	ZONMW-74_00677	msrB_2
msrB_2	ZONMW-74_00676	msrB_2
msrB_2	ZONMW-74_02651	msrB_2
msrB_2	ZONMW-74_00680	msrB_2
msrB_2	ZONMW-75_03434	msrB_2
msrB_2	ZONMW-75_01286	msrB_2
msrB_2	ZONMW-75_01289	msrB_2
msrB_2	ZONMW-75_02504	msrB_2
msrB_2	ZONMW-75_01285	msrB_2
msrB_2	da5A-8_03549	msrB_2
msrB_2	da5A-8_01689	msrB_2
msrB_2	da5A-8_02628	msrB_2
msrB_2	da5A-8_01688	msrB_2
rpe	18_01726	rpe
rpe	ACAM48_01676	rpe
rpe	ACAM48_01751	rpe
rpe	CAM030_01374	rpe
rpe	CECT7946_00859	rpe
rpe	CGMCC1.8863_00640	rpe
rpe	CL8139_03356	rpe
rpe	DAU203_02154	rpe
rpe	DD5b_02719	rpe
rpe	DSM1208_00750	rpe
rpe	DSM12212_02698	rpe
rpe	DSM15343_02061	rpe
rpe	DSM15361_02390	rpe
rpe	DSM15365_00774	rpe
rpe	DSM15718_00443	rpe
rpe	DSM16505T_00080	rpe
rpe	DSM18103_01399	rpe
rpe	DSM18293_00521	rpe
rpe	DSM18841_02582	rpe
rpe	DSM18842_02482	rpe
rpe	DSM19866_02205	rpe
rpe	DSM2041_03584	rpe
rpe	DSM2061_02249	rpe
rpe	DSM2064_02443	rpe
rpe	DSM21164_00147	rpe
rpe	DSM23404_01001	rpe
rpe	DSM23424_02433	rpe
rpe	DSM24786_01891	rpe
rpe	DSM7489_02066	rpe
rpe	DSW1_01367	rpe
rpe	DSW5_00626	rpe
rpe	DSW6_01863	rpe
rpe	DSW8_03792	rpe
rpe	F52_02850	rpe

rpe	GCA_001027945.1_00957	rpe
rpe	HI1_01993	rpe
rpe	IR1_00430	rpe
rpe	KCOM3131_01582	rpe
rpe	KCTC12974_02424	rpe
rpe	KL-A_00204	rpe
rpe	L12_00396	rpe
rpe	LL0412.1.7_00079	rpe
rpe	MAR_2009_71_00336	rpe
rpe	NN016038_01747	rpe
rpe	PAO1_00622	rpe
rpe	UW101_01497	rpe
rpe	V4-33_01964	rpe
rpe	W5C_02992	rpe
rpe	ZONMW-01_00515	rpe
rpe	ZONMW-02_02295	rpe
rpe	ZONMW-03_01241	rpe
rpe	ZONMW-04_00430	rpe
rpe	ZONMW-05_01395	rpe
rpe	ZONMW-06_00119	rpe
rpe	ZONMW-07_01132	rpe
rpe	ZONMW-08_03268	rpe
rpe	ZONMW-09_01946	rpe
rpe	ZONMW-10_01119	rpe
rpe	ZONMW-11_02899	rpe
rpe	ZONMW-12_02168	rpe
rpe	ZONMW-13_02642	rpe
rpe	ZONMW-14_01591	rpe
rpe	ZONMW-15_01266	rpe
rpe	ZONMW-19_03980	rpe
rpe	ZONMW-20_03404	rpe
rpe	ZONMW-20_03742	rpe
rpe	ZONMW-21_03147	rpe
rpe	ZONMW-22_03172	rpe
rpe	ZONMW-23_03193	rpe
rpe	ZONMW-24_03194	rpe
rpe	ZONMW-25_01923	rpe
rpe	ZONMW-26_01448	rpe
rpe	ZONMW-27_00424	rpe
rpe	ZONMW-28_01313	rpe
rpe	ZONMW-29_02542	rpe
rpe	ZONMW-30_00910	rpe
rpe	ZONMW-31_00453	rpe
rpe	ZONMW-32_01721	rpe
rpe	ZONMW-33_01711	rpe
rpe	ZONMW-34_00094	rpe
rpe	ZONMW-35_01297	rpe
rpe	ZONMW-36_01645	rpe

rpe	ZONMW-37_03181	rpe
rpe	ZONMW-38_03259	rpe
rpe	ZONMW-39_02433	rpe
rpe	ZONMW-40_01026	rpe
rpe	ZONMW-41_00439	rpe
rpe	ZONMW-42_00063	rpe
rpe	ZONMW-43_00855	rpe
rpe	ZONMW-44_01927	rpe
rpe	ZONMW-45_02456	rpe
rpe	ZONMW-46_02449	rpe
rpe	ZONMW-47_02439	rpe
rpe	ZONMW-48_02430	rpe
rpe	ZONMW-49_00848	rpe
rpe	ZONMW-50_00849	rpe
rpe	ZONMW-51_00863	rpe
rpe	ZONMW-52_01463	rpe
rpe	ZONMW-53_00364	rpe
rpe	ZONMW-54_00670	rpe
rpe	ZONMW-55_00357	rpe
rpe	ZONMW-56_00958	rpe
rpe	ZONMW-57_01258	rpe
rpe	ZONMW-58_01326	rpe
rpe	ZONMW-59_01259	rpe
rpe	ZONMW-60_01311	rpe
rpe	ZONMW-61_03863	rpe
rpe	ZONMW-62_03665	rpe
rpe	ZONMW-63_01307	rpe
rpe	ZONMW-64_01140	rpe
rpe	ZONMW-65_01459	rpe
rpe	ZONMW-66_01661	rpe
rpe	ZONMW-67_00570	rpe
rpe	ZONMW-68_01621	rpe
rpe	ZONMW-69_00317	rpe
rpe	ZONMW-72_00082	rpe
rpe	ZONMW-73_02090	rpe
rpe	ZONMW-74_01139	rpe
rpe	ZONMW-75_03779	rpe
rpe	da5A-8_01007	rpe
mnmA	18_02111	mnmA
mnmA	ACAM48_01198	mnmA
mnmA	CAM030_01656	mnmA
mnmA	CECT7946_02227	mnmA
mnmA	CGMCC1.8863_03355	mnmA
mnmA	CL8139_01200	mnmA
mnmA	DAU203_00225	mnmA
mnmA	DD5b_00803	mnmA
mnmA	DSM1208_00530	mnmA
mnmA	DSM12212_03436	mnmA

mnmA	DSM15343_00388	mnmA
mnmA	DSM15361_02269	mnmA
mnmA	DSM15365_01630	mnmA
mnmA	DSM15718_02074	mnmA
mnmA	DSM16505T_02695	mnmA
mnmA	DSM18103_00667	mnmA
mnmA	DSM18293_03828	mnmA
mnmA	DSM18841_01399	mnmA
mnmA	DSM18842_01973	mnmA
mnmA	DSM19866_00797	mnmA
mnmA	DSM2041_02613	mnmA
mnmA	DSM2061_02021	mnmA
mnmA	DSM2064_03300	mnmA
mnmA	DSM21164_03053	mnmA
mnmA	DSM23404_02700	mnmA
mnmA	DSM23424_01820	mnmA
mnmA	DSM24786_01169	mnmA
mnmA	DSM7489_00218	mnmA
mnmA	DSW1_02393	mnmA
mnmA	DSW5_02042	mnmA
mnmA	DSW6_00030	mnmA
mnmA	DSW8_03463	mnmA
mnmA	F52_00382	mnmA
mnmA	GCA_001027945.1_02177	mnmA
mnmA	HI1_00150	mnmA
mnmA	IR1_02509	mnmA
mnmA	KCOM3131_01247	mnmA
mnmA	KCTC12974_01470	mnmA
mnmA	KL-A_01214	mnmA
mnmA	L12_00313	mnmA
mnmA	LL0412.1.7_01529	mnmA
mnmA	MAR_2009_71_00123	mnmA
mnmA	NN016038_02129	mnmA
mnmA	PAO1_02705	mnmA
mnmA	UW101_02500	mnmA
mnmA	V4-33_01683	mnmA
mnmA	W5C_00127	mnmA
mnmA	ZONMW-01_02102	mnmA
mnmA	ZONMW-02_02245	mnmA
mnmA	ZONMW-03_02608	mnmA
mnmA	ZONMW-04_01947	mnmA
mnmA	ZONMW-05_02422	mnmA
mnmA	ZONMW-06_03162	mnmA
mnmA	ZONMW-07_02268	mnmA
mnmA	ZONMW-08_02929	mnmA
mnmA	ZONMW-09_01441	mnmA
mnmA	ZONMW-10_01912	mnmA
mnmA	ZONMW-11_04377	mnmA

mnmA	ZONMW-12_03810	mnmA
mnmA	ZONMW-13_01393	mnmA
mnmA	ZONMW-14_02397	mnmA
mnmA	ZONMW-15_03140	mnmA
mnmA	ZONMW-19_00234	mnmA
mnmA	ZONMW-20_01714	mnmA
mnmA	ZONMW-21_03278	mnmA
mnmA	ZONMW-22_03238	mnmA
mnmA	ZONMW-23_03260	mnmA
mnmA	ZONMW-24_03261	mnmA
mnmA	ZONMW-25_01309	mnmA
mnmA	ZONMW-26_02694	mnmA
mnmA	ZONMW-27_02118	mnmA
mnmA	ZONMW-28_02972	mnmA
mnmA	ZONMW-29_01709	mnmA
mnmA	ZONMW-30_02444	mnmA
mnmA	ZONMW-31_01630	mnmA
mnmA	ZONMW-32_01456	mnmA
mnmA	ZONMW-33_01446	mnmA
mnmA	ZONMW-34_01634	mnmA
mnmA	ZONMW-35_03598	mnmA
mnmA	ZONMW-36_00665	mnmA
mnmA	ZONMW-37_03247	mnmA
mnmA	ZONMW-38_02342	mnmA
mnmA	ZONMW-39_00065	mnmA
mnmA	ZONMW-40_02826	mnmA
mnmA	ZONMW-41_00887	mnmA
mnmA	ZONMW-42_03268	mnmA
mnmA	ZONMW-43_01634	mnmA
mnmA	ZONMW-44_02835	mnmA
mnmA	ZONMW-45_01244	mnmA
mnmA	ZONMW-46_02628	mnmA
mnmA	ZONMW-47_02618	mnmA
mnmA	ZONMW-48_02909	mnmA
mnmA	ZONMW-49_02459	mnmA
mnmA	ZONMW-50_02253	mnmA
mnmA	ZONMW-51_02436	mnmA
mnmA	ZONMW-52_02419	mnmA
mnmA	ZONMW-53_02214	mnmA
mnmA	ZONMW-54_02704	mnmA
mnmA	ZONMW-55_02527	mnmA
mnmA	ZONMW-56_01702	mnmA
mnmA	ZONMW-57_02049	mnmA
mnmA	ZONMW-58_02029	mnmA
mnmA	ZONMW-59_02248	mnmA
mnmA	ZONMW-60_02544	mnmA
mnmA	ZONMW-61_02772	mnmA
mnmA	ZONMW-62_03462	mnmA

mnmA	ZONMW-63_01634	mnmA
mnmA	ZONMW-64_03060	mnmA
mnmA	ZONMW-65_02488	mnmA
mnmA	ZONMW-66_00957	mnmA
mnmA	ZONMW-67_01568	mnmA
mnmA	ZONMW-68_02192	mnmA
mnmA	ZONMW-69_01433	mnmA
mnmA	ZONMW-72_02537	mnmA
mnmA	ZONMW-73_01390	mnmA
mnmA	ZONMW-74_02624	mnmA
mnmA	ZONMW-75_03297	mnmA
mnmA	da5A-8_02030	mnmA
tyrS1	18_00603	tyrS1
tyrS1	ACAM48_02049	tyrS1
tyrS1	CAM030_02130	tyrS1
tyrS1	CECT7946_00445	tyrS1
tyrS1	CGMCC1.8863_00190	tyrS1
tyrS1	CGMCC1.8863_01040	tyrS1
tyrS1	CL8139_01369	tyrS1
tyrS1	DAU203_01088	tyrS1
tyrS1	DD5b_01875	tyrS1
tyrS1	DSM1208_01262	tyrS1
tyrS1	DSM12212_03111	tyrS1
tyrS1	DSM15343_00789	tyrS1
tyrS1	DSM15361_00155	tyrS1
tyrS1	DSM15365_00885	tyrS1
tyrS1	DSM15718_03006	tyrS1
tyrS1	DSM16505T_00676	tyrS1
tyrS1	DSM18103_01104	tyrS1
tyrS1	DSM18293_00926	tyrS1
tyrS1	DSM18841_00552	tyrS1
tyrS1	DSM18842_00675	tyrS1
tyrS1	DSM19866_03659	tyrS1
tyrS1	DSM2041_00651	tyrS1
tyrS1	DSM2061_03627	tyrS1
tyrS1	DSM2064_05141	tyrS1
tyrS1	DSM2064_00819	tyrS1
tyrS1	DSM21164_01214	tyrS1
tyrS1	DSM23404_01851	tyrS1
tyrS1	DSM23424_00694	tyrS1
tyrS1	DSM24786_03316	tyrS1
tyrS1	DSM7489_01009	tyrS1
tyrS1	DSW1_00952	tyrS1
tyrS1	DSW5_01318	tyrS1
tyrS1	DSW6_01025	tyrS1
tyrS1	DSW8_02669	tyrS1
tyrS1	F52_02014	tyrS1
tyrS1	GCA_001027945.1_00049	tyrS1

tyrS1	HI1_00947	tyrS1
tyrS1	IR1_01213	tyrS1
tyrS1	KCOM3131_01244	tyrS1
tyrS1	KCTC12974_00824	tyrS1
tyrS1	KL-A_02253	tyrS1
tyrS1	L12_00491	tyrS1
tyrS1	LL0412.1.7_00261	tyrS1
tyrS1	MAR_2009_71_00785	tyrS1
tyrS1	NN016038_00617	tyrS1
tyrS1	PAO1_04269	tyrS1
tyrS1	PAO1_00682	tyrS1
tyrS1	UW101_00581	tyrS1
tyrS1	UW101_03530	tyrS1
tyrS1	V4-33_00144	tyrS1
tyrS1	W5C_03053	tyrS1
tyrS1	ZONMW-01_02935	tyrS1
tyrS1	ZONMW-02_01601	tyrS1
tyrS1	ZONMW-03_01921	tyrS1
tyrS1	ZONMW-04_02938	tyrS1
tyrS1	ZONMW-05_02987	tyrS1
tyrS1	ZONMW-06_01977	tyrS1
tyrS1	ZONMW-07_00581	tyrS1
tyrS1	ZONMW-08_00303	tyrS1
tyrS1	ZONMW-09_00088	tyrS1
tyrS1	ZONMW-10_00114	tyrS1
tyrS1	ZONMW-11_00427	tyrS1
tyrS1	ZONMW-12_00614	tyrS1
tyrS1	ZONMW-13_00933	tyrS1
tyrS1	ZONMW-14_01171	tyrS1
tyrS1	ZONMW-15_00933	tyrS1
tyrS1	ZONMW-19_03212	tyrS1
tyrS1	ZONMW-20_01543	tyrS1
tyrS1	ZONMW-21_01226	tyrS1
tyrS1	ZONMW-22_01228	tyrS1
tyrS1	ZONMW-23_02340	tyrS1
tyrS1	ZONMW-24_00233	tyrS1
tyrS1	ZONMW-25_03243	tyrS1
tyrS1	ZONMW-26_01929	tyrS1
tyrS1	ZONMW-27_03007	tyrS1
tyrS1	ZONMW-28_02606	tyrS1
tyrS1	ZONMW-29_00811	tyrS1
tyrS1	ZONMW-30_00494	tyrS1
tyrS1	ZONMW-31_01878	tyrS1
tyrS1	ZONMW-32_03270	tyrS1
tyrS1	ZONMW-33_03004	tyrS1
tyrS1	ZONMW-34_02648	tyrS1
tyrS1	ZONMW-35_02612	tyrS1
tyrS1	ZONMW-36_02998	tyrS1

tyrS1	ZONMW-37_01136	tyrS1
tyrS1	ZONMW-38_00788	tyrS1
tyrS1	ZONMW-39_03981	tyrS1
tyrS1	ZONMW-40_00055	tyrS1
tyrS1	ZONMW-41_01302	tyrS1
tyrS1	ZONMW-42_04056	tyrS1
tyrS1	ZONMW-43_01276	tyrS1
tyrS1	ZONMW-44_00002	tyrS1
tyrS1	ZONMW-45_00693	tyrS1
tyrS1	ZONMW-46_00766	tyrS1
tyrS1	ZONMW-47_00766	tyrS1
tyrS1	ZONMW-48_00347	tyrS1
tyrS1	ZONMW-49_02200	tyrS1
tyrS1	ZONMW-50_01994	tyrS1
tyrS1	ZONMW-51_03117	tyrS1
tyrS1	ZONMW-52_02002	tyrS1
tyrS1	ZONMW-53_01746	tyrS1
tyrS1	ZONMW-54_02290	tyrS1
tyrS1	ZONMW-55_03084	tyrS1
tyrS1	ZONMW-56_01258	tyrS1
tyrS1	ZONMW-57_02987	tyrS1
tyrS1	ZONMW-58_03006	tyrS1
tyrS1	ZONMW-59_01813	tyrS1
tyrS1	ZONMW-60_02165	tyrS1
tyrS1	ZONMW-61_01683	tyrS1
tyrS1	ZONMW-62_01429	tyrS1
tyrS1	ZONMW-63_03000	tyrS1
tyrS1	ZONMW-64_04208	tyrS1
tyrS1	ZONMW-65_00104	tyrS1
tyrS1	ZONMW-66_03836	tyrS1
tyrS1	ZONMW-67_03275	tyrS1
tyrS1	ZONMW-68_00781	tyrS1
tyrS1	ZONMW-69_03276	tyrS1
tyrS1	ZONMW-72_01571	tyrS1
tyrS1	ZONMW-73_02997	tyrS1
tyrS1	ZONMW-74_02076	tyrS1
tyrS1	ZONMW-75_00399	tyrS1
tyrS1	da5A-8_01584	tyrS1
thiL	18_01155	thiL
thiL	ACAM48_02139	thiL
thiL	CAM030_00845	thiL
thiL	CECT7946_01952	thiL
thiL	CGMCC1.8863_00888	thiL
thiL	CL8139_00812	thiL
thiL	DAU203_01609	thiL
thiL	DD5b_01974	thiL
thiL	DSM1208_01771	thiL
thiL	DSM12212_02091	thiL

thiL	DSM15343_02414	thiL
thiL	DSM15361_01449	thiL
thiL	DSM15365_00922	thiL
thiL	DSM15718_02907	thiL
thiL	DSM16505T_00770	thiL
thiL	DSM18103_01229	thiL
thiL	DSM18293_00753	thiL
thiL	DSM18841_00648	thiL
thiL	DSM18842_03007	thiL
thiL	DSM19866_02008	thiL
thiL	DSM2041_00309	thiL
thiL	DSM2061_03298	thiL
thiL	DSM2064_01778	thiL
thiL	DSM21164_00711	thiL
thiL	DSM23404_01936	thiL
thiL	DSM23424_00157	thiL
thiL	DSM24786_02383	thiL
thiL	DSM7489_01535	thiL
thiL	DSW1_00994	thiL
thiL	DSW5_01370	thiL
thiL	DSW6_01272	thiL
thiL	DSW8_02168	thiL
thiL	F52_02223	thiL
thiL	GCA_001027945.1_03068	thiL
thiL	HI1_01460	thiL
thiL	IR1_04066	thiL
thiL	KCOM3131_00208	thiL
thiL	KCTC12974_00395	thiL
thiL	KL-A_03260	thiL
thiL	L12_00061	thiL
thiL	LL0412.1.7_00776	thiL
thiL	MAR_2009_71_01272	thiL
thiL	NN016038_01176	thiL
thiL	PAO1_04179	thiL
thiL	UW101_00832	thiL
thiL	V4-33_02247	thiL
thiL	W5C_00217	thiL
thiL	ZONMW-01_02999	thiL
thiL	ZONMW-02_01639	thiL
thiL	ZONMW-03_03283	thiL
thiL	ZONMW-04_03245	thiL
thiL	ZONMW-05_02717	thiL
thiL	ZONMW-06_03941	thiL
thiL	ZONMW-07_01426	thiL
thiL	ZONMW-08_00791	thiL
thiL	ZONMW-09_00500	thiL
thiL	ZONMW-10_00211	thiL
thiL	ZONMW-11_04116	thiL

thiL	ZONMW-12_00090	thiL
thiL	ZONMW-13_00221	thiL
thiL	ZONMW-14_00373	thiL
thiL	ZONMW-15_00010	thiL
thiL	ZONMW-19_01941	thiL
thiL	ZONMW-20_01360	thiL
thiL	ZONMW-21_00695	thiL
thiL	ZONMW-22_00696	thiL
thiL	ZONMW-23_01810	thiL
thiL	ZONMW-24_00765	thiL
thiL	ZONMW-25_01640	thiL
thiL	ZONMW-26_03339	thiL
thiL	ZONMW-27_02591	thiL
thiL	ZONMW-28_03917	thiL
thiL	ZONMW-29_00348	thiL
thiL	ZONMW-30_02357	thiL
thiL	ZONMW-31_03201	thiL
thiL	ZONMW-32_03969	thiL
thiL	ZONMW-33_03893	thiL
thiL	ZONMW-34_03815	thiL
thiL	ZONMW-35_03810	thiL
thiL	ZONMW-36_03912	thiL
thiL	ZONMW-37_00695	thiL
thiL	ZONMW-38_03580	thiL
thiL	ZONMW-39_04083	thiL
thiL	ZONMW-40_01277	thiL
thiL	ZONMW-41_02410	thiL
thiL	ZONMW-42_02342	thiL
thiL	ZONMW-43_03768	thiL
thiL	ZONMW-44_03155	thiL
thiL	ZONMW-45_01000	thiL
thiL	ZONMW-46_00667	thiL
thiL	ZONMW-47_00667	thiL
thiL	ZONMW-48_00671	thiL
thiL	ZONMW-49_01073	thiL
thiL	ZONMW-50_01074	thiL
thiL	ZONMW-51_03277	thiL
thiL	ZONMW-52_03256	thiL
thiL	ZONMW-53_03286	thiL
thiL	ZONMW-54_00150	thiL
thiL	ZONMW-55_03244	thiL
thiL	ZONMW-56_03248	thiL
thiL	ZONMW-57_03296	thiL
thiL	ZONMW-58_03287	thiL
thiL	ZONMW-59_02140	thiL
thiL	ZONMW-60_01827	thiL
thiL	ZONMW-61_00387	thiL
thiL	ZONMW-62_01933	thiL

thiL	ZONMW-63_03372	thiL
thiL	ZONMW-64_04088	thiL
thiL	ZONMW-65_01785	thiL
thiL	ZONMW-66_01499	thiL
thiL	ZONMW-67_03592	thiL
thiL	ZONMW-68_00152	thiL
thiL	ZONMW-69_03594	thiL
thiL	ZONMW-72_02785	thiL
thiL	ZONMW-73_01843	thiL
thiL	ZONMW-74_01386	thiL
thiL	ZONMW-75_00435	thiL
thiL	da5A-8_01480	thiL
wbpE	18_01508	wbpE
wbpE	18_02987	wbpE
wbpE	18_02497	wbpE
wbpE	ACAM48_00449	wbpE
wbpE	ACAM48_01717	wbpE
wbpE	ACAM48_00077	wbpE
wbpE	CAM030_02369	wbpE
wbpE	CAM030_02581	wbpE
wbpE	CAM030_00417	wbpE
wbpE	CAM030_01009	wbpE
wbpE	CAM030_01020	wbpE
wbpE	CECT7946_02639	wbpE
wbpE	CECT7946_03657	wbpE
wbpE	CECT7946_03594	wbpE
wbpE	CECT7946_03572	wbpE
wbpE	CECT7946_01387	wbpE
wbpE	CECT7946_03600	wbpE
wbpE	CGMCC1.8863_01044	wbpE
wbpE	CGMCC1.8863_00812	wbpE
wbpE	CGMCC1.8863_00270	wbpE
wbpE	CGMCC1.8863_01506	wbpE
wbpE	CL8139_00949	wbpE
wbpE	CL8139_01943	wbpE
wbpE	CL8139_02972	wbpE
wbpE	DAU203_01726	wbpE
wbpE	DAU203_03253	wbpE
wbpE	DAU203_02818	wbpE
wbpE	DD5b_02643	wbpE
wbpE	DD5b_00727	wbpE
wbpE	DD5b_02368	wbpE
wbpE	DD5b_02383	wbpE
wbpE	DSM1208_02235	wbpE
wbpE	DSM1208_01681	wbpE
wbpE	DSM1208_04441	wbpE
wbpE	DSM1208_03633	wbpE
wbpE	DSM12212_01644	wbpE

wbpE	DSM12212_00997	wbpE
wbpE	DSM12212_00081	wbpE
wbpE	DSM12212_00750	wbpE
wbpE	DSM12212_00339	wbpE
wbpE	DSM12212_00967	wbpE
wbpE	DSM15343_01956	wbpE
wbpE	DSM15343_02879	wbpE
wbpE	DSM15343_01626	wbpE
wbpE	DSM15361_01142	wbpE
wbpE	DSM15361_02635	wbpE
wbpE	DSM15361_02170	wbpE
wbpE	DSM15365_02250	wbpE
wbpE	DSM15365_03587	wbpE
wbpE	DSM15365_02396	wbpE
wbpE	DSM15365_03046	wbpE
wbpE	DSM15365_01865	wbpE
wbpE	DSM15718_00515	wbpE
wbpE	DSM15718_01159	wbpE
wbpE	DSM15718_00715	wbpE
wbpE	DSM15718_00731	wbpE
wbpE	DSM16505T_00128	wbpE
wbpE	DSM16505T_01543	wbpE
wbpE	DSM18103_01430	wbpE
wbpE	DSM18103_03397	wbpE
wbpE	DSM18103_03385	wbpE
wbpE	DSM18103_03386	wbpE
wbpE	DSM18293_02013	wbpE
wbpE	DSM18293_03680	wbpE
wbpE	DSM18293_02480	wbpE
wbpE	DSM18293_02467	wbpE
wbpE	DSM18841_02713	wbpE
wbpE	DSM18841_02742	wbpE
wbpE	DSM18841_02753	wbpE
wbpE	DSM18842_02533	wbpE
wbpE	DSM18842_02686	wbpE
wbpE	DSM19866_00670	wbpE
wbpE	DSM19866_01462	wbpE
wbpE	DSM19866_04492	wbpE
wbpE	DSM19866_01556	wbpE
wbpE	DSM19866_01908	wbpE
wbpE	DSM2041_04205	wbpE
wbpE	DSM2041_05001	wbpE
wbpE	DSM2041_00221	wbpE
wbpE	DSM2041_03999	wbpE
wbpE	DSM2041_01931	wbpE
wbpE	DSM2061_03765	wbpE
wbpE	DSM2061_03216	wbpE
wbpE	DSM2061_00242	wbpE

wbpE	DSM2061_01426	wbpE
wbpE	DSM2061_00715	wbpE
wbpE	DSM2064_02746	wbpE
wbpE	DSM2064_03453	wbpE
wbpE	DSM2064_04757	wbpE
wbpE	DSM2064_04746	wbpE
wbpE	DSM21164_00372	wbpE
wbpE	DSM21164_00740	wbpE
wbpE	DSM21164_02149	wbpE
wbpE	DSM21164_01494	wbpE
wbpE	DSM21164_01716	wbpE
wbpE	DSM23404_01126	wbpE
wbpE	DSM23404_01392	wbpE
wbpE	DSM23424_02327	wbpE
wbpE	DSM23424_03157	wbpE
wbpE	DSM23424_00333	wbpE
wbpE	DSM23424_02688	wbpE
wbpE	DSM23424_02095	wbpE
wbpE	DSM23424_02261	wbpE
wbpE	DSM24786_02255	wbpE
wbpE	DSM24786_02955	wbpE
wbpE	DSM24786_00698	wbpE
wbpE	DSM24786_00276	wbpE
wbpE	DSM7489_01645	wbpE
wbpE	DSM7489_03085	wbpE
wbpE	DSM7489_02689	wbpE
wbpE	DSW1_00262	wbpE
wbpE	DSW1_01560	wbpE
wbpE	DSW1_02177	wbpE
wbpE	DSW5_01642	wbpE
wbpE	DSW5_00239	wbpE
wbpE	DSW6_01133	wbpE
wbpE	DSW6_02294	wbpE
wbpE	DSW6_03422	wbpE
wbpE	DSW6_03445	wbpE
wbpE	DSW8_01713	wbpE
wbpE	DSW8_03563	wbpE
wbpE	DSW8_00874	wbpE
wbpE	F52_03337	wbpE
wbpE	F52_00221	wbpE
wbpE	F52_01556	wbpE
wbpE	F52_01576	wbpE
wbpE	F52_01568	wbpE
wbpE	GCA_001027945.1_01688	wbpE
wbpE	GCA_001027945.1_00811	wbpE
wbpE	HI1_01566	wbpE
wbpE	HI1_03026	wbpE
wbpE	HI1_02628	wbpE

wbpE	IR1_00150	wbpE
wbpE	IR1_02639	wbpE
wbpE	IR1_03016	wbpE
wbpE	KCOM3131_01789	wbpE
wbpE	KCTC12974_02307	wbpE
wbpE	KCTC12974_00120	wbpE
wbpE	KCTC12974_00133	wbpE
wbpE	KCTC12974_00090	wbpE
wbpE	KL-A_01447	wbpE
wbpE	KL-A_02941	wbpE
wbpE	KL-A_01369	wbpE
wbpE	L12_00467	wbpE
wbpE	LL0412.1.7_00105	wbpE
wbpE	MAR_2009_71_01704	wbpE
wbpE	MAR_2009_71_02720	wbpE
wbpE	MAR_2009_71_02570	wbpE
wbpE	NN016038_01515	wbpE
wbpE	NN016038_03022	wbpE
wbpE	NN016038_02530	wbpE
wbpE	PAO1_03265	wbpE
wbpE	PAO1_03674	wbpE
wbpE	UW101_01800	wbpE
wbpE	UW101_02347	wbpE
wbpE	UW101_00345	wbpE
wbpE	UW101_00356	wbpE
wbpE	V4-33_00383	wbpE
wbpE	V4-33_01780	wbpE
wbpE	V4-33_01186	wbpE
wbpE	W5C_00339	wbpE
wbpE	W5C_02292	wbpE
wbpE	W5C_00742	wbpE
wbpE	ZONMW-01_00092	wbpE
wbpE	ZONMW-01_00689	wbpE
wbpE	ZONMW-01_01733	wbpE
wbpE	ZONMW-02_02421	wbpE
wbpE	ZONMW-02_03187	wbpE
wbpE	ZONMW-02_02283	wbpE
wbpE	ZONMW-02_03333	wbpE
wbpE	ZONMW-03_00818	wbpE
wbpE	ZONMW-03_00067	wbpE
wbpE	ZONMW-03_00474	wbpE
wbpE	ZONMW-04_02128	wbpE
wbpE	ZONMW-04_02472	wbpE
wbpE	ZONMW-04_01074	wbpE
wbpE	ZONMW-04_01413	wbpE
wbpE	ZONMW-05_01007	wbpE
wbpE	ZONMW-05_00327	wbpE
wbpE	ZONMW-05_00739	wbpE

wbpE	ZONMW-05_00737	wbpE
wbpE	ZONMW-06_01046	wbpE
wbpE	ZONMW-06_01049	wbpE
wbpE	ZONMW-06_02042	wbpE
wbpE	ZONMW-07_00310	wbpE
wbpE	ZONMW-07_02065	wbpE
wbpE	ZONMW-07_02469	wbpE
wbpE	ZONMW-07_03053	wbpE
wbpE	ZONMW-08_01530	wbpE
wbpE	ZONMW-08_03571	wbpE
wbpE	ZONMW-08_01931	wbpE
wbpE	ZONMW-09_01711	wbpE
wbpE	ZONMW-09_02661	wbpE
wbpE	ZONMW-09_02297	wbpE
wbpE	ZONMW-10_01293	wbpE
wbpE	ZONMW-10_01006	wbpE
wbpE	ZONMW-10_00374	wbpE
wbpE	ZONMW-10_01585	wbpE
wbpE	ZONMW-10_01575	wbpE
wbpE	ZONMW-10_02442	wbpE
wbpE	ZONMW-11_00806	wbpE
wbpE	ZONMW-11_01481	wbpE
wbpE	ZONMW-11_00123	wbpE
wbpE	ZONMW-11_01510	wbpE
wbpE	ZONMW-11_03493	wbpE
wbpE	ZONMW-11_04310	wbpE
wbpE	ZONMW-12_02122	wbpE
wbpE	ZONMW-12_01464	wbpE
wbpE	ZONMW-12_01599	wbpE
wbpE	ZONMW-13_01105	wbpE
wbpE	ZONMW-13_02781	wbpE
wbpE	ZONMW-13_01217	wbpE
wbpE	ZONMW-13_01211	wbpE
wbpE	ZONMW-14_01491	wbpE
wbpE	ZONMW-14_01936	wbpE
wbpE	ZONMW-14_02072	wbpE
wbpE	ZONMW-15_00365	wbpE
wbpE	ZONMW-15_01401	wbpE
wbpE	ZONMW-19_04201	wbpE
wbpE	ZONMW-19_02223	wbpE
wbpE	ZONMW-19_00179	wbpE
wbpE	ZONMW-20_03301	wbpE
wbpE	ZONMW-20_00847	wbpE
wbpE	ZONMW-21_00258	wbpE
wbpE	ZONMW-21_01736	wbpE
wbpE	ZONMW-21_02199	wbpE
wbpE	ZONMW-22_00258	wbpE
wbpE	ZONMW-22_01988	wbpE

wbpE	ZONMW-22_01526	wbpE
wbpE	ZONMW-23_01373	wbpE
wbpE	ZONMW-23_00563	wbpE
wbpE	ZONMW-23_01025	wbpE
wbpE	ZONMW-24_01201	wbpE
wbpE	ZONMW-24_02613	wbpE
wbpE	ZONMW-24_01550	wbpE
wbpE	ZONMW-25_02935	wbpE
wbpE	ZONMW-25_00805	wbpE
wbpE	ZONMW-25_00335	wbpE
wbpE	ZONMW-26_01051	wbpE
wbpE	ZONMW-26_00640	wbpE
wbpE	ZONMW-26_00232	wbpE
wbpE	ZONMW-27_00821	wbpE
wbpE	ZONMW-27_01149	wbpE
wbpE	ZONMW-27_01889	wbpE
wbpE	ZONMW-27_01891	wbpE
wbpE	ZONMW-28_03535	wbpE
wbpE	ZONMW-28_00542	wbpE
wbpE	ZONMW-29_02949	wbpE
wbpE	ZONMW-29_03070	wbpE
wbpE	ZONMW-29_01401	wbpE
wbpE	ZONMW-30_01462	wbpE
wbpE	ZONMW-30_01117	wbpE
wbpE	ZONMW-30_03278	wbpE
wbpE	ZONMW-31_01317	wbpE
wbpE	ZONMW-31_01286	wbpE
wbpE	ZONMW-31_01494	wbpE
wbpE	ZONMW-32_00620	wbpE
wbpE	ZONMW-32_01333	wbpE
wbpE	ZONMW-33_00488	wbpE
wbpE	ZONMW-33_01322	wbpE
wbpE	ZONMW-34_00844	wbpE
wbpE	ZONMW-34_01512	wbpE
wbpE	ZONMW-35_02619	wbpE
wbpE	ZONMW-35_00585	wbpE
wbpE	ZONMW-36_00883	wbpE
wbpE	ZONMW-36_00542	wbpE
wbpE	ZONMW-37_00258	wbpE
wbpE	ZONMW-37_02114	wbpE
wbpE	ZONMW-37_01549	wbpE
wbpE	ZONMW-38_01515	wbpE
wbpE	ZONMW-38_02630	wbpE
wbpE	ZONMW-38_02628	wbpE
wbpE	ZONMW-39_01317	wbpE
wbpE	ZONMW-39_01306	wbpE
wbpE	ZONMW-39_00186	wbpE
wbpE	ZONMW-40_02668	wbpE

wbpE	ZONMW-40_03241	wbpE
wbpE	ZONMW-40_00613	wbpE
wbpE	ZONMW-40_00614	wbpE
wbpE	ZONMW-41_02453	wbpE
wbpE	ZONMW-42_03980	wbpE
wbpE	ZONMW-43_00388	wbpE
wbpE	ZONMW-44_03966	wbpE
wbpE	ZONMW-44_02228	wbpE
wbpE	ZONMW-44_02724	wbpE
wbpE	ZONMW-45_02825	wbpE
wbpE	ZONMW-45_02718	wbpE
wbpE	ZONMW-45_02704	wbpE
wbpE	ZONMW-46_02389	wbpE
wbpE	ZONMW-46_03071	wbpE
wbpE	ZONMW-46_03085	wbpE
wbpE	ZONMW-47_02379	wbpE
wbpE	ZONMW-47_03062	wbpE
wbpE	ZONMW-47_03076	wbpE
wbpE	ZONMW-48_01002	wbpE
wbpE	ZONMW-48_00641	wbpE
wbpE	ZONMW-48_01761	wbpE
wbpE	ZONMW-49_00957	wbpE
wbpE	ZONMW-49_01223	wbpE
wbpE	ZONMW-49_01767	wbpE
wbpE	ZONMW-49_00243	wbpE
wbpE	ZONMW-50_00958	wbpE
wbpE	ZONMW-50_01224	wbpE
wbpE	ZONMW-50_01647	wbpE
wbpE	ZONMW-50_00242	wbpE
wbpE	ZONMW-51_01307	wbpE
wbpE	ZONMW-51_02825	wbpE
wbpE	ZONMW-51_00663	wbpE
wbpE	ZONMW-51_00231	wbpE
wbpE	ZONMW-52_01034	wbpE
wbpE	ZONMW-52_02220	wbpE
wbpE	ZONMW-52_00622	wbpE
wbpE	ZONMW-52_00215	wbpE
wbpE	ZONMW-53_00800	wbpE
wbpE	ZONMW-53_01377	wbpE
wbpE	ZONMW-53_00952	wbpE
wbpE	ZONMW-54_00269	wbpE
wbpE	ZONMW-54_01340	wbpE
wbpE	ZONMW-54_01775	wbpE
wbpE	ZONMW-55_00769	wbpE
wbpE	ZONMW-55_02816	wbpE
wbpE	ZONMW-55_00861	wbpE
wbpE	ZONMW-55_01295	wbpE
wbpE	ZONMW-56_02310	wbpE

wbpE	ZONMW-56_02572	wbpE
wbpE	ZONMW-56_00097	wbpE
wbpE	ZONMW-56_00524	wbpE
wbpE	ZONMW-57_02438	wbpE
wbpE	ZONMW-57_02721	wbpE
wbpE	ZONMW-57_00308	wbpE
wbpE	ZONMW-57_00737	wbpE
wbpE	ZONMW-58_02463	wbpE
wbpE	ZONMW-58_02745	wbpE
wbpE	ZONMW-58_00663	wbpE
wbpE	ZONMW-58_00234	wbpE
wbpE	ZONMW-59_02028	wbpE
wbpE	ZONMW-59_02989	wbpE
wbpE	ZONMW-59_00324	wbpE
wbpE	ZONMW-59_00726	wbpE
wbpE	ZONMW-60_01711	wbpE
wbpE	ZONMW-60_00335	wbpE
wbpE	ZONMW-60_00739	wbpE
wbpE	ZONMW-61_02865	wbpE
wbpE	ZONMW-61_02337	wbpE
wbpE	ZONMW-62_02363	wbpE
wbpE	ZONMW-62_00830	wbpE
wbpE	ZONMW-63_00605	wbpE
wbpE	ZONMW-63_02353	wbpE
wbpE	ZONMW-63_01947	wbpE
wbpE	ZONMW-63_00410	wbpE
wbpE	ZONMW-64_00406	wbpE
wbpE	ZONMW-64_02857	wbpE
wbpE	ZONMW-65_00967	wbpE
wbpE	ZONMW-66_02457	wbpE
wbpE	ZONMW-66_01414	wbpE
wbpE	ZONMW-66_00731	wbpE
wbpE	ZONMW-66_03934	wbpE
wbpE	ZONMW-67_01461	wbpE
wbpE	ZONMW-68_03929	wbpE
wbpE	ZONMW-68_01837	wbpE
wbpE	ZONMW-68_01260	wbpE
wbpE	ZONMW-68_04265	wbpE
wbpE	ZONMW-68_00041	wbpE
wbpE	ZONMW-68_01942	wbpE
wbpE	ZONMW-69_01539	wbpE
wbpE	ZONMW-72_00533	wbpE
wbpE	ZONMW-72_03387	wbpE
wbpE	ZONMW-72_01204	wbpE
wbpE	ZONMW-73_00330	wbpE
wbpE	ZONMW-73_03866	wbpE
wbpE	ZONMW-73_00518	wbpE
wbpE	ZONMW-73_03318	wbpE

wbpE	ZONMW-73_02501	wbpE
wbpE	ZONMW-74_01252	wbpE
wbpE	ZONMW-74_01527	wbpE
wbpE	ZONMW-74_00311	wbpE
wbpE	ZONMW-74_03091	wbpE
wbpE	ZONMW-75_00197	wbpE
wbpE	ZONMW-75_03259	wbpE
wbpE	ZONMW-75_02044	wbpE
wbpE	da5A-8_00972	wbpE
wbpE	da5A-8_00273	wbpE
rpsP	18_01425	rpsP
rpsP	ACAM48_01994	rpsP
rpsP	CAM030_02161	rpsP
rpsP	CECT7946_00465	rpsP
rpsP	CGMCC1.8863_00575	rpsP
rpsP	CL8139_01030	rpsP
rpsP	DAU203_01807	rpsP
rpsP	DD5b_01645	rpsP
rpsP	DSM1208_02094	rpsP
rpsP	DSM12212_01376	rpsP
rpsP	DSM15343_00749	rpsP
rpsP	DSM15361_00133	rpsP
rpsP	DSM15365_02235	rpsP
rpsP	DSM15718_02349	rpsP
rpsP	DSM16505T_00177	rpsP
rpsP	DSM18103_01622	rpsP
rpsP	DSM18293_00386	rpsP
rpsP	DSM18841_02671	rpsP
rpsP	DSM18842_02571	rpsP
rpsP	DSM19866_03071	rpsP
rpsP	DSM2041_00692	rpsP
rpsP	DSM2061_03652	rpsP
rpsP	DSM2064_02308	rpsP
rpsP	DSM21164_00476	rpsP
rpsP	DSM23404_01800	rpsP
rpsP	DSM23424_00719	rpsP
rpsP	DSM24786_02173	rpsP
rpsP	DSM7489_01726	rpsP
rpsP	DSW1_00319	rpsP
rpsP	DSW5_00613	rpsP
rpsP	DSW6_00858	rpsP
rpsP	DSW8_01793	rpsP
rpsP	F52_02704	rpsP
rpsP	GCA_001027945.1_00855	rpsP
rpsP	HI1_01647	rpsP
rpsP	IR1_00555	rpsP
rpsP	KCOM3131_01055	rpsP
rpsP	KCTC12974_00770	rpsP

rpsP	KL-A_01532	rpsP
rpsP	L12_00506	rpsP
rpsP	LL0412.1.7_00133	rpsP
rpsP	MAR_2009_71_01627	rpsP
rpsP	NN016038_01434	rpsP
rpsP	PAO1_03868	rpsP
rpsP	UW101_01362	rpsP
rpsP	V4-33_00321	rpsP
rpsP	W5C_00428	rpsP
rpsP	ZONMW-01_00173	rpsP
rpsP	ZONMW-02_02436	rpsP
rpsP	ZONMW-03_00899	rpsP
rpsP	ZONMW-04_02047	rpsP
rpsP	ZONMW-05_01088	rpsP
rpsP	ZONMW-06_02900	rpsP
rpsP	ZONMW-07_00114	rpsP
rpsP	ZONMW-08_01606	rpsP
rpsP	ZONMW-09_00060	rpsP
rpsP	ZONMW-10_00079	rpsP
rpsP	ZONMW-11_00385	rpsP
rpsP	ZONMW-12_03187	rpsP
rpsP	ZONMW-13_00134	rpsP
rpsP	ZONMW-14_02677	rpsP
rpsP	ZONMW-15_00225	rpsP
rpsP	ZONMW-19_00412	rpsP
rpsP	ZONMW-20_02721	rpsP
rpsP	ZONMW-21_00323	rpsP
rpsP	ZONMW-22_00323	rpsP
rpsP	ZONMW-23_01438	rpsP
rpsP	ZONMW-24_01136	rpsP
rpsP	ZONMW-25_02544	rpsP
rpsP	ZONMW-26_01132	rpsP
rpsP	ZONMW-27_00740	rpsP
rpsP	ZONMW-28_02006	rpsP
rpsP	ZONMW-29_00026	rpsP
rpsP	ZONMW-30_01531	rpsP
rpsP	ZONMW-31_03008	rpsP
rpsP	ZONMW-32_02412	rpsP
rpsP	ZONMW-33_02706	rpsP
rpsP	ZONMW-34_02000	rpsP
rpsP	ZONMW-35_01692	rpsP
rpsP	ZONMW-36_02546	rpsP
rpsP	ZONMW-37_00323	rpsP
rpsP	ZONMW-38_00801	rpsP
rpsP	ZONMW-39_02192	rpsP
rpsP	ZONMW-40_01526	rpsP
rpsP	ZONMW-41_01307	rpsP
rpsP	ZONMW-42_00419	rpsP

rpsP	ZONMW-43_01504	rpsP
rpsP	ZONMW-44_00878	rpsP
rpsP	ZONMW-45_01987	rpsP
rpsP	ZONMW-46_02336	rpsP
rpsP	ZONMW-47_02196	rpsP
rpsP	ZONMW-48_00871	rpsP
rpsP	ZONMW-49_01273	rpsP
rpsP	ZONMW-50_01275	rpsP
rpsP	ZONMW-51_01388	rpsP
rpsP	ZONMW-52_01130	rpsP
rpsP	ZONMW-53_00717	rpsP
rpsP	ZONMW-54_00350	rpsP
rpsP	ZONMW-55_00688	rpsP
rpsP	ZONMW-56_02391	rpsP
rpsP	ZONMW-57_02520	rpsP
rpsP	ZONMW-58_02545	rpsP
rpsP	ZONMW-59_01947	rpsP
rpsP	ZONMW-60_01630	rpsP
rpsP	ZONMW-61_00736	rpsP
rpsP	ZONMW-62_02441	rpsP
rpsP	ZONMW-63_00686	rpsP
rpsP	ZONMW-64_02081	rpsP
rpsP	ZONMW-65_01170	rpsP
rpsP	ZONMW-66_02239	rpsP
rpsP	ZONMW-67_02399	rpsP
rpsP	ZONMW-68_00506	rpsP
rpsP	ZONMW-69_02447	rpsP
rpsP	ZONMW-72_00207	rpsP
rpsP	ZONMW-73_01334	rpsP
rpsP	ZONMW-74_01943	rpsP
rpsP	ZONMW-75_00229	rpsP
rpsP	da5A-8_00685	rpsP
purM	18_03932	purM
purM	ACAM48_03314	purM
purM	CAM030_01539	purM
purM	CECT7946_01059	purM
purM	CGMCC1.8863_03490	purM
purM	CL8139_00606	purM
purM	DAU203_00415	purM
purM	DD5b_00786	purM
purM	DSM1208_02698	purM
purM	DSM12212_02000	purM
purM	DSM15343_01144	purM
purM	DSM15361_01962	purM
purM	DSM15365_00511	purM
purM	DSM15718_02097	purM
purM	DSM16505T_01180	purM
purM	DSM18103_01998	purM

purM	DSM18293_03777	purM
purM	DSM18841_01100	purM
purM	DSM18842_00203	purM
purM	DSM19866_00209	purM
purM	DSM2041_02837	purM
purM	DSM2061_04186	purM
purM	DSM2064_03335	purM
purM	DSM21164_02048	purM
purM	DSM23404_00843	purM
purM	DSM23424_02600	purM
purM	DSM24786_02487	purM
purM	DSM7489_00399	purM
purM	DSW1_02479	purM
purM	DSW5_01763	purM
purM	DSW6_02689	purM
purM	DSW8_01260	purM
purM	F52_00324	purM
purM	GCA_001027945.1_01345	purM
purM	HI1_00331	purM
purM	IR1_02543	purM
purM	KCOM3131_00871	purM
purM	KCTC12974_00311	purM
purM	KL-A_02593	purM
purM	L12_00360	purM
purM	LL0412.1.7_01157	purM
purM	MAR_2009_71_02176	purM
purM	NN016038_03988	purM
purM	PAO1_00977	purM
purM	UW101_02465	purM
purM	V4-33_00657	purM
purM	W5C_03201	purM
purM	ZONMW-01_01851	purM
purM	ZONMW-02_00958	purM
purM	ZONMW-03_02836	purM
purM	ZONMW-04_02694	purM
purM	ZONMW-05_02243	purM
purM	ZONMW-06_03876	purM
purM	ZONMW-07_01764	purM
purM	ZONMW-08_01068	purM
purM	ZONMW-09_01250	purM
purM	ZONMW-10_01928	purM
purM	ZONMW-11_03969	purM
purM	ZONMW-12_00869	purM
purM	ZONMW-13_02873	purM
purM	ZONMW-14_01659	purM
purM	ZONMW-15_00570	purM
purM	ZONMW-19_00016	purM
purM	ZONMW-20_03046	purM

purM	ZONMW-21_02572	purM
purM	ZONMW-22_02537	purM
purM	ZONMW-23_00013	purM
purM	ZONMW-24_02302	purM
purM	ZONMW-25_00792	purM
purM	ZONMW-26_02911	purM
purM	ZONMW-27_02428	purM
purM	ZONMW-28_03819	purM
purM	ZONMW-29_02028	purM
purM	ZONMW-30_00319	purM
purM	ZONMW-31_02599	purM
purM	ZONMW-32_03757	purM
purM	ZONMW-33_03790	purM
purM	ZONMW-34_03688	purM
purM	ZONMW-35_00013	purM
purM	ZONMW-36_03739	purM
purM	ZONMW-37_02239	purM
purM	ZONMW-38_00749	purM
purM	ZONMW-39_03552	purM
purM	ZONMW-40_01826	purM
purM	ZONMW-41_02866	purM
purM	ZONMW-42_03210	purM
purM	ZONMW-43_02554	purM
purM	ZONMW-44_03850	purM
purM	ZONMW-45_03009	purM
purM	ZONMW-46_03219	purM
purM	ZONMW-47_03211	purM
purM	ZONMW-48_02231	purM
purM	ZONMW-49_01925	purM
purM	ZONMW-50_02521	purM
purM	ZONMW-51_02230	purM
purM	ZONMW-52_02642	purM
purM	ZONMW-53_02395	purM
purM	ZONMW-54_02437	purM
purM	ZONMW-55_01758	purM
purM	ZONMW-56_01912	purM
purM	ZONMW-57_02226	purM
purM	ZONMW-58_02250	purM
purM	ZONMW-59_02465	purM
purM	ZONMW-60_02318	purM
purM	ZONMW-61_01026	purM
purM	ZONMW-62_02837	purM
purM	ZONMW-63_02398	purM
purM	ZONMW-64_03735	purM
purM	ZONMW-65_00561	purM
purM	ZONMW-66_01283	purM
purM	ZONMW-67_01770	purM
purM	ZONMW-68_04945	purM

purM	ZONMW-69_02173	purM
purM	ZONMW-72_02503	purM
purM	ZONMW-73_02542	purM
purM	ZONMW-74_02339	purM
purM	ZONMW-75_00852	purM
purM	da5A-8_02860	purM
lysC	18_01382	lysC
lysC	18_01653	lysC
lysC	18_02354	lysC
lysC	ACAM48_02584	lysC
lysC	ACAM48_00709	lysC
lysC	ACAM48_00116	lysC
lysC	ACAM48_00499	lysC
lysC	ACAM48_00498	lysC
lysC	CAM030_01237	lysC
lysC	CAM030_00838	lysC
lysC	CAM030_00760	lysC
lysC	CAM030_01404	lysC
lysC	CECT7946_01866	lysC
lysC	CECT7946_01488	lysC
lysC	CECT7946_00640	lysC
lysC	CGMCC1.8863_00566	lysC
lysC	CGMCC1.8863_03221	lysC
lysC	CGMCC1.8863_01285	lysC
lysC	CL8139_01082	lysC
lysC	CL8139_00416	lysC
lysC	CL8139_03288	lysC
lysC	DAU203_01848	lysC
lysC	DAU203_02564	lysC
lysC	DAU203_02086	lysC
lysC	DD5b_01860	lysC
lysC	DD5b_00926	lysC
lysC	DD5b_01797	lysC
lysC	DD5b_01796	lysC
lysC	DSM1208_02038	lysC
lysC	DSM1208_00453	lysC
lysC	DSM1208_00828	lysC
lysC	DSM12212_01658	lysC
lysC	DSM12212_00531	lysC
lysC	DSM12212_01604	lysC
lysC	DSM15343_02510	lysC
lysC	DSM15343_00327	lysC
lysC	DSM15361_00617	lysC
lysC	DSM15361_01453	lysC
lysC	DSM15365_03293	lysC
lysC	DSM15365_00918	lysC
lysC	DSM15365_01682	lysC
lysC	DSM15365_00644	lysC

lysC	DSM15718_01228	lysC
lysC	DSM15718_01816	lysC
lysC	DSM15718_02160	lysC
lysC	DSM16505T_00368	lysC
lysC	DSM16505T_01374	lysC
lysC	DSM16505T_01663	lysC
lysC	DSM18103_03507	lysC
lysC	DSM18103_00389	lysC
lysC	DSM18103_03598	lysC
lysC	DSM18103_02900	lysC
lysC	DSM18103_01592	lysC
lysC	DSM18293_00942	lysC
lysC	DSM18293_04015	lysC
lysC	DSM18293_00580	lysC
lysC	DSM18293_00577	lysC
lysC	DSM18293_00579	lysC
lysC	DSM18841_00184	lysC
lysC	DSM18841_02383	lysC
lysC	DSM18841_03121	lysC
lysC	DSM18842_01029	lysC
lysC	DSM18842_01290	lysC
lysC	DSM18842_03140	lysC
lysC	DSM19866_03003	lysC
lysC	DSM19866_03250	lysC
lysC	DSM19866_02303	lysC
lysC	DSM2041_04516	lysC
lysC	DSM2041_05146	lysC
lysC	DSM2041_04237	lysC
lysC	DSM2041_01590	lysC
lysC	DSM2041_04236	lysC
lysC	DSM2061_02811	lysC
lysC	DSM2061_01942	lysC
lysC	DSM2061_02330	lysC
lysC	DSM2064_05126	lysC
lysC	DSM2064_03110	lysC
lysC	DSM2064_02507	lysC
lysC	DSM2064_02504	lysC
lysC	DSM2064_02506	lysC
lysC	DSM21164_00515	lysC
lysC	DSM21164_01805	lysC
lysC	DSM21164_00216	lysC
lysC	DSM23404_02370	lysC
lysC	DSM23404_02611	lysC
lysC	DSM23404_01377	lysC
lysC	DSM23404_01084	lysC
lysC	DSM23404_01085	lysC
lysC	DSM23424_00535	lysC
lysC	DSM23424_01000	lysC

lysC	DSM23424_03014	lysC
lysC	DSM23424_03015	lysC
lysC	DSM24786_03253	lysC
lysC	DSM24786_01959	lysC
lysC	DSM24786_00031	lysC
lysC	DSM7489_01777	lysC
lysC	DSM7489_02455	lysC
lysC	DSM7489_01998	lysC
lysC	DSW1_00445	lysC
lysC	DSW1_00999	lysC
lysC	DSW1_02326	lysC
lysC	DSW1_00218	lysC
lysC	DSW1_00219	lysC
lysC	DSW5_01428	lysC
lysC	DSW5_00933	lysC
lysC	DSW5_01707	lysC
lysC	DSW6_01751	lysC
lysC	DSW6_03156	lysC
lysC	DSW6_03185	lysC
lysC	DSW8_01870	lysC
lysC	DSW8_03043	lysC
lysC	DSW8_03099	lysC
lysC	F52_01990	lysC
lysC	F52_00552	lysC
lysC	F52_02910	lysC
lysC	F52_02907	lysC
lysC	F52_02909	lysC
lysC	GCA_001027945.1_00423	lysC
lysC	GCA_001027945.1_00005	lysC
lysC	GCA_001027945.1_02665	lysC
lysC	HI1_01703	lysC
lysC	HI1_02387	lysC
lysC	HI1_01925	lysC
lysC	IR1_01224	lysC
lysC	IR1_02326	lysC
lysC	IR1_00374	lysC
lysC	IR1_00377	lysC
lysC	IR1_00375	lysC
lysC	KCOM3131_00989	lysC
lysC	KCTC12974_01955	lysC
lysC	KCTC12974_03007	lysC
lysC	KCTC12974_00075	lysC
lysC	KCTC12974_02350	lysC
lysC	KCTC12974_02349	lysC
lysC	KL-A_02902	lysC
lysC	KL-A_03102	lysC
lysC	KL-A_00272	lysC
lysC	L12_00039	lysC

lysC	L12_00040	lysC
lysC	LL0412.1.7_00609	lysC
lysC	LL0412.1.7_02116	lysC
lysC	LL0412.1.7_02669	lysC
lysC	MAR_2009_71_01509	lysC
lysC	MAR_2009_71_00411	lysC
lysC	MAR_2009_71_00035	lysC
lysC	NN016038_01394	lysC
lysC	NN016038_01661	lysC
lysC	NN016038_02364	lysC
lysC	PAO1_00931	lysC
lysC	PAO1_03859	lysC
lysC	PAO1_00400	lysC
lysC	UW101_00566	lysC
lysC	UW101_02690	lysC
lysC	UW101_01561	lysC
lysC	UW101_01558	lysC
lysC	UW101_01560	lysC
lysC	V4-33_00280	lysC
lysC	V4-33_00232	lysC
lysC	V4-33_00233	lysC
lysC	W5C_00465	lysC
lysC	W5C_00685	lysC
lysC	W5C_00966	lysC
lysC	W5C_00918	lysC
lysC	ZONMW-01_00214	lysC
lysC	ZONMW-01_03354	lysC
lysC	ZONMW-01_00450	lysC
lysC	ZONMW-02_00226	lysC
lysC	ZONMW-02_01635	lysC
lysC	ZONMW-02_02194	lysC
lysC	ZONMW-02_01087	lysC
lysC	ZONMW-03_00940	lysC
lysC	ZONMW-03_00723	lysC
lysC	ZONMW-03_01176	lysC
lysC	ZONMW-04_00705	lysC
lysC	ZONMW-04_03407	lysC
lysC	ZONMW-04_00498	lysC
lysC	ZONMW-05_01119	lysC
lysC	ZONMW-05_02864	lysC
lysC	ZONMW-05_01327	lysC
lysC	ZONMW-06_03856	lysC
lysC	ZONMW-06_01426	lysC
lysC	ZONMW-06_02894	lysC
lysC	ZONMW-06_01901	lysC
lysC	ZONMW-06_00234	lysC
lysC	ZONMW-07_00095	lysC
lysC	ZONMW-07_03623	lysC

lysC	ZONMW-07_01529	lysC
lysC	ZONMW-08_01696	lysC
lysC	ZONMW-08_03733	lysC
lysC	ZONMW-08_03019	lysC
lysC	ZONMW-09_00162	lysC
lysC	ZONMW-09_01038	lysC
lysC	ZONMW-09_02310	lysC
lysC	ZONMW-09_01748	lysC
lysC	ZONMW-10_00581	lysC
lysC	ZONMW-10_02408	lysC
lysC	ZONMW-10_02409	lysC
lysC	ZONMW-11_01476	lysC
lysC	ZONMW-11_00788	lysC
lysC	ZONMW-11_02232	lysC
lysC	ZONMW-11_02948	lysC
lysC	ZONMW-12_00587	lysC
lysC	ZONMW-12_03106	lysC
lysC	ZONMW-12_02058	lysC
lysC	ZONMW-12_01607	lysC
lysC	ZONMW-13_00681	lysC
lysC	ZONMW-13_02003	lysC
lysC	ZONMW-13_02793	lysC
lysC	ZONMW-13_01148	lysC
lysC	ZONMW-13_01147	lysC
lysC	ZONMW-14_01360	lysC
lysC	ZONMW-14_00692	lysC
lysC	ZONMW-14_02061	lysC
lysC	ZONMW-14_01445	lysC
lysC	ZONMW-14_01446	lysC
lysC	ZONMW-15_00259	lysC
lysC	ZONMW-15_01509	lysC
lysC	ZONMW-15_01338	lysC
lysC	ZONMW-19_03242	lysC
lysC	ZONMW-19_04744	lysC
lysC	ZONMW-19_00404	lysC
lysC	ZONMW-19_00931	lysC
lysC	ZONMW-20_03680	lysC
lysC	ZONMW-20_01055	lysC
lysC	ZONMW-20_03574	lysC
lysC	ZONMW-21_00452	lysC
lysC	ZONMW-21_03202	lysC
lysC	ZONMW-21_00080	lysC
lysC	ZONMW-22_00452	lysC
lysC	ZONMW-22_03314	lysC
lysC	ZONMW-22_00080	lysC
lysC	ZONMW-23_01567	lysC
lysC	ZONMW-23_03335	lysC
lysC	ZONMW-23_01195	lysC

lysC	ZONMW-24_01007	lysC
lysC	ZONMW-24_03337	lysC
lysC	ZONMW-24_01380	lysC
lysC	ZONMW-25_02491	lysC
lysC	ZONMW-25_01230	lysC
lysC	ZONMW-25_03178	lysC
lysC	ZONMW-26_01171	lysC
lysC	ZONMW-26_03239	lysC
lysC	ZONMW-26_01381	lysC
lysC	ZONMW-27_00699	lysC
lysC	ZONMW-27_03282	lysC
lysC	ZONMW-27_00492	lysC
lysC	ZONMW-28_03799	lysC
lysC	ZONMW-28_02012	lysC
lysC	ZONMW-28_01618	lysC
lysC	ZONMW-28_02681	lysC
lysC	ZONMW-28_01473	lysC
lysC	ZONMW-29_00111	lysC
lysC	ZONMW-29_02268	lysC
lysC	ZONMW-29_02468	lysC
lysC	ZONMW-29_00865	lysC
lysC	ZONMW-30_01577	lysC
lysC	ZONMW-30_02523	lysC
lysC	ZONMW-30_00834	lysC
lysC	ZONMW-31_02618	lysC
lysC	ZONMW-31_03002	lysC
lysC	ZONMW-31_03477	lysC
lysC	ZONMW-31_01948	lysC
lysC	ZONMW-32_03737	lysC
lysC	ZONMW-32_02418	lysC
lysC	ZONMW-32_02353	lysC
lysC	ZONMW-32_03195	lysC
lysC	ZONMW-32_01880	lysC
lysC	ZONMW-33_04040	lysC
lysC	ZONMW-33_02700	lysC
lysC	ZONMW-33_02345	lysC
lysC	ZONMW-33_03079	lysC
lysC	ZONMW-33_01870	lysC
lysC	ZONMW-34_02655	lysC
lysC	ZONMW-34_02303	lysC
lysC	ZONMW-34_01994	lysC
lysC	ZONMW-34_03110	lysC
lysC	ZONMW-34_02575	lysC
lysC	ZONMW-35_00032	lysC
lysC	ZONMW-35_01946	lysC
lysC	ZONMW-35_01686	lysC
lysC	ZONMW-35_03127	lysC
lysC	ZONMW-35_02539	lysC

lysC	ZONMW-36_04041	lysC
lysC	ZONMW-36_02552	lysC
lysC	ZONMW-36_01891	lysC
lysC	ZONMW-36_02923	lysC
lysC	ZONMW-36_01485	lysC
lysC	ZONMW-37_00452	lysC
lysC	ZONMW-37_03323	lysC
lysC	ZONMW-37_00079	lysC
lysC	ZONMW-38_00880	lysC
lysC	ZONMW-38_00955	lysC
lysC	ZONMW-38_02475	lysC
lysC	ZONMW-38_00991	lysC
lysC	ZONMW-38_01176	lysC
lysC	ZONMW-39_03533	lysC
lysC	ZONMW-39_01798	lysC
lysC	ZONMW-39_02198	lysC
lysC	ZONMW-39_02118	lysC
lysC	ZONMW-40_01474	lysC
lysC	ZONMW-40_00951	lysC
lysC	ZONMW-40_02902	lysC
lysC	ZONMW-41_00780	lysC
lysC	ZONMW-41_00631	lysC
lysC	ZONMW-41_01888	lysC
lysC	ZONMW-42_03230	lysC
lysC	ZONMW-42_02473	lysC
lysC	ZONMW-42_00425	lysC
lysC	ZONMW-42_01097	lysC
lysC	ZONMW-42_03357	lysC
lysC	ZONMW-43_02535	lysC
lysC	ZONMW-43_03436	lysC
lysC	ZONMW-43_01498	lysC
lysC	ZONMW-43_01202	lysC
lysC	ZONMW-44_03870	lysC
lysC	ZONMW-44_00663	lysC
lysC	ZONMW-44_00872	lysC
lysC	ZONMW-44_00119	lysC
lysC	ZONMW-44_01812	lysC
lysC	ZONMW-45_00322	lysC
lysC	ZONMW-45_02576	lysC
lysC	ZONMW-45_02295	lysC
lysC	ZONMW-46_01517	lysC
lysC	ZONMW-46_02982	lysC
lysC	ZONMW-46_03120	lysC
lysC	ZONMW-47_01335	lysC
lysC	ZONMW-47_02891	lysC
lysC	ZONMW-47_03112	lysC
lysC	ZONMW-48_00905	lysC
lysC	ZONMW-48_01882	lysC

lysC	ZONMW-48_02358	lysC
lysC	ZONMW-49_01321	lysC
lysC	ZONMW-49_00003	lysC
lysC	ZONMW-49_01540	lysC
lysC	ZONMW-50_01323	lysC
lysC	ZONMW-50_00483	lysC
lysC	ZONMW-50_01542	lysC
lysC	ZONMW-51_01435	lysC
lysC	ZONMW-51_00003	lysC
lysC	ZONMW-51_00795	lysC
lysC	ZONMW-52_01177	lysC
lysC	ZONMW-52_03242	lysC
lysC	ZONMW-52_01395	lysC
lysC	ZONMW-53_00673	lysC
lysC	ZONMW-53_03256	lysC
lysC	ZONMW-53_00430	lysC
lysC	ZONMW-54_00388	lysC
lysC	ZONMW-54_03225	lysC
lysC	ZONMW-54_00604	lysC
lysC	ZONMW-55_00645	lysC
lysC	ZONMW-55_01535	lysC
lysC	ZONMW-55_00425	lysC
lysC	ZONMW-56_03158	lysC
lysC	ZONMW-56_00761	lysC
lysC	ZONMW-56_00890	lysC
lysC	ZONMW-57_00970	lysC
lysC	ZONMW-57_03242	lysC
lysC	ZONMW-57_01190	lysC
lysC	ZONMW-58_01614	lysC
lysC	ZONMW-58_00003	lysC
lysC	ZONMW-58_01394	lysC
lysC	ZONMW-59_01901	lysC
lysC	ZONMW-59_01002	lysC
lysC	ZONMW-59_01191	lysC
lysC	ZONMW-60_01584	lysC
lysC	ZONMW-60_03309	lysC
lysC	ZONMW-60_01377	lysC
lysC	ZONMW-60_00949	lysC
lysC	ZONMW-61_00626	lysC
lysC	ZONMW-61_03461	lysC
lysC	ZONMW-61_02688	lysC
lysC	ZONMW-62_02511	lysC
lysC	ZONMW-62_03585	lysC
lysC	ZONMW-62_03375	lysC
lysC	ZONMW-63_00728	lysC
lysC	ZONMW-63_02102	lysC
lysC	ZONMW-63_03354	lysC
lysC	ZONMW-63_00951	lysC

lysC	ZONMW-64_03716	lysC
lysC	ZONMW-64_01758	lysC
lysC	ZONMW-64_02075	lysC
lysC	ZONMW-64_03552	lysC
lysC	ZONMW-65_00322	lysC
lysC	ZONMW-65_00054	lysC
lysC	ZONMW-65_03423	lysC
lysC	ZONMW-66_03507	lysC
lysC	ZONMW-66_04128	lysC
lysC	ZONMW-66_02231	lysC
lysC	ZONMW-66_00230	lysC
lysC	ZONMW-67_01790	lysC
lysC	ZONMW-67_02393	lysC
lysC	ZONMW-67_00001	lysC
lysC	ZONMW-67_03343	lysC
lysC	ZONMW-67_02628	lysC
lysC	ZONMW-68_00425	lysC
lysC	ZONMW-68_03844	lysC
lysC	ZONMW-68_01522	lysC
lysC	ZONMW-69_02153	lysC
lysC	ZONMW-69_02441	lysC
lysC	ZONMW-69_02610	lysC
lysC	ZONMW-69_03344	lysC
lysC	ZONMW-69_00425	lysC
lysC	ZONMW-72_01560	lysC
lysC	ZONMW-72_02888	lysC
lysC	ZONMW-72_00026	lysC
lysC	ZONMW-72_00029	lysC
lysC	ZONMW-72_00027	lysC
lysC	ZONMW-73_01794	lysC
lysC	ZONMW-73_01840	lysC
lysC	ZONMW-73_02301	lysC
lysC	ZONMW-73_02487	lysC
lysC	ZONMW-74_01909	lysC
lysC	ZONMW-74_01693	lysC
lysC	ZONMW-74_03221	lysC
lysC	ZONMW-75_01093	lysC
lysC	ZONMW-75_00430	lysC
lysC	ZONMW-75_03343	lysC
lysC	ZONMW-75_00603	lysC
lysC	da5A-8_03108	lysC
lysC	da5A-8_03222	lysC
lysC	da5A-8_02582	lysC
lysC	da5A-8_00651	lysC
trxA_1	18_01427	trxA_1
trxA_1	18_02389	trxA_1
trxA_1	18_01540	trxA_1
trxA_1	18_02995	trxA_1

trxA_1	ACAM48_01990	trxA_1
trxA_1	ACAM48_02278	trxA_1
trxA_1	ACAM48_02020	trxA_1
trxA_1	ACAM48_01138	trxA_1
trxA_1	CAM030_02165	trxA_1
trxA_1	CAM030_01934	trxA_1
trxA_1	CAM030_02281	trxA_1
trxA_1	CAM030_00430	trxA_1
trxA_1	CECT7946_00475	trxA_1
trxA_1	CECT7946_02467	trxA_1
trxA_1	CECT7946_02505	trxA_1
trxA_1	CECT7946_01586	trxA_1
trxA_1	CECT7946_01722	trxA_1
trxA_1	CGMCC1.8863_00577	trxA_1
trxA_1	CGMCC1.8863_01671	trxA_1
trxA_1	CGMCC1.8863_01076	trxA_1
trxA_1	CGMCC1.8863_00278	trxA_1
trxA_1	CL8139_02152	trxA_1
trxA_1	CL8139_03063	trxA_1
trxA_1	CL8139_00155	trxA_1
trxA_1	CL8139_01933	trxA_1
trxA_1	DAU203_00937	trxA_1
trxA_1	DAU203_03398	trxA_1
trxA_1	DAU203_01966	trxA_1
trxA_1	DAU203_03243	trxA_1
trxA_1	DD5b_00479	trxA_1
trxA_1	DD5b_01657	trxA_1
trxA_1	DD5b_00379	trxA_1
trxA_1	DD5b_00837	trxA_1
trxA_1	DSM1208_02096	trxA_1
trxA_1	DSM1208_03340	trxA_1
trxA_1	DSM1208_02266	trxA_1
trxA_1	DSM1208_04426	trxA_1
trxA_1	DSM12212_01380	trxA_1
trxA_1	DSM12212_02353	trxA_1
trxA_1	DSM12212_01739	trxA_1
trxA_1	DSM12212_01834	trxA_1
trxA_1	DSM15343_00745	trxA_1
trxA_1	DSM15343_01365	trxA_1
trxA_1	DSM15343_02606	trxA_1
trxA_1	DSM15343_01734	trxA_1
trxA_1	DSM15343_00524	trxA_1
trxA_1	DSM15361_00130	trxA_1
trxA_1	DSM15361_00654	trxA_1
trxA_1	DSM15361_01395	trxA_1
trxA_1	DSM15365_02239	trxA_1
trxA_1	DSM15365_02965	trxA_1
trxA_1	DSM15365_03418	trxA_1

trxA_1	DSM15365_01621	trxA_1
trxA_1	DSM15718_02334	trxA_1
trxA_1	DSM15718_01708	trxA_1
trxA_1	DSM15718_01292	trxA_1
trxA_1	DSM15718_01528	trxA_1
trxA_1	DSM16505T_00982	trxA_1
trxA_1	DSM16505T_00085	trxA_1
trxA_1	DSM16505T_00466	trxA_1
trxA_1	DSM16505T_00380	trxA_1
trxA_1	DSM16505T_02532	trxA_1
trxA_1	DSM18103_02591	trxA_1
trxA_1	DSM18103_01206	trxA_1
trxA_1	DSM18103_02796	trxA_1
trxA_1	DSM18103_01338	trxA_1
trxA_1	DSM18103_02977	trxA_1
trxA_1	DSM18293_02589	trxA_1
trxA_1	DSM18293_00426	trxA_1
trxA_1	DSM18293_00711	trxA_1
trxA_1	DSM18293_03547	trxA_1
trxA_1	DSM18293_01141	trxA_1
trxA_1	DSM18841_03157	trxA_1
trxA_1	DSM18841_00309	trxA_1
trxA_1	DSM18841_02928	trxA_1
trxA_1	DSM18841_00205	trxA_1
trxA_1	DSM18842_00444	trxA_1
trxA_1	DSM18842_02487	trxA_1
trxA_1	DSM18842_00926	trxA_1
trxA_1	DSM18842_02767	trxA_1
trxA_1	DSM18842_01014	trxA_1
trxA_1	DSM19866_03074	trxA_1
trxA_1	DSM19866_02517	trxA_1
trxA_1	DSM19866_00635	trxA_1
trxA_1	DSM19866_01547	trxA_1
trxA_1	DSM2041_00684	trxA_1
trxA_1	DSM2041_03607	trxA_1
trxA_1	DSM2041_02313	trxA_1
trxA_1	DSM2041_04642	trxA_1
trxA_1	DSM2041_04008	trxA_1
trxA_1	DSM2061_03649	trxA_1
trxA_1	DSM2061_01715	trxA_1
trxA_1	DSM2061_03796	trxA_1
trxA_1	DSM2061_00730	trxA_1
trxA_1	DSM2064_02349	trxA_1
trxA_1	DSM2064_02636	trxA_1
trxA_1	DSM2064_03577	trxA_1
trxA_1	DSM2064_04042	trxA_1
trxA_1	DSM21164_00470	trxA_1
trxA_1	DSM21164_01914	trxA_1

trxA_1	DSM21164_00336	trxA_1
trxA_1	DSM21164_01486	trxA_1
trxA_1	DSM23404_01796	trxA_1
trxA_1	DSM23404_02998	trxA_1
trxA_1	DSM23404_01823	trxA_1
trxA_1	DSM23404_02758	trxA_1
trxA_1	DSM23424_00735	trxA_1
trxA_1	DSM23424_02060	trxA_1
trxA_1	DSM23424_00089	trxA_1
trxA_1	DSM24786_03098	trxA_1
trxA_1	DSM24786_02082	trxA_1
trxA_1	DSM24786_00688	trxA_1
trxA_1	DSM7489_00872	trxA_1
trxA_1	DSM7489_03237	trxA_1
trxA_1	DSM7489_01877	trxA_1
trxA_1	DSM7489_03075	trxA_1
trxA_1	DSW1_00821	trxA_1
trxA_1	DSW1_00376	trxA_1
trxA_1	DSW1_01033	trxA_1
trxA_1	DSW1_02563	trxA_1
trxA_1	DSW5_01726	trxA_1
trxA_1	DSW5_00861	trxA_1
trxA_1	DSW5_01289	trxA_1
trxA_1	DSW5_00143	trxA_1
trxA_1	DSW5_00947	trxA_1
trxA_1	DSW6_00961	trxA_1
trxA_1	DSW6_00868	trxA_1
trxA_1	DSW6_02901	trxA_1
trxA_1	DSW6_02361	trxA_1
trxA_1	DSW6_01690	trxA_1
trxA_1	DSW6_02723	trxA_1
trxA_1	DSW8_01795	trxA_1
trxA_1	DSW8_02521	trxA_1
trxA_1	DSW8_03267	trxA_1
trxA_1	DSW8_01678	trxA_1
trxA_1	DSW8_03571	trxA_1
trxA_1	F52_04198	trxA_1
trxA_1	F52_02747	trxA_1
trxA_1	F52_03040	trxA_1
trxA_1	F52_00086	trxA_1
trxA_1	GCA_001027945.1_01564	trxA_1
trxA_1	GCA_001027945.1_00953	trxA_1
trxA_1	GCA_001027945.1_00296	trxA_1
trxA_1	GCA_001027945.1_00406	trxA_1
trxA_1	GCA_001027945.1_00609	trxA_1
trxA_1	HI1_00804	trxA_1
trxA_1	HI1_03181	trxA_1
trxA_1	HI1_01805	trxA_1

trxA_1	HI1_03016	trxA_1
trxA_1	IR1_00509	trxA_1
trxA_1	IR1_00250	trxA_1
trxA_1	IR1_04507	trxA_1
trxA_1	KCOM3131_00977	trxA_1
trxA_1	KCOM3131_01255	trxA_1
trxA_1	KCTC12974_00766	trxA_1
trxA_1	KCTC12974_01847	trxA_1
trxA_1	KCTC12974_00797	trxA_1
trxA_1	KCTC12974_02671	trxA_1
trxA_1	KL-A_02399	trxA_1
trxA_1	KL-A_01938	trxA_1
trxA_1	KL-A_00394	trxA_1
trxA_1	KL-A_02951	trxA_1
trxA_1	L12_00377	trxA_1
trxA_1	LL0412.1.7_01017	trxA_1
trxA_1	LL0412.1.7_00430	trxA_1
trxA_1	LL0412.1.7_00598	trxA_1
trxA_1	LL0412.1.7_01804	trxA_1
trxA_1	MAR_2009_71_01625	trxA_1
trxA_1	MAR_2009_71_00931	trxA_1
trxA_1	MAR_2009_71_01741	trxA_1
trxA_1	MAR_2009_71_03657	trxA_1
trxA_1	MAR_2009_71_02728	trxA_1
trxA_1	NN016038_01436	trxA_1
trxA_1	NN016038_02400	trxA_1
trxA_1	NN016038_01547	trxA_1
trxA_1	NN016038_03030	trxA_1
trxA_1	PAO1_05416	trxA_1
trxA_1	PAO1_04189	trxA_1
trxA_1	PAO1_02775	trxA_1
trxA_1	UW101_01403	trxA_1
trxA_1	UW101_01690	trxA_1
trxA_1	UW101_02223	trxA_1
trxA_1	UW101_04845	trxA_1
trxA_1	V4-33_01915	trxA_1
trxA_1	V4-33_00237	trxA_1
trxA_1	V4-33_01721	trxA_1
trxA_1	V4-33_00270	trxA_1
trxA_1	V4-33_01636	trxA_1
trxA_1	W5C_01515	trxA_1
trxA_1	W5C_01784	trxA_1
trxA_1	W5C_00563	trxA_1
trxA_1	W5C_02282	trxA_1
trxA_1	ZONMW-01_02508	trxA_1
trxA_1	ZONMW-01_00550	trxA_1
trxA_1	ZONMW-01_00331	trxA_1
trxA_1	ZONMW-01_00699	trxA_1

trxA_1	ZONMW-02_02428	trxA_1
trxA_1	ZONMW-02_00723	trxA_1
trxA_1	ZONMW-02_00732	trxA_1
trxA_1	ZONMW-02_00344	trxA_1
trxA_1	ZONMW-02_02254	trxA_1
trxA_1	ZONMW-03_01789	trxA_1
trxA_1	ZONMW-03_02444	trxA_1
trxA_1	ZONMW-03_01057	trxA_1
trxA_1	ZONMW-03_00077	trxA_1
trxA_1	ZONMW-04_02994	trxA_1
trxA_1	ZONMW-04_00925	trxA_1
trxA_1	ZONMW-04_00615	trxA_1
trxA_1	ZONMW-04_01084	trxA_1
trxA_1	ZONMW-05_03238	trxA_1
trxA_1	ZONMW-05_00177	trxA_1
trxA_1	ZONMW-05_01210	trxA_1
trxA_1	ZONMW-05_00337	trxA_1
trxA_1	ZONMW-06_01846	trxA_1
trxA_1	ZONMW-06_00693	trxA_1
trxA_1	ZONMW-06_02308	trxA_1
trxA_1	ZONMW-06_02321	trxA_1
trxA_1	ZONMW-06_00333	trxA_1
trxA_1	ZONMW-07_00116	trxA_1
trxA_1	ZONMW-07_02916	trxA_1
trxA_1	ZONMW-07_00342	trxA_1
trxA_1	ZONMW-07_03039	trxA_1
trxA_1	ZONMW-08_01608	trxA_1
trxA_1	ZONMW-08_00451	trxA_1
trxA_1	ZONMW-08_02257	trxA_1
trxA_1	ZONMW-08_01493	trxA_1
trxA_1	ZONMW-08_01939	trxA_1
trxA_1	ZONMW-09_00057	trxA_1
trxA_1	ZONMW-09_00026	trxA_1
trxA_1	ZONMW-09_00031	trxA_1
trxA_1	ZONMW-09_02119	trxA_1
trxA_1	ZONMW-10_00075	trxA_1
trxA_1	ZONMW-10_00495	trxA_1
trxA_1	ZONMW-10_00647	trxA_1
trxA_1	ZONMW-10_00728	trxA_1
trxA_1	ZONMW-11_02338	trxA_1
trxA_1	ZONMW-11_00722	trxA_1
trxA_1	ZONMW-11_02754	trxA_1
trxA_1	ZONMW-11_00828	trxA_1
trxA_1	ZONMW-12_03191	trxA_1
trxA_1	ZONMW-12_00025	trxA_1
trxA_1	ZONMW-12_00644	trxA_1
trxA_1	ZONMW-12_01887	trxA_1
trxA_1	ZONMW-13_00131	trxA_1

trxA_1	ZONMW-13_00469	trxA_1
trxA_1	ZONMW-13_00970	trxA_1
trxA_1	ZONMW-13_01348	trxA_1
trxA_1	ZONMW-14_02674	trxA_1
trxA_1	ZONMW-14_00093	trxA_1
trxA_1	ZONMW-14_01138	trxA_1
trxA_1	ZONMW-14_02830	trxA_1
trxA_1	ZONMW-14_01814	trxA_1
trxA_1	ZONMW-15_00222	trxA_1
trxA_1	ZONMW-15_00395	trxA_1
trxA_1	ZONMW-15_03082	trxA_1
trxA_1	ZONMW-19_01138	trxA_1
trxA_1	ZONMW-19_00070	trxA_1
trxA_1	ZONMW-20_00840	trxA_1
trxA_1	ZONMW-20_02957	trxA_1
trxA_1	ZONMW-20_01264	trxA_1
trxA_1	ZONMW-21_00325	trxA_1
trxA_1	ZONMW-21_00208	trxA_1
trxA_1	ZONMW-21_01746	trxA_1
trxA_1	ZONMW-22_00325	trxA_1
trxA_1	ZONMW-22_00208	trxA_1
trxA_1	ZONMW-22_01978	trxA_1
trxA_1	ZONMW-23_01440	trxA_1
trxA_1	ZONMW-23_01323	trxA_1
trxA_1	ZONMW-23_00573	trxA_1
trxA_1	ZONMW-24_01134	trxA_1
trxA_1	ZONMW-24_01252	trxA_1
trxA_1	ZONMW-24_02603	trxA_1
trxA_1	ZONMW-25_02541	trxA_1
trxA_1	ZONMW-25_02578	trxA_1
trxA_1	ZONMW-25_03087	trxA_1
trxA_1	ZONMW-25_00813	trxA_1
trxA_1	ZONMW-26_02060	trxA_1
trxA_1	ZONMW-26_00780	trxA_1
trxA_1	ZONMW-26_01264	trxA_1
trxA_1	ZONMW-26_00630	trxA_1
trxA_1	ZONMW-27_02860	trxA_1
trxA_1	ZONMW-27_01000	trxA_1
trxA_1	ZONMW-27_00609	trxA_1
trxA_1	ZONMW-27_01159	trxA_1
trxA_1	ZONMW-28_02736	trxA_1
trxA_1	ZONMW-28_00886	trxA_1
trxA_1	ZONMW-28_01564	trxA_1
trxA_1	ZONMW-29_00030	trxA_1
trxA_1	ZONMW-29_02867	trxA_1
trxA_1	ZONMW-29_02349	trxA_1
trxA_1	ZONMW-29_02996	trxA_1
trxA_1	ZONMW-30_01535	trxA_1

trxA_1	ZONMW-30_00161	trxA_1
trxA_1	ZONMW-30_01417	trxA_1
trxA_1	ZONMW-30_01125	trxA_1
trxA_1	ZONMW-31_02001	trxA_1
trxA_1	ZONMW-31_00295	trxA_1
trxA_1	ZONMW-31_00649	trxA_1
trxA_1	ZONMW-31_02754	trxA_1
trxA_1	ZONMW-32_03139	trxA_1
trxA_1	ZONMW-32_00205	trxA_1
trxA_1	ZONMW-32_01971	trxA_1
trxA_1	ZONMW-33_03134	trxA_1
trxA_1	ZONMW-33_00072	trxA_1
trxA_1	ZONMW-33_01962	trxA_1
trxA_1	ZONMW-34_02522	trxA_1
trxA_1	ZONMW-34_01293	trxA_1
trxA_1	ZONMW-34_00297	trxA_1
trxA_1	ZONMW-35_02486	trxA_1
trxA_1	ZONMW-35_00366	trxA_1
trxA_1	ZONMW-35_01093	trxA_1
trxA_1	ZONMW-36_02867	trxA_1
trxA_1	ZONMW-36_01297	trxA_1
trxA_1	ZONMW-36_01394	trxA_1
trxA_1	ZONMW-37_00325	trxA_1
trxA_1	ZONMW-37_00207	trxA_1
trxA_1	ZONMW-37_02124	trxA_1
trxA_1	ZONMW-38_00187	trxA_1
trxA_1	ZONMW-38_02873	trxA_1
trxA_1	ZONMW-38_00290	trxA_1
trxA_1	ZONMW-39_02063	trxA_1
trxA_1	ZONMW-39_00409	trxA_1
trxA_1	ZONMW-39_04000	trxA_1
trxA_1	ZONMW-40_01523	trxA_1
trxA_1	ZONMW-40_02263	trxA_1
trxA_1	ZONMW-40_02622	trxA_1
trxA_1	ZONMW-40_03233	trxA_1
trxA_1	ZONMW-41_02183	trxA_1
trxA_1	ZONMW-41_02219	trxA_1
trxA_1	ZONMW-41_00702	trxA_1
trxA_1	ZONMW-42_01153	trxA_1
trxA_1	ZONMW-42_01504	trxA_1
trxA_1	ZONMW-42_03585	trxA_1
trxA_1	ZONMW-43_01149	trxA_1
trxA_1	ZONMW-43_03902	trxA_1
trxA_1	ZONMW-43_00655	trxA_1
trxA_1	ZONMW-44_00174	trxA_1
trxA_1	ZONMW-44_01170	trxA_1
trxA_1	ZONMW-44_01511	trxA_1
trxA_1	ZONMW-44_03523	trxA_1

trxA_1	ZONMW-44_01157	trxA_1
trxA_1	ZONMW-44_03106	trxA_1
trxA_1	ZONMW-45_01407	trxA_1
trxA_1	ZONMW-45_00459	trxA_1
trxA_1	ZONMW-45_02514	trxA_1
trxA_1	ZONMW-45_00355	trxA_1
trxA_1	ZONMW-46_01662	trxA_1
trxA_1	ZONMW-46_01380	trxA_1
trxA_1	ZONMW-46_02736	trxA_1
trxA_1	ZONMW-46_01484	trxA_1
trxA_1	ZONMW-47_01827	trxA_1
trxA_1	ZONMW-47_01472	trxA_1
trxA_1	ZONMW-47_02727	trxA_1
trxA_1	ZONMW-47_01368	trxA_1
trxA_1	ZONMW-48_00868	trxA_1
trxA_1	ZONMW-48_01032	trxA_1
trxA_1	ZONMW-48_03449	trxA_1
trxA_1	ZONMW-49_02066	trxA_1
trxA_1	ZONMW-49_02970	trxA_1
trxA_1	ZONMW-49_01419	trxA_1
trxA_1	ZONMW-49_01757	trxA_1
trxA_1	ZONMW-50_01860	trxA_1
trxA_1	ZONMW-50_02885	trxA_1
trxA_1	ZONMW-50_01421	trxA_1
trxA_1	ZONMW-50_01657	trxA_1
trxA_1	ZONMW-51_01724	trxA_1
trxA_1	ZONMW-51_01989	trxA_1
trxA_1	ZONMW-51_00675	trxA_1
trxA_1	ZONMW-51_00653	trxA_1
trxA_1	ZONMW-52_01867	trxA_1
trxA_1	ZONMW-52_00783	trxA_1
trxA_1	ZONMW-52_01275	trxA_1
trxA_1	ZONMW-52_00612	trxA_1
trxA_1	ZONMW-53_01889	trxA_1
trxA_1	ZONMW-53_01517	trxA_1
trxA_1	ZONMW-53_00550	trxA_1
trxA_1	ZONMW-53_01367	trxA_1
trxA_1	ZONMW-54_02168	trxA_1
trxA_1	ZONMW-54_01203	trxA_1
trxA_1	ZONMW-54_00485	trxA_1
trxA_1	ZONMW-54_01350	trxA_1
trxA_1	ZONMW-55_02046	trxA_1
trxA_1	ZONMW-55_02311	trxA_1
trxA_1	ZONMW-55_00545	trxA_1
trxA_1	ZONMW-55_00871	trxA_1
trxA_1	ZONMW-56_01388	trxA_1
trxA_1	ZONMW-56_02045	trxA_1
trxA_1	ZONMW-56_00768	trxA_1

trxA_1	ZONMW-56_00107	trxA_1
trxA_1	ZONMW-57_01836	trxA_1
trxA_1	ZONMW-57_00171	trxA_1
trxA_1	ZONMW-57_01069	trxA_1
trxA_1	ZONMW-57_00318	trxA_1
trxA_1	ZONMW-58_01860	trxA_1
trxA_1	ZONMW-58_00800	trxA_1
trxA_1	ZONMW-58_01515	trxA_1
trxA_1	ZONMW-58_00653	trxA_1
trxA_1	ZONMW-59_01673	trxA_1
trxA_1	ZONMW-59_00164	trxA_1
trxA_1	ZONMW-59_01071	trxA_1
trxA_1	ZONMW-59_00334	trxA_1
trxA_1	ZONMW-60_02051	trxA_1
trxA_1	ZONMW-60_00197	trxA_1
trxA_1	ZONMW-60_01496	trxA_1
trxA_1	ZONMW-60_00345	trxA_1
trxA_1	ZONMW-61_00734	trxA_1
trxA_1	ZONMW-61_00022	trxA_1
trxA_1	ZONMW-61_02904	trxA_1
trxA_1	ZONMW-61_03115	trxA_1
trxA_1	ZONMW-61_02345	trxA_1
trxA_1	ZONMW-62_02443	trxA_1
trxA_1	ZONMW-62_01575	trxA_1
trxA_1	ZONMW-62_03190	trxA_1
trxA_1	ZONMW-62_02325	trxA_1
trxA_1	ZONMW-62_00822	trxA_1
trxA_1	ZONMW-63_02755	trxA_1
trxA_1	ZONMW-63_02568	trxA_1
trxA_1	ZONMW-63_00825	trxA_1
trxA_1	ZONMW-63_01957	trxA_1
trxA_1	ZONMW-64_03499	trxA_1
trxA_1	ZONMW-64_02165	trxA_1
trxA_1	ZONMW-64_00917	trxA_1
trxA_1	ZONMW-65_01306	trxA_1
trxA_1	ZONMW-65_01291	trxA_1
trxA_1	ZONMW-66_00546	trxA_1
trxA_1	ZONMW-66_00568	trxA_1
trxA_1	ZONMW-67_03397	trxA_1
trxA_1	ZONMW-67_02091	trxA_1
trxA_1	ZONMW-67_00675	trxA_1
trxA_1	ZONMW-67_02707	trxA_1
trxA_1	ZONMW-67_02114	trxA_1
trxA_1	ZONMW-68_00509	trxA_1
trxA_1	ZONMW-68_02350	trxA_1
trxA_1	ZONMW-68_03894	trxA_1
trxA_1	ZONMW-68_01933	trxA_1
trxA_1	ZONMW-69_03398	trxA_1

trxA_1	ZONMW-69_01852	trxA_1
trxA_1	ZONMW-69_00722	trxA_1
trxA_1	ZONMW-69_00504	trxA_1
trxA_1	ZONMW-69_01829	trxA_1
trxA_1	ZONMW-72_00161	trxA_1
trxA_1	ZONMW-72_00433	trxA_1
trxA_1	ZONMW-72_04263	trxA_1
trxA_1	ZONMW-73_00354	trxA_1
trxA_1	ZONMW-73_00918	trxA_1
trxA_1	ZONMW-73_02435	trxA_1
trxA_1	ZONMW-73_03798	trxA_1
trxA_1	ZONMW-74_03351	trxA_1
trxA_1	ZONMW-74_00787	trxA_1
trxA_1	ZONMW-74_01826	trxA_1
trxA_1	ZONMW-74_00301	trxA_1
trxA_1	ZONMW-75_00226	trxA_1
trxA_1	ZONMW-75_02771	trxA_1
trxA_1	ZONMW-75_03289	trxA_1
trxA_1	ZONMW-75_01205	trxA_1
trxA_1	da5A-8_00403	trxA_1
trxA_1	da5A-8_01506	trxA_1
trxA_1	da5A-8_01891	trxA_1
trxA_1	da5A-8_01064	trxA_1
trxA_1	da5A-8_01992	trxA_1
hflX	18_01166	hflX
hflX	ACAM48_02151	hflX
hflX	CAM030_00867	hflX
hflX	CECT7946_01971	hflX
hflX	CGMCC1.8863_00899	hflX
hflX	CL8139_00899	hflX
hflX	DAU203_01675	hflX
hflX	DD5b_02168	hflX
hflX	DSM1208_01782	hflX
hflX	DSM12212_02076	hflX
hflX	DSM15343_03055	hflX
hflX	DSM15361_00640	hflX
hflX	DSM15365_00936	hflX
hflX	DSM15718_02664	hflX
hflX	DSM16505T_00242	hflX
hflX	DSM18103_01704	hflX
hflX	DSM18293_00269	hflX
hflX	DSM18841_00041	hflX
hflX	DSM18842_01168	hflX
hflX	DSM19866_02023	hflX
hflX	DSM2041_00285	hflX
hflX	DSM2061_03310	hflX
hflX	DSM2064_02174	hflX
hflX	DSM21164_00698	hflX

hflX	DSM23404_01948	hflX
hflX	DSM23424_00176	hflX
hflX	DSM24786_02314	hflX
hflX	DSM7489_01594	hflX
hflX	DSW1_00336	hflX
hflX	DSW5_01416	hflX
hflX	DSW6_01643	hflX
hflX	DSW8_02153	hflX
hflX	F52_02596	hflX
hflX	GCA_001027945.1_00530	hflX
hflX	HI1_01514	hflX
hflX	IR1_00679	hflX
hflX	KCOM3131_00687	hflX
hflX	KCTC12974_00407	hflX
hflX	KL-A_02837	hflX
hflX	L12_00287	hflX
hflX	LL0412.1.7_00210	hflX
hflX	MAR_2009_71_01287	hflX
hflX	NN016038_01187	hflX
hflX	PAO1_05112	hflX
hflX	UW101_01228	hflX
hflX	V4-33_02335	hflX
hflX	W5C_00282	hflX
hflX	ZONMW-01_00040	hflX
hflX	ZONMW-02_01653	hflX
hflX	ZONMW-03_00765	hflX
hflX	ZONMW-04_02177	hflX
hflX	ZONMW-05_00958	hflX
hflX	ZONMW-06_00928	hflX
hflX	ZONMW-07_01414	hflX
hflX	ZONMW-08_00806	hflX
hflX	ZONMW-09_00488	hflX
hflX	ZONMW-10_00323	hflX
hflX	ZONMW-11_02839	hflX
hflX	ZONMW-12_00103	hflX
hflX	ZONMW-13_00207	hflX
hflX	ZONMW-14_00385	hflX
hflX	ZONMW-15_00023	hflX
hflX	ZONMW-19_00312	hflX
hflX	ZONMW-20_03154	hflX
hflX	ZONMW-21_00682	hflX
hflX	ZONMW-22_00683	hflX
hflX	ZONMW-23_01797	hflX
hflX	ZONMW-24_00778	hflX
hflX	ZONMW-25_01650	hflX
hflX	ZONMW-26_01001	hflX
hflX	ZONMW-27_02662	hflX
hflX	ZONMW-28_00596	hflX

hflX	ZONMW-29_00335	hflX
hflX	ZONMW-30_02347	hflX
hflX	ZONMW-31_01185	hflX
hflX	ZONMW-32_00496	hflX
hflX	ZONMW-33_00364	hflX
hflX	ZONMW-34_00740	hflX
hflX	ZONMW-35_02722	hflX
hflX	ZONMW-36_01007	hflX
hflX	ZONMW-37_00682	hflX
hflX	ZONMW-38_03471	hflX
hflX	ZONMW-39_01204	hflX
hflX	ZONMW-40_01289	hflX
hflX	ZONMW-41_01053	hflX
hflX	ZONMW-41_00864	hflX
hflX	ZONMW-42_01691	hflX
hflX	ZONMW-43_00279	hflX
hflX	ZONMW-44_02125	hflX
hflX	ZONMW-45_02050	hflX
hflX	ZONMW-46_02232	hflX
hflX	ZONMW-47_02300	hflX
hflX	ZONMW-48_00684	hflX
hflX	ZONMW-49_01013	hflX
hflX	ZONMW-50_01014	hflX
hflX	ZONMW-51_01254	hflX
hflX	ZONMW-52_00983	hflX
hflX	ZONMW-53_00853	hflX
hflX	ZONMW-54_00211	hflX
hflX	ZONMW-55_00819	hflX
hflX	ZONMW-56_02253	hflX
hflX	ZONMW-57_02384	hflX
hflX	ZONMW-58_02409	hflX
hflX	ZONMW-59_02084	hflX
hflX	ZONMW-60_01763	hflX
hflX	ZONMW-61_00403	hflX
hflX	ZONMW-62_01948	hflX
hflX	ZONMW-63_00547	hflX
hflX	ZONMW-64_00296	hflX
hflX	ZONMW-65_01872	hflX
hflX	ZONMW-66_01053	hflX
hflX	ZONMW-67_00963	hflX
hflX	ZONMW-68_00164	hflX
hflX	ZONMW-69_01010	hflX
hflX	ZONMW-72_00331	hflX
hflX	ZONMW-73_01857	hflX
hflX	ZONMW-74_01429	hflX
hflX	ZONMW-75_00451	hflX
hflX	da5A-8_00768	hflX
tsf	18_01532	tsf

tsf	ACAM48_02031	tsf
tsf	CAM030_01325	tsf
tsf	CECT7946_01761	tsf
tsf	CGMCC1.8863_01067	tsf
tsf	CL8139_00145	tsf
tsf	DAU203_01956	tsf
tsf	DD5b_01828	tsf
tsf	DSM1208_02257	tsf
tsf	DSM12212_01708	tsf
tsf	DSM15343_02585	tsf
tsf	DSM15361_01403	tsf
tsf	DSM15365_03401	tsf
tsf	DSM15718_01285	tsf
tsf	DSM16505T_00865	tsf
tsf	DSM18103_03171	tsf
tsf	DSM18293_00698	tsf
tsf	DSM18841_00732	tsf
tsf	DSM18842_00588	tsf
tsf	DSM19866_00645	tsf
tsf	DSM2041_04632	tsf
tsf	DSM2061_03787	tsf
tsf	DSM2064_02621	tsf
tsf	DSM21164_00345	tsf
tsf	DSM23404_01832	tsf
tsf	DSM23424_00633	tsf
tsf	DSM24786_02092	tsf
tsf	DSM7489_01867	tsf
tsf	DSW1_00163	tsf
tsf	DSW5_00908	tsf
tsf	DSW6_01016	tsf
tsf	DSW8_01689	tsf
tsf	F52_03027	tsf
tsf	GCA_001027945.1_03158	tsf
tsf	HI1_01795	tsf
tsf	IR1_00260	tsf
tsf	KCOM3131_00436	tsf
tsf	KCTC12974_00806	tsf
tsf	KL-A_00404	tsf
tsf	L12_00272	tsf
tsf	LL0412.1.7_00865	tsf
tsf	MAR_2009_71_01728	tsf
tsf	NN016038_01539	tsf
tsf	PAO1_03778	tsf
tsf	UW101_01675	tsf
tsf	V4-33_00433	tsf
tsf	W5C_00553	tsf
tsf	ZONMW-01_00321	tsf
tsf	ZONMW-02_00327	tsf

tsf	ZONMW-03_01047	tsf
tsf	ZONMW-04_00625	tsf
tsf	ZONMW-05_01200	tsf
tsf	ZONMW-06_03529	tsf
tsf	ZONMW-07_00333	tsf
tsf	ZONMW-08_01506	tsf
tsf	ZONMW-09_00040	tsf
tsf	ZONMW-10_00640	tsf
tsf	ZONMW-11_01522	tsf
tsf	ZONMW-12_00634	tsf
tsf	ZONMW-13_00953	tsf
tsf	ZONMW-14_01153	tsf
tsf	ZONMW-15_00388	tsf
tsf	ZONMW-19_00795	tsf
tsf	ZONMW-20_04124	tsf
tsf	ZONMW-21_00234	tsf
tsf	ZONMW-22_00234	tsf
tsf	ZONMW-23_01349	tsf
tsf	ZONMW-24_01225	tsf
tsf	ZONMW-25_03063	tsf
tsf	ZONMW-26_01254	tsf
tsf	ZONMW-27_00619	tsf
tsf	ZONMW-28_03402	tsf
tsf	ZONMW-29_02970	tsf
tsf	ZONMW-30_01441	tsf
tsf	ZONMW-31_02386	tsf
tsf	ZONMW-32_03579	tsf
tsf	ZONMW-33_03929	tsf
tsf	ZONMW-34_03407	tsf
tsf	ZONMW-35_03408	tsf
tsf	ZONMW-36_03878	tsf
tsf	ZONMW-37_00234	tsf
tsf	ZONMW-38_00856	tsf
tsf	ZONMW-39_03269	tsf
tsf	ZONMW-40_02647	tsf
tsf	ZONMW-41_02497	tsf
tsf	ZONMW-42_02873	tsf
tsf	ZONMW-43_02630	tsf
tsf	ZONMW-44_02581	tsf
tsf	ZONMW-45_01091	tsf
tsf	ZONMW-46_00576	tsf
tsf	ZONMW-47_00576	tsf
tsf	ZONMW-48_01025	tsf
tsf	ZONMW-49_01409	tsf
tsf	ZONMW-50_01411	tsf
tsf	ZONMW-51_01525	tsf
tsf	ZONMW-52_01265	tsf
tsf	ZONMW-53_00560	tsf

tsf	ZONMW-54_00475	tsf
tsf	ZONMW-55_00555	tsf
tsf	ZONMW-56_03013	tsf
tsf	ZONMW-57_01059	tsf
tsf	ZONMW-58_01525	tsf
tsf	ZONMW-59_01061	tsf
tsf	ZONMW-60_01506	tsf
tsf	ZONMW-61_02889	tsf
tsf	ZONMW-62_02338	tsf
tsf	ZONMW-63_00815	tsf
tsf	ZONMW-64_03283	tsf
tsf	ZONMW-65_00905	tsf
tsf	ZONMW-66_00180	tsf
tsf	ZONMW-67_03172	tsf
tsf	ZONMW-68_03904	tsf
tsf	ZONMW-69_03173	tsf
tsf	ZONMW-72_00424	tsf
tsf	ZONMW-73_03186	tsf
tsf	ZONMW-74_01835	tsf
tsf	ZONMW-75_01185	tsf
tsf	da5A-8_01747	tsf
uppS	18_01627	uppS
uppS	ACAM48_02729	uppS
uppS	CAM030_02351	uppS
uppS	CECT7946_02560	uppS
uppS	CGMCC1.8863_01259	uppS
uppS	CL8139_03268	uppS
uppS	DAU203_02066	uppS
uppS	DD5b_02612	uppS
uppS	DSM1208_02371	uppS
uppS	DSM12212_03403	uppS
uppS	DSM15343_01921	uppS
uppS	DSM15361_01338	uppS
uppS	DSM15365_01102	uppS
uppS	DSM15718_03271	uppS
uppS	DSM16505T_00941	uppS
uppS	DSM18103_02641	uppS
uppS	DSM18293_01978	uppS
uppS	DSM18841_00830	uppS
uppS	DSM18842_00488	uppS
uppS	DSM19866_01034	uppS
uppS	DSM2041_04703	uppS
uppS	DSM2061_03886	uppS
uppS	DSM2064_02708	uppS
uppS	DSM21164_00241	uppS
uppS	DSM23404_02227	uppS
uppS	DSM23424_03287	uppS
uppS	DSM24786_01979	uppS

uppS	DSM7489_01978	uppS
uppS	DSW1_00305	uppS
uppS	DSW5_01530	uppS
uppS	DSW6_01579	uppS
uppS	DSW8_01598	uppS
uppS	F52_03372	uppS
uppS	GCA_001027945.1_01607	uppS
uppS	HI1_01905	uppS
uppS	IR1_00183	uppS
uppS	KCOM3131_01178	uppS
uppS	KCTC12974_01220	uppS
uppS	KL-A_00294	uppS
uppS	L12_00275	uppS
uppS	LL0412.1.7_00966	uppS
uppS	MAR_2009_71_01827	uppS
uppS	NN016038_01635	uppS
uppS	PAO1_03775	uppS
uppS	UW101_01762	uppS
uppS	V4-33_02010	uppS
uppS	W5C_00665	uppS
uppS	ZONMW-01_00429	uppS
uppS	ZONMW-02_02657	uppS
uppS	ZONMW-03_01155	uppS
uppS	ZONMW-04_00518	uppS
uppS	ZONMW-05_01306	uppS
uppS	ZONMW-06_03532	uppS
uppS	ZONMW-07_01192	uppS
uppS	ZONMW-08_01397	uppS
uppS	ZONMW-09_01638	uppS
uppS	ZONMW-10_01260	uppS
uppS	ZONMW-11_02098	uppS
uppS	ZONMW-12_01070	uppS
uppS	ZONMW-13_01051	uppS
uppS	ZONMW-14_01541	uppS
uppS	ZONMW-15_01361	uppS
uppS	ZONMW-19_00792	uppS
uppS	ZONMW-20_04120	uppS
uppS	ZONMW-21_00119	uppS
uppS	ZONMW-22_00119	uppS
uppS	ZONMW-23_01234	uppS
uppS	ZONMW-24_01341	uppS
uppS	ZONMW-25_03393	uppS
uppS	ZONMW-26_01361	uppS
uppS	ZONMW-27_00512	uppS
uppS	ZONMW-28_03405	uppS
uppS	ZONMW-29_02431	uppS
uppS	ZONMW-30_00805	uppS
uppS	ZONMW-31_02383	uppS

uppS	ZONMW-32_03576	uppS
uppS	ZONMW-33_03926	uppS
uppS	ZONMW-34_03410	uppS
uppS	ZONMW-35_03405	uppS
uppS	ZONMW-36_03875	uppS
uppS	ZONMW-37_00118	uppS
uppS	ZONMW-38_00859	uppS
uppS	ZONMW-39_03266	uppS
uppS	ZONMW-40_00924	uppS
uppS	ZONMW-41_02494	uppS
uppS	ZONMW-42_02870	uppS
uppS	ZONMW-43_02633	uppS
uppS	ZONMW-44_02578	uppS
uppS	ZONMW-45_01360	uppS
uppS	ZONMW-46_01615	uppS
uppS	ZONMW-47_01874	uppS
uppS	ZONMW-48_02335	uppS
uppS	ZONMW-49_01520	uppS
uppS	ZONMW-50_01522	uppS
uppS	ZONMW-51_00775	uppS
uppS	ZONMW-52_01375	uppS
uppS	ZONMW-53_00450	uppS
uppS	ZONMW-54_00584	uppS
uppS	ZONMW-55_00445	uppS
uppS	ZONMW-56_00870	uppS
uppS	ZONMW-57_01170	uppS
uppS	ZONMW-58_01414	uppS
uppS	ZONMW-59_01171	uppS
uppS	ZONMW-60_01397	uppS
uppS	ZONMW-61_02990	uppS
uppS	ZONMW-62_02245	uppS
uppS	ZONMW-63_00925	uppS
uppS	ZONMW-64_03280	uppS
uppS	ZONMW-65_00908	uppS
uppS	ZONMW-66_00183	uppS
uppS	ZONMW-67_03169	uppS
uppS	ZONMW-68_04637	uppS
uppS	ZONMW-69_03170	uppS
uppS	ZONMW-72_00500	uppS
uppS	ZONMW-73_02008	uppS
uppS	ZONMW-74_01722	uppS
uppS	ZONMW-75_02969	uppS
uppS	da5A-8_00352	uppS
frr	18_01529	frr
frr	ACAM48_02064	frr
frr	CAM030_01323	frr
frr	CECT7946_01763	frr
frr	CGMCC1.8863_01062	frr

frr	CL8139_00143	frr
frr	DAU203_01954	frr
frr	DD5b_01895	frr
frr	DSM1208_02254	frr
frr	DSM12212_01697	frr
frr	DSM15343_00814	frr
frr	DSM15361_01406	frr
frr	DSM15365_03399	frr
frr	DSM15718_02988	frr
frr	DSM16505T_00862	frr
frr	DSM18103_03169	frr
frr	DSM18293_00906	frr
frr	DSM18841_00730	frr
frr	DSM18842_00590	frr
frr	DSM19866_00649	frr
frr	DSM2041_04630	frr
frr	DSM2061_03784	frr
frr	DSM2064_05166	frr
frr	DSM21164_00348	frr
frr	DSM23404_01866	frr
frr	DSM23424_00631	frr
frr	DSM24786_02094	frr
frr	DSM7489_01865	frr
frr	DSW1_00166	frr
frr	DSW5_00769	frr
frr	DSW6_00989	frr
frr	DSW8_01692	frr
frr	F52_02037	frr
frr	GCA_001027945.1_03156	frr
frr	HI1_01793	frr
frr	IR1_01189	frr
frr	IR1_04176	frr
frr	KCOM3131_00706	frr
frr	KCTC12974_02070	frr
frr	KL-A_00406	frr
frr	L12_00274	frr
frr	LL0412.1.7_00863	frr
frr	MAR_2009_71_01725	frr
frr	NN016038_01536	frr
frr	PAO1_03776	frr
frr	UW101_00606	frr
frr	V4-33_00416	frr
frr	W5C_00551	frr
frr	ZONMW-01_00319	frr
frr	ZONMW-02_00325	frr
frr	ZONMW-03_01045	frr
frr	ZONMW-04_00627	frr
frr	ZONMW-05_01198	frr

frr	ZONMW-06_03531	frr
frr	ZONMW-07_00330	frr
frr	ZONMW-08_01509	frr
frr	ZONMW-09_00101	frr
frr	ZONMW-10_00556	frr
frr	ZONMW-11_03047	frr
frr	ZONMW-12_00426	frr
frr	ZONMW-13_00920	frr
frr	ZONMW-14_01184	frr
frr	ZONMW-15_00385	frr
frr	ZONMW-19_00793	frr
frr	ZONMW-20_04122	frr
frr	ZONMW-21_00236	frr
frr	ZONMW-22_00236	frr
frr	ZONMW-23_01351	frr
frr	ZONMW-24_01223	frr
frr	ZONMW-25_03061	frr
frr	ZONMW-26_01252	frr
frr	ZONMW-27_00621	frr
frr	ZONMW-28_03404	frr
frr	ZONMW-29_02968	frr
frr	ZONMW-30_01443	frr
frr	ZONMW-31_02384	frr
frr	ZONMW-32_03577	frr
frr	ZONMW-33_03927	frr
frr	ZONMW-34_03409	frr
frr	ZONMW-35_03406	frr
frr	ZONMW-36_03876	frr
frr	ZONMW-37_00236	frr
frr	ZONMW-38_00858	frr
frr	ZONMW-39_03267	frr
frr	ZONMW-40_02649	frr
frr	ZONMW-41_02495	frr
frr	ZONMW-42_02871	frr
frr	ZONMW-43_02632	frr
frr	ZONMW-44_02579	frr
frr	ZONMW-45_01088	frr
frr	ZONMW-46_00579	frr
frr	ZONMW-47_00579	frr
frr	ZONMW-48_01022	frr
frr	ZONMW-49_01407	frr
frr	ZONMW-50_01409	frr
frr	ZONMW-51_01523	frr
frr	ZONMW-52_01263	frr
frr	ZONMW-53_00562	frr
frr	ZONMW-54_00473	frr
frr	ZONMW-55_00557	frr
frr	ZONMW-56_03015	frr

frr	ZONMW-57_01057	frr
frr	ZONMW-58_01527	frr
frr	ZONMW-59_01059	frr
frr	ZONMW-60_01508	frr
frr	ZONMW-61_02886	frr
frr	ZONMW-62_02341	frr
frr	ZONMW-63_00813	frr
frr	ZONMW-64_03281	frr
frr	ZONMW-65_00907	frr
frr	ZONMW-66_00182	frr
frr	ZONMW-67_03170	frr
frr	ZONMW-68_03908	frr
frr	ZONMW-69_03171	frr
frr	ZONMW-72_01595	frr
frr	ZONMW-73_03190	frr
frr	ZONMW-74_01837	frr
frr	ZONMW-75_01181	frr
frr	da5A-8_01749	frr
copA_1	18_00134	copA_1
copA_1	18_00816	copA_1
copA_1	18_01262	copA_1
copA_1	18_02869	copA_1
copA_1	18_02866	copA_1
copA_1	18_01456	copA_1
copA_1	18_02878	copA_1
copA_1	18_01581	copA_1
copA_1	18_01585	copA_1
copA_1	18_03356	copA_1
copA_1	18_02396	copA_1
copA_1	18_02406	copA_1
copA_1	ACAM48_02275	copA_1
copA_1	ACAM48_02086	copA_1
copA_1	ACAM48_01741	copA_1
copA_1	ACAM48_03288	copA_1
copA_1	ACAM48_01525	copA_1
copA_1	ACAM48_00187	copA_1
copA_1	ACAM48_02717	copA_1
copA_1	ACAM48_00424	copA_1
copA_1	ACAM48_02912	copA_1
copA_1	ACAM48_01814	copA_1
copA_1	ACAM48_02927	copA_1
copA_1	ACAM48_00422	copA_1
copA_1	ACAM48_02715	copA_1
copA_1	ACAM48_02989	copA_1
copA_1	ACAM48_01944	copA_1
copA_1	ACAM48_02918	copA_1
copA_1	ACAM48_02913	copA_1
copA_1	CAM030_01134	copA_1

copA_1	CAM030_02915	copA_1
copA_1	CAM030_01288	copA_1
copA_1	CAM030_01945	copA_1
copA_1	CAM030_00812	copA_1
copA_1	CAM030_02444	copA_1
copA_1	CAM030_02457	copA_1
copA_1	CAM030_00124	copA_1
copA_1	CAM030_01911	copA_1
copA_1	CAM030_02455	copA_1
copA_1	CAM030_00120	copA_1
copA_1	CAM030_00114	copA_1
copA_1	CAM030_02447	copA_1
copA_1	CAM030_00813	copA_1
copA_1	CAM030_02327	copA_1
copA_1	CAM030_01832	copA_1
copA_1	CAM030_02452	copA_1
copA_1	CECT7946_01879	copA_1
copA_1	CECT7946_01806	copA_1
copA_1	CECT7946_02441	copA_1
copA_1	CECT7946_01675	copA_1
copA_1	CECT7946_00428	copA_1
copA_1	CECT7946_02010	copA_1
copA_1	CECT7946_01260	copA_1
copA_1	CECT7946_03131	copA_1
copA_1	CECT7946_02017	copA_1
copA_1	CECT7946_01677	copA_1
copA_1	CECT7946_01008	copA_1
copA_1	CECT7946_02486	copA_1
copA_1	CGMCC1.8863_01018	copA_1
copA_1	CGMCC1.8863_02142	copA_1
copA_1	CGMCC1.8863_00985	copA_1
copA_1	CGMCC1.8863_00193	copA_1
copA_1	CGMCC1.8863_00727	copA_1
copA_1	CGMCC1.8863_02994	copA_1
copA_1	CGMCC1.8863_02072	copA_1
copA_1	CGMCC1.8863_00482	copA_1
copA_1	CGMCC1.8863_02070	copA_1
copA_1	CGMCC1.8863_02998	copA_1
copA_1	CGMCC1.8863_02715	copA_1
copA_1	CGMCC1.8863_01113	copA_1
copA_1	CGMCC1.8863_00218	copA_1
copA_1	CGMCC1.8863_00229	copA_1
copA_1	CGMCC1.8863_02068	copA_1
copA_1	CL8139_00945	copA_1
copA_1	CL8139_03181	copA_1
copA_1	CL8139_01009	copA_1
copA_1	CL8139_01678	copA_1
copA_1	CL8139_01549	copA_1

copA_1	CL8139_01538	copA_1
copA_1	CL8139_00632	copA_1
copA_1	CL8139_00201	copA_1
copA_1	DAU203_01722	copA_1
copA_1	DAU203_02472	copA_1
copA_1	DAU203_01786	copA_1
copA_1	DAU203_03113	copA_1
copA_1	DAU203_03122	copA_1
copA_1	DAU203_01284	copA_1
copA_1	DAU203_02170	copA_1
copA_1	DAU203_03120	copA_1
copA_1	DAU203_02181	copA_1
copA_1	DAU203_02965	copA_1
copA_1	DAU203_03116	copA_1
copA_1	DAU203_02012	copA_1
copA_1	DD5b_02033	copA_1
copA_1	DD5b_00393	copA_1
copA_1	DD5b_01544	copA_1
copA_1	DD5b_02601	copA_1
copA_1	DD5b_01335	copA_1
copA_1	DD5b_00644	copA_1
copA_1	DD5b_01217	copA_1
copA_1	DD5b_00897	copA_1
copA_1	DD5b_01208	copA_1
copA_1	DD5b_00165	copA_1
copA_1	DD5b_02599	copA_1
copA_1	DD5b_01205	copA_1
copA_1	DD5b_01631	copA_1
copA_1	DD5b_01214	copA_1
copA_1	DD5b_00623	copA_1
copA_1	DSM1208_02814	copA_1
copA_1	DSM1208_01466	copA_1
copA_1	DSM1208_01875	copA_1
copA_1	DSM1208_03291	copA_1
copA_1	DSM1208_04114	copA_1
copA_1	DSM1208_03934	copA_1
copA_1	DSM1208_02308	copA_1
copA_1	DSM1208_02173	copA_1
copA_1	DSM1208_01124	copA_1
copA_1	DSM1208_00757	copA_1
copA_1	DSM1208_02304	copA_1
copA_1	DSM1208_03703	copA_1
copA_1	DSM1208_03345	copA_1
copA_1	DSM1208_03344	copA_1
copA_1	DSM12212_02606	copA_1
copA_1	DSM12212_01666	copA_1
copA_1	DSM12212_02386	copA_1
copA_1	DSM12212_01782	copA_1

copA_1	DSM12212_02933	copA_1
copA_1	DSM12212_03171	copA_1
copA_1	DSM12212_00422	copA_1
copA_1	DSM12212_02930	copA_1
copA_1	DSM12212_02391	copA_1
copA_1	DSM12212_01780	copA_1
copA_1	DSM12212_02356	copA_1
copA_1	DSM12212_02518	copA_1
copA_1	DSM15343_02048	copA_1
copA_1	DSM15343_02457	copA_1
copA_1	DSM15343_00420	copA_1
copA_1	DSM15343_00849	copA_1
copA_1	DSM15343_01756	copA_1
copA_1	DSM15343_02646	copA_1
copA_1	DSM15343_00270	copA_1
copA_1	DSM15343_00250	copA_1
copA_1	DSM15343_01356	copA_1
copA_1	DSM15343_02233	copA_1
copA_1	DSM15343_02679	copA_1
copA_1	DSM15343_00100	copA_1
copA_1	DSM15343_00176	copA_1
copA_1	DSM15343_02658	copA_1
copA_1	DSM15343_01378	copA_1
copA_1	DSM15343_02663	copA_1
copA_1	DSM15343_00774	copA_1
copA_1	DSM15343_02659	copA_1
copA_1	DSM15361_00348	copA_1
copA_1	DSM15361_00933	copA_1
copA_1	DSM15361_01431	copA_1
copA_1	DSM15361_00759	copA_1
copA_1	DSM15361_01814	copA_1
copA_1	DSM15361_01353	copA_1
copA_1	DSM15361_01351	copA_1
copA_1	DSM15361_00928	copA_1
copA_1	DSM15361_01775	copA_1
copA_1	DSM15361_01766	copA_1
copA_1	DSM15361_01828	copA_1
copA_1	DSM15361_01819	copA_1
copA_1	DSM15361_01832	copA_1
copA_1	DSM15361_01769	copA_1
copA_1	DSM15361_01826	copA_1
copA_1	DSM15365_02246	copA_1
copA_1	DSM15365_03264	copA_1
copA_1	DSM15365_03363	copA_1
copA_1	DSM15365_03766	copA_1
copA_1	DSM15365_01737	copA_1
copA_1	DSM15365_00722	copA_1
copA_1	DSM15365_03767	copA_1

copA_1	DSM15365_02264	copA_1
copA_1	DSM15365_01271	copA_1
copA_1	DSM15365_01095	copA_1
copA_1	DSM15718_01796	copA_1
copA_1	DSM15718_01382	copA_1
copA_1	DSM15718_01429	copA_1
copA_1	DSM15718_01690	copA_1
copA_1	DSM15718_02394	copA_1
copA_1	DSM15718_03290	copA_1
copA_1	DSM15718_00952	copA_1
copA_1	DSM15718_01352	copA_1
copA_1	DSM15718_00348	copA_1
copA_1	DSM15718_03258	copA_1
copA_1	DSM15718_02093	copA_1
copA_1	DSM15718_03292	copA_1
copA_1	DSM15718_03055	copA_1
copA_1	DSM15718_01947	copA_1
copA_1	DSM15718_02804	copA_1
copA_1	DSM15718_02805	copA_1
copA_1	DSM15718_00886	copA_1
copA_1	DSM15718_00301	copA_1
copA_1	DSM15718_03260	copA_1
copA_1	DSM16505T_01831	copA_1
copA_1	DSM16505T_00145	copA_1
copA_1	DSM16505T_00829	copA_1
copA_1	DSM16505T_00090	copA_1
copA_1	DSM16505T_01294	copA_1
copA_1	DSM16505T_01639	copA_1
copA_1	DSM16505T_02424	copA_1
copA_1	DSM16505T_01167	copA_1
copA_1	DSM16505T_02480	copA_1
copA_1	DSM16505T_02551	copA_1
copA_1	DSM16505T_02420	copA_1
copA_1	DSM16505T_02417	copA_1
copA_1	DSM16505T_01564	copA_1
copA_1	DSM18103_03130	copA_1
copA_1	DSM18103_01212	copA_1
copA_1	DSM18103_00683	copA_1
copA_1	DSM18103_02448	copA_1
copA_1	DSM18103_03247	copA_1
copA_1	DSM18103_03319	copA_1
copA_1	DSM18103_00238	copA_1
copA_1	DSM18103_00031	copA_1
copA_1	DSM18103_02449	copA_1
copA_1	DSM18293_01406	copA_1
copA_1	DSM18293_00076	copA_1
copA_1	DSM18293_04278	copA_1
copA_1	DSM18293_03170	copA_1

copA_1	DSM18293_01707	copA_1
copA_1	DSM18293_03370	copA_1
copA_1	DSM18293_01959	copA_1
copA_1	DSM18293_00051	copA_1
copA_1	DSM18293_00353	copA_1
copA_1	DSM18293_03977	copA_1
copA_1	DSM18293_02617	copA_1
copA_1	DSM18293_02267	copA_1
copA_1	DSM18293_04255	copA_1
copA_1	DSM18293_01957	copA_1
copA_1	DSM18293_00412	copA_1
copA_1	DSM18293_04254	copA_1
copA_1	DSM18293_04356	copA_1
copA_1	DSM18293_00437	copA_1
copA_1	DSM18293_04513	copA_1
copA_1	DSM18293_01448	copA_1
copA_1	DSM18293_02621	copA_1
copA_1	DSM18841_01557	copA_1
copA_1	DSM18841_02696	copA_1
copA_1	DSM18841_00695	copA_1
copA_1	DSM18841_01068	copA_1
copA_1	DSM18841_02860	copA_1
copA_1	DSM18841_01275	copA_1
copA_1	DSM18841_02311	copA_1
copA_1	DSM18841_02185	copA_1
copA_1	DSM18841_02310	copA_1
copA_1	DSM18842_02247	copA_1
copA_1	DSM18842_02548	copA_1
copA_1	DSM18842_00632	copA_1
copA_1	DSM18842_02494	copA_1
copA_1	DSM18842_01763	copA_1
copA_1	DSM18842_01201	copA_1
copA_1	DSM18842_03177	copA_1
copA_1	DSM18842_00254	copA_1
copA_1	DSM18842_02842	copA_1
copA_1	DSM18842_02909	copA_1
copA_1	DSM18842_01529	copA_1
copA_1	DSM19866_03463	copA_1
copA_1	DSM19866_03460	copA_1
copA_1	DSM19866_02129	copA_1
copA_1	DSM19866_04374	copA_1
copA_1	DSM19866_04041	copA_1
copA_1	DSM19866_02535	copA_1
copA_1	DSM19866_02351	copA_1
copA_1	DSM19866_03360	copA_1
copA_1	DSM19866_02791	copA_1
copA_1	DSM19866_04413	copA_1
copA_1	DSM19866_04809	copA_1

copA_1	DSM19866_03797	copA_1
copA_1	DSM19866_04065	copA_1
copA_1	DSM19866_02497	copA_1
copA_1	DSM19866_00511	copA_1
copA_1	DSM19866_03410	copA_1
copA_1	DSM19866_03408	copA_1
copA_1	DSM2041_05204	copA_1
copA_1	DSM2041_03904	copA_1
copA_1	DSM2041_04688	copA_1
copA_1	DSM2041_01818	copA_1
copA_1	DSM2041_03907	copA_1
copA_1	DSM2041_01082	copA_1
copA_1	DSM2041_01069	copA_1
copA_1	DSM2041_03670	copA_1
copA_1	DSM2041_01085	copA_1
copA_1	DSM2041_03902	copA_1
copA_1	DSM2041_01018	copA_1
copA_1	DSM2041_03918	copA_1
copA_1	DSM2041_03922	copA_1
copA_1	DSM2041_01084	copA_1
copA_1	DSM2041_04686	copA_1
copA_1	DSM2041_02270	copA_1
copA_1	DSM2041_00795	copA_1
copA_1	DSM2041_00793	copA_1
copA_1	DSM2041_01063	copA_1
copA_1	DSM2041_00792	copA_1
copA_1	DSM2041_03900	copA_1
copA_1	DSM2041_01061	copA_1
copA_1	DSM2061_04298	copA_1
copA_1	DSM2061_02989	copA_1
copA_1	DSM2061_03429	copA_1
copA_1	DSM2061_03756	copA_1
copA_1	DSM2061_03133	copA_1
copA_1	DSM2061_02257	copA_1
copA_1	DSM2061_02763	copA_1
copA_1	DSM2061_03837	copA_1
copA_1	DSM2061_03718	copA_1
copA_1	DSM2061_03135	copA_1
copA_1	DSM2061_02256	copA_1
copA_1	DSM2061_03833	copA_1
copA_1	DSM2061_00936	copA_1
copA_1	DSM2061_03137	copA_1
copA_1	DSM2064_04292	copA_1
copA_1	DSM2064_03814	copA_1
copA_1	DSM2064_00668	copA_1
copA_1	DSM2064_01175	copA_1
copA_1	DSM2064_04409	copA_1
copA_1	DSM2064_00658	copA_1

copA_1	DSM2064_01895	copA_1
copA_1	DSM2064_00362	copA_1
copA_1	DSM2064_04415	copA_1
copA_1	DSM2064_05241	copA_1
copA_1	DSM2064_04423	copA_1
copA_1	DSM2064_00775	copA_1
copA_1	DSM2064_02688	copA_1
copA_1	DSM2064_02271	copA_1
copA_1	DSM2064_00501	copA_1
copA_1	DSM2064_03151	copA_1
copA_1	DSM2064_04895	copA_1
copA_1	DSM2064_00503	copA_1
copA_1	DSM2064_03008	copA_1
copA_1	DSM2064_00774	copA_1
copA_1	DSM2064_04222	copA_1
copA_1	DSM2064_02686	copA_1
copA_1	DSM2064_02337	copA_1
copA_1	DSM2064_04234	copA_1
copA_1	DSM2064_01379	copA_1
copA_1	DSM2064_02362	copA_1
copA_1	DSM2064_03774	copA_1
copA_1	DSM2064_00500	copA_1
copA_1	DSM2064_04897	copA_1
copA_1	DSM2064_00502	copA_1
copA_1	DSM2064_00495	copA_1
copA_1	DSM2064_00494	copA_1
copA_1	DSM2064_04213	copA_1
copA_1	DSM2064_01517	copA_1
copA_1	DSM21164_02777	copA_1
copA_1	DSM21164_00607	copA_1
copA_1	DSM21164_01506	copA_1
copA_1	DSM21164_01510	copA_1
copA_1	DSM21164_00291	copA_1
copA_1	DSM21164_00421	copA_1
copA_1	DSM21164_01906	copA_1
copA_1	DSM21164_00295	copA_1
copA_1	DSM21164_01900	copA_1
copA_1	DSM21164_01343	copA_1
copA_1	DSM21164_02579	copA_1
copA_1	DSM23404_02995	copA_1
copA_1	DSM23404_01888	copA_1
copA_1	DSM23404_00868	copA_1
copA_1	DSM23404_02239	copA_1
copA_1	DSM23404_01320	copA_1
copA_1	DSM23404_02141	copA_1
copA_1	DSM23404_03243	copA_1
copA_1	DSM23404_01743	copA_1
copA_1	DSM23404_02429	copA_1

copA_1	DSM23404_01732	copA_1
copA_1	DSM23404_03213	copA_1
copA_1	DSM23404_02135	copA_1
copA_1	DSM23404_02241	copA_1
copA_1	DSM23404_01788	copA_1
copA_1	DSM23404_02875	copA_1
copA_1	DSM23404_00373	copA_1
copA_1	DSM23404_01782	copA_1
copA_1	DSM23404_03247	copA_1
copA_1	DSM23404_03244	copA_1
copA_1	DSM23404_01736	copA_1
copA_1	DSM23404_03251	copA_1
copA_1	DSM23404_01741	copA_1
copA_1	DSM23404_03264	copA_1
copA_1	DSM23424_00456	copA_1
copA_1	DSM23424_00608	copA_1
copA_1	DSM23424_02077	copA_1
copA_1	DSM23424_00007	copA_1
copA_1	DSM23424_00867	copA_1
copA_1	DSM23424_01130	copA_1
copA_1	DSM23424_03088	copA_1
copA_1	DSM23424_02017	copA_1
copA_1	DSM23424_00866	copA_1
copA_1	DSM23424_03206	copA_1
copA_1	DSM23424_03086	copA_1
copA_1	DSM23424_00009	copA_1
copA_1	DSM23424_01577	copA_1
copA_1	DSM23424_02053	copA_1
copA_1	DSM23424_01183	copA_1
copA_1	DSM23424_00858	copA_1
copA_1	DSM23424_00863	copA_1
copA_1	DSM24786_02260	copA_1
copA_1	DSM24786_01536	copA_1
copA_1	DSM24786_02196	copA_1
copA_1	DSM24786_00545	copA_1
copA_1	DSM24786_00427	copA_1
copA_1	DSM24786_02726	copA_1
copA_1	DSM24786_01875	copA_1
copA_1	DSM24786_01858	copA_1
copA_1	DSM24786_00429	copA_1
copA_1	DSM24786_02038	copA_1
copA_1	DSM24786_01857	copA_1
copA_1	DSM7489_01640	copA_1
copA_1	DSM7489_02387	copA_1
copA_1	DSM7489_01705	copA_1
copA_1	DSM7489_03269	copA_1
copA_1	DSM7489_01193	copA_1
copA_1	DSM7489_02082	copA_1

copA_1	DSM7489_02093	copA_1
copA_1	DSM7489_02835	copA_1
copA_1	DSM7489_03272	copA_1
copA_1	DSM7489_01923	copA_1
copA_1	DSW1_00277	copA_1
copA_1	DSW1_01085	copA_1
copA_1	DSW1_01822	copA_1
copA_1	DSW1_01083	copA_1
copA_1	DSW1_00033	copA_1
copA_1	DSW1_00040	copA_1
copA_1	DSW5_01254	copA_1
copA_1	DSW5_01805	copA_1
copA_1	DSW5_02452	copA_1
copA_1	DSW5_01809	copA_1
copA_1	DSW5_01813	copA_1
copA_1	DSW5_02376	copA_1
copA_1	DSW5_00734	copA_1
copA_1	DSW6_01736	copA_1
copA_1	DSW6_00712	copA_1
copA_1	DSW6_01543	copA_1
copA_1	DSW6_00710	copA_1
copA_1	DSW6_00188	copA_1
copA_1	DSW6_00706	copA_1
copA_1	DSW6_00716	copA_1
copA_1	DSW6_01545	copA_1
copA_1	DSW6_00147	copA_1
copA_1	DSW6_02085	copA_1
copA_1	DSW6_02462	copA_1
copA_1	DSW6_00703	copA_1
copA_1	DSW8_02469	copA_1
copA_1	DSW8_02023	copA_1
copA_1	DSW8_02477	copA_1
copA_1	DSW8_03221	copA_1
copA_1	DSW8_02052	copA_1
copA_1	DSW8_03647	copA_1
copA_1	DSW8_00309	copA_1
copA_1	DSW8_02686	copA_1
copA_1	DSW8_03314	copA_1
copA_1	DSW8_00433	copA_1
copA_1	DSW8_03261	copA_1
copA_1	DSW8_03552	copA_1
copA_1	DSW8_01640	copA_1
copA_1	DSW8_01636	copA_1
copA_1	DSW8_03258	copA_1
copA_1	DSW8_00430	copA_1
copA_1	DSW8_00434	copA_1
copA_1	F52_03961	copA_1
copA_1	F52_00948	copA_1

copA_1	F52_03618	copA_1
copA_1	F52_03502	copA_1
copA_1	F52_02326	copA_1
copA_1	F52_01323	copA_1
copA_1	F52_04407	copA_1
copA_1	F52_01742	copA_1
copA_1	F52_03390	copA_1
copA_1	F52_03610	copA_1
copA_1	F52_02661	copA_1
copA_1	F52_01756	copA_1
copA_1	F52_01173	copA_1
copA_1	F52_00508	copA_1
copA_1	F52_01773	copA_1
copA_1	F52_01171	copA_1
copA_1	F52_01206	copA_1
copA_1	F52_03073	copA_1
copA_1	F52_00871	copA_1
copA_1	F52_03392	copA_1
copA_1	F52_02733	copA_1
copA_1	F52_01202	copA_1
copA_1	F52_00870	copA_1
copA_1	F52_02759	copA_1
copA_1	F52_04051	copA_1
copA_1	F52_01174	copA_1
copA_1	F52_03922	copA_1
copA_1	F52_01172	copA_1
copA_1	F52_01775	copA_1
copA_1	F52_01179	copA_1
copA_1	F52_01180	copA_1
copA_1	F52_04064	copA_1
copA_1	F52_01033	copA_1
copA_1	GCA_001027945.1_02522	copA_1
copA_1	GCA_001027945.1_01643	copA_1
copA_1	GCA_001027945.1_01341	copA_1
copA_1	GCA_001027945.1_03119	copA_1
copA_1	GCA_001027945.1_00949	copA_1
copA_1	GCA_001027945.1_00661	copA_1
copA_1	GCA_001027945.1_02919	copA_1
copA_1	GCA_001027945.1_01760	copA_1
copA_1	GCA_001027945.1_01077	copA_1
copA_1	GCA_001027945.1_01362	copA_1
copA_1	HI1_01561	copA_1
copA_1	HI1_02320	copA_1
copA_1	HI1_01626	copA_1
copA_1	HI1_03233	copA_1
copA_1	HI1_03213	copA_1
copA_1	HI1_03042	copA_1
copA_1	HI1_01135	copA_1

copA_1	HI1_02009	copA_1
copA_1	HI1_02020	copA_1
copA_1	HI1_02775	copA_1
copA_1	HI1_03216	copA_1
copA_1	HI1_01851	copA_1
copA_1	HI1_03039	copA_1
copA_1	IR1_01505	copA_1
copA_1	IR1_01926	copA_1
copA_1	IR1_01320	copA_1
copA_1	IR1_03259	copA_1
copA_1	IR1_03571	copA_1
copA_1	IR1_03405	copA_1
copA_1	IR1_01969	copA_1
copA_1	IR1_02193	copA_1
copA_1	IR1_03567	copA_1
copA_1	IR1_00203	copA_1
copA_1	IR1_00598	copA_1
copA_1	IR1_02375	copA_1
copA_1	IR1_02873	copA_1
copA_1	IR1_00781	copA_1
copA_1	IR1_03254	copA_1
copA_1	IR1_00928	copA_1
copA_1	IR1_03635	copA_1
copA_1	IR1_00205	copA_1
copA_1	IR1_00528	copA_1
copA_1	IR1_03252	copA_1
copA_1	IR1_03854	copA_1
copA_1	IR1_03372	copA_1
copA_1	IR1_01448	copA_1
copA_1	IR1_00783	copA_1
copA_1	IR1_00784	copA_1
copA_1	IR1_00502	copA_1
copA_1	IR1_00782	copA_1
copA_1	IR1_00789	copA_1
copA_1	IR1_02871	copA_1
copA_1	KCOM3131_00389	copA_1
copA_1	KCOM3131_00291	copA_1
copA_1	KCOM3131_01503	copA_1
copA_1	KCOM3131_01458	copA_1
copA_1	KCOM3131_00004	copA_1
copA_1	KCOM3131_01930	copA_1
copA_1	KCOM3131_00172	copA_1
copA_1	KCOM3131_00862	copA_1
copA_1	KCOM3131_00519	copA_1
copA_1	KCOM3131_00884	copA_1
copA_1	KCTC12974_01850	copA_1
copA_1	KCTC12974_02092	copA_1
copA_1	KCTC12974_00295	copA_1

copA_1	KCTC12974_01232	copA_1
copA_1	KCTC12974_00019	copA_1
copA_1	KCTC12974_01383	copA_1
copA_1	KCTC12974_02786	copA_1
copA_1	KCTC12974_01391	copA_1
copA_1	KCTC12974_01234	copA_1
copA_1	KCTC12974_02113	copA_1
copA_1	KCTC12974_02583	copA_1
copA_1	KCTC12974_01738	copA_1
copA_1	KCTC12974_00288	copA_1
copA_1	KCTC12974_02114	copA_1
copA_1	KCTC12974_00657	copA_1
copA_1	KCTC12974_02112	copA_1
copA_1	KCTC12974_00664	copA_1
copA_1	KCTC12974_02242	copA_1
copA_1	KCTC12974_01388	copA_1
copA_1	KCTC12974_01384	copA_1
copA_1	KL-A_01442	copA_1
copA_1	KL-A_01667	copA_1
copA_1	KL-A_01507	copA_1
copA_1	KL-A_00541	copA_1
copA_1	KL-A_02703	copA_1
copA_1	KL-A_00532	copA_1
copA_1	KL-A_03021	copA_1
copA_1	KL-A_00188	copA_1
copA_1	KL-A_00534	copA_1
copA_1	KL-A_00177	copA_1
copA_1	KL-A_00680	copA_1
copA_1	KL-A_00538	copA_1
copA_1	KL-A_00348	copA_1
copA_1	KL-A_02700	copA_1
copA_1	L12_00414	copA_1
copA_1	LL0412.1.7_01423	copA_1
copA_1	LL0412.1.7_00828	copA_1
copA_1	LL0412.1.7_00761	copA_1
copA_1	LL0412.1.7_01834	copA_1
copA_1	LL0412.1.7_01830	copA_1
copA_1	LL0412.1.7_02409	copA_1
copA_1	LL0412.1.7_01754	copA_1
copA_1	LL0412.1.7_01829	copA_1
copA_1	LL0412.1.7_01838	copA_1
copA_1	LL0412.1.7_02468	copA_1
copA_1	LL0412.1.7_01827	copA_1
copA_1	MAR_2009_71_00984	copA_1
copA_1	MAR_2009_71_00976	copA_1
copA_1	MAR_2009_71_03698	copA_1
copA_1	MAR_2009_71_01379	copA_1
copA_1	MAR_2009_71_02799	copA_1

copA_1	MAR_2009_71_03112	copA_1
copA_1	MAR_2009_71_00767	copA_1
copA_1	MAR_2009_71_02033	copA_1
copA_1	MAR_2009_71_03608	copA_1
copA_1	MAR_2009_71_03230	copA_1
copA_1	MAR_2009_71_02027	copA_1
copA_1	MAR_2009_71_02709	copA_1
copA_1	MAR_2009_71_01785	copA_1
copA_1	MAR_2009_71_01789	copA_1
copA_1	MAR_2009_71_03651	copA_1
copA_1	MAR_2009_71_03227	copA_1
copA_1	MAR_2009_71_03232	copA_1
copA_1	NN016038_00137	copA_1
copA_1	NN016038_00836	copA_1
copA_1	NN016038_01286	copA_1
copA_1	NN016038_02921	copA_1
copA_1	NN016038_02918	copA_1
copA_1	NN016038_01464	copA_1
copA_1	NN016038_02930	copA_1
copA_1	NN016038_02462	copA_1
copA_1	NN016038_01588	copA_1
copA_1	NN016038_01592	copA_1
copA_1	NN016038_03401	copA_1
copA_1	NN016038_02458	copA_1
copA_1	NN016038_02407	copA_1
copA_1	NN016038_02417	copA_1
copA_1	PAO1_02563	copA_1
copA_1	PAO1_03645	copA_1
copA_1	PAO1_00436	copA_1
copA_1	PAO1_02599	copA_1
copA_1	PAO1_04749	copA_1
copA_1	PAO1_04516	copA_1
copA_1	PAO1_02082	copA_1
copA_1	PAO1_03800	copA_1
copA_1	PAO1_01475	copA_1
copA_1	PAO1_00163	copA_1
copA_1	PAO1_02591	copA_1
copA_1	PAO1_04337	copA_1
copA_1	PAO1_01590	copA_1
copA_1	PAO1_04048	copA_1
copA_1	PAO1_03813	copA_1
copA_1	PAO1_02507	copA_1
copA_1	PAO1_01678	copA_1
copA_1	PAO1_01469	copA_1
copA_1	PAO1_02461	copA_1
copA_1	PAO1_04991	copA_1
copA_1	PAO1_00162	copA_1
copA_1	PAO1_05332	copA_1

copA_1	PAO1_03248	copA_1
copA_1	PAO1_03521	copA_1
copA_1	PAO1_02501	copA_1
copA_1	PAO1_04740	copA_1
copA_1	PAO1_04019	copA_1
copA_1	PAO1_02937	copA_1
copA_1	PAO1_01274	copA_1
copA_1	PAO1_03478	copA_1
copA_1	PAO1_03419	copA_1
copA_1	PAO1_01268	copA_1
copA_1	UW101_04594	copA_1
copA_1	UW101_05072	copA_1
copA_1	UW101_03379	copA_1
copA_1	UW101_04304	copA_1
copA_1	UW101_04477	copA_1
copA_1	UW101_03369	copA_1
copA_1	UW101_00949	copA_1
copA_1	UW101_03073	copA_1
copA_1	UW101_04471	copA_1
copA_1	UW101_02850	copA_1
copA_1	UW101_04463	copA_1
copA_1	UW101_03486	copA_1
copA_1	UW101_01742	copA_1
copA_1	UW101_01325	copA_1
copA_1	UW101_03212	copA_1
copA_1	UW101_02649	copA_1
copA_1	UW101_00207	copA_1
copA_1	UW101_03214	copA_1
copA_1	UW101_02062	copA_1
copA_1	UW101_03485	copA_1
copA_1	UW101_04663	copA_1
copA_1	UW101_01740	copA_1
copA_1	UW101_01391	copA_1
copA_1	UW101_04651	copA_1
copA_1	UW101_04100	copA_1
copA_1	UW101_01416	copA_1
copA_1	UW101_05112	copA_1
copA_1	UW101_03211	copA_1
copA_1	UW101_00205	copA_1
copA_1	UW101_03213	copA_1
copA_1	UW101_03206	copA_1
copA_1	UW101_03205	copA_1
copA_1	UW101_04672	copA_1
copA_1	UW101_03962	copA_1
copA_1	V4-33_00843	copA_1
copA_1	V4-33_02323	copA_1
copA_1	V4-33_01718	copA_1
copA_1	V4-33_02031	copA_1

copA_1	V4-33_01063	copA_1
copA_1	V4-33_00406	copA_1
copA_1	V4-33_01597	copA_1
copA_1	V4-33_00835	copA_1
copA_1	V4-33_00481	copA_1
copA_1	V4-33_00796	copA_1
copA_1	V4-33_00273	copA_1
copA_1	W5C_00334	copA_1
copA_1	W5C_02707	copA_1
copA_1	W5C_00401	copA_1
copA_1	W5C_02143	copA_1
copA_1	W5C_02962	copA_1
copA_1	W5C_02034	copA_1
copA_1	W5C_02977	copA_1
copA_1	W5C_02963	copA_1
copA_1	W5C_02519	copA_1
copA_1	W5C_00609	copA_1
copA_1	ZONMW-01_00088	copA_1
copA_1	ZONMW-01_02824	copA_1
copA_1	ZONMW-01_00152	copA_1
copA_1	ZONMW-01_01074	copA_1
copA_1	ZONMW-01_01504	copA_1
copA_1	ZONMW-01_01493	copA_1
copA_1	ZONMW-01_02320	copA_1
copA_1	ZONMW-01_00376	copA_1
copA_1	ZONMW-02_00197	copA_1
copA_1	ZONMW-02_00295	copA_1
copA_1	ZONMW-02_00740	copA_1
copA_1	ZONMW-02_02117	copA_1
copA_1	ZONMW-02_02069	copA_1
copA_1	ZONMW-02_01165	copA_1
copA_1	ZONMW-02_02407	copA_1
copA_1	ZONMW-02_02953	copA_1
copA_1	ZONMW-02_02650	copA_1
copA_1	ZONMW-03_00813	copA_1
copA_1	ZONMW-03_01587	copA_1
copA_1	ZONMW-03_00878	copA_1
copA_1	ZONMW-03_02143	copA_1
copA_1	ZONMW-03_01276	copA_1
copA_1	ZONMW-03_01287	copA_1
copA_1	ZONMW-03_00328	copA_1
copA_1	ZONMW-03_01102	copA_1
copA_1	ZONMW-04_02132	copA_1
copA_1	ZONMW-04_00095	copA_1
copA_1	ZONMW-04_02068	copA_1
copA_1	ZONMW-04_00892	copA_1
copA_1	ZONMW-04_01675	copA_1
copA_1	ZONMW-04_00414	copA_1

copA_1	ZONMW-04_00403	copA_1
copA_1	ZONMW-04_01262	copA_1
copA_1	ZONMW-04_00889	copA_1
copA_1	ZONMW-04_00570	copA_1
copA_1	ZONMW-05_01003	copA_1
copA_1	ZONMW-05_01735	copA_1
copA_1	ZONMW-05_01067	copA_1
copA_1	ZONMW-05_01954	copA_1
copA_1	ZONMW-05_01411	copA_1
copA_1	ZONMW-05_01422	copA_1
copA_1	ZONMW-05_00587	copA_1
copA_1	ZONMW-05_01255	copA_1
copA_1	ZONMW-06_00145	copA_1
copA_1	ZONMW-06_02722	copA_1
copA_1	ZONMW-06_01255	copA_1
copA_1	ZONMW-06_01563	copA_1
copA_1	ZONMW-06_03428	copA_1
copA_1	ZONMW-06_00346	copA_1
copA_1	ZONMW-06_03189	copA_1
copA_1	ZONMW-06_01122	copA_1
copA_1	ZONMW-06_00041	copA_1
copA_1	ZONMW-06_01567	copA_1
copA_1	ZONMW-06_01446	copA_1
copA_1	ZONMW-07_00748	copA_1
copA_1	ZONMW-07_01329	copA_1
copA_1	ZONMW-07_02991	copA_1
copA_1	ZONMW-07_00255	copA_1
copA_1	ZONMW-07_02057	copA_1
copA_1	ZONMW-07_02583	copA_1
copA_1	ZONMW-07_02933	copA_1
copA_1	ZONMW-07_00381	copA_1
copA_1	ZONMW-07_00149	copA_1
copA_1	ZONMW-07_00158	copA_1
copA_1	ZONMW-07_00154	copA_1
copA_1	ZONMW-07_02426	copA_1
copA_1	ZONMW-07_02428	copA_1
copA_1	ZONMW-08_00503	copA_1
copA_1	ZONMW-08_00495	copA_1
copA_1	ZONMW-08_03817	copA_1
copA_1	ZONMW-08_01815	copA_1
copA_1	ZONMW-08_02016	copA_1
copA_1	ZONMW-08_02628	copA_1
copA_1	ZONMW-08_00285	copA_1
copA_1	ZONMW-08_02247	copA_1
copA_1	ZONMW-08_02307	copA_1
copA_1	ZONMW-08_02745	copA_1
copA_1	ZONMW-08_02251	copA_1
copA_1	ZONMW-08_01920	copA_1

copA_1	ZONMW-08_01439	copA_1
copA_1	ZONMW-08_01435	copA_1
copA_1	ZONMW-08_02263	copA_1
copA_1	ZONMW-08_02742	copA_1
copA_1	ZONMW-08_02747	copA_1
copA_1	ZONMW-09_00124	copA_1
copA_1	ZONMW-09_02056	copA_1
copA_1	ZONMW-09_01627	copA_1
copA_1	ZONMW-09_00687	copA_1
copA_1	ZONMW-09_00259	copA_1
copA_1	ZONMW-09_00799	copA_1
copA_1	ZONMW-09_01555	copA_1
copA_1	ZONMW-09_00802	copA_1
copA_1	ZONMW-09_01559	copA_1
copA_1	ZONMW-09_00679	copA_1
copA_1	ZONMW-09_00681	copA_1
copA_1	ZONMW-09_00685	copA_1
copA_1	ZONMW-10_00246	copA_1
copA_1	ZONMW-10_02191	copA_1
copA_1	ZONMW-10_02496	copA_1
copA_1	ZONMW-10_01846	copA_1
copA_1	ZONMW-10_00143	copA_1
copA_1	ZONMW-10_00687	copA_1
copA_1	ZONMW-10_00769	copA_1
copA_1	ZONMW-10_01029	copA_1
copA_1	ZONMW-10_00310	copA_1
copA_1	ZONMW-10_00768	copA_1
copA_1	ZONMW-10_01530	copA_1
copA_1	ZONMW-10_01872	copA_1
copA_1	ZONMW-11_04570	copA_1
copA_1	ZONMW-11_02864	copA_1
copA_1	ZONMW-11_00926	copA_1
copA_1	ZONMW-11_00662	copA_1
copA_1	ZONMW-11_03202	copA_1
copA_1	ZONMW-11_00333	copA_1
copA_1	ZONMW-11_00472	copA_1
copA_1	ZONMW-11_04383	copA_1
copA_1	ZONMW-11_00672	copA_1
copA_1	ZONMW-11_03788	copA_1
copA_1	ZONMW-11_01663	copA_1
copA_1	ZONMW-11_00888	copA_1
copA_1	ZONMW-11_03531	copA_1
copA_1	ZONMW-11_02426	copA_1
copA_1	ZONMW-11_00356	copA_1
copA_1	ZONMW-11_04614	copA_1
copA_1	ZONMW-11_03284	copA_1
copA_1	ZONMW-11_01360	copA_1
copA_1	ZONMW-11_01759	copA_1

copA_1	ZONMW-11_00076	copA_1
copA_1	ZONMW-11_04123	copA_1
copA_1	ZONMW-11_02919	copA_1
copA_1	ZONMW-12_03925	copA_1
copA_1	ZONMW-12_00021	copA_1
copA_1	ZONMW-12_04237	copA_1
copA_1	ZONMW-12_02872	copA_1
copA_1	ZONMW-12_00402	copA_1
copA_1	ZONMW-12_00992	copA_1
copA_1	ZONMW-12_02499	copA_1
copA_1	ZONMW-12_02937	copA_1
copA_1	ZONMW-12_01444	copA_1
copA_1	ZONMW-12_01081	copA_1
copA_1	ZONMW-12_01256	copA_1
copA_1	ZONMW-12_00557	copA_1
copA_1	ZONMW-12_00332	copA_1
copA_1	ZONMW-12_01021	copA_1
copA_1	ZONMW-12_02481	copA_1
copA_1	ZONMW-12_04343	copA_1
copA_1	ZONMW-12_01640	copA_1
copA_1	ZONMW-12_04334	copA_1
copA_1	ZONMW-12_01151	copA_1
copA_1	ZONMW-12_01017	copA_1
copA_1	ZONMW-12_01157	copA_1
copA_1	ZONMW-12_03826	copA_1
copA_1	ZONMW-12_01266	copA_1
copA_1	ZONMW-12_00544	copA_1
copA_1	ZONMW-12_01439	copA_1
copA_1	ZONMW-12_01083	copA_1
copA_1	ZONMW-12_01154	copA_1
copA_1	ZONMW-12_03663	copA_1
copA_1	ZONMW-12_02431	copA_1
copA_1	ZONMW-12_04464	copA_1
copA_1	ZONMW-12_01020	copA_1
copA_1	ZONMW-13_00472	copA_1
copA_1	ZONMW-13_00182	copA_1
copA_1	ZONMW-13_00864	copA_1
copA_1	ZONMW-13_01040	copA_1
copA_1	ZONMW-13_02846	copA_1
copA_1	ZONMW-13_00023	copA_1
copA_1	ZONMW-13_00078	copA_1
copA_1	ZONMW-13_00016	copA_1
copA_1	ZONMW-13_01100	copA_1
copA_1	ZONMW-13_01038	copA_1
copA_1	ZONMW-13_03128	copA_1
copA_1	ZONMW-13_00020	copA_1
copA_1	ZONMW-14_00089	copA_1
copA_1	ZONMW-14_00657	copA_1

copA_1	ZONMW-14_00412	copA_1
copA_1	ZONMW-14_01230	copA_1
copA_1	ZONMW-14_01552	copA_1
copA_1	ZONMW-14_01387	copA_1
copA_1	ZONMW-14_02416	copA_1
copA_1	ZONMW-14_00157	copA_1
copA_1	ZONMW-14_02410	copA_1
copA_1	ZONMW-14_01496	copA_1
copA_1	ZONMW-14_01554	copA_1
copA_1	ZONMW-14_00662	copA_1
copA_1	ZONMW-14_02414	copA_1
copA_1	ZONMW-15_01171	copA_1
copA_1	ZONMW-15_01764	copA_1
copA_1	ZONMW-15_00112	copA_1
copA_1	ZONMW-15_00321	copA_1
copA_1	ZONMW-15_01047	copA_1
copA_1	ZONMW-15_01461	copA_1
copA_1	ZONMW-15_02899	copA_1
copA_1	ZONMW-15_00434	copA_1
copA_1	ZONMW-15_00438	copA_1
copA_1	ZONMW-19_03450	copA_1
copA_1	ZONMW-19_01686	copA_1
copA_1	ZONMW-19_03644	copA_1
copA_1	ZONMW-19_03611	copA_1
copA_1	ZONMW-19_03738	copA_1
copA_1	ZONMW-19_03694	copA_1
copA_1	ZONMW-19_03377	copA_1
copA_1	ZONMW-19_04465	copA_1
copA_1	ZONMW-19_00147	copA_1
copA_1	ZONMW-19_03270	copA_1
copA_1	ZONMW-19_04072	copA_1
copA_1	ZONMW-19_02693	copA_1
copA_1	ZONMW-19_03368	copA_1
copA_1	ZONMW-19_03130	copA_1
copA_1	ZONMW-19_02532	copA_1
copA_1	ZONMW-19_00274	copA_1
copA_1	ZONMW-19_04234	copA_1
copA_1	ZONMW-20_03033	copA_1
copA_1	ZONMW-20_02781	copA_1
copA_1	ZONMW-20_01290	copA_1
copA_1	ZONMW-20_04078	copA_1
copA_1	ZONMW-20_00732	copA_1
copA_1	ZONMW-20_02207	copA_1
copA_1	ZONMW-20_02399	copA_1
copA_1	ZONMW-20_03939	copA_1
copA_1	ZONMW-20_02489	copA_1
copA_1	ZONMW-20_01271	copA_1
copA_1	ZONMW-20_03332	copA_1

copA_1	ZONMW-20_02349	copA_1
copA_1	ZONMW-20_02206	copA_1
copA_1	ZONMW-20_01551	copA_1
copA_1	ZONMW-20_03942	copA_1
copA_1	ZONMW-20_03234	copA_1
copA_1	ZONMW-20_02498	copA_1
copA_1	ZONMW-20_00414	copA_1
copA_1	ZONMW-20_02289	copA_1
copA_1	ZONMW-20_02523	copA_1
copA_1	ZONMW-20_01131	copA_1
copA_1	ZONMW-20_02610	copA_1
copA_1	ZONMW-20_02807	copA_1
copA_1	ZONMW-20_02287	copA_1
copA_1	ZONMW-21_01013	copA_1
copA_1	ZONMW-21_03362	copA_1
copA_1	ZONMW-21_00590	copA_1
copA_1	ZONMW-21_00576	copA_1
copA_1	ZONMW-21_01368	copA_1
copA_1	ZONMW-21_00940	copA_1
copA_1	ZONMW-21_00276	copA_1
copA_1	ZONMW-21_01366	copA_1
copA_1	ZONMW-21_00282	copA_1
copA_1	ZONMW-21_00170	copA_1
copA_1	ZONMW-21_00166	copA_1
copA_1	ZONMW-21_02216	copA_1
copA_1	ZONMW-22_01014	copA_1
copA_1	ZONMW-22_03363	copA_1
copA_1	ZONMW-22_00590	copA_1
copA_1	ZONMW-22_00576	copA_1
copA_1	ZONMW-22_01370	copA_1
copA_1	ZONMW-22_00941	copA_1
copA_1	ZONMW-22_00276	copA_1
copA_1	ZONMW-22_01368	copA_1
copA_1	ZONMW-22_00282	copA_1
copA_1	ZONMW-22_00170	copA_1
copA_1	ZONMW-22_00166	copA_1
copA_1	ZONMW-22_01509	copA_1
copA_1	ZONMW-23_02127	copA_1
copA_1	ZONMW-23_03408	copA_1
copA_1	ZONMW-23_01705	copA_1
copA_1	ZONMW-23_01691	copA_1
copA_1	ZONMW-23_02483	copA_1
copA_1	ZONMW-23_02054	copA_1
copA_1	ZONMW-23_01391	copA_1
copA_1	ZONMW-23_02481	copA_1
copA_1	ZONMW-23_01397	copA_1
copA_1	ZONMW-23_01285	copA_1
copA_1	ZONMW-23_01281	copA_1

copA_1	ZONMW-23_01042	copA_1
copA_1	ZONMW-24_00447	copA_1
copA_1	ZONMW-24_03410	copA_1
copA_1	ZONMW-24_00870	copA_1
copA_1	ZONMW-24_00883	copA_1
copA_1	ZONMW-24_00091	copA_1
copA_1	ZONMW-24_00520	copA_1
copA_1	ZONMW-24_01183	copA_1
copA_1	ZONMW-24_00093	copA_1
copA_1	ZONMW-24_01177	copA_1
copA_1	ZONMW-24_01290	copA_1
copA_1	ZONMW-24_01294	copA_1
copA_1	ZONMW-24_01533	copA_1
copA_1	ZONMW-25_01359	copA_1
copA_1	ZONMW-25_02017	copA_1
copA_1	ZONMW-25_02240	copA_1
copA_1	ZONMW-25_02177	copA_1
copA_1	ZONMW-25_01203	copA_1
copA_1	ZONMW-25_02229	copA_1
copA_1	ZONMW-25_01205	copA_1
copA_1	ZONMW-25_03018	copA_1
copA_1	ZONMW-25_02910	copA_1
copA_1	ZONMW-25_01210	copA_1
copA_1	ZONMW-25_02847	copA_1
copA_1	ZONMW-25_01199	copA_1
copA_1	ZONMW-25_02289	copA_1
copA_1	ZONMW-25_02293	copA_1
copA_1	ZONMW-25_01564	copA_1
copA_1	ZONMW-25_00019	copA_1
copA_1	ZONMW-25_01559	copA_1
copA_1	ZONMW-25_02845	copA_1
copA_1	ZONMW-25_01213	copA_1
copA_1	ZONMW-26_01047	copA_1
copA_1	ZONMW-26_01558	copA_1
copA_1	ZONMW-26_01111	copA_1
copA_1	ZONMW-26_02444	copA_1
copA_1	ZONMW-26_01858	copA_1
copA_1	ZONMW-26_01847	copA_1
copA_1	ZONMW-26_00380	copA_1
copA_1	ZONMW-26_01309	copA_1
copA_1	ZONMW-27_00826	copA_1
copA_1	ZONMW-27_00092	copA_1
copA_1	ZONMW-27_00761	copA_1
copA_1	ZONMW-27_01624	copA_1
copA_1	ZONMW-27_00408	copA_1
copA_1	ZONMW-27_00397	copA_1
copA_1	ZONMW-27_01411	copA_1
copA_1	ZONMW-27_00564	copA_1

copA_1	ZONMW-28_01909	copA_1
copA_1	ZONMW-28_01337	copA_1
copA_1	ZONMW-28_02398	copA_1
copA_1	ZONMW-28_01755	copA_1
copA_1	ZONMW-28_02252	copA_1
copA_1	ZONMW-28_02535	copA_1
copA_1	ZONMW-28_02539	copA_1
copA_1	ZONMW-28_02480	copA_1
copA_1	ZONMW-28_01580	copA_1
copA_1	ZONMW-28_03000	copA_1
copA_1	ZONMW-28_02508	copA_1
copA_1	ZONMW-28_02520	copA_1
copA_1	ZONMW-28_01873	copA_1
copA_1	ZONMW-28_02370	copA_1
copA_1	ZONMW-28_01475	copA_1
copA_1	ZONMW-28_00866	copA_1
copA_1	ZONMW-28_02479	copA_1
copA_1	ZONMW-29_00585	copA_1
copA_1	ZONMW-29_02864	copA_1
copA_1	ZONMW-29_00256	copA_1
copA_1	ZONMW-29_00189	copA_1
copA_1	ZONMW-29_00242	copA_1
copA_1	ZONMW-29_02285	copA_1
copA_1	ZONMW-29_00523	copA_1
copA_1	ZONMW-29_03044	copA_1
copA_1	ZONMW-29_00915	copA_1
copA_1	ZONMW-29_02928	copA_1
copA_1	ZONMW-29_00913	copA_1
copA_1	ZONMW-29_01221	copA_1
copA_1	ZONMW-29_02283	copA_1
copA_1	ZONMW-29_03040	copA_1
copA_1	ZONMW-29_02156	copA_1
copA_1	ZONMW-30_02108	copA_1
copA_1	ZONMW-30_01780	copA_1
copA_1	ZONMW-30_01699	copA_1
copA_1	ZONMW-30_01639	copA_1
copA_1	ZONMW-30_02968	copA_1
copA_1	ZONMW-30_01687	copA_1
copA_1	ZONMW-30_01861	copA_1
copA_1	ZONMW-30_02970	copA_1
copA_1	ZONMW-30_01487	copA_1
copA_1	ZONMW-30_02974	copA_1
copA_1	ZONMW-30_00646	copA_1
copA_1	ZONMW-30_02964	copA_1
copA_1	ZONMW-30_00753	copA_1
copA_1	ZONMW-30_00757	copA_1
copA_1	ZONMW-30_01111	copA_1
copA_1	ZONMW-30_01106	copA_1

copA_1	ZONMW-30_00648	copA_1
copA_1	ZONMW-30_02977	copA_1
copA_1	ZONMW-31_02537	copA_1
copA_1	ZONMW-31_00473	copA_1
copA_1	ZONMW-31_00055	copA_1
copA_1	ZONMW-31_02482	copA_1
copA_1	ZONMW-31_00662	copA_1
copA_1	ZONMW-31_01656	copA_1
copA_1	ZONMW-31_00779	copA_1
copA_1	ZONMW-31_00219	copA_1
copA_1	ZONMW-31_01707	copA_1
copA_1	ZONMW-31_03039	copA_1
copA_1	ZONMW-31_01931	copA_1
copA_1	ZONMW-31_00120	copA_1
copA_1	ZONMW-31_02214	copA_1
copA_1	ZONMW-31_02211	copA_1
copA_1	ZONMW-32_02063	copA_1
copA_1	ZONMW-32_01745	copA_1
copA_1	ZONMW-32_02952	copA_1
copA_1	ZONMW-32_02217	copA_1
copA_1	ZONMW-32_02657	copA_1
copA_1	ZONMW-32_02815	copA_1
copA_1	ZONMW-32_02811	copA_1
copA_1	ZONMW-32_02870	copA_1
copA_1	ZONMW-32_01987	copA_1
copA_1	ZONMW-32_01484	copA_1
copA_1	ZONMW-32_02842	copA_1
copA_1	ZONMW-32_02830	copA_1
copA_1	ZONMW-32_02099	copA_1
copA_1	ZONMW-32_02980	copA_1
copA_1	ZONMW-32_01882	copA_1
copA_1	ZONMW-32_00225	copA_1
copA_1	ZONMW-32_02871	copA_1
copA_1	ZONMW-33_02054	copA_1
copA_1	ZONMW-33_01735	copA_1
copA_1	ZONMW-33_02796	copA_1
copA_1	ZONMW-33_02208	copA_1
copA_1	ZONMW-33_02461	copA_1
copA_1	ZONMW-33_02933	copA_1
copA_1	ZONMW-33_02937	copA_1
copA_1	ZONMW-33_02878	copA_1
copA_1	ZONMW-33_01978	copA_1
copA_1	ZONMW-33_01474	copA_1
copA_1	ZONMW-33_02906	copA_1
copA_1	ZONMW-33_02918	copA_1
copA_1	ZONMW-33_02090	copA_1
copA_1	ZONMW-33_02768	copA_1
copA_1	ZONMW-33_01872	copA_1

copA_1	ZONMW-33_00092	copA_1
copA_1	ZONMW-33_02877	copA_1
copA_1	ZONMW-34_02172	copA_1
copA_1	ZONMW-34_00118	copA_1
copA_1	ZONMW-34_02360	copA_1
copA_1	ZONMW-34_01784	copA_1
copA_1	ZONMW-34_03366	copA_1
copA_1	ZONMW-34_03370	copA_1
copA_1	ZONMW-34_03453	copA_1
copA_1	ZONMW-34_00313	copA_1
copA_1	ZONMW-34_01662	copA_1
copA_1	ZONMW-34_02208	copA_1
copA_1	ZONMW-34_02874	copA_1
copA_1	ZONMW-34_03441	copA_1
copA_1	ZONMW-34_00217	copA_1
copA_1	ZONMW-35_02077	copA_1
copA_1	ZONMW-35_01273	copA_1
copA_1	ZONMW-35_01889	copA_1
copA_1	ZONMW-35_01475	copA_1
copA_1	ZONMW-35_03268	copA_1
copA_1	ZONMW-35_03264	copA_1
copA_1	ZONMW-35_03362	copA_1
copA_1	ZONMW-35_01077	copA_1
copA_1	ZONMW-35_03626	copA_1
copA_1	ZONMW-35_02041	copA_1
copA_1	ZONMW-35_02841	copA_1
copA_1	ZONMW-35_03374	copA_1
copA_1	ZONMW-35_01173	copA_1
copA_1	ZONMW-36_02182	copA_1
copA_1	ZONMW-36_01621	copA_1
copA_1	ZONMW-36_02308	copA_1
copA_1	ZONMW-36_02028	copA_1
copA_1	ZONMW-36_02791	copA_1
copA_1	ZONMW-36_02445	copA_1
copA_1	ZONMW-36_02449	copA_1
copA_1	ZONMW-36_02390	copA_1
copA_1	ZONMW-36_01378	copA_1
copA_1	ZONMW-36_00693	copA_1
copA_1	ZONMW-36_02418	copA_1
copA_1	ZONMW-36_02430	copA_1
copA_1	ZONMW-36_02146	copA_1
copA_1	ZONMW-36_02280	copA_1
copA_1	ZONMW-36_01483	copA_1
copA_1	ZONMW-36_01277	copA_1
copA_1	ZONMW-36_02389	copA_1
copA_1	ZONMW-37_01349	copA_1
copA_1	ZONMW-37_03372	copA_1
copA_1	ZONMW-37_00590	copA_1

copA_1	ZONMW-37_00576	copA_1
copA_1	ZONMW-37_00994	copA_1
copA_1	ZONMW-37_01422	copA_1
copA_1	ZONMW-37_00276	copA_1
copA_1	ZONMW-37_00996	copA_1
copA_1	ZONMW-37_00282	copA_1
copA_1	ZONMW-37_00169	copA_1
copA_1	ZONMW-37_00165	copA_1
copA_1	ZONMW-37_01532	copA_1
copA_1	ZONMW-38_02934	copA_1
copA_1	ZONMW-38_03169	copA_1
copA_1	ZONMW-38_01045	copA_1
copA_1	ZONMW-38_01854	copA_1
copA_1	ZONMW-38_01055	copA_1
copA_1	ZONMW-38_00633	copA_1
copA_1	ZONMW-38_00730	copA_1
copA_1	ZONMW-38_02001	copA_1
copA_1	ZONMW-38_03271	copA_1
copA_1	ZONMW-39_01452	copA_1
copA_1	ZONMW-39_02409	copA_1
copA_1	ZONMW-39_01653	copA_1
copA_1	ZONMW-39_03698	copA_1
copA_1	ZONMW-39_03221	copA_1
copA_1	ZONMW-39_02998	copA_1
copA_1	ZONMW-39_03225	copA_1
copA_1	ZONMW-39_00037	copA_1
copA_1	ZONMW-39_01488	copA_1
copA_1	ZONMW-39_03339	copA_1
copA_1	ZONMW-39_00428	copA_1
copA_1	ZONMW-39_00690	copA_1
copA_1	ZONMW-40_03519	copA_1
copA_1	ZONMW-40_00245	copA_1
copA_1	ZONMW-40_03049	copA_1
copA_1	ZONMW-40_02260	copA_1
copA_1	ZONMW-40_01370	copA_1
copA_1	ZONMW-40_01932	copA_1
copA_1	ZONMW-40_01416	copA_1
copA_1	ZONMW-40_02736	copA_1
copA_1	ZONMW-40_01382	copA_1
copA_1	ZONMW-40_00337	copA_1
copA_1	ZONMW-40_02691	copA_1
copA_1	ZONMW-40_02734	copA_1
copA_1	ZONMW-40_02577	copA_1
copA_1	ZONMW-40_02573	copA_1
copA_1	ZONMW-41_02950	copA_1
copA_1	ZONMW-41_00240	copA_1
copA_1	ZONMW-41_03159	copA_1
copA_1	ZONMW-41_01540	copA_1

copA_1	ZONMW-41_00994	copA_1
copA_1	ZONMW-41_01710	copA_1
copA_1	ZONMW-41_01998	copA_1
copA_1	ZONMW-41_01581	copA_1
copA_1	ZONMW-41_03129	copA_1
copA_1	ZONMW-41_00980	copA_1
copA_1	ZONMW-41_00326	copA_1
copA_1	ZONMW-41_00536	copA_1
copA_1	ZONMW-41_02572	copA_1
copA_1	ZONMW-41_00522	copA_1
copA_1	ZONMW-42_00586	copA_1
copA_1	ZONMW-42_00040	copA_1
copA_1	ZONMW-42_02746	copA_1
copA_1	ZONMW-42_01334	copA_1
copA_1	ZONMW-42_00679	copA_1
copA_1	ZONMW-42_03725	copA_1
copA_1	ZONMW-42_02933	copA_1
copA_1	ZONMW-42_00650	copA_1
copA_1	ZONMW-42_03571	copA_1
copA_1	ZONMW-42_00750	copA_1
copA_1	ZONMW-42_00315	copA_1
copA_1	ZONMW-42_01719	copA_1
copA_1	ZONMW-42_00142	copA_1
copA_1	ZONMW-42_01330	copA_1
copA_1	ZONMW-42_02454	copA_1
copA_1	ZONMW-42_01425	copA_1
copA_1	ZONMW-42_01423	copA_1
copA_1	ZONMW-43_03246	copA_1
copA_1	ZONMW-43_00831	copA_1
copA_1	ZONMW-43_01871	copA_1
copA_1	ZONMW-43_04385	copA_1
copA_1	ZONMW-43_03949	copA_1
copA_1	ZONMW-43_01295	copA_1
copA_1	ZONMW-43_04375	copA_1
copA_1	ZONMW-43_02678	copA_1
copA_1	ZONMW-43_02674	copA_1
copA_1	ZONMW-43_00632	copA_1
copA_1	ZONMW-43_01031	copA_1
copA_1	ZONMW-43_01606	copA_1
copA_1	ZONMW-43_03282	copA_1
copA_1	ZONMW-43_02689	copA_1
copA_1	ZONMW-43_00731	copA_1
copA_1	ZONMW-44_01901	copA_1
copA_1	ZONMW-44_02472	copA_1
copA_1	ZONMW-44_00708	copA_1
copA_1	ZONMW-44_00083	copA_1
copA_1	ZONMW-44_00073	copA_1
copA_1	ZONMW-44_00525	copA_1

copA_1	ZONMW-44_02682	copA_1
copA_1	ZONMW-44_03537	copA_1
copA_1	ZONMW-44_00336	copA_1
copA_1	ZONMW-44_00342	copA_1
copA_1	ZONMW-44_00274	copA_1
copA_1	ZONMW-44_02862	copA_1
copA_1	ZONMW-44_00304	copA_1
copA_1	ZONMW-44_00316	copA_1
copA_1	ZONMW-44_03108	copA_1
copA_1	ZONMW-44_02005	copA_1
copA_1	ZONMW-44_00521	copA_1
copA_1	ZONMW-44_00643	copA_1
copA_1	ZONMW-44_00273	copA_1
copA_1	ZONMW-45_00897	copA_1
copA_1	ZONMW-45_03196	copA_1
copA_1	ZONMW-45_01050	copA_1
copA_1	ZONMW-45_02449	copA_1
copA_1	ZONMW-45_02198	copA_1
copA_1	ZONMW-45_03180	copA_1
copA_1	ZONMW-45_03132	copA_1
copA_1	ZONMW-45_03090	copA_1
copA_1	ZONMW-45_03179	copA_1
copA_1	ZONMW-45_01618	copA_1
copA_1	ZONMW-46_00977	copA_1
copA_1	ZONMW-46_02372	copA_1
copA_1	ZONMW-46_00617	copA_1
copA_1	ZONMW-46_02442	copA_1
copA_1	ZONMW-46_01874	copA_1
copA_1	ZONMW-46_02920	copA_1
copA_1	ZONMW-46_02806	copA_1
copA_1	ZONMW-46_00145	copA_1
copA_1	ZONMW-46_02921	copA_1
copA_1	ZONMW-46_02129	copA_1
copA_1	ZONMW-47_01196	copA_1
copA_1	ZONMW-47_03235	copA_1
copA_1	ZONMW-47_00617	copA_1
copA_1	ZONMW-47_02432	copA_1
copA_1	ZONMW-47_01615	copA_1
copA_1	ZONMW-47_02953	copA_1
copA_1	ZONMW-47_02797	copA_1
copA_1	ZONMW-47_00360	copA_1
copA_1	ZONMW-47_02952	copA_1
copA_1	ZONMW-47_01932	copA_1
copA_1	ZONMW-48_02522	copA_1
copA_1	ZONMW-48_00151	copA_1
copA_1	ZONMW-48_00773	copA_1
copA_1	ZONMW-48_00958	copA_1
copA_1	ZONMW-48_00463	copA_1

copA_1	ZONMW-48_01836	copA_1
copA_1	ZONMW-48_00003	copA_1
copA_1	ZONMW-48_01071	copA_1
copA_1	ZONMW-48_01075	copA_1
copA_1	ZONMW-49_00961	copA_1
copA_1	ZONMW-49_00522	copA_1
copA_1	ZONMW-49_01252	copA_1
copA_1	ZONMW-49_01626	copA_1
copA_1	ZONMW-49_01635	copA_1
copA_1	ZONMW-49_01781	copA_1
copA_1	ZONMW-49_03120	copA_1
copA_1	ZONMW-49_00832	copA_1
copA_1	ZONMW-49_01633	copA_1
copA_1	ZONMW-49_00821	copA_1
copA_1	ZONMW-49_00393	copA_1
copA_1	ZONMW-49_01629	copA_1
copA_1	ZONMW-49_01465	copA_1
copA_1	ZONMW-50_00962	copA_1
copA_1	ZONMW-50_00523	copA_1
copA_1	ZONMW-50_01254	copA_1
copA_1	ZONMW-50_01788	copA_1
copA_1	ZONMW-50_01779	copA_1
copA_1	ZONMW-50_01633	copA_1
copA_1	ZONMW-50_03116	copA_1
copA_1	ZONMW-50_00833	copA_1
copA_1	ZONMW-50_01781	copA_1
copA_1	ZONMW-50_00822	copA_1
copA_1	ZONMW-50_00092	copA_1
copA_1	ZONMW-50_01785	copA_1
copA_1	ZONMW-50_01467	copA_1
copA_1	ZONMW-51_01302	copA_1
copA_1	ZONMW-51_01183	copA_1
copA_1	ZONMW-51_01367	copA_1
copA_1	ZONMW-51_00525	copA_1
copA_1	ZONMW-51_00534	copA_1
copA_1	ZONMW-51_03176	copA_1
copA_1	ZONMW-51_00879	copA_1
copA_1	ZONMW-51_00532	copA_1
copA_1	ZONMW-51_00890	copA_1
copA_1	ZONMW-51_00381	copA_1
copA_1	ZONMW-51_00528	copA_1
copA_1	ZONMW-51_00721	copA_1
copA_1	ZONMW-52_01029	copA_1
copA_1	ZONMW-52_02876	copA_1
copA_1	ZONMW-52_01109	copA_1
copA_1	ZONMW-52_00635	copA_1
copA_1	ZONMW-52_03135	copA_1
copA_1	ZONMW-52_01479	copA_1

copA_1	ZONMW-52_00637	copA_1
copA_1	ZONMW-52_01490	copA_1
copA_1	ZONMW-52_00366	copA_1
copA_1	ZONMW-52_00641	copA_1
copA_1	ZONMW-52_01321	copA_1
copA_1	ZONMW-53_00805	copA_1
copA_1	ZONMW-53_00039	copA_1
copA_1	ZONMW-53_00740	copA_1
copA_1	ZONMW-53_01237	copA_1
copA_1	ZONMW-53_01246	copA_1
copA_1	ZONMW-53_03152	copA_1
copA_1	ZONMW-53_00348	copA_1
copA_1	ZONMW-53_01244	copA_1
copA_1	ZONMW-53_00337	copA_1
copA_1	ZONMW-53_01098	copA_1
copA_1	ZONMW-53_01240	copA_1
copA_1	ZONMW-53_00504	copA_1
copA_1	ZONMW-54_00264	copA_1
copA_1	ZONMW-54_00989	copA_1
copA_1	ZONMW-54_00329	copA_1
copA_1	ZONMW-54_01480	copA_1
copA_1	ZONMW-54_01471	copA_1
copA_1	ZONMW-54_03146	copA_1
copA_1	ZONMW-54_00686	copA_1
copA_1	ZONMW-54_01473	copA_1
copA_1	ZONMW-54_00697	copA_1
copA_1	ZONMW-54_01624	copA_1
copA_1	ZONMW-54_01477	copA_1
copA_1	ZONMW-54_00531	copA_1
copA_1	ZONMW-55_00773	copA_1
copA_1	ZONMW-55_00037	copA_1
copA_1	ZONMW-55_00709	copA_1
copA_1	ZONMW-55_00999	copA_1
copA_1	ZONMW-55_00990	copA_1
copA_1	ZONMW-55_03150	copA_1
copA_1	ZONMW-55_00341	copA_1
copA_1	ZONMW-55_00992	copA_1
copA_1	ZONMW-55_00330	copA_1
copA_1	ZONMW-55_01145	copA_1
copA_1	ZONMW-55_00996	copA_1
copA_1	ZONMW-55_00499	copA_1
copA_1	ZONMW-56_02306	copA_1
copA_1	ZONMW-56_02770	copA_1
copA_1	ZONMW-56_02370	copA_1
copA_1	ZONMW-56_00235	copA_1
copA_1	ZONMW-56_00226	copA_1
copA_1	ZONMW-56_03110	copA_1
copA_1	ZONMW-56_00974	copA_1

copA_1	ZONMW-56_00228	copA_1
copA_1	ZONMW-56_00985	copA_1
copA_1	ZONMW-56_00374	copA_1
copA_1	ZONMW-56_00232	copA_1
copA_1	ZONMW-56_00814	copA_1
copA_1	ZONMW-57_02433	copA_1
copA_1	ZONMW-57_01576	copA_1
copA_1	ZONMW-57_02499	copA_1
copA_1	ZONMW-57_00448	copA_1
copA_1	ZONMW-57_00439	copA_1
copA_1	ZONMW-57_03159	copA_1
copA_1	ZONMW-57_01274	copA_1
copA_1	ZONMW-57_00441	copA_1
copA_1	ZONMW-57_01285	copA_1
copA_1	ZONMW-57_00587	copA_1
copA_1	ZONMW-57_00445	copA_1
copA_1	ZONMW-57_01115	copA_1
copA_1	ZONMW-58_02458	copA_1
copA_1	ZONMW-58_01008	copA_1
copA_1	ZONMW-58_02524	copA_1
copA_1	ZONMW-58_00523	copA_1
copA_1	ZONMW-58_00532	copA_1
copA_1	ZONMW-58_03188	copA_1
copA_1	ZONMW-58_01310	copA_1
copA_1	ZONMW-58_00530	copA_1
copA_1	ZONMW-58_01299	copA_1
copA_1	ZONMW-58_00384	copA_1
copA_1	ZONMW-58_00526	copA_1
copA_1	ZONMW-58_01469	copA_1
copA_1	ZONMW-59_02032	copA_1
copA_1	ZONMW-59_02756	copA_1
copA_1	ZONMW-59_01968	copA_1
copA_1	ZONMW-59_00312	copA_1
copA_1	ZONMW-59_00916	copA_1
copA_1	ZONMW-59_03245	copA_1
copA_1	ZONMW-59_01275	copA_1
copA_1	ZONMW-59_00310	copA_1
copA_1	ZONMW-59_01286	copA_1
copA_1	ZONMW-59_00920	copA_1
copA_1	ZONMW-59_00574	copA_1
copA_1	ZONMW-59_00926	copA_1
copA_1	ZONMW-59_00306	copA_1
copA_1	ZONMW-59_01117	copA_1
copA_1	ZONMW-60_01716	copA_1
copA_1	ZONMW-60_00982	copA_1
copA_1	ZONMW-60_01651	copA_1
copA_1	ZONMW-60_00165	copA_1
copA_1	ZONMW-60_00157	copA_1

copA_1	ZONMW-60_03170	copA_1
copA_1	ZONMW-60_01295	copA_1
copA_1	ZONMW-60_00158	copA_1
copA_1	ZONMW-60_01284	copA_1
copA_1	ZONMW-60_00589	copA_1
copA_1	ZONMW-60_00162	copA_1
copA_1	ZONMW-60_01450	copA_1
copA_1	ZONMW-61_00073	copA_1
copA_1	ZONMW-61_00066	copA_1
copA_1	ZONMW-61_00517	copA_1
copA_1	ZONMW-61_02617	copA_1
copA_1	ZONMW-61_01372	copA_1
copA_1	ZONMW-61_03144	copA_1
copA_1	ZONMW-61_02616	copA_1
copA_1	ZONMW-61_03320	copA_1
copA_1	ZONMW-61_01411	copA_1
copA_1	ZONMW-61_03937	copA_1
copA_1	ZONMW-61_01665	copA_1
copA_1	ZONMW-61_03184	copA_1
copA_1	ZONMW-61_01535	copA_1
copA_1	ZONMW-61_01367	copA_1
copA_1	ZONMW-61_01408	copA_1
copA_1	ZONMW-61_02324	copA_1
copA_1	ZONMW-61_02596	copA_1
copA_1	ZONMW-61_02948	copA_1
copA_1	ZONMW-61_02952	copA_1
copA_1	ZONMW-61_03104	copA_1
copA_1	ZONMW-61_03121	copA_1
copA_1	ZONMW-61_03509	copA_1
copA_1	ZONMW-61_01361	copA_1
copA_1	ZONMW-62_01628	copA_1
copA_1	ZONMW-62_01620	copA_1
copA_1	ZONMW-62_03143	copA_1
copA_1	ZONMW-62_02047	copA_1
copA_1	ZONMW-62_00744	copA_1
copA_1	ZONMW-62_00456	copA_1
copA_1	ZONMW-62_01411	copA_1
copA_1	ZONMW-62_03540	copA_1
copA_1	ZONMW-62_03239	copA_1
copA_1	ZONMW-62_00327	copA_1
copA_1	ZONMW-62_03184	copA_1
copA_1	ZONMW-62_00841	copA_1
copA_1	ZONMW-62_02287	copA_1
copA_1	ZONMW-62_02283	copA_1
copA_1	ZONMW-62_03196	copA_1
copA_1	ZONMW-62_03181	copA_1
copA_1	ZONMW-62_00330	copA_1
copA_1	ZONMW-62_00326	copA_1

copA_1	ZONMW-63_00600	copA_1
copA_1	ZONMW-63_00981	copA_1
copA_1	ZONMW-63_00665	copA_1
copA_1	ZONMW-63_00118	copA_1
copA_1	ZONMW-63_00109	copA_1
copA_1	ZONMW-63_03162	copA_1
copA_1	ZONMW-63_01291	copA_1
copA_1	ZONMW-63_00111	copA_1
copA_1	ZONMW-63_01280	copA_1
copA_1	ZONMW-63_00260	copA_1
copA_1	ZONMW-63_00115	copA_1
copA_1	ZONMW-63_00871	copA_1
copA_1	ZONMW-64_01374	copA_1
copA_1	ZONMW-64_01116	copA_1
copA_1	ZONMW-64_01608	copA_1
copA_1	ZONMW-64_01876	copA_1
copA_1	ZONMW-64_03237	copA_1
copA_1	ZONMW-64_03201	copA_1
copA_1	ZONMW-64_03205	copA_1
copA_1	ZONMW-64_00894	copA_1
copA_1	ZONMW-64_02545	copA_1
copA_1	ZONMW-64_03088	copA_1
copA_1	ZONMW-64_01410	copA_1
copA_1	ZONMW-64_03250	copA_1
copA_1	ZONMW-64_01016	copA_1
copA_1	ZONMW-64_01863	copA_1
copA_1	ZONMW-65_01577	copA_1
copA_1	ZONMW-65_00368	copA_1
copA_1	ZONMW-65_02014	copA_1
copA_1	ZONMW-65_00487	copA_1
copA_1	ZONMW-65_01168	copA_1
copA_1	ZONMW-65_03584	copA_1
copA_1	ZONMW-65_02056	copA_1
copA_1	ZONMW-65_03511	copA_1
copA_1	ZONMW-65_03513	copA_1
copA_1	ZONMW-65_01471	copA_1
copA_1	ZONMW-65_02272	copA_1
copA_1	ZONMW-65_00633	copA_1
copA_1	ZONMW-65_01323	copA_1
copA_1	ZONMW-65_02841	copA_1
copA_1	ZONMW-65_00193	copA_1
copA_1	ZONMW-65_02026	copA_1
copA_1	ZONMW-65_00418	copA_1
copA_1	ZONMW-65_00235	copA_1
copA_1	ZONMW-65_03628	copA_1
copA_1	ZONMW-65_03589	copA_1
copA_1	ZONMW-65_00541	copA_1
copA_1	ZONMW-65_01364	copA_1

copA_1	ZONMW-66_00105	copA_1
copA_1	ZONMW-66_03616	copA_1
copA_1	ZONMW-66_02933	copA_1
copA_1	ZONMW-66_03489	copA_1
copA_1	ZONMW-66_03852	copA_1
copA_1	ZONMW-66_03878	copA_1
copA_1	ZONMW-66_00507	copA_1
copA_1	ZONMW-66_02171	copA_1
copA_1	ZONMW-66_00763	copA_1
copA_1	ZONMW-66_03872	copA_1
copA_1	ZONMW-66_03951	copA_1
copA_1	ZONMW-66_04435	copA_1
copA_1	ZONMW-66_00498	copA_1
copA_1	ZONMW-66_00891	copA_1
copA_1	ZONMW-66_01487	copA_1
copA_1	ZONMW-66_00916	copA_1
copA_1	ZONMW-66_01678	copA_1
copA_1	ZONMW-67_02205	copA_1
copA_1	ZONMW-67_00595	copA_1
copA_1	ZONMW-67_00151	copA_1
copA_1	ZONMW-67_02880	copA_1
copA_1	ZONMW-67_03271	copA_1
copA_1	ZONMW-67_02722	copA_1
copA_1	ZONMW-67_01596	copA_1
copA_1	ZONMW-67_00310	copA_1
copA_1	ZONMW-67_00245	copA_1
copA_1	ZONMW-67_02990	copA_1
copA_1	ZONMW-68_04929	copA_1
copA_1	ZONMW-68_00584	copA_1
copA_1	ZONMW-68_00581	copA_1
copA_1	ZONMW-68_00269	copA_1
copA_1	ZONMW-68_04528	copA_1
copA_1	ZONMW-68_04581	copA_1
copA_1	ZONMW-68_02023	copA_1
copA_1	ZONMW-68_03647	copA_1
copA_1	ZONMW-68_02100	copA_1
copA_1	ZONMW-68_01474	copA_1
copA_1	ZONMW-68_02366	copA_1
copA_1	ZONMW-68_03243	copA_1
copA_1	ZONMW-68_04282	copA_1
copA_1	ZONMW-68_03433	copA_1
copA_1	ZONMW-68_02016	copA_1
copA_1	ZONMW-68_00914	copA_1
copA_1	ZONMW-68_02066	copA_1
copA_1	ZONMW-68_02093	copA_1
copA_1	ZONMW-68_03645	copA_1
copA_1	ZONMW-68_02334	copA_1
copA_1	ZONMW-68_02021	copA_1

copA_1	ZONMW-68_02035	copA_1
copA_1	ZONMW-68_04583	copA_1
copA_1	ZONMW-68_02033	copA_1
copA_1	ZONMW-69_02253	copA_1
copA_1	ZONMW-69_00342	copA_1
copA_1	ZONMW-69_02760	copA_1
copA_1	ZONMW-69_03074	copA_1
copA_1	ZONMW-69_03272	copA_1
copA_1	ZONMW-69_00519	copA_1
copA_1	ZONMW-69_01405	copA_1
copA_1	ZONMW-69_00057	copA_1
copA_1	ZONMW-69_02854	copA_1
copA_1	ZONMW-69_02964	copA_1
copA_1	ZONMW-72_02011	copA_1
copA_1	ZONMW-72_01022	copA_1
copA_1	ZONMW-72_00913	copA_1
copA_1	ZONMW-72_01724	copA_1
copA_1	ZONMW-72_04056	copA_1
copA_1	ZONMW-72_03484	copA_1
copA_1	ZONMW-72_04007	copA_1
copA_1	ZONMW-72_03686	copA_1
copA_1	ZONMW-72_04114	copA_1
copA_1	ZONMW-72_00480	copA_1
copA_1	ZONMW-72_00250	copA_1
copA_1	ZONMW-72_02908	copA_1
copA_1	ZONMW-72_01440	copA_1
copA_1	ZONMW-72_02670	copA_1
copA_1	ZONMW-72_01729	copA_1
copA_1	ZONMW-72_02341	copA_1
copA_1	ZONMW-72_03416	copA_1
copA_1	ZONMW-72_00478	copA_1
copA_1	ZONMW-72_00180	copA_1
copA_1	ZONMW-72_01731	copA_1
copA_1	ZONMW-72_00689	copA_1
copA_1	ZONMW-72_03451	copA_1
copA_1	ZONMW-72_01954	copA_1
copA_1	ZONMW-72_02668	copA_1
copA_1	ZONMW-72_02667	copA_1
copA_1	ZONMW-72_00154	copA_1
copA_1	ZONMW-72_02669	copA_1
copA_1	ZONMW-72_02662	copA_1
copA_1	ZONMW-72_01438	copA_1
copA_1	ZONMW-73_03630	copA_1
copA_1	ZONMW-73_01811	copA_1
copA_1	ZONMW-73_02796	copA_1
copA_1	ZONMW-73_03385	copA_1
copA_1	ZONMW-73_03752	copA_1
copA_1	ZONMW-73_00264	copA_1

copA_1	ZONMW-73_00268	copA_1
copA_1	ZONMW-73_00725	copA_1
copA_1	ZONMW-73_03254	copA_1
copA_1	ZONMW-73_00262	copA_1
copA_1	ZONMW-73_00271	copA_1
copA_1	ZONMW-73_03661	copA_1
copA_1	ZONMW-73_00673	copA_1
copA_1	ZONMW-74_00804	copA_1
copA_1	ZONMW-74_01280	copA_1
copA_1	ZONMW-74_01961	copA_1
copA_1	ZONMW-74_00790	copA_1
copA_1	ZONMW-74_02231	copA_1
copA_1	ZONMW-74_01096	copA_1
copA_1	ZONMW-74_01123	copA_1
copA_1	ZONMW-74_01098	copA_1
copA_1	ZONMW-74_00083	copA_1
copA_1	ZONMW-74_00776	copA_1
copA_1	ZONMW-74_01782	copA_1
copA_1	ZONMW-74_00081	copA_1
copA_1	ZONMW-75_00201	copA_1
copA_1	ZONMW-75_01065	copA_1
copA_1	ZONMW-75_02121	copA_1
copA_1	ZONMW-75_01158	copA_1
copA_1	ZONMW-75_02158	copA_1
copA_1	ZONMW-75_01232	copA_1
copA_1	ZONMW-75_01228	copA_1
copA_1	ZONMW-75_01834	copA_1
copA_1	ZONMW-75_00183	copA_1
copA_1	ZONMW-75_01633	copA_1
copA_1	ZONMW-75_01226	copA_1
copA_1	ZONMW-75_01235	copA_1
copA_1	ZONMW-75_02976	copA_1
copA_1	ZONMW-75_01223	copA_1
copA_1	da5A-8_01802	copA_1
copA_1	da5A-8_01500	copA_1
copA_1	da5A-8_02049	copA_1
copA_1	da5A-8_00606	copA_1
copA_1	da5A-8_03429	copA_1
copA_1	da5A-8_00326	copA_1
copA_1	da5A-8_03787	copA_1
copA_1	da5A-8_03538	copA_1
purN	18_00832	purN
purN	18_03921	purN
purN	18_02712	purN
purN	ACAM48_02289	purN
purN	ACAM48_03322	purN
purN	ACAM48_00649	purN
purN	CAM030_01168	purN

purN	CAM030_01548	purN
purN	CAM030_00295	purN
purN	CECT7946_00011	purN
purN	CECT7946_01070	purN
purN	CECT7946_02859	purN
purN	CGMCC1.8863_02131	purN
purN	CGMCC1.8863_01524	purN
purN	CGMCC1.8863_03480	purN
purN	CGMCC1.8863_01508	purN
purN	CGMCC1.8863_02601	purN
purN	CL8139_02282	purN
purN	CL8139_00564	purN
purN	CL8139_03069	purN
purN	DAU203_01362	purN
purN	DAU203_00374	purN
purN	DAU203_03392	purN
purN	DD5b_01978	purN
purN	DD5b_00768	purN
purN	DD5b_00570	purN
purN	DD5b_02823	purN
purN	DSM1208_01543	purN
purN	DSM1208_02707	purN
purN	DSM1208_03803	purN
purN	DSM12212_02121	purN
purN	DSM12212_00088	purN
purN	DSM12212_03039	purN
purN	DSM12212_00083	purN
purN	DSM15343_02444	purN
purN	DSM15343_02767	purN
purN	DSM15361_00363	purN
purN	DSM15361_02027	purN
purN	DSM15365_01028	purN
purN	DSM15365_01394	purN
purN	DSM15365_02490	purN
purN	DSM15718_02761	purN
purN	DSM15718_00034	purN
purN	DSM15718_02846	purN
purN	DSM15718_03396	purN
purN	DSM16505T_00312	purN
purN	DSM16505T_01807	purN
purN	DSM18103_01788	purN
purN	DSM18103_00798	purN
purN	DSM18293_02953	purN
purN	DSM18293_03751	purN
purN	DSM18293_02677	purN
purN	DSM18841_00117	purN
purN	DSM18841_01540	purN
purN	DSM18842_01088	purN

purN	DSM18842_02226	purN
purN	DSM19866_03430	purN
purN	DSM19866_00221	purN
purN	DSM19866_04295	purN
purN	DSM2041_00350	purN
purN	DSM2041_03189	purN
purN	DSM2041_01409	purN
purN	DSM2041_04584	purN
purN	DSM2041_01356	purN
purN	DSM2041_00213	purN
purN	DSM2061_03071	purN
purN	DSM2061_04195	purN
purN	DSM2061_01227	purN
purN	DSM2064_02135	purN
purN	DSM2064_03363	purN
purN	DSM2064_04952	purN
purN	DSM2064_01260	purN
purN	DSM21164_01009	purN
purN	DSM21164_02033	purN
purN	DSM21164_01649	purN
purN	DSM23404_03009	purN
purN	DSM23404_00834	purN
purN	DSM23404_02672	purN
purN	DSM23404_00336	purN
purN	DSM23424_00439	purN
purN	DSM23424_01391	purN
purN	DSM23424_00327	purN
purN	DSM24786_02804	purN
purN	DSM24786_02447	purN
purN	DSM24786_00870	purN
purN	DSM7489_01272	purN
purN	DSM7489_00359	purN
purN	DSM7489_03228	purN
purN	DSW1_00577	purN
purN	DSW1_02471	purN
purN	DSW1_01640	purN
purN	DSW5_01343	purN
purN	DSW5_01829	purN
purN	DSW5_02609	purN
purN	DSW6_01232	purN
purN	DSW6_02762	purN
purN	DSW6_03424	purN
purN	DSW8_02460	purN
purN	DSW8_00897	purN
purN	DSW8_01252	purN
purN	DSW8_00080	purN
purN	F52_02559	purN
purN	F52_00297	purN

purN	F52_01812	purN
purN	GCA_001027945.1_00479	purN
purN	GCA_001027945.1_02123	purN
purN	HI1_01214	purN
purN	HI1_00292	purN
purN	HI1_03175	purN
purN	IR1_00706	purN
purN	IR1_02566	purN
purN	IR1_04597	purN
purN	IR1_02839	purN
purN	KCOM3131_00542	purN
purN	KCOM3131_00812	purN
purN	KCTC12974_01835	purN
purN	KCTC12974_02946	purN
purN	KCTC12974_00114	purN
purN	KL-A_02161	purN
purN	KL-A_02634	purN
purN	KL-A_01944	purN
purN	L12_00383	purN
purN	LL0412.1.7_00669	purN
purN	LL0412.1.7_02255	purN
purN	MAR_2009_71_00993	purN
purN	MAR_2009_71_02545	purN
purN	MAR_2009_71_02184	purN
purN	MAR_2009_71_02881	purN
purN	NN016038_00852	purN
purN	NN016038_03974	purN
purN	NN016038_02761	purN
purN	PAO1_00976	purN
purN	PAO1_04454	purN
purN	PAO1_03676	purN
purN	PAO1_00018	purN
purN	PAO1_05602	purN
purN	UW101_01189	purN
purN	UW101_02437	purN
purN	UW101_00150	purN
purN	UW101_04219	purN
purN	V4-33_00174	purN
purN	V4-33_01924	purN
purN	W5C_01648	purN
purN	W5C_02745	purN
purN	W5C_01778	purN
purN	ZONMW-01_01002	purN
purN	ZONMW-01_01802	purN
purN	ZONMW-01_00557	purN
purN	ZONMW-02_01747	purN
purN	ZONMW-02_01849	purN
purN	ZONMW-02_00610	purN

purN	ZONMW-03_02071	purN
purN	ZONMW-03_02787	purN
purN	ZONMW-03_02451	purN
purN	ZONMW-04_01748	purN
purN	ZONMW-04_02736	purN
purN	ZONMW-04_02469	purN
purN	ZONMW-04_00932	purN
purN	ZONMW-05_02027	purN
purN	ZONMW-05_02286	purN
purN	ZONMW-05_00184	purN
purN	ZONMW-06_03877	purN
purN	ZONMW-06_02614	purN
purN	ZONMW-06_01315	purN
purN	ZONMW-06_02054	purN
purN	ZONMW-07_00760	purN
purN	ZONMW-07_01773	purN
purN	ZONMW-07_02490	purN
purN	ZONMW-07_03970	purN
purN	ZONMW-08_00512	purN
purN	ZONMW-08_01060	purN
purN	ZONMW-08_02080	purN
purN	ZONMW-09_00448	purN
purN	ZONMW-09_00791	purN
purN	ZONMW-09_01186	purN
purN	ZONMW-10_00232	purN
purN	ZONMW-10_02460	purN
purN	ZONMW-10_01628	purN
purN	ZONMW-11_01375	purN
purN	ZONMW-11_02200	purN
purN	ZONMW-11_02562	purN
purN	ZONMW-12_00034	purN
purN	ZONMW-12_00860	purN
purN	ZONMW-12_03781	purN
purN	ZONMW-13_00458	purN
purN	ZONMW-13_02881	purN
purN	ZONMW-13_01210	purN
purN	ZONMW-13_01897	purN
purN	ZONMW-14_00104	purN
purN	ZONMW-14_00795	purN
purN	ZONMW-15_01749	purN
purN	ZONMW-15_00559	purN
purN	ZONMW-15_02921	purN
purN	ZONMW-19_00015	purN
purN	ZONMW-19_03171	purN
purN	ZONMW-19_02664	purN
purN	ZONMW-20_03045	purN
purN	ZONMW-20_03547	purN
purN	ZONMW-20_03303	purN

purN	ZONMW-20_04245	purN
purN	ZONMW-21_00996	purN
purN	ZONMW-21_02590	purN
purN	ZONMW-21_02150	purN
purN	ZONMW-22_00997	purN
purN	ZONMW-22_02519	purN
purN	ZONMW-22_01575	purN
purN	ZONMW-23_02110	purN
purN	ZONMW-23_00031	purN
purN	ZONMW-23_00976	purN
purN	ZONMW-24_00464	purN
purN	ZONMW-24_02284	purN
purN	ZONMW-24_01599	purN
purN	ZONMW-25_01996	purN
purN	ZONMW-25_00778	purN
purN	ZONMW-25_00388	purN
purN	ZONMW-26_02373	purN
purN	ZONMW-26_02951	purN
purN	ZONMW-26_00773	purN
purN	ZONMW-27_01553	purN
purN	ZONMW-27_03278	purN
purN	ZONMW-27_01007	purN
purN	ZONMW-28_03820	purN
purN	ZONMW-28_00019	purN
purN	ZONMW-28_01727	purN
purN	ZONMW-28_00528	purN
purN	ZONMW-29_00571	purN
purN	ZONMW-29_01788	purN
purN	ZONMW-29_01352	purN
purN	ZONMW-30_01802	purN
purN	ZONMW-30_00333	purN
purN	ZONMW-30_02687	purN
purN	ZONMW-31_02598	purN
purN	ZONMW-31_02687	purN
purN	ZONMW-31_00014	purN
purN	ZONMW-31_01480	purN
purN	ZONMW-32_03758	purN
purN	ZONMW-32_00810	purN
purN	ZONMW-32_02245	purN
purN	ZONMW-32_01319	purN
purN	ZONMW-33_03789	purN
purN	ZONMW-33_00800	purN
purN	ZONMW-33_02236	purN
purN	ZONMW-33_01308	purN
purN	ZONMW-34_03687	purN
purN	ZONMW-34_02701	purN
purN	ZONMW-34_02390	purN
purN	ZONMW-34_01500	purN

purN	ZONMW-34_00847	purN
purN	ZONMW-35_00012	purN
purN	ZONMW-35_00078	purN
purN	ZONMW-35_01859	purN
purN	ZONMW-35_00573	purN
purN	ZONMW-35_02616	purN
purN	ZONMW-36_03738	purN
purN	ZONMW-36_00019	purN
purN	ZONMW-36_02000	purN
purN	ZONMW-36_00528	purN
purN	ZONMW-37_01366	purN
purN	ZONMW-37_02257	purN
purN	ZONMW-37_01598	purN
purN	ZONMW-38_00750	purN
purN	ZONMW-38_03232	purN
purN	ZONMW-38_03264	purN
purN	ZONMW-39_03553	purN
purN	ZONMW-39_03495	purN
purN	ZONMW-39_01682	purN
purN	ZONMW-39_00198	purN
purN	ZONMW-40_00262	purN
purN	ZONMW-40_01814	purN
purN	ZONMW-40_00487	purN
purN	ZONMW-41_02867	purN
purN	ZONMW-41_02804	purN
purN	ZONMW-41_00413	purN
purN	ZONMW-42_03209	purN
purN	ZONMW-42_02569	purN
purN	ZONMW-42_02808	purN
purN	ZONMW-42_03979	purN
purN	ZONMW-43_02555	purN
purN	ZONMW-43_02483	purN
purN	ZONMW-43_01842	purN
purN	ZONMW-44_03849	purN
purN	ZONMW-44_03911	purN
purN	ZONMW-44_02421	purN
purN	ZONMW-44_01389	purN
purN	ZONMW-45_03034	purN
purN	ZONMW-45_00915	purN
purN	ZONMW-46_01586	purN
purN	ZONMW-46_00959	purN
purN	ZONMW-47_01266	purN
purN	ZONMW-47_01214	purN
purN	ZONMW-48_00136	purN
purN	ZONMW-48_02242	purN
purN	ZONMW-48_01673	purN
purN	ZONMW-49_02665	purN
purN	ZONMW-49_01888	purN

purN	ZONMW-49_02976	purN
purN	ZONMW-50_02459	purN
purN	ZONMW-50_03069	purN
purN	ZONMW-50_02879	purN
purN	ZONMW-51_02613	purN
purN	ZONMW-51_02171	purN
purN	ZONMW-51_01995	purN
purN	ZONMW-52_02106	purN
purN	ZONMW-52_02600	purN
purN	ZONMW-52_00777	purN
purN	ZONMW-53_02733	purN
purN	ZONMW-53_02352	purN
purN	ZONMW-53_01511	purN
purN	ZONMW-54_02876	purN
purN	ZONMW-54_02393	purN
purN	ZONMW-54_01212	purN
purN	ZONMW-55_02733	purN
purN	ZONMW-55_01801	purN
purN	ZONMW-55_02317	purN
purN	ZONMW-56_02462	purN
purN	ZONMW-56_01949	purN
purN	ZONMW-56_02039	purN
purN	ZONMW-57_02637	purN
purN	ZONMW-57_02185	purN
purN	ZONMW-57_00177	purN
purN	ZONMW-58_02662	purN
purN	ZONMW-58_02209	purN
purN	ZONMW-58_00794	purN
purN	ZONMW-59_02670	purN
purN	ZONMW-59_02428	purN
purN	ZONMW-59_00170	purN
purN	ZONMW-60_02724	purN
purN	ZONMW-60_02273	purN
purN	ZONMW-60_00203	purN
purN	ZONMW-61_00082	purN
purN	ZONMW-61_01015	purN
purN	ZONMW-61_02439	purN
purN	ZONMW-62_01637	purN
purN	ZONMW-62_02829	purN
purN	ZONMW-62_00685	purN
purN	ZONMW-63_02183	purN
purN	ZONMW-63_03081	purN
purN	ZONMW-63_02575	purN
purN	ZONMW-64_03736	purN
purN	ZONMW-64_02460	purN
purN	ZONMW-64_01637	purN
purN	ZONMW-64_02844	purN
purN	ZONMW-65_00560	purN

purN	ZONMW-65_01499	purN
purN	ZONMW-65_02389	purN
purN	ZONMW-65_01182	purN
purN	ZONMW-66_01284	purN
purN	ZONMW-66_02330	purN
purN	ZONMW-66_03070	purN
purN	ZONMW-67_01769	purN
purN	ZONMW-67_01845	purN
purN	ZONMW-67_00118	purN
purN	ZONMW-67_01449	purN
purN	ZONMW-68_00551	purN
purN	ZONMW-68_04889	purN
purN	ZONMW-68_04816	purN
purN	ZONMW-69_02174	purN
purN	ZONMW-69_02098	purN
purN	ZONMW-69_02727	purN
purN	ZONMW-69_01551	purN
purN	ZONMW-72_00358	purN
purN	ZONMW-72_02480	purN
purN	ZONMW-72_01406	purN
purN	ZONMW-73_00161	purN
purN	ZONMW-73_03380	purN
purN	ZONMW-73_00499	purN
purN	ZONMW-74_01652	purN
purN	ZONMW-74_02318	purN
purN	ZONMW-74_00472	purN
purN	ZONMW-75_02247	purN
purN	ZONMW-75_00815	purN
purN	ZONMW-75_01810	purN
purN	da5A-8_02416	purN
purN	da5A-8_03154	purN
purN	da5A-8_00270	purN
purA	18_02210	purA
purA	ACAM48_01340	purA
purA	CAM030_01588	purA
purA	CECT7946_03196	purA
purA	CGMCC1.8863_01131	purA
purA	CL8139_01139	purA
purA	DAU203_00285	purA
purA	DD5b_00720	purA
purA	DSM1208_03524	purA
purA	DSM12212_02447	purA
purA	DSM15343_01909	purA
purA	DSM15361_01545	purA
purA	DSM15361_00790	purA
purA	DSM15365_00073	purA
purA	DSM15718_01152	purA
purA	DSM16505T_01381	purA

purA	DSM18103_00917	purA
purA	DSM18293_03663	purA
purA	DSM18841_02394	purA
purA	DSM18842_01308	purA
purA	DSM19866_04618	purA
purA	DSM2041_05494	purA
purA	DSM2061_01539	purA
purA	DSM2064_03469	purA
purA	DSM2064_01736	purA
purA	DSM21164_03098	purA
purA	DSM23404_01638	purA
purA	DSM23424_01878	purA
purA	DSM24786_01086	purA
purA	DSM7489_00279	purA
purA	DSW1_02496	purA
purA	DSW5_02186	purA
purA	DSW6_03295	purA
purA	DSW8_00983	purA
purA	F52_00205	purA
purA	GCA_001027945.1_01971	purA
purA	HI1_00211	purA
purA	IR1_04409	purA
purA	IR1_04105	purA
purA	KCOM3131_00358	purA
purA	KCTC12974_01831	purA
purA	KL-A_01275	purA
purA	L12_00280	purA
purA	LL0412.1.7_02029	purA
purA	MAR_2009_71_02458	purA
purA	NN016038_02226	purA
purA	PAO1_05107	purA
purA	UW101_02331	purA
purA	UW101_00790	purA
purA	V4-33_01813	purA
purA	W5C_00188	purA
purA	ZONMW-01_02163	purA
purA	ZONMW-02_00917	purA
purA	ZONMW-03_02547	purA
purA	ZONMW-04_02008	purA
purA	ZONMW-05_02361	purA
purA	ZONMW-06_00932	purA
purA	ZONMW-07_01747	purA
purA	ZONMW-08_03457	purA
purA	ZONMW-09_02070	purA
purA	ZONMW-10_01015	purA
purA	ZONMW-11_02022	purA
purA	ZONMW-12_01771	purA
purA	ZONMW-13_02474	purA

purA	ZONMW-14_02529	purA
purA	ZONMW-15_02674	purA
purA	ZONMW-19_00307	purA
purA	ZONMW-20_03158	purA
purA	ZONMW-21_02741	purA
purA	ZONMW-22_02368	purA
purA	ZONMW-23_00182	purA
purA	ZONMW-24_02133	purA
purA	ZONMW-25_00659	purA
purA	ZONMW-26_02755	purA
purA	ZONMW-27_02179	purA
purA	ZONMW-28_00592	purA
purA	ZONMW-28_00894	purA
purA	ZONMW-29_01905	purA
purA	ZONMW-30_02857	purA
purA	ZONMW-31_01189	purA
purA	ZONMW-32_00500	purA
purA	ZONMW-32_00197	purA
purA	ZONMW-33_00368	purA
purA	ZONMW-33_00064	purA
purA	ZONMW-34_00744	purA
purA	ZONMW-35_02718	purA
purA	ZONMW-36_01003	purA
purA	ZONMW-36_01305	purA
purA	ZONMW-37_02408	purA
purA	ZONMW-38_02805	purA
purA	ZONMW-39_01208	purA
purA	ZONMW-40_01696	purA
purA	ZONMW-41_01057	purA
purA	ZONMW-42_01695	purA
purA	ZONMW-43_00283	purA
purA	ZONMW-44_02129	purA
purA	ZONMW-45_01807	purA
purA	ZONMW-46_01907	purA
purA	ZONMW-47_02154	purA
purA	ZONMW-48_02851	purA
purA	ZONMW-49_03223	purA
purA	ZONMW-50_03211	purA
purA	ZONMW-51_02374	purA
purA	ZONMW-52_02358	purA
purA	ZONMW-53_02275	purA
purA	ZONMW-54_02765	purA
purA	ZONMW-55_02465	purA
purA	ZONMW-56_01763	purA
purA	ZONMW-57_02110	purA
purA	ZONMW-58_01968	purA
purA	ZONMW-59_02187	purA
purA	ZONMW-60_02484	purA

purA	ZONMW-61_03626	purA
purA	ZONMW-62_01080	purA
purA	ZONMW-63_01573	purA
purA	ZONMW-64_00300	purA
purA	ZONMW-65_01286	purA
purA	ZONMW-66_01058	purA
purA	ZONMW-67_00967	purA
purA	ZONMW-68_01134	purA
purA	ZONMW-69_01014	purA
purA	ZONMW-72_03182	purA
purA	ZONMW-72_02746	purA
purA	ZONMW-73_00746	purA
purA	ZONMW-74_02543	purA
purA	ZONMW-75_01504	purA
purA	da5A-8_01370	purA
trmD	18_03614	trmD
trmD	ACAM48_01427	trmD
trmD	CAM030_01804	trmD
trmD	CECT7946_03503	trmD
trmD	CGMCC1.8863_02971	trmD
trmD	CL8139_01253	trmD
trmD	DAU203_00012	trmD
trmD	DD5b_00141	trmD
trmD	DSM1208_00012	trmD
trmD	DSM12212_01939	trmD
trmD	DSM15343_00343	trmD
trmD	DSM15361_01874	trmD
trmD	DSM15365_00102	trmD
trmD	DSM15718_03045	trmD
trmD	DSM16505T_01918	trmD
trmD	DSM18103_00552	trmD
trmD	DSM18293_03622	trmD
trmD	DSM18841_02016	trmD
trmD	DSM18842_02329	trmD
trmD	DSM19866_04724	trmD
trmD	DSM2041_04798	trmD
trmD	DSM2061_00542	trmD
trmD	DSM2064_03506	trmD
trmD	DSM21164_02828	trmD
trmD	DSM23404_01712	trmD
trmD	DSM23424_01736	trmD
trmD	DSM24786_01006	trmD
trmD	DSM7489_00012	trmD
trmD	DSW1_02556	trmD
trmD	DSW5_02748	trmD
trmD	DSW6_00120	trmD
trmD	DSW8_00584	trmD
trmD	F52_00169	trmD

trmD	GCA_001027945.1_02853	trmD
trmD	HI1_03311	trmD
trmD	IR1_04439	trmD
trmD	KCOM3131_00824	trmD
trmD	KCTC12974_02238	trmD
trmD	KL-A_01827	trmD
trmD	L12_00508	trmD
trmD	LL0412.1.7_01982	trmD
trmD	MAR_2009_71_03381	trmD
trmD	NN016038_03654	trmD
trmD	PAO1_03866	trmD
trmD	UW101_02294	trmD
trmD	V4-33_01784	trmD
trmD	W5C_00859	trmD
trmD	ZONMW-01_03103	trmD
trmD	ZONMW-02_00816	trmD
trmD	ZONMW-03_02338	trmD
trmD	ZONMW-04_00802	trmD
trmD	ZONMW-05_00072	trmD
trmD	ZONMW-06_02898	trmD
trmD	ZONMW-07_03416	trmD
trmD	ZONMW-08_02546	trmD
trmD	ZONMW-09_02198	trmD
trmD	ZONMW-10_01849	trmD
trmD	ZONMW-11_00383	trmD
trmD	ZONMW-12_03631	trmD
trmD	ZONMW-13_02344	trmD
trmD	ZONMW-14_02637	trmD
trmD	ZONMW-15_02972	trmD
trmD	ZONMW-19_00410	trmD
trmD	ZONMW-20_02723	trmD
trmD	ZONMW-21_01630	trmD
trmD	ZONMW-22_02094	trmD
trmD	ZONMW-23_00457	trmD
trmD	ZONMW-24_02719	trmD
trmD	ZONMW-25_01423	trmD
trmD	ZONMW-26_00888	trmD
trmD	ZONMW-27_00899	trmD
trmD	ZONMW-28_02008	trmD
trmD	ZONMW-29_03160	trmD
trmD	ZONMW-30_02171	trmD
trmD	ZONMW-31_03006	trmD
trmD	ZONMW-32_02414	trmD
trmD	ZONMW-33_02704	trmD
trmD	ZONMW-34_01998	trmD
trmD	ZONMW-35_01690	trmD
trmD	ZONMW-36_02548	trmD
trmD	ZONMW-37_02008	trmD

trmD	ZONMW-38_00805	trmD
trmD	ZONMW-39_02194	trmD
trmD	ZONMW-40_02461	trmD
trmD	ZONMW-41_01309	trmD
trmD	ZONMW-42_00421	trmD
trmD	ZONMW-43_01502	trmD
trmD	ZONMW-44_00876	trmD
trmD	ZONMW-45_00053	trmD
trmD	ZONMW-46_00268	trmD
trmD	ZONMW-47_00237	trmD
trmD	ZONMW-48_03091	trmD
trmD	ZONMW-49_03065	trmD
trmD	ZONMW-50_02967	trmD
trmD	ZONMW-51_01895	trmD
trmD	ZONMW-52_00878	trmD
trmD	ZONMW-53_01621	trmD
trmD	ZONMW-54_01104	trmD
trmD	ZONMW-55_02217	trmD
trmD	ZONMW-56_02151	trmD
trmD	ZONMW-57_00069	trmD
trmD	ZONMW-58_00897	trmD
trmD	ZONMW-59_00069	trmD
trmD	ZONMW-60_00070	trmD
trmD	ZONMW-61_02068	trmD
trmD	ZONMW-62_00178	trmD
trmD	ZONMW-63_01833	trmD
trmD	ZONMW-64_02079	trmD
trmD	ZONMW-65_01172	trmD
trmD	ZONMW-66_02237	trmD
trmD	ZONMW-67_02397	trmD
trmD	ZONMW-68_01029	trmD
trmD	ZONMW-69_02445	trmD
trmD	ZONMW-72_03152	trmD
trmD	ZONMW-73_00772	trmD
trmD	ZONMW-74_00593	trmD
trmD	ZONMW-75_01478	trmD
trmD	da5A-8_02199	trmD
thrS	18_02059	thrS
thrS	ACAM48_01439	thrS
thrS	CAM030_01795	thrS
thrS	CECT7946_02772	thrS
thrS	CGMCC1.8863_02818	thrS
thrS	CL8139_03221	thrS
thrS	DAU203_02556	thrS
thrS	DD5b_01124	thrS
thrS	DSM1208_02930	thrS
thrS	DSM12212_03452	thrS
thrS	DSM15343_02866	thrS

thrS	DSM15361_02238	thrS
thrS	DSM15365_01298	thrS
thrS	DSM15718_03367	thrS
thrS	DSM16505T_00001	thrS
thrS	DSM18103_01546	thrS
thrS	DSM18293_02793	thrS
thrS	DSM18841_02472	thrS
thrS	DSM18842_02379	thrS
thrS	DSM19866_02699	thrS
thrS	DSM2041_05502	thrS
thrS	DSM2061_04413	thrS
thrS	DSM2064_05072	thrS
thrS	DSM21164_03005	thrS
thrS	DSM23404_00001	thrS
thrS	DSM23424_03340	thrS
thrS	DSM24786_01470	thrS
thrS	DSM7489_02447	thrS
thrS	DSW1_00061	thrS
thrS	DSW5_01778	thrS
thrS	DSW6_00222	thrS
thrS	DSW8_01024	thrS
thrS	F52_01927	thrS
thrS	GCA_001027945.1_01089	thrS
thrS	HI1_02379	thrS
thrS	IR1_02713	thrS
thrS	KCOM3131_01693	thrS
thrS	KCTC12974_00683	thrS
thrS	KL-A_03251	thrS
thrS	L12_00025	thrS
thrS	LL0412.1.7_00001	thrS
thrS	MAR_2009_71_02417	thrS
thrS	NN016038_02077	thrS
thrS	PAO1_02827	thrS
thrS	UW101_00030	thrS
thrS	V4-33_00810	thrS
thrS	W5C_02093	thrS
thrS	ZONMW-01_03266	thrS
thrS	ZONMW-02_00853	thrS
thrS	ZONMW-03_03396	thrS
thrS	ZONMW-04_00001	thrS
thrS	ZONMW-05_01827	thrS
thrS	ZONMW-06_03482	thrS
thrS	ZONMW-07_03775	thrS
thrS	ZONMW-08_00820	thrS
thrS	ZONMW-09_01788	thrS
thrS	ZONMW-10_01432	thrS
thrS	ZONMW-11_00951	thrS
thrS	ZONMW-12_04456	thrS

thrS	ZONMW-13_03140	thrS
thrS	ZONMW-14_01778	thrS
thrS	ZONMW-15_02036	thrS
thrS	ZONMW-19_02927	thrS
thrS	ZONMW-20_01640	thrS
thrS	ZONMW-21_03393	thrS
thrS	ZONMW-22_03332	thrS
thrS	ZONMW-23_03439	thrS
thrS	ZONMW-24_03441	thrS
thrS	ZONMW-25_03129	thrS
thrS	ZONMW-26_01460	thrS
thrS	ZONMW-27_00001	thrS
thrS	ZONMW-28_02944	thrS
thrS	ZONMW-28_02302	thrS
thrS	ZONMW-29_02905	thrS
thrS	ZONMW-30_02868	thrS
thrS	ZONMW-31_02432	thrS
thrS	ZONMW-32_01428	thrS
thrS	ZONMW-32_02708	thrS
thrS	ZONMW-33_01418	thrS
thrS	ZONMW-33_02410	thrS
thrS	ZONMW-34_01732	thrS
thrS	ZONMW-35_01423	thrS
thrS	ZONMW-36_00637	thrS
thrS	ZONMW-36_03841	thrS
thrS	ZONMW-37_03341	thrS
thrS	ZONMW-38_00217	thrS
thrS	ZONMW-38_01985	thrS
thrS	ZONMW-39_03752	thrS
thrS	ZONMW-40_03465	thrS
thrS	ZONMW-41_01490	thrS
thrS	ZONMW-42_00715	thrS
thrS	ZONMW-43_03348	thrS
thrS	ZONMW-44_02628	thrS
thrS	ZONMW-45_02683	thrS
thrS	ZONMW-46_02546	thrS
thrS	ZONMW-47_02536	thrS
thrS	ZONMW-48_03339	thrS
thrS	ZONMW-49_00485	thrS
thrS	ZONMW-50_00486	thrS
thrS	ZONMW-51_01219	thrS
thrS	ZONMW-52_02913	thrS
thrS	ZONMW-53_00001	thrS
thrS	ZONMW-54_01035	thrS
thrS	ZONMW-55_00001	thrS
thrS	ZONMW-56_02809	thrS
thrS	ZONMW-57_01617	thrS
thrS	ZONMW-58_00967	thrS

thrS	ZONMW-59_02719	thrS
thrS	ZONMW-60_03257	thrS
thrS	ZONMW-61_04010	thrS
thrS	ZONMW-62_02592	thrS
thrS	ZONMW-63_03290	thrS
thrS	ZONMW-64_01824	thrS
thrS	ZONMW-65_03080	thrS
thrS	ZONMW-66_00860	thrS
thrS	ZONMW-67_03221	thrS
thrS	ZONMW-68_02499	thrS
thrS	ZONMW-69_03222	thrS
thrS	ZONMW-72_01280	thrS
thrS	ZONMW-73_02291	thrS
thrS	ZONMW-74_00692	thrS
thrS	ZONMW-75_00938	thrS
thrS	da5A-8_00207	thrS
radA	18_00731	radA
radA	ACAM48_02606	radA
radA	CAM030_01187	radA
radA	CECT7946_00355	radA
radA	CGMCC1.8863_02218	radA
radA	CL8139_01560	radA
radA	DAU203_01158	radA
radA	DD5b_02298	radA
radA	DSM1208_01369	radA
radA	DSM12212_00290	radA
radA	DSM15343_00925	radA
radA	DSM15361_00301	radA
radA	DSM15365_00990	radA
radA	DSM15718_02449	radA
radA	DSM16505T_00338	radA
radA	DSM18103_03473	radA
radA	DSM18293_03248	radA
radA	DSM18841_00146	radA
radA	DSM18842_01059	radA
radA	DSM19866_03558	radA
radA	DSM2041_00537	radA
radA	DSM2061_02891	radA
radA	DSM2064_01815	radA
radA	DSM21164_01119	radA
radA	DSM23404_02349	radA
radA	DSM23424_00128	radA
radA	DSM24786_02637	radA
radA	DSM7489_01075	radA
radA	DSW1_00410	radA
radA	DSW5_01325	radA
radA	DSW6_01180	radA
radA	DSW8_02566	radA

radA	F52_02252	radA
radA	GCA_001027945.1_00449	radA
radA	HI1_01014	radA
radA	IR1_04016	radA
radA	KCOM3131_01213	radA
radA	KCTC12974_01976	radA
radA	KL-A_02021	radA
radA	L12_00109	radA
radA	LL0412.1.7_00637	radA
radA	MAR_2009_71_00886	radA
radA	NN016038_00750	radA
radA	PAO1_04759	radA
radA	UW101_00869	radA
radA	V4-33_00089	radA
radA	W5C_01921	radA
radA	ZONMW-01_01190	radA
radA	ZONMW-02_01709	radA
radA	ZONMW-03_02259	radA
radA	ZONMW-04_01554	radA
radA	ZONMW-05_01835	radA
radA	ZONMW-06_03933	radA
radA	ZONMW-07_03134	radA
radA	ZONMW-08_00406	radA
radA	ZONMW-09_00519	radA
radA	ZONMW-10_00516	radA
radA	ZONMW-11_04411	radA
radA	ZONMW-12_00436	radA
radA	ZONMW-13_00703	radA
radA	ZONMW-14_01341	radA
radA	ZONMW-15_00830	radA
radA	ZONMW-19_01255	radA
radA	ZONMW-20_01068	radA
radA	ZONMW-21_01125	radA
radA	ZONMW-22_01127	radA
radA	ZONMW-23_02239	radA
radA	ZONMW-24_00334	radA
radA	ZONMW-25_02116	radA
radA	ZONMW-26_02558	radA
radA	ZONMW-27_01741	radA
radA	ZONMW-28_03909	radA
radA	ZONMW-29_00720	radA
radA	ZONMW-30_00411	radA
radA	ZONMW-31_03193	radA
radA	ZONMW-32_03977	radA
radA	ZONMW-33_03901	radA
radA	ZONMW-34_03806	radA
radA	ZONMW-35_03733	radA
radA	ZONMW-36_03951	radA

radA	ZONMW-37_01237	radA
radA	ZONMW-38_01941	radA
radA	ZONMW-39_04208	radA
radA	ZONMW-40_00143	radA
radA	ZONMW-41_02422	radA
radA	ZONMW-42_02334	radA
radA	ZONMW-43_03760	radA
radA	ZONMW-44_03163	radA
radA	ZONMW-45_03063	radA
radA	ZONMW-46_01557	radA
radA	ZONMW-47_01295	radA
radA	ZONMW-48_00244	radA
radA	ZONMW-49_02823	radA
radA	ZONMW-50_02833	radA
radA	ZONMW-51_02952	radA
radA	ZONMW-52_02921	radA
radA	ZONMW-53_02929	radA
radA	ZONMW-54_02932	radA
radA	ZONMW-55_02936	radA
radA	ZONMW-56_02817	radA
radA	ZONMW-57_02925	radA
radA	ZONMW-58_02949	radA
radA	ZONMW-59_03024	radA
radA	ZONMW-60_03043	radA
radA	ZONMW-61_01789	radA
radA	ZONMW-62_01530	radA
radA	ZONMW-63_02785	radA
radA	ZONMW-64_04129	radA
radA	ZONMW-65_01773	radA
radA	ZONMW-66_02275	radA
radA	ZONMW-67_03584	radA
radA	ZONMW-68_00679	radA
radA	ZONMW-69_03586	radA
radA	ZONMW-72_02330	radA
radA	ZONMW-73_00245	radA
radA	ZONMW-74_02115	radA
radA	ZONMW-75_00506	radA
radA	da5A-8_02450	radA
pheS	18_00813	pheS
pheS	ACAM48_02249	pheS
pheS	CAM030_02913	pheS
pheS	CECT7946_01881	pheS
pheS	CGMCC1.8863_02145	pheS
pheS	CL8139_01655	pheS
pheS	DAU203_01262	pheS
pheS	DD5b_02274	pheS
pheS	DSM1208_01463	pheS
pheS	DSM12212_02608	pheS

pheS	DSM15343_02534	pheS
pheS	DSM15361_00318	pheS
pheS	DSM15365_03194	pheS
pheS	DSM15718_02714	pheS
pheS	DSM16505T_00347	pheS
pheS	DSM18103_03482	pheS
pheS	DSM18293_00777	pheS
pheS	DSM18841_00156	pheS
pheS	DSM18842_01049	pheS
pheS	DSM19866_03465	pheS
pheS	DSM2041_00065	pheS
pheS	DSM2061_02986	pheS
pheS	DSM2064_01754	pheS
pheS	DSM21164_01024	pheS
pheS	DSM23404_02971	pheS
pheS	DSM23424_00484	pheS
pheS	DSM24786_02705	pheS
pheS	DSM7489_01171	pheS
pheS	DSW1_00774	pheS
pheS	DSW5_01227	pheS
pheS	DSW6_01657	pheS
pheS	DSW8_02481	pheS
pheS	F52_02203	pheS
pheS	GCA_001027945.1_00439	pheS
pheS	HI1_01113	pheS
pheS	IR1_04090	pheS
pheS	KCOM3131_00546	pheS
pheS	KCTC12974_01875	pheS
pheS	KL-A_02998	pheS
pheS	L12_00029	pheS
pheS	LL0412.1.7_00626	pheS
pheS	MAR_2009_71_00972	pheS
pheS	NN016038_00833	pheS
pheS	PAO1_02823	pheS
pheS	UW101_00808	pheS
pheS	V4-33_00107	pheS
pheS	W5C_02012	pheS
pheS	ZONMW-01_01095	pheS
pheS	ZONMW-02_00097	pheS
pheS	ZONMW-03_02164	pheS
pheS	ZONMW-04_01654	pheS
pheS	ZONMW-05_01932	pheS
pheS	ZONMW-06_03486	pheS
pheS	ZONMW-07_00745	pheS
pheS	ZONMW-08_00491	pheS
pheS	ZONMW-09_00290	pheS
pheS	ZONMW-10_00285	pheS
pheS	ZONMW-11_02770	pheS

pheS	ZONMW-12_03364	pheS
pheS	ZONMW-13_00499	pheS
pheS	ZONMW-14_00063	pheS
pheS	ZONMW-15_01767	pheS
pheS	ZONMW-19_02931	pheS
pheS	ZONMW-20_01636	pheS
pheS	ZONMW-21_01015	pheS
pheS	ZONMW-22_01016	pheS
pheS	ZONMW-23_02129	pheS
pheS	ZONMW-24_00445	pheS
pheS	ZONMW-25_02020	pheS
pheS	ZONMW-26_02464	pheS
pheS	ZONMW-27_01644	pheS
pheS	ZONMW-28_02306	pheS
pheS	ZONMW-29_00587	pheS
pheS	ZONMW-30_01777	pheS
pheS	ZONMW-31_02428	pheS
pheS	ZONMW-32_02712	pheS
pheS	ZONMW-33_02406	pheS
pheS	ZONMW-34_01728	pheS
pheS	ZONMW-35_01419	pheS
pheS	ZONMW-36_03837	pheS
pheS	ZONMW-37_01347	pheS
pheS	ZONMW-38_00200	pheS
pheS	ZONMW-39_03756	pheS
pheS	ZONMW-40_00242	pheS
pheS	ZONMW-41_03049	pheS
pheS	ZONMW-42_00719	pheS
pheS	ZONMW-43_03344	pheS
pheS	ZONMW-44_02624	pheS
pheS	ZONMW-45_00292	pheS
pheS	ZONMW-46_01547	pheS
pheS	ZONMW-47_01305	pheS
pheS	ZONMW-48_00154	pheS
pheS	ZONMW-49_03098	pheS
pheS	ZONMW-50_03094	pheS
pheS	ZONMW-51_03153	pheS
pheS	ZONMW-52_03112	pheS
pheS	ZONMW-53_03174	pheS
pheS	ZONMW-54_03124	pheS
pheS	ZONMW-55_03173	pheS
pheS	ZONMW-56_03132	pheS
pheS	ZONMW-57_03137	pheS
pheS	ZONMW-58_03210	pheS
pheS	ZONMW-59_03267	pheS
pheS	ZONMW-60_03148	pheS
pheS	ZONMW-61_00063	pheS
pheS	ZONMW-62_01616	pheS

pheS	ZONMW-63_03184	pheS
pheS	ZONMW-64_01820	pheS
pheS	ZONMW-65_03098	pheS
pheS	ZONMW-66_00856	pheS
pheS	ZONMW-67_03217	pheS
pheS	ZONMW-68_00586	pheS
pheS	ZONMW-69_03218	pheS
pheS	ZONMW-72_02761	pheS
pheS	ZONMW-73_01815	pheS
pheS	ZONMW-74_02198	pheS
pheS	ZONMW-75_00985	pheS
pheS	da5A-8_02459	pheS
oprF_1	18_00097	oprF_1
oprF_1	18_00840	oprF_1
oprF_1	18_00735	oprF_1
oprF_1	18_03176	oprF_1
oprF_1	18_00230	oprF_1
oprF_1	18_03845	oprF_1
oprF_1	18_03822	oprF_1
oprF_1	18_03079	oprF_1
oprF_1	18_02510	oprF_1
oprF_1	18_01878	oprF_1
oprF_1	18_02642	oprF_1
oprF_1	18_01140	oprF_1
oprF_1	18_01599	oprF_1
oprF_1	18_00329	oprF_1
oprF_1	18_02808	oprF_1
oprF_1	18_03747	oprF_1
oprF_1	18_01196	oprF_1
oprF_1	18_00792	oprF_1
oprF_1	18_03434	oprF_1
oprF_1	18_03112	oprF_1
oprF_1	18_03121	oprF_1
oprF_1	ACAM48_02296	oprF_1
oprF_1	ACAM48_01986	oprF_1
oprF_1	ACAM48_00838	oprF_1
oprF_1	ACAM48_00282	oprF_1
oprF_1	ACAM48_03108	oprF_1
oprF_1	ACAM48_00998	oprF_1
oprF_1	ACAM48_03151	oprF_1
oprF_1	ACAM48_02601	oprF_1
oprF_1	ACAM48_00725	oprF_1
oprF_1	CAM030_00715	oprF_1
oprF_1	CAM030_01222	oprF_1
oprF_1	CAM030_01161	oprF_1
oprF_1	CAM030_02075	oprF_1
oprF_1	CAM030_01177	oprF_1
oprF_1	CAM030_01211	oprF_1

oprF_1	CAM030_00806	oprF_1
oprF_1	CAM030_00162	oprF_1
oprF_1	CAM030_00655	oprF_1
oprF_1	CAM030_02071	oprF_1
oprF_1	CAM030_00003	oprF_1
oprF_1	CAM030_00517	oprF_1
oprF_1	CAM030_02891	oprF_1
oprF_1	CAM030_01272	oprF_1
oprF_1	CAM030_01857	oprF_1
oprF_1	CAM030_01063	oprF_1
oprF_1	CAM030_01561	oprF_1
oprF_1	CECT7946_00030	oprF_1
oprF_1	CECT7946_00018	oprF_1
oprF_1	CECT7946_01088	oprF_1
oprF_1	CECT7946_00302	oprF_1
oprF_1	CECT7946_03240	oprF_1
oprF_1	CECT7946_03700	oprF_1
oprF_1	CECT7946_03517	oprF_1
oprF_1	CECT7946_00001	oprF_1
oprF_1	CECT7946_01069	oprF_1
oprF_1	CECT7946_02955	oprF_1
oprF_1	CECT7946_00591	oprF_1
oprF_1	CECT7946_01907	oprF_1
oprF_1	CECT7946_00433	oprF_1
oprF_1	CECT7946_00105	oprF_1
oprF_1	CECT7946_03435	oprF_1
oprF_1	CECT7946_01825	oprF_1
oprF_1	CECT7946_00821	oprF_1
oprF_1	CECT7946_02907	oprF_1
oprF_1	CGMCC1.8863_02124	oprF_1
oprF_1	CGMCC1.8863_02681	oprF_1
oprF_1	CGMCC1.8863_00781	oprF_1
oprF_1	CGMCC1.8863_03208	oprF_1
oprF_1	CGMCC1.8863_00586	oprF_1
oprF_1	CGMCC1.8863_02259	oprF_1
oprF_1	CGMCC1.8863_00666	oprF_1
oprF_1	CGMCC1.8863_02900	oprF_1
oprF_1	CGMCC1.8863_02267	oprF_1
oprF_1	CGMCC1.8863_02440	oprF_1
oprF_1	CGMCC1.8863_02436	oprF_1
oprF_1	CGMCC1.8863_00334	oprF_1
oprF_1	CL8139_01876	oprF_1
oprF_1	CL8139_01706	oprF_1
oprF_1	CL8139_02274	oprF_1
oprF_1	CL8139_02615	oprF_1
oprF_1	CL8139_01739	oprF_1
oprF_1	CL8139_01055	oprF_1
oprF_1	CL8139_02559	oprF_1

oprF_1	CL8139_01310	oprF_1
oprF_1	CL8139_00441	oprF_1
oprF_1	CL8139_00250	oprF_1
oprF_1	CL8139_00077	oprF_1
oprF_1	CL8139_03228	oprF_1
oprF_1	CL8139_02799	oprF_1
oprF_1	CL8139_00853	oprF_1
oprF_1	CL8139_01108	oprF_1
oprF_1	CL8139_01633	oprF_1
oprF_1	CL8139_02001	oprF_1
oprF_1	CL8139_00765	oprF_1
oprF_1	DAU203_01518	oprF_1
oprF_1	DAU203_01311	oprF_1
oprF_1	DAU203_01354	oprF_1
oprF_1	DAU203_02343	oprF_1
oprF_1	DAU203_00086	oprF_1
oprF_1	DAU203_00090	oprF_1
oprF_1	DAU203_01833	oprF_1
oprF_1	DAU203_01455	oprF_1
oprF_1	DAU203_03443	oprF_1
oprF_1	DAU203_02588	oprF_1
oprF_1	DAU203_00539	oprF_1
oprF_1	DAU203_03329	oprF_1
oprF_1	DAU203_02026	oprF_1
oprF_1	DAU203_02785	oprF_1
oprF_1	DAU203_02894	oprF_1
oprF_1	DAU203_01891	oprF_1
oprF_1	DAU203_01240	oprF_1
oprF_1	DAU203_02942	oprF_1
oprF_1	DAU203_02951	oprF_1
oprF_1	DAU203_01404	oprF_1
oprF_1	DD5b_00434	oprF_1
oprF_1	DD5b_01083	oprF_1
oprF_1	DD5b_00078	oprF_1
oprF_1	DD5b_01094	oprF_1
oprF_1	DD5b_01032	oprF_1
oprF_1	DD5b_01058	oprF_1
oprF_1	DD5b_01834	oprF_1
oprF_1	DD5b_01994	oprF_1
oprF_1	DD5b_01964	oprF_1
oprF_1	DD5b_01963	oprF_1
oprF_1	DD5b_01059	oprF_1
oprF_1	DD5b_01118	oprF_1
oprF_1	DD5b_02753	oprF_1
oprF_1	DSM1208_02644	oprF_1
oprF_1	DSM1208_02356	oprF_1
oprF_1	DSM1208_01534	oprF_1
oprF_1	DSM1208_04222	oprF_1

oprF_1	DSM1208_02399	oprF_1
oprF_1	DSM1208_03503	oprF_1
oprF_1	DSM1208_03499	oprF_1
oprF_1	DSM1208_03892	oprF_1
oprF_1	DSM1208_01670	oprF_1
oprF_1	DSM1208_01438	oprF_1
oprF_1	DSM1208_02973	oprF_1
oprF_1	DSM1208_03600	oprF_1
oprF_1	DSM1208_02789	oprF_1
oprF_1	DSM1208_00610	oprF_1
oprF_1	DSM1208_01947	oprF_1
oprF_1	DSM1208_02322	oprF_1
oprF_1	DSM1208_04448	oprF_1
oprF_1	DSM1208_03926	oprF_1
oprF_1	DSM1208_00906	oprF_1
oprF_1	DSM1208_04370	oprF_1
oprF_1	DSM1208_04321	oprF_1
oprF_1	DSM1208_04017	oprF_1
oprF_1	DSM12212_02129	oprF_1
oprF_1	DSM12212_02794	oprF_1
oprF_1	DSM12212_00236	oprF_1
oprF_1	DSM12212_02072	oprF_1
oprF_1	DSM12212_00775	oprF_1
oprF_1	DSM12212_02285	oprF_1
oprF_1	DSM12212_00472	oprF_1
oprF_1	DSM12212_03176	oprF_1
oprF_1	DSM12212_00688	oprF_1
oprF_1	DSM12212_00939	oprF_1
oprF_1	DSM12212_00036	oprF_1
oprF_1	DSM12212_02673	oprF_1
oprF_1	DSM12212_03477	oprF_1
oprF_1	DSM15343_00862	oprF_1
oprF_1	DSM15343_01198	oprF_1
oprF_1	DSM15343_02800	oprF_1
oprF_1	DSM15343_01489	oprF_1
oprF_1	DSM15343_02437	oprF_1
oprF_1	DSM15343_01856	oprF_1
oprF_1	DSM15343_02924	oprF_1
oprF_1	DSM15343_02516	oprF_1
oprF_1	DSM15343_02517	oprF_1
oprF_1	DSM15343_01849	oprF_1
oprF_1	DSM15343_01143	oprF_1
oprF_1	DSM15343_00931	oprF_1
oprF_1	DSM15343_00410	oprF_1
oprF_1	DSM15343_00495	oprF_1
oprF_1	DSM15343_02064	oprF_1
oprF_1	DSM15361_00860	oprF_1
oprF_1	DSM15361_01480	oprF_1

oprF_1	DSM15361_02837	oprF_1
oprF_1	DSM15361_01474	oprF_1
oprF_1	DSM15361_00058	oprF_1
oprF_1	DSM15361_02512	oprF_1
oprF_1	DSM15361_02234	oprF_1
oprF_1	DSM15361_00066	oprF_1
oprF_1	DSM15361_00238	oprF_1
oprF_1	DSM15361_02208	oprF_1
oprF_1	DSM15361_00945	oprF_1
oprF_1	DSM15361_00057	oprF_1
oprF_1	DSM15365_01034	oprF_1
oprF_1	DSM15365_00260	oprF_1
oprF_1	DSM15365_00975	oprF_1
oprF_1	DSM15365_02336	oprF_1
oprF_1	DSM15365_00302	oprF_1
oprF_1	DSM15365_01646	oprF_1
oprF_1	DSM15365_01395	oprF_1
oprF_1	DSM15365_00767	oprF_1
oprF_1	DSM15365_01069	oprF_1
oprF_1	DSM15365_01647	oprF_1
oprF_1	DSM15365_01499	oprF_1
oprF_1	DSM15365_03251	oprF_1
oprF_1	DSM15365_03650	oprF_1
oprF_1	DSM15365_00892	oprF_1
oprF_1	DSM15365_03092	oprF_1
oprF_1	DSM15718_03348	oprF_1
oprF_1	DSM15718_00800	oprF_1
oprF_1	DSM15718_02534	oprF_1
oprF_1	DSM15718_02916	oprF_1
oprF_1	DSM15718_01539	oprF_1
oprF_1	DSM15718_02455	oprF_1
oprF_1	DSM15718_01226	oprF_1
oprF_1	DSM15718_02472	oprF_1
oprF_1	DSM15718_01411	oprF_1
oprF_1	DSM16505T_01783	oprF_1
oprF_1	DSM16505T_00150	oprF_1
oprF_1	DSM16505T_00715	oprF_1
oprF_1	DSM16505T_02691	oprF_1
oprF_1	DSM16505T_01285	oprF_1
oprF_1	DSM16505T_02128	oprF_1
oprF_1	DSM16505T_02127	oprF_1
oprF_1	DSM16505T_00724	oprF_1
oprF_1	DSM16505T_00869	oprF_1
oprF_1	DSM16505T_00515	oprF_1
oprF_1	DSM16505T_00481	oprF_1
oprF_1	DSM18103_02302	oprF_1
oprF_1	DSM18103_01179	oprF_1
oprF_1	DSM18103_03703	oprF_1

oprF_1	DSM18103_01363	oprF_1
oprF_1	DSM18103_00937	oprF_1
oprF_1	DSM18103_03640	oprF_1
oprF_1	DSM18103_00745	oprF_1
oprF_1	DSM18103_01916	oprF_1
oprF_1	DSM18103_02010	oprF_1
oprF_1	DSM18103_00810	oprF_1
oprF_1	DSM18103_02815	oprF_1
oprF_1	DSM18103_01186	oprF_1
oprF_1	DSM18103_01545	oprF_1
oprF_1	DSM18103_02865	oprF_1
oprF_1	DSM18103_01838	oprF_1
oprF_1	DSM18103_00459	oprF_1
oprF_1	DSM18293_02547	oprF_1
oprF_1	DSM18293_01004	oprF_1
oprF_1	DSM18293_02880	oprF_1
oprF_1	DSM18293_03208	oprF_1
oprF_1	DSM18293_03711	oprF_1
oprF_1	DSM18293_00831	oprF_1
oprF_1	DSM18293_04187	oprF_1
oprF_1	DSM18293_03129	oprF_1
oprF_1	DSM18293_01936	oprF_1
oprF_1	DSM18293_04127	oprF_1
oprF_1	DSM18293_03491	oprF_1
oprF_1	DSM18293_03035	oprF_1
oprF_1	DSM18293_03245	oprF_1
oprF_1	DSM18293_01853	oprF_1
oprF_1	DSM18293_02931	oprF_1
oprF_1	DSM18293_03759	oprF_1
oprF_1	DSM18293_01723	oprF_1
oprF_1	DSM18841_01515	oprF_1
oprF_1	DSM18841_00595	oprF_1
oprF_1	DSM18841_02281	oprF_1
oprF_1	DSM18841_00808	oprF_1
oprF_1	DSM18841_01490	oprF_1
oprF_1	DSM18841_02692	oprF_1
oprF_1	DSM18841_00667	oprF_1
oprF_1	DSM18841_00604	oprF_1
oprF_1	DSM18841_00736	oprF_1
oprF_1	DSM18841_00369	oprF_1
oprF_1	DSM18841_01489	oprF_1
oprF_1	DSM18842_02203	oprF_1
oprF_1	DSM18842_02952	oprF_1
oprF_1	DSM18842_01192	oprF_1
oprF_1	DSM18842_02552	oprF_1
oprF_1	DSM18842_01889	oprF_1
oprF_1	DSM18842_03066	oprF_1
oprF_1	DSM18842_00167	oprF_1

oprF_1	DSM18842_00511	oprF_1
oprF_1	DSM18842_02961	oprF_1
oprF_1	DSM18842_00584	oprF_1
oprF_1	DSM18842_00865	oprF_1
oprF_1	DSM18842_01890	oprF_1
oprF_1	DSM18842_01427	oprF_1
oprF_1	DSM19866_00436	oprF_1
oprF_1	DSM19866_01014	oprF_1
oprF_1	DSM19866_03422	oprF_1
oprF_1	DSM19866_03947	oprF_1
oprF_1	DSM19866_03203	oprF_1
oprF_1	DSM19866_00882	oprF_1
oprF_1	DSM19866_00297	oprF_1
oprF_1	DSM19866_01893	oprF_1
oprF_1	DSM19866_02805	oprF_1
oprF_1	DSM19866_01577	oprF_1
oprF_1	DSM19866_04533	oprF_1
oprF_1	DSM19866_04768	oprF_1
oprF_1	DSM19866_03882	oprF_1
oprF_1	DSM19866_03896	oprF_1
oprF_1	DSM19866_04093	oprF_1
oprF_1	DSM19866_03265	oprF_1
oprF_1	DSM19866_03126	oprF_1
oprF_1	DSM2041_00358	oprF_1
oprF_1	DSM2041_03245	oprF_1
oprF_1	DSM2041_01707	oprF_1
oprF_1	DSM2041_05227	oprF_1
oprF_1	DSM2041_02758	oprF_1
oprF_1	DSM2041_05332	oprF_1
oprF_1	DSM2041_01487	oprF_1
oprF_1	DSM2041_04396	oprF_1
oprF_1	DSM2041_01476	oprF_1
oprF_1	DSM2041_01183	oprF_1
oprF_1	DSM2041_01463	oprF_1
oprF_1	DSM2041_04105	oprF_1
oprF_1	DSM2041_01327	oprF_1
oprF_1	DSM2041_04924	oprF_1
oprF_1	DSM2041_02479	oprF_1
oprF_1	DSM2041_01709	oprF_1
oprF_1	DSM2041_00531	oprF_1
oprF_1	DSM2041_03744	oprF_1
oprF_1	DSM2041_05488	oprF_1
oprF_1	DSM2041_04902	oprF_1
oprF_1	DSM2041_01916	oprF_1
oprF_1	DSM2041_01693	oprF_1
oprF_1	DSM2061_04126	oprF_1
oprF_1	DSM2061_03869	oprF_1
oprF_1	DSM2061_01005	oprF_1

oprF_1	DSM2061_03910	oprF_1
oprF_1	DSM2061_02960	oprF_1
oprF_1	DSM2061_03498	oprF_1
oprF_1	DSM2061_03205	oprF_1
oprF_1	DSM2061_01460	oprF_1
oprF_1	DSM2061_02412	oprF_1
oprF_1	DSM2061_02106	oprF_1
oprF_1	DSM2061_04277	oprF_1
oprF_1	DSM2061_03851	oprF_1
oprF_1	DSM2061_00708	oprF_1
oprF_1	DSM2061_01140	oprF_1
oprF_1	DSM2061_00765	oprF_1
oprF_1	DSM2061_01072	oprF_1
oprF_1	DSM2061_00871	oprF_1
oprF_1	DSM2061_00332	oprF_1
oprF_1	DSM2064_04825	oprF_1
oprF_1	DSM2064_04157	oprF_1
oprF_1	DSM2064_03368	oprF_1
oprF_1	DSM2064_01681	oprF_1
oprF_1	DSM2064_03416	oprF_1
oprF_1	DSM2064_01361	oprF_1
oprF_1	DSM2064_01818	oprF_1
oprF_1	DSM2064_02663	oprF_1
oprF_1	DSM2064_01337	oprF_1
oprF_1	DSM2064_00912	oprF_1
oprF_1	DSM2064_00204	oprF_1
oprF_1	DSM2064_00849	oprF_1
oprF_1	DSM2064_01851	oprF_1
oprF_1	DSM2064_02155	oprF_1
oprF_1	DSM2064_01967	oprF_1
oprF_1	DSM2064_00342	oprF_1
oprF_1	DSM2064_03380	oprF_1
oprF_1	DSM21164_02120	oprF_1
oprF_1	DSM21164_01002	oprF_1
oprF_1	DSM21164_02540	oprF_1
oprF_1	DSM21164_01745	oprF_1
oprF_1	DSM21164_01958	oprF_1
oprF_1	DSM21164_02490	oprF_1
oprF_1	DSM21164_01413	oprF_1
oprF_1	DSM21164_01893	oprF_1
oprF_1	DSM21164_00729	oprF_1
oprF_1	DSM21164_00003	oprF_1
oprF_1	DSM21164_00277	oprF_1
oprF_1	DSM21164_02299	oprF_1
oprF_1	DSM21164_01567	oprF_1
oprF_1	DSM21164_01049	oprF_1
oprF_1	DSM21164_02483	oprF_1
oprF_1	DSM21164_02491	oprF_1

oprF_1	DSM21164_02611	oprF_1
oprF_1	DSM23404_03016	oprF_1
oprF_1	DSM23404_02490	oprF_1
oprF_1	DSM23404_02883	oprF_1
oprF_1	DSM23404_00509	oprF_1
oprF_1	DSM23404_00263	oprF_1
oprF_1	DSM23404_02352	oprF_1
oprF_1	DSM23404_00222	oprF_1
oprF_1	DSM23404_02595	oprF_1
oprF_1	DSM23424_00431	oprF_1
oprF_1	DSM23424_01061	oprF_1
oprF_1	DSM23424_01525	oprF_1
oprF_1	DSM23424_00559	oprF_1
oprF_1	DSM23424_00180	oprF_1
oprF_1	DSM23424_00132	oprF_1
oprF_1	DSM23424_01750	oprF_1
oprF_1	DSM23424_01754	oprF_1
oprF_1	DSM23424_01753	oprF_1
oprF_1	DSM23424_02317	oprF_1
oprF_1	DSM23424_02151	oprF_1
oprF_1	DSM23424_01335	oprF_1
oprF_1	DSM23424_03345	oprF_1
oprF_1	DSM23424_01522	oprF_1
oprF_1	DSM23424_01228	oprF_1
oprF_1	DSM23424_00465	oprF_1
oprF_1	DSM23424_01244	oprF_1
oprF_1	DSM23424_00675	oprF_1
oprF_1	DSM24786_03007	oprF_1
oprF_1	DSM24786_02752	oprF_1
oprF_1	DSM24786_02796	oprF_1
oprF_1	DSM24786_03274	oprF_1
oprF_1	DSM24786_02943	oprF_1
oprF_1	DSM24786_00946	oprF_1
oprF_1	DSM24786_00014	oprF_1
oprF_1	DSM24786_01040	oprF_1
oprF_1	DSM24786_00804	oprF_1
oprF_1	DSM24786_02024	oprF_1
oprF_1	DSM24786_00301	oprF_1
oprF_1	DSM24786_02464	oprF_1
oprF_1	DSM24786_02888	oprF_1
oprF_1	DSM24786_00404	oprF_1
oprF_1	DSM24786_00413	oprF_1
oprF_1	DSM24786_02861	oprF_1
oprF_1	DSM7489_01422	oprF_1
oprF_1	DSM7489_01221	oprF_1
oprF_1	DSM7489_01264	oprF_1
oprF_1	DSM7489_02260	oprF_1
oprF_1	DSM7489_00087	oprF_1

oprF_1	DSM7489_01754	oprF_1
oprF_1	DSM7489_01367	oprF_1
oprF_1	DSM7489_03308	oprF_1
oprF_1	DSM7489_02479	oprF_1
oprF_1	DSM7489_00526	oprF_1
oprF_1	DSM7489_03165	oprF_1
oprF_1	DSM7489_01937	oprF_1
oprF_1	DSM7489_02657	oprF_1
oprF_1	DSM7489_02765	oprF_1
oprF_1	DSM7489_01804	oprF_1
oprF_1	DSM7489_01149	oprF_1
oprF_1	DSM7489_02812	oprF_1
oprF_1	DSM7489_02821	oprF_1
oprF_1	DSM7489_01316	oprF_1
oprF_1	DSW1_00590	oprF_1
oprF_1	DSW1_00293	oprF_1
oprF_1	DSW1_01942	oprF_1
oprF_1	DSW1_00729	oprF_1
oprF_1	DSW1_00340	oprF_1
oprF_1	DSW1_00866	oprF_1
oprF_1	DSW1_02503	oprF_1
oprF_1	DSW1_01755	oprF_1
oprF_1	DSW1_01331	oprF_1
oprF_1	DSW1_00264	oprF_1
oprF_1	DSW1_00267	oprF_1
oprF_1	DSW1_01693	oprF_1
oprF_1	DSW1_00412	oprF_1
oprF_1	DSW1_01993	oprF_1
oprF_1	DSW1_01899	oprF_1
oprF_1	DSW1_02452	oprF_1
oprF_1	DSW1_00313	oprF_1
oprF_1	DSW1_00608	oprF_1
oprF_1	DSW5_00811	oprF_1
oprF_1	DSW5_01382	oprF_1
oprF_1	DSW5_01136	oprF_1
oprF_1	DSW5_00007	oprF_1
oprF_1	DSW5_02560	oprF_1
oprF_1	DSW5_01452	oprF_1
oprF_1	DSW5_02721	oprF_1
oprF_1	DSW5_01677	oprF_1
oprF_1	DSW5_00818	oprF_1
oprF_1	DSW6_01062	oprF_1
oprF_1	DSW6_03041	oprF_1
oprF_1	DSW6_03452	oprF_1
oprF_1	DSW6_01264	oprF_1
oprF_1	DSW6_02036	oprF_1
oprF_1	DSW6_02912	oprF_1
oprF_1	DSW6_03397	oprF_1

oprF_1	DSW6_01348	oprF_1
oprF_1	DSW6_02419	oprF_1
oprF_1	DSW6_02346	oprF_1
oprF_1	DSW8_01353	oprF_1
oprF_1	DSW8_03067	oprF_1
oprF_1	DSW8_02453	oprF_1
oprF_1	DSW8_00457	oprF_1
oprF_1	DSW8_03681	oprF_1
oprF_1	DSW8_01050	oprF_1
oprF_1	DSW8_01553	oprF_1
oprF_1	DSW8_01942	oprF_1
oprF_1	DSW8_01976	oprF_1
oprF_1	DSW8_02503	oprF_1
oprF_1	DSW8_01965	oprF_1
oprF_1	DSW8_02264	oprF_1
oprF_1	DSW8_01621	oprF_1
oprF_1	DSW8_03241	oprF_1
oprF_1	DSW8_00899	oprF_1
oprF_1	DSW8_00810	oprF_1
oprF_1	DSW8_03617	oprF_1
oprF_1	DSW8_03622	oprF_1
oprF_1	DSW8_00263	oprF_1
oprF_1	DSW8_00419	oprF_1
oprF_1	F52_01634	oprF_1
oprF_1	F52_00258	oprF_1
oprF_1	F52_02136	oprF_1
oprF_1	F52_04305	oprF_1
oprF_1	F52_02285	oprF_1
oprF_1	F52_02256	oprF_1
oprF_1	F52_03415	oprF_1
oprF_1	F52_00915	oprF_1
oprF_1	F52_00750	oprF_1
oprF_1	F52_00310	oprF_1
oprF_1	F52_00811	oprF_1
oprF_1	F52_02577	oprF_1
oprF_1	F52_02372	oprF_1
oprF_1	F52_01341	oprF_1
oprF_1	GCA_001027945.1_02099	oprF_1
oprF_1	GCA_001027945.1_01237	oprF_1
oprF_1	GCA_001027945.1_01260	oprF_1
oprF_1	GCA_001027945.1_01648	oprF_1
oprF_1	GCA_001027945.1_01144	oprF_1
oprF_1	GCA_001027945.1_03090	oprF_1
oprF_1	GCA_001027945.1_02254	oprF_1
oprF_1	GCA_001027945.1_00832	oprF_1
oprF_1	GCA_001027945.1_01701	oprF_1
oprF_1	GCA_001027945.1_01272	oprF_1
oprF_1	GCA_001027945.1_02253	oprF_1

oprF_1	GCA_001027945.1_03163	oprF_1
oprF_1	GCA_001027945.1_00245	oprF_1
oprF_1	HI1_01363	oprF_1
oprF_1	HI1_01163	oprF_1
oprF_1	HI1_01206	oprF_1
oprF_1	HI1_02187	oprF_1
oprF_1	HI1_00017	oprF_1
oprF_1	HI1_00021	oprF_1
oprF_1	HI1_01680	oprF_1
oprF_1	HI1_01308	oprF_1
oprF_1	HI1_03254	oprF_1
oprF_1	HI1_02411	oprF_1
oprF_1	HI1_00458	oprF_1
oprF_1	HI1_03112	oprF_1
oprF_1	HI1_01865	oprF_1
oprF_1	HI1_02591	oprF_1
oprF_1	HI1_02704	oprF_1
oprF_1	HI1_01729	oprF_1
oprF_1	HI1_01091	oprF_1
oprF_1	HI1_02752	oprF_1
oprF_1	HI1_02761	oprF_1
oprF_1	HI1_01256	oprF_1
oprF_1	IR1_02950	oprF_1
oprF_1	IR1_02611	oprF_1
oprF_1	IR1_00227	oprF_1
oprF_1	IR1_02572	oprF_1
oprF_1	IR1_04162	oprF_1
oprF_1	IR1_04614	oprF_1
oprF_1	IR1_04007	oprF_1
oprF_1	IR1_01393	oprF_1
oprF_1	IR1_02099	oprF_1
oprF_1	IR1_03418	oprF_1
oprF_1	IR1_02038	oprF_1
oprF_1	KCOM3131_00840	oprF_1
oprF_1	KCOM3131_01752	oprF_1
oprF_1	KCOM3131_01659	oprF_1
oprF_1	KCOM3131_00160	oprF_1
oprF_1	KCOM3131_00159	oprF_1
oprF_1	KCTC12974_01677	oprF_1
oprF_1	KCTC12974_02729	oprF_1
oprF_1	KCTC12974_02445	oprF_1
oprF_1	KCTC12974_01096	oprF_1
oprF_1	KCTC12974_02476	oprF_1
oprF_1	KCTC12974_01972	oprF_1
oprF_1	KCTC12974_00646	oprF_1
oprF_1	KCTC12974_03022	oprF_1
oprF_1	KL-A_00804	oprF_1
oprF_1	KL-A_03048	oprF_1

oprF_1	KL-A_02153	oprF_1
oprF_1	KL-A_00013	oprF_1
oprF_1	KL-A_03503	oprF_1
oprF_1	KL-A_02876	oprF_1
oprF_1	KL-A_00862	oprF_1
oprF_1	KL-A_01885	oprF_1
oprF_1	KL-A_03126	oprF_1
oprF_1	KL-A_03375	oprF_1
oprF_1	KL-A_02007	oprF_1
oprF_1	KL-A_00334	oprF_1
oprF_1	KL-A_01404	oprF_1
oprF_1	KL-A_01293	oprF_1
oprF_1	KL-A_02928	oprF_1
oprF_1	KL-A_02094	oprF_1
oprF_1	KL-A_00703	oprF_1
oprF_1	KL-A_00694	oprF_1
oprF_1	KL-A_00917	oprF_1
oprF_1	LL0412.1.7_00730	oprF_1
oprF_1	LL0412.1.7_01150	oprF_1
oprF_1	LL0412.1.7_02525	oprF_1
oprF_1	LL0412.1.7_01730	oprF_1
oprF_1	LL0412.1.7_00737	oprF_1
oprF_1	LL0412.1.7_00870	oprF_1
oprF_1	LL0412.1.7_00474	oprF_1
oprF_1	LL0412.1.7_02184	oprF_1
oprF_1	MAR_2009_71_02098	oprF_1
oprF_1	MAR_2009_71_00387	oprF_1
oprF_1	MAR_2009_71_01000	oprF_1
oprF_1	MAR_2009_71_03255	oprF_1
oprF_1	MAR_2009_71_00275	oprF_1
oprF_1	MAR_2009_71_01872	oprF_1
oprF_1	MAR_2009_71_02391	oprF_1
oprF_1	MAR_2009_71_01439	oprF_1
oprF_1	MAR_2009_71_00184	oprF_1
oprF_1	MAR_2009_71_00950	oprF_1
oprF_1	MAR_2009_71_01416	oprF_1
oprF_1	MAR_2009_71_01175	oprF_1
oprF_1	MAR_2009_71_02250	oprF_1
oprF_1	MAR_2009_71_01804	oprF_1
oprF_1	MAR_2009_71_03678	oprF_1
oprF_1	MAR_2009_71_02251	oprF_1
oprF_1	MAR_2009_71_02543	oprF_1
oprF_1	MAR_2009_71_02636	oprF_1
oprF_1	MAR_2009_71_02767	oprF_1
oprF_1	MAR_2009_71_02772	oprF_1
oprF_1	MAR_2009_71_03068	oprF_1
oprF_1	MAR_2009_71_03818	oprF_1
oprF_1	NN016038_00103	oprF_1

oprF_1	NN016038_00860	oprF_1
oprF_1	NN016038_03898	oprF_1
oprF_1	NN016038_03214	oprF_1
oprF_1	NN016038_00239	oprF_1
oprF_1	NN016038_00755	oprF_1
oprF_1	NN016038_03875	oprF_1
oprF_1	NN016038_01901	oprF_1
oprF_1	NN016038_02717	oprF_1
oprF_1	NN016038_01154	oprF_1
oprF_1	NN016038_01606	oprF_1
oprF_1	NN016038_00333	oprF_1
oprF_1	NN016038_02857	oprF_1
oprF_1	NN016038_03798	oprF_1
oprF_1	NN016038_01216	oprF_1
oprF_1	NN016038_00812	oprF_1
oprF_1	NN016038_03470	oprF_1
oprF_1	NN016038_03148	oprF_1
oprF_1	NN016038_03157	oprF_1
oprF_1	NN016038_03874	oprF_1
oprF_1	PAO1_03815	oprF_1
oprF_1	PAO1_01076	oprF_1
oprF_1	PAO1_00859	oprF_1
oprF_1	PAO1_03002	oprF_1
oprF_1	PAO1_01824	oprF_1
oprF_1	PAO1_01157	oprF_1
oprF_1	PAO1_01083	oprF_1
oprF_1	PAO1_03648	oprF_1
oprF_1	PAO1_01006	oprF_1
oprF_1	PAO1_01501	oprF_1
oprF_1	UW101_00277	oprF_1
oprF_1	UW101_04729	oprF_1
oprF_1	UW101_02432	oprF_1
oprF_1	UW101_00735	oprF_1
oprF_1	UW101_02384	oprF_1
oprF_1	UW101_04118	oprF_1
oprF_1	UW101_00872	oprF_1
oprF_1	UW101_01717	oprF_1
oprF_1	UW101_04142	oprF_1
oprF_1	UW101_03623	oprF_1
oprF_1	UW101_02915	oprF_1
oprF_1	UW101_03560	oprF_1
oprF_1	UW101_00905	oprF_1
oprF_1	UW101_01209	oprF_1
oprF_1	UW101_01022	oprF_1
oprF_1	UW101_03053	oprF_1
oprF_1	UW101_02420	oprF_1
oprF_1	V4-33_01416	oprF_1
oprF_1	V4-33_02332	oprF_1

oprF_1	V4-33_01831	oprF_1
oprF_1	V4-33_00139	oprF_1
oprF_1	V4-33_00298	oprF_1
oprF_1	V4-33_00525	oprF_1
oprF_1	V4-33_00656	oprF_1
oprF_1	V4-33_00047	oprF_1
oprF_1	V4-33_00007	oprF_1
oprF_1	W5C_01121	oprF_1
oprF_1	W5C_02060	oprF_1
oprF_1	W5C_01640	oprF_1
oprF_1	W5C_02594	oprF_1
oprF_1	W5C_00450	oprF_1
oprF_1	W5C_02381	oprF_1
oprF_1	W5C_01868	oprF_1
oprF_1	W5C_01832	oprF_1
oprF_1	W5C_00990	oprF_1
oprF_1	W5C_01824	oprF_1
oprF_1	W5C_00624	oprF_1
oprF_1	W5C_02362	oprF_1
oprF_1	W5C_00783	oprF_1
oprF_1	W5C_02444	oprF_1
oprF_1	W5C_00491	oprF_1
oprF_1	W5C_01992	oprF_1
oprF_1	W5C_02496	oprF_1
oprF_1	W5C_02505	oprF_1
oprF_1	W5C_01697	oprF_1
oprF_1	ZONMW-01_02419	oprF_1
oprF_1	ZONMW-01_01048	oprF_1
oprF_1	ZONMW-01_01010	oprF_1
oprF_1	ZONMW-01_03458	oprF_1
oprF_1	ZONMW-01_00192	oprF_1
oprF_1	ZONMW-01_03390	oprF_1
oprF_1	ZONMW-01_03436	oprF_1
oprF_1	ZONMW-01_03297	oprF_1
oprF_1	ZONMW-01_03229	oprF_1
oprF_1	ZONMW-01_00620	oprF_1
oprF_1	ZONMW-01_00390	oprF_1
oprF_1	ZONMW-01_01710	oprF_1
oprF_1	ZONMW-01_02249	oprF_1
oprF_1	ZONMW-01_00261	oprF_1
oprF_1	ZONMW-01_01116	oprF_1
oprF_1	ZONMW-01_02297	oprF_1
oprF_1	ZONMW-01_02306	oprF_1
oprF_1	ZONMW-01_00951	oprF_1
oprF_1	ZONMW-02_01753	oprF_1
oprF_1	ZONMW-02_03253	oprF_1
oprF_1	ZONMW-02_02337	oprF_1
oprF_1	ZONMW-02_01694	oprF_1

oprF_1	ZONMW-02_02232	oprF_1
oprF_1	ZONMW-02_02894	oprF_1
oprF_1	ZONMW-02_02623	oprF_1
oprF_1	ZONMW-02_01850	oprF_1
oprF_1	ZONMW-02_02288	oprF_1
oprF_1	ZONMW-02_02231	oprF_1
oprF_1	ZONMW-02_01959	oprF_1
oprF_1	ZONMW-02_00184	oprF_1
oprF_1	ZONMW-02_01525	oprF_1
oprF_1	ZONMW-02_01607	oprF_1
oprF_1	ZONMW-02_00005	oprF_1
oprF_1	ZONMW-03_03022	oprF_1
oprF_1	ZONMW-03_02117	oprF_1
oprF_1	ZONMW-03_02079	oprF_1
oprF_1	ZONMW-03_02734	oprF_1
oprF_1	ZONMW-03_00918	oprF_1
oprF_1	ZONMW-03_03441	oprF_1
oprF_1	ZONMW-03_03184	oprF_1
oprF_1	ZONMW-03_00696	oprF_1
oprF_1	ZONMW-03_02395	oprF_1
oprF_1	ZONMW-03_02514	oprF_1
oprF_1	ZONMW-03_01116	oprF_1
oprF_1	ZONMW-03_00497	oprF_1
oprF_1	ZONMW-03_00399	oprF_1
oprF_1	ZONMW-03_00987	oprF_1
oprF_1	ZONMW-03_02185	oprF_1
oprF_1	ZONMW-03_00351	oprF_1
oprF_1	ZONMW-03_00342	oprF_1
oprF_1	ZONMW-03_02020	oprF_1
oprF_1	ZONMW-04_02419	oprF_1
oprF_1	ZONMW-04_01700	oprF_1
oprF_1	ZONMW-04_01740	oprF_1
oprF_1	ZONMW-04_03423	oprF_1
oprF_1	ZONMW-04_00729	oprF_1
oprF_1	ZONMW-04_03377	oprF_1
oprF_1	ZONMW-04_03396	oprF_1
oprF_1	ZONMW-04_03276	oprF_1
oprF_1	ZONMW-04_00860	oprF_1
oprF_1	ZONMW-04_00995	oprF_1
oprF_1	ZONMW-04_00556	oprF_1
oprF_1	ZONMW-04_01338	oprF_1
oprF_1	ZONMW-04_01436	oprF_1
oprF_1	ZONMW-04_01633	oprF_1
oprF_1	ZONMW-04_03031	oprF_1
oprF_1	ZONMW-04_01286	oprF_1
oprF_1	ZONMW-04_01277	oprF_1
oprF_1	ZONMW-04_01803	oprF_1
oprF_1	ZONMW-05_02604	oprF_1

oprF_1	ZONMW-05_01981	oprF_1
oprF_1	ZONMW-05_02019	oprF_1
oprF_1	ZONMW-05_03383	oprF_1
oprF_1	ZONMW-05_01105	oprF_1
oprF_1	ZONMW-05_03370	oprF_1
oprF_1	ZONMW-05_03141	oprF_1
oprF_1	ZONMW-05_02891	oprF_1
oprF_1	ZONMW-05_00129	oprF_1
oprF_1	ZONMW-05_00247	oprF_1
oprF_1	ZONMW-05_01269	oprF_1
oprF_1	ZONMW-05_00760	oprF_1
oprF_1	ZONMW-05_00662	oprF_1
oprF_1	ZONMW-05_01911	oprF_1
oprF_1	ZONMW-05_03274	oprF_1
oprF_1	ZONMW-05_00610	oprF_1
oprF_1	ZONMW-05_00601	oprF_1
oprF_1	ZONMW-05_02078	oprF_1
oprF_1	ZONMW-06_00876	oprF_1
oprF_1	ZONMW-06_00584	oprF_1
oprF_1	ZONMW-06_03831	oprF_1
oprF_1	ZONMW-06_03110	oprF_1
oprF_1	ZONMW-06_03806	oprF_1
oprF_1	ZONMW-06_03578	oprF_1
oprF_1	ZONMW-06_01575	oprF_1
oprF_1	ZONMW-06_03374	oprF_1
oprF_1	ZONMW-06_01323	oprF_1
oprF_1	ZONMW-06_01584	oprF_1
oprF_1	ZONMW-07_01051	oprF_1
oprF_1	ZONMW-07_01211	oprF_1
oprF_1	ZONMW-07_03076	oprF_1
oprF_1	ZONMW-07_03200	oprF_1
oprF_1	ZONMW-07_03512	oprF_1
oprF_1	ZONMW-07_03724	oprF_1
oprF_1	ZONMW-07_04174	oprF_1
oprF_1	ZONMW-07_01481	oprF_1
oprF_1	ZONMW-07_04183	oprF_1
oprF_1	ZONMW-07_02319	oprF_1
oprF_1	ZONMW-07_01263	oprF_1
oprF_1	ZONMW-07_02360	oprF_1
oprF_1	ZONMW-07_02504	oprF_1
oprF_1	ZONMW-07_04224	oprF_1
oprF_1	ZONMW-07_04146	oprF_1
oprF_1	ZONMW-07_03698	oprF_1
oprF_1	ZONMW-07_03704	oprF_1
oprF_1	ZONMW-07_03230	oprF_1
oprF_1	ZONMW-07_02648	oprF_1
oprF_1	ZONMW-08_01160	oprF_1
oprF_1	ZONMW-08_03708	oprF_1

oprF_1	ZONMW-08_00519	oprF_1
oprF_1	ZONMW-08_02770	oprF_1
oprF_1	ZONMW-08_03329	oprF_1
oprF_1	ZONMW-08_01354	oprF_1
oprF_1	ZONMW-08_00847	oprF_1
oprF_1	ZONMW-08_03419	oprF_1
oprF_1	ZONMW-08_00469	oprF_1
oprF_1	ZONMW-08_01777	oprF_1
oprF_1	ZONMW-08_00697	oprF_1
oprF_1	ZONMW-08_00994	oprF_1
oprF_1	ZONMW-08_01420	oprF_1
oprF_1	ZONMW-08_03797	oprF_1
oprF_1	ZONMW-08_03542	oprF_1
oprF_1	ZONMW-08_03640	oprF_1
oprF_1	ZONMW-08_01984	oprF_1
oprF_1	ZONMW-08_01989	oprF_1
oprF_1	ZONMW-08_02582	oprF_1
oprF_1	ZONMW-08_02729	oprF_1
oprF_1	ZONMW-09_00441	oprF_1
oprF_1	ZONMW-09_01374	oprF_1
oprF_1	ZONMW-09_01122	oprF_1
oprF_1	ZONMW-09_02665	oprF_1
oprF_1	ZONMW-09_00188	oprF_1
oprF_1	ZONMW-09_01483	oprF_1
oprF_1	ZONMW-09_00197	oprF_1
oprF_1	ZONMW-09_01051	oprF_1
oprF_1	ZONMW-10_00173	oprF_1
oprF_1	ZONMW-10_00320	oprF_1
oprF_1	ZONMW-10_01251	oprF_1
oprF_1	ZONMW-10_01930	oprF_1
oprF_1	ZONMW-10_01361	oprF_1
oprF_1	ZONMW-10_00225	oprF_1
oprF_1	ZONMW-10_02157	oprF_1
oprF_1	ZONMW-10_01929	oprF_1
oprF_1	ZONMW-10_00512	oprF_1
oprF_1	ZONMW-10_02200	oprF_1
oprF_1	ZONMW-10_02071	oprF_1
oprF_1	ZONMW-10_00340	oprF_1
oprF_1	ZONMW-11_02036	oprF_1
oprF_1	ZONMW-11_03968	oprF_1
oprF_1	ZONMW-11_00344	oprF_1
oprF_1	ZONMW-11_04025	oprF_1
oprF_1	ZONMW-11_02178	oprF_1
oprF_1	ZONMW-11_03739	oprF_1
oprF_1	ZONMW-11_02633	oprF_1
oprF_1	ZONMW-11_02591	oprF_1
oprF_1	ZONMW-12_00041	oprF_1
oprF_1	ZONMW-12_04121	oprF_1

oprF_1	ZONMW-12_02597	oprF_1
oprF_1	ZONMW-12_02692	oprF_1
oprF_1	ZONMW-12_00431	oprF_1
oprF_1	ZONMW-12_02591	oprF_1
oprF_1	ZONMW-12_00710	oprF_1
oprF_1	ZONMW-12_00815	oprF_1
oprF_1	ZONMW-13_00451	oprF_1
oprF_1	ZONMW-13_03293	oprF_1
oprF_1	ZONMW-13_01527	oprF_1
oprF_1	ZONMW-13_01850	oprF_1
oprF_1	ZONMW-13_02061	oprF_1
oprF_1	ZONMW-13_00699	oprF_1
oprF_1	ZONMW-13_01482	oprF_1
oprF_1	ZONMW-13_02015	oprF_1
oprF_1	ZONMW-13_02815	oprF_1
oprF_1	ZONMW-13_00008	oprF_1
oprF_1	ZONMW-14_00111	oprF_1
oprF_1	ZONMW-14_00631	oprF_1
oprF_1	ZONMW-14_00460	oprF_1
oprF_1	ZONMW-14_01082	oprF_1
oprF_1	ZONMW-14_01344	oprF_1
oprF_1	ZONMW-14_02304	oprF_1
oprF_1	ZONMW-14_00687	oprF_1
oprF_1	ZONMW-15_00714	oprF_1
oprF_1	ZONMW-15_01742	oprF_1
oprF_1	ZONMW-15_01953	oprF_1
oprF_1	ZONMW-15_00797	oprF_1
oprF_1	ZONMW-15_03192	oprF_1
oprF_1	ZONMW-15_01594	oprF_1
oprF_1	ZONMW-15_03368	oprF_1
oprF_1	ZONMW-15_00491	oprF_1
oprF_1	ZONMW-15_02523	oprF_1
oprF_1	ZONMW-15_01388	oprF_1
oprF_1	ZONMW-15_00453	oprF_1
oprF_1	ZONMW-15_02773	oprF_1
oprF_1	ZONMW-15_02334	oprF_1
oprF_1	ZONMW-15_02814	oprF_1
oprF_1	ZONMW-15_01790	oprF_1
oprF_1	ZONMW-15_01837	oprF_1
oprF_1	ZONMW-15_01940	oprF_1
oprF_1	ZONMW-15_02008	oprF_1
oprF_1	ZONMW-15_01998	oprF_1
oprF_1	ZONMW-19_04416	oprF_1
oprF_1	ZONMW-19_00485	oprF_1
oprF_1	ZONMW-19_00571	oprF_1
oprF_1	ZONMW-19_00011	oprF_1
oprF_1	ZONMW-19_04424	oprF_1
oprF_1	ZONMW-19_03935	oprF_1

oprF_1	ZONMW-19_00059	oprF_1
oprF_1	ZONMW-19_01756	oprF_1
oprF_1	ZONMW-19_01952	oprF_1
oprF_1	ZONMW-19_02012	oprF_1
oprF_1	ZONMW-19_02035	oprF_1
oprF_1	ZONMW-19_04855	oprF_1
oprF_1	ZONMW-19_00530	oprF_1
oprF_1	ZONMW-20_00903	oprF_1
oprF_1	ZONMW-20_02379	oprF_1
oprF_1	ZONMW-20_02727	oprF_1
oprF_1	ZONMW-20_03450	oprF_1
oprF_1	ZONMW-20_03819	oprF_1
oprF_1	ZONMW-20_00209	oprF_1
oprF_1	ZONMW-21_02352	oprF_1
oprF_1	ZONMW-21_00989	oprF_1
oprF_1	ZONMW-21_01854	oprF_1
oprF_1	ZONMW-21_02893	oprF_1
oprF_1	ZONMW-21_03447	oprF_1
oprF_1	ZONMW-21_02218	oprF_1
oprF_1	ZONMW-21_02061	oprF_1
oprF_1	ZONMW-21_02247	oprF_1
oprF_1	ZONMW-21_00806	oprF_1
oprF_1	ZONMW-21_03005	oprF_1
oprF_1	ZONMW-21_00150	oprF_1
oprF_1	ZONMW-21_01717	oprF_1
oprF_1	ZONMW-21_02042	oprF_1
oprF_1	ZONMW-21_01455	oprF_1
oprF_1	ZONMW-21_02548	oprF_1
oprF_1	ZONMW-21_01791	oprF_1
oprF_1	ZONMW-21_02921	oprF_1
oprF_1	ZONMW-21_01784	oprF_1
oprF_1	ZONMW-21_01960	oprF_1
oprF_1	ZONMW-21_02931	oprF_1
oprF_1	ZONMW-22_02756	oprF_1
oprF_1	ZONMW-22_00990	oprF_1
oprF_1	ZONMW-22_01872	oprF_1
oprF_1	ZONMW-22_02931	oprF_1
oprF_1	ZONMW-22_03460	oprF_1
oprF_1	ZONMW-22_01507	oprF_1
oprF_1	ZONMW-22_01664	oprF_1
oprF_1	ZONMW-22_01478	oprF_1
oprF_1	ZONMW-22_00807	oprF_1
oprF_1	ZONMW-22_03029	oprF_1
oprF_1	ZONMW-22_00150	oprF_1
oprF_1	ZONMW-22_02007	oprF_1
oprF_1	ZONMW-22_01684	oprF_1
oprF_1	ZONMW-22_01457	oprF_1
oprF_1	ZONMW-22_02561	oprF_1

oprF_1	ZONMW-22_01933	oprF_1
oprF_1	ZONMW-22_02903	oprF_1
oprF_1	ZONMW-22_01940	oprF_1
oprF_1	ZONMW-22_01766	oprF_1
oprF_1	ZONMW-22_02893	oprF_1
oprF_1	ZONMW-23_02780	oprF_1
oprF_1	ZONMW-23_02103	oprF_1
oprF_1	ZONMW-23_00680	oprF_1
oprF_1	ZONMW-23_02939	oprF_1
oprF_1	ZONMW-23_03454	oprF_1
oprF_1	ZONMW-23_01044	oprF_1
oprF_1	ZONMW-23_00887	oprF_1
oprF_1	ZONMW-23_01073	oprF_1
oprF_1	ZONMW-23_01921	oprF_1
oprF_1	ZONMW-23_03051	oprF_1
oprF_1	ZONMW-23_01265	oprF_1
oprF_1	ZONMW-23_00544	oprF_1
oprF_1	ZONMW-23_00868	oprF_1
oprF_1	ZONMW-23_02570	oprF_1
oprF_1	ZONMW-23_02585	oprF_1
oprF_1	ZONMW-23_00618	oprF_1
oprF_1	ZONMW-23_02967	oprF_1
oprF_1	ZONMW-23_00611	oprF_1
oprF_1	ZONMW-23_00786	oprF_1
oprF_1	ZONMW-23_02977	oprF_1
oprF_1	ZONMW-24_02376	oprF_1
oprF_1	ZONMW-24_00471	oprF_1
oprF_1	ZONMW-24_01895	oprF_1
oprF_1	ZONMW-24_02954	oprF_1
oprF_1	ZONMW-24_03456	oprF_1
oprF_1	ZONMW-24_01531	oprF_1
oprF_1	ZONMW-24_01688	oprF_1
oprF_1	ZONMW-24_01502	oprF_1
oprF_1	ZONMW-24_00654	oprF_1
oprF_1	ZONMW-24_03051	oprF_1
oprF_1	ZONMW-24_01310	oprF_1
oprF_1	ZONMW-24_02632	oprF_1
oprF_1	ZONMW-24_01707	oprF_1
oprF_1	ZONMW-24_00004	oprF_1
oprF_1	ZONMW-24_02572	oprF_1
oprF_1	ZONMW-24_01956	oprF_1
oprF_1	ZONMW-24_02926	oprF_1
oprF_1	ZONMW-24_01963	oprF_1
oprF_1	ZONMW-24_01789	oprF_1
oprF_1	ZONMW-24_02916	oprF_1
oprF_1	ZONMW-25_02640	oprF_1
oprF_1	ZONMW-25_01989	oprF_1
oprF_1	ZONMW-25_00942	oprF_1

oprF_1	ZONMW-25_02530	oprF_1
oprF_1	ZONMW-25_01084	oprF_1
oprF_1	ZONMW-25_01732	oprF_1
oprF_1	ZONMW-25_00304	oprF_1
oprF_1	ZONMW-25_01783	oprF_1
oprF_1	ZONMW-25_02308	oprF_1
oprF_1	ZONMW-25_01506	oprF_1
oprF_1	ZONMW-25_00046	oprF_1
oprF_1	ZONMW-25_00082	oprF_1
oprF_1	ZONMW-25_03332	oprF_1
oprF_1	ZONMW-25_00855	oprF_1
oprF_1	ZONMW-25_00847	oprF_1
oprF_1	ZONMW-25_00514	oprF_1
oprF_1	ZONMW-25_00235	oprF_1
oprF_1	ZONMW-25_00081	oprF_1
oprF_1	ZONMW-26_03041	oprF_1
oprF_1	ZONMW-26_02419	oprF_1
oprF_1	ZONMW-26_02381	oprF_1
oprF_1	ZONMW-26_03405	oprF_1
oprF_1	ZONMW-26_01151	oprF_1
oprF_1	ZONMW-26_03311	oprF_1
oprF_1	ZONMW-26_03208	oprF_1
oprF_1	ZONMW-26_03212	oprF_1
oprF_1	ZONMW-26_00828	oprF_1
oprF_1	ZONMW-26_00710	oprF_1
oprF_1	ZONMW-26_01323	oprF_1
oprF_1	ZONMW-26_00307	oprF_1
oprF_1	ZONMW-26_00199	oprF_1
oprF_1	ZONMW-26_02485	oprF_1
oprF_1	ZONMW-26_00356	oprF_1
oprF_1	ZONMW-26_00365	oprF_1
oprF_1	ZONMW-26_02319	oprF_1
oprF_1	ZONMW-27_02761	oprF_1
oprF_1	ZONMW-27_01599	oprF_1
oprF_1	ZONMW-27_01561	oprF_1
oprF_1	ZONMW-27_03414	oprF_1
oprF_1	ZONMW-27_00720	oprF_1
oprF_1	ZONMW-27_03362	oprF_1
oprF_1	ZONMW-27_03395	oprF_1
oprF_1	ZONMW-27_03309	oprF_1
oprF_1	ZONMW-27_00956	oprF_1
oprF_1	ZONMW-27_01070	oprF_1
oprF_1	ZONMW-27_00550	oprF_1
oprF_1	ZONMW-27_01868	oprF_1
oprF_1	ZONMW-27_01965	oprF_1
oprF_1	ZONMW-27_01664	oprF_1
oprF_1	ZONMW-27_01434	oprF_1
oprF_1	ZONMW-27_01425	oprF_1

oprF_1	ZONMW-27_01494	oprF_1
oprF_1	ZONMW-28_00650	oprF_1
oprF_1	ZONMW-28_02077	oprF_1
oprF_1	ZONMW-28_02919	oprF_1
oprF_1	ZONMW-28_01157	oprF_1
oprF_1	ZONMW-28_01136	oprF_1
oprF_1	ZONMW-28_01719	oprF_1
oprF_1	ZONMW-28_02577	oprF_1
oprF_1	ZONMW-28_03438	oprF_1
oprF_1	ZONMW-28_00725	oprF_1
oprF_1	ZONMW-28_03196	oprF_1
oprF_1	ZONMW-29_00558	oprF_1
oprF_1	ZONMW-29_01152	oprF_1
oprF_1	ZONMW-29_00068	oprF_1
oprF_1	ZONMW-29_02750	oprF_1
oprF_1	ZONMW-29_01193	oprF_1
oprF_1	ZONMW-29_01432	oprF_1
oprF_1	ZONMW-29_02679	oprF_1
oprF_1	ZONMW-29_00446	oprF_1
oprF_1	ZONMW-29_03059	oprF_1
oprF_1	ZONMW-29_01469	oprF_1
oprF_1	ZONMW-29_01012	oprF_1
oprF_1	ZONMW-29_01073	oprF_1
oprF_1	ZONMW-29_01065	oprF_1
oprF_1	ZONMW-30_00257	oprF_1
oprF_1	ZONMW-30_01809	oprF_1
oprF_1	ZONMW-30_01260	oprF_1
oprF_1	ZONMW-30_01400	oprF_1
oprF_1	ZONMW-30_02264	oprF_1
oprF_1	ZONMW-30_03072	oprF_1
oprF_1	ZONMW-30_03401	oprF_1
oprF_1	ZONMW-30_00772	oprF_1
oprF_1	ZONMW-30_01052	oprF_1
oprF_1	ZONMW-30_03132	oprF_1
oprF_1	ZONMW-30_03327	oprF_1
oprF_1	ZONMW-30_01168	oprF_1
oprF_1	ZONMW-30_01160	oprF_1
oprF_1	ZONMW-30_01987	oprF_1
oprF_1	ZONMW-30_03373	oprF_1
oprF_1	ZONMW-31_01120	oprF_1
oprF_1	ZONMW-31_01136	oprF_1
oprF_1	ZONMW-31_00322	oprF_1
oprF_1	ZONMW-31_01563	oprF_1
oprF_1	ZONMW-31_00127	oprF_1
oprF_1	ZONMW-31_00306	oprF_1
oprF_1	ZONMW-31_02177	oprF_1
oprF_1	ZONMW-31_03747	oprF_1
oprF_1	ZONMW-32_00441	oprF_1

oprF_1	ZONMW-32_02482	oprF_1
oprF_1	ZONMW-32_01403	oprF_1
oprF_1	ZONMW-32_01565	oprF_1
oprF_1	ZONMW-32_01544	oprF_1
oprF_1	ZONMW-32_02253	oprF_1
oprF_1	ZONMW-32_02773	oprF_1
oprF_1	ZONMW-32_03543	oprF_1
oprF_1	ZONMW-32_00366	oprF_1
oprF_1	ZONMW-32_00721	oprF_1
oprF_1	ZONMW-33_00308	oprF_1
oprF_1	ZONMW-33_02636	oprF_1
oprF_1	ZONMW-33_01393	oprF_1
oprF_1	ZONMW-33_01555	oprF_1
oprF_1	ZONMW-33_01534	oprF_1
oprF_1	ZONMW-33_02244	oprF_1
oprF_1	ZONMW-33_02975	oprF_1
oprF_1	ZONMW-33_03843	oprF_1
oprF_1	ZONMW-33_00233	oprF_1
oprF_1	ZONMW-33_00589	oprF_1
oprF_1	ZONMW-34_00686	oprF_1
oprF_1	ZONMW-34_01931	oprF_1
oprF_1	ZONMW-34_01581	oprF_1
oprF_1	ZONMW-34_02905	oprF_1
oprF_1	ZONMW-34_02924	oprF_1
oprF_1	ZONMW-34_02398	oprF_1
oprF_1	ZONMW-34_00953	oprF_1
oprF_1	ZONMW-34_03334	oprF_1
oprF_1	ZONMW-34_00608	oprF_1
oprF_1	ZONMW-34_03051	oprF_1
oprF_1	ZONMW-35_00703	oprF_1
oprF_1	ZONMW-35_01623	oprF_1
oprF_1	ZONMW-35_00652	oprF_1
oprF_1	ZONMW-35_02872	oprF_1
oprF_1	ZONMW-35_02891	oprF_1
oprF_1	ZONMW-35_01851	oprF_1
oprF_1	ZONMW-35_02290	oprF_1
oprF_1	ZONMW-35_03300	oprF_1
oprF_1	ZONMW-35_00781	oprF_1
oprF_1	ZONMW-35_02941	oprF_1
oprF_1	ZONMW-36_01061	oprF_1
oprF_1	ZONMW-36_02616	oprF_1
oprF_1	ZONMW-36_00612	oprF_1
oprF_1	ZONMW-36_01801	oprF_1
oprF_1	ZONMW-36_01822	oprF_1
oprF_1	ZONMW-36_01992	oprF_1
oprF_1	ZONMW-36_02487	oprF_1
oprF_1	ZONMW-36_03763	oprF_1
oprF_1	ZONMW-36_01136	oprF_1

oprF_1	ZONMW-36_00782	oprF_1
oprF_1	ZONMW-37_02622	oprF_1
oprF_1	ZONMW-37_01373	oprF_1
oprF_1	ZONMW-37_03476	oprF_1
oprF_1	ZONMW-37_02941	oprF_1
oprF_1	ZONMW-37_03441	oprF_1
oprF_1	ZONMW-37_01530	oprF_1
oprF_1	ZONMW-37_01687	oprF_1
oprF_1	ZONMW-37_01501	oprF_1
oprF_1	ZONMW-37_00806	oprF_1
oprF_1	ZONMW-37_03038	oprF_1
oprF_1	ZONMW-37_00149	oprF_1
oprF_1	ZONMW-37_02095	oprF_1
oprF_1	ZONMW-37_01707	oprF_1
oprF_1	ZONMW-37_00907	oprF_1
oprF_1	ZONMW-37_02818	oprF_1
oprF_1	ZONMW-37_02169	oprF_1
oprF_1	ZONMW-37_02913	oprF_1
oprF_1	ZONMW-37_02162	oprF_1
oprF_1	ZONMW-37_01789	oprF_1
oprF_1	ZONMW-37_02903	oprF_1
oprF_1	ZONMW-38_00277	oprF_1
oprF_1	ZONMW-39_01149	oprF_1
oprF_1	ZONMW-39_02271	oprF_1
oprF_1	ZONMW-39_00119	oprF_1
oprF_1	ZONMW-39_02583	oprF_1
oprF_1	ZONMW-39_02602	oprF_1
oprF_1	ZONMW-39_03144	oprF_1
oprF_1	ZONMW-39_01690	oprF_1
oprF_1	ZONMW-39_02824	oprF_1
oprF_1	ZONMW-39_03189	oprF_1
oprF_1	ZONMW-39_01072	oprF_1
oprF_1	ZONMW-39_02713	oprF_1
oprF_1	ZONMW-40_03117	oprF_1
oprF_1	ZONMW-40_00269	oprF_1
oprF_1	ZONMW-40_03157	oprF_1
oprF_1	ZONMW-40_02937	oprF_1
oprF_1	ZONMW-40_02143	oprF_1
oprF_1	ZONMW-40_01177	oprF_1
oprF_1	ZONMW-40_00400	oprF_1
oprF_1	ZONMW-40_02558	oprF_1
oprF_1	ZONMW-40_02378	oprF_1
oprF_1	ZONMW-40_00649	oprF_1
oprF_1	ZONMW-40_03443	oprF_1
oprF_1	ZONMW-40_03307	oprF_1
oprF_1	ZONMW-40_00542	oprF_1
oprF_1	ZONMW-40_00551	oprF_1
oprF_1	ZONMW-40_01505	oprF_1

oprF_1	ZONMW-40_02039	oprF_1
oprF_1	ZONMW-41_01333	oprF_1
oprF_1	ZONMW-41_02636	oprF_1
oprF_1	ZONMW-41_02878	oprF_1
oprF_1	ZONMW-41_03091	oprF_1
oprF_1	ZONMW-42_02782	oprF_1
oprF_1	ZONMW-42_01640	oprF_1
oprF_1	ZONMW-42_03922	oprF_1
oprF_1	ZONMW-42_02195	oprF_1
oprF_1	ZONMW-42_03318	oprF_1
oprF_1	ZONMW-42_00209	oprF_1
oprF_1	ZONMW-42_00235	oprF_1
oprF_1	ZONMW-42_00933	oprF_1
oprF_1	ZONMW-42_02816	oprF_1
oprF_1	ZONMW-42_01325	oprF_1
oprF_1	ZONMW-42_01293	oprF_1
oprF_1	ZONMW-42_03637	oprF_1
oprF_1	ZONMW-43_00226	oprF_1
oprF_1	ZONMW-43_01436	oprF_1
oprF_1	ZONMW-43_01688	oprF_1
oprF_1	ZONMW-43_04010	oprF_1
oprF_1	ZONMW-43_03990	oprF_1
oprF_1	ZONMW-43_02404	oprF_1
oprF_1	ZONMW-43_01834	oprF_1
oprF_1	ZONMW-43_00486	oprF_1
oprF_1	ZONMW-43_02713	oprF_1
oprF_1	ZONMW-43_00150	oprF_1
oprF_1	ZONMW-43_04095	oprF_1
oprF_1	ZONMW-44_02073	oprF_1
oprF_1	ZONMW-44_03935	oprF_1
oprF_1	ZONMW-44_01614	oprF_1
oprF_1	ZONMW-44_02979	oprF_1
oprF_1	ZONMW-44_02783	oprF_1
oprF_1	ZONMW-44_04115	oprF_1
oprF_1	ZONMW-44_03004	oprF_1
oprF_1	ZONMW-44_02545	oprF_1
oprF_1	ZONMW-44_00515	oprF_1
oprF_1	ZONMW-44_03700	oprF_1
oprF_1	ZONMW-44_02413	oprF_1
oprF_1	ZONMW-44_00483	oprF_1
oprF_1	ZONMW-45_00938	oprF_1
oprF_1	ZONMW-45_00745	oprF_1
oprF_1	ZONMW-45_03170	oprF_1
oprF_1	ZONMW-45_01334	oprF_1
oprF_1	ZONMW-45_02016	oprF_1
oprF_1	ZONMW-45_03218	oprF_1
oprF_1	ZONMW-45_01022	oprF_1
oprF_1	ZONMW-45_00962	oprF_1

oprF_1	ZONMW-45_01095	oprF_1
oprF_1	ZONMW-45_00523	oprF_1
oprF_1	ZONMW-45_01333	oprF_1
oprF_1	ZONMW-45_01515	oprF_1
oprF_1	ZONMW-46_00936	oprF_1
oprF_1	ZONMW-46_00715	oprF_1
oprF_1	ZONMW-46_02892	oprF_1
oprF_1	ZONMW-46_02718	oprF_1
oprF_1	ZONMW-46_02365	oprF_1
oprF_1	ZONMW-46_03247	oprF_1
oprF_1	ZONMW-46_00645	oprF_1
oprF_1	ZONMW-46_00705	oprF_1
oprF_1	ZONMW-46_00572	oprF_1
oprF_1	ZONMW-46_01316	oprF_1
oprF_1	ZONMW-46_02717	oprF_1
oprF_1	ZONMW-46_02026	oprF_1
oprF_1	ZONMW-47_01237	oprF_1
oprF_1	ZONMW-47_00715	oprF_1
oprF_1	ZONMW-47_02981	oprF_1
oprF_1	ZONMW-47_02708	oprF_1
oprF_1	ZONMW-47_02167	oprF_1
oprF_1	ZONMW-47_03257	oprF_1
oprF_1	ZONMW-47_00645	oprF_1
oprF_1	ZONMW-47_00705	oprF_1
oprF_1	ZONMW-47_00572	oprF_1
oprF_1	ZONMW-47_01536	oprF_1
oprF_1	ZONMW-47_02707	oprF_1
oprF_1	ZONMW-47_02035	oprF_1
oprF_1	ZONMW-48_02088	oprF_1
oprF_1	ZONMW-48_00129	oprF_1
oprF_1	ZONMW-48_01541	oprF_1
oprF_1	ZONMW-48_02008	oprF_1
oprF_1	ZONMW-48_01965	oprF_1
oprF_1	ZONMW-48_03477	oprF_1
oprF_1	ZONMW-48_00550	oprF_1
oprF_1	ZONMW-48_02310	oprF_1
oprF_1	ZONMW-48_00658	oprF_1
oprF_1	ZONMW-48_01747	oprF_1
oprF_1	ZONMW-48_01090	oprF_1
oprF_1	ZONMW-48_03255	oprF_1
oprF_1	ZONMW-48_01277	oprF_1
oprF_1	ZONMW-48_03214	oprF_1
oprF_1	ZONMW-48_00177	oprF_1
oprF_1	ZONMW-48_01421	oprF_1
oprF_1	ZONMW-48_01596	oprF_1
oprF_1	ZONMW-48_01586	oprF_1
oprF_1	ZONMW-49_01175	oprF_1
oprF_1	ZONMW-49_03148	oprF_1

oprF_1	ZONMW-49_02673	oprF_1
oprF_1	ZONMW-49_00651	oprF_1
oprF_1	ZONMW-49_03329	oprF_1
oprF_1	ZONMW-49_01298	oprF_1
oprF_1	ZONMW-49_01235	oprF_1
oprF_1	ZONMW-49_02924	oprF_1
oprF_1	ZONMW-49_00028	oprF_1
oprF_1	ZONMW-49_02814	oprF_1
oprF_1	ZONMW-49_03263	oprF_1
oprF_1	ZONMW-49_01479	oprF_1
oprF_1	ZONMW-49_00209	oprF_1
oprF_1	ZONMW-49_00319	oprF_1
oprF_1	ZONMW-49_01347	oprF_1
oprF_1	ZONMW-49_02895	oprF_1
oprF_1	ZONMW-49_00371	oprF_1
oprF_1	ZONMW-49_00380	oprF_1
oprF_1	ZONMW-49_02623	oprF_1
oprF_1	ZONMW-50_01176	oprF_1
oprF_1	ZONMW-50_03144	oprF_1
oprF_1	ZONMW-50_02467	oprF_1
oprF_1	ZONMW-50_00652	oprF_1
oprF_1	ZONMW-50_03323	oprF_1
oprF_1	ZONMW-50_01300	oprF_1
oprF_1	ZONMW-50_01236	oprF_1
oprF_1	ZONMW-50_02931	oprF_1
oprF_1	ZONMW-50_00458	oprF_1
oprF_1	ZONMW-50_02740	oprF_1
oprF_1	ZONMW-50_03246	oprF_1
oprF_1	ZONMW-50_01481	oprF_1
oprF_1	ZONMW-50_00276	oprF_1
oprF_1	ZONMW-50_00166	oprF_1
oprF_1	ZONMW-50_01349	oprF_1
oprF_1	ZONMW-50_02761	oprF_1
oprF_1	ZONMW-50_00114	oprF_1
oprF_1	ZONMW-50_00105	oprF_1
oprF_1	ZONMW-50_02417	oprF_1
oprF_1	ZONMW-51_02781	oprF_1
oprF_1	ZONMW-51_03205	oprF_1
oprF_1	ZONMW-51_02605	oprF_1
oprF_1	ZONMW-51_01053	oprF_1
oprF_1	ZONMW-51_03301	oprF_1
oprF_1	ZONMW-51_01413	oprF_1
oprF_1	ZONMW-51_02845	oprF_1
oprF_1	ZONMW-51_01951	oprF_1
oprF_1	ZONMW-51_00027	oprF_1
oprF_1	ZONMW-51_02961	oprF_1
oprF_1	ZONMW-51_02058	oprF_1
oprF_1	ZONMW-51_00735	oprF_1

oprF_1	ZONMW-51_00207	oprF_1
oprF_1	ZONMW-51_00307	oprF_1
oprF_1	ZONMW-51_01461	oprF_1
oprF_1	ZONMW-51_02879	oprF_1
oprF_1	ZONMW-51_00358	oprF_1
oprF_1	ZONMW-51_00367	oprF_1
oprF_1	ZONMW-51_02677	oprF_1
oprF_1	ZONMW-52_02265	oprF_1
oprF_1	ZONMW-52_03164	oprF_1
oprF_1	ZONMW-52_02098	oprF_1
oprF_1	ZONMW-52_01654	oprF_1
oprF_1	ZONMW-52_03292	oprF_1
oprF_1	ZONMW-52_01155	oprF_1
oprF_1	ZONMW-52_02200	oprF_1
oprF_1	ZONMW-52_00821	oprF_1
oprF_1	ZONMW-52_03218	oprF_1
oprF_1	ZONMW-52_03282	oprF_1
oprF_1	ZONMW-52_00714	oprF_1
oprF_1	ZONMW-52_01335	oprF_1
oprF_1	ZONMW-52_00178	oprF_1
oprF_1	ZONMW-52_00291	oprF_1
oprF_1	ZONMW-52_01203	oprF_1
oprF_1	ZONMW-52_02995	oprF_1
oprF_1	ZONMW-52_00343	oprF_1
oprF_1	ZONMW-52_00352	oprF_1
oprF_1	ZONMW-52_02148	oprF_1
oprF_1	ZONMW-53_02854	oprF_1
oprF_1	ZONMW-53_03124	oprF_1
oprF_1	ZONMW-53_02741	oprF_1
oprF_1	ZONMW-53_00171	oprF_1
oprF_1	ZONMW-53_03319	oprF_1
oprF_1	ZONMW-53_00696	oprF_1
oprF_1	ZONMW-53_02794	oprF_1
oprF_1	ZONMW-53_01555	oprF_1
oprF_1	ZONMW-53_03232	oprF_1
oprF_1	ZONMW-53_03023	oprF_1
oprF_1	ZONMW-53_01446	oprF_1
oprF_1	ZONMW-53_00490	oprF_1
oprF_1	ZONMW-53_00925	oprF_1
oprF_1	ZONMW-53_01028	oprF_1
oprF_1	ZONMW-53_00623	oprF_1
oprF_1	ZONMW-53_03002	oprF_1
oprF_1	ZONMW-53_01076	oprF_1
oprF_1	ZONMW-53_01085	oprF_1
oprF_1	ZONMW-53_02691	oprF_1
oprF_1	ZONMW-54_00070	oprF_1
oprF_1	ZONMW-54_03172	oprF_1
oprF_1	ZONMW-54_02884	oprF_1

oprF_1	ZONMW-54_00860	oprF_1
oprF_1	ZONMW-54_00365	oprF_1
oprF_1	ZONMW-54_00016	oprF_1
oprF_1	ZONMW-54_03269	oprF_1
oprF_1	ZONMW-54_01163	oprF_1
oprF_1	ZONMW-54_03249	oprF_1
oprF_1	ZONMW-54_03025	oprF_1
oprF_1	ZONMW-54_01275	oprF_1
oprF_1	ZONMW-54_00545	oprF_1
oprF_1	ZONMW-54_01798	oprF_1
oprF_1	ZONMW-54_01699	oprF_1
oprF_1	ZONMW-54_00414	oprF_1
oprF_1	ZONMW-54_03003	oprF_1
oprF_1	ZONMW-54_01647	oprF_1
oprF_1	ZONMW-54_01638	oprF_1
oprF_1	ZONMW-54_02830	oprF_1
oprF_1	ZONMW-55_02860	oprF_1
oprF_1	ZONMW-55_03121	oprF_1
oprF_1	ZONMW-55_02741	oprF_1
oprF_1	ZONMW-55_00167	oprF_1
oprF_1	ZONMW-55_03273	oprF_1
oprF_1	ZONMW-55_00667	oprF_1
oprF_1	ZONMW-55_02796	oprF_1
oprF_1	ZONMW-55_02273	oprF_1
oprF_1	ZONMW-55_01511	oprF_1
oprF_1	ZONMW-55_01635	oprF_1
oprF_1	ZONMW-55_02380	oprF_1
oprF_1	ZONMW-55_00485	oprF_1
oprF_1	ZONMW-55_01330	oprF_1
oprF_1	ZONMW-55_01219	oprF_1
oprF_1	ZONMW-55_00619	oprF_1
oprF_1	ZONMW-55_03009	oprF_1
oprF_1	ZONMW-55_01168	oprF_1
oprF_1	ZONMW-55_01159	oprF_1
oprF_1	ZONMW-55_02691	oprF_1
oprF_1	ZONMW-56_02620	oprF_1
oprF_1	ZONMW-56_03082	oprF_1
oprF_1	ZONMW-56_02454	oprF_1
oprF_1	ZONMW-56_01153	oprF_1
oprF_1	ZONMW-56_03287	oprF_1
oprF_1	ZONMW-56_03182	oprF_1
oprF_1	ZONMW-56_02560	oprF_1
oprF_1	ZONMW-56_02094	oprF_1
oprF_1	ZONMW-56_00737	oprF_1
oprF_1	ZONMW-56_02909	oprF_1
oprF_1	ZONMW-56_00025	oprF_1
oprF_1	ZONMW-56_00828	oprF_1
oprF_1	ZONMW-56_00557	oprF_1

oprF_1	ZONMW-56_00448	oprF_1
oprF_1	ZONMW-56_03078	oprF_1
oprF_1	ZONMW-56_02888	oprF_1
oprF_1	ZONMW-56_00396	oprF_1
oprF_1	ZONMW-56_00387	oprF_1
oprF_1	ZONMW-56_02510	oprF_1
oprF_1	ZONMW-57_02764	oprF_1
oprF_1	ZONMW-57_03188	oprF_1
oprF_1	ZONMW-57_02645	oprF_1
oprF_1	ZONMW-57_01450	oprF_1
oprF_1	ZONMW-57_03325	oprF_1
oprF_1	ZONMW-57_00948	oprF_1
oprF_1	ZONMW-57_02701	oprF_1
oprF_1	ZONMW-57_00133	oprF_1
oprF_1	ZONMW-57_03266	oprF_1
oprF_1	ZONMW-57_03312	oprF_1
oprF_1	ZONMW-57_00240	oprF_1
oprF_1	ZONMW-57_01129	oprF_1
oprF_1	ZONMW-57_00764	oprF_1
oprF_1	ZONMW-57_00660	oprF_1
oprF_1	ZONMW-57_00996	oprF_1
oprF_1	ZONMW-57_02853	oprF_1
oprF_1	ZONMW-57_00609	oprF_1
oprF_1	ZONMW-57_00600	oprF_1
oprF_1	ZONMW-57_02580	oprF_1
oprF_1	ZONMW-58_02788	oprF_1
oprF_1	ZONMW-58_03159	oprF_1
oprF_1	ZONMW-58_02670	oprF_1
oprF_1	ZONMW-58_01134	oprF_1
oprF_1	ZONMW-58_03314	oprF_1
oprF_1	ZONMW-58_01636	oprF_1
oprF_1	ZONMW-58_02725	oprF_1
oprF_1	ZONMW-58_00838	oprF_1
oprF_1	ZONMW-58_00027	oprF_1
oprF_1	ZONMW-58_03304	oprF_1
oprF_1	ZONMW-58_00731	oprF_1
oprF_1	ZONMW-58_01455	oprF_1
oprF_1	ZONMW-58_00207	oprF_1
oprF_1	ZONMW-58_00311	oprF_1
oprF_1	ZONMW-58_01588	oprF_1
oprF_1	ZONMW-58_02877	oprF_1
oprF_1	ZONMW-58_00362	oprF_1
oprF_1	ZONMW-58_00371	oprF_1
oprF_1	ZONMW-58_02605	oprF_1
oprF_1	ZONMW-59_02940	oprF_1
oprF_1	ZONMW-59_03217	oprF_1
oprF_1	ZONMW-59_02678	oprF_1
oprF_1	ZONMW-59_01455	oprF_1

oprF_1	ZONMW-59_03334	oprF_1
oprF_1	ZONMW-59_01923	oprF_1
oprF_1	ZONMW-59_03001	oprF_1
oprF_1	ZONMW-59_00126	oprF_1
oprF_1	ZONMW-59_00978	oprF_1
oprF_1	ZONMW-59_03213	oprF_1
oprF_1	ZONMW-59_00233	oprF_1
oprF_1	ZONMW-59_01131	oprF_1
oprF_1	ZONMW-59_00760	oprF_1
oprF_1	ZONMW-59_00649	oprF_1
oprF_1	ZONMW-59_01875	oprF_1
oprF_1	ZONMW-59_03097	oprF_1
oprF_1	ZONMW-59_00597	oprF_1
oprF_1	ZONMW-59_00588	oprF_1
oprF_1	ZONMW-59_02628	oprF_1
oprF_1	ZONMW-60_02883	oprF_1
oprF_1	ZONMW-60_03197	oprF_1
oprF_1	ZONMW-60_02716	oprF_1
oprF_1	ZONMW-60_01122	oprF_1
oprF_1	ZONMW-60_01610	oprF_1
oprF_1	ZONMW-60_03427	oprF_1
oprF_1	ZONMW-60_02829	oprF_1
oprF_1	ZONMW-60_00132	oprF_1
oprF_1	ZONMW-60_03416	oprF_1
oprF_1	ZONMW-60_03333	oprF_1
oprF_1	ZONMW-60_00265	oprF_1
oprF_1	ZONMW-60_01436	oprF_1
oprF_1	ZONMW-60_00763	oprF_1
oprF_1	ZONMW-60_00663	oprF_1
oprF_1	ZONMW-60_01558	oprF_1
oprF_1	ZONMW-60_02971	oprF_1
oprF_1	ZONMW-60_00612	oprF_1
oprF_1	ZONMW-60_00603	oprF_1
oprF_1	ZONMW-60_02778	oprF_1
oprF_1	ZONMW-61_01174	oprF_1
oprF_1	ZONMW-61_03437	oprF_1
oprF_1	ZONMW-61_00089	oprF_1
oprF_1	ZONMW-61_01346	oprF_1
oprF_1	ZONMW-61_03679	oprF_1
oprF_1	ZONMW-61_01933	oprF_1
oprF_1	ZONMW-61_03772	oprF_1
oprF_1	ZONMW-61_03947	oprF_1
oprF_1	ZONMW-61_03983	oprF_1
oprF_1	ZONMW-61_00040	oprF_1
oprF_1	ZONMW-61_00280	oprF_1
oprF_1	ZONMW-61_00554	oprF_1
oprF_1	ZONMW-61_02967	oprF_1
oprF_1	ZONMW-61_03920	oprF_1

oprF_1	ZONMW-61_03545	oprF_1
oprF_1	ZONMW-61_02261	oprF_1
oprF_1	ZONMW-61_02897	oprF_1
oprF_1	ZONMW-61_02383	oprF_1
oprF_1	ZONMW-61_02388	oprF_1
oprF_1	ZONMW-61_03280	oprF_1
oprF_1	ZONMW-62_02928	oprF_1
oprF_1	ZONMW-62_03609	oprF_1
oprF_1	ZONMW-62_01644	oprF_1
oprF_1	ZONMW-62_00303	oprF_1
oprF_1	ZONMW-62_03726	oprF_1
oprF_1	ZONMW-62_02202	oprF_1
oprF_1	ZONMW-62_02615	oprF_1
oprF_1	ZONMW-62_03816	oprF_1
oprF_1	ZONMW-62_01594	oprF_1
oprF_1	ZONMW-62_02589	oprF_1
oprF_1	ZONMW-62_01833	oprF_1
oprF_1	ZONMW-62_02763	oprF_1
oprF_1	ZONMW-62_02268	oprF_1
oprF_1	ZONMW-62_03163	oprF_1
oprF_1	ZONMW-62_00995	oprF_1
oprF_1	ZONMW-62_00905	oprF_1
oprF_1	ZONMW-62_00776	oprF_1
oprF_1	ZONMW-62_00771	oprF_1
oprF_1	ZONMW-62_00498	oprF_1
oprF_1	ZONMW-63_02309	oprF_1
oprF_1	ZONMW-63_03134	oprF_1
oprF_1	ZONMW-63_02191	oprF_1
oprF_1	ZONMW-63_01112	oprF_1
oprF_1	ZONMW-63_03416	oprF_1
oprF_1	ZONMW-63_00707	oprF_1
oprF_1	ZONMW-63_02373	oprF_1
oprF_1	ZONMW-63_02530	oprF_1
oprF_1	ZONMW-63_02078	oprF_1
oprF_1	ZONMW-63_03401	oprF_1
oprF_1	ZONMW-63_02640	oprF_1
oprF_1	ZONMW-63_00885	oprF_1
oprF_1	ZONMW-63_00439	oprF_1
oprF_1	ZONMW-63_00334	oprF_1
oprF_1	ZONMW-63_00754	oprF_1
oprF_1	ZONMW-63_02859	oprF_1
oprF_1	ZONMW-63_00282	oprF_1
oprF_1	ZONMW-63_00273	oprF_1
oprF_1	ZONMW-63_02141	oprF_1
oprF_1	ZONMW-64_00242	oprF_1
oprF_1	ZONMW-64_02013	oprF_1
oprF_1	ZONMW-64_03006	oprF_1
oprF_1	ZONMW-64_01274	oprF_1

oprF_1	ZONMW-64_01294	oprF_1
oprF_1	ZONMW-64_00668	oprF_1
oprF_1	ZONMW-64_01645	oprF_1
oprF_1	ZONMW-64_00504	oprF_1
oprF_1	ZONMW-64_03164	oprF_1
oprF_1	ZONMW-64_00165	oprF_1
oprF_1	ZONMW-64_02774	oprF_1
oprF_1	ZONMW-65_00934	oprF_1
oprF_1	ZONMW-65_03604	oprF_1
oprF_1	ZONMW-65_02343	oprF_1
oprF_1	ZONMW-65_01098	oprF_1
oprF_1	ZONMW-65_01887	oprF_1
oprF_1	ZONMW-65_01800	oprF_1
oprF_1	ZONMW-65_02858	oprF_1
oprF_1	ZONMW-66_03040	oprF_1
oprF_1	ZONMW-66_00083	oprF_1
oprF_1	ZONMW-66_02547	oprF_1
oprF_1	ZONMW-66_01288	oprF_1
oprF_1	ZONMW-66_01332	oprF_1
oprF_1	ZONMW-66_00557	oprF_1
oprF_1	ZONMW-66_00266	oprF_1
oprF_1	ZONMW-66_01510	oprF_1
oprF_1	ZONMW-66_02781	oprF_1
oprF_1	ZONMW-66_04109	oprF_1
oprF_1	ZONMW-66_00698	oprF_1
oprF_1	ZONMW-66_01598	oprF_1
oprF_1	ZONMW-67_00912	oprF_1
oprF_1	ZONMW-67_01839	oprF_1
oprF_1	ZONMW-67_01518	oprF_1
oprF_1	ZONMW-67_00444	oprF_1
oprF_1	ZONMW-67_00423	oprF_1
oprF_1	ZONMW-67_00110	oprF_1
oprF_1	ZONMW-67_03009	oprF_1
oprF_1	ZONMW-67_03000	oprF_1
oprF_1	ZONMW-67_01169	oprF_1
oprF_1	ZONMW-67_03117	oprF_1
oprF_1	ZONMW-68_03343	oprF_1
oprF_1	ZONMW-68_03285	oprF_1
oprF_1	ZONMW-68_00540	oprF_1
oprF_1	ZONMW-68_01690	oprF_1
oprF_1	ZONMW-68_03512	oprF_1
oprF_1	ZONMW-68_00028	oprF_1
oprF_1	ZONMW-68_03258	oprF_1
oprF_1	ZONMW-68_01966	oprF_1
oprF_1	ZONMW-68_01220	oprF_1
oprF_1	ZONMW-68_04129	oprF_1
oprF_1	ZONMW-68_00977	oprF_1
oprF_1	ZONMW-68_01756	oprF_1

oprF_1	ZONMW-68_01740	oprF_1
oprF_1	ZONMW-68_03673	oprF_1
oprF_1	ZONMW-69_00959	oprF_1
oprF_1	ZONMW-69_02104	oprF_1
oprF_1	ZONMW-69_01483	oprF_1
oprF_1	ZONMW-69_00191	oprF_1
oprF_1	ZONMW-69_00170	oprF_1
oprF_1	ZONMW-69_02719	oprF_1
oprF_1	ZONMW-69_02945	oprF_1
oprF_1	ZONMW-69_02954	oprF_1
oprF_1	ZONMW-69_01215	oprF_1
oprF_1	ZONMW-69_03118	oprF_1
oprF_1	ZONMW-72_01269	oprF_1
oprF_1	ZONMW-72_03359	oprF_1
oprF_1	ZONMW-72_00456	oprF_1
oprF_1	ZONMW-72_02474	oprF_1
oprF_1	ZONMW-72_04447	oprF_1
oprF_1	ZONMW-72_03806	oprF_1
oprF_1	ZONMW-72_02321	oprF_1
oprF_1	ZONMW-72_00986	oprF_1
oprF_1	ZONMW-72_03260	oprF_1
oprF_1	ZONMW-72_03497	oprF_1
oprF_1	ZONMW-72_03199	oprF_1
oprF_1	ZONMW-73_00155	oprF_1
oprF_1	ZONMW-73_00223	oprF_1
oprF_1	ZONMW-73_03169	oprF_1
oprF_1	ZONMW-73_01725	oprF_1
oprF_1	ZONMW-73_03088	oprF_1
oprF_1	ZONMW-73_01874	oprF_1
oprF_1	ZONMW-73_03379	oprF_1
oprF_1	ZONMW-73_00999	oprF_1
oprF_1	ZONMW-73_00889	oprF_1
oprF_1	ZONMW-73_01831	oprF_1
oprF_1	ZONMW-73_03611	oprF_1
oprF_1	ZONMW-73_01031	oprF_1
oprF_1	ZONMW-73_01998	oprF_1
oprF_1	ZONMW-74_01471	oprF_1
oprF_1	ZONMW-74_02257	oprF_1
oprF_1	ZONMW-74_01660	oprF_1
oprF_1	ZONMW-74_01927	oprF_1
oprF_1	ZONMW-74_01540	oprF_1
oprF_1	ZONMW-74_02429	oprF_1
oprF_1	ZONMW-74_00528	oprF_1
oprF_1	ZONMW-74_01768	oprF_1
oprF_1	ZONMW-74_00424	oprF_1
oprF_1	ZONMW-74_00065	oprF_1
oprF_1	ZONMW-74_03214	oprF_1
oprF_1	ZONMW-74_01596	oprF_1

oprF_1	ZONMW-74_00054	oprF_1
oprF_1	ZONMW-74_00066	oprF_1
oprF_1	ZONMW-75_02253	oprF_1
oprF_1	ZONMW-75_01696	oprF_1
oprF_1	ZONMW-75_00491	oprF_1
oprF_1	ZONMW-75_00108	oprF_1
oprF_1	ZONMW-75_03309	oprF_1
oprF_1	ZONMW-75_03004	oprF_1
oprF_1	ZONMW-75_03252	oprF_1
oprF_1	ZONMW-75_03782	oprF_1
oprF_1	ZONMW-75_03310	oprF_1
oprF_1	ZONMW-75_00591	oprF_1
oprF_1	ZONMW-75_00814	oprF_1
oprF_1	ZONMW-75_01050	oprF_1
oprF_1	ZONMW-75_02609	oprF_1
oprF_1	ZONMW-75_00404	oprF_1
oprF_1	ZONMW-75_02534	oprF_1
oprF_1	ZONMW-75_02376	oprF_1
oprF_1	da5A-8_02981	oprF_1
oprF_1	da5A-8_01533	oprF_1
oprF_1	da5A-8_00484	oprF_1
oprF_1	da5A-8_01390	oprF_1
oprF_1	da5A-8_01852	oprF_1
oprF_1	da5A-8_01395	oprF_1
oprF_1	da5A-8_00853	oprF_1
oprF_1	da5A-8_02847	oprF_1
oprF_1	da5A-8_03166	oprF_1
oprF_1	da5A-8_01871	oprF_1
oprF_1	da5A-8_01526	oprF_1
oprF_1	da5A-8_01743	oprF_1
oprF_1	da5A-8_02472	oprF_1
oprF_1	da5A-8_00926	oprF_1
rpsO	18_01736	rpsO
rpsO	ACAM48_01671	rpsO
rpsO	CAM030_01384	rpsO
rpsO	CECT7946_00864	rpsO
rpsO	CGMCC1.8863_00633	rpsO
rpsO	CL8139_02991	rpsO
rpsO	DAU203_00677	rpsO
rpsO	DD5b_02726	rpsO
rpsO	DSM1208_00744	rpsO
rpsO	DSM12212_02708	rpsO
rpsO	DSM15343_02076	rpsO
rpsO	DSM15361_02398	rpsO
rpsO	DSM15365_03893	rpsO
rpsO	DSM15718_00439	rpsO
rpsO	DSM16505T_00961	rpsO
rpsO	DSM18103_02619	rpsO

rpsO	DSM18293_00532	rpsO
rpsO	DSM18841_00853	rpsO
rpsO	DSM18842_00466	rpsO
rpsO	DSM19866_02198	rpsO
rpsO	DSM2041_03570	rpsO
rpsO	DSM2061_02243	rpsO
rpsO	DSM2064_02456	rpsO
rpsO	DSM21164_00142	rpsO
rpsO	DSM23404_00997	rpsO
rpsO	DSM23424_02442	rpsO
rpsO	DSM24786_03484	rpsO
rpsO	DSM7489_00663	rpsO
rpsO	DSW1_02835	rpsO
rpsO	DSW5_00757	rpsO
rpsO	DSW6_01844	rpsO
rpsO	DSW8_03799	rpsO
rpsO	F52_02860	rpsO
rpsO	GCA_001027945.1_01584	rpsO
rpsO	HI1_00595	rpsO
rpsO	IR1_00423	rpsO
rpsO	KCOM3131_00534	rpsO
rpsO	KCTC12974_02428	rpsO
rpsO	KL-A_03059	rpsO
rpsO	LL0412.1.7_00984	rpsO
rpsO	MAR_2009_71_00329	rpsO
rpsO	NN016038_01757	rpsO
rpsO	PAO1_04902	rpsO
rpsO	UW101_01510	rpsO
rpsO	V4-33_01960	rpsO
rpsO	W5C_01178	rpsO
rpsO	ZONMW-01_03195	rpsO
rpsO	ZONMW-02_03785	rpsO
rpsO	ZONMW-03_03323	rpsO
rpsO	ZONMW-04_03213	rpsO
rpsO	ZONMW-05_03344	rpsO
rpsO	ZONMW-06_00320	rpsO
rpsO	ZONMW-07_01126	rpsO
rpsO	ZONMW-08_03275	rpsO
rpsO	ZONMW-09_01943	rpsO
rpsO	ZONMW-10_01127	rpsO
rpsO	ZONMW-11_00944	rpsO
rpsO	ZONMW-12_02173	rpsO
rpsO	ZONMW-13_02639	rpsO
rpsO	ZONMW-14_01597	rpsO
rpsO	ZONMW-15_01261	rpsO
rpsO	ZONMW-19_00666	rpsO
rpsO	ZONMW-20_02886	rpsO
rpsO	ZONMW-21_03140	rpsO

rpsO	ZONMW-22_03165	rpsO
rpsO	ZONMW-23_03186	rpsO
rpsO	ZONMW-24_03187	rpsO
rpsO	ZONMW-25_01920	rpsO
rpsO	ZONMW-26_03249	rpsO
rpsO	ZONMW-27_03319	rpsO
rpsO	ZONMW-28_01550	rpsO
rpsO	ZONMW-29_02545	rpsO
rpsO	ZONMW-30_00913	rpsO
rpsO	ZONMW-31_00634	rpsO
rpsO	ZONMW-32_01957	rpsO
rpsO	ZONMW-33_01948	rpsO
rpsO	ZONMW-34_00283	rpsO
rpsO	ZONMW-35_01107	rpsO
rpsO	ZONMW-36_01408	rpsO
rpsO	ZONMW-37_03174	rpsO
rpsO	ZONMW-38_00872	rpsO
rpsO	ZONMW-39_04014	rpsO
rpsO	ZONMW-40_01034	rpsO
rpsO	ZONMW-41_02529	rpsO
rpsO	ZONMW-42_03598	rpsO
rpsO	ZONMW-43_00669	rpsO
rpsO	ZONMW-44_03510	rpsO
rpsO	ZONMW-45_01383	rpsO
rpsO	ZONMW-46_01638	rpsO
rpsO	ZONMW-47_01851	rpsO
rpsO	ZONMW-48_02435	rpsO
rpsO	ZONMW-49_03159	rpsO
rpsO	ZONMW-50_03155	rpsO
rpsO	ZONMW-51_03216	rpsO
rpsO	ZONMW-52_03207	rpsO
rpsO	ZONMW-53_03185	rpsO
rpsO	ZONMW-54_03182	rpsO
rpsO	ZONMW-55_03183	rpsO
rpsO	ZONMW-56_03227	rpsO
rpsO	ZONMW-57_03199	rpsO
rpsO	ZONMW-58_03252	rpsO
rpsO	ZONMW-59_03309	rpsO
rpsO	ZONMW-60_03298	rpsO
rpsO	ZONMW-61_03870	rpsO
rpsO	ZONMW-62_03672	rpsO
rpsO	ZONMW-63_03226	rpsO
rpsO	ZONMW-64_00931	rpsO
rpsO	ZONMW-65_02536	rpsO
rpsO	ZONMW-66_00475	rpsO
rpsO	ZONMW-67_02694	rpsO
rpsO	ZONMW-68_01627	rpsO
rpsO	ZONMW-69_00491	rpsO

rpsO	ZONMW-72_00075	rpsO
rpsO	ZONMW-73_03226	rpsO
rpsO	ZONMW-74_03406	rpsO
rpsO	ZONMW-75_03801	rpsO
rpsO	da5A-8_00374	rpsO
murA	18_02717	murA
murA	ACAM48_00654	murA
murA	CAM030_00300	murA
murA	CECT7946_02854	murA
murA	CGMCC1.8863_02606	murA
murA	CL8139_03074	murA
murA	DAU203_03387	murA
murA	DD5b_00575	murA
murA	DSM1208_03808	murA
murA	DSM12212_03034	murA
murA	DSM12212_02678	murA
murA	DSM15343_02762	murA
murA	DSM15361_02032	murA
murA	DSM15365_02484	murA
murA	DSM15718_02841	murA
murA	DSM16505T_01703	murA
murA	DSM18103_00695	murA
murA	DSM18293_02683	murA
murA	DSM18841_01797	murA
murA	DSM18842_02092	murA
murA	DSM19866_04290	murA
murA	DSM2041_01418	murA
murA	DSM2061_01222	murA
murA	DSM2064_04958	murA
murA	DSM21164_01644	murA
murA	DSM23404_02667	murA
murA	DSM23424_01386	murA
murA	DSM24786_00865	murA
murA	DSM7489_03223	murA
murA	DSW1_01645	murA
murA	DSW5_02119	murA
murA	DSW6_02817	murA
murA	DSW8_00085	murA
murA	F52_01818	murA
murA	GCA_001027945.1_02131	murA
murA	HI1_03170	murA
murA	IR1_02833	murA
murA	KCOM3131_01665	murA
murA	KCTC12974_02951	murA
murA	KL-A_01949	murA
murA	LL0412.1.7_02353	murA
murA	MAR_2009_71_02886	murA
murA	NN016038_02766	murA

murA	PAO1_04593	murA
murA	UW101_00144	murA
murA	V4-33_01929	murA
murA	W5C_01773	murA
murA	ZONMW-01_00562	murA
murA	ZONMW-02_00604	murA
murA	ZONMW-03_02456	murA
murA	ZONMW-04_00937	murA
murA	ZONMW-05_00189	murA
murA	ZONMW-06_00994	murA
murA	ZONMW-07_03965	murA
murA	ZONMW-08_02085	murA
murA	ZONMW-09_00786	murA
murA	ZONMW-10_01633	murA
murA	ZONMW-11_03938	murA
murA	ZONMW-12_03786	murA
murA	ZONMW-13_01902	murA
murA	ZONMW-14_00790	murA
murA	ZONMW-15_02926	murA
murA	ZONMW-19_01389	murA
murA	ZONMW-20_02861	murA
murA	ZONMW-21_02145	murA
murA	ZONMW-22_01580	murA
murA	ZONMW-23_00971	murA
murA	ZONMW-24_01604	murA
murA	ZONMW-25_00393	murA
murA	ZONMW-26_00768	murA
murA	ZONMW-27_01012	murA
murA	ZONMW-28_04062	murA
murA	ZONMW-29_01347	murA
murA	ZONMW-30_02692	murA
murA	ZONMW-31_01250	murA
murA	ZONMW-32_00563	murA
murA	ZONMW-33_00431	murA
murA	ZONMW-34_00808	murA
murA	ZONMW-35_02655	murA
murA	ZONMW-36_00940	murA
murA	ZONMW-37_01603	murA
murA	ZONMW-38_02291	murA
murA	ZONMW-38_02915	murA
murA	ZONMW-39_01272	murA
murA	ZONMW-40_00482	murA
murA	ZONMW-41_01188	murA
murA	ZONMW-42_03015	murA
murA	ZONMW-43_00347	murA
murA	ZONMW-44_02191	murA
murA	ZONMW-45_00255	murA
murA	ZONMW-46_00470	murA

murA	ZONMW-47_00035	murA
murA	ZONMW-48_01668	murA
murA	ZONMW-49_02981	murA
murA	ZONMW-50_02874	murA
murA	ZONMW-51_02000	murA
murA	ZONMW-52_00772	murA
murA	ZONMW-53_01506	murA
murA	ZONMW-54_01217	murA
murA	ZONMW-55_02322	murA
murA	ZONMW-56_02034	murA
murA	ZONMW-57_00182	murA
murA	ZONMW-58_00789	murA
murA	ZONMW-59_00175	murA
murA	ZONMW-60_00208	murA
murA	ZONMW-61_02444	murA
murA	ZONMW-62_00680	murA
murA	ZONMW-63_02580	murA
murA	ZONMW-64_00364	murA
murA	ZONMW-65_00263	murA
murA	ZONMW-66_01345	murA
murA	ZONMW-67_01030	murA
murA	ZONMW-68_04884	murA
murA	ZONMW-69_01077	murA
murA	ZONMW-72_01400	murA
murA	ZONMW-73_00504	murA
murA	ZONMW-74_00467	murA
murA	ZONMW-75_01816	murA
murA	da5A-8_02937	murA
rplR	18_00191	rplR
rplR	ACAM48_00320	rplR
rplR	CAM030_02835	rplR
rplR	CECT7946_03317	rplR
rplR	CGMCC1.8863_01429	rplR
rplR	CL8139_01434	rplR
rplR	DAU203_02282	rplR
rplR	DD5b_02404	rplR
rplR	DSM1208_02439	rplR
rplR	DSM12212_01099	rplR
rplR	DSM15343_01579	rplR
rplR	DSM15361_02578	rplR
rplR	DSM15365_01150	rplR
rplR	DSM15718_00664	rplR
rplR	DSM16505T_01247	rplR
rplR	DSM18103_01870	rplR
rplR	DSM18293_02430	rplR
rplR	DSM18841_01180	rplR
rplR	DSM18842_00124	rplR
rplR	DSM19866_01093	rplR

rplR	DSM2041_02962	rplR
rplR	DSM2061_03950	rplR
rplR	DSM2064_04700	rplR
rplR	DSM21164_02205	rplR
rplR	DSM23404_00470	rplR
rplR	DSM23424_02871	rplR
rplR	DSM24786_01749	rplR
rplR	DSM7489_02198	rplR
rplR	DSW1_01279	rplR
rplR	DSW5_00459	rplR
rplR	DSW6_00504	rplR
rplR	DSW8_01514	rplR
rplR	F52_01521	rplR
rplR	GCA_001027945.1_02975	rplR
rplR	HI1_02124	rplR
rplR	IR1_03057	rplR
rplR	KCOM3131_00726	rplR
rplR	KCTC12974_01060	rplR
rplR	KL-A_00075	rplR
rplR	LL0412.1.7_01243	rplR
rplR	MAR_2009_71_01911	rplR
rplR	NN016038_00198	rplR
rplR	PAO1_04378	rplR
rplR	UW101_00402	rplR
rplR	V4-33_01231	rplR
rplR	W5C_02863	rplR
rplR	ZONMW-01_01391	rplR
rplR	ZONMW-02_02702	rplR
rplR	ZONMW-03_01389	rplR
rplR	ZONMW-04_00303	rplR
rplR	ZONMW-05_01522	rplR
rplR	ZONMW-06_03647	rplR
rplR	ZONMW-07_01582	rplR
rplR	ZONMW-08_01315	rplR
rplR	ZONMW-09_01334	rplR
rplR	ZONMW-10_01398	rplR
rplR	ZONMW-11_03084	rplR
rplR	ZONMW-12_01403	rplR
rplR	ZONMW-13_01807	rplR
rplR	ZONMW-14_01043	rplR
rplR	ZONMW-15_00756	rplR
rplR	ZONMW-19_01647	rplR
rplR	ZONMW-20_04261	rplR
rplR	ZONMW-21_02451	rplR
rplR	ZONMW-22_02658	rplR
rplR	ZONMW-23_02682	rplR
rplR	ZONMW-24_02475	rplR
rplR	ZONMW-25_03475	rplR

rplR	ZONMW-26_01748	rplR
rplR	ZONMW-27_00297	rplR
rplR	ZONMW-28_03619	rplR
rplR	ZONMW-29_02790	rplR
rplR	ZONMW-30_00094	rplR
rplR	ZONMW-31_03099	rplR
rplR	ZONMW-32_03647	rplR
rplR	ZONMW-33_03574	rplR
rplR	ZONMW-34_03516	rplR
rplR	ZONMW-35_03477	rplR
rplR	ZONMW-36_03404	rplR
rplR	ZONMW-37_02721	rplR
rplR	ZONMW-38_01899	rplR
rplR	ZONMW-39_03863	rplR
rplR	ZONMW-40_02182	rplR
rplR	ZONMW-41_02966	rplR
rplR	ZONMW-42_02241	rplR
rplR	ZONMW-43_03684	rplR
rplR	ZONMW-44_03253	rplR
rplR	ZONMW-45_01751	rplR
rplR	ZONMW-46_00046	rplR
rplR	ZONMW-47_00459	rplR
rplR	ZONMW-48_02046	rplR
rplR	ZONMW-49_00714	rplR
rplR	ZONMW-50_00715	rplR
rplR	ZONMW-51_00993	rplR
rplR	ZONMW-52_01593	rplR
rplR	ZONMW-53_00232	rplR
rplR	ZONMW-54_00798	rplR
rplR	ZONMW-55_00227	rplR
rplR	ZONMW-56_01091	rplR
rplR	ZONMW-57_01390	rplR
rplR	ZONMW-58_01194	rplR
rplR	ZONMW-59_01392	rplR
rplR	ZONMW-60_01183	rplR
rplR	ZONMW-61_01304	rplR
rplR	ZONMW-62_02163	rplR
rplR	ZONMW-63_01175	rplR
rplR	ZONMW-64_03966	rplR
rplR	ZONMW-65_02959	rplR
rplR	ZONMW-66_01176	rplR
rplR	ZONMW-67_03492	rplR
rplR	ZONMW-68_04446	rplR
rplR	ZONMW-69_03494	rplR
rplR	ZONMW-72_01163	rplR
rplR	ZONMW-73_02137	rplR
rplR	ZONMW-74_01007	rplR
rplR	ZONMW-75_02923	rplR

rplR	da5A-8_00896	rplR
rluE	18_00552	rluE
rluE	18_03776	rluE
rluE	18_03412	rluE
rluE	ACAM48_02756	rluE
rluE	ACAM48_00349	rluE
rluE	ACAM48_00149	rluE
rluE	CAM030_01354	rluE
rluE	CAM030_02862	rluE
rluE	CAM030_00683	rluE
rluE	CECT7946_00583	rluE
rluE	CECT7946_03268	rluE
rluE	CECT7946_01191	rluE
rluE	CGMCC1.8863_01211	rluE
rluE	CGMCC1.8863_03370	rluE
rluE	CGMCC1.8863_02717	rluE
rluE	CL8139_00413	rluE
rluE	CL8139_02678	rluE
rluE	CL8139_00688	rluE
rluE	DAU203_00912	rluE
rluE	DAU203_02688	rluE
rluE	DAU203_03028	rluE
rluE	DD5b_01665	rluE
rluE	DD5b_00463	rluE
rluE	DD5b_02828	rluE
rluE	DSM1208_01198	rluE
rluE	DSM1208_02852	rluE
rluE	DSM1208_04033	rluE
rluE	DSM12212_01454	rluE
rluE	DSM12212_01148	rluE
rluE	DSM12212_00759	rluE
rluE	DSM15343_00683	rluE
rluE	DSM15343_01779	rluE
rluE	DSM15343_02811	rluE
rluE	DSM15361_02003	rluE
rluE	DSM15361_02545	rluE
rluE	DSM15361_00667	rluE
rluE	DSM15365_02302	rluE
rluE	DSM15365_02320	rluE
rluE	DSM15365_00223	rluE
rluE	DSM15718_02255	rluE
rluE	DSM15718_00811	rluE
rluE	DSM15718_02783	rluE
rluE	DSM16505T_00053	rluE
rluE	DSM16505T_01837	rluE
rluE	DSM16505T_00497	rluE
rluE	DSM18103_01373	rluE
rluE	DSM18103_02255	rluE

rluE	DSM18103_02845	rluE
rluE	DSM18293_00472	rluE
rluE	DSM18293_02568	rluE
rluE	DSM18293_02731	rluE
rluE	DSM18841_02520	rluE
rluE	DSM18841_01564	rluE
rluE	DSM18841_00345	rluE
rluE	DSM18842_02429	rluE
rluE	DSM18842_02253	rluE
rluE	DSM18842_00886	rluE
rluE	DSM19866_03718	rluE
rluE	DSM19866_00073	rluE
rluE	DSM19866_04052	rluE
rluE	DSM2041_00708	rluE
rluE	DSM2041_04968	rluE
rluE	DSM2041_01671	rluE
rluE	DSM2061_02714	rluE
rluE	DSM2061_04336	rluE
rluE	DSM2061_00887	rluE
rluE	DSM2064_02393	rluE
rluE	DSM2064_04848	rluE
rluE	DSM2064_05012	rluE
rluE	DSM21164_01281	rluE
rluE	DSM21164_02457	rluE
rluE	DSM21164_02590	rluE
rluE	DSM23404_02204	rluE
rluE	DSM23404_00441	rluE
rluE	DSM23404_01344	rluE
rluE	DSM23424_00792	rluE
rluE	DSM23424_01840	rluE
rluE	DSM23424_01220	rluE
rluE	DSM24786_01278	rluE
rluE	DSM24786_00148	rluE
rluE	DSM24786_00488	rluE
rluE	DSM7489_00846	rluE
rluE	DSM7489_02562	rluE
rluE	DSM7489_02892	rluE
rluE	DSW1_01020	rluE
rluE	DSW1_02215	rluE
rluE	DSW1_02119	rluE
rluE	DSW5_00699	rluE
rluE	DSW5_02501	rluE
rluE	DSW5_01211	rluE
rluE	DSW6_01909	rluE
rluE	DSW6_00038	rluE
rluE	DSW6_02820	rluE
rluE	DSW8_02739	rluE
rluE	DSW8_01107	rluE

rluE	DSW8_00277	rluE
rluE	F52_02800	rluE
rluE	F52_01656	rluE
rluE	F52_01871	rluE
rluE	GCA_001027945.1_01137	rluE
rluE	GCA_001027945.1_02516	rluE
rluE	GCA_001027945.1_00267	rluE
rluE	HI1_00781	rluE
rluE	HI1_02495	rluE
rluE	HI1_02831	rluE
rluE	IR1_00474	rluE
rluE	IR1_02924	rluE
rluE	IR1_02782	rluE
rluE	KCOM3131_01806	rluE
rluE	KCOM3131_01773	rluE
rluE	KCTC12974_01196	rluE
rluE	KCTC12974_00550	rluE
rluE	KCTC12974_00043	rluE
rluE	KL-A_03219	rluE
rluE	KL-A_00993	rluE
rluE	KL-A_00624	rluE
rluE	LL0412.1.7_00054	rluE
rluE	LL0412.1.7_01955	rluE
rluE	LL0412.1.7_00456	rluE
rluE	MAR_2009_71_00716	rluE
rluE	MAR_2009_71_02338	rluE
rluE	MAR_2009_71_03081	rluE
rluE	NN016038_00561	rluE
rluE	NN016038_03828	rluE
rluE	NN016038_03447	rluE
rluE	PAO1_00755	rluE
rluE	PAO1_03289	rluE
rluE	PAO1_04096	rluE
rluE	PAO1_01146	rluE
rluE	UW101_01447	rluE
rluE	UW101_00254	rluE
rluE	UW101_00090	rluE
rluE	V4-33_00291	rluE
rluE	V4-33_01131	rluE
rluE	V4-33_01908	rluE
rluE	W5C_01472	rluE
rluE	W5C_01065	rluE
rluE	W5C_02573	rluE
rluE	ZONMW-01_02486	rluE
rluE	ZONMW-01_01612	rluE
rluE	ZONMW-01_00896	rluE
rluE	ZONMW-02_02370	rluE
rluE	ZONMW-02_02353	rluE

rluE	ZONMW-02_03281	rluE
rluE	ZONMW-03_01767	rluE
rluE	ZONMW-03_00595	rluE
rluE	ZONMW-03_00274	rluE
rluE	ZONMW-04_02972	rluE
rluE	ZONMW-04_01533	rluE
rluE	ZONMW-04_03067	rluE
rluE	ZONMW-05_03215	rluE
rluE	ZONMW-05_00857	rluE
rluE	ZONMW-05_00532	rluE
rluE	ZONMW-06_02762	rluE
rluE	ZONMW-06_00760	rluE
rluE	ZONMW-06_02443	rluE
rluE	ZONMW-06_00047	rluE
rluE	ZONMW-06_03160	rluE
rluE	ZONMW-07_00551	rluE
rluE	ZONMW-07_02253	rluE
rluE	ZONMW-07_02580	rluE
rluE	ZONMW-08_00232	rluE
rluE	ZONMW-08_00908	rluE
rluE	ZONMW-08_02596	rluE
rluE	ZONMW-09_00601	rluE
rluE	ZONMW-09_01307	rluE
rluE	ZONMW-09_02488	rluE
rluE	ZONMW-10_00040	rluE
rluE	ZONMW-10_01643	rluE
rluE	ZONMW-10_01486	rluE
rluE	ZONMW-11_00625	rluE
rluE	ZONMW-11_02823	rluE
rluE	ZONMW-11_02632	rluE
rluE	ZONMW-12_01183	rluE
rluE	ZONMW-12_01374	rluE
rluE	ZONMW-12_01625	rluE
rluE	ZONMW-13_00781	rluE
rluE	ZONMW-13_01773	rluE
rluE	ZONMW-13_02808	rluE
rluE	ZONMW-14_01263	rluE
rluE	ZONMW-14_01013	rluE
rluE	ZONMW-14_02047	rluE
rluE	ZONMW-15_00982	rluE
rluE	ZONMW-15_03033	rluE
rluE	ZONMW-15_01856	rluE
rluE	ZONMW-19_00100	rluE
rluE	ZONMW-19_01882	rluE
rluE	ZONMW-20_03677	rluE
rluE	ZONMW-20_02102	rluE
rluE	ZONMW-20_03508	rluE
rluE	ZONMW-20_01712	rluE

rluE	ZONMW-21_01274	rluE
rluE	ZONMW-22_01276	rluE
rluE	ZONMW-23_02388	rluE
rluE	ZONMW-24_00185	rluE
rluE	ZONMW-25_02440	rluE
rluE	ZONMW-26_02082	rluE
rluE	ZONMW-26_00103	rluE
rluE	ZONMW-26_00435	rluE
rluE	ZONMW-27_02838	rluE
rluE	ZONMW-27_01771	rluE
rluE	ZONMW-27_01356	rluE
rluE	ZONMW-28_01844	rluE
rluE	ZONMW-28_00188	rluE
rluE	ZONMW-28_00745	rluE
rluE	ZONMW-28_01228	rluE
rluE	ZONMW-28_02970	rluE
rluE	ZONMW-29_00862	rluE
rluE	ZONMW-30_00546	rluE
rluE	ZONMW-31_03651	rluE
rluE	ZONMW-31_01015	rluE
rluE	ZONMW-31_00381	rluE
rluE	ZONMW-31_01628	rluE
rluE	ZONMW-32_02128	rluE
rluE	ZONMW-32_00979	rluE
rluE	ZONMW-32_00346	rluE
rluE	ZONMW-32_01636	rluE
rluE	ZONMW-32_01454	rluE
rluE	ZONMW-33_02119	rluE
rluE	ZONMW-33_00968	rluE
rluE	ZONMW-33_00213	rluE
rluE	ZONMW-33_01626	rluE
rluE	ZONMW-33_01444	rluE
rluE	ZONMW-34_02252	rluE
rluE	ZONMW-34_01173	rluE
rluE	ZONMW-34_00591	rluE
rluE	ZONMW-34_00012	rluE
rluE	ZONMW-34_01632	rluE
rluE	ZONMW-35_01997	rluE
rluE	ZONMW-35_00246	rluE
rluE	ZONMW-35_00798	rluE
rluE	ZONMW-35_01378	rluE
rluE	ZONMW-35_03596	rluE
rluE	ZONMW-36_02117	rluE
rluE	ZONMW-36_00188	rluE
rluE	ZONMW-36_01156	rluE
rluE	ZONMW-36_01730	rluE
rluE	ZONMW-36_00663	rluE
rluE	ZONMW-37_01088	rluE

rluE	ZONMW-38_02410	rluE
rluE	ZONMW-38_00301	rluE
rluE	ZONMW-39_01528	rluE
rluE	ZONMW-39_00832	rluE
rluE	ZONMW-39_01055	rluE
rluE	ZONMW-39_02522	rluE
rluE	ZONMW-39_00067	rluE
rluE	ZONMW-40_00006	rluE
rluE	ZONMW-41_01937	rluE
rluE	ZONMW-41_01483	rluE
rluE	ZONMW-41_03190	rluE
rluE	ZONMW-41_00883	rluE
rluE	ZONMW-42_00546	rluE
rluE	ZONMW-42_03685	rluE
rluE	ZONMW-42_01546	rluE
rluE	ZONMW-42_00135	rluE
rluE	ZONMW-42_03270	rluE
rluE	ZONMW-43_01980	rluE
rluE	ZONMW-43_03128	rluE
rluE	ZONMW-43_00131	rluE
rluE	ZONMW-43_00936	rluE
rluE	ZONMW-43_01636	rluE
rluE	ZONMW-44_00750	rluE
rluE	ZONMW-44_01466	rluE
rluE	ZONMW-44_03319	rluE
rluE	ZONMW-44_01999	rluE
rluE	ZONMW-44_02833	rluE
rluE	ZONMW-45_02636	rluE
rluE	ZONMW-45_00890	rluE
rluE	ZONMW-45_00499	rluE
rluE	ZONMW-46_02499	rluE
rluE	ZONMW-46_00984	rluE
rluE	ZONMW-46_01340	rluE
rluE	ZONMW-47_02489	rluE
rluE	ZONMW-47_01189	rluE
rluE	ZONMW-47_01512	rluE
rluE	ZONMW-48_00396	rluE
rluE	ZONMW-48_03152	rluE
rluE	ZONMW-48_01440	rluE
rluE	ZONMW-49_02254	rluE
rluE	ZONMW-49_00113	rluE
rluE	ZONMW-49_00449	rluE
rluE	ZONMW-50_02206	rluE
rluE	ZONMW-50_00373	rluE
rluE	ZONMW-50_00036	rluE
rluE	ZONMW-51_01702	rluE
rluE	ZONMW-51_00110	rluE
rluE	ZONMW-51_00438	rluE

rluE	ZONMW-52_01845	rluE
rluE	ZONMW-52_00082	rluE
rluE	ZONMW-52_00422	rluE
rluE	ZONMW-53_01911	rluE
rluE	ZONMW-53_02572	rluE
rluE	ZONMW-53_01154	rluE
rluE	ZONMW-54_02143	rluE
rluE	ZONMW-54_01895	rluE
rluE	ZONMW-54_01567	rluE
rluE	ZONMW-55_02024	rluE
rluE	ZONMW-55_01428	rluE
rluE	ZONMW-55_01088	rluE
rluE	ZONMW-56_01412	rluE
rluE	ZONMW-56_00654	rluE
rluE	ZONMW-56_00318	rluE
rluE	ZONMW-57_01815	rluE
rluE	ZONMW-57_00860	rluE
rluE	ZONMW-57_00530	rluE
rluE	ZONMW-58_01839	rluE
rluE	ZONMW-58_00111	rluE
rluE	ZONMW-58_00441	rluE
rluE	ZONMW-59_01651	rluE
rluE	ZONMW-59_00855	rluE
rluE	ZONMW-59_00518	rluE
rluE	ZONMW-60_02032	rluE
rluE	ZONMW-60_00859	rluE
rluE	ZONMW-60_00532	rluE
rluE	ZONMW-61_01617	rluE
rluE	ZONMW-61_00861	rluE
rluE	ZONMW-61_03292	rluE
rluE	ZONMW-62_01357	rluE
rluE	ZONMW-62_02677	rluE
rluE	ZONMW-62_00485	rluE
rluE	ZONMW-63_02777	rluE
rluE	ZONMW-63_01994	rluE
rluE	ZONMW-63_00204	rluE
rluE	ZONMW-64_01454	rluE
rluE	ZONMW-64_02298	rluE
rluE	ZONMW-64_00148	rluE
rluE	ZONMW-64_01221	rluE
rluE	ZONMW-64_03058	rluE
rluE	ZONMW-65_03486	rluE
rluE	ZONMW-65_03437	rluE
rluE	ZONMW-65_02487	rluE
rluE	ZONMW-66_00598	rluE
rluE	ZONMW-66_00621	rluE
rluE	ZONMW-67_02248	rluE
rluE	ZONMW-67_00807	rluE

rluE	ZONMW-67_01976	rluE
rluE	ZONMW-67_00490	rluE
rluE	ZONMW-67_01566	rluE
rluE	ZONMW-68_00839	rluE
rluE	ZONMW-68_02656	rluE
rluE	ZONMW-68_03631	rluE
rluE	ZONMW-69_02296	rluE
rluE	ZONMW-69_00854	rluE
rluE	ZONMW-69_01967	rluE
rluE	ZONMW-69_00237	rluE
rluE	ZONMW-69_01435	rluE
rluE	ZONMW-72_00126	rluE
rluE	ZONMW-72_01491	rluE
rluE	ZONMW-72_01349	rluE
rluE	ZONMW-73_02956	rluE
rluE	ZONMW-73_01278	rluE
rluE	ZONMW-73_01535	rluE
rluE	ZONMW-74_03326	rluE
rluE	ZONMW-74_02971	rluE
rluE	ZONMW-74_00105	rluE
rluE	ZONMW-75_00145	rluE
rluE	ZONMW-75_00125	rluE
rluE	ZONMW-75_03735	rluE
rluE	da5A-8_01033	rluE
rluE	da5A-8_00118	rluE
rluE	da5A-8_03603	rluE
rplD	18_00206	rplD
rplD	ACAM48_00305	rplD
rplD	CAM030_02820	rplD
rplD	CECT7946_03332	rplD
rplD	CGMCC1.8863_01414	rplD
rplD	CL8139_01449	rplD
rplD	DAU203_02267	rplD
rplD	DD5b_02419	rplD
rplD	DSM1208_02424	rplD
rplD	DSM12212_01084	rplD
rplD	DSM15343_01564	rplD
rplD	DSM15361_02593	rplD
rplD	DSM15365_01135	rplD
rplD	DSM15718_00649	rplD
rplD	DSM16505T_01232	rplD
rplD	DSM18103_01886	rplD
rplD	DSM18293_02415	rplD
rplD	DSM18841_01165	rplD
rplD	DSM18842_00139	rplD
rplD	DSM19866_01078	rplD
rplD	DSM2041_02977	rplD
rplD	DSM2061_03935	rplD

rplD	DSM2064_04685	rplD
rplD	DSM21164_02220	rplD
rplD	DSM23404_00485	rplD
rplD	DSM23424_02886	rplD
rplD	DSM24786_01764	rplD
rplD	DSM7489_02183	rplD
rplD	DSW1_01264	rplD
rplD	DSW5_00474	rplD
rplD	DSW6_00519	rplD
rplD	DSW8_01529	rplD
rplD	F52_01506	rplD
rplD	GCA_001027945.1_02990	rplD
rplD	HI1_02109	rplD
rplD	IR1_03072	rplD
rplD	KCOM3131_00741	rplD
rplD	KCTC12974_01075	rplD
rplD	KL-A_00090	rplD
rplD	LL0412.1.7_01228	rplD
rplD	MAR_2009_71_01896	rplD
rplD	NN016038_00213	rplD
rplD	PAO1_04393	rplD
rplD	UW101_00417	rplD
rplD	V4-33_01246	rplD
rplD	W5C_02878	rplD
rplD	ZONMW-01_01406	rplD
rplD	ZONMW-02_02687	rplD
rplD	ZONMW-03_01374	rplD
rplD	ZONMW-04_00318	rplD
rplD	ZONMW-05_01507	rplD
rplD	ZONMW-06_03632	rplD
rplD	ZONMW-07_01567	rplD
rplD	ZONMW-08_01330	rplD
rplD	ZONMW-09_01349	rplD
rplD	ZONMW-10_01383	rplD
rplD	ZONMW-11_03069	rplD
rplD	ZONMW-12_01418	rplD
rplD	ZONMW-13_01822	rplD
rplD	ZONMW-14_01058	rplD
rplD	ZONMW-15_00771	rplD
rplD	ZONMW-19_01632	rplD
rplD	ZONMW-20_04276	rplD
rplD	ZONMW-21_02466	rplD
rplD	ZONMW-22_02643	rplD
rplD	ZONMW-23_02667	rplD
rplD	ZONMW-24_02490	rplD
rplD	ZONMW-25_03460	rplD
rplD	ZONMW-26_01763	rplD
rplD	ZONMW-27_00312	rplD

rplD	ZONMW-28_03604	rplD
rplD	ZONMW-29_02775	rplD
rplD	ZONMW-30_00079	rplD
rplD	ZONMW-31_03084	rplD
rplD	ZONMW-32_03632	rplD
rplD	ZONMW-33_03589	rplD
rplD	ZONMW-34_03501	rplD
rplD	ZONMW-35_03462	rplD
rplD	ZONMW-36_03389	rplD
rplD	ZONMW-37_02736	rplD
rplD	ZONMW-38_01914	rplD
rplD	ZONMW-39_03878	rplD
rplD	ZONMW-40_02167	rplD
rplD	ZONMW-41_02981	rplD
rplD	ZONMW-42_02226	rplD
rplD	ZONMW-43_03699	rplD
rplD	ZONMW-44_03268	rplD
rplD	ZONMW-45_01766	rplD
rplD	ZONMW-46_00031	rplD
rplD	ZONMW-47_00474	rplD
rplD	ZONMW-48_02031	rplD
rplD	ZONMW-49_00729	rplD
rplD	ZONMW-50_00730	rplD
rplD	ZONMW-51_00978	rplD
rplD	ZONMW-52_01578	rplD
rplD	ZONMW-53_00247	rplD
rplD	ZONMW-54_00783	rplD
rplD	ZONMW-55_00242	rplD
rplD	ZONMW-56_01076	rplD
rplD	ZONMW-57_01375	rplD
rplD	ZONMW-58_01209	rplD
rplD	ZONMW-59_01377	rplD
rplD	ZONMW-60_01198	rplD
rplD	ZONMW-61_01319	rplD
rplD	ZONMW-62_02178	rplD
rplD	ZONMW-63_01190	rplD
rplD	ZONMW-64_03951	rplD
rplD	ZONMW-65_01518	rplD
rplD	ZONMW-66_01161	rplD
rplD	ZONMW-67_03477	rplD
rplD	ZONMW-68_04461	rplD
rplD	ZONMW-69_03479	rplD
rplD	ZONMW-72_01148	rplD
rplD	ZONMW-73_02122	rplD
rplD	ZONMW-74_01022	rplD
rplD	ZONMW-75_02938	rplD
rplD	da5A-8_00881	rplD
pbpC	18_00782	pbpC

pbpC	18_01720	pbpC
pbpC	18_01064	pbpC
pbpC	18_01891	pbpC
pbpC	ACAM48_02488	pbpC
pbpC	ACAM48_01780	pbpC
pbpC	ACAM48_02452	pbpC
pbpC	CAM030_02886	pbpC
pbpC	CAM030_00866	pbpC
pbpC	CAM030_00952	pbpC
pbpC	CAM030_01129	pbpC
pbpC	CECT7946_01926	pbpC
pbpC	CECT7946_01970	pbpC
pbpC	CECT7946_00216	pbpC
pbpC	CECT7946_00051	pbpC
pbpC	CGMCC1.8863_02171	pbpC
pbpC	CGMCC1.8863_00868	pbpC
pbpC	CGMCC1.8863_02759	pbpC
pbpC	CL8139_01624	pbpC
pbpC	CL8139_00060	pbpC
pbpC	CL8139_00387	pbpC
pbpC	DAU203_01231	pbpC
pbpC	DAU203_00525	pbpC
pbpC	DAU203_01533	pbpC
pbpC	DD5b_01840	pbpC
pbpC	DD5b_02041	pbpC
pbpC	DD5b_02014	pbpC
pbpC	DD5b_01926	pbpC
pbpC	DSM1208_01426	pbpC
pbpC	DSM1208_01746	pbpC
pbpC	DSM1208_00593	pbpC
pbpC	DSM12212_02065	pbpC
pbpC	DSM12212_02333	pbpC
pbpC	DSM12212_00144	pbpC
pbpC	DSM12212_03432	pbpC
pbpC	DSM15343_02553	pbpC
pbpC	DSM15343_00978	pbpC
pbpC	DSM15343_02392	pbpC
pbpC	DSM15361_00427	pbpC
pbpC	DSM15361_00952	pbpC
pbpC	DSM15361_00467	pbpC
pbpC	DSM15365_03214	pbpC
pbpC	DSM15365_00935	pbpC
pbpC	DSM15365_02198	pbpC
pbpC	DSM15718_02452	pbpC
pbpC	DSM15718_02504	pbpC
pbpC	DSM15718_02942	pbpC
pbpC	DSM16505T_01295	pbpC
pbpC	DSM16505T_00337	pbpC

pbpC	DSM16505T_00428	pbpC
pbpC	DSM18103_03352	pbpC
pbpC	DSM18103_03467	pbpC
pbpC	DSM18103_03026	pbpC
pbpC	DSM18293_00615	pbpC
pbpC	DSM18293_03180	pbpC
pbpC	DSM18293_03247	pbpC
pbpC	DSM18293_00873	pbpC
pbpC	DSM18841_02289	pbpC
pbpC	DSM18841_00145	pbpC
pbpC	DSM18841_00258	pbpC
pbpC	DSM18842_01202	pbpC
pbpC	DSM18842_01061	pbpC
pbpC	DSM18842_00965	pbpC
pbpC	DSM19866_03498	pbpC
pbpC	DSM19866_01981	pbpC
pbpC	DSM19866_00865	pbpC
pbpC	DSM2041_04463	pbpC
pbpC	DSM2041_03249	pbpC
pbpC	DSM2041_00048	pbpC
pbpC	DSM2041_00049	pbpC
pbpC	DSM2041_00464	pbpC
pbpC	DSM2061_02947	pbpC
pbpC	DSM2061_03277	pbpC
pbpC	DSM2061_02089	pbpC
pbpC	DSM2064_02543	pbpC
pbpC	DSM2064_01877	pbpC
pbpC	DSM2064_01816	pbpC
pbpC	DSM2064_01614	pbpC
pbpC	DSM21164_01060	pbpC
pbpC	DSM21164_00152	pbpC
pbpC	DSM21164_00807	pbpC
pbpC	DSM21164_02847	pbpC
pbpC	DSM23404_02032	pbpC
pbpC	DSM23404_02460	pbpC
pbpC	DSM23404_02068	pbpC
pbpC	DSM23424_00458	pbpC
pbpC	DSM23424_00268	pbpC
pbpC	DSM23424_00398	pbpC
pbpC	DSM24786_02879	pbpC
pbpC	DSM24786_03022	pbpC
pbpC	DSM24786_02602	pbpC
pbpC	DSM7489_01139	pbpC
pbpC	DSM7489_00512	pbpC
pbpC	DSM7489_01437	pbpC
pbpC	DSW1_00880	pbpC
pbpC	DSW1_00543	pbpC
pbpC	DSW5_00885	pbpC

pbpC	DSW6_01245	pbpC
pbpC	DSW6_01212	pbpC
pbpC	DSW8_02191	pbpC
pbpC	DSW8_03413	pbpC
pbpC	F52_02941	pbpC
pbpC	F52_02313	pbpC
pbpC	F52_02253	pbpC
pbpC	F52_02070	pbpC
pbpC	GCA_001027945.1_01149	pbpC
pbpC	GCA_001027945.1_00451	pbpC
pbpC	GCA_001027945.1_00354	pbpC
pbpC	HI1_01082	pbpC
pbpC	HI1_00444	pbpC
pbpC	HI1_01378	pbpC
pbpC	IR1_00335	pbpC
pbpC	IR1_03933	pbpC
pbpC	IR1_01150	pbpC
pbpC	IR1_04015	pbpC
pbpC	KCOM3131_00070	pbpC
pbpC	KCTC12974_02871	pbpC
pbpC	KCTC12974_02753	pbpC
pbpC	KCTC12974_02908	pbpC
pbpC	KL-A_02085	pbpC
pbpC	KL-A_01694	pbpC
pbpC	KL-A_00789	pbpC
pbpC	LL0412.1.7_02194	pbpC
pbpC	LL0412.1.7_00639	pbpC
pbpC	LL0412.1.7_00362	pbpC
pbpC	MAR_2009_71_01249	pbpC
pbpC	MAR_2009_71_00170	pbpC
pbpC	NN016038_00802	pbpC
pbpC	NN016038_01728	pbpC
pbpC	NN016038_01741	pbpC
pbpC	NN016038_01080	pbpC
pbpC	NN016038_01917	pbpC
pbpC	PAO1_04860	pbpC
pbpC	PAO1_05215	pbpC
pbpC	PAO1_00388	pbpC
pbpC	UW101_01597	pbpC
pbpC	UW101_00931	pbpC
pbpC	UW101_00870	pbpC
pbpC	UW101_00668	pbpC
pbpC	V4-33_02156	pbpC
pbpC	V4-33_02268	pbpC
pbpC	V4-33_00011	pbpC
pbpC	V4-33_00085	pbpC
pbpC	W5C_01983	pbpC
pbpC	W5C_01136	pbpC

pbpC	W5C_02790	pbpC
pbpC	ZONMW-01_01126	pbpC
pbpC	ZONMW-01_01965	pbpC
pbpC	ZONMW-01_02434	pbpC
pbpC	ZONMW-02_00144	pbpC
pbpC	ZONMW-02_01652	pbpC
pbpC	ZONMW-02_02777	pbpC
pbpC	ZONMW-03_02195	pbpC
pbpC	ZONMW-03_02949	pbpC
pbpC	ZONMW-03_03037	pbpC
pbpC	ZONMW-04_01623	pbpC
pbpC	ZONMW-04_02498	pbpC
pbpC	ZONMW-04_02406	pbpC
pbpC	ZONMW-05_01901	pbpC
pbpC	ZONMW-05_02129	pbpC
pbpC	ZONMW-05_02618	pbpC
pbpC	ZONMW-06_01958	pbpC
pbpC	ZONMW-06_03725	pbpC
pbpC	ZONMW-06_00632	pbpC
pbpC	ZONMW-07_00716	pbpC
pbpC	ZONMW-07_01109	pbpC
pbpC	ZONMW-07_01443	pbpC
pbpC	ZONMW-07_04073	pbpC
pbpC	ZONMW-08_00769	pbpC
pbpC	ZONMW-08_02880	pbpC
pbpC	ZONMW-09_01023	pbpC
pbpC	ZONMW-09_00315	pbpC
pbpC	ZONMW-09_00418	pbpC
pbpC	ZONMW-10_00454	pbpC
pbpC	ZONMW-10_00186	pbpC
pbpC	ZONMW-10_00515	pbpC
pbpC	ZONMW-11_00519	pbpC
pbpC	ZONMW-11_02838	pbpC
pbpC	ZONMW-12_00208	pbpC
pbpC	ZONMW-12_03085	pbpC
pbpC	ZONMW-12_00344	pbpC
pbpC	ZONMW-13_00315	pbpC
pbpC	ZONMW-13_01988	pbpC
pbpC	ZONMW-13_00427	pbpC
pbpC	ZONMW-14_00277	pbpC
pbpC	ZONMW-14_00706	pbpC
pbpC	ZONMW-14_00137	pbpC
pbpC	ZONMW-15_01801	pbpC
pbpC	ZONMW-15_02597	pbpC
pbpC	ZONMW-15_02198	pbpC
pbpC	ZONMW-19_03037	pbpC
pbpC	ZONMW-19_02418	pbpC
pbpC	ZONMW-19_01032	pbpC

pbpC	ZONMW-19_03841	pbpC
pbpC	ZONMW-20_03011	pbpC
pbpC	ZONMW-20_00921	pbpC
pbpC	ZONMW-20_03394	pbpC
pbpC	ZONMW-20_02843	pbpC
pbpC	ZONMW-21_01408	pbpC
pbpC	ZONMW-21_00728	pbpC
pbpC	ZONMW-22_01410	pbpC
pbpC	ZONMW-22_00729	pbpC
pbpC	ZONMW-23_02523	pbpC
pbpC	ZONMW-23_01843	pbpC
pbpC	ZONMW-24_00051	pbpC
pbpC	ZONMW-24_00732	pbpC
pbpC	ZONMW-25_03116	pbpC
pbpC	ZONMW-25_01660	pbpC
pbpC	ZONMW-26_02495	pbpC
pbpC	ZONMW-26_02798	pbpC
pbpC	ZONMW-26_03055	pbpC
pbpC	ZONMW-27_01674	pbpC
pbpC	ZONMW-27_02543	pbpC
pbpC	ZONMW-27_02775	pbpC
pbpC	ZONMW-28_02625	pbpC
pbpC	ZONMW-28_03896	pbpC
pbpC	ZONMW-28_00744	pbpC
pbpC	ZONMW-29_00962	pbpC
pbpC	ZONMW-29_00371	pbpC
pbpC	ZONMW-30_00685	pbpC
pbpC	ZONMW-30_02336	pbpC
pbpC	ZONMW-31_01896	pbpC
pbpC	ZONMW-31_03181	pbpC
pbpC	ZONMW-31_01017	pbpC
pbpC	ZONMW-32_03251	pbpC
pbpC	ZONMW-32_04005	pbpC
pbpC	ZONMW-32_00347	pbpC
pbpC	ZONMW-33_03023	pbpC
pbpC	ZONMW-33_03962	pbpC
pbpC	ZONMW-33_00214	pbpC
pbpC	ZONMW-34_02629	pbpC
pbpC	ZONMW-34_03793	pbpC
pbpC	ZONMW-34_00592	pbpC
pbpC	ZONMW-35_02593	pbpC
pbpC	ZONMW-35_03747	pbpC
pbpC	ZONMW-35_00797	pbpC
pbpC	ZONMW-36_02979	pbpC
pbpC	ZONMW-36_03964	pbpC
pbpC	ZONMW-36_01155	pbpC
pbpC	ZONMW-37_00954	pbpC
pbpC	ZONMW-37_00728	pbpC

pbpC	ZONMW-38_01717	pbpC
pbpC	ZONMW-38_02269	pbpC
pbpC	ZONMW-38_03419	pbpC
pbpC	ZONMW-38_00273	pbpC
pbpC	ZONMW-39_03962	pbpC
pbpC	ZONMW-39_04150	pbpC
pbpC	ZONMW-39_01056	pbpC
pbpC	ZONMW-40_02778	pbpC
pbpC	ZONMW-40_00472	pbpC
pbpC	ZONMW-41_00961	pbpC
pbpC	ZONMW-41_00427	pbpC
pbpC	ZONMW-41_00444	pbpC
pbpC	ZONMW-41_00716	pbpC
pbpC	ZONMW-42_04070	pbpC
pbpC	ZONMW-42_02321	pbpC
pbpC	ZONMW-43_01257	pbpC
pbpC	ZONMW-43_03746	pbpC
pbpC	ZONMW-43_00132	pbpC
pbpC	ZONMW-44_00021	pbpC
pbpC	ZONMW-44_03176	pbpC
pbpC	ZONMW-44_01569	pbpC
pbpC	ZONMW-45_03162	pbpC
pbpC	ZONMW-45_03062	pbpC
pbpC	ZONMW-45_00408	pbpC
pbpC	ZONMW-46_02900	pbpC
pbpC	ZONMW-46_01558	pbpC
pbpC	ZONMW-46_01431	pbpC
pbpC	ZONMW-47_02973	pbpC
pbpC	ZONMW-47_01294	pbpC
pbpC	ZONMW-47_01421	pbpC
pbpC	ZONMW-48_00188	pbpC
pbpC	ZONMW-48_00584	pbpC
pbpC	ZONMW-48_02609	pbpC
pbpC	ZONMW-49_02886	pbpC
pbpC	ZONMW-49_02033	pbpC
pbpC	ZONMW-49_01160	pbpC
pbpC	ZONMW-50_02770	pbpC
pbpC	ZONMW-50_02629	pbpC
pbpC	ZONMW-50_01161	pbpC
pbpC	ZONMW-51_02888	pbpC
pbpC	ZONMW-51_02339	pbpC
pbpC	ZONMW-51_02766	pbpC
pbpC	ZONMW-52_02986	pbpC
pbpC	ZONMW-52_02754	pbpC
pbpC	ZONMW-52_02280	pbpC
pbpC	ZONMW-53_02993	pbpC
pbpC	ZONMW-53_02504	pbpC
pbpC	ZONMW-53_02869	pbpC

pbpC	ZONMW-54_02994	pbpC
pbpC	ZONMW-54_02564	pbpC
pbpC	ZONMW-54_00085	pbpC
pbpC	ZONMW-55_03000	pbpC
pbpC	ZONMW-55_01649	pbpC
pbpC	ZONMW-55_02875	pbpC
pbpC	ZONMW-56_02879	pbpC
pbpC	ZONMW-56_01799	pbpC
pbpC	ZONMW-56_02635	pbpC
pbpC	ZONMW-57_02862	pbpC
pbpC	ZONMW-57_02341	pbpC
pbpC	ZONMW-57_02779	pbpC
pbpC	ZONMW-58_02886	pbpC
pbpC	ZONMW-58_02365	pbpC
pbpC	ZONMW-58_02803	pbpC
pbpC	ZONMW-59_03088	pbpC
pbpC	ZONMW-59_02579	pbpC
pbpC	ZONMW-59_02925	pbpC
pbpC	ZONMW-60_02980	pbpC
pbpC	ZONMW-60_02443	pbpC
pbpC	ZONMW-60_02898	pbpC
pbpC	ZONMW-61_00363	pbpC
pbpC	ZONMW-61_02824	pbpC
pbpC	ZONMW-62_01910	pbpC
pbpC	ZONMW-62_03509	pbpC
pbpC	ZONMW-63_02850	pbpC
pbpC	ZONMW-63_02510	pbpC
pbpC	ZONMW-63_02294	pbpC
pbpC	ZONMW-64_04189	pbpC
pbpC	ZONMW-64_04142	pbpC
pbpC	ZONMW-64_00149	pbpC
pbpC	ZONMW-65_01487	pbpC
pbpC	ZONMW-65_01446	pbpC
pbpC	ZONMW-66_03592	pbpC
pbpC	ZONMW-66_00310	pbpC
pbpC	ZONMW-66_01117	pbpC
pbpC	ZONMW-66_01890	pbpC
pbpC	ZONMW-67_03294	pbpC
pbpC	ZONMW-67_03571	pbpC
pbpC	ZONMW-67_00819	pbpC
pbpC	ZONMW-68_00619	pbpC
pbpC	ZONMW-68_00124	pbpC
pbpC	ZONMW-68_02260	pbpC
pbpC	ZONMW-69_03295	pbpC
pbpC	ZONMW-69_03573	pbpC
pbpC	ZONMW-69_00866	pbpC
pbpC	ZONMW-72_03776	pbpC
pbpC	ZONMW-72_02247	pbpC

pbpC	ZONMW-72_01634	pbpC
pbpC	ZONMW-72_02329	pbpC
pbpC	ZONMW-73_01829	pbpC
pbpC	ZONMW-73_02219	pbpC
pbpC	ZONMW-74_02168	pbpC
pbpC	ZONMW-74_01449	pbpC
pbpC	ZONMW-74_02411	pbpC
pbpC	ZONMW-75_01014	pbpC
pbpC	ZONMW-75_00450	pbpC
pbpC	ZONMW-75_00266	pbpC
pbpC	da5A-8_03559	pbpC
pbpC	da5A-8_02447	pbpC
pbpC	da5A-8_01941	pbpC
lipA	18_01798	lipA
lipA	ACAM48_03413	lipA
lipA	CAM030_02667	lipA
lipA	CECT7946_00956	lipA
lipA	CGMCC1.8863_01607	lipA
lipA	CL8139_02930	lipA
lipA	DAU203_00614	lipA
lipA	DD5b_00472	lipA
lipA	DSM1208_00685	lipA
lipA	DSM12212_02903	lipA
lipA	DSM15343_01786	lipA
lipA	DSM15361_02648	lipA
lipA	DSM15365_03809	lipA
lipA	DSM15718_00819	lipA
lipA	DSM16505T_01761	lipA
lipA	DSM18103_00785	lipA
lipA	DSM18293_02578	lipA
lipA	DSM18841_01862	lipA
lipA	DSM18842_02155	lipA
lipA	DSM19866_00540	lipA
lipA	DSM2041_02811	lipA
lipA	DSM2061_02179	lipA
lipA	DSM2064_04857	lipA
lipA	DSM21164_00082	lipA
lipA	DSM23404_00751	lipA
lipA	DSM23424_02536	lipA
lipA	DSM24786_01147	lipA
lipA	DSM7489_00600	lipA
lipA	DSW1_02883	lipA
lipA	DSW5_02579	lipA
lipA	DSW6_00562	lipA
lipA	DSW8_03694	lipA
lipA	F52_01664	lipA
lipA	GCA_001027945.1_02633	lipA
lipA	HI1_00532	lipA

lipA	IR1_02913	lipA
lipA	KCOM3131_00325	lipA
lipA	KCTC12974_01011	lipA
lipA	KL-A_02559	lipA
lipA	LL0412.1.7_02073	lipA
lipA	MAR_2009_71_00262	lipA
lipA	NN016038_01819	lipA
lipA	PAO1_04124	lipA
lipA	UW101_00245	lipA
lipA	V4-33_01377	lipA
lipA	W5C_01242	lipA
lipA	ZONMW-01_02796	lipA
lipA	ZONMW-02_03055	lipA
lipA	ZONMW-03_03110	lipA
lipA	ZONMW-04_02810	lipA
lipA	ZONMW-05_03067	lipA
lipA	ZONMW-06_02961	lipA
lipA	ZONMW-07_02786	lipA
lipA	ZONMW-08_03342	lipA
lipA	ZONMW-09_01878	lipA
lipA	ZONMW-10_01651	lipA
lipA	ZONMW-11_02186	lipA
lipA	ZONMW-12_01295	lipA
lipA	ZONMW-13_01691	lipA
lipA	ZONMW-14_00934	lipA
lipA	ZONMW-15_01200	lipA
lipA	ZONMW-19_01236	lipA
lipA	ZONMW-20_02296	lipA
lipA	ZONMW-21_03079	lipA
lipA	ZONMW-22_03104	lipA
lipA	ZONMW-23_03125	lipA
lipA	ZONMW-24_03126	lipA
lipA	ZONMW-25_01860	lipA
lipA	ZONMW-26_03134	lipA
lipA	ZONMW-27_03156	lipA
lipA	ZONMW-28_03274	lipA
lipA	ZONMW-29_02606	lipA
lipA	ZONMW-30_00974	lipA
lipA	ZONMW-31_03519	lipA
lipA	ZONMW-32_03399	lipA
lipA	ZONMW-33_03402	lipA
lipA	ZONMW-34_02070	lipA
lipA	ZONMW-35_01762	lipA
lipA	ZONMW-36_03259	lipA
lipA	ZONMW-37_03113	lipA
lipA	ZONMW-38_02950	lipA
lipA	ZONMW-39_04159	lipA
lipA	ZONMW-40_01099	lipA

lipA	ZONMW-41_02893	lipA
lipA	ZONMW-42_00365	lipA
lipA	ZONMW-43_02878	lipA
lipA	ZONMW-44_00947	lipA
lipA	ZONMW-45_00190	lipA
lipA	ZONMW-46_00405	lipA
lipA	ZONMW-47_00100	lipA
lipA	ZONMW-48_02493	lipA
lipA	ZONMW-49_02739	lipA
lipA	ZONMW-50_02665	lipA
lipA	ZONMW-51_03036	lipA
lipA	ZONMW-52_03038	lipA
lipA	ZONMW-53_03098	lipA
lipA	ZONMW-54_03100	lipA
lipA	ZONMW-55_01560	lipA
lipA	ZONMW-56_02985	lipA
lipA	ZONMW-57_03063	lipA
lipA	ZONMW-58_03084	lipA
lipA	ZONMW-59_03139	lipA
lipA	ZONMW-60_03075	lipA
lipA	ZONMW-61_03694	lipA
lipA	ZONMW-62_03739	lipA
lipA	ZONMW-63_02951	lipA
lipA	ZONMW-64_03596	lipA
lipA	ZONMW-65_02445	lipA
lipA	ZONMW-66_03441	lipA
lipA	ZONMW-67_02459	lipA
lipA	ZONMW-68_03604	lipA
lipA	ZONMW-69_02507	lipA
lipA	ZONMW-72_01480	lipA
lipA	ZONMW-73_01228	lipA
lipA	ZONMW-74_02504	lipA
lipA	ZONMW-75_03440	lipA
lipA	da5A-8_01447	lipA
infC	18_02058	infC
infC	ACAM48_01440	infC
infC	CAM030_01794	infC
infC	CECT7946_02773	infC
infC	CGMCC1.8863_02817	infC
infC	CL8139_03220	infC
infC	DAU203_02555	infC
infC	DD5b_01123	infC
infC	DSM1208_02929	infC
infC	DSM12212_03453	infC
infC	DSM15343_02865	infC
infC	DSM15361_02237	infC
infC	DSM15365_01299	infC
infC	DSM15718_03368	infC

infC	DSM18103_01547	infC
infC	DSM18293_02792	infC
infC	DSM18841_02473	infC
infC	DSM18842_02380	infC
infC	DSM19866_02698	infC
infC	DSM2041_05501	infC
infC	DSM2061_04412	infC
infC	DSM2064_05071	infC
infC	DSM21164_03004	infC
infC	DSM23404_00002	infC
infC	DSM23424_03341	infC
infC	DSM24786_01471	infC
infC	DSM7489_02446	infC
infC	DSW1_00062	infC
infC	DSW5_01777	infC
infC	DSW6_00221	infC
infC	DSW8_01025	infC
infC	F52_01926	infC
infC	GCA_001027945.1_01090	infC
infC	HI1_02378	infC
infC	IR1_02714	infC
infC	KCOM3131_01692	infC
infC	KCTC12974_00684	infC
infC	KL-A_03250	infC
infC	L12_00026	infC
infC	LL0412.1.7_00002	infC
infC	MAR_2009_71_02416	infC
infC	NN016038_02076	infC
infC	PAO1_02826	infC
infC	UW101_00031	infC
infC	V4-33_00809	infC
infC	W5C_02094	infC
infC	ZONMW-01_03267	infC
infC	ZONMW-02_00854	infC
infC	ZONMW-03_03397	infC
infC	ZONMW-04_00002	infC
infC	ZONMW-05_01826	infC
infC	ZONMW-06_03483	infC
infC	ZONMW-07_03776	infC
infC	ZONMW-08_00821	infC
infC	ZONMW-09_01789	infC
infC	ZONMW-10_01433	infC
infC	ZONMW-11_00950	infC
infC	ZONMW-12_04457	infC
infC	ZONMW-13_03141	infC
infC	ZONMW-14_01777	infC
infC	ZONMW-15_02037	infC
infC	ZONMW-19_02928	infC

infC	ZONMW-20_01639	infC
infC	ZONMW-21_03392	infC
infC	ZONMW-22_03333	infC
infC	ZONMW-23_03438	infC
infC	ZONMW-24_03440	infC
infC	ZONMW-25_03130	infC
infC	ZONMW-26_01461	infC
infC	ZONMW-27_00002	infC
infC	ZONMW-28_02303	infC
infC	ZONMW-29_02904	infC
infC	ZONMW-30_02869	infC
infC	ZONMW-31_02431	infC
infC	ZONMW-32_02709	infC
infC	ZONMW-33_02409	infC
infC	ZONMW-34_01731	infC
infC	ZONMW-35_01422	infC
infC	ZONMW-36_03840	infC
infC	ZONMW-37_03342	infC
infC	ZONMW-38_00215	infC
infC	ZONMW-39_03753	infC
infC	ZONMW-40_03466	infC
infC	ZONMW-41_01491	infC
infC	ZONMW-42_00716	infC
infC	ZONMW-43_03347	infC
infC	ZONMW-44_02627	infC
infC	ZONMW-45_02682	infC
infC	ZONMW-46_02545	infC
infC	ZONMW-47_02535	infC
infC	ZONMW-48_03340	infC
infC	ZONMW-49_00486	infC
infC	ZONMW-50_00487	infC
infC	ZONMW-51_01218	infC
infC	ZONMW-52_02912	infC
infC	ZONMW-53_00002	infC
infC	ZONMW-54_01034	infC
infC	ZONMW-55_00002	infC
infC	ZONMW-56_02808	infC
infC	ZONMW-57_01616	infC
infC	ZONMW-58_00968	infC
infC	ZONMW-59_02720	infC
infC	ZONMW-60_03256	infC
infC	ZONMW-61_04009	infC
infC	ZONMW-62_02593	infC
infC	ZONMW-63_03289	infC
infC	ZONMW-64_01823	infC
infC	ZONMW-65_03081	infC
infC	ZONMW-66_00859	infC
infC	ZONMW-67_03220	infC

infC	ZONMW-68_02498	infC
infC	ZONMW-69_03221	infC
infC	ZONMW-72_01281	infC
infC	ZONMW-73_02290	infC
infC	ZONMW-74_00693	infC
infC	ZONMW-75_00937	infC
infC	da5A-8_00208	infC
groL	18_00479	groL
groL	ACAM48_02775	groL
groL	CAM030_02404	groL
groL	CECT7946_00981	groL
groL	CGMCC1.8863_02507	groL
groL	CL8139_02419	groL
groL	DAU203_00852	groL
groL	DD5b_02736	groL
groL	DSM1208_01121	groL
groL	DSM12212_02937	groL
groL	DSM15343_02094	groL
groL	DSM15361_01326	groL
groL	DSM15365_00415	groL
groL	DSM15718_00427	groL
groL	DSM16505T_01022	groL
groL	DSM18103_02531	groL
groL	DSM18103_02533	groL
groL	DSM18293_02129	groL
groL	DSM18841_00903	groL
groL	DSM18842_00404	groL
groL	DSM19866_03802	groL
groL	DSM2041_00801	groL
groL	DSM2061_02638	groL
groL	DSM2064_02868	groL
groL	DSM21164_01347	groL
groL	DSM23404_02164	groL
groL	DSM23424_00874	groL
groL	DSM24786_01334	groL
groL	DSM7489_00785	groL
groL	DSW1_00032	groL
groL	DSW5_00732	groL
groL	DSW6_00843	groL
groL	DSW8_02814	groL
groL	F52_03236	groL
groL	GCA_001027945.1_01521	groL
groL	HI1_00720	groL
groL	IR1_00023	groL
groL	KCOM3131_00311	groL
groL	KCTC12974_01157	groL
groL	KL-A_02441	groL
groL	LL0412.1.7_01050	groL

groL	MAR_2009_71_00640	groL
groL	NN016038_00488	groL
groL	PAO1_04528	groL
groL	UW101_01922	groL
groL	V4-33_01900	groL
groL	W5C_01411	groL
groL	ZONMW-01_02678	groL
groL	ZONMW-02_03650	groL
groL	ZONMW-03_01704	groL
groL	ZONMW-04_02288	groL
groL	ZONMW-05_02804	groL
groL	ZONMW-06_01076	groL
groL	ZONMW-07_00485	groL
groL	ZONMW-08_00156	groL
groL	ZONMW-09_01651	groL
groL	ZONMW-10_01135	groL
groL	ZONMW-11_01005	groL
groL	ZONMW-12_01233	groL
groL	ZONMW-13_00759	groL
groL	ZONMW-14_01287	groL
groL	ZONMW-15_01051	groL
groL	ZONMW-19_02997	groL
groL	ZONMW-20_03120	groL
groL	ZONMW-21_01369	groL
groL	ZONMW-22_01371	groL
groL	ZONMW-23_02484	groL
groL	ZONMW-24_00090	groL
groL	ZONMW-25_02844	groL
groL	ZONMW-26_02144	groL
groL	ZONMW-27_02267	groL
groL	ZONMW-28_03564	groL
groL	ZONMW-29_00916	groL
groL	ZONMW-30_00649	groL
groL	ZONMW-31_01868	groL
groL	ZONMW-32_00649	groL
groL	ZONMW-33_00517	groL
groL	ZONMW-34_00881	groL
groL	ZONMW-35_02362	groL
groL	ZONMW-36_00854	groL
groL	ZONMW-37_00993	groL
groL	ZONMW-38_03569	groL
groL	ZONMW-39_01344	groL
groL	ZONMW-40_02737	groL
groL	ZONMW-41_03012	groL
groL	ZONMW-42_00999	groL
groL	ZONMW-43_00414	groL
groL	ZONMW-44_02265	groL
groL	ZONMW-45_01454	groL

groL	ZONMW-46_01709	groL
groL	ZONMW-47_01780	groL
groL	ZONMW-48_00467	groL
groL	ZONMW-49_02314	groL
groL	ZONMW-50_02146	groL
groL	ZONMW-51_01642	groL
groL	ZONMW-52_01785	groL
groL	ZONMW-53_01971	groL
groL	ZONMW-54_02083	groL
groL	ZONMW-55_01964	groL
groL	ZONMW-56_01472	groL
groL	ZONMW-57_01754	groL
groL	ZONMW-58_01778	groL
groL	ZONMW-59_01590	groL
groL	ZONMW-60_01972	groL
groL	ZONMW-61_01531	groL
groL	ZONMW-62_01283	groL
groL	ZONMW-63_01490	groL
groL	ZONMW-64_00432	groL
groL	ZONMW-65_02177	groL
groL	ZONMW-66_02480	groL
groL	ZONMW-67_01104	groL
groL	ZONMW-68_00919	groL
groL	ZONMW-69_01151	groL
groL	ZONMW-72_00659	groL
groL	ZONMW-73_00608	groL
groL	ZONMW-74_02790	groL
groL	ZONMW-75_03160	groL
groL	da5A-8_00079	groL
btsR_2	18_02419	btsR_2
btsR_2	18_00243	btsR_2
btsR_2	18_03423	btsR_2
btsR_2	18_03634	btsR_2
btsR_2	18_01229	btsR_2
btsR_2	18_01732	btsR_2
btsR_2	18_01594	btsR_2
btsR_2	18_03352	btsR_2
btsR_2	18_03320	btsR_2
btsR_2	18_02856	btsR_2
btsR_2	18_03766	btsR_2
btsR_2	18_00924	btsR_2
btsR_2	18_02780	btsR_2
btsR_2	18_03045	btsR_2
btsR_2	18_03772	btsR_2
btsR_2	18_03382	btsR_2
btsR_2	ACAM48_01179	btsR_2
btsR_2	ACAM48_00217	btsR_2
btsR_2	ACAM48_00892	btsR_2

btsR_2	ACAM48_01116	btsR_2
btsR_2	ACAM48_02420	btsR_2
btsR_2	ACAM48_02802	btsR_2
btsR_2	ACAM48_01962	btsR_2
btsR_2	ACAM48_01273	btsR_2
btsR_2	ACAM48_01613	btsR_2
btsR_2	ACAM48_02418	btsR_2
btsR_2	CAM030_00274	btsR_2
btsR_2	CAM030_01217	btsR_2
btsR_2	CAM030_01459	btsR_2
btsR_2	CAM030_02196	btsR_2
btsR_2	CAM030_00181	btsR_2
btsR_2	CAM030_02336	btsR_2
btsR_2	CAM030_00823	btsR_2
btsR_2	CAM030_01787	btsR_2
btsR_2	CECT7946_01830	btsR_2
btsR_2	CECT7946_00914	btsR_2
btsR_2	CECT7946_03372	btsR_2
btsR_2	CECT7946_00787	btsR_2
btsR_2	CECT7946_02910	btsR_2
btsR_2	CECT7946_00592	btsR_2
btsR_2	CECT7946_00796	btsR_2
btsR_2	CECT7946_02902	btsR_2
btsR_2	CECT7946_01265	btsR_2
btsR_2	CECT7946_00505	btsR_2
btsR_2	CECT7946_02546	btsR_2
btsR_2	CECT7946_01572	btsR_2
btsR_2	CECT7946_00801	btsR_2
btsR_2	CECT7946_03163	btsR_2
btsR_2	CECT7946_03072	btsR_2
btsR_2	CECT7946_01252	btsR_2
btsR_2	CECT7946_01728	btsR_2
btsR_2	CGMCC1.8863_03574	btsR_2
btsR_2	CGMCC1.8863_01805	btsR_2
btsR_2	CGMCC1.8863_03067	btsR_2
btsR_2	CGMCC1.8863_03200	btsR_2
btsR_2	CGMCC1.8863_00949	btsR_2
btsR_2	CGMCC1.8863_01841	btsR_2
btsR_2	CGMCC1.8863_03119	btsR_2
btsR_2	CGMCC1.8863_01714	btsR_2
btsR_2	CGMCC1.8863_02867	btsR_2
btsR_2	CGMCC1.8863_01838	btsR_2
btsR_2	CGMCC1.8863_02666	btsR_2
btsR_2	CGMCC1.8863_03240	btsR_2
btsR_2	CGMCC1.8863_02911	btsR_2
btsR_2	CGMCC1.8863_01702	btsR_2
btsR_2	CL8139_02266	btsR_2
btsR_2	CL8139_00692	btsR_2

btsR_2	CL8139_01477	btsR_2
btsR_2	CL8139_03165	btsR_2
btsR_2	CL8139_03043	btsR_2
btsR_2	CL8139_01814	btsR_2
btsR_2	CL8139_01984	btsR_2
btsR_2	CL8139_03223	btsR_2
btsR_2	CL8139_01003	btsR_2
btsR_2	CL8139_02673	btsR_2
btsR_2	CL8139_02194	btsR_2
btsR_2	CL8139_02306	btsR_2
btsR_2	CL8139_00356	btsR_2
btsR_2	CL8139_00767	btsR_2
btsR_2	CL8139_00272	btsR_2
btsR_2	CL8139_02491	btsR_2
btsR_2	CL8139_02521	btsR_2
btsR_2	CL8139_00076	btsR_2
btsR_2	DAU203_01346	btsR_2
btsR_2	DAU203_03032	btsR_2
btsR_2	DAU203_02242	btsR_2
btsR_2	DAU203_02456	btsR_2
btsR_2	DAU203_03098	btsR_2
btsR_2	DAU203_03418	btsR_2
btsR_2	DAU203_00329	btsR_2
btsR_2	DAU203_03287	btsR_2
btsR_2	DAU203_02021	btsR_2
btsR_2	DAU203_01780	btsR_2
btsR_2	DAU203_00074	btsR_2
btsR_2	DAU203_02693	btsR_2
btsR_2	DAU203_00980	btsR_2
btsR_2	DAU203_00168	btsR_2
btsR_2	DAU203_01564	btsR_2
btsR_2	DAU203_01406	btsR_2
btsR_2	DAU203_00814	btsR_2
btsR_2	DAU203_02838	btsR_2
btsR_2	DAU203_02868	btsR_2
btsR_2	DAU203_03328	btsR_2
btsR_2	DD5b_01018	btsR_2
btsR_2	DD5b_01254	btsR_2
btsR_2	DD5b_01409	btsR_2
btsR_2	DD5b_01042	btsR_2
btsR_2	DD5b_01309	btsR_2
btsR_2	DD5b_01988	btsR_2
btsR_2	DD5b_02722	btsR_2
btsR_2	DSM1208_02470	btsR_2
btsR_2	DSM1208_03178	btsR_2
btsR_2	DSM1208_03128	btsR_2
btsR_2	DSM1208_00116	btsR_2
btsR_2	DSM1208_00027	btsR_2

btsR_2	DSM1208_04122	btsR_2
btsR_2	DSM1208_00152	btsR_2
btsR_2	DSM1208_00536	btsR_2
btsR_2	DSM1208_02461	btsR_2
btsR_2	DSM1208_02317	btsR_2
btsR_2	DSM1208_01828	btsR_2
btsR_2	DSM1208_02874	btsR_2
btsR_2	DSM1208_01579	btsR_2
btsR_2	DSM1208_03516	btsR_2
btsR_2	DSM1208_03867	btsR_2
btsR_2	DSM1208_04547	btsR_2
btsR_2	DSM1208_01087	btsR_2
btsR_2	DSM12212_02576	btsR_2
btsR_2	DSM12212_02504	btsR_2
btsR_2	DSM12212_01957	btsR_2
btsR_2	DSM12212_01408	btsR_2
btsR_2	DSM12212_03397	btsR_2
btsR_2	DSM12212_01817	btsR_2
btsR_2	DSM12212_00623	btsR_2
btsR_2	DSM12212_03582	btsR_2
btsR_2	DSM12212_01288	btsR_2
btsR_2	DSM12212_03420	btsR_2
btsR_2	DSM12212_00387	btsR_2
btsR_2	DSM12212_03471	btsR_2
btsR_2	DSM15343_01593	btsR_2
btsR_2	DSM15343_02299	btsR_2
btsR_2	DSM15343_00246	btsR_2
btsR_2	DSM15343_00720	btsR_2
btsR_2	DSM15343_01876	btsR_2
btsR_2	DSM15343_01372	btsR_2
btsR_2	DSM15343_02419	btsR_2
btsR_2	DSM15343_02513	btsR_2
btsR_2	DSM15361_00342	btsR_2
btsR_2	DSM15361_02096	btsR_2
btsR_2	DSM15361_02332	btsR_2
btsR_2	DSM15361_00938	btsR_2
btsR_2	DSM15361_02710	btsR_2
btsR_2	DSM15361_02768	btsR_2
btsR_2	DSM15365_00863	btsR_2
btsR_2	DSM15365_00086	btsR_2
btsR_2	DSM15365_03469	btsR_2
btsR_2	DSM15365_00769	btsR_2
btsR_2	DSM15365_01742	btsR_2
btsR_2	DSM15365_02073	btsR_2
btsR_2	DSM15365_02274	btsR_2
btsR_2	DSM15365_03713	btsR_2
btsR_2	DSM15365_01779	btsR_2
btsR_2	DSM15365_00288	btsR_2

btsR_2	DSM15365_02043	btsR_2
btsR_2	DSM15365_03562	btsR_2
btsR_2	DSM15365_00938	btsR_2
btsR_2	DSM15365_03674	btsR_2
btsR_2	DSM15365_03114	btsR_2
btsR_2	DSM15365_02632	btsR_2
btsR_2	DSM15718_03181	btsR_2
btsR_2	DSM15718_03282	btsR_2
btsR_2	DSM15718_00248	btsR_2
btsR_2	DSM15718_03192	btsR_2
btsR_2	DSM15718_02281	btsR_2
btsR_2	DSM15718_00369	btsR_2
btsR_2	DSM15718_02903	btsR_2
btsR_2	DSM16505T_00048	btsR_2
btsR_2	DSM16505T_01367	btsR_2
btsR_2	DSM16505T_01046	btsR_2
btsR_2	DSM16505T_01138	btsR_2
btsR_2	DSM16505T_01994	btsR_2
btsR_2	DSM16505T_00905	btsR_2
btsR_2	DSM16505T_00017	btsR_2
btsR_2	DSM16505T_01813	btsR_2
btsR_2	DSM16505T_00592	btsR_2
btsR_2	DSM16505T_02241	btsR_2
btsR_2	DSM16505T_02641	btsR_2
btsR_2	DSM16505T_02166	btsR_2
btsR_2	DSM16505T_02679	btsR_2
btsR_2	DSM16505T_01626	btsR_2
btsR_2	DSM16505T_01737	btsR_2
btsR_2	DSM18103_02507	btsR_2
btsR_2	DSM18103_00143	btsR_2
btsR_2	DSM18103_02176	btsR_2
btsR_2	DSM18103_01366	btsR_2
btsR_2	DSM18103_00680	btsR_2
btsR_2	DSM18103_02075	btsR_2
btsR_2	DSM18103_00139	btsR_2
btsR_2	DSM18103_00380	btsR_2
btsR_2	DSM18103_01006	btsR_2
btsR_2	DSM18103_00599	btsR_2
btsR_2	DSM18103_03134	btsR_2
btsR_2	DSM18103_01501	btsR_2
btsR_2	DSM18103_00399	btsR_2
btsR_2	DSM18103_02785	btsR_2
btsR_2	DSM18103_01561	btsR_2
btsR_2	DSM18103_00457	btsR_2
btsR_2	DSM18103_03472	btsR_2
btsR_2	DSM18103_01708	btsR_2
btsR_2	DSM18103_00026	btsR_2
btsR_2	DSM18103_03683	btsR_2

btsR_2	DSM18103_02553	btsR_2
btsR_2	DSM18103_00778	btsR_2
btsR_2	DSM18103_01245	btsR_2
btsR_2	DSM18103_02747	btsR_2
btsR_2	DSM18103_00133	btsR_2
btsR_2	DSM18103_00101	btsR_2
btsR_2	DSM18293_02823	btsR_2
btsR_2	DSM18293_04276	btsR_2
btsR_2	DSM18293_01031	btsR_2
btsR_2	DSM18293_02037	btsR_2
btsR_2	DSM18293_02041	btsR_2
btsR_2	DSM18293_04119	btsR_2
btsR_2	DSM18293_03312	btsR_2
btsR_2	DSM18293_00047	btsR_2
btsR_2	DSM18293_01966	btsR_2
btsR_2	DSM18293_01456	btsR_2
btsR_2	DSM18293_00428	btsR_2
btsR_2	DSM18293_00903	btsR_2
btsR_2	DSM18293_00749	btsR_2
btsR_2	DSM18293_02606	btsR_2
btsR_2	DSM18841_00932	btsR_2
btsR_2	DSM18841_02973	btsR_2
btsR_2	DSM18841_02101	btsR_2
btsR_2	DSM18841_02376	btsR_2
btsR_2	DSM18841_01735	btsR_2
btsR_2	DSM18841_01038	btsR_2
btsR_2	DSM18841_00776	btsR_2
btsR_2	DSM18841_00448	btsR_2
btsR_2	DSM18841_01461	btsR_2
btsR_2	DSM18841_02488	btsR_2
btsR_2	DSM18841_01664	btsR_2
btsR_2	DSM18841_01910	btsR_2
btsR_2	DSM18841_00301	btsR_2
btsR_2	DSM18841_00886	btsR_2
btsR_2	DSM18841_01721	btsR_2
btsR_2	DSM18841_01839	btsR_2
btsR_2	DSM18842_00628	btsR_2
btsR_2	DSM18842_00382	btsR_2
btsR_2	DSM18842_02424	btsR_2
btsR_2	DSM18842_02020	btsR_2
btsR_2	DSM18842_01759	btsR_2
btsR_2	DSM18842_01283	btsR_2
btsR_2	DSM18842_01440	btsR_2
btsR_2	DSM18842_03158	btsR_2
btsR_2	DSM18842_02075	btsR_2
btsR_2	DSM18842_03109	btsR_2
btsR_2	DSM18842_00537	btsR_2
btsR_2	DSM18842_00280	btsR_2

btsR_2	DSM18842_00781	btsR_2
btsR_2	DSM18842_02395	btsR_2
btsR_2	DSM18842_02043	btsR_2
btsR_2	DSM18842_01848	btsR_2
btsR_2	DSM18842_01984	btsR_2
btsR_2	DSM18842_02132	btsR_2
btsR_2	DSM18842_02504	btsR_2
btsR_2	DSM19866_02660	btsR_2
btsR_2	DSM19866_01817	btsR_2
btsR_2	DSM19866_02584	btsR_2
btsR_2	DSM19866_04739	btsR_2
btsR_2	DSM19866_02512	btsR_2
btsR_2	DSM19866_04034	btsR_2
btsR_2	DSM19866_02074	btsR_2
btsR_2	DSM19866_01736	btsR_2
btsR_2	DSM19866_02800	btsR_2
btsR_2	DSM19866_02591	btsR_2
btsR_2	DSM19866_04805	btsR_2
btsR_2	DSM19866_03586	btsR_2
btsR_2	DSM19866_04795	btsR_2
btsR_2	DSM19866_03059	btsR_2
btsR_2	DSM19866_00036	btsR_2
btsR_2	DSM19866_01744	btsR_2
btsR_2	DSM19866_01642	btsR_2
btsR_2	DSM19866_01746	btsR_2
btsR_2	DSM19866_04657	btsR_2
btsR_2	DSM19866_04195	btsR_2
btsR_2	DSM2041_00568	btsR_2
btsR_2	DSM2041_01631	btsR_2
btsR_2	DSM2041_02655	btsR_2
btsR_2	DSM2041_05432	btsR_2
btsR_2	DSM2041_01834	btsR_2
btsR_2	DSM2041_04897	btsR_2
btsR_2	DSM2041_00092	btsR_2
btsR_2	DSM2041_01860	btsR_2
btsR_2	DSM2041_01887	btsR_2
btsR_2	DSM2041_00830	btsR_2
btsR_2	DSM2041_01395	btsR_2
btsR_2	DSM2041_00478	btsR_2
btsR_2	DSM2041_01861	btsR_2
btsR_2	DSM2041_04880	btsR_2
btsR_2	DSM2041_01576	btsR_2
btsR_2	DSM2041_01178	btsR_2
btsR_2	DSM2041_03530	btsR_2
btsR_2	DSM2041_04697	btsR_2
btsR_2	DSM2041_01794	btsR_2
btsR_2	DSM2041_02233	btsR_2
btsR_2	DSM2041_02628	btsR_2

btsR_2	DSM2041_01804	btsR_2
btsR_2	DSM2041_00299	btsR_2
btsR_2	DSM2041_01743	btsR_2
btsR_2	DSM2041_02558	btsR_2
btsR_2	DSM2041_02703	btsR_2
btsR_2	DSM2041_01741	btsR_2
btsR_2	DSM2041_04196	btsR_2
btsR_2	DSM2041_04770	btsR_2
btsR_2	DSM2041_05077	btsR_2
btsR_2	DSM2041_04775	btsR_2
btsR_2	DSM2041_03649	btsR_2
btsR_2	DSM2041_03021	btsR_2
btsR_2	DSM2041_01875	btsR_2
btsR_2	DSM2041_04870	btsR_2
btsR_2	DSM2061_03981	btsR_2
btsR_2	DSM2061_00526	btsR_2
btsR_2	DSM2061_00382	btsR_2
btsR_2	DSM2061_02027	btsR_2
btsR_2	DSM2061_04358	btsR_2
btsR_2	DSM2061_03972	btsR_2
btsR_2	DSM2061_00940	btsR_2
btsR_2	DSM2061_03846	btsR_2
btsR_2	DSM2061_03355	btsR_2
btsR_2	DSM2061_00384	btsR_2
btsR_2	DSM2061_00418	btsR_2
btsR_2	DSM2061_03106	btsR_2
btsR_2	DSM2061_00606	btsR_2
btsR_2	DSM2061_00976	btsR_2
btsR_2	DSM2061_01165	btsR_2
btsR_2	DSM2061_01561	btsR_2
btsR_2	DSM2061_02603	btsR_2
btsR_2	DSM2061_01782	btsR_2
btsR_2	DSM2061_00202	btsR_2
btsR_2	DSM2064_03526	btsR_2
btsR_2	DSM2064_01173	btsR_2
btsR_2	DSM2064_01986	btsR_2
btsR_2	DSM2064_03644	btsR_2
btsR_2	DSM2064_02860	btsR_2
btsR_2	DSM2064_00920	btsR_2
btsR_2	DSM2064_04427	btsR_2
btsR_2	DSM2064_04132	btsR_2
btsR_2	DSM2064_01334	btsR_2
btsR_2	DSM2064_01342	btsR_2
btsR_2	DSM2064_02695	btsR_2
btsR_2	DSM2064_04511	btsR_2
btsR_2	DSM2064_03769	btsR_2
btsR_2	DSM2064_00659	btsR_2
btsR_2	DSM2064_02351	btsR_2

btsR_2	DSM2064_01309	btsR_2
btsR_2	DSM2064_04017	btsR_2
btsR_2	DSM2064_05170	btsR_2
btsR_2	DSM2064_04202	btsR_2
btsR_2	DSM2064_01782	btsR_2
btsR_2	DSM2064_02452	btsR_2
btsR_2	DSM2064_01423	btsR_2
btsR_2	DSM2064_04240	btsR_2
btsR_2	DSM21164_01782	btsR_2
btsR_2	DSM21164_02246	btsR_2
btsR_2	DSM21164_02599	btsR_2
btsR_2	DSM21164_02344	btsR_2
btsR_2	DSM21164_01521	btsR_2
btsR_2	DSM21164_00642	btsR_2
btsR_2	DSM21164_00282	btsR_2
btsR_2	DSM21164_02451	btsR_2
btsR_2	DSM21164_00811	btsR_2
btsR_2	DSM21164_01595	btsR_2
btsR_2	DSM21164_01448	btsR_2
btsR_2	DSM23404_02719	btsR_2
btsR_2	DSM23404_02189	btsR_2
btsR_2	DSM23404_03199	btsR_2
btsR_2	DSM23404_00545	btsR_2
btsR_2	DSM23404_02771	btsR_2
btsR_2	DSM23404_00170	btsR_2
btsR_2	DSM23404_00566	btsR_2
btsR_2	DSM23404_00083	btsR_2
btsR_2	DSM23424_01565	btsR_2
btsR_2	DSM23424_00765	btsR_2
btsR_2	DSM23424_01126	btsR_2
btsR_2	DSM23424_03085	btsR_2
btsR_2	DSM23424_01404	btsR_2
btsR_2	DSM23424_03293	btsR_2
btsR_2	DSM23424_01941	btsR_2
btsR_2	DSM23424_01511	btsR_2
btsR_2	DSM23424_03361	btsR_2
btsR_2	DSM23424_02828	btsR_2
btsR_2	DSM23424_02964	btsR_2
btsR_2	DSM24786_01790	btsR_2
btsR_2	DSM24786_00499	btsR_2
btsR_2	DSM24786_01553	btsR_2
btsR_2	DSM24786_03487	btsR_2
btsR_2	DSM24786_00549	btsR_2
btsR_2	DSM24786_02202	btsR_2
btsR_2	DSM24786_02029	btsR_2
btsR_2	DSM24786_00756	btsR_2
btsR_2	DSM24786_02788	btsR_2
btsR_2	DSM24786_00153	btsR_2

btsR_2	DSM24786_00078	btsR_2
btsR_2	DSM24786_02402	btsR_2
btsR_2	DSM24786_03053	btsR_2
btsR_2	DSM24786_02890	btsR_2
btsR_2	DSM24786_00344	btsR_2
btsR_2	DSM24786_00314	btsR_2
btsR_2	DSM24786_02403	btsR_2
btsR_2	DSM24786_00803	btsR_2
btsR_2	DSM7489_01256	btsR_2
btsR_2	DSM7489_02896	btsR_2
btsR_2	DSM7489_02155	btsR_2
btsR_2	DSM7489_02371	btsR_2
btsR_2	DSM7489_03286	btsR_2
btsR_2	DSM7489_00317	btsR_2
btsR_2	DSM7489_03125	btsR_2
btsR_2	DSM7489_01932	btsR_2
btsR_2	DSM7489_01699	btsR_2
btsR_2	DSM7489_00737	btsR_2
btsR_2	DSM7489_00073	btsR_2
btsR_2	DSM7489_02567	btsR_2
btsR_2	DSM7489_00162	btsR_2
btsR_2	DSM7489_01467	btsR_2
btsR_2	DSM7489_00747	btsR_2
btsR_2	DSM7489_02709	btsR_2
btsR_2	DSM7489_02739	btsR_2
btsR_2	DSM7489_03164	btsR_2
btsR_2	DSM7489_01318	btsR_2
btsR_2	DSW1_02714	btsR_2
btsR_2	DSW1_00352	btsR_2
btsR_2	DSW1_00310	btsR_2
btsR_2	DSW1_02039	btsR_2
btsR_2	DSW1_01855	btsR_2
btsR_2	DSW1_00917	btsR_2
btsR_2	DSW1_02278	btsR_2
btsR_2	DSW5_00704	btsR_2
btsR_2	DSW5_02701	btsR_2
btsR_2	DSW5_00366	btsR_2
btsR_2	DSW5_01281	btsR_2
btsR_2	DSW5_02686	btsR_2
btsR_2	DSW5_01110	btsR_2
btsR_2	DSW5_02325	btsR_2
btsR_2	DSW5_02272	btsR_2
btsR_2	DSW5_02146	btsR_2
btsR_2	DSW5_02676	btsR_2
btsR_2	DSW5_02028	btsR_2
btsR_2	DSW5_00789	btsR_2
btsR_2	DSW5_01346	btsR_2
btsR_2	DSW5_01799	btsR_2

btsR_2	DSW5_01046	btsR_2
btsR_2	DSW5_02150	btsR_2
btsR_2	DSW5_02681	btsR_2
btsR_2	DSW5_00292	btsR_2
btsR_2	DSW5_00775	btsR_2
btsR_2	DSW6_00913	btsR_2
btsR_2	DSW6_00380	btsR_2
btsR_2	DSW6_02903	btsR_2
btsR_2	DSW6_00744	btsR_2
btsR_2	DSW6_03219	btsR_2
btsR_2	DSW6_01222	btsR_2
btsR_2	DSW6_00377	btsR_2
btsR_2	DSW6_02423	btsR_2
btsR_2	DSW6_00020	btsR_2
btsR_2	DSW6_01075	btsR_2
btsR_2	DSW8_01082	btsR_2
btsR_2	DSW8_01779	btsR_2
btsR_2	DSW8_00318	btsR_2
btsR_2	DSW8_00601	btsR_2
btsR_2	DSW8_00313	btsR_2
btsR_2	DSW8_00729	btsR_2
btsR_2	DSW8_02088	btsR_2
btsR_2	DSW8_03280	btsR_2
btsR_2	DSW8_03335	btsR_2
btsR_2	DSW8_01626	btsR_2
btsR_2	DSW8_01717	btsR_2
btsR_2	DSW8_03515	btsR_2
btsR_2	DSW8_02843	btsR_2
btsR_2	DSW8_00839	btsR_2
btsR_2	DSW8_00376	btsR_2
btsR_2	DSW8_00519	btsR_2
btsR_2	F52_00150	btsR_2
btsR_2	F52_03504	btsR_2
btsR_2	F52_04285	btsR_2
btsR_2	F52_04539	btsR_2
btsR_2	F52_03797	btsR_2
btsR_2	F52_03246	btsR_2
btsR_2	F52_00742	btsR_2
btsR_2	F52_03383	btsR_2
btsR_2	F52_00904	btsR_2
btsR_2	F52_03606	btsR_2
btsR_2	F52_03446	btsR_2
btsR_2	F52_02534	btsR_2
btsR_2	F52_03917	btsR_2
btsR_2	F52_00991	btsR_2
btsR_2	F52_00980	btsR_2
btsR_2	F52_02040	btsR_2
btsR_2	F52_04178	btsR_2

btsR_2	F52_02227	btsR_2
btsR_2	GCA_001027945.1_01832	btsR_2
btsR_2	GCA_001027945.1_00012	btsR_2
btsR_2	GCA_001027945.1_01495	btsR_2
btsR_2	GCA_001027945.1_02344	btsR_2
btsR_2	GCA_001027945.1_01300	btsR_2
btsR_2	GCA_001027945.1_00159	btsR_2
btsR_2	GCA_001027945.1_01106	btsR_2
btsR_2	GCA_001027945.1_01389	btsR_2
btsR_2	GCA_001027945.1_00946	btsR_2
btsR_2	GCA_001027945.1_02053	btsR_2
btsR_2	GCA_001027945.1_02454	btsR_2
btsR_2	GCA_001027945.1_02782	btsR_2
btsR_2	GCA_001027945.1_00287	btsR_2
btsR_2	GCA_001027945.1_02166	btsR_2
btsR_2	GCA_001027945.1_02656	btsR_2
btsR_2	HI1_01198	btsR_2
btsR_2	HI1_02835	btsR_2
btsR_2	HI1_02081	btsR_2
btsR_2	HI1_02304	btsR_2
btsR_2	HI1_02901	btsR_2
btsR_2	HI1_03229	btsR_2
btsR_2	HI1_00249	btsR_2
btsR_2	HI1_03074	btsR_2
btsR_2	HI1_01860	btsR_2
btsR_2	HI1_01620	btsR_2
btsR_2	HI1_03372	btsR_2
btsR_2	HI1_02500	btsR_2
btsR_2	HI1_00094	btsR_2
btsR_2	HI1_01408	btsR_2
btsR_2	HI1_01258	btsR_2
btsR_2	HI1_00682	btsR_2
btsR_2	HI1_02648	btsR_2
btsR_2	HI1_02678	btsR_2
btsR_2	HI1_00003	btsR_2
btsR_2	HI1_03111	btsR_2
btsR_2	IR1_04451	btsR_2
btsR_2	IR1_01318	btsR_2
btsR_2	IR1_03718	btsR_2
btsR_2	IR1_02107	btsR_2
btsR_2	IR1_04014	btsR_2
btsR_2	IR1_03788	btsR_2
btsR_2	IR1_03563	btsR_2
btsR_2	IR1_00029	btsR_2
btsR_2	IR1_03350	btsR_2
btsR_2	IR1_00196	btsR_2
btsR_2	IR1_03351	btsR_2
btsR_2	IR1_01328	btsR_2

btsR_2	IR1_01923	btsR_2
btsR_2	IR1_04578	btsR_2
btsR_2	IR1_03651	btsR_2
btsR_2	IR1_00126	btsR_2
btsR_2	IR1_01440	btsR_2
btsR_2	IR1_03258	btsR_2
btsR_2	IR1_01186	btsR_2
btsR_2	IR1_01060	btsR_2
btsR_2	IR1_04053	btsR_2
btsR_2	IR1_01203	btsR_2
btsR_2	IR1_03515	btsR_2
btsR_2	IR1_02927	btsR_2
btsR_2	IR1_04379	btsR_2
btsR_2	KCTC12974_02623	btsR_2
btsR_2	KCTC12974_01180	btsR_2
btsR_2	KCTC12974_00454	btsR_2
btsR_2	KCTC12974_01135	btsR_2
btsR_2	KCTC12974_02691	btsR_2
btsR_2	KCTC12974_00871	btsR_2
btsR_2	KL-A_02145	btsR_2
btsR_2	KL-A_00620	btsR_2
btsR_2	KL-A_00116	btsR_2
btsR_2	KL-A_01651	btsR_2
btsR_2	KL-A_00556	btsR_2
btsR_2	KL-A_01907	btsR_2
btsR_2	KL-A_02680	btsR_2
btsR_2	KL-A_02736	btsR_2
btsR_2	KL-A_00339	btsR_2
btsR_2	KL-A_01501	btsR_2
btsR_2	KL-A_03383	btsR_2
btsR_2	KL-A_03411	btsR_2
btsR_2	KL-A_00988	btsR_2
btsR_2	KL-A_02357	btsR_2
btsR_2	KL-A_01158	btsR_2
btsR_2	KL-A_00759	btsR_2
btsR_2	KL-A_02479	btsR_2
btsR_2	KL-A_01349	btsR_2
btsR_2	KL-A_01319	btsR_2
btsR_2	KL-A_02008	btsR_2
btsR_2	L12_00196	btsR_2
btsR_2	LL0412.1.7_01437	btsR_2
btsR_2	LL0412.1.7_01074	btsR_2
btsR_2	LL0412.1.7_00379	btsR_2
btsR_2	LL0412.1.7_02385	btsR_2
btsR_2	LL0412.1.7_02225	btsR_2
btsR_2	LL0412.1.7_02309	btsR_2
btsR_2	LL0412.1.7_00049	btsR_2
btsR_2	LL0412.1.7_02664	btsR_2

btsR_2	LL0412.1.7_01415	btsR_2
btsR_2	LL0412.1.7_01582	btsR_2
btsR_2	LL0412.1.7_02149	btsR_2
btsR_2	LL0412.1.7_00355	btsR_2
btsR_2	LL0412.1.7_01637	btsR_2
btsR_2	LL0412.1.7_00914	btsR_2
btsR_2	LL0412.1.7_01471	btsR_2
btsR_2	LL0412.1.7_02586	btsR_2
btsR_2	LL0412.1.7_00418	btsR_2
btsR_2	LL0412.1.7_00017	btsR_2
btsR_2	LL0412.1.7_01638	btsR_2
btsR_2	LL0412.1.7_00847	btsR_2
btsR_2	LL0412.1.7_00786	btsR_2
btsR_2	MAR_2009_71_02363	btsR_2
btsR_2	MAR_2009_71_03828	btsR_2
btsR_2	MAR_2009_71_01641	btsR_2
btsR_2	MAR_2009_71_03120	btsR_2
btsR_2	MAR_2009_71_03398	btsR_2
btsR_2	MAR_2009_71_03116	btsR_2
btsR_2	MAR_2009_71_03525	btsR_2
btsR_2	MAR_2009_71_03833	btsR_2
btsR_2	MAR_2009_71_01342	btsR_2
btsR_2	MAR_2009_71_03642	btsR_2
btsR_2	MAR_2009_71_03594	btsR_2
btsR_2	MAR_2009_71_01799	btsR_2
btsR_2	MAR_2009_71_01700	btsR_2
btsR_2	MAR_2009_71_00071	btsR_2
btsR_2	MAR_2009_71_00611	btsR_2
btsR_2	MAR_2009_71_02608	btsR_2
btsR_2	MAR_2009_71_03175	btsR_2
btsR_2	MAR_2009_71_03317	btsR_2
btsR_2	MAR_2009_71_03978	btsR_2
btsR_2	NN016038_02430	btsR_2
btsR_2	NN016038_00253	btsR_2
btsR_2	NN016038_03459	btsR_2
btsR_2	NN016038_03674	btsR_2
btsR_2	NN016038_01753	btsR_2
btsR_2	NN016038_01254	btsR_2
btsR_2	NN016038_01601	btsR_2
btsR_2	NN016038_03397	btsR_2
btsR_2	NN016038_03360	btsR_2
btsR_2	NN016038_02908	btsR_2
btsR_2	NN016038_01013	btsR_2
btsR_2	NN016038_03817	btsR_2
btsR_2	NN016038_00934	btsR_2
btsR_2	NN016038_02827	btsR_2
btsR_2	NN016038_03077	btsR_2
btsR_2	NN016038_03823	btsR_2

btsR_2	NN016038_03426	btsR_2
btsR_2	PAO1_05438	btsR_2
btsR_2	PAO1_00178	btsR_2
btsR_2	UW101_02274	btsR_2
btsR_2	UW101_04306	btsR_2
btsR_2	UW101_01041	btsR_2
btsR_2	UW101_04754	btsR_2
btsR_2	UW101_05242	btsR_2
btsR_2	UW101_01914	btsR_2
btsR_2	UW101_03631	btsR_2
btsR_2	UW101_04459	btsR_2
btsR_2	UW101_04145	btsR_2
btsR_2	UW101_04137	btsR_2
btsR_2	UW101_01749	btsR_2
btsR_2	UW101_02124	btsR_2
btsR_2	UW101_05117	btsR_2
btsR_2	UW101_03370	btsR_2
btsR_2	UW101_01405	btsR_2
btsR_2	UW101_04170	btsR_2
btsR_2	UW101_04870	btsR_2
btsR_2	UW101_00610	btsR_2
btsR_2	UW101_04683	btsR_2
btsR_2	UW101_00836	btsR_2
btsR_2	UW101_01506	btsR_2
btsR_2	UW101_04056	btsR_2
btsR_2	UW101_04645	btsR_2
btsR_2	V4-33_01762	btsR_2
btsR_2	V4-33_01427	btsR_2
btsR_2	V4-33_01554	btsR_2
btsR_2	V4-33_00931	btsR_2
btsR_2	V4-33_02083	btsR_2
btsR_2	W5C_01632	btsR_2
btsR_2	W5C_01584	btsR_2
btsR_2	W5C_02904	btsR_2
btsR_2	W5C_01853	btsR_2
btsR_2	W5C_02147	btsR_2
btsR_2	W5C_02328	btsR_2
btsR_2	W5C_01603	btsR_2
btsR_2	W5C_02577	btsR_2
btsR_2	W5C_00619	btsR_2
btsR_2	W5C_01070	btsR_2
btsR_2	W5C_00395	btsR_2
btsR_2	W5C_03362	btsR_2
btsR_2	W5C_00071	btsR_2
btsR_2	W5C_01164	btsR_2
btsR_2	W5C_01699	btsR_2
btsR_2	W5C_02418	btsR_2
btsR_2	W5C_02388	btsR_2

btsR_2	W5C_02361	btsR_2
btsR_2	ZONMW-01_02551	btsR_2
btsR_2	ZONMW-01_00892	btsR_2
btsR_2	ZONMW-01_01018	btsR_2
btsR_2	ZONMW-01_01431	btsR_2
btsR_2	ZONMW-01_00647	btsR_2
btsR_2	ZONMW-01_01761	btsR_2
btsR_2	ZONMW-01_00385	btsR_2
btsR_2	ZONMW-01_00146	btsR_2
btsR_2	ZONMW-01_01617	btsR_2
btsR_2	ZONMW-01_02049	btsR_2
btsR_2	ZONMW-01_02464	btsR_2
btsR_2	ZONMW-01_00949	btsR_2
btsR_2	ZONMW-01_02223	btsR_2
btsR_2	ZONMW-01_02194	btsR_2
btsR_2	ZONMW-01_00621	btsR_2
btsR_2	ZONMW-02_02887	btsR_2
btsR_2	ZONMW-02_00905	btsR_2
btsR_2	ZONMW-02_03478	btsR_2
btsR_2	ZONMW-02_00401	btsR_2
btsR_2	ZONMW-02_02113	btsR_2
btsR_2	ZONMW-02_03449	btsR_2
btsR_2	ZONMW-02_01491	btsR_2
btsR_2	ZONMW-02_02397	btsR_2
btsR_2	ZONMW-02_02290	btsR_2
btsR_2	ZONMW-02_03222	btsR_2
btsR_2	ZONMW-02_00236	btsR_2
btsR_2	ZONMW-02_01655	btsR_2
btsR_2	ZONMW-02_00025	btsR_2
btsR_2	ZONMW-03_01832	btsR_2
btsR_2	ZONMW-03_00270	btsR_2
btsR_2	ZONMW-03_02087	btsR_2
btsR_2	ZONMW-03_01349	btsR_2
btsR_2	ZONMW-03_00025	btsR_2
btsR_2	ZONMW-03_02746	btsR_2
btsR_2	ZONMW-03_01111	btsR_2
btsR_2	ZONMW-03_00872	btsR_2
btsR_2	ZONMW-03_00590	btsR_2
btsR_2	ZONMW-03_02662	btsR_2
btsR_2	ZONMW-03_03067	btsR_2
btsR_2	ZONMW-03_02018	btsR_2
btsR_2	ZONMW-03_00425	btsR_2
btsR_2	ZONMW-03_00454	btsR_2
btsR_2	ZONMW-03_02515	btsR_2
btsR_2	ZONMW-04_03039	btsR_2
btsR_2	ZONMW-04_03071	btsR_2
btsR_2	ZONMW-04_01732	btsR_2
btsR_2	ZONMW-04_00344	btsR_2

btsR_2	ZONMW-04_00110	btsR_2
btsR_2	ZONMW-04_01037	btsR_2
btsR_2	ZONMW-04_02777	btsR_2
btsR_2	ZONMW-04_00561	btsR_2
btsR_2	ZONMW-04_02074	btsR_2
btsR_2	ZONMW-04_01528	btsR_2
btsR_2	ZONMW-04_01892	btsR_2
btsR_2	ZONMW-04_02378	btsR_2
btsR_2	ZONMW-04_01805	btsR_2
btsR_2	ZONMW-04_01364	btsR_2
btsR_2	ZONMW-04_01393	btsR_2
btsR_2	ZONMW-04_00996	btsR_2
btsR_2	ZONMW-05_00528	btsR_2
btsR_2	ZONMW-05_02011	btsR_2
btsR_2	ZONMW-05_01481	btsR_2
btsR_2	ZONMW-05_01720	btsR_2
btsR_2	ZONMW-05_00285	btsR_2
btsR_2	ZONMW-05_02327	btsR_2
btsR_2	ZONMW-05_01264	btsR_2
btsR_2	ZONMW-05_01061	btsR_2
btsR_2	ZONMW-05_00852	btsR_2
btsR_2	ZONMW-05_02478	btsR_2
btsR_2	ZONMW-05_02646	btsR_2
btsR_2	ZONMW-05_02080	btsR_2
btsR_2	ZONMW-05_00688	btsR_2
btsR_2	ZONMW-05_00717	btsR_2
btsR_2	ZONMW-05_00248	btsR_2
btsR_2	ZONMW-06_02565	btsR_2
btsR_2	ZONMW-06_01858	btsR_2
btsR_2	ZONMW-07_04052	btsR_2
btsR_2	ZONMW-07_00122	btsR_2
btsR_2	ZONMW-07_02988	btsR_2
btsR_2	ZONMW-07_04095	btsR_2
btsR_2	ZONMW-07_00814	btsR_2
btsR_2	ZONMW-07_01850	btsR_2
btsR_2	ZONMW-07_00390	btsR_2
btsR_2	ZONMW-07_01363	btsR_2
btsR_2	ZONMW-07_00145	btsR_2
btsR_2	ZONMW-07_03732	btsR_2
btsR_2	ZONMW-07_00138	btsR_2
btsR_2	ZONMW-07_03162	btsR_2
btsR_2	ZONMW-07_02813	btsR_2
btsR_2	ZONMW-07_04046	btsR_2
btsR_2	ZONMW-07_02357	btsR_2
btsR_2	ZONMW-07_01834	btsR_2
btsR_2	ZONMW-07_03351	btsR_2
btsR_2	ZONMW-08_00883	btsR_2
btsR_2	ZONMW-08_01592	btsR_2

btsR_2	ZONMW-08_02636	btsR_2
btsR_2	ZONMW-08_02529	btsR_2
btsR_2	ZONMW-08_02632	btsR_2
btsR_2	ZONMW-08_02401	btsR_2
btsR_2	ZONMW-08_01851	btsR_2
btsR_2	ZONMW-08_02273	btsR_2
btsR_2	ZONMW-08_02330	btsR_2
btsR_2	ZONMW-08_01425	btsR_2
btsR_2	ZONMW-08_01536	btsR_2
btsR_2	ZONMW-08_02980	btsR_2
btsR_2	ZONMW-08_00127	btsR_2
btsR_2	ZONMW-08_03612	btsR_2
btsR_2	ZONMW-08_02689	btsR_2
btsR_2	ZONMW-08_02831	btsR_2
btsR_2	ZONMW-09_01526	btsR_2
btsR_2	ZONMW-09_01582	btsR_2
btsR_2	ZONMW-09_00862	btsR_2
btsR_2	ZONMW-09_02230	btsR_2
btsR_2	ZONMW-09_02444	btsR_2
btsR_2	ZONMW-10_00958	btsR_2
btsR_2	ZONMW-10_01842	btsR_2
btsR_2	ZONMW-10_00780	btsR_2
btsR_2	ZONMW-11_04022	btsR_2
btsR_2	ZONMW-11_02629	btsR_2
btsR_2	ZONMW-11_02478	btsR_2
btsR_2	ZONMW-11_03784	btsR_2
btsR_2	ZONMW-11_00468	btsR_2
btsR_2	ZONMW-11_02906	btsR_2
btsR_2	ZONMW-11_00473	btsR_2
btsR_2	ZONMW-11_03207	btsR_2
btsR_2	ZONMW-11_04067	btsR_2
btsR_2	ZONMW-11_03221	btsR_2
btsR_2	ZONMW-11_02628	btsR_2
btsR_2	ZONMW-11_04731	btsR_2
btsR_2	ZONMW-11_04738	btsR_2
btsR_2	ZONMW-12_04100	btsR_2
btsR_2	ZONMW-12_00011	btsR_2
btsR_2	ZONMW-12_01523	btsR_2
btsR_2	ZONMW-12_03922	btsR_2
btsR_2	ZONMW-12_03655	btsR_2
btsR_2	ZONMW-12_00301	btsR_2
btsR_2	ZONMW-12_00746	btsR_2
btsR_2	ZONMW-12_01204	btsR_2
btsR_2	ZONMW-12_01893	btsR_2
btsR_2	ZONMW-12_04127	btsR_2
btsR_2	ZONMW-12_00814	btsR_2
btsR_2	ZONMW-12_03143	btsR_2
btsR_2	ZONMW-12_04314	btsR_2

btsR_2	ZONMW-12_00006	btsR_2
btsR_2	ZONMW-12_00007	btsR_2
btsR_2	ZONMW-12_04369	btsR_2
btsR_2	ZONMW-12_02900	btsR_2
btsR_2	ZONMW-13_02438	btsR_2
btsR_2	ZONMW-13_02994	btsR_2
btsR_2	ZONMW-13_02185	btsR_2
btsR_2	ZONMW-13_02276	btsR_2
btsR_2	ZONMW-13_02423	btsR_2
btsR_2	ZONMW-13_02425	btsR_2
btsR_2	ZONMW-13_02306	btsR_2
btsR_2	ZONMW-13_01339	btsR_2
btsR_2	ZONMW-13_02536	btsR_2
btsR_2	ZONMW-14_02556	btsR_2
btsR_2	ZONMW-14_02889	btsR_2
btsR_2	ZONMW-14_00648	btsR_2
btsR_2	ZONMW-14_02723	btsR_2
btsR_2	ZONMW-14_02106	btsR_2
btsR_2	ZONMW-14_02195	btsR_2
btsR_2	ZONMW-14_01982	btsR_2
btsR_2	ZONMW-14_02227	btsR_2
btsR_2	ZONMW-15_01480	btsR_2
btsR_2	ZONMW-15_00804	btsR_2
btsR_2	ZONMW-15_02412	btsR_2
btsR_2	ZONMW-15_00080	btsR_2
btsR_2	ZONMW-15_03132	btsR_2
btsR_2	ZONMW-15_02884	btsR_2
btsR_2	ZONMW-15_00448	btsR_2
btsR_2	ZONMW-15_02601	btsR_2
btsR_2	ZONMW-15_03157	btsR_2
btsR_2	ZONMW-15_02360	btsR_2
btsR_2	ZONMW-15_02399	btsR_2
btsR_2	ZONMW-15_02890	btsR_2
btsR_2	ZONMW-15_03284	btsR_2
btsR_2	ZONMW-19_00426	btsR_2
btsR_2	ZONMW-19_01748	btsR_2
btsR_2	ZONMW-19_02014	btsR_2
btsR_2	ZONMW-20_02154	btsR_2
btsR_2	ZONMW-20_01980	btsR_2
btsR_2	ZONMW-20_02658	btsR_2
btsR_2	ZONMW-20_01875	btsR_2
btsR_2	ZONMW-20_00113	btsR_2
btsR_2	ZONMW-20_03966	btsR_2
btsR_2	ZONMW-21_00339	btsR_2
btsR_2	ZONMW-21_00623	btsR_2
btsR_2	ZONMW-21_02494	btsR_2
btsR_2	ZONMW-21_02996	btsR_2
btsR_2	ZONMW-21_03176	btsR_2

btsR_2	ZONMW-21_03144	btsR_2
btsR_2	ZONMW-21_00155	btsR_2
btsR_2	ZONMW-21_01612	btsR_2
btsR_2	ZONMW-21_02395	btsR_2
btsR_2	ZONMW-21_01514	btsR_2
btsR_2	ZONMW-21_01174	btsR_2
btsR_2	ZONMW-21_00539	btsR_2
btsR_2	ZONMW-21_00374	btsR_2
btsR_2	ZONMW-21_02913	btsR_2
btsR_2	ZONMW-21_03434	btsR_2
btsR_2	ZONMW-21_01692	btsR_2
btsR_2	ZONMW-21_02108	btsR_2
btsR_2	ZONMW-21_02290	btsR_2
btsR_2	ZONMW-21_00651	btsR_2
btsR_2	ZONMW-22_00339	btsR_2
btsR_2	ZONMW-22_00623	btsR_2
btsR_2	ZONMW-22_02615	btsR_2
btsR_2	ZONMW-22_02828	btsR_2
btsR_2	ZONMW-22_03201	btsR_2
btsR_2	ZONMW-22_03169	btsR_2
btsR_2	ZONMW-22_00155	btsR_2
btsR_2	ZONMW-22_02112	btsR_2
btsR_2	ZONMW-22_02713	btsR_2
btsR_2	ZONMW-22_02209	btsR_2
btsR_2	ZONMW-22_01176	btsR_2
btsR_2	ZONMW-22_00539	btsR_2
btsR_2	ZONMW-22_00374	btsR_2
btsR_2	ZONMW-22_02911	btsR_2
btsR_2	ZONMW-22_03447	btsR_2
btsR_2	ZONMW-22_02032	btsR_2
btsR_2	ZONMW-22_01617	btsR_2
btsR_2	ZONMW-22_02819	btsR_2
btsR_2	ZONMW-22_00652	btsR_2
btsR_2	ZONMW-23_01454	btsR_2
btsR_2	ZONMW-23_01738	btsR_2
btsR_2	ZONMW-23_02639	btsR_2
btsR_2	ZONMW-23_03042	btsR_2
btsR_2	ZONMW-23_03222	btsR_2
btsR_2	ZONMW-23_03190	btsR_2
btsR_2	ZONMW-23_01270	btsR_2
btsR_2	ZONMW-23_00439	btsR_2
btsR_2	ZONMW-23_02737	btsR_2
btsR_2	ZONMW-23_00342	btsR_2
btsR_2	ZONMW-23_02288	btsR_2
btsR_2	ZONMW-23_01654	btsR_2
btsR_2	ZONMW-23_01489	btsR_2
btsR_2	ZONMW-23_02959	btsR_2
btsR_2	ZONMW-23_03441	btsR_2

btsR_2	ZONMW-23_00519	btsR_2
btsR_2	ZONMW-23_00934	btsR_2
btsR_2	ZONMW-23_02841	btsR_2
btsR_2	ZONMW-23_01766	btsR_2
btsR_2	ZONMW-24_01120	btsR_2
btsR_2	ZONMW-24_00837	btsR_2
btsR_2	ZONMW-24_02518	btsR_2
btsR_2	ZONMW-24_02851	btsR_2
btsR_2	ZONMW-24_03223	btsR_2
btsR_2	ZONMW-24_03191	btsR_2
btsR_2	ZONMW-24_01305	btsR_2
btsR_2	ZONMW-24_02737	btsR_2
btsR_2	ZONMW-24_02419	btsR_2
btsR_2	ZONMW-24_02834	btsR_2
btsR_2	ZONMW-24_00285	btsR_2
btsR_2	ZONMW-24_00920	btsR_2
btsR_2	ZONMW-24_01085	btsR_2
btsR_2	ZONMW-24_02934	btsR_2
btsR_2	ZONMW-24_03443	btsR_2
btsR_2	ZONMW-24_02657	btsR_2
btsR_2	ZONMW-24_01641	btsR_2
btsR_2	ZONMW-24_02315	btsR_2
btsR_2	ZONMW-24_00809	btsR_2
btsR_2	ZONMW-25_01580	btsR_2
btsR_2	ZONMW-25_02399	btsR_2
btsR_2	ZONMW-25_02759	btsR_2
btsR_2	ZONMW-25_00023	btsR_2
btsR_2	ZONMW-25_03168	btsR_2
btsR_2	ZONMW-25_01407	btsR_2
btsR_2	ZONMW-25_02206	btsR_2
btsR_2	ZONMW-25_02617	btsR_2
btsR_2	ZONMW-25_00053	btsR_2
btsR_2	ZONMW-25_02522	btsR_2
btsR_2	ZONMW-25_00078	btsR_2
btsR_2	ZONMW-25_02303	btsR_2
btsR_2	ZONMW-25_03509	btsR_2
btsR_2	ZONMW-25_01484	btsR_2
btsR_2	ZONMW-25_00428	btsR_2
btsR_2	ZONMW-26_02017	btsR_2
btsR_2	ZONMW-26_00439	btsR_2
btsR_2	ZONMW-26_02389	btsR_2
btsR_2	ZONMW-26_01788	btsR_2
btsR_2	ZONMW-26_00683	btsR_2
btsR_2	ZONMW-26_02993	btsR_2
btsR_2	ZONMW-26_01318	btsR_2
btsR_2	ZONMW-26_01105	btsR_2
btsR_2	ZONMW-26_00108	btsR_2
btsR_2	ZONMW-26_02638	btsR_2

btsR_2	ZONMW-26_03084	btsR_2
btsR_2	ZONMW-26_02317	btsR_2
btsR_2	ZONMW-26_00281	btsR_2
btsR_2	ZONMW-26_00252	btsR_2
btsR_2	ZONMW-26_00709	btsR_2
btsR_2	ZONMW-27_02912	btsR_2
btsR_2	ZONMW-27_01352	btsR_2
btsR_2	ZONMW-27_01569	btsR_2
btsR_2	ZONMW-27_00338	btsR_2
btsR_2	ZONMW-27_00107	btsR_2
btsR_2	ZONMW-27_01109	btsR_2
btsR_2	ZONMW-27_03237	btsR_2
btsR_2	ZONMW-27_00555	btsR_2
btsR_2	ZONMW-27_00767	btsR_2
btsR_2	ZONMW-27_01776	btsR_2
btsR_2	ZONMW-27_02063	btsR_2
btsR_2	ZONMW-27_02803	btsR_2
btsR_2	ZONMW-27_01492	btsR_2
btsR_2	ZONMW-27_01939	btsR_2
btsR_2	ZONMW-27_01910	btsR_2
btsR_2	ZONMW-27_01071	btsR_2
btsR_2	ZONMW-28_02724	btsR_2
btsR_2	ZONMW-28_03738	btsR_2
btsR_2	ZONMW-29_00303	btsR_2
btsR_2	ZONMW-29_02251	btsR_2
btsR_2	ZONMW-29_02746	btsR_2
btsR_2	ZONMW-29_03319	btsR_2
btsR_2	ZONMW-29_03328	btsR_2
btsR_2	ZONMW-29_03054	btsR_2
btsR_2	ZONMW-29_03177	btsR_2
btsR_2	ZONMW-29_00949	btsR_2
btsR_2	ZONMW-29_00222	btsR_2
btsR_2	ZONMW-29_02001	btsR_2
btsR_2	ZONMW-29_03310	btsR_2
btsR_2	ZONMW-29_00076	btsR_2
btsR_2	ZONMW-29_03099	btsR_2
btsR_2	ZONMW-29_01306	btsR_2
btsR_2	ZONMW-29_01166	btsR_2
btsR_2	ZONMW-30_02418	btsR_2
btsR_2	ZONMW-30_00054	btsR_2
btsR_2	ZONMW-30_03389	btsR_2
btsR_2	ZONMW-30_02155	btsR_2
btsR_2	ZONMW-30_01938	btsR_2
btsR_2	ZONMW-30_03383	btsR_2
btsR_2	ZONMW-30_01664	btsR_2
btsR_2	ZONMW-30_00194	btsR_2
btsR_2	ZONMW-30_03136	btsR_2
btsR_2	ZONMW-30_00767	btsR_2

btsR_2	ZONMW-30_02039	btsR_2
btsR_2	ZONMW-30_02232	btsR_2
btsR_2	ZONMW-30_02726	btsR_2
btsR_2	ZONMW-31_01989	btsR_2
btsR_2	ZONMW-31_01037	btsR_2
btsR_2	ZONMW-32_03152	btsR_2
btsR_2	ZONMW-32_03927	btsR_2
btsR_2	ZONMW-33_03122	btsR_2
btsR_2	ZONMW-33_03732	btsR_2
btsR_2	ZONMW-34_02534	btsR_2
btsR_2	ZONMW-34_02034	btsR_2
btsR_2	ZONMW-35_02498	btsR_2
btsR_2	ZONMW-35_01726	btsR_2
btsR_2	ZONMW-36_02880	btsR_2
btsR_2	ZONMW-36_03681	btsR_2
btsR_2	ZONMW-37_00339	btsR_2
btsR_2	ZONMW-37_00623	btsR_2
btsR_2	ZONMW-37_02764	btsR_2
btsR_2	ZONMW-37_02838	btsR_2
btsR_2	ZONMW-37_03210	btsR_2
btsR_2	ZONMW-37_03178	btsR_2
btsR_2	ZONMW-37_00154	btsR_2
btsR_2	ZONMW-37_01990	btsR_2
btsR_2	ZONMW-37_02665	btsR_2
btsR_2	ZONMW-37_01892	btsR_2
btsR_2	ZONMW-37_01188	btsR_2
btsR_2	ZONMW-37_00539	btsR_2
btsR_2	ZONMW-37_00374	btsR_2
btsR_2	ZONMW-37_02921	btsR_2
btsR_2	ZONMW-37_03454	btsR_2
btsR_2	ZONMW-37_02070	btsR_2
btsR_2	ZONMW-37_01640	btsR_2
btsR_2	ZONMW-37_02561	btsR_2
btsR_2	ZONMW-37_00651	btsR_2
btsR_2	ZONMW-38_00439	btsR_2
btsR_2	ZONMW-38_00529	btsR_2
btsR_2	ZONMW-38_00328	btsR_2
btsR_2	ZONMW-38_03606	btsR_2
btsR_2	ZONMW-38_03107	btsR_2
btsR_2	ZONMW-38_00937	btsR_2
btsR_2	ZONMW-38_00925	btsR_2
btsR_2	ZONMW-38_03004	btsR_2
btsR_2	ZONMW-39_02076	btsR_2
btsR_2	ZONMW-39_02157	btsR_2
btsR_2	ZONMW-40_01340	btsR_2
btsR_2	ZONMW-40_02139	btsR_2
btsR_2	ZONMW-40_03247	btsR_2
btsR_2	ZONMW-40_02478	btsR_2

btsR_2	ZONMW-40_01245	btsR_2
btsR_2	ZONMW-40_00868	btsR_2
btsR_2	ZONMW-40_01408	btsR_2
btsR_2	ZONMW-40_02026	btsR_2
btsR_2	ZONMW-40_02563	btsR_2
btsR_2	ZONMW-40_01265	btsR_2
btsR_2	ZONMW-40_03289	btsR_2
btsR_2	ZONMW-40_02048	btsR_2
btsR_2	ZONMW-40_02400	btsR_2
btsR_2	ZONMW-40_03319	btsR_2
btsR_2	ZONMW-41_02385	btsR_2
btsR_2	ZONMW-41_01454	btsR_2
btsR_2	ZONMW-41_01536	btsR_2
btsR_2	ZONMW-41_03089	btsR_2
btsR_2	ZONMW-41_01167	btsR_2
btsR_2	ZONMW-42_01141	btsR_2
btsR_2	ZONMW-42_01557	btsR_2
btsR_2	ZONMW-42_03471	btsR_2
btsR_2	ZONMW-43_01161	btsR_2
btsR_2	ZONMW-43_01538	btsR_2
btsR_2	ZONMW-44_00162	btsR_2
btsR_2	ZONMW-44_03442	btsR_2
btsR_2	ZONMW-45_02584	btsR_2
btsR_2	ZONMW-45_01532	btsR_2
btsR_2	ZONMW-45_01055	btsR_2
btsR_2	ZONMW-45_01480	btsR_2
btsR_2	ZONMW-45_02750	btsR_2
btsR_2	ZONMW-45_02169	btsR_2
btsR_2	ZONMW-45_02248	btsR_2
btsR_2	ZONMW-45_01133	btsR_2
btsR_2	ZONMW-45_00587	btsR_2
btsR_2	ZONMW-45_01305	btsR_2
btsR_2	ZONMW-45_02667	btsR_2
btsR_2	ZONMW-45_00144	btsR_2
btsR_2	ZONMW-45_01438	btsR_2
btsR_2	ZONMW-45_00213	btsR_2
btsR_2	ZONMW-45_02770	btsR_2
btsR_2	ZONMW-45_02440	btsR_2
btsR_2	ZONMW-46_02974	btsR_2
btsR_2	ZONMW-46_02043	btsR_2
btsR_2	ZONMW-46_00612	btsR_2
btsR_2	ZONMW-46_01735	btsR_2
btsR_2	ZONMW-46_01161	btsR_2
btsR_2	ZONMW-46_01845	btsR_2
btsR_2	ZONMW-46_03167	btsR_2
btsR_2	ZONMW-46_00534	btsR_2
btsR_2	ZONMW-46_00872	btsR_2
btsR_2	ZONMW-46_02689	btsR_2

btsR_2	ZONMW-46_02530	btsR_2
btsR_2	ZONMW-46_00359	btsR_2
btsR_2	ZONMW-46_01693	btsR_2
btsR_2	ZONMW-46_00428	btsR_2
btsR_2	ZONMW-46_01141	btsR_2
btsR_2	ZONMW-46_02433	btsR_2
btsR_2	ZONMW-47_02899	btsR_2
btsR_2	ZONMW-47_02018	btsR_2
btsR_2	ZONMW-47_00612	btsR_2
btsR_2	ZONMW-47_01754	btsR_2
btsR_2	ZONMW-47_01012	btsR_2
btsR_2	ZONMW-47_01644	btsR_2
btsR_2	ZONMW-47_03159	btsR_2
btsR_2	ZONMW-47_00534	btsR_2
btsR_2	ZONMW-47_00872	btsR_2
btsR_2	ZONMW-47_02679	btsR_2
btsR_2	ZONMW-47_02520	btsR_2
btsR_2	ZONMW-47_00146	btsR_2
btsR_2	ZONMW-47_01796	btsR_2
btsR_2	ZONMW-47_00077	btsR_2
btsR_2	ZONMW-47_01032	btsR_2
btsR_2	ZONMW-47_02423	btsR_2
btsR_2	ZONMW-48_01853	btsR_2
btsR_2	ZONMW-48_02001	btsR_2
btsR_2	ZONMW-48_01720	btsR_2
btsR_2	ZONMW-48_01711	btsR_2
btsR_2	ZONMW-48_03075	btsR_2
btsR_2	ZONMW-48_00741	btsR_2
btsR_2	ZONMW-48_02917	btsR_2
btsR_2	ZONMW-48_01706	btsR_2
btsR_2	ZONMW-48_00511	btsR_2
btsR_2	ZONMW-48_01156	btsR_2
btsR_2	ZONMW-48_01085	btsR_2
btsR_2	ZONMW-48_00580	btsR_2
btsR_2	ZONMW-48_01251	btsR_2
btsR_2	ZONMW-48_01213	btsR_2
btsR_2	ZONMW-48_01150	btsR_2
btsR_2	ZONMW-49_02681	btsR_2
btsR_2	ZONMW-49_00454	btsR_2
btsR_2	ZONMW-49_00757	btsR_2
btsR_2	ZONMW-49_00538	btsR_2
btsR_2	ZONMW-49_01846	btsR_2
btsR_2	ZONMW-49_01814	btsR_2
btsR_2	ZONMW-49_01474	btsR_2
btsR_2	ZONMW-49_00886	btsR_2
btsR_2	ZONMW-49_00118	btsR_2
btsR_2	ZONMW-49_02515	btsR_2
btsR_2	ZONMW-49_01129	btsR_2

btsR_2	ZONMW-49_02621	btsR_2
btsR_2	ZONMW-49_02352	btsR_2
btsR_2	ZONMW-49_00263	btsR_2
btsR_2	ZONMW-49_00293	btsR_2
btsR_2	ZONMW-49_03264	btsR_2
btsR_2	ZONMW-50_02475	btsR_2
btsR_2	ZONMW-50_00031	btsR_2
btsR_2	ZONMW-50_00758	btsR_2
btsR_2	ZONMW-50_00539	btsR_2
btsR_2	ZONMW-50_03027	btsR_2
btsR_2	ZONMW-50_01600	btsR_2
btsR_2	ZONMW-50_01476	btsR_2
btsR_2	ZONMW-50_00887	btsR_2
btsR_2	ZONMW-50_00368	btsR_2
btsR_2	ZONMW-50_02309	btsR_2
btsR_2	ZONMW-50_01130	btsR_2
btsR_2	ZONMW-50_02415	btsR_2
btsR_2	ZONMW-50_02108	btsR_2
btsR_2	ZONMW-50_00222	btsR_2
btsR_2	ZONMW-50_00192	btsR_2
btsR_2	ZONMW-50_03245	btsR_2
btsR_2	ZONMW-51_02597	btsR_2
btsR_2	ZONMW-51_00442	btsR_2
btsR_2	ZONMW-51_00951	btsR_2
btsR_2	ZONMW-51_01167	btsR_2
btsR_2	ZONMW-51_00510	btsR_2
btsR_2	ZONMW-51_02129	btsR_2
btsR_2	ZONMW-51_02090	btsR_2
btsR_2	ZONMW-51_00730	btsR_2
btsR_2	ZONMW-51_01361	btsR_2
btsR_2	ZONMW-51_00115	btsR_2
btsR_2	ZONMW-51_01766	btsR_2
btsR_2	ZONMW-51_02492	btsR_2
btsR_2	ZONMW-51_02736	btsR_2
btsR_2	ZONMW-51_03146	btsR_2
btsR_2	ZONMW-51_02679	btsR_2
btsR_2	ZONMW-51_01604	btsR_2
btsR_2	ZONMW-51_00251	btsR_2
btsR_2	ZONMW-51_00281	btsR_2
btsR_2	ZONMW-51_03147	btsR_2
btsR_2	ZONMW-51_02059	btsR_2
btsR_2	ZONMW-52_02090	btsR_2
btsR_2	ZONMW-52_00426	btsR_2
btsR_2	ZONMW-52_01551	btsR_2
btsR_2	ZONMW-52_02860	btsR_2
btsR_2	ZONMW-52_01973	btsR_2
btsR_2	ZONMW-52_02558	btsR_2
btsR_2	ZONMW-52_00679	btsR_2

btsR_2	ZONMW-52_01330	btsR_2
btsR_2	ZONMW-52_01103	btsR_2
btsR_2	ZONMW-52_00087	btsR_2
btsR_2	ZONMW-52_01909	btsR_2
btsR_2	ZONMW-52_02475	btsR_2
btsR_2	ZONMW-52_02310	btsR_2
btsR_2	ZONMW-52_02150	btsR_2
btsR_2	ZONMW-52_01747	btsR_2
btsR_2	ZONMW-52_00235	btsR_2
btsR_2	ZONMW-52_00265	btsR_2
btsR_2	ZONMW-52_00713	btsR_2
btsR_2	ZONMW-53_02749	btsR_2
btsR_2	ZONMW-53_01158	btsR_2
btsR_2	ZONMW-53_00276	btsR_2
btsR_2	ZONMW-53_00055	btsR_2
btsR_2	ZONMW-53_02308	btsR_2
btsR_2	ZONMW-53_01414	btsR_2
btsR_2	ZONMW-53_00495	btsR_2
btsR_2	ZONMW-53_00746	btsR_2
btsR_2	ZONMW-53_02019	btsR_2
btsR_2	ZONMW-53_02567	btsR_2
btsR_2	ZONMW-53_01847	btsR_2
btsR_2	ZONMW-53_02158	btsR_2
btsR_2	ZONMW-53_02899	btsR_2
btsR_2	ZONMW-53_02689	btsR_2
btsR_2	ZONMW-53_02009	btsR_2
btsR_2	ZONMW-53_00972	btsR_2
btsR_2	ZONMW-53_01002	btsR_2
btsR_2	ZONMW-53_01445	btsR_2
btsR_2	ZONMW-54_02892	btsR_2
btsR_2	ZONMW-54_01563	btsR_2
btsR_2	ZONMW-54_02224	btsR_2
btsR_2	ZONMW-54_00755	btsR_2
btsR_2	ZONMW-54_00973	btsR_2
btsR_2	ZONMW-54_01495	btsR_2
btsR_2	ZONMW-54_01309	btsR_2
btsR_2	ZONMW-54_02351	btsR_2
btsR_2	ZONMW-54_00540	btsR_2
btsR_2	ZONMW-54_00323	btsR_2
btsR_2	ZONMW-54_01890	btsR_2
btsR_2	ZONMW-54_02648	btsR_2
btsR_2	ZONMW-54_00115	btsR_2
btsR_2	ZONMW-54_02828	btsR_2
btsR_2	ZONMW-54_02045	btsR_2
btsR_2	ZONMW-54_01755	btsR_2
btsR_2	ZONMW-54_01725	btsR_2
btsR_2	ZONMW-54_01276	btsR_2
btsR_2	ZONMW-55_02749	btsR_2

btsR_2	ZONMW-55_01084	btsR_2
btsR_2	ZONMW-55_00269	btsR_2
btsR_2	ZONMW-55_00053	btsR_2
btsR_2	ZONMW-55_01014	btsR_2
btsR_2	ZONMW-55_01843	btsR_2
btsR_2	ZONMW-55_02412	btsR_2
btsR_2	ZONMW-55_00490	btsR_2
btsR_2	ZONMW-55_00715	btsR_2
btsR_2	ZONMW-55_01423	btsR_2
btsR_2	ZONMW-55_02088	btsR_2
btsR_2	ZONMW-55_02583	btsR_2
btsR_2	ZONMW-55_02905	btsR_2
btsR_2	ZONMW-55_03113	btsR_2
btsR_2	ZONMW-55_02689	btsR_2
btsR_2	ZONMW-55_01926	btsR_2
btsR_2	ZONMW-55_01275	btsR_2
btsR_2	ZONMW-55_01245	btsR_2
btsR_2	ZONMW-55_03114	btsR_2
btsR_2	ZONMW-55_02381	btsR_2
btsR_2	ZONMW-56_02446	btsR_2
btsR_2	ZONMW-56_00312	btsR_2
btsR_2	ZONMW-56_01048	btsR_2
btsR_2	ZONMW-56_02754	btsR_2
btsR_2	ZONMW-56_01991	btsR_2
btsR_2	ZONMW-56_00060	btsR_2
btsR_2	ZONMW-56_00823	btsR_2
btsR_2	ZONMW-56_02364	btsR_2
btsR_2	ZONMW-56_00649	btsR_2
btsR_2	ZONMW-56_01646	btsR_2
btsR_2	ZONMW-56_02666	btsR_2
btsR_2	ZONMW-56_02512	btsR_2
btsR_2	ZONMW-56_01510	btsR_2
btsR_2	ZONMW-56_00504	btsR_2
btsR_2	ZONMW-56_00474	btsR_2
btsR_2	ZONMW-56_00026	btsR_2
btsR_2	ZONMW-57_02653	btsR_2
btsR_2	ZONMW-57_00525	btsR_2
btsR_2	ZONMW-57_01348	btsR_2
btsR_2	ZONMW-57_01560	btsR_2
btsR_2	ZONMW-57_02143	btsR_2
btsR_2	ZONMW-57_00272	btsR_2
btsR_2	ZONMW-57_01124	btsR_2
btsR_2	ZONMW-57_02493	btsR_2
btsR_2	ZONMW-57_01706	btsR_2
btsR_2	ZONMW-57_00855	btsR_2
btsR_2	ZONMW-57_01878	btsR_2
btsR_2	ZONMW-57_01993	btsR_2
btsR_2	ZONMW-57_02810	btsR_2

btsR_2	ZONMW-57_02578	btsR_2
btsR_2	ZONMW-57_01716	btsR_2
btsR_2	ZONMW-57_00717	btsR_2
btsR_2	ZONMW-57_00686	btsR_2
btsR_2	ZONMW-57_00241	btsR_2
btsR_2	ZONMW-58_02678	btsR_2
btsR_2	ZONMW-58_00446	btsR_2
btsR_2	ZONMW-58_01236	btsR_2
btsR_2	ZONMW-58_01024	btsR_2
btsR_2	ZONMW-58_02167	btsR_2
btsR_2	ZONMW-58_00699	btsR_2
btsR_2	ZONMW-58_01460	btsR_2
btsR_2	ZONMW-58_02518	btsR_2
btsR_2	ZONMW-58_01730	btsR_2
btsR_2	ZONMW-58_00116	btsR_2
btsR_2	ZONMW-58_01902	btsR_2
btsR_2	ZONMW-58_02085	btsR_2
btsR_2	ZONMW-58_02834	btsR_2
btsR_2	ZONMW-58_02603	btsR_2
btsR_2	ZONMW-58_01740	btsR_2
btsR_2	ZONMW-58_00254	btsR_2
btsR_2	ZONMW-58_00285	btsR_2
btsR_2	ZONMW-58_00730	btsR_2
btsR_2	ZONMW-59_02686	btsR_2
btsR_2	ZONMW-59_00514	btsR_2
btsR_2	ZONMW-59_01349	btsR_2
btsR_2	ZONMW-59_02772	btsR_2
btsR_2	ZONMW-59_02386	btsR_2
btsR_2	ZONMW-59_00269	btsR_2
btsR_2	ZONMW-59_01126	btsR_2
btsR_2	ZONMW-59_01974	btsR_2
btsR_2	ZONMW-59_00850	btsR_2
btsR_2	ZONMW-59_01715	btsR_2
btsR_2	ZONMW-59_02304	btsR_2
btsR_2	ZONMW-59_02894	btsR_2
btsR_2	ZONMW-59_02626	btsR_2
btsR_2	ZONMW-59_01552	btsR_2
btsR_2	ZONMW-59_00706	btsR_2
btsR_2	ZONMW-59_00676	btsR_2
btsR_2	ZONMW-59_00234	btsR_2
btsR_2	ZONMW-60_02708	btsR_2
btsR_2	ZONMW-60_00528	btsR_2
btsR_2	ZONMW-60_02098	btsR_2
btsR_2	ZONMW-60_01226	btsR_2
btsR_2	ZONMW-60_00998	btsR_2
btsR_2	ZONMW-60_00460	btsR_2
btsR_2	ZONMW-60_02231	btsR_2
btsR_2	ZONMW-60_00299	btsR_2

btsR_2	ZONMW-60_01441	btsR_2
btsR_2	ZONMW-60_01657	btsR_2
btsR_2	ZONMW-60_03225	btsR_2
btsR_2	ZONMW-60_00854	btsR_2
btsR_2	ZONMW-60_02600	btsR_2
btsR_2	ZONMW-60_02928	btsR_2
btsR_2	ZONMW-60_02780	btsR_2
btsR_2	ZONMW-60_01933	btsR_2
btsR_2	ZONMW-60_00719	btsR_2
btsR_2	ZONMW-60_00689	btsR_2
btsR_2	ZONMW-60_00266	btsR_2
btsR_2	ZONMW-61_00835	btsR_2
btsR_2	ZONMW-61_00739	btsR_2
btsR_2	ZONMW-61_03326	btsR_2
btsR_2	ZONMW-61_03329	btsR_2
btsR_2	ZONMW-61_02085	btsR_2
btsR_2	ZONMW-61_02210	btsR_2
btsR_2	ZONMW-61_00483	btsR_2
btsR_2	ZONMW-61_03130	btsR_2
btsR_2	ZONMW-61_03076	btsR_2
btsR_2	ZONMW-61_03204	btsR_2
btsR_2	ZONMW-61_02723	btsR_2
btsR_2	ZONMW-61_02962	btsR_2
btsR_2	ZONMW-61_01502	btsR_2
btsR_2	ZONMW-61_00228	btsR_2
btsR_2	ZONMW-61_03785	btsR_2
btsR_2	ZONMW-61_01865	btsR_2
btsR_2	ZONMW-61_01999	btsR_2
btsR_2	ZONMW-61_00423	btsR_2
btsR_2	ZONMW-62_02651	btsR_2
btsR_2	ZONMW-62_02427	btsR_2
btsR_2	ZONMW-62_00448	btsR_2
btsR_2	ZONMW-62_00161	btsR_2
btsR_2	ZONMW-62_00452	btsR_2
btsR_2	ZONMW-62_00029	btsR_2
btsR_2	ZONMW-62_02010	btsR_2
btsR_2	ZONMW-62_03205	btsR_2
btsR_2	ZONMW-62_03256	btsR_2
btsR_2	ZONMW-62_02273	btsR_2
btsR_2	ZONMW-62_02369	btsR_2
btsR_2	ZONMW-62_03410	btsR_2
btsR_2	ZONMW-62_01254	btsR_2
btsR_2	ZONMW-62_00933	btsR_2
btsR_2	ZONMW-62_00391	btsR_2
btsR_2	ZONMW-62_00243	btsR_2
btsR_2	ZONMW-63_02199	btsR_2
btsR_2	ZONMW-63_00198	btsR_2
btsR_2	ZONMW-63_01217	btsR_2

btsR_2	ZONMW-63_00997	btsR_2
btsR_2	ZONMW-63_03122	btsR_2
btsR_2	ZONMW-63_01910	btsR_2
btsR_2	ZONMW-63_00880	btsR_2
btsR_2	ZONMW-63_00659	btsR_2
btsR_2	ZONMW-63_01442	btsR_2
btsR_2	ZONMW-63_03258	btsR_2
btsR_2	ZONMW-63_01989	btsR_2
btsR_2	ZONMW-63_02713	btsR_2
btsR_2	ZONMW-63_01690	btsR_2
btsR_2	ZONMW-63_02263	btsR_2
btsR_2	ZONMW-63_02139	btsR_2
btsR_2	ZONMW-63_01452	btsR_2
btsR_2	ZONMW-63_00390	btsR_2
btsR_2	ZONMW-63_00360	btsR_2
btsR_2	ZONMW-63_02641	btsR_2
btsR_2	ZONMW-64_03511	btsR_2
btsR_2	ZONMW-64_02115	btsR_2
btsR_2	ZONMW-65_01260	btsR_2
btsR_2	ZONMW-65_00851	btsR_2
btsR_2	ZONMW-65_00737	btsR_2
btsR_2	ZONMW-65_01009	btsR_2
btsR_2	ZONMW-65_01671	btsR_2
btsR_2	ZONMW-65_00826	btsR_2
btsR_2	ZONMW-66_02253	btsR_2
btsR_2	ZONMW-66_00274	btsR_2
btsR_2	ZONMW-66_02783	btsR_2
btsR_2	ZONMW-67_03385	btsR_2
btsR_2	ZONMW-67_00820	btsR_2
btsR_2	ZONMW-68_00548	btsR_2
btsR_2	ZONMW-68_04813	btsR_2
btsR_2	ZONMW-68_02798	btsR_2
btsR_2	ZONMW-68_02395	btsR_2
btsR_2	ZONMW-68_01014	btsR_2
btsR_2	ZONMW-68_02380	btsR_2
btsR_2	ZONMW-68_04532	btsR_2
btsR_2	ZONMW-68_00218	btsR_2
btsR_2	ZONMW-68_02402	btsR_2
btsR_2	ZONMW-68_00707	btsR_2
btsR_2	ZONMW-68_03253	btsR_2
btsR_2	ZONMW-68_02717	btsR_2
btsR_2	ZONMW-68_02346	btsR_2
btsR_2	ZONMW-68_03429	btsR_2
btsR_2	ZONMW-68_00953	btsR_2
btsR_2	ZONMW-68_02725	btsR_2
btsR_2	ZONMW-68_00493	btsR_2
btsR_2	ZONMW-68_04009	btsR_2
btsR_2	ZONMW-68_04183	btsR_2

btsR_2	ZONMW-68_02727	btsR_2
btsR_2	ZONMW-68_01096	btsR_2
btsR_2	ZONMW-68_02929	btsR_2
btsR_2	ZONMW-68_01785	btsR_2
btsR_2	ZONMW-69_03680	btsR_2
btsR_2	ZONMW-69_03386	btsR_2
btsR_2	ZONMW-69_00867	btsR_2
btsR_2	ZONMW-72_03140	btsR_2
btsR_2	ZONMW-72_00911	btsR_2
btsR_2	ZONMW-72_00825	btsR_2
btsR_2	ZONMW-72_03907	btsR_2
btsR_2	ZONMW-72_02328	btsR_2
btsR_2	ZONMW-72_00755	btsR_2
btsR_2	ZONMW-72_04118	btsR_2
btsR_2	ZONMW-72_00653	btsR_2
btsR_2	ZONMW-72_03744	btsR_2
btsR_2	ZONMW-72_00487	btsR_2
btsR_2	ZONMW-72_03745	btsR_2
btsR_2	ZONMW-72_00921	btsR_2
btsR_2	ZONMW-72_01019	btsR_2
btsR_2	ZONMW-72_04230	btsR_2
btsR_2	ZONMW-72_03400	btsR_2
btsR_2	ZONMW-72_00557	btsR_2
btsR_2	ZONMW-72_01946	btsR_2
btsR_2	ZONMW-72_01725	btsR_2
btsR_2	ZONMW-72_01598	btsR_2
btsR_2	ZONMW-72_03889	btsR_2
btsR_2	ZONMW-72_03961	btsR_2
btsR_2	ZONMW-72_01581	btsR_2
btsR_2	ZONMW-72_01494	btsR_2
btsR_2	ZONMW-72_04080	btsR_2
btsR_2	ZONMW-73_00814	btsR_2
btsR_2	ZONMW-73_01010	btsR_2
btsR_2	ZONMW-73_00758	btsR_2
btsR_2	ZONMW-73_03763	btsR_2
btsR_2	ZONMW-73_03082	btsR_2
btsR_2	ZONMW-73_00349	btsR_2
btsR_2	ZONMW-73_01006	btsR_2
btsR_2	ZONMW-73_01653	btsR_2
btsR_2	ZONMW-73_02707	btsR_2
btsR_2	ZONMW-73_02284	btsR_2
btsR_2	ZONMW-73_00603	btsR_2
btsR_2	ZONMW-73_03840	btsR_2
btsR_2	ZONMW-74_01593	btsR_2
btsR_2	ZONMW-74_01044	btsR_2
btsR_2	ZONMW-74_01595	btsR_2
btsR_2	ZONMW-74_01590	btsR_2
btsR_2	ZONMW-74_00820	btsR_2

btsR_2	ZONMW-74_02282	btsR_2
btsR_2	ZONMW-74_02092	btsR_2
btsR_2	ZONMW-74_01773	btsR_2
btsR_2	ZONMW-74_01972	btsR_2
btsR_2	ZONMW-74_03208	btsR_2
btsR_2	ZONMW-74_02884	btsR_2
btsR_2	ZONMW-74_00139	btsR_2
btsR_2	ZONMW-74_02966	btsR_2
btsR_2	ZONMW-74_01454	btsR_2
btsR_2	ZONMW-74_01613	btsR_2
btsR_2	ZONMW-74_01470	btsR_2
btsR_2	ZONMW-74_03370	btsR_2
btsR_2	ZONMW-74_03113	btsR_2
btsR_2	ZONMW-74_02974	btsR_2
btsR_2	ZONMW-75_00376	btsR_2
btsR_2	ZONMW-75_01492	btsR_2
btsR_2	ZONMW-75_01316	btsR_2
btsR_2	ZONMW-75_02154	btsR_2
btsR_2	ZONMW-75_00057	btsR_2
btsR_2	ZONMW-75_02445	btsR_2
btsR_2	ZONMW-75_00172	btsR_2
btsR_2	ZONMW-75_00453	btsR_2
btsR_2	ZONMW-75_01667	btsR_2
btsR_2	ZONMW-75_01103	btsR_2
btsR_2	ZONMW-75_03603	btsR_2
btsR_2	ZONMW-75_01721	btsR_2
btsR_2	ZONMW-75_02397	btsR_2
btsR_2	da5A-8_00850	btsR_2
btsR_2	da5A-8_00056	btsR_2
btsR_2	da5A-8_03385	btsR_2
btsR_2	da5A-8_02759	btsR_2
btsR_2	da5A-8_02046	btsR_2
btsR_2	da5A-8_03389	btsR_2
btsR_2	da5A-8_03492	btsR_2
btsR_2	da5A-8_01228	btsR_2
btsR_2	da5A-8_02123	btsR_2
btsR_2	da5A-8_03563	btsR_2
btsR_2	da5A-8_01702	btsR_2
btsR_2	da5A-8_03232	btsR_2
btsR_2	da5A-8_02316	btsR_2
btsR_2	da5A-8_03097	btsR_2
btsR_2	da5A-8_00738	btsR_2
btsR_2	da5A-8_01902	btsR_2
btsR_2	da5A-8_00774	btsR_2
btsR_2	da5A-8_00197	btsR_2
btsR_2	da5A-8_00097	btsR_2
btsR_2	da5A-8_03776	btsR_2
btsR_2	da5A-8_01440	btsR_2

btsR_2	da5A-8_01469	btsR_2
btsR_2	da5A-8_03208	btsR_2
btsR_2	da5A-8_02491	btsR_2
btsR_2	da5A-8_01798	btsR_2
phaA	18_02362	phaA
phaA	18_03707	phaA
phaA	ACAM48_00785	phaA
phaA	ACAM48_01081	phaA
phaA	CAM030_00751	phaA
phaA	CAM030_00197	phaA
phaA	CECT7946_01479	phaA
phaA	CECT7946_02713	phaA
phaA	CECT7946_03116	phaA
phaA	CGMCC1.8863_03214	phaA
phaA	CGMCC1.8863_01733	phaA
phaA	CL8139_00424	phaA
phaA	CL8139_02648	phaA
phaA	DAU203_02571	phaA
phaA	DAU203_02718	phaA
phaA	DD5b_00877	phaA
phaA	DD5b_01378	phaA
phaA	DSM1208_00446	phaA
phaA	DSM1208_00097	phaA
phaA	DSM12212_00541	phaA
phaA	DSM12212_01557	phaA
phaA	DSM12212_00448	phaA
phaA	DSM15343_00316	phaA
phaA	DSM15343_02147	phaA
phaA	DSM15343_02228	phaA
phaA	DSM15361_00967	phaA
phaA	DSM15361_01184	phaA
phaA	DSM15361_01853	phaA
phaA	DSM15365_02084	phaA
phaA	DSM15365_01537	phaA
phaA	DSM15718_01808	phaA
phaA	DSM15718_01879	phaA
phaA	DSM15718_01756	phaA
phaA	DSM16505T_02028	phaA
phaA	DSM16505T_02542	phaA
phaA	DSM18103_02019	phaA
phaA	DSM18103_01348	phaA
phaA	DSM18293_01089	phaA
phaA	DSM18293_01248	phaA
phaA	DSM18293_04268	phaA
phaA	DSM18293_04510	phaA
phaA	DSM18841_01282	phaA
phaA	DSM18841_02938	phaA
phaA	DSM18842_02917	phaA

phaA	DSM18842_02757	phaA
phaA	DSM19866_03257	phaA
phaA	DSM19866_01697	phaA
phaA	DSM2041_01637	phaA
phaA	DSM2041_01214	phaA
phaA	DSM2041_02447	phaA
phaA	DSM2061_01935	phaA
phaA	DSM2061_00453	phaA
phaA	DSM2064_04080	phaA
phaA	DSM2064_03932	phaA
phaA	DSM2064_00751	phaA
phaA	DSM21164_01797	phaA
phaA	DSM21164_02421	phaA
phaA	DSM23404_02537	phaA
phaA	DSM23404_02801	phaA
phaA	DSM23424_01013	phaA
phaA	DSM23424_02367	phaA
phaA	DSM23424_01471	phaA
phaA	DSM24786_01200	phaA
phaA	DSM24786_00177	phaA
phaA	DSM7489_02462	phaA
phaA	DSM7489_02592	phaA
phaA	DSW1_02091	phaA
phaA	DSW1_01112	phaA
phaA	DSW1_02233	phaA
phaA	DSW5_00047	phaA
phaA	DSW5_00154	phaA
phaA	DSW6_02527	phaA
phaA	DSW6_02135	phaA
phaA	DSW6_02582	phaA
phaA	DSW8_03107	phaA
phaA	DSW8_00678	phaA
phaA	F52_04236	phaA
phaA	F52_04104	phaA
phaA	F52_00888	phaA
phaA	GCA_001027945.1_02913	phaA
phaA	GCA_001027945.1_00596	phaA
phaA	HI1_02394	phaA
phaA	HI1_02525	phaA
phaA	IR1_04195	phaA
phaA	IR1_04297	phaA
phaA	IR1_01765	phaA
phaA	KCTC12974_01704	phaA
phaA	KCTC12974_01534	phaA
phaA	KL-A_03109	phaA
phaA	KL-A_00963	phaA
phaA	L12_00222	phaA
phaA	LL0412.1.7_01340	phaA

phaA	LL0412.1.7_01817	phaA
phaA	MAR_2009_71_00027	phaA
phaA	MAR_2009_71_03473	phaA
phaA	NN016038_02373	phaA
phaA	NN016038_03748	phaA
phaA	PAO1_02063	phaA
phaA	PAO1_02625	phaA
phaA	PAO1_04053	phaA
phaA	PAO1_00234	phaA
phaA	PAO1_03118	phaA
phaA	PAO1_03712	phaA
phaA	PAO1_03576	phaA
phaA	PAO1_01783	phaA
phaA	PAO1_03043	phaA
phaA	PAO1_04948	phaA
phaA	PAO1_01050	phaA
phaA	UW101_04807	phaA
phaA	UW101_04955	phaA
phaA	UW101_03462	phaA
phaA	V4-33_00744	phaA
phaA	V4-33_01492	phaA
phaA	V4-33_01633	phaA
phaA	W5C_00973	phaA
phaA	W5C_01296	phaA
phaA	ZONMW-01_03347	phaA
phaA	ZONMW-01_01641	phaA
phaA	ZONMW-02_03438	phaA
phaA	ZONMW-02_02000	phaA
phaA	ZONMW-03_00716	phaA
phaA	ZONMW-03_00566	phaA
phaA	ZONMW-04_03296	phaA
phaA	ZONMW-04_01504	phaA
phaA	ZONMW-05_02871	phaA
phaA	ZONMW-05_00828	phaA
phaA	ZONMW-06_01001	phaA
phaA	ZONMW-06_00384	phaA
phaA	ZONMW-06_02649	phaA
phaA	ZONMW-06_00738	phaA
phaA	ZONMW-06_02459	phaA
phaA	ZONMW-06_03593	phaA
phaA	ZONMW-06_01507	phaA
phaA	ZONMW-06_03422	phaA
phaA	ZONMW-07_03631	phaA
phaA	ZONMW-07_03171	phaA
phaA	ZONMW-08_03027	phaA
phaA	ZONMW-08_02454	phaA
phaA	ZONMW-09_00729	phaA
phaA	ZONMW-09_00847	phaA

phaA	ZONMW-10_00813	phaA
phaA	ZONMW-10_01193	phaA
phaA	ZONMW-10_02007	phaA
phaA	ZONMW-11_02011	phaA
phaA	ZONMW-11_01456	phaA
phaA	ZONMW-12_03125	phaA
phaA	ZONMW-12_01489	phaA
phaA	ZONMW-13_01960	phaA
phaA	ZONMW-13_02245	phaA
phaA	ZONMW-14_00733	phaA
phaA	ZONMW-14_02163	phaA
phaA	ZONMW-15_01502	phaA
phaA	ZONMW-15_02078	phaA
phaA	ZONMW-15_02856	phaA
phaA	ZONMW-19_00488	phaA
phaA	ZONMW-19_00732	phaA
phaA	ZONMW-20_00230	phaA
phaA	ZONMW-20_00387	phaA
phaA	ZONMW-20_03903	phaA
phaA	ZONMW-20_01991	phaA
phaA	ZONMW-21_03195	phaA
phaA	ZONMW-21_01540	phaA
phaA	ZONMW-22_03321	phaA
phaA	ZONMW-22_02183	phaA
phaA	ZONMW-23_03342	phaA
phaA	ZONMW-23_00368	phaA
phaA	ZONMW-24_03344	phaA
phaA	ZONMW-24_02808	phaA
phaA	ZONMW-25_01223	phaA
phaA	ZONMW-25_02730	phaA
phaA	ZONMW-26_03232	phaA
phaA	ZONMW-26_00132	phaA
phaA	ZONMW-27_03289	phaA
phaA	ZONMW-27_01800	phaA
phaA	ZONMW-28_02831	phaA
phaA	ZONMW-28_03485	phaA
phaA	ZONMW-28_01062	phaA
phaA	ZONMW-28_02773	phaA
phaA	ZONMW-28_00172	phaA
phaA	ZONMW-28_03453	phaA
phaA	ZONMW-28_04006	phaA
phaA	ZONMW-28_04011	phaA
phaA	ZONMW-28_01837	phaA
phaA	ZONMW-28_03141	phaA
phaA	ZONMW-29_02276	phaA
phaA	ZONMW-29_02019	phaA
phaA	ZONMW-30_02530	phaA
phaA	ZONMW-30_02068	phaA

phaA	ZONMW-31_00721	phaA
phaA	ZONMW-31_01257	phaA
phaA	ZONMW-31_02490	phaA
phaA	ZONMW-31_03577	phaA
phaA	ZONMW-31_02816	phaA
phaA	ZONMW-31_02285	phaA
phaA	ZONMW-31_02252	phaA
phaA	ZONMW-31_00160	phaA
phaA	ZONMW-31_01852	phaA
phaA	ZONMW-32_03044	phaA
phaA	ZONMW-32_00570	phaA
phaA	ZONMW-32_00029	phaA
phaA	ZONMW-32_03102	phaA
phaA	ZONMW-32_00963	phaA
phaA	ZONMW-32_03528	phaA
phaA	ZONMW-32_03863	phaA
phaA	ZONMW-32_03868	phaA
phaA	ZONMW-32_02135	phaA
phaA	ZONMW-32_00666	phaA
phaA	ZONMW-33_03229	phaA
phaA	ZONMW-33_00438	phaA
phaA	ZONMW-33_03290	phaA
phaA	ZONMW-33_03171	phaA
phaA	ZONMW-33_00952	phaA
phaA	ZONMW-33_03828	phaA
phaA	ZONMW-33_03641	phaA
phaA	ZONMW-33_03636	phaA
phaA	ZONMW-33_02126	phaA
phaA	ZONMW-33_00534	phaA
phaA	ZONMW-34_03208	phaA
phaA	ZONMW-34_00351	phaA
phaA	ZONMW-34_00815	phaA
phaA	ZONMW-34_01157	phaA
phaA	ZONMW-34_03319	phaA
phaA	ZONMW-34_03003	phaA
phaA	ZONMW-34_02259	phaA
phaA	ZONMW-34_03008	phaA
phaA	ZONMW-34_00898	phaA
phaA	ZONMW-34_03874	phaA
phaA	ZONMW-35_03169	phaA
phaA	ZONMW-35_01039	phaA
phaA	ZONMW-35_02648	phaA
phaA	ZONMW-35_00230	phaA
phaA	ZONMW-35_03315	phaA
phaA	ZONMW-35_02989	phaA
phaA	ZONMW-35_01990	phaA
phaA	ZONMW-35_02984	phaA
phaA	ZONMW-35_02345	phaA

phaA	ZONMW-35_03892	phaA
phaA	ZONMW-36_03530	phaA
phaA	ZONMW-36_00933	phaA
phaA	ZONMW-36_03027	phaA
phaA	ZONMW-36_02830	phaA
phaA	ZONMW-36_00172	phaA
phaA	ZONMW-36_03778	phaA
phaA	ZONMW-36_03614	phaA
phaA	ZONMW-36_03619	phaA
phaA	ZONMW-36_02110	phaA
phaA	ZONMW-36_00837	phaA
phaA	ZONMW-37_03330	phaA
phaA	ZONMW-37_01918	phaA
phaA	ZONMW-38_02281	phaA
phaA	ZONMW-38_01173	phaA
phaA	ZONMW-38_00057	phaA
phaA	ZONMW-38_00317	phaA
phaA	ZONMW-38_03093	phaA
phaA	ZONMW-38_00974	phaA
phaA	ZONMW-39_01932	phaA
phaA	ZONMW-39_03040	phaA
phaA	ZONMW-39_01279	phaA
phaA	ZONMW-39_02645	phaA
phaA	ZONMW-39_03642	phaA
phaA	ZONMW-39_00848	phaA
phaA	ZONMW-39_03174	phaA
phaA	ZONMW-39_01535	phaA
phaA	ZONMW-39_02751	phaA
phaA	ZONMW-39_01361	phaA
phaA	ZONMW-40_02910	phaA
phaA	ZONMW-40_03263	phaA
phaA	ZONMW-41_00405	phaA
phaA	ZONMW-41_01426	phaA
phaA	ZONMW-42_03022	phaA
phaA	ZONMW-42_03448	phaA
phaA	ZONMW-42_00640	phaA
phaA	ZONMW-42_01526	phaA
phaA	ZONMW-42_03701	phaA
phaA	ZONMW-42_03622	phaA
phaA	ZONMW-42_02395	phaA
phaA	ZONMW-42_00981	phaA
phaA	ZONMW-43_01005	phaA
phaA	ZONMW-43_02216	phaA
phaA	ZONMW-43_02313	phaA
phaA	ZONMW-43_00354	phaA
phaA	ZONMW-43_02275	phaA
phaA	ZONMW-43_03401	phaA
phaA	ZONMW-43_02823	phaA

phaA	ZONMW-43_03588	phaA
phaA	ZONMW-43_03144	phaA
phaA	ZONMW-43_02728	phaA
phaA	ZONMW-43_03862	phaA
phaA	ZONMW-43_04431	phaA
phaA	ZONMW-43_01973	phaA
phaA	ZONMW-43_03867	phaA
phaA	ZONMW-43_00431	phaA
phaA	ZONMW-43_02262	phaA
phaA	ZONMW-44_02198	phaA
phaA	ZONMW-44_03574	phaA
phaA	ZONMW-44_02691	phaA
phaA	ZONMW-44_00242	phaA
phaA	ZONMW-44_01486	phaA
phaA	ZONMW-44_03335	phaA
phaA	ZONMW-44_02530	phaA
phaA	ZONMW-44_00583	phaA
phaA	ZONMW-44_03652	phaA
phaA	ZONMW-45_03083	phaA
phaA	ZONMW-45_02505	phaA
phaA	ZONMW-46_00152	phaA
phaA	ZONMW-46_02727	phaA
phaA	ZONMW-47_00353	phaA
phaA	ZONMW-47_02718	phaA
phaA	ZONMW-48_01875	phaA
phaA	ZONMW-48_02740	phaA
phaA	ZONMW-48_01184	phaA
phaA	ZONMW-49_00011	phaA
phaA	ZONMW-49_00143	phaA
phaA	ZONMW-50_00475	phaA
phaA	ZONMW-50_00343	phaA
phaA	ZONMW-51_00010	phaA
phaA	ZONMW-51_00140	phaA
phaA	ZONMW-52_03235	phaA
phaA	ZONMW-52_00112	phaA
phaA	ZONMW-53_03249	phaA
phaA	ZONMW-53_02542	phaA
phaA	ZONMW-54_03232	phaA
phaA	ZONMW-54_01864	phaA
phaA	ZONMW-55_01528	phaA
phaA	ZONMW-55_01398	phaA
phaA	ZONMW-56_00754	phaA
phaA	ZONMW-56_00624	phaA
phaA	ZONMW-57_03249	phaA
phaA	ZONMW-57_00830	phaA
phaA	ZONMW-58_00010	phaA
phaA	ZONMW-58_00141	phaA
phaA	ZONMW-59_00995	phaA

phaA	ZONMW-59_00825	phaA
phaA	ZONMW-60_03316	phaA
phaA	ZONMW-60_00829	phaA
phaA	ZONMW-61_02681	phaA
phaA	ZONMW-61_02159	phaA
phaA	ZONMW-61_02581	phaA
phaA	ZONMW-62_03367	phaA
phaA	ZONMW-62_00085	phaA
phaA	ZONMW-63_02095	phaA
phaA	ZONMW-63_00505	phaA
phaA	ZONMW-64_02571	phaA
phaA	ZONMW-64_00868	phaA
phaA	ZONMW-64_00770	phaA
phaA	ZONMW-64_00371	phaA
phaA	ZONMW-64_00808	phaA
phaA	ZONMW-64_02722	phaA
phaA	ZONMW-64_02314	phaA
phaA	ZONMW-64_03149	phaA
phaA	ZONMW-64_03820	phaA
phaA	ZONMW-64_01461	phaA
phaA	ZONMW-64_03815	phaA
phaA	ZONMW-64_00449	phaA
phaA	ZONMW-64_04077	phaA
phaA	ZONMW-64_04068	phaA
phaA	ZONMW-64_00821	phaA
phaA	ZONMW-65_03134	phaA
phaA	ZONMW-65_01230	phaA
phaA	ZONMW-65_02566	phaA
phaA	ZONMW-65_01142	phaA
phaA	ZONMW-66_00086	phaA
phaA	ZONMW-66_00983	phaA
phaA	ZONMW-67_02762	phaA
phaA	ZONMW-67_01037	phaA
phaA	ZONMW-67_02180	phaA
phaA	ZONMW-67_03021	phaA
phaA	ZONMW-67_01960	phaA
phaA	ZONMW-67_02952	phaA
phaA	ZONMW-67_00270	phaA
phaA	ZONMW-67_03102	phaA
phaA	ZONMW-67_02957	phaA
phaA	ZONMW-67_01121	phaA
phaA	ZONMW-68_03837	phaA
phaA	ZONMW-68_02671	phaA
phaA	ZONMW-69_00559	phaA
phaA	ZONMW-69_01084	phaA
phaA	ZONMW-69_02227	phaA
phaA	ZONMW-69_02933	phaA
phaA	ZONMW-69_01983	phaA

phaA	ZONMW-69_03002	phaA
phaA	ZONMW-69_00017	phaA
phaA	ZONMW-69_03103	phaA
phaA	ZONMW-69_02997	phaA
phaA	ZONMW-69_01167	phaA
phaA	ZONMW-72_01916	phaA
phaA	ZONMW-72_01814	phaA
phaA	ZONMW-72_03618	phaA
phaA	ZONMW-73_01112	phaA
phaA	ZONMW-73_01596	phaA
phaA	ZONMW-74_03228	phaA
phaA	ZONMW-74_02941	phaA
phaA	ZONMW-75_02494	phaA
phaA	ZONMW-75_00555	phaA
phaA	da5A-8_00530	phaA
phaA	da5A-8_01055	phaA
rplJ	18_01870	rplJ
rplJ	ACAM48_03100	rplJ
rplJ	CAM030_02539	rplJ
rplJ	CECT7946_01160	rplJ
rplJ	CGMCC1.8863_01537	rplJ
rplJ	CL8139_00317	rplJ
rplJ	DAU203_00547	rplJ
rplJ	DD5b_02802	rplJ
rplJ	DSM1208_00618	rplJ
rplJ	DSM12212_03228	rplJ
rplJ	DSM15343_02195	rplJ
rplJ	DSM15361_02504	rplJ
rplJ	DSM15365_03626	rplJ
rplJ	DSM15718_01066	rplJ
rplJ	DSM16505T_02486	rplJ
rplJ	DSM18103_03253	rplJ
rplJ	DSM18293_02231	rplJ
rplJ	DSM18841_02866	rplJ
rplJ	DSM18842_02836	rplJ
rplJ	DSM19866_00620	rplJ
rplJ	DSM2041_05063	rplJ
rplJ	DSM2061_02114	rplJ
rplJ	DSM2064_02974	rplJ
rplJ	DSM21164_00012	rplJ
rplJ	DSM23404_00271	rplJ
rplJ	DSM23424_03111	rplJ
rplJ	DSM24786_01464	rplJ
rplJ	DSM7489_00534	rplJ
rplJ	DSW1_02686	rplJ
rplJ	DSW5_00056	rplJ
rplJ	DSW6_02029	rplJ
rplJ	DSW8_03764	rplJ

rplJ	F52_03105	rplJ
rplJ	GCA_001027945.1_00655	rplJ
rplJ	HI1_00466	rplJ
rplJ	IR1_00956	rplJ
rplJ	KCOM3131_00421	rplJ
rplJ	KCTC12974_02484	rplJ
rplJ	KL-A_02492	rplJ
rplJ	LL0412.1.7_01760	rplJ
rplJ	MAR_2009_71_00192	rplJ
rplJ	NN016038_01893	rplJ
rplJ	PAO1_04403	rplJ
rplJ	UW101_02028	rplJ
rplJ	V4-33_01087	rplJ
rplJ	W5C_03274	rplJ
rplJ	ZONMW-01_02730	rplJ
rplJ	ZONMW-02_03764	rplJ
rplJ	ZONMW-03_03176	rplJ
rplJ	ZONMW-04_02876	rplJ
rplJ	ZONMW-05_03133	rplJ
rplJ	ZONMW-06_03622	rplJ
rplJ	ZONMW-07_02868	rplJ
rplJ	ZONMW-08_03411	rplJ
rplJ	ZONMW-09_01987	rplJ
rplJ	ZONMW-10_01235	rplJ
rplJ	ZONMW-11_03059	rplJ
rplJ	ZONMW-12_03401	rplJ
rplJ	ZONMW-13_01535	rplJ
rplJ	ZONMW-14_02858	rplJ
rplJ	ZONMW-15_01101	rplJ
rplJ	ZONMW-19_01622	rplJ
rplJ	ZONMW-20_04214	rplJ
rplJ	ZONMW-21_03015	rplJ
rplJ	ZONMW-22_03040	rplJ
rplJ	ZONMW-23_03061	rplJ
rplJ	ZONMW-24_03062	rplJ
rplJ	ZONMW-25_01792	rplJ
rplJ	ZONMW-26_03200	rplJ
rplJ	ZONMW-27_03222	rplJ
rplJ	ZONMW-28_03593	rplJ
rplJ	ZONMW-29_02670	rplJ
rplJ	ZONMW-30_01042	rplJ
rplJ	ZONMW-31_03074	rplJ
rplJ	ZONMW-32_03621	rplJ
rplJ	ZONMW-33_03600	rplJ
rplJ	ZONMW-34_03491	rplJ
rplJ	ZONMW-35_03452	rplJ
rplJ	ZONMW-36_03378	rplJ
rplJ	ZONMW-37_03049	rplJ

rplJ	ZONMW-38_01926	rplJ
rplJ	ZONMW-39_03888	rplJ
rplJ	ZONMW-40_01167	rplJ
rplJ	ZONMW-41_01948	rplJ
rplJ	ZONMW-42_02216	rplJ
rplJ	ZONMW-43_03709	rplJ
rplJ	ZONMW-44_03278	rplJ
rplJ	ZONMW-45_02571	rplJ
rplJ	ZONMW-46_02793	rplJ
rplJ	ZONMW-47_02784	rplJ
rplJ	ZONMW-48_02592	rplJ
rplJ	ZONMW-49_02806	rplJ
rplJ	ZONMW-50_02732	rplJ
rplJ	ZONMW-51_02969	rplJ
rplJ	ZONMW-52_03105	rplJ
rplJ	ZONMW-53_03031	rplJ
rplJ	ZONMW-54_03033	rplJ
rplJ	ZONMW-55_01627	rplJ
rplJ	ZONMW-56_02917	rplJ
rplJ	ZONMW-57_03130	rplJ
rplJ	ZONMW-58_03151	rplJ
rplJ	ZONMW-59_03205	rplJ
rplJ	ZONMW-60_03141	rplJ
rplJ	ZONMW-61_03764	rplJ
rplJ	ZONMW-62_03808	rplJ
rplJ	ZONMW-63_02884	rplJ
rplJ	ZONMW-64_04215	rplJ
rplJ	ZONMW-65_01822	rplJ
rplJ	ZONMW-66_01151	rplJ
rplJ	ZONMW-67_03467	rplJ
rplJ	ZONMW-68_03520	rplJ
rplJ	ZONMW-69_03469	rplJ
rplJ	ZONMW-72_02369	rplJ
rplJ	ZONMW-73_01887	rplJ
rplJ	ZONMW-74_02437	rplJ
rplJ	ZONMW-75_03791	rplJ
rplJ	da5A-8_00612	rplJ
iscS	18_01161	iscS
iscS	18_02088	iscS
iscS	ACAM48_02145	iscS
iscS	ACAM48_02233	iscS
iscS	ACAM48_00946	iscS
iscS	ACAM48_01955	iscS
iscS	CAM030_00858	iscS
iscS	CAM030_02025	iscS
iscS	CAM030_01877	iscS
iscS	CECT7946_01963	iscS
iscS	CECT7946_02413	iscS

iscS	CECT7946_03032	iscS
iscS	CGMCC1.8863_00894	iscS
iscS	CGMCC1.8863_00109	iscS
iscS	CGMCC1.8863_03156	iscS
iscS	CL8139_00807	iscS
iscS	CL8139_02864	iscS
iscS	CL8139_03140	iscS
iscS	DAU203_01614	iscS
iscS	DAU203_00024	iscS
iscS	DAU203_02599	iscS
iscS	DD5b_02175	iscS
iscS	DD5b_00973	iscS
iscS	DSM1208_01777	iscS
iscS	DSM1208_03029	iscS
iscS	DSM12212_02082	iscS
iscS	DSM12212_00910	iscS
iscS	DSM12212_00817	iscS
iscS	DSM15343_03047	iscS
iscS	DSM15343_00078	iscS
iscS	DSM15343_00466	iscS
iscS	DSM15361_01442	iscS
iscS	DSM15361_02373	iscS
iscS	DSM15365_00929	iscS
iscS	DSM15365_00383	iscS
iscS	DSM15365_02109	iscS
iscS	DSM15718_02674	iscS
iscS	DSM15718_01966	iscS
iscS	DSM15718_00179	iscS
iscS	DSM16505T_00361	iscS
iscS	DSM16505T_02131	iscS
iscS	DSM16505T_01472	iscS
iscS	DSM18103_03500	iscS
iscS	DSM18103_00185	iscS
iscS	DSM18103_02249	iscS
iscS	DSM18293_00278	iscS
iscS	DSM18293_01154	iscS
iscS	DSM18293_01848	iscS
iscS	DSM18293_03343	iscS
iscS	DSM18293_02896	iscS
iscS	DSM18293_03955	iscS
iscS	DSM18293_03342	iscS
iscS	DSM18841_00177	iscS
iscS	DSM18841_01493	iscS
iscS	DSM18841_02072	iscS
iscS	DSM18842_01035	iscS
iscS	DSM18842_01886	iscS
iscS	DSM18842_01404	iscS
iscS	DSM19866_02014	iscS

iscS	DSM19866_01839	iscS
iscS	DSM2041_00292	iscS
iscS	DSM2041_04084	iscS
iscS	DSM2041_04503	iscS
iscS	DSM2061_03305	iscS
iscS	DSM2061_00049	iscS
iscS	DSM2064_02182	iscS
iscS	DSM2064_04036	iscS
iscS	DSM2064_00209	iscS
iscS	DSM2064_01320	iscS
iscS	DSM2064_03176	iscS
iscS	DSM2064_04494	iscS
iscS	DSM2064_01103	iscS
iscS	DSM2064_00433	iscS
iscS	DSM21164_00705	iscS
iscS	DSM21164_03023	iscS
iscS	DSM23404_01942	iscS
iscS	DSM23404_02956	iscS
iscS	DSM23404_03152	iscS
iscS	DSM23424_00170	iscS
iscS	DSM23424_02229	iscS
iscS	DSM23424_00942	iscS
iscS	DSM24786_02378	iscS
iscS	DSM24786_01208	iscS
iscS	DSM24786_01033	iscS
iscS	DSM7489_01540	iscS
iscS	DSM7489_00024	iscS
iscS	DSM7489_02482	iscS
iscS	DSW1_00977	iscS
iscS	DSW1_01622	iscS
iscS	DSW1_02220	iscS
iscS	DSW5_01386	iscS
iscS	DSW5_01848	iscS
iscS	DSW5_01792	iscS
iscS	DSW6_01279	iscS
iscS	DSW6_00345	iscS
iscS	DSW8_02158	iscS
iscS	DSW8_01003	iscS
iscS	F52_02602	iscS
iscS	F52_04381	iscS
iscS	F52_04046	iscS
iscS	F52_03544	iscS
iscS	F52_00486	iscS
iscS	GCA_001027945.1_00429	iscS
iscS	GCA_001027945.1_02817	iscS
iscS	GCA_001027945.1_01882	iscS
iscS	HI1_01465	iscS
iscS	HI1_03323	iscS

iscS	HI1_02414	iscS
iscS	IR1_00673	iscS
iscS	IR1_02669	iscS
iscS	IR1_02391	iscS
iscS	IR1_01830	iscS
iscS	IR1_01722	iscS
iscS	KCOM3131_00122	iscS
iscS	KCOM3131_00528	iscS
iscS	KCTC12974_00401	iscS
iscS	KCTC12974_01890	iscS
iscS	KCTC12974_00501	iscS
iscS	KL-A_03265	iscS
iscS	KL-A_01815	iscS
iscS	KL-A_01073	iscS
iscS	LL0412.1.7_00616	iscS
iscS	LL0412.1.7_01464	iscS
iscS	LL0412.1.7_02512	iscS
iscS	MAR_2009_71_01282	iscS
iscS	MAR_2009_71_02438	iscS
iscS	NN016038_01182	iscS
iscS	NN016038_02106	iscS
iscS	PAO1_03790	iscS
iscS	PAO1_03938	iscS
iscS	PAO1_02125	iscS
iscS	PAO1_02466	iscS
iscS	PAO1_00839	iscS
iscS	UW101_01236	iscS
iscS	UW101_04851	iscS
iscS	UW101_02920	iscS
iscS	UW101_04159	iscS
iscS	UW101_02624	iscS
iscS	UW101_02107	iscS
iscS	UW101_04376	iscS
iscS	UW101_03144	iscS
iscS	V4-33_02310	iscS
iscS	V4-33_00681	iscS
iscS	V4-33_00775	iscS
iscS	W5C_00222	iscS
iscS	W5C_00847	iscS
iscS	W5C_00993	iscS
iscS	ZONMW-01_03004	iscS
iscS	ZONMW-01_03321	iscS
iscS	ZONMW-01_03091	iscS
iscS	ZONMW-02_01646	iscS
iscS	ZONMW-02_02972	iscS
iscS	ZONMW-02_03414	iscS
iscS	ZONMW-03_03288	iscS
iscS	ZONMW-03_00685	iscS

iscS	ZONMW-03_02326	iscS
iscS	ZONMW-04_03240	iscS
iscS	ZONMW-04_02672	iscS
iscS	ZONMW-04_00790	iscS
iscS	ZONMW-05_02722	iscS
iscS	ZONMW-05_02902	iscS
iscS	ZONMW-05_00060	iscS
iscS	ZONMW-06_00393	iscS
iscS	ZONMW-06_02395	iscS
iscS	ZONMW-06_02486	iscS
iscS	ZONMW-07_01419	iscS
iscS	ZONMW-07_01732	iscS
iscS	ZONMW-08_00801	iscS
iscS	ZONMW-08_03437	iscS
iscS	ZONMW-09_00494	iscS
iscS	ZONMW-09_00231	iscS
iscS	ZONMW-09_02243	iscS
iscS	ZONMW-10_00329	iscS
iscS	ZONMW-10_01867	iscS
iscS	ZONMW-10_02072	iscS
iscS	ZONMW-11_04653	iscS
iscS	ZONMW-11_00985	iscS
iscS	ZONMW-11_02194	iscS
iscS	ZONMW-11_02945	iscS
iscS	ZONMW-12_00097	iscS
iscS	ZONMW-12_03347	iscS
iscS	ZONMW-12_03446	iscS
iscS	ZONMW-12_00484	iscS
iscS	ZONMW-12_04385	iscS
iscS	ZONMW-13_00214	iscS
iscS	ZONMW-13_00518	iscS
iscS	ZONMW-13_03078	iscS
iscS	ZONMW-14_00379	iscS
iscS	ZONMW-14_00044	iscS
iscS	ZONMW-14_02033	iscS
iscS	ZONMW-14_02915	iscS
iscS	ZONMW-15_00016	iscS
iscS	ZONMW-15_03113	iscS
iscS	ZONMW-19_00993	iscS
iscS	ZONMW-19_02463	iscS
iscS	ZONMW-20_01595	iscS
iscS	ZONMW-20_00274	iscS
iscS	ZONMW-20_02997	iscS
iscS	ZONMW-21_00689	iscS
iscS	ZONMW-21_02725	iscS
iscS	ZONMW-22_00690	iscS
iscS	ZONMW-22_02384	iscS
iscS	ZONMW-23_01804	iscS

iscS	ZONMW-23_00166	iscS
iscS	ZONMW-24_00771	iscS
iscS	ZONMW-24_02149	iscS
iscS	ZONMW-25_01645	iscS
iscS	ZONMW-25_02467	iscS
iscS	ZONMW-25_00675	iscS
iscS	ZONMW-25_01516	iscS
iscS	ZONMW-26_03344	iscS
iscS	ZONMW-26_00012	iscS
iscS	ZONMW-26_00900	iscS
iscS	ZONMW-27_02596	iscS
iscS	ZONMW-27_03125	iscS
iscS	ZONMW-27_00887	iscS
iscS	ZONMW-28_01053	iscS
iscS	ZONMW-28_00145	iscS
iscS	ZONMW-29_00342	iscS
iscS	ZONMW-29_01890	iscS
iscS	ZONMW-30_02352	iscS
iscS	ZONMW-30_00519	iscS
iscS	ZONMW-30_03005	iscS
iscS	ZONMW-30_01062	iscS
iscS	ZONMW-31_00728	iscS
iscS	ZONMW-31_02791	iscS
iscS	ZONMW-32_00038	iscS
iscS	ZONMW-32_00936	iscS
iscS	ZONMW-33_03299	iscS
iscS	ZONMW-33_00926	iscS
iscS	ZONMW-34_00360	iscS
iscS	ZONMW-34_01133	iscS
iscS	ZONMW-35_01030	iscS
iscS	ZONMW-35_00206	iscS
iscS	ZONMW-36_03036	iscS
iscS	ZONMW-36_00145	iscS
iscS	ZONMW-37_00689	iscS
iscS	ZONMW-37_02392	iscS
iscS	ZONMW-38_03081	iscS
iscS	ZONMW-38_02341	iscS
iscS	ZONMW-38_00265	iscS
iscS	ZONMW-38_02537	iscS
iscS	ZONMW-39_03049	iscS
iscS	ZONMW-39_00872	iscS
iscS	ZONMW-40_01283	iscS
iscS	ZONMW-40_03312	iscS
iscS	ZONMW-40_01713	iscS
iscS	ZONMW-40_02367	iscS
iscS	ZONMW-41_00901	iscS
iscS	ZONMW-41_01413	iscS
iscS	ZONMW-41_02849	iscS

iscS	ZONMW-41_00870	iscS
iscS	ZONMW-42_03457	iscS
iscS	ZONMW-42_02691	iscS
iscS	ZONMW-43_02322	iscS
iscS	ZONMW-43_03168	iscS
iscS	ZONMW-44_03583	iscS
iscS	ZONMW-44_03362	iscS
iscS	ZONMW-45_00315	iscS
iscS	ZONMW-45_00960	iscS
iscS	ZONMW-45_01901	iscS
iscS	ZONMW-46_01524	iscS
iscS	ZONMW-46_00914	iscS
iscS	ZONMW-46_02001	iscS
iscS	ZONMW-47_01328	iscS
iscS	ZONMW-47_01259	iscS
iscS	ZONMW-47_02060	iscS
iscS	ZONMW-48_00677	iscS
iscS	ZONMW-48_02936	iscS
iscS	ZONMW-49_01068	iscS
iscS	ZONMW-49_03077	iscS
iscS	ZONMW-49_00033	iscS
iscS	ZONMW-50_01069	iscS
iscS	ZONMW-50_02955	iscS
iscS	ZONMW-50_00453	iscS
iscS	ZONMW-51_03272	iscS
iscS	ZONMW-51_01883	iscS
iscS	ZONMW-51_00030	iscS
iscS	ZONMW-52_03261	iscS
iscS	ZONMW-52_00890	iscS
iscS	ZONMW-52_00003	iscS
iscS	ZONMW-53_03281	iscS
iscS	ZONMW-53_01633	iscS
iscS	ZONMW-53_02653	iscS
iscS	ZONMW-54_00155	iscS
iscS	ZONMW-54_01092	iscS
iscS	ZONMW-54_01973	iscS
iscS	ZONMW-55_03239	iscS
iscS	ZONMW-55_02205	iscS
iscS	ZONMW-55_01508	iscS
iscS	ZONMW-56_03253	iscS
iscS	ZONMW-56_02163	iscS
iscS	ZONMW-56_00734	iscS
iscS	ZONMW-57_03291	iscS
iscS	ZONMW-57_00057	iscS
iscS	ZONMW-57_00941	iscS
iscS	ZONMW-58_03282	iscS
iscS	ZONMW-58_00909	iscS
iscS	ZONMW-58_00030	iscS

iscS	ZONMW-59_02135	iscS
iscS	ZONMW-59_00057	iscS
iscS	ZONMW-59_00975	iscS
iscS	ZONMW-60_01822	iscS
iscS	ZONMW-60_00058	iscS
iscS	ZONMW-60_00935	iscS
iscS	ZONMW-61_00396	iscS
iscS	ZONMW-61_01641	iscS
iscS	ZONMW-61_03644	iscS
iscS	ZONMW-62_01943	iscS
iscS	ZONMW-62_01100	iscS
iscS	ZONMW-63_03377	iscS
iscS	ZONMW-63_01821	iscS
iscS	ZONMW-63_02075	iscS
iscS	ZONMW-64_00761	iscS
iscS	ZONMW-64_02338	iscS
iscS	ZONMW-65_01686	iscS
iscS	ZONMW-65_01682	iscS
iscS	ZONMW-66_02963	iscS
iscS	ZONMW-66_00667	iscS
iscS	ZONMW-66_02356	iscS
iscS	ZONMW-67_02771	iscS
iscS	ZONMW-67_01934	iscS
iscS	ZONMW-68_00159	iscS
iscS	ZONMW-68_02825	iscS
iscS	ZONMW-69_00568	iscS
iscS	ZONMW-69_02009	iscS
iscS	ZONMW-72_00325	iscS
iscS	ZONMW-72_04375	iscS
iscS	ZONMW-72_02924	iscS
iscS	ZONMW-72_03070	iscS
iscS	ZONMW-72_03661	iscS
iscS	ZONMW-73_01849	iscS
iscS	ZONMW-73_01123	iscS
iscS	ZONMW-74_01394	iscS
iscS	ZONMW-74_00609	iscS
iscS	ZONMW-75_00442	iscS
iscS	ZONMW-75_03191	iscS
iscS	ZONMW-75_02518	iscS
iscS	da5A-8_03116	iscS
iscS	da5A-8_03087	iscS
iscS	da5A-8_02905	iscS
rplO	18_00188	rplO
rplO	ACAM48_00323	rplO
rplO	CAM030_02838	rplO
rplO	CECT7946_03314	rplO
rplO	CGMCC1.8863_01432	rplO
rplO	CL8139_01431	rplO

rplO	DAU203_02285	rplO
rplO	DD5b_02401	rplO
rplO	DSM1208_02442	rplO
rplO	DSM12212_01102	rplO
rplO	DSM15343_01582	rplO
rplO	DSM15361_02575	rplO
rplO	DSM15365_01153	rplO
rplO	DSM15718_00667	rplO
rplO	DSM16505T_01250	rplO
rplO	DSM18103_01867	rplO
rplO	DSM18293_02433	rplO
rplO	DSM18841_01183	rplO
rplO	DSM18842_00121	rplO
rplO	DSM19866_01096	rplO
rplO	DSM2041_02959	rplO
rplO	DSM2061_03953	rplO
rplO	DSM2064_04703	rplO
rplO	DSM21164_02202	rplO
rplO	DSM23404_00467	rplO
rplO	DSM23424_02868	rplO
rplO	DSM24786_01746	rplO
rplO	DSM7489_02201	rplO
rplO	DSW1_01282	rplO
rplO	DSW5_00456	rplO
rplO	DSW6_00501	rplO
rplO	DSW8_01511	rplO
rplO	F52_01524	rplO
rplO	GCA_001027945.1_02972	rplO
rplO	HI1_02127	rplO
rplO	IR1_03054	rplO
rplO	KCOM3131_00724	rplO
rplO	KCTC12974_01057	rplO
rplO	KL-A_00072	rplO
rplO	LL0412.1.7_01246	rplO
rplO	MAR_2009_71_01914	rplO
rplO	NN016038_00195	rplO
rplO	PAO1_04375	rplO
rplO	UW101_00399	rplO
rplO	V4-33_01228	rplO
rplO	W5C_02860	rplO
rplO	ZONMW-01_01388	rplO
rplO	ZONMW-02_02705	rplO
rplO	ZONMW-03_01392	rplO
rplO	ZONMW-04_00300	rplO
rplO	ZONMW-05_01525	rplO
rplO	ZONMW-06_03650	rplO
rplO	ZONMW-07_01585	rplO
rplO	ZONMW-08_01312	rplO

rplO	ZONMW-09_01331	rplO
rplO	ZONMW-10_01401	rplO
rplO	ZONMW-11_03087	rplO
rplO	ZONMW-12_01400	rplO
rplO	ZONMW-13_01804	rplO
rplO	ZONMW-14_01040	rplO
rplO	ZONMW-15_00753	rplO
rplO	ZONMW-19_01650	rplO
rplO	ZONMW-20_04258	rplO
rplO	ZONMW-21_02448	rplO
rplO	ZONMW-22_02661	rplO
rplO	ZONMW-23_02685	rplO
rplO	ZONMW-24_02472	rplO
rplO	ZONMW-25_03478	rplO
rplO	ZONMW-26_01745	rplO
rplO	ZONMW-27_00294	rplO
rplO	ZONMW-28_03622	rplO
rplO	ZONMW-29_02793	rplO
rplO	ZONMW-30_00097	rplO
rplO	ZONMW-31_03102	rplO
rplO	ZONMW-32_03650	rplO
rplO	ZONMW-33_03571	rplO
rplO	ZONMW-34_03519	rplO
rplO	ZONMW-35_03480	rplO
rplO	ZONMW-36_03407	rplO
rplO	ZONMW-37_02718	rplO
rplO	ZONMW-38_01896	rplO
rplO	ZONMW-39_03860	rplO
rplO	ZONMW-40_02185	rplO
rplO	ZONMW-41_02963	rplO
rplO	ZONMW-42_02244	rplO
rplO	ZONMW-43_03681	rplO
rplO	ZONMW-44_03250	rplO
rplO	ZONMW-45_01748	rplO
rplO	ZONMW-46_00049	rplO
rplO	ZONMW-47_00456	rplO
rplO	ZONMW-48_02049	rplO
rplO	ZONMW-49_00711	rplO
rplO	ZONMW-50_00712	rplO
rplO	ZONMW-51_00996	rplO
rplO	ZONMW-52_01596	rplO
rplO	ZONMW-53_00229	rplO
rplO	ZONMW-54_00801	rplO
rplO	ZONMW-55_00224	rplO
rplO	ZONMW-56_01094	rplO
rplO	ZONMW-57_01393	rplO
rplO	ZONMW-58_01191	rplO
rplO	ZONMW-59_01395	rplO

rplO	ZONMW-60_01180	rplO
rplO	ZONMW-61_01301	rplO
rplO	ZONMW-62_02160	rplO
rplO	ZONMW-63_01172	rplO
rplO	ZONMW-64_03969	rplO
rplO	ZONMW-65_02962	rplO
rplO	ZONMW-66_01179	rplO
rplO	ZONMW-67_03495	rplO
rplO	ZONMW-68_04443	rplO
rplO	ZONMW-69_03497	rplO
rplO	ZONMW-72_01166	rplO
rplO	ZONMW-73_02140	rplO
rplO	ZONMW-74_01004	rplO
rplO	ZONMW-75_02920	rplO
rplO	da5A-8_00899	rplO
pabB	18_01847	pabB
pabB	18_03006	pabB
pabB	18_02774	pabB
pabB	ACAM48_03445	pabB
pabB	ACAM48_03498	pabB
pabB	ACAM48_00020	pabB
pabB	ACAM48_03573	pabB
pabB	CAM030_02612	pabB
pabB	CAM030_00440	pabB
pabB	CAM030_00467	pabB
pabB	CECT7946_01974	pabB
pabB	CECT7946_01563	pabB
pabB	CECT7946_01519	pabB
pabB	CGMCC1.8863_01563	pabB
pabB	CGMCC1.8863_00287	pabB
pabB	CGMCC1.8863_02660	pabB
pabB	CL8139_02886	pabB
pabB	CL8139_01923	pabB
pabB	CL8139_02515	pabB
pabB	DAU203_00569	pabB
pabB	DAU203_03233	pabB
pabB	DAU203_02862	pabB
pabB	DD5b_01145	pabB
pabB	DD5b_01368	pabB
pabB	DSM1208_00640	pabB
pabB	DSM1208_04420	pabB
pabB	DSM1208_03861	pabB
pabB	DSM12212_02320	pabB
pabB	DSM12212_01812	pabB
pabB	DSM12212_01195	pabB
pabB	DSM15343_02308	pabB
pabB	DSM15343_00513	pabB
pabB	DSM15343_02219	pabB

pabB	DSM15361_01933	pabB
pabB	DSM15361_02291	pabB
pabB	DSM15361_01605	pabB
pabB	DSM15365_03870	pabB
pabB	DSM15365_02067	pabB
pabB	DSM15365_03947	pabB
pabB	DSM15718_01617	pabB
pabB	DSM15718_01505	pabB
pabB	DSM15718_01854	pabB
pabB	DSM16505T_01115	pabB
pabB	DSM16505T_00193	pabB
pabB	DSM16505T_02284	pabB
pabB	DSM18103_01330	pabB
pabB	DSM18103_01656	pabB
pabB	DSM18103_02719	pabB
pabB	DSM18293_01431	pabB
pabB	DSM18293_03571	pabB
pabB	DSM18293_01220	pabB
pabB	DSM18841_01009	pabB
pabB	DSM18841_02624	pabB
pabB	DSM18841_01343	pabB
pabB	DSM18842_00308	pabB
pabB	DSM18842_02606	pabB
pabB	DSM18842_01687	pabB
pabB	DSM19866_00596	pabB
pabB	DSM19866_01527	pabB
pabB	DSM19866_01648	pabB
pabB	DSM2041_03156	pabB
pabB	DSM2041_03478	pabB
pabB	DSM2041_02016	pabB
pabB	DSM2061_02136	pabB
pabB	DSM2061_00738	pabB
pabB	DSM2061_01171	pabB
pabB	DSM2064_03787	pabB
pabB	DSM2064_03555	pabB
pabB	DSM2064_03972	pabB
pabB	DSM21164_00036	pabB
pabB	DSM21164_01477	pabB
pabB	DSM21164_01601	pabB
pabB	DSM23404_00706	pabB
pabB	DSM23404_01534	pabB
pabB	DSM23404_01463	pabB
pabB	DSM23424_03219	pabB
pabB	DSM23424_02124	pabB
pabB	DSM23424_02208	pabB
pabB	DSM24786_03389	pabB
pabB	DSM24786_00678	pabB
pabB	DSM24786_00338	pabB

pabB	DSM7489_00556	pabB
pabB	DSM7489_03065	pabB
pabB	DSM7489_02733	pabB
pabB	DSW1_01445	pabB
pabB	DSW1_02590	pabB
pabB	DSW1_01591	pabB
pabB	DSW5_00536	pabB
pabB	DSW5_00974	pabB
pabB	DSW5_01904	pabB
pabB	DSW6_00571	pabB
pabB	DSW6_03455	pabB
pabB	DSW6_03279	pabB
pabB	DSW8_03740	pabB
pabB	DSW8_03031	pabB
pabB	DSW8_00845	pabB
pabB	F52_03936	pabB
pabB	F52_00106	pabB
pabB	F52_04131	pabB
pabB	GCA_001027945.1_01417	pabB
pabB	GCA_001027945.1_00895	pabB
pabB	GCA_001027945.1_02386	pabB
pabB	HI1_00488	pabB
pabB	HI1_03006	pabB
pabB	HI1_02672	pabB
pabB	IR1_01464	pabB
pabB	IR1_04481	pabB
pabB	IR1_04271	pabB
pabB	KCOM3131_01723	pabB
pabB	KCTC12974_01413	pabB
pabB	KCTC12974_00240	pabB
pabB	KCTC12974_00169	pabB
pabB	KL-A_02515	pabB
pabB	KL-A_02961	pabB
pabB	KL-A_01325	pabB
pabB	LL0412.1.7_01123	pabB
pabB	LL0412.1.7_00150	pabB
pabB	LL0412.1.7_01652	pabB
pabB	MAR_2009_71_00216	pabB
pabB	MAR_2009_71_00423	pabB
pabB	MAR_2009_71_02602	pabB
pabB	NN016038_01868	pabB
pabB	NN016038_03041	pabB
pabB	NN016038_02821	pabB
pabB	PAO1_00624	pabB
pabB	PAO1_01033	pabB
pabB	PAO1_01805	pabB
pabB	PAO1_04362	pabB
pabB	UW101_05099	pabB

pabB	UW101_02245	pabB
pabB	UW101_04915	pabB
pabB	V4-33_00491	pabB
pabB	V4-33_01532	pabB
pabB	V4-33_00630	pabB
pabB	W5C_03251	pabB
pabB	W5C_02272	pabB
pabB	W5C_02412	pabB
pabB	ZONMW-01_02753	pabB
pabB	ZONMW-01_00707	pabB
pabB	ZONMW-01_02217	pabB
pabB	ZONMW-02_03115	pabB
pabB	ZONMW-02_03455	pabB
pabB	ZONMW-02_01414	pabB
pabB	ZONMW-03_03153	pabB
pabB	ZONMW-03_00085	pabB
pabB	ZONMW-03_00431	pabB
pabB	ZONMW-04_02853	pabB
pabB	ZONMW-04_01093	pabB
pabB	ZONMW-04_01370	pabB
pabB	ZONMW-05_03110	pabB
pabB	ZONMW-05_00346	pabB
pabB	ZONMW-05_00694	pabB
pabB	ZONMW-06_00117	pabB
pabB	ZONMW-06_02098	pabB
pabB	ZONMW-07_02845	pabB
pabB	ZONMW-07_03030	pabB
pabB	ZONMW-07_02351	pabB
pabB	ZONMW-08_03387	pabB
pabB	ZONMW-08_03745	pabB
pabB	ZONMW-08_03606	pabB
pabB	ZONMW-09_01840	pabB
pabB	ZONMW-09_02108	pabB
pabB	ZONMW-09_02617	pabB
pabB	ZONMW-10_00829	pabB
pabB	ZONMW-10_00722	pabB
pabB	ZONMW-10_01996	pabB
pabB	ZONMW-11_04315	pabB
pabB	ZONMW-11_01020	pabB
pabB	ZONMW-11_00921	pabB
pabB	ZONMW-12_01940	pabB
pabB	ZONMW-12_01900	pabB
pabB	ZONMW-12_01810	pabB
pabB	ZONMW-13_01654	pabB
pabB	ZONMW-13_01334	pabB
pabB	ZONMW-13_01262	pabB
pabB	ZONMW-14_00898	pabB
pabB	ZONMW-14_01826	pabB

pabB	ZONMW-14_01898	pabB
pabB	ZONMW-15_01125	pabB
pabB	ZONMW-15_03092	pabB
pabB	ZONMW-15_02366	pabB
pabB	ZONMW-19_03982	pabB
pabB	ZONMW-19_02203	pabB
pabB	ZONMW-19_02565	pabB
pabB	ZONMW-19_02090	pabB
pabB	ZONMW-20_03511	pabB
pabB	ZONMW-20_01808	pabB
pabB	ZONMW-20_02247	pabB
pabB	ZONMW-20_02060	pabB
pabB	ZONMW-21_03041	pabB
pabB	ZONMW-21_01755	pabB
pabB	ZONMW-21_02114	pabB
pabB	ZONMW-22_03066	pabB
pabB	ZONMW-22_01969	pabB
pabB	ZONMW-22_01611	pabB
pabB	ZONMW-23_03087	pabB
pabB	ZONMW-23_00582	pabB
pabB	ZONMW-23_00940	pabB
pabB	ZONMW-24_03088	pabB
pabB	ZONMW-24_02594	pabB
pabB	ZONMW-24_01635	pabB
pabB	ZONMW-25_01818	pabB
pabB	ZONMW-25_00821	pabB
pabB	ZONMW-25_00422	pabB
pabB	ZONMW-26_03177	pabB
pabB	ZONMW-26_00621	pabB
pabB	ZONMW-26_00275	pabB
pabB	ZONMW-27_03199	pabB
pabB	ZONMW-27_01168	pabB
pabB	ZONMW-27_01933	pabB
pabB	ZONMW-28_01311	pabB
pabB	ZONMW-28_00485	pabB
pabB	ZONMW-29_02645	pabB
pabB	ZONMW-29_01032	pabB
pabB	ZONMW-29_01312	pabB
pabB	ZONMW-30_01016	pabB
pabB	ZONMW-30_01133	pabB
pabB	ZONMW-30_02720	pabB
pabB	ZONMW-31_00451	pabB
pabB	ZONMW-31_01440	pabB
pabB	ZONMW-32_01719	pabB
pabB	ZONMW-32_01276	pabB
pabB	ZONMW-33_01709	pabB
pabB	ZONMW-33_01265	pabB
pabB	ZONMW-34_00092	pabB

pabB	ZONMW-34_01457	pabB
pabB	ZONMW-35_01299	pabB
pabB	ZONMW-35_00530	pabB
pabB	ZONMW-36_01647	pabB
pabB	ZONMW-36_00485	pabB
pabB	ZONMW-37_03075	pabB
pabB	ZONMW-37_02133	pabB
pabB	ZONMW-37_01634	pabB
pabB	ZONMW-38_01523	pabB
pabB	ZONMW-38_01425	pabB
pabB	ZONMW-38_00468	pabB
pabB	ZONMW-39_02435	pabB
pabB	ZONMW-39_00242	pabB
pabB	ZONMW-40_01140	pabB
pabB	ZONMW-40_03225	pabB
pabB	ZONMW-40_03325	pabB
pabB	ZONMW-41_01478	pabB
pabB	ZONMW-41_03058	pabB
pabB	ZONMW-42_00065	pabB
pabB	ZONMW-42_01881	pabB
pabB	ZONMW-43_00857	pabB
pabB	ZONMW-43_01797	pabB
pabB	ZONMW-44_01929	pabB
pabB	ZONMW-44_01345	pabB
pabB	ZONMW-45_02143	pabB
pabB	ZONMW-45_01948	pabB
pabB	ZONMW-45_02339	pabB
pabB	ZONMW-46_01819	pabB
pabB	ZONMW-46_02297	pabB
pabB	ZONMW-46_01201	pabB
pabB	ZONMW-47_01670	pabB
pabB	ZONMW-47_02235	pabB
pabB	ZONMW-47_00972	pabB
pabB	ZONMW-48_02568	pabB
pabB	ZONMW-48_03439	pabB
pabB	ZONMW-48_01245	pabB
pabB	ZONMW-49_02783	pabB
pabB	ZONMW-49_01747	pabB
pabB	ZONMW-49_00287	pabB
pabB	ZONMW-50_02709	pabB
pabB	ZONMW-50_01667	pabB
pabB	ZONMW-50_00198	pabB
pabB	ZONMW-51_02992	pabB
pabB	ZONMW-51_00643	pabB
pabB	ZONMW-51_00275	pabB
pabB	ZONMW-52_03082	pabB
pabB	ZONMW-52_00602	pabB
pabB	ZONMW-52_00259	pabB

pabB	ZONMW-53_03054	pabB
pabB	ZONMW-53_01357	pabB
pabB	ZONMW-53_00996	pabB
pabB	ZONMW-54_03056	pabB
pabB	ZONMW-54_01360	pabB
pabB	ZONMW-54_01731	pabB
pabB	ZONMW-55_01604	pabB
pabB	ZONMW-55_00881	pabB
pabB	ZONMW-55_01251	pabB
pabB	ZONMW-56_02940	pabB
pabB	ZONMW-56_00117	pabB
pabB	ZONMW-56_00480	pabB
pabB	ZONMW-57_03107	pabB
pabB	ZONMW-57_00328	pabB
pabB	ZONMW-57_00692	pabB
pabB	ZONMW-58_03128	pabB
pabB	ZONMW-58_00643	pabB
pabB	ZONMW-58_00279	pabB
pabB	ZONMW-59_03183	pabB
pabB	ZONMW-59_00344	pabB
pabB	ZONMW-59_00682	pabB
pabB	ZONMW-60_03119	pabB
pabB	ZONMW-60_00355	pabB
pabB	ZONMW-60_00695	pabB
pabB	ZONMW-61_03740	pabB
pabB	ZONMW-61_03474	pabB
pabB	ZONMW-61_03791	pabB
pabB	ZONMW-62_03784	pabB
pabB	ZONMW-62_03574	pabB
pabB	ZONMW-62_00939	pabB
pabB	ZONMW-63_02907	pabB
pabB	ZONMW-63_01967	pabB
pabB	ZONMW-63_00366	pabB
pabB	ZONMW-64_01142	pabB
pabB	ZONMW-64_02800	pabB
pabB	ZONMW-65_03442	pabB
pabB	ZONMW-65_00718	pabB
pabB	ZONMW-66_01663	pabB
pabB	ZONMW-66_01394	pabB
pabB	ZONMW-66_02759	pabB
pabB	ZONMW-66_02416	pabB
pabB	ZONMW-67_00568	pabB
pabB	ZONMW-67_01407	pabB
pabB	ZONMW-68_03544	pabB
pabB	ZONMW-68_01903	pabB
pabB	ZONMW-68_04177	pabB
pabB	ZONMW-69_00315	pabB
pabB	ZONMW-69_01593	pabB

pabB	ZONMW-72_01970	pabB
pabB	ZONMW-72_03110	pabB
pabB	ZONMW-72_01840	pabB
pabB	ZONMW-73_01160	pabB
pabB	ZONMW-73_01501	pabB
pabB	ZONMW-73_03413	pabB
pabB	ZONMW-74_02460	pabB
pabB	ZONMW-74_00286	pabB
pabB	ZONMW-74_03376	pabB
pabB	ZONMW-75_03499	pabB
pabB	ZONMW-75_02439	pabB
pabB	ZONMW-75_03026	pabB
pabB	da5A-8_01073	pabB
pabB	da5A-8_00720	pabB
pabB	da5A-8_01125	pabB
rplW	18_00205	rplW
rplW	ACAM48_00306	rplW
rplW	CAM030_02821	rplW
rplW	CECT7946_03331	rplW
rplW	CGMCC1.8863_01415	rplW
rplW	CL8139_01448	rplW
rplW	DAU203_02268	rplW
rplW	DD5b_02418	rplW
rplW	DSM1208_02425	rplW
rplW	DSM12212_01085	rplW
rplW	DSM15343_01565	rplW
rplW	DSM15361_02592	rplW
rplW	DSM15365_01136	rplW
rplW	DSM15718_00650	rplW
rplW	DSM16505T_01233	rplW
rplW	DSM18103_01885	rplW
rplW	DSM18293_02416	rplW
rplW	DSM18841_01166	rplW
rplW	DSM18842_00138	rplW
rplW	DSM19866_01079	rplW
rplW	DSM2041_02976	rplW
rplW	DSM2061_03936	rplW
rplW	DSM2064_04686	rplW
rplW	DSM21164_02219	rplW
rplW	DSM23404_00484	rplW
rplW	DSM23424_02885	rplW
rplW	DSM24786_01763	rplW
rplW	DSM7489_02184	rplW
rplW	DSW1_01265	rplW
rplW	DSW5_00473	rplW
rplW	DSW6_00518	rplW
rplW	DSW8_01528	rplW
rplW	F52_01507	rplW

rplW	GCA_001027945.1_02989	rplW
rplW	HI1_02110	rplW
rplW	IR1_03071	rplW
rplW	KCOM3131_00740	rplW
rplW	KCTC12974_01074	rplW
rplW	KL-A_00089	rplW
rplW	LL0412.1.7_01229	rplW
rplW	MAR_2009_71_01897	rplW
rplW	NN016038_00212	rplW
rplW	PAO1_04392	rplW
rplW	UW101_00416	rplW
rplW	V4-33_01245	rplW
rplW	W5C_02877	rplW
rplW	ZONMW-01_01405	rplW
rplW	ZONMW-02_02688	rplW
rplW	ZONMW-03_01375	rplW
rplW	ZONMW-04_00317	rplW
rplW	ZONMW-05_01508	rplW
rplW	ZONMW-06_03633	rplW
rplW	ZONMW-07_01568	rplW
rplW	ZONMW-08_01329	rplW
rplW	ZONMW-09_01348	rplW
rplW	ZONMW-10_01384	rplW
rplW	ZONMW-11_03070	rplW
rplW	ZONMW-12_01417	rplW
rplW	ZONMW-13_01821	rplW
rplW	ZONMW-14_01057	rplW
rplW	ZONMW-15_00770	rplW
rplW	ZONMW-19_01633	rplW
rplW	ZONMW-20_04275	rplW
rplW	ZONMW-21_02465	rplW
rplW	ZONMW-22_02644	rplW
rplW	ZONMW-23_02668	rplW
rplW	ZONMW-24_02489	rplW
rplW	ZONMW-25_03461	rplW
rplW	ZONMW-26_01762	rplW
rplW	ZONMW-27_00311	rplW
rplW	ZONMW-28_03605	rplW
rplW	ZONMW-29_02776	rplW
rplW	ZONMW-30_00080	rplW
rplW	ZONMW-31_03085	rplW
rplW	ZONMW-32_03633	rplW
rplW	ZONMW-33_03588	rplW
rplW	ZONMW-34_03502	rplW
rplW	ZONMW-35_03463	rplW
rplW	ZONMW-36_03390	rplW
rplW	ZONMW-37_02735	rplW
rplW	ZONMW-38_01913	rplW

rplW	ZONMW-39_03877	rplW
rplW	ZONMW-40_02168	rplW
rplW	ZONMW-41_02980	rplW
rplW	ZONMW-42_02227	rplW
rplW	ZONMW-43_03698	rplW
rplW	ZONMW-44_03267	rplW
rplW	ZONMW-45_01765	rplW
rplW	ZONMW-46_00032	rplW
rplW	ZONMW-47_00473	rplW
rplW	ZONMW-48_02032	rplW
rplW	ZONMW-49_00728	rplW
rplW	ZONMW-50_00729	rplW
rplW	ZONMW-51_00979	rplW
rplW	ZONMW-52_01579	rplW
rplW	ZONMW-53_00246	rplW
rplW	ZONMW-54_00784	rplW
rplW	ZONMW-55_00241	rplW
rplW	ZONMW-56_01077	rplW
rplW	ZONMW-57_01376	rplW
rplW	ZONMW-58_01208	rplW
rplW	ZONMW-59_01378	rplW
rplW	ZONMW-60_01197	rplW
rplW	ZONMW-61_01318	rplW
rplW	ZONMW-62_02177	rplW
rplW	ZONMW-63_01189	rplW
rplW	ZONMW-64_03952	rplW
rplW	ZONMW-65_01517	rplW
rplW	ZONMW-66_01162	rplW
rplW	ZONMW-67_03478	rplW
rplW	ZONMW-68_04460	rplW
rplW	ZONMW-69_03480	rplW
rplW	ZONMW-72_01149	rplW
rplW	ZONMW-73_02123	rplW
rplW	ZONMW-74_01021	rplW
rplW	ZONMW-75_02937	rplW
rplW	da5A-8_00882	rplW
recA	18_01068	recA
recA	ACAM48_02484	recA
recA	CAM030_00956	recA
recA	CECT7946_00207	recA
recA	CGMCC1.8863_00864	recA
recA	CL8139_00383	recA
recA	DAU203_01537	recA
recA	DD5b_02093	recA
recA	DSM1208_01741	recA
recA	DSM12212_00140	recA
recA	DSM15343_00982	recA
recA	DSM15361_00433	recA

recA	DSM15365_03138	recA
recA	DSM15718_02542	recA
recA	DSM16505T_00496	recA
recA	DSM18103_02840	recA
recA	DSM18293_03111	recA
recA	DSM18841_00344	recA
recA	DSM18842_00887	recA
recA	DSM19866_01976	recA
recA	DSM2041_00053	recA
recA	DSM2061_03272	recA
recA	DSM2064_01982	recA
recA	DSM21164_00802	recA
recA	DSM23404_02036	recA
recA	DSM23424_00272	recA
recA	DSM24786_03026	recA
recA	DSM7489_01441	recA
recA	DSW1_00547	recA
recA	DSW5_01213	recA
recA	DSW6_01443	recA
recA	DSW8_02195	recA
recA	F52_02388	recA
recA	GCA_001027945.1_00268	recA
recA	HI1_01382	recA
recA	IR1_04767	recA
recA	KCOM3131_01982	recA
recA	KCTC12974_02875	recA
recA	KL-A_00785	recA
recA	LL0412.1.7_00454	recA
recA	MAR_2009_71_01245	recA
recA	NN016038_01084	recA
recA	PAO1_03740	recA
recA	UW101_01037	recA
recA	V4-33_02166	recA
recA	W5C_01140	recA
recA	ZONMW-01_02438	recA
recA	ZONMW-02_00047	recA
recA	ZONMW-03_03041	recA
recA	ZONMW-04_02402	recA
recA	ZONMW-05_02622	recA
recA	ZONMW-06_03457	recA
recA	ZONMW-07_01447	recA
recA	ZONMW-08_00765	recA
recA	ZONMW-09_00320	recA
recA	ZONMW-10_00424	recA
recA	ZONMW-11_01054	recA
recA	ZONMW-12_00214	recA
recA	ZONMW-13_00319	recA
recA	ZONMW-14_00273	recA

recA	ZONMW-15_02593	recA
recA	ZONMW-19_00762	recA
recA	ZONMW-20_02595	recA
recA	ZONMW-21_00732	recA
recA	ZONMW-22_00733	recA
recA	ZONMW-23_01847	recA
recA	ZONMW-24_00728	recA
recA	ZONMW-25_01664	recA
recA	ZONMW-26_03059	recA
recA	ZONMW-27_02779	recA
recA	ZONMW-28_02277	recA
recA	ZONMW-29_00375	recA
recA	ZONMW-30_02332	recA
recA	ZONMW-31_02456	recA
recA	ZONMW-32_02682	recA
recA	ZONMW-33_02436	recA
recA	ZONMW-34_01756	recA
recA	ZONMW-35_01447	recA
recA	ZONMW-36_02816	recA
recA	ZONMW-37_00732	recA
recA	ZONMW-38_00898	recA
recA	ZONMW-39_03727	recA
recA	ZONMW-40_00468	recA
recA	ZONMW-41_01253	recA
recA	ZONMW-42_00691	recA
recA	ZONMW-43_03372	recA
recA	ZONMW-43_02139	recA
recA	ZONMW-44_02653	recA
recA	ZONMW-45_00498	recA
recA	ZONMW-46_01341	recA
recA	ZONMW-47_01511	recA
recA	ZONMW-48_00588	recA
recA	ZONMW-49_01156	recA
recA	ZONMW-50_01157	recA
recA	ZONMW-51_02762	recA
recA	ZONMW-52_02284	recA
recA	ZONMW-53_02873	recA
recA	ZONMW-54_00089	recA
recA	ZONMW-55_02879	recA
recA	ZONMW-56_02639	recA
recA	ZONMW-57_02783	recA
recA	ZONMW-58_02807	recA
recA	ZONMW-59_02921	recA
recA	ZONMW-60_02902	recA
recA	ZONMW-61_00359	recA
recA	ZONMW-62_01906	recA
recA	ZONMW-63_02290	recA
recA	ZONMW-64_01848	recA

recA	ZONMW-65_00849	recA
recA	ZONMW-66_00213	recA
recA	ZONMW-67_03244	recA
recA	ZONMW-68_00120	recA
recA	ZONMW-69_03245	recA
recA	ZONMW-72_04147	recA
recA	ZONMW-73_00122	recA
recA	ZONMW-74_01445	recA
recA	ZONMW-75_02421	recA
recA	da5A-8_03608	recA
lpxA	18_03425	lpxA
lpxA	18_03490	lpxA
lpxA	18_03486	lpxA
lpxA	18_03488	lpxA
lpxA	ACAM48_01853	lpxA
lpxA	ACAM48_01849	lpxA
lpxA	ACAM48_01851	lpxA
lpxA	CAM030_00576	lpxA
lpxA	CAM030_00580	lpxA
lpxA	CAM030_00578	lpxA
lpxA	CECT7946_01328	lpxA
lpxA	CECT7946_01362	lpxA
lpxA	CECT7946_01358	lpxA
lpxA	CECT7946_01360	lpxA
lpxA	CGMCC1.8863_02694	lpxA
lpxA	CGMCC1.8863_00383	lpxA
lpxA	CGMCC1.8863_00379	lpxA
lpxA	CGMCC1.8863_00381	lpxA
lpxA	CL8139_02166	lpxA
lpxA	CL8139_02573	lpxA
lpxA	CL8139_02577	lpxA
lpxA	CL8139_02575	lpxA
lpxA	DAU203_00952	lpxA
lpxA	DAU203_03184	lpxA
lpxA	DAU203_03180	lpxA
lpxA	DAU203_03182	lpxA
lpxA	DD5b_00863	lpxA
lpxA	DD5b_00057	lpxA
lpxA	DD5b_00061	lpxA
lpxA	DD5b_00059	lpxA
lpxA	DSM1208_04349	lpxA
lpxA	DSM1208_04345	lpxA
lpxA	DSM1208_04347	lpxA
lpxA	DSM12212_00682	lpxA
lpxA	DSM12212_00726	lpxA
lpxA	DSM12212_00722	lpxA
lpxA	DSM12212_00724	lpxA
lpxA	DSM15343_02347	lpxA

lpxA	DSM15343_00304	lpxA
lpxA	DSM15343_00300	lpxA
lpxA	DSM15343_00302	lpxA
lpxA	DSM15361_00697	lpxA
lpxA	DSM15361_00701	lpxA
lpxA	DSM15361_00699	lpxA
lpxA	DSM15365_00740	lpxA
lpxA	DSM15365_01847	lpxA
lpxA	DSM15365_01843	lpxA
lpxA	DSM15365_01845	lpxA
lpxA	DSM15718_01841	lpxA
lpxA	DSM15718_01379	lpxA
lpxA	DSM15718_01375	lpxA
lpxA	DSM15718_01377	lpxA
lpxA	DSM16505T_01518	lpxA
lpxA	DSM16505T_01514	lpxA
lpxA	DSM16505T_01516	lpxA
lpxA	DSM18103_00713	lpxA
lpxA	DSM18103_00249	lpxA
lpxA	DSM18103_00245	lpxA
lpxA	DSM18103_00247	lpxA
lpxA	DSM18293_03990	lpxA
lpxA	DSM18293_01740	lpxA
lpxA	DSM18293_01744	lpxA
lpxA	DSM18293_01742	lpxA
lpxA	DSM18293_04621	lpxA
lpxA	DSM18841_02142	lpxA
lpxA	DSM18841_02138	lpxA
lpxA	DSM18841_02140	lpxA
lpxA	DSM18842_01485	lpxA
lpxA	DSM18842_01481	lpxA
lpxA	DSM18842_01483	lpxA
lpxA	DSM19866_04166	lpxA
lpxA	DSM19866_04162	lpxA
lpxA	DSM19866_04164	lpxA
lpxA	DSM2041_01986	lpxA
lpxA	DSM2041_01982	lpxA
lpxA	DSM2041_01984	lpxA
lpxA	DSM2061_00711	lpxA
lpxA	DSM2061_00787	lpxA
lpxA	DSM2061_00791	lpxA
lpxA	DSM2061_00789	lpxA
lpxA	DSM2064_03134	lpxA
lpxA	DSM2064_00325	lpxA
lpxA	DSM2064_00321	lpxA
lpxA	DSM2064_00323	lpxA
lpxA	DSM21164_02601	lpxA
lpxA	DSM21164_02667	lpxA

lpxA	DSM21164_02663	lpxA
lpxA	DSM21164_02665	lpxA
lpxA	DSM23404_01223	lpxA
lpxA	DSM23404_01219	lpxA
lpxA	DSM23404_01221	lpxA
lpxA	DSM23424_01257	lpxA
lpxA	DSM23424_01253	lpxA
lpxA	DSM23424_01255	lpxA
lpxA	DSM24786_03117	lpxA
lpxA	DSM24786_00635	lpxA
lpxA	DSM24786_00631	lpxA
lpxA	DSM24786_00633	lpxA
lpxA	DSM7489_00886	lpxA
lpxA	DSM7489_03016	lpxA
lpxA	DSM7489_03012	lpxA
lpxA	DSM7489_03014	lpxA
lpxA	DSW1_01980	lpxA
lpxA	DSW1_01984	lpxA
lpxA	DSW1_01982	lpxA
lpxA	DSW5_02260	lpxA
lpxA	DSW5_02256	lpxA
lpxA	DSW5_02258	lpxA
lpxA	DSW6_03119	lpxA
lpxA	DSW6_02793	lpxA
lpxA	DSW6_02797	lpxA
lpxA	DSW6_02795	lpxA
lpxA	DSW8_00161	lpxA
lpxA	DSW8_00165	lpxA
lpxA	DSW8_00163	lpxA
lpxA	F52_00521	lpxA
lpxA	F52_01355	lpxA
lpxA	F52_01359	lpxA
lpxA	F52_01357	lpxA
lpxA	GCA_001027945.1_01799	lpxA
lpxA	GCA_001027945.1_01803	lpxA
lpxA	GCA_001027945.1_01801	lpxA
lpxA	HI1_00818	lpxA
lpxA	HI1_02957	lpxA
lpxA	HI1_02953	lpxA
lpxA	HI1_02955	lpxA
lpxA	IR1_02362	lpxA
lpxA	IR1_03433	lpxA
lpxA	IR1_03437	lpxA
lpxA	IR1_03435	lpxA
lpxA	IR1_03171	lpxA
lpxA	KCOM3131_00868	lpxA
lpxA	KCOM3131_00866	lpxA
lpxA	KCTC12974_02820	lpxA

lpxA	KCTC12974_02816	lpxA
lpxA	KCTC12974_02818	lpxA
lpxA	KL-A_02385	lpxA
lpxA	KL-A_00470	lpxA
lpxA	KL-A_00474	lpxA
lpxA	KL-A_00472	lpxA
lpxA	L12_00013	lpxA
lpxA	LL0412.1.7_01541	lpxA
lpxA	LL0412.1.7_02451	lpxA
lpxA	LL0412.1.7_02455	lpxA
lpxA	LL0412.1.7_02453	lpxA
lpxA	MAR_2009_71_02962	lpxA
lpxA	MAR_2009_71_02966	lpxA
lpxA	MAR_2009_71_02964	lpxA
lpxA	NN016038_03461	lpxA
lpxA	NN016038_03527	lpxA
lpxA	NN016038_03523	lpxA
lpxA	NN016038_03525	lpxA
lpxA	PAO1_03769	lpxA
lpxA	PAO1_03767	lpxA
lpxA	UW101_02666	lpxA
lpxA	UW101_03036	lpxA
lpxA	UW101_03032	lpxA
lpxA	UW101_03034	lpxA
lpxA	V4-33_01507	lpxA
lpxA	V4-33_00889	lpxA
lpxA	V4-33_00885	lpxA
lpxA	V4-33_00887	lpxA
lpxA	W5C_01529	lpxA
lpxA	W5C_02225	lpxA
lpxA	W5C_02221	lpxA
lpxA	W5C_02223	lpxA
lpxA	ZONMW-01_02523	lpxA
lpxA	ZONMW-01_00756	lpxA
lpxA	ZONMW-01_00760	lpxA
lpxA	ZONMW-01_00758	lpxA
lpxA	ZONMW-02_01183	lpxA
lpxA	ZONMW-02_03350	lpxA
lpxA	ZONMW-02_03354	lpxA
lpxA	ZONMW-02_03352	lpxA
lpxA	ZONMW-03_01804	lpxA
lpxA	ZONMW-03_00134	lpxA
lpxA	ZONMW-03_00138	lpxA
lpxA	ZONMW-03_00136	lpxA
lpxA	ZONMW-04_03009	lpxA
lpxA	ZONMW-04_01138	lpxA
lpxA	ZONMW-04_01142	lpxA
lpxA	ZONMW-04_01140	lpxA

lpxA	ZONMW-05_03253	lpxA
lpxA	ZONMW-05_00389	lpxA
lpxA	ZONMW-05_00393	lpxA
lpxA	ZONMW-05_00391	lpxA
lpxA	ZONMW-06_03538	lpxA
lpxA	ZONMW-06_03540	lpxA
lpxA	ZONMW-07_02535	lpxA
lpxA	ZONMW-07_02539	lpxA
lpxA	ZONMW-07_02537	lpxA
lpxA	ZONMW-08_02161	lpxA
lpxA	ZONMW-08_02165	lpxA
lpxA	ZONMW-08_02163	lpxA
lpxA	ZONMW-09_01082	lpxA
lpxA	ZONMW-09_01002	lpxA
lpxA	ZONMW-09_01006	lpxA
lpxA	ZONMW-09_01004	lpxA
lpxA	ZONMW-10_02263	lpxA
lpxA	ZONMW-10_00870	lpxA
lpxA	ZONMW-10_00866	lpxA
lpxA	ZONMW-10_00868	lpxA
lpxA	ZONMW-11_00235	lpxA
lpxA	ZONMW-11_00233	lpxA
lpxA	ZONMW-12_04267	lpxA
lpxA	ZONMW-12_04263	lpxA
lpxA	ZONMW-12_04265	lpxA
lpxA	ZONMW-13_02707	lpxA
lpxA	ZONMW-13_02711	lpxA
lpxA	ZONMW-13_02709	lpxA
lpxA	ZONMW-14_00527	lpxA
lpxA	ZONMW-14_00531	lpxA
lpxA	ZONMW-14_00529	lpxA
lpxA	ZONMW-15_01848	lpxA
lpxA	ZONMW-15_02248	lpxA
lpxA	ZONMW-15_02244	lpxA
lpxA	ZONMW-15_02246	lpxA
lpxA	ZONMW-19_00786	lpxA
lpxA	ZONMW-19_00784	lpxA
lpxA	ZONMW-20_04115	lpxA
lpxA	ZONMW-20_04113	lpxA
lpxA	ZONMW-21_01997	lpxA
lpxA	ZONMW-21_01993	lpxA
lpxA	ZONMW-21_01995	lpxA
lpxA	ZONMW-22_01729	lpxA
lpxA	ZONMW-22_01733	lpxA
lpxA	ZONMW-22_01731	lpxA
lpxA	ZONMW-23_00823	lpxA
lpxA	ZONMW-23_00819	lpxA
lpxA	ZONMW-23_00821	lpxA

lpxA	ZONMW-24_01752	lpxA
lpxA	ZONMW-24_01756	lpxA
lpxA	ZONMW-24_01754	lpxA
lpxA	ZONMW-25_01021	lpxA
lpxA	ZONMW-25_01017	lpxA
lpxA	ZONMW-25_01019	lpxA
lpxA	ZONMW-26_02045	lpxA
lpxA	ZONMW-26_00576	lpxA
lpxA	ZONMW-26_00572	lpxA
lpxA	ZONMW-26_00574	lpxA
lpxA	ZONMW-27_02884	lpxA
lpxA	ZONMW-27_01215	lpxA
lpxA	ZONMW-27_01219	lpxA
lpxA	ZONMW-27_01217	lpxA
lpxA	ZONMW-28_03411	lpxA
lpxA	ZONMW-28_03413	lpxA
lpxA	ZONMW-29_01269	lpxA
lpxA	ZONMW-29_01265	lpxA
lpxA	ZONMW-29_01267	lpxA
lpxA	ZONMW-30_01339	lpxA
lpxA	ZONMW-30_01335	lpxA
lpxA	ZONMW-30_01337	lpxA
lpxA	ZONMW-31_02377	lpxA
lpxA	ZONMW-31_02375	lpxA
lpxA	ZONMW-32_03570	lpxA
lpxA	ZONMW-32_03568	lpxA
lpxA	ZONMW-33_03920	lpxA
lpxA	ZONMW-33_03918	lpxA
lpxA	ZONMW-34_03416	lpxA
lpxA	ZONMW-34_03418	lpxA
lpxA	ZONMW-35_03399	lpxA
lpxA	ZONMW-35_03397	lpxA
lpxA	ZONMW-36_03869	lpxA
lpxA	ZONMW-36_03867	lpxA
lpxA	ZONMW-37_01752	lpxA
lpxA	ZONMW-37_01756	lpxA
lpxA	ZONMW-37_01754	lpxA
lpxA	ZONMW-39_03260	lpxA
lpxA	ZONMW-39_03258	lpxA
lpxA	ZONMW-40_03194	lpxA
lpxA	ZONMW-40_03190	lpxA
lpxA	ZONMW-40_03192	lpxA
lpxA	ZONMW-41_02488	lpxA
lpxA	ZONMW-41_02486	lpxA
lpxA	ZONMW-42_02864	lpxA
lpxA	ZONMW-42_02862	lpxA
lpxA	ZONMW-43_02639	lpxA
lpxA	ZONMW-43_02641	lpxA

lpxA	ZONMW-44_02572	lpxA
lpxA	ZONMW-44_02570	lpxA
lpxA	ZONMW-45_01575	lpxA
lpxA	ZONMW-45_01571	lpxA
lpxA	ZONMW-45_01573	lpxA
lpxA	ZONMW-46_02086	lpxA
lpxA	ZONMW-46_02082	lpxA
lpxA	ZONMW-46_02084	lpxA
lpxA	ZONMW-47_01975	lpxA
lpxA	ZONMW-47_01979	lpxA
lpxA	ZONMW-47_01977	lpxA
lpxA	ZONMW-48_01432	lpxA
lpxA	ZONMW-48_01362	lpxA
lpxA	ZONMW-48_01366	lpxA
lpxA	ZONMW-48_01364	lpxA
lpxA	ZONMW-49_02080	lpxA
lpxA	ZONMW-49_01698	lpxA
lpxA	ZONMW-49_01694	lpxA
lpxA	ZONMW-49_01696	lpxA
lpxA	ZONMW-50_01874	lpxA
lpxA	ZONMW-50_01716	lpxA
lpxA	ZONMW-50_01720	lpxA
lpxA	ZONMW-50_01718	lpxA
lpxA	ZONMW-51_01738	lpxA
lpxA	ZONMW-51_00596	lpxA
lpxA	ZONMW-51_00592	lpxA
lpxA	ZONMW-51_00594	lpxA
lpxA	ZONMW-52_01881	lpxA
lpxA	ZONMW-52_00553	lpxA
lpxA	ZONMW-52_00549	lpxA
lpxA	ZONMW-52_00551	lpxA
lpxA	ZONMW-53_01875	lpxA
lpxA	ZONMW-53_01308	lpxA
lpxA	ZONMW-53_01304	lpxA
lpxA	ZONMW-53_01306	lpxA
lpxA	ZONMW-54_02196	lpxA
lpxA	ZONMW-54_01409	lpxA
lpxA	ZONMW-54_01413	lpxA
lpxA	ZONMW-54_01411	lpxA
lpxA	ZONMW-55_02060	lpxA
lpxA	ZONMW-55_00928	lpxA
lpxA	ZONMW-55_00932	lpxA
lpxA	ZONMW-55_00930	lpxA
lpxA	ZONMW-56_01374	lpxA
lpxA	ZONMW-56_00164	lpxA
lpxA	ZONMW-56_00168	lpxA
lpxA	ZONMW-56_00166	lpxA
lpxA	ZONMW-57_01850	lpxA

lpxA	ZONMW-57_00377	lpxA
lpxA	ZONMW-57_00381	lpxA
lpxA	ZONMW-57_00379	lpxA
lpxA	ZONMW-58_01874	lpxA
lpxA	ZONMW-58_00594	lpxA
lpxA	ZONMW-58_00590	lpxA
lpxA	ZONMW-58_00592	lpxA
lpxA	ZONMW-59_01687	lpxA
lpxA	ZONMW-59_00393	lpxA
lpxA	ZONMW-59_00397	lpxA
lpxA	ZONMW-59_00395	lpxA
lpxA	ZONMW-60_02070	lpxA
lpxA	ZONMW-60_00404	lpxA
lpxA	ZONMW-60_00408	lpxA
lpxA	ZONMW-60_00406	lpxA
lpxA	ZONMW-61_02522	lpxA
lpxA	ZONMW-61_02526	lpxA
lpxA	ZONMW-61_02524	lpxA
lpxA	ZONMW-62_00605	lpxA
lpxA	ZONMW-62_00601	lpxA
lpxA	ZONMW-62_00603	lpxA
lpxA	ZONMW-63_02741	lpxA
lpxA	ZONMW-63_00045	lpxA
lpxA	ZONMW-63_00049	lpxA
lpxA	ZONMW-63_00047	lpxA
lpxA	ZONMW-64_03274	lpxA
lpxA	ZONMW-64_03272	lpxA
lpxA	ZONMW-65_01777	lpxA
lpxA	ZONMW-65_00915	lpxA
lpxA	ZONMW-66_00189	lpxA
lpxA	ZONMW-66_00191	lpxA
lpxA	ZONMW-67_03163	lpxA
lpxA	ZONMW-67_03161	lpxA
lpxA	ZONMW-68_02893	lpxA
lpxA	ZONMW-68_02889	lpxA
lpxA	ZONMW-68_02891	lpxA
lpxA	ZONMW-69_03164	lpxA
lpxA	ZONMW-69_03162	lpxA
lpxA	ZONMW-72_04335	lpxA
lpxA	ZONMW-72_04185	lpxA
lpxA	ZONMW-72_04181	lpxA
lpxA	ZONMW-72_04183	lpxA
lpxA	ZONMW-72_01049	lpxA
lpxA	ZONMW-73_00607	lpxA
lpxA	ZONMW-73_02451	lpxA
lpxA	ZONMW-73_02455	lpxA
lpxA	ZONMW-73_02453	lpxA
lpxA	ZONMW-74_02857	lpxA

lpxA	ZONMW-74_00241	lpxA
lpxA	ZONMW-74_00237	lpxA
lpxA	ZONMW-74_00239	lpxA
lpxA	ZONMW-75_01653	lpxA
lpxA	ZONMW-75_02060	lpxA
lpxA	ZONMW-75_02064	lpxA
lpxA	ZONMW-75_02062	lpxA
lpxA	da5A-8_02954	lpxA
lpxA	da5A-8_02701	lpxA
lpxA	da5A-8_02697	lpxA
lpxA	da5A-8_02699	lpxA
pcrA	18_00838	pcrA
pcrA	18_02963	pcrA
pcrA	18_03116	pcrA
pcrA	18_03389	pcrA
pcrA	ACAM48_02294	pcrA
pcrA	ACAM48_03147	pcrA
pcrA	CAM030_01163	pcrA
pcrA	CAM030_01855	pcrA
pcrA	CAM030_00108	pcrA
pcrA	CECT7946_00016	pcrA
pcrA	CECT7946_02290	pcrA
pcrA	CECT7946_03048	pcrA
pcrA	CGMCC1.8863_02126	pcrA
pcrA	CGMCC1.8863_02438	pcrA
pcrA	CGMCC1.8863_02039	pcrA
pcrA	CGMCC1.8863_00046	pcrA
pcrA	CGMCC1.8863_00054	pcrA
pcrA	CL8139_02276	pcrA
pcrA	CL8139_01995	pcrA
pcrA	DAU203_01356	pcrA
pcrA	DAU203_02948	pcrA
pcrA	DD5b_01966	pcrA
pcrA	DSM1208_01536	pcrA
pcrA	DSM1208_03965	pcrA
pcrA	DSM12212_02127	pcrA
pcrA	DSM12212_00942	pcrA
pcrA	DSM15343_02439	pcrA
pcrA	DSM15343_00415	pcrA
pcrA	DSM15361_01476	pcrA
pcrA	DSM15361_02352	pcrA
pcrA	DSM15361_02832	pcrA
pcrA	DSM15365_01033	pcrA
pcrA	DSM15365_03671	pcrA
pcrA	DSM15365_00530	pcrA
pcrA	DSM15718_02914	pcrA
pcrA	DSM15718_01413	pcrA
pcrA	DSM15718_01465	pcrA

pcrA	DSM16505T_00713	pcrA
pcrA	DSM16505T_01705	pcrA
pcrA	DSM18103_01177	pcrA
pcrA	DSM18103_00697	pcrA
pcrA	DSM18103_00131	pcrA
pcrA	DSM18293_00829	pcrA
pcrA	DSM18293_01721	pcrA
pcrA	DSM18293_00175	pcrA
pcrA	DSM18841_00593	pcrA
pcrA	DSM18841_01799	pcrA
pcrA	DSM18841_01118	pcrA
pcrA	DSM18842_02950	pcrA
pcrA	DSM18842_02094	pcrA
pcrA	DSM19866_03424	pcrA
pcrA	DSM19866_03886	pcrA
pcrA	DSM2041_00357	pcrA
pcrA	DSM2041_05465	pcrA
pcrA	DSM2041_00876	pcrA
pcrA	DSM2061_03066	pcrA
pcrA	DSM2061_01283	pcrA
pcrA	DSM2061_01923	pcrA
pcrA	DSM2061_01922	pcrA
pcrA	DSM2061_01075	pcrA
pcrA	DSM2064_01683	pcrA
pcrA	DSM2064_00345	pcrA
pcrA	DSM2064_01544	pcrA
pcrA	DSM2064_00069	pcrA
pcrA	DSM21164_01004	pcrA
pcrA	DSM21164_02487	pcrA
pcrA	DSM23404_03014	pcrA
pcrA	DSM23404_00226	pcrA
pcrA	DSM23424_00433	pcrA
pcrA	DSM23424_01944	pcrA
pcrA	DSM24786_02798	pcrA
pcrA	DSM24786_01391	pcrA
pcrA	DSM24786_00410	pcrA
pcrA	DSM7489_01266	pcrA
pcrA	DSM7489_02818	pcrA
pcrA	DSW1_00588	pcrA
pcrA	DSW1_02457	pcrA
pcrA	DSW5_00813	pcrA
pcrA	DSW5_02117	pcrA
pcrA	DSW6_01064	pcrA
pcrA	DSW6_00249	pcrA
pcrA	DSW8_02455	pcrA
pcrA	DSW8_03620	pcrA
pcrA	DSW8_00036	pcrA
pcrA	F52_02138	pcrA

pcrA	F52_01339	pcrA
pcrA	F52_00692	pcrA
pcrA	GCA_001027945.1_01262	pcrA
pcrA	GCA_001027945.1_02133	pcrA
pcrA	HI1_01208	pcrA
pcrA	HI1_02758	pcrA
pcrA	IR1_04160	pcrA
pcrA	IR1_03416	pcrA
pcrA	IR1_03361	pcrA
pcrA	KCOM3131_00899	pcrA
pcrA	KCOM3131_01734	pcrA
pcrA	KCTC12974_01679	pcrA
pcrA	KCTC12974_01339	pcrA
pcrA	KCTC12974_00642	pcrA
pcrA	KCTC12974_00279	pcrA
pcrA	KL-A_02155	pcrA
pcrA	KL-A_00697	pcrA
pcrA	KL-A_03430	pcrA
pcrA	LL0412.1.7_00728	pcrA
pcrA	LL0412.1.7_02351	pcrA
pcrA	MAR_2009_71_00998	pcrA
pcrA	MAR_2009_71_02770	pcrA
pcrA	MAR_2009_71_02834	pcrA
pcrA	MAR_2009_71_01563	pcrA
pcrA	NN016038_00858	pcrA
pcrA	NN016038_03152	pcrA
pcrA	NN016038_03001	pcrA
pcrA	PAO1_05627	pcrA
pcrA	PAO1_05473	pcrA
pcrA	PAO1_04423	pcrA
pcrA	PAO1_01445	pcrA
pcrA	PAO1_01996	pcrA
pcrA	PAO1_01999	pcrA
pcrA	UW101_00737	pcrA
pcrA	UW101_03056	pcrA
pcrA	UW101_03935	pcrA
pcrA	UW101_03814	pcrA
pcrA	V4-33_00142	pcrA
pcrA	V4-33_00783	pcrA
pcrA	V4-33_01467	pcrA
pcrA	W5C_01642	pcrA
pcrA	W5C_02502	pcrA
pcrA	ZONMW-01_01008	pcrA
pcrA	ZONMW-01_02303	pcrA
pcrA	ZONMW-02_01752	pcrA
pcrA	ZONMW-02_01519	pcrA
pcrA	ZONMW-02_01427	pcrA
pcrA	ZONMW-02_01430	pcrA

pcrA	ZONMW-02_03790	pcrA
pcrA	ZONMW-03_02077	pcrA
pcrA	ZONMW-03_00345	pcrA
pcrA	ZONMW-04_01742	pcrA
pcrA	ZONMW-04_01280	pcrA
pcrA	ZONMW-05_02021	pcrA
pcrA	ZONMW-05_00604	pcrA
pcrA	ZONMW-06_01833	pcrA
pcrA	ZONMW-06_00001	pcrA
pcrA	ZONMW-06_03832	pcrA
pcrA	ZONMW-06_01278	pcrA
pcrA	ZONMW-07_03074	pcrA
pcrA	ZONMW-07_03702	pcrA
pcrA	ZONMW-08_00517	pcrA
pcrA	ZONMW-08_01987	pcrA
pcrA	ZONMW-08_02067	pcrA
pcrA	ZONMW-08_02737	pcrA
pcrA	ZONMW-09_00443	pcrA
pcrA	ZONMW-09_01488	pcrA
pcrA	ZONMW-09_02048	pcrA
pcrA	ZONMW-10_00227	pcrA
pcrA	ZONMW-10_02195	pcrA
pcrA	ZONMW-11_00771	pcrA
pcrA	ZONMW-11_03940	pcrA
pcrA	ZONMW-11_04664	pcrA
pcrA	ZONMW-12_00039	pcrA
pcrA	ZONMW-12_03991	pcrA
pcrA	ZONMW-13_00453	pcrA
pcrA	ZONMW-13_01487	pcrA
pcrA	ZONMW-14_00109	pcrA
pcrA	ZONMW-14_02299	pcrA
pcrA	ZONMW-15_01744	pcrA
pcrA	ZONMW-15_02005	pcrA
pcrA	ZONMW-15_00145	pcrA
pcrA	ZONMW-19_03871	pcrA
pcrA	ZONMW-19_02067	pcrA
pcrA	ZONMW-19_02212	pcrA
pcrA	ZONMW-20_00837	pcrA
pcrA	ZONMW-20_00868	pcrA
pcrA	ZONMW-20_00334	pcrA
pcrA	ZONMW-20_03824	pcrA
pcrA	ZONMW-20_00266	pcrA
pcrA	ZONMW-21_00991	pcrA
pcrA	ZONMW-21_01788	pcrA
pcrA	ZONMW-22_00992	pcrA
pcrA	ZONMW-22_01936	pcrA
pcrA	ZONMW-23_02105	pcrA
pcrA	ZONMW-23_00615	pcrA

pcrA	ZONMW-24_00469	pcrA
pcrA	ZONMW-24_01959	pcrA
pcrA	ZONMW-25_01991	pcrA
pcrA	ZONMW-25_00851	pcrA
pcrA	ZONMW-26_02379	pcrA
pcrA	ZONMW-26_00362	pcrA
pcrA	ZONMW-27_01559	pcrA
pcrA	ZONMW-27_01428	pcrA
pcrA	ZONMW-28_01173	pcrA
pcrA	ZONMW-28_02749	pcrA
pcrA	ZONMW-28_01158	pcrA
pcrA	ZONMW-28_02428	pcrA
pcrA	ZONMW-29_00560	pcrA
pcrA	ZONMW-29_01069	pcrA
pcrA	ZONMW-30_01807	pcrA
pcrA	ZONMW-30_01164	pcrA
pcrA	ZONMW-31_00333	pcrA
pcrA	ZONMW-31_02013	pcrA
pcrA	ZONMW-31_00323	pcrA
pcrA	ZONMW-31_00034	pcrA
pcrA	ZONMW-32_01581	pcrA
pcrA	ZONMW-32_03126	pcrA
pcrA	ZONMW-32_01566	pcrA
pcrA	ZONMW-32_02922	pcrA
pcrA	ZONMW-33_01571	pcrA
pcrA	ZONMW-33_03147	pcrA
pcrA	ZONMW-33_01556	pcrA
pcrA	ZONMW-33_02826	pcrA
pcrA	ZONMW-34_02509	pcrA
pcrA	ZONMW-34_02889	pcrA
pcrA	ZONMW-34_02904	pcrA
pcrA	ZONMW-34_03588	pcrA
pcrA	ZONMW-34_01355	pcrA
pcrA	ZONMW-35_02473	pcrA
pcrA	ZONMW-35_02856	pcrA
pcrA	ZONMW-35_02871	pcrA
pcrA	ZONMW-35_03576	pcrA
pcrA	ZONMW-35_00428	pcrA
pcrA	ZONMW-36_01785	pcrA
pcrA	ZONMW-36_02854	pcrA
pcrA	ZONMW-36_01800	pcrA
pcrA	ZONMW-36_02338	pcrA
pcrA	ZONMW-37_01371	pcrA
pcrA	ZONMW-37_02166	pcrA
pcrA	ZONMW-38_03032	pcrA
pcrA	ZONMW-38_00757	pcrA
pcrA	ZONMW-38_01044	pcrA
pcrA	ZONMW-39_02050	pcrA

pcrA	ZONMW-39_02570	pcrA
pcrA	ZONMW-39_02582	pcrA
pcrA	ZONMW-39_01847	pcrA
pcrA	ZONMW-40_00267	pcrA
pcrA	ZONMW-40_00546	pcrA
pcrA	ZONMW-41_02994	pcrA
pcrA	ZONMW-41_00341	pcrA
pcrA	ZONMW-41_00541	pcrA
pcrA	ZONMW-41_01636	pcrA
pcrA	ZONMW-42_01166	pcrA
pcrA	ZONMW-42_00197	pcrA
pcrA	ZONMW-42_00208	pcrA
pcrA	ZONMW-42_02765	pcrA
pcrA	ZONMW-42_03742	pcrA
pcrA	ZONMW-43_01136	pcrA
pcrA	ZONMW-43_00977	pcrA
pcrA	ZONMW-43_04011	pcrA
pcrA	ZONMW-43_03533	pcrA
pcrA	ZONMW-44_00187	pcrA
pcrA	ZONMW-44_02968	pcrA
pcrA	ZONMW-44_02978	pcrA
pcrA	ZONMW-44_02451	pcrA
pcrA	ZONMW-45_00742	pcrA
pcrA	ZONMW-45_00253	pcrA
pcrA	ZONMW-46_00717	pcrA
pcrA	ZONMW-46_00468	pcrA
pcrA	ZONMW-47_00717	pcrA
pcrA	ZONMW-47_00037	pcrA
pcrA	ZONMW-48_00131	pcrA
pcrA	ZONMW-48_01593	pcrA
pcrA	ZONMW-48_03382	pcrA
pcrA	ZONMW-49_02671	pcrA
pcrA	ZONMW-49_00377	pcrA
pcrA	ZONMW-50_02465	pcrA
pcrA	ZONMW-50_00108	pcrA
pcrA	ZONMW-51_02607	pcrA
pcrA	ZONMW-51_00364	pcrA
pcrA	ZONMW-52_02100	pcrA
pcrA	ZONMW-52_00349	pcrA
pcrA	ZONMW-53_02739	pcrA
pcrA	ZONMW-53_01082	pcrA
pcrA	ZONMW-54_02882	pcrA
pcrA	ZONMW-54_01641	pcrA
pcrA	ZONMW-55_02739	pcrA
pcrA	ZONMW-55_01162	pcrA
pcrA	ZONMW-56_02456	pcrA
pcrA	ZONMW-56_00390	pcrA
pcrA	ZONMW-57_02643	pcrA

pcrA	ZONMW-57_00603	pcrA
pcrA	ZONMW-58_02668	pcrA
pcrA	ZONMW-58_00368	pcrA
pcrA	ZONMW-59_02676	pcrA
pcrA	ZONMW-59_00591	pcrA
pcrA	ZONMW-59_00932	pcrA
pcrA	ZONMW-60_02718	pcrA
pcrA	ZONMW-60_00606	pcrA
pcrA	ZONMW-61_00087	pcrA
pcrA	ZONMW-61_02386	pcrA
pcrA	ZONMW-61_01418	pcrA
pcrA	ZONMW-62_01642	pcrA
pcrA	ZONMW-62_00773	pcrA
pcrA	ZONMW-62_00698	pcrA
pcrA	ZONMW-63_02189	pcrA
pcrA	ZONMW-63_00276	pcrA
pcrA	ZONMW-64_03486	pcrA
pcrA	ZONMW-64_01261	pcrA
pcrA	ZONMW-64_01273	pcrA
pcrA	ZONMW-64_02667	pcrA
pcrA	ZONMW-65_03706	pcrA
pcrA	ZONMW-65_01991	pcrA
pcrA	ZONMW-65_02659	pcrA
pcrA	ZONMW-65_02686	pcrA
pcrA	ZONMW-65_02589	pcrA
pcrA	ZONMW-66_03002	pcrA
pcrA	ZONMW-66_02601	pcrA
pcrA	ZONMW-66_01403	pcrA
pcrA	ZONMW-67_00458	pcrA
pcrA	ZONMW-67_03410	pcrA
pcrA	ZONMW-67_00445	pcrA
pcrA	ZONMW-67_00135	pcrA
pcrA	ZONMW-68_00542	pcrA
pcrA	ZONMW-68_01752	pcrA
pcrA	ZONMW-68_04478	pcrA
pcrA	ZONMW-68_04490	pcrA
pcrA	ZONMW-68_01450	pcrA
pcrA	ZONMW-69_00205	pcrA
pcrA	ZONMW-69_03411	pcrA
pcrA	ZONMW-69_00192	pcrA
pcrA	ZONMW-69_02744	pcrA
pcrA	ZONMW-72_04449	pcrA
pcrA	ZONMW-72_03495	pcrA
pcrA	ZONMW-72_03755	pcrA
pcrA	ZONMW-73_00156	pcrA
pcrA	ZONMW-73_01388	pcrA
pcrA	ZONMW-74_01658	pcrA
pcrA	ZONMW-74_00063	pcrA

pcrA	ZONMW-74_02744	pcrA
pcrA	ZONMW-74_02745	pcrA
pcrA	ZONMW-75_02252	pcrA
pcrA	ZONMW-75_02617	pcrA
pcrA	da5A-8_01535	pcrA
pcrA	da5A-8_02939	pcrA
rpsH	18_00193	rpsH
rpsH	ACAM48_00318	rpsH
rpsH	CAM030_02833	rpsH
rpsH	CECT7946_03319	rpsH
rpsH	CGMCC1.8863_01427	rpsH
rpsH	CL8139_01436	rpsH
rpsH	DAU203_02280	rpsH
rpsH	DD5b_02406	rpsH
rpsH	DSM1208_02437	rpsH
rpsH	DSM12212_01097	rpsH
rpsH	DSM15343_01577	rpsH
rpsH	DSM15361_02580	rpsH
rpsH	DSM15365_01148	rpsH
rpsH	DSM15718_00662	rpsH
rpsH	DSM16505T_01245	rpsH
rpsH	DSM18103_01872	rpsH
rpsH	DSM18293_02428	rpsH
rpsH	DSM18841_01178	rpsH
rpsH	DSM18842_00126	rpsH
rpsH	DSM19866_01091	rpsH
rpsH	DSM2041_02964	rpsH
rpsH	DSM2061_03948	rpsH
rpsH	DSM2064_04698	rpsH
rpsH	DSM21164_02207	rpsH
rpsH	DSM23404_00472	rpsH
rpsH	DSM23424_02873	rpsH
rpsH	DSM24786_01751	rpsH
rpsH	DSM7489_02196	rpsH
rpsH	DSW1_01277	rpsH
rpsH	DSW5_00461	rpsH
rpsH	DSW6_00506	rpsH
rpsH	DSW8_01516	rpsH
rpsH	F52_01519	rpsH
rpsH	GCA_001027945.1_02977	rpsH
rpsH	HI1_02122	rpsH
rpsH	IR1_03059	rpsH
rpsH	KCOM3131_00728	rpsH
rpsH	KCTC12974_01062	rpsH
rpsH	KL-A_00077	rpsH
rpsH	LL0412.1.7_01241	rpsH
rpsH	MAR_2009_71_01909	rpsH
rpsH	NN016038_00200	rpsH

rpsH	PAO1_04380	rpsH
rpsH	UW101_00404	rpsH
rpsH	V4-33_01233	rpsH
rpsH	W5C_02865	rpsH
rpsH	ZONMW-01_01393	rpsH
rpsH	ZONMW-02_02700	rpsH
rpsH	ZONMW-03_01387	rpsH
rpsH	ZONMW-04_00305	rpsH
rpsH	ZONMW-05_01520	rpsH
rpsH	ZONMW-06_03645	rpsH
rpsH	ZONMW-07_01580	rpsH
rpsH	ZONMW-08_01317	rpsH
rpsH	ZONMW-09_01336	rpsH
rpsH	ZONMW-10_01396	rpsH
rpsH	ZONMW-11_03082	rpsH
rpsH	ZONMW-12_01405	rpsH
rpsH	ZONMW-13_01809	rpsH
rpsH	ZONMW-14_01045	rpsH
rpsH	ZONMW-15_00758	rpsH
rpsH	ZONMW-19_01645	rpsH
rpsH	ZONMW-20_04263	rpsH
rpsH	ZONMW-21_02453	rpsH
rpsH	ZONMW-22_02656	rpsH
rpsH	ZONMW-23_02680	rpsH
rpsH	ZONMW-24_02477	rpsH
rpsH	ZONMW-25_03473	rpsH
rpsH	ZONMW-26_01750	rpsH
rpsH	ZONMW-27_00299	rpsH
rpsH	ZONMW-28_03617	rpsH
rpsH	ZONMW-29_02788	rpsH
rpsH	ZONMW-30_00092	rpsH
rpsH	ZONMW-31_03097	rpsH
rpsH	ZONMW-32_03645	rpsH
rpsH	ZONMW-33_03576	rpsH
rpsH	ZONMW-34_03514	rpsH
rpsH	ZONMW-35_03475	rpsH
rpsH	ZONMW-36_03402	rpsH
rpsH	ZONMW-37_02723	rpsH
rpsH	ZONMW-38_01901	rpsH
rpsH	ZONMW-39_03865	rpsH
rpsH	ZONMW-40_02180	rpsH
rpsH	ZONMW-41_02968	rpsH
rpsH	ZONMW-42_02239	rpsH
rpsH	ZONMW-43_03686	rpsH
rpsH	ZONMW-44_03255	rpsH
rpsH	ZONMW-45_01753	rpsH
rpsH	ZONMW-46_00044	rpsH
rpsH	ZONMW-47_00461	rpsH

rpsH	ZONMW-48_02044	rpsH
rpsH	ZONMW-49_00716	rpsH
rpsH	ZONMW-50_00717	rpsH
rpsH	ZONMW-51_00991	rpsH
rpsH	ZONMW-52_01591	rpsH
rpsH	ZONMW-53_00234	rpsH
rpsH	ZONMW-54_00796	rpsH
rpsH	ZONMW-55_00229	rpsH
rpsH	ZONMW-56_01089	rpsH
rpsH	ZONMW-57_01388	rpsH
rpsH	ZONMW-58_01196	rpsH
rpsH	ZONMW-59_01390	rpsH
rpsH	ZONMW-60_01185	rpsH
rpsH	ZONMW-61_01306	rpsH
rpsH	ZONMW-62_02165	rpsH
rpsH	ZONMW-63_01177	rpsH
rpsH	ZONMW-64_03964	rpsH
rpsH	ZONMW-65_02957	rpsH
rpsH	ZONMW-66_01174	rpsH
rpsH	ZONMW-67_03490	rpsH
rpsH	ZONMW-68_04448	rpsH
rpsH	ZONMW-69_03492	rpsH
rpsH	ZONMW-72_01161	rpsH
rpsH	ZONMW-73_02135	rpsH
rpsH	ZONMW-74_01009	rpsH
rpsH	ZONMW-75_02925	rpsH
rpsH	da5A-8_00894	rpsH
purE	18_00261	purE
purE	ACAM48_00276	purE
purE	CAM030_02640	purE
purE	CECT7946_03382	purE
purE	CGMCC1.8863_01387	purE
purE	CL8139_01485	purE
purE	DAU203_02234	purE
purE	DD5b_02456	purE
purE	DSM1208_02390	purE
purE	DSM12212_01049	purE
purE	DSM15343_01534	purE
purE	DSM15361_02619	purE
purE	DSM15365_02289	purE
purE	DSM15718_00615	purE
purE	DSM16505T_01072	purE
purE	DSM18103_01450	purE
purE	DSM18293_02382	purE
purE	DSM18841_00958	purE
purE	DSM18842_00360	purE
purE	DSM19866_01047	purE
purE	DSM2041_03037	purE

purE	DSM2061_03901	purE
purE	DSM2064_04655	purE
purE	DSM21164_02258	purE
purE	DSM23404_00515	purE
purE	DSM23424_02915	purE
purE	DSM24786_01798	purE
purE	DSM7489_02147	purE
purE	DSW1_01218	purE
purE	DSW5_00967	purE
purE	DSW6_02414	purE
purE	DSW8_01581	purE
purE	F52_01474	purE
purE	GCA_001027945.1_01469	purE
purE	HI1_02073	purE
purE	IR1_03099	purE
purE	KCOM3131_01920	purE
purE	KCTC12974_01102	purE
purE	KL-A_00124	purE
purE	LL0412.1.7_01098	purE
purE	MAR_2009_71_01844	purE
purE	NN016038_00271	purE
purE	PAO1_05608	purE
purE	UW101_00447	purE
purE	V4-33_01271	purE
purE	W5C_02912	purE
purE	ZONMW-01_01439	purE
purE	ZONMW-02_02383	purE
purE	ZONMW-03_01341	purE
purE	ZONMW-04_00352	purE
purE	ZONMW-05_01473	purE
purE	ZONMW-06_01085	purE
purE	ZONMW-07_02013	purE
purE	ZONMW-08_01380	purE
purE	ZONMW-09_01379	purE
purE	ZONMW-10_01352	purE
purE	ZONMW-11_01419	purE
purE	ZONMW-12_02699	purE
purE	ZONMW-13_01855	purE
purE	ZONMW-14_01087	purE
purE	ZONMW-15_00816	purE
purE	ZONMW-19_02660	purE
purE	ZONMW-20_01256	purE
purE	ZONMW-21_02502	purE
purE	ZONMW-22_02607	purE
purE	ZONMW-23_02631	purE
purE	ZONMW-24_02526	purE
purE	ZONMW-25_02392	purE
purE	ZONMW-26_01796	purE

purE	ZONMW-27_00346	purE
purE	ZONMW-28_03573	purE
purE	ZONMW-29_02739	purE
purE	ZONMW-30_00047	purE
purE	ZONMW-31_01860	purE
purE	ZONMW-32_00658	purE
purE	ZONMW-33_00526	purE
purE	ZONMW-34_00890	purE
purE	ZONMW-35_02353	purE
purE	ZONMW-36_00845	purE
purE	ZONMW-37_02772	purE
purE	ZONMW-38_00741	purE
purE	ZONMW-39_01353	purE
purE	ZONMW-40_02132	purE
purE	ZONMW-41_02707	purE
purE	ZONMW-42_00990	purE
purE	ZONMW-43_00423	purE
purE	ZONMW-44_02274	purE
purE	ZONMW-45_02952	purE
purE	ZONMW-46_01762	purE
purE	ZONMW-47_01727	purE
purE	ZONMW-48_01989	purE
purE	ZONMW-49_00765	purE
purE	ZONMW-50_00766	purE
purE	ZONMW-51_00943	purE
purE	ZONMW-52_01543	purE
purE	ZONMW-53_00284	purE
purE	ZONMW-54_00747	purE
purE	ZONMW-55_00277	purE
purE	ZONMW-56_01040	purE
purE	ZONMW-57_01340	purE
purE	ZONMW-58_01244	purE
purE	ZONMW-59_01341	purE
purE	ZONMW-60_01234	purE
purE	ZONMW-61_03005	purE
purE	ZONMW-62_02229	purE
purE	ZONMW-63_01225	purE
purE	ZONMW-64_00441	purE
purE	ZONMW-65_01302	purE
purE	ZONMW-66_03066	purE
purE	ZONMW-67_01113	purE
purE	ZONMW-68_04651	purE
purE	ZONMW-69_01160	purE
purE	ZONMW-72_01121	purE
purE	ZONMW-73_03041	purE
purE	ZONMW-74_01052	purE
purE	ZONMW-75_00157	purE
purE	da5A-8_02273	purE

ypdA_2	18_00244	ypdA_2
ypdA_2	18_00245	ypdA_2
ypdA_2	18_03770	ypdA_2
ypdA_2	18_03321	ypdA_2
ypdA_2	18_03422	ypdA_2
ypdA_2	18_03353	ypdA_2
ypdA_2	18_02855	ypdA_2
ypdA_2	18_01593	ypdA_2
ypdA_2	18_01228	ypdA_2
ypdA_2	18_01733	ypdA_2
ypdA_2	18_03767	ypdA_2
ypdA_2	18_03768	ypdA_2
ypdA_2	18_03769	ypdA_2
ypdA_2	ACAM48_01178	ypdA_2
ypdA_2	ACAM48_02421	ypdA_2
ypdA_2	ACAM48_00218	ypdA_2
ypdA_2	ACAM48_01118	ypdA_2
ypdA_2	ACAM48_01117	ypdA_2
ypdA_2	CAM030_01458	ypdA_2
ypdA_2	CAM030_00179	ypdA_2
ypdA_2	CAM030_00824	ypdA_2
ypdA_2	CAM030_01216	ypdA_2
ypdA_2	CAM030_02335	ypdA_2
ypdA_2	CAM030_00180	ypdA_2
ypdA_2	CECT7946_00913	ypdA_2
ypdA_2	CECT7946_03373	ypdA_2
ypdA_2	CECT7946_00786	ypdA_2
ypdA_2	CECT7946_00802	ypdA_2
ypdA_2	CECT7946_03167	ypdA_2
ypdA_2	CECT7946_00795	ypdA_2
ypdA_2	CECT7946_01571	ypdA_2
ypdA_2	CECT7946_00593	ypdA_2
ypdA_2	CECT7946_01264	ypdA_2
ypdA_2	CECT7946_01253	ypdA_2
ypdA_2	CECT7946_03071	ypdA_2
ypdA_2	CECT7946_02909	ypdA_2
ypdA_2	CECT7946_02545	ypdA_2
ypdA_2	CECT7946_01831	ypdA_2
ypdA_2	CECT7946_03166	ypdA_2
ypdA_2	CECT7946_03165	ypdA_2
ypdA_2	CECT7946_03164	ypdA_2
ypdA_2	CGMCC1.8863_03573	ypdA_2
ypdA_2	CGMCC1.8863_01710	ypdA_2
ypdA_2	CGMCC1.8863_01846	ypdA_2
ypdA_2	CGMCC1.8863_02866	ypdA_2
ypdA_2	CGMCC1.8863_00948	ypdA_2
ypdA_2	CGMCC1.8863_03120	ypdA_2
ypdA_2	CGMCC1.8863_01712	ypdA_2

ypdA_2	CGMCC1.8863_01713	ypdA_2
ypdA_2	CGMCC1.8863_01711	ypdA_2
ypdA_2	CL8139_01478	ypdA_2
ypdA_2	CL8139_03166	ypdA_2
ypdA_2	CL8139_00693	ypdA_2
ypdA_2	CL8139_02193	ypdA_2
ypdA_2	CL8139_02677	ypdA_2
ypdA_2	CL8139_03042	ypdA_2
ypdA_2	CL8139_00768	ypdA_2
ypdA_2	CL8139_01985	ypdA_2
ypdA_2	CL8139_02307	ypdA_2
ypdA_2	CL8139_00360	ypdA_2
ypdA_2	CL8139_01004	ypdA_2
ypdA_2	CL8139_03222	ypdA_2
ypdA_2	CL8139_02674	ypdA_2
ypdA_2	CL8139_02675	ypdA_2
ypdA_2	CL8139_02676	ypdA_2
ypdA_2	DAU203_02241	ypdA_2
ypdA_2	DAU203_03099	ypdA_2
ypdA_2	DAU203_02457	ypdA_2
ypdA_2	DAU203_03033	ypdA_2
ypdA_2	DAU203_00979	ypdA_2
ypdA_2	DAU203_02689	ypdA_2
ypdA_2	DAU203_03419	ypdA_2
ypdA_2	DAU203_01407	ypdA_2
ypdA_2	DAU203_03288	ypdA_2
ypdA_2	DAU203_00073	ypdA_2
ypdA_2	DAU203_00167	ypdA_2
ypdA_2	DAU203_01560	ypdA_2
ypdA_2	DAU203_01781	ypdA_2
ypdA_2	DAU203_02020	ypdA_2
ypdA_2	DAU203_02692	ypdA_2
ypdA_2	DAU203_02691	ypdA_2
ypdA_2	DAU203_02690	ypdA_2
ypdA_2	DD5b_01253	ypdA_2
ypdA_2	DD5b_01019	ypdA_2
ypdA_2	DD5b_01413	ypdA_2
ypdA_2	DD5b_01411	ypdA_2
ypdA_2	DD5b_01410	ypdA_2
ypdA_2	DSM1208_00119	ypdA_2
ypdA_2	DSM1208_03127	ypdA_2
ypdA_2	DSM1208_02469	ypdA_2
ypdA_2	DSM1208_04117	ypdA_2
ypdA_2	DSM1208_02460	ypdA_2
ypdA_2	DSM1208_03177	ypdA_2
ypdA_2	DSM1208_02316	ypdA_2
ypdA_2	DSM1208_01827	ypdA_2
ypdA_2	DSM1208_00537	ypdA_2

ypdA_2	DSM1208_00118	ypdA_2
ypdA_2	DSM1208_00117	ypdA_2
ypdA_2	DSM12212_03419	ypdA_2
ypdA_2	DSM12212_01289	ypdA_2
ypdA_2	DSM12212_01816	ypdA_2
ypdA_2	DSM12212_02503	ypdA_2
ypdA_2	DSM12212_00383	ypdA_2
ypdA_2	DSM12212_03583	ypdA_2
ypdA_2	DSM12212_03472	ypdA_2
ypdA_2	DSM12212_03396	ypdA_2
ypdA_2	DSM12212_02575	ypdA_2
ypdA_2	DSM12212_00622	ypdA_2
ypdA_2	DSM12212_00386	ypdA_2
ypdA_2	DSM12212_00385	ypdA_2
ypdA_2	DSM12212_00384	ypdA_2
ypdA_2	DSM12212_00620	ypdA_2
ypdA_2	DSM12212_00621	ypdA_2
ypdA_2	DSM12212_00618	ypdA_2
ypdA_2	DSM12212_00619	ypdA_2
ypdA_2	DSM15343_00247	ypdA_2
ypdA_2	DSM15343_01872	ypdA_2
ypdA_2	DSM15343_01592	ypdA_2
ypdA_2	DSM15343_01873	ypdA_2
ypdA_2	DSM15343_01874	ypdA_2
ypdA_2	DSM15361_00937	ypdA_2
ypdA_2	DSM15361_00341	ypdA_2
ypdA_2	DSM15361_02770	ypdA_2
ypdA_2	DSM15361_02097	ypdA_2
ypdA_2	DSM15361_02769	ypdA_2
ypdA_2	DSM15365_01778	ypdA_2
ypdA_2	DSM15365_02072	ypdA_2
ypdA_2	DSM15365_03470	ypdA_2
ypdA_2	DSM15365_01741	ypdA_2
ypdA_2	DSM15365_00286	ypdA_2
ypdA_2	DSM15365_03714	ypdA_2
ypdA_2	DSM15365_03113	ypdA_2
ypdA_2	DSM15365_03675	ypdA_2
ypdA_2	DSM15365_00937	ypdA_2
ypdA_2	DSM15365_00862	ypdA_2
ypdA_2	DSM15365_02273	ypdA_2
ypdA_2	DSM15365_00768	ypdA_2
ypdA_2	DSM15365_03563	ypdA_2
ypdA_2	DSM15365_00287	ypdA_2
ypdA_2	DSM15718_03180	ypdA_2
ypdA_2	DSM15718_00373	ypdA_2
ypdA_2	DSM15718_03281	ypdA_2
ypdA_2	DSM15718_03191	ypdA_2
ypdA_2	DSM15718_03166	ypdA_2

ypdA_2	DSM15718_01602	ypdA_2
ypdA_2	DSM15718_03167	ypdA_2
ypdA_2	DSM15718_00370	ypdA_2
ypdA_2	DSM15718_00371	ypdA_2
ypdA_2	DSM15718_00372	ypdA_2
ypdA_2	DSM16505T_00047	ypdA_2
ypdA_2	DSM16505T_01368	ypdA_2
ypdA_2	DSM16505T_02170	ypdA_2
ypdA_2	DSM16505T_01993	ypdA_2
ypdA_2	DSM16505T_02242	ypdA_2
ypdA_2	DSM16505T_00904	ypdA_2
ypdA_2	DSM16505T_02678	ypdA_2
ypdA_2	DSM16505T_01627	ypdA_2
ypdA_2	DSM16505T_01814	ypdA_2
ypdA_2	DSM16505T_00016	ypdA_2
ypdA_2	DSM16505T_01045	ypdA_2
ypdA_2	DSM16505T_02640	ypdA_2
ypdA_2	DSM16505T_00593	ypdA_2
ypdA_2	DSM16505T_02168	ypdA_2
ypdA_2	DSM16505T_02167	ypdA_2
ypdA_2	DSM16505T_02169	ypdA_2
ypdA_2	DSM18103_02174	ypdA_2
ypdA_2	DSM18103_03133	ypdA_2
ypdA_2	DSM18103_01365	ypdA_2
ypdA_2	DSM18103_00140	ypdA_2
ypdA_2	DSM18103_00679	ypdA_2
ypdA_2	DSM18103_00144	ypdA_2
ypdA_2	DSM18103_00404	ypdA_2
ypdA_2	DSM18103_02784	ypdA_2
ypdA_2	DSM18103_02077	ypdA_2
ypdA_2	DSM18103_03471	ypdA_2
ypdA_2	DSM18103_00598	ypdA_2
ypdA_2	DSM18103_01007	ypdA_2
ypdA_2	DSM18103_01502	ypdA_2
ypdA_2	DSM18103_00456	ypdA_2
ypdA_2	DSM18103_01560	ypdA_2
ypdA_2	DSM18103_02175	ypdA_2
ypdA_2	DSM18103_02508	ypdA_2
ypdA_2	DSM18103_00027	ypdA_2
ypdA_2	DSM18103_00401	ypdA_2
ypdA_2	DSM18103_00402	ypdA_2
ypdA_2	DSM18103_00400	ypdA_2
ypdA_2	DSM18103_00403	ypdA_2
ypdA_2	DSM18293_02040	ypdA_2
ypdA_2	DSM18293_02038	ypdA_2
ypdA_2	DSM18293_00048	ypdA_2
ypdA_2	DSM18293_02822	ypdA_2
ypdA_2	DSM18293_04277	ypdA_2

ypdA_2	DSM18293_01030	ypdA_2
ypdA_2	DSM18293_01462	ypdA_2
ypdA_2	DSM18293_01968	ypdA_2
ypdA_2	DSM18293_03311	ypdA_2
ypdA_2	DSM18293_01460	ypdA_2
ypdA_2	DSM18293_01461	ypdA_2
ypdA_2	DSM18293_00902	ypdA_2
ypdA_2	DSM18293_01458	ypdA_2
ypdA_2	DSM18293_01457	ypdA_2
ypdA_2	DSM18841_02100	ypdA_2
ypdA_2	DSM18841_01460	ypdA_2
ypdA_2	DSM18841_02377	ypdA_2
ypdA_2	DSM18841_00300	ypdA_2
ypdA_2	DSM18841_01915	ypdA_2
ypdA_2	DSM18841_01734	ypdA_2
ypdA_2	DSM18841_00775	ypdA_2
ypdA_2	DSM18841_01665	ypdA_2
ypdA_2	DSM18841_02487	ypdA_2
ypdA_2	DSM18841_00449	ypdA_2
ypdA_2	DSM18841_00931	ypdA_2
ypdA_2	DSM18841_01911	ypdA_2
ypdA_2	DSM18841_01912	ypdA_2
ypdA_2	DSM18841_01914	ypdA_2
ypdA_2	DSM18841_01913	ypdA_2
ypdA_2	DSM18842_00629	ypdA_2
ypdA_2	DSM18842_02074	ypdA_2
ypdA_2	DSM18842_03157	ypdA_2
ypdA_2	DSM18842_01439	ypdA_2
ypdA_2	DSM18842_02423	ypdA_2
ypdA_2	DSM18842_01284	ypdA_2
ypdA_2	DSM18842_01760	ypdA_2
ypdA_2	DSM18842_03110	ypdA_2
ypdA_2	DSM18842_01843	ypdA_2
ypdA_2	DSM18842_00538	ypdA_2
ypdA_2	DSM18842_02042	ypdA_2
ypdA_2	DSM18842_01983	ypdA_2
ypdA_2	DSM18842_00383	ypdA_2
ypdA_2	DSM18842_00780	ypdA_2
ypdA_2	DSM18842_02394	ypdA_2
ypdA_2	DSM18842_01846	ypdA_2
ypdA_2	DSM18842_01847	ypdA_2
ypdA_2	DSM18842_01844	ypdA_2
ypdA_2	DSM18842_01845	ypdA_2
ypdA_2	DSM19866_04804	ypdA_2
ypdA_2	DSM19866_02583	ypdA_2
ypdA_2	DSM19866_02590	ypdA_2
ypdA_2	DSM19866_04038	ypdA_2
ypdA_2	DSM19866_01743	ypdA_2

ypdA_2	DSM19866_01739	ypdA_2
ypdA_2	DSM19866_02659	ypdA_2
ypdA_2	DSM19866_03587	ypdA_2
ypdA_2	DSM19866_02799	ypdA_2
ypdA_2	DSM19866_02073	ypdA_2
ypdA_2	DSM19866_01737	ypdA_2
ypdA_2	DSM19866_01738	ypdA_2
ypdA_2	DSM2041_01179	ypdA_2
ypdA_2	DSM2041_01859	ypdA_2
ypdA_2	DSM2041_04881	ypdA_2
ypdA_2	DSM2041_01630	ypdA_2
ypdA_2	DSM2041_01577	ypdA_2
ypdA_2	DSM2041_05431	ypdA_2
ypdA_2	DSM2041_00567	ypdA_2
ypdA_2	DSM2041_01835	ypdA_2
ypdA_2	DSM2041_02654	ypdA_2
ypdA_2	DSM2041_01744	ypdA_2
ypdA_2	DSM2041_04898	ypdA_2
ypdA_2	DSM2041_00300	ypdA_2
ypdA_2	DSM2041_01862	ypdA_2
ypdA_2	DSM2041_00477	ypdA_2
ypdA_2	DSM2041_03529	ypdA_2
ypdA_2	DSM2041_02627	ypdA_2
ypdA_2	DSM2041_01805	ypdA_2
ypdA_2	DSM2041_02551	ypdA_2
ypdA_2	DSM2041_01886	ypdA_2
ypdA_2	DSM2041_04195	ypdA_2
ypdA_2	DSM2041_01793	ypdA_2
ypdA_2	DSM2041_02232	ypdA_2
ypdA_2	DSM2041_02702	ypdA_2
ypdA_2	DSM2041_01740	ypdA_2
ypdA_2	DSM2041_00093	ypdA_2
ypdA_2	DSM2041_04696	ypdA_2
ypdA_2	DSM2041_02554	ypdA_2
ypdA_2	DSM2041_02555	ypdA_2
ypdA_2	DSM2041_02556	ypdA_2
ypdA_2	DSM2041_02552	ypdA_2
ypdA_2	DSM2041_02553	ypdA_2
ypdA_2	DSM2061_00415	ypdA_2
ypdA_2	DSM2061_00939	ypdA_2
ypdA_2	DSM2061_03980	ypdA_2
ypdA_2	DSM2061_03971	ypdA_2
ypdA_2	DSM2061_03354	ypdA_2
ypdA_2	DSM2061_02028	ypdA_2
ypdA_2	DSM2061_03845	ypdA_2
ypdA_2	DSM2061_00416	ypdA_2
ypdA_2	DSM2061_00417	ypdA_2
ypdA_2	DSM2064_01987	ypdA_2

ypdA_2	DSM2064_04426	ypdA_2
ypdA_2	DSM2064_01308	ypdA_2
ypdA_2	DSM2064_00660	ypdA_2
ypdA_2	DSM2064_01341	ypdA_2
ypdA_2	DSM2064_04512	ypdA_2
ypdA_2	DSM2064_01174	ypdA_2
ypdA_2	DSM2064_04133	ypdA_2
ypdA_2	DSM2064_03643	ypdA_2
ypdA_2	DSM2064_02861	ypdA_2
ypdA_2	DSM2064_03527	ypdA_2
ypdA_2	DSM2064_02697	ypdA_2
ypdA_2	DSM2064_03764	ypdA_2
ypdA_2	DSM2064_04022	ypdA_2
ypdA_2	DSM2064_01333	ypdA_2
ypdA_2	DSM2064_03765	ypdA_2
ypdA_2	DSM2064_05171	ypdA_2
ypdA_2	DSM2064_03768	ypdA_2
ypdA_2	DSM2064_03767	ypdA_2
ypdA_2	DSM2064_04203	ypdA_2
ypdA_2	DSM21164_02247	ypdA_2
ypdA_2	DSM21164_02248	ypdA_2
ypdA_2	DSM21164_00812	ypdA_2
ypdA_2	DSM21164_02455	ypdA_2
ypdA_2	DSM21164_02598	ypdA_2
ypdA_2	DSM21164_01522	ypdA_2
ypdA_2	DSM21164_00643	ypdA_2
ypdA_2	DSM21164_00283	ypdA_2
ypdA_2	DSM21164_02452	ypdA_2
ypdA_2	DSM21164_02453	ypdA_2
ypdA_2	DSM21164_02454	ypdA_2
ypdA_2	DSM23404_02720	ypdA_2
ypdA_2	DSM23404_02188	ypdA_2
ypdA_2	DSM23404_02769	ypdA_2
ypdA_2	DSM23404_00544	ypdA_2
ypdA_2	DSM23404_02770	ypdA_2
ypdA_2	DSM23424_03360	ypdA_2
ypdA_2	DSM23424_03084	ypdA_2
ypdA_2	DSM23424_01127	ypdA_2
ypdA_2	DSM23424_01566	ypdA_2
ypdA_2	DSM23424_01515	ypdA_2
ypdA_2	DSM23424_01940	ypdA_2
ypdA_2	DSM23424_03294	ypdA_2
ypdA_2	DSM23424_00711	ypdA_2
ypdA_2	DSM23424_01513	ypdA_2
ypdA_2	DSM23424_01514	ypdA_2
ypdA_2	DSM24786_01791	ypdA_2
ypdA_2	DSM24786_00548	ypdA_2
ypdA_2	DSM24786_01552	ypdA_2

ypdA_2	DSM24786_00500	ypdA_2
ypdA_2	DSM24786_00149	ypdA_2
ypdA_2	DSM24786_02891	ypdA_2
ypdA_2	DSM24786_00757	ypdA_2
ypdA_2	DSM24786_00079	ypdA_2
ypdA_2	DSM24786_02201	ypdA_2
ypdA_2	DSM24786_03486	ypdA_2
ypdA_2	DSM24786_03049	ypdA_2
ypdA_2	DSM24786_02030	ypdA_2
ypdA_2	DSM24786_00152	ypdA_2
ypdA_2	DSM24786_00151	ypdA_2
ypdA_2	DSM24786_00150	ypdA_2
ypdA_2	DSM7489_02154	ypdA_2
ypdA_2	DSM7489_02372	ypdA_2
ypdA_2	DSM7489_02897	ypdA_2
ypdA_2	DSM7489_02563	ypdA_2
ypdA_2	DSM7489_03287	ypdA_2
ypdA_2	DSM7489_00736	ypdA_2
ypdA_2	DSM7489_01319	ypdA_2
ypdA_2	DSM7489_03126	ypdA_2
ypdA_2	DSM7489_00072	ypdA_2
ypdA_2	DSM7489_00161	ypdA_2
ypdA_2	DSM7489_01463	ypdA_2
ypdA_2	DSM7489_01700	ypdA_2
ypdA_2	DSM7489_01931	ypdA_2
ypdA_2	DSM7489_02566	ypdA_2
ypdA_2	DSM7489_02565	ypdA_2
ypdA_2	DSM7489_02564	ypdA_2
ypdA_2	DSW1_00916	ypdA_2
ypdA_2	DSW1_02713	ypdA_2
ypdA_2	DSW1_01737	ypdA_2
ypdA_2	DSW1_00311	ypdA_2
ypdA_2	DSW1_00351	ypdA_2
ypdA_2	DSW1_02280	ypdA_2
ypdA_2	DSW1_02282	ypdA_2
ypdA_2	DSW1_02281	ypdA_2
ypdA_2	DSW5_02702	ypdA_2
ypdA_2	DSW5_02675	ypdA_2
ypdA_2	DSW5_00367	ypdA_2
ypdA_2	DSW5_02687	ypdA_2
ypdA_2	DSW5_01280	ypdA_2
ypdA_2	DSW5_02026	ypdA_2
ypdA_2	DSW5_00705	ypdA_2
ypdA_2	DSW5_00788	ypdA_2
ypdA_2	DSW5_02329	ypdA_2
ypdA_2	DSW5_02147	ypdA_2
ypdA_2	DSW5_02326	ypdA_2
ypdA_2	DSW5_02328	ypdA_2

ypdA_2	DSW5_02027	ypdA_2
ypdA_2	DSW5_02327	ypdA_2
ypdA_2	DSW6_00379	ypdA_2
ypdA_2	DSW6_00912	ypdA_2
ypdA_2	DSW6_00743	ypdA_2
ypdA_2	DSW6_03222	ypdA_2
ypdA_2	DSW6_00376	ypdA_2
ypdA_2	DSW6_02424	ypdA_2
ypdA_2	DSW6_01221	ypdA_2
ypdA_2	DSW6_03220	ypdA_2
ypdA_2	DSW6_03221	ypdA_2
ypdA_2	DSW8_03279	ypdA_2
ypdA_2	DSW8_01718	ypdA_2
ypdA_2	DSW8_03514	ypdA_2
ypdA_2	DSW8_01780	ypdA_2
ypdA_2	DSW8_00312	ypdA_2
ypdA_2	DSW8_00735	ypdA_2
ypdA_2	DSW8_00319	ypdA_2
ypdA_2	DSW8_02842	ypdA_2
ypdA_2	DSW8_01627	ypdA_2
ypdA_2	DSW8_02089	ypdA_2
ypdA_2	DSW8_00730	ypdA_2
ypdA_2	DSW8_00731	ypdA_2
ypdA_2	DSW8_00733	ypdA_2
ypdA_2	DSW8_00732	ypdA_2
ypdA_2	F52_03607	ypdA_2
ypdA_2	F52_00979	ypdA_2
ypdA_2	F52_04286	ypdA_2
ypdA_2	F52_00903	ypdA_2
ypdA_2	F52_00990	ypdA_2
ypdA_2	F52_03445	ypdA_2
ypdA_2	F52_03503	ypdA_2
ypdA_2	F52_03796	ypdA_2
ypdA_2	F52_00149	ypdA_2
ypdA_2	F52_03382	ypdA_2
ypdA_2	F52_03912	ypdA_2
ypdA_2	F52_03245	ypdA_2
ypdA_2	F52_04538	ypdA_2
ypdA_2	F52_04182	ypdA_2
ypdA_2	F52_02535	ypdA_2
ypdA_2	F52_03913	ypdA_2
ypdA_2	F52_02041	ypdA_2
ypdA_2	F52_03915	ypdA_2
ypdA_2	F52_03916	ypdA_2
ypdA_2	GCA_001027945.1_00945	ypdA_2
ypdA_2	GCA_001027945.1_01833	ypdA_2
ypdA_2	GCA_001027945.1_00011	ypdA_2
ypdA_2	GCA_001027945.1_00286	ypdA_2

ypdA_2	GCA_001027945.1_02343	ypdA_2
ypdA_2	GCA_001027945.1_02777	ypdA_2
ypdA_2	GCA_001027945.1_01299	ypdA_2
ypdA_2	GCA_001027945.1_02455	ypdA_2
ypdA_2	GCA_001027945.1_02167	ypdA_2
ypdA_2	GCA_001027945.1_01105	ypdA_2
ypdA_2	GCA_001027945.1_01496	ypdA_2
ypdA_2	GCA_001027945.1_00158	ypdA_2
ypdA_2	GCA_001027945.1_02780	ypdA_2
ypdA_2	GCA_001027945.1_02781	ypdA_2
ypdA_2	GCA_001027945.1_02778	ypdA_2
ypdA_2	GCA_001027945.1_02779	ypdA_2
ypdA_2	HI1_02080	ypdA_2
ypdA_2	HI1_02902	ypdA_2
ypdA_2	HI1_02305	ypdA_2
ypdA_2	HI1_02836	ypdA_2
ypdA_2	HI1_02496	ypdA_2
ypdA_2	HI1_03230	ypdA_2
ypdA_2	HI1_01259	ypdA_2
ypdA_2	HI1_03075	ypdA_2
ypdA_2	HI1_00002	ypdA_2
ypdA_2	HI1_03371	ypdA_2
ypdA_2	HI1_00093	ypdA_2
ypdA_2	HI1_01404	ypdA_2
ypdA_2	HI1_01621	ypdA_2
ypdA_2	HI1_01859	ypdA_2
ypdA_2	HI1_02499	ypdA_2
ypdA_2	HI1_02498	ypdA_2
ypdA_2	HI1_02497	ypdA_2
ypdA_2	HI1_00001	ypdA_2
ypdA_2	IR1_00124	ypdA_2
ypdA_2	IR1_03564	ypdA_2
ypdA_2	IR1_03349	ypdA_2
ypdA_2	IR1_03352	ypdA_2
ypdA_2	IR1_03787	ypdA_2
ypdA_2	IR1_01319	ypdA_2
ypdA_2	IR1_01329	ypdA_2
ypdA_2	IR1_01437	ypdA_2
ypdA_2	IR1_03257	ypdA_2
ypdA_2	IR1_04452	ypdA_2
ypdA_2	IR1_00028	ypdA_2
ypdA_2	IR1_00194	ypdA_2
ypdA_2	IR1_04577	ypdA_2
ypdA_2	IR1_04013	ypdA_2
ypdA_2	IR1_01922	ypdA_2
ypdA_2	IR1_03717	ypdA_2
ypdA_2	IR1_03650	ypdA_2
ypdA_2	IR1_01185	ypdA_2

ypdA_2	IR1_01439	ypdA_2
ypdA_2	IR1_01438	ypdA_2
ypdA_2	IR1_01059	ypdA_2
ypdA_2	KCTC12974_02625	ypdA_2
ypdA_2	KCTC12974_01179	ypdA_2
ypdA_2	KCTC12974_02689	ypdA_2
ypdA_2	KCTC12974_01134	ypdA_2
ypdA_2	KCTC12974_02690	ypdA_2
ypdA_2	KCTC12974_02624	ypdA_2
ypdA_2	KL-A_00117	ypdA_2
ypdA_2	KL-A_00555	ypdA_2
ypdA_2	KL-A_01652	ypdA_2
ypdA_2	KL-A_00619	ypdA_2
ypdA_2	KL-A_02358	ypdA_2
ypdA_2	KL-A_00992	ypdA_2
ypdA_2	KL-A_01906	ypdA_2
ypdA_2	KL-A_03382	ypdA_2
ypdA_2	KL-A_02737	ypdA_2
ypdA_2	KL-A_03412	ypdA_2
ypdA_2	KL-A_01157	ypdA_2
ypdA_2	KL-A_00763	ypdA_2
ypdA_2	KL-A_01502	ypdA_2
ypdA_2	KL-A_00340	ypdA_2
ypdA_2	KL-A_00989	ypdA_2
ypdA_2	KL-A_00990	ypdA_2
ypdA_2	KL-A_00991	ypdA_2
ypdA_2	L12_00197	ypdA_2
ypdA_2	LL0412.1.7_02148	ypdA_2
ypdA_2	LL0412.1.7_01417	ypdA_2
ypdA_2	LL0412.1.7_00380	ypdA_2
ypdA_2	LL0412.1.7_02665	ypdA_2
ypdA_2	LL0412.1.7_00417	ypdA_2
ypdA_2	LL0412.1.7_02226	ypdA_2
ypdA_2	LL0412.1.7_00048	ypdA_2
ypdA_2	LL0412.1.7_02310	ypdA_2
ypdA_2	LL0412.1.7_01476	ypdA_2
ypdA_2	LL0412.1.7_01581	ypdA_2
ypdA_2	LL0412.1.7_02384	ypdA_2
ypdA_2	LL0412.1.7_02585	ypdA_2
ypdA_2	LL0412.1.7_00848	ypdA_2
ypdA_2	LL0412.1.7_00913	ypdA_2
ypdA_2	LL0412.1.7_00016	ypdA_2
ypdA_2	LL0412.1.7_01073	ypdA_2
ypdA_2	LL0412.1.7_00354	ypdA_2
ypdA_2	LL0412.1.7_01473	ypdA_2
ypdA_2	LL0412.1.7_01474	ypdA_2
ypdA_2	LL0412.1.7_01472	ypdA_2
ypdA_2	LL0412.1.7_01475	ypdA_2

ypdA_2	MAR_2009_71_03643	ypdA_2
ypdA_2	MAR_2009_71_03829	ypdA_2
ypdA_2	MAR_2009_71_03832	ypdA_2
ypdA_2	MAR_2009_71_01699	ypdA_2
ypdA_2	MAR_2009_71_00072	ypdA_2
ypdA_2	MAR_2009_71_01640	ypdA_2
ypdA_2	MAR_2009_71_03115	ypdA_2
ypdA_2	MAR_2009_71_03531	ypdA_2
ypdA_2	MAR_2009_71_03121	ypdA_2
ypdA_2	MAR_2009_71_00612	ypdA_2
ypdA_2	MAR_2009_71_01798	ypdA_2
ypdA_2	MAR_2009_71_01341	ypdA_2
ypdA_2	MAR_2009_71_03526	ypdA_2
ypdA_2	MAR_2009_71_03527	ypdA_2
ypdA_2	MAR_2009_71_03529	ypdA_2
ypdA_2	MAR_2009_71_03528	ypdA_2
ypdA_2	NN016038_00254	ypdA_2
ypdA_2	NN016038_00255	ypdA_2
ypdA_2	NN016038_03361	ypdA_2
ypdA_2	NN016038_03821	ypdA_2
ypdA_2	NN016038_01012	ypdA_2
ypdA_2	NN016038_03458	ypdA_2
ypdA_2	NN016038_03398	ypdA_2
ypdA_2	NN016038_02907	ypdA_2
ypdA_2	NN016038_01600	ypdA_2
ypdA_2	NN016038_01754	ypdA_2
ypdA_2	NN016038_01253	ypdA_2
ypdA_2	NN016038_03818	ypdA_2
ypdA_2	NN016038_03819	ypdA_2
ypdA_2	NN016038_03820	ypdA_2
ypdA_2	PAO1_05439	ypdA_2
ypdA_2	UW101_01042	ypdA_2
ypdA_2	UW101_04460	ypdA_2
ypdA_2	UW101_04171	ypdA_2
ypdA_2	UW101_03371	ypdA_2
ypdA_2	UW101_04138	ypdA_2
ypdA_2	UW101_02125	ypdA_2
ypdA_2	UW101_04305	ypdA_2
ypdA_2	UW101_04753	ypdA_2
ypdA_2	UW101_05243	ypdA_2
ypdA_2	UW101_01915	ypdA_2
ypdA_2	UW101_02273	ypdA_2
ypdA_2	UW101_01751	ypdA_2
ypdA_2	UW101_05122	ypdA_2
ypdA_2	UW101_04865	ypdA_2
ypdA_2	UW101_04146	ypdA_2
ypdA_2	UW101_05121	ypdA_2
ypdA_2	UW101_00611	ypdA_2

ypdA_2	UW101_05118	ypdA_2
ypdA_2	UW101_05119	ypdA_2
ypdA_2	UW101_04682	ypdA_2
ypdA_2	V4-33_01558	ypdA_2
ypdA_2	V4-33_01426	ypdA_2
ypdA_2	V4-33_01556	ypdA_2
ypdA_2	V4-33_01555	ypdA_2
ypdA_2	W5C_02905	ypdA_2
ypdA_2	W5C_01854	ypdA_2
ypdA_2	W5C_03363	ypdA_2
ypdA_2	W5C_01066	ypdA_2
ypdA_2	W5C_02146	ypdA_2
ypdA_2	W5C_01585	ypdA_2
ypdA_2	W5C_02578	ypdA_2
ypdA_2	W5C_01700	ypdA_2
ypdA_2	W5C_02329	ypdA_2
ypdA_2	W5C_00070	ypdA_2
ypdA_2	W5C_00396	ypdA_2
ypdA_2	W5C_01160	ypdA_2
ypdA_2	W5C_00618	ypdA_2
ypdA_2	W5C_01069	ypdA_2
ypdA_2	W5C_01068	ypdA_2
ypdA_2	W5C_01067	ypdA_2
ypdA_2	ZONMW-01_01432	ypdA_2
ypdA_2	ZONMW-01_01613	ypdA_2
ypdA_2	ZONMW-01_02550	ypdA_2
ypdA_2	ZONMW-01_00891	ypdA_2
ypdA_2	ZONMW-01_00948	ypdA_2
ypdA_2	ZONMW-01_02048	ypdA_2
ypdA_2	ZONMW-01_00646	ypdA_2
ypdA_2	ZONMW-01_00147	ypdA_2
ypdA_2	ZONMW-01_02460	ypdA_2
ypdA_2	ZONMW-01_00384	ypdA_2
ypdA_2	ZONMW-01_01616	ypdA_2
ypdA_2	ZONMW-01_01615	ypdA_2
ypdA_2	ZONMW-01_01614	ypdA_2
ypdA_2	ZONMW-02_00402	ypdA_2
ypdA_2	ZONMW-02_03450	ypdA_2
ypdA_2	ZONMW-02_02114	ypdA_2
ypdA_2	ZONMW-02_03224	ypdA_2
ypdA_2	ZONMW-02_00024	ypdA_2
ypdA_2	ZONMW-02_01490	ypdA_2
ypdA_2	ZONMW-02_00235	ypdA_2
ypdA_2	ZONMW-02_01654	ypdA_2
ypdA_2	ZONMW-02_02886	ypdA_2
ypdA_2	ZONMW-02_02398	ypdA_2
ypdA_2	ZONMW-02_02289	ypdA_2
ypdA_2	ZONMW-02_03223	ypdA_2

ypdA_2	ZONMW-03_01348	ypdA_2
ypdA_2	ZONMW-03_00594	ypdA_2
ypdA_2	ZONMW-03_01831	ypdA_2
ypdA_2	ZONMW-03_00269	ypdA_2
ypdA_2	ZONMW-03_02017	ypdA_2
ypdA_2	ZONMW-03_02663	ypdA_2
ypdA_2	ZONMW-03_00024	ypdA_2
ypdA_2	ZONMW-03_00873	ypdA_2
ypdA_2	ZONMW-03_03063	ypdA_2
ypdA_2	ZONMW-03_01110	ypdA_2
ypdA_2	ZONMW-03_00591	ypdA_2
ypdA_2	ZONMW-03_00592	ypdA_2
ypdA_2	ZONMW-03_00593	ypdA_2
ypdA_2	ZONMW-04_00345	ypdA_2
ypdA_2	ZONMW-04_00109	ypdA_2
ypdA_2	ZONMW-04_01532	ypdA_2
ypdA_2	ZONMW-04_03038	ypdA_2
ypdA_2	ZONMW-04_03072	ypdA_2
ypdA_2	ZONMW-04_01806	ypdA_2
ypdA_2	ZONMW-04_01891	ypdA_2
ypdA_2	ZONMW-04_01036	ypdA_2
ypdA_2	ZONMW-04_02382	ypdA_2
ypdA_2	ZONMW-04_02073	ypdA_2
ypdA_2	ZONMW-04_00562	ypdA_2
ypdA_2	ZONMW-04_01529	ypdA_2
ypdA_2	ZONMW-04_01530	ypdA_2
ypdA_2	ZONMW-04_01531	ypdA_2
ypdA_2	ZONMW-05_01480	ypdA_2
ypdA_2	ZONMW-05_01721	ypdA_2
ypdA_2	ZONMW-05_00856	ypdA_2
ypdA_2	ZONMW-05_00527	ypdA_2
ypdA_2	ZONMW-05_02081	ypdA_2
ypdA_2	ZONMW-05_02479	ypdA_2
ypdA_2	ZONMW-05_00284	ypdA_2
ypdA_2	ZONMW-05_01062	ypdA_2
ypdA_2	ZONMW-05_02642	ypdA_2
ypdA_2	ZONMW-05_01263	ypdA_2
ypdA_2	ZONMW-05_00853	ypdA_2
ypdA_2	ZONMW-05_00854	ypdA_2
ypdA_2	ZONMW-05_00855	ypdA_2
ypdA_2	ZONMW-06_02566	ypdA_2
ypdA_2	ZONMW-06_01859	ypdA_2
ypdA_2	ZONMW-07_03163	ypdA_2
ypdA_2	ZONMW-07_00139	ypdA_2
ypdA_2	ZONMW-07_01846	ypdA_2
ypdA_2	ZONMW-07_00123	ypdA_2
ypdA_2	ZONMW-07_00146	ypdA_2
ypdA_2	ZONMW-07_00815	ypdA_2

ypdA_2	ZONMW-07_00389	ypdA_2
ypdA_2	ZONMW-07_01364	ypdA_2
ypdA_2	ZONMW-07_01849	ypdA_2
ypdA_2	ZONMW-07_01848	ypdA_2
ypdA_2	ZONMW-07_01847	ypdA_2
ypdA_2	ZONMW-08_02272	ypdA_2
ypdA_2	ZONMW-08_01537	ypdA_2
ypdA_2	ZONMW-08_02979	ypdA_2
ypdA_2	ZONMW-08_01593	ypdA_2
ypdA_2	ZONMW-08_02631	ypdA_2
ypdA_2	ZONMW-08_02395	ypdA_2
ypdA_2	ZONMW-08_02637	ypdA_2
ypdA_2	ZONMW-08_00128	ypdA_2
ypdA_2	ZONMW-08_01426	ypdA_2
ypdA_2	ZONMW-08_01852	ypdA_2
ypdA_2	ZONMW-08_02400	ypdA_2
ypdA_2	ZONMW-08_02399	ypdA_2
ypdA_2	ZONMW-08_02397	ypdA_2
ypdA_2	ZONMW-08_02398	ypdA_2
ypdA_2	ZONMW-09_01527	ypdA_2
ypdA_2	ZONMW-09_00864	ypdA_2
ypdA_2	ZONMW-09_01583	ypdA_2
ypdA_2	ZONMW-09_00863	ypdA_2
ypdA_2	ZONMW-10_01843	ypdA_2
ypdA_2	ZONMW-11_02630	ypdA_2
ypdA_2	ZONMW-11_02479	ypdA_2
ypdA_2	ZONMW-11_04023	ypdA_2
ypdA_2	ZONMW-11_03785	ypdA_2
ypdA_2	ZONMW-11_03206	ypdA_2
ypdA_2	ZONMW-11_00469	ypdA_2
ypdA_2	ZONMW-11_00474	ypdA_2
ypdA_2	ZONMW-11_02907	ypdA_2
ypdA_2	ZONMW-12_04101	ypdA_2
ypdA_2	ZONMW-12_00010	ypdA_2
ypdA_2	ZONMW-12_01895	ypdA_2
ypdA_2	ZONMW-12_01205	ypdA_2
ypdA_2	ZONMW-12_01526	ypdA_2
ypdA_2	ZONMW-12_04126	ypdA_2
ypdA_2	ZONMW-12_00302	ypdA_2
ypdA_2	ZONMW-12_00813	ypdA_2
ypdA_2	ZONMW-12_01524	ypdA_2
ypdA_2	ZONMW-12_01525	ypdA_2
ypdA_2	ZONMW-13_02439	ypdA_2
ypdA_2	ZONMW-13_02278	ypdA_2
ypdA_2	ZONMW-13_02422	ypdA_2
ypdA_2	ZONMW-13_02277	ypdA_2
ypdA_2	ZONMW-14_00649	ypdA_2
ypdA_2	ZONMW-14_02555	ypdA_2

ypdA_2	ZONMW-14_02724	ypdA_2
ypdA_2	ZONMW-14_01983	ypdA_2
ypdA_2	ZONMW-14_02199	ypdA_2
ypdA_2	ZONMW-14_02196	ypdA_2
ypdA_2	ZONMW-14_02197	ypdA_2
ypdA_2	ZONMW-14_02198	ypdA_2
ypdA_2	ZONMW-15_00805	ypdA_2
ypdA_2	ZONMW-15_00806	ypdA_2
ypdA_2	ZONMW-15_02888	ypdA_2
ypdA_2	ZONMW-15_02602	ypdA_2
ypdA_2	ZONMW-15_01095	ypdA_2
ypdA_2	ZONMW-15_00447	ypdA_2
ypdA_2	ZONMW-15_00079	ypdA_2
ypdA_2	ZONMW-15_03131	ypdA_2
ypdA_2	ZONMW-15_02885	ypdA_2
ypdA_2	ZONMW-15_02886	ypdA_2
ypdA_2	ZONMW-15_02887	ypdA_2
ypdA_2	ZONMW-19_01749	ypdA_2
ypdA_2	ZONMW-19_00425	ypdA_2
ypdA_2	ZONMW-20_01981	ypdA_2
ypdA_2	ZONMW-20_02153	ypdA_2
ypdA_2	ZONMW-21_02495	ypdA_2
ypdA_2	ZONMW-21_00540	ypdA_2
ypdA_2	ZONMW-21_03143	ypdA_2
ypdA_2	ZONMW-21_02396	ypdA_2
ypdA_2	ZONMW-21_01173	ypdA_2
ypdA_2	ZONMW-21_01509	ypdA_2
ypdA_2	ZONMW-21_03175	ypdA_2
ypdA_2	ZONMW-21_00338	ypdA_2
ypdA_2	ZONMW-21_02995	ypdA_2
ypdA_2	ZONMW-21_00736	ypdA_2
ypdA_2	ZONMW-21_00737	ypdA_2
ypdA_2	ZONMW-21_02914	ypdA_2
ypdA_2	ZONMW-21_03433	ypdA_2
ypdA_2	ZONMW-21_00570	ypdA_2
ypdA_2	ZONMW-21_00624	ypdA_2
ypdA_2	ZONMW-21_00156	ypdA_2
ypdA_2	ZONMW-21_01511	ypdA_2
ypdA_2	ZONMW-21_01513	ypdA_2
ypdA_2	ZONMW-21_01512	ypdA_2
ypdA_2	ZONMW-21_01510	ypdA_2
ypdA_2	ZONMW-22_02614	ypdA_2
ypdA_2	ZONMW-22_00540	ypdA_2
ypdA_2	ZONMW-22_03168	ypdA_2
ypdA_2	ZONMW-22_02712	ypdA_2
ypdA_2	ZONMW-22_01175	ypdA_2
ypdA_2	ZONMW-22_02214	ypdA_2
ypdA_2	ZONMW-22_03200	ypdA_2

ypdA_2	ZONMW-22_00338	ypdA_2
ypdA_2	ZONMW-22_02829	ypdA_2
ypdA_2	ZONMW-22_00737	ypdA_2
ypdA_2	ZONMW-22_00738	ypdA_2
ypdA_2	ZONMW-22_02910	ypdA_2
ypdA_2	ZONMW-22_03446	ypdA_2
ypdA_2	ZONMW-22_00570	ypdA_2
ypdA_2	ZONMW-22_00624	ypdA_2
ypdA_2	ZONMW-22_00156	ypdA_2
ypdA_2	ZONMW-22_02212	ypdA_2
ypdA_2	ZONMW-22_02210	ypdA_2
ypdA_2	ZONMW-22_02211	ypdA_2
ypdA_2	ZONMW-22_02213	ypdA_2
ypdA_2	ZONMW-23_02638	ypdA_2
ypdA_2	ZONMW-23_01655	ypdA_2
ypdA_2	ZONMW-23_03189	ypdA_2
ypdA_2	ZONMW-23_02736	ypdA_2
ypdA_2	ZONMW-23_02287	ypdA_2
ypdA_2	ZONMW-23_00337	ypdA_2
ypdA_2	ZONMW-23_03221	ypdA_2
ypdA_2	ZONMW-23_01453	ypdA_2
ypdA_2	ZONMW-23_03041	ypdA_2
ypdA_2	ZONMW-23_01851	ypdA_2
ypdA_2	ZONMW-23_01852	ypdA_2
ypdA_2	ZONMW-23_02960	ypdA_2
ypdA_2	ZONMW-23_03440	ypdA_2
ypdA_2	ZONMW-23_01685	ypdA_2
ypdA_2	ZONMW-23_01739	ypdA_2
ypdA_2	ZONMW-23_01271	ypdA_2
ypdA_2	ZONMW-23_00339	ypdA_2
ypdA_2	ZONMW-23_00341	ypdA_2
ypdA_2	ZONMW-23_00340	ypdA_2
ypdA_2	ZONMW-23_00338	ypdA_2
ypdA_2	ZONMW-24_02519	ypdA_2
ypdA_2	ZONMW-24_00919	ypdA_2
ypdA_2	ZONMW-24_03190	ypdA_2
ypdA_2	ZONMW-24_02420	ypdA_2
ypdA_2	ZONMW-24_00286	ypdA_2
ypdA_2	ZONMW-24_02839	ypdA_2
ypdA_2	ZONMW-24_03222	ypdA_2
ypdA_2	ZONMW-24_01121	ypdA_2
ypdA_2	ZONMW-24_02852	ypdA_2
ypdA_2	ZONMW-24_00724	ypdA_2
ypdA_2	ZONMW-24_00723	ypdA_2
ypdA_2	ZONMW-24_02933	ypdA_2
ypdA_2	ZONMW-24_03442	ypdA_2
ypdA_2	ZONMW-24_00889	ypdA_2
ypdA_2	ZONMW-24_00836	ypdA_2

ypdA_2	ZONMW-24_01304	ypdA_2
ypdA_2	ZONMW-24_02837	ypdA_2
ypdA_2	ZONMW-24_02835	ypdA_2
ypdA_2	ZONMW-24_02836	ypdA_2
ypdA_2	ZONMW-24_02838	ypdA_2
ypdA_2	ZONMW-25_03169	ypdA_2
ypdA_2	ZONMW-25_02398	ypdA_2
ypdA_2	ZONMW-25_02758	ypdA_2
ypdA_2	ZONMW-25_00054	ypdA_2
ypdA_2	ZONMW-25_02207	ypdA_2
ypdA_2	ZONMW-25_00024	ypdA_2
ypdA_2	ZONMW-25_00468	ypdA_2
ypdA_2	ZONMW-25_00079	ypdA_2
ypdA_2	ZONMW-25_02616	ypdA_2
ypdA_2	ZONMW-25_02523	ypdA_2
ypdA_2	ZONMW-25_01668	ypdA_2
ypdA_2	ZONMW-25_02302	ypdA_2
ypdA_2	ZONMW-25_00394	ypdA_2
ypdA_2	ZONMW-25_01581	ypdA_2
ypdA_2	ZONMW-25_03511	ypdA_2
ypdA_2	ZONMW-25_03510	ypdA_2
ypdA_2	ZONMW-25_03512	ypdA_2
ypdA_2	ZONMW-25_03513	ypdA_2
ypdA_2	ZONMW-25_03514	ypdA_2
ypdA_2	ZONMW-26_01789	ypdA_2
ypdA_2	ZONMW-26_00104	ypdA_2
ypdA_2	ZONMW-26_02018	ypdA_2
ypdA_2	ZONMW-26_00440	ypdA_2
ypdA_2	ZONMW-26_02316	ypdA_2
ypdA_2	ZONMW-26_02637	ypdA_2
ypdA_2	ZONMW-26_00684	ypdA_2
ypdA_2	ZONMW-26_01106	ypdA_2
ypdA_2	ZONMW-26_03080	ypdA_2
ypdA_2	ZONMW-26_01317	ypdA_2
ypdA_2	ZONMW-26_00107	ypdA_2
ypdA_2	ZONMW-26_00106	ypdA_2
ypdA_2	ZONMW-26_00105	ypdA_2
ypdA_2	ZONMW-27_00339	ypdA_2
ypdA_2	ZONMW-27_00106	ypdA_2
ypdA_2	ZONMW-27_01772	ypdA_2
ypdA_2	ZONMW-27_02911	ypdA_2
ypdA_2	ZONMW-27_01351	ypdA_2
ypdA_2	ZONMW-27_01491	ypdA_2
ypdA_2	ZONMW-27_02062	ypdA_2
ypdA_2	ZONMW-27_01108	ypdA_2
ypdA_2	ZONMW-27_02799	ypdA_2
ypdA_2	ZONMW-27_00766	ypdA_2
ypdA_2	ZONMW-27_00556	ypdA_2

ypdA_2	ZONMW-27_01775	ypdA_2
ypdA_2	ZONMW-27_01774	ypdA_2
ypdA_2	ZONMW-27_01773	ypdA_2
ypdA_2	ZONMW-28_02723	ypdA_2
ypdA_2	ZONMW-29_02745	ypdA_2
ypdA_2	ZONMW-29_03318	ypdA_2
ypdA_2	ZONMW-29_00223	ypdA_2
ypdA_2	ZONMW-29_03309	ypdA_2
ypdA_2	ZONMW-29_02252	ypdA_2
ypdA_2	ZONMW-29_01996	ypdA_2
ypdA_2	ZONMW-29_00948	ypdA_2
ypdA_2	ZONMW-29_00075	ypdA_2
ypdA_2	ZONMW-29_03327	ypdA_2
ypdA_2	ZONMW-29_00379	ypdA_2
ypdA_2	ZONMW-29_01346	ypdA_2
ypdA_2	ZONMW-29_00302	ypdA_2
ypdA_2	ZONMW-29_03053	ypdA_2
ypdA_2	ZONMW-29_02000	ypdA_2
ypdA_2	ZONMW-29_01999	ypdA_2
ypdA_2	ZONMW-29_01998	ypdA_2
ypdA_2	ZONMW-29_01997	ypdA_2
ypdA_2	ZONMW-30_00053	ypdA_2
ypdA_2	ZONMW-30_03390	ypdA_2
ypdA_2	ZONMW-30_03382	ypdA_2
ypdA_2	ZONMW-30_01665	ypdA_2
ypdA_2	ZONMW-30_01939	ypdA_2
ypdA_2	ZONMW-30_02033	ypdA_2
ypdA_2	ZONMW-30_03135	ypdA_2
ypdA_2	ZONMW-30_00193	ypdA_2
ypdA_2	ZONMW-30_02328	ypdA_2
ypdA_2	ZONMW-30_00766	ypdA_2
ypdA_2	ZONMW-30_02693	ypdA_2
ypdA_2	ZONMW-30_02417	ypdA_2
ypdA_2	ZONMW-30_02694	ypdA_2
ypdA_2	ZONMW-30_02037	ypdA_2
ypdA_2	ZONMW-30_02038	ypdA_2
ypdA_2	ZONMW-30_02036	ypdA_2
ypdA_2	ZONMW-30_02035	ypdA_2
ypdA_2	ZONMW-30_02034	ypdA_2
ypdA_2	ZONMW-31_01988	ypdA_2
ypdA_2	ZONMW-32_03153	ypdA_2
ypdA_2	ZONMW-33_03121	ypdA_2
ypdA_2	ZONMW-34_02535	ypdA_2
ypdA_2	ZONMW-35_02499	ypdA_2
ypdA_2	ZONMW-36_02881	ypdA_2
ypdA_2	ZONMW-37_02765	ypdA_2
ypdA_2	ZONMW-37_00540	ypdA_2
ypdA_2	ZONMW-37_03177	ypdA_2

ypdA_2	ZONMW-37_02666	ypdA_2
ypdA_2	ZONMW-37_01189	ypdA_2
ypdA_2	ZONMW-37_01887	ypdA_2
ypdA_2	ZONMW-37_03209	ypdA_2
ypdA_2	ZONMW-37_00338	ypdA_2
ypdA_2	ZONMW-37_02839	ypdA_2
ypdA_2	ZONMW-37_00736	ypdA_2
ypdA_2	ZONMW-37_00737	ypdA_2
ypdA_2	ZONMW-37_02920	ypdA_2
ypdA_2	ZONMW-37_03455	ypdA_2
ypdA_2	ZONMW-37_00570	ypdA_2
ypdA_2	ZONMW-37_00624	ypdA_2
ypdA_2	ZONMW-37_00155	ypdA_2
ypdA_2	ZONMW-37_01889	ypdA_2
ypdA_2	ZONMW-37_01891	ypdA_2
ypdA_2	ZONMW-37_01890	ypdA_2
ypdA_2	ZONMW-37_01888	ypdA_2
ypdA_2	ZONMW-38_01032	ypdA_2
ypdA_2	ZONMW-38_00440	ypdA_2
ypdA_2	ZONMW-39_02077	ypdA_2
ypdA_2	ZONMW-40_02138	ypdA_2
ypdA_2	ZONMW-40_03248	ypdA_2
ypdA_2	ZONMW-40_02027	ypdA_2
ypdA_2	ZONMW-40_01407	ypdA_2
ypdA_2	ZONMW-40_00867	ypdA_2
ypdA_2	ZONMW-40_03295	ypdA_2
ypdA_2	ZONMW-40_01246	ypdA_2
ypdA_2	ZONMW-40_01266	ypdA_2
ypdA_2	ZONMW-40_00464	ypdA_2
ypdA_2	ZONMW-40_02564	ypdA_2
ypdA_2	ZONMW-40_00481	ypdA_2
ypdA_2	ZONMW-40_01339	ypdA_2
ypdA_2	ZONMW-40_03290	ypdA_2
ypdA_2	ZONMW-40_03291	ypdA_2
ypdA_2	ZONMW-40_03292	ypdA_2
ypdA_2	ZONMW-40_03293	ypdA_2
ypdA_2	ZONMW-40_03294	ypdA_2
ypdA_2	ZONMW-40_03352	ypdA_2
ypdA_2	ZONMW-41_01453	ypdA_2
ypdA_2	ZONMW-41_01452	ypdA_2
ypdA_2	ZONMW-41_02386	ypdA_2
ypdA_2	ZONMW-42_01140	ypdA_2
ypdA_2	ZONMW-43_01162	ypdA_2
ypdA_2	ZONMW-44_03443	ypdA_2
ypdA_2	ZONMW-44_00161	ypdA_2
ypdA_2	ZONMW-45_01054	ypdA_2
ypdA_2	ZONMW-45_01531	ypdA_2
ypdA_2	ZONMW-45_01304	ypdA_2

ypdA_2	ZONMW-45_02583	ypdA_2
ypdA_2	ZONMW-45_02751	ypdA_2
ypdA_2	ZONMW-45_00139	ypdA_2
ypdA_2	ZONMW-45_01132	ypdA_2
ypdA_2	ZONMW-45_01479	ypdA_2
ypdA_2	ZONMW-45_02668	ypdA_2
ypdA_2	ZONMW-45_00588	ypdA_2
ypdA_2	ZONMW-45_00143	ypdA_2
ypdA_2	ZONMW-45_00142	ypdA_2
ypdA_2	ZONMW-45_00140	ypdA_2
ypdA_2	ZONMW-45_00141	ypdA_2
ypdA_2	ZONMW-46_00613	ypdA_2
ypdA_2	ZONMW-46_02042	ypdA_2
ypdA_2	ZONMW-46_02688	ypdA_2
ypdA_2	ZONMW-46_02975	ypdA_2
ypdA_2	ZONMW-46_01160	ypdA_2
ypdA_2	ZONMW-46_00354	ypdA_2
ypdA_2	ZONMW-46_00535	ypdA_2
ypdA_2	ZONMW-46_01734	ypdA_2
ypdA_2	ZONMW-46_02531	ypdA_2
ypdA_2	ZONMW-46_00871	ypdA_2
ypdA_2	ZONMW-46_00358	ypdA_2
ypdA_2	ZONMW-46_00357	ypdA_2
ypdA_2	ZONMW-46_00355	ypdA_2
ypdA_2	ZONMW-46_00356	ypdA_2
ypdA_2	ZONMW-47_00613	ypdA_2
ypdA_2	ZONMW-47_02019	ypdA_2
ypdA_2	ZONMW-47_02678	ypdA_2
ypdA_2	ZONMW-47_02898	ypdA_2
ypdA_2	ZONMW-47_01013	ypdA_2
ypdA_2	ZONMW-47_00151	ypdA_2
ypdA_2	ZONMW-47_00535	ypdA_2
ypdA_2	ZONMW-47_01755	ypdA_2
ypdA_2	ZONMW-47_02521	ypdA_2
ypdA_2	ZONMW-47_00871	ypdA_2
ypdA_2	ZONMW-47_00147	ypdA_2
ypdA_2	ZONMW-47_00148	ypdA_2
ypdA_2	ZONMW-47_00150	ypdA_2
ypdA_2	ZONMW-47_00149	ypdA_2
ypdA_2	ZONMW-48_02000	ypdA_2
ypdA_2	ZONMW-48_01999	ypdA_2
ypdA_2	ZONMW-48_01721	ypdA_2
ypdA_2	ZONMW-48_01705	ypdA_2
ypdA_2	ZONMW-48_01152	ypdA_2
ypdA_2	ZONMW-48_01712	ypdA_2
ypdA_2	ZONMW-48_00579	ypdA_2
ypdA_2	ZONMW-48_00510	ypdA_2
ypdA_2	ZONMW-48_01084	ypdA_2

ypdA_2	ZONMW-48_00740	ypdA_2
ypdA_2	ZONMW-48_02918	ypdA_2
ypdA_2	ZONMW-48_01155	ypdA_2
ypdA_2	ZONMW-48_01154	ypdA_2
ypdA_2	ZONMW-48_01153	ypdA_2
ypdA_2	ZONMW-49_00758	ypdA_2
ypdA_2	ZONMW-49_00537	ypdA_2
ypdA_2	ZONMW-49_00455	ypdA_2
ypdA_2	ZONMW-49_00114	ypdA_2
ypdA_2	ZONMW-49_02620	ypdA_2
ypdA_2	ZONMW-49_01815	ypdA_2
ypdA_2	ZONMW-49_02516	ypdA_2
ypdA_2	ZONMW-49_01133	ypdA_2
ypdA_2	ZONMW-49_00885	ypdA_2
ypdA_2	ZONMW-49_01473	ypdA_2
ypdA_2	ZONMW-49_00117	ypdA_2
ypdA_2	ZONMW-49_00116	ypdA_2
ypdA_2	ZONMW-49_00115	ypdA_2
ypdA_2	ZONMW-50_00759	ypdA_2
ypdA_2	ZONMW-50_00538	ypdA_2
ypdA_2	ZONMW-50_00030	ypdA_2
ypdA_2	ZONMW-50_00372	ypdA_2
ypdA_2	ZONMW-50_02414	ypdA_2
ypdA_2	ZONMW-50_01599	ypdA_2
ypdA_2	ZONMW-50_02310	ypdA_2
ypdA_2	ZONMW-50_01134	ypdA_2
ypdA_2	ZONMW-50_00886	ypdA_2
ypdA_2	ZONMW-50_01475	ypdA_2
ypdA_2	ZONMW-50_00369	ypdA_2
ypdA_2	ZONMW-50_00370	ypdA_2
ypdA_2	ZONMW-50_00371	ypdA_2
ypdA_2	ZONMW-51_00950	ypdA_2
ypdA_2	ZONMW-51_00511	ypdA_2
ypdA_2	ZONMW-51_03145	ypdA_2
ypdA_2	ZONMW-51_01168	ypdA_2
ypdA_2	ZONMW-51_00443	ypdA_2
ypdA_2	ZONMW-51_01765	ypdA_2
ypdA_2	ZONMW-51_00111	ypdA_2
ypdA_2	ZONMW-51_02680	ypdA_2
ypdA_2	ZONMW-51_02089	ypdA_2
ypdA_2	ZONMW-51_02493	ypdA_2
ypdA_2	ZONMW-51_02740	ypdA_2
ypdA_2	ZONMW-51_01362	ypdA_2
ypdA_2	ZONMW-51_00729	ypdA_2
ypdA_2	ZONMW-51_00114	ypdA_2
ypdA_2	ZONMW-51_00113	ypdA_2
ypdA_2	ZONMW-51_00112	ypdA_2
ypdA_2	ZONMW-52_01550	ypdA_2

ypdA_2	ZONMW-52_01974	ypdA_2
ypdA_2	ZONMW-52_02861	ypdA_2
ypdA_2	ZONMW-52_00427	ypdA_2
ypdA_2	ZONMW-52_01908	ypdA_2
ypdA_2	ZONMW-52_00083	ypdA_2
ypdA_2	ZONMW-52_02151	ypdA_2
ypdA_2	ZONMW-52_00680	ypdA_2
ypdA_2	ZONMW-52_02476	ypdA_2
ypdA_2	ZONMW-52_02306	ypdA_2
ypdA_2	ZONMW-52_01104	ypdA_2
ypdA_2	ZONMW-52_01329	ypdA_2
ypdA_2	ZONMW-52_00086	ypdA_2
ypdA_2	ZONMW-52_00085	ypdA_2
ypdA_2	ZONMW-52_00084	ypdA_2
ypdA_2	ZONMW-53_00277	ypdA_2
ypdA_2	ZONMW-53_00054	ypdA_2
ypdA_2	ZONMW-53_01159	ypdA_2
ypdA_2	ZONMW-53_01848	ypdA_2
ypdA_2	ZONMW-53_02571	ypdA_2
ypdA_2	ZONMW-53_02020	ypdA_2
ypdA_2	ZONMW-53_02688	ypdA_2
ypdA_2	ZONMW-53_01415	ypdA_2
ypdA_2	ZONMW-53_02157	ypdA_2
ypdA_2	ZONMW-53_02895	ypdA_2
[truncated: 566,185 more chars]
