# Supplementary material for: Structural color in the bacterial domain: The ecogenomics of a 2-dimensional optical phenotype
Source: Proc Natl Acad Sci U S A. 2024 Jul 11;121(29):e2309757121. doi: 10.1073/pnas.2309757121 (PMC11260094; doi:10.1073/pnas.2309757121)
Supplement: Supplementary file 8 — Dataset S02 (PDF) [file pnas.2309757121.sd02.pdf]

PATRIC_id	SC_score	Species	Genus	Family	Order	Class	Phylum	Superkingdom	CheckM_completenes	CheckM_contaminatio	PATRIC_coarse_consisten	PATRIC_fine_consistenc	Status
9.107	0.0294	Buchnera aphidicola (32199)	Buchnera (1903409)	Erwiniaceae (91347)	Enterobacterales (1236)	Gammaproteobacteria (122	Proteobacteria (2)	Bacteria (1)					Plasmid
9.108	0.03	Buchnera aphidicola (32199)	Buchnera (1903409)	Erwiniaceae (91347)	Enterobacterales (1236)	Gammaproteobacteria (122	Proteobacteria (2)	Bacteria (1)					Plasmid
9.109	0.0294	Buchnera aphidicola (32199)	Buchnera (1903409)	Erwiniaceae (91347)	Enterobacterales (1236)	Gammaproteobacteria (122	Proteobacteria (2)	Bacteria (1)					Plasmid
9.110	0.0294	Buchnera aphidicola (32199)	Buchnera (1903409)	Erwiniaceae (91347)	Enterobacterales (1236)	Gammaproteobacteria (122	Proteobacteria (2)	Bacteria (1)					Plasmid
9.111	0.0294	Buchnera aphidicola (32199)	Buchnera (1903409)	Erwiniaceae (91347)	Enterobacterales (1236)	Gammaproteobacteria (122	Proteobacteria (2)	Bacteria (1)					Plasmid
9.112	0.03	Buchnera aphidicola (32199)	Buchnera (1903409)	Erwiniaceae (91347)	Enterobacterales (1236)	Gammaproteobacteria (122	Proteobacteria (2)	Bacteria (1)					Plasmid
9.164	0.0294	Buchnera aphidicola (32199)	Buchnera (1903409)	Erwiniaceae (91347)	Enterobacterales (1236)	Gammaproteobacteria (122	Proteobacteria (2)	Bacteria (1)	100.00	0.00	99.20	99.00	WGS
9.55	0.0512	Buchnera aphidicola (32199)	Buchnera (1903409)	Erwiniaceae (91347)	Enterobacterales (1236)	Gammaproteobacteria (122	Proteobacteria (2)	Bacteria (1)	82.40	0.00	96.50	96.40	Complete
23.9	0.243	Shewanella colwelliana (22)	Shewanella (267890)	Shewanellaceae (135622)	Alteromonadales (1236)	Gammaproteobacteria (122	Proteobacteria (2)	Bacteria (1)			98.90	96.40	WGS
24.12	0.4158	Shewanella putrefaciens (22)	Shewanella (267890)	Shewanellaceae (135622)	Alteromonadales (1236)	Gammaproteobacteria (122	Proteobacteria (2)	Bacteria (1)	100.00	1.20	99.50	98.40	WGS
24.13	0.3106	Shewanella putrefaciens (22)	Shewanella (267890)	Shewanellaceae (135622)	Alteromonadales (1236)	Gammaproteobacteria (122	Proteobacteria (2)	Bacteria (1)	100.00	1.70	99.50	97.90	WGS
24.14	0.4338	Shewanella putrefaciens (22)	Shewanella (267890)	Shewanellaceae (135622)	Alteromonadales (1236)	Gammaproteobacteria (122	Proteobacteria (2)	Bacteria (1)	100.00	0.00	99.60	98.40	WGS
24.15	0.3932	Shewanella putrefaciens (22)	Shewanella (267890)	Shewanellaceae (135622)	Alteromonadales (1236)	Gammaproteobacteria (122	Proteobacteria (2)	Bacteria (1)	100.00	0.00	98.70	97.20	WGS
24.16	0.419	Shewanella putrefaciens (22)	Shewanella (267890)	Shewanellaceae (135622)	Alteromonadales (1236)	Gammaproteobacteria (122	Proteobacteria (2)	Bacteria (1)			99.40	98.20	Complete
24.17	0.4272	Shewanella putrefaciens (22)	Shewanella (267890)	Shewanellaceae (135622)	Alteromonadales (1236)	Gammaproteobacteria (122	Proteobacteria (2)	Bacteria (1)			99.70	98.70	Complete
24.6	0.421	Shewanella putrefaciens (22)	Shewanella (267890)	Shewanellaceae (135622)	Alteromonadales (1236)	Gammaproteobacteria (122	Proteobacteria (2)	Bacteria (1)	100.00	0.00	99.70	98.40	WGS
24.8	0.3662	Shewanella putrefaciens (22)	Shewanella (267890)	Shewanellaceae (135622)	Alteromonadales (1236)	Gammaproteobacteria (122	Proteobacteria (2)	Bacteria (1)	99.40	14.70	99.60	92.10	Complete
33.10	0.0294	Myxococcus fulvus (80811)	Myxococcus (29)	Myxococcaceae (28221)	Myxococcales (68525)	Deltaproteobacteria (1224)	Proteobacteria (2)	Bacteria (1)					Plasmid
33.18	0.3346	Myxococcus fulvus (80811)	Myxococcus (29)	Myxococcaceae (28221)	Myxococcales (68525)	Deltaproteobacteria (1224)	Proteobacteria (2)	Bacteria (1)			96.80	93.60	WGS
34.40	0.3314	Myxococcus xanthus (80811)	Myxococcus (29)	Myxococcaceae (28221)	Myxococcales (68525)	Deltaproteobacteria (1224)	Proteobacteria (2)	Bacteria (1)			99.10	98.80	WGS
41.8	0.413	Stigmatella aurantiaca (80811)	Stigmatella (29)	Archangiaceae (28221)	Myxococcales (68525)	Deltaproteobacteria (1224)	Proteobacteria (2)	Bacteria (1)			98.80	97.00	WGS
43.3	0.4354	Cystobacter fuscus (80811)	Cystobacter (29)	Archangiaceae (28221)	Myxococcales (68525)	Deltaproteobacteria (1224)	Proteobacteria (2)	Bacteria (1)	98.30	9.00	96.20	92.10	Complete
48.18	0.4098	Archangium gephyra (80811)	Archangium (29)	Archangiaceae (28221)	Myxococcales (68525)	Deltaproteobacteria (1224)	Proteobacteria (2)	Bacteria (1)					Complete
48.7	0.2252	Archangium gephyra (80811)	Archangium (29)	Archangiaceae (28221)	Myxococcales (68525)	Deltaproteobacteria (1224)	Proteobacteria (2)	Bacteria (1)	95.40	12.60	90.50	86.80	WGS
48.8	0.4098	Archangium gephyra (80811)	Archangium (29)	Archangiaceae (28221)	Myxococcales (68525)	Deltaproteobacteria (1224)	Proteobacteria (2)	Bacteria (1)	98.30	12.80	96.20	92.10	WGS
52.12	0.4516	Chondromyces crocatus (80812)	Chondromyces (29)	Polyangiaceae (28221)	Myxococcales (68525)	Deltaproteobacteria (1224)	Proteobacteria (2)	Bacteria (1)			92.60	89.30	Complete
54.3	0.367	Nannocystis exedens (224462)	Nannocystis (29)	Nannocystaceae (28221)	Myxococcales (68525)	Deltaproteobacteria (1224)	Proteobacteria (2)	Bacteria (1)	89.10	7.30	87.40	85.10	WGS
54.5	0.3716	Nannocystis exedens (224462)	Nannocystis (29)	Nannocystaceae (28221)	Myxococcales (68525)	Deltaproteobacteria (1224)	Proteobacteria (2)	Bacteria (1)	89.10	7.30	87.50	85.00	WGS
56.43	0.4054	Sorangium cellulosum (80812)	Sorangium (29)	Polyangiaceae (28221)	Myxococcales (68525)	Deltaproteobacteria (1224)	Proteobacteria (2)	Bacteria (1)	95.40	13.70	97.80	93.10	WGS
56.44	0.4946	Sorangium cellulosum (80812)	Sorangium (29)	Polyangiaceae (28221)	Myxococcales (68525)	Deltaproteobacteria (1224)	Proteobacteria (2)	Bacteria (1)	95.40	15.50	98.10	91.60	WGS
56.45	0.034	Sorangium cellulosum (80812)	Sorangium (29)	Polyangiaceae (28221)	Myxococcales (68525)	Deltaproteobacteria (1224)	Proteobacteria (2)	Bacteria (1)	37.50	7.80	88.00	86.80	WGS
56.48	0.4774	Sorangium cellulosum (80812)	Sorangium (29)	Polyangiaceae (28221)	Myxococcales (68525)	Deltaproteobacteria (1224)	Proteobacteria (2)	Bacteria (1)	95.40	13.50	96.50	93.00	Complete
56.50	0.3676	Sorangium cellulosum (80812)	Sorangium (29)	Polyangiaceae (28221)	Myxococcales (68525)	Deltaproteobacteria (1224)	Proteobacteria (2)	Bacteria (1)					WGS
56.51	0.3764	Sorangium cellulosum (80812)	Sorangium (29)	Polyangiaceae (28221)	Myxococcales (68525)	Deltaproteobacteria (1224)	Proteobacteria (2)	Bacteria (1)					WGS
56.52	0.3982	Sorangium cellulosum (80812)	Sorangium (29)	Polyangiaceae (28221)	Myxococcales (68525)	Deltaproteobacteria (1224)	Proteobacteria (2)	Bacteria (1)					WGS
56.53	0.498	Sorangium cellulosum (80812)	Sorangium (29)	Polyangiaceae (28221)	Myxococcales (68525)	Deltaproteobacteria (1224)	Proteobacteria (2)	Bacteria (1)					WGS
56.54	0.3434	Sorangium cellulosum (80812)	Sorangium (29)	Polyangiaceae (28221)	Myxococcales (68525)	Deltaproteobacteria (1224)	Proteobacteria (2)	Bacteria (1)					WGS
56.55	0.3434	Sorangium cellulosum (80812)	Sorangium (29)	Polyangiaceae (28221)	Myxococcales (68525)	Deltaproteobacteria (1224)	Proteobacteria (2)	Bacteria (1)					WGS
56.56	0.3608	Sorangium cellulosum (80812)	Sorangium (29)	Polyangiaceae (28221)	Myxococcales (68525)	Deltaproteobacteria (1224)	Proteobacteria (2)	Bacteria (1)					Complete
56.57	0.4622	Sorangium cellulosum (80812)	Sorangium (29)	Polyangiaceae (28221)	Myxococcales (68525)	Deltaproteobacteria (1224)	Proteobacteria (2)	Bacteria (1)					Complete
63.3	0.312	Vitreoscilla filiformis (59)	Vitreoscilla (481)	Neisseriaceae (206351)	Neisseriales (28216)	Betaproteobacteria (1224)	Proteobacteria (2)	Bacteria (1)	91.00	4.70	95.70	94.50	Complete
63.4	0.0294	Vitreoscilla filiformis (59)	Vitreoscilla (481)	Neisseriaceae (206351)	Neisseriales (28216)	Betaproteobacteria (1224)	Proteobacteria (2)	Bacteria (1)					Plasmid
63.5	0.0506	Vitreoscilla filiformis (59)	Vitreoscilla (481)	Neisseriaceae (206351)	Neisseriales (28216)	Betaproteobacteria (1224)	Proteobacteria (2)	Bacteria (1)					Plasmid
69.16	0.3918	Lysobacter enzymogenes (68)	Lysobacter (32033)	Xanthomonadaceae (1356	Xanthomonadales (1236)	Gammaproteobacteria (122	Proteobacteria (2)	Bacteria (1)	98.40	1.90	97.00	95.80	WGS
69.17	0.418	Lysobacter enzymogenes (68)	Lysobacter (32033)	Xanthomonadaceae (1356	Xanthomonadales (1236)	Gammaproteobacteria (122	Proteobacteria (2)	Bacteria (1)	98.10	1.30	96.70	95.00	WGS
69.24	0.4136	Lysobacter enzymogenes (68)	Lysobacter (32033)	Xanthomonadaceae (1356	Xanthomonadales (1236)	Gammaproteobacteria (122	Proteobacteria (2)	Bacteria (1)	98.40	1.00	96.70	95.30	Complete
69.33	0.4202	Lysobacter enzymogenes (68)	Lysobacter (32033)	Xanthomonadaceae (1356	Xanthomonadales (1236)	Gammaproteobacteria (122	Proteobacteria (2)	Bacteria (1)			96.80	95.00	WGS
69.39	0.3652	Lysobacter enzymogenes (68)	Lysobacter (32033)	Xanthomonadaceae (1356	Xanthomonadales (1236)	Gammaproteobacteria (122	Proteobacteria (2)	Bacteria (1)			96.80	93.40	Complete
69.6	0.4208	Lysobacter enzymogenes (68)	Lysobacter (32033)	Xanthomonadaceae (1356	Xanthomonadales (1236)	Gammaproteobacteria (122	Proteobacteria (2)	Bacteria (1)	98.10	1.00	97.00	95.70	Complete
82.11	0.3658	Hyphomicrobium sp. (81)	Hyphomicrobium (45401)	Hyphomicrobiaceae (356)	Rhizobiales (28211)	Alphaproteobacteria (1224	Proteobacteria (2)	Bacteria (1)			98.20	96.20	WGS
82.12	0.3378	Hyphomicrobium sp. (81)	Hyphomicrobium (45401)	Hyphomicrobiaceae (356)	Rhizobiales (28211)	Alphaproteobacteria (1224	Proteobacteria (2)	Bacteria (1)			96.30	94.80	WGS
82.3	0.341	Hyphomicrobium sp. MC1 (81)	Hyphomicrobium (45401)	Hyphomicrobiaceae (356)	Rhizobiales (28211)	Alphaproteobacteria (1224	Proteobacteria (2)	Bacteria (1)	98.00	2.20	96.70	94.10	Complete
82.5	0.2004	Hyphomicrobium sp. (81)	Hyphomicrobium (45401)	Hyphomicrobiaceae (356)	Rhizobiales (28211)	Alphaproteobacteria (1224	Proteobacteria (2)	Bacteria (1)	77.20	2.10	89.80	88.10	WGS
82.6	0.2352	Hyphomicrobium sp. (81)	Hyphomicrobium (45401)	Hyphomicrobiaceae (356)	Rhizobiales (28211)	Alphaproteobacteria (1224	Proteobacteria (2)	Bacteria (1)	81.90	6.10	88.80	86.10	WGS
82.7	0.3486	Hyphomicrobium sp. (81)	Hyphomicrobium (45401)	Hyphomicrobiaceae (356)	Rhizobiales (28211)	Alphaproteobacteria (1224	Proteobacteria (2)	Bacteria (1)	99.20	0.40	97.90	97.50	WGS
82.8	0.3326	Hyphomicrobium sp. (81)	Hyphomicrobium (45401)	Hyphomicrobiaceae (356)	Rhizobiales (28211)	Alphaproteobacteria (1224	Proteobacteria (2)	Bacteria (1)	95.90	1.30	95.60	94.60	WGS
82.9	0.2692	Hyphomicrobium sp. (81)	Hyphomicrobium (45401)	Hyphomicrobiaceae (356)	Rhizobiales (28211)	Alphaproteobacteria (1224	Proteobacteria (2)	Bacteria (1)	94.50	0.40	95.70	94.60	WGS
86.3	0.1938	Hyphomonas jannaschiana (85)	Hyphomonas (69657)	Hyphomonadaceae (20445	Rhodobacterales (28211)	Alphaproteobacteria (1224	Proteobacteria (2)	Bacteria (1)	73.50	14.40	84.80	81.90	WGS
87.10	0.4368	Hyphomonas sp. (85)	Hyphomonas (69657)	Hyphomonadaceae (20445	Rhodobacterales (28211)	Alphaproteobacteria (1224	Proteobacteria (2)	Bacteria (1)	97.30	0.00	95.80	94.30	WGS
87.11	0.3698	Hyphomonas sp. (85)	Hyphomonas (69657)	Hyphomonadaceae (20445	Rhodobacterales (28211)	Alphaproteobacteria (1224	Proteobacteria (2)	Bacteria (1)	99.10	0.00	97.50	95.50	WGS
87.12	0.2004	Hyphomonas sp. (85)	Hyphomonas (69657)	Hyphomonadaceae (20445	Rhodobacterales (28211)	Alphaproteobacteria (1224	Proteobacteria (2)	Bacteria (1)	92.00	0.00	93.30	92.90	WGS
87.13	0.1194	Hyphomonas sp. (85)	Hyphomonas (69657)	Hyphomonadaceae (20445	Rhodobacterales (28211)	Alphaproteobacteria (1224	Proteobacteria (2)	Bacteria (1)	75.20	1.20	87.10	86.30	WGS
87.14	0.3948	Hyphomonas sp. (85)	Hyphomonas (69657)	Hyphomonadaceae (20445	Rhodobacterales (28211)	Alphaproteobacteria (1224	Proteobacteria (2)	Bacteria (1)	100.00	0.90	97.60	94.50	WGS
87.17	0.1358	Hyphomonas sp. (85)	Hyphomonas (69657)	Hyphomonadaceae (20445	Rhodobacterales (28211)	Alphaproteobacteria (1224	Proteobacteria (2)	Bacteria (1)	95.60	2.70	96.70	94.50	WGS
87.18	0.2488	Hyphomonas sp. (85)	Hyphomonas (69657)	Hyphomonadaceae (20445	Rhodobacterales (28211)	Alphaproteobacteria (1224	Proteobacteria (2)	Bacteria (1)	99.10	7.80	95.90	88.00	WGS
87.19	0.2458	Hyphomonas sp. (85)	Hyphomonas (69657)	Hyphomonadaceae (20445	Rhodobacterales (28211)	Alphaproteobacteria (1224	Proteobacteria (2)	Bacteria (1)	95.60	17.20	94.80	84.30	WGS
87.20	0.1774	Hyphomonas sp. (85)	Hyphomonas (69657)	Hyphomonadaceae (20445	Rhodobacterales (28211)	Alphaproteobacteria (1224	Proteobacteria (2)	Bacteria (1)	75.20	9.60	89.80	85.10	WGS
87.21	0.249	Hyphomonas sp. (85)	Hyphomonas (69657)	Hyphomonadaceae (20445	Rhodobacterales (28211)	Alphaproteobacteria (1224	Proteobacteria (2)	Bacteria (1)	97.30	22.00	95.90	84.60	WGS
87.22	0.2546	Hyphomonas sp. (85)	Hyphomonas (69657)	Hyphomonadaceae (20445	Rhodobacterales (28211)	Alphaproteobacteria (1224	Proteobacteria (2)	Bacteria (1)	94.70	11.20	92.60	84.70	WGS
87.23	0.209	Hyphomonas sp. (85)	Hyphomonas (69657)	Hyphomonadaceae (20445	Rhodobacterales (28211)	Alphaproteobacteria (1224	Proteobacteria (2)	Bacteria (1)	97.30	14.10	95.70	87.80	WGS
87.24	0.2566	Hyphomonas sp. (85)	Hyphomonas (69657)	Hyphomonadaceae (20445	Rhodobacterales (28211)	Alphaproteobacteria (1224	Proteobacteria (2)	Bacteria (1)	97.30	11.30	96.50	88.80	WGS
87.25	0.2568	Hyphomonas sp. (85)	Hyphomonas (69657)	Hyphomonadaceae (20445	Rhodobacterales (28211)	Alphaproteobacteria (1224	Proteobacteria (2)	Bacteria (1)	100.00	7.40	96.90	89.50	WGS
87.26	0.2202	Hyphomonas sp. (85)	Hyphomonas (69657)	Hyphomonadaceae (20445	Rhodobacterales (28211)	Alphaproteobacteria (1224	Proteobacteria (2)	Bacteria (1)	95.60	16.30	96.70	85.40	WGS
87.27	0.2322	Hyphomonas sp. (85)	Hyphomonas (69657)	Hyphomonadaceae (20445	Rhodobacterales (28211)	Alphaproteobacteria (1224	Proteobacteria (2)	Bacteria (1)	96.50	12.80	95.00	86.70	WGS
87.28	0.2502	Hyphomonas sp. (85)	Hyphomonas (69657)	Hyphomonadaceae (20445	Rhodobacterales (28211)	Alphaproteobacteria (1224	Proteobacteria (2)	Bacteria (1)	95.60	12.90	96.20	87.70	WGS
87.29	0.2416	Hyphomonas sp. (85)	Hyphomonas (69657)	Hyphomonadaceae (20445	Rhodobacterales (28211)	Alphaproteobacteria (1224	Proteobacteria (2)	Bacteria (1)	95.60	27.80	93.90	79.30	WGS
87.30	0.249	Hyphomonas sp. (85)	Hyphomonas (69657)	Hyphomonadaceae (20445	Rhodobacterales (28211)	Alphaproteobacteria (1224	Proteobacteria (2)	Bacteria (1)	98.20	17.20	92.80	81.40	WGS
87.31	0.2278	Hyphomonas sp. (85)	Hyphomonas (69657)	Hyphomonadaceae (20445	Rhodobacterales (28211)	Alphaproteobacteria (1224	Proteobacteria (2)	Bacteria (1)	95.60	13.60	93.30	85.50	WGS
87.32	0.2258	Hyphomonas sp. (85)	Hyphomonas (69657)	Hyphomonadaceae (20445	Rhodobacterales (28211)	Alphaproteobacteria (1224	Proteobacteria (2)	Bacteria (1)	81.40	34.50	88.70	74.50	WGS
87.4	0.2548	Hyphomonas sp. (85)	Hyphomonas (69657)	Hyphomonadaceae (20445	Rhodobacterales (28211)	Alphaproteobacteria (1224	Proteobacteria (2)	Bacteria (1)	99.10	1.80	96.90	94.80	WGS
87.5	0.2052	Hyphomonas sp. (85)	Hyphomonas (69657)	Hyphomonadaceae (20445	Rhodobacterales (28211)	Alphaproteobacteria (1224	Proteobacteria (2)	Bacteria (1)	80.50	5.20	87.70	86.20	WGS
87.6	0.1176	Hyphomonas sp. (85)	Hyphomonas (69657)	Hyphomonadaceae (20445	Rhodobacterales (28211)	Alphaproteobacteria (1224	Proteobacteria (2)	Bacteria (1)	99.10	0.00	96.00	92.80	WGS
87.7	0.2476	Hyphomonas sp. (85)	Hyphomonas (69657)	Hyphomonadaceae (20445	Rhodobacterales (28211)	Alphaproteobacteria (1224	Proteobacteria (2)	Bacteria (1)	100.00	0.00	97.10	95.90	WGS
87.8	0.0952	Hyphomonas sp. (85)	Hyphomonas (69657)	Hyphomonadaceae (20445	Rhodobacterales (28211)	Alphaproteobacteria (1224	Proteobacteria (2)	Bacteria (1)	93.80	19.10	94.30	88.90	WGS
87.9	0.1374	Hyphomonas sp. (85)	Hyphomonas (69657)	Hyphomonadaceae (20445	Rhodobacterales (28211)	Alphaproteobacteria (1224	Proteobacteria (2)	Bacteria (1)	95.60	3.10	93.60	90.50	WGS
94.4	0.3736	Stella humosa (93)	Stella (433)	Acetobacteraceae (204441	Rhodospirillales (28211)	Alphaproteobacteria (1224	Proteobacteria (2)	Bacteria (1)			95.60	92.90	WGS
100.11	0.4036	Ancylobacter aquaticus (99)	Ancylobacter (335928)	Xanthobacteraceae (356)	Rhizobiales (28211)	Alphaproteobacteria (1224	Proteobacteria (2)	Bacteria (1)			98.30	96.20	WGS
100.9	0.3962	Ancylobacter aquaticus (99)	Ancylobacter (335928)	Xanthobacteraceae (356)	Rhizobiales (28211)	Alphaproteobacteria (1224	Proteobacteria (2)	Bacteria (1)			98.10	94.80	WGS
102.4	0.6164	Polaribacter glomeratus (200644)	Polaribacter (117743)	Flavobacteriaceae (976)	Flavobacteriales (68336)	Flavobacteriia (1783270)	Bacteroidetes (2)	Bacteria (1)	100.00	0.00	98.70	98.20	WGS
106.10	0.2936	Runella slithyformis (768507)	Runella (768503)	Cytophagaceae (976)	Cytophagales (68336)	Cytophagia (1783270)	Bacteroidetes (2)	Bacteria (1)			90.80	90.00	WGS
106.11	0.2962	Runella slithyformis (768507)	Runella (768503)	Cytophagaceae (976)	Cytophagales (68336)	Cytophagia (1783270)	Bacteroidetes (2)	Bacteria (1)			92.60	91.80	WGS
106.12	0.1794	Runella slithyformis (768507)	Runella (768503)	Cytophagaceae (976)	Cytophagales (68336)	Cytophagia (1783270)	Bacteroidetes (2)	Bacteria (1)			92.30	88.00	WGS
106.13	0.2536	Runella slithyformis (768507)	Runella (768503)	Cytophagaceae (976)	Cytophagales (68336)	Cytophagia (1783270)	Bacteroidetes (2)	Bacteria (1)			90.20	86.00	WGS
106.6	0.2816	Runella slithyformis (768507)	Runella (768503)	Cytophagaceae (976)	Cytophagales (68336)	Cytophagia (1783270)	Bacteroidetes (2)	Bacteria (1)			91.60	90.80	WGS
106.7	0.2346	Runella slithyformis (768507)	Runella (768503)	Cytophagaceae (976)	Cytophagales (68336)	Cytophagia (1783270)	Bacteroidetes (2)	Bacteria (1)			90.90	87.20	WGS
106.8	0.3018	Runella slithyformis (768507)	Runella (768503)	Cytophagaceae (976)	Cytophagales (68336)	Cytophagia (1783270)	Bacteroidetes (2)	Bacteria (1)			90.10	89.30	WGS
106.9	0.267	Runella slithyformis (768507)	Runella (768503)	Cytophagaceae (976)	Cytophagales (68336)	Cytophagia (1783270)	Bacteroidetes (2)	Bacteria (1)			93.10	90.30	WGS
114.8	0.2468	Gemmata obscuriglobus (1914233	Gemmata (112)	Gemmataceae (203683)	Planctomycetales (203682)	Planctomycetia (1783257)	Planctomycetes (2)	Bacteria (1)	90.60	7.80	91.60	89.20	Complete
122.4	0.287	Gimesia maris (126)	Gimesia (112)	Planctomycetaceae (20368	Planctomycetales (203682)	Planctomycetia (1783257)	Planctomycetes (2)	Bacteria (1)	93.60	1.40	94.10	92.80	WGS
122.6	0.2554	Gimesia maris (126)	Gimesia (112)	Planctomycetaceae (20368	Planctomycetales (203682)	Planctomycetia (1783257)	Planctomycetes (2)	Bacteria (1)	94.90	13.50	94.70	88.70	WGS
124.10	0.36	Blastopirellula marina (126)	Blastopirellula (112)	Planctomycetaceae (20368	Planctomycetales (203682)	Planctomycetia (1783257)	Planctomycetes (2)	Bacteria (1)	79.50	42.10	91.90	81.00	WGS
124.11	0.372	Blastopirellula marina (126)	Blastopirellula (112)	Planctomycetaceae (20368	Planctomycetales (203682)	Planctomycetia (1783257)	Planctomycetes (2)	Bacteria (1)	92.30	1.40	95.90	94.60	WGS
124.12	0.231	Blastopirellula marina (126)	Blastopirellula (112)	Planctomycetaceae (20368	Planctomycetales (203682)	Planctomycetia (1783257)	Planctomycetes (2)	Bacteria (1)	87.20	31.70	93.80	82.20	WGS
124.8	0.3252	Blastopirellula marina (126)	Blastopirellula (112)	Planctomycetaceae (20368	Planctomycetales (203682)	Planctomycetia (1783257)	Planctomycetes (2)	Bacteria (1)	93.60	3.90	96.40	94.10	WGS
124.9	0.231	Blastopirellula marina (126)	Blastopirellula (112)	Planctomycetaceae (20368	Planctomycetales (203682)	Planctomycetia (1783257)	Planctomycetes (2)	Bacteria (1)	87.20	31.70	93.80	82.20	WGS
139.100	0.0294	Borreliella burgdorferi (64895)	Borreliella (1643685)	Borreliaceae (136)	Spirochaetales (203692)	Spirochaetia (203691)	Spirochaetes (2)	Bacteria (1)					Plasmid
139.101	0.0294	Borreliella burgdorferi (64895)	Borreliella (1643685)	Borreliaceae (136)	Spirochaetales (203692)	Spirochaetia (203691)	Spirochaetes (2)	Bacteria (1)					Plasmid
139.102	0.0294	Borreliella burgdorferi (64895)	Borreliella (1643685)	Borreliaceae (136)	Spirochaetales (203692)	Spirochaetia (203691)	Spirochaetes (2)	Bacteria (1)					Plasmid
139.103	0.0294	Borreliella burgdorferi (64895)	Borreliella (1643685)	Borreliaceae (136)	Spirochaetales (203692)	Spirochaetia (203691)	Spirochaetes (2)	Bacteria (1)					Plasmid
139.104	0.0294	Borreliella burgdorferi (64895)	Borreliella (1643685)	Borreliaceae (136)	Spirochaetales (203692)	Spirochaetia (203691)	Spirochaetes (2)	Bacteria (1)					Plasmid
139.105	0.0294	Borreliella burgdorferi (64895)	Borreliella (1643685)	Borreliaceae (136)	Spirochaetales (203692)	Spirochaetia (203691)	Spirochaetes (2)	Bacteria (1)					Plasmid
139.106	0.0294	Borreliella burgdorferi (64895)	Borreliella (1643685)	Borreliaceae (136)	Spirochaetales (203692)	Spirochaetia (203691)	Spirochaetes (2)	Bacteria (1)					Plasmid
139.107	0.0294	Borreliella burgdorferi (64895)	Borreliella (1643685)	Borreliaceae (136)	Spirochaetales (203692)	Spirochaetia (203691)	Spirochaetes (2)	Bacteria (1)					Plasmid
139.108	0.0294	Borreliella burgdorferi (64895)	Borreliella (1643685)	Borreliaceae (136)	Spirochaetales (203692)	Spirochaetia (203691)	Spirochaetes (2)	Bacteria (1)					Plasmid
139.109	0.0294	Borreliella burgdorferi (64895)	Borreliella (1643685)	Borreliaceae (136)	Spirochaetales (203692)	Spirochaetia (203691)	Spirochaetes (2)	Bacteria (1)					Plasmid
139.110	0.0294	Borreliella burgdorferi (64895)	Borreliella (1643685)	Borreliaceae (136)	Spirochaetales (203692)	Spirochaetia (203691)	Spirochaetes (2)	Bacteria (1)					Plasmid
139.111	0.0294	Borreliella burgdorferi (64895)	Borreliella (1643685)	Borreliaceae (136)	Spirochaetales (203692)	Spirochaetia (203691)	Spirochaetes (2)	Bacteria (1)					Plasmid
139.112	0.03	Borreliella burgdorferi (64895)	Borreliella (1643685)	Borreliaceae (136)	Spirochaetales (203692)	Spirochaetia (203691)	Spirochaetes (2)	Bacteria (1)					Plasmid
139.113	0.0294	Borreliella burgdorferi (64895)	Borreliella (1643685)	Borreliaceae (136)	Spirochaetales (203692)	Spirochaetia (203691)	Spirochaetes (2)	Bacteria (1)					Plasmid
139.114	0.0294	Borreliella burgdorferi (64895)	Borreliella (1643685)	Borreliaceae (136)	Spirochaetales (203692)	Spirochaetia (203691)	Spirochaetes (2)	Bacteria (1)					Plasmid
139.115	0.0294	Borreliella burgdorferi (64895)	Borreliella (1643685)	Borreliaceae (136)	Spirochaetales (203692)	Spirochaetia (203691)	Spirochaetes (2)	Bacteria (1)					Plasmid
139.116	0.0294	Borreliella burgdorferi (64895)	Borreliella (1643685)	Borreliaceae (136)	Spirochaetales (203692)	Spirochaetia (203691)	Spirochaetes (2)	Bacteria (1)					Plasmid
139.117	0.03	Borreliella burgdorferi (64895)	Borreliella (1643685)	Borreliaceae (136)	Spirochaetales (203692)	Spirochaetia (203691)	Spirochaetes (2)	Bacteria (1)					Plasmid
139.118	0.0294	Borreliella burgdorferi (64895)	Borreliella (1643685)	Borreliaceae (136)	Spirochaetales (203692)	Spirochaetia (203691)	Spirochaetes (2)	Bacteria (1)					Plasmid
139.119	0.0294	Borreliella burgdorferi (64895)	Borreliella (1643685)	Borreliaceae (136)	Spirochaetales (203692)	Spirochaetia (203691)	Spirochaetes (2)	Bacteria (1)					Plasmid
139.120	0.0294	Borreliella burgdorferi (64895)	Borreliella (1643685)	Borreliaceae (136)	Spirochaetales (203692)	Spirochaetia (203691)	Spirochaetes (2)	Bacteria (1)					Plasmid
139.121	0.0294	Borreliella burgdorferi (64895)	Borreliella (1643685)	Borreliaceae (136)	Spirochaetales (203692)	Spirochaetia (203691)	Spirochaetes (2)	Bacteria (1)					Plasmid
139.122	0.0294	Borreliella burgdorferi (64895)	Borreliella (1643685)	Borreliaceae (136)	Spirochaetales (203692)	Spirochaetia (203691)	Spirochaetes (2)	Bacteria (1)					Plasmid
139.123	0.0294	Borreliella burgdorferi (64895)	Borreliella (1643685)	Borreliaceae (136)	Spirochaetales (203692)	Spirochaetia (203691)	Spirochaetes (2)	Bacteria (1)					Plasmid
139.124	0.0294	Borreliella burgdorferi (64895)	Borreliella (1643685)	Borreliaceae (136)	Spirochaetales (203692)	Spirochaetia (203691)	Spirochaetes (2)	Bacteria (1)					Plasmid
139.125	0.0294	Borreliella burgdorferi (64895)	Borreliella (1643685)	Borreliaceae (136)	Spirochaetales (203692)	Spirochaetia (203691)	Spirochaetes (2)	Bacteria (1)					Plasmid
139.126	0.0294	Borreliella burgdorferi (64895)	Borreliella (1643685)	Borreliaceae (136)	Spirochaetales (203692)	Spirochaetia (203691)	Spirochaetes (2)	Bacteria (1)					Plasmid
139.127	0.0294	Borreliella burgdorferi (64895)	Borreliella (1643685)	Borreliaceae (136)	Spirochaetales (203692)	Spirochaetia (203691)	Spirochaetes (2)	Bacteria (1)					Plasmid
139.128	0.0294	Borreliella burgdorferi (64895)	Borreliella (1643685)	Borreliaceae (136)	Spirochaetales (203692)	Spirochaetia (203691)	Spirochaetes (2)	Bacteria (1)					Plasmid
139.129	0.0294	Borreliella burgdorferi (64895)	Borreliella (1643685)	Borreliaceae (136)	Spirochaetales (203692)	Spirochaetia (203691)	Spirochaetes (2)	Bacteria (1)					Plasmid
139.130	0.0294	Borreliella burgdorferi (64895)	Borreliella (1643685)	Borreliaceae (136)	Spirochaetales (203692)	Spirochaetia (203691)	Spirochaetes (2)	Bacteria (1)					Plasmid
139.131	0.0294	Borreliella burgdorferi (64895)	Borreliella (1643685)	Borreliaceae (136)	Spirochaetales (203692)	Spirochaetia (203691)	Spirochaetes (2)	Bacteria (1)					Plasmid
139.132	0.0294	Borreliella burgdorferi (64895)	Borreliella (1643685)	Borreliaceae (136)	Spirochaetales (203692)	Spirochaetia (203691)	Spirochaetes (2)	Bacteria (1)					Plasmid
139.133	0.0294	Borreliella burgdorferi (64895)	Borreliella (1643685)	Borreliaceae (136)	Spirochaetales (203692)	Spirochaetia (203691)	Spirochaetes (2)	Bacteria (1)					Plasmid
139.134	0.0294	Borreliella burgdorferi (64895)	Borreliella (1643685)	Borreliaceae (136)	Spirochaetales (203692)	Spirochaetia (203691)	Spirochaetes (2)	Bacteria (1)					Plasmid
139.135	0.03	Borreliella burgdorferi (64895)	Borreliella (1643685)	Borreliaceae (136)	Spirochaetales (203692)	Spirochaetia (203691)	Spirochaetes (2)	Bacteria (1)					Plasmid
139.136	0.0294	Borreliella burgdorferi (64895)	Borreliella (1643685)	Borreliaceae (136)	Spirochaetales (203692)	Spirochaetia (203691)	Spirochaetes (2)	Bacteria (1)					Plasmid
139.137	0.0294	Borreliella burgdorferi (64895)	Borreliella (1643685)	Borreliaceae (136)	Spirochaetales (203692)	Spirochaetia (203691)	Spirochaetes (2)	Bacteria (1)					Plasmid
139.138	0.0294	Borreliella burgdorferi (64895)	Borreliella (1643685)	Borreliaceae (136)	Spirochaetales (203692)	Spirochaetia (203691)	Spirochaetes (2)	Bacteria (1)					Plasmid
139.139	0.0294	Borreliella burgdorferi (64895)	Borreliella (1643685)	Borreliaceae (136)	Spirochaetales (203692)	Spirochaetia (203691)	Spirochaetes (2)	Bacteria (1)					Plasmid
139.140	0.0294	Borreliella burgdorferi (64895)	Borreliella (1643685)	Borreliaceae (136)	Spirochaetales (203692)	Spirochaetia (203691)	Spirochaetes (2)	Bacteria (1)					Plasmid
139.141	0.0294	Borreliella burgdorferi (64895)	Borreliella (1643685)	Borreliaceae (136)	Spirochaetales (203692)	Spirochaetia (203691)	Spirochaetes (2)	Bacteria (1)					Plasmid
139.142	0.0294	Borreliella burgdorferi (64895)	Borreliella (1643685)	Borreliaceae (136)	Spirochaetales (203692)	Spirochaetia (203691)	Spirochaetes (2)	Bacteria (1)					Plasmid
139.143	0.0294	Borreliella burgdorferi (64895)	Borreliella (1643685)	Borreliaceae (136)	Spirochaetales (203692)	Spirochaetia (203691)	Spirochaetes (2)	Bacteria (1)					Plasmid
139.144	0.0294	Borreliella burgdorferi (64895)	Borreliella (1643685)	Borreliaceae (136)	Spirochaetales (203692)	Spirochaetia (203691)	Spirochaetes (2)	Bacteria (1)					Plasmid
139.145	0.0294	Borreliella burgdorferi (64895)	Borreliella (1643685)	Borreliaceae (136)	Spirochaetales (203692)	Spirochaetia (203691)	Spirochaetes (2)	Bacteria (1)					Plasmid
139.146	0.0294	Borreliella burgdorferi (64895)	Borreliella (1643685)	Borreliaceae (136)	Spirochaetales (203692)	Spirochaetia (203691)	Spirochaetes (2)	Bacteria (1)					Plasmid
139.147	0.03	Borreliella burgdorferi (64895)	Borreliella (1643685)	Borreliaceae (136)	Spirochaetales (203692)	Spirochaetia (203691)	Spirochaetes (2)	Bacteria (1)					Plasmid
139.148	0.0294	Borreliella burgdorferi (64895)	Borreliella (1643685)	Borreliaceae (136)	Spirochaetales (203692)	Spirochaetia (203691)	Spirochaetes (2)	Bacteria (1)					Plasmid
139.149	0.0294	Borreliella burgdorferi (64895)	Borreliella (1643685)	Borreliaceae (136)	Spirochaetales (203692)	Spirochaetia (203691)	Spirochaetes (2)	Bacteria (1)					Plasmid
139.150	0.03	Borreliella burgdorferi (64895)	Borreliella (1643685)	Borreliaceae (136)	Spirochaetales (203692)	Spirochaetia (203691)	Spirochaetes (2)	Bacteria (1)					Plasmid
139.151	0.0294	Borreliella burgdorferi (64895)	Borreliella (1643685)	Borreliaceae (136)	Spirochaetales (203692)	Spirochaetia (203691)	Spirochaetes (2)	Bacteria (1)					Plasmid
139.152	0.0294	Borreliella burgdorferi (64895)	Borreliella (1643685)	Borreliaceae (136)	Spirochaetales (203692)	Spirochaetia (203691)	Spirochaetes (2)	Bacteria (1)					Plasmid
139.153	0.0294	Borreliella burgdorferi (64895)	Borreliella (1643685)	Borreliaceae (136)	Spirochaetales (203692)	Spirochaetia (203691)	Spirochaetes (2)	Bacteria (1)					Plasmid
139.154	0.0294	Borreliella burgdorferi (64895)	Borreliella (1643685)	Borreliaceae (136)	Spirochaetales (203692)	Spirochaetia (203691)	Spirochaetes (2)	Bacteria (1)					Plasmid
139.155	0.0294	Borreliella burgdorferi (64895)	Borreliella (1643685)	Borreliaceae (136)	Spirochaetales (203692)	Spirochaetia (203691)	Spirochaetes (2)	Bacteria (1)					Plasmid
139.156	0.0294	Borreliella burgdorferi (64895)	Borreliella (1643685)	Borreliaceae (136)	Spirochaetales (203692)	Spirochaetia (203691)	Spirochaetes (2)	Bacteria (1)					Plasmid
139.157	0.0294	Borreliella burgdorferi (64895)	Borreliella (1643685)	Borreliaceae (136)	Spirochaetales (203692)	Spirochaetia (203691)	Spirochaetes (2)	Bacteria (1)					Plasmid
139.158	0.0294	Borreliella burgdorferi (64895)	Borreliella (1643685)	Borreliaceae (136)	Spirochaetales (203692)	Spirochaetia (203691)	Spirochaetes (2)	Bacteria (1)					Plasmid
139.159	0.0294	Borreliella burgdorferi (64895)	Borreliella (1643685)	Borreliaceae (136)	Spirochaetales (203692)	Spirochaetia (203691)	Spirochaetes (2)	Bacteria (1)					Plasmid
139.160	0.0294	Borreliella burgdorferi (64895)	Borreliella (1643685)	Borreliaceae (136)	Spirochaetales (203692)	Spirochaetia (203691)	Spirochaetes (2)	Bacteria (1)					Plasmid
139.161	0.0294	Borreliella burgdorferi (64895)	Borreliella (1643685)	Borreliaceae (136)	Spirochaetales (203692)	Spirochaetia (203691)	Spirochaetes (2)	Bacteria (1)					Plasmid
139.162	0.0294	Borreliella burgdorferi (64895)	Borreliella (1643685)	Borreliaceae (136)	Spirochaetales (203692)	Spirochaetia (203691)	Spirochaetes (2)	Bacteria (1)					Plasmid
139.163	0.0294	Borreliella burgdorferi (64895)	Borreliella (1643685)	Borreliaceae (136)	Spirochaetales (203692)	Spirochaetia (203691)	Spirochaetes (2)	Bacteria (1)					Plasmid
139.164	0.0294	Borreliella burgdorferi (64895)	Borreliella (1643685)	Borreliaceae (136)	Spirochaetales (203692)	Spirochaetia (203691)	Spirochaetes (2)	Bacteria (1)					Plasmid
139.165	0.0294	Borreliella burgdorferi (64895)	Borreliella (1643685)	Borreliaceae (136)	Spirochaetales (203692)	Spirochaetia (203691)	Spirochaetes (2)	Bacteria (1)					Plasmid
139.166	0.0294	Borreliella burgdorferi (64895)	Borreliella (1643685)	Borreliaceae (136)	Spirochaetales (203692)	Spirochaetia (203691)	Spirochaetes (2)	Bacteria (1)					Plasmid
139.167	0.0294	Borreliella burgdorferi (64895)	Borreliella (1643685)	Borreliaceae (136)	Spirochaetales (203692)	Spirochaetia (203691)	Spirochaetes (2)	Bacteria (1)					Plasmid
139.168	0.03	Borreliella burgdorferi (64895)	Borreliella (1643685)	Borreliaceae (136)	Spirochaetales (203692)	Spirochaetia (203691)	Spirochaetes (2)	Bacteria (1)					Plasmid
139.169	0.0294	Borreliella burgdorferi (64895)	Borreliella (1643685)	Borreliaceae (136)	Spirochaetales (203692)	Spirochaetia (203691)	Spirochaetes (2)	Bacteria (1)					Plasmid
139.170	0.0294	Borreliella burgdorferi (64895)	Borreliella (1643685)	Borreliaceae (136)	Spirochaetales (203692)	Spirochaetia (203691)	Spirochaetes (2)	Bacteria (1)					Plasmid
139.18	0.037	Borreliella afzelii (64895)	Borreliella (1643685)	Borreliaceae (136)	Spirochaetales (203692)	Spirochaetia (203691)	Spirochaetes (2)	Bacteria (1)	20.60	21.40	97.00	88.90	WGS
139.19	0.0388	Borreliella afzelii (64895)	Borreliella (1643685)	Borreliaceae (136)	Spirochaetales (203692)	Spirochaetia (203691)	Spirochaetes (2)	Bacteria (1)	15.70	32.90	94.10	88.40	WGS
139.196	0.0416	Borreliella burgdorferi (64895)	Borreliella (1643685)	Borreliaceae (136)	Spirochaetales (203692)	Spirochaetia (203691)	Spirochaetes (2)	Bacteria (1)	23.10	0.10	100.00	99.80	WGS
139.197	0.0416	Borreliella burgdorferi (64895)	Borreliella (1643685)	Borreliaceae (136)	Spirochaetales (203692)	Spirochaetia (203691)	Spirochaetes (2)	Bacteria (1)	23.10	0.10	100.00	99.70	WGS
139.198	0.0416	Borreliella burgdorferi (64895)	Borreliella (1643685)	Borreliaceae (136)	Spirochaetales (203692)	Spirochaetia (203691)	Spirochaetes (2)	Bacteria (1)	23.10	0.10	100.00	99.70	WGS
139.199	0.0416	Borreliella burgdorferi (64895)	Borreliella (1643685)	Borreliaceae (136)	Spirochaetales (203692)	Spirochaetia (203691)	Spirochaetes (2)	Bacteria (1)	23.10	0.10	100.00	99.80	WGS
139.20	0.0396	Borreliella afzelii (64895)	Borreliella (1643685)	Borreliaceae (136)	Spirochaetales (203692)	Spirochaetia (203691)	Spirochaetes (2)	Bacteria (1)	23.10	18.90	99.40	92.10	WGS
139.200	0.0416	Borreliella burgdorferi (64895)	Borreliella (1643685)	Borreliaceae (136)	Spirochaetales (203692)	Spirochaetia (203691)	Spirochaetes (2)	Bacteria (1)	23.10	0.20	100.00	99.70	WGS
139.201	0.0416	Borreliella burgdorferi (64895)	Borreliella (1643685)	Borreliaceae (136)	Spirochaetales (203692)	Spirochaetia (203691)	Spirochaetes (2)	Bacteria (1)	23.10	0.10	100.00	99.70	WGS
139.202	0.0416	Borreliella burgdorferi (64895)	Borreliella (1643685)	Borreliaceae (136)	Spirochaetales (203692)	Spirochaetia (203691)	Spirochaetes (2)	Bacteria (1)	23.10	0.20	100.00	99.70	WGS
139.203	0.0416	Borreliella burgdorferi (64895)	Borreliella (1643685)	Borreliaceae (136)	Spirochaetales (203692)	Spirochaetia (203691)	Spirochaetes (2)	Bacteria (1)	23.10	0.20	100.00	99.70	WGS
139.204	0.0416	Borreliella burgdorferi (64895)	Borreliella (1643685)	Borreliaceae (136)	Spirochaetales (203692)	Spirochaetia (203691)	Spirochaetes (2)	Bacteria (1)	23.10	0.10	100.00	99.80	WGS
139.205	0.0416	Borreliella burgdorferi (64895)	Borreliella (1643685)	Borreliaceae (136)	Spirochaetales (203692)	Spirochaetia (203691)	Spirochaetes (2)	Bacteria (1)	23.10	0.10	100.00	99.80	WGS
139.206	0.0416	Borreliella burgdorferi (64895)	Borreliella (1643685)	Borreliaceae (136)	Spirochaetales (203692)	Spirochaetia (203691)	Spirochaetes (2)	Bacteria (1)	23.10	0.20	100.00	99.70	WGS
139.207	0.0416	Borreliella burgdorferi (64895)	Borreliella (1643685)	Borreliaceae (136)	Spirochaetales (203692)	Spirochaetia (203691)	Spirochaetes (2)	Bacteria (1)	23.10	0.20	100.00	99.70	WGS
139.208	0.0416	Borreliella burgdorferi (64895)	Borreliella (1643685)	Borreliaceae (136)	Spirochaetales (203692)	Spirochaetia (203691)	Spirochaetes (2)	Bacteria (1)	23.10	0.20	100.00	99.60	WGS
139.209	0.0416	Borreliella burgdorferi (64895)	Borreliella (1643685)	Borreliaceae (136)	Spirochaetales (203692)	Spirochaetia (203691)	Spirochaetes (2)	Bacteria (1)	23.10	0.10	100.00	99.80	WGS
139.21	0.0392	Borreliella afzelii (64895)	Borreliella (1643685)	Borreliaceae (136)	Spirochaetales (203692)	Spirochaetia (203691)	Spirochaetes (2)	Bacteria (1)	18.10	14.20	96.70	89.20	WGS
139.210	0.0416	Borreliella burgdorferi (64895)	Borreliella (1643685)	Borreliaceae (136)	Spirochaetales (203692)	Spirochaetia (203691)	Spirochaetes (2)	Bacteria (1)	23.10	0.20	99.90	99.60	WGS
139.211	0.0416	Borreliella burgdorferi (64895)	Borreliella (1643685)	Borreliaceae (136)	Spirochaetales (203692)	Spirochaetia (203691)	Spirochaetes (2)	Bacteria (1)	23.10	0.20	100.00	99.70	WGS
139.212	0.0416	Borreliella burgdorferi (64895)	Borreliella (1643685)	Borreliaceae (136)	Spirochaetales (203692)	Spirochaetia (203691)	Spirochaetes (2)	Bacteria (1)	23.10	0.10	99.90	99.60	WGS
139.213	0.0416	Borreliella burgdorferi (64895)	Borreliella (1643685)	Borreliaceae (136)	Spirochaetales (203692)	Spirochaetia (203691)	Spirochaetes (2)	Bacteria (1)	23.10	0.20	100.00	99.80	WGS
139.214	0.0416	Borreliella burgdorferi (64895)	Borreliella (1643685)	Borreliaceae (136)	Spirochaetales (203692)	Spirochaetia (203691)	Spirochaetes (2)	Bacteria (1)	23.10	0.20	100.00	99.70	WGS
139.215	0.0418	Borreliella burgdorferi (64895)	Borreliella (1643685)	Borreliaceae (136)	Spirochaetales (203692)	Spirochaetia (203691)	Spirochaetes (2)	Bacteria (1)	23.10	0.20	100.00	99.70	WGS
139.216	0.0416	Borreliella burgdorferi (64895)	Borreliella (1643685)	Borreliaceae (136)	Spirochaetales (203692)	Spirochaetia (203691)	Spirochaetes (2)	Bacteria (1)	23.10	0.20	100.00	99.70	WGS
139.217	0.0416	Borreliella burgdorferi (64895)	Borreliella (1643685)	Borreliaceae (136)	Spirochaetales (203692)	Spirochaetia (203691)	Spirochaetes (2)	Bacteria (1)	23.10	0.20	100.00	99.70	WGS
139.218	0.0416	Borreliella burgdorferi (64895)	Borreliella (1643685)	Borreliaceae (136)	Spirochaetales (203692)	Spirochaetia (203691)	Spirochaetes (2)	Bacteria (1)	23.10	0.10	100.00	99.90	WGS
139.219	0.0416	Borreliella burgdorferi (64895)	Borreliella (1643685)	Borreliaceae (136)	Spirochaetales (203692)	Spirochaetia (203691)	Spirochaetes (2)	Bacteria (1)	23.10	0.20	100.00	99.60	WGS
139.220	0.0416	Borreliella burgdorferi (64895)	Borreliella (1643685)	Borreliaceae (136)	Spirochaetales (203692)	Spirochaetia (203691)	Spirochaetes (2)	Bacteria (1)	23.10	0.10	100.00	99.70	WGS
139.22	0.0378	Borreliella burgdorferi (64895)	Borreliella (1643685)	Borreliaceae (136)	Spirochaetales (203692)	Spirochaetia (203691)	Spirochaetes (2)	Bacteria (1)	15.60	25.20	93.60	88.00	WGS
139.221	0.0416	Borreliella burgdorferi (64895)	Borreliella (1643685)	Borreliaceae (136)	Spirochaetales (203692)	Spirochaetia (203691)	Spirochaetes (2)	Bacteria (1)	23.10	0.10	100.00	99.80	WGS
139.222	0.0416	Borreliella burgdorferi (64895)	Borreliella (1643685)	Borreliaceae (136)	Spirochaetales (203692)	Spirochaetia (203691)	Spirochaetes (2)	Bacteria (1)	23.10	0.20	100.00	99.70	WGS
139.223	0.0416	Borreliella burgdorferi (64895)	Borreliella (1643685)	Borreliaceae (136)	Spirochaetales (203692)	Spirochaetia (203691)	Spirochaetes (2)	Bacteria (1)	23.10	0.10	100.00	99.80	WGS
139.224	0.0416	Borreliella burgdorferi (64895)	Borreliella (1643685)	Borreliaceae (136)	Spirochaetales (203692)	Spirochaetia (203691)	Spirochaetes (2)	Bacteria (1)	23.10	0.20	100.00	99.70	WGS
139.225	0.0416	Borreliella burgdorferi (64895)	Borreliella (1643685)	Borreliaceae (136)	Spirochaetales (203692)	Spirochaetia (203691)	Spirochaetes (2)	Bacteria (1)	23.10	0.20	100.00	99.70	WGS
139.226	0.0416	Borreliella burgdorferi (64895)	Borreliella (1643685)	Borreliaceae (136)	Spirochaetales (203692)	Spirochaetia (203691)	Spirochaetes (2)	Bacteria (1)	23.10	0.20	100.00	99.70	WGS
139.227	0.0416	Borreliella burgdorferi (64895)	Borreliella (1643685)	Borreliaceae (136)	Spirochaetales (203692)	Spirochaetia (203691)	Spirochaetes (2)	Bacteria (1)	23.10	0.20	100.00	99.60	WGS
139.228	0.0416	Borreliella burgdorferi (64895)	Borreliella (1643685)	Borreliaceae (136)	Spirochaetales (203692)	Spirochaetia (203691)	Spirochaetes (2)	Bacteria (1)	23.10	0.10	100.00	99.80	WGS
139.229	0.0416	Borreliella burgdorferi (64895)	Borreliella (1643685)	Borreliaceae (136)	Spirochaetales (203692)	Spirochaetia (203691)	Spirochaetes (2)	Bacteria (1)	23.10	0.20	100.00	99.60	WGS
139.23	0.0396	Borreliella burgdorferi (64895)	Borreliella (1643685)	Borreliaceae (136)	Spirochaetales (203692)	Spirochaetia (203691)	Spirochaetes (2)	Bacteria (1)	20.60	20.90	97.50	90.20	WGS
139.230	0.0416	Borreliella burgdorferi (64895)	Borreliella (1643685)	Borreliaceae (136)	Spirochaetales (203692)	Spirochaetia (203691)	Spirochaetes (2)	Bacteria (1)	23.10	0.20	100.00	99.80	WGS
139.231	0.0416	Borreliella burgdorferi (64895)	Borreliella (1643685)	Borreliaceae (136)	Spirochaetales (203692)	Spirochaetia (203691)	Spirochaetes (2)	Bacteria (1)	23.10	0.20	100.00	99.70	WGS
139.232	0.0416	Borreliella burgdorferi (64895)	Borreliella (1643685)	Borreliaceae (136)	Spirochaetales (203692)	Spirochaetia (203691)	Spirochaetes (2)	Bacteria (1)	23.10	0.20	100.00	99.70	WGS
139.233	0.0416	Borreliella burgdorferi (64895)	Borreliella (1643685)	Borreliaceae (136)	Spirochaetales (203692)	Spirochaetia (203691)	Spirochaetes (2)	Bacteria (1)	23.10	0.20	100.00	99.70	WGS
139.234	0.0416	Borreliella burgdorferi (64895)	Borreliella (1643685)	Borreliaceae (136)	Spirochaetales (203692)	Spirochaetia (203691)	Spirochaetes (2)	Bacteria (1)	23.10	0.20	100.00	99.70	WGS
139.235	0.0416	Borreliella burgdorferi (64895)	Borreliella (1643685)	Borreliaceae (136)	Spirochaetales (203692)	Spirochaetia (203691)	Spirochaetes (2)	Bacteria (1)	23.10	0.20	100.00	99.70	WGS
139.236	0.0416	Borreliella burgdorferi (64895)	Borreliella (1643685)	Borreliaceae (136)	Spirochaetales (203692)	Spirochaetia (203691)	Spirochaetes (2)	Bacteria (1)	23.10	0.10	100.00	99.80	WGS
139.237	0.0416	Borreliella burgdorferi (64895)	Borreliella (1643685)	Borreliaceae (136)	Spirochaetales (203692)	Spirochaetia (203691)	Spirochaetes (2)	Bacteria (1)	23.10	0.10	100.00	99.80	WGS
139.238	0.0416	Borreliella burgdorferi (64895)	Borreliella (1643685)	Borreliaceae (136)	Spirochaetales (203692)	Spirochaetia (203691)	Spirochaetes (2)	Bacteria (1)	23.10	0.20	100.00	99.60	WGS
139.239	0.0416	Borreliella burgdorferi (64895)	Borreliella (1643685)	Borreliaceae (136)	Spirochaetales (203692)	Spirochaetia (203691)	Spirochaetes (2)	Bacteria (1)	23.10	0.20	100.00	99.70	WGS
139.24	0.0416	Borreliella burgdorferi (64895)	Borreliella (1643685)	Borreliaceae (136)	Spirochaetales (203692)	Spirochaetia (203691)	Spirochaetes (2)	Bacteria (1)	23.10	25.10	99.30	92.50	WGS
139.240	0.0416	Borreliella burgdorferi (64895)	Borreliella (1643685)	Borreliaceae (136)	Spirochaetales (203692)	Spirochaetia (203691)	Spirochaetes (2)	Bacteria (1)	23.10	0.20	99.90	99.60	WGS
139.241	0.0418	Borreliella burgdorferi (64895)	Borreliella (1643685)	Borreliaceae (136)	Spirochaetales (203692)	Spirochaetia (203691)	Spirochaetes (2)	Bacteria (1)	23.10	0.20	100.00	99.70	WGS
139.242	0.0416	Borreliella burgdorferi (64895)	Borreliella (1643685)	Borreliaceae (136)	Spirochaetales (203692)	Spirochaetia (203691)	Spirochaetes (2)	Bacteria (1)	23.10	0.20	100.00	99.70	WGS
139.243	0.0416	Borreliella burgdorferi (64895)	Borreliella (1643685)	Borreliaceae (136)	Spirochaetales (203692)	Spirochaetia (203691)	Spirochaetes (2)	Bacteria (1)	23.10	0.10	100.00	99.70	WGS
139.244	0.0416	Borreliella burgdorferi (64895)	Borreliella (1643685)	Borreliaceae (136)	Spirochaetales (203692)	Spirochaetia (203691)	Spirochaetes (2)	Bacteria (1)	23.10	0.10	100.00	99.70	WGS
139.245	0.0416	Borreliella burgdorferi (64895)	Borreliella (1643685)	Borreliaceae (136)	Spirochaetales (203692)	Spirochaetia (203691)	Spirochaetes (2)	Bacteria (1)	23.10	0.20	100.00	99.70	WGS
139.246	0.0416	Borreliella burgdorferi (64895)	Borreliella (1643685)	Borreliaceae (136)	Spirochaetales (203692)	Spirochaetia (203691)	Spirochaetes (2)	Bacteria (1)	23.10	0.20	100.00	99.70	WGS
139.247	0.0416	Borreliella burgdorferi (64895)	Borreliella (1643685)	Borreliaceae (136)	Spirochaetales (203692)	Spirochaetia (203691)	Spirochaetes (2)	Bacteria (1)	23.10	0.10	100.00	99.80	WGS
139.248	0.0418	Borreliella burgdorferi (64895)	Borreliella (1643685)	Borreliaceae (136)	Spirochaetales (203692)	Spirochaetia (203691)	Spirochaetes (2)	Bacteria (1)	23.10	0.20	100.00	99.70	WGS
139.249	0.0418	Borreliella burgdorferi (64895)	Borreliella (1643685)	Borreliaceae (136)	Spirochaetales (203692)	Spirochaetia (203691)	Spirochaetes (2)	Bacteria (1)	23.10	0.20	100.00	99.70	WGS
139.250	0.0416	Borreliella burgdorferi (64895)	Borreliella (1643685)	Borreliaceae (136)	Spirochaetales (203692)	Spirochaetia (203691)	Spirochaetes (2)	Bacteria (1)	23.10	0.20	100.00	99.80	WGS
139.25	0.0396	Borreliella burgdorferi (64895)	Borreliella (1643685)	Borreliaceae (136)	Spirochaetales (203692)	Spirochaetia (203691)	Spirochaetes (2)	Bacteria (1)	23.10	30.10	99.00	93.10	WGS
139.251	0.0416	Borreliella burgdorferi (64895)	Borreliella (1643685)	Borreliaceae (136)	Spirochaetales (203692)	Spirochaetia (203691)	Spirochaetes (2)	Bacteria (1)	23.10	0.20	99.90	99.80	WGS
139.252	0.0416	Borreliella burgdorferi (64895)	Borreliella (1643685)	Borreliaceae (136)	Spirochaetales (203692)	Spirochaetia (203691)	Spirochaetes (2)	Bacteria (1)	23.10	0.20	100.00	99.70	WGS
139.253	0.0416	Borreliella burgdorferi (64895)	Borreliella (1643685)	Borreliaceae (136)	Spirochaetales (203692)	Spirochaetia (203691)	Spirochaetes (2)	Bacteria (1)	23.10	0.20	100.00	99.70	WGS
139.254	0.0416	Borreliella burgdorferi (64895)	Borreliella (1643685)	Borreliaceae (136)	Spirochaetales (203692)	Spirochaetia (203691)	Spirochaetes (2)	Bacteria (1)	23.10	0.10	100.00	99.80	WGS
139.255	0.0416	Borreliella burgdorferi (64895)	Borreliella (1643685)	Borreliaceae (136)	Spirochaetales (203692)	Spirochaetia (203691)	Spirochaetes (2)	Bacteria (1)	23.10	0.10	100.00	99.80	WGS
139.256	0.0416	Borreliella burgdorferi (64895)	Borreliella (1643685)	Borreliaceae (136)	Spirochaetales (203692)	Spirochaetia (203691)	Spirochaetes (2)	Bacteria (1)	23.10	0.20	100.00	99.60	WGS
139.257	0.0416	Borreliella burgdorferi (64895)	Borreliella (1643685)	Borreliaceae (136)	Spirochaetales (203692)	Spirochaetia (203691)	Spirochaetes (2)	Bacteria (1)	23.10	0.10	100.00	99.80	WGS
139.258	0.0416	Borreliella burgdorferi (64895)	Borreliella (1643685)	Borreliaceae (136)	Spirochaetales (203692)	Spirochaetia (203691)	Spirochaetes (2)	Bacteria (1)	23.10	0.10	100.00	99.80	WGS
139.259	0.0416	Borreliella burgdorferi (64895)	Borreliella (1643685)	Borreliaceae (136)	Spirochaetales (203692)	Spirochaetia (203691)	Spirochaetes (2)	Bacteria (1)	23.10	0.10	100.00	99.70	WGS
139.26	0.0416	Borreliella burgdorferi (64895)	Borreliella (1643685)	Borreliaceae (136)	Spirochaetales (203692)	Spirochaetia (203691)	Spirochaetes (2)	Bacteria (1)	23.10	10.10	99.60	96.70	WGS
139.268	0.0416	Borreliella burgdorferi (64895)	Borreliella (1643685)	Borreliaceae (136)	Spirochaetales (203692)	Spirochaetia (203691)	Spirochaetes (2)	Bacteria (1)	25.50	34.20	99.40	85.40	Complete
139.27	0.037	Borreliella burgdorferi (64895)	Borreliella (1643685)	Borreliaceae (136)	Spirochaetales (203692)	Spirochaetia (203691)	Spirochaetes (2)	Bacteria (1)	18.20	30.20	98.00	90.20	WGS
139.28	0.0396	Borreliella burgdorferi (64895)	Borreliella (1643685)	Borreliaceae (136)	Spirochaetales (203692)	Spirochaetia (203691)	Spirochaetes (2)	Bacteria (1)	23.10	25.10	99.40	91.50	WGS
139.29	0.0404	Borreliella burgdorferi (64895)	Borreliella (1643685)	Borreliaceae (136)	Spirochaetales (203692)	Spirochaetia (203691)	Spirochaetes (2)	Bacteria (1)	23.10	25.10	98.90	91.40	WGS
139.30	0.0396	Borreliella burgdorferi (64895)	Borreliella (1643685)	Borreliaceae (136)	Spirochaetales (203692)	Spirochaetia (203691)	Spirochaetes (2)	Bacteria (1)	23.10	25.00	98.70	92.20	WGS
139.31	0.0416	Borreliella burgdorferi (64895)	Borreliella (1643685)	Borreliaceae (136)	Spirochaetales (203692)	Spirochaetia (203691)	Spirochaetes (2)	Bacteria (1)	23.10	25.10	99.50	93.10	WGS
139.32	0.0388	Borreliella burgdorferi (64895)	Borreliella (1643685)	Borreliaceae (136)	Spirochaetales (203692)	Spirochaetia (203691)	Spirochaetes (2)	Bacteria (1)	7.80	24.40	90.20	89.80	WGS
139.33	0.0396	Borreliella burgdorferi (64895)	Borreliella (1643685)	Borreliaceae (136)	Spirochaetales (203692)	Spirochaetia (203691)	Spirochaetes (2)	Bacteria (1)	23.10	24.70	99.30	93.50	WGS
139.34	0.038	Borreliella burgdorferi (64895)	Borreliella (1643685)	Borreliaceae (136)	Spirochaetales (203692)	Spirochaetia (203691)	Spirochaetes (2)	Bacteria (1)	13.40	5.10	96.30	88.60	WGS
139.35	0.0404	Borreliella burgdorferi (64895)	Borreliella (1643685)	Borreliaceae (136)	Spirochaetales (203692)	Spirochaetia (203691)	Spirochaetes (2)	Bacteria (1)	18.30	22.70	99.00	92.00	WGS
139.36	0.0412	Borreliella burgdorferi (64895)	Borreliella (1643685)	Borreliaceae (136)	Spirochaetales (203692)	Spirochaetia (203691)	Spirochaetes (2)	Bacteria (1)	20.50	45.80	96.90	88.60	WGS
139.37	0.0416	Borreliella burgdorferi (64895)	Borreliella (1643685)	Borreliaceae (136)	Spirochaetales (203692)	Spirochaetia (203691)	Spirochaetes (2)	Bacteria (1)	23.10	10.60	99.00	93.40	WGS
139.38	0.038	Borreliella burgdorferi (64895)	Borreliella (1643685)	Borreliaceae (136)	Spirochaetales (203692)	Spirochaetia (203691)	Spirochaetes (2)	Bacteria (1)	20.60	41.90	97.60	90.00	WGS
139.39	0.039	Borreliella burgdorferi (64895)	Borreliella (1643685)	Borreliaceae (136)	Spirochaetales (203692)	Spirochaetia (203691)	Spirochaetes (2)	Bacteria (1)	23.10	35.30	99.20	91.10	WGS
139.40	0.0376	Borreliella burgdorferi (64895)	Borreliella (1643685)	Borreliaceae (136)	Spirochaetales (203692)	Spirochaetia (203691)	Spirochaetes (2)	Bacteria (1)	15.70	33.10	95.10	89.00	WGS
139.41	0.0294	Borreliella burgdorferi (64895)	Borreliella (1643685)	Borreliaceae (136)	Spirochaetales (203692)	Spirochaetia (203691)	Spirochaetes (2)	Bacteria (1)	7.70	1.90	90.00	89.50	WGS
139.42	0.031	Borreliella burgdorferi (64895)	Borreliella (1643685)	Borreliaceae (136)	Spirochaetales (203692)	Spirochaetia (203691)	Spirochaetes (2)	Bacteria (1)	10.50	32.80	89.90	87.00	WGS
139.43	0.0404	Borreliella burgdorferi (64895)	Borreliella (1643685)	Borreliaceae (136)	Spirochaetales (203692)	Spirochaetia (203691)	Spirochaetes (2)	Bacteria (1)	18.10	41.50	95.00	86.70	WGS
139.44	0.0416	Borreliella burgdorferi (64895)	Borreliella (1643685)	Borreliaceae (136)	Spirochaetales (203692)	Spirochaetia (203691)	Spirochaetes (2)	Bacteria (1)	23.10	35.20	99.50	91.40	WGS
139.45	0.0416	Borreliella burgdorferi (64895)	Borreliella (1643685)	Borreliaceae (136)	Spirochaetales (203692)	Spirochaetia (203691)	Spirochaetes (2)	Bacteria (1)	18.30	35.40	99.40	92.80	WGS
139.46	0.039	Borreliella burgdorferi (64895)	Borreliella (1643685)	Borreliaceae (136)	Spirochaetales (203692)	Spirochaetia (203691)	Spirochaetes (2)	Bacteria (1)	23.10	24.90	99.90	93.50	WGS
139.47	0.0284	Borreliella garinii (64895)	Borreliella (1643685)	Borreliaceae (136)	Spirochaetales (203692)	Spirochaetia (203691)	Spirochaetes (2)	Bacteria (1)	17.90	41.00	93.30	88.40	WGS
139.48	0.0396	Borreliella garinii (64895)	Borreliella (1643685)	Borreliaceae (136)	Spirochaetales (203692)	Spirochaetia (203691)	Spirochaetes (2)	Bacteria (1)	20.70	20.30	98.90	91.90	WGS
139.49	0.0404	Borreliella garinii (64895)	Borreliella (1643685)	Borreliaceae (136)	Spirochaetales (203692)	Spirochaetia (203691)	Spirochaetes (2)	Bacteria (1)	23.10	35.00	99.70	93.20	WGS
139.50	0.0284	Borreliella garinii (64895)	Borreliella (1643685)	Borreliaceae (136)	Spirochaetales (203692)	Spirochaetia (203691)	Spirochaetes (2)	Bacteria (1)	10.20	32.30	89.00	88.00	WGS
139.51	0.0396	Borreliella garinii (64895)	Borreliella (1643685)	Borreliaceae (136)	Spirochaetales (203692)	Spirochaetia (203691)	Spirochaetes (2)	Bacteria (1)	23.10	24.50	99.50	96.10	WGS
139.52	0.0378	Borreliella garinii (64895)	Borreliella (1643685)	Borreliaceae (136)	Spirochaetales (203692)	Spirochaetia (203691)	Spirochaetes (2)	Bacteria (1)	18.30	52.10	97.90	90.10	WGS
139.53	0.037	Borreliella garinii (64895)	Borreliella (1643685)	Borreliaceae (136)	Spirochaetales (203692)	Spirochaetia (203691)	Spirochaetes (2)	Bacteria (1)	20.60	33.10	97.30	89.80	WGS
139.54	0.0396	Borreliella garinii (64895)	Borreliella (1643685)	Borreliaceae (136)	Spirochaetales (203692)	Spirochaetia (203691)	Spirochaetes (2)	Bacteria (1)	15.80	25.70	97.20	91.00	WGS
139.55	0.0388	Borreliella garinii (64895)	Borreliella (1643685)	Borreliaceae (136)	Spirochaetales (203692)	Spirochaetia (203691)	Spirochaetes (2)	Bacteria (1)	13.00	17.60	90.80	86.80	WGS
139.56	0.0416	Borreliella garinii (64895)	Borreliella (1643685)	Borreliaceae (136)	Spirochaetales (203692)	Spirochaetia (203691)	Spirochaetes (2)	Bacteria (1)	20.70	12.00	99.10	94.30	WGS
139.57	0.0416	Borreliella garinii (64895)	Borreliella (1643685)	Borreliaceae (136)	Spirochaetales (203692)	Spirochaetia (203691)	Spirochaetes (2)	Bacteria (1)	23.10	39.20	99.40	92.10	WGS
139.58	0.0388	Borreliella garinii (64895)	Borreliella (1643685)	Borreliaceae (136)	Spirochaetales (203692)	Spirochaetia (203691)	Spirochaetes (2)	Bacteria (1)	15.60	26.20	93.60	86.90	WGS
139.59	0.0388	Borreliella garinii (64895)	Borreliella (1643685)	Borreliaceae (136)	Spirochaetales (203692)	Spirochaetia (203691)	Spirochaetes (2)	Bacteria (1)	18.20	23.20	97.20	89.20	WGS
139.60	0.0388	Borreliella garinii (64895)	Borreliella (1643685)	Borreliaceae (136)	Spirochaetales (203692)	Spirochaetia (203691)	Spirochaetes (2)	Bacteria (1)	18.10	41.10	96.50	88.80	WGS
139.61	0.0396	Borreliella garinii (64895)	Borreliella (1643685)	Borreliaceae (136)	Spirochaetales (203692)	Spirochaetia (203691)	Spirochaetes (2)	Bacteria (1)	23.10	25.10	99.10	91.80	WGS
139.62	0.0396	Borreliella garinii (64895)	Borreliella (1643685)	Borreliaceae (136)	Spirochaetales (203692)	Spirochaetia (203691)	Spirochaetes (2)	Bacteria (1)	20.70	20.40	97.60	91.90	WGS
139.63	0.0424	Borreliella garinii (64895)	Borreliella (1643685)	Borreliaceae (136)	Spirochaetales (203692)	Spirochaetia (203691)	Spirochaetes (2)	Bacteria (1)	20.60	20.40	97.20	90.80	WGS
139.64	0.0416	Borreliella garinii (64895)	Borreliella (1643685)	Borreliaceae (136)	Spirochaetales (203692)	Spirochaetia (203691)	Spirochaetes (2)	Bacteria (1)	23.10	18.40	99.70	94.20	WGS
139.65	0.038	Borreliella garinii (64895)	Borreliella (1643685)	Borreliaceae (136)	Spirochaetales (203692)	Spirochaetia (203691)	Spirochaetes (2)	Bacteria (1)	22.90	30.80	96.50	89.50	WGS
139.66	0.0378	Borreliella garinii (64895)	Borreliella (1643685)	Borreliaceae (136)	Spirochaetales (203692)	Spirochaetia (203691)	Spirochaetes (2)	Bacteria (1)	23.10	18.80	98.80	91.60	WGS
139.67	0.0424	Borreliella garinii (64895)	Borreliella (1643685)	Borreliaceae (136)	Spirochaetales (203692)	Spirochaetia (203691)	Spirochaetes (2)	Bacteria (1)	18.20	30.00	97.60	90.10	WGS
139.68	0.0396	Borreliella garinii (64895)	Borreliella (1643685)	Borreliaceae (136)	Spirochaetales (203692)	Spirochaetia (203691)	Spirochaetes (2)	Bacteria (1)	23.10	30.70	99.30	91.70	WGS
139.69	0.037	Borreliella garinii (64895)	Borreliella (1643685)	Borreliaceae (136)	Spirochaetales (203692)	Spirochaetia (203691)	Spirochaetes (2)	Bacteria (1)	23.10	35.10	99.10	92.80	WGS
139.70	0.0404	Borreliella garinii (64895)	Borreliella (1643685)	Borreliaceae (136)	Spirochaetales (203692)	Spirochaetia (203691)	Spirochaetes (2)	Bacteria (1)	23.00	18.80	98.40	92.20	WGS
139.71	0.04	Borreliella garinii (64895)	Borreliella (1643685)	Borreliaceae (136)	Spirochaetales (203692)	Spirochaetia (203691)	Spirochaetes (2)	Bacteria (1)	18.10	36.00	95.40	87.20	WGS
139.72	0.0396	Borreliella garinii (64895)	Borreliella (1643685)	Borreliaceae (136)	Spirochaetales (203692)	Spirochaetia (203691)	Spirochaetes (2)	Bacteria (1)	23.10	39.50	98.50	91.10	WGS
139.73	0.0396	Borreliella garinii (64895)	Borreliella (1643685)	Borreliaceae (136)	Spirochaetales (203692)	Spirochaetia (203691)	Spirochaetes (2)	Bacteria (1)	18.30	29.90	99.60	91.60	WGS
139.74	0.04	Borreliella garinii (64895)	Borreliella (1643685)	Borreliaceae (136)	Spirochaetales (203692)	Spirochaetia (203691)	Spirochaetes (2)	Bacteria (1)	18.20	36.20	97.30	89.90	WGS
139.75	0.0378	Borreliella garinii (64895)	Borreliella (1643685)	Borreliaceae (136)	Spirochaetales (203692)	Spirochaetia (203691)	Spirochaetes (2)	Bacteria (1)	20.60	20.90	96.50	90.50	WGS
139.76	0.0392	Borreliella garinii (64895)	Borreliella (1643685)	Borreliaceae (136)	Spirochaetales (203692)	Spirochaetia (203691)	Spirochaetes (2)	Bacteria (1)	23.00	39.20	99.00	92.20	WGS
139.78	0.0416	Borreliella garinii (64895)	Borreliella (1643685)	Borreliaceae (136)	Spirochaetales (203692)	Spirochaetia (203691)	Spirochaetes (2)	Bacteria (1)	23.10	39.00	99.50	93.20	WGS
139.79	0.0404	Borreliella garinii (64895)	Borreliella (1643685)	Borreliaceae (136)	Spirochaetales (203692)	Spirochaetia (203691)	Spirochaetes (2)	Bacteria (1)	20.50	21.40	95.30	88.00	WGS
139.80	0.0378	Borreliella garinii (64895)	Borreliella (1643685)	Borreliaceae (136)	Spirochaetales (203692)	Spirochaetia (203691)	Spirochaetes (2)	Bacteria (1)	20.60	27.20	98.70	91.90	WGS
139.81	0.0416	Borreliella burgdorferi (64895)	Borreliella (1643685)	Borreliaceae (136)	Spirochaetales (203692)	Spirochaetia (203691)	Spirochaetes (2)	Bacteria (1)	23.10	0.20	99.60	99.30	Complete
139.87	0.0416	Borreliella burgdorferi (64895)	Borreliella (1643685)	Borreliaceae (136)	Spirochaetales (203692)	Spirochaetia (203691)	Spirochaetes (2)	Bacteria (1)	23.10	0.70	100.00	98.40	WGS
139.88	0.0416	Borreliella burgdorferi (64895)	Borreliella (1643685)	Borreliaceae (136)	Spirochaetales (203692)	Spirochaetia (203691)	Spirochaetes (2)	Bacteria (1)	23.10	0.10	99.90	99.90	Complete
139.89	0.0416	Borreliella burgdorferi (64895)	Borreliella (1643685)	Borreliaceae (136)	Spirochaetales (203692)	Spirochaetia (203691)	Spirochaetes (2)	Bacteria (1)	23.10	0.10	99.90	99.80	Complete
139.90	0.0294	Borreliella burgdorferi (64895)	Borreliella (1643685)	Borreliaceae (136)	Spirochaetales (203692)	Spirochaetia (203691)	Spirochaetes (2)	Bacteria (1)	23.10	0.10	99.90	99.90	WGS
139.96	0.0416	Borreliella burgdorferi (64895)	Borreliella (1643685)	Borreliaceae (136)	Spirochaetales (203692)	Spirochaetia (203691)	Spirochaetes (2)	Bacteria (1)	23.10	0.20	99.90	99.80	Complete
139.97	0.0294	Borreliella burgdorferi (64895)	Borreliella (1643685)	Borreliaceae (136)	Spirochaetales (203692)	Spirochaetia (203691)	Spirochaetes (2)	Bacteria (1)					Plasmid
139.98	0.0294	Borreliella burgdorferi (64895)	Borreliella (1643685)	Borreliaceae (136)	Spirochaetales (203692)	Spirochaetia (203691)	Spirochaetes (2)	Bacteria (1)					Plasmid
139.99	0.0294	Borreliella burgdorferi (64895)	Borreliella (1643685)	Borreliaceae (136)	Spirochaetales (203692)	Spirochaetia (203691)	Spirochaetes (2)	Bacteria (1)					Plasmid
140.10	0.0294	Borrelia hermsii (138)	Borrelia (1643685)	Borreliaceae (136)	Spirochaetales (203692)	Spirochaetia (203691)	Spirochaetes (2)	Bacteria (1)					Plasmid
140.11	0.0294	Borrelia hermsii (138)	Borrelia (1643685)	Borreliaceae (136)	Spirochaetales (203692)	Spirochaetia (203691)	Spirochaetes (2)	Bacteria (1)					Plasmid
140.12	0.0294	Borrelia hermsii (138)	Borrelia (1643685)	Borreliaceae (136)	Spirochaetales (203692)	Spirochaetia (203691)	Spirochaetes (2)	Bacteria (1)					Plasmid
140.13	0.0294	Borrelia hermsii (138)	Borrelia (1643685)	Borreliaceae (136)	Spirochaetales (203692)	Spirochaetia (203691)	Spirochaetes (2)	Bacteria (1)					Plasmid
140.14	0.0294	Borrelia hermsii (138)	Borrelia (1643685)	Borreliaceae (136)	Spirochaetales (203692)	Spirochaetia (203691)	Spirochaetes (2)	Bacteria (1)					Plasmid
140.15	0.0294	Borrelia hermsii (138)	Borrelia (1643685)	Borreliaceae (136)	Spirochaetales (203692)	Spirochaetia (203691)	Spirochaetes (2)	Bacteria (1)					Plasmid
140.16	0.0294	Borrelia hermsii (138)	Borrelia (1643685)	Borreliaceae (136)	Spirochaetales (203692)	Spirochaetia (203691)	Spirochaetes (2)	Bacteria (1)					Plasmid
140.17	0.0294	Borrelia hermsii (138)	Borrelia (1643685)	Borreliaceae (136)	Spirochaetales (203692)	Spirochaetia (203691)	Spirochaetes (2)	Bacteria (1)					Plasmid
140.18	0.0294	Borrelia hermsii (138)	Borrelia (1643685)	Borreliaceae (136)	Spirochaetales (203692)	Spirochaetia (203691)	Spirochaetes (2)	Bacteria (1)					Plasmid
140.19	0.0294	Borrelia hermsii (138)	Borrelia (1643685)	Borreliaceae (136)	Spirochaetales (203692)	Spirochaetia (203691)	Spirochaetes (2)	Bacteria (1)					Plasmid
140.20	0.0294	Borrelia hermsii (138)	Borrelia (1643685)	Borreliaceae (136)	Spirochaetales (203692)	Spirochaetia (203691)	Spirochaetes (2)	Bacteria (1)					Plasmid
140.21	0.0294	Borrelia hermsii (138)	Borrelia (1643685)	Borreliaceae (136)	Spirochaetales (203692)	Spirochaetia (203691)	Spirochaetes (2)	Bacteria (1)					Plasmid
140.22	0.0294	Borrelia hermsii (138)	Borrelia (1643685)	Borreliaceae (136)	Spirochaetales (203692)	Spirochaetia (203691)	Spirochaetes (2)	Bacteria (1)					Plasmid
140.23	0.0294	Borrelia hermsii (138)	Borrelia (1643685)	Borreliaceae (136)	Spirochaetales (203692)	Spirochaetia (203691)	Spirochaetes (2)	Bacteria (1)					Plasmid
140.24	0.0294	Borrelia hermsii (138)	Borrelia (1643685)	Borreliaceae (136)	Spirochaetales (203692)	Spirochaetia (203691)	Spirochaetes (2)	Bacteria (1)					Plasmid
140.25	0.0294	Borrelia hermsii (138)	Borrelia (1643685)	Borreliaceae (136)	Spirochaetales (203692)	Spirochaetia (203691)	Spirochaetes (2)	Bacteria (1)					Plasmid
140.26	0.0294	Borrelia hermsii (138)	Borrelia (1643685)	Borreliaceae (136)	Spirochaetales (203692)	Spirochaetia (203691)	Spirochaetes (2)	Bacteria (1)					Plasmid
140.27	0.0294	Borrelia hermsii (138)	Borrelia (1643685)	Borreliaceae (136)	Spirochaetales (203692)	Spirochaetia (203691)	Spirochaetes (2)	Bacteria (1)					Plasmid
140.28	0.0294	Borrelia hermsii (138)	Borrelia (1643685)	Borreliaceae (136)	Spirochaetales (203692)	Spirochaetia (203691)	Spirochaetes (2)	Bacteria (1)					Plasmid
140.29	0.0294	Borrelia hermsii (138)	Borrelia (1643685)	Borreliaceae (136)	Spirochaetales (203692)	Spirochaetia (203691)	Spirochaetes (2)	Bacteria (1)					Plasmid
140.3	0.0494	Borrelia hermsii (138)	Borrelia (1643685)	Borreliaceae (136)	Spirochaetales (203692)	Spirochaetia (203691)	Spirochaetes (2)	Bacteria (1)	30.30	7.40	99.80	99.70	Complete
140.4	0.0294	Borrelia hermsii (138)	Borrelia (1643685)	Borreliaceae (136)	Spirochaetales (203692)	Spirochaetia (203691)	Spirochaetes (2)	Bacteria (1)					Plasmid
140.5	0.03	Borrelia hermsii (138)	Borrelia (1643685)	Borreliaceae (136)	Spirochaetales (203692)	Spirochaetia (203691)	Spirochaetes (2)	Bacteria (1)					Plasmid
140.6	0.0294	Borrelia hermsii (138)	Borrelia (1643685)	Borreliaceae (136)	Spirochaetales (203692)	Spirochaetia (203691)	Spirochaetes (2)	Bacteria (1)					Plasmid
140.7	0.0294	Borrelia hermsii (138)	Borrelia (1643685)	Borreliaceae (136)	Spirochaetales (203692)	Spirochaetia (203691)	Spirochaetes (2)	Bacteria (1)					Plasmid
140.8	0.0294	Borrelia hermsii (138)	Borrelia (1643685)	Borreliaceae (136)	Spirochaetales (203692)	Spirochaetia (203691)	Spirochaetes (2)	Bacteria (1)					Plasmid
140.9	0.0294	Borrelia hermsii (138)	Borrelia (1643685)	Borreliaceae (136)	Spirochaetales (203692)	Spirochaetia (203691)	Spirochaetes (2)	Bacteria (1)					Plasmid
142.3	0.0512	Borrelia turicatae (138)	Borrelia (1643685)	Borreliaceae (136)	Spirochaetales (203692)	Spirochaetia (203691)	Spirochaetes (2)	Bacteria (1)	30.30	39.00	99.70	99.30	Complete
158.10	0.0294	Treponema denticola (157)	Treponema (137)	Spirochaetaceae (136)	Spirochaetales (203692)	Spirochaetia (203691)	Spirochaetes (2)	Bacteria (1)					Plasmid
159.146	0.1844	Brachyspira hyodysenteriae (2952	Brachyspira (143786)	Brachyspiraceae (1643686	Brachyspirales (203692)	Spirochaetia (203691)	Spirochaetes (2)	Bacteria (1)	100.00	10.80	99.30	96.40	WGS
159.147	0.1816	Brachyspira hyodysenteriae (2952	Brachyspira (143786)	Brachyspiraceae (1643686	Brachyspirales (203692)	Spirochaetia (203691)	Spirochaetes (2)	Bacteria (1)	100.00	10.80	99.10	96.10	WGS
159.148	0.1844	Brachyspira hyodysenteriae (2952	Brachyspira (143786)	Brachyspiraceae (1643686	Brachyspirales (203692)	Spirochaetia (203691)	Spirochaetes (2)	Bacteria (1)	100.00	6.90	99.00	96.20	WGS
159.151	0.1834	Brachyspira hyodysenteriae (2952	Brachyspira (143786)	Brachyspiraceae (1643686	Brachyspirales (203692)	Spirochaetia (203691)	Spirochaetes (2)	Bacteria (1)	100.00	0.00	99.50	98.90	
159.53	0.1816	Brachyspira hyodysenteriae (2952	Brachyspira (143786)	Brachyspiraceae (1643686	Brachyspirales (203692)	Spirochaetia (203691)	Spirochaetes (2)	Bacteria (1)	100.00	0.00	99.50	98.80	WGS
159.54	0.1806	Brachyspira hyodysenteriae (2952	Brachyspira (143786)	Brachyspiraceae (1643686	Brachyspirales (203692)	Spirochaetia (203691)	Spirochaetes (2)	Bacteria (1)	100.00	0.00	99.50	99.00	WGS
159.55	0.1834	Brachyspira hyodysenteriae (2952	Brachyspira (143786)	Brachyspiraceae (1643686	Brachyspirales (203692)	Spirochaetia (203691)	Spirochaetes (2)	Bacteria (1)	100.00	0.00	99.50	98.40	WGS
159.56	0.1834	Brachyspira hyodysenteriae (2952	Brachyspira (143786)	Brachyspiraceae (1643686	Brachyspirales (203692)	Spirochaetia (203691)	Spirochaetes (2)	Bacteria (1)	100.00	0.00	99.50	98.80	WGS
159.57	0.1816	Brachyspira hyodysenteriae (2952	Brachyspira (143786)	Brachyspiraceae (1643686	Brachyspirales (203692)	Spirochaetia (203691)	Spirochaetes (2)	Bacteria (1)	100.00	0.00	99.50	98.90	WGS
159.58	0.1862	Brachyspira hyodysenteriae (2952	Brachyspira (143786)	Brachyspiraceae (1643686	Brachyspirales (203692)	Spirochaetia (203691)	Spirochaetes (2)	Bacteria (1)	100.00	0.00	99.50	99.00	WGS
159.59	0.1834	Brachyspira hyodysenteriae (2952	Brachyspira (143786)	Brachyspiraceae (1643686	Brachyspirales (203692)	Spirochaetia (203691)	Spirochaetes (2)	Bacteria (1)	100.00	0.00	99.50	98.70	WGS
159.60	0.1816	Brachyspira hyodysenteriae (2952	Brachyspira (143786)	Brachyspiraceae (1643686	Brachyspirales (203692)	Spirochaetia (203691)	Spirochaetes (2)	Bacteria (1)	100.00	0.00	99.50	98.70	WGS
159.61	0.1844	Brachyspira hyodysenteriae (2952	Brachyspira (143786)	Brachyspiraceae (1643686	Brachyspirales (203692)	Spirochaetia (203691)	Spirochaetes (2)	Bacteria (1)	100.00	0.00	99.50	98.90	WGS
159.62	0.1834	Brachyspira hyodysenteriae (2952	Brachyspira (143786)	Brachyspiraceae (1643686	Brachyspirales (203692)	Spirochaetia (203691)	Spirochaetes (2)	Bacteria (1)	100.00	0.00	99.50	98.90	WGS
159.63	0.1844	Brachyspira hyodysenteriae (2952	Brachyspira (143786)	Brachyspiraceae (1643686	Brachyspirales (203692)	Spirochaetia (203691)	Spirochaetes (2)	Bacteria (1)	100.00	0.00	99.50	98.90	WGS
159.64	0.1816	Brachyspira hyodysenteriae (2952	Brachyspira (143786)	Brachyspiraceae (1643686	Brachyspirales (203692)	Spirochaetia (203691)	Spirochaetes (2)	Bacteria (1)	100.00	0.00	99.40	98.50	WGS
159.65	0.1834	Brachyspira hyodysenteriae (2952	Brachyspira (143786)	Brachyspiraceae (1643686	Brachyspirales (203692)	Spirochaetia (203691)	Spirochaetes (2)	Bacteria (1)	100.00	0.00	99.50	98.50	WGS
159.66	0.1816	Brachyspira hyodysenteriae (2952	Brachyspira (143786)	Brachyspiraceae (1643686	Brachyspirales (203692)	Spirochaetia (203691)	Spirochaetes (2)	Bacteria (1)	100.00	0.00	99.50	98.80	WGS
159.67	0.1816	Brachyspira hyodysenteriae (2952	Brachyspira (143786)	Brachyspiraceae (1643686	Brachyspirales (203692)	Spirochaetia (203691)	Spirochaetes (2)	Bacteria (1)	100.00	0.00	99.50	98.80	WGS
159.68	0.1816	Brachyspira hyodysenteriae (2952	Brachyspira (143786)	Brachyspiraceae (1643686	Brachyspirales (203692)	Spirochaetia (203691)	Spirochaetes (2)	Bacteria (1)	100.00	0.00	99.50	98.70	WGS
159.69	0.1862	Brachyspira hyodysenteriae (2952	Brachyspira (143786)	Brachyspiraceae (1643686	Brachyspirales (203692)	Spirochaetia (203691)	Spirochaetes (2)	Bacteria (1)	100.00	0.00	99.50	98.90	WGS
159.70	0.1816	Brachyspira hyodysenteriae (2952	Brachyspira (143786)	Brachyspiraceae (1643686	Brachyspirales (203692)	Spirochaetia (203691)	Spirochaetes (2)	Bacteria (1)	100.00	0.00	99.50	98.90	WGS
159.71	0.1816	Brachyspira hyodysenteriae (2952	Brachyspira (143786)	Brachyspiraceae (1643686	Brachyspirales (203692)	Spirochaetia (203691)	Spirochaetes (2)	Bacteria (1)	100.00	0.00	99.50	98.70	WGS
161.10	0.0438	Treponema pallidum (157)	Treponema (137)	Spirochaetaceae (136)	Spirochaetales (203692)	Spirochaetia (203691)	Spirochaetes (2)	Bacteria (1)	37.60	0.00	99.70	99.60	Complete
161.11	0.0438	Treponema pallidum (157)	Treponema (137)	Spirochaetaceae (136)	Spirochaetales (203692)	Spirochaetia (203691)	Spirochaetes (2)	Bacteria (1)	37.60	0.00	99.70	99.60	Complete
161.12	0.0438	Treponema pallidum (157)	Treponema (137)	Spirochaetaceae (136)	Spirochaetales (203692)	Spirochaetia (203691)	Spirochaetes (2)	Bacteria (1)	37.60	0.00	99.70	99.60	Complete
161.13	0.0438	Treponema pallidum (157)	Treponema (137)	Spirochaetaceae (136)	Spirochaetales (203692)	Spirochaetia (203691)	Spirochaetes (2)	Bacteria (1)	37.60	0.00	99.70	99.60	Complete
161.14	0.0438	Treponema pallidum (157)	Treponema (137)	Spirochaetaceae (136)	Spirochaetales (203692)	Spirochaetia (203691)	Spirochaetes (2)	Bacteria (1)	37.60	0.00	99.70	99.60	Complete
161.15	0.0438	Treponema pallidum (157)	Treponema (137)	Spirochaetaceae (136)	Spirochaetales (203692)	Spirochaetia (203691)	Spirochaetes (2)	Bacteria (1)	37.60	0.00	99.70	99.60	Complete
161.16	0.0438	Treponema pallidum (157)	Treponema (137)	Spirochaetaceae (136)	Spirochaetales (203692)	Spirochaetia (203691)	Spirochaetes (2)	Bacteria (1)	37.60	0.00	99.70	99.60	Complete
161.17	0.0432	Treponema pallidum (157)	Treponema (137)	Spirochaetaceae (136)	Spirochaetales (203692)	Spirochaetia (203691)	Spirochaetes (2)	Bacteria (1)	37.50	0.00	99.70	99.60	Complete
161.18	0.0438	Treponema pallidum (157)	Treponema (137)	Spirochaetaceae (136)	Spirochaetales (203692)	Spirochaetia (203691)	Spirochaetes (2)	Bacteria (1)	37.60	0.00	99.70	99.60	Complete
161.19	0.0438	Treponema pallidum (157)	Treponema (137)	Spirochaetaceae (136)	Spirochaetales (203692)	Spirochaetia (203691)	Spirochaetes (2)	Bacteria (1)	37.60	0.00	99.70	99.60	Complete
161.20	0.0438	Treponema pallidum (157)	Treponema (137)	Spirochaetaceae (136)	Spirochaetales (203692)	Spirochaetia (203691)	Spirochaetes (2)	Bacteria (1)	37.60	0.00	99.70	99.60	Complete
161.21	0.0438	Treponema pallidum (157)	Treponema (137)	Spirochaetaceae (136)	Spirochaetales (203692)	Spirochaetia (203691)	Spirochaetes (2)	Bacteria (1)	37.60	0.00	99.70	99.60	Complete
161.22	0.0424	Treponema pallidum (157)	Treponema (137)	Spirochaetaceae (136)	Spirochaetales (203692)	Spirochaetia (203691)	Spirochaetes (2)	Bacteria (1)	37.60	0.00	99.60	99.50	Complete
161.23	0.0438	Treponema pallidum (157)	Treponema (137)	Spirochaetaceae (136)	Spirochaetales (203692)	Spirochaetia (203691)	Spirochaetes (2)	Bacteria (1)	37.60	0.00	99.70	99.60	Complete
161.24	0.0438	Treponema pallidum (157)	Treponema (137)	Spirochaetaceae (136)	Spirochaetales (203692)	Spirochaetia (203691)	Spirochaetes (2)	Bacteria (1)	37.50	0.00	99.30	99.20	Complete
161.25	0.0438	Treponema pallidum (157)	Treponema (137)	Spirochaetaceae (136)	Spirochaetales (203692)	Spirochaetia (203691)	Spirochaetes (2)	Bacteria (1)	37.60	0.00	99.70	99.60	Complete
161.26	0.0438	Treponema pallidum (157)	Treponema (137)	Spirochaetaceae (136)	Spirochaetales (203692)	Spirochaetia (203691)	Spirochaetes (2)	Bacteria (1)	37.60	0.00	99.70	99.60	Complete
161.27	0.0438	Treponema pallidum (157)	Treponema (137)	Spirochaetaceae (136)	Spirochaetales (203692)	Spirochaetia (203691)	Spirochaetes (2)	Bacteria (1)	37.60	0.00	99.70	99.60	Complete
161.28	0.0438	Treponema pallidum (157)	Treponema (137)	Spirochaetaceae (136)	Spirochaetales (203692)	Spirochaetia (203691)	Spirochaetes (2)	Bacteria (1)	37.60	0.00	99.70	99.60	Complete
161.29	0.0438	Treponema pallidum (157)	Treponema (137)	Spirochaetaceae (136)	Spirochaetales (203692)	Spirochaetia (203691)	Spirochaetes (2)	Bacteria (1)	37.60	0.00	99.70	99.60	Complete
161.30	0.0438	Treponema pallidum (157)	Treponema (137)	Spirochaetaceae (136)	Spirochaetales (203692)	Spirochaetia (203691)	Spirochaetes (2)	Bacteria (1)	37.60	0.00	99.70	99.60	Complete
161.31	0.0438	Treponema pallidum (157)	Treponema (137)	Spirochaetaceae (136)	Spirochaetales (203692)	Spirochaetia (203691)	Spirochaetes (2)	Bacteria (1)	37.60	0.00	99.70	99.60	Complete
161.33	0.0438	Treponema pallidum (157)	Treponema (137)	Spirochaetaceae (136)	Spirochaetales (203692)	Spirochaetia (203691)	Spirochaetes (2)	Bacteria (1)	37.60	0.00	99.70	99.60	Complete
161.34	0.0438	Treponema pallidum (157)	Treponema (137)	Spirochaetaceae (136)	Spirochaetales (203692)	Spirochaetia (203691)	Spirochaetes (2)	Bacteria (1)	37.60	0.00	99.70	99.60	Complete
161.35	0.0438	Treponema pallidum (157)	Treponema (137)	Spirochaetaceae (136)	Spirochaetales (203692)	Spirochaetia (203691)	Spirochaetes (2)	Bacteria (1)	37.60	0.00	99.70	99.60	Complete
161.36	0.0438	Treponema pallidum (157)	Treponema (137)	Spirochaetaceae (136)	Spirochaetales (203692)	Spirochaetia (203691)	Spirochaetes (2)	Bacteria (1)	37.60	0.00	99.70	99.60	Complete
161.37	0.0438	Treponema pallidum (157)	Treponema (137)	Spirochaetaceae (136)	Spirochaetales (203692)	Spirochaetia (203691)	Spirochaetes (2)	Bacteria (1)	37.60	0.00	99.70	99.60	Complete
161.38	0.0438	Treponema pallidum (157)	Treponema (137)	Spirochaetaceae (136)	Spirochaetales (203692)	Spirochaetia (203691)	Spirochaetes (2)	Bacteria (1)	37.60	0.00	99.70	99.60	Complete
161.39	0.0438	Treponema pallidum (157)	Treponema (137)	Spirochaetaceae (136)	Spirochaetales (203692)	Spirochaetia (203691)	Spirochaetes (2)	Bacteria (1)	37.60	0.00	99.70	99.60	Complete
161.40	0.0438	Treponema pallidum (157)	Treponema (137)	Spirochaetaceae (136)	Spirochaetales (203692)	Spirochaetia (203691)	Spirochaetes (2)	Bacteria (1)	37.60	0.00	99.70	99.60	Complete
161.42	0.0438	Treponema pallidum (157)	Treponema (137)	Spirochaetaceae (136)	Spirochaetales (203692)	Spirochaetia (203691)	Spirochaetes (2)	Bacteria (1)	37.60	0.00	99.70	99.60	Complete
161.45	0.0438	Treponema pallidum (157)	Treponema (137)	Spirochaetaceae (136)	Spirochaetales (203692)	Spirochaetia (203691)	Spirochaetes (2)	Bacteria (1)	37.60	0.00	99.90	99.70	Complete
161.5	0.0438	Treponema pallidum (157)	Treponema (137)	Spirochaetaceae (136)	Spirochaetales (203692)	Spirochaetia (203691)	Spirochaetes (2)	Bacteria (1)	39.90	0.00	99.70	99.60	Complete
161.6	0.0438	Treponema pallidum (157)	Treponema (137)	Spirochaetaceae (136)	Spirochaetales (203692)	Spirochaetia (203691)	Spirochaetes (2)	Bacteria (1)	37.60	0.00	99.70	99.60	Complete
161.67	0.0438	Treponema pallidum (157)	Treponema (137)	Spirochaetaceae (136)	Spirochaetales (203692)	Spirochaetia (203691)	Spirochaetes (2)	Bacteria (1)			99.70	99.60	Complete
161.68	0.0438	Treponema pallidum (157)	Treponema (137)	Spirochaetaceae (136)	Spirochaetales (203692)	Spirochaetia (203691)	Spirochaetes (2)	Bacteria (1)					Complete
161.69	0.0438	Treponema pallidum (157)	Treponema (137)	Spirochaetaceae (136)	Spirochaetales (203692)	Spirochaetia (203691)	Spirochaetes (2)	Bacteria (1)					Complete
161.70	0.0438	Treponema pallidum (157)	Treponema (137)	Spirochaetaceae (136)	Spirochaetales (203692)	Spirochaetia (203691)	Spirochaetes (2)	Bacteria (1)					Complete
161.7	0.0438	Treponema pallidum (157)	Treponema (137)	Spirochaetaceae (136)	Spirochaetales (203692)	Spirochaetia (203691)	Spirochaetes (2)	Bacteria (1)	37.60	0.00	99.70	99.60	Complete
161.71	0.0438	Treponema pallidum (157)	Treponema (137)	Spirochaetaceae (136)	Spirochaetales (203692)	Spirochaetia (203691)	Spirochaetes (2)	Bacteria (1)					Complete
161.72	0.0438	Treponema pallidum (157)	Treponema (137)	Spirochaetaceae (136)	Spirochaetales (203692)	Spirochaetia (203691)	Spirochaetes (2)	Bacteria (1)					Complete
161.73	0.0438	Treponema pallidum (157)	Treponema (137)	Spirochaetaceae (136)	Spirochaetales (203692)	Spirochaetia (203691)	Spirochaetes (2)	Bacteria (1)					Complete
161.74	0.0438	Treponema pallidum (157)	Treponema (137)	Spirochaetaceae (136)	Spirochaetales (203692)	Spirochaetia (203691)	Spirochaetes (2)	Bacteria (1)					Complete
161.75	0.0438	Treponema pallidum (157)	Treponema (137)	Spirochaetaceae (136)	Spirochaetales (203692)	Spirochaetia (203691)	Spirochaetes (2)	Bacteria (1)					Complete
161.76	0.0438	Treponema pallidum (157)	Treponema (137)	Spirochaetaceae (136)	Spirochaetales (203692)	Spirochaetia (203691)	Spirochaetes (2)	Bacteria (1)					Complete
161.77	0.0438	Treponema pallidum (157)	Treponema (137)	Spirochaetaceae (136)	Spirochaetales (203692)	Spirochaetia (203691)	Spirochaetes (2)	Bacteria (1)					Complete
161.78	0.0438	Treponema pallidum (157)	Treponema (137)	Spirochaetaceae (136)	Spirochaetales (203692)	Spirochaetia (203691)	Spirochaetes (2)	Bacteria (1)					Complete
161.79	0.0438	Treponema pallidum (157)	Treponema (137)	Spirochaetaceae (136)	Spirochaetales (203692)	Spirochaetia (203691)	Spirochaetes (2)	Bacteria (1)					Complete
161.80	0.0438	Treponema pallidum (157)	Treponema (137)	Spirochaetaceae (136)	Spirochaetales (203692)	Spirochaetia (203691)	Spirochaetes (2)	Bacteria (1)					Complete
161.8	0.0438	Treponema pallidum (157)	Treponema (137)	Spirochaetaceae (136)	Spirochaetales (203692)	Spirochaetia (203691)	Spirochaetes (2)	Bacteria (1)	37.60	0.00	99.70	99.50	Complete
161.81	0.0438	Treponema pallidum (157)	Treponema (137)	Spirochaetaceae (136)	Spirochaetales (203692)	Spirochaetia (203691)	Spirochaetes (2)	Bacteria (1)					Complete
161.9	0.0438	Treponema pallidum (157)	Treponema (137)	Spirochaetaceae (136)	Spirochaetales (203692)	Spirochaetia (203691)	Spirochaetes (2)	Bacteria (1)	37.60	0.00	99.70	99.60	Complete
162.3	0.1848	Treponema phagedenis (157)	Treponema (137)	Spirochaetaceae (136)	Spirochaetales (203692)	Spirochaetia (203691)	Spirochaetes (2)	Bacteria (1)	52.00	0.10	96.10	94.80	WGS
163.6	0.1242	Treponema bryantii (157)	Treponema (137)	Spirochaetaceae (136)	Spirochaetales (203692)	Spirochaetia (203691)	Spirochaetes (2)	Bacteria (1)					WGS
163.7	0.0974	Treponema bryantii (157)	Treponema (137)	Spirochaetaceae (136)	Spirochaetales (203692)	Spirochaetia (203691)	Spirochaetes (2)	Bacteria (1)					WGS
166.15	0.0748	Treponema sp. (157)	Treponema (137)	Spirochaetaceae (136)	Spirochaetales (203692)	Spirochaetia (203691)	Spirochaetes (2)	Bacteria (1)	66.10	18.10	92.10	88.80	WGS
166.16	0.1174	Treponema sp. (157)	Treponema (137)	Spirochaetaceae (136)	Spirochaetales (203692)	Spirochaetia (203691)	Spirochaetes (2)	Bacteria (1)	66.40	26.90	94.80	88.10	WGS
166.17	0.0996	Treponema sp. (157)	Treponema (137)	Spirochaetaceae (136)	Spirochaetales (203692)	Spirochaetia (203691)	Spirochaetes (2)	Bacteria (1)	61.20	10.60	92.30	91.10	WGS
166.18	0.0934	Treponema sp. (157)	Treponema (137)	Spirochaetaceae (136)	Spirochaetales (203692)	Spirochaetia (203691)	Spirochaetes (2)	Bacteria (1)	56.10	4.40	89.40	87.60	WGS
166.19	0.1134	Treponema sp. (157)	Treponema (137)	Spirochaetaceae (136)	Spirochaetales (203692)	Spirochaetia (203691)	Spirochaetes (2)	Bacteria (1)	61.10	13.60	92.70	90.70	WGS
166.20	0.0766	Treponema sp. (157)	Treponema (137)	Spirochaetaceae (136)	Spirochaetales (203692)	Spirochaetia (203691)	Spirochaetes (2)	Bacteria (1)	65.90	9.90	90.80	89.70	WGS
166.21	0.1386	Treponema sp. (157)	Treponema (137)	Spirochaetaceae (136)	Spirochaetales (203692)	Spirochaetia (203691)	Spirochaetes (2)	Bacteria (1)	66.10	12.90	92.60	89.10	WGS
166.22	0.1118	Treponema sp. (157)	Treponema (137)	Spirochaetaceae (136)	Spirochaetales (203692)	Spirochaetia (203691)	Spirochaetes (2)	Bacteria (1)	61.00	7.40	89.90	88.30	WGS
166.23	0.0676	Treponema sp. (157)	Treponema (137)	Spirochaetaceae (136)	Spirochaetales (203692)	Spirochaetia (203691)	Spirochaetes (2)	Bacteria (1)	63.90	29.40	95.30	90.70	WGS
166.24	0.0958	Treponema sp. (157)	Treponema (137)	Spirochaetaceae (136)	Spirochaetales (203692)	Spirochaetia (203691)	Spirochaetes (2)	Bacteria (1)	63.60	15.90	91.00	88.30	WGS
166.25	0.182	Treponema sp. (157)	Treponema (137)	Spirochaetaceae (136)	Spirochaetales (203692)	Spirochaetia (203691)	Spirochaetes (2)	Bacteria (1)	78.20	13.50	94.30	91.00	WGS
166.26	0.0928	Treponema sp. (157)	Treponema (137)	Spirochaetaceae (136)	Spirochaetales (203692)	Spirochaetia (203691)	Spirochaetes (2)	Bacteria (1)	65.90	12.80	92.10	90.70	WGS
166.27	0.1034	Treponema sp. (157)	Treponema (137)	Spirochaetaceae (136)	Spirochaetales (203692)	Spirochaetia (203691)	Spirochaetes (2)	Bacteria (1)	71.10	27.10	92.20	89.40	WGS
166.28	0.0846	Treponema sp. (157)	Treponema (137)	Spirochaetaceae (136)	Spirochaetales (203692)	Spirochaetia (203691)	Spirochaetes (2)	Bacteria (1)	66.10	33.80	94.50	89.30	WGS
166.29	0.0734	Treponema sp. (157)	Treponema (137)	Spirochaetaceae (136)	Spirochaetales (203692)	Spirochaetia (203691)	Spirochaetes (2)	Bacteria (1)	54.40	0.10	99.30	98.90	WGS
166.3	0.0574	Treponema sp. (157)	Treponema (137)	Spirochaetaceae (136)	Spirochaetales (203692)	Spirochaetia (203691)	Spirochaetes (2)	Bacteria (1)	44.70	5.10	94.90	94.30	WGS
166.30	0.0938	Treponema sp. (157)	Treponema (137)	Spirochaetaceae (136)	Spirochaetales (203692)	Spirochaetia (203691)	Spirochaetes (2)	Bacteria (1)	66.40	15.60	98.70	95.10	WGS
166.4	0.1796	Treponema sp. (157)	Treponema (137)	Spirochaetaceae (136)	Spirochaetales (203692)	Spirochaetia (203691)	Spirochaetes (2)	Bacteria (1)	70.80	16.90	90.60	88.80	WGS
166.41	0.1246	Treponema sp. (157)	Treponema (137)	Spirochaetaceae (136)	Spirochaetales (203692)	Spirochaetia (203691)	Spirochaetes (2)	Bacteria (1)					WGS
166.42	0.0848	Treponema sp. (157)	Treponema (137)	Spirochaetaceae (136)	Spirochaetales (203692)	Spirochaetia (203691)	Spirochaetes (2)	Bacteria (1)					WGS
166.43	0.0858	Treponema sp. (157)	Treponema (137)	Spirochaetaceae (136)	Spirochaetales (203692)	Spirochaetia (203691)	Spirochaetes (2)	Bacteria (1)					WGS
166.44	0.0608	Treponema sp. (157)	Treponema (137)	Spirochaetaceae (136)	Spirochaetales (203692)	Spirochaetia (203691)	Spirochaetes (2)	Bacteria (1)					WGS
166.45	0.1166	Treponema sp. (157)	Treponema (137)	Spirochaetaceae (136)	Spirochaetales (203692)	Spirochaetia (203691)	Spirochaetes (2)	Bacteria (1)					WGS
166.5	0.1302	Treponema sp. (157)	Treponema (137)	Spirochaetaceae (136)	Spirochaetales (203692)	Spirochaetia (203691)	Spirochaetes (2)	Bacteria (1)	36.40	11.70	88.40	87.40	WGS
166.6	0.1838	Treponema sp. (157)	Treponema (137)	Spirochaetaceae (136)	Spirochaetales (203692)	Spirochaetia (203691)	Spirochaetes (2)	Bacteria (1)	77.60	27.10	88.20	83.20	WGS
168.10	0.0438	Treponema pallidum (157)	Treponema (137)	Spirochaetaceae (136)	Spirochaetales (203692)	Spirochaetia (203691)	Spirochaetes (2)	Bacteria (1)			99.70	99.60	Complete
168.11	0.0438	Treponema pallidum (157)	Treponema (137)	Spirochaetaceae (136)	Spirochaetales (203692)	Spirochaetia (203691)	Spirochaetes (2)	Bacteria (1)			99.60	99.50	Complete
168.12	0.0438	Treponema pallidum (157)	Treponema (137)	Spirochaetaceae (136)	Spirochaetales (203692)	Spirochaetia (203691)	Spirochaetes (2)	Bacteria (1)			99.60	99.50	Complete
168.4	0.0438	Treponema pallidum (157)	Treponema (137)	Spirochaetaceae (136)	Spirochaetales (203692)	Spirochaetia (203691)	Spirochaetes (2)	Bacteria (1)	37.60	0.00	99.90	99.70	WGS
168.5	0.0438	Treponema pallidum (157)	Treponema (137)	Spirochaetaceae (136)	Spirochaetales (203692)	Spirochaetia (203691)	Spirochaetes (2)	Bacteria (1)	37.60	0.00	99.90	99.70	WGS
168.8	0.0438	Treponema pallidum (157)	Treponema (137)	Spirochaetaceae (136)	Spirochaetales (203692)	Spirochaetia (203691)	Spirochaetes (2)	Bacteria (1)	37.60	0.00	99.90	99.80	WGS
172.13	0.2462	Leptospira biflexa (171)	Leptospira (170)	Leptospiraceae (1643688)	Leptospirales (203692)	Spirochaetia (203691)	Spirochaetes (2)	Bacteria (1)			99.40	99.10	WGS
172.14	0.2462	Leptospira biflexa (171)	Leptospira (170)	Leptospiraceae (1643688)	Leptospirales (203692)	Spirochaetia (203691)	Spirochaetes (2)	Bacteria (1)			99.40	99.00	WGS
172.15	0.2356	Leptospira biflexa (171)	Leptospira (170)	Leptospiraceae (1643688)	Leptospirales (203692)	Spirochaetia (203691)	Spirochaetes (2)	Bacteria (1)			99.50	99.20	WGS
172.16	0.2512	Leptospira biflexa (171)	Leptospira (170)	Leptospiraceae (1643688)	Leptospirales (203692)	Spirochaetia (203691)	Spirochaetes (2)	Bacteria (1)			99.50	99.20	WGS
172.17	0.2356	Leptospira biflexa (171)	Leptospira (170)	Leptospiraceae (1643688)	Leptospirales (203692)	Spirochaetia (203691)	Spirochaetes (2)	Bacteria (1)			99.50	99.20	WGS
173.10	0.1044	Leptospira interrogans (171)	Leptospira (170)	Leptospiraceae (1643688)	Leptospirales (203692)	Spirochaetia (203691)	Spirochaetes (2)	Bacteria (1)	91.70	10.70	96.00	91.80	WGS
173.11	0.1186	Leptospira interrogans (171)	Leptospira (170)	Leptospiraceae (1643688)	Leptospirales (203692)	Spirochaetia (203691)	Spirochaetes (2)	Bacteria (1)	98.60	14.90	98.40	91.70	WGS
173.12	0.13	Leptospira interrogans (171)	Leptospira (170)	Leptospiraceae (1643688)	Leptospirales (203692)	Spirochaetia (203691)	Spirochaetes (2)	Bacteria (1)	100.00	1.40	99.50	99.00	WGS
173.13	0.13	Leptospira interrogans (171)	Leptospira (170)	Leptospiraceae (1643688)	Leptospirales (203692)	Spirochaetia (203691)	Spirochaetes (2)	Bacteria (1)	100.00	1.40	99.50	99.00	WGS
173.14	0.1254	Leptospira interrogans (171)	Leptospira (170)	Leptospiraceae (1643688)	Leptospirales (203692)	Spirochaetia (203691)	Spirochaetes (2)	Bacteria (1)	100.00	0.00	99.50	99.00	WGS
173.142	0.1292	Leptospira interrogans (171)	Leptospira (170)	Leptospiraceae (1643688)	Leptospirales (203692)	Spirochaetia (203691)	Spirochaetes (2)	Bacteria (1)	100.00	0.00	99.50	99.30	WGS
173.147	0.1254	Leptospira interrogans (171)	Leptospira (170)	Leptospiraceae (1643688)	Leptospirales (203692)	Spirochaetia (203691)	Spirochaetes (2)	Bacteria (1)	100.00	0.00	99.60	99.30	WGS
173.15	0.1254	Leptospira interrogans (171)	Leptospira (170)	Leptospiraceae (1643688)	Leptospirales (203692)	Spirochaetia (203691)	Spirochaetes (2)	Bacteria (1)	100.00	0.00	99.50	99.00	WGS
173.16	0.1254	Leptospira interrogans (171)	Leptospira (170)	Leptospiraceae (1643688)	Leptospirales (203692)	Spirochaetia (203691)	Spirochaetes (2)	Bacteria (1)	100.00	0.00	99.50	99.10	WGS
173.164	0.1292	Leptospira interrogans (171)	Leptospira (170)	Leptospiraceae (1643688)	Leptospirales (203692)	Spirochaetia (203691)	Spirochaetes (2)	Bacteria (1)	100.00	0.00	99.60	99.20	WGS
173.165	0.1332	Leptospira interrogans (171)	Leptospira (170)	Leptospiraceae (1643688)	Leptospirales (203692)	Spirochaetia (203691)	Spirochaetes (2)	Bacteria (1)	100.00	1.40	99.70	98.90	WGS
173.17	0.1292	Leptospira interrogans (171)	Leptospira (170)	Leptospiraceae (1643688)	Leptospirales (203692)	Spirochaetia (203691)	Spirochaetes (2)	Bacteria (1)	100.00	0.00	99.50	99.40	WGS
173.18	0.1152	Leptospira interrogans (171)	Leptospira (170)	Leptospiraceae (1643688)	Leptospirales (203692)	Spirochaetia (203691)	Spirochaetes (2)	Bacteria (1)	100.00	0.00	99.50	99.10	WGS
173.19	0.1254	Leptospira interrogans (171)	Leptospira (170)	Leptospiraceae (1643688)	Leptospirales (203692)	Spirochaetia (203691)	Spirochaetes (2)	Bacteria (1)	100.00	0.00	99.50	99.10	WGS
173.20	0.129	Leptospira interrogans (171)	Leptospira (170)	Leptospiraceae (1643688)	Leptospirales (203692)	Spirochaetia (203691)	Spirochaetes (2)	Bacteria (1)	99.30	0.00	99.50	99.00	WGS
173.208	0.119	Leptospira interrogans (171)	Leptospira (170)	Leptospiraceae (1643688)	Leptospirales (203692)	Spirochaetia (203691)	Spirochaetes (2)	Bacteria (1)			99.70	98.80	Complete
173.209	0.1192	Leptospira interrogans (171)	Leptospira (170)	Leptospiraceae (1643688)	Leptospirales (203692)	Spirochaetia (203691)	Spirochaetes (2)	Bacteria (1)			99.70	99.20	Complete
173.210	0.1324	Leptospira interrogans (171)	Leptospira (170)	Leptospiraceae (1643688)	Leptospirales (203692)	Spirochaetia (203691)	Spirochaetes (2)	Bacteria (1)			99.60	98.80	Complete
173.21	0.121	Leptospira interrogans (171)	Leptospira (170)	Leptospiraceae (1643688)	Leptospirales (203692)	Spirochaetia (203691)	Spirochaetes (2)	Bacteria (1)	100.00	0.00	99.50	99.20	WGS
173.22	0.119	Leptospira interrogans (171)	Leptospira (170)	Leptospiraceae (1643688)	Leptospirales (203692)	Spirochaetia (203691)	Spirochaetes (2)	Bacteria (1)	100.00	2.00	99.50	98.40	WGS
173.23	0.1292	Leptospira interrogans (171)	Leptospira (170)	Leptospiraceae (1643688)	Leptospirales (203692)	Spirochaetia (203691)	Spirochaetes (2)	Bacteria (1)	100.00	1.40	99.50	98.30	WGS
173.24	0.1254	Leptospira interrogans (171)	Leptospira (170)	Leptospiraceae (1643688)	Leptospirales (203692)	Spirochaetia (203691)	Spirochaetes (2)	Bacteria (1)	100.00	0.00	99.50	99.30	WGS
173.25	0.119	Leptospira interrogans (171)	Leptospira (170)	Leptospiraceae (1643688)	Leptospirales (203692)	Spirochaetia (203691)	Spirochaetes (2)	Bacteria (1)	100.00	0.00	99.50	99.00	WGS
173.26	0.1292	Leptospira interrogans (171)	Leptospira (170)	Leptospiraceae (1643688)	Leptospirales (203692)	Spirochaetia (203691)	Spirochaetes (2)	Bacteria (1)	100.00	0.70	99.50	99.30	WGS
173.27	0.1292	Leptospira interrogans (171)	Leptospira (170)	Leptospiraceae (1643688)	Leptospirales (203692)	Spirochaetia (203691)	Spirochaetes (2)	Bacteria (1)	100.00	0.00	99.50	99.10	WGS
173.28	0.1252	Leptospira interrogans (171)	Leptospira (170)	Leptospiraceae (1643688)	Leptospirales (203692)	Spirochaetia (203691)	Spirochaetes (2)	Bacteria (1)	100.00	0.00	99.50	99.20	WGS
173.29	0.1292	Leptospira interrogans (171)	Leptospira (170)	Leptospiraceae (1643688)	Leptospirales (203692)	Spirochaetia (203691)	Spirochaetes (2)	Bacteria (1)	100.00	0.00	99.50	99.00	WGS
173.30	0.1312	Leptospira interrogans (171)	Leptospira (170)	Leptospiraceae (1643688)	Leptospirales (203692)	Spirochaetia (203691)	Spirochaetes (2)	Bacteria (1)	100.00	0.00	99.50	99.10	WGS
173.31	0.1324	Leptospira interrogans (171)	Leptospira (170)	Leptospiraceae (1643688)	Leptospirales (203692)	Spirochaetia (203691)	Spirochaetes (2)	Bacteria (1)	100.00	0.00	99.40	98.90	WGS
173.32	0.1252	Leptospira interrogans (171)	Leptospira (170)	Leptospiraceae (1643688)	Leptospirales (203692)	Spirochaetia (203691)	Spirochaetes (2)	Bacteria (1)	100.00	0.70	99.50	99.00	WGS
173.33	0.1152	Leptospira interrogans (171)	Leptospira (170)	Leptospiraceae (1643688)	Leptospirales (203692)	Spirochaetia (203691)	Spirochaetes (2)	Bacteria (1)	100.00	1.40	99.50	99.10	WGS
173.34	0.13	Leptospira interrogans (171)	Leptospira (170)	Leptospiraceae (1643688)	Leptospirales (203692)	Spirochaetia (203691)	Spirochaetes (2)	Bacteria (1)	100.00	0.00	99.30	98.80	WGS
173.35	0.1254	Leptospira interrogans (171)	Leptospira (170)	Leptospiraceae (1643688)	Leptospirales (203692)	Spirochaetia (203691)	Spirochaetes (2)	Bacteria (1)	100.00	0.00	99.50	99.20	WGS
173.36	0.1292	Leptospira interrogans (171)	Leptospira (170)	Leptospiraceae (1643688)	Leptospirales (203692)	Spirochaetia (203691)	Spirochaetes (2)	Bacteria (1)	100.00	0.00	99.50	99.30	WGS
173.37	0.1292	Leptospira interrogans (171)	Leptospira (170)	Leptospiraceae (1643688)	Leptospirales (203692)	Spirochaetia (203691)	Spirochaetes (2)	Bacteria (1)	100.00	0.00	99.20	98.80	WGS
173.38	0.1254	Leptospira interrogans (171)	Leptospira (170)	Leptospiraceae (1643688)	Leptospirales (203692)	Spirochaetia (203691)	Spirochaetes (2)	Bacteria (1)	100.00	0.00	99.50	99.10	WGS
173.39	0.1254	Leptospira interrogans (171)	Leptospira (170)	Leptospiraceae (1643688)	Leptospirales (203692)	Spirochaetia (203691)	Spirochaetes (2)	Bacteria (1)	100.00	0.00	99.50	99.10	WGS
173.40	0.1152	Leptospira interrogans (171)	Leptospira (170)	Leptospiraceae (1643688)	Leptospirales (203692)	Spirochaetia (203691)	Spirochaetes (2)	Bacteria (1)	100.00	0.00	99.50	99.20	WGS
173.41	0.127	Leptospira interrogans (171)	Leptospira (170)	Leptospiraceae (1643688)	Leptospirales (203692)	Spirochaetia (203691)	Spirochaetes (2)	Bacteria (1)	100.00	0.00	99.50	99.10	WGS
173.42	0.1234	Leptospira interrogans (171)	Leptospira (170)	Leptospiraceae (1643688)	Leptospirales (203692)	Spirochaetia (203691)	Spirochaetes (2)	Bacteria (1)	100.00	0.00	99.30	98.90	WGS
173.43	0.1258	Leptospira interrogans (171)	Leptospira (170)	Leptospiraceae (1643688)	Leptospirales (203692)	Spirochaetia (203691)	Spirochaetes (2)	Bacteria (1)	100.00	0.00	99.50	99.10	WGS
173.44	0.1254	Leptospira interrogans (171)	Leptospira (170)	Leptospiraceae (1643688)	Leptospirales (203692)	Spirochaetia (203691)	Spirochaetes (2)	Bacteria (1)	100.00	0.00	99.50	99.00	WGS
173.45	0.119	Leptospira interrogans (171)	Leptospira (170)	Leptospiraceae (1643688)	Leptospirales (203692)	Spirochaetia (203691)	Spirochaetes (2)	Bacteria (1)	100.00	0.00	99.50	99.00	WGS
173.46	0.119	Leptospira interrogans (171)	Leptospira (170)	Leptospiraceae (1643688)	Leptospirales (203692)	Spirochaetia (203691)	Spirochaetes (2)	Bacteria (1)	100.00	0.00	99.50	99.00	WGS
173.47	0.13	Leptospira interrogans (171)	Leptospira (170)	Leptospiraceae (1643688)	Leptospirales (203692)	Spirochaetia (203691)	Spirochaetes (2)	Bacteria (1)	100.00	0.70	99.30	98.90	WGS
173.48	0.1292	Leptospira interrogans (171)	Leptospira (170)	Leptospiraceae (1643688)	Leptospirales (203692)	Spirochaetia (203691)	Spirochaetes (2)	Bacteria (1)	100.00	0.00	99.50	99.00	WGS
173.49	0.1254	Leptospira interrogans (171)	Leptospira (170)	Leptospiraceae (1643688)	Leptospirales (203692)	Spirochaetia (203691)	Spirochaetes (2)	Bacteria (1)	100.00	0.00	99.40	99.00	WGS
173.50	0.123	Leptospira interrogans (171)	Leptospira (170)	Leptospiraceae (1643688)	Leptospirales (203692)	Spirochaetia (203691)	Spirochaetes (2)	Bacteria (1)	100.00	0.00	99.50	99.30	WGS
173.51	0.1254	Leptospira interrogans (171)	Leptospira (170)	Leptospiraceae (1643688)	Leptospirales (203692)	Spirochaetia (203691)	Spirochaetes (2)	Bacteria (1)	100.00	0.00	99.50	99.30	WGS
173.52	0.1286	Leptospira interrogans (171)	Leptospira (170)	Leptospiraceae (1643688)	Leptospirales (203692)	Spirochaetia (203691)	Spirochaetes (2)	Bacteria (1)	100.00	0.00	99.30	99.00	WGS
173.53	0.121	Leptospira interrogans (171)	Leptospira (170)	Leptospiraceae (1643688)	Leptospirales (203692)	Spirochaetia (203691)	Spirochaetes (2)	Bacteria (1)	100.00	0.00	99.30	98.70	WGS
173.54	0.1254	Leptospira interrogans (171)	Leptospira (170)	Leptospiraceae (1643688)	Leptospirales (203692)	Spirochaetia (203691)	Spirochaetes (2)	Bacteria (1)	100.00	0.00	99.50	99.00	WGS
173.55	0.1254	Leptospira interrogans (171)	Leptospira (170)	Leptospiraceae (1643688)	Leptospirales (203692)	Spirochaetia (203691)	Spirochaetes (2)	Bacteria (1)	100.00	0.00	99.50	99.20	WGS
173.56	0.1284	Leptospira interrogans (171)	Leptospira (170)	Leptospiraceae (1643688)	Leptospirales (203692)	Spirochaetia (203691)	Spirochaetes (2)	Bacteria (1)	100.00	0.70	99.40	99.00	WGS
173.57	0.1262	Leptospira interrogans (171)	Leptospira (170)	Leptospiraceae (1643688)	Leptospirales (203692)	Spirochaetia (203691)	Spirochaetes (2)	Bacteria (1)	100.00	0.00	99.50	99.20	WGS
173.58	0.1252	Leptospira interrogans (171)	Leptospira (170)	Leptospiraceae (1643688)	Leptospirales (203692)	Spirochaetia (203691)	Spirochaetes (2)	Bacteria (1)	100.00	0.00	99.30	98.70	WGS
173.59	0.1252	Leptospira interrogans (171)	Leptospira (170)	Leptospiraceae (1643688)	Leptospirales (203692)	Spirochaetia (203691)	Spirochaetes (2)	Bacteria (1)	100.00	0.00	99.30	98.70	WGS
173.60	0.1286	Leptospira interrogans (171)	Leptospira (170)	Leptospiraceae (1643688)	Leptospirales (203692)	Spirochaetia (203691)	Spirochaetes (2)	Bacteria (1)	100.00	0.00	99.50	99.00	WGS
173.61	0.1332	Leptospira interrogans (171)	Leptospira (170)	Leptospiraceae (1643688)	Leptospirales (203692)	Spirochaetia (203691)	Spirochaetes (2)	Bacteria (1)	100.00	0.00	99.40	99.00	WGS
173.62	0.1322	Leptospira interrogans (171)	Leptospira (170)	Leptospiraceae (1643688)	Leptospirales (203692)	Spirochaetia (203691)	Spirochaetes (2)	Bacteria (1)	100.00	0.00	99.50	99.20	WGS
173.63	0.1246	Leptospira interrogans (171)	Leptospira (170)	Leptospiraceae (1643688)	Leptospirales (203692)	Spirochaetia (203691)	Spirochaetes (2)	Bacteria (1)	100.00	0.70	99.50	98.80	WGS
173.64	0.1286	Leptospira interrogans (171)	Leptospira (170)	Leptospiraceae (1643688)	Leptospirales (203692)	Spirochaetia (203691)	Spirochaetes (2)	Bacteria (1)	100.00	0.00	99.50	99.20	WGS
173.65	0.1254	Leptospira interrogans (171)	Leptospira (170)	Leptospiraceae (1643688)	Leptospirales (203692)	Spirochaetia (203691)	Spirochaetes (2)	Bacteria (1)	100.00	0.00	99.50	99.20	WGS
173.66	0.1292	Leptospira interrogans (171)	Leptospira (170)	Leptospiraceae (1643688)	Leptospirales (203692)	Spirochaetia (203691)	Spirochaetes (2)	Bacteria (1)	100.00	0.70	99.50	98.70	WGS
173.67	0.1152	Leptospira interrogans (171)	Leptospira (170)	Leptospiraceae (1643688)	Leptospirales (203692)	Spirochaetia (203691)	Spirochaetes (2)	Bacteria (1)	100.00	0.00	99.50	99.20	WGS
173.68	0.1292	Leptospira interrogans (171)	Leptospira (170)	Leptospiraceae (1643688)	Leptospirales (203692)	Spirochaetia (203691)	Spirochaetes (2)	Bacteria (1)	100.00	0.00	99.50	99.00	WGS
173.69	0.119	Leptospira interrogans (171)	Leptospira (170)	Leptospiraceae (1643688)	Leptospirales (203692)	Spirochaetia (203691)	Spirochaetes (2)	Bacteria (1)	100.00	0.00	99.50	99.00	WGS
173.70	0.1254	Leptospira interrogans (171)	Leptospira (170)	Leptospiraceae (1643688)	Leptospirales (203692)	Spirochaetia (203691)	Spirochaetes (2)	Bacteria (1)	100.00	0.00	99.50	99.20	WGS
173.71	0.1268	Leptospira interrogans (171)	Leptospira (170)	Leptospiraceae (1643688)	Leptospirales (203692)	Spirochaetia (203691)	Spirochaetes (2)	Bacteria (1)	98.60	0.00	98.80	98.50	WGS
173.72	0.1292	Leptospira interrogans (171)	Leptospira (170)	Leptospiraceae (1643688)	Leptospirales (203692)	Spirochaetia (203691)	Spirochaetes (2)	Bacteria (1)	100.00	0.70	99.50	98.70	WGS
173.73	0.1254	Leptospira interrogans (171)	Leptospira (170)	Leptospiraceae (1643688)	Leptospirales (203692)	Spirochaetia (203691)	Spirochaetes (2)	Bacteria (1)	100.00	0.70	99.30	98.70	WGS
173.74	0.1254	Leptospira interrogans (171)	Leptospira (170)	Leptospiraceae (1643688)	Leptospirales (203692)	Spirochaetia (203691)	Spirochaetes (2)	Bacteria (1)	100.00	0.00	99.30	99.00	WGS
173.75	0.1286	Leptospira interrogans (171)	Leptospira (170)	Leptospiraceae (1643688)	Leptospirales (203692)	Spirochaetia (203691)	Spirochaetes (2)	Bacteria (1)	100.00	0.00	99.50	99.10	WGS
173.76	0.1292	Leptospira interrogans (171)	Leptospira (170)	Leptospiraceae (1643688)	Leptospirales (203692)	Spirochaetia (203691)	Spirochaetes (2)	Bacteria (1)	100.00	0.00	99.80	99.60	WGS
173.9	0.1166	Leptospira interrogans (171)	Leptospira (170)	Leptospiraceae (1643688)	Leptospirales (203692)	Spirochaetia (203691)	Spirochaetes (2)	Bacteria (1)	89.70	11.00	97.30	92.70	WGS
174.12	0.1164	Leptospira borgpetersenii (171)	Leptospira (170)	Leptospiraceae (1643688)	Leptospirales (203692)	Spirochaetia (203691)	Spirochaetes (2)	Bacteria (1)	100.00	0.00	99.50	99.20	WGS
174.13	0.1188	Leptospira borgpetersenii (171)	Leptospira (170)	Leptospiraceae (1643688)	Leptospirales (203692)	Spirochaetia (203691)	Spirochaetes (2)	Bacteria (1)	100.00	0.00	99.30	99.00	WGS
174.14	0.1168	Leptospira borgpetersenii (171)	Leptospira (170)	Leptospiraceae (1643688)	Leptospirales (203692)	Spirochaetia (203691)	Spirochaetes (2)	Bacteria (1)	100.00	0.00	99.50	99.30	WGS
174.15	0.1188	Leptospira borgpetersenii (171)	Leptospira (170)	Leptospiraceae (1643688)	Leptospirales (203692)	Spirochaetia (203691)	Spirochaetes (2)	Bacteria (1)	99.00	0.00	99.40	99.10	WGS
174.16	0.1188	Leptospira borgpetersenii (171)	Leptospira (170)	Leptospiraceae (1643688)	Leptospirales (203692)	Spirochaetia (203691)	Spirochaetes (2)	Bacteria (1)	100.00	0.00	99.50	99.30	WGS
174.17	0.1188	Leptospira borgpetersenii (171)	Leptospira (170)	Leptospiraceae (1643688)	Leptospirales (203692)	Spirochaetia (203691)	Spirochaetes (2)	Bacteria (1)	100.00	0.00	99.50	99.40	WGS
174.18	0.1188	Leptospira borgpetersenii (171)	Leptospira (170)	Leptospiraceae (1643688)	Leptospirales (203692)	Spirochaetia (203691)	Spirochaetes (2)	Bacteria (1)	100.00	0.00	99.30	99.00	WGS
174.19	0.1188	Leptospira borgpetersenii (171)	Leptospira (170)	Leptospiraceae (1643688)	Leptospirales (203692)	Spirochaetia (203691)	Spirochaetes (2)	Bacteria (1)	100.00	0.00	99.50	99.40	WGS
174.20	0.1188	Leptospira borgpetersenii (171)	Leptospira (170)	Leptospiraceae (1643688)	Leptospirales (203692)	Spirochaetia (203691)	Spirochaetes (2)	Bacteria (1)	100.00	0.00	99.50	99.20	WGS
174.21	0.1188	Leptospira borgpetersenii (171)	Leptospira (170)	Leptospiraceae (1643688)	Leptospirales (203692)	Spirochaetia (203691)	Spirochaetes (2)	Bacteria (1)	100.00	0.00	99.30	98.70	WGS
176.4	0.1292	Leptospira interrogans (171)	Leptospira (170)	Leptospiraceae (1643688)	Leptospirales (203692)	Spirochaetia (203691)	Spirochaetes (2)	Bacteria (1)	100.00	0.00	99.70	99.30	WGS
178.4	0.1064	Leptospira sp. (171)	Leptospira (170)	Leptospiraceae (1643688)	Leptospirales (203692)	Spirochaetia (203691)	Spirochaetes (2)	Bacteria (1)	88.50	2.20	94.80	93.90	WGS
180.8	0.0294	Leptospirillum ferrooxidans (179)	Leptospirillum (189779)	Nitrospiraceae (189778)	Nitrospirales (203693)	Nitrospira (40117)	Nitrospirae (2)	Bacteria (1)					Plasmid
180.9	0.0294	Leptospirillum ferrooxidans (179)	Leptospirillum (189779)	Nitrospiraceae (189778)	Nitrospirales (203693)	Nitrospira (40117)	Nitrospirae (2)	Bacteria (1)					Plasmid
183.6	0.2866	Leptonema illini (32205)	Leptonema (170)	Leptospiraceae (1643688)	Leptospirales (203692)	Spirochaetia (203691)	Spirochaetes (2)	Bacteria (1)	92.30	5.30	99.80	99.60	WGS
192.125	0.4508	Azospirillum brasilense (191)	Azospirillum (41295)	Rhodospirillaceae (204441)	Rhodospirillales (28211)	Alphaproteobacteria (1224	Proteobacteria (2)	Bacteria (1)			98.00	93.00	Complete
192.126	0.4538	Azospirillum brasilense (191)	Azospirillum (41295)	Rhodospirillaceae (204441)	Rhodospirillales (28211)	Alphaproteobacteria (1224	Proteobacteria (2)	Bacteria (1)			96.50	90.80	Complete
192.127	0.3892	Azospirillum brasilense (191)	Azospirillum (41295)	Rhodospirillaceae (204441)	Rhodospirillales (28211)	Alphaproteobacteria (1224	Proteobacteria (2)	Bacteria (1)			96.80	90.80	Complete
192.128	0.4464	Azospirillum brasilense (191)	Azospirillum (41295)	Rhodospirillaceae (204441)	Rhodospirillales (28211)	Alphaproteobacteria (1224	Proteobacteria (2)	Bacteria (1)			97.80	93.10	Complete
192.45	0.4346	Azospirillum brasilense (191)	Azospirillum (41295)	Rhodospirillaceae (204441)	Rhodospirillales (28211)	Alphaproteobacteria (1224	Proteobacteria (2)	Bacteria (1)	100.00	3.80	96.90	92.10	Complete
192.50	0.4108	Azospirillum brasilense (191)	Azospirillum (41295)	Rhodospirillaceae (204441)	Rhodospirillales (28211)	Alphaproteobacteria (1224	Proteobacteria (2)	Bacteria (1)	100.00	2.00	97.30	93.40	Complete
192.56	0.448	Azospirillum brasilense (191)	Azospirillum (41295)	Rhodospirillaceae (204441)	Rhodospirillales (28211)	Alphaproteobacteria (1224	Proteobacteria (2)	Bacteria (1)	100.00	11.90	96.20	88.90	WGS
192.57	0.448	Azospirillum brasilense (191)	Azospirillum (41295)	Rhodospirillaceae (204441)	Rhodospirillales (28211)	Alphaproteobacteria (1224	Proteobacteria (2)	Bacteria (1)	100.00	14.10	96.30	88.20	WGS
192.61	0.425	Azospirillum brasilense (191)	Azospirillum (41295)	Rhodospirillaceae (204441)	Rhodospirillales (28211)	Alphaproteobacteria (1224	Proteobacteria (2)	Bacteria (1)	100.00	3.80	97.00	92.90	WGS
192.68	0.2166	Azospirillum brasilense (191)	Azospirillum (41295)	Rhodospirillaceae (204441)	Rhodospirillales (28211)	Alphaproteobacteria (1224	Proteobacteria (2)	Bacteria (1)					Plasmid
192.69	0.0496	Azospirillum brasilense (191)	Azospirillum (41295)	Rhodospirillaceae (204441)	Rhodospirillales (28211)	Alphaproteobacteria (1224	Proteobacteria (2)	Bacteria (1)					Plasmid
192.7	0.4334	Azospirillum brasilense (191)	Azospirillum (41295)	Rhodospirillaceae (204441)	Rhodospirillales (28211)	Alphaproteobacteria (1224	Proteobacteria (2)	Bacteria (1)	100.00	2.90	96.60	91.90	Complete

192.70	0.0966	Azospirillum brasilense (191)	Azospirillum (41295)	Rhodospirillaceae (204441)	Rhodospirillales (28211)	Alphaproteobacteria (1224	Proteobacteria (2)	Bacteria (1)					Plasmid
192.71	0.0708	Azospirillum brasilense (191)	Azospirillum (41295)	Rhodospirillaceae (204441)	Rhodospirillales (28211)	Alphaproteobacteria (1224	Proteobacteria (2)	Bacteria (1)					Plasmid
192.72	0.0322	Azospirillum brasilense (191)	Azospirillum (41295)	Rhodospirillaceae (204441)	Rhodospirillales (28211)	Alphaproteobacteria (1224	Proteobacteria (2)	Bacteria (1)					Plasmid
192.73	0.0496	Azospirillum brasilense (191)	Azospirillum (41295)	Rhodospirillaceae (204441)	Rhodospirillales (28211)	Alphaproteobacteria (1224	Proteobacteria (2)	Bacteria (1)					Plasmid
192.74	0.1988	Azospirillum brasilense (191)	Azospirillum (41295)	Rhodospirillaceae (204441)	Rhodospirillales (28211)	Alphaproteobacteria (1224	Proteobacteria (2)	Bacteria (1)					Plasmid
192.75	0.0708	Azospirillum brasilense (191)	Azospirillum (41295)	Rhodospirillaceae (204441)	Rhodospirillales (28211)	Alphaproteobacteria (1224	Proteobacteria (2)	Bacteria (1)					Plasmid
192.76	0.0944	Azospirillum brasilense (191)	Azospirillum (41295)	Rhodospirillaceae (204441)	Rhodospirillales (28211)	Alphaproteobacteria (1224	Proteobacteria (2)	Bacteria (1)					Plasmid
192.77	0.0294	Azospirillum brasilense (191)	Azospirillum (41295)	Rhodospirillaceae (204441)	Rhodospirillales (28211)	Alphaproteobacteria (1224	Proteobacteria (2)	Bacteria (1)					Plasmid
192.81	0.4366	Azospirillum brasilense (191)	Azospirillum (41295)	Rhodospirillaceae (204441)	Rhodospirillales (28211)	Alphaproteobacteria (1224	Proteobacteria (2)	Bacteria (1)	100.00	3.80	96.70	91.50	Complete
192.82	0.448	Azospirillum brasilense (191)	Azospirillum (41295)	Rhodospirillaceae (204441)	Rhodospirillales (28211)	Alphaproteobacteria (1224	Proteobacteria (2)	Bacteria (1)	100.00	2.40	97.10	93.30	WGS
192.83	0.448	Azospirillum brasilense (191)	Azospirillum (41295)	Rhodospirillaceae (204441)	Rhodospirillales (28211)	Alphaproteobacteria (1224	Proteobacteria (2)	Bacteria (1)	100.00	2.40	97.10	92.80	WGS
192.87	0.0902	Azospirillum brasilense (191)	Azospirillum (41295)	Rhodospirillaceae (204441)	Rhodospirillales (28211)	Alphaproteobacteria (1224	Proteobacteria (2)	Bacteria (1)	97.00	2.00	93.70	92.50	WGS
192.88	0.3342	Azospirillum brasilense (191)	Azospirillum (41295)	Rhodospirillaceae (204441)	Rhodospirillales (28211)	Alphaproteobacteria (1224	Proteobacteria (2)	Bacteria (1)	99.00	2.00	93.80	91.30	WGS
192.92	0.3658	Azospirillum brasilense (191)	Azospirillum (41295)	Rhodospirillaceae (204441)	Rhodospirillales (28211)	Alphaproteobacteria (1224	Proteobacteria (2)	Bacteria (1)	99.00	3.90	94.20	90.50	WGS
192.93	0.4372	Azospirillum brasilense (191)	Azospirillum (41295)	Rhodospirillaceae (204441)	Rhodospirillales (28211)	Alphaproteobacteria (1224	Proteobacteria (2)	Bacteria (1)	100.00	7.40	96.50	92.50	WGS
192.95	0.47	Azospirillum brasilense (191)	Azospirillum (41295)	Rhodospirillaceae (204441)	Rhodospirillales (28211)	Alphaproteobacteria (1224	Proteobacteria (2)	Bacteria (1)	100.00	9.40	97.90	93.20	WGS
193.3	0.4132	Azospirillum lipoferum (191)	Azospirillum (41295)	Rhodospirillaceae (204441)	Rhodospirillales (28211)	Alphaproteobacteria (1224	Proteobacteria (2)	Bacteria (1)	100.00	3.80	97.70	94.30	WGS
195.1000	0.11	Campylobacter coli (72294)	Campylobacter (213849)	Campylobacteraceae (2954	Campylobacterales (68525)	Epsilonproteobacteria (122	Proteobacteria (2)	Bacteria (1)	100.00	3.50	99.60	98.90	WGS
195.1001	0.11	Campylobacter coli (72294)	Campylobacter (213849)	Campylobacteraceae (2954	Campylobacterales (68525)	Epsilonproteobacteria (122	Proteobacteria (2)	Bacteria (1)	100.00	0.00	99.60	99.60	WGS
195.1002	0.1144	Campylobacter coli (72294)	Campylobacter (213849)	Campylobacteraceae (2954	Campylobacterales (68525)	Epsilonproteobacteria (122	Proteobacteria (2)	Bacteria (1)	100.00	0.00	99.60	99.50	WGS
195.1003	0.11	Campylobacter coli (72294)	Campylobacter (213849)	Campylobacteraceae (2954	Campylobacterales (68525)	Epsilonproteobacteria (122	Proteobacteria (2)	Bacteria (1)	100.00	0.00	99.60	99.60	WGS
195.1004	0.11	Campylobacter coli (72294)	Campylobacter (213849)	Campylobacteraceae (2954	Campylobacterales (68525)	Epsilonproteobacteria (122	Proteobacteria (2)	Bacteria (1)	100.00	0.00	99.60	99.60	WGS
195.1005	0.1228	Campylobacter coli (72294)	Campylobacter (213849)	Campylobacteraceae (2954	Campylobacterales (68525)	Epsilonproteobacteria (122	Proteobacteria (2)	Bacteria (1)	100.00	0.00	99.60	99.60	WGS
195.1006	0.11	Campylobacter coli (72294)	Campylobacter (213849)	Campylobacteraceae (2954	Campylobacterales (68525)	Epsilonproteobacteria (122	Proteobacteria (2)	Bacteria (1)	100.00	0.00	99.60	99.60	WGS
195.1007	0.1144	Campylobacter coli (72294)	Campylobacter (213849)	Campylobacteraceae (2954	Campylobacterales (68525)	Epsilonproteobacteria (122	Proteobacteria (2)	Bacteria (1)	100.00	0.00	99.60	99.50	WGS
195.1008	0.11	Campylobacter coli (72294)	Campylobacter (213849)	Campylobacteraceae (2954	Campylobacterales (68525)	Epsilonproteobacteria (122	Proteobacteria (2)	Bacteria (1)	100.00	0.00	99.80	99.80	WGS
195.1009	0.116	Campylobacter coli (72294)	Campylobacter (213849)	Campylobacteraceae (2954	Campylobacterales (68525)	Epsilonproteobacteria (122	Proteobacteria (2)	Bacteria (1)	100.00	0.00	99.60	99.60	WGS
195.1010	0.1132	Campylobacter coli (72294)	Campylobacter (213849)	Campylobacteraceae (2954	Campylobacterales (68525)	Epsilonproteobacteria (122	Proteobacteria (2)	Bacteria (1)	100.00	0.00	99.80	99.70	WGS
195.1011	0.1132	Campylobacter coli (72294)	Campylobacter (213849)	Campylobacteraceae (2954	Campylobacterales (68525)	Epsilonproteobacteria (122	Proteobacteria (2)	Bacteria (1)	100.00	0.00	99.80	99.80	WGS
195.1012	0.1132	Campylobacter coli (72294)	Campylobacter (213849)	Campylobacteraceae (2954	Campylobacterales (68525)	Epsilonproteobacteria (122	Proteobacteria (2)	Bacteria (1)	99.40	0.00	99.70	99.70	WGS
195.1013	0.1132	Campylobacter coli (72294)	Campylobacter (213849)	Campylobacteraceae (2954	Campylobacterales (68525)	Epsilonproteobacteria (122	Proteobacteria (2)	Bacteria (1)	100.00	0.00	99.60	99.50	WGS
195.1014	0.116	Campylobacter coli (72294)	Campylobacter (213849)	Campylobacteraceae (2954	Campylobacterales (68525)	Epsilonproteobacteria (122	Proteobacteria (2)	Bacteria (1)	100.00	0.00	99.60	99.60	WGS
195.1015	0.116	Campylobacter coli (72294)	Campylobacter (213849)	Campylobacteraceae (2954	Campylobacterales (68525)	Epsilonproteobacteria (122	Proteobacteria (2)	Bacteria (1)	100.00	0.00	99.60	99.60	WGS
195.1016	0.1132	Campylobacter coli (72294)	Campylobacter (213849)	Campylobacteraceae (2954	Campylobacterales (68525)	Epsilonproteobacteria (122	Proteobacteria (2)	Bacteria (1)	100.00	0.00	99.60	99.60	WGS
195.1017	0.1132	Campylobacter coli (72294)	Campylobacter (213849)	Campylobacteraceae (2954	Campylobacterales (68525)	Epsilonproteobacteria (122	Proteobacteria (2)	Bacteria (1)	100.00	0.00	99.60	99.60	WGS
195.1018	0.1172	Campylobacter coli (72294)	Campylobacter (213849)	Campylobacteraceae (2954	Campylobacterales (68525)	Epsilonproteobacteria (122	Proteobacteria (2)	Bacteria (1)	100.00	0.00	99.60	99.60	WGS
195.1019	0.116	Campylobacter coli (72294)	Campylobacter (213849)	Campylobacteraceae (2954	Campylobacterales (68525)	Epsilonproteobacteria (122	Proteobacteria (2)	Bacteria (1)	99.40	0.00	99.60	99.30	WGS
195.1020	0.109	Campylobacter coli (72294)	Campylobacter (213849)	Campylobacteraceae (2954	Campylobacterales (68525)	Epsilonproteobacteria (122	Proteobacteria (2)	Bacteria (1)	99.40	1.80	99.70	99.30	WGS
195.1021	0.1142	Campylobacter coli (72294)	Campylobacter (213849)	Campylobacteraceae (2954	Campylobacterales (68525)	Epsilonproteobacteria (122	Proteobacteria (2)	Bacteria (1)	100.00	1.20	99.60	99.20	WGS
195.1022	0.114	Campylobacter coli (72294)	Campylobacter (213849)	Campylobacteraceae (2954	Campylobacterales (68525)	Epsilonproteobacteria (122	Proteobacteria (2)	Bacteria (1)	99.40	12.70	99.60	96.50	WGS
195.1023	0.11	Campylobacter coli (72294)	Campylobacter (213849)	Campylobacteraceae (2954	Campylobacterales (68525)	Epsilonproteobacteria (122	Proteobacteria (2)	Bacteria (1)	100.00	2.40	99.70	99.20	Complete
195.1029	0.1144	Campylobacter coli (72294)	Campylobacter (213849)	Campylobacteraceae (2954	Campylobacterales (68525)	Epsilonproteobacteria (122	Proteobacteria (2)	Bacteria (1)	100.00	17.00	99.30	93.10	WGS
195.1030	0.1132	Campylobacter coli (72294)	Campylobacter (213849)	Campylobacteraceae (2954	Campylobacterales (68525)	Epsilonproteobacteria (122	Proteobacteria (2)	Bacteria (1)	100.00	4.70	99.60	97.20	WGS
195.1031	0.1154	Campylobacter coli (72294)	Campylobacter (213849)	Campylobacteraceae (2954	Campylobacterales (68525)	Epsilonproteobacteria (122	Proteobacteria (2)	Bacteria (1)	100.00	0.00	99.60	99.60	Complete
195.1032	0.1172	Campylobacter coli (72294)	Campylobacter (213849)	Campylobacteraceae (2954	Campylobacterales (68525)	Epsilonproteobacteria (122	Proteobacteria (2)	Bacteria (1)	100.00	0.00	99.80	99.70	WGS
195.1033	0.1172	Campylobacter coli (72294)	Campylobacter (213849)	Campylobacteraceae (2954	Campylobacterales (68525)	Epsilonproteobacteria (122	Proteobacteria (2)	Bacteria (1)	100.00	0.00	99.80	99.70	WGS
195.1034	0.1172	Campylobacter coli (72294)	Campylobacter (213849)	Campylobacteraceae (2954	Campylobacterales (68525)	Epsilonproteobacteria (122	Proteobacteria (2)	Bacteria (1)	100.00	0.00	99.80	99.70	WGS
195.1035	0.11	Campylobacter coli (72294)	Campylobacter (213849)	Campylobacteraceae (2954	Campylobacterales (68525)	Epsilonproteobacteria (122	Proteobacteria (2)	Bacteria (1)	100.00	0.00	99.90	99.70	WGS
195.1036	0.11	Campylobacter coli (72294)	Campylobacter (213849)	Campylobacteraceae (2954	Campylobacterales (68525)	Epsilonproteobacteria (122	Proteobacteria (2)	Bacteria (1)	100.00	0.00	99.90	99.70	WGS
195.1037	0.11	Campylobacter coli (72294)	Campylobacter (213849)	Campylobacteraceae (2954	Campylobacterales (68525)	Epsilonproteobacteria (122	Proteobacteria (2)	Bacteria (1)	100.00	0.00	99.90	99.70	WGS
195.1038	0.1144	Campylobacter coli (72294)	Campylobacter (213849)	Campylobacteraceae (2954	Campylobacterales (68525)	Epsilonproteobacteria (122	Proteobacteria (2)	Bacteria (1)	100.00	0.00	99.80	99.60	WGS
195.1039	0.1144	Campylobacter coli (72294)	Campylobacter (213849)	Campylobacteraceae (2954	Campylobacterales (68525)	Epsilonproteobacteria (122	Proteobacteria (2)	Bacteria (1)	100.00	0.00	99.80	99.80	WGS
195.1040	0.116	Campylobacter coli (72294)	Campylobacter (213849)	Campylobacteraceae (2954	Campylobacterales (68525)	Epsilonproteobacteria (122	Proteobacteria (2)	Bacteria (1)	100.00	0.00	99.70	99.60	WGS
195.1041	0.116	Campylobacter coli (72294)	Campylobacter (213849)	Campylobacteraceae (2954	Campylobacterales (68525)	Epsilonproteobacteria (122	Proteobacteria (2)	Bacteria (1)	100.00	0.00	99.70	99.60	WGS
195.1042	0.1132	Campylobacter coli (72294)	Campylobacter (213849)	Campylobacteraceae (2954	Campylobacterales (68525)	Epsilonproteobacteria (122	Proteobacteria (2)	Bacteria (1)	100.00	0.00	99.80	99.50	WGS
195.1043	0.11	Campylobacter coli (72294)	Campylobacter (213849)	Campylobacteraceae (2954	Campylobacterales (68525)	Epsilonproteobacteria (122	Proteobacteria (2)	Bacteria (1)	100.00	0.00	99.90	99.70	WGS
195.1044	0.1132	Campylobacter coli (72294)	Campylobacter (213849)	Campylobacteraceae (2954	Campylobacterales (68525)	Epsilonproteobacteria (122	Proteobacteria (2)	Bacteria (1)	100.00	0.00	99.80	99.60	WGS
195.1045	0.11	Campylobacter coli (72294)	Campylobacter (213849)	Campylobacteraceae (2954	Campylobacterales (68525)	Epsilonproteobacteria (122	Proteobacteria (2)	Bacteria (1)	100.00	0.00	99.90	99.60	WGS
195.1046	0.1132	Campylobacter coli (72294)	Campylobacter (213849)	Campylobacteraceae (2954	Campylobacterales (68525)	Epsilonproteobacteria (122	Proteobacteria (2)	Bacteria (1)	100.00	0.60	99.80	99.50	WGS
195.1047	0.11	Campylobacter coli (72294)	Campylobacter (213849)	Campylobacteraceae (2954	Campylobacterales (68525)	Epsilonproteobacteria (122	Proteobacteria (2)	Bacteria (1)	100.00	0.00	99.90	99.60	WGS
195.1048	0.11	Campylobacter coli (72294)	Campylobacter (213849)	Campylobacteraceae (2954	Campylobacterales (68525)	Epsilonproteobacteria (122	Proteobacteria (2)	Bacteria (1)	100.00	0.00	99.90	99.60	WGS
195.1049	0.1172	Campylobacter coli (72294)	Campylobacter (213849)	Campylobacteraceae (2954	Campylobacterales (68525)	Epsilonproteobacteria (122	Proteobacteria (2)	Bacteria (1)	100.00	0.00	99.80	99.70	WGS
195.1050	0.1144	Campylobacter coli (72294)	Campylobacter (213849)	Campylobacteraceae (2954	Campylobacterales (68525)	Epsilonproteobacteria (122	Proteobacteria (2)	Bacteria (1)	100.00	0.00	99.80	99.80	WGS
195.1051	0.1144	Campylobacter coli (72294)	Campylobacter (213849)	Campylobacteraceae (2954	Campylobacterales (68525)	Epsilonproteobacteria (122	Proteobacteria (2)	Bacteria (1)	100.00	0.00	99.80	99.60	WGS
195.1052	0.1132	Campylobacter coli (72294)	Campylobacter (213849)	Campylobacteraceae (2954	Campylobacterales (68525)	Epsilonproteobacteria (122	Proteobacteria (2)	Bacteria (1)	100.00	0.00	99.80	99.50	WGS
195.1053	0.116	Campylobacter coli (72294)	Campylobacter (213849)	Campylobacteraceae (2954	Campylobacterales (68525)	Epsilonproteobacteria (122	Proteobacteria (2)	Bacteria (1)	100.00	0.00	99.70	99.60	WGS
195.1054	0.1132	Campylobacter coli (72294)	Campylobacter (213849)	Campylobacteraceae (2954	Campylobacterales (68525)	Epsilonproteobacteria (122	Proteobacteria (2)	Bacteria (1)	100.00	0.00	99.80	99.60	WGS
195.1055	0.1132	Campylobacter coli (72294)	Campylobacter (213849)	Campylobacteraceae (2954	Campylobacterales (68525)	Epsilonproteobacteria (122	Proteobacteria (2)	Bacteria (1)	100.00	0.00	99.80	99.60	WGS
195.1056	0.116	Campylobacter coli (72294)	Campylobacter (213849)	Campylobacteraceae (2954	Campylobacterales (68525)	Epsilonproteobacteria (122	Proteobacteria (2)	Bacteria (1)	100.00	0.00	99.70	99.60	WGS
195.1057	0.11	Campylobacter coli (72294)	Campylobacter (213849)	Campylobacteraceae (2954	Campylobacterales (68525)	Epsilonproteobacteria (122	Proteobacteria (2)	Bacteria (1)	100.00	0.00	99.90	99.60	WGS
195.1058	0.11	Campylobacter coli (72294)	Campylobacter (213849)	Campylobacteraceae (2954	Campylobacterales (68525)	Epsilonproteobacteria (122	Proteobacteria (2)	Bacteria (1)	100.00	0.00	99.90	99.70	WGS
195.1059	0.1112	Campylobacter coli (72294)	Campylobacter (213849)	Campylobacteraceae (2954	Campylobacterales (68525)	Epsilonproteobacteria (122	Proteobacteria (2)	Bacteria (1)	100.00	0.00	99.80	99.60	WGS
195.1060	0.1128	Campylobacter coli (72294)	Campylobacter (213849)	Campylobacteraceae (2954	Campylobacterales (68525)	Epsilonproteobacteria (122	Proteobacteria (2)	Bacteria (1)	100.00	0.00	99.80	99.60	WGS
195.1061	0.1132	Campylobacter coli (72294)	Campylobacter (213849)	Campylobacteraceae (2954	Campylobacterales (68525)	Epsilonproteobacteria (122	Proteobacteria (2)	Bacteria (1)	100.00	0.60	99.80	99.60	Complete
195.1062	0.116	Campylobacter coli (72294)	Campylobacter (213849)	Campylobacteraceae (2954	Campylobacterales (68525)	Epsilonproteobacteria (122	Proteobacteria (2)	Bacteria (1)	100.00	0.00	99.80	99.60	Complete
195.1063	0.1126	Campylobacter coli (72294)	Campylobacter (213849)	Campylobacteraceae (2954	Campylobacterales (68525)	Epsilonproteobacteria (122	Proteobacteria (2)	Bacteria (1)	100.00	0.00	99.80	99.70	Complete
195.1064	0.1144	Campylobacter coli (72294)	Campylobacter (213849)	Campylobacteraceae (2954	Campylobacterales (68525)	Epsilonproteobacteria (122	Proteobacteria (2)	Bacteria (1)	100.00	9.40	99.80	96.50	Complete
195.1065	0.118	Campylobacter coli (72294)	Campylobacter (213849)	Campylobacteraceae (2954	Campylobacterales (68525)	Epsilonproteobacteria (122	Proteobacteria (2)	Bacteria (1)	100.00	5.80	99.60	96.70	Complete
195.1066	0.1132	Campylobacter coli (72294)	Campylobacter (213849)	Campylobacteraceae (2954	Campylobacterales (68525)	Epsilonproteobacteria (122	Proteobacteria (2)	Bacteria (1)	100.00	0.00	99.80	99.80	Complete
195.1086	0.11	Campylobacter coli (72294)	Campylobacter (213849)	Campylobacteraceae (2954	Campylobacterales (68525)	Epsilonproteobacteria (122	Proteobacteria (2)	Bacteria (1)	100.00	0.00	99.90	99.80	WGS
195.1088	0.1172	Campylobacter coli (72294)	Campylobacter (213849)	Campylobacteraceae (2954	Campylobacterales (68525)	Epsilonproteobacteria (122	Proteobacteria (2)	Bacteria (1)	100.00	0.00	99.60	99.60	Complete
195.1091	0.116	Campylobacter coli (72294)	Campylobacter (213849)	Campylobacteraceae (2954	Campylobacterales (68525)	Epsilonproteobacteria (122	Proteobacteria (2)	Bacteria (1)	100.00	0.00	99.60	99.40	WGS
195.1092	0.1078	Campylobacter coli (72294)	Campylobacter (213849)	Campylobacteraceae (2954	Campylobacterales (68525)	Epsilonproteobacteria (122	Proteobacteria (2)	Bacteria (1)	100.00	3.50	99.70	98.80	WGS
195.1093	0.1138	Campylobacter coli (72294)	Campylobacter (213849)	Campylobacteraceae (2954	Campylobacterales (68525)	Epsilonproteobacteria (122	Proteobacteria (2)	Bacteria (1)	100.00	1.80	99.70	98.30	WGS
195.1094	0.1146	Campylobacter coli (72294)	Campylobacter (213849)	Campylobacteraceae (2954	Campylobacterales (68525)	Epsilonproteobacteria (122	Proteobacteria (2)	Bacteria (1)	100.00	5.20	99.60	97.40	WGS
195.1095	0.1096	Campylobacter coli (72294)	Campylobacter (213849)	Campylobacteraceae (2954	Campylobacterales (68525)	Epsilonproteobacteria (122	Proteobacteria (2)	Bacteria (1)	100.00	7.90	99.70	97.20	WGS
195.1096	0.1112	Campylobacter coli (72294)	Campylobacter (213849)	Campylobacteraceae (2954	Campylobacterales (68525)	Epsilonproteobacteria (122	Proteobacteria (2)	Bacteria (1)	99.40	11.00	99.70	96.50	WGS
195.1097	0.1108	Campylobacter coli (72294)	Campylobacter (213849)	Campylobacteraceae (2954	Campylobacterales (68525)	Epsilonproteobacteria (122	Proteobacteria (2)	Bacteria (1)	100.00	2.40	99.80	99.00	WGS
195.1098	0.11	Campylobacter coli (72294)	Campylobacter (213849)	Campylobacteraceae (2954	Campylobacterales (68525)	Epsilonproteobacteria (122	Proteobacteria (2)	Bacteria (1)	100.00	1.80	99.70	99.10	WGS
195.1099	0.109	Campylobacter coli (72294)	Campylobacter (213849)	Campylobacteraceae (2954	Campylobacterales (68525)	Epsilonproteobacteria (122	Proteobacteria (2)	Bacteria (1)	100.00	6.80	99.80	98.10	WGS
195.1100	0.1172	Campylobacter coli (72294)	Campylobacter (213849)	Campylobacteraceae (2954	Campylobacterales (68525)	Epsilonproteobacteria (122	Proteobacteria (2)	Bacteria (1)	100.00	0.00	99.40	99.30	Complete
195.1108	0.1128	Campylobacter coli (72294)	Campylobacter (213849)	Campylobacteraceae (2954	Campylobacterales (68525)	Epsilonproteobacteria (122	Proteobacteria (2)	Bacteria (1)	100.00	0.00	99.60	99.60	WGS
195.1166	0.116	Campylobacter coli (72294)	Campylobacter (213849)	Campylobacteraceae (2954	Campylobacterales (68525)	Epsilonproteobacteria (122	Proteobacteria (2)	Bacteria (1)	100.00	0.00	99.80	99.70	WGS
195.1167	0.1112	Campylobacter coli (72294)	Campylobacter (213849)	Campylobacteraceae (2954	Campylobacterales (68525)	Epsilonproteobacteria (122	Proteobacteria (2)	Bacteria (1)	100.00	0.00	99.60	99.60	WGS
195.1168	0.11	Campylobacter coli (72294)	Campylobacter (213849)	Campylobacteraceae (2954	Campylobacterales (68525)	Epsilonproteobacteria (122	Proteobacteria (2)	Bacteria (1)	100.00	4.70	99.60	97.90	WGS
195.1169	0.11	Campylobacter coli (72294)	Campylobacter (213849)	Campylobacteraceae (2954	Campylobacterales (68525)	Epsilonproteobacteria (122	Proteobacteria (2)	Bacteria (1)	100.00	0.60	99.60	99.60	WGS
195.1170	0.11	Campylobacter coli (72294)	Campylobacter (213849)	Campylobacteraceae (2954	Campylobacterales (68525)	Epsilonproteobacteria (122	Proteobacteria (2)	Bacteria (1)	100.00	0.00	99.60	99.60	WGS
195.1171	0.11	Campylobacter coli (72294)	Campylobacter (213849)	Campylobacteraceae (2954	Campylobacterales (68525)	Epsilonproteobacteria (122	Proteobacteria (2)	Bacteria (1)	100.00	0.00	99.60	99.60	WGS
195.1176	0.1112	Campylobacter coli (72294)	Campylobacter (213849)	Campylobacteraceae (2954	Campylobacterales (68525)	Epsilonproteobacteria (122	Proteobacteria (2)	Bacteria (1)	100.00	0.00	99.80	99.70	Complete
195.1197	0.0294	Campylobacter coli (72294)	Campylobacter (213849)	Campylobacteraceae (2954	Campylobacterales (68525)	Epsilonproteobacteria (122	Proteobacteria (2)	Bacteria (1)					Plasmid
195.1198	0.0294	Campylobacter coli (72294)	Campylobacter (213849)	Campylobacteraceae (2954	Campylobacterales (68525)	Epsilonproteobacteria (122	Proteobacteria (2)	Bacteria (1)					Plasmid
195.1199	0.0294	Campylobacter coli (72294)	Campylobacter (213849)	Campylobacteraceae (2954	Campylobacterales (68525)	Epsilonproteobacteria (122	Proteobacteria (2)	Bacteria (1)					Plasmid
195.1201	0.0294	Campylobacter coli (72294)	Campylobacter (213849)	Campylobacteraceae (2954	Campylobacterales (68525)	Epsilonproteobacteria (122	Proteobacteria (2)	Bacteria (1)					Plasmid
195.1202	0.0294	Campylobacter coli (72294)	Campylobacter (213849)	Campylobacteraceae (2954	Campylobacterales (68525)	Epsilonproteobacteria (122	Proteobacteria (2)	Bacteria (1)					Plasmid
195.1203	0.0294	Campylobacter coli (72294)	Campylobacter (213849)	Campylobacteraceae (2954	Campylobacterales (68525)	Epsilonproteobacteria (122	Proteobacteria (2)	Bacteria (1)					Plasmid
195.1204	0.0294	Campylobacter coli (72294)	Campylobacter (213849)	Campylobacteraceae (2954	Campylobacterales (68525)	Epsilonproteobacteria (122	Proteobacteria (2)	Bacteria (1)					Plasmid
195.1205	0.0294	Campylobacter coli (72294)	Campylobacter (213849)	Campylobacteraceae (2954	Campylobacterales (68525)	Epsilonproteobacteria (122	Proteobacteria (2)	Bacteria (1)					Plasmid
195.1206	0.0294	Campylobacter coli (72294)	Campylobacter (213849)	Campylobacteraceae (2954	Campylobacterales (68525)	Epsilonproteobacteria (122	Proteobacteria (2)	Bacteria (1)					Plasmid
195.1207	0.0294	Campylobacter coli (72294)	Campylobacter (213849)	Campylobacteraceae (2954	Campylobacterales (68525)	Epsilonproteobacteria (122	Proteobacteria (2)	Bacteria (1)					Plasmid
195.1208	0.0294	Campylobacter coli (72294)	Campylobacter (213849)	Campylobacteraceae (2954	Campylobacterales (68525)	Epsilonproteobacteria (122	Proteobacteria (2)	Bacteria (1)					Plasmid
195.1209	0.0294	Campylobacter coli (72294)	Campylobacter (213849)	Campylobacteraceae (2954	Campylobacterales (68525)	Epsilonproteobacteria (122	Proteobacteria (2)	Bacteria (1)					Plasmid
195.1210	0.0294	Campylobacter coli (72294)	Campylobacter (213849)	Campylobacteraceae (2954	Campylobacterales (68525)	Epsilonproteobacteria (122	Proteobacteria (2)	Bacteria (1)					Plasmid
195.1211	0.0294	Campylobacter coli (72294)	Campylobacter (213849)	Campylobacteraceae (2954	Campylobacterales (68525)	Epsilonproteobacteria (122	Proteobacteria (2)	Bacteria (1)					Plasmid
195.1212	0.0294	Campylobacter coli (72294)	Campylobacter (213849)	Campylobacteraceae (2954	Campylobacterales (68525)	Epsilonproteobacteria (122	Proteobacteria (2)	Bacteria (1)					Plasmid
195.1213	0.0294	Campylobacter coli (72294)	Campylobacter (213849)	Campylobacteraceae (2954	Campylobacterales (68525)	Epsilonproteobacteria (122	Proteobacteria (2)	Bacteria (1)					Plasmid
195.1214	0.0294	Campylobacter coli (72294)	Campylobacter (213849)	Campylobacteraceae (2954	Campylobacterales (68525)	Epsilonproteobacteria (122	Proteobacteria (2)	Bacteria (1)					Plasmid
195.1215	0.0294	Campylobacter coli (72294)	Campylobacter (213849)	Campylobacteraceae (2954	Campylobacterales (68525)	Epsilonproteobacteria (122	Proteobacteria (2)	Bacteria (1)					Plasmid
195.1216	0.0294	Campylobacter coli (72294)	Campylobacter (213849)	Campylobacteraceae (2954	Campylobacterales (68525)	Epsilonproteobacteria (122	Proteobacteria (2)	Bacteria (1)					Plasmid
195.1217	0.0294	Campylobacter coli (72294)	Campylobacter (213849)	Campylobacteraceae (2954	Campylobacterales (68525)	Epsilonproteobacteria (122	Proteobacteria (2)	Bacteria (1)					Plasmid
195.1218	0.0294	Campylobacter coli (72294)	Campylobacter (213849)	Campylobacteraceae (2954	Campylobacterales (68525)	Epsilonproteobacteria (122	Proteobacteria (2)	Bacteria (1)					Plasmid
195.1219	0.0298	Campylobacter coli (72294)	Campylobacter (213849)	Campylobacteraceae (2954	Campylobacterales (68525)	Epsilonproteobacteria (122	Proteobacteria (2)	Bacteria (1)					Plasmid
195.1220	0.0294	Campylobacter coli (72294)	Campylobacter (213849)	Campylobacteraceae (2954	Campylobacterales (68525)	Epsilonproteobacteria (122	Proteobacteria (2)	Bacteria (1)					Plasmid
195.1221	0.0294	Campylobacter coli (72294)	Campylobacter (213849)	Campylobacteraceae (2954	Campylobacterales (68525)	Epsilonproteobacteria (122	Proteobacteria (2)	Bacteria (1)					Plasmid
195.1224	0.0294	Campylobacter coli (72294)	Campylobacter (213849)	Campylobacteraceae (2954	Campylobacterales (68525)	Epsilonproteobacteria (122	Proteobacteria (2)	Bacteria (1)					Plasmid
195.1225	0.0294	Campylobacter coli (72294)	Campylobacter (213849)	Campylobacteraceae (2954	Campylobacterales (68525)	Epsilonproteobacteria (122	Proteobacteria (2)	Bacteria (1)					Plasmid
195.1226	0.0294	Campylobacter coli (72294)	Campylobacter (213849)	Campylobacteraceae (2954	Campylobacterales (68525)	Epsilonproteobacteria (122	Proteobacteria (2)	Bacteria (1)					Plasmid
195.1228	0.1144	Campylobacter coli (72294)	Campylobacter (213849)	Campylobacteraceae (2954	Campylobacterales (68525)	Epsilonproteobacteria (122	Proteobacteria (2)	Bacteria (1)	100.00	1.40	99.60	99.40	Complete
195.1282	0.1148	Campylobacter coli (72294)	Campylobacter (213849)	Campylobacteraceae (2954	Campylobacterales (68525)	Epsilonproteobacteria (122	Proteobacteria (2)	Bacteria (1)	100.00	0.00	99.80	99.40	Complete
195.1500	0.1152	Campylobacter coli (72294)	Campylobacter (213849)	Campylobacteraceae (2954	Campylobacterales (68525)	Epsilonproteobacteria (122	Proteobacteria (2)	Bacteria (1)	99.40	30.30	99.40	87.90	WGS
195.1501	0.1152	Campylobacter coli (72294)	Campylobacter (213849)	Campylobacteraceae (2954	Campylobacterales (68525)	Epsilonproteobacteria (122	Proteobacteria (2)	Bacteria (1)	98.80	5.30	99.00	96.60	WGS
195.1502	0.1152	Campylobacter coli (72294)	Campylobacter (213849)	Campylobacteraceae (2954	Campylobacterales (68525)	Epsilonproteobacteria (122	Proteobacteria (2)	Bacteria (1)	99.40	13.80	99.00	94.20	WGS
195.1503	0.1112	Campylobacter coli (72294)	Campylobacter (213849)	Campylobacteraceae (2954	Campylobacterales (68525)	Epsilonproteobacteria (122	Proteobacteria (2)	Bacteria (1)	97.60	20.00	98.70	89.70	WGS
195.1504	0.1168	Campylobacter coli (72294)	Campylobacter (213849)	Campylobacteraceae (2954	Campylobacterales (68525)	Epsilonproteobacteria (122	Proteobacteria (2)	Bacteria (1)	100.00	1.20	99.60	99.30	WGS
195.1505	0.116	Campylobacter coli (72294)	Campylobacter (213849)	Campylobacteraceae (2954	Campylobacterales (68525)	Epsilonproteobacteria (122	Proteobacteria (2)	Bacteria (1)	100.00	1.80	99.60	99.10	WGS
195.1506	0.11	Campylobacter coli (72294)	Campylobacter (213849)	Campylobacteraceae (2954	Campylobacterales (68525)	Epsilonproteobacteria (122	Proteobacteria (2)	Bacteria (1)	100.00	1.20	99.70	99.50	WGS
195.1507	0.1076	Campylobacter coli (72294)	Campylobacter (213849)	Campylobacteraceae (2954	Campylobacterales (68525)	Epsilonproteobacteria (122	Proteobacteria (2)	Bacteria (1)	100.00	1.20	99.60	99.20	WGS
195.1508	0.1112	Campylobacter coli (72294)	Campylobacter (213849)	Campylobacteraceae (2954	Campylobacterales (68525)	Epsilonproteobacteria (122	Proteobacteria (2)	Bacteria (1)	100.00	1.20	99.60	99.30	WGS
195.1509	0.1132	Campylobacter coli (72294)	Campylobacter (213849)	Campylobacteraceae (2954	Campylobacterales (68525)	Epsilonproteobacteria (122	Proteobacteria (2)	Bacteria (1)	100.00	1.20	99.60	99.40	WGS
195.1510	0.1132	Campylobacter coli (72294)	Campylobacter (213849)	Campylobacteraceae (2954	Campylobacterales (68525)	Epsilonproteobacteria (122	Proteobacteria (2)	Bacteria (1)	88.30	18.30	93.30	89.80	WGS
195.1511	0.116	Campylobacter coli (72294)	Campylobacter (213849)	Campylobacteraceae (2954	Campylobacterales (68525)	Epsilonproteobacteria (122	Proteobacteria (2)	Bacteria (1)	100.00	3.00	99.30	98.90	WGS
195.1512	0.1132	Campylobacter coli (72294)	Campylobacter (213849)	Campylobacteraceae (2954	Campylobacterales (68525)	Epsilonproteobacteria (122	Proteobacteria (2)	Bacteria (1)	100.00	0.60	99.60	99.50	WGS
195.1513	0.1112	Campylobacter coli (72294)	Campylobacter (213849)	Campylobacteraceae (2954	Campylobacterales (68525)	Epsilonproteobacteria (122	Proteobacteria (2)	Bacteria (1)	100.00	1.20	99.60	99.30	WGS
195.1514	0.1144	Campylobacter coli (72294)	Campylobacter (213849)	Campylobacteraceae (2954	Campylobacterales (68525)	Epsilonproteobacteria (122	Proteobacteria (2)	Bacteria (1)	100.00	0.60	99.40	99.30	WGS
195.1515	0.116	Campylobacter coli (72294)	Campylobacter (213849)	Campylobacteraceae (2954	Campylobacterales (68525)	Epsilonproteobacteria (122	Proteobacteria (2)	Bacteria (1)	100.00	1.20	99.40	99.20	WGS
195.1544	0.11	Campylobacter coli (72294)	Campylobacter (213849)	Campylobacteraceae (2954	Campylobacterales (68525)	Epsilonproteobacteria (122	Proteobacteria (2)	Bacteria (1)			99.80	98.50	WGS
195.1545	0.118	Campylobacter coli (72294)	Campylobacter (213849)	Campylobacteraceae (2954	Campylobacterales (68525)	Epsilonproteobacteria (122	Proteobacteria (2)	Bacteria (1)			99.80	98.40	WGS
195.1546	0.1164	Campylobacter coli (72294)	Campylobacter (213849)	Campylobacteraceae (2954	Campylobacterales (68525)	Epsilonproteobacteria (122	Proteobacteria (2)	Bacteria (1)			99.80	99.60	WGS
195.1547	0.116	Campylobacter coli (72294)	Campylobacter (213849)	Campylobacteraceae (2954	Campylobacterales (68525)	Epsilonproteobacteria (122	Proteobacteria (2)	Bacteria (1)			99.80	99.50	WGS
195.1548	0.1128	Campylobacter coli (72294)	Campylobacter (213849)	Campylobacteraceae (2954	Campylobacterales (68525)	Epsilonproteobacteria (122	Proteobacteria (2)	Bacteria (1)			99.80	99.50	WGS
195.1549	0.1112	Campylobacter coli (72294)	Campylobacter (213849)	Campylobacteraceae (2954	Campylobacterales (68525)	Epsilonproteobacteria (122	Proteobacteria (2)	Bacteria (1)			99.80	99.10	WGS
195.1550	0.116	Campylobacter coli (72294)	Campylobacter (213849)	Campylobacteraceae (2954	Campylobacterales (68525)	Epsilonproteobacteria (122	Proteobacteria (2)	Bacteria (1)			99.80	98.50	WGS
195.1551	0.11	Campylobacter coli (72294)	Campylobacter (213849)	Campylobacteraceae (2954	Campylobacterales (68525)	Epsilonproteobacteria (122	Proteobacteria (2)	Bacteria (1)			99.80	98.50	WGS
195.1552	0.1132	Campylobacter coli (72294)	Campylobacter (213849)	Campylobacteraceae (2954	Campylobacterales (68525)	Epsilonproteobacteria (122	Proteobacteria (2)	Bacteria (1)			99.80	98.80	WGS
195.1553	0.114	Campylobacter coli (72294)	Campylobacter (213849)	Campylobacteraceae (2954	Campylobacterales (68525)	Epsilonproteobacteria (122	Proteobacteria (2)	Bacteria (1)			99.80	99.80	WGS
195.1554	0.1132	Campylobacter coli (72294)	Campylobacter (213849)	Campylobacteraceae (2954	Campylobacterales (68525)	Epsilonproteobacteria (122	Proteobacteria (2)	Bacteria (1)			99.80	96.60	WGS
195.1555	0.1132	Campylobacter coli (72294)	Campylobacter (213849)	Campylobacteraceae (2954	Campylobacterales (68525)	Epsilonproteobacteria (122	Proteobacteria (2)	Bacteria (1)			99.30	98.80	WGS
195.1556	0.1144	Campylobacter coli (72294)	Campylobacter (213849)	Campylobacteraceae (2954	Campylobacterales (68525)	Epsilonproteobacteria (122	Proteobacteria (2)	Bacteria (1)			99.70	99.40	WGS
195.1557	0.1172	Campylobacter coli (72294)	Campylobacter (213849)	Campylobacteraceae (2954	Campylobacterales (68525)	Epsilonproteobacteria (122	Proteobacteria (2)	Bacteria (1)			99.80	99.70	WGS
195.1558	0.116	Campylobacter coli (72294)	Campylobacter (213849)	Campylobacteraceae (2954	Campylobacterales (68525)	Epsilonproteobacteria (122	Proteobacteria (2)	Bacteria (1)			99.80	98.50	WGS
195.1559	0.1132	Campylobacter coli (72294)	Campylobacter (213849)	Campylobacteraceae (2954	Campylobacterales (68525)	Epsilonproteobacteria (122	Proteobacteria (2)	Bacteria (1)			99.80	98.20	WGS
195.1560	0.1164	Campylobacter coli (72294)	Campylobacter (213849)	Campylobacteraceae (2954	Campylobacterales (68525)	Epsilonproteobacteria (122	Proteobacteria (2)	Bacteria (1)			99.80	94.00	WGS
195.1561	0.1132	Campylobacter coli (72294)	Campylobacter (213849)	Campylobacteraceae (2954	Campylobacterales (68525)	Epsilonproteobacteria (122	Proteobacteria (2)	Bacteria (1)			99.80	97.60	WGS
195.1562	0.1132	Campylobacter coli (72294)	Campylobacter (213849)	Campylobacteraceae (2954	Campylobacterales (68525)	Epsilonproteobacteria (122	Proteobacteria (2)	Bacteria (1)			99.80	98.00	WGS
195.1563	0.114	Campylobacter coli (72294)	Campylobacter (213849)	Campylobacteraceae (2954	Campylobacterales (68525)	Epsilonproteobacteria (122	Proteobacteria (2)	Bacteria (1)			99.80	99.80	WGS
195.1564	0.1132	Campylobacter coli (72294)	Campylobacter (213849)	Campylobacteraceae (2954	Campylobacterales (68525)	Epsilonproteobacteria (122	Proteobacteria (2)	Bacteria (1)			99.40	98.50	WGS
195.1565	0.1112	Campylobacter coli (72294)	Campylobacter (213849)	Campylobacteraceae (2954	Campylobacterales (68525)	Epsilonproteobacteria (122	Proteobacteria (2)	Bacteria (1)			99.60	99.00	WGS
195.1566	0.116	Campylobacter coli (72294)	Campylobacter (213849)	Campylobacteraceae (2954	Campylobacterales (68525)	Epsilonproteobacteria (122	Proteobacteria (2)	Bacteria (1)			99.70	98.70	WGS
195.1567	0.1164	Campylobacter coli (72294)	Campylobacter (213849)	Campylobacteraceae (2954	Campylobacterales (68525)	Epsilonproteobacteria (122	Proteobacteria (2)	Bacteria (1)			99.80	98.20	WGS
195.1568	0.114	Campylobacter coli (72294)	Campylobacter (213849)	Campylobacteraceae (2954	Campylobacterales (68525)	Epsilonproteobacteria (122	Proteobacteria (2)	Bacteria (1)			99.80	99.70	WGS
195.1569	0.1172	Campylobacter coli (72294)	Campylobacter (213849)	Campylobacteraceae (2954	Campylobacterales (68525)	Epsilonproteobacteria (122	Proteobacteria (2)	Bacteria (1)			99.80	99.70	WGS
195.1570	0.1172	Campylobacter coli (72294)	Campylobacter (213849)	Campylobacteraceae (2954	Campylobacterales (68525)	Epsilonproteobacteria (122	Proteobacteria (2)	Bacteria (1)			99.80	99.70	WGS
195.1571	0.116	Campylobacter coli (72294)	Campylobacter (213849)	Campylobacteraceae (2954	Campylobacterales (68525)	Epsilonproteobacteria (122	Proteobacteria (2)	Bacteria (1)			99.80	98.20	WGS
195.1572	0.1144	Campylobacter coli (72294)	Campylobacter (213849)	Campylobacteraceae (2954	Campylobacterales (68525)	Epsilonproteobacteria (122	Proteobacteria (2)	Bacteria (1)			99.80	99.70	WGS
195.1573	0.1132	Campylobacter coli (72294)	Campylobacter (213849)	Campylobacteraceae (2954	Campylobacterales (68525)	Epsilonproteobacteria (122	Proteobacteria (2)	Bacteria (1)			99.80	98.70	WGS
195.1574	0.1172	Campylobacter coli (72294)	Campylobacter (213849)	Campylobacteraceae (2954	Campylobacterales (68525)	Epsilonproteobacteria (122	Proteobacteria (2)	Bacteria (1)			99.50	98.40	WGS
195.1575	0.1132	Campylobacter coli (72294)	Campylobacter (213849)	Campylobacteraceae (2954	Campylobacterales (68525)	Epsilonproteobacteria (122	Proteobacteria (2)	Bacteria (1)			99.40	98.60	WGS
195.1576	0.116	Campylobacter coli (72294)	Campylobacter (213849)	Campylobacteraceae (2954	Campylobacterales (68525)	Epsilonproteobacteria (122	Proteobacteria (2)	Bacteria (1)			99.80	99.70	WGS
195.1577	0.1132	Campylobacter coli (72294)	Campylobacter (213849)	Campylobacteraceae (2954	Campylobacterales (68525)	Epsilonproteobacteria (122	Proteobacteria (2)	Bacteria (1)			99.40	98.70	WGS
195.1578	0.1172	Campylobacter coli (72294)	Campylobacter (213849)	Campylobacteraceae (2954	Campylobacterales (68525)	Epsilonproteobacteria (122	Proteobacteria (2)	Bacteria (1)			99.80	99.70	WGS
195.1579	0.1144	Campylobacter coli (72294)	Campylobacter (213849)	Campylobacteraceae (2954	Campylobacterales (68525)	Epsilonproteobacteria (122	Proteobacteria (2)	Bacteria (1)			99.80	99.70	WGS
195.1580	0.116	Campylobacter coli (72294)	Campylobacter (213849)	Campylobacteraceae (2954	Campylobacterales (68525)	Epsilonproteobacteria (122	Proteobacteria (2)	Bacteria (1)			99.80	99.80	WGS
195.1581	0.11	Campylobacter coli (72294)	Campylobacter (213849)	Campylobacteraceae (2954	Campylobacterales (68525)	Epsilonproteobacteria (122	Proteobacteria (2)	Bacteria (1)			99.80	99.50	WGS
195.1582	0.1172	Campylobacter coli (72294)	Campylobacter (213849)	Campylobacteraceae (2954	Campylobacterales (68525)	Epsilonproteobacteria (122	Proteobacteria (2)	Bacteria (1)			99.80	99.70	WGS
195.1583	0.1132	Campylobacter coli (72294)	Campylobacter (213849)	Campylobacteraceae (2954	Campylobacterales (68525)	Epsilonproteobacteria (122	Proteobacteria (2)	Bacteria (1)			99.40	98.80	WGS
195.1584	0.1132	Campylobacter coli (72294)	Campylobacter (213849)	Campylobacteraceae (2954	Campylobacterales (68525)	Epsilonproteobacteria (122	Proteobacteria (2)	Bacteria (1)			99.50	98.10	WGS
195.1585	0.116	Campylobacter coli (72294)	Campylobacter (213849)	Campylobacteraceae (2954	Campylobacterales (68525)	Epsilonproteobacteria (122	Proteobacteria (2)	Bacteria (1)			99.60	98.50	WGS
195.1586	0.1172	Campylobacter coli (72294)	Campylobacter (213849)	Campylobacteraceae (2954	Campylobacterales (68525)	Epsilonproteobacteria (122	Proteobacteria (2)	Bacteria (1)			99.80	99.70	WGS
195.1587	0.116	Campylobacter coli (72294)	Campylobacter (213849)	Campylobacteraceae (2954	Campylobacterales (68525)	Epsilonproteobacteria (122	Proteobacteria (2)	Bacteria (1)			99.70	99.50	WGS
195.1588	0.1132	Campylobacter coli (72294)	Campylobacter (213849)	Campylobacteraceae (2954	Campylobacterales (68525)	Epsilonproteobacteria (122	Proteobacteria (2)	Bacteria (1)			99.30	98.70	WGS
195.1589	0.1132	Campylobacter coli (72294)	Campylobacter (213849)	Campylobacteraceae (2954	Campylobacterales (68525)	Epsilonproteobacteria (122	Proteobacteria (2)	Bacteria (1)			99.30	99.20	WGS
195.1590	0.1112	Campylobacter coli (72294)	Campylobacter (213849)	Campylobacteraceae (2954	Campylobacterales (68525)	Epsilonproteobacteria (122	Proteobacteria (2)	Bacteria (1)			99.80	99.80	WGS
195.1591	0.116	Campylobacter coli (72294)	Campylobacter (213849)	Campylobacteraceae (2954	Campylobacterales (68525)	Epsilonproteobacteria (122	Proteobacteria (2)	Bacteria (1)			99.80	99.70	WGS
195.1592	0.1128	Campylobacter coli (72294)	Campylobacter (213849)	Campylobacteraceae (2954	Campylobacterales (68525)	Epsilonproteobacteria (122	Proteobacteria (2)	Bacteria (1)			99.80	99.30	WGS
195.1593	0.116	Campylobacter coli (72294)	Campylobacter (213849)	Campylobacteraceae (2954	Campylobacterales (68525)	Epsilonproteobacteria (122	Proteobacteria (2)	Bacteria (1)			99.80	98.20	WGS
195.1594	0.1132	Campylobacter coli (72294)	Campylobacter (213849)	Campylobacteraceae (2954	Campylobacterales (68525)	Epsilonproteobacteria (122	Proteobacteria (2)	Bacteria (1)			99.70	98.50	WGS
195.1595	0.1078	Campylobacter coli (72294)	Campylobacter (213849)	Campylobacteraceae (2954	Campylobacterales (68525)	Epsilonproteobacteria (122	Proteobacteria (2)	Bacteria (1)			99.90	99.20	WGS
195.1596	0.1172	Campylobacter coli (72294)	Campylobacter (213849)	Campylobacteraceae (2954	Campylobacterales (68525)	Epsilonproteobacteria (122	Proteobacteria (2)	Bacteria (1)			99.80	99.00	WGS
195.1597	0.1112	Campylobacter coli (72294)	Campylobacter (213849)	Campylobacteraceae (2954	Campylobacterales (68525)	Epsilonproteobacteria (122	Proteobacteria (2)	Bacteria (1)			99.80	99.00	WGS
195.1598	0.116	Campylobacter coli (72294)	Campylobacter (213849)	Campylobacteraceae (2954	Campylobacterales (68525)	Epsilonproteobacteria (122	Proteobacteria (2)	Bacteria (1)			99.60	99.50	WGS
195.1599	0.1172	Campylobacter coli (72294)	Campylobacter (213849)	Campylobacteraceae (2954	Campylobacterales (68525)	Epsilonproteobacteria (122	Proteobacteria (2)	Bacteria (1)			99.80	99.30	WGS
195.1600	0.116	Campylobacter coli (72294)	Campylobacter (213849)	Campylobacteraceae (2954	Campylobacterales (68525)	Epsilonproteobacteria (122	Proteobacteria (2)	Bacteria (1)			99.70	99.70	WGS
195.1601	0.1144	Campylobacter coli (72294)	Campylobacter (213849)	Campylobacteraceae (2954	Campylobacterales (68525)	Epsilonproteobacteria (122	Proteobacteria (2)	Bacteria (1)			99.60	99.30	WGS
195.1602	0.1148	Campylobacter coli (72294)	Campylobacter (213849)	Campylobacteraceae (2954	Campylobacterales (68525)	Epsilonproteobacteria (122	Proteobacteria (2)	Bacteria (1)			99.70	97.50	WGS
195.1603	0.116	Campylobacter coli (72294)	Campylobacter (213849)	Campylobacteraceae (2954	Campylobacterales (68525)	Epsilonproteobacteria (122	Proteobacteria (2)	Bacteria (1)			99.80	96.70	WGS
195.1604	0.1128	Campylobacter coli (72294)	Campylobacter (213849)	Campylobacteraceae (2954	Campylobacterales (68525)	Epsilonproteobacteria (122	Proteobacteria (2)	Bacteria (1)			99.80	98.80	WGS
195.1605	0.1128	Campylobacter coli (72294)	Campylobacter (213849)	Campylobacteraceae (2954	Campylobacterales (68525)	Epsilonproteobacteria (122	Proteobacteria (2)	Bacteria (1)			99.80	98.50	WGS
195.1606	0.1172	Campylobacter coli (72294)	Campylobacter (213849)	Campylobacteraceae (2954	Campylobacterales (68525)	Epsilonproteobacteria (122	Proteobacteria (2)	Bacteria (1)			99.60	99.60	WGS
195.1607	0.1144	Campylobacter coli (72294)	Campylobacter (213849)	Campylobacteraceae (2954	Campylobacterales (68525)	Epsilonproteobacteria (122	Proteobacteria (2)	Bacteria (1)			99.40	99.30	WGS
195.1608	0.1112	Campylobacter coli (72294)	Campylobacter (213849)	Campylobacteraceae (2954	Campylobacterales (68525)	Epsilonproteobacteria (122	Proteobacteria (2)	Bacteria (1)			99.80	99.80	WGS
195.1609	0.1132	Campylobacter coli (72294)	Campylobacter (213849)	Campylobacteraceae (2954	Campylobacterales (68525)	Epsilonproteobacteria (122	Proteobacteria (2)	Bacteria (1)			99.50	99.30	WGS
195.1610	0.11	Campylobacter coli (72294)	Campylobacter (213849)	Campylobacteraceae (2954	Campylobacterales (68525)	Epsilonproteobacteria (122	Proteobacteria (2)	Bacteria (1)			99.80	99.60	WGS
195.1611	0.1128	Campylobacter coli (72294)	Campylobacter (213849)	Campylobacteraceae (2954	Campylobacterales (68525)	Epsilonproteobacteria (122	Proteobacteria (2)	Bacteria (1)			99.80	99.60	WGS
195.1612	0.1172	Campylobacter coli (72294)	Campylobacter (213849)	Campylobacteraceae (2954	Campylobacterales (68525)	Epsilonproteobacteria (122	Proteobacteria (2)	Bacteria (1)			99.60	99.00	WGS
195.1613	0.1132	Campylobacter coli (72294)	Campylobacter (213849)	Campylobacteraceae (2954	Campylobacterales (68525)	Epsilonproteobacteria (122	Proteobacteria (2)	Bacteria (1)			99.80	98.90	WGS
195.1614	0.1128	Campylobacter coli (72294)	Campylobacter (213849)	Campylobacteraceae (2954	Campylobacterales (68525)	Epsilonproteobacteria (122	Proteobacteria (2)	Bacteria (1)			99.70	99.60	WGS
195.1615	0.11	Campylobacter coli (72294)	Campylobacter (213849)	Campylobacteraceae (2954	Campylobacterales (68525)	Epsilonproteobacteria (122	Proteobacteria (2)	Bacteria (1)			99.70	97.50	WGS
195.1616	0.1172	Campylobacter coli (72294)	Campylobacter (213849)	Campylobacteraceae (2954	Campylobacterales (68525)	Epsilonproteobacteria (122	Proteobacteria (2)	Bacteria (1)			99.70	98.70	WGS
195.1617	0.11	Campylobacter coli (72294)	Campylobacter (213849)	Campylobacteraceae (2954	Campylobacterales (68525)	Epsilonproteobacteria (122	Proteobacteria (2)	Bacteria (1)			99.70	99.60	WGS
195.1618	0.11	Campylobacter coli (72294)	Campylobacter (213849)	Campylobacteraceae (2954	Campylobacterales (68525)	Epsilonproteobacteria (122	Proteobacteria (2)	Bacteria (1)			99.80	99.60	WGS
195.1619	0.1132	Campylobacter coli (72294)	Campylobacter (213849)	Campylobacteraceae (2954	Campylobacterales (68525)	Epsilonproteobacteria (122	Proteobacteria (2)	Bacteria (1)			99.30	99.10	WGS
195.1620	0.1128	Campylobacter coli (72294)	Campylobacter (213849)	Campylobacteraceae (2954	Campylobacterales (68525)	Epsilonproteobacteria (122	Proteobacteria (2)	Bacteria (1)			99.70	99.70	WGS
195.1621	0.116	Campylobacter coli (72294)	Campylobacter (213849)	Campylobacteraceae (2954	Campylobacterales (68525)	Epsilonproteobacteria (122	Proteobacteria (2)	Bacteria (1)			99.80	99.30	WGS
195.1622	0.11	Campylobacter coli (72294)	Campylobacter (213849)	Campylobacteraceae (2954	Campylobacterales (68525)	Epsilonproteobacteria (122	Proteobacteria (2)	Bacteria (1)			99.70	99.70	WGS
195.1623	0.1132	Campylobacter coli (72294)	Campylobacter (213849)	Campylobacteraceae (2954	Campylobacterales (68525)	Epsilonproteobacteria (122	Proteobacteria (2)	Bacteria (1)			99.70	99.40	WGS
195.1624	0.1112	Campylobacter coli (72294)	Campylobacter (213849)	Campylobacteraceae (2954	Campylobacterales (68525)	Epsilonproteobacteria (122	Proteobacteria (2)	Bacteria (1)			99.80	99.60	WGS
195.1625	0.116	Campylobacter coli (72294)	Campylobacter (213849)	Campylobacteraceae (2954	Campylobacterales (68525)	Epsilonproteobacteria (122	Proteobacteria (2)	Bacteria (1)			99.60	98.50	WGS
195.1626	0.1172	Campylobacter coli (72294)	Campylobacter (213849)	Campylobacteraceae (2954	Campylobacterales (68525)	Epsilonproteobacteria (122	Proteobacteria (2)	Bacteria (1)			99.60	98.40	WGS
195.1627	0.1132	Campylobacter coli (72294)	Campylobacter (213849)	Campylobacteraceae (2954	Campylobacterales (68525)	Epsilonproteobacteria (122	Proteobacteria (2)	Bacteria (1)			99.40	99.00	WGS
195.1628	0.1144	Campylobacter coli (72294)	Campylobacter (213849)	Campylobacteraceae (2954	Campylobacterales (68525)	Epsilonproteobacteria (122	Proteobacteria (2)	Bacteria (1)			99.60	98.80	WGS
195.1629	0.11	Campylobacter coli (72294)	Campylobacter (213849)	Campylobacteraceae (2954	Campylobacterales (68525)	Epsilonproteobacteria (122	Proteobacteria (2)	Bacteria (1)			99.80	98.80	WGS
195.1630	0.1172	Campylobacter coli (72294)	Campylobacter (213849)	Campylobacteraceae (2954	Campylobacterales (68525)	Epsilonproteobacteria (122	Proteobacteria (2)	Bacteria (1)			99.80	99.70	WGS
195.1631	0.1112	Campylobacter coli (72294)	Campylobacter (213849)	Campylobacteraceae (2954	Campylobacterales (68525)	Epsilonproteobacteria (122	Proteobacteria (2)	Bacteria (1)			99.80	99.80	WGS
195.1632	0.1172	Campylobacter coli (72294)	Campylobacter (213849)	Campylobacteraceae (2954	Campylobacterales (68525)	Epsilonproteobacteria (122	Proteobacteria (2)	Bacteria (1)			99.80	99.70	WGS
195.1633	0.1172	Campylobacter coli (72294)	Campylobacter (213849)	Campylobacteraceae (2954	Campylobacterales (68525)	Epsilonproteobacteria (122	Proteobacteria (2)	Bacteria (1)			99.60	99.60	WGS
195.1634	0.1154	Campylobacter coli (72294)	Campylobacter (213849)	Campylobacteraceae (2954	Campylobacterales (68525)	Epsilonproteobacteria (122	Proteobacteria (2)	Bacteria (1)			99.60	98.70	WGS
195.1635	0.1132	Campylobacter coli (72294)	Campylobacter (213849)	Campylobacteraceae (2954	Campylobacterales (68525)	Epsilonproteobacteria (122	Proteobacteria (2)	Bacteria (1)			99.60	98.60	WGS
195.1636	0.1112	Campylobacter coli (72294)	Campylobacter (213849)	Campylobacteraceae (2954	Campylobacterales (68525)	Epsilonproteobacteria (122	Proteobacteria (2)	Bacteria (1)			99.70	99.10	WGS
195.1637	0.1078	Campylobacter coli (72294)	Campylobacter (213849)	Campylobacteraceae (2954	Campylobacterales (68525)	Epsilonproteobacteria (122	Proteobacteria (2)	Bacteria (1)			99.90	98.60	WGS
195.1638	0.1128	Campylobacter coli (72294)	Campylobacter (213849)	Campylobacteraceae (2954	Campylobacterales (68525)	Epsilonproteobacteria (122	Proteobacteria (2)	Bacteria (1)			99.70	99.70	WGS
195.1639	0.11	Campylobacter coli (72294)	Campylobacter (213849)	Campylobacteraceae (2954	Campylobacterales (68525)	Epsilonproteobacteria (122	Proteobacteria (2)	Bacteria (1)			99.80	98.60	WGS
195.1640	0.114	Campylobacter coli (72294)	Campylobacter (213849)	Campylobacteraceae (2954	Campylobacterales (68525)	Epsilonproteobacteria (122	Proteobacteria (2)	Bacteria (1)			99.80	99.80	WGS
195.1641	0.1164	Campylobacter coli (72294)	Campylobacter (213849)	Campylobacteraceae (2954	Campylobacterales (68525)	Epsilonproteobacteria (122	Proteobacteria (2)	Bacteria (1)			99.60	97.90	WGS
195.1642	0.1172	Campylobacter coli (72294)	Campylobacter (213849)	Campylobacteraceae (2954	Campylobacterales (68525)	Epsilonproteobacteria (122	Proteobacteria (2)	Bacteria (1)			99.70	98.30	WGS
195.1643	0.1144	Campylobacter coli (72294)	Campylobacter (213849)	Campylobacteraceae (2954	Campylobacterales (68525)	Epsilonproteobacteria (122	Proteobacteria (2)	Bacteria (1)			99.80	99.60	WGS
195.1644	0.1172	Campylobacter coli (72294)	Campylobacter (213849)	Campylobacteraceae (2954	Campylobacterales (68525)	Epsilonproteobacteria (122	Proteobacteria (2)	Bacteria (1)			99.60	99.10	WGS
195.1645	0.1172	Campylobacter coli (72294)	Campylobacter (213849)	Campylobacteraceae (2954	Campylobacterales (68525)	Epsilonproteobacteria (122	Proteobacteria (2)	Bacteria (1)			99.80	99.70	WGS
195.1646	0.11	Campylobacter coli (72294)	Campylobacter (213849)	Campylobacteraceae (2954	Campylobacterales (68525)	Epsilonproteobacteria (122	Proteobacteria (2)	Bacteria (1)			99.80	99.80	WGS
195.1647	0.11	Campylobacter coli (72294)	Campylobacter (213849)	Campylobacteraceae (2954	Campylobacterales (68525)	Epsilonproteobacteria (122	Proteobacteria (2)	Bacteria (1)			99.70	99.70	WGS
195.1648	0.1128	Campylobacter coli (72294)	Campylobacter (213849)	Campylobacteraceae (2954	Campylobacterales (68525)	Epsilonproteobacteria (122	Proteobacteria (2)	Bacteria (1)			99.80	98.20	WGS
195.1649	0.116	Campylobacter coli (72294)	Campylobacter (213849)	Campylobacteraceae (2954	Campylobacterales (68525)	Epsilonproteobacteria (122	Proteobacteria (2)	Bacteria (1)			99.60	99.50	WGS
195.1650	0.1128	Campylobacter coli (72294)	Campylobacter (213849)	Campylobacteraceae (2954	Campylobacterales (68525)	Epsilonproteobacteria (122	Proteobacteria (2)	Bacteria (1)			99.70	99.60	WGS
195.1651	0.1172	Campylobacter coli (72294)	Campylobacter (213849)	Campylobacteraceae (2954	Campylobacterales (68525)	Epsilonproteobacteria (122	Proteobacteria (2)	Bacteria (1)			99.80	99.20	WGS
195.1652	0.1144	Campylobacter coli (72294)	Campylobacter (213849)	Campylobacteraceae (2954	Campylobacterales (68525)	Epsilonproteobacteria (122	Proteobacteria (2)	Bacteria (1)			99.30	99.00	WGS
195.1653	0.1132	Campylobacter coli (72294)	Campylobacter (213849)	Campylobacteraceae (2954	Campylobacterales (68525)	Epsilonproteobacteria (122	Proteobacteria (2)	Bacteria (1)			99.80	99.70	WGS
195.1654	0.1132	Campylobacter coli (72294)	Campylobacter (213849)	Campylobacteraceae (2954	Campylobacterales (68525)	Epsilonproteobacteria (122	Proteobacteria (2)	Bacteria (1)			99.50	98.60	WGS
195.1655	0.116	Campylobacter coli (72294)	Campylobacter (213849)	Campylobacteraceae (2954	Campylobacterales (68525)	Epsilonproteobacteria (122	Proteobacteria (2)	Bacteria (1)			99.30	98.90	WGS
195.1656	0.1096	Campylobacter coli (72294)	Campylobacter (213849)	Campylobacteraceae (2954	Campylobacterales (68525)	Epsilonproteobacteria (122	Proteobacteria (2)	Bacteria (1)			99.80	99.60	WGS
195.1657	0.116	Campylobacter coli (72294)	Campylobacter (213849)	Campylobacteraceae (2954	Campylobacterales (68525)	Epsilonproteobacteria (122	Proteobacteria (2)	Bacteria (1)			99.80	99.00	WGS
195.1658	0.118	Campylobacter coli (72294)	Campylobacter (213849)	Campylobacteraceae (2954	Campylobacterales (68525)	Epsilonproteobacteria (122	Proteobacteria (2)	Bacteria (1)			99.80	98.60	WGS
195.1659	0.11	Campylobacter coli (72294)	Campylobacter (213849)	Campylobacteraceae (2954	Campylobacterales (68525)	Epsilonproteobacteria (122	Proteobacteria (2)	Bacteria (1)			99.80	99.00	WGS
195.1660	0.1132	Campylobacter coli (72294)	Campylobacter (213849)	Campylobacteraceae (2954	Campylobacterales (68525)	Epsilonproteobacteria (122	Proteobacteria (2)	Bacteria (1)			99.80	99.00	WGS
195.1681	0.1132	Campylobacter coli (72294)	Campylobacter (213849)	Campylobacteraceae (2954	Campylobacterales (68525)	Epsilonproteobacteria (122	Proteobacteria (2)	Bacteria (1)			99.70	98.70	WGS
195.1682	0.116	Campylobacter coli (72294)	Campylobacter (213849)	Campylobacteraceae (2954	Campylobacterales (68525)	Epsilonproteobacteria (122	Proteobacteria (2)	Bacteria (1)			99.70	99.00	WGS
195.1683	0.1172	Campylobacter coli (72294)	Campylobacter (213849)	Campylobacteraceae (2954	Campylobacterales (68525)	Epsilonproteobacteria (122	Proteobacteria (2)	Bacteria (1)			99.80	99.10	WGS
195.1684	0.1132	Campylobacter coli (72294)	Campylobacter (213849)	Campylobacteraceae (2954	Campylobacterales (68525)	Epsilonproteobacteria (122	Proteobacteria (2)	Bacteria (1)			99.70	98.80	WGS
195.1685	0.1132	Campylobacter coli (72294)	Campylobacter (213849)	Campylobacteraceae (2954	Campylobacterales (68525)	Epsilonproteobacteria (122	Proteobacteria (2)	Bacteria (1)			99.50	98.40	WGS
195.1686	0.1132	Campylobacter coli (72294)	Campylobacter (213849)	Campylobacteraceae (2954	Campylobacterales (68525)	Epsilonproteobacteria (122	Proteobacteria (2)	Bacteria (1)			99.80	99.60	WGS
195.1687	0.1172	Campylobacter coli (72294)	Campylobacter (213849)	Campylobacteraceae (2954	Campylobacterales (68525)	Epsilonproteobacteria (122	Proteobacteria (2)	Bacteria (1)			99.60	98.60	WGS
195.1688	0.116	Campylobacter coli (72294)	Campylobacter (213849)	Campylobacteraceae (2954	Campylobacterales (68525)	Epsilonproteobacteria (122	Proteobacteria (2)	Bacteria (1)			99.70	99.70	WGS
195.1689	0.1132	Campylobacter coli (72294)	Campylobacter (213849)	Campylobacteraceae (2954	Campylobacterales (68525)	Epsilonproteobacteria (122	Proteobacteria (2)	Bacteria (1)			99.00	98.20	WGS
195.1690	0.116	Campylobacter coli (72294)	Campylobacter (213849)	Campylobacteraceae (2954	Campylobacterales (68525)	Epsilonproteobacteria (122	Proteobacteria (2)	Bacteria (1)			99.70	99.60	WGS
195.1691	0.1128	Campylobacter coli (72294)	Campylobacter (213849)	Campylobacteraceae (2954	Campylobacterales (68525)	Epsilonproteobacteria (122	Proteobacteria (2)	Bacteria (1)			99.70	98.60	WGS
195.1692	0.11	Campylobacter coli (72294)	Campylobacter (213849)	Campylobacteraceae (2954	Campylobacterales (68525)	Epsilonproteobacteria (122	Proteobacteria (2)	Bacteria (1)			99.80	99.30	WGS
195.1693	0.1132	Campylobacter coli (72294)	Campylobacter (213849)	Campylobacteraceae (2954	Campylobacterales (68525)	Epsilonproteobacteria (122	Proteobacteria (2)	Bacteria (1)			99.80	97.60	WGS
195.1694	0.1172	Campylobacter coli (72294)	Campylobacter (213849)	Campylobacteraceae (2954	Campylobacterales (68525)	Epsilonproteobacteria (122	Proteobacteria (2)	Bacteria (1)			99.60	98.60	WGS
195.1695	0.1128	Campylobacter coli (72294)	Campylobacter (213849)	Campylobacteraceae (2954	Campylobacterales (68525)	Epsilonproteobacteria (122	Proteobacteria (2)	Bacteria (1)			99.70	99.60	WGS
195.1696	0.1112	Campylobacter coli (72294)	Campylobacter (213849)	Campylobacteraceae (2954	Campylobacterales (68525)	Epsilonproteobacteria (122	Proteobacteria (2)	Bacteria (1)			99.80	99.80	WGS
195.1697	0.1112	Campylobacter coli (72294)	Campylobacter (213849)	Campylobacteraceae (2954	Campylobacterales (68525)	Epsilonproteobacteria (122	Proteobacteria (2)	Bacteria (1)			99.80	99.80	WGS
195.1698	0.1132	Campylobacter coli (72294)	Campylobacter (213849)	Campylobacteraceae (2954	Campylobacterales (68525)	Epsilonproteobacteria (122	Proteobacteria (2)	Bacteria (1)			99.80	98.70	WGS
195.1699	0.1128	Campylobacter coli (72294)	Campylobacter (213849)	Campylobacteraceae (2954	Campylobacterales (68525)	Epsilonproteobacteria (122	Proteobacteria (2)	Bacteria (1)			99.80	99.00	WGS
195.1700	0.1172	Campylobacter coli (72294)	Campylobacter (213849)	Campylobacteraceae (2954	Campylobacterales (68525)	Epsilonproteobacteria (122	Proteobacteria (2)	Bacteria (1)			99.80	99.70	WGS
195.1701	0.1128	Campylobacter coli (72294)	Campylobacter (213849)	Campylobacteraceae (2954	Campylobacterales (68525)	Epsilonproteobacteria (122	Proteobacteria (2)	Bacteria (1)			99.80	99.80	WGS
195.1702	0.1172	Campylobacter coli (72294)	Campylobacter (213849)	Campylobacteraceae (2954	Campylobacterales (68525)	Epsilonproteobacteria (122	Proteobacteria (2)	Bacteria (1)			99.60	97.80	WGS
195.1703	0.11	Campylobacter coli (72294)	Campylobacter (213849)	Campylobacteraceae (2954	Campylobacterales (68525)	Epsilonproteobacteria (122	Proteobacteria (2)	Bacteria (1)			99.80	99.80	WGS
195.1704	0.1132	Campylobacter coli (72294)	Campylobacter (213849)	Campylobacteraceae (2954	Campylobacterales (68525)	Epsilonproteobacteria (122	Proteobacteria (2)	Bacteria (1)			99.80	97.60	WGS
195.1705	0.1144	Campylobacter coli (72294)	Campylobacter (213849)	Campylobacteraceae (2954	Campylobacterales (68525)	Epsilonproteobacteria (122	Proteobacteria (2)	Bacteria (1)			99.60	99.00	WGS
195.1706	0.11	Campylobacter coli (72294)	Campylobacter (213849)	Campylobacteraceae (2954	Campylobacterales (68525)	Epsilonproteobacteria (122	Proteobacteria (2)	Bacteria (1)			99.70	99.70	WGS
195.1707	0.11	Campylobacter coli (72294)	Campylobacter (213849)	Campylobacteraceae (2954	Campylobacterales (68525)	Epsilonproteobacteria (122	Proteobacteria (2)	Bacteria (1)			99.70	99.50	WGS
195.1708	0.1154	Campylobacter coli (72294)	Campylobacter (213849)	Campylobacteraceae (2954	Campylobacterales (68525)	Epsilonproteobacteria (122	Proteobacteria (2)	Bacteria (1)			99.60	99.60	WGS
195.1709	0.1154	Campylobacter coli (72294)	Campylobacter (213849)	Campylobacteraceae (2954	Campylobacterales (68525)	Epsilonproteobacteria (122	Proteobacteria (2)	Bacteria (1)			99.60	99.60	WGS
195.1710	0.1154	Campylobacter coli (72294)	Campylobacter (213849)	Campylobacteraceae (2954	Campylobacterales (68525)	Epsilonproteobacteria (122	Proteobacteria (2)	Bacteria (1)			99.60	99.60	WGS
195.1711	0.1172	Campylobacter coli (72294)	Campylobacter (213849)	Campylobacteraceae (2954	Campylobacterales (68525)	Epsilonproteobacteria (122	Proteobacteria (2)	Bacteria (1)			99.60	99.60	WGS
195.1712	0.1172	Campylobacter coli (72294)	Campylobacter (213849)	Campylobacteraceae (2954	Campylobacterales (68525)	Epsilonproteobacteria (122	Proteobacteria (2)	Bacteria (1)			99.60	99.60	WGS
195.1713	0.1172	Campylobacter coli (72294)	Campylobacter (213849)	Campylobacteraceae (2954	Campylobacterales (68525)	Epsilonproteobacteria (122	Proteobacteria (2)	Bacteria (1)			99.60	99.60	WGS
195.1714	0.11	Campylobacter coli (72294)	Campylobacter (213849)	Campylobacteraceae (2954	Campylobacterales (68525)	Epsilonproteobacteria (122	Proteobacteria (2)	Bacteria (1)			99.00	98.70	WGS
195.2024	0.1132	Campylobacter coli (72294)	Campylobacter (213849)	Campylobacteraceae (2954	Campylobacterales (68525)	Epsilonproteobacteria (122	Proteobacteria (2)	Bacteria (1)			99.60	99.60	WGS
195.2025	0.1132	Campylobacter coli (72294)	Campylobacter (213849)	Campylobacteraceae (2954	Campylobacterales (68525)	Epsilonproteobacteria (122	Proteobacteria (2)	Bacteria (1)			99.60	99.50	WGS
195.2026	0.112	Campylobacter coli (72294)	Campylobacter (213849)	Campylobacteraceae (2954	Campylobacterales (68525)	Epsilonproteobacteria (122	Proteobacteria (2)	Bacteria (1)			99.80	99.70	WGS
195.2027	0.1132	Campylobacter coli (72294)	Campylobacter (213849)	Campylobacteraceae (2954	Campylobacterales (68525)	Epsilonproteobacteria (122	Proteobacteria (2)	Bacteria (1)			99.60	99.30	WGS
195.2028	0.1128	Campylobacter coli (72294)	Campylobacter (213849)	Campylobacteraceae (2954	Campylobacterales (68525)	Epsilonproteobacteria (122	Proteobacteria (2)	Bacteria (1)			99.60	99.40	WGS
195.2029	0.116	Campylobacter coli (72294)	Campylobacter (213849)	Campylobacteraceae (2954	Campylobacterales (68525)	Epsilonproteobacteria (122	Proteobacteria (2)	Bacteria (1)			99.60	99.40	WGS
195.2030	0.1132	Campylobacter coli (72294)	Campylobacter (213849)	Campylobacteraceae (2954	Campylobacterales (68525)	Epsilonproteobacteria (122	Proteobacteria (2)	Bacteria (1)			99.60	99.50	WGS
195.2031	0.1132	Campylobacter coli (72294)	Campylobacter (213849)	Campylobacteraceae (2954	Campylobacterales (68525)	Epsilonproteobacteria (122	Proteobacteria (2)	Bacteria (1)			99.60	99.20	WGS
195.2032	0.1132	Campylobacter coli (72294)	Campylobacter (213849)	Campylobacteraceae (2954	Campylobacterales (68525)	Epsilonproteobacteria (122	Proteobacteria (2)	Bacteria (1)			99.60	99.20	WGS
195.2033	0.116	Campylobacter coli (72294)	Campylobacter (213849)	Campylobacteraceae (2954	Campylobacterales (68525)	Epsilonproteobacteria (122	Proteobacteria (2)	Bacteria (1)			99.70	99.40	WGS
195.2034	0.1112	Campylobacter coli (72294)	Campylobacter (213849)	Campylobacteraceae (2954	Campylobacterales (68525)	Epsilonproteobacteria (122	Proteobacteria (2)	Bacteria (1)			99.60	99.50	WGS
195.2035	0.1132	Campylobacter coli (72294)	Campylobacter (213849)	Campylobacteraceae (2954	Campylobacterales (68525)	Epsilonproteobacteria (122	Proteobacteria (2)	Bacteria (1)			99.60	99.50	WGS
195.2036	0.1132	Campylobacter coli (72294)	Campylobacter (213849)	Campylobacteraceae (2954	Campylobacterales (68525)	Epsilonproteobacteria (122	Proteobacteria (2)	Bacteria (1)			99.60	99.50	WGS
195.2037	0.116	Campylobacter coli (72294)	Campylobacter (213849)	Campylobacteraceae (2954	Campylobacterales (68525)	Epsilonproteobacteria (122	Proteobacteria (2)	Bacteria (1)			99.60	99.50	WGS
195.2038	0.114	Campylobacter coli (72294)	Campylobacter (213849)	Campylobacteraceae (2954	Campylobacterales (68525)	Epsilonproteobacteria (122	Proteobacteria (2)	Bacteria (1)			99.60	99.00	WGS
195.2039	0.1128	Campylobacter coli (72294)	Campylobacter (213849)	Campylobacteraceae (2954	Campylobacterales (68525)	Epsilonproteobacteria (122	Proteobacteria (2)	Bacteria (1)			99.60	99.60	WGS
195.2040	0.116	Campylobacter coli (72294)	Campylobacter (213849)	Campylobacteraceae (2954	Campylobacterales (68525)	Epsilonproteobacteria (122	Proteobacteria (2)	Bacteria (1)			99.60	99.40	WGS
195.2041	0.1132	Campylobacter coli (72294)	Campylobacter (213849)	Campylobacteraceae (2954	Campylobacterales (68525)	Epsilonproteobacteria (122	Proteobacteria (2)	Bacteria (1)			99.60	99.60	WGS
195.2042	0.1112	Campylobacter coli (72294)	Campylobacter (213849)	Campylobacteraceae (2954	Campylobacterales (68525)	Epsilonproteobacteria (122	Proteobacteria (2)	Bacteria (1)			99.60	99.10	WGS
195.2043	0.1132	Campylobacter coli (72294)	Campylobacter (213849)	Campylobacteraceae (2954	Campylobacterales (68525)	Epsilonproteobacteria (122	Proteobacteria (2)	Bacteria (1)			99.60	99.60	WGS
195.2044	0.11	Campylobacter coli (72294)	Campylobacter (213849)	Campylobacteraceae (2954	Campylobacterales (68525)	Epsilonproteobacteria (122	Proteobacteria (2)	Bacteria (1)			99.60	99.50	WGS
195.2045	0.1152	Campylobacter coli (72294)	Campylobacter (213849)	Campylobacteraceae (2954	Campylobacterales (68525)	Epsilonproteobacteria (122	Proteobacteria (2)	Bacteria (1)			99.60	99.30	WGS
195.2046	0.1132	Campylobacter coli (72294)	Campylobacter (213849)	Campylobacteraceae (2954	Campylobacterales (68525)	Epsilonproteobacteria (122	Proteobacteria (2)	Bacteria (1)			99.60	99.30	WGS
195.2047	0.116	Campylobacter coli (72294)	Campylobacter (213849)	Campylobacteraceae (2954	Campylobacterales (68525)	Epsilonproteobacteria (122	Proteobacteria (2)	Bacteria (1)			99.60	99.50	WGS
195.2048	0.11	Campylobacter coli (72294)	Campylobacter (213849)	Campylobacteraceae (2954	Campylobacterales (68525)	Epsilonproteobacteria (122	Proteobacteria (2)	Bacteria (1)			99.60	99.50	WGS
195.2049	0.1172	Campylobacter coli (72294)	Campylobacter (213849)	Campylobacteraceae (2954	Campylobacterales (68525)	Epsilonproteobacteria (122	Proteobacteria (2)	Bacteria (1)			99.60	99.60	WGS
195.2050	0.118	Campylobacter coli (72294)	Campylobacter (213849)	Campylobacteraceae (2954	Campylobacterales (68525)	Epsilonproteobacteria (122	Proteobacteria (2)	Bacteria (1)			99.50	98.60	WGS
195.2051	0.116	Campylobacter coli (72294)	Campylobacter (213849)	Campylobacteraceae (2954	Campylobacterales (68525)	Epsilonproteobacteria (122	Proteobacteria (2)	Bacteria (1)			99.60	99.60	WGS
195.2052	0.11	Campylobacter coli (72294)	Campylobacter (213849)	Campylobacteraceae (2954	Campylobacterales (68525)	Epsilonproteobacteria (122	Proteobacteria (2)	Bacteria (1)			99.60	99.30	WGS
195.2053	0.112	Campylobacter coli (72294)	Campylobacter (213849)	Campylobacteraceae (2954	Campylobacterales (68525)	Epsilonproteobacteria (122	Proteobacteria (2)	Bacteria (1)			99.60	99.30	WGS
195.2054	0.1144	Campylobacter coli (72294)	Campylobacter (213849)	Campylobacteraceae (2954	Campylobacterales (68525)	Epsilonproteobacteria (122	Proteobacteria (2)	Bacteria (1)			99.60	99.40	WGS
195.2055	0.11	Campylobacter coli (72294)	Campylobacter (213849)	Campylobacteraceae (2954	Campylobacterales (68525)	Epsilonproteobacteria (122	Proteobacteria (2)	Bacteria (1)			99.60	99.40	WGS
195.2056	0.1132	Campylobacter coli (72294)	Campylobacter (213849)	Campylobacteraceae (2954	Campylobacterales (68525)	Epsilonproteobacteria (122	Proteobacteria (2)	Bacteria (1)			99.60	99.30	WGS
195.2057	0.1132	Campylobacter coli (72294)	Campylobacter (213849)	Campylobacteraceae (2954	Campylobacterales (68525)	Epsilonproteobacteria (122	Proteobacteria (2)	Bacteria (1)			99.60	99.60	WGS
195.2058	0.1132	Campylobacter coli (72294)	Campylobacter (213849)	Campylobacteraceae (2954	Campylobacterales (68525)	Epsilonproteobacteria (122	Proteobacteria (2)	Bacteria (1)			99.60	99.40	WGS
195.2059	0.1112	Campylobacter coli (72294)	Campylobacter (213849)	Campylobacteraceae (2954	Campylobacterales (68525)	Epsilonproteobacteria (122	Proteobacteria (2)	Bacteria (1)			99.60	99.40	WGS
195.2060	0.1172	Campylobacter coli (72294)	Campylobacter (213849)	Campylobacteraceae (2954	Campylobacterales (68525)	Epsilonproteobacteria (122	Proteobacteria (2)	Bacteria (1)			99.60	99.00	WGS
195.2061	0.1132	Campylobacter coli (72294)	Campylobacter (213849)	Campylobacteraceae (2954	Campylobacterales (68525)	Epsilonproteobacteria (122	Proteobacteria (2)	Bacteria (1)			99.60	99.60	WGS
195.2062	0.1164	Campylobacter coli (72294)	Campylobacter (213849)	Campylobacteraceae (2954	Campylobacterales (68525)	Epsilonproteobacteria (122	Proteobacteria (2)	Bacteria (1)			99.60	98.70	WGS
195.2063	0.118	Campylobacter coli (72294)	Campylobacter (213849)	Campylobacteraceae (2954	Campylobacterales (68525)	Epsilonproteobacteria (122	Proteobacteria (2)	Bacteria (1)			99.60	99.30	WGS
195.2064	0.1132	Campylobacter coli (72294)	Campylobacter (213849)	Campylobacteraceae (2954	Campylobacterales (68525)	Epsilonproteobacteria (122	Proteobacteria (2)	Bacteria (1)			99.60	99.40	WGS
195.2065	0.112	Campylobacter coli (72294)	Campylobacter (213849)	Campylobacteraceae (2954	Campylobacterales (68525)	Epsilonproteobacteria (122	Proteobacteria (2)	Bacteria (1)			99.60	99.30	WGS
195.2066	0.116	Campylobacter coli (72294)	Campylobacter (213849)	Campylobacteraceae (2954	Campylobacterales (68525)	Epsilonproteobacteria (122	Proteobacteria (2)	Bacteria (1)			99.60	99.50	WGS
195.2067	0.1148	Campylobacter coli (72294)	Campylobacter (213849)	Campylobacteraceae (2954	Campylobacterales (68525)	Epsilonproteobacteria (122	Proteobacteria (2)	Bacteria (1)			99.60	99.50	WGS
195.2068	0.1112	Campylobacter coli (72294)	Campylobacter (213849)	Campylobacteraceae (2954	Campylobacterales (68525)	Epsilonproteobacteria (122	Proteobacteria (2)	Bacteria (1)			99.60	99.00	WGS
195.2069	0.1132	Campylobacter coli (72294)	Campylobacter (213849)	Campylobacteraceae (2954	Campylobacterales (68525)	Epsilonproteobacteria (122	Proteobacteria (2)	Bacteria (1)			99.60	99.30	WGS
195.2070	0.116	Campylobacter coli (72294)	Campylobacter (213849)	Campylobacteraceae (2954	Campylobacterales (68525)	Epsilonproteobacteria (122	Proteobacteria (2)	Bacteria (1)			99.60	99.10	WGS
195.2071	0.116	Campylobacter coli (72294)	Campylobacter (213849)	Campylobacteraceae (2954	Campylobacterales (68525)	Epsilonproteobacteria (122	Proteobacteria (2)	Bacteria (1)			99.60	99.40	WGS
195.2072	0.1132	Campylobacter coli (72294)	Campylobacter (213849)	Campylobacteraceae (2954	Campylobacterales (68525)	Epsilonproteobacteria (122	Proteobacteria (2)	Bacteria (1)			99.60	99.50	WGS
195.2073	0.1132	Campylobacter coli (72294)	Campylobacter (213849)	Campylobacteraceae (2954	Campylobacterales (68525)	Epsilonproteobacteria (122	Proteobacteria (2)	Bacteria (1)			99.80	98.70	WGS
195.2074	0.116	Campylobacter coli (72294)	Campylobacter (213849)	Campylobacteraceae (2954	Campylobacterales (68525)	Epsilonproteobacteria (122	Proteobacteria (2)	Bacteria (1)			99.60	99.30	WGS
195.2075	0.114	Campylobacter coli (72294)	Campylobacter (213849)	Campylobacteraceae (2954	Campylobacterales (68525)	Epsilonproteobacteria (122	Proteobacteria (2)	Bacteria (1)			99.60	99.00	WGS
195.2076	0.11	Campylobacter coli (72294)	Campylobacter (213849)	Campylobacteraceae (2954	Campylobacterales (68525)	Epsilonproteobacteria (122	Proteobacteria (2)	Bacteria (1)			99.40	98.70	WGS
195.2077	0.1132	Campylobacter coli (72294)	Campylobacter (213849)	Campylobacteraceae (2954	Campylobacterales (68525)	Epsilonproteobacteria (122	Proteobacteria (2)	Bacteria (1)			99.60	99.00	WGS
195.2078	0.1132	Campylobacter coli (72294)	Campylobacter (213849)	Campylobacteraceae (2954	Campylobacterales (68525)	Epsilonproteobacteria (122	Proteobacteria (2)	Bacteria (1)			99.60	99.00	WGS
195.2079	0.116	Campylobacter coli (72294)	Campylobacter (213849)	Campylobacteraceae (2954	Campylobacterales (68525)	Epsilonproteobacteria (122	Proteobacteria (2)	Bacteria (1)			99.60	98.40	WGS
195.2080	0.1144	Campylobacter coli (72294)	Campylobacter (213849)	Campylobacteraceae (2954	Campylobacterales (68525)	Epsilonproteobacteria (122	Proteobacteria (2)	Bacteria (1)			99.60	99.40	WGS
195.2081	0.1172	Campylobacter coli (72294)	Campylobacter (213849)	Campylobacteraceae (2954	Campylobacterales (68525)	Epsilonproteobacteria (122	Proteobacteria (2)	Bacteria (1)			99.80	99.10	WGS
195.2082	0.11	Campylobacter coli (72294)	Campylobacter (213849)	Campylobacteraceae (2954	Campylobacterales (68525)	Epsilonproteobacteria (122	Proteobacteria (2)	Bacteria (1)			99.60	99.50	WGS
195.2083	0.112	Campylobacter coli (72294)	Campylobacter (213849)	Campylobacteraceae (2954	Campylobacterales (68525)	Epsilonproteobacteria (122	Proteobacteria (2)	Bacteria (1)			99.60	99.30	WGS
195.2084	0.1132	Campylobacter coli (72294)	Campylobacter (213849)	Campylobacteraceae (2954	Campylobacterales (68525)	Epsilonproteobacteria (122	Proteobacteria (2)	Bacteria (1)			99.80	99.60	WGS
195.2085	0.116	Campylobacter coli (72294)	Campylobacter (213849)	Campylobacteraceae (2954	Campylobacterales (68525)	Epsilonproteobacteria (122	Proteobacteria (2)	Bacteria (1)			99.50	99.30	WGS
195.2086	0.112	Campylobacter coli (72294)	Campylobacter (213849)	Campylobacteraceae (2954	Campylobacterales (68525)	Epsilonproteobacteria (122	Proteobacteria (2)	Bacteria (1)			99.60	99.20	WGS
195.2087	0.1128	Campylobacter coli (72294)	Campylobacter (213849)	Campylobacteraceae (2954	Campylobacterales (68525)	Epsilonproteobacteria (122	Proteobacteria (2)	Bacteria (1)			99.50	99.40	WGS
195.2088	0.118	Campylobacter coli (72294)	Campylobacter (213849)	Campylobacteraceae (2954	Campylobacterales (68525)	Epsilonproteobacteria (122	Proteobacteria (2)	Bacteria (1)			99.70	98.30	WGS
195.2089	0.1132	Campylobacter coli (72294)	Campylobacter (213849)	Campylobacteraceae (2954	Campylobacterales (68525)	Epsilonproteobacteria (122	Proteobacteria (2)	Bacteria (1)			99.60	99.40	WGS
195.2090	0.1132	Campylobacter coli (72294)	Campylobacter (213849)	Campylobacteraceae (2954	Campylobacterales (68525)	Epsilonproteobacteria (122	Proteobacteria (2)	Bacteria (1)			99.60	99.50	WGS
195.2091	0.118	Campylobacter coli (72294)	Campylobacter (213849)	Campylobacteraceae (2954	Campylobacterales (68525)	Epsilonproteobacteria (122	Proteobacteria (2)	Bacteria (1)			99.70	98.70	WGS
195.2092	0.11	Campylobacter coli (72294)	Campylobacter (213849)	Campylobacteraceae (2954	Campylobacterales (68525)	Epsilonproteobacteria (122	Proteobacteria (2)	Bacteria (1)			99.60	99.50	WGS
195.2093	0.11	Campylobacter coli (72294)	Campylobacter (213849)	Campylobacteraceae (2954	Campylobacterales (68525)	Epsilonproteobacteria (122	Proteobacteria (2)	Bacteria (1)			99.60	99.50	WGS
195.2094	0.1132	Campylobacter coli (72294)	Campylobacter (213849)	Campylobacteraceae (2954	Campylobacterales (68525)	Epsilonproteobacteria (122	Proteobacteria (2)	Bacteria (1)			99.60	99.20	WGS
195.2095	0.1152	Campylobacter coli (72294)	Campylobacter (213849)	Campylobacteraceae (2954	Campylobacterales (68525)	Epsilonproteobacteria (122	Proteobacteria (2)	Bacteria (1)			99.60	99.50	WGS
195.2096	0.11	Campylobacter coli (72294)	Campylobacter (213849)	Campylobacteraceae (2954	Campylobacterales (68525)	Epsilonproteobacteria (122	Proteobacteria (2)	Bacteria (1)			99.60	99.30	WGS
195.2097	0.1112	Campylobacter coli (72294)	Campylobacter (213849)	Campylobacteraceae (2954	Campylobacterales (68525)	Epsilonproteobacteria (122	Proteobacteria (2)	Bacteria (1)			99.60	99.60	WGS
195.2098	0.116	Campylobacter coli (72294)	Campylobacter (213849)	Campylobacteraceae (2954	Campylobacterales (68525)	Epsilonproteobacteria (122	Proteobacteria (2)	Bacteria (1)			99.60	99.40	WGS
195.2099	0.1128	Campylobacter coli (72294)	Campylobacter (213849)	Campylobacteraceae (2954	Campylobacterales (68525)	Epsilonproteobacteria (122	Proteobacteria (2)	Bacteria (1)			99.60	99.50	WGS
195.2100	0.1132	Campylobacter coli (72294)	Campylobacter (213849)	Campylobacteraceae (2954	Campylobacterales (68525)	Epsilonproteobacteria (122	Proteobacteria (2)	Bacteria (1)			99.60	99.10	WGS
195.2101	0.1144	Campylobacter coli (72294)	Campylobacter (213849)	Campylobacteraceae (2954	Campylobacterales (68525)	Epsilonproteobacteria (122	Proteobacteria (2)	Bacteria (1)			99.60	99.00	WGS
195.2102	0.116	Campylobacter coli (72294)	Campylobacter (213849)	Campylobacteraceae (2954	Campylobacterales (68525)	Epsilonproteobacteria (122	Proteobacteria (2)	Bacteria (1)			99.80	99.60	WGS
195.2103	0.114	Campylobacter coli (72294)	Campylobacter (213849)	Campylobacteraceae (2954	Campylobacterales (68525)	Epsilonproteobacteria (122	Proteobacteria (2)	Bacteria (1)			99.50	99.30	WGS
195.2104	0.116	Campylobacter coli (72294)	Campylobacter (213849)	Campylobacteraceae (2954	Campylobacterales (68525)	Epsilonproteobacteria (122	Proteobacteria (2)	Bacteria (1)			99.60	99.50	WGS
195.2105	0.116	Campylobacter coli (72294)	Campylobacter (213849)	Campylobacteraceae (2954	Campylobacterales (68525)	Epsilonproteobacteria (122	Proteobacteria (2)	Bacteria (1)			99.60	99.30	WGS
195.2106	0.1112	Campylobacter coli (72294)	Campylobacter (213849)	Campylobacteraceae (2954	Campylobacterales (68525)	Epsilonproteobacteria (122	Proteobacteria (2)	Bacteria (1)			99.60	99.60	WGS
195.2107	0.116	Campylobacter coli (72294)	Campylobacter (213849)	Campylobacteraceae (2954	Campylobacterales (68525)	Epsilonproteobacteria (122	Proteobacteria (2)	Bacteria (1)			99.60	99.60	WGS
195.2108	0.1132	Campylobacter coli (72294)	Campylobacter (213849)	Campylobacteraceae (2954	Campylobacterales (68525)	Epsilonproteobacteria (122	Proteobacteria (2)	Bacteria (1)			99.60	99.40	WGS
195.2109	0.116	Campylobacter coli (72294)	Campylobacter (213849)	Campylobacteraceae (2954	Campylobacterales (68525)	Epsilonproteobacteria (122	Proteobacteria (2)	Bacteria (1)			99.60	99.60	WGS
195.2110	0.1132	Campylobacter coli (72294)	Campylobacter (213849)	Campylobacteraceae (2954	Campylobacterales (68525)	Epsilonproteobacteria (122	Proteobacteria (2)	Bacteria (1)			99.50	99.50	WGS
195.2111	0.1128	Campylobacter coli (72294)	Campylobacter (213849)	Campylobacteraceae (2954	Campylobacterales (68525)	Epsilonproteobacteria (122	Proteobacteria (2)	Bacteria (1)			99.60	99.30	WGS
195.2112	0.116	Campylobacter coli (72294)	Campylobacter (213849)	Campylobacteraceae (2954	Campylobacterales (68525)	Epsilonproteobacteria (122	Proteobacteria (2)	Bacteria (1)			99.60	99.50	WGS
195.2113	0.1112	Campylobacter coli (72294)	Campylobacter (213849)	Campylobacteraceae (2954	Campylobacterales (68525)	Epsilonproteobacteria (122	Proteobacteria (2)	Bacteria (1)			99.60	99.50	WGS
195.2114	0.118	Campylobacter coli (72294)	Campylobacter (213849)	Campylobacteraceae (2954	Campylobacterales (68525)	Epsilonproteobacteria (122	Proteobacteria (2)	Bacteria (1)			99.60	99.30	WGS
195.2115	0.1128	Campylobacter coli (72294)	Campylobacter (213849)	Campylobacteraceae (2954	Campylobacterales (68525)	Epsilonproteobacteria (122	Proteobacteria (2)	Bacteria (1)			99.60	99.40	WGS
195.2116	0.11	Campylobacter coli (72294)	Campylobacter (213849)	Campylobacteraceae (2954	Campylobacterales (68525)	Epsilonproteobacteria (122	Proteobacteria (2)	Bacteria (1)			99.60	99.40	WGS
195.2117	0.1132	Campylobacter coli (72294)	Campylobacter (213849)	Campylobacteraceae (2954	Campylobacterales (68525)	Epsilonproteobacteria (122	Proteobacteria (2)	Bacteria (1)			99.60	99.40	WGS
195.2118	0.118	Campylobacter coli (72294)	Campylobacter (213849)	Campylobacteraceae (2954	Campylobacterales (68525)	Epsilonproteobacteria (122	Proteobacteria (2)	Bacteria (1)			99.80	99.70	WGS
195.2119	0.1172	Campylobacter coli (72294)	Campylobacter (213849)	Campylobacteraceae (2954	Campylobacterales (68525)	Epsilonproteobacteria (122	Proteobacteria (2)	Bacteria (1)			99.60	99.60	WGS
195.2120	0.118	Campylobacter coli (72294)	Campylobacter (213849)	Campylobacteraceae (2954	Campylobacterales (68525)	Epsilonproteobacteria (122	Proteobacteria (2)	Bacteria (1)			99.70	99.50	WGS
195.2121	0.11	Campylobacter coli (72294)	Campylobacter (213849)	Campylobacteraceae (2954	Campylobacterales (68525)	Epsilonproteobacteria (122	Proteobacteria (2)	Bacteria (1)			99.60	99.60	WGS
195.2122	0.1132	Campylobacter coli (72294)	Campylobacter (213849)	Campylobacteraceae (2954	Campylobacterales (68525)	Epsilonproteobacteria (122	Proteobacteria (2)	Bacteria (1)			99.60	99.50	WGS
195.2123	0.1132	Campylobacter coli (72294)	Campylobacter (213849)	Campylobacteraceae (2954	Campylobacterales (68525)	Epsilonproteobacteria (122	Proteobacteria (2)	Bacteria (1)			99.60	99.50	WGS
195.2124	0.116	Campylobacter coli (72294)	Campylobacter (213849)	Campylobacteraceae (2954	Campylobacterales (68525)	Epsilonproteobacteria (122	Proteobacteria (2)	Bacteria (1)			99.60	99.50	WGS
195.2125	0.11	Campylobacter coli (72294)	Campylobacter (213849)	Campylobacteraceae (2954	Campylobacterales (68525)	Epsilonproteobacteria (122	Proteobacteria (2)	Bacteria (1)			99.60	99.20	WGS
195.2126	0.116	Campylobacter coli (72294)	Campylobacter (213849)	Campylobacteraceae (2954	Campylobacterales (68525)	Epsilonproteobacteria (122	Proteobacteria (2)	Bacteria (1)			99.60	99.30	WGS
195.2127	0.1144	Campylobacter coli (72294)	Campylobacter (213849)	Campylobacteraceae (2954	Campylobacterales (68525)	Epsilonproteobacteria (122	Proteobacteria (2)	Bacteria (1)			99.60	99.60	WGS
195.2128	0.1128	Campylobacter coli (72294)	Campylobacter (213849)	Campylobacteraceae (2954	Campylobacterales (68525)	Epsilonproteobacteria (122	Proteobacteria (2)	Bacteria (1)			99.60	99.60	WGS
195.2129	0.116	Campylobacter coli (72294)	Campylobacter (213849)	Campylobacteraceae (2954	Campylobacterales (68525)	Epsilonproteobacteria (122	Proteobacteria (2)	Bacteria (1)			99.60	99.50	WGS
195.2130	0.1132	Campylobacter coli (72294)	Campylobacter (213849)	Campylobacteraceae (2954	Campylobacterales (68525)	Epsilonproteobacteria (122	Proteobacteria (2)	Bacteria (1)			99.70	99.50	WGS
195.2131	0.112	Campylobacter coli (72294)	Campylobacter (213849)	Campylobacteraceae (2954	Campylobacterales (68525)	Epsilonproteobacteria (122	Proteobacteria (2)	Bacteria (1)			99.60	99.60	WGS
195.2132	0.116	Campylobacter coli (72294)	Campylobacter (213849)	Campylobacteraceae (2954	Campylobacterales (68525)	Epsilonproteobacteria (122	Proteobacteria (2)	Bacteria (1)			99.60	99.40	WGS
195.2133	0.116	Campylobacter coli (72294)	Campylobacter (213849)	Campylobacteraceae (2954	Campylobacterales (68525)	Epsilonproteobacteria (122	Proteobacteria (2)	Bacteria (1)			99.60	99.40	WGS
195.2134	0.1132	Campylobacter coli (72294)	Campylobacter (213849)	Campylobacteraceae (2954	Campylobacterales (68525)	Epsilonproteobacteria (122	Proteobacteria (2)	Bacteria (1)			99.60	99.60	WGS
195.2135	0.116	Campylobacter coli (72294)	Campylobacter (213849)	Campylobacteraceae (2954	Campylobacterales (68525)	Epsilonproteobacteria (122	Proteobacteria (2)	Bacteria (1)			99.60	99.40	WGS
195.2136	0.1128	Campylobacter coli (72294)	Campylobacter (213849)	Campylobacteraceae (2954	Campylobacterales (68525)	Epsilonproteobacteria (122	Proteobacteria (2)	Bacteria (1)			99.60	99.50	WGS
195.2137	0.116	Campylobacter coli (72294)	Campylobacter (213849)	Campylobacteraceae (2954	Campylobacterales (68525)	Epsilonproteobacteria (122	Proteobacteria (2)	Bacteria (1)			99.60	99.40	WGS
195.2138	0.116	Campylobacter coli (72294)	Campylobacter (213849)	Campylobacteraceae (2954	Campylobacterales (68525)	Epsilonproteobacteria (122	Proteobacteria (2)	Bacteria (1)			99.60	99.50	WGS
195.2139	0.1132	Campylobacter coli (72294)	Campylobacter (213849)	Campylobacteraceae (2954	Campylobacterales (68525)	Epsilonproteobacteria (122	Proteobacteria (2)	Bacteria (1)			99.60	99.60	WGS
195.2140	0.1132	Campylobacter coli (72294)	Campylobacter (213849)	Campylobacteraceae (2954	Campylobacterales (68525)	Epsilonproteobacteria (122	Proteobacteria (2)	Bacteria (1)			99.60	99.50	WGS
195.2141	0.118	Campylobacter coli (72294)	Campylobacter (213849)	Campylobacteraceae (2954	Campylobacterales (68525)	Epsilonproteobacteria (122	Proteobacteria (2)	Bacteria (1)			99.60	99.50	WGS
195.2142	0.116	Campylobacter coli (72294)	Campylobacter (213849)	Campylobacteraceae (2954	Campylobacterales (68525)	Epsilonproteobacteria (122	Proteobacteria (2)	Bacteria (1)			99.60	99.50	WGS
195.2143	0.1128	Campylobacter coli (72294)	Campylobacter (213849)	Campylobacteraceae (2954	Campylobacterales (68525)	Epsilonproteobacteria (122	Proteobacteria (2)	Bacteria (1)			99.60	99.50	WGS
195.2144	0.11	Campylobacter coli (72294)	Campylobacter (213849)	Campylobacteraceae (2954	Campylobacterales (68525)	Epsilonproteobacteria (122	Proteobacteria (2)	Bacteria (1)			99.60	99.30	WGS
195.2145	0.118	Campylobacter coli (72294)	Campylobacter (213849)	Campylobacteraceae (2954	Campylobacterales (68525)	Epsilonproteobacteria (122	Proteobacteria (2)	Bacteria (1)			99.60	99.50	WGS
195.2146	0.1128	Campylobacter coli (72294)	Campylobacter (213849)	Campylobacteraceae (2954	Campylobacterales (68525)	Epsilonproteobacteria (122	Proteobacteria (2)	Bacteria (1)			99.60	99.20	WGS
195.2147	0.11	Campylobacter coli (72294)	Campylobacter (213849)	Campylobacteraceae (2954	Campylobacterales (68525)	Epsilonproteobacteria (122	Proteobacteria (2)	Bacteria (1)			99.60	99.40	WGS
195.2148	0.118	Campylobacter coli (72294)	Campylobacter (213849)	Campylobacteraceae (2954	Campylobacterales (68525)	Epsilonproteobacteria (122	Proteobacteria (2)	Bacteria (1)			99.60	99.20	WGS
195.2149	0.1144	Campylobacter coli (72294)	Campylobacter (213849)	Campylobacteraceae (2954	Campylobacterales (68525)	Epsilonproteobacteria (122	Proteobacteria (2)	Bacteria (1)			99.60	99.20	WGS
195.2150	0.116	Campylobacter coli (72294)	Campylobacter (213849)	Campylobacteraceae (2954	Campylobacterales (68525)	Epsilonproteobacteria (122	Proteobacteria (2)	Bacteria (1)			99.60	99.00	WGS
195.2151	0.1144	Campylobacter coli (72294)	Campylobacter (213849)	Campylobacteraceae (2954	Campylobacterales (68525)	Epsilonproteobacteria (122	Proteobacteria (2)	Bacteria (1)			99.80	99.00	WGS
195.2152	0.116	Campylobacter coli (72294)	Campylobacter (213849)	Campylobacteraceae (2954	Campylobacterales (68525)	Epsilonproteobacteria (122	Proteobacteria (2)	Bacteria (1)			99.60	99.30	WGS
195.2153	0.116	Campylobacter coli (72294)	Campylobacter (213849)	Campylobacteraceae (2954	Campylobacterales (68525)	Epsilonproteobacteria (122	Proteobacteria (2)	Bacteria (1)			99.60	99.50	WGS
195.2154	0.1132	Campylobacter coli (72294)	Campylobacter (213849)	Campylobacteraceae (2954	Campylobacterales (68525)	Epsilonproteobacteria (122	Proteobacteria (2)	Bacteria (1)			99.60	99.40	WGS
195.2155	0.1148	Campylobacter coli (72294)	Campylobacter (213849)	Campylobacteraceae (2954	Campylobacterales (68525)	Epsilonproteobacteria (122	Proteobacteria (2)	Bacteria (1)			99.60	99.50	WGS
195.2156	0.11	Campylobacter coli (72294)	Campylobacter (213849)	Campylobacteraceae (2954	Campylobacterales (68525)	Epsilonproteobacteria (122	Proteobacteria (2)	Bacteria (1)			99.60	99.30	WGS
195.2157	0.114	Campylobacter coli (72294)	Campylobacter (213849)	Campylobacteraceae (2954	Campylobacterales (68525)	Epsilonproteobacteria (122	Proteobacteria (2)	Bacteria (1)			99.60	99.30	WGS
195.2158	0.116	Campylobacter coli (72294)	Campylobacter (213849)	Campylobacteraceae (2954	Campylobacterales (68525)	Epsilonproteobacteria (122	Proteobacteria (2)	Bacteria (1)			99.60	99.50	WGS
195.2159	0.1132	Campylobacter coli (72294)	Campylobacter (213849)	Campylobacteraceae (2954	Campylobacterales (68525)	Epsilonproteobacteria (122	Proteobacteria (2)	Bacteria (1)			99.60	99.50	WGS
195.2160	0.116	Campylobacter coli (72294)	Campylobacter (213849)	Campylobacteraceae (2954	Campylobacterales (68525)	Epsilonproteobacteria (122	Proteobacteria (2)	Bacteria (1)			99.60	99.30	WGS
195.2161	0.116	Campylobacter coli (72294)	Campylobacter (213849)	Campylobacteraceae (2954	Campylobacterales (68525)	Epsilonproteobacteria (122	Proteobacteria (2)	Bacteria (1)			99.60	99.40	WGS
195.2162	0.1128	Campylobacter coli (72294)	Campylobacter (213849)	Campylobacteraceae (2954	Campylobacterales (68525)	Epsilonproteobacteria (122	Proteobacteria (2)	Bacteria (1)			99.60	99.40	WGS
195.2163	0.1128	Campylobacter coli (72294)	Campylobacter (213849)	Campylobacteraceae (2954	Campylobacterales (68525)	Epsilonproteobacteria (122	Proteobacteria (2)	Bacteria (1)			99.60	99.00	WGS
195.2164	0.1132	Campylobacter coli (72294)	Campylobacter (213849)	Campylobacteraceae (2954	Campylobacterales (68525)	Epsilonproteobacteria (122	Proteobacteria (2)	Bacteria (1)			99.60	99.30	WGS
195.2165	0.1132	Campylobacter coli (72294)	Campylobacter (213849)	Campylobacteraceae (2954	Campylobacterales (68525)	Epsilonproteobacteria (122	Proteobacteria (2)	Bacteria (1)			99.60	99.50	WGS
195.2166	0.11	Campylobacter coli (72294)	Campylobacter (213849)	Campylobacteraceae (2954	Campylobacterales (68525)	Epsilonproteobacteria (122	Proteobacteria (2)	Bacteria (1)			99.60	99.00	WGS
195.2167	0.1144	Campylobacter coli (72294)	Campylobacter (213849)	Campylobacteraceae (2954	Campylobacterales (68525)	Epsilonproteobacteria (122	Proteobacteria (2)	Bacteria (1)			99.60	98.60	WGS
195.2168	0.1112	Campylobacter coli (72294)	Campylobacter (213849)	Campylobacteraceae (2954	Campylobacterales (68525)	Epsilonproteobacteria (122	Proteobacteria (2)	Bacteria (1)			99.60	98.70	WGS
195.2169	0.116	Campylobacter coli (72294)	Campylobacter (213849)	Campylobacteraceae (2954	Campylobacterales (68525)	Epsilonproteobacteria (122	Proteobacteria (2)	Bacteria (1)			99.60	99.40	WGS
195.2170	0.1132	Campylobacter coli (72294)	Campylobacter (213849)	Campylobacteraceae (2954	Campylobacterales (68525)	Epsilonproteobacteria (122	Proteobacteria (2)	Bacteria (1)			99.60	99.20	WGS
195.2171	0.114	Campylobacter coli (72294)	Campylobacter (213849)	Campylobacteraceae (2954	Campylobacterales (68525)	Epsilonproteobacteria (122	Proteobacteria (2)	Bacteria (1)			99.60	99.30	WGS
195.2172	0.1172	Campylobacter coli (72294)	Campylobacter (213849)	Campylobacteraceae (2954	Campylobacterales (68525)	Epsilonproteobacteria (122	Proteobacteria (2)	Bacteria (1)			99.80	99.30	WGS
195.2173	0.1192	Campylobacter coli (72294)	Campylobacter (213849)	Campylobacteraceae (2954	Campylobacterales (68525)	Epsilonproteobacteria (122	Proteobacteria (2)	Bacteria (1)			99.60	99.30	WGS
195.2174	0.1112	Campylobacter coli (72294)	Campylobacter (213849)	Campylobacteraceae (2954	Campylobacterales (68525)	Epsilonproteobacteria (122	Proteobacteria (2)	Bacteria (1)			99.60	99.40	WGS
195.2175	0.116	Campylobacter coli (72294)	Campylobacter (213849)	Campylobacteraceae (2954	Campylobacterales (68525)	Epsilonproteobacteria (122	Proteobacteria (2)	Bacteria (1)			99.60	99.50	WGS
195.2176	0.116	Campylobacter coli (72294)	Campylobacter (213849)	Campylobacteraceae (2954	Campylobacterales (68525)	Epsilonproteobacteria (122	Proteobacteria (2)	Bacteria (1)			99.60	99.60	WGS
195.2177	0.1172	Campylobacter coli (72294)	Campylobacter (213849)	Campylobacteraceae (2954	Campylobacterales (68525)	Epsilonproteobacteria (122	Proteobacteria (2)	Bacteria (1)			99.30	99.20	WGS
195.2178	0.1112	Campylobacter coli (72294)	Campylobacter (213849)	Campylobacteraceae (2954	Campylobacterales (68525)	Epsilonproteobacteria (122	Proteobacteria (2)	Bacteria (1)			99.60	99.60	WGS
195.2179	0.1152	Campylobacter coli (72294)	Campylobacter (213849)	Campylobacteraceae (2954	Campylobacterales (68525)	Epsilonproteobacteria (122	Proteobacteria (2)	Bacteria (1)			99.60	99.50	WGS
195.2180	0.1132	Campylobacter coli (72294)	Campylobacter (213849)	Campylobacteraceae (2954	Campylobacterales (68525)	Epsilonproteobacteria (122	Proteobacteria (2)	Bacteria (1)			99.60	99.40	WGS
195.2181	0.1128	Campylobacter coli (72294)	Campylobacter (213849)	Campylobacteraceae (2954	Campylobacterales (68525)	Epsilonproteobacteria (122	Proteobacteria (2)	Bacteria (1)			99.80	99.60	WGS
195.2182	0.1152	Campylobacter coli (72294)	Campylobacter (213849)	Campylobacteraceae (2954	Campylobacterales (68525)	Epsilonproteobacteria (122	Proteobacteria (2)	Bacteria (1)			99.60	99.60	WGS
195.2183	0.1144	Campylobacter coli (72294)	Campylobacter (213849)	Campylobacteraceae (2954	Campylobacterales (68525)	Epsilonproteobacteria (122	Proteobacteria (2)	Bacteria (1)			99.40	99.00	WGS
195.2184	0.1152	Campylobacter coli (72294)	Campylobacter (213849)	Campylobacteraceae (2954	Campylobacterales (68525)	Epsilonproteobacteria (122	Proteobacteria (2)	Bacteria (1)			99.60	99.20	WGS
195.2185	0.1112	Campylobacter coli (72294)	Campylobacter (213849)	Campylobacteraceae (2954	Campylobacterales (68525)	Epsilonproteobacteria (122	Proteobacteria (2)	Bacteria (1)			99.60	99.60	WGS
195.2186	0.116	Campylobacter coli (72294)	Campylobacter (213849)	Campylobacteraceae (2954	Campylobacterales (68525)	Epsilonproteobacteria (122	Proteobacteria (2)	Bacteria (1)			99.60	99.60	WGS
195.2187	0.11	Campylobacter coli (72294)	Campylobacter (213849)	Campylobacteraceae (2954	Campylobacterales (68525)	Epsilonproteobacteria (122	Proteobacteria (2)	Bacteria (1)					WGS
195.2188	0.116	Campylobacter coli (72294)	Campylobacter (213849)	Campylobacteraceae (2954	Campylobacterales (68525)	Epsilonproteobacteria (122	Proteobacteria (2)	Bacteria (1)			99.60	99.40	WGS
195.2189	0.116	Campylobacter coli (72294)	Campylobacter (213849)	Campylobacteraceae (2954	Campylobacterales (68525)	Epsilonproteobacteria (122	Proteobacteria (2)	Bacteria (1)			99.60	99.50	WGS
195.2190	0.116	Campylobacter coli (72294)	Campylobacter (213849)	Campylobacteraceae (2954	Campylobacterales (68525)	Epsilonproteobacteria (122	Proteobacteria (2)	Bacteria (1)					WGS
195.2191	0.1132	Campylobacter coli (72294)	Campylobacter (213849)	Campylobacteraceae (2954	Campylobacterales (68525)	Epsilonproteobacteria (122	Proteobacteria (2)	Bacteria (1)			99.60	99.60	WGS
195.2192	0.1144	Campylobacter coli (72294)	Campylobacter (213849)	Campylobacteraceae (2954	Campylobacterales (68525)	Epsilonproteobacteria (122	Proteobacteria (2)	Bacteria (1)			99.60	99.00	WGS
195.2193	0.116	Campylobacter coli (72294)	Campylobacter (213849)	Campylobacteraceae (2954	Campylobacterales (68525)	Epsilonproteobacteria (122	Proteobacteria (2)	Bacteria (1)			99.60	99.20	WGS
195.2194	0.1132	Campylobacter coli (72294)	Campylobacter (213849)	Campylobacteraceae (2954	Campylobacterales (68525)	Epsilonproteobacteria (122	Proteobacteria (2)	Bacteria (1)			99.80	99.60	WGS
195.2195	0.11	Campylobacter coli (72294)	Campylobacter (213849)	Campylobacteraceae (2954	Campylobacterales (68525)	Epsilonproteobacteria (122	Proteobacteria (2)	Bacteria (1)			99.60	99.30	WGS
195.2196	0.1144	Campylobacter coli (72294)	Campylobacter (213849)	Campylobacteraceae (2954	Campylobacterales (68525)	Epsilonproteobacteria (122	Proteobacteria (2)	Bacteria (1)			99.60	99.40	WGS
195.2197	0.1132	Campylobacter coli (72294)	Campylobacter (213849)	Campylobacteraceae (2954	Campylobacterales (68525)	Epsilonproteobacteria (122	Proteobacteria (2)	Bacteria (1)			99.60	99.40	WGS
195.2198	0.11	Campylobacter coli (72294)	Campylobacter (213849)	Campylobacteraceae (2954	Campylobacterales (68525)	Epsilonproteobacteria (122	Proteobacteria (2)	Bacteria (1)			99.60	99.40	WGS
195.2199	0.1112	Campylobacter coli (72294)	Campylobacter (213849)	Campylobacteraceae (2954	Campylobacterales (68525)	Epsilonproteobacteria (122	Proteobacteria (2)	Bacteria (1)			99.60	99.50	WGS
195.2200	0.1132	Campylobacter coli (72294)	Campylobacter (213849)	Campylobacteraceae (2954	Campylobacterales (68525)	Epsilonproteobacteria (122	Proteobacteria (2)	Bacteria (1)			99.60	99.40	WGS
195.2201	0.116	Campylobacter coli (72294)	Campylobacter (213849)	Campylobacteraceae (2954	Campylobacterales (68525)	Epsilonproteobacteria (122	Proteobacteria (2)	Bacteria (1)			99.60	99.50	WGS
195.2202	0.118	Campylobacter coli (72294)	Campylobacter (213849)	Campylobacteraceae (2954	Campylobacterales (68525)	Epsilonproteobacteria (122	Proteobacteria (2)	Bacteria (1)			99.80	99.70	WGS
195.2203	0.1172	Campylobacter coli (72294)	Campylobacter (213849)	Campylobacteraceae (2954	Campylobacterales (68525)	Epsilonproteobacteria (122	Proteobacteria (2)	Bacteria (1)			99.60	99.40	WGS
195.2204	0.116	Campylobacter coli (72294)	Campylobacter (213849)	Campylobacteraceae (2954	Campylobacterales (68525)	Epsilonproteobacteria (122	Proteobacteria (2)	Bacteria (1)			99.60	99.60	WGS
195.2205	0.1132	Campylobacter coli (72294)	Campylobacter (213849)	Campylobacteraceae (2954	Campylobacterales (68525)	Epsilonproteobacteria (122	Proteobacteria (2)	Bacteria (1)			99.60	99.60	WGS
195.2206	0.1152	Campylobacter coli (72294)	Campylobacter (213849)	Campylobacteraceae (2954	Campylobacterales (68525)	Epsilonproteobacteria (122	Proteobacteria (2)	Bacteria (1)			99.60	99.30	WGS
195.2207	0.11	Campylobacter coli (72294)	Campylobacter (213849)	Campylobacteraceae (2954	Campylobacterales (68525)	Epsilonproteobacteria (122	Proteobacteria (2)	Bacteria (1)			99.60	99.40	WGS
195.2208	0.116	Campylobacter coli (72294)	Campylobacter (213849)	Campylobacteraceae (2954	Campylobacterales (68525)	Epsilonproteobacteria (122	Proteobacteria (2)	Bacteria (1)			99.60	99.60	WGS
195.2209	0.116	Campylobacter coli (72294)	Campylobacter (213849)	Campylobacteraceae (2954	Campylobacterales (68525)	Epsilonproteobacteria (122	Proteobacteria (2)	Bacteria (1)			99.60	99.50	WGS
195.2210	0.11	Campylobacter coli (72294)	Campylobacter (213849)	Campylobacteraceae (2954	Campylobacterales (68525)	Epsilonproteobacteria (122	Proteobacteria (2)	Bacteria (1)			99.60	99.60	WGS
195.2211	0.1132	Campylobacter coli (72294)	Campylobacter (213849)	Campylobacteraceae (2954	Campylobacterales (68525)	Epsilonproteobacteria (122	Proteobacteria (2)	Bacteria (1)			99.60	99.60	WGS
195.2212	0.112	Campylobacter coli (72294)	Campylobacter (213849)	Campylobacteraceae (2954	Campylobacterales (68525)	Epsilonproteobacteria (122	Proteobacteria (2)	Bacteria (1)			99.60	99.10	WGS
195.2213	0.116	Campylobacter coli (72294)	Campylobacter (213849)	Campylobacteraceae (2954	Campylobacterales (68525)	Epsilonproteobacteria (122	Proteobacteria (2)	Bacteria (1)			99.60	99.40	WGS
195.2214	0.116	Campylobacter coli (72294)	Campylobacter (213849)	Campylobacteraceae (2954	Campylobacterales (68525)	Epsilonproteobacteria (122	Proteobacteria (2)	Bacteria (1)			99.60	99.10	WGS
195.2215	0.116	Campylobacter coli (72294)	Campylobacter (213849)	Campylobacteraceae (2954	Campylobacterales (68525)	Epsilonproteobacteria (122	Proteobacteria (2)	Bacteria (1)			99.60	99.50	WGS
195.2216	0.1132	Campylobacter coli (72294)	Campylobacter (213849)	Campylobacteraceae (2954	Campylobacterales (68525)	Epsilonproteobacteria (122	Proteobacteria (2)	Bacteria (1)			99.60	99.60	WGS
195.2217	0.1114	Campylobacter coli (72294)	Campylobacter (213849)	Campylobacteraceae (2954	Campylobacterales (68525)	Epsilonproteobacteria (122	Proteobacteria (2)	Bacteria (1)			99.60	99.50	WGS
195.2218	0.11	Campylobacter coli (72294)	Campylobacter (213849)	Campylobacteraceae (2954	Campylobacterales (68525)	Epsilonproteobacteria (122	Proteobacteria (2)	Bacteria (1)			99.60	99.60	WGS
195.2219	0.11	Campylobacter coli (72294)	Campylobacter (213849)	Campylobacteraceae (2954	Campylobacterales (68525)	Epsilonproteobacteria (122	Proteobacteria (2)	Bacteria (1)			99.60	99.40	WGS
195.2220	0.1148	Campylobacter coli (72294)	Campylobacter (213849)	Campylobacteraceae (2954	Campylobacterales (68525)	Epsilonproteobacteria (122	Proteobacteria (2)	Bacteria (1)			99.60	99.00	WGS
195.2221	0.1132	Campylobacter coli (72294)	Campylobacter (213849)	Campylobacteraceae (2954	Campylobacterales (68525)	Epsilonproteobacteria (122	Proteobacteria (2)	Bacteria (1)			99.60	99.40	WGS
195.2222	0.11	Campylobacter coli (72294)	Campylobacter (213849)	Campylobacteraceae (2954	Campylobacterales (68525)	Epsilonproteobacteria (122	Proteobacteria (2)	Bacteria (1)			99.60	99.40	WGS
195.2223	0.1132	Campylobacter coli (72294)	Campylobacter (213849)	Campylobacteraceae (2954	Campylobacterales (68525)	Epsilonproteobacteria (122	Proteobacteria (2)	Bacteria (1)			99.60	99.40	WGS
195.2224	0.1132	Campylobacter coli (72294)	Campylobacter (213849)	Campylobacteraceae (2954	Campylobacterales (68525)	Epsilonproteobacteria (122	Proteobacteria (2)	Bacteria (1)			99.60	99.00	WGS
195.2225	0.116	Campylobacter coli (72294)	Campylobacter (213849)	Campylobacteraceae (2954	Campylobacterales (68525)	Epsilonproteobacteria (122	Proteobacteria (2)	Bacteria (1)			99.60	99.60	WGS
195.2226	0.1132	Campylobacter coli (72294)	Campylobacter (213849)	Campylobacteraceae (2954	Campylobacterales (68525)	Epsilonproteobacteria (122	Proteobacteria (2)	Bacteria (1)			99.60	99.20	WGS
195.2227	0.116	Campylobacter coli (72294)	Campylobacter (213849)	Campylobacteraceae (2954	Campylobacterales (68525)	Epsilonproteobacteria (122	Proteobacteria (2)	Bacteria (1)			99.60	99.30	WGS
195.2228	0.1132	Campylobacter coli (72294)	Campylobacter (213849)	Campylobacteraceae (2954	Campylobacterales (68525)	Epsilonproteobacteria (122	Proteobacteria (2)	Bacteria (1)			99.60	99.50	WGS
195.2229	0.116	Campylobacter coli (72294)	Campylobacter (213849)	Campylobacteraceae (2954	Campylobacterales (68525)	Epsilonproteobacteria (122	Proteobacteria (2)	Bacteria (1)			99.60	99.40	WGS
195.2230	0.1112	Campylobacter coli (72294)	Campylobacter (213849)	Campylobacteraceae (2954	Campylobacterales (68525)	Epsilonproteobacteria (122	Proteobacteria (2)	Bacteria (1)			99.60	99.60	WGS
195.2231	0.1172	Campylobacter coli (72294)	Campylobacter (213849)	Campylobacteraceae (2954	Campylobacterales (68525)	Epsilonproteobacteria (122	Proteobacteria (2)	Bacteria (1)			99.60	99.30	WGS
195.2232	0.116	Campylobacter coli (72294)	Campylobacter (213849)	Campylobacteraceae (2954	Campylobacterales (68525)	Epsilonproteobacteria (122	Proteobacteria (2)	Bacteria (1)			99.60	99.30	WGS
195.2233	0.112	Campylobacter coli (72294)	Campylobacter (213849)	Campylobacteraceae (2954	Campylobacterales (68525)	Epsilonproteobacteria (122	Proteobacteria (2)	Bacteria (1)			99.60	98.40	WGS
195.2234	0.116	Campylobacter coli (72294)	Campylobacter (213849)	Campylobacteraceae (2954	Campylobacterales (68525)	Epsilonproteobacteria (122	Proteobacteria (2)	Bacteria (1)			99.60	99.50	WGS
195.2235	0.118	Campylobacter coli (72294)	Campylobacter (213849)	Campylobacteraceae (2954	Campylobacterales (68525)	Epsilonproteobacteria (122	Proteobacteria (2)	Bacteria (1)			99.80	99.30	WGS
195.2236	0.1132	Campylobacter coli (72294)	Campylobacter (213849)	Campylobacteraceae (2954	Campylobacterales (68525)	Epsilonproteobacteria (122	Proteobacteria (2)	Bacteria (1)			99.60	99.30	WGS
195.2237	0.1172	Campylobacter coli (72294)	Campylobacter (213849)	Campylobacteraceae (2954	Campylobacterales (68525)	Epsilonproteobacteria (122	Proteobacteria (2)	Bacteria (1)			99.60	99.30	WGS
195.2238	0.11	Campylobacter coli (72294)	Campylobacter (213849)	Campylobacteraceae (2954	Campylobacterales (68525)	Epsilonproteobacteria (122	Proteobacteria (2)	Bacteria (1)			99.60	99.40	WGS
195.2239	0.1152	Campylobacter coli (72294)	Campylobacter (213849)	Campylobacteraceae (2954	Campylobacterales (68525)	Epsilonproteobacteria (122	Proteobacteria (2)	Bacteria (1)			99.60	99.30	WGS
195.2240	0.11	Campylobacter coli (72294)	Campylobacter (213849)	Campylobacteraceae (2954	Campylobacterales (68525)	Epsilonproteobacteria (122	Proteobacteria (2)	Bacteria (1)			99.60	99.60	WGS
195.2241	0.1128	Campylobacter coli (72294)	Campylobacter (213849)	Campylobacteraceae (2954	Campylobacterales (68525)	Epsilonproteobacteria (122	Proteobacteria (2)	Bacteria (1)			99.60	99.20	WGS
195.2242	0.116	Campylobacter coli (72294)	Campylobacter (213849)	Campylobacteraceae (2954	Campylobacterales (68525)	Epsilonproteobacteria (122	Proteobacteria (2)	Bacteria (1)			99.60	99.60	WGS
195.2243	0.116	Campylobacter coli (72294)	Campylobacter (213849)	Campylobacteraceae (2954	Campylobacterales (68525)	Epsilonproteobacteria (122	Proteobacteria (2)	Bacteria (1)			99.60	99.00	WGS
195.2244	0.1132	Campylobacter coli (72294)	Campylobacter (213849)	Campylobacteraceae (2954	Campylobacterales (68525)	Epsilonproteobacteria (122	Proteobacteria (2)	Bacteria (1)			99.40	99.00	WGS
195.2245	0.116	Campylobacter coli (72294)	Campylobacter (213849)	Campylobacteraceae (2954	Campylobacterales (68525)	Epsilonproteobacteria (122	Proteobacteria (2)	Bacteria (1)			99.60	99.60	WGS
195.2246	0.116	Campylobacter coli (72294)	Campylobacter (213849)	Campylobacteraceae (2954	Campylobacterales (68525)	Epsilonproteobacteria (122	Proteobacteria (2)	Bacteria (1)			99.60	99.10	WGS
195.2247	0.1152	Campylobacter coli (72294)	Campylobacter (213849)	Campylobacteraceae (2954	Campylobacterales (68525)	Epsilonproteobacteria (122	Proteobacteria (2)	Bacteria (1)			99.60	99.30	WGS
195.2248	0.116	Campylobacter coli (72294)	Campylobacter (213849)	Campylobacteraceae (2954	Campylobacterales (68525)	Epsilonproteobacteria (122	Proteobacteria (2)	Bacteria (1)			99.60	99.00	WGS
195.2249	0.116	Campylobacter coli (72294)	Campylobacter (213849)	Campylobacteraceae (2954	Campylobacterales (68525)	Epsilonproteobacteria (122	Proteobacteria (2)	Bacteria (1)			99.60	99.10	WGS
195.2250	0.1132	Campylobacter coli (72294)	Campylobacter (213849)	Campylobacteraceae (2954	Campylobacterales (68525)	Epsilonproteobacteria (122	Proteobacteria (2)	Bacteria (1)			99.60	99.40	WGS
195.2251	0.1132	Campylobacter coli (72294)	Campylobacter (213849)	Campylobacteraceae (2954	Campylobacterales (68525)	Epsilonproteobacteria (122	Proteobacteria (2)	Bacteria (1)			99.60	99.60	WGS
195.2252	0.1132	Campylobacter coli (72294)	Campylobacter (213849)	Campylobacteraceae (2954	Campylobacterales (68525)	Epsilonproteobacteria (122	Proteobacteria (2)	Bacteria (1)			99.60	99.60	WGS
195.2253	0.11	Campylobacter coli (72294)	Campylobacter (213849)	Campylobacteraceae (2954	Campylobacterales (68525)	Epsilonproteobacteria (122	Proteobacteria (2)	Bacteria (1)			99.60	99.30	WGS
195.2254	0.1132	Campylobacter coli (72294)	Campylobacter (213849)	Campylobacteraceae (2954	Campylobacterales (68525)	Epsilonproteobacteria (122	Proteobacteria (2)	Bacteria (1)			99.50	99.30	WGS
195.2255	0.1128	Campylobacter coli (72294)	Campylobacter (213849)	Campylobacteraceae (2954	Campylobacterales (68525)	Epsilonproteobacteria (122	Proteobacteria (2)	Bacteria (1)			99.60	99.60	WGS
195.2256	0.1132	Campylobacter coli (72294)	Campylobacter (213849)	Campylobacteraceae (2954	Campylobacterales (68525)	Epsilonproteobacteria (122	Proteobacteria (2)	Bacteria (1)			99.60	99.60	WGS
195.2257	0.116	Campylobacter coli (72294)	Campylobacter (213849)	Campylobacteraceae (2954	Campylobacterales (68525)	Epsilonproteobacteria (122	Proteobacteria (2)	Bacteria (1)			99.60	99.60	WGS
195.2258	0.116	Campylobacter coli (72294)	Campylobacter (213849)	Campylobacteraceae (2954	Campylobacterales (68525)	Epsilonproteobacteria (122	Proteobacteria (2)	Bacteria (1)			99.60	99.60	WGS
195.2259	0.11	Campylobacter coli (72294)	Campylobacter (213849)	Campylobacteraceae (2954	Campylobacterales (68525)	Epsilonproteobacteria (122	Proteobacteria (2)	Bacteria (1)			99.60	99.40	WGS
195.2260	0.1172	Campylobacter coli (72294)	Campylobacter (213849)	Campylobacteraceae (2954	Campylobacterales (68525)	Epsilonproteobacteria (122	Proteobacteria (2)	Bacteria (1)			99.60	99.60	WGS
195.2261	0.116	Campylobacter coli (72294)	Campylobacter (213849)	Campylobacteraceae (2954	Campylobacterales (68525)	Epsilonproteobacteria (122	Proteobacteria (2)	Bacteria (1)			99.60	99.60	WGS
195.2262	0.116	Campylobacter coli (72294)	Campylobacter (213849)	Campylobacteraceae (2954	Campylobacterales (68525)	Epsilonproteobacteria (122	Proteobacteria (2)	Bacteria (1)			99.60	99.20	WGS
195.2263	0.1132	Campylobacter coli (72294)	Campylobacter (213849)	Campylobacteraceae (2954	Campylobacterales (68525)	Epsilonproteobacteria (122	Proteobacteria (2)	Bacteria (1)			99.60	99.60	WGS
195.2264	0.1144	Campylobacter coli (72294)	Campylobacter (213849)	Campylobacteraceae (2954	Campylobacterales (68525)	Epsilonproteobacteria (122	Proteobacteria (2)	Bacteria (1)			99.60	99.60	WGS
195.2265	0.116	Campylobacter coli (72294)	Campylobacter (213849)	Campylobacteraceae (2954	Campylobacterales (68525)	Epsilonproteobacteria (122	Proteobacteria (2)	Bacteria (1)			99.60	99.60	WGS
195.2266	0.1128	Campylobacter coli (72294)	Campylobacter (213849)	Campylobacteraceae (2954	Campylobacterales (68525)	Epsilonproteobacteria (122	Proteobacteria (2)	Bacteria (1)			99.60	99.60	WGS
195.2267	0.116	Campylobacter coli (72294)	Campylobacter (213849)	Campylobacteraceae (2954	Campylobacterales (68525)	Epsilonproteobacteria (122	Proteobacteria (2)	Bacteria (1)			99.60	99.60	WGS
195.2268	0.1132	Campylobacter coli (72294)	Campylobacter (213849)	Campylobacteraceae (2954	Campylobacterales (68525)	Epsilonproteobacteria (122	Proteobacteria (2)	Bacteria (1)			99.60	99.60	WGS
195.2269	0.1112	Campylobacter coli (72294)	Campylobacter (213849)	Campylobacteraceae (2954	Campylobacterales (68525)	Epsilonproteobacteria (122	Proteobacteria (2)	Bacteria (1)			99.60	99.50	WGS
195.2270	0.11	Campylobacter coli (72294)	Campylobacter (213849)	Campylobacteraceae (2954	Campylobacterales (68525)	Epsilonproteobacteria (122	Proteobacteria (2)	Bacteria (1)			99.60	99.60	WGS
195.2271	0.1132	Campylobacter coli (72294)	Campylobacter (213849)	Campylobacteraceae (2954	Campylobacterales (68525)	Epsilonproteobacteria (122	Proteobacteria (2)	Bacteria (1)			99.60	99.40	WGS
195.2272	0.11	Campylobacter coli (72294)	Campylobacter (213849)	Campylobacteraceae (2954	Campylobacterales (68525)	Epsilonproteobacteria (122	Proteobacteria (2)	Bacteria (1)			99.60	99.30	WGS
195.2273	0.1132	Campylobacter coli (72294)	Campylobacter (213849)	Campylobacteraceae (2954	Campylobacterales (68525)	Epsilonproteobacteria (122	Proteobacteria (2)	Bacteria (1)			99.60	99.60	WGS
195.2274	0.1172	Campylobacter coli (72294)	Campylobacter (213849)	Campylobacteraceae (2954	Campylobacterales (68525)	Epsilonproteobacteria (122	Proteobacteria (2)	Bacteria (1)			99.50	99.30	WGS
195.2275	0.1172	Campylobacter coli (72294)	Campylobacter (213849)	Campylobacteraceae (2954	Campylobacterales (68525)	Epsilonproteobacteria (122	Proteobacteria (2)	Bacteria (1)			99.50	99.20	WGS
195.2276	0.118	Campylobacter coli (72294)	Campylobacter (213849)	Campylobacteraceae (2954	Campylobacterales (68525)	Epsilonproteobacteria (122	Proteobacteria (2)	Bacteria (1)			99.60	99.50	WGS
195.2277	0.116	Campylobacter coli (72294)	Campylobacter (213849)	Campylobacteraceae (2954	Campylobacterales (68525)	Epsilonproteobacteria (122	Proteobacteria (2)	Bacteria (1)			99.50	99.40	WGS
195.2278	0.11	Campylobacter coli (72294)	Campylobacter (213849)	Campylobacteraceae (2954	Campylobacterales (68525)	Epsilonproteobacteria (122	Proteobacteria (2)	Bacteria (1)			99.60	99.60	WGS
195.2279	0.1112	Campylobacter coli (72294)	Campylobacter (213849)	Campylobacteraceae (2954	Campylobacterales (68525)	Epsilonproteobacteria (122	Proteobacteria (2)	Bacteria (1)			99.60	99.50	WGS
195.2280	0.116	Campylobacter coli (72294)	Campylobacter (213849)	Campylobacteraceae (2954	Campylobacterales (68525)	Epsilonproteobacteria (122	Proteobacteria (2)	Bacteria (1)			99.60	99.60	WGS
195.2281	0.116	Campylobacter coli (72294)	Campylobacter (213849)	Campylobacteraceae (2954	Campylobacterales (68525)	Epsilonproteobacteria (122	Proteobacteria (2)	Bacteria (1)			99.60	99.40	WGS
195.2282	0.1132	Campylobacter coli (72294)	Campylobacter (213849)	Campylobacteraceae (2954	Campylobacterales (68525)	Epsilonproteobacteria (122	Proteobacteria (2)	Bacteria (1)			99.60	99.50	WGS
195.2283	0.1132	Campylobacter coli (72294)	Campylobacter (213849)	Campylobacteraceae (2954	Campylobacterales (68525)	Epsilonproteobacteria (122	Proteobacteria (2)	Bacteria (1)			99.60	99.40	WGS
195.2284	0.11	Campylobacter coli (72294)	Campylobacter (213849)	Campylobacteraceae (2954	Campylobacterales (68525)	Epsilonproteobacteria (122	Proteobacteria (2)	Bacteria (1)			99.60	99.40	WGS
195.2285	0.1192	Campylobacter coli (72294)	Campylobacter (213849)	Campylobacteraceae (2954	Campylobacterales (68525)	Epsilonproteobacteria (122	Proteobacteria (2)	Bacteria (1)			99.60	99.50	WGS
195.2286	0.116	Campylobacter coli (72294)	Campylobacter (213849)	Campylobacteraceae (2954	Campylobacterales (68525)	Epsilonproteobacteria (122	Proteobacteria (2)	Bacteria (1)			99.50	99.20	WGS
195.2287	0.116	Campylobacter coli (72294)	Campylobacter (213849)	Campylobacteraceae (2954	Campylobacterales (68525)	Epsilonproteobacteria (122	Proteobacteria (2)	Bacteria (1)			99.60	99.50	WGS
195.2288	0.116	Campylobacter coli (72294)	Campylobacter (213849)	Campylobacteraceae (2954	Campylobacterales (68525)	Epsilonproteobacteria (122	Proteobacteria (2)	Bacteria (1)			99.60	99.50	WGS
195.2289	0.116	Campylobacter coli (72294)	Campylobacter (213849)	Campylobacteraceae (2954	Campylobacterales (68525)	Epsilonproteobacteria (122	Proteobacteria (2)	Bacteria (1)			99.60	99.40	WGS
195.2290	0.118	Campylobacter coli (72294)	Campylobacter (213849)	Campylobacteraceae (2954	Campylobacterales (68525)	Epsilonproteobacteria (122	Proteobacteria (2)	Bacteria (1)			99.60	99.50	WGS
195.2291	0.1132	Campylobacter coli (72294)	Campylobacter (213849)	Campylobacteraceae (2954	Campylobacterales (68525)	Epsilonproteobacteria (122	Proteobacteria (2)	Bacteria (1)			99.60	99.60	WGS
195.2292	0.116	Campylobacter coli (72294)	Campylobacter (213849)	Campylobacteraceae (2954	Campylobacterales (68525)	Epsilonproteobacteria (122	Proteobacteria (2)	Bacteria (1)			99.60	99.30	WGS

195.2293	0.116	Campylobacter coli (72294)	Campylobacter (213849)	Campylobacteraceae (2954	Campylobacterales (68525)	Epsilonproteobacteria (122	Proteobacteria (2)	Bacteria (1)			99.60	99.30	WGS
195.2294	0.1164	Campylobacter coli (72294)	Campylobacter (213849)	Campylobacteraceae (2954	Campylobacterales (68525)	Epsilonproteobacteria (122	Proteobacteria (2)	Bacteria (1)			99.60	99.30	WGS
195.2295	0.1132	Campylobacter coli (72294)	Campylobacter (213849)	Campylobacteraceae (2954	Campylobacterales (68525)	Epsilonproteobacteria (122	Proteobacteria (2)	Bacteria (1)			99.60	99.20	WGS
195.2296	0.1132	Campylobacter coli (72294)	Campylobacter (213849)	Campylobacteraceae (2954	Campylobacterales (68525)	Epsilonproteobacteria (122	Proteobacteria (2)	Bacteria (1)			99.70	99.60	WGS
195.2297	0.1132	Campylobacter coli (72294)	Campylobacter (213849)	Campylobacteraceae (2954	Campylobacterales (68525)	Epsilonproteobacteria (122	Proteobacteria (2)	Bacteria (1)			99.60	99.30	WGS
195.2298	0.116	Campylobacter coli (72294)	Campylobacter (213849)	Campylobacteraceae (2954	Campylobacterales (68525)	Epsilonproteobacteria (122	Proteobacteria (2)	Bacteria (1)			99.60	99.60	WGS
195.2299	0.1192	Campylobacter coli (72294)	Campylobacter (213849)	Campylobacteraceae (2954	Campylobacterales (68525)	Epsilonproteobacteria (122	Proteobacteria (2)	Bacteria (1)			99.60	99.20	WGS
195.2300	0.1132	Campylobacter coli (72294)	Campylobacter (213849)	Campylobacteraceae (2954	Campylobacterales (68525)	Epsilonproteobacteria (122	Proteobacteria (2)	Bacteria (1)			99.60	98.90	WGS
195.2301	0.1172	Campylobacter coli (72294)	Campylobacter (213849)	Campylobacteraceae (2954	Campylobacterales (68525)	Epsilonproteobacteria (122	Proteobacteria (2)	Bacteria (1)			99.60	99.50	WGS
195.2302	0.1132	Campylobacter coli (72294)	Campylobacter (213849)	Campylobacteraceae (2954	Campylobacterales (68525)	Epsilonproteobacteria (122	Proteobacteria (2)	Bacteria (1)			99.60	99.60	WGS
195.2303	0.116	Campylobacter coli (72294)	Campylobacter (213849)	Campylobacteraceae (2954	Campylobacterales (68525)	Epsilonproteobacteria (122	Proteobacteria (2)	Bacteria (1)			99.60	99.40	WGS
195.2304	0.1144	Campylobacter coli (72294)	Campylobacter (213849)	Campylobacteraceae (2954	Campylobacterales (68525)	Epsilonproteobacteria (122	Proteobacteria (2)	Bacteria (1)			99.60	99.60	WGS
195.2305	0.116	Campylobacter coli (72294)	Campylobacter (213849)	Campylobacteraceae (2954	Campylobacterales (68525)	Epsilonproteobacteria (122	Proteobacteria (2)	Bacteria (1)			99.60	99.60	WGS
195.2306	0.116	Campylobacter coli (72294)	Campylobacter (213849)	Campylobacteraceae (2954	Campylobacterales (68525)	Epsilonproteobacteria (122	Proteobacteria (2)	Bacteria (1)			99.60	99.60	WGS
195.2307	0.1132	Campylobacter coli (72294)	Campylobacter (213849)	Campylobacteraceae (2954	Campylobacterales (68525)	Epsilonproteobacteria (122	Proteobacteria (2)	Bacteria (1)			99.60	99.30	WGS
195.2308	0.1132	Campylobacter coli (72294)	Campylobacter (213849)	Campylobacteraceae (2954	Campylobacterales (68525)	Epsilonproteobacteria (122	Proteobacteria (2)	Bacteria (1)			99.60	99.00	WGS
195.2309	0.116	Campylobacter coli (72294)	Campylobacter (213849)	Campylobacteraceae (2954	Campylobacterales (68525)	Epsilonproteobacteria (122	Proteobacteria (2)	Bacteria (1)			99.60	99.40	WGS
195.2310	0.116	Campylobacter coli (72294)	Campylobacter (213849)	Campylobacteraceae (2954	Campylobacterales (68525)	Epsilonproteobacteria (122	Proteobacteria (2)	Bacteria (1)			99.60	99.60	WGS
195.2311	0.114	Campylobacter coli (72294)	Campylobacter (213849)	Campylobacteraceae (2954	Campylobacterales (68525)	Epsilonproteobacteria (122	Proteobacteria (2)	Bacteria (1)			99.80	99.10	WGS
195.2312	0.11	Campylobacter coli (72294)	Campylobacter (213849)	Campylobacteraceae (2954	Campylobacterales (68525)	Epsilonproteobacteria (122	Proteobacteria (2)	Bacteria (1)			99.60	99.50	WGS
195.2313	0.1132	Campylobacter coli (72294)	Campylobacter (213849)	Campylobacteraceae (2954	Campylobacterales (68525)	Epsilonproteobacteria (122	Proteobacteria (2)	Bacteria (1)			99.40	99.30	WGS
195.2314	0.11	Campylobacter coli (72294)	Campylobacter (213849)	Campylobacteraceae (2954	Campylobacterales (68525)	Epsilonproteobacteria (122	Proteobacteria (2)	Bacteria (1)			99.60	99.30	WGS
195.2315	0.116	Campylobacter coli (72294)	Campylobacter (213849)	Campylobacteraceae (2954	Campylobacterales (68525)	Epsilonproteobacteria (122	Proteobacteria (2)	Bacteria (1)			99.80	99.70	WGS
195.2316	0.11	Campylobacter coli (72294)	Campylobacter (213849)	Campylobacteraceae (2954	Campylobacterales (68525)	Epsilonproteobacteria (122	Proteobacteria (2)	Bacteria (1)			99.60	99.30	WGS
195.2317	0.1112	Campylobacter coli (72294)	Campylobacter (213849)	Campylobacteraceae (2954	Campylobacterales (68525)	Epsilonproteobacteria (122	Proteobacteria (2)	Bacteria (1)			99.60	99.30	WGS
195.2344	0.11	Campylobacter coli (72294)	Campylobacter (213849)	Campylobacteraceae (2954	Campylobacterales (68525)	Epsilonproteobacteria (122	Proteobacteria (2)	Bacteria (1)			99.80	99.70	Complete
195.2345	0.1144	Campylobacter coli (72294)	Campylobacter (213849)	Campylobacteraceae (2954	Campylobacterales (68525)	Epsilonproteobacteria (122	Proteobacteria (2)	Bacteria (1)			99.60	99.50	WGS
195.2346	0.1172	Campylobacter coli (72294)	Campylobacter (213849)	Campylobacteraceae (2954	Campylobacterales (68525)	Epsilonproteobacteria (122	Proteobacteria (2)	Bacteria (1)			99.60	99.60	WGS
195.2347	0.116	Campylobacter coli (72294)	Campylobacter (213849)	Campylobacteraceae (2954	Campylobacterales (68525)	Epsilonproteobacteria (122	Proteobacteria (2)	Bacteria (1)			99.60	99.50	WGS
195.2348	0.116	Campylobacter coli (72294)	Campylobacter (213849)	Campylobacteraceae (2954	Campylobacterales (68525)	Epsilonproteobacteria (122	Proteobacteria (2)	Bacteria (1)			99.60	99.20	WGS
195.2349	0.1132	Campylobacter coli (72294)	Campylobacter (213849)	Campylobacteraceae (2954	Campylobacterales (68525)	Epsilonproteobacteria (122	Proteobacteria (2)	Bacteria (1)			99.50	99.30	WGS
195.2350	0.1144	Campylobacter coli (72294)	Campylobacter (213849)	Campylobacteraceae (2954	Campylobacterales (68525)	Epsilonproteobacteria (122	Proteobacteria (2)	Bacteria (1)			99.60	99.40	WGS
195.2351	0.11	Campylobacter coli (72294)	Campylobacter (213849)	Campylobacteraceae (2954	Campylobacterales (68525)	Epsilonproteobacteria (122	Proteobacteria (2)	Bacteria (1)			99.60	99.60	WGS
195.2352	0.115	Campylobacter coli (72294)	Campylobacter (213849)	Campylobacteraceae (2954	Campylobacterales (68525)	Epsilonproteobacteria (122	Proteobacteria (2)	Bacteria (1)			99.60	99.00	WGS
195.2353	0.112	Campylobacter coli (72294)	Campylobacter (213849)	Campylobacteraceae (2954	Campylobacterales (68525)	Epsilonproteobacteria (122	Proteobacteria (2)	Bacteria (1)			99.30	98.40	WGS
195.2354	0.1172	Campylobacter coli (72294)	Campylobacter (213849)	Campylobacteraceae (2954	Campylobacterales (68525)	Epsilonproteobacteria (122	Proteobacteria (2)	Bacteria (1)			99.70	99.60	WGS
195.2355	0.114	Campylobacter coli (72294)	Campylobacter (213849)	Campylobacteraceae (2954	Campylobacterales (68525)	Epsilonproteobacteria (122	Proteobacteria (2)	Bacteria (1)			99.80	99.70	Complete
195.282	0.11	Campylobacter coli (72294)	Campylobacter (213849)	Campylobacteraceae (2954	Campylobacterales (68525)	Epsilonproteobacteria (122	Proteobacteria (2)	Bacteria (1)	100.00	0.00	99.60	99.60	Complete
195.283	0.11	Campylobacter coli (72294)	Campylobacter (213849)	Campylobacteraceae (2954	Campylobacterales (68525)	Epsilonproteobacteria (122	Proteobacteria (2)	Bacteria (1)	100.00	0.00	99.60	99.60	WGS
195.284	0.11	Campylobacter coli (72294)	Campylobacter (213849)	Campylobacteraceae (2954	Campylobacterales (68525)	Epsilonproteobacteria (122	Proteobacteria (2)	Bacteria (1)	100.00	0.00	99.60	99.60	WGS
195.285	0.116	Campylobacter coli (72294)	Campylobacter (213849)	Campylobacteraceae (2954	Campylobacterales (68525)	Epsilonproteobacteria (122	Proteobacteria (2)	Bacteria (1)	100.00	0.00	99.60	99.60	WGS
195.286	0.11	Campylobacter coli (72294)	Campylobacter (213849)	Campylobacteraceae (2954	Campylobacterales (68525)	Epsilonproteobacteria (122	Proteobacteria (2)	Bacteria (1)	100.00	0.00	99.60	99.60	WGS
195.287	0.11	Campylobacter coli (72294)	Campylobacter (213849)	Campylobacteraceae (2954	Campylobacterales (68525)	Epsilonproteobacteria (122	Proteobacteria (2)	Bacteria (1)	100.00	0.00	99.80	99.80	WGS
195.288	0.11	Campylobacter coli (72294)	Campylobacter (213849)	Campylobacteraceae (2954	Campylobacterales (68525)	Epsilonproteobacteria (122	Proteobacteria (2)	Bacteria (1)	100.00	0.00	99.80	99.80	WGS
195.289	0.116	Campylobacter coli (72294)	Campylobacter (213849)	Campylobacteraceae (2954	Campylobacterales (68525)	Epsilonproteobacteria (122	Proteobacteria (2)	Bacteria (1)	100.00	0.00	99.60	99.50	WGS
195.290	0.1132	Campylobacter coli (72294)	Campylobacter (213849)	Campylobacteraceae (2954	Campylobacterales (68525)	Epsilonproteobacteria (122	Proteobacteria (2)	Bacteria (1)	100.00	0.00	99.40	99.30	WGS
195.291	0.11	Campylobacter coli (72294)	Campylobacter (213849)	Campylobacteraceae (2954	Campylobacterales (68525)	Epsilonproteobacteria (122	Proteobacteria (2)	Bacteria (1)	100.00	0.00	99.60	99.60	WGS
195.292	0.1132	Campylobacter coli (72294)	Campylobacter (213849)	Campylobacteraceae (2954	Campylobacterales (68525)	Epsilonproteobacteria (122	Proteobacteria (2)	Bacteria (1)	99.40	1.20	99.60	99.60	WGS
195.293	0.11	Campylobacter coli (72294)	Campylobacter (213849)	Campylobacteraceae (2954	Campylobacterales (68525)	Epsilonproteobacteria (122	Proteobacteria (2)	Bacteria (1)	100.00	0.00	99.60	99.60	WGS
195.294	0.11	Campylobacter coli (72294)	Campylobacter (213849)	Campylobacteraceae (2954	Campylobacterales (68525)	Epsilonproteobacteria (122	Proteobacteria (2)	Bacteria (1)	100.00	0.00	99.60	99.60	WGS
195.295	0.11	Campylobacter coli (72294)	Campylobacter (213849)	Campylobacteraceae (2954	Campylobacterales (68525)	Epsilonproteobacteria (122	Proteobacteria (2)	Bacteria (1)	100.00	0.00	99.60	99.60	WGS
195.296	0.1132	Campylobacter coli (72294)	Campylobacter (213849)	Campylobacteraceae (2954	Campylobacterales (68525)	Epsilonproteobacteria (122	Proteobacteria (2)	Bacteria (1)	100.00	35.10	99.80	84.40	WGS
195.297	0.1112	Campylobacter coli (72294)	Campylobacter (213849)	Campylobacteraceae (2954	Campylobacterales (68525)	Epsilonproteobacteria (122	Proteobacteria (2)	Bacteria (1)	100.00	0.60	99.80	99.70	WGS
195.298	0.11	Campylobacter coli (72294)	Campylobacter (213849)	Campylobacteraceae (2954	Campylobacterales (68525)	Epsilonproteobacteria (122	Proteobacteria (2)	Bacteria (1)	100.00	0.00	99.60	99.60	WGS
195.299	0.1112	Campylobacter coli (72294)	Campylobacter (213849)	Campylobacteraceae (2954	Campylobacterales (68525)	Epsilonproteobacteria (122	Proteobacteria (2)	Bacteria (1)	100.00	0.00	99.60	99.60	WGS
195.300	0.116	Campylobacter coli (72294)	Campylobacter (213849)	Campylobacteraceae (2954	Campylobacterales (68525)	Epsilonproteobacteria (122	Proteobacteria (2)	Bacteria (1)	100.00	0.00	99.40	99.30	WGS
195.301	0.116	Campylobacter coli (72294)	Campylobacter (213849)	Campylobacteraceae (2954	Campylobacterales (68525)	Epsilonproteobacteria (122	Proteobacteria (2)	Bacteria (1)	100.00	0.00	99.50	99.30	WGS
195.302	0.11	Campylobacter coli (72294)	Campylobacter (213849)	Campylobacteraceae (2954	Campylobacterales (68525)	Epsilonproteobacteria (122	Proteobacteria (2)	Bacteria (1)	100.00	0.00	99.60	99.60	WGS
195.303	0.1132	Campylobacter coli (72294)	Campylobacter (213849)	Campylobacteraceae (2954	Campylobacterales (68525)	Epsilonproteobacteria (122	Proteobacteria (2)	Bacteria (1)	100.00	0.00	99.60	99.30	WGS
195.304	0.1128	Campylobacter coli (72294)	Campylobacter (213849)	Campylobacteraceae (2954	Campylobacterales (68525)	Epsilonproteobacteria (122	Proteobacteria (2)	Bacteria (1)	100.00	0.00	99.60	99.60	WGS
195.305	0.11	Campylobacter coli (72294)	Campylobacter (213849)	Campylobacteraceae (2954	Campylobacterales (68525)	Epsilonproteobacteria (122	Proteobacteria (2)	Bacteria (1)	100.00	0.00	99.60	99.60	WGS
195.306	0.1132	Campylobacter coli (72294)	Campylobacter (213849)	Campylobacteraceae (2954	Campylobacterales (68525)	Epsilonproteobacteria (122	Proteobacteria (2)	Bacteria (1)	100.00	0.00	99.60	99.60	WGS
195.307	0.11	Campylobacter coli (72294)	Campylobacter (213849)	Campylobacteraceae (2954	Campylobacterales (68525)	Epsilonproteobacteria (122	Proteobacteria (2)	Bacteria (1)	100.00	0.00	99.80	99.70	WGS
195.308	0.1128	Campylobacter coli (72294)	Campylobacter (213849)	Campylobacteraceae (2954	Campylobacterales (68525)	Epsilonproteobacteria (122	Proteobacteria (2)	Bacteria (1)	100.00	0.00	99.60	99.60	WGS
195.309	0.11	Campylobacter coli (72294)	Campylobacter (213849)	Campylobacteraceae (2954	Campylobacterales (68525)	Epsilonproteobacteria (122	Proteobacteria (2)	Bacteria (1)	100.00	0.00	99.60	99.60	WGS
195.310	0.1112	Campylobacter coli (72294)	Campylobacter (213849)	Campylobacteraceae (2954	Campylobacterales (68525)	Epsilonproteobacteria (122	Proteobacteria (2)	Bacteria (1)	100.00	0.00	99.60	99.60	WGS
195.311	0.1128	Campylobacter coli (72294)	Campylobacter (213849)	Campylobacteraceae (2954	Campylobacterales (68525)	Epsilonproteobacteria (122	Proteobacteria (2)	Bacteria (1)	100.00	0.00	99.60	99.60	WGS
195.312	0.11	Campylobacter coli (72294)	Campylobacter (213849)	Campylobacteraceae (2954	Campylobacterales (68525)	Epsilonproteobacteria (122	Proteobacteria (2)	Bacteria (1)	100.00	0.00	99.60	99.60	WGS
195.313	0.116	Campylobacter coli (72294)	Campylobacter (213849)	Campylobacteraceae (2954	Campylobacterales (68525)	Epsilonproteobacteria (122	Proteobacteria (2)	Bacteria (1)	100.00	0.00	99.60	99.60	WGS
195.314	0.11	Campylobacter coli (72294)	Campylobacter (213849)	Campylobacteraceae (2954	Campylobacterales (68525)	Epsilonproteobacteria (122	Proteobacteria (2)	Bacteria (1)	100.00	0.00	99.80	99.80	WGS
195.315	0.11	Campylobacter coli (72294)	Campylobacter (213849)	Campylobacteraceae (2954	Campylobacterales (68525)	Epsilonproteobacteria (122	Proteobacteria (2)	Bacteria (1)	100.00	0.00	99.60	99.60	WGS
195.316	0.116	Campylobacter coli (72294)	Campylobacter (213849)	Campylobacteraceae (2954	Campylobacterales (68525)	Epsilonproteobacteria (122	Proteobacteria (2)	Bacteria (1)	100.00	0.00	99.50	99.40	WGS
195.317	0.114	Campylobacter coli (72294)	Campylobacter (213849)	Campylobacteraceae (2954	Campylobacterales (68525)	Epsilonproteobacteria (122	Proteobacteria (2)	Bacteria (1)	100.00	0.00	99.60	99.60	WGS
195.318	0.11	Campylobacter coli (72294)	Campylobacter (213849)	Campylobacteraceae (2954	Campylobacterales (68525)	Epsilonproteobacteria (122	Proteobacteria (2)	Bacteria (1)	100.00	0.00	99.60	99.60	WGS
195.319	0.116	Campylobacter coli (72294)	Campylobacter (213849)	Campylobacteraceae (2954	Campylobacterales (68525)	Epsilonproteobacteria (122	Proteobacteria (2)	Bacteria (1)	100.00	0.00	99.60	99.60	WGS
195.320	0.1112	Campylobacter coli (72294)	Campylobacter (213849)	Campylobacteraceae (2954	Campylobacterales (68525)	Epsilonproteobacteria (122	Proteobacteria (2)	Bacteria (1)	100.00	0.00	99.60	99.60	WGS
195.321	0.1132	Campylobacter coli (72294)	Campylobacter (213849)	Campylobacteraceae (2954	Campylobacterales (68525)	Epsilonproteobacteria (122	Proteobacteria (2)	Bacteria (1)	100.00	0.00	99.50	99.30	WGS
195.322	0.1128	Campylobacter coli (72294)	Campylobacter (213849)	Campylobacteraceae (2954	Campylobacterales (68525)	Epsilonproteobacteria (122	Proteobacteria (2)	Bacteria (1)	100.00	0.00	99.60	99.60	WGS
195.323	0.1132	Campylobacter coli (72294)	Campylobacter (213849)	Campylobacteraceae (2954	Campylobacterales (68525)	Epsilonproteobacteria (122	Proteobacteria (2)	Bacteria (1)	100.00	0.00	99.40	99.30	WGS
195.324	0.1128	Campylobacter coli (72294)	Campylobacter (213849)	Campylobacteraceae (2954	Campylobacterales (68525)	Epsilonproteobacteria (122	Proteobacteria (2)	Bacteria (1)	100.00	0.00	99.60	99.60	WGS
195.325	0.11	Campylobacter coli (72294)	Campylobacter (213849)	Campylobacteraceae (2954	Campylobacterales (68525)	Epsilonproteobacteria (122	Proteobacteria (2)	Bacteria (1)	100.00	0.00	99.60	99.50	WGS
195.326	0.11	Campylobacter coli (72294)	Campylobacter (213849)	Campylobacteraceae (2954	Campylobacterales (68525)	Epsilonproteobacteria (122	Proteobacteria (2)	Bacteria (1)	100.00	0.00	99.80	99.80	WGS
195.327	0.11	Campylobacter coli (72294)	Campylobacter (213849)	Campylobacteraceae (2954	Campylobacterales (68525)	Epsilonproteobacteria (122	Proteobacteria (2)	Bacteria (1)	100.00	0.00	99.60	99.60	WGS
195.328	0.11	Campylobacter coli (72294)	Campylobacter (213849)	Campylobacteraceae (2954	Campylobacterales (68525)	Epsilonproteobacteria (122	Proteobacteria (2)	Bacteria (1)	100.00	0.00	99.60	99.60	WGS
195.329	0.116	Campylobacter coli (72294)	Campylobacter (213849)	Campylobacteraceae (2954	Campylobacterales (68525)	Epsilonproteobacteria (122	Proteobacteria (2)	Bacteria (1)	100.00	0.00	99.60	99.60	WGS
195.330	0.1112	Campylobacter coli (72294)	Campylobacter (213849)	Campylobacteraceae (2954	Campylobacterales (68525)	Epsilonproteobacteria (122	Proteobacteria (2)	Bacteria (1)	100.00	0.00	99.60	99.60	WGS
195.331	0.1132	Campylobacter coli (72294)	Campylobacter (213849)	Campylobacteraceae (2954	Campylobacterales (68525)	Epsilonproteobacteria (122	Proteobacteria (2)	Bacteria (1)	100.00	0.60	99.40	99.20	WGS
195.332	0.1132	Campylobacter coli (72294)	Campylobacter (213849)	Campylobacteraceae (2954	Campylobacterales (68525)	Epsilonproteobacteria (122	Proteobacteria (2)	Bacteria (1)	100.00	1.20	99.50	99.30	WGS
195.333	0.116	Campylobacter coli (72294)	Campylobacter (213849)	Campylobacteraceae (2954	Campylobacterales (68525)	Epsilonproteobacteria (122	Proteobacteria (2)	Bacteria (1)	100.00	1.80	99.40	98.50	WGS
195.334	0.1132	Campylobacter coli (72294)	Campylobacter (213849)	Campylobacteraceae (2954	Campylobacterales (68525)	Epsilonproteobacteria (122	Proteobacteria (2)	Bacteria (1)	100.00	0.60	99.50	98.40	WGS
195.335	0.1132	Campylobacter coli (72294)	Campylobacter (213849)	Campylobacteraceae (2954	Campylobacterales (68525)	Epsilonproteobacteria (122	Proteobacteria (2)	Bacteria (1)	100.00	6.80	99.30	97.60	WGS
195.336	0.1132	Campylobacter coli (72294)	Campylobacter (213849)	Campylobacteraceae (2954	Campylobacterales (68525)	Epsilonproteobacteria (122	Proteobacteria (2)	Bacteria (1)	99.40	7.40	99.40	97.50	WGS
195.337	0.1132	Campylobacter coli (72294)	Campylobacter (213849)	Campylobacteraceae (2954	Campylobacterales (68525)	Epsilonproteobacteria (122	Proteobacteria (2)	Bacteria (1)	100.00	3.50	99.30	98.30	WGS
195.338	0.1132	Campylobacter coli (72294)	Campylobacter (213849)	Campylobacteraceae (2954	Campylobacterales (68525)	Epsilonproteobacteria (122	Proteobacteria (2)	Bacteria (1)	100.00	1.80	99.30	98.20	WGS
195.339	0.1132	Campylobacter coli (72294)	Campylobacter (213849)	Campylobacteraceae (2954	Campylobacterales (68525)	Epsilonproteobacteria (122	Proteobacteria (2)	Bacteria (1)	100.00	1.20	99.40	98.90	WGS
195.340	0.1132	Campylobacter coli (72294)	Campylobacter (213849)	Campylobacteraceae (2954	Campylobacterales (68525)	Epsilonproteobacteria (122	Proteobacteria (2)	Bacteria (1)	100.00	0.60	99.40	99.30	WGS
195.341	0.1132	Campylobacter coli (72294)	Campylobacter (213849)	Campylobacteraceae (2954	Campylobacterales (68525)	Epsilonproteobacteria (122	Proteobacteria (2)	Bacteria (1)	100.00	9.90	99.00	96.50	WGS
195.342	0.1132	Campylobacter coli (72294)	Campylobacter (213849)	Campylobacteraceae (2954	Campylobacterales (68525)	Epsilonproteobacteria (122	Proteobacteria (2)	Bacteria (1)	100.00	1.20	99.40	98.90	WGS
195.343	0.1122	Campylobacter coli (72294)	Campylobacter (213849)	Campylobacteraceae (2954	Campylobacterales (68525)	Epsilonproteobacteria (122	Proteobacteria (2)	Bacteria (1)	99.40	0.00	99.20	98.70	WGS
195.344	0.116	Campylobacter coli (72294)	Campylobacter (213849)	Campylobacteraceae (2954	Campylobacterales (68525)	Epsilonproteobacteria (122	Proteobacteria (2)	Bacteria (1)	100.00	1.20	99.40	99.00	WGS
195.345	0.1172	Campylobacter coli (72294)	Campylobacter (213849)	Campylobacteraceae (2954	Campylobacterales (68525)	Epsilonproteobacteria (122	Proteobacteria (2)	Bacteria (1)	100.00	0.00	99.60	99.30	WGS
195.346	0.1132	Campylobacter coli (72294)	Campylobacter (213849)	Campylobacteraceae (2954	Campylobacterales (68525)	Epsilonproteobacteria (122	Proteobacteria (2)	Bacteria (1)	100.00	1.80	99.40	99.00	WGS
195.347	0.1144	Campylobacter coli (72294)	Campylobacter (213849)	Campylobacteraceae (2954	Campylobacterales (68525)	Epsilonproteobacteria (122	Proteobacteria (2)	Bacteria (1)	100.00	0.60	99.40	99.30	WGS
195.348	0.1132	Campylobacter coli (72294)	Campylobacter (213849)	Campylobacteraceae (2954	Campylobacterales (68525)	Epsilonproteobacteria (122	Proteobacteria (2)	Bacteria (1)	100.00	3.00	99.40	98.50	WGS
195.349	0.1132	Campylobacter coli (72294)	Campylobacter (213849)	Campylobacteraceae (2954	Campylobacterales (68525)	Epsilonproteobacteria (122	Proteobacteria (2)	Bacteria (1)	100.00	3.50	99.40	98.30	WGS
195.350	0.1132	Campylobacter coli (72294)	Campylobacter (213849)	Campylobacteraceae (2954	Campylobacterales (68525)	Epsilonproteobacteria (122	Proteobacteria (2)	Bacteria (1)	100.00	0.60	99.40	99.00	WGS
195.351	0.109	Campylobacter coli (72294)	Campylobacter (213849)	Campylobacteraceae (2954	Campylobacterales (68525)	Epsilonproteobacteria (122	Proteobacteria (2)	Bacteria (1)	99.40	2.40	99.90	99.50	WGS
195.352	0.116	Campylobacter coli (72294)	Campylobacter (213849)	Campylobacteraceae (2954	Campylobacterales (68525)	Epsilonproteobacteria (122	Proteobacteria (2)	Bacteria (1)	100.00	3.60	99.30	97.80	WGS
195.353	0.1078	Campylobacter coli (72294)	Campylobacter (213849)	Campylobacteraceae (2954	Campylobacterales (68525)	Epsilonproteobacteria (122	Proteobacteria (2)	Bacteria (1)	100.00	0.60	100.00	99.30	WGS
195.354	0.116	Campylobacter coli (72294)	Campylobacter (213849)	Campylobacteraceae (2954	Campylobacterales (68525)	Epsilonproteobacteria (122	Proteobacteria (2)	Bacteria (1)	100.00	5.20	99.30	98.70	WGS
195.355	0.1132	Campylobacter coli (72294)	Campylobacter (213849)	Campylobacteraceae (2954	Campylobacterales (68525)	Epsilonproteobacteria (122	Proteobacteria (2)	Bacteria (1)	100.00	0.00	99.40	99.20	WGS
195.356	0.1132	Campylobacter coli (72294)	Campylobacter (213849)	Campylobacteraceae (2954	Campylobacterales (68525)	Epsilonproteobacteria (122	Proteobacteria (2)	Bacteria (1)	100.00	0.20	99.30	99.10	WGS
195.357	0.1132	Campylobacter coli (72294)	Campylobacter (213849)	Campylobacteraceae (2954	Campylobacterales (68525)	Epsilonproteobacteria (122	Proteobacteria (2)	Bacteria (1)	100.00	2.40	99.40	98.70	WGS
195.358	0.1132	Campylobacter coli (72294)	Campylobacter (213849)	Campylobacteraceae (2954	Campylobacterales (68525)	Epsilonproteobacteria (122	Proteobacteria (2)	Bacteria (1)	99.40	2.40	99.40	99.10	WGS
195.359	0.1132	Campylobacter coli (72294)	Campylobacter (213849)	Campylobacteraceae (2954	Campylobacterales (68525)	Epsilonproteobacteria (122	Proteobacteria (2)	Bacteria (1)	100.00	6.00	99.30	97.50	WGS
195.360	0.1132	Campylobacter coli (72294)	Campylobacter (213849)	Campylobacteraceae (2954	Campylobacterales (68525)	Epsilonproteobacteria (122	Proteobacteria (2)	Bacteria (1)	100.00	0.60	99.40	99.30	WGS
195.361	0.116	Campylobacter coli (72294)	Campylobacter (213849)	Campylobacteraceae (2954	Campylobacterales (68525)	Epsilonproteobacteria (122	Proteobacteria (2)	Bacteria (1)	100.00	0.20	99.90	99.80	WGS
195.362	0.1126	Campylobacter coli (72294)	Campylobacter (213849)	Campylobacteraceae (2954	Campylobacterales (68525)	Epsilonproteobacteria (122	Proteobacteria (2)	Bacteria (1)	100.00	35.50	99.60	83.60	WGS
195.363	0.1128	Campylobacter coli (72294)	Campylobacter (213849)	Campylobacteraceae (2954	Campylobacterales (68525)	Epsilonproteobacteria (122	Proteobacteria (2)	Bacteria (1)	100.00	4.70	99.80	98.50	Complete
195.364	0.1172	Campylobacter coli (72294)	Campylobacter (213849)	Campylobacteraceae (2954	Campylobacterales (68525)	Epsilonproteobacteria (122	Proteobacteria (2)	Bacteria (1)	100.00	0.00	99.60	99.50	WGS
195.365	0.1144	Campylobacter coli (72294)	Campylobacter (213849)	Campylobacteraceae (2954	Campylobacterales (68525)	Epsilonproteobacteria (122	Proteobacteria (2)	Bacteria (1)	100.00	0.00	99.60	99.50	WGS
195.366	0.1144	Campylobacter coli (72294)	Campylobacter (213849)	Campylobacteraceae (2954	Campylobacterales (68525)	Epsilonproteobacteria (122	Proteobacteria (2)	Bacteria (1)	100.00	0.60	99.60	99.60	WGS
195.367	0.1144	Campylobacter coli (72294)	Campylobacter (213849)	Campylobacteraceae (2954	Campylobacterales (68525)	Epsilonproteobacteria (122	Proteobacteria (2)	Bacteria (1)	100.00	1.80	99.80	99.60	WGS
195.368	0.1112	Campylobacter coli (72294)	Campylobacter (213849)	Campylobacteraceae (2954	Campylobacterales (68525)	Epsilonproteobacteria (122	Proteobacteria (2)	Bacteria (1)	100.00	0.00	99.80	99.70	WGS
195.369	0.1144	Campylobacter coli (72294)	Campylobacter (213849)	Campylobacteraceae (2954	Campylobacterales (68525)	Epsilonproteobacteria (122	Proteobacteria (2)	Bacteria (1)	100.00	0.60	99.80	99.40	WGS
195.370	0.1112	Campylobacter coli (72294)	Campylobacter (213849)	Campylobacteraceae (2954	Campylobacterales (68525)	Epsilonproteobacteria (122	Proteobacteria (2)	Bacteria (1)	100.00	0.60	99.80	99.30	WGS
195.371	0.1112	Campylobacter coli (72294)	Campylobacter (213849)	Campylobacteraceae (2954	Campylobacterales (68525)	Epsilonproteobacteria (122	Proteobacteria (2)	Bacteria (1)	100.00	0.00	99.80	99.80	WGS
195.372	0.1172	Campylobacter coli (72294)	Campylobacter (213849)	Campylobacteraceae (2954	Campylobacterales (68525)	Epsilonproteobacteria (122	Proteobacteria (2)	Bacteria (1)	100.00	0.00	99.60	99.50	WGS
195.373	0.1112	Campylobacter coli (72294)	Campylobacter (213849)	Campylobacteraceae (2954	Campylobacterales (68525)	Epsilonproteobacteria (122	Proteobacteria (2)	Bacteria (1)	100.00	0.00	99.80	99.80	WGS
195.374	0.1172	Campylobacter coli (72294)	Campylobacter (213849)	Campylobacteraceae (2954	Campylobacterales (68525)	Epsilonproteobacteria (122	Proteobacteria (2)	Bacteria (1)	100.00	0.00	99.60	99.20	WGS
195.375	0.1144	Campylobacter coli (72294)	Campylobacter (213849)	Campylobacteraceae (2954	Campylobacterales (68525)	Epsilonproteobacteria (122	Proteobacteria (2)	Bacteria (1)	99.40	4.10	99.70	98.70	WGS
195.376	0.1144	Campylobacter coli (72294)	Campylobacter (213849)	Campylobacteraceae (2954	Campylobacterales (68525)	Epsilonproteobacteria (122	Proteobacteria (2)	Bacteria (1)	100.00	0.60	99.80	99.40	WGS
195.377	0.1144	Campylobacter coli (72294)	Campylobacter (213849)	Campylobacteraceae (2954	Campylobacterales (68525)	Epsilonproteobacteria (122	Proteobacteria (2)	Bacteria (1)	100.00	2.40	99.80	98.70	WGS
195.378	0.1144	Campylobacter coli (72294)	Campylobacter (213849)	Campylobacteraceae (2954	Campylobacterales (68525)	Epsilonproteobacteria (122	Proteobacteria (2)	Bacteria (1)	100.00	3.50	99.80	99.00	WGS
195.379	0.1112	Campylobacter coli (72294)	Campylobacter (213849)	Campylobacteraceae (2954	Campylobacterales (68525)	Epsilonproteobacteria (122	Proteobacteria (2)	Bacteria (1)	100.00	0.00	99.80	99.80	WGS
195.380	0.1144	Campylobacter coli (72294)	Campylobacter (213849)	Campylobacteraceae (2954	Campylobacterales (68525)	Epsilonproteobacteria (122	Proteobacteria (2)	Bacteria (1)	100.00	0.00	99.80	99.70	WGS
195.381	0.1112	Campylobacter coli (72294)	Campylobacter (213849)	Campylobacteraceae (2954	Campylobacterales (68525)	Epsilonproteobacteria (122	Proteobacteria (2)	Bacteria (1)	100.00	0.00	99.80	99.80	WGS
195.382	0.1112	Campylobacter coli (72294)	Campylobacter (213849)	Campylobacteraceae (2954	Campylobacterales (68525)	Epsilonproteobacteria (122	Proteobacteria (2)	Bacteria (1)	100.00	0.00	99.80	99.70	WGS
195.383	0.1144	Campylobacter coli (72294)	Campylobacter (213849)	Campylobacteraceae (2954	Campylobacterales (68525)	Epsilonproteobacteria (122	Proteobacteria (2)	Bacteria (1)	100.00	0.00	99.80	99.80	WGS
195.384	0.1078	Campylobacter coli (72294)	Campylobacter (213849)	Campylobacteraceae (2954	Campylobacterales (68525)	Epsilonproteobacteria (122	Proteobacteria (2)	Bacteria (1)	98.80	0.00	99.70	99.70	WGS
195.385	0.1144	Campylobacter coli (72294)	Campylobacter (213849)	Campylobacteraceae (2954	Campylobacterales (68525)	Epsilonproteobacteria (122	Proteobacteria (2)	Bacteria (1)	100.00	0.00	99.80	99.80	WGS
195.386	0.1112	Campylobacter coli (72294)	Campylobacter (213849)	Campylobacteraceae (2954	Campylobacterales (68525)	Epsilonproteobacteria (122	Proteobacteria (2)	Bacteria (1)	100.00	0.60	99.90	99.70	WGS
195.387	0.1144	Campylobacter coli (72294)	Campylobacter (213849)	Campylobacteraceae (2954	Campylobacterales (68525)	Epsilonproteobacteria (122	Proteobacteria (2)	Bacteria (1)	99.40	3.90	99.80	98.80	WGS
195.388	0.114	Campylobacter coli (72294)	Campylobacter (213849)	Campylobacteraceae (2954	Campylobacterales (68525)	Epsilonproteobacteria (122	Proteobacteria (2)	Bacteria (1)	100.00	0.00	99.80	99.70	WGS
195.389	0.1144	Campylobacter coli (72294)	Campylobacter (213849)	Campylobacteraceae (2954	Campylobacterales (68525)	Epsilonproteobacteria (122	Proteobacteria (2)	Bacteria (1)	100.00	0.60	99.80	99.20	WGS
195.390	0.1144	Campylobacter coli (72294)	Campylobacter (213849)	Campylobacteraceae (2954	Campylobacterales (68525)	Epsilonproteobacteria (122	Proteobacteria (2)	Bacteria (1)	100.00	7.40	99.80	99.00	WGS
195.391	0.1144	Campylobacter coli (72294)	Campylobacter (213849)	Campylobacteraceae (2954	Campylobacterales (68525)	Epsilonproteobacteria (122	Proteobacteria (2)	Bacteria (1)	100.00	0.60	99.80	99.60	WGS
195.392	0.1132	Campylobacter coli (72294)	Campylobacter (213849)	Campylobacteraceae (2954	Campylobacterales (68525)	Epsilonproteobacteria (122	Proteobacteria (2)	Bacteria (1)	100.00	0.60	99.80	99.30	WGS
195.393	0.1154	Campylobacter coli (72294)	Campylobacter (213849)	Campylobacteraceae (2954	Campylobacterales (68525)	Epsilonproteobacteria (122	Proteobacteria (2)	Bacteria (1)	100.00	2.00	99.60	99.30	WGS
195.394	0.1144	Campylobacter coli (72294)	Campylobacter (213849)	Campylobacteraceae (2954	Campylobacterales (68525)	Epsilonproteobacteria (122	Proteobacteria (2)	Bacteria (1)	100.00	1.20	99.80	99.30	WGS
195.395	0.1144	Campylobacter coli (72294)	Campylobacter (213849)	Campylobacteraceae (2954	Campylobacterales (68525)	Epsilonproteobacteria (122	Proteobacteria (2)	Bacteria (1)	100.00	1.80	99.80	99.00	WGS
195.396	0.1112	Campylobacter coli (72294)	Campylobacter (213849)	Campylobacteraceae (2954	Campylobacterales (68525)	Epsilonproteobacteria (122	Proteobacteria (2)	Bacteria (1)	100.00	0.00	99.80	99.80	WGS
195.397	0.1172	Campylobacter coli (72294)	Campylobacter (213849)	Campylobacteraceae (2954	Campylobacterales (68525)	Epsilonproteobacteria (122	Proteobacteria (2)	Bacteria (1)	100.00	0.00	99.80	99.80	WGS
195.398	0.1132	Campylobacter coli (72294)	Campylobacter (213849)	Campylobacteraceae (2954	Campylobacterales (68525)	Epsilonproteobacteria (122	Proteobacteria (2)	Bacteria (1)	100.00	1.80	99.80	99.10	Complete
195.399	0.1132	Campylobacter coli (72294)	Campylobacter (213849)	Campylobacteraceae (2954	Campylobacterales (68525)	Epsilonproteobacteria (122	Proteobacteria (2)	Bacteria (1)	100.00	0.00	99.70	99.50	Complete
195.400	0.116	Campylobacter coli (72294)	Campylobacter (213849)	Campylobacteraceae (2954	Campylobacterales (68525)	Epsilonproteobacteria (122	Proteobacteria (2)	Bacteria (1)	100.00	0.00	99.80	99.60	Complete
195.401	0.1172	Campylobacter coli (72294)	Campylobacter (213849)	Campylobacteraceae (2954	Campylobacterales (68525)	Epsilonproteobacteria (122	Proteobacteria (2)	Bacteria (1)	100.00	0.00	99.60	99.20	WGS
195.402	0.1132	Campylobacter coli (72294)	Campylobacter (213849)	Campylobacteraceae (2954	Campylobacterales (68525)	Epsilonproteobacteria (122	Proteobacteria (2)	Bacteria (1)	100.00	1.20	99.60	99.20	WGS
195.403	0.1144	Campylobacter coli (72294)	Campylobacter (213849)	Campylobacteraceae (2954	Campylobacterales (68525)	Epsilonproteobacteria (122	Proteobacteria (2)	Bacteria (1)	99.40	0.00	99.60	99.50	WGS
195.404	0.1132	Campylobacter coli (72294)	Campylobacter (213849)	Campylobacteraceae (2954	Campylobacterales (68525)	Epsilonproteobacteria (122	Proteobacteria (2)	Bacteria (1)	98.20	16.20	99.00	95.60	WGS
195.405	0.116	Campylobacter coli (72294)	Campylobacter (213849)	Campylobacteraceae (2954	Campylobacterales (68525)	Epsilonproteobacteria (122	Proteobacteria (2)	Bacteria (1)	100.00	0.00	99.80	99.80	WGS
195.406	0.1142	Campylobacter coli (72294)	Campylobacter (213849)	Campylobacteraceae (2954	Campylobacterales (68525)	Epsilonproteobacteria (122	Proteobacteria (2)	Bacteria (1)	100.00	0.00	99.60	99.60	WGS
195.407	0.1164	Campylobacter coli (72294)	Campylobacter (213849)	Campylobacteraceae (2954	Campylobacterales (68525)	Epsilonproteobacteria (122	Proteobacteria (2)	Bacteria (1)	100.00	0.00	99.60	99.50	WGS
195.408	0.11	Campylobacter coli (72294)	Campylobacter (213849)	Campylobacteraceae (2954	Campylobacterales (68525)	Epsilonproteobacteria (122	Proteobacteria (2)	Bacteria (1)	100.00	0.00	99.80	99.80	WGS
195.409	0.1172	Campylobacter coli (72294)	Campylobacter (213849)	Campylobacteraceae (2954	Campylobacterales (68525)	Epsilonproteobacteria (122	Proteobacteria (2)	Bacteria (1)	100.00	1.80	99.60	99.10	WGS
195.410	0.11	Campylobacter coli (72294)	Campylobacter (213849)	Campylobacteraceae (2954	Campylobacterales (68525)	Epsilonproteobacteria (122	Proteobacteria (2)	Bacteria (1)	100.00	0.00	99.80	99.60	WGS
195.411	0.116	Campylobacter coli (72294)	Campylobacter (213849)	Campylobacteraceae (2954	Campylobacterales (68525)	Epsilonproteobacteria (122	Proteobacteria (2)	Bacteria (1)	100.00	0.00	99.70	99.40	WGS
195.412	0.1144	Campylobacter coli (72294)	Campylobacter (213849)	Campylobacteraceae (2954	Campylobacterales (68525)	Epsilonproteobacteria (122	Proteobacteria (2)	Bacteria (1)	100.00	3.00	99.60	98.80	WGS
195.413	0.1152	Campylobacter coli (72294)	Campylobacter (213849)	Campylobacteraceae (2954	Campylobacterales (68525)	Epsilonproteobacteria (122	Proteobacteria (2)	Bacteria (1)	99.40	1.20	99.60	99.00	WGS
195.414	0.116	Campylobacter coli (72294)	Campylobacter (213849)	Campylobacteraceae (2954	Campylobacterales (68525)	Epsilonproteobacteria (122	Proteobacteria (2)	Bacteria (1)	100.00	0.00	99.60	99.30	WGS
195.415	0.116	Campylobacter coli (72294)	Campylobacter (213849)	Campylobacteraceae (2954	Campylobacterales (68525)	Epsilonproteobacteria (122	Proteobacteria (2)	Bacteria (1)	100.00	0.00	99.60	99.50	WGS
195.416	0.116	Campylobacter coli (72294)	Campylobacter (213849)	Campylobacteraceae (2954	Campylobacterales (68525)	Epsilonproteobacteria (122	Proteobacteria (2)	Bacteria (1)	100.00	0.00	99.80	99.70	WGS
195.417	0.1132	Campylobacter coli (72294)	Campylobacter (213849)	Campylobacteraceae (2954	Campylobacterales (68525)	Epsilonproteobacteria (122	Proteobacteria (2)	Bacteria (1)	100.00	1.20	99.70	99.30	WGS
195.418	0.1126	Campylobacter coli (72294)	Campylobacter (213849)	Campylobacteraceae (2954	Campylobacterales (68525)	Epsilonproteobacteria (122	Proteobacteria (2)	Bacteria (1)	100.00	0.00	99.60	99.60	WGS
195.419	0.1112	Campylobacter coli (72294)	Campylobacter (213849)	Campylobacteraceae (2954	Campylobacterales (68525)	Epsilonproteobacteria (122	Proteobacteria (2)	Bacteria (1)	100.00	0.00	99.90	99.70	WGS
195.420	0.1144	Campylobacter coli (72294)	Campylobacter (213849)	Campylobacteraceae (2954	Campylobacterales (68525)	Epsilonproteobacteria (122	Proteobacteria (2)	Bacteria (1)	100.00	1.20	99.60	99.40	WGS
195.421	0.11	Campylobacter coli (72294)	Campylobacter (213849)	Campylobacteraceae (2954	Campylobacterales (68525)	Epsilonproteobacteria (122	Proteobacteria (2)	Bacteria (1)	100.00	2.40	99.80	99.30	WGS
195.422	0.116	Campylobacter coli (72294)	Campylobacter (213849)	Campylobacteraceae (2954	Campylobacterales (68525)	Epsilonproteobacteria (122	Proteobacteria (2)	Bacteria (1)	100.00	0.00	99.60	99.50	WGS
195.423	0.1172	Campylobacter coli (72294)	Campylobacter (213849)	Campylobacteraceae (2954	Campylobacterales (68525)	Epsilonproteobacteria (122	Proteobacteria (2)	Bacteria (1)	100.00	0.00	99.60	99.30	WGS
195.424	0.1172	Campylobacter coli (72294)	Campylobacter (213849)	Campylobacteraceae (2954	Campylobacterales (68525)	Epsilonproteobacteria (122	Proteobacteria (2)	Bacteria (1)	100.00	0.60	99.60	99.60	WGS
195.425	0.1132	Campylobacter coli (72294)	Campylobacter (213849)	Campylobacteraceae (2954	Campylobacterales (68525)	Epsilonproteobacteria (122	Proteobacteria (2)	Bacteria (1)	100.00	0.00	99.60	99.40	WGS
195.426	0.1112	Campylobacter coli (72294)	Campylobacter (213849)	Campylobacteraceae (2954	Campylobacterales (68525)	Epsilonproteobacteria (122	Proteobacteria (2)	Bacteria (1)	100.00	3.00	99.80	99.40	WGS
195.429	0.1172	Campylobacter coli (72294)	Campylobacter (213849)	Campylobacteraceae (2954	Campylobacterales (68525)	Epsilonproteobacteria (122	Proteobacteria (2)	Bacteria (1)	100.00	0.00	99.50	99.30	Complete
195.430	0.11	Campylobacter coli (72294)	Campylobacter (213849)	Campylobacteraceae (2954	Campylobacterales (68525)	Epsilonproteobacteria (122	Proteobacteria (2)	Bacteria (1)	100.00	0.00	99.60	99.60	WGS
195.431	0.11	Campylobacter coli (72294)	Campylobacter (213849)	Campylobacteraceae (2954	Campylobacterales (68525)	Epsilonproteobacteria (122	Proteobacteria (2)	Bacteria (1)	100.00	0.00	99.60	99.60	WGS
195.432	0.1128	Campylobacter coli (72294)	Campylobacter (213849)	Campylobacteraceae (2954	Campylobacterales (68525)	Epsilonproteobacteria (122	Proteobacteria (2)	Bacteria (1)	100.00	0.00	99.60	99.60	WGS
195.433	0.11	Campylobacter coli (72294)	Campylobacter (213849)	Campylobacteraceae (2954	Campylobacterales (68525)	Epsilonproteobacteria (122	Proteobacteria (2)	Bacteria (1)	100.00	0.00	99.60	99.60	WGS
195.434	0.1136	Campylobacter coli (72294)	Campylobacter (213849)	Campylobacteraceae (2954	Campylobacterales (68525)	Epsilonproteobacteria (122	Proteobacteria (2)	Bacteria (1)	100.00	0.00	99.50	99.50	WGS
195.436	0.116	Campylobacter coli (72294)	Campylobacter (213849)	Campylobacteraceae (2954	Campylobacterales (68525)	Epsilonproteobacteria (122	Proteobacteria (2)	Bacteria (1)	100.00	0.00	99.60	99.60	WGS
195.437	0.1078	Campylobacter coli (72294)	Campylobacter (213849)	Campylobacteraceae (2954	Campylobacterales (68525)	Epsilonproteobacteria (122	Proteobacteria (2)	Bacteria (1)	100.00	0.00	99.60	99.60	WGS
195.438	0.11	Campylobacter coli (72294)	Campylobacter (213849)	Campylobacteraceae (2954	Campylobacterales (68525)	Epsilonproteobacteria (122	Proteobacteria (2)	Bacteria (1)	100.00	0.00	99.60	99.60	WGS
195.439	0.11	Campylobacter coli (72294)	Campylobacter (213849)	Campylobacteraceae (2954	Campylobacterales (68525)	Epsilonproteobacteria (122	Proteobacteria (2)	Bacteria (1)	100.00	0.00	99.60	99.60	WGS
195.440	0.116	Campylobacter coli (72294)	Campylobacter (213849)	Campylobacteraceae (2954	Campylobacterales (68525)	Epsilonproteobacteria (122	Proteobacteria (2)	Bacteria (1)	100.00	0.00	99.50	99.30	WGS
195.442	0.112	Campylobacter coli (72294)	Campylobacter (213849)	Campylobacteraceae (2954	Campylobacterales (68525)	Epsilonproteobacteria (122	Proteobacteria (2)	Bacteria (1)	100.00	0.00	99.60	99.60	WGS
195.444	0.1078	Campylobacter coli (72294)	Campylobacter (213849)	Campylobacteraceae (2954	Campylobacterales (68525)	Epsilonproteobacteria (122	Proteobacteria (2)	Bacteria (1)	100.00	0.00	99.70	99.70	WGS
195.445	0.1172	Campylobacter coli (72294)	Campylobacter (213849)	Campylobacteraceae (2954	Campylobacterales (68525)	Epsilonproteobacteria (122	Proteobacteria (2)	Bacteria (1)	100.00	0.00	99.60	99.50	WGS
195.446	0.1112	Campylobacter coli (72294)	Campylobacter (213849)	Campylobacteraceae (2954	Campylobacterales (68525)	Epsilonproteobacteria (122	Proteobacteria (2)	Bacteria (1)	100.00	0.00	99.60	99.40	WGS
195.447	0.1096	Campylobacter coli (72294)	Campylobacter (213849)	Campylobacteraceae (2954	Campylobacterales (68525)	Epsilonproteobacteria (122	Proteobacteria (2)	Bacteria (1)	100.00	0.00	99.50	99.40	WGS
195.448	0.11	Campylobacter coli (72294)	Campylobacter (213849)	Campylobacteraceae (2954	Campylobacterales (68525)	Epsilonproteobacteria (122	Proteobacteria (2)	Bacteria (1)	100.00	0.00	99.60	99.60	WGS
195.450	0.11	Campylobacter coli (72294)	Campylobacter (213849)	Campylobacteraceae (2954	Campylobacterales (68525)	Epsilonproteobacteria (122	Proteobacteria (2)	Bacteria (1)	100.00	0.00	99.60	99.60	WGS
195.451	0.11	Campylobacter coli (72294)	Campylobacter (213849)	Campylobacteraceae (2954	Campylobacterales (68525)	Epsilonproteobacteria (122	Proteobacteria (2)	Bacteria (1)	100.00	0.00	99.60	99.60	WGS
195.452	0.11	Campylobacter coli (72294)	Campylobacter (213849)	Campylobacteraceae (2954	Campylobacterales (68525)	Epsilonproteobacteria (122	Proteobacteria (2)	Bacteria (1)	100.00	0.00	99.60	99.60	WGS
195.454	0.11	Campylobacter coli (72294)	Campylobacter (213849)	Campylobacteraceae (2954	Campylobacterales (68525)	Epsilonproteobacteria (122	Proteobacteria (2)	Bacteria (1)	100.00	0.00	99.60	99.60	WGS
195.455	0.1172	Campylobacter coli (72294)	Campylobacter (213849)	Campylobacteraceae (2954	Campylobacterales (68525)	Epsilonproteobacteria (122	Proteobacteria (2)	Bacteria (1)	100.00	0.00	99.80	99.60	WGS
195.456	0.11	Campylobacter coli (72294)	Campylobacter (213849)	Campylobacteraceae (2954	Campylobacterales (68525)	Epsilonproteobacteria (122	Proteobacteria (2)	Bacteria (1)	100.00	0.00	99.60	99.50	WGS
195.457	0.1112	Campylobacter coli (72294)	Campylobacter (213849)	Campylobacteraceae (2954	Campylobacterales (68525)	Epsilonproteobacteria (122	Proteobacteria (2)	Bacteria (1)	100.00	0.00	99.60	99.30	WGS
195.458	0.11	Campylobacter coli (72294)	Campylobacter (213849)	Campylobacteraceae (2954	Campylobacterales (68525)	Epsilonproteobacteria (122	Proteobacteria (2)	Bacteria (1)	100.00	0.00	99.60	99.50	WGS
195.459	0.1132	Campylobacter coli (72294)	Campylobacter (213849)	Campylobacteraceae (2954	Campylobacterales (68525)	Epsilonproteobacteria (122	Proteobacteria (2)	Bacteria (1)	100.00	0.00	99.60	99.60	WGS
195.461	0.116	Campylobacter coli (72294)	Campylobacter (213849)	Campylobacteraceae (2954	Campylobacterales (68525)	Epsilonproteobacteria (122	Proteobacteria (2)	Bacteria (1)	100.00	0.60	99.60	99.60	WGS
195.462	0.1078	Campylobacter coli (72294)	Campylobacter (213849)	Campylobacteraceae (2954	Campylobacterales (68525)	Epsilonproteobacteria (122	Proteobacteria (2)	Bacteria (1)	100.00	0.00	99.60	99.50	Complete
195.463	0.1132	Campylobacter coli (72294)	Campylobacter (213849)	Campylobacteraceae (2954	Campylobacterales (68525)	Epsilonproteobacteria (122	Proteobacteria (2)	Bacteria (1)	100.00	0.00	99.60	99.60	WGS
195.464	0.11	Campylobacter coli (72294)	Campylobacter (213849)	Campylobacteraceae (2954	Campylobacterales (68525)	Epsilonproteobacteria (122	Proteobacteria (2)	Bacteria (1)	100.00	0.00	99.60	99.60	WGS
195.465	0.11	Campylobacter coli (72294)	Campylobacter (213849)	Campylobacteraceae (2954	Campylobacterales (68525)	Epsilonproteobacteria (122	Proteobacteria (2)	Bacteria (1)	100.00	0.00	99.60	99.60	WGS
195.466	0.11	Campylobacter coli (72294)	Campylobacter (213849)	Campylobacteraceae (2954	Campylobacterales (68525)	Epsilonproteobacteria (122	Proteobacteria (2)	Bacteria (1)	100.00	0.00	99.60	99.60	WGS
195.467	0.1098	Campylobacter coli (72294)	Campylobacter (213849)	Campylobacteraceae (2954	Campylobacterales (68525)	Epsilonproteobacteria (122	Proteobacteria (2)	Bacteria (1)	100.00	0.00	99.60	99.60	WGS
195.468	0.1144	Campylobacter coli (72294)	Campylobacter (213849)	Campylobacteraceae (2954	Campylobacterales (68525)	Epsilonproteobacteria (122	Proteobacteria (2)	Bacteria (1)	100.00	0.00	99.40	99.30	WGS
195.469	0.11	Campylobacter coli (72294)	Campylobacter (213849)	Campylobacteraceae (2954	Campylobacterales (68525)	Epsilonproteobacteria (122	Proteobacteria (2)	Bacteria (1)	100.00	0.00	99.60	99.60	WGS
195.470	0.11	Campylobacter coli (72294)	Campylobacter (213849)	Campylobacteraceae (2954	Campylobacterales (68525)	Epsilonproteobacteria (122	Proteobacteria (2)	Bacteria (1)	100.00	0.00	99.60	99.60	WGS
195.471	0.1142	Campylobacter coli (72294)	Campylobacter (213849)	Campylobacteraceae (2954	Campylobacterales (68525)	Epsilonproteobacteria (122	Proteobacteria (2)	Bacteria (1)	100.00	0.00	99.60	99.60	WGS
195.472	0.1128	Campylobacter coli (72294)	Campylobacter (213849)	Campylobacteraceae (2954	Campylobacterales (68525)	Epsilonproteobacteria (122	Proteobacteria (2)	Bacteria (1)	100.00	0.00	99.60	99.60	WGS
195.473	0.1112	Campylobacter coli (72294)	Campylobacter (213849)	Campylobacteraceae (2954	Campylobacterales (68525)	Epsilonproteobacteria (122	Proteobacteria (2)	Bacteria (1)	100.00	0.60	99.60	99.60	WGS
195.474	0.1146	Campylobacter coli (72294)	Campylobacter (213849)	Campylobacteraceae (2954	Campylobacterales (68525)	Epsilonproteobacteria (122	Proteobacteria (2)	Bacteria (1)	100.00	0.00	99.40	99.30	WGS
195.475	0.117	Campylobacter coli (72294)	Campylobacter (213849)	Campylobacteraceae (2954	Campylobacterales (68525)	Epsilonproteobacteria (122	Proteobacteria (2)	Bacteria (1)	100.00	2.70	99.60	98.70	WGS
195.476	0.112	Campylobacter coli (72294)	Campylobacter (213849)	Campylobacteraceae (2954	Campylobacterales (68525)	Epsilonproteobacteria (122	Proteobacteria (2)	Bacteria (1)	100.00	0.00	99.50	99.40	WGS
195.477	0.1096	Campylobacter coli (72294)	Campylobacter (213849)	Campylobacteraceae (2954	Campylobacterales (68525)	Epsilonproteobacteria (122	Proteobacteria (2)	Bacteria (1)	100.00	0.00	99.70	99.70	WGS
195.478	0.112	Campylobacter coli (72294)	Campylobacter (213849)	Campylobacteraceae (2954	Campylobacterales (68525)	Epsilonproteobacteria (122	Proteobacteria (2)	Bacteria (1)	100.00	0.00	99.60	99.60	WGS
195.479	0.11	Campylobacter coli (72294)	Campylobacter (213849)	Campylobacteraceae (2954	Campylobacterales (68525)	Epsilonproteobacteria (122	Proteobacteria (2)	Bacteria (1)	100.00	0.00	99.60	99.60	WGS
195.480	0.11	Campylobacter coli (72294)	Campylobacter (213849)	Campylobacteraceae (2954	Campylobacterales (68525)	Epsilonproteobacteria (122	Proteobacteria (2)	Bacteria (1)	100.00	0.00	99.80	99.80	WGS
195.481	0.1096	Campylobacter coli (72294)	Campylobacter (213849)	Campylobacteraceae (2954	Campylobacterales (68525)	Epsilonproteobacteria (122	Proteobacteria (2)	Bacteria (1)	100.00	0.00	99.60	99.60	WGS
195.482	0.1132	Campylobacter coli (72294)	Campylobacter (213849)	Campylobacteraceae (2954	Campylobacterales (68525)	Epsilonproteobacteria (122	Proteobacteria (2)	Bacteria (1)	100.00	0.00	99.60	99.60	WGS
195.483	0.11	Campylobacter coli (72294)	Campylobacter (213849)	Campylobacteraceae (2954	Campylobacterales (68525)	Epsilonproteobacteria (122	Proteobacteria (2)	Bacteria (1)	100.00	0.00	99.60	99.60	WGS
195.484	0.11	Campylobacter coli (72294)	Campylobacter (213849)	Campylobacteraceae (2954	Campylobacterales (68525)	Epsilonproteobacteria (122	Proteobacteria (2)	Bacteria (1)	100.00	0.00	99.60	99.60	WGS
195.485	0.1132	Campylobacter coli (72294)	Campylobacter (213849)	Campylobacteraceae (2954	Campylobacterales (68525)	Epsilonproteobacteria (122	Proteobacteria (2)	Bacteria (1)	100.00	1.20	99.80	99.80	WGS
195.486	0.11	Campylobacter coli (72294)	Campylobacter (213849)	Campylobacteraceae (2954	Campylobacterales (68525)	Epsilonproteobacteria (122	Proteobacteria (2)	Bacteria (1)	100.00	0.00	99.60	99.60	WGS
195.487	0.11	Campylobacter coli (72294)	Campylobacter (213849)	Campylobacteraceae (2954	Campylobacterales (68525)	Epsilonproteobacteria (122	Proteobacteria (2)	Bacteria (1)	100.00	0.00	99.60	99.60	WGS
195.488	0.11	Campylobacter coli (72294)	Campylobacter (213849)	Campylobacteraceae (2954	Campylobacterales (68525)	Epsilonproteobacteria (122	Proteobacteria (2)	Bacteria (1)	100.00	0.00	99.60	99.60	WGS
195.489	0.1118	Campylobacter coli (72294)	Campylobacter (213849)	Campylobacteraceae (2954	Campylobacterales (68525)	Epsilonproteobacteria (122	Proteobacteria (2)	Bacteria (1)	100.00	0.00	99.50	99.30	WGS
195.490	0.1138	Campylobacter coli (72294)	Campylobacter (213849)	Campylobacteraceae (2954	Campylobacterales (68525)	Epsilonproteobacteria (122	Proteobacteria (2)	Bacteria (1)	100.00	0.00	99.50	99.50	WGS
195.491	0.116	Campylobacter coli (72294)	Campylobacter (213849)	Campylobacteraceae (2954	Campylobacterales (68525)	Epsilonproteobacteria (122	Proteobacteria (2)	Bacteria (1)	100.00	0.60	99.60	99.60	WGS
195.492	0.11	Campylobacter coli (72294)	Campylobacter (213849)	Campylobacteraceae (2954	Campylobacterales (68525)	Epsilonproteobacteria (122	Proteobacteria (2)	Bacteria (1)	100.00	0.00	99.60	99.60	WGS
195.493	0.1132	Campylobacter coli (72294)	Campylobacter (213849)	Campylobacteraceae (2954	Campylobacterales (68525)	Epsilonproteobacteria (122	Proteobacteria (2)	Bacteria (1)	100.00	0.00	99.40	99.30	WGS
195.494	0.11	Campylobacter coli (72294)	Campylobacter (213849)	Campylobacteraceae (2954	Campylobacterales (68525)	Epsilonproteobacteria (122	Proteobacteria (2)	Bacteria (1)	100.00	0.00	99.80	99.70	WGS
195.495	0.1132	Campylobacter coli (72294)	Campylobacter (213849)	Campylobacteraceae (2954	Campylobacterales (68525)	Epsilonproteobacteria (122	Proteobacteria (2)	Bacteria (1)	100.00	0.00	99.60	99.60	WGS
195.496	0.1132	Campylobacter coli (72294)	Campylobacter (213849)	Campylobacteraceae (2954	Campylobacterales (68525)	Epsilonproteobacteria (122	Proteobacteria (2)	Bacteria (1)	100.00	0.00	99.60	99.60	WGS
195.497	0.1132	Campylobacter coli (72294)	Campylobacter (213849)	Campylobacteraceae (2954	Campylobacterales (68525)	Epsilonproteobacteria (122	Proteobacteria (2)	Bacteria (1)	100.00	0.00	99.60	99.60	WGS
195.498	0.1078	Campylobacter coli (72294)	Campylobacter (213849)	Campylobacteraceae (2954	Campylobacterales (68525)	Epsilonproteobacteria (122	Proteobacteria (2)	Bacteria (1)	100.00	0.00	99.40	99.30	WGS
195.499	0.1098	Campylobacter coli (72294)	Campylobacter (213849)	Campylobacteraceae (2954	Campylobacterales (68525)	Epsilonproteobacteria (122	Proteobacteria (2)	Bacteria (1)	100.00	0.00	99.70	99.60	WGS
195.500	0.1078	Campylobacter coli (72294)	Campylobacter (213849)	Campylobacteraceae (2954	Campylobacterales (68525)	Epsilonproteobacteria (122	Proteobacteria (2)	Bacteria (1)	100.00	0.00	99.70	99.70	WGS
195.501	0.11	Campylobacter coli (72294)	Campylobacter (213849)	Campylobacteraceae (2954	Campylobacterales (68525)	Epsilonproteobacteria (122	Proteobacteria (2)	Bacteria (1)	100.00	0.00	99.60	99.60	WGS
195.502	0.1132	Campylobacter coli (72294)	Campylobacter (213849)	Campylobacteraceae (2954	Campylobacterales (68525)	Epsilonproteobacteria (122	Proteobacteria (2)	Bacteria (1)	100.00	0.00	99.60	99.60	WGS
195.503	0.11	Campylobacter coli (72294)	Campylobacter (213849)	Campylobacteraceae (2954	Campylobacterales (68525)	Epsilonproteobacteria (122	Proteobacteria (2)	Bacteria (1)	100.00	0.00	99.60	99.60	WGS
195.504	0.1098	Campylobacter coli (72294)	Campylobacter (213849)	Campylobacteraceae (2954	Campylobacterales (68525)	Epsilonproteobacteria (122	Proteobacteria (2)	Bacteria (1)	100.00	0.00	99.30	99.30	WGS
195.505	0.1152	Campylobacter coli (72294)	Campylobacter (213849)	Campylobacteraceae (2954	Campylobacterales (68525)	Epsilonproteobacteria (122	Proteobacteria (2)	Bacteria (1)	100.00	0.00	99.60	99.50	WGS
195.506	0.1118	Campylobacter coli (72294)	Campylobacter (213849)	Campylobacteraceae (2954	Campylobacterales (68525)	Epsilonproteobacteria (122	Proteobacteria (2)	Bacteria (1)	100.00	0.00	99.60	99.50	WGS
195.507	0.11	Campylobacter coli (72294)	Campylobacter (213849)	Campylobacteraceae (2954	Campylobacterales (68525)	Epsilonproteobacteria (122	Proteobacteria (2)	Bacteria (1)	100.00	0.00	99.60	99.60	WGS
195.508	0.1172	Campylobacter coli (72294)	Campylobacter (213849)	Campylobacteraceae (2954	Campylobacterales (68525)	Epsilonproteobacteria (122	Proteobacteria (2)	Bacteria (1)	100.00	0.00	99.60	99.50	WGS
195.509	0.11	Campylobacter coli (72294)	Campylobacter (213849)	Campylobacteraceae (2954	Campylobacterales (68525)	Epsilonproteobacteria (122	Proteobacteria (2)	Bacteria (1)	100.00	0.00	99.60	99.60	WGS
195.510	0.11	Campylobacter coli (72294)	Campylobacter (213849)	Campylobacteraceae (2954	Campylobacterales (68525)	Epsilonproteobacteria (122	Proteobacteria (2)	Bacteria (1)	100.00	0.00	99.60	99.60	WGS
195.511	0.1124	Campylobacter coli (72294)	Campylobacter (213849)	Campylobacteraceae (2954	Campylobacterales (68525)	Epsilonproteobacteria (122	Proteobacteria (2)	Bacteria (1)	100.00	0.00	99.50	99.50	WGS
195.512	0.1132	Campylobacter coli (72294)	Campylobacter (213849)	Campylobacteraceae (2954	Campylobacterales (68525)	Epsilonproteobacteria (122	Proteobacteria (2)	Bacteria (1)	100.00	0.00	99.60	99.60	WGS
195.514	0.1128	Campylobacter coli (72294)	Campylobacter (213849)	Campylobacteraceae (2954	Campylobacterales (68525)	Epsilonproteobacteria (122	Proteobacteria (2)	Bacteria (1)	100.00	0.00	99.60	99.60	WGS
195.515	0.1132	Campylobacter coli (72294)	Campylobacter (213849)	Campylobacteraceae (2954	Campylobacterales (68525)	Epsilonproteobacteria (122	Proteobacteria (2)	Bacteria (1)	100.00	0.60	99.40	99.30	WGS
195.516	0.11	Campylobacter coli (72294)	Campylobacter (213849)	Campylobacteraceae (2954	Campylobacterales (68525)	Epsilonproteobacteria (122	Proteobacteria (2)	Bacteria (1)	100.00	0.00	99.60	99.60	WGS
195.517	0.1172	Campylobacter coli (72294)	Campylobacter (213849)	Campylobacteraceae (2954	Campylobacterales (68525)	Epsilonproteobacteria (122	Proteobacteria (2)	Bacteria (1)	100.00	0.00	99.60	99.50	WGS
195.519	0.11	Campylobacter coli (72294)	Campylobacter (213849)	Campylobacteraceae (2954	Campylobacterales (68525)	Epsilonproteobacteria (122	Proteobacteria (2)	Bacteria (1)	100.00	0.60	99.60	99.60	WGS
195.520	0.11	Campylobacter coli (72294)	Campylobacter (213849)	Campylobacteraceae (2954	Campylobacterales (68525)	Epsilonproteobacteria (122	Proteobacteria (2)	Bacteria (1)	100.00	0.00	99.60	99.60	WGS
195.522	0.11	Campylobacter coli (72294)	Campylobacter (213849)	Campylobacteraceae (2954	Campylobacterales (68525)	Epsilonproteobacteria (122	Proteobacteria (2)	Bacteria (1)	100.00	0.60	99.60	99.60	WGS
195.524	0.11	Campylobacter coli (72294)	Campylobacter (213849)	Campylobacteraceae (2954	Campylobacterales (68525)	Epsilonproteobacteria (122	Proteobacteria (2)	Bacteria (1)	100.00	0.00	99.60	99.60	WGS
195.525	0.1108	Campylobacter coli (72294)	Campylobacter (213849)	Campylobacteraceae (2954	Campylobacterales (68525)	Epsilonproteobacteria (122	Proteobacteria (2)	Bacteria (1)	100.00	0.00	99.60	99.60	WGS
195.526	0.1128	Campylobacter coli (72294)	Campylobacter (213849)	Campylobacteraceae (2954	Campylobacterales (68525)	Epsilonproteobacteria (122	Proteobacteria (2)	Bacteria (1)	100.00	0.00	99.50	99.40	WGS
195.527	0.11	Campylobacter coli (72294)	Campylobacter (213849)	Campylobacteraceae (2954	Campylobacterales (68525)	Epsilonproteobacteria (122	Proteobacteria (2)	Bacteria (1)	100.00	0.00	99.60	99.60	WGS
195.528	0.11	Campylobacter coli (72294)	Campylobacter (213849)	Campylobacteraceae (2954	Campylobacterales (68525)	Epsilonproteobacteria (122	Proteobacteria (2)	Bacteria (1)	100.00	0.00	99.60	99.60	WGS
195.529	0.1128	Campylobacter coli (72294)	Campylobacter (213849)	Campylobacteraceae (2954	Campylobacterales (68525)	Epsilonproteobacteria (122	Proteobacteria (2)	Bacteria (1)	100.00	0.00	99.60	99.50	WGS
195.530	0.1172	Campylobacter coli (72294)	Campylobacter (213849)	Campylobacteraceae (2954	Campylobacterales (68525)	Epsilonproteobacteria (122	Proteobacteria (2)	Bacteria (1)	100.00	0.00	99.80	99.60	WGS
195.531	0.11	Campylobacter coli (72294)	Campylobacter (213849)	Campylobacteraceae (2954	Campylobacterales (68525)	Epsilonproteobacteria (122	Proteobacteria (2)	Bacteria (1)	100.00	0.00	99.60	99.60	WGS
195.533	0.11	Campylobacter coli (72294)	Campylobacter (213849)	Campylobacteraceae (2954	Campylobacterales (68525)	Epsilonproteobacteria (122	Proteobacteria (2)	Bacteria (1)	100.00	0.00	99.60	99.50	WGS
195.534	0.1172	Campylobacter coli (72294)	Campylobacter (213849)	Campylobacteraceae (2954	Campylobacterales (68525)	Epsilonproteobacteria (122	Proteobacteria (2)	Bacteria (1)	100.00	0.00	99.60	99.50	WGS
195.535	0.1172	Campylobacter coli (72294)	Campylobacter (213849)	Campylobacteraceae (2954	Campylobacterales (68525)	Epsilonproteobacteria (122	Proteobacteria (2)	Bacteria (1)	100.00	14.10	99.80	94.80	WGS
195.536	0.1096	Campylobacter coli (72294)	Campylobacter (213849)	Campylobacteraceae (2954	Campylobacterales (68525)	Epsilonproteobacteria (122	Proteobacteria (2)	Bacteria (1)	100.00	0.00	99.60	99.60	WGS
195.537	0.11	Campylobacter coli (72294)	Campylobacter (213849)	Campylobacteraceae (2954	Campylobacterales (68525)	Epsilonproteobacteria (122	Proteobacteria (2)	Bacteria (1)	100.00	0.00	99.60	99.60	WGS
195.538	0.1132	Campylobacter coli (72294)	Campylobacter (213849)	Campylobacteraceae (2954	Campylobacterales (68525)	Epsilonproteobacteria (122	Proteobacteria (2)	Bacteria (1)	100.00	0.00	99.60	99.60	WGS
195.539	0.1172	Campylobacter coli (72294)	Campylobacter (213849)	Campylobacteraceae (2954	Campylobacterales (68525)	Epsilonproteobacteria (122	Proteobacteria (2)	Bacteria (1)	100.00	0.00	99.60	99.50	WGS
195.541	0.1118	Campylobacter coli (72294)	Campylobacter (213849)	Campylobacteraceae (2954	Campylobacterales (68525)	Epsilonproteobacteria (122	Proteobacteria (2)	Bacteria (1)	100.00	0.00	99.50	99.50	WGS
195.543	0.11	Campylobacter coli (72294)	Campylobacter (213849)	Campylobacteraceae (2954	Campylobacterales (68525)	Epsilonproteobacteria (122	Proteobacteria (2)	Bacteria (1)	100.00	0.00	99.60	99.60	WGS
195.544	0.11	Campylobacter coli (72294)	Campylobacter (213849)	Campylobacteraceae (2954	Campylobacterales (68525)	Epsilonproteobacteria (122	Proteobacteria (2)	Bacteria (1)	100.00	0.00	99.60	99.60	WGS
195.545	0.112	Campylobacter coli (72294)	Campylobacter (213849)	Campylobacteraceae (2954	Campylobacterales (68525)	Epsilonproteobacteria (122	Proteobacteria (2)	Bacteria (1)	100.00	0.00	99.60	99.50	WGS
195.546	0.1132	Campylobacter coli (72294)	Campylobacter (213849)	Campylobacteraceae (2954	Campylobacterales (68525)	Epsilonproteobacteria (122	Proteobacteria (2)	Bacteria (1)	100.00	0.00	99.60	99.60	WGS
195.547	0.11	Campylobacter coli (72294)	Campylobacter (213849)	Campylobacteraceae (2954	Campylobacterales (68525)	Epsilonproteobacteria (122	Proteobacteria (2)	Bacteria (1)	100.00	0.00	99.60	99.60	WGS
195.548	0.1144	Campylobacter coli (72294)	Campylobacter (213849)	Campylobacteraceae (2954	Campylobacterales (68525)	Epsilonproteobacteria (122	Proteobacteria (2)	Bacteria (1)	100.00	0.00	99.60	99.40	WGS
195.549	0.11	Campylobacter coli (72294)	Campylobacter (213849)	Campylobacteraceae (2954	Campylobacterales (68525)	Epsilonproteobacteria (122	Proteobacteria (2)	Bacteria (1)	100.00	0.00	99.60	99.60	WGS
195.550	0.11	Campylobacter coli (72294)	Campylobacter (213849)	Campylobacteraceae (2954	Campylobacterales (68525)	Epsilonproteobacteria (122	Proteobacteria (2)	Bacteria (1)	100.00	0.00	99.60	99.60	WGS
195.553	0.1112	Campylobacter coli (72294)	Campylobacter (213849)	Campylobacteraceae (2954	Campylobacterales (68525)	Epsilonproteobacteria (122	Proteobacteria (2)	Bacteria (1)	100.00	0.00	99.60	99.60	WGS
195.554	0.116	Campylobacter coli (72294)	Campylobacter (213849)	Campylobacteraceae (2954	Campylobacterales (68525)	Epsilonproteobacteria (122	Proteobacteria (2)	Bacteria (1)	100.00	0.60	99.60	99.60	WGS
195.555	0.11	Campylobacter coli (72294)	Campylobacter (213849)	Campylobacteraceae (2954	Campylobacterales (68525)	Epsilonproteobacteria (122	Proteobacteria (2)	Bacteria (1)	100.00	0.00	99.60	99.60	WGS
195.556	0.1118	Campylobacter coli (72294)	Campylobacter (213849)	Campylobacteraceae (2954	Campylobacterales (68525)	Epsilonproteobacteria (122	Proteobacteria (2)	Bacteria (1)	100.00	0.00	99.50	99.50	WGS
195.557	0.11	Campylobacter coli (72294)	Campylobacter (213849)	Campylobacteraceae (2954	Campylobacterales (68525)	Epsilonproteobacteria (122	Proteobacteria (2)	Bacteria (1)	100.00	0.00	99.60	99.60	WGS
195.558	0.116	Campylobacter coli (72294)	Campylobacter (213849)	Campylobacteraceae (2954	Campylobacterales (68525)	Epsilonproteobacteria (122	Proteobacteria (2)	Bacteria (1)	100.00	0.60	99.60	99.50	WGS
195.559	0.11	Campylobacter coli (72294)	Campylobacter (213849)	Campylobacteraceae (2954	Campylobacterales (68525)	Epsilonproteobacteria (122	Proteobacteria (2)	Bacteria (1)	100.00	0.00	99.60	99.60	WGS
195.560	0.1096	Campylobacter coli (72294)	Campylobacter (213849)	Campylobacteraceae (2954	Campylobacterales (68525)	Epsilonproteobacteria (122	Proteobacteria (2)	Bacteria (1)	99.40	0.00	99.50	99.50	WGS
195.561	0.1132	Campylobacter coli (72294)	Campylobacter (213849)	Campylobacteraceae (2954	Campylobacterales (68525)	Epsilonproteobacteria (122	Proteobacteria (2)	Bacteria (1)	100.00	0.00	99.50	99.40	WGS
195.562	0.11	Campylobacter coli (72294)	Campylobacter (213849)	Campylobacteraceae (2954	Campylobacterales (68525)	Epsilonproteobacteria (122	Proteobacteria (2)	Bacteria (1)	100.00	0.00	99.60	99.60	WGS
195.564	0.11	Campylobacter coli (72294)	Campylobacter (213849)	Campylobacteraceae (2954	Campylobacterales (68525)	Epsilonproteobacteria (122	Proteobacteria (2)	Bacteria (1)	100.00	0.00	99.60	99.60	WGS
195.565	0.1142	Campylobacter coli (72294)	Campylobacter (213849)	Campylobacteraceae (2954	Campylobacterales (68525)	Epsilonproteobacteria (122	Proteobacteria (2)	Bacteria (1)	100.00	0.00	99.60	99.60	WGS
195.567	0.11	Campylobacter coli (72294)	Campylobacter (213849)	Campylobacteraceae (2954	Campylobacterales (68525)	Epsilonproteobacteria (122	Proteobacteria (2)	Bacteria (1)	100.00	0.00	99.60	99.60	WGS
195.569	0.11	Campylobacter coli (72294)	Campylobacter (213849)	Campylobacteraceae (2954	Campylobacterales (68525)	Epsilonproteobacteria (122	Proteobacteria (2)	Bacteria (1)	100.00	0.00	99.60	99.60	WGS
195.570	0.11	Campylobacter coli (72294)	Campylobacter (213849)	Campylobacteraceae (2954	Campylobacterales (68525)	Epsilonproteobacteria (122	Proteobacteria (2)	Bacteria (1)	100.00	0.00	99.80	99.80	WGS
195.571	0.11	Campylobacter coli (72294)	Campylobacter (213849)	Campylobacteraceae (2954	Campylobacterales (68525)	Epsilonproteobacteria (122	Proteobacteria (2)	Bacteria (1)	100.00	0.00	99.50	99.50	WGS
195.572	0.1128	Campylobacter coli (72294)	Campylobacter (213849)	Campylobacteraceae (2954	Campylobacterales (68525)	Epsilonproteobacteria (122	Proteobacteria (2)	Bacteria (1)	100.00	0.00	99.60	99.60	WGS
195.574	0.11	Campylobacter coli (72294)	Campylobacter (213849)	Campylobacteraceae (2954	Campylobacterales (68525)	Epsilonproteobacteria (122	Proteobacteria (2)	Bacteria (1)	100.00	0.00	99.60	99.60	WGS
195.575	0.1078	Campylobacter coli (72294)	Campylobacter (213849)	Campylobacteraceae (2954	Campylobacterales (68525)	Epsilonproteobacteria (122	Proteobacteria (2)	Bacteria (1)	100.00	0.00	99.70	99.60	WGS
195.576	0.1132	Campylobacter coli (72294)	Campylobacter (213849)	Campylobacteraceae (2954	Campylobacterales (68525)	Epsilonproteobacteria (122	Proteobacteria (2)	Bacteria (1)	100.00	0.00	99.40	99.30	WGS
195.577	0.11	Campylobacter coli (72294)	Campylobacter (213849)	Campylobacteraceae (2954	Campylobacterales (68525)	Epsilonproteobacteria (122	Proteobacteria (2)	Bacteria (1)	100.00	0.00	99.60	99.60	WGS
195.578	0.116	Campylobacter coli (72294)	Campylobacter (213849)	Campylobacteraceae (2954	Campylobacterales (68525)	Epsilonproteobacteria (122	Proteobacteria (2)	Bacteria (1)	100.00	0.00	99.60	99.60	WGS
195.579	0.11	Campylobacter coli (72294)	Campylobacter (213849)	Campylobacteraceae (2954	Campylobacterales (68525)	Epsilonproteobacteria (122	Proteobacteria (2)	Bacteria (1)	100.00	0.00	99.60	99.60	WGS
195.580	0.1238	Campylobacter coli (72294)	Campylobacter (213849)	Campylobacteraceae (2954	Campylobacterales (68525)	Epsilonproteobacteria (122	Proteobacteria (2)	Bacteria (1)	100.00	0.00	99.50	99.40	WGS
195.581	0.11	Campylobacter coli (72294)	Campylobacter (213849)	Campylobacteraceae (2954	Campylobacterales (68525)	Epsilonproteobacteria (122	Proteobacteria (2)	Bacteria (1)	100.00	0.00	99.80	99.60	WGS
195.582	0.1166	Campylobacter coli (72294)	Campylobacter (213849)	Campylobacteraceae (2954	Campylobacterales (68525)	Epsilonproteobacteria (122	Proteobacteria (2)	Bacteria (1)	100.00	0.00	99.60	99.50	WGS
195.583	0.1152	Campylobacter coli (72294)	Campylobacter (213849)	Campylobacteraceae (2954	Campylobacterales (68525)	Epsilonproteobacteria (122	Proteobacteria (2)	Bacteria (1)	100.00	0.00	99.60	99.60	WGS
195.584	0.11	Campylobacter coli (72294)	Campylobacter (213849)	Campylobacteraceae (2954	Campylobacterales (68525)	Epsilonproteobacteria (122	Proteobacteria (2)	Bacteria (1)	100.00	0.00	99.60	99.60	WGS
195.585	0.1126	Campylobacter coli (72294)	Campylobacter (213849)	Campylobacteraceae (2954	Campylobacterales (68525)	Epsilonproteobacteria (122	Proteobacteria (2)	Bacteria (1)	100.00	0.00	99.50	99.40	WGS
195.586	0.1128	Campylobacter coli (72294)	Campylobacter (213849)	Campylobacteraceae (2954	Campylobacterales (68525)	Epsilonproteobacteria (122	Proteobacteria (2)	Bacteria (1)	100.00	0.00	99.60	99.60	WGS
195.587	0.11	Campylobacter coli (72294)	Campylobacter (213849)	Campylobacteraceae (2954	Campylobacterales (68525)	Epsilonproteobacteria (122	Proteobacteria (2)	Bacteria (1)	100.00	0.00	99.60	99.60	WGS
195.588	0.11	Campylobacter coli (72294)	Campylobacter (213849)	Campylobacteraceae (2954	Campylobacterales (68525)	Epsilonproteobacteria (122	Proteobacteria (2)	Bacteria (1)	100.00	0.00	99.60	99.60	WGS
195.589	0.1132	Campylobacter coli (72294)	Campylobacter (213849)	Campylobacteraceae (2954	Campylobacterales (68525)	Epsilonproteobacteria (122	Proteobacteria (2)	Bacteria (1)	100.00	0.00	99.60	99.60	WGS
195.591	0.11	Campylobacter coli (72294)	Campylobacter (213849)	Campylobacteraceae (2954	Campylobacterales (68525)	Epsilonproteobacteria (122	Proteobacteria (2)	Bacteria (1)	100.00	0.00	99.40	99.30	WGS
195.592	0.1132	Campylobacter coli (72294)	Campylobacter (213849)	Campylobacteraceae (2954	Campylobacterales (68525)	Epsilonproteobacteria (122	Proteobacteria (2)	Bacteria (1)	100.00	0.00	99.60	99.40	WGS
195.593	0.1124	Campylobacter coli (72294)	Campylobacter (213849)	Campylobacteraceae (2954	Campylobacterales (68525)	Epsilonproteobacteria (122	Proteobacteria (2)	Bacteria (1)	100.00	0.00	99.30	99.30	WGS
195.594	0.1132	Campylobacter coli (72294)	Campylobacter (213849)	Campylobacteraceae (2954	Campylobacterales (68525)	Epsilonproteobacteria (122	Proteobacteria (2)	Bacteria (1)	100.00	0.00	99.60	99.60	WGS
195.595	0.1132	Campylobacter coli (72294)	Campylobacter (213849)	Campylobacteraceae (2954	Campylobacterales (68525)	Epsilonproteobacteria (122	Proteobacteria (2)	Bacteria (1)	100.00	0.00	99.30	99.20	WGS
195.596	0.11	Campylobacter coli (72294)	Campylobacter (213849)	Campylobacteraceae (2954	Campylobacterales (68525)	Epsilonproteobacteria (122	Proteobacteria (2)	Bacteria (1)	100.00	0.00	99.60	99.60	WGS
195.597	0.1128	Campylobacter coli (72294)	Campylobacter (213849)	Campylobacteraceae (2954	Campylobacterales (68525)	Epsilonproteobacteria (122	Proteobacteria (2)	Bacteria (1)	100.00	0.00	99.60	99.50	WGS
195.598	0.1112	Campylobacter coli (72294)	Campylobacter (213849)	Campylobacteraceae (2954	Campylobacterales (68525)	Epsilonproteobacteria (122	Proteobacteria (2)	Bacteria (1)	100.00	0.00	99.60	99.60	WGS
195.599	0.1132	Campylobacter coli (72294)	Campylobacter (213849)	Campylobacteraceae (2954	Campylobacterales (68525)	Epsilonproteobacteria (122	Proteobacteria (2)	Bacteria (1)	100.00	0.00	99.30	99.20	WGS
195.600	0.11	Campylobacter coli (72294)	Campylobacter (213849)	Campylobacteraceae (2954	Campylobacterales (68525)	Epsilonproteobacteria (122	Proteobacteria (2)	Bacteria (1)	100.00	0.00	99.60	99.60	WGS
195.601	0.1172	Campylobacter coli (72294)	Campylobacter (213849)	Campylobacteraceae (2954	Campylobacterales (68525)	Epsilonproteobacteria (122	Proteobacteria (2)	Bacteria (1)	100.00	0.00	99.80	99.60	WGS
195.602	0.11	Campylobacter coli (72294)	Campylobacter (213849)	Campylobacteraceae (2954	Campylobacterales (68525)	Epsilonproteobacteria (122	Proteobacteria (2)	Bacteria (1)	100.00	0.00	99.60	99.60	WGS
195.603	0.116	Campylobacter coli (72294)	Campylobacter (213849)	Campylobacteraceae (2954	Campylobacterales (68525)	Epsilonproteobacteria (122	Proteobacteria (2)	Bacteria (1)	100.00	0.60	99.80	99.80	WGS
195.605	0.11	Campylobacter coli (72294)	Campylobacter (213849)	Campylobacteraceae (2954	Campylobacterales (68525)	Epsilonproteobacteria (122	Proteobacteria (2)	Bacteria (1)	100.00	0.60	99.60	99.60	WGS
195.607	0.1118	Campylobacter coli (72294)	Campylobacter (213849)	Campylobacteraceae (2954	Campylobacterales (68525)	Epsilonproteobacteria (122	Proteobacteria (2)	Bacteria (1)	100.00	0.00	99.70	99.60	WGS
195.608	0.1132	Campylobacter coli (72294)	Campylobacter (213849)	Campylobacteraceae (2954	Campylobacterales (68525)	Epsilonproteobacteria (122	Proteobacteria (2)	Bacteria (1)	100.00	0.00	99.60	99.60	WGS
195.609	0.11	Campylobacter coli (72294)	Campylobacter (213849)	Campylobacteraceae (2954	Campylobacterales (68525)	Epsilonproteobacteria (122	Proteobacteria (2)	Bacteria (1)	100.00	0.00	99.60	99.60	WGS
195.610	0.11	Campylobacter coli (72294)	Campylobacter (213849)	Campylobacteraceae (2954	Campylobacterales (68525)	Epsilonproteobacteria (122	Proteobacteria (2)	Bacteria (1)	100.00	0.00	99.60	99.60	WGS
195.611	0.11	Campylobacter coli (72294)	Campylobacter (213849)	Campylobacteraceae (2954	Campylobacterales (68525)	Epsilonproteobacteria (122	Proteobacteria (2)	Bacteria (1)	100.00	0.00	99.60	99.60	WGS
195.612	0.116	Campylobacter coli (72294)	Campylobacter (213849)	Campylobacteraceae (2954	Campylobacterales (68525)	Epsilonproteobacteria (122	Proteobacteria (2)	Bacteria (1)	100.00	0.00	99.60	99.60	WGS
195.613	0.1132	Campylobacter coli (72294)	Campylobacter (213849)	Campylobacteraceae (2954	Campylobacterales (68525)	Epsilonproteobacteria (122	Proteobacteria (2)	Bacteria (1)	100.00	0.00	99.60	99.60	WGS
195.614	0.11	Campylobacter coli (72294)	Campylobacter (213849)	Campylobacteraceae (2954	Campylobacterales (68525)	Epsilonproteobacteria (122	Proteobacteria (2)	Bacteria (1)	100.00	0.00	99.80	99.80	WGS
195.615	0.11	Campylobacter coli (72294)	Campylobacter (213849)	Campylobacteraceae (2954	Campylobacterales (68525)	Epsilonproteobacteria (122	Proteobacteria (2)	Bacteria (1)	100.00	0.00	99.60	99.60	WGS
195.616	0.11	Campylobacter coli (72294)	Campylobacter (213849)	Campylobacteraceae (2954	Campylobacterales (68525)	Epsilonproteobacteria (122	Proteobacteria (2)	Bacteria (1)	100.00	0.00	99.60	99.60	WGS
195.617	0.11	Campylobacter coli (72294)	Campylobacter (213849)	Campylobacteraceae (2954	Campylobacterales (68525)	Epsilonproteobacteria (122	Proteobacteria (2)	Bacteria (1)	100.00	0.00	99.60	99.60	WGS
195.618	0.11	Campylobacter coli (72294)	Campylobacter (213849)	Campylobacteraceae (2954	Campylobacterales (68525)	Epsilonproteobacteria (122	Proteobacteria (2)	Bacteria (1)	100.00	0.00	99.60	99.60	WGS
195.620	0.1132	Campylobacter coli (72294)	Campylobacter (213849)	Campylobacteraceae (2954	Campylobacterales (68525)	Epsilonproteobacteria (122	Proteobacteria (2)	Bacteria (1)	99.40	1.20	99.60	99.60	WGS
195.621	0.1132	Campylobacter coli (72294)	Campylobacter (213849)	Campylobacteraceae (2954	Campylobacterales (68525)	Epsilonproteobacteria (122	Proteobacteria (2)	Bacteria (1)	100.00	0.00	99.60	99.60	WGS
195.622	0.11	Campylobacter coli (72294)	Campylobacter (213849)	Campylobacteraceae (2954	Campylobacterales (68525)	Epsilonproteobacteria (122	Proteobacteria (2)	Bacteria (1)	100.00	0.00	99.60	99.60	WGS
195.624	0.11	Campylobacter coli (72294)	Campylobacter (213849)	Campylobacteraceae (2954	Campylobacterales (68525)	Epsilonproteobacteria (122	Proteobacteria (2)	Bacteria (1)	100.00	0.00	99.60	99.60	WGS
195.626	0.116	Campylobacter coli (72294)	Campylobacter (213849)	Campylobacteraceae (2954	Campylobacterales (68525)	Epsilonproteobacteria (122	Proteobacteria (2)	Bacteria (1)	100.00	0.00	99.40	99.20	WGS
195.628	0.11	Campylobacter coli (72294)	Campylobacter (213849)	Campylobacteraceae (2954	Campylobacterales (68525)	Epsilonproteobacteria (122	Proteobacteria (2)	Bacteria (1)	100.00	0.00	99.60	99.60	WGS
195.630	0.1138	Campylobacter coli (72294)	Campylobacter (213849)	Campylobacteraceae (2954	Campylobacterales (68525)	Epsilonproteobacteria (122	Proteobacteria (2)	Bacteria (1)	100.00	0.00	99.50	99.40	WGS
195.631	0.1096	Campylobacter coli (72294)	Campylobacter (213849)	Campylobacteraceae (2954	Campylobacterales (68525)	Epsilonproteobacteria (122	Proteobacteria (2)	Bacteria (1)	100.00	0.00	99.60	99.50	WGS
195.633	0.11	Campylobacter coli (72294)	Campylobacter (213849)	Campylobacteraceae (2954	Campylobacterales (68525)	Epsilonproteobacteria (122	Proteobacteria (2)	Bacteria (1)	100.00	0.00	99.60	99.60	WGS
195.634	0.11	Campylobacter coli (72294)	Campylobacter (213849)	Campylobacteraceae (2954	Campylobacterales (68525)	Epsilonproteobacteria (122	Proteobacteria (2)	Bacteria (1)	100.00	0.00	99.60	99.60	WGS
195.636	0.112	Campylobacter coli (72294)	Campylobacter (213849)	Campylobacteraceae (2954	Campylobacterales (68525)	Epsilonproteobacteria (122	Proteobacteria (2)	Bacteria (1)	100.00	0.00	99.60	99.40	WGS
195.637	0.11	Campylobacter coli (72294)	Campylobacter (213849)	Campylobacteraceae (2954	Campylobacterales (68525)	Epsilonproteobacteria (122	Proteobacteria (2)	Bacteria (1)	100.00	0.00	99.60	99.60	WGS
195.638	0.1118	Campylobacter coli (72294)	Campylobacter (213849)	Campylobacteraceae (2954	Campylobacterales (68525)	Epsilonproteobacteria (122	Proteobacteria (2)	Bacteria (1)	100.00	0.60	99.40	99.40	WGS
195.639	0.1078	Campylobacter coli (72294)	Campylobacter (213849)	Campylobacteraceae (2954	Campylobacterales (68525)	Epsilonproteobacteria (122	Proteobacteria (2)	Bacteria (1)	100.00	0.00	99.60	99.60	WGS
195.641	0.1132	Campylobacter coli (72294)	Campylobacter (213849)	Campylobacteraceae (2954	Campylobacterales (68525)	Epsilonproteobacteria (122	Proteobacteria (2)	Bacteria (1)	100.00	0.00	99.30	99.10	WGS
195.642	0.1116	Campylobacter coli (72294)	Campylobacter (213849)	Campylobacteraceae (2954	Campylobacterales (68525)	Epsilonproteobacteria (122	Proteobacteria (2)	Bacteria (1)	100.00	0.00	99.70	99.60	WGS
195.643	0.115	Campylobacter coli (72294)	Campylobacter (213849)	Campylobacteraceae (2954	Campylobacterales (68525)	Epsilonproteobacteria (122	Proteobacteria (2)	Bacteria (1)	99.40	0.00	99.50	99.30	WGS
195.644	0.11	Campylobacter coli (72294)	Campylobacter (213849)	Campylobacteraceae (2954	Campylobacterales (68525)	Epsilonproteobacteria (122	Proteobacteria (2)	Bacteria (1)	100.00	0.00	99.60	99.60	WGS
195.645	0.1132	Campylobacter coli (72294)	Campylobacter (213849)	Campylobacteraceae (2954	Campylobacterales (68525)	Epsilonproteobacteria (122	Proteobacteria (2)	Bacteria (1)	100.00	0.00	99.30	99.20	WGS
195.646	0.11	Campylobacter coli (72294)	Campylobacter (213849)	Campylobacteraceae (2954	Campylobacterales (68525)	Epsilonproteobacteria (122	Proteobacteria (2)	Bacteria (1)	100.00	0.00	99.60	99.60	WGS
195.647	0.11	Campylobacter coli (72294)	Campylobacter (213849)	Campylobacteraceae (2954	Campylobacterales (68525)	Epsilonproteobacteria (122	Proteobacteria (2)	Bacteria (1)	100.00	0.00	99.60	99.60	WGS
195.649	0.1132	Campylobacter coli (72294)	Campylobacter (213849)	Campylobacteraceae (2954	Campylobacterales (68525)	Epsilonproteobacteria (122	Proteobacteria (2)	Bacteria (1)	100.00	0.00	99.60	99.60	WGS
195.650	0.11	Campylobacter coli (72294)	Campylobacter (213849)	Campylobacteraceae (2954	Campylobacterales (68525)	Epsilonproteobacteria (122	Proteobacteria (2)	Bacteria (1)	100.00	0.00	99.60	99.60	WGS
195.651	0.116	Campylobacter coli (72294)	Campylobacter (213849)	Campylobacteraceae (2954	Campylobacterales (68525)	Epsilonproteobacteria (122	Proteobacteria (2)	Bacteria (1)	100.00	13.60	99.40	95.40	WGS
195.652	0.11	Campylobacter coli (72294)	Campylobacter (213849)	Campylobacteraceae (2954	Campylobacterales (68525)	Epsilonproteobacteria (122	Proteobacteria (2)	Bacteria (1)	100.00	0.00	99.60	99.60	WGS
195.653	0.1128	Campylobacter coli (72294)	Campylobacter (213849)	Campylobacteraceae (2954	Campylobacterales (68525)	Epsilonproteobacteria (122	Proteobacteria (2)	Bacteria (1)	100.00	0.00	99.60	99.60	WGS
195.654	0.11	Campylobacter coli (72294)	Campylobacter (213849)	Campylobacteraceae (2954	Campylobacterales (68525)	Epsilonproteobacteria (122	Proteobacteria (2)	Bacteria (1)	100.00	0.00	99.60	99.60	WGS
195.655	0.11	Campylobacter coli (72294)	Campylobacter (213849)	Campylobacteraceae (2954	Campylobacterales (68525)	Epsilonproteobacteria (122	Proteobacteria (2)	Bacteria (1)	100.00	0.00	99.60	99.50	WGS
195.657	0.11	Campylobacter coli (72294)	Campylobacter (213849)	Campylobacteraceae (2954	Campylobacterales (68525)	Epsilonproteobacteria (122	Proteobacteria (2)	Bacteria (1)	100.00	0.00	99.60	99.60	WGS
195.658	0.11	Campylobacter coli (72294)	Campylobacter (213849)	Campylobacteraceae (2954	Campylobacterales (68525)	Epsilonproteobacteria (122	Proteobacteria (2)	Bacteria (1)	100.00	0.00	99.60	99.60	WGS
195.660	0.1132	Campylobacter coli (72294)	Campylobacter (213849)	Campylobacteraceae (2954	Campylobacterales (68525)	Epsilonproteobacteria (122	Proteobacteria (2)	Bacteria (1)	100.00	0.00	99.60	99.50	WGS
195.661	0.1144	Campylobacter coli (72294)	Campylobacter (213849)	Campylobacteraceae (2954	Campylobacterales (68525)	Epsilonproteobacteria (122	Proteobacteria (2)	Bacteria (1)	100.00	0.00	99.40	99.40	WGS
195.662	0.1128	Campylobacter coli (72294)	Campylobacter (213849)	Campylobacteraceae (2954	Campylobacterales (68525)	Epsilonproteobacteria (122	Proteobacteria (2)	Bacteria (1)	100.00	0.00	99.40	99.30	WGS
195.663	0.1124	Campylobacter coli (72294)	Campylobacter (213849)	Campylobacteraceae (2954	Campylobacterales (68525)	Epsilonproteobacteria (122	Proteobacteria (2)	Bacteria (1)	100.00	0.60	99.20	99.00	WGS
195.665	0.112	Campylobacter coli (72294)	Campylobacter (213849)	Campylobacteraceae (2954	Campylobacterales (68525)	Epsilonproteobacteria (122	Proteobacteria (2)	Bacteria (1)	100.00	0.00	99.60	99.60	WGS
195.666	0.1128	Campylobacter coli (72294)	Campylobacter (213849)	Campylobacteraceae (2954	Campylobacterales (68525)	Epsilonproteobacteria (122	Proteobacteria (2)	Bacteria (1)	100.00	0.00	99.60	99.60	WGS
195.667	0.1132	Campylobacter coli (72294)	Campylobacter (213849)	Campylobacteraceae (2954	Campylobacterales (68525)	Epsilonproteobacteria (122	Proteobacteria (2)	Bacteria (1)	100.00	0.00	99.80	99.60	WGS
195.668	0.1132	Campylobacter coli (72294)	Campylobacter (213849)	Campylobacteraceae (2954	Campylobacterales (68525)	Epsilonproteobacteria (122	Proteobacteria (2)	Bacteria (1)	100.00	0.00	99.60	99.60	WGS
195.669	0.1132	Campylobacter coli (72294)	Campylobacter (213849)	Campylobacteraceae (2954	Campylobacterales (68525)	Epsilonproteobacteria (122	Proteobacteria (2)	Bacteria (1)	100.00	0.60	99.60	99.30	WGS
195.671	0.112	Campylobacter coli (72294)	Campylobacter (213849)	Campylobacteraceae (2954	Campylobacterales (68525)	Epsilonproteobacteria (122	Proteobacteria (2)	Bacteria (1)	100.00	0.00	99.60	99.50	WGS
195.672	0.11	Campylobacter coli (72294)	Campylobacter (213849)	Campylobacteraceae (2954	Campylobacterales (68525)	Epsilonproteobacteria (122	Proteobacteria (2)	Bacteria (1)	100.00	0.00	99.60	99.60	WGS
195.673	0.11	Campylobacter coli (72294)	Campylobacter (213849)	Campylobacteraceae (2954	Campylobacterales (68525)	Epsilonproteobacteria (122	Proteobacteria (2)	Bacteria (1)	100.00	0.00	99.60	99.60	WGS
195.674	0.11	Campylobacter coli (72294)	Campylobacter (213849)	Campylobacteraceae (2954	Campylobacterales (68525)	Epsilonproteobacteria (122	Proteobacteria (2)	Bacteria (1)	100.00	0.00	99.60	99.60	WGS
195.676	0.1172	Campylobacter coli (72294)	Campylobacter (213849)	Campylobacteraceae (2954	Campylobacterales (68525)	Epsilonproteobacteria (122	Proteobacteria (2)	Bacteria (1)	100.00	0.00	99.80	99.60	WGS
195.678	0.1128	Campylobacter coli (72294)	Campylobacter (213849)	Campylobacteraceae (2954	Campylobacterales (68525)	Epsilonproteobacteria (122	Proteobacteria (2)	Bacteria (1)	100.00	0.00	99.60	99.50	WGS
195.680	0.11	Campylobacter coli (72294)	Campylobacter (213849)	Campylobacteraceae (2954	Campylobacterales (68525)	Epsilonproteobacteria (122	Proteobacteria (2)	Bacteria (1)	100.00	0.00	99.60	99.60	WGS
195.681	0.11	Campylobacter coli (72294)	Campylobacter (213849)	Campylobacteraceae (2954	Campylobacterales (68525)	Epsilonproteobacteria (122	Proteobacteria (2)	Bacteria (1)	100.00	0.00	99.60	99.60	WGS
195.683	0.11	Campylobacter coli (72294)	Campylobacter (213849)	Campylobacteraceae (2954	Campylobacterales (68525)	Epsilonproteobacteria (122	Proteobacteria (2)	Bacteria (1)	100.00	0.00	99.60	99.60	WGS
195.685	0.1118	Campylobacter coli (72294)	Campylobacter (213849)	Campylobacteraceae (2954	Campylobacterales (68525)	Epsilonproteobacteria (122	Proteobacteria (2)	Bacteria (1)	100.00	0.00	99.60	99.50	WGS
195.686	0.11	Campylobacter coli (72294)	Campylobacter (213849)	Campylobacteraceae (2954	Campylobacterales (68525)	Epsilonproteobacteria (122	Proteobacteria (2)	Bacteria (1)	100.00	0.00	99.60	99.60	WGS
195.688	0.1128	Campylobacter coli (72294)	Campylobacter (213849)	Campylobacteraceae (2954	Campylobacterales (68525)	Epsilonproteobacteria (122	Proteobacteria (2)	Bacteria (1)	100.00	0.00	99.60	99.50	WGS
195.690	0.1132	Campylobacter coli (72294)	Campylobacter (213849)	Campylobacteraceae (2954	Campylobacterales (68525)	Epsilonproteobacteria (122	Proteobacteria (2)	Bacteria (1)	100.00	0.00	99.60	99.60	WGS
195.691	0.11	Campylobacter coli (72294)	Campylobacter (213849)	Campylobacteraceae (2954	Campylobacterales (68525)	Epsilonproteobacteria (122	Proteobacteria (2)	Bacteria (1)	100.00	0.00	99.60	99.60	WGS
195.692	0.1128	Campylobacter coli (72294)	Campylobacter (213849)	Campylobacteraceae (2954	Campylobacterales (68525)	Epsilonproteobacteria (122	Proteobacteria (2)	Bacteria (1)	100.00	0.00	99.60	99.60	WGS
195.693	0.1146	Campylobacter coli (72294)	Campylobacter (213849)	Campylobacteraceae (2954	Campylobacterales (68525)	Epsilonproteobacteria (122	Proteobacteria (2)	Bacteria (1)	100.00	0.00	99.50	99.50	WGS
195.694	0.1132	Campylobacter coli (72294)	Campylobacter (213849)	Campylobacteraceae (2954	Campylobacterales (68525)	Epsilonproteobacteria (122	Proteobacteria (2)	Bacteria (1)	100.00	0.00	99.60	99.60	WGS
195.695	0.1142	Campylobacter coli (72294)	Campylobacter (213849)	Campylobacteraceae (2954	Campylobacterales (68525)	Epsilonproteobacteria (122	Proteobacteria (2)	Bacteria (1)	100.00	0.00	99.60	99.60	WGS
195.696	0.11	Campylobacter coli (72294)	Campylobacter (213849)	Campylobacteraceae (2954	Campylobacterales (68525)	Epsilonproteobacteria (122	Proteobacteria (2)	Bacteria (1)	100.00	0.00	99.60	99.50	WGS
195.697	0.1128	Campylobacter coli (72294)	Campylobacter (213849)	Campylobacteraceae (2954	Campylobacterales (68525)	Epsilonproteobacteria (122	Proteobacteria (2)	Bacteria (1)	100.00	0.00	99.50	99.40	WGS
195.699	0.1118	Campylobacter coli (72294)	Campylobacter (213849)	Campylobacteraceae (2954	Campylobacterales (68525)	Epsilonproteobacteria (122	Proteobacteria (2)	Bacteria (1)	100.00	0.00	99.60	99.60	WGS
195.700	0.1132	Campylobacter coli (72294)	Campylobacter (213849)	Campylobacteraceae (2954	Campylobacterales (68525)	Epsilonproteobacteria (122	Proteobacteria (2)	Bacteria (1)	100.00	0.00	99.60	99.60	WGS
195.701	0.11	Campylobacter coli (72294)	Campylobacter (213849)	Campylobacteraceae (2954	Campylobacterales (68525)	Epsilonproteobacteria (122	Proteobacteria (2)	Bacteria (1)	100.00	0.00	99.60	99.60	WGS
195.702	0.1138	Campylobacter coli (72294)	Campylobacter (213849)	Campylobacteraceae (2954	Campylobacterales (68525)	Epsilonproteobacteria (122	Proteobacteria (2)	Bacteria (1)	100.00	0.00	99.60	99.50	WGS
195.704	0.11	Campylobacter coli (72294)	Campylobacter (213849)	Campylobacteraceae (2954	Campylobacterales (68525)	Epsilonproteobacteria (122	Proteobacteria (2)	Bacteria (1)	100.00	0.00	99.60	99.60	WGS
195.705	0.1144	Campylobacter coli (72294)	Campylobacter (213849)	Campylobacteraceae (2954	Campylobacterales (68525)	Epsilonproteobacteria (122	Proteobacteria (2)	Bacteria (1)	100.00	1.20	99.60	99.00	WGS
195.707	0.1148	Campylobacter coli (72294)	Campylobacter (213849)	Campylobacteraceae (2954	Campylobacterales (68525)	Epsilonproteobacteria (122	Proteobacteria (2)	Bacteria (1)	100.00	0.00	99.60	99.50	WGS
195.708	0.1096	Campylobacter coli (72294)	Campylobacter (213849)	Campylobacteraceae (2954	Campylobacterales (68525)	Epsilonproteobacteria (122	Proteobacteria (2)	Bacteria (1)	99.40	0.00	99.50	99.50	WGS
195.709	0.11	Campylobacter coli (72294)	Campylobacter (213849)	Campylobacteraceae (2954	Campylobacterales (68525)	Epsilonproteobacteria (122	Proteobacteria (2)	Bacteria (1)	100.00	0.00	99.60	99.60	WGS
195.710	0.1096	Campylobacter coli (72294)	Campylobacter (213849)	Campylobacteraceae (2954	Campylobacterales (68525)	Epsilonproteobacteria (122	Proteobacteria (2)	Bacteria (1)	99.40	1.20	99.50	99.10	WGS
195.711	0.11	Campylobacter coli (72294)	Campylobacter (213849)	Campylobacteraceae (2954	Campylobacterales (68525)	Epsilonproteobacteria (122	Proteobacteria (2)	Bacteria (1)	100.00	0.00	99.60	99.60	WGS
195.713	0.1144	Campylobacter coli (72294)	Campylobacter (213849)	Campylobacteraceae (2954	Campylobacterales (68525)	Epsilonproteobacteria (122	Proteobacteria (2)	Bacteria (1)	100.00	0.00	99.40	99.30	WGS
195.714	0.11	Campylobacter coli (72294)	Campylobacter (213849)	Campylobacteraceae (2954	Campylobacterales (68525)	Epsilonproteobacteria (122	Proteobacteria (2)	Bacteria (1)	100.00	0.00	99.60	99.50	WGS
195.715	0.116	Campylobacter coli (72294)	Campylobacter (213849)	Campylobacteraceae (2954	Campylobacterales (68525)	Epsilonproteobacteria (122	Proteobacteria (2)	Bacteria (1)	100.00	0.60	99.80	99.80	WGS
195.716	0.112	Campylobacter coli (72294)	Campylobacter (213849)	Campylobacteraceae (2954	Campylobacterales (68525)	Epsilonproteobacteria (122	Proteobacteria (2)	Bacteria (1)	100.00	0.00	99.60	99.40	WGS
195.717	0.11	Campylobacter coli (72294)	Campylobacter (213849)	Campylobacteraceae (2954	Campylobacterales (68525)	Epsilonproteobacteria (122	Proteobacteria (2)	Bacteria (1)	100.00	0.00	99.60	99.60	WGS
195.718	0.11	Campylobacter coli (72294)	Campylobacter (213849)	Campylobacteraceae (2954	Campylobacterales (68525)	Epsilonproteobacteria (122	Proteobacteria (2)	Bacteria (1)	100.00	0.00	99.60	99.60	WGS
195.719	0.1112	Campylobacter coli (72294)	Campylobacter (213849)	Campylobacteraceae (2954	Campylobacterales (68525)	Epsilonproteobacteria (122	Proteobacteria (2)	Bacteria (1)	100.00	0.00	99.50	99.30	WGS
195.720	0.1096	Campylobacter coli (72294)	Campylobacter (213849)	Campylobacteraceae (2954	Campylobacterales (68525)	Epsilonproteobacteria (122	Proteobacteria (2)	Bacteria (1)	100.00	0.00	99.50	99.50	WGS
195.721	0.11	Campylobacter coli (72294)	Campylobacter (213849)	Campylobacteraceae (2954	Campylobacterales (68525)	Epsilonproteobacteria (122	Proteobacteria (2)	Bacteria (1)	100.00	0.00	99.60	99.60	WGS
195.722	0.1112	Campylobacter coli (72294)	Campylobacter (213849)	Campylobacteraceae (2954	Campylobacterales (68525)	Epsilonproteobacteria (122	Proteobacteria (2)	Bacteria (1)	100.00	0.00	99.80	99.70	WGS
195.723	0.11	Campylobacter coli (72294)	Campylobacter (213849)	Campylobacteraceae (2954	Campylobacterales (68525)	Epsilonproteobacteria (122	Proteobacteria (2)	Bacteria (1)	100.00	0.00	99.60	99.50	WGS
195.724	0.11	Campylobacter coli (72294)	Campylobacter (213849)	Campylobacteraceae (2954	Campylobacterales (68525)	Epsilonproteobacteria (122	Proteobacteria (2)	Bacteria (1)	100.00	0.00	99.60	99.60	WGS
195.725	0.112	Campylobacter coli (72294)	Campylobacter (213849)	Campylobacteraceae (2954	Campylobacterales (68525)	Epsilonproteobacteria (122	Proteobacteria (2)	Bacteria (1)	100.00	0.00	99.80	99.70	WGS
195.726	0.11	Campylobacter coli (72294)	Campylobacter (213849)	Campylobacteraceae (2954	Campylobacterales (68525)	Epsilonproteobacteria (122	Proteobacteria (2)	Bacteria (1)	100.00	0.00	99.60	99.60	WGS
195.727	0.112	Campylobacter coli (72294)	Campylobacter (213849)	Campylobacteraceae (2954	Campylobacterales (68525)	Epsilonproteobacteria (122	Proteobacteria (2)	Bacteria (1)	100.00	0.00	99.70	99.60	WGS
195.728	0.11	Campylobacter coli (72294)	Campylobacter (213849)	Campylobacteraceae (2954	Campylobacterales (68525)	Epsilonproteobacteria (122	Proteobacteria (2)	Bacteria (1)	100.00	0.00	99.60	99.60	WGS
195.729	0.11	Campylobacter coli (72294)	Campylobacter (213849)	Campylobacteraceae (2954	Campylobacterales (68525)	Epsilonproteobacteria (122	Proteobacteria (2)	Bacteria (1)	100.00	0.00	99.60	99.60	WGS
195.730	0.1138	Campylobacter coli (72294)	Campylobacter (213849)	Campylobacteraceae (2954	Campylobacterales (68525)	Epsilonproteobacteria (122	Proteobacteria (2)	Bacteria (1)	100.00	0.00	99.50	99.40	WGS
195.732	0.1128	Campylobacter coli (72294)	Campylobacter (213849)	Campylobacteraceae (2954	Campylobacterales (68525)	Epsilonproteobacteria (122	Proteobacteria (2)	Bacteria (1)	100.00	0.00	99.60	99.50	WGS
195.733	0.11	Campylobacter coli (72294)	Campylobacter (213849)	Campylobacteraceae (2954	Campylobacterales (68525)	Epsilonproteobacteria (122	Proteobacteria (2)	Bacteria (1)	100.00	0.00	99.60	99.60	WGS
195.734	0.1146	Campylobacter coli (72294)	Campylobacter (213849)	Campylobacteraceae (2954	Campylobacterales (68525)	Epsilonproteobacteria (122	Proteobacteria (2)	Bacteria (1)	100.00	0.60	99.50	99.40	WGS
195.735	0.11	Campylobacter coli (72294)	Campylobacter (213849)	Campylobacteraceae (2954	Campylobacterales (68525)	Epsilonproteobacteria (122	Proteobacteria (2)	Bacteria (1)	100.00	0.00	99.60	99.60	WGS
195.737	0.11	Campylobacter coli (72294)	Campylobacter (213849)	Campylobacteraceae (2954	Campylobacterales (68525)	Epsilonproteobacteria (122	Proteobacteria (2)	Bacteria (1)	100.00	0.00	99.60	99.60	WGS
195.738	0.11	Campylobacter coli (72294)	Campylobacter (213849)	Campylobacteraceae (2954	Campylobacterales (68525)	Epsilonproteobacteria (122	Proteobacteria (2)	Bacteria (1)	100.00	0.00	99.60	99.60	WGS
195.739	0.11	Campylobacter coli (72294)	Campylobacter (213849)	Campylobacteraceae (2954	Campylobacterales (68525)	Epsilonproteobacteria (122	Proteobacteria (2)	Bacteria (1)	100.00	0.00	99.60	99.60	WGS
195.741	0.1172	Campylobacter coli (72294)	Campylobacter (213849)	Campylobacteraceae (2954	Campylobacterales (68525)	Epsilonproteobacteria (122	Proteobacteria (2)	Bacteria (1)	100.00	0.00	99.80	99.60	WGS
195.742	0.112	Campylobacter coli (72294)	Campylobacter (213849)	Campylobacteraceae (2954	Campylobacterales (68525)	Epsilonproteobacteria (122	Proteobacteria (2)	Bacteria (1)	100.00	0.00	99.50	99.40	WGS
195.743	0.1112	Campylobacter coli (72294)	Campylobacter (213849)	Campylobacteraceae (2954	Campylobacterales (68525)	Epsilonproteobacteria (122	Proteobacteria (2)	Bacteria (1)	100.00	0.00	99.60	99.60	WGS
195.744	0.116	Campylobacter coli (72294)	Campylobacter (213849)	Campylobacteraceae (2954	Campylobacterales (68525)	Epsilonproteobacteria (122	Proteobacteria (2)	Bacteria (1)	100.00	1.20	99.80	99.70	WGS
195.745	0.113	Campylobacter coli (72294)	Campylobacter (213849)	Campylobacteraceae (2954	Campylobacterales (68525)	Epsilonproteobacteria (122	Proteobacteria (2)	Bacteria (1)	100.00	0.00	99.50	99.50	WGS
195.746	0.11	Campylobacter coli (72294)	Campylobacter (213849)	Campylobacteraceae (2954	Campylobacterales (68525)	Epsilonproteobacteria (122	Proteobacteria (2)	Bacteria (1)	100.00	0.00	99.60	99.60	WGS
195.747	0.116	Campylobacter coli (72294)	Campylobacter (213849)	Campylobacteraceae (2954	Campylobacterales (68525)	Epsilonproteobacteria (122	Proteobacteria (2)	Bacteria (1)	100.00	0.00	99.60	99.50	WGS
195.748	0.1126	Campylobacter coli (72294)	Campylobacter (213849)	Campylobacteraceae (2954	Campylobacterales (68525)	Epsilonproteobacteria (122	Proteobacteria (2)	Bacteria (1)	100.00	0.60	99.30	99.30	WGS
195.749	0.11	Campylobacter coli (72294)	Campylobacter (213849)	Campylobacteraceae (2954	Campylobacterales (68525)	Epsilonproteobacteria (122	Proteobacteria (2)	Bacteria (1)	100.00	0.00	99.60	99.60	WGS
195.750	0.1132	Campylobacter coli (72294)	Campylobacter (213849)	Campylobacteraceae (2954	Campylobacterales (68525)	Epsilonproteobacteria (122	Proteobacteria (2)	Bacteria (1)	100.00	0.00	99.60	99.60	WGS
195.751	0.1096	Campylobacter coli (72294)	Campylobacter (213849)	Campylobacteraceae (2954	Campylobacterales (68525)	Epsilonproteobacteria (122	Proteobacteria (2)	Bacteria (1)	100.00	0.00	99.50	99.50	WGS
195.752	0.1132	Campylobacter coli (72294)	Campylobacter (213849)	Campylobacteraceae (2954	Campylobacterales (68525)	Epsilonproteobacteria (122	Proteobacteria (2)	Bacteria (1)	100.00	0.00	99.60	99.40	WGS
195.754	0.1132	Campylobacter coli (72294)	Campylobacter (213849)	Campylobacteraceae (2954	Campylobacterales (68525)	Epsilonproteobacteria (122	Proteobacteria (2)	Bacteria (1)	100.00	0.00	99.60	99.30	WGS
195.756	0.1132	Campylobacter coli (72294)	Campylobacter (213849)	Campylobacteraceae (2954	Campylobacterales (68525)	Epsilonproteobacteria (122	Proteobacteria (2)	Bacteria (1)	100.00	0.00	99.60	99.60	WGS
195.757	0.1128	Campylobacter coli (72294)	Campylobacter (213849)	Campylobacteraceae (2954	Campylobacterales (68525)	Epsilonproteobacteria (122	Proteobacteria (2)	Bacteria (1)	100.00	0.00	99.60	99.60	WGS
195.758	0.11	Campylobacter coli (72294)	Campylobacter (213849)	Campylobacteraceae (2954	Campylobacterales (68525)	Epsilonproteobacteria (122	Proteobacteria (2)	Bacteria (1)	100.00	0.00	99.60	99.60	WGS
195.759	0.112	Campylobacter coli (72294)	Campylobacter (213849)	Campylobacteraceae (2954	Campylobacterales (68525)	Epsilonproteobacteria (122	Proteobacteria (2)	Bacteria (1)	100.00	0.00	99.50	99.30	WGS
195.760	0.112	Campylobacter coli (72294)	Campylobacter (213849)	Campylobacteraceae (2954	Campylobacterales (68525)	Epsilonproteobacteria (122	Proteobacteria (2)	Bacteria (1)	100.00	0.00	99.60	99.60	WGS
195.761	0.11	Campylobacter coli (72294)	Campylobacter (213849)	Campylobacteraceae (2954	Campylobacterales (68525)	Epsilonproteobacteria (122	Proteobacteria (2)	Bacteria (1)	100.00	0.00	99.60	99.50	WGS
195.762	0.11	Campylobacter coli (72294)	Campylobacter (213849)	Campylobacteraceae (2954	Campylobacterales (68525)	Epsilonproteobacteria (122	Proteobacteria (2)	Bacteria (1)	100.00	0.00	99.60	99.60	WGS
195.763	0.1172	Campylobacter coli (72294)	Campylobacter (213849)	Campylobacteraceae (2954	Campylobacterales (68525)	Epsilonproteobacteria (122	Proteobacteria (2)	Bacteria (1)	100.00	0.00	99.80	99.60	WGS
195.764	0.11	Campylobacter coli (72294)	Campylobacter (213849)	Campylobacteraceae (2954	Campylobacterales (68525)	Epsilonproteobacteria (122	Proteobacteria (2)	Bacteria (1)	100.00	0.00	99.60	99.60	WGS
195.765	0.1132	Campylobacter coli (72294)	Campylobacter (213849)	Campylobacteraceae (2954	Campylobacterales (68525)	Epsilonproteobacteria (122	Proteobacteria (2)	Bacteria (1)	100.00	0.00	99.60	99.30	WGS
195.766	0.11	Campylobacter coli (72294)	Campylobacter (213849)	Campylobacteraceae (2954	Campylobacterales (68525)	Epsilonproteobacteria (122	Proteobacteria (2)	Bacteria (1)	100.00	0.00	99.60	99.60	WGS
195.768	0.11	Campylobacter coli (72294)	Campylobacter (213849)	Campylobacteraceae (2954	Campylobacterales (68525)	Epsilonproteobacteria (122	Proteobacteria (2)	Bacteria (1)	100.00	0.00	99.60	99.60	WGS
195.769	0.1172	Campylobacter coli (72294)	Campylobacter (213849)	Campylobacteraceae (2954	Campylobacterales (68525)	Epsilonproteobacteria (122	Proteobacteria (2)	Bacteria (1)	100.00	0.00	99.80	99.60	WGS
195.770	0.11	Campylobacter coli (72294)	Campylobacter (213849)	Campylobacteraceae (2954	Campylobacterales (68525)	Epsilonproteobacteria (122	Proteobacteria (2)	Bacteria (1)	100.00	0.00	99.60	99.60	WGS
195.771	0.1128	Campylobacter coli (72294)	Campylobacter (213849)	Campylobacteraceae (2954	Campylobacterales (68525)	Epsilonproteobacteria (122	Proteobacteria (2)	Bacteria (1)	100.00	0.00	99.60	99.60	WGS
195.772	0.1172	Campylobacter coli (72294)	Campylobacter (213849)	Campylobacteraceae (2954	Campylobacterales (68525)	Epsilonproteobacteria (122	Proteobacteria (2)	Bacteria (1)	100.00	0.00	99.60	99.50	WGS
195.773	0.11	Campylobacter coli (72294)	Campylobacter (213849)	Campylobacteraceae (2954	Campylobacterales (68525)	Epsilonproteobacteria (122	Proteobacteria (2)	Bacteria (1)	100.00	0.00	99.60	99.60	WGS
195.774	0.11	Campylobacter coli (72294)	Campylobacter (213849)	Campylobacteraceae (2954	Campylobacterales (68525)	Epsilonproteobacteria (122	Proteobacteria (2)	Bacteria (1)	100.00	0.00	99.60	99.60	WGS
195.775	0.11	Campylobacter coli (72294)	Campylobacter (213849)	Campylobacteraceae (2954	Campylobacterales (68525)	Epsilonproteobacteria (122	Proteobacteria (2)	Bacteria (1)	100.00	0.00	99.60	99.60	WGS
195.776	0.1132	Campylobacter coli (72294)	Campylobacter (213849)	Campylobacteraceae (2954	Campylobacterales (68525)	Epsilonproteobacteria (122	Proteobacteria (2)	Bacteria (1)	100.00	0.00	99.60	99.60	WGS
195.777	0.11	Campylobacter coli (72294)	Campylobacter (213849)	Campylobacteraceae (2954	Campylobacterales (68525)	Epsilonproteobacteria (122	Proteobacteria (2)	Bacteria (1)	100.00	0.60	99.60	99.60	WGS
195.778	0.1078	Campylobacter coli (72294)	Campylobacter (213849)	Campylobacteraceae (2954	Campylobacterales (68525)	Epsilonproteobacteria (122	Proteobacteria (2)	Bacteria (1)	100.00	0.00	99.30	99.20	WGS
195.779	0.11	Campylobacter coli (72294)	Campylobacter (213849)	Campylobacteraceae (2954	Campylobacterales (68525)	Epsilonproteobacteria (122	Proteobacteria (2)	Bacteria (1)	100.00	0.00	99.60	99.60	WGS
195.781	0.11	Campylobacter coli (72294)	Campylobacter (213849)	Campylobacteraceae (2954	Campylobacterales (68525)	Epsilonproteobacteria (122	Proteobacteria (2)	Bacteria (1)	100.00	0.00	99.60	99.60	WGS
195.782	0.11	Campylobacter coli (72294)	Campylobacter (213849)	Campylobacteraceae (2954	Campylobacterales (68525)	Epsilonproteobacteria (122	Proteobacteria (2)	Bacteria (1)	100.00	0.60	99.60	99.60	WGS
195.783	0.11	Campylobacter coli (72294)	Campylobacter (213849)	Campylobacteraceae (2954	Campylobacterales (68525)	Epsilonproteobacteria (122	Proteobacteria (2)	Bacteria (1)	100.00	0.00	99.60	99.60	WGS
195.784	0.11	Campylobacter coli (72294)	Campylobacter (213849)	Campylobacteraceae (2954	Campylobacterales (68525)	Epsilonproteobacteria (122	Proteobacteria (2)	Bacteria (1)	100.00	0.00	99.60	99.60	WGS
195.785	0.1096	Campylobacter coli (72294)	Campylobacter (213849)	Campylobacteraceae (2954	Campylobacterales (68525)	Epsilonproteobacteria (122	Proteobacteria (2)	Bacteria (1)	100.00	0.00	99.60	99.60	WGS
195.786	0.11	Campylobacter coli (72294)	Campylobacter (213849)	Campylobacteraceae (2954	Campylobacterales (68525)	Epsilonproteobacteria (122	Proteobacteria (2)	Bacteria (1)	100.00	0.00	99.60	99.60	WGS
195.787	0.11	Campylobacter coli (72294)	Campylobacter (213849)	Campylobacteraceae (2954	Campylobacterales (68525)	Epsilonproteobacteria (122	Proteobacteria (2)	Bacteria (1)	100.00	0.00	99.60	99.60	WGS
195.788	0.112	Campylobacter coli (72294)	Campylobacter (213849)	Campylobacteraceae (2954	Campylobacterales (68525)	Epsilonproteobacteria (122	Proteobacteria (2)	Bacteria (1)	100.00	0.00	99.60	99.40	WGS
195.789	0.1132	Campylobacter coli (72294)	Campylobacter (213849)	Campylobacteraceae (2954	Campylobacterales (68525)	Epsilonproteobacteria (122	Proteobacteria (2)	Bacteria (1)	100.00	0.00	99.80	99.60	WGS
195.791	0.1128	Campylobacter coli (72294)	Campylobacter (213849)	Campylobacteraceae (2954	Campylobacterales (68525)	Epsilonproteobacteria (122	Proteobacteria (2)	Bacteria (1)	100.00	0.00	99.60	99.60	WGS
195.792	0.1172	Campylobacter coli (72294)	Campylobacter (213849)	Campylobacteraceae (2954	Campylobacterales (68525)	Epsilonproteobacteria (122	Proteobacteria (2)	Bacteria (1)	100.00	0.00	99.60	99.50	WGS
195.793	0.11	Campylobacter coli (72294)	Campylobacter (213849)	Campylobacteraceae (2954	Campylobacterales (68525)	Epsilonproteobacteria (122	Proteobacteria (2)	Bacteria (1)	100.00	0.00	99.60	99.60	WGS
195.794	0.1172	Campylobacter coli (72294)	Campylobacter (213849)	Campylobacteraceae (2954	Campylobacterales (68525)	Epsilonproteobacteria (122	Proteobacteria (2)	Bacteria (1)	100.00	0.00	99.60	99.50	WGS
195.795	0.112	Campylobacter coli (72294)	Campylobacter (213849)	Campylobacteraceae (2954	Campylobacterales (68525)	Epsilonproteobacteria (122	Proteobacteria (2)	Bacteria (1)	100.00	0.00	99.60	99.60	WGS
195.797	0.1172	Campylobacter coli (72294)	Campylobacter (213849)	Campylobacteraceae (2954	Campylobacterales (68525)	Epsilonproteobacteria (122	Proteobacteria (2)	Bacteria (1)	100.00	0.00	99.60	99.50	WGS
195.798	0.11	Campylobacter coli (72294)	Campylobacter (213849)	Campylobacteraceae (2954	Campylobacterales (68525)	Epsilonproteobacteria (122	Proteobacteria (2)	Bacteria (1)	100.00	0.00	99.60	99.60	WGS
195.799	0.1152	Campylobacter coli (72294)	Campylobacter (213849)	Campylobacteraceae (2954	Campylobacterales (68525)	Epsilonproteobacteria (122	Proteobacteria (2)	Bacteria (1)	100.00	0.00	99.60	99.60	WGS
195.800	0.11	Campylobacter coli (72294)	Campylobacter (213849)	Campylobacteraceae (2954	Campylobacterales (68525)	Epsilonproteobacteria (122	Proteobacteria (2)	Bacteria (1)	100.00	0.00	99.50	99.50	WGS
195.801	0.11	Campylobacter coli (72294)	Campylobacter (213849)	Campylobacteraceae (2954	Campylobacterales (68525)	Epsilonproteobacteria (122	Proteobacteria (2)	Bacteria (1)	100.00	0.00	99.60	99.60	WGS
195.802	0.112	Campylobacter coli (72294)	Campylobacter (213849)	Campylobacteraceae (2954	Campylobacterales (68525)	Epsilonproteobacteria (122	Proteobacteria (2)	Bacteria (1)	100.00	0.00	99.60	99.40	WGS
195.803	0.11	Campylobacter coli (72294)	Campylobacter (213849)	Campylobacteraceae (2954	Campylobacterales (68525)	Epsilonproteobacteria (122	Proteobacteria (2)	Bacteria (1)	100.00	0.00	99.60	99.60	WGS
195.804	0.1096	Campylobacter coli (72294)	Campylobacter (213849)	Campylobacteraceae (2954	Campylobacterales (68525)	Epsilonproteobacteria (122	Proteobacteria (2)	Bacteria (1)	99.40	0.00	99.50	99.50	WGS
195.805	0.1112	Campylobacter coli (72294)	Campylobacter (213849)	Campylobacteraceae (2954	Campylobacterales (68525)	Epsilonproteobacteria (122	Proteobacteria (2)	Bacteria (1)	100.00	0.00	99.80	99.80	WGS
195.806	0.1128	Campylobacter coli (72294)	Campylobacter (213849)	Campylobacteraceae (2954	Campylobacterales (68525)	Epsilonproteobacteria (122	Proteobacteria (2)	Bacteria (1)	100.00	0.00	99.60	99.50	WGS
195.807	0.1144	Campylobacter coli (72294)	Campylobacter (213849)	Campylobacteraceae (2954	Campylobacterales (68525)	Epsilonproteobacteria (122	Proteobacteria (2)	Bacteria (1)	100.00	0.00	99.40	99.30	WGS
195.808	0.11	Campylobacter coli (72294)	Campylobacter (213849)	Campylobacteraceae (2954	Campylobacterales (68525)	Epsilonproteobacteria (122	Proteobacteria (2)	Bacteria (1)	100.00	0.00	99.60	99.60	WGS
195.810	0.11	Campylobacter coli (72294)	Campylobacter (213849)	Campylobacteraceae (2954	Campylobacterales (68525)	Epsilonproteobacteria (122	Proteobacteria (2)	Bacteria (1)	100.00	0.00	99.60	99.60	WGS
195.811	0.1142	Campylobacter coli (72294)	Campylobacter (213849)	Campylobacteraceae (2954	Campylobacterales (68525)	Epsilonproteobacteria (122	Proteobacteria (2)	Bacteria (1)	100.00	0.00	99.60	99.60	WGS
195.812	0.1144	Campylobacter coli (72294)	Campylobacter (213849)	Campylobacteraceae (2954	Campylobacterales (68525)	Epsilonproteobacteria (122	Proteobacteria (2)	Bacteria (1)	100.00	0.00	99.60	99.40	WGS
195.813	0.11	Campylobacter coli (72294)	Campylobacter (213849)	Campylobacteraceae (2954	Campylobacterales (68525)	Epsilonproteobacteria (122	Proteobacteria (2)	Bacteria (1)	100.00	0.00	99.60	99.60	WGS
195.814	0.11	Campylobacter coli (72294)	Campylobacter (213849)	Campylobacteraceae (2954	Campylobacterales (68525)	Epsilonproteobacteria (122	Proteobacteria (2)	Bacteria (1)	100.00	0.00	99.60	99.60	WGS
195.816	0.11	Campylobacter coli (72294)	Campylobacter (213849)	Campylobacteraceae (2954	Campylobacterales (68525)	Epsilonproteobacteria (122	Proteobacteria (2)	Bacteria (1)	100.00	0.00	99.60	99.60	WGS
195.818	0.112	Campylobacter coli (72294)	Campylobacter (213849)	Campylobacteraceae (2954	Campylobacterales (68525)	Epsilonproteobacteria (122	Proteobacteria (2)	Bacteria (1)	100.00	0.00	99.60	99.60	WGS
195.819	0.11	Campylobacter coli (72294)	Campylobacter (213849)	Campylobacteraceae (2954	Campylobacterales (68525)	Epsilonproteobacteria (122	Proteobacteria (2)	Bacteria (1)	100.00	0.00	99.60	99.60	WGS
195.820	0.1146	Campylobacter coli (72294)	Campylobacter (213849)	Campylobacteraceae (2954	Campylobacterales (68525)	Epsilonproteobacteria (122	Proteobacteria (2)	Bacteria (1)	100.00	0.00	99.40	99.40	WGS
195.821	0.1132	Campylobacter coli (72294)	Campylobacter (213849)	Campylobacteraceae (2954	Campylobacterales (68525)	Epsilonproteobacteria (122	Proteobacteria (2)	Bacteria (1)	100.00	0.00	99.60	99.60	WGS
195.822	0.11	Campylobacter coli (72294)	Campylobacter (213849)	Campylobacteraceae (2954	Campylobacterales (68525)	Epsilonproteobacteria (122	Proteobacteria (2)	Bacteria (1)	100.00	0.00	99.60	99.60	WGS
195.823	0.11	Campylobacter coli (72294)	Campylobacter (213849)	Campylobacteraceae (2954	Campylobacterales (68525)	Epsilonproteobacteria (122	Proteobacteria (2)	Bacteria (1)	100.00	0.00	99.60	99.60	WGS
195.825	0.11	Campylobacter coli (72294)	Campylobacter (213849)	Campylobacteraceae (2954	Campylobacterales (68525)	Epsilonproteobacteria (122	Proteobacteria (2)	Bacteria (1)	100.00	0.00	99.60	99.60	WGS
195.826	0.1098	Campylobacter coli (72294)	Campylobacter (213849)	Campylobacteraceae (2954	Campylobacterales (68525)	Epsilonproteobacteria (122	Proteobacteria (2)	Bacteria (1)	100.00	0.00	99.60	99.60	WGS
195.827	0.1118	Campylobacter coli (72294)	Campylobacter (213849)	Campylobacteraceae (2954	Campylobacterales (68525)	Epsilonproteobacteria (122	Proteobacteria (2)	Bacteria (1)	100.00	0.00	99.40	99.30	WGS
195.828	0.11	Campylobacter coli (72294)	Campylobacter (213849)	Campylobacteraceae (2954	Campylobacterales (68525)	Epsilonproteobacteria (122	Proteobacteria (2)	Bacteria (1)	100.00	0.00	99.60	99.60	WGS
195.829	0.1118	Campylobacter coli (72294)	Campylobacter (213849)	Campylobacteraceae (2954	Campylobacterales (68525)	Epsilonproteobacteria (122	Proteobacteria (2)	Bacteria (1)	100.00	0.00	99.50	99.30	WGS
195.830	0.1116	Campylobacter coli (72294)	Campylobacter (213849)	Campylobacteraceae (2954	Campylobacterales (68525)	Epsilonproteobacteria (122	Proteobacteria (2)	Bacteria (1)	100.00	0.00	99.50	99.50	WGS
195.831	0.1078	Campylobacter coli (72294)	Campylobacter (213849)	Campylobacteraceae (2954	Campylobacterales (68525)	Epsilonproteobacteria (122	Proteobacteria (2)	Bacteria (1)	100.00	0.00	99.30	99.10	WGS
195.833	0.1132	Campylobacter coli (72294)	Campylobacter (213849)	Campylobacteraceae (2954	Campylobacterales (68525)	Epsilonproteobacteria (122	Proteobacteria (2)	Bacteria (1)	100.00	0.00	99.60	99.60	WGS
195.834	0.1138	Campylobacter coli (72294)	Campylobacter (213849)	Campylobacteraceae (2954	Campylobacterales (68525)	Epsilonproteobacteria (122	Proteobacteria (2)	Bacteria (1)	100.00	0.00	99.60	99.60	WGS
195.835	0.1126	Campylobacter coli (72294)	Campylobacter (213849)	Campylobacteraceae (2954	Campylobacterales (68525)	Epsilonproteobacteria (122	Proteobacteria (2)	Bacteria (1)	100.00	0.60	99.50	99.40	WGS
195.836	0.1096	Campylobacter coli (72294)	Campylobacter (213849)	Campylobacteraceae (2954	Campylobacterales (68525)	Epsilonproteobacteria (122	Proteobacteria (2)	Bacteria (1)	100.00	11.40	99.60	96.10	WGS
195.837	0.1078	Campylobacter coli (72294)	Campylobacter (213849)	Campylobacteraceae (2954	Campylobacterales (68525)	Epsilonproteobacteria (122	Proteobacteria (2)	Bacteria (1)	100.00	0.00	99.70	99.60	WGS
195.839	0.11	Campylobacter coli (72294)	Campylobacter (213849)	Campylobacteraceae (2954	Campylobacterales (68525)	Epsilonproteobacteria (122	Proteobacteria (2)	Bacteria (1)	100.00	0.00	99.60	99.60	WGS
195.840	0.1132	Campylobacter coli (72294)	Campylobacter (213849)	Campylobacteraceae (2954	Campylobacterales (68525)	Epsilonproteobacteria (122	Proteobacteria (2)	Bacteria (1)	100.00	0.00	99.60	99.60	WGS
195.841	0.1132	Campylobacter coli (72294)	Campylobacter (213849)	Campylobacteraceae (2954	Campylobacterales (68525)	Epsilonproteobacteria (122	Proteobacteria (2)	Bacteria (1)	100.00	0.00	99.40	99.30	WGS
195.842	0.1144	Campylobacter coli (72294)	Campylobacter (213849)	Campylobacteraceae (2954	Campylobacterales (68525)	Epsilonproteobacteria (122	Proteobacteria (2)	Bacteria (1)	100.00	0.00	99.60	99.30	WGS
195.843	0.1132	Campylobacter coli (72294)	Campylobacter (213849)	Campylobacteraceae (2954	Campylobacterales (68525)	Epsilonproteobacteria (122	Proteobacteria (2)	Bacteria (1)	100.00	0.00	99.60	99.60	WGS
195.845	0.11	Campylobacter coli (72294)	Campylobacter (213849)	Campylobacteraceae (2954	Campylobacterales (68525)	Epsilonproteobacteria (122	Proteobacteria (2)	Bacteria (1)	100.00	0.00	99.60	99.60	WGS
195.846	0.1132	Campylobacter coli (72294)	Campylobacter (213849)	Campylobacteraceae (2954	Campylobacterales (68525)	Epsilonproteobacteria (122	Proteobacteria (2)	Bacteria (1)	100.00	0.00	99.30	99.20	WGS
195.848	0.11	Campylobacter coli (72294)	Campylobacter (213849)	Campylobacteraceae (2954	Campylobacterales (68525)	Epsilonproteobacteria (122	Proteobacteria (2)	Bacteria (1)	100.00	0.60	99.60	99.60	WGS
195.849	0.1144	Campylobacter coli (72294)	Campylobacter (213849)	Campylobacteraceae (2954	Campylobacterales (68525)	Epsilonproteobacteria (122	Proteobacteria (2)	Bacteria (1)	100.00	0.00	99.50	99.40	WGS
195.850	0.1132	Campylobacter coli (72294)	Campylobacter (213849)	Campylobacteraceae (2954	Campylobacterales (68525)	Epsilonproteobacteria (122	Proteobacteria (2)	Bacteria (1)	100.00	0.00	99.30	99.10	WGS
195.851	0.1144	Campylobacter coli (72294)	Campylobacter (213849)	Campylobacteraceae (2954	Campylobacterales (68525)	Epsilonproteobacteria (122	Proteobacteria (2)	Bacteria (1)	100.00	0.00	99.60	99.30	WGS
195.852	0.11	Campylobacter coli (72294)	Campylobacter (213849)	Campylobacteraceae (2954	Campylobacterales (68525)	Epsilonproteobacteria (122	Proteobacteria (2)	Bacteria (1)	100.00	0.00	99.60	99.60	WGS
195.853	0.1108	Campylobacter coli (72294)	Campylobacter (213849)	Campylobacteraceae (2954	Campylobacterales (68525)	Epsilonproteobacteria (122	Proteobacteria (2)	Bacteria (1)	100.00	0.60	99.60	99.30	WGS
195.854	0.1112	Campylobacter coli (72294)	Campylobacter (213849)	Campylobacteraceae (2954	Campylobacterales (68525)	Epsilonproteobacteria (122	Proteobacteria (2)	Bacteria (1)	100.00	0.00	99.60	99.50	WGS
195.855	0.1128	Campylobacter coli (72294)	Campylobacter (213849)	Campylobacteraceae (2954	Campylobacterales (68525)	Epsilonproteobacteria (122	Proteobacteria (2)	Bacteria (1)	100.00	0.00	99.60	99.50	WGS
195.856	0.11	Campylobacter coli (72294)	Campylobacter (213849)	Campylobacteraceae (2954	Campylobacterales (68525)	Epsilonproteobacteria (122	Proteobacteria (2)	Bacteria (1)	100.00	0.00	99.60	99.60	WGS
195.858	0.116	Campylobacter coli (72294)	Campylobacter (213849)	Campylobacteraceae (2954	Campylobacterales (68525)	Epsilonproteobacteria (122	Proteobacteria (2)	Bacteria (1)	100.00	0.00	99.50	99.40	WGS
195.859	0.11	Campylobacter coli (72294)	Campylobacter (213849)	Campylobacteraceae (2954	Campylobacterales (68525)	Epsilonproteobacteria (122	Proteobacteria (2)	Bacteria (1)	100.00	0.00	99.60	99.60	WGS
195.860	0.11	Campylobacter coli (72294)	Campylobacter (213849)	Campylobacteraceae (2954	Campylobacterales (68525)	Epsilonproteobacteria (122	Proteobacteria (2)	Bacteria (1)	100.00	0.00	99.60	99.60	WGS
195.861	0.1078	Campylobacter coli (72294)	Campylobacter (213849)	Campylobacteraceae (2954	Campylobacterales (68525)	Epsilonproteobacteria (122	Proteobacteria (2)	Bacteria (1)	100.00	0.00	99.30	99.20	WGS
195.862	0.1112	Campylobacter coli (72294)	Campylobacter (213849)	Campylobacteraceae (2954	Campylobacterales (68525)	Epsilonproteobacteria (122	Proteobacteria (2)	Bacteria (1)	100.00	0.00	99.60	99.60	WGS
195.863	0.11	Campylobacter coli (72294)	Campylobacter (213849)	Campylobacteraceae (2954	Campylobacterales (68525)	Epsilonproteobacteria (122	Proteobacteria (2)	Bacteria (1)	100.00	0.00	99.60	99.60	WGS
195.864	0.1128	Campylobacter coli (72294)	Campylobacter (213849)	Campylobacteraceae (2954	Campylobacterales (68525)	Epsilonproteobacteria (122	Proteobacteria (2)	Bacteria (1)	100.00	0.00	99.60	99.60	WGS
195.867	0.11	Campylobacter coli (72294)	Campylobacter (213849)	Campylobacteraceae (2954	Campylobacterales (68525)	Epsilonproteobacteria (122	Proteobacteria (2)	Bacteria (1)	100.00	0.00	99.60	99.60	WGS
195.868	0.114	Campylobacter coli (72294)	Campylobacter (213849)	Campylobacteraceae (2954	Campylobacterales (68525)	Epsilonproteobacteria (122	Proteobacteria (2)	Bacteria (1)	100.00	0.00	99.60	99.60	WGS
195.869	0.11	Campylobacter coli (72294)	Campylobacter (213849)	Campylobacteraceae (2954	Campylobacterales (68525)	Epsilonproteobacteria (122	Proteobacteria (2)	Bacteria (1)	100.00	2.40	99.60	99.50	WGS
195.870	0.1144	Campylobacter coli (72294)	Campylobacter (213849)	Campylobacteraceae (2954	Campylobacterales (68525)	Epsilonproteobacteria (122	Proteobacteria (2)	Bacteria (1)	100.00	17.70	99.60	94.50	WGS
195.871	0.11	Campylobacter coli (72294)	Campylobacter (213849)	Campylobacteraceae (2954	Campylobacterales (68525)	Epsilonproteobacteria (122	Proteobacteria (2)	Bacteria (1)	100.00	0.00	99.60	99.60	WGS
195.872	0.1128	Campylobacter coli (72294)	Campylobacter (213849)	Campylobacteraceae (2954	Campylobacterales (68525)	Epsilonproteobacteria (122	Proteobacteria (2)	Bacteria (1)	100.00	0.00	99.60	99.60	WGS
195.873	0.1128	Campylobacter coli (72294)	Campylobacter (213849)	Campylobacteraceae (2954	Campylobacterales (68525)	Epsilonproteobacteria (122	Proteobacteria (2)	Bacteria (1)	100.00	0.00	99.50	99.50	WGS
195.875	0.11	Campylobacter coli (72294)	Campylobacter (213849)	Campylobacteraceae (2954	Campylobacterales (68525)	Epsilonproteobacteria (122	Proteobacteria (2)	Bacteria (1)	100.00	0.00	99.80	99.60	WGS
195.877	0.11	Campylobacter coli (72294)	Campylobacter (213849)	Campylobacteraceae (2954	Campylobacterales (68525)	Epsilonproteobacteria (122	Proteobacteria (2)	Bacteria (1)	100.00	0.00	99.60	99.60	WGS
195.879	0.1132	Campylobacter coli (72294)	Campylobacter (213849)	Campylobacteraceae (2954	Campylobacterales (68525)	Epsilonproteobacteria (122	Proteobacteria (2)	Bacteria (1)	100.00	0.00	99.40	99.30	WGS

195.880	0.1118	Campylobacter coli (72294)	Campylobacter (213849)	Campylobacteraceae (2954	Campylobacterales (68525)	Epsilonproteobacteria (122	Proteobacteria (2)	Bacteria (1)	100.00	0.60	99.50	99.40	WGS
195.881	0.11	Campylobacter coli (72294)	Campylobacter (213849)	Campylobacteraceae (2954	Campylobacterales (68525)	Epsilonproteobacteria (122	Proteobacteria (2)	Bacteria (1)	100.00	0.00	99.60	99.60	WGS
195.884	0.112	Campylobacter coli (72294)	Campylobacter (213849)	Campylobacteraceae (2954	Campylobacterales (68525)	Epsilonproteobacteria (122	Proteobacteria (2)	Bacteria (1)	100.00	0.00	99.60	99.40	WGS
195.885	0.1116	Campylobacter coli (72294)	Campylobacter (213849)	Campylobacteraceae (2954	Campylobacterales (68525)	Epsilonproteobacteria (122	Proteobacteria (2)	Bacteria (1)	100.00	0.00	99.60	99.60	WGS
195.886	0.11	Campylobacter coli (72294)	Campylobacter (213849)	Campylobacteraceae (2954	Campylobacterales (68525)	Epsilonproteobacteria (122	Proteobacteria (2)	Bacteria (1)	100.00	0.00	99.60	99.60	WGS
195.888	0.11	Campylobacter coli (72294)	Campylobacter (213849)	Campylobacteraceae (2954	Campylobacterales (68525)	Epsilonproteobacteria (122	Proteobacteria (2)	Bacteria (1)	100.00	0.00	99.60	99.60	WGS
195.890	0.11	Campylobacter coli (72294)	Campylobacter (213849)	Campylobacteraceae (2954	Campylobacterales (68525)	Epsilonproteobacteria (122	Proteobacteria (2)	Bacteria (1)	100.00	0.00	99.60	99.60	WGS
195.891	0.1172	Campylobacter coli (72294)	Campylobacter (213849)	Campylobacteraceae (2954	Campylobacterales (68525)	Epsilonproteobacteria (122	Proteobacteria (2)	Bacteria (1)	100.00	0.00	99.60	99.50	WGS
195.893	0.11	Campylobacter coli (72294)	Campylobacter (213849)	Campylobacteraceae (2954	Campylobacterales (68525)	Epsilonproteobacteria (122	Proteobacteria (2)	Bacteria (1)	100.00	0.00	99.60	99.60	WGS
195.894	0.116	Campylobacter coli (72294)	Campylobacter (213849)	Campylobacteraceae (2954	Campylobacterales (68525)	Epsilonproteobacteria (122	Proteobacteria (2)	Bacteria (1)	100.00	0.00	99.60	99.60	WGS
195.895	0.11	Campylobacter coli (72294)	Campylobacter (213849)	Campylobacteraceae (2954	Campylobacterales (68525)	Epsilonproteobacteria (122	Proteobacteria (2)	Bacteria (1)	100.00	0.00	99.60	99.60	WGS
195.896	0.1132	Campylobacter coli (72294)	Campylobacter (213849)	Campylobacteraceae (2954	Campylobacterales (68525)	Epsilonproteobacteria (122	Proteobacteria (2)	Bacteria (1)	100.00	0.00	99.60	99.60	WGS
195.897	0.1112	Campylobacter coli (72294)	Campylobacter (213849)	Campylobacteraceae (2954	Campylobacterales (68525)	Epsilonproteobacteria (122	Proteobacteria (2)	Bacteria (1)	100.00	0.00	99.60	99.50	WGS
195.898	0.1078	Campylobacter coli (72294)	Campylobacter (213849)	Campylobacteraceae (2954	Campylobacterales (68525)	Epsilonproteobacteria (122	Proteobacteria (2)	Bacteria (1)	100.00	0.00	99.50	99.50	WGS
195.899	0.1098	Campylobacter coli (72294)	Campylobacter (213849)	Campylobacteraceae (2954	Campylobacterales (68525)	Epsilonproteobacteria (122	Proteobacteria (2)	Bacteria (1)	100.00	0.00	99.30	99.30	Complete
195.900	0.1132	Campylobacter coli (72294)	Campylobacter (213849)	Campylobacteraceae (2954	Campylobacterales (68525)	Epsilonproteobacteria (122	Proteobacteria (2)	Bacteria (1)	100.00	0.00	99.60	99.60	WGS
195.901	0.112	Campylobacter coli (72294)	Campylobacter (213849)	Campylobacteraceae (2954	Campylobacterales (68525)	Epsilonproteobacteria (122	Proteobacteria (2)	Bacteria (1)	100.00	0.00	99.60	99.50	WGS
195.903	0.1172	Campylobacter coli (72294)	Campylobacter (213849)	Campylobacteraceae (2954	Campylobacterales (68525)	Epsilonproteobacteria (122	Proteobacteria (2)	Bacteria (1)	100.00	0.00	99.80	99.60	WGS
195.904	0.1128	Campylobacter coli (72294)	Campylobacter (213849)	Campylobacteraceae (2954	Campylobacterales (68525)	Epsilonproteobacteria (122	Proteobacteria (2)	Bacteria (1)	100.00	0.00	99.60	99.60	WGS
195.905	0.1096	Campylobacter coli (72294)	Campylobacter (213849)	Campylobacteraceae (2954	Campylobacterales (68525)	Epsilonproteobacteria (122	Proteobacteria (2)	Bacteria (1)	100.00	0.00	99.50	99.50	WGS
195.906	0.11	Campylobacter coli (72294)	Campylobacter (213849)	Campylobacteraceae (2954	Campylobacterales (68525)	Epsilonproteobacteria (122	Proteobacteria (2)	Bacteria (1)	100.00	0.00	99.60	99.60	WGS
195.908	0.1132	Campylobacter coli (72294)	Campylobacter (213849)	Campylobacteraceae (2954	Campylobacterales (68525)	Epsilonproteobacteria (122	Proteobacteria (2)	Bacteria (1)	100.00	0.00	99.60	99.60	WGS
195.909	0.11	Campylobacter coli (72294)	Campylobacter (213849)	Campylobacteraceae (2954	Campylobacterales (68525)	Epsilonproteobacteria (122	Proteobacteria (2)	Bacteria (1)	100.00	0.00	99.60	99.60	WGS
195.910	0.1132	Campylobacter coli (72294)	Campylobacter (213849)	Campylobacteraceae (2954	Campylobacterales (68525)	Epsilonproteobacteria (122	Proteobacteria (2)	Bacteria (1)	100.00	0.00	99.60	99.60	WGS
195.911	0.114	Campylobacter coli (72294)	Campylobacter (213849)	Campylobacteraceae (2954	Campylobacterales (68525)	Epsilonproteobacteria (122	Proteobacteria (2)	Bacteria (1)	100.00	0.00	99.50	99.30	WGS
195.912	0.11	Campylobacter coli (72294)	Campylobacter (213849)	Campylobacteraceae (2954	Campylobacterales (68525)	Epsilonproteobacteria (122	Proteobacteria (2)	Bacteria (1)	100.00	0.00	99.60	99.60	WGS
195.914	0.1118	Campylobacter coli (72294)	Campylobacter (213849)	Campylobacteraceae (2954	Campylobacterales (68525)	Epsilonproteobacteria (122	Proteobacteria (2)	Bacteria (1)	100.00	0.00	99.70	99.60	WGS
195.915	0.11	Campylobacter coli (72294)	Campylobacter (213849)	Campylobacteraceae (2954	Campylobacterales (68525)	Epsilonproteobacteria (122	Proteobacteria (2)	Bacteria (1)	100.00	0.00	99.60	99.60	WGS
195.916	0.121	Campylobacter coli (72294)	Campylobacter (213849)	Campylobacteraceae (2954	Campylobacterales (68525)	Epsilonproteobacteria (122	Proteobacteria (2)	Bacteria (1)	100.00	0.00	99.50	99.40	WGS
195.917	0.112	Campylobacter coli (72294)	Campylobacter (213849)	Campylobacteraceae (2954	Campylobacterales (68525)	Epsilonproteobacteria (122	Proteobacteria (2)	Bacteria (1)	100.00	0.00	99.60	99.50	WGS
195.918	0.11	Campylobacter coli (72294)	Campylobacter (213849)	Campylobacteraceae (2954	Campylobacterales (68525)	Epsilonproteobacteria (122	Proteobacteria (2)	Bacteria (1)	100.00	0.00	99.60	99.60	WGS
195.919	0.11	Campylobacter coli (72294)	Campylobacter (213849)	Campylobacteraceae (2954	Campylobacterales (68525)	Epsilonproteobacteria (122	Proteobacteria (2)	Bacteria (1)	100.00	0.00	99.50	99.50	WGS
195.921	0.1132	Campylobacter coli (72294)	Campylobacter (213849)	Campylobacteraceae (2954	Campylobacterales (68525)	Epsilonproteobacteria (122	Proteobacteria (2)	Bacteria (1)	100.00	0.00	99.40	99.40	WGS
195.922	0.11	Campylobacter coli (72294)	Campylobacter (213849)	Campylobacteraceae (2954	Campylobacterales (68525)	Epsilonproteobacteria (122	Proteobacteria (2)	Bacteria (1)	100.00	0.00	99.60	99.60	WGS
195.923	0.1144	Campylobacter coli (72294)	Campylobacter (213849)	Campylobacteraceae (2954	Campylobacterales (68525)	Epsilonproteobacteria (122	Proteobacteria (2)	Bacteria (1)	100.00	0.00	99.60	99.40	WGS
195.925	0.1152	Campylobacter coli (72294)	Campylobacter (213849)	Campylobacteraceae (2954	Campylobacterales (68525)	Epsilonproteobacteria (122	Proteobacteria (2)	Bacteria (1)	100.00	0.00	99.50	99.30	WGS
195.926	0.116	Campylobacter coli (72294)	Campylobacter (213849)	Campylobacteraceae (2954	Campylobacterales (68525)	Epsilonproteobacteria (122	Proteobacteria (2)	Bacteria (1)	100.00	0.60	99.80	99.70	WGS
195.927	0.116	Campylobacter coli (72294)	Campylobacter (213849)	Campylobacteraceae (2954	Campylobacterales (68525)	Epsilonproteobacteria (122	Proteobacteria (2)	Bacteria (1)	100.00	0.00	99.80	99.70	WGS
195.928	0.11	Campylobacter coli (72294)	Campylobacter (213849)	Campylobacteraceae (2954	Campylobacterales (68525)	Epsilonproteobacteria (122	Proteobacteria (2)	Bacteria (1)	100.00	0.00	99.60	99.60	WGS
195.929	0.11	Campylobacter coli (72294)	Campylobacter (213849)	Campylobacteraceae (2954	Campylobacterales (68525)	Epsilonproteobacteria (122	Proteobacteria (2)	Bacteria (1)	100.00	0.00	99.60	99.60	WGS
195.930	0.11	Campylobacter coli (72294)	Campylobacter (213849)	Campylobacteraceae (2954	Campylobacterales (68525)	Epsilonproteobacteria (122	Proteobacteria (2)	Bacteria (1)	100.00	0.00	99.60	99.60	WGS
195.931	0.1128	Campylobacter coli (72294)	Campylobacter (213849)	Campylobacteraceae (2954	Campylobacterales (68525)	Epsilonproteobacteria (122	Proteobacteria (2)	Bacteria (1)	100.00	0.00	99.60	99.50	WGS
195.932	0.112	Campylobacter coli (72294)	Campylobacter (213849)	Campylobacteraceae (2954	Campylobacterales (68525)	Epsilonproteobacteria (122	Proteobacteria (2)	Bacteria (1)	100.00	0.00	99.60	99.60	WGS
195.933	0.1132	Campylobacter coli (72294)	Campylobacter (213849)	Campylobacteraceae (2954	Campylobacterales (68525)	Epsilonproteobacteria (122	Proteobacteria (2)	Bacteria (1)	100.00	0.00	99.60	99.60	WGS
195.934	0.1144	Campylobacter coli (72294)	Campylobacter (213849)	Campylobacteraceae (2954	Campylobacterales (68525)	Epsilonproteobacteria (122	Proteobacteria (2)	Bacteria (1)	100.00	0.00	99.60	99.30	WGS
195.935	0.1132	Campylobacter coli (72294)	Campylobacter (213849)	Campylobacteraceae (2954	Campylobacterales (68525)	Epsilonproteobacteria (122	Proteobacteria (2)	Bacteria (1)	100.00	0.00	99.50	99.30	WGS
195.936	0.11	Campylobacter coli (72294)	Campylobacter (213849)	Campylobacteraceae (2954	Campylobacterales (68525)	Epsilonproteobacteria (122	Proteobacteria (2)	Bacteria (1)	100.00	0.00	99.60	99.60	WGS
195.937	0.11	Campylobacter coli (72294)	Campylobacter (213849)	Campylobacteraceae (2954	Campylobacterales (68525)	Epsilonproteobacteria (122	Proteobacteria (2)	Bacteria (1)	100.00	0.00	99.60	99.60	WGS
195.938	0.1132	Campylobacter coli (72294)	Campylobacter (213849)	Campylobacteraceae (2954	Campylobacterales (68525)	Epsilonproteobacteria (122	Proteobacteria (2)	Bacteria (1)	100.00	0.00	99.60	99.60	WGS
195.939	0.1128	Campylobacter coli (72294)	Campylobacter (213849)	Campylobacteraceae (2954	Campylobacterales (68525)	Epsilonproteobacteria (122	Proteobacteria (2)	Bacteria (1)	100.00	0.00	99.60	99.60	WGS
195.940	0.11	Campylobacter coli (72294)	Campylobacter (213849)	Campylobacteraceae (2954	Campylobacterales (68525)	Epsilonproteobacteria (122	Proteobacteria (2)	Bacteria (1)	100.00	0.00	99.60	99.60	WGS
195.941	0.1128	Campylobacter coli (72294)	Campylobacter (213849)	Campylobacteraceae (2954	Campylobacterales (68525)	Epsilonproteobacteria (122	Proteobacteria (2)	Bacteria (1)	100.00	0.00	99.60	99.60	WGS
195.942	0.11	Campylobacter coli (72294)	Campylobacter (213849)	Campylobacteraceae (2954	Campylobacterales (68525)	Epsilonproteobacteria (122	Proteobacteria (2)	Bacteria (1)	100.00	0.00	99.60	99.60	WGS
195.943	0.1078	Campylobacter coli (72294)	Campylobacter (213849)	Campylobacteraceae (2954	Campylobacterales (68525)	Epsilonproteobacteria (122	Proteobacteria (2)	Bacteria (1)	100.00	0.00	99.60	99.50	WGS
195.944	0.11	Campylobacter coli (72294)	Campylobacter (213849)	Campylobacteraceae (2954	Campylobacterales (68525)	Epsilonproteobacteria (122	Proteobacteria (2)	Bacteria (1)	100.00	0.60	99.60	99.40	WGS
195.945	0.112	Campylobacter coli (72294)	Campylobacter (213849)	Campylobacteraceae (2954	Campylobacterales (68525)	Epsilonproteobacteria (122	Proteobacteria (2)	Bacteria (1)	100.00	0.00	99.60	99.60	WGS
195.946	0.11	Campylobacter coli (72294)	Campylobacter (213849)	Campylobacteraceae (2954	Campylobacterales (68525)	Epsilonproteobacteria (122	Proteobacteria (2)	Bacteria (1)	100.00	0.00	99.60	99.60	WGS
195.947	0.1132	Campylobacter coli (72294)	Campylobacter (213849)	Campylobacteraceae (2954	Campylobacterales (68525)	Epsilonproteobacteria (122	Proteobacteria (2)	Bacteria (1)	100.00	0.00	99.60	99.30	WGS
195.948	0.1118	Campylobacter coli (72294)	Campylobacter (213849)	Campylobacteraceae (2954	Campylobacterales (68525)	Epsilonproteobacteria (122	Proteobacteria (2)	Bacteria (1)	100.00	0.00	99.50	99.40	WGS
195.949	0.1128	Campylobacter coli (72294)	Campylobacter (213849)	Campylobacteraceae (2954	Campylobacterales (68525)	Epsilonproteobacteria (122	Proteobacteria (2)	Bacteria (1)	100.00	0.00	99.40	99.40	WGS
195.950	0.1128	Campylobacter coli (72294)	Campylobacter (213849)	Campylobacteraceae (2954	Campylobacterales (68525)	Epsilonproteobacteria (122	Proteobacteria (2)	Bacteria (1)	100.00	0.00	99.60	99.50	WGS
195.951	0.1128	Campylobacter coli (72294)	Campylobacter (213849)	Campylobacteraceae (2954	Campylobacterales (68525)	Epsilonproteobacteria (122	Proteobacteria (2)	Bacteria (1)	100.00	0.60	99.60	99.60	WGS
195.952	0.11	Campylobacter coli (72294)	Campylobacter (213849)	Campylobacteraceae (2954	Campylobacterales (68525)	Epsilonproteobacteria (122	Proteobacteria (2)	Bacteria (1)	100.00	0.00	99.60	99.60	WGS
195.953	0.112	Campylobacter coli (72294)	Campylobacter (213849)	Campylobacteraceae (2954	Campylobacterales (68525)	Epsilonproteobacteria (122	Proteobacteria (2)	Bacteria (1)	100.00	0.00	99.60	99.50	WGS
195.954	0.11	Campylobacter coli (72294)	Campylobacter (213849)	Campylobacteraceae (2954	Campylobacterales (68525)	Epsilonproteobacteria (122	Proteobacteria (2)	Bacteria (1)	100.00	0.00	99.60	99.60	WGS
195.955	0.1138	Campylobacter coli (72294)	Campylobacter (213849)	Campylobacteraceae (2954	Campylobacterales (68525)	Epsilonproteobacteria (122	Proteobacteria (2)	Bacteria (1)	99.40	0.00	99.60	99.50	WGS
195.956	0.1132	Campylobacter coli (72294)	Campylobacter (213849)	Campylobacteraceae (2954	Campylobacterales (68525)	Epsilonproteobacteria (122	Proteobacteria (2)	Bacteria (1)	100.00	0.00	99.60	99.60	WGS
195.957	0.11	Campylobacter coli (72294)	Campylobacter (213849)	Campylobacteraceae (2954	Campylobacterales (68525)	Epsilonproteobacteria (122	Proteobacteria (2)	Bacteria (1)	100.00	0.00	99.60	99.60	WGS
195.958	0.1132	Campylobacter coli (72294)	Campylobacter (213849)	Campylobacteraceae (2954	Campylobacterales (68525)	Epsilonproteobacteria (122	Proteobacteria (2)	Bacteria (1)	100.00	0.00	99.60	99.60	WGS
195.959	0.11	Campylobacter coli (72294)	Campylobacter (213849)	Campylobacteraceae (2954	Campylobacterales (68525)	Epsilonproteobacteria (122	Proteobacteria (2)	Bacteria (1)	100.00	0.00	99.60	99.60	WGS
195.960	0.11	Campylobacter coli (72294)	Campylobacter (213849)	Campylobacteraceae (2954	Campylobacterales (68525)	Epsilonproteobacteria (122	Proteobacteria (2)	Bacteria (1)	100.00	0.00	99.60	99.60	WGS
195.961	0.11	Campylobacter coli (72294)	Campylobacter (213849)	Campylobacteraceae (2954	Campylobacterales (68525)	Epsilonproteobacteria (122	Proteobacteria (2)	Bacteria (1)	100.00	0.00	99.60	99.60	WGS
195.962	0.1132	Campylobacter coli (72294)	Campylobacter (213849)	Campylobacteraceae (2954	Campylobacterales (68525)	Epsilonproteobacteria (122	Proteobacteria (2)	Bacteria (1)	100.00	0.00	99.60	99.60	WGS
195.963	0.1118	Campylobacter coli (72294)	Campylobacter (213849)	Campylobacteraceae (2954	Campylobacterales (68525)	Epsilonproteobacteria (122	Proteobacteria (2)	Bacteria (1)	100.00	0.00	99.60	99.50	WGS
195.964	0.1132	Campylobacter coli (72294)	Campylobacter (213849)	Campylobacteraceae (2954	Campylobacterales (68525)	Epsilonproteobacteria (122	Proteobacteria (2)	Bacteria (1)	100.00	0.00	99.60	99.60	WGS
195.965	0.1144	Campylobacter coli (72294)	Campylobacter (213849)	Campylobacteraceae (2954	Campylobacterales (68525)	Epsilonproteobacteria (122	Proteobacteria (2)	Bacteria (1)	100.00	0.00	99.60	99.30	WGS
195.966	0.11	Campylobacter coli (72294)	Campylobacter (213849)	Campylobacteraceae (2954	Campylobacterales (68525)	Epsilonproteobacteria (122	Proteobacteria (2)	Bacteria (1)	100.00	0.00	99.60	99.60	WGS
195.967	0.11	Campylobacter coli (72294)	Campylobacter (213849)	Campylobacteraceae (2954	Campylobacterales (68525)	Epsilonproteobacteria (122	Proteobacteria (2)	Bacteria (1)	100.00	0.00	99.60	99.60	WGS
195.968	0.11	Campylobacter coli (72294)	Campylobacter (213849)	Campylobacteraceae (2954	Campylobacterales (68525)	Epsilonproteobacteria (122	Proteobacteria (2)	Bacteria (1)	100.00	0.00	99.60	99.50	WGS
195.969	0.111	Campylobacter coli (72294)	Campylobacter (213849)	Campylobacteraceae (2954	Campylobacterales (68525)	Epsilonproteobacteria (122	Proteobacteria (2)	Bacteria (1)	100.00	24.90	99.70	92.90	WGS
195.970	0.1098	Campylobacter coli (72294)	Campylobacter (213849)	Campylobacteraceae (2954	Campylobacterales (68525)	Epsilonproteobacteria (122	Proteobacteria (2)	Bacteria (1)	100.00	0.00	99.60	99.60	WGS
195.971	0.11	Campylobacter coli (72294)	Campylobacter (213849)	Campylobacteraceae (2954	Campylobacterales (68525)	Epsilonproteobacteria (122	Proteobacteria (2)	Bacteria (1)	100.00	0.00	99.60	99.60	WGS
195.972	0.1132	Campylobacter coli (72294)	Campylobacter (213849)	Campylobacteraceae (2954	Campylobacterales (68525)	Epsilonproteobacteria (122	Proteobacteria (2)	Bacteria (1)	100.00	0.00	99.60	99.50	WGS
195.973	0.11	Campylobacter coli (72294)	Campylobacter (213849)	Campylobacteraceae (2954	Campylobacterales (68525)	Epsilonproteobacteria (122	Proteobacteria (2)	Bacteria (1)	100.00	0.00	99.60	99.60	WGS
195.974	0.112	Campylobacter coli (72294)	Campylobacter (213849)	Campylobacteraceae (2954	Campylobacterales (68525)	Epsilonproteobacteria (122	Proteobacteria (2)	Bacteria (1)	100.00	0.00	99.80	99.70	WGS
195.975	0.113	Campylobacter coli (72294)	Campylobacter (213849)	Campylobacteraceae (2954	Campylobacterales (68525)	Epsilonproteobacteria (122	Proteobacteria (2)	Bacteria (1)	99.40	0.60	99.60	99.50	WGS
195.976	0.1172	Campylobacter coli (72294)	Campylobacter (213849)	Campylobacteraceae (2954	Campylobacterales (68525)	Epsilonproteobacteria (122	Proteobacteria (2)	Bacteria (1)	100.00	0.00	99.60	99.50	WGS
195.977	0.11	Campylobacter coli (72294)	Campylobacter (213849)	Campylobacteraceae (2954	Campylobacterales (68525)	Epsilonproteobacteria (122	Proteobacteria (2)	Bacteria (1)	100.00	0.00	99.60	99.60	WGS
195.978	0.1132	Campylobacter coli (72294)	Campylobacter (213849)	Campylobacteraceae (2954	Campylobacterales (68525)	Epsilonproteobacteria (122	Proteobacteria (2)	Bacteria (1)	100.00	0.00	99.60	99.40	WGS
195.979	0.11	Campylobacter coli (72294)	Campylobacter (213849)	Campylobacteraceae (2954	Campylobacterales (68525)	Epsilonproteobacteria (122	Proteobacteria (2)	Bacteria (1)	100.00	0.00	99.60	99.60	WGS
195.980	0.1112	Campylobacter coli (72294)	Campylobacter (213849)	Campylobacteraceae (2954	Campylobacterales (68525)	Epsilonproteobacteria (122	Proteobacteria (2)	Bacteria (1)	100.00	0.00	99.80	99.70	WGS
195.981	0.11	Campylobacter coli (72294)	Campylobacter (213849)	Campylobacteraceae (2954	Campylobacterales (68525)	Epsilonproteobacteria (122	Proteobacteria (2)	Bacteria (1)	100.00	0.00	99.60	99.60	WGS
195.982	0.1132	Campylobacter coli (72294)	Campylobacter (213849)	Campylobacteraceae (2954	Campylobacterales (68525)	Epsilonproteobacteria (122	Proteobacteria (2)	Bacteria (1)	100.00	0.00	99.30	99.20	WGS
195.983	0.1172	Campylobacter coli (72294)	Campylobacter (213849)	Campylobacteraceae (2954	Campylobacterales (68525)	Epsilonproteobacteria (122	Proteobacteria (2)	Bacteria (1)	100.00	0.00	99.60	99.50	WGS
195.984	0.11	Campylobacter coli (72294)	Campylobacter (213849)	Campylobacteraceae (2954	Campylobacterales (68525)	Epsilonproteobacteria (122	Proteobacteria (2)	Bacteria (1)	100.00	0.00	99.60	99.60	WGS
195.985	0.1078	Campylobacter coli (72294)	Campylobacter (213849)	Campylobacteraceae (2954	Campylobacterales (68525)	Epsilonproteobacteria (122	Proteobacteria (2)	Bacteria (1)	100.00	0.00	99.60	99.60	WGS
195.986	0.1172	Campylobacter coli (72294)	Campylobacter (213849)	Campylobacteraceae (2954	Campylobacterales (68525)	Epsilonproteobacteria (122	Proteobacteria (2)	Bacteria (1)	100.00	0.00	99.60	99.60	WGS
195.987	0.11	Campylobacter coli (72294)	Campylobacter (213849)	Campylobacteraceae (2954	Campylobacterales (68525)	Epsilonproteobacteria (122	Proteobacteria (2)	Bacteria (1)	100.00	0.00	99.60	99.60	WGS
195.988	0.11	Campylobacter coli (72294)	Campylobacter (213849)	Campylobacteraceae (2954	Campylobacterales (68525)	Epsilonproteobacteria (122	Proteobacteria (2)	Bacteria (1)	100.00	0.00	99.60	99.60	WGS
195.989	0.11	Campylobacter coli (72294)	Campylobacter (213849)	Campylobacteraceae (2954	Campylobacterales (68525)	Epsilonproteobacteria (122	Proteobacteria (2)	Bacteria (1)	100.00	0.00	99.60	99.50	WGS
195.990	0.1118	Campylobacter coli (72294)	Campylobacter (213849)	Campylobacteraceae (2954	Campylobacterales (68525)	Epsilonproteobacteria (122	Proteobacteria (2)	Bacteria (1)	100.00	0.00	99.60	99.30	WGS
195.991	0.1172	Campylobacter coli (72294)	Campylobacter (213849)	Campylobacteraceae (2954	Campylobacterales (68525)	Epsilonproteobacteria (122	Proteobacteria (2)	Bacteria (1)	100.00	0.00	99.60	99.60	WGS
195.992	0.1126	Campylobacter coli (72294)	Campylobacter (213849)	Campylobacteraceae (2954	Campylobacterales (68525)	Epsilonproteobacteria (122	Proteobacteria (2)	Bacteria (1)	100.00	0.00	99.50	99.40	WGS
195.993	0.11	Campylobacter coli (72294)	Campylobacter (213849)	Campylobacteraceae (2954	Campylobacterales (68525)	Epsilonproteobacteria (122	Proteobacteria (2)	Bacteria (1)	100.00	0.00	99.60	99.60	WGS
195.994	0.11	Campylobacter coli (72294)	Campylobacter (213849)	Campylobacteraceae (2954	Campylobacterales (68525)	Epsilonproteobacteria (122	Proteobacteria (2)	Bacteria (1)	100.00	0.00	99.60	99.50	WGS
195.995	0.1144	Campylobacter coli (72294)	Campylobacter (213849)	Campylobacteraceae (2954	Campylobacterales (68525)	Epsilonproteobacteria (122	Proteobacteria (2)	Bacteria (1)	100.00	0.00	99.40	99.30	WGS
195.996	0.1128	Campylobacter coli (72294)	Campylobacter (213849)	Campylobacteraceae (2954	Campylobacterales (68525)	Epsilonproteobacteria (122	Proteobacteria (2)	Bacteria (1)	100.00	0.00	99.60	99.60	WGS
195.997	0.11	Campylobacter coli (72294)	Campylobacter (213849)	Campylobacteraceae (2954	Campylobacterales (68525)	Epsilonproteobacteria (122	Proteobacteria (2)	Bacteria (1)	100.00	0.00	99.60	99.60	WGS
195.998	0.1118	Campylobacter coli (72294)	Campylobacter (213849)	Campylobacteraceae (2954	Campylobacterales (68525)	Epsilonproteobacteria (122	Proteobacteria (2)	Bacteria (1)	100.00	0.00	99.70	99.60	WGS
195.999	0.1096	Campylobacter coli (72294)	Campylobacter (213849)	Campylobacteraceae (2954	Campylobacterales (68525)	Epsilonproteobacteria (122	Proteobacteria (2)	Bacteria (1)	100.00	0.60	99.70	99.60	Complete
196.40	0.1266	Campylobacter fetus (72294)	Campylobacter (213849)	Campylobacteraceae (2954	Campylobacterales (68525)	Epsilonproteobacteria (122	Proteobacteria (2)	Bacteria (1)	97.40	25.80	98.50	89.70	WGS
196.79	0.1226	Campylobacter fetus (72294)	Campylobacter (213849)	Campylobacteraceae (2954	Campylobacterales (68525)	Epsilonproteobacteria (122	Proteobacteria (2)	Bacteria (1)			99.80	99.70	WGS
196.92	0.1228	Campylobacter fetus (72294)	Campylobacter (213849)	Campylobacteraceae (2954	Campylobacterales (68525)	Epsilonproteobacteria (122	Proteobacteria (2)	Bacteria (1)			99.80	99.70	WGS
196.93	0.1228	Campylobacter fetus (72294)	Campylobacter (213849)	Campylobacteraceae (2954	Campylobacterales (68525)	Epsilonproteobacteria (122	Proteobacteria (2)	Bacteria (1)			99.80	99.60	WGS
197.10101	0.1106	Campylobacter jejuni (72294)	Campylobacter (213849)	Campylobacteraceae (2954	Campylobacterales (68525)	Epsilonproteobacteria (122	Proteobacteria (2)	Bacteria (1)	100.00	0.00	99.90	99.80	Complete
197.10102	0.1118	Campylobacter jejuni (72294)	Campylobacter (213849)	Campylobacteraceae (2954	Campylobacterales (68525)	Epsilonproteobacteria (122	Proteobacteria (2)	Bacteria (1)	100.00	0.00	100.00	99.90	Complete
197.10103	0.1118	Campylobacter jejuni (72294)	Campylobacter (213849)	Campylobacteraceae (2954	Campylobacterales (68525)	Epsilonproteobacteria (122	Proteobacteria (2)	Bacteria (1)	100.00	0.00	100.00	99.90	Complete
197.10104	0.1078	Campylobacter jejuni (72294)	Campylobacter (213849)	Campylobacteraceae (2954	Campylobacterales (68525)	Epsilonproteobacteria (122	Proteobacteria (2)	Bacteria (1)	100.00	0.00	99.90	99.90	Complete
197.10105	0.1078	Campylobacter jejuni (72294)	Campylobacter (213849)	Campylobacteraceae (2954	Campylobacterales (68525)	Epsilonproteobacteria (122	Proteobacteria (2)	Bacteria (1)	100.00	0.00	99.90	99.90	Complete
197.10106	0.1078	Campylobacter jejuni (72294)	Campylobacter (213849)	Campylobacteraceae (2954	Campylobacterales (68525)	Epsilonproteobacteria (122	Proteobacteria (2)	Bacteria (1)	100.00	0.00	99.90	99.90	Complete
197.10107	0.1078	Campylobacter jejuni (72294)	Campylobacter (213849)	Campylobacteraceae (2954	Campylobacterales (68525)	Epsilonproteobacteria (122	Proteobacteria (2)	Bacteria (1)	100.00	0.00	99.90	99.80	WGS
197.10112	0.1096	Campylobacter jejuni (72294)	Campylobacter (213849)	Campylobacteraceae (2954	Campylobacterales (68525)	Epsilonproteobacteria (122	Proteobacteria (2)	Bacteria (1)	100.00	0.00	100.00	100.00	Complete
197.11403	0.1106	Campylobacter jejuni (72294)	Campylobacter (213849)	Campylobacteraceae (2954	Campylobacterales (68525)	Epsilonproteobacteria (122	Proteobacteria (2)	Bacteria (1)	100.00	0.00	99.90	99.80	Complete
197.11407	0.1144	Campylobacter jejuni (72294)	Campylobacter (213849)	Campylobacteraceae (2954	Campylobacterales (68525)	Epsilonproteobacteria (122	Proteobacteria (2)	Bacteria (1)	100.00	0.70	99.60	99.20	WGS
197.11408	0.0976	Campylobacter jejuni (72294)	Campylobacter (213849)	Campylobacteraceae (2954	Campylobacterales (68525)	Epsilonproteobacteria (122	Proteobacteria (2)	Bacteria (1)	100.00	1.90	99.70	99.00	WGS
197.11409	0.1078	Campylobacter jejuni (72294)	Campylobacter (213849)	Campylobacteraceae (2954	Campylobacterales (68525)	Epsilonproteobacteria (122	Proteobacteria (2)	Bacteria (1)	100.00	0.00	100.00	99.60	WGS
197.11410	0.1078	Campylobacter jejuni (72294)	Campylobacter (213849)	Campylobacteraceae (2954	Campylobacterales (68525)	Epsilonproteobacteria (122	Proteobacteria (2)	Bacteria (1)	100.00	6.20	99.90	98.10	WGS
197.11576	0.1106	Campylobacter jejuni (72294)	Campylobacter (213849)	Campylobacteraceae (2954	Campylobacterales (68525)	Epsilonproteobacteria (122	Proteobacteria (2)	Bacteria (1)	100.00	0.00	99.90	99.60	WGS
197.11577	0.11	Campylobacter jejuni (72294)	Campylobacter (213849)	Campylobacteraceae (2954	Campylobacterales (68525)	Epsilonproteobacteria (122	Proteobacteria (2)	Bacteria (1)	100.00	0.40	98.40	98.20	WGS
197.11578	0.1118	Campylobacter jejuni (72294)	Campylobacter (213849)	Campylobacteraceae (2954	Campylobacterales (68525)	Epsilonproteobacteria (122	Proteobacteria (2)	Bacteria (1)	100.00	0.40	98.40	98.30	WGS
197.11579	0.11	Campylobacter jejuni (72294)	Campylobacter (213849)	Campylobacteraceae (2954	Campylobacterales (68525)	Epsilonproteobacteria (122	Proteobacteria (2)	Bacteria (1)	100.00	0.40	98.40	98.40	WGS
197.11580	0.1106	Campylobacter jejuni (72294)	Campylobacter (213849)	Campylobacteraceae (2954	Campylobacterales (68525)	Epsilonproteobacteria (122	Proteobacteria (2)	Bacteria (1)	100.00	0.40	98.40	98.40	WGS
197.11581	0.1078	Campylobacter jejuni (72294)	Campylobacter (213849)	Campylobacteraceae (2954	Campylobacterales (68525)	Epsilonproteobacteria (122	Proteobacteria (2)	Bacteria (1)	100.00	5.00	100.00	99.30	WGS
197.11582	0.1128	Campylobacter jejuni (72294)	Campylobacter (213849)	Campylobacteraceae (2954	Campylobacterales (68525)	Epsilonproteobacteria (122	Proteobacteria (2)	Bacteria (1)	100.00	0.40	98.40	98.30	WGS
197.11583	0.1128	Campylobacter jejuni (72294)	Campylobacter (213849)	Campylobacteraceae (2954	Campylobacterales (68525)	Epsilonproteobacteria (122	Proteobacteria (2)	Bacteria (1)	100.00	3.40	97.90	95.90	WGS
197.11584	0.1118	Campylobacter jejuni (72294)	Campylobacter (213849)	Campylobacteraceae (2954	Campylobacterales (68525)	Epsilonproteobacteria (122	Proteobacteria (2)	Bacteria (1)	100.00	0.70	99.90	99.70	WGS
197.11585	0.0942	Campylobacter jejuni (72294)	Campylobacter (213849)	Campylobacteraceae (2954	Campylobacterales (68525)	Epsilonproteobacteria (122	Proteobacteria (2)	Bacteria (1)	91.80	0.00	98.20	98.10	WGS
197.11586	0.1138	Campylobacter jejuni (72294)	Campylobacter (213849)	Campylobacteraceae (2954	Campylobacterales (68525)	Epsilonproteobacteria (122	Proteobacteria (2)	Bacteria (1)	100.00	4.40	99.70	98.80	WGS
197.11587	0.0942	Campylobacter jejuni (72294)	Campylobacter (213849)	Campylobacteraceae (2954	Campylobacterales (68525)	Epsilonproteobacteria (122	Proteobacteria (2)	Bacteria (1)	91.80	0.00	98.20	98.00	WGS
197.11588	0.1098	Campylobacter jejuni (72294)	Campylobacter (213849)	Campylobacteraceae (2954	Campylobacterales (68525)	Epsilonproteobacteria (122	Proteobacteria (2)	Bacteria (1)	100.00	0.00	99.60	99.30	WGS
197.11589	0.1096	Campylobacter jejuni (72294)	Campylobacter (213849)	Campylobacteraceae (2954	Campylobacterales (68525)	Epsilonproteobacteria (122	Proteobacteria (2)	Bacteria (1)	100.00	0.00	99.90	99.90	WGS
197.11590	0.1078	Campylobacter jejuni (72294)	Campylobacter (213849)	Campylobacteraceae (2954	Campylobacterales (68525)	Epsilonproteobacteria (122	Proteobacteria (2)	Bacteria (1)	100.00	0.00	99.90	99.60	WGS
197.11591	0.1132	Campylobacter jejuni (72294)	Campylobacter (213849)	Campylobacteraceae (2954	Campylobacterales (68525)	Epsilonproteobacteria (122	Proteobacteria (2)	Bacteria (1)	96.00	6.40	98.50	97.30	WGS
197.11592	0.1096	Campylobacter jejuni (72294)	Campylobacter (213849)	Campylobacteraceae (2954	Campylobacterales (68525)	Epsilonproteobacteria (122	Proteobacteria (2)	Bacteria (1)	100.00	0.00	99.40	99.30	WGS
197.11593	0.1096	Campylobacter jejuni (72294)	Campylobacter (213849)	Campylobacteraceae (2954	Campylobacterales (68525)	Epsilonproteobacteria (122	Proteobacteria (2)	Bacteria (1)	100.00	0.00	99.90	99.90	WGS
197.11594	0.1146	Campylobacter jejuni (72294)	Campylobacter (213849)	Campylobacteraceae (2954	Campylobacterales (68525)	Epsilonproteobacteria (122	Proteobacteria (2)	Bacteria (1)	100.00	0.00	99.60	99.50	WGS
197.11595	0.1138	Campylobacter jejuni (72294)	Campylobacter (213849)	Campylobacteraceae (2954	Campylobacterales (68525)	Epsilonproteobacteria (122	Proteobacteria (2)	Bacteria (1)	100.00	0.00	100.00	99.90	WGS
197.11596	0.1138	Campylobacter jejuni (72294)	Campylobacter (213849)	Campylobacteraceae (2954	Campylobacterales (68525)	Epsilonproteobacteria (122	Proteobacteria (2)	Bacteria (1)	100.00	0.00	100.00	99.90	WGS
197.11597	0.1118	Campylobacter jejuni (72294)	Campylobacter (213849)	Campylobacteraceae (2954	Campylobacterales (68525)	Epsilonproteobacteria (122	Proteobacteria (2)	Bacteria (1)	100.00	0.00	99.60	99.50	WGS
197.11598	0.1138	Campylobacter jejuni (72294)	Campylobacter (213849)	Campylobacteraceae (2954	Campylobacterales (68525)	Epsilonproteobacteria (122	Proteobacteria (2)	Bacteria (1)	100.00	0.00	100.00	99.70	WGS
197.11599	0.1166	Campylobacter jejuni (72294)	Campylobacter (213849)	Campylobacteraceae (2954	Campylobacterales (68525)	Epsilonproteobacteria (122	Proteobacteria (2)	Bacteria (1)	100.00	0.00	99.90	99.90	WGS
197.11600	0.11	Campylobacter jejuni (72294)	Campylobacter (213849)	Campylobacteraceae (2954	Campylobacterales (68525)	Epsilonproteobacteria (122	Proteobacteria (2)	Bacteria (1)	100.00	0.00	99.90	99.80	WGS
197.11601	0.0964	Campylobacter jejuni (72294)	Campylobacter (213849)	Campylobacteraceae (2954	Campylobacterales (68525)	Epsilonproteobacteria (122	Proteobacteria (2)	Bacteria (1)	91.80	0.00	98.10	97.90	WGS
197.11602	0.1132	Campylobacter jejuni (72294)	Campylobacter (213849)	Campylobacteraceae (2954	Campylobacterales (68525)	Epsilonproteobacteria (122	Proteobacteria (2)	Bacteria (1)	100.00	0.00	99.90	99.50	WGS
197.11603	0.1138	Campylobacter jejuni (72294)	Campylobacter (213849)	Campylobacteraceae (2954	Campylobacterales (68525)	Epsilonproteobacteria (122	Proteobacteria (2)	Bacteria (1)	100.00	0.00	100.00	99.90	WGS
197.11604	0.116	Campylobacter jejuni (72294)	Campylobacter (213849)	Campylobacteraceae (2954	Campylobacterales (68525)	Epsilonproteobacteria (122	Proteobacteria (2)	Bacteria (1)	100.00	0.00	99.60	99.40	WGS
197.11605	0.1144	Campylobacter jejuni (72294)	Campylobacter (213849)	Campylobacteraceae (2954	Campylobacterales (68525)	Epsilonproteobacteria (122	Proteobacteria (2)	Bacteria (1)	100.00	6.20	99.30	97.50	WGS
197.11606	0.1146	Campylobacter jejuni (72294)	Campylobacter (213849)	Campylobacteraceae (2954	Campylobacterales (68525)	Epsilonproteobacteria (122	Proteobacteria (2)	Bacteria (1)	98.70	42.10	99.30	86.60	WGS
197.11607	0.1112	Campylobacter jejuni (72294)	Campylobacter (213849)	Campylobacteraceae (2954	Campylobacterales (68525)	Epsilonproteobacteria (122	Proteobacteria (2)	Bacteria (1)	100.00	0.00	99.90	99.90	WGS
197.11608	0.1132	Campylobacter jejuni (72294)	Campylobacter (213849)	Campylobacteraceae (2954	Campylobacterales (68525)	Epsilonproteobacteria (122	Proteobacteria (2)	Bacteria (1)	100.00	0.00	99.90	99.70	WGS
197.11609	0.1096	Campylobacter jejuni (72294)	Campylobacter (213849)	Campylobacteraceae (2954	Campylobacterales (68525)	Epsilonproteobacteria (122	Proteobacteria (2)	Bacteria (1)	100.00	0.00	99.90	99.90	WGS
197.11610	0.1094	Campylobacter jejuni (72294)	Campylobacter (213849)	Campylobacteraceae (2954	Campylobacterales (68525)	Epsilonproteobacteria (122	Proteobacteria (2)	Bacteria (1)	99.70	24.30	99.90	89.10	WGS
197.11611	0.11	Campylobacter jejuni (72294)	Campylobacter (213849)	Campylobacteraceae (2954	Campylobacterales (68525)	Epsilonproteobacteria (122	Proteobacteria (2)	Bacteria (1)	100.00	0.00	99.90	99.80	WGS
197.11612	0.1132	Campylobacter jejuni (72294)	Campylobacter (213849)	Campylobacteraceae (2954	Campylobacterales (68525)	Epsilonproteobacteria (122	Proteobacteria (2)	Bacteria (1)	100.00	0.00	99.90	99.60	WGS
197.11613	0.1078	Campylobacter jejuni (72294)	Campylobacter (213849)	Campylobacteraceae (2954	Campylobacterales (68525)	Epsilonproteobacteria (122	Proteobacteria (2)	Bacteria (1)	100.00	0.00	99.90	99.80	WGS
197.11614	0.1078	Campylobacter jejuni (72294)	Campylobacter (213849)	Campylobacteraceae (2954	Campylobacterales (68525)	Epsilonproteobacteria (122	Proteobacteria (2)	Bacteria (1)	100.00	0.00	99.90	99.90	WGS
197.11615	0.1126	Campylobacter jejuni (72294)	Campylobacter (213849)	Campylobacteraceae (2954	Campylobacterales (68525)	Epsilonproteobacteria (122	Proteobacteria (2)	Bacteria (1)	100.00	0.70	99.50	99.10	WGS
197.11616	0.1078	Campylobacter jejuni (72294)	Campylobacter (213849)	Campylobacteraceae (2954	Campylobacterales (68525)	Epsilonproteobacteria (122	Proteobacteria (2)	Bacteria (1)	100.00	0.70	99.90	99.30	WGS
197.11617	0.109	Campylobacter jejuni (72294)	Campylobacter (213849)	Campylobacteraceae (2954	Campylobacterales (68525)	Epsilonproteobacteria (122	Proteobacteria (2)	Bacteria (1)	100.00	0.00	99.90	99.70	WGS
197.11618	0.11	Campylobacter jejuni (72294)	Campylobacter (213849)	Campylobacteraceae (2954	Campylobacterales (68525)	Epsilonproteobacteria (122	Proteobacteria (2)	Bacteria (1)	100.00	0.00	99.90	99.80	WGS
197.11619	0.1166	Campylobacter jejuni (72294)	Campylobacter (213849)	Campylobacteraceae (2954	Campylobacterales (68525)	Epsilonproteobacteria (122	Proteobacteria (2)	Bacteria (1)	100.00	0.00	100.00	100.00	WGS
197.11620	0.1118	Campylobacter jejuni (72294)	Campylobacter (213849)	Campylobacteraceae (2954	Campylobacterales (68525)	Epsilonproteobacteria (122	Proteobacteria (2)	Bacteria (1)	100.00	0.00	99.60	99.50	WGS
197.11621	0.11	Campylobacter jejuni (72294)	Campylobacter (213849)	Campylobacteraceae (2954	Campylobacterales (68525)	Epsilonproteobacteria (122	Proteobacteria (2)	Bacteria (1)	100.00	0.00	99.90	99.90	WGS
197.11622	0.1078	Campylobacter jejuni (72294)	Campylobacter (213849)	Campylobacteraceae (2954	Campylobacterales (68525)	Epsilonproteobacteria (122	Proteobacteria (2)	Bacteria (1)	100.00	0.00	100.00	100.00	WGS
197.11623	0.1118	Campylobacter jejuni (72294)	Campylobacter (213849)	Campylobacteraceae (2954	Campylobacterales (68525)	Epsilonproteobacteria (122	Proteobacteria (2)	Bacteria (1)	100.00	3.80	99.90	99.00	WGS
197.11624	0.1144	Campylobacter jejuni (72294)	Campylobacter (213849)	Campylobacteraceae (2954	Campylobacterales (68525)	Epsilonproteobacteria (122	Proteobacteria (2)	Bacteria (1)	100.00	7.30	99.90	97.10	WGS
197.11625	0.1226	Campylobacter jejuni (72294)	Campylobacter (213849)	Campylobacteraceae (2954	Campylobacterales (68525)	Epsilonproteobacteria (122	Proteobacteria (2)	Bacteria (1)	99.30	55.40	99.50	80.00	WGS
197.11626	0.1106	Campylobacter jejuni (72294)	Campylobacter (213849)	Campylobacteraceae (2954	Campylobacterales (68525)	Epsilonproteobacteria (122	Proteobacteria (2)	Bacteria (1)	100.00	0.00	99.90	99.80	WGS
197.11627	0.1106	Campylobacter jejuni (72294)	Campylobacter (213849)	Campylobacteraceae (2954	Campylobacterales (68525)	Epsilonproteobacteria (122	Proteobacteria (2)	Bacteria (1)	100.00	0.00	99.90	99.80	WGS
197.11628	0.1106	Campylobacter jejuni (72294)	Campylobacter (213849)	Campylobacteraceae (2954	Campylobacterales (68525)	Epsilonproteobacteria (122	Proteobacteria (2)	Bacteria (1)	100.00	0.00	100.00	99.90	WGS
197.11629	0.1078	Campylobacter jejuni (72294)	Campylobacter (213849)	Campylobacteraceae (2954	Campylobacterales (68525)	Epsilonproteobacteria (122	Proteobacteria (2)	Bacteria (1)	100.00	0.00	100.00	99.80	WGS
197.11630	0.1078	Campylobacter jejuni (72294)	Campylobacter (213849)	Campylobacteraceae (2954	Campylobacterales (68525)	Epsilonproteobacteria (122	Proteobacteria (2)	Bacteria (1)	100.00	0.70	100.00	100.00	WGS
197.11631	0.1118	Campylobacter jejuni (72294)	Campylobacter (213849)	Campylobacteraceae (2954	Campylobacterales (68525)	Epsilonproteobacteria (122	Proteobacteria (2)	Bacteria (1)	100.00	0.00	99.90	99.90	WGS
197.11632	0.1106	Campylobacter jejuni (72294)	Campylobacter (213849)	Campylobacteraceae (2954	Campylobacterales (68525)	Epsilonproteobacteria (122	Proteobacteria (2)	Bacteria (1)	100.00	0.00	99.90	99.80	WGS
197.11633	0.1106	Campylobacter jejuni (72294)	Campylobacter (213849)	Campylobacteraceae (2954	Campylobacterales (68525)	Epsilonproteobacteria (122	Proteobacteria (2)	Bacteria (1)	100.00	4.40	100.00	99.20	WGS
197.11634	0.11	Campylobacter jejuni (72294)	Campylobacter (213849)	Campylobacteraceae (2954	Campylobacterales (68525)	Epsilonproteobacteria (122	Proteobacteria (2)	Bacteria (1)	100.00	6.20	99.90	98.00	WGS
197.11635	0.109	Campylobacter jejuni (72294)	Campylobacter (213849)	Campylobacteraceae (2954	Campylobacterales (68525)	Epsilonproteobacteria (122	Proteobacteria (2)	Bacteria (1)	100.00	5.00	99.90	99.00	WGS
197.11636	0.1072	Campylobacter jejuni (72294)	Campylobacter (213849)	Campylobacteraceae (2954	Campylobacterales (68525)	Epsilonproteobacteria (122	Proteobacteria (2)	Bacteria (1)	99.30	27.90	99.80	88.90	WGS
197.11637	0.1078	Campylobacter jejuni (72294)	Campylobacter (213849)	Campylobacteraceae (2954	Campylobacterales (68525)	Epsilonproteobacteria (122	Proteobacteria (2)	Bacteria (1)	100.00	0.00	100.00	99.90	WGS
197.11638	0.1096	Campylobacter jejuni (72294)	Campylobacter (213849)	Campylobacteraceae (2954	Campylobacterales (68525)	Epsilonproteobacteria (122	Proteobacteria (2)	Bacteria (1)	100.00	0.00	100.00	100.00	WGS
197.11639	0.1078	Campylobacter jejuni (72294)	Campylobacter (213849)	Campylobacteraceae (2954	Campylobacterales (68525)	Epsilonproteobacteria (122	Proteobacteria (2)	Bacteria (1)	100.00	0.70	99.90	99.80	WGS
197.11640	0.1148	Campylobacter jejuni (72294)	Campylobacter (213849)	Campylobacteraceae (2954	Campylobacterales (68525)	Epsilonproteobacteria (122	Proteobacteria (2)	Bacteria (1)	100.00	0.00	99.90	99.70	WGS
197.11641	0.1106	Campylobacter jejuni (72294)	Campylobacter (213849)	Campylobacteraceae (2954	Campylobacterales (68525)	Epsilonproteobacteria (122	Proteobacteria (2)	Bacteria (1)	100.00	0.70	99.90	99.90	WGS
197.11642	0.1118	Campylobacter jejuni (72294)	Campylobacter (213849)	Campylobacteraceae (2954	Campylobacterales (68525)	Epsilonproteobacteria (122	Proteobacteria (2)	Bacteria (1)	100.00	0.00	100.00	99.90	WGS
197.11643	0.1114	Campylobacter jejuni (72294)	Campylobacter (213849)	Campylobacteraceae (2954	Campylobacterales (68525)	Epsilonproteobacteria (122	Proteobacteria (2)	Bacteria (1)	100.00	0.00	99.90	99.90	WGS
197.11644	0.1106	Campylobacter jejuni (72294)	Campylobacter (213849)	Campylobacteraceae (2954	Campylobacterales (68525)	Epsilonproteobacteria (122	Proteobacteria (2)	Bacteria (1)	100.00	1.90	100.00	99.50	WGS
197.11645	0.11	Campylobacter jejuni (72294)	Campylobacter (213849)	Campylobacteraceae (2954	Campylobacterales (68525)	Epsilonproteobacteria (122	Proteobacteria (2)	Bacteria (1)	100.00	0.00	100.00	99.90	WGS
197.11646	0.116	Campylobacter jejuni (72294)	Campylobacter (213849)	Campylobacteraceae (2954	Campylobacterales (68525)	Epsilonproteobacteria (122	Proteobacteria (2)	Bacteria (1)	100.00	0.00	99.60	99.30	WGS
197.11647	0.11	Campylobacter jejuni (72294)	Campylobacter (213849)	Campylobacteraceae (2954	Campylobacterales (68525)	Epsilonproteobacteria (122	Proteobacteria (2)	Bacteria (1)	100.00	0.70	99.90	99.80	WGS
197.11648	0.1172	Campylobacter jejuni (72294)	Campylobacter (213849)	Campylobacteraceae (2954	Campylobacterales (68525)	Epsilonproteobacteria (122	Proteobacteria (2)	Bacteria (1)	100.00	0.70	99.70	99.60	WGS
197.11649	0.1132	Campylobacter jejuni (72294)	Campylobacter (213849)	Campylobacteraceae (2954	Campylobacterales (68525)	Epsilonproteobacteria (122	Proteobacteria (2)	Bacteria (1)	100.00	0.00	100.00	100.00	WGS
197.11650	0.1172	Campylobacter jejuni (72294)	Campylobacter (213849)	Campylobacteraceae (2954	Campylobacterales (68525)	Epsilonproteobacteria (122	Proteobacteria (2)	Bacteria (1)	100.00	0.00	99.70	99.60	WGS
197.11651	0.116	Campylobacter jejuni (72294)	Campylobacter (213849)	Campylobacteraceae (2954	Campylobacterales (68525)	Epsilonproteobacteria (122	Proteobacteria (2)	Bacteria (1)	100.00	1.90	99.60	99.10	WGS
197.11652	0.1106	Campylobacter jejuni (72294)	Campylobacter (213849)	Campylobacteraceae (2954	Campylobacterales (68525)	Epsilonproteobacteria (122	Proteobacteria (2)	Bacteria (1)	100.00	0.70	99.90	99.90	WGS
197.11653	0.1128	Campylobacter jejuni (72294)	Campylobacter (213849)	Campylobacteraceae (2954	Campylobacterales (68525)	Epsilonproteobacteria (122	Proteobacteria (2)	Bacteria (1)	100.00	0.00	100.00	100.00	WGS
197.11654	0.1078	Campylobacter jejuni (72294)	Campylobacter (213849)	Campylobacteraceae (2954	Campylobacterales (68525)	Epsilonproteobacteria (122	Proteobacteria (2)	Bacteria (1)	100.00	0.00	100.00	99.90	WGS
197.11655	0.1078	Campylobacter jejuni (72294)	Campylobacter (213849)	Campylobacteraceae (2954	Campylobacterales (68525)	Epsilonproteobacteria (122	Proteobacteria (2)	Bacteria (1)	100.00	2.90	99.90	98.80	WGS
197.11656	0.11	Campylobacter jejuni (72294)	Campylobacter (213849)	Campylobacteraceae (2954	Campylobacterales (68525)	Epsilonproteobacteria (122	Proteobacteria (2)	Bacteria (1)	99.30	1.30	99.90	99.90	WGS
197.11657	0.1106	Campylobacter jejuni (72294)	Campylobacter (213849)	Campylobacteraceae (2954	Campylobacterales (68525)	Epsilonproteobacteria (122	Proteobacteria (2)	Bacteria (1)	100.00	0.70	99.70	99.50	WGS
197.11658	0.11	Campylobacter jejuni (72294)	Campylobacter (213849)	Campylobacteraceae (2954	Campylobacterales (68525)	Epsilonproteobacteria (122	Proteobacteria (2)	Bacteria (1)	100.00	0.40	98.40	98.20	WGS
197.11659	0.1144	Campylobacter jejuni (72294)	Campylobacter (213849)	Campylobacteraceae (2954	Campylobacterales (68525)	Epsilonproteobacteria (122	Proteobacteria (2)	Bacteria (1)	100.00	1.10	98.40	98.20	WGS
197.11660	0.1126	Campylobacter jejuni (72294)	Campylobacter (213849)	Campylobacteraceae (2954	Campylobacterales (68525)	Epsilonproteobacteria (122	Proteobacteria (2)	Bacteria (1)	100.00	7.30	98.00	95.60	WGS
197.11661	0.1138	Campylobacter jejuni (72294)	Campylobacter (213849)	Campylobacteraceae (2954	Campylobacterales (68525)	Epsilonproteobacteria (122	Proteobacteria (2)	Bacteria (1)	100.00	0.00	100.00	99.90	WGS
197.11662	0.1118	Campylobacter jejuni (72294)	Campylobacter (213849)	Campylobacteraceae (2954	Campylobacterales (68525)	Epsilonproteobacteria (122	Proteobacteria (2)	Bacteria (1)	100.00	2.60	99.80	99.10	WGS
197.11663	0.121	Campylobacter jejuni (72294)	Campylobacter (213849)	Campylobacteraceae (2954	Campylobacterales (68525)	Epsilonproteobacteria (122	Proteobacteria (2)	Bacteria (1)	99.30	66.20	99.70	75.30	WGS
197.11664	0.1138	Campylobacter jejuni (72294)	Campylobacter (213849)	Campylobacteraceae (2954	Campylobacterales (68525)	Epsilonproteobacteria (122	Proteobacteria (2)	Bacteria (1)	100.00	0.60	100.00	99.90	WGS
197.11665	0.11	Campylobacter jejuni (72294)	Campylobacter (213849)	Campylobacteraceae (2954	Campylobacterales (68525)	Epsilonproteobacteria (122	Proteobacteria (2)	Bacteria (1)	100.00	0.00	100.00	100.00	WGS
197.11666	0.1166	Campylobacter jejuni (72294)	Campylobacter (213849)	Campylobacteraceae (2954	Campylobacterales (68525)	Epsilonproteobacteria (122	Proteobacteria (2)	Bacteria (1)	100.00	0.00	99.90	99.90	WGS
197.11667	0.1114	Campylobacter jejuni (72294)	Campylobacter (213849)	Campylobacteraceae (2954	Campylobacterales (68525)	Epsilonproteobacteria (122	Proteobacteria (2)	Bacteria (1)	98.70	8.40	99.80	97.30	WGS
197.11668	0.11	Campylobacter jejuni (72294)	Campylobacter (213849)	Campylobacteraceae (2954	Campylobacterales (68525)	Epsilonproteobacteria (122	Proteobacteria (2)	Bacteria (1)	100.00	2.60	99.90	99.20	WGS
197.11669	0.11	Campylobacter jejuni (72294)	Campylobacter (213849)	Campylobacteraceae (2954	Campylobacterales (68525)	Epsilonproteobacteria (122	Proteobacteria (2)	Bacteria (1)	100.00	0.70	100.00	100.00	WGS
197.11670	0.1114	Campylobacter jejuni (72294)	Campylobacter (213849)	Campylobacteraceae (2954	Campylobacterales (68525)	Epsilonproteobacteria (122	Proteobacteria (2)	Bacteria (1)	100.00	0.00	99.60	99.50	WGS
197.11671	0.1132	Campylobacter jejuni (72294)	Campylobacter (213849)	Campylobacteraceae (2954	Campylobacterales (68525)	Epsilonproteobacteria (122	Proteobacteria (2)	Bacteria (1)	100.00	0.70	99.90	99.60	WGS
197.11672	0.11	Campylobacter jejuni (72294)	Campylobacter (213849)	Campylobacteraceae (2954	Campylobacterales (68525)	Epsilonproteobacteria (122	Proteobacteria (2)	Bacteria (1)	100.00	1.90	99.90	99.30	WGS
197.11673	0.1078	Campylobacter jejuni (72294)	Campylobacter (213849)	Campylobacteraceae (2954	Campylobacterales (68525)	Epsilonproteobacteria (122	Proteobacteria (2)	Bacteria (1)	100.00	0.00	100.00	99.90	WGS
197.11674	0.1078	Campylobacter jejuni (72294)	Campylobacter (213849)	Campylobacteraceae (2954	Campylobacterales (68525)	Epsilonproteobacteria (122	Proteobacteria (2)	Bacteria (1)	100.00	0.00	100.00	99.90	WGS
197.11675	0.1106	Campylobacter jejuni (72294)	Campylobacter (213849)	Campylobacteraceae (2954	Campylobacterales (68525)	Epsilonproteobacteria (122	Proteobacteria (2)	Bacteria (1)	100.00	0.00	99.90	99.70	WGS
197.11676	0.109	Campylobacter jejuni (72294)	Campylobacter (213849)	Campylobacteraceae (2954	Campylobacterales (68525)	Epsilonproteobacteria (122	Proteobacteria (2)	Bacteria (1)	100.00	0.00	100.00	99.80	WGS
197.11677	0.1098	Campylobacter jejuni (72294)	Campylobacter (213849)	Campylobacteraceae (2954	Campylobacterales (68525)	Epsilonproteobacteria (122	Proteobacteria (2)	Bacteria (1)	88.10	0.00	94.70	94.50	WGS
197.11678	0.112	Campylobacter jejuni (72294)	Campylobacter (213849)	Campylobacteraceae (2954	Campylobacterales (68525)	Epsilonproteobacteria (122	Proteobacteria (2)	Bacteria (1)	100.00	0.00	99.60	99.40	WGS
197.11679	0.116	Campylobacter jejuni (72294)	Campylobacter (213849)	Campylobacteraceae (2954	Campylobacterales (68525)	Epsilonproteobacteria (122	Proteobacteria (2)	Bacteria (1)	100.00	0.70	99.60	99.30	WGS
197.11680	0.1106	Campylobacter jejuni (72294)	Campylobacter (213849)	Campylobacteraceae (2954	Campylobacterales (68525)	Epsilonproteobacteria (122	Proteobacteria (2)	Bacteria (1)	97.40	2.00	98.80	96.70	WGS
197.11681	0.11	Campylobacter jejuni (72294)	Campylobacter (213849)	Campylobacteraceae (2954	Campylobacterales (68525)	Epsilonproteobacteria (122	Proteobacteria (2)	Bacteria (1)	100.00	2.60	99.90	98.40	WGS
197.11682	0.1128	Campylobacter jejuni (72294)	Campylobacter (213849)	Campylobacteraceae (2954	Campylobacterales (68525)	Epsilonproteobacteria (122	Proteobacteria (2)	Bacteria (1)	100.00	18.50	99.80	94.50	WGS
197.11683	0.1172	Campylobacter jejuni (72294)	Campylobacter (213849)	Campylobacteraceae (2954	Campylobacterales (68525)	Epsilonproteobacteria (122	Proteobacteria (2)	Bacteria (1)	100.00	0.00	99.70	99.60	WGS
197.11684	0.1106	Campylobacter jejuni (72294)	Campylobacter (213849)	Campylobacteraceae (2954	Campylobacterales (68525)	Epsilonproteobacteria (122	Proteobacteria (2)	Bacteria (1)	100.00	0.60	100.00	99.90	WGS
197.11685	0.11	Campylobacter jejuni (72294)	Campylobacter (213849)	Campylobacteraceae (2954	Campylobacterales (68525)	Epsilonproteobacteria (122	Proteobacteria (2)	Bacteria (1)	97.70	0.00	98.20	98.10	WGS
197.11686	0.118	Campylobacter jejuni (72294)	Campylobacter (213849)	Campylobacteraceae (2954	Campylobacterales (68525)	Epsilonproteobacteria (122	Proteobacteria (2)	Bacteria (1)	100.00	0.60	99.70	99.60	WGS
197.11687	0.11	Campylobacter jejuni (72294)	Campylobacter (213849)	Campylobacteraceae (2954	Campylobacterales (68525)	Epsilonproteobacteria (122	Proteobacteria (2)	Bacteria (1)	100.00	1.30	99.90	99.50	WGS
197.11688	0.1028	Campylobacter jejuni (72294)	Campylobacter (213849)	Campylobacteraceae (2954	Campylobacterales (68525)	Epsilonproteobacteria (122	Proteobacteria (2)	Bacteria (1)	91.10	0.00	97.90	97.70	WGS
197.11689	0.1114	Campylobacter jejuni (72294)	Campylobacter (213849)	Campylobacteraceae (2954	Campylobacterales (68525)	Epsilonproteobacteria (122	Proteobacteria (2)	Bacteria (1)	100.00	2.60	99.90	99.30	WGS
197.11690	0.0926	Campylobacter jejuni (72294)	Campylobacter (213849)	Campylobacteraceae (2954	Campylobacterales (68525)	Epsilonproteobacteria (122	Proteobacteria (2)	Bacteria (1)	100.00	5.60	99.60	97.50	WGS
197.11691	0.0944	Campylobacter jejuni (72294)	Campylobacter (213849)	Campylobacteraceae (2954	Campylobacterales (68525)	Epsilonproteobacteria (122	Proteobacteria (2)	Bacteria (1)	100.00	4.70	99.40	97.00	WGS
197.11692	0.116	Campylobacter jejuni (72294)	Campylobacter (213849)	Campylobacteraceae (2954	Campylobacterales (68525)	Epsilonproteobacteria (122	Proteobacteria (2)	Bacteria (1)	100.00	0.00	99.90	99.90	Complete
197.11720	0.1128	Campylobacter jejuni (72294)	Campylobacter (213849)	Campylobacteraceae (2954	Campylobacterales (68525)	Epsilonproteobacteria (122	Proteobacteria (2)	Bacteria (1)	100.00	0.00	99.90	99.90	Complete
197.11722	0.1132	Campylobacter jejuni (72294)	Campylobacter (213849)	Campylobacteraceae (2954	Campylobacterales (68525)	Epsilonproteobacteria (122	Proteobacteria (2)	Bacteria (1)	97.40	5.10	99.10	97.90	Complete
197.11723	0.1114	Campylobacter jejuni (72294)	Campylobacter (213849)	Campylobacteraceae (2954	Campylobacterales (68525)	Epsilonproteobacteria (122	Proteobacteria (2)	Bacteria (1)	100.00	3.80	99.90	98.40	Complete
197.11724	0.1096	Campylobacter jejuni (72294)	Campylobacter (213849)	Campylobacteraceae (2954	Campylobacterales (68525)	Epsilonproteobacteria (122	Proteobacteria (2)	Bacteria (1)	100.00	6.70	99.90	98.60	Complete
197.11725	0.1096	Campylobacter jejuni (72294)	Campylobacter (213849)	Campylobacteraceae (2954	Campylobacterales (68525)	Epsilonproteobacteria (122	Proteobacteria (2)	Bacteria (1)	100.00	2.60	100.00	99.60	Complete
197.11726	0.1078	Campylobacter jejuni (72294)	Campylobacter (213849)	Campylobacteraceae (2954	Campylobacterales (68525)	Epsilonproteobacteria (122	Proteobacteria (2)	Bacteria (1)	100.00	0.70	100.00	99.80	Complete
197.11727	0.1114	Campylobacter jejuni (72294)	Campylobacter (213849)	Campylobacteraceae (2954	Campylobacterales (68525)	Epsilonproteobacteria (122	Proteobacteria (2)	Bacteria (1)	100.00	3.80	99.90	98.40	Complete
197.11728	0.1096	Campylobacter jejuni (72294)	Campylobacter (213849)	Campylobacteraceae (2954	Campylobacterales (68525)	Epsilonproteobacteria (122	Proteobacteria (2)	Bacteria (1)	100.00	6.70	99.90	98.60	Complete
197.11826	0.11	Campylobacter jejuni (72294)	Campylobacter (213849)	Campylobacteraceae (2954	Campylobacterales (68525)	Epsilonproteobacteria (122	Proteobacteria (2)	Bacteria (1)	100.00	0.70	99.90	99.60	WGS
197.11827	0.1114	Campylobacter jejuni (72294)	Campylobacter (213849)	Campylobacteraceae (2954	Campylobacterales (68525)	Epsilonproteobacteria (122	Proteobacteria (2)	Bacteria (1)	100.00	15.10	99.80	95.00	Complete
197.11828	0.0294	Campylobacter jejuni (72294)	Campylobacter (213849)	Campylobacteraceae (2954	Campylobacterales (68525)	Epsilonproteobacteria (122	Proteobacteria (2)	Bacteria (1)	100.00	0.00	99.90	99.60	Complete
197.11830	0.1106	Campylobacter jejuni (72294)	Campylobacter (213849)	Campylobacteraceae (2954	Campylobacterales (68525)	Epsilonproteobacteria (122	Proteobacteria (2)	Bacteria (1)	100.00	0.00	100.00	99.90	Complete
197.11831	0.114	Campylobacter jejuni (72294)	Campylobacter (213849)	Campylobacteraceae (2954	Campylobacterales (68525)	Epsilonproteobacteria (122	Proteobacteria (2)	Bacteria (1)	100.00	0.00	99.90	99.90	Complete
197.11844	0.0294	Campylobacter jejuni (72294)	Campylobacter (213849)	Campylobacteraceae (2954	Campylobacterales (68525)	Epsilonproteobacteria (122	Proteobacteria (2)	Bacteria (1)					Plasmid
197.11845	0.0294	Campylobacter jejuni (72294)	Campylobacter (213849)	Campylobacteraceae (2954	Campylobacterales (68525)	Epsilonproteobacteria (122	Proteobacteria (2)	Bacteria (1)					Plasmid
197.11846	0.0294	Campylobacter jejuni (72294)	Campylobacter (213849)	Campylobacteraceae (2954	Campylobacterales (68525)	Epsilonproteobacteria (122	Proteobacteria (2)	Bacteria (1)					Plasmid
197.11847	0.0294	Campylobacter jejuni (72294)	Campylobacter (213849)	Campylobacteraceae (2954	Campylobacterales (68525)	Epsilonproteobacteria (122	Proteobacteria (2)	Bacteria (1)					Plasmid
197.11848	0.0294	Campylobacter jejuni (72294)	Campylobacter (213849)	Campylobacteraceae (2954	Campylobacterales (68525)	Epsilonproteobacteria (122	Proteobacteria (2)	Bacteria (1)					Plasmid
197.11849	0.0294	Campylobacter jejuni (72294)	Campylobacter (213849)	Campylobacteraceae (2954	Campylobacterales (68525)	Epsilonproteobacteria (122	Proteobacteria (2)	Bacteria (1)					Plasmid
197.11852	0.0316	Campylobacter jejuni (72294)	Campylobacter (213849)	Campylobacteraceae (2954	Campylobacterales (68525)	Epsilonproteobacteria (122	Proteobacteria (2)	Bacteria (1)					Plasmid
197.11853	0.0294	Campylobacter jejuni (72294)	Campylobacter (213849)	Campylobacteraceae (2954	Campylobacterales (68525)	Epsilonproteobacteria (122	Proteobacteria (2)	Bacteria (1)					Plasmid
197.11854	0.0294	Campylobacter jejuni (72294)	Campylobacter (213849)	Campylobacteraceae (2954	Campylobacterales (68525)	Epsilonproteobacteria (122	Proteobacteria (2)	Bacteria (1)					Plasmid
197.11855	0.0294	Campylobacter jejuni (72294)	Campylobacter (213849)	Campylobacteraceae (2954	Campylobacterales (68525)	Epsilonproteobacteria (122	Proteobacteria (2)	Bacteria (1)					Plasmid
197.11856	0.0294	Campylobacter jejuni (72294)	Campylobacter (213849)	Campylobacteraceae (2954	Campylobacterales (68525)	Epsilonproteobacteria (122	Proteobacteria (2)	Bacteria (1)					Plasmid
197.11857	0.0294	Campylobacter jejuni (72294)	Campylobacter (213849)	Campylobacteraceae (2954	Campylobacterales (68525)	Epsilonproteobacteria (122	Proteobacteria (2)	Bacteria (1)					Plasmid
197.11858	0.0294	Campylobacter jejuni (72294)	Campylobacter (213849)	Campylobacteraceae (2954	Campylobacterales (68525)	Epsilonproteobacteria (122	Proteobacteria (2)	Bacteria (1)					Plasmid
197.11859	0.0294	Campylobacter jejuni (72294)	Campylobacter (213849)	Campylobacteraceae (2954	Campylobacterales (68525)	Epsilonproteobacteria (122	Proteobacteria (2)	Bacteria (1)					Plasmid
197.11860	0.0294	Campylobacter jejuni (72294)	Campylobacter (213849)	Campylobacteraceae (2954	Campylobacterales (68525)	Epsilonproteobacteria (122	Proteobacteria (2)	Bacteria (1)					Plasmid
197.11861	0.0294	Campylobacter jejuni (72294)	Campylobacter (213849)	Campylobacteraceae (2954	Campylobacterales (68525)	Epsilonproteobacteria (122	Proteobacteria (2)	Bacteria (1)					Plasmid
197.11862	0.0294	Campylobacter jejuni (72294)	Campylobacter (213849)	Campylobacteraceae (2954	Campylobacterales (68525)	Epsilonproteobacteria (122	Proteobacteria (2)	Bacteria (1)					Plasmid
197.11863	0.0294	Campylobacter jejuni (72294)	Campylobacter (213849)	Campylobacteraceae (2954	Campylobacterales (68525)	Epsilonproteobacteria (122	Proteobacteria (2)	Bacteria (1)					Plasmid
197.11864	0.0294	Campylobacter jejuni (72294)	Campylobacter (213849)	Campylobacteraceae (2954	Campylobacterales (68525)	Epsilonproteobacteria (122	Proteobacteria (2)	Bacteria (1)					Plasmid
197.11866	0.0294	Campylobacter jejuni (72294)	Campylobacter (213849)	Campylobacteraceae (2954	Campylobacterales (68525)	Epsilonproteobacteria (122	Proteobacteria (2)	Bacteria (1)					Plasmid
197.11867	0.0294	Campylobacter jejuni (72294)	Campylobacter (213849)	Campylobacteraceae (2954	Campylobacterales (68525)	Epsilonproteobacteria (122	Proteobacteria (2)	Bacteria (1)					Plasmid
197.11868	0.0312	Campylobacter jejuni (72294)	Campylobacter (213849)	Campylobacteraceae (2954	Campylobacterales (68525)	Epsilonproteobacteria (122	Proteobacteria (2)	Bacteria (1)					Plasmid
197.11869	0.0294	Campylobacter jejuni (72294)	Campylobacter (213849)	Campylobacteraceae (2954	Campylobacterales (68525)	Epsilonproteobacteria (122	Proteobacteria (2)	Bacteria (1)					Plasmid
197.11870	0.0294	Campylobacter jejuni (72294)	Campylobacter (213849)	Campylobacteraceae (2954	Campylobacterales (68525)	Epsilonproteobacteria (122	Proteobacteria (2)	Bacteria (1)					Plasmid
197.11871	0.0294	Campylobacter jejuni (72294)	Campylobacter (213849)	Campylobacteraceae (2954	Campylobacterales (68525)	Epsilonproteobacteria (122	Proteobacteria (2)	Bacteria (1)					Plasmid
197.13032	0.1162	Campylobacter jejuni (72294)	Campylobacter (213849)	Campylobacteraceae (2954	Campylobacterales (68525)	Epsilonproteobacteria (122	Proteobacteria (2)	Bacteria (1)	90.10	0.70	95.50	95.30	WGS
197.13033	0.1116	Campylobacter jejuni (72294)	Campylobacter (213849)	Campylobacteraceae (2954	Campylobacterales (68525)	Epsilonproteobacteria (122	Proteobacteria (2)	Bacteria (1)	84.60	0.00	96.50	96.30	WGS
197.13034	0.1088	Campylobacter jejuni (72294)	Campylobacter (213849)	Campylobacteraceae (2954	Campylobacterales (68525)	Epsilonproteobacteria (122	Proteobacteria (2)	Bacteria (1)	100.00	1.90	99.90	99.00	WGS
197.13035	0.1078	Campylobacter jejuni (72294)	Campylobacter (213849)	Campylobacteraceae (2954	Campylobacterales (68525)	Epsilonproteobacteria (122	Proteobacteria (2)	Bacteria (1)	97.40	2.30	98.70	97.40	WGS
197.13036	0.1088	Campylobacter jejuni (72294)	Campylobacter (213849)	Campylobacteraceae (2954	Campylobacterales (68525)	Epsilonproteobacteria (122	Proteobacteria (2)	Bacteria (1)	100.00	0.70	99.90	99.50	WGS
197.13037	0.1088	Campylobacter jejuni (72294)	Campylobacter (213849)	Campylobacteraceae (2954	Campylobacterales (68525)	Epsilonproteobacteria (122	Proteobacteria (2)	Bacteria (1)	99.70	1.60	99.40	98.90	WGS
197.13038	0.1088	Campylobacter jejuni (72294)	Campylobacter (213849)	Campylobacteraceae (2954	Campylobacterales (68525)	Epsilonproteobacteria (122	Proteobacteria (2)	Bacteria (1)	100.00	1.60	99.90	99.10	WGS
197.13039	0.1046	Campylobacter jejuni (72294)	Campylobacter (213849)	Campylobacteraceae (2954	Campylobacterales (68525)	Epsilonproteobacteria (122	Proteobacteria (2)	Bacteria (1)	100.00	10.50	99.30	95.20	WGS
197.13040	0.1088	Campylobacter jejuni (72294)	Campylobacter (213849)	Campylobacteraceae (2954	Campylobacterales (68525)	Epsilonproteobacteria (122	Proteobacteria (2)	Bacteria (1)	100.00	1.30	99.90	99.30	WGS
197.13041	0.1106	Campylobacter jejuni (72294)	Campylobacter (213849)	Campylobacteraceae (2954	Campylobacterales (68525)	Epsilonproteobacteria (122	Proteobacteria (2)	Bacteria (1)	100.00	14.10	99.90	94.20	WGS
197.13042	0.1114	Campylobacter jejuni (72294)	Campylobacter (213849)	Campylobacteraceae (2954	Campylobacterales (68525)	Epsilonproteobacteria (122	Proteobacteria (2)	Bacteria (1)	100.00	21.60	99.80	92.40	WGS
197.13043	0.11	Campylobacter jejuni (72294)	Campylobacter (213849)	Campylobacteraceae (2954	Campylobacterales (68525)	Epsilonproteobacteria (122	Proteobacteria (2)	Bacteria (1)	100.00	5.50	99.80	97.30	WGS
197.13044	0.1142	Campylobacter jejuni (72294)	Campylobacter (213849)	Campylobacteraceae (2954	Campylobacterales (68525)	Epsilonproteobacteria (122	Proteobacteria (2)	Bacteria (1)	100.00	4.40	99.80	98.20	WGS
197.13045	0.1078	Campylobacter jejuni (72294)	Campylobacter (213849)	Campylobacteraceae (2954	Campylobacterales (68525)	Epsilonproteobacteria (122	Proteobacteria (2)	Bacteria (1)	100.00	0.00	99.90	99.90	WGS
197.13046	0.11	Campylobacter jejuni (72294)	Campylobacter (213849)	Campylobacteraceae (2954	Campylobacterales (68525)	Epsilonproteobacteria (122	Proteobacteria (2)	Bacteria (1)	100.00	1.30	99.90	99.60	WGS
197.13047	0.1078	Campylobacter jejuni (72294)	Campylobacter (213849)	Campylobacteraceae (2954	Campylobacterales (68525)	Epsilonproteobacteria (122	Proteobacteria (2)	Bacteria (1)	100.00	0.00	100.00	99.60	WGS
197.13048	0.116	Campylobacter jejuni (72294)	Campylobacter (213849)	Campylobacteraceae (2954	Campylobacterales (68525)	Epsilonproteobacteria (122	Proteobacteria (2)	Bacteria (1)	100.00	24.60	99.90	94.90	WGS
197.13049	0.1106	Campylobacter jejuni (72294)	Campylobacter (213849)	Campylobacteraceae (2954	Campylobacterales (68525)	Epsilonproteobacteria (122	Proteobacteria (2)	Bacteria (1)	100.00	0.00	100.00	99.90	WGS
197.13050	0.109	Campylobacter jejuni (72294)	Campylobacter (213849)	Campylobacteraceae (2954	Campylobacterales (68525)	Epsilonproteobacteria (122	Proteobacteria (2)	Bacteria (1)	100.00	0.00	99.90	99.20	WGS
197.13051	0.1106	Campylobacter jejuni (72294)	Campylobacter (213849)	Campylobacteraceae (2954	Campylobacterales (68525)	Epsilonproteobacteria (122	Proteobacteria (2)	Bacteria (1)	100.00	0.00	100.00	99.60	WGS
197.13052	0.1106	Campylobacter jejuni (72294)	Campylobacter (213849)	Campylobacteraceae (2954	Campylobacterales (68525)	Epsilonproteobacteria (122	Proteobacteria (2)	Bacteria (1)	100.00	0.70	100.00	99.60	WGS
197.13053	0.116	Campylobacter jejuni (72294)	Campylobacter (213849)	Campylobacteraceae (2954	Campylobacterales (68525)	Epsilonproteobacteria (122	Proteobacteria (2)	Bacteria (1)	100.00	14.60	99.90	96.50	WGS
197.13054	0.1146	Campylobacter jejuni (72294)	Campylobacter (213849)	Campylobacteraceae (2954	Campylobacterales (68525)	Epsilonproteobacteria (122	Proteobacteria (2)	Bacteria (1)	100.00	1.90	99.90	99.30	WGS
197.13055	0.1134	Campylobacter jejuni (72294)	Campylobacter (213849)	Campylobacteraceae (2954	Campylobacterales (68525)	Epsilonproteobacteria (122	Proteobacteria (2)	Bacteria (1)	100.00	0.30	99.80	99.20	WGS
197.13056	0.1118	Campylobacter jejuni (72294)	Campylobacter (213849)	Campylobacteraceae (2954	Campylobacterales (68525)	Epsilonproteobacteria (122	Proteobacteria (2)	Bacteria (1)	100.00	8.40	99.80	97.60	WGS
197.13057	0.111	Campylobacter jejuni (72294)	Campylobacter (213849)	Campylobacteraceae (2954	Campylobacterales (68525)	Epsilonproteobacteria (122	Proteobacteria (2)	Bacteria (1)	100.00	21.80	99.90	94.60	WGS
197.13058	0.1118	Campylobacter jejuni (72294)	Campylobacter (213849)	Campylobacteraceae (2954	Campylobacterales (68525)	Epsilonproteobacteria (122	Proteobacteria (2)	Bacteria (1)	100.00	0.00	99.90	99.40	WGS
197.13059	0.1152	Campylobacter jejuni (72294)	Campylobacter (213849)	Campylobacteraceae (2954	Campylobacterales (68525)	Epsilonproteobacteria (122	Proteobacteria (2)	Bacteria (1)	100.00	0.00	99.90	99.80	WGS
197.13060	0.1132	Campylobacter jejuni (72294)	Campylobacter (213849)	Campylobacteraceae (2954	Campylobacterales (68525)	Epsilonproteobacteria (122	Proteobacteria (2)	Bacteria (1)	100.00	0.00	99.80	99.80	WGS
197.13061	0.1126	Campylobacter jejuni (72294)	Campylobacter (213849)	Campylobacteraceae (2954	Campylobacterales (68525)	Epsilonproteobacteria (122	Proteobacteria (2)	Bacteria (1)	100.00	0.00	99.70	99.60	WGS
197.13062	0.1132	Campylobacter jejuni (72294)	Campylobacter (213849)	Campylobacteraceae (2954	Campylobacterales (68525)	Epsilonproteobacteria (122	Proteobacteria (2)	Bacteria (1)	100.00	0.00	99.80	99.60	Complete
197.13316	0.1096	Campylobacter jejuni (72294)	Campylobacter (213849)	Campylobacteraceae (2954	Campylobacterales (68525)	Epsilonproteobacteria (122	Proteobacteria (2)	Bacteria (1)	100.00	0.00	100.00	99.90	Complete
197.13317	0.11	Campylobacter jejuni (72294)	Campylobacter (213849)	Campylobacteraceae (2954	Campylobacterales (68525)	Epsilonproteobacteria (122	Proteobacteria (2)	Bacteria (1)	100.00	0.00	100.00	99.90	Complete
197.13318	0.1166	Campylobacter jejuni (72294)	Campylobacter (213849)	Campylobacteraceae (2954	Campylobacterales (68525)	Epsilonproteobacteria (122	Proteobacteria (2)	Bacteria (1)	100.00	0.00	99.90	99.80	Complete
197.13319	0.0994	Campylobacter jejuni (72294)	Campylobacter (213849)	Campylobacteraceae (2954	Campylobacterales (68525)	Epsilonproteobacteria (122	Proteobacteria (2)	Bacteria (1)	100.00	0.00	99.80	99.60	Complete
197.13320	0.11	Campylobacter jejuni (72294)	Campylobacter (213849)	Campylobacteraceae (2954	Campylobacterales (68525)	Epsilonproteobacteria (122	Proteobacteria (2)	Bacteria (1)	100.00	0.00	100.00	100.00	Complete
197.13321	0.11	Campylobacter jejuni (72294)	Campylobacter (213849)	Campylobacteraceae (2954	Campylobacterales (68525)	Epsilonproteobacteria (122	Proteobacteria (2)	Bacteria (1)	100.00	0.00	99.90	99.90	Complete
197.13322	0.112	Campylobacter jejuni (72294)	Campylobacter (213849)	Campylobacteraceae (2954	Campylobacterales (68525)	Epsilonproteobacteria (122	Proteobacteria (2)	Bacteria (1)	100.00	0.00	99.80	99.70	Complete
197.13323	0.1172	Campylobacter jejuni (72294)	Campylobacter (213849)	Campylobacteraceae (2954	Campylobacterales (68525)	Epsilonproteobacteria (122	Proteobacteria (2)	Bacteria (1)	100.00	0.00	99.70	99.60	Complete
197.13324	0.0948	Campylobacter jejuni (72294)	Campylobacter (213849)	Campylobacteraceae (2954	Campylobacterales (68525)	Epsilonproteobacteria (122	Proteobacteria (2)	Bacteria (1)	100.00	0.00	99.90	99.90	Complete
197.13325	0.1248	Campylobacter jejuni (72294)	Campylobacter (213849)	Campylobacteraceae (2954	Campylobacterales (68525)	Epsilonproteobacteria (122	Proteobacteria (2)	Bacteria (1)	100.00	0.00	99.80	99.60	Complete
197.13326	0.1116	Campylobacter jejuni (72294)	Campylobacter (213849)	Campylobacteraceae (2954	Campylobacterales (68525)	Epsilonproteobacteria (122	Proteobacteria (2)	Bacteria (1)	100.00	1.30	99.90	99.70	Complete
197.13330	0.11	Campylobacter jejuni (72294)	Campylobacter (213849)	Campylobacteraceae (2954	Campylobacterales (68525)	Epsilonproteobacteria (122	Proteobacteria (2)	Bacteria (1)	100.00	0.00	99.70	99.60	WGS
197.13331	0.1118	Campylobacter jejuni (72294)	Campylobacter (213849)	Campylobacteraceae (2954	Campylobacterales (68525)	Epsilonproteobacteria (122	Proteobacteria (2)	Bacteria (1)	98.70	4.60	99.00	98.20	WGS
197.13332	0.1106	Campylobacter jejuni (72294)	Campylobacter (213849)	Campylobacteraceae (2954	Campylobacterales (68525)	Epsilonproteobacteria (122	Proteobacteria (2)	Bacteria (1)	100.00	0.00	99.90	99.80	WGS
197.13333	0.11	Campylobacter jejuni (72294)	Campylobacter (213849)	Campylobacteraceae (2954	Campylobacterales (68525)	Epsilonproteobacteria (122	Proteobacteria (2)	Bacteria (1)	100.00	0.00	99.80	99.60	WGS
197.14641	0.106	Campylobacter jejuni (72294)	Campylobacter (213849)	Campylobacteraceae (2954	Campylobacterales (68525)	Epsilonproteobacteria (122	Proteobacteria (2)	Bacteria (1)			99.90	95.50	Complete
197.14642	0.1106	Campylobacter jejuni (72294)	Campylobacter (213849)	Campylobacteraceae (2954	Campylobacterales (68525)	Epsilonproteobacteria (122	Proteobacteria (2)	Bacteria (1)			99.80	97.30	WGS
197.14643	0.116	Campylobacter jejuni (72294)	Campylobacter (213849)	Campylobacteraceae (2954	Campylobacterales (68525)	Epsilonproteobacteria (122	Proteobacteria (2)	Bacteria (1)			99.70	85.70	WGS
197.14644	0.1106	Campylobacter jejuni (72294)	Campylobacter (213849)	Campylobacteraceae (2954	Campylobacterales (68525)	Epsilonproteobacteria (122	Proteobacteria (2)	Bacteria (1)			99.70	95.10	WGS
197.14645	0.116	Campylobacter jejuni (72294)	Campylobacter (213849)	Campylobacteraceae (2954	Campylobacterales (68525)	Epsilonproteobacteria (122	Proteobacteria (2)	Bacteria (1)			99.80	97.70	WGS
197.14646	0.1078	Campylobacter jejuni (72294)	Campylobacter (213849)	Campylobacteraceae (2954	Campylobacterales (68525)	Epsilonproteobacteria (122	Proteobacteria (2)	Bacteria (1)			99.90	98.50	WGS
197.14647	0.1078	Campylobacter jejuni (72294)	Campylobacter (213849)	Campylobacteraceae (2954	Campylobacterales (68525)	Epsilonproteobacteria (122	Proteobacteria (2)	Bacteria (1)			99.90	98.50	WGS
197.14648	0.1128	Campylobacter jejuni (72294)	Campylobacter (213849)	Campylobacteraceae (2954	Campylobacterales (68525)	Epsilonproteobacteria (122	Proteobacteria (2)	Bacteria (1)			99.90	98.70	WGS
197.14649	0.1106	Campylobacter jejuni (72294)	Campylobacter (213849)	Campylobacteraceae (2954	Campylobacterales (68525)	Epsilonproteobacteria (122	Proteobacteria (2)	Bacteria (1)			99.90	98.20	WGS
197.14650	0.1128	Campylobacter jejuni (72294)	Campylobacter (213849)	Campylobacteraceae (2954	Campylobacterales (68525)	Epsilonproteobacteria (122	Proteobacteria (2)	Bacteria (1)			99.90	98.40	WGS
197.14651	0.1078	Campylobacter jejuni (72294)	Campylobacter (213849)	Campylobacteraceae (2954	Campylobacterales (68525)	Epsilonproteobacteria (122	Proteobacteria (2)	Bacteria (1)			99.90	98.40	WGS
197.14652	0.1174	Campylobacter jejuni (72294)	Campylobacter (213849)	Campylobacteraceae (2954	Campylobacterales (68525)	Epsilonproteobacteria (122	Proteobacteria (2)	Bacteria (1)			99.60	98.40	WGS
197.14653	0.1078	Campylobacter jejuni (72294)	Campylobacter (213849)	Campylobacteraceae (2954	Campylobacterales (68525)	Epsilonproteobacteria (122	Proteobacteria (2)	Bacteria (1)			100.00	98.00	WGS
197.14654	0.1118	Campylobacter jejuni (72294)	Campylobacter (213849)	Campylobacteraceae (2954	Campylobacterales (68525)	Epsilonproteobacteria (122	Proteobacteria (2)	Bacteria (1)			100.00	99.90	WGS
197.14655	0.1078	Campylobacter jejuni (72294)	Campylobacter (213849)	Campylobacteraceae (2954	Campylobacterales (68525)	Epsilonproteobacteria (122	Proteobacteria (2)	Bacteria (1)			99.90	98.40	WGS
197.14656	0.1078	Campylobacter jejuni (72294)	Campylobacter (213849)	Campylobacteraceae (2954	Campylobacterales (68525)	Epsilonproteobacteria (122	Proteobacteria (2)	Bacteria (1)			99.90	98.50	WGS
197.14657	0.1154	Campylobacter jejuni (72294)	Campylobacter (213849)	Campylobacteraceae (2954	Campylobacterales (68525)	Epsilonproteobacteria (122	Proteobacteria (2)	Bacteria (1)			99.60	99.60	WGS
197.14658	0.116	Campylobacter jejuni (72294)	Campylobacter (213849)	Campylobacteraceae (2954	Campylobacterales (68525)	Epsilonproteobacteria (122	Proteobacteria (2)	Bacteria (1)			99.80	98.70	WGS
197.14659	0.109	Campylobacter jejuni (72294)	Campylobacter (213849)	Campylobacteraceae (2954	Campylobacterales (68525)	Epsilonproteobacteria (122	Proteobacteria (2)	Bacteria (1)			99.90	99.00	WGS
197.14660	0.1078	Campylobacter jejuni (72294)	Campylobacter (213849)	Campylobacteraceae (2954	Campylobacterales (68525)	Epsilonproteobacteria (122	Proteobacteria (2)	Bacteria (1)			99.90	99.00	WGS
197.14661	0.1078	Campylobacter jejuni (72294)	Campylobacter (213849)	Campylobacteraceae (2954	Campylobacterales (68525)	Epsilonproteobacteria (122	Proteobacteria (2)	Bacteria (1)			99.90	99.10	WGS
197.14662	0.1114	Campylobacter jejuni (72294)	Campylobacter (213849)	Campylobacteraceae (2954	Campylobacterales (68525)	Epsilonproteobacteria (122	Proteobacteria (2)	Bacteria (1)			99.60	99.60	WGS
197.14663	0.1142	Campylobacter jejuni (72294)	Campylobacter (213849)	Campylobacteraceae (2954	Campylobacterales (68525)	Epsilonproteobacteria (122	Proteobacteria (2)	Bacteria (1)			99.60	99.30	WGS
197.14664	0.1152	Campylobacter jejuni (72294)	Campylobacter (213849)	Campylobacteraceae (2954	Campylobacterales (68525)	Epsilonproteobacteria (122	Proteobacteria (2)	Bacteria (1)			99.80	99.00	WGS
197.14665	0.1106	Campylobacter jejuni (72294)	Campylobacter (213849)	Campylobacteraceae (2954	Campylobacterales (68525)	Epsilonproteobacteria (122	Proteobacteria (2)	Bacteria (1)			99.80	98.30	WGS
197.14666	0.11	Campylobacter jejuni (72294)	Campylobacter (213849)	Campylobacteraceae (2954	Campylobacterales (68525)	Epsilonproteobacteria (122	Proteobacteria (2)	Bacteria (1)			99.90	98.80	WGS
197.14667	0.1142	Campylobacter jejuni (72294)	Campylobacter (213849)	Campylobacteraceae (2954	Campylobacterales (68525)	Epsilonproteobacteria (122	Proteobacteria (2)	Bacteria (1)			99.50	99.10	WGS
197.14668	0.1152	Campylobacter jejuni (72294)	Campylobacter (213849)	Campylobacteraceae (2954	Campylobacterales (68525)	Epsilonproteobacteria (122	Proteobacteria (2)	Bacteria (1)			99.80	99.00	WGS
197.14669	0.1146	Campylobacter jejuni (72294)	Campylobacter (213849)	Campylobacteraceae (2954	Campylobacterales (68525)	Epsilonproteobacteria (122	Proteobacteria (2)	Bacteria (1)			99.90	98.60	WGS
197.14670	0.1078	Campylobacter jejuni (72294)	Campylobacter (213849)	Campylobacteraceae (2954	Campylobacterales (68525)	Epsilonproteobacteria (122	Proteobacteria (2)	Bacteria (1)			99.90	99.00	WGS
197.14671	0.1106	Campylobacter jejuni (72294)	Campylobacter (213849)	Campylobacteraceae (2954	Campylobacterales (68525)	Epsilonproteobacteria (122	Proteobacteria (2)	Bacteria (1)			99.90	98.20	WGS
197.14672	0.1078	Campylobacter jejuni (72294)	Campylobacter (213849)	Campylobacteraceae (2954	Campylobacterales (68525)	Epsilonproteobacteria (122	Proteobacteria (2)	Bacteria (1)			99.90	99.00	WGS
197.14673	0.1114	Campylobacter jejuni (72294)	Campylobacter (213849)	Campylobacteraceae (2954	Campylobacterales (68525)	Epsilonproteobacteria (122	Proteobacteria (2)	Bacteria (1)			99.60	98.60	WGS
197.14674	0.109	Campylobacter jejuni (72294)	Campylobacter (213849)	Campylobacteraceae (2954	Campylobacterales (68525)	Epsilonproteobacteria (122	Proteobacteria (2)	Bacteria (1)			99.90	99.40	WGS
197.14675	0.1106	Campylobacter jejuni (72294)	Campylobacter (213849)	Campylobacteraceae (2954	Campylobacterales (68525)	Epsilonproteobacteria (122	Proteobacteria (2)	Bacteria (1)			99.90	99.50	WGS
197.14676	0.1172	Campylobacter jejuni (72294)	Campylobacter (213849)	Campylobacteraceae (2954	Campylobacterales (68525)	Epsilonproteobacteria (122	Proteobacteria (2)	Bacteria (1)			99.80	99.70	WGS
197.14677	0.1128	Campylobacter jejuni (72294)	Campylobacter (213849)	Campylobacteraceae (2954	Campylobacterales (68525)	Epsilonproteobacteria (122	Proteobacteria (2)	Bacteria (1)			99.90	99.00	WGS
197.14678	0.1078	Campylobacter jejuni (72294)	Campylobacter (213849)	Campylobacteraceae (2954	Campylobacterales (68525)	Epsilonproteobacteria (122	Proteobacteria (2)	Bacteria (1)			99.90	99.90	WGS
197.14679	0.1098	Campylobacter jejuni (72294)	Campylobacter (213849)	Campylobacteraceae (2954	Campylobacterales (68525)	Epsilonproteobacteria (122	Proteobacteria (2)	Bacteria (1)			99.90	99.60	WGS
197.14680	0.1118	Campylobacter jejuni (72294)	Campylobacter (213849)	Campylobacteraceae (2954	Campylobacterales (68525)	Epsilonproteobacteria (122	Proteobacteria (2)	Bacteria (1)			99.90	99.90	WGS
197.14681	0.1164	Campylobacter jejuni (72294)	Campylobacter (213849)	Campylobacteraceae (2954	Campylobacterales (68525)	Epsilonproteobacteria (122	Proteobacteria (2)	Bacteria (1)			99.80	98.90	WGS
197.14682	0.1128	Campylobacter jejuni (72294)	Campylobacter (213849)	Campylobacteraceae (2954	Campylobacterales (68525)	Epsilonproteobacteria (122	Proteobacteria (2)	Bacteria (1)			99.70	99.30	WGS
197.14683	0.1078	Campylobacter jejuni (72294)	Campylobacter (213849)	Campylobacteraceae (2954	Campylobacterales (68525)	Epsilonproteobacteria (122	Proteobacteria (2)	Bacteria (1)			99.90	98.50	WGS
197.14684	0.1078	Campylobacter jejuni (72294)	Campylobacter (213849)	Campylobacteraceae (2954	Campylobacterales (68525)	Epsilonproteobacteria (122	Proteobacteria (2)	Bacteria (1)			100.00	99.20	WGS
197.14685	0.1106	Campylobacter jejuni (72294)	Campylobacter (213849)	Campylobacteraceae (2954	Campylobacterales (68525)	Epsilonproteobacteria (122	Proteobacteria (2)	Bacteria (1)			99.60	98.50	WGS
197.14686	0.11	Campylobacter jejuni (72294)	Campylobacter (213849)	Campylobacteraceae (2954	Campylobacterales (68525)	Epsilonproteobacteria (122	Proteobacteria (2)	Bacteria (1)			100.00	98.60	WGS
197.14687	0.1136	Campylobacter jejuni (72294)	Campylobacter (213849)	Campylobacteraceae (2954	Campylobacterales (68525)	Epsilonproteobacteria (122	Proteobacteria (2)	Bacteria (1)			99.90	99.90	WGS
197.14688	0.1142	Campylobacter jejuni (72294)	Campylobacter (213849)	Campylobacteraceae (2954	Campylobacterales (68525)	Epsilonproteobacteria (122	Proteobacteria (2)	Bacteria (1)			99.60	98.40	WGS
197.14689	0.1128	Campylobacter jejuni (72294)	Campylobacter (213849)	Campylobacteraceae (2954	Campylobacterales (68525)	Epsilonproteobacteria (122	Proteobacteria (2)	Bacteria (1)			99.90	99.30	WGS
197.14690	0.1126	Campylobacter jejuni (72294)	Campylobacter (213849)	Campylobacteraceae (2954	Campylobacterales (68525)	Epsilonproteobacteria (122	Proteobacteria (2)	Bacteria (1)			99.90	99.40	WGS
197.14691	0.1106	Campylobacter jejuni (72294)	Campylobacter (213849)	Campylobacteraceae (2954	Campylobacterales (68525)	Epsilonproteobacteria (122	Proteobacteria (2)	Bacteria (1)			99.90	99.50	WGS
197.14692	0.1132	Campylobacter jejuni (72294)	Campylobacter (213849)	Campylobacteraceae (2954	Campylobacterales (68525)	Epsilonproteobacteria (122	Proteobacteria (2)	Bacteria (1)			99.80	99.60	WGS
197.14693	0.1142	Campylobacter jejuni (72294)	Campylobacter (213849)	Campylobacteraceae (2954	Campylobacterales (68525)	Epsilonproteobacteria (122	Proteobacteria (2)	Bacteria (1)			99.80	99.60	WGS
197.14694	0.109	Campylobacter jejuni (72294)	Campylobacter (213849)	Campylobacteraceae (2954	Campylobacterales (68525)	Epsilonproteobacteria (122	Proteobacteria (2)	Bacteria (1)			99.90	99.00	WGS
197.14695	0.1106	Campylobacter jejuni (72294)	Campylobacter (213849)	Campylobacteraceae (2954	Campylobacterales (68525)	Epsilonproteobacteria (122	Proteobacteria (2)	Bacteria (1)			99.80	99.20	WGS
197.14696	0.1078	Campylobacter jejuni (72294)	Campylobacter (213849)	Campylobacteraceae (2954	Campylobacterales (68525)	Epsilonproteobacteria (122	Proteobacteria (2)	Bacteria (1)			99.90	99.60	WGS
197.14697	0.1078	Campylobacter jejuni (72294)	Campylobacter (213849)	Campylobacteraceae (2954	Campylobacterales (68525)	Epsilonproteobacteria (122	Proteobacteria (2)	Bacteria (1)			99.90	99.30	WGS
197.14698	0.1132	Campylobacter jejuni (72294)	Campylobacter (213849)	Campylobacteraceae (2954	Campylobacterales (68525)	Epsilonproteobacteria (122	Proteobacteria (2)	Bacteria (1)			99.90	99.20	WGS
197.14699	0.1078	Campylobacter jejuni (72294)	Campylobacter (213849)	Campylobacteraceae (2954	Campylobacterales (68525)	Epsilonproteobacteria (122	Proteobacteria (2)	Bacteria (1)			99.90	99.60	WGS
197.14700	0.1144	Campylobacter jejuni (72294)	Campylobacter (213849)	Campylobacteraceae (2954	Campylobacterales (68525)	Epsilonproteobacteria (122	Proteobacteria (2)	Bacteria (1)			99.70	99.60	WGS
197.14701	0.1136	Campylobacter jejuni (72294)	Campylobacter (213849)	Campylobacteraceae (2954	Campylobacterales (68525)	Epsilonproteobacteria (122	Proteobacteria (2)	Bacteria (1)			99.90	99.80	WGS
197.14702	0.1152	Campylobacter jejuni (72294)	Campylobacter (213849)	Campylobacteraceae (2954	Campylobacterales (68525)	Epsilonproteobacteria (122	Proteobacteria (2)	Bacteria (1)			99.90	99.60	WGS
197.14703	0.1114	Campylobacter jejuni (72294)	Campylobacter (213849)	Campylobacteraceae (2954	Campylobacterales (68525)	Epsilonproteobacteria (122	Proteobacteria (2)	Bacteria (1)			100.00	99.60	WGS
197.14704	0.1078	Campylobacter jejuni (72294)	Campylobacter (213849)	Campylobacteraceae (2954	Campylobacterales (68525)	Epsilonproteobacteria (122	Proteobacteria (2)	Bacteria (1)			99.90	98.50	WGS
197.14705	0.1078	Campylobacter jejuni (72294)	Campylobacter (213849)	Campylobacteraceae (2954	Campylobacterales (68525)	Epsilonproteobacteria (122	Proteobacteria (2)	Bacteria (1)			99.70	99.00	WGS
197.14706	0.114	Campylobacter jejuni (72294)	Campylobacter (213849)	Campylobacteraceae (2954	Campylobacterales (68525)	Epsilonproteobacteria (122	Proteobacteria (2)	Bacteria (1)			99.90	99.30	WGS
197.14707	0.1126	Campylobacter jejuni (72294)	Campylobacter (213849)	Campylobacteraceae (2954	Campylobacterales (68525)	Epsilonproteobacteria (122	Proteobacteria (2)	Bacteria (1)			99.60	99.60	WGS
197.14708	0.1132	Campylobacter jejuni (72294)	Campylobacter (213849)	Campylobacteraceae (2954	Campylobacterales (68525)	Epsilonproteobacteria (122	Proteobacteria (2)	Bacteria (1)			99.80	99.50	WGS
197.14709	0.1118	Campylobacter jejuni (72294)	Campylobacter (213849)	Campylobacteraceae (2954	Campylobacterales (68525)	Epsilonproteobacteria (122	Proteobacteria (2)	Bacteria (1)			99.90	99.30	WGS
197.14710	0.1138	Campylobacter jejuni (72294)	Campylobacter (213849)	Campylobacteraceae (2954	Campylobacterales (68525)	Epsilonproteobacteria (122	Proteobacteria (2)	Bacteria (1)			100.00	99.70	WGS
197.14711	0.1078	Campylobacter jejuni (72294)	Campylobacter (213849)	Campylobacteraceae (2954	Campylobacterales (68525)	Epsilonproteobacteria (122	Proteobacteria (2)	Bacteria (1)			99.90	99.40	WGS
197.14712	0.1126	Campylobacter jejuni (72294)	Campylobacter (213849)	Campylobacteraceae (2954	Campylobacterales (68525)	Epsilonproteobacteria (122	Proteobacteria (2)	Bacteria (1)			99.70	99.00	WGS
197.14713	0.1078	Campylobacter jejuni (72294)	Campylobacter (213849)	Campylobacteraceae (2954	Campylobacterales (68525)	Epsilonproteobacteria (122	Proteobacteria (2)	Bacteria (1)			99.90	99.00	WGS
197.14714	0.1098	Campylobacter jejuni (72294)	Campylobacter (213849)	Campylobacteraceae (2954	Campylobacterales (68525)	Epsilonproteobacteria (122	Proteobacteria (2)	Bacteria (1)			99.60	99.10	WGS
197.14715	0.1118	Campylobacter jejuni (72294)	Campylobacter (213849)	Campylobacteraceae (2954	Campylobacterales (68525)	Epsilonproteobacteria (122	Proteobacteria (2)	Bacteria (1)			99.90	99.60	WGS
197.14716	0.1078	Campylobacter jejuni (72294)	Campylobacter (213849)	Campylobacteraceae (2954	Campylobacterales (68525)	Epsilonproteobacteria (122	Proteobacteria (2)	Bacteria (1)			99.90	98.50	WGS
197.14717	0.1078	Campylobacter jejuni (72294)	Campylobacter (213849)	Campylobacteraceae (2954	Campylobacterales (68525)	Epsilonproteobacteria (122	Proteobacteria (2)	Bacteria (1)			100.00	99.60	WGS
197.14718	0.1106	Campylobacter jejuni (72294)	Campylobacter (213849)	Campylobacteraceae (2954	Campylobacterales (68525)	Epsilonproteobacteria (122	Proteobacteria (2)	Bacteria (1)			99.90	97.30	WGS
197.14719	0.11	Campylobacter jejuni (72294)	Campylobacter (213849)	Campylobacteraceae (2954	Campylobacterales (68525)	Epsilonproteobacteria (122	Proteobacteria (2)	Bacteria (1)			99.90	99.60	WGS
197.14720	0.1136	Campylobacter jejuni (72294)	Campylobacter (213849)	Campylobacteraceae (2954	Campylobacterales (68525)	Epsilonproteobacteria (122	Proteobacteria (2)	Bacteria (1)			99.90	99.00	WGS
197.14721	0.1146	Campylobacter jejuni (72294)	Campylobacter (213849)	Campylobacteraceae (2954	Campylobacterales (68525)	Epsilonproteobacteria (122	Proteobacteria (2)	Bacteria (1)			99.80	99.40	WGS
197.14722	0.1096	Campylobacter jejuni (72294)	Campylobacter (213849)	Campylobacteraceae (2954	Campylobacterales (68525)	Epsilonproteobacteria (122	Proteobacteria (2)	Bacteria (1)			99.70	99.20	WGS
197.14723	0.109	Campylobacter jejuni (72294)	Campylobacter (213849)	Campylobacteraceae (2954	Campylobacterales (68525)	Epsilonproteobacteria (122	Proteobacteria (2)	Bacteria (1)			99.70	99.10	WGS
197.14724	0.1106	Campylobacter jejuni (72294)	Campylobacter (213849)	Campylobacteraceae (2954	Campylobacterales (68525)	Epsilonproteobacteria (122	Proteobacteria (2)	Bacteria (1)			99.90	99.20	WGS
197.14725	0.1118	Campylobacter jejuni (72294)	Campylobacter (213849)	Campylobacteraceae (2954	Campylobacterales (68525)	Epsilonproteobacteria (122	Proteobacteria (2)	Bacteria (1)			99.60	98.20	WGS
197.14726	0.1106	Campylobacter jejuni (72294)	Campylobacter (213849)	Campylobacteraceae (2954	Campylobacterales (68525)	Epsilonproteobacteria (122	Proteobacteria (2)	Bacteria (1)			100.00	99.70	WGS
197.14727	0.116	Campylobacter jejuni (72294)	Campylobacter (213849)	Campylobacteraceae (2954	Campylobacterales (68525)	Epsilonproteobacteria (122	Proteobacteria (2)	Bacteria (1)			99.90	99.30	WGS
197.14728	0.108	Campylobacter jejuni (72294)	Campylobacter (213849)	Campylobacteraceae (2954	Campylobacterales (68525)	Epsilonproteobacteria (122	Proteobacteria (2)	Bacteria (1)			99.90	99.90	WGS
197.14729	0.1132	Campylobacter jejuni (72294)	Campylobacter (213849)	Campylobacteraceae (2954	Campylobacterales (68525)	Epsilonproteobacteria (122	Proteobacteria (2)	Bacteria (1)			99.80	98.70	WGS
197.14730	0.1126	Campylobacter jejuni (72294)	Campylobacter (213849)	Campylobacteraceae (2954	Campylobacterales (68525)	Epsilonproteobacteria (122	Proteobacteria (2)	Bacteria (1)			99.70	99.20	WGS
197.14731	0.112	Campylobacter jejuni (72294)	Campylobacter (213849)	Campylobacteraceae (2954	Campylobacterales (68525)	Epsilonproteobacteria (122	Proteobacteria (2)	Bacteria (1)			99.90	98.20	WGS
197.14732	0.1172	Campylobacter jejuni (72294)	Campylobacter (213849)	Campylobacteraceae (2954	Campylobacterales (68525)	Epsilonproteobacteria (122	Proteobacteria (2)	Bacteria (1)			99.60	99.40	WGS
197.14733	0.1098	Campylobacter jejuni (72294)	Campylobacter (213849)	Campylobacteraceae (2954	Campylobacterales (68525)	Epsilonproteobacteria (122	Proteobacteria (2)	Bacteria (1)			99.70	99.50	WGS
197.14734	0.1078	Campylobacter jejuni (72294)	Campylobacter (213849)	Campylobacteraceae (2954	Campylobacterales (68525)	Epsilonproteobacteria (122	Proteobacteria (2)	Bacteria (1)			99.90	99.90	WGS
197.14735	0.1142	Campylobacter jejuni (72294)	Campylobacter (213849)	Campylobacteraceae (2954	Campylobacterales (68525)	Epsilonproteobacteria (122	Proteobacteria (2)	Bacteria (1)			99.60	99.60	WGS
197.14736	0.11	Campylobacter jejuni (72294)	Campylobacter (213849)	Campylobacteraceae (2954	Campylobacterales (68525)	Epsilonproteobacteria (122	Proteobacteria (2)	Bacteria (1)			99.90	99.90	WGS
197.14737	0.1132	Campylobacter jejuni (72294)	Campylobacter (213849)	Campylobacteraceae (2954	Campylobacterales (68525)	Epsilonproteobacteria (122	Proteobacteria (2)	Bacteria (1)			99.80	99.00	WGS
197.14738	0.1132	Campylobacter jejuni (72294)	Campylobacter (213849)	Campylobacteraceae (2954	Campylobacterales (68525)	Epsilonproteobacteria (122	Proteobacteria (2)	Bacteria (1)			99.80	99.50	WGS
197.14739	0.1154	Campylobacter jejuni (72294)	Campylobacter (213849)	Campylobacteraceae (2954	Campylobacterales (68525)	Epsilonproteobacteria (122	Proteobacteria (2)	Bacteria (1)			99.30	99.00	WGS
197.14740	0.1078	Campylobacter jejuni (72294)	Campylobacter (213849)	Campylobacteraceae (2954	Campylobacterales (68525)	Epsilonproteobacteria (122	Proteobacteria (2)	Bacteria (1)			99.90	99.20	WGS
197.14741	0.1078	Campylobacter jejuni (72294)	Campylobacter (213849)	Campylobacteraceae (2954	Campylobacterales (68525)	Epsilonproteobacteria (122	Proteobacteria (2)	Bacteria (1)			99.90	99.30	WGS
197.14742	0.1118	Campylobacter jejuni (72294)	Campylobacter (213849)	Campylobacteraceae (2954	Campylobacterales (68525)	Epsilonproteobacteria (122	Proteobacteria (2)	Bacteria (1)			99.90	99.70	WGS
197.14743	0.1114	Campylobacter jejuni (72294)	Campylobacter (213849)	Campylobacteraceae (2954	Campylobacterales (68525)	Epsilonproteobacteria (122	Proteobacteria (2)	Bacteria (1)			99.80	99.60	WGS
197.14744	0.1142	Campylobacter jejuni (72294)	Campylobacter (213849)	Campylobacteraceae (2954	Campylobacterales (68525)	Epsilonproteobacteria (122	Proteobacteria (2)	Bacteria (1)			99.70	99.60	WGS
197.14745	0.1096	Campylobacter jejuni (72294)	Campylobacter (213849)	Campylobacteraceae (2954	Campylobacterales (68525)	Epsilonproteobacteria (122	Proteobacteria (2)	Bacteria (1)			99.90	99.90	WGS
197.14746	0.1126	Campylobacter jejuni (72294)	Campylobacter (213849)	Campylobacteraceae (2954	Campylobacterales (68525)	Epsilonproteobacteria (122	Proteobacteria (2)	Bacteria (1)			99.80	99.60	WGS
197.14747	0.1154	Campylobacter jejuni (72294)	Campylobacter (213849)	Campylobacteraceae (2954	Campylobacterales (68525)	Epsilonproteobacteria (122	Proteobacteria (2)	Bacteria (1)			99.70	99.30	WGS
197.14748	0.1078	Campylobacter jejuni (72294)	Campylobacter (213849)	Campylobacteraceae (2954	Campylobacterales (68525)	Epsilonproteobacteria (122	Proteobacteria (2)	Bacteria (1)			99.90	98.20	WGS
197.14749	0.1106	Campylobacter jejuni (72294)	Campylobacter (213849)	Campylobacteraceae (2954	Campylobacterales (68525)	Epsilonproteobacteria (122	Proteobacteria (2)	Bacteria (1)			99.90	98.90	WGS
197.14750	0.1096	Campylobacter jejuni (72294)	Campylobacter (213849)	Campylobacteraceae (2954	Campylobacterales (68525)	Epsilonproteobacteria (122	Proteobacteria (2)	Bacteria (1)			99.70	99.40	WGS
197.14751	0.1132	Campylobacter jejuni (72294)	Campylobacter (213849)	Campylobacteraceae (2954	Campylobacterales (68525)	Epsilonproteobacteria (122	Proteobacteria (2)	Bacteria (1)			99.90	99.30	WGS
197.14752	0.1126	Campylobacter jejuni (72294)	Campylobacter (213849)	Campylobacteraceae (2954	Campylobacterales (68525)	Epsilonproteobacteria (122	Proteobacteria (2)	Bacteria (1)			99.70	99.60	WGS
197.14753	0.1078	Campylobacter jejuni (72294)	Campylobacter (213849)	Campylobacteraceae (2954	Campylobacterales (68525)	Epsilonproteobacteria (122	Proteobacteria (2)	Bacteria (1)			99.90	99.10	WGS
197.14754	0.1078	Campylobacter jejuni (72294)	Campylobacter (213849)	Campylobacteraceae (2954	Campylobacterales (68525)	Epsilonproteobacteria (122	Proteobacteria (2)	Bacteria (1)			99.90	98.40	WGS
197.14755	0.1106	Campylobacter jejuni (72294)	Campylobacter (213849)	Campylobacteraceae (2954	Campylobacterales (68525)	Epsilonproteobacteria (122	Proteobacteria (2)	Bacteria (1)			99.90	99.50	WGS
197.14756	0.112	Campylobacter jejuni (72294)	Campylobacter (213849)	Campylobacteraceae (2954	Campylobacterales (68525)	Epsilonproteobacteria (122	Proteobacteria (2)	Bacteria (1)			99.90	98.40	WGS
197.14757	0.109	Campylobacter jejuni (72294)	Campylobacter (213849)	Campylobacteraceae (2954	Campylobacterales (68525)	Epsilonproteobacteria (122	Proteobacteria (2)	Bacteria (1)			99.90	99.00	WGS
197.14758	0.1106	Campylobacter jejuni (72294)	Campylobacter (213849)	Campylobacteraceae (2954	Campylobacterales (68525)	Epsilonproteobacteria (122	Proteobacteria (2)	Bacteria (1)			100.00	99.90	WGS
197.14759	0.11	Campylobacter jejuni (72294)	Campylobacter (213849)	Campylobacteraceae (2954	Campylobacterales (68525)	Epsilonproteobacteria (122	Proteobacteria (2)	Bacteria (1)			99.90	99.60	WGS
197.14760	0.1132	Campylobacter jejuni (72294)	Campylobacter (213849)	Campylobacteraceae (2954	Campylobacterales (68525)	Epsilonproteobacteria (122	Proteobacteria (2)	Bacteria (1)			99.70	99.50	WGS
197.14761	0.1132	Campylobacter jejuni (72294)	Campylobacter (213849)	Campylobacteraceae (2954	Campylobacterales (68525)	Epsilonproteobacteria (122	Proteobacteria (2)	Bacteria (1)			99.80	99.60	WGS
197.14762	0.1162	Campylobacter jejuni (72294)	Campylobacter (213849)	Campylobacteraceae (2954	Campylobacterales (68525)	Epsilonproteobacteria (122	Proteobacteria (2)	Bacteria (1)			99.80	99.70	WGS
197.14763	0.116	Campylobacter jejuni (72294)	Campylobacter (213849)	Campylobacteraceae (2954	Campylobacterales (68525)	Epsilonproteobacteria (122	Proteobacteria (2)	Bacteria (1)			99.60	99.50	WGS
197.14764	0.1118	Campylobacter jejuni (72294)	Campylobacter (213849)	Campylobacteraceae (2954	Campylobacterales (68525)	Epsilonproteobacteria (122	Proteobacteria (2)	Bacteria (1)			99.90	99.90	WGS
197.14765	0.1078	Campylobacter jejuni (72294)	Campylobacter (213849)	Campylobacteraceae (2954	Campylobacterales (68525)	Epsilonproteobacteria (122	Proteobacteria (2)	Bacteria (1)			99.90	99.40	WGS
197.14766	0.1012	Campylobacter jejuni (72294)	Campylobacter (213849)	Campylobacteraceae (2954	Campylobacterales (68525)	Epsilonproteobacteria (122	Proteobacteria (2)	Bacteria (1)			99.50	99.30	WGS
197.14767	0.1106	Campylobacter jejuni (72294)	Campylobacter (213849)	Campylobacteraceae (2954	Campylobacterales (68525)	Epsilonproteobacteria (122	Proteobacteria (2)	Bacteria (1)			99.90	98.10	WGS
197.14768	0.1126	Campylobacter jejuni (72294)	Campylobacter (213849)	Campylobacteraceae (2954	Campylobacterales (68525)	Epsilonproteobacteria (122	Proteobacteria (2)	Bacteria (1)			99.70	98.70	WGS
197.14769	0.1126	Campylobacter jejuni (72294)	Campylobacter (213849)	Campylobacteraceae (2954	Campylobacterales (68525)	Epsilonproteobacteria (122	Proteobacteria (2)	Bacteria (1)			99.90	99.00	WGS
197.14770	0.1078	Campylobacter jejuni (72294)	Campylobacter (213849)	Campylobacteraceae (2954	Campylobacterales (68525)	Epsilonproteobacteria (122	Proteobacteria (2)	Bacteria (1)			99.90	99.30	WGS
197.14771	0.1126	Campylobacter jejuni (72294)	Campylobacter (213849)	Campylobacteraceae (2954	Campylobacterales (68525)	Epsilonproteobacteria (122	Proteobacteria (2)	Bacteria (1)			100.00	99.10	WGS
197.14772	0.1118	Campylobacter jejuni (72294)	Campylobacter (213849)	Campylobacteraceae (2954	Campylobacterales (68525)	Epsilonproteobacteria (122	Proteobacteria (2)	Bacteria (1)			100.00	99.70	WGS
197.14773	0.116	Campylobacter jejuni (72294)	Campylobacter (213849)	Campylobacteraceae (2954	Campylobacterales (68525)	Epsilonproteobacteria (122	Proteobacteria (2)	Bacteria (1)			99.80	99.60	WGS
197.14774	0.1142	Campylobacter jejuni (72294)	Campylobacter (213849)	Campylobacteraceae (2954	Campylobacterales (68525)	Epsilonproteobacteria (122	Proteobacteria (2)	Bacteria (1)			99.70	99.50	WGS
197.14775	0.1114	Campylobacter jejuni (72294)	Campylobacter (213849)	Campylobacteraceae (2954	Campylobacterales (68525)	Epsilonproteobacteria (122	Proteobacteria (2)	Bacteria (1)			99.80	99.20	WGS
197.14776	0.1126	Campylobacter jejuni (72294)	Campylobacter (213849)	Campylobacteraceae (2954	Campylobacterales (68525)	Epsilonproteobacteria (122	Proteobacteria (2)	Bacteria (1)			99.90	97.40	WGS
197.14777	0.1118	Campylobacter jejuni (72294)	Campylobacter (213849)	Campylobacteraceae (2954	Campylobacterales (68525)	Epsilonproteobacteria (122	Proteobacteria (2)	Bacteria (1)			100.00	99.90	WGS
197.14778	0.109	Campylobacter jejuni (72294)	Campylobacter (213849)	Campylobacteraceae (2954	Campylobacterales (68525)	Epsilonproteobacteria (122	Proteobacteria (2)	Bacteria (1)			99.90	99.60	WGS
197.14779	0.112	Campylobacter jejuni (72294)	Campylobacter (213849)	Campylobacteraceae (2954	Campylobacterales (68525)	Epsilonproteobacteria (122	Proteobacteria (2)	Bacteria (1)			99.90	99.40	WGS
197.14780	0.116	Campylobacter jejuni (72294)	Campylobacter (213849)	Campylobacteraceae (2954	Campylobacterales (68525)	Epsilonproteobacteria (122	Proteobacteria (2)	Bacteria (1)			99.90	99.60	WGS
197.14781	0.1106	Campylobacter jejuni (72294)	Campylobacter (213849)	Campylobacteraceae (2954	Campylobacterales (68525)	Epsilonproteobacteria (122	Proteobacteria (2)	Bacteria (1)			99.90	98.60	WGS
197.14782	0.1114	Campylobacter jejuni (72294)	Campylobacter (213849)	Campylobacteraceae (2954	Campylobacterales (68525)	Epsilonproteobacteria (122	Proteobacteria (2)	Bacteria (1)			99.70	99.50	WGS
197.14783	0.1154	Campylobacter jejuni (72294)	Campylobacter (213849)	Campylobacteraceae (2954	Campylobacterales (68525)	Epsilonproteobacteria (122	Proteobacteria (2)	Bacteria (1)			99.60	99.50	WGS
197.14784	0.1114	Campylobacter jejuni (72294)	Campylobacter (213849)	Campylobacteraceae (2954	Campylobacterales (68525)	Epsilonproteobacteria (122	Proteobacteria (2)	Bacteria (1)			99.80	99.30	WGS
197.14785	0.1132	Campylobacter jejuni (72294)	Campylobacter (213849)	Campylobacteraceae (2954	Campylobacterales (68525)	Epsilonproteobacteria (122	Proteobacteria (2)	Bacteria (1)			99.90	99.80	WGS
197.14786	0.1078	Campylobacter jejuni (72294)	Campylobacter (213849)	Campylobacteraceae (2954	Campylobacterales (68525)	Epsilonproteobacteria (122	Proteobacteria (2)	Bacteria (1)			99.90	98.40	WGS
197.14787	0.1078	Campylobacter jejuni (72294)	Campylobacter (213849)	Campylobacteraceae (2954	Campylobacterales (68525)	Epsilonproteobacteria (122	Proteobacteria (2)	Bacteria (1)			99.90	99.00	WGS
197.14788	0.0976	Campylobacter jejuni (72294)	Campylobacter (213849)	Campylobacteraceae (2954	Campylobacterales (68525)	Epsilonproteobacteria (122	Proteobacteria (2)	Bacteria (1)			99.80	99.60	WGS
197.14789	0.1106	Campylobacter jejuni (72294)	Campylobacter (213849)	Campylobacteraceae (2954	Campylobacterales (68525)	Epsilonproteobacteria (122	Proteobacteria (2)	Bacteria (1)			100.00	99.90	WGS
197.14790	0.1078	Campylobacter jejuni (72294)	Campylobacter (213849)	Campylobacteraceae (2954	Campylobacterales (68525)	Epsilonproteobacteria (122	Proteobacteria (2)	Bacteria (1)			99.90	98.70	WGS
197.14791	0.1144	Campylobacter jejuni (72294)	Campylobacter (213849)	Campylobacteraceae (2954	Campylobacterales (68525)	Epsilonproteobacteria (122	Proteobacteria (2)	Bacteria (1)			99.60	99.40	WGS
197.14792	0.11	Campylobacter jejuni (72294)	Campylobacter (213849)	Campylobacteraceae (2954	Campylobacterales (68525)	Epsilonproteobacteria (122	Proteobacteria (2)	Bacteria (1)			99.90	99.10	WGS
197.14793	0.1078	Campylobacter jejuni (72294)	Campylobacter (213849)	Campylobacteraceae (2954	Campylobacterales (68525)	Epsilonproteobacteria (122	Proteobacteria (2)	Bacteria (1)			99.90	99.70	WGS
197.14794	0.1152	Campylobacter jejuni (72294)	Campylobacter (213849)	Campylobacteraceae (2954	Campylobacterales (68525)	Epsilonproteobacteria (122	Proteobacteria (2)	Bacteria (1)			100.00	99.80	WGS
197.14795	0.1172	Campylobacter jejuni (72294)	Campylobacter (213849)	Campylobacteraceae (2954	Campylobacterales (68525)	Epsilonproteobacteria (122	Proteobacteria (2)	Bacteria (1)			99.70	99.60	WGS
197.14796	0.11	Campylobacter jejuni (72294)	Campylobacter (213849)	Campylobacteraceae (2954	Campylobacterales (68525)	Epsilonproteobacteria (122	Proteobacteria (2)	Bacteria (1)			99.90	97.40	WGS
197.14797	0.1078	Campylobacter jejuni (72294)	Campylobacter (213849)	Campylobacteraceae (2954	Campylobacterales (68525)	Epsilonproteobacteria (122	Proteobacteria (2)	Bacteria (1)			99.90	98.90	WGS
197.14798	0.1078	Campylobacter jejuni (72294)	Campylobacter (213849)	Campylobacteraceae (2954	Campylobacterales (68525)	Epsilonproteobacteria (122	Proteobacteria (2)	Bacteria (1)			99.90	99.30	WGS
197.14799	0.1126	Campylobacter jejuni (72294)	Campylobacter (213849)	Campylobacteraceae (2954	Campylobacterales (68525)	Epsilonproteobacteria (122	Proteobacteria (2)	Bacteria (1)			99.60	97.60	WGS
197.14800	0.1124	Campylobacter jejuni (72294)	Campylobacter (213849)	Campylobacteraceae (2954	Campylobacterales (68525)	Epsilonproteobacteria (122	Proteobacteria (2)	Bacteria (1)			99.90	99.70	WGS
197.14801	0.1114	Campylobacter jejuni (72294)	Campylobacter (213849)	Campylobacteraceae (2954	Campylobacterales (68525)	Epsilonproteobacteria (122	Proteobacteria (2)	Bacteria (1)			99.70	99.60	WGS
197.14802	0.112	Campylobacter jejuni (72294)	Campylobacter (213849)	Campylobacteraceae (2954	Campylobacterales (68525)	Epsilonproteobacteria (122	Proteobacteria (2)	Bacteria (1)			99.90	98.40	WGS
197.14803	0.1144	Campylobacter jejuni (72294)	Campylobacter (213849)	Campylobacteraceae (2954	Campylobacterales (68525)	Epsilonproteobacteria (122	Proteobacteria (2)	Bacteria (1)			99.70	99.60	WGS
197.14804	0.116	Campylobacter jejuni (72294)	Campylobacter (213849)	Campylobacteraceae (2954	Campylobacterales (68525)	Epsilonproteobacteria (122	Proteobacteria (2)	Bacteria (1)			99.90	98.90	WGS
197.14805	0.11	Campylobacter jejuni (72294)	Campylobacter (213849)	Campylobacteraceae (2954	Campylobacterales (68525)	Epsilonproteobacteria (122	Proteobacteria (2)	Bacteria (1)			99.80	98.40	WGS
197.14806	0.1154	Campylobacter jejuni (72294)	Campylobacter (213849)	Campylobacteraceae (2954	Campylobacterales (68525)	Epsilonproteobacteria (122	Proteobacteria (2)	Bacteria (1)			99.80	99.70	WGS
197.14807	0.1144	Campylobacter jejuni (72294)	Campylobacter (213849)	Campylobacteraceae (2954	Campylobacterales (68525)	Epsilonproteobacteria (122	Proteobacteria (2)	Bacteria (1)			99.70	99.30	WGS
197.14808	0.1142	Campylobacter jejuni (72294)	Campylobacter (213849)	Campylobacteraceae (2954	Campylobacterales (68525)	Epsilonproteobacteria (122	Proteobacteria (2)	Bacteria (1)			99.60	98.40	WGS
197.14809	0.1096	Campylobacter jejuni (72294)	Campylobacter (213849)	Campylobacteraceae (2954	Campylobacterales (68525)	Epsilonproteobacteria (122	Proteobacteria (2)	Bacteria (1)			99.60	99.30	WGS
197.14810	0.1106	Campylobacter jejuni (72294)	Campylobacter (213849)	Campylobacteraceae (2954	Campylobacterales (68525)	Epsilonproteobacteria (122	Proteobacteria (2)	Bacteria (1)			99.80	99.50	WGS
197.14811	0.109	Campylobacter jejuni (72294)	Campylobacter (213849)	Campylobacteraceae (2954	Campylobacterales (68525)	Epsilonproteobacteria (122	Proteobacteria (2)	Bacteria (1)			99.40	98.50	WGS
197.14812	0.1142	Campylobacter jejuni (72294)	Campylobacter (213849)	Campylobacteraceae (2954	Campylobacterales (68525)	Epsilonproteobacteria (122	Proteobacteria (2)	Bacteria (1)			99.70	99.60	WGS
197.14813	0.1118	Campylobacter jejuni (72294)	Campylobacter (213849)	Campylobacteraceae (2954	Campylobacterales (68525)	Epsilonproteobacteria (122	Proteobacteria (2)	Bacteria (1)			99.90	99.60	WGS
197.14814	0.1078	Campylobacter jejuni (72294)	Campylobacter (213849)	Campylobacteraceae (2954	Campylobacterales (68525)	Epsilonproteobacteria (122	Proteobacteria (2)	Bacteria (1)			99.90	99.60	WGS
197.14815	0.1132	Campylobacter jejuni (72294)	Campylobacter (213849)	Campylobacteraceae (2954	Campylobacterales (68525)	Epsilonproteobacteria (122	Proteobacteria (2)	Bacteria (1)			99.90	99.90	WGS
197.14816	0.1166	Campylobacter jejuni (72294)	Campylobacter (213849)	Campylobacteraceae (2954	Campylobacterales (68525)	Epsilonproteobacteria (122	Proteobacteria (2)	Bacteria (1)			99.70	99.20	WGS
197.14817	0.1078	Campylobacter jejuni (72294)	Campylobacter (213849)	Campylobacteraceae (2954	Campylobacterales (68525)	Epsilonproteobacteria (122	Proteobacteria (2)	Bacteria (1)			99.90	99.30	WGS
197.14818	0.1106	Campylobacter jejuni (72294)	Campylobacter (213849)	Campylobacteraceae (2954	Campylobacterales (68525)	Epsilonproteobacteria (122	Proteobacteria (2)	Bacteria (1)			99.90	99.10	WGS
197.14819	0.1172	Campylobacter jejuni (72294)	Campylobacter (213849)	Campylobacteraceae (2954	Campylobacterales (68525)	Epsilonproteobacteria (122	Proteobacteria (2)	Bacteria (1)			99.70	99.60	WGS
197.14820	0.1118	Campylobacter jejuni (72294)	Campylobacter (213849)	Campylobacteraceae (2954	Campylobacterales (68525)	Epsilonproteobacteria (122	Proteobacteria (2)	Bacteria (1)			100.00	99.60	WGS
197.14821	0.1078	Campylobacter jejuni (72294)	Campylobacter (213849)	Campylobacteraceae (2954	Campylobacterales (68525)	Epsilonproteobacteria (122	Proteobacteria (2)	Bacteria (1)			99.90	99.30	WGS
197.14822	0.1126	Campylobacter jejuni (72294)	Campylobacter (213849)	Campylobacteraceae (2954	Campylobacterales (68525)	Epsilonproteobacteria (122	Proteobacteria (2)	Bacteria (1)			99.90	99.70	WGS
197.14823	0.1106	Campylobacter jejuni (72294)	Campylobacter (213849)	Campylobacteraceae (2954	Campylobacterales (68525)	Epsilonproteobacteria (122	Proteobacteria (2)	Bacteria (1)			99.90	99.00	WGS
197.14824	0.1064	Campylobacter jejuni (72294)	Campylobacter (213849)	Campylobacteraceae (2954	Campylobacterales (68525)	Epsilonproteobacteria (122	Proteobacteria (2)	Bacteria (1)			99.60	98.70	WGS
197.14825	0.1106	Campylobacter jejuni (72294)	Campylobacter (213849)	Campylobacteraceae (2954	Campylobacterales (68525)	Epsilonproteobacteria (122	Proteobacteria (2)	Bacteria (1)			99.90	98.30	WGS
197.14826	0.1078	Campylobacter jejuni (72294)	Campylobacter (213849)	Campylobacteraceae (2954	Campylobacterales (68525)	Epsilonproteobacteria (122	Proteobacteria (2)	Bacteria (1)			100.00	99.60	WGS
197.14827	0.1106	Campylobacter jejuni (72294)	Campylobacter (213849)	Campylobacteraceae (2954	Campylobacterales (68525)	Epsilonproteobacteria (122	Proteobacteria (2)	Bacteria (1)			99.90	98.70	WGS
197.14828	0.116	Campylobacter jejuni (72294)	Campylobacter (213849)	Campylobacteraceae (2954	Campylobacterales (68525)	Epsilonproteobacteria (122	Proteobacteria (2)	Bacteria (1)			99.90	99.70	WGS
197.14829	0.1106	Campylobacter jejuni (72294)	Campylobacter (213849)	Campylobacteraceae (2954	Campylobacterales (68525)	Epsilonproteobacteria (122	Proteobacteria (2)	Bacteria (1)			100.00	99.90	WGS
197.14830	0.1114	Campylobacter jejuni (72294)	Campylobacter (213849)	Campylobacteraceae (2954	Campylobacterales (68525)	Epsilonproteobacteria (122	Proteobacteria (2)	Bacteria (1)			99.70	99.60	WGS
197.14831	0.112	Campylobacter jejuni (72294)	Campylobacter (213849)	Campylobacteraceae (2954	Campylobacterales (68525)	Epsilonproteobacteria (122	Proteobacteria (2)	Bacteria (1)			99.90	98.70	WGS
197.14832	0.1126	Campylobacter jejuni (72294)	Campylobacter (213849)	Campylobacteraceae (2954	Campylobacterales (68525)	Epsilonproteobacteria (122	Proteobacteria (2)	Bacteria (1)			99.80	99.70	WGS
197.14833	0.1132	Campylobacter jejuni (72294)	Campylobacter (213849)	Campylobacteraceae (2954	Campylobacterales (68525)	Epsilonproteobacteria (122	Proteobacteria (2)	Bacteria (1)			99.90	99.70	WGS
197.14834	0.116	Campylobacter jejuni (72294)	Campylobacter (213849)	Campylobacteraceae (2954	Campylobacterales (68525)	Epsilonproteobacteria (122	Proteobacteria (2)	Bacteria (1)			99.80	99.50	WGS
197.14835	0.1078	Campylobacter jejuni (72294)	Campylobacter (213849)	Campylobacteraceae (2954	Campylobacterales (68525)	Epsilonproteobacteria (122	Proteobacteria (2)	Bacteria (1)			99.90	99.30	WGS
197.14836	0.112	Campylobacter jejuni (72294)	Campylobacter (213849)	Campylobacteraceae (2954	Campylobacterales (68525)	Epsilonproteobacteria (122	Proteobacteria (2)	Bacteria (1)			99.90	97.70	WGS
197.14837	0.1116	Campylobacter jejuni (72294)	Campylobacter (213849)	Campylobacteraceae (2954	Campylobacterales (68525)	Epsilonproteobacteria (122	Proteobacteria (2)	Bacteria (1)			99.90	99.70	WGS
197.14838	0.1128	Campylobacter jejuni (72294)	Campylobacter (213849)	Campylobacteraceae (2954	Campylobacterales (68525)	Epsilonproteobacteria (122	Proteobacteria (2)	Bacteria (1)			99.70	98.30	WGS
197.14839	0.116	Campylobacter jejuni (72294)	Campylobacter (213849)	Campylobacteraceae (2954	Campylobacterales (68525)	Epsilonproteobacteria (122	Proteobacteria (2)	Bacteria (1)			99.80	99.30	WGS
197.14840	0.112	Campylobacter jejuni (72294)	Campylobacter (213849)	Campylobacteraceae (2954	Campylobacterales (68525)	Epsilonproteobacteria (122	Proteobacteria (2)	Bacteria (1)			99.90	99.30	WGS
197.14841	0.116	Campylobacter jejuni (72294)	Campylobacter (213849)	Campylobacteraceae (2954	Campylobacterales (68525)	Epsilonproteobacteria (122	Proteobacteria (2)	Bacteria (1)			99.70	99.30	WGS
197.14842	0.1142	Campylobacter jejuni (72294)	Campylobacter (213849)	Campylobacteraceae (2954	Campylobacterales (68525)	Epsilonproteobacteria (122	Proteobacteria (2)	Bacteria (1)			99.60	99.40	WGS
197.14843	0.11	Campylobacter jejuni (72294)	Campylobacter (213849)	Campylobacteraceae (2954	Campylobacterales (68525)	Epsilonproteobacteria (122	Proteobacteria (2)	Bacteria (1)			100.00	99.70	WGS
197.14844	0.1128	Campylobacter jejuni (72294)	Campylobacter (213849)	Campylobacteraceae (2954	Campylobacterales (68525)	Epsilonproteobacteria (122	Proteobacteria (2)	Bacteria (1)			99.90	99.40	WGS
197.14845	0.1078	Campylobacter jejuni (72294)	Campylobacter (213849)	Campylobacteraceae (2954	Campylobacterales (68525)	Epsilonproteobacteria (122	Proteobacteria (2)	Bacteria (1)			100.00	99.20	WGS
197.14846	0.116	Campylobacter jejuni (72294)	Campylobacter (213849)	Campylobacteraceae (2954	Campylobacterales (68525)	Epsilonproteobacteria (122	Proteobacteria (2)	Bacteria (1)			99.90	98.30	WGS
197.14847	0.1078	Campylobacter jejuni (72294)	Campylobacter (213849)	Campylobacteraceae (2954	Campylobacterales (68525)	Epsilonproteobacteria (122	Proteobacteria (2)	Bacteria (1)			99.90	98.50	WGS
197.14848	0.1154	Campylobacter jejuni (72294)	Campylobacter (213849)	Campylobacteraceae (2954	Campylobacterales (68525)	Epsilonproteobacteria (122	Proteobacteria (2)	Bacteria (1)			99.80	99.60	WGS
197.14849	0.1118	Campylobacter jejuni (72294)	Campylobacter (213849)	Campylobacteraceae (2954	Campylobacterales (68525)	Epsilonproteobacteria (122	Proteobacteria (2)	Bacteria (1)			99.90	99.10	WGS
197.14850	0.1116	Campylobacter jejuni (72294)	Campylobacter (213849)	Campylobacteraceae (2954	Campylobacterales (68525)	Epsilonproteobacteria (122	Proteobacteria (2)	Bacteria (1)			99.90	99.10	WGS
197.14851	0.116	Campylobacter jejuni (72294)	Campylobacter (213849)	Campylobacteraceae (2954	Campylobacterales (68525)	Epsilonproteobacteria (122	Proteobacteria (2)	Bacteria (1)			99.80	99.60	WGS
197.14852	0.112	Campylobacter jejuni (72294)	Campylobacter (213849)	Campylobacteraceae (2954	Campylobacterales (68525)	Epsilonproteobacteria (122	Proteobacteria (2)	Bacteria (1)			99.90	98.00	WGS
197.14853	0.1106	Campylobacter jejuni (72294)	Campylobacter (213849)	Campylobacteraceae (2954	Campylobacterales (68525)	Epsilonproteobacteria (122	Proteobacteria (2)	Bacteria (1)			99.90	99.00	WGS
197.14854	0.1114	Campylobacter jejuni (72294)	Campylobacter (213849)	Campylobacteraceae (2954	Campylobacterales (68525)	Epsilonproteobacteria (122	Proteobacteria (2)	Bacteria (1)			99.80	99.00	WGS
197.14855	0.1142	Campylobacter jejuni (72294)	Campylobacter (213849)	Campylobacteraceae (2954	Campylobacterales (68525)	Epsilonproteobacteria (122	Proteobacteria (2)	Bacteria (1)			99.60	98.80	WGS
197.14856	0.1078	Campylobacter jejuni (72294)	Campylobacter (213849)	Campylobacteraceae (2954	Campylobacterales (68525)	Epsilonproteobacteria (122	Proteobacteria (2)	Bacteria (1)			99.90	98.70	WGS
197.14857	0.1116	Campylobacter jejuni (72294)	Campylobacter (213849)	Campylobacteraceae (2954	Campylobacterales (68525)	Epsilonproteobacteria (122	Proteobacteria (2)	Bacteria (1)			99.90	98.90	WGS
197.14858	0.1132	Campylobacter jejuni (72294)	Campylobacter (213849)	Campylobacteraceae (2954	Campylobacterales (68525)	Epsilonproteobacteria (122	Proteobacteria (2)	Bacteria (1)			99.50	98.70	WGS
197.14859	0.1096	Campylobacter jejuni (72294)	Campylobacter (213849)	Campylobacteraceae (2954	Campylobacterales (68525)	Epsilonproteobacteria (122	Proteobacteria (2)	Bacteria (1)			99.90	99.70	WGS
197.14860	0.1118	Campylobacter jejuni (72294)	Campylobacter (213849)	Campylobacteraceae (2954	Campylobacterales (68525)	Epsilonproteobacteria (122	Proteobacteria (2)	Bacteria (1)			99.90	99.50	WGS
197.14861	0.1106	Campylobacter jejuni (72294)	Campylobacter (213849)	Campylobacteraceae (2954	Campylobacterales (68525)	Epsilonproteobacteria (122	Proteobacteria (2)	Bacteria (1)			100.00	96.40	WGS
197.14862	0.1078	Campylobacter jejuni (72294)	Campylobacter (213849)	Campylobacteraceae (2954	Campylobacterales (68525)	Epsilonproteobacteria (122	Proteobacteria (2)	Bacteria (1)			99.90	99.60	WGS
197.14863	0.1078	Campylobacter jejuni (72294)	Campylobacter (213849)	Campylobacteraceae (2954	Campylobacterales (68525)	Epsilonproteobacteria (122	Proteobacteria (2)	Bacteria (1)			99.90	98.70	WGS
197.14864	0.1118	Campylobacter jejuni (72294)	Campylobacter (213849)	Campylobacteraceae (2954	Campylobacterales (68525)	Epsilonproteobacteria (122	Proteobacteria (2)	Bacteria (1)			99.90	99.70	WGS
197.14865	0.116	Campylobacter jejuni (72294)	Campylobacter (213849)	Campylobacteraceae (2954	Campylobacterales (68525)	Epsilonproteobacteria (122	Proteobacteria (2)	Bacteria (1)			99.80	99.60	WGS
197.14866	0.114	Campylobacter jejuni (72294)	Campylobacter (213849)	Campylobacteraceae (2954	Campylobacterales (68525)	Epsilonproteobacteria (122	Proteobacteria (2)	Bacteria (1)			99.90	98.80	WGS
197.14867	0.1114	Campylobacter jejuni (72294)	Campylobacter (213849)	Campylobacteraceae (2954	Campylobacterales (68525)	Epsilonproteobacteria (122	Proteobacteria (2)	Bacteria (1)			99.60	99.30	WGS
197.14868	0.1078	Campylobacter jejuni (72294)	Campylobacter (213849)	Campylobacteraceae (2954	Campylobacterales (68525)	Epsilonproteobacteria (122	Proteobacteria (2)	Bacteria (1)			99.90	99.20	WGS
197.14869	0.1078	Campylobacter jejuni (72294)	Campylobacter (213849)	Campylobacteraceae (2954	Campylobacterales (68525)	Epsilonproteobacteria (122	Proteobacteria (2)	Bacteria (1)			99.90	99.30	WGS
197.14870	0.116	Campylobacter jejuni (72294)	Campylobacter (213849)	Campylobacteraceae (2954	Campylobacterales (68525)	Epsilonproteobacteria (122	Proteobacteria (2)	Bacteria (1)			99.90	99.30	WGS
197.14871	0.099	Campylobacter jejuni (72294)	Campylobacter (213849)	Campylobacteraceae (2954	Campylobacterales (68525)	Epsilonproteobacteria (122	Proteobacteria (2)	Bacteria (1)			99.60	98.80	WGS
197.14872	0.114	Campylobacter jejuni (72294)	Campylobacter (213849)	Campylobacteraceae (2954	Campylobacterales (68525)	Epsilonproteobacteria (122	Proteobacteria (2)	Bacteria (1)			99.90	99.00	WGS
197.14873	0.1132	Campylobacter jejuni (72294)	Campylobacter (213849)	Campylobacteraceae (2954	Campylobacterales (68525)	Epsilonproteobacteria (122	Proteobacteria (2)	Bacteria (1)			99.90	99.80	WGS
197.14874	0.1078	Campylobacter jejuni (72294)	Campylobacter (213849)	Campylobacteraceae (2954	Campylobacterales (68525)	Epsilonproteobacteria (122	Proteobacteria (2)	Bacteria (1)			100.00	99.60	WGS
197.14875	0.1132	Campylobacter jejuni (72294)	Campylobacter (213849)	Campylobacteraceae (2954	Campylobacterales (68525)	Epsilonproteobacteria (122	Proteobacteria (2)	Bacteria (1)			99.80	99.60	WGS
197.14876	0.1142	Campylobacter jejuni (72294)	Campylobacter (213849)	Campylobacteraceae (2954	Campylobacterales (68525)	Epsilonproteobacteria (122	Proteobacteria (2)	Bacteria (1)			99.60	99.50	WGS

197.14877	0.1078	Campylobacter jejuni (72294)	Campylobacter (213849)	Campylobacteraceae (2954	Campylobacterales (68525)	Epsilonproteobacteria (122	Proteobacteria (2)	Bacteria (1)	100.00	98.00	WGS
197.14878	0.109	Campylobacter jejuni (72294)	Campylobacter (213849)	Campylobacteraceae (2954	Campylobacterales (68525)	Epsilonproteobacteria (122	Proteobacteria (2)	Bacteria (1)	99.90	98.80	WGS
197.14879	0.1132	Campylobacter jejuni (72294)	Campylobacter (213849)	Campylobacteraceae (2954	Campylobacterales (68525)	Epsilonproteobacteria (122	Proteobacteria (2)	Bacteria (1)	99.80	99.00	WGS
197.14880	0.1126	Campylobacter jejuni (72294)	Campylobacter (213849)	Campylobacteraceae (2954	Campylobacterales (68525)	Epsilonproteobacteria (122	Proteobacteria (2)	Bacteria (1)	99.70	99.40	WGS
197.14881	0.1154	Campylobacter jejuni (72294)	Campylobacter (213849)	Campylobacteraceae (2954	Campylobacterales (68525)	Epsilonproteobacteria (122	Proteobacteria (2)	Bacteria (1)	99.80	98.30	WGS
197.14882	0.1142	Campylobacter jejuni (72294)	Campylobacter (213849)	Campylobacteraceae (2954	Campylobacterales (68525)	Epsilonproteobacteria (122	Proteobacteria (2)	Bacteria (1)	99.70	99.50	WGS
197.14883	0.1098	Campylobacter jejuni (72294)	Campylobacter (213849)	Campylobacteraceae (2954	Campylobacterales (68525)	Epsilonproteobacteria (122	Proteobacteria (2)	Bacteria (1)	99.70	99.40	WGS
197.14884	0.1166	Campylobacter jejuni (72294)	Campylobacter (213849)	Campylobacteraceae (2954	Campylobacterales (68525)	Epsilonproteobacteria (122	Proteobacteria (2)	Bacteria (1)	99.70	99.60	WGS
197.14885	0.1106	Campylobacter jejuni (72294)	Campylobacter (213849)	Campylobacteraceae (2954	Campylobacterales (68525)	Epsilonproteobacteria (122	Proteobacteria (2)	Bacteria (1)	99.90	99.60	WGS
197.14886	0.1078	Campylobacter jejuni (72294)	Campylobacter (213849)	Campylobacteraceae (2954	Campylobacterales (68525)	Epsilonproteobacteria (122	Proteobacteria (2)	Bacteria (1)	100.00	99.60	WGS
197.14887	0.1116	Campylobacter jejuni (72294)	Campylobacter (213849)	Campylobacteraceae (2954	Campylobacterales (68525)	Epsilonproteobacteria (122	Proteobacteria (2)	Bacteria (1)	99.90	99.60	WGS
197.14888	0.1106	Campylobacter jejuni (72294)	Campylobacter (213849)	Campylobacteraceae (2954	Campylobacterales (68525)	Epsilonproteobacteria (122	Proteobacteria (2)	Bacteria (1)	100.00	99.20	WGS
197.14889	0.1078	Campylobacter jejuni (72294)	Campylobacter (213849)	Campylobacteraceae (2954	Campylobacterales (68525)	Epsilonproteobacteria (122	Proteobacteria (2)	Bacteria (1)	99.90	98.10	WGS
197.14890	0.1126	Campylobacter jejuni (72294)	Campylobacter (213849)	Campylobacteraceae (2954	Campylobacterales (68525)	Epsilonproteobacteria (122	Proteobacteria (2)	Bacteria (1)	99.60	99.30	WGS
197.14891	0.116	Campylobacter jejuni (72294)	Campylobacter (213849)	Campylobacteraceae (2954	Campylobacterales (68525)	Epsilonproteobacteria (122	Proteobacteria (2)	Bacteria (1)	99.90	99.60	WGS
197.14892	0.1118	Campylobacter jejuni (72294)	Campylobacter (213849)	Campylobacteraceae (2954	Campylobacterales (68525)	Epsilonproteobacteria (122	Proteobacteria (2)	Bacteria (1)	99.90	98.90	WGS
197.14893	0.1096	Campylobacter jejuni (72294)	Campylobacter (213849)	Campylobacteraceae (2954	Campylobacterales (68525)	Epsilonproteobacteria (122	Proteobacteria (2)	Bacteria (1)	99.90	98.50	WGS
197.14894	0.1124	Campylobacter jejuni (72294)	Campylobacter (213849)	Campylobacteraceae (2954	Campylobacterales (68525)	Epsilonproteobacteria (122	Proteobacteria (2)	Bacteria (1)	99.90	99.00	WGS
197.14895	0.11	Campylobacter jejuni (72294)	Campylobacter (213849)	Campylobacteraceae (2954	Campylobacterales (68525)	Epsilonproteobacteria (122	Proteobacteria (2)	Bacteria (1)	99.90	99.00	WGS
197.14896	0.1138	Campylobacter jejuni (72294)	Campylobacter (213849)	Campylobacteraceae (2954	Campylobacterales (68525)	Epsilonproteobacteria (122	Proteobacteria (2)	Bacteria (1)	99.90	99.60	WGS
197.14897	0.116	Campylobacter jejuni (72294)	Campylobacter (213849)	Campylobacteraceae (2954	Campylobacterales (68525)	Epsilonproteobacteria (122	Proteobacteria (2)	Bacteria (1)	99.90	98.50	WGS
197.14898	0.1138	Campylobacter jejuni (72294)	Campylobacter (213849)	Campylobacteraceae (2954	Campylobacterales (68525)	Epsilonproteobacteria (122	Proteobacteria (2)	Bacteria (1)	99.90	99.20	WGS
197.14899	0.1078	Campylobacter jejuni (72294)	Campylobacter (213849)	Campylobacteraceae (2954	Campylobacterales (68525)	Epsilonproteobacteria (122	Proteobacteria (2)	Bacteria (1)	99.90	99.50	WGS
197.14900	0.1106	Campylobacter jejuni (72294)	Campylobacter (213849)	Campylobacteraceae (2954	Campylobacterales (68525)	Epsilonproteobacteria (122	Proteobacteria (2)	Bacteria (1)	100.00	99.70	WGS
197.14901	0.1078	Campylobacter jejuni (72294)	Campylobacter (213849)	Campylobacteraceae (2954	Campylobacterales (68525)	Epsilonproteobacteria (122	Proteobacteria (2)	Bacteria (1)	100.00	99.60	WGS
197.14902	0.1116	Campylobacter jejuni (72294)	Campylobacter (213849)	Campylobacteraceae (2954	Campylobacterales (68525)	Epsilonproteobacteria (122	Proteobacteria (2)	Bacteria (1)	99.90	99.50	WGS
197.14903	0.1098	Campylobacter jejuni (72294)	Campylobacter (213849)	Campylobacteraceae (2954	Campylobacterales (68525)	Epsilonproteobacteria (122	Proteobacteria (2)	Bacteria (1)	99.90	99.30	WGS
197.14904	0.11	Campylobacter jejuni (72294)	Campylobacter (213849)	Campylobacteraceae (2954	Campylobacterales (68525)	Epsilonproteobacteria (122	Proteobacteria (2)	Bacteria (1)	99.90	99.10	WGS
197.14905	0.1078	Campylobacter jejuni (72294)	Campylobacter (213849)	Campylobacteraceae (2954	Campylobacterales (68525)	Epsilonproteobacteria (122	Proteobacteria (2)	Bacteria (1)	99.90	96.60	WGS
197.14906	0.1128	Campylobacter jejuni (72294)	Campylobacter (213849)	Campylobacteraceae (2954	Campylobacterales (68525)	Epsilonproteobacteria (122	Proteobacteria (2)	Bacteria (1)	99.90	97.80	WGS
197.14907	0.1078	Campylobacter jejuni (72294)	Campylobacter (213849)	Campylobacteraceae (2954	Campylobacterales (68525)	Epsilonproteobacteria (122	Proteobacteria (2)	Bacteria (1)	99.90	98.40	WGS
197.14908	0.1078	Campylobacter jejuni (72294)	Campylobacter (213849)	Campylobacteraceae (2954	Campylobacterales (68525)	Epsilonproteobacteria (122	Proteobacteria (2)	Bacteria (1)	99.90	99.00	WGS
197.14909	0.1124	Campylobacter jejuni (72294)	Campylobacter (213849)	Campylobacteraceae (2954	Campylobacterales (68525)	Epsilonproteobacteria (122	Proteobacteria (2)	Bacteria (1)	99.90	99.60	WGS
197.14910	0.1078	Campylobacter jejuni (72294)	Campylobacter (213849)	Campylobacteraceae (2954	Campylobacterales (68525)	Epsilonproteobacteria (122	Proteobacteria (2)	Bacteria (1)	99.90	98.70	WGS
197.14911	0.1126	Campylobacter jejuni (72294)	Campylobacter (213849)	Campylobacteraceae (2954	Campylobacterales (68525)	Epsilonproteobacteria (122	Proteobacteria (2)	Bacteria (1)	99.70	99.60	WGS
197.15030	0.1152	Campylobacter jejuni (72294)	Campylobacter (213849)	Campylobacteraceae (2954	Campylobacterales (68525)	Epsilonproteobacteria (122	Proteobacteria (2)	Bacteria (1)	99.80	99.30	WGS
197.15031	0.1148	Campylobacter jejuni (72294)	Campylobacter (213849)	Campylobacteraceae (2954	Campylobacterales (68525)	Epsilonproteobacteria (122	Proteobacteria (2)	Bacteria (1)	99.90	98.70	WGS
197.15032	0.1078	Campylobacter jejuni (72294)	Campylobacter (213849)	Campylobacteraceae (2954	Campylobacterales (68525)	Epsilonproteobacteria (122	Proteobacteria (2)	Bacteria (1)	100.00	99.70	WGS
197.15033	0.1174	Campylobacter jejuni (72294)	Campylobacter (213849)	Campylobacteraceae (2954	Campylobacterales (68525)	Epsilonproteobacteria (122	Proteobacteria (2)	Bacteria (1)	99.60	99.50	WGS
197.15034	0.1162	Campylobacter jejuni (72294)	Campylobacter (213849)	Campylobacteraceae (2954	Campylobacterales (68525)	Epsilonproteobacteria (122	Proteobacteria (2)	Bacteria (1)	99.70	99.60	WGS
197.15035	0.1154	Campylobacter jejuni (72294)	Campylobacter (213849)	Campylobacteraceae (2954	Campylobacterales (68525)	Epsilonproteobacteria (122	Proteobacteria (2)	Bacteria (1)	99.50	98.80	WGS
197.15036	0.11	Campylobacter jejuni (72294)	Campylobacter (213849)	Campylobacteraceae (2954	Campylobacterales (68525)	Epsilonproteobacteria (122	Proteobacteria (2)	Bacteria (1)	99.90	99.30	WGS
197.15037	0.1142	Campylobacter jejuni (72294)	Campylobacter (213849)	Campylobacteraceae (2954	Campylobacterales (68525)	Epsilonproteobacteria (122	Proteobacteria (2)	Bacteria (1)	99.30	98.70	WGS
197.15038	0.1144	Campylobacter jejuni (72294)	Campylobacter (213849)	Campylobacteraceae (2954	Campylobacterales (68525)	Epsilonproteobacteria (122	Proteobacteria (2)	Bacteria (1)	99.90	99.90	WGS
197.15039	0.1142	Campylobacter jejuni (72294)	Campylobacter (213849)	Campylobacteraceae (2954	Campylobacterales (68525)	Epsilonproteobacteria (122	Proteobacteria (2)	Bacteria (1)	99.60	99.40	WGS
197.15040	0.1114	Campylobacter jejuni (72294)	Campylobacter (213849)	Campylobacteraceae (2954	Campylobacterales (68525)	Epsilonproteobacteria (122	Proteobacteria (2)	Bacteria (1)	99.80	99.70	WGS
197.15041	0.1078	Campylobacter jejuni (72294)	Campylobacter (213849)	Campylobacteraceae (2954	Campylobacterales (68525)	Epsilonproteobacteria (122	Proteobacteria (2)	Bacteria (1)	99.90	99.70	WGS
197.15042	0.1106	Campylobacter jejuni (72294)	Campylobacter (213849)	Campylobacteraceae (2954	Campylobacterales (68525)	Epsilonproteobacteria (122	Proteobacteria (2)	Bacteria (1)	99.90	98.70	WGS
197.15043	0.1142	Campylobacter jejuni (72294)	Campylobacter (213849)	Campylobacteraceae (2954	Campylobacterales (68525)	Epsilonproteobacteria (122	Proteobacteria (2)	Bacteria (1)	99.60	99.50	WGS
197.15044	0.1096	Campylobacter jejuni (72294)	Campylobacter (213849)	Campylobacteraceae (2954	Campylobacterales (68525)	Epsilonproteobacteria (122	Proteobacteria (2)	Bacteria (1)	99.90	99.90	WGS
197.15045	0.1078	Campylobacter jejuni (72294)	Campylobacter (213849)	Campylobacteraceae (2954	Campylobacterales (68525)	Epsilonproteobacteria (122	Proteobacteria (2)	Bacteria (1)	99.90	98.80	WGS
197.15046	0.1146	Campylobacter jejuni (72294)	Campylobacter (213849)	Campylobacteraceae (2954	Campylobacterales (68525)	Epsilonproteobacteria (122	Proteobacteria (2)	Bacteria (1)	99.60	98.50	WGS
197.15047	0.1078	Campylobacter jejuni (72294)	Campylobacter (213849)	Campylobacteraceae (2954	Campylobacterales (68525)	Epsilonproteobacteria (122	Proteobacteria (2)	Bacteria (1)	99.90	99.00	WGS
197.15048	0.1106	Campylobacter jejuni (72294)	Campylobacter (213849)	Campylobacteraceae (2954	Campylobacterales (68525)	Epsilonproteobacteria (122	Proteobacteria (2)	Bacteria (1)	99.90	99.20	WGS
197.15049	0.1154	Campylobacter jejuni (72294)	Campylobacter (213849)	Campylobacteraceae (2954	Campylobacterales (68525)	Epsilonproteobacteria (122	Proteobacteria (2)	Bacteria (1)	99.60	99.40	WGS
197.15050	0.1078	Campylobacter jejuni (72294)	Campylobacter (213849)	Campylobacteraceae (2954	Campylobacterales (68525)	Epsilonproteobacteria (122	Proteobacteria (2)	Bacteria (1)	99.90	98.70	WGS
197.15051	0.1114	Campylobacter jejuni (72294)	Campylobacter (213849)	Campylobacteraceae (2954	Campylobacterales (68525)	Epsilonproteobacteria (122	Proteobacteria (2)	Bacteria (1)	99.90	99.30	WGS
197.15052	0.1106	Campylobacter jejuni (72294)	Campylobacter (213849)	Campylobacteraceae (2954	Campylobacterales (68525)	Epsilonproteobacteria (122	Proteobacteria (2)	Bacteria (1)	100.00	100.00	WGS
197.15053	0.1078	Campylobacter jejuni (72294)	Campylobacter (213849)	Campylobacteraceae (2954	Campylobacterales (68525)	Epsilonproteobacteria (122	Proteobacteria (2)	Bacteria (1)	99.90	99.10	WGS
197.15054	0.1078	Campylobacter jejuni (72294)	Campylobacter (213849)	Campylobacteraceae (2954	Campylobacterales (68525)	Epsilonproteobacteria (122	Proteobacteria (2)	Bacteria (1)	99.90	99.50	WGS
197.15055	0.11	Campylobacter jejuni (72294)	Campylobacter (213849)	Campylobacteraceae (2954	Campylobacterales (68525)	Epsilonproteobacteria (122	Proteobacteria (2)	Bacteria (1)	99.90	98.70	WGS
197.15056	0.1128	Campylobacter jejuni (72294)	Campylobacter (213849)	Campylobacteraceae (2954	Campylobacterales (68525)	Epsilonproteobacteria (122	Proteobacteria (2)	Bacteria (1)	99.90	97.60	WGS
197.15057	0.1146	Campylobacter jejuni (72294)	Campylobacter (213849)	Campylobacteraceae (2954	Campylobacterales (68525)	Epsilonproteobacteria (122	Proteobacteria (2)	Bacteria (1)	99.70	99.20	WGS
197.15058	0.1126	Campylobacter jejuni (72294)	Campylobacter (213849)	Campylobacteraceae (2954	Campylobacterales (68525)	Epsilonproteobacteria (122	Proteobacteria (2)	Bacteria (1)	99.90	98.80	WGS
197.15059	0.112	Campylobacter jejuni (72294)	Campylobacter (213849)	Campylobacteraceae (2954	Campylobacterales (68525)	Epsilonproteobacteria (122	Proteobacteria (2)	Bacteria (1)	99.90	99.00	WGS
197.15060	0.116	Campylobacter jejuni (72294)	Campylobacter (213849)	Campylobacteraceae (2954	Campylobacterales (68525)	Epsilonproteobacteria (122	Proteobacteria (2)	Bacteria (1)	99.80	98.40	WGS
197.15061	0.1078	Campylobacter jejuni (72294)	Campylobacter (213849)	Campylobacteraceae (2954	Campylobacterales (68525)	Epsilonproteobacteria (122	Proteobacteria (2)	Bacteria (1)	99.90	99.70	WGS
197.15062	0.1154	Campylobacter jejuni (72294)	Campylobacter (213849)	Campylobacteraceae (2954	Campylobacterales (68525)	Epsilonproteobacteria (122	Proteobacteria (2)	Bacteria (1)	99.80	99.60	WGS
197.15063	0.116	Campylobacter jejuni (72294)	Campylobacter (213849)	Campylobacteraceae (2954	Campylobacterales (68525)	Epsilonproteobacteria (122	Proteobacteria (2)	Bacteria (1)	99.90	98.20	WGS
197.15064	0.1172	Campylobacter jejuni (72294)	Campylobacter (213849)	Campylobacteraceae (2954	Campylobacterales (68525)	Epsilonproteobacteria (122	Proteobacteria (2)	Bacteria (1)	99.60	99.50	WGS
197.15065	0.1154	Campylobacter jejuni (72294)	Campylobacter (213849)	Campylobacteraceae (2954	Campylobacterales (68525)	Epsilonproteobacteria (122	Proteobacteria (2)	Bacteria (1)	99.80	99.40	WGS
197.15066	0.1132	Campylobacter jejuni (72294)	Campylobacter (213849)	Campylobacteraceae (2954	Campylobacterales (68525)	Epsilonproteobacteria (122	Proteobacteria (2)	Bacteria (1)	99.60	98.40	WGS
197.15067	0.1118	Campylobacter jejuni (72294)	Campylobacter (213849)	Campylobacteraceae (2954	Campylobacterales (68525)	Epsilonproteobacteria (122	Proteobacteria (2)	Bacteria (1)	99.80	98.90	WGS
197.15068	0.1078	Campylobacter jejuni (72294)	Campylobacter (213849)	Campylobacteraceae (2954	Campylobacterales (68525)	Epsilonproteobacteria (122	Proteobacteria (2)	Bacteria (1)	100.00	99.40	WGS
197.15069	0.1106	Campylobacter jejuni (72294)	Campylobacter (213849)	Campylobacteraceae (2954	Campylobacterales (68525)	Epsilonproteobacteria (122	Proteobacteria (2)	Bacteria (1)	99.90	98.80	WGS
197.15070	0.115	Campylobacter jejuni (72294)	Campylobacter (213849)	Campylobacteraceae (2954	Campylobacterales (68525)	Epsilonproteobacteria (122	Proteobacteria (2)	Bacteria (1)	100.00	99.80	WGS
197.15071	0.1106	Campylobacter jejuni (72294)	Campylobacter (213849)	Campylobacteraceae (2954	Campylobacterales (68525)	Epsilonproteobacteria (122	Proteobacteria (2)	Bacteria (1)	99.90	98.70	WGS
197.15072	0.1142	Campylobacter jejuni (72294)	Campylobacter (213849)	Campylobacteraceae (2954	Campylobacterales (68525)	Epsilonproteobacteria (122	Proteobacteria (2)	Bacteria (1)	99.60	99.40	WGS
197.15073	0.1078	Campylobacter jejuni (72294)	Campylobacter (213849)	Campylobacteraceae (2954	Campylobacterales (68525)	Epsilonproteobacteria (122	Proteobacteria (2)	Bacteria (1)	99.90	98.50	WGS
197.15074	0.1154	Campylobacter jejuni (72294)	Campylobacter (213849)	Campylobacteraceae (2954	Campylobacterales (68525)	Epsilonproteobacteria (122	Proteobacteria (2)	Bacteria (1)	99.60	99.50	WGS
197.15075	0.1142	Campylobacter jejuni (72294)	Campylobacter (213849)	Campylobacteraceae (2954	Campylobacterales (68525)	Epsilonproteobacteria (122	Proteobacteria (2)	Bacteria (1)	99.40	99.30	WGS
197.15076	0.1106	Campylobacter jejuni (72294)	Campylobacter (213849)	Campylobacteraceae (2954	Campylobacterales (68525)	Epsilonproteobacteria (122	Proteobacteria (2)	Bacteria (1)	99.90	99.50	WGS
197.15077	0.116	Campylobacter jejuni (72294)	Campylobacter (213849)	Campylobacteraceae (2954	Campylobacterales (68525)	Epsilonproteobacteria (122	Proteobacteria (2)	Bacteria (1)	99.60	99.40	WGS
197.15078	0.109	Campylobacter jejuni (72294)	Campylobacter (213849)	Campylobacteraceae (2954	Campylobacterales (68525)	Epsilonproteobacteria (122	Proteobacteria (2)	Bacteria (1)	99.90	98.50	WGS
197.15079	0.1146	Campylobacter jejuni (72294)	Campylobacter (213849)	Campylobacteraceae (2954	Campylobacterales (68525)	Epsilonproteobacteria (122	Proteobacteria (2)	Bacteria (1)	99.60	99.60	WGS
197.15080	0.1078	Campylobacter jejuni (72294)	Campylobacter (213849)	Campylobacteraceae (2954	Campylobacterales (68525)	Epsilonproteobacteria (122	Proteobacteria (2)	Bacteria (1)	100.00	99.20	WGS
197.15081	0.1106	Campylobacter jejuni (72294)	Campylobacter (213849)	Campylobacteraceae (2954	Campylobacterales (68525)	Epsilonproteobacteria (122	Proteobacteria (2)	Bacteria (1)	99.90	98.50	WGS
197.15082	0.1106	Campylobacter jejuni (72294)	Campylobacter (213849)	Campylobacteraceae (2954	Campylobacterales (68525)	Epsilonproteobacteria (122	Proteobacteria (2)	Bacteria (1)	100.00	99.80	WGS
197.15083	0.1106	Campylobacter jejuni (72294)	Campylobacter (213849)	Campylobacteraceae (2954	Campylobacterales (68525)	Epsilonproteobacteria (122	Proteobacteria (2)	Bacteria (1)	99.90	99.00	WGS
197.15084	0.1114	Campylobacter jejuni (72294)	Campylobacter (213849)	Campylobacteraceae (2954	Campylobacterales (68525)	Epsilonproteobacteria (122	Proteobacteria (2)	Bacteria (1)	99.70	99.60	WGS
197.15085	0.1128	Campylobacter jejuni (72294)	Campylobacter (213849)	Campylobacteraceae (2954	Campylobacterales (68525)	Epsilonproteobacteria (122	Proteobacteria (2)	Bacteria (1)	99.90	99.20	WGS
197.15086	0.1124	Campylobacter jejuni (72294)	Campylobacter (213849)	Campylobacteraceae (2954	Campylobacterales (68525)	Epsilonproteobacteria (122	Proteobacteria (2)	Bacteria (1)	100.00	99.70	WGS
197.15087	0.1114	Campylobacter jejuni (72294)	Campylobacter (213849)	Campylobacteraceae (2954	Campylobacterales (68525)	Epsilonproteobacteria (122	Proteobacteria (2)	Bacteria (1)	99.60	99.30	WGS
197.15088	0.1078	Campylobacter jejuni (72294)	Campylobacter (213849)	Campylobacteraceae (2954	Campylobacterales (68525)	Epsilonproteobacteria (122	Proteobacteria (2)	Bacteria (1)	99.90	98.70	WGS
197.15089	0.1154	Campylobacter jejuni (72294)	Campylobacter (213849)	Campylobacteraceae (2954	Campylobacterales (68525)	Epsilonproteobacteria (122	Proteobacteria (2)	Bacteria (1)	99.70	99.30	WGS
197.15090	0.1142	Campylobacter jejuni (72294)	Campylobacter (213849)	Campylobacteraceae (2954	Campylobacterales (68525)	Epsilonproteobacteria (122	Proteobacteria (2)	Bacteria (1)	99.70	99.30	WGS
197.15091	0.1142	Campylobacter jejuni (72294)	Campylobacter (213849)	Campylobacteraceae (2954	Campylobacterales (68525)	Epsilonproteobacteria (122	Proteobacteria (2)	Bacteria (1)	99.60	98.50	WGS
197.15092	0.1096	Campylobacter jejuni (72294)	Campylobacter (213849)	Campylobacteraceae (2954	Campylobacterales (68525)	Epsilonproteobacteria (122	Proteobacteria (2)	Bacteria (1)	99.90	99.00	WGS
197.15093	0.1078	Campylobacter jejuni (72294)	Campylobacter (213849)	Campylobacteraceae (2954	Campylobacterales (68525)	Epsilonproteobacteria (122	Proteobacteria (2)	Bacteria (1)	100.00	99.40	WGS
197.15094	0.1144	Campylobacter jejuni (72294)	Campylobacter (213849)	Campylobacteraceae (2954	Campylobacterales (68525)	Epsilonproteobacteria (122	Proteobacteria (2)	Bacteria (1)	99.60	99.40	WGS
197.15095	0.1114	Campylobacter jejuni (72294)	Campylobacter (213849)	Campylobacteraceae (2954	Campylobacterales (68525)	Epsilonproteobacteria (122	Proteobacteria (2)	Bacteria (1)	99.80	99.50	WGS
197.15096	0.11	Campylobacter jejuni (72294)	Campylobacter (213849)	Campylobacteraceae (2954	Campylobacterales (68525)	Epsilonproteobacteria (122	Proteobacteria (2)	Bacteria (1)	99.80	99.40	WGS
197.15097	0.1078	Campylobacter jejuni (72294)	Campylobacter (213849)	Campylobacteraceae (2954	Campylobacterales (68525)	Epsilonproteobacteria (122	Proteobacteria (2)	Bacteria (1)	99.90	98.40	WGS
197.15098	0.1146	Campylobacter jejuni (72294)	Campylobacter (213849)	Campylobacteraceae (2954	Campylobacterales (68525)	Epsilonproteobacteria (122	Proteobacteria (2)	Bacteria (1)	100.00	99.60	WGS
197.15099	0.1078	Campylobacter jejuni (72294)	Campylobacter (213849)	Campylobacteraceae (2954	Campylobacterales (68525)	Epsilonproteobacteria (122	Proteobacteria (2)	Bacteria (1)	99.90	99.90	WGS
197.15100	0.1078	Campylobacter jejuni (72294)	Campylobacter (213849)	Campylobacteraceae (2954	Campylobacterales (68525)	Epsilonproteobacteria (122	Proteobacteria (2)	Bacteria (1)	99.90	99.90	WGS
197.15101	0.1078	Campylobacter jejuni (72294)	Campylobacter (213849)	Campylobacteraceae (2954	Campylobacterales (68525)	Epsilonproteobacteria (122	Proteobacteria (2)	Bacteria (1)	99.90	99.90	WGS
197.15102	0.1078	Campylobacter jejuni (72294)	Campylobacter (213849)	Campylobacteraceae (2954	Campylobacterales (68525)	Epsilonproteobacteria (122	Proteobacteria (2)	Bacteria (1)	99.90	99.90	WGS
197.15103	0.0958	Campylobacter jejuni (72294)	Campylobacter (213849)	Campylobacteraceae (2954	Campylobacterales (68525)	Epsilonproteobacteria (122	Proteobacteria (2)	Bacteria (1)	99.60	99.60	WGS
197.15104	0.1132	Campylobacter jejuni (72294)	Campylobacter (213849)	Campylobacteraceae (2954	Campylobacterales (68525)	Epsilonproteobacteria (122	Proteobacteria (2)	Bacteria (1)	99.60	99.30	WGS
197.15105	0.1132	Campylobacter jejuni (72294)	Campylobacter (213849)	Campylobacteraceae (2954	Campylobacterales (68525)	Epsilonproteobacteria (122	Proteobacteria (2)	Bacteria (1)	99.60	99.30	WGS
197.15106	0.1152	Campylobacter jejuni (72294)	Campylobacter (213849)	Campylobacteraceae (2954	Campylobacterales (68525)	Epsilonproteobacteria (122	Proteobacteria (2)	Bacteria (1)	99.50	99.30	WGS
197.15107	0.1126	Campylobacter jejuni (72294)	Campylobacter (213849)	Campylobacteraceae (2954	Campylobacterales (68525)	Epsilonproteobacteria (122	Proteobacteria (2)	Bacteria (1)	99.50	99.10	WGS
197.15108	0.1126	Campylobacter jejuni (72294)	Campylobacter (213849)	Campylobacteraceae (2954	Campylobacterales (68525)	Epsilonproteobacteria (122	Proteobacteria (2)	Bacteria (1)	99.50	99.30	WGS
197.15109	0.1142	Campylobacter jejuni (72294)	Campylobacter (213849)	Campylobacteraceae (2954	Campylobacterales (68525)	Epsilonproteobacteria (122	Proteobacteria (2)	Bacteria (1)	99.50	99.20	WGS
197.15110	0.1114	Campylobacter jejuni (72294)	Campylobacter (213849)	Campylobacteraceae (2954	Campylobacterales (68525)	Epsilonproteobacteria (122	Proteobacteria (2)	Bacteria (1)	99.80	99.60	WGS
197.15111	0.1132	Campylobacter jejuni (72294)	Campylobacter (213849)	Campylobacteraceae (2954	Campylobacterales (68525)	Epsilonproteobacteria (122	Proteobacteria (2)	Bacteria (1)	99.60	99.60	WGS
197.15112	0.1114	Campylobacter jejuni (72294)	Campylobacter (213849)	Campylobacteraceae (2954	Campylobacterales (68525)	Epsilonproteobacteria (122	Proteobacteria (2)	Bacteria (1)	99.80	99.60	WGS
197.15113	0.1132	Campylobacter jejuni (72294)	Campylobacter (213849)	Campylobacteraceae (2954	Campylobacterales (68525)	Epsilonproteobacteria (122	Proteobacteria (2)	Bacteria (1)	99.60	99.10	WGS
197.15114	0.1132	Campylobacter jejuni (72294)	Campylobacter (213849)	Campylobacteraceae (2954	Campylobacterales (68525)	Epsilonproteobacteria (122	Proteobacteria (2)	Bacteria (1)	99.50	99.30	WGS
197.15115	0.1114	Campylobacter jejuni (72294)	Campylobacter (213849)	Campylobacteraceae (2954	Campylobacterales (68525)	Epsilonproteobacteria (122	Proteobacteria (2)	Bacteria (1)	99.80	99.30	WGS
197.15116	0.1132	Campylobacter jejuni (72294)	Campylobacter (213849)	Campylobacteraceae (2954	Campylobacterales (68525)	Epsilonproteobacteria (122	Proteobacteria (2)	Bacteria (1)	99.60	99.30	WGS
197.15117	0.1126	Campylobacter jejuni (72294)	Campylobacter (213849)	Campylobacteraceae (2954	Campylobacterales (68525)	Epsilonproteobacteria (122	Proteobacteria (2)	Bacteria (1)	99.50	99.30	WGS
197.15118	0.1132	Campylobacter jejuni (72294)	Campylobacter (213849)	Campylobacteraceae (2954	Campylobacterales (68525)	Epsilonproteobacteria (122	Proteobacteria (2)	Bacteria (1)	99.60	99.30	WGS
197.15119	0.1132	Campylobacter jejuni (72294)	Campylobacter (213849)	Campylobacteraceae (2954	Campylobacterales (68525)	Epsilonproteobacteria (122	Proteobacteria (2)	Bacteria (1)	99.60	99.30	WGS
197.15120	0.1114	Campylobacter jejuni (72294)	Campylobacter (213849)	Campylobacteraceae (2954	Campylobacterales (68525)	Epsilonproteobacteria (122	Proteobacteria (2)	Bacteria (1)	99.80	99.60	WGS
197.15121	0.1132	Campylobacter jejuni (72294)	Campylobacter (213849)	Campylobacteraceae (2954	Campylobacterales (68525)	Epsilonproteobacteria (122	Proteobacteria (2)	Bacteria (1)	99.60	99.30	WGS
197.15122	0.0976	Campylobacter jejuni (72294)	Campylobacter (213849)	Campylobacteraceae (2954	Campylobacterales (68525)	Epsilonproteobacteria (122	Proteobacteria (2)	Bacteria (1)	99.60	99.30	WGS
197.15123	0.0976	Campylobacter jejuni (72294)	Campylobacter (213849)	Campylobacteraceae (2954	Campylobacterales (68525)	Epsilonproteobacteria (122	Proteobacteria (2)	Bacteria (1)	99.60	99.00	WGS
197.15124	0.1132	Campylobacter jejuni (72294)	Campylobacter (213849)	Campylobacteraceae (2954	Campylobacterales (68525)	Epsilonproteobacteria (122	Proteobacteria (2)	Bacteria (1)	99.60	99.30	WGS
197.15125	0.1006	Campylobacter jejuni (72294)	Campylobacter (213849)	Campylobacteraceae (2954	Campylobacterales (68525)	Epsilonproteobacteria (122	Proteobacteria (2)	Bacteria (1)	99.40	99.00	WGS
197.15126	0.1078	Campylobacter jejuni (72294)	Campylobacter (213849)	Campylobacteraceae (2954	Campylobacterales (68525)	Epsilonproteobacteria (122	Proteobacteria (2)	Bacteria (1)	99.90	99.70	WGS
197.15127	0.119	Campylobacter jejuni (72294)	Campylobacter (213849)	Campylobacteraceae (2954	Campylobacterales (68525)	Epsilonproteobacteria (122	Proteobacteria (2)	Bacteria (1)	99.90	99.50	WGS
197.15128	0.0966	Campylobacter jejuni (72294)	Campylobacter (213849)	Campylobacteraceae (2954	Campylobacterales (68525)	Epsilonproteobacteria (122	Proteobacteria (2)	Bacteria (1)	99.80	99.60	WGS
197.15129	0.1134	Campylobacter jejuni (72294)	Campylobacter (213849)	Campylobacteraceae (2954	Campylobacterales (68525)	Epsilonproteobacteria (122	Proteobacteria (2)	Bacteria (1)	99.60	99.60	WGS
197.15130	0.1084	Campylobacter jejuni (72294)	Campylobacter (213849)	Campylobacteraceae (2954	Campylobacterales (68525)	Epsilonproteobacteria (122	Proteobacteria (2)	Bacteria (1)	99.90	99.90	WGS
197.15131	0.1106	Campylobacter jejuni (72294)	Campylobacter (213849)	Campylobacteraceae (2954	Campylobacterales (68525)	Epsilonproteobacteria (122	Proteobacteria (2)	Bacteria (1)	99.80	99.80	WGS
197.15132	0.1084	Campylobacter jejuni (72294)	Campylobacter (213849)	Campylobacteraceae (2954	Campylobacterales (68525)	Epsilonproteobacteria (122	Proteobacteria (2)	Bacteria (1)	99.90	99.90	WGS
197.15133	0.0906	Campylobacter jejuni (72294)	Campylobacter (213849)	Campylobacteraceae (2954	Campylobacterales (68525)	Epsilonproteobacteria (122	Proteobacteria (2)	Bacteria (1)	99.50	99.30	WGS
197.15134	0.1078	Campylobacter jejuni (72294)	Campylobacter (213849)	Campylobacteraceae (2954	Campylobacterales (68525)	Epsilonproteobacteria (122	Proteobacteria (2)	Bacteria (1)	99.80	99.60	WGS
197.15135	0.1084	Campylobacter jejuni (72294)	Campylobacter (213849)	Campylobacteraceae (2954	Campylobacterales (68525)	Epsilonproteobacteria (122	Proteobacteria (2)	Bacteria (1)	99.90	99.90	WGS
197.15136	0.124	Campylobacter jejuni (72294)	Campylobacter (213849)	Campylobacteraceae (2954	Campylobacterales (68525)	Epsilonproteobacteria (122	Proteobacteria (2)	Bacteria (1)	99.90	99.50	WGS
197.15137	0.1078	Campylobacter jejuni (72294)	Campylobacter (213849)	Campylobacteraceae (2954	Campylobacterales (68525)	Epsilonproteobacteria (122	Proteobacteria (2)	Bacteria (1)	99.80	99.80	WGS
[illegible]	[illegible]	[illegible]	[illegible]	[illegible]	[illegible]	[illegible]	[illegible]	[illegible]	[illegible]	[illegible]	[illegible]

197.16244	0.1078	Campylobacter jejuni (72294)	Campylobacter (213849)	Campylobacteraceae (2954	Campylobacterales (68525)	Epsilonproteobacteria (122	Proteobacteria (2)	Bacteria (1)			99.70	99.40	WGS
197.16245	0.1144	Campylobacter jejuni (72294)	Campylobacter (213849)	Campylobacteraceae (2954	Campylobacterales (68525)	Epsilonproteobacteria (122	Proteobacteria (2)	Bacteria (1)			99.90	99.70	WGS
197.16246	0.1078	Campylobacter jejuni (72294)	Campylobacter (213849)	Campylobacteraceae (2954	Campylobacterales (68525)	Epsilonproteobacteria (122	Proteobacteria (2)	Bacteria (1)			99.90	99.40	WGS
197.16247	0.11	Campylobacter jejuni (72294)	Campylobacter (213849)	Campylobacteraceae (2954	Campylobacterales (68525)	Epsilonproteobacteria (122	Proteobacteria (2)	Bacteria (1)			99.70	99.00	WGS
197.16248	0.1078	Campylobacter jejuni (72294)	Campylobacter (213849)	Campylobacteraceae (2954	Campylobacterales (68525)	Epsilonproteobacteria (122	Proteobacteria (2)	Bacteria (1)			99.90	99.70	WGS
197.16249	0.1078	Campylobacter jejuni (72294)	Campylobacter (213849)	Campylobacteraceae (2954	Campylobacterales (68525)	Epsilonproteobacteria (122	Proteobacteria (2)	Bacteria (1)			99.90	99.80	WGS
197.16250	0.1078	Campylobacter jejuni (72294)	Campylobacter (213849)	Campylobacteraceae (2954	Campylobacterales (68525)	Epsilonproteobacteria (122	Proteobacteria (2)	Bacteria (1)			99.90	99.70	WGS
197.16251	0.1106	Campylobacter jejuni (72294)	Campylobacter (213849)	Campylobacteraceae (2954	Campylobacterales (68525)	Epsilonproteobacteria (122	Proteobacteria (2)	Bacteria (1)			99.90	99.50	WGS
197.16252	0.1106	Campylobacter jejuni (72294)	Campylobacter (213849)	Campylobacteraceae (2954	Campylobacterales (68525)	Epsilonproteobacteria (122	Proteobacteria (2)	Bacteria (1)			100.00	99.90	WGS
197.16253	0.1078	Campylobacter jejuni (72294)	Campylobacter (213849)	Campylobacteraceae (2954	Campylobacterales (68525)	Epsilonproteobacteria (122	Proteobacteria (2)	Bacteria (1)			99.90	99.60	WGS
197.16254	0.1078	Campylobacter jejuni (72294)	Campylobacter (213849)	Campylobacteraceae (2954	Campylobacterales (68525)	Epsilonproteobacteria (122	Proteobacteria (2)	Bacteria (1)			99.90	99.60	WGS
197.16255	0.1106	Campylobacter jejuni (72294)	Campylobacter (213849)	Campylobacteraceae (2954	Campylobacterales (68525)	Epsilonproteobacteria (122	Proteobacteria (2)	Bacteria (1)			99.90	99.80	WGS
197.16256	0.1078	Campylobacter jejuni (72294)	Campylobacter (213849)	Campylobacteraceae (2954	Campylobacterales (68525)	Epsilonproteobacteria (122	Proteobacteria (2)	Bacteria (1)			99.90	99.70	WGS
197.16257	0.1078	Campylobacter jejuni (72294)	Campylobacter (213849)	Campylobacteraceae (2954	Campylobacterales (68525)	Epsilonproteobacteria (122	Proteobacteria (2)	Bacteria (1)			99.90	99.80	WGS
197.16258	0.1118	Campylobacter jejuni (72294)	Campylobacter (213849)	Campylobacteraceae (2954	Campylobacterales (68525)	Epsilonproteobacteria (122	Proteobacteria (2)	Bacteria (1)			99.90	99.90	WGS
197.16259	0.1078	Campylobacter jejuni (72294)	Campylobacter (213849)	Campylobacteraceae (2954	Campylobacterales (68525)	Epsilonproteobacteria (122	Proteobacteria (2)	Bacteria (1)			99.90	99.60	WGS
197.16260	0.1078	Campylobacter jejuni (72294)	Campylobacter (213849)	Campylobacteraceae (2954	Campylobacterales (68525)	Epsilonproteobacteria (122	Proteobacteria (2)	Bacteria (1)			99.90	99.50	WGS
197.16261	0.1128	Campylobacter jejuni (72294)	Campylobacter (213849)	Campylobacteraceae (2954	Campylobacterales (68525)	Epsilonproteobacteria (122	Proteobacteria (2)	Bacteria (1)			99.90	99.80	WGS
197.16262	0.1106	Campylobacter jejuni (72294)	Campylobacter (213849)	Campylobacteraceae (2954	Campylobacterales (68525)	Epsilonproteobacteria (122	Proteobacteria (2)	Bacteria (1)			99.90	99.90	WGS
197.16263	0.1078	Campylobacter jejuni (72294)	Campylobacter (213849)	Campylobacteraceae (2954	Campylobacterales (68525)	Epsilonproteobacteria (122	Proteobacteria (2)	Bacteria (1)			100.00	99.90	WGS
197.16264	0.1078	Campylobacter jejuni (72294)	Campylobacter (213849)	Campylobacteraceae (2954	Campylobacterales (68525)	Epsilonproteobacteria (122	Proteobacteria (2)	Bacteria (1)			100.00	99.90	WGS
197.16265	0.1078	Campylobacter jejuni (72294)	Campylobacter (213849)	Campylobacteraceae (2954	Campylobacterales (68525)	Epsilonproteobacteria (122	Proteobacteria (2)	Bacteria (1)			100.00	99.90	WGS
197.16266	0.11	Campylobacter jejuni (72294)	Campylobacter (213849)	Campylobacteraceae (2954	Campylobacterales (68525)	Epsilonproteobacteria (122	Proteobacteria (2)	Bacteria (1)			100.00	100.00	WGS
197.16267	0.1078	Campylobacter jejuni (72294)	Campylobacter (213849)	Campylobacteraceae (2954	Campylobacterales (68525)	Epsilonproteobacteria (122	Proteobacteria (2)	Bacteria (1)			99.90	99.60	WGS
197.16268	0.1106	Campylobacter jejuni (72294)	Campylobacter (213849)	Campylobacteraceae (2954	Campylobacterales (68525)	Epsilonproteobacteria (122	Proteobacteria (2)	Bacteria (1)			99.90	99.40	WGS
197.16269	0.1078	Campylobacter jejuni (72294)	Campylobacter (213849)	Campylobacteraceae (2954	Campylobacterales (68525)	Epsilonproteobacteria (122	Proteobacteria (2)	Bacteria (1)			99.90	99.70	WGS
197.16270	0.1078	Campylobacter jejuni (72294)	Campylobacter (213849)	Campylobacteraceae (2954	Campylobacterales (68525)	Epsilonproteobacteria (122	Proteobacteria (2)	Bacteria (1)			99.90	99.70	WGS
197.16271	0.1148	Campylobacter jejuni (72294)	Campylobacter (213849)	Campylobacteraceae (2954	Campylobacterales (68525)	Epsilonproteobacteria (122	Proteobacteria (2)	Bacteria (1)			99.80	99.40	WGS
197.16272	0.109	Campylobacter jejuni (72294)	Campylobacter (213849)	Campylobacteraceae (2954	Campylobacterales (68525)	Epsilonproteobacteria (122	Proteobacteria (2)	Bacteria (1)			99.80	99.00	WGS
197.16273	0.1078	Campylobacter jejuni (72294)	Campylobacter (213849)	Campylobacteraceae (2954	Campylobacterales (68525)	Epsilonproteobacteria (122	Proteobacteria (2)	Bacteria (1)			99.90	99.40	WGS
197.16274	0.1106	Campylobacter jejuni (72294)	Campylobacter (213849)	Campylobacteraceae (2954	Campylobacterales (68525)	Epsilonproteobacteria (122	Proteobacteria (2)	Bacteria (1)			99.90	99.60	WGS
197.16275	0.1078	Campylobacter jejuni (72294)	Campylobacter (213849)	Campylobacteraceae (2954	Campylobacterales (68525)	Epsilonproteobacteria (122	Proteobacteria (2)	Bacteria (1)			99.90	99.60	WGS
197.16276	0.1152	Campylobacter jejuni (72294)	Campylobacter (213849)	Campylobacteraceae (2954	Campylobacterales (68525)	Epsilonproteobacteria (122	Proteobacteria (2)	Bacteria (1)			99.90	99.70	WGS
197.16277	0.1078	Campylobacter jejuni (72294)	Campylobacter (213849)	Campylobacteraceae (2954	Campylobacterales (68525)	Epsilonproteobacteria (122	Proteobacteria (2)	Bacteria (1)			99.60	99.30	WGS
197.16278	0.1078	Campylobacter jejuni (72294)	Campylobacter (213849)	Campylobacteraceae (2954	Campylobacterales (68525)	Epsilonproteobacteria (122	Proteobacteria (2)	Bacteria (1)			99.70	99.30	WGS
197.16279	0.1152	Campylobacter jejuni (72294)	Campylobacter (213849)	Campylobacteraceae (2954	Campylobacterales (68525)	Epsilonproteobacteria (122	Proteobacteria (2)	Bacteria (1)			99.70	98.40	WGS
197.16280	0.1096	Campylobacter jejuni (72294)	Campylobacter (213849)	Campylobacteraceae (2954	Campylobacterales (68525)	Epsilonproteobacteria (122	Proteobacteria (2)	Bacteria (1)			100.00	100.00	WGS
197.16281	0.1078	Campylobacter jejuni (72294)	Campylobacter (213849)	Campylobacteraceae (2954	Campylobacterales (68525)	Epsilonproteobacteria (122	Proteobacteria (2)	Bacteria (1)			99.90	99.80	WGS
197.16282	0.1106	Campylobacter jejuni (72294)	Campylobacter (213849)	Campylobacteraceae (2954	Campylobacterales (68525)	Epsilonproteobacteria (122	Proteobacteria (2)	Bacteria (1)			99.90	99.50	WGS
197.16283	0.1078	Campylobacter jejuni (72294)	Campylobacter (213849)	Campylobacteraceae (2954	Campylobacterales (68525)	Epsilonproteobacteria (122	Proteobacteria (2)	Bacteria (1)			99.90	99.70	WGS
197.16284	0.1126	Campylobacter jejuni (72294)	Campylobacter (213849)	Campylobacteraceae (2954	Campylobacterales (68525)	Epsilonproteobacteria (122	Proteobacteria (2)	Bacteria (1)			99.80	99.40	WGS
197.16285	0.1078	Campylobacter jejuni (72294)	Campylobacter (213849)	Campylobacteraceae (2954	Campylobacterales (68525)	Epsilonproteobacteria (122	Proteobacteria (2)	Bacteria (1)			99.90	99.90	WGS
197.16286	0.11	Campylobacter jejuni (72294)	Campylobacter (213849)	Campylobacteraceae (2954	Campylobacterales (68525)	Epsilonproteobacteria (122	Proteobacteria (2)	Bacteria (1)			99.90	99.80	WGS
197.16287	0.1106	Campylobacter jejuni (72294)	Campylobacter (213849)	Campylobacteraceae (2954	Campylobacterales (68525)	Epsilonproteobacteria (122	Proteobacteria (2)	Bacteria (1)			99.90	99.90	WGS
197.16288	0.1096	Campylobacter jejuni (72294)	Campylobacter (213849)	Campylobacteraceae (2954	Campylobacterales (68525)	Epsilonproteobacteria (122	Proteobacteria (2)	Bacteria (1)			99.90	99.40	WGS
197.16289	0.11	Campylobacter jejuni (72294)	Campylobacter (213849)	Campylobacteraceae (2954	Campylobacterales (68525)	Epsilonproteobacteria (122	Proteobacteria (2)	Bacteria (1)			99.90	99.70	WGS
197.16290	0.1078	Campylobacter jejuni (72294)	Campylobacter (213849)	Campylobacteraceae (2954	Campylobacterales (68525)	Epsilonproteobacteria (122	Proteobacteria (2)	Bacteria (1)			99.70	99.40	WGS
197.16291	0.1078	Campylobacter jejuni (72294)	Campylobacter (213849)	Campylobacteraceae (2954	Campylobacterales (68525)	Epsilonproteobacteria (122	Proteobacteria (2)	Bacteria (1)			99.90	99.80	WGS
197.16292	0.1078	Campylobacter jejuni (72294)	Campylobacter (213849)	Campylobacteraceae (2954	Campylobacterales (68525)	Epsilonproteobacteria (122	Proteobacteria (2)	Bacteria (1)			99.90	99.70	WGS
197.16293	0.109	Campylobacter jejuni (72294)	Campylobacter (213849)	Campylobacteraceae (2954	Campylobacterales (68525)	Epsilonproteobacteria (122	Proteobacteria (2)	Bacteria (1)			99.90	99.60	WGS
197.16294	0.1106	Campylobacter jejuni (72294)	Campylobacter (213849)	Campylobacteraceae (2954	Campylobacterales (68525)	Epsilonproteobacteria (122	Proteobacteria (2)	Bacteria (1)			100.00	99.60	WGS
197.16295	0.1124	Campylobacter jejuni (72294)	Campylobacter (213849)	Campylobacteraceae (2954	Campylobacterales (68525)	Epsilonproteobacteria (122	Proteobacteria (2)	Bacteria (1)			99.90	99.60	WGS
197.16296	0.1098	Campylobacter jejuni (72294)	Campylobacter (213849)	Campylobacteraceae (2954	Campylobacterales (68525)	Epsilonproteobacteria (122	Proteobacteria (2)	Bacteria (1)			99.70	99.50	WGS
197.16297	0.1078	Campylobacter jejuni (72294)	Campylobacter (213849)	Campylobacteraceae (2954	Campylobacterales (68525)	Epsilonproteobacteria (122	Proteobacteria (2)	Bacteria (1)			99.80	99.60	WGS
197.16298	0.1078	Campylobacter jejuni (72294)	Campylobacter (213849)	Campylobacteraceae (2954	Campylobacterales (68525)	Epsilonproteobacteria (122	Proteobacteria (2)	Bacteria (1)			100.00	99.90	WGS
197.16299	0.1078	Campylobacter jejuni (72294)	Campylobacter (213849)	Campylobacteraceae (2954	Campylobacterales (68525)	Epsilonproteobacteria (122	Proteobacteria (2)	Bacteria (1)			99.90	99.80	WGS
197.16300	0.1078	Campylobacter jejuni (72294)	Campylobacter (213849)	Campylobacteraceae (2954	Campylobacterales (68525)	Epsilonproteobacteria (122	Proteobacteria (2)	Bacteria (1)			99.90	99.70	WGS
197.16301	0.1078	Campylobacter jejuni (72294)	Campylobacter (213849)	Campylobacteraceae (2954	Campylobacterales (68525)	Epsilonproteobacteria (122	Proteobacteria (2)	Bacteria (1)			99.80	99.60	WGS
197.16302	0.1106	Campylobacter jejuni (72294)	Campylobacter (213849)	Campylobacteraceae (2954	Campylobacterales (68525)	Epsilonproteobacteria (122	Proteobacteria (2)	Bacteria (1)			99.90	99.80	WGS
197.16303	0.1106	Campylobacter jejuni (72294)	Campylobacter (213849)	Campylobacteraceae (2954	Campylobacterales (68525)	Epsilonproteobacteria (122	Proteobacteria (2)	Bacteria (1)			99.90	99.60	WGS
197.16304	0.108	Campylobacter jejuni (72294)	Campylobacter (213849)	Campylobacteraceae (2954	Campylobacterales (68525)	Epsilonproteobacteria (122	Proteobacteria (2)	Bacteria (1)			99.90	99.70	WGS
197.16305	0.1098	Campylobacter jejuni (72294)	Campylobacter (213849)	Campylobacteraceae (2954	Campylobacterales (68525)	Epsilonproteobacteria (122	Proteobacteria (2)	Bacteria (1)			99.50	99.20	WGS
197.16306	0.1166	Campylobacter jejuni (72294)	Campylobacter (213849)	Campylobacteraceae (2954	Campylobacterales (68525)	Epsilonproteobacteria (122	Proteobacteria (2)	Bacteria (1)			99.70	99.50	WGS
197.16307	0.1078	Campylobacter jejuni (72294)	Campylobacter (213849)	Campylobacteraceae (2954	Campylobacterales (68525)	Epsilonproteobacteria (122	Proteobacteria (2)	Bacteria (1)			99.90	99.70	WGS
197.16308	0.1118	Campylobacter jejuni (72294)	Campylobacter (213849)	Campylobacteraceae (2954	Campylobacterales (68525)	Epsilonproteobacteria (122	Proteobacteria (2)	Bacteria (1)			99.90	99.60	WGS
197.16309	0.1078	Campylobacter jejuni (72294)	Campylobacter (213849)	Campylobacteraceae (2954	Campylobacterales (68525)	Epsilonproteobacteria (122	Proteobacteria (2)	Bacteria (1)			100.00	100.00	WGS
197.16310	0.11	Campylobacter jejuni (72294)	Campylobacter (213849)	Campylobacteraceae (2954	Campylobacterales (68525)	Epsilonproteobacteria (122	Proteobacteria (2)	Bacteria (1)			99.90	99.70	WGS
197.16311	0.1114	Campylobacter jejuni (72294)	Campylobacter (213849)	Campylobacteraceae (2954	Campylobacterales (68525)	Epsilonproteobacteria (122	Proteobacteria (2)	Bacteria (1)			99.90	99.20	WGS
197.16312	0.1078	Campylobacter jejuni (72294)	Campylobacter (213849)	Campylobacteraceae (2954	Campylobacterales (68525)	Epsilonproteobacteria (122	Proteobacteria (2)	Bacteria (1)			99.90	99.90	WGS
197.16313	0.1106	Campylobacter jejuni (72294)	Campylobacter (213849)	Campylobacteraceae (2954	Campylobacterales (68525)	Epsilonproteobacteria (122	Proteobacteria (2)	Bacteria (1)			99.90	99.80	WGS
197.16314	0.1078	Campylobacter jejuni (72294)	Campylobacter (213849)	Campylobacteraceae (2954	Campylobacterales (68525)	Epsilonproteobacteria (122	Proteobacteria (2)	Bacteria (1)			99.90	99.50	WGS
197.16315	0.1078	Campylobacter jejuni (72294)	Campylobacter (213849)	Campylobacteraceae (2954	Campylobacterales (68525)	Epsilonproteobacteria (122	Proteobacteria (2)	Bacteria (1)			99.90	99.50	WGS
197.16316	0.1096	Campylobacter jejuni (72294)	Campylobacter (213849)	Campylobacteraceae (2954	Campylobacterales (68525)	Epsilonproteobacteria (122	Proteobacteria (2)	Bacteria (1)			99.90	99.60	WGS
197.16317	0.1078	Campylobacter jejuni (72294)	Campylobacter (213849)	Campylobacteraceae (2954	Campylobacterales (68525)	Epsilonproteobacteria (122	Proteobacteria (2)	Bacteria (1)			99.90	99.80	WGS
197.16318	0.1078	Campylobacter jejuni (72294)	Campylobacter (213849)	Campylobacteraceae (2954	Campylobacterales (68525)	Epsilonproteobacteria (122	Proteobacteria (2)	Bacteria (1)			99.90	99.90	WGS
197.16319	0.1078	Campylobacter jejuni (72294)	Campylobacter (213849)	Campylobacteraceae (2954	Campylobacterales (68525)	Epsilonproteobacteria (122	Proteobacteria (2)	Bacteria (1)			99.90	99.90	WGS
197.16320	0.1078	Campylobacter jejuni (72294)	Campylobacter (213849)	Campylobacteraceae (2954	Campylobacterales (68525)	Epsilonproteobacteria (122	Proteobacteria (2)	Bacteria (1)			100.00	100.00	WGS
197.16321	0.1124	Campylobacter jejuni (72294)	Campylobacter (213849)	Campylobacteraceae (2954	Campylobacterales (68525)	Epsilonproteobacteria (122	Proteobacteria (2)	Bacteria (1)			99.90	99.80	WGS
197.16322	0.11	Campylobacter jejuni (72294)	Campylobacter (213849)	Campylobacteraceae (2954	Campylobacterales (68525)	Epsilonproteobacteria (122	Proteobacteria (2)	Bacteria (1)			99.90	99.90	WGS
197.16323	0.1124	Campylobacter jejuni (72294)	Campylobacter (213849)	Campylobacteraceae (2954	Campylobacterales (68525)	Epsilonproteobacteria (122	Proteobacteria (2)	Bacteria (1)			99.90	99.90	WGS
197.16324	0.109	Campylobacter jejuni (72294)	Campylobacter (213849)	Campylobacteraceae (2954	Campylobacterales (68525)	Epsilonproteobacteria (122	Proteobacteria (2)	Bacteria (1)			99.90	99.60	WGS
197.16325	0.11	Campylobacter jejuni (72294)	Campylobacter (213849)	Campylobacteraceae (2954	Campylobacterales (68525)	Epsilonproteobacteria (122	Proteobacteria (2)	Bacteria (1)			99.90	99.90	WGS
197.16326	0.1118	Campylobacter jejuni (72294)	Campylobacter (213849)	Campylobacteraceae (2954	Campylobacterales (68525)	Epsilonproteobacteria (122	Proteobacteria (2)	Bacteria (1)			99.90	99.60	WGS
197.16327	0.1106	Campylobacter jejuni (72294)	Campylobacter (213849)	Campylobacteraceae (2954	Campylobacterales (68525)	Epsilonproteobacteria (122	Proteobacteria (2)	Bacteria (1)			99.90	99.50	WGS
197.16328	0.1078	Campylobacter jejuni (72294)	Campylobacter (213849)	Campylobacteraceae (2954	Campylobacterales (68525)	Epsilonproteobacteria (122	Proteobacteria (2)	Bacteria (1)			100.00	99.90	WGS
197.16329	0.108	Campylobacter jejuni (72294)	Campylobacter (213849)	Campylobacteraceae (2954	Campylobacterales (68525)	Epsilonproteobacteria (122	Proteobacteria (2)	Bacteria (1)			99.90	99.30	WGS
197.16330	0.1124	Campylobacter jejuni (72294)	Campylobacter (213849)	Campylobacteraceae (2954	Campylobacterales (68525)	Epsilonproteobacteria (122	Proteobacteria (2)	Bacteria (1)			99.90	99.70	WGS
197.16331	0.11	Campylobacter jejuni (72294)	Campylobacter (213849)	Campylobacteraceae (2954	Campylobacterales (68525)	Epsilonproteobacteria (122	Proteobacteria (2)	Bacteria (1)			99.90	99.50	WGS
197.16332	0.1106	Campylobacter jejuni (72294)	Campylobacter (213849)	Campylobacteraceae (2954	Campylobacterales (68525)	Epsilonproteobacteria (122	Proteobacteria (2)	Bacteria (1)			99.90	99.90	WGS
197.16333	0.1126	Campylobacter jejuni (72294)	Campylobacter (213849)	Campylobacteraceae (2954	Campylobacterales (68525)	Epsilonproteobacteria (122	Proteobacteria (2)	Bacteria (1)			99.60	99.00	WGS
197.16334	0.11	Campylobacter jejuni (72294)	Campylobacter (213849)	Campylobacteraceae (2954	Campylobacterales (68525)	Epsilonproteobacteria (122	Proteobacteria (2)	Bacteria (1)			99.90	99.40	WGS
197.16335	0.11	Campylobacter jejuni (72294)	Campylobacter (213849)	Campylobacteraceae (2954	Campylobacterales (68525)	Epsilonproteobacteria (122	Proteobacteria (2)	Bacteria (1)			99.90	99.60	WGS
197.16336	0.1106	Campylobacter jejuni (72294)	Campylobacter (213849)	Campylobacteraceae (2954	Campylobacterales (68525)	Epsilonproteobacteria (122	Proteobacteria (2)	Bacteria (1)			100.00	100.00	WGS
197.16337	0.1078	Campylobacter jejuni (72294)	Campylobacter (213849)	Campylobacteraceae (2954	Campylobacterales (68525)	Epsilonproteobacteria (122	Proteobacteria (2)	Bacteria (1)			100.00	99.90	WGS
197.16338	0.1126	Campylobacter jejuni (72294)	Campylobacter (213849)	Campylobacteraceae (2954	Campylobacterales (68525)	Epsilonproteobacteria (122	Proteobacteria (2)	Bacteria (1)			99.60	99.50	WGS
197.16339	0.1126	Campylobacter jejuni (72294)	Campylobacter (213849)	Campylobacteraceae (2954	Campylobacterales (68525)	Epsilonproteobacteria (122	Proteobacteria (2)	Bacteria (1)			99.60	99.60	WGS
197.16340	0.1098	Campylobacter jejuni (72294)	Campylobacter (213849)	Campylobacteraceae (2954	Campylobacterales (68525)	Epsilonproteobacteria (122	Proteobacteria (2)	Bacteria (1)			99.60	99.30	WGS
197.16341	0.1144	Campylobacter jejuni (72294)	Campylobacter (213849)	Campylobacteraceae (2954	Campylobacterales (68525)	Epsilonproteobacteria (122	Proteobacteria (2)	Bacteria (1)			100.00	99.90	WGS
197.16342	0.1124	Campylobacter jejuni (72294)	Campylobacter (213849)	Campylobacteraceae (2954	Campylobacterales (68525)	Epsilonproteobacteria (122	Proteobacteria (2)	Bacteria (1)			100.00	99.90	WGS
197.16343	0.1124	Campylobacter jejuni (72294)	Campylobacter (213849)	Campylobacteraceae (2954	Campylobacterales (68525)	Epsilonproteobacteria (122	Proteobacteria (2)	Bacteria (1)			100.00	99.80	WGS
197.16344	0.1078	Campylobacter jejuni (72294)	Campylobacter (213849)	Campylobacteraceae (2954	Campylobacterales (68525)	Epsilonproteobacteria (122	Proteobacteria (2)	Bacteria (1)			99.90	99.40	WGS
197.16345	0.11	Campylobacter jejuni (72294)	Campylobacter (213849)	Campylobacteraceae (2954	Campylobacterales (68525)	Epsilonproteobacteria (122	Proteobacteria (2)	Bacteria (1)			99.90	99.60	WGS
197.16346	0.11	Campylobacter jejuni (72294)	Campylobacter (213849)	Campylobacteraceae (2954	Campylobacterales (68525)	Epsilonproteobacteria (122	Proteobacteria (2)	Bacteria (1)			99.90	99.20	WGS
197.16347	0.108	Campylobacter jejuni (72294)	Campylobacter (213849)	Campylobacteraceae (2954	Campylobacterales (68525)	Epsilonproteobacteria (122	Proteobacteria (2)	Bacteria (1)			99.90	99.70	WGS
197.16348	0.1152	Campylobacter jejuni (72294)	Campylobacter (213849)	Campylobacteraceae (2954	Campylobacterales (68525)	Epsilonproteobacteria (122	Proteobacteria (2)	Bacteria (1)			99.90	99.70	WGS
197.16349	0.1078	Campylobacter jejuni (72294)	Campylobacter (213849)	Campylobacteraceae (2954	Campylobacterales (68525)	Epsilonproteobacteria (122	Proteobacteria (2)	Bacteria (1)			99.80	99.60	WGS
197.16350	0.1078	Campylobacter jejuni (72294)	Campylobacter (213849)	Campylobacteraceae (2954	Campylobacterales (68525)	Epsilonproteobacteria (122	Proteobacteria (2)	Bacteria (1)			99.80	99.60	WGS
197.16351	0.1078	Campylobacter jejuni (72294)	Campylobacter (213849)	Campylobacteraceae (2954	Campylobacterales (68525)	Epsilonproteobacteria (122	Proteobacteria (2)	Bacteria (1)			99.90	99.90	WGS
197.16352	0.1096	Campylobacter jejuni (72294)	Campylobacter (213849)	Campylobacteraceae (2954	Campylobacterales (68525)	Epsilonproteobacteria (122	Proteobacteria (2)	Bacteria (1)			99.90	99.80	WGS
197.16353	0.116	Campylobacter jejuni (72294)	Campylobacter (213849)	Campylobacteraceae (2954	Campylobacterales (68525)	Epsilonproteobacteria (122	Proteobacteria (2)	Bacteria (1)			99.90	99.80	WGS
197.16354	0.1078	Campylobacter jejuni (72294)	Campylobacter (213849)	Campylobacteraceae (2954	Campylobacterales (68525)	Epsilonproteobacteria (122	Proteobacteria (2)	Bacteria (1)			99.90	99.70	WGS
197.16355	0.1098	Campylobacter jejuni (72294)	Campylobacter (213849)	Campylobacteraceae (2954	Campylobacterales (68525)	Epsilonproteobacteria (122	Proteobacteria (2)	Bacteria (1)			99.90	99.90	WGS
197.16356	0.1078	Campylobacter jejuni (72294)	Campylobacter (213849)	Campylobacteraceae (2954	Campylobacterales (68525)	Epsilonproteobacteria (122	Proteobacteria (2)	Bacteria (1)			99.90	99.80	WGS
197.16357	0.1078	Campylobacter jejuni (72294)	Campylobacter (213849)	Campylobacteraceae (2954	Campylobacterales (68525)	Epsilonproteobacteria (122	Proteobacteria (2)	Bacteria (1)			99.70	99.60	WGS
197.16358	0.11	Campylobacter jejuni (72294)	Campylobacter (213849)	Campylobacteraceae (2954	Campylobacterales (68525)	Epsilonproteobacteria (122	Proteobacteria (2)	Bacteria (1)			99.90	99.90	WGS
197.16359	0.1078	Campylobacter jejuni (72294)	Campylobacter (213849)	Campylobacteraceae (2954	Campylobacterales (68525)	Epsilonproteobacteria (122	Proteobacteria (2)	Bacteria (1)			99.90	99.90	WGS
197.16360	0.1138	Campylobacter jejuni (72294)	Campylobacter (213849)	Campylobacteraceae (2954	Campylobacterales (68525)	Epsilonproteobacteria (122	Proteobacteria (2)	Bacteria (1)			99.90	99.30	WGS
197.16361	0.1078	Campylobacter jejuni (72294)	Campylobacter (213849)	Campylobacteraceae (2954	Campylobacterales (68525)	Epsilonproteobacteria (122	Proteobacteria (2)	Bacteria (1)			99.90	99.70	WGS
197.16362	0.1078	Campylobacter jejuni (72294)	Campylobacter (213849)	Campylobacteraceae (2954	Campylobacterales (68525)	Epsilonproteobacteria (122	Proteobacteria (2)	Bacteria (1)			100.00	99.70	WGS
197.16363	0.1078	Campylobacter jejuni (72294)	Campylobacter (213849)	Campylobacteraceae (2954	Campylobacterales (68525)	Epsilonproteobacteria (122	Proteobacteria (2)	Bacteria (1)			99.90	99.70	WGS
197.16364	0.116	Campylobacter jejuni (72294)	Campylobacter (213849)	Campylobacteraceae (2954	Campylobacterales (68525)	Epsilonproteobacteria (122	Proteobacteria (2)	Bacteria (1)			99.90	99.70	WGS
197.16365	0.1078	Campylobacter jejuni (72294)	Campylobacter (213849)	Campylobacteraceae (2954	Campylobacterales (68525)	Epsilonproteobacteria (122	Proteobacteria (2)	Bacteria (1)			99.90	99.40	WGS
197.16366	0.1106	Campylobacter jejuni (72294)	Campylobacter (213849)	Campylobacteraceae (2954	Campylobacterales (68525)	Epsilonproteobacteria (122	Proteobacteria (2)	Bacteria (1)			99.90	99.90	WGS
197.16367	0.1078	Campylobacter jejuni (72294)	Campylobacter (213849)	Campylobacteraceae (2954	Campylobacterales (68525)	Epsilonproteobacteria (122	Proteobacteria (2)	Bacteria (1)			99.90	99.50	WGS
197.16368	0.1078	Campylobacter jejuni (72294)	Campylobacter (213849)	Campylobacteraceae (2954	Campylobacterales (68525)	Epsilonproteobacteria (122	Proteobacteria (2)	Bacteria (1)			99.90	99.30	WGS
197.16369	0.108	Campylobacter jejuni (72294)	Campylobacter (213849)	Campylobacteraceae (2954	Campylobacterales (68525)	Epsilonproteobacteria (122	Proteobacteria (2)	Bacteria (1)			99.90	99.80	WGS
197.16370	0.11	Campylobacter jejuni (72294)	Campylobacter (213849)	Campylobacteraceae (2954	Campylobacterales (68525)	Epsilonproteobacteria (122	Proteobacteria (2)	Bacteria (1)			99.90	99.50	WGS
197.16371	0.1124	Campylobacter jejuni (72294)	Campylobacter (213849)	Campylobacteraceae (2954	Campylobacterales (68525)	Epsilonproteobacteria (122	Proteobacteria (2)	Bacteria (1)			99.90	99.60	WGS
197.16372	0.1106	Campylobacter jejuni (72294)	Campylobacter (213849)	Campylobacteraceae (2954	Campylobacterales (68525)	Epsilonproteobacteria (122	Proteobacteria (2)	Bacteria (1)			99.90	99.80	WGS
197.16373	0.1078	Campylobacter jejuni (72294)	Campylobacter (213849)	Campylobacteraceae (2954	Campylobacterales (68525)	Epsilonproteobacteria (122	Proteobacteria (2)	Bacteria (1)			99.80	99.20	WGS
197.16374	0.1078	Campylobacter jejuni (72294)	Campylobacter (213849)	Campylobacteraceae (2954	Campylobacterales (68525)	Epsilonproteobacteria (122	Proteobacteria (2)	Bacteria (1)			99.90	99.30	WGS
197.16375	0.112	Campylobacter jejuni (72294)	Campylobacter (213849)	Campylobacteraceae (2954	Campylobacterales (68525)	Epsilonproteobacteria (122	Proteobacteria (2)	Bacteria (1)			99.60	99.00	WGS
197.16376	0.1106	Campylobacter jejuni (72294)	Campylobacter (213849)	Campylobacteraceae (2954	Campylobacterales (68525)	Epsilonproteobacteria (122	Proteobacteria (2)	Bacteria (1)			99.90	99.70	WGS
197.16377	0.11	Campylobacter jejuni (72294)	Campylobacter (213849)	Campylobacteraceae (2954	Campylobacterales (68525)	Epsilonproteobacteria (122	Proteobacteria (2)	Bacteria (1)			99.90	99.60	WGS
197.16378	0.1078	Campylobacter jejuni (72294)	Campylobacter (213849)	Campylobacteraceae (2954	Campylobacterales (68525)	Epsilonproteobacteria (122	Proteobacteria (2)	Bacteria (1)			99.90	99.60	WGS
197.16379	0.1118	Campylobacter jejuni (72294)	Campylobacter (213849)	Campylobacteraceae (2954	Campylobacterales (68525)	Epsilonproteobacteria (122	Proteobacteria (2)	Bacteria (1)			99.80	99.50	WGS
197.16380	0.11	Campylobacter jejuni (72294)	Campylobacter (213849)	Campylobacteraceae (2954	Campylobacterales (68525)	Epsilonproteobacteria (122	Proteobacteria (2)	Bacteria (1)			99.90	99.80	WGS
197.16381	0.11	Campylobacter jejuni (72294)	Campylobacter (213849)	Campylobacteraceae (2954	Campylobacterales (68525)	Epsilonproteobacteria (122	Proteobacteria (2)	Bacteria (1)			99.80	99.70	WGS
197.16382	0.1124	Campylobacter jejuni (72294)	Campylobacter (213849)	Campylobacteraceae (2954	Campylobacterales (68525)	Epsilonproteobacteria (122	Proteobacteria (2)	Bacteria (1)			99.90	99.60	WGS
[illegible]	[illegible]	[illegible]	[illegible]	[illegible]	[illegible]	[illegible]	[illegible]	[illegible]	[illegible]	[illegible]	[illegible]	[illegible]	[illegible]

197.5202	0.114	Campylobacter jejuni (72294)	Campylobacter (213849)	Campylobacteraceae (2954	Campylobacterales (68525)	Epsilonproteobacteria (122	Proteobacteria (2)	Bacteria (1)	100.00	0.70	99.70	99.30	WGS
197.5203	0.109	Campylobacter jejuni (72294)	Campylobacter (213849)	Campylobacteraceae (2954	Campylobacterales (68525)	Epsilonproteobacteria (122	Proteobacteria (2)	Bacteria (1)	100.00	0.00	99.90	99.60	WGS
197.5204	0.1106	Campylobacter jejuni (72294)	Campylobacter (213849)	Campylobacteraceae (2954	Campylobacterales (68525)	Epsilonproteobacteria (122	Proteobacteria (2)	Bacteria (1)	99.30	3.20	99.90	99.30	WGS
197.5205	0.1106	Campylobacter jejuni (72294)	Campylobacter (213849)	Campylobacteraceae (2954	Campylobacterales (68525)	Epsilonproteobacteria (122	Proteobacteria (2)	Bacteria (1)	100.00	0.00	99.80	99.70	WGS
197.5206	0.116	Campylobacter jejuni (72294)	Campylobacter (213849)	Campylobacteraceae (2954	Campylobacterales (68525)	Epsilonproteobacteria (122	Proteobacteria (2)	Bacteria (1)	100.00	0.00	99.90	99.60	WGS
197.5207	0.1128	Campylobacter jejuni (72294)	Campylobacter (213849)	Campylobacteraceae (2954	Campylobacterales (68525)	Epsilonproteobacteria (122	Proteobacteria (2)	Bacteria (1)	100.00	0.00	99.90	99.20	WGS
197.5208	0.118	Campylobacter jejuni (72294)	Campylobacter (213849)	Campylobacteraceae (2954	Campylobacterales (68525)	Epsilonproteobacteria (122	Proteobacteria (2)	Bacteria (1)	100.00	0.00	99.60	99.10	WGS
197.5209	0.1078	Campylobacter jejuni (72294)	Campylobacter (213849)	Campylobacteraceae (2954	Campylobacterales (68525)	Epsilonproteobacteria (122	Proteobacteria (2)	Bacteria (1)	100.00	0.00	99.90	99.80	WGS
197.5210	0.2128	Campylobacter jejuni (72294)	Campylobacter (213849)	Campylobacteraceae (2954	Campylobacterales (68525)	Epsilonproteobacteria (122	Proteobacteria (2)	Bacteria (1)	100.00	40.60	96.10	71.20	WGS
197.5211	0.11	Campylobacter jejuni (72294)	Campylobacter (213849)	Campylobacteraceae (2954	Campylobacterales (68525)	Epsilonproteobacteria (122	Proteobacteria (2)	Bacteria (1)	100.00	2.60	99.90	99.70	WGS
197.5212	0.1138	Campylobacter jejuni (72294)	Campylobacter (213849)	Campylobacteraceae (2954	Campylobacterales (68525)	Epsilonproteobacteria (122	Proteobacteria (2)	Bacteria (1)	100.00	0.00	100.00	99.90	WGS
197.5213	0.1078	Campylobacter jejuni (72294)	Campylobacter (213849)	Campylobacteraceae (2954	Campylobacterales (68525)	Epsilonproteobacteria (122	Proteobacteria (2)	Bacteria (1)	100.00	0.00	100.00	99.90	WGS
197.5214	0.1078	Campylobacter jejuni (72294)	Campylobacter (213849)	Campylobacteraceae (2954	Campylobacterales (68525)	Epsilonproteobacteria (122	Proteobacteria (2)	Bacteria (1)	100.00	0.00	99.90	99.80	WGS
197.5215	0.1118	Campylobacter jejuni (72294)	Campylobacter (213849)	Campylobacteraceae (2954	Campylobacterales (68525)	Epsilonproteobacteria (122	Proteobacteria (2)	Bacteria (1)	100.00	0.70	100.00	99.70	WGS
197.5216	0.1078	Campylobacter jejuni (72294)	Campylobacter (213849)	Campylobacteraceae (2954	Campylobacterales (68525)	Epsilonproteobacteria (122	Proteobacteria (2)	Bacteria (1)	100.00	0.00	100.00	100.00	WGS
197.5217	0.1078	Campylobacter jejuni (72294)	Campylobacter (213849)	Campylobacteraceae (2954	Campylobacterales (68525)	Epsilonproteobacteria (122	Proteobacteria (2)	Bacteria (1)	100.00	1.30	99.90	99.60	WGS
197.5218	0.116	Campylobacter jejuni (72294)	Campylobacter (213849)	Campylobacteraceae (2954	Campylobacterales (68525)	Epsilonproteobacteria (122	Proteobacteria (2)	Bacteria (1)	100.00	0.70	99.60	99.50	WGS
197.5219	0.1132	Campylobacter jejuni (72294)	Campylobacter (213849)	Campylobacteraceae (2954	Campylobacterales (68525)	Epsilonproteobacteria (122	Proteobacteria (2)	Bacteria (1)	100.00	2.20	99.30	98.60	WGS
197.5220	0.1078	Campylobacter jejuni (72294)	Campylobacter (213849)	Campylobacteraceae (2954	Campylobacterales (68525)	Epsilonproteobacteria (122	Proteobacteria (2)	Bacteria (1)	99.30	5.60	99.70	98.00	WGS
197.5221	0.1078	Campylobacter jejuni (72294)	Campylobacter (213849)	Campylobacteraceae (2954	Campylobacterales (68525)	Epsilonproteobacteria (122	Proteobacteria (2)	Bacteria (1)	100.00	0.00	100.00	100.00	WGS
197.5222	0.1128	Campylobacter jejuni (72294)	Campylobacter (213849)	Campylobacteraceae (2954	Campylobacterales (68525)	Epsilonproteobacteria (122	Proteobacteria (2)	Bacteria (1)	100.00	1.30	99.40	98.80	WGS
197.5223	0.11	Campylobacter jejuni (72294)	Campylobacter (213849)	Campylobacteraceae (2954	Campylobacterales (68525)	Epsilonproteobacteria (122	Proteobacteria (2)	Bacteria (1)	99.30	1.30	99.90	99.00	WGS
197.5224	0.1172	Campylobacter jejuni (72294)	Campylobacter (213849)	Campylobacteraceae (2954	Campylobacterales (68525)	Epsilonproteobacteria (122	Proteobacteria (2)	Bacteria (1)	100.00	0.00	99.90	99.70	WGS
197.5225	0.1128	Campylobacter jejuni (72294)	Campylobacter (213849)	Campylobacteraceae (2954	Campylobacterales (68525)	Epsilonproteobacteria (122	Proteobacteria (2)	Bacteria (1)	100.00	1.90	99.80	99.30	WGS
197.5226	0.1144	Campylobacter jejuni (72294)	Campylobacter (213849)	Campylobacteraceae (2954	Campylobacterales (68525)	Epsilonproteobacteria (122	Proteobacteria (2)	Bacteria (1)	98.70	2.20	99.10	98.00	WGS
197.5227	0.1118	Campylobacter jejuni (72294)	Campylobacter (213849)	Campylobacteraceae (2954	Campylobacterales (68525)	Epsilonproteobacteria (122	Proteobacteria (2)	Bacteria (1)	100.00	0.70	99.80	99.30	WGS
197.5228	0.1078	Campylobacter jejuni (72294)	Campylobacter (213849)	Campylobacteraceae (2954	Campylobacterales (68525)	Epsilonproteobacteria (122	Proteobacteria (2)	Bacteria (1)	100.00	1.30	99.90	99.30	WGS
197.5229	0.1118	Campylobacter jejuni (72294)	Campylobacter (213849)	Campylobacteraceae (2954	Campylobacterales (68525)	Epsilonproteobacteria (122	Proteobacteria (2)	Bacteria (1)	100.00	0.70	99.90	99.60	Complete
197.5230	0.1128	Campylobacter jejuni (72294)	Campylobacter (213849)	Campylobacteraceae (2954	Campylobacterales (68525)	Epsilonproteobacteria (122	Proteobacteria (2)	Bacteria (1)	100.00	0.00	99.60	99.40	WGS
197.5231	0.112	Campylobacter jejuni (72294)	Campylobacter (213849)	Campylobacteraceae (2954	Campylobacterales (68525)	Epsilonproteobacteria (122	Proteobacteria (2)	Bacteria (1)	100.00	0.00	99.90	99.60	WGS
197.5232	0.1128	Campylobacter jejuni (72294)	Campylobacter (213849)	Campylobacteraceae (2954	Campylobacterales (68525)	Epsilonproteobacteria (122	Proteobacteria (2)	Bacteria (1)	100.00	0.00	99.50	99.00	WGS
197.5236	0.1128	Campylobacter jejuni (72294)	Campylobacter (213849)	Campylobacteraceae (2954	Campylobacterales (68525)	Epsilonproteobacteria (122	Proteobacteria (2)	Bacteria (1)	98.00	0.00	99.30	99.30	Complete
197.5237	0.1124	Campylobacter jejuni (72294)	Campylobacter (213849)	Campylobacteraceae (2954	Campylobacterales (68525)	Epsilonproteobacteria (122	Proteobacteria (2)	Bacteria (1)	100.00	0.00	99.90	99.60	WGS
197.5238	0.1098	Campylobacter jejuni (72294)	Campylobacter (213849)	Campylobacteraceae (2954	Campylobacterales (68525)	Epsilonproteobacteria (122	Proteobacteria (2)	Bacteria (1)	100.00	2.20	99.90	99.30	WGS
197.5239	0.1114	Campylobacter jejuni (72294)	Campylobacter (213849)	Campylobacteraceae (2954	Campylobacterales (68525)	Epsilonproteobacteria (122	Proteobacteria (2)	Bacteria (1)	100.00	0.70	99.70	99.40	WGS
197.5240	0.1126	Campylobacter jejuni (72294)	Campylobacter (213849)	Campylobacteraceae (2954	Campylobacterales (68525)	Epsilonproteobacteria (122	Proteobacteria (2)	Bacteria (1)	100.00	1.30	99.60	99.20	WGS
197.5241	0.1114	Campylobacter jejuni (72294)	Campylobacter (213849)	Campylobacteraceae (2954	Campylobacterales (68525)	Epsilonproteobacteria (122	Proteobacteria (2)	Bacteria (1)	100.00	0.00	99.60	99.30	WGS
197.5242	0.1078	Campylobacter jejuni (72294)	Campylobacter (213849)	Campylobacteraceae (2954	Campylobacterales (68525)	Epsilonproteobacteria (122	Proteobacteria (2)	Bacteria (1)	100.00	0.00	99.90	99.80	WGS
197.5243	0.1134	Campylobacter jejuni (72294)	Campylobacter (213849)	Campylobacteraceae (2954	Campylobacterales (68525)	Epsilonproteobacteria (122	Proteobacteria (2)	Bacteria (1)	100.00	0.70	99.60	99.60	WGS
197.5244	0.1154	Campylobacter jejuni (72294)	Campylobacter (213849)	Campylobacteraceae (2954	Campylobacterales (68525)	Epsilonproteobacteria (122	Proteobacteria (2)	Bacteria (1)	100.00	0.00	99.60	99.00	WGS
197.5245	0.116	Campylobacter jejuni (72294)	Campylobacter (213849)	Campylobacteraceae (2954	Campylobacterales (68525)	Epsilonproteobacteria (122	Proteobacteria (2)	Bacteria (1)	100.00	0.00	99.80	99.40	WGS
197.5246	0.116	Campylobacter jejuni (72294)	Campylobacter (213849)	Campylobacteraceae (2954	Campylobacterales (68525)	Epsilonproteobacteria (122	Proteobacteria (2)	Bacteria (1)	100.00	0.70	99.70	99.30	WGS
197.5247	0.109	Campylobacter jejuni (72294)	Campylobacter (213849)	Campylobacteraceae (2954	Campylobacterales (68525)	Epsilonproteobacteria (122	Proteobacteria (2)	Bacteria (1)	100.00	10.80	99.90	96.90	WGS
197.5249	0.1098	Campylobacter jejuni (72294)	Campylobacter (213849)	Campylobacteraceae (2954	Campylobacterales (68525)	Epsilonproteobacteria (122	Proteobacteria (2)	Bacteria (1)	100.00	0.00	99.80	99.60	Complete
197.5250	0.1078	Campylobacter jejuni (72294)	Campylobacter (213849)	Campylobacteraceae (2954	Campylobacterales (68525)	Epsilonproteobacteria (122	Proteobacteria (2)	Bacteria (1)	100.00	0.00	100.00	99.90	WGS
197.5252	0.1078	Campylobacter jejuni (72294)	Campylobacter (213849)	Campylobacteraceae (2954	Campylobacterales (68525)	Epsilonproteobacteria (122	Proteobacteria (2)	Bacteria (1)	100.00	0.00	99.90	99.70	WGS
197.5253	0.1162	Campylobacter jejuni (72294)	Campylobacter (213849)	Campylobacteraceae (2954	Campylobacterales (68525)	Epsilonproteobacteria (122	Proteobacteria (2)	Bacteria (1)	100.00	0.70	99.90	99.80	WGS
197.5255	0.1106	Campylobacter jejuni (72294)	Campylobacter (213849)	Campylobacteraceae (2954	Campylobacterales (68525)	Epsilonproteobacteria (122	Proteobacteria (2)	Bacteria (1)	100.00	0.00	99.60	99.60	WGS
197.5256	0.1078	Campylobacter jejuni (72294)	Campylobacter (213849)	Campylobacteraceae (2954	Campylobacterales (68525)	Epsilonproteobacteria (122	Proteobacteria (2)	Bacteria (1)	100.00	0.70	99.90	99.90	WGS
197.5257	0.1132	Campylobacter jejuni (72294)	Campylobacter (213849)	Campylobacteraceae (2954	Campylobacterales (68525)	Epsilonproteobacteria (122	Proteobacteria (2)	Bacteria (1)	100.00	1.30	99.90	99.80	WGS
197.5258	0.1132	Campylobacter jejuni (72294)	Campylobacter (213849)	Campylobacteraceae (2954	Campylobacterales (68525)	Epsilonproteobacteria (122	Proteobacteria (2)	Bacteria (1)	100.00	0.70	99.90	99.20	WGS
197.5259	0.1146	Campylobacter jejuni (72294)	Campylobacter (213849)	Campylobacteraceae (2954	Campylobacterales (68525)	Epsilonproteobacteria (122	Proteobacteria (2)	Bacteria (1)	100.00	1.90	99.90	99.90	WGS
197.5260	0.1142	Campylobacter jejuni (72294)	Campylobacter (213849)	Campylobacteraceae (2954	Campylobacterales (68525)	Epsilonproteobacteria (122	Proteobacteria (2)	Bacteria (1)	100.00	0.00	99.90	99.90	Complete
197.5261	0.1142	Campylobacter jejuni (72294)	Campylobacter (213849)	Campylobacteraceae (2954	Campylobacterales (68525)	Epsilonproteobacteria (122	Proteobacteria (2)	Bacteria (1)	100.00	0.70	99.90	99.90	Complete
197.5262	0.1162	Campylobacter jejuni (72294)	Campylobacter (213849)	Campylobacteraceae (2954	Campylobacterales (68525)	Epsilonproteobacteria (122	Proteobacteria (2)	Bacteria (1)	100.00	0.00	99.90	99.70	Complete
197.5263	0.1142	Campylobacter jejuni (72294)	Campylobacter (213849)	Campylobacteraceae (2954	Campylobacterales (68525)	Epsilonproteobacteria (122	Proteobacteria (2)	Bacteria (1)	100.00	0.00	99.90	99.90	Complete
197.5264	0.1142	Campylobacter jejuni (72294)	Campylobacter (213849)	Campylobacteraceae (2954	Campylobacterales (68525)	Epsilonproteobacteria (122	Proteobacteria (2)	Bacteria (1)	100.00	0.00	99.90	99.90	Complete
197.5265	0.1142	Campylobacter jejuni (72294)	Campylobacter (213849)	Campylobacteraceae (2954	Campylobacterales (68525)	Epsilonproteobacteria (122	Proteobacteria (2)	Bacteria (1)	100.00	0.00	99.90	99.70	Complete
197.5266	0.1142	Campylobacter jejuni (72294)	Campylobacter (213849)	Campylobacteraceae (2954	Campylobacterales (68525)	Epsilonproteobacteria (122	Proteobacteria (2)	Bacteria (1)	100.00	0.00	99.90	99.90	Complete
197.5267	0.1142	Campylobacter jejuni (72294)	Campylobacter (213849)	Campylobacteraceae (2954	Campylobacterales (68525)	Epsilonproteobacteria (122	Proteobacteria (2)	Bacteria (1)	100.00	0.00	99.90	99.90	Complete
197.5268	0.1142	Campylobacter jejuni (72294)	Campylobacter (213849)	Campylobacteraceae (2954	Campylobacterales (68525)	Epsilonproteobacteria (122	Proteobacteria (2)	Bacteria (1)	100.00	0.00	99.90	99.90	Complete
197.5269	0.1142	Campylobacter jejuni (72294)	Campylobacter (213849)	Campylobacteraceae (2954	Campylobacterales (68525)	Epsilonproteobacteria (122	Proteobacteria (2)	Bacteria (1)	100.00	3.20	99.90	99.10	Complete
197.5270	0.1162	Campylobacter jejuni (72294)	Campylobacter (213849)	Campylobacteraceae (2954	Campylobacterales (68525)	Epsilonproteobacteria (122	Proteobacteria (2)	Bacteria (1)	100.00	0.00	99.90	99.90	Complete
197.5271	0.1142	Campylobacter jejuni (72294)	Campylobacter (213849)	Campylobacteraceae (2954	Campylobacterales (68525)	Epsilonproteobacteria (122	Proteobacteria (2)	Bacteria (1)	100.00	0.00	99.90	99.90	Complete
197.5272	0.1162	Campylobacter jejuni (72294)	Campylobacter (213849)	Campylobacteraceae (2954	Campylobacterales (68525)	Epsilonproteobacteria (122	Proteobacteria (2)	Bacteria (1)	100.00	0.00	99.90	99.90	Complete
197.5273	0.1162	Campylobacter jejuni (72294)	Campylobacter (213849)	Campylobacteraceae (2954	Campylobacterales (68525)	Epsilonproteobacteria (122	Proteobacteria (2)	Bacteria (1)	100.00	0.00	99.90	99.90	Complete
197.5274	0.1114	Campylobacter jejuni (72294)	Campylobacter (213849)	Campylobacteraceae (2954	Campylobacterales (68525)	Epsilonproteobacteria (122	Proteobacteria (2)	Bacteria (1)	100.00	0.00	99.90	99.90	Complete
197.5275	0.1142	Campylobacter jejuni (72294)	Campylobacter (213849)	Campylobacteraceae (2954	Campylobacterales (68525)	Epsilonproteobacteria (122	Proteobacteria (2)	Bacteria (1)	100.00	0.00	99.90	99.90	Complete
197.5276	0.1162	Campylobacter jejuni (72294)	Campylobacter (213849)	Campylobacteraceae (2954	Campylobacterales (68525)	Epsilonproteobacteria (122	Proteobacteria (2)	Bacteria (1)	100.00	0.00	99.90	99.90	Complete
197.5277	0.1142	Campylobacter jejuni (72294)	Campylobacter (213849)	Campylobacteraceae (2954	Campylobacterales (68525)	Epsilonproteobacteria (122	Proteobacteria (2)	Bacteria (1)	100.00	0.00	99.90	99.90	Complete
197.5278	0.1142	Campylobacter jejuni (72294)	Campylobacter (213849)	Campylobacteraceae (2954	Campylobacterales (68525)	Epsilonproteobacteria (122	Proteobacteria (2)	Bacteria (1)	100.00	0.00	99.90	99.90	Complete
197.5279	0.1162	Campylobacter jejuni (72294)	Campylobacter (213849)	Campylobacteraceae (2954	Campylobacterales (68525)	Epsilonproteobacteria (122	Proteobacteria (2)	Bacteria (1)	100.00	0.00	99.90	99.90	Complete
197.5280	0.1142	Campylobacter jejuni (72294)	Campylobacter (213849)	Campylobacteraceae (2954	Campylobacterales (68525)	Epsilonproteobacteria (122	Proteobacteria (2)	Bacteria (1)	100.00	0.00	99.90	99.90	Complete
197.5281	0.1142	Campylobacter jejuni (72294)	Campylobacter (213849)	Campylobacteraceae (2954	Campylobacterales (68525)	Epsilonproteobacteria (122	Proteobacteria (2)	Bacteria (1)	100.00	0.00	99.90	99.90	Complete
197.5282	0.1162	Campylobacter jejuni (72294)	Campylobacter (213849)	Campylobacteraceae (2954	Campylobacterales (68525)	Epsilonproteobacteria (122	Proteobacteria (2)	Bacteria (1)	100.00	0.00	99.90	99.90	Complete
197.5283	0.1142	Campylobacter jejuni (72294)	Campylobacter (213849)	Campylobacteraceae (2954	Campylobacterales (68525)	Epsilonproteobacteria (122	Proteobacteria (2)	Bacteria (1)	100.00	0.00	99.90	99.90	Complete
197.5284	0.1142	Campylobacter jejuni (72294)	Campylobacter (213849)	Campylobacteraceae (2954	Campylobacterales (68525)	Epsilonproteobacteria (122	Proteobacteria (2)	Bacteria (1)	100.00	0.00	99.90	99.90	Complete
197.5285	0.1162	Campylobacter jejuni (72294)	Campylobacter (213849)	Campylobacteraceae (2954	Campylobacterales (68525)	Epsilonproteobacteria (122	Proteobacteria (2)	Bacteria (1)	100.00	0.00	99.90	99.90	Complete
197.5286	0.1142	Campylobacter jejuni (72294)	Campylobacter (213849)	Campylobacteraceae (2954	Campylobacterales (68525)	Epsilonproteobacteria (122	Proteobacteria (2)	Bacteria (1)	100.00	0.00	99.90	99.90	Complete
197.5287	0.1142	Campylobacter jejuni (72294)	Campylobacter (213849)	Campylobacteraceae (2954	Campylobacterales (68525)	Epsilonproteobacteria (122	Proteobacteria (2)	Bacteria (1)	100.00	0.00	99.90	99.90	Complete
197.5288	0.1162	Campylobacter jejuni (72294)	Campylobacter (213849)	Campylobacteraceae (2954	Campylobacterales (68525)	Epsilonproteobacteria (122	Proteobacteria (2)	Bacteria (1)	100.00	0.00	99.90	99.90	Complete
197.5289	0.1142	Campylobacter jejuni (72294)	Campylobacter (213849)	Campylobacteraceae (2954	Campylobacterales (68525)	Epsilonproteobacteria (122	Proteobacteria (2)	Bacteria (1)	100.00	0.00	99.90	99.90	Complete
197.5290	0.1142	Campylobacter jejuni (72294)	Campylobacter (213849)	Campylobacteraceae (2954	Campylobacterales (68525)	Epsilonproteobacteria (122	Proteobacteria (2)	Bacteria (1)	100.00	0.00	99.90	99.90	Complete
197.5291	0.1142	Campylobacter jejuni (72294)	Campylobacter (213849)	Campylobacteraceae (2954	Campylobacterales (68525)	Epsilonproteobacteria (122	Proteobacteria (2)	Bacteria (1)	100.00	0.00	99.90	99.90	Complete
197.5292	0.1162	Campylobacter jejuni (72294)	Campylobacter (213849)	Campylobacteraceae (2954	Campylobacterales (68525)	Epsilonproteobacteria (122	Proteobacteria (2)	Bacteria (1)	100.00	0.00	99.90	99.80	Complete
197.5293	0.1142	Campylobacter jejuni (72294)	Campylobacter (213849)	Campylobacteraceae (2954	Campylobacterales (68525)	Epsilonproteobacteria (122	Proteobacteria (2)	Bacteria (1)	100.00	0.00	99.90	99.90	Complete
197.5294	0.1142	Campylobacter jejuni (72294)	Campylobacter (213849)	Campylobacteraceae (2954	Campylobacterales (68525)	Epsilonproteobacteria (122	Proteobacteria (2)	Bacteria (1)	100.00	0.00	99.90	99.70	Complete
197.5295	0.1142	Campylobacter jejuni (72294)	Campylobacter (213849)	Campylobacteraceae (2954	Campylobacterales (68525)	Epsilonproteobacteria (122	Proteobacteria (2)	Bacteria (1)	100.00	0.00	99.90	99.90	Complete
197.5296	0.1162	Campylobacter jejuni (72294)	Campylobacter (213849)	Campylobacteraceae (2954	Campylobacterales (68525)	Epsilonproteobacteria (122	Proteobacteria (2)	Bacteria (1)	100.00	0.00	99.90	99.90	Complete
197.5297	0.1142	Campylobacter jejuni (72294)	Campylobacter (213849)	Campylobacteraceae (2954	Campylobacterales (68525)	Epsilonproteobacteria (122	Proteobacteria (2)	Bacteria (1)	100.00	0.00	99.90	99.90	Complete
197.5298	0.1142	Campylobacter jejuni (72294)	Campylobacter (213849)	Campylobacteraceae (2954	Campylobacterales (68525)	Epsilonproteobacteria (122	Proteobacteria (2)	Bacteria (1)	100.00	0.00	99.90	99.90	Complete
197.5299	0.1162	Campylobacter jejuni (72294)	Campylobacter (213849)	Campylobacteraceae (2954	Campylobacterales (68525)	Epsilonproteobacteria (122	Proteobacteria (2)	Bacteria (1)	100.00	0.00	99.90	99.90	Complete
197.5300	0.1162	Campylobacter jejuni (72294)	Campylobacter (213849)	Campylobacteraceae (2954	Campylobacterales (68525)	Epsilonproteobacteria (122	Proteobacteria (2)	Bacteria (1)	100.00	0.00	99.90	99.90	Complete
197.5301	0.1142	Campylobacter jejuni (72294)	Campylobacter (213849)	Campylobacteraceae (2954	Campylobacterales (68525)	Epsilonproteobacteria (122	Proteobacteria (2)	Bacteria (1)	100.00	0.00	99.90	99.90	Complete
197.5302	0.1142	Campylobacter jejuni (72294)	Campylobacter (213849)	Campylobacteraceae (2954	Campylobacterales (68525)	Epsilonproteobacteria (122	Proteobacteria (2)	Bacteria (1)	100.00	0.00	99.90	99.90	Complete
197.5303	0.1142	Campylobacter jejuni (72294)	Campylobacter (213849)	Campylobacteraceae (2954	Campylobacterales (68525)	Epsilonproteobacteria (122	Proteobacteria (2)	Bacteria (1)	100.00	0.00	99.90	99.90	Complete
197.5304	0.1142	Campylobacter jejuni (72294)	Campylobacter (213849)	Campylobacteraceae (2954	Campylobacterales (68525)	Epsilonproteobacteria (122	Proteobacteria (2)	Bacteria (1)	100.00	0.00	99.90	99.90	Complete
197.5305	0.1142	Campylobacter jejuni (72294)	Campylobacter (213849)	Campylobacteraceae (2954	Campylobacterales (68525)	Epsilonproteobacteria (122	Proteobacteria (2)	Bacteria (1)	100.00	0.00	99.90	99.90	Complete
197.5306	0.1142	Campylobacter jejuni (72294)	Campylobacter (213849)	Campylobacteraceae (2954	Campylobacterales (68525)	Epsilonproteobacteria (122	Proteobacteria (2)	Bacteria (1)	100.00	0.00	99.90	99.90	Complete
197.5307	0.1142	Campylobacter jejuni (72294)	Campylobacter (213849)	Campylobacteraceae (2954	Campylobacterales (68525)	Epsilonproteobacteria (122	Proteobacteria (2)	Bacteria (1)	100.00	0.00	99.90	99.90	Complete
197.5308	0.1142	Campylobacter jejuni (72294)	Campylobacter (213849)	Campylobacteraceae (2954	Campylobacterales (68525)	Epsilonproteobacteria (122	Proteobacteria (2)	Bacteria (1)	100.00	0.00	99.90	99.90	Complete
197.5309	0.1142	Campylobacter jejuni (72294)	Campylobacter (213849)	Campylobacteraceae (2954	Campylobacterales (68525)	Epsilonproteobacteria (122	Proteobacteria (2)	Bacteria (1)	100.00	0.00	99.90	99.90	Complete
197.5310	0.1142	Campylobacter jejuni (72294)	Campylobacter (213849)	Campylobacteraceae (2954	Campylobacterales (68525)	Epsilonproteobacteria (122	Proteobacteria (2)	Bacteria (1)	100.00	0.00	99.90	99.90	Complete
197.5311	0.1142	Campylobacter jejuni (72294)	Campylobacter (213849)	Campylobacteraceae (2954	Campylobacterales (68525)	Epsilonproteobacteria (122	Proteobacteria (2)	Bacteria (1)	100.00	0.00	99.90	99.90	Complete
197.5312	0.1142	Campylobacter jejuni (72294)	Campylobacter (213849)	Campylobacteraceae (2954	Campylobacterales (68525)	Epsilonproteobacteria (122	Proteobacteria (2)	Bacteria (1)	100.00	0.00	99.90	99.90	Complete
197.5313	0.1142	Campylobacter jejuni (72294)	Campylobacter (213849)	Campylobacteraceae (2954	Campylobacterales (68525)	Epsilonproteobacteria (122	Proteobacteria (2)	Bacteria (1)	100.00	0.00	99.90	99.90	Complete
197.5314	0.1142	Campylobacter jejuni (72294)	Campylobacter (213849)	Campylobacteraceae (2954	Campylobacterales (68525)	Epsilonproteobacteria (122	Proteobacteria (2)	Bacteria (1)	100.00	0.00	99.90	99.90	Complete
197.5316	0.1106	Campylobacter jejuni (72294)	Campylobacter (213849)	Campylobacteraceae (2954	Campylobacterales (68525)	Epsilonproteobacteria (122	Proteobacteria (2)	Bacteria (1)	100.00	0.00	99.60	99.60	WGS
197.5317	0.109	Campylobacter jejuni (72294)	Campylobacter (213849)	Campylobacteraceae (2954	Campylobacterales (68525)	Epsilonproteobacteria (122	Proteobacteria (2)	Bacteria (1)	100.00	0.00	99.80	99.70	WGS
197.5318	0.1132	Campylobacter jejuni (72294)	Campylobacter (213849)	Campylobacteraceae (2954	Campylobacterales (68525)	Epsilonproteobacteria (122	Proteobacteria (2)	Bacteria (1)	100.00	0.00	99.90	99.90	WGS
197.5319	0.1114	Campylobacter jejuni (72294)	Campylobacter (213849)	Campylobacteraceae (2954	Campylobacterales (68525)	Epsilonproteobacteria (122	Proteobacteria (2)	Bacteria (1)	100.00	0.00	99.80	99.60	WGS
197.5320	0.1144	Campylobacter jejuni (72294)	Campylobacter (213849)	Campylobacteraceae (2954	Campylobacterales (68525)	Epsilonproteobacteria (122	Proteobacteria (2)	Bacteria (1)	100.00	0.70	99.80	99.60	WGS
197.5321	0.1078	Campylobacter jejuni (72294)	Campylobacter (213849)	Campylobacteraceae (2954	Campylobacterales (68525)	Epsilonproteobacteria (122	Proteobacteria (2)	Bacteria (1)	100.00	0.00	99.90	99.80	WGS
197.5322	0.1154	Campylobacter jejuni (72294)	Campylobacter (213849)	Campylobacteraceae (2954	Campylobacterales (68525)	Epsilonproteobacteria (122	Proteobacteria (2)	Bacteria (1)	100.00	0.00	99.80	99.60	WGS
197.5323	0.1144	Campylobacter jejuni (72294)	Campylobacter (213849)	Campylobacteraceae (2954	Campylobacterales (68525)	Epsilonproteobacteria (122	Proteobacteria (2)	Bacteria (1)	100.00	0.00	99.70	99.60	Complete
197.5324	0.1078	Campylobacter jejuni (72294)	Campylobacter (213849)	Campylobacteraceae (2954	Campylobacterales (68525)	Epsilonproteobacteria (122	Proteobacteria (2)	Bacteria (1)	100.00	0.00	99.80	99.50	WGS
197.5325	0.1114	Campylobacter jejuni (72294)	Campylobacter (213849)	Campylobacteraceae (2954	Campylobacterales (68525)	Epsilonproteobacteria (122	Proteobacteria (2)	Bacteria (1)	100.00	1.30	99.80	99.00	WGS
197.5326	0.11	Campylobacter jejuni (72294)	Campylobacter (213849)	Campylobacteraceae (2954	Campylobacterales (68525)	Epsilonproteobacteria (122	Proteobacteria (2)	Bacteria (1)	100.00	1.30	99.70	99.00	WGS
197.5327	0.1078	Campylobacter jejuni (72294)	Campylobacter (213849)	Campylobacteraceae (2954	Campylobacterales (68525)	Epsilonproteobacteria (122	Proteobacteria (2)	Bacteria (1)	97.40	1.30	99.20	99.00	Complete
197.5328	0.1106	Campylobacter jejuni (72294)	Campylobacter (213849)	Campylobacteraceae (2954	Campylobacterales (68525)	Epsilonproteobacteria (122	Proteobacteria (2)	Bacteria (1)	100.00	0.00	99.70	99.60	Complete
197.5329	0.1108	Campylobacter jejuni (72294)	Campylobacter (213849)	Campylobacteraceae (2954	Campylobacterales (68525)	Epsilonproteobacteria (122	Proteobacteria (2)	Bacteria (1)	96.00	2.70	96.50	94.90	WGS
197.6642	0.1144	Campylobacter jejuni (72294)	Campylobacter (213849)	Campylobacteraceae (2954	Campylobacterales (68525)	Epsilonproteobacteria (122	Proteobacteria (2)	Bacteria (1)	100.00	0.00	99.60	99.40	WGS
197.6643	0.0944	Campylobacter jejuni (72294)	Campylobacter (213849)	Campylobacteraceae (2954	Campylobacterales (68525)	Epsilonproteobacteria (122	Proteobacteria (2)	Bacteria (1)	100.00	0.00	99.40	99.30	WGS
197.6644	0.1206	Campylobacter jejuni (72294)	Campylobacter (213849)	Campylobacteraceae (2954	Campylobacterales (68525)	Epsilonproteobacteria (122	Proteobacteria (2)	Bacteria (1)	100.00	0.00	99.90	99.60	WGS
197.6645	0.1132	Campylobacter jejuni (72294)	Campylobacter (213849)	Campylobacteraceae (2954	Campylobacterales (68525)	Epsilonproteobacteria (122	Proteobacteria (2)	Bacteria (1)	100.00	0.60	99.60	99.60	WGS
197.6646	0.0944	Campylobacter jejuni (72294)	Campylobacter (213849)	Campylobacteraceae (2954	Campylobacterales (68525)	Epsilonproteobacteria (122	Proteobacteria (2)	Bacteria (1)	100.00	0.00	99.40	99.30	WGS
197.6647	0.117	Campylobacter jejuni (72294)	Campylobacter (213849)	Campylobacteraceae (2954	Campylobacterales (68525)	Epsilonproteobacteria (122	Proteobacteria (2)	Bacteria (1)	100.00	0.00	99.80	99.60	WGS
197.6648	0.1116	Campylobacter jejuni (72294)	Campylobacter (213849)	Campylobacteraceae (2954	Campylobacterales (68525)	Epsilonproteobacteria (122	Proteobacteria (2)	Bacteria (1)	100.00	1.30	99.90	99.80	WGS
197.6649	0.0984	Campylobacter jejuni (72294)	Campylobacter (213849)	Campylobacteraceae (2954	Campylobacterales (68525)	Epsilonproteobacteria (122	Proteobacteria (2)	Bacteria (1)	100.00	0.60	99.60	99.30	WGS
197.6650	0.106	Campylobacter jejuni (72294)	Campylobacter (213849)	Campylobacteraceae (2954	Campylobacterales (68525)	Epsilonproteobacteria (122	Proteobacteria (2)	Bacteria (1)	100.00	0.00	99.60	99.30	WGS
197.6651	0.117	Campylobacter jejuni (72294)	Campylobacter (213849)	Campylobacteraceae (2954	Campylobacterales (68525)	Epsilonproteobacteria (122	Proteobacteria (2)	Bacteria (1)	100.00	1.30	99.70	99.60	WGS
197.6652	0.117	Campylobacter jejuni (72294)	Campylobacter (213849)	Campylobacteraceae (2954	Campylobacterales (68525)	Epsilonproteobacteria (122	Proteobacteria (2)	Bacteria (1)	100.00	1.30	99.70	99.60	WGS
197.6653	0.1124	Campylobacter jejuni (72294)	Campylobacter (213849)	Campylobacteraceae (2954	Campylobacterales (68525)	Epsilonproteobacteria (122	Proteobacteria (2)	Bacteria (1)	100.00	0.00	100.00	100.00	WGS
197.6654	0.0944	Campylobacter jejuni (72294)	Campylobacter (213849)	Campylobacteraceae (2954	Campylobacterales (68525)	Epsilonproteobacteria (122	Proteobacteria (2)	Bacteria (1)	100.00	0.00	99.40	99.30	WGS
197.6656	0.1206	Campylobacter jejuni (72294)	Campylobacter (213849)	Campylobacteraceae (2954	Campylobacterales (68525)	Epsilonproteobacteria (122	Proteobacteria (2)	Bacteria (1)	100.00	0.00	99.90	99.60	WGS
197.6657	0.0944	Campylobacter jejuni (72294)	Campylobacter (213849)	Campylobacteraceae (2954	Campylobacterales (68525)	Epsilonproteobacteria (122	Proteobacteria (2)	Bacteria (1)	100.00	0.00	99.40	99.30	WGS
197.6658	0.117	Campylobacter jejuni (72294)	Campylobacter (213849)	Campylobacteraceae (2954	Campylobacterales (68525)	Epsilonproteobacteria (122	Proteobacteria (2)	Bacteria (1)	100.00	1.30	99.70	99.60	WGS
197.6659	0.1142	Campylobacter jejuni (72294)	Campylobacter (213849)	Campylobacteraceae (2954	Campylobacterales (68525)	Epsilonproteobacteria (122	Proteobacteria (2)	Bacteria (1)	100.00	0.00	99.90	99.90	WGS
197.6660	0.1128	Campylobacter jejuni (72294)	Campylobacter (213849)	Campylobacteraceae (2954	Campylobacterales (68525)	Epsilonproteobacteria (122	Proteobacteria (2)	Bacteria (1)	100.00	0.00	100.00	100.00	WGS
197.6661	0.11	Campylobacter jejuni (72294)	Campylobacter (213849)	Campylobacteraceae (2954	Campylobacterales (68525)	Epsilonproteobacteria (122	Proteobacteria (2)	Bacteria (1)	100.00	0.00	100.00	100.00	WGS
197.6662	0.11	Campylobacter jejuni (72294)	Campylobacter (213849)	Campylobacteraceae (2954	Campylobacterales (68525)	Epsilonproteobacteria (122	Proteobacteria (2)	Bacteria (1)	100.00	0.00	99.90	99.90	WGS
197.6663	0.11	Campylobacter jejuni (72294)	Campylobacter (213849)	Campylobacteraceae (2954	Campylobacterales (68525)	Epsilonproteobacteria (122	Proteobacteria (2)	Bacteria (1)	100.00	0.00	99.90	99.90	WGS
197.6664	0.1126	Campylobacter jejuni (72294)	Campylobacter (213849)	Campylobacteraceae (2954	Campylobacterales (68525)	Epsilonproteobacteria (122	Proteobacteria (2)	Bacteria (1)	100.00	0.00	99.60	99.40	WGS
197.6665	0.1206	Campylobacter jejuni (72294)	Campylobacter (213849)	Campylobacteraceae (2954	Campylobacterales (68525)	Epsilonproteobacteria (122	Proteobacteria (2)	Bacteria (1)	100.00	0.00	99.90	99.60	WGS
197.6666	0.0944	Campylobacter jejuni (72294)	Campylobacter (213849)	Campylobacteraceae (2954	Campylobacterales (68525)	Epsilonproteobacteria (122	Proteobacteria (2)	Bacteria (1)	100.00	0.00	99.40	99.30	WGS
197.6667	0.11	Campylobacter jejuni (72294)	Campylobacter (213849)	Campylobacteraceae (2954	Campylobacterales (68525)	Epsilonproteobacteria (122	Proteobacteria (2)	Bacteria (1)	100.00	0.00	99.90	99.70	WGS
197.6668	0.11	Campylobacter jejuni (72294)	Campylobacter (213849)	Campylobacteraceae (2954	Campylobacterales (68525)	Epsilonproteobacteria (122	Proteobacteria (2)	Bacteria (1)	100.00	0.00	99.90	99.70	WGS
197.6669	0.0954	Campylobacter jejuni (72294)	Campylobacter (213849)	Campylobacteraceae (2954	Campylobacterales (68525)	Epsilonproteobacteria (122	Proteobacteria (2)	Bacteria (1)	100.00	0.00	99.60	99.30	WGS
197.6670	0.0972	Campylobacter jejuni (72294)	Campylobacter (213849)	Campylobacteraceae (2954	Campylobacterales (68525)	Epsilonproteobacteria (122	Proteobacteria (2)	Bacteria (1)	100.00	0.00	99.50	99.30	WGS
197.6671	0.0984	Campylobacter jejuni (72294)	Campylobacter (213849)	Campylobacteraceae (2954	Campylobacterales (68525)	Epsilonproteobacteria (122	Proteobacteria (2)	Bacteria (1)	100.00	0.00	99.80	99.50	WGS
197.6672	0.0944	Campylobacter jejuni (72294)	Campylobacter (213849)	Campylobacteraceae (2954	Campylobacterales (68525)	Epsilonproteobacteria (122	Proteobacteria (2)	Bacteria (1)	100.00	0.60	99.60	99.40	WGS
197.6673	0.106	Campylobacter jejuni (72294)	Campylobacter (213849)	Campylobacteraceae (2954	Campylobacterales (68525)	Epsilonproteobacteria (122	Proteobacteria (2)	Bacteria (1)	100.00	0.00	99.60	99.30	WGS
197.6674	0.104	Campylobacter jejuni (72294)	Campylobacter (213849)	Campylobacteraceae (2954	Campylobacterales (68525)	Epsilonproteobacteria (122	Proteobacteria (2)	Bacteria (1)	100.00	0.00	99.70	99.60	WGS
197.6675	0.0944	Campylobacter jejuni (72294)	Campylobacter (213849)	Campylobacteraceae (2954	Campylobacterales (68525)	Epsilonproteobacteria (122	Proteobacteria (2)	Bacteria (1)	100.00	0.00	99.40	99.30	WGS
197.6676	0.1142	Campylobacter jejuni (72294)	Campylobacter (213849)	Campylobacteraceae (2954	Campylobacterales (68525)	Epsilonproteobacteria (122	Proteobacteria (2)	Bacteria (1)	100.00	0.00	99.90	99.90	WGS
197.6677	0.0944	Campylobacter jejuni (72294)	Campylobacter (213849)	Campylobacteraceae (2954	Campylobacterales (68525)	Epsilonproteobacteria (122	Proteobacteria (2)	Bacteria (1)	100.00	0.00	99.40	99.30	WGS
197.6678	0.0984	Campylobacter jejuni (72294)	Campylobacter (213849)	Campylobacteraceae (2954	Campylobacterales (68525)	Epsilonproteobacteria (122	Proteobacteria (2)	Bacteria (1)	100.00	0.00	99.50	99.50	WGS
197.6679	0.0944	Campylobacter jejuni (72294)	Campylobacter (213849)	Campylobacteraceae (2954	Campylobacterales (68525)	Epsilonproteobacteria (122	Proteobacteria (2)	Bacteria (1)	100.00	0.00	99.40	99.30	WGS
197.6680	0.1078	Campylobacter jejuni (72294)	Campylobacter (213849)	Campylobacteraceae (2954	Campylobacterales (68525)	Epsilonproteobacteria (122	Proteobacteria (2)	Bacteria (1)	100.00	0.00	99.90	99.90	WGS
197.6681	0.0944	Campylobacter jejuni (72294)	Campylobacter (213849)	Campylobacteraceae (2954	Campylobacterales (68525)	Epsilonproteobacteria (122	Proteobacteria (2)	Bacteria (1)	100.00	0.00	99.40	99.30	WGS
197.6682	0.0944	Campylobacter jejuni (72294)	Campylobacter (213849)	Campylobacteraceae (2954	Campylobacterales (68525)	Epsilonproteobacteria (122	Proteobacteria (2)	Bacteria (1)	100.00	0.00	99.40	99.30	WGS
197.6683	0.117	Campylobacter jejuni (72294)	Campylobacter (213849)	Campylobacteraceae (2954	Campylobacterales (68525)	Epsilonproteobacteria (122	Proteobacteria (2)	Bacteria (1)	100.00	1.30	99.70	99.60	WGS
197.6684	0.11	Campylobacter jejuni (72294)	Campylobacter (213849)	Campylobacteraceae (2954	Campylobacterales (68525)	Epsilonproteobacteria (122	Proteobacteria (2)	Bacteria (1)	100.00	0.00	100.00	99.90	WGS
197.6685	0.0994	Campylobacter jejuni (72294)	Campylobacter (213849)	Campylobacteraceae (2954	Campylobacterales (68525)	Epsilonproteobacteria (122	Proteobacteria (2)	Bacteria (1)	100.00	5.00	99.80	98.70	WGS
197.6686	0.1072	Campylobacter jejuni (72294)	Campylobacter (213849)	Campylobacteraceae (2954	Campylobacterales (68525)	Epsilonproteobacteria (122	Proteobacteria (2)	Bacteria (1)	100.00	0.00	99.90	99.70	WGS
197.6687	0.114	Campylobacter jejuni (72294)	Campylobacter (213849)	Campylobacteraceae (2954	Campylobacterales (68525)	Epsilonproteobacteria (122	Proteobacteria (2)	Bacteria (1)	100.00	1.30	99.90	99.50	WGS
197.6688	0.0966	Campylobacter jejuni (72294)	Campylobacter (213849)	Campylobacteraceae (2954	Campylobacterales (68525)	Epsilonproteobacteria (122	Proteobacteria (2)	Bacteria (1)	100.00	0.00	99.60	99.40	WGS
197.6689	0.0974	Campylobacter jejuni (72294)	Campylobacter (213849)	Campylobacteraceae (2954	Campylobacterales (68525)	Epsilonproteobacteria (122	Proteobacteria (2)	Bacteria (1)	100.00	0.00	99.70	99.50	WGS
197.6690	0.0964	Campylobacter jejuni (72294)	Campylobacter (213849)	Campylobacteraceae (2954	Campylobacterales (68525)	Epsilonproteobacteria (122	Proteobacteria (2)	Bacteria (1)	100.00	0.00	99.70	99.60	WGS
197.6691	0.0946	Campylobacter jejuni (72294)	Campylobacter (213849)	Campylobacteraceae (2954	Campylobacterales (68525)	Epsilonproteobacteria (122	Proteobacteria (2)	Bacteria (1)	100.00	0.00	99.60	99.30	WGS
197.6692	0.0974	Campylobacter jejuni (72294)	Campylobacter (213849)	Campylobacteraceae (2954	Campylobacterales (68525)	Epsilonproteobacteria (122	Proteobacteria (2)	Bacteria (1)	100.00	0.00	99.70	99.50	WGS
197.6693	0.1012	Campylobacter jejuni (72294)	Campylobacter (213849)	Campylobacteraceae (2954	Campylobacterales (68525)	Epsilonproteobacteria (122	Proteobacteria (2)	Bacteria (1)	100.00	0.30	99.80	99.40	WGS
197.6694	0.1144	Campylobacter jejuni (72294)	Campylobacter (213849)	Campylobacteraceae (2954	Campylobacterales (68525)	Epsilonproteobacteria (122	Proteobacteria (2)	Bacteria (1)	100.00	0.70	99.70	99.30	WGS
197.6695	0.106	Campylobacter jejuni (72294)	Campylobacter (213849)	Campylobacteraceae (2954	Campylobacterales (68525)	Epsilonproteobacteria (122	Proteobacteria (2)	Bacteria (1)	100.00	0.00	99.60	99.30	WGS
197.6696	0.1238	Campylobacter jejuni (72294)	Campylobacter (213849)	Campylobacteraceae (2954	Campylobacterales (68525)	Epsilonproteobacteria (122	Proteobacteria (2)	Bacteria (1)	100.00	3.80	99.90	98.70	WGS
197.6697	0.11	Campylobacter jejuni (72294)	Campylobacter (213849)	Campylobacteraceae (2954	Campylobacterales (68525)	Epsilonproteobacteria (122	Proteobacteria (2)	Bacteria (1)	100.00	0.60	99.90	99.90	WGS
197.6698	0.1118	Campylobacter jejuni (72294)	Campylobacter (213849)	Campylobacteraceae (2954	Campylobacterales (68525)	Epsilonproteobacteria (122	Proteobacteria (2)	Bacteria (1)	100.00	0.00	99.90	99.90	WGS
197.6699	0.1118	Campylobacter jejuni (72294)	Campylobacter (213849)	Campylobacteraceae (2954	Campylobacterales (68525)	Epsilonproteobacteria (122	Proteobacteria (2)	Bacteria (1)	100.00	0.00	99.90	99.90	WGS
197.6700	0.1012	Campylobacter jejuni (72294)	Campylobacter (213849)	Campylobacteraceae (2954	Campylobacterales (68525)	Epsilonproteobacteria (122	Proteobacteria (2)	Bacteria (1)	100.00	4.10	99.80	97.90	WGS
197.6701	0.1012	Campylobacter jejuni (72294)	Campylobacter (213849)	Campylobacteraceae (2954	Campylobacterales (68525)	Epsilonproteobacteria (122	Proteobacteria (2)	Bacteria (1)	100.00	7.60	99.80	97.60	WGS
197.6702	0.0934	Campylobacter jejuni (72294)	Campylobacter (213849)	Campylobacteraceae (2954	Campylobacterales (68525)	Epsilonproteobacteria (122	Proteobacteria (2)	Bacteria (1)	100.00	0.00	99.70	99.40	WGS
197.6703	0.1126	Campylobacter jejuni (72294)	Campylobacter (213849)	Campylobacteraceae (2954	Campylobacterales (68525)	Epsilonproteobacteria (122	Proteobacteria (2)	Bacteria (1)	100.00	0.00	99.70	99.50	WGS
197.6704	0.1106	Campylobacter jejuni (72294)	Campylobacter (213849)	Campylobacteraceae (2954	Campylobacterales (68525)	Epsilonproteobacteria (122	Proteobacteria (2)	Bacteria (1)	100.00	0.00	100.00	99.90	WGS
197.6705	0.118	Campylobacter jejuni (72294)	Campylobacter (213849)	Campylobacteraceae (2954	Campylobacterales (68525)	Epsilonproteobacteria (122	Proteobacteria (2)	Bacteria (1)	100.00	0.00	99.90	99.90	WGS
197.6706	0.111	Campylobacter jejuni (72294)	Campylobacter (213849)	Campylobacteraceae (2954	Campylobacterales (68525)	Epsilonproteobacteria (122	Proteobacteria (2)	Bacteria (1)	100.00	0.00	99.70	99.40	WGS
197.6707	0.1098	Campylobacter jejuni (72294)	Campylobacter (213849)	Campylobacteraceae (2954	Campylobacterales (68525)	Epsilonproteobacteria (122	Proteobacteria (2)	Bacteria (1)	100.00	0.00	99.70	99.40	WGS
197.6708	0.118	Campylobacter jejuni (72294)	Campylobacter (213849)	Campylobacteraceae (2954	Campylobacterales (68525)	Epsilonproteobacteria (122	Proteobacteria (2)	Bacteria (1)	100.00	0.00	99.90	99.90	WGS
197.6709	0.1126	Campylobacter jejuni (72294)	Campylobacter (213849)	Campylobacteraceae (2954	Campylobacterales (68525)	Epsilonproteobacteria (122	Proteobacteria (2)	Bacteria (1)	100.00	0.00	99.60	99.40	WGS
197.6710	0.1126	Campylobacter jejuni (72294)	Campylobacter (213849)	Campylobacteraceae (2954	Campylobacterales (68525)	Epsilonproteobacteria (122	Proteobacteria (2)	Bacteria (1)	100.00	0.00	99.80	99.70	WGS
197.6711	0.1078	Campylobacter jejuni (72294)	Campylobacter (213849)	Campylobacteraceae (2954	Campylobacterales (68525)	Epsilonproteobacteria (122	Proteobacteria (2)	Bacteria (1)	100.00	0.00	99.90	99.90	WGS
197.6712	0.1078	Campylobacter jejuni (72294)	Campylobacter (213849)	Campylobacteraceae (2954	Campylobacterales (68525)	Epsilonproteobacteria (122	Proteobacteria (2)	Bacteria (1)	100.00	0.00	99.90	99.90	WGS
197.6713	0.1078	Campylobacter jejuni (72294)	Campylobacter (213849)	Campylobacteraceae (2954	Campylobacterales (68525)	Epsilonproteobacteria (122	Proteobacteria (2)	Bacteria (1)	100.00	0.00	99.90	99.90	WGS
197.6714	0.1078	Campylobacter jejuni (72294)	Campylobacter (213849)	Campylobacteraceae (2954	Campylobacterales (68525)	Epsilonproteobacteria (122	Proteobacteria (2)	Bacteria (1)	100.00	0.00	99.90	99.90	WGS
197.6715	0.1078	Campylobacter jejuni (72294)	Campylobacter (213849)	Campylobacteraceae (2954	Campylobacterales (68525)	Epsilonproteobacteria (122	Proteobacteria (2)	Bacteria (1)	100.00	0.00	99.90	99.90	WGS
197.6716	0.1078	Campylobacter jejuni (72294)	Campylobacter (213849)	Campylobacteraceae (2954	Campylobacterales (68525)	Epsilonproteobacteria (122	Proteobacteria (2)	Bacteria (1)	100.00	0.00	100.00	99.90	WGS
197.6717	0.1078	Campylobacter jejuni (72294)	Campylobacter (213849)	Campylobacteraceae (2954	Campylobacterales (68525)	Epsilonproteobacteria (122	Proteobacteria (2)	Bacteria (1)	100.00	0.00	99.90	99.90	WGS
197.6718	0.1078	Campylobacter jejuni (72294)	Campylobacter (213849)	Campylobacteraceae (2954	Campylobacterales (68525)	Epsilonproteobacteria (122	Proteobacteria (2)	Bacteria (1)	100.00	0.00	99.90	99.90	WGS
197.6719	0.1116	Campylobacter jejuni (72294)	Campylobacter (213849)	Campylobacteraceae (2954	Campylobacterales (68525)	Epsilonproteobacteria (122	Proteobacteria (2)	Bacteria (1)	100.00	1.30	99.90	99.80	WGS
197.6720	0.1142	Campylobacter jejuni (72294)	Campylobacter (213849)	Campylobacteraceae (2954	Campylobacterales (68525)	Epsilonproteobacteria (122	Proteobacteria (2)	Bacteria (1)	100.00	0.00	99.90	99.90	WGS
197.6721	0.109	Campylobacter jejuni (72294)	Campylobacter (213849)	Campylobacteraceae (2954	Campylobacterales (68525)	Epsilonproteobacteria (122	Proteobacteria (2)	Bacteria (1)	100.00	0.70	99.60	99.40	WGS
197.6722	0.1132	Campylobacter jejuni (72294)	Campylobacter (213849)	Campylobacteraceae (2954	Campylobacterales (68525)	Epsilonproteobacteria (122	Proteobacteria (2)	Bacteria (1)	100.00	0.00	99.70	99.60	WGS
197.6723	0.1078	Campylobacter jejuni (72294)	Campylobacter (213849)	Campylobacteraceae (2954	Campylobacterales (68525)	Epsilonproteobacteria (122	Proteobacteria (2)	Bacteria (1)	100.00	0.00	99.90	99.90	WGS
197.6724	0.1078	Campylobacter jejuni (72294)	Campylobacter (213849)	Campylobacteraceae (2954	Campylobacterales (68525)	Epsilonproteobacteria (122	Proteobacteria (2)	Bacteria (1)	100.00	0.00	99.90	99.90	WGS
197.6725	0.1078	Campylobacter jejuni (72294)	Campylobacter (213849)	Campylobacteraceae (2954	Campylobacterales (68525)	Epsilonproteobacteria (122	Proteobacteria (2)	Bacteria (1)	100.00	0.00	99.90	99.90	WGS
197.6726	0.1144	Campylobacter jejuni (72294)	Campylobacter (213849)	Campylobacteraceae (2954	Campylobacterales (68525)	Epsilonproteobacteria (122	Proteobacteria (2)	Bacteria (1)	100.00	0.00	99.70	99.60	WGS
197.6727	0.1098	Campylobacter jejuni (72294)	Campylobacter (213849)	Campylobacteraceae (2954	Campylobacterales (68525)	Epsilonproteobacteria (122	Proteobacteria (2)	Bacteria (1)	100.00	0.00	99.90	99.90	WGS
197.6728	0.1078	Campylobacter jejuni (72294)	Campylobacter (213849)	Campylobacteraceae (2954	Campylobacterales (68525)	Epsilonproteobacteria (122	Proteobacteria (2)	Bacteria (1)	100.00	0.60	99.90	99.80	WGS
197.6729	0.1078	Campylobacter jejuni (72294)	Campylobacter (213849)	Campylobacteraceae (2954	Campylobacterales (68525)	Epsilonproteobacteria (122	Proteobacteria (2)	Bacteria (1)	100.00	0.00	99.90	99.70	WGS
197.6730	0.1078	Campylobacter jejuni (72294)	Campylobacter (213849)	Campylobacteraceae (2954	Campylobacterales (68525)	Epsilonproteobacteria (122	Proteobacteria (2)	Bacteria (1)	100.00	0.00	99.90	99.90	WGS
197.6731	0.1118	Campylobacter jejuni (72294)	Campylobacter (213849)	Campylobacteraceae (2954	Campylobacterales (68525)	Epsilonproteobacteria (122	Proteobacteria (2)	Bacteria (1)	100.00	0.00	100.00	99.90	WGS
197.6732	0.1106	Campylobacter jejuni (72294)	Campylobacter (213849)	Campylobacteraceae (2954	Campylobacterales (68525)	Epsilonproteobacteria (122	Proteobacteria (2)	Bacteria (1)	100.00	0.00	100.00	99.90	WGS
197.6733	0.1078	Campylobacter jejuni (72294)	Campylobacter (213849)	Campylobacteraceae (2954	Campylobacterales (68525)	Epsilonproteobacteria (122	Proteobacteria (2)	Bacteria (1)	100.00	0.00	99.90	99.80	WGS
197.6734	0.1106	Campylobacter jejuni (72294)	Campylobacter (213849)	Campylobacteraceae (2954	Campylobacterales (68525)	Epsilonproteobacteria (122	Proteobacteria (2)	Bacteria (1)	99.30	0.70	99.90	99.90	WGS
197.6735	0.1106	Campylobacter jejuni (72294)	Campylobacter (213849)	Campylobacteraceae (2954	Campylobacterales (68525)	Epsilonproteobacteria (122	Proteobacteria (2)	Bacteria (1)	100.00	4.40	99.90	98.30	WGS
197.6736	0.117	Campylobacter jejuni (72294)	Campylobacter (213849)	Campylobacteraceae (2954	Campylobacterales (68525)	Epsilonproteobacteria (122	Proteobacteria (2)	Bacteria (1)	100.00	0.00	99.90	99.80	WGS
197.6737	0.11	Campylobacter jejuni (72294)	Campylobacter (213849)	Campylobacteraceae (2954	Campylobacterales (68525)	Epsilonproteobacteria (122	Proteobacteria (2)	Bacteria (1)	100.00	0.70	99.90	99.70	WGS
197.6738	0.1106	Campylobacter jejuni (72294)	Campylobacter (213849)	Campylobacteraceae (2954	Campylobacterales (68525)	Epsilonproteobacteria (122	Proteobacteria (2)	Bacteria (1)	100.00	0.00	99.90	99.90	WGS
197.6739	0.1118	Campylobacter jejuni (72294)	Campylobacter (213849)	Campylobacteraceae (2954	Campylobacterales (68525)	Epsilonproteobacteria (122	Proteobacteria (2)	Bacteria (1)	100.00	0.70	100.00	99.90	WGS
197.6740	0.1132	Campylobacter jejuni (72294)	Campylobacter (213849)	Campylobacteraceae (2954	Campylobacterales (68525)	Epsilonproteobacteria (122	Proteobacteria (2)	Bacteria (1)	100.00	0.00	99.90	99.90	WGS
197.6741	0.1146	Campylobacter jejuni (72294)	Campylobacter (213849)	Campylobacteraceae (2954	Campylobacterales (68525)	Epsilonproteobacteria (122	Proteobacteria (2)	Bacteria (1)	100.00	1.30	99.90	99.70	WGS
197.6742	0.1078	Campylobacter jejuni (72294)	Campylobacter (213849)	Campylobacteraceae (2954	Campylobacterales (68525)	Epsilonproteobacteria (122	Proteobacteria (2)	Bacteria (1)	100.00	0.00	100.00	99.90	WGS
197.6743	0.114	Campylobacter jejuni (72294)	Campylobacter (213849)	Campylobacteraceae (2954	Campylobacterales (68525)	Epsilonproteobacteria (122	Proteobacteria (2)	Bacteria (1)	100.00	0.00	100.00	100.00	WGS
197.6744	0.1114	Campylobacter jejuni (72294)	Campylobacter (213849)	Campylobacteraceae (2954	Campylobacterales (68525)	Epsilonproteobacteria (122	Proteobacteria (2)	Bacteria (1)	100.00	0.00	99.60	99.40	WGS
197.6745	0.116	Campylobacter jejuni (72294)	Campylobacter (213849)	Campylobacteraceae (2954	Campylobacterales (68525)	Epsilonproteobacteria (122	Proteobacteria (2)	Bacteria (1)	100.00	0.00	99.80	99.60	WGS
197.6746	0.1106	Campylobacter jejuni (72294)	Campylobacter (213849)	Campylobacteraceae (2954	Campylobacterales (68525)	Epsilonproteobacteria (122	Proteobacteria (2)	Bacteria (1)	100.00	0.00	99.90	99.90	WGS
197.6747	0.1112	Campylobacter jejuni (72294)	Campylobacter (213849)	Campylobacteraceae (2954	Campylobacterales (68525)	Epsilonproteobacteria (122	Proteobacteria (2)	Bacteria (1)	100.00	0.00	99.90	99.90	WGS
197.6748	0.1078	Campylobacter jejuni (72294)	Campylobacter (213849)	Campylobacteraceae (2954	Campylobacterales (68525)	Epsilonproteobacteria (122	Proteobacteria (2)	Bacteria (1)	100.00	0.00	99.70	99.60	WGS
197.6749	0.116	Campylobacter jejuni (72294)	Campylobacter (213849)	Campylobacteraceae (2954	Campylobacterales (68525)	Epsilonproteobacteria (122	Proteobacteria (2)	Bacteria (1)	100.00	0.00	99.80	99.60	WGS
197.6750	0.1144	Campylobacter jejuni (72294)	Campylobacter (213849)	Campylobacteraceae (2954	Campylobacterales (68525)	Epsilonproteobacteria (122	Proteobacteria (2)	Bacteria (1)	100.00	0.00	99.70	99.60	WGS
197.6751	0.1118	Campylobacter jejuni (72294)	Campylobacter (213849)	Campylobacteraceae (2954	Campylobacterales (68525)	Epsilonproteobacteria (122	Proteobacteria (2)	Bacteria (1)	100.00	0.00	99.90	99.80	WGS
197.6752	0.1172	Campylobacter jejuni (72294)	Campylobacter (213849)	Campylobacteraceae (2954	Campylobacterales (68525)	Epsilonproteobacteria (122	Proteobacteria (2)	Bacteria (1)	100.00	0.00	99.80	99.60	WGS
197.6753	0.112	Campylobacter jejuni (72294)	Campylobacter (213849)	Campylobacteraceae (2954	Campylobacterales (68525)	Epsilonproteobacteria (122	Proteobacteria (2)	Bacteria (1)	100.00	0.00	99.90	99.70	WGS
197.6754	0.116	Campylobacter jejuni (72294)	Campylobacter (213849)	Campylobacteraceae (2954	Campylobacterales (68525)	Epsilonproteobacteria (122	Proteobacteria (2)	Bacteria (1)	100.00	0.60	99.80	99.60	WGS
197.6755	0.1128	Campylobacter jejuni (72294)	Campylobacter (213849)	Campylobacteraceae (2954	Campylobacterales (68525)	Epsilonproteobacteria (122	Proteobacteria (2)	Bacteria (1)	100.00	0.00	99.90	99.70	WGS
197.6756	0.1114	Campylobacter jejuni (72294)	Campylobacter (213849)	Campylobacteraceae (2954	Campylobacterales (68525)	Epsilonproteobacteria (122	Proteobacteria (2)	Bacteria (1)	100.00	0.00	99.80	99.70	WGS
197.6757	0.1078	Campylobacter jejuni (72294)	Campylobacter (213849)	Campylobacteraceae (2954	Campylobacterales (68525)	Epsilonproteobacteria (122	Proteobacteria (2)	Bacteria (1)	100.00	0.00	99.90	99.90	WGS
197.6758	0.1078	Campylobacter jejuni (72294)	Campylobacter (213849)	Campylobacteraceae (2954	Campylobacterales (68525)	Epsilonproteobacteria (122	Proteobacteria (2)	Bacteria (1)	100.00	0.00	99.90	99.70	WGS
197.6759	0.1144	Campylobacter jejuni (72294)	Campylobacter (213849)	Campylobacteraceae (2954	Campylobacterales (68525)	Epsilonproteobacteria (122	Proteobacteria (2)	Bacteria (1)	100.00	0.00	99.80	99.60	WGS
197.6760	0.116	Campylobacter jejuni (72294)	Campylobacter (213849)	Campylobacteraceae (2954	Campylobacterales (68525)	Epsilonproteobacteria (122	Proteobacteria (2)	Bacteria (1)	100.00	0.00	99.70	99.60	WGS
197.6761	0.1114	Campylobacter jejuni (72294)	Campylobacter (213849)	Campylobacteraceae (2954	Campylobacterales (68525)	Epsilonproteobacteria (122	Proteobacteria (2)	Bacteria (1)	100.00	0.00	99.70	99.60	WGS
197.6762	0.1142	Campylobacter jejuni (72294)	Campylobacter (213849)	Campylobacteraceae (2954	Campylobacterales (68525)	Epsilonproteobacteria (122	Proteobacteria (2)	Bacteria (1)	100.00	0.00	99.80	99.70	WGS
197.6763	0.1144	Campylobacter jejuni (72294)	Campylobacter (213849)	Campylobacteraceae (2954	Campylobacterales (68525)	Epsilonproteobacteria (122	Proteobacteria (2)	Bacteria (1)	100.00	0.00	99.80	99.50	WGS
197.6764	0.109	Campylobacter jejuni (72294)	Campylobacter (213849)	Campylobacteraceae (2954	Campylobacterales (68525)	Epsilonproteobacteria (122	Proteobacteria (2)	Bacteria (1)	100.00	0.00	99.90	99.90	WGS
197.6765	0.1118	Campylobacter jejuni (72294)	Campylobacter (213849)	Campylobacteraceae (2954	Campylobacterales (68525)	Epsilonproteobacteria (122	Proteobacteria (2)	Bacteria (1)	100.00	0.00	99.80	99.60	WGS
197.6766	0.11	Campylobacter jejuni (72294)	Campylobacter (213849)	Campylobacteraceae (2954	Campylobacterales (68525)	Epsilonproteobacteria (122	Proteobacteria (2)	Bacteria (1)	100.00	0.00	99.90	99.80	WGS
197.7742	0.1172	Campylobacter jejuni (72294)	Campylobacter (213849)	Campylobacteraceae (2954	Campylobacterales (68525)	Epsilonproteobacteria (122	Proteobacteria (2)	Bacteria (1)	100.00	0.00	99.80	99.70	WGS
197.7743	0.1132	Campylobacter jejuni (72294)	Campylobacter (213849)	Campylobacteraceae (2954	Campylobacterales (68525)	Epsilonproteobacteria (122	Proteobacteria (2)	Bacteria (1)	100.00	0.00	99.80	99.60	WGS
197.7744	0.1126	Campylobacter jejuni (72294)	Campylobacter (213849)	Campylobacteraceae (2954	Campylobacterales (68525)	Epsilonproteobacteria (122	Proteobacteria (2)	Bacteria (1)	100.00	0.00	99.70	99.60	WGS
197.7745	0.1132	Campylobacter jejuni (72294)	Campylobacter (213849)	Campylobacteraceae (2954	Campylobacterales (68525)	Epsilonproteobacteria (122	Proteobacteria (2)	Bacteria (1)	100.00	0.00	99.90	99.80	WGS
197.7746	0.1132	Campylobacter jejuni (72294)	Campylobacter (213849)	Campylobacteraceae (2954	Campylobacterales (68525)	Epsilonproteobacteria (122	Proteobacteria (2)	Bacteria (1)	100.00	0.00	99.80	99.60	WGS
197.7747	0.1126	Campylobacter jejuni (72294)	Campylobacter (213849)	Campylobacteraceae (2954	Campylobacterales (68525)	Epsilonproteobacteria (122	Proteobacteria (2)	Bacteria (1)	100.00	0.00	99.60	99.40	WGS
197.7748	0.1118	Campylobacter jejuni (72294)	Campylobacter (213849)	Campylobacteraceae (2954	Campylobacterales (68525)	Epsilonproteobacteria (122	Proteobacteria (2)	Bacteria (1)	100.00	0.00	100.00	99.90	WGS
197.7749	0.116	Campylobacter jejuni (72294)	Campylobacter (213849)	Campylobacteraceae (2954	Campylobacterales (68525)	Epsilonproteobacteria (122	Proteobacteria (2)	Bacteria (1)	100.00	0.00	99.80	99.60	WGS
197.7750	0.1078	Campylobacter jejuni (72294)	Campylobacter (213849)	Campylobacteraceae (2954	Campylobacterales (68525)	Epsilonproteobacteria (122	Proteobacteria (2)	Bacteria (1)	100.00	0.00	99.90	99.90	WGS
197.7751	0.1078	Campylobacter jejuni (72294)	Campylobacter (213849)	Campylobacteraceae (2954	Campylobacterales (68525)	Epsilonproteobacteria (122	Proteobacteria (2)	Bacteria (1)	100.00	0.00	99.90	99.90	WGS
197.7752	0.1078	Campylobacter jejuni (72294)	Campylobacter (213849)	Campylobacteraceae (2954	Campylobacterales (68525)	Epsilonproteobacteria (122	Proteobacteria (2)	Bacteria (1)	100.00	0.00	99.90	99.90	WGS
197.7753	0.1078	Campylobacter jejuni (72294)	Campylobacter (213849)	Campylobacteraceae (2954	Campylobacterales (68525)	Epsilonproteobacteria (122	Proteobacteria (2)	Bacteria (1)	100.00	0.00	99.90	99.90	WGS
197.7754	0.1128	Campylobacter jejuni (72294)	Campylobacter (213849)	Campylobacteraceae (2954	Campylobacterales (68525)	Epsilonproteobacteria (122	Proteobacteria (2)	Bacteria (1)	100.00	6.70	99.90	97.90	Complete
197.7755	0.1132	Campylobacter jejuni (72294)	Campylobacter (213849)	Campylobacteraceae (2954	Campylobacterales (68525)	Epsilonproteobacteria (122	Proteobacteria (2)	Bacteria (1)	100.00	0.00	99.70	99.60	Complete
197.7756	0.1142	Campylobacter jejuni (72294)	Campylobacter (213849)	Campylobacteraceae (2954	Campylobacterales (68525)	Epsilonproteobacteria (122	Proteobacteria (2)	Bacteria (1)	100.00	0.00	99.80	99.60	Complete
197.7757	0.1168	Campylobacter jejuni (72294)	Campylobacter (213849)	Campylobacteraceae (2954	Campylobacterales (68525)	Epsilonproteobacteria (122	Proteobacteria (2)	Bacteria (1)	100.00	0.00	99.70	99.50	Complete
197.7758	0.1132	Campylobacter jejuni (72294)	Campylobacter (213849)	Campylobacteraceae (2954	Campylobacterales (68525)	Epsilonproteobacteria (122	Proteobacteria (2)	Bacteria (1)	100.00	0.70	99.60	99.30	Complete
197.7759	0.0964	Campylobacter jejuni (72294)	Campylobacter (213849)	Campylobacteraceae (2954	Campylobacterales (68525)	Epsilonproteobacteria (122	Proteobacteria (2)	Bacteria (1)	100.00	0.00	99.70	99.60	WGS
197.7760	0.1158	Campylobacter jejuni (72294)	Campylobacter (213849)	Campylobacteraceae (2954	Campylobacterales (68525)	Epsilonproteobacteria (122	Proteobacteria (2)	Bacteria (1)	100.00	0.00	99.90	99.60	WGS
197.7875	0.1078	Campylobacter jejuni (72294)	Campylobacter (213849)	Campylobacteraceae (2954	Campylobacterales (68525)	Epsilonproteobacteria (122	Proteobacteria (2)	Bacteria (1)	100.00	0.00	100.00	99.90	WGS
197.7876	0.1078	Campylobacter jejuni (72294)	Campylobacter (213849)	Campylobacteraceae (2954	Campylobacterales (68525)	Epsilonproteobacteria (122	Proteobacteria (2)	Bacteria (1)	100.00	0.00	100.00	100.00	WGS
197.7877	0.1078	Campylobacter jejuni (72294)	Campylobacter (213849)	Campylobacteraceae (2954	Campylobacterales (68525)	Epsilonproteobacteria (122	Proteobacteria (2)	Bacteria (1)	100.00	0.00	100.00	100.00	WGS
197.7878	0.1078	Campylobacter jejuni (72294)	Campylobacter (213849)	Campylobacteraceae (2954	Campylobacterales (68525)	Epsilonproteobacteria (122	Proteobacteria (2)	Bacteria (1)	100.00	1.30	99.90	99.70	WGS
197.7879	0.1078	Campylobacter jejuni (72294)	Campylobacter (213849)	Campylobacteraceae (2954	Campylobacterales (68525)	Epsilonproteobacteria (122	Proteobacteria (2)	Bacteria (1)	100.00	0.00	100.00	99.90	WGS
197.7880	0.1078	Campylobacter jejuni (72294)	Campylobacter (213849)	Campylobacteraceae (2954	Campylobacterales (68525)	Epsilonproteobacteria (122	Proteobacteria (2)	Bacteria (1)	100.00	0.00	100.00	99.70	WGS
197.7881	0.1126	Campylobacter jejuni (72294)	Campylobacter (213849)	Campylobacteraceae (2954	Campylobacterales (68525)	Epsilonproteobacteria (122	Proteobacteria (2)	Bacteria (1)	100.00	1.30	99.60	99.10	WGS
197.7882	0.1078	Campylobacter jejuni (72294)	Campylobacter (213849)	Campylobacteraceae (2954	Campylobacterales (68525)	Epsilonproteobacteria (122	Proteobacteria (2)	Bacteria (1)	100.00	0.00	100.00	100.00	WGS
197.7883	0.1126	Campylobacter jejuni (72294)	Campylobacter (213849)	Campylobacteraceae (2954	Campylobacterales (68525)	Epsilonproteobacteria (122	Proteobacteria (2)	Bacteria (1)	100.00	0.70	99.60	99.20	WGS
197.7884	0.1096	Campylobacter jejuni (72294)	Campylobacter (213849)	Campylobacteraceae (2954	Campylobacterales (68525)	Epsilonproteobacteria (122	Proteobacteria (2)	Bacteria (1)	100.00	0.70	99.90	99.90	WGS
197.7885	0.1096	Campylobacter jejuni (72294)	Campylobacter (213849)	Campylobacteraceae (2954	Campylobacterales (68525)	Epsilonproteobacteria (122	Proteobacteria (2)	Bacteria (1)	100.00	2.90	99.90	99.10	WGS
197.7886	0.1128	Campylobacter jejuni (72294)	Campylobacter (213849)	Campylobacteraceae (2954	Campylobacterales (68525)	Epsilonproteobacteria (122	Proteobacteria (2)	Bacteria (1)	100.00	0.00	100.00	99.90	WGS
197.7887	0.1096	Campylobacter jejuni (72294)	Campylobacter (213849)	Campylobacteraceae (2954	Campylobacterales (68525)	Epsilonproteobacteria (122	Proteobacteria (2)	Bacteria (1)	100.00	0.00	99.90	99.90	WGS
197.7888	0.116	Campylobacter jejuni (72294)	Campylobacter (213849)	Campylobacteraceae (2954	Campylobacterales (68525)	Epsilonproteobacteria (122	Proteobacteria (2)	Bacteria (1)	100.00	0.00	100.00	99.90	WGS
197.7889	0.1078	Campylobacter jejuni (72294)	Campylobacter (213849)	Campylobacteraceae (2954	Campylobacterales (68525)	Epsilonproteobacteria (122	Proteobacteria (2)	Bacteria (1)	100.00	0.00	99.90	99.90	WGS
197.7890	0.1096	Campylobacter jejuni (72294)	Campylobacter (213849)	Campylobacteraceae (2954	Campylobacterales (68525)	Epsilonproteobacteria (122	Proteobacteria (2)	Bacteria (1)	100.00	0.00	99.90	99.90	WGS
197.7891	0.1096	Campylobacter jejuni (72294)	Campylobacter (213849)	Campylobacteraceae (2954	Campylobacterales (68525)	Epsilonproteobacteria (122	Proteobacteria (2)	Bacteria (1)	100.00	0.00	99.90	99.90	WGS
197.7892	0.1096	Campylobacter jejuni (72294)	Campylobacter (213849)	Campylobacteraceae (2954	Campylobacterales (68525)	Epsilonproteobacteria (122	Proteobacteria (2)	Bacteria (1)	100.00	0.00	99.90	99.90	WGS
197.7893	0.1078	Campylobacter jejuni (72294)	Campylobacter (213849)	Campylobacteraceae (2954	Campylobacterales (68525)	Epsilonproteobacteria (122	Proteobacteria (2)	Bacteria (1)	100.00	0.00	100.00	99.90	WGS
197.7894	0.1078	Campylobacter jejuni (72294)	Campylobacter (213849)	Campylobacteraceae (2954	Campylobacterales (68525)	Epsilonproteobacteria (122	Proteobacteria (2)	Bacteria (1)	100.00	1.60	100.00	99.70	WGS
197.7895	0.1078	Campylobacter jejuni (72294)	Campylobacter (213849)	Campylobacteraceae (2954	Campylobacterales (68525)	Epsilonproteobacteria (122	Proteobacteria (2)	Bacteria (1)	100.00	0.00	100.00	99.90	WGS
197.7896	0.1078	Campylobacter jejuni (72294)	Campylobacter (213849)	Campylobacteraceae (2954	Campylobacterales (68525)	Epsilonproteobacteria (122	Proteobacteria (2)	Bacteria (1)	100.00	1.30	100.00	99.80	WGS
197.7897	0.1078	Campylobacter jejuni (72294)	Campylobacter (213849)	Campylobacteraceae (2954	Campylobacterales (68525)	Epsilonproteobacteria (122	Proteobacteria (2)	Bacteria (1)	100.00	0.00	100.00	99.90	WGS
197.7898	0.1114	Campylobacter jejuni (72294)	Campylobacter (213849)	Campylobacteraceae (2954	Campylobacterales (68525)	Epsilonproteobacteria (122	Proteobacteria (2)	Bacteria (1)	100.00	0.00	100.00	99.90	WGS
197.7899	0.1078	Campylobacter jejuni (72294)	Campylobacter (213849)	Campylobacteraceae (2954	Campylobacterales (68525)	Epsilonproteobacteria (122	Proteobacteria (2)	Bacteria (1)	100.00	0.70	99.90	99.60	WGS
197.7900	0.1078	Campylobacter jejuni (72294)	Campylobacter (213849)	Campylobacteraceae (2954	Campylobacterales (68525)	Epsilonproteobacteria (122	Proteobacteria (2)	Bacteria (1)	100.00	0.00	100.00	99.80	WGS
197.7901	0.1078	Campylobacter jejuni (72294)	Campylobacter (213849)	Campylobacteraceae (2954	Campylobacterales (68525)	Epsilonproteobacteria (122	Proteobacteria (2)	Bacteria (1)	100.00	0.00	99.90	99.70	WGS
197.7902	0.1078	Campylobacter jejuni (72294)	Campylobacter (213849)	Campylobacteraceae (2954	Campylobacterales (68525)	Epsilonproteobacteria (122	Proteobacteria (2)	Bacteria (1)	100.00	0.70	99.90	99.80	WGS
197.7903	0.1078	Campylobacter jejuni (72294)	Campylobacter (213849)	Campylobacteraceae (2954	Campylobacterales (68525)	Epsilonproteobacteria (122	Proteobacteria (2)	Bacteria (1)	100.00	0.00	99.90	99.90	WGS
197.7904	0.1078	Campylobacter jejuni (72294)	Campylobacter (213849)	Campylobacteraceae (2954	Campylobacterales (68525)	Epsilonproteobacteria (122	Proteobacteria (2)	Bacteria (1)	100.00	0.00	99.90	99.50	WGS
197.7905	0.1078	Campylobacter jejuni (72294)	Campylobacter (213849)	Campylobacteraceae (2954	Campylobacterales (68525)	Epsilonproteobacteria (122	Proteobacteria (2)	Bacteria (1)	100.00	0.30	99.90	99.30	WGS
197.7906	0.1078	Campylobacter jejuni (72294)	Campylobacter (213849)	Campylobacteraceae (2954	Campylobacterales (68525)	Epsilonproteobacteria (122	Proteobacteria (2)	Bacteria (1)	100.00	1.60	99.80	99.20	WGS
197.7907	0.1078	Campylobacter jejuni (72294)	Campylobacter (213849)	Campylobacteraceae (2954	Campylobacterales (68525)	Epsilonproteobacteria (122	Proteobacteria (2)	Bacteria (1)	100.00	1.00	99.90	99.70	WGS
197.7908	0.1078	Campylobacter jejuni (72294)	Campylobacter (213849)	Campylobacteraceae (2954	Campylobacterales (68525)	Epsilonproteobacteria (122	Proteobacteria (2)	Bacteria (1)	100.00	0.00	99.90	99.80	WGS
197.7909	0.1078	Campylobacter jejuni (72294)	Campylobacter (213849)	Campylobacteraceae (2954	Campylobacterales (68525)	Epsilonproteobacteria (122	Proteobacteria (2)	Bacteria (1)	100.00	0.00	100.00	100.00	WGS
197.7910	0.1078	Campylobacter jejuni (72294)	Campylobacter (213849)	Campylobacteraceae (2954	Campylobacterales (68525)	Epsilonproteobacteria (122	Proteobacteria (2)	Bacteria (1)	100.00	0.00	100.00	100.00	WGS
197.7911	0.1078	Campylobacter jejuni (72294)	Campylobacter (213849)	Campylobacteraceae (2954	Campylobacterales (68525)	Epsilonproteobacteria (122	Proteobacteria (2)	Bacteria (1)	100.00	0.00	100.00	100.00	WGS
197.7912	0.1078	Campylobacter jejuni (72294)	Campylobacter (213849)	Campylobacteraceae (2954	Campylobacterales (68525)	Epsilonproteobacteria (122	Proteobacteria (2)	Bacteria (1)	100.00	0.00	100.00	100.00	WGS
197.7913	0.1078	Campylobacter jejuni (72294)	Campylobacter (213849)	Campylobacteraceae (2954	Campylobacterales (68525)	Epsilonproteobacteria (122	Proteobacteria (2)	Bacteria (1)	100.00	0.00	100.00	100.00	WGS
197.7914	0.1078	Campylobacter jejuni (72294)	Campylobacter (213849)	Campylobacteraceae (2954	Campylobacterales (68525)	Epsilonproteobacteria (122	Proteobacteria (2)	Bacteria (1)	100.00	0.00	100.00	100.00	WGS
197.7915	0.1078	Campylobacter jejuni (72294)	Campylobacter (213849)	Campylobacteraceae (2954	Campylobacterales (68525)	Epsilonproteobacteria (122	Proteobacteria (2)	Bacteria (1)	100.00	0.00	100.00	100.00	WGS
197.7916	0.1078	Campylobacter jejuni (72294)	Campylobacter (213849)	Campylobacteraceae (2954	Campylobacterales (68525)	Epsilonproteobacteria (122	Proteobacteria (2)	Bacteria (1)	100.00	0.00	100.00	100.00	WGS
197.7917	0.1078	Campylobacter jejuni (72294)	Campylobacter (213849)	Campylobacteraceae (2954	Campylobacterales (68525)	Epsilonproteobacteria (122	Proteobacteria (2)	Bacteria (1)	100.00	0.00	100.00	100.00	WGS
197.7918	0.1078	Campylobacter jejuni (72294)	Campylobacter (213849)	Campylobacteraceae (2954	Campylobacterales (68525)	Epsilonproteobacteria (122	Proteobacteria (2)	Bacteria (1)	100.00	0.00	100.00	100.00	WGS
197.7919	0.1078	Campylobacter jejuni (72294)	Campylobacter (213849)	Campylobacteraceae (2954	Campylobacterales (68525)	Epsilonproteobacteria (122	Proteobacteria (2)	Bacteria (1)	100.00	0.00	100.00	99.90	WGS
197.7920	0.116	Campylobacter jejuni (72294)	Campylobacter (213849)	Campylobacteraceae (2954	Campylobacterales (68525)	Epsilonproteobacteria (122	Proteobacteria (2)	Bacteria (1)	100.00	0.00	99.90	99.60	WGS
197.7921	0.116	Campylobacter jejuni (72294)	Campylobacter (213849)	Campylobacteraceae (2954	Campylobacterales (68525)	Epsilonproteobacteria (122	Proteobacteria (2)	Bacteria (1)	100.00	0.00	99.90	99.70	WGS
197.7922	0.11	Campylobacter jejuni (72294)	Campylobacter (213849)	Campylobacteraceae (2954	Campylobacterales (68525)	Epsilonproteobacteria (122	Proteobacteria (2)	Bacteria (1)	100.00	0.00	100.00	99.90	WGS
197.7923	0.1124	Campylobacter jejuni (72294)	Campylobacter (213849)	Campylobacteraceae (2954	Campylobacterales (68525)	Epsilonproteobacteria (122	Proteobacteria (2)	Bacteria (1)	100.00	0.00	99.90	99.90	WGS
197.7924	0.1124	Campylobacter jejuni (72294)	Campylobacter (213849)	Campylobacteraceae (2954	Campylobacterales (68525)	Epsilonproteobacteria (122	Proteobacteria (2)	Bacteria (1)	100.00	0.00	99.90	99.90	WGS
197.7925	0.116	Campylobacter jejuni (72294)	Campylobacter (213849)	Campylobacteraceae (2954	Campylobacterales (68525)	Epsilonproteobacteria (122	Proteobacteria (2)	Bacteria (1)	100.00	0.00	99.90	99.90	WGS
197.7926	0.116	Campylobacter jejuni (72294)	Campylobacter (213849)	Campylobacteraceae (2954	Campylobacterales (68525)	Epsilonproteobacteria (122	Proteobacteria (2)	Bacteria (1)	100.00	0.00	99.90	99.90	WGS
197.7927	0.1078	Campylobacter jejuni (72294)	Campylobacter (213849)	Campylobacteraceae (2954	Campylobacterales (68525)	Epsilonproteobacteria (122	Proteobacteria (2)	Bacteria (1)	100.00	0.00	100.00	100.00	WGS
197.7928	0.1078	Campylobacter jejuni (72294)	Campylobacter (213849)	Campylobacteraceae (2954	Campylobacterales (68525)	Epsilonproteobacteria (122	Proteobacteria (2)	Bacteria (1)	100.00	0.00	99.90	99.70	WGS
197.7929	0.1078	Campylobacter jejuni (72294)	Campylobacter (213849)	Campylobacteraceae (2954	Campylobacterales (68525)	Epsilonproteobacteria (122	Proteobacteria (2)	Bacteria (1)	100.00	0.00	100.00	99.60	WGS
197.7930	0.1124	Campylobacter jejuni (72294)	Campylobacter (213849)	Campylobacteraceae (2954	Campylobacterales (68525)	Epsilonproteobacteria (122	Proteobacteria (2)	Bacteria (1)	100.00	0.00	99.90	99.90	WGS
197.7931	0.116	Campylobacter jejuni (72294)	Campylobacter (213849)	Campylobacteraceae (2954	Campylobacterales (68525)	Epsilonproteobacteria (122	Proteobacteria (2)	Bacteria (1)	100.00	0.70	99.90	99.70	WGS
197.7932	0.116	Campylobacter jejuni (72294)	Campylobacter (213849)	Campylobacteraceae (2954	Campylobacterales (68525)	Epsilonproteobacteria (122	Proteobacteria (2)	Bacteria (1)	100.00	1.30	99.90	99.10	WGS
197.7933	0.1124	Campylobacter jejuni (72294)	Campylobacter (213849)	Campylobacteraceae (2954	Campylobacterales (68525)	Epsilonproteobacteria (122	Proteobacteria (2)	Bacteria (1)	100.00	0.00	99.90	99.90	WGS
197.7934	0.1128	Campylobacter jejuni (72294)	Campylobacter (213849)	Campylobacteraceae (2954	Campylobacterales (68525)	Epsilonproteobacteria (122	Proteobacteria (2)	Bacteria (1)	100.00	0.00	99.90	99.90	WGS
197.7935	0.1078	Campylobacter jejuni (72294)	Campylobacter (213849)	Campylobacteraceae (2954	Campylobacterales (68525)	Epsilonproteobacteria (122	Proteobacteria (2)	Bacteria (1)	100.00	1.00	99.90	99.60	WGS
197.7936	0.116	Campylobacter jejuni (72294)	Campylobacter (213849)	Campylobacteraceae (2954	Campylobacterales (68525)	Epsilonproteobacteria (122	Proteobacteria (2)	Bacteria (1)	100.00	0.30	99.90	99.80	WGS
197.7937	0.1078	Campylobacter jejuni (72294)	Campylobacter (213849)	Campylobacteraceae (2954	Campylobacterales (68525)	Epsilonproteobacteria (122	Proteobacteria (2)	Bacteria (1)	100.00	0.00	100.00	100.00	WGS
197.7938	0.1078	Campylobacter jejuni (72294)	Campylobacter (213849)	Campylobacteraceae (2954	Campylobacterales (68525)	Epsilonproteobacteria (122	Proteobacteria (2)	Bacteria (1)	100.00	0.00	99.90	99.90	WGS
197.7939	0.1078	Campylobacter jejuni (72294)	Campylobacter (213849)	Campylobacteraceae (2954	Campylobacterales (68525)	Epsilonproteobacteria (122	Proteobacteria (2)	Bacteria (1)	100.00	0.70	99.90	99.80	WGS
197.7940	0.1078	Campylobacter jejuni (72294)	Campylobacter (213849)	Campylobacteraceae (2954	Campylobacterales (68525)	Epsilonproteobacteria (122	Proteobacteria (2)	Bacteria (1)	100.00	0.30	99.90	99.70	WGS
197.7941	0.1078	Campylobacter jejuni (72294)	Campylobacter (213849)	Campylobacteraceae (2954	Campylobacterales (68525)	Epsilonproteobacteria (122	Proteobacteria (2)	Bacteria (1)	100.00	0.70	100.00	99.80	WGS
197.7942	0.1078	Campylobacter jejuni (72294)	Campylobacter (213849)	Campylobacteraceae (2954	Campylobacterales (68525)	Epsilonproteobacteria (122	Proteobacteria (2)	Bacteria (1)	100.00	1.90	99.40	99.10	WGS
197.7943	0.1078	Campylobacter jejuni (72294)	Campylobacter (213849)	Campylobacteraceae (2954	Campylobacterales (68525)	Epsilonproteobacteria (122	Proteobacteria (2)	Bacteria (1)	100.00	1.30	99.70	99.00	WGS
197.7944	0.1078	Campylobacter jejuni (72294)	Campylobacter (213849)	Campylobacteraceae (2954	Campylobacterales (68525)	Epsilonproteobacteria (122	Proteobacteria (2)	Bacteria (1)	100.00	0.70	99.70	99.40	WGS
197.7945	0.1078	Campylobacter jejuni (72294)	Campylobacter (213849)	Campylobacteraceae (2954	Campylobacterales (68525)	Epsilonproteobacteria (122	Proteobacteria (2)	Bacteria (1)	100.00	0.70	99.90	99.90	WGS
197.7946	0.1078	Campylobacter jejuni (72294)	Campylobacter (213849)	Campylobacteraceae (2954	Campylobacterales (68525)	Epsilonproteobacteria (122	Proteobacteria (2)	Bacteria (1)	100.00	0.00	99.90	99.90	WGS
197.7947	0.1078	Campylobacter jejuni (72294)	Campylobacter (213849)	Campylobacteraceae (2954	Campylobacterales (68525)	Epsilonproteobacteria (122	Proteobacteria (2)	Bacteria (1)	100.00	0.00	99.90	99.80	WGS
197.7948	0.1078	Campylobacter jejuni (72294)	Campylobacter (213849)	Campylobacteraceae (2954	Campylobacterales (68525)	Epsilonproteobacteria (122	Proteobacteria (2)	Bacteria (1)	100.00	0.00	99.90	99.60	WGS
197.7949	0.1078	Campylobacter jejuni (72294)	Campylobacter (213849)	Campylobacteraceae (2954	Campylobacterales (68525)	Epsilonproteobacteria (122	Proteobacteria (2)	Bacteria (1)	100.00	0.00	99.90	99.80	WGS
197.7950	0.1078	Campylobacter jejuni (72294)	Campylobacter (213849)	Campylobacteraceae (2954	Campylobacterales (68525)	Epsilonproteobacteria (122	Proteobacteria (2)	Bacteria (1)	100.00	0.00	99.90	99.70	WGS
197.7951	0.1078	Campylobacter jejuni (72294)	Campylobacter (213849)	Campylobacteraceae (2954	Campylobacterales (68525)	Epsilonproteobacteria (122	Proteobacteria (2)	Bacteria (1)	100.00	0.00	99.90	99.90	WGS
197.7953	0.1078	Campylobacter jejuni (72294)	Campylobacter (213849)	Campylobacteraceae (2954	Campylobacterales (68525)	Epsilonproteobacteria (122	Proteobacteria (2)	Bacteria (1)	100.00	0.00	99.80	99.70	WGS
197.7954	0.1118	Campylobacter jejuni (72294)	Campylobacter (213849)	Campylobacteraceae (2954	Campylobacterales (68525)	Epsilonproteobacteria (122	Proteobacteria (2)	Bacteria (1)	100.00	0.70	99.70	99.60	WGS
197.7955	0.118	Campylobacter jejuni (72294)	Campylobacter (213849)	Campylobacteraceae (2954	Campylobacterales (68525)	Epsilonproteobacteria (122	Proteobacteria (2)	Bacteria (1)	100.00	1.30	99.90	99.80	WGS
197.7956	0.104	Campylobacter jejuni (72294)	Campylobacter (213849)	Campylobacteraceae (2954	Campylobacterales (68525)	Epsilonproteobacteria (122	Proteobacteria (2)	Bacteria (1)	100.00	0.00	99.60	99.10	WGS
197.7957	0.1172	Campylobacter jejuni (72294)	Campylobacter (213849)	Campylobacteraceae (2954	Campylobacterales (68525)	Epsilonproteobacteria (122	Proteobacteria (2)	Bacteria (1)	100.00	0.00	99.90	99.60	WGS
197.7958	0.1112	Campylobacter jejuni (72294)	Campylobacter (213849)	Campylobacteraceae (2954	Campylobacterales (68525)	Epsilonproteobacteria (122	Proteobacteria (2)	Bacteria (1)	100.00	0.00	99.70	99.50	WGS
197.7959	0.109	Campylobacter jejuni (72294)	Campylobacter (213849)	Campylobacteraceae (2954	Campylobacterales (68525)	Epsilonproteobacteria (122	Proteobacteria (2)	Bacteria (1)	100.00	0.00	99.90	99.90	WGS
197.7960	0.116	Campylobacter jejuni (72294)	Campylobacter (213849)	Campylobacteraceae (2954	Campylobacterales (68525)	Epsilonproteobacteria (122	Proteobacteria (2)	Bacteria (1)	100.00	0.00	99.90	99.80	WGS
197.7961	0.1266	Campylobacter jejuni (72294)	Campylobacter (213849)	Campylobacteraceae (2954	Campylobacterales (68525)	Epsilonproteobacteria (122	Proteobacteria (2)	Bacteria (1)	100.00	0.00	99.50	99.30	WGS
197.7962	0.1112	Campylobacter jejuni (72294)	Campylobacter (213849)	Campylobacteraceae (2954	Campylobacterales (68525)	Epsilonproteobacteria (122	Proteobacteria (2)	Bacteria (1)	100.00	0.00	99.90	99.80	WGS
197.7963	0.1132	Campylobacter jejuni (72294)	Campylobacter (213849)	Campylobacteraceae (2954	Campylobacterales (68525)	Epsilonproteobacteria (122	Proteobacteria (2)	Bacteria (1)	100.00	0.00	99.70	99.60	WGS
197.7964	0.1132	Campylobacter jejuni (72294)	Campylobacter (213849)	Campylobacteraceae (2954	Campylobacterales (68525)	Epsilonproteobacteria (122	Proteobacteria (2)	Bacteria (1)	100.00	0.00	99.80	99.60	WGS
197.7965	0.1132	Campylobacter jejuni (72294)	Campylobacter (213849)	Campylobacteraceae (2954	Campylobacterales (68525)	Epsilonproteobacteria (122	Proteobacteria (2)	Bacteria (1)	100.00	0.00	99.80	99.60	WGS
197.7966	0.1078	Campylobacter jejuni (72294)	Campylobacter (213849)	Campylobacteraceae (2954	Campylobacterales (68525)	Epsilonproteobacteria (122	Proteobacteria (2)	Bacteria (1)	100.00	0.00	99.90	99.60	WGS
198.15	0.123	Campylobacter hyointestinalis (722	Campylobacter (213849)	Campylobacteraceae (2954	Campylobacterales (68525)	Epsilonproteobacteria (122	Proteobacteria (2)	Bacteria (1)	100.00	2.50	98.80	98.20	WGS
198.16	0.115	Campylobacter hyointestinalis (722	Campylobacter (213849)	Campylobacteraceae (2954	Campylobacterales (68525)	Epsilonproteobacteria (122	Proteobacteria (2)	Bacteria (1)	100.00	2.50	98.90	98.30	WGS
198.4	0.115	Campylobacter hyointestinalis (722	Campylobacter (213849)	Campylobacteraceae (2954	Campylobacterales (68525)	Epsilonproteobacteria (122	Proteobacteria (2)	Bacteria (1)	100.00	0.00	98.80	98.50	WGS
199.248	0.1266	Campylobacter concisus (72294)	Campylobacter (213849)	Campylobacteraceae (2954	Campylobacterales (68525)	Epsilonproteobacteria (122	Proteobacteria (2)	Bacteria (1)	100.00	0.00	96.10	95.90	Complete
199.447	0.1352	Campylobacter concisus (72294)	Campylobacter (213849)	Campylobacteraceae (2954	Campylobacterales (68525)	Epsilonproteobacteria (122	Proteobacteria (2)	Bacteria (1)	100.00	0.00	96.30	95.60	WGS
199.475	0.13	Campylobacter concisus (72294)	Campylobacter (213849)	Campylobacteraceae (2954	Campylobacterales (68525)	Epsilonproteobacteria (122	Proteobacteria (2)	Bacteria (1)	99.50	6.90	96.70	93.60	WGS
199.476	0.1284	Campylobacter concisus (72294)	Campylobacter (213849)	Campylobacteraceae (2954	Campylobacterales (68525)	Epsilonproteobacteria (122	Proteobacteria (2)	Bacteria (1)	100.00	1.30	96.70	95.60	WGS
199.477	0.1282	Campylobacter concisus (72294)	Campylobacter (213849)	Campylobacteraceae (2954	Campylobacterales (68525)	Epsilonproteobacteria (122	Proteobacteria (2)	Bacteria (1)	100.00	0.00	96.70	96.20	WGS
199.478	0.1296	Campylobacter concisus (72294)	Campylobacter (213849)	Campylobacteraceae (2954	Campylobacterales (68525)	Epsilonproteobacteria (122	Proteobacteria (2)	Bacteria (1)	100.00	0.50	96.60	96.20	WGS
199.585	0.127	Campylobacter concisus (72294)	Campylobacter (213849)	Campylobacteraceae (2954	Campylobacterales (68525)	Epsilonproteobacteria (122	Proteobacteria (2)	Bacteria (1)	100.00	0.00	96.40	95.80	WGS
199.586	0.1364	Campylobacter concisus (72294)	Campylobacter (213849)	Campylobacteraceae (2954	Campylobacterales (68525)	Epsilonproteobacteria (122	Proteobacteria (2)	Bacteria (1)	100.00	0.00	96.70	95.90	WGS
199.587	0.1404	Campylobacter concisus (72294)	Campylobacter (213849)	Campylobacteraceae (2954	Campylobacterales (68525)	Epsilonproteobacteria (122	Proteobacteria (2)	Bacteria (1)	100.00	0.00	96.70	96.10	WGS
199.588	0.1304	Campylobacter concisus (72294)	Campylobacter (213849)	Campylobacteraceae (2954	Campylobacterales (68525)	Epsilonproteobacteria (122	Proteobacteria (2)	Bacteria (1)	100.00	0.00	97.20	96.60	WGS
199.589	0.1284	Campylobacter concisus (72294)	Campylobacter (213849)	Campylobacteraceae (2954	Campylobacterales (68525)	Epsilonproteobacteria (122	Proteobacteria (2)	Bacteria (1)	100.00	0.00	97.00	96.70	WGS
199.590	0.1272	Campylobacter concisus (72294)	Campylobacter (213849)	Campylobacteraceae (2954	Campylobacterales (68525)	Epsilonproteobacteria (122	Proteobacteria (2)	Bacteria (1)	100.00	0.50	97.00	96.60	WGS
199.591	0.1358	Campylobacter concisus (72294)	Campylobacter (213849)	Campylobacteraceae (2954	Campylobacterales (68525)	Epsilonproteobacteria (122	Proteobacteria (2)	Bacteria (1)	100.00	0.00	97.40	96.70	WGS
199.653	0.0294	Campylobacter concisus (72294)	Campylobacter (213849)	Campylobacteraceae (2954	Campylobacterales (68525)	Epsilonproteobacteria (122	Proteobacteria (2)	Bacteria (1)					Plasmid
199.654	0.0294	Campylobacter concisus (72294)	Campylobacter (213849)	Campylobacteraceae (2954	Campylobacterales (68525)	Epsilonproteobacteria (122	Proteobacteria (2)	Bacteria (1)					Plasmid
199.761	0.1338	Campylobacter concisus (72294)	Campylobacter (213849)	Campylobacteraceae (2954	Campylobacterales (68525)	Epsilonproteobacteria (122	Proteobacteria (2)	Bacteria (1)	100.00	21.00	95.90	86.90	WGS
199.762	0.1318	Campylobacter concisus (72294)	Campylobacter (213849)	Campylobacteraceae (2954	Campylobacterales (68525)	Epsilonproteobacteria (122	Proteobacteria (2)	Bacteria (1)	100.00	7.60	96.80	94.30	WGS
199.763	0.1318	Campylobacter concisus (72294)	Campylobacter (213849)	Campylobacteraceae (2954	Campylobacterales (68525)	Epsilonproteobacteria (122	Proteobacteria (2)	Bacteria (1)	100.00	6.70	96.80	93.90	WGS
199.764	0.1318	Campylobacter concisus (72294)	Campylobacter (213849)	Campylobacteraceae (2954	Campylobacterales (68525)	Epsilonproteobacteria (122	Proteobacteria (2)	Bacteria (1)	99.70	23.10	96.40	87.80	WGS
199.765	0.1242	Campylobacter concisus (72294)	Campylobacter (213849)	Campylobacteraceae (2954	Campylobacterales (68525)	Epsilonproteobacteria (122	Proteobacteria (2)	Bacteria (1)	96.40	24.70	95.60	88.00	WGS
199.766	0.1366	Campylobacter concisus (72294)	Campylobacter (213849)	Campylobacteraceae (2954	Campylobacterales (68525)	Epsilonproteobacteria (122	Proteobacteria (2)	Bacteria (1)	100.00	1.30	96.70	95.20	WGS
199.767	0.1406	Campylobacter concisus (72294)	Campylobacter (213849)	Campylobacteraceae (2954	Campylobacterales (68525)	Epsilonproteobacteria (122	Proteobacteria (2)	Bacteria (1)	100.00	2.60	96.90	95.60	WGS
199.768	0.1318	Campylobacter concisus (72294)	Campylobacter (213849)	Campylobacteraceae (2954	Campylobacterales (68525)	Epsilonproteobacteria (122	Proteobacteria (2)	Bacteria (1)	100.00	1.30	95.90	95.00	WGS
199.769	0.1318	Campylobacter concisus (72294)	Campylobacter (213849)	Campylobacteraceae (2954	Campylobacterales (68525)	Epsilonproteobacteria (122	Proteobacteria (2)	Bacteria (1)	100.00	0.00	96.70	95.70	WGS
199.770	0.1408	Campylobacter concisus (72294)	Campylobacter (213849)	Campylobacteraceae (2954	Campylobacterales (68525)	Epsilonproteobacteria (122	Proteobacteria (2)	Bacteria (1)	100.00	0.00	96.90	96.30	WGS
199.771	0.1338	Campylobacter concisus (72294)	Campylobacter (213849)	Campylobacteraceae (2954	Campylobacterales (68525)	Epsilonproteobacteria (122	Proteobacteria (2)	Bacteria (1)	100.00	0.00	97.00	96.30	WGS
199.772	0.1318	Campylobacter concisus (72294)	Campylobacter (213849)	Campylobacteraceae (2954	Campylobacterales (68525)	Epsilonproteobacteria (122	Proteobacteria (2)	Bacteria (1)	100.00	1.50	96.90	94.50	WGS
199.773	0.1318	Campylobacter concisus (72294)	Campylobacter (213849)	Campylobacteraceae (2954	Campylobacterales (68525)	Epsilonproteobacteria (122	Proteobacteria (2)	Bacteria (1)	100.00	0.00	96.90	96.20	WGS
199.774	0.1318	Campylobacter concisus (72294)	Campylobacter (213849)	Campylobacteraceae (2954	Campylobacterales (68525)	Epsilonproteobacteria (122	Proteobacteria (2)	Bacteria (1)	100.00	1.00	96.70	96.00	WGS
199.775	0.13	Campylobacter concisus (72294)	Campylobacter (213849)	Campylobacteraceae (2954	Campylobacterales (68525)	Epsilonproteobacteria (122	Proteobacteria (2)	Bacteria (1)	100.00	0.00	96.50	95.90	WGS
199.776	0.1318	Campylobacter concisus (72294)	Campylobacter (213849)	Campylobacteraceae (2954	Campylobacterales (68525)	Epsilonproteobacteria (122	Proteobacteria (2)	Bacteria (1)	100.00	0.00	96.90	96.20	WGS
199.777	0.1318	Campylobacter concisus (72294)	Campylobacter (213849)	Campylobacteraceae (2954	Campylobacterales (68525)	Epsilonproteobacteria (122	Proteobacteria (2)	Bacteria (1)	98.50	1.50	96.30	95.00	WGS
199.778	0.1318	Campylobacter concisus (72294)	Campylobacter (213849)	Campylobacteraceae (2954	Campylobacterales (68525)	Epsilonproteobacteria (122	Proteobacteria (2)	Bacteria (1)	100.00	0.00	96.70	96.20	WGS
199.779	0.1318	Campylobacter concisus (72294)	Campylobacter (213849)	Campylobacteraceae (2954	Campylobacterales (68525)	Epsilonproteobacteria (122	Proteobacteria (2)	Bacteria (1)	99.00	0.00	96.70	96.00	WGS
199.780	0.1322	Campylobacter concisus (72294)	Campylobacter (213849)	Campylobacteraceae (2954	Campylobacterales (68525)	Epsilonproteobacteria (122	Proteobacteria (2)	Bacteria (1)	100.00	0.00	96.80	96.20	WGS
199.781	0.1318	Campylobacter concisus (72294)	Campylobacter (213849)	Campylobacteraceae (2954	Campylobacterales (68525)	Epsilonproteobacteria (122	Proteobacteria (2)	Bacteria (1)	96.90	0.00	96.20	95.60	WGS
199.782	0.1432	Campylobacter concisus (72294)	Campylobacter (213849)	Campylobacteraceae (2954	Campylobacterales (68525)	Epsilonproteobacteria (122	Proteobacteria (2)	Bacteria (1)	99.50	3.20	96.50	95.70	WGS
199.783	0.1306	Campylobacter concisus (72294)	Campylobacter (213849)	Campylobacteraceae (2954	Campylobacterales (68525)	Epsilonproteobacteria (122	Proteobacteria (2)	Bacteria (1)	100.00	0.00	96.70	96.20	WGS
199.784	0.1318	Campylobacter concisus (72294)	Campylobacter (213849)	Campylobacteraceae (2954	Campylobacterales (68525)	Epsilonproteobacteria (122	Proteobacteria (2)	Bacteria (1)	100.00	0.10	96.80	96.10	WGS
199.785	0.1318	Campylobacter concisus (72294)	Campylobacter (213849)	Campylobacteraceae (2954	Campylobacterales (68525)	Epsilonproteobacteria (122	Proteobacteria (2)	Bacteria (1)	99.00	0.30	95.40	94.80	WGS
199.786	0.1318	Campylobacter concisus (72294)	Campylobacter (213849)	Campylobacteraceae (2954	Campylobacterales (68525)	Epsilonproteobacteria (122	Proteobacteria (2)	Bacteria (1)	95.80	0.00	95.40	94.70	WGS
199.787	0.1294	Campylobacter concisus (72294)	Campylobacter (213849)	Campylobacteraceae (2954	Campylobacterales (68525)	Epsilonproteobacteria (122	Proteobacteria (2)	Bacteria (1)	98.20	0.00	95.90	95.10	WGS
199.788	0.1272	Campylobacter concisus (72294)	Campylobacter (213849)	Campylobacteraceae (2954	Campylobacterales (68525)	Epsilonproteobacteria (122	Proteobacteria (2)	Bacteria (1)	99.50	0.00	96.20	95.40	WGS
199.789	0.131	Campylobacter concisus (72294)	Campylobacter (213849)	Campylobacteraceae (2954	Campylobacterales (68525)	Epsilonproteobacteria (122	Proteobacteria (2)	Bacteria (1)	100.00	1.00	96.20	95.50	WGS
199.790	0.139	Campylobacter concisus (72294)	Campylobacter (213849)	Campylobacteraceae (2954	Campylobacterales (68525)	Epsilonproteobacteria (122	Proteobacteria (2)	Bacteria (1)	100.00	0.00	96.00	95.30	WGS
199.791	0.1288	Campylobacter concisus (72294)	Campylobacter (213849)	Campylobacteraceae (2954	Campylobacterales (68525)	Epsilonproteobacteria (122	Proteobacteria (2)	Bacteria (1)	98.90	1.00	94.20	93.40	WGS
199.792	0.1266	Campylobacter concisus (72294)	Campylobacter (213849)	Campylobacteraceae (2954	Campylobacterales (68525)	Epsilonproteobacteria (122	Proteobacteria (2)	Bacteria (1)	99.50	1.50	95.60	94.90	WGS
199.793	0.1284	Campylobacter concisus (72294)	Campylobacter (213849)	Campylobacteraceae (2954	Campylobacterales (68525)	Epsilonproteobacteria (122	Proteobacteria (2)	Bacteria (1)	99.00	0.00	96.00	95.60	WGS
199.794	0.1296	Campylobacter concisus (72294)	Campylobacter (213849)	Campylobacteraceae (2954	Campylobacterales (68525)	Epsilonproteobacteria (122	Proteobacteria (2)	Bacteria (1)	100.00	0.50	96.50	95.90	WGS
199.795	0.107	Campylobacter concisus (72294)	Campylobacter (213849)	Campylobacteraceae (2954	Campylobacterales (68525)	Epsilonproteobacteria (122	Proteobacteria (2)	Bacteria (1)	89.70	0.00	92.40	91.90	WGS
199.796	0.124	Campylobacter concisus (72294)	Campylobacter (213849)	Campylobacteraceae (2954	Campylobacterales (68525)	Epsilonproteobacteria (122	Proteobacteria (2)	Bacteria (1)	100.00	1.50	96.50	95.60	WGS
199.797	0.1296	Campylobacter concisus (72294)	Campylobacter (213849)	Campylobacteraceae (2954	Campylobacterales (68525)	Epsilonproteobacteria (122	Proteobacteria (2)	Bacteria (1)	100.00	0.00	96.50	96.20	WGS
199.798	0.1318	Campylobacter concisus (72294)	Campylobacter (213849)	Campylobacteraceae (2954	Campylobacterales (68525)	Epsilonproteobacteria (122	Proteobacteria (2)	Bacteria (1)	100.00	0.00	97.00	96.00	WGS
199.799	0.1296	Campylobacter concisus (72294)	Campylobacter (213849)	Campylobacteraceae (2954	Campylobacterales (68525)	Epsilonproteobacteria (122	Proteobacteria (2)	Bacteria (1)	97.90	0.50	94.70	94.20	WGS
199.800	0.1266	Campylobacter concisus (72294)	Campylobacter (213849)	Campylobacteraceae (2954	Campylobacterales (68525)	Epsilonproteobacteria (122	Proteobacteria (2)	Bacteria (1)	96.90	0.50	94.90	94.30	WGS
199.801	0.1296	Campylobacter concisus (72294)	Campylobacter (213849)	Campylobacteraceae (2954	Campylobacterales (68525)	Epsilonproteobacteria (122	Proteobacteria (2)	Bacteria (1)	100.00	1.00	96.50	95.90	WGS
199.802	0.1254	Campylobacter concisus (72294)	Campylobacter (213849)	Campylobacteraceae (2954	Campylobacterales (68525)	Epsilonproteobacteria (122	Proteobacteria (2)	Bacteria (1)	86.20	1.20	93.80	93.30	WGS
199.803	0.1274	Campylobacter concisus (72294)	Campylobacter (213849)	Campylobacteraceae (2954	Campylobacterales (68525)	Epsilonproteobacteria (122	Proteobacteria (2)	Bacteria (1)	100.00	0.00	96.70	96.10	WGS
199.804	0.1338	Campylobacter concisus (72294)	Campylobacter (213849)	Campylobacteraceae (2954	Campylobacterales (68525)	Epsilonproteobacteria (122	Proteobacteria (2)	Bacteria (1)	100.00	0.80	96.60	95.60	WGS
199.805	0.1404	Campylobacter concisus (72294)	Campylobacter (213849)	Campylobacteraceae (2954	Campylobacterales (68525)	Epsilonproteobacteria (122	Proteobacteria (2)	Bacteria (1)	99.70	0.00	97.00	96.20	WGS
199.806	0.1454	Campylobacter concisus (72294)	Campylobacter (213849)	Campylobacteraceae (2954	Campylobacterales (68525)	Epsilonproteobacteria (122	Proteobacteria (2)	Bacteria (1)	100.00	1.00	97.00	96.20	WGS
199.807	0.1454	Campylobacter concisus (72294)	Campylobacter (213849)	Campylobacteraceae (2954	Campylobacterales (68525)	Epsilonproteobacteria (122	Proteobacteria (2)	Bacteria (1)	100.00	0.50	97.00	96.30	WGS
199.808	0.1338	Campylobacter concisus (72294)	Campylobacter (213849)	Campylobacteraceae (2954	Campylobacterales (68525)	Epsilonproteobacteria (122	Proteobacteria (2)	Bacteria (1)	100.00	2.70	96.60	95.30	WGS
199.809	0.1454	Campylobacter concisus (72294)	Campylobacter (213849)	Campylobacteraceae (2954	Campylobacterales (68525)	Epsilonproteobacteria (122	Proteobacteria (2)	Bacteria (1)	100.00	0.50	96.30	95.40	WGS
199.810	0.1454	Campylobacter concisus (72294)	Campylobacter (213849)	Campylobacteraceae (2954	Campylobacterales (68525)	Epsilonproteobacteria (122	Proteobacteria (2)	Bacteria (1)	100.00	1.10	97.00	96.20	WGS
199.811	0.1454	Campylobacter concisus (72294)	Campylobacter (213849)	Campylobacteraceae (2954	Campylobacterales (68525)	Epsilonproteobacteria (122	Proteobacteria (2)	Bacteria (1)	100.00	1.00	96.90	95.40	WGS
199.812	0.1454	Campylobacter concisus (72294)	Campylobacter (213849)	Campylobacteraceae (2954	Campylobacterales (68525)	Epsilonproteobacteria (122	Proteobacteria (2)	Bacteria (1)	100.00	0.00	96.30	95.00	WGS
199.813	0.139	Campylobacter concisus (72294)	Campylobacter (213849)	Campylobacteraceae (2954	Campylobacterales (68525)	Epsilonproteobacteria (122	Proteobacteria (2)	Bacteria (1)	95.90	0.00	95.70	95.10	WGS
199.814	0.1454	Campylobacter concisus (72294)	Campylobacter (213849)	Campylobacteraceae (2954	Campylobacterales (68525)	Epsilonproteobacteria (122	Proteobacteria (2)	Bacteria (1)	100.00	1.30	96.90	95.30	WGS
199.815	0.13	Campylobacter concisus (72294)	Campylobacter (213849)	Campylobacteraceae (2954	Campylobacterales (68525)	Epsilonproteobacteria (122	Proteobacteria (2)	Bacteria (1)	98.80	0.30	96.40	95.30	WGS
199.816	0.1454	Campylobacter concisus (72294)	Campylobacter (213849)	Campylobacteraceae (2954	Campylobacterales (68525)	Epsilonproteobacteria (122	Proteobacteria (2)	Bacteria (1)	100.00	3.50	97.00	94.60	WGS
199.817	0.1114	Campylobacter concisus (72294)	Campylobacter (213849)	Campylobacteraceae (2954	Campylobacterales (68525)	Epsilonproteobacteria (122	Proteobacteria (2)	Bacteria (1)	93.90	0.00	93.90	93.20	WGS
199.818	0.128	Campylobacter concisus (72294)	Campylobacter (213849)	Campylobacteraceae (2954	Campylobacterales (68525)	Epsilonproteobacteria (122	Proteobacteria (2)	Bacteria (1)	97.90	1.00	95.00	94.00	WGS
199.819	0.1344	Campylobacter concisus (72294)	Campylobacter (213849)	Campylobacteraceae (2954	Campylobacterales (68525)	Epsilonproteobacteria (122	Proteobacteria (2)	Bacteria (1)	98.00	0.00	96.60	95.30	WGS
199.820	0.1396	Campylobacter concisus (72294)	Campylobacter (213849)	Campylobacteraceae (2954	Campylobacterales (68525)	Epsilonproteobacteria (122	Proteobacteria (2)	Bacteria (1)	100.00	0.00	96.70	95.70	WGS
199.821	0.13	Campylobacter concisus (72294)	Campylobacter (213849)	Campylobacteraceae (2954	Campylobacterales (68525)	Epsilonproteobacteria (122	Proteobacteria (2)	Bacteria (1)	94.00	5.70	94.30	91.40	WGS
199.822	0.1212	Campylobacter concisus (72294)	Campylobacter (213849)	Campylobacteraceae (2954	Campylobacterales (68525)	Epsilonproteobacteria (122	Proteobacteria (2)	Bacteria (1)	98.00	0.00	95.40	94.90	WGS
199.823	0.1318	Campylobacter concisus (72294)	Campylobacter (213849)	Campylobacteraceae (2954	Campylobacterales (68525)	Epsilonproteobacteria (122	Proteobacteria (2)	Bacteria (1)	100.00	0.00	97.00	96.00	WGS
199.824	0.1306	Campylobacter concisus (72294)	Campylobacter (213849)	Campylobacteraceae (2954	Campylobacterales (68525)	Epsilonproteobacteria (122	Proteobacteria (2)	Bacteria (1)	98.50	2.50	96.40	94.90	WGS
199.825	0.129	Campylobacter concisus (72294)	Campylobacter (213849)	Campylobacteraceae (2954	Campylobacterales (68525)	Epsilonproteobacteria (122	Proteobacteria (2)	Bacteria (1)	100.00	1.00	96.70	95.90	WGS
199.826	0.1424	Campylobacter concisus (72294)	Campylobacter (213849)	Campylobacteraceae (2954	Campylobacterales (68525)	Epsilonproteobacteria (122	Proteobacteria (2)	Bacteria (1)	100.00	1.30	96.10	94.80	WGS
199.827	0.1318	Campylobacter concisus (72294)	Campylobacter (213849)	Campylobacteraceae (2954	Campylobacterales (68525)	Epsilonproteobacteria (122	Proteobacteria (2)	Bacteria (1)	99.50	6.30	96.70	94.40	WGS
199.828	0.1346	Campylobacter concisus (72294)	Campylobacter (213849)	Campylobacteraceae (2954	Campylobacterales (68525)	Epsilonproteobacteria (122	Proteobacteria (2)	Bacteria (1)	100.00	9.20	95.70	91.10	WGS
199.829	0.1384	Campylobacter concisus (72294)	Campylobacter (213849)	Campylobacteraceae (2954	Campylobacterales (68525)	Epsilonproteobacteria (122	Proteobacteria (2)	Bacteria (1)	100.00	0.00	96.90	96.00	WGS
199.830	0.1296	Campylobacter concisus (72294)	Campylobacter (213849)	Campylobacteraceae (2954	Campylobacterales (68525)	Epsilonproteobacteria (122	Proteobacteria (2)	Bacteria (1)	100.00	0.00	96.50	96.20	WGS
199.831	0.1318	Campylobacter concisus (72294)	Campylobacter (213849)	Campylobacteraceae (2954	Campylobacterales (68525)	Epsilonproteobacteria (122	Proteobacteria (2)	Bacteria (1)	100.00	0.00	96.90	96.20	WGS
199.832	0.1338	Campylobacter concisus (72294)	Campylobacter (213849)	Campylobacteraceae (2954	Campylobacterales (68525)	Epsilonproteobacteria (122	Proteobacteria (2)	Bacteria (1)	100.00	0.00	96.50	95.80	WGS
199.833	0.13	Campylobacter concisus (72294)	Campylobacter (213849)	Campylobacteraceae (2954	Campylobacterales (68525)	Epsilonproteobacteria (122	Proteobacteria (2)	Bacteria (1)	100.00	0.00	96.70	96.10	WGS
199.834	0.1318	Campylobacter concisus (72294)	Campylobacter (213849)	Campylobacteraceae (2954	Campylobacterales (68525)	Epsilonproteobacteria (122	Proteobacteria (2)	Bacteria (1)	99.40	13.00	93.80	89.20	WGS
199.835	0.138	Campylobacter concisus (72294)	Campylobacter (213849)	Campylobacteraceae (2954	Campylobacterales (68525)	Epsilonproteobacteria (122	Proteobacteria (2)	Bacteria (1)	100.00	2.00	95.20	92.50	WGS
199.836	0.129	Campylobacter concisus (72294)	Campylobacter (213849)	Campylobacteraceae (2954	Campylobacterales (68525)	Epsilonproteobacteria (122	Proteobacteria (2)	Bacteria (1)	100.00	1.00	97.00	96.40	WGS
199.837	0.13	Campylobacter concisus (72294)	Campylobacter (213849)	Campylobacteraceae (2954	Campylobacterales (68525)	Epsilonproteobacteria (122	Proteobacteria (2)	Bacteria (1)	97.40	0.00	95.70	95.10	WGS
199.838	0.1426	Campylobacter concisus (72294)	Campylobacter (213849)	Campylobacteraceae (2954	Campylobacterales (68525)	Epsilonproteobacteria (122	Proteobacteria (2)	Bacteria (1)	100.00	0.50	96.80	96.00	WGS
199.839	0.1318	Campylobacter concisus (72294)	Campylobacter (213849)	Campylobacteraceae (2954	Campylobacterales (68525)	Epsilonproteobacteria (122	Proteobacteria (2)	Bacteria (1)	99.00	0.50	96.70	95.90	WGS
199.840	0.1318	Campylobacter concisus (72294)	Campylobacter (213849)	Campylobacteraceae (2954	Campylobacterales (68525)	Epsilonproteobacteria (122	Proteobacteria (2)	Bacteria (1)	99.50	0.50	96.20	95.60	WGS
199.841	0.1292	Campylobacter concisus (72294)	Campylobacter (213849)	Campylobacteraceae (2954	Campylobacterales (68525)	Epsilonproteobacteria (122	Proteobacteria (2)	Bacteria (1)	100.00	1.00	96.50	95.90	WGS
199.842	0.1376	Campylobacter concisus (72294)	Campylobacter (213849)	Campylobacteraceae (2954	Campylobacterales (68525)	Epsilonproteobacteria (122	Proteobacteria (2)	Bacteria (1)	100.00	1.30	96.30	95.40	WGS
199.843	0.1356	Campylobacter concisus (72294)	Campylobacter (213849)	Campylobacteraceae (2954	Campylobacterales (68525)	Epsilonproteobacteria (122	Proteobacteria (2)	Bacteria (1)	99.50	0.30	97.10	96.10	WGS
199.844	0.1318	Campylobacter concisus (72294)	Campylobacter (213849)	Campylobacteraceae (2954	Campylobacterales (68525)	Epsilonproteobacteria (122	Proteobacteria (2)	Bacteria (1)	100.00	0.00	96.80	96.50	WGS
199.845	0.1266	Campylobacter concisus (72294)	Campylobacter (213849)	Campylobacteraceae (2954	Campylobacterales (68525)	Epsilonproteobacteria (122	Proteobacteria (2)	Bacteria (1)	100.00	1.50	96.30	95.60	WGS
199.846	0.1318	Campylobacter concisus (72294)	Campylobacter (213849)	Campylobacteraceae (2954	Campylobacterales (68525)	Epsilonproteobacteria (122	Proteobacteria (2)	Bacteria (1)	100.00	0.00	97.30	96.20	WGS
199.847	0.1296	Campylobacter concisus (72294)	Campylobacter (213849)	Campylobacteraceae (2954	Campylobacterales (68525)	Epsilonproteobacteria (122	Proteobacteria (2)	Bacteria (1)	100.00	0.00	96.50	96.10	WGS
199.848	0.1326	Campylobacter concisus (72294)	Campylobacter (213849)	Campylobacteraceae (2954	Campylobacterales (68525)	Epsilonproteobacteria (122	Proteobacteria (2)	Bacteria (1)	94.90	0.00	95.90	95.10	WGS
199.849	0.1296	Campylobacter concisus (72294)	Campylobacter (213849)	Campylobacteraceae (2954	Campylobacterales (68525)	Epsilonproteobacteria (122	Proteobacteria (2)	Bacteria (1)	100.00	0.30	96.50	96.00	WGS
199.850	0.1266	Campylobacter concisus (72294)	Campylobacter (213849)	Campylobacteraceae (2954	Campylobacterales (68525)	Epsilonproteobacteria (122	Proteobacteria (2)	Bacteria (1)	100.00	0.50	96.20	95.30	WGS
199.851	0.1296	Campylobacter concisus (72294)	Campylobacter (213849)	Campylobacteraceae (2954	Campylobacterales (68525)	Epsilonproteobacteria (122	Proteobacteria (2)	Bacteria (1)	100.00	0.00	96.50	96.00	WGS
199.852	0.1266	Campylobacter concisus (72294)	Campylobacter (213849)	Campylobacteraceae (2954	Campylobacterales (68525)	Epsilonproteobacteria (122	Proteobacteria (2)	Bacteria (1)	100.00	1.90	96.10	95.20	WGS
199.853	0.1344	Campylobacter concisus (72294)	Campylobacter (213849)	Campylobacteraceae (2954	Campylobacterales (68525)	Epsilonproteobacteria (122	Proteobacteria (2)	Bacteria (1)	100.00	0.50	97.20	96.20	WGS
199.854	0.128	Campylobacter concisus (72294)	Campylobacter (213849)	Campylobacteraceae (2954	Campylobacterales (68525)	Epsilonproteobacteria (122	Proteobacteria (2)	Bacteria (1)	100.00	1.50	96.70	95.90	WGS
199.855	0.1454	Campylobacter concisus (72294)	Campylobacter (213849)	Campylobacteraceae (2954	Campylobacterales (68525)	Epsilonproteobacteria (122	Proteobacteria (2)	Bacteria (1)	100.00	0.50	97.00	96.40	WGS
199.856	0.1322	Campylobacter concisus (72294)	Campylobacter (213849)	Campylobacteraceae (2954	Campylobacterales (68525)	Epsilonproteobacteria (122	Proteobacteria (2)	Bacteria (1)	100.00	0.50	96.60	95.60	WGS
199.857	0.0674	Campylobacter concisus (72294)	Campylobacter (213849)	Campylobacteraceae (2954	Campylobacterales (68525)	Epsilonproteobacteria (122	Proteobacteria (2)	Bacteria (1)	87.90	0.60	92.90	92.20	WGS
199.858	0.129	Campylobacter concisus (72294)	Campylobacter (213849)	Campylobacteraceae (2954	Campylobacterales (68525)	Epsilonproteobacteria (122	Proteobacteria (2)	Bacteria (1)	100.00	1.00	96.70	95.90	WGS
199.859	0.1318	Campylobacter concisus (72294)	Campylobacter (213849)	Campylobacteraceae (2954	Campylobacterales (68525)	Epsilonproteobacteria (122	Proteobacteria (2)	Bacteria (1)	100.00	1.30	96.60	95.70	WGS
199.860	0.1344	Campylobacter concisus (72294)	Campylobacter (213849)	Campylobacteraceae (2954	Campylobacterales (68525)	Epsilonproteobacteria (122	Proteobacteria (2)	Bacteria (1)	100.00	0.50	96.80	95.80	WGS
199.861	0.1338	Campylobacter concisus (72294)	Campylobacter (213849)	Campylobacteraceae (2954	Campylobacterales (68525)	Epsilonproteobacteria (122	Proteobacteria (2)	Bacteria (1)	100.00	1.00	96.50	95.80	WGS
199.862	0.1296	Campylobacter concisus (72294)	Campylobacter (213849)	Campylobacteraceae (2954	Campylobacterales (68525)	Epsilonproteobacteria (122	Proteobacteria (2)	Bacteria (1)	100.00	0.00	96.50	96.20	WGS
199.863	0.1266	Campylobacter concisus (72294)	Campylobacter (213849)	Campylobacteraceae (2954	Campylobacterales (68525)	Epsilonproteobacteria (122	Proteobacteria (2)	Bacteria (1)	99.50	4.70	96.10	94.20	WGS
199.864	0.1364	Campylobacter concisus (72294)	Campylobacter (213849)	Campylobacteraceae (2954	Campylobacterales (68525)	Epsilonproteobacteria (122	Proteobacteria (2)	Bacteria (1)	100.00	0.00	96.70	96.30	WGS
199.865	0.1288	Campylobacter concisus (72294)	Campylobacter (213849)	Campylobacteraceae (2954	Campylobacterales (68525)	Epsilonproteobacteria (122	Proteobacteria (2)	Bacteria (1)	100.00	0.00	96.70	96.50	WGS
199.866	0.1378	Campylobacter concisus (72294)	Campylobacter (213849)	Campylobacteraceae (2954	Campylobacterales (68525)	Epsilonproteobacteria (122	Proteobacteria (2)	Bacteria (1)	100.00	0.00	96.30	96.00	WGS
199.867	0.1292	Campylobacter concisus (72294)	Campylobacter (213849)	Campylobacteraceae (2954	Campylobacterales (68525)	Epsilonproteobacteria (122	Proteobacteria (2)	Bacteria (1)	100.00	0.00	96.50	96.20	WGS
199.868	0.1364	Campylobacter concisus (72294)	Campylobacter (213849)	Campylobacteraceae (2954	Campylobacterales (68525)	Epsilonproteobacteria (122	Proteobacteria (2)	Bacteria (1)	100.00	0.00	96.70	96.30	WGS
199.869	0.1426	Campylobacter concisus (72294)	Campylobacter (213849)	Campylobacteraceae (2954	Campylobacterales (68525)	Epsilonproteobacteria (122	Proteobacteria (2)	Bacteria (1)	100.00	0.00	96.50	95.90	WGS
199.870	0.1384	Campylobacter concisus (72294)	Campylobacter (213849)	Campylobacteraceae (2954	Campylobacterales (68525)	Epsilonproteobacteria (122	Proteobacteria (2)	Bacteria (1)	100.00	0.00	97.10	96.00	WGS
199.871	0.1338	Campylobacter concisus (72294)	Campylobacter (213849)	Campylobacteraceae (2954	Campylobacterales (68525)	Epsilonproteobacteria (122	Proteobacteria (2)	Bacteria (1)	100.00	0.00	97.00	96.40	WGS
199.872	0.1266	Campylobacter concisus (72294)	Campylobacter (213849)	Campylobacteraceae (2954	Campylobacterales (68525)	Epsilonproteobacteria (122	Proteobacteria (2)	Bacteria (1)	100.00	0.00	96.10	95.90	WGS
199.873	0.1408	Campylobacter concisus (72294)	Campylobacter (213849)	Campylobacteraceae (2954	Campylobacterales (68525)	Epsilonproteobacteria (122	Proteobacteria (2)	Bacteria (1)	100.00	0.10	97.00	96.20	WGS
199.874	0.1268	Campylobacter concisus (72294)	Campylobacter (213849)	Campylobacteraceae (2954	Campylobacterales (68525)	Epsilonproteobacteria (122	Proteobacteria (2)	Bacteria (1)	100.00	0.00	96.90	96.60	WGS
199.875	0.1288	Campylobacter concisus (72294)	Campylobacter (213849)	Campylobacteraceae (2954	Campylobacterales (68525)	Epsilonproteobacteria (122	Proteobacteria (2)	Bacteria (1)	100.00	0.00	96.70	96.50	WGS
199.876	0.1318	Campylobacter concisus (72294)	Campylobacter (213849)	Campylobacteraceae (2954	Campylobacterales (68525)	Epsilonproteobacteria (122	Proteobacteria (2)	Bacteria (1)	100.00	0.00	96.60	96.20	WGS
199.877	0.1338	Campylobacter concisus (72294)	Campylobacter (213849)	Campylobacteraceae (2954	Campylobacterales (68525)	Epsilonproteobacteria (122	Proteobacteria (2)	Bacteria (1)	100.00	0.00	96.50	96.20	WGS
199.878	0.133	Campylobacter concisus (72294)	Campylobacter (213849)	Campylobacteraceae (2954	Campylobacterales (68525)	Epsilonproteobacteria (122	Proteobacteria (2)	Bacteria (1)	99.70	0.00	97.00	96.30	WGS
199.879	0.1338	Campylobacter concisus (72294)	Campylobacter (213849)	Campylobacteraceae (2954	Campylobacterales (68525)	Epsilonproteobacteria (122	Proteobacteria (2)	Bacteria (1)	100.00	0.00	96.70	96.20	WGS
199.880	0.1318	Campylobacter concisus (72294)	Campylobacter (213849)	Campylobacteraceae (2954	Campylobacterales (68525)	Epsilonproteobacteria (122	Proteobacteria (2)	Bacteria (1)	100.00	0.00	96.70	96.20	WGS
199.881	0.126	Campylobacter concisus (72294)	Campylobacter (213849)	Campylobacteraceae (2954	Campylobacterales (68525)	Epsilonproteobacteria (122	Proteobacteria (2)	Bacteria (1)	100.00	0.00	96.80	96.50	WGS
199.882	0.1338	Campylobacter concisus (72294)	Campylobacter (213849)	Campylobacteraceae (2954	Campylobacterales (68525)	Epsilonproteobacteria (122	Proteobacteria (2)	Bacteria (1)	100.00	0.00	96.70	96.20	WGS
199.883	0.1318	Campylobacter concisus (72294)	Campylobacter (213849)	Campylobacteraceae (2954	Campylobacterales (68525)	Epsilonproteobacteria (122	Proteobacteria (2)	Bacteria (1)	100.00	0.00	96.70	96.10	WGS
199.884	0.126	Campylobacter concisus (72294)	Campylobacter (213849)	Campylobacteraceae (2954	Campylobacterales (68525)	Epsilonproteobacteria (122	Proteobacteria (2)	Bacteria (1)	100.00	0.00	96.70	96.30	WGS
199.885	0.1318	Campylobacter concisus (72294)	Campylobacter (213849)	Campylobacteraceae (2954	Campylobacterales (68525)	Epsilonproteobacteria (122	Proteobacteria (2)	Bacteria (1)	100.00	0.00	96.70	96.10	WGS
199.886	0.131	Campylobacter concisus (72294)	Campylobacter (213849)	Campylobacteraceae (2954	Campylobacterales (68525)	Epsilonproteobacteria (122	Proteobacteria (2)	Bacteria (1)	100.00	0.00	96.40	96.10	WGS
199.887	0.143	Campylobacter concisus (72294)	Campylobacter (213849)	Campylobacteraceae (2954	Campylobacterales (68525)	Epsilonproteobacteria (122	Proteobacteria (2)	Bacteria (1)	100.00	0.00	96.40	95.60	WGS
199.888	0.129	Campylobacter concisus (72294)	Campylobacter (213849)	Campylobacteraceae (2954	Campylobacterales (68525)	Epsilonproteobacteria (122	Proteobacteria (2)	Bacteria (1)	100.00	0.00	97.00	96.50	WGS
199.889	0.1318	Campylobacter concisus (72294)	Campylobacter (213849)	Campylobacteraceae (2954	Campylobacterales (68525)	Epsilonproteobacteria (122	Proteobacteria (2)	Bacteria (1)	100.00	0.00	96.60	96.10	WGS
199.890	0.1338	Campylobacter concisus (72294)	Campylobacter (213849)	Campylobacteraceae (2954	Campylobacterales (68525)	Epsilonproteobacteria (122	Proteobacteria (2)	Bacteria (1)	100.00	0.00	96.70	96.10	WGS
199.891	0.137	Campylobacter concisus (72294)	Campylobacter (213849)	Campylobacteraceae (2954	Campylobacterales (68525)	Epsilonproteobacteria (122	Proteobacteria (2)	Bacteria (1)	100.00	0.00	96.80	96.20	WGS
199.892	0.1364	Campylobacter concisus (72294)	Campylobacter (213849)	Campylobacteraceae (2954	Campylobacterales (68525)	Epsilonproteobacteria (122	Proteobacteria (2)	Bacteria (1)	100.00	0.00	97.00	96.60	WGS
199.893	0.1244	Campylobacter concisus (72294)	Campylobacter (213849)	Campylobacteraceae (2954	Campylobacterales (68525)	Epsilonproteobacteria (122	Proteobacteria (2)	Bacteria (1)	100.00	0.00	96.80	96.50	WGS
199.894	0.125	Campylobacter concisus (72294)	Campylobacter (213849)	Campylobacteraceae (2954	Campylobacterales (68525)	Epsilonproteobacteria (122	Proteobacteria (2)	Bacteria (1)	100.00	0.00	96.20	95.90	WGS
199.895	0.1284	Campylobacter concisus (72294)	Campylobacter (213849)	Campylobacteraceae (2954	Campylobacterales (68525)	Epsilonproteobacteria (122	Proteobacteria (2)	Bacteria (1)	100.00	0.00	96.50	96.20	WGS
199.896	0.1318	Campylobacter concisus (72294)	Campylobacter (213849)	Campylobacteraceae (2954	Campylobacterales (68525)	Epsilonproteobacteria (122	Proteobacteria (2)	Bacteria (1)	100.00	0.00	96.70	95.90	WGS
199.897	0.123	Campylobacter concisus (72294)	Campylobacter (213849)	Campylobacteraceae (2954	Campylobacterales (68525)	Epsilonproteobacteria (122	Proteobacteria (2)	Bacteria (1)	100.00	0.00	96.50	96.20	WGS
199.898	0.1318	Campylobacter concisus (72294)	Campylobacter (213849)	Campylobacteraceae (2954	Campylobacterales (68525)	Epsilonproteobacteria (122	Proteobacteria (2)	Bacteria (1)	100.00	0.00	96.70	96.20	WGS
199.899	0.1318	Campylobacter concisus (72294)	Campylobacter (213849)	Campylobacteraceae (2954	Campylobacterales (68525)	Epsilonproteobacteria (122	Proteobacteria (2)	Bacteria (1)	100.00	0.00	96.50	95.90	WGS
199.900	0.1252	Campylobacter concisus (72294)	Campylobacter (213849)	Campylobacteraceae (2954	Campylobacterales (68525)	Epsilonproteobacteria (122	Proteobacteria (2)	Bacteria (1)	100.00	0.00	96.50	96.00	WGS
199.901	0.1318	Campylobacter concisus (72294)	Campylobacter (213849)	Campylobacteraceae (2954	Campylobacterales (68525)	Epsilonproteobacteria (122	Proteobacteria (2)	Bacteria (1)	100.00	0.00	96.50	95.70	Complete
201.18	0.0984	Campylobacter lari (72294)	Campylobacter (213849)	Campylobacteraceae (2954	Campylobacterales (68525)	Epsilonproteobacteria (122	Proteobacteria (2)	Bacteria (1)	95.70	0.00	98.20	98.00	Complete
201.26	0.1022	Campylobacter lari (72294)	Campylobacter (213849)	Campylobacteraceae (2954	Campylobacterales (68525)	Epsilonproteobacteria (122	Proteobacteria (2)	Bacteria (1)	94.80	0.00	98.60	98.40	WGS
201.27	0.1022	Campylobacter lari (72294)	Campylobacter (213849)	Campylobacteraceae (2954	Campylobacterales (68525)	Epsilonproteobacteria (122	Proteobacteria (2)	Bacteria (1)	94.80	0.00	98.60	98.40	WGS
201.28	0.1022	Campylobacter lari (72294)	Campylobacter (213849)	Campylobacteraceae (2954	Campylobacterales (68525)	Epsilonproteobacteria (122	Proteobacteria (2)	Bacteria (1)	94.80	0.00	98.60	98.40	WGS
201.29	0.0294	Campylobacter lari (72294)	Campylobacter (213849)	Campylobacteraceae (2954	Campylobacterales (68525)	Epsilonproteobacteria (122	Proteobacteria (2)	Bacteria (1)					Plasmid
201.30	0.0294	Campylobacter lari (72294)	Campylobacter (213849)	Campylobacteraceae (2954	Campylobacterales (68525)	Epsilonproteobacteria (122	Proteobacteria (2)	Bacteria (1)					Plasmid
201.32	0.1008	Campylobacter lari (72294)	Campylobacter (213849)	Campylobacteraceae (2954	Campylobacterales (68525)	Epsilonproteobacteria (122	Proteobacteria (2)	Bacteria (1)	95.70	4.70	98.40	98.10	WGS
201.33	0.1012	Campylobacter lari (72294)	Campylobacter (213849)	Campylobacteraceae (2954	Campylobacterales (68525)	Epsilonproteobacteria (122	Proteobacteria (2)	Bacteria (1)	94.80	0.00	98.70	98.70	WGS
201.34	0.0984	Campylobacter lari (72294)	Campylobacter (213849)	Campylobacteraceae (2954	Campylobacterales (68525)	Epsilonproteobacteria (122	Proteobacteria (2)	Bacteria (1)	95.70	0.00	98.60	98.40	WGS
201.35	0.0812	Campylobacter lari (72294)	Campylobacter (213849)	Campylobacteraceae (2954	Campylobacterales (68525)	Epsilonproteobacteria (122	Proteobacteria (2)	Bacteria (1)			99.50	99.30	WGS
201.36	0.1046	Campylobacter lari (72294)	Campylobacter (213849)	Campylobacteraceae (2954	Campylobacterales (68525)	Epsilonproteobacteria (122	Proteobacteria (2)	Bacteria (1)			98.70	98.40	Complete
201.55	0.0916	Campylobacter lari (72294)	Campylobacter (213849)	Campylobacteraceae (2954	Campylobacterales (68525)	Epsilonproteobacteria (122	Proteobacteria (2)	Bacteria (1)			98.60	98.30	WGS
202.4	0.1202	Campylobacter mucosalis (72294)	Campylobacter (213849)	Campylobacteraceae (2954	Campylobacterales (68525)	Epsilonproteobacteria (122	Proteobacteria (2)	Bacteria (1)	96.50	9.70	96.70	94.10	WGS
203.53	0.1052	Campylobacter rectus (72294)	Campylobacter (213849)	Campylobacteraceae (2954	Campylobacterales (68525)	Epsilonproteobacteria (122	Proteobacteria (2)	Bacteria (1)			97.70	97.00	WGS
204.123	0.143	Campylobacter showae (72294)	Campylobacter (213849)	Campylobacteraceae (2954	Campylobacterales (68525)	Epsilonproteobacteria (122	Proteobacteria (2)	Bacteria (1)			97.70	97.00	Complete
204.16	0.145	Campylobacter showae (72294)	Campylobacter (213849)	Campylobacteraceae (2954	Campylobacterales (68525)	Epsilonproteobacteria (122	Proteobacteria (2)	Bacteria (1)			97.70	96.90	WGS
204.17	0.143	Campylobacter showae (72294)	Campylobacter (213849)	Campylobacteraceae (2954	Campylobacterales (68525)	Epsilonproteobacteria (122	Proteobacteria (2)	Bacteria (1)			97.70	97.20	WGS
204.18	0.143	Campylobacter showae (72294)	Campylobacter (213849)	Campylobacteraceae (2954	Campylobacterales (68525)	Epsilonproteobacteria (122	Proteobacteria (2)	Bacteria (1)			97.60	97.00	WGS
204.19	0.141	Campylobacter showae (72294)	Campylobacter (213849)	Campylobacteraceae (2954	Campylobacterales (68525)	Epsilonproteobacteria (122	Proteobacteria (2)	Bacteria (1)			98.00	92.30	WGS
204.20	0.141	Campylobacter showae (72294)	Campylobacter (213849)	Campylobacteraceae (2954	Campylobacterales (68525)	Epsilonproteobacteria (122	Proteobacteria (2)	Bacteria (1)			98.10	97.50	WGS
204.21	0.1494	Campylobacter showae (72294)	Campylobacter (213849)	Campylobacteraceae (2954	Campylobacterales (68525)	Epsilonproteobacteria (122	Proteobacteria (2)	Bacteria (1)			97.30	96.10	WGS
204.22	0.141	Campylobacter showae (72294)	Campylobacter (213849)	Campylobacteraceae (2954	Campylobacterales (68525)	Epsilonproteobacteria (122	Proteobacteria (2)	Bacteria (1)			98.10	97.50	WGS
205.10	0.131	Campylobacter sp. (72294)	Campylobacter (213849)	Campylobacteraceae (2954	Campylobacterales (68525)	Epsilonproteobacteria (122	Proteobacteria (2)	Bacteria (1)			87.90	86.40	WGS
205.11	0.1384	Campylobacter sp. (72294)	Campylobacter (213849)	Campylobacteraceae (2954	Campylobacterales (68525)	Epsilonproteobacteria (122	Proteobacteria (2)	Bacteria (1)			94.00	77.20	WGS
206.3	0.1026	Campylobacter sputorum (72294)	Campylobacter (213849)	Campylobacteraceae (2954	Campylobacterales (68525)	Epsilonproteobacteria (122	Proteobacteria (2)	Bacteria (1)	98.20	0.90	96.70	96.70	Complete
210.1338	0.091	Helicobacter pylori (72293)	Helicobacter (213849)	Helicobacteraceae (29547)	Campylobacterales (68525)	Epsilonproteobacteria (122	Proteobacteria (2)	Bacteria (1)	99.60	6.10	99.50	96.50	WGS
210.1339	0.092	Helicobacter pylori (72293)	Helicobacter (213849)	Helicobacteraceae (29547)	Campylobacterales (68525)	Epsilonproteobacteria (122	Proteobacteria (2)	Bacteria (1)	100.00	0.00	99.70	99.30	WGS
210.1340	0.092	Helicobacter pylori (72293)	Helicobacter (213849)	Helicobacteraceae (29547)	Campylobacterales (68525)	Epsilonproteobacteria (122	Proteobacteria (2)	Bacteria (1)	100.00	11.60	99.60	95.00	WGS
210.1341	0.092	Helicobacter pylori (72293)	Helicobacter (213849)	Helicobacteraceae (29547)	Campylobacterales (68525)	Epsilonproteobacteria (122	Proteobacteria (2)	Bacteria (1)	100.00	0.00	99.70	99.30	WGS
210.1342	0.0814	Helicobacter pylori (72293)	Helicobacter (213849)	Helicobacteraceae (29547)	Campylobacterales (68525)	Epsilonproteobacteria (122	Proteobacteria (2)	Bacteria (1)	100.00	0.00	99.90	99.60	WGS
210.1343	0.0872	Helicobacter pylori (72293)	Helicobacter (213849)	Helicobacteraceae (29547)	Campylobacterales (68525)	Epsilonproteobacteria (122	Proteobacteria (2)	Bacteria (1)	100.00	0.00	99.80	99.60	WGS
210.1344	0.0948	Helicobacter pylori (72293)	Helicobacter (213849)	Helicobacteraceae (29547)	Campylobacterales (68525)	Epsilonproteobacteria (122	Proteobacteria (2)	Bacteria (1)	99.70	0.00	99.70	99.40	WGS
210.1345	0.0948	Helicobacter pylori (72293)	Helicobacter (213849)	Helicobacteraceae (29547)	Campylobacterales (68525)	Epsilonproteobacteria (122	Proteobacteria (2)	Bacteria (1)	99.40	0.30	99.60	99.00	WGS
210.1346	0.1028	Helicobacter pylori (72293)	Helicobacter (213849)	Helicobacteraceae (29547)	Campylobacterales (68525)	Epsilonproteobacteria (122	Proteobacteria (2)	Bacteria (1)	100.00	0.00	99.80	99.70	WGS
210.1347	0.0978	Helicobacter pylori (72293)	Helicobacter (213849)	Helicobacteraceae (29547)	Campylobacterales (68525)	Epsilonproteobacteria (122	Proteobacteria (2)	Bacteria (1)	100.00	0.00	99.60	99.30	WGS
210.1348	0.1016	Helicobacter pylori (72293)	Helicobacter (213849)	Helicobacteraceae (29547)	Campylobacterales (68525)	Epsilonproteobacteria (122	Proteobacteria (2)	Bacteria (1)	99.40	0.00	99.50	99.30	WGS
210.1349	0.092	Helicobacter pylori (72293)	Helicobacter (213849)	Helicobacteraceae (29547)	Campylobacterales (68525)	Epsilonproteobacteria (122	Proteobacteria (2)	Bacteria (1)	100.00	0.00	99.80	99.50	WGS
210.1380	0.0864	Helicobacter pylori (72293)	Helicobacter (213849)	Helicobacteraceae (29547)	Campylobacterales (68525)	Epsilonproteobacteria (122	Proteobacteria (2)	Bacteria (1)	100.00	0.00	99.80	99.40	Complete
210.1381	0.0988	Helicobacter pylori (72293)	Helicobacter (213849)	Helicobacteraceae (29547)	Campylobacterales (68525)	Epsilonproteobacteria (122	Proteobacteria (2)	Bacteria (1)	100.00	0.00	99.80	99.40	Complete
210.1916	0.0948	Helicobacter pylori (72293)	Helicobacter (213849)	Helicobacteraceae (29547)	Campylobacterales (68525)	Epsilonproteobacteria (122	Proteobacteria (2)	Bacteria (1)	100.00	0.00	99.90	99.50	Complete
210.1917	0.0978	Helicobacter pylori (72293)	Helicobacter (213849)	Helicobacteraceae (29547)	Campylobacterales (68525)	Epsilonproteobacteria (122	Proteobacteria (2)	Bacteria (1)	100.00	0.30	99.90	99.60	Complete
210.1918	0.0948	Helicobacter pylori (72293)	Helicobacter (213849)	Helicobacteraceae (29547)	Campylobacterales (68525)	Epsilonproteobacteria (122	Proteobacteria (2)	Bacteria (1)	100.00	0.00	99.90	99.60	Complete
210.1919	0.0938	Helicobacter pylori (72293)	Helicobacter (213849)	Helicobacteraceae (29547)	Campylobacterales (68525)	Epsilonproteobacteria (122	Proteobacteria (2)	Bacteria (1)	99.70	0.00	99.60	99.10	WGS
210.1920	0.0938	Helicobacter pylori (72293)	Helicobacter (213849)	Helicobacteraceae (29547)	Campylobacterales (68525)	Epsilonproteobacteria (122	Proteobacteria (2)	Bacteria (1)	100.00	0.60	99.70	99.50	WGS
210.1921	0.0938	Helicobacter pylori (72293)	Helicobacter (213849)	Helicobacteraceae (29547)	Campylobacterales (68525)	Epsilonproteobacteria (122	Proteobacteria (2)	Bacteria (1)	100.00	0.00	99.70	99.30	WGS
210.1922	0.0938	Helicobacter pylori (72293)	Helicobacter (213849)	Helicobacteraceae (29547)	Campylobacterales (68525)	Epsilonproteobacteria (122	Proteobacteria (2)	Bacteria (1)	100.00	0.00	99.70	99.30	WGS
210.1923	0.0938	Helicobacter pylori (72293)	Helicobacter (213849)	Helicobacteraceae (29547)	Campylobacterales (68525)	Epsilonproteobacteria (122	Proteobacteria (2)	Bacteria (1)	100.00	0.00	99.70	99.10	WGS
210.1924	0.0938	Helicobacter pylori (72293)	Helicobacter (213849)	Helicobacteraceae (29547)	Campylobacterales (68525)	Epsilonproteobacteria (122	Proteobacteria (2)	Bacteria (1)	100.00	0.00	99.70	99.30	WGS
210.1925	0.0938	Helicobacter pylori (72293)	Helicobacter (213849)	Helicobacteraceae (29547)	Campylobacterales (68525)	Epsilonproteobacteria (122	Proteobacteria (2)	Bacteria (1)	100.00	0.00	99.60	99.30	WGS
210.1926	0.0938	Helicobacter pylori (72293)	Helicobacter (213849)	Helicobacteraceae (29547)	Campylobacterales (68525)	Epsilonproteobacteria (122	Proteobacteria (2)	Bacteria (1)	100.00	0.00	99.70	99.30	WGS

210.1927	0.0938	Helicobacter pylori (72293)	Helicobacter (213849)	Helicobacteraceae (29547)	Campylobacterales (68525)	Epsilonproteobacteria (122	Proteobacteria (2)	Bacteria (1)	100.00	0.00	99.70	99.40	WGS
210.1928	0.0938	Helicobacter pylori (72293)	Helicobacter (213849)	Helicobacteraceae (29547)	Campylobacterales (68525)	Epsilonproteobacteria (122	Proteobacteria (2)	Bacteria (1)	100.00	0.60	99.70	99.40	WGS
210.1929	0.0938	Helicobacter pylori (72293)	Helicobacter (213849)	Helicobacteraceae (29547)	Campylobacterales (68525)	Epsilonproteobacteria (122	Proteobacteria (2)	Bacteria (1)	100.00	0.60	99.70	99.00	WGS
210.1930	0.0938	Helicobacter pylori (72293)	Helicobacter (213849)	Helicobacteraceae (29547)	Campylobacterales (68525)	Epsilonproteobacteria (122	Proteobacteria (2)	Bacteria (1)	100.00	0.30	99.70	99.40	WGS
210.1931	0.0988	Helicobacter pylori (72293)	Helicobacter (213849)	Helicobacteraceae (29547)	Campylobacterales (68525)	Epsilonproteobacteria (122	Proteobacteria (2)	Bacteria (1)	100.00	0.60	99.80	99.50	WGS
210.1932	0.0622	Helicobacter pylori (72293)	Helicobacter (213849)	Helicobacteraceae (29547)	Campylobacterales (68525)	Epsilonproteobacteria (122	Proteobacteria (2)	Bacteria (1)	100.00	0.30	99.90	99.50	WGS
210.1933	0.0622	Helicobacter pylori (72293)	Helicobacter (213849)	Helicobacteraceae (29547)	Campylobacterales (68525)	Epsilonproteobacteria (122	Proteobacteria (2)	Bacteria (1)	100.00	0.30	99.90	99.70	WGS
210.1934	0.1016	Helicobacter pylori (72293)	Helicobacter (213849)	Helicobacteraceae (29547)	Campylobacterales (68525)	Epsilonproteobacteria (122	Proteobacteria (2)	Bacteria (1)	100.00	0.60	99.80	99.40	WGS
210.1935	0.1028	Helicobacter pylori (72293)	Helicobacter (213849)	Helicobacteraceae (29547)	Campylobacterales (68525)	Epsilonproteobacteria (122	Proteobacteria (2)	Bacteria (1)	100.00	0.00	99.90	99.60	WGS
210.1936	0.092	Helicobacter pylori (72293)	Helicobacter (213849)	Helicobacteraceae (29547)	Campylobacterales (68525)	Epsilonproteobacteria (122	Proteobacteria (2)	Bacteria (1)	100.00	0.90	99.90	99.30	WGS
210.1937	0.0938	Helicobacter pylori (72293)	Helicobacter (213849)	Helicobacteraceae (29547)	Campylobacterales (68525)	Epsilonproteobacteria (122	Proteobacteria (2)	Bacteria (1)	100.00	0.00	99.90	99.30	WGS
210.1938	0.0938	Helicobacter pylori (72293)	Helicobacter (213849)	Helicobacteraceae (29547)	Campylobacterales (68525)	Epsilonproteobacteria (122	Proteobacteria (2)	Bacteria (1)	100.00	0.00	99.70	99.30	WGS
210.1940	0.0834	Helicobacter pylori (72293)	Helicobacter (213849)	Helicobacteraceae (29547)	Campylobacterales (68525)	Epsilonproteobacteria (122	Proteobacteria (2)	Bacteria (1)	100.00	4.00	99.90	98.50	WGS
210.1941	0.1016	Helicobacter pylori (72293)	Helicobacter (213849)	Helicobacteraceae (29547)	Campylobacterales (68525)	Epsilonproteobacteria (122	Proteobacteria (2)	Bacteria (1)	100.00	0.30	99.80	99.30	WGS
210.1942	0.091	Helicobacter pylori (72293)	Helicobacter (213849)	Helicobacteraceae (29547)	Campylobacterales (68525)	Epsilonproteobacteria (122	Proteobacteria (2)	Bacteria (1)	99.70	0.30	99.90	99.60	WGS
210.1943	0.091	Helicobacter pylori (72293)	Helicobacter (213849)	Helicobacteraceae (29547)	Campylobacterales (68525)	Epsilonproteobacteria (122	Proteobacteria (2)	Bacteria (1)	100.00	0.30	99.90	99.60	WGS
210.1944	0.09	Helicobacter pylori (72293)	Helicobacter (213849)	Helicobacteraceae (29547)	Campylobacterales (68525)	Epsilonproteobacteria (122	Proteobacteria (2)	Bacteria (1)	100.00	0.60	99.90	99.40	WGS
210.1945	0.0988	Helicobacter pylori (72293)	Helicobacter (213849)	Helicobacteraceae (29547)	Campylobacterales (68525)	Epsilonproteobacteria (122	Proteobacteria (2)	Bacteria (1)	100.00	0.30	99.70	99.40	WGS
210.1946	0.0948	Helicobacter pylori (72293)	Helicobacter (213849)	Helicobacteraceae (29547)	Campylobacterales (68525)	Epsilonproteobacteria (122	Proteobacteria (2)	Bacteria (1)	100.00	0.30	99.90	99.70	WGS
210.1947	0.0938	Helicobacter pylori (72293)	Helicobacter (213849)	Helicobacteraceae (29547)	Campylobacterales (68525)	Epsilonproteobacteria (122	Proteobacteria (2)	Bacteria (1)	100.00	0.00	99.90	99.70	WGS
210.1948	0.0948	Helicobacter pylori (72293)	Helicobacter (213849)	Helicobacteraceae (29547)	Campylobacterales (68525)	Epsilonproteobacteria (122	Proteobacteria (2)	Bacteria (1)	100.00	1.90	99.90	99.00	WGS
210.1949	0.0864	Helicobacter pylori (72293)	Helicobacter (213849)	Helicobacteraceae (29547)	Campylobacterales (68525)	Epsilonproteobacteria (122	Proteobacteria (2)	Bacteria (1)	85.40	0.70	95.10	94.80	WGS
210.1950	0.1006	Helicobacter pylori (72293)	Helicobacter (213849)	Helicobacteraceae (29547)	Campylobacterales (68525)	Epsilonproteobacteria (122	Proteobacteria (2)	Bacteria (1)	100.00	0.60	99.90	99.60	WGS
210.1951	0.0978	Helicobacter pylori (72293)	Helicobacter (213849)	Helicobacteraceae (29547)	Campylobacterales (68525)	Epsilonproteobacteria (122	Proteobacteria (2)	Bacteria (1)	100.00	0.30	99.90	99.50	WGS
210.1952	0.091	Helicobacter pylori (72293)	Helicobacter (213849)	Helicobacteraceae (29547)	Campylobacterales (68525)	Epsilonproteobacteria (122	Proteobacteria (2)	Bacteria (1)	100.00	0.00	99.90	99.50	WGS
210.1953	0.092	Helicobacter pylori (72293)	Helicobacter (213849)	Helicobacteraceae (29547)	Campylobacterales (68525)	Epsilonproteobacteria (122	Proteobacteria (2)	Bacteria (1)	99.70	0.90	99.90	99.60	WGS
210.1954	0.0978	Helicobacter pylori (72293)	Helicobacter (213849)	Helicobacteraceae (29547)	Campylobacterales (68525)	Epsilonproteobacteria (122	Proteobacteria (2)	Bacteria (1)	100.00	0.30	99.90	99.60	WGS
210.1955	0.0692	Helicobacter pylori (72293)	Helicobacter (213849)	Helicobacteraceae (29547)	Campylobacterales (68525)	Epsilonproteobacteria (122	Proteobacteria (2)	Bacteria (1)	100.00	0.00	99.90	99.60	WGS
210.1956	0.0842	Helicobacter pylori (72293)	Helicobacter (213849)	Helicobacteraceae (29547)	Campylobacterales (68525)	Epsilonproteobacteria (122	Proteobacteria (2)	Bacteria (1)	100.00	0.00	99.90	99.40	WGS
210.1957	0.091	Helicobacter pylori (72293)	Helicobacter (213849)	Helicobacteraceae (29547)	Campylobacterales (68525)	Epsilonproteobacteria (122	Proteobacteria (2)	Bacteria (1)	100.00	0.00	99.90	99.40	WGS
210.1958	0.0682	Helicobacter pylori (72293)	Helicobacter (213849)	Helicobacteraceae (29547)	Campylobacterales (68525)	Epsilonproteobacteria (122	Proteobacteria (2)	Bacteria (1)	100.00	0.00	99.80	99.50	WGS
210.1959	0.1006	Helicobacter pylori (72293)	Helicobacter (213849)	Helicobacteraceae (29547)	Campylobacterales (68525)	Epsilonproteobacteria (122	Proteobacteria (2)	Bacteria (1)	100.00	2.60	99.40	98.30	WGS
210.1960	0.1006	Helicobacter pylori (72293)	Helicobacter (213849)	Helicobacteraceae (29547)	Campylobacterales (68525)	Epsilonproteobacteria (122	Proteobacteria (2)	Bacteria (1)	100.00	0.60	99.90	99.30	WGS
210.1961	0.0948	Helicobacter pylori (72293)	Helicobacter (213849)	Helicobacteraceae (29547)	Campylobacterales (68525)	Epsilonproteobacteria (122	Proteobacteria (2)	Bacteria (1)	100.00	0.00	99.90	99.60	WGS
210.1962	0.091	Helicobacter pylori (72293)	Helicobacter (213849)	Helicobacteraceae (29547)	Campylobacterales (68525)	Epsilonproteobacteria (122	Proteobacteria (2)	Bacteria (1)	100.00	0.00	99.90	99.70	WGS
210.1963	0.0948	Helicobacter pylori (72293)	Helicobacter (213849)	Helicobacteraceae (29547)	Campylobacterales (68525)	Epsilonproteobacteria (122	Proteobacteria (2)	Bacteria (1)	100.00	0.60	99.90	99.50	WGS
210.1964	0.074	Helicobacter pylori (72293)	Helicobacter (213849)	Helicobacteraceae (29547)	Campylobacterales (68525)	Epsilonproteobacteria (122	Proteobacteria (2)	Bacteria (1)	100.00	0.30	99.70	99.20	WGS
210.1965	0.1016	Helicobacter pylori (72293)	Helicobacter (213849)	Helicobacteraceae (29547)	Campylobacterales (68525)	Epsilonproteobacteria (122	Proteobacteria (2)	Bacteria (1)	100.00	0.00	99.90	99.40	WGS
210.1966	0.0988	Helicobacter pylori (72293)	Helicobacter (213849)	Helicobacteraceae (29547)	Campylobacterales (68525)	Epsilonproteobacteria (122	Proteobacteria (2)	Bacteria (1)	100.00	0.00	99.80	99.30	WGS
210.1967	0.092	Helicobacter pylori (72293)	Helicobacter (213849)	Helicobacteraceae (29547)	Campylobacterales (68525)	Epsilonproteobacteria (122	Proteobacteria (2)	Bacteria (1)	100.00	0.00	99.90	99.50	WGS
210.1969	0.092	Helicobacter pylori (72293)	Helicobacter (213849)	Helicobacteraceae (29547)	Campylobacterales (68525)	Epsilonproteobacteria (122	Proteobacteria (2)	Bacteria (1)	100.00	0.60	99.90	99.30	Complete
210.1970	0.092	Helicobacter pylori (72293)	Helicobacter (213849)	Helicobacteraceae (29547)	Campylobacterales (68525)	Epsilonproteobacteria (122	Proteobacteria (2)	Bacteria (1)	100.00	0.60	99.90	99.40	Complete
210.1972	0.0892	Helicobacter pylori (72293)	Helicobacter (213849)	Helicobacteraceae (29547)	Campylobacterales (68525)	Epsilonproteobacteria (122	Proteobacteria (2)	Bacteria (1)	100.00	12.80	99.60	94.50	WGS
210.1973	0.0892	Helicobacter pylori (72293)	Helicobacter (213849)	Helicobacteraceae (29547)	Campylobacterales (68525)	Epsilonproteobacteria (122	Proteobacteria (2)	Bacteria (1)	100.00	5.60	99.90	98.10	WGS
210.1974	0.0842	Helicobacter pylori (72293)	Helicobacter (213849)	Helicobacteraceae (29547)	Campylobacterales (68525)	Epsilonproteobacteria (122	Proteobacteria (2)	Bacteria (1)	100.00	2.50	99.90	99.10	WGS
210.1975	0.0842	Helicobacter pylori (72293)	Helicobacter (213849)	Helicobacteraceae (29547)	Campylobacterales (68525)	Epsilonproteobacteria (122	Proteobacteria (2)	Bacteria (1)	100.00	1.40	99.90	99.10	WGS
210.1976	0.091	Helicobacter pylori (72293)	Helicobacter (213849)	Helicobacteraceae (29547)	Campylobacterales (68525)	Epsilonproteobacteria (122	Proteobacteria (2)	Bacteria (1)	100.00	18.50	99.90	94.00	WGS
210.1977	0.091	Helicobacter pylori (72293)	Helicobacter (213849)	Helicobacteraceae (29547)	Campylobacterales (68525)	Epsilonproteobacteria (122	Proteobacteria (2)	Bacteria (1)	100.00	1.50	99.90	99.60	WGS
210.1978	0.0892	Helicobacter pylori (72293)	Helicobacter (213849)	Helicobacteraceae (29547)	Campylobacterales (68525)	Epsilonproteobacteria (122	Proteobacteria (2)	Bacteria (1)	100.00	10.20	99.70	97.10	WGS
210.1979	0.091	Helicobacter pylori (72293)	Helicobacter (213849)	Helicobacteraceae (29547)	Campylobacterales (68525)	Epsilonproteobacteria (122	Proteobacteria (2)	Bacteria (1)	100.00	0.90	99.90	99.40	WGS
210.1980	0.0842	Helicobacter pylori (72293)	Helicobacter (213849)	Helicobacteraceae (29547)	Campylobacterales (68525)	Epsilonproteobacteria (122	Proteobacteria (2)	Bacteria (1)	100.00	2.20	99.90	99.10	WGS
210.2016	0.0714	Helicobacter pylori (72293)	Helicobacter (213849)	Helicobacteraceae (29547)	Campylobacterales (68525)	Epsilonproteobacteria (122	Proteobacteria (2)	Bacteria (1)	100.00	4.50	99.70	98.60	WGS
210.2017	0.074	Helicobacter pylori (72293)	Helicobacter (213849)	Helicobacteraceae (29547)	Campylobacterales (68525)	Epsilonproteobacteria (122	Proteobacteria (2)	Bacteria (1)	100.00	2.60	99.70	98.50	WGS
210.2018	0.0938	Helicobacter pylori (72293)	Helicobacter (213849)	Helicobacteraceae (29547)	Campylobacterales (68525)	Epsilonproteobacteria (122	Proteobacteria (2)	Bacteria (1)	100.00	0.80	99.90	98.70	WGS
210.2027	0.091	Helicobacter pylori (72293)	Helicobacter (213849)	Helicobacteraceae (29547)	Campylobacterales (68525)	Epsilonproteobacteria (122	Proteobacteria (2)	Bacteria (1)	100.00	4.20	99.90	99.20	WGS
210.2065	0.0978	Helicobacter pylori (72293)	Helicobacter (213849)	Helicobacteraceae (29547)	Campylobacterales (68525)	Epsilonproteobacteria (122	Proteobacteria (2)	Bacteria (1)	100.00	2.60	100.00	99.00	WGS
210.2066	0.0978	Helicobacter pylori (72293)	Helicobacter (213849)	Helicobacteraceae (29547)	Campylobacterales (68525)	Epsilonproteobacteria (122	Proteobacteria (2)	Bacteria (1)	100.00	0.70	100.00	99.50	WGS
210.2067	0.0938	Helicobacter pylori (72293)	Helicobacter (213849)	Helicobacteraceae (29547)	Campylobacterales (68525)	Epsilonproteobacteria (122	Proteobacteria (2)	Bacteria (1)	100.00	0.70	99.90	98.80	WGS
210.2068	0.0638	Helicobacter pylori (72293)	Helicobacter (213849)	Helicobacteraceae (29547)	Campylobacterales (68525)	Epsilonproteobacteria (122	Proteobacteria (2)	Bacteria (1)	100.00	0.60	99.80	99.30	WGS
210.2069	0.0638	Helicobacter pylori (72293)	Helicobacter (213849)	Helicobacteraceae (29547)	Campylobacterales (68525)	Epsilonproteobacteria (122	Proteobacteria (2)	Bacteria (1)	100.00	2.30	99.80	99.00	WGS
210.2070	0.0656	Helicobacter pylori (72293)	Helicobacter (213849)	Helicobacteraceae (29547)	Campylobacterales (68525)	Epsilonproteobacteria (122	Proteobacteria (2)	Bacteria (1)	100.00	1.40	99.70	98.00	Complete
210.2071	0.0752	Helicobacter pylori (72293)	Helicobacter (213849)	Helicobacteraceae (29547)	Campylobacterales (68525)	Epsilonproteobacteria (122	Proteobacteria (2)	Bacteria (1)	100.00	9.60	99.80	95.50	Complete
210.2072	0.0814	Helicobacter pylori (72293)	Helicobacter (213849)	Helicobacteraceae (29547)	Campylobacterales (68525)	Epsilonproteobacteria (122	Proteobacteria (2)	Bacteria (1)	100.00	2.90	99.80	97.30	Complete
210.2437	0.1016	Helicobacter pylori (72293)	Helicobacter (213849)	Helicobacteraceae (29547)	Campylobacterales (68525)	Epsilonproteobacteria (122	Proteobacteria (2)	Bacteria (1)	100.00	0.00	99.90	99.30	Complete
210.2438	0.0656	Helicobacter pylori (72293)	Helicobacter (213849)	Helicobacteraceae (29547)	Campylobacterales (68525)	Epsilonproteobacteria (122	Proteobacteria (2)	Bacteria (1)	99.70	0.00	99.90	99.50	Complete
210.2439	0.0948	Helicobacter pylori (72293)	Helicobacter (213849)	Helicobacteraceae (29547)	Campylobacterales (68525)	Epsilonproteobacteria (122	Proteobacteria (2)	Bacteria (1)	100.00	0.90	99.80	99.10	Complete
210.2440	0.1016	Helicobacter pylori (72293)	Helicobacter (213849)	Helicobacteraceae (29547)	Campylobacterales (68525)	Epsilonproteobacteria (122	Proteobacteria (2)	Bacteria (1)	100.00	0.00	99.90	99.40	Complete
210.2441	0.0948	Helicobacter pylori (72293)	Helicobacter (213849)	Helicobacteraceae (29547)	Campylobacterales (68525)	Epsilonproteobacteria (122	Proteobacteria (2)	Bacteria (1)	100.00	0.90	99.90	99.50	Complete
210.2442	0.0806	Helicobacter pylori (72293)	Helicobacter (213849)	Helicobacteraceae (29547)	Campylobacterales (68525)	Epsilonproteobacteria (122	Proteobacteria (2)	Bacteria (1)	99.70	0.60	99.90	99.80	Complete
210.2443	0.0988	Helicobacter pylori (72293)	Helicobacter (213849)	Helicobacteraceae (29547)	Campylobacterales (68525)	Epsilonproteobacteria (122	Proteobacteria (2)	Bacteria (1)	100.00	0.30	99.90	99.60	WGS
210.2444	0.0988	Helicobacter pylori (72293)	Helicobacter (213849)	Helicobacteraceae (29547)	Campylobacterales (68525)	Epsilonproteobacteria (122	Proteobacteria (2)	Bacteria (1)	100.00	1.90	99.90	99.00	WGS
210.2445	0.0988	Helicobacter pylori (72293)	Helicobacter (213849)	Helicobacteraceae (29547)	Campylobacterales (68525)	Epsilonproteobacteria (122	Proteobacteria (2)	Bacteria (1)	100.00	2.00	99.90	98.90	WGS
210.2446	0.0988	Helicobacter pylori (72293)	Helicobacter (213849)	Helicobacteraceae (29547)	Campylobacterales (68525)	Epsilonproteobacteria (122	Proteobacteria (2)	Bacteria (1)	100.00	3.10	99.90	98.70	WGS
210.2447	0.0988	Helicobacter pylori (72293)	Helicobacter (213849)	Helicobacteraceae (29547)	Campylobacterales (68525)	Epsilonproteobacteria (122	Proteobacteria (2)	Bacteria (1)	100.00	0.00	99.90	99.60	WGS
210.2448	0.0988	Helicobacter pylori (72293)	Helicobacter (213849)	Helicobacteraceae (29547)	Campylobacterales (68525)	Epsilonproteobacteria (122	Proteobacteria (2)	Bacteria (1)	100.00	1.20	99.90	99.30	WGS
210.2449	0.0988	Helicobacter pylori (72293)	Helicobacter (213849)	Helicobacteraceae (29547)	Campylobacterales (68525)	Epsilonproteobacteria (122	Proteobacteria (2)	Bacteria (1)	100.00	0.00	99.90	99.40	WGS
210.2450	0.0988	Helicobacter pylori (72293)	Helicobacter (213849)	Helicobacteraceae (29547)	Campylobacterales (68525)	Epsilonproteobacteria (122	Proteobacteria (2)	Bacteria (1)	100.00	0.00	99.90	99.50	WGS
210.2451	0.0988	Helicobacter pylori (72293)	Helicobacter (213849)	Helicobacteraceae (29547)	Campylobacterales (68525)	Epsilonproteobacteria (122	Proteobacteria (2)	Bacteria (1)	100.00	1.00	99.90	99.00	WGS
210.2452	0.0988	Helicobacter pylori (72293)	Helicobacter (213849)	Helicobacteraceae (29547)	Campylobacterales (68525)	Epsilonproteobacteria (122	Proteobacteria (2)	Bacteria (1)	100.00	1.00	99.90	98.70	WGS
210.2453	0.0988	Helicobacter pylori (72293)	Helicobacter (213849)	Helicobacteraceae (29547)	Campylobacterales (68525)	Epsilonproteobacteria (122	Proteobacteria (2)	Bacteria (1)	100.00	0.00	99.90	99.60	WGS
210.2454	0.0988	Helicobacter pylori (72293)	Helicobacter (213849)	Helicobacteraceae (29547)	Campylobacterales (68525)	Epsilonproteobacteria (122	Proteobacteria (2)	Bacteria (1)	100.00	0.00	99.90	99.60	WGS
210.2455	0.0988	Helicobacter pylori (72293)	Helicobacter (213849)	Helicobacteraceae (29547)	Campylobacterales (68525)	Epsilonproteobacteria (122	Proteobacteria (2)	Bacteria (1)	100.00	0.00	99.90	99.60	WGS
210.2456	0.0988	Helicobacter pylori (72293)	Helicobacter (213849)	Helicobacteraceae (29547)	Campylobacterales (68525)	Epsilonproteobacteria (122	Proteobacteria (2)	Bacteria (1)	100.00	0.00	99.90	99.60	WGS
210.2457	0.0988	Helicobacter pylori (72293)	Helicobacter (213849)	Helicobacteraceae (29547)	Campylobacterales (68525)	Epsilonproteobacteria (122	Proteobacteria (2)	Bacteria (1)	100.00	0.00	99.90	99.60	WGS
210.2458	0.0988	Helicobacter pylori (72293)	Helicobacter (213849)	Helicobacteraceae (29547)	Campylobacterales (68525)	Epsilonproteobacteria (122	Proteobacteria (2)	Bacteria (1)	100.00	0.30	99.90	99.60	WGS
210.2459	0.0988	Helicobacter pylori (72293)	Helicobacter (213849)	Helicobacteraceae (29547)	Campylobacterales (68525)	Epsilonproteobacteria (122	Proteobacteria (2)	Bacteria (1)	100.00	2.00	99.90	99.20	WGS
210.2460	0.092	Helicobacter pylori (72293)	Helicobacter (213849)	Helicobacteraceae (29547)	Campylobacterales (68525)	Epsilonproteobacteria (122	Proteobacteria (2)	Bacteria (1)	100.00	0.00	99.90	99.60	WGS
210.2461	0.0988	Helicobacter pylori (72293)	Helicobacter (213849)	Helicobacteraceae (29547)	Campylobacterales (68525)	Epsilonproteobacteria (122	Proteobacteria (2)	Bacteria (1)	100.00	0.10	99.90	99.60	WGS
210.2462	0.0988	Helicobacter pylori (72293)	Helicobacter (213849)	Helicobacteraceae (29547)	Campylobacterales (68525)	Epsilonproteobacteria (122	Proteobacteria (2)	Bacteria (1)	100.00	0.90	99.90	99.60	WGS
210.2463	0.0988	Helicobacter pylori (72293)	Helicobacter (213849)	Helicobacteraceae (29547)	Campylobacterales (68525)	Epsilonproteobacteria (122	Proteobacteria (2)	Bacteria (1)	100.00	0.00	99.90	99.60	WGS
210.2464	0.0988	Helicobacter pylori (72293)	Helicobacter (213849)	Helicobacteraceae (29547)	Campylobacterales (68525)	Epsilonproteobacteria (122	Proteobacteria (2)	Bacteria (1)	100.00	0.90	99.90	99.30	WGS
210.2465	0.0988	Helicobacter pylori (72293)	Helicobacter (213849)	Helicobacteraceae (29547)	Campylobacterales (68525)	Epsilonproteobacteria (122	Proteobacteria (2)	Bacteria (1)	100.00	0.00	99.90	99.60	WGS
210.2466	0.0988	Helicobacter pylori (72293)	Helicobacter (213849)	Helicobacteraceae (29547)	Campylobacterales (68525)	Epsilonproteobacteria (122	Proteobacteria (2)	Bacteria (1)	100.00	0.30	99.90	99.50	WGS
210.2467	0.092	Helicobacter pylori (72293)	Helicobacter (213849)	Helicobacteraceae (29547)	Campylobacterales (68525)	Epsilonproteobacteria (122	Proteobacteria (2)	Bacteria (1)	100.00	0.00	99.90	99.60	WGS
210.2468	0.0988	Helicobacter pylori (72293)	Helicobacter (213849)	Helicobacteraceae (29547)	Campylobacterales (68525)	Epsilonproteobacteria (122	Proteobacteria (2)	Bacteria (1)	100.00	0.70	99.90	99.60	WGS
210.2469	0.092	Helicobacter pylori (72293)	Helicobacter (213849)	Helicobacteraceae (29547)	Campylobacterales (68525)	Epsilonproteobacteria (122	Proteobacteria (2)	Bacteria (1)	100.00	0.00	99.90	99.40	WGS
210.2470	0.0988	Helicobacter pylori (72293)	Helicobacter (213849)	Helicobacteraceae (29547)	Campylobacterales (68525)	Epsilonproteobacteria (122	Proteobacteria (2)	Bacteria (1)	100.00	0.00	99.90	99.50	WGS
210.2471	0.0988	Helicobacter pylori (72293)	Helicobacter (213849)	Helicobacteraceae (29547)	Campylobacterales (68525)	Epsilonproteobacteria (122	Proteobacteria (2)	Bacteria (1)	100.00	0.90	99.90	99.50	WGS
210.2472	0.0988	Helicobacter pylori (72293)	Helicobacter (213849)	Helicobacteraceae (29547)	Campylobacterales (68525)	Epsilonproteobacteria (122	Proteobacteria (2)	Bacteria (1)	100.00	0.00	99.90	99.50	WGS
210.2473	0.0988	Helicobacter pylori (72293)	Helicobacter (213849)	Helicobacteraceae (29547)	Campylobacterales (68525)	Epsilonproteobacteria (122	Proteobacteria (2)	Bacteria (1)	100.00	0.00	99.90	99.60	WGS
210.2474	0.092	Helicobacter pylori (72293)	Helicobacter (213849)	Helicobacteraceae (29547)	Campylobacterales (68525)	Epsilonproteobacteria (122	Proteobacteria (2)	Bacteria (1)	100.00	0.90	99.90	99.30	WGS
210.2475	0.0988	Helicobacter pylori (72293)	Helicobacter (213849)	Helicobacteraceae (29547)	Campylobacterales (68525)	Epsilonproteobacteria (122	Proteobacteria (2)	Bacteria (1)	100.00	2.30	99.80	99.10	WGS
210.2476	0.0988	Helicobacter pylori (72293)	Helicobacter (213849)	Helicobacteraceae (29547)	Campylobacterales (68525)	Epsilonproteobacteria (122	Proteobacteria (2)	Bacteria (1)	100.00	0.00	99.90	99.50	WGS
210.2477	0.0988	Helicobacter pylori (72293)	Helicobacter (213849)	Helicobacteraceae (29547)	Campylobacterales (68525)	Epsilonproteobacteria (122	Proteobacteria (2)	Bacteria (1)	100.00	0.60	99.90	99.40	WGS
210.2478	0.0988	Helicobacter pylori (72293)	Helicobacter (213849)	Helicobacteraceae (29547)	Campylobacterales (68525)	Epsilonproteobacteria (122	Proteobacteria (2)	Bacteria (1)	100.00	0.00	99.90	99.60	WGS
210.2479	0.0988	Helicobacter pylori (72293)	Helicobacter (213849)	Helicobacteraceae (29547)	Campylobacterales (68525)	Epsilonproteobacteria (122	Proteobacteria (2)	Bacteria (1)	100.00	0.30	99.90	99.30	WGS
210.2480	0.0988	Helicobacter pylori (72293)	Helicobacter (213849)	Helicobacteraceae (29547)	Campylobacterales (68525)	Epsilonproteobacteria (122	Proteobacteria (2)	Bacteria (1)	100.00	0.10	99.90	99.50	WGS
210.2481	0.0988	Helicobacter pylori (72293)	Helicobacter (213849)	Helicobacteraceae (29547)	Campylobacterales (68525)	Epsilonproteobacteria (122	Proteobacteria (2)	Bacteria (1)	100.00	0.00	99.90	99.60	WGS
210.2482	0.0988	Helicobacter pylori (72293)	Helicobacter (213849)	Helicobacteraceae (29547)	Campylobacterales (68525)	Epsilonproteobacteria (122	Proteobacteria (2)	Bacteria (1)	100.00	0.30	99.90	99.50	WGS
210.2483	0.0988	Helicobacter pylori (72293)	Helicobacter (213849)	Helicobacteraceae (29547)	Campylobacterales (68525)	Epsilonproteobacteria (122	Proteobacteria (2)	Bacteria (1)	100.00	16.90	99.90	94.20	WGS
210.2484	0.0988	Helicobacter pylori (72293)	Helicobacter (213849)	Helicobacteraceae (29547)	Campylobacterales (68525)	Epsilonproteobacteria (122	Proteobacteria (2)	Bacteria (1)	100.00	0.00	99.90	99.40	WGS
210.2485	0.0988	Helicobacter pylori (72293)	Helicobacter (213849)	Helicobacteraceae (29547)	Campylobacterales (68525)	Epsilonproteobacteria (122	Proteobacteria (2)	Bacteria (1)	100.00	0.00	99.90	99.50	WGS
210.2486	0.0988	Helicobacter pylori (72293)	Helicobacter (213849)	Helicobacteraceae (29547)	Campylobacterales (68525)	Epsilonproteobacteria (122	Proteobacteria (2)	Bacteria (1)	100.00	0.30	99.90	99.30	WGS
210.2487	0.0988	Helicobacter pylori (72293)	Helicobacter (213849)	Helicobacteraceae (29547)	Campylobacterales (68525)	Epsilonproteobacteria (122	Proteobacteria (2)	Bacteria (1)	100.00	0.60	99.90	99.40	WGS
210.2488	0.0988	Helicobacter pylori (72293)	Helicobacter (213849)	Helicobacteraceae (29547)	Campylobacterales (68525)	Epsilonproteobacteria (122	Proteobacteria (2)	Bacteria (1)	100.00	1.20	99.90	98.90	WGS
210.2489	0.0988	Helicobacter pylori (72293)	Helicobacter (213849)	Helicobacteraceae (29547)	Campylobacterales (68525)	Epsilonproteobacteria (122	Proteobacteria (2)	Bacteria (1)	100.00	0.00	99.90	99.60	WGS
210.2490	0.0988	Helicobacter pylori (72293)	Helicobacter (213849)	Helicobacteraceae (29547)	Campylobacterales (68525)	Epsilonproteobacteria (122	Proteobacteria (2)	Bacteria (1)	100.00	0.90	99.70	99.00	WGS
210.2491	0.0988	Helicobacter pylori (72293)	Helicobacter (213849)	Helicobacteraceae (29547)	Campylobacterales (68525)	Epsilonproteobacteria (122	Proteobacteria (2)	Bacteria (1)	100.00	0.60	99.90	99.50	WGS
210.2492	0.0988	Helicobacter pylori (72293)	Helicobacter (213849)	Helicobacteraceae (29547)	Campylobacterales (68525)	Epsilonproteobacteria (122	Proteobacteria (2)	Bacteria (1)	100.00	0.00	99.90	99.40	WGS
210.2493	0.0988	Helicobacter pylori (72293)	Helicobacter (213849)	Helicobacteraceae (29547)	Campylobacterales (68525)	Epsilonproteobacteria (122	Proteobacteria (2)	Bacteria (1)	100.00	0.40	99.90	99.30	WGS
210.2494	0.0988	Helicobacter pylori (72293)	Helicobacter (213849)	Helicobacteraceae (29547)	Campylobacterales (68525)	Epsilonproteobacteria (122	Proteobacteria (2)	Bacteria (1)	100.00	0.00	99.90	99.40	WGS
210.2495	0.0988	Helicobacter pylori (72293)	Helicobacter (213849)	Helicobacteraceae (29547)	Campylobacterales (68525)	Epsilonproteobacteria (122	Proteobacteria (2)	Bacteria (1)	100.00	0.00	99.90	99.40	WGS
210.2496	0.0988	Helicobacter pylori (72293)	Helicobacter (213849)	Helicobacteraceae (29547)	Campylobacterales (68525)	Epsilonproteobacteria (122	Proteobacteria (2)	Bacteria (1)	100.00	1.30	99.90	99.30	WGS
210.2497	0.0988	Helicobacter pylori (72293)	Helicobacter (213849)	Helicobacteraceae (29547)	Campylobacterales (68525)	Epsilonproteobacteria (122	Proteobacteria (2)	Bacteria (1)	100.00	0.90	99.90	99.40	WGS
210.2498	0.0988	Helicobacter pylori (72293)	Helicobacter (213849)	Helicobacteraceae (29547)	Campylobacterales (68525)	Epsilonproteobacteria (122	Proteobacteria (2)	Bacteria (1)	100.00	1.80	99.90	99.10	WGS
210.2499	0.0988	Helicobacter pylori (72293)	Helicobacter (213849)	Helicobacteraceae (29547)	Campylobacterales (68525)	Epsilonproteobacteria (122	Proteobacteria (2)	Bacteria (1)	100.00	0.60	99.90	99.30	WGS
210.2500	0.0988	Helicobacter pylori (72293)	Helicobacter (213849)	Helicobacteraceae (29547)	Campylobacterales (68525)	Epsilonproteobacteria (122	Proteobacteria (2)	Bacteria (1)	100.00	0.00	99.90	99.60	WGS
210.2501	0.0988	Helicobacter pylori (72293)	Helicobacter (213849)	Helicobacteraceae (29547)	Campylobacterales (68525)	Epsilonproteobacteria (122	Proteobacteria (2)	Bacteria (1)	100.00	0.40	99.90	99.30	WGS
210.2502	0.0988	Helicobacter pylori (72293)	Helicobacter (213849)	Helicobacteraceae (29547)	Campylobacterales (68525)	Epsilonproteobacteria (122	Proteobacteria (2)	Bacteria (1)	100.00	1.20	99.90	99.50	WGS
210.2503	0.0988	Helicobacter pylori (72293)	Helicobacter (213849)	Helicobacteraceae (29547)	Campylobacterales (68525)	Epsilonproteobacteria (122	Proteobacteria (2)	Bacteria (1)	100.00	0.00	99.80	99.40	WGS
210.2536	0.0638	Helicobacter pylori (72293)	Helicobacter (213849)	Helicobacteraceae (29547)	Campylobacterales (68525)	Epsilonproteobacteria (122	Proteobacteria (2)	Bacteria (1)	100.00	0.00	99.90	99.80	WGS
210.2549	0.0408	Helicobacter pylori (72293)	Helicobacter (213849)	Helicobacteraceae (29547)	Campylobacterales (68525)	Epsilonproteobacteria (122	Proteobacteria (2)	Bacteria (1)	99.70	0.30	99.80	99.40	WGS
210.2550	0.0806	Helicobacter pylori (72293)	Helicobacter (213849)	Helicobacteraceae (29547)	Campylobacterales (68525)	Epsilonproteobacteria (122	Proteobacteria (2)	Bacteria (1)	100.00	0.00	99.90	99.50	WGS
210.2551	0.09	Helicobacter pylori (72293)	Helicobacter (213849)	Helicobacteraceae (29547)	Campylobacterales (68525)	Epsilonproteobacteria (122	Proteobacteria (2)	Bacteria (1)	100.00	0.30	99.80	99.50	WGS
210.2552	0.0842	Helicobacter pylori (72293)	Helicobacter (213849)	Helicobacteraceae (29547)	Campylobacterales (68525)	Epsilonproteobacteria (122	Proteobacteria (2)	Bacteria (1)	100.00	0.00	99.90	99.90	WGS
210.2553	0.0612	Helicobacter pylori (72293)	Helicobacter (213849)	Helicobacteraceae (29547)	Campylobacterales (68525)	Epsilonproteobacteria (122	Proteobacteria (2)	Bacteria (1)	100.00	0.90	99.90	99.60	WGS
210.2554	0.0638	Helicobacter pylori (72293)	Helicobacter (213849)	Helicobacteraceae (29547)	Campylobacterales (68525)	Epsilonproteobacteria (122	Proteobacteria (2)	Bacteria (1)	100.00	0.60	99.90	99.70	WGS
210.2555	0.1016	Helicobacter pylori (72293)	Helicobacter (213849)	Helicobacteraceae (29547)	Campylobacterales (68525)	Epsilonproteobacteria (122	Proteobacteria (2)	Bacteria (1)	100.00	0.10	99.80	99.40	WGS
210.2556	0.091	Helicobacter pylori (72293)	Helicobacter (213849)	Helicobacteraceae (29547)	Campylobacterales (68525)	Epsilonproteobacteria (122	Proteobacteria (2)	Bacteria (1)	100.00	0.00	99.90	99.70	WGS
210.2557	0.0842	Helicobacter pylori (72293)	Helicobacter (213849)	Helicobacteraceae (29547)	Campylobacterales (68525)	Epsilonproteobacteria (122	Proteobacteria (2)	Bacteria (1)	100.00	0.00	99.90	99.90	WGS
210.2558	0.1016	Helicobacter pylori (72293)	Helicobacter (213849)	Helicobacteraceae (29547)	Campylobacterales (68525)	Epsilonproteobacteria (122	Proteobacteria (2)	Bacteria (1)	100.00	0.00	99.80	99.50	WGS
210.2559	0.09	Helicobacter pylori (72293)	Helicobacter (213849)	Helicobacteraceae (29547)	Campylobacterales (68525)	Epsilonproteobacteria (122	Proteobacteria (2)	Bacteria (1)	100.00	0.30	99.80	99.60	WGS
210.2560	0.0692	Helicobacter pylori (72293)	Helicobacter (213849)	Helicobacteraceae (29547)	Campylobacterales (68525)	Epsilonproteobacteria (122	Proteobacteria (2)	Bacteria (1)	100.00	0.00	99.90	99.70	WGS
210.2569	0.0938	Helicobacter pylori (72293)	Helicobacter (213849)	Helicobacteraceae (29547)	Campylobacterales (68525)	Epsilonproteobacteria (122	Proteobacteria (2)	Bacteria (1)	100.00	0.00	99.90	99.60	WGS
210.2570	0.0938	Helicobacter pylori (72293)	Helicobacter (213849)	Helicobacteraceae (29547)	Campylobacterales (68525)	Epsilonproteobacteria (122	Proteobacteria (2)	Bacteria (1)	100.00	0.00	99.90	99.60	WGS
210.2571	0.092	Helicobacter pylori (72293)	Helicobacter (213849)	Helicobacteraceae (29547)	Campylobacterales (68525)	Epsilonproteobacteria (122	Proteobacteria (2)	Bacteria (1)	100.00	0.00	99.80	99.40	WGS
210.2572	0.0938	Helicobacter pylori (72293)	Helicobacter (213849)	Helicobacteraceae (29547)	Campylobacterales (68525)	Epsilonproteobacteria (122	Proteobacteria (2)	Bacteria (1)	100.00	0.00	99.90	99.40	WGS
210.2573	0.091	Helicobacter pylori (72293)	Helicobacter (213849)	Helicobacteraceae (29547)	Campylobacterales (68525)	Epsilonproteobacteria (122	Proteobacteria (2)	Bacteria (1)	100.00	0.00	99.90	99.50	WGS
210.2574	0.0938	Helicobacter pylori (72293)	Helicobacter (213849)	Helicobacteraceae (29547)	Campylobacterales (68525)	Epsilonproteobacteria (122	Proteobacteria (2)	Bacteria (1)	100.00	0.60	99.90	99.40	WGS
210.2575	0.091	Helicobacter pylori (72293)	Helicobacter (213849)	Helicobacteraceae (29547)	Campylobacterales (68525)	Epsilonproteobacteria (122	Proteobacteria (2)	Bacteria (1)	100.00	10.20	99.90	95.70	WGS
210.2576	0.091	Helicobacter pylori (72293)	Helicobacter (213849)	Helicobacteraceae (29547)	Campylobacterales (68525)	Epsilonproteobacteria (122	Proteobacteria (2)	Bacteria (1)	100.00	0.00	99.80	99.30	WGS
210.2577	0.0948	Helicobacter pylori (72293)	Helicobacter (213849)	Helicobacteraceae (29547)	Campylobacterales (68525)	Epsilonproteobacteria (122	Proteobacteria (2)	Bacteria (1)	100.00	0.30	99.80	99.60	WGS
210.2578	0.0948	Helicobacter pylori (72293)	Helicobacter (213849)	Helicobacteraceae (29547)	Campylobacterales (68525)	Epsilonproteobacteria (122	Proteobacteria (2)	Bacteria (1)	100.00	0.30	99.90	99.30	WGS
210.2579	0.092	Helicobacter pylori (72293)	Helicobacter (213849)	Helicobacteraceae (29547)	Campylobacterales (68525)	Epsilonproteobacteria (122	Proteobacteria (2)	Bacteria (1)	100.00	0.30	99.90	99.30	WGS
210.2580	0.091	Helicobacter pylori (72293)	Helicobacter (213849)	Helicobacteraceae (29547)	Campylobacterales (68525)	Epsilonproteobacteria (122	Proteobacteria (2)	Bacteria (1)	100.00	1.20	99.90	99.50	WGS
210.2581	0.091	Helicobacter pylori (72293)	Helicobacter (213849)	Helicobacteraceae (29547)	Campylobacterales (68525)	Epsilonproteobacteria (122	Proteobacteria (2)	Bacteria (1)	100.00	1.20	99.90	99.30	WGS
210.2582	0.0938	Helicobacter pylori (72293)	Helicobacter (213849)	Helicobacteraceae (29547)	Campylobacterales (68525)	Epsilonproteobacteria (122	Proteobacteria (2)	Bacteria (1)	100.00	1.20	99.90	99.30	WGS
210.2583	0.091	Helicobacter pylori (72293)	Helicobacter (213849)	Helicobacteraceae (29547)	Campylobacterales (68525)	Epsilonproteobacteria (122	Proteobacteria (2)	Bacteria (1)	99.70	0.00	99.90	99.30	WGS
210.2584	0.0978	Helicobacter pylori (72293)	Helicobacter (213849)	Helicobacteraceae (29547)	Campylobacterales (68525)	Epsilonproteobacteria (122	Proteobacteria (2)	Bacteria (1)	100.00	0.30	99.90	99.30	WGS
210.2585	0.091	Helicobacter pylori (72293)	Helicobacter (213849)	Helicobacteraceae (29547)	Campylobacterales (68525)	Epsilonproteobacteria (122	Proteobacteria (2)	Bacteria (1)	100.00	0.60	99.90	99.30	WGS
210.2586	0.091	Helicobacter pylori (72293)	Helicobacter (213849)	Helicobacteraceae (29547)	Campylobacterales (68525)	Epsilonproteobacteria (122	Proteobacteria (2)	Bacteria (1)	100.00	1.70	99.90	99.10	WGS
210.2587	0.092	Helicobacter pylori (72293)	Helicobacter (213849)	Helicobacteraceae (29547)	Campylobacterales (68525)	Epsilonproteobacteria (122	Proteobacteria (2)	Bacteria (1)	100.00	2.60	99.90	99.20	WGS
210.2588	0.092	Helicobacter pylori (72293)	Helicobacter (213849)	Helicobacteraceae (29547)	Campylobacterales (68525)	Epsilonproteobacteria (122	Proteobacteria (2)	Bacteria (1)	100.00	8.20	99.90	97.50	WGS
210.2589	0.092	Helicobacter pylori (72293)	Helicobacter (213849)	Helicobacteraceae (29547)	Campylobacterales (68525)	Epsilonproteobacteria (122	Proteobacteria (2)	Bacteria (1)	100.00	1.40	99.90	99.00	WGS
210.2590	0.092	Helicobacter pylori (72293)	Helicobacter (213849)	Helicobacteraceae (29547)	Campylobacterales (68525)	Epsilonproteobacteria (122	Proteobacteria (2)	Bacteria (1)	100.00	6.30	99.90	97.60	WGS
210.2591	0.0948	Helicobacter pylori (72293)	Helicobacter (213849)	Helicobacteraceae (29547)	Campylobacterales (68525)	Epsilonproteobacteria (122	Proteobacteria (2)	Bacteria (1)	100.00	0.90	99.70	99.00	WGS
210.2592	0.0938	Helicobacter pylori (72293)	Helicobacter (213849)	Helicobacteraceae (29547)	Campylobacterales (68525)	Epsilonproteobacteria (122	Proteobacteria (2)	Bacteria (1)	100.00	5.40	99.90	97.60	WGS
210.2593	0.0948	Helicobacter pylori (72293)	Helicobacter (213849)	Helicobacteraceae (29547)	Campylobacterales (68525)	Epsilonproteobacteria (122	Proteobacteria (2)	Bacteria (1)	100.00	0.90	99.90	99.10	WGS
210.2594	0.0938	Helicobacter pylori (72293)	Helicobacter (213849)	Helicobacteraceae (29547)	Campylobacterales (68525)	Epsilonproteobacteria (122	Proteobacteria (2)	Bacteria (1)	100.00	0.60	99.90	99.50	WGS
210.2595	0.0988	Helicobacter pylori (72293)	Helicobacter (213849)	Helicobacteraceae (29547)	Campylobacterales (68525)	Epsilonproteobacteria (122	Proteobacteria (2)	Bacteria (1)	100.00	3.70	99.90	98.70	WGS
210.2596	0.0948	Helicobacter pylori (72293)	Helicobacter (213849)	Helicobacteraceae (29547)	Campylobacterales (68525)	Epsilonproteobacteria (122	Proteobacteria (2)	Bacteria (1)	100.00	0.60	99.90	98.80	WGS
210.2597	0.0294	Helicobacter pylori (72293)	Helicobacter (213849)	Helicobacteraceae (29547)	Campylobacterales (68525)	Epsilonproteobacteria (122	Proteobacteria (2)	Bacteria (1)	100.00	6.10	99.90	97.00	WGS
210.2598	0.0938	Helicobacter pylori (72293)	Helicobacter (213849)	Helicobacteraceae (29547)	Campylobacterales (68525)	Epsilonproteobacteria (122	Proteobacteria (2)	Bacteria (1)	100.00	2.30	99.80	98.00	WGS
210.2599	0.092	Helicobacter pylori (72293)	Helicobacter (213849)	Helicobacteraceae (29547)	Campylobacterales (68525)	Epsilonproteobacteria (122	Proteobacteria (2)	Bacteria (1)	100.00	0.70	99.90	99.30	WGS
210.2600	0.091	Helicobacter pylori (72293)	Helicobacter (213849)	Helicobacteraceae (29547)	Campylobacterales (68525)	Epsilonproteobacteria (122	Proteobacteria (2)	Bacteria (1)	100.00	0.30	99.90	99.50	WGS
210.2601	0.0988	Helicobacter pylori (72293)	Helicobacter (213849)	Helicobacteraceae (29547)	Campylobacterales (68525)	Epsilonproteobacteria (122	Proteobacteria (2)	Bacteria (1)	100.00	0.90	99.90	99.50	WGS
210.2602	0.092	Helicobacter pylori (72293)	Helicobacter (213849)	Helicobacteraceae (29547)	Campylobacterales (68525)	Epsilonproteobacteria (122	Proteobacteria (2)	Bacteria (1)	100.00	1.80	99.80	99.10	WGS
210.2603	0.0938	Helicobacter pylori (72293)	Helicobacter (213849)	Helicobacteraceae (29547)	Campylobacterales (68525)	Epsilonproteobacteria (122	Proteobacteria (2)	Bacteria (1)	100.00	2.40	99.90	99.30	WGS
210.2604	0.0988	Helicobacter pylori (72293)	Helicobacter (213849)	Helicobacteraceae (29547)	Campylobacterales (68525)	Epsilonproteobacteria (122	Proteobacteria (2)	Bacteria (1)	100.00	0.30	99.80	99.30	WGS
210.2605	0.091	Helicobacter pylori (72293)	Helicobacter (213849)	Helicobacteraceae (29547)	Campylobacterales (68525)	Epsilonproteobacteria (122	Proteobacteria (2)	Bacteria (1)	100.00	5.00	99.90	97.70	WGS
210.2606	0.0938	Helicobacter pylori (72293)	Helicobacter (213849)	Helicobacteraceae (29547)	Campylobacterales (68525)	Epsilonproteobacteria (122	Proteobacteria (2)	Bacteria (1)	100.00	1.50	99.90	98.60	WGS
210.2607	0.091	Helicobacter pylori (72293)	Helicobacter (213849)	Helicobacteraceae (29547)	Campylobacterales (68525)	Epsilonproteobacteria (122	Proteobacteria (2)	Bacteria (1)	100.00	0.30	99.90	99.60	WGS
210.2608	0.091	Helicobacter pylori (72293)	Helicobacter (213849)	Helicobacteraceae (29547)	Campylobacterales (68525)	Epsilonproteobacteria (122	Proteobacteria (2)	Bacteria (1)	100.00	6.50	99.90	96.30	WGS
210.2609	0.1006	Helicobacter pylori (72293)	Helicobacter (213849)	Helicobacteraceae (29547)	Campylobacterales (68525)	Epsilonproteobacteria (122	Proteobacteria (2)	Bacteria (1)	99.70	3.80	99.90	98.20	WGS
210.2610	0.1016	Helicobacter pylori (72293)	Helicobacter (213849)	Helicobacteraceae (29547)	Campylobacterales (68525)	Epsilonproteobacteria (122	Proteobacteria (2)	Bacteria (1)	100.00	0.70	99.90	99.10	WGS
210.2611	0.0988	Helicobacter pylori (72293)	Helicobacter (213849)	Helicobacteraceae (29547)	Campylobacterales (68525)	Epsilonproteobacteria (122	Proteobacteria (2)	Bacteria (1)	100.00	2.40	99.90	98.70	WGS
210.2612	0.091	Helicobacter pylori (72293)	Helicobacter (213849)	Helicobacteraceae (29547)	Campylobacterales (68525)	Epsilonproteobacteria (122	Proteobacteria (2)	Bacteria (1)	100.00	0.00	99.90	99.50	WGS
210.2613	0.0938	Helicobacter pylori (72293)	Helicobacter (213849)	Helicobacteraceae (29547)	Campylobacterales (68525)	Epsilonproteobacteria (122	Proteobacteria (2)	Bacteria (1)	100.00	0.60	99.80	99.00	WGS
210.2614	0.092	Helicobacter pylori (72293)	Helicobacter (213849)	Helicobacteraceae (29547)	Campylobacterales (68525)	Epsilonproteobacteria (122	Proteobacteria (2)	Bacteria (1)	100.00	0.00	99.80	99.40	WGS
210.2615	0.0978	Helicobacter pylori (72293)	Helicobacter (213849)	Helicobacteraceae (29547)	Campylobacterales (68525)	Epsilonproteobacteria (122	Proteobacteria (2)	Bacteria (1)	99.70	1.50	99.80	99.00	WGS
210.2616	0.0948	Helicobacter pylori (72293)	Helicobacter (213849)	Helicobacteraceae (29547)	Campylobacterales (68525)	Epsilonproteobacteria (122	Proteobacteria (2)	Bacteria (1)	100.00	0.00	99.90	99.40	WGS
210.2617	0.091	Helicobacter pylori (72293)	Helicobacter (213849)	Helicobacteraceae (29547)	Campylobacterales (68525)	Epsilonproteobacteria (122	Proteobacteria (2)	Bacteria (1)	100.00	0.20	99.90	99.00	WGS
210.2618	0.1016	Helicobacter pylori (72293)	Helicobacter (213849)	Helicobacteraceae (29547)	Campylobacterales (68525)	Epsilonproteobacteria (122	Proteobacteria (2)	Bacteria (1)	100.00	0.00	99.90	99.50	WGS
210.2619	0.092	Helicobacter pylori (72293)	Helicobacter (213849)	Helicobacteraceae (29547)	Campylobacterales (68525)	Epsilonproteobacteria (122	Proteobacteria (2)	Bacteria (1)	100.00	0.00	99.90	99.50	WGS
210.2620	0.0978	Helicobacter pylori (72293)	Helicobacter (213849)	Helicobacteraceae (29547)	Campylobacterales (68525)	Epsilonproteobacteria (122	Proteobacteria (2)	Bacteria (1)	100.00	11.00	99.70	96.60	WGS
210.2621	0.0938	Helicobacter pylori (72293)	Helicobacter (213849)	Helicobacteraceae (29547)	Campylobacterales (68525)	Epsilonproteobacteria (122	Proteobacteria (2)	Bacteria (1)	100.00	21.20	99.80	94.50	WGS
210.2622	0.0978	Helicobacter pylori (72293)	Helicobacter (213849)	Helicobacteraceae (29547)	Campylobacterales (68525)	Epsilonproteobacteria (122	Proteobacteria (2)	Bacteria (1)	100.00	1.20	99.80	98.80	WGS
210.2623	0.0988	Helicobacter pylori (72293)	Helicobacter (213849)	Helicobacteraceae (29547)	Campylobacterales (68525)	Epsilonproteobacteria (122	Proteobacteria (2)	Bacteria (1)	100.00	0.60	99.80	99.30	WGS
210.2624	0.092	Helicobacter pylori (72293)	Helicobacter (213849)	Helicobacteraceae (29547)	Campylobacterales (68525)	Epsilonproteobacteria (122	Proteobacteria (2)	Bacteria (1)	100.00	12.10	99.90	97.20	WGS
210.2625	0.0978	Helicobacter pylori (72293)	Helicobacter (213849)	Helicobacteraceae (29547)	Campylobacterales (68525)	Epsilonproteobacteria (122	Proteobacteria (2)	Bacteria (1)	100.00	0.30	99.90	99.60	WGS
210.2641	0.0938	Helicobacter pylori (72293)	Helicobacter (213849)	Helicobacteraceae (29547)	Campylobacterales (68525)	Epsilonproteobacteria (122	Proteobacteria (2)	Bacteria (1)	100.00	0.60	99.90	99.30	WGS
210.2642	0.0978	Helicobacter pylori (72293)	Helicobacter (213849)	Helicobacteraceae (29547)	Campylobacterales (68525)	Epsilonproteobacteria (122	Proteobacteria (2)	Bacteria (1)	100.00	0.00	99.90	99.30	WGS
210.2643	0.0938	Helicobacter pylori (72293)	Helicobacter (213849)	Helicobacteraceae (29547)	Campylobacterales (68525)	Epsilonproteobacteria (122	Proteobacteria (2)	Bacteria (1)	100.00	0.00	99.80	99.30	WGS
210.2644	0.092	Helicobacter pylori (72293)	Helicobacter (213849)	Helicobacteraceae (29547)	Campylobacterales (68525)	Epsilonproteobacteria (122	Proteobacteria (2)	Bacteria (1)	100.00	1.50	99.90	99.00	WGS
210.2645	0.0938	Helicobacter pylori (72293)	Helicobacter (213849)	Helicobacteraceae (29547)	Campylobacterales (68525)	Epsilonproteobacteria (122	Proteobacteria (2)	Bacteria (1)	100.00	0.90	99.90	99.10	WGS
210.2646	0.092	Helicobacter pylori (72293)	Helicobacter (213849)	Helicobacteraceae (29547)	Campylobacterales (68525)	Epsilonproteobacteria (122	Proteobacteria (2)	Bacteria (1)	100.00	0.30	99.90	99.20	WGS
210.2647	0.1006	Helicobacter pylori (72293)	Helicobacter (213849)	Helicobacteraceae (29547)	Campylobacterales (68525)	Epsilonproteobacteria (122	Proteobacteria (2)	Bacteria (1)	100.00	2.90	99.90	95.80	WGS
210.2648	0.0978	Helicobacter pylori (72293)	Helicobacter (213849)	Helicobacteraceae (29547)	Campylobacterales (68525)	Epsilonproteobacteria (122	Proteobacteria (2)	Bacteria (1)	100.00	5.10	99.60	97.50	WGS
210.2649	0.1006	Helicobacter pylori (72293)	Helicobacter (213849)	Helicobacteraceae (29547)	Campylobacterales (68525)	Epsilonproteobacteria (122	Proteobacteria (2)	Bacteria (1)	100.00	0.00	99.90	99.50	WGS
210.2650	0.1028	Helicobacter pylori (72293)	Helicobacter (213849)	Helicobacteraceae (29547)	Campylobacterales (68525)	Epsilonproteobacteria (122	Proteobacteria (2)	Bacteria (1)	100.00	11.50	99.90	96.40	WGS
210.2651	0.0988	Helicobacter pylori (72293)	Helicobacter (213849)	Helicobacteraceae (29547)	Campylobacterales (68525)	Epsilonproteobacteria (122	Proteobacteria (2)	Bacteria (1)	100.00	2.60	99.90	98.70	WGS
210.2652	0.0988	Helicobacter pylori (72293)	Helicobacter (213849)	Helicobacteraceae (29547)	Campylobacterales (68525)	Epsilonproteobacteria (122	Proteobacteria (2)	Bacteria (1)	100.00	0.30	99.90	99.50	WGS
210.2653	0.0948	Helicobacter pylori (72293)	Helicobacter (213849)	Helicobacteraceae (29547)	Campylobacterales (68525)	Epsilonproteobacteria (122	Proteobacteria (2)	Bacteria (1)	100.00	0.00	99.90	99.30	WGS
210.2654	0.1016	Helicobacter pylori (72293)	Helicobacter (213849)	Helicobacteraceae (29547)	Campylobacterales (68525)	Epsilonproteobacteria (122	Proteobacteria (2)	Bacteria (1)	100.00	0.00	99.90	99.60	WGS
210.2655	0.0948	Helicobacter pylori (72293)	Helicobacter (213849)	Helicobacteraceae (29547)	Campylobacterales (68525)	Epsilonproteobacteria (122	Proteobacteria (2)	Bacteria (1)	100.00	2.90	99.90	98.70	WGS
210.2656	0.0938	Helicobacter pylori (72293)	Helicobacter (213849)	Helicobacteraceae (29547)	Campylobacterales (68525)	Epsilonproteobacteria (122	Proteobacteria (2)	Bacteria (1)	100.00	0.90	99.90	99.60	WGS
210.2657	0.0978	Helicobacter pylori (72293)	Helicobacter (213849)	Helicobacteraceae (29547)	Campylobacterales (68525)	Epsilonproteobacteria (122	Proteobacteria (2)	Bacteria (1)	100.00	0.90	99.90	99.30	WGS
210.2658	0.0978	Helicobacter pylori (72293)	Helicobacter (213849)	Helicobacteraceae (29547)	Campylobacterales (68525)	Epsilonproteobacteria (122	Proteobacteria (2)	Bacteria (1)	100.00	0.00	99.90	99.60	WGS
210.2659	0.092	Helicobacter pylori (72293)	Helicobacter (213849)	Helicobacteraceae (29547)	Campylobacterales (68525)	Epsilonproteobacteria (122	Proteobacteria (2)	Bacteria (1)	100.00	0.60	99.90	99.40	WGS
210.2660	0.091	Helicobacter pylori (72293)	Helicobacter (213849)	Helicobacteraceae (29547)	Campylobacterales (68525)	Epsilonproteobacteria (122	Proteobacteria (2)	Bacteria (1)	100.00	0.60	99.90	99.00	WGS
210.2661	0.0938	Helicobacter pylori (72293)	Helicobacter (213849)	Helicobacteraceae (29547)	Campylobacterales (68525)	Epsilonproteobacteria (122	Proteobacteria (2)	Bacteria (1)	100.00	0.30	99.90	99.40	WGS
210.2662	0.0948	Helicobacter pylori (72293)	Helicobacter (213849)	Helicobacteraceae (29547)	Campylobacterales (68525)	Epsilonproteobacteria (122	Proteobacteria (2)	Bacteria (1)	100.00	0.00	99.90	99.30	WGS
210.2663	0.092	Helicobacter pylori (72293)	Helicobacter (213849)	Helicobacteraceae (29547)	Campylobacterales (68525)	Epsilonproteobacteria (122	Proteobacteria (2)	Bacteria (1)	100.00	0.00	99.90	99.30	WGS
210.2664	0.0938	Helicobacter pylori (72293)	Helicobacter (213849)	Helicobacteraceae (29547)	Campylobacterales (68525)	Epsilonproteobacteria (122	Proteobacteria (2)	Bacteria (1)	100.00	0.90	99.80	99.30	WGS
210.2665	0.091	Helicobacter pylori (72293)	Helicobacter (213849)	Helicobacteraceae (29547)	Campylobacterales (68525)	Epsilonproteobacteria (122	Proteobacteria (2)	Bacteria (1)	100.00	0.30	99.90	99.50	WGS
210.2666	0.092	Helicobacter pylori (72293)	Helicobacter (213849)	Helicobacteraceae (29547)	Campylobacterales (68525)	Epsilonproteobacteria (122	Proteobacteria (2)	Bacteria (1)	100.00	1.80	99.90	99.10	WGS
210.2667	0.092	Helicobacter pylori (72293)	Helicobacter (213849)	Helicobacteraceae (29547)	Campylobacterales (68525)	Epsilonproteobacteria (122	Proteobacteria (2)	Bacteria (1)	100.00	0.60	99.80	99.00	WGS
210.2668	0.0948	Helicobacter pylori (72293)	Helicobacter (213849)	Helicobacteraceae (29547)	Campylobacterales (68525)	Epsilonproteobacteria (122	Proteobacteria (2)	Bacteria (1)	100.00	0.30	99.90	99.30	WGS
210.2669	0.092	Helicobacter pylori (72293)	Helicobacter (213849)	Helicobacteraceae (29547)	Campylobacterales (68525)	Epsilonproteobacteria (122	Proteobacteria (2)	Bacteria (1)	100.00	0.00	99.90	99.60	WGS
210.2670	0.092	Helicobacter pylori (72293)	Helicobacter (213849)	Helicobacteraceae (29547)	Campylobacterales (68525)	Epsilonproteobacteria (122	Proteobacteria (2)	Bacteria (1)	100.00	0.30	99.90	99.60	WGS
210.2671	0.0938	Helicobacter pylori (72293)	Helicobacter (213849)	Helicobacteraceae (29547)	Campylobacterales (68525)	Epsilonproteobacteria (122	Proteobacteria (2)	Bacteria (1)	100.00	0.00	99.80	99.30	WGS
210.2672	0.092	Helicobacter pylori (72293)	Helicobacter (213849)	Helicobacteraceae (29547)	Campylobacterales (68525)	Epsilonproteobacteria (122	Proteobacteria (2)	Bacteria (1)	100.00	0.00	99.90	99.60	WGS
210.2673	0.0948	Helicobacter pylori (72293)	Helicobacter (213849)	Helicobacteraceae (29547)	Campylobacterales (68525)	Epsilonproteobacteria (122	Proteobacteria (2)	Bacteria (1)	100.00	1.40	99.90	99.20	WGS
210.2674	0.0948	Helicobacter pylori (72293)	Helicobacter (213849)	Helicobacteraceae (29547)	Campylobacterales (68525)	Epsilonproteobacteria (122	Proteobacteria (2)	Bacteria (1)	100.00	2.60	99.80	98.80	WGS
210.2675	0.092	Helicobacter pylori (72293)	Helicobacter (213849)	Helicobacteraceae (29547)	Campylobacterales (68525)	Epsilonproteobacteria (122	Proteobacteria (2)	Bacteria (1)	100.00	8.00	99.60	96.80	WGS
210.2676	0.092	Helicobacter pylori (72293)	Helicobacter (213849)	Helicobacteraceae (29547)	Campylobacterales (68525)	Epsilonproteobacteria (122	Proteobacteria (2)	Bacteria (1)	100.00	1.40	99.90	99.10	WGS
210.2677	0.092	Helicobacter pylori (72293)	Helicobacter (213849)	Helicobacteraceae (29547)	Campylobacterales (68525)	Epsilonproteobacteria (122	Proteobacteria (2)	Bacteria (1)	100.00	1.20	99.90	99.10	WGS
210.2678	0.092	Helicobacter pylori (72293)	Helicobacter (213849)	Helicobacteraceae (29547)	Campylobacterales (68525)	Epsilonproteobacteria (122	Proteobacteria (2)	Bacteria (1)	100.00	2.60	99.90	99.00	WGS
210.2679	0.0988	Helicobacter pylori (72293)	Helicobacter (213849)	Helicobacteraceae (29547)	Campylobacterales (68525)	Epsilonproteobacteria (122	Proteobacteria (2)	Bacteria (1)	100.00	0.30	99.60	99.10	WGS
210.2680	0.0988	Helicobacter pylori (72293)	Helicobacter (213849)	Helicobacteraceae (29547)	Campylobacterales (68525)	Epsilonproteobacteria (122	Proteobacteria (2)	Bacteria (1)	100.00	40.60	99.90	86.00	WGS
210.2681	0.0938	Helicobacter pylori (72293)	Helicobacter (213849)	Helicobacteraceae (29547)	Campylobacterales (68525)	Epsilonproteobacteria (122	Proteobacteria (2)	Bacteria (1)	100.00	1.50	99.70	99.20	Complete
210.2712	0.074	Helicobacter pylori (72293)	Helicobacter (213849)	Helicobacteraceae (29547)	Campylobacterales (68525)	Epsilonproteobacteria (122	Proteobacteria (2)	Bacteria (1)	100.00	2.10	99.90	99.40	Complete
210.2901	0.0726	Helicobacter pylori (72293)	Helicobacter (213849)	Helicobacteraceae (29547)	Campylobacterales (68525)	Epsilonproteobacteria (122	Proteobacteria (2)	Bacteria (1)	100.00	4.10	99.70	98.90	Complete
210.2902	0.0806	Helicobacter pylori (72293)	Helicobacter (213849)	Helicobacteraceae (29547)	Campylobacterales (68525)	Epsilonproteobacteria (122	Proteobacteria (2)	Bacteria (1)	100.00	0.60	99.60	99.30	Complete
210.2903	0.0612	Helicobacter pylori (72293)	Helicobacter (213849)	Helicobacteraceae (29547)	Campylobacterales (68525)	Epsilonproteobacteria (122	Proteobacteria (2)	Bacteria (1)	100.00	0.00	99.80	99.30	Complete
210.2904	0.0656	Helicobacter pylori (72293)	Helicobacter (213849)	Helicobacteraceae (29547)	Campylobacterales (68525)	Epsilonproteobacteria (122	Proteobacteria (2)	Bacteria (1)	100.00	0.00	99.60	99.10	Complete
210.2905	0.0842	Helicobacter pylori (72293)	Helicobacter (213849)	Helicobacteraceae (29547)	Campylobacterales (68525)	Epsilonproteobacteria (122	Proteobacteria (2)	Bacteria (1)	100.00	0.30	99.70	99.20	Complete
210.2906	0.0656	Helicobacter pylori (72293)	Helicobacter (213849)	Helicobacteraceae (29547)	Campylobacterales (68525)	Epsilonproteobacteria (122	Proteobacteria (2)	Bacteria (1)	100.00	0.60	99.80	99.40	Complete
210.2907	0.0682	Helicobacter pylori (72293)	Helicobacter (213849)	Helicobacteraceae (29547)	Campylobacterales (68525)	Epsilonproteobacteria (122	Proteobacteria (2)	Bacteria (1)	100.00	0.30	99.60	99.30	Complete
210.2908	0.0656	Helicobacter pylori (72293)	Helicobacter (213849)	Helicobacteraceae (29547)	Campylobacterales (68525)	Epsilonproteobacteria (122	Proteobacteria (2)	Bacteria (1)	100.00	0.00	99.80	99.30	Complete
210.2909	0.0648	Helicobacter pylori (72293)	Helicobacter (213849)	Helicobacteraceae (29547)	Campylobacterales (68525)	Epsilonproteobacteria (122	Proteobacteria (2)	Bacteria (1)	100.00	0.00	99.70	99.30	Complete
210.2910	0.0444	Helicobacter pylori (72293)	Helicobacter (213849)	Helicobacteraceae (29547)	Campylobacterales (68525)	Epsilonproteobacteria (122	Proteobacteria (2)	Bacteria (1)	100.00	0.60	99.50	99.00	Complete
210.2911	0.0692	Helicobacter pylori (72293)	Helicobacter (213849)	Helicobacteraceae (29547)	Campylobacterales (68525)	Epsilonproteobacteria (122	Proteobacteria (2)	Bacteria (1)	100.00	0.00	99.40	99.00	Complete
210.2912	0.0648	Helicobacter pylori (72293)	Helicobacter (213849)	Helicobacteraceae (29547)	Campylobacterales (68525)	Epsilonproteobacteria (122	Proteobacteria (2)	Bacteria (1)	100.00	0.30	99.70	99.30	Complete
210.2913	0.0892	Helicobacter pylori (72293)	Helicobacter (213849)	Helicobacteraceae (29547)	Campylobacterales (68525)	Epsilonproteobacteria (122	Proteobacteria (2)	Bacteria (1)	100.00	0.30	99.60	99.20	Complete
210.2914	0.0656	Helicobacter pylori (72293)	Helicobacter (213849)	Helicobacteraceae (29547)	Campylobacterales (68525)	Epsilonproteobacteria (122	Proteobacteria (2)	Bacteria (1)	100.00	0.00	99.80	99.40	Complete
210.2915	0.0388	Helicobacter pylori (72293)	Helicobacter (213849)	Helicobacteraceae (29547)	Campylobacterales (68525)	Epsilonproteobacteria (122	Proteobacteria (2)	Bacteria (1)	100.00	0.30	99.90	99.50	Complete
210.2916	0.0656	Helicobacter pylori (72293)	Helicobacter (213849)	Helicobacteraceae (29547)	Campylobacterales (68525)	Epsilonproteobacteria (122	Proteobacteria (2)	Bacteria (1)	100.00	0.00	99.50	99.10	Complete
210.2917	0.0666	Helicobacter pylori (72293)	Helicobacter (213849)	Helicobacteraceae (29547)	Campylobacterales (68525)	Epsilonproteobacteria (122	Proteobacteria (2)	Bacteria (1)	100.00	0.30	99.70	99.10	Complete
210.2918	0.0682	Helicobacter pylori (72293)	Helicobacter (213849)	Helicobacteraceae (29547)	Campylobacterales (68525)	Epsilonproteobacteria (122	Proteobacteria (2)	Bacteria (1)	100.00	0.90	99.60	98.70	Complete
210.2919	0.0612	Helicobacter pylori (72293)	Helicobacter (213849)	Helicobacteraceae (29547)	Campylobacterales (68525)	Epsilonproteobacteria (122	Proteobacteria (2)	Bacteria (1)	99.70	2.00	99.80	99.20	Complete
210.2920	0.0648	Helicobacter pylori (72293)	Helicobacter (213849)	Helicobacteraceae (29547)	Campylobacterales (68525)	Epsilonproteobacteria (122	Proteobacteria (2)	Bacteria (1)	100.00	2.60	99.70	98.30	Complete
210.2921	0.0692	Helicobacter pylori (72293)	Helicobacter (213849)	Helicobacteraceae (29547)	Campylobacterales (68525)	Epsilonproteobacteria (122	Proteobacteria (2)	Bacteria (1)	100.00	0.00	99.60	99.40	Complete
210.2922	0.0656	Helicobacter pylori (72293)	Helicobacter (213849)	Helicobacteraceae (29547)	Campylobacterales (68525)	Epsilonproteobacteria (122	Proteobacteria (2)	Bacteria (1)	100.00	0.00	99.60	99.20	Complete
210.2923	0.0612	Helicobacter pylori (72293)	Helicobacter (213849)	Helicobacteraceae (29547)	Campylobacterales (68525)	Epsilonproteobacteria (122	Proteobacteria (2)	Bacteria (1)	100.00	0.00	99.80	99.30	Complete
210.2924	0.0656	Helicobacter pylori (72293)	Helicobacter (213849)	Helicobacteraceae (29547)	Campylobacterales (68525)	Epsilonproteobacteria (122	Proteobacteria (2)	Bacteria (1)	100.00	0.00	99.80	99.30	Complete
210.2925	0.0648	Helicobacter pylori (72293)	Helicobacter (213849)	Helicobacteraceae (29547)	Campylobacterales (68525)	Epsilonproteobacteria (122	Proteobacteria (2)	Bacteria (1)	100.00	0.30	99.70	99.30	Complete
210.2926	0.0692	Helicobacter pylori (72293)	Helicobacter (213849)	Helicobacteraceae (29547)	Campylobacterales (68525)	Epsilonproteobacteria (122	Proteobacteria (2)	Bacteria (1)	100.00	0.30	99.40	99.00	Complete
210.2927	0.0692	Helicobacter pylori (72293)	Helicobacter (213849)	Helicobacteraceae (29547)	Campylobacterales (68525)	Epsilonproteobacteria (122	Proteobacteria (2)	Bacteria (1)	100.00	0.00	99.50	99.00	Complete
210.2928	0.0638	Helicobacter pylori (72293)	Helicobacter (213849)	Helicobacteraceae (29547)	Campylobacterales (68525)	Epsilonproteobacteria (122	Proteobacteria (2)	Bacteria (1)	100.00	0.60	99.70	99.20	Complete
210.2929	0.0814	Helicobacter pylori (72293)	Helicobacter (213849)	Helicobacteraceae (29547)	Campylobacterales (68525)	Epsilonproteobacteria (122	Proteobacteria (2)	Bacteria (1)	100.00	0.00	99.60	99.30	Complete
210.2930	0.092	Helicobacter pylori (72293)	Helicobacter (213849)	Helicobacteraceae (29547)	Campylobacterales (68525)	Epsilonproteobacteria (122	Proteobacteria (2)	Bacteria (1)	100.00	0.60	99.90	99.60	WGS
210.2931	0.0948	Helicobacter pylori (72293)	Helicobacter (213849)	Helicobacteraceae (29547)	Campylobacterales (68525)	Epsilonproteobacteria (122	Proteobacteria (2)	Bacteria (1)	99.70	0.00	99.90	99.00	WGS
210.2932	0.0948	Helicobacter pylori (72293)	Helicobacter (213849)	Helicobacteraceae (29547)	Campylobacterales (68525)	Epsilonproteobacteria (122	Proteobacteria (2)	Bacteria (1)	100.00	0.30	99.90	99.30	WGS
210.2933	0.0988	Helicobacter pylori (72293)	Helicobacter (213849)	Helicobacteraceae (29547)	Campylobacterales (68525)	Epsilonproteobacteria (122	Proteobacteria (2)	Bacteria (1)	100.00	0.00	99.90	99.50	WGS
210.2934	0.0938	Helicobacter pylori (72293)	Helicobacter (213849)	Helicobacteraceae (29547)	Campylobacterales (68525)	Epsilonproteobacteria (122	Proteobacteria (2)	Bacteria (1)	100.00	0.00	99.90	99.30	WGS
210.2935	0.0988	Helicobacter pylori (72293)	Helicobacter (213849)	Helicobacteraceae (29547)	Campylobacterales (68525)	Epsilonproteobacteria (122	Proteobacteria (2)	Bacteria (1)	100.00	0.00	99.90	99.70	WGS
210.2936	0.091	Helicobacter pylori (72293)	Helicobacter (213849)	Helicobacteraceae (29547)	Campylobacterales (68525)	Epsilonproteobacteria (122	Proteobacteria (2)	Bacteria (1)	100.00	0.00	99.90	99.60	WGS
210.2937	0.0978	Helicobacter pylori (72293)	Helicobacter (213849)	Helicobacteraceae (29547)	Campylobacterales (68525)	Epsilonproteobacteria (122	Proteobacteria (2)	Bacteria (1)	100.00	0.00	99.80	99.40	WGS
210.2938	0.0948	Helicobacter pylori (72293)	Helicobacter (213849)	Helicobacteraceae (29547)	Campylobacterales (68525)	Epsilonproteobacteria (122	Proteobacteria (2)	Bacteria (1)	100.00	0.30	99.80	99.40	WGS
210.2939	0.0938	Helicobacter pylori (72293)	Helicobacter (213849)	Helicobacteraceae (29547)	Campylobacterales (68525)	Epsilonproteobacteria (122	Proteobacteria (2)	Bacteria (1)	100.00	0.20	99.90	99.40	WGS
210.2940	0.092	Helicobacter pylori (72293)	Helicobacter (213849)	Helicobacteraceae (29547)	Campylobacterales (68525)	Epsilonproteobacteria (122	Proteobacteria (2)	Bacteria (1)	100.00	0.30	99.90	99.20	WGS
210.2941	0.0948	Helicobacter pylori (72293)	Helicobacter (213849)	Helicobacteraceae (29547)	Campylobacterales (68525)	Epsilonproteobacteria (122	Proteobacteria (2)	Bacteria (1)	100.00	0.30	99.70	99.40	WGS
210.2942	0.092	Helicobacter pylori (72293)	Helicobacter (213849)	Helicobacteraceae (29547)	Campylobacterales (68525)	Epsilonproteobacteria (122	Proteobacteria (2)	Bacteria (1)	99.70	4.50	99.90	97.90	WGS
210.2943	0.092	Helicobacter pylori (72293)	Helicobacter (213849)	Helicobacteraceae (29547)	Campylobacterales (68525)	Epsilonproteobacteria (122	Proteobacteria (2)	Bacteria (1)	100.00	0.00	99.90	99.60	WGS
210.2944	0.0978	Helicobacter pylori (72293)	Helicobacter (213849)	Helicobacteraceae (29547)	Campylobacterales (68525)	Epsilonproteobacteria (122	Proteobacteria (2)	Bacteria (1)	100.00	0.00	99.90	99.70	WGS
210.2945	0.092	Helicobacter pylori (72293)	Helicobacter (213849)	Helicobacteraceae (29547)	Campylobacterales (68525)	Epsilonproteobacteria (122	Proteobacteria (2)	Bacteria (1)	100.00	0.00	99.80	99.30	WGS
210.2946	0.0948	Helicobacter pylori (72293)	Helicobacter (213849)	Helicobacteraceae (29547)	Campylobacterales (68525)	Epsilonproteobacteria (122	Proteobacteria (2)	Bacteria (1)	100.00	0.90	99.90	99.40	WGS
210.2947	0.0938	Helicobacter pylori (72293)	Helicobacter (213849)	Helicobacteraceae (29547)	Campylobacterales (68525)	Epsilonproteobacteria (122	Proteobacteria (2)	Bacteria (1)	100.00	0.00	99.90	99.50	WGS
210.2948	0.0978	Helicobacter pylori (72293)	Helicobacter (213849)	Helicobacteraceae (29547)	Campylobacterales (68525)	Epsilonproteobacteria (122	Proteobacteria (2)	Bacteria (1)	100.00	0.00	99.90	99.60	WGS
210.2949	0.092	Helicobacter pylori (72293)	Helicobacter (213849)	Helicobacteraceae (29547)	Campylobacterales (68525)	Epsilonproteobacteria (122	Proteobacteria (2)	Bacteria (1)	100.00	0.00	99.90	99.60	WGS
210.2950	0.1006	Helicobacter pylori (72293)	Helicobacter (213849)	Helicobacteraceae (29547)	Campylobacterales (68525)	Epsilonproteobacteria (122	Proteobacteria (2)	Bacteria (1)	100.00	0.00	99.80	99.40	WGS
210.2951	0.092	Helicobacter pylori (72293)	Helicobacter (213849)	Helicobacteraceae (29547)	Campylobacterales (68525)	Epsilonproteobacteria (122	Proteobacteria (2)	Bacteria (1)	100.00	0.00	99.80	99.60	WGS
210.2952	0.091	Helicobacter pylori (72293)	Helicobacter (213849)	Helicobacteraceae (29547)	Campylobacterales (68525)	Epsilonproteobacteria (122	Proteobacteria (2)	Bacteria (1)	100.00	0.00	99.90	99.80	WGS
210.2953	0.0948	Helicobacter pylori (72293)	Helicobacter (213849)	Helicobacteraceae (29547)	Campylobacterales (68525)	Epsilonproteobacteria (122	Proteobacteria (2)	Bacteria (1)	100.00	0.00	99.80	99.40	WGS
210.2954	0.0938	Helicobacter pylori (72293)	Helicobacter (213849)	Helicobacteraceae (29547)	Campylobacterales (68525)	Epsilonproteobacteria (122	Proteobacteria (2)	Bacteria (1)	100.00	0.60	99.90	99.60	WGS
210.2955	0.1006	Helicobacter pylori (72293)	Helicobacter (213849)	Helicobacteraceae (29547)	Campylobacterales (68525)	Epsilonproteobacteria (122	Proteobacteria (2)	Bacteria (1)	100.00	0.00	99.90	99.60	WGS
210.2956	0.092	Helicobacter pylori (72293)	Helicobacter (213849)	Helicobacteraceae (29547)	Campylobacterales (68525)	Epsilonproteobacteria (122	Proteobacteria (2)	Bacteria (1)	100.00	0.30	99.90	99.60	WGS
210.2957	0.0948	Helicobacter pylori (72293)	Helicobacter (213849)	Helicobacteraceae (29547)	Campylobacterales (68525)	Epsilonproteobacteria (122	Proteobacteria (2)	Bacteria (1)	100.00	0.00	99.90	99.40	WGS
210.2958	0.0948	Helicobacter pylori (72293)	Helicobacter (213849)	Helicobacteraceae (29547)	Campylobacterales (68525)	Epsilonproteobacteria (122	Proteobacteria (2)	Bacteria (1)	100.00	0.00	99.90	99.30	WGS
210.2959	0.091	Helicobacter pylori (72293)	Helicobacter (213849)	Helicobacteraceae (29547)	Campylobacterales (68525)	Epsilonproteobacteria (122	Proteobacteria (2)	Bacteria (1)	100.00	12.50	99.90	95.90	WGS
210.2960	0.0938	Helicobacter pylori (72293)	Helicobacter (213849)	Helicobacteraceae (29547)	Campylobacterales (68525)	Epsilonproteobacteria (122	Proteobacteria (2)	Bacteria (1)	100.00	0.30	99.80	99.50	WGS
210.2961	0.1016	Helicobacter pylori (72293)	Helicobacter (213849)	Helicobacteraceae (29547)	Campylobacterales (68525)	Epsilonproteobacteria (122	Proteobacteria (2)	Bacteria (1)	100.00	0.00	99.90	99.60	WGS
210.2962	0.0978	Helicobacter pylori (72293)	Helicobacter (213849)	Helicobacteraceae (29547)	Campylobacterales (68525)	Epsilonproteobacteria (122	Proteobacteria (2)	Bacteria (1)	100.00	0.00	99.90	99.60	WGS
210.2963	0.091	Helicobacter pylori (72293)	Helicobacter (213849)	Helicobacteraceae (29547)	Campylobacterales (68525)	Epsilonproteobacteria (122	Proteobacteria (2)	Bacteria (1)	100.00	0.00	99.80	99.60	WGS
210.2964	0.1016	Helicobacter pylori (72293)	Helicobacter (213849)	Helicobacteraceae (29547)	Campylobacterales (68525)	Epsilonproteobacteria (122	Proteobacteria (2)	Bacteria (1)	100.00	13.20	99.90	94.80	WGS
210.2965	0.0988	Helicobacter pylori (72293)	Helicobacter (213849)	Helicobacteraceae (29547)	Campylobacterales (68525)	Epsilonproteobacteria (122	Proteobacteria (2)	Bacteria (1)	100.00	0.00	99.80	99.30	WGS
210.2966	0.0948	Helicobacter pylori (72293)	Helicobacter (213849)	Helicobacteraceae (29547)	Campylobacterales (68525)	Epsilonproteobacteria (122	Proteobacteria (2)	Bacteria (1)	100.00	0.30	99.90	99.00	WGS
210.2967	0.091	Helicobacter pylori (72293)	Helicobacter (213849)	Helicobacteraceae (29547)	Campylobacterales (68525)	Epsilonproteobacteria (122	Proteobacteria (2)	Bacteria (1)	99.70	8.10	99.80	96.20	WGS
210.2968	0.0948	Helicobacter pylori (72293)	Helicobacter (213849)	Helicobacteraceae (29547)	Campylobacterales (68525)	Epsilonproteobacteria (122	Proteobacteria (2)	Bacteria (1)	100.00	0.60	99.90	99.40	WGS
210.2969	0.0938	Helicobacter pylori (72293)	Helicobacter (213849)	Helicobacteraceae (29547)	Campylobacterales (68525)	Epsilonproteobacteria (122	Proteobacteria (2)	Bacteria (1)	100.00	0.00	99.90	99.40	WGS
210.2970	0.0948	Helicobacter pylori (72293)	Helicobacter (213849)	Helicobacteraceae (29547)	Campylobacterales (68525)	Epsilonproteobacteria (122	Proteobacteria (2)	Bacteria (1)	100.00	1.40	99.90	99.30	WGS
210.2971	0.0988	Helicobacter pylori (72293)	Helicobacter (213849)	Helicobacteraceae (29547)	Campylobacterales (68525)	Epsilonproteobacteria (122	Proteobacteria (2)	Bacteria (1)	100.00	0.00	99.80	99.20	WGS
210.2972	0.1016	Helicobacter pylori (72293)	Helicobacter (213849)	Helicobacteraceae (29547)	Campylobacterales (68525)	Epsilonproteobacteria (122	Proteobacteria (2)	Bacteria (1)	100.00	7.00	99.90	98.00	WGS
210.2973	0.0938	Helicobacter pylori (72293)	Helicobacter (213849)	Helicobacteraceae (29547)	Campylobacterales (68525)	Epsilonproteobacteria (122	Proteobacteria (2)	Bacteria (1)	100.00	0.00	99.90	99.60	WGS
210.2974	0.1016	Helicobacter pylori (72293)	Helicobacter (213849)	Helicobacteraceae (29547)	Campylobacterales (68525)	Epsilonproteobacteria (122	Proteobacteria (2)	Bacteria (1)	100.00	0.00	99.80	99.00	WGS
210.2975	0.092	Helicobacter pylori (72293)	Helicobacter (213849)	Helicobacteraceae (29547)	Campylobacterales (68525)	Epsilonproteobacteria (122	Proteobacteria (2)	Bacteria (1)	100.00	0.60	99.90	99.00	WGS
210.2976	0.0938	Helicobacter pylori (72293)	Helicobacter (213849)	Helicobacteraceae (29547)	Campylobacterales (68525)	Epsilonproteobacteria (122	Proteobacteria (2)	Bacteria (1)	100.00	5.30	99.90	98.80	WGS
210.2977	0.0948	Helicobacter pylori (72293)	Helicobacter (213849)	Helicobacteraceae (29547)	Campylobacterales (68525)	Epsilonproteobacteria (122	Proteobacteria (2)	Bacteria (1)	100.00	9.40	99.90	97.40	WGS
210.2978	0.1006	Helicobacter pylori (72293)	Helicobacter (213849)	Helicobacteraceae (29547)	Campylobacterales (68525)	Epsilonproteobacteria (122	Proteobacteria (2)	Bacteria (1)	100.00	0.00	99.90	99.40	WGS
210.2979	0.0948	Helicobacter pylori (72293)	Helicobacter (213849)	Helicobacteraceae (29547)	Campylobacterales (68525)	Epsilonproteobacteria (122	Proteobacteria (2)	Bacteria (1)	100.00	3.70	99.90	99.00	WGS
210.2980	0.091	Helicobacter pylori (72293)	Helicobacter (213849)	Helicobacteraceae (29547)	Campylobacterales (68525)	Epsilonproteobacteria (122	Proteobacteria (2)	Bacteria (1)	100.00	17.80	99.90	93.70	WGS
210.2981	0.0948	Helicobacter pylori (72293)	Helicobacter (213849)	Helicobacteraceae (29547)	Campylobacterales (68525)	Epsilonproteobacteria (122	Proteobacteria (2)	Bacteria (1)	100.00	0.00	99.90	99.40	WGS
210.2982	0.0948	Helicobacter pylori (72293)	Helicobacter (213849)	Helicobacteraceae (29547)	Campylobacterales (68525)	Epsilonproteobacteria (122	Proteobacteria (2)	Bacteria (1)	100.00	16.00	99.60	92.90	WGS
210.2983	0.092	Helicobacter pylori (72293)	Helicobacter (213849)	Helicobacteraceae (29547)	Campylobacterales (68525)	Epsilonproteobacteria (122	Proteobacteria (2)	Bacteria (1)	100.00	3.30	99.90	98.80	WGS
210.2984	0.091	Helicobacter pylori (72293)	Helicobacter (213849)	Helicobacteraceae (29547)	Campylobacterales (68525)	Epsilonproteobacteria (122	Proteobacteria (2)	Bacteria (1)	100.00	0.30	99.90	99.10	WGS
210.2985	0.091	Helicobacter pylori (72293)	Helicobacter (213849)	Helicobacteraceae (29547)	Campylobacterales (68525)	Epsilonproteobacteria (122	Proteobacteria (2)	Bacteria (1)	100.00	0.00	99.90	99.60	WGS
210.2986	0.0948	Helicobacter pylori (72293)	Helicobacter (213849)	Helicobacteraceae (29547)	Campylobacterales (68525)	Epsilonproteobacteria (122	Proteobacteria (2)	Bacteria (1)	100.00	5.00	99.90	98.70	WGS
210.2987	0.1006	Helicobacter pylori (72293)	Helicobacter (213849)	Helicobacteraceae (29547)	Campylobacterales (68525)	Epsilonproteobacteria (122	Proteobacteria (2)	Bacteria (1)	100.00	0.00	99.90	99.40	WGS
210.2988	0.0938	Helicobacter pylori (72293)	Helicobacter (213849)	Helicobacteraceae (29547)	Campylobacterales (68525)	Epsilonproteobacteria (122	Proteobacteria (2)	Bacteria (1)	100.00	0.00	99.90	99.50	WGS
210.2989	0.091	Helicobacter pylori (72293)	Helicobacter (213849)	Helicobacteraceae (29547)	Campylobacterales (68525)	Epsilonproteobacteria (122	Proteobacteria (2)	Bacteria (1)	100.00	0.60	99.90	99.50	WGS
210.2990	0.1006	Helicobacter pylori (72293)	Helicobacter (213849)	Helicobacteraceae (29547)	Campylobacterales (68525)	Epsilonproteobacteria (122	Proteobacteria (2)	Bacteria (1)	99.70	0.30	99.80	99.60	WGS
210.2991	0.0988	Helicobacter pylori (72293)	Helicobacter (213849)	Helicobacteraceae (29547)	Campylobacterales (68525)	Epsilonproteobacteria (122	Proteobacteria (2)	Bacteria (1)	100.00	4.80	99.90	98.50	WGS
210.2992	0.0948	Helicobacter pylori (72293)	Helicobacter (213849)	Helicobacteraceae (29547)	Campylobacterales (68525)	Epsilonproteobacteria (122	Proteobacteria (2)	Bacteria (1)	100.00	0.00	99.90	99.30	WGS
210.2993	0.092	Helicobacter pylori (72293)	Helicobacter (213849)	Helicobacteraceae (29547)	Campylobacterales (68525)	Epsilonproteobacteria (122	Proteobacteria (2)	Bacteria (1)	100.00	0.30	99.90	99.60	WGS
210.2994	0.091	Helicobacter pylori (72293)	Helicobacter (213849)	Helicobacteraceae (29547)	Campylobacterales (68525)	Epsilonproteobacteria (122	Proteobacteria (2)	Bacteria (1)	100.00	1.20	99.90	99.60	WGS
210.2995	0.092	Helicobacter pylori (72293)	Helicobacter (213849)	Helicobacteraceae (29547)	Campylobacterales (68525)	Epsilonproteobacteria (122	Proteobacteria (2)	Bacteria (1)	100.00	0.30	99.90	99.50	WGS
210.2996	0.092	Helicobacter pylori (72293)	Helicobacter (213849)	Helicobacteraceae (29547)	Campylobacterales (68525)	Epsilonproteobacteria (122	Proteobacteria (2)	Bacteria (1)	100.00	0.00	99.90	99.60	WGS
210.2997	0.0938	Helicobacter pylori (72293)	Helicobacter (213849)	Helicobacteraceae (29547)	Campylobacterales (68525)	Epsilonproteobacteria (122	Proteobacteria (2)	Bacteria (1)	100.00	0.40	99.90	99.30	WGS
210.2998	0.091	Helicobacter pylori (72293)	Helicobacter (213849)	Helicobacteraceae (29547)	Campylobacterales (68525)	Epsilonproteobacteria (122	Proteobacteria (2)	Bacteria (1)	100.00	0.90	99.80	99.30	WGS
210.2999	0.0978	Helicobacter pylori (72293)	Helicobacter (213849)	Helicobacteraceae (29547)	Campylobacterales (68525)	Epsilonproteobacteria (122	Proteobacteria (2)	Bacteria (1)	100.00	0.90	99.90	99.00	WGS
210.3000	0.092	Helicobacter pylori (72293)	Helicobacter (213849)	Helicobacteraceae (29547)	Campylobacterales (68525)	Epsilonproteobacteria (122	Proteobacteria (2)	Bacteria (1)	100.00	3.30	99.90	98.40	WGS
210.3001	0.092	Helicobacter pylori (72293)	Helicobacter (213849)	Helicobacteraceae (29547)	Campylobacterales (68525)	Epsilonproteobacteria (122	Proteobacteria (2)	Bacteria (1)	100.00	0.30	99.90	99.80	WGS
210.3002	0.0948	Helicobacter pylori (72293)	Helicobacter (213849)	Helicobacteraceae (29547)	Campylobacterales (68525)	Epsilonproteobacteria (122	Proteobacteria (2)	Bacteria (1)	100.00	0.00	99.70	99.50	WGS
210.3003	0.0988	Helicobacter pylori (72293)	Helicobacter (213849)	Helicobacteraceae (29547)	Campylobacterales (68525)	Epsilonproteobacteria (122	Proteobacteria (2)	Bacteria (1)	100.00	0.00	99.90	99.30	WGS
210.3004	0.0948	Helicobacter pylori (72293)	Helicobacter (213849)	Helicobacteraceae (29547)	Campylobacterales (68525)	Epsilonproteobacteria (122	Proteobacteria (2)	Bacteria (1)	99.70	0.00	99.70	99.00	WGS
210.3005	0.0988	Helicobacter pylori (72293)	Helicobacter (213849)	Helicobacteraceae (29547)	Campylobacterales (68525)	Epsilonproteobacteria (122	Proteobacteria (2)	Bacteria (1)	100.00	0.60	99.90	99.30	WGS
210.3006	0.0938	Helicobacter pylori (72293)	Helicobacter (213849)	Helicobacteraceae (29547)	Campylobacterales (68525)	Epsilonproteobacteria (122	Proteobacteria (2)	Bacteria (1)	100.00	0.90	99.90	99.50	WGS
210.3007	0.091	Helicobacter pylori (72293)	Helicobacter (213849)	Helicobacteraceae (29547)	Campylobacterales (68525)	Epsilonproteobacteria (122	Proteobacteria (2)	Bacteria (1)	100.00	0.00	99.90	99.50	WGS
210.3008	0.1006	Helicobacter pylori (72293)	Helicobacter (213849)	Helicobacteraceae (29547)	Campylobacterales (68525)	Epsilonproteobacteria (122	Proteobacteria (2)	Bacteria (1)	100.00	0.30	99.80	99.50	WGS
210.3009	0.0938	Helicobacter pylori (72293)	Helicobacter (213849)	Helicobacteraceae (29547)	Campylobacterales (68525)	Epsilonproteobacteria (122	Proteobacteria (2)	Bacteria (1)	100.00	0.40	99.90	99.20	WGS
210.3010	0.0938	Helicobacter pylori (72293)	Helicobacter (213849)	Helicobacteraceae (29547)	Campylobacterales (68525)	Epsilonproteobacteria (122	Proteobacteria (2)	Bacteria (1)	100.00	0.90	99.80	99.50	WGS
210.3011	0.092	Helicobacter pylori (72293)	Helicobacter (213849)	Helicobacteraceae (29547)	Campylobacterales (68525)	Epsilonproteobacteria (122	Proteobacteria (2)	Bacteria (1)	100.00	0.00	99.90	99.80	WGS
210.3012	0.0948	Helicobacter pylori (72293)	Helicobacter (213849)	Helicobacteraceae (29547)	Campylobacterales (68525)	Epsilonproteobacteria (122	Proteobacteria (2)	Bacteria (1)	100.00	0.90	99.80	99.20	WGS
210.3013	0.091	Helicobacter pylori (72293)	Helicobacter (213849)	Helicobacteraceae (29547)	Campylobacterales (68525)	Epsilonproteobacteria (122	Proteobacteria (2)	Bacteria (1)	100.00	0.00	99.90	99.30	WGS
210.3014	0.0988	Helicobacter pylori (72293)	Helicobacter (213849)	Helicobacteraceae (29547)	Campylobacterales (68525)	Epsilonproteobacteria (122	Proteobacteria (2)	Bacteria (1)	100.00	2.00	99.90	98.70	WGS
210.3015	0.092	Helicobacter pylori (72293)	Helicobacter (213849)	Helicobacteraceae (29547)	Campylobacterales (68525)	Epsilonproteobacteria (122	Proteobacteria (2)	Bacteria (1)	100.00	13.20	99.90	96.90	WGS
210.3016	0.091	Helicobacter pylori (72293)	Helicobacter (213849)	Helicobacteraceae (29547)	Campylobacterales (68525)	Epsilonproteobacteria (122	Proteobacteria (2)	Bacteria (1)	100.00	1.00	99.90	99.10	WGS
210.3017	0.091	Helicobacter pylori (72293)	Helicobacter (213849)	Helicobacteraceae (29547)	Campylobacterales (68525)	Epsilonproteobacteria (122	Proteobacteria (2)	Bacteria (1)	100.00	2.90	99.90	98.00	WGS
210.3018	0.092	Helicobacter pylori (72293)	Helicobacter (213849)	Helicobacteraceae (29547)	Campylobacterales (68525)	Epsilonproteobacteria (122	Proteobacteria (2)	Bacteria (1)	100.00	0.00	99.90	99.30	WGS
210.3019	0.091	Helicobacter pylori (72293)	Helicobacter (213849)	Helicobacteraceae (29547)	Campylobacterales (68525)	Epsilonproteobacteria (122	Proteobacteria (2)	Bacteria (1)	100.00	0.00	99.90	99.40	WGS
210.3020	0.0938	Helicobacter pylori (72293)	Helicobacter (213849)	Helicobacteraceae (29547)	Campylobacterales (68525)	Epsilonproteobacteria (122	Proteobacteria (2)	Bacteria (1)	100.00	0.60	99.90	99.50	WGS
210.3021	0.0938	Helicobacter pylori (72293)	Helicobacter (213849)	Helicobacteraceae (29547)	Campylobacterales (68525)	Epsilonproteobacteria (122	Proteobacteria (2)	Bacteria (1)	99.70	0.00	99.80	99.50	WGS
210.3022	0.091	Helicobacter pylori (72293)	Helicobacter (213849)	Helicobacteraceae (29547)	Campylobacterales (68525)	Epsilonproteobacteria (122	Proteobacteria (2)	Bacteria (1)	100.00	0.00	99.80	99.30	WGS
210.3023	0.092	Helicobacter pylori (72293)	Helicobacter (213849)	Helicobacteraceae (29547)	Campylobacterales (68525)	Epsilonproteobacteria (122	Proteobacteria (2)	Bacteria (1)	100.00	0.60	99.90	99.40	WGS
210.3024	0.1016	Helicobacter pylori (72293)	Helicobacter (213849)	Helicobacteraceae (29547)	Campylobacterales (68525)	Epsilonproteobacteria (122	Proteobacteria (2)	Bacteria (1)	100.00	3.80	99.80	98.20	WGS
210.3025	0.0938	Helicobacter pylori (72293)	Helicobacter (213849)	Helicobacteraceae (29547)	Campylobacterales (68525)	Epsilonproteobacteria (122	Proteobacteria (2)	Bacteria (1)	100.00	0.00	99.80	99.60	WGS
210.3026	0.0938	Helicobacter pylori (72293)	Helicobacter (213849)	Helicobacteraceae (29547)	Campylobacterales (68525)	Epsilonproteobacteria (122	Proteobacteria (2)	Bacteria (1)	100.00	0.00	99.90	99.60	WGS
210.3027	0.1016	Helicobacter pylori (72293)	Helicobacter (213849)	Helicobacteraceae (29547)	Campylobacterales (68525)	Epsilonproteobacteria (122	Proteobacteria (2)	Bacteria (1)	100.00	4.60	99.90	97.90	WGS
210.3028	0.0938	Helicobacter pylori (72293)	Helicobacter (213849)	Helicobacteraceae (29547)	Campylobacterales (68525)	Epsilonproteobacteria (122	Proteobacteria (2)	Bacteria (1)	100.00	0.40	99.90	99.10	WGS
210.3029	0.0948	Helicobacter pylori (72293)	Helicobacter (213849)	Helicobacteraceae (29547)	Campylobacterales (68525)	Epsilonproteobacteria (122	Proteobacteria (2)	Bacteria (1)	100.00	0.30	99.90	99.30	WGS
210.3030	0.0938	Helicobacter pylori (72293)	Helicobacter (213849)	Helicobacteraceae (29547)	Campylobacterales (68525)	Epsilonproteobacteria (122	Proteobacteria (2)	Bacteria (1)	100.00	0.00	99.90	99.50	Complete
210.3031	0.0938	Helicobacter pylori (72293)	Helicobacter (213849)	Helicobacteraceae (29547)	Campylobacterales (68525)	Epsilonproteobacteria (122	Proteobacteria (2)	Bacteria (1)	100.00	1.80	99.90	99.10	Complete
210.3032	0.0938	Helicobacter pylori (72293)	Helicobacter (213849)	Helicobacteraceae (29547)	Campylobacterales (68525)	Epsilonproteobacteria (122	Proteobacteria (2)	Bacteria (1)	100.00	0.40	99.90	99.20	Complete
210.3033	0.0938	Helicobacter pylori (72293)	Helicobacter (213849)	Helicobacteraceae (29547)	Campylobacterales (68525)	Epsilonproteobacteria (122	Proteobacteria (2)	Bacteria (1)	100.00	0.00	99.70	99.30	Complete
210.3034	0.0938	Helicobacter pylori (72293)	Helicobacter (213849)	Helicobacteraceae (29547)	Campylobacterales (68525)	Epsilonproteobacteria (122	Proteobacteria (2)	Bacteria (1)	100.00	0.00	99.90	99.40	Complete
210.3035	0.0938	Helicobacter pylori (72293)	Helicobacter (213849)	Helicobacteraceae (29547)	Campylobacterales (68525)	Epsilonproteobacteria (122	Proteobacteria (2)	Bacteria (1)	100.00	0.30	99.90	99.40	Complete
210.3036	0.0938	Helicobacter pylori (72293)	Helicobacter (213849)	Helicobacteraceae (29547)	Campylobacterales (68525)	Epsilonproteobacteria (122	Proteobacteria (2)	Bacteria (1)	100.00	0.90	99.90	99.30	Complete
210.3037	0.0938	Helicobacter pylori (72293)	Helicobacter (213849)	Helicobacteraceae (29547)	Campylobacterales (68525)	Epsilonproteobacteria (122	Proteobacteria (2)	Bacteria (1)	100.00	0.60	99.70	99.10	Complete
210.3038	0.0938	Helicobacter pylori (72293)	Helicobacter (213849)	Helicobacteraceae (29547)	Campylobacterales (68525)	Epsilonproteobacteria (122	Proteobacteria (2)	Bacteria (1)	100.00	0.00	99.90	99.50	Complete
210.3039	0.0938	Helicobacter pylori (72293)	Helicobacter (213849)	Helicobacteraceae (29547)	Campylobacterales (68525)	Epsilonproteobacteria (122	Proteobacteria (2)	Bacteria (1)	100.00	0.00	99.90	99.50	Complete
210.3040	0.0938	Helicobacter pylori (72293)	Helicobacter (213849)	Helicobacteraceae (29547)	Campylobacterales (68525)	Epsilonproteobacteria (122	Proteobacteria (2)	Bacteria (1)	100.00	0.90	99.70	99.10	Complete
210.3041	0.0938	Helicobacter pylori (72293)	Helicobacter (213849)	Helicobacteraceae (29547)	Campylobacterales (68525)	Epsilonproteobacteria (122	Proteobacteria (2)	Bacteria (1)	100.00	0.90	99.70	99.30	Complete
210.3073	0.0294	Helicobacter pylori (72293)	Helicobacter (213849)	Helicobacteraceae (29547)	Campylobacterales (68525)	Epsilonproteobacteria (122	Proteobacteria (2)	Bacteria (1)					Plasmid
210.3074	0.0294	Helicobacter pylori (72293)	Helicobacter (213849)	Helicobacteraceae (29547)	Campylobacterales (68525)	Epsilonproteobacteria (122	Proteobacteria (2)	Bacteria (1)					Plasmid
210.3075	0.0294	Helicobacter pylori (72293)	Helicobacter (213849)	Helicobacteraceae (29547)	Campylobacterales (68525)	Epsilonproteobacteria (122	Proteobacteria (2)	Bacteria (1)					Plasmid
210.3076	0.0294	Helicobacter pylori (72293)	Helicobacter (213849)	Helicobacteraceae (29547)	Campylobacterales (68525)	Epsilonproteobacteria (122	Proteobacteria (2)	Bacteria (1)					Plasmid
210.3077	0.0294	Helicobacter pylori (72293)	Helicobacter (213849)	Helicobacteraceae (29547)	Campylobacterales (68525)	Epsilonproteobacteria (122	Proteobacteria (2)	Bacteria (1)					Plasmid
210.3078	0.0294	Helicobacter pylori (72293)	Helicobacter (213849)	Helicobacteraceae (29547)	Campylobacterales (68525)	Epsilonproteobacteria (122	Proteobacteria (2)	Bacteria (1)					Plasmid
210.3079	0.0294	Helicobacter pylori (72293)	Helicobacter (213849)	Helicobacteraceae (29547)	Campylobacterales (68525)	Epsilonproteobacteria (122	Proteobacteria (2)	Bacteria (1)					Plasmid
210.3080	0.0294	Helicobacter pylori (72293)	Helicobacter (213849)	Helicobacteraceae (29547)	Campylobacterales (68525)	Epsilonproteobacteria (122	Proteobacteria (2)	Bacteria (1)					Plasmid
210.3081	0.0294	Helicobacter pylori (72293)	Helicobacter (213849)	Helicobacteraceae (29547)	Campylobacterales (68525)	Epsilonproteobacteria (122	Proteobacteria (2)	Bacteria (1)					Plasmid
210.3082	0.0294	Helicobacter pylori (72293)	Helicobacter (213849)	Helicobacteraceae (29547)	Campylobacterales (68525)	Epsilonproteobacteria (122	Proteobacteria (2)	Bacteria (1)					Plasmid
210.3083	0.0294	Helicobacter pylori (72293)	Helicobacter (213849)	Helicobacteraceae (29547)	Campylobacterales (68525)	Epsilonproteobacteria (122	Proteobacteria (2)	Bacteria (1)					Plasmid
210.3084	0.0294	Helicobacter pylori (72293)	Helicobacter (213849)	Helicobacteraceae (29547)	Campylobacterales (68525)	Epsilonproteobacteria (122	Proteobacteria (2)	Bacteria (1)					Plasmid
210.3085	0.0294	Helicobacter pylori (72293)	Helicobacter (213849)	Helicobacteraceae (29547)	Campylobacterales (68525)	Epsilonproteobacteria (122	Proteobacteria (2)	Bacteria (1)					Plasmid
210.3086	0.0294	Helicobacter pylori (72293)	Helicobacter (213849)	Helicobacteraceae (29547)	Campylobacterales (68525)	Epsilonproteobacteria (122	Proteobacteria (2)	Bacteria (1)					Plasmid
210.3087	0.0294	Helicobacter pylori (72293)	Helicobacter (213849)	Helicobacteraceae (29547)	Campylobacterales (68525)	Epsilonproteobacteria (122	Proteobacteria (2)	Bacteria (1)					Plasmid
210.3088	0.0294	Helicobacter pylori (72293)	Helicobacter (213849)	Helicobacteraceae (29547)	Campylobacterales (68525)	Epsilonproteobacteria (122	Proteobacteria (2)	Bacteria (1)					Plasmid
210.3089	0.0294	Helicobacter pylori (72293)	Helicobacter (213849)	Helicobacteraceae (29547)	Campylobacterales (68525)	Epsilonproteobacteria (122	Proteobacteria (2)	Bacteria (1)					Plasmid
210.3090	0.0294	Helicobacter pylori (72293)	Helicobacter (213849)	Helicobacteraceae (29547)	Campylobacterales (68525)	Epsilonproteobacteria (122	Proteobacteria (2)	Bacteria (1)					Plasmid
210.3091	0.0294	Helicobacter pylori (72293)	Helicobacter (213849)	Helicobacteraceae (29547)	Campylobacterales (68525)	Epsilonproteobacteria (122	Proteobacteria (2)	Bacteria (1)					Plasmid
210.3092	0.0294	Helicobacter pylori (72293)	Helicobacter (213849)	Helicobacteraceae (29547)	Campylobacterales (68525)	Epsilonproteobacteria (122	Proteobacteria (2)	Bacteria (1)					Plasmid
210.3093	0.0294	Helicobacter pylori (72293)	Helicobacter (213849)	Helicobacteraceae (29547)	Campylobacterales (68525)	Epsilonproteobacteria (122	Proteobacteria (2)	Bacteria (1)					Plasmid
210.3094	0.0294	Helicobacter pylori (72293)	Helicobacter (213849)	Helicobacteraceae (29547)	Campylobacterales (68525)	Epsilonproteobacteria (122	Proteobacteria (2)	Bacteria (1)					Plasmid
210.3095	0.0294	Helicobacter pylori (72293)	Helicobacter (213849)	Helicobacteraceae (29547)	Campylobacterales (68525)	Epsilonproteobacteria (122	Proteobacteria (2)	Bacteria (1)					Plasmid
210.3096	0.0294	Helicobacter pylori (72293)	Helicobacter (213849)	Helicobacteraceae (29547)	Campylobacterales (68525)	Epsilonproteobacteria (122	Proteobacteria (2)	Bacteria (1)					Plasmid
210.3098	0.0294	Helicobacter pylori (72293)	Helicobacter (213849)	Helicobacteraceae (29547)	Campylobacterales (68525)	Epsilonproteobacteria (122	Proteobacteria (2)	Bacteria (1)					Plasmid
210.3099	0.0294	Helicobacter pylori (72293)	Helicobacter (213849)	Helicobacteraceae (29547)	Campylobacterales (68525)	Epsilonproteobacteria (122	Proteobacteria (2)	Bacteria (1)					Plasmid
210.3100	0.0294	Helicobacter pylori (72293)	Helicobacter (213849)	Helicobacteraceae (29547)	Campylobacterales (68525)	Epsilonproteobacteria (122	Proteobacteria (2)	Bacteria (1)					Plasmid
210.3101	0.0294	Helicobacter pylori (72293)	Helicobacter (213849)	Helicobacteraceae (29547)	Campylobacterales (68525)	Epsilonproteobacteria (122	Proteobacteria (2)	Bacteria (1)					Plasmid
210.3102	0.078	Helicobacter pylori (72293)	Helicobacter (213849)	Helicobacteraceae (29547)	Campylobacterales (68525)	Epsilonproteobacteria (122	Proteobacteria (2)	Bacteria (1)	100.00	0.00	99.70	99.40	WGS
210.3103	0.0938	Helicobacter pylori (72293)	Helicobacter (213849)	Helicobacteraceae (29547)	Campylobacterales (68525)	Epsilonproteobacteria (122	Proteobacteria (2)	Bacteria (1)	100.00	0.00	99.70	99.50	WGS
210.3104	0.092	Helicobacter pylori (72293)	Helicobacter (213849)	Helicobacteraceae (29547)	Campylobacterales (68525)	Epsilonproteobacteria (122	Proteobacteria (2)	Bacteria (1)	100.00	0.40	99.80	99.30	WGS
210.3105	0.092	Helicobacter pylori (72293)	Helicobacter (213849)	Helicobacteraceae (29547)	Campylobacterales (68525)	Epsilonproteobacteria (122	Proteobacteria (2)	Bacteria (1)	100.00	0.60	99.60	99.30	WGS
210.3106	0.0948	Helicobacter pylori (72293)	Helicobacter (213849)	Helicobacteraceae (29547)	Campylobacterales (68525)	Epsilonproteobacteria (122	Proteobacteria (2)	Bacteria (1)	100.00	0.00	99.60	99.50	WGS
210.3107	0.0938	Helicobacter pylori (72293)	Helicobacter (213849)	Helicobacteraceae (29547)	Campylobacterales (68525)	Epsilonproteobacteria (122	Proteobacteria (2)	Bacteria (1)	100.00	0.00	99.50	99.10	WGS
210.3108	0.0938	Helicobacter pylori (72293)	Helicobacter (213849)	Helicobacteraceae (29547)	Campylobacterales (68525)	Epsilonproteobacteria (122	Proteobacteria (2)	Bacteria (1)	100.00	0.00	99.40	99.30	WGS
210.3109	0.0948	Helicobacter pylori (72293)	Helicobacter (213849)	Helicobacteraceae (29547)	Campylobacterales (68525)	Epsilonproteobacteria (122	Proteobacteria (2)	Bacteria (1)	100.00	0.00	99.50	99.00	WGS
210.3110	0.0938	Helicobacter pylori (72293)	Helicobacter (213849)	Helicobacteraceae (29547)	Campylobacterales (68525)	Epsilonproteobacteria (122	Proteobacteria (2)	Bacteria (1)	100.00	0.30	99.50	99.20	WGS
210.3111	0.092	Helicobacter pylori (72293)	Helicobacter (213849)	Helicobacteraceae (29547)	Campylobacterales (68525)	Epsilonproteobacteria (122	Proteobacteria (2)	Bacteria (1)	100.00	0.30	99.50	99.00	WGS
210.3112	0.092	Helicobacter pylori (72293)	Helicobacter (213849)	Helicobacteraceae (29547)	Campylobacterales (68525)	Epsilonproteobacteria (122	Proteobacteria (2)	Bacteria (1)	100.00	0.00	99.60	99.00	WGS
210.3113	0.089	Helicobacter pylori (72293)	Helicobacter (213849)	Helicobacteraceae (29547)	Campylobacterales (68525)	Epsilonproteobacteria (122	Proteobacteria (2)	Bacteria (1)	100.00	0.00	99.60	99.40	WGS
210.3114	0.0938	Helicobacter pylori (72293)	Helicobacter (213849)	Helicobacteraceae (29547)	Campylobacterales (68525)	Epsilonproteobacteria (122	Proteobacteria (2)	Bacteria (1)	100.00	0.00	99.50	99.30	WGS
210.3115	0.092	Helicobacter pylori (72293)	Helicobacter (213849)	Helicobacteraceae (29547)	Campylobacterales (68525)	Epsilonproteobacteria (122	Proteobacteria (2)	Bacteria (1)	100.00	0.00	99.70	99.30	WGS
210.3116	0.0978	Helicobacter pylori (72293)	Helicobacter (213849)	Helicobacteraceae (29547)	Campylobacterales (68525)	Epsilonproteobacteria (122	Proteobacteria (2)	Bacteria (1)	100.00	0.30	99.80	99.80	WGS
210.3117	0.1016	Helicobacter pylori (72293)	Helicobacter (213849)	Helicobacteraceae (29547)	Campylobacterales (68525)	Epsilonproteobacteria (122	Proteobacteria (2)	Bacteria (1)	100.00	0.00	99.50	99.00	WGS
210.3118	0.0948	Helicobacter pylori (72293)	Helicobacter (213849)	Helicobacteraceae (29547)	Campylobacterales (68525)	Epsilonproteobacteria (122	Proteobacteria (2)	Bacteria (1)	100.00	0.60	99.40	99.00	WGS
210.3119	0.1056	Helicobacter pylori (72293)	Helicobacter (213849)	Helicobacteraceae (29547)	Campylobacterales (68525)	Epsilonproteobacteria (122	Proteobacteria (2)	Bacteria (1)	100.00	0.30	99.50	99.00	WGS
210.3120	0.1016	Helicobacter pylori (72293)	Helicobacter (213849)	Helicobacteraceae (29547)	Campylobacterales (68525)	Epsilonproteobacteria (122	Proteobacteria (2)	Bacteria (1)	100.00	0.60	99.40	99.00	WGS
210.3121	0.0948	Helicobacter pylori (72293)	Helicobacter (213849)	Helicobacteraceae (29547)	Campylobacterales (68525)	Epsilonproteobacteria (122	Proteobacteria (2)	Bacteria (1)	100.00	0.00	99.50	99.20	WGS
210.3122	0.1016	Helicobacter pylori (72293)	Helicobacter (213849)	Helicobacteraceae (29547)	Campylobacterales (68525)	Epsilonproteobacteria (122	Proteobacteria (2)	Bacteria (1)	100.00	0.00	99.70	99.60	WGS
210.3123	0.092	Helicobacter pylori (72293)	Helicobacter (213849)	Helicobacteraceae (29547)	Campylobacterales (68525)	Epsilonproteobacteria (122	Proteobacteria (2)	Bacteria (1)	100.00	0.00	99.70	99.30	WGS
210.3124	0.0988	Helicobacter pylori (72293)	Helicobacter (213849)	Helicobacteraceae (29547)	Campylobacterales (68525)	Epsilonproteobacteria (122	Proteobacteria (2)	Bacteria (1)	100.00	0.30	99.80	99.70	WGS
210.3125	0.092	Helicobacter pylori (72293)	Helicobacter (213849)	Helicobacteraceae (29547)	Campylobacterales (68525)	Epsilonproteobacteria (122	Proteobacteria (2)	Bacteria (1)	100.00	0.00	99.30	99.00	WGS
210.3126	0.0988	Helicobacter pylori (72293)	Helicobacter (213849)	Helicobacteraceae (29547)	Campylobacterales (68525)	Epsilonproteobacteria (122	Proteobacteria (2)	Bacteria (1)	100.00	0.00	99.60	99.50	WGS
210.3127	0.092	Helicobacter pylori (72293)	Helicobacter (213849)	Helicobacteraceae (29547)	Campylobacterales (68525)	Epsilonproteobacteria (122	Proteobacteria (2)	Bacteria (1)	100.00	0.00	99.60	99.10	WGS
210.3128	0.091	Helicobacter pylori (72293)	Helicobacter (213849)	Helicobacteraceae (29547)	Campylobacterales (68525)	Epsilonproteobacteria (122	Proteobacteria (2)	Bacteria (1)	100.00	0.00	99.80	99.40	WGS
210.3129	0.0948	Helicobacter pylori (72293)	Helicobacter (213849)	Helicobacteraceae (29547)	Campylobacterales (68525)	Epsilonproteobacteria (122	Proteobacteria (2)	Bacteria (1)	100.00	0.60	99.30	98.40	WGS
210.3130	0.0948	Helicobacter pylori (72293)	Helicobacter (213849)	Helicobacteraceae (29547)	Campylobacterales (68525)	Epsilonproteobacteria (122	Proteobacteria (2)	Bacteria (1)	100.00	0.60	99.70	99.60	WGS
210.3131	0.1028	Helicobacter pylori (72293)	Helicobacter (213849)	Helicobacteraceae (29547)	Campylobacterales (68525)	Epsilonproteobacteria (122	Proteobacteria (2)	Bacteria (1)	100.00	0.30	99.60	99.20	WGS
210.3132	0.091	Helicobacter pylori (72293)	Helicobacter (213849)	Helicobacteraceae (29547)	Campylobacterales (68525)	Epsilonproteobacteria (122	Proteobacteria (2)	Bacteria (1)	100.00	1.40	99.80	99.30	WGS
210.3133	0.0988	Helicobacter pylori (72293)	Helicobacter (213849)	Helicobacteraceae (29547)	Campylobacterales (68525)	Epsilonproteobacteria (122	Proteobacteria (2)	Bacteria (1)	100.00	0.00	99.70	99.10	WGS
210.3134	0.092	Helicobacter pylori (72293)	Helicobacter (213849)	Helicobacteraceae (29547)	Campylobacterales (68525)	Epsilonproteobacteria (122	Proteobacteria (2)	Bacteria (1)	100.00	2.80	99.80	99.40	WGS
210.3135	0.091	Helicobacter pylori (72293)	Helicobacter (213849)	Helicobacteraceae (29547)	Campylobacterales (68525)	Epsilonproteobacteria (122	Proteobacteria (2)	Bacteria (1)	100.00	0.00	99.80	99.60	WGS
210.3136	0.0948	Helicobacter pylori (72293)	Helicobacter (213849)	Helicobacteraceae (29547)	Campylobacterales (68525)	Epsilonproteobacteria (122	Proteobacteria (2)	Bacteria (1)	100.00	0.60	99.50	99.00	WGS
210.3137	0.1016	Helicobacter pylori (72293)	Helicobacter (213849)	Helicobacteraceae (29547)	Campylobacterales (68525)	Epsilonproteobacteria (122	Proteobacteria (2)	Bacteria (1)	100.00	0.00	99.50	99.30	WGS
210.3138	0.0948	Helicobacter pylori (72293)	Helicobacter (213849)	Helicobacteraceae (29547)	Campylobacterales (68525)	Epsilonproteobacteria (122	Proteobacteria (2)	Bacteria (1)	100.00	0.00	99.70	99.40	WGS
210.3139	0.0948	Helicobacter pylori (72293)	Helicobacter (213849)	Helicobacteraceae (29547)	Campylobacterales (68525)	Epsilonproteobacteria (122	Proteobacteria (2)	Bacteria (1)	100.00	1.40	99.50	98.90	WGS
210.3140	0.0978	Helicobacter pylori (72293)	Helicobacter (213849)	Helicobacteraceae (29547)	Campylobacterales (68525)	Epsilonproteobacteria (122	Proteobacteria (2)	Bacteria (1)	100.00	0.00	99.50	99.00	WGS
210.3141	0.092	Helicobacter pylori (72293)	Helicobacter (213849)	Helicobacteraceae (29547)	Campylobacterales (68525)	Epsilonproteobacteria (122	Proteobacteria (2)	Bacteria (1)	100.00	0.00	99.90	99.90	WGS
210.3142	0.0948	Helicobacter pylori (72293)	Helicobacter (213849)	Helicobacteraceae (29547)	Campylobacterales (68525)	Epsilonproteobacteria (122	Proteobacteria (2)	Bacteria (1)	100.00	0.00	99.50	99.30	WGS
210.3143	0.091	Helicobacter pylori (72293)	Helicobacter (213849)	Helicobacteraceae (29547)	Campylobacterales (68525)	Epsilonproteobacteria (122	Proteobacteria (2)	Bacteria (1)	100.00	0.00	99.60	99.00	WGS
210.4121	0.0988	Helicobacter pylori (72293)	Helicobacter (213849)	Helicobacteraceae (29547)	Campylobacterales (68525)	Epsilonproteobacteria (122	Proteobacteria (2)	Bacteria (1)	100.00	0.00	100.00	99.70	Complete
210.4122	0.0988	Helicobacter pylori (72293)	Helicobacter (213849)	Helicobacteraceae (29547)	Campylobacterales (68525)	Epsilonproteobacteria (122	Proteobacteria (2)	Bacteria (1)	100.00	2.30	100.00	99.00	Complete
210.4123	0.0988	Helicobacter pylori (72293)	Helicobacter (213849)	Helicobacteraceae (29547)	Campylobacterales (68525)	Epsilonproteobacteria (122	Proteobacteria (2)	Bacteria (1)	100.00	0.90	100.00	99.40	Complete
210.4124	0.0988	Helicobacter pylori (72293)	Helicobacter (213849)	Helicobacteraceae (29547)	Campylobacterales (68525)	Epsilonproteobacteria (122	Proteobacteria (2)	Bacteria (1)	100.00	0.60	100.00	99.70	Complete
210.4125	0.0938	Helicobacter pylori (72293)	Helicobacter (213849)	Helicobacteraceae (29547)	Campylobacterales (68525)	Epsilonproteobacteria (122	Proteobacteria (2)	Bacteria (1)	100.00	0.00	99.90	99.50	Complete
210.4126	0.0938	Helicobacter pylori (72293)	Helicobacter (213849)	Helicobacteraceae (29547)	Campylobacterales (68525)	Epsilonproteobacteria (122	Proteobacteria (2)	Bacteria (1)	100.00	1.20	99.90	99.20	Complete
210.4127	0.0938	Helicobacter pylori (72293)	Helicobacter (213849)	Helicobacteraceae (29547)	Campylobacterales (68525)	Epsilonproteobacteria (122	Proteobacteria (2)	Bacteria (1)	100.00	0.00	99.90	99.50	Complete
210.4128	0.0938	Helicobacter pylori (72293)	Helicobacter (213849)	Helicobacteraceae (29547)	Campylobacterales (68525)	Epsilonproteobacteria (122	Proteobacteria (2)	Bacteria (1)	100.00	7.70	99.90	97.00	WGS
210.4129	0.0938	Helicobacter pylori (72293)	Helicobacter (213849)	Helicobacteraceae (29547)	Campylobacterales (68525)	Epsilonproteobacteria (122	Proteobacteria (2)	Bacteria (1)	100.00	2.70	99.90	98.00	WGS
210.4130	0.0938	Helicobacter pylori (72293)	Helicobacter (213849)	Helicobacteraceae (29547)	Campylobacterales (68525)	Epsilonproteobacteria (122	Proteobacteria (2)	Bacteria (1)	100.00	0.30	99.90	99.50	WGS
210.4131	0.0938	Helicobacter pylori (72293)	Helicobacter (213849)	Helicobacteraceae (29547)	Campylobacterales (68525)	Epsilonproteobacteria (122	Proteobacteria (2)	Bacteria (1)	100.00	4.40	99.90	98.20	Complete
210.4132	0.1006	Helicobacter pylori (72293)	Helicobacter (213849)	Helicobacteraceae (29547)	Campylobacterales (68525)	Epsilonproteobacteria (122	Proteobacteria (2)	Bacteria (1)	100.00	2.50	99.90	99.00	Complete
210.4133	0.091	Helicobacter pylori (72293)	Helicobacter (213849)	Helicobacteraceae (29547)	Campylobacterales (68525)	Epsilonproteobacteria (122	Proteobacteria (2)	Bacteria (1)	99.70	2.40	99.80	98.50	Complete
210.4134	0.0938	Helicobacter pylori (72293)	Helicobacter (213849)	Helicobacteraceae (29547)	Campylobacterales (68525)	Epsilonproteobacteria (122	Proteobacteria (2)	Bacteria (1)	100.00	0.00	99.90	99.50	Complete
210.4135	0.0938	Helicobacter pylori (72293)	Helicobacter (213849)	Helicobacteraceae (29547)	Campylobacterales (68525)	Epsilonproteobacteria (122	Proteobacteria (2)	Bacteria (1)	100.00	0.00	99.90	99.50	Complete
210.4136	0.0938	Helicobacter pylori (72293)	Helicobacter (213849)	Helicobacteraceae (29547)	Campylobacterales (68525)	Epsilonproteobacteria (122	Proteobacteria (2)	Bacteria (1)	100.00	0.00	99.90	99.50	Complete
210.4137	0.0938	Helicobacter pylori (72293)	Helicobacter (213849)	Helicobacteraceae (29547)	Campylobacterales (68525)	Epsilonproteobacteria (122	Proteobacteria (2)	Bacteria (1)	100.00	0.00	99.90	99.50	Complete
210.4138	0.0938	Helicobacter pylori (72293)	Helicobacter (213849)	Helicobacteraceae (29547)	Campylobacterales (68525)	Epsilonproteobacteria (122	Proteobacteria (2)	Bacteria (1)	100.00	0.00	99.90	99.50	Complete
210.4139	0.0938	Helicobacter pylori (72293)	Helicobacter (213849)	Helicobacteraceae (29547)	Campylobacterales (68525)	Epsilonproteobacteria (122	Proteobacteria (2)	Bacteria (1)	100.00	0.00	99.90	99.50	Complete
210.4140	0.0938	Helicobacter pylori (72293)	Helicobacter (213849)	Helicobacteraceae (29547)	Campylobacterales (68525)	Epsilonproteobacteria (122	Proteobacteria (2)	Bacteria (1)	100.00	0.00	99.90	99.50	Complete
210.4141	0.0938	Helicobacter pylori (72293)	Helicobacter (213849)	Helicobacteraceae (29547)	Campylobacterales (68525)	Epsilonproteobacteria (122	Proteobacteria (2)	Bacteria (1)	100.00	0.00	99.90	99.50	Complete
210.4142	0.0938	Helicobacter pylori (72293)	Helicobacter (213849)	Helicobacteraceae (29547)	Campylobacterales (68525)	Epsilonproteobacteria (122	Proteobacteria (2)	Bacteria (1)	100.00	0.00	99.90	99.50	Complete
210.4143	0.0938	Helicobacter pylori (72293)	Helicobacter (213849)	Helicobacteraceae (29547)	Campylobacterales (68525)	Epsilonproteobacteria (122	Proteobacteria (2)	Bacteria (1)	100.00	0.00	99.90	99.50	Complete
210.4144	0.0938	Helicobacter pylori (72293)	Helicobacter (213849)	Helicobacteraceae (29547)	Campylobacterales (68525)	Epsilonproteobacteria (122	Proteobacteria (2)	Bacteria (1)	100.00	0.00	99.90	99.50	Complete
210.4145	0.0938	Helicobacter pylori (72293)	Helicobacter (213849)	Helicobacteraceae (29547)	Campylobacterales (68525)	Epsilonproteobacteria (122	Proteobacteria (2)	Bacteria (1)	100.00	0.00	99.90	99.50	Complete
210.4146	0.0938	Helicobacter pylori (72293)	Helicobacter (213849)	Helicobacteraceae (29547)	Campylobacterales (68525)	Epsilonproteobacteria (122	Proteobacteria (2)	Bacteria (1)	100.00	0.00	99.90	99.50	Complete
210.4147	0.0938	Helicobacter pylori (72293)	Helicobacter (213849)	Helicobacteraceae (29547)	Campylobacterales (68525)	Epsilonproteobacteria (122	Proteobacteria (2)	Bacteria (1)	100.00	0.00	99.90	99.50	Complete
210.4148	0.0938	Helicobacter pylori (72293)	Helicobacter (213849)	Helicobacteraceae (29547)	Campylobacterales (68525)	Epsilonproteobacteria (122	Proteobacteria (2)	Bacteria (1)	100.00	0.00	99.90	99.50	Complete
210.4149	0.0988	Helicobacter pylori (72293)	Helicobacter (213849)	Helicobacteraceae (29547)	Campylobacterales (68525)	Epsilonproteobacteria (122	Proteobacteria (2)	Bacteria (1)	100.00	0.30	100.00	99.70	Complete
210.4150	0.092	Helicobacter pylori (72293)	Helicobacter (213849)	Helicobacteraceae (29547)	Campylobacterales (68525)	Epsilonproteobacteria (122	Proteobacteria (2)	Bacteria (1)	100.00	0.60	100.00	99.80	Complete
210.4153	0.092	Helicobacter pylori (72293)	Helicobacter (213849)	Helicobacteraceae (29547)	Campylobacterales (68525)	Epsilonproteobacteria (122	Proteobacteria (2)	Bacteria (1)	100.00	0.00	99.90	99.10	WGS
210.4154	0.0948	Helicobacter pylori (72293)	Helicobacter (213849)	Helicobacteraceae (29547)	Campylobacterales (68525)	Epsilonproteobacteria (122	Proteobacteria (2)	Bacteria (1)	100.00	0.60	99.80	99.10	WGS
210.4155	0.0938	Helicobacter pylori (72293)	Helicobacter (213849)	Helicobacteraceae (29547)	Campylobacterales (68525)	Epsilonproteobacteria (122	Proteobacteria (2)	Bacteria (1)	100.00	0.90	99.70	99.20	WGS
210.4156	0.0988	Helicobacter pylori (72293)	Helicobacter (213849)	Helicobacteraceae (29547)	Campylobacterales (68525)	Epsilonproteobacteria (122	Proteobacteria (2)	Bacteria (1)	100.00	0.30	99.90	99.40	WGS
210.4157	0.0938	Helicobacter pylori (72293)	Helicobacter (213849)	Helicobacteraceae (29547)	Campylobacterales (68525)	Epsilonproteobacteria (122	Proteobacteria (2)	Bacteria (1)	100.00	0.60	99.90	99.40	WGS
210.4158	0.091	Helicobacter pylori (72293)	Helicobacter (213849)	Helicobacteraceae (29547)	Campylobacterales (68525)	Epsilonproteobacteria (122	Proteobacteria (2)	Bacteria (1)	100.00	0.00	99.90	99.60	WGS
210.4159	0.1006	Helicobacter pylori (72293)	Helicobacter (213849)	Helicobacteraceae (29547)	Campylobacterales (68525)	Epsilonproteobacteria (122	Proteobacteria (2)	Bacteria (1)	100.00	0.00	99.90	99.40	WGS
210.4160	0.0938	Helicobacter pylori (72293)	Helicobacter (213849)	Helicobacteraceae (29547)	Campylobacterales (68525)	Epsilonproteobacteria (122	Proteobacteria (2)	Bacteria (1)	100.00	0.00	99.90	99.50	WGS
210.4161	0.0938	Helicobacter pylori (72293)	Helicobacter (213849)	Helicobacteraceae (29547)	Campylobacterales (68525)	Epsilonproteobacteria (122	Proteobacteria (2)	Bacteria (1)	100.00	0.00	99.90	99.60	WGS
210.4162	0.1016	Helicobacter pylori (72293)	Helicobacter (213849)	Helicobacteraceae (29547)	Campylobacterales (68525)	Epsilonproteobacteria (122	Proteobacteria (2)	Bacteria (1)	100.00	0.40	99.90	99.60	WGS
210.4163	0.091	Helicobacter pylori (72293)	Helicobacter (213849)	Helicobacteraceae (29547)	Campylobacterales (68525)	Epsilonproteobacteria (122	Proteobacteria (2)	Bacteria (1)	100.00	0.30	99.90	99.50	WGS
210.4164	0.091	Helicobacter pylori (72293)	Helicobacter (213849)	Helicobacteraceae (29547)	Campylobacterales (68525)	Epsilonproteobacteria (122	Proteobacteria (2)	Bacteria (1)	99.70	4.10	99.90	98.10	WGS
210.4165	0.0988	Helicobacter pylori (72293)	Helicobacter (213849)	Helicobacteraceae (29547)	Campylobacterales (68525)	Epsilonproteobacteria (122	Proteobacteria (2)	Bacteria (1)	100.00	2.60	99.80	99.00	WGS
210.4166	0.0938	Helicobacter pylori (72293)	Helicobacter (213849)	Helicobacteraceae (29547)	Campylobacterales (68525)	Epsilonproteobacteria (122	Proteobacteria (2)	Bacteria (1)	100.00	0.30	99.90	99.60	WGS
210.4167	0.0948	Helicobacter pylori (72293)	Helicobacter (213849)	Helicobacteraceae (29547)	Campylobacterales (68525)	Epsilonproteobacteria (122	Proteobacteria (2)	Bacteria (1)	100.00	0.30	99.90	99.40	WGS
210.4168	0.0938	Helicobacter pylori (72293)	Helicobacter (213849)	Helicobacteraceae (29547)	Campylobacterales (68525)	Epsilonproteobacteria (122	Proteobacteria (2)	Bacteria (1)	100.00	0.00	99.80	99.20	WGS
210.4169	0.0948	Helicobacter pylori (72293)	Helicobacter (213849)	Helicobacteraceae (29547)	Campylobacterales (68525)	Epsilonproteobacteria (122	Proteobacteria (2)	Bacteria (1)	99.70	3.50	99.90	98.40	WGS
210.4170	0.0938	Helicobacter pylori (72293)	Helicobacter (213849)	Helicobacteraceae (29547)	Campylobacterales (68525)	Epsilonproteobacteria (122	Proteobacteria (2)	Bacteria (1)	100.00	3.60	99.80	98.30	WGS
210.4171	0.0948	Helicobacter pylori (72293)	Helicobacter (213849)	Helicobacteraceae (29547)	Campylobacterales (68525)	Epsilonproteobacteria (122	Proteobacteria (2)	Bacteria (1)	100.00	3.20	99.90	98.40	WGS
210.4172	0.0948	Helicobacter pylori (72293)	Helicobacter (213849)	Helicobacteraceae (29547)	Campylobacterales (68525)	Epsilonproteobacteria (122	Proteobacteria (2)	Bacteria (1)	100.00	0.30	99.90	99.30	WGS
210.4173	0.1006	Helicobacter pylori (72293)	Helicobacter (213849)	Helicobacteraceae (29547)	Campylobacterales (68525)	Epsilonproteobacteria (122	Proteobacteria (2)	Bacteria (1)	100.00	0.00	99.90	99.70	WGS
210.4174	0.091	Helicobacter pylori (72293)	Helicobacter (213849)	Helicobacteraceae (29547)	Campylobacterales (68525)	Epsilonproteobacteria (122	Proteobacteria (2)	Bacteria (1)	97.90	15.20	99.20	93.60	WGS
210.4175	0.0978	Helicobacter pylori (72293)	Helicobacter (213849)	Helicobacteraceae (29547)	Campylobacterales (68525)	Epsilonproteobacteria (122	Proteobacteria (2)	Bacteria (1)	100.00	11.10	99.90	96.90	WGS
210.4176	0.091	Helicobacter pylori (72293)	Helicobacter (213849)	Helicobacteraceae (29547)	Campylobacterales (68525)	Epsilonproteobacteria (122	Proteobacteria (2)	Bacteria (1)	99.10	8.60	99.60	97.00	WGS
210.4177	0.092	Helicobacter pylori (72293)	Helicobacter (213849)	Helicobacteraceae (29547)	Campylobacterales (68525)	Epsilonproteobacteria (122	Proteobacteria (2)	Bacteria (1)	100.00	0.00	99.90	99.80	WGS
210.4178	0.092	Helicobacter pylori (72293)	Helicobacter (213849)	Helicobacteraceae (29547)	Campylobacterales (68525)	Epsilonproteobacteria (122	Proteobacteria (2)	Bacteria (1)	100.00	0.00	99.80	99.50	WGS
210.4179	0.091	Helicobacter pylori (72293)	Helicobacter (213849)	Helicobacteraceae (29547)	Campylobacterales (68525)	Epsilonproteobacteria (122	Proteobacteria (2)	Bacteria (1)	100.00	0.30	99.90	99.30	WGS
210.4180	0.0948	Helicobacter pylori (72293)	Helicobacter (213849)	Helicobacteraceae (29547)	Campylobacterales (68525)	Epsilonproteobacteria (122	Proteobacteria (2)	Bacteria (1)	100.00	0.70	99.90	99.10	WGS
210.4181	0.0948	Helicobacter pylori (72293)	Helicobacter (213849)	Helicobacteraceae (29547)	Campylobacterales (68525)	Epsilonproteobacteria (122	Proteobacteria (2)	Bacteria (1)	100.00	0.00	99.90	99.60	WGS
210.4182	0.092	Helicobacter pylori (72293)	Helicobacter (213849)	Helicobacteraceae (29547)	Campylobacterales (68525)	Epsilonproteobacteria (122	Proteobacteria (2)	Bacteria (1)	99.70	5.60	99.80	98.10	WGS
210.4183	0.1006	Helicobacter pylori (72293)	Helicobacter (213849)	Helicobacteraceae (29547)	Campylobacterales (68525)	Epsilonproteobacteria (122	Proteobacteria (2)	Bacteria (1)	100.00	0.00	99.90	99.50	WGS
210.4184	0.091	Helicobacter pylori (72293)	Helicobacter (213849)	Helicobacteraceae (29547)	Campylobacterales (68525)	Epsilonproteobacteria (122	Proteobacteria (2)	Bacteria (1)	100.00	0.00	99.90	99.50	WGS
210.4185	0.0948	Helicobacter pylori (72293)	Helicobacter (213849)	Helicobacteraceae (29547)	Campylobacterales (68525)	Epsilonproteobacteria (122	Proteobacteria (2)	Bacteria (1)	100.00	2.50	99.90	98.10	WGS
210.4186	0.0948	Helicobacter pylori (72293)	Helicobacter (213849)	Helicobacteraceae (29547)	Campylobacterales (68525)	Epsilonproteobacteria (122	Proteobacteria (2)	Bacteria (1)	99.40	0.60	99.60	99.30	WGS
210.4187	0.0938	Helicobacter pylori (72293)	Helicobacter (213849)	Helicobacteraceae (29547)	Campylobacterales (68525)	Epsilonproteobacteria (122	Proteobacteria (2)	Bacteria (1)	100.00	0.00	99.80	99.60	WGS
210.4188	0.0948	Helicobacter pylori (72293)	Helicobacter (213849)	Helicobacteraceae (29547)	Campylobacterales (68525)	Epsilonproteobacteria (122	Proteobacteria (2)	Bacteria (1)	100.00	0.60	99.90	99.60	WGS
210.4189	0.0938	Helicobacter pylori (72293)	Helicobacter (213849)	Helicobacteraceae (29547)	Campylobacterales (68525)	Epsilonproteobacteria (122	Proteobacteria (2)	Bacteria (1)	100.00	0.00	99.80	99.50	WGS
210.4190	0.0948	Helicobacter pylori (72293)	Helicobacter (213849)	Helicobacteraceae (29547)	Campylobacterales (68525)	Epsilonproteobacteria (122	Proteobacteria (2)	Bacteria (1)	100.00	0.00	99.80	99.60	WGS
210.4191	0.0948	Helicobacter pylori (72293)	Helicobacter (213849)	Helicobacteraceae (29547)	Campylobacterales (68525)	Epsilonproteobacteria (122	Proteobacteria (2)	Bacteria (1)	100.00	0.00	99.90	99.30	WGS
210.4192	0.0988	Helicobacter pylori (72293)	Helicobacter (213849)	Helicobacteraceae (29547)	Campylobacterales (68525)	Epsilonproteobacteria (122	Proteobacteria (2)	Bacteria (1)	100.00	0.60	100.00	99.70	WGS
210.4193	0.092	Helicobacter pylori (72293)	Helicobacter (213849)	Helicobacteraceae (29547)	Campylobacterales (68525)	Epsilonproteobacteria (122	Proteobacteria (2)	Bacteria (1)	100.00	17.20	99.90	93.60	WGS
210.4194	0.1006	Helicobacter pylori (72293)	Helicobacter (213849)	Helicobacteraceae (29547)	Campylobacterales (68525)	Epsilonproteobacteria (122	Proteobacteria (2)	Bacteria (1)	100.00	0.60	99.90	99.30	WGS
210.4195	0.0938	Helicobacter pylori (72293)	Helicobacter (213849)	Helicobacteraceae (29547)	Campylobacterales (68525)	Epsilonproteobacteria (122	Proteobacteria (2)	Bacteria (1)	100.00	0.30	99.90	99.50	WGS
210.4196	0.1006	Helicobacter pylori (72293)	Helicobacter (213849)	Helicobacteraceae (29547)	Campylobacterales (68525)	Epsilonproteobacteria (122	Proteobacteria (2)	Bacteria (1)	100.00	2.30	99.70	99.20	WGS
210.4197	0.091	Helicobacter pylori (72293)	Helicobacter (213849)	Helicobacteraceae (29547)	Campylobacterales (68525)	Epsilonproteobacteria (122	Proteobacteria (2)	Bacteria (1)	99.70	5.50	99.80	97.30	WGS
210.4198	0.1016	Helicobacter pylori (72293)	Helicobacter (213849)	Helicobacteraceae (29547)	Campylobacterales (68525)	Epsilonproteobacteria (122	Proteobacteria (2)	Bacteria (1)	100.00	0.30	99.90	99.50	WGS
210.4199	0.1016	Helicobacter pylori (72293)	Helicobacter (213849)	Helicobacteraceae (29547)	Campylobacterales (68525)	Epsilonproteobacteria (122	Proteobacteria (2)	Bacteria (1)	100.00	0.30	99.90	99.20	WGS
210.4200	0.0938	Helicobacter pylori (72293)	Helicobacter (213849)	Helicobacteraceae (29547)	Campylobacterales (68525)	Epsilonproteobacteria (122	Proteobacteria (2)	Bacteria (1)	100.00	1.40	99.90	99.30	WGS
210.4201	0.0978	Helicobacter pylori (72293)	Helicobacter (213849)	Helicobacteraceae (29547)	Campylobacterales (68525)	Epsilonproteobacteria (122	Proteobacteria (2)	Bacteria (1)	100.00	0.00	99.90	99.30	WGS
210.4202	0.0948	Helicobacter pylori (72293)	Helicobacter (213849)	Helicobacteraceae (29547)	Campylobacterales (68525)	Epsilonproteobacteria (122	Proteobacteria (2)	Bacteria (1)	100.00	0.30	99.80	99.00	WGS
210.4203	0.1016	Helicobacter pylori (72293)	Helicobacter (213849)	Helicobacteraceae (29547)	Campylobacterales (68525)	Epsilonproteobacteria (122	Proteobacteria (2)	Bacteria (1)	100.00	9.10	99.90	97.10	WGS
210.4204	0.0638	Helicobacter pylori (72293)	Helicobacter (213849)	Helicobacteraceae (29547)	Campylobacterales (68525)	Epsilonproteobacteria (122	Proteobacteria (2)	Bacteria (1)	100.00	0.00	99.70	99.40	WGS
210.4205	0.092	Helicobacter pylori (72293)	Helicobacter (213849)	Helicobacteraceae (29547)	Campylobacterales (68525)	Epsilonproteobacteria (122	Proteobacteria (2)	Bacteria (1)	100.00	0.00	99.90	99.50	WGS
210.4206	0.0294	Helicobacter pylori (72293)	Helicobacter (213849)	Helicobacteraceae (29547)	Campylobacterales (68525)	Epsilonproteobacteria (122	Proteobacteria (2)	Bacteria (1)	100.00	0.00	99.90	99.60	WGS
210.4207	0.091	Helicobacter pylori (72293)	Helicobacter (213849)	Helicobacteraceae (29547)	Campylobacterales (68525)	Epsilonproteobacteria (122	Proteobacteria (2)	Bacteria (1)	100.00	10.30	99.90	96.40	WGS
210.4208	0.0938	Helicobacter pylori (72293)	Helicobacter (213849)	Helicobacteraceae (29547)	Campylobacterales (68525)	Epsilonproteobacteria (122	Proteobacteria (2)	Bacteria (1)	100.00	0.50	99.90	98.90	WGS
210.4209	0.0938	Helicobacter pylori (72293)	Helicobacter (213849)	Helicobacteraceae (29547)	Campylobacterales (68525)	Epsilonproteobacteria (122	Proteobacteria (2)	Bacteria (1)	100.00	0.90	99.90	99.50	WGS
210.4210	0.091	Helicobacter pylori (72293)	Helicobacter (213849)	Helicobacteraceae (29547)	Campylobacterales (68525)	Epsilonproteobacteria (122	Proteobacteria (2)	Bacteria (1)	100.00	0.00	99.90	99.50	WGS
210.4211	0.0988	Helicobacter pylori (72293)	Helicobacter (213849)	Helicobacteraceae (29547)	Campylobacterales (68525)	Epsilonproteobacteria (122	Proteobacteria (2)	Bacteria (1)	100.00	0.60	99.90	99.40	WGS
210.4212	0.091	Helicobacter pylori (72293)	Helicobacter (213849)	Helicobacteraceae (29547)	Campylobacterales (68525)	Epsilonproteobacteria (122	Proteobacteria (2)	Bacteria (1)	100.00	0.60	99.90	99.00	WGS
210.4213	0.0938	Helicobacter pylori (72293)	Helicobacter (213849)	Helicobacteraceae (29547)	Campylobacterales (68525)	Epsilonproteobacteria (122	Proteobacteria (2)	Bacteria (1)	100.00	5.30	99.90	97.90	WGS
210.4214	0.0938	Helicobacter pylori (72293)	Helicobacter (213849)	Helicobacteraceae (29547)	Campylobacterales (68525)	Epsilonproteobacteria (122	Proteobacteria (2)	Bacteria (1)	100.00	0.60	99.90	99.50	WGS
210.4215	0.1016	Helicobacter pylori (72293)	Helicobacter (213849)	Helicobacteraceae (29547)	Campylobacterales (68525)	Epsilonproteobacteria (122	Proteobacteria (2)	Bacteria (1)	100.00	0.00	99.70	99.30	WGS
210.4216	0.0938	Helicobacter pylori (72293)	Helicobacter (213849)	Helicobacteraceae (29547)	Campylobacterales (68525)	Epsilonproteobacteria (122	Proteobacteria (2)	Bacteria (1)	100.00	0.30	99.80	99.30	WGS
210.4217	0.0978	Helicobacter pylori (72293)	Helicobacter (213849)	Helicobacteraceae (29547)	Campylobacterales (68525)	Epsilonproteobacteria (122	Proteobacteria (2)	Bacteria (1)	100.00	0.30	99.80	99.30	WGS
210.4218	0.0978	Helicobacter pylori (72293)	Helicobacter (213849)	Helicobacteraceae (29547)	Campylobacterales (68525)	Epsilonproteobacteria (122	Proteobacteria (2)	Bacteria (1)	100.00	0.30	99.90	99.60	WGS
210.4219	0.0948	Helicobacter pylori (72293)	Helicobacter (213849)	Helicobacteraceae (29547)	Campylobacterales (68525)	Epsilonproteobacteria (122	Proteobacteria (2)	Bacteria (1)	100.00	1.00	99.90	99.00	WGS
210.4220	0.1016	Helicobacter pylori (72293)	Helicobacter (213849)	Helicobacteraceae (29547)	Campylobacterales (68525)	Epsilonproteobacteria (122	Proteobacteria (2)	Bacteria (1)	100.00	0.00	99.90	99.60	Complete
210.4852	0.092	Helicobacter pylori (72293)	Helicobacter (213849)	Helicobacteraceae (29547)	Campylobacterales (68525)	Epsilonproteobacteria (122	Proteobacteria (2)	Bacteria (1)	100.00	0.00	99.90	99.60	WGS
210.4853	0.092	Helicobacter pylori (72293)	Helicobacter (213849)	Helicobacteraceae (29547)	Campylobacterales (68525)	Epsilonproteobacteria (122	Proteobacteria (2)	Bacteria (1)	100.00	0.30	99.90	99.60	WGS
210.4854	0.092	Helicobacter pylori (72293)	Helicobacter (213849)	Helicobacteraceae (29547)	Campylobacterales (68525)	Epsilonproteobacteria (122	Proteobacteria (2)	Bacteria (1)	100.00	0.30	99.90	99.60	WGS
210.4856	0.0948	Helicobacter pylori (72293)	Helicobacter (213849)	Helicobacteraceae (29547)	Campylobacterales (68525)	Epsilonproteobacteria (122	Proteobacteria (2)	Bacteria (1)	100.00	0.30	99.60	99.30	WGS
210.4857	0.0938	Helicobacter pylori (72293)	Helicobacter (213849)	Helicobacteraceae (29547)	Campylobacterales (68525)	Epsilonproteobacteria (122	Proteobacteria (2)	Bacteria (1)	99.70	1.30	99.30	98.30	WGS
210.4858	0.0948	Helicobacter pylori (72293)	Helicobacter (213849)	Helicobacteraceae (29547)	Campylobacterales (68525)	Epsilonproteobacteria (122	Proteobacteria (2)	Bacteria (1)	100.00	1.60	99.30	98.80	WGS
210.4975	0.0948	Helicobacter pylori (72293)	Helicobacter (213849)	Helicobacteraceae (29547)	Campylobacterales (68525)	Epsilonproteobacteria (122	Proteobacteria (2)	Bacteria (1)			99.50	99.00	WGS
210.4976	0.0948	Helicobacter pylori (72293)	Helicobacter (213849)	Helicobacteraceae (29547)	Campylobacterales (68525)	Epsilonproteobacteria (122	Proteobacteria (2)	Bacteria (1)			99.30	98.90	WGS
210.4977	0.0938	Helicobacter pylori (72293)	Helicobacter (213849)	Helicobacteraceae (29547)	Campylobacterales (68525)	Epsilonproteobacteria (122	Proteobacteria (2)	Bacteria (1)			99.70	99.30	WGS
210.4978	0.0948	Helicobacter pylori (72293)	Helicobacter (213849)	Helicobacteraceae (29547)	Campylobacterales (68525)	Epsilonproteobacteria (122	Proteobacteria (2)	Bacteria (1)			99.10	98.60	WGS
210.4979	0.091	Helicobacter pylori (72293)	Helicobacter (213849)	Helicobacteraceae (29547)	Campylobacterales (68525)	Epsilonproteobacteria (122	Proteobacteria (2)	Bacteria (1)			99.80	99.50	WGS
210.4980	0.0938	Helicobacter pylori (72293)	Helicobacter (213849)	Helicobacteraceae (29547)	Campylobacterales (68525)	Epsilonproteobacteria (122	Proteobacteria (2)	Bacteria (1)			99.80	99.10	WGS
210.4981	0.0938	Helicobacter pylori (72293)	Helicobacter (213849)	Helicobacteraceae (29547)	Campylobacterales (68525)	Epsilonproteobacteria (122	Proteobacteria (2)	Bacteria (1)			99.40	99.10	WGS

210.4982	0.092	Helicobacter pylori (72293)	Helicobacter (213849)	Helicobacteraceae (29547)	Campylobacterales (68525)	Epsilonproteobacteria (122	Proteobacteria (2)	Bacteria (1)			99.60	99.30	WGS
210.4983	0.092	Helicobacter pylori (72293)	Helicobacter (213849)	Helicobacteraceae (29547)	Campylobacterales (68525)	Epsilonproteobacteria (122	Proteobacteria (2)	Bacteria (1)			99.60	99.20	WGS
210.4984	0.092	Helicobacter pylori (72293)	Helicobacter (213849)	Helicobacteraceae (29547)	Campylobacterales (68525)	Epsilonproteobacteria (122	Proteobacteria (2)	Bacteria (1)			99.60	99.00	WGS
210.4985	0.0988	Helicobacter pylori (72293)	Helicobacter (213849)	Helicobacteraceae (29547)	Campylobacterales (68525)	Epsilonproteobacteria (122	Proteobacteria (2)	Bacteria (1)			99.90	99.50	WGS
210.4986	0.09	Helicobacter pylori (72293)	Helicobacter (213849)	Helicobacteraceae (29547)	Campylobacterales (68525)	Epsilonproteobacteria (122	Proteobacteria (2)	Bacteria (1)			99.50	99.00	WGS
210.4987	0.0948	Helicobacter pylori (72293)	Helicobacter (213849)	Helicobacteraceae (29547)	Campylobacterales (68525)	Epsilonproteobacteria (122	Proteobacteria (2)	Bacteria (1)			99.40	98.90	WGS
210.4988	0.1016	Helicobacter pylori (72293)	Helicobacter (213849)	Helicobacteraceae (29547)	Campylobacterales (68525)	Epsilonproteobacteria (122	Proteobacteria (2)	Bacteria (1)			99.60	99.30	WGS
210.4989	0.0938	Helicobacter pylori (72293)	Helicobacter (213849)	Helicobacteraceae (29547)	Campylobacterales (68525)	Epsilonproteobacteria (122	Proteobacteria (2)	Bacteria (1)			99.70	99.40	WGS
210.4990	0.092	Helicobacter pylori (72293)	Helicobacter (213849)	Helicobacteraceae (29547)	Campylobacterales (68525)	Epsilonproteobacteria (122	Proteobacteria (2)	Bacteria (1)			99.40	98.90	WGS
210.4991	0.091	Helicobacter pylori (72293)	Helicobacter (213849)	Helicobacteraceae (29547)	Campylobacterales (68525)	Epsilonproteobacteria (122	Proteobacteria (2)	Bacteria (1)			99.60	99.00	WGS
210.4992	0.0892	Helicobacter pylori (72293)	Helicobacter (213849)	Helicobacteraceae (29547)	Campylobacterales (68525)	Epsilonproteobacteria (122	Proteobacteria (2)	Bacteria (1)			99.70	99.50	WGS
210.4993	0.0948	Helicobacter pylori (72293)	Helicobacter (213849)	Helicobacteraceae (29547)	Campylobacterales (68525)	Epsilonproteobacteria (122	Proteobacteria (2)	Bacteria (1)			99.80	99.30	WGS
210.4994	0.091	Helicobacter pylori (72293)	Helicobacter (213849)	Helicobacteraceae (29547)	Campylobacterales (68525)	Epsilonproteobacteria (122	Proteobacteria (2)	Bacteria (1)			99.70	99.30	WGS
210.4995	0.0814	Helicobacter pylori (72293)	Helicobacter (213849)	Helicobacteraceae (29547)	Campylobacterales (68525)	Epsilonproteobacteria (122	Proteobacteria (2)	Bacteria (1)			99.80	99.30	WGS
210.4996	0.0938	Helicobacter pylori (72293)	Helicobacter (213849)	Helicobacteraceae (29547)	Campylobacterales (68525)	Epsilonproteobacteria (122	Proteobacteria (2)	Bacteria (1)			99.60	99.20	WGS
210.4997	0.0814	Helicobacter pylori (72293)	Helicobacter (213849)	Helicobacteraceae (29547)	Campylobacterales (68525)	Epsilonproteobacteria (122	Proteobacteria (2)	Bacteria (1)			99.80	99.60	WGS
210.4998	0.09	Helicobacter pylori (72293)	Helicobacter (213849)	Helicobacteraceae (29547)	Campylobacterales (68525)	Epsilonproteobacteria (122	Proteobacteria (2)	Bacteria (1)			99.80	99.20	WGS
210.4999	0.0814	Helicobacter pylori (72293)	Helicobacter (213849)	Helicobacteraceae (29547)	Campylobacterales (68525)	Epsilonproteobacteria (122	Proteobacteria (2)	Bacteria (1)			99.60	99.20	WGS
210.5000	0.091	Helicobacter pylori (72293)	Helicobacter (213849)	Helicobacteraceae (29547)	Campylobacterales (68525)	Epsilonproteobacteria (122	Proteobacteria (2)	Bacteria (1)			99.70	99.30	WGS
210.5001	0.1016	Helicobacter pylori (72293)	Helicobacter (213849)	Helicobacteraceae (29547)	Campylobacterales (68525)	Epsilonproteobacteria (122	Proteobacteria (2)	Bacteria (1)			99.60	99.30	WGS
210.5002	0.0714	Helicobacter pylori (72293)	Helicobacter (213849)	Helicobacteraceae (29547)	Campylobacterales (68525)	Epsilonproteobacteria (122	Proteobacteria (2)	Bacteria (1)			99.80	99.60	WGS
210.5003	0.0948	Helicobacter pylori (72293)	Helicobacter (213849)	Helicobacteraceae (29547)	Campylobacterales (68525)	Epsilonproteobacteria (122	Proteobacteria (2)	Bacteria (1)			99.70	99.30	WGS
210.5004	0.1016	Helicobacter pylori (72293)	Helicobacter (213849)	Helicobacteraceae (29547)	Campylobacterales (68525)	Epsilonproteobacteria (122	Proteobacteria (2)	Bacteria (1)			99.50	99.10	WGS
210.5005	0.092	Helicobacter pylori (72293)	Helicobacter (213849)	Helicobacteraceae (29547)	Campylobacterales (68525)	Epsilonproteobacteria (122	Proteobacteria (2)	Bacteria (1)			99.30	98.70	WGS
210.5006	0.0892	Helicobacter pylori (72293)	Helicobacter (213849)	Helicobacteraceae (29547)	Campylobacterales (68525)	Epsilonproteobacteria (122	Proteobacteria (2)	Bacteria (1)			99.70	99.20	WGS
210.5007	0.092	Helicobacter pylori (72293)	Helicobacter (213849)	Helicobacteraceae (29547)	Campylobacterales (68525)	Epsilonproteobacteria (122	Proteobacteria (2)	Bacteria (1)			99.50	99.00	WGS
210.5008	0.0988	Helicobacter pylori (72293)	Helicobacter (213849)	Helicobacteraceae (29547)	Campylobacterales (68525)	Epsilonproteobacteria (122	Proteobacteria (2)	Bacteria (1)			99.60	99.40	WGS
210.5009	0.1016	Helicobacter pylori (72293)	Helicobacter (213849)	Helicobacteraceae (29547)	Campylobacterales (68525)	Epsilonproteobacteria (122	Proteobacteria (2)	Bacteria (1)			99.70	99.30	WGS
210.5010	0.0948	Helicobacter pylori (72293)	Helicobacter (213849)	Helicobacteraceae (29547)	Campylobacterales (68525)	Epsilonproteobacteria (122	Proteobacteria (2)	Bacteria (1)			99.90	99.40	WGS
210.5011	0.0948	Helicobacter pylori (72293)	Helicobacter (213849)	Helicobacteraceae (29547)	Campylobacterales (68525)	Epsilonproteobacteria (122	Proteobacteria (2)	Bacteria (1)			99.70	99.40	WGS
210.5012	0.0948	Helicobacter pylori (72293)	Helicobacter (213849)	Helicobacteraceae (29547)	Campylobacterales (68525)	Epsilonproteobacteria (122	Proteobacteria (2)	Bacteria (1)			99.50	99.00	WGS
210.5013	0.0948	Helicobacter pylori (72293)	Helicobacter (213849)	Helicobacteraceae (29547)	Campylobacterales (68525)	Epsilonproteobacteria (122	Proteobacteria (2)	Bacteria (1)			99.90	99.60	WGS
210.5014	0.0938	Helicobacter pylori (72293)	Helicobacter (213849)	Helicobacteraceae (29547)	Campylobacterales (68525)	Epsilonproteobacteria (122	Proteobacteria (2)	Bacteria (1)			99.90	99.50	WGS
210.5015	0.0948	Helicobacter pylori (72293)	Helicobacter (213849)	Helicobacteraceae (29547)	Campylobacterales (68525)	Epsilonproteobacteria (122	Proteobacteria (2)	Bacteria (1)			99.50	99.30	WGS
210.5016	0.0938	Helicobacter pylori (72293)	Helicobacter (213849)	Helicobacteraceae (29547)	Campylobacterales (68525)	Epsilonproteobacteria (122	Proteobacteria (2)	Bacteria (1)			99.40	99.20	WGS
210.5017	0.1006	Helicobacter pylori (72293)	Helicobacter (213849)	Helicobacteraceae (29547)	Campylobacterales (68525)	Epsilonproteobacteria (122	Proteobacteria (2)	Bacteria (1)			99.60	99.00	WGS
210.5018	0.0948	Helicobacter pylori (72293)	Helicobacter (213849)	Helicobacteraceae (29547)	Campylobacterales (68525)	Epsilonproteobacteria (122	Proteobacteria (2)	Bacteria (1)			99.70	99.30	WGS
210.5019	0.092	Helicobacter pylori (72293)	Helicobacter (213849)	Helicobacteraceae (29547)	Campylobacterales (68525)	Epsilonproteobacteria (122	Proteobacteria (2)	Bacteria (1)			99.70	99.60	WGS
210.5020	0.092	Helicobacter pylori (72293)	Helicobacter (213849)	Helicobacteraceae (29547)	Campylobacterales (68525)	Epsilonproteobacteria (122	Proteobacteria (2)	Bacteria (1)			99.80	99.60	WGS
210.5021	0.09	Helicobacter pylori (72293)	Helicobacter (213849)	Helicobacteraceae (29547)	Campylobacterales (68525)	Epsilonproteobacteria (122	Proteobacteria (2)	Bacteria (1)			99.80	99.20	WGS
210.5022	0.0948	Helicobacter pylori (72293)	Helicobacter (213849)	Helicobacteraceae (29547)	Campylobacterales (68525)	Epsilonproteobacteria (122	Proteobacteria (2)	Bacteria (1)			99.60	99.10	WGS
210.5023	0.0976	Helicobacter pylori (72293)	Helicobacter (213849)	Helicobacteraceae (29547)	Campylobacterales (68525)	Epsilonproteobacteria (122	Proteobacteria (2)	Bacteria (1)			99.90	99.50	WGS
210.5024	0.0988	Helicobacter pylori (72293)	Helicobacter (213849)	Helicobacteraceae (29547)	Campylobacterales (68525)	Epsilonproteobacteria (122	Proteobacteria (2)	Bacteria (1)			99.60	99.00	WGS
210.5025	0.0938	Helicobacter pylori (72293)	Helicobacter (213849)	Helicobacteraceae (29547)	Campylobacterales (68525)	Epsilonproteobacteria (122	Proteobacteria (2)	Bacteria (1)			99.50	99.20	WGS
210.5026	0.074	Helicobacter pylori (72293)	Helicobacter (213849)	Helicobacteraceae (29547)	Campylobacterales (68525)	Epsilonproteobacteria (122	Proteobacteria (2)	Bacteria (1)			99.60	99.10	WGS
210.5027	0.1016	Helicobacter pylori (72293)	Helicobacter (213849)	Helicobacteraceae (29547)	Campylobacterales (68525)	Epsilonproteobacteria (122	Proteobacteria (2)	Bacteria (1)			99.50	99.20	WGS
210.5028	0.0638	Helicobacter pylori (72293)	Helicobacter (213849)	Helicobacteraceae (29547)	Campylobacterales (68525)	Epsilonproteobacteria (122	Proteobacteria (2)	Bacteria (1)			99.60	99.40	WGS
210.5029	0.0842	Helicobacter pylori (72293)	Helicobacter (213849)	Helicobacteraceae (29547)	Campylobacterales (68525)	Epsilonproteobacteria (122	Proteobacteria (2)	Bacteria (1)			99.60	99.10	WGS
210.5030	0.0892	Helicobacter pylori (72293)	Helicobacter (213849)	Helicobacteraceae (29547)	Campylobacterales (68525)	Epsilonproteobacteria (122	Proteobacteria (2)	Bacteria (1)			99.60	99.30	WGS
210.5031	0.0814	Helicobacter pylori (72293)	Helicobacter (213849)	Helicobacteraceae (29547)	Campylobacterales (68525)	Epsilonproteobacteria (122	Proteobacteria (2)	Bacteria (1)			99.60	99.20	WGS
210.5032	0.0814	Helicobacter pylori (72293)	Helicobacter (213849)	Helicobacteraceae (29547)	Campylobacterales (68525)	Epsilonproteobacteria (122	Proteobacteria (2)	Bacteria (1)			99.60	98.90	WGS
210.5033	0.1016	Helicobacter pylori (72293)	Helicobacter (213849)	Helicobacteraceae (29547)	Campylobacterales (68525)	Epsilonproteobacteria (122	Proteobacteria (2)	Bacteria (1)			99.70	99.30	WGS
210.5034	0.092	Helicobacter pylori (72293)	Helicobacter (213849)	Helicobacteraceae (29547)	Campylobacterales (68525)	Epsilonproteobacteria (122	Proteobacteria (2)	Bacteria (1)			99.80	99.40	WGS
210.5035	0.0814	Helicobacter pylori (72293)	Helicobacter (213849)	Helicobacteraceae (29547)	Campylobacterales (68525)	Epsilonproteobacteria (122	Proteobacteria (2)	Bacteria (1)			99.70	99.40	WGS
210.5036	0.0988	Helicobacter pylori (72293)	Helicobacter (213849)	Helicobacteraceae (29547)	Campylobacterales (68525)	Epsilonproteobacteria (122	Proteobacteria (2)	Bacteria (1)			99.80	99.40	WGS
210.5037	0.0714	Helicobacter pylori (72293)	Helicobacter (213849)	Helicobacteraceae (29547)	Campylobacterales (68525)	Epsilonproteobacteria (122	Proteobacteria (2)	Bacteria (1)			99.70	99.40	WGS
210.5038	0.0806	Helicobacter pylori (72293)	Helicobacter (213849)	Helicobacteraceae (29547)	Campylobacterales (68525)	Epsilonproteobacteria (122	Proteobacteria (2)	Bacteria (1)			99.70	99.50	WGS
210.5039	0.0948	Helicobacter pylori (72293)	Helicobacter (213849)	Helicobacteraceae (29547)	Campylobacterales (68525)	Epsilonproteobacteria (122	Proteobacteria (2)	Bacteria (1)			99.50	99.20	WGS
210.5040	0.092	Helicobacter pylori (72293)	Helicobacter (213849)	Helicobacteraceae (29547)	Campylobacterales (68525)	Epsilonproteobacteria (122	Proteobacteria (2)	Bacteria (1)			99.60	99.50	WGS
210.5041	0.1016	Helicobacter pylori (72293)	Helicobacter (213849)	Helicobacteraceae (29547)	Campylobacterales (68525)	Epsilonproteobacteria (122	Proteobacteria (2)	Bacteria (1)			99.60	99.00	WGS
210.5042	0.091	Helicobacter pylori (72293)	Helicobacter (213849)	Helicobacteraceae (29547)	Campylobacterales (68525)	Epsilonproteobacteria (122	Proteobacteria (2)	Bacteria (1)			99.80	99.30	WGS
210.5043	0.0938	Helicobacter pylori (72293)	Helicobacter (213849)	Helicobacteraceae (29547)	Campylobacterales (68525)	Epsilonproteobacteria (122	Proteobacteria (2)	Bacteria (1)			99.60	99.40	WGS
210.5044	0.0988	Helicobacter pylori (72293)	Helicobacter (213849)	Helicobacteraceae (29547)	Campylobacterales (68525)	Epsilonproteobacteria (122	Proteobacteria (2)	Bacteria (1)			99.50	99.30	WGS
210.5045	0.092	Helicobacter pylori (72293)	Helicobacter (213849)	Helicobacteraceae (29547)	Campylobacterales (68525)	Epsilonproteobacteria (122	Proteobacteria (2)	Bacteria (1)			99.80	99.30	WGS
210.5046	0.09	Helicobacter pylori (72293)	Helicobacter (213849)	Helicobacteraceae (29547)	Campylobacterales (68525)	Epsilonproteobacteria (122	Proteobacteria (2)	Bacteria (1)			99.70	99.60	WGS
210.5047	0.091	Helicobacter pylori (72293)	Helicobacter (213849)	Helicobacteraceae (29547)	Campylobacterales (68525)	Epsilonproteobacteria (122	Proteobacteria (2)	Bacteria (1)			99.70	99.30	WGS
210.5048	0.0938	Helicobacter pylori (72293)	Helicobacter (213849)	Helicobacteraceae (29547)	Campylobacterales (68525)	Epsilonproteobacteria (122	Proteobacteria (2)	Bacteria (1)			99.70	99.10	WGS
210.5049	0.0682	Helicobacter pylori (72293)	Helicobacter (213849)	Helicobacteraceae (29547)	Campylobacterales (68525)	Epsilonproteobacteria (122	Proteobacteria (2)	Bacteria (1)			99.60	99.20	WGS
210.5050	0.1016	Helicobacter pylori (72293)	Helicobacter (213849)	Helicobacteraceae (29547)	Campylobacterales (68525)	Epsilonproteobacteria (122	Proteobacteria (2)	Bacteria (1)			99.60	99.30	WGS
210.5051	0.0978	Helicobacter pylori (72293)	Helicobacter (213849)	Helicobacteraceae (29547)	Campylobacterales (68525)	Epsilonproteobacteria (122	Proteobacteria (2)	Bacteria (1)			99.50	99.20	WGS
210.5052	0.092	Helicobacter pylori (72293)	Helicobacter (213849)	Helicobacteraceae (29547)	Campylobacterales (68525)	Epsilonproteobacteria (122	Proteobacteria (2)	Bacteria (1)			99.60	99.30	WGS
210.5053	0.0948	Helicobacter pylori (72293)	Helicobacter (213849)	Helicobacteraceae (29547)	Campylobacterales (68525)	Epsilonproteobacteria (122	Proteobacteria (2)	Bacteria (1)			99.70	99.40	WGS
210.5054	0.0938	Helicobacter pylori (72293)	Helicobacter (213849)	Helicobacteraceae (29547)	Campylobacterales (68525)	Epsilonproteobacteria (122	Proteobacteria (2)	Bacteria (1)			99.70	99.40	WGS
210.5055	0.0938	Helicobacter pylori (72293)	Helicobacter (213849)	Helicobacteraceae (29547)	Campylobacterales (68525)	Epsilonproteobacteria (122	Proteobacteria (2)	Bacteria (1)			99.60	99.20	WGS
210.5056	0.1016	Helicobacter pylori (72293)	Helicobacter (213849)	Helicobacteraceae (29547)	Campylobacterales (68525)	Epsilonproteobacteria (122	Proteobacteria (2)	Bacteria (1)			99.70	99.30	WGS
210.5057	0.092	Helicobacter pylori (72293)	Helicobacter (213849)	Helicobacteraceae (29547)	Campylobacterales (68525)	Epsilonproteobacteria (122	Proteobacteria (2)	Bacteria (1)			99.50	99.10	WGS
210.5058	0.0988	Helicobacter pylori (72293)	Helicobacter (213849)	Helicobacteraceae (29547)	Campylobacterales (68525)	Epsilonproteobacteria (122	Proteobacteria (2)	Bacteria (1)			99.30	99.10	WGS
210.5059	0.0948	Helicobacter pylori (72293)	Helicobacter (213849)	Helicobacteraceae (29547)	Campylobacterales (68525)	Epsilonproteobacteria (122	Proteobacteria (2)	Bacteria (1)			99.60	99.10	WGS
210.5060	0.1016	Helicobacter pylori (72293)	Helicobacter (213849)	Helicobacteraceae (29547)	Campylobacterales (68525)	Epsilonproteobacteria (122	Proteobacteria (2)	Bacteria (1)			99.60	99.20	WGS
210.5061	0.0938	Helicobacter pylori (72293)	Helicobacter (213849)	Helicobacteraceae (29547)	Campylobacterales (68525)	Epsilonproteobacteria (122	Proteobacteria (2)	Bacteria (1)			99.70	99.30	WGS
210.5062	0.0834	Helicobacter pylori (72293)	Helicobacter (213849)	Helicobacteraceae (29547)	Campylobacterales (68525)	Epsilonproteobacteria (122	Proteobacteria (2)	Bacteria (1)			99.60	99.10	WGS
210.5063	0.0948	Helicobacter pylori (72293)	Helicobacter (213849)	Helicobacteraceae (29547)	Campylobacterales (68525)	Epsilonproteobacteria (122	Proteobacteria (2)	Bacteria (1)			99.80	99.30	WGS
210.5064	0.0814	Helicobacter pylori (72293)	Helicobacter (213849)	Helicobacteraceae (29547)	Campylobacterales (68525)	Epsilonproteobacteria (122	Proteobacteria (2)	Bacteria (1)			99.70	99.30	WGS
210.5065	0.1122	Helicobacter pylori (72293)	Helicobacter (213849)	Helicobacteraceae (29547)	Campylobacterales (68525)	Epsilonproteobacteria (122	Proteobacteria (2)	Bacteria (1)			99.70	99.30	WGS
210.5066	0.091	Helicobacter pylori (72293)	Helicobacter (213849)	Helicobacteraceae (29547)	Campylobacterales (68525)	Epsilonproteobacteria (122	Proteobacteria (2)	Bacteria (1)			99.60	99.30	WGS
210.5067	0.0978	Helicobacter pylori (72293)	Helicobacter (213849)	Helicobacteraceae (29547)	Campylobacterales (68525)	Epsilonproteobacteria (122	Proteobacteria (2)	Bacteria (1)			99.50	99.30	WGS
210.5068	0.1016	Helicobacter pylori (72293)	Helicobacter (213849)	Helicobacteraceae (29547)	Campylobacterales (68525)	Epsilonproteobacteria (122	Proteobacteria (2)	Bacteria (1)			99.60	99.30	WGS
210.5069	0.091	Helicobacter pylori (72293)	Helicobacter (213849)	Helicobacteraceae (29547)	Campylobacterales (68525)	Epsilonproteobacteria (122	Proteobacteria (2)	Bacteria (1)			99.60	99.50	WGS
210.5070	0.0948	Helicobacter pylori (72293)	Helicobacter (213849)	Helicobacteraceae (29547)	Campylobacterales (68525)	Epsilonproteobacteria (122	Proteobacteria (2)	Bacteria (1)			99.50	99.10	WGS
210.5071	0.09	Helicobacter pylori (72293)	Helicobacter (213849)	Helicobacteraceae (29547)	Campylobacterales (68525)	Epsilonproteobacteria (122	Proteobacteria (2)	Bacteria (1)			99.60	99.10	WGS
210.5072	0.091	Helicobacter pylori (72293)	Helicobacter (213849)	Helicobacteraceae (29547)	Campylobacterales (68525)	Epsilonproteobacteria (122	Proteobacteria (2)	Bacteria (1)			99.30	99.20	WGS
210.5073	0.092	Helicobacter pylori (72293)	Helicobacter (213849)	Helicobacteraceae (29547)	Campylobacterales (68525)	Epsilonproteobacteria (122	Proteobacteria (2)	Bacteria (1)			99.60	99.30	WGS
210.5074	0.0648	Helicobacter pylori (72293)	Helicobacter (213849)	Helicobacteraceae (29547)	Campylobacterales (68525)	Epsilonproteobacteria (122	Proteobacteria (2)	Bacteria (1)			99.60	99.30	WGS
210.5075	0.1028	Helicobacter pylori (72293)	Helicobacter (213849)	Helicobacteraceae (29547)	Campylobacterales (68525)	Epsilonproteobacteria (122	Proteobacteria (2)	Bacteria (1)			99.40	99.10	WGS
210.5076	0.091	Helicobacter pylori (72293)	Helicobacter (213849)	Helicobacteraceae (29547)	Campylobacterales (68525)	Epsilonproteobacteria (122	Proteobacteria (2)	Bacteria (1)			99.80	99.70	WGS
210.5077	0.0872	Helicobacter pylori (72293)	Helicobacter (213849)	Helicobacteraceae (29547)	Campylobacterales (68525)	Epsilonproteobacteria (122	Proteobacteria (2)	Bacteria (1)			99.60	99.30	WGS
210.5078	0.0948	Helicobacter pylori (72293)	Helicobacter (213849)	Helicobacteraceae (29547)	Campylobacterales (68525)	Epsilonproteobacteria (122	Proteobacteria (2)	Bacteria (1)			99.60	99.30	WGS
210.5079	0.0948	Helicobacter pylori (72293)	Helicobacter (213849)	Helicobacteraceae (29547)	Campylobacterales (68525)	Epsilonproteobacteria (122	Proteobacteria (2)	Bacteria (1)			99.60	99.20	WGS
210.5080	0.091	Helicobacter pylori (72293)	Helicobacter (213849)	Helicobacteraceae (29547)	Campylobacterales (68525)	Epsilonproteobacteria (122	Proteobacteria (2)	Bacteria (1)			99.60	99.10	WGS
210.5081	0.0988	Helicobacter pylori (72293)	Helicobacter (213849)	Helicobacteraceae (29547)	Campylobacterales (68525)	Epsilonproteobacteria (122	Proteobacteria (2)	Bacteria (1)			99.50	98.80	WGS
210.5082	0.1028	Helicobacter pylori (72293)	Helicobacter (213849)	Helicobacteraceae (29547)	Campylobacterales (68525)	Epsilonproteobacteria (122	Proteobacteria (2)	Bacteria (1)			99.60	99.30	WGS
210.5083	0.0948	Helicobacter pylori (72293)	Helicobacter (213849)	Helicobacteraceae (29547)	Campylobacterales (68525)	Epsilonproteobacteria (122	Proteobacteria (2)	Bacteria (1)			99.80	99.40	WGS
210.5084	0.0938	Helicobacter pylori (72293)	Helicobacter (213849)	Helicobacteraceae (29547)	Campylobacterales (68525)	Epsilonproteobacteria (122	Proteobacteria (2)	Bacteria (1)			99.60	99.00	WGS
210.5085	0.091	Helicobacter pylori (72293)	Helicobacter (213849)	Helicobacteraceae (29547)	Campylobacterales (68525)	Epsilonproteobacteria (122	Proteobacteria (2)	Bacteria (1)			99.50	99.20	WGS
210.5086	0.1016	Helicobacter pylori (72293)	Helicobacter (213849)	Helicobacteraceae (29547)	Campylobacterales (68525)	Epsilonproteobacteria (122	Proteobacteria (2)	Bacteria (1)			99.70	99.20	WGS
210.5087	0.092	Helicobacter pylori (72293)	Helicobacter (213849)	Helicobacteraceae (29547)	Campylobacterales (68525)	Epsilonproteobacteria (122	Proteobacteria (2)	Bacteria (1)			99.50	99.00	WGS
210.5088	0.0988	Helicobacter pylori (72293)	Helicobacter (213849)	Helicobacteraceae (29547)	Campylobacterales (68525)	Epsilonproteobacteria (122	Proteobacteria (2)	Bacteria (1)			99.70	99.60	WGS
210.5089	0.091	Helicobacter pylori (72293)	Helicobacter (213849)	Helicobacteraceae (29547)	Campylobacterales (68525)	Epsilonproteobacteria (122	Proteobacteria (2)	Bacteria (1)			99.70	99.30	WGS
210.5090	0.0938	Helicobacter pylori (72293)	Helicobacter (213849)	Helicobacteraceae (29547)	Campylobacterales (68525)	Epsilonproteobacteria (122	Proteobacteria (2)	Bacteria (1)			99.60	99.10	WGS
210.5091	0.0938	Helicobacter pylori (72293)	Helicobacter (213849)	Helicobacteraceae (29547)	Campylobacterales (68525)	Epsilonproteobacteria (122	Proteobacteria (2)	Bacteria (1)			99.60	99.20	WGS
210.5092	0.0948	Helicobacter pylori (72293)	Helicobacter (213849)	Helicobacteraceae (29547)	Campylobacterales (68525)	Epsilonproteobacteria (122	Proteobacteria (2)	Bacteria (1)			99.50	99.00	WGS
210.5093	0.092	Helicobacter pylori (72293)	Helicobacter (213849)	Helicobacteraceae (29547)	Campylobacterales (68525)	Epsilonproteobacteria (122	Proteobacteria (2)	Bacteria (1)			99.70	99.30	WGS
210.5094	0.0948	Helicobacter pylori (72293)	Helicobacter (213849)	Helicobacteraceae (29547)	Campylobacterales (68525)	Epsilonproteobacteria (122	Proteobacteria (2)	Bacteria (1)			99.40	99.10	WGS
210.5095	0.0948	Helicobacter pylori (72293)	Helicobacter (213849)	Helicobacteraceae (29547)	Campylobacterales (68525)	Epsilonproteobacteria (122	Proteobacteria (2)	Bacteria (1)			99.60	99.00	WGS
210.5096	0.091	Helicobacter pylori (72293)	Helicobacter (213849)	Helicobacteraceae (29547)	Campylobacterales (68525)	Epsilonproteobacteria (122	Proteobacteria (2)	Bacteria (1)			99.80	99.30	WGS
210.5097	0.0814	Helicobacter pylori (72293)	Helicobacter (213849)	Helicobacteraceae (29547)	Campylobacterales (68525)	Epsilonproteobacteria (122	Proteobacteria (2)	Bacteria (1)			99.70	99.30	WGS
210.5098	0.091	Helicobacter pylori (72293)	Helicobacter (213849)	Helicobacteraceae (29547)	Campylobacterales (68525)	Epsilonproteobacteria (122	Proteobacteria (2)	Bacteria (1)			99.80	99.10	WGS
210.5099	0.0938	Helicobacter pylori (72293)	Helicobacter (213849)	Helicobacteraceae (29547)	Campylobacterales (68525)	Epsilonproteobacteria (122	Proteobacteria (2)	Bacteria (1)			99.70	99.40	WGS
210.5100	0.092	Helicobacter pylori (72293)	Helicobacter (213849)	Helicobacteraceae (29547)	Campylobacterales (68525)	Epsilonproteobacteria (122	Proteobacteria (2)	Bacteria (1)			99.60	99.00	WGS
210.5101	0.1016	Helicobacter pylori (72293)	Helicobacter (213849)	Helicobacteraceae (29547)	Campylobacterales (68525)	Epsilonproteobacteria (122	Proteobacteria (2)	Bacteria (1)			99.70	99.30	WGS
210.5102	0.092	Helicobacter pylori (72293)	Helicobacter (213849)	Helicobacteraceae (29547)	Campylobacterales (68525)	Epsilonproteobacteria (122	Proteobacteria (2)	Bacteria (1)			99.60	99.20	WGS
210.5103	0.1006	Helicobacter pylori (72293)	Helicobacter (213849)	Helicobacteraceae (29547)	Campylobacterales (68525)	Epsilonproteobacteria (122	Proteobacteria (2)	Bacteria (1)			99.70	99.20	WGS
210.5104	0.092	Helicobacter pylori (72293)	Helicobacter (213849)	Helicobacteraceae (29547)	Campylobacterales (68525)	Epsilonproteobacteria (122	Proteobacteria (2)	Bacteria (1)			99.60	99.10	WGS
210.5105	0.0948	Helicobacter pylori (72293)	Helicobacter (213849)	Helicobacteraceae (29547)	Campylobacterales (68525)	Epsilonproteobacteria (122	Proteobacteria (2)	Bacteria (1)			99.70	99.30	WGS
210.5106	0.092	Helicobacter pylori (72293)	Helicobacter (213849)	Helicobacteraceae (29547)	Campylobacterales (68525)	Epsilonproteobacteria (122	Proteobacteria (2)	Bacteria (1)			99.80	99.20	WGS
210.5107	0.092	Helicobacter pylori (72293)	Helicobacter (213849)	Helicobacteraceae (29547)	Campylobacterales (68525)	Epsilonproteobacteria (122	Proteobacteria (2)	Bacteria (1)			99.90	99.60	WGS
210.5108	0.0948	Helicobacter pylori (72293)	Helicobacter (213849)	Helicobacteraceae (29547)	Campylobacterales (68525)	Epsilonproteobacteria (122	Proteobacteria (2)	Bacteria (1)			99.70	99.30	WGS
210.5109	0.092	Helicobacter pylori (72293)	Helicobacter (213849)	Helicobacteraceae (29547)	Campylobacterales (68525)	Epsilonproteobacteria (122	Proteobacteria (2)	Bacteria (1)			99.50	99.30	WGS
210.5110	0.0988	Helicobacter pylori (72293)	Helicobacter (213849)	Helicobacteraceae (29547)	Campylobacterales (68525)	Epsilonproteobacteria (122	Proteobacteria (2)	Bacteria (1)			99.60	99.30	WGS
210.5111	0.091	Helicobacter pylori (72293)	Helicobacter (213849)	Helicobacteraceae (29547)	Campylobacterales (68525)	Epsilonproteobacteria (122	Proteobacteria (2)	Bacteria (1)			99.80	99.30	WGS
210.5112	0.1016	Helicobacter pylori (72293)	Helicobacter (213849)	Helicobacteraceae (29547)	Campylobacterales (68525)	Epsilonproteobacteria (122	Proteobacteria (2)	Bacteria (1)			99.70	99.30	WGS
210.5113	0.0938	Helicobacter pylori (72293)	Helicobacter (213849)	Helicobacteraceae (29547)	Campylobacterales (68525)	Epsilonproteobacteria (122	Proteobacteria (2)	Bacteria (1)			99.50	98.80	WGS
210.5114	0.0948	Helicobacter pylori (72293)	Helicobacter (213849)	Helicobacteraceae (29547)	Campylobacterales (68525)	Epsilonproteobacteria (122	Proteobacteria (2)	Bacteria (1)			99.70	99.30	WGS
210.5115	0.0948	Helicobacter pylori (72293)	Helicobacter (213849)	Helicobacteraceae (29547)	Campylobacterales (68525)	Epsilonproteobacteria (122	Proteobacteria (2)	Bacteria (1)			99.60	99.30	WGS
210.5116	0.091	Helicobacter pylori (72293)	Helicobacter (213849)	Helicobacteraceae (29547)	Campylobacterales (68525)	Epsilonproteobacteria (122	Proteobacteria (2)	Bacteria (1)			99.80	99.60	WGS
210.5117	0.0938	Helicobacter pylori (72293)	Helicobacter (213849)	Helicobacteraceae (29547)	Campylobacterales (68525)	Epsilonproteobacteria (122	Proteobacteria (2)	Bacteria (1)			99.60	99.20	WGS
210.5118	0.0978	Helicobacter pylori (72293)	Helicobacter (213849)	Helicobacteraceae (29547)	Campylobacterales (68525)	Epsilonproteobacteria (122	Proteobacteria (2)	Bacteria (1)			99.80	99.50	WGS
210.5119	0.091	Helicobacter pylori (72293)	Helicobacter (213849)	Helicobacteraceae (29547)	Campylobacterales (68525)	Epsilonproteobacteria (122	Proteobacteria (2)	Bacteria (1)			99.40	99.20	WGS
210.5120	0.0948	Helicobacter pylori (72293)	Helicobacter (213849)	Helicobacteraceae (29547)	Campylobacterales (68525)	Epsilonproteobacteria (122	Proteobacteria (2)	Bacteria (1)			99.40	99.00	WGS
210.5121	0.0938	Helicobacter pylori (72293)	Helicobacter (213849)	Helicobacteraceae (29547)	Campylobacterales (68525)	Epsilonproteobacteria (122	Proteobacteria (2)	Bacteria (1)			99.60	99.20	WGS
210.5122	0.092	Helicobacter pylori (72293)	Helicobacter (213849)	Helicobacteraceae (29547)	Campylobacterales (68525)	Epsilonproteobacteria (122	Proteobacteria (2)	Bacteria (1)			99.90	99.40	WGS
210.5123	0.092	Helicobacter pylori (72293)	Helicobacter (213849)	Helicobacteraceae (29547)	Campylobacterales (68525)	Epsilonproteobacteria (122	Proteobacteria (2)	Bacteria (1)			99.50	99.30	WGS
210.5124	0.0938	Helicobacter pylori (72293)	Helicobacter (213849)	Helicobacteraceae (29547)	Campylobacterales (68525)	Epsilonproteobacteria (122	Proteobacteria (2)	Bacteria (1)			99.20	98.70	WGS
210.5125	0.0938	Helicobacter pylori (72293)	Helicobacter (213849)	Helicobacteraceae (29547)	Campylobacterales (68525)	Epsilonproteobacteria (122	Proteobacteria (2)	Bacteria (1)			99.50	99.10	WGS
210.5126	0.0988	Helicobacter pylori (72293)	Helicobacter (213849)	Helicobacteraceae (29547)	Campylobacterales (68525)	Epsilonproteobacteria (122	Proteobacteria (2)	Bacteria (1)			99.80	99.60	WGS
210.5127	0.0938	Helicobacter pylori (72293)	Helicobacter (213849)	Helicobacteraceae (29547)	Campylobacterales (68525)	Epsilonproteobacteria (122	Proteobacteria (2)	Bacteria (1)			99.60	99.30	WGS
210.5128	0.0948	Helicobacter pylori (72293)	Helicobacter (213849)	Helicobacteraceae (29547)	Campylobacterales (68525)	Epsilonproteobacteria (122	Proteobacteria (2)	Bacteria (1)			99.30	98.80	WGS
210.5129	0.0938	Helicobacter pylori (72293)	Helicobacter (213849)	Helicobacteraceae (29547)	Campylobacterales (68525)	Epsilonproteobacteria (122	Proteobacteria (2)	Bacteria (1)			99.80	99.30	WGS
210.5130	0.0948	Helicobacter pylori (72293)	Helicobacter (213849)	Helicobacteraceae (29547)	Campylobacterales (68525)	Epsilonproteobacteria (122	Proteobacteria (2)	Bacteria (1)			99.90	95.10	WGS
210.5131	0.092	Helicobacter pylori (72293)	Helicobacter (213849)	Helicobacteraceae (29547)	Campylobacterales (68525)	Epsilonproteobacteria (122	Proteobacteria (2)	Bacteria (1)			99.90	99.60	WGS
210.5132	0.0988	Helicobacter pylori (72293)	Helicobacter (213849)	Helicobacteraceae (29547)	Campylobacterales (68525)	Epsilonproteobacteria (122	Proteobacteria (2)	Bacteria (1)			99.90	99.60	WGS
210.5133	0.0978	Helicobacter pylori (72293)	Helicobacter (213849)	Helicobacteraceae (29547)	Campylobacterales (68525)	Epsilonproteobacteria (122	Proteobacteria (2)	Bacteria (1)			99.90	99.60	WGS
210.5134	0.0978	Helicobacter pylori (72293)	Helicobacter (213849)	Helicobacteraceae (29547)	Campylobacterales (68525)	Epsilonproteobacteria (122	Proteobacteria (2)	Bacteria (1)			99.90	99.80	WGS
210.5135	0.0948	Helicobacter pylori (72293)	Helicobacter (213849)	Helicobacteraceae (29547)	Campylobacterales (68525)	Epsilonproteobacteria (122	Proteobacteria (2)	Bacteria (1)			99.80	99.40	WGS
210.5136	0.0988	Helicobacter pylori (72293)	Helicobacter (213849)	Helicobacteraceae (29547)	Campylobacterales (68525)	Epsilonproteobacteria (122	Proteobacteria (2)	Bacteria (1)			99.70	99.20	WGS
210.5137	0.0948	Helicobacter pylori (72293)	Helicobacter (213849)	Helicobacteraceae (29547)	Campylobacterales (68525)	Epsilonproteobacteria (122	Proteobacteria (2)	Bacteria (1)			99.80	99.30	WGS
210.5138	0.092	Helicobacter pylori (72293)	Helicobacter (213849)	Helicobacteraceae (29547)	Campylobacterales (68525)	Epsilonproteobacteria (122	Proteobacteria (2)	Bacteria (1)			99.90	99.60	WGS
210.5139	0.0938	Helicobacter pylori (72293)	Helicobacter (213849)	Helicobacteraceae (29547)	Campylobacterales (68525)	Epsilonproteobacteria (122	Proteobacteria (2)	Bacteria (1)			99.90	99.50	WGS
210.5140	0.0978	Helicobacter pylori (72293)	Helicobacter (213849)	Helicobacteraceae (29547)	Campylobacterales (68525)	Epsilonproteobacteria (122	Proteobacteria (2)	Bacteria (1)			99.90	99.70	WGS
210.5141	0.0948	Helicobacter pylori (72293)	Helicobacter (213849)	Helicobacteraceae (29547)	Campylobacterales (68525)	Epsilonproteobacteria (122	Proteobacteria (2)	Bacteria (1)			99.90	99.40	WGS
210.5142	0.0948	Helicobacter pylori (72293)	Helicobacter (213849)	Helicobacteraceae (29547)	Campylobacterales (68525)	Epsilonproteobacteria (122	Proteobacteria (2)	Bacteria (1)			99.70	99.30	WGS
210.5143	0.0988	Helicobacter pylori (72293)	Helicobacter (213849)	Helicobacteraceae (29547)	Campylobacterales (68525)	Epsilonproteobacteria (122	Proteobacteria (2)	Bacteria (1)			99.90	99.80	WGS
210.5144	0.091	Helicobacter pylori (72293)	Helicobacter (213849)	Helicobacteraceae (29547)	Campylobacterales (68525)	Epsilonproteobacteria (122	Proteobacteria (2)	Bacteria (1)			99.90	99.80	WGS
210.5145	0.0978	Helicobacter pylori (72293)	Helicobacter (213849)	Helicobacteraceae (29547)	Campylobacterales (68525)	Epsilonproteobacteria (122	Proteobacteria (2)	Bacteria (1)			99.90	99.50	WGS
210.5146	0.092	Helicobacter pylori (72293)	Helicobacter (213849)	Helicobacteraceae (29547)	Campylobacterales (68525)	Epsilonproteobacteria (122	Proteobacteria (2)	Bacteria (1)			100.00	99.50	WGS
210.5147	0.092	Helicobacter pylori (72293)	Helicobacter (213849)	Helicobacteraceae (29547)	Campylobacterales (68525)	Epsilonproteobacteria (122	Proteobacteria (2)	Bacteria (1)			99.90	97.90	WGS
210.5148	0.0988	Helicobacter pylori (72293)	Helicobacter (213849)	Helicobacteraceae (29547)	Campylobacterales (68525)	Epsilonproteobacteria (122	Proteobacteria (2)	Bacteria (1)			99.90	99.60	WGS
210.5149	0.092	Helicobacter pylori (72293)	Helicobacter (213849)	Helicobacteraceae (29547)	Campylobacterales (68525)	Epsilonproteobacteria (122	Proteobacteria (2)	Bacteria (1)			99.90	99.60	WGS
210.5150	0.0948	Helicobacter pylori (72293)	Helicobacter (213849)	Helicobacteraceae (29547)	Campylobacterales (68525)	Epsilonproteobacteria (122	Proteobacteria (2)	Bacteria (1)			99.90	99.40	WGS
210.5151	0.0948	Helicobacter pylori (72293)	Helicobacter (213849)	Helicobacteraceae (29547)	Campylobacterales (68525)	Epsilonproteobacteria (122	Proteobacteria (2)	Bacteria (1)			99.90	99.00	WGS
210.5152	0.1016	Helicobacter pylori (72293)	Helicobacter (213849)	Helicobacteraceae (29547)	Campylobacterales (68525)	Epsilonproteobacteria (122	Proteobacteria (2)	Bacteria (1)			99.80	99.00	WGS
210.5153	0.0938	Helicobacter pylori (72293)	Helicobacter (213849)	Helicobacteraceae (29547)	Campylobacterales (68525)	Epsilonproteobacteria (122	Proteobacteria (2)	Bacteria (1)			99.90	99.10	WGS
210.5154	0.091	Helicobacter pylori (72293)	Helicobacter (213849)	Helicobacteraceae (29547)	Campylobacterales (68525)	Epsilonproteobacteria (122	Proteobacteria (2)	Bacteria (1)			99.90	99.40	WGS
210.5155	0.092	Helicobacter pylori (72293)	Helicobacter (213849)	Helicobacteraceae (29547)	Campylobacterales (68525)	Epsilonproteobacteria (122	Proteobacteria (2)	Bacteria (1)			99.90	99.60	WGS
210.5156	0.092	Helicobacter pylori (72293)	Helicobacter (213849)	Helicobacteraceae (29547)	Campylobacterales (68525)	Epsilonproteobacteria (122	Proteobacteria (2)	Bacteria (1)			99.90	99.60	WGS
210.5157	0.0948	Helicobacter pylori (72293)	Helicobacter (213849)	Helicobacteraceae (29547)	Campylobacterales (68525)	Epsilonproteobacteria (122	Proteobacteria (2)	Bacteria (1)			99.70	99.40	WGS
210.5158	0.091	Helicobacter pylori (72293)	Helicobacter (213849)	Helicobacteraceae (29547)	Campylobacterales (68525)	Epsilonproteobacteria (122	Proteobacteria (2)	Bacteria (1)			99.90	99.40	WGS
210.5159	0.0948	Helicobacter pylori (72293)	Helicobacter (213849)	Helicobacteraceae (29547)	Campylobacterales (68525)	Epsilonproteobacteria (122	Proteobacteria (2)	Bacteria (1)			99.90	99.50	WGS
210.5160	0.0948	Helicobacter pylori (72293)	Helicobacter (213849)	Helicobacteraceae (29547)	Campylobacterales (68525)	Epsilonproteobacteria (122	Proteobacteria (2)	Bacteria (1)			99.90	99.20	WGS
210.5161	0.092	Helicobacter pylori (72293)	Helicobacter (213849)	Helicobacteraceae (29547)	Campylobacterales (68525)	Epsilonproteobacteria (122	Proteobacteria (2)	Bacteria (1)			99.90	99.60	WGS
210.5162	0.0948	Helicobacter pylori (72293)	Helicobacter (213849)	Helicobacteraceae (29547)	Campylobacterales (68525)	Epsilonproteobacteria (122	Proteobacteria (2)	Bacteria (1)			99.90	99.60	WGS
210.5163	0.0938	Helicobacter pylori (72293)	Helicobacter (213849)	Helicobacteraceae (29547)	Campylobacterales (68525)	Epsilonproteobacteria (122	Proteobacteria (2)	Bacteria (1)			99.60	99.20	WGS
210.5164	0.1028	Helicobacter pylori (72293)	Helicobacter (213849)	Helicobacteraceae (29547)	Campylobacterales (68525)	Epsilonproteobacteria (122	Proteobacteria (2)	Bacteria (1)			99.90	99.30	WGS
210.5165	0.0988	Helicobacter pylori (72293)	Helicobacter (213849)	Helicobacteraceae (29547)	Campylobacterales (68525)	Epsilonproteobacteria (122	Proteobacteria (2)	Bacteria (1)			99.90	99.50	WGS
210.5166	0.092	Helicobacter pylori (72293)	Helicobacter (213849)	Helicobacteraceae (29547)	Campylobacterales (68525)	Epsilonproteobacteria (122	Proteobacteria (2)	Bacteria (1)			99.80	99.30	WGS
210.5167	0.091	Helicobacter pylori (72293)	Helicobacter (213849)	Helicobacteraceae (29547)	Campylobacterales (68525)	Epsilonproteobacteria (122	Proteobacteria (2)	Bacteria (1)			99.80	99.40	WGS
210.5168	0.092	Helicobacter pylori (72293)	Helicobacter (213849)	Helicobacteraceae (29547)	Campylobacterales (68525)	Epsilonproteobacteria (122	Proteobacteria (2)	Bacteria (1)			99.90	99.30	WGS
210.5169	0.1006	Helicobacter pylori (72293)	Helicobacter (213849)	Helicobacteraceae (29547)	Campylobacterales (68525)	Epsilonproteobacteria (122	Proteobacteria (2)	Bacteria (1)			99.90	99.50	WGS
210.5170	0.0948	Helicobacter pylori (72293)	Helicobacter (213849)	Helicobacteraceae (29547)	Campylobacterales (68525)	Epsilonproteobacteria (122	Proteobacteria (2)	Bacteria (1)			99.90	99.40	WGS
210.5171	0.092	Helicobacter pylori (72293)	Helicobacter (213849)	Helicobacteraceae (29547)	Campylobacterales (68525)	Epsilonproteobacteria (122	Proteobacteria (2)	Bacteria (1)			99.90	99.30	WGS
210.5172	0.091	Helicobacter pylori (72293)	Helicobacter (213849)	Helicobacteraceae (29547)	Campylobacterales (68525)	Epsilonproteobacteria (122	Proteobacteria (2)	Bacteria (1)			99.80	99.30	WGS
210.5173	0.0948	Helicobacter pylori (72293)	Helicobacter (213849)	Helicobacteraceae (29547)	Campylobacterales (68525)	Epsilonproteobacteria (122	Proteobacteria (2)	Bacteria (1)			99.90	99.40	WGS
210.5174	0.0978	Helicobacter pylori (72293)	Helicobacter (213849)	Helicobacteraceae (29547)	Campylobacterales (68525)	Epsilonproteobacteria (122	Proteobacteria (2)	Bacteria (1)			99.90	99.60	WGS
210.5175	0.092	Helicobacter pylori (72293)	Helicobacter (213849)	Helicobacteraceae (29547)	Campylobacterales (68525)	Epsilonproteobacteria (122	Proteobacteria (2)	Bacteria (1)			99.90	99.50	WGS
210.5176	0.0948	Helicobacter pylori (72293)	Helicobacter (213849)	Helicobacteraceae (29547)	Campylobacterales (68525)	Epsilonproteobacteria (122	Proteobacteria (2)	Bacteria (1)			99.90	99.50	WGS
210.5177	0.0938	Helicobacter pylori (72293)	Helicobacter (213849)	Helicobacteraceae (29547)	Campylobacterales (68525)	Epsilonproteobacteria (122	Proteobacteria (2)	Bacteria (1)			99.90	99.40	WGS
210.5178	0.0948	Helicobacter pylori (72293)	Helicobacter (213849)	Helicobacteraceae (29547)	Campylobacterales (68525)	Epsilonproteobacteria (122	Proteobacteria (2)	Bacteria (1)			99.90	99.30	WGS
210.5179	0.092	Helicobacter pylori (72293)	Helicobacter (213849)	Helicobacteraceae (29547)	Campylobacterales (68525)	Epsilonproteobacteria (122	Proteobacteria (2)	Bacteria (1)			99.80	98.50	WGS
210.5180	0.0864	Helicobacter pylori (72293)	Helicobacter (213849)	Helicobacteraceae (29547)	Campylobacterales (68525)	Epsilonproteobacteria (122	Proteobacteria (2)	Bacteria (1)			100.00	98.90	Complete
210.5181	0.091	Helicobacter pylori (72293)	Helicobacter (213849)	Helicobacteraceae (29547)	Campylobacterales (68525)	Epsilonproteobacteria (122	Proteobacteria (2)	Bacteria (1)			99.90	99.20	Complete
210.5244	0.1016	Helicobacter pylori (72293)	Helicobacter (213849)	Helicobacteraceae (29547)	Campylobacterales (68525)	Epsilonproteobacteria (122	Proteobacteria (2)	Bacteria (1)			99.90	99.50	WGS
210.5245	0.092	Helicobacter pylori (72293)	Helicobacter (213849)	Helicobacteraceae (29547)	Campylobacterales (68525)	Epsilonproteobacteria (122	Proteobacteria (2)	Bacteria (1)			99.80	92.90	WGS
210.5246	0.0948	Helicobacter pylori (72293)	Helicobacter (213849)	Helicobacteraceae (29547)	Campylobacterales (68525)	Epsilonproteobacteria (122	Proteobacteria (2)	Bacteria (1)			99.80	99.00	WGS
210.5247	0.092	Helicobacter pylori (72293)	Helicobacter (213849)	Helicobacteraceae (29547)	Campylobacterales (68525)	Epsilonproteobacteria (122	Proteobacteria (2)	Bacteria (1)			99.90	99.70	WGS
210.5248	0.1006	Helicobacter pylori (72293)	Helicobacter (213849)	Helicobacteraceae (29547)	Campylobacterales (68525)	Epsilonproteobacteria (122	Proteobacteria (2)	Bacteria (1)			99.80	99.40	WGS
210.5249	0.091	Helicobacter pylori (72293)	Helicobacter (213849)	Helicobacteraceae (29547)	Campylobacterales (68525)	Epsilonproteobacteria (122	Proteobacteria (2)	Bacteria (1)			99.90	99.60	WGS
210.5250	0.0814	Helicobacter pylori (72293)	Helicobacter (213849)	Helicobacteraceae (29547)	Campylobacterales (68525)	Epsilonproteobacteria (122	Proteobacteria (2)	Bacteria (1)			99.80	99.50	Complete
210.5255	0.0948	Helicobacter pylori (72293)	Helicobacter (213849)	Helicobacteraceae (29547)	Campylobacterales (68525)	Epsilonproteobacteria (122	Proteobacteria (2)	Bacteria (1)			99.90	98.90	Complete
210.5258	0.0892	Helicobacter pylori (72293)	Helicobacter (213849)	Helicobacteraceae (29547)	Campylobacterales (68525)	Epsilonproteobacteria (122	Proteobacteria (2)	Bacteria (1)			99.90	99.10	Complete
210.5261	0.0988	Helicobacter pylori (72293)	Helicobacter (213849)	Helicobacteraceae (29547)	Campylobacterales (68525)	Epsilonproteobacteria (122	Proteobacteria (2)	Bacteria (1)			99.90	99.30	WGS
210.5262	0.092	Helicobacter pylori (72293)	Helicobacter (213849)	Helicobacteraceae (29547)	Campylobacterales (68525)	Epsilonproteobacteria (122	Proteobacteria (2)	Bacteria (1)			99.90	99.70	WGS
210.5263	0.0988	Helicobacter pylori (72293)	Helicobacter (213849)	Helicobacteraceae (29547)	Campylobacterales (68525)	Epsilonproteobacteria (122	Proteobacteria (2)	Bacteria (1)			99.90	99.60	WGS
210.5264	0.0988	Helicobacter pylori (72293)	Helicobacter (213849)	Helicobacteraceae (29547)	Campylobacterales (68525)	Epsilonproteobacteria (122	Proteobacteria (2)	Bacteria (1)			99.90	99.30	WGS
210.5265	0.0988	Helicobacter pylori (72293)	Helicobacter (213849)	Helicobacteraceae (29547)	Campylobacterales (68525)	Epsilonproteobacteria (122	Proteobacteria (2)	Bacteria (1)			99.90	99.30	WGS
210.5266	0.0988	Helicobacter pylori (72293)	Helicobacter (213849)	Helicobacteraceae (29547)	Campylobacterales (68525)	Epsilonproteobacteria (122	Proteobacteria (2)	Bacteria (1)			99.90	99.30	WGS
210.5267	0.091	Helicobacter pylori (72293)	Helicobacter (213849)	Helicobacteraceae (29547)	Campylobacterales (68525)	Epsilonproteobacteria (122	Proteobacteria (2)	Bacteria (1)			99.90	99.60	WGS
210.5268	0.0948	Helicobacter pylori (72293)	Helicobacter (213849)	Helicobacteraceae (29547)	Campylobacterales (68525)	Epsilonproteobacteria (122	Proteobacteria (2)	Bacteria (1)			99.90	99.60	WGS
210.5269	0.1006	Helicobacter pylori (72293)	Helicobacter (213849)	Helicobacteraceae (29547)	Campylobacterales (68525)	Epsilonproteobacteria (122	Proteobacteria (2)	Bacteria (1)			99.80	99.50	WGS
210.5270	0.0978	Helicobacter pylori (72293)	Helicobacter (213849)	Helicobacteraceae (29547)	Campylobacterales (68525)	Epsilonproteobacteria (122	Proteobacteria (2)	Bacteria (1)			99.90	99.60	WGS
210.5271	0.0948	Helicobacter pylori (72293)	Helicobacter (213849)	Helicobacteraceae (29547)	Campylobacterales (68525)	Epsilonproteobacteria (122	Proteobacteria (2)	Bacteria (1)			99.70	99.10	WGS
210.5272	0.0938	Helicobacter pylori (72293)	Helicobacter (213849)	Helicobacteraceae (29547)	Campylobacterales (68525)	Epsilonproteobacteria (122	Proteobacteria (2)	Bacteria (1)			99.90	99.50	WGS
210.5273	0.091	Helicobacter pylori (72293)	Helicobacter (213849)	Helicobacteraceae (29547)	Campylobacterales (68525)	Epsilonproteobacteria (122	Proteobacteria (2)	Bacteria (1)			99.90	99.90	WGS
210.5274	0.0978	Helicobacter pylori (72293)	Helicobacter (213849)	Helicobacteraceae (29547)	Campylobacterales (68525)	Epsilonproteobacteria (122	Proteobacteria (2)	Bacteria (1)			97.00	96.90	WGS
210.5275	0.0938	Helicobacter pylori (72293)	Helicobacter (213849)	Helicobacteraceae (29547)	Campylobacterales (68525)	Epsilonproteobacteria (122	Proteobacteria (2)	Bacteria (1)			99.90	99.30	WGS
210.5276	0.0948	Helicobacter pylori (72293)	Helicobacter (213849)	Helicobacteraceae (29547)	Campylobacterales (68525)	Epsilonproteobacteria (122	Proteobacteria (2)	Bacteria (1)			99.90	99.30	WGS
210.5277	0.092	Helicobacter pylori (72293)	Helicobacter (213849)	Helicobacteraceae (29547)	Campylobacterales (68525)	Epsilonproteobacteria (122	Proteobacteria (2)	Bacteria (1)			99.90	99.60	WGS
210.5278	0.091	Helicobacter pylori (72293)	Helicobacter (213849)	Helicobacteraceae (29547)	Campylobacterales (68525)	Epsilonproteobacteria (122	Proteobacteria (2)	Bacteria (1)			99.90	99.80	WGS
210.5279	0.091	Helicobacter pylori (72293)	Helicobacter (213849)	Helicobacteraceae (29547)	Campylobacterales (68525)	Epsilonproteobacteria (122	Proteobacteria (2)	Bacteria (1)			99.90	99.40	WGS
210.5280	0.0948	Helicobacter pylori (72293)	Helicobacter (213849)	Helicobacteraceae (29547)	Campylobacterales (68525)	Epsilonproteobacteria (122	Proteobacteria (2)	Bacteria (1)			99.80	99.50	WGS
210.5281	0.1016	Helicobacter pylori (72293)	Helicobacter (213849)	Helicobacteraceae (29547)	Campylobacterales (68525)	Epsilonproteobacteria (122	Proteobacteria (2)	Bacteria (1)			99.90	99.70	WGS
210.5282	0.0978	Helicobacter pylori (72293)	Helicobacter (213849)	Helicobacteraceae (29547)	Campylobacterales (68525)	Epsilonproteobacteria (122	Proteobacteria (2)	Bacteria (1)			99.80	99.40	WGS
210.5283	0.0948	Helicobacter pylori (72293)	Helicobacter (213849)	Helicobacteraceae (29547)	Campylobacterales (68525)	Epsilonproteobacteria (122	Proteobacteria (2)	Bacteria (1)			99.70	99.00	WGS
210.5284	0.0948	Helicobacter pylori (72293)	Helicobacter (213849)	Helicobacteraceae (29547)	Campylobacterales (68525)	Epsilonproteobacteria (122	Proteobacteria (2)	Bacteria (1)			99.70	99.40	WGS
210.5285	0.0948	Helicobacter pylori (72293)	Helicobacter (213849)	Helicobacteraceae (29547)	Campylobacterales (68525)	Epsilonproteobacteria (122	Proteobacteria (2)	Bacteria (1)			99.60	97.90	WGS
210.5286	0.092	Helicobacter pylori (72293)	Helicobacter (213849)	Helicobacteraceae (29547)	Campylobacterales (68525)	Epsilonproteobacteria (122	Proteobacteria (2)	Bacteria (1)			99.90	99.60	WGS
210.5287	0.0988	Helicobacter pylori (72293)	Helicobacter (213849)	Helicobacteraceae (29547)	Campylobacterales (68525)	Epsilonproteobacteria (122	Proteobacteria (2)	Bacteria (1)			99.80	99.50	WGS
210.5288	0.092	Helicobacter pylori (72293)	Helicobacter (213849)	Helicobacteraceae (29547)	Campylobacterales (68525)	Epsilonproteobacteria (122	Proteobacteria (2)	Bacteria (1)			99.90	99.40	WGS
210.5289	0.0958	Helicobacter pylori (72293)	Helicobacter (213849)	Helicobacteraceae (29547)	Campylobacterales (68525)	Epsilonproteobacteria (122	Proteobacteria (2)	Bacteria (1)			99.90	99.90	WGS
210.5290	0.0948	Helicobacter pylori (72293)	Helicobacter (213849)	Helicobacteraceae (29547)	Campylobacterales (68525)	Epsilonproteobacteria (122	Proteobacteria (2)	Bacteria (1)			99.80	99.30	WGS
210.5291	0.0948	Helicobacter pylori (72293)	Helicobacter (213849)	Helicobacteraceae (29547)	Campylobacterales (68525)	Epsilonproteobacteria (122	Proteobacteria (2)	Bacteria (1)			99.00	98.50	WGS
210.5292	0.0978	Helicobacter pylori (72293)	Helicobacter (213849)	Helicobacteraceae (29547)	Campylobacterales (68525)	Epsilonproteobacteria (122	Proteobacteria (2)	Bacteria (1)			99.90	99.70	WGS
210.5293	0.0948	Helicobacter pylori (72293)	Helicobacter (213849)	Helicobacteraceae (29547)	Campylobacterales (68525)	Epsilonproteobacteria (122	Proteobacteria (2)	Bacteria (1)			99.90	99.60	WGS
210.5294	0.091	Helicobacter pylori (72293)	Helicobacter (213849)	Helicobacteraceae (29547)	Campylobacterales (68525)	Epsilonproteobacteria (122	Proteobacteria (2)	Bacteria (1)			99.60	99.40	WGS
210.5295	0.1006	Helicobacter pylori (72293)	Helicobacter (213849)	Helicobacteraceae (29547)	Campylobacterales (68525)	Epsilonproteobacteria (122	Proteobacteria (2)	Bacteria (1)			99.90	99.50	WGS
210.5296	0.092	Helicobacter pylori (72293)	Helicobacter (213849)	Helicobacteraceae (29547)	Campylobacterales (68525)	Epsilonproteobacteria (122	Proteobacteria (2)	Bacteria (1)			99.90	99.30	WGS
210.5297	0.092	Helicobacter pylori (72293)	Helicobacter (213849)	Helicobacteraceae (29547)	Campylobacterales (68525)	Epsilonproteobacteria (122	Proteobacteria (2)	Bacteria (1)			99.90	99.60	WGS
210.5298	0.091	Helicobacter pylori (72293)	Helicobacter (213849)	Helicobacteraceae (29547)	Campylobacterales (68525)	Epsilonproteobacteria (122	Proteobacteria (2)	Bacteria (1)			99.90	98.90	WGS
210.5299	0.092	Helicobacter pylori (72293)	Helicobacter (213849)	Helicobacteraceae (29547)	Campylobacterales (68525)	Epsilonproteobacteria (122	Proteobacteria (2)	Bacteria (1)			99.90	99.40	WGS
210.5300	0.0938	Helicobacter pylori (72293)	Helicobacter (213849)	Helicobacteraceae (29547)	Campylobacterales (68525)	Epsilonproteobacteria (122	Proteobacteria (2)	Bacteria (1)			99.90	99.80	WGS
210.5301	0.092	Helicobacter pylori (72293)	Helicobacter (213849)	Helicobacteraceae (29547)	Campylobacterales (68525)	Epsilonproteobacteria (122	Proteobacteria (2)	Bacteria (1)			99.90	99.50	WGS
210.5302	0.092	Helicobacter pylori (72293)	Helicobacter (213849)	Helicobacteraceae (29547)	Campylobacterales (68525)	Epsilonproteobacteria (122	Proteobacteria (2)	Bacteria (1)			99.90	99.50	WGS
210.5303	0.092	Helicobacter pylori (72293)	Helicobacter (213849)	Helicobacteraceae (29547)	Campylobacterales (68525)	Epsilonproteobacteria (122	Proteobacteria (2)	Bacteria (1)			99.70	99.70	WGS
210.5304	0.0988	Helicobacter pylori (72293)	Helicobacter (213849)	Helicobacteraceae (29547)	Campylobacterales (68525)	Epsilonproteobacteria (122	Proteobacteria (2)	Bacteria (1)			98.80	98.30	WGS
210.5305	0.091	Helicobacter pylori (72293)	Helicobacter (213849)	Helicobacteraceae (29547)	Campylobacterales (68525)	Epsilonproteobacteria (122	Proteobacteria (2)	Bacteria (1)			99.90	99.50	WGS
210.5306	0.1006	Helicobacter pylori (72293)	Helicobacter (213849)	Helicobacteraceae (29547)	Campylobacterales (68525)	Epsilonproteobacteria (122	Proteobacteria (2)	Bacteria (1)			99.90	99.60	WGS
210.5307	0.0666	Helicobacter pylori (72293)	Helicobacter (213849)	Helicobacteraceae (29547)	Campylobacterales (68525)	Epsilonproteobacteria (122	Proteobacteria (2)	Bacteria (1)			99.90	99.80	WGS
210.5308	0.1016	Helicobacter pylori (72293)	Helicobacter (213849)	Helicobacteraceae (29547)	Campylobacterales (68525)	Epsilonproteobacteria (122	Proteobacteria (2)	Bacteria (1)			99.90	99.60	WGS
210.5309	0.1006	Helicobacter pylori (72293)	Helicobacter (213849)	Helicobacteraceae (29547)	Campylobacterales (68525)	Epsilonproteobacteria (122	Proteobacteria (2)	Bacteria (1)			99.90	99.50	WGS
210.5310	0.092	Helicobacter pylori (72293)	Helicobacter (213849)	Helicobacteraceae (29547)	Campylobacterales (68525)	Epsilonproteobacteria (122	Proteobacteria (2)	Bacteria (1)			99.90	99.70	WGS
210.5311	0.0714	Helicobacter pylori (72293)	Helicobacter (213849)	Helicobacteraceae (29547)	Campylobacterales (68525)	Epsilonproteobacteria (122	Proteobacteria (2)	Bacteria (1)			100.00	99.60	WGS
210.5312	0.0938	Helicobacter pylori (72293)	Helicobacter (213849)	Helicobacteraceae (29547)	Campylobacterales (68525)	Epsilonproteobacteria (122	Proteobacteria (2)	Bacteria (1)			99.90	99.60	WGS
210.5313	0.1016	Helicobacter pylori (72293)	Helicobacter (213849)	Helicobacteraceae (29547)	Campylobacterales (68525)	Epsilonproteobacteria (122	Proteobacteria (2)	Bacteria (1)			99.90	99.50	WGS
210.5314	0.091	Helicobacter pylori (72293)	Helicobacter (213849)	Helicobacteraceae (29547)	Campylobacterales (68525)	Epsilonproteobacteria (122	Proteobacteria (2)	Bacteria (1)			99.90	99.50	WGS
210.5315	0.092	Helicobacter pylori (72293)	Helicobacter (213849)	Helicobacteraceae (29547)	Campylobacterales (68525)	Epsilonproteobacteria (122	Proteobacteria (2)	Bacteria (1)			99.90	95.60	WGS
210.5316	0.0938	Helicobacter pylori (72293)	Helicobacter (213849)	Helicobacteraceae (29547)	Campylobacterales (68525)	Epsilonproteobacteria (122	Proteobacteria (2)	Bacteria (1)			99.90	99.70	WGS
210.5317	0.0938	Helicobacter pylori (72293)	Helicobacter (213849)	Helicobacteraceae (29547)	Campylobacterales (68525)	Epsilonproteobacteria (122	Proteobacteria (2)	Bacteria (1)			99.80	97.30	WGS
210.5318	0.091	Helicobacter pylori (72293)	Helicobacter (213849)	Helicobacteraceae (29547)	Campylobacterales (68525)	Epsilonproteobacteria (122	Proteobacteria (2)	Bacteria (1)			99.90	99.50	WGS
210.5319	0.0834	Helicobacter pylori (72293)	Helicobacter (213849)	Helicobacteraceae (29547)	Campylobacterales (68525)	Epsilonproteobacteria (122	Proteobacteria (2)	Bacteria (1)			99.90	99.40	WGS
210.5320	0.0948	Helicobacter pylori (72293)	Helicobacter (213849)	Helicobacteraceae (29547)	Campylobacterales (68525)	Epsilonproteobacteria (122	Proteobacteria (2)	Bacteria (1)			99.70	99.60	WGS
210.5321	0.091	Helicobacter pylori (72293)	Helicobacter (213849)	Helicobacteraceae (29547)	Campylobacterales (68525)	Epsilonproteobacteria (122	Proteobacteria (2)	Bacteria (1)			99.90	99.60	WGS
210.5322	0.0948	Helicobacter pylori (72293)	Helicobacter (213849)	Helicobacteraceae (29547)	Campylobacterales (68525)	Epsilonproteobacteria (122	Proteobacteria (2)	Bacteria (1)			99.80	99.50	WGS
210.5323	0.09	Helicobacter pylori (72293)	Helicobacter (213849)	Helicobacteraceae (29547)	Campylobacterales (68525)	Epsilonproteobacteria (122	Proteobacteria (2)	Bacteria (1)			99.90	99.60	WGS
210.5324	0.092	Helicobacter pylori (72293)	Helicobacter (213849)	Helicobacteraceae (29547)	Campylobacterales (68525)	Epsilonproteobacteria (122	Proteobacteria (2)	Bacteria (1)			99.80	99.50	WGS
210.5325	0.0948	Helicobacter pylori (72293)	Helicobacter (213849)	Helicobacteraceae (29547)	Campylobacterales (68525)	Epsilonproteobacteria (122	Proteobacteria (2)	Bacteria (1)			99.90	99.30	WGS
210.5326	0.0938	Helicobacter pylori (72293)	Helicobacter (213849)	Helicobacteraceae (29547)	Campylobacterales (68525)	Epsilonproteobacteria (122	Proteobacteria (2)	Bacteria (1)			99.90	99.70	WGS
210.5327	0.091	Helicobacter pylori (72293)	Helicobacter (213849)	Helicobacteraceae (29547)	Campylobacterales (68525)	Epsilonproteobacteria (122	Proteobacteria (2)	Bacteria (1)			99.90	99.40	WGS
210.5328	0.0948	Helicobacter pylori (72293)	Helicobacter (213849)	Helicobacteraceae (29547)	Campylobacterales (68525)	Epsilonproteobacteria (122	Proteobacteria (2)	Bacteria (1)			99.90	99.20	WGS
210.5329	0.0948	Helicobacter pylori (72293)	Helicobacter (213849)	Helicobacteraceae (29547)	Campylobacterales (68525)	Epsilonproteobacteria (122	Proteobacteria (2)	Bacteria (1)			99.80	99.50	WGS
210.5330	0.0936	Helicobacter pylori (72293)	Helicobacter (213849)	Helicobacteraceae (29547)	Campylobacterales (68525)	Epsilonproteobacteria (122	Proteobacteria (2)	Bacteria (1)			97.40	97.10	WGS
210.5331	0.0988	Helicobacter pylori (72293)	Helicobacter (213849)	Helicobacteraceae (29547)	Campylobacterales (68525)	Epsilonproteobacteria (122	Proteobacteria (2)	Bacteria (1)			99.90	99.50	WGS
210.5332	0.0948	Helicobacter pylori (72293)	Helicobacter (213849)	Helicobacteraceae (29547)	Campylobacterales (68525)	Epsilonproteobacteria (122	Proteobacteria (2)	Bacteria (1)			99.80	99.50	WGS
210.5333	0.0948	Helicobacter pylori (72293)	Helicobacter (213849)	Helicobacteraceae (29547)	Campylobacterales (68525)	Epsilonproteobacteria (122	Proteobacteria (2)	Bacteria (1)			99.90	99.40	WGS
210.5334	0.0948	Helicobacter pylori (72293)	Helicobacter (213849)	Helicobacteraceae (29547)	Campylobacterales (68525)	Epsilonproteobacteria (122	Proteobacteria (2)	Bacteria (1)			99.90	99.60	WGS
210.5335	0.1006	Helicobacter pylori (72293)	Helicobacter (213849)	Helicobacteraceae (29547)	Campylobacterales (68525)	Epsilonproteobacteria (122	Proteobacteria (2)	Bacteria (1)			99.90	99.50	WGS
210.5336	0.0948	Helicobacter pylori (72293)	Helicobacter (213849)	Helicobacteraceae (29547)	Campylobacterales (68525)	Epsilonproteobacteria (122	Proteobacteria (2)	Bacteria (1)			99.90	99.70	WGS
210.5337	0.0988	Helicobacter pylori (72293)	Helicobacter (213849)	Helicobacteraceae (29547)	Campylobacterales (68525)	Epsilonproteobacteria (122	Proteobacteria (2)	Bacteria (1)			99.90	99.30	WGS
210.5338	0.0988	Helicobacter pylori (72293)	Helicobacter (213849)	Helicobacteraceae (29547)	Campylobacterales (68525)	Epsilonproteobacteria (122	Proteobacteria (2)	Bacteria (1)			100.00	99.50	WGS
210.5339	0.1006	Helicobacter pylori (72293)	Helicobacter (213849)	Helicobacteraceae (29547)	Campylobacterales (68525)	Epsilonproteobacteria (122	Proteobacteria (2)	Bacteria (1)			99.90	99.60	WGS
210.5340	0.0948	Helicobacter pylori (72293)	Helicobacter (213849)	Helicobacteraceae (29547)	Campylobacterales (68525)	Epsilonproteobacteria (122	Proteobacteria (2)	Bacteria (1)			99.90	99.30	WGS
210.5341	0.0948	Helicobacter pylori (72293)	Helicobacter (213849)	Helicobacteraceae (29547)	Campylobacterales (68525)	Epsilonproteobacteria (122	Proteobacteria (2)	Bacteria (1)			99.90	99.60	WGS
210.5342	0.0892	Helicobacter pylori (72293)	Helicobacter (213849)	Helicobacteraceae (29547)	Campylobacterales (68525)	Epsilonproteobacteria (122	Proteobacteria (2)	Bacteria (1)			99.90	81.80	WGS
210.5343	0.092	Helicobacter pylori (72293)	Helicobacter (213849)	Helicobacteraceae (29547)	Campylobacterales (68525)	Epsilonproteobacteria (122	Proteobacteria (2)	Bacteria (1)			99.90	99.40	WGS
210.5344	0.0948	Helicobacter pylori (72293)	Helicobacter (213849)	Helicobacteraceae (29547)	Campylobacterales (68525)	Epsilonproteobacteria (122	Proteobacteria (2)	Bacteria (1)			99.80	99.30	WGS
210.5345	0.0948	Helicobacter pylori (72293)	Helicobacter (213849)	Helicobacteraceae (29547)	Campylobacterales (68525)	Epsilonproteobacteria (122	Proteobacteria (2)	Bacteria (1)			99.80	99.60	WGS
210.5346	0.0948	Helicobacter pylori (72293)	Helicobacter (213849)	Helicobacteraceae (29547)	Campylobacterales (68525)	Epsilonproteobacteria (122	Proteobacteria (2)	Bacteria (1)			99.60	99.00	WGS
210.5347	0.092	Helicobacter pylori (72293)	Helicobacter (213849)	Helicobacteraceae (29547)	Campylobacterales (68525)	Epsilonproteobacteria (122	Proteobacteria (2)	Bacteria (1)			99.90	99.50	WGS
210.5348	0.0988	Helicobacter pylori (72293)	Helicobacter (213849)	Helicobacteraceae (29547)	Campylobacterales (68525)	Epsilonproteobacteria (122	Proteobacteria (2)	Bacteria (1)			99.90	99.60	WGS
210.5349	0.1006	Helicobacter pylori (72293)	Helicobacter (213849)	Helicobacteraceae (29547)	Campylobacterales (68525)	Epsilonproteobacteria (122	Proteobacteria (2)	Bacteria (1)			98.30	98.00	WGS
210.5350	0.0842	Helicobacter pylori (72293)	Helicobacter (213849)	Helicobacteraceae (29547)	Campylobacterales (68525)	Epsilonproteobacteria (122	Proteobacteria (2)	Bacteria (1)			99.90	99.40	WGS
210.5351	0.092	Helicobacter pylori (72293)	Helicobacter (213849)	Helicobacteraceae (29547)	Campylobacterales (68525)	Epsilonproteobacteria (122	Proteobacteria (2)	Bacteria (1)			99.90	99.60	WGS
210.5352	0.091	Helicobacter pylori (72293)	Helicobacter (213849)	Helicobacteraceae (29547)	Campylobacterales (68525)	Epsilonproteobacteria (122	Proteobacteria (2)	Bacteria (1)			97.30	96.80	WGS
210.5353	0.0892	Helicobacter pylori (72293)	Helicobacter (213849)	Helicobacteraceae (29547)	Campylobacterales (68525)	Epsilonproteobacteria (122	Proteobacteria (2)	Bacteria (1)			99.90	99.60	WGS
210.5354	0.092	Helicobacter pylori (72293)	Helicobacter (213849)	Helicobacteraceae (29547)	Campylobacterales (68525)	Epsilonproteobacteria (122	Proteobacteria (2)	Bacteria (1)			99.90	99.30	WGS
210.5355	0.091	Helicobacter pylori (72293)	Helicobacter (213849)	Helicobacteraceae (29547)	Campylobacterales (68525)	Epsilonproteobacteria (122	Proteobacteria (2)	Bacteria (1)			100.00	99.70	WGS
210.5356	0.092	Helicobacter pylori (72293)	Helicobacter (213849)	Helicobacteraceae (29547)	Campylobacterales (68525)	Epsilonproteobacteria (122	Proteobacteria (2)	Bacteria (1)			99.20	98.70	WGS
210.5357	0.092	Helicobacter pylori (72293)	Helicobacter (213849)	Helicobacteraceae (29547)	Campylobacterales (68525)	Epsilonproteobacteria (122	Proteobacteria (2)	Bacteria (1)			99.90	99.20	WGS
210.5358	0.0948	Helicobacter pylori (72293)	Helicobacter (213849)	Helicobacteraceae (29547)	Campylobacterales (68525)	Epsilonproteobacteria (122	Proteobacteria (2)	Bacteria (1)			99.80	99.50	WGS
210.5359	0.0948	Helicobacter pylori (72293)	Helicobacter (213849)	Helicobacteraceae (29547)	Campylobacterales (68525)	Epsilonproteobacteria (122	Proteobacteria (2)	Bacteria (1)			99.90	99.30	WGS
210.5360	0.092	Helicobacter pylori (72293)	Helicobacter (213849)	Helicobacteraceae (29547)	Campylobacterales (68525)	Epsilonproteobacteria (122	Proteobacteria (2)	Bacteria (1)			99.90	99.60	WGS
210.5361	0.0948	Helicobacter pylori (72293)	Helicobacter (213849)	Helicobacteraceae (29547)	Campylobacterales (68525)	Epsilonproteobacteria (122	Proteobacteria (2)	Bacteria (1)			99.90	99.60	WGS
210.5362	0.0948	Helicobacter pylori (72293)	Helicobacter (213849)	Helicobacteraceae (29547)	Campylobacterales (68525)	Epsilonproteobacteria (122	Proteobacteria (2)	Bacteria (1)			99.90	99.60	WGS
210.5363	0.0948	Helicobacter pylori (72293)	Helicobacter (213849)	Helicobacteraceae (29547)	Campylobacterales (68525)	Epsilonproteobacteria (122	Proteobacteria (2)	Bacteria (1)			99.90	99.30	WGS
210.5364	0.1006	Helicobacter pylori (72293)	Helicobacter (213849)	Helicobacteraceae (29547)	Campylobacterales (68525)	Epsilonproteobacteria (122	Proteobacteria (2)	Bacteria (1)			99.90	99.50	WGS
210.5365	0.1016	Helicobacter pylori (72293)	Helicobacter (213849)	Helicobacteraceae (29547)	Campylobacterales (68525)	Epsilonproteobacteria (122	Proteobacteria (2)	Bacteria (1)			99.90	99.70	WGS
210.5366	0.0948	Helicobacter pylori (72293)	Helicobacter (213849)	Helicobacteraceae (29547)	Campylobacterales (68525)	Epsilonproteobacteria (122	Proteobacteria (2)	Bacteria (1)			99.90	99.60	WGS
210.5367	0.092	Helicobacter pylori (72293)	Helicobacter (213849)	Helicobacteraceae (29547)	Campylobacterales (68525)	Epsilonproteobacteria (122	Proteobacteria (2)	Bacteria (1)			99.90	99.80	WGS
210.5368	0.091	Helicobacter pylori (72293)	Helicobacter (213849)	Helicobacteraceae (29547)	Campylobacterales (68525)	Epsilonproteobacteria (122	Proteobacteria (2)	Bacteria (1)			99.30	98.80	WGS
210.5369	0.1006	Helicobacter pylori (72293)	Helicobacter (213849)	Helicobacteraceae (29547)	Campylobacterales (68525)	Epsilonproteobacteria (122	Proteobacteria (2)	Bacteria (1)			99.90	98.30	WGS
210.5370	0.1028	Helicobacter pylori (72293)	Helicobacter (213849)	Helicobacteraceae (29547)	Campylobacterales (68525)	Epsilonproteobacteria (122	Proteobacteria (2)	Bacteria (1)			99.90	99.40	WGS
210.5371	0.0988	Helicobacter pylori (72293)	Helicobacter (213849)	Helicobacteraceae (29547)	Campylobacterales (68525)	Epsilonproteobacteria (122	Proteobacteria (2)	Bacteria (1)			99.90	99.70	WGS
210.5372	0.0938	Helicobacter pylori (72293)	Helicobacter (213849)	Helicobacteraceae (29547)	Campylobacterales (68525)	Epsilonproteobacteria (122	Proteobacteria (2)	Bacteria (1)			99.80	99.50	WGS
210.5373	0.0938	Helicobacter pylori (72293)	Helicobacter (213849)	Helicobacteraceae (29547)	Campylobacterales (68525)	Epsilonproteobacteria (122	Proteobacteria (2)	Bacteria (1)			99.90	99.50	WGS
210.5374	0.0948	Helicobacter pylori (72293)	Helicobacter (213849)	Helicobacteraceae (29547)	Campylobacterales (68525)	Epsilonproteobacteria (122	Proteobacteria (2)	Bacteria (1)			99.90	99.60	WGS
210.5375	0.0948	Helicobacter pylori (72293)	Helicobacter (213849)	Helicobacteraceae (29547)	Campylobacterales (68525)	Epsilonproteobacteria (122	Proteobacteria (2)	Bacteria (1)			99.90	99.30	WGS
210.5376	0.092	Helicobacter pylori (72293)	Helicobacter (213849)	Helicobacteraceae (29547)	Campylobacterales (68525)	Epsilonproteobacteria (122	Proteobacteria (2)	Bacteria (1)			99.90	99.50	WGS
210.5377	0.0948	Helicobacter pylori (72293)	Helicobacter (213849)	Helicobacteraceae (29547)	Campylobacterales (68525)	Epsilonproteobacteria (122	Proteobacteria (2)	Bacteria (1)			99.90	99.50	WGS
210.5378	0.1122	Helicobacter pylori (72293)	Helicobacter (213849)	Helicobacteraceae (29547)	Campylobacterales (68525)	Epsilonproteobacteria (122	Proteobacteria (2)	Bacteria (1)			99.40	99.10	WGS
210.5379	0.0978	Helicobacter pylori (72293)	Helicobacter (213849)	Helicobacteraceae (29547)	Campylobacterales (68525)	Epsilonproteobacteria (122	Proteobacteria (2)	Bacteria (1)			99.90	99.40	WGS
210.5380	0.092	Helicobacter pylori (72293)	Helicobacter (213849)	Helicobacteraceae (29547)	Campylobacterales (68525)	Epsilonproteobacteria (122	Proteobacteria (2)	Bacteria (1)			99.80	99.40	WGS
210.5381	0.0938	Helicobacter pylori (72293)	Helicobacter (213849)	Helicobacteraceae (29547)	Campylobacterales (68525)	Epsilonproteobacteria (122	Proteobacteria (2)	Bacteria (1)			99.70	99.50	WGS
210.5382	0.0948	Helicobacter pylori (72293)	Helicobacter (213849)	Helicobacteraceae (29547)	Campylobacterales (68525)	Epsilonproteobacteria (122	Proteobacteria (2)	Bacteria (1)			99.90	99.60	WGS
210.5383	0.1016	Helicobacter pylori (72293)	Helicobacter (213849)	Helicobacteraceae (29547)	Campylobacterales (68525)	Epsilonproteobacteria (122	Proteobacteria (2)	Bacteria (1)			99.90	99.60	WGS
210.5384	0.0938	Helicobacter pylori (72293)	Helicobacter (213849)	Helicobacteraceae (29547)	Campylobacterales (68525)	Epsilonproteobacteria (122	Proteobacteria (2)	Bacteria (1)			99.80	98.60	WGS
210.5385	0.091	Helicobacter pylori (72293)	Helicobacter (213849)	Helicobacteraceae (29547)	Campylobacterales (68525)	Epsilonproteobacteria (122	Proteobacteria (2)	Bacteria (1)			99.90	99.30	WGS
210.5386	0.091	Helicobacter pylori (72293)	Helicobacter (213849)	Helicobacteraceae (29547)	Campylobacterales (68525)	Epsilonproteobacteria (122	Proteobacteria (2)	Bacteria (1)			99.90	99.50	WGS
210.5387	0.0988	Helicobacter pylori (72293)	Helicobacter (213849)	Helicobacteraceae (29547)	Campylobacterales (68525)	Epsilonproteobacteria (122	Proteobacteria (2)	Bacteria (1)			99.90	99.60	WGS
210.5388	0.092	Helicobacter pylori (72293)	Helicobacter (213849)	Helicobacteraceae (29547)	Campylobacterales (68525)	Epsilonproteobacteria (122	Proteobacteria (2)	Bacteria (1)			99.90	99.10	WGS
210.5389	0.0988	Helicobacter pylori (72293)	Helicobacter (213849)	Helicobacteraceae (29547)	Campylobacterales (68525)	Epsilonproteobacteria (122	Proteobacteria (2)	Bacteria (1)			99.90	99.50	WGS
210.5390	0.1006	Helicobacter pylori (72293)	Helicobacter (213849)	Helicobacteraceae (29547)	Campylobacterales (68525)	Epsilonproteobacteria (122	Proteobacteria (2)	Bacteria (1)			99.80	98.50	WGS
210.5391	0.0948	Helicobacter pylori (72293)	Helicobacter (213849)	Helicobacteraceae (29547)	Campylobacterales (68525)	Epsilonproteobacteria (122	Proteobacteria (2)	Bacteria (1)			99.50	99.30	Complete
210.5392	0.091	Helicobacter pylori (72293)	Helicobacter (213849)	Helicobacteraceae (29547)	Campylobacterales (68525)	Epsilonproteobacteria (122	Proteobacteria (2)	Bacteria (1)			99.70	98.90	Complete
210.5625	0.0692	Helicobacter pylori (72293)	Helicobacter (213849)	Helicobacteraceae (29547)	Campylobacterales (68525)	Epsilonproteobacteria (122	Proteobacteria (2)	Bacteria (1)			99.60	99.30	Complete
210.5626	0.0714	Helicobacter pylori (72293)	Helicobacter (213849)	Helicobacteraceae (29547)	Campylobacterales (68525)	Epsilonproteobacteria (122	Proteobacteria (2)	Bacteria (1)			99.90	94.50	WGS
210.5627	0.0752	Helicobacter pylori (72293)	Helicobacter (213849)	Helicobacteraceae (29547)	Campylobacterales (68525)	Epsilonproteobacteria (122	Proteobacteria (2)	Bacteria (1)			99.70	99.20	Complete
210.5628	0.0864	Helicobacter pylori (72293)	Helicobacter (213849)	Helicobacteraceae (29547)	Campylobacterales (68525)	Epsilonproteobacteria (122	Proteobacteria (2)	Bacteria (1)			99.50	97.00	WGS
210.5629	0.0948	Helicobacter pylori (72293)	Helicobacter (213849)	Helicobacteraceae (29547)	Campylobacterales (68525)	Epsilonproteobacteria (122	Proteobacteria (2)	Bacteria (1)			99.70	93.30	WGS
210.5630	0.1016	Helicobacter pylori (72293)	Helicobacter (213849)	Helicobacteraceae (29547)	Campylobacterales (68525)	Epsilonproteobacteria (122	Proteobacteria (2)	Bacteria (1)			99.70	99.10	Complete
210.5631	0.0948	Helicobacter pylori (72293)	Helicobacter (213849)	Helicobacteraceae (29547)	Campylobacterales (68525)	Epsilonproteobacteria (122	Proteobacteria (2)	Bacteria (1)			99.40	98.80	Complete
210.5632	0.0794	Helicobacter pylori (72293)	Helicobacter (213849)	Helicobacteraceae (29547)	Campylobacterales (68525)	Epsilonproteobacteria (122	Proteobacteria (2)	Bacteria (1)			99.70	99.30	WGS
210.5633	0.0656	Helicobacter pylori (72293)	Helicobacter (213849)	Helicobacteraceae (29547)	Campylobacterales (68525)	Epsilonproteobacteria (122	Proteobacteria (2)	Bacteria (1)			99.90	96.50	WGS
210.5634	0.0814	Helicobacter pylori (72293)	Helicobacter (213849)	Helicobacteraceae (29547)	Campylobacterales (68525)	Epsilonproteobacteria (122	Proteobacteria (2)	Bacteria (1)			99.60	99.10	WGS
210.5635	0.0842	Helicobacter pylori (72293)	Helicobacter (213849)	Helicobacteraceae (29547)	Campylobacterales (68525)	Epsilonproteobacteria (122	Proteobacteria (2)	Bacteria (1)			99.50	99.00	WGS
210.5636	0.0978	Helicobacter pylori (72293)	Helicobacter (213849)	Helicobacteraceae (29547)	Campylobacterales (68525)	Epsilonproteobacteria (122	Proteobacteria (2)	Bacteria (1)			99.60	99.30	WGS
210.5637	0.1006	Helicobacter pylori (72293)	Helicobacter (213849)	Helicobacteraceae (29547)	Campylobacterales (68525)	Epsilonproteobacteria (122	Proteobacteria (2)	Bacteria (1)			99.70	99.30	WGS
210.5638	0.091	Helicobacter pylori (72293)	Helicobacter (213849)	Helicobacteraceae (29547)	Campylobacterales (68525)	Epsilonproteobacteria (122	Proteobacteria (2)	Bacteria (1)			99.80	99.50	WGS
210.5639	0.091	Helicobacter pylori (72293)	Helicobacter (213849)	Helicobacteraceae (29547)	Campylobacterales (68525)	Epsilonproteobacteria (122	Proteobacteria (2)	Bacteria (1)			99.80	99.40	WGS
210.5640	0.1006	Helicobacter pylori (72293)	Helicobacter (213849)	Helicobacteraceae (29547)	Campylobacterales (68525)	Epsilonproteobacteria (122	Proteobacteria (2)	Bacteria (1)			99.50	99.30	WGS
210.5641	0.091	Helicobacter pylori (72293)	Helicobacter (213849)	Helicobacteraceae (29547)	Campylobacterales (68525)	Epsilonproteobacteria (122	Proteobacteria (2)	Bacteria (1)			99.50	99.00	WGS
210.5642	0.0692	Helicobacter pylori (72293)	Helicobacter (213849)	Helicobacteraceae (29547)	Campylobacterales (68525)	Epsilonproteobacteria (122	Proteobacteria (2)	Bacteria (1)			99.70	99.40	WGS
210.5643	0.09	Helicobacter pylori (72293)	Helicobacter (213849)	Helicobacteraceae (29547)	Campylobacterales (68525)	Epsilonproteobacteria (122	Proteobacteria (2)	Bacteria (1)			99.80	99.20	WGS
210.5644	0.092	Helicobacter pylori (72293)	Helicobacter (213849)	Helicobacteraceae (29547)	Campylobacterales (68525)	Epsilonproteobacteria (122	Proteobacteria (2)	Bacteria (1)			99.80	99.30	WGS
210.5645	0.092	Helicobacter pylori (72293)	Helicobacter (213849)	Helicobacteraceae (29547)	Campylobacterales (68525)	Epsilonproteobacteria (122	Proteobacteria (2)	Bacteria (1)			99.80	99.40	WGS
210.5646	0.0682	Helicobacter pylori (72293)	Helicobacter (213849)	Helicobacteraceae (29547)	Campylobacterales (68525)	Epsilonproteobacteria (122	Proteobacteria (2)	Bacteria (1)			99.60	99.30	WGS
210.5647	0.0948	Helicobacter pylori (72293)	Helicobacter (213849)	Helicobacteraceae (29547)	Campylobacterales (68525)	Epsilonproteobacteria (122	Proteobacteria (2)	Bacteria (1)			99.60	98.70	WGS
210.5648	0.1006	Helicobacter pylori (72293)	Helicobacter (213849)	Helicobacteraceae (29547)	Campylobacterales (68525)	Epsilonproteobacteria (122	Proteobacteria (2)	Bacteria (1)			99.70	99.40	WGS
210.5649	0.0938	Helicobacter pylori (72293)	Helicobacter (213849)	Helicobacteraceae (29547)	Campylobacterales (68525)	Epsilonproteobacteria (122	Proteobacteria (2)	Bacteria (1)			99.50	98.80	WGS
210.5650	0.092	Helicobacter pylori (72293)	Helicobacter (213849)	Helicobacteraceae (29547)	Campylobacterales (68525)	Epsilonproteobacteria (122	Proteobacteria (2)	Bacteria (1)			99.70	99.00	WGS
210.5651	0.0948	Helicobacter pylori (72293)	Helicobacter (213849)	Helicobacteraceae (29547)	Campylobacterales (68525)	Epsilonproteobacteria (122	Proteobacteria (2)	Bacteria (1)			99.50	99.20	WGS
210.5652	0.1006	Helicobacter pylori (72293)	Helicobacter (213849)	Helicobacteraceae (29547)	Campylobacterales (68525)	Epsilonproteobacteria (122	Proteobacteria (2)	Bacteria (1)			99.60	98.40	WGS
210.5653	0.091	Helicobacter pylori (72293)	Helicobacter (213849)	Helicobacteraceae (29547)	Campylobacterales (68525)	Epsilonproteobacteria (122	Proteobacteria (2)	Bacteria (1)			99.90	99.40	WGS
210.5654	0.091	Helicobacter pylori (72293)	Helicobacter (213849)	Helicobacteraceae (29547)	Campylobacterales (68525)	Epsilonproteobacteria (122	Proteobacteria (2)	Bacteria (1)			99.90	99.40	WGS
210.5655	0.091	Helicobacter pylori (72293)	Helicobacter (213849)	Helicobacteraceae (29547)	Campylobacterales (68525)	Epsilonproteobacteria (122	Proteobacteria (2)	Bacteria (1)			99.90	99.40	WGS
211.4	0.0618	Helicobacter sp. CLO-3 (72293)	Helicobacter (213849)	Helicobacteraceae (29547)	Campylobacterales (68525)	Epsilonproteobacteria (122	Proteobacteria (2)	Bacteria (1)	98.80	0.00	97.60	97.50	WGS
211.6	0.0618	Helicobacter sp. CLO-3 (72293)	Helicobacter (213849)	Helicobacteraceae (29547)	Campylobacterales (68525)	Epsilonproteobacteria (122	Proteobacteria (2)	Bacteria (1)			98.10	97.90	WGS
212.40	0.0386	Helicobacter acinonychis (72293)	Helicobacter (213849)	Helicobacteraceae (29547)	Campylobacterales (68525)	Epsilonproteobacteria (122	Proteobacteria (2)	Bacteria (1)	100.00	0.80	99.40	99.30	Complete
212.41	0.0386	Helicobacter acinonychis (72293)	Helicobacter (213849)	Helicobacteraceae (29547)	Campylobacterales (68525)	Epsilonproteobacteria (122	Proteobacteria (2)	Bacteria (1)	100.00	0.40	99.40	99.30	WGS
212.42	0.0386	Helicobacter acinonychis (72293)	Helicobacter (213849)	Helicobacteraceae (29547)	Campylobacterales (68525)	Epsilonproteobacteria (122	Proteobacteria (2)	Bacteria (1)	100.00	0.40	99.40	99.30	WGS
212.43	0.0386	Helicobacter acinonychis (72293)	Helicobacter (213849)	Helicobacteraceae (29547)	Campylobacterales (68525)	Epsilonproteobacteria (122	Proteobacteria (2)	Bacteria (1)	100.00	0.40	99.70	99.70	WGS
212.44	0.0386	Helicobacter acinonychis (72293)	Helicobacter (213849)	Helicobacteraceae (29547)	Campylobacterales (68525)	Epsilonproteobacteria (122	Proteobacteria (2)	Bacteria (1)	100.00	0.80	99.70	99.70	WGS
212.45	0.0406	Helicobacter acinonychis (72293)	Helicobacter (213849)	Helicobacteraceae (29547)	Campylobacterales (68525)	Epsilonproteobacteria (122	Proteobacteria (2)	Bacteria (1)	99.80	0.80	99.40	99.00	WGS
212.46	0.0386	Helicobacter acinonychis (72293)	Helicobacter (213849)	Helicobacteraceae (29547)	Campylobacterales (68525)	Epsilonproteobacteria (122	Proteobacteria (2)	Bacteria (1)	100.00	5.80	99.60	98.20	WGS
213.21	0.1256	Helicobacter cinaedi (72293)	Helicobacter (213849)	Helicobacteraceae (29547)	Campylobacterales (68525)	Epsilonproteobacteria (122	Proteobacteria (2)	Bacteria (1)	99.60	0.40	99.30	98.80	WGS
213.22	0.122	Helicobacter cinaedi (72293)	Helicobacter (213849)	Helicobacteraceae (29547)	Campylobacterales (68525)	Epsilonproteobacteria (122	Proteobacteria (2)	Bacteria (1)	100.00	0.00	99.30	98.60	WGS
213.23	0.1192	Helicobacter cinaedi (72293)	Helicobacter (213849)	Helicobacteraceae (29547)	Campylobacterales (68525)	Epsilonproteobacteria (122	Proteobacteria (2)	Bacteria (1)	100.00	0.00	99.40	98.60	WGS
213.24	0.122	Helicobacter cinaedi (72293)	Helicobacter (213849)	Helicobacteraceae (29547)	Campylobacterales (68525)	Epsilonproteobacteria (122	Proteobacteria (2)	Bacteria (1)	100.00	0.80	99.00	98.00	WGS
213.25	0.122	Helicobacter cinaedi (72293)	Helicobacter (213849)	Helicobacteraceae (29547)	Campylobacterales (68525)	Epsilonproteobacteria (122	Proteobacteria (2)	Bacteria (1)	100.00	1.20	99.30	97.70	WGS
213.26	0.122	Helicobacter cinaedi (72293)	Helicobacter (213849)	Helicobacteraceae (29547)	Campylobacterales (68525)	Epsilonproteobacteria (122	Proteobacteria (2)	Bacteria (1)	100.00	2.40	99.00	97.30	WGS
213.27	0.122	Helicobacter cinaedi (72293)	Helicobacter (213849)	Helicobacteraceae (29547)	Campylobacterales (68525)	Epsilonproteobacteria (122	Proteobacteria (2)	Bacteria (1)	100.00	2.40	99.00	97.60	WGS
213.28	0.1202	Helicobacter cinaedi (72293)	Helicobacter (213849)	Helicobacteraceae (29547)	Campylobacterales (68525)	Epsilonproteobacteria (122	Proteobacteria (2)	Bacteria (1)	100.00	2.40	99.50	98.50	WGS
213.29	0.122	Helicobacter cinaedi (72293)	Helicobacter (213849)	Helicobacteraceae (29547)	Campylobacterales (68525)	Epsilonproteobacteria (122	Proteobacteria (2)	Bacteria (1)	100.00	1.70	99.00	97.40	WGS
213.30	0.122	Helicobacter cinaedi (72293)	Helicobacter (213849)	Helicobacteraceae (29547)	Campylobacterales (68525)	Epsilonproteobacteria (122	Proteobacteria (2)	Bacteria (1)	100.00	2.40	99.00	97.20	WGS
213.31	0.122	Helicobacter cinaedi (72293)	Helicobacter (213849)	Helicobacteraceae (29547)	Campylobacterales (68525)	Epsilonproteobacteria (122	Proteobacteria (2)	Bacteria (1)	100.00	2.40	99.30	97.60	WGS
213.32	0.122	Helicobacter cinaedi (72293)	Helicobacter (213849)	Helicobacteraceae (29547)	Campylobacterales (68525)	Epsilonproteobacteria (122	Proteobacteria (2)	Bacteria (1)	100.00	3.60	99.30	97.50	WGS
213.33	0.122	Helicobacter cinaedi (72293)	Helicobacter (213849)	Helicobacteraceae (29547)	Campylobacterales (68525)	Epsilonproteobacteria (122	Proteobacteria (2)	Bacteria (1)	100.00	1.20	99.30	97.80	WGS
213.34	0.122	Helicobacter cinaedi (72293)	Helicobacter (213849)	Helicobacteraceae (29547)	Campylobacterales (68525)	Epsilonproteobacteria (122	Proteobacteria (2)	Bacteria (1)	100.00	1.60	99.00	97.40	WGS
213.35	0.122	Helicobacter cinaedi (72293)	Helicobacter (213849)	Helicobacteraceae (29547)	Campylobacterales (68525)	Epsilonproteobacteria (122	Proteobacteria (2)	Bacteria (1)	100.00	1.20	99.20	97.80	WGS
213.36	0.122	Helicobacter cinaedi (72293)	Helicobacter (213849)	Helicobacteraceae (29547)	Campylobacterales (68525)	Epsilonproteobacteria (122	Proteobacteria (2)	Bacteria (1)	100.00	1.20	99.00	97.80	WGS
213.37	0.1202	Helicobacter cinaedi (72293)	Helicobacter (213849)	Helicobacteraceae (29547)	Campylobacterales (68525)	Epsilonproteobacteria (122	Proteobacteria (2)	Bacteria (1)	100.00	0.80	99.50	98.60	WGS
213.38	0.1202	Helicobacter cinaedi (72293)	Helicobacter (213849)	Helicobacteraceae (29547)	Campylobacterales (68525)	Epsilonproteobacteria (122	Proteobacteria (2)	Bacteria (1)	100.00	1.20	99.30	98.40	WGS
213.39	0.122	Helicobacter cinaedi (72293)	Helicobacter (213849)	Helicobacteraceae (29547)	Campylobacterales (68525)	Epsilonproteobacteria (122	Proteobacteria (2)	Bacteria (1)	100.00	2.00	99.30	97.70	WGS
213.40	0.122	Helicobacter cinaedi (72293)	Helicobacter (213849)	Helicobacteraceae (29547)	Campylobacterales (68525)	Epsilonproteobacteria (122	Proteobacteria (2)	Bacteria (1)	100.00	1.20	99.30	97.90	WGS
213.41	0.1202	Helicobacter cinaedi (72293)	Helicobacter (213849)	Helicobacteraceae (29547)	Campylobacterales (68525)	Epsilonproteobacteria (122	Proteobacteria (2)	Bacteria (1)	100.00	3.10	99.30	97.60	WGS
213.42	0.122	Helicobacter cinaedi (72293)	Helicobacter (213849)	Helicobacteraceae (29547)	Campylobacterales (68525)	Epsilonproteobacteria (122	Proteobacteria (2)	Bacteria (1)	100.00	3.30	99.00	97.00	WGS
213.43	0.122	Helicobacter cinaedi (72293)	Helicobacter (213849)	Helicobacteraceae (29547)	Campylobacterales (68525)	Epsilonproteobacteria (122	Proteobacteria (2)	Bacteria (1)	100.00	3.50	99.30	97.20	WGS
213.44	0.122	Helicobacter cinaedi (72293)	Helicobacter (213849)	Helicobacteraceae (29547)	Campylobacterales (68525)	Epsilonproteobacteria (122	Proteobacteria (2)	Bacteria (1)	100.00	1.20	99.30	97.90	WGS
213.45	0.122	Helicobacter cinaedi (72293)	Helicobacter (213849)	Helicobacteraceae (29547)	Campylobacterales (68525)	Epsilonproteobacteria (122	Proteobacteria (2)	Bacteria (1)	100.00	1.20	99.30	98.00	WGS
213.46	0.122	Helicobacter cinaedi (72293)	Helicobacter (213849)	Helicobacteraceae (29547)	Campylobacterales (68525)	Epsilonproteobacteria (122	Proteobacteria (2)	Bacteria (1)	100.00	1.60	99.00	97.60	WGS
213.47	0.122	Helicobacter cinaedi (72293)	Helicobacter (213849)	Helicobacteraceae (29547)	Campylobacterales (68525)	Epsilonproteobacteria (122	Proteobacteria (2)	Bacteria (1)	100.00	0.80	99.50	98.40	WGS
213.48	0.122	Helicobacter cinaedi (72293)	Helicobacter (213849)	Helicobacteraceae (29547)	Campylobacterales (68525)	Epsilonproteobacteria (122	Proteobacteria (2)	Bacteria (1)	100.00	0.80	99.50	98.20	WGS
213.49	0.1192	Helicobacter cinaedi (72293)	Helicobacter (213849)	Helicobacteraceae (29547)	Campylobacterales (68525)	Epsilonproteobacteria (122	Proteobacteria (2)	Bacteria (1)	100.00	1.00	99.40	98.20	WGS
213.50	0.122	Helicobacter cinaedi (72293)	Helicobacter (213849)	Helicobacteraceae (29547)	Campylobacterales (68525)	Epsilonproteobacteria (122	Proteobacteria (2)	Bacteria (1)	100.00	0.80	99.30	98.10	WGS
213.51	0.122	Helicobacter cinaedi (72293)	Helicobacter (213849)	Helicobacteraceae (29547)	Campylobacterales (68525)	Epsilonproteobacteria (122	Proteobacteria (2)	Bacteria (1)	100.00	1.60	99.30	97.60	Complete
213.52	0.1164	Helicobacter cinaedi (72293)	Helicobacter (213849)	Helicobacteraceae (29547)	Campylobacterales (68525)	Epsilonproteobacteria (122	Proteobacteria (2)	Bacteria (1)	99.60	1.20	99.60	99.10	WGS
213.53	0.1192	Helicobacter cinaedi (72293)	Helicobacter (213849)	Helicobacteraceae (29547)	Campylobacterales (68525)	Epsilonproteobacteria (122	Proteobacteria (2)	Bacteria (1)	99.60	0.80	99.60	99.30	WGS
214.31	0.1062	Helicobacter felis (72293)	Helicobacter (213849)	Helicobacteraceae (29547)	Campylobacterales (68525)	Epsilonproteobacteria (122	Proteobacteria (2)	Bacteria (1)	100.00	0.40	96.20	95.50	WGS
214.32	0.1062	Helicobacter felis (72293)	Helicobacter (213849)	Helicobacteraceae (29547)	Campylobacterales (68525)	Epsilonproteobacteria (122	Proteobacteria (2)	Bacteria (1)	100.00	1.20	96.10	95.50	WGS
214.33	0.1042	Helicobacter felis (72293)	Helicobacter (213849)	Helicobacteraceae (29547)	Campylobacterales (68525)	Epsilonproteobacteria (122	Proteobacteria (2)	Bacteria (1)	100.00	1.20	96.00	95.50	WGS
214.34	0.1042	Helicobacter felis (72293)	Helicobacter (213849)	Helicobacteraceae (29547)	Campylobacterales (68525)	Epsilonproteobacteria (122	Proteobacteria (2)	Bacteria (1)	99.60	0.40	96.10	95.60	WGS
214.35	0.1074	Helicobacter felis (72293)	Helicobacter (213849)	Helicobacteraceae (29547)	Campylobacterales (68525)	Epsilonproteobacteria (122	Proteobacteria (2)	Bacteria (1)	100.00	2.20	96.40	95.60	WGS
214.36	0.1042	Helicobacter felis (72293)	Helicobacter (213849)	Helicobacteraceae (29547)	Campylobacterales (68525)	Epsilonproteobacteria (122	Proteobacteria (2)	Bacteria (1)	99.60	0.40	96.20	95.80	WGS
214.37	0.1062	Helicobacter felis (72293)	Helicobacter (213849)	Helicobacteraceae (29547)	Campylobacterales (68525)	Epsilonproteobacteria (122	Proteobacteria (2)	Bacteria (1)	100.00	0.00	96.30	95.90	WGS
214.38	0.1042	Helicobacter felis (72293)	Helicobacter (213849)	Helicobacteraceae (29547)	Campylobacterales (68525)	Epsilonproteobacteria (122	Proteobacteria (2)	Bacteria (1)	100.00	0.80	96.10	95.50	WGS
214.39	0.1114	Helicobacter felis (72293)	Helicobacter (213849)	Helicobacteraceae (29547)	Campylobacterales (68525)	Epsilonproteobacteria (122	Proteobacteria (2)	Bacteria (1)	100.00	0.80	96.30	95.80	WGS
214.40	0.1042	Helicobacter felis (72293)	Helicobacter (213849)	Helicobacteraceae (29547)	Campylobacterales (68525)	Epsilonproteobacteria (122	Proteobacteria (2)	Bacteria (1)	99.60	0.40	96.10	95.40	WGS
214.41	0.1042	Helicobacter felis (72293)	Helicobacter (213849)	Helicobacteraceae (29547)	Campylobacterales (68525)	Epsilonproteobacteria (122	Proteobacteria (2)	Bacteria (1)	100.00	0.80	96.10	95.60	WGS
214.42	0.1074	Helicobacter felis (72293)	Helicobacter (213849)	Helicobacteraceae (29547)	Campylobacterales (68525)	Epsilonproteobacteria (122	Proteobacteria (2)	Bacteria (1)	100.00	0.40	96.20	95.40	WGS
214.43	0.1082	Helicobacter felis (72293)	Helicobacter (213849)	Helicobacteraceae (29547)	Campylobacterales (68525)	Epsilonproteobacteria (122	Proteobacteria (2)	Bacteria (1)	100.00	0.60	96.30	95.70	WGS
214.44	0.1074	Helicobacter felis (72293)	Helicobacter (213849)	Helicobacteraceae (29547)	Campylobacterales (68525)	Epsilonproteobacteria (122	Proteobacteria (2)	Bacteria (1)	100.00	2.80	96.30	95.90	WGS
214.45	0.1042	Helicobacter felis (72293)	Helicobacter (213849)	Helicobacteraceae (29547)	Campylobacterales (68525)	Epsilonproteobacteria (122	Proteobacteria (2)	Bacteria (1)	100.00	1.20	96.10	95.50	WGS
214.46	0.1062	Helicobacter felis (72293)	Helicobacter (213849)	Helicobacteraceae (29547)	Campylobacterales (68525)	Epsilonproteobacteria (122	Proteobacteria (2)	Bacteria (1)	100.00	0.80	96.10	95.60	WGS
214.47	0.1042	Helicobacter felis (72293)	Helicobacter (213849)	Helicobacteraceae (29547)	Campylobacterales (68525)	Epsilonproteobacteria (122	Proteobacteria (2)	Bacteria (1)	100.00	0.40	96.10	95.30	WGS
214.48	0.1062	Helicobacter felis (72293)	Helicobacter (213849)	Helicobacteraceae (29547)	Campylobacterales (68525)	Epsilonproteobacteria (122	Proteobacteria (2)	Bacteria (1)	100.00	1.60	95.90	95.00	WGS
214.49	0.1042	Helicobacter felis (72293)	Helicobacter (213849)	Helicobacteraceae (29547)	Campylobacterales (68525)	Epsilonproteobacteria (122	Proteobacteria (2)	Bacteria (1)	100.00	2.00	96.20	95.30	WGS
214.50	0.1042	Helicobacter felis (72293)	Helicobacter (213849)	Helicobacteraceae (29547)	Campylobacterales (68525)	Epsilonproteobacteria (122	Proteobacteria (2)	Bacteria (1)	100.00	0.00	96.30	95.60	WGS
214.51	0.1042	Helicobacter felis (72293)	Helicobacter (213849)	Helicobacteraceae (29547)	Campylobacterales (68525)	Epsilonproteobacteria (122	Proteobacteria (2)	Bacteria (1)	99.20	0.40	96.30	95.80	WGS
214.52	0.1074	Helicobacter felis (72293)	Helicobacter (213849)	Helicobacteraceae (29547)	Campylobacterales (68525)	Epsilonproteobacteria (122	Proteobacteria (2)	Bacteria (1)			97.30	96.40	WGS
215.17	0.057	Helicobacter fennelliae (72293)	Helicobacter (213849)	Helicobacteraceae (29547)	Campylobacterales (68525)	Epsilonproteobacteria (122	Proteobacteria (2)	Bacteria (1)	99.60	1.20	98.20	97.60	WGS
215.18	0.0558	Helicobacter fennelliae (72293)	Helicobacter (213849)	Helicobacteraceae (29547)	Campylobacterales (68525)	Epsilonproteobacteria (122	Proteobacteria (2)	Bacteria (1)	98.30	1.60	97.90	97.30	WGS
215.25	0.0652	Helicobacter fennelliae (72293)	Helicobacter (213849)	Helicobacteraceae (29547)	Campylobacterales (68525)	Epsilonproteobacteria (122	Proteobacteria (2)	Bacteria (1)			98.20	96.20	WGS
216.12	0.0692	Helicobacter muridarum (72293)	Helicobacter (213849)	Helicobacteraceae (29547)	Campylobacterales (68525)	Epsilonproteobacteria (122	Proteobacteria (2)	Bacteria (1)	96.50	0.90	95.40	95.10	WGS
216.13	0.0692	Helicobacter muridarum (72293)	Helicobacter (213849)	Helicobacteraceae (29547)	Campylobacterales (68525)	Epsilonproteobacteria (122	Proteobacteria (2)	Bacteria (1)	96.50	0.90	95.40	95.10	WGS
216.14	0.0692	Helicobacter muridarum (72293)	Helicobacter (213849)	Helicobacteraceae (29547)	Campylobacterales (68525)	Epsilonproteobacteria (122	Proteobacteria (2)	Bacteria (1)	96.50	0.90	95.30	95.00	WGS
216.15	0.0692	Helicobacter muridarum (72293)	Helicobacter (213849)	Helicobacteraceae (29547)	Campylobacterales (68525)	Epsilonproteobacteria (122	Proteobacteria (2)	Bacteria (1)	98.10	0.80	96.80	96.50	WGS
216.5	0.0728	Helicobacter muridarum (72293)	Helicobacter (213849)	Helicobacteraceae (29547)	Campylobacterales (68525)	Epsilonproteobacteria (122	Proteobacteria (2)	Bacteria (1)	96.10	10.20	94.90	90.40	WGS
217.6	0.103	Helicobacter mustelae (72293)	Helicobacter (213849)	Helicobacteraceae (29547)	Campylobacterales (68525)	Epsilonproteobacteria (122	Proteobacteria (2)	Bacteria (1)	98.70	0.40	99.30	99.10	Complete
217.7	0.103	Helicobacter mustelae (72293)	Helicobacter (213849)	Helicobacteraceae (29547)	Campylobacterales (68525)	Epsilonproteobacteria (122	Proteobacteria (2)	Bacteria (1)	98.70	5.90	99.40	98.10	WGS
218.4	0.085	Helicobacter sp. (72293)	Helicobacter (213849)	Helicobacteraceae (29547)	Campylobacterales (68525)	Epsilonproteobacteria (122	Proteobacteria (2)	Bacteria (1)	70.90	12.40	86.30	84.60	WGS
218.5	0.1512	Helicobacter sp. (72293)	Helicobacter (213849)	Helicobacteraceae (29547)	Campylobacterales (68525)	Epsilonproteobacteria (122	Proteobacteria (2)	Bacteria (1)	98.40	3.40	99.90	99.50	WGS
227.4	0.6272	Pseudoalteromonas carrageenovo	Pseudoalteromonas (26788	Pseudoalteromonadaceae (	Alteromonadales (1236)	Gammaproteobacteria (122	Proteobacteria (2)	Bacteria (1)			98.00	96.60	Complete
228.7	0.1912	Pseudoalteromonas haloplanktis (	Pseudoalteromonas (26788	Pseudoalteromonadaceae (	Alteromonadales (1236)	Gammaproteobacteria (122	Proteobacteria (2)	Bacteria (1)	100.00	0.00	98.90	97.30	WGS
228.8	0.5752	Pseudoalteromonas haloplanktis (	Pseudoalteromonas (26788	Pseudoalteromonadaceae (	Alteromonadales (1236)	Gammaproteobacteria (122	Proteobacteria (2)	Bacteria (1)	100.00	0.00	98.20	96.90	WGS
232.10	0.4312	Alteromonas sp. (226)	Alteromonas (72275)	Alteromonadaceae (135622	Alteromonadales (1236)	Gammaproteobacteria (122	Proteobacteria (2)	Bacteria (1)	95.80	2.90	93.20	90.80	WGS
232.11	0.5116	Alteromonas sp. (226)	Alteromonas (72275)	Alteromonadaceae (135622	Alteromonadales (1236)	Gammaproteobacteria (122	Proteobacteria (2)	Bacteria (1)	90.10	5.90	91.40	87.10	WGS
232.12	0.0676	Alteromonas sp. (226)	Alteromonas (72275)	Alteromonadaceae (135622	Alteromonadales (1236)	Gammaproteobacteria (122	Proteobacteria (2)	Bacteria (1)	54.90	0.10	93.60	92.00	WGS
232.13	0.3292	Alteromonas sp. (226)	Alteromonas (72275)	Alteromonadaceae (135622	Alteromonadales (1236)	Gammaproteobacteria (122	Proteobacteria (2)	Bacteria (1)	74.70	3.70	86.00	81.70	WGS
232.14	0.2582	Alteromonas sp. (226)	Alteromonas (72275)	Alteromonadaceae (135622	Alteromonadales (1236)	Gammaproteobacteria (122	Proteobacteria (2)	Bacteria (1)	55.00	2.60	83.10	80.30	WGS
232.15	0.2464	Alteromonas sp. (226)	Alteromonas (72275)	Alteromonadaceae (135622	Alteromonadales (1236)	Gammaproteobacteria (122	Proteobacteria (2)	Bacteria (1)	70.50	7.40	84.00	80.90	WGS
232.16	0.4502	Alteromonas sp. (226)	Alteromonas (72275)	Alteromonadaceae (135622	Alteromonadales (1236)	Gammaproteobacteria (122	Proteobacteria (2)	Bacteria (1)	87.30	3.20	90.70	88.40	WGS
232.17	0.4408	Alteromonas sp. (226)	Alteromonas (72275)	Alteromonadaceae (135622	Alteromonadales (1236)	Gammaproteobacteria (122	Proteobacteria (2)	Bacteria (1)	95.70	4.20	94.30	91.70	WGS
232.3	0.1436	Alteromonas sp. (226)	Alteromonas (72275)	Alteromonadaceae (135622	Alteromonadales (1236)	Gammaproteobacteria (122	Proteobacteria (2)	Bacteria (1)	32.60	8.00	82.60	81.00	WGS
232.4	0.4402	Alteromonas sp. (226)	Alteromonas (72275)	Alteromonadaceae (135622	Alteromonadales (1236)	Gammaproteobacteria (122	Proteobacteria (2)	Bacteria (1)	80.30	1.80	89.00	85.90	WGS
232.42	0.3246	Alteromonas sp. (226)	Alteromonas (72275)	Alteromonadaceae (135622	Alteromonadales (1236)	Gammaproteobacteria (122	Proteobacteria (2)	Bacteria (1)	91.40	13.30	84.90	79.30	WGS
232.43	0.5352	Alteromonas sp. (226)	Alteromonas (72275)	Alteromonadaceae (135622	Alteromonadales (1236)	Gammaproteobacteria (122	Proteobacteria (2)	Bacteria (1)	91.50	16.60	90.50	83.80	WGS
232.44	0.4762	Alteromonas sp. (226)	Alteromonas (72275)	Alteromonadaceae (135622	Alteromonadales (1236)	Gammaproteobacteria (122	Proteobacteria (2)	Bacteria (1)	85.90	7.60	86.00	80.90	WGS
232.45	0.4404	Alteromonas sp. (226)	Alteromonas (72275)	Alteromonadaceae (135622	Alteromonadales (1236)	Gammaproteobacteria (122	Proteobacteria (2)	Bacteria (1)	85.90	17.50	88.00	82.50	WGS
232.46	0.4342	Alteromonas sp. (226)	Alteromonas (72275)	Alteromonadaceae (135622	Alteromonadales (1236)	Gammaproteobacteria (122	Proteobacteria (2)	Bacteria (1)	73.20	13.30	81.30	77.50	WGS
232.47	0.3654	Alteromonas sp. (226)	Alteromonas (72275)	Alteromonadaceae (135622	Alteromonadales (1236)	Gammaproteobacteria (122	Proteobacteria (2)	Bacteria (1)	77.50	16.60	84.00	79.20	WGS
232.48	0.5008	Alteromonas sp. (226)	Alteromonas (72275)	Alteromonadaceae (135622	Alteromonadales (1236)	Gammaproteobacteria (122	Proteobacteria (2)	Bacteria (1)	84.50	4.80	88.10	83.10	WGS
232.49	0.3906	Alteromonas sp. (226)	Alteromonas (72275)	Alteromonadaceae (135622	Alteromonadales (1236)	Gammaproteobacteria (122	Proteobacteria (2)	Bacteria (1)			85.00	82.60	WGS
232.50	0.3758	Alteromonas sp. (226)	Alteromonas (72275)	Alteromonadaceae (135622	Alteromonadales (1236)	Gammaproteobacteria (122	Proteobacteria (2)	Bacteria (1)			82.80	81.40	WGS
232.5	0.365	Alteromonas sp. (226)	Alteromonas (72275)	Alteromonadaceae (135622	Alteromonadales (1236)	Gammaproteobacteria (122	Proteobacteria (2)	Bacteria (1)	81.70	3.30	89.80	87.50	WGS
232.6	0.2622	Alteromonas sp. (226)	Alteromonas (72275)	Alteromonadaceae (135622	Alteromonadales (1236)	Gammaproteobacteria (122	Proteobacteria (2)	Bacteria (1)	66.20	6.00	85.60	82.30	WGS
232.7	0.3616	Alteromonas sp. (226)	Alteromonas (72275)	Alteromonadaceae (135622	Alteromonadales (1236)	Gammaproteobacteria (122	Proteobacteria (2)	Bacteria (1)	84.40	4.80	87.00	84.10	WGS
232.8	0.3946	Alteromonas sp. (226)	Alteromonas (72275)	Alteromonadaceae (135622	Alteromonadales (1236)	Gammaproteobacteria (122	Proteobacteria (2)	Bacteria (1)	98.60	1.40	94.90	93.90	WGS
232.9	0.2916	Alteromonas sp. (226)	Alteromonas (72275)	Alteromonadaceae (135622	Alteromonadales (1236)	Gammaproteobacteria (122	Proteobacteria (2)	Bacteria (1)	60.70	8.40	81.90	79.30	WGS
235.100	0.2618	Brucella abortus (234)	Brucella (118882)	Brucellaceae (356)	Rhizobiales (28211)	Alphaproteobacteria (1224	Proteobacteria (2)	Bacteria (1)	98.30	2.20	98.80	96.60	Complete
235.101	0.25	Brucella abortus (234)	Brucella (118882)	Brucellaceae (356)	Rhizobiales (28211)	Alphaproteobacteria (1224	Proteobacteria (2)	Bacteria (1)	95.50	0.60	99.60	97.40	Complete
235.102	0.2454	Brucella abortus (234)	Brucella (118882)	Brucellaceae (356)	Rhizobiales (28211)	Alphaproteobacteria (1224	Proteobacteria (2)	Bacteria (1)	92.20	10.80	93.30	87.70	Complete
235.103	0.265	Brucella abortus (234)	Brucella (118882)	Brucellaceae (356)	Rhizobiales (28211)	Alphaproteobacteria (1224	Proteobacteria (2)	Bacteria (1)	100.00	1.30	99.90	96.00	Complete
235.104	0.265	Brucella abortus (234)	Brucella (118882)	Brucellaceae (356)	Rhizobiales (28211)	Alphaproteobacteria (1224	Proteobacteria (2)	Bacteria (1)	100.00	1.10	99.90	96.60	Complete
235.105	0.2646	Brucella abortus (234)	Brucella (118882)	Brucellaceae (356)	Rhizobiales (28211)	Alphaproteobacteria (1224	Proteobacteria (2)	Bacteria (1)	100.00	2.40	99.90	97.00	Complete
235.106	0.259	Brucella abortus (234)	Brucella (118882)	Brucellaceae (356)	Rhizobiales (28211)	Alphaproteobacteria (1224	Proteobacteria (2)	Bacteria (1)	95.00	18.70	97.90	86.00	Complete
235.107	0.2618	Brucella abortus (234)	Brucella (118882)	Brucellaceae (356)	Rhizobiales (28211)	Alphaproteobacteria (1224	Proteobacteria (2)	Bacteria (1)	98.30	1.10	98.70	95.80	Complete
235.108	0.2444	Brucella abortus (234)	Brucella (118882)	Brucellaceae (356)	Rhizobiales (28211)	Alphaproteobacteria (1224	Proteobacteria (2)	Bacteria (1)	92.20	19.50	95.90	84.40	Complete
235.109	0.2532	Brucella abortus (234)	Brucella (118882)	Brucellaceae (356)	Rhizobiales (28211)	Alphaproteobacteria (1224	Proteobacteria (2)	Bacteria (1)	100.00	0.60	99.90	97.40	Complete
235.110	0.265	Brucella abortus (234)	Brucella (118882)	Brucellaceae (356)	Rhizobiales (28211)	Alphaproteobacteria (1224	Proteobacteria (2)	Bacteria (1)	100.00	5.80	99.70	95.90	Complete
235.111	0.2128	Brucella abortus (234)	Brucella (118882)	Brucellaceae (356)	Rhizobiales (28211)	Alphaproteobacteria (1224	Proteobacteria (2)	Bacteria (1)	96.70	1.70	99.10	96.20	Complete
235.126	0.265	Brucella abortus (234)	Brucella (118882)	Brucellaceae (356)	Rhizobiales (28211)	Alphaproteobacteria (1224	Proteobacteria (2)	Bacteria (1)	100.00	0.00	99.90	98.90	Complete
235.127	0.265	Brucella abortus (234)	Brucella (118882)	Brucellaceae (356)	Rhizobiales (28211)	Alphaproteobacteria (1224	Proteobacteria (2)	Bacteria (1)	99.40	0.00	99.90	99.00	Complete
235.138	0.265	Brucella abortus (234)	Brucella (118882)	Brucellaceae (356)	Rhizobiales (28211)	Alphaproteobacteria (1224	Proteobacteria (2)	Bacteria (1)	100.00	0.00	99.90	98.70	WGS
235.139	0.265	Brucella abortus (234)	Brucella (118882)	Brucellaceae (356)	Rhizobiales (28211)	Alphaproteobacteria (1224	Proteobacteria (2)	Bacteria (1)	100.00	0.00	99.90	98.70	WGS
235.140	0.265	Brucella abortus (234)	Brucella (118882)	Brucellaceae (356)	Rhizobiales (28211)	Alphaproteobacteria (1224	Proteobacteria (2)	Bacteria (1)	100.00	0.00	99.90	98.70	WGS
235.14	0.2388	Brucella abortus (234)	Brucella (118882)	Brucellaceae (356)	Rhizobiales (28211)	Alphaproteobacteria (1224	Proteobacteria (2)	Bacteria (1)	99.40	3.30	99.60	96.90	WGS
235.141	0.265	Brucella abortus (234)	Brucella (118882)	Brucellaceae (356)	Rhizobiales (28211)	Alphaproteobacteria (1224	Proteobacteria (2)	Bacteria (1)	100.00	0.00	99.90	98.70	WGS
235.142	0.2798	Brucella abortus (234)	Brucella (118882)	Brucellaceae (356)	Rhizobiales (28211)	Alphaproteobacteria (1224	Proteobacteria (2)	Bacteria (1)	100.00	7.50	99.60	92.80	WGS
235.143	0.2616	Brucella abortus (234)	Brucella (118882)	Brucellaceae (356)	Rhizobiales (28211)	Alphaproteobacteria (1224	Proteobacteria (2)	Bacteria (1)	99.40	3.00	99.70	94.80	WGS
235.144	0.2578	Brucella abortus (234)	Brucella (118882)	Brucellaceae (356)	Rhizobiales (28211)	Alphaproteobacteria (1224	Proteobacteria (2)	Bacteria (1)	100.00	6.80	99.80	95.70	WGS
235.145	0.265	Brucella abortus (234)	Brucella (118882)	Brucellaceae (356)	Rhizobiales (28211)	Alphaproteobacteria (1224	Proteobacteria (2)	Bacteria (1)	100.00	5.30	99.80	95.80	WGS
235.146	0.265	Brucella abortus (234)	Brucella (118882)	Brucellaceae (356)	Rhizobiales (28211)	Alphaproteobacteria (1224	Proteobacteria (2)	Bacteria (1)	100.00	3.20	99.80	96.70	WGS
235.147	0.2646	Brucella abortus (234)	Brucella (118882)	Brucellaceae (356)	Rhizobiales (28211)	Alphaproteobacteria (1224	Proteobacteria (2)	Bacteria (1)	100.00	2.50	99.80	96.00	WGS
235.148	0.265	Brucella abortus (234)	Brucella (118882)	Brucellaceae (356)	Rhizobiales (28211)	Alphaproteobacteria (1224	Proteobacteria (2)	Bacteria (1)	100.00	0.00	99.90	98.80	Complete
235.15	0.265	Brucella abortus (234)	Brucella (118882)	Brucellaceae (356)	Rhizobiales (28211)	Alphaproteobacteria (1224	Proteobacteria (2)	Bacteria (1)	100.00	0.00	99.60	98.60	Complete
235.151	0.2578	Brucella abortus (234)	Brucella (118882)	Brucellaceae (356)	Rhizobiales (28211)	Alphaproteobacteria (1224	Proteobacteria (2)	Bacteria (1)	100.00	0.00	99.90	98.50	WGS
235.152	0.265	Brucella abortus (234)	Brucella (118882)	Brucellaceae (356)	Rhizobiales (28211)	Alphaproteobacteria (1224	Proteobacteria (2)	Bacteria (1)	99.40	0.60	99.90	97.30	WGS
235.153	0.265	Brucella abortus (234)	Brucella (118882)	Brucellaceae (356)	Rhizobiales (28211)	Alphaproteobacteria (1224	Proteobacteria (2)	Bacteria (1)	100.00	0.00	99.90	98.60	WGS
235.154	0.265	Brucella abortus (234)	Brucella (118882)	Brucellaceae (356)	Rhizobiales (28211)	Alphaproteobacteria (1224	Proteobacteria (2)	Bacteria (1)	100.00	0.00	99.90	98.40	WGS
235.155	0.265	Brucella abortus (234)	Brucella (118882)	Brucellaceae (356)	Rhizobiales (28211)	Alphaproteobacteria (1224	Proteobacteria (2)	Bacteria (1)	100.00	0.00	99.90	98.70	WGS
235.156	0.265	Brucella abortus (234)	Brucella (118882)	Brucellaceae (356)	Rhizobiales (28211)	Alphaproteobacteria (1224	Proteobacteria (2)	Bacteria (1)	100.00	1.60	99.90	98.30	WGS
235.16	0.265	Brucella abortus (234)	Brucella (118882)	Brucellaceae (356)	Rhizobiales (28211)	Alphaproteobacteria (1224	Proteobacteria (2)	Bacteria (1)	100.00	0.00	99.60	98.50	Complete
235.169	0.265	Brucella abortus (234)	Brucella (118882)	Brucellaceae (356)	Rhizobiales (28211)	Alphaproteobacteria (1224	Proteobacteria (2)	Bacteria (1)			99.90	98.60	Complete
235.17	0.265	Brucella abortus (234)	Brucella (118882)	Brucellaceae (356)	Rhizobiales (28211)	Alphaproteobacteria (1224	Proteobacteria (2)	Bacteria (1)	100.00	0.00	99.90	99.30	Complete
235.171	0.265	Brucella abortus (234)	Brucella (118882)	Brucellaceae (356)	Rhizobiales (28211)	Alphaproteobacteria (1224	Proteobacteria (2)	Bacteria (1)			99.90	98.20	WGS
235.172	0.265	Brucella abortus (234)	Brucella (118882)	Brucellaceae (356)	Rhizobiales (28211)	Alphaproteobacteria (1224	Proteobacteria (2)	Bacteria (1)			99.90	98.40	WGS
235.173	0.2648	Brucella abortus (234)	Brucella (118882)	Brucellaceae (356)	Rhizobiales (28211)	Alphaproteobacteria (1224	Proteobacteria (2)	Bacteria (1)			99.90	98.90	WGS
235.174	0.265	Brucella abortus (234)	Brucella (118882)	Brucellaceae (356)	Rhizobiales (28211)	Alphaproteobacteria (1224	Proteobacteria (2)	Bacteria (1)			99.90	98.40	WGS
235.175	0.265	Brucella abortus (234)	Brucella (118882)	Brucellaceae (356)	Rhizobiales (28211)	Alphaproteobacteria (1224	Proteobacteria (2)	Bacteria (1)			99.90	98.30	WGS
235.176	0.2532	Brucella abortus (234)	Brucella (118882)	Brucellaceae (356)	Rhizobiales (28211)	Alphaproteobacteria (1224	Proteobacteria (2)	Bacteria (1)			99.80	95.10	WGS
235.177	0.2686	Brucella abortus (234)	Brucella (118882)	Brucellaceae (356)	Rhizobiales (28211)	Alphaproteobacteria (1224	Proteobacteria (2)	Bacteria (1)			99.90	96.80	WGS
235.178	0.2686	Brucella abortus (234)	Brucella (118882)	Brucellaceae (356)	Rhizobiales (28211)	Alphaproteobacteria (1224	Proteobacteria (2)	Bacteria (1)			99.80	96.90	WGS
235.179	0.265	Brucella abortus (234)	Brucella (118882)	Brucellaceae (356)	Rhizobiales (28211)	Alphaproteobacteria (1224	Proteobacteria (2)	Bacteria (1)			99.70	97.20	WGS
235.18	0.265	Brucella abortus (234)	Brucella (118882)	Brucellaceae (356)	Rhizobiales (28211)	Alphaproteobacteria (1224	Proteobacteria (2)	Bacteria (1)	100.00	0.00	99.60	98.50	Complete
235.180	0.257	Brucella abortus (234)	Brucella (118882)	Brucellaceae (356)	Rhizobiales (28211)	Alphaproteobacteria (1224	Proteobacteria (2)	Bacteria (1)			99.90	95.10	WGS
235.181	0.2616	Brucella abortus (234)	Brucella (118882)	Brucellaceae (356)	Rhizobiales (28211)	Alphaproteobacteria (1224	Proteobacteria (2)	Bacteria (1)			99.90	90.00	WGS
235.182	0.265	Brucella abortus (234)	Brucella (118882)	Brucellaceae (356)	Rhizobiales (28211)	Alphaproteobacteria (1224	Proteobacteria (2)	Bacteria (1)			99.80	98.70	WGS
235.183	0.265	Brucella abortus (234)	Brucella (118882)	Brucellaceae (356)	Rhizobiales (28211)	Alphaproteobacteria (1224	Proteobacteria (2)	Bacteria (1)			99.90	98.80	Complete
235.188	0.265	Brucella abortus (234)	Brucella (118882)	Brucellaceae (356)	Rhizobiales (28211)	Alphaproteobacteria (1224	Proteobacteria (2)	Bacteria (1)			99.80	97.70	WGS
235.189	0.265	Brucella abortus (234)	Brucella (118882)	Brucellaceae (356)	Rhizobiales (28211)	Alphaproteobacteria (1224	Proteobacteria (2)	Bacteria (1)			99.90	98.00	WGS
235.19	0.2152	Brucella abortus (234)	Brucella (118882)	Brucellaceae (356)	Rhizobiales (28211)	Alphaproteobacteria (1224	Proteobacteria (2)	Bacteria (1)	92.20	10.80	96.20	88.70	Complete
235.190	0.265	Brucella abortus (234)	Brucella (118882)	Brucellaceae (356)	Rhizobiales (28211)	Alphaproteobacteria (1224	Proteobacteria (2)	Bacteria (1)			99.70	95.60	WGS
235.191	0.264	Brucella abortus (234)	Brucella (118882)	Brucellaceae (356)	Rhizobiales (28211)	Alphaproteobacteria (1224	Proteobacteria (2)	Bacteria (1)			99.60	94.80	WGS
235.192	0.2578	Brucella abortus (234)	Brucella (118882)	Brucellaceae (356)	Rhizobiales (28211)	Alphaproteobacteria (1224	Proteobacteria (2)	Bacteria (1)			99.90	94.30	WGS
235.193	0.265	Brucella abortus (234)	Brucella (118882)	Brucellaceae (356)	Rhizobiales (28211)	Alphaproteobacteria (1224	Proteobacteria (2)	Bacteria (1)			99.90	98.50	WGS
235.20	0.265	Brucella abortus (234)	Brucella (118882)	Brucellaceae (356)	Rhizobiales (28211)	Alphaproteobacteria (1224	Proteobacteria (2)	Bacteria (1)	100.00	0.00	99.90	99.30	Complete
235.21	0.265	Brucella abortus (234)	Brucella (118882)	Brucellaceae (356)	Rhizobiales (28211)	Alphaproteobacteria (1224	Proteobacteria (2)	Bacteria (1)	100.00	1.10	99.60	98.50	Complete
235.28	0.264	Brucella abortus (234)	Brucella (118882)	Brucellaceae (356)	Rhizobiales (28211)	Alphaproteobacteria (1224	Proteobacteria (2)	Bacteria (1)	98.90	7.80	99.40	94.80	WGS
235.29	0.265	Brucella abortus (234)	Brucella (118882)	Brucellaceae (356)	Rhizobiales (28211)	Alphaproteobacteria (1224	Proteobacteria (2)	Bacteria (1)	100.00	0.00	99.80	98.70	Complete
235.30	0.265	Brucella abortus (234)	Brucella (118882)	Brucellaceae (356)	Rhizobiales (28211)	Alphaproteobacteria (1224	Proteobacteria (2)	Bacteria (1)	100.00	0.60	99.80	97.70	WGS
235.31	0.265	Brucella abortus (234)	Brucella (118882)	Brucellaceae (356)	Rhizobiales (28211)	Alphaproteobacteria (1224	Proteobacteria (2)	Bacteria (1)	100.00	0.60	99.90	98.80	WGS
235.32	0.265	Brucella abortus (234)	Brucella (118882)	Brucellaceae (356)	Rhizobiales (28211)	Alphaproteobacteria (1224	Proteobacteria (2)	Bacteria (1)	100.00	0.00	99.90	98.60	WGS
235.33	0.265	Brucella abortus (234)	Brucella (118882)	Brucellaceae (356)	Rhizobiales (28211)	Alphaproteobacteria (1224	Proteobacteria (2)	Bacteria (1)	100.00	0.60	99.90	98.70	WGS
235.34	0.265	Brucella abortus (234)	Brucella (118882)	Brucellaceae (356)	Rhizobiales (28211)	Alphaproteobacteria (1224	Proteobacteria (2)	Bacteria (1)	100.00	0.00	99.90	98.70	WGS
235.35	0.265	Brucella abortus (234)	Brucella (118882)	Brucellaceae (356)	Rhizobiales (28211)	Alphaproteobacteria (1224	Proteobacteria (2)	Bacteria (1)	100.00	0.00	99.90	98.80	WGS
235.36	0.265	Brucella abortus (234)	Brucella (118882)	Brucellaceae (356)	Rhizobiales (28211)	Alphaproteobacteria (1224	Proteobacteria (2)	Bacteria (1)	100.00	0.60	99.90	98.90	WGS
235.37	0.265	Brucella abortus (234)	Brucella (118882)	Brucellaceae (356)	Rhizobiales (28211)	Alphaproteobacteria (1224	Proteobacteria (2)	Bacteria (1)	100.00	0.00	99.90	98.80	WGS
235.38	0.265	Brucella abortus (234)	Brucella (118882)	Brucellaceae (356)	Rhizobiales (28211)	Alphaproteobacteria (1224	Proteobacteria (2)	Bacteria (1)	100.00	0.00	99.90	98.70	WGS
235.39	0.265	Brucella abortus (234)	Brucella (118882)	Brucellaceae (356)	Rhizobiales (28211)	Alphaproteobacteria (1224	Proteobacteria (2)	Bacteria (1)	100.00	0.00	99.90	98.70	WGS
235.40	0.265	Brucella abortus (234)	Brucella (118882)	Brucellaceae (356)	Rhizobiales (28211)	Alphaproteobacteria (1224	Proteobacteria (2)	Bacteria (1)	100.00	0.60	99.90	98.70	WGS
235.41	0.265	Brucella abortus (234)	Brucella (118882)	Brucellaceae (356)	Rhizobiales (28211)	Alphaproteobacteria (1224	Proteobacteria (2)	Bacteria (1)	100.00	0.00	99.90	98.90	WGS
235.72	0.265	Brucella abortus (234)	Brucella (118882)	Brucellaceae (356)	Rhizobiales (28211)	Alphaproteobacteria (1224	Proteobacteria (2)	Bacteria (1)	100.00	7.00	99.80	94.30	WGS
235.74	0.265	Brucella abortus (234)	Brucella (118882)	Brucellaceae (356)	Rhizobiales (28211)	Alphaproteobacteria (1224	Proteobacteria (2)	Bacteria (1)	100.00	0.00	99.90	98.90	WGS
235.75	0.265	Brucella abortus (234)	Brucella (118882)	Brucellaceae (356)	Rhizobiales (28211)	Alphaproteobacteria (1224	Proteobacteria (2)	Bacteria (1)	100.00	0.00	99.90	98.70	WGS
235.76	0.265	Brucella abortus (234)	Brucella (118882)	Brucellaceae (356)	Rhizobiales (28211)	Alphaproteobacteria (1224	Proteobacteria (2)	Bacteria (1)	100.00	0.00	99.90	98.70	WGS
235.77	0.265	Brucella abortus (234)	Brucella (118882)	Brucellaceae (356)	Rhizobiales (28211)	Alphaproteobacteria (1224	Proteobacteria (2)	Bacteria (1)	100.00	0.00	99.90	98.80	WGS
235.78	0.265	Brucella abortus (234)	Brucella (118882)	Brucellaceae (356)	Rhizobiales (28211)	Alphaproteobacteria (1224	Proteobacteria (2)	Bacteria (1)	100.00	0.00	99.90	98.70	WGS
235.79	0.265	Brucella abortus (234)	Brucella (118882)	Brucellaceae (356)	Rhizobiales (28211)	Alphaproteobacteria (1224	Proteobacteria (2)	Bacteria (1)	100.00	0.00	99.90	98.80	WGS
235.80	0.265	Brucella abortus (234)	Brucella (118882)	Brucellaceae (356)	Rhizobiales (28211)	Alphaproteobacteria (1224	Proteobacteria (2)	Bacteria (1)	100.00	0.00	99.90	98.60	WGS
235.82	0.265	Brucella abortus (234)	Brucella (118882)	Brucellaceae (356)	Rhizobiales (28211)	Alphaproteobacteria (1224	Proteobacteria (2)	Bacteria (1)	100.00	3.40	99.90	97.30	WGS
235.92	0.265	Brucella abortus (234)	Brucella (118882)	Brucellaceae (356)	Rhizobiales (28211)	Alphaproteobacteria (1224	Proteobacteria (2)	Bacteria (1)	100.00	0.00	99.90	98.70	Complete
235.93	0.265	Brucella abortus (234)	Brucella (118882)	Brucellaceae (356)	Rhizobiales (28211)	Alphaproteobacteria (1224	Proteobacteria (2)	Bacteria (1)	100.00	0.00	99.90	98.70	Complete
235.94	0.265	Brucella abortus (234)	Brucella (118882)	Brucellaceae (356)	Rhizobiales (28211)	Alphaproteobacteria (1224	Proteobacteria (2)	Bacteria (1)	100.00	1.90	99.80	96.60	Complete
235.95	0.2622	Brucella abortus (234)	Brucella (118882)	Brucellaceae (356)	Rhizobiales (28211)	Alphaproteobacteria (1224	Proteobacteria (2)	Bacteria (1)	99.40	0.60	99.70	96.50	Complete
235.96	0.2468	Brucella abortus (234)	Brucella (118882)	Brucellaceae (356)	Rhizobiales (28211)	Alphaproteobacteria (1224	Proteobacteria (2)	Bacteria (1)	93.90	0.00	98.50	96.20	Complete
235.97	0.25	Brucella abortus (234)	Brucella (118882)	Brucellaceae (356)	Rhizobiales (28211)	Alphaproteobacteria (1224	Proteobacteria (2)	Bacteria (1)	95.50	0.30	99.60	97.50	Complete
235.98	0.265	Brucella abortus (234)	Brucella (118882)	Brucellaceae (356)	Rhizobiales (28211)	Alphaproteobacteria (1224	Proteobacteria (2)	Bacteria (1)	100.00	2.20	99.80	96.50	Complete

235.99	0.265	Brucella abortus (234)	Brucella (118882)	Brucellaceae (356)	Rhizobiales (28211)	Alphaproteobacteria (1224	Proteobacteria (2)	Bacteria (1)	100.00	0.60	99.90	97.40	Complete
236.3	0.2132	Brucella ovis (234)	Brucella (118882)	Brucellaceae (356)	Rhizobiales (28211)	Alphaproteobacteria (1224	Proteobacteria (2)	Bacteria (1)	100.00	0.00	99.90	98.00	WGS
238.17	0.2962	Elizabethkingia meningoseptica (2	Elizabethkingia (117743)	Flavobacteriaceae (976)	Flavobacteriales (68336)	Flavobacteriia (1783270)	Bacteroidetes (2)	Bacteria (1)	100.00	0.00	97.50	96.70	WGS
238.22	0.2832	Elizabethkingia meningoseptica (2	Elizabethkingia (117743)	Flavobacteriaceae (976)	Flavobacteriales (68336)	Flavobacteriia (1783270)	Bacteroidetes (2)	Bacteria (1)	100.00	19.10	97.40	89.00	WGS
238.24	0.149	Elizabethkingia meningoseptica (2	Elizabethkingia (117743)	Flavobacteriaceae (976)	Flavobacteriales (68336)	Flavobacteriia (1783270)	Bacteroidetes (2)	Bacteria (1)	100.00	0.00	98.00	97.40	WGS
238.33	0.1554	Elizabethkingia meningoseptica (2	Elizabethkingia (117743)	Flavobacteriaceae (976)	Flavobacteriales (68336)	Flavobacteriia (1783270)	Bacteroidetes (2)	Bacteria (1)	100.00	0.00	97.90	97.20	WGS
238.36	0.1554	Elizabethkingia meningoseptica (2	Elizabethkingia (117743)	Flavobacteriaceae (976)	Flavobacteriales (68336)	Flavobacteriia (1783270)	Bacteroidetes (2)	Bacteria (1)	100.00	0.00	97.90	97.20	WGS
238.44	0.1488	Elizabethkingia meningoseptica (2	Elizabethkingia (117743)	Flavobacteriaceae (976)	Flavobacteriales (68336)	Flavobacteriia (1783270)	Bacteroidetes (2)	Bacteria (1)	100.00	0.70	98.00	97.30	WGS
238.45	0.1462	Elizabethkingia meningoseptica (2	Elizabethkingia (117743)	Flavobacteriaceae (976)	Flavobacteriales (68336)	Flavobacteriia (1783270)	Bacteroidetes (2)	Bacteria (1)	100.00	0.00	97.90	97.20	WGS
238.46	0.149	Elizabethkingia meningoseptica (2	Elizabethkingia (117743)	Flavobacteriaceae (976)	Flavobacteriales (68336)	Flavobacteriia (1783270)	Bacteroidetes (2)	Bacteria (1)	100.00	0.00	98.00	97.40	Complete
238.47	0.1552	Elizabethkingia meningoseptica (2	Elizabethkingia (117743)	Flavobacteriaceae (976)	Flavobacteriales (68336)	Flavobacteriia (1783270)	Bacteroidetes (2)	Bacteria (1)	100.00	0.00	97.90	97.20	Complete
238.48	0.149	Elizabethkingia meningoseptica (2	Elizabethkingia (117743)	Flavobacteriaceae (976)	Flavobacteriales (68336)	Flavobacteriia (1783270)	Bacteroidetes (2)	Bacteria (1)	100.00	0.00	98.00	97.40	WGS
238.49	0.1552	Elizabethkingia meningoseptica (2	Elizabethkingia (117743)	Flavobacteriaceae (976)	Flavobacteriales (68336)	Flavobacteriia (1783270)	Bacteroidetes (2)	Bacteria (1)	100.00	0.00	97.90	97.20	WGS
238.50	0.1472	Elizabethkingia meningoseptica (2	Elizabethkingia (117743)	Flavobacteriaceae (976)	Flavobacteriales (68336)	Flavobacteriia (1783270)	Bacteroidetes (2)	Bacteria (1)	100.00	0.00	98.00	97.40	WGS
238.51	0.1336	Elizabethkingia meningoseptica (2	Elizabethkingia (117743)	Flavobacteriaceae (976)	Flavobacteriales (68336)	Flavobacteriia (1783270)	Bacteroidetes (2)	Bacteria (1)	100.00	0.00	97.90	97.20	WGS
238.52	0.1472	Elizabethkingia meningoseptica (2	Elizabethkingia (117743)	Flavobacteriaceae (976)	Flavobacteriales (68336)	Flavobacteriia (1783270)	Bacteroidetes (2)	Bacteria (1)	100.00	0.00	97.90	97.20	WGS
238.53	0.152	Elizabethkingia meningoseptica (2	Elizabethkingia (117743)	Flavobacteriaceae (976)	Flavobacteriales (68336)	Flavobacteriia (1783270)	Bacteroidetes (2)	Bacteria (1)	100.00	0.00	97.90	97.30	WGS
238.54	0.1604	Elizabethkingia meningoseptica (2	Elizabethkingia (117743)	Flavobacteriaceae (976)	Flavobacteriales (68336)	Flavobacteriia (1783270)	Bacteroidetes (2)	Bacteria (1)	100.00	0.00	98.00	97.40	WGS
238.66	0.149	Elizabethkingia meningoseptica (2	Elizabethkingia (117743)	Flavobacteriaceae (976)	Flavobacteriales (68336)	Flavobacteriia (1783270)	Bacteroidetes (2)	Bacteria (1)	100.00	0.00	98.00	97.30	WGS
238.67	0.1552	Elizabethkingia meningoseptica (2	Elizabethkingia (117743)	Flavobacteriaceae (976)	Flavobacteriales (68336)	Flavobacteriia (1783270)	Bacteroidetes (2)	Bacteria (1)	100.00	0.00	97.90	97.20	WGS
238.68	0.1546	Elizabethkingia meningoseptica (2	Elizabethkingia (117743)	Flavobacteriaceae (976)	Flavobacteriales (68336)	Flavobacteriia (1783270)	Bacteroidetes (2)	Bacteria (1)	100.00	0.00	97.80	97.10	WGS
238.73	0.1424	Elizabethkingia meningoseptica (2	Elizabethkingia (117743)	Flavobacteriaceae (976)	Flavobacteriales (68336)	Flavobacteriia (1783270)	Bacteroidetes (2)	Bacteria (1)	100.00	0.00	97.90	97.20	WGS
238.80	0.149	Elizabethkingia meningoseptica (2	Elizabethkingia (117743)	Flavobacteriaceae (976)	Flavobacteriales (68336)	Flavobacteriia (1783270)	Bacteroidetes (2)	Bacteria (1)	100.00	0.70	98.40	97.70	Complete
238.86	0.3246	Elizabethkingia meningoseptica (2	Elizabethkingia (117743)	Flavobacteriaceae (976)	Flavobacteriales (68336)	Flavobacteriia (1783270)	Bacteroidetes (2)	Bacteria (1)	100.00	5.30	98.20	95.80	WGS
238.87	0.3072	Elizabethkingia meningoseptica (2	Elizabethkingia (117743)	Flavobacteriaceae (976)	Flavobacteriales (68336)	Flavobacteriia (1783270)	Bacteroidetes (2)	Bacteria (1)	100.00	0.00	97.90	96.80	WGS
238.88	0.3174	Elizabethkingia meningoseptica (2	Elizabethkingia (117743)	Flavobacteriaceae (976)	Flavobacteriales (68336)	Flavobacteriia (1783270)	Bacteroidetes (2)	Bacteria (1)	100.00	1.40	98.10	96.90	WGS
238.96	0.1336	Elizabethkingia meningoseptica (2	Elizabethkingia (117743)	Flavobacteriaceae (976)	Flavobacteriales (68336)	Flavobacteriia (1783270)	Bacteroidetes (2)	Bacteria (1)			98.40	97.80	WGS
239.10	0.285	Flavobacterium sp. (200644)	Flavobacterium (117743)	Flavobacteriaceae (976)	Flavobacteriales (68336)	Flavobacteriia (1783270)	Bacteroidetes (2)	Bacteria (1)	85.70	0.00	97.00	96.20	WGS
239.11	0.0262	Flavobacterium sp. (200644)	Flavobacterium (117743)	Flavobacteriaceae (976)	Flavobacteriales (68336)	Flavobacteriia (1783270)	Bacteroidetes (2)	Bacteria (1)	100.00	0.00	99.30	99.00	WGS
239.14	0.196	Flavobacterium sp. (200644)	Flavobacterium (117743)	Flavobacteriaceae (976)	Flavobacteriales (68336)	Flavobacteriia (1783270)	Bacteroidetes (2)	Bacteria (1)	89.30	11.70	94.70	91.40	WGS
239.15	0.0758	Flavobacterium sp. (200644)	Flavobacterium (117743)	Flavobacteriaceae (976)	Flavobacteriales (68336)	Flavobacteriia (1783270)	Bacteroidetes (2)	Bacteria (1)	68.40	17.20	86.00	83.80	WGS
239.16	0.1424	Flavobacterium sp. (200644)	Flavobacterium (117743)	Flavobacteriaceae (976)	Flavobacteriales (68336)	Flavobacteriia (1783270)	Bacteroidetes (2)	Bacteria (1)	84.50	10.70	90.70	86.80	WGS
239.17	0.0318	Flavobacterium sp. (200644)	Flavobacterium (117743)	Flavobacteriaceae (976)	Flavobacteriales (68336)	Flavobacteriia (1783270)	Bacteroidetes (2)	Bacteria (1)	97.60	4.60	99.00	97.80	WGS
239.18	0.1852	Flavobacterium sp. (200644)	Flavobacterium (117743)	Flavobacteriaceae (976)	Flavobacteriales (68336)	Flavobacteriia (1783270)	Bacteroidetes (2)	Bacteria (1)	96.30	31.10	95.10	85.30	WGS
239.19	0.052	Flavobacterium sp. (200644)	Flavobacterium (117743)	Flavobacteriaceae (976)	Flavobacteriales (68336)	Flavobacteriia (1783270)	Bacteroidetes (2)	Bacteria (1)	73.30	13.90	91.60	89.70	WGS
239.20	0.0702	Flavobacterium sp. (200644)	Flavobacterium (117743)	Flavobacteriaceae (976)	Flavobacteriales (68336)	Flavobacteriia (1783270)	Bacteroidetes (2)	Bacteria (1)	77.50	7.20	95.20	91.40	WGS
239.21	0.0278	Flavobacterium sp. (200644)	Flavobacterium (117743)	Flavobacteriaceae (976)	Flavobacteriales (68336)	Flavobacteriia (1783270)	Bacteroidetes (2)	Bacteria (1)	97.60	6.80	98.70	96.20	WGS
239.22	0.0694	Flavobacterium sp. (200644)	Flavobacterium (117743)	Flavobacteriaceae (976)	Flavobacteriales (68336)	Flavobacteriia (1783270)	Bacteroidetes (2)	Bacteria (1)	98.80	1.20	99.30	98.90	WGS
239.34	0.0458	Flavobacterium sp. (200644)	Flavobacterium (117743)	Flavobacteriaceae (976)	Flavobacteriales (68336)	Flavobacteriia (1783270)	Bacteroidetes (2)	Bacteria (1)			98.90	93.90	WGS
239.35	0.0474	Flavobacterium sp. (200644)	Flavobacterium (117743)	Flavobacteriaceae (976)	Flavobacteriales (68336)	Flavobacteriia (1783270)	Bacteroidetes (2)	Bacteria (1)			93.40	86.30	WGS
239.36	0.332	Flavobacterium sp. (200644)	Flavobacterium (117743)	Flavobacteriaceae (976)	Flavobacteriales (68336)	Flavobacteriia (1783270)	Bacteroidetes (2)	Bacteria (1)			95.30	73.50	WGS
239.37	0.334	Flavobacterium sp. (200644)	Flavobacterium (117743)	Flavobacteriaceae (976)	Flavobacteriales (68336)	Flavobacteriia (1783270)	Bacteroidetes (2)	Bacteria (1)			95.70	94.20	WGS
239.38	0.2118	Flavobacterium sp. (200644)	Flavobacterium (117743)	Flavobacteriaceae (976)	Flavobacteriales (68336)	Flavobacteriia (1783270)	Bacteroidetes (2)	Bacteria (1)			91.80	77.00	WGS
239.39	0.1142	Flavobacterium sp. (200644)	Flavobacterium (117743)	Flavobacteriaceae (976)	Flavobacteriales (68336)	Flavobacteriia (1783270)	Bacteroidetes (2)	Bacteria (1)			83.80	79.00	WGS
239.40	0.3074	Flavobacterium sp. (200644)	Flavobacterium (117743)	Flavobacteriaceae (976)	Flavobacteriales (68336)	Flavobacteriia (1783270)	Bacteroidetes (2)	Bacteria (1)			96.90	91.40	WGS
239.41	0.1018	Flavobacterium sp. (200644)	Flavobacterium (117743)	Flavobacteriaceae (976)	Flavobacteriales (68336)	Flavobacteriia (1783270)	Bacteroidetes (2)	Bacteria (1)			97.60	84.30	WGS
239.42	0.372	Flavobacterium sp. (200644)	Flavobacterium (117743)	Flavobacteriaceae (976)	Flavobacteriales (68336)	Flavobacteriia (1783270)	Bacteroidetes (2)	Bacteria (1)			98.50	98.10	WGS
239.43	0.5168	Flavobacterium sp. (200644)	Flavobacterium (117743)	Flavobacteriaceae (976)	Flavobacteriales (68336)	Flavobacteriia (1783270)	Bacteroidetes (2)	Bacteria (1)			92.30	90.40	WGS
239.44	0.0498	Flavobacterium sp. (200644)	Flavobacterium (117743)	Flavobacteriaceae (976)	Flavobacteriales (68336)	Flavobacteriia (1783270)	Bacteroidetes (2)	Bacteria (1)			89.10	88.00	WGS
239.8	0.0332	Flavobacterium sp. (200644)	Flavobacterium (117743)	Flavobacteriaceae (976)	Flavobacteriales (68336)	Flavobacteriia (1783270)	Bacteroidetes (2)	Bacteria (1)	100.00	4.50	99.00	97.40	WGS
239.9	0.1298	Flavobacterium sp. (200644)	Flavobacterium (117743)	Flavobacteriaceae (976)	Flavobacteriales (68336)	Flavobacteriia (1783270)	Bacteroidetes (2)	Bacteria (1)	95.20	2.50	97.00	94.90	WGS
244.3	0.1478	Planomicrobium okeanokoites (186	Planomicrobium (1385)	Planococcaceae (91061)	Bacillales (1239)	Bacilli (1783272)	Firmicutes (2)	Bacteria (1)	99.50	0.00	97.10	96.60	WGS
244.8	0.1476	Planomicrobium okeanokoites (186	Planomicrobium (1385)	Planococcaceae (91061)	Bacillales (1239)	Bacilli (1783272)	Firmicutes (2)	Bacteria (1)	99.50	0.00	97.30	96.50	WGS
245.10	0.1324	Flavobacterium aquatile (200644)	Flavobacterium (117743)	Flavobacteriaceae (976)	Flavobacteriales (68336)	Flavobacteriia (1783270)	Bacteroidetes (2)	Bacteria (1)	100.00	1.20	99.20	98.50	WGS
246.10	0.2768	Chryseobacterium balustinum (200	Chryseobacterium (117743	Flavobacteriaceae (976)	Flavobacteriales (68336)	Flavobacteriia (1783270)	Bacteroidetes (2)	Bacteria (1)	94.60	0.00	97.10	96.30	WGS
246.12	0.305	Chryseobacterium balustinum (200	Chryseobacterium (117743	Flavobacteriaceae (976)	Flavobacteriales (68336)	Flavobacteriia (1783270)	Bacteroidetes (2)	Bacteria (1)			98.10	96.70	Complete
246.6	0.3026	Chryseobacterium balustinum (200	Chryseobacterium (117743	Flavobacteriaceae (976)	Flavobacteriales (68336)	Flavobacteriia (1783270)	Bacteroidetes (2)	Bacteria (1)	100.00	0.00	97.60	96.90	WGS
247.3	0.0604	Empedobacter brevis (200644)	Empedobacter (117743)	Flavobacteriaceae (976)	Flavobacteriales (68336)	Flavobacteriia (1783270)	Bacteroidetes (2)	Bacteria (1)	56.00	0.00	91.90	91.60	WGS
247.4	0.057	Empedobacter brevis (200644)	Empedobacter (117743)	Flavobacteriaceae (976)	Flavobacteriales (68336)	Flavobacteriia (1783270)	Bacteroidetes (2)	Bacteria (1)	100.00	0.00	99.90	99.20	WGS
247.5	0.0724	Empedobacter brevis (200644)	Empedobacter (117743)	Flavobacteriaceae (976)	Flavobacteriales (68336)	Flavobacteriia (1783270)	Bacteroidetes (2)	Bacteria (1)			99.00	98.00	WGS
250.33	0.3296	Chryseobacterium gleum (200644)	Chryseobacterium (117743	Flavobacteriaceae (976)	Flavobacteriales (68336)	Flavobacteriia (1783270)	Bacteroidetes (2)	Bacteria (1)			98.00	96.60	Complete
[illegible]	[illegible]	[illegible]	[illegible]	[illegible]	[illegible]	[illegible]	[illegible]	[illegible]	[illegible]	[illegible]	[illegible]	[illegible]	[illegible]

287.1588	0.3636	Pseudomonas aeruginosa (286)	Pseudomonas (135621)	Pseudomonadaceae (7227	Pseudomonadales (1236)	Gammaproteobacteria (122	Proteobacteria (2)	Bacteria (1)	100.00	1.20	99.90	98.20	WGS
287.1589	0.3636	Pseudomonas aeruginosa (286)	Pseudomonas (135621)	Pseudomonadaceae (7227	Pseudomonadales (1236)	Gammaproteobacteria (122	Proteobacteria (2)	Bacteria (1)	100.00	0.60	99.90	99.10	WGS
287.1590	0.3636	Pseudomonas aeruginosa (286)	Pseudomonas (135621)	Pseudomonadaceae (7227	Pseudomonadales (1236)	Gammaproteobacteria (122	Proteobacteria (2)	Bacteria (1)	100.00	0.90	99.90	98.70	WGS
287.1591	0.3636	Pseudomonas aeruginosa (286)	Pseudomonas (135621)	Pseudomonadaceae (7227	Pseudomonadales (1236)	Gammaproteobacteria (122	Proteobacteria (2)	Bacteria (1)	100.00	1.80	99.90	98.90	WGS
287.1592	0.3636	Pseudomonas aeruginosa (286)	Pseudomonas (135621)	Pseudomonadaceae (7227	Pseudomonadales (1236)	Gammaproteobacteria (122	Proteobacteria (2)	Bacteria (1)	100.00	0.90	99.80	99.30	WGS
287.1593	0.3834	Pseudomonas aeruginosa (286)	Pseudomonas (135621)	Pseudomonadaceae (7227	Pseudomonadales (1236)	Gammaproteobacteria (122	Proteobacteria (2)	Bacteria (1)	100.00	0.30	99.80	98.50	WGS
287.1594	0.3636	Pseudomonas aeruginosa (286)	Pseudomonas (135621)	Pseudomonadaceae (7227	Pseudomonadales (1236)	Gammaproteobacteria (122	Proteobacteria (2)	Bacteria (1)	100.00	0.30	99.90	99.00	WGS
287.1595	0.3636	Pseudomonas aeruginosa (286)	Pseudomonas (135621)	Pseudomonadaceae (7227	Pseudomonadales (1236)	Gammaproteobacteria (122	Proteobacteria (2)	Bacteria (1)	100.00	0.60	99.70	99.00	WGS
287.1596	0.3636	Pseudomonas aeruginosa (286)	Pseudomonas (135621)	Pseudomonadaceae (7227	Pseudomonadales (1236)	Gammaproteobacteria (122	Proteobacteria (2)	Bacteria (1)	100.00	0.30	99.80	99.10	WGS
287.1597	0.3636	Pseudomonas aeruginosa (286)	Pseudomonas (135621)	Pseudomonadaceae (7227	Pseudomonadales (1236)	Gammaproteobacteria (122	Proteobacteria (2)	Bacteria (1)	99.70	0.00	99.80	99.00	WGS
287.1598	0.3636	Pseudomonas aeruginosa (286)	Pseudomonas (135621)	Pseudomonadaceae (7227	Pseudomonadales (1236)	Gammaproteobacteria (122	Proteobacteria (2)	Bacteria (1)	100.00	2.20	99.60	97.10	WGS
287.1599	0.3636	Pseudomonas aeruginosa (286)	Pseudomonas (135621)	Pseudomonadaceae (7227	Pseudomonadales (1236)	Gammaproteobacteria (122	Proteobacteria (2)	Bacteria (1)	100.00	0.30	99.80	99.00	WGS
287.1600	0.3636	Pseudomonas aeruginosa (286)	Pseudomonas (135621)	Pseudomonadaceae (7227	Pseudomonadales (1236)	Gammaproteobacteria (122	Proteobacteria (2)	Bacteria (1)	99.40	1.30	99.00	97.40	WGS
287.1601	0.3636	Pseudomonas aeruginosa (286)	Pseudomonas (135621)	Pseudomonadaceae (7227	Pseudomonadales (1236)	Gammaproteobacteria (122	Proteobacteria (2)	Bacteria (1)	100.00	0.00	99.80	99.20	WGS
287.1602	0.3636	Pseudomonas aeruginosa (286)	Pseudomonas (135621)	Pseudomonadaceae (7227	Pseudomonadales (1236)	Gammaproteobacteria (122	Proteobacteria (2)	Bacteria (1)	99.70	0.70	99.50	98.20	WGS
287.1603	0.3636	Pseudomonas aeruginosa (286)	Pseudomonas (135621)	Pseudomonadaceae (7227	Pseudomonadales (1236)	Gammaproteobacteria (122	Proteobacteria (2)	Bacteria (1)	100.00	0.30	99.90	99.00	WGS
287.1604	0.3636	Pseudomonas aeruginosa (286)	Pseudomonas (135621)	Pseudomonadaceae (7227	Pseudomonadales (1236)	Gammaproteobacteria (122	Proteobacteria (2)	Bacteria (1)	100.00	0.00	99.80	98.90	WGS
287.1605	0.3636	Pseudomonas aeruginosa (286)	Pseudomonas (135621)	Pseudomonadaceae (7227	Pseudomonadales (1236)	Gammaproteobacteria (122	Proteobacteria (2)	Bacteria (1)	99.70	0.30	99.70	98.90	WGS
287.1606	0.3636	Pseudomonas aeruginosa (286)	Pseudomonas (135621)	Pseudomonadaceae (7227	Pseudomonadales (1236)	Gammaproteobacteria (122	Proteobacteria (2)	Bacteria (1)	100.00	0.00	99.70	99.00	WGS
287.1607	0.3636	Pseudomonas aeruginosa (286)	Pseudomonas (135621)	Pseudomonadaceae (7227	Pseudomonadales (1236)	Gammaproteobacteria (122	Proteobacteria (2)	Bacteria (1)	99.70	0.30	99.70	98.90	WGS
287.1608	0.3636	Pseudomonas aeruginosa (286)	Pseudomonas (135621)	Pseudomonadaceae (7227	Pseudomonadales (1236)	Gammaproteobacteria (122	Proteobacteria (2)	Bacteria (1)	100.00	0.70	99.70	98.80	WGS
287.1609	0.3636	Pseudomonas aeruginosa (286)	Pseudomonas (135621)	Pseudomonadaceae (7227	Pseudomonadales (1236)	Gammaproteobacteria (122	Proteobacteria (2)	Bacteria (1)	100.00	1.00	99.70	98.30	WGS
287.1610	0.3636	Pseudomonas aeruginosa (286)	Pseudomonas (135621)	Pseudomonadaceae (7227	Pseudomonadales (1236)	Gammaproteobacteria (122	Proteobacteria (2)	Bacteria (1)	99.40	1.60	99.60	97.70	WGS
287.1611	0.3636	Pseudomonas aeruginosa (286)	Pseudomonas (135621)	Pseudomonadaceae (7227	Pseudomonadales (1236)	Gammaproteobacteria (122	Proteobacteria (2)	Bacteria (1)	99.70	1.60	99.50	97.70	WGS
287.1612	0.3636	Pseudomonas aeruginosa (286)	Pseudomonas (135621)	Pseudomonadaceae (7227	Pseudomonadales (1236)	Gammaproteobacteria (122	Proteobacteria (2)	Bacteria (1)	100.00	0.40	99.90	99.10	WGS
287.1613	0.3636	Pseudomonas aeruginosa (286)	Pseudomonas (135621)	Pseudomonadaceae (7227	Pseudomonadales (1236)	Gammaproteobacteria (122	Proteobacteria (2)	Bacteria (1)	99.70	1.60	99.60	98.50	WGS
287.1614	0.3636	Pseudomonas aeruginosa (286)	Pseudomonas (135621)	Pseudomonadaceae (7227	Pseudomonadales (1236)	Gammaproteobacteria (122	Proteobacteria (2)	Bacteria (1)	100.00	1.60	99.80	98.30	WGS
287.1615	0.3636	Pseudomonas aeruginosa (286)	Pseudomonas (135621)	Pseudomonadaceae (7227	Pseudomonadales (1236)	Gammaproteobacteria (122	Proteobacteria (2)	Bacteria (1)	100.00	2.10	99.80	98.20	WGS
287.1616	0.3636	Pseudomonas aeruginosa (286)	Pseudomonas (135621)	Pseudomonadaceae (7227	Pseudomonadales (1236)	Gammaproteobacteria (122	Proteobacteria (2)	Bacteria (1)	100.00	0.60	99.80	99.30	WGS
287.1617	0.3636	Pseudomonas aeruginosa (286)	Pseudomonas (135621)	Pseudomonadaceae (7227	Pseudomonadales (1236)	Gammaproteobacteria (122	Proteobacteria (2)	Bacteria (1)	100.00	0.00	99.80	98.30	WGS
287.1618	0.3636	Pseudomonas aeruginosa (286)	Pseudomonas (135621)	Pseudomonadaceae (7227	Pseudomonadales (1236)	Gammaproteobacteria (122	Proteobacteria (2)	Bacteria (1)	100.00	0.00	99.80	99.10	WGS
287.1619	0.3834	Pseudomonas aeruginosa (286)	Pseudomonas (135621)	Pseudomonadaceae (7227	Pseudomonadales (1236)	Gammaproteobacteria (122	Proteobacteria (2)	Bacteria (1)	100.00	0.10	99.80	99.30	WGS
287.1620	0.3636	Pseudomonas aeruginosa (286)	Pseudomonas (135621)	Pseudomonadaceae (7227	Pseudomonadales (1236)	Gammaproteobacteria (122	Proteobacteria (2)	Bacteria (1)	100.00	1.20	99.90	98.70	WGS
287.1621	0.3636	Pseudomonas aeruginosa (286)	Pseudomonas (135621)	Pseudomonadaceae (7227	Pseudomonadales (1236)	Gammaproteobacteria (122	Proteobacteria (2)	Bacteria (1)	100.00	0.60	99.90	99.00	WGS
287.1622	0.3636	Pseudomonas aeruginosa (286)	Pseudomonas (135621)	Pseudomonadaceae (7227	Pseudomonadales (1236)	Gammaproteobacteria (122	Proteobacteria (2)	Bacteria (1)	100.00	0.60	99.90	99.40	WGS
287.1623	0.3636	Pseudomonas aeruginosa (286)	Pseudomonas (135621)	Pseudomonadaceae (7227	Pseudomonadales (1236)	Gammaproteobacteria (122	Proteobacteria (2)	Bacteria (1)	100.00	0.00	99.90	99.40	WGS
287.1624	0.3636	Pseudomonas aeruginosa (286)	Pseudomonas (135621)	Pseudomonadaceae (7227	Pseudomonadales (1236)	Gammaproteobacteria (122	Proteobacteria (2)	Bacteria (1)	100.00	0.30	99.90	99.00	WGS
287.1625	0.3636	Pseudomonas aeruginosa (286)	Pseudomonas (135621)	Pseudomonadaceae (7227	Pseudomonadales (1236)	Gammaproteobacteria (122	Proteobacteria (2)	Bacteria (1)	100.00	1.20	99.90	99.10	WGS
287.1626	0.3636	Pseudomonas aeruginosa (286)	Pseudomonas (135621)	Pseudomonadaceae (7227	Pseudomonadales (1236)	Gammaproteobacteria (122	Proteobacteria (2)	Bacteria (1)	100.00	0.30	99.70	98.60	WGS
287.1627	0.3636	Pseudomonas aeruginosa (286)	Pseudomonas (135621)	Pseudomonadaceae (7227	Pseudomonadales (1236)	Gammaproteobacteria (122	Proteobacteria (2)	Bacteria (1)	100.00	0.60	99.80	98.80	WGS
287.1628	0.3636	Pseudomonas aeruginosa (286)	Pseudomonas (135621)	Pseudomonadaceae (7227	Pseudomonadales (1236)	Gammaproteobacteria (122	Proteobacteria (2)	Bacteria (1)	100.00	0.40	99.80	99.00	WGS
287.1629	0.3636	Pseudomonas aeruginosa (286)	Pseudomonas (135621)	Pseudomonadaceae (7227	Pseudomonadales (1236)	Gammaproteobacteria (122	Proteobacteria (2)	Bacteria (1)	100.00	0.90	99.80	98.70	WGS
287.1630	0.3636	Pseudomonas aeruginosa (286)	Pseudomonas (135621)	Pseudomonadaceae (7227	Pseudomonadales (1236)	Gammaproteobacteria (122	Proteobacteria (2)	Bacteria (1)	100.00	1.20	99.90	99.20	WGS
287.1631	0.3636	Pseudomonas aeruginosa (286)	Pseudomonas (135621)	Pseudomonadaceae (7227	Pseudomonadales (1236)	Gammaproteobacteria (122	Proteobacteria (2)	Bacteria (1)	100.00	0.30	99.80	99.30	WGS
287.1632	0.3636	Pseudomonas aeruginosa (286)	Pseudomonas (135621)	Pseudomonadaceae (7227	Pseudomonadales (1236)	Gammaproteobacteria (122	Proteobacteria (2)	Bacteria (1)	100.00	2.40	99.80	98.20	WGS
287.1633	0.3636	Pseudomonas aeruginosa (286)	Pseudomonas (135621)	Pseudomonadaceae (7227	Pseudomonadales (1236)	Gammaproteobacteria (122	Proteobacteria (2)	Bacteria (1)	100.00	1.50	99.90	98.70	WGS
287.1634	0.3636	Pseudomonas aeruginosa (286)	Pseudomonas (135621)	Pseudomonadaceae (7227	Pseudomonadales (1236)	Gammaproteobacteria (122	Proteobacteria (2)	Bacteria (1)	100.00	0.90	99.90	99.40	WGS
287.1635	0.3636	Pseudomonas aeruginosa (286)	Pseudomonas (135621)	Pseudomonadaceae (7227	Pseudomonadales (1236)	Gammaproteobacteria (122	Proteobacteria (2)	Bacteria (1)	100.00	0.30	99.90	98.90	WGS
287.1636	0.3636	Pseudomonas aeruginosa (286)	Pseudomonas (135621)	Pseudomonadaceae (7227	Pseudomonadales (1236)	Gammaproteobacteria (122	Proteobacteria (2)	Bacteria (1)	100.00	0.00	99.70	98.70	WGS
287.1637	0.3636	Pseudomonas aeruginosa (286)	Pseudomonas (135621)	Pseudomonadaceae (7227	Pseudomonadales (1236)	Gammaproteobacteria (122	Proteobacteria (2)	Bacteria (1)	100.00	0.00	99.80	99.00	WGS
287.1638	0.3636	Pseudomonas aeruginosa (286)	Pseudomonas (135621)	Pseudomonadaceae (7227	Pseudomonadales (1236)	Gammaproteobacteria (122	Proteobacteria (2)	Bacteria (1)	100.00	0.00	99.80	99.10	WGS
287.1639	0.3636	Pseudomonas aeruginosa (286)	Pseudomonas (135621)	Pseudomonadaceae (7227	Pseudomonadales (1236)	Gammaproteobacteria (122	Proteobacteria (2)	Bacteria (1)	100.00	0.00	99.80	99.10	WGS
287.1640	0.3636	Pseudomonas aeruginosa (286)	Pseudomonas (135621)	Pseudomonadaceae (7227	Pseudomonadales (1236)	Gammaproteobacteria (122	Proteobacteria (2)	Bacteria (1)	100.00	0.30	99.70	98.80	WGS
287.1641	0.3636	Pseudomonas aeruginosa (286)	Pseudomonas (135621)	Pseudomonadaceae (7227	Pseudomonadales (1236)	Gammaproteobacteria (122	Proteobacteria (2)	Bacteria (1)	100.00	0.70	99.90	99.00	WGS
287.1642	0.3636	Pseudomonas aeruginosa (286)	Pseudomonas (135621)	Pseudomonadaceae (7227	Pseudomonadales (1236)	Gammaproteobacteria (122	Proteobacteria (2)	Bacteria (1)	100.00	0.00	99.80	99.30	WGS
287.1643	0.3636	Pseudomonas aeruginosa (286)	Pseudomonas (135621)	Pseudomonadaceae (7227	Pseudomonadales (1236)	Gammaproteobacteria (122	Proteobacteria (2)	Bacteria (1)	100.00	0.90	99.80	99.30	WGS
287.1644	0.3636	Pseudomonas aeruginosa (286)	Pseudomonas (135621)	Pseudomonadaceae (7227	Pseudomonadales (1236)	Gammaproteobacteria (122	Proteobacteria (2)	Bacteria (1)	100.00	0.90	99.80	98.80	WGS
287.1645	0.3636	Pseudomonas aeruginosa (286)	Pseudomonas (135621)	Pseudomonadaceae (7227	Pseudomonadales (1236)	Gammaproteobacteria (122	Proteobacteria (2)	Bacteria (1)	100.00	1.20	99.90	99.10	WGS
287.1646	0.3636	Pseudomonas aeruginosa (286)	Pseudomonas (135621)	Pseudomonadaceae (7227	Pseudomonadales (1236)	Gammaproteobacteria (122	Proteobacteria (2)	Bacteria (1)	100.00	0.00	99.80	99.30	WGS
287.1647	0.3636	Pseudomonas aeruginosa (286)	Pseudomonas (135621)	Pseudomonadaceae (7227	Pseudomonadales (1236)	Gammaproteobacteria (122	Proteobacteria (2)	Bacteria (1)	100.00	0.60	99.90	99.10	WGS
287.1648	0.3636	Pseudomonas aeruginosa (286)	Pseudomonas (135621)	Pseudomonadaceae (7227	Pseudomonadales (1236)	Gammaproteobacteria (122	Proteobacteria (2)	Bacteria (1)	100.00	1.20	99.90	98.90	WGS
287.1649	0.3636	Pseudomonas aeruginosa (286)	Pseudomonas (135621)	Pseudomonadaceae (7227	Pseudomonadales (1236)	Gammaproteobacteria (122	Proteobacteria (2)	Bacteria (1)	100.00	0.00	99.80	99.30	WGS
287.1650	0.3636	Pseudomonas aeruginosa (286)	Pseudomonas (135621)	Pseudomonadaceae (7227	Pseudomonadales (1236)	Gammaproteobacteria (122	Proteobacteria (2)	Bacteria (1)	100.00	2.50	99.70	99.00	WGS
287.1651	0.3636	Pseudomonas aeruginosa (286)	Pseudomonas (135621)	Pseudomonadaceae (7227	Pseudomonadales (1236)	Gammaproteobacteria (122	Proteobacteria (2)	Bacteria (1)	100.00	0.00	99.90	99.30	WGS
287.1652	0.3636	Pseudomonas aeruginosa (286)	Pseudomonas (135621)	Pseudomonadaceae (7227	Pseudomonadales (1236)	Gammaproteobacteria (122	Proteobacteria (2)	Bacteria (1)	100.00	0.00	99.70	99.20	WGS
287.1653	0.3834	Pseudomonas aeruginosa (286)	Pseudomonas (135621)	Pseudomonadaceae (7227	Pseudomonadales (1236)	Gammaproteobacteria (122	Proteobacteria (2)	Bacteria (1)	100.00	0.30	99.80	99.10	WGS
287.1654	0.3926	Pseudomonas aeruginosa (286)	Pseudomonas (135621)	Pseudomonadaceae (7227	Pseudomonadales (1236)	Gammaproteobacteria (122	Proteobacteria (2)	Bacteria (1)	100.00	14.40	99.80	94.40	WGS
287.1655	0.3636	Pseudomonas aeruginosa (286)	Pseudomonas (135621)	Pseudomonadaceae (7227	Pseudomonadales (1236)	Gammaproteobacteria (122	Proteobacteria (2)	Bacteria (1)	97.90	3.70	98.50	93.70	WGS
287.1656	0.3636	Pseudomonas aeruginosa (286)	Pseudomonas (135621)	Pseudomonadaceae (7227	Pseudomonadales (1236)	Gammaproteobacteria (122	Proteobacteria (2)	Bacteria (1)	100.00	0.60	99.80	98.40	WGS
287.1657	0.3636	Pseudomonas aeruginosa (286)	Pseudomonas (135621)	Pseudomonadaceae (7227	Pseudomonadales (1236)	Gammaproteobacteria (122	Proteobacteria (2)	Bacteria (1)	99.00	2.10	99.30	97.60	WGS
287.1658	0.3636	Pseudomonas aeruginosa (286)	Pseudomonas (135621)	Pseudomonadaceae (7227	Pseudomonadales (1236)	Gammaproteobacteria (122	Proteobacteria (2)	Bacteria (1)	100.00	1.20	99.80	98.40	WGS
287.1659	0.3636	Pseudomonas aeruginosa (286)	Pseudomonas (135621)	Pseudomonadaceae (7227	Pseudomonadales (1236)	Gammaproteobacteria (122	Proteobacteria (2)	Bacteria (1)	100.00	0.90	99.80	98.70	WGS
287.1660	0.3636	Pseudomonas aeruginosa (286)	Pseudomonas (135621)	Pseudomonadaceae (7227	Pseudomonadales (1236)	Gammaproteobacteria (122	Proteobacteria (2)	Bacteria (1)	100.00	0.40	99.80	99.00	WGS
287.1661	0.3636	Pseudomonas aeruginosa (286)	Pseudomonas (135621)	Pseudomonadaceae (7227	Pseudomonadales (1236)	Gammaproteobacteria (122	Proteobacteria (2)	Bacteria (1)	99.70	0.90	99.60	98.80	WGS
287.1662	0.3636	Pseudomonas aeruginosa (286)	Pseudomonas (135621)	Pseudomonadaceae (7227	Pseudomonadales (1236)	Gammaproteobacteria (122	Proteobacteria (2)	Bacteria (1)	100.00	1.50	99.80	98.60	WGS
287.1663	0.3636	Pseudomonas aeruginosa (286)	Pseudomonas (135621)	Pseudomonadaceae (7227	Pseudomonadales (1236)	Gammaproteobacteria (122	Proteobacteria (2)	Bacteria (1)	100.00	0.90	99.90	98.20	WGS
287.1664	0.3636	Pseudomonas aeruginosa (286)	Pseudomonas (135621)	Pseudomonadaceae (7227	Pseudomonadales (1236)	Gammaproteobacteria (122	Proteobacteria (2)	Bacteria (1)	100.00	0.00	99.80	99.20	WGS
287.1665	0.3636	Pseudomonas aeruginosa (286)	Pseudomonas (135621)	Pseudomonadaceae (7227	Pseudomonadales (1236)	Gammaproteobacteria (122	Proteobacteria (2)	Bacteria (1)	100.00	0.00	99.80	99.00	WGS
287.1666	0.3636	Pseudomonas aeruginosa (286)	Pseudomonas (135621)	Pseudomonadaceae (7227	Pseudomonadales (1236)	Gammaproteobacteria (122	Proteobacteria (2)	Bacteria (1)	98.60	2.10	99.30	96.20	WGS
287.1668	0.3636	Pseudomonas aeruginosa (286)	Pseudomonas (135621)	Pseudomonadaceae (7227	Pseudomonadales (1236)	Gammaproteobacteria (122	Proteobacteria (2)	Bacteria (1)	100.00	1.20	99.80	98.20	WGS
287.1669	0.3636	Pseudomonas aeruginosa (286)	Pseudomonas (135621)	Pseudomonadaceae (7227	Pseudomonadales (1236)	Gammaproteobacteria (122	Proteobacteria (2)	Bacteria (1)	100.00	0.00	99.90	99.00	WGS
287.1670	0.3636	Pseudomonas aeruginosa (286)	Pseudomonas (135621)	Pseudomonadaceae (7227	Pseudomonadales (1236)	Gammaproteobacteria (122	Proteobacteria (2)	Bacteria (1)	100.00	0.00	99.80	99.30	WGS
287.1671	0.3636	Pseudomonas aeruginosa (286)	Pseudomonas (135621)	Pseudomonadaceae (7227	Pseudomonadales (1236)	Gammaproteobacteria (122	Proteobacteria (2)	Bacteria (1)	100.00	0.50	99.80	98.70	WGS
287.1672	0.3636	Pseudomonas aeruginosa (286)	Pseudomonas (135621)	Pseudomonadaceae (7227	Pseudomonadales (1236)	Gammaproteobacteria (122	Proteobacteria (2)	Bacteria (1)	100.00	0.00	99.90	99.40	WGS
287.1673	0.3558	Pseudomonas aeruginosa (286)	Pseudomonas (135621)	Pseudomonadaceae (7227	Pseudomonadales (1236)	Gammaproteobacteria (122	Proteobacteria (2)	Bacteria (1)	100.00	0.60	99.90	99.00	WGS
287.1674	0.3636	Pseudomonas aeruginosa (286)	Pseudomonas (135621)	Pseudomonadaceae (7227	Pseudomonadales (1236)	Gammaproteobacteria (122	Proteobacteria (2)	Bacteria (1)	100.00	0.60	99.90	99.30	WGS
287.1675	0.3636	Pseudomonas aeruginosa (286)	Pseudomonas (135621)	Pseudomonadaceae (7227	Pseudomonadales (1236)	Gammaproteobacteria (122	Proteobacteria (2)	Bacteria (1)	100.00	0.30	99.90	99.30	WGS
287.1676	0.3636	Pseudomonas aeruginosa (286)	Pseudomonas (135621)	Pseudomonadaceae (7227	Pseudomonadales (1236)	Gammaproteobacteria (122	Proteobacteria (2)	Bacteria (1)	100.00	0.60	99.90	98.90	WGS
287.1677	0.3636	Pseudomonas aeruginosa (286)	Pseudomonas (135621)	Pseudomonadaceae (7227	Pseudomonadales (1236)	Gammaproteobacteria (122	Proteobacteria (2)	Bacteria (1)	100.00	0.60	99.90	99.00	WGS
287.1678	0.3636	Pseudomonas aeruginosa (286)	Pseudomonas (135621)	Pseudomonadaceae (7227	Pseudomonadales (1236)	Gammaproteobacteria (122	Proteobacteria (2)	Bacteria (1)	100.00	0.90	99.70	98.50	WGS
287.1679	0.3636	Pseudomonas aeruginosa (286)	Pseudomonas (135621)	Pseudomonadaceae (7227	Pseudomonadales (1236)	Gammaproteobacteria (122	Proteobacteria (2)	Bacteria (1)	100.00	2.50	99.90	99.10	WGS
287.1680	0.3636	Pseudomonas aeruginosa (286)	Pseudomonas (135621)	Pseudomonadaceae (7227	Pseudomonadales (1236)	Gammaproteobacteria (122	Proteobacteria (2)	Bacteria (1)	100.00	0.60	99.90	99.10	WGS
287.1681	0.3636	Pseudomonas aeruginosa (286)	Pseudomonas (135621)	Pseudomonadaceae (7227	Pseudomonadales (1236)	Gammaproteobacteria (122	Proteobacteria (2)	Bacteria (1)	100.00	0.30	99.80	99.00	WGS
287.1682	0.3636	Pseudomonas aeruginosa (286)	Pseudomonas (135621)	Pseudomonadaceae (7227	Pseudomonadales (1236)	Gammaproteobacteria (122	Proteobacteria (2)	Bacteria (1)	100.00	0.00	99.80	99.00	WGS
287.1683	0.3636	Pseudomonas aeruginosa (286)	Pseudomonas (135621)	Pseudomonadaceae (7227	Pseudomonadales (1236)	Gammaproteobacteria (122	Proteobacteria (2)	Bacteria (1)	100.00	0.00	99.80	99.20	WGS
287.1684	0.3636	Pseudomonas aeruginosa (286)	Pseudomonas (135621)	Pseudomonadaceae (7227	Pseudomonadales (1236)	Gammaproteobacteria (122	Proteobacteria (2)	Bacteria (1)	100.00	2.10	99.90	98.90	WGS
287.1685	0.3636	Pseudomonas aeruginosa (286)	Pseudomonas (135621)	Pseudomonadaceae (7227	Pseudomonadales (1236)	Gammaproteobacteria (122	Proteobacteria (2)	Bacteria (1)	100.00	1.80	99.70	98.70	WGS
287.1686	0.3636	Pseudomonas aeruginosa (286)	Pseudomonas (135621)	Pseudomonadaceae (7227	Pseudomonadales (1236)	Gammaproteobacteria (122	Proteobacteria (2)	Bacteria (1)	100.00	0.30	99.80	99.00	WGS
287.1687	0.3636	Pseudomonas aeruginosa (286)	Pseudomonas (135621)	Pseudomonadaceae (7227	Pseudomonadales (1236)	Gammaproteobacteria (122	Proteobacteria (2)	Bacteria (1)	100.00	1.20	99.90	98.70	WGS
287.1688	0.3636	Pseudomonas aeruginosa (286)	Pseudomonas (135621)	Pseudomonadaceae (7227	Pseudomonadales (1236)	Gammaproteobacteria (122	Proteobacteria (2)	Bacteria (1)	100.00	0.00	99.90	99.30	WGS
287.1689	0.3636	Pseudomonas aeruginosa (286)	Pseudomonas (135621)	Pseudomonadaceae (7227	Pseudomonadales (1236)	Gammaproteobacteria (122	Proteobacteria (2)	Bacteria (1)	100.00	1.20	99.90	98.70	WGS
287.1690	0.3636	Pseudomonas aeruginosa (286)	Pseudomonas (135621)	Pseudomonadaceae (7227	Pseudomonadales (1236)	Gammaproteobacteria (122	Proteobacteria (2)	Bacteria (1)	100.00	1.20	99.90	98.90	WGS
287.1691	0.3636	Pseudomonas aeruginosa (286)	Pseudomonas (135621)	Pseudomonadaceae (7227	Pseudomonadales (1236)	Gammaproteobacteria (122	Proteobacteria (2)	Bacteria (1)	100.00	0.60	99.80	99.20	WGS
287.1692	0.3636	Pseudomonas aeruginosa (286)	Pseudomonas (135621)	Pseudomonadaceae (7227	Pseudomonadales (1236)	Gammaproteobacteria (122	Proteobacteria (2)	Bacteria (1)	100.00	1.20	99.90	98.60	WGS
287.1693	0.3636	Pseudomonas aeruginosa (286)	Pseudomonas (135621)	Pseudomonadaceae (7227	Pseudomonadales (1236)	Gammaproteobacteria (122	Proteobacteria (2)	Bacteria (1)	100.00	0.60	99.80	98.60	WGS
287.1694	0.3636	Pseudomonas aeruginosa (286)	Pseudomonas (135621)	Pseudomonadaceae (7227	Pseudomonadales (1236)	Gammaproteobacteria (122	Proteobacteria (2)	Bacteria (1)	100.00	0.90	99.90	99.00	WGS
287.1695	0.3558	Pseudomonas aeruginosa (286)	Pseudomonas (135621)	Pseudomonadaceae (7227	Pseudomonadales (1236)	Gammaproteobacteria (122	Proteobacteria (2)	Bacteria (1)	100.00	1.00	99.70	98.40	WGS
287.1696	0.3636	Pseudomonas aeruginosa (286)	Pseudomonas (135621)	Pseudomonadaceae (7227	Pseudomonadales (1236)	Gammaproteobacteria (122	Proteobacteria (2)	Bacteria (1)	99.70	0.60	99.70	98.10	WGS
287.1697	0.3636	Pseudomonas aeruginosa (286)	Pseudomonas (135621)	Pseudomonadaceae (7227	Pseudomonadales (1236)	Gammaproteobacteria (122	Proteobacteria (2)	Bacteria (1)	99.40	2.50	99.60	97.90	WGS
287.1698	0.3258	Pseudomonas aeruginosa (286)	Pseudomonas (135621)	Pseudomonadaceae (7227	Pseudomonadales (1236)	Gammaproteobacteria (122	Proteobacteria (2)	Bacteria (1)	98.50	3.90	99.30	96.10	WGS
287.1699	0.3636	Pseudomonas aeruginosa (286)	Pseudomonas (135621)	Pseudomonadaceae (7227	Pseudomonadales (1236)	Gammaproteobacteria (122	Proteobacteria (2)	Bacteria (1)	100.00	0.90	99.60	98.00	WGS
287.1700	0.3636	Pseudomonas aeruginosa (286)	Pseudomonas (135621)	Pseudomonadaceae (7227	Pseudomonadales (1236)	Gammaproteobacteria (122	Proteobacteria (2)	Bacteria (1)	99.70	2.90	99.70	98.20	WGS
287.1701	0.3636	Pseudomonas aeruginosa (286)	Pseudomonas (135621)	Pseudomonadaceae (7227	Pseudomonadales (1236)	Gammaproteobacteria (122	Proteobacteria (2)	Bacteria (1)	99.40	3.40	99.20	95.80	WGS
287.1702	0.3636	Pseudomonas aeruginosa (286)	Pseudomonas (135621)	Pseudomonadaceae (7227	Pseudomonadales (1236)	Gammaproteobacteria (122	Proteobacteria (2)	Bacteria (1)	100.00	1.20	99.60	97.60	WGS
287.1703	0.3636	Pseudomonas aeruginosa (286)	Pseudomonas (135621)	Pseudomonadaceae (7227	Pseudomonadales (1236)	Gammaproteobacteria (122	Proteobacteria (2)	Bacteria (1)	99.70	0.90	99.50	98.00	WGS
287.1704	0.3636	Pseudomonas aeruginosa (286)	Pseudomonas (135621)	Pseudomonadaceae (7227	Pseudomonadales (1236)	Gammaproteobacteria (122	Proteobacteria (2)	Bacteria (1)	99.40	0.90	99.70	97.90	WGS
287.1705	0.3334	Pseudomonas aeruginosa (286)	Pseudomonas (135621)	Pseudomonadaceae (7227	Pseudomonadales (1236)	Gammaproteobacteria (122	Proteobacteria (2)	Bacteria (1)	99.40	2.40	99.10	96.10	WGS
287.1706	0.3636	Pseudomonas aeruginosa (286)	Pseudomonas (135621)	Pseudomonadaceae (7227	Pseudomonadales (1236)	Gammaproteobacteria (122	Proteobacteria (2)	Bacteria (1)	100.00	1.80	99.20	95.30	WGS
287.1707	0.3334	Pseudomonas aeruginosa (286)	Pseudomonas (135621)	Pseudomonadaceae (7227	Pseudomonadales (1236)	Gammaproteobacteria (122	Proteobacteria (2)	Bacteria (1)	98.60	1.80	99.60	96.80	WGS
287.1708	0.3636	Pseudomonas aeruginosa (286)	Pseudomonas (135621)	Pseudomonadaceae (7227	Pseudomonadales (1236)	Gammaproteobacteria (122	Proteobacteria (2)	Bacteria (1)	98.80	2.10	98.90	95.50	WGS
287.1709	0.3636	Pseudomonas aeruginosa (286)	Pseudomonas (135621)	Pseudomonadaceae (7227	Pseudomonadales (1236)	Gammaproteobacteria (122	Proteobacteria (2)	Bacteria (1)	100.00	1.80	99.80	98.50	WGS
287.1710	0.3636	Pseudomonas aeruginosa (286)	Pseudomonas (135621)	Pseudomonadaceae (7227	Pseudomonadales (1236)	Gammaproteobacteria (122	Proteobacteria (2)	Bacteria (1)	100.00	1.80	99.80	98.90	WGS
287.1711	0.3636	Pseudomonas aeruginosa (286)	Pseudomonas (135621)	Pseudomonadaceae (7227	Pseudomonadales (1236)	Gammaproteobacteria (122	Proteobacteria (2)	Bacteria (1)	100.00	0.90	99.90	99.30	WGS
287.1712	0.3636	Pseudomonas aeruginosa (286)	Pseudomonas (135621)	Pseudomonadaceae (7227	Pseudomonadales (1236)	Gammaproteobacteria (122	Proteobacteria (2)	Bacteria (1)	100.00	0.60	99.90	99.00	WGS
287.1713	0.3558	Pseudomonas aeruginosa (286)	Pseudomonas (135621)	Pseudomonadaceae (7227	Pseudomonadales (1236)	Gammaproteobacteria (122	Proteobacteria (2)	Bacteria (1)	100.00	0.00	99.80	99.30	WGS
287.1714	0.3558	Pseudomonas aeruginosa (286)	Pseudomonas (135621)	Pseudomonadaceae (7227	Pseudomonadales (1236)	Gammaproteobacteria (122	Proteobacteria (2)	Bacteria (1)	99.70	1.60	99.50	97.30	WGS
287.1715	0.3636	Pseudomonas aeruginosa (286)	Pseudomonas (135621)	Pseudomonadaceae (7227	Pseudomonadales (1236)	Gammaproteobacteria (122	Proteobacteria (2)	Bacteria (1)	99.70	4.70	99.60	96.20	WGS
287.1716	0.3558	Pseudomonas aeruginosa (286)	Pseudomonas (135621)	Pseudomonadaceae (7227	Pseudomonadales (1236)	Gammaproteobacteria (122	Proteobacteria (2)	Bacteria (1)	100.00	0.00	99.90	99.10	WGS
287.1717	0.3636	Pseudomonas aeruginosa (286)	Pseudomonas (135621)	Pseudomonadaceae (7227	Pseudomonadales (1236)	Gammaproteobacteria (122	Proteobacteria (2)	Bacteria (1)	100.00	0.00	99.90	99.30	WGS
287.1718	0.3636	Pseudomonas aeruginosa (286)	Pseudomonas (135621)	Pseudomonadaceae (7227	Pseudomonadales (1236)	Gammaproteobacteria (122	Proteobacteria (2)	Bacteria (1)	100.00	0.60	99.80	99.00	WGS
287.1719	0.3636	Pseudomonas aeruginosa (286)	Pseudomonas (135621)	Pseudomonadaceae (7227	Pseudomonadales (1236)	Gammaproteobacteria (122	Proteobacteria (2)	Bacteria (1)	100.00	0.90	99.80	99.10	WGS
287.1720	0.3636	Pseudomonas aeruginosa (286)	Pseudomonas (135621)	Pseudomonadaceae (7227	Pseudomonadales (1236)	Gammaproteobacteria (122	Proteobacteria (2)	Bacteria (1)	100.00	0.30	99.80	98.40	WGS
287.1721	0.3636	Pseudomonas aeruginosa (286)	Pseudomonas (135621)	Pseudomonadaceae (7227	Pseudomonadales (1236)	Gammaproteobacteria (122	Proteobacteria (2)	Bacteria (1)	100.00	0.60	99.90	99.30	WGS
287.1722	0.373	Pseudomonas aeruginosa (286)	Pseudomonas (135621)	Pseudomonadaceae (7227	Pseudomonadales (1236)	Gammaproteobacteria (122	Proteobacteria (2)	Bacteria (1)	98.20	4.00	98.90	94.30	WGS
287.1723	0.3688	Pseudomonas aeruginosa (286)	Pseudomonas (135621)	Pseudomonadaceae (7227	Pseudomonadales (1236)	Gammaproteobacteria (122	Proteobacteria (2)	Bacteria (1)	97.40	5.50	98.10	91.10	WGS
287.1724	0.3636	Pseudomonas aeruginosa (286)	Pseudomonas (135621)	Pseudomonadaceae (7227	Pseudomonadales (1236)	Gammaproteobacteria (122	Proteobacteria (2)	Bacteria (1)	100.00	0.90	99.90	99.10	WGS
287.1725	0.3614	Pseudomonas aeruginosa (286)	Pseudomonas (135621)	Pseudomonadaceae (7227	Pseudomonadales (1236)	Gammaproteobacteria (122	Proteobacteria (2)	Bacteria (1)	100.00	0.00	99.90	99.60	WGS
287.1726	0.3118	Pseudomonas aeruginosa (286)	Pseudomonas (135621)	Pseudomonadaceae (7227	Pseudomonadales (1236)	Gammaproteobacteria (122	Proteobacteria (2)	Bacteria (1)	96.40	7.10	95.80	89.70	WGS
287.1727	0.3738	Pseudomonas aeruginosa (286)	Pseudomonas (135621)	Pseudomonadaceae (7227	Pseudomonadales (1236)	Gammaproteobacteria (122	Proteobacteria (2)	Bacteria (1)	99.70	2.20	99.60	97.50	WGS
287.1728	0.3622	Pseudomonas aeruginosa (286)	Pseudomonas (135621)	Pseudomonadaceae (7227	Pseudomonadales (1236)	Gammaproteobacteria (122	Proteobacteria (2)	Bacteria (1)	100.00	0.30	99.50	97.80	WGS
287.1729	0.3658	Pseudomonas aeruginosa (286)	Pseudomonas (135621)	Pseudomonadaceae (7227	Pseudomonadales (1236)	Gammaproteobacteria (122	Proteobacteria (2)	Bacteria (1)	100.00	6.70	99.80	94.00	WGS
287.1730	0.37	Pseudomonas aeruginosa (286)	Pseudomonas (135621)	Pseudomonadaceae (7227	Pseudomonadales (1236)	Gammaproteobacteria (122	Proteobacteria (2)	Bacteria (1)	100.00	1.00	99.90	98.00	WGS
287.1731	0.3336	Pseudomonas aeruginosa (286)	Pseudomonas (135621)	Pseudomonadaceae (7227	Pseudomonadales (1236)	Gammaproteobacteria (122	Proteobacteria (2)	Bacteria (1)	100.00	0.30	99.90	98.80	WGS
287.1732	0.3184	Pseudomonas aeruginosa (286)	Pseudomonas (135621)	Pseudomonadaceae (7227	Pseudomonadales (1236)	Gammaproteobacteria (122	Proteobacteria (2)	Bacteria (1)	100.00	7.50	99.80	91.40	WGS
287.1733	0.4122	Pseudomonas aeruginosa (286)	Pseudomonas (135621)	Pseudomonadaceae (7227	Pseudomonadales (1236)	Gammaproteobacteria (122	Proteobacteria (2)	Bacteria (1)	99.40	3.50	99.50	97.00	WGS
287.1734	0.4122	Pseudomonas aeruginosa (286)	Pseudomonas (135621)	Pseudomonadaceae (7227	Pseudomonadales (1236)	Gammaproteobacteria (122	Proteobacteria (2)	Bacteria (1)	99.40	2.10	99.50	97.20	WGS
287.1735	0.3292	Pseudomonas aeruginosa (286)	Pseudomonas (135621)	Pseudomonadaceae (7227	Pseudomonadales (1236)	Gammaproteobacteria (122	Proteobacteria (2)	Bacteria (1)	99.40	0.00	99.70	98.70	WGS
287.1736	0.3938	Pseudomonas aeruginosa (286)	Pseudomonas (135621)	Pseudomonadaceae (7227	Pseudomonadales (1236)	Gammaproteobacteria (122	Proteobacteria (2)	Bacteria (1)	100.00	1.50	99.70	96.70	WGS
287.1737	0.3334	Pseudomonas aeruginosa (286)	Pseudomonas (135621)	Pseudomonadaceae (7227	Pseudomonadales (1236)	Gammaproteobacteria (122	Proteobacteria (2)	Bacteria (1)	100.00	9.00	99.80	93.00	WGS
287.1738	0.3324	Pseudomonas aeruginosa (286)	Pseudomonas (135621)	Pseudomonadaceae (7227	Pseudomonadales (1236)	Gammaproteobacteria (122	Proteobacteria (2)	Bacteria (1)	100.00	11.70	99.70	88.60	WGS
287.1739	0.3628	Pseudomonas aeruginosa (286)	Pseudomonas (135621)	Pseudomonadaceae (7227	Pseudomonadales (1236)	Gammaproteobacteria (122	Proteobacteria (2)	Bacteria (1)	100.00	1.80	99.90	96.70	WGS
287.1740	0.369	Pseudomonas aeruginosa (286)	Pseudomonas (135621)	Pseudomonadaceae (7227	Pseudomonadales (1236)	Gammaproteobacteria (122	Proteobacteria (2)	Bacteria (1)	100.00	0.00	99.80	99.10	WGS
287.1741	0.3324	Pseudomonas aeruginosa (286)	Pseudomonas (135621)	Pseudomonadaceae (7227	Pseudomonadales (1236)	Gammaproteobacteria (122	Proteobacteria (2)	Bacteria (1)	100.00	0.30	99.70	98.40	WGS
287.1742	0.3324	Pseudomonas aeruginosa (286)	Pseudomonas (135621)	Pseudomonadaceae (7227	Pseudomonadales (1236)	Gammaproteobacteria (122	Proteobacteria (2)	Bacteria (1)	100.00	0.30	99.80	97.90	WGS
287.1743	0.3376	Pseudomonas aeruginosa (286)	Pseudomonas (135621)	Pseudomonadaceae (7227	Pseudomonadales (1236)	Gammaproteobacteria (122	Proteobacteria (2)	Bacteria (1)	98.50	0.40	99.60	98.20	WGS
287.1744	0.3424	Pseudomonas aeruginosa (286)	Pseudomonas (135621)	Pseudomonadaceae (7227	Pseudomonadales (1236)	Gammaproteobacteria (122	Proteobacteria (2)	Bacteria (1)	99.10	4.50	99.50	95.40	WGS
287.1745	0.311	Pseudomonas aeruginosa (286)	Pseudomonas (135621)	Pseudomonadaceae (7227	Pseudomonadales (1236)	Gammaproteobacteria (122	Proteobacteria (2)	Bacteria (1)	100.00	10.30	99.70	90.80	WGS
287.1746	0.3288	Pseudomonas aeruginosa (286)	Pseudomonas (135621)	Pseudomonadaceae (7227	Pseudomonadales (1236)	Gammaproteobacteria (122	Proteobacteria (2)	Bacteria (1)	100.00	0.60	99.80	96.90	WGS
287.1747	0.331	Pseudomonas aeruginosa (286)	Pseudomonas (135621)	Pseudomonadaceae (7227	Pseudomonadales (1236)	Gammaproteobacteria (122	Proteobacteria (2)	Bacteria (1)	100.00	3.10	99.90	95.80	WGS
287.1748	0.371	Pseudomonas aeruginosa (286)	Pseudomonas (135621)	Pseudomonadaceae (7227	Pseudomonadales (1236)	Gammaproteobacteria (122	Proteobacteria (2)	Bacteria (1)	100.00	1.20	99.90	96.60	WGS
287.1749	0.3628	Pseudomonas aeruginosa (286)	Pseudomonas (135621)	Pseudomonadaceae (7227	Pseudomonadales (1236)	Gammaproteobacteria (122	Proteobacteria (2)	Bacteria (1)	100.00	3.80	99.90	96.80	WGS
287.1750	0.3748	Pseudomonas aeruginosa (286)	Pseudomonas (135621)	Pseudomonadaceae (7227	Pseudomonadales (1236)	Gammaproteobacteria (122	Proteobacteria (2)	Bacteria (1)	100.00	3.50	99.80	96.50	WGS
287.1751	0.3386	Pseudomonas aeruginosa (286)	Pseudomonas (135621)	Pseudomonadaceae (7227	Pseudomonadales (1236)	Gammaproteobacteria (122	Proteobacteria (2)	Bacteria (1)	100.00	9.00	99.70	93.90	WGS
287.1752	0.3288	Pseudomonas aeruginosa (286)	Pseudomonas (135621)	Pseudomonadaceae (7227	Pseudomonadales (1236)	Gammaproteobacteria (122	Proteobacteria (2)	Bacteria (1)	100.00	2.10	99.80	96.50	WGS
287.1753	0.3288	Pseudomonas aeruginosa (286)	Pseudomonas (135621)	Pseudomonadaceae (7227	Pseudomonadales (1236)	Gammaproteobacteria (122	Proteobacteria (2)	Bacteria (1)	100.00	0.30	99.80	97.70	WGS
287.1754	0.331	Pseudomonas aeruginosa (286)	Pseudomonas (135621)	Pseudomonadaceae (7227	Pseudomonadales (1236)	Gammaproteobacteria (122	Proteobacteria (2)	Bacteria (1)	100.00	1.80	99.90	97.60	WGS
287.1755	0.368	Pseudomonas aeruginosa (286)	Pseudomonas (135621)	Pseudomonadaceae (7227	Pseudomonadales (1236)	Gammaproteobacteria (122	Proteobacteria (2)	Bacteria (1)	100.00	3.30	99.70	97.10	WGS
287.1756	0.413	Pseudomonas aeruginosa (286)	Pseudomonas (135621)	Pseudomonadaceae (7227	Pseudomonadales (1236)	Gammaproteobacteria (122	Proteobacteria (2)	Bacteria (1)	100.00	47.60	96.70	50.10	WGS
287.1757	0.3376	Pseudomonas aeruginosa (286)	Pseudomonas (135621)	Pseudomonadaceae (7227	Pseudomonadales (1236)	Gammaproteobacteria (122	Proteobacteria (2)	Bacteria (1)	100.00	5.00	99.80	93.90	WGS
287.1758	0.3754	Pseudomonas aeruginosa (286)	Pseudomonas (135621)	Pseudomonadaceae (7227	Pseudomonadales (1236)	Gammaproteobacteria (122	Proteobacteria (2)	Bacteria (1)	100.00	2.30	99.90	97.60	WGS
287.1759	0.371	Pseudomonas aeruginosa (286)	Pseudomonas (135621)	Pseudomonadaceae (7227	Pseudomonadales (1236)	Gammaproteobacteria (122	Proteobacteria (2)	Bacteria (1)	100.00	2.10	99.90	97.50	WGS
287.1760	0.3324	Pseudomonas aeruginosa (286)	Pseudomonas (135621)	Pseudomonadaceae (7227	Pseudomonadales (1236)	Gammaproteobacteria (122	Proteobacteria (2)	Bacteria (1)	99.10	6.10	99.60	93.40	WGS
287.1761	0.3306	Pseudomonas aeruginosa (286)	Pseudomonas (135621)	Pseudomonadaceae (7227	Pseudomonadales (1236)	Gammaproteobacteria (122	Proteobacteria (2)	Bacteria (1)	100.00	0.60	99.90	97.40	WGS
287.1762	0.3754	Pseudomonas aeruginosa (286)	Pseudomonas (135621)	Pseudomonadaceae (7227	Pseudomonadales (1236)	Gammaproteobacteria (122	Proteobacteria (2)	Bacteria (1)	100.00	1.50	99.90	98.40	WGS
287.1763	0.3324	Pseudomonas aeruginosa (286)	Pseudomonas (135621)	Pseudomonadaceae (7227	Pseudomonadales (1236)	Gammaproteobacteria (122	Proteobacteria (2)	Bacteria (1)	99.10	0.90	99.60	97.60	WGS
287.1764	0.3306	Pseudomonas aeruginosa (286)	Pseudomonas (135621)	Pseudomonadaceae (7227	Pseudomonadales (1236)	Gammaproteobacteria (122	Proteobacteria (2)	Bacteria (1)	100.00	1.20	99.90	96.70	WGS
287.1765	0.3754	Pseudomonas aeruginosa (286)	Pseudomonas (135621)	Pseudomonadaceae (7227	Pseudomonadales (1236)	Gammaproteobacteria (122	Proteobacteria (2)	Bacteria (1)	100.00	0.30	99.90	98.40	WGS
287.1766	0.3306	Pseudomonas aeruginosa (286)	Pseudomonas (135621)	Pseudomonadaceae (7227	Pseudomonadales (1236)	Gammaproteobacteria (122	Proteobacteria (2)	Bacteria (1)	100.00	0.30	99.90	97.40	WGS
287.1767	0.3738	Pseudomonas aeruginosa (286)	Pseudomonas (135621)	Pseudomonadaceae (7227	Pseudomonadales (1236)	Gammaproteobacteria (122	Proteobacteria (2)	Bacteria (1)	100.00	1.80	99.80	96.70	WGS
287.1768	0.357	Pseudomonas aeruginosa (286)	Pseudomonas (135621)	Pseudomonadaceae (7227	Pseudomonadales (1236)	Gammaproteobacteria (122	Proteobacteria (2)	Bacteria (1)	100.00	4.30	99.90	95.80	WGS
287.1769	0.368	Pseudomonas aeruginosa (286)	Pseudomonas (135621)	Pseudomonadaceae (7227	Pseudomonadales (1236)	Gammaproteobacteria (122	Proteobacteria (2)	Bacteria (1)	97.00	0.00	99.80	98.80	WGS
287.1770	0.3672	Pseudomonas aeruginosa (286)	Pseudomonas (135621)	Pseudomonadaceae (7227	Pseudomonadales (1236)	Gammaproteobacteria (122	Proteobacteria (2)	Bacteria (1)	100.00	0.30	99.90	98.90	WGS
287.1771	0.3672	Pseudomonas aeruginosa (286)	Pseudomonas (135621)	Pseudomonadaceae (7227	Pseudomonadales (1236)	Gammaproteobacteria (122	Proteobacteria (2)	Bacteria (1)	100.00	0.30	99.90	98.70	WGS
287.1772	0.3748	Pseudomonas aeruginosa (286)	Pseudomonas (135621)	Pseudomonadaceae (7227	Pseudomonadales (1236)	Gammaproteobacteria (122	Proteobacteria (2)	Bacteria (1)	100.00	0.60	99.80	99.00	WGS
287.1773	0.3672	Pseudomonas aeruginosa (286)	Pseudomonas (135621)	Pseudomonadaceae (7227	Pseudomonadales (1236)	Gammaproteobacteria (122	Proteobacteria (2)	Bacteria (1)	100.00	0.30	99.90	98.90	Complete
287.1774	0.3672	Pseudomonas aeruginosa (286)	Pseudomonas (135621)	Pseudomonadaceae (7227	Pseudomonadales (1236)	Gammaproteobacteria (122	Proteobacteria (2)	Bacteria (1)	100.00	0.30	99.90	98.90	WGS
287.1775	0.3672	Pseudomonas aeruginosa (286)	Pseudomonas (135621)	Pseudomonadaceae (7227	Pseudomonadales (1236)	Gammaproteobacteria (122	Proteobacteria (2)	Bacteria (1)	100.00	0.30	99.90	98.90	WGS
287.1776	0.3188	Pseudomonas aeruginosa (286)	Pseudomonas (135621)	Pseudomonadaceae (7227	Pseudomonadales (1236)	Gammaproteobacteria (122	Proteobacteria (2)	Bacteria (1)	100.00	0.60	99.80	99.30	WGS
287.1777	0.337	Pseudomonas aeruginosa (286)	Pseudomonas (135621)	Pseudomonadaceae (7227	Pseudomonadales (1236)	Gammaproteobacteria (122	Proteobacteria (2)	Bacteria (1)	100.00	0.90	99.80	97.60	WGS
287.1778	0.3304	Pseudomonas aeruginosa (286)	Pseudomonas (135621)	Pseudomonadaceae (7227	Pseudomonadales (1236)	Gammaproteobacteria (122	Proteobacteria (2)	Bacteria (1)	100.00	0.70	99.70	97.10	WGS
287.1779	0.3314	Pseudomonas aeruginosa (286)	Pseudomonas (135621)	Pseudomonadaceae (7227	Pseudomonadales (1236)	Gammaproteobacteria (122	Proteobacteria (2)	Bacteria (1)	100.00	1.20	99.60	97.70	WGS
287.1780	0.3054	Pseudomonas aeruginosa (286)	Pseudomonas (135621)	Pseudomonadaceae (7227	Pseudomonadales (1236)	Gammaproteobacteria (122	Proteobacteria (2)	Bacteria (1)	100.00	6.30	99.80	93.80	WGS
287.1781	0.3314	Pseudomonas aeruginosa (286)	Pseudomonas (135621)	Pseudomonadaceae (7227	Pseudomonadales (1236)	Gammaproteobacteria (122	Proteobacteria (2)	Bacteria (1)	100.00	0.90	99.80	97.60	WGS
287.1782	0.3314	Pseudomonas aeruginosa (286)	Pseudomonas (135621)	Pseudomonadaceae (7227	Pseudomonadales (1236)	Gammaproteobacteria (122	Proteobacteria (2)	Bacteria (1)	100.00	0.60	99.80	97.60	WGS
287.1783	0.3314	Pseudomonas aeruginosa (286)	Pseudomonas (135621)	Pseudomonadaceae (7227	Pseudomonadales (1236)	Gammaproteobacteria (122	Proteobacteria (2)	Bacteria (1)	100.00	0.60	99.80	97.90	WGS
287.1784	0.3314	Pseudomonas aeruginosa (286)	Pseudomonas (135621)	Pseudomonadaceae (7227	Pseudomonadales (1236)	Gammaproteobacteria (122	Proteobacteria (2)	Bacteria (1)	100.00	0.90	99.80	98.40	WGS
287.1785	0.3314	Pseudomonas aeruginosa (286)	Pseudomonas (135621)	Pseudomonadaceae (7227	Pseudomonadales (1236)	Gammaproteobacteria (122	Proteobacteria (2)	Bacteria (1)	100.00	0.60	99.80	99.00	WGS
287.1786	0.337	Pseudomonas aeruginosa (286)	Pseudomonas (135621)	Pseudomonadaceae (7227	Pseudomonadales (1236)	Gammaproteobacteria (122	Proteobacteria (2)	Bacteria (1)	100.00	1.90	99.80	96.50	WGS
287.1787	0.3314	Pseudomonas aeruginosa (286)	Pseudomonas (135621)	Pseudomonadaceae (7227	Pseudomonadales (1236)	Gammaproteobacteria (122	Proteobacteria (2)	Bacteria (1)	100.00	0.60	99.80	97.90	WGS
287.1788	0.3314	Pseudomonas aeruginosa (286)	Pseudomonas (135621)	Pseudomonadaceae (7227	Pseudomonadales (1236)	Gammaproteobacteria (122	Proteobacteria (2)	Bacteria (1)	100.00	0.60	99.60	97.80	WGS
287.1789	0.337	Pseudomonas aeruginosa (286)	Pseudomonas (135621)	Pseudomonadaceae (7227	Pseudomonadales (1236)	Gammaproteobacteria (122	Proteobacteria (2)	Bacteria (1)	100.00	0.00	99.80	98.20	WGS
287.1790	0.3314	Pseudomonas aeruginosa (286)	Pseudomonas (135621)	Pseudomonadaceae (7227	Pseudomonadales (1236)	Gammaproteobacteria (122	Proteobacteria (2)	Bacteria (1)	99.70	0.30	99.80	98.80	WGS
287.1791	0.3314	Pseudomonas aeruginosa (286)	Pseudomonas (135621)	Pseudomonadaceae (7227	Pseudomonadales (1236)	Gammaproteobacteria (122	Proteobacteria (2)	Bacteria (1)	99.70	0.30	99.80	98.80	WGS
287.1792	0.3314	Pseudomonas aeruginosa (286)	Pseudomonas (135621)	Pseudomonadaceae (7227	Pseudomonadales (1236)	Gammaproteobacteria (122	Proteobacteria (2)	Bacteria (1)	100.00	0.30	99.80	98.80	WGS
287.1793	0.3314	Pseudomonas aeruginosa (286)	Pseudomonas (135621)	Pseudomonadaceae (7227	Pseudomonadales (1236)	Gammaproteobacteria (122	Proteobacteria (2)	Bacteria (1)	100.00	0.60	99.70	98.70	WGS
287.1794	0.3304	Pseudomonas aeruginosa (286)	Pseudomonas (135621)	Pseudomonadaceae (7227	Pseudomonadales (1236)	Gammaproteobacteria (122	Proteobacteria (2)	Bacteria (1)	100.00	1.20	99.80	98.40	WGS
287.1795	0.3314	Pseudomonas aeruginosa (286)	Pseudomonas (135621)	Pseudomonadaceae (7227	Pseudomonadales (1236)	Gammaproteobacteria (122	Proteobacteria (2)	Bacteria (1)	99.70	0.90	99.80	98.70	WGS
287.1796	0.3314	Pseudomonas aeruginosa (286)	Pseudomonas (135621)	Pseudomonadaceae (7227	Pseudomonadales (1236)	Gammaproteobacteria (122	Proteobacteria (2)	Bacteria (1)	100.00	1.50	99.80	98.00	WGS
287.1797	0.3314	Pseudomonas aeruginosa (286)	Pseudomonas (135621)	Pseudomonadaceae (7227	Pseudomonadales (1236)	Gammaproteobacteria (122	Proteobacteria (2)	Bacteria (1)	100.00	0.60	99.80	98.20	WGS
287.1798	0.3314	Pseudomonas aeruginosa (286)	Pseudomonas (135621)	Pseudomonadaceae (7227	Pseudomonadales (1236)	Gammaproteobacteria (122	Proteobacteria (2)	Bacteria (1)	100.00	4.30	99.80	96.20	WGS
287.1799	0.3314	Pseudomonas aeruginosa (286)	Pseudomonas (135621)	Pseudomonadaceae (7227	Pseudomonadales (1236)	Gammaproteobacteria (122	Proteobacteria (2)	Bacteria (1)	100.00	0.30	99.80	98.40	WGS
287.1800	0.3314	Pseudomonas aeruginosa (286)	Pseudomonas (135621)	Pseudomonadaceae (7227	Pseudomonadales (1236)	Gammaproteobacteria (122	Proteobacteria (2)	Bacteria (1)	100.00	0.30	99.80	98.70	WGS
287.1801	0.3314	Pseudomonas aeruginosa (286)	Pseudomonas (135621)	Pseudomonadaceae (7227	Pseudomonadales (1236)	Gammaproteobacteria (122	Proteobacteria (2)	Bacteria (1)	100.00	0.30	99.80	98.70	WGS
287.1802	0.3314	Pseudomonas aeruginosa (286)	Pseudomonas (135621)	Pseudomonadaceae (7227	Pseudomonadales (1236)	Gammaproteobacteria (122	Proteobacteria (2)	Bacteria (1)	100.00	0.60	99.80	98.90	WGS
287.1803	0.337	Pseudomonas aeruginosa (286)	Pseudomonas (135621)	Pseudomonadaceae (7227	Pseudomonadales (1236)	Gammaproteobacteria (122	Proteobacteria (2)	Bacteria (1)	100.00	0.00	99.80	98.40	WGS
287.1804	0.3314	Pseudomonas aeruginosa (286)	Pseudomonas (135621)	Pseudomonadaceae (7227	Pseudomonadales (1236)	Gammaproteobacteria (122	Proteobacteria (2)	Bacteria (1)	100.00	0.30	99.90	98.70	WGS
287.1805	0.3314	Pseudomonas aeruginosa (286)	Pseudomonas (135621)	Pseudomonadaceae (7227	Pseudomonadales (1236)	Gammaproteobacteria (122	Proteobacteria (2)	Bacteria (1)	100.00	0.30	99.80	98.70	WGS
287.1806	0.325	Pseudomonas aeruginosa (286)	Pseudomonas (135621)	Pseudomonadaceae (7227	Pseudomonadales (1236)	Gammaproteobacteria (122	Proteobacteria (2)	Bacteria (1)	100.00	0.00	99.80	98.50	WGS
287.1807	0.3316	Pseudomonas aeruginosa (286)	Pseudomonas (135621)	Pseudomonadaceae (7227	Pseudomonadales (1236)	Gammaproteobacteria (122	Proteobacteria (2)	Bacteria (1)	100.00	0.00	99.80	98.70	WGS
287.1808	0.3304	Pseudomonas aeruginosa (286)	Pseudomonas (135621)	Pseudomonadaceae (7227	Pseudomonadales (1236)	Gammaproteobacteria (122	Proteobacteria (2)	Bacteria (1)	100.00	0.90	99.80	98.40	WGS
287.1809	0.3314	Pseudomonas aeruginosa (286)	Pseudomonas (135621)	Pseudomonadaceae (7227	Pseudomonadales (1236)	Gammaproteobacteria (122	Proteobacteria (2)	Bacteria (1)	100.00	0.30	99.80	98.70	WGS
287.1810	0.3314	Pseudomonas aeruginosa (286)	Pseudomonas (135621)	Pseudomonadaceae (7227	Pseudomonadales (1236)	Gammaproteobacteria (122	Proteobacteria (2)	Bacteria (1)	100.00	0.00	99.80	98.70	WGS
287.1811	0.325	Pseudomonas aeruginosa (286)	Pseudomonas (135621)	Pseudomonadaceae (7227	Pseudomonadales (1236)	Gammaproteobacteria (122	Proteobacteria (2)	Bacteria (1)	100.00	0.00	99.80	98.70	WGS
287.1812	0.3314	Pseudomonas aeruginosa (286)	Pseudomonas (135621)	Pseudomonadaceae (7227	Pseudomonadales (1236)	Gammaproteobacteria (122	Proteobacteria (2)	Bacteria (1)	100.00	0.90	99.80	98.60	WGS
287.1813	0.338	Pseudomonas aeruginosa (286)	Pseudomonas (135621)	Pseudomonadaceae (7227	Pseudomonadales (1236)	Gammaproteobacteria (122	Proteobacteria (2)	Bacteria (1)	100.00	0.00	99.80	98.70	WGS
287.1814	0.3314	Pseudomonas aeruginosa (286)	Pseudomonas (135621)	Pseudomonadaceae (7227	Pseudomonadales (1236)	Gammaproteobacteria (122	Proteobacteria (2)	Bacteria (1)	100.00	0.00	99.80	98.70	WGS
287.1815	0.3314	Pseudomonas aeruginosa (286)	Pseudomonas (135621)	Pseudomonadaceae (7227	Pseudomonadales (1236)	Gammaproteobacteria (122	Proteobacteria (2)	Bacteria (1)	100.00	0.30	99.80	97.90	WGS
287.1816	0.3314	Pseudomonas aeruginosa (286)	Pseudomonas (135621)	Pseudomonadaceae (7227	Pseudomonadales (1236)	Gammaproteobacteria (122	Proteobacteria (2)	Bacteria (1)	100.00	0.30	99.80	98.70	WGS
287.1817	0.3314	Pseudomonas aeruginosa (286)	Pseudomonas (135621)	Pseudomonadaceae (7227	Pseudomonadales (1236)	Gammaproteobacteria (122	Proteobacteria (2)	Bacteria (1)	100.00	0.60	99.80	99.00	WGS
287.1818	0.3314	Pseudomonas aeruginosa (286)	Pseudomonas (135621)	Pseudomonadaceae (7227	Pseudomonadales (1236)	Gammaproteobacteria (122	Proteobacteria (2)	Bacteria (1)	100.00	0.30	99.80	98.90	WGS
287.1819	0.3304	Pseudomonas aeruginosa (286)	Pseudomonas (135621)	Pseudomonadaceae (7227	Pseudomonadales (1236)	Gammaproteobacteria (122	Proteobacteria (2)	Bacteria (1)	100.00	0.00	99.80	98.70	WGS
287.1820	0.338	Pseudomonas aeruginosa (286)	Pseudomonas (135621)	Pseudomonadaceae (7227	Pseudomonadales (1236)	Gammaproteobacteria (122	Proteobacteria (2)	Bacteria (1)	100.00	0.00	99.90	98.60	WGS
287.1821	0.3304	Pseudomonas aeruginosa (286)	Pseudomonas (135621)	Pseudomonadaceae (7227	Pseudomonadales (1236)	Gammaproteobacteria (122	Proteobacteria (2)	Bacteria (1)	100.00	0.00	99.80	98.50	WGS
287.1822	0.3314	Pseudomonas aeruginosa (286)	Pseudomonas (135621)	Pseudomonadaceae (7227	Pseudomonadales (1236)	Gammaproteobacteria (122	Proteobacteria (2)	Bacteria (1)	100.00	2.30	99.80	97.70	WGS
287.1823	0.338	Pseudomonas aeruginosa (286)	Pseudomonas (135621)	Pseudomonadaceae (7227	Pseudomonadales (1236)	Gammaproteobacteria (122	Proteobacteria (2)	Bacteria (1)	100.00	1.20	99.80	97.80	WGS
287.1824	0.3314	Pseudomonas aeruginosa (286)	Pseudomonas (135621)	Pseudomonadaceae (7227	Pseudomonadales (1236)	Gammaproteobacteria (122	Proteobacteria (2)	Bacteria (1)	100.00	0.30	99.80	98.80	WGS
287.1825	0.331	Pseudomonas aeruginosa (286)	Pseudomonas (135621)	Pseudomonadaceae (7227	Pseudomonadales (1236)	Gammaproteobacteria (122	Proteobacteria (2)	Bacteria (1)	100.00	5.70	99.80	94.20	WGS
287.1826	0.3314	Pseudomonas aeruginosa (286)	Pseudomonas (135621)	Pseudomonadaceae (7227	Pseudomonadales (1236)	Gammaproteobacteria (122	Proteobacteria (2)	Bacteria (1)	100.00	7.60	99.80	92.50	WGS
287.1827	0.3314	Pseudomonas aeruginosa (286)	Pseudomonas (135621)	Pseudomonadaceae (7227	Pseudomonadales (1236)	Gammaproteobacteria (122	Proteobacteria (2)	Bacteria (1)	100.00	1.80	99.80	97.00	WGS
287.1828	0.3304	Pseudomonas aeruginosa (286)	Pseudomonas (135621)	Pseudomonadaceae (7227	Pseudomonadales (1236)	Gammaproteobacteria (122	Proteobacteria (2)	Bacteria (1)	100.00	0.00	99.80	98.40	WGS
287.1829	0.3314	Pseudomonas aeruginosa (286)	Pseudomonas (135621)	Pseudomonadaceae (7227	Pseudomonadales (1236)	Gammaproteobacteria (122	Proteobacteria (2)	Bacteria (1)	99.70	3.20	99.70	95.00	WGS
287.1830	0.3054	Pseudomonas aeruginosa (286)	Pseudomonas (135621)	Pseudomonadaceae (7227	Pseudomonadales (1236)	Gammaproteobacteria (122	Proteobacteria (2)	Bacteria (1)	99.40	7.30	99.60	92.20	WGS
287.1831	0.3304	Pseudomonas aeruginosa (286)	Pseudomonas (135621)	Pseudomonadaceae (7227	Pseudomonadales (1236)	Gammaproteobacteria (122	Proteobacteria (2)	Bacteria (1)	100.00	1.20	99.80	97.60	WGS
287.1832	0.338	Pseudomonas aeruginosa (286)	Pseudomonas (135621)	Pseudomonadaceae (7227	Pseudomonadales (1236)	Gammaproteobacteria (122	Proteobacteria (2)	Bacteria (1)	100.00	0.00	99.80	98.60	WGS
287.1833	0.338	Pseudomonas aeruginosa (286)	Pseudomonas (135621)	Pseudomonadaceae (7227	Pseudomonadales (1236)	Gammaproteobacteria (122	Proteobacteria (2)	Bacteria (1)	100.00	0.00	99.80	98.70	WGS
287.1834	0.338	Pseudomonas aeruginosa (286)	Pseudomonas (135621)	Pseudomonadaceae (7227	Pseudomonadales (1236)	Gammaproteobacteria (122	Proteobacteria (2)	Bacteria (1)	100.00	0.60	99.80	97.90	WGS
287.1835	0.338	Pseudomonas aeruginosa (286)	Pseudomonas (135621)	Pseudomonadaceae (7227	Pseudomonadales (1236)	Gammaproteobacteria (122	Proteobacteria (2)	Bacteria (1)	100.00	0.90	99.80	97.00	WGS
287.1836	0.3314	Pseudomonas aeruginosa (286)	Pseudomonas (135621)	Pseudomonadaceae (7227	Pseudomonadales (1236)	Gammaproteobacteria (122	Proteobacteria (2)	Bacteria (1)	100.00	0.90	99.80	98.30	WGS
287.1837	0.338	Pseudomonas aeruginosa (286)	Pseudomonas (135621)	Pseudomonadaceae (7227	Pseudomonadales (1236)	Gammaproteobacteria (122	Proteobacteria (2)	Bacteria (1)	100.00	0.00	99.80	98.60	WGS
287.1838	0.3314	Pseudomonas aeruginosa (286)	Pseudomonas (135621)	Pseudomonadaceae (7227	Pseudomonadales (1236)	Gammaproteobacteria (122	Proteobacteria (2)	Bacteria (1)	100.00	0.90	99.80	98.10	WGS
287.1839	0.338	Pseudomonas aeruginosa (286)	Pseudomonas (135621)	Pseudomonadaceae (7227	Pseudomonadales (1236)	Gammaproteobacteria (122	Proteobacteria (2)	Bacteria (1)	100.00	2.30	99.80	97.20	WGS
287.1840	0.338	Pseudomonas aeruginosa (286)	Pseudomonas (135621)	Pseudomonadaceae (7227	Pseudomonadales (1236)	Gammaproteobacteria (122	Proteobacteria (2)	Bacteria (1)	100.00	7.00	99.90	93.20	WGS
287.1841	0.3314	Pseudomonas aeruginosa (286)	Pseudomonas (135621)	Pseudomonadaceae (7227	Pseudomonadales (1236)	Gammaproteobacteria (122	Proteobacteria (2)	Bacteria (1)	100.00	0.30	99.80	98.40	WGS
287.1842	0.338	Pseudomonas aeruginosa (286)	Pseudomonas (135621)	Pseudomonadaceae (7227	Pseudomonadales (1236)	Gammaproteobacteria (122	Proteobacteria (2)	Bacteria (1)	100.00	1.50	99.80	97.80	WGS
287.1843	0.3314	Pseudomonas aeruginosa (286)	Pseudomonas (135621)	Pseudomonadaceae (7227	Pseudomonadales (1236)	Gammaproteobacteria (122	Proteobacteria (2)	Bacteria (1)	100.00	0.30	99.70	98.70	WGS
287.1844	0.3314	Pseudomonas aeruginosa (286)	Pseudomonas (135621)	Pseudomonadaceae (7227	Pseudomonadales (1236)	Gammaproteobacteria (122	Proteobacteria (2)	Bacteria (1)	100.00	0.60	99.80	98.70	WGS
287.1845	0.334	Pseudomonas aeruginosa (286)	Pseudomonas (135621)	Pseudomonadaceae (7227	Pseudomonadales (1236)	Gammaproteobacteria (122	Proteobacteria (2)	Bacteria (1)	100.00	1.00	99.80	97.60	WGS
287.1846	0.3054	Pseudomonas aeruginosa (286)	Pseudomonas (135621)	Pseudomonadaceae (7227	Pseudomonadales (1236)	Gammaproteobacteria (122	Proteobacteria (2)	Bacteria (1)	100.00	0.60	99.80	98.80	WGS
287.1847	0.3314	Pseudomonas aeruginosa (286)	Pseudomonas (135621)	Pseudomonadaceae (7227	Pseudomonadales (1236)	Gammaproteobacteria (122	Proteobacteria (2)	Bacteria (1)	100.00	2.30	99.80	97.60	WGS
287.1848	0.3054	Pseudomonas aeruginosa (286)	Pseudomonas (135621)	Pseudomonadaceae (7227	Pseudomonadales (1236)	Gammaproteobacteria (122	Proteobacteria (2)	Bacteria (1)	100.00	0.30	99.80	97.60	WGS
287.1849	0.3314	Pseudomonas aeruginosa (286)	Pseudomonas (135621)	Pseudomonadaceae (7227	Pseudomonadales (1236)	Gammaproteobacteria (122	Proteobacteria (2)	Bacteria (1)	100.00	0.30	99.80	98.80	WGS
287.1850	0.3314	Pseudomonas aeruginosa (286)	Pseudomonas (135621)	Pseudomonadaceae (7227	Pseudomonadales (1236)	Gammaproteobacteria (122	Proteobacteria (2)	Bacteria (1)	100.00	0.30	99.80	98.70	WGS
287.1851	0.3314	Pseudomonas aeruginosa (286)	Pseudomonas (135621)	Pseudomonadaceae (7227	Pseudomonadales (1236)	Gammaproteobacteria (122	Proteobacteria (2)	Bacteria (1)	100.00	0.30	99.60	98.60	WGS
287.1852	0.3314	Pseudomonas aeruginosa (286)	Pseudomonas (135621)	Pseudomonadaceae (7227	Pseudomonadales (1236)	Gammaproteobacteria (122	Proteobacteria (2)	Bacteria (1)	100.00	0.60	99.80	98.70	WGS
287.1853	0.3304	Pseudomonas aeruginosa (286)	Pseudomonas (135621)	Pseudomonadaceae (7227	Pseudomonadales (1236)	Gammaproteobacteria (122	Proteobacteria (2)	Bacteria (1)	100.00	0.30	99.80	98.40	WGS
287.1854	0.3316	Pseudomonas aeruginosa (286)	Pseudomonas (135621)	Pseudomonadaceae (7227	Pseudomonadales (1236)	Gammaproteobacteria (122	Proteobacteria (2)	Bacteria (1)	100.00	0.00	99.70	98.60	WGS
287.1855	0.338	Pseudomonas aeruginosa (286)	Pseudomonas (135621)	Pseudomonadaceae (7227	Pseudomonadales (1236)	Gammaproteobacteria (122	Proteobacteria (2)	Bacteria (1)	100.00	0.60	99.80	98.70	WGS
287.1856	0.338	Pseudomonas aeruginosa (286)	Pseudomonas (135621)	Pseudomonadaceae (7227	Pseudomonadales (1236)	Gammaproteobacteria (122	Proteobacteria (2)	Bacteria (1)	100.00	0.90	99.80	98.50	WGS
287.1857	0.3304	Pseudomonas aeruginosa (286)	Pseudomonas (135621)	Pseudomonadaceae (7227	Pseudomonadales (1236)	Gammaproteobacteria (122	Proteobacteria (2)	Bacteria (1)	100.00	0.60	99.80	98.30	WGS
287.1858	0.338	Pseudomonas aeruginosa (286)	Pseudomonas (135621)	Pseudomonadaceae (7227	Pseudomonadales (1236)	Gammaproteobacteria (122	Proteobacteria (2)	Bacteria (1)	100.00	1.50	99.80	97.50	WGS
287.1859	0.338	Pseudomonas aeruginosa (286)	Pseudomonas (135621)	Pseudomonadaceae (7227	Pseudomonadales (1236)	Gammaproteobacteria (122	Proteobacteria (2)	Bacteria (1)	100.00	0.30	99.80	98.20	WGS
287.1860	0.4024	Pseudomonas aeruginosa (286)	Pseudomonas (135621)	Pseudomonadaceae (7227	Pseudomonadales (1236)	Gammaproteobacteria (122	Proteobacteria (2)	Bacteria (1)	99.70	39.40	97.90	56.00	WGS
287.1861	0.322	Pseudomonas aeruginosa (286)	Pseudomonas (135621)	Pseudomonadaceae (7227	Pseudomonadales (1236)	Gammaproteobacteria (122	Proteobacteria (2)	Bacteria (1)	100.00	14.70	99.90	87.70	WGS
287.1862	0.325	Pseudomonas aeruginosa (286)	Pseudomonas (135621)	Pseudomonadaceae (7227	Pseudomonadales (1236)	Gammaproteobacteria (122	Proteobacteria (2)	Bacteria (1)	100.00	0.00	99.80	98.50	WGS
287.1863	0.3604	Pseudomonas aeruginosa (286)	Pseudomonas (135621)	Pseudomonadaceae (7227	Pseudomonadales (1236)	Gammaproteobacteria (122	Proteobacteria (2)	Bacteria (1)	98.10	1.20	99.60	98.10	WGS
287.1864	0.368	Pseudomonas aeruginosa (286)	Pseudomonas (135621)	Pseudomonadaceae (7227	Pseudomonadales (1236)	Gammaproteobacteria (122	Proteobacteria (2)	Bacteria (1)	98.70	1.20	99.70	98.20	WGS
287.1865	0.368	Pseudomonas aeruginosa (286)	Pseudomonas (135621)	Pseudomonadaceae (7227	Pseudomonadales (1236)	Gammaproteobacteria (122	Proteobacteria (2)	Bacteria (1)	98.40	1.50	99.60	97.90	WGS
287.1866	0.3694	Pseudomonas aeruginosa (286)	Pseudomonas (135621)	Pseudomonadaceae (7227	Pseudomonadales (1236)	Gammaproteobacteria (122	Proteobacteria (2)	Bacteria (1)	99.40	0.90	99.70	98.40	Complete
287.1867	0.3696	Pseudomonas aeruginosa (286)	Pseudomonas (135621)	Pseudomonadaceae (7227	Pseudomonadales (1236)	Gammaproteobacteria (122	Proteobacteria (2)	Bacteria (1)	98.30	3.00	99.60	98.40	WGS
287.1868	0.3636	Pseudomonas aeruginosa (286)	Pseudomonas (135621)	Pseudomonadaceae (7227	Pseudomonadales (1236)	Gammaproteobacteria (122	Proteobacteria (2)	Bacteria (1)	99.10	1.60	99.60	97.90	WGS
287.1869	0.3324	Pseudomonas aeruginosa (286)	Pseudomonas (135621)	Pseudomonadaceae (7227	Pseudomonadales (1236)	Gammaproteobacteria (122	Proteobacteria (2)	Bacteria (1)	100.00	0.00	99.90	98.70	WGS
287.1870	0.3322	Pseudomonas aeruginosa (286)	Pseudomonas (135621)	Pseudomonadaceae (7227	Pseudomonadales (1236)	Gammaproteobacteria (122	Proteobacteria (2)	Bacteria (1)	100.00	4.30	99.90	97.10	WGS
287.1871	0.3322	Pseudomonas aeruginosa (286)	Pseudomonas (135621)	Pseudomonadaceae (7227	Pseudomonadales (1236)	Gammaproteobacteria (122	Proteobacteria (2)	Bacteria (1)	100.00	4.70	99.90	95.90	WGS
287.1874	0.3268	Pseudomonas aeruginosa (286)	Pseudomonas (135621)	Pseudomonadaceae (7227	Pseudomonadales (1236)	Gammaproteobacteria (122	Proteobacteria (2)	Bacteria (1)	100.00	0.00	99.90	99.30	WGS
287.1875	0.3268	Pseudomonas aeruginosa (286)	Pseudomonas (135621)	Pseudomonadaceae (7227	Pseudomonadales (1236)	Gammaproteobacteria (122	Proteobacteria (2)	Bacteria (1)	100.00	0.00	99.90	99.30	WGS
287.1876	0.3268	Pseudomonas aeruginosa (286)	Pseudomonas (135621)	Pseudomonadaceae (7227	Pseudomonadales (1236)	Gammaproteobacteria (122	Proteobacteria (2)	Bacteria (1)	100.00	0.60	99.90	98.80	WGS
287.1877	0.3268	Pseudomonas aeruginosa (286)	Pseudomonas (135621)	Pseudomonadaceae (7227	Pseudomonadales (1236)	Gammaproteobacteria (122	Proteobacteria (2)	Bacteria (1)	100.00	0.30	99.90	99.10	WGS
287.1878	0.3268	Pseudomonas aeruginosa (286)	Pseudomonas (135621)	Pseudomonadaceae (7227	Pseudomonadales (1236)	Gammaproteobacteria (122	Proteobacteria (2)	Bacteria (1)	100.00	0.00	99.90	99.20	WGS
287.1879	0.3268	Pseudomonas aeruginosa (286)	Pseudomonas (135621)	Pseudomonadaceae (7227	Pseudomonadales (1236)	Gammaproteobacteria (122	Proteobacteria (2)	Bacteria (1)	100.00	0.30	99.90	99.10	WGS
287.1880	0.3268	Pseudomonas aeruginosa (286)	Pseudomonas (135621)	Pseudomonadaceae (7227	Pseudomonadales (1236)	Gammaproteobacteria (122	Proteobacteria (2)	Bacteria (1)	100.00	0.30	99.90	99.10	WGS
287.1881	0.3268	Pseudomonas aeruginosa (286)	Pseudomonas (135621)	Pseudomonadaceae (7227	Pseudomonadales (1236)	Gammaproteobacteria (122	Proteobacteria (2)	Bacteria (1)	100.00	0.30	99.80	99.00	WGS
287.1882	0.3268	Pseudomonas aeruginosa (286)	Pseudomonas (135621)	Pseudomonadaceae (7227	Pseudomonadales (1236)	Gammaproteobacteria (122	Proteobacteria (2)	Bacteria (1)	100.00	0.00	99.90	99.30	WGS
287.1883	0.3268	Pseudomonas aeruginosa (286)	Pseudomonas (135621)	Pseudomonadaceae (7227	Pseudomonadales (1236)	Gammaproteobacteria (122	Proteobacteria (2)	Bacteria (1)	100.00	0.00	99.90	99.30	WGS
287.1884	0.3268	Pseudomonas aeruginosa (286)	Pseudomonas (135621)	Pseudomonadaceae (7227	Pseudomonadales (1236)	Gammaproteobacteria (122	Proteobacteria (2)	Bacteria (1)	100.00	0.00	99.90	99.30	WGS
287.1885	0.3268	Pseudomonas aeruginosa (286)	Pseudomonas (135621)	Pseudomonadaceae (7227	Pseudomonadales (1236)	Gammaproteobacteria (122	Proteobacteria (2)	Bacteria (1)	100.00	0.00	99.90	99.30	WGS
287.1886	0.3268	Pseudomonas aeruginosa (286)	Pseudomonas (135621)	Pseudomonadaceae (7227	Pseudomonadales (1236)	Gammaproteobacteria (122	Proteobacteria (2)	Bacteria (1)	100.00	0.00	99.90	99.30	WGS
287.1887	0.3268	Pseudomonas aeruginosa (286)	Pseudomonas (135621)	Pseudomonadaceae (7227	Pseudomonadales (1236)	Gammaproteobacteria (122	Proteobacteria (2)	Bacteria (1)	100.00	0.00	99.90	99.20	WGS
287.1888	0.3268	Pseudomonas aeruginosa (286)	Pseudomonas (135621)	Pseudomonadaceae (7227	Pseudomonadales (1236)	Gammaproteobacteria (122	Proteobacteria (2)	Bacteria (1)	100.00	0.00	99.90	99.30	WGS
287.1889	0.3268	Pseudomonas aeruginosa (286)	Pseudomonas (135621)	Pseudomonadaceae (7227	Pseudomonadales (1236)	Gammaproteobacteria (122	Proteobacteria (2)	Bacteria (1)	100.00	0.00	99.90	99.20	WGS
287.1890	0.3268	Pseudomonas aeruginosa (286)	Pseudomonas (135621)	Pseudomonadaceae (7227	Pseudomonadales (1236)	Gammaproteobacteria (122	Proteobacteria (2)	Bacteria (1)	100.00	0.00	99.90	99.30	WGS
287.1891	0.3268	Pseudomonas aeruginosa (286)	Pseudomonas (135621)	Pseudomonadaceae (7227	Pseudomonadales (1236)	Gammaproteobacteria (122	Proteobacteria (2)	Bacteria (1)	100.00	0.00	99.90	99.30	WGS
287.1892	0.3268	Pseudomonas aeruginosa (286)	Pseudomonas (135621)	Pseudomonadaceae (7227	Pseudomonadales (1236)	Gammaproteobacteria (122	Proteobacteria (2)	Bacteria (1)	100.00	0.00	99.90	99.20	WGS
287.1893	0.3268	Pseudomonas aeruginosa (286)	Pseudomonas (135621)	Pseudomonadaceae (7227	Pseudomonadales (1236)	Gammaproteobacteria (122	Proteobacteria (2)	Bacteria (1)	100.00	0.00	99.90	99.30	WGS
287.1894	0.3268	Pseudomonas aeruginosa (286)	Pseudomonas (135621)	Pseudomonadaceae (7227	Pseudomonadales (1236)	Gammaproteobacteria (122	Proteobacteria (2)	Bacteria (1)	100.00	0.00	99.90	99.30	WGS
287.1895	0.3268	Pseudomonas aeruginosa (286)	Pseudomonas (135621)	Pseudomonadaceae (7227	Pseudomonadales (1236)	Gammaproteobacteria (122	Proteobacteria (2)	Bacteria (1)	100.00	0.00	99.90	99.30	WGS
287.1896	0.3754	Pseudomonas aeruginosa (286)	Pseudomonas (135621)	Pseudomonadaceae (7227	Pseudomonadales (1236)	Gammaproteobacteria (122	Proteobacteria (2)	Bacteria (1)	100.00	0.90	99.80	98.10	WGS
287.1897	0.3306	Pseudomonas aeruginosa (286)	Pseudomonas (135621)	Pseudomonadaceae (7227	Pseudomonadales (1236)	Gammaproteobacteria (122	Proteobacteria (2)	Bacteria (1)	99.70	2.60	99.90	98.20	WGS
287.1898	0.3678	Pseudomonas aeruginosa (286)	Pseudomonas (135621)	Pseudomonadaceae (7227	Pseudomonadales (1236)	Gammaproteobacteria (122	Proteobacteria (2)	Bacteria (1)	100.00	0.30	99.70	99.00	WGS
287.1899	0.3754	Pseudomonas aeruginosa (286)	Pseudomonas (135621)	Pseudomonadaceae (7227	Pseudomonadales (1236)	Gammaproteobacteria (122	Proteobacteria (2)	Bacteria (1)	100.00	0.90	99.90	98.10	WGS
287.1900	0.3254	Pseudomonas aeruginosa (286)	Pseudomonas (135621)	Pseudomonadaceae (7227	Pseudomonadales (1236)	Gammaproteobacteria (122	Proteobacteria (2)	Bacteria (1)	100.00	0.30	99.90	99.00	WGS
287.1901	0.3754	Pseudomonas aeruginosa (286)	Pseudomonas (135621)	Pseudomonadaceae (7227	Pseudomonadales (1236)	Gammaproteobacteria (122	Proteobacteria (2)	Bacteria (1)	100.00	0.90	99.80	98.00	WGS
287.1902	0.3254	Pseudomonas aeruginosa (286)	Pseudomonas (135621)	Pseudomonadaceae (7227	Pseudomonadales (1236)	Gammaproteobacteria (122	Proteobacteria (2)	Bacteria (1)	100.00	0.00	99.80	98.60	WGS
287.1903	0.3748	Pseudomonas aeruginosa (286)	Pseudomonas (135621)	Pseudomonadaceae (7227	Pseudomonadales (1236)	Gammaproteobacteria (122	Proteobacteria (2)	Bacteria (1)	100.00	0.00	99.90	98.80	WGS
287.1904	0.3618	Pseudomonas aeruginosa (286)	Pseudomonas (135621)	Pseudomonadaceae (7227	Pseudomonadales (1236)	Gammaproteobacteria (122	Proteobacteria (2)	Bacteria (1)	100.00	0.00	99.80	99.40	WGS
287.1905	0.3292	Pseudomonas aeruginosa (286)	Pseudomonas (135621)	Pseudomonadaceae (7227	Pseudomonadales (1236)	Gammaproteobacteria (122	Proteobacteria (2)	Bacteria (1)	100.00	0.30	99.70	98.00	WGS
287.1906	0.3176	Pseudomonas aeruginosa (286)	Pseudomonas (135621)	Pseudomonadaceae (7227	Pseudomonadales (1236)	Gammaproteobacteria (122	Proteobacteria (2)	Bacteria (1)	100.00	1.20	100.00	98.40	WGS
287.1907	0.3738	Pseudomonas aeruginosa (286)	Pseudomonas (135621)	Pseudomonadaceae (7227	Pseudomonadales (1236)	Gammaproteobacteria (122	Proteobacteria (2)	Bacteria (1)	99.10	0.20	99.10	97.90	WGS
287.1908	0.3878	Pseudomonas aeruginosa (286)	Pseudomonas (135621)	Pseudomonadaceae (7227	Pseudomonadales (1236)	Gammaproteobacteria (122	Proteobacteria (2)	Bacteria (1)	99.70	0.00	99.90	99.00	WGS
287.1909	0.325	Pseudomonas aeruginosa (286)	Pseudomonas (135621)	Pseudomonadaceae (7227	Pseudomonadales (1236)	Gammaproteobacteria (122	Proteobacteria (2)	Bacteria (1)	100.00	0.00	99.90	99.20	WGS
287.1910	0.3376	Pseudomonas aeruginosa (286)	Pseudomonas (135621)	Pseudomonadaceae (7227	Pseudomonadales (1236)	Gammaproteobacteria (122	Proteobacteria (2)	Bacteria (1)	100.00	0.00	99.90	98.60	WGS
287.1911	0.3292	Pseudomonas aeruginosa (286)	Pseudomonas (135621)	Pseudomonadaceae (7227	Pseudomonadales (1236)	Gammaproteobacteria (122	Proteobacteria (2)	Bacteria (1)	100.00	0.90	99.70	98.40	WGS
287.1912	0.3254	Pseudomonas aeruginosa (286)	Pseudomonas (135621)	Pseudomonadaceae (7227	Pseudomonadales (1236)	Gammaproteobacteria (122	Proteobacteria (2)	Bacteria (1)	100.00	0.00	99.90	99.00	WGS
287.1913	0.3678	Pseudomonas aeruginosa (286)	Pseudomonas (135621)	Pseudomonadaceae (7227	Pseudomonadales (1236)	Gammaproteobacteria (122	Proteobacteria (2)	Bacteria (1)	100.00	0.30	99.60	98.70	WGS
287.1914	0.331	Pseudomonas aeruginosa (286)	Pseudomonas (135621)	Pseudomonadaceae (7227	Pseudomonadales (1236)	Gammaproteobacteria (122	Proteobacteria (2)	Bacteria (1)	100.00	0.30	99.60	98.50	WGS
287.1915	0.3268	Pseudomonas aeruginosa (286)	Pseudomonas (135621)	Pseudomonadaceae (7227	Pseudomonadales (1236)	Gammaproteobacteria (122	Proteobacteria (2)	Bacteria (1)	100.00	0.00	99.90	99.20	WGS
287.1916	0.3268	Pseudomonas aeruginosa (286)	Pseudomonas (135621)	Pseudomonadaceae (7227	Pseudomonadales (1236)	Gammaproteobacteria (122	Proteobacteria (2)	Bacteria (1)	100.00	0.00	99.90	99.30	WGS
287.1917	0.3268	Pseudomonas aeruginosa (286)	Pseudomonas (135621)	Pseudomonadaceae (7227	Pseudomonadales (1236)	Gammaproteobacteria (122	Proteobacteria (2)	Bacteria (1)	100.00	0.00	99.90	99.30	WGS
287.1918	0.3268	Pseudomonas aeruginosa (286)	Pseudomonas (135621)	Pseudomonadaceae (7227	Pseudomonadales (1236)	Gammaproteobacteria (122	Proteobacteria (2)	Bacteria (1)	100.00	0.00	99.90	99.30	WGS
287.1919	0.3268	Pseudomonas aeruginosa (286)	Pseudomonas (135621)	Pseudomonadaceae (7227	Pseudomonadales (1236)	Gammaproteobacteria (122	Proteobacteria (2)	Bacteria (1)	100.00	0.00	99.90	99.30	WGS
287.1920	0.3268	Pseudomonas aeruginosa (286)	Pseudomonas (135621)	Pseudomonadaceae (7227	Pseudomonadales (1236)	Gammaproteobacteria (122	Proteobacteria (2)	Bacteria (1)	100.00	0.00	99.90	99.30	WGS
287.1921	0.3268	Pseudomonas aeruginosa (286)	Pseudomonas (135621)	Pseudomonadaceae (7227	Pseudomonadales (1236)	Gammaproteobacteria (122	Proteobacteria (2)	Bacteria (1)	100.00	0.00	99.90	99.20	WGS
287.1922	0.3268	Pseudomonas aeruginosa (286)	Pseudomonas (135621)	Pseudomonadaceae (7227	Pseudomonadales (1236)	Gammaproteobacteria (122	Proteobacteria (2)	Bacteria (1)	100.00	0.00	99.90	99.30	WGS
287.1923	0.3268	Pseudomonas aeruginosa (286)	Pseudomonas (135621)	Pseudomonadaceae (7227	Pseudomonadales (1236)	Gammaproteobacteria (122	Proteobacteria (2)	Bacteria (1)	100.00	0.00	99.90	99.30	WGS
287.1924	0.3268	Pseudomonas aeruginosa (286)	Pseudomonas (135621)	Pseudomonadaceae (7227	Pseudomonadales (1236)	Gammaproteobacteria (122	Proteobacteria (2)	Bacteria (1)	100.00	0.00	99.90	99.30	WGS
287.1925	0.3268	Pseudomonas aeruginosa (286)	Pseudomonas (135621)	Pseudomonadaceae (7227	Pseudomonadales (1236)	Gammaproteobacteria (122	Proteobacteria (2)	Bacteria (1)	100.00	0.00	99.90	99.30	WGS
287.1926	0.3268	Pseudomonas aeruginosa (286)	Pseudomonas (135621)	Pseudomonadaceae (7227	Pseudomonadales (1236)	Gammaproteobacteria (122	Proteobacteria (2)	Bacteria (1)	100.00	0.00	99.90	99.30	WGS
287.1927	0.3268	Pseudomonas aeruginosa (286)	Pseudomonas (135621)	Pseudomonadaceae (7227	Pseudomonadales (1236)	Gammaproteobacteria (122	Proteobacteria (2)	Bacteria (1)	100.00	0.00	99.90	99.30	WGS
287.1928	0.3268	Pseudomonas aeruginosa (286)	Pseudomonas (135621)	Pseudomonadaceae (7227	Pseudomonadales (1236)	Gammaproteobacteria (122	Proteobacteria (2)	Bacteria (1)	100.00	0.00	99.90	99.30	WGS
287.1929	0.3268	Pseudomonas aeruginosa (286)	Pseudomonas (135621)	Pseudomonadaceae (7227	Pseudomonadales (1236)	Gammaproteobacteria (122	Proteobacteria (2)	Bacteria (1)	100.00	0.00	99.90	99.30	WGS
287.1930	0.3268	Pseudomonas aeruginosa (286)	Pseudomonas (135621)	Pseudomonadaceae (7227	Pseudomonadales (1236)	Gammaproteobacteria (122	Proteobacteria (2)	Bacteria (1)	100.00	0.00	99.90	99.30	WGS
287.1931	0.3268	Pseudomonas aeruginosa (286)	Pseudomonas (135621)	Pseudomonadaceae (7227	Pseudomonadales (1236)	Gammaproteobacteria (122	Proteobacteria (2)	Bacteria (1)	100.00	0.00	99.90	99.30	WGS
287.1932	0.3268	Pseudomonas aeruginosa (286)	Pseudomonas (135621)	Pseudomonadaceae (7227	Pseudomonadales (1236)	Gammaproteobacteria (122	Proteobacteria (2)	Bacteria (1)	100.00	0.00	99.90	99.30	WGS
287.1933	0.3268	Pseudomonas aeruginosa (286)	Pseudomonas (135621)	Pseudomonadaceae (7227	Pseudomonadales (1236)	Gammaproteobacteria (122	Proteobacteria (2)	Bacteria (1)	100.00	0.30	99.90	99.10	WGS
287.1934	0.3268	Pseudomonas aeruginosa (286)	Pseudomonas (135621)	Pseudomonadaceae (7227	Pseudomonadales (1236)	Gammaproteobacteria (122	Proteobacteria (2)	Bacteria (1)	100.00	0.00	99.90	99.20	WGS
287.1935	0.3268	Pseudomonas aeruginosa (286)	Pseudomonas (135621)	Pseudomonadaceae (7227	Pseudomonadales (1236)	Gammaproteobacteria (122	Proteobacteria (2)	Bacteria (1)	100.00	0.30	99.90	99.20	WGS
287.1936	0.3268	Pseudomonas aeruginosa (286)	Pseudomonas (135621)	Pseudomonadaceae (7227	Pseudomonadales (1236)	Gammaproteobacteria (122	Proteobacteria (2)	Bacteria (1)	100.00	0.30	99.90	99.00	WGS
287.1937	0.3268	Pseudomonas aeruginosa (286)	Pseudomonas (135621)	Pseudomonadaceae (7227	Pseudomonadales (1236)	Gammaproteobacteria (122	Proteobacteria (2)	Bacteria (1)	100.00	0.00	99.90	99.30	WGS
287.1938	0.3268	Pseudomonas aeruginosa (286)	Pseudomonas (135621)	Pseudomonadaceae (7227	Pseudomonadales (1236)	Gammaproteobacteria (122	Proteobacteria (2)	Bacteria (1)	100.00	0.00	99.90	99.30	WGS
287.1939	0.3268	Pseudomonas aeruginosa (286)	Pseudomonas (135621)	Pseudomonadaceae (7227	Pseudomonadales (1236)	Gammaproteobacteria (122	Proteobacteria (2)	Bacteria (1)	100.00	0.00	99.90	99.30	WGS
287.1940	0.3268	Pseudomonas aeruginosa (286)	Pseudomonas (135621)	Pseudomonadaceae (7227	Pseudomonadales (1236)	Gammaproteobacteria (122	Proteobacteria (2)	Bacteria (1)	100.00	0.00	99.90	99.30	WGS
287.1941	0.3268	Pseudomonas aeruginosa (286)	Pseudomonas (135621)	Pseudomonadaceae (7227	Pseudomonadales (1236)	Gammaproteobacteria (122	Proteobacteria (2)	Bacteria (1)	100.00	0.00	99.90	99.10	WGS
287.1942	0.3268	Pseudomonas aeruginosa (286)	Pseudomonas (135621)	Pseudomonadaceae (7227	Pseudomonadales (1236)	Gammaproteobacteria (122	Proteobacteria (2)	Bacteria (1)	100.00	0.00	99.90	99.30	WGS
287.1943	0.3268	Pseudomonas aeruginosa (286)	Pseudomonas (135621)	Pseudomonadaceae (7227	Pseudomonadales (1236)	Gammaproteobacteria (122	Proteobacteria (2)	Bacteria (1)	100.00	0.00	99.90	99.30	WGS
287.1944	0.3268	Pseudomonas aeruginosa (286)	Pseudomonas (135621)	Pseudomonadaceae (7227	Pseudomonadales (1236)	Gammaproteobacteria (122	Proteobacteria (2)	Bacteria (1)	100.00	0.00	99.90	99.30	WGS
287.1945	0.3268	Pseudomonas aeruginosa (286)	Pseudomonas (135621)	Pseudomonadaceae (7227	Pseudomonadales (1236)	Gammaproteobacteria (122	Proteobacteria (2)	Bacteria (1)	100.00	0.00	99.80	99.10	WGS
287.1946	0.3268	Pseudomonas aeruginosa (286)	Pseudomonas (135621)	Pseudomonadaceae (7227	Pseudomonadales (1236)	Gammaproteobacteria (122	Proteobacteria (2)	Bacteria (1)	100.00	0.00	99.90	99.30	WGS
287.1947	0.3268	Pseudomonas aeruginosa (286)	Pseudomonas (135621)	Pseudomonadaceae (7227	Pseudomonadales (1236)	Gammaproteobacteria (122	Proteobacteria (2)	Bacteria (1)	100.00	0.00	99.90	99.30	WGS
287.1948	0.3268	Pseudomonas aeruginosa (286)	Pseudomonas (135621)	Pseudomonadaceae (7227	Pseudomonadales (1236)	Gammaproteobacteria (122	Proteobacteria (2)	Bacteria (1)	100.00	0.00	99.90	99.30	WGS
287.1949	0.3268	Pseudomonas aeruginosa (286)	Pseudomonas (135621)	Pseudomonadaceae (7227	Pseudomonadales (1236)	Gammaproteobacteria (122	Proteobacteria (2)	Bacteria (1)	100.00	0.00	99.90	99.30	WGS
287.1950	0.3268	Pseudomonas aeruginosa (286)	Pseudomonas (135621)	Pseudomonadaceae (7227	Pseudomonadales (1236)	Gammaproteobacteria (122	Proteobacteria (2)	Bacteria (1)	100.00	0.00	99.90	99.30	WGS
287.1951	0.3268	Pseudomonas aeruginosa (286)	Pseudomonas (135621)	Pseudomonadaceae (7227	Pseudomonadales (1236)	Gammaproteobacteria (122	Proteobacteria (2)	Bacteria (1)	100.00	0.00	99.90	99.30	WGS
287.1952	0.3268	Pseudomonas aeruginosa (286)	Pseudomonas (135621)	Pseudomonadaceae (7227	Pseudomonadales (1236)	Gammaproteobacteria (122	Proteobacteria (2)	Bacteria (1)	100.00	0.00	99.90	99.30	WGS
287.1953	0.3268	Pseudomonas aeruginosa (286)	Pseudomonas (135621)	Pseudomonadaceae (7227	Pseudomonadales (1236)	Gammaproteobacteria (122	Proteobacteria (2)	Bacteria (1)	100.00	0.00	99.90	99.30	WGS
287.1954	0.3268	Pseudomonas aeruginosa (286)	Pseudomonas (135621)	Pseudomonadaceae (7227	Pseudomonadales (1236)	Gammaproteobacteria (122	Proteobacteria (2)	Bacteria (1)	100.00	0.00	99.90	99.30	WGS
287.1955	0.3268	Pseudomonas aeruginosa (286)	Pseudomonas (135621)	Pseudomonadaceae (7227	Pseudomonadales (1236)	Gammaproteobacteria (122	Proteobacteria (2)	Bacteria (1)	100.00	0.00	99.90	99.30	WGS
287.1956	0.3268	Pseudomonas aeruginosa (286)	Pseudomonas (135621)	Pseudomonadaceae (7227	Pseudomonadales (1236)	Gammaproteobacteria (122	Proteobacteria (2)	Bacteria (1)	100.00	0.00	99.90	99.30	WGS
287.1957	0.368	Pseudomonas aeruginosa (286)	Pseudomonas (135621)	Pseudomonadaceae (7227	Pseudomonadales (1236)	Gammaproteobacteria (122	Proteobacteria (2)	Bacteria (1)	97.00	0.00	99.80	98.70	WGS
287.1958	0.3268	Pseudomonas aeruginosa (286)	Pseudomonas (135621)	Pseudomonadaceae (7227	Pseudomonadales (1236)	Gammaproteobacteria (122	Proteobacteria (2)	Bacteria (1)	100.00	0.00	99.80	99.30	WGS
287.1959	0.324	Pseudomonas aeruginosa (286)	Pseudomonas (135621)	Pseudomonadaceae (7227	Pseudomonadales (1236)	Gammaproteobacteria (122	Proteobacteria (2)	Bacteria (1)	99.70	0.00	99.80	99.30	WGS
287.1960	0.3128	Pseudomonas aeruginosa (286)	Pseudomonas (135621)	Pseudomonadaceae (7227	Pseudomonadales (1236)	Gammaproteobacteria (122	Proteobacteria (2)	Bacteria (1)	99.10	0.00	99.60	99.20	WGS
287.1961	0.3376	Pseudomonas aeruginosa (286)	Pseudomonas (135621)	Pseudomonadaceae (7227	Pseudomonadales (1236)	Gammaproteobacteria (122	Proteobacteria (2)	Bacteria (1)	100.00	0.00	99.90	99.00	WGS
287.1962	0.3268	Pseudomonas aeruginosa (286)	Pseudomonas (135621)	Pseudomonadaceae (7227	Pseudomonadales (1236)	Gammaproteobacteria (122	Proteobacteria (2)	Bacteria (1)	100.00	0.00	99.90	99.30	WGS
287.1963	0.3268	Pseudomonas aeruginosa (286)	Pseudomonas (135621)	Pseudomonadaceae (7227	Pseudomonadales (1236)	Gammaproteobacteria (122	Proteobacteria (2)	Bacteria (1)	100.00	0.00	99.90	99.30	WGS
287.1964	0.3268	Pseudomonas aeruginosa (286)	Pseudomonas (135621)	Pseudomonadaceae (7227	Pseudomonadales (1236)	Gammaproteobacteria (122	Proteobacteria (2)	Bacteria (1)	100.00	0.00	99.90	99.30	WGS
287.1965	0.3268	Pseudomonas aeruginosa (286)	Pseudomonas (135621)	Pseudomonadaceae (7227	Pseudomonadales (1236)	Gammaproteobacteria (122	Proteobacteria (2)	Bacteria (1)	100.00	0.00	99.90	99.30	WGS
287.1966	0.3268	Pseudomonas aeruginosa (286)	Pseudomonas (135621)	Pseudomonadaceae (7227	Pseudomonadales (1236)	Gammaproteobacteria (122	Proteobacteria (2)	Bacteria (1)	100.00	0.00	99.90	99.20	WGS
287.1967	0.3268	Pseudomonas aeruginosa (286)	Pseudomonas (135621)	Pseudomonadaceae (7227	Pseudomonadales (1236)	Gammaproteobacteria (122	Proteobacteria (2)	Bacteria (1)	100.00	0.00	99.90	99.30	WGS
287.1968	0.3268	Pseudomonas aeruginosa (286)	Pseudomonas (135621)	Pseudomonadaceae (7227	Pseudomonadales (1236)	Gammaproteobacteria (122	Proteobacteria (2)	Bacteria (1)	100.00	0.00	99.90	99.30	WGS
287.1969	0.3696	Pseudomonas aeruginosa (286)	Pseudomonas (135621)	Pseudomonadaceae (7227	Pseudomonadales (1236)	Gammaproteobacteria (122	Proteobacteria (2)	Bacteria (1)	98.40	1.50	99.60	97.60	WGS
287.1970	0.3694	Pseudomonas aeruginosa (286)	Pseudomonas (135621)	Pseudomonadaceae (7227	Pseudomonadales (1236)	Gammaproteobacteria (122	Proteobacteria (2)	Bacteria (1)	100.00	0.00	99.80	99.40	WGS
287.1971	0.3694	Pseudomonas aeruginosa (286)	Pseudomonas (135621)	Pseudomonadaceae (7227	Pseudomonadales (1236)	Gammaproteobacteria (122	Proteobacteria (2)	Bacteria (1)	100.00	0.00	99.70	99.30	WGS
287.1972	0.3738	Pseudomonas aeruginosa (286)	Pseudomonas (135621)	Pseudomonadaceae (7227	Pseudomonadales (1236)	Gammaproteobacteria (122	Proteobacteria (2)	Bacteria (1)	100.00	0.60	99.80	99.00	WGS
287.1973	0.3694	Pseudomonas aeruginosa (286)	Pseudomonas (135621)	Pseudomonadaceae (7227	Pseudomonadales (1236)	Gammaproteobacteria (122	Proteobacteria (2)	Bacteria (1)	100.00	0.30	99.60	99.10	WGS
287.1974	0.3694	Pseudomonas aeruginosa (286)	Pseudomonas (135621)	Pseudomonadaceae (7227	Pseudomonadales (1236)	Gammaproteobacteria (122	Proteobacteria (2)	Bacteria (1)	100.00	0.00	99.60	99.30	WGS
287.1982	0.369	Pseudomonas aeruginosa (286)	Pseudomonas (135621)	Pseudomonadaceae (7227	Pseudomonadales (1236)	Gammaproteobacteria (122	Proteobacteria (2)	Bacteria (1)	100.00	0.30	99.90	98.70	WGS
287.1983	0.3376	Pseudomonas aeruginosa (286)	Pseudomonas (135621)	Pseudomonadaceae (7227	Pseudomonadales (1236)	Gammaproteobacteria (122	Proteobacteria (2)	Bacteria (1)	97.00	0.30	99.60	98.70	WGS
287.1984	0.3618	Pseudomonas aeruginosa (286)	Pseudomonas (135621)	Pseudomonadaceae (7227	Pseudomonadales (1236)	Gammaproteobacteria (122	Proteobacteria (2)	Bacteria (1)	100.00	0.00	99.90	98.90	WGS
287.1985	0.3324	Pseudomonas aeruginosa (286)	Pseudomonas (135621)	Pseudomonadaceae (7227	Pseudomonadales (1236)	Gammaproteobacteria (122	Proteobacteria (2)	Bacteria (1)	99.70	0.30	99.90	98.50	WGS
287.1986	0.332	Pseudomonas aeruginosa (286)	Pseudomonas (135621)	Pseudomonadaceae (7227	Pseudomonadales (1236)	Gammaproteobacteria (122	Proteobacteria (2)	Bacteria (1)	98.80	0.60	99.80	97.70	WGS
287.1987	0.3252	Pseudomonas aeruginosa (286)	Pseudomonas (135621)	Pseudomonadaceae (7227	Pseudomonadales (1236)	Gammaproteobacteria (122	Proteobacteria (2)	Bacteria (1)	98.80	1.30	99.90	97.50	WGS
287.1988	0.308	Pseudomonas aeruginosa (286)	Pseudomonas (135621)	Pseudomonadaceae (7227	Pseudomonadales (1236)	Gammaproteobacteria (122	Proteobacteria (2)	Bacteria (1)	99.10	0.00	99.70	98.90	WGS
287.1989	0.356	Pseudomonas aeruginosa (286)	Pseudomonas (135621)	Pseudomonadaceae (7227	Pseudomonadales (1236)	Gammaproteobacteria (122	Proteobacteria (2)	Bacteria (1)	100.00	0.60	99.70	98.30	WGS
287.1990	0.3302	Pseudomonas aeruginosa (286)	Pseudomonas (135621)	Pseudomonadaceae (7227	Pseudomonadales (1236)	Gammaproteobacteria (122	Proteobacteria (2)	Bacteria (1)	100.00	0.70	99.90	98.00	WGS
287.1991	0.3302	Pseudomonas aeruginosa (286)	Pseudomonas (135621)	Pseudomonadaceae (7227	Pseudomonadales (1236)	Gammaproteobacteria (122	Proteobacteria (2)	Bacteria (1)	100.00	0.30	99.70	98.10	WGS
287.1992	0.3376	Pseudomonas aeruginosa (286)	Pseudomonas (135621)	Pseudomonadaceae (7227	Pseudomonadales (1236)	Gammaproteobacteria (122	Proteobacteria (2)	Bacteria (1)	99.70	0.30	99.90	98.40	WGS
287.1993	0.3604	Pseudomonas aeruginosa (286)	Pseudomonas (135621)	Pseudomonadaceae (7227	Pseudomonadales (1236)	Gammaproteobacteria (122	Proteobacteria (2)	Bacteria (1)	98.40	1.80	99.80	97.30	WGS
287.1994	0.3252	Pseudomonas aeruginosa (286)	Pseudomonas (135621)	Pseudomonadaceae (7227	Pseudomonadales (1236)	Gammaproteobacteria (122	Proteobacteria (2)	Bacteria (1)	98.80	1.30	99.90	97.30	WGS
287.1995	0.3268	Pseudomonas aeruginosa (286)	Pseudomonas (135621)	Pseudomonadaceae (7227	Pseudomonadales (1236)	Gammaproteobacteria (122	Proteobacteria (2)	Bacteria (1)	99.70	0.30	99.80	97.90	WGS
287.1996	0.3648	Pseudomonas aeruginosa (286)	Pseudomonas (135621)	Pseudomonadaceae (7227	Pseudomonadales (1236)	Gammaproteobacteria (122	Proteobacteria (2)	Bacteria (1)	99.10	0.00	100.00	98.50	WGS
287.1997	0.3636	Pseudomonas aeruginosa (286)	Pseudomonas (135621)	Pseudomonadaceae (7227	Pseudomonadales (1236)	Gammaproteobacteria (122	Proteobacteria (2)	Bacteria (1)	100.00	1.80	99.80	98.70	Complete
287.1998	0.331	Pseudomonas aeruginosa (286)	Pseudomonas (135621)	Pseudomonadaceae (7227	Pseudomonadales (1236)	Gammaproteobacteria (122	Proteobacteria (2)	Bacteria (1)	99.70	1.60	99.80	98.90	WGS
287.1999	0.331	Pseudomonas aeruginosa (286)	Pseudomonas (135621)	Pseudomonadaceae (7227	Pseudomonadales (1236)	Gammaproteobacteria (122	Proteobacteria (2)	Bacteria (1)	99.70	1.90	99.80	98.80	WGS
287.2000	0.3188	Pseudomonas aeruginosa (286)	Pseudomonas (135621)	Pseudomonadaceae (7227	Pseudomonadales (1236)	Gammaproteobacteria (122	Proteobacteria (2)	Bacteria (1)	99.70	0.00	100.00	98.10	WGS
287.2001	0.324	Pseudomonas aeruginosa (286)	Pseudomonas (135621)	Pseudomonadaceae (7227	Pseudomonadales (1236)	Gammaproteobacteria (122	Proteobacteria (2)	Bacteria (1)	100.00	0.00	99.90	98.20	WGS
287.2002	0.331	Pseudomonas aeruginosa (286)	Pseudomonas (135621)	Pseudomonadaceae (7227	Pseudomonadales (1236)	Gammaproteobacteria (122	Proteobacteria (2)	Bacteria (1)	100.00	0.00	99.90	97.80	WGS
287.2003	0.3636	Pseudomonas aeruginosa (286)	Pseudomonas (135621)	Pseudomonadaceae (7227	Pseudomonadales (1236)	Gammaproteobacteria (122	Proteobacteria (2)	Bacteria (1)	99.70	0.00	99.90	98.60	WGS
287.2004	0.3236	Pseudomonas aeruginosa (286)	Pseudomonas (135621)	Pseudomonadaceae (7227	Pseudomonadales (1236)	Gammaproteobacteria (122	Proteobacteria (2)	Bacteria (1)	100.00	0.00	99.70	98.20	WGS
287.2005	0.3324	Pseudomonas aeruginosa (286)	Pseudomonas (135621)	Pseudomonadaceae (7227	Pseudomonadales (1236)	Gammaproteobacteria (122	Proteobacteria (2)	Bacteria (1)	100.00	0.00	99.90	99.60	Complete
287.2006	0.3936	Pseudomonas aeruginosa (286)	Pseudomonas (135621)	Pseudomonadaceae (7227	Pseudomonadales (1236)	Gammaproteobacteria (122	Proteobacteria (2)	Bacteria (1)	99.70	3.00	99.80	95.80	WGS
287.2007	0.371	Pseudomonas aeruginosa (286)	Pseudomonas (135621)	Pseudomonadaceae (7227	Pseudomonadales (1236)	Gammaproteobacteria (122	Proteobacteria (2)	Bacteria (1)	99.70	3.10	99.60	97.70	WGS
287.2008	0.3268	Pseudomonas aeruginosa (286)	Pseudomonas (135621)	Pseudomonadaceae (7227	Pseudomonadales (1236)	Gammaproteobacteria (122	Proteobacteria (2)	Bacteria (1)	100.00	0.60	99.80	98.40	WGS
287.2009	0.3172	Pseudomonas aeruginosa (286)	Pseudomonas (135621)	Pseudomonadaceae (7227	Pseudomonadales (1236)	Gammaproteobacteria (122	Proteobacteria (2)	Bacteria (1)	99.40	0.90	99.30	98.40	WGS
287.2010	0.331	Pseudomonas aeruginosa (286)	Pseudomonas (135621)	Pseudomonadaceae (7227	Pseudomonadales (1236)	Gammaproteobacteria (122	Proteobacteria (2)	Bacteria (1)	100.00	0.00	99.90	99.70	WGS
287.2011	0.3636	Pseudomonas aeruginosa (286)	Pseudomonas (135621)	Pseudomonadaceae (7227	Pseudomonadales (1236)	Gammaproteobacteria (122	Proteobacteria (2)	Bacteria (1)	99.90	1.30	99.70	98.30	WGS
287.2012	0.3636	Pseudomonas aeruginosa (286)	Pseudomonas (135621)	Pseudomonadaceae (7227	Pseudomonadales (1236)	Gammaproteobacteria (122	Proteobacteria (2)	Bacteria (1)	99.70	1.80	99.30	98.20	WGS
287.2013	0.3636	Pseudomonas aeruginosa (286)	Pseudomonas (135621)	Pseudomonadaceae (7227	Pseudomonadales (1236)	Gammaproteobacteria (122	Proteobacteria (2)	Bacteria (1)	99.40	3.10	99.40	95.60	WGS
287.2252	0.3636	Pseudomonas aeruginosa (286)	Pseudomonas (135621)	Pseudomonadaceae (7227	Pseudomonadales (1236)	Gammaproteobacteria (122	Proteobacteria (2)	Bacteria (1)	100.00	0.90	99.80	99.30	WGS
287.2253	0.3636	Pseudomonas aeruginosa (286)	Pseudomonas (135621)	Pseudomonadaceae (7227	Pseudomonadales (1236)	Gammaproteobacteria (122	Proteobacteria (2)	Bacteria (1)	100.00	1.20	99.80	99.20	WGS
287.2254	0.3636	Pseudomonas aeruginosa (286)	Pseudomonas (135621)	Pseudomonadaceae (7227	Pseudomonadales (1236)	Gammaproteobacteria (122	Proteobacteria (2)	Bacteria (1)	100.00	1.20	99.70	99.00	WGS
287.2255	0.3694	Pseudomonas aeruginosa (286)	Pseudomonas (135621)	Pseudomonadaceae (7227	Pseudomonadales (1236)	Gammaproteobacteria (122	Proteobacteria (2)	Bacteria (1)	100.00	0.00	99.90	99.50	WGS
287.2256	0.3636	Pseudomonas aeruginosa (286)	Pseudomonas (135621)	Pseudomonadaceae (7227	Pseudomonadales (1236)	Gammaproteobacteria (122	Proteobacteria (2)	Bacteria (1)	100.00	0.00	99.80	99.60	WGS
287.2257	0.3306	Pseudomonas aeruginosa (286)	Pseudomonas (135621)	Pseudomonadaceae (7227	Pseudomonadales (1236)	Gammaproteobacteria (122	Proteobacteria (2)	Bacteria (1)	100.00	0.60	99.90	98.70	WGS
287.2258	0.368	Pseudomonas aeruginosa (286)	Pseudomonas (135621)	Pseudomonadaceae (7227	Pseudomonadales (1236)	Gammaproteobacteria (122	Proteobacteria (2)	Bacteria (1)	100.00	0.90	99.80	98.80	WGS
287.2259	0.324	Pseudomonas aeruginosa (286)	Pseudomonas (135621)	Pseudomonadaceae (7227	Pseudomonadales (1236)	Gammaproteobacteria (122	Proteobacteria (2)	Bacteria (1)	100.00	0.00	99.90	99.10	WGS
287.2260	0.3174	Pseudomonas aeruginosa (286)	Pseudomonas (135621)	Pseudomonadaceae (7227	Pseudomonadales (1236)	Gammaproteobacteria (122	Proteobacteria (2)	Bacteria (1)	100.00	0.00	99.80	99.30	WGS
287.2261	0.3694	Pseudomonas aeruginosa (286)	Pseudomonas (135621)	Pseudomonadaceae (7227	Pseudomonadales (1236)	Gammaproteobacteria (122	Proteobacteria (2)	Bacteria (1)	100.00	0.00	99.80	99.30	WGS
287.2262	0.3694	Pseudomonas aeruginosa (286)	Pseudomonas (135621)	Pseudomonadaceae (7227	Pseudomonadales (1236)	Gammaproteobacteria (122	Proteobacteria (2)	Bacteria (1)	100.00	0.00	99.80	99.30	WGS
287.2263	0.3636	Pseudomonas aeruginosa (286)	Pseudomonas (135621)	Pseudomonadaceae (7227	Pseudomonadales (1236)	Gammaproteobacteria (122	Proteobacteria (2)	Bacteria (1)	100.00	0.00	99.90	99.70	WGS
287.2264	0.3258	Pseudomonas aeruginosa (286)	Pseudomonas (135621)	Pseudomonadaceae (7227	Pseudomonadales (1236)	Gammaproteobacteria (122	Proteobacteria (2)	Bacteria (1)	100.00	0.60	99.80	99.20	WGS
287.2265	0.3422	Pseudomonas aeruginosa (286)	Pseudomonas (135621)	Pseudomonadaceae (7227	Pseudomonadales (1236)	Gammaproteobacteria (122	Proteobacteria (2)	Bacteria (1)	98.80	0.60	99.90	99.00	WGS
287.2266	0.3398	Pseudomonas aeruginosa (286)	Pseudomonas (135621)	Pseudomonadaceae (7227	Pseudomonadales (1236)	Gammaproteobacteria (122	Proteobacteria (2)	Bacteria (1)	99.70	0.00	99.80	99.30	WGS
287.2267	0.3694	Pseudomonas aeruginosa (286)	Pseudomonas (135621)	Pseudomonadaceae (7227	Pseudomonadales (1236)	Gammaproteobacteria (122	Proteobacteria (2)	Bacteria (1)	98.60	1.30	99.60	98.60	WGS
287.2268	0.331	Pseudomonas aeruginosa (286)	Pseudomonas (135621)	Pseudomonadaceae (7227	Pseudomonadales (1236)	Gammaproteobacteria (122	Proteobacteria (2)	Bacteria (1)	100.00	0.00	99.90	99.00	WGS
287.2269	0.3636	Pseudomonas aeruginosa (286)	Pseudomonas (135621)	Pseudomonadaceae (7227	Pseudomonadales (1236)	Gammaproteobacteria (122	Proteobacteria (2)	Bacteria (1)	100.00	0.00	99.90	99.40	WGS
287.2270	0.3618	Pseudomonas aeruginosa (286)	Pseudomonas (135621)	Pseudomonadaceae (7227	Pseudomonadales (1236)	Gammaproteobacteria (122	Proteobacteria (2)	Bacteria (1)	99.10	0.30	99.80	99.30	WGS
287.2271	0.3738	Pseudomonas aeruginosa (286)	Pseudomonas (135621)	Pseudomonadaceae (7227	Pseudomonadales (1236)	Gammaproteobacteria (122	Proteobacteria (2)	Bacteria (1)	98.40	1.20	99.60	97.00	WGS
287.2272	0.3268	Pseudomonas aeruginosa (286)	Pseudomonas (135621)	Pseudomonadaceae (7227	Pseudomonadales (1236)	Gammaproteobacteria (122	Proteobacteria (2)	Bacteria (1)	100.00	0.90	99.90	99.30	WGS
287.2273	0.3672	Pseudomonas aeruginosa (286)	Pseudomonas (135621)	Pseudomonadaceae (7227	Pseudomonadales (1236)	Gammaproteobacteria (122	Proteobacteria (2)	Bacteria (1)	100.00	0.30	99.70	98.70	WGS
287.2274	0.3314	Pseudomonas aeruginosa (286)	Pseudomonas (135621)	Pseudomonadaceae (7227	Pseudomonadales (1236)	Gammaproteobacteria (122	Proteobacteria (2)	Bacteria (1)	100.00	0.00	99.80	99.10	WGS
287.2275	0.3748	Pseudomonas aeruginosa (286)	Pseudomonas (135621)	Pseudomonadaceae (7227	Pseudomonadales (1236)	Gammaproteobacteria (122	Proteobacteria (2)	Bacteria (1)	100.00	0.30	99.80	98.50	WGS
287.2276	0.3314	Pseudomonas aeruginosa (286)	Pseudomonas (135621)	Pseudomonadaceae (7227	Pseudomonadales (1236)	Gammaproteobacteria (122	Proteobacteria (2)	Bacteria (1)	100.00	0.00	99.80	99.00	WGS
287.2277	0.3386	Pseudomonas aeruginosa (286)	Pseudomonas (135621)	Pseudomonadaceae (7227	Pseudomonadales (1236)	Gammaproteobacteria (122	Proteobacteria (2)	Bacteria (1)	100.00	0.00	99.80	98.70	WGS
287.2278	0.3314	Pseudomonas aeruginosa (286)	Pseudomonas (135621)	Pseudomonadaceae (7227	Pseudomonadales (1236)	Gammaproteobacteria (122	Proteobacteria (2)	Bacteria (1)	100.00	0.00	99.80	99.00	WGS
287.2279	0.3314	Pseudomonas aeruginosa (286)	Pseudomonas (135621)	Pseudomonadaceae (7227	Pseudomonadales (1236)	Gammaproteobacteria (122	Proteobacteria (2)	Bacteria (1)	100.00	0.00	99.90	99.10	WGS
287.2280	0.3628	Pseudomonas aeruginosa (286)	Pseudomonas (135621)	Pseudomonadaceae (7227	Pseudomonadales (1236)	Gammaproteobacteria (122	Proteobacteria (2)	Bacteria (1)	100.00	0.00	99.90	99.10	WGS
287.2281	0.332	Pseudomonas aeruginosa (286)	Pseudomonas (135621)	Pseudomonadaceae (7227	Pseudomonadales (1236)	Gammaproteobacteria (122	Proteobacteria (2)	Bacteria (1)	100.00	0.30	100.00	99.20	WGS
287.2282	0.3314	Pseudomonas aeruginosa (286)	Pseudomonas (135621)	Pseudomonadaceae (7227	Pseudomonadales (1236)	Gammaproteobacteria (122	Proteobacteria (2)	Bacteria (1)	100.00	0.00	99.80	99.00	WGS
287.2283	0.3314	Pseudomonas aeruginosa (286)	Pseudomonas (135621)	Pseudomonadaceae (7227	Pseudomonadales (1236)	Gammaproteobacteria (122	Proteobacteria (2)	Bacteria (1)	100.00	0.00	99.80	99.10	WGS
287.2284	0.3358	Pseudomonas aeruginosa (286)	Pseudomonas (135621)	Pseudomonadaceae (7227	Pseudomonadales (1236)	Gammaproteobacteria (122	Proteobacteria (2)	Bacteria (1)	99.40	0.00	99.80	98.90	WGS
287.2285	0.3578	Pseudomonas aeruginosa (286)	Pseudomonas (135621)	Pseudomonadaceae (7227	Pseudomonadales (1236)	Gammaproteobacteria (122	Proteobacteria (2)	Bacteria (1)	100.00	0.60	99.90	99.00	WGS
287.2286	0.3628	Pseudomonas aeruginosa (286)	Pseudomonas (135621)	Pseudomonadaceae (7227	Pseudomonadales (1236)	Gammaproteobacteria (122	Proteobacteria (2)	Bacteria (1)	100.00	0.00	99.90	99.10	WGS
287.2287	0.3258	Pseudomonas aeruginosa (286)	Pseudomonas (135621)	Pseudomonadaceae (7227	Pseudomonadales (1236)	Gammaproteobacteria (122	Proteobacteria (2)	Bacteria (1)	100.00	0.30	99.90	99.60	WGS
287.2288	0.368	Pseudomonas aeruginosa (286)	Pseudomonas (135621)	Pseudomonadaceae (7227	Pseudomonadales (1236)	Gammaproteobacteria (122	Proteobacteria (2)	Bacteria (1)	100.00	0.00	99.90	99.00	WGS
287.2289	0.3258	Pseudomonas aeruginosa (286)	Pseudomonas (135621)	Pseudomonadaceae (7227	Pseudomonadales (1236)	Gammaproteobacteria (122	Proteobacteria (2)	Bacteria (1)	100.00	0.90	99.80	98.90	WGS
287.2290	0.3872	Pseudomonas aeruginosa (286)	Pseudomonas (135621)	Pseudomonadaceae (7227	Pseudomonadales (1236)	Gammaproteobacteria (122	Proteobacteria (2)	Bacteria (1)	100.00	0.30	99.90	99.10	WGS
287.2291	0.3258	Pseudomonas aeruginosa (286)	Pseudomonas (135621)	Pseudomonadaceae (7227	Pseudomonadales (1236)	Gammaproteobacteria (122	Proteobacteria (2)	Bacteria (1)	100.00	1.20	99.90	99.20	WGS
287.2292	0.3636	Pseudomonas aeruginosa (286)	Pseudomonas (135621)	Pseudomonadaceae (7227	Pseudomonadales (1236)	Gammaproteobacteria (122	Proteobacteria (2)	Bacteria (1)	100.00	0.00	99.80	99.30	WGS
287.2293	0.318	Pseudomonas aeruginosa (286)	Pseudomonas (135621)	Pseudomonadaceae (7227	Pseudomonadales (1236)	Gammaproteobacteria (122	Proteobacteria (2)	Bacteria (1)	99.70	0.60	99.60	98.50	WGS
287.2294	0.3376	Pseudomonas aeruginosa (286)	Pseudomonas (135621)	Pseudomonadaceae (7227	Pseudomonadales (1236)	Gammaproteobacteria (122	Proteobacteria (2)	Bacteria (1)	100.00	0.00	99.90	99.30	WGS
287.2295	0.368	Pseudomonas aeruginosa (286)	Pseudomonas (135621)	Pseudomonadaceae (7227	Pseudomonadales (1236)	Gammaproteobacteria (122	Proteobacteria (2)	Bacteria (1)	100.00	0.00	99.80	99.30	WGS
287.2296	0.3738	Pseudomonas aeruginosa (286)	Pseudomonas (135621)	Pseudomonadaceae (7227	Pseudomonadales (1236)	Gammaproteobacteria (122	Proteobacteria (2)	Bacteria (1)	98.10	0.90	99.70	98.70	WGS
287.2297	0.3376	Pseudomonas aeruginosa (286)	Pseudomonas (135621)	Pseudomonadaceae (7227	Pseudomonadales (1236)	Gammaproteobacteria (122	Proteobacteria (2)	Bacteria (1)	100.00	0.00	99.90	98.70	WGS
287.2298	0.3336	Pseudomonas aeruginosa (286)	Pseudomonas (135621)	Pseudomonadaceae (7227	Pseudomonadales (1236)	Gammaproteobacteria (122	Proteobacteria (2)	Bacteria (1)	100.00	0.90	99.80	98.40	WGS
287.2299	0.3376	Pseudomonas aeruginosa (286)	Pseudomonas (135621)	Pseudomonadaceae (7227	Pseudomonadales (1236)	Gammaproteobacteria (122	Proteobacteria (2)	Bacteria (1)	100.00	0.00	99.80	98.90	WGS
287.2300	0.3376	Pseudomonas aeruginosa (286)	Pseudomonas (135621)	Pseudomonadaceae (7227	Pseudomonadales (1236)	Gammaproteobacteria (122	Proteobacteria (2)	Bacteria (1)	100.00	0.00	99.80	97.90	WGS
287.2301	0.4056	Pseudomonas aeruginosa (286)	Pseudomonas (135621)	Pseudomonadaceae (7227	Pseudomonadales (1236)	Gammaproteobacteria (122	Proteobacteria (2)	Bacteria (1)	99.10	0.00	99.30	98.60	WGS
287.2302	0.3336	Pseudomonas aeruginosa (286)	Pseudomonas (135621)	Pseudomonadaceae (7227	Pseudomonadales (1236)	Gammaproteobacteria (122	Proteobacteria (2)	Bacteria (1)	100.00	0.00	99.90	99.10	WGS
287.2303	0.371	Pseudomonas aeruginosa (286)	Pseudomonas (135621)	Pseudomonadaceae (7227	Pseudomonadales (1236)	Gammaproteobacteria (122	Proteobacteria (2)	Bacteria (1)	100.00	0.00	99.90	99.10	WGS
287.2304	0.3628	Pseudomonas aeruginosa (286)	Pseudomonas (135621)	Pseudomonadaceae (7227	Pseudomonadales (1236)	Gammaproteobacteria (122	Proteobacteria (2)	Bacteria (1)	99.70	0.00	99.90	99.00	WGS
287.2305	0.3704	Pseudomonas aeruginosa (286)	Pseudomonas (135621)	Pseudomonadaceae (7227	Pseudomonadales (1236)	Gammaproteobacteria (122	Proteobacteria (2)	Bacteria (1)	99.70	0.30	99.70	98.50	WGS
287.2306	0.3738	Pseudomonas aeruginosa (286)	Pseudomonas (135621)	Pseudomonadaceae (7227	Pseudomonadales (1236)	Gammaproteobacteria (122	Proteobacteria (2)	Bacteria (1)	98.90	0.90	99.70	99.00	WGS
287.2307	0.3324	Pseudomonas aeruginosa (286)	Pseudomonas (135621)	Pseudomonadaceae (7227	Pseudomonadales (1236)	Gammaproteobacteria (122	Proteobacteria (2)	Bacteria (1)	100.00	0.00	99.90	99.30	WGS
287.2308	0.3236	Pseudomonas aeruginosa (286)	Pseudomonas (135621)	Pseudomonadaceae (7227	Pseudomonadales (1236)	Gammaproteobacteria (122	Proteobacteria (2)	Bacteria (1)	100.00	0.60	99.60	98.70	WGS
287.2309	0.3258	Pseudomonas aeruginosa (286)	Pseudomonas (135621)	Pseudomonadaceae (7227	Pseudomonadales (1236)	Gammaproteobacteria (122	Proteobacteria (2)	Bacteria (1)	100.00	0.00	99.80	99.30	WGS
287.2310	0.3704	Pseudomonas aeruginosa (286)	Pseudomonas (135621)	Pseudomonadaceae (7227	Pseudomonadales (1236)	Gammaproteobacteria (122	Proteobacteria (2)	Bacteria (1)	100.00	0.00	99.80	99.40	WGS
287.2311	0.369	Pseudomonas aeruginosa (286)	Pseudomonas (135621)	Pseudomonadaceae (7227	Pseudomonadales (1236)	Gammaproteobacteria (122	Proteobacteria (2)	Bacteria (1)	100.00	0.90	99.70	98.20	WGS
287.2312	0.3748	Pseudomonas aeruginosa (286)	Pseudomonas (135621)	Pseudomonadaceae (7227	Pseudomonadales (1236)	Gammaproteobacteria (122	Proteobacteria (2)	Bacteria (1)	100.00	0.00	99.80	99.00	WGS
287.2313	0.3184	Pseudomonas aeruginosa (286)	Pseudomonas (135621)	Pseudomonadaceae (7227	Pseudomonadales (1236)	Gammaproteobacteria (122	Proteobacteria (2)	Bacteria (1)	100.00	0.00	99.90	99.40	WGS
287.2314	0.3636	Pseudomonas aeruginosa (286)	Pseudomonas (135621)	Pseudomonadaceae (7227	Pseudomonadales (1236)	Gammaproteobacteria (122	Proteobacteria (2)	Bacteria (1)	100.00	0.00	99.80	99.40	WGS
287.2315	0.3324	Pseudomonas aeruginosa (286)	Pseudomonas (135621)	Pseudomonadaceae (7227	Pseudomonadales (1236)	Gammaproteobacteria (122	Proteobacteria (2)	Bacteria (1)	100.00	0.00	99.70	99.50	WGS
287.2316	0.3604	Pseudomonas aeruginosa (286)	Pseudomonas (135621)	Pseudomonadaceae (7227	Pseudomonadales (1236)	Gammaproteobacteria (122	Proteobacteria (2)	Bacteria (1)	98.70	1.50	99.40	97.70	WGS
287.2317	0.3316	Pseudomonas aeruginosa (286)	Pseudomonas (135621)	Pseudomonadaceae (7227	Pseudomonadales (1236)	Gammaproteobacteria (122	Proteobacteria (2)	Bacteria (1)	100.00	0.00	99.90	99.20	WGS
287.2318	0.3324	Pseudomonas aeruginosa (286)	Pseudomonas (135621)	Pseudomonadaceae (7227	Pseudomonadales (1236)	Gammaproteobacteria (122	Proteobacteria (2)	Bacteria (1)	100.00	0.60	99.90	98.70	WGS
287.2319	0.3636	Pseudomonas aeruginosa (286)	Pseudomonas (135621)	Pseudomonadaceae (7227	Pseudomonadales (1236)	Gammaproteobacteria (122	Proteobacteria (2)	Bacteria (1)	100.00	0.30	99.80	99.30	WGS
287.2320	0.3422	Pseudomonas aeruginosa (286)	Pseudomonas (135621)	Pseudomonadaceae (7227	Pseudomonadales (1236)	Gammaproteobacteria (122	Proteobacteria (2)	Bacteria (1)	98.80	0.60	99.90	99.10	WGS
287.2321	0.3738	Pseudomonas aeruginosa (286)	Pseudomonas (135621)	Pseudomonadaceae (7227	Pseudomonadales (1236)	Gammaproteobacteria (122	Proteobacteria (2)	Bacteria (1)	98.90	1.20	99.90	99.00	WGS
287.2322	0.3938	Pseudomonas aeruginosa (286)	Pseudomonas (135621)	Pseudomonadaceae (7227	Pseudomonadales (1236)	Gammaproteobacteria (122	Proteobacteria (2)	Bacteria (1)	99.70	0.60	99.80	97.90	WGS
287.2323	0.3672	Pseudomonas aeruginosa (286)	Pseudomonas (135621)	Pseudomonadaceae (7227	Pseudomonadales (1236)	Gammaproteobacteria (122	Proteobacteria (2)	Bacteria (1)	100.00	0.60	99.90	98.90	WGS
287.2324	0.3314	Pseudomonas aeruginosa (286)	Pseudomonas (135621)	Pseudomonadaceae (7227	Pseudomonadales (1236)	Gammaproteobacteria (122	Proteobacteria (2)	Bacteria (1)	100.00	0.00	99.80	99.00	WGS
287.2325	0.3628	Pseudomonas aeruginosa (286)	Pseudomonas (135621)	Pseudomonadaceae (7227	Pseudomonadales (1236)	Gammaproteobacteria (122	Proteobacteria (2)	Bacteria (1)	99.70	0.00	99.80	99.00	WGS
287.2326	0.368	Pseudomonas aeruginosa (286)	Pseudomonas (135621)	Pseudomonadaceae (7227	Pseudomonadales (1236)	Gammaproteobacteria (122	Proteobacteria (2)	Bacteria (1)	100.00	0.30	99.80	98.40	WGS
287.2327	0.3306	Pseudomonas aeruginosa (286)	Pseudomonas (135621)	Pseudomonadaceae (7227	Pseudomonadales (1236)	Gammaproteobacteria (122	Proteobacteria (2)	Bacteria (1)	100.00	0.30	99.90	99.20	WGS
287.2328	0.3938	Pseudomonas aeruginosa (286)	Pseudomonas (135621)	Pseudomonadaceae (7227	Pseudomonadales (1236)	Gammaproteobacteria (122	Proteobacteria (2)	Bacteria (1)	99.70	0.60	99.80	97.90	WGS
287.2329	0.3578	Pseudomonas aeruginosa (286)	Pseudomonas (135621)	Pseudomonadaceae (7227	Pseudomonadales (1236)	Gammaproteobacteria (122	Proteobacteria (2)	Bacteria (1)	100.00	0.30	99.80	98.80	WGS
287.2330	0.3376	Pseudomonas aeruginosa (286)	Pseudomonas (135621)	Pseudomonadaceae (7227	Pseudomonadales (1236)	Gammaproteobacteria (122	Proteobacteria (2)	Bacteria (1)	99.70	0.00	99.90	99.00	WGS
287.2331	0.3306	Pseudomonas aeruginosa (286)	Pseudomonas (135621)	Pseudomonadaceae (7227	Pseudomonadales (1236)	Gammaproteobacteria (122	Proteobacteria (2)	Bacteria (1)	100.00	0.60	99.90	98.90	WGS
287.2332	0.3324	Pseudomonas aeruginosa (286)	Pseudomonas (135621)	Pseudomonadaceae (7227	Pseudomonadales (1236)	Gammaproteobacteria (122	Proteobacteria (2)	Bacteria (1)	100.00	0.30	99.80	98.70	WGS
287.2333	0.3174	Pseudomonas aeruginosa (286)	Pseudomonas (135621)	Pseudomonadaceae (7227	Pseudomonadales (1236)	Gammaproteobacteria (122	Proteobacteria (2)	Bacteria (1)	100.00	0.30	99.80	99.00	WGS
287.2334	0.324	Pseudomonas aeruginosa (286)	Pseudomonas (135621)	Pseudomonadaceae (7227	Pseudomonadales (1236)	Gammaproteobacteria (122	Proteobacteria (2)	Bacteria (1)	98.50	0.00	99.90	99.30	WGS
287.2335	0.3258	Pseudomonas aeruginosa (286)	Pseudomonas (135621)	Pseudomonadaceae (7227	Pseudomonadales (1236)	Gammaproteobacteria (122	Proteobacteria (2)	Bacteria (1)	100.00	0.30	99.90	99.40	WGS
287.2336	0.3618	Pseudomonas aeruginosa (286)	Pseudomonas (135621)	Pseudomonadaceae (7227	Pseudomonadales (1236)	Gammaproteobacteria (122	Proteobacteria (2)	Bacteria (1)	100.00	0.30	99.70	98.30	WGS
287.2337	0.3704	Pseudomonas aeruginosa (286)	Pseudomonas (135621)	Pseudomonadaceae (7227	Pseudomonadales (1236)	Gammaproteobacteria (122	Proteobacteria (2)	Bacteria (1)	100.00	0.00	99.80	99.10	WGS
287.2338	0.3398	Pseudomonas aeruginosa (286)	Pseudomonas (135621)	Pseudomonadaceae (7227	Pseudomonadales (1236)	Gammaproteobacteria (122	Proteobacteria (2)	Bacteria (1)	100.00	0.00	99.90	98.90	WGS
287.2339	0.3346	Pseudomonas aeruginosa (286)	Pseudomonas (135621)	Pseudomonadaceae (7227	Pseudomonadales (1236)	Gammaproteobacteria (122	Proteobacteria (2)	Bacteria (1)	100.00	0.30	99.90	98.70	WGS
287.2340	0.371	Pseudomonas aeruginosa (286)	Pseudomonas (135621)	Pseudomonadaceae (7227	Pseudomonadales (1236)	Gammaproteobacteria (122	Proteobacteria (2)	Bacteria (1)	100.00	0.00	99.90	99.60	WGS
287.2341	0.327	Pseudomonas aeruginosa (286)	Pseudomonas (135621)	Pseudomonadaceae (7227	Pseudomonadales (1236)	Gammaproteobacteria (122	Proteobacteria (2)	Bacteria (1)	100.00	0.30	99.80	99.00	WGS
287.2342	0.3754	Pseudomonas aeruginosa (286)	Pseudomonas (135621)	Pseudomonadaceae (7227	Pseudomonadales (1236)	Gammaproteobacteria (122	Proteobacteria (2)	Bacteria (1)	100.00	0.00	99.90	99.10	WGS
287.2343	0.332	Pseudomonas aeruginosa (286)	Pseudomonas (135621)	Pseudomonadaceae (7227	Pseudomonadales (1236)	Gammaproteobacteria (122	Proteobacteria (2)	Bacteria (1)	100.00	0.00	99.80	98.60	WGS
287.2344	0.3292	Pseudomonas aeruginosa (286)	Pseudomonas (135621)	Pseudomonadaceae (7227	Pseudomonadales (1236)	Gammaproteobacteria (122	Proteobacteria (2)	Bacteria (1)	100.00	0.00	99.60	98.40	WGS
287.2345	0.368	Pseudomonas aeruginosa (286)	Pseudomonas (135621)	Pseudomonadaceae (7227	Pseudomonadales (1236)	Gammaproteobacteria (122	Proteobacteria (2)	Bacteria (1)	100.00	0.00	99.80	99.20	WGS
287.2346	0.3738	Pseudomonas aeruginosa (286)	Pseudomonas (135621)	Pseudomonadaceae (7227	Pseudomonadales (1236)	Gammaproteobacteria (122	Proteobacteria (2)	Bacteria (1)	100.00	0.00	99.90	99.10	WGS
287.2347	0.368	Pseudomonas aeruginosa (286)	Pseudomonas (135621)	Pseudomonadaceae (7227	Pseudomonadales (1236)	Gammaproteobacteria (122	Proteobacteria (2)	Bacteria (1)	97.00	0.00	99.80	98.80	WGS
287.2348	0.3628	Pseudomonas aeruginosa (286)	Pseudomonas (135621)	Pseudomonadaceae (7227	Pseudomonadales (1236)	Gammaproteobacteria (122	Proteobacteria (2)	Bacteria (1)	100.00	0.00	99.90	99.20	WGS
287.2349	0.3336	Pseudomonas aeruginosa (286)	Pseudomonas (135621)	Pseudomonadaceae (7227	Pseudomonadales (1236)	Gammaproteobacteria (122	Proteobacteria (2)	Bacteria (1)	100.00	0.00	99.90	99.00	WGS
287.2350	0.3336	Pseudomonas aeruginosa (286)	Pseudomonas (135621)	Pseudomonadaceae (7227	Pseudomonadales (1236)	Gammaproteobacteria (122	Proteobacteria (2)	Bacteria (1)	100.00	0.00	99.90	98.90	WGS
287.2351	0.3304	Pseudomonas aeruginosa (286)	Pseudomonas (135621)	Pseudomonadaceae (7227	Pseudomonadales (1236)	Gammaproteobacteria (122	Proteobacteria (2)	Bacteria (1)	100.00	0.00	99.80	98.90	WGS
287.2352	0.3314	Pseudomonas aeruginosa (286)	Pseudomonas (135621)	Pseudomonadaceae (7227	Pseudomonadales (1236)	Gammaproteobacteria (122	Proteobacteria (2)	Bacteria (1)	100.00	0.00	99.80	99.10	WGS
287.2353	0.3258	Pseudomonas aeruginosa (286)	Pseudomonas (135621)	Pseudomonadaceae (7227	Pseudomonadales (1236)	Gammaproteobacteria (122	Proteobacteria (2)	Bacteria (1)	100.00	0.00	99.90	99.60	WGS
287.2354	0.3628	Pseudomonas aeruginosa (286)	Pseudomonas (135621)	Pseudomonadaceae (7227	Pseudomonadales (1236)	Gammaproteobacteria (122	Proteobacteria (2)	Bacteria (1)	100.00	0.00	99.90	98.90	WGS
287.2355	0.3246	Pseudomonas aeruginosa (286)	Pseudomonas (135621)	Pseudomonadaceae (7227	Pseudomonadales (1236)	Gammaproteobacteria (122	Proteobacteria (2)	Bacteria (1)	100.00	0.00	99.80	99.20	WGS
287.2356	0.368	Pseudomonas aeruginosa (286)	Pseudomonas (135621)	Pseudomonadaceae (7227	Pseudomonadales (1236)	Gammaproteobacteria (122	Proteobacteria (2)	Bacteria (1)	100.00	1.50	99.90	98.80	WGS
287.2357	0.3258	Pseudomonas aeruginosa (286)	Pseudomonas (135621)	Pseudomonadaceae (7227	Pseudomonadales (1236)	Gammaproteobacteria (122	Proteobacteria (2)	Bacteria (1)	100.00	0.60	99.90	99.50	WGS
287.2358	0.368	Pseudomonas aeruginosa (286)	Pseudomonas (135621)	Pseudomonadaceae (7227	Pseudomonadales (1236)	Gammaproteobacteria (122	Proteobacteria (2)	Bacteria (1)	96.70	0.00	99.80	98.80	WGS
287.2359	0.386	Pseudomonas aeruginosa (286)	Pseudomonas (135621)	Pseudomonadaceae (7227	Pseudomonadales (1236)	Gammaproteobacteria (122	Proteobacteria (2)	Bacteria (1)	97.00	0.30	98.40	97.30	WGS
287.2360	0.3682	Pseudomonas aeruginosa (286)	Pseudomonas (135621)	Pseudomonadaceae (7227	Pseudomonadales (1236)	Gammaproteobacteria (122	Proteobacteria (2)	Bacteria (1)	100.00	0.00	99.90	98.50	WGS
287.2361	0.3398	Pseudomonas aeruginosa (286)	Pseudomonas (135621)	Pseudomonadaceae (7227	Pseudomonadales (1236)	Gammaproteobacteria (122	Proteobacteria (2)	Bacteria (1)	100.00	0.00	99.90	99.00	WGS
287.2362	0.3346	Pseudomonas aeruginosa (286)	Pseudomonas (135621)	Pseudomonadaceae (7227	Pseudomonadales (1236)	Gammaproteobacteria (122	Proteobacteria (2)	Bacteria (1)	100.00	0.30	99.90	99.30	WGS
287.2363	0.3246	Pseudomonas aeruginosa (286)	Pseudomonas (135621)	Pseudomonadaceae (7227	Pseudomonadales (1236)	Gammaproteobacteria (122	Proteobacteria (2)	Bacteria (1)	100.00	0.00	99.80	99.20	WGS
287.2364	0.3306	Pseudomonas aeruginosa (286)	Pseudomonas (135621)	Pseudomonadaceae (7227	Pseudomonadales (1236)	Gammaproteobacteria (122	Proteobacteria (2)	Bacteria (1)	100.00	0.00	100.00	99.20	WGS
287.2365	0.3226	Pseudomonas aeruginosa (286)	Pseudomonas (135621)	Pseudomonadaceae (7227	Pseudomonadales (1236)	Gammaproteobacteria (122	Proteobacteria (2)	Bacteria (1)	100.00	0.40	99.90	99.10	WGS
287.2366	0.3258	Pseudomonas aeruginosa (286)	Pseudomonas (135621)	Pseudomonadaceae (7227	Pseudomonadales (1236)	Gammaproteobacteria (122	Proteobacteria (2)	Bacteria (1)	100.00	0.00	99.80	99.30	WGS
287.2367	0.3292	Pseudomonas aeruginosa (286)	Pseudomonas (135621)	Pseudomonadaceae (7227	Pseudomonadales (1236)	Gammaproteobacteria (122	Proteobacteria (2)	Bacteria (1)	100.00	0.30	99.90	99.00	WGS
287.2368	0.3376	Pseudomonas aeruginosa (286)	Pseudomonas (135621)	Pseudomonadaceae (7227	Pseudomonadales (1236)	Gammaproteobacteria (122	Proteobacteria (2)	Bacteria (1)	99.70	0.00	99.70	98.70	WGS
287.2369	0.3376	Pseudomonas aeruginosa (286)	Pseudomonas (135621)	Pseudomonadaceae (7227	Pseudomonadales (1236)	Gammaproteobacteria (122	Proteobacteria (2)	Bacteria (1)	100.00	0.00	99.80	99.00	WGS
287.2370	0.3578	Pseudomonas aeruginosa (286)	Pseudomonas (135621)	Pseudomonadaceae (7227	Pseudomonadales (1236)	Gammaproteobacteria (122	Proteobacteria (2)	Bacteria (1)	100.00	0.00	99.00	98.80	WGS
287.2371	0.3748	Pseudomonas aeruginosa (286)	Pseudomonas (135621)	Pseudomonadaceae (7227	Pseudomonadales (1236)	Gammaproteobacteria (122	Proteobacteria (2)	Bacteria (1)	99.70	0.00	99.80	98.70	WGS
287.2372	0.3628	Pseudomonas aeruginosa (286)	Pseudomonas (135621)	Pseudomonadaceae (7227	Pseudomonadales (1236)	Gammaproteobacteria (122	Proteobacteria (2)	Bacteria (1)	100.00	0.00	99.90	99.00	WGS
287.2373	0.3388	Pseudomonas aeruginosa (286)	Pseudomonas (135621)	Pseudomonadaceae (7227	Pseudomonadales (1236)	Gammaproteobacteria (122	Proteobacteria (2)	Bacteria (1)	100.00	0.00	99.60	98.80	WGS
287.2374	0.3618	Pseudomonas aeruginosa (286)	Pseudomonas (135621)	Pseudomonadaceae (7227	Pseudomonadales (1236)	Gammaproteobacteria (122	Proteobacteria (2)	Bacteria (1)	100.00	0.30	99.80	99.30	WGS
287.2375	0.3694	Pseudomonas aeruginosa (286)	Pseudomonas (135621)	Pseudomonadaceae (7227	Pseudomonadales (1236)	Gammaproteobacteria (122	Proteobacteria (2)	Bacteria (1)	100.00	0.30	99.80	99.00	WGS
287.2376	0.3306	Pseudomonas aeruginosa (286)	Pseudomonas (135621)	Pseudomonadaceae (7227	Pseudomonadales (1236)	Gammaproteobacteria (122	Proteobacteria (2)	Bacteria (1)	100.00	0.00	99.80	98.70	WGS
287.2377	0.331	Pseudomonas aeruginosa (286)	Pseudomonas (135621)	Pseudomonadaceae (7227	Pseudomonadales (1236)	Gammaproteobacteria (122	Proteobacteria (2)	Bacteria (1)	100.00	0.00	99.90	99.60	WGS
287.2378	0.3672	Pseudomonas aeruginosa (286)	Pseudomonas (135621)	Pseudomonadaceae (7227	Pseudomonadales (1236)	Gammaproteobacteria (122	Proteobacteria (2)	Bacteria (1)	100.00	0.30	99.90	99.00	WGS
287.2379	0.3804	Pseudomonas aeruginosa (286)	Pseudomonas (135621)	Pseudomonadaceae (7227	Pseudomonadales (1236)	Gammaproteobacteria (122	Proteobacteria (2)	Bacteria (1)	93.40	0.00	97.20	96.00	WGS
287.2380	0.312	Pseudomonas aeruginosa (286)	Pseudomonas (135621)	Pseudomonadaceae (7227	Pseudomonadales (1236)	Gammaproteobacteria (122	Proteobacteria (2)	Bacteria (1)	89.50	0.00	93.30	91.70	WGS
287.2381	0.3302	Pseudomonas aeruginosa (286)	Pseudomonas (135621)	Pseudomonadaceae (7227	Pseudomonadales (1236)	Gammaproteobacteria (122	Proteobacteria (2)	Bacteria (1)	100.00	0.00	99.90	99.70	WGS
287.2382	0.3636	Pseudomonas aeruginosa (286)	Pseudomonas (135621)	Pseudomonadaceae (7227	Pseudomonadales (1236)	Gammaproteobacteria (122	Proteobacteria (2)	Bacteria (1)	100.00	0.00	99.80	99.60	WGS
287.2383	0.356	Pseudomonas aeruginosa (286)	Pseudomonas (135621)	Pseudomonadaceae (7227	Pseudomonadales (1236)	Gammaproteobacteria (122	Proteobacteria (2)	Bacteria (1)	100.00	0.30	99.70	99.10	WGS
287.2384	0.328	Pseudomonas aeruginosa (286)	Pseudomonas (135621)	Pseudomonadaceae (7227	Pseudomonadales (1236)	Gammaproteobacteria (122	Proteobacteria (2)	Bacteria (1)	99.70	0.00	99.90	99.20	WGS
287.2385	0.3284	Pseudomonas aeruginosa (286)	Pseudomonas (135621)	Pseudomonadaceae (7227	Pseudomonadales (1236)	Gammaproteobacteria (122	Proteobacteria (2)	Bacteria (1)	95.80	0.30	98.40	97.50	WGS
287.2386	0.3386	Pseudomonas aeruginosa (286)	Pseudomonas (135621)	Pseudomonadaceae (7227	Pseudomonadales (1236)	Gammaproteobacteria (122	Proteobacteria (2)	Bacteria (1)	100.00	0.00	99.80	98.70	WGS
287.2387	0.357	Pseudomonas aeruginosa (286)	Pseudomonas (135621)	Pseudomonadaceae (7227	Pseudomonadales (1236)	Gammaproteobacteria (122	Proteobacteria (2)	Bacteria (1)	100.00	0.10	99.90	99.30	WGS
287.2388	0.3198	Pseudomonas aeruginosa (286)	Pseudomonas (135621)	Pseudomonadaceae (7227	Pseudomonadales (1236)	Gammaproteobacteria (122	Proteobacteria (2)	Bacteria (1)	99.70	0.30	99.40	99.00	WGS
287.2389	0.359	Pseudomonas aeruginosa (286)	Pseudomonas (135621)	Pseudomonadaceae (7227	Pseudomonadales (1236)	Gammaproteobacteria (122	Proteobacteria (2)	Bacteria (1)	100.00	0.00	99.80	99.10	WGS
287.2390	0.3324	Pseudomonas aeruginosa (286)	Pseudomonas (135621)	Pseudomonadaceae (7227	Pseudomonadales (1236)	Gammaproteobacteria (122	Proteobacteria (2)	Bacteria (1)	98.80	0.00	99.80	98.50	WGS
287.2391	0.3174	Pseudomonas aeruginosa (286)	Pseudomonas (135621)	Pseudomonadaceae (7227	Pseudomonadales (1236)	Gammaproteobacteria (122	Proteobacteria (2)	Bacteria (1)	100.00	0.00	99.90	99.50	WGS
287.2392	0.3628	Pseudomonas aeruginosa (286)	Pseudomonas (135621)	Pseudomonadaceae (7227	Pseudomonadales (1236)	Gammaproteobacteria (122	Proteobacteria (2)	Bacteria (1)	100.00	0.00	99.80	99.00	WGS
287.2393	0.3386	Pseudomonas aeruginosa (286)	Pseudomonas (135621)	Pseudomonadaceae (7227	Pseudomonadales (1236)	Gammaproteobacteria (122	Proteobacteria (2)	Bacteria (1)	99.70	0.00	99.80	98.70	WGS
287.2394	0.3354	Pseudomonas aeruginosa (286)	Pseudomonas (135621)	Pseudomonadaceae (7227	Pseudomonadales (1236)	Gammaproteobacteria (122	Proteobacteria (2)	Bacteria (1)	100.00	0.90	99.80	98.70	WGS
287.2395	0.356	Pseudomonas aeruginosa (286)	Pseudomonas (135621)	Pseudomonadaceae (7227	Pseudomonadales (1236)	Gammaproteobacteria (122	Proteobacteria (2)	Bacteria (1)	98.40	0.90	99.80	98.00	WGS
287.2396	0.368	Pseudomonas aeruginosa (286)	Pseudomonas (135621)	Pseudomonadaceae (7227	Pseudomonadales (1236)	Gammaproteobacteria (122	Proteobacteria (2)	Bacteria (1)	100.00	0.30	99.70	99.10	WGS
287.2397	0.3748	Pseudomonas aeruginosa (286)	Pseudomonas (135621)	Pseudomonadaceae (7227	Pseudomonadales (1236)	Gammaproteobacteria (122	Proteobacteria (2)	Bacteria (1)	100.00	0.00	99.80	99.10	WGS
287.2398	0.368	Pseudomonas aeruginosa (286)	Pseudomonas (135621)	Pseudomonadaceae (7227	Pseudomonadales (1236)	Gammaproteobacteria (122	Proteobacteria (2)	Bacteria (1)	96.70	0.00	98.20	96.80	WGS
287.2399	0.3628	Pseudomonas aeruginosa (286)	Pseudomonas (135621)	Pseudomonadaceae (7227	Pseudomonadales (1236)	Gammaproteobacteria (122	Proteobacteria (2)	Bacteria (1)	100.00	0.00	99.90	99.20	WGS

287.2400	0.3672	Pseudomonas aeruginosa (286)	Pseudomonas (135621)	Pseudomonadaceae (7227	Pseudomonadales (1236)	Gammaproteobacteria (122	Proteobacteria (2)	Bacteria (1)	100.00	0.00	99.90	99.00	WGS
287.2401	0.357	Pseudomonas aeruginosa (286)	Pseudomonas (135621)	Pseudomonadaceae (7227	Pseudomonadales (1236)	Gammaproteobacteria (122	Proteobacteria (2)	Bacteria (1)	100.00	0.10	99.90	99.30	WGS
287.2402	0.3636	Pseudomonas aeruginosa (286)	Pseudomonas (135621)	Pseudomonadaceae (7227	Pseudomonadales (1236)	Gammaproteobacteria (122	Proteobacteria (2)	Bacteria (1)	100.00	0.00	99.90	99.50	WGS
287.2403	0.3376	Pseudomonas aeruginosa (286)	Pseudomonas (135621)	Pseudomonadaceae (7227	Pseudomonadales (1236)	Gammaproteobacteria (122	Proteobacteria (2)	Bacteria (1)	100.00	0.60	99.80	99.20	WGS
287.2404	0.331	Pseudomonas aeruginosa (286)	Pseudomonas (135621)	Pseudomonadaceae (7227	Pseudomonadales (1236)	Gammaproteobacteria (122	Proteobacteria (2)	Bacteria (1)	99.40	0.00	99.90	99.00	WGS
287.2405	0.3306	Pseudomonas aeruginosa (286)	Pseudomonas (135621)	Pseudomonadaceae (7227	Pseudomonadales (1236)	Gammaproteobacteria (122	Proteobacteria (2)	Bacteria (1)	100.00	0.30	99.80	98.90	WGS
287.2406	0.3376	Pseudomonas aeruginosa (286)	Pseudomonas (135621)	Pseudomonadaceae (7227	Pseudomonadales (1236)	Gammaproteobacteria (122	Proteobacteria (2)	Bacteria (1)	100.00	0.90	99.90	98.90	WGS
287.2407	0.3306	Pseudomonas aeruginosa (286)	Pseudomonas (135621)	Pseudomonadaceae (7227	Pseudomonadales (1236)	Gammaproteobacteria (122	Proteobacteria (2)	Bacteria (1)	100.00	0.00	99.90	99.60	WGS
287.2408	0.3236	Pseudomonas aeruginosa (286)	Pseudomonas (135621)	Pseudomonadaceae (7227	Pseudomonadales (1236)	Gammaproteobacteria (122	Proteobacteria (2)	Bacteria (1)	100.00	0.00	99.90	99.30	WGS
287.2409	0.3268	Pseudomonas aeruginosa (286)	Pseudomonas (135621)	Pseudomonadaceae (7227	Pseudomonadales (1236)	Gammaproteobacteria (122	Proteobacteria (2)	Bacteria (1)	100.00	0.00	99.60	98.40	WGS
287.2410	0.3174	Pseudomonas aeruginosa (286)	Pseudomonas (135621)	Pseudomonadaceae (7227	Pseudomonadales (1236)	Gammaproteobacteria (122	Proteobacteria (2)	Bacteria (1)	99.70	0.00	99.80	99.00	WGS
287.2411	0.3306	Pseudomonas aeruginosa (286)	Pseudomonas (135621)	Pseudomonadaceae (7227	Pseudomonadales (1236)	Gammaproteobacteria (122	Proteobacteria (2)	Bacteria (1)	100.00	0.00	99.80	99.00	WGS
287.2412	0.3618	Pseudomonas aeruginosa (286)	Pseudomonas (135621)	Pseudomonadaceae (7227	Pseudomonadales (1236)	Gammaproteobacteria (122	Proteobacteria (2)	Bacteria (1)	100.00	0.00	99.90	99.00	WGS
287.2413	0.3662	Pseudomonas aeruginosa (286)	Pseudomonas (135621)	Pseudomonadaceae (7227	Pseudomonadales (1236)	Gammaproteobacteria (122	Proteobacteria (2)	Bacteria (1)	100.00	0.00	99.90	99.30	WGS
287.2414	0.3672	Pseudomonas aeruginosa (286)	Pseudomonas (135621)	Pseudomonadaceae (7227	Pseudomonadales (1236)	Gammaproteobacteria (122	Proteobacteria (2)	Bacteria (1)	99.40	0.60	99.90	98.20	WGS
287.2415	0.356	Pseudomonas aeruginosa (286)	Pseudomonas (135621)	Pseudomonadaceae (7227	Pseudomonadales (1236)	Gammaproteobacteria (122	Proteobacteria (2)	Bacteria (1)	100.00	0.00	99.90	99.70	WGS
287.2416	0.3314	Pseudomonas aeruginosa (286)	Pseudomonas (135621)	Pseudomonadaceae (7227	Pseudomonadales (1236)	Gammaproteobacteria (122	Proteobacteria (2)	Bacteria (1)	100.00	0.00	99.80	99.00	WGS
287.2417	0.3306	Pseudomonas aeruginosa (286)	Pseudomonas (135621)	Pseudomonadaceae (7227	Pseudomonadales (1236)	Gammaproteobacteria (122	Proteobacteria (2)	Bacteria (1)	100.00	0.00	99.90	98.80	WGS
287.2418	0.368	Pseudomonas aeruginosa (286)	Pseudomonas (135621)	Pseudomonadaceae (7227	Pseudomonadales (1236)	Gammaproteobacteria (122	Proteobacteria (2)	Bacteria (1)	100.00	0.00	99.80	99.10	WGS
287.2419	0.331	Pseudomonas aeruginosa (286)	Pseudomonas (135621)	Pseudomonadaceae (7227	Pseudomonadales (1236)	Gammaproteobacteria (122	Proteobacteria (2)	Bacteria (1)	100.00	0.00	99.90	99.20	WGS
287.2420	0.3292	Pseudomonas aeruginosa (286)	Pseudomonas (135621)	Pseudomonadaceae (7227	Pseudomonadales (1236)	Gammaproteobacteria (122	Proteobacteria (2)	Bacteria (1)	100.00	0.00	99.80	99.00	WGS
287.2421	0.3604	Pseudomonas aeruginosa (286)	Pseudomonas (135621)	Pseudomonadaceae (7227	Pseudomonadales (1236)	Gammaproteobacteria (122	Proteobacteria (2)	Bacteria (1)	100.00	0.00	99.60	97.90	WGS
287.2422	0.3314	Pseudomonas aeruginosa (286)	Pseudomonas (135621)	Pseudomonadaceae (7227	Pseudomonadales (1236)	Gammaproteobacteria (122	Proteobacteria (2)	Bacteria (1)	100.00	0.30	99.80	98.80	WGS
287.2423	0.3704	Pseudomonas aeruginosa (286)	Pseudomonas (135621)	Pseudomonadaceae (7227	Pseudomonadales (1236)	Gammaproteobacteria (122	Proteobacteria (2)	Bacteria (1)	100.00	0.00	99.80	99.10	WGS
287.2424	0.356	Pseudomonas aeruginosa (286)	Pseudomonas (135621)	Pseudomonadaceae (7227	Pseudomonadales (1236)	Gammaproteobacteria (122	Proteobacteria (2)	Bacteria (1)	100.00	0.60	99.40	98.20	WGS
287.2425	0.3292	Pseudomonas aeruginosa (286)	Pseudomonas (135621)	Pseudomonadaceae (7227	Pseudomonadales (1236)	Gammaproteobacteria (122	Proteobacteria (2)	Bacteria (1)	100.00	0.00	99.90	99.30	WGS
287.2426	0.3604	Pseudomonas aeruginosa (286)	Pseudomonas (135621)	Pseudomonadaceae (7227	Pseudomonadales (1236)	Gammaproteobacteria (122	Proteobacteria (2)	Bacteria (1)	100.00	0.00	99.90	99.00	WGS
287.2427	0.3628	Pseudomonas aeruginosa (286)	Pseudomonas (135621)	Pseudomonadaceae (7227	Pseudomonadales (1236)	Gammaproteobacteria (122	Proteobacteria (2)	Bacteria (1)	100.00	0.00	99.90	99.30	WGS
287.2428	0.3324	Pseudomonas aeruginosa (286)	Pseudomonas (135621)	Pseudomonadaceae (7227	Pseudomonadales (1236)	Gammaproteobacteria (122	Proteobacteria (2)	Bacteria (1)	100.00	0.60	99.70	99.20	WGS
287.2429	0.3306	Pseudomonas aeruginosa (286)	Pseudomonas (135621)	Pseudomonadaceae (7227	Pseudomonadales (1236)	Gammaproteobacteria (122	Proteobacteria (2)	Bacteria (1)	100.00	0.90	99.90	98.80	WGS
287.2430	0.3304	Pseudomonas aeruginosa (286)	Pseudomonas (135621)	Pseudomonadaceae (7227	Pseudomonadales (1236)	Gammaproteobacteria (122	Proteobacteria (2)	Bacteria (1)	100.00	0.00	99.80	99.10	WGS
287.2431	0.3314	Pseudomonas aeruginosa (286)	Pseudomonas (135621)	Pseudomonadaceae (7227	Pseudomonadales (1236)	Gammaproteobacteria (122	Proteobacteria (2)	Bacteria (1)	100.00	0.00	99.80	98.80	WGS
287.2432	0.3628	Pseudomonas aeruginosa (286)	Pseudomonas (135621)	Pseudomonadaceae (7227	Pseudomonadales (1236)	Gammaproteobacteria (122	Proteobacteria (2)	Bacteria (1)	100.00	0.00	99.90	99.00	WGS
287.2433	0.3704	Pseudomonas aeruginosa (286)	Pseudomonas (135621)	Pseudomonadaceae (7227	Pseudomonadales (1236)	Gammaproteobacteria (122	Proteobacteria (2)	Bacteria (1)	94.90	0.30	95.30	94.50	WGS
287.2434	0.3388	Pseudomonas aeruginosa (286)	Pseudomonas (135621)	Pseudomonadaceae (7227	Pseudomonadales (1236)	Gammaproteobacteria (122	Proteobacteria (2)	Bacteria (1)	100.00	0.00	99.90	98.80	WGS
287.2435	0.3292	Pseudomonas aeruginosa (286)	Pseudomonas (135621)	Pseudomonadaceae (7227	Pseudomonadales (1236)	Gammaproteobacteria (122	Proteobacteria (2)	Bacteria (1)	100.00	0.00	99.90	99.00	WGS
287.2436	0.3332	Pseudomonas aeruginosa (286)	Pseudomonas (135621)	Pseudomonadaceae (7227	Pseudomonadales (1236)	Gammaproteobacteria (122	Proteobacteria (2)	Bacteria (1)	100.00	0.00	99.90	99.00	WGS
287.2437	0.332	Pseudomonas aeruginosa (286)	Pseudomonas (135621)	Pseudomonadaceae (7227	Pseudomonadales (1236)	Gammaproteobacteria (122	Proteobacteria (2)	Bacteria (1)	100.00	0.30	99.60	98.20	WGS
287.2438	0.3618	Pseudomonas aeruginosa (286)	Pseudomonas (135621)	Pseudomonadaceae (7227	Pseudomonadales (1236)	Gammaproteobacteria (122	Proteobacteria (2)	Bacteria (1)	100.00	0.00	99.90	98.90	WGS
287.2439	0.3636	Pseudomonas aeruginosa (286)	Pseudomonas (135621)	Pseudomonadaceae (7227	Pseudomonadales (1236)	Gammaproteobacteria (122	Proteobacteria (2)	Bacteria (1)	100.00	0.60	99.80	99.10	WGS
287.2440	0.3386	Pseudomonas aeruginosa (286)	Pseudomonas (135621)	Pseudomonadaceae (7227	Pseudomonadales (1236)	Gammaproteobacteria (122	Proteobacteria (2)	Bacteria (1)	100.00	0.00	99.90	98.40	WGS
287.2441	0.368	Pseudomonas aeruginosa (286)	Pseudomonas (135621)	Pseudomonadaceae (7227	Pseudomonadales (1236)	Gammaproteobacteria (122	Proteobacteria (2)	Bacteria (1)	97.00	0.00	99.80	98.70	WGS
287.2442	0.3738	Pseudomonas aeruginosa (286)	Pseudomonas (135621)	Pseudomonadaceae (7227	Pseudomonadales (1236)	Gammaproteobacteria (122	Proteobacteria (2)	Bacteria (1)	100.00	0.00	99.60	99.30	WGS
287.2443	0.3322	Pseudomonas aeruginosa (286)	Pseudomonas (135621)	Pseudomonadaceae (7227	Pseudomonadales (1236)	Gammaproteobacteria (122	Proteobacteria (2)	Bacteria (1)	100.00	1.50	99.90	98.40	WGS
287.2444	0.3388	Pseudomonas aeruginosa (286)	Pseudomonas (135621)	Pseudomonadaceae (7227	Pseudomonadales (1236)	Gammaproteobacteria (122	Proteobacteria (2)	Bacteria (1)	100.00	0.70	99.90	98.70	WGS
287.2445	0.3946	Pseudomonas aeruginosa (286)	Pseudomonas (135621)	Pseudomonadaceae (7227	Pseudomonadales (1236)	Gammaproteobacteria (122	Proteobacteria (2)	Bacteria (1)	100.00	0.90	99.80	98.70	WGS
287.2446	0.3636	Pseudomonas aeruginosa (286)	Pseudomonas (135621)	Pseudomonadaceae (7227	Pseudomonadales (1236)	Gammaproteobacteria (122	Proteobacteria (2)	Bacteria (1)	100.00	0.90	99.80	98.80	WGS
287.2447	0.3662	Pseudomonas aeruginosa (286)	Pseudomonas (135621)	Pseudomonadaceae (7227	Pseudomonadales (1236)	Gammaproteobacteria (122	Proteobacteria (2)	Bacteria (1)	98.10	1.50	99.80	98.60	WGS
287.2448	0.3258	Pseudomonas aeruginosa (286)	Pseudomonas (135621)	Pseudomonadaceae (7227	Pseudomonadales (1236)	Gammaproteobacteria (122	Proteobacteria (2)	Bacteria (1)	100.00	0.00	99.70	99.30	WGS
287.2449	0.3662	Pseudomonas aeruginosa (286)	Pseudomonas (135621)	Pseudomonadaceae (7227	Pseudomonadales (1236)	Gammaproteobacteria (122	Proteobacteria (2)	Bacteria (1)	100.00	1.20	99.90	97.70	WGS
287.2450	0.3524	Pseudomonas aeruginosa (286)	Pseudomonas (135621)	Pseudomonadaceae (7227	Pseudomonadales (1236)	Gammaproteobacteria (122	Proteobacteria (2)	Bacteria (1)	94.90	0.00	97.30	96.00	WGS
287.2451	0.371	Pseudomonas aeruginosa (286)	Pseudomonas (135621)	Pseudomonadaceae (7227	Pseudomonadales (1236)	Gammaproteobacteria (122	Proteobacteria (2)	Bacteria (1)	99.70	0.00	99.90	98.80	WGS
287.2452	0.3636	Pseudomonas aeruginosa (286)	Pseudomonas (135621)	Pseudomonadaceae (7227	Pseudomonadales (1236)	Gammaproteobacteria (122	Proteobacteria (2)	Bacteria (1)	100.00	0.00	99.90	99.60	WGS
287.2453	0.331	Pseudomonas aeruginosa (286)	Pseudomonas (135621)	Pseudomonadaceae (7227	Pseudomonadales (1236)	Gammaproteobacteria (122	Proteobacteria (2)	Bacteria (1)	100.00	0.00	99.70	99.10	WGS
287.2454	0.3292	Pseudomonas aeruginosa (286)	Pseudomonas (135621)	Pseudomonadaceae (7227	Pseudomonadales (1236)	Gammaproteobacteria (122	Proteobacteria (2)	Bacteria (1)	100.00	0.30	99.80	99.00	WGS
287.2455	0.3258	Pseudomonas aeruginosa (286)	Pseudomonas (135621)	Pseudomonadaceae (7227	Pseudomonadales (1236)	Gammaproteobacteria (122	Proteobacteria (2)	Bacteria (1)	99.40	0.60	99.90	99.50	WGS
287.2456	0.3738	Pseudomonas aeruginosa (286)	Pseudomonas (135621)	Pseudomonadaceae (7227	Pseudomonadales (1236)	Gammaproteobacteria (122	Proteobacteria (2)	Bacteria (1)	99.40	0.90	99.90	98.40	WGS
287.2457	0.369	Pseudomonas aeruginosa (286)	Pseudomonas (135621)	Pseudomonadaceae (7227	Pseudomonadales (1236)	Gammaproteobacteria (122	Proteobacteria (2)	Bacteria (1)	100.00	0.30	99.70	98.40	WGS
287.2458	0.3694	Pseudomonas aeruginosa (286)	Pseudomonas (135621)	Pseudomonadaceae (7227	Pseudomonadales (1236)	Gammaproteobacteria (122	Proteobacteria (2)	Bacteria (1)	100.00	0.00	99.90	99.40	WGS
287.2459	0.324	Pseudomonas aeruginosa (286)	Pseudomonas (135621)	Pseudomonadaceae (7227	Pseudomonadales (1236)	Gammaproteobacteria (122	Proteobacteria (2)	Bacteria (1)	100.00	0.00	99.90	99.00	WGS
287.2460	0.371	Pseudomonas aeruginosa (286)	Pseudomonas (135621)	Pseudomonadaceae (7227	Pseudomonadales (1236)	Gammaproteobacteria (122	Proteobacteria (2)	Bacteria (1)	100.00	0.00	99.90	98.80	WGS
287.2461	0.3704	Pseudomonas aeruginosa (286)	Pseudomonas (135621)	Pseudomonadaceae (7227	Pseudomonadales (1236)	Gammaproteobacteria (122	Proteobacteria (2)	Bacteria (1)	100.00	0.60	99.60	99.20	WGS
287.2462	0.3312	Pseudomonas aeruginosa (286)	Pseudomonas (135621)	Pseudomonadaceae (7227	Pseudomonadales (1236)	Gammaproteobacteria (122	Proteobacteria (2)	Bacteria (1)	98.80	1.20	99.60	98.50	WGS
287.2463	0.3696	Pseudomonas aeruginosa (286)	Pseudomonas (135621)	Pseudomonadaceae (7227	Pseudomonadales (1236)	Gammaproteobacteria (122	Proteobacteria (2)	Bacteria (1)	100.00	0.30	99.90	99.20	WGS
287.2464	0.3382	Pseudomonas aeruginosa (286)	Pseudomonas (135621)	Pseudomonadaceae (7227	Pseudomonadales (1236)	Gammaproteobacteria (122	Proteobacteria (2)	Bacteria (1)	100.00	0.00	99.90	99.30	WGS
287.2465	0.3628	Pseudomonas aeruginosa (286)	Pseudomonas (135621)	Pseudomonadaceae (7227	Pseudomonadales (1236)	Gammaproteobacteria (122	Proteobacteria (2)	Bacteria (1)	100.00	0.10	99.90	99.30	WGS
287.2466	0.368	Pseudomonas aeruginosa (286)	Pseudomonas (135621)	Pseudomonadaceae (7227	Pseudomonadales (1236)	Gammaproteobacteria (122	Proteobacteria (2)	Bacteria (1)	100.00	0.00	99.90	98.90	WGS
287.2467	0.3332	Pseudomonas aeruginosa (286)	Pseudomonas (135621)	Pseudomonadaceae (7227	Pseudomonadales (1236)	Gammaproteobacteria (122	Proteobacteria (2)	Bacteria (1)	98.50	0.00	99.90	98.80	WGS
287.2468	0.3268	Pseudomonas aeruginosa (286)	Pseudomonas (135621)	Pseudomonadaceae (7227	Pseudomonadales (1236)	Gammaproteobacteria (122	Proteobacteria (2)	Bacteria (1)	99.70	0.30	99.80	99.10	WGS
287.2469	0.3672	Pseudomonas aeruginosa (286)	Pseudomonas (135621)	Pseudomonadaceae (7227	Pseudomonadales (1236)	Gammaproteobacteria (122	Proteobacteria (2)	Bacteria (1)	99.40	0.60	99.90	98.40	WGS
287.2470	0.327	Pseudomonas aeruginosa (286)	Pseudomonas (135621)	Pseudomonadaceae (7227	Pseudomonadales (1236)	Gammaproteobacteria (122	Proteobacteria (2)	Bacteria (1)	100.00	0.00	99.80	99.10	WGS
287.2471	0.4158	Pseudomonas aeruginosa (286)	Pseudomonas (135621)	Pseudomonadaceae (7227	Pseudomonadales (1236)	Gammaproteobacteria (122	Proteobacteria (2)	Bacteria (1)	99.40	0.30	99.50	98.20	WGS
287.2472	0.368	Pseudomonas aeruginosa (286)	Pseudomonas (135621)	Pseudomonadaceae (7227	Pseudomonadales (1236)	Gammaproteobacteria (122	Proteobacteria (2)	Bacteria (1)	100.00	0.00	99.90	99.00	WGS
287.2473	0.328	Pseudomonas aeruginosa (286)	Pseudomonas (135621)	Pseudomonadaceae (7227	Pseudomonadales (1236)	Gammaproteobacteria (122	Proteobacteria (2)	Bacteria (1)	100.00	0.00	99.80	99.10	WGS
287.2474	0.33	Pseudomonas aeruginosa (286)	Pseudomonas (135621)	Pseudomonadaceae (7227	Pseudomonadales (1236)	Gammaproteobacteria (122	Proteobacteria (2)	Bacteria (1)	100.00	0.90	99.80	98.80	WGS
287.2475	0.3226	Pseudomonas aeruginosa (286)	Pseudomonas (135621)	Pseudomonadaceae (7227	Pseudomonadales (1236)	Gammaproteobacteria (122	Proteobacteria (2)	Bacteria (1)	100.00	0.60	99.90	99.70	WGS
287.2476	0.4126	Pseudomonas aeruginosa (286)	Pseudomonas (135621)	Pseudomonadaceae (7227	Pseudomonadales (1236)	Gammaproteobacteria (122	Proteobacteria (2)	Bacteria (1)	98.20	0.00	99.30	98.10	WGS
287.2477	0.3336	Pseudomonas aeruginosa (286)	Pseudomonas (135621)	Pseudomonadaceae (7227	Pseudomonadales (1236)	Gammaproteobacteria (122	Proteobacteria (2)	Bacteria (1)	100.00	0.00	99.90	99.00	WGS
287.2478	0.3628	Pseudomonas aeruginosa (286)	Pseudomonas (135621)	Pseudomonadaceae (7227	Pseudomonadales (1236)	Gammaproteobacteria (122	Proteobacteria (2)	Bacteria (1)	100.00	0.30	99.70	98.70	WGS
287.2479	0.3614	Pseudomonas aeruginosa (286)	Pseudomonas (135621)	Pseudomonadaceae (7227	Pseudomonadales (1236)	Gammaproteobacteria (122	Proteobacteria (2)	Bacteria (1)	100.00	0.00	99.90	99.30	WGS
287.2480	0.3634	Pseudomonas aeruginosa (286)	Pseudomonas (135621)	Pseudomonadaceae (7227	Pseudomonadales (1236)	Gammaproteobacteria (122	Proteobacteria (2)	Bacteria (1)	99.70	0.30	99.90	99.00	WGS
287.2481	0.3174	Pseudomonas aeruginosa (286)	Pseudomonas (135621)	Pseudomonadaceae (7227	Pseudomonadales (1236)	Gammaproteobacteria (122	Proteobacteria (2)	Bacteria (1)	99.70	0.00	99.70	99.00	WGS
287.2482	0.3324	Pseudomonas aeruginosa (286)	Pseudomonas (135621)	Pseudomonadaceae (7227	Pseudomonadales (1236)	Gammaproteobacteria (122	Proteobacteria (2)	Bacteria (1)	100.00	0.00	99.70	99.60	WGS
287.2483	0.328	Pseudomonas aeruginosa (286)	Pseudomonas (135621)	Pseudomonadaceae (7227	Pseudomonadales (1236)	Gammaproteobacteria (122	Proteobacteria (2)	Bacteria (1)	99.40	0.00	99.40	98.70	WGS
287.2484	0.332	Pseudomonas aeruginosa (286)	Pseudomonas (135621)	Pseudomonadaceae (7227	Pseudomonadales (1236)	Gammaproteobacteria (122	Proteobacteria (2)	Bacteria (1)	100.00	0.30	99.60	98.50	WGS
287.2485	0.3346	Pseudomonas aeruginosa (286)	Pseudomonas (135621)	Pseudomonadaceae (7227	Pseudomonadales (1236)	Gammaproteobacteria (122	Proteobacteria (2)	Bacteria (1)	100.00	0.00	99.90	99.00	WGS
287.2486	0.3292	Pseudomonas aeruginosa (286)	Pseudomonas (135621)	Pseudomonadaceae (7227	Pseudomonadales (1236)	Gammaproteobacteria (122	Proteobacteria (2)	Bacteria (1)	100.00	0.00	99.90	99.30	WGS
287.2487	0.3268	Pseudomonas aeruginosa (286)	Pseudomonas (135621)	Pseudomonadaceae (7227	Pseudomonadales (1236)	Gammaproteobacteria (122	Proteobacteria (2)	Bacteria (1)	100.00	0.00	99.80	99.30	WGS
287.2488	0.2918	Pseudomonas aeruginosa (286)	Pseudomonas (135621)	Pseudomonadaceae (7227	Pseudomonadales (1236)	Gammaproteobacteria (122	Proteobacteria (2)	Bacteria (1)	98.50	0.00	99.30	99.20	WGS
287.2489	0.3268	Pseudomonas aeruginosa (286)	Pseudomonas (135621)	Pseudomonadaceae (7227	Pseudomonadales (1236)	Gammaproteobacteria (122	Proteobacteria (2)	Bacteria (1)	100.00	0.00	99.80	98.60	WGS
287.2490	0.3576	Pseudomonas aeruginosa (286)	Pseudomonas (135621)	Pseudomonadaceae (7227	Pseudomonadales (1236)	Gammaproteobacteria (122	Proteobacteria (2)	Bacteria (1)	98.20	1.80	98.60	97.60	WGS
287.2491	0.3314	Pseudomonas aeruginosa (286)	Pseudomonas (135621)	Pseudomonadaceae (7227	Pseudomonadales (1236)	Gammaproteobacteria (122	Proteobacteria (2)	Bacteria (1)	100.00	0.00	99.80	99.00	WGS
287.2492	0.3314	Pseudomonas aeruginosa (286)	Pseudomonas (135621)	Pseudomonadaceae (7227	Pseudomonadales (1236)	Gammaproteobacteria (122	Proteobacteria (2)	Bacteria (1)	100.00	0.00	99.80	99.00	WGS
287.2493	0.371	Pseudomonas aeruginosa (286)	Pseudomonas (135621)	Pseudomonadaceae (7227	Pseudomonadales (1236)	Gammaproteobacteria (122	Proteobacteria (2)	Bacteria (1)	100.00	0.30	99.90	98.70	WGS
287.2494	0.3672	Pseudomonas aeruginosa (286)	Pseudomonas (135621)	Pseudomonadaceae (7227	Pseudomonadales (1236)	Gammaproteobacteria (122	Proteobacteria (2)	Bacteria (1)	100.00	0.30	99.90	99.00	WGS
287.2495	0.3754	Pseudomonas aeruginosa (286)	Pseudomonas (135621)	Pseudomonadaceae (7227	Pseudomonadales (1236)	Gammaproteobacteria (122	Proteobacteria (2)	Bacteria (1)	100.00	0.00	99.90	99.30	WGS
287.2496	0.3188	Pseudomonas aeruginosa (286)	Pseudomonas (135621)	Pseudomonadaceae (7227	Pseudomonadales (1236)	Gammaproteobacteria (122	Proteobacteria (2)	Bacteria (1)	100.00	0.30	100.00	99.30	WGS
287.2497	0.3628	Pseudomonas aeruginosa (286)	Pseudomonas (135621)	Pseudomonadaceae (7227	Pseudomonadales (1236)	Gammaproteobacteria (122	Proteobacteria (2)	Bacteria (1)	100.00	0.00	99.90	99.00	WGS
287.2498	0.332	Pseudomonas aeruginosa (286)	Pseudomonas (135621)	Pseudomonadaceae (7227	Pseudomonadales (1236)	Gammaproteobacteria (122	Proteobacteria (2)	Bacteria (1)	100.00	0.00	99.70	98.80	WGS
287.2499	0.3302	Pseudomonas aeruginosa (286)	Pseudomonas (135621)	Pseudomonadaceae (7227	Pseudomonadales (1236)	Gammaproteobacteria (122	Proteobacteria (2)	Bacteria (1)	100.00	0.30	99.90	98.40	WGS
287.2500	0.3258	Pseudomonas aeruginosa (286)	Pseudomonas (135621)	Pseudomonadaceae (7227	Pseudomonadales (1236)	Gammaproteobacteria (122	Proteobacteria (2)	Bacteria (1)	100.00	0.00	99.80	99.50	WGS
287.2501	0.3226	Pseudomonas aeruginosa (286)	Pseudomonas (135621)	Pseudomonadaceae (7227	Pseudomonadales (1236)	Gammaproteobacteria (122	Proteobacteria (2)	Bacteria (1)	100.00	0.00	99.60	99.20	WGS
287.2502	0.3622	Pseudomonas aeruginosa (286)	Pseudomonas (135621)	Pseudomonadaceae (7227	Pseudomonadales (1236)	Gammaproteobacteria (122	Proteobacteria (2)	Bacteria (1)	100.00	0.00	99.60	99.20	WGS
287.2503	0.3704	Pseudomonas aeruginosa (286)	Pseudomonas (135621)	Pseudomonadaceae (7227	Pseudomonadales (1236)	Gammaproteobacteria (122	Proteobacteria (2)	Bacteria (1)	100.00	0.00	99.90	99.00	WGS
287.2504	0.324	Pseudomonas aeruginosa (286)	Pseudomonas (135621)	Pseudomonadaceae (7227	Pseudomonadales (1236)	Gammaproteobacteria (122	Proteobacteria (2)	Bacteria (1)	100.00	0.00	99.90	99.30	WGS
287.2505	0.3662	Pseudomonas aeruginosa (286)	Pseudomonas (135621)	Pseudomonadaceae (7227	Pseudomonadales (1236)	Gammaproteobacteria (122	Proteobacteria (2)	Bacteria (1)	99.40	0.30	99.80	98.70	WGS
287.2506	0.3314	Pseudomonas aeruginosa (286)	Pseudomonas (135621)	Pseudomonadaceae (7227	Pseudomonadales (1236)	Gammaproteobacteria (122	Proteobacteria (2)	Bacteria (1)	100.00	0.00	99.80	99.10	WGS
287.2507	0.331	Pseudomonas aeruginosa (286)	Pseudomonas (135621)	Pseudomonadaceae (7227	Pseudomonadales (1236)	Gammaproteobacteria (122	Proteobacteria (2)	Bacteria (1)	100.00	0.90	99.60	98.40	WGS
287.2508	0.3558	Pseudomonas aeruginosa (286)	Pseudomonas (135621)	Pseudomonadaceae (7227	Pseudomonadales (1236)	Gammaproteobacteria (122	Proteobacteria (2)	Bacteria (1)	90.40	2.00	95.80	93.60	WGS
287.2509	0.3906	Pseudomonas aeruginosa (286)	Pseudomonas (135621)	Pseudomonadaceae (7227	Pseudomonadales (1236)	Gammaproteobacteria (122	Proteobacteria (2)	Bacteria (1)	92.20	1.30	97.80	96.20	WGS
287.2510	0.3618	Pseudomonas aeruginosa (286)	Pseudomonas (135621)	Pseudomonadaceae (7227	Pseudomonadales (1236)	Gammaproteobacteria (122	Proteobacteria (2)	Bacteria (1)	99.40	0.00	99.80	98.70	WGS
287.2511	0.324	Pseudomonas aeruginosa (286)	Pseudomonas (135621)	Pseudomonadaceae (7227	Pseudomonadales (1236)	Gammaproteobacteria (122	Proteobacteria (2)	Bacteria (1)	100.00	0.60	99.90	99.60	WGS
287.2512	0.3664	Pseudomonas aeruginosa (286)	Pseudomonas (135621)	Pseudomonadaceae (7227	Pseudomonadales (1236)	Gammaproteobacteria (122	Proteobacteria (2)	Bacteria (1)	99.70	0.60	99.60	98.70	WGS
287.2513	0.3376	Pseudomonas aeruginosa (286)	Pseudomonas (135621)	Pseudomonadaceae (7227	Pseudomonadales (1236)	Gammaproteobacteria (122	Proteobacteria (2)	Bacteria (1)	100.00	0.30	99.70	99.00	WGS
287.2514	0.3694	Pseudomonas aeruginosa (286)	Pseudomonas (135621)	Pseudomonadaceae (7227	Pseudomonadales (1236)	Gammaproteobacteria (122	Proteobacteria (2)	Bacteria (1)	100.00	0.00	99.70	99.40	WGS
287.2515	0.368	Pseudomonas aeruginosa (286)	Pseudomonas (135621)	Pseudomonadaceae (7227	Pseudomonadales (1236)	Gammaproteobacteria (122	Proteobacteria (2)	Bacteria (1)	100.00	0.00	99.90	99.60	WGS
287.2516	0.3254	Pseudomonas aeruginosa (286)	Pseudomonas (135621)	Pseudomonadaceae (7227	Pseudomonadales (1236)	Gammaproteobacteria (122	Proteobacteria (2)	Bacteria (1)	100.00	0.30	99.80	99.40	WGS
287.2517	0.3314	Pseudomonas aeruginosa (286)	Pseudomonas (135621)	Pseudomonadaceae (7227	Pseudomonadales (1236)	Gammaproteobacteria (122	Proteobacteria (2)	Bacteria (1)	98.80	0.00	99.40	98.30	WGS
287.2518	0.3314	Pseudomonas aeruginosa (286)	Pseudomonas (135621)	Pseudomonadaceae (7227	Pseudomonadales (1236)	Gammaproteobacteria (122	Proteobacteria (2)	Bacteria (1)	96.70	0.00	97.80	97.00	WGS
287.2519	0.3244	Pseudomonas aeruginosa (286)	Pseudomonas (135621)	Pseudomonadaceae (7227	Pseudomonadales (1236)	Gammaproteobacteria (122	Proteobacteria (2)	Bacteria (1)	98.80	0.60	98.90	97.70	WGS
287.2520	0.3314	Pseudomonas aeruginosa (286)	Pseudomonas (135621)	Pseudomonadaceae (7227	Pseudomonadales (1236)	Gammaproteobacteria (122	Proteobacteria (2)	Bacteria (1)	99.40	0.00	99.30	98.40	WGS
287.2521	0.3356	Pseudomonas aeruginosa (286)	Pseudomonas (135621)	Pseudomonadaceae (7227	Pseudomonadales (1236)	Gammaproteobacteria (122	Proteobacteria (2)	Bacteria (1)	98.80	0.00	99.60	98.70	WGS
287.2522	0.309	Pseudomonas aeruginosa (286)	Pseudomonas (135621)	Pseudomonadaceae (7227	Pseudomonadales (1236)	Gammaproteobacteria (122	Proteobacteria (2)	Bacteria (1)	99.40	0.30	99.90	98.80	WGS
287.2523	0.3314	Pseudomonas aeruginosa (286)	Pseudomonas (135621)	Pseudomonadaceae (7227	Pseudomonadales (1236)	Gammaproteobacteria (122	Proteobacteria (2)	Bacteria (1)	100.00	0.00	99.80	99.00	WGS
287.2524	0.331	Pseudomonas aeruginosa (286)	Pseudomonas (135621)	Pseudomonadaceae (7227	Pseudomonadales (1236)	Gammaproteobacteria (122	Proteobacteria (2)	Bacteria (1)	100.00	0.30	99.90	99.40	WGS
287.2525	0.3618	Pseudomonas aeruginosa (286)	Pseudomonas (135621)	Pseudomonadaceae (7227	Pseudomonadales (1236)	Gammaproteobacteria (122	Proteobacteria (2)	Bacteria (1)	100.00	0.00	99.90	99.00	WGS
287.2526	0.3754	Pseudomonas aeruginosa (286)	Pseudomonas (135621)	Pseudomonadaceae (7227	Pseudomonadales (1236)	Gammaproteobacteria (122	Proteobacteria (2)	Bacteria (1)	99.70	0.00	99.60	98.80	WGS
287.2527	0.331	Pseudomonas aeruginosa (286)	Pseudomonas (135621)	Pseudomonadaceae (7227	Pseudomonadales (1236)	Gammaproteobacteria (122	Proteobacteria (2)	Bacteria (1)	99.70	1.20	99.70	98.60	WGS
287.2528	0.356	Pseudomonas aeruginosa (286)	Pseudomonas (135621)	Pseudomonadaceae (7227	Pseudomonadales (1236)	Gammaproteobacteria (122	Proteobacteria (2)	Bacteria (1)	100.00	0.60	99.80	98.60	WGS
287.2529	0.368	Pseudomonas aeruginosa (286)	Pseudomonas (135621)	Pseudomonadaceae (7227	Pseudomonadales (1236)	Gammaproteobacteria (122	Proteobacteria (2)	Bacteria (1)	98.40	1.20	99.80	98.30	WGS
287.2530	0.3376	Pseudomonas aeruginosa (286)	Pseudomonas (135621)	Pseudomonadaceae (7227	Pseudomonadales (1236)	Gammaproteobacteria (122	Proteobacteria (2)	Bacteria (1)	100.00	0.00	99.90	98.70	WGS
287.2531	0.377	Pseudomonas aeruginosa (286)	Pseudomonas (135621)	Pseudomonadaceae (7227	Pseudomonadales (1236)	Gammaproteobacteria (122	Proteobacteria (2)	Bacteria (1)	100.00	0.30	99.90	99.00	WGS
287.2532	0.3226	Pseudomonas aeruginosa (286)	Pseudomonas (135621)	Pseudomonadaceae (7227	Pseudomonadales (1236)	Gammaproteobacteria (122	Proteobacteria (2)	Bacteria (1)	100.00	0.60	99.90	99.30	WGS
287.2533	0.3622	Pseudomonas aeruginosa (286)	Pseudomonas (135621)	Pseudomonadaceae (7227	Pseudomonadales (1236)	Gammaproteobacteria (122	Proteobacteria (2)	Bacteria (1)	96.70	0.30	99.60	98.70	WGS
287.2534	0.3368	Pseudomonas aeruginosa (286)	Pseudomonas (135621)	Pseudomonadaceae (7227	Pseudomonadales (1236)	Gammaproteobacteria (122	Proteobacteria (2)	Bacteria (1)	99.10	0.00	99.60	98.70	WGS
287.2535	0.3314	Pseudomonas aeruginosa (286)	Pseudomonas (135621)	Pseudomonadaceae (7227	Pseudomonadales (1236)	Gammaproteobacteria (122	Proteobacteria (2)	Bacteria (1)	100.00	0.00	99.80	99.10	WGS
287.2536	0.3314	Pseudomonas aeruginosa (286)	Pseudomonas (135621)	Pseudomonadaceae (7227	Pseudomonadales (1236)	Gammaproteobacteria (122	Proteobacteria (2)	Bacteria (1)	100.00	0.00	99.80	98.90	WGS
287.2537	0.3614	Pseudomonas aeruginosa (286)	Pseudomonas (135621)	Pseudomonadaceae (7227	Pseudomonadales (1236)	Gammaproteobacteria (122	Proteobacteria (2)	Bacteria (1)	100.00	0.90	99.80	98.70	Complete
287.2538	0.3636	Pseudomonas aeruginosa (286)	Pseudomonas (135621)	Pseudomonadaceae (7227	Pseudomonadales (1236)	Gammaproteobacteria (122	Proteobacteria (2)	Bacteria (1)	99.10	3.20	99.50	97.20	Complete
287.2539	0.3276	Pseudomonas aeruginosa (286)	Pseudomonas (135621)	Pseudomonadaceae (7227	Pseudomonadales (1236)	Gammaproteobacteria (122	Proteobacteria (2)	Bacteria (1)	100.00	2.80	99.80	93.20	WGS
287.2540	0.3314	Pseudomonas aeruginosa (286)	Pseudomonas (135621)	Pseudomonadaceae (7227	Pseudomonadales (1236)	Gammaproteobacteria (122	Proteobacteria (2)	Bacteria (1)	100.00	0.90	99.80	98.70	WGS
287.2541	0.2986	Pseudomonas aeruginosa (286)	Pseudomonas (135621)	Pseudomonadaceae (7227	Pseudomonadales (1236)	Gammaproteobacteria (122	Proteobacteria (2)	Bacteria (1)	98.20	8.50	99.60	91.70	WGS
287.2542	0.368	Pseudomonas aeruginosa (286)	Pseudomonas (135621)	Pseudomonadaceae (7227	Pseudomonadales (1236)	Gammaproteobacteria (122	Proteobacteria (2)	Bacteria (1)	100.00	0.00	99.90	99.60	Complete
287.2543	0.3636	Pseudomonas aeruginosa (286)	Pseudomonas (135621)	Pseudomonadaceae (7227	Pseudomonadales (1236)	Gammaproteobacteria (122	Proteobacteria (2)	Bacteria (1)	98.80	1.30	99.70	98.20	WGS
287.2544	0.3254	Pseudomonas aeruginosa (286)	Pseudomonas (135621)	Pseudomonadaceae (7227	Pseudomonadales (1236)	Gammaproteobacteria (122	Proteobacteria (2)	Bacteria (1)	100.00	1.30	99.80	98.20	WGS
287.2545	0.3636	Pseudomonas aeruginosa (286)	Pseudomonas (135621)	Pseudomonadaceae (7227	Pseudomonadales (1236)	Gammaproteobacteria (122	Proteobacteria (2)	Bacteria (1)	100.00	0.60	99.80	98.70	WGS
287.2546	0.3284	Pseudomonas aeruginosa (286)	Pseudomonas (135621)	Pseudomonadaceae (7227	Pseudomonadales (1236)	Gammaproteobacteria (122	Proteobacteria (2)	Bacteria (1)	100.00	0.60	100.00	98.90	WGS
287.2547	0.3258	Pseudomonas aeruginosa (286)	Pseudomonas (135621)	Pseudomonadaceae (7227	Pseudomonadales (1236)	Gammaproteobacteria (122	Proteobacteria (2)	Bacteria (1)	100.00	0.30	99.80	98.60	WGS
287.2548	0.3738	Pseudomonas aeruginosa (286)	Pseudomonas (135621)	Pseudomonadaceae (7227	Pseudomonadales (1236)	Gammaproteobacteria (122	Proteobacteria (2)	Bacteria (1)	100.00	0.00	99.60	98.60	Complete
287.2549	0.3738	Pseudomonas aeruginosa (286)	Pseudomonas (135621)	Pseudomonadaceae (7227	Pseudomonadales (1236)	Gammaproteobacteria (122	Proteobacteria (2)	Bacteria (1)	100.00	0.30	99.70	98.00	Complete
287.2550	0.3696	Pseudomonas aeruginosa (286)	Pseudomonas (135621)	Pseudomonadaceae (7227	Pseudomonadales (1236)	Gammaproteobacteria (122	Proteobacteria (2)	Bacteria (1)	100.00	1.20	99.80	99.00	Complete
287.2551	0.324	Pseudomonas aeruginosa (286)	Pseudomonas (135621)	Pseudomonadaceae (7227	Pseudomonadales (1236)	Gammaproteobacteria (122	Proteobacteria (2)	Bacteria (1)	100.00	0.00	99.90	98.50	Complete
287.2552	0.3236	Pseudomonas aeruginosa (286)	Pseudomonas (135621)	Pseudomonadaceae (7227	Pseudomonadales (1236)	Gammaproteobacteria (122	Proteobacteria (2)	Bacteria (1)	100.00	1.80	99.90	98.40	Complete
287.2553	0.3634	Pseudomonas aeruginosa (286)	Pseudomonas (135621)	Pseudomonadaceae (7227	Pseudomonadales (1236)	Gammaproteobacteria (122	Proteobacteria (2)	Bacteria (1)	100.00	0.30	99.70	98.90	Complete
287.2554	0.331	Pseudomonas aeruginosa (286)	Pseudomonas (135621)	Pseudomonadaceae (7227	Pseudomonadales (1236)	Gammaproteobacteria (122	Proteobacteria (2)	Bacteria (1)	100.00	0.00	99.90	98.90	Complete
287.2555	0.3292	Pseudomonas aeruginosa (286)	Pseudomonas (135621)	Pseudomonadaceae (7227	Pseudomonadales (1236)	Gammaproteobacteria (122	Proteobacteria (2)	Bacteria (1)	100.00	1.50	99.80	98.40	Complete
287.2556	0.3226	Pseudomonas aeruginosa (286)	Pseudomonas (135621)	Pseudomonadaceae (7227	Pseudomonadales (1236)	Gammaproteobacteria (122	Proteobacteria (2)	Bacteria (1)	100.00	0.00	99.90	99.30	Complete
287.2557	0.324	Pseudomonas aeruginosa (286)	Pseudomonas (135621)	Pseudomonadaceae (7227	Pseudomonadales (1236)	Gammaproteobacteria (122	Proteobacteria (2)	Bacteria (1)	100.00	1.20	99.90	98.90	Complete
287.2558	0.3376	Pseudomonas aeruginosa (286)	Pseudomonas (135621)	Pseudomonadaceae (7227	Pseudomonadales (1236)	Gammaproteobacteria (122	Proteobacteria (2)	Bacteria (1)	100.00	0.30	99.90	98.90	Complete
287.2559	0.3174	Pseudomonas aeruginosa (286)	Pseudomonas (135621)	Pseudomonadaceae (7227	Pseudomonadales (1236)	Gammaproteobacteria (122	Proteobacteria (2)	Bacteria (1)	100.00	0.60	99.80	98.90	Complete
287.2560	0.3704	Pseudomonas aeruginosa (286)	Pseudomonas (135621)	Pseudomonadaceae (7227	Pseudomonadales (1236)	Gammaproteobacteria (122	Proteobacteria (2)	Bacteria (1)	100.00	0.00	99.90	98.50	Complete
287.2561	0.372	Pseudomonas aeruginosa (286)	Pseudomonas (135621)	Pseudomonadaceae (7227	Pseudomonadales (1236)	Gammaproteobacteria (122	Proteobacteria (2)	Bacteria (1)	97.60	0.60	99.10	97.90	Complete
287.2562	0.3268	Pseudomonas aeruginosa (286)	Pseudomonas (135621)	Pseudomonadaceae (7227	Pseudomonadales (1236)	Gammaproteobacteria (122	Proteobacteria (2)	Bacteria (1)	100.00	0.00	99.80	99.10	Complete
287.2563	0.327	Pseudomonas aeruginosa (286)	Pseudomonas (135621)	Pseudomonadaceae (7227	Pseudomonadales (1236)	Gammaproteobacteria (122	Proteobacteria (2)	Bacteria (1)	100.00	0.00	99.90	99.70	Complete
287.2564	0.255	Pseudomonas aeruginosa (286)	Pseudomonas (135621)	Pseudomonadaceae (7227	Pseudomonadales (1236)	Gammaproteobacteria (122	Proteobacteria (2)	Bacteria (1)	97.60	19.10	98.50	82.20	WGS
287.2570	0.3258	Pseudomonas aeruginosa (286)	Pseudomonas (135621)	Pseudomonadaceae (7227	Pseudomonadales (1236)	Gammaproteobacteria (122	Proteobacteria (2)	Bacteria (1)	100.00	0.60	99.90	99.00	Complete
287.2571	0.3438	Pseudomonas aeruginosa (286)	Pseudomonas (135621)	Pseudomonadaceae (7227	Pseudomonadales (1236)	Gammaproteobacteria (122	Proteobacteria (2)	Bacteria (1)	100.00	3.20	99.80	96.80	Complete
287.2572	0.3324	Pseudomonas aeruginosa (286)	Pseudomonas (135621)	Pseudomonadaceae (7227	Pseudomonadales (1236)	Gammaproteobacteria (122	Proteobacteria (2)	Bacteria (1)	99.40	1.60	99.90	98.50	WGS
287.2573	0.3324	Pseudomonas aeruginosa (286)	Pseudomonas (135621)	Pseudomonadaceae (7227	Pseudomonadales (1236)	Gammaproteobacteria (122	Proteobacteria (2)	Bacteria (1)	98.50	1.60	99.80	96.90	WGS
287.2574	0.3324	Pseudomonas aeruginosa (286)	Pseudomonas (135621)	Pseudomonadaceae (7227	Pseudomonadales (1236)	Gammaproteobacteria (122	Proteobacteria (2)	Bacteria (1)	98.50	1.60	99.80	97.80	WGS
287.2575	0.338	Pseudomonas aeruginosa (286)	Pseudomonas (135621)	Pseudomonadaceae (7227	Pseudomonadales (1236)	Gammaproteobacteria (122	Proteobacteria (2)	Bacteria (1)	100.00	1.50	99.90	98.00	WGS
287.2576	0.3324	Pseudomonas aeruginosa (286)	Pseudomonas (135621)	Pseudomonadaceae (7227	Pseudomonadales (1236)	Gammaproteobacteria (122	Proteobacteria (2)	Bacteria (1)	99.40	1.30	99.90	98.20	WGS
287.2577	0.3226	Pseudomonas aeruginosa (286)	Pseudomonas (135621)	Pseudomonadaceae (7227	Pseudomonadales (1236)	Gammaproteobacteria (122	Proteobacteria (2)	Bacteria (1)	98.50	9.50	99.20	92.30	WGS
287.2578	0.368	Pseudomonas aeruginosa (286)	Pseudomonas (135621)	Pseudomonadaceae (7227	Pseudomonadales (1236)	Gammaproteobacteria (122	Proteobacteria (2)	Bacteria (1)	100.00	0.90	99.80	99.00	Complete
287.2579	0.3268	Pseudomonas aeruginosa (286)	Pseudomonas (135621)	Pseudomonadaceae (7227	Pseudomonadales (1236)	Gammaproteobacteria (122	Proteobacteria (2)	Bacteria (1)	100.00	0.00	99.90	99.40	Complete
287.2580	0.356	Pseudomonas aeruginosa (286)	Pseudomonas (135621)	Pseudomonadaceae (7227	Pseudomonadales (1236)	Gammaproteobacteria (122	Proteobacteria (2)	Bacteria (1)	100.00	0.90	99.60	98.80	Complete
287.2581	0.3142	Pseudomonas aeruginosa (286)	Pseudomonas (135621)	Pseudomonadaceae (7227	Pseudomonadales (1236)	Gammaproteobacteria (122	Proteobacteria (2)	Bacteria (1)	99.70	0.30	99.90	98.50	WGS
287.2582	0.3324	Pseudomonas aeruginosa (286)	Pseudomonas (135621)	Pseudomonadaceae (7227	Pseudomonadales (1236)	Gammaproteobacteria (122	Proteobacteria (2)	Bacteria (1)	99.70	0.30	99.90	98.80	WGS
287.2583	0.324	Pseudomonas aeruginosa (286)	Pseudomonas (135621)	Pseudomonadaceae (7227	Pseudomonadales (1236)	Gammaproteobacteria (122	Proteobacteria (2)	Bacteria (1)	100.00	0.90	99.90	98.20	WGS
287.2584	0.3264	Pseudomonas aeruginosa (286)	Pseudomonas (135621)	Pseudomonadaceae (7227	Pseudomonadales (1236)	Gammaproteobacteria (122	Proteobacteria (2)	Bacteria (1)	99.70	0.90	99.80	98.70	WGS
287.2585	0.326	Pseudomonas aeruginosa (286)	Pseudomonas (135621)	Pseudomonadaceae (7227	Pseudomonadales (1236)	Gammaproteobacteria (122	Proteobacteria (2)	Bacteria (1)	99.70	1.20	99.80	98.40	WGS
287.2586	0.3174	Pseudomonas aeruginosa (286)	Pseudomonas (135621)	Pseudomonadaceae (7227	Pseudomonadales (1236)	Gammaproteobacteria (122	Proteobacteria (2)	Bacteria (1)	100.00	0.60	99.90	98.90	WGS
287.2587	0.3292	Pseudomonas aeruginosa (286)	Pseudomonas (135621)	Pseudomonadaceae (7227	Pseudomonadales (1236)	Gammaproteobacteria (122	Proteobacteria (2)	Bacteria (1)	100.00	0.30	99.90	98.20	WGS
287.2588	0.3174	Pseudomonas aeruginosa (286)	Pseudomonas (135621)	Pseudomonadaceae (7227	Pseudomonadales (1236)	Gammaproteobacteria (122	Proteobacteria (2)	Bacteria (1)	100.00	0.30	99.90	98.50	WGS
287.2589	0.3264	Pseudomonas aeruginosa (286)	Pseudomonas (135621)	Pseudomonadaceae (7227	Pseudomonadales (1236)	Gammaproteobacteria (122	Proteobacteria (2)	Bacteria (1)	99.70	0.90	99.80	98.40	WGS
287.2590	0.3738	Pseudomonas aeruginosa (286)	Pseudomonas (135621)	Pseudomonadaceae (7227	Pseudomonadales (1236)	Gammaproteobacteria (122	Proteobacteria (2)	Bacteria (1)	100.00	0.90	99.60	98.20	WGS
287.2591	0.3738	Pseudomonas aeruginosa (286)	Pseudomonas (135621)	Pseudomonadaceae (7227	Pseudomonadales (1236)	Gammaproteobacteria (122	Proteobacteria (2)	Bacteria (1)	100.00	0.30	99.90	98.60	WGS
287.2592	0.3332	Pseudomonas aeruginosa (286)	Pseudomonas (135621)	Pseudomonadaceae (7227	Pseudomonadales (1236)	Gammaproteobacteria (122	Proteobacteria (2)	Bacteria (1)	99.40	0.60	99.80	97.90	WGS
287.2593	0.3332	Pseudomonas aeruginosa (286)	Pseudomonas (135621)	Pseudomonadaceae (7227	Pseudomonadales (1236)	Gammaproteobacteria (122	Proteobacteria (2)	Bacteria (1)	99.40	0.30	99.80	98.20	WGS
287.2594	0.324	Pseudomonas aeruginosa (286)	Pseudomonas (135621)	Pseudomonadaceae (7227	Pseudomonadales (1236)	Gammaproteobacteria (122	Proteobacteria (2)	Bacteria (1)	100.00	0.10	99.90	98.70	WGS
287.2595	0.3268	Pseudomonas aeruginosa (286)	Pseudomonas (135621)	Pseudomonadaceae (7227	Pseudomonadales (1236)	Gammaproteobacteria (122	Proteobacteria (2)	Bacteria (1)	99.40	1.60	99.80	97.80	WGS
287.2596	0.3332	Pseudomonas aeruginosa (286)	Pseudomonas (135621)	Pseudomonadaceae (7227	Pseudomonadales (1236)	Gammaproteobacteria (122	Proteobacteria (2)	Bacteria (1)	99.40	1.20	99.80	98.40	WGS
287.2597	0.3332	Pseudomonas aeruginosa (286)	Pseudomonas (135621)	Pseudomonadaceae (7227	Pseudomonadales (1236)	Gammaproteobacteria (122	Proteobacteria (2)	Bacteria (1)	99.40	0.60	99.80	98.50	WGS
287.2598	0.3258	Pseudomonas aeruginosa (286)	Pseudomonas (135621)	Pseudomonadaceae (7227	Pseudomonadales (1236)	Gammaproteobacteria (122	Proteobacteria (2)	Bacteria (1)	100.00	0.00	99.90	98.70	WGS
287.2599	0.3258	Pseudomonas aeruginosa (286)	Pseudomonas (135621)	Pseudomonadaceae (7227	Pseudomonadales (1236)	Gammaproteobacteria (122	Proteobacteria (2)	Bacteria (1)	100.00	0.30	99.90	99.00	WGS
287.2600	0.3324	Pseudomonas aeruginosa (286)	Pseudomonas (135621)	Pseudomonadaceae (7227	Pseudomonadales (1236)	Gammaproteobacteria (122	Proteobacteria (2)	Bacteria (1)	99.70	0.00	99.90	99.00	WGS
287.2601	0.3236	Pseudomonas aeruginosa (286)	Pseudomonas (135621)	Pseudomonadaceae (7227	Pseudomonadales (1236)	Gammaproteobacteria (122	Proteobacteria (2)	Bacteria (1)	100.00	1.20	99.80	98.80	WGS
287.2602	0.3324	Pseudomonas aeruginosa (286)	Pseudomonas (135621)	Pseudomonadaceae (7227	Pseudomonadales (1236)	Gammaproteobacteria (122	Proteobacteria (2)	Bacteria (1)	100.00	1.20	99.80	98.80	WGS
287.2603	0.3324	Pseudomonas aeruginosa (286)	Pseudomonas (135621)	Pseudomonadaceae (7227	Pseudomonadales (1236)	Gammaproteobacteria (122	Proteobacteria (2)	Bacteria (1)	99.70	1.20	99.80	98.80	WGS
287.2604	0.368	Pseudomonas aeruginosa (286)	Pseudomonas (135621)	Pseudomonadaceae (7227	Pseudomonadales (1236)	Gammaproteobacteria (122	Proteobacteria (2)	Bacteria (1)	100.00	0.30	100.00	99.30	WGS
287.2605	0.368	Pseudomonas aeruginosa (286)	Pseudomonas (135621)	Pseudomonadaceae (7227	Pseudomonadales (1236)	Gammaproteobacteria (122	Proteobacteria (2)	Bacteria (1)	100.00	0.00	99.90	99.40	WGS
287.2606	0.368	Pseudomonas aeruginosa (286)	Pseudomonas (135621)	Pseudomonadaceae (7227	Pseudomonadales (1236)	Gammaproteobacteria (122	Proteobacteria (2)	Bacteria (1)	99.70	0.00	99.90	98.90	WGS
287.2607	0.371	Pseudomonas aeruginosa (286)	Pseudomonas (135621)	Pseudomonadaceae (7227	Pseudomonadales (1236)	Gammaproteobacteria (122	Proteobacteria (2)	Bacteria (1)	100.00	0.30	99.80	99.30	WGS
287.2608	0.371	Pseudomonas aeruginosa (286)	Pseudomonas (135621)	Pseudomonadaceae (7227	Pseudomonadales (1236)	Gammaproteobacteria (122	Proteobacteria (2)	Bacteria (1)	100.00	0.00	99.80	98.70	WGS
287.2609	0.371	Pseudomonas aeruginosa (286)	Pseudomonas (135621)	Pseudomonadaceae (7227	Pseudomonadales (1236)	Gammaproteobacteria (122	Proteobacteria (2)	Bacteria (1)	100.00	0.60	99.80	99.00	WGS
287.2610	0.371	Pseudomonas aeruginosa (286)	Pseudomonas (135621)	Pseudomonadaceae (7227	Pseudomonadales (1236)	Gammaproteobacteria (122	Proteobacteria (2)	Bacteria (1)	99.70	0.00	99.70	98.70	WGS
287.2611	0.371	Pseudomonas aeruginosa (286)	Pseudomonas (135621)	Pseudomonadaceae (7227	Pseudomonadales (1236)	Gammaproteobacteria (122	Proteobacteria (2)	Bacteria (1)	100.00	0.60	99.80	98.70	WGS
287.2612	0.3636	Pseudomonas aeruginosa (286)	Pseudomonas (135621)	Pseudomonadaceae (7227	Pseudomonadales (1236)	Gammaproteobacteria (122	Proteobacteria (2)	Bacteria (1)	99.70	1.20	99.90	98.70	WGS
287.2613	0.3636	Pseudomonas aeruginosa (286)	Pseudomonas (135621)	Pseudomonadaceae (7227	Pseudomonadales (1236)	Gammaproteobacteria (122	Proteobacteria (2)	Bacteria (1)	99.70	1.80	99.80	98.70	WGS
287.2614	0.3324	Pseudomonas aeruginosa (286)	Pseudomonas (135621)	Pseudomonadaceae (7227	Pseudomonadales (1236)	Gammaproteobacteria (122	Proteobacteria (2)	Bacteria (1)	99.70	0.30	99.90	98.20	WGS
287.2615	0.3636	Pseudomonas aeruginosa (286)	Pseudomonas (135621)	Pseudomonadaceae (7227	Pseudomonadales (1236)	Gammaproteobacteria (122	Proteobacteria (2)	Bacteria (1)	99.70	0.90	99.90	98.60	WGS
287.2616	0.356	Pseudomonas aeruginosa (286)	Pseudomonas (135621)	Pseudomonadaceae (7227	Pseudomonadales (1236)	Gammaproteobacteria (122	Proteobacteria (2)	Bacteria (1)	100.00	0.30	99.70	98.70	WGS
287.2617	0.3324	Pseudomonas aeruginosa (286)	Pseudomonas (135621)	Pseudomonadaceae (7227	Pseudomonadales (1236)	Gammaproteobacteria (122	Proteobacteria (2)	Bacteria (1)	100.00	0.30	99.90	99.20	WGS
287.2618	0.3324	Pseudomonas aeruginosa (286)	Pseudomonas (135621)	Pseudomonadaceae (7227	Pseudomonadales (1236)	Gammaproteobacteria (122	Proteobacteria (2)	Bacteria (1)	99.70	0.30	99.90	99.00	WGS
287.2619	0.356	Pseudomonas aeruginosa (286)	Pseudomonas (135621)	Pseudomonadaceae (7227	Pseudomonadales (1236)	Gammaproteobacteria (122	Proteobacteria (2)	Bacteria (1)	100.00	0.00	99.80	98.90	WGS
287.2620	0.356	Pseudomonas aeruginosa (286)	Pseudomonas (135621)	Pseudomonadaceae (7227	Pseudomonadales (1236)	Gammaproteobacteria (122	Proteobacteria (2)	Bacteria (1)	100.00	0.60	99.80	98.30	WGS
287.2621	0.3578	Pseudomonas aeruginosa (286)	Pseudomonas (135621)	Pseudomonadaceae (7227	Pseudomonadales (1236)	Gammaproteobacteria (122	Proteobacteria (2)	Bacteria (1)	100.00	0.30	99.80	98.80	WGS
287.2622	0.3268	Pseudomonas aeruginosa (286)	Pseudomonas (135621)	Pseudomonadaceae (7227	Pseudomonadales (1236)	Gammaproteobacteria (122	Proteobacteria (2)	Bacteria (1)	100.00	1.90	99.90	98.20	WGS
287.2623	0.3332	Pseudomonas aeruginosa (286)	Pseudomonas (135621)	Pseudomonadaceae (7227	Pseudomonadales (1236)	Gammaproteobacteria (122	Proteobacteria (2)	Bacteria (1)	99.40	0.00	99.80	98.30	WGS
287.2624	0.3258	Pseudomonas aeruginosa (286)	Pseudomonas (135621)	Pseudomonadaceae (7227	Pseudomonadales (1236)	Gammaproteobacteria (122	Proteobacteria (2)	Bacteria (1)	100.00	0.30	99.70	98.50	WGS
287.2625	0.3738	Pseudomonas aeruginosa (286)	Pseudomonas (135621)	Pseudomonadaceae (7227	Pseudomonadales (1236)	Gammaproteobacteria (122	Proteobacteria (2)	Bacteria (1)	100.00	0.30	99.80	98.60	WGS
287.2626	0.3258	Pseudomonas aeruginosa (286)	Pseudomonas (135621)	Pseudomonadaceae (7227	Pseudomonadales (1236)	Gammaproteobacteria (122	Proteobacteria (2)	Bacteria (1)	100.00	0.30	99.70	98.60	WGS
287.2627	0.3324	Pseudomonas aeruginosa (286)	Pseudomonas (135621)	Pseudomonadaceae (7227	Pseudomonadales (1236)	Gammaproteobacteria (122	Proteobacteria (2)	Bacteria (1)	99.70	1.20	99.80	98.30	WGS
287.2628	0.3324	Pseudomonas aeruginosa (286)	Pseudomonas (135621)	Pseudomonadaceae (7227	Pseudomonadales (1236)	Gammaproteobacteria (122	Proteobacteria (2)	Bacteria (1)	100.00	1.20	99.80	97.50	WGS
287.2629	0.371	Pseudomonas aeruginosa (286)	Pseudomonas (135621)	Pseudomonadaceae (7227	Pseudomonadales (1236)	Gammaproteobacteria (122	Proteobacteria (2)	Bacteria (1)	100.00	0.60	99.80	98.90	WGS
287.2630	0.371	Pseudomonas aeruginosa (286)	Pseudomonas (135621)	Pseudomonadaceae (7227	Pseudomonadales (1236)	Gammaproteobacteria (122	Proteobacteria (2)	Bacteria (1)	100.00	0.70	99.70	98.30	WGS
287.2631	0.356	Pseudomonas aeruginosa (286)	Pseudomonas (135621)	Pseudomonadaceae (7227	Pseudomonadales (1236)	Gammaproteobacteria (122	Proteobacteria (2)	Bacteria (1)	100.00	0.60	99.70	98.60	WGS
287.2632	0.3636	Pseudomonas aeruginosa (286)	Pseudomonas (135621)	Pseudomonadaceae (7227	Pseudomonadales (1236)	Gammaproteobacteria (122	Proteobacteria (2)	Bacteria (1)	99.40	0.90	99.90	98.40	WGS
287.2633	0.3618	Pseudomonas aeruginosa (286)	Pseudomonas (135621)	Pseudomonadaceae (7227	Pseudomonadales (1236)	Gammaproteobacteria (122	Proteobacteria (2)	Bacteria (1)	100.00	1.80	99.90	98.80	WGS
287.2634	0.3618	Pseudomonas aeruginosa (286)	Pseudomonas (135621)	Pseudomonadaceae (7227	Pseudomonadales (1236)	Gammaproteobacteria (122	Proteobacteria (2)	Bacteria (1)	100.00	1.50	99.80	98.10	WGS
287.2635	0.3618	Pseudomonas aeruginosa (286)	Pseudomonas (135621)	Pseudomonadaceae (7227	Pseudomonadales (1236)	Gammaproteobacteria (122	Proteobacteria (2)	Bacteria (1)	100.00	1.30	99.90	98.20	WGS
287.2636	0.371	Pseudomonas aeruginosa (286)	Pseudomonas (135621)	Pseudomonadaceae (7227	Pseudomonadales (1236)	Gammaproteobacteria (122	Proteobacteria (2)	Bacteria (1)	100.00	0.00	99.80	98.30	WGS
287.2637	0.371	Pseudomonas aeruginosa (286)	Pseudomonas (135621)	Pseudomonadaceae (7227	Pseudomonadales (1236)	Gammaproteobacteria (122	Proteobacteria (2)	Bacteria (1)	100.00	0.00	99.70	98.40	WGS
287.2638	0.371	Pseudomonas aeruginosa (286)	Pseudomonas (135621)	Pseudomonadaceae (7227	Pseudomonadales (1236)	Gammaproteobacteria (122	Proteobacteria (2)	Bacteria (1)	99.70	0.00	99.70	98.40	WGS
287.2639	0.3296	Pseudomonas aeruginosa (286)	Pseudomonas (135621)	Pseudomonadaceae (7227	Pseudomonadales (1236)	Gammaproteobacteria (122	Proteobacteria (2)	Bacteria (1)	100.00	0.00	99.80	98.60	WGS
287.2645	0.3268	Pseudomonas aeruginosa (286)	Pseudomonas (135621)	Pseudomonadaceae (7227	Pseudomonadales (1236)	Gammaproteobacteria (122	Proteobacteria (2)	Bacteria (1)	100.00	0.00	99.90	99.30	WGS
287.2658	0.331	Pseudomonas aeruginosa (286)	Pseudomonas (135621)	Pseudomonadaceae (7227	Pseudomonadales (1236)	Gammaproteobacteria (122	Proteobacteria (2)	Bacteria (1)	100.00	0.00	99.80	99.00	Complete
287.2659	0.368	Pseudomonas aeruginosa (286)	Pseudomonas (135621)	Pseudomonadaceae (7227	Pseudomonadales (1236)	Gammaproteobacteria (122	Proteobacteria (2)	Bacteria (1)	99.70	0.00	99.70	99.00	WGS
287.2660	0.327	Pseudomonas aeruginosa (286)	Pseudomonas (135621)	Pseudomonadaceae (7227	Pseudomonadales (1236)	Gammaproteobacteria (122	Proteobacteria (2)	Bacteria (1)	100.00	0.10	99.90	99.30	WGS
287.2661	0.331	Pseudomonas aeruginosa (286)	Pseudomonas (135621)	Pseudomonadaceae (7227	Pseudomonadales (1236)	Gammaproteobacteria (122	Proteobacteria (2)	Bacteria (1)	100.00	0.30	100.00	99.30	WGS
287.2662	0.3324	Pseudomonas aeruginosa (286)	Pseudomonas (135621)	Pseudomonadaceae (7227	Pseudomonadales (1236)	Gammaproteobacteria (122	Proteobacteria (2)	Bacteria (1)	100.00	1.50	99.80	97.90	WGS
287.2663	0.304	Pseudomonas aeruginosa (286)	Pseudomonas (135621)	Pseudomonadaceae (7227	Pseudomonadales (1236)	Gammaproteobacteria (122	Proteobacteria (2)	Bacteria (1)	98.60	24.50	98.90	81.50	WGS
287.2664	0.3258	Pseudomonas aeruginosa (286)	Pseudomonas (135621)	Pseudomonadaceae (7227	Pseudomonadales (1236)	Gammaproteobacteria (122	Proteobacteria (2)	Bacteria (1)	100.00	0.00	99.90	99.30	Complete
287.2665	0.368	Pseudomonas aeruginosa (286)	Pseudomonas (135621)	Pseudomonadaceae (7227	Pseudomonadales (1236)	Gammaproteobacteria (122	Proteobacteria (2)	Bacteria (1)	97.00	0.00	99.80	98.80	Complete
287.2666	0.331	Pseudomonas aeruginosa (286)	Pseudomonas (135621)	Pseudomonadaceae (7227	Pseudomonadales (1236)	Gammaproteobacteria (122	Proteobacteria (2)	Bacteria (1)	100.00	0.00	99.80	99.70	WGS
287.2667	0.3636	Pseudomonas aeruginosa (286)	Pseudomonas (135621)	Pseudomonadaceae (7227	Pseudomonadales (1236)	Gammaproteobacteria (122	Proteobacteria (2)	Bacteria (1)	100.00	0.30	99.90	98.70	WGS
287.2668	0.368	Pseudomonas aeruginosa (286)	Pseudomonas (135621)	Pseudomonadaceae (7227	Pseudomonadales (1236)	Gammaproteobacteria (122	Proteobacteria (2)	Bacteria (1)	100.00	0.30	99.80	98.40	WGS
287.2669	0.3636	Pseudomonas aeruginosa (286)	Pseudomonas (135621)	Pseudomonadaceae (7227	Pseudomonadales (1236)	Gammaproteobacteria (122	Proteobacteria (2)	Bacteria (1)	100.00	0.30	99.90	98.40	WGS
287.2670	0.3188	Pseudomonas aeruginosa (286)	Pseudomonas (135621)	Pseudomonadaceae (7227	Pseudomonadales (1236)	Gammaproteobacteria (122	Proteobacteria (2)	Bacteria (1)	100.00	0.00	100.00	99.90	Complete
287.2692	0.3376	Pseudomonas aeruginosa (286)	Pseudomonas (135621)	Pseudomonadaceae (7227	Pseudomonadales (1236)	Gammaproteobacteria (122	Proteobacteria (2)	Bacteria (1)	100.00	0.90	99.90	98.60	Complete
287.2816	0.3258	Pseudomonas aeruginosa (286)	Pseudomonas (135621)	Pseudomonadaceae (7227	Pseudomonadales (1236)	Gammaproteobacteria (122	Proteobacteria (2)	Bacteria (1)	100.00	0.00	99.60	98.90	Complete
287.2825	0.371	Pseudomonas aeruginosa (286)	Pseudomonas (135621)	Pseudomonadaceae (7227	Pseudomonadales (1236)	Gammaproteobacteria (122	Proteobacteria (2)	Bacteria (1)	100.00	0.00	99.90	99.10	WGS
287.2826	0.3628	Pseudomonas aeruginosa (286)	Pseudomonas (135621)	Pseudomonadaceae (7227	Pseudomonadales (1236)	Gammaproteobacteria (122	Proteobacteria (2)	Bacteria (1)	100.00	0.60	99.90	98.60	WGS
287.2899	0.3662	Pseudomonas aeruginosa (286)	Pseudomonas (135621)	Pseudomonadaceae (7227	Pseudomonadales (1236)	Gammaproteobacteria (122	Proteobacteria (2)	Bacteria (1)	100.00	0.00	99.80	99.50	Complete
287.2900	0.3662	Pseudomonas aeruginosa (286)	Pseudomonas (135621)	Pseudomonadaceae (7227	Pseudomonadales (1236)	Gammaproteobacteria (122	Proteobacteria (2)	Bacteria (1)	100.00	0.00	99.80	99.50	Complete
287.2901	0.3662	Pseudomonas aeruginosa (286)	Pseudomonas (135621)	Pseudomonadaceae (7227	Pseudomonadales (1236)	Gammaproteobacteria (122	Proteobacteria (2)	Bacteria (1)	100.00	0.00	99.80	99.40	Complete
287.2902	0.3662	Pseudomonas aeruginosa (286)	Pseudomonas (135621)	Pseudomonadaceae (7227	Pseudomonadales (1236)	Gammaproteobacteria (122	Proteobacteria (2)	Bacteria (1)	100.00	0.00	99.80	99.40	Complete
287.2903	0.3662	Pseudomonas aeruginosa (286)	Pseudomonas (135621)	Pseudomonadaceae (7227	Pseudomonadales (1236)	Gammaproteobacteria (122	Proteobacteria (2)	Bacteria (1)	100.00	0.00	99.80	99.40	Complete
287.2904	0.3662	Pseudomonas aeruginosa (286)	Pseudomonas (135621)	Pseudomonadaceae (7227	Pseudomonadales (1236)	Gammaproteobacteria (122	Proteobacteria (2)	Bacteria (1)	100.00	0.00	99.80	99.40	Complete
287.2905	0.3292	Pseudomonas aeruginosa (286)	Pseudomonas (135621)	Pseudomonadaceae (7227	Pseudomonadales (1236)	Gammaproteobacteria (122	Proteobacteria (2)	Bacteria (1)	100.00	0.00	99.70	99.00	Complete
287.2906	0.3662	Pseudomonas aeruginosa (286)	Pseudomonas (135621)	Pseudomonadaceae (7227	Pseudomonadales (1236)	Gammaproteobacteria (122	Proteobacteria (2)	Bacteria (1)	100.00	0.00	99.80	99.40	Complete
287.2907	0.3662	Pseudomonas aeruginosa (286)	Pseudomonas (135621)	Pseudomonadaceae (7227	Pseudomonadales (1236)	Gammaproteobacteria (122	Proteobacteria (2)	Bacteria (1)	99.70	0.00	99.80	99.50	Complete
287.2908	0.3188	Pseudomonas aeruginosa (286)	Pseudomonas (135621)	Pseudomonadaceae (7227	Pseudomonadales (1236)	Gammaproteobacteria (122	Proteobacteria (2)	Bacteria (1)	100.00	0.10	100.00	99.70	Complete
287.2914	0.3376	Pseudomonas aeruginosa (286)	Pseudomonas (135621)	Pseudomonadaceae (7227	Pseudomonadales (1236)	Gammaproteobacteria (122	Proteobacteria (2)	Bacteria (1)	99.70	1.20	99.80	96.70	Complete
287.2915	0.3226	Pseudomonas aeruginosa (286)	Pseudomonas (135621)	Pseudomonadaceae (7227	Pseudomonadales (1236)	Gammaproteobacteria (122	Proteobacteria (2)	Bacteria (1)	100.00	0.00	99.60	99.20	WGS
287.2916	0.3314	Pseudomonas aeruginosa (286)	Pseudomonas (135621)	Pseudomonadaceae (7227	Pseudomonadales (1236)	Gammaproteobacteria (122	Proteobacteria (2)	Bacteria (1)	100.00	0.00	99.80	99.00	WGS
287.2917	0.3388	Pseudomonas aeruginosa (286)	Pseudomonas (135621)	Pseudomonadaceae (7227	Pseudomonadales (1236)	Gammaproteobacteria (122	Proteobacteria (2)	Bacteria (1)	100.00	0.60	99.60	97.80	WGS
287.2918	0.3618	Pseudomonas aeruginosa (286)	Pseudomonas (135621)	Pseudomonadaceae (7227	Pseudomonadales (1236)	Gammaproteobacteria (122	Proteobacteria (2)	Bacteria (1)	100.00	1.90	99.90	98.70	WGS
287.2919	0.3258	Pseudomonas aeruginosa (286)	Pseudomonas (135621)	Pseudomonadaceae (7227	Pseudomonadales (1236)	Gammaproteobacteria (122	Proteobacteria (2)	Bacteria (1)	100.00	0.30	99.90	99.60	WGS
287.2920	0.3766	Pseudomonas aeruginosa (286)	Pseudomonas (135621)	Pseudomonadaceae (7227	Pseudomonadales (1236)	Gammaproteobacteria (122	Proteobacteria (2)	Bacteria (1)	100.00	2.10	99.60	98.70	WGS
287.2921	0.3314	Pseudomonas aeruginosa (286)	Pseudomonas (135621)	Pseudomonadaceae (7227	Pseudomonadales (1236)	Gammaproteobacteria (122	Proteobacteria (2)	Bacteria (1)	100.00	0.00	99.80	99.00	WGS
287.2922	0.3304	Pseudomonas aeruginosa (286)	Pseudomonas (135621)	Pseudomonadaceae (7227	Pseudomonadales (1236)	Gammaproteobacteria (122	Proteobacteria (2)	Bacteria (1)	100.00	0.00	99.90	99.10	WGS
287.2923	0.331	Pseudomonas aeruginosa (286)	Pseudomonas (135621)	Pseudomonadaceae (7227	Pseudomonadales (1236)	Gammaproteobacteria (122	Proteobacteria (2)	Bacteria (1)	99.70	1.20	99.70	98.70	WGS
287.2924	0.3304	Pseudomonas aeruginosa (286)	Pseudomonas (135621)	Pseudomonadaceae (7227	Pseudomonadales (1236)	Gammaproteobacteria (122	Proteobacteria (2)	Bacteria (1)	100.00	0.00	99.80	98.80	WGS
287.2925	0.3646	Pseudomonas aeruginosa (286)	Pseudomonas (135621)	Pseudomonadaceae (7227	Pseudomonadales (1236)	Gammaproteobacteria (122	Proteobacteria (2)	Bacteria (1)	100.00	0.30	99.90	98.10	WGS
287.2926	0.3646	Pseudomonas aeruginosa (286)	Pseudomonas (135621)	Pseudomonadaceae (7227	Pseudomonadales (1236)	Gammaproteobacteria (122	Proteobacteria (2)	Bacteria (1)	100.00	0.30	99.80	98.40	WGS
287.2927	0.3646	Pseudomonas aeruginosa (286)	Pseudomonas (135621)	Pseudomonadaceae (7227	Pseudomonadales (1236)	Gammaproteobacteria (122	Proteobacteria (2)	Bacteria (1)	99.70	2.20	99.70	97.30	WGS
287.2928	0.3646	Pseudomonas aeruginosa (286)	Pseudomonas (135621)	Pseudomonadaceae (7227	Pseudomonadales (1236)	Gammaproteobacteria (122	Proteobacteria (2)	Bacteria (1)	100.00	0.30	99.90	98.60	WGS
287.2929	0.3646	Pseudomonas aeruginosa (286)	Pseudomonas (135621)	Pseudomonadaceae (7227	Pseudomonadales (1236)	Gammaproteobacteria (122	Proteobacteria (2)	Bacteria (1)	100.00	2.10	99.90	96.50	WGS
287.2930	0.3646	Pseudomonas aeruginosa (286)	Pseudomonas (135621)	Pseudomonadaceae (7227	Pseudomonadales (1236)	Gammaproteobacteria (122	Proteobacteria (2)	Bacteria (1)	100.00	1.50	99.70	98.00	WGS
287.2931	0.355	Pseudomonas aeruginosa (286)	Pseudomonas (135621)	Pseudomonadaceae (7227	Pseudomonadales (1236)	Gammaproteobacteria (122	Proteobacteria (2)	Bacteria (1)	100.00	0.30	99.90	98.30	WGS
287.2932	0.3646	Pseudomonas aeruginosa (286)	Pseudomonas (135621)	Pseudomonadaceae (7227	Pseudomonadales (1236)	Gammaproteobacteria (122	Proteobacteria (2)	Bacteria (1)	100.00	0.30	99.90	98.70	WGS
287.2933	0.3646	Pseudomonas aeruginosa (286)	Pseudomonas (135621)	Pseudomonadaceae (7227	Pseudomonadales (1236)	Gammaproteobacteria (122	Proteobacteria (2)	Bacteria (1)	100.00	0.30	99.80	98.30	WGS
287.2934	0.3646	Pseudomonas aeruginosa (286)	Pseudomonas (135621)	Pseudomonadaceae (7227	Pseudomonadales (1236)	Gammaproteobacteria (122	Proteobacteria (2)	Bacteria (1)	100.00	0.30	99.90	98.70	WGS
287.2935	0.3646	Pseudomonas aeruginosa (286)	Pseudomonas (135621)	Pseudomonadaceae (7227	Pseudomonadales (1236)	Gammaproteobacteria (122	Proteobacteria (2)	Bacteria (1)	100.00	0.90	99.90	98.50	WGS
287.2936	0.3646	Pseudomonas aeruginosa (286)	Pseudomonas (135621)	Pseudomonadaceae (7227	Pseudomonadales (1236)	Gammaproteobacteria (122	Proteobacteria (2)	Bacteria (1)	100.00	0.30	99.90	98.80	WGS
287.2937	0.3646	Pseudomonas aeruginosa (286)	Pseudomonas (135621)	Pseudomonadaceae (7227	Pseudomonadales (1236)	Gammaproteobacteria (122	Proteobacteria (2)	Bacteria (1)	100.00	0.30	99.90	98.60	WGS
287.2938	0.3646	Pseudomonas aeruginosa (286)	Pseudomonas (135621)	Pseudomonadaceae (7227	Pseudomonadales (1236)	Gammaproteobacteria (122	Proteobacteria (2)	Bacteria (1)	100.00	0.30	99.90	98.70	WGS
287.2939	0.3646	Pseudomonas aeruginosa (286)	Pseudomonas (135621)	Pseudomonadaceae (7227	Pseudomonadales (1236)	Gammaproteobacteria (122	Proteobacteria (2)	Bacteria (1)	100.00	0.30	99.90	98.70	WGS
287.2940	0.3646	Pseudomonas aeruginosa (286)	Pseudomonas (135621)	Pseudomonadaceae (7227	Pseudomonadales (1236)	Gammaproteobacteria (122	Proteobacteria (2)	Bacteria (1)	100.00	0.60	99.90	98.40	WGS
287.2941	0.355	Pseudomonas aeruginosa (286)	Pseudomonas (135621)	Pseudomonadaceae (7227	Pseudomonadales (1236)	Gammaproteobacteria (122	Proteobacteria (2)	Bacteria (1)	100.00	1.30	99.90	98.10	WGS
287.2942	0.3646	Pseudomonas aeruginosa (286)	Pseudomonas (135621)	Pseudomonadaceae (7227	Pseudomonadales (1236)	Gammaproteobacteria (122	Proteobacteria (2)	Bacteria (1)	100.00	0.60	99.90	98.70	WGS
287.2943	0.3646	Pseudomonas aeruginosa (286)	Pseudomonas (135621)	Pseudomonadaceae (7227	Pseudomonadales (1236)	Gammaproteobacteria (122	Proteobacteria (2)	Bacteria (1)	100.00	1.50	99.70	97.90	WGS
287.2944	0.3646	Pseudomonas aeruginosa (286)	Pseudomonas (135621)	Pseudomonadaceae (7227	Pseudomonadales (1236)	Gammaproteobacteria (122	Proteobacteria (2)	Bacteria (1)	100.00	0.30	99.90	98.50	WGS
287.2945	0.3646	Pseudomonas aeruginosa (286)	Pseudomonas (135621)	Pseudomonadaceae (7227	Pseudomonadales (1236)	Gammaproteobacteria (122	Proteobacteria (2)	Bacteria (1)	100.00	0.60	99.70	97.80	WGS
287.2946	0.3646	Pseudomonas aeruginosa (286)	Pseudomonas (135621)	Pseudomonadaceae (7227	Pseudomonadales (1236)	Gammaproteobacteria (122	Proteobacteria (2)	Bacteria (1)	100.00	0.40	99.70	98.50	WGS
287.2947	0.355	Pseudomonas aeruginosa (286)	Pseudomonas (135621)	Pseudomonadaceae (7227	Pseudomonadales (1236)	Gammaproteobacteria (122	Proteobacteria (2)	Bacteria (1)	100.00	0.60	99.90	98.00	WGS
287.2948	0.355	Pseudomonas aeruginosa (286)	Pseudomonas (135621)	Pseudomonadaceae (7227	Pseudomonadales (1236)	Gammaproteobacteria (122	Proteobacteria (2)	Bacteria (1)	100.00	0.60	99.90	98.40	WGS
287.2949	0.3646	Pseudomonas aeruginosa (286)	Pseudomonas (135621)	Pseudomonadaceae (7227	Pseudomonadales (1236)	Gammaproteobacteria (122	Proteobacteria (2)	Bacteria (1)	100.00	0.60	99.90	98.30	WGS
287.2950	0.3646	Pseudomonas aeruginosa (286)	Pseudomonas (135621)	Pseudomonadaceae (7227	Pseudomonadales (1236)	Gammaproteobacteria (122	Proteobacteria (2)	Bacteria (1)	100.00	0.90	99.70	98.10	WGS
287.2951	0.3646	Pseudomonas aeruginosa (286)	Pseudomonas (135621)	Pseudomonadaceae (7227	Pseudomonadales (1236)	Gammaproteobacteria (122	Proteobacteria (2)	Bacteria (1)	100.00	0.60	99.90	98.20	WGS
287.2952	0.355	Pseudomonas aeruginosa (286)	Pseudomonas (135621)	Pseudomonadaceae (7227	Pseudomonadales (1236)	Gammaproteobacteria (122	Proteobacteria (2)	Bacteria (1)	100.00	1.20	99.90	98.10	WGS
287.2953	0.355	Pseudomonas aeruginosa (286)	Pseudomonas (135621)	Pseudomonadaceae (7227	Pseudomonadales (1236)	Gammaproteobacteria (122	Proteobacteria (2)	Bacteria (1)	100.00	0.60	99.90	98.40	WGS
287.2954	0.355	Pseudomonas aeruginosa (286)	Pseudomonas (135621)	Pseudomonadaceae (7227	Pseudomonadales (1236)	Gammaproteobacteria (122	Proteobacteria (2)	Bacteria (1)	100.00	0.30	99.90	98.40	WGS
287.2955	0.355	Pseudomonas aeruginosa (286)	Pseudomonas (135621)	Pseudomonadaceae (7227	Pseudomonadales (1236)	Gammaproteobacteria (122	Proteobacteria (2)	Bacteria (1)	100.00	0.90	99.80	98.10	WGS
287.2956	0.355	Pseudomonas aeruginosa (286)	Pseudomonas (135621)	Pseudomonadaceae (7227	Pseudomonadales (1236)	Gammaproteobacteria (122	Proteobacteria (2)	Bacteria (1)	100.00	0.30	99.90	98.10	WGS
287.2957	0.355	Pseudomonas aeruginosa (286)	Pseudomonas (135621)	Pseudomonadaceae (7227	Pseudomonadales (1236)	Gammaproteobacteria (122	Proteobacteria (2)	Bacteria (1)	100.00	0.60	99.90	98.40	WGS
287.2958	0.3572	Pseudomonas aeruginosa (286)	Pseudomonas (135621)	Pseudomonadaceae (7227	Pseudomonadales (1236)	Gammaproteobacteria (122	Proteobacteria (2)	Bacteria (1)	100.00	2.70	99.90	97.70	WGS
287.2959	0.3646	Pseudomonas aeruginosa (286)	Pseudomonas (135621)	Pseudomonadaceae (7227	Pseudomonadales (1236)	Gammaproteobacteria (122	Proteobacteria (2)	Bacteria (1)	100.00	0.90	99.90	98.40	WGS
287.2960	0.3646	Pseudomonas aeruginosa (286)	Pseudomonas (135621)	Pseudomonadaceae (7227	Pseudomonadales (1236)	Gammaproteobacteria (122	Proteobacteria (2)	Bacteria (1)	100.00	1.20	99.90	97.90	WGS
287.2961	0.355	Pseudomonas aeruginosa (286)	Pseudomonas (135621)	Pseudomonadaceae (7227	Pseudomonadales (1236)	Gammaproteobacteria (122	Proteobacteria (2)	Bacteria (1)	99.70	1.20	99.80	98.20	WGS
287.2962	0.3646	Pseudomonas aeruginosa (286)	Pseudomonas (135621)	Pseudomonadaceae (7227	Pseudomonadales (1236)	Gammaproteobacteria (122	Proteobacteria (2)	Bacteria (1)	100.00	0.30	99.90	98.70	WGS
287.2963	0.3646	Pseudomonas aeruginosa (286)	Pseudomonas (135621)	Pseudomonadaceae (7227	Pseudomonadales (1236)	Gammaproteobacteria (122	Proteobacteria (2)	Bacteria (1)	100.00	0.30	99.90	98.70	WGS
287.2964	0.3844	Pseudomonas aeruginosa (286)	Pseudomonas (135621)	Pseudomonadaceae (7227	Pseudomonadales (1236)	Gammaproteobacteria (122	Proteobacteria (2)	Bacteria (1)	100.00	2.10	99.90	97.70	WGS
287.2965	0.3306	Pseudomonas aeruginosa (286)	Pseudomonas (135621)	Pseudomonadaceae (7227	Pseudomonadales (1236)	Gammaproteobacteria (122	Proteobacteria (2)	Bacteria (1)	100.00	0.00	99.80	99.00	Complete
287.2966	0.3306	Pseudomonas aeruginosa (286)	Pseudomonas (135621)	Pseudomonadaceae (7227	Pseudomonadales (1236)	Gammaproteobacteria (122	Proteobacteria (2)	Bacteria (1)	100.00	0.00	99.80	99.00	Complete
287.2967	0.3306	Pseudomonas aeruginosa (286)	Pseudomonas (135621)	Pseudomonadaceae (7227	Pseudomonadales (1236)	Gammaproteobacteria (122	Proteobacteria (2)	Bacteria (1)	100.00	0.00	99.90	99.30	Complete
287.2968	0.3306	Pseudomonas aeruginosa (286)	Pseudomonas (135621)	Pseudomonadaceae (7227	Pseudomonadales (1236)	Gammaproteobacteria (122	Proteobacteria (2)	Bacteria (1)	100.00	0.00	99.80	99.10	WGS
287.2969	0.3306	Pseudomonas aeruginosa (286)	Pseudomonas (135621)	Pseudomonadaceae (7227	Pseudomonadales (1236)	Gammaproteobacteria (122	Proteobacteria (2)	Bacteria (1)	100.00	0.00	99.90	99.40	WGS
287.2970	0.3306	Pseudomonas aeruginosa (286)	Pseudomonas (135621)	Pseudomonadaceae (7227	Pseudomonadales (1236)	Gammaproteobacteria (122	Proteobacteria (2)	Bacteria (1)	100.00	0.00	99.80	99.00	Complete
287.2971	0.3618	Pseudomonas aeruginosa (286)	Pseudomonas (135621)	Pseudomonadaceae (7227	Pseudomonadales (1236)	Gammaproteobacteria (122	Proteobacteria (2)	Bacteria (1)	100.00	1.00	99.80	98.70	Complete
287.2972	0.3254	Pseudomonas aeruginosa (286)	Pseudomonas (135621)	Pseudomonadaceae (7227	Pseudomonadales (1236)	Gammaproteobacteria (122	Proteobacteria (2)	Bacteria (1)	100.00	0.90	99.80	99.30	WGS
287.2973	0.357	Pseudomonas aeruginosa (286)	Pseudomonas (135621)	Pseudomonadaceae (7227	Pseudomonadales (1236)	Gammaproteobacteria (122	Proteobacteria (2)	Bacteria (1)	100.00	0.00	99.90	99.30	Complete
287.2975	0.357	Pseudomonas aeruginosa (286)	Pseudomonas (135621)	Pseudomonadaceae (7227	Pseudomonadales (1236)	Gammaproteobacteria (122	Proteobacteria (2)	Bacteria (1)	100.00	0.60	99.90	99.00	WGS
287.2976	0.3608	Pseudomonas aeruginosa (286)	Pseudomonas (135621)	Pseudomonadaceae (7227	Pseudomonadales (1236)	Gammaproteobacteria (122	Proteobacteria (2)	Bacteria (1)	99.10	1.50	99.90	99.10	WGS
287.2977	0.3254	Pseudomonas aeruginosa (286)	Pseudomonas (135621)	Pseudomonadaceae (7227	Pseudomonadales (1236)	Gammaproteobacteria (122	Proteobacteria (2)	Bacteria (1)	100.00	0.60	99.90	98.70	WGS
287.2978	0.3306	Pseudomonas aeruginosa (286)	Pseudomonas (135621)	Pseudomonadaceae (7227	Pseudomonadales (1236)	Gammaproteobacteria (122	Proteobacteria (2)	Bacteria (1)	100.00	1.20	99.80	98.50	WGS
287.2979	0.3738	Pseudomonas aeruginosa (286)	Pseudomonas (135621)	Pseudomonadaceae (7227	Pseudomonadales (1236)	Gammaproteobacteria (122	Proteobacteria (2)	Bacteria (1)	100.00	0.30	99.50	98.30	WGS
287.2980	0.368	Pseudomonas aeruginosa (286)	Pseudomonas (135621)	Pseudomonadaceae (7227	Pseudomonadales (1236)	Gammaproteobacteria (122	Proteobacteria (2)	Bacteria (1)	100.00	0.00	99.70	98.80	WGS
287.2981	0.3268	Pseudomonas aeruginosa (286)	Pseudomonas (135621)	Pseudomonadaceae (7227	Pseudomonadales (1236)	Gammaproteobacteria (122	Proteobacteria (2)	Bacteria (1)	100.00	0.00	99.90	99.30	Complete
287.3101	0.3636	Pseudomonas aeruginosa (286)	Pseudomonas (135621)	Pseudomonadaceae (7227	Pseudomonadales (1236)	Gammaproteobacteria (122	Proteobacteria (2)	Bacteria (1)	100.00	0.00	99.70	99.50	Complete
287.3102	0.3636	Pseudomonas aeruginosa (286)	Pseudomonas (135621)	Pseudomonadaceae (7227	Pseudomonadales (1236)	Gammaproteobacteria (122	Proteobacteria (2)	Bacteria (1)	100.00	0.00	99.90	99.70	Complete
287.3103	0.3636	Pseudomonas aeruginosa (286)	Pseudomonas (135621)	Pseudomonadaceae (7227	Pseudomonadales (1236)	Gammaproteobacteria (122	Proteobacteria (2)	Bacteria (1)	100.00	0.00	99.90	99.70	Complete
287.3104	0.3636	Pseudomonas aeruginosa (286)	Pseudomonas (135621)	Pseudomonadaceae (7227	Pseudomonadales (1236)	Gammaproteobacteria (122	Proteobacteria (2)	Bacteria (1)	100.00	0.00	99.90	99.70	Complete
287.3105	0.3662	Pseudomonas aeruginosa (286)	Pseudomonas (135621)	Pseudomonadaceae (7227	Pseudomonadales (1236)	Gammaproteobacteria (122	Proteobacteria (2)	Bacteria (1)	98.70	1.30	99.90	97.60	WGS
287.3106	0.3738	Pseudomonas aeruginosa (286)	Pseudomonas (135621)	Pseudomonadaceae (7227	Pseudomonadales (1236)	Gammaproteobacteria (122	Proteobacteria (2)	Bacteria (1)	100.00	0.40	99.80	99.00	WGS
287.3107	0.3376	Pseudomonas aeruginosa (286)	Pseudomonas (135621)	Pseudomonadaceae (7227	Pseudomonadales (1236)	Gammaproteobacteria (122	Proteobacteria (2)	Bacteria (1)	100.00	0.40	99.80	98.00	WGS
287.3108	0.3604	Pseudomonas aeruginosa (286)	Pseudomonas (135621)	Pseudomonadaceae (7227	Pseudomonadales (1236)	Gammaproteobacteria (122	Proteobacteria (2)	Bacteria (1)	100.00	0.20	99.80	98.10	WGS
287.3109	0.3694	Pseudomonas aeruginosa (286)	Pseudomonas (135621)	Pseudomonadaceae (7227	Pseudomonadales (1236)	Gammaproteobacteria (122	Proteobacteria (2)	Bacteria (1)	100.00	0.10	99.90	99.30	WGS
287.3110	0.3304	Pseudomonas aeruginosa (286)	Pseudomonas (135621)	Pseudomonadaceae (7227	Pseudomonadales (1236)	Gammaproteobacteria (122	Proteobacteria (2)	Bacteria (1)	100.00	0.10	99.80	98.60	WGS
287.3111	0.3268	Pseudomonas aeruginosa (286)	Pseudomonas (135621)	Pseudomonadaceae (7227	Pseudomonadales (1236)	Gammaproteobacteria (122	Proteobacteria (2)	Bacteria (1)	98.80	1.10	99.70	98.20	WGS
287.3112	0.3236	Pseudomonas aeruginosa (286)	Pseudomonas (135621)	Pseudomonadaceae (7227	Pseudomonadales (1236)	Gammaproteobacteria (122	Proteobacteria (2)	Bacteria (1)	100.00	0.40	99.60	98.50	WGS
287.3113	0.3292	Pseudomonas aeruginosa (286)	Pseudomonas (135621)	Pseudomonadaceae (7227	Pseudomonadales (1236)	Gammaproteobacteria (122	Proteobacteria (2)	Bacteria (1)	100.00	0.70	99.90	98.50	WGS
287.3114	0.3618	Pseudomonas aeruginosa (286)	Pseudomonas (135621)	Pseudomonadaceae (7227	Pseudomonadales (1236)	Gammaproteobacteria (122	Proteobacteria (2)	Bacteria (1)	100.00	0.40	99.90	98.50	WGS
287.3115	0.3268	Pseudomonas aeruginosa (286)	Pseudomonas (135621)	Pseudomonadaceae (7227	Pseudomonadales (1236)	Gammaproteobacteria (122	Proteobacteria (2)	Bacteria (1)	100.00	1.00	99.90	98.40	WGS
287.3116	0.3292	Pseudomonas aeruginosa (286)	Pseudomonas (135621)	Pseudomonadaceae (7227	Pseudomonadales (1236)	Gammaproteobacteria (122	Proteobacteria (2)	Bacteria (1)	98.80	0.80	99.90	98.40	WGS
287.3117	0.3628	Pseudomonas aeruginosa (286)	Pseudomonas (135621)	Pseudomonadaceae (7227	Pseudomonadales (1236)	Gammaproteobacteria (122	Proteobacteria (2)	Bacteria (1)	99.70	0.40	99.90	98.50	WGS
287.3118	0.3268	Pseudomonas aeruginosa (286)	Pseudomonas (135621)	Pseudomonadaceae (7227	Pseudomonadales (1236)	Gammaproteobacteria (122	Proteobacteria (2)	Bacteria (1)	100.00	0.40	99.60	97.90	WGS
287.3119	0.3628	Pseudomonas aeruginosa (286)	Pseudomonas (135621)	Pseudomonadaceae (7227	Pseudomonadales (1236)	Gammaproteobacteria (122	Proteobacteria (2)	Bacteria (1)	100.00	0.40	99.90	98.20	WGS
287.3120	0.3618	Pseudomonas aeruginosa (286)	Pseudomonas (135621)	Pseudomonadaceae (7227	Pseudomonadales (1236)	Gammaproteobacteria (122	Proteobacteria (2)	Bacteria (1)	100.00	0.40	99.90	99.20	WGS
287.3121	0.3174	Pseudomonas aeruginosa (286)	Pseudomonas (135621)	Pseudomonadaceae (7227	Pseudomonadales (1236)	Gammaproteobacteria (122	Proteobacteria (2)	Bacteria (1)	99.70	0.40	99.80	98.50	WGS
287.3122	0.3184	Pseudomonas aeruginosa (286)	Pseudomonas (135621)	Pseudomonadaceae (7227	Pseudomonadales (1236)	Gammaproteobacteria (122	Proteobacteria (2)	Bacteria (1)	100.00	0.40	99.80	98.90	WGS
287.3123	0.3546	Pseudomonas aeruginosa (286)	Pseudomonas (135621)	Pseudomonadaceae (7227	Pseudomonadales (1236)	Gammaproteobacteria (122	Proteobacteria (2)	Bacteria (1)	100.00	0.70	99.90	98.70	WGS
287.3124	0.356	Pseudomonas aeruginosa (286)	Pseudomonas (135621)	Pseudomonadaceae (7227	Pseudomonadales (1236)	Gammaproteobacteria (122	Proteobacteria (2)	Bacteria (1)	100.00	0.20	99.90	99.30	WGS
287.3125	0.3618	Pseudomonas aeruginosa (286)	Pseudomonas (135621)	Pseudomonadaceae (7227	Pseudomonadales (1236)	Gammaproteobacteria (122	Proteobacteria (2)	Bacteria (1)	100.00	0.20	99.90	98.80	WGS
287.3126	0.3236	Pseudomonas aeruginosa (286)	Pseudomonas (135621)	Pseudomonadaceae (7227	Pseudomonadales (1236)	Gammaproteobacteria (122	Proteobacteria (2)	Bacteria (1)	100.00	0.70	99.70	98.80	WGS
287.3127	0.3662	Pseudomonas aeruginosa (286)	Pseudomonas (135621)	Pseudomonadaceae (7227	Pseudomonadales (1236)	Gammaproteobacteria (122	Proteobacteria (2)	Bacteria (1)	100.00	0.70	99.90	98.40	WGS
287.3128	0.3628	Pseudomonas aeruginosa (286)	Pseudomonas (135621)	Pseudomonadaceae (7227	Pseudomonadales (1236)	Gammaproteobacteria (122	Proteobacteria (2)	Bacteria (1)	100.00	0.40	99.90	98.30	WGS
287.3129	0.357	Pseudomonas aeruginosa (286)	Pseudomonas (135621)	Pseudomonadaceae (7227	Pseudomonadales (1236)	Gammaproteobacteria (122	Proteobacteria (2)	Bacteria (1)	100.00	0.40	99.70	98.40	WGS
287.3130	0.3662	Pseudomonas aeruginosa (286)	Pseudomonas (135621)	Pseudomonadaceae (7227	Pseudomonadales (1236)	Gammaproteobacteria (122	Proteobacteria (2)	Bacteria (1)	100.00	0.70	99.90	98.70	WGS
287.3131	0.3268	Pseudomonas aeruginosa (286)	Pseudomonas (135621)	Pseudomonadaceae (7227	Pseudomonadales (1236)	Gammaproteobacteria (122	Proteobacteria (2)	Bacteria (1)	100.00	0.60	99.90	98.70	WGS
287.3134	0.3614	Pseudomonas aeruginosa (286)	Pseudomonas (135621)	Pseudomonadaceae (7227	Pseudomonadales (1236)	Gammaproteobacteria (122	Proteobacteria (2)	Bacteria (1)	98.20	6.80	98.70	93.10	WGS
287.3135	0.3636	Pseudomonas aeruginosa (286)	Pseudomonas (135621)	Pseudomonadaceae (7227	Pseudomonadales (1236)	Gammaproteobacteria (122	Proteobacteria (2)	Bacteria (1)	99.70	0.60	99.60	98.20	WGS
287.3136	0.3662	Pseudomonas aeruginosa (286)	Pseudomonas (135621)	Pseudomonadaceae (7227	Pseudomonadales (1236)	Gammaproteobacteria (122	Proteobacteria (2)	Bacteria (1)	100.00	0.00	99.80	98.40	WGS
287.3137	0.3672	Pseudomonas aeruginosa (286)	Pseudomonas (135621)	Pseudomonadaceae (7227	Pseudomonadales (1236)	Gammaproteobacteria (122	Proteobacteria (2)	Bacteria (1)	99.70	0.60	99.60	98.10	WGS
287.3138	0.3614	Pseudomonas aeruginosa (286)	Pseudomonas (135621)	Pseudomonadaceae (7227	Pseudomonadales (1236)	Gammaproteobacteria (122	Proteobacteria (2)	Bacteria (1)	100.00	0.60	99.80	99.00	WGS
287.3139	0.3604	Pseudomonas aeruginosa (286)	Pseudomonas (135621)	Pseudomonadaceae (7227	Pseudomonadales (1236)	Gammaproteobacteria (122	Proteobacteria (2)	Bacteria (1)	100.00	0.00	99.60	98.00	WGS
287.3140	0.3604	Pseudomonas aeruginosa (286)	Pseudomonas (135621)	Pseudomonadaceae (7227	Pseudomonadales (1236)	Gammaproteobacteria (122	Proteobacteria (2)	Bacteria (1)	100.00	0.60	99.70	98.70	WGS
287.3141	0.3614	Pseudomonas aeruginosa (286)	Pseudomonas (135621)	Pseudomonadaceae (7227	Pseudomonadales (1236)	Gammaproteobacteria (122	Proteobacteria (2)	Bacteria (1)	100.00	2.30	99.30	97.60	WGS
287.3142	0.3614	Pseudomonas aeruginosa (286)	Pseudomonas (135621)	Pseudomonadaceae (7227	Pseudomonadales (1236)	Gammaproteobacteria (122	Proteobacteria (2)	Bacteria (1)	99.70	0.90	99.30	97.90	WGS
287.3143	0.3724	Pseudomonas aeruginosa (286)	Pseudomonas (135621)	Pseudomonadaceae (7227	Pseudomonadales (1236)	Gammaproteobacteria (122	Proteobacteria (2)	Bacteria (1)	99.70	2.90	99.30	96.80	WGS
287.3144	0.3604	Pseudomonas aeruginosa (286)	Pseudomonas (135621)	Pseudomonadaceae (7227	Pseudomonadales (1236)	Gammaproteobacteria (122	Proteobacteria (2)	Bacteria (1)	100.00	1.20	99.30	98.10	WGS
287.3147	0.325	Pseudomonas aeruginosa (286)	Pseudomonas (135621)	Pseudomonadaceae (7227	Pseudomonadales (1236)	Gammaproteobacteria (122	Proteobacteria (2)	Bacteria (1)	100.00	0.30	99.90	99.50	WGS
287.3148	0.3636	Pseudomonas aeruginosa (286)	Pseudomonas (135621)	Pseudomonadaceae (7227	Pseudomonadales (1236)	Gammaproteobacteria (122	Proteobacteria (2)	Bacteria (1)	100.00	0.00	99.80	98.20	WGS
287.3149	0.3636	Pseudomonas aeruginosa (286)	Pseudomonas (135621)	Pseudomonadaceae (7227	Pseudomonadales (1236)	Gammaproteobacteria (122	Proteobacteria (2)	Bacteria (1)	100.00	0.00	99.90	98.70	WGS
287.3150	0.3636	Pseudomonas aeruginosa (286)	Pseudomonas (135621)	Pseudomonadaceae (7227	Pseudomonadales (1236)	Gammaproteobacteria (122	Proteobacteria (2)	Bacteria (1)	100.00	0.00	99.90	98.70	WGS
287.3151	0.3694	Pseudomonas aeruginosa (286)	Pseudomonas (135621)	Pseudomonadaceae (7227	Pseudomonadales (1236)	Gammaproteobacteria (122	Proteobacteria (2)	Bacteria (1)	100.00	0.00	99.90	99.50	WGS
287.3152	0.3636	Pseudomonas aeruginosa (286)	Pseudomonas (135621)	Pseudomonadaceae (7227	Pseudomonadales (1236)	Gammaproteobacteria (122	Proteobacteria (2)	Bacteria (1)	100.00	0.00	99.90	98.90	WGS
287.3153	0.3636	Pseudomonas aeruginosa (286)	Pseudomonas (135621)	Pseudomonadaceae (7227	Pseudomonadales (1236)	Gammaproteobacteria (122	Proteobacteria (2)	Bacteria (1)	100.00	0.00	99.90	97.80	WGS
287.3154	0.368	Pseudomonas aeruginosa (286)	Pseudomonas (135621)	Pseudomonadaceae (7227	Pseudomonadales (1236)	Gammaproteobacteria (122	Proteobacteria (2)	Bacteria (1)	100.00	0.00	99.70	97.60	WGS
287.3155	0.325	Pseudomonas aeruginosa (286)	Pseudomonas (135621)	Pseudomonadaceae (7227	Pseudomonadales (1236)	Gammaproteobacteria (122	Proteobacteria (2)	Bacteria (1)	100.00	0.30	99.90	99.00	WGS
287.3156	0.368	Pseudomonas aeruginosa (286)	Pseudomonas (135621)	Pseudomonadaceae (7227	Pseudomonadales (1236)	Gammaproteobacteria (122	Proteobacteria (2)	Bacteria (1)	100.00	0.00	99.70	98.30	WGS
287.3157	0.3694	Pseudomonas aeruginosa (286)	Pseudomonas (135621)	Pseudomonadaceae (7227	Pseudomonadales (1236)	Gammaproteobacteria (122	Proteobacteria (2)	Bacteria (1)	100.00	0.00	99.90	98.30	WGS
287.3158	0.3636	Pseudomonas aeruginosa (286)	Pseudomonas (135621)	Pseudomonadaceae (7227	Pseudomonadales (1236)	Gammaproteobacteria (122	Proteobacteria (2)	Bacteria (1)	100.00	0.00	99.90	98.70	WGS
287.3159	0.368	Pseudomonas aeruginosa (286)	Pseudomonas (135621)	Pseudomonadaceae (7227	Pseudomonadales (1236)	Gammaproteobacteria (122	Proteobacteria (2)	Bacteria (1)	100.00	0.00	99.70	97.40	WGS
287.3160	0.325	Pseudomonas aeruginosa (286)	Pseudomonas (135621)	Pseudomonadaceae (7227	Pseudomonadales (1236)	Gammaproteobacteria (122	Proteobacteria (2)	Bacteria (1)	100.00	0.60	99.90	98.70	WGS
287.3161	0.368	Pseudomonas aeruginosa (286)	Pseudomonas (135621)	Pseudomonadaceae (7227	Pseudomonadales (1236)	Gammaproteobacteria (122	Proteobacteria (2)	Bacteria (1)	100.00	0.00	99.70	98.20	WGS
287.3162	0.368	Pseudomonas aeruginosa (286)	Pseudomonas (135621)	Pseudomonadaceae (7227	Pseudomonadales (1236)	Gammaproteobacteria (122	Proteobacteria (2)	Bacteria (1)	100.00	0.60	99.70	98.40	WGS
287.3163	0.3636	Pseudomonas aeruginosa (286)	Pseudomonas (135621)	Pseudomonadaceae (7227	Pseudomonadales (1236)	Gammaproteobacteria (122	Proteobacteria (2)	Bacteria (1)	100.00	0.00	99.90	98.60	WGS
287.3164	0.3292	Pseudomonas aeruginosa (286)	Pseudomonas (135621)	Pseudomonadaceae (7227	Pseudomonadales (1236)	Gammaproteobacteria (122	Proteobacteria (2)	Bacteria (1)	99.40	0.00	99.90	99.00	WGS
287.3165	0.3738	Pseudomonas aeruginosa (286)	Pseudomonas (135621)	Pseudomonadaceae (7227	Pseudomonadales (1236)	Gammaproteobacteria (122	Proteobacteria (2)	Bacteria (1)	100.00	0.30	100.00	98.00	WGS
287.3166	0.325	Pseudomonas aeruginosa (286)	Pseudomonas (135621)	Pseudomonadaceae (7227	Pseudomonadales (1236)	Gammaproteobacteria (122	Proteobacteria (2)	Bacteria (1)	100.00	0.30	99.90	99.50	WGS
287.3167	0.325	Pseudomonas aeruginosa (286)	Pseudomonas (135621)	Pseudomonadaceae (7227	Pseudomonadales (1236)	Gammaproteobacteria (122	Proteobacteria (2)	Bacteria (1)	100.00	0.30	99.90	98.20	WGS
287.3168	0.368	Pseudomonas aeruginosa (286)	Pseudomonas (135621)	Pseudomonadaceae (7227	Pseudomonadales (1236)	Gammaproteobacteria (122	Proteobacteria (2)	Bacteria (1)	100.00	0.00	99.70	98.70	WGS
287.3169	0.3738	Pseudomonas aeruginosa (286)	Pseudomonas (135621)	Pseudomonadaceae (7227	Pseudomonadales (1236)	Gammaproteobacteria (122	Proteobacteria (2)	Bacteria (1)	100.00	0.30	100.00	97.30	WGS
287.3170	0.325	Pseudomonas aeruginosa (286)	Pseudomonas (135621)	Pseudomonadaceae (7227	Pseudomonadales (1236)	Gammaproteobacteria (122	Proteobacteria (2)	Bacteria (1)	100.00	0.30	99.90	99.40	WGS
287.3171	0.368	Pseudomonas aeruginosa (286)	Pseudomonas (135621)	Pseudomonadaceae (7227	Pseudomonadales (1236)	Gammaproteobacteria (122	Proteobacteria (2)	Bacteria (1)	100.00	0.60	99.70	98.40	WGS
287.3172	0.325	Pseudomonas aeruginosa (286)	Pseudomonas (135621)	Pseudomonadaceae (7227	Pseudomonadales (1236)	Gammaproteobacteria (122	Proteobacteria (2)	Bacteria (1)	100.00	0.30	99.90	99.30	WGS
287.3173	0.368	Pseudomonas aeruginosa (286)	Pseudomonas (135621)	Pseudomonadaceae (7227	Pseudomonadales (1236)	Gammaproteobacteria (122	Proteobacteria (2)	Bacteria (1)	100.00	0.00	99.70	98.20	WGS
287.3174	0.3694	Pseudomonas aeruginosa (286)	Pseudomonas (135621)	Pseudomonadaceae (7227	Pseudomonadales (1236)	Gammaproteobacteria (122	Proteobacteria (2)	Bacteria (1)	100.00	0.00	99.90	99.50	WGS
287.3175	0.3694	Pseudomonas aeruginosa (286)	Pseudomonas (135621)	Pseudomonadaceae (7227	Pseudomonadales (1236)	Gammaproteobacteria (122	Proteobacteria (2)	Bacteria (1)	100.00	0.00	99.90	99.30	WGS
287.3176	0.368	Pseudomonas aeruginosa (286)	Pseudomonas (135621)	Pseudomonadaceae (7227	Pseudomonadales (1236)	Gammaproteobacteria (122	Proteobacteria (2)	Bacteria (1)	100.00	0.00	99.70	97.20	WGS
287.3177	0.325	Pseudomonas aeruginosa (286)	Pseudomonas (135621)	Pseudomonadaceae (7227	Pseudomonadales (1236)	Gammaproteobacteria (122	Proteobacteria (2)	Bacteria (1)	100.00	0.60	99.90	98.50	WGS
287.3178	0.3738	Pseudomonas aeruginosa (286)	Pseudomonas (135621)	Pseudomonadaceae (7227	Pseudomonadales (1236)	Gammaproteobacteria (122	Proteobacteria (2)	Bacteria (1)	100.00	0.00	100.00	97.70	WGS
287.3179	0.325	Pseudomonas aeruginosa (286)	Pseudomonas (135621)	Pseudomonadaceae (7227	Pseudomonadales (1236)	Gammaproteobacteria (122	Proteobacteria (2)	Bacteria (1)	100.00	0.30	99.90	99.50	WGS
287.3180	0.325	Pseudomonas aeruginosa (286)	Pseudomonas (135621)	Pseudomonadaceae (7227	Pseudomonadales (1236)	Gammaproteobacteria (122	Proteobacteria (2)	Bacteria (1)	100.00	0.30	99.90	98.10	WGS
287.3181	0.325	Pseudomonas aeruginosa (286)	Pseudomonas (135621)	Pseudomonadaceae (7227	Pseudomonadales (1236)	Gammaproteobacteria (122	Proteobacteria (2)	Bacteria (1)	100.00	0.30	99.90	99.60	WGS
287.3182	0.3636	Pseudomonas aeruginosa (286)	Pseudomonas (135621)	Pseudomonadaceae (7227	Pseudomonadales (1236)	Gammaproteobacteria (122	Proteobacteria (2)	Bacteria (1)	100.00	0.00	99.90	98.70	WGS
287.3183	0.3738	Pseudomonas aeruginosa (286)	Pseudomonas (135621)	Pseudomonadaceae (7227	Pseudomonadales (1236)	Gammaproteobacteria (122	Proteobacteria (2)	Bacteria (1)	100.00	0.00	100.00	98.00	WGS
287.3184	0.325	Pseudomonas aeruginosa (286)	Pseudomonas (135621)	Pseudomonadaceae (7227	Pseudomonadales (1236)	Gammaproteobacteria (122	Proteobacteria (2)	Bacteria (1)	100.00	0.30	99.90	99.50	WGS
287.3185	0.3738	Pseudomonas aeruginosa (286)	Pseudomonas (135621)	Pseudomonadaceae (7227	Pseudomonadales (1236)	Gammaproteobacteria (122	Proteobacteria (2)	Bacteria (1)	100.00	0.00	100.00	97.50	WGS
287.3186	0.368	Pseudomonas aeruginosa (286)	Pseudomonas (135621)	Pseudomonadaceae (7227	Pseudomonadales (1236)	Gammaproteobacteria (122	Proteobacteria (2)	Bacteria (1)	100.00	0.00	99.70	98.00	WGS
287.3187	0.368	Pseudomonas aeruginosa (286)	Pseudomonas (135621)	Pseudomonadaceae (7227	Pseudomonadales (1236)	Gammaproteobacteria (122	Proteobacteria (2)	Bacteria (1)	100.00	0.00	99.70	98.30	WGS
287.3188	0.325	Pseudomonas aeruginosa (286)	Pseudomonas (135621)	Pseudomonadaceae (7227	Pseudomonadales (1236)	Gammaproteobacteria (122	Proteobacteria (2)	Bacteria (1)	100.00	0.30	99.90	99.20	WGS
287.3189	0.3694	Pseudomonas aeruginosa (286)	Pseudomonas (135621)	Pseudomonadaceae (7227	Pseudomonadales (1236)	Gammaproteobacteria (122	Proteobacteria (2)	Bacteria (1)	100.00	0.00	99.90	99.30	WGS
287.3190	0.368	Pseudomonas aeruginosa (286)	Pseudomonas (135621)	Pseudomonadaceae (7227	Pseudomonadales (1236)	Gammaproteobacteria (122	Proteobacteria (2)	Bacteria (1)	100.00	0.90	99.70	97.00	WGS
287.3191	0.368	Pseudomonas aeruginosa (286)	Pseudomonas (135621)	Pseudomonadaceae (7227	Pseudomonadales (1236)	Gammaproteobacteria (122	Proteobacteria (2)	Bacteria (1)	100.00	0.30	99.70	97.80	WGS
287.3192	0.368	Pseudomonas aeruginosa (286)	Pseudomonas (135621)	Pseudomonadaceae (7227	Pseudomonadales (1236)	Gammaproteobacteria (122	Proteobacteria (2)	Bacteria (1)	100.00	0.60	99.70	97.00	WGS
287.3193	0.3636	Pseudomonas aeruginosa (286)	Pseudomonas (135621)	Pseudomonadaceae (7227	Pseudomonadales (1236)	Gammaproteobacteria (122	Proteobacteria (2)	Bacteria (1)	100.00	0.00	99.90	99.00	WGS
287.3194	0.368	Pseudomonas aeruginosa (286)	Pseudomonas (135621)	Pseudomonadaceae (7227	Pseudomonadales (1236)	Gammaproteobacteria (122	Proteobacteria (2)	Bacteria (1)	100.00	0.60	99.70	98.90	WGS
287.3195	0.368	Pseudomonas aeruginosa (286)	Pseudomonas (135621)	Pseudomonadaceae (7227	Pseudomonadales (1236)	Gammaproteobacteria (122	Proteobacteria (2)	Bacteria (1)	100.00	0.90	99.70	97.30	WGS
287.3196	0.368	Pseudomonas aeruginosa (286)	Pseudomonas (135621)	Pseudomonadaceae (7227	Pseudomonadales (1236)	Gammaproteobacteria (122	Proteobacteria (2)	Bacteria (1)	98.80	0.90	99.60	98.10	WGS
287.3197	0.368	Pseudomonas aeruginosa (286)	Pseudomonas (135621)	Pseudomonadaceae (7227	Pseudomonadales (1236)	Gammaproteobacteria (122	Proteobacteria (2)	Bacteria (1)	100.00	0.30	99.70	98.30	WGS
287.3198	0.368	Pseudomonas aeruginosa (286)	Pseudomonas (135621)	Pseudomonadaceae (7227	Pseudomonadales (1236)	Gammaproteobacteria (122	Proteobacteria (2)	Bacteria (1)	98.80	0.60	99.60	98.20	WGS
287.3199	0.368	Pseudomonas aeruginosa (286)	Pseudomonas (135621)	Pseudomonadaceae (7227	Pseudomonadales (1236)	Gammaproteobacteria (122	Proteobacteria (2)	Bacteria (1)	99.40	0.60	99.60	98.30	WGS
287.3200	0.368	Pseudomonas aeruginosa (286)	Pseudomonas (135621)	Pseudomonadaceae (7227	Pseudomonadales (1236)	Gammaproteobacteria (122	Proteobacteria (2)	Bacteria (1)	99.40	0.90	99.50	98.10	WGS
287.3201	0.368	Pseudomonas aeruginosa (286)	Pseudomonas (135621)	Pseudomonadaceae (7227	Pseudomonadales (1236)	Gammaproteobacteria (122	Proteobacteria (2)	Bacteria (1)	100.00	0.30	99.70	98.70	WGS
287.3202	0.368	Pseudomonas aeruginosa (286)	Pseudomonas (135621)	Pseudomonadaceae (7227	Pseudomonadales (1236)	Gammaproteobacteria (122	Proteobacteria (2)	Bacteria (1)	100.00	0.60	99.70	98.90	WGS
287.3203	0.368	Pseudomonas aeruginosa (286)	Pseudomonas (135621)	Pseudomonadaceae (7227	Pseudomonadales (1236)	Gammaproteobacteria (122	Proteobacteria (2)	Bacteria (1)	100.00	0.30	99.70	98.90	WGS
287.3204	0.368	Pseudomonas aeruginosa (286)	Pseudomonas (135621)	Pseudomonadaceae (7227	Pseudomonadales (1236)	Gammaproteobacteria (122	Proteobacteria (2)	Bacteria (1)	100.00	0.60	99.70	98.20	WGS
287.3205	0.371	Pseudomonas aeruginosa (286)	Pseudomonas (135621)	Pseudomonadaceae (7227	Pseudomonadales (1236)	Gammaproteobacteria (122	Proteobacteria (2)	Bacteria (1)	100.00	0.00	99.60	98.20	WGS
287.3206	0.371	Pseudomonas aeruginosa (286)	Pseudomonas (135621)	Pseudomonadaceae (7227	Pseudomonadales (1236)	Gammaproteobacteria (122	Proteobacteria (2)	Bacteria (1)	100.00	0.00	99.60	98.60	WGS
287.3207	0.3918	Pseudomonas aeruginosa (286)	Pseudomonas (135621)	Pseudomonadaceae (7227	Pseudomonadales (1236)	Gammaproteobacteria (122	Proteobacteria (2)	Bacteria (1)	100.00	1.20	99.90	97.40	WGS
287.3208	0.356	Pseudomonas aeruginosa (286)	Pseudomonas (135621)	Pseudomonadaceae (7227	Pseudomonadales (1236)	Gammaproteobacteria (122	Proteobacteria (2)	Bacteria (1)	100.00	0.00	99.80	99.40	WGS
287.3209	0.3376	Pseudomonas aeruginosa (286)	Pseudomonas (135621)	Pseudomonadaceae (7227	Pseudomonadales (1236)	Gammaproteobacteria (122	Proteobacteria (2)	Bacteria (1)	100.00	0.60	99.80	97.70	WGS
287.3210	0.3918	Pseudomonas aeruginosa (286)	Pseudomonas (135621)	Pseudomonadaceae (7227	Pseudomonadales (1236)	Gammaproteobacteria (122	Proteobacteria (2)	Bacteria (1)	100.00	1.20	99.90	98.40	WGS
287.3211	0.3918	Pseudomonas aeruginosa (286)	Pseudomonas (135621)	Pseudomonadaceae (7227	Pseudomonadales (1236)	Gammaproteobacteria (122	Proteobacteria (2)	Bacteria (1)	100.00	1.20	99.80	98.20	WGS
287.3212	0.3918	Pseudomonas aeruginosa (286)	Pseudomonas (135621)	Pseudomonadaceae (7227	Pseudomonadales (1236)	Gammaproteobacteria (122	Proteobacteria (2)	Bacteria (1)	100.00	1.20	99.80	98.10	WGS
287.3213	0.3918	Pseudomonas aeruginosa (286)	Pseudomonas (135621)	Pseudomonadaceae (7227	Pseudomonadales (1236)	Gammaproteobacteria (122	Proteobacteria (2)	Bacteria (1)	100.00	1.20	99.80	98.20	WGS
287.3214	0.3918	Pseudomonas aeruginosa (286)	Pseudomonas (135621)	Pseudomonadaceae (7227	Pseudomonadales (1236)	Gammaproteobacteria (122	Proteobacteria (2)	Bacteria (1)	100.00	1.20	99.80	97.40	WGS
287.3215	0.3918	Pseudomonas aeruginosa (286)	Pseudomonas (135621)	Pseudomonadaceae (7227	Pseudomonadales (1236)	Gammaproteobacteria (122	Proteobacteria (2)	Bacteria (1)	100.00	1.20	99.80	97.90	WGS
287.3216	0.3918	Pseudomonas aeruginosa (286)	Pseudomonas (135621)	Pseudomonadaceae (7227	Pseudomonadales (1236)	Gammaproteobacteria (122	Proteobacteria (2)	Bacteria (1)	100.00	1.20	99.80	98.20	WGS
287.3217	0.3918	Pseudomonas aeruginosa (286)	Pseudomonas (135621)	Pseudomonadaceae (7227	Pseudomonadales (1236)	Gammaproteobacteria (122	Proteobacteria (2)	Bacteria (1)	100.00	1.20	99.90	97.90	WGS
287.3218	0.3918	Pseudomonas aeruginosa (286)	Pseudomonas (135621)	Pseudomonadaceae (7227	Pseudomonadales (1236)	Gammaproteobacteria (122	Proteobacteria (2)	Bacteria (1)	100.00	1.20	99.70	97.40	WGS
287.3219	0.3918	Pseudomonas aeruginosa (286)	Pseudomonas (135621)	Pseudomonadaceae (7227	Pseudomonadales (1236)	Gammaproteobacteria (122	Proteobacteria (2)	Bacteria (1)	100.00	1.20	99.80	98.20	WGS
287.3220	0.3918	Pseudomonas aeruginosa (286)	Pseudomonas (135621)	Pseudomonadaceae (7227	Pseudomonadales (1236)	Gammaproteobacteria (122	Proteobacteria (2)	Bacteria (1)	100.00	1.20	99.70	97.60	WGS
287.3221	0.3918	Pseudomonas aeruginosa (286)	Pseudomonas (135621)	Pseudomonadaceae (7227	Pseudomonadales (1236)	Gammaproteobacteria (122	Proteobacteria (2)	Bacteria (1)	100.00	1.20	99.90	98.10	WGS
287.3222	0.3918	Pseudomonas aeruginosa (286)	Pseudomonas (135621)	Pseudomonadaceae (7227	Pseudomonadales (1236)	Gammaproteobacteria (122	Proteobacteria (2)	Bacteria (1)	100.00	1.20	99.80	97.20	WGS
287.3223	0.3258	Pseudomonas aeruginosa (286)	Pseudomonas (135621)	Pseudomonadaceae (7227	Pseudomonadales (1236)	Gammaproteobacteria (122	Proteobacteria (2)	Bacteria (1)	100.00	0.30	99.90	98.60	WGS
287.3224	0.3258	Pseudomonas aeruginosa (286)	Pseudomonas (135621)	Pseudomonadaceae (7227	Pseudomonadales (1236)	Gammaproteobacteria (122	Proteobacteria (2)	Bacteria (1)	100.00	0.30	99.90	99.20	WGS
287.3225	0.3258	Pseudomonas aeruginosa (286)	Pseudomonas (135621)	Pseudomonadaceae (7227	Pseudomonadales (1236)	Gammaproteobacteria (122	Proteobacteria (2)	Bacteria (1)	100.00	0.30	99.90	98.60	WGS
287.3226	0.356	Pseudomonas aeruginosa (286)	Pseudomonas (135621)	Pseudomonadaceae (7227	Pseudomonadales (1236)	Gammaproteobacteria (122	Proteobacteria (2)	Bacteria (1)	97.30	0.00	99.30	98.30	WGS
287.3227	0.3258	Pseudomonas aeruginosa (286)	Pseudomonas (135621)	Pseudomonadaceae (7227	Pseudomonadales (1236)	Gammaproteobacteria (122	Proteobacteria (2)	Bacteria (1)	100.00	0.30	99.90	98.90	WGS
287.3228	0.3258	Pseudomonas aeruginosa (286)	Pseudomonas (135621)	Pseudomonadaceae (7227	Pseudomonadales (1236)	Gammaproteobacteria (122	Proteobacteria (2)	Bacteria (1)	100.00	0.30	99.90	99.10	WGS
287.3229	0.3258	Pseudomonas aeruginosa (286)	Pseudomonas (135621)	Pseudomonadaceae (7227	Pseudomonadales (1236)	Gammaproteobacteria (122	Proteobacteria (2)	Bacteria (1)	100.00	0.00	99.90	99.00	WGS
287.3230	0.3526	Pseudomonas aeruginosa (286)	Pseudomonas (135621)	Pseudomonadaceae (7227	Pseudomonadales (1236)	Gammaproteobacteria (122	Proteobacteria (2)	Bacteria (1)	98.50	0.00	99.10	96.80	WGS
287.3231	0.3688	Pseudomonas aeruginosa (286)	Pseudomonas (135621)	Pseudomonadaceae (7227	Pseudomonadales (1236)	Gammaproteobacteria (122	Proteobacteria (2)	Bacteria (1)	99.10	0.00	99.30	97.00	WGS
287.3232	0.3258	Pseudomonas aeruginosa (286)	Pseudomonas (135621)	Pseudomonadaceae (7227	Pseudomonadales (1236)	Gammaproteobacteria (122	Proteobacteria (2)	Bacteria (1)	100.00	0.30	99.90	98.40	WGS
287.3233	0.3254	Pseudomonas aeruginosa (286)	Pseudomonas (135621)	Pseudomonadaceae (7227	Pseudomonadales (1236)	Gammaproteobacteria (122	Proteobacteria (2)	Bacteria (1)	99.40	0.00	99.30	97.50	WGS
287.3234	0.3302	Pseudomonas aeruginosa (286)	Pseudomonas (135621)	Pseudomonadaceae (7227	Pseudomonadales (1236)	Gammaproteobacteria (122	Proteobacteria (2)	Bacteria (1)	98.80	0.00	99.60	98.00	WGS
287.3235	0.3258	Pseudomonas aeruginosa (286)	Pseudomonas (135621)	Pseudomonadaceae (7227	Pseudomonadales (1236)	Gammaproteobacteria (122	Proteobacteria (2)	Bacteria (1)	100.00	0.30	99.90	98.70	WGS
287.3236	0.3188	Pseudomonas aeruginosa (286)	Pseudomonas (135621)	Pseudomonadaceae (7227	Pseudomonadales (1236)	Gammaproteobacteria (122	Proteobacteria (2)	Bacteria (1)	100.00	0.00	99.90	98.70	WGS
287.3237	0.3188	Pseudomonas aeruginosa (286)	Pseudomonas (135621)	Pseudomonadaceae (7227	Pseudomonadales (1236)	Gammaproteobacteria (122	Proteobacteria (2)	Bacteria (1)	100.00	0.30	99.90	98.90	WGS
287.3238	0.3188	Pseudomonas aeruginosa (286)	Pseudomonas (135621)	Pseudomonadaceae (7227	Pseudomonadales (1236)	Gammaproteobacteria (122	Proteobacteria (2)	Bacteria (1)	100.00	0.30	99.90	98.50	WGS
287.3239	0.3188	Pseudomonas aeruginosa (286)	Pseudomonas (135621)	Pseudomonadaceae (7227	Pseudomonadales (1236)	Gammaproteobacteria (122	Proteobacteria (2)	Bacteria (1)	100.00	0.00	99.90	97.20	WGS
287.3240	0.3188	Pseudomonas aeruginosa (286)	Pseudomonas (135621)	Pseudomonadaceae (7227	Pseudomonadales (1236)	Gammaproteobacteria (122	Proteobacteria (2)	Bacteria (1)	100.00	0.30	99.90	97.90	WGS
287.3241	0.3188	Pseudomonas aeruginosa (286)	Pseudomonas (135621)	Pseudomonadaceae (7227	Pseudomonadales (1236)	Gammaproteobacteria (122	Proteobacteria (2)	Bacteria (1)	100.00	0.30	99.90	98.20	WGS
287.3242	0.3188	Pseudomonas aeruginosa (286)	Pseudomonas (135621)	Pseudomonadaceae (7227	Pseudomonadales (1236)	Gammaproteobacteria (122	Proteobacteria (2)	Bacteria (1)	100.00	0.00	99.90	98.20	WGS
287.3243	0.3188	Pseudomonas aeruginosa (286)	Pseudomonas (135621)	Pseudomonadaceae (7227	Pseudomonadales (1236)	Gammaproteobacteria (122	Proteobacteria (2)	Bacteria (1)	100.00	0.30	99.90	98.30	WGS
287.3244	0.3188	Pseudomonas aeruginosa (286)	Pseudomonas (135621)	Pseudomonadaceae (7227	Pseudomonadales (1236)	Gammaproteobacteria (122	Proteobacteria (2)	Bacteria (1)	100.00	0.30	99.90	98.20	WGS
287.3245	0.3188	Pseudomonas aeruginosa (286)	Pseudomonas (135621)	Pseudomonadaceae (7227	Pseudomonadales (1236)	Gammaproteobacteria (122	Proteobacteria (2)	Bacteria (1)	100.00	0.00	99.90	97.90	WGS
287.3246	0.3188	Pseudomonas aeruginosa (286)	Pseudomonas (135621)	Pseudomonadaceae (7227	Pseudomonadales (1236)	Gammaproteobacteria (122	Proteobacteria (2)	Bacteria (1)	100.00	0.30	99.90	97.80	WGS
287.3247	0.368	Pseudomonas aeruginosa (286)	Pseudomonas (135621)	Pseudomonadaceae (7227	Pseudomonadales (1236)	Gammaproteobacteria (122	Proteobacteria (2)	Bacteria (1)	99.10	1.20	99.90	97.50	WGS
287.3248	0.368	Pseudomonas aeruginosa (286)	Pseudomonas (135621)	Pseudomonadaceae (7227	Pseudomonadales (1236)	Gammaproteobacteria (122	Proteobacteria (2)	Bacteria (1)	99.10	0.90	99.90	97.50	WGS
287.3249	0.2796	Pseudomonas aeruginosa (286)	Pseudomonas (135621)	Pseudomonadaceae (7227	Pseudomonadales (1236)	Gammaproteobacteria (122	Proteobacteria (2)	Bacteria (1)	87.30	13.10	94.20	83.30	WGS
287.3250	0.273	Pseudomonas aeruginosa (286)	Pseudomonas (135621)	Pseudomonadaceae (7227	Pseudomonadales (1236)	Gammaproteobacteria (122	Proteobacteria (2)	Bacteria (1)	90.70	13.30	96.20	85.80	WGS
287.3251	0.2692	Pseudomonas aeruginosa (286)	Pseudomonas (135621)	Pseudomonadaceae (7227	Pseudomonadales (1236)	Gammaproteobacteria (122	Proteobacteria (2)	Bacteria (1)	91.00	13.00	96.40	85.80	WGS
287.3252	0.3292	Pseudomonas aeruginosa (286)	Pseudomonas (135621)	Pseudomonadaceae (7227	Pseudomonadales (1236)	Gammaproteobacteria (122	Proteobacteria (2)	Bacteria (1)	97.60	8.10	99.20	91.60	WGS
287.3253	0.2768	Pseudomonas aeruginosa (286)	Pseudomonas (135621)	Pseudomonadaceae (7227	Pseudomonadales (1236)	Gammaproteobacteria (122	Proteobacteria (2)	Bacteria (1)	91.90	13.10	96.60	85.30	WGS
287.3254	0.2692	Pseudomonas aeruginosa (286)	Pseudomonas (135621)	Pseudomonadaceae (7227	Pseudomonadales (1236)	Gammaproteobacteria (122	Proteobacteria (2)	Bacteria (1)	89.50	11.70	94.50	84.20	WGS
287.3255	0.2634	Pseudomonas aeruginosa (286)	Pseudomonas (135621)	Pseudomonadaceae (7227	Pseudomonadales (1236)	Gammaproteobacteria (122	Proteobacteria (2)	Bacteria (1)	87.30	11.60	94.20	83.10	WGS
287.3256	0.2956	Pseudomonas aeruginosa (286)	Pseudomonas (135621)	Pseudomonadaceae (7227	Pseudomonadales (1236)	Gammaproteobacteria (122	Proteobacteria (2)	Bacteria (1)	90.40	13.80	96.70	86.30	WGS
287.3257	0.3274	Pseudomonas aeruginosa (286)	Pseudomonas (135621)	Pseudomonadaceae (7227	Pseudomonadales (1236)	Gammaproteobacteria (122	Proteobacteria (2)	Bacteria (1)	97.00	9.50	98.70	90.00	WGS
287.3258	0.2702	Pseudomonas aeruginosa (286)	Pseudomonas (135621)	Pseudomonadaceae (7227	Pseudomonadales (1236)	Gammaproteobacteria (122	Proteobacteria (2)	Bacteria (1)	87.60	13.00	94.20	82.10	WGS
287.3259	0.2768	Pseudomonas aeruginosa (286)	Pseudomonas (135621)	Pseudomonadaceae (7227	Pseudomonadales (1236)	Gammaproteobacteria (122	Proteobacteria (2)	Bacteria (1)	90.10	12.30	95.50	85.10	WGS
287.3260	0.2656	Pseudomonas aeruginosa (286)	Pseudomonas (135621)	Pseudomonadaceae (7227	Pseudomonadales (1236)	Gammaproteobacteria (122	Proteobacteria (2)	Bacteria (1)	87.10	13.40	93.80	81.90	WGS
287.3261	0.368	Pseudomonas aeruginosa (286)	Pseudomonas (135621)	Pseudomonadaceae (7227	Pseudomonadales (1236)	Gammaproteobacteria (122	Proteobacteria (2)	Bacteria (1)	99.10	0.90	99.90	97.60	WGS
287.3262	0.332	Pseudomonas aeruginosa (286)	Pseudomonas (135621)	Pseudomonadaceae (7227	Pseudomonadales (1236)	Gammaproteobacteria (122	Proteobacteria (2)	Bacteria (1)	100.00	0.60	99.80	98.80	WGS
287.3263	0.332	Pseudomonas aeruginosa (286)	Pseudomonas (135621)	Pseudomonadaceae (7227	Pseudomonadales (1236)	Gammaproteobacteria (122	Proteobacteria (2)	Bacteria (1)	100.00	0.00	99.80	97.90	WGS
287.3264	0.332	Pseudomonas aeruginosa (286)	Pseudomonas (135621)	Pseudomonadaceae (7227	Pseudomonadales (1236)	Gammaproteobacteria (122	Proteobacteria (2)	Bacteria (1)	100.00	0.00	99.80	97.70	WGS
287.3265	0.304	Pseudomonas aeruginosa (286)	Pseudomonas (135621)	Pseudomonadaceae (7227	Pseudomonadales (1236)	Gammaproteobacteria (122	Proteobacteria (2)	Bacteria (1)	100.00	1.80	99.80	96.30	WGS
287.3266	0.332	Pseudomonas aeruginosa (286)	Pseudomonas (135621)	Pseudomonadaceae (7227	Pseudomonadales (1236)	Gammaproteobacteria (122	Proteobacteria (2)	Bacteria (1)	100.00	0.00	99.80	97.30	WGS
287.3267	0.332	Pseudomonas aeruginosa (286)	Pseudomonas (135621)	Pseudomonadaceae (7227	Pseudomonadales (1236)	Gammaproteobacteria (122	Proteobacteria (2)	Bacteria (1)	100.00	0.00	99.80	97.70	WGS
287.3268	0.332	Pseudomonas aeruginosa (286)	Pseudomonas (135621)	Pseudomonadaceae (7227	Pseudomonadales (1236)	Gammaproteobacteria (122	Proteobacteria (2)	Bacteria (1)	100.00	0.00	99.80	97.30	WGS
287.3269	0.332	Pseudomonas aeruginosa (286)	Pseudomonas (135621)	Pseudomonadaceae (7227	Pseudomonadales (1236)	Gammaproteobacteria (122	Proteobacteria (2)	Bacteria (1)	100.00	0.00	99.80	98.60	WGS
287.3270	0.332	Pseudomonas aeruginosa (286)	Pseudomonas (135621)	Pseudomonadaceae (7227	Pseudomonadales (1236)	Gammaproteobacteria (122	Proteobacteria (2)	Bacteria (1)	100.00	0.00	99.80	98.70	WGS
287.3271	0.332	Pseudomonas aeruginosa (286)	Pseudomonas (135621)	Pseudomonadaceae (7227	Pseudomonadales (1236)	Gammaproteobacteria (122	Proteobacteria (2)	Bacteria (1)	100.00	0.00	99.80	98.70	WGS
287.3272	0.332	Pseudomonas aeruginosa (286)	Pseudomonas (135621)	Pseudomonadaceae (7227	Pseudomonadales (1236)	Gammaproteobacteria (122	Proteobacteria (2)	Bacteria (1)	100.00	0.30	99.80	98.40	WGS
287.3273	0.332	Pseudomonas aeruginosa (286)	Pseudomonas (135621)	Pseudomonadaceae (7227	Pseudomonadales (1236)	Gammaproteobacteria (122	Proteobacteria (2)	Bacteria (1)	100.00	0.30	99.70	98.20	WGS
287.3274	0.332	Pseudomonas aeruginosa (286)	Pseudomonas (135621)	Pseudomonadaceae (7227	Pseudomonadales (1236)	Gammaproteobacteria (122	Proteobacteria (2)	Bacteria (1)	100.00	0.30	99.80	98.40	WGS
287.3275	0.332	Pseudomonas aeruginosa (286)	Pseudomonas (135621)	Pseudomonadaceae (7227	Pseudomonadales (1236)	Gammaproteobacteria (122	Proteobacteria (2)	Bacteria (1)	100.00	0.00	99.80	98.70	WGS
287.3276	0.332	Pseudomonas aeruginosa (286)	Pseudomonas (135621)	Pseudomonadaceae (7227	Pseudomonadales (1236)	Gammaproteobacteria (122	Proteobacteria (2)	Bacteria (1)	100.00	0.30	99.80	98.70	WGS
287.3277	0.332	Pseudomonas aeruginosa (286)	Pseudomonas (135621)	Pseudomonadaceae (7227	Pseudomonadales (1236)	Gammaproteobacteria (122	Proteobacteria (2)	Bacteria (1)	100.00	0.00	99.80	98.50	WGS
287.3278	0.368	Pseudomonas aeruginosa (286)	Pseudomonas (135621)	Pseudomonadaceae (7227	Pseudomonadales (1236)	Gammaproteobacteria (122	Proteobacteria (2)	Bacteria (1)	96.70	0.30	99.90	98.30	WGS
287.3279	0.332	Pseudomonas aeruginosa (286)	Pseudomonas (135621)	Pseudomonadaceae (7227	Pseudomonadales (1236)	Gammaproteobacteria (122	Proteobacteria (2)	Bacteria (1)	100.00	0.30	99.80	97.90	WGS
287.3280	0.3618	Pseudomonas aeruginosa (286)	Pseudomonas (135621)	Pseudomonadaceae (7227	Pseudomonadales (1236)	Gammaproteobacteria (122	Proteobacteria (2)	Bacteria (1)	100.00	0.30	99.90	98.00	WGS
287.3281	0.332	Pseudomonas aeruginosa (286)	Pseudomonas (135621)	Pseudomonadaceae (7227	Pseudomonadales (1236)	Gammaproteobacteria (122	Proteobacteria (2)	Bacteria (1)	100.00	0.00	99.80	98.90	WGS
287.3282	0.332	Pseudomonas aeruginosa (286)	Pseudomonas (135621)	Pseudomonadaceae (7227	Pseudomonadales (1236)	Gammaproteobacteria (122	Proteobacteria (2)	Bacteria (1)	100.00	0.30	99.80	98.70	WGS
287.3283	0.332	Pseudomonas aeruginosa (286)	Pseudomonas (135621)	Pseudomonadaceae (7227	Pseudomonadales (1236)	Gammaproteobacteria (122	Proteobacteria (2)	Bacteria (1)	100.00	0.30	99.80	98.80	WGS
287.3284	0.332	Pseudomonas aeruginosa (286)	Pseudomonas (135621)	Pseudomonadaceae (7227	Pseudomonadales (1236)	Gammaproteobacteria (122	Proteobacteria (2)	Bacteria (1)	100.00	0.30	99.80	98.10	WGS
287.3285	0.332	Pseudomonas aeruginosa (286)	Pseudomonas (135621)	Pseudomonadaceae (7227	Pseudomonadales (1236)	Gammaproteobacteria (122	Proteobacteria (2)	Bacteria (1)	100.00	0.30	99.80	98.60	WGS
287.3286	0.368	Pseudomonas aeruginosa (286)	Pseudomonas (135621)	Pseudomonadaceae (7227	Pseudomonadales (1236)	Gammaproteobacteria (122	Proteobacteria (2)	Bacteria (1)	100.00	0.00	99.90	99.20	WGS
287.3287	0.368	Pseudomonas aeruginosa (286)	Pseudomonas (135621)	Pseudomonadaceae (7227	Pseudomonadales (1236)	Gammaproteobacteria (122	Proteobacteria (2)	Bacteria (1)	100.00	0.60	99.90	99.20	WGS
287.3288	0.368	Pseudomonas aeruginosa (286)	Pseudomonas (135621)	Pseudomonadaceae (7227	Pseudomonadales (1236)	Gammaproteobacteria (122	Proteobacteria (2)	Bacteria (1)	100.00	0.30	99.90	99.00	WGS
287.3289	0.368	Pseudomonas aeruginosa (286)	Pseudomonas (135621)	Pseudomonadaceae (7227	Pseudomonadales (1236)	Gammaproteobacteria (122	Proteobacteria (2)	Bacteria (1)	100.00	0.00	99.90	99.30	WGS
287.3290	0.3652	Pseudomonas aeruginosa (286)	Pseudomonas (135621)	Pseudomonadaceae (7227	Pseudomonadales (1236)	Gammaproteobacteria (122	Proteobacteria (2)	Bacteria (1)	100.00	0.00	99.80	99.20	WGS
287.3291	0.331	Pseudomonas aeruginosa (286)	Pseudomonas (135621)	Pseudomonadaceae (7227	Pseudomonadales (1236)	Gammaproteobacteria (122	Proteobacteria (2)	Bacteria (1)	100.00	0.30	99.90	98.40	WGS
287.3292	0.3604	Pseudomonas aeruginosa (286)	Pseudomonas (135621)	Pseudomonadaceae (7227	Pseudomonadales (1236)	Gammaproteobacteria (122	Proteobacteria (2)	Bacteria (1)	100.00	0.60	99.90	98.70	WGS
287.3293	0.3604	Pseudomonas aeruginosa (286)	Pseudomonas (135621)	Pseudomonadaceae (7227	Pseudomonadales (1236)	Gammaproteobacteria (122	Proteobacteria (2)	Bacteria (1)	100.00	0.30	99.90	98.80	WGS
287.3294	0.3604	Pseudomonas aeruginosa (286)	Pseudomonas (135621)	Pseudomonadaceae (7227	Pseudomonadales (1236)	Gammaproteobacteria (122	Proteobacteria (2)	Bacteria (1)	100.00	0.60	99.90	98.30	WGS
287.3295	0.3604	Pseudomonas aeruginosa (286)	Pseudomonas (135621)	Pseudomonadaceae (7227	Pseudomonadales (1236)	Gammaproteobacteria (122	Proteobacteria (2)	Bacteria (1)	100.00	0.30	99.70	98.10	WGS
287.3296	0.3636	Pseudomonas aeruginosa (286)	Pseudomonas (135621)	Pseudomonadaceae (7227	Pseudomonadales (1236)	Gammaproteobacteria (122	Proteobacteria (2)	Bacteria (1)	100.00	0.00	99.90	99.30	WGS
287.3297	0.368	Pseudomonas aeruginosa (286)	Pseudomonas (135621)	Pseudomonadaceae (7227	Pseudomonadales (1236)	Gammaproteobacteria (122	Proteobacteria (2)	Bacteria (1)	100.00	0.30	99.90	99.00	WGS
287.3298	0.331	Pseudomonas aeruginosa (286)	Pseudomonas (135621)	Pseudomonadaceae (7227	Pseudomonadales (1236)	Gammaproteobacteria (122	Proteobacteria (2)	Bacteria (1)	100.00	0.00	99.60	98.50	WGS
287.3299	0.3174	Pseudomonas aeruginosa (286)	Pseudomonas (135621)	Pseudomonadaceae (7227	Pseudomonadales (1236)	Gammaproteobacteria (122	Proteobacteria (2)	Bacteria (1)	100.00	0.30	99.90	98.80	WGS
287.3300	0.331	Pseudomonas aeruginosa (286)	Pseudomonas (135621)	Pseudomonadaceae (7227	Pseudomonadales (1236)	Gammaproteobacteria (122	Proteobacteria (2)	Bacteria (1)	100.00	0.00	99.60	98.60	WGS
287.3301	0.331	Pseudomonas aeruginosa (286)	Pseudomonas (135621)	Pseudomonadaceae (7227	Pseudomonadales (1236)	Gammaproteobacteria (122	Proteobacteria (2)	Bacteria (1)	100.00	0.30	99.60	97.90	WGS
287.3302	0.3258	Pseudomonas aeruginosa (286)	Pseudomonas (135621)	Pseudomonadaceae (7227	Pseudomonadales (1236)	Gammaproteobacteria (122	Proteobacteria (2)	Bacteria (1)	100.00	0.00	99.90	98.60	WGS
287.3303	0.331	Pseudomonas aeruginosa (286)	Pseudomonas (135621)	Pseudomonadaceae (7227	Pseudomonadales (1236)	Gammaproteobacteria (122	Proteobacteria (2)	Bacteria (1)	100.00	0.00	99.60	97.00	WGS
287.3304	0.331	Pseudomonas aeruginosa (286)	Pseudomonas (135621)	Pseudomonadaceae (7227	Pseudomonadales (1236)	Gammaproteobacteria (122	Proteobacteria (2)	Bacteria (1)	100.00	0.00	99.60	98.70	WGS
287.3305	0.331	Pseudomonas aeruginosa (286)	Pseudomonas (135621)	Pseudomonadaceae (7227	Pseudomonadales (1236)	Gammaproteobacteria (122	Proteobacteria (2)	Bacteria (1)	100.00	0.00	99.60	98.30	WGS
287.3306	0.3258	Pseudomonas aeruginosa (286)	Pseudomonas (135621)	Pseudomonadaceae (7227	Pseudomonadales (1236)	Gammaproteobacteria (122	Proteobacteria (2)	Bacteria (1)	100.00	0.00	99.90	99.60	WGS
287.3307	0.3258	Pseudomonas aeruginosa (286)	Pseudomonas (135621)	Pseudomonadaceae (7227	Pseudomonadales (1236)	Gammaproteobacteria (122	Proteobacteria (2)	Bacteria (1)	100.00	0.00	99.90	98.40	WGS
287.3308	0.3258	Pseudomonas aeruginosa (286)	Pseudomonas (135621)	Pseudomonadaceae (7227	Pseudomonadales (1236)	Gammaproteobacteria (122	Proteobacteria (2)	Bacteria (1)	100.00	0.00	99.90	99.00	WGS
287.3309	0.368	Pseudomonas aeruginosa (286)	Pseudomonas (135621)	Pseudomonadaceae (7227	Pseudomonadales (1236)	Gammaproteobacteria (122	Proteobacteria (2)	Bacteria (1)	100.00	0.00	99.90	98.70	WGS
287.3310	0.368	Pseudomonas aeruginosa (286)	Pseudomonas (135621)	Pseudomonadaceae (7227	Pseudomonadales (1236)	Gammaproteobacteria (122	Proteobacteria (2)	Bacteria (1)	100.00	0.00	99.90	98.70	WGS
287.3311	0.3636	Pseudomonas aeruginosa (286)	Pseudomonas (135621)	Pseudomonadaceae (7227	Pseudomonadales (1236)	Gammaproteobacteria (122	Proteobacteria (2)	Bacteria (1)	100.00	0.90	99.90	97.90	WGS
287.3312	0.368	Pseudomonas aeruginosa (286)	Pseudomonas (135621)	Pseudomonadaceae (7227	Pseudomonadales (1236)	Gammaproteobacteria (122	Proteobacteria (2)	Bacteria (1)	100.00	0.00	99.90	98.00	WGS
287.3313	0.368	Pseudomonas aeruginosa (286)	Pseudomonas (135621)	Pseudomonadaceae (7227	Pseudomonadales (1236)	Gammaproteobacteria (122	Proteobacteria (2)	Bacteria (1)	100.00	0.00	99.90	99.00	WGS
287.3314	0.3316	Pseudomonas aeruginosa (286)	Pseudomonas (135621)	Pseudomonadaceae (7227	Pseudomonadales (1236)	Gammaproteobacteria (122	Proteobacteria (2)	Bacteria (1)	100.00	0.00	99.90	98.60	WGS
287.3315	0.3316	Pseudomonas aeruginosa (286)	Pseudomonas (135621)	Pseudomonadaceae (7227	Pseudomonadales (1236)	Gammaproteobacteria (122	Proteobacteria (2)	Bacteria (1)	100.00	0.30	99.90	97.90	WGS
287.3316	0.3738	Pseudomonas aeruginosa (286)	Pseudomonas (135621)	Pseudomonadaceae (7227	Pseudomonadales (1236)	Gammaproteobacteria (122	Proteobacteria (2)	Bacteria (1)	100.00	1.20	99.80	97.90	WGS
287.3317	0.3316	Pseudomonas aeruginosa (286)	Pseudomonas (135621)	Pseudomonadaceae (7227	Pseudomonadales (1236)	Gammaproteobacteria (122	Proteobacteria (2)	Bacteria (1)	100.00	0.00	99.90	98.40	WGS
287.3318	0.3316	Pseudomonas aeruginosa (286)	Pseudomonas (135621)	Pseudomonadaceae (7227	Pseudomonadales (1236)	Gammaproteobacteria (122	Proteobacteria (2)	Bacteria (1)	100.00	0.00	99.90	98.50	WGS
287.3319	0.368	Pseudomonas aeruginosa (286)	Pseudomonas (135621)	Pseudomonadaceae (7227	Pseudomonadales (1236)	Gammaproteobacteria (122	Proteobacteria (2)	Bacteria (1)	100.00	0.30	99.90	99.20	WGS
287.3320	0.368	Pseudomonas aeruginosa (286)	Pseudomonas (135621)	Pseudomonadaceae (7227	Pseudomonadales (1236)	Gammaproteobacteria (122	Proteobacteria (2)	Bacteria (1)	100.00	0.00	99.90	99.50	WGS
287.3321	0.368	Pseudomonas aeruginosa (286)	Pseudomonas (135621)	Pseudomonadaceae (7227	Pseudomonadales (1236)	Gammaproteobacteria (122	Proteobacteria (2)	Bacteria (1)	100.00	0.00	99.80	99.40	WGS
287.3322	0.368	Pseudomonas aeruginosa (286)	Pseudomonas (135621)	Pseudomonadaceae (7227	Pseudomonadales (1236)	Gammaproteobacteria (122	Proteobacteria (2)	Bacteria (1)	100.00	0.00	99.90	99.30	WGS
287.3323	0.368	Pseudomonas aeruginosa (286)	Pseudomonas (135621)	Pseudomonadaceae (7227	Pseudomonadales (1236)	Gammaproteobacteria (122	Proteobacteria (2)	Bacteria (1)	100.00	0.00	99.90	99.60	WGS
287.3324	0.368	Pseudomonas aeruginosa (286)	Pseudomonas (135621)	Pseudomonadaceae (7227	Pseudomonadales (1236)	Gammaproteobacteria (122	Proteobacteria (2)	Bacteria (1)	100.00	0.00	99.90	99.10	WGS
287.3325	0.368	Pseudomonas aeruginosa (286)	Pseudomonas (135621)	Pseudomonadaceae (7227	Pseudomonadales (1236)	Gammaproteobacteria (122	Proteobacteria (2)	Bacteria (1)	100.00	0.00	99.90	99.40	WGS
287.3326	0.368	Pseudomonas aeruginosa (286)	Pseudomonas (135621)	Pseudomonadaceae (7227	Pseudomonadales (1236)	Gammaproteobacteria (122	Proteobacteria (2)	Bacteria (1)	100.00	0.00	99.90	99.50	WGS
287.3327	0.3316	Pseudomonas aeruginosa (286)	Pseudomonas (135621)	Pseudomonadaceae (7227	Pseudomonadales (1236)	Gammaproteobacteria (122	Proteobacteria (2)	Bacteria (1)	100.00	0.00	99.90	98.60	WGS
287.3328	0.368	Pseudomonas aeruginosa (286)	Pseudomonas (135621)	Pseudomonadaceae (7227	Pseudomonadales (1236)	Gammaproteobacteria (122	Proteobacteria (2)	Bacteria (1)	100.00	0.00	99.90	99.60	WGS
287.3329	0.3376	Pseudomonas aeruginosa (286)	Pseudomonas (135621)	Pseudomonadaceae (7227	Pseudomonadales (1236)	Gammaproteobacteria (122	Proteobacteria (2)	Bacteria (1)	100.00	0.00	99.60	98.90	WGS
287.3330	0.3376	Pseudomonas aeruginosa (286)	Pseudomonas (135621)	Pseudomonadaceae (7227	Pseudomonadales (1236)	Gammaproteobacteria (122	Proteobacteria (2)	Bacteria (1)	100.00	0.00	99.60	98.90	WGS
287.3331	0.3376	Pseudomonas aeruginosa (286)	Pseudomonas (135621)	Pseudomonadaceae (7227	Pseudomonadales (1236)	Gammaproteobacteria (122	Proteobacteria (2)	Bacteria (1)	100.00	0.00	99.60	98.80	WGS
287.3332	0.3376	Pseudomonas aeruginosa (286)	Pseudomonas (135621)	Pseudomonadaceae (7227	Pseudomonadales (1236)	Gammaproteobacteria (122	Proteobacteria (2)	Bacteria (1)	100.00	0.00	99.80	98.70	WGS
287.3333	0.3376	Pseudomonas aeruginosa (286)	Pseudomonas (135621)	Pseudomonadaceae (7227	Pseudomonadales (1236)	Gammaproteobacteria (122	Proteobacteria (2)	Bacteria (1)	100.00	0.00	99.60	99.00	WGS
287.3334	0.3376	Pseudomonas aeruginosa (286)	Pseudomonas (135621)	Pseudomonadaceae (7227	Pseudomonadales (1236)	Gammaproteobacteria (122	Proteobacteria (2)	Bacteria (1)	100.00	0.00	99.60	98.80	WGS
287.3335	0.3376	Pseudomonas aeruginosa (286)	Pseudomonas (135621)	Pseudomonadaceae (7227	Pseudomonadales (1236)	Gammaproteobacteria (122	Proteobacteria (2)	Bacteria (1)	100.00	0.00	99.80	99.00	WGS
287.3336	0.3316	Pseudomonas aeruginosa (286)	Pseudomonas (135621)	Pseudomonadaceae (7227	Pseudomonadales (1236)	Gammaproteobacteria (122	Proteobacteria (2)	Bacteria (1)	100.00	0.30	99.90	97.00	WGS
287.3337	0.324	Pseudomonas aeruginosa (286)	Pseudomonas (135621)	Pseudomonadaceae (7227	Pseudomonadales (1236)	Gammaproteobacteria (122	Proteobacteria (2)	Bacteria (1)	100.00	0.00	99.80	99.10	WGS
287.3338	0.324	Pseudomonas aeruginosa (286)	Pseudomonas (135621)	Pseudomonadaceae (7227	Pseudomonadales (1236)	Gammaproteobacteria (122	Proteobacteria (2)	Bacteria (1)	100.00	0.00	99.90	99.30	WGS

287.3339	0.324	Pseudomonas aeruginosa (286)	Pseudomonas (135621)	Pseudomonadaceae (7227	Pseudomonadales (1236)	Gammaproteobacteria (122	Proteobacteria (2)	Bacteria (1)	100.00	0.00	99.80	99.10	WGS
287.3340	0.324	Pseudomonas aeruginosa (286)	Pseudomonas (135621)	Pseudomonadaceae (7227	Pseudomonadales (1236)	Gammaproteobacteria (122	Proteobacteria (2)	Bacteria (1)	100.00	0.00	99.90	98.90	WGS
287.3341	0.324	Pseudomonas aeruginosa (286)	Pseudomonas (135621)	Pseudomonadaceae (7227	Pseudomonadales (1236)	Gammaproteobacteria (122	Proteobacteria (2)	Bacteria (1)	100.00	0.00	99.90	99.00	WGS
287.3342	0.324	Pseudomonas aeruginosa (286)	Pseudomonas (135621)	Pseudomonadaceae (7227	Pseudomonadales (1236)	Gammaproteobacteria (122	Proteobacteria (2)	Bacteria (1)	100.00	0.00	99.90	98.90	WGS
287.3343	0.324	Pseudomonas aeruginosa (286)	Pseudomonas (135621)	Pseudomonadaceae (7227	Pseudomonadales (1236)	Gammaproteobacteria (122	Proteobacteria (2)	Bacteria (1)	100.00	0.00	99.90	99.20	WGS
287.3344	0.324	Pseudomonas aeruginosa (286)	Pseudomonas (135621)	Pseudomonadaceae (7227	Pseudomonadales (1236)	Gammaproteobacteria (122	Proteobacteria (2)	Bacteria (1)	100.00	0.00	99.90	99.20	WGS
287.3345	0.324	Pseudomonas aeruginosa (286)	Pseudomonas (135621)	Pseudomonadaceae (7227	Pseudomonadales (1236)	Gammaproteobacteria (122	Proteobacteria (2)	Bacteria (1)	100.00	0.00	99.90	99.20	WGS
287.3346	0.324	Pseudomonas aeruginosa (286)	Pseudomonas (135621)	Pseudomonadaceae (7227	Pseudomonadales (1236)	Gammaproteobacteria (122	Proteobacteria (2)	Bacteria (1)	100.00	0.00	99.80	99.20	WGS
287.3347	0.324	Pseudomonas aeruginosa (286)	Pseudomonas (135621)	Pseudomonadaceae (7227	Pseudomonadales (1236)	Gammaproteobacteria (122	Proteobacteria (2)	Bacteria (1)	100.00	0.00	99.90	99.00	WGS
287.3348	0.324	Pseudomonas aeruginosa (286)	Pseudomonas (135621)	Pseudomonadaceae (7227	Pseudomonadales (1236)	Gammaproteobacteria (122	Proteobacteria (2)	Bacteria (1)	100.00	0.00	99.90	99.00	WGS
287.3349	0.324	Pseudomonas aeruginosa (286)	Pseudomonas (135621)	Pseudomonadaceae (7227	Pseudomonadales (1236)	Gammaproteobacteria (122	Proteobacteria (2)	Bacteria (1)	100.00	0.30	99.90	98.80	WGS
287.3350	0.324	Pseudomonas aeruginosa (286)	Pseudomonas (135621)	Pseudomonadaceae (7227	Pseudomonadales (1236)	Gammaproteobacteria (122	Proteobacteria (2)	Bacteria (1)	100.00	0.00	99.90	98.40	WGS
287.3351	0.324	Pseudomonas aeruginosa (286)	Pseudomonas (135621)	Pseudomonadaceae (7227	Pseudomonadales (1236)	Gammaproteobacteria (122	Proteobacteria (2)	Bacteria (1)	100.00	0.30	99.90	98.40	WGS
287.3352	0.324	Pseudomonas aeruginosa (286)	Pseudomonas (135621)	Pseudomonadaceae (7227	Pseudomonadales (1236)	Gammaproteobacteria (122	Proteobacteria (2)	Bacteria (1)	100.00	0.00	99.90	98.10	WGS
287.3353	0.324	Pseudomonas aeruginosa (286)	Pseudomonas (135621)	Pseudomonadaceae (7227	Pseudomonadales (1236)	Gammaproteobacteria (122	Proteobacteria (2)	Bacteria (1)	100.00	0.00	99.90	98.70	WGS
287.3354	0.369	Pseudomonas aeruginosa (286)	Pseudomonas (135621)	Pseudomonadaceae (7227	Pseudomonadales (1236)	Gammaproteobacteria (122	Proteobacteria (2)	Bacteria (1)	100.00	0.30	99.90	98.80	WGS
287.3355	0.3738	Pseudomonas aeruginosa (286)	Pseudomonas (135621)	Pseudomonadaceae (7227	Pseudomonadales (1236)	Gammaproteobacteria (122	Proteobacteria (2)	Bacteria (1)	100.00	0.30	99.80	98.40	WGS
287.3356	0.3738	Pseudomonas aeruginosa (286)	Pseudomonas (135621)	Pseudomonadaceae (7227	Pseudomonadales (1236)	Gammaproteobacteria (122	Proteobacteria (2)	Bacteria (1)	100.00	0.00	99.80	98.70	WGS
287.3357	0.3738	Pseudomonas aeruginosa (286)	Pseudomonas (135621)	Pseudomonadaceae (7227	Pseudomonadales (1236)	Gammaproteobacteria (122	Proteobacteria (2)	Bacteria (1)	100.00	0.00	99.80	98.30	WGS
287.3358	0.3738	Pseudomonas aeruginosa (286)	Pseudomonas (135621)	Pseudomonadaceae (7227	Pseudomonadales (1236)	Gammaproteobacteria (122	Proteobacteria (2)	Bacteria (1)	100.00	0.00	99.80	98.00	WGS
287.3359	0.3738	Pseudomonas aeruginosa (286)	Pseudomonas (135621)	Pseudomonadaceae (7227	Pseudomonadales (1236)	Gammaproteobacteria (122	Proteobacteria (2)	Bacteria (1)	100.00	0.00	99.80	97.90	WGS
287.3360	0.3738	Pseudomonas aeruginosa (286)	Pseudomonas (135621)	Pseudomonadaceae (7227	Pseudomonadales (1236)	Gammaproteobacteria (122	Proteobacteria (2)	Bacteria (1)	100.00	0.60	99.80	96.80	WGS
287.3361	0.3738	Pseudomonas aeruginosa (286)	Pseudomonas (135621)	Pseudomonadaceae (7227	Pseudomonadales (1236)	Gammaproteobacteria (122	Proteobacteria (2)	Bacteria (1)	100.00	0.00	99.80	97.60	WGS
287.3362	0.3738	Pseudomonas aeruginosa (286)	Pseudomonas (135621)	Pseudomonadaceae (7227	Pseudomonadales (1236)	Gammaproteobacteria (122	Proteobacteria (2)	Bacteria (1)	100.00	0.00	99.80	98.20	WGS
287.3363	0.3738	Pseudomonas aeruginosa (286)	Pseudomonas (135621)	Pseudomonadaceae (7227	Pseudomonadales (1236)	Gammaproteobacteria (122	Proteobacteria (2)	Bacteria (1)	100.00	0.30	99.80	98.70	WGS
287.3364	0.3738	Pseudomonas aeruginosa (286)	Pseudomonas (135621)	Pseudomonadaceae (7227	Pseudomonadales (1236)	Gammaproteobacteria (122	Proteobacteria (2)	Bacteria (1)	100.00	0.00	99.80	98.40	WGS
287.3365	0.3738	Pseudomonas aeruginosa (286)	Pseudomonas (135621)	Pseudomonadaceae (7227	Pseudomonadales (1236)	Gammaproteobacteria (122	Proteobacteria (2)	Bacteria (1)	100.00	0.60	99.70	97.90	WGS
287.3366	0.3738	Pseudomonas aeruginosa (286)	Pseudomonas (135621)	Pseudomonadaceae (7227	Pseudomonadales (1236)	Gammaproteobacteria (122	Proteobacteria (2)	Bacteria (1)	100.00	0.00	99.70	97.60	WGS
287.3367	0.3738	Pseudomonas aeruginosa (286)	Pseudomonas (135621)	Pseudomonadaceae (7227	Pseudomonadales (1236)	Gammaproteobacteria (122	Proteobacteria (2)	Bacteria (1)	100.00	0.00	99.70	97.90	WGS
287.3368	0.3376	Pseudomonas aeruginosa (286)	Pseudomonas (135621)	Pseudomonadaceae (7227	Pseudomonadales (1236)	Gammaproteobacteria (122	Proteobacteria (2)	Bacteria (1)	100.00	0.00	99.90	98.40	WGS
287.3369	0.3376	Pseudomonas aeruginosa (286)	Pseudomonas (135621)	Pseudomonadaceae (7227	Pseudomonadales (1236)	Gammaproteobacteria (122	Proteobacteria (2)	Bacteria (1)	100.00	0.00	99.90	97.30	WGS
287.3370	0.331	Pseudomonas aeruginosa (286)	Pseudomonas (135621)	Pseudomonadaceae (7227	Pseudomonadales (1236)	Gammaproteobacteria (122	Proteobacteria (2)	Bacteria (1)	100.00	0.00	99.90	97.30	WGS
287.3371	0.3376	Pseudomonas aeruginosa (286)	Pseudomonas (135621)	Pseudomonadaceae (7227	Pseudomonadales (1236)	Gammaproteobacteria (122	Proteobacteria (2)	Bacteria (1)	100.00	0.00	99.90	98.60	WGS
287.3372	0.3376	Pseudomonas aeruginosa (286)	Pseudomonas (135621)	Pseudomonadaceae (7227	Pseudomonadales (1236)	Gammaproteobacteria (122	Proteobacteria (2)	Bacteria (1)	100.00	0.00	99.90	98.60	WGS
287.3373	0.331	Pseudomonas aeruginosa (286)	Pseudomonas (135621)	Pseudomonadaceae (7227	Pseudomonadales (1236)	Gammaproteobacteria (122	Proteobacteria (2)	Bacteria (1)	100.00	0.00	99.90	98.80	WGS
287.3374	0.3376	Pseudomonas aeruginosa (286)	Pseudomonas (135621)	Pseudomonadaceae (7227	Pseudomonadales (1236)	Gammaproteobacteria (122	Proteobacteria (2)	Bacteria (1)	100.00	0.00	99.90	97.80	WGS
287.3375	0.331	Pseudomonas aeruginosa (286)	Pseudomonas (135621)	Pseudomonadaceae (7227	Pseudomonadales (1236)	Gammaproteobacteria (122	Proteobacteria (2)	Bacteria (1)	100.00	0.00	99.90	98.70	WGS
287.3376	0.331	Pseudomonas aeruginosa (286)	Pseudomonas (135621)	Pseudomonadaceae (7227	Pseudomonadales (1236)	Gammaproteobacteria (122	Proteobacteria (2)	Bacteria (1)	100.00	0.30	99.90	98.50	WGS
287.3377	0.3376	Pseudomonas aeruginosa (286)	Pseudomonas (135621)	Pseudomonadaceae (7227	Pseudomonadales (1236)	Gammaproteobacteria (122	Proteobacteria (2)	Bacteria (1)	100.00	0.30	99.80	98.20	WGS
287.3378	0.3376	Pseudomonas aeruginosa (286)	Pseudomonas (135621)	Pseudomonadaceae (7227	Pseudomonadales (1236)	Gammaproteobacteria (122	Proteobacteria (2)	Bacteria (1)	100.00	0.30	99.80	98.00	WGS
287.3379	0.3376	Pseudomonas aeruginosa (286)	Pseudomonas (135621)	Pseudomonadaceae (7227	Pseudomonadales (1236)	Gammaproteobacteria (122	Proteobacteria (2)	Bacteria (1)	97.00	0.00	99.70	98.20	WGS
287.3380	0.3376	Pseudomonas aeruginosa (286)	Pseudomonas (135621)	Pseudomonadaceae (7227	Pseudomonadales (1236)	Gammaproteobacteria (122	Proteobacteria (2)	Bacteria (1)	100.00	0.30	99.80	98.00	WGS
287.3381	0.3376	Pseudomonas aeruginosa (286)	Pseudomonas (135621)	Pseudomonadaceae (7227	Pseudomonadales (1236)	Gammaproteobacteria (122	Proteobacteria (2)	Bacteria (1)	100.00	0.00	99.80	98.30	WGS
287.3382	0.3376	Pseudomonas aeruginosa (286)	Pseudomonas (135621)	Pseudomonadaceae (7227	Pseudomonadales (1236)	Gammaproteobacteria (122	Proteobacteria (2)	Bacteria (1)	100.00	0.00	99.80	98.70	WGS
287.3383	0.3376	Pseudomonas aeruginosa (286)	Pseudomonas (135621)	Pseudomonadaceae (7227	Pseudomonadales (1236)	Gammaproteobacteria (122	Proteobacteria (2)	Bacteria (1)	100.00	0.00	99.80	98.20	WGS
287.3384	0.3376	Pseudomonas aeruginosa (286)	Pseudomonas (135621)	Pseudomonadaceae (7227	Pseudomonadales (1236)	Gammaproteobacteria (122	Proteobacteria (2)	Bacteria (1)	100.00	0.00	99.80	98.40	WGS
287.3385	0.3376	Pseudomonas aeruginosa (286)	Pseudomonas (135621)	Pseudomonadaceae (7227	Pseudomonadales (1236)	Gammaproteobacteria (122	Proteobacteria (2)	Bacteria (1)	100.00	0.00	99.80	98.30	WGS
287.3386	0.365	Pseudomonas aeruginosa (286)	Pseudomonas (135621)	Pseudomonadaceae (7227	Pseudomonadales (1236)	Gammaproteobacteria (122	Proteobacteria (2)	Bacteria (1)	98.50	0.00	99.40	98.00	WGS
287.3387	0.3376	Pseudomonas aeruginosa (286)	Pseudomonas (135621)	Pseudomonadaceae (7227	Pseudomonadales (1236)	Gammaproteobacteria (122	Proteobacteria (2)	Bacteria (1)	100.00	0.00	99.80	98.50	WGS
287.3388	0.3376	Pseudomonas aeruginosa (286)	Pseudomonas (135621)	Pseudomonadaceae (7227	Pseudomonadales (1236)	Gammaproteobacteria (122	Proteobacteria (2)	Bacteria (1)	100.00	0.00	99.80	98.10	WGS
287.3389	0.3376	Pseudomonas aeruginosa (286)	Pseudomonas (135621)	Pseudomonadaceae (7227	Pseudomonadales (1236)	Gammaproteobacteria (122	Proteobacteria (2)	Bacteria (1)	100.00	0.30	99.80	98.40	WGS
287.3390	0.3376	Pseudomonas aeruginosa (286)	Pseudomonas (135621)	Pseudomonadaceae (7227	Pseudomonadales (1236)	Gammaproteobacteria (122	Proteobacteria (2)	Bacteria (1)	100.00	0.00	99.80	98.00	WGS
287.3391	0.3376	Pseudomonas aeruginosa (286)	Pseudomonas (135621)	Pseudomonadaceae (7227	Pseudomonadales (1236)	Gammaproteobacteria (122	Proteobacteria (2)	Bacteria (1)	100.00	0.00	99.80	98.40	WGS
287.3392	0.3376	Pseudomonas aeruginosa (286)	Pseudomonas (135621)	Pseudomonadaceae (7227	Pseudomonadales (1236)	Gammaproteobacteria (122	Proteobacteria (2)	Bacteria (1)	100.00	0.00	99.80	98.70	WGS
287.3394	0.3376	Pseudomonas aeruginosa (286)	Pseudomonas (135621)	Pseudomonadaceae (7227	Pseudomonadales (1236)	Gammaproteobacteria (122	Proteobacteria (2)	Bacteria (1)	100.00	0.00	99.80	98.20	WGS
287.3395	0.3604	Pseudomonas aeruginosa (286)	Pseudomonas (135621)	Pseudomonadaceae (7227	Pseudomonadales (1236)	Gammaproteobacteria (122	Proteobacteria (2)	Bacteria (1)	100.00	0.00	99.90	99.30	WGS
287.3396	0.37	Pseudomonas aeruginosa (286)	Pseudomonas (135621)	Pseudomonadaceae (7227	Pseudomonadales (1236)	Gammaproteobacteria (122	Proteobacteria (2)	Bacteria (1)	100.00	0.00	99.90	98.70	WGS
287.3397	0.37	Pseudomonas aeruginosa (286)	Pseudomonas (135621)	Pseudomonadaceae (7227	Pseudomonadales (1236)	Gammaproteobacteria (122	Proteobacteria (2)	Bacteria (1)	100.00	0.00	99.90	98.40	WGS
287.3398	0.37	Pseudomonas aeruginosa (286)	Pseudomonas (135621)	Pseudomonadaceae (7227	Pseudomonadales (1236)	Gammaproteobacteria (122	Proteobacteria (2)	Bacteria (1)	100.00	0.00	99.90	98.90	WGS
287.3399	0.37	Pseudomonas aeruginosa (286)	Pseudomonas (135621)	Pseudomonadaceae (7227	Pseudomonadales (1236)	Gammaproteobacteria (122	Proteobacteria (2)	Bacteria (1)	100.00	0.00	99.90	98.20	WGS
287.3400	0.37	Pseudomonas aeruginosa (286)	Pseudomonas (135621)	Pseudomonadaceae (7227	Pseudomonadales (1236)	Gammaproteobacteria (122	Proteobacteria (2)	Bacteria (1)	100.00	0.00	99.90	98.70	WGS
287.3401	0.37	Pseudomonas aeruginosa (286)	Pseudomonas (135621)	Pseudomonadaceae (7227	Pseudomonadales (1236)	Gammaproteobacteria (122	Proteobacteria (2)	Bacteria (1)	100.00	0.00	99.90	98.60	WGS
287.3402	0.37	Pseudomonas aeruginosa (286)	Pseudomonas (135621)	Pseudomonadaceae (7227	Pseudomonadales (1236)	Gammaproteobacteria (122	Proteobacteria (2)	Bacteria (1)	100.00	0.00	99.90	98.70	WGS
287.3403	0.37	Pseudomonas aeruginosa (286)	Pseudomonas (135621)	Pseudomonadaceae (7227	Pseudomonadales (1236)	Gammaproteobacteria (122	Proteobacteria (2)	Bacteria (1)	100.00	0.00	99.90	98.30	WGS
287.3404	0.37	Pseudomonas aeruginosa (286)	Pseudomonas (135621)	Pseudomonadaceae (7227	Pseudomonadales (1236)	Gammaproteobacteria (122	Proteobacteria (2)	Bacteria (1)	100.00	0.00	99.70	98.20	WGS
287.3405	0.37	Pseudomonas aeruginosa (286)	Pseudomonas (135621)	Pseudomonadaceae (7227	Pseudomonadales (1236)	Gammaproteobacteria (122	Proteobacteria (2)	Bacteria (1)	100.00	0.30	99.90	98.40	WGS
287.3406	0.37	Pseudomonas aeruginosa (286)	Pseudomonas (135621)	Pseudomonadaceae (7227	Pseudomonadales (1236)	Gammaproteobacteria (122	Proteobacteria (2)	Bacteria (1)	100.00	0.00	99.70	98.80	WGS
287.3407	0.37	Pseudomonas aeruginosa (286)	Pseudomonas (135621)	Pseudomonadaceae (7227	Pseudomonadales (1236)	Gammaproteobacteria (122	Proteobacteria (2)	Bacteria (1)	100.00	0.60	99.80	98.30	WGS
287.3408	0.37	Pseudomonas aeruginosa (286)	Pseudomonas (135621)	Pseudomonadaceae (7227	Pseudomonadales (1236)	Gammaproteobacteria (122	Proteobacteria (2)	Bacteria (1)	100.00	0.00	99.70	98.20	WGS
287.3409	0.37	Pseudomonas aeruginosa (286)	Pseudomonas (135621)	Pseudomonadaceae (7227	Pseudomonadales (1236)	Gammaproteobacteria (122	Proteobacteria (2)	Bacteria (1)	100.00	0.00	99.70	98.40	WGS
287.3410	0.3662	Pseudomonas aeruginosa (286)	Pseudomonas (135621)	Pseudomonadaceae (7227	Pseudomonadales (1236)	Gammaproteobacteria (122	Proteobacteria (2)	Bacteria (1)	100.00	0.60	99.90	98.20	WGS
287.3411	0.3662	Pseudomonas aeruginosa (286)	Pseudomonas (135621)	Pseudomonadaceae (7227	Pseudomonadales (1236)	Gammaproteobacteria (122	Proteobacteria (2)	Bacteria (1)	100.00	0.60	99.90	98.40	WGS
287.3412	0.3662	Pseudomonas aeruginosa (286)	Pseudomonas (135621)	Pseudomonadaceae (7227	Pseudomonadales (1236)	Gammaproteobacteria (122	Proteobacteria (2)	Bacteria (1)	100.00	0.60	99.90	98.70	WGS
287.3413	0.3662	Pseudomonas aeruginosa (286)	Pseudomonas (135621)	Pseudomonadaceae (7227	Pseudomonadales (1236)	Gammaproteobacteria (122	Proteobacteria (2)	Bacteria (1)	100.00	0.60	99.90	98.40	WGS
287.3414	0.3662	Pseudomonas aeruginosa (286)	Pseudomonas (135621)	Pseudomonadaceae (7227	Pseudomonadales (1236)	Gammaproteobacteria (122	Proteobacteria (2)	Bacteria (1)	100.00	0.60	99.90	98.40	WGS
287.3415	0.3662	Pseudomonas aeruginosa (286)	Pseudomonas (135621)	Pseudomonadaceae (7227	Pseudomonadales (1236)	Gammaproteobacteria (122	Proteobacteria (2)	Bacteria (1)	100.00	0.60	99.90	99.00	WGS
287.3416	0.3662	Pseudomonas aeruginosa (286)	Pseudomonas (135621)	Pseudomonadaceae (7227	Pseudomonadales (1236)	Gammaproteobacteria (122	Proteobacteria (2)	Bacteria (1)	100.00	0.60	99.90	98.80	WGS
287.3417	0.3662	Pseudomonas aeruginosa (286)	Pseudomonas (135621)	Pseudomonadaceae (7227	Pseudomonadales (1236)	Gammaproteobacteria (122	Proteobacteria (2)	Bacteria (1)	100.00	0.60	99.90	98.70	WGS
287.3418	0.3662	Pseudomonas aeruginosa (286)	Pseudomonas (135621)	Pseudomonadaceae (7227	Pseudomonadales (1236)	Gammaproteobacteria (122	Proteobacteria (2)	Bacteria (1)	100.00	0.60	99.90	98.80	WGS
287.3419	0.3662	Pseudomonas aeruginosa (286)	Pseudomonas (135621)	Pseudomonadaceae (7227	Pseudomonadales (1236)	Gammaproteobacteria (122	Proteobacteria (2)	Bacteria (1)	100.00	0.60	99.90	99.00	WGS
287.3420	0.3662	Pseudomonas aeruginosa (286)	Pseudomonas (135621)	Pseudomonadaceae (7227	Pseudomonadales (1236)	Gammaproteobacteria (122	Proteobacteria (2)	Bacteria (1)	100.00	0.60	99.90	98.40	WGS
287.3421	0.3662	Pseudomonas aeruginosa (286)	Pseudomonas (135621)	Pseudomonadaceae (7227	Pseudomonadales (1236)	Gammaproteobacteria (122	Proteobacteria (2)	Bacteria (1)	100.00	0.60	99.90	98.40	WGS
287.3422	0.3662	Pseudomonas aeruginosa (286)	Pseudomonas (135621)	Pseudomonadaceae (7227	Pseudomonadales (1236)	Gammaproteobacteria (122	Proteobacteria (2)	Bacteria (1)	100.00	0.60	99.90	98.90	WGS
287.3423	0.3662	Pseudomonas aeruginosa (286)	Pseudomonas (135621)	Pseudomonadaceae (7227	Pseudomonadales (1236)	Gammaproteobacteria (122	Proteobacteria (2)	Bacteria (1)	100.00	0.60	99.90	98.70	WGS
287.3424	0.3604	Pseudomonas aeruginosa (286)	Pseudomonas (135621)	Pseudomonadaceae (7227	Pseudomonadales (1236)	Gammaproteobacteria (122	Proteobacteria (2)	Bacteria (1)	100.00	0.00	99.90	98.90	WGS
287.3425	0.3704	Pseudomonas aeruginosa (286)	Pseudomonas (135621)	Pseudomonadaceae (7227	Pseudomonadales (1236)	Gammaproteobacteria (122	Proteobacteria (2)	Bacteria (1)	99.40	0.90	99.90	98.10	WGS
287.3426	0.3376	Pseudomonas aeruginosa (286)	Pseudomonas (135621)	Pseudomonadaceae (7227	Pseudomonadales (1236)	Gammaproteobacteria (122	Proteobacteria (2)	Bacteria (1)	100.00	0.00	99.80	98.60	WGS
287.3427	0.3376	Pseudomonas aeruginosa (286)	Pseudomonas (135621)	Pseudomonadaceae (7227	Pseudomonadales (1236)	Gammaproteobacteria (122	Proteobacteria (2)	Bacteria (1)	100.00	0.00	99.80	98.70	WGS
287.3428	0.3376	Pseudomonas aeruginosa (286)	Pseudomonas (135621)	Pseudomonadaceae (7227	Pseudomonadales (1236)	Gammaproteobacteria (122	Proteobacteria (2)	Bacteria (1)	100.00	0.00	99.80	98.60	WGS
287.3429	0.3704	Pseudomonas aeruginosa (286)	Pseudomonas (135621)	Pseudomonadaceae (7227	Pseudomonadales (1236)	Gammaproteobacteria (122	Proteobacteria (2)	Bacteria (1)	99.40	1.20	99.90	98.00	WGS
287.3430	0.3754	Pseudomonas aeruginosa (286)	Pseudomonas (135621)	Pseudomonadaceae (7227	Pseudomonadales (1236)	Gammaproteobacteria (122	Proteobacteria (2)	Bacteria (1)	100.00	0.00	99.80	99.40	WGS
287.3431	0.3754	Pseudomonas aeruginosa (286)	Pseudomonas (135621)	Pseudomonadaceae (7227	Pseudomonadales (1236)	Gammaproteobacteria (122	Proteobacteria (2)	Bacteria (1)	100.00	0.00	99.60	99.30	WGS
287.3432	0.3376	Pseudomonas aeruginosa (286)	Pseudomonas (135621)	Pseudomonadaceae (7227	Pseudomonadales (1236)	Gammaproteobacteria (122	Proteobacteria (2)	Bacteria (1)	100.00	0.00	99.80	98.70	WGS
287.3433	0.3754	Pseudomonas aeruginosa (286)	Pseudomonas (135621)	Pseudomonadaceae (7227	Pseudomonadales (1236)	Gammaproteobacteria (122	Proteobacteria (2)	Bacteria (1)	100.00	0.00	99.90	99.30	WGS
287.3434	0.3754	Pseudomonas aeruginosa (286)	Pseudomonas (135621)	Pseudomonadaceae (7227	Pseudomonadales (1236)	Gammaproteobacteria (122	Proteobacteria (2)	Bacteria (1)	100.00	0.00	99.80	98.80	WGS
287.3435	0.3754	Pseudomonas aeruginosa (286)	Pseudomonas (135621)	Pseudomonadaceae (7227	Pseudomonadales (1236)	Gammaproteobacteria (122	Proteobacteria (2)	Bacteria (1)	100.00	0.00	99.70	99.30	WGS
287.3436	0.3754	Pseudomonas aeruginosa (286)	Pseudomonas (135621)	Pseudomonadaceae (7227	Pseudomonadales (1236)	Gammaproteobacteria (122	Proteobacteria (2)	Bacteria (1)	100.00	0.00	99.80	99.30	WGS
287.3437	0.3754	Pseudomonas aeruginosa (286)	Pseudomonas (135621)	Pseudomonadaceae (7227	Pseudomonadales (1236)	Gammaproteobacteria (122	Proteobacteria (2)	Bacteria (1)	100.00	0.00	99.60	99.20	WGS
287.3438	0.3268	Pseudomonas aeruginosa (286)	Pseudomonas (135621)	Pseudomonadaceae (7227	Pseudomonadales (1236)	Gammaproteobacteria (122	Proteobacteria (2)	Bacteria (1)	100.00	0.00	99.80	99.00	WGS
287.3439	0.3376	Pseudomonas aeruginosa (286)	Pseudomonas (135621)	Pseudomonadaceae (7227	Pseudomonadales (1236)	Gammaproteobacteria (122	Proteobacteria (2)	Bacteria (1)	100.00	0.60	99.70	98.70	WGS
287.3440	0.3268	Pseudomonas aeruginosa (286)	Pseudomonas (135621)	Pseudomonadaceae (7227	Pseudomonadales (1236)	Gammaproteobacteria (122	Proteobacteria (2)	Bacteria (1)	100.00	0.00	99.80	99.00	WGS
287.3441	0.2906	Pseudomonas aeruginosa (286)	Pseudomonas (135621)	Pseudomonadaceae (7227	Pseudomonadales (1236)	Gammaproteobacteria (122	Proteobacteria (2)	Bacteria (1)	98.20	8.60	98.70	90.20	WGS
287.3442	0.3268	Pseudomonas aeruginosa (286)	Pseudomonas (135621)	Pseudomonadaceae (7227	Pseudomonadales (1236)	Gammaproteobacteria (122	Proteobacteria (2)	Bacteria (1)	100.00	0.00	99.80	99.00	WGS
287.3443	0.3268	Pseudomonas aeruginosa (286)	Pseudomonas (135621)	Pseudomonadaceae (7227	Pseudomonadales (1236)	Gammaproteobacteria (122	Proteobacteria (2)	Bacteria (1)	100.00	0.00	99.80	98.80	WGS
287.3444	0.3268	Pseudomonas aeruginosa (286)	Pseudomonas (135621)	Pseudomonadaceae (7227	Pseudomonadales (1236)	Gammaproteobacteria (122	Proteobacteria (2)	Bacteria (1)	100.00	0.00	99.80	99.00	WGS
287.3445	0.3268	Pseudomonas aeruginosa (286)	Pseudomonas (135621)	Pseudomonadaceae (7227	Pseudomonadales (1236)	Gammaproteobacteria (122	Proteobacteria (2)	Bacteria (1)	100.00	0.00	99.80	99.00	WGS
287.3446	0.3268	Pseudomonas aeruginosa (286)	Pseudomonas (135621)	Pseudomonadaceae (7227	Pseudomonadales (1236)	Gammaproteobacteria (122	Proteobacteria (2)	Bacteria (1)	100.00	0.00	99.80	99.00	WGS
287.3447	0.3268	Pseudomonas aeruginosa (286)	Pseudomonas (135621)	Pseudomonadaceae (7227	Pseudomonadales (1236)	Gammaproteobacteria (122	Proteobacteria (2)	Bacteria (1)	100.00	0.00	99.80	99.00	WGS
287.3448	0.3268	Pseudomonas aeruginosa (286)	Pseudomonas (135621)	Pseudomonadaceae (7227	Pseudomonadales (1236)	Gammaproteobacteria (122	Proteobacteria (2)	Bacteria (1)	100.00	0.00	99.80	99.00	WGS
287.3449	0.331	Pseudomonas aeruginosa (286)	Pseudomonas (135621)	Pseudomonadaceae (7227	Pseudomonadales (1236)	Gammaproteobacteria (122	Proteobacteria (2)	Bacteria (1)	100.00	0.00	99.90	99.40	WGS
287.3450	0.3636	Pseudomonas aeruginosa (286)	Pseudomonas (135621)	Pseudomonadaceae (7227	Pseudomonadales (1236)	Gammaproteobacteria (122	Proteobacteria (2)	Bacteria (1)	100.00	0.00	99.80	99.60	WGS
287.3451	0.3636	Pseudomonas aeruginosa (286)	Pseudomonas (135621)	Pseudomonadaceae (7227	Pseudomonadales (1236)	Gammaproteobacteria (122	Proteobacteria (2)	Bacteria (1)	100.00	0.30	99.90	99.40	WGS
287.3452	0.3636	Pseudomonas aeruginosa (286)	Pseudomonas (135621)	Pseudomonadaceae (7227	Pseudomonadales (1236)	Gammaproteobacteria (122	Proteobacteria (2)	Bacteria (1)	100.00	0.00	99.90	99.60	WGS
287.3453	0.3636	Pseudomonas aeruginosa (286)	Pseudomonas (135621)	Pseudomonadaceae (7227	Pseudomonadales (1236)	Gammaproteobacteria (122	Proteobacteria (2)	Bacteria (1)	100.00	0.00	99.80	99.60	WGS
287.3454	0.3334	Pseudomonas aeruginosa (286)	Pseudomonas (135621)	Pseudomonadaceae (7227	Pseudomonadales (1236)	Gammaproteobacteria (122	Proteobacteria (2)	Bacteria (1)	100.00	0.00	99.60	99.20	WGS
287.3455	0.332	Pseudomonas aeruginosa (286)	Pseudomonas (135621)	Pseudomonadaceae (7227	Pseudomonadales (1236)	Gammaproteobacteria (122	Proteobacteria (2)	Bacteria (1)	100.00	1.20	99.80	98.20	WGS
287.3456	0.332	Pseudomonas aeruginosa (286)	Pseudomonas (135621)	Pseudomonadaceae (7227	Pseudomonadales (1236)	Gammaproteobacteria (122	Proteobacteria (2)	Bacteria (1)	100.00	1.20	99.80	98.30	WGS
287.3457	0.3258	Pseudomonas aeruginosa (286)	Pseudomonas (135621)	Pseudomonadaceae (7227	Pseudomonadales (1236)	Gammaproteobacteria (122	Proteobacteria (2)	Bacteria (1)	100.00	0.30	99.70	99.10	WGS
287.3458	0.3258	Pseudomonas aeruginosa (286)	Pseudomonas (135621)	Pseudomonadaceae (7227	Pseudomonadales (1236)	Gammaproteobacteria (122	Proteobacteria (2)	Bacteria (1)	99.70	0.30	99.60	99.20	WGS
287.3459	0.3258	Pseudomonas aeruginosa (286)	Pseudomonas (135621)	Pseudomonadaceae (7227	Pseudomonadales (1236)	Gammaproteobacteria (122	Proteobacteria (2)	Bacteria (1)	100.00	0.30	99.70	99.00	WGS
287.3460	0.3258	Pseudomonas aeruginosa (286)	Pseudomonas (135621)	Pseudomonadaceae (7227	Pseudomonadales (1236)	Gammaproteobacteria (122	Proteobacteria (2)	Bacteria (1)	99.70	0.30	99.60	99.30	WGS
287.3461	0.3258	Pseudomonas aeruginosa (286)	Pseudomonas (135621)	Pseudomonadaceae (7227	Pseudomonadales (1236)	Gammaproteobacteria (122	Proteobacteria (2)	Bacteria (1)	100.00	0.60	99.70	99.20	WGS
287.3462	0.3258	Pseudomonas aeruginosa (286)	Pseudomonas (135621)	Pseudomonadaceae (7227	Pseudomonadales (1236)	Gammaproteobacteria (122	Proteobacteria (2)	Bacteria (1)	100.00	0.30	99.50	98.80	WGS
287.3463	0.3258	Pseudomonas aeruginosa (286)	Pseudomonas (135621)	Pseudomonadaceae (7227	Pseudomonadales (1236)	Gammaproteobacteria (122	Proteobacteria (2)	Bacteria (1)	100.00	0.30	99.70	99.00	WGS
287.3464	0.3258	Pseudomonas aeruginosa (286)	Pseudomonas (135621)	Pseudomonadaceae (7227	Pseudomonadales (1236)	Gammaproteobacteria (122	Proteobacteria (2)	Bacteria (1)	100.00	0.30	99.70	99.00	WGS
287.3465	0.3258	Pseudomonas aeruginosa (286)	Pseudomonas (135621)	Pseudomonadaceae (7227	Pseudomonadales (1236)	Gammaproteobacteria (122	Proteobacteria (2)	Bacteria (1)	100.00	0.30	99.70	99.00	WGS
287.3466	0.3258	Pseudomonas aeruginosa (286)	Pseudomonas (135621)	Pseudomonadaceae (7227	Pseudomonadales (1236)	Gammaproteobacteria (122	Proteobacteria (2)	Bacteria (1)	100.00	0.30	99.70	99.30	WGS
287.3467	0.3258	Pseudomonas aeruginosa (286)	Pseudomonas (135621)	Pseudomonadaceae (7227	Pseudomonadales (1236)	Gammaproteobacteria (122	Proteobacteria (2)	Bacteria (1)	100.00	0.30	99.70	99.20	WGS
287.3468	0.369	Pseudomonas aeruginosa (286)	Pseudomonas (135621)	Pseudomonadaceae (7227	Pseudomonadales (1236)	Gammaproteobacteria (122	Proteobacteria (2)	Bacteria (1)	100.00	0.30	99.60	98.80	WGS
287.3469	0.3258	Pseudomonas aeruginosa (286)	Pseudomonas (135621)	Pseudomonadaceae (7227	Pseudomonadales (1236)	Gammaproteobacteria (122	Proteobacteria (2)	Bacteria (1)	100.00	0.30	99.70	99.30	WGS
287.3470	0.369	Pseudomonas aeruginosa (286)	Pseudomonas (135621)	Pseudomonadaceae (7227	Pseudomonadales (1236)	Gammaproteobacteria (122	Proteobacteria (2)	Bacteria (1)	100.00	0.30	99.60	98.80	WGS
287.3471	0.3336	Pseudomonas aeruginosa (286)	Pseudomonas (135621)	Pseudomonadaceae (7227	Pseudomonadales (1236)	Gammaproteobacteria (122	Proteobacteria (2)	Bacteria (1)	100.00	0.00	99.90	98.40	WGS
287.3472	0.3792	Pseudomonas aeruginosa (286)	Pseudomonas (135621)	Pseudomonadaceae (7227	Pseudomonadales (1236)	Gammaproteobacteria (122	Proteobacteria (2)	Bacteria (1)	98.20	0.30	99.30	97.60	WGS
287.3473	0.3258	Pseudomonas aeruginosa (286)	Pseudomonas (135621)	Pseudomonadaceae (7227	Pseudomonadales (1236)	Gammaproteobacteria (122	Proteobacteria (2)	Bacteria (1)	100.00	0.30	99.70	99.00	WGS
287.3474	0.3258	Pseudomonas aeruginosa (286)	Pseudomonas (135621)	Pseudomonadaceae (7227	Pseudomonadales (1236)	Gammaproteobacteria (122	Proteobacteria (2)	Bacteria (1)	100.00	0.30	99.70	99.30	WGS
287.3475	0.3258	Pseudomonas aeruginosa (286)	Pseudomonas (135621)	Pseudomonadaceae (7227	Pseudomonadales (1236)	Gammaproteobacteria (122	Proteobacteria (2)	Bacteria (1)	100.00	0.60	99.70	99.00	WGS
287.3476	0.3258	Pseudomonas aeruginosa (286)	Pseudomonas (135621)	Pseudomonadaceae (7227	Pseudomonadales (1236)	Gammaproteobacteria (122	Proteobacteria (2)	Bacteria (1)	100.00	0.30	99.70	99.30	WGS
287.3477	0.3258	Pseudomonas aeruginosa (286)	Pseudomonas (135621)	Pseudomonadaceae (7227	Pseudomonadales (1236)	Gammaproteobacteria (122	Proteobacteria (2)	Bacteria (1)	100.00	0.30	99.70	99.30	WGS
287.3478	0.3344	Pseudomonas aeruginosa (286)	Pseudomonas (135621)	Pseudomonadaceae (7227	Pseudomonadales (1236)	Gammaproteobacteria (122	Proteobacteria (2)	Bacteria (1)	100.00	0.60	99.90	98.50	WGS
287.3479	0.3258	Pseudomonas aeruginosa (286)	Pseudomonas (135621)	Pseudomonadaceae (7227	Pseudomonadales (1236)	Gammaproteobacteria (122	Proteobacteria (2)	Bacteria (1)	100.00	0.30	99.70	99.00	WGS
287.3480	0.3376	Pseudomonas aeruginosa (286)	Pseudomonas (135621)	Pseudomonadaceae (7227	Pseudomonadales (1236)	Gammaproteobacteria (122	Proteobacteria (2)	Bacteria (1)	100.00	0.00	99.90	98.70	WGS
287.3481	0.3324	Pseudomonas aeruginosa (286)	Pseudomonas (135621)	Pseudomonadaceae (7227	Pseudomonadales (1236)	Gammaproteobacteria (122	Proteobacteria (2)	Bacteria (1)	100.00	0.00	99.90	98.80	WGS
287.3482	0.3324	Pseudomonas aeruginosa (286)	Pseudomonas (135621)	Pseudomonadaceae (7227	Pseudomonadales (1236)	Gammaproteobacteria (122	Proteobacteria (2)	Bacteria (1)	100.00	0.00	99.90	98.70	WGS
287.3483	0.3324	Pseudomonas aeruginosa (286)	Pseudomonas (135621)	Pseudomonadaceae (7227	Pseudomonadales (1236)	Gammaproteobacteria (122	Proteobacteria (2)	Bacteria (1)	100.00	0.00	99.90	98.70	WGS
287.3484	0.3324	Pseudomonas aeruginosa (286)	Pseudomonas (135621)	Pseudomonadaceae (7227	Pseudomonadales (1236)	Gammaproteobacteria (122	Proteobacteria (2)	Bacteria (1)	100.00	0.10	99.90	99.20	WGS
287.3485	0.3324	Pseudomonas aeruginosa (286)	Pseudomonas (135621)	Pseudomonadaceae (7227	Pseudomonadales (1236)	Gammaproteobacteria (122	Proteobacteria (2)	Bacteria (1)	100.00	0.10	99.90	99.30	WGS
287.3486	0.37	Pseudomonas aeruginosa (286)	Pseudomonas (135621)	Pseudomonadaceae (7227	Pseudomonadales (1236)	Gammaproteobacteria (122	Proteobacteria (2)	Bacteria (1)	100.00	0.30	99.90	96.90	WGS
287.3487	0.37	Pseudomonas aeruginosa (286)	Pseudomonas (135621)	Pseudomonadaceae (7227	Pseudomonadales (1236)	Gammaproteobacteria (122	Proteobacteria (2)	Bacteria (1)	100.00	0.00	99.90	97.50	WGS
287.3488	0.3258	Pseudomonas aeruginosa (286)	Pseudomonas (135621)	Pseudomonadaceae (7227	Pseudomonadales (1236)	Gammaproteobacteria (122	Proteobacteria (2)	Bacteria (1)	100.00	0.00	99.80	99.30	WGS
287.3489	0.37	Pseudomonas aeruginosa (286)	Pseudomonas (135621)	Pseudomonadaceae (7227	Pseudomonadales (1236)	Gammaproteobacteria (122	Proteobacteria (2)	Bacteria (1)	100.00	0.00	99.90	97.80	WGS
287.3490	0.326	Pseudomonas aeruginosa (286)	Pseudomonas (135621)	Pseudomonadaceae (7227	Pseudomonadales (1236)	Gammaproteobacteria (122	Proteobacteria (2)	Bacteria (1)	100.00	0.00	99.90	97.10	WGS
287.3491	0.37	Pseudomonas aeruginosa (286)	Pseudomonas (135621)	Pseudomonadaceae (7227	Pseudomonadales (1236)	Gammaproteobacteria (122	Proteobacteria (2)	Bacteria (1)	100.00	0.00	99.90	98.20	WGS
287.3492	0.356	Pseudomonas aeruginosa (286)	Pseudomonas (135621)	Pseudomonadaceae (7227	Pseudomonadales (1236)	Gammaproteobacteria (122	Proteobacteria (2)	Bacteria (1)	100.00	0.00	99.90	97.50	WGS
287.3493	0.326	Pseudomonas aeruginosa (286)	Pseudomonas (135621)	Pseudomonadaceae (7227	Pseudomonadales (1236)	Gammaproteobacteria (122	Proteobacteria (2)	Bacteria (1)	100.00	0.30	99.90	97.10	WGS
287.3494	0.356	Pseudomonas aeruginosa (286)	Pseudomonas (135621)	Pseudomonadaceae (7227	Pseudomonadales (1236)	Gammaproteobacteria (122	Proteobacteria (2)	Bacteria (1)	100.00	0.00	99.90	97.90	WGS
287.3495	0.3376	Pseudomonas aeruginosa (286)	Pseudomonas (135621)	Pseudomonadaceae (7227	Pseudomonadales (1236)	Gammaproteobacteria (122	Proteobacteria (2)	Bacteria (1)	99.70	1.80	99.90	96.20	WGS
287.3496	0.37	Pseudomonas aeruginosa (286)	Pseudomonas (135621)	Pseudomonadaceae (7227	Pseudomonadales (1236)	Gammaproteobacteria (122	Proteobacteria (2)	Bacteria (1)	100.00	0.00	99.90	97.40	WGS
287.3497	0.3376	Pseudomonas aeruginosa (286)	Pseudomonas (135621)	Pseudomonadaceae (7227	Pseudomonadales (1236)	Gammaproteobacteria (122	Proteobacteria (2)	Bacteria (1)	98.80	2.70	99.90	95.30	WGS
287.3498	0.342	Pseudomonas aeruginosa (286)	Pseudomonas (135621)	Pseudomonadaceae (7227	Pseudomonadales (1236)	Gammaproteobacteria (122	Proteobacteria (2)	Bacteria (1)	99.40	[illegible]	99.90	96.50	WGS
287.3499	0.3288	Pseudomonas aeruginosa (286)	Pseudomonas (135621)	Pseudomonadaceae (7227	Pseudomonadales (1236)	Gammaproteobacteria (122	Proteobacteria (2)	Bacteria (1)	99.10	2.90	99.90	95.40	WGS
287.3500	0.3376	Pseudomonas aeruginosa (286)	Pseudomonas (135621)	Pseudomonadaceae (7227	Pseudomonadales (1236)	Gammaproteobacteria (122	Proteobacteria (2)	Bacteria (1)	98.80	1.50	99.80	95.70	WGS
287.3501	0.3376	Pseudomonas aeruginosa (286)	Pseudomonas (135621)	Pseudomonadaceae (7227	Pseudomonadales (1236)	Gammaproteobacteria (122	Proteobacteria (2)	Bacteria (1)	99.70	1.20	99.90	96.50	WGS
287.3502	0.3376	Pseudomonas aeruginosa (286)	Pseudomonas (135621)	Pseudomonadaceae (7227	Pseudomonadales (1236)	Gammaproteobacteria (122	Proteobacteria (2)	Bacteria (1)	99.40	0.90	99.90	96.70	WGS
287.3503	0.3386	Pseudomonas aeruginosa (286)	Pseudomonas (135621)	Pseudomonadaceae (7227	Pseudomonadales (1236)	Gammaproteobacteria (122	Proteobacteria (2)	Bacteria (1)	100.00	0.30	99.90	97.50	WGS
287.3504	0.3386	Pseudomonas aeruginosa (286)	Pseudomonas (135621)	Pseudomonadaceae (7227	Pseudomonadales (1236)	Gammaproteobacteria (122	Proteobacteria (2)	Bacteria (1)	100.00	0.30	99.90	97.50	WGS
287.3505	0.3376	Pseudomonas aeruginosa (286)	Pseudomonas (135621)	Pseudomonadaceae (7227	Pseudomonadales (1236)	Gammaproteobacteria (122	Proteobacteria (2)	Bacteria (1)	99.70	0.90	99.90	96.70	WGS
287.3506	0.3376	Pseudomonas aeruginosa (286)	Pseudomonas (135621)	Pseudomonadaceae (7227	Pseudomonadales (1236)	Gammaproteobacteria (122	Proteobacteria (2)	Bacteria (1)	99.40	1.50	99.90	96.40	WGS
287.3507	0.3376	Pseudomonas aeruginosa (286)	Pseudomonas (135621)	Pseudomonadaceae (7227	Pseudomonadales (1236)	Gammaproteobacteria (122	Proteobacteria (2)	Bacteria (1)	99.70	0.30	99.90	97.10	WGS
287.3508	0.3098	Pseudomonas aeruginosa (286)	Pseudomonas (135621)	Pseudomonadaceae (7227	Pseudomonadales (1236)	Gammaproteobacteria (122	Proteobacteria (2)	Bacteria (1)	99.70	1.50	99.90	95.90	WGS
287.3509	0.3214	Pseudomonas aeruginosa (286)	Pseudomonas (135621)	Pseudomonadaceae (7227	Pseudomonadales (1236)	Gammaproteobacteria (122	Proteobacteria (2)	Bacteria (1)	100.00	1.20	99.60	97.00	WGS
287.3510	0.3214	Pseudomonas aeruginosa (286)	Pseudomonas (135621)	Pseudomonadaceae (7227	Pseudomonadales (1236)	Gammaproteobacteria (122	Proteobacteria (2)	Bacteria (1)	100.00	1.20	99.60	96.30	WGS
287.3511	0.3214	Pseudomonas aeruginosa (286)	Pseudomonas (135621)	Pseudomonadaceae (7227	Pseudomonadales (1236)	Gammaproteobacteria (122	Proteobacteria (2)	Bacteria (1)	100.00	1.20	99.60	96.00	WGS
287.3512	0.3214	Pseudomonas aeruginosa (286)	Pseudomonas (135621)	Pseudomonadaceae (7227	Pseudomonadales (1236)	Gammaproteobacteria (122	Proteobacteria (2)	Bacteria (1)	100.00	1.50	99.60	96.60	WGS
287.3513	0.2988	Pseudomonas aeruginosa (286)	Pseudomonas (135621)	Pseudomonadaceae (7227	Pseudomonadales (1236)	Gammaproteobacteria (122	Proteobacteria (2)	Bacteria (1)	100.00	1.50	99.80	95.60	WGS
287.3514	0.3268	Pseudomonas aeruginosa (286)	Pseudomonas (135621)	Pseudomonadaceae (7227	Pseudomonadales (1236)	Gammaproteobacteria (122	Proteobacteria (2)	Bacteria (1)	100.00	0.00	99.80	97.60	WGS
287.3515	0.2988	Pseudomonas aeruginosa (286)	Pseudomonas (135621)	Pseudomonadaceae (7227	Pseudomonadales (1236)	Gammaproteobacteria (122	Proteobacteria (2)	Bacteria (1)	100.00	1.20	99.80	95.70	WGS
287.3516	0.3636	Pseudomonas aeruginosa (286)	Pseudomonas (135621)	Pseudomonadaceae (7227	Pseudomonadales (1236)	Gammaproteobacteria (122	Proteobacteria (2)	Bacteria (1)	100.00	0.90	99.90	97.00	WGS
287.3517	0.3694	Pseudomonas aeruginosa (286)	Pseudomonas (135621)	Pseudomonadaceae (7227	Pseudomonadales (1236)	Gammaproteobacteria (122	Proteobacteria (2)	Bacteria (1)	100.00	0.30	99.80	97.60	WGS
287.3518	0.3636	Pseudomonas aeruginosa (286)	Pseudomonas (135621)	Pseudomonadaceae (7227	Pseudomonadales (1236)	Gammaproteobacteria (122	Proteobacteria (2)	Bacteria (1)	100.00	1.20	99.90	97.00	WGS
287.3519	0.3636	Pseudomonas aeruginosa (286)	Pseudomonas (135621)	Pseudomonadaceae (7227	Pseudomonadales (1236)	Gammaproteobacteria (122	Proteobacteria (2)	Bacteria (1)	99.70	1.50	99.80	96.60	WGS
287.3520	0.3694	Pseudomonas aeruginosa (286)	Pseudomonas (135621)	Pseudomonadaceae (7227	Pseudomonadales (1236)	Gammaproteobacteria (122	Proteobacteria (2)	Bacteria (1)	100.00	0.60	99.70	97.60	WGS
287.3521	0.368	Pseudomonas aeruginosa (286)	Pseudomonas (135621)	Pseudomonadaceae (7227	Pseudomonadales (1236)	Gammaproteobacteria (122	Proteobacteria (2)	Bacteria (1)	99.10	0.60	99.90	97.70	WGS
287.3522	0.3694	Pseudomonas aeruginosa (286)	Pseudomonas (135621)	Pseudomonadaceae (7227	Pseudomonadales (1236)	Gammaproteobacteria (122	Proteobacteria (2)	Bacteria (1)	100.00	0.90	99.80	96.60	WGS
287.3523	0.3636	Pseudomonas aeruginosa (286)	Pseudomonas (135621)	Pseudomonadaceae (7227	Pseudomonadales (1236)	Gammaproteobacteria (122	Proteobacteria (2)	Bacteria (1)	100.00	1.20	99.80	96.70	WGS
287.3524	0.3636	Pseudomonas aeruginosa (286)	Pseudomonas (135621)	Pseudomonadaceae (7227	Pseudomonadales (1236)	Gammaproteobacteria (122	Proteobacteria (2)	Bacteria (1)	100.00	2.10	99.70	97.00	WGS
287.3525	0.3694	Pseudomonas aeruginosa (286)	Pseudomonas (135621)	Pseudomonadaceae (7227	Pseudomonadales (1236)	Gammaproteobacteria (122	Proteobacteria (2)	Bacteria (1)	100.00	0.30	99.80	97.50	WGS
287.3526	0.368	Pseudomonas aeruginosa (286)	Pseudomonas (135621)	Pseudomonadaceae (7227	Pseudomonadales (1236)	Gammaproteobacteria (122	Proteobacteria (2)	Bacteria (1)	99.10	0.30	99.90	98.20	WGS
287.3527	0.3694	Pseudomonas aeruginosa (286)	Pseudomonas (135621)	Pseudomonadaceae (7227	Pseudomonadales (1236)	Gammaproteobacteria (122	Proteobacteria (2)	Bacteria (1)	100.00	0.60	99.70	97.50	WGS
287.3528	0.3394	Pseudomonas aeruginosa (286)	Pseudomonas (135621)	Pseudomonadaceae (7227	Pseudomonadales (1236)	Gammaproteobacteria (122	Proteobacteria (2)	Bacteria (1)	100.00	1.20	99.70	96.20	WGS
287.3529	0.3636	Pseudomonas aeruginosa (286)	Pseudomonas (135621)	Pseudomonadaceae (7227	Pseudomonadales (1236)	Gammaproteobacteria (122	Proteobacteria (2)	Bacteria (1)	100.00	1.50	99.70	97.30	WGS
287.3530	0.3636	Pseudomonas aeruginosa (286)	Pseudomonas (135621)	Pseudomonadaceae (7227	Pseudomonadales (1236)	Gammaproteobacteria (122	Proteobacteria (2)	Bacteria (1)	100.00	0.90	99.80	97.30	WGS
287.3531	0.3694	Pseudomonas aeruginosa (286)	Pseudomonas (135621)	Pseudomonadaceae (7227	Pseudomonadales (1236)	Gammaproteobacteria (122	Proteobacteria (2)	Bacteria (1)	100.00	0.60	99.70	96.70	WGS
287.3532	0.3636	Pseudomonas aeruginosa (286)	Pseudomonas (135621)	Pseudomonadaceae (7227	Pseudomonadales (1236)	Gammaproteobacteria (122	Proteobacteria (2)	Bacteria (1)	100.00	1.20	99.80	96.80	WGS
287.3533	0.368	Pseudomonas aeruginosa (286)	Pseudomonas (135621)	Pseudomonadaceae (7227	Pseudomonadales (1236)	Gammaproteobacteria (122	Proteobacteria (2)	Bacteria (1)	99.10	0.30	99.90	98.20	WGS
287.3534	0.3694	Pseudomonas aeruginosa (286)	Pseudomonas (135621)	Pseudomonadaceae (7227	Pseudomonadales (1236)	Gammaproteobacteria (122	Proteobacteria (2)	Bacteria (1)	100.00	0.30	99.70	97.30	WGS
287.3535	0.3636	Pseudomonas aeruginosa (286)	Pseudomonas (135621)	Pseudomonadaceae (7227	Pseudomonadales (1236)	Gammaproteobacteria (122	Proteobacteria (2)	Bacteria (1)	100.00	1.80	99.80	96.90	WGS
287.3536	0.3594	Pseudomonas aeruginosa (286)	Pseudomonas (135621)	Pseudomonadaceae (7227	Pseudomonadales (1236)	Gammaproteobacteria (122	Proteobacteria (2)	Bacteria (1)	100.00	1.20	99.80	95.90	WGS
287.3537	0.3214	Pseudomonas aeruginosa (286)	Pseudomonas (135621)	Pseudomonadaceae (7227	Pseudomonadales (1236)	Gammaproteobacteria (122	Proteobacteria (2)	Bacteria (1)	100.00	1.20	99.60	97.00	WGS
287.3538	0.3438	Pseudomonas aeruginosa (286)	Pseudomonas (135621)	Pseudomonadaceae (7227	Pseudomonadales (1236)	Gammaproteobacteria (122	Proteobacteria (2)	Bacteria (1)	100.00	2.60	100.00	95.40	WGS
287.3539	0.3636	Pseudomonas aeruginosa (286)	Pseudomonas (135621)	Pseudomonadaceae (7227	Pseudomonadales (1236)	Gammaproteobacteria (122	Proteobacteria (2)	Bacteria (1)	100.00	0.00	99.90	97.40	WGS
287.3540	0.3188	Pseudomonas aeruginosa (286)	Pseudomonas (135621)	Pseudomonadaceae (7227	Pseudomonadales (1236)	Gammaproteobacteria (122	Proteobacteria (2)	Bacteria (1)	100.00	0.30	99.90	98.70	WGS
287.3541	0.3188	Pseudomonas aeruginosa (286)	Pseudomonas (135621)	Pseudomonadaceae (7227	Pseudomonadales (1236)	Gammaproteobacteria (122	Proteobacteria (2)	Bacteria (1)	100.00	0.90	99.90	97.00	WGS
287.3542	0.3188	Pseudomonas aeruginosa (286)	Pseudomonas (135621)	Pseudomonadaceae (7227	Pseudomonadales (1236)	Gammaproteobacteria (122	Proteobacteria (2)	Bacteria (1)	100.00	0.30	99.90	97.90	WGS
287.3543	0.3636	Pseudomonas aeruginosa (286)	Pseudomonas (135621)	Pseudomonadaceae (7227	Pseudomonadales (1236)	Gammaproteobacteria (122	Proteobacteria (2)	Bacteria (1)	100.00	0.00	99.90	97.40	WGS
287.3544	0.2692	Pseudomonas aeruginosa (286)	Pseudomonas (135621)	Pseudomonadaceae (7227	Pseudomonadales (1236)	Gammaproteobacteria (122	Proteobacteria (2)	Bacteria (1)	89.50	14.40	95.40	84.50	WGS
287.3545	0.368	Pseudomonas aeruginosa (286)	Pseudomonas (135621)	Pseudomonadaceae (7227	Pseudomonadales (1236)	Gammaproteobacteria (122	Proteobacteria (2)	Bacteria (1)	99.10	0.60	99.90	97.80	WGS
287.3546	0.3636	Pseudomonas aeruginosa (286)	Pseudomonas (135621)	Pseudomonadaceae (7227	Pseudomonadales (1236)	Gammaproteobacteria (122	Proteobacteria (2)	Bacteria (1)	100.00	0.00	99.80	98.40	WGS
287.3547	0.2692	Pseudomonas aeruginosa (286)	Pseudomonas (135621)	Pseudomonadaceae (7227	Pseudomonadales (1236)	Gammaproteobacteria (122	Proteobacteria (2)	Bacteria (1)	90.70	13.00	95.50	85.10	WGS
287.3548	0.332	Pseudomonas aeruginosa (286)	Pseudomonas (135621)	Pseudomonadaceae (7227	Pseudomonadales (1236)	Gammaproteobacteria (122	Proteobacteria (2)	Bacteria (1)	100.00	0.00	99.80	97.30	WGS
287.3549	0.3636	Pseudomonas aeruginosa (286)	Pseudomonas (135621)	Pseudomonadaceae (7227	Pseudomonadales (1236)	Gammaproteobacteria (122	Proteobacteria (2)	Bacteria (1)	100.00	0.70	99.90	96.50	WGS
287.3550	0.332	Pseudomonas aeruginosa (286)	Pseudomonas (135621)	Pseudomonadaceae (7227	Pseudomonadales (1236)	Gammaproteobacteria (122	Proteobacteria (2)	Bacteria (1)	100.00	0.30	99.80	98.70	WGS
287.3551	0.332	Pseudomonas aeruginosa (286)	Pseudomonas (135621)	Pseudomonadaceae (7227	Pseudomonadales (1236)	Gammaproteobacteria (122	Proteobacteria (2)	Bacteria (1)	100.00	0.00	99.80	98.20	WGS
287.3552	0.3604	Pseudomonas aeruginosa (286)	Pseudomonas (135621)	Pseudomonadaceae (7227	Pseudomonadales (1236)	Gammaproteobacteria (122	Proteobacteria (2)	Bacteria (1)	100.00	0.00	99.90	98.40	WGS
287.3553	0.3738	Pseudomonas aeruginosa (286)	Pseudomonas (135621)	Pseudomonadaceae (7227	Pseudomonadales (1236)	Gammaproteobacteria (122	Proteobacteria (2)	Bacteria (1)	100.00	0.60	100.00	95.90	WGS
287.3554	0.3316	Pseudomonas aeruginosa (286)	Pseudomonas (135621)	Pseudomonadaceae (7227	Pseudomonadales (1236)	Gammaproteobacteria (122	Proteobacteria (2)	Bacteria (1)	100.00	0.00	99.90	98.20	WGS
287.3555	0.3316	Pseudomonas aeruginosa (286)	Pseudomonas (135621)	Pseudomonadaceae (7227	Pseudomonadales (1236)	Gammaproteobacteria (122	Proteobacteria (2)	Bacteria (1)	100.00	0.30	99.90	98.20	WGS
287.3556	0.324	Pseudomonas aeruginosa (286)	Pseudomonas (135621)	Pseudomonadaceae (7227	Pseudomonadales (1236)	Gammaproteobacteria (122	Proteobacteria (2)	Bacteria (1)	100.00	0.00	99.90	99.00	WGS
287.3557	0.3738	Pseudomonas aeruginosa (286)	Pseudomonas (135621)	Pseudomonadaceae (7227	Pseudomonadales (1236)	Gammaproteobacteria (122	Proteobacteria (2)	Bacteria (1)	100.00	0.00	99.80	98.20	WGS
287.3558	0.3738	Pseudomonas aeruginosa (286)	Pseudomonas (135621)	Pseudomonadaceae (7227	Pseudomonadales (1236)	Gammaproteobacteria (122	Proteobacteria (2)	Bacteria (1)	100.00	0.30	100.00	97.00	WGS
287.3559	0.325	Pseudomonas aeruginosa (286)	Pseudomonas (135621)	Pseudomonadaceae (7227	Pseudomonadales (1236)	Gammaproteobacteria (122	Proteobacteria (2)	Bacteria (1)	100.00	0.60	99.90	99.00	WGS
287.3560	0.3662	Pseudomonas aeruginosa (286)	Pseudomonas (135621)	Pseudomonadaceae (7227	Pseudomonadales (1236)	Gammaproteobacteria (122	Proteobacteria (2)	Bacteria (1)	100.00	0.60	99.90	98.80	WGS
287.3561	0.3662	Pseudomonas aeruginosa (286)	Pseudomonas (135621)	Pseudomonadaceae (7227	Pseudomonadales (1236)	Gammaproteobacteria (122	Proteobacteria (2)	Bacteria (1)	100.00	0.60	99.90	98.40	WGS
287.3562	0.3662	Pseudomonas aeruginosa (286)	Pseudomonas (135621)	Pseudomonadaceae (7227	Pseudomonadales (1236)	Gammaproteobacteria (122	Proteobacteria (2)	Bacteria (1)	100.00	0.60	99.90	98.40	WGS
287.3563	0.3738	Pseudomonas aeruginosa (286)	Pseudomonas (135621)	Pseudomonadaceae (7227	Pseudomonadales (1236)	Gammaproteobacteria (122	Proteobacteria (2)	Bacteria (1)	100.00	0.00	100.00	97.00	WGS
287.3564	0.3376	Pseudomonas aeruginosa (286)	Pseudomonas (135621)	Pseudomonadaceae (7227	Pseudomonadales (1236)	Gammaproteobacteria (122	Proteobacteria (2)	Bacteria (1)	99.70	0.90	99.90	96.20	WGS
287.3565	0.3254	Pseudomonas aeruginosa (286)	Pseudomonas (135621)	Pseudomonadaceae (7227	Pseudomonadales (1236)	Gammaproteobacteria (122	Proteobacteria (2)	Bacteria (1)	98.80	1.20	99.40	95.90	WGS
287.3566	0.3694	Pseudomonas aeruginosa (286)	Pseudomonas (135621)	Pseudomonadaceae (7227	Pseudomonadales (1236)	Gammaproteobacteria (122	Proteobacteria (2)	Bacteria (1)	100.00	0.30	99.70	97.30	WGS
287.3567	0.3214	Pseudomonas aeruginosa (286)	Pseudomonas (135621)	Pseudomonadaceae (7227	Pseudomonadales (1236)	Gammaproteobacteria (122	Proteobacteria (2)	Bacteria (1)	100.00	1.20	99.60	97.30	WGS
287.3568	0.3636	Pseudomonas aeruginosa (286)	Pseudomonas (135621)	Pseudomonadaceae (7227	Pseudomonadales (1236)	Gammaproteobacteria (122	Proteobacteria (2)	Bacteria (1)	100.00	0.90	99.90	98.60	WGS
287.3569	0.3694	Pseudomonas aeruginosa (286)	Pseudomonas (135621)	Pseudomonadaceae (7227	Pseudomonadales (1236)	Gammaproteobacteria (122	Proteobacteria (2)	Bacteria (1)	100.00	0.30	99.70	97.90	WGS
287.3570	0.368	Pseudomonas aeruginosa (286)	Pseudomonas (135621)	Pseudomonadaceae (7227	Pseudomonadales (1236)	Gammaproteobacteria (122	Proteobacteria (2)	Bacteria (1)	99.10	0.90	99.90	97.60	WGS
287.3571	0.368	Pseudomonas aeruginosa (286)	Pseudomonas (135621)	Pseudomonadaceae (7227	Pseudomonadales (1236)	Gammaproteobacteria (122	Proteobacteria (2)	Bacteria (1)	99.70	0.00	99.70	98.50	WGS
287.3572	0.325	Pseudomonas aeruginosa (286)	Pseudomonas (135621)	Pseudomonadaceae (7227	Pseudomonadales (1236)	Gammaproteobacteria (122	Proteobacteria (2)	Bacteria (1)	100.00	1.80	99.90	96.50	WGS
287.3573	0.3636	Pseudomonas aeruginosa (286)	Pseudomonas (135621)	Pseudomonadaceae (7227	Pseudomonadales (1236)	Gammaproteobacteria (122	Proteobacteria (2)	Bacteria (1)	99.70	2.50	99.90	95.10	WGS
287.3574	0.368	Pseudomonas aeruginosa (286)	Pseudomonas (135621)	Pseudomonadaceae (7227	Pseudomonadales (1236)	Gammaproteobacteria (122	Proteobacteria (2)	Bacteria (1)	100.00	0.90	99.70	98.20	WGS
287.3575	0.368	Pseudomonas aeruginosa (286)	Pseudomonas (135621)	Pseudomonadaceae (7227	Pseudomonadales (1236)	Gammaproteobacteria (122	Proteobacteria (2)	Bacteria (1)	100.00	0.90	99.70	98.70	WGS
287.3576	0.29	Pseudomonas aeruginosa (286)	Pseudomonas (135621)	Pseudomonadaceae (7227	Pseudomonadales (1236)	Gammaproteobacteria (122	Proteobacteria (2)	Bacteria (1)	100.00	2.10	99.70	95.90	WGS
287.3577	0.2974	Pseudomonas aeruginosa (286)	Pseudomonas (135621)	Pseudomonadaceae (7227	Pseudomonadales (1236)	Gammaproteobacteria (122	Proteobacteria (2)	Bacteria (1)	100.00	1.50	99.70	96.20	WGS
287.3578	0.324	Pseudomonas aeruginosa (286)	Pseudomonas (135621)	Pseudomonadaceae (7227	Pseudomonadales (1236)	Gammaproteobacteria (122	Proteobacteria (2)	Bacteria (1)	100.00	0.90	99.70	96.40	WGS
287.3579	0.324	Pseudomonas aeruginosa (286)	Pseudomonas (135621)	Pseudomonadaceae (7227	Pseudomonadales (1236)	Gammaproteobacteria (122	Proteobacteria (2)	Bacteria (1)	100.00	1.60	99.70	96.50	WGS
287.3580	0.324	Pseudomonas aeruginosa (286)	Pseudomonas (135621)	Pseudomonadaceae (7227	Pseudomonadales (1236)	Gammaproteobacteria (122	Proteobacteria (2)	Bacteria (1)	100.00	0.00	99.70	98.50	WGS
287.3581	0.324	Pseudomonas aeruginosa (286)	Pseudomonas (135621)	Pseudomonadaceae (7227	Pseudomonadales (1236)	Gammaproteobacteria (122	Proteobacteria (2)	Bacteria (1)	100.00	0.60	99.70	96.80	WGS
287.3582	0.29	Pseudomonas aeruginosa (286)	Pseudomonas (135621)	Pseudomonadaceae (7227	Pseudomonadales (1236)	Gammaproteobacteria (122	Proteobacteria (2)	Bacteria (1)	100.00	2.10	99.70	96.30	WGS
287.3583	0.324	Pseudomonas aeruginosa (286)	Pseudomonas (135621)	Pseudomonadaceae (7227	Pseudomonadales (1236)	Gammaproteobacteria (122	Proteobacteria (2)	Bacteria (1)	100.00	0.90	99.70	96.70	WGS
287.3584	0.324	Pseudomonas aeruginosa (286)	Pseudomonas (135621)	Pseudomonadaceae (7227	Pseudomonadales (1236)	Gammaproteobacteria (122	Proteobacteria (2)	Bacteria (1)	100.00	1.80	99.70	96.40	WGS
287.3585	0.324	Pseudomonas aeruginosa (286)	Pseudomonas (135621)	Pseudomonadaceae (7227	Pseudomonadales (1236)	Gammaproteobacteria (122	Proteobacteria (2)	Bacteria (1)	100.00	1.50	99.70	96.50	WGS
287.3586	0.2974	Pseudomonas aeruginosa (286)	Pseudomonas (135621)	Pseudomonadaceae (7227	Pseudomonadales (1236)	Gammaproteobacteria (122	Proteobacteria (2)	Bacteria (1)	100.00	1.50	99.70	96.20	WGS
287.3587	0.324	Pseudomonas aeruginosa (286)	Pseudomonas (135621)	Pseudomonadaceae (7227	Pseudomonadales (1236)	Gammaproteobacteria (122	Proteobacteria (2)	Bacteria (1)	100.00	0.00	99.70	97.40	WGS
287.3588	0.29	Pseudomonas aeruginosa (286)	Pseudomonas (135621)	Pseudomonadaceae (7227	Pseudomonadales (1236)	Gammaproteobacteria (122	Proteobacteria (2)	Bacteria (1)	100.00	4.10	99.80	94.50	WGS
287.3589	0.29	Pseudomonas aeruginosa (286)	Pseudomonas (135621)	Pseudomonadaceae (7227	Pseudomonadales (1236)	Gammaproteobacteria (122	Proteobacteria (2)	Bacteria (1)	99.50	8.50	99.60	92.50	WGS
287.3590	0.3248	Pseudomonas aeruginosa (286)	Pseudomonas (135621)	Pseudomonadaceae (7227	Pseudomonadales (1236)	Gammaproteobacteria (122	Proteobacteria (2)	Bacteria (1)	98.80	7.20	99.70	91.60	WGS
287.3591	0.3248	Pseudomonas aeruginosa (286)	Pseudomonas (135621)	Pseudomonadaceae (7227	Pseudomonadales (1236)	Gammaproteobacteria (122	Proteobacteria (2)	Bacteria (1)	99.70	3.60	99.80	93.50	WGS
287.3592	0.3302	Pseudomonas aeruginosa (286)	Pseudomonas (135621)	Pseudomonadaceae (7227	Pseudomonadales (1236)	Gammaproteobacteria (122	Proteobacteria (2)	Bacteria (1)	99.40	6.60	99.70	93.10	WGS
287.3593	0.2906	Pseudomonas aeruginosa (286)	Pseudomonas (135621)	Pseudomonadaceae (7227	Pseudomonadales (1236)	Gammaproteobacteria (122	Proteobacteria (2)	Bacteria (1)	97.90	8.80	99.20	91.90	WGS
287.3594	0.2906	Pseudomonas aeruginosa (286)	Pseudomonas (135621)	Pseudomonadaceae (7227	Pseudomonadales (1236)	Gammaproteobacteria (122	Proteobacteria (2)	Bacteria (1)	99.30	5.40	99.60	92.50	WGS
287.3595	0.362	Pseudomonas aeruginosa (286)	Pseudomonas (135621)	Pseudomonadaceae (7227	Pseudomonadales (1236)	Gammaproteobacteria (122	Proteobacteria (2)	Bacteria (1)	97.90	9.90	98.70	90.10	WGS
287.3596	0.3296	Pseudomonas aeruginosa (286)	Pseudomonas (135621)	Pseudomonadaceae (7227	Pseudomonadales (1236)	Gammaproteobacteria (122	Proteobacteria (2)	Bacteria (1)	94.60	12.20	97.30	87.00	WGS
287.3597	0.368	Pseudomonas aeruginosa (286)	Pseudomonas (135621)	Pseudomonadaceae (7227	Pseudomonadales (1236)	Gammaproteobacteria (122	Proteobacteria (2)	Bacteria (1)	100.00	0.30	99.70	98.00	WGS
287.3598	0.368	Pseudomonas aeruginosa (286)	Pseudomonas (135621)	Pseudomonadaceae (7227	Pseudomonadales (1236)	Gammaproteobacteria (122	Proteobacteria (2)	Bacteria (1)	100.00	0.30	99.70	98.60	WGS
287.3599	0.3636	Pseudomonas aeruginosa (286)	Pseudomonas (135621)	Pseudomonadaceae (7227	Pseudomonadales (1236)	Gammaproteobacteria (122	Proteobacteria (2)	Bacteria (1)	99.10	0.30	99.80	99.10	WGS
287.3600	0.3918	Pseudomonas aeruginosa (286)	Pseudomonas (135621)	Pseudomonadaceae (7227	Pseudomonadales (1236)	Gammaproteobacteria (122	Proteobacteria (2)	Bacteria (1)	100.00	1.20	99.80	98.20	WGS
287.3601	0.368	Pseudomonas aeruginosa (286)	Pseudomonas (135621)	Pseudomonadaceae (7227	Pseudomonadales (1236)	Gammaproteobacteria (122	Proteobacteria (2)	Bacteria (1)	99.10	0.30	99.90	99.20	WGS
287.3602	0.3918	Pseudomonas aeruginosa (286)	Pseudomonas (135621)	Pseudomonadaceae (7227	Pseudomonadales (1236)	Gammaproteobacteria (122	Proteobacteria (2)	Bacteria (1)	100.00	1.20	99.80	98.40	WGS
287.3603	0.3918	Pseudomonas aeruginosa (286)	Pseudomonas (135621)	Pseudomonadaceae (7227	Pseudomonadales (1236)	Gammaproteobacteria (122	Proteobacteria (2)	Bacteria (1)	100.00	1.20	99.70	98.20	WGS
287.3604	0.324	Pseudomonas aeruginosa (286)	Pseudomonas (135621)	Pseudomonadaceae (7227	Pseudomonadales (1236)	Gammaproteobacteria (122	Proteobacteria (2)	Bacteria (1)	100.00	0.30	99.70	96.80	WGS
287.3605	0.2974	Pseudomonas aeruginosa (286)	Pseudomonas (135621)	Pseudomonadaceae (7227	Pseudomonadales (1236)	Gammaproteobacteria (122	Proteobacteria (2)	Bacteria (1)	100.00	1.80	99.70	96.20	WGS
287.3606	0.324	Pseudomonas aeruginosa (286)	Pseudomonas (135621)	Pseudomonadaceae (7227	Pseudomonadales (1236)	Gammaproteobacteria (122	Proteobacteria (2)	Bacteria (1)	100.00	0.60	99.70	96.80	WGS
287.3607	0.324	Pseudomonas aeruginosa (286)	Pseudomonas (135621)	Pseudomonadaceae (7227	Pseudomonadales (1236)	Gammaproteobacteria (122	Proteobacteria (2)	Bacteria (1)	100.00	1.20	99.70	96.70	WGS
287.3608	0.324	Pseudomonas aeruginosa (286)	Pseudomonas (135621)	Pseudomonadaceae (7227	Pseudomonadales (1236)	Gammaproteobacteria (122	Proteobacteria (2)	Bacteria (1)	100.00	0.00	99.70	97.00	WGS
287.3609	0.324	Pseudomonas aeruginosa (286)	Pseudomonas (135621)	Pseudomonadaceae (7227	Pseudomonadales (1236)	Gammaproteobacteria (122	Proteobacteria (2)	Bacteria (1)	100.00	1.60	99.70	95.90	WGS
287.3610	0.324	Pseudomonas aeruginosa (286)	Pseudomonas (135621)	Pseudomonadaceae (7227	Pseudomonadales (1236)	Gammaproteobacteria (122	Proteobacteria (2)	Bacteria (1)	100.00	0.30	99.70	96.30	WGS
287.3611	0.29	Pseudomonas aeruginosa (286)	Pseudomonas (135621)	Pseudomonadaceae (7227	Pseudomonadales (1236)	Gammaproteobacteria (122	Proteobacteria (2)	Bacteria (1)	100.00	2.40	99.70	95.60	WGS
287.3612	0.324	Pseudomonas aeruginosa (286)	Pseudomonas (135621)	Pseudomonadaceae (7227	Pseudomonadales (1236)	Gammaproteobacteria (122	Proteobacteria (2)	Bacteria (1)	100.00	1.60	99.70	96.20	WGS
287.3613	0.324	Pseudomonas aeruginosa (286)	Pseudomonas (135621)	Pseudomonadaceae (7227	Pseudomonadales (1236)	Gammaproteobacteria (122	Proteobacteria (2)	Bacteria (1)	100.00	1.80	99.70	96.10	WGS
287.3614	0.2974	Pseudomonas aeruginosa (286)	Pseudomonas (135621)	Pseudomonadaceae (7227	Pseudomonadales (1236)	Gammaproteobacteria (122	Proteobacteria (2)	Bacteria (1)	100.00	1.60	99.70	96.20	WGS
287.3615	0.324	Pseudomonas aeruginosa (286)	Pseudomonas (135621)	Pseudomonadaceae (7227	Pseudomonadales (1236)	Gammaproteobacteria (122	Proteobacteria (2)	Bacteria (1)	100.00	0.30	99.70	96.90	WGS
287.3616	0.324	Pseudomonas aeruginosa (286)	Pseudomonas (135621)	Pseudomonadaceae (7227	Pseudomonadales (1236)	Gammaproteobacteria (122	Proteobacteria (2)	Bacteria (1)	100.00	0.60	99.70	96.70	WGS
287.3617	0.324	Pseudomonas aeruginosa (286)	Pseudomonas (135621)	Pseudomonadaceae (7227	Pseudomonadales (1236)	Gammaproteobacteria (122	Proteobacteria (2)	Bacteria (1)	100.00	0.30	99.70	96.90	WGS
287.3618	0.324	Pseudomonas aeruginosa (286)	Pseudomonas (135621)	Pseudomonadaceae (7227	Pseudomonadales (1236)	Gammaproteobacteria (122	Proteobacteria (2)	Bacteria (1)	100.00	0.90	99.70	97.00	WGS
287.3619	0.324	Pseudomonas aeruginosa (286)	Pseudomonas (135621)	Pseudomonadaceae (7227	Pseudomonadales (1236)	Gammaproteobacteria (122	Proteobacteria (2)	Bacteria (1)	100.00	0.00	99.70	97.00	WGS
287.3622	0.2488	Pseudomonas aeruginosa (286)	Pseudomonas (135621)	Pseudomonadaceae (7227	Pseudomonadales (1236)	Gammaproteobacteria (122	Proteobacteria (2)	Bacteria (1)	93.80	36.90	96.20	69.80	WGS
287.3623	0.324	Pseudomonas aeruginosa (286)	Pseudomonas (135621)	Pseudomonadaceae (7227	Pseudomonadales (1236)	Gammaproteobacteria (122	Proteobacteria (2)	Bacteria (1)	100.00	0.60	99.90	99.10	WGS
287.3713	0.3498	Pseudomonas aeruginosa (286)	Pseudomonas (135621)	Pseudomonadaceae (7227	Pseudomonadales (1236)	Gammaproteobacteria (122	Proteobacteria (2)	Bacteria (1)	99.70	0.30	99.60	98.40	WGS
287.3714	0.3258	Pseudomonas aeruginosa (286)	Pseudomonas (135621)	Pseudomonadaceae (7227	Pseudomonadales (1236)	Gammaproteobacteria (122	Proteobacteria (2)	Bacteria (1)	100.00	0.00	99.90	99.40	WGS
287.3715	0.3258	Pseudomonas aeruginosa (286)	Pseudomonas (135621)	Pseudomonadaceae (7227	Pseudomonadales (1236)	Gammaproteobacteria (122	Proteobacteria (2)	Bacteria (1)	100.00	0.00	99.90	99.60	WGS
287.3716	0.3174	Pseudomonas aeruginosa (286)	Pseudomonas (135621)	Pseudomonadaceae (7227	Pseudomonadales (1236)	Gammaproteobacteria (122	Proteobacteria (2)	Bacteria (1)	99.70	0.90	99.70	98.40	WGS
287.3717	0.3258	Pseudomonas aeruginosa (286)	Pseudomonas (135621)	Pseudomonadaceae (7227	Pseudomonadales (1236)	Gammaproteobacteria (122	Proteobacteria (2)	Bacteria (1)	100.00	0.00	99.90	99.30	WGS
287.3718	0.3258	Pseudomonas aeruginosa (286)	Pseudomonas (135621)	Pseudomonadaceae (7227	Pseudomonadales (1236)	Gammaproteobacteria (122	Proteobacteria (2)	Bacteria (1)	100.00	0.90	99.90	98.80	WGS
287.3719	0.3636	Pseudomonas aeruginosa (286)	Pseudomonas (135621)	Pseudomonadaceae (7227	Pseudomonadales (1236)	Gammaproteobacteria (122	Proteobacteria (2)	Bacteria (1)	100.00	0.30	99.70	99.00	WGS
287.3720	0.3376	Pseudomonas aeruginosa (286)	Pseudomonas (135621)	Pseudomonadaceae (7227	Pseudomonadales (1236)	Gammaproteobacteria (122	Proteobacteria (2)	Bacteria (1)	100.00	0.00	99.90	98.80	WGS
287.3721	0.371	Pseudomonas aeruginosa (286)	Pseudomonas (135621)	Pseudomonadaceae (7227	Pseudomonadales (1236)	Gammaproteobacteria (122	Proteobacteria (2)	Bacteria (1)	100.00	0.00	99.90	99.10	WGS
287.3722	0.371	Pseudomonas aeruginosa (286)	Pseudomonas (135621)	Pseudomonadaceae (7227	Pseudomonadales (1236)	Gammaproteobacteria (122	Proteobacteria (2)	Bacteria (1)	100.00	0.00	99.90	99.10	WGS
287.3723	0.368	Pseudomonas aeruginosa (286)	Pseudomonas (135621)	Pseudomonadaceae (7227	Pseudomonadales (1236)	Gammaproteobacteria (122	Proteobacteria (2)	Bacteria (1)	97.00	0.30	99.80	98.40	WGS
287.3724	0.3258	Pseudomonas aeruginosa (286)	Pseudomonas (135621)	Pseudomonadaceae (7227	Pseudomonadales (1236)	Gammaproteobacteria (122	Proteobacteria (2)	Bacteria (1)	100.00	0.00	99.90	99.30	WGS
287.3725	0.332	Pseudomonas aeruginosa (286)	Pseudomonas (135621)	Pseudomonadaceae (7227	Pseudomonadales (1236)	Gammaproteobacteria (122	Proteobacteria (2)	Bacteria (1)	100.00	0.00	99.90	99.00	WGS
287.3726	0.3324	Pseudomonas aeruginosa (286)	Pseudomonas (135621)	Pseudomonadaceae (7227	Pseudomonadales (1236)	Gammaproteobacteria (122	Proteobacteria (2)	Bacteria (1)	100.00	0.30	99.70	99.00	WGS
287.3727	0.3324	Pseudomonas aeruginosa (286)	Pseudomonas (135621)	Pseudomonadaceae (7227	Pseudomonadales (1236)	Gammaproteobacteria (122	Proteobacteria (2)	Bacteria (1)	100.00	0.30	99.70	99.00	WGS
287.3728	0.3268	Pseudomonas aeruginosa (286)	Pseudomonas (135621)	Pseudomonadaceae (7227	Pseudomonadales (1236)	Gammaproteobacteria (122	Proteobacteria (2)	Bacteria (1)	100.00	0.00	99.80	98.80	WGS
287.3729	0.3268	Pseudomonas aeruginosa (286)	Pseudomonas (135621)	Pseudomonadaceae (7227	Pseudomonadales (1236)	Gammaproteobacteria (122	Proteobacteria (2)	Bacteria (1)	100.00	0.00	99.90	99.10	WGS
287.3730	0.3258	Pseudomonas aeruginosa (286)	Pseudomonas (135621)	Pseudomonadaceae (7227	Pseudomonadales (1236)	Gammaproteobacteria (122	Proteobacteria (2)	Bacteria (1)	100.00	0.30	99.90	99.00	WGS
287.3731	0.3694	Pseudomonas aeruginosa (286)	Pseudomonas (135621)	Pseudomonadaceae (7227	Pseudomonadales (1236)	Gammaproteobacteria (122	Proteobacteria (2)	Bacteria (1)	100.00	0.90	99.60	98.60	WGS
287.3732	0.3646	Pseudomonas aeruginosa (286)	Pseudomonas (135621)	Pseudomonadaceae (7227	Pseudomonadales (1236)	Gammaproteobacteria (122	Proteobacteria (2)	Bacteria (1)	100.00	0.60	99.90	98.90	WGS
287.3733	0.3628	Pseudomonas aeruginosa (286)	Pseudomonas (135621)	Pseudomonadaceae (7227	Pseudomonadales (1236)	Gammaproteobacteria (122	Proteobacteria (2)	Bacteria (1)	100.00	1.80	99.90	97.80	WGS
287.3734	0.332	Pseudomonas aeruginosa (286)	Pseudomonas (135621)	Pseudomonadaceae (7227	Pseudomonadales (1236)	Gammaproteobacteria (122	Proteobacteria (2)	Bacteria (1)	100.00	0.60	99.80	98.60	WGS
287.3735	0.3376	Pseudomonas aeruginosa (286)	Pseudomonas (135621)	Pseudomonadaceae (7227	Pseudomonadales (1236)	Gammaproteobacteria (122	Proteobacteria (2)	Bacteria (1)	100.00	0.00	99.90	98.80	WGS
287.3736	0.3334	Pseudomonas aeruginosa (286)	Pseudomonas (135621)	Pseudomonadaceae (7227	Pseudomonadales (1236)	Gammaproteobacteria (122	Proteobacteria (2)	Bacteria (1)	100.00	0.60	99.80	97.80	WGS
287.3737	0.3714	Pseudomonas aeruginosa (286)	Pseudomonas (135621)	Pseudomonadaceae (7227	Pseudomonadales (1236)	Gammaproteobacteria (122	Proteobacteria (2)	Bacteria (1)	98.80	0.90	99.40	97.40	WGS
287.3738	0.3184	Pseudomonas aeruginosa (286)	Pseudomonas (135621)	Pseudomonadaceae (7227	Pseudomonadales (1236)	Gammaproteobacteria (122	Proteobacteria (2)	Bacteria (1)	100.00	0.60	99.90	97.20	WGS
287.3739	0.327	Pseudomonas aeruginosa (286)	Pseudomonas (135621)	Pseudomonadaceae (7227	Pseudomonadales (1236)	Gammaproteobacteria (122	Proteobacteria (2)	Bacteria (1)	100.00	1.20	99.90	97.90	WGS
287.3740	0.325	Pseudomonas aeruginosa (286)	Pseudomonas (135621)	Pseudomonadaceae (7227	Pseudomonadales (1236)	Gammaproteobacteria (122	Proteobacteria (2)	Bacteria (1)	100.00	0.30	99.90	98.20	WGS
287.3741	0.3258	Pseudomonas aeruginosa (286)	Pseudomonas (135621)	Pseudomonadaceae (7227	Pseudomonadales (1236)	Gammaproteobacteria (122	Proteobacteria (2)	Bacteria (1)	100.00	0.00	99.80	99.40	Complete
287.3865	0.2818	Pseudomonas aeruginosa (286)	Pseudomonas (135621)	Pseudomonadaceae (7227	Pseudomonadales (1236)	Gammaproteobacteria (122	Proteobacteria (2)	Bacteria (1)	98.80	6.50	99.50	93.00	WGS
287.3866	0.3394	Pseudomonas aeruginosa (286)	Pseudomonas (135621)	Pseudomonadaceae (7227	Pseudomonadales (1236)	Gammaproteobacteria (122	Proteobacteria (2)	Bacteria (1)	100.00	4.20	99.80	95.50	WGS
287.3867	0.3694	Pseudomonas aeruginosa (286)	Pseudomonas (135621)	Pseudomonadaceae (7227	Pseudomonadales (1236)	Gammaproteobacteria (122	Proteobacteria (2)	Bacteria (1)	100.00	0.00	99.70	99.10	Complete
287.3868	0.3314	Pseudomonas aeruginosa (286)	Pseudomonas (135621)	Pseudomonadaceae (7227	Pseudomonadales (1236)	Gammaproteobacteria (122	Proteobacteria (2)	Bacteria (1)	100.00	0.00	99.80	99.00	Complete
287.3869	0.3618	Pseudomonas aeruginosa (286)	Pseudomonas (135621)	Pseudomonadaceae (7227	Pseudomonadales (1236)	Gammaproteobacteria (122	Proteobacteria (2)	Bacteria (1)	99.40	0.30	99.80	98.70	Complete
287.3870	0.3004	Pseudomonas aeruginosa (286)	Pseudomonas (135621)	Pseudomonadaceae (7227	Pseudomonadales (1236)	Gammaproteobacteria (122	Proteobacteria (2)	Bacteria (1)	86.80	1.70	92.80	89.70	WGS
287.3871	0.327	Pseudomonas aeruginosa (286)	Pseudomonas (135621)	Pseudomonadaceae (7227	Pseudomonadales (1236)	Gammaproteobacteria (122	Proteobacteria (2)	Bacteria (1)	99.70	3.80	99.80	96.30	WGS
287.3872	0.2998	Pseudomonas aeruginosa (286)	Pseudomonas (135621)	Pseudomonadaceae (7227	Pseudomonadales (1236)	Gammaproteobacteria (122	Proteobacteria (2)	Bacteria (1)	60.50	0.00	80.00	78.90	WGS
287.3873	0.3704	Pseudomonas aeruginosa (286)	Pseudomonas (135621)	Pseudomonadaceae (7227	Pseudomonadales (1236)	Gammaproteobacteria (122	Proteobacteria (2)	Bacteria (1)	100.00	2.30	99.90	96.20	WGS
287.3874	0.368	Pseudomonas aeruginosa (286)	Pseudomonas (135621)	Pseudomonadaceae (7227	Pseudomonadales (1236)	Gammaproteobacteria (122	Proteobacteria (2)	Bacteria (1)	100.00	0.30	99.80	98.70	WGS
287.3875	0.3376	Pseudomonas aeruginosa (286)	Pseudomonas (135621)	Pseudomonadaceae (7227	Pseudomonadales (1236)	Gammaproteobacteria (122	Proteobacteria (2)	Bacteria (1)	99.10	1.60	99.80	96.90	WGS
287.3876	0.324	Pseudomonas aeruginosa (286)	Pseudomonas (135621)	Pseudomonadaceae (7227	Pseudomonadales (1236)	Gammaproteobacteria (122	Proteobacteria (2)	Bacteria (1)	100.00	0.60	99.70	97.90	WGS
287.3877	0.356	Pseudomonas aeruginosa (286)	Pseudomonas (135621)	Pseudomonadaceae (7227	Pseudomonadales (1236)	Gammaproteobacteria (122	Proteobacteria (2)	Bacteria (1)	100.00	0.00	99.90	99.00	Complete
287.3878	0.3604	Pseudomonas aeruginosa (286)	Pseudomonas (135621)	Pseudomonadaceae (7227	Pseudomonadales (1236)	Gammaproteobacteria (122	Proteobacteria (2)	Bacteria (1)	100.00	0.00	99.90	99.00	Complete
287.3879	0.332	Pseudomonas aeruginosa (286)	Pseudomonas (135621)	Pseudomonadaceae (7227	Pseudomonadales (1236)	Gammaproteobacteria (122	Proteobacteria (2)	Bacteria (1)	100.00	0.60	99.80	99.50	WGS
287.3880	0.3258	Pseudomonas aeruginosa (286)	Pseudomonas (135621)	Pseudomonadaceae (7227	Pseudomonadales (1236)	Gammaproteobacteria (122	Proteobacteria (2)	Bacteria (1)	100.00	0.00	99.90	99.10	WGS
287.3881	0.3174	Pseudomonas aeruginosa (286)	Pseudomonas (135621)	Pseudomonadaceae (7227	Pseudomonadales (1236)	Gammaproteobacteria (122	Proteobacteria (2)	Bacteria (1)	100.00	0.00	99.90	98.90	WGS
287.3882	0.324	Pseudomonas aeruginosa (286)	Pseudomonas (135621)	Pseudomonadaceae (7227	Pseudomonadales (1236)	Gammaproteobacteria (122	Proteobacteria (2)	Bacteria (1)	100.00	0.00	99.90	99.20	WGS
287.3883	0.338	Pseudomonas aeruginosa (286)	Pseudomonas (135621)	Pseudomonadaceae (7227	Pseudomonadales (1236)	Gammaproteobacteria (122	Proteobacteria (2)	Bacteria (1)	98.80	12.30	99.20	88.10	WGS
287.3884	0.356	Pseudomonas aeruginosa (286)	Pseudomonas (135621)	Pseudomonadaceae (7227	Pseudomonadales (1236)	Gammaproteobacteria (122	Proteobacteria (2)	Bacteria (1)	100.00	0.00	99.90	98.70	WGS
287.3885	0.356	Pseudomonas aeruginosa (286)	Pseudomonas (135621)	Pseudomonadaceae (7227	Pseudomonadales (1236)	Gammaproteobacteria (122	Proteobacteria (2)	Bacteria (1)	100.00	0.30	99.90	98.50	WGS
287.3886	0.356	Pseudomonas aeruginosa (286)	Pseudomonas (135621)	Pseudomonadaceae (7227	Pseudomonadales (1236)	Gammaproteobacteria (122	Proteobacteria (2)	Bacteria (1)	100.00	0.00	99.90	98.60	WGS
287.3887	0.356	Pseudomonas aeruginosa (286)	Pseudomonas (135621)	Pseudomonadaceae (7227	Pseudomonadales (1236)	Gammaproteobacteria (122	Proteobacteria (2)	Bacteria (1)	99.70	0.30	99.60	98.40	WGS
287.3888	0.356	Pseudomonas aeruginosa (286)	Pseudomonas (135621)	Pseudomonadaceae (7227	Pseudomonadales (1236)	Gammaproteobacteria (122	Proteobacteria (2)	Bacteria (1)	100.00	0.10	99.90	98.80	WGS
287.3889	0.3552	Pseudomonas aeruginosa (286)	Pseudomonas (135621)	Pseudomonadaceae (7227	Pseudomonadales (1236)	Gammaproteobacteria (122	Proteobacteria (2)	Bacteria (1)	99.40	0.50	99.60	98.30	WGS
287.3890	0.356	Pseudomonas aeruginosa (286)	Pseudomonas (135621)	Pseudomonadaceae (7227	Pseudomonadales (1236)	Gammaproteobacteria (122	Proteobacteria (2)	Bacteria (1)	99.40	0.60	99.90	98.70	WGS
287.3891	0.356	Pseudomonas aeruginosa (286)	Pseudomonas (135621)	Pseudomonadaceae (7227	Pseudomonadales (1236)	Gammaproteobacteria (122	Proteobacteria (2)	Bacteria (1)	100.00	0.60	99.90	98.70	WGS
287.3892	0.3548	Pseudomonas aeruginosa (286)	Pseudomonas (135621)	Pseudomonadaceae (7227	Pseudomonadales (1236)	Gammaproteobacteria (122	Proteobacteria (2)	Bacteria (1)	99.70	0.00	99.80	98.80	WGS
287.3893	0.3388	Pseudomonas aeruginosa (286)	Pseudomonas (135621)	Pseudomonadaceae (7227	Pseudomonadales (1236)	Gammaproteobacteria (122	Proteobacteria (2)	Bacteria (1)	100.00	0.00	99.90	99.20	WGS
287.3894	0.3388	Pseudomonas aeruginosa (286)	Pseudomonas (135621)	Pseudomonadaceae (7227	Pseudomonadales (1236)	Gammaproteobacteria (122	Proteobacteria (2)	Bacteria (1)	100.00	0.30	99.90	99.00	WGS
287.3895	0.3662	Pseudomonas aeruginosa (286)	Pseudomonas (135621)	Pseudomonadaceae (7227	Pseudomonadales (1236)	Gammaproteobacteria (122	Proteobacteria (2)	Bacteria (1)	100.00	0.30	99.90	98.60	WGS
287.3896	0.0294	Pseudomonas aeruginosa (286)	Pseudomonas (135621)	Pseudomonadaceae (7227	Pseudomonadales (1236)	Gammaproteobacteria (122	Proteobacteria (2)	Bacteria (1)	100.00	0.00	99.70	98.40	WGS
287.3897	0.3672	Pseudomonas aeruginosa (286)	Pseudomonas (135621)	Pseudomonadaceae (7227	Pseudomonadales (1236)	Gammaproteobacteria (122	Proteobacteria (2)	Bacteria (1)	100.00	0.00	99.90	98.70	WGS
287.3898	0.3336	Pseudomonas aeruginosa (286)	Pseudomonas (135621)	Pseudomonadaceae (7227	Pseudomonadales (1236)	Gammaproteobacteria (122	Proteobacteria (2)	Bacteria (1)	100.00	0.00	99.90	99.20	WGS
287.3899	0.3336	Pseudomonas aeruginosa (286)	Pseudomonas (135621)	Pseudomonadaceae (7227	Pseudomonadales (1236)	Gammaproteobacteria (122	Proteobacteria (2)	Bacteria (1)	100.00	0.00	99.90	99.20	WGS
287.3900	0.3314	Pseudomonas aeruginosa (286)	Pseudomonas (135621)	Pseudomonadaceae (7227	Pseudomonadales (1236)	Gammaproteobacteria (122	Proteobacteria (2)	Bacteria (1)	100.00	0.00	99.90	99.00	WGS
287.3901	0.3254	Pseudomonas aeruginosa (286)	Pseudomonas (135621)	Pseudomonadaceae (7227	Pseudomonadales (1236)	Gammaproteobacteria (122	Proteobacteria (2)	Bacteria (1)	100.00	5.70	99.80	94.80	WGS
287.3902	0.368	Pseudomonas aeruginosa (286)	Pseudomonas (135621)	Pseudomonadaceae (7227	Pseudomonadales (1236)	Gammaproteobacteria (122	Proteobacteria (2)	Bacteria (1)	99.70	0.00	99.70	98.90	WGS
287.3903	0.3314	Pseudomonas aeruginosa (286)	Pseudomonas (135621)	Pseudomonadaceae (7227	Pseudomonadales (1236)	Gammaproteobacteria (122	Proteobacteria (2)	Bacteria (1)	99.40	0.00	99.80	98.90	WGS
287.3904	0.3336	Pseudomonas aeruginosa (286)	Pseudomonas (135621)	Pseudomonadaceae (7227	Pseudomonadales (1236)	Gammaproteobacteria (122	Proteobacteria (2)	Bacteria (1)	100.00	0.30	99.90	98.70	WGS
287.3905	0.3184	Pseudomonas aeruginosa (286)	Pseudomonas (135621)	Pseudomonadaceae (7227	Pseudomonadales (1236)	Gammaproteobacteria (122	Proteobacteria (2)	Bacteria (1)	99.70	0.00	99.80	99.00	WGS
287.3906	0.372	Pseudomonas aeruginosa (286)	Pseudomonas (135621)	Pseudomonadaceae (7227	Pseudomonadales (1236)	Gammaproteobacteria (122	Proteobacteria (2)	Bacteria (1)	100.00	0.40	99.80	99.10	WGS
287.3907	0.3672	Pseudomonas aeruginosa (286)	Pseudomonas (135621)	Pseudomonadaceae (7227	Pseudomonadales (1236)	Gammaproteobacteria (122	Proteobacteria (2)	Bacteria (1)	100.00	0.40	99.70	98.70	WGS
287.3908	0.372	Pseudomonas aeruginosa (286)	Pseudomonas (135621)	Pseudomonadaceae (7227	Pseudomonadales (1236)	Gammaproteobacteria (122	Proteobacteria (2)	Bacteria (1)	100.00	0.30	99.80	99.00	WGS
287.3909	0.372	Pseudomonas aeruginosa (286)	Pseudomonas (135621)	Pseudomonadaceae (7227	Pseudomonadales (1236)	Gammaproteobacteria (122	Proteobacteria (2)	Bacteria (1)	100.00	0.30	99.90	98.70	WGS
287.3910	0.3314	Pseudomonas aeruginosa (286)	Pseudomonas (135621)	Pseudomonadaceae (7227	Pseudomonadales (1236)	Gammaproteobacteria (122	Proteobacteria (2)	Bacteria (1)	100.00	0.10	99.80	99.00	WGS
287.3911	0.3386	Pseudomonas aeruginosa (286)	Pseudomonas (135621)	Pseudomonadaceae (7227	Pseudomonadales (1236)	Gammaproteobacteria (122	Proteobacteria (2)	Bacteria (1)	100.00	0.60	99.90	98.50	WGS
287.3912	0.3174	Pseudomonas aeruginosa (286)	Pseudomonas (135621)	Pseudomonadaceae (7227	Pseudomonadales (1236)	Gammaproteobacteria (122	Proteobacteria (2)	Bacteria (1)	99.70	0.30	99.80	99.00	WGS
287.3913	0.3314	Pseudomonas aeruginosa (286)	Pseudomonas (135621)	Pseudomonadaceae (7227	Pseudomonadales (1236)	Gammaproteobacteria (122	Proteobacteria (2)	Bacteria (1)	99.70	0.30	99.90	98.70	WGS
287.3914	0.3188	Pseudomonas aeruginosa (286)	Pseudomonas (135621)	Pseudomonadaceae (7227	Pseudomonadales (1236)	Gammaproteobacteria (122	Proteobacteria (2)	Bacteria (1)	99.70	0.00	99.80	99.00	WGS
287.3915	0.3188	Pseudomonas aeruginosa (286)	Pseudomonas (135621)	Pseudomonadaceae (7227	Pseudomonadales (1236)	Gammaproteobacteria (122	Proteobacteria (2)	Bacteria (1)	99.70	0.00	99.80	99.00	WGS
287.3916	0.3188	Pseudomonas aeruginosa (286)	Pseudomonas (135621)	Pseudomonadaceae (7227	Pseudomonadales (1236)	Gammaproteobacteria (122	Proteobacteria (2)	Bacteria (1)	99.70	0.30	99.80	99.00	WGS
287.3917	0.3188	Pseudomonas aeruginosa (286)	Pseudomonas (135621)	Pseudomonadaceae (7227	Pseudomonadales (1236)	Gammaproteobacteria (122	Proteobacteria (2)	Bacteria (1)	99.70	0.00	99.80	99.00	WGS
287.3918	0.3188	Pseudomonas aeruginosa (286)	Pseudomonas (135621)	Pseudomonadaceae (7227	Pseudomonadales (1236)	Gammaproteobacteria (122	Proteobacteria (2)	Bacteria (1)	99.70	0.00	99.80	98.80	WGS
287.3919	0.3188	Pseudomonas aeruginosa (286)	Pseudomonas (135621)	Pseudomonadaceae (7227	Pseudomonadales (1236)	Gammaproteobacteria (122	Proteobacteria (2)	Bacteria (1)	99.70	0.00	99.80	99.00	WGS
287.3920	0.3188	Pseudomonas aeruginosa (286)	Pseudomonas (135621)	Pseudomonadaceae (7227	Pseudomonadales (1236)	Gammaproteobacteria (122	Proteobacteria (2)	Bacteria (1)	99.70	0.00	99.80	99.00	WGS
287.3921	0.3174	Pseudomonas aeruginosa (286)	Pseudomonas (135621)	Pseudomonadaceae (7227	Pseudomonadales (1236)	Gammaproteobacteria (122	Proteobacteria (2)	Bacteria (1)	99.70	0.00	99.80	99.00	WGS
287.3922	0.3336	Pseudomonas aeruginosa (286)	Pseudomonas (135621)	Pseudomonadaceae (7227	Pseudomonadales (1236)	Gammaproteobacteria (122	Proteobacteria (2)	Bacteria (1)	100.00	0.30	99.90	98.80	WGS
287.3923	0.3388	Pseudomonas aeruginosa (286)	Pseudomonas (135621)	Pseudomonadaceae (7227	Pseudomonadales (1236)	Gammaproteobacteria (122	Proteobacteria (2)	Bacteria (1)	100.00	0.00	99.90	98.90	WGS
287.3924	0.3174	Pseudomonas aeruginosa (286)	Pseudomonas (135621)	Pseudomonadaceae (7227	Pseudomonadales (1236)	Gammaproteobacteria (122	Proteobacteria (2)	Bacteria (1)	99.70	0.30	99.80	99.00	WGS
287.3925	0.3636	Pseudomonas aeruginosa (286)	Pseudomonas (135621)	Pseudomonadaceae (7227	Pseudomonadales (1236)	Gammaproteobacteria (122	Proteobacteria (2)	Bacteria (1)	100.00	0.30	99.90	98.50	WGS
287.3926	0.3314	Pseudomonas aeruginosa (286)	Pseudomonas (135621)	Pseudomonadaceae (7227	Pseudomonadales (1236)	Gammaproteobacteria (122	Proteobacteria (2)	Bacteria (1)	100.00	0.00	99.80	99.00	WGS
287.3927	0.3636	Pseudomonas aeruginosa (286)	Pseudomonas (135621)	Pseudomonadaceae (7227	Pseudomonadales (1236)	Gammaproteobacteria (122	Proteobacteria (2)	Bacteria (1)	100.00	0.30	99.90	98.90	WGS
287.3928	0.3738	Pseudomonas aeruginosa (286)	Pseudomonas (135621)	Pseudomonadaceae (7227	Pseudomonadales (1236)	Gammaproteobacteria (122	Proteobacteria (2)	Bacteria (1)	99.70	0.40	99.60	98.70	WGS
287.3929	0.3618	Pseudomonas aeruginosa (286)	Pseudomonas (135621)	Pseudomonadaceae (7227	Pseudomonadales (1236)	Gammaproteobacteria (122	Proteobacteria (2)	Bacteria (1)	100.00	0.90	99.90	98.40	WGS
287.3930	0.0294	Pseudomonas aeruginosa (286)	Pseudomonas (135621)	Pseudomonadaceae (7227	Pseudomonadales (1236)	Gammaproteobacteria (122	Proteobacteria (2)	Bacteria (1)	100.00	0.00	99.90	98.70	WGS
287.3931	0.3672	Pseudomonas aeruginosa (286)	Pseudomonas (135621)	Pseudomonadaceae (7227	Pseudomonadales (1236)	Gammaproteobacteria (122	Proteobacteria (2)	Bacteria (1)	100.00	0.00	99.90	98.50	WGS
287.3932	0.3336	Pseudomonas aeruginosa (286)	Pseudomonas (135621)	Pseudomonadaceae (7227	Pseudomonadales (1236)	Gammaproteobacteria (122	Proteobacteria (2)	Bacteria (1)	100.00	0.00	99.90	99.20	WGS
287.3933	0.3314	Pseudomonas aeruginosa (286)	Pseudomonas (135621)	Pseudomonadaceae (7227	Pseudomonadales (1236)	Gammaproteobacteria (122	Proteobacteria (2)	Bacteria (1)	100.00	0.30	99.80	98.20	WGS
287.3934	0.3578	Pseudomonas aeruginosa (286)	Pseudomonas (135621)	Pseudomonadaceae (7227	Pseudomonadales (1236)	Gammaproteobacteria (122	Proteobacteria (2)	Bacteria (1)	100.00	5.30	99.80	94.60	WGS
287.3935	0.3188	Pseudomonas aeruginosa (286)	Pseudomonas (135621)	Pseudomonadaceae (7227	Pseudomonadales (1236)	Gammaproteobacteria (122	Proteobacteria (2)	Bacteria (1)	99.70	0.00	99.80	98.90	WGS
287.3936	0.3628	Pseudomonas aeruginosa (286)	Pseudomonas (135621)	Pseudomonadaceae (7227	Pseudomonadales (1236)	Gammaproteobacteria (122	Proteobacteria (2)	Bacteria (1)	100.00	0.60	99.90	98.70	WGS
287.3937	0.3388	Pseudomonas aeruginosa (286)	Pseudomonas (135621)	Pseudomonadaceae (7227	Pseudomonadales (1236)	Gammaproteobacteria (122	Proteobacteria (2)	Bacteria (1)	100.00	0.00	99.90	99.00	WGS
287.3938	0.3336	Pseudomonas aeruginosa (286)	Pseudomonas (135621)	Pseudomonadaceae (7227	Pseudomonadales (1236)	Gammaproteobacteria (122	Proteobacteria (2)	Bacteria (1)	100.00	0.00	99.90	99.00	WGS
287.3939	0.3636	Pseudomonas aeruginosa (286)	Pseudomonas (135621)	Pseudomonadaceae (7227	Pseudomonadales (1236)	Gammaproteobacteria (122	Proteobacteria (2)	Bacteria (1)	100.00	0.30	99.90	98.90	WGS
287.3940	0.3662	Pseudomonas aeruginosa (286)	Pseudomonas (135621)	Pseudomonadaceae (7227	Pseudomonadales (1236)	Gammaproteobacteria (122	Proteobacteria (2)	Bacteria (1)	100.00	0.20	99.70	98.60	WGS
287.3967	0.3604	Pseudomonas aeruginosa (286)	Pseudomonas (135621)	Pseudomonadaceae (7227	Pseudomonadales (1236)	Gammaproteobacteria (122	Proteobacteria (2)	Bacteria (1)	100.00	0.00	99.70	99.40	WGS
287.4036	0.3498	Pseudomonas aeruginosa (286)	Pseudomonas (135621)	Pseudomonadaceae (7227	Pseudomonadales (1236)	Gammaproteobacteria (122	Proteobacteria (2)	Bacteria (1)	99.70	0.30	99.60	98.50	WGS
287.4037	0.3354	Pseudomonas aeruginosa (286)	Pseudomonas (135621)	Pseudomonadaceae (7227	Pseudomonadales (1236)	Gammaproteobacteria (122	Proteobacteria (2)	Bacteria (1)	99.70	4.60	99.20	95.60	WGS
287.4038	0.3292	Pseudomonas aeruginosa (286)	Pseudomonas (135621)	Pseudomonadaceae (7227	Pseudomonadales (1236)	Gammaproteobacteria (122	Proteobacteria (2)	Bacteria (1)	98.80	0.60	99.90	98.70	WGS
287.4039	0.3662	Pseudomonas aeruginosa (286)	Pseudomonas (135621)	Pseudomonadaceae (7227	Pseudomonadales (1236)	Gammaproteobacteria (122	Proteobacteria (2)	Bacteria (1)	100.00	0.60	99.90	98.40	WGS
287.4040	0.3318	Pseudomonas aeruginosa (286)	Pseudomonas (135621)	Pseudomonadaceae (7227	Pseudomonadales (1236)	Gammaproteobacteria (122	Proteobacteria (2)	Bacteria (1)	100.00	2.10	99.60	98.40	WGS
287.4041	0.3226	Pseudomonas aeruginosa (286)	Pseudomonas (135621)	Pseudomonadaceae (7227	Pseudomonadales (1236)	Gammaproteobacteria (122	Proteobacteria (2)	Bacteria (1)	99.70	4.70	99.70	94.10	WGS
287.4042	0.3314	Pseudomonas aeruginosa (286)	Pseudomonas (135621)	Pseudomonadaceae (7227	Pseudomonadales (1236)	Gammaproteobacteria (122	Proteobacteria (2)	Bacteria (1)	100.00	0.30	99.80	98.20	WGS
287.4043	0.0294	Pseudomonas aeruginosa (286)	Pseudomonas (135621)	Pseudomonadaceae (7227	Pseudomonadales (1236)	Gammaproteobacteria (122	Proteobacteria (2)	Bacteria (1)	100.00	0.90	99.60	98.30	WGS
287.4044	0.3268	Pseudomonas aeruginosa (286)	Pseudomonas (135621)	Pseudomonadaceae (7227	Pseudomonadales (1236)	Gammaproteobacteria (122	Proteobacteria (2)	Bacteria (1)	100.00	1.50	99.60	95.20	WGS
287.4045	0.0294	Pseudomonas aeruginosa (286)	Pseudomonas (135621)	Pseudomonadaceae (7227	Pseudomonadales (1236)	Gammaproteobacteria (122	Proteobacteria (2)	Bacteria (1)	99.70	15.30	99.90	87.00	WGS
287.4046	0.324	Pseudomonas aeruginosa (286)	Pseudomonas (135621)	Pseudomonadaceae (7227	Pseudomonadales (1236)	Gammaproteobacteria (122	Proteobacteria (2)	Bacteria (1)	100.00	2.10	99.60	97.30	WGS
287.4047	0.3226	Pseudomonas aeruginosa (286)	Pseudomonas (135621)	Pseudomonadaceae (7227	Pseudomonadales (1236)	Gammaproteobacteria (122	Proteobacteria (2)	Bacteria (1)	100.00	0.70	99.70	98.30	WGS
287.4048	0.3636	Pseudomonas aeruginosa (286)	Pseudomonas (135621)	Pseudomonadaceae (7227	Pseudomonadales (1236)	Gammaproteobacteria (122	Proteobacteria (2)	Bacteria (1)	100.00	0.00	99.90	98.80	WGS
287.4049	0.0294	Pseudomonas aeruginosa (286)	Pseudomonas (135621)	Pseudomonadaceae (7227	Pseudomonadales (1236)	Gammaproteobacteria (122	Proteobacteria (2)	Bacteria (1)	98.80	0.30	99.60	98.80	WGS
287.4050	0.325	Pseudomonas aeruginosa (286)	Pseudomonas (135621)	Pseudomonadaceae (7227	Pseudomonadales (1236)	Gammaproteobacteria (122	Proteobacteria (2)	Bacteria (1)	100.00	0.30	99.80	99.00	Complete
287.4051	0.3672	Pseudomonas aeruginosa (286)	Pseudomonas (135621)	Pseudomonadaceae (7227	Pseudomonadales (1236)	Gammaproteobacteria (122	Proteobacteria (2)	Bacteria (1)	99.40	0.90	99.80	97.50	Complete
287.4052	0.3704	Pseudomonas aeruginosa (286)	Pseudomonas (135621)	Pseudomonadaceae (7227	Pseudomonadales (1236)	Gammaproteobacteria (122	Proteobacteria (2)	Bacteria (1)	100.00	0.00	99.90	97.20	WGS
287.4053	0.3704	Pseudomonas aeruginosa (286)	Pseudomonas (135621)	Pseudomonadaceae (7227	Pseudomonadales (1236)	Gammaproteobacteria (122	Proteobacteria (2)	Bacteria (1)	99.40	0.60	99.90	98.70	WGS
287.4054	0.3298	Pseudomonas aeruginosa (286)	Pseudomonas (135621)	Pseudomonadaceae (7227	Pseudomonadales (1236)	Gammaproteobacteria (122	Proteobacteria (2)	Bacteria (1)	100.00	0.30	99.80	99.00	WGS
287.4055	0.3604	Pseudomonas aeruginosa (286)	Pseudomonas (135621)	Pseudomonadaceae (7227	Pseudomonadales (1236)	Gammaproteobacteria (122	Proteobacteria (2)	Bacteria (1)	100.00	0.30	99.90	99.40	WGS
287.4056	0.3704	Pseudomonas aeruginosa (286)	Pseudomonas (135621)	Pseudomonadaceae (7227	Pseudomonadales (1236)	Gammaproteobacteria (122	Proteobacteria (2)	Bacteria (1)	100.00	0.60	99.80	98.90	WGS
287.4057	0.331	Pseudomonas aeruginosa (286)	Pseudomonas (135621)	Pseudomonadaceae (7227	Pseudomonadales (1236)	Gammaproteobacteria (122	Proteobacteria (2)	Bacteria (1)	100.00	0.00	99.90	98.40	WGS
287.4058	0.3236	Pseudomonas aeruginosa (286)	Pseudomonas (135621)	Pseudomonadaceae (7227	Pseudomonadales (1236)	Gammaproteobacteria (122	Proteobacteria (2)	Bacteria (1)	100.00	0.00	99.70	99.20	WGS
287.4059	0.3604	Pseudomonas aeruginosa (286)	Pseudomonas (135621)	Pseudomonadaceae (7227	Pseudomonadales (1236)	Gammaproteobacteria (122	Proteobacteria (2)	Bacteria (1)	100.00	0.00	99.80	99.50	WGS
287.4060	0.3236	Pseudomonas aeruginosa (286)	Pseudomonas (135621)	Pseudomonadaceae (7227	Pseudomonadales (1236)	Gammaproteobacteria (122	Proteobacteria (2)	Bacteria (1)	100.00	0.00	99.70	99.20	WGS
287.4061	0.369	Pseudomonas aeruginosa (286)	Pseudomonas (135621)	Pseudomonadaceae (7227	Pseudomonadales (1236)	Gammaproteobacteria (122	Proteobacteria (2)	Bacteria (1)	100.00	0.70	99.60	98.70	WGS
287.4062	0.3376	Pseudomonas aeruginosa (286)	Pseudomonas (135621)	Pseudomonadaceae (7227	Pseudomonadales (1236)	Gammaproteobacteria (122	Proteobacteria (2)	Bacteria (1)	100.00	2.10	99.60	98.20	WGS
287.4063	0.3748	Pseudomonas aeruginosa (286)	Pseudomonas (135621)	Pseudomonadaceae (7227	Pseudomonadales (1236)	Gammaproteobacteria (122	Proteobacteria (2)	Bacteria (1)	100.00	0.30	99.80	98.10	WGS
287.4064	0.385	Pseudomonas aeruginosa (286)	Pseudomonas (135621)	Pseudomonadaceae (7227	Pseudomonadales (1236)	Gammaproteobacteria (122	Proteobacteria (2)	Bacteria (1)	100.00	0.00	99.90	98.30	WGS
287.4065	0.3226	Pseudomonas aeruginosa (286)	Pseudomonas (135621)	Pseudomonadaceae (7227	Pseudomonadales (1236)	Gammaproteobacteria (122	Proteobacteria (2)	Bacteria (1)	100.00	0.00	99.60	98.70	WGS
287.4066	0.3346	Pseudomonas aeruginosa (286)	Pseudomonas (135621)	Pseudomonadaceae (7227	Pseudomonadales (1236)	Gammaproteobacteria (122	Proteobacteria (2)	Bacteria (1)	100.00	0.00	99.90	98.10	WGS
287.4067	0.3628	Pseudomonas aeruginosa (286)	Pseudomonas (135621)	Pseudomonadaceae (7227	Pseudomonadales (1236)	Gammaproteobacteria (122	Proteobacteria (2)	Bacteria (1)	100.00	0.00	99.90	98.70	WGS
287.4068	0.3258	Pseudomonas aeruginosa (286)	Pseudomonas (135621)	Pseudomonadaceae (7227	Pseudomonadales (1236)	Gammaproteobacteria (122	Proteobacteria (2)	Bacteria (1)	100.00	0.00	99.90	99.00	WGS
287.4069	0.3346	Pseudomonas aeruginosa (286)	Pseudomonas (135621)	Pseudomonadaceae (7227	Pseudomonadales (1236)	Gammaproteobacteria (122	Proteobacteria (2)	Bacteria (1)	100.00	0.00	99.90	98.80	WGS
287.4070	0.3618	Pseudomonas aeruginosa (286)	Pseudomonas (135621)	Pseudomonadaceae (7227	Pseudomonadales (1236)	Gammaproteobacteria (122	Proteobacteria (2)	Bacteria (1)	100.00	0.00	99.90	99.00	WGS
287.4071	0.3236	Pseudomonas aeruginosa (286)	Pseudomonas (135621)	Pseudomonadaceae (7227	Pseudomonadales (1236)	Gammaproteobacteria (122	Proteobacteria (2)	Bacteria (1)	100.00	0.00	99.60	99.00	WGS
287.4072	0.3694	Pseudomonas aeruginosa (286)	Pseudomonas (135621)	Pseudomonadaceae (7227	Pseudomonadales (1236)	Gammaproteobacteria (122	Proteobacteria (2)	Bacteria (1)	100.00	0.00	99.70	98.70	WGS
287.4073	0.3174	Pseudomonas aeruginosa (286)	Pseudomonas (135621)	Pseudomonadaceae (7227	Pseudomonadales (1236)	Gammaproteobacteria (122	Proteobacteria (2)	Bacteria (1)	100.00	0.00	99.80	99.10	WGS
287.4074	0.3636	Pseudomonas aeruginosa (286)	Pseudomonas (135621)	Pseudomonadaceae (7227	Pseudomonadales (1236)	Gammaproteobacteria (122	Proteobacteria (2)	Bacteria (1)	100.00	0.30	99.80	99.00	WGS
287.4075	0.3388	Pseudomonas aeruginosa (286)	Pseudomonas (135621)	Pseudomonadaceae (7227	Pseudomonadales (1236)	Gammaproteobacteria (122	Proteobacteria (2)	Bacteria (1)	100.00	0.30	99.90	98.80	WGS
287.4076	0.3226	Pseudomonas aeruginosa (286)	Pseudomonas (135621)	Pseudomonadaceae (7227	Pseudomonadales (1236)	Gammaproteobacteria (122	Proteobacteria (2)	Bacteria (1)	100.00	0.30	99.90	99.10	WGS
287.4077	0.3268	Pseudomonas aeruginosa (286)	Pseudomonas (135621)	Pseudomonadaceae (7227	Pseudomonadales (1236)	Gammaproteobacteria (122	Proteobacteria (2)	Bacteria (1)	98.80	0.90	99.70	98.50	WGS
287.4078	0.3292	Pseudomonas aeruginosa (286)	Pseudomonas (135621)	Pseudomonadaceae (7227	Pseudomonadales (1236)	Gammaproteobacteria (122	Proteobacteria (2)	Bacteria (1)	100.00	0.30	100.00	98.40	WGS
287.4079	0.3578	Pseudomonas aeruginosa (286)	Pseudomonas (135621)	Pseudomonadaceae (7227	Pseudomonadales (1236)	Gammaproteobacteria (122	Proteobacteria (2)	Bacteria (1)	100.00	0.30	99.90	98.90	WGS
287.4080	0.3578	Pseudomonas aeruginosa (286)	Pseudomonas (135621)	Pseudomonadaceae (7227	Pseudomonadales (1236)	Gammaproteobacteria (122	Proteobacteria (2)	Bacteria (1)	100.00	0.90	99.90	97.90	WGS
287.4081	0.3636	Pseudomonas aeruginosa (286)	Pseudomonas (135621)	Pseudomonadaceae (7227	Pseudomonadales (1236)	Gammaproteobacteria (122	Proteobacteria (2)	Bacteria (1)	100.00	0.00	99.50	98.60	WGS
287.4082	0.3646	Pseudomonas aeruginosa (286)	Pseudomonas (135621)	Pseudomonadaceae (7227	Pseudomonadales (1236)	Gammaproteobacteria (122	Proteobacteria (2)	Bacteria (1)	100.00	0.30	99.50	98.10	WGS
287.4083	0.3578	Pseudomonas aeruginosa (286)	Pseudomonas (135621)	Pseudomonadaceae (7227	Pseudomonadales (1236)	Gammaproteobacteria (122	Proteobacteria (2)	Bacteria (1)	100.00	0.30	99.90	98.70	WGS
287.4084	0.3578	Pseudomonas aeruginosa (286)	Pseudomonas (135621)	Pseudomonadaceae (7227	Pseudomonadales (1236)	Gammaproteobacteria (122	Proteobacteria (2)	Bacteria (1)	100.00	0.90	99.90	98.10	WGS
287.4085	0.3626	Pseudomonas aeruginosa (286)	Pseudomonas (135621)	Pseudomonadaceae (7227	Pseudomonadales (1236)	Gammaproteobacteria (122	Proteobacteria (2)	Bacteria (1)	100.00	0.60	99.50	98.20	WGS
287.4086	0.3622	Pseudomonas aeruginosa (286)	Pseudomonas (135621)	Pseudomonadaceae (7227	Pseudomonadales (1236)	Gammaproteobacteria (122	Proteobacteria (2)	Bacteria (1)	100.00	0.00	99.60	98.40	WGS
287.4087	0.3578	Pseudomonas aeruginosa (286)	Pseudomonas (135621)	Pseudomonadaceae (7227	Pseudomonadales (1236)	Gammaproteobacteria (122	Proteobacteria (2)	Bacteria (1)	100.00	0.00	99.90	98.60	WGS
287.4088	0.3622	Pseudomonas aeruginosa (286)	Pseudomonas (135621)	Pseudomonadaceae (7227	Pseudomonadales (1236)	Gammaproteobacteria (122	Proteobacteria (2)	Bacteria (1)	100.00	0.30	99.70	98.40	WGS
287.4089	0.3324	Pseudomonas aeruginosa (286)	Pseudomonas (135621)	Pseudomonadaceae (7227	Pseudomonadales (1236)	Gammaproteobacteria (122	Proteobacteria (2)	Bacteria (1)	100.00	0.00	99.80	99.30	WGS
287.4090	0.3306	Pseudomonas aeruginosa (286)	Pseudomonas (135621)	Pseudomonadaceae (7227	Pseudomonadales (1236)	Gammaproteobacteria (122	Proteobacteria (2)	Bacteria (1)	100.00	0.00	99.80	98.80	Complete
287.4091	0.325	Pseudomonas aeruginosa (286)	Pseudomonas (135621)	Pseudomonadaceae (7227	Pseudomonadales (1236)	Gammaproteobacteria (122	Proteobacteria (2)	Bacteria (1)	100.00	3.20	99.80	98.20	Complete
287.4092	0.3322	Pseudomonas aeruginosa (286)	Pseudomonas (135621)	Pseudomonadaceae (7227	Pseudomonadales (1236)	Gammaproteobacteria (122	Proteobacteria (2)	Bacteria (1)	99.40	2.20	99.60	97.20	Complete
287.4093	0.0294	Pseudomonas aeruginosa (286)	Pseudomonas (135621)	Pseudomonadaceae (7227	Pseudomonadales (1236)	Gammaproteobacteria (122	Proteobacteria (2)	Bacteria (1)	100.00	2.60	99.70	98.60	WGS
287.4094	0.3576	Pseudomonas aeruginosa (286)	Pseudomonas (135621)	Pseudomonadaceae (7227	Pseudomonadales (1236)	Gammaproteobacteria (122	Proteobacteria (2)	Bacteria (1)	100.00	3.30	99.70	97.10	Complete
287.4095	0.3614	Pseudomonas aeruginosa (286)	Pseudomonas (135621)	Pseudomonadaceae (7227	Pseudomonadales (1236)	Gammaproteobacteria (122	Proteobacteria (2)	Bacteria (1)	100.00	1.50	99.80	98.70	WGS
287.4096	0.3704	Pseudomonas aeruginosa (286)	Pseudomonas (135621)	Pseudomonadaceae (7227	Pseudomonadales (1236)	Gammaproteobacteria (122	Proteobacteria (2)	Bacteria (1)	100.00	0.00	99.80	99.20	WGS
287.4097	0.3258	Pseudomonas aeruginosa (286)	Pseudomonas (135621)	Pseudomonadaceae (7227	Pseudomonadales (1236)	Gammaproteobacteria (122	Proteobacteria (2)	Bacteria (1)	100.00	0.00	99.80	99.30	WGS
287.4098	0.368	Pseudomonas aeruginosa (286)	Pseudomonas (135621)	Pseudomonadaceae (7227	Pseudomonadales (1236)	Gammaproteobacteria (122	Proteobacteria (2)	Bacteria (1)	100.00	0.30	99.90	99.30	WGS
287.4099	0.3628	Pseudomonas aeruginosa (286)	Pseudomonas (135621)	Pseudomonadaceae (7227	Pseudomonadales (1236)	Gammaproteobacteria (122	Proteobacteria (2)	Bacteria (1)	100.00	0.00	99.80	99.30	WGS
287.4100	0.3302	Pseudomonas aeruginosa (286)	Pseudomonas (135621)	Pseudomonadaceae (7227	Pseudomonadales (1236)	Gammaproteobacteria (122	Proteobacteria (2)	Bacteria (1)	100.00	0.60	99.90	98.60	WGS
287.4102	0.368	Pseudomonas aeruginosa (286)	Pseudomonas (135621)	Pseudomonadaceae (7227	Pseudomonadales (1236)	Gammaproteobacteria (122	Proteobacteria (2)	Bacteria (1)	100.00	0.30	99.90	99.50	WGS
287.4103	0.3748	Pseudomonas aeruginosa (286)	Pseudomonas (135621)	Pseudomonadaceae (7227	Pseudomonadales (1236)	Gammaproteobacteria (122	Proteobacteria (2)	Bacteria (1)	100.00	0.00	99.80	99.40	WGS
287.4104	0.3302	Pseudomonas aeruginosa (286)	Pseudomonas (135621)	Pseudomonadaceae (7227	Pseudomonadales (1236)	Gammaproteobacteria (122	Proteobacteria (2)	Bacteria (1)	100.00	0.60	99.60	98.60	WGS
287.4105	0.368	Pseudomonas aeruginosa (286)	Pseudomonas (135621)	Pseudomonadaceae (7227	Pseudomonadales (1236)	Gammaproteobacteria (122	Proteobacteria (2)	Bacteria (1)	100.00	0.30	99.90	99.50	WGS
287.4106	0.3636	Pseudomonas aeruginosa (286)	Pseudomonas (135621)	Pseudomonadaceae (7227	Pseudomonadales (1236)	Gammaproteobacteria (122	Proteobacteria (2)	Bacteria (1)	100.00	0.00	99.90	99.40	WGS
287.4258	0.3324	Pseudomonas aeruginosa (286)	Pseudomonas (135621)	Pseudomonadaceae (7227	Pseudomonadales (1236)	Gammaproteobacteria (122	Proteobacteria (2)	Bacteria (1)	99.40	0.90	99.60	98.40	WGS
287.4259	0.3694	Pseudomonas aeruginosa (286)	Pseudomonas (135621)	Pseudomonadaceae (7227	Pseudomonadales (1236)	Gammaproteobacteria (122	Proteobacteria (2)	Bacteria (1)	100.00	0.00	99.90	99.50	WGS
287.4260	0.331	Pseudomonas aeruginosa (286)	Pseudomonas (135621)	Pseudomonadaceae (7227	Pseudomonadales (1236)	Gammaproteobacteria (122	Proteobacteria (2)	Bacteria (1)	99.70	0.00	99.80	99.30	WGS
287.4261	0.3636	Pseudomonas aeruginosa (286)	Pseudomonas (135621)	Pseudomonadaceae (7227	Pseudomonadales (1236)	Gammaproteobacteria (122	Proteobacteria (2)	Bacteria (1)	100.00	0.00	99.90	99.70	WGS
287.4262	0.3694	Pseudomonas aeruginosa (286)	Pseudomonas (135621)	Pseudomonadaceae (7227	Pseudomonadales (1236)	Gammaproteobacteria (122	Proteobacteria (2)	Bacteria (1)	100.00	0.30	99.90	99.30	WGS
287.4263	0.327	Pseudomonas aeruginosa (286)	Pseudomonas (135621)	Pseudomonadaceae (7227	Pseudomonadales (1236)	Gammaproteobacteria (122	Proteobacteria (2)	Bacteria (1)	100.00	0.00	99.90	99.60	WGS
287.4264	0.3334	Pseudomonas aeruginosa (286)	Pseudomonas (135621)	Pseudomonadaceae (7227	Pseudomonadales (1236)	Gammaproteobacteria (122	Proteobacteria (2)	Bacteria (1)	99.40	0.00	99.90	99.80	WGS
287.4265	0.3436	Pseudomonas aeruginosa (286)	Pseudomonas (135621)	Pseudomonadaceae (7227	Pseudomonadales (1236)	Gammaproteobacteria (122	Proteobacteria (2)	Bacteria (1)	97.90	0.60	99.50	98.50	WGS
287.4266	0.331	Pseudomonas aeruginosa (286)	Pseudomonas (135621)	Pseudomonadaceae (7227	Pseudomonadales (1236)	Gammaproteobacteria (122	Proteobacteria (2)	Bacteria (1)	100.00	0.00	100.00	99.90	WGS
287.4267	0.3334	Pseudomonas aeruginosa (286)	Pseudomonas (135621)	Pseudomonadaceae (7227	Pseudomonadales (1236)	Gammaproteobacteria (122	Proteobacteria (2)	Bacteria (1)	100.00	0.00	99.80	99.50	WGS
287.4268	0.3026	Pseudomonas aeruginosa (286)	Pseudomonas (135621)	Pseudomonadaceae (7227	Pseudomonadales (1236)	Gammaproteobacteria (122	Proteobacteria (2)	Bacteria (1)	99.40	0.00	99.90	98.90	WGS
287.4269	0.3174	Pseudomonas aeruginosa (286)	Pseudomonas (135621)	Pseudomonadaceae (7227	Pseudomonadales (1236)	Gammaproteobacteria (122	Proteobacteria (2)	Bacteria (1)	100.00	0.00	99.90	99.20	WGS
287.4270	0.373	Pseudomonas aeruginosa (286)	Pseudomonas (135621)	Pseudomonadaceae (7227	Pseudomonadales (1236)	Gammaproteobacteria (122	Proteobacteria (2)	Bacteria (1)	97.00	0.00	98.40	97.60	WGS
287.4271	0.3662	Pseudomonas aeruginosa (286)	Pseudomonas (135621)	Pseudomonadaceae (7227	Pseudomonadales (1236)	Gammaproteobacteria (122	Proteobacteria (2)	Bacteria (1)	100.00	0.00	99.90	99.30	WGS
287.4272	0.331	Pseudomonas aeruginosa (286)	Pseudomonas (135621)	Pseudomonadaceae (7227	Pseudomonadales (1236)	Gammaproteobacteria (122	Proteobacteria (2)	Bacteria (1)	100.00	0.00	99.70	99.20	WGS
287.4273	0.3628	Pseudomonas aeruginosa (286)	Pseudomonas (135621)	Pseudomonadaceae (7227	Pseudomonadales (1236)	Gammaproteobacteria (122	Proteobacteria (2)	Bacteria (1)	99.70	0.30	99.90	98.60	WGS
287.4274	0.3614	Pseudomonas aeruginosa (286)	Pseudomonas (135621)	Pseudomonadaceae (7227	Pseudomonadales (1236)	Gammaproteobacteria (122	Proteobacteria (2)	Bacteria (1)	100.00	0.60	99.60	98.70	WGS
287.4275	0.327	Pseudomonas aeruginosa (286)	Pseudomonas (135621)	Pseudomonadaceae (7227	Pseudomonadales (1236)	Gammaproteobacteria (122	Proteobacteria (2)	Bacteria (1)	100.00	0.60	99.80	99.40	WGS
287.4276	0.3258	Pseudomonas aeruginosa (286)	Pseudomonas (135621)	Pseudomonadaceae (7227	Pseudomonadales (1236)	Gammaproteobacteria (122	Proteobacteria (2)	Bacteria (1)	100.00	0.00	99.90	99.90	WGS
287.4277	0.0294	Pseudomonas aeruginosa (286)	Pseudomonas (135621)	Pseudomonadaceae (7227	Pseudomonadales (1236)	Gammaproteobacteria (122	Proteobacteria (2)	Bacteria (1)	100.00	0.00	99.80	99.40	WGS
287.4283	0.331	Pseudomonas aeruginosa (286)	Pseudomonas (135621)	Pseudomonadaceae (7227	Pseudomonadales (1236)	Gammaproteobacteria (122	Proteobacteria (2)	Bacteria (1)	100.00	1.50	99.80	98.30	WGS
287.4284	0.0294	Pseudomonas aeruginosa (286)	Pseudomonas (135621)	Pseudomonadaceae (7227	Pseudomonadales (1236)	Gammaproteobacteria (122	Proteobacteria (2)	Bacteria (1)	100.00	2.30	99.60	95.90	WGS
287.4285	0.3268	Pseudomonas aeruginosa (286)	Pseudomonas (135621)	Pseudomonadaceae (7227	Pseudomonadales (1236)	Gammaproteobacteria (122	Proteobacteria (2)	Bacteria (1)	100.00	1.00	99.80	97.40	WGS
287.4286	0.0294	Pseudomonas aeruginosa (286)	Pseudomonas (135621)	Pseudomonadaceae (7227	Pseudomonadales (1236)	Gammaproteobacteria (122	Proteobacteria (2)	Bacteria (1)	100.00	6.10	99.70	93.70	WGS
287.4287	0.332	Pseudomonas aeruginosa (286)	Pseudomonas (135621)	Pseudomonadaceae (7227	Pseudomonadales (1236)	Gammaproteobacteria (122	Proteobacteria (2)	Bacteria (1)	100.00	3.30	99.80	96.30	WGS
287.4288	0.3306	Pseudomonas aeruginosa (286)	Pseudomonas (135621)	Pseudomonadaceae (7227	Pseudomonadales (1236)	Gammaproteobacteria (122	Proteobacteria (2)	Bacteria (1)	100.00	0.30	99.80	99.00	WGS
287.4289	0.3292	Pseudomonas aeruginosa (286)	Pseudomonas (135621)	Pseudomonadaceae (7227	Pseudomonadales (1236)	Gammaproteobacteria (122	Proteobacteria (2)	Bacteria (1)	100.00	0.60	99.60	98.90	WGS
287.4290	0.3376	Pseudomonas aeruginosa (286)	Pseudomonas (135621)	Pseudomonadaceae (7227	Pseudomonadales (1236)	Gammaproteobacteria (122	Proteobacteria (2)	Bacteria (1)	100.00	0.90	99.90	98.70	WGS
287.4291	0.3292	Pseudomonas aeruginosa (286)	Pseudomonas (135621)	Pseudomonadaceae (7227	Pseudomonadales (1236)	Gammaproteobacteria (122	Proteobacteria (2)	Bacteria (1)	100.00	0.30	99.70	99.00	WGS
287.4292	0.3292	Pseudomonas aeruginosa (286)	Pseudomonas (135621)	Pseudomonadaceae (7227	Pseudomonadales (1236)	Gammaproteobacteria (122	Proteobacteria (2)	Bacteria (1)	100.00	0.30	99.70	99.10	WGS
287.4293	0.331	Pseudomonas aeruginosa (286)	Pseudomonas (135621)	Pseudomonadaceae (7227	Pseudomonadales (1236)	Gammaproteobacteria (122	Proteobacteria (2)	Bacteria (1)	100.00	0.00	99.90	99.50	WGS
287.4294	0.0294	Pseudomonas aeruginosa (286)	Pseudomonas (135621)	Pseudomonadaceae (7227	Pseudomonadales (1236)	Gammaproteobacteria (122	Proteobacteria (2)	Bacteria (1)	100.00	0.30	99.90	98.90	WGS
287.4295	0.3174	Pseudomonas aeruginosa (286)	Pseudomonas (135621)	Pseudomonadaceae (7227	Pseudomonadales (1236)	Gammaproteobacteria (122	Proteobacteria (2)	Bacteria (1)	100.00	0.00	99.80	99.30	WGS
287.4296	0.3306	Pseudomonas aeruginosa (286)	Pseudomonas (135621)	Pseudomonadaceae (7227	Pseudomonadales (1236)	Gammaproteobacteria (122	Proteobacteria (2)	Bacteria (1)	100.00	0.30	99.80	99.00	WGS
287.4297	0.368	Pseudomonas aeruginosa (286)	Pseudomonas (135621)	Pseudomonadaceae (7227	Pseudomonadales (1236)	Gammaproteobacteria (122	Proteobacteria (2)	Bacteria (1)	99.70	0.70	99.90	98.70	WGS
287.4298	0.371	Pseudomonas aeruginosa (286)	Pseudomonas (135621)	Pseudomonadaceae (7227	Pseudomonadales (1236)	Gammaproteobacteria (122	Proteobacteria (2)	Bacteria (1)	100.00	0.00	99.80	99.30	WGS
287.4299	0.3694	Pseudomonas aeruginosa (286)	Pseudomonas (135621)	Pseudomonadaceae (7227	Pseudomonadales (1236)	Gammaproteobacteria (122	Proteobacteria (2)	Bacteria (1)	100.00	0.00	99.90	99.20	WGS
287.4300	0.327	Pseudomonas aeruginosa (286)	Pseudomonas (135621)	Pseudomonadaceae (7227	Pseudomonadales (1236)	Gammaproteobacteria (122	Proteobacteria (2)	Bacteria (1)	100.00	0.00	99.90	99.60	WGS
287.4301	0.3334	Pseudomonas aeruginosa (286)	Pseudomonas (135621)	Pseudomonadaceae (7227	Pseudomonadales (1236)	Gammaproteobacteria (122	Proteobacteria (2)	Bacteria (1)	100.00	0.00	99.80	99.30	WGS
287.4302	0.3338	Pseudomonas aeruginosa (286)	Pseudomonas (135621)	Pseudomonadaceae (7227	Pseudomonadales (1236)	Gammaproteobacteria (122	Proteobacteria (2)	Bacteria (1)	99.40	1.80	99.70	97.60	WGS
287.4303	0.3302	Pseudomonas aeruginosa (286)	Pseudomonas (135621)	Pseudomonadaceae (7227	Pseudomonadales (1236)	Gammaproteobacteria (122	Proteobacteria (2)	Bacteria (1)	100.00	0.30	99.90	98.40	WGS
287.4304	0.3178	Pseudomonas aeruginosa (286)	Pseudomonas (135621)	Pseudomonadaceae (7227	Pseudomonadales (1236)	Gammaproteobacteria (122	Proteobacteria (2)	Bacteria (1)	99.10	9.60	99.20	90.70	WGS
287.4305	0.3694	Pseudomonas aeruginosa (286)	Pseudomonas (135621)	Pseudomonadaceae (7227	Pseudomonadales (1236)	Gammaproteobacteria (122	Proteobacteria (2)	Bacteria (1)	99.40	0.00	98.30	97.60	WGS
287.4306	0.3428	Pseudomonas aeruginosa (286)	Pseudomonas (135621)	Pseudomonadaceae (7227	Pseudomonadales (1236)	Gammaproteobacteria (122	Proteobacteria (2)	Bacteria (1)	99.40	27.20	99.70	78.50	WGS
287.4307	0.371	Pseudomonas aeruginosa (286)	Pseudomonas (135621)	Pseudomonadaceae (7227	Pseudomonadales (1236)	Gammaproteobacteria (122	Proteobacteria (2)	Bacteria (1)	100.00	0.30	99.80	99.40	WGS
287.4308	0.371	Pseudomonas aeruginosa (286)	Pseudomonas (135621)	Pseudomonadaceae (7227	Pseudomonadales (1236)	Gammaproteobacteria (122	Proteobacteria (2)	Bacteria (1)	100.00	0.60	99.90	99.10	WGS
287.4309	0.371	Pseudomonas aeruginosa (286)	Pseudomonas (135621)	Pseudomonadaceae (7227	Pseudomonadales (1236)	Gammaproteobacteria (122	Proteobacteria (2)	Bacteria (1)	100.00	0.00	99.90	99.30	WGS
287.431	0.3818	Pseudomonas aeruginosa (286)	Pseudomonas (135621)	Pseudomonadaceae (7227	Pseudomonadales (1236)	Gammaproteobacteria (122	Proteobacteria (2)	Bacteria (1)	97.60	0.00	98.20	97.40	WGS
287.4310	0.3174	Pseudomonas aeruginosa (286)	Pseudomonas (135621)	Pseudomonadaceae (7227	Pseudomonadales (1236)	Gammaproteobacteria (122	Proteobacteria (2)	Bacteria (1)	100.00	0.00	99.80	99.40	WGS
287.4311	0.331	Pseudomonas aeruginosa (286)	Pseudomonas (135621)	Pseudomonadaceae (7227	Pseudomonadales (1236)	Gammaproteobacteria (122	Proteobacteria (2)	Bacteria (1)	100.00	0.70	99.70	98.60	WGS
287.4312	0.331	Pseudomonas aeruginosa (286)	Pseudomonas (135621)	Pseudomonadaceae (7227	Pseudomonadales (1236)	Gammaproteobacteria (122	Proteobacteria (2)	Bacteria (1)	100.00	0.00	99.70	98.70	WGS
287.4313	0.3302	Pseudomonas aeruginosa (286)	Pseudomonas (135621)	Pseudomonadaceae (7227	Pseudomonadales (1236)	Gammaproteobacteria (122	Proteobacteria (2)	Bacteria (1)	100.00	0.90	99.70	98.20	WGS
287.4314	0.3302	Pseudomonas aeruginosa (286)	Pseudomonas (135621)	Pseudomonadaceae (7227	Pseudomonadales (1236)	Gammaproteobacteria (122	Proteobacteria (2)	Bacteria (1)	100.00	0.30	99.90	98.30	WGS
287.4315	0.3302	Pseudomonas aeruginosa (286)	Pseudomonas (135621)	Pseudomonadaceae (7227	Pseudomonadales (1236)	Gammaproteobacteria (122	Proteobacteria (2)	Bacteria (1)	100.00	0.30	99.90	98.30	WGS
287.4316	0.368	Pseudomonas aeruginosa (286)	Pseudomonas (135621)	Pseudomonadaceae (7227	Pseudomonadales (1236)	Gammaproteobacteria (122	Proteobacteria (2)	Bacteria (1)	100.00	0.30	99.80	98.60	WGS
287.4317	0.3302	Pseudomonas aeruginosa (286)	Pseudomonas (135621)	Pseudomonadaceae (7227	Pseudomonadales (1236)	Gammaproteobacteria (122	Proteobacteria (2)	Bacteria (1)	100.00	0.30	99.90	98.00	WGS
287.4318	0.3506	Pseudomonas aeruginosa (286)	Pseudomonas (135621)	Pseudomonadaceae (7227	Pseudomonadales (1236)	Gammaproteobacteria (122	Proteobacteria (2)	Bacteria (1)	100.00	1.20	99.70	98.50	WGS
287.4319	0.331	Pseudomonas aeruginosa (286)	Pseudomonas (135621)	Pseudomonadaceae (7227	Pseudomonadales (1236)	Gammaproteobacteria (122	Proteobacteria (2)	Bacteria (1)	100.00	0.60	99.70	98.80	WGS
287.4320	0.331	Pseudomonas aeruginosa (286)	Pseudomonas (135621)	Pseudomonadaceae (7227	Pseudomonadales (1236)	Gammaproteobacteria (122	Proteobacteria (2)	Bacteria (1)	100.00	0.60	99.70	98.90	WGS
287.432	0.334	Pseudomonas aeruginosa (286)	Pseudomonas (135621)	Pseudomonadaceae (7227	Pseudomonadales (1236)	Gammaproteobacteria (122	Proteobacteria (2)	Bacteria (1)	97.30	0.00	97.90	97.00	WGS
287.4321	0.331	Pseudomonas aeruginosa (286)	Pseudomonas (135621)	Pseudomonadaceae (7227	Pseudomonadales (1236)	Gammaproteobacteria (122	Proteobacteria (2)	Bacteria (1)	100.00	0.60	99.70	98.90	WGS
287.4322	0.371	Pseudomonas aeruginosa (286)	Pseudomonas (135621)	Pseudomonadaceae (7227	Pseudomonadales (1236)	Gammaproteobacteria (122	Proteobacteria (2)	Bacteria (1)	99.70	0.00	99.70	98.40	WGS
287.4323	0.371	Pseudomonas aeruginosa (286)	Pseudomonas (135621)	Pseudomonadaceae (7227	Pseudomonadales (1236)	Gammaproteobacteria (122	Proteobacteria (2)	Bacteria (1)	100.00	0.00	99.90	99.10	WGS
287.4324	0.371	Pseudomonas aeruginosa (286)	Pseudomonas (135621)	Pseudomonadaceae (7227	Pseudomonadales (1236)	Gammaproteobacteria (122	Proteobacteria (2)	Bacteria (1)	100.00	0.30	99.90	99.20	WGS
287.4325	0.3618	Pseudomonas aeruginosa (286)	Pseudomonas (135621)	Pseudomonadaceae (7227	Pseudomonadales (1236)	Gammaproteobacteria (122	Proteobacteria (2)	Bacteria (1)	100.00	0.00	99.80	99.10	WGS
287.4326	0.368	Pseudomonas aeruginosa (286)	Pseudomonas (135621)	Pseudomonadaceae (7227	Pseudomonadales (1236)	Gammaproteobacteria (122	Proteobacteria (2)	Bacteria (1)	100.00	0.00	99.90	99.20	WGS
287.4327	0.3258	Pseudomonas aeruginosa (286)	Pseudomonas (135621)	Pseudomonadaceae (7227	Pseudomonadales (1236)	Gammaproteobacteria (122	Proteobacteria (2)	Bacteria (1)	100.00	0.00	99.90	99.10	WGS
287.4328	0.368	Pseudomonas aeruginosa (286)	Pseudomonas (135621)	Pseudomonadaceae (7227	Pseudomonadales (1236)	Gammaproteobacteria (122	Proteobacteria (2)	Bacteria (1)	100.00	0.30	99.80	99.60	WGS
287.4329	0.3174	Pseudomonas aeruginosa (286)	Pseudomonas (135621)	Pseudomonadaceae (7227	Pseudomonadales (1236)	Gammaproteobacteria (122	Proteobacteria (2)	Bacteria (1)	100.00	0.60	99.80	99.30	WGS
287.4330	0.3174	Pseudomonas aeruginosa (286)	Pseudomonas (135621)	Pseudomonadaceae (7227	Pseudomonadales (1236)	Gammaproteobacteria (122	Proteobacteria (2)	Bacteria (1)	100.00	0.00	99.80	99.30	WGS
287.433	0.4014	Pseudomonas aeruginosa (286)	Pseudomonas (135621)	Pseudomonadaceae (7227	Pseudomonadales (1236)	Gammaproteobacteria (122	Proteobacteria (2)	Bacteria (1)	95.90	0.30	96.80	95.60	WGS
287.4331	0.3636	Pseudomonas aeruginosa (286)	Pseudomonas (135621)	Pseudomonadaceae (7227	Pseudomonadales (1236)	Gammaproteobacteria (122	Proteobacteria (2)	Bacteria (1)	100.00	0.30	99.70	99.20	WGS
287.4332	0.3748	Pseudomonas aeruginosa (286)	Pseudomonas (135621)	Pseudomonadaceae (7227	Pseudomonadales (1236)	Gammaproteobacteria (122	Proteobacteria (2)	Bacteria (1)	100.00	0.00	99.80	99.10	WGS
287.4333	0.371	Pseudomonas aeruginosa (286)	Pseudomonas (135621)	Pseudomonadaceae (7227	Pseudomonadales (1236)	Gammaproteobacteria (122	Proteobacteria (2)	Bacteria (1)	99.70	0.30	99.70	98.30	WGS
287.4334	0.3302	Pseudomonas aeruginosa (286)	Pseudomonas (135621)	Pseudomonadaceae (7227	Pseudomonadales (1236)	Gammaproteobacteria (122	Proteobacteria (2)	Bacteria (1)	100.00	0.30	99.90	98.30	WGS
287.4335	0.3284	Pseudomonas aeruginosa (286)	Pseudomonas (135621)	Pseudomonadaceae (7227	Pseudomonadales (1236)	Gammaproteobacteria (122	Proteobacteria (2)	Bacteria (1)	100.00	0.60	99.70	98.40	WGS
287.4336	0.3514	Pseudomonas aeruginosa (286)	Pseudomonas (135621)	Pseudomonadaceae (7227	Pseudomonadales (1236)	Gammaproteobacteria (122	Proteobacteria (2)	Bacteria (1)	100.00	1.20	99.70	98.30	WGS
287.4337	0.331	Pseudomonas aeruginosa (286)	Pseudomonas (135621)	Pseudomonadaceae (7227	Pseudomonadales (1236)	Gammaproteobacteria (122	Proteobacteria (2)	Bacteria (1)	100.00	0.90	99.60	98.80	WGS
287.4338	0.331	Pseudomonas aeruginosa (286)	Pseudomonas (135621)	Pseudomonadaceae (7227	Pseudomonadales (1236)	Gammaproteobacteria (122	Proteobacteria (2)	Bacteria (1)	100.00	0.30	99.70	98.70	WGS
287.4339	0.3506	Pseudomonas aeruginosa (286)	Pseudomonas (135621)	Pseudomonadaceae (7227	Pseudomonadales (1236)	Gammaproteobacteria (122	Proteobacteria (2)	Bacteria (1)	100.00	1.20	99.70	98.50	WGS
287.4340	0.3662	Pseudomonas aeruginosa (286)	Pseudomonas (135621)	Pseudomonadaceae (7227	Pseudomonadales (1236)	Gammaproteobacteria (122	Proteobacteria (2)	Bacteria (1)	100.00	0.00	99.90	99.60	WGS
287.434	0.3474	Pseudomonas aeruginosa (286)	Pseudomonas (135621)	Pseudomonadaceae (7227	Pseudomonadales (1236)	Gammaproteobacteria (122	Proteobacteria (2)	Bacteria (1)	98.30	0.00	98.20	97.00	WGS
287.4341	0.3174	Pseudomonas aeruginosa (286)	Pseudomonas (135621)	Pseudomonadaceae (7227	Pseudomonadales (1236)	Gammaproteobacteria (122	Proteobacteria (2)	Bacteria (1)	100.00	0.00	99.80	99.30	WGS
287.4342	0.3334	Pseudomonas aeruginosa (286)	Pseudomonas (135621)	Pseudomonadaceae (7227	Pseudomonadales (1236)	Gammaproteobacteria (122	Proteobacteria (2)	Bacteria (1)	100.00	0.00	99.70	99.30	WGS
287.4343	0.3174	Pseudomonas aeruginosa (286)	Pseudomonas (135621)	Pseudomonadaceae (7227	Pseudomonadales (1236)	Gammaproteobacteria (122	Proteobacteria (2)	Bacteria (1)	100.00	0.00	99.80	99.30	WGS
287.4344	0.3174	Pseudomonas aeruginosa (286)	Pseudomonas (135621)	Pseudomonadaceae (7227	Pseudomonadales (1236)	Gammaproteobacteria (122	Proteobacteria (2)	Bacteria (1)	100.00	0.30	99.80	99.00	WGS
287.4345	0.3336	Pseudomonas aeruginosa (286)	Pseudomonas (135621)	Pseudomonadaceae (7227	Pseudomonadales (1236)	Gammaproteobacteria (122	Proteobacteria (2)	Bacteria (1)	100.00	0.30	99.90	99.60	WGS
287.4346	0.3268	Pseudomonas aeruginosa (286)	Pseudomonas (135621)	Pseudomonadaceae (7227	Pseudomonadales (1236)	Gammaproteobacteria (122	Proteobacteria (2)	Bacteria (1)	100.00	1.80	99.80	98.50	WGS
287.4347	0.3684	Pseudomonas aeruginosa (286)	Pseudomonas (135621)	Pseudomonadaceae (7227	Pseudomonadales (1236)	Gammaproteobacteria (122	Proteobacteria (2)	Bacteria (1)	100.00	10.60	99.60	89.30	WGS
287.4348	0.3236	Pseudomonas aeruginosa (286)	Pseudomonas (135621)	Pseudomonadaceae (7227	Pseudomonadales (1236)	Gammaproteobacteria (122	Proteobacteria (2)	Bacteria (1)	100.00	3.80	99.90	96.50	WGS
287.4349	0.3324	Pseudomonas aeruginosa (286)	Pseudomonas (135621)	Pseudomonadaceae (7227	Pseudomonadales (1236)	Gammaproteobacteria (122	Proteobacteria (2)	Bacteria (1)	100.00	0.30	99.90	99.20	WGS
287.435	0.3892	Pseudomonas aeruginosa (286)	Pseudomonas (135621)	Pseudomonadaceae (7227	Pseudomonadales (1236)	Gammaproteobacteria (122	Proteobacteria (2)	Bacteria (1)	97.30	0.00	98.40	96.80	WGS
287.4350	0.3324	Pseudomonas aeruginosa (286)	Pseudomonas (135621)	Pseudomonadaceae (7227	Pseudomonadales (1236)	Gammaproteobacteria (122	Proteobacteria (2)	Bacteria (1)	100.00	0.60	99.60	99.10	WGS
287.4351	0.3268	Pseudomonas aeruginosa (286)	Pseudomonas (135621)	Pseudomonadaceae (7227	Pseudomonadales (1236)	Gammaproteobacteria (122	Proteobacteria (2)	Bacteria (1)	100.00	0.00	99.80	99.30	WGS
287.4352	0.3268	Pseudomonas aeruginosa (286)	Pseudomonas (135621)	Pseudomonadaceae (7227	Pseudomonadales (1236)	Gammaproteobacteria (122	Proteobacteria (2)	Bacteria (1)	100.00	0.00	99.80	99.30	WGS
287.4353	0.331	Pseudomonas aeruginosa (286)	Pseudomonas (135621)	Pseudomonadaceae (7227	Pseudomonadales (1236)	Gammaproteobacteria (122	Proteobacteria (2)	Bacteria (1)	100.00	0.00	99.70	99.20	WGS
287.4354	0.3258	Pseudomonas aeruginosa (286)	Pseudomonas (135621)	Pseudomonadaceae (7227	Pseudomonadales (1236)	Gammaproteobacteria (122	Proteobacteria (2)	Bacteria (1)	100.00	0.00	99.90	98.80	WGS
287.4355	0.3694	Pseudomonas aeruginosa (286)	Pseudomonas (135621)	Pseudomonadaceae (7227	Pseudomonadales (1236)	Gammaproteobacteria (122	Proteobacteria (2)	Bacteria (1)	100.00	0.30	99.70	99.00	WGS
287.4356	0.3174	Pseudomonas aeruginosa (286)	Pseudomonas (135621)	Pseudomonadaceae (7227	Pseudomonadales (1236)	Gammaproteobacteria (122	Proteobacteria (2)	Bacteria (1)	100.00	0.00	99.70	99.00	WGS
287.4357	0.3628	Pseudomonas aeruginosa (286)	Pseudomonas (135621)	Pseudomonadaceae (7227	Pseudomonadales (1236)	Gammaproteobacteria (122	Proteobacteria (2)	Bacteria (1)	100.00	0.00	99.90	99.00	WGS
287.4358	0.0294	Pseudomonas aeruginosa (286)	Pseudomonas (135621)	Pseudomonadaceae (7227	Pseudomonadales (1236)	Gammaproteobacteria (122	Proteobacteria (2)	Bacteria (1)	100.00	0.60	99.80	98.20	WGS
287.4359	0.3258	Pseudomonas aeruginosa (286)	Pseudomonas (135621)	Pseudomonadaceae (7227	Pseudomonadales (1236)	Gammaproteobacteria (122	Proteobacteria (2)	Bacteria (1)	100.00	0.30	99.90	99.60	WGS
287.436	0.3854	Pseudomonas aeruginosa (286)	Pseudomonas (135621)	Pseudomonadaceae (7227	Pseudomonadales (1236)	Gammaproteobacteria (122	Proteobacteria (2)	Bacteria (1)	96.80	0.30	97.40	96.30	WGS
287.4360	0.3336	Pseudomonas aeruginosa (286)	Pseudomonas (135621)	Pseudomonadaceae (7227	Pseudomonadales (1236)	Gammaproteobacteria (122	Proteobacteria (2)	Bacteria (1)	100.00	0.00	99.90	99.30	WGS
287.4361	0.3336	Pseudomonas aeruginosa (286)	Pseudomonas (135621)	Pseudomonadaceae (7227	Pseudomonadales (1236)	Gammaproteobacteria (122	Proteobacteria (2)	Bacteria (1)	100.00	0.00	99.90	99.50	WGS
287.4362	0.3258	Pseudomonas aeruginosa (286)	Pseudomonas (135621)	Pseudomonadaceae (7227	Pseudomonadales (1236)	Gammaproteobacteria (122	Proteobacteria (2)	Bacteria (1)	100.00	0.00	99.80	99.40	WGS

287.4363	0.3258	Pseudomonas aeruginosa (286)	Pseudomonas (135621)	Pseudomonadaceae (7227	Pseudomonadales (1236)	Gammaproteobacteria (122	Proteobacteria (2)	Bacteria (1)	100.00	0.00	99.80	99.40	WGS
287.4364	0.3258	Pseudomonas aeruginosa (286)	Pseudomonas (135621)	Pseudomonadaceae (7227	Pseudomonadales (1236)	Gammaproteobacteria (122	Proteobacteria (2)	Bacteria (1)	100.00	0.00	99.80	99.40	WGS
287.4365	0.371	Pseudomonas aeruginosa (286)	Pseudomonas (135621)	Pseudomonadaceae (7227	Pseudomonadales (1236)	Gammaproteobacteria (122	Proteobacteria (2)	Bacteria (1)	100.00	0.00	99.80	99.30	WGS
287.4366	0.3636	Pseudomonas aeruginosa (286)	Pseudomonas (135621)	Pseudomonadaceae (7227	Pseudomonadales (1236)	Gammaproteobacteria (122	Proteobacteria (2)	Bacteria (1)	99.40	1.20	99.70	99.00	WGS
287.4367	0.3268	Pseudomonas aeruginosa (286)	Pseudomonas (135621)	Pseudomonadaceae (7227	Pseudomonadales (1236)	Gammaproteobacteria (122	Proteobacteria (2)	Bacteria (1)	100.00	0.30	99.80	98.40	WGS
287.4368	0.3184	Pseudomonas aeruginosa (286)	Pseudomonas (135621)	Pseudomonadaceae (7227	Pseudomonadales (1236)	Gammaproteobacteria (122	Proteobacteria (2)	Bacteria (1)	100.00	0.00	99.90	99.00	WGS
287.4369	0.3694	Pseudomonas aeruginosa (286)	Pseudomonas (135621)	Pseudomonadaceae (7227	Pseudomonadales (1236)	Gammaproteobacteria (122	Proteobacteria (2)	Bacteria (1)	100.00	0.00	99.60	99.20	WGS
287.4370	0.3302	Pseudomonas aeruginosa (286)	Pseudomonas (135621)	Pseudomonadaceae (7227	Pseudomonadales (1236)	Gammaproteobacteria (122	Proteobacteria (2)	Bacteria (1)	100.00	0.70	99.60	98.60	WGS
287.437	0.3496	Pseudomonas aeruginosa (286)	Pseudomonas (135621)	Pseudomonadaceae (7227	Pseudomonadales (1236)	Gammaproteobacteria (122	Proteobacteria (2)	Bacteria (1)	95.20	0.30	97.40	95.90	WGS
287.4371	0.3398	Pseudomonas aeruginosa (286)	Pseudomonas (135621)	Pseudomonadaceae (7227	Pseudomonadales (1236)	Gammaproteobacteria (122	Proteobacteria (2)	Bacteria (1)	100.00	0.20	99.90	99.10	WGS
287.4372	0.3636	Pseudomonas aeruginosa (286)	Pseudomonas (135621)	Pseudomonadaceae (7227	Pseudomonadales (1236)	Gammaproteobacteria (122	Proteobacteria (2)	Bacteria (1)	100.00	0.00	99.90	99.30	WGS
287.4373	0.3174	Pseudomonas aeruginosa (286)	Pseudomonas (135621)	Pseudomonadaceae (7227	Pseudomonadales (1236)	Gammaproteobacteria (122	Proteobacteria (2)	Bacteria (1)	100.00	0.00	99.70	99.30	WGS
287.4374	0.3258	Pseudomonas aeruginosa (286)	Pseudomonas (135621)	Pseudomonadaceae (7227	Pseudomonadales (1236)	Gammaproteobacteria (122	Proteobacteria (2)	Bacteria (1)	100.00	0.00	99.90	99.60	WGS
287.4375	0.331	Pseudomonas aeruginosa (286)	Pseudomonas (135621)	Pseudomonadaceae (7227	Pseudomonadales (1236)	Gammaproteobacteria (122	Proteobacteria (2)	Bacteria (1)	100.00	0.00	99.90	99.30	WGS
287.4376	0.3376	Pseudomonas aeruginosa (286)	Pseudomonas (135621)	Pseudomonadaceae (7227	Pseudomonadales (1236)	Gammaproteobacteria (122	Proteobacteria (2)	Bacteria (1)	100.00	0.00	99.80	99.50	WGS
287.4377	0.371	Pseudomonas aeruginosa (286)	Pseudomonas (135621)	Pseudomonadaceae (7227	Pseudomonadales (1236)	Gammaproteobacteria (122	Proteobacteria (2)	Bacteria (1)	100.00	0.00	99.80	99.30	WGS
287.4378	0.3174	Pseudomonas aeruginosa (286)	Pseudomonas (135621)	Pseudomonadaceae (7227	Pseudomonadales (1236)	Gammaproteobacteria (122	Proteobacteria (2)	Bacteria (1)	100.00	0.30	99.90	98.80	WGS
287.4379	0.3646	Pseudomonas aeruginosa (286)	Pseudomonas (135621)	Pseudomonadaceae (7227	Pseudomonadales (1236)	Gammaproteobacteria (122	Proteobacteria (2)	Bacteria (1)	100.00	0.00	99.90	99.60	WGS
287.438	0.3394	Pseudomonas aeruginosa (286)	Pseudomonas (135621)	Pseudomonadaceae (7227	Pseudomonadales (1236)	Gammaproteobacteria (122	Proteobacteria (2)	Bacteria (1)	97.60	0.00	98.50	97.70	WGS
287.4380	0.332	Pseudomonas aeruginosa (286)	Pseudomonas (135621)	Pseudomonadaceae (7227	Pseudomonadales (1236)	Gammaproteobacteria (122	Proteobacteria (2)	Bacteria (1)	100.00	1.20	99.80	98.30	WGS
287.4381	0.3636	Pseudomonas aeruginosa (286)	Pseudomonas (135621)	Pseudomonadaceae (7227	Pseudomonadales (1236)	Gammaproteobacteria (122	Proteobacteria (2)	Bacteria (1)	100.00	0.00	100.00	99.90	WGS
287.4382	0.3636	Pseudomonas aeruginosa (286)	Pseudomonas (135621)	Pseudomonadaceae (7227	Pseudomonadales (1236)	Gammaproteobacteria (122	Proteobacteria (2)	Bacteria (1)	100.00	0.00	99.90	99.80	WGS
287.4383	0.331	Pseudomonas aeruginosa (286)	Pseudomonas (135621)	Pseudomonadaceae (7227	Pseudomonadales (1236)	Gammaproteobacteria (122	Proteobacteria (2)	Bacteria (1)	100.00	0.30	99.80	99.40	WGS
287.4384	0.3174	Pseudomonas aeruginosa (286)	Pseudomonas (135621)	Pseudomonadaceae (7227	Pseudomonadales (1236)	Gammaproteobacteria (122	Proteobacteria (2)	Bacteria (1)	100.00	0.00	99.70	99.30	WGS
287.4385	0.3302	Pseudomonas aeruginosa (286)	Pseudomonas (135621)	Pseudomonadaceae (7227	Pseudomonadales (1236)	Gammaproteobacteria (122	Proteobacteria (2)	Bacteria (1)	100.00	0.30	99.90	98.90	WGS
287.4386	0.3652	Pseudomonas aeruginosa (286)	Pseudomonas (135621)	Pseudomonadaceae (7227	Pseudomonadales (1236)	Gammaproteobacteria (122	Proteobacteria (2)	Bacteria (1)	100.00	0.00	99.90	98.70	WGS
287.4387	0.3334	Pseudomonas aeruginosa (286)	Pseudomonas (135621)	Pseudomonadaceae (7227	Pseudomonadales (1236)	Gammaproteobacteria (122	Proteobacteria (2)	Bacteria (1)	100.00	0.00	99.80	99.30	WGS
287.4388	0.3334	Pseudomonas aeruginosa (286)	Pseudomonas (135621)	Pseudomonadaceae (7227	Pseudomonadales (1236)	Gammaproteobacteria (122	Proteobacteria (2)	Bacteria (1)	100.00	0.00	99.80	99.30	WGS
287.4389	0.3332	Pseudomonas aeruginosa (286)	Pseudomonas (135621)	Pseudomonadaceae (7227	Pseudomonadales (1236)	Gammaproteobacteria (122	Proteobacteria (2)	Bacteria (1)	100.00	0.00	99.80	99.30	WGS
287.4390	0.3332	Pseudomonas aeruginosa (286)	Pseudomonas (135621)	Pseudomonadaceae (7227	Pseudomonadales (1236)	Gammaproteobacteria (122	Proteobacteria (2)	Bacteria (1)	100.00	0.00	99.80	99.40	WGS
287.439	0.3688	Pseudomonas aeruginosa (286)	Pseudomonas (135621)	Pseudomonadaceae (7227	Pseudomonadales (1236)	Gammaproteobacteria (122	Proteobacteria (2)	Bacteria (1)	97.20	0.30	97.90	96.30	WGS
287.4391	0.3324	Pseudomonas aeruginosa (286)	Pseudomonas (135621)	Pseudomonadaceae (7227	Pseudomonadales (1236)	Gammaproteobacteria (122	Proteobacteria (2)	Bacteria (1)	100.00	0.00	99.90	99.30	WGS
287.4392	0.3324	Pseudomonas aeruginosa (286)	Pseudomonas (135621)	Pseudomonadaceae (7227	Pseudomonadales (1236)	Gammaproteobacteria (122	Proteobacteria (2)	Bacteria (1)	100.00	0.00	99.90	99.30	WGS
287.4393	0.3324	Pseudomonas aeruginosa (286)	Pseudomonas (135621)	Pseudomonadaceae (7227	Pseudomonadales (1236)	Gammaproteobacteria (122	Proteobacteria (2)	Bacteria (1)	100.00	0.00	99.90	99.30	WGS
287.4394	0.327	Pseudomonas aeruginosa (286)	Pseudomonas (135621)	Pseudomonadaceae (7227	Pseudomonadales (1236)	Gammaproteobacteria (122	Proteobacteria (2)	Bacteria (1)	100.00	0.00	99.90	99.70	WGS
287.4395	0.327	Pseudomonas aeruginosa (286)	Pseudomonas (135621)	Pseudomonadaceae (7227	Pseudomonadales (1236)	Gammaproteobacteria (122	Proteobacteria (2)	Bacteria (1)	100.00	0.00	99.90	99.60	WGS
287.4396	0.327	Pseudomonas aeruginosa (286)	Pseudomonas (135621)	Pseudomonadaceae (7227	Pseudomonadales (1236)	Gammaproteobacteria (122	Proteobacteria (2)	Bacteria (1)	100.00	0.00	99.90	99.70	WGS
287.4397	0.3694	Pseudomonas aeruginosa (286)	Pseudomonas (135621)	Pseudomonadaceae (7227	Pseudomonadales (1236)	Gammaproteobacteria (122	Proteobacteria (2)	Bacteria (1)	100.00	0.00	99.90	99.20	WGS
287.4398	0.3694	Pseudomonas aeruginosa (286)	Pseudomonas (135621)	Pseudomonadaceae (7227	Pseudomonadales (1236)	Gammaproteobacteria (122	Proteobacteria (2)	Bacteria (1)	100.00	0.00	99.90	99.20	WGS
287.4399	0.3226	Pseudomonas aeruginosa (286)	Pseudomonas (135621)	Pseudomonadaceae (7227	Pseudomonadales (1236)	Gammaproteobacteria (122	Proteobacteria (2)	Bacteria (1)	100.00	0.00	99.90	99.70	WGS
287.4400	0.3226	Pseudomonas aeruginosa (286)	Pseudomonas (135621)	Pseudomonadaceae (7227	Pseudomonadales (1236)	Gammaproteobacteria (122	Proteobacteria (2)	Bacteria (1)	100.00	0.00	99.90	99.70	WGS
287.440	0.3344	Pseudomonas aeruginosa (286)	Pseudomonas (135621)	Pseudomonadaceae (7227	Pseudomonadales (1236)	Gammaproteobacteria (122	Proteobacteria (2)	Bacteria (1)	98.30	0.30	98.40	97.00	WGS
287.4401	0.3332	Pseudomonas aeruginosa (286)	Pseudomonas (135621)	Pseudomonadaceae (7227	Pseudomonadales (1236)	Gammaproteobacteria (122	Proteobacteria (2)	Bacteria (1)	100.00	0.00	99.80	99.30	WGS
287.4402	0.327	Pseudomonas aeruginosa (286)	Pseudomonas (135621)	Pseudomonadaceae (7227	Pseudomonadales (1236)	Gammaproteobacteria (122	Proteobacteria (2)	Bacteria (1)	100.00	0.00	99.90	99.70	WGS
287.4403	0.327	Pseudomonas aeruginosa (286)	Pseudomonas (135621)	Pseudomonadaceae (7227	Pseudomonadales (1236)	Gammaproteobacteria (122	Proteobacteria (2)	Bacteria (1)	100.00	0.00	99.90	99.60	WGS
287.4404	0.3258	Pseudomonas aeruginosa (286)	Pseudomonas (135621)	Pseudomonadaceae (7227	Pseudomonadales (1236)	Gammaproteobacteria (122	Proteobacteria (2)	Bacteria (1)	100.00	0.00	99.80	99.30	WGS
287.4405	0.331	Pseudomonas aeruginosa (286)	Pseudomonas (135621)	Pseudomonadaceae (7227	Pseudomonadales (1236)	Gammaproteobacteria (122	Proteobacteria (2)	Bacteria (1)	100.00	0.30	99.90	98.90	WGS
287.4406	0.331	Pseudomonas aeruginosa (286)	Pseudomonas (135621)	Pseudomonadaceae (7227	Pseudomonadales (1236)	Gammaproteobacteria (122	Proteobacteria (2)	Bacteria (1)	100.00	0.30	99.90	98.90	WGS
287.4407	0.3346	Pseudomonas aeruginosa (286)	Pseudomonas (135621)	Pseudomonadaceae (7227	Pseudomonadales (1236)	Gammaproteobacteria (122	Proteobacteria (2)	Bacteria (1)	100.00	0.00	99.90	99.00	WGS
287.4408	0.3376	Pseudomonas aeruginosa (286)	Pseudomonas (135621)	Pseudomonadaceae (7227	Pseudomonadales (1236)	Gammaproteobacteria (122	Proteobacteria (2)	Bacteria (1)	100.00	0.00	99.90	99.00	WGS
287.4409	0.332	Pseudomonas aeruginosa (286)	Pseudomonas (135621)	Pseudomonadaceae (7227	Pseudomonadales (1236)	Gammaproteobacteria (122	Proteobacteria (2)	Bacteria (1)	100.00	1.20	99.80	98.20	WGS
287.441	0.3356	Pseudomonas aeruginosa (286)	Pseudomonas (135621)	Pseudomonadaceae (7227	Pseudomonadales (1236)	Gammaproteobacteria (122	Proteobacteria (2)	Bacteria (1)	96.70	0.30	97.70	97.00	WGS
287.4410	0.3376	Pseudomonas aeruginosa (286)	Pseudomonas (135621)	Pseudomonadaceae (7227	Pseudomonadales (1236)	Gammaproteobacteria (122	Proteobacteria (2)	Bacteria (1)	100.00	0.30	99.90	99.40	WGS
287.4411	0.324	Pseudomonas aeruginosa (286)	Pseudomonas (135621)	Pseudomonadaceae (7227	Pseudomonadales (1236)	Gammaproteobacteria (122	Proteobacteria (2)	Bacteria (1)	100.00	0.30	99.80	98.90	WGS
287.4412	0.3258	Pseudomonas aeruginosa (286)	Pseudomonas (135621)	Pseudomonadaceae (7227	Pseudomonadales (1236)	Gammaproteobacteria (122	Proteobacteria (2)	Bacteria (1)	100.00	0.00	99.80	99.10	WGS
287.4413	0.3636	Pseudomonas aeruginosa (286)	Pseudomonas (135621)	Pseudomonadaceae (7227	Pseudomonadales (1236)	Gammaproteobacteria (122	Proteobacteria (2)	Bacteria (1)	100.00	0.00	99.90	99.80	WGS
287.4414	0.3322	Pseudomonas aeruginosa (286)	Pseudomonas (135621)	Pseudomonadaceae (7227	Pseudomonadales (1236)	Gammaproteobacteria (122	Proteobacteria (2)	Bacteria (1)	100.00	2.90	99.60	98.40	WGS
287.4415	0.3652	Pseudomonas aeruginosa (286)	Pseudomonas (135621)	Pseudomonadaceae (7227	Pseudomonadales (1236)	Gammaproteobacteria (122	Proteobacteria (2)	Bacteria (1)	100.00	0.00	99.90	99.30	WGS
287.4416	0.332	Pseudomonas aeruginosa (286)	Pseudomonas (135621)	Pseudomonadaceae (7227	Pseudomonadales (1236)	Gammaproteobacteria (122	Proteobacteria (2)	Bacteria (1)	100.00	1.20	99.80	98.40	WGS
287.4417	0.3292	Pseudomonas aeruginosa (286)	Pseudomonas (135621)	Pseudomonadaceae (7227	Pseudomonadales (1236)	Gammaproteobacteria (122	Proteobacteria (2)	Bacteria (1)	100.00	0.90	99.90	98.40	WGS
287.4418	0.3652	Pseudomonas aeruginosa (286)	Pseudomonas (135621)	Pseudomonadaceae (7227	Pseudomonadales (1236)	Gammaproteobacteria (122	Proteobacteria (2)	Bacteria (1)	100.00	1.00	99.90	98.70	WGS
287.4419	0.0294	Pseudomonas aeruginosa (286)	Pseudomonas (135621)	Pseudomonadaceae (7227	Pseudomonadales (1236)	Gammaproteobacteria (122	Proteobacteria (2)	Bacteria (1)	100.00	0.00	99.90	99.50	WGS
287.442	0.399	Pseudomonas aeruginosa (286)	Pseudomonas (135621)	Pseudomonadaceae (7227	Pseudomonadales (1236)	Gammaproteobacteria (122	Proteobacteria (2)	Bacteria (1)	96.40	0.30	98.20	96.50	WGS
287.4420	0.3258	Pseudomonas aeruginosa (286)	Pseudomonas (135621)	Pseudomonadaceae (7227	Pseudomonadales (1236)	Gammaproteobacteria (122	Proteobacteria (2)	Bacteria (1)	100.00	1.20	99.80	98.90	WGS
287.4421	0.3324	Pseudomonas aeruginosa (286)	Pseudomonas (135621)	Pseudomonadaceae (7227	Pseudomonadales (1236)	Gammaproteobacteria (122	Proteobacteria (2)	Bacteria (1)	99.70	0.30	99.70	99.00	WGS
287.4422	0.357	Pseudomonas aeruginosa (286)	Pseudomonas (135621)	Pseudomonadaceae (7227	Pseudomonadales (1236)	Gammaproteobacteria (122	Proteobacteria (2)	Bacteria (1)	100.00	0.00	99.90	98.80	WGS
287.4423	0.3376	Pseudomonas aeruginosa (286)	Pseudomonas (135621)	Pseudomonadaceae (7227	Pseudomonadales (1236)	Gammaproteobacteria (122	Proteobacteria (2)	Bacteria (1)	100.00	0.00	99.90	98.80	WGS
287.4424	0.331	Pseudomonas aeruginosa (286)	Pseudomonas (135621)	Pseudomonadaceae (7227	Pseudomonadales (1236)	Gammaproteobacteria (122	Proteobacteria (2)	Bacteria (1)	100.00	0.00	99.90	99.50	WGS
287.4425	0.3636	Pseudomonas aeruginosa (286)	Pseudomonas (135621)	Pseudomonadaceae (7227	Pseudomonadales (1236)	Gammaproteobacteria (122	Proteobacteria (2)	Bacteria (1)	100.00	0.30	99.90	99.40	WGS
287.4426	0.3334	Pseudomonas aeruginosa (286)	Pseudomonas (135621)	Pseudomonadaceae (7227	Pseudomonadales (1236)	Gammaproteobacteria (122	Proteobacteria (2)	Bacteria (1)	100.00	0.00	99.80	98.90	WGS
287.4427	0.3302	Pseudomonas aeruginosa (286)	Pseudomonas (135621)	Pseudomonadaceae (7227	Pseudomonadales (1236)	Gammaproteobacteria (122	Proteobacteria (2)	Bacteria (1)	100.00	0.30	99.90	99.00	WGS
287.4428	0.0294	Pseudomonas aeruginosa (286)	Pseudomonas (135621)	Pseudomonadaceae (7227	Pseudomonadales (1236)	Gammaproteobacteria (122	Proteobacteria (2)	Bacteria (1)	100.00	0.30	99.80	99.30	WGS
287.4429	0.371	Pseudomonas aeruginosa (286)	Pseudomonas (135621)	Pseudomonadaceae (7227	Pseudomonadales (1236)	Gammaproteobacteria (122	Proteobacteria (2)	Bacteria (1)	100.00	0.60	99.70	98.70	WGS
287.443	0.3394	Pseudomonas aeruginosa (286)	Pseudomonas (135621)	Pseudomonadaceae (7227	Pseudomonadales (1236)	Gammaproteobacteria (122	Proteobacteria (2)	Bacteria (1)	96.70	0.30	98.30	97.50	WGS
287.4430	0.3258	Pseudomonas aeruginosa (286)	Pseudomonas (135621)	Pseudomonadaceae (7227	Pseudomonadales (1236)	Gammaproteobacteria (122	Proteobacteria (2)	Bacteria (1)	100.00	0.00	99.80	99.40	WGS
287.4431	0.3646	Pseudomonas aeruginosa (286)	Pseudomonas (135621)	Pseudomonadaceae (7227	Pseudomonadales (1236)	Gammaproteobacteria (122	Proteobacteria (2)	Bacteria (1)	100.00	0.30	99.70	99.20	WGS
287.4432	0.3694	Pseudomonas aeruginosa (286)	Pseudomonas (135621)	Pseudomonadaceae (7227	Pseudomonadales (1236)	Gammaproteobacteria (122	Proteobacteria (2)	Bacteria (1)	100.00	1.30	99.80	97.50	WGS
287.4433	0.356	Pseudomonas aeruginosa (286)	Pseudomonas (135621)	Pseudomonadaceae (7227	Pseudomonadales (1236)	Gammaproteobacteria (122	Proteobacteria (2)	Bacteria (1)	100.00	0.30	99.80	99.30	WGS
287.4434	0.356	Pseudomonas aeruginosa (286)	Pseudomonas (135621)	Pseudomonadaceae (7227	Pseudomonadales (1236)	Gammaproteobacteria (122	Proteobacteria (2)	Bacteria (1)	100.00	0.00	99.90	99.60	WGS
287.4435	0.368	Pseudomonas aeruginosa (286)	Pseudomonas (135621)	Pseudomonadaceae (7227	Pseudomonadales (1236)	Gammaproteobacteria (122	Proteobacteria (2)	Bacteria (1)	100.00	1.20	99.80	98.00	WGS
287.4436	0.3292	Pseudomonas aeruginosa (286)	Pseudomonas (135621)	Pseudomonadaceae (7227	Pseudomonadales (1236)	Gammaproteobacteria (122	Proteobacteria (2)	Bacteria (1)	100.00	0.60	99.90	99.00	WGS
287.4437	0.3324	Pseudomonas aeruginosa (286)	Pseudomonas (135621)	Pseudomonadaceae (7227	Pseudomonadales (1236)	Gammaproteobacteria (122	Proteobacteria (2)	Bacteria (1)	100.00	0.60	99.60	98.50	WGS
287.4438	0.3636	Pseudomonas aeruginosa (286)	Pseudomonas (135621)	Pseudomonadaceae (7227	Pseudomonadales (1236)	Gammaproteobacteria (122	Proteobacteria (2)	Bacteria (1)	100.00	0.00	99.70	99.20	WGS
287.4439	0.3324	Pseudomonas aeruginosa (286)	Pseudomonas (135621)	Pseudomonadaceae (7227	Pseudomonadales (1236)	Gammaproteobacteria (122	Proteobacteria (2)	Bacteria (1)	100.00	0.00	99.90	99.50	WGS
287.4440	0.3694	Pseudomonas aeruginosa (286)	Pseudomonas (135621)	Pseudomonadaceae (7227	Pseudomonadales (1236)	Gammaproteobacteria (122	Proteobacteria (2)	Bacteria (1)	100.00	0.00	99.90	99.50	WGS
287.444	0.4264	Pseudomonas aeruginosa (286)	Pseudomonas (135621)	Pseudomonadaceae (7227	Pseudomonadales (1236)	Gammaproteobacteria (122	Proteobacteria (2)	Bacteria (1)	97.60	0.00	97.60	96.00	WGS
287.4441	0.3748	Pseudomonas aeruginosa (286)	Pseudomonas (135621)	Pseudomonadaceae (7227	Pseudomonadales (1236)	Gammaproteobacteria (122	Proteobacteria (2)	Bacteria (1)	100.00	0.30	99.80	98.30	WGS
287.4442	0.3258	Pseudomonas aeruginosa (286)	Pseudomonas (135621)	Pseudomonadaceae (7227	Pseudomonadales (1236)	Gammaproteobacteria (122	Proteobacteria (2)	Bacteria (1)	100.00	0.30	99.90	99.00	WGS
287.4443	0.3376	Pseudomonas aeruginosa (286)	Pseudomonas (135621)	Pseudomonadaceae (7227	Pseudomonadales (1236)	Gammaproteobacteria (122	Proteobacteria (2)	Bacteria (1)	100.00	0.00	99.80	99.00	WGS
287.4444	0.324	Pseudomonas aeruginosa (286)	Pseudomonas (135621)	Pseudomonadaceae (7227	Pseudomonadales (1236)	Gammaproteobacteria (122	Proteobacteria (2)	Bacteria (1)	100.00	0.00	99.90	99.30	WGS
287.4445	0.3738	Pseudomonas aeruginosa (286)	Pseudomonas (135621)	Pseudomonadaceae (7227	Pseudomonadales (1236)	Gammaproteobacteria (122	Proteobacteria (2)	Bacteria (1)	99.70	0.00	99.90	98.90	WGS
287.4446	0.331	Pseudomonas aeruginosa (286)	Pseudomonas (135621)	Pseudomonadaceae (7227	Pseudomonadales (1236)	Gammaproteobacteria (122	Proteobacteria (2)	Bacteria (1)	99.70	0.60	99.60	99.00	WGS
287.4447	0.3694	Pseudomonas aeruginosa (286)	Pseudomonas (135621)	Pseudomonadaceae (7227	Pseudomonadales (1236)	Gammaproteobacteria (122	Proteobacteria (2)	Bacteria (1)	100.00	0.00	99.80	99.00	WGS
287.4448	0.331	Pseudomonas aeruginosa (286)	Pseudomonas (135621)	Pseudomonadaceae (7227	Pseudomonadales (1236)	Gammaproteobacteria (122	Proteobacteria (2)	Bacteria (1)	100.00	0.30	99.60	98.80	WGS
287.4449	0.3268	Pseudomonas aeruginosa (286)	Pseudomonas (135621)	Pseudomonadaceae (7227	Pseudomonadales (1236)	Gammaproteobacteria (122	Proteobacteria (2)	Bacteria (1)	100.00	0.00	99.80	99.20	WGS
287.445	0.3502	Pseudomonas aeruginosa (286)	Pseudomonas (135621)	Pseudomonadaceae (7227	Pseudomonadales (1236)	Gammaproteobacteria (122	Proteobacteria (2)	Bacteria (1)	97.30	0.90	97.70	96.20	WGS
287.4450	0.3618	Pseudomonas aeruginosa (286)	Pseudomonas (135621)	Pseudomonadaceae (7227	Pseudomonadales (1236)	Gammaproteobacteria (122	Proteobacteria (2)	Bacteria (1)	100.00	0.60	99.90	98.70	WGS
287.4451	0.3652	Pseudomonas aeruginosa (286)	Pseudomonas (135621)	Pseudomonadaceae (7227	Pseudomonadales (1236)	Gammaproteobacteria (122	Proteobacteria (2)	Bacteria (1)	100.00	0.00	99.90	99.10	WGS
287.4452	0.3652	Pseudomonas aeruginosa (286)	Pseudomonas (135621)	Pseudomonadaceae (7227	Pseudomonadales (1236)	Gammaproteobacteria (122	Proteobacteria (2)	Bacteria (1)	100.00	0.00	99.90	99.30	WGS
287.4453	0.3672	Pseudomonas aeruginosa (286)	Pseudomonas (135621)	Pseudomonadaceae (7227	Pseudomonadales (1236)	Gammaproteobacteria (122	Proteobacteria (2)	Bacteria (1)	100.00	0.00	99.90	99.10	WGS
287.4454	0.3292	Pseudomonas aeruginosa (286)	Pseudomonas (135621)	Pseudomonadaceae (7227	Pseudomonadales (1236)	Gammaproteobacteria (122	Proteobacteria (2)	Bacteria (1)	100.00	0.60	99.90	98.80	WGS
287.4455	0.3652	Pseudomonas aeruginosa (286)	Pseudomonas (135621)	Pseudomonadaceae (7227	Pseudomonadales (1236)	Gammaproteobacteria (122	Proteobacteria (2)	Bacteria (1)	100.00	0.00	99.90	99.20	WGS
287.4456	0.3604	Pseudomonas aeruginosa (286)	Pseudomonas (135621)	Pseudomonadaceae (7227	Pseudomonadales (1236)	Gammaproteobacteria (122	Proteobacteria (2)	Bacteria (1)	100.00	1.60	99.70	98.00	WGS
287.4457	0.356	Pseudomonas aeruginosa (286)	Pseudomonas (135621)	Pseudomonadaceae (7227	Pseudomonadales (1236)	Gammaproteobacteria (122	Proteobacteria (2)	Bacteria (1)	100.00	0.00	99.80	99.40	WGS
287.4458	0.3376	Pseudomonas aeruginosa (286)	Pseudomonas (135621)	Pseudomonadaceae (7227	Pseudomonadales (1236)	Gammaproteobacteria (122	Proteobacteria (2)	Bacteria (1)	99.70	0.00	99.80	98.90	WGS
287.4459	0.3258	Pseudomonas aeruginosa (286)	Pseudomonas (135621)	Pseudomonadaceae (7227	Pseudomonadales (1236)	Gammaproteobacteria (122	Proteobacteria (2)	Bacteria (1)	100.00	1.50	99.80	98.20	WGS
287.446	0.3744	Pseudomonas aeruginosa (286)	Pseudomonas (135621)	Pseudomonadaceae (7227	Pseudomonadales (1236)	Gammaproteobacteria (122	Proteobacteria (2)	Bacteria (1)	98.20	0.00	97.50	96.60	WGS
287.4460	0.3636	Pseudomonas aeruginosa (286)	Pseudomonas (135621)	Pseudomonadaceae (7227	Pseudomonadales (1236)	Gammaproteobacteria (122	Proteobacteria (2)	Bacteria (1)	100.00	0.60	99.90	99.60	WGS
287.4461	0.3292	Pseudomonas aeruginosa (286)	Pseudomonas (135621)	Pseudomonadaceae (7227	Pseudomonadales (1236)	Gammaproteobacteria (122	Proteobacteria (2)	Bacteria (1)	100.00	0.30	99.70	99.10	Complete
287.4462	0.3292	Pseudomonas aeruginosa (286)	Pseudomonas (135621)	Pseudomonadaceae (7227	Pseudomonadales (1236)	Gammaproteobacteria (122	Proteobacteria (2)	Bacteria (1)	100.00	0.90	99.70	99.00	Complete
287.4463	0.3246	Pseudomonas aeruginosa (286)	Pseudomonas (135621)	Pseudomonadaceae (7227	Pseudomonadales (1236)	Gammaproteobacteria (122	Proteobacteria (2)	Bacteria (1)	100.00	0.00	99.80	99.50	Complete
287.4464	0.369	Pseudomonas aeruginosa (286)	Pseudomonas (135621)	Pseudomonadaceae (7227	Pseudomonadales (1236)	Gammaproteobacteria (122	Proteobacteria (2)	Bacteria (1)	100.00	0.30	99.70	99.00	Complete
287.4467	0.3636	Pseudomonas aeruginosa (286)	Pseudomonas (135621)	Pseudomonadaceae (7227	Pseudomonadales (1236)	Gammaproteobacteria (122	Proteobacteria (2)	Bacteria (1)	100.00	0.60	99.90	99.60	WGS
287.4468	0.3292	Pseudomonas aeruginosa (286)	Pseudomonas (135621)	Pseudomonadaceae (7227	Pseudomonadales (1236)	Gammaproteobacteria (122	Proteobacteria (2)	Bacteria (1)	100.00	0.30	99.70	99.10	Complete
287.4469	0.3292	Pseudomonas aeruginosa (286)	Pseudomonas (135621)	Pseudomonadaceae (7227	Pseudomonadales (1236)	Gammaproteobacteria (122	Proteobacteria (2)	Bacteria (1)	100.00	0.90	99.70	99.00	Complete
287.447	0.3344	Pseudomonas aeruginosa (286)	Pseudomonas (135621)	Pseudomonadaceae (7227	Pseudomonadales (1236)	Gammaproteobacteria (122	Proteobacteria (2)	Bacteria (1)	98.60	0.00	98.10	97.20	WGS
287.448	0.3834	Pseudomonas aeruginosa (286)	Pseudomonas (135621)	Pseudomonadaceae (7227	Pseudomonadales (1236)	Gammaproteobacteria (122	Proteobacteria (2)	Bacteria (1)	99.70	0.30	98.00	96.60	WGS
287.449	0.3516	Pseudomonas aeruginosa (286)	Pseudomonas (135621)	Pseudomonadaceae (7227	Pseudomonadales (1236)	Gammaproteobacteria (122	Proteobacteria (2)	Bacteria (1)	97.20	0.00	98.10	97.20	WGS
287.450	0.3668	Pseudomonas aeruginosa (286)	Pseudomonas (135621)	Pseudomonadaceae (7227	Pseudomonadales (1236)	Gammaproteobacteria (122	Proteobacteria (2)	Bacteria (1)	95.20	0.30	96.60	94.50	WGS
287.451	0.3834	Pseudomonas aeruginosa (286)	Pseudomonas (135621)	Pseudomonadaceae (7227	Pseudomonadales (1236)	Gammaproteobacteria (122	Proteobacteria (2)	Bacteria (1)	97.30	0.00	97.90	96.70	WGS
287.452	0.3636	Pseudomonas aeruginosa (286)	Pseudomonas (135621)	Pseudomonadaceae (7227	Pseudomonadales (1236)	Gammaproteobacteria (122	Proteobacteria (2)	Bacteria (1)	97.40	0.30	97.50	96.50	WGS
287.453	0.376	Pseudomonas aeruginosa (286)	Pseudomonas (135621)	Pseudomonadaceae (7227	Pseudomonadales (1236)	Gammaproteobacteria (122	Proteobacteria (2)	Bacteria (1)	96.60	0.30	97.80	95.90	WGS
287.454	0.3344	Pseudomonas aeruginosa (286)	Pseudomonas (135621)	Pseudomonadaceae (7227	Pseudomonadales (1236)	Gammaproteobacteria (122	Proteobacteria (2)	Bacteria (1)	98.60	0.00	98.20	97.50	WGS
287.4540	0.371	Pseudomonas aeruginosa (286)	Pseudomonas (135621)	Pseudomonadaceae (7227	Pseudomonadales (1236)	Gammaproteobacteria (122	Proteobacteria (2)	Bacteria (1)	100.00	0.00	99.90	99.10	WGS
287.4541	0.3258	Pseudomonas aeruginosa (286)	Pseudomonas (135621)	Pseudomonadaceae (7227	Pseudomonadales (1236)	Gammaproteobacteria (122	Proteobacteria (2)	Bacteria (1)	99.70	0.00	99.00	98.70	WGS
287.4542	0.3636	Pseudomonas aeruginosa (286)	Pseudomonas (135621)	Pseudomonadaceae (7227	Pseudomonadales (1236)	Gammaproteobacteria (122	Proteobacteria (2)	Bacteria (1)	100.00	0.30	99.80	98.80	WGS
287.4543	0.3554	Pseudomonas aeruginosa (286)	Pseudomonas (135621)	Pseudomonadaceae (7227	Pseudomonadales (1236)	Gammaproteobacteria (122	Proteobacteria (2)	Bacteria (1)	100.00	0.90	99.80	98.40	WGS
287.4544	0.3258	Pseudomonas aeruginosa (286)	Pseudomonas (135621)	Pseudomonadaceae (7227	Pseudomonadales (1236)	Gammaproteobacteria (122	Proteobacteria (2)	Bacteria (1)	99.70	0.00	98.90	98.60	WGS
287.4545	0.368	Pseudomonas aeruginosa (286)	Pseudomonas (135621)	Pseudomonadaceae (7227	Pseudomonadales (1236)	Gammaproteobacteria (122	Proteobacteria (2)	Bacteria (1)	99.90	1.60	99.90	96.70	WGS
287.4546	0.3256	Pseudomonas aeruginosa (286)	Pseudomonas (135621)	Pseudomonadaceae (7227	Pseudomonadales (1236)	Gammaproteobacteria (122	Proteobacteria (2)	Bacteria (1)	100.00	3.90	99.60	94.80	WGS
287.4547	0.3628	Pseudomonas aeruginosa (286)	Pseudomonas (135621)	Pseudomonadaceae (7227	Pseudomonadales (1236)	Gammaproteobacteria (122	Proteobacteria (2)	Bacteria (1)	100.00	0.00	99.90	98.00	WGS
287.4548	0.3636	Pseudomonas aeruginosa (286)	Pseudomonas (135621)	Pseudomonadaceae (7227	Pseudomonadales (1236)	Gammaproteobacteria (122	Proteobacteria (2)	Bacteria (1)	100.00	0.90	99.90	97.30	WGS
287.4549	0.324	Pseudomonas aeruginosa (286)	Pseudomonas (135621)	Pseudomonadaceae (7227	Pseudomonadales (1236)	Gammaproteobacteria (122	Proteobacteria (2)	Bacteria (1)	100.00	1.20	99.90	97.00	WGS
287.4550	0.324	Pseudomonas aeruginosa (286)	Pseudomonas (135621)	Pseudomonadaceae (7227	Pseudomonadales (1236)	Gammaproteobacteria (122	Proteobacteria (2)	Bacteria (1)	100.00	1.20	99.90	97.60	WGS
287.4551	0.324	Pseudomonas aeruginosa (286)	Pseudomonas (135621)	Pseudomonadaceae (7227	Pseudomonadales (1236)	Gammaproteobacteria (122	Proteobacteria (2)	Bacteria (1)	100.00	0.60	99.90	97.70	WGS
287.4552	0.324	Pseudomonas aeruginosa (286)	Pseudomonas (135621)	Pseudomonadaceae (7227	Pseudomonadales (1236)	Gammaproteobacteria (122	Proteobacteria (2)	Bacteria (1)	100.00	0.00	99.90	99.10	WGS
287.4553	0.3578	Pseudomonas aeruginosa (286)	Pseudomonas (135621)	Pseudomonadaceae (7227	Pseudomonadales (1236)	Gammaproteobacteria (122	Proteobacteria (2)	Bacteria (1)	100.00	0.60	99.80	98.70	WGS
287.4554	0.3646	Pseudomonas aeruginosa (286)	Pseudomonas (135621)	Pseudomonadaceae (7227	Pseudomonadales (1236)	Gammaproteobacteria (122	Proteobacteria (2)	Bacteria (1)	100.00	0.30	99.90	99.30	Complete
287.4556	0.289	Pseudomonas aeruginosa (286)	Pseudomonas (135621)	Pseudomonadaceae (7227	Pseudomonadales (1236)	Gammaproteobacteria (122	Proteobacteria (2)	Bacteria (1)	88.00	2.20	95.60	92.50	WGS
287.4557	0.3672	Pseudomonas aeruginosa (286)	Pseudomonas (135621)	Pseudomonadaceae (7227	Pseudomonadales (1236)	Gammaproteobacteria (122	Proteobacteria (2)	Bacteria (1)	99.70	1.80	98.10	96.20	WGS
287.4558	0.331	Pseudomonas aeruginosa (286)	Pseudomonas (135621)	Pseudomonadaceae (7227	Pseudomonadales (1236)	Gammaproteobacteria (122	Proteobacteria (2)	Bacteria (1)	100.00	0.30	99.90	98.90	WGS
287.4560	0.3696	Pseudomonas aeruginosa (286)	Pseudomonas (135621)	Pseudomonadaceae (7227	Pseudomonadales (1236)	Gammaproteobacteria (122	Proteobacteria (2)	Bacteria (1)	98.40	1.80	99.40	98.40	WGS
287.4561	0.3226	Pseudomonas aeruginosa (286)	Pseudomonas (135621)	Pseudomonadaceae (7227	Pseudomonadales (1236)	Gammaproteobacteria (122	Proteobacteria (2)	Bacteria (1)	100.00	0.00	99.90	99.30	WGS
287.4562	0.3422	Pseudomonas aeruginosa (286)	Pseudomonas (135621)	Pseudomonadaceae (7227	Pseudomonadales (1236)	Gammaproteobacteria (122	Proteobacteria (2)	Bacteria (1)	98.30	7.10	98.80	91.20	WGS
287.4563	0.3696	Pseudomonas aeruginosa (286)	Pseudomonas (135621)	Pseudomonadaceae (7227	Pseudomonadales (1236)	Gammaproteobacteria (122	Proteobacteria (2)	Bacteria (1)	98.40	1.50	99.60	98.60	WGS
287.4564	0.3636	Pseudomonas aeruginosa (286)	Pseudomonas (135621)	Pseudomonadaceae (7227	Pseudomonadales (1236)	Gammaproteobacteria (122	Proteobacteria (2)	Bacteria (1)	100.00	0.90	99.80	99.20	WGS
287.4565	0.3636	Pseudomonas aeruginosa (286)	Pseudomonas (135621)	Pseudomonadaceae (7227	Pseudomonadales (1236)	Gammaproteobacteria (122	Proteobacteria (2)	Bacteria (1)	100.00	0.00	99.90	99.00	WGS
287.4566	0.368	Pseudomonas aeruginosa (286)	Pseudomonas (135621)	Pseudomonadaceae (7227	Pseudomonadales (1236)	Gammaproteobacteria (122	Proteobacteria (2)	Bacteria (1)	100.00	0.60	99.90	99.00	Complete
287.4598	0.0294	Pseudomonas aeruginosa (286)	Pseudomonas (135621)	Pseudomonadaceae (7227	Pseudomonadales (1236)	Gammaproteobacteria (122	Proteobacteria (2)	Bacteria (1)					Plasmid
287.4599	0.0284	Pseudomonas aeruginosa (286)	Pseudomonas (135621)	Pseudomonadaceae (7227	Pseudomonadales (1236)	Gammaproteobacteria (122	Proteobacteria (2)	Bacteria (1)					Plasmid
287.4600	0.0294	Pseudomonas aeruginosa (286)	Pseudomonas (135621)	Pseudomonadaceae (7227	Pseudomonadales (1236)	Gammaproteobacteria (122	Proteobacteria (2)	Bacteria (1)					Plasmid
287.4601	0.0294	Pseudomonas aeruginosa (286)	Pseudomonas (135621)	Pseudomonadaceae (7227	Pseudomonadales (1236)	Gammaproteobacteria (122	Proteobacteria (2)	Bacteria (1)					Plasmid
287.4602	0.0294	Pseudomonas aeruginosa (286)	Pseudomonas (135621)	Pseudomonadaceae (7227	Pseudomonadales (1236)	Gammaproteobacteria (122	Proteobacteria (2)	Bacteria (1)					Plasmid
287.4603	0.0294	Pseudomonas aeruginosa (286)	Pseudomonas (135621)	Pseudomonadaceae (7227	Pseudomonadales (1236)	Gammaproteobacteria (122	Proteobacteria (2)	Bacteria (1)					Plasmid
287.4604	0.0294	Pseudomonas aeruginosa (286)	Pseudomonas (135621)	Pseudomonadaceae (7227	Pseudomonadales (1236)	Gammaproteobacteria (122	Proteobacteria (2)	Bacteria (1)					Plasmid
287.4605	0.0294	Pseudomonas aeruginosa (286)	Pseudomonas (135621)	Pseudomonadaceae (7227	Pseudomonadales (1236)	Gammaproteobacteria (122	Proteobacteria (2)	Bacteria (1)					Plasmid
287.4606	0.0296	Pseudomonas aeruginosa (286)	Pseudomonas (135621)	Pseudomonadaceae (7227	Pseudomonadales (1236)	Gammaproteobacteria (122	Proteobacteria (2)	Bacteria (1)					Plasmid
287.4607	0.034	Pseudomonas aeruginosa (286)	Pseudomonas (135621)	Pseudomonadaceae (7227	Pseudomonadales (1236)	Gammaproteobacteria (122	Proteobacteria (2)	Bacteria (1)					Plasmid
287.4608	0.0298	Pseudomonas aeruginosa (286)	Pseudomonas (135621)	Pseudomonadaceae (7227	Pseudomonadales (1236)	Gammaproteobacteria (122	Proteobacteria (2)	Bacteria (1)					Plasmid
287.4609	0.0294	Pseudomonas aeruginosa (286)	Pseudomonas (135621)	Pseudomonadaceae (7227	Pseudomonadales (1236)	Gammaproteobacteria (122	Proteobacteria (2)	Bacteria (1)					Plasmid
287.4610	0.0294	Pseudomonas aeruginosa (286)	Pseudomonas (135621)	Pseudomonadaceae (7227	Pseudomonadales (1236)	Gammaproteobacteria (122	Proteobacteria (2)	Bacteria (1)					Plasmid
287.4611	0.0294	Pseudomonas aeruginosa (286)	Pseudomonas (135621)	Pseudomonadaceae (7227	Pseudomonadales (1236)	Gammaproteobacteria (122	Proteobacteria (2)	Bacteria (1)					Plasmid
287.4612	0.0294	Pseudomonas aeruginosa (286)	Pseudomonas (135621)	Pseudomonadaceae (7227	Pseudomonadales (1236)	Gammaproteobacteria (122	Proteobacteria (2)	Bacteria (1)					Plasmid
287.4613	0.0324	Pseudomonas aeruginosa (286)	Pseudomonas (135621)	Pseudomonadaceae (7227	Pseudomonadales (1236)	Gammaproteobacteria (122	Proteobacteria (2)	Bacteria (1)					Plasmid
287.4614	0.0294	Pseudomonas aeruginosa (286)	Pseudomonas (135621)	Pseudomonadaceae (7227	Pseudomonadales (1236)	Gammaproteobacteria (122	Proteobacteria (2)	Bacteria (1)					Plasmid
287.4615	0.0294	Pseudomonas aeruginosa (286)	Pseudomonas (135621)	Pseudomonadaceae (7227	Pseudomonadales (1236)	Gammaproteobacteria (122	Proteobacteria (2)	Bacteria (1)					Plasmid
287.4616	0.0294	Pseudomonas aeruginosa (286)	Pseudomonas (135621)	Pseudomonadaceae (7227	Pseudomonadales (1236)	Gammaproteobacteria (122	Proteobacteria (2)	Bacteria (1)					Plasmid
287.4617	0.0294	Pseudomonas aeruginosa (286)	Pseudomonas (135621)	Pseudomonadaceae (7227	Pseudomonadales (1236)	Gammaproteobacteria (122	Proteobacteria (2)	Bacteria (1)					Plasmid
287.4618	0.04	Pseudomonas aeruginosa (286)	Pseudomonas (135621)	Pseudomonadaceae (7227	Pseudomonadales (1236)	Gammaproteobacteria (122	Proteobacteria (2)	Bacteria (1)					Plasmid
287.4620	0.3258	Pseudomonas aeruginosa (286)	Pseudomonas (135621)	Pseudomonadaceae (7227	Pseudomonadales (1236)	Gammaproteobacteria (122	Proteobacteria (2)	Bacteria (1)	100.00	0.30	99.70	98.20	WGS
287.4621	0.3742	Pseudomonas aeruginosa (286)	Pseudomonas (135621)	Pseudomonadaceae (7227	Pseudomonadales (1236)	Gammaproteobacteria (122	Proteobacteria (2)	Bacteria (1)	97.30	0.00	98.60	97.50	Complete
287.4622	0.3352	Pseudomonas aeruginosa (286)	Pseudomonas (135621)	Pseudomonadaceae (7227	Pseudomonadales (1236)	Gammaproteobacteria (122	Proteobacteria (2)	Bacteria (1)	98.20	0.00	99.00	98.20	Complete
287.4623	0.3618	Pseudomonas aeruginosa (286)	Pseudomonas (135621)	Pseudomonadaceae (7227	Pseudomonadales (1236)	Gammaproteobacteria (122	Proteobacteria (2)	Bacteria (1)	100.00	0.00	99.90	99.00	Complete
287.4624	0.3618	Pseudomonas aeruginosa (286)	Pseudomonas (135621)	Pseudomonadaceae (7227	Pseudomonadales (1236)	Gammaproteobacteria (122	Proteobacteria (2)	Bacteria (1)	100.00	0.00	99.90	99.00	Complete
287.4625	0.3306	Pseudomonas aeruginosa (286)	Pseudomonas (135621)	Pseudomonadaceae (7227	Pseudomonadales (1236)	Gammaproteobacteria (122	Proteobacteria (2)	Bacteria (1)	100.00	4.40	99.70	96.60	WGS
287.4626	0.3306	Pseudomonas aeruginosa (286)	Pseudomonas (135621)	Pseudomonadaceae (7227	Pseudomonadales (1236)	Gammaproteobacteria (122	Proteobacteria (2)	Bacteria (1)	100.00	1.50	99.90	97.60	WGS
287.5660	0.3658	Pseudomonas aeruginosa (286)	Pseudomonas (135621)	Pseudomonadaceae (7227	Pseudomonadales (1236)	Gammaproteobacteria (122	Proteobacteria (2)	Bacteria (1)	98.80	0.00	99.30	98.80	
287.5661	0.3632	Pseudomonas aeruginosa (286)	Pseudomonas (135621)	Pseudomonadaceae (7227	Pseudomonadales (1236)	Gammaproteobacteria (122	Proteobacteria (2)	Bacteria (1)	100.00	0.30	99.60	99.00	
287.5662	0.3672	Pseudomonas aeruginosa (286)	Pseudomonas (135621)	Pseudomonadaceae (7227	Pseudomonadales (1236)	Gammaproteobacteria (122	Proteobacteria (2)	Bacteria (1)	100.00	0.00	99.80	98.70	
287.5663	0.3226	Pseudomonas aeruginosa (286)	Pseudomonas (135621)	Pseudomonadaceae (7227	Pseudomonadales (1236)	Gammaproteobacteria (122	Proteobacteria (2)	Bacteria (1)	100.00	0.30	99.40	98.80	
287.5664	0.3632	Pseudomonas aeruginosa (286)	Pseudomonas (135621)	Pseudomonadaceae (7227	Pseudomonadales (1236)	Gammaproteobacteria (122	Proteobacteria (2)	Bacteria (1)	100.00	0.00	99.60	99.00	
287.5665	0.3704	Pseudomonas aeruginosa (286)	Pseudomonas (135621)	Pseudomonadaceae (7227	Pseudomonadales (1236)	Gammaproteobacteria (122	Proteobacteria (2)	Bacteria (1)	100.00	0.60	99.70	98.60	
287.5666	0.3628	Pseudomonas aeruginosa (286)	Pseudomonas (135621)	Pseudomonadaceae (7227	Pseudomonadales (1236)	Gammaproteobacteria (122	Proteobacteria (2)	Bacteria (1)	100.00	0.70	99.90	99.00	
287.5667	0.3258	Pseudomonas aeruginosa (286)	Pseudomonas (135621)	Pseudomonadaceae (7227	Pseudomonadales (1236)	Gammaproteobacteria (122	Proteobacteria (2)	Bacteria (1)	100.00	0.60	99.70	99.00	WGS
287.5668	0.3738	Pseudomonas aeruginosa (286)	Pseudomonas (135621)	Pseudomonadaceae (7227	Pseudomonadales (1236)	Gammaproteobacteria (122	Proteobacteria (2)	Bacteria (1)	100.00	0.00	99.90	99.30	WGS
287.5669	0.368	Pseudomonas aeruginosa (286)	Pseudomonas (135621)	Pseudomonadaceae (7227	Pseudomonadales (1236)	Gammaproteobacteria (122	Proteobacteria (2)	Bacteria (1)	100.00	0.30	99.80	99.30	WGS
287.5670	0.3754	Pseudomonas aeruginosa (286)	Pseudomonas (135621)	Pseudomonadaceae (7227	Pseudomonadales (1236)	Gammaproteobacteria (122	Proteobacteria (2)	Bacteria (1)	99.70	0.90	99.60	97.00	WGS
287.5671	0.324	Pseudomonas aeruginosa (286)	Pseudomonas (135621)	Pseudomonadaceae (7227	Pseudomonadales (1236)	Gammaproteobacteria (122	Proteobacteria (2)	Bacteria (1)	100.00	0.90	99.90	98.50	WGS
287.5672	0.3738	Pseudomonas aeruginosa (286)	Pseudomonas (135621)	Pseudomonadaceae (7227	Pseudomonadales (1236)	Gammaproteobacteria (122	Proteobacteria (2)	Bacteria (1)	98.80	0.90	99.70	99.00	WGS
287.5673	0.3188	Pseudomonas aeruginosa (286)	Pseudomonas (135621)	Pseudomonadaceae (7227	Pseudomonadales (1236)	Gammaproteobacteria (122	Proteobacteria (2)	Bacteria (1)	100.00	1.30	99.90	99.00	WGS
287.5674	0.3748	Pseudomonas aeruginosa (286)	Pseudomonas (135621)	Pseudomonadaceae (7227	Pseudomonadales (1236)	Gammaproteobacteria (122	Proteobacteria (2)	Bacteria (1)	100.00	0.00	99.70	99.00	WGS
287.5675	0.0294	Pseudomonas aeruginosa (286)	Pseudomonas (135621)	Pseudomonadaceae (7227	Pseudomonadales (1236)	Gammaproteobacteria (122	Proteobacteria (2)	Bacteria (1)	79.00	7.40	99.50	97.60	WGS
287.5676	0.3174	Pseudomonas aeruginosa (286)	Pseudomonas (135621)	Pseudomonadaceae (7227	Pseudomonadales (1236)	Gammaproteobacteria (122	Proteobacteria (2)	Bacteria (1)	99.70	0.00	99.80	99.00	WGS
287.5677	0.3324	Pseudomonas aeruginosa (286)	Pseudomonas (135621)	Pseudomonadaceae (7227	Pseudomonadales (1236)	Gammaproteobacteria (122	Proteobacteria (2)	Bacteria (1)	100.00	0.70	99.80	98.60	WGS
287.5678	0.324	Pseudomonas aeruginosa (286)	Pseudomonas (135621)	Pseudomonadaceae (7227	Pseudomonadales (1236)	Gammaproteobacteria (122	Proteobacteria (2)	Bacteria (1)	100.00	0.90	99.90	99.20	WGS
287.5679	0.3188	Pseudomonas aeruginosa (286)	Pseudomonas (135621)	Pseudomonadaceae (7227	Pseudomonadales (1236)	Gammaproteobacteria (122	Proteobacteria (2)	Bacteria (1)	100.00	0.00	99.90	99.60	WGS
287.5680	0.3244	Pseudomonas aeruginosa (286)	Pseudomonas (135621)	Pseudomonadaceae (7227	Pseudomonadales (1236)	Gammaproteobacteria (122	Proteobacteria (2)	Bacteria (1)	100.00	3.80	99.10	96.20	WGS
287.5681	0.3488	Pseudomonas aeruginosa (286)	Pseudomonas (135621)	Pseudomonadaceae (7227	Pseudomonadales (1236)	Gammaproteobacteria (122	Proteobacteria (2)	Bacteria (1)	99.70	0.30	99.70	99.00	Complete
287.5682	0.3372	Pseudomonas aeruginosa (286)	Pseudomonas (135621)	Pseudomonadaceae (7227	Pseudomonadales (1236)	Gammaproteobacteria (122	Proteobacteria (2)	Bacteria (1)	100.00	1.20	100.00	99.30	WGS
287.5683	0.3748	Pseudomonas aeruginosa (286)	Pseudomonas (135621)	Pseudomonadaceae (7227	Pseudomonadales (1236)	Gammaproteobacteria (122	Proteobacteria (2)	Bacteria (1)	100.00	0.00	99.90	99.00	WGS
287.5684	0.3754	Pseudomonas aeruginosa (286)	Pseudomonas (135621)	Pseudomonadaceae (7227	Pseudomonadales (1236)	Gammaproteobacteria (122	Proteobacteria (2)	Bacteria (1)	100.00	0.10	99.80	99.30	WGS
287.5685	0.3636	Pseudomonas aeruginosa (286)	Pseudomonas (135621)	Pseudomonadaceae (7227	Pseudomonadales (1236)	Gammaproteobacteria (122	Proteobacteria (2)	Bacteria (1)	100.00	0.00	99.80	99.30	Complete
287.5686	0.3672	Pseudomonas aeruginosa (286)	Pseudomonas (135621)	Pseudomonadaceae (7227	Pseudomonadales (1236)	Gammaproteobacteria (122	Proteobacteria (2)	Bacteria (1)	100.00	0.30	99.90	98.70	Complete
287.5687	0.3672	Pseudomonas aeruginosa (286)	Pseudomonas (135621)	Pseudomonadaceae (7227	Pseudomonadales (1236)	Gammaproteobacteria (122	Proteobacteria (2)	Bacteria (1)	100.00	0.30	99.90	98.80	Complete
287.5688	0.368	Pseudomonas aeruginosa (286)	Pseudomonas (135621)	Pseudomonadaceae (7227	Pseudomonadales (1236)	Gammaproteobacteria (122	Proteobacteria (2)	Bacteria (1)	100.00	0.00	99.80	99.10	Complete
287.5689	0.367	Pseudomonas aeruginosa (286)	Pseudomonas (135621)	Pseudomonadaceae (7227	Pseudomonadales (1236)	Gammaproteobacteria (122	Proteobacteria (2)	Bacteria (1)	98.40	1.50	99.60	97.60	Complete
287.5690	0.3618	Pseudomonas aeruginosa (286)	Pseudomonas (135621)	Pseudomonadaceae (7227	Pseudomonadales (1236)	Gammaproteobacteria (122	Proteobacteria (2)	Bacteria (1)	100.00	0.00	99.90	98.90	Complete
287.5691	0.3292	Pseudomonas aeruginosa (286)	Pseudomonas (135621)	Pseudomonadaceae (7227	Pseudomonadales (1236)	Gammaproteobacteria (122	Proteobacteria (2)	Bacteria (1)	100.00	0.00	99.90	99.80	WGS
287.5692	0.3292	Pseudomonas aeruginosa (286)	Pseudomonas (135621)	Pseudomonadaceae (7227	Pseudomonadales (1236)	Gammaproteobacteria (122	Proteobacteria (2)	Bacteria (1)	100.00	0.00	99.90	99.80	WGS
287.5693	0.3636	Pseudomonas aeruginosa (286)	Pseudomonas (135621)	Pseudomonadaceae (7227	Pseudomonadales (1236)	Gammaproteobacteria (122	Proteobacteria (2)	Bacteria (1)	100.00	0.00	99.90	99.60	WGS
287.5694	0.331	Pseudomonas aeruginosa (286)	Pseudomonas (135621)	Pseudomonadaceae (7227	Pseudomonadales (1236)	Gammaproteobacteria (122	Proteobacteria (2)	Bacteria (1)	100.00	0.00	99.90	99.60	WGS
287.5695	0.3188	Pseudomonas aeruginosa (286)	Pseudomonas (135621)	Pseudomonadaceae (7227	Pseudomonadales (1236)	Gammaproteobacteria (122	Proteobacteria (2)	Bacteria (1)	100.00	0.30	99.90	99.30	WGS
287.5696	0.3188	Pseudomonas aeruginosa (286)	Pseudomonas (135621)	Pseudomonadaceae (7227	Pseudomonadales (1236)	Gammaproteobacteria (122	Proteobacteria (2)	Bacteria (1)	100.00	0.30	99.90	99.30	WGS
287.5697	0.3188	Pseudomonas aeruginosa (286)	Pseudomonas (135621)	Pseudomonadaceae (7227	Pseudomonadales (1236)	Gammaproteobacteria (122	Proteobacteria (2)	Bacteria (1)	100.00	0.30	99.90	99.30	WGS
287.5698	0.3636	Pseudomonas aeruginosa (286)	Pseudomonas (135621)	Pseudomonadaceae (7227	Pseudomonadales (1236)	Gammaproteobacteria (122	Proteobacteria (2)	Bacteria (1)	100.00	0.00	99.90	99.60	WGS
287.5699	0.3636	Pseudomonas aeruginosa (286)	Pseudomonas (135621)	Pseudomonadaceae (7227	Pseudomonadales (1236)	Gammaproteobacteria (122	Proteobacteria (2)	Bacteria (1)	100.00	0.00	99.70	99.30	WGS
287.5700	0.3636	Pseudomonas aeruginosa (286)	Pseudomonas (135621)	Pseudomonadaceae (7227	Pseudomonadales (1236)	Gammaproteobacteria (122	Proteobacteria (2)	Bacteria (1)	100.00	0.60	100.00	99.40	WGS
287.5701	0.3672	Pseudomonas aeruginosa (286)	Pseudomonas (135621)	Pseudomonadaceae (7227	Pseudomonadales (1236)	Gammaproteobacteria (122	Proteobacteria (2)	Bacteria (1)	100.00	0.30	99.70	98.70	WGS
287.5702	0.3376	Pseudomonas aeruginosa (286)	Pseudomonas (135621)	Pseudomonadaceae (7227	Pseudomonadales (1236)	Gammaproteobacteria (122	Proteobacteria (2)	Bacteria (1)	100.00	0.00	99.90	98.50	WGS
287.5703	0.3386	Pseudomonas aeruginosa (286)	Pseudomonas (135621)	Pseudomonadaceae (7227	Pseudomonadales (1236)	Gammaproteobacteria (122	Proteobacteria (2)	Bacteria (1)	100.00	0.00	99.70	98.70	WGS
287.5704	0.3258	Pseudomonas aeruginosa (286)	Pseudomonas (135621)	Pseudomonadaceae (7227	Pseudomonadales (1236)	Gammaproteobacteria (122	Proteobacteria (2)	Bacteria (1)	100.00	0.00	99.80	99.30	WGS
287.5705	0.3258	Pseudomonas aeruginosa (286)	Pseudomonas (135621)	Pseudomonadaceae (7227	Pseudomonadales (1236)	Gammaproteobacteria (122	Proteobacteria (2)	Bacteria (1)	100.00	0.20	99.70	99.30	WGS
287.5706	0.3636	Pseudomonas aeruginosa (286)	Pseudomonas (135621)	Pseudomonadaceae (7227	Pseudomonadales (1236)	Gammaproteobacteria (122	Proteobacteria (2)	Bacteria (1)	98.40	1.80	99.80	98.90	Complete
287.5707	0.3672	Pseudomonas aeruginosa (286)	Pseudomonas (135621)	Pseudomonadaceae (7227	Pseudomonadales (1236)	Gammaproteobacteria (122	Proteobacteria (2)	Bacteria (1)	100.00	0.20	99.90	98.70	Complete
287.5708	0.3268	Pseudomonas aeruginosa (286)	Pseudomonas (135621)	Pseudomonadaceae (7227	Pseudomonadales (1236)	Gammaproteobacteria (122	Proteobacteria (2)	Bacteria (1)	100.00	3.70	99.90	95.90	WGS
287.5709	0.327	Pseudomonas aeruginosa (286)	Pseudomonas (135621)	Pseudomonadaceae (7227	Pseudomonadales (1236)	Gammaproteobacteria (122	Proteobacteria (2)	Bacteria (1)	100.00	0.30	99.60	99.30	WGS
287.5710	0.3738	Pseudomonas aeruginosa (286)	Pseudomonas (135621)	Pseudomonadaceae (7227	Pseudomonadales (1236)	Gammaproteobacteria (122	Proteobacteria (2)	Bacteria (1)	100.00	0.30	99.90	99.00	WGS
287.5711	0.3174	Pseudomonas aeruginosa (286)	Pseudomonas (135621)	Pseudomonadaceae (7227	Pseudomonadales (1236)	Gammaproteobacteria (122	Proteobacteria (2)	Bacteria (1)	100.00	0.00	99.90	99.10	WGS
287.5728	0.3268	Pseudomonas aeruginosa (286)	Pseudomonas (135621)	Pseudomonadaceae (7227	Pseudomonadales (1236)	Gammaproteobacteria (122	Proteobacteria (2)	Bacteria (1)	100.00	5.90	99.90	95.60	WGS
287.5729	0.3636	Pseudomonas aeruginosa (286)	Pseudomonas (135621)	Pseudomonadaceae (7227	Pseudomonadales (1236)	Gammaproteobacteria (122	Proteobacteria (2)	Bacteria (1)	100.00	0.90	99.90	99.10	WGS
287.5730	0.3694	Pseudomonas aeruginosa (286)	Pseudomonas (135621)	Pseudomonadaceae (7227	Pseudomonadales (1236)	Gammaproteobacteria (122	Proteobacteria (2)	Bacteria (1)	100.00	0.00	99.40	99.10	WGS
287.5731	0.3636	Pseudomonas aeruginosa (286)	Pseudomonas (135621)	Pseudomonadaceae (7227	Pseudomonadales (1236)	Gammaproteobacteria (122	Proteobacteria (2)	Bacteria (1)	100.00	2.60	99.70	97.90	WGS
287.5732	0.3254	Pseudomonas aeruginosa (286)	Pseudomonas (135621)	Pseudomonadaceae (7227	Pseudomonadales (1236)	Gammaproteobacteria (122	Proteobacteria (2)	Bacteria (1)	100.00	0.90	99.60	98.80	WGS
287.5733	0.324	Pseudomonas aeruginosa (286)	Pseudomonas (135621)	Pseudomonadaceae (7227	Pseudomonadales (1236)	Gammaproteobacteria (122	Proteobacteria (2)	Bacteria (1)	100.00	0.90	99.80	98.30	WGS
287.5734	0.368	Pseudomonas aeruginosa (286)	Pseudomonas (135621)	Pseudomonadaceae (7227	Pseudomonadales (1236)	Gammaproteobacteria (122	Proteobacteria (2)	Bacteria (1)	100.00	0.00	99.90	99.60	WGS
287.5735	0.3632	Pseudomonas aeruginosa (286)	Pseudomonas (135621)	Pseudomonadaceae (7227	Pseudomonadales (1236)	Gammaproteobacteria (122	Proteobacteria (2)	Bacteria (1)	100.00	0.30	99.50	98.60	WGS
287.5736	0.3636	Pseudomonas aeruginosa (286)	Pseudomonas (135621)	Pseudomonadaceae (7227	Pseudomonadales (1236)	Gammaproteobacteria (122	Proteobacteria (2)	Bacteria (1)	100.00	0.60	99.90	98.20	WGS
287.5737	0.3694	Pseudomonas aeruginosa (286)	Pseudomonas (135621)	Pseudomonadaceae (7227	Pseudomonadales (1236)	Gammaproteobacteria (122	Proteobacteria (2)	Bacteria (1)	100.00	0.60	99.70	99.20	WGS
287.5738	0.324	Pseudomonas aeruginosa (286)	Pseudomonas (135621)	Pseudomonadaceae (7227	Pseudomonadales (1236)	Gammaproteobacteria (122	Proteobacteria (2)	Bacteria (1)	100.00	0.00	99.90	99.10	WGS
287.5739	0.3646	Pseudomonas aeruginosa (286)	Pseudomonas (135621)	Pseudomonadaceae (7227	Pseudomonadales (1236)	Gammaproteobacteria (122	Proteobacteria (2)	Bacteria (1)	100.00	0.00	99.80	99.60	WGS
287.5742	0.3694	Pseudomonas aeruginosa (286)	Pseudomonas (135621)	Pseudomonadaceae (7227	Pseudomonadales (1236)	Gammaproteobacteria (122	Proteobacteria (2)	Bacteria (1)	100.00	0.60	99.70	97.00	WGS
287.5743	0.3628	Pseudomonas aeruginosa (286)	Pseudomonas (135621)	Pseudomonadaceae (7227	Pseudomonadales (1236)	Gammaproteobacteria (122	Proteobacteria (2)	Bacteria (1)	100.00	0.00	99.90	99.10	WGS
287.5744	0.3314	Pseudomonas aeruginosa (286)	Pseudomonas (135621)	Pseudomonadaceae (7227	Pseudomonadales (1236)	Gammaproteobacteria (122	Proteobacteria (2)	Bacteria (1)	100.00	0.30	99.80	99.00	WGS
287.5746	0.3632	Pseudomonas aeruginosa (286)	Pseudomonas (135621)	Pseudomonadaceae (7227	Pseudomonadales (1236)	Gammaproteobacteria (122	Proteobacteria (2)	Bacteria (1)	100.00	0.00	99.60	99.30	Complete
287.5747	0.3672	Pseudomonas aeruginosa (286)	Pseudomonas (135621)	Pseudomonadaceae (7227	Pseudomonadales (1236)	Gammaproteobacteria (122	Proteobacteria (2)	Bacteria (1)	100.00	0.60	99.70	98.70	Complete
287.5748	0.3672	Pseudomonas aeruginosa (286)	Pseudomonas (135621)	Pseudomonadaceae (7227	Pseudomonadales (1236)	Gammaproteobacteria (122	Proteobacteria (2)	Bacteria (1)	100.00	0.30	99.60	98.50	Complete
287.5749	0.3386	Pseudomonas aeruginosa (286)	Pseudomonas (135621)	Pseudomonadaceae (7227	Pseudomonadales (1236)	Gammaproteobacteria (122	Proteobacteria (2)	Bacteria (1)	100.00	0.00	99.80	98.80	Complete
287.5750	0.338	Pseudomonas aeruginosa (286)	Pseudomonas (135621)	Pseudomonadaceae (7227	Pseudomonadales (1236)	Gammaproteobacteria (122	Proteobacteria (2)	Bacteria (1)	100.00	0.00	99.80	99.10	Complete
287.5751	0.367	Pseudomonas aeruginosa (286)	Pseudomonas (135621)	Pseudomonadaceae (7227	Pseudomonadales (1236)	Gammaproteobacteria (122	Proteobacteria (2)	Bacteria (1)	98.10	1.90	99.50	96.90	Complete
287.5752	0.3622	Pseudomonas aeruginosa (286)	Pseudomonas (135621)	Pseudomonadaceae (7227	Pseudomonadales (1236)	Gammaproteobacteria (122	Proteobacteria (2)	Bacteria (1)	100.00	0.00	99.80	99.40	Complete
287.5753	0.3354	Pseudomonas aeruginosa (286)	Pseudomonas (135621)	Pseudomonadaceae (7227	Pseudomonadales (1236)	Gammaproteobacteria (122	Proteobacteria (2)	Bacteria (1)	99.40	0.30	99.60	98.20	Complete
287.5754	0.3694	Pseudomonas aeruginosa (286)	Pseudomonas (135621)	Pseudomonadaceae (7227	Pseudomonadales (1236)	Gammaproteobacteria (122	Proteobacteria (2)	Bacteria (1)	98.40	1.50	99.80	97.60	WGS
287.5756	0.0294	Pseudomonas aeruginosa (286)	Pseudomonas (135621)	Pseudomonadaceae (7227	Pseudomonadales (1236)	Gammaproteobacteria (122	Proteobacteria (2)	Bacteria (1)	100.00	0.00	99.90	98.70	WGS
287.5757	0.3386	Pseudomonas aeruginosa (286)	Pseudomonas (135621)	Pseudomonadaceae (7227	Pseudomonadales (1236)	Gammaproteobacteria (122	Proteobacteria (2)	Bacteria (1)	100.00	0.00	99.90	99.00	WGS
287.5758	0.338	Pseudomonas aeruginosa (286)	Pseudomonas (135621)	Pseudomonadaceae (7227	Pseudomonadales (1236)	Gammaproteobacteria (122	Proteobacteria (2)	Bacteria (1)	100.00	0.00	99.90	99.50	WGS
287.5759	0.331	Pseudomonas aeruginosa (286)	Pseudomonas (135621)	Pseudomonadaceae (7227	Pseudomonadales (1236)	Gammaproteobacteria (122	Proteobacteria (2)	Bacteria (1)	100.00	0.00	99.80	99.30	WGS
287.5760	0.3618	Pseudomonas aeruginosa (286)	Pseudomonas (135621)	Pseudomonadaceae (7227	Pseudomonadales (1236)	Gammaproteobacteria (122	Proteobacteria (2)	Bacteria (1)	100.00	0.00	99.90	99.60	WGS
287.5761	0.3618	Pseudomonas aeruginosa (286)	Pseudomonas (135621)	Pseudomonadaceae (7227	Pseudomonadales (1236)	Gammaproteobacteria (122	Proteobacteria (2)	Bacteria (1)	100.00	0.00	99.90	98.80	WGS
287.5762	0.3324	Pseudomonas aeruginosa (286)	Pseudomonas (135621)	Pseudomonadaceae (7227	Pseudomonadales (1236)	Gammaproteobacteria (122	Proteobacteria (2)	Bacteria (1)	100.00	0.00	99.80	99.70	WGS
287.5763	0.3388	Pseudomonas aeruginosa (286)	Pseudomonas (135621)	Pseudomonadaceae (7227	Pseudomonadales (1236)	Gammaproteobacteria (122	Proteobacteria (2)	Bacteria (1)	100.00	0.30	99.90	98.90	WGS
287.5764	0.3748	Pseudomonas aeruginosa (286)	Pseudomonas (135621)	Pseudomonadaceae (7227	Pseudomonadales (1236)	Gammaproteobacteria (122	Proteobacteria (2)	Bacteria (1)	100.00	0.30	99.90	98.70	WGS
287.5765	0.3334	Pseudomonas aeruginosa (286)	Pseudomonas (135621)	Pseudomonadaceae (7227	Pseudomonadales (1236)	Gammaproteobacteria (122	Proteobacteria (2)	Bacteria (1)	100.00	0.00	99.90	99.60	WGS
287.5766	0.3268	Pseudomonas aeruginosa (286)	Pseudomonas (135621)	Pseudomonadaceae (7227	Pseudomonadales (1236)	Gammaproteobacteria (122	Proteobacteria (2)	Bacteria (1)	100.00	0.00	99.90	99.40	WGS
287.5767	0.3618	Pseudomonas aeruginosa (286)	Pseudomonas (135621)	Pseudomonadaceae (7227	Pseudomonadales (1236)	Gammaproteobacteria (122	Proteobacteria (2)	Bacteria (1)	100.00	0.00	99.90	98.80	WGS
287.5768	0.368	Pseudomonas aeruginosa (286)	Pseudomonas (135621)	Pseudomonadaceae (7227	Pseudomonadales (1236)	Gammaproteobacteria (122	Proteobacteria (2)	Bacteria (1)	100.00	0.00	99.90	98.70	WGS
287.5769	0.324	Pseudomonas aeruginosa (286)	Pseudomonas (135621)	Pseudomonadaceae (7227	Pseudomonadales (1236)	Gammaproteobacteria (122	Proteobacteria (2)	Bacteria (1)	100.00	1.20	99.90	99.40	WGS
287.5770	0.3268	Pseudomonas aeruginosa (286)	Pseudomonas (135621)	Pseudomonadaceae (7227	Pseudomonadales (1236)	Gammaproteobacteria (122	Proteobacteria (2)	Bacteria (1)	99.40	0.10	99.90	99.00	WGS
287.5771	0.3258	Pseudomonas aeruginosa (286)	Pseudomonas (135621)	Pseudomonadaceae (7227	Pseudomonadales (1236)	Gammaproteobacteria (122	Proteobacteria (2)	Bacteria (1)	100.00	0.00	99.90	99.00	WGS
287.5772	0.3226	Pseudomonas aeruginosa (286)	Pseudomonas (135621)	Pseudomonadaceae (7227	Pseudomonadales (1236)	Gammaproteobacteria (122	Proteobacteria (2)	Bacteria (1)	100.00	0.30	99.90	99.30	WGS
287.5773	0.3316	Pseudomonas aeruginosa (286)	Pseudomonas (135621)	Pseudomonadaceae (7227	Pseudomonadales (1236)	Gammaproteobacteria (122	Proteobacteria (2)	Bacteria (1)	100.00	0.00	99.80	99.10	WGS
287.5774	0.3268	Pseudomonas aeruginosa (286)	Pseudomonas (135621)	Pseudomonadaceae (7227	Pseudomonadales (1236)	Gammaproteobacteria (122	Proteobacteria (2)	Bacteria (1)	100.00	0.90	99.90	98.90	WGS
287.5775	0.3268	Pseudomonas aeruginosa (286)	Pseudomonas (135621)	Pseudomonadaceae (7227	Pseudomonadales (1236)	Gammaproteobacteria (122	Proteobacteria (2)	Bacteria (1)	100.00	0.00	99.90	99.60	WGS
287.5776	0.3226	Pseudomonas aeruginosa (286)	Pseudomonas (135621)	Pseudomonadaceae (7227	Pseudomonadales (1236)	Gammaproteobacteria (122	Proteobacteria (2)	Bacteria (1)	99.40	1.20	99.60	97.50	Complete
287.5777	0.3394	Pseudomonas aeruginosa (286)	Pseudomonas (135621)	Pseudomonadaceae (7227	Pseudomonadales (1236)	Gammaproteobacteria (122	Proteobacteria (2)	Bacteria (1)	98.60	38.90	98.30	64.20	WGS
287.5778	0.3618	Pseudomonas aeruginosa (286)	Pseudomonas (135621)	Pseudomonadaceae (7227	Pseudomonadales (1236)	Gammaproteobacteria (122	Proteobacteria (2)	Bacteria (1)	100.00	0.00	99.80	98.30	Complete
287.5948	0.3594	Pseudomonas aeruginosa (286)	Pseudomonas (135621)	Pseudomonadaceae (7227	Pseudomonadales (1236)	Gammaproteobacteria (122	Proteobacteria (2)	Bacteria (1)	100.00	1.20	99.80	97.90	WGS
287.5949	0.3266	Pseudomonas aeruginosa (286)	Pseudomonas (135621)	Pseudomonadaceae (7227	Pseudomonadales (1236)	Gammaproteobacteria (122	Proteobacteria (2)	Bacteria (1)	94.60	35.20	97.30	69.50	WGS
287.5950	0.0294	Pseudomonas aeruginosa (286)	Pseudomonas (135621)	Pseudomonadaceae (7227	Pseudomonadales (1236)	Gammaproteobacteria (122	Proteobacteria (2)	Bacteria (1)	98.80	31.60	99.60	72.50	WGS
287.5951	0.2932	Pseudomonas aeruginosa (286)	Pseudomonas (135621)	Pseudomonadaceae (7227	Pseudomonadales (1236)	Gammaproteobacteria (122	Proteobacteria (2)	Bacteria (1)	100.00	33.90	99.40	72.80	WGS
287.5952	0.3662	Pseudomonas aeruginosa (286)	Pseudomonas (135621)	Pseudomonadaceae (7227	Pseudomonadales (1236)	Gammaproteobacteria (122	Proteobacteria (2)	Bacteria (1)	100.00	1.50	99.80	97.70	WGS
287.5953	0.2948	Pseudomonas aeruginosa (286)	Pseudomonas (135621)	Pseudomonadaceae (7227	Pseudomonadales (1236)	Gammaproteobacteria (122	Proteobacteria (2)	Bacteria (1)	100.00	7.20	99.80	92.80	WGS
287.5954	0.3046	Pseudomonas aeruginosa (286)	Pseudomonas (135621)	Pseudomonadaceae (7227	Pseudomonadales (1236)	Gammaproteobacteria (122	Proteobacteria (2)	Bacteria (1)	98.20	27.50	98.70	76.90	WGS
287.5955	0.3652	Pseudomonas aeruginosa (286)	Pseudomonas (135621)	Pseudomonadaceae (7227	Pseudomonadales (1236)	Gammaproteobacteria (122	Proteobacteria (2)	Bacteria (1)	99.10	0.30	99.90	98.80	Complete
287.5956	0.3376	Pseudomonas aeruginosa (286)	Pseudomonas (135621)	Pseudomonadaceae (7227	Pseudomonadales (1236)	Gammaproteobacteria (122	Proteobacteria (2)	Bacteria (1)	99.70	0.00	99.90	99.00	Complete
287.5957	0.3254	Pseudomonas aeruginosa (286)	Pseudomonas (135621)	Pseudomonadaceae (7227	Pseudomonadales (1236)	Gammaproteobacteria (122	Proteobacteria (2)	Bacteria (1)	100.00	2.40	99.70	96.20	WGS
287.5958	0.3324	Pseudomonas aeruginosa (286)	Pseudomonas (135621)	Pseudomonadaceae (7227	Pseudomonadales (1236)	Gammaproteobacteria (122	Proteobacteria (2)	Bacteria (1)	100.00	5.20	99.70	92.90	WGS
287.5959	0.368	Pseudomonas aeruginosa (286)	Pseudomonas (135621)	Pseudomonadaceae (7227	Pseudomonadales (1236)	Gammaproteobacteria (122	Proteobacteria (2)	Bacteria (1)	99.10	0.00	99.70	98.50	Complete
287.5960	0.3254	Pseudomonas aeruginosa (286)	Pseudomonas (135621)	Pseudomonadaceae (7227	Pseudomonadales (1236)	Gammaproteobacteria (122	Proteobacteria (2)	Bacteria (1)	100.00	1.50	99.70	97.30	WGS
287.5961	0.3306	Pseudomonas aeruginosa (286)	Pseudomonas (135621)	Pseudomonadaceae (7227	Pseudomonadales (1236)	Gammaproteobacteria (122	Proteobacteria (2)	Bacteria (1)	100.00	0.90	99.70	98.20	WGS
287.5962	0.3324	Pseudomonas aeruginosa (286)	Pseudomonas (135621)	Pseudomonadaceae (7227	Pseudomonadales (1236)	Gammaproteobacteria (122	Proteobacteria (2)	Bacteria (1)	100.00	12.40	99.50	88.70	WGS
287.5963	0.3188	Pseudomonas aeruginosa (286)	Pseudomonas (135621)	Pseudomonadaceae (7227	Pseudomonadales (1236)	Gammaproteobacteria (122	Proteobacteria (2)	Bacteria (1)	100.00	2.60	99.60	94.50	WGS
287.5964	0.3306	Pseudomonas aeruginosa (286)	Pseudomonas (135621)	Pseudomonadaceae (7227	Pseudomonadales (1236)	Gammaproteobacteria (122	Proteobacteria (2)	Bacteria (1)	99.70	2.50	99.80	95.30	WGS
287.5965	0.3306	Pseudomonas aeruginosa (286)	Pseudomonas (135621)	Pseudomonadaceae (7227	Pseudomonadales (1236)	Gammaproteobacteria (122	Proteobacteria (2)	Bacteria (1)	100.00	2.10	99.80	97.20	WGS
287.5966	0.3306	Pseudomonas aeruginosa (286)	Pseudomonas (135621)	Pseudomonadaceae (7227	Pseudomonadales (1236)	Gammaproteobacteria (122	Proteobacteria (2)	Bacteria (1)	100.00	0.00	99.80	98.80	WGS
287.5967	0.3254	Pseudomonas aeruginosa (286)	Pseudomonas (135621)	Pseudomonadaceae (7227	Pseudomonadales (1236)	Gammaproteobacteria (122	Proteobacteria (2)	Bacteria (1)	99.70	8.30	99.70	90.20	WGS
287.5968	0.3632	Pseudomonas aeruginosa (286)	Pseudomonas (135621)	Pseudomonadaceae (7227	Pseudomonadales (1236)	Gammaproteobacteria (122	Proteobacteria (2)	Bacteria (1)	100.00	0.00	99.80	99.00	Complete
287.5969	0.3704	Pseudomonas aeruginosa (286)	Pseudomonas (135621)	Pseudomonadaceae (7227	Pseudomonadales (1236)	Gammaproteobacteria (122	Proteobacteria (2)	Bacteria (1)	100.00	0.60	99.80	98.70	WGS
287.5970	0.3184	Pseudomonas aeruginosa (286)	Pseudomonas (135621)	Pseudomonadaceae (7227	Pseudomonadales (1236)	Gammaproteobacteria (122	Proteobacteria (2)	Bacteria (1)	100.00	4.00	99.50	95.30	WGS
287.5971	0.3644	Pseudomonas aeruginosa (286)	Pseudomonas (135621)	Pseudomonadaceae (7227	Pseudomonadales (1236)	Gammaproteobacteria (122	Proteobacteria (2)	Bacteria (1)	100.00	1.20	99.70	98.70	Complete
287.5972	0.3734	Pseudomonas aeruginosa (286)	Pseudomonas (135621)	Pseudomonadaceae (7227	Pseudomonadales (1236)	Gammaproteobacteria (122	Proteobacteria (2)	Bacteria (1)	97.00	0.00	99.20	97.90	Complete
287.5973	0.3694	Pseudomonas aeruginosa (286)	Pseudomonas (135621)	Pseudomonadaceae (7227	Pseudomonadales (1236)	Gammaproteobacteria (122	Proteobacteria (2)	Bacteria (1)	100.00	0.00	99.90	99.30	Complete
287.5974	0.3544	Pseudomonas aeruginosa (286)	Pseudomonas (135621)	Pseudomonadaceae (7227	Pseudomonadales (1236)	Gammaproteobacteria (122	Proteobacteria (2)	Bacteria (1)	96.70	0.00	99.00	97.40	WGS
287.5978	0.3304	Pseudomonas aeruginosa (286)	Pseudomonas (135621)	Pseudomonadaceae (7227	Pseudomonadales (1236)	Gammaproteobacteria (122	Proteobacteria (2)	Bacteria (1)	100.00	6.90	99.70	93.50	WGS
287.5979	0.3696	Pseudomonas aeruginosa (286)	Pseudomonas (135621)	Pseudomonadaceae (7227	Pseudomonadales (1236)	Gammaproteobacteria (122	Proteobacteria (2)	Bacteria (1)	100.00	0.00	99.90	99.00	WGS
287.5980	0.3636	Pseudomonas aeruginosa (286)	Pseudomonas (135621)	Pseudomonadaceae (7227	Pseudomonadales (1236)	Gammaproteobacteria (122	Proteobacteria (2)	Bacteria (1)	100.00	0.30	100.00	99.40	WGS
287.5995	0.0786	Pseudomonas aeruginosa (286)	Pseudomonas (135621)	Pseudomonadaceae (7227	Pseudomonadales (1236)	Gammaproteobacteria (122	Proteobacteria (2)	Bacteria (1)	35.70	3.30	98.80	98.20	Complete
287.5996	0.3294	Pseudomonas aeruginosa (286)	Pseudomonas (135621)	Pseudomonadaceae (7227	Pseudomonadales (1236)	Gammaproteobacteria (122	Proteobacteria (2)	Bacteria (1)	99.10	5.40	99.70	95.90	Complete
287.6323	0.368	Pseudomonas aeruginosa (286)	Pseudomonas (135621)	Pseudomonadaceae (7227	Pseudomonadales (1236)	Gammaproteobacteria (122	Proteobacteria (2)	Bacteria (1)	97.00	0.00	99.80	98.60	Complete
287.6324	0.3376	Pseudomonas aeruginosa (286)	Pseudomonas (135621)	Pseudomonadaceae (7227	Pseudomonadales (1236)	Gammaproteobacteria (122	Proteobacteria (2)	Bacteria (1)	100.00	0.30	99.90	98.50	Complete
287.6325	0.3174	Pseudomonas aeruginosa (286)	Pseudomonas (135621)	Pseudomonadaceae (7227	Pseudomonadales (1236)	Gammaproteobacteria (122	Proteobacteria (2)	Bacteria (1)	100.00	0.00	99.70	98.90	WGS
287.6326	0.324	Pseudomonas aeruginosa (286)	Pseudomonas (135621)	Pseudomonadaceae (7227	Pseudomonadales (1236)	Gammaproteobacteria (122	Proteobacteria (2)	Bacteria (1)	100.00	0.00	99.70	98.60	WGS
287.6327	0.3672	Pseudomonas aeruginosa (286)	Pseudomonas (135621)	Pseudomonadaceae (7227	Pseudomonadales (1236)	Gammaproteobacteria (122	Proteobacteria (2)	Bacteria (1)	100.00	0.50	99.80	97.10	WGS
287.6328	0.356	Pseudomonas aeruginosa (286)	Pseudomonas (135621)	Pseudomonadaceae (7227	Pseudomonadales (1236)	Gammaproteobacteria (122	Proteobacteria (2)	Bacteria (1)	100.00	0.40	99.90	98.60	WGS
287.6329	0.3674	Pseudomonas aeruginosa (286)	Pseudomonas (135621)	Pseudomonadaceae (7227	Pseudomonadales (1236)	Gammaproteobacteria (122	Proteobacteria (2)	Bacteria (1)	98.50	0.80	99.70	97.20	WGS
287.6330	0.3304	Pseudomonas aeruginosa (286)	Pseudomonas (135621)	Pseudomonadaceae (7227	Pseudomonadales (1236)	Gammaproteobacteria (122	Proteobacteria (2)	Bacteria (1)	100.00	0.00	99.90	98.30	WGS
287.6331	0.368	Pseudomonas aeruginosa (286)	Pseudomonas (135621)	Pseudomonadaceae (7227	Pseudomonadales (1236)	Gammaproteobacteria (122	Proteobacteria (2)	Bacteria (1)	100.00	0.00	99.80	99.40	WGS
287.6332	0.3614	Pseudomonas aeruginosa (286)	Pseudomonas (135621)	Pseudomonadaceae (7227	Pseudomonadales (1236)	Gammaproteobacteria (122	Proteobacteria (2)	Bacteria (1)	100.00	0.00	99.70	98.50	WGS
287.6333	0.3292	Pseudomonas aeruginosa (286)	Pseudomonas (135621)	Pseudomonadaceae (7227	Pseudomonadales (1236)	Gammaproteobacteria (122	Proteobacteria (2)	Bacteria (1)	100.00	1.20	99.90	98.20	WGS
287.6334	0.368	Pseudomonas aeruginosa (286)	Pseudomonas (135621)	Pseudomonadaceae (7227	Pseudomonadales (1236)	Gammaproteobacteria (122	Proteobacteria (2)	Bacteria (1)	100.00	1.30	99.50	97.30	WGS
287.6335	0.368	Pseudomonas aeruginosa (286)	Pseudomonas (135621)	Pseudomonadaceae (7227	Pseudomonadales (1236)	Gammaproteobacteria (122	Proteobacteria (2)	Bacteria (1)	100.00	1.80	99.60	96.80	WGS
287.6336	0.3018	Pseudomonas aeruginosa (286)	Pseudomonas (135621)	Pseudomonadaceae (7227	Pseudomonadales (1236)	Gammaproteobacteria (122	Proteobacteria (2)	Bacteria (1)	98.50	34.70	98.80	73.20	WGS
287.6337	0.331	Pseudomonas aeruginosa (286)	Pseudomonas (135621)	Pseudomonadaceae (7227	Pseudomonadales (1236)	Gammaproteobacteria (122	Proteobacteria (2)	Bacteria (1)	99.70	1.20	99.10	97.60	WGS
287.6338	0.367	Pseudomonas aeruginosa (286)	Pseudomonas (135621)	Pseudomonadaceae (7227	Pseudomonadales (1236)	Gammaproteobacteria (122	Proteobacteria (2)	Bacteria (1)	100.00	0.30	99.80	98.60	WGS
287.6339	0.3324	Pseudomonas aeruginosa (286)	Pseudomonas (135621)	Pseudomonadaceae (7227	Pseudomonadales (1236)	Gammaproteobacteria (122	Proteobacteria (2)	Bacteria (1)	100.00	0.00	99.90	98.80	WGS
287.6340	0.3174	Pseudomonas aeruginosa (286)	Pseudomonas (135621)	Pseudomonadaceae (7227	Pseudomonadales (1236)	Gammaproteobacteria (122	Proteobacteria (2)	Bacteria (1)	100.00	0.00	99.70	98.70	WGS
287.6341	0.3324	Pseudomonas aeruginosa (286)	Pseudomonas (135621)	Pseudomonadaceae (7227	Pseudomonadales (1236)	Gammaproteobacteria (122	Proteobacteria (2)	Bacteria (1)	100.00	0.00	99.90	99.00	WGS
287.6342	0.3188	Pseudomonas aeruginosa (286)	Pseudomonas (135621)	Pseudomonadaceae (7227	Pseudomonadales (1236)	Gammaproteobacteria (122	Proteobacteria (2)	Bacteria (1)	100.00	0.00	99.90	99.50	WGS
287.6343	0.3302	Pseudomonas aeruginosa (286)	Pseudomonas (135621)	Pseudomonadaceae (7227	Pseudomonadales (1236)	Gammaproteobacteria (122	Proteobacteria (2)	Bacteria (1)	100.00	0.30	99.70	99.00	WGS
287.6344	0.3174	Pseudomonas aeruginosa (286)	Pseudomonas (135621)	Pseudomonadaceae (7227	Pseudomonadales (1236)	Gammaproteobacteria (122	Proteobacteria (2)	Bacteria (1)	100.00	0.00	99.80	98.70	WGS
287.6345	0.3324	Pseudomonas aeruginosa (286)	Pseudomonas (135621)	Pseudomonadaceae (7227	Pseudomonadales (1236)	Gammaproteobacteria (122	Proteobacteria (2)	Bacteria (1)	100.00	0.00	99.80	98.20	WGS
287.6346	0.3694	Pseudomonas aeruginosa (286)	Pseudomonas (135621)	Pseudomonadaceae (7227	Pseudomonadales (1236)	Gammaproteobacteria (122	Proteobacteria (2)	Bacteria (1)	99.10	2.90	99.90	98.90	WGS
287.6347	0.3334	Pseudomonas aeruginosa (286)	Pseudomonas (135621)	Pseudomonadaceae (7227	Pseudomonadales (1236)	Gammaproteobacteria (122	Proteobacteria (2)	Bacteria (1)	100.00	0.30	99.70	99.00	WGS
287.6348	0.3258	Pseudomonas aeruginosa (286)	Pseudomonas (135621)	Pseudomonadaceae (7227	Pseudomonadales (1236)	Gammaproteobacteria (122	Proteobacteria (2)	Bacteria (1)	100.00	0.00	99.80	99.00	WGS
287.6349	0.3636	Pseudomonas aeruginosa (286)	Pseudomonas (135621)	Pseudomonadaceae (7227	Pseudomonadales (1236)	Gammaproteobacteria (122	Proteobacteria (2)	Bacteria (1)	100.00	0.00	99.90	99.00	WGS
287.6350	0.3324	Pseudomonas aeruginosa (286)	Pseudomonas (135621)	Pseudomonadaceae (7227	Pseudomonadales (1236)	Gammaproteobacteria (122	Proteobacteria (2)	Bacteria (1)	100.00	0.30	99.90	99.20	WGS
287.6351	0.3324	Pseudomonas aeruginosa (286)	Pseudomonas (135621)	Pseudomonadaceae (7227	Pseudomonadales (1236)	Gammaproteobacteria (122	Proteobacteria (2)	Bacteria (1)	100.00	0.00	99.80	99.60	WGS
287.6352	0.3376	Pseudomonas aeruginosa (286)	Pseudomonas (135621)	Pseudomonadaceae (7227	Pseudomonadales (1236)	Gammaproteobacteria (122	Proteobacteria (2)	Bacteria (1)	100.00	0.30	99.60	98.80	WGS
287.6353	0.3188	Pseudomonas aeruginosa (286)	Pseudomonas (135621)	Pseudomonadaceae (7227	Pseudomonadales (1236)	Gammaproteobacteria (122	Proteobacteria (2)	Bacteria (1)	100.00	0.00	99.80	98.50	WGS
287.6354	0.3672	Pseudomonas aeruginosa (286)	Pseudomonas (135621)	Pseudomonadaceae (7227	Pseudomonadales (1236)	Gammaproteobacteria (122	Proteobacteria (2)	Bacteria (1)	100.00	0.00	99.90	97.80	WGS
287.6355	0.431	Pseudomonas aeruginosa (286)	Pseudomonas (135621)	Pseudomonadaceae (7227	Pseudomonadales (1236)	Gammaproteobacteria (122	Proteobacteria (2)	Bacteria (1)	99.70	0.00	99.80	98.40	WGS
287.6356	0.3672	Pseudomonas aeruginosa (286)	Pseudomonas (135621)	Pseudomonadaceae (7227	Pseudomonadales (1236)	Gammaproteobacteria (122	Proteobacteria (2)	Bacteria (1)	100.00	0.00	99.80	98.20	WGS
287.6357	0.3672	Pseudomonas aeruginosa (286)	Pseudomonas (135621)	Pseudomonadaceae (7227	Pseudomonadales (1236)	Gammaproteobacteria (122	Proteobacteria (2)	Bacteria (1)	100.00	0.00	99.80	98.30	WGS
287.6358	0.3672	Pseudomonas aeruginosa (286)	Pseudomonas (135621)	Pseudomonadaceae (7227	Pseudomonadales (1236)	Gammaproteobacteria (122	Proteobacteria (2)	Bacteria (1)	100.00	0.00	99.90	98.20	WGS
287.6359	0.3258	Pseudomonas aeruginosa (286)	Pseudomonas (135621)	Pseudomonadaceae (7227	Pseudomonadales (1236)	Gammaproteobacteria (122	Proteobacteria (2)	Bacteria (1)	100.00	0.30	99.90	98.80	WGS
287.6360	0.3672	Pseudomonas aeruginosa (286)	Pseudomonas (135621)	Pseudomonadaceae (7227	Pseudomonadales (1236)	Gammaproteobacteria (122	Proteobacteria (2)	Bacteria (1)	100.00	0.00	99.80	98.40	WGS
287.6361	0.3324	Pseudomonas aeruginosa (286)	Pseudomonas (135621)	Pseudomonadaceae (7227	Pseudomonadales (1236)	Gammaproteobacteria (122	Proteobacteria (2)	Bacteria (1)	100.00	0.90	99.70	98.50	WGS
287.6362	0.3292	Pseudomonas aeruginosa (286)	Pseudomonas (135621)	Pseudomonadaceae (7227	Pseudomonadales (1236)	Gammaproteobacteria (122	Proteobacteria (2)	Bacteria (1)	100.00	0.00	99.90	99.50	WGS
287.6364	0.3388	Pseudomonas aeruginosa (286)	Pseudomonas (135621)	Pseudomonadaceae (7227	Pseudomonadales (1236)	Gammaproteobacteria (122	Proteobacteria (2)	Bacteria (1)	100.00	0.00	99.90	98.50	Complete
287.6365	0.3314	Pseudomonas aeruginosa (286)	Pseudomonas (135621)	Pseudomonadaceae (7227	Pseudomonadales (1236)	Gammaproteobacteria (122	Proteobacteria (2)	Bacteria (1)	99.70	0.00	99.90	99.00	Complete
287.6366	0.429	Pseudomonas aeruginosa (286)	Pseudomonas (135621)	Pseudomonadaceae (7227	Pseudomonadales (1236)	Gammaproteobacteria (122	Proteobacteria (2)	Bacteria (1)	100.00	1.20	99.40	98.10	Complete
287.6367	0.3632	Pseudomonas aeruginosa (286)	Pseudomonas (135621)	Pseudomonadaceae (7227	Pseudomonadales (1236)	Gammaproteobacteria (122	Proteobacteria (2)	Bacteria (1)	100.00	0.00	99.80	98.60	WGS
287.6368	0.3632	Pseudomonas aeruginosa (286)	Pseudomonas (135621)	Pseudomonadaceae (7227	Pseudomonadales (1236)	Gammaproteobacteria (122	Proteobacteria (2)	Bacteria (1)	100.00	0.30	99.80	98.60	WGS
287.6369	0.3662	Pseudomonas aeruginosa (286)	Pseudomonas (135621)	Pseudomonadaceae (7227	Pseudomonadales (1236)	Gammaproteobacteria (122	Proteobacteria (2)	Bacteria (1)	100.00	0.00	99.80	99.00	WGS
287.6370	0.3632	Pseudomonas aeruginosa (286)	Pseudomonas (135621)	Pseudomonadaceae (7227	Pseudomonadales (1236)	Gammaproteobacteria (122	Proteobacteria (2)	Bacteria (1)	100.00	0.20	99.80	98.60	WGS
287.6371	0.3336	Pseudomonas aeruginosa (286)	Pseudomonas (135621)	Pseudomonadaceae (7227	Pseudomonadales (1236)	Gammaproteobacteria (122	Proteobacteria (2)	Bacteria (1)	100.00	0.00	99.70	98.80	WGS
287.6372	0.3618	Pseudomonas aeruginosa (286)	Pseudomonas (135621)	Pseudomonadaceae (7227	Pseudomonadales (1236)	Gammaproteobacteria (122	Proteobacteria (2)	Bacteria (1)	100.00	0.60	99.60	98.00	WGS
287.6373	0.3632	Pseudomonas aeruginosa (286)	Pseudomonas (135621)	Pseudomonadaceae (7227	Pseudomonadales (1236)	Gammaproteobacteria (122	Proteobacteria (2)	Bacteria (1)	100.00	0.20	99.80	98.60	WGS
287.6374	0.3292	Pseudomonas aeruginosa (286)	Pseudomonas (135621)	Pseudomonadaceae (7227	Pseudomonadales (1236)	Gammaproteobacteria (122	Proteobacteria (2)	Bacteria (1)	98.80	2.80	98.40	95.70	WGS
287.6375	0.3292	Pseudomonas aeruginosa (286)	Pseudomonas (135621)	Pseudomonadaceae (7227	Pseudomonadales (1236)	Gammaproteobacteria (122	Proteobacteria (2)	Bacteria (1)	100.00	0.00	99.80	99.10	WGS
287.6376	0.3322	Pseudomonas aeruginosa (286)	Pseudomonas (135621)	Pseudomonadaceae (7227	Pseudomonadales (1236)	Gammaproteobacteria (122	Proteobacteria (2)	Bacteria (1)	100.00	0.30	99.90	98.30	Complete
287.6377	0.3292	Pseudomonas aeruginosa (286)	Pseudomonas (135621)	Pseudomonadaceae (7227	Pseudomonadales (1236)	Gammaproteobacteria (122	Proteobacteria (2)	Bacteria (1)	100.00	0.30	99.90	98.70	WGS
287.6378	0.3634	Pseudomonas aeruginosa (286)	Pseudomonas (135621)	Pseudomonadaceae (7227	Pseudomonadales (1236)	Gammaproteobacteria (122	Proteobacteria (2)	Bacteria (1)	100.00	0.00	99.60	98.20	WGS
287.6379	0.3264	Pseudomonas aeruginosa (286)	Pseudomonas (135621)	Pseudomonadaceae (7227	Pseudomonadales (1236)	Gammaproteobacteria (122	Proteobacteria (2)	Bacteria (1)	100.00	0.60	99.60	97.50	WGS
287.6380	0.368	Pseudomonas aeruginosa (286)	Pseudomonas (135621)	Pseudomonadaceae (7227	Pseudomonadales (1236)	Gammaproteobacteria (122	Proteobacteria (2)	Bacteria (1)	100.00	0.00	99.80	97.80	WGS
287.6381	0.3662	Pseudomonas aeruginosa (286)	Pseudomonas (135621)	Pseudomonadaceae (7227	Pseudomonadales (1236)	Gammaproteobacteria (122	Proteobacteria (2)	Bacteria (1)	100.00	0.00	99.80	98.70	WGS
287.6382	0.3258	Pseudomonas aeruginosa (286)	Pseudomonas (135621)	Pseudomonadaceae (7227	Pseudomonadales (1236)	Gammaproteobacteria (122	Proteobacteria (2)	Bacteria (1)	100.00	0.30	99.70	97.60	WGS
287.6383	0.3662	Pseudomonas aeruginosa (286)	Pseudomonas (135621)	Pseudomonadaceae (7227	Pseudomonadales (1236)	Gammaproteobacteria (122	Proteobacteria (2)	Bacteria (1)	100.00	0.30	99.90	98.60	WGS
287.6384	0.3292	Pseudomonas aeruginosa (286)	Pseudomonas (135621)	Pseudomonadaceae (7227	Pseudomonadales (1236)	Gammaproteobacteria (122	Proteobacteria (2)	Bacteria (1)	100.00	0.30	99.90	99.10	WGS
287.6385	0.368	Pseudomonas aeruginosa (286)	Pseudomonas (135621)	Pseudomonadaceae (7227	Pseudomonadales (1236)	Gammaproteobacteria (122	Proteobacteria (2)	Bacteria (1)	100.00	0.00	99.70	98.30	WGS
287.6386	0.3098	Pseudomonas aeruginosa (286)	Pseudomonas (135621)	Pseudomonadaceae (7227	Pseudomonadales (1236)	Gammaproteobacteria (122	Proteobacteria (2)	Bacteria (1)	100.00	0.70	99.80	96.70	WGS
287.6387	0.3302	Pseudomonas aeruginosa (286)	Pseudomonas (135621)	Pseudomonadaceae (7227	Pseudomonadales (1236)	Gammaproteobacteria (122	Proteobacteria (2)	Bacteria (1)	100.00	0.60	99.70	96.40	WGS
287.6388	0.324	Pseudomonas aeruginosa (286)	Pseudomonas (135621)	Pseudomonadaceae (7227	Pseudomonadales (1236)	Gammaproteobacteria (122	Proteobacteria (2)	Bacteria (1)	100.00	0.40	99.80	98.00	WGS
287.6389	0.3306	Pseudomonas aeruginosa (286)	Pseudomonas (135621)	Pseudomonadaceae (7227	Pseudomonadales (1236)	Gammaproteobacteria (122	Proteobacteria (2)	Bacteria (1)	100.00	0.60	99.80	97.60	WGS
287.6390	0.2948	Pseudomonas aeruginosa (286)	Pseudomonas (135621)	Pseudomonadaceae (7227	Pseudomonadales (1236)	Gammaproteobacteria (122	Proteobacteria (2)	Bacteria (1)	99.70	1.30	99.60	96.10	WGS
287.6391	0.332	Pseudomonas aeruginosa (286)	Pseudomonas (135621)	Pseudomonadaceae (7227	Pseudomonadales (1236)	Gammaproteobacteria (122	Proteobacteria (2)	Bacteria (1)	100.00	0.00	99.60	97.50	WGS
287.6392	0.3636	Pseudomonas aeruginosa (286)	Pseudomonas (135621)	Pseudomonadaceae (7227	Pseudomonadales (1236)	Gammaproteobacteria (122	Proteobacteria (2)	Bacteria (1)	100.00	0.00	99.90	98.20	WGS
287.6393	0.3226	Pseudomonas aeruginosa (286)	Pseudomonas (135621)	Pseudomonadaceae (7227	Pseudomonadales (1236)	Gammaproteobacteria (122	Proteobacteria (2)	Bacteria (1)	99.70	0.20	99.90	97.70	WGS
287.6394	0.3324	Pseudomonas aeruginosa (286)	Pseudomonas (135621)	Pseudomonadaceae (7227	Pseudomonadales (1236)	Gammaproteobacteria (122	Proteobacteria (2)	Bacteria (1)	100.00	0.00	99.90	98.40	WGS
287.6395	0.3704	Pseudomonas aeruginosa (286)	Pseudomonas (135621)	Pseudomonadaceae (7227	Pseudomonadales (1236)	Gammaproteobacteria (122	Proteobacteria (2)	Bacteria (1)	100.00	0.60	99.80	97.30	WGS
287.6396	0.3322	Pseudomonas aeruginosa (286)	Pseudomonas (135621)	Pseudomonadaceae (7227	Pseudomonadales (1236)	Gammaproteobacteria (122	Proteobacteria (2)	Bacteria (1)	100.00	0.90	99.80	97.80	WGS
287.6397	0.325	Pseudomonas aeruginosa (286)	Pseudomonas (135621)	Pseudomonadaceae (7227	Pseudomonadales (1236)	Gammaproteobacteria (122	Proteobacteria (2)	Bacteria (1)	100.00	0.00	99.90	97.90	WGS
287.6398	0.3258	Pseudomonas aeruginosa (286)	Pseudomonas (135621)	Pseudomonadaceae (7227	Pseudomonadales (1236)	Gammaproteobacteria (122	Proteobacteria (2)	Bacteria (1)	100.00	0.60	99.70	97.30	WGS
287.6399	0.3306	Pseudomonas aeruginosa (286)	Pseudomonas (135621)	Pseudomonadaceae (7227	Pseudomonadales (1236)	Gammaproteobacteria (122	Proteobacteria (2)	Bacteria (1)	100.00	0.60	99.70	97.10	WGS
287.6400	0.3324	Pseudomonas aeruginosa (286)	Pseudomonas (135621)	Pseudomonadaceae (7227	Pseudomonadales (1236)	Gammaproteobacteria (122	Proteobacteria (2)	Bacteria (1)	100.00	0.00	99.60	98.10	WGS
287.6401	0.3376	Pseudomonas aeruginosa (286)	Pseudomonas (135621)	Pseudomonadaceae (7227	Pseudomonadales (1236)	Gammaproteobacteria (122	Proteobacteria (2)	Bacteria (1)	99.40	0.60	99.70	97.30	WGS
287.6402	0.3314	Pseudomonas aeruginosa (286)	Pseudomonas (135621)	Pseudomonadaceae (7227	Pseudomonadales (1236)	Gammaproteobacteria (122	Proteobacteria (2)	Bacteria (1)	100.00	0.50	99.90	97.30	WGS
287.6403	0.3258	Pseudomonas aeruginosa (286)	Pseudomonas (135621)	Pseudomonadaceae (7227	Pseudomonadales (1236)	Gammaproteobacteria (122	Proteobacteria (2)	Bacteria (1)	100.00	0.30	99.70	97.40	WGS
287.6404	0.338	Pseudomonas aeruginosa (286)	Pseudomonas (135621)	Pseudomonadaceae (7227	Pseudomonadales (1236)	Gammaproteobacteria (122	Proteobacteria (2)	Bacteria (1)	100.00	0.00	99.90	97.60	WGS
287.6405	0.3622	Pseudomonas aeruginosa (286)	Pseudomonas (135621)	Pseudomonadaceae (7227	Pseudomonadales (1236)	Gammaproteobacteria (122	Proteobacteria (2)	Bacteria (1)	100.00	16.70	99.10	84.20	WGS
287.6406	0.3662	Pseudomonas aeruginosa (286)	Pseudomonas (135621)	Pseudomonadaceae (7227	Pseudomonadales (1236)	Gammaproteobacteria (122	Proteobacteria (2)	Bacteria (1)	100.00	0.00	99.90	98.20	WGS
287.6407	0.3236	Pseudomonas aeruginosa (286)	Pseudomonas (135621)	Pseudomonadaceae (7227	Pseudomonadales (1236)	Gammaproteobacteria (122	Proteobacteria (2)	Bacteria (1)	100.00	0.30	99.70	97.30	WGS
287.6408	0.3306	Pseudomonas aeruginosa (286)	Pseudomonas (135621)	Pseudomonadaceae (7227	Pseudomonadales (1236)	Gammaproteobacteria (122	Proteobacteria (2)	Bacteria (1)	100.00	0.60	99.80	97.90	WGS
287.6409	0.3378	Pseudomonas aeruginosa (286)	Pseudomonas (135621)	Pseudomonadaceae (7227	Pseudomonadales (1236)	Gammaproteobacteria (122	Proteobacteria (2)	Bacteria (1)	100.00	3.40	99.90	94.60	WGS
287.6410	0.3346	Pseudomonas aeruginosa (286)	Pseudomonas (135621)	Pseudomonadaceae (7227	Pseudomonadales (1236)	Gammaproteobacteria (122	Proteobacteria (2)	Bacteria (1)	100.00	0.00	99.90	97.70	WGS
287.6411	0.3226	Pseudomonas aeruginosa (286)	Pseudomonas (135621)	Pseudomonadaceae (7227	Pseudomonadales (1236)	Gammaproteobacteria (122	Proteobacteria (2)	Bacteria (1)	100.00	0.00	99.90	98.20	WGS
287.6412	0.3304	Pseudomonas aeruginosa (286)	Pseudomonas (135621)	Pseudomonadaceae (7227	Pseudomonadales (1236)	Gammaproteobacteria (122	Proteobacteria (2)	Bacteria (1)	100.00	0.00	99.90	97.40	WGS
287.6413	0.2916	Pseudomonas aeruginosa (286)	Pseudomonas (135621)	Pseudomonadaceae (7227	Pseudomonadales (1236)	Gammaproteobacteria (122	Proteobacteria (2)	Bacteria (1)	100.00	7.00	99.90	92.20	WGS
287.6414	0.3324	Pseudomonas aeruginosa (286)	Pseudomonas (135621)	Pseudomonadaceae (7227	Pseudomonadales (1236)	Gammaproteobacteria (122	Proteobacteria (2)	Bacteria (1)	100.00	0.30	99.90	98.50	WGS
287.6415	0.3694	Pseudomonas aeruginosa (286)	Pseudomonas (135621)	Pseudomonadaceae (7227	Pseudomonadales (1236)	Gammaproteobacteria (122	Proteobacteria (2)	Bacteria (1)	99.70	0.00	99.80	97.60	WGS
287.6416	0.3174	Pseudomonas aeruginosa (286)	Pseudomonas (135621)	Pseudomonadaceae (7227	Pseudomonadales (1236)	Gammaproteobacteria (122	Proteobacteria (2)	Bacteria (1)	100.00	0.20	99.60	96.40	WGS
287.6417	0.3628	Pseudomonas aeruginosa (286)	Pseudomonas (135621)	Pseudomonadaceae (7227	Pseudomonadales (1236)	Gammaproteobacteria (122	Proteobacteria (2)	Bacteria (1)	100.00	0.00	99.80	97.30	WGS
287.6418	0.3376	Pseudomonas aeruginosa (286)	Pseudomonas (135621)	Pseudomonadaceae (7227	Pseudomonadales (1236)	Gammaproteobacteria (122	Proteobacteria (2)	Bacteria (1)	100.00	0.60	99.40	95.80	WGS
287.6419	0.332	Pseudomonas aeruginosa (286)	Pseudomonas (135621)	Pseudomonadaceae (7227	Pseudomonadales (1236)	Gammaproteobacteria (122	Proteobacteria (2)	Bacteria (1)	100.00	0.00	99.80	97.80	WGS
287.6420	0.3258	Pseudomonas aeruginosa (286)	Pseudomonas (135621)	Pseudomonadaceae (7227	Pseudomonadales (1236)	Gammaproteobacteria (122	Proteobacteria (2)	Bacteria (1)	100.00	0.00	99.70	98.40	WGS
287.6421	0.3398	Pseudomonas aeruginosa (286)	Pseudomonas (135621)	Pseudomonadaceae (7227	Pseudomonadales (1236)	Gammaproteobacteria (122	Proteobacteria (2)	Bacteria (1)	99.40	0.00	99.90	98.00	WGS
287.6422	0.3376	Pseudomonas aeruginosa (286)	Pseudomonas (135621)	Pseudomonadaceae (7227	Pseudomonadales (1236)	Gammaproteobacteria (122	Proteobacteria (2)	Bacteria (1)	100.00	1.20	99.30	96.80	WGS
287.6423	0.3324	Pseudomonas aeruginosa (286)	Pseudomonas (135621)	Pseudomonadaceae (7227	Pseudomonadales (1236)	Gammaproteobacteria (122	Proteobacteria (2)	Bacteria (1)	100.00	0.30	99.90	98.40	WGS
287.6424	0.3694	Pseudomonas aeruginosa (286)	Pseudomonas (135621)	Pseudomonadaceae (7227	Pseudomonadales (1236)	Gammaproteobacteria (122	Proteobacteria (2)	Bacteria (1)	100.00	0.00	99.90	98.40	WGS
287.6425	0.3132	Pseudomonas aeruginosa (286)	Pseudomonas (135621)	Pseudomonadaceae (7227	Pseudomonadales (1236)	Gammaproteobacteria (122	Proteobacteria (2)	Bacteria (1)	100.00	0.40	99.90	96.70	WGS
287.6426	0.3398	Pseudomonas aeruginosa (286)	Pseudomonas (135621)	Pseudomonadaceae (7227	Pseudomonadales (1236)	Gammaproteobacteria (122	Proteobacteria (2)	Bacteria (1)	100.00	0.30	99.90	96.90	WGS
287.6427	0.2978	Pseudomonas aeruginosa (286)	Pseudomonas (135621)	Pseudomonadaceae (7227	Pseudomonadales (1236)	Gammaproteobacteria (122	Proteobacteria (2)	Bacteria (1)	100.00	2.00	99.70	95.00	WGS
287.6428	0.3314	Pseudomonas aeruginosa (286)	Pseudomonas (135621)	Pseudomonadaceae (7227	Pseudomonadales (1236)	Gammaproteobacteria (122	Proteobacteria (2)	Bacteria (1)	100.00	1.30	99.90	95.60	WGS
287.6429	0.3268	Pseudomonas aeruginosa (286)	Pseudomonas (135621)	Pseudomonadaceae (7227	Pseudomonadales (1236)	Gammaproteobacteria (122	Proteobacteria (2)	Bacteria (1)	100.00	0.00	99.90	97.90	WGS
287.6430	0.291	Pseudomonas aeruginosa (286)	Pseudomonas (135621)	Pseudomonadaceae (7227	Pseudomonadales (1236)	Gammaproteobacteria (122	Proteobacteria (2)	Bacteria (1)	100.00	3.40	99.90	95.30	WGS
287.6431	0.3738	Pseudomonas aeruginosa (286)	Pseudomonas (135621)	Pseudomonadaceae (7227	Pseudomonadales (1236)	Gammaproteobacteria (122	Proteobacteria (2)	Bacteria (1)	98.90	1.90	99.90	97.00	WGS
287.6432	0.3324	Pseudomonas aeruginosa (286)	Pseudomonas (135621)	Pseudomonadaceae (7227	Pseudomonadales (1236)	Gammaproteobacteria (122	Proteobacteria (2)	Bacteria (1)	100.00	0.30	99.70	98.50	WGS
287.6433	0.325	Pseudomonas aeruginosa (286)	Pseudomonas (135621)	Pseudomonadaceae (7227	Pseudomonadales (1236)	Gammaproteobacteria (122	Proteobacteria (2)	Bacteria (1)	100.00	1.00	99.80	97.40	WGS
287.6434	0.3662	Pseudomonas aeruginosa (286)	Pseudomonas (135621)	Pseudomonadaceae (7227	Pseudomonadales (1236)	Gammaproteobacteria (122	Proteobacteria (2)	Bacteria (1)	100.00	0.30	99.90	97.00	WGS
287.6435	0.3604	Pseudomonas aeruginosa (286)	Pseudomonas (135621)	Pseudomonadaceae (7227	Pseudomonadales (1236)	Gammaproteobacteria (122	Proteobacteria (2)	Bacteria (1)	99.40	1.80	99.90	97.80	WGS
287.6436	0.3314	Pseudomonas aeruginosa (286)	Pseudomonas (135621)	Pseudomonadaceae (7227	Pseudomonadales (1236)	Gammaproteobacteria (122	Proteobacteria (2)	Bacteria (1)	100.00	0.60	99.90	98.00	WGS
287.6437	0.3618	Pseudomonas aeruginosa (286)	Pseudomonas (135621)	Pseudomonadaceae (7227	Pseudomonadales (1236)	Gammaproteobacteria (122	Proteobacteria (2)	Bacteria (1)	100.00	0.60	99.90	97.00	WGS
287.6438	0.3384	Pseudomonas aeruginosa (286)	Pseudomonas (135621)	Pseudomonadaceae (7227	Pseudomonadales (1236)	Gammaproteobacteria (122	Proteobacteria (2)	Bacteria (1)	100.00	1.60	99.70	95.60	WGS
287.6439	0.3376	Pseudomonas aeruginosa (286)	Pseudomonas (135621)	Pseudomonadaceae (7227	Pseudomonadales (1236)	Gammaproteobacteria (122	Proteobacteria (2)	Bacteria (1)	99.40	1.00	99.80	97.00	WGS
287.6440	0.3254	Pseudomonas aeruginosa (286)	Pseudomonas (135621)	Pseudomonadaceae (7227	Pseudomonadales (1236)	Gammaproteobacteria (122	Proteobacteria (2)	Bacteria (1)	100.00	0.00	99.80	97.00	WGS
287.6441	0.3292	Pseudomonas aeruginosa (286)	Pseudomonas (135621)	Pseudomonadaceae (7227	Pseudomonadales (1236)	Gammaproteobacteria (122	Proteobacteria (2)	Bacteria (1)	100.00	0.00	99.80	98.00	WGS
287.6442	0.3292	Pseudomonas aeruginosa (286)	Pseudomonas (135621)	Pseudomonadaceae (7227	Pseudomonadales (1236)	Gammaproteobacteria (122	Proteobacteria (2)	Bacteria (1)	100.00	0.00	99.60	97.30	WGS
287.6443	0.3236	Pseudomonas aeruginosa (286)	Pseudomonas (135621)	Pseudomonadaceae (7227	Pseudomonadales (1236)	Gammaproteobacteria (122	Proteobacteria (2)	Bacteria (1)	100.00	0.30	99.90	98.40	WGS
287.6444	0.3376	Pseudomonas aeruginosa (286)	Pseudomonas (135621)	Pseudomonadaceae (7227	Pseudomonadales (1236)	Gammaproteobacteria (122	Proteobacteria (2)	Bacteria (1)	100.00	0.30	99.60	98.20	WGS
287.6445	0.3376	Pseudomonas aeruginosa (286)	Pseudomonas (135621)	Pseudomonadaceae (7227	Pseudomonadales (1236)	Gammaproteobacteria (122	Proteobacteria (2)	Bacteria (1)	99.40	1.20	99.60	96.20	WGS
287.6446	0.3226	Pseudomonas aeruginosa (286)	Pseudomonas (135621)	Pseudomonadaceae (7227	Pseudomonadales (1236)	Gammaproteobacteria (122	Proteobacteria (2)	Bacteria (1)	100.00	0.00	99.80	98.20	WGS
287.6447	0.324	Pseudomonas aeruginosa (286)	Pseudomonas (135621)	Pseudomonadaceae (7227	Pseudomonadales (1236)	Gammaproteobacteria (122	Proteobacteria (2)	Bacteria (1)	100.00	0.60	99.80	97.00	WGS
287.6448	0.3254	Pseudomonas aeruginosa (286)	Pseudomonas (135621)	Pseudomonadaceae (7227	Pseudomonadales (1236)	Gammaproteobacteria (122	Proteobacteria (2)	Bacteria (1)	100.00	0.00	99.90	99.30	WGS
287.6449	0.3314	Pseudomonas aeruginosa (286)	Pseudomonas (135621)	Pseudomonadaceae (7227	Pseudomonadales (1236)	Gammaproteobacteria (122	Proteobacteria (2)	Bacteria (1)	100.00	0.00	99.90	98.70	WGS
287.6450	0.3376	Pseudomonas aeruginosa (286)	Pseudomonas (135621)	Pseudomonadaceae (7227	Pseudomonadales (1236)	Gammaproteobacteria (122	Proteobacteria (2)	Bacteria (1)	99.40	0.60	99.70	96.60	WGS
287.6451	0.3738	Pseudomonas aeruginosa (286)	Pseudomonas (135621)	Pseudomonadaceae (7227	Pseudomonadales (1236)	Gammaproteobacteria (122	Proteobacteria (2)	Bacteria (1)	99.70	0.60	99.90	96.90	WGS
287.6452	0.3398	Pseudomonas aeruginosa (286)	Pseudomonas (135621)	Pseudomonadaceae (7227	Pseudomonadales (1236)	Gammaproteobacteria (122	Proteobacteria (2)	Bacteria (1)	100.00	0.00	99.90	98.20	WGS
287.6453	0.3662	Pseudomonas aeruginosa (286)	Pseudomonas (135621)	Pseudomonadaceae (7227	Pseudomonadales (1236)	Gammaproteobacteria (122	Proteobacteria (2)	Bacteria (1)	100.00	2.30	99.90	95.80	WGS
287.6454	0.3388	Pseudomonas aeruginosa (286)	Pseudomonas (135621)	Pseudomonadaceae (7227	Pseudomonadales (1236)	Gammaproteobacteria (122	Proteobacteria (2)	Bacteria (1)	100.00	0.60	99.60	97.60	WGS
287.6455	0.3592	Pseudomonas aeruginosa (286)	Pseudomonas (135621)	Pseudomonadaceae (7227	Pseudomonadales (1236)	Gammaproteobacteria (122	Proteobacteria (2)	Bacteria (1)	100.00	0.30	99.70	98.20	WGS
287.6456	0.3258	Pseudomonas aeruginosa (286)	Pseudomonas (135621)	Pseudomonadaceae (7227	Pseudomonadales (1236)	Gammaproteobacteria (122	Proteobacteria (2)	Bacteria (1)	99.70	0.60	100.00	98.90	WGS
287.6457	0.332	Pseudomonas aeruginosa (286)	Pseudomonas (135621)	Pseudomonadaceae (7227	Pseudomonadales (1236)	Gammaproteobacteria (122	Proteobacteria (2)	Bacteria (1)	100.00	1.00	99.80	95.40	WGS
287.6458	0.2978	Pseudomonas aeruginosa (286)	Pseudomonas (135621)	Pseudomonadaceae (7227	Pseudomonadales (1236)	Gammaproteobacteria (122	Proteobacteria (2)	Bacteria (1)	100.00	0.70	99.80	96.90	WGS
287.6459	0.3814	Pseudomonas aeruginosa (286)	Pseudomonas (135621)	Pseudomonadaceae (7227	Pseudomonadales (1236)	Gammaproteobacteria (122	Proteobacteria (2)	Bacteria (1)	98.20	1.70	99.20	94.50	WGS
287.6460	0.3054	Pseudomonas aeruginosa (286)	Pseudomonas (135621)	Pseudomonadaceae (7227	Pseudomonadales (1236)	Gammaproteobacteria (122	Proteobacteria (2)	Bacteria (1)	100.00	3.30	99.90	93.90	WGS
287.6461	0.3334	Pseudomonas aeruginosa (286)	Pseudomonas (135621)	Pseudomonadaceae (7227	Pseudomonadales (1236)	Gammaproteobacteria (122	Proteobacteria (2)	Bacteria (1)	100.00	0.00	99.60	98.20	WGS
287.6462	0.3254	Pseudomonas aeruginosa (286)	Pseudomonas (135621)	Pseudomonadaceae (7227	Pseudomonadales (1236)	Gammaproteobacteria (122	Proteobacteria (2)	Bacteria (1)	100.00	0.00	99.60	97.50	WGS
287.6463	0.3376	Pseudomonas aeruginosa (286)	Pseudomonas (135621)	Pseudomonadaceae (7227	Pseudomonadales (1236)	Gammaproteobacteria (122	Proteobacteria (2)	Bacteria (1)	99.40	1.80	99.70	95.40	WGS
287.6464	0.3604	Pseudomonas aeruginosa (286)	Pseudomonas (135621)	Pseudomonadaceae (7227	Pseudomonadales (1236)	Gammaproteobacteria (122	Proteobacteria (2)	Bacteria (1)	100.00	0.20	99.90	97.60	WGS
287.6465	0.3292	Pseudomonas aeruginosa (286)	Pseudomonas (135621)	Pseudomonadaceae (7227	Pseudomonadales (1236)	Gammaproteobacteria (122	Proteobacteria (2)	Bacteria (1)	100.00	0.00	99.60	98.10	WGS
287.6466	0.368	Pseudomonas aeruginosa (286)	Pseudomonas (135621)	Pseudomonadaceae (7227	Pseudomonadales (1236)	Gammaproteobacteria (122	Proteobacteria (2)	Bacteria (1)	100.00	1.60	99.60	96.20	WGS
287.6467	0.331	Pseudomonas aeruginosa (286)	Pseudomonas (135621)	Pseudomonadaceae (7227	Pseudomonadales (1236)	Gammaproteobacteria (122	Proteobacteria (2)	Bacteria (1)	100.00	2.50	99.80	95.90	WGS
287.6468	0.3288	Pseudomonas aeruginosa (286)	Pseudomonas (135621)	Pseudomonadaceae (7227	Pseudomonadales (1236)	Gammaproteobacteria (122	Proteobacteria (2)	Bacteria (1)	100.00	0.30	99.80	97.20	WGS
287.6469	0.368	Pseudomonas aeruginosa (286)	Pseudomonas (135621)	Pseudomonadaceae (7227	Pseudomonadales (1236)	Gammaproteobacteria (122	Proteobacteria (2)	Bacteria (1)	100.00	0.40	99.90	97.30	WGS
287.6470	0.3268	Pseudomonas aeruginosa (286)	Pseudomonas (135621)	Pseudomonadaceae (7227	Pseudomonadales (1236)	Gammaproteobacteria (122	Proteobacteria (2)	Bacteria (1)	100.00	0.00	99.90	98.70	WGS
287.6471	0.3324	Pseudomonas aeruginosa (286)	Pseudomonas (135621)	Pseudomonadaceae (7227	Pseudomonadales (1236)	Gammaproteobacteria (122	Proteobacteria (2)	Bacteria (1)	100.00	0.30	99.90	97.80	WGS
287.6472	0.3662	Pseudomonas aeruginosa (286)	Pseudomonas (135621)	Pseudomonadaceae (7227	Pseudomonadales (1236)	Gammaproteobacteria (122	Proteobacteria (2)	Bacteria (1)	100.00	0.30	99.90	98.70	WGS
287.6473	0.3376	Pseudomonas aeruginosa (286)	Pseudomonas (135621)	Pseudomonadaceae (7227	Pseudomonadales (1236)	Gammaproteobacteria (122	Proteobacteria (2)	Bacteria (1)	100.00	0.00	99.90	97.90	WGS
287.6474	0.3292	Pseudomonas aeruginosa (286)	Pseudomonas (135621)	Pseudomonadaceae (7227	Pseudomonadales (1236)	Gammaproteobacteria (122	Proteobacteria (2)	Bacteria (1)	100.00	0.30	99.90	97.00	WGS
287.6475	0.3636	Pseudomonas aeruginosa (286)	Pseudomonas (135621)	Pseudomonadaceae (7227	Pseudomonadales (1236)	Gammaproteobacteria (122	Proteobacteria (2)	Bacteria (1)	100.00	0.00	99.80	99.30	WGS
287.6476	0.3226	Pseudomonas aeruginosa (286)	Pseudomonas (135621)	Pseudomonadaceae (7227	Pseudomonadales (1236)	Gammaproteobacteria (122	Proteobacteria (2)	Bacteria (1)	100.00	8.80	99.80	93.00	WGS
287.6479	0.3302	Pseudomonas aeruginosa (286)	Pseudomonas (135621)	Pseudomonadaceae (7227	Pseudomonadales (1236)	Gammaproteobacteria (122	Proteobacteria (2)	Bacteria (1)	99.70	0.60	99.60	98.70	WGS
287.6480	0.368	Pseudomonas aeruginosa (286)	Pseudomonas (135621)	Pseudomonadaceae (7227	Pseudomonadales (1236)	Gammaproteobacteria (122	Proteobacteria (2)	Bacteria (1)	100.00	0.00	99.90	99.50	Complete
287.6481	0.3292	Pseudomonas aeruginosa (286)	Pseudomonas (135621)	Pseudomonadaceae (7227	Pseudomonadales (1236)	Gammaproteobacteria (122	Proteobacteria (2)	Bacteria (1)	100.00	0.00	99.60	99.00	Complete
287.6482	0.3584	Pseudomonas aeruginosa (286)	Pseudomonas (135621)	Pseudomonadaceae (7227	Pseudomonadales (1236)	Gammaproteobacteria (122	Proteobacteria (2)	Bacteria (1)	100.00	1.20	99.80	98.60	Complete
287.6483	0.356	Pseudomonas aeruginosa (286)	Pseudomonas (135621)	Pseudomonadaceae (7227	Pseudomonadales (1236)	Gammaproteobacteria (122	Proteobacteria (2)	Bacteria (1)	100.00	0.00	99.80	98.80	Complete
287.6484	0.356	Pseudomonas aeruginosa (286)	Pseudomonas (135621)	Pseudomonadaceae (7227	Pseudomonadales (1236)	Gammaproteobacteria (122	Proteobacteria (2)	Bacteria (1)	100.00	0.00	99.80	98.70	Complete
287.6485	0.356	Pseudomonas aeruginosa (286)	Pseudomonas (135621)	Pseudomonadaceae (7227	Pseudomonadales (1236)	Gammaproteobacteria (122	Proteobacteria (2)	Bacteria (1)	100.00	0.00	99.80	98.80	Complete
287.6486	0.324	Pseudomonas aeruginosa (286)	Pseudomonas (135621)	Pseudomonadaceae (7227	Pseudomonadales (1236)	Gammaproteobacteria (122	Proteobacteria (2)	Bacteria (1)	100.00	0.30	99.90	99.60	Complete
287.6491	0.3188	Pseudomonas aeruginosa (286)	Pseudomonas (135621)	Pseudomonadaceae (7227	Pseudomonadales (1236)	Gammaproteobacteria (122	Proteobacteria (2)	Bacteria (1)	100.00	0.90	99.70	98.80	WGS
287.6492	0.368	Pseudomonas aeruginosa (286)	Pseudomonas (135621)	Pseudomonadaceae (7227	Pseudomonadales (1236)	Gammaproteobacteria (122	Proteobacteria (2)	Bacteria (1)	100.00	0.00	99.70	98.60	Complete
287.6494	0.3796	Pseudomonas aeruginosa (286)	Pseudomonas (135621)	Pseudomonadaceae (7227	Pseudomonadales (1236)	Gammaproteobacteria (122	Proteobacteria (2)	Bacteria (1)	100.00	1.20	99.40	98.10	WGS
287.6495	0.3614	Pseudomonas aeruginosa (286)	Pseudomonas (135621)	Pseudomonadaceae (7227	Pseudomonadales (1236)	Gammaproteobacteria (122	Proteobacteria (2)	Bacteria (1)	99.40	0.60	99.40	98.30	WGS
287.6496	0.3656	Pseudomonas aeruginosa (286)	Pseudomonas (135621)	Pseudomonadaceae (7227	Pseudomonadales (1236)	Gammaproteobacteria (122	Proteobacteria (2)	Bacteria (1)	100.00	0.90	99.90	98.70	WGS
287.6497	0.368	Pseudomonas aeruginosa (286)	Pseudomonas (135621)	Pseudomonadaceae (7227	Pseudomonadales (1236)	Gammaproteobacteria (122	Proteobacteria (2)	Bacteria (1)	97.00	0.00	99.80	98.70	WGS
287.6498	0.3336	Pseudomonas aeruginosa (286)	Pseudomonas (135621)	Pseudomonadaceae (7227	Pseudomonadales (1236)	Gammaproteobacteria (122	Proteobacteria (2)	Bacteria (1)	100.00	0.00	99.70	98.90	WGS
287.6499	0.324	Pseudomonas aeruginosa (286)	Pseudomonas (135621)	Pseudomonadaceae (7227	Pseudomonadales (1236)	Gammaproteobacteria (122	Proteobacteria (2)	Bacteria (1)	100.00	0.30	99.60	98.90	WGS
287.6500	0.3618	Pseudomonas aeruginosa (286)	Pseudomonas (135621)	Pseudomonadaceae (7227	Pseudomonadales (1236)	Gammaproteobacteria (122	Proteobacteria (2)	Bacteria (1)	100.00	0.30	99.80	98.40	WGS
287.6501	0.4066	Pseudomonas aeruginosa (286)	Pseudomonas (135621)	Pseudomonadaceae (7227	Pseudomonadales (1236)	Gammaproteobacteria (122	Proteobacteria (2)	Bacteria (1)	99.40	0.40	99.50	98.70	WGS
287.6502	0.324	Pseudomonas aeruginosa (286)	Pseudomonas (135621)	Pseudomonadaceae (7227	Pseudomonadales (1236)	Gammaproteobacteria (122	Proteobacteria (2)	Bacteria (1)	100.00	0.00	99.90	99.50	WGS
287.6503	0.368	Pseudomonas aeruginosa (286)	Pseudomonas (135621)	Pseudomonadaceae (7227	Pseudomonadales (1236)	Gammaproteobacteria (122	Proteobacteria (2)	Bacteria (1)	100.00	0.00	99.90	99.20	WGS
287.6504	0.3268	Pseudomonas aeruginosa (286)	Pseudomonas (135621)	Pseudomonadaceae (7227	Pseudomonadales (1236)	Gammaproteobacteria (122	Proteobacteria (2)	Bacteria (1)	100.00	0.00	99.90	99.00	WGS
287.6505	0.3604	Pseudomonas aeruginosa (286)	Pseudomonas (135621)	Pseudomonadaceae (7227	Pseudomonadales (1236)	Gammaproteobacteria (122	Proteobacteria (2)	Bacteria (1)	100.00	0.30	99.90	98.90	WGS
287.6506	0.3174	Pseudomonas aeruginosa (286)	Pseudomonas (135621)	Pseudomonadaceae (7227	Pseudomonadales (1236)	Gammaproteobacteria (122	Proteobacteria (2)	Bacteria (1)	99.10	0.30	99.90	99.00	WGS
287.6507	0.3174	Pseudomonas aeruginosa (286)	Pseudomonas (135621)	Pseudomonadaceae (7227	Pseudomonadales (1236)	Gammaproteobacteria (122	Proteobacteria (2)	Bacteria (1)	100.00	0.30	99.80	98.80	WGS
287.6508	0.3614	Pseudomonas aeruginosa (286)	Pseudomonas (135621)	Pseudomonadaceae (7227	Pseudomonadales (1236)	Gammaproteobacteria (122	Proteobacteria (2)	Bacteria (1)	99.40	0.60	99.60	98.40	WGS
287.6509	0.368	Pseudomonas aeruginosa (286)	Pseudomonas (135621)	Pseudomonadaceae (7227	Pseudomonadales (1236)	Gammaproteobacteria (122	Proteobacteria (2)	Bacteria (1)	97.00	0.00	99.80	98.70	WGS
287.6510	0.3738	Pseudomonas aeruginosa (286)	Pseudomonas (135621)	Pseudomonadaceae (7227	Pseudomonadales (1236)	Gammaproteobacteria (122	Proteobacteria (2)	Bacteria (1)	99.70	0.00	99.90	99.20	WGS
287.6512	0.3324	Pseudomonas aeruginosa (286)	Pseudomonas (135621)	Pseudomonadaceae (7227	Pseudomonadales (1236)	Gammaproteobacteria (122	Proteobacteria (2)	Bacteria (1)	100.00	0.30	99.70	98.50	WGS
287.6513	0.332	Pseudomonas aeruginosa (286)	Pseudomonas (135621)	Pseudomonadaceae (7227	Pseudomonadales (1236)	Gammaproteobacteria (122	Proteobacteria (2)	Bacteria (1)	99.10	1.40	99.90	97.60	WGS
287.6514	0.3376	Pseudomonas aeruginosa (286)	Pseudomonas (135621)	Pseudomonadaceae (7227	Pseudomonadales (1236)	Gammaproteobacteria (122	Proteobacteria (2)	Bacteria (1)	96.70	4.20	99.30	95.60	WGS
287.6515	0.3258	Pseudomonas aeruginosa (286)	Pseudomonas (135621)	Pseudomonadaceae (7227	Pseudomonadales (1236)	Gammaproteobacteria (122	Proteobacteria (2)	Bacteria (1)	100.00	0.00	99.70	99.30	WGS
287.6516	0.3688	Pseudomonas aeruginosa (286)	Pseudomonas (135621)	Pseudomonadaceae (7227	Pseudomonadales (1236)	Gammaproteobacteria (122	Proteobacteria (2)	Bacteria (1)	96.70	0.90	98.90	97.60	WGS
287.6517	0.3322	Pseudomonas aeruginosa (286)	Pseudomonas (135621)	Pseudomonadaceae (7227	Pseudomonadales (1236)	Gammaproteobacteria (122	Proteobacteria (2)	Bacteria (1)	100.00	0.00	99.90	98.90	WGS
287.6518	0.3618	Pseudomonas aeruginosa (286)	Pseudomonas (135621)	Pseudomonadaceae (7227	Pseudomonadales (1236)	Gammaproteobacteria (122	Proteobacteria (2)	Bacteria (1)	100.00	1.50	99.90	98.70	WGS
287.6519	0.3632	Pseudomonas aeruginosa (286)	Pseudomonas (135621)	Pseudomonadaceae (7227	Pseudomonadales (1236)	Gammaproteobacteria (122	Proteobacteria (2)	Bacteria (1)	100.00	0.90	99.80	98.90	WGS
287.6520	0.3322	Pseudomonas aeruginosa (286)	Pseudomonas (135621)	Pseudomonadaceae (7227	Pseudomonadales (1236)	Gammaproteobacteria (122	Proteobacteria (2)	Bacteria (1)	100.00	0.30	99.90	99.30	WGS
287.6521	0.325	Pseudomonas aeruginosa (286)	Pseudomonas (135621)	Pseudomonadaceae (7227	Pseudomonadales (1236)	Gammaproteobacteria (122	Proteobacteria (2)	Bacteria (1)	100.00	0.30	99.90	98.50	WGS
287.6523	0.3376	Pseudomonas aeruginosa (286)	Pseudomonas (135621)	Pseudomonadaceae (7227	Pseudomonadales (1236)	Gammaproteobacteria (122	Proteobacteria (2)	Bacteria (1)	99.40	0.00	99.10	98.50	WGS
287.6794	0.324	Pseudomonas aeruginosa (286)	Pseudomonas (135621)	Pseudomonadaceae (7227	Pseudomonadales (1236)	Gammaproteobacteria (122	Proteobacteria (2)	Bacteria (1)			99.90	98.70	WGS
287.6795	0.3302	Pseudomonas aeruginosa (286)	Pseudomonas (135621)	Pseudomonadaceae (7227	Pseudomonadales (1236)	Gammaproteobacteria (122	Proteobacteria (2)	Bacteria (1)			99.40	97.70	WGS
287.6796	0.324	Pseudomonas aeruginosa (286)	Pseudomonas (135621)	Pseudomonadaceae (7227	Pseudomonadales (1236)	Gammaproteobacteria (122	Proteobacteria (2)	Bacteria (1)			99.90	99.10	WGS
287.6797	0.324	Pseudomonas aeruginosa (286)	Pseudomonas (135621)	Pseudomonadaceae (7227	Pseudomonadales (1236)	Gammaproteobacteria (122	Proteobacteria (2)	Bacteria (1)			99.90	99.00	WGS
287.6798	0.3302	Pseudomonas aeruginosa (286)	Pseudomonas (135621)	Pseudomonadaceae (7227	Pseudomonadales (1236)	Gammaproteobacteria (122	Proteobacteria (2)	Bacteria (1)			99.60	97.90	WGS
287.6799	0.3744	Pseudomonas aeruginosa (286)	Pseudomonas (135621)	Pseudomonadaceae (7227	Pseudomonadales (1236)	Gammaproteobacteria (122	Proteobacteria (2)	Bacteria (1)			97.90	85.60	Complete
287.6800	0.3188	Pseudomonas aeruginosa (286)	Pseudomonas (135621)	Pseudomonadaceae (7227	Pseudomonadales (1236)	Gammaproteobacteria (122	Proteobacteria (2)	Bacteria (1)			99.90	99.60	WGS
287.6801	0.3236	Pseudomonas aeruginosa (286)	Pseudomonas (135621)	Pseudomonadaceae (7227	Pseudomonadales (1236)	Gammaproteobacteria (122	Proteobacteria (2)	Bacteria (1)			99.90	98.40	WGS
287.6802	0.3376	Pseudomonas aeruginosa (286)	Pseudomonas (135621)	Pseudomonadaceae (7227	Pseudomonadales (1236)	Gammaproteobacteria (122	Proteobacteria (2)	Bacteria (1)			99.90	99.30	WGS
287.6803	0.3094	Pseudomonas aeruginosa (286)	Pseudomonas (135621)	Pseudomonadaceae (7227	Pseudomonadales (1236)	Gammaproteobacteria (122	Proteobacteria (2)	Bacteria (1)			99.80	96.00	WGS
287.6804	0.3174	Pseudomonas aeruginosa (286)	Pseudomonas (135621)	Pseudomonadaceae (7227	Pseudomonadales (1236)	Gammaproteobacteria (122	Proteobacteria (2)	Bacteria (1)			99.80	98.60	WGS
287.6805	0.3622	Pseudomonas aeruginosa (286)	Pseudomonas (135621)	Pseudomonadaceae (7227	Pseudomonadales (1236)	Gammaproteobacteria (122	Proteobacteria (2)	Bacteria (1)			99.10	94.20	WGS
287.6806	0.3236	Pseudomonas aeruginosa (286)	Pseudomonas (135621)	Pseudomonadaceae (7227	Pseudomonadales (1236)	Gammaproteobacteria (122	Proteobacteria (2)	Bacteria (1)			100.00	98.60	WGS
287.6807	0.3376	Pseudomonas aeruginosa (286)	Pseudomonas (135621)	Pseudomonadaceae (7227	Pseudomonadales (1236)	Gammaproteobacteria (122	Proteobacteria (2)	Bacteria (1)			99.90	98.70	WGS
287.6808	0.3376	Pseudomonas aeruginosa (286)	Pseudomonas (135621)	Pseudomonadaceae (7227	Pseudomonadales (1236)	Gammaproteobacteria (122	Proteobacteria (2)	Bacteria (1)			99.90	98.20	WGS
287.6809	0.3376	Pseudomonas aeruginosa (286)	Pseudomonas (135621)	Pseudomonadaceae (7227	Pseudomonadales (1236)	Gammaproteobacteria (122	Proteobacteria (2)	Bacteria (1)			99.90	96.60	WGS
287.6810	0.3618	Pseudomonas aeruginosa (286)	Pseudomonas (135621)	Pseudomonadaceae (7227	Pseudomonadales (1236)	Gammaproteobacteria (122	Proteobacteria (2)	Bacteria (1)			100.00	99.30	WGS
287.6811	0.3618	Pseudomonas aeruginosa (286)	Pseudomonas (135621)	Pseudomonadaceae (7227	Pseudomonadales (1236)	Gammaproteobacteria (122	Proteobacteria (2)	Bacteria (1)			99.90	93.10	WGS
287.6812	0.3636	Pseudomonas aeruginosa (286)	Pseudomonas (135621)	Pseudomonadaceae (7227	Pseudomonadales (1236)	Gammaproteobacteria (122	Proteobacteria (2)	Bacteria (1)			99.50	98.70	WGS
287.6813	0.3636	Pseudomonas aeruginosa (286)	Pseudomonas (135621)	Pseudomonadaceae (7227	Pseudomonadales (1236)	Gammaproteobacteria (122	Proteobacteria (2)	Bacteria (1)			100.00	92.90	WGS
287.6814	0.324	Pseudomonas aeruginosa (286)	Pseudomonas (135621)	Pseudomonadaceae (7227	Pseudomonadales (1236)	Gammaproteobacteria (122	Proteobacteria (2)	Bacteria (1)			99.50	98.20	WGS
287.6815	0.3188	Pseudomonas aeruginosa (286)	Pseudomonas (135621)	Pseudomonadaceae (7227	Pseudomonadales (1236)	Gammaproteobacteria (122	Proteobacteria (2)	Bacteria (1)			99.90	99.20	WGS
287.6816	0.3346	Pseudomonas aeruginosa (286)	Pseudomonas (135621)	Pseudomonadaceae (7227	Pseudomonadales (1236)	Gammaproteobacteria (122	Proteobacteria (2)	Bacteria (1)			99.90	93.10	WGS
287.6817	0.3376	Pseudomonas aeruginosa (286)	Pseudomonas (135621)	Pseudomonadaceae (7227	Pseudomonadales (1236)	Gammaproteobacteria (122	Proteobacteria (2)	Bacteria (1)			99.70	98.40	WGS
287.6818	0.368	Pseudomonas aeruginosa (286)	Pseudomonas (135621)	Pseudomonadaceae (7227	Pseudomonadales (1236)	Gammaproteobacteria (122	Proteobacteria (2)	Bacteria (1)			99.80	98.40	WGS
287.6819	0.368	Pseudomonas aeruginosa (286)	Pseudomonas (135621)	Pseudomonadaceae (7227	Pseudomonadales (1236)	Gammaproteobacteria (122	Proteobacteria (2)	Bacteria (1)			99.80	98.60	WGS
287.6820	0.3636	Pseudomonas aeruginosa (286)	Pseudomonas (135621)	Pseudomonadaceae (7227	Pseudomonadales (1236)	Gammaproteobacteria (122	Proteobacteria (2)	Bacteria (1)			99.80	98.00	WGS
287.6821	0.3666	Pseudomonas aeruginosa (286)	Pseudomonas (135621)	Pseudomonadaceae (7227	Pseudomonadales (1236)	Gammaproteobacteria (122	Proteobacteria (2)	Bacteria (1)			99.50	97.60	WGS
287.6822	0.3636	Pseudomonas aeruginosa (286)	Pseudomonas (135621)	Pseudomonadaceae (7227	Pseudomonadales (1236)	Gammaproteobacteria (122	Proteobacteria (2)	Bacteria (1)			99.80	98.70	WGS
287.6823	0.3636	Pseudomonas aeruginosa (286)	Pseudomonas (135621)	Pseudomonadaceae (7227	Pseudomonadales (1236)	Gammaproteobacteria (122	Proteobacteria (2)	Bacteria (1)			99.80	97.90	WGS
287.6824	0.3636	Pseudomonas aeruginosa (286)	Pseudomonas (135621)	Pseudomonadaceae (7227	Pseudomonadales (1236)	Gammaproteobacteria (122	Proteobacteria (2)	Bacteria (1)			98.90	94.30	WGS
287.6825	0.3646	Pseudomonas aeruginosa (286)	Pseudomonas (135621)	Pseudomonadaceae (7227	Pseudomonadales (1236)	Gammaproteobacteria (122	Proteobacteria (2)	Bacteria (1)			99.80	92.40	WGS
287.6826	0.3188	Pseudomonas aeruginosa (286)	Pseudomonas (135621)	Pseudomonadaceae (7227	Pseudomonadales (1236)	Gammaproteobacteria (122	Proteobacteria (2)	Bacteria (1)			99.80	93.90	WGS
287.6827	0.332	Pseudomonas aeruginosa (286)	Pseudomonas (135621)	Pseudomonadaceae (7227	Pseudomonadales (1236)	Gammaproteobacteria (122	Proteobacteria (2)	Bacteria (1)			99.90	98.00	WGS
287.6828	0.3258	Pseudomonas aeruginosa (286)	Pseudomonas (135621)	Pseudomonadaceae (7227	Pseudomonadales (1236)	Gammaproteobacteria (122	Proteobacteria (2)	Bacteria (1)			99.90	99.60	WGS
287.6829	0.3696	Pseudomonas aeruginosa (286)	Pseudomonas (135621)	Pseudomonadaceae (7227	Pseudomonadales (1236)	Gammaproteobacteria (122	Proteobacteria (2)	Bacteria (1)			99.90	97.30	WGS
287.6830	0.3662	Pseudomonas aeruginosa (286)	Pseudomonas (135621)	Pseudomonadaceae (7227	Pseudomonadales (1236)	Gammaproteobacteria (122	Proteobacteria (2)	Bacteria (1)			99.80	93.30	WGS
287.6831	0.3258	Pseudomonas aeruginosa (286)	Pseudomonas (135621)	Pseudomonadaceae (7227	Pseudomonadales (1236)	Gammaproteobacteria (122	Proteobacteria (2)	Bacteria (1)			99.80	98.90	WGS
287.6832	0.3738	Pseudomonas aeruginosa (286)	Pseudomonas (135621)	Pseudomonadaceae (7227	Pseudomonadales (1236)	Gammaproteobacteria (122	Proteobacteria (2)	Bacteria (1)			99.90	96.60	WGS
287.6833	0.3324	Pseudomonas aeruginosa (286)	Pseudomonas (135621)	Pseudomonadaceae (7227	Pseudomonadales (1236)	Gammaproteobacteria (122	Proteobacteria (2)	Bacteria (1)			99.80	93.00	WGS
287.6834	0.331	Pseudomonas aeruginosa (286)	Pseudomonas (135621)	Pseudomonadaceae (7227	Pseudomonadales (1236)	Gammaproteobacteria (122	Proteobacteria (2)	Bacteria (1)			99.30	97.60	WGS
287.6835	0.332	Pseudomonas aeruginosa (286)	Pseudomonas (135621)	Pseudomonadaceae (7227	Pseudomonadales (1236)	Gammaproteobacteria (122	Proteobacteria (2)	Bacteria (1)			99.90	98.40	WGS
287.6836	0.331	Pseudomonas aeruginosa (286)	Pseudomonas (135621)	Pseudomonadaceae (7227	Pseudomonadales (1236)	Gammaproteobacteria (122	Proteobacteria (2)	Bacteria (1)			99.90	98.30	WGS
287.6837	0.3334	Pseudomonas aeruginosa (286)	Pseudomonas (135621)	Pseudomonadaceae (7227	Pseudomonadales (1236)	Gammaproteobacteria (122	Proteobacteria (2)	Bacteria (1)			99.90	97.70	WGS
287.6838	0.3334	Pseudomonas aeruginosa (286)	Pseudomonas (135621)	Pseudomonadaceae (7227	Pseudomonadales (1236)	Gammaproteobacteria (122	Proteobacteria (2)	Bacteria (1)			99.80	97.20	WGS
287.6839	0.3334	Pseudomonas aeruginosa (286)	Pseudomonas (135621)	Pseudomonadaceae (7227	Pseudomonadales (1236)	Gammaproteobacteria (122	Proteobacteria (2)	Bacteria (1)			99.80	95.30	WGS
287.6840	0.3334	Pseudomonas aeruginosa (286)	Pseudomonas (135621)	Pseudomonadaceae (7227	Pseudomonadales (1236)	Gammaproteobacteria (122	Proteobacteria (2)	Bacteria (1)			99.80	96.20	WGS
287.6841	0.342	Pseudomonas aeruginosa (286)	Pseudomonas (135621)	Pseudomonadaceae (7227	Pseudomonadales (1236)	Gammaproteobacteria (122	Proteobacteria (2)	Bacteria (1)			99.70	93.10	WGS
287.6842	0.3334	Pseudomonas aeruginosa (286)	Pseudomonas (135621)	Pseudomonadaceae (7227	Pseudomonadales (1236)	Gammaproteobacteria (122	Proteobacteria (2)	Bacteria (1)			99.90	94.50	WGS
287.6843	0.3646	Pseudomonas aeruginosa (286)	Pseudomonas (135621)	Pseudomonadaceae (7227	Pseudomonadales (1236)	Gammaproteobacteria (122	Proteobacteria (2)	Bacteria (1)			99.80	99.00	WGS
287.6844	0.3682	Pseudomonas aeruginosa (286)	Pseudomonas (135621)	Pseudomonadaceae (7227	Pseudomonadales (1236)	Gammaproteobacteria (122	Proteobacteria (2)	Bacteria (1)			99.40	96.60	WGS
287.6845	0.356	Pseudomonas aeruginosa (286)	Pseudomonas (135621)	Pseudomonadaceae (7227	Pseudomonadales (1236)	Gammaproteobacteria (122	Proteobacteria (2)	Bacteria (1)			99.70	98.60	WGS
287.6846	0.356	Pseudomonas aeruginosa (286)	Pseudomonas (135621)	Pseudomonadaceae (7227	Pseudomonadales (1236)	Gammaproteobacteria (122	Proteobacteria (2)	Bacteria (1)			99.80	98.30	WGS
287.6847	0.369	Pseudomonas aeruginosa (286)	Pseudomonas (135621)	Pseudomonadaceae (7227	Pseudomonadales (1236)	Gammaproteobacteria (122	Proteobacteria (2)	Bacteria (1)			99.80	99.40	WGS
287.6848	0.3258	Pseudomonas aeruginosa (286)	Pseudomonas (135621)	Pseudomonadaceae (7227	Pseudomonadales (1236)	Gammaproteobacteria (122	Proteobacteria (2)	Bacteria (1)			99.90	99.40	WGS
287.6849	0.369	Pseudomonas aeruginosa (286)	Pseudomonas (135621)	Pseudomonadaceae (7227	Pseudomonadales (1236)	Gammaproteobacteria (122	Proteobacteria (2)	Bacteria (1)			99.80	99.40	WGS
287.6850	0.3738	Pseudomonas aeruginosa (286)	Pseudomonas (135621)	Pseudomonadaceae (7227	Pseudomonadales (1236)	Gammaproteobacteria (122	Proteobacteria (2)	Bacteria (1)			99.70	98.10	WGS
287.6851	0.3646	Pseudomonas aeruginosa (286)	Pseudomonas (135621)	Pseudomonadaceae (7227	Pseudomonadales (1236)	Gammaproteobacteria (122	Proteobacteria (2)	Bacteria (1)			99.50	97.30	WGS
287.6852	0.368	Pseudomonas aeruginosa (286)	Pseudomonas (135621)	Pseudomonadaceae (7227	Pseudomonadales (1236)	Gammaproteobacteria (122	Proteobacteria (2)	Bacteria (1)			99.90	99.30	WGS
287.6853	0.356	Pseudomonas aeruginosa (286)	Pseudomonas (135621)	Pseudomonadaceae (7227	Pseudomonadales (1236)	Gammaproteobacteria (122	Proteobacteria (2)	Bacteria (1)			99.70	98.40	WGS
287.6854	0.3722	Pseudomonas aeruginosa (286)	Pseudomonas (135621)	Pseudomonadaceae (7227	Pseudomonadales (1236)	Gammaproteobacteria (122	Proteobacteria (2)	Bacteria (1)			99.60	93.90	WGS
287.6855	0.3614	Pseudomonas aeruginosa (286)	Pseudomonas (135621)	Pseudomonadaceae (7227	Pseudomonadales (1236)	Gammaproteobacteria (122	Proteobacteria (2)	Bacteria (1)			99.60	98.40	WGS
287.6856	0.357	Pseudomonas aeruginosa (286)	Pseudomonas (135621)	Pseudomonadaceae (7227	Pseudomonadales (1236)	Gammaproteobacteria (122	Proteobacteria (2)	Bacteria (1)			99.60	97.30	WGS
287.6857	0.368	Pseudomonas aeruginosa (286)	Pseudomonas (135621)	Pseudomonadaceae (7227	Pseudomonadales (1236)	Gammaproteobacteria (122	Proteobacteria (2)	Bacteria (1)			99.90	98.80	WGS
287.6858	0.3604	Pseudomonas aeruginosa (286)	Pseudomonas (135621)	Pseudomonadaceae (7227	Pseudomonadales (1236)	Gammaproteobacteria (122	Proteobacteria (2)	Bacteria (1)			99.70	98.80	WGS
287.6859	0.3604	Pseudomonas aeruginosa (286)	Pseudomonas (135621)	Pseudomonadaceae (7227	Pseudomonadales (1236)	Gammaproteobacteria (122	Proteobacteria (2)	Bacteria (1)			99.40	96.50	WGS

287.6860	0.356	Pseudomonas aeruginosa (286)	Pseudomonas (135621)	Pseudomonadaceae (7227	Pseudomonadales (1236)	Gammaproteobacteria (122	Proteobacteria (2)	Bacteria (1)	99.90	98.50	WGS
287.6861	0.324	Pseudomonas aeruginosa (286)	Pseudomonas (135621)	Pseudomonadaceae (7227	Pseudomonadales (1236)	Gammaproteobacteria (122	Proteobacteria (2)	Bacteria (1)	99.90	98.80	WGS
287.6862	0.3226	Pseudomonas aeruginosa (286)	Pseudomonas (135621)	Pseudomonadaceae (7227	Pseudomonadales (1236)	Gammaproteobacteria (122	Proteobacteria (2)	Bacteria (1)	99.60	91.20	WGS
287.6863	0.3636	Pseudomonas aeruginosa (286)	Pseudomonas (135621)	Pseudomonadaceae (7227	Pseudomonadales (1236)	Gammaproteobacteria (122	Proteobacteria (2)	Bacteria (1)	99.80	94.80	WGS
287.6864	0.3834	Pseudomonas aeruginosa (286)	Pseudomonas (135621)	Pseudomonadaceae (7227	Pseudomonadales (1236)	Gammaproteobacteria (122	Proteobacteria (2)	Bacteria (1)	99.80	93.00	WGS
287.6865	0.3386	Pseudomonas aeruginosa (286)	Pseudomonas (135621)	Pseudomonadaceae (7227	Pseudomonadales (1236)	Gammaproteobacteria (122	Proteobacteria (2)	Bacteria (1)	99.60	93.80	WGS
287.6866	0.331	Pseudomonas aeruginosa (286)	Pseudomonas (135621)	Pseudomonadaceae (7227	Pseudomonadales (1236)	Gammaproteobacteria (122	Proteobacteria (2)	Bacteria (1)	99.80	97.40	WGS
287.6867	0.3358	Pseudomonas aeruginosa (286)	Pseudomonas (135621)	Pseudomonadaceae (7227	Pseudomonadales (1236)	Gammaproteobacteria (122	Proteobacteria (2)	Bacteria (1)	99.90	94.00	WGS
287.6868	0.3324	Pseudomonas aeruginosa (286)	Pseudomonas (135621)	Pseudomonadaceae (7227	Pseudomonadales (1236)	Gammaproteobacteria (122	Proteobacteria (2)	Bacteria (1)	99.90	98.80	WGS
287.6869	0.3636	Pseudomonas aeruginosa (286)	Pseudomonas (135621)	Pseudomonadaceae (7227	Pseudomonadales (1236)	Gammaproteobacteria (122	Proteobacteria (2)	Bacteria (1)	99.60	97.90	WGS
287.6870	0.3636	Pseudomonas aeruginosa (286)	Pseudomonas (135621)	Pseudomonadaceae (7227	Pseudomonadales (1236)	Gammaproteobacteria (122	Proteobacteria (2)	Bacteria (1)	99.30	91.20	WGS
287.6871	0.327	Pseudomonas aeruginosa (286)	Pseudomonas (135621)	Pseudomonadaceae (7227	Pseudomonadales (1236)	Gammaproteobacteria (122	Proteobacteria (2)	Bacteria (1)	99.90	99.00	WGS
287.6872	0.3324	Pseudomonas aeruginosa (286)	Pseudomonas (135621)	Pseudomonadaceae (7227	Pseudomonadales (1236)	Gammaproteobacteria (122	Proteobacteria (2)	Bacteria (1)	99.70	93.90	WGS
287.6873	0.3704	Pseudomonas aeruginosa (286)	Pseudomonas (135621)	Pseudomonadaceae (7227	Pseudomonadales (1236)	Gammaproteobacteria (122	Proteobacteria (2)	Bacteria (1)	99.20	95.90	WGS
287.6874	0.368	Pseudomonas aeruginosa (286)	Pseudomonas (135621)	Pseudomonadaceae (7227	Pseudomonadales (1236)	Gammaproteobacteria (122	Proteobacteria (2)	Bacteria (1)	99.90	98.70	WGS
287.6875	0.3636	Pseudomonas aeruginosa (286)	Pseudomonas (135621)	Pseudomonadaceae (7227	Pseudomonadales (1236)	Gammaproteobacteria (122	Proteobacteria (2)	Bacteria (1)	99.50	97.20	WGS
287.6876	0.3704	Pseudomonas aeruginosa (286)	Pseudomonas (135621)	Pseudomonadaceae (7227	Pseudomonadales (1236)	Gammaproteobacteria (122	Proteobacteria (2)	Bacteria (1)	99.90	94.40	WGS
287.6877	0.356	Pseudomonas aeruginosa (286)	Pseudomonas (135621)	Pseudomonadaceae (7227	Pseudomonadales (1236)	Gammaproteobacteria (122	Proteobacteria (2)	Bacteria (1)	99.60	98.50	WGS
287.6878	0.3174	Pseudomonas aeruginosa (286)	Pseudomonas (135621)	Pseudomonadaceae (7227	Pseudomonadales (1236)	Gammaproteobacteria (122	Proteobacteria (2)	Bacteria (1)	99.80	96.80	WGS
287.6879	0.3694	Pseudomonas aeruginosa (286)	Pseudomonas (135621)	Pseudomonadaceae (7227	Pseudomonadales (1236)	Gammaproteobacteria (122	Proteobacteria (2)	Bacteria (1)	99.70	98.90	WGS
287.6880	0.3636	Pseudomonas aeruginosa (286)	Pseudomonas (135621)	Pseudomonadaceae (7227	Pseudomonadales (1236)	Gammaproteobacteria (122	Proteobacteria (2)	Bacteria (1)	99.60	95.70	WGS
287.6881	0.368	Pseudomonas aeruginosa (286)	Pseudomonas (135621)	Pseudomonadaceae (7227	Pseudomonadales (1236)	Gammaproteobacteria (122	Proteobacteria (2)	Bacteria (1)	99.90	98.70	WGS
287.6882	0.3694	Pseudomonas aeruginosa (286)	Pseudomonas (135621)	Pseudomonadaceae (7227	Pseudomonadales (1236)	Gammaproteobacteria (122	Proteobacteria (2)	Bacteria (1)	99.70	98.50	WGS
287.6883	0.331	Pseudomonas aeruginosa (286)	Pseudomonas (135621)	Pseudomonadaceae (7227	Pseudomonadales (1236)	Gammaproteobacteria (122	Proteobacteria (2)	Bacteria (1)	99.90	98.90	WGS
287.6884	0.331	Pseudomonas aeruginosa (286)	Pseudomonas (135621)	Pseudomonadaceae (7227	Pseudomonadales (1236)	Gammaproteobacteria (122	Proteobacteria (2)	Bacteria (1)	99.60	97.00	WGS
287.6885	0.331	Pseudomonas aeruginosa (286)	Pseudomonas (135621)	Pseudomonadaceae (7227	Pseudomonadales (1236)	Gammaproteobacteria (122	Proteobacteria (2)	Bacteria (1)	99.90	91.40	WGS
287.6886	0.331	Pseudomonas aeruginosa (286)	Pseudomonas (135621)	Pseudomonadaceae (7227	Pseudomonadales (1236)	Gammaproteobacteria (122	Proteobacteria (2)	Bacteria (1)	99.80	93.10	WGS
287.6887	0.3306	Pseudomonas aeruginosa (286)	Pseudomonas (135621)	Pseudomonadaceae (7227	Pseudomonadales (1236)	Gammaproteobacteria (122	Proteobacteria (2)	Bacteria (1)	99.80	97.30	WGS
287.6888	0.3628	Pseudomonas aeruginosa (286)	Pseudomonas (135621)	Pseudomonadaceae (7227	Pseudomonadales (1236)	Gammaproteobacteria (122	Proteobacteria (2)	Bacteria (1)	99.90	98.50	WGS
287.6889	0.3628	Pseudomonas aeruginosa (286)	Pseudomonas (135621)	Pseudomonadaceae (7227	Pseudomonadales (1236)	Gammaproteobacteria (122	Proteobacteria (2)	Bacteria (1)	99.90	99.30	WGS
287.6890	0.3628	Pseudomonas aeruginosa (286)	Pseudomonas (135621)	Pseudomonadaceae (7227	Pseudomonadales (1236)	Gammaproteobacteria (122	Proteobacteria (2)	Bacteria (1)	98.10	97.30	WGS
287.6891	0.3628	Pseudomonas aeruginosa (286)	Pseudomonas (135621)	Pseudomonadaceae (7227	Pseudomonadales (1236)	Gammaproteobacteria (122	Proteobacteria (2)	Bacteria (1)	99.90	99.20	WGS
287.6892	0.3376	Pseudomonas aeruginosa (286)	Pseudomonas (135621)	Pseudomonadaceae (7227	Pseudomonadales (1236)	Gammaproteobacteria (122	Proteobacteria (2)	Bacteria (1)	99.90	98.90	WGS
287.6893	0.3628	Pseudomonas aeruginosa (286)	Pseudomonas (135621)	Pseudomonadaceae (7227	Pseudomonadales (1236)	Gammaproteobacteria (122	Proteobacteria (2)	Bacteria (1)	99.90	97.60	WGS
287.6894	0.3376	Pseudomonas aeruginosa (286)	Pseudomonas (135621)	Pseudomonadaceae (7227	Pseudomonadales (1236)	Gammaproteobacteria (122	Proteobacteria (2)	Bacteria (1)	99.70	96.20	WGS
287.6895	0.3376	Pseudomonas aeruginosa (286)	Pseudomonas (135621)	Pseudomonadaceae (7227	Pseudomonadales (1236)	Gammaproteobacteria (122	Proteobacteria (2)	Bacteria (1)	99.80	98.40	WGS
287.6896	0.2964	Pseudomonas aeruginosa (286)	Pseudomonas (135621)	Pseudomonadaceae (7227	Pseudomonadales (1236)	Gammaproteobacteria (122	Proteobacteria (2)	Bacteria (1)	99.90	93.60	WGS
287.6897	0.369	Pseudomonas aeruginosa (286)	Pseudomonas (135621)	Pseudomonadaceae (7227	Pseudomonadales (1236)	Gammaproteobacteria (122	Proteobacteria (2)	Bacteria (1)	99.60	98.00	WGS
287.6898	0.369	Pseudomonas aeruginosa (286)	Pseudomonas (135621)	Pseudomonadaceae (7227	Pseudomonadales (1236)	Gammaproteobacteria (122	Proteobacteria (2)	Bacteria (1)	99.80	98.70	WGS
287.6899	0.369	Pseudomonas aeruginosa (286)	Pseudomonas (135621)	Pseudomonadaceae (7227	Pseudomonadales (1236)	Gammaproteobacteria (122	Proteobacteria (2)	Bacteria (1)	99.80	97.70	WGS
287.6900	0.369	Pseudomonas aeruginosa (286)	Pseudomonas (135621)	Pseudomonadaceae (7227	Pseudomonadales (1236)	Gammaproteobacteria (122	Proteobacteria (2)	Bacteria (1)	99.70	91.10	WGS
287.6901	0.369	Pseudomonas aeruginosa (286)	Pseudomonas (135621)	Pseudomonadaceae (7227	Pseudomonadales (1236)	Gammaproteobacteria (122	Proteobacteria (2)	Bacteria (1)	99.80	98.30	WGS
287.6902	0.369	Pseudomonas aeruginosa (286)	Pseudomonas (135621)	Pseudomonadaceae (7227	Pseudomonadales (1236)	Gammaproteobacteria (122	Proteobacteria (2)	Bacteria (1)	99.60	98.50	WGS
287.6903	0.369	Pseudomonas aeruginosa (286)	Pseudomonas (135621)	Pseudomonadaceae (7227	Pseudomonadales (1236)	Gammaproteobacteria (122	Proteobacteria (2)	Bacteria (1)	99.70	98.00	WGS
287.6904	0.368	Pseudomonas aeruginosa (286)	Pseudomonas (135621)	Pseudomonadaceae (7227	Pseudomonadales (1236)	Gammaproteobacteria (122	Proteobacteria (2)	Bacteria (1)	99.80	98.90	WGS
287.6905	0.369	Pseudomonas aeruginosa (286)	Pseudomonas (135621)	Pseudomonadaceae (7227	Pseudomonadales (1236)	Gammaproteobacteria (122	Proteobacteria (2)	Bacteria (1)	99.80	98.40	WGS
287.6906	0.368	Pseudomonas aeruginosa (286)	Pseudomonas (135621)	Pseudomonadaceae (7227	Pseudomonadales (1236)	Gammaproteobacteria (122	Proteobacteria (2)	Bacteria (1)	99.70	93.90	WGS
287.6907	0.368	Pseudomonas aeruginosa (286)	Pseudomonas (135621)	Pseudomonadaceae (7227	Pseudomonadales (1236)	Gammaproteobacteria (122	Proteobacteria (2)	Bacteria (1)	99.80	92.50	WGS
287.6908	0.368	Pseudomonas aeruginosa (286)	Pseudomonas (135621)	Pseudomonadaceae (7227	Pseudomonadales (1236)	Gammaproteobacteria (122	Proteobacteria (2)	Bacteria (1)	99.50	97.10	WGS
287.6909	0.369	Pseudomonas aeruginosa (286)	Pseudomonas (135621)	Pseudomonadaceae (7227	Pseudomonadales (1236)	Gammaproteobacteria (122	Proteobacteria (2)	Bacteria (1)	99.40	97.70	WGS
287.6910	0.369	Pseudomonas aeruginosa (286)	Pseudomonas (135621)	Pseudomonadaceae (7227	Pseudomonadales (1236)	Gammaproteobacteria (122	Proteobacteria (2)	Bacteria (1)	98.80	93.70	WGS
287.6911	0.3636	Pseudomonas aeruginosa (286)	Pseudomonas (135621)	Pseudomonadaceae (7227	Pseudomonadales (1236)	Gammaproteobacteria (122	Proteobacteria (2)	Bacteria (1)	99.80	98.70	WGS
287.6912	0.368	Pseudomonas aeruginosa (286)	Pseudomonas (135621)	Pseudomonadaceae (7227	Pseudomonadales (1236)	Gammaproteobacteria (122	Proteobacteria (2)	Bacteria (1)	99.80	94.20	WGS
287.6913	0.3636	Pseudomonas aeruginosa (286)	Pseudomonas (135621)	Pseudomonadaceae (7227	Pseudomonadales (1236)	Gammaproteobacteria (122	Proteobacteria (2)	Bacteria (1)	99.80	98.90	WGS
287.6914	0.368	Pseudomonas aeruginosa (286)	Pseudomonas (135621)	Pseudomonadaceae (7227	Pseudomonadales (1236)	Gammaproteobacteria (122	Proteobacteria (2)	Bacteria (1)	99.60	93.30	WGS
287.6915	0.368	Pseudomonas aeruginosa (286)	Pseudomonas (135621)	Pseudomonadaceae (7227	Pseudomonadales (1236)	Gammaproteobacteria (122	Proteobacteria (2)	Bacteria (1)	99.80	94.50	WGS
287.6916	0.368	Pseudomonas aeruginosa (286)	Pseudomonas (135621)	Pseudomonadaceae (7227	Pseudomonadales (1236)	Gammaproteobacteria (122	Proteobacteria (2)	Bacteria (1)	99.80	99.00	WGS
287.6917	0.3662	Pseudomonas aeruginosa (286)	Pseudomonas (135621)	Pseudomonadaceae (7227	Pseudomonadales (1236)	Gammaproteobacteria (122	Proteobacteria (2)	Bacteria (1)	99.80	99.00	WGS
287.6918	0.3636	Pseudomonas aeruginosa (286)	Pseudomonas (135621)	Pseudomonadaceae (7227	Pseudomonadales (1236)	Gammaproteobacteria (122	Proteobacteria (2)	Bacteria (1)	99.70	96.50	WGS
287.6919	0.324	Pseudomonas aeruginosa (286)	Pseudomonas (135621)	Pseudomonadaceae (7227	Pseudomonadales (1236)	Gammaproteobacteria (122	Proteobacteria (2)	Bacteria (1)	99.80	97.90	WGS
287.6920	0.368	Pseudomonas aeruginosa (286)	Pseudomonas (135621)	Pseudomonadaceae (7227	Pseudomonadales (1236)	Gammaproteobacteria (122	Proteobacteria (2)	Bacteria (1)	99.60	95.00	WGS
287.6921	0.324	Pseudomonas aeruginosa (286)	Pseudomonas (135621)	Pseudomonadaceae (7227	Pseudomonadales (1236)	Gammaproteobacteria (122	Proteobacteria (2)	Bacteria (1)	99.70	98.70	WGS
287.6922	0.3316	Pseudomonas aeruginosa (286)	Pseudomonas (135621)	Pseudomonadaceae (7227	Pseudomonadales (1236)	Gammaproteobacteria (122	Proteobacteria (2)	Bacteria (1)	99.90	96.50	WGS
287.6923	0.327	Pseudomonas aeruginosa (286)	Pseudomonas (135621)	Pseudomonadaceae (7227	Pseudomonadales (1236)	Gammaproteobacteria (122	Proteobacteria (2)	Bacteria (1)	99.80	98.70	WGS
287.6924	0.327	Pseudomonas aeruginosa (286)	Pseudomonas (135621)	Pseudomonadaceae (7227	Pseudomonadales (1236)	Gammaproteobacteria (122	Proteobacteria (2)	Bacteria (1)	99.80	98.80	WGS
287.6925	0.3636	Pseudomonas aeruginosa (286)	Pseudomonas (135621)	Pseudomonadaceae (7227	Pseudomonadales (1236)	Gammaproteobacteria (122	Proteobacteria (2)	Bacteria (1)	99.80	98.30	WGS
287.6926	0.3748	Pseudomonas aeruginosa (286)	Pseudomonas (135621)	Pseudomonadaceae (7227	Pseudomonadales (1236)	Gammaproteobacteria (122	Proteobacteria (2)	Bacteria (1)	99.70	97.40	WGS
287.6927	0.324	Pseudomonas aeruginosa (286)	Pseudomonas (135621)	Pseudomonadaceae (7227	Pseudomonadales (1236)	Gammaproteobacteria (122	Proteobacteria (2)	Bacteria (1)	99.80	98.20	WGS
287.6928	0.3174	Pseudomonas aeruginosa (286)	Pseudomonas (135621)	Pseudomonadaceae (7227	Pseudomonadales (1236)	Gammaproteobacteria (122	Proteobacteria (2)	Bacteria (1)	99.90	98.00	WGS
287.6929	0.3174	Pseudomonas aeruginosa (286)	Pseudomonas (135621)	Pseudomonadaceae (7227	Pseudomonadales (1236)	Gammaproteobacteria (122	Proteobacteria (2)	Bacteria (1)	99.90	99.40	WGS
287.6930	0.3174	Pseudomonas aeruginosa (286)	Pseudomonas (135621)	Pseudomonadaceae (7227	Pseudomonadales (1236)	Gammaproteobacteria (122	Proteobacteria (2)	Bacteria (1)	99.90	95.00	WGS
287.6931	0.3258	Pseudomonas aeruginosa (286)	Pseudomonas (135621)	Pseudomonadaceae (7227	Pseudomonadales (1236)	Gammaproteobacteria (122	Proteobacteria (2)	Bacteria (1)	99.90	99.30	WGS
287.6932	0.3226	Pseudomonas aeruginosa (286)	Pseudomonas (135621)	Pseudomonadaceae (7227	Pseudomonadales (1236)	Gammaproteobacteria (122	Proteobacteria (2)	Bacteria (1)	99.90	99.10	WGS
287.6933	0.3226	Pseudomonas aeruginosa (286)	Pseudomonas (135621)	Pseudomonadaceae (7227	Pseudomonadales (1236)	Gammaproteobacteria (122	Proteobacteria (2)	Bacteria (1)	99.60	97.90	WGS
287.6934	0.3226	Pseudomonas aeruginosa (286)	Pseudomonas (135621)	Pseudomonadaceae (7227	Pseudomonadales (1236)	Gammaproteobacteria (122	Proteobacteria (2)	Bacteria (1)	99.80	97.60	WGS
287.6935	0.3258	Pseudomonas aeruginosa (286)	Pseudomonas (135621)	Pseudomonadaceae (7227	Pseudomonadales (1236)	Gammaproteobacteria (122	Proteobacteria (2)	Bacteria (1)	99.70	93.90	WGS
287.6936	0.3258	Pseudomonas aeruginosa (286)	Pseudomonas (135621)	Pseudomonadaceae (7227	Pseudomonadales (1236)	Gammaproteobacteria (122	Proteobacteria (2)	Bacteria (1)	99.10	96.50	WGS
287.6937	0.3258	Pseudomonas aeruginosa (286)	Pseudomonas (135621)	Pseudomonadaceae (7227	Pseudomonadales (1236)	Gammaproteobacteria (122	Proteobacteria (2)	Bacteria (1)	99.80	99.20	WGS
287.6938	0.3694	Pseudomonas aeruginosa (286)	Pseudomonas (135621)	Pseudomonadaceae (7227	Pseudomonadales (1236)	Gammaproteobacteria (122	Proteobacteria (2)	Bacteria (1)	99.90	97.60	WGS
287.6939	0.3694	Pseudomonas aeruginosa (286)	Pseudomonas (135621)	Pseudomonadaceae (7227	Pseudomonadales (1236)	Gammaproteobacteria (122	Proteobacteria (2)	Bacteria (1)	99.90	98.80	WGS
287.6940	0.3694	Pseudomonas aeruginosa (286)	Pseudomonas (135621)	Pseudomonadaceae (7227	Pseudomonadales (1236)	Gammaproteobacteria (122	Proteobacteria (2)	Bacteria (1)	99.90	98.20	WGS
287.6941	0.3694	Pseudomonas aeruginosa (286)	Pseudomonas (135621)	Pseudomonadaceae (7227	Pseudomonadales (1236)	Gammaproteobacteria (122	Proteobacteria (2)	Bacteria (1)	99.90	98.80	WGS
287.6942	0.3694	Pseudomonas aeruginosa (286)	Pseudomonas (135621)	Pseudomonadaceae (7227	Pseudomonadales (1236)	Gammaproteobacteria (122	Proteobacteria (2)	Bacteria (1)	99.70	98.00	WGS
287.6943	0.3694	Pseudomonas aeruginosa (286)	Pseudomonas (135621)	Pseudomonadaceae (7227	Pseudomonadales (1236)	Gammaproteobacteria (122	Proteobacteria (2)	Bacteria (1)	99.90	98.50	WGS
287.6944	0.3934	Pseudomonas aeruginosa (286)	Pseudomonas (135621)	Pseudomonadaceae (7227	Pseudomonadales (1236)	Gammaproteobacteria (122	Proteobacteria (2)	Bacteria (1)	99.90	94.20	WGS
287.6945	0.371	Pseudomonas aeruginosa (286)	Pseudomonas (135621)	Pseudomonadaceae (7227	Pseudomonadales (1236)	Gammaproteobacteria (122	Proteobacteria (2)	Bacteria (1)	99.90	92.20	WGS
287.6946	0.3694	Pseudomonas aeruginosa (286)	Pseudomonas (135621)	Pseudomonadaceae (7227	Pseudomonadales (1236)	Gammaproteobacteria (122	Proteobacteria (2)	Bacteria (1)	99.70	91.10	WGS
287.6947	0.3694	Pseudomonas aeruginosa (286)	Pseudomonas (135621)	Pseudomonadaceae (7227	Pseudomonadales (1236)	Gammaproteobacteria (122	Proteobacteria (2)	Bacteria (1)	99.90	98.40	WGS
287.6948	0.3694	Pseudomonas aeruginosa (286)	Pseudomonas (135621)	Pseudomonadaceae (7227	Pseudomonadales (1236)	Gammaproteobacteria (122	Proteobacteria (2)	Bacteria (1)	99.90	97.90	WGS
287.6949	0.3694	Pseudomonas aeruginosa (286)	Pseudomonas (135621)	Pseudomonadaceae (7227	Pseudomonadales (1236)	Gammaproteobacteria (122	Proteobacteria (2)	Bacteria (1)	99.90	98.70	WGS
287.6950	0.3694	Pseudomonas aeruginosa (286)	Pseudomonas (135621)	Pseudomonadaceae (7227	Pseudomonadales (1236)	Gammaproteobacteria (122	Proteobacteria (2)	Bacteria (1)	99.90	97.30	WGS
287.6951	0.3888	Pseudomonas aeruginosa (286)	Pseudomonas (135621)	Pseudomonadaceae (7227	Pseudomonadales (1236)	Gammaproteobacteria (122	Proteobacteria (2)	Bacteria (1)	99.90	94.20	WGS
287.6952	0.3694	Pseudomonas aeruginosa (286)	Pseudomonas (135621)	Pseudomonadaceae (7227	Pseudomonadales (1236)	Gammaproteobacteria (122	Proteobacteria (2)	Bacteria (1)	99.90	99.00	WGS
287.6953	0.3694	Pseudomonas aeruginosa (286)	Pseudomonas (135621)	Pseudomonadaceae (7227	Pseudomonadales (1236)	Gammaproteobacteria (122	Proteobacteria (2)	Bacteria (1)	99.90	98.70	WGS
287.6954	0.3652	Pseudomonas aeruginosa (286)	Pseudomonas (135621)	Pseudomonadaceae (7227	Pseudomonadales (1236)	Gammaproteobacteria (122	Proteobacteria (2)	Bacteria (1)	99.90	98.60	WGS
287.6955	0.3694	Pseudomonas aeruginosa (286)	Pseudomonas (135621)	Pseudomonadaceae (7227	Pseudomonadales (1236)	Gammaproteobacteria (122	Proteobacteria (2)	Bacteria (1)	99.90	98.90	WGS
287.6956	0.3694	Pseudomonas aeruginosa (286)	Pseudomonas (135621)	Pseudomonadaceae (7227	Pseudomonadales (1236)	Gammaproteobacteria (122	Proteobacteria (2)	Bacteria (1)	99.90	98.20	WGS
287.6957	0.371	Pseudomonas aeruginosa (286)	Pseudomonas (135621)	Pseudomonadaceae (7227	Pseudomonadales (1236)	Gammaproteobacteria (122	Proteobacteria (2)	Bacteria (1)	99.90	98.70	WGS
287.6958	0.3694	Pseudomonas aeruginosa (286)	Pseudomonas (135621)	Pseudomonadaceae (7227	Pseudomonadales (1236)	Gammaproteobacteria (122	Proteobacteria (2)	Bacteria (1)	99.90	98.70	WGS
287.6959	0.3694	Pseudomonas aeruginosa (286)	Pseudomonas (135621)	Pseudomonadaceae (7227	Pseudomonadales (1236)	Gammaproteobacteria (122	Proteobacteria (2)	Bacteria (1)	99.90	98.70	WGS
287.6960	0.3694	Pseudomonas aeruginosa (286)	Pseudomonas (135621)	Pseudomonadaceae (7227	Pseudomonadales (1236)	Gammaproteobacteria (122	Proteobacteria (2)	Bacteria (1)	99.80	96.70	WGS
287.6961	0.3636	Pseudomonas aeruginosa (286)	Pseudomonas (135621)	Pseudomonadaceae (7227	Pseudomonadales (1236)	Gammaproteobacteria (122	Proteobacteria (2)	Bacteria (1)	99.80	91.90	WGS
287.6962	0.3336	Pseudomonas aeruginosa (286)	Pseudomonas (135621)	Pseudomonadaceae (7227	Pseudomonadales (1236)	Gammaproteobacteria (122	Proteobacteria (2)	Bacteria (1)	99.70	98.50	WGS
287.6963	0.3694	Pseudomonas aeruginosa (286)	Pseudomonas (135621)	Pseudomonadaceae (7227	Pseudomonadales (1236)	Gammaproteobacteria (122	Proteobacteria (2)	Bacteria (1)	99.90	98.30	WGS
287.6964	0.3578	Pseudomonas aeruginosa (286)	Pseudomonas (135621)	Pseudomonadaceae (7227	Pseudomonadales (1236)	Gammaproteobacteria (122	Proteobacteria (2)	Bacteria (1)	99.90	93.30	WGS
287.6965	0.331	Pseudomonas aeruginosa (286)	Pseudomonas (135621)	Pseudomonadaceae (7227	Pseudomonadales (1236)	Gammaproteobacteria (122	Proteobacteria (2)	Bacteria (1)	99.90	93.90	WGS
287.6966	0.3258	Pseudomonas aeruginosa (286)	Pseudomonas (135621)	Pseudomonadaceae (7227	Pseudomonadales (1236)	Gammaproteobacteria (122	Proteobacteria (2)	Bacteria (1)	99.80	96.60	WGS
287.6967	0.332	Pseudomonas aeruginosa (286)	Pseudomonas (135621)	Pseudomonadaceae (7227	Pseudomonadales (1236)	Gammaproteobacteria (122	Proteobacteria (2)	Bacteria (1)	99.60	98.20	WGS
287.6968	0.3386	Pseudomonas aeruginosa (286)	Pseudomonas (135621)	Pseudomonadaceae (7227	Pseudomonadales (1236)	Gammaproteobacteria (122	Proteobacteria (2)	Bacteria (1)	99.80	98.20	WGS
287.6969	0.331	Pseudomonas aeruginosa (286)	Pseudomonas (135621)	Pseudomonadaceae (7227	Pseudomonadales (1236)	Gammaproteobacteria (122	Proteobacteria (2)	Bacteria (1)	99.60	98.60	WGS
287.6970	0.332	Pseudomonas aeruginosa (286)	Pseudomonas (135621)	Pseudomonadaceae (7227	Pseudomonadales (1236)	Gammaproteobacteria (122	Proteobacteria (2)	Bacteria (1)	99.60	98.80	WGS
287.6971	0.3268	Pseudomonas aeruginosa (286)	Pseudomonas (135621)	Pseudomonadaceae (7227	Pseudomonadales (1236)	Gammaproteobacteria (122	Proteobacteria (2)	Bacteria (1)	99.80	96.40	WGS
287.6972	0.3696	Pseudomonas aeruginosa (286)	Pseudomonas (135621)	Pseudomonadaceae (7227	Pseudomonadales (1236)	Gammaproteobacteria (122	Proteobacteria (2)	Bacteria (1)	99.80	98.50	WGS
287.6973	0.3744	Pseudomonas aeruginosa (286)	Pseudomonas (135621)	Pseudomonadaceae (7227	Pseudomonadales (1236)	Gammaproteobacteria (122	Proteobacteria (2)	Bacteria (1)	99.80	96.70	WGS
287.6974	0.3744	Pseudomonas aeruginosa (286)	Pseudomonas (135621)	Pseudomonadaceae (7227	Pseudomonadales (1236)	Gammaproteobacteria (122	Proteobacteria (2)	Bacteria (1)	99.90	97.30	WGS
287.6975	0.3744	Pseudomonas aeruginosa (286)	Pseudomonas (135621)	Pseudomonadaceae (7227	Pseudomonadales (1236)	Gammaproteobacteria (122	Proteobacteria (2)	Bacteria (1)	99.90	95.70	WGS
287.6976	0.3268	Pseudomonas aeruginosa (286)	Pseudomonas (135621)	Pseudomonadaceae (7227	Pseudomonadales (1236)	Gammaproteobacteria (122	Proteobacteria (2)	Bacteria (1)	99.90	97.90	WGS
287.6977	0.3268	Pseudomonas aeruginosa (286)	Pseudomonas (135621)	Pseudomonadaceae (7227	Pseudomonadales (1236)	Gammaproteobacteria (122	Proteobacteria (2)	Bacteria (1)	99.90	96.00	WGS
287.6978	0.3268	Pseudomonas aeruginosa (286)	Pseudomonas (135621)	Pseudomonadaceae (7227	Pseudomonadales (1236)	Gammaproteobacteria (122	Proteobacteria (2)	Bacteria (1)	99.90	95.20	WGS
287.6979	0.332	Pseudomonas aeruginosa (286)	Pseudomonas (135621)	Pseudomonadaceae (7227	Pseudomonadales (1236)	Gammaproteobacteria (122	Proteobacteria (2)	Bacteria (1)	99.70	96.20	WGS
287.6980	0.332	Pseudomonas aeruginosa (286)	Pseudomonas (135621)	Pseudomonadaceae (7227	Pseudomonadales (1236)	Gammaproteobacteria (122	Proteobacteria (2)	Bacteria (1)	99.90	98.70	WGS
287.6981	0.3246	Pseudomonas aeruginosa (286)	Pseudomonas (135621)	Pseudomonadaceae (7227	Pseudomonadales (1236)	Gammaproteobacteria (122	Proteobacteria (2)	Bacteria (1)	99.70	95.20	WGS
287.6982	0.332	Pseudomonas aeruginosa (286)	Pseudomonas (135621)	Pseudomonadaceae (7227	Pseudomonadales (1236)	Gammaproteobacteria (122	Proteobacteria (2)	Bacteria (1)	99.90	98.70	WGS
287.6983	0.3258	Pseudomonas aeruginosa (286)	Pseudomonas (135621)	Pseudomonadaceae (7227	Pseudomonadales (1236)	Gammaproteobacteria (122	Proteobacteria (2)	Bacteria (1)	99.90	97.10	WGS
287.6984	0.332	Pseudomonas aeruginosa (286)	Pseudomonas (135621)	Pseudomonadaceae (7227	Pseudomonadales (1236)	Gammaproteobacteria (122	Proteobacteria (2)	Bacteria (1)	99.80	98.60	WGS
287.6985	0.3694	Pseudomonas aeruginosa (286)	Pseudomonas (135621)	Pseudomonadaceae (7227	Pseudomonadales (1236)	Gammaproteobacteria (122	Proteobacteria (2)	Bacteria (1)	99.90	93.20	WGS
287.6986	0.3738	Pseudomonas aeruginosa (286)	Pseudomonas (135621)	Pseudomonadaceae (7227	Pseudomonadales (1236)	Gammaproteobacteria (122	Proteobacteria (2)	Bacteria (1)	99.90	95.00	WGS
287.6987	0.331	Pseudomonas aeruginosa (286)	Pseudomonas (135621)	Pseudomonadaceae (7227	Pseudomonadales (1236)	Gammaproteobacteria (122	Proteobacteria (2)	Bacteria (1)	99.30	96.10	WGS
287.6988	0.371	Pseudomonas aeruginosa (286)	Pseudomonas (135621)	Pseudomonadaceae (7227	Pseudomonadales (1236)	Gammaproteobacteria (122	Proteobacteria (2)	Bacteria (1)	99.50	96.60	WGS
287.6989	0.3388	Pseudomonas aeruginosa (286)	Pseudomonas (135621)	Pseudomonadaceae (7227	Pseudomonadales (1236)	Gammaproteobacteria (122	Proteobacteria (2)	Bacteria (1)	99.40	97.10	WGS
287.6990	0.331	Pseudomonas aeruginosa (286)	Pseudomonas (135621)	Pseudomonadaceae (7227	Pseudomonadales (1236)	Gammaproteobacteria (122	Proteobacteria (2)	Bacteria (1)	99.70	98.30	WGS
287.6991	0.3356	Pseudomonas aeruginosa (286)	Pseudomonas (135621)	Pseudomonadaceae (7227	Pseudomonadales (1236)	Gammaproteobacteria (122	Proteobacteria (2)	Bacteria (1)	99.90	96.50	WGS
287.6992	0.3356	Pseudomonas aeruginosa (286)	Pseudomonas (135621)	Pseudomonadaceae (7227	Pseudomonadales (1236)	Gammaproteobacteria (122	Proteobacteria (2)	Bacteria (1)	99.90	94.70	WGS
287.6993	0.3902	Pseudomonas aeruginosa (286)	Pseudomonas (135621)	Pseudomonadaceae (7227	Pseudomonadales (1236)	Gammaproteobacteria (122	Proteobacteria (2)	Bacteria (1)	99.70	98.10	WGS
287.6994	0.3694	Pseudomonas aeruginosa (286)	Pseudomonas (135621)	Pseudomonadaceae (7227	Pseudomonadales (1236)	Gammaproteobacteria (122	Proteobacteria (2)	Bacteria (1)	99.80	98.40	WGS
287.6995	0.3694	Pseudomonas aeruginosa (286)	Pseudomonas (135621)	Pseudomonadaceae (7227	Pseudomonadales (1236)	Gammaproteobacteria (122	Proteobacteria (2)	Bacteria (1)	99.30	96.50	WGS
287.6996	0.3694	Pseudomonas aeruginosa (286)	Pseudomonas (135621)	Pseudomonadaceae (7227	Pseudomonadales (1236)	Gammaproteobacteria (122	Proteobacteria (2)	Bacteria (1)	99.80	98.60	WGS
287.6997	0.3738	Pseudomonas aeruginosa (286)	Pseudomonas (135621)	Pseudomonadaceae (7227	Pseudomonadales (1236)	Gammaproteobacteria (122	Proteobacteria (2)	Bacteria (1)	99.70	94.70	WGS
287.6998	0.3694	Pseudomonas aeruginosa (286)	Pseudomonas (135621)	Pseudomonadaceae (7227	Pseudomonadales (1236)	Gammaproteobacteria (122	Proteobacteria (2)	Bacteria (1)	99.80	98.60	WGS
287.6999	0.3268	Pseudomonas aeruginosa (286)	Pseudomonas (135621)	Pseudomonadaceae (7227	Pseudomonadales (1236)	Gammaproteobacteria (122	Proteobacteria (2)	Bacteria (1)	99.70	91.10	WGS
287.7000	0.3694	Pseudomonas aeruginosa (286)	Pseudomonas (135621)	Pseudomonadaceae (7227	Pseudomonadales (1236)	Gammaproteobacteria (122	Proteobacteria (2)	Bacteria (1)	99.60	97.30	WGS
287.7001	0.3694	Pseudomonas aeruginosa (286)	Pseudomonas (135621)	Pseudomonadaceae (7227	Pseudomonadales (1236)	Gammaproteobacteria (122	Proteobacteria (2)	Bacteria (1)	99.90	94.50	WGS
287.7002	0.4156	Pseudomonas aeruginosa (286)	Pseudomonas (135621)	Pseudomonadaceae (7227	Pseudomonadales (1236)	Gammaproteobacteria (122	Proteobacteria (2)	Bacteria (1)	99.40	98.00	WGS
287.7003	0.371	Pseudomonas aeruginosa (286)	Pseudomonas (135621)	Pseudomonadaceae (7227	Pseudomonadales (1236)	Gammaproteobacteria (122	Proteobacteria (2)	Bacteria (1)	99.90	98.00	WGS
287.7004	0.3694	Pseudomonas aeruginosa (286)	Pseudomonas (135621)	Pseudomonadaceae (7227	Pseudomonadales (1236)	Gammaproteobacteria (122	Proteobacteria (2)	Bacteria (1)	99.90	98.70	WGS
287.7005	0.3694	Pseudomonas aeruginosa (286)	Pseudomonas (135621)	Pseudomonadaceae (7227	Pseudomonadales (1236)	Gammaproteobacteria (122	Proteobacteria (2)	Bacteria (1)	99.90	98.60	WGS
287.7006	0.3694	Pseudomonas aeruginosa (286)	Pseudomonas (135621)	Pseudomonadaceae (7227	Pseudomonadales (1236)	Gammaproteobacteria (122	Proteobacteria (2)	Bacteria (1)	99.90	98.10	WGS
287.7007	0.3694	Pseudomonas aeruginosa (286)	Pseudomonas (135621)	Pseudomonadaceae (7227	Pseudomonadales (1236)	Gammaproteobacteria (122	Proteobacteria (2)	Bacteria (1)	99.90	98.70	WGS
287.7008	0.3394	Pseudomonas aeruginosa (286)	Pseudomonas (135621)	Pseudomonadaceae (7227	Pseudomonadales (1236)	Gammaproteobacteria (122	Proteobacteria (2)	Bacteria (1)	99.90	94.50	WGS
287.7009	0.3646	Pseudomonas aeruginosa (286)	Pseudomonas (135621)	Pseudomonadaceae (7227	Pseudomonadales (1236)	Gammaproteobacteria (122	Proteobacteria (2)	Bacteria (1)	99.90	98.50	WGS
287.7010	0.3694	Pseudomonas aeruginosa (286)	Pseudomonas (135621)	Pseudomonadaceae (7227	Pseudomonadales (1236)	Gammaproteobacteria (122	Proteobacteria (2)	Bacteria (1)	99.60	98.20	WGS
287.7011	0.3694	Pseudomonas aeruginosa (286)	Pseudomonas (135621)	Pseudomonadaceae (7227	Pseudomonadales (1236)	Gammaproteobacteria (122	Proteobacteria (2)	Bacteria (1)	99.80	94.50	WGS
287.7012	0.3328	Pseudomonas aeruginosa (286)	Pseudomonas (135621)	Pseudomonadaceae (7227	Pseudomonadales (1236)	Gammaproteobacteria (122	Proteobacteria (2)	Bacteria (1)	99.80	93.90	WGS
287.7013	0.3608	Pseudomonas aeruginosa (286)	Pseudomonas (135621)	Pseudomonadaceae (7227	Pseudomonadales (1236)	Gammaproteobacteria (122	Proteobacteria (2)	Bacteria (1)	99.80	94.50	WGS
287.7014	0.327	Pseudomonas aeruginosa (286)	Pseudomonas (135621)	Pseudomonadaceae (7227	Pseudomonadales (1236)	Gammaproteobacteria (122	Proteobacteria (2)	Bacteria (1)	99.60	99.00	WGS
287.7015	0.3258	Pseudomonas aeruginosa (286)	Pseudomonas (135621)	Pseudomonadaceae (7227	Pseudomonadales (1236)	Gammaproteobacteria (122	Proteobacteria (2)	Bacteria (1)	99.90	99.00	WGS
287.7016	0.3292	Pseudomonas aeruginosa (286)	Pseudomonas (135621)	Pseudomonadaceae (7227	Pseudomonadales (1236)	Gammaproteobacteria (122	Proteobacteria (2)	Bacteria (1)	99.70	97.80	WGS
287.7017	0.3386	Pseudomonas aeruginosa (286)	Pseudomonas (135621)	Pseudomonadaceae (7227	Pseudomonadales (1236)	Gammaproteobacteria (122	Proteobacteria (2)	Bacteria (1)	99.70	98.50	WGS
287.7018	0.3386	Pseudomonas aeruginosa (286)	Pseudomonas (135621)	Pseudomonadaceae (7227	Pseudomonadales (1236)	Gammaproteobacteria (122	Proteobacteria (2)	Bacteria (1)	99.90	97.80	WGS
287.7019	0.332	Pseudomonas aeruginosa (286)	Pseudomonas (135621)	Pseudomonadaceae (7227	Pseudomonadales (1236)	Gammaproteobacteria (122	Proteobacteria (2)	Bacteria (1)	99.60	97.30	WGS
287.7020	0.3268	Pseudomonas aeruginosa (286)	Pseudomonas (135621)	Pseudomonadaceae (7227	Pseudomonadales (1236)	Gammaproteobacteria (122	Proteobacteria (2)	Bacteria (1)	99.80	98.50	WGS
287.7021	0.3696	Pseudomonas aeruginosa (286)	Pseudomonas (135621)	Pseudomonadaceae (7227	Pseudomonadales (1236)	Gammaproteobacteria (122	Proteobacteria (2)	Bacteria (1)	99.70	98.70	WGS
287.7022	0.3386	Pseudomonas aeruginosa (286)	Pseudomonas (135621)	Pseudomonadaceae (7227	Pseudomonadales (1236)	Gammaproteobacteria (122	Proteobacteria (2)	Bacteria (1)	99.90	92.40	WGS
287.7023	0.332	Pseudomonas aeruginosa (286)	Pseudomonas (135621)	Pseudomonadaceae (7227	Pseudomonadales (1236)	Gammaproteobacteria (122	Proteobacteria (2)	Bacteria (1)	99.80	98.70	WGS
287.7024	0.3284	Pseudomonas aeruginosa (286)	Pseudomonas (135621)	Pseudomonadaceae (7227	Pseudomonadales (1236)	Gammaproteobacteria (122	Proteobacteria (2)	Bacteria (1)	99.90	98.70	WGS
287.7025	0.3936	Pseudomonas aeruginosa (286)	Pseudomonas (135621)	Pseudomonadaceae (7227	Pseudomonadales (1236)	Gammaproteobacteria (122	Proteobacteria (2)	Bacteria (1)	99.70	97.60	WGS
287.7026	0.3936	Pseudomonas aeruginosa (286)	Pseudomonas (135621)	Pseudomonadaceae (7227	Pseudomonadales (1236)	Gammaproteobacteria (122	Proteobacteria (2)	Bacteria (1)	99.70	97.80	WGS
287.7027	0.3376	Pseudomonas aeruginosa (286)	Pseudomonas (135621)	Pseudomonadaceae (7227	Pseudomonadales (1236)	Gammaproteobacteria (122	Proteobacteria (2)	Bacteria (1)	99.80	96.70	WGS
287.7028	0.303	Pseudomonas aeruginosa (286)	Pseudomonas (135621)	Pseudomonadaceae (7227	Pseudomonadales (1236)	Gammaproteobacteria (122	Proteobacteria (2)	Bacteria (1)	99.50	93.90	WGS
287.7029	0.331	Pseudomonas aeruginosa (286)	Pseudomonas (135621)	Pseudomonadaceae (7227	Pseudomonadales (1236)	Gammaproteobacteria (122	Proteobacteria (2)	Bacteria (1)	99.60	98.80	WGS
287.7030	0.331	Pseudomonas aeruginosa (286)	Pseudomonas (135621)	Pseudomonadaceae (7227	Pseudomonadales (1236)	Gammaproteobacteria (122	Proteobacteria (2)	Bacteria (1)	99.70	98.20	WGS
287.7031	0.331	Pseudomonas aeruginosa (286)	Pseudomonas (135621)	Pseudomonadaceae (7227	Pseudomonadales (1236)	Gammaproteobacteria (122	Proteobacteria (2)	Bacteria (1)	99.70	98.20	WGS
287.7032	0.3292	Pseudomonas aeruginosa (286)	Pseudomonas (135621)	Pseudomonadaceae (7227	Pseudomonadales (1236)	Gammaproteobacteria (122	Proteobacteria (2)	Bacteria (1)	99.80	98.30	WGS
287.7033	0.3292	Pseudomonas aeruginosa (286)	Pseudomonas (135621)	Pseudomonadaceae (7227	Pseudomonadales (1236)	Gammaproteobacteria (122	Proteobacteria (2)	Bacteria (1)	99.80	98.60	WGS
287.7034	0.3226	Pseudomonas aeruginosa (286)	Pseudomonas (135621)	Pseudomonadaceae (7227	Pseudomonadales (1236)	Gammaproteobacteria (122	Proteobacteria (2)	Bacteria (1)	99.60	98.70	WGS
287.7035	0.3694	Pseudomonas aeruginosa (286)	Pseudomonas (135621)	Pseudomonadaceae (7227	Pseudomonadales (1236)	Gammaproteobacteria (122	Proteobacteria (2)	Bacteria (1)	99.80	98.60	WGS
287.7036	0.356	Pseudomonas aeruginosa (286)	Pseudomonas (135621)	Pseudomonadaceae (7227	Pseudomonadales (1236)	Gammaproteobacteria (122	Proteobacteria (2)	Bacteria (1)	99.60	96.90	WGS
287.7037	0.3618	Pseudomonas aeruginosa (286)	Pseudomonas (135621)	Pseudomonadaceae (7227	Pseudomonadales (1236)	Gammaproteobacteria (122	Proteobacteria (2)	Bacteria (1)	99.70	98.70	Complete
287.7038	0.3322	Pseudomonas aeruginosa (286)	Pseudomonas (135621)	Pseudomonadaceae (7227	Pseudomonadales (1236)	Gammaproteobacteria (122	Proteobacteria (2)	Bacteria (1)	99.90	99.20	Complete
287.7039	0.3302	Pseudomonas aeruginosa (286)	Pseudomonas (135621)	Pseudomonadaceae (7227	Pseudomonadales (1236)	Gammaproteobacteria (122	Proteobacteria (2)	Bacteria (1)	99.40	97.80	WGS
287.7040	0.3302	Pseudomonas aeruginosa (286)	Pseudomonas (135621)	Pseudomonadaceae (7227	Pseudomonadales (1236)	Gammaproteobacteria (122	Proteobacteria (2)	Bacteria (1)	99.50	97.60	WGS
287.7041	0.3914	Pseudomonas aeruginosa (286)	Pseudomonas (135621)	Pseudomonadaceae (7227	Pseudomonadales (1236)	Gammaproteobacteria (122	Proteobacteria (2)	Bacteria (1)	99.60	97.40	WGS
287.7042	0.3866	Pseudomonas aeruginosa (286)	Pseudomonas (135621)	Pseudomonadaceae (7227	Pseudomonadales (1236)	Gammaproteobacteria (122	Proteobacteria (2)	Bacteria (1)	98.00	80.90	WGS
287.7043	0.3632	Pseudomonas aeruginosa (286)	Pseudomonas (135621)	Pseudomonadaceae (7227	Pseudomonadales (1236)	Gammaproteobacteria (122	Proteobacteria (2)	Bacteria (1)	99.80	98.40	WGS
287.7044	0.3632	Pseudomonas aeruginosa (286)	Pseudomonas (135621)	Pseudomonadaceae (7227	Pseudomonadales (1236)	Gammaproteobacteria (122	Proteobacteria (2)	Bacteria (1)	99.60	98.70	WGS
287.7045	0.3632	Pseudomonas aeruginosa (286)	Pseudomonas (135621)	Pseudomonadaceae (7227	Pseudomonadales (1236)	Gammaproteobacteria (122	Proteobacteria (2)	Bacteria (1)	99.80	98.60	WGS
287.7046	0.3632	Pseudomonas aeruginosa (286)	Pseudomonas (135621)	Pseudomonadaceae (7227	Pseudomonadales (1236)	Gammaproteobacteria (122	Proteobacteria (2)	Bacteria (1)	99.80	99.00	WGS
287.7047	0.3632	Pseudomonas aeruginosa (286)	Pseudomonas (135621)	Pseudomonadaceae (7227	Pseudomonadales (1236)	Gammaproteobacteria (122	Proteobacteria (2)	Bacteria (1)	99.80	99.00	WGS
287.7048	0.3632	Pseudomonas aeruginosa (286)	Pseudomonas (135621)	Pseudomonadaceae (7227	Pseudomonadales (1236)	Gammaproteobacteria (122	Proteobacteria (2)	Bacteria (1)	99.80	99.00	WGS
287.7049	0.3632	Pseudomonas aeruginosa (286)	Pseudomonas (135621)	Pseudomonadaceae (7227	Pseudomonadales (1236)	Gammaproteobacteria (122	Proteobacteria (2)	Bacteria (1)	99.80	98.80	WGS
287.7050	0.3632	Pseudomonas aeruginosa (286)	Pseudomonas (135621)	Pseudomonadaceae (7227	Pseudomonadales (1236)	Gammaproteobacteria (122	Proteobacteria (2)	Bacteria (1)	99.80	99.00	WGS
287.7051	0.3632	Pseudomonas aeruginosa (286)	Pseudomonas (135621)	Pseudomonadaceae (7227	Pseudomonadales (1236)	Gammaproteobacteria (122	Proteobacteria (2)	Bacteria (1)	99.80	98.60	WGS
287.7052	0.3602	Pseudomonas aeruginosa (286)	Pseudomonas (135621)	Pseudomonadaceae (7227	Pseudomonadales (1236)	Gammaproteobacteria (122	Proteobacteria (2)	Bacteria (1)	99.00	90.50	Complete
287.7053	0.3694	Pseudomonas aeruginosa (286)	Pseudomonas (135621)	Pseudomonadaceae (7227	Pseudomonadales (1236)	Gammaproteobacteria (122	Proteobacteria (2)	Bacteria (1)	99.70	99.30	WGS
287.7054	0.3254	Pseudomonas aeruginosa (286)	Pseudomonas (135621)	Pseudomonadaceae (7227	Pseudomonadales (1236)	Gammaproteobacteria (122	Proteobacteria (2)	Bacteria (1)	99.90	98.90	WGS
287.7055	0.338	Pseudomonas aeruginosa (286)	Pseudomonas (135621)	Pseudomonadaceae (7227	Pseudomonadales (1236)	Gammaproteobacteria (122	Proteobacteria (2)	Bacteria (1)	99.90	98.50	WGS
287.7056	0.3636	Pseudomonas aeruginosa (286)	Pseudomonas (135621)	Pseudomonadaceae (7227	Pseudomonadales (1236)	Gammaproteobacteria (122	Proteobacteria (2)	Bacteria (1)	99.90	99.60	WGS
287.7057	0.3376	Pseudomonas aeruginosa (286)	Pseudomonas (135621)	Pseudomonadaceae (7227	Pseudomonadales (1236)	Gammaproteobacteria (122	Proteobacteria (2)	Bacteria (1)	99.90	99.10	WGS
287.7058	0.327	Pseudomonas aeruginosa (286)	Pseudomonas (135621)	Pseudomonadaceae (7227	Pseudomonadales (1236)	Gammaproteobacteria (122	Proteobacteria (2)	Bacteria (1)	99.90	99.60	WGS
287.7059	0.3258	Pseudomonas aeruginosa (286)	Pseudomonas (135621)	Pseudomonadaceae (7227	Pseudomonadales (1236)	Gammaproteobacteria (122	Proteobacteria (2)	Bacteria (1)	99.80	99.10	WGS
287.7060	0.3324	Pseudomonas aeruginosa (286)	Pseudomonas (135621)	Pseudomonadaceae (7227	Pseudomonadales (1236)	Gammaproteobacteria (122	Proteobacteria (2)	Bacteria (1)	99.90	99.20	WGS
287.7061	0.3636	Pseudomonas aeruginosa (286)	Pseudomonas (135621)	Pseudomonadaceae (7227	Pseudomonadales (1236)	Gammaproteobacteria (122	Proteobacteria (2)	Bacteria (1)	99.70	98.70	WGS
287.7062	0.3258	Pseudomonas aeruginosa (286)	Pseudomonas (135621)	Pseudomonadaceae (7227	Pseudomonadales (1236)	Gammaproteobacteria (122	Proteobacteria (2)	Bacteria (1)	99.70	98.70	WGS
287.7063	0.3324	Pseudomonas aeruginosa (286)	Pseudomonas (135621)	Pseudomonadaceae (7227	Pseudomonadales (1236)	Gammaproteobacteria (122	Proteobacteria (2)	Bacteria (1)	99.70	98.40	WGS
287.7064	0.368	Pseudomonas aeruginosa (286)	Pseudomonas (135621)	Pseudomonadaceae (7227	Pseudomonadales (1236)	Gammaproteobacteria (122	Proteobacteria (2)	Bacteria (1)	99.60	99.00	WGS
287.7065	0.3376	Pseudomonas aeruginosa (286)	Pseudomonas (135621)	Pseudomonadaceae (7227	Pseudomonadales (1236)	Gammaproteobacteria (122	Proteobacteria (2)	Bacteria (1)	99.90	99.00	WGS
287.7066	0.3336	Pseudomonas aeruginosa (286)	Pseudomonas (135621)	Pseudomonadaceae (7227	Pseudomonadales (1236)	Gammaproteobacteria (122	Proteobacteria (2)	Bacteria (1)	99.90	98.50	WGS
287.7067	0.3738	Pseudomonas aeruginosa (286)	Pseudomonas (135621)	Pseudomonadaceae (7227	Pseudomonadales (1236)	Gammaproteobacteria (122	Proteobacteria (2)	Bacteria (1)	99.90	98.90	WGS
287.7068	0.368	Pseudomonas aeruginosa (286)	Pseudomonas (135621)	Pseudomonadaceae (7227	Pseudomonadales (1236)	Gammaproteobacteria (122	Proteobacteria (2)	Bacteria (1)	99.80	99.20	WGS
287.7069	0.338	Pseudomonas aeruginosa (286)	Pseudomonas (135621)	Pseudomonadaceae (7227	Pseudomonadales (1236)	Gammaproteobacteria (122	Proteobacteria (2)	Bacteria (1)	99.90	98.60	WGS
287.7070	0.338	Pseudomonas aeruginosa (286)	Pseudomonas (135621)	Pseudomonadaceae (7227	Pseudomonadales (1236)	Gammaproteobacteria (122	Proteobacteria (2)	Bacteria (1)	99.90	98.70	WGS
287.7071	0.3324	Pseudomonas aeruginosa (286)	Pseudomonas (135621)	Pseudomonadaceae (7227	Pseudomonadales (1236)	Gammaproteobacteria (122	Proteobacteria (2)	Bacteria (1)	99.90	99.30	WGS
287.7072	0.3324	Pseudomonas aeruginosa (286)	Pseudomonas (135621)	Pseudomonadaceae (7227	Pseudomonadales (1236)	Gammaproteobacteria (122	Proteobacteria (2)	Bacteria (1)	99.90	99.30	WGS
287.7073	0.3322	Pseudomonas aeruginosa (286)	Pseudomonas (135621)	Pseudomonadaceae (7227	Pseudomonadales (1236)	Gammaproteobacteria (122	Proteobacteria (2)	Bacteria (1)	99.90	98.90	WGS
287.7074	0.3704	Pseudomonas aeruginosa (286)	Pseudomonas (135621)	Pseudomonadaceae (7227	Pseudomonadales (1236)	Gammaproteobacteria (122	Proteobacteria (2)	Bacteria (1)	99.80	99.30	WGS
287.7075	0.3302	Pseudomonas aeruginosa (286)	Pseudomonas (135621)	Pseudomonadaceae (7227	Pseudomonadales (1236)	Gammaproteobacteria (122	Proteobacteria (2)	Bacteria (1)	99.90	98.20	WGS
287.7076	0.3552	Pseudomonas aeruginosa (286)	Pseudomonas (135621)	Pseudomonadaceae (7227	Pseudomonadales (1236)	Gammaproteobacteria (122	Proteobacteria (2)	Bacteria (1)	99.70	99.30	WGS
287.7077	0.3388	Pseudomonas aeruginosa (286)	Pseudomonas (135621)	Pseudomonadaceae (7227	Pseudomonadales (1236)	Gammaproteobacteria (122	Proteobacteria (2)	Bacteria (1)	99.90	99.40	WGS
287.7078	0.3376	Pseudomonas aeruginosa (286)	Pseudomonas (135621)	Pseudomonadaceae (7227	Pseudomonadales (1236)	Gammaproteobacteria (122	Proteobacteria (2)	Bacteria (1)	99.90	98.70	WGS
287.7079	0.368	Pseudomonas aeruginosa (286)	Pseudomonas (135621)	Pseudomonadaceae (7227	Pseudomonadales (1236)	Gammaproteobacteria (122	Proteobacteria (2)	Bacteria (1)	99.90	99.10	WGS
287.7080	0.3662	Pseudomonas aeruginosa (286)	Pseudomonas (135621)	Pseudomonadaceae (7227	Pseudomonadales (1236)	Gammaproteobacteria (122	Proteobacteria (2)	Bacteria (1)	99.50	98.10	WGS
287.7081	0.3636	Pseudomonas aeruginosa (286)	Pseudomonas (135621)	Pseudomonadaceae (7227	Pseudomonadales (1236)	Gammaproteobacteria (122	Proteobacteria (2)	Bacteria (1)	100.00	99.60	WGS
287.7082	0.316	Pseudomonas aeruginosa (286)	Pseudomonas (135621)	Pseudomonadaceae (7227	Pseudomonadales (1236)	Gammaproteobacteria (122	Proteobacteria (2)	Bacteria (1)	93.40	78.30	WGS
287.7083	0.3388	Pseudomonas aeruginosa (286)	Pseudomonas (135621)	Pseudomonadaceae (7227	Pseudomonadales (1236)	Gammaproteobacteria (122	Proteobacteria (2)	Bacteria (1)	99.60	98.20	WGS
287.7084	0.3388	Pseudomonas aeruginosa (286)	Pseudomonas (135621)	Pseudomonadaceae (7227	Pseudomonadales (1236)	Gammaproteobacteria (122	Proteobacteria (2)	Bacteria (1)	99.60	98.20	WGS
287.7085	0.2472	Pseudomonas aeruginosa (286)	Pseudomonas (135621)	Pseudomonadaceae (7227	Pseudomonadales (1236)	Gammaproteobacteria (122	Proteobacteria (2)	Bacteria (1)	97.00	58.90	WGS
287.7086	0.3632	Pseudomonas aeruginosa (286)	Pseudomonas (135621)	Pseudomonadaceae (7227	Pseudomonadales (1236)	Gammaproteobacteria (122	Proteobacteria (2)	Bacteria (1)	99.50	95.60	Complete
287.7087	0.3632	Pseudomonas aeruginosa (286)	Pseudomonas (135621)	Pseudomonadaceae (7227	Pseudomonadales (1236)	Gammaproteobacteria (122	Proteobacteria (2)	Bacteria (1)	99.60	96.90	Complete
287.7088	0.3188	Pseudomonas aeruginosa (286)	Pseudomonas (135621)	Pseudomonadaceae (7227	Pseudomonadales (1236)	Gammaproteobacteria (122	Proteobacteria (2)	Bacteria (1)	100.00	99.10	WGS
287.7089	0.299	Pseudomonas aeruginosa (286)	Pseudomonas (135621)	Pseudomonadaceae (7227	Pseudomonadales (1236)	Gammaproteobacteria (122	Proteobacteria (2)	Bacteria (1)	99.30	84.70	WGS
287.7090	0.3652	Pseudomonas aeruginosa (286)	Pseudomonas (135621)	Pseudomonadaceae (7227	Pseudomonadales (1236)	Gammaproteobacteria (122	Proteobacteria (2)	Bacteria (1)	99.80	99.20	WGS
287.7091	0.3376	Pseudomonas aeruginosa (286)	Pseudomonas (135621)	Pseudomonadaceae (7227	Pseudomonadales (1236)	Gammaproteobacteria (122	Proteobacteria (2)	Bacteria (1)	99.70	95.50	Complete
287.7092	0.371	Pseudomonas aeruginosa (286)	Pseudomonas (135621)	Pseudomonadaceae (7227	Pseudomonadales (1236)	Gammaproteobacteria (122	Proteobacteria (2)	Bacteria (1)	99.90	99.00	Complete
287.7093	0.3636	Pseudomonas aeruginosa (286)	Pseudomonas (135621)	Pseudomonadaceae (7227	Pseudomonadales (1236)	Gammaproteobacteria (122	Proteobacteria (2)	Bacteria (1)	99.70	98.90	Complete
287.7094	0.3386	Pseudomonas aeruginosa (286)	Pseudomonas (135621)	Pseudomonadaceae (7227	Pseudomonadales (1236)	Gammaproteobacteria (122	Proteobacteria (2)	Bacteria (1)	99.90	99.20	Complete
287.7095	0.3748	Pseudomonas aeruginosa (286)	Pseudomonas (135621)	Pseudomonadaceae (7227	Pseudomonadales (1236)	Gammaproteobacteria (122	Proteobacteria (2)	Bacteria (1)	99.80	98.70	Complete
287.7096	0.3628	Pseudomonas aeruginosa (286)	Pseudomonas (135621)	Pseudomonadaceae (7227	Pseudomonadales (1236)	Gammaproteobacteria (122	Proteobacteria (2)	Bacteria (1)	99.90	98.80	Complete
287.7097	0.3672	Pseudomonas aeruginosa (286)	Pseudomonas (135621)	Pseudomonadaceae (7227	Pseudomonadales (1236)	Gammaproteobacteria (122	Proteobacteria (2)	Bacteria (1)	99.90	98.70	Complete
287.7098	0.3694	Pseudomonas aeruginosa (286)	Pseudomonas (135621)	Pseudomonadaceae (7227	Pseudomonadales (1236)	Gammaproteobacteria (122	Proteobacteria (2)	Bacteria (1)	99.90	99.00	Complete
287.7099	0.331	Pseudomonas aeruginosa (286)	Pseudomonas (135621)	Pseudomonadaceae (7227	Pseudomonadales (1236)	Gammaproteobacteria (122	Proteobacteria (2)	Bacteria (1)	100.00	99.30	Complete
287.7265	0.3388	Pseudomonas aeruginosa (286)	Pseudomonas (135621)	Pseudomonadaceae (7227	Pseudomonadales (1236)	Gammaproteobacteria (122	Proteobacteria (2)	Bacteria (1)	99.90	98.70	WGS
287.7266	0.3376	Pseudomonas aeruginosa (286)	Pseudomonas (135621)	Pseudomonadaceae (7227	Pseudomonadales (1236)	Gammaproteobacteria (122	Proteobacteria (2)	Bacteria (1)	99.90	98.90	WGS
287.7267	0.3694	Pseudomonas aeruginosa (286)	Pseudomonas (135621)	Pseudomonadaceae (7227	Pseudomonadales (1236)	Gammaproteobacteria (122	Proteobacteria (2)	Bacteria (1)	99.80	99.00	WGS
287.7268	0.356	Pseudomonas aeruginosa (286)	Pseudomonas (135621)	Pseudomonadaceae (7227	Pseudomonadales (1236)	Gammaproteobacteria (122	Proteobacteria (2)	Bacteria (1)	99.90	99.00	WGS
287.7269	0.3374	Pseudomonas aeruginosa (286)	Pseudomonas (135621)	Pseudomonadaceae (7227	Pseudomonadales (1236)	Gammaproteobacteria (122	Proteobacteria (2)	Bacteria (1)	99.90	98.70	WGS
287.7270	0.368	Pseudomonas aeruginosa (286)	Pseudomonas (135621)	Pseudomonadaceae (7227	Pseudomonadales (1236)	Gammaproteobacteria (122	Proteobacteria (2)	Bacteria (1)	99.80	99.00	WGS
287.7271	0.3636	Pseudomonas aeruginosa (286)	Pseudomonas (135621)	Pseudomonadaceae (7227	Pseudomonadales (1236)	Gammaproteobacteria (122	Proteobacteria (2)	Bacteria (1)	99.80	99.20	WGS
287.7272	0.331	Pseudomonas aeruginosa (286)	Pseudomonas (135621)	Pseudomonadaceae (7227	Pseudomonadales (1236)	Gammaproteobacteria (122	Proteobacteria (2)	Bacteria (1)	99.90	98.70	WGS
287.7273	0.3812	Pseudomonas aeruginosa (286)	Pseudomonas (135621)	Pseudomonadaceae (7227	Pseudomonadales (1236)	Gammaproteobacteria (122	Proteobacteria (2)	Bacteria (1)	99.90	98.40	WGS
287.7274	0.3662	Pseudomonas aeruginosa (286)	Pseudomonas (135621)	Pseudomonadaceae (7227	Pseudomonadales (1236)	Gammaproteobacteria (122	Proteobacteria (2)	Bacteria (1)	99.70	98.20	WGS
287.7275	0.3604	Pseudomonas aeruginosa (286)	Pseudomonas (135621)	Pseudomonadaceae (7227	Pseudomonadales (1236)	Gammaproteobacteria (122	Proteobacteria (2)	Bacteria (1)	99.90	98.90	WGS
287.7276	0.3386	Pseudomonas aeruginosa (286)	Pseudomonas (135621)	Pseudomonadaceae (7227	Pseudomonadales (1236)	Gammaproteobacteria (122	Proteobacteria (2)	Bacteria (1)	99.70	98.60	WGS
287.7277	0.356	Pseudomonas aeruginosa (286)	Pseudomonas (135621)	Pseudomonadaceae (7227	Pseudomonadales (1236)	Gammaproteobacteria (122	Proteobacteria (2)	Bacteria (1)	99.70	98.50	WGS
287.7278	0.3382	Pseudomonas aeruginosa (286)	Pseudomonas (135621)	Pseudomonadaceae (7227	Pseudomonadales (1236)	Gammaproteobacteria (122	Proteobacteria (2)	Bacteria (1)	99.60	98.50	WGS
287.7279	0.3662	Pseudomonas aeruginosa (286)	Pseudomonas (135621)	Pseudomonadaceae (7227	Pseudomonadales (1236)	Gammaproteobacteria (122	Proteobacteria (2)	Bacteria (1)	99.70	98.60	WGS
287.7280	0.3748	Pseudomonas aeruginosa (286)	Pseudomonas (135621)	Pseudomonadaceae (7227	Pseudomonadales (1236)	Gammaproteobacteria (122	Proteobacteria (2)	Bacteria (1)	99.70	98.50	WGS
287.7281	0.3432	Pseudomonas aeruginosa (286)	Pseudomonas (135621)	Pseudomonadaceae (7227	Pseudomonadales (1236)	Gammaproteobacteria (122	Proteobacteria (2)	Bacteria (1)	99.90	98.90	WGS
287.7282	0.3738	Pseudomonas aeruginosa (286)	Pseudomonas (135621)	Pseudomonadaceae (7227	Pseudomonadales (1236)	Gammaproteobacteria (122	Proteobacteria (2)	Bacteria (1)	99.80	99.00	WGS
287.7283	0.368	Pseudomonas aeruginosa (286)	Pseudomonas (135621)	Pseudomonadaceae (7227	Pseudomonadales (1236)	Gammaproteobacteria (122	Proteobacteria (2)	Bacteria (1)	99.90	99.40	WGS
287.7284	0.3386	Pseudomonas aeruginosa (286)	Pseudomonas (135621)	Pseudomonadaceae (7227	Pseudomonadales (1236)	Gammaproteobacteria (122	Proteobacteria (2)	Bacteria (1)	99.90	98.70	WGS
287.7285	0.3636	Pseudomonas aeruginosa (286)	Pseudomonas (135621)	Pseudomonadaceae (7227	Pseudomonadales (1236)	Gammaproteobacteria (122	Proteobacteria (2)	Bacteria (1)	99.90	99.50	WGS
287.7286	0.3302	Pseudomonas aeruginosa (286)	Pseudomonas (135621)	Pseudomonadaceae (7227	Pseudomonadales (1236)	Gammaproteobacteria (122	Proteobacteria (2)	Bacteria (1)	99.80	99.00	WGS
287.7287	0.327	Pseudomonas aeruginosa (286)	Pseudomonas (135621)	Pseudomonadaceae (7227	Pseudomonadales (1236)	Gammaproteobacteria (122	Proteobacteria (2)	Bacteria (1)	99.90	99.00	WGS
287.7288	0.368	Pseudomonas aeruginosa (286)	Pseudomonas (135621)	Pseudomonadaceae (7227	Pseudomonadales (1236)	Gammaproteobacteria (122	Proteobacteria (2)	Bacteria (1)	99.90	99.30	WGS
287.7289	0.3306	Pseudomonas aeruginosa (286)	Pseudomonas (135621)	Pseudomonadaceae (7227	Pseudomonadales (1236)	Gammaproteobacteria (122	Proteobacteria (2)	Bacteria (1)	99.80	98.70	WGS
287.7290	0.3306	Pseudomonas aeruginosa (286)	Pseudomonas (135621)	Pseudomonadaceae (7227	Pseudomonadales (1236)	Gammaproteobacteria (122	Proteobacteria (2)	Bacteria (1)	99.80	98.90	WGS
287.7291	0.3226	Pseudomonas aeruginosa (286)	Pseudomonas (135621)	Pseudomonadaceae (7227	Pseudomonadales (1236)	Gammaproteobacteria (122	Proteobacteria (2)	Bacteria (1)	99.90	99.20	WGS
287.7292	0.3738	Pseudomonas aeruginosa (286)	Pseudomonas (135621)	Pseudomonadaceae (7227	Pseudomonadales (1236)	Gammaproteobacteria (122	Proteobacteria (2)	Bacteria (1)	99.90	98.90	WGS
287.7293	0.3324	Pseudomonas aeruginosa (286)	Pseudomonas (135621)	Pseudomonadaceae (7227	Pseudomonadales (1236)	Gammaproteobacteria (122	Proteobacteria (2)	Bacteria (1)	99.90	98.70	WGS
287.7294	0.3254	Pseudomonas aeruginosa (286)	Pseudomonas (135621)	Pseudomonadaceae (7227	Pseudomonadales (1236)	Gammaproteobacteria (122	Proteobacteria (2)	Bacteria (1)	99.90	99.60	WGS
287.7295	0.3386	Pseudomonas aeruginosa (286)	Pseudomonas (135621)	Pseudomonadaceae (7227	Pseudomonadales (1236)	Gammaproteobacteria (122	Proteobacteria (2)	Bacteria (1)	99.90	98.70	WGS
287.7296	0.3386	Pseudomonas aeruginosa (286)	Pseudomonas (135621)	Pseudomonadaceae (7227	Pseudomonadales (1236)	Gammaproteobacteria (122	Proteobacteria (2)	Bacteria (1)	99.90	98.80	WGS
287.7297	0.3386	Pseudomonas aeruginosa (286)	Pseudomonas (135621)	Pseudomonadaceae (7227	Pseudomonadales (1236)	Gammaproteobacteria (122	Proteobacteria (2)	Bacteria (1)	99.60	98.40	WGS
287.7298	0.3184	Pseudomonas aeruginosa (286)	Pseudomonas (135621)	Pseudomonadaceae (7227	Pseudomonadales (1236)	Gammaproteobacteria (122	Proteobacteria (2)	Bacteria (1)	99.90	98.60	WGS
287.7299	0.3694	Pseudomonas aeruginosa (286)	Pseudomonas (135621)	Pseudomonadaceae (7227	Pseudomonadales (1236)	Gammaproteobacteria (122	Proteobacteria (2)	Bacteria (1)	99.80	98.90	WGS
287.7300	0.3376	Pseudomonas aeruginosa (286)	Pseudomonas (135621)	Pseudomonadaceae (7227	Pseudomonadales (1236)	Gammaproteobacteria (122	Proteobacteria (2)	Bacteria (1)	99.80	98.20	WGS
287.7301	0.3346	Pseudomonas aeruginosa (286)	Pseudomonas (135621)	Pseudomonadaceae (7227	Pseudomonadales (1236)	Gammaproteobacteria (122	Proteobacteria (2)	Bacteria (1)	99.90	98.30	WGS
287.7302	0.3376	Pseudomonas aeruginosa (286)	Pseudomonas (135621)	Pseudomonadaceae (7227	Pseudomonadales (1236)	Gammaproteobacteria (122	Proteobacteria (2)	Bacteria (1)	99.70	98.30	WGS
287.7303	0.3306	Pseudomonas aeruginosa (286)	Pseudomonas (135621)	Pseudomonadaceae (7227	Pseudomonadales (1236)	Gammaproteobacteria (122	Proteobacteria (2)	Bacteria (1)	99.80	98.60	WGS
287.7304	0.3376	Pseudomonas aeruginosa (286)	Pseudomonas (135621)	Pseudomonadaceae (7227	Pseudomonadales (1236)	Gammaproteobacteria (122	Proteobacteria (2)	Bacteria (1)	99.80	98.80	WGS
287.7305	0.3306	Pseudomonas aeruginosa (286)	Pseudomonas (135621)	Pseudomonadaceae (7227	Pseudomonadales (1236)	Gammaproteobacteria (122	Proteobacteria (2)	Bacteria (1)	99.80	98.50	WGS
287.7306	0.3306	Pseudomonas aeruginosa (286)	Pseudomonas (135621)	Pseudomonadaceae (7227	Pseudomonadales (1236)	Gammaproteobacteria (122	Proteobacteria (2)	Bacteria (1)	99.80	98.70	WGS
287.7307	0.3324	Pseudomonas aeruginosa (286)	Pseudomonas (135621)	Pseudomonadaceae (7227	Pseudomonadales (1236)	Gammaproteobacteria (122	Proteobacteria (2)	Bacteria (1)	99.90	99.40	WGS
287.7308	0.3306	Pseudomonas aeruginosa (286)	Pseudomonas (135621)	Pseudomonadaceae (7227	Pseudomonadales (1236)	Gammaproteobacteria (122	Proteobacteria (2)	Bacteria (1)	99.80	98.70	WGS
287.7309	0.3236	Pseudomonas aeruginosa (286)	Pseudomonas (135621)	Pseudomonadaceae (7227	Pseudomonadales (1236)	Gammaproteobacteria (122	Proteobacteria (2)	Bacteria (1)	99.60	98.80	WGS
287.7310	0.3376	Pseudomonas aeruginosa (286)	Pseudomonas (135621)	Pseudomonadaceae (7227	Pseudomonadales (1236)	Gammaproteobacteria (122	Proteobacteria (2)	Bacteria (1)	99.80	99.00	WGS
287.7311	0.3184	Pseudomonas aeruginosa (286)	Pseudomonas (135621)	Pseudomonadaceae (7227	Pseudomonadales (1236)	Gammaproteobacteria (122	Proteobacteria (2)	Bacteria (1)	99.90	99.00	WGS
287.7312	0.3614	Pseudomonas aeruginosa (286)	Pseudomonas (135621)	Pseudomonadaceae (7227	Pseudomonadales (1236)	Gammaproteobacteria (122	Proteobacteria (2)	Bacteria (1)	99.50	91.40	WGS
287.7313	0.327	Pseudomonas aeruginosa (286)	Pseudomonas (135621)	Pseudomonadaceae (7227	Pseudomonadales (1236)	Gammaproteobacteria (122	Proteobacteria (2)	Bacteria (1)	99.90	99.30	WGS
287.7314	0.3258	Pseudomonas aeruginosa (286)	Pseudomonas (135621)	Pseudomonadaceae (7227	Pseudomonadales (1236)	Gammaproteobacteria (122	Proteobacteria (2)	Bacteria (1)	99.80	99.30	WGS
287.7315	0.3388	Pseudomonas aeruginosa (286)	Pseudomonas (135621)	Pseudomonadaceae (7227	Pseudomonadales (1236)	Gammaproteobacteria (122	Proteobacteria (2)	Bacteria (1)	99.80	98.70	WGS
287.7316	0.3334	Pseudomonas aeruginosa (286)	Pseudomonas (135621)	Pseudomonadaceae (7227	Pseudomonadales (1236)	Gammaproteobacteria (122	Proteobacteria (2)	Bacteria (1)	99.80	99.20	WGS
287.7317	0.368	Pseudomonas aeruginosa (286)	Pseudomonas (135621)	Pseudomonadaceae (7227	Pseudomonadales (1236)	Gammaproteobacteria (122	Proteobacteria (2)	Bacteria (1)	99.90	98.80	WGS
287.7318	0.3346	Pseudomonas aeruginosa (286)	Pseudomonas (135621)	Pseudomonadaceae (7227	Pseudomonadales (1236)	Gammaproteobacteria (122	Proteobacteria (2)	Bacteria (1)	99.90	98.50	WGS
287.7319	0.3672	Pseudomonas aeruginosa (286)	Pseudomonas (135621)	Pseudomonadaceae (7227	Pseudomonadales (1236)	Gammaproteobacteria (122	Proteobacteria (2)	Bacteria (1)	99.90	99.00	WGS
287.7320	0.3324	Pseudomonas aeruginosa (286)	Pseudomonas (135621)	Pseudomonadaceae (7227	Pseudomonadales (1236)	Gammaproteobacteria (122	Proteobacteria (2)	Bacteria (1)	99.90	98.80	WGS
287.7321	0.3324	Pseudomonas aeruginosa (286)	Pseudomonas (135621)	Pseudomonadaceae (7227	Pseudomonadales (1236)	Gammaproteobacteria (122	Proteobacteria (2)	Bacteria (1)	99.90	98.80	WGS
287.7322	0.3628	Pseudomonas aeruginosa (286)	Pseudomonas (135621)	Pseudomonadaceae (7227	Pseudomonadales (1236)	Gammaproteobacteria (122	Proteobacteria (2)	Bacteria (1)	99.90	99.00	WGS
287.7323	0.3324	Pseudomonas aeruginosa (286)	Pseudomonas (135621)	Pseudomonadaceae (7227	Pseudomonadales (1236)	Gammaproteobacteria (122	Proteobacteria (2)	Bacteria (1)	99.90	98.80	WGS
287.7324	0.3324	Pseudomonas aeruginosa (286)	Pseudomonas (135621)	Pseudomonadaceae (7227	Pseudomonadales (1236)	Gammaproteobacteria (122	Proteobacteria (2)	Bacteria (1)	99.90	98.10	WGS
287.7325	0.3324	Pseudomonas aeruginosa (286)	Pseudomonas (135621)	Pseudomonadaceae (7227	Pseudomonadales (1236)	Gammaproteobacteria (122	Proteobacteria (2)	Bacteria (1)	99.80	98.70	WGS
287.7326	0.3748	Pseudomonas aeruginosa (286)	Pseudomonas (135621)	Pseudomonadaceae (7227	Pseudomonadales (1236)	Gammaproteobacteria (122	Proteobacteria (2)	Bacteria (1)	99.80	98.90	WGS
287.7327	0.3324	Pseudomonas aeruginosa (286)	Pseudomonas (135621)	Pseudomonadaceae (7227	Pseudomonadales (1236)	Gammaproteobacteria (122	Proteobacteria (2)	Bacteria (1)	99.90	98.80	WGS
287.7328	0.3324	Pseudomonas aeruginosa (286)	Pseudomonas (135621)	Pseudomonadaceae (7227	Pseudomonadales (1236)	Gammaproteobacteria (122	Proteobacteria (2)	Bacteria (1)	99.90	98.70	WGS
287.7329	0.3258	Pseudomonas aeruginosa (286)	Pseudomonas (135621)	Pseudomonadaceae (7227	Pseudomonadales (1236)	Gammaproteobacteria (122	Proteobacteria (2)	Bacteria (1)	99.90	99.30	WGS
287.7330	0.368	Pseudomonas aeruginosa (286)	Pseudomonas (135621)	Pseudomonadaceae (7227	Pseudomonadales (1236)	Gammaproteobacteria (122	Proteobacteria (2)	Bacteria (1)	99.60	98.20	WGS
287.7331	0.3738	Pseudomonas aeruginosa (286)	Pseudomonas (135621)	Pseudomonadaceae (7227	Pseudomonadales (1236)	Gammaproteobacteria (122	Proteobacteria (2)	Bacteria (1)	99.60	98.40	WGS
287.7332	0.3258	Pseudomonas aeruginosa (286)	Pseudomonas (135621)	Pseudomonadaceae (7227	Pseudomonadales (1236)	Gammaproteobacteria (122	Proteobacteria (2)	Bacteria (1)	99.90	99.30	WGS
287.7333	0.3258	Pseudomonas aeruginosa (286)	Pseudomonas (135621)	Pseudomonadaceae (7227	Pseudomonadales (1236)	Gammaproteobacteria (122	Proteobacteria (2)	Bacteria (1)	99.90	99.40	WGS
287.7334	0.3258	Pseudomonas aeruginosa (286)	Pseudomonas (135621)	Pseudomonadaceae (7227	Pseudomonadales (1236)	Gammaproteobacteria (122	Proteobacteria (2)	Bacteria (1)	99.90	99.40	WGS
287.7335	0.3258	Pseudomonas aeruginosa (286)	Pseudomonas (135621)	Pseudomonadaceae (7227	Pseudomonadales (1236)	Gammaproteobacteria (122	Proteobacteria (2)	Bacteria (1)	99.90	99.10	WGS
287.7336	0.3392	Pseudomonas aeruginosa (286)	Pseudomonas (135621)	Pseudomonadaceae (7227	Pseudomonadales (1236)	Gammaproteobacteria (122	Proteobacteria (2)	Bacteria (1)	99.80	98.10	WGS
287.7337	0.3258	Pseudomonas aeruginosa (286)	Pseudomonas (135621)	Pseudomonadaceae (7227	Pseudomonadales (1236)	Gammaproteobacteria (122	Proteobacteria (2)	Bacteria (1)	99.90	99.20	WGS
287.7338	0.3604	Pseudomonas aeruginosa (286)	Pseudomonas (135621)	Pseudomonadaceae (7227	Pseudomonadales (1236)	Gammaproteobacteria (122	Proteobacteria (2)	Bacteria (1)	99.80	99.00	WGS
287.7339	0.3174	Pseudomonas aeruginosa (286)	Pseudomonas (135621)	Pseudomonadaceae (7227	Pseudomonadales (1236)	Gammaproteobacteria (122	Proteobacteria (2)	Bacteria (1)	99.90	99.30	WGS
287.7340	0.3258	Pseudomonas aeruginosa (286)	Pseudomonas (135621)	Pseudomonadaceae (7227	Pseudomonadales (1236)	Gammaproteobacteria (122	Proteobacteria (2)	Bacteria (1)	99.90	99.30	WGS
287.7341	0.3258	Pseudomonas aeruginosa (286)	Pseudomonas (135621)	Pseudomonadaceae (7227	Pseudomonadales (1236)	Gammaproteobacteria (122	Proteobacteria (2)	Bacteria (1)	99.60	99.20	WGS
287.7342	0.3376	Pseudomonas aeruginosa (286)	Pseudomonas (135621)	Pseudomonadaceae (7227	Pseudomonadales (1236)	Gammaproteobacteria (122	Proteobacteria (2)	Bacteria (1)	99.60	98.20	WGS
287.7343	0.3258	Pseudomonas aeruginosa (286)	Pseudomonas (135621)	Pseudomonadaceae (7227	Pseudomonadales (1236)	Gammaproteobacteria (122	Proteobacteria (2)	Bacteria (1)	99.90	99.30	WGS
287.7344	0.3258	Pseudomonas aeruginosa (286)	Pseudomonas (135621)	Pseudomonadaceae (7227	Pseudomonadales (1236)	Gammaproteobacteria (122	Proteobacteria (2)	Bacteria (1)	99.90	99.30	WGS
287.7345	0.3258	Pseudomonas aeruginosa (286)	Pseudomonas (135621)	Pseudomonadaceae (7227	Pseudomonadales (1236)	Gammaproteobacteria (122	Proteobacteria (2)	Bacteria (1)	99.90	99.30	WGS
287.7346	0.3324	Pseudomonas aeruginosa (286)	Pseudomonas (135621)	Pseudomonadaceae (7227	Pseudomonadales (1236)	Gammaproteobacteria (122	Proteobacteria (2)	Bacteria (1)	99.70	98.50	WGS
287.7347	0.3314	Pseudomonas aeruginosa (286)	Pseudomonas (135621)	Pseudomonadaceae (7227	Pseudomonadales (1236)	Gammaproteobacteria (122	Proteobacteria (2)	Bacteria (1)	99.80	98.70	WGS
287.7348	0.371	Pseudomonas aeruginosa (286)	Pseudomonas (135621)	Pseudomonadaceae (7227	Pseudomonadales (1236)	Gammaproteobacteria (122	Proteobacteria (2)	Bacteria (1)	99.90	98.80	WGS
287.7349	0.3376	Pseudomonas aeruginosa (286)	Pseudomonas (135621)	Pseudomonadaceae (7227	Pseudomonadales (1236)	Gammaproteobacteria (122	Proteobacteria (2)	Bacteria (1)	99.70	98.40	WGS
287.7350	0.3694	Pseudomonas aeruginosa (286)	Pseudomonas (135621)	Pseudomonadaceae (7227	Pseudomonadales (1236)	Gammaproteobacteria (122	Proteobacteria (2)	Bacteria (1)	99.90	98.80	WGS
287.7351	0.324	Pseudomonas aeruginosa (286)	Pseudomonas (135621)	Pseudomonadaceae (7227	Pseudomonadales (1236)	Gammaproteobacteria (122	Proteobacteria (2)	Bacteria (1)	99.80	98.90	WGS
287.7352	0.3388	Pseudomonas aeruginosa (286)	Pseudomonas (135621)	Pseudomonadaceae (7227	Pseudomonadales (1236)	Gammaproteobacteria (122	Proteobacteria (2)	Bacteria (1)	99.90	99.00	WGS
287.7353	0.324	Pseudomonas aeruginosa (286)	Pseudomonas (135621)	Pseudomonadaceae (7227	Pseudomonadales (1236)	Gammaproteobacteria (122	Proteobacteria (2)	Bacteria (1)	99.90	98.70	WGS
287.7354	0.3376	Pseudomonas aeruginosa (286)	Pseudomonas (135621)	Pseudomonadaceae (7227	Pseudomonadales (1236)	Gammaproteobacteria (122	Proteobacteria (2)	Bacteria (1)	99.70	98.40	WGS
287.7355	0.3334	Pseudomonas aeruginosa (286)	Pseudomonas (135621)	Pseudomonadaceae (7227	Pseudomonadales (1236)	Gammaproteobacteria (122	Proteobacteria (2)	Bacteria (1)	99.70	98.40	WGS
287.7356	0.3246	Pseudomonas aeruginosa (286)	Pseudomonas (135621)	Pseudomonadaceae (7227	Pseudomonadales (1236)	Gammaproteobacteria (122	Proteobacteria (2)	Bacteria (1)	99.80	99.10	WGS
287.7357	0.3324	Pseudomonas aeruginosa (286)	Pseudomonas (135621)	Pseudomonadaceae (7227	Pseudomonadales (1236)	Gammaproteobacteria (122	Proteobacteria (2)	Bacteria (1)	99.70	98.90	WGS
287.7358	0.331	Pseudomonas aeruginosa (286)	Pseudomonas (135621)	Pseudomonadaceae (7227	Pseudomonadales (1236)	Gammaproteobacteria (122	Proteobacteria (2)	Bacteria (1)	99.40	97.60	WGS
287.7359	0.368	Pseudomonas aeruginosa (286)	Pseudomonas (135621)	Pseudomonadaceae (7227	Pseudomonadales (1236)	Gammaproteobacteria (122	Proteobacteria (2)	Bacteria (1)	99.90	99.60	WGS
287.7360	0.324	Pseudomonas aeruginosa (286)	Pseudomonas (135621)	Pseudomonadaceae (7227	Pseudomonadales (1236)	Gammaproteobacteria (122	Proteobacteria (2)	Bacteria (1)	99.80	98.90	WGS
287.7361	0.324	Pseudomonas aeruginosa (286)	Pseudomonas (135621)	Pseudomonadaceae (7227	Pseudomonadales (1236)	Gammaproteobacteria (122	Proteobacteria (2)	Bacteria (1)	99.80	99.00	WGS
287.7362	0.3694	Pseudomonas aeruginosa (286)	Pseudomonas (135621)	Pseudomonadaceae (7227	Pseudomonadales (1236)	Gammaproteobacteria (122	Proteobacteria (2)	Bacteria (1)	99.80	98.90	WGS
287.7363	0.3694	Pseudomonas aeruginosa (286)	Pseudomonas (135621)	Pseudomonadaceae (7227	Pseudomonadales (1236)	Gammaproteobacteria (122	Proteobacteria (2)	Bacteria (1)	99.80	98.70	WGS
287.7364	0.3662	Pseudomonas aeruginosa (286)	Pseudomonas (135621)	Pseudomonadaceae (7227	Pseudomonadales (1236)	Gammaproteobacteria (122	Proteobacteria (2)	Bacteria (1)	99.70	98.20	WGS
287.7365	0.3324	Pseudomonas aeruginosa (286)	Pseudomonas (135621)	Pseudomonadaceae (7227	Pseudomonadales (1236)	Gammaproteobacteria (122	Proteobacteria (2)	Bacteria (1)	99.90	98.80	WGS
287.7366	0.368	Pseudomonas aeruginosa (286)	Pseudomonas (135621)	Pseudomonadaceae (7227	Pseudomonadales (1236)	Gammaproteobacteria (122	Proteobacteria (2)	Bacteria (1)	99.60	98.20	WGS
287.7367	0.342	Pseudomonas aeruginosa (286)	Pseudomonas (135621)	Pseudomonadaceae (7227	Pseudomonadales (1236)	Gammaproteobacteria (122	Proteobacteria (2)	Bacteria (1)	99.90	98.90	WGS
287.7368	0.3738	Pseudomonas aeruginosa (286)	Pseudomonas (135621)	Pseudomonadaceae (7227	Pseudomonadales (1236)	Gammaproteobacteria (122	Proteobacteria (2)	Bacteria (1)	99.50	98.00	WGS
287.7369	0.3226	Pseudomonas aeruginosa (286)	Pseudomonas (135621)	Pseudomonadaceae (7227	Pseudomonadales (1236)	Gammaproteobacteria (122	Proteobacteria (2)	Bacteria (1)	99.90	99.20	WGS
287.7370	0.3614	Pseudomonas aeruginosa (286)	Pseudomonas (135621)	Pseudomonadaceae (7227	Pseudomonadales (1236)	Gammaproteobacteria (122	Proteobacteria (2)	Bacteria (1)	99.80	98.90	WGS
287.7371	0.3258	Pseudomonas aeruginosa (286)	Pseudomonas (135621)	Pseudomonadaceae (7227	Pseudomonadales (1236)	Gammaproteobacteria (122	Proteobacteria (2)	Bacteria (1)	99.90	99.30	WGS
287.7372	0.3258	Pseudomonas aeruginosa (286)	Pseudomonas (135621)	Pseudomonadaceae (7227	Pseudomonadales (1236)	Gammaproteobacteria (122	Proteobacteria (2)	Bacteria (1)	99.90	99.30	WGS
287.7373	0.3258	Pseudomonas aeruginosa (286)	Pseudomonas (135621)	Pseudomonadaceae (7227	Pseudomonadales (1236)	Gammaproteobacteria (122	Proteobacteria (2)	Bacteria (1)	99.80	99.20	WGS
287.7374	0.3258	Pseudomonas aeruginosa (286)	Pseudomonas (135621)	Pseudomonadaceae (7227	Pseudomonadales (1236)	Gammaproteobacteria (122	Proteobacteria (2)	Bacteria (1)	99.90	99.20	WGS
287.7375	0.3258	Pseudomonas aeruginosa (286)	Pseudomonas (135621)	Pseudomonadaceae (7227	Pseudomonadales (1236)	Gammaproteobacteria (122	Proteobacteria (2)	Bacteria (1)	99.90	98.70	WGS
287.7376	0.3258	Pseudomonas aeruginosa (286)	Pseudomonas (135621)	Pseudomonadaceae (7227	Pseudomonadales (1236)	Gammaproteobacteria (122	Proteobacteria (2)	Bacteria (1)	99.90	99.50	WGS
287.7377	0.3342	Pseudomonas aeruginosa (286)	Pseudomonas (135621)	Pseudomonadaceae (7227	Pseudomonadales (1236)	Gammaproteobacteria (122	Proteobacteria (2)	Bacteria (1)	99.70	99.10	WGS
287.7378	0.3268	Pseudomonas aeruginosa (286)	Pseudomonas (135621)	Pseudomonadaceae (7227	Pseudomonadales (1236)	Gammaproteobacteria (122	Proteobacteria (2)	Bacteria (1)	99.70	98.80	WGS
287.7379	0.3748	Pseudomonas aeruginosa (286)	Pseudomonas (135621)	Pseudomonadaceae (7227	Pseudomonadales (1236)	Gammaproteobacteria (122	Proteobacteria (2)	Bacteria (1)	99.60	97.90	WGS
287.7380	0.3324	Pseudomonas aeruginosa (286)	Pseudomonas (135621)	Pseudomonadaceae (7227	Pseudomonadales (1236)	Gammaproteobacteria (122	Proteobacteria (2)	Bacteria (1)	99.90	98.70	WGS
287.7381	0.3978	Pseudomonas aeruginosa (286)	Pseudomonas (135621)	Pseudomonadaceae (7227	Pseudomonadales (1236)	Gammaproteobacteria (122	Proteobacteria (2)	Bacteria (1)	99.70	98.70	WGS
287.7382	0.368	Pseudomonas aeruginosa (286)	Pseudomonas (135621)	Pseudomonadaceae (7227	Pseudomonadales (1236)	Gammaproteobacteria (122	Proteobacteria (2)	Bacteria (1)	99.90	98.90	WGS
287.7383	0.3376	Pseudomonas aeruginosa (286)	Pseudomonas (135621)	Pseudomonadaceae (7227	Pseudomonadales (1236)	Gammaproteobacteria (122	Proteobacteria (2)	Bacteria (1)	99.90	99.30	WGS
287.7384	0.324	Pseudomonas aeruginosa (286)	Pseudomonas (135621)	Pseudomonadaceae (7227	Pseudomonadales (1236)	Gammaproteobacteria (122	Proteobacteria (2)	Bacteria (1)	99.80	98.90	WGS
287.7385	0.3302	Pseudomonas aeruginosa (286)	Pseudomonas (135621)	Pseudomonadaceae (7227	Pseudomonadales (1236)	Gammaproteobacteria (122	Proteobacteria (2)	Bacteria (1)	99.60	97.90	WGS
287.7386	0.324	Pseudomonas aeruginosa (286)	Pseudomonas (135621)	Pseudomonadaceae (7227	Pseudomonadales (1236)	Gammaproteobacteria (122	Proteobacteria (2)	Bacteria (1)	99.80	99.00	WGS
287.7387	0.324	Pseudomonas aeruginosa (286)	Pseudomonas (135621)	Pseudomonadaceae (7227	Pseudomonadales (1236)	Gammaproteobacteria (122	Proteobacteria (2)	Bacteria (1)	99.80	98.90	WGS
287.7388	0.327	Pseudomonas aeruginosa (286)	Pseudomonas (135621)	Pseudomonadaceae (7227	Pseudomonadales (1236)	Gammaproteobacteria (122	Proteobacteria (2)	Bacteria (1)	99.80	99.30	WGS
287.7389	0.324	Pseudomonas aeruginosa (286)	Pseudomonas (135621)	Pseudomonadaceae (7227	Pseudomonadales (1236)	Gammaproteobacteria (122	Proteobacteria (2)	Bacteria (1)	99.80	99.00	WGS
287.7390	0.324	Pseudomonas aeruginosa (286)	Pseudomonas (135621)	Pseudomonadaceae (7227	Pseudomonadales (1236)	Gammaproteobacteria (122	Proteobacteria (2)	Bacteria (1)	99.60	98.70	WGS
287.7391	0.3324	Pseudomonas aeruginosa (286)	Pseudomonas (135621)	Pseudomonadaceae (7227	Pseudomonadales (1236)	Gammaproteobacteria (122	Proteobacteria (2)	Bacteria (1)	99.90	99.10	WGS
287.7392	0.324	Pseudomonas aeruginosa (286)	Pseudomonas (135621)	Pseudomonadaceae (7227	Pseudomonadales (1236)	Gammaproteobacteria (122	Proteobacteria (2)	Bacteria (1)	99.80	98.90	WGS
287.7393	0.324	Pseudomonas aeruginosa (286)	Pseudomonas (135621)	Pseudomonadaceae (7227	Pseudomonadales (1236)	Gammaproteobacteria (122	Proteobacteria (2)	Bacteria (1)	99.90	99.60	WGS
287.7394	0.324	Pseudomonas aeruginosa (286)	Pseudomonas (135621)	Pseudomonadaceae (7227	Pseudomonadales (1236)	Gammaproteobacteria (122	Proteobacteria (2)	Bacteria (1)	99.80	99.00	WGS
287.7395	0.3636	Pseudomonas aeruginosa (286)	Pseudomonas (135621)	Pseudomonadaceae (7227	Pseudomonadales (1236)	Gammaproteobacteria (122	Proteobacteria (2)	Bacteria (1)	99.90	99.60	WGS
287.7396	0.3694	Pseudomonas aeruginosa (286)	Pseudomonas (135621)	Pseudomonadaceae (7227	Pseudomonadales (1236)	Gammaproteobacteria (122	Proteobacteria (2)	Bacteria (1)	99.70	98.90	WGS
287.7397	0.3694	Pseudomonas aeruginosa (286)	Pseudomonas (135621)	Pseudomonadaceae (7227	Pseudomonadales (1236)	Gammaproteobacteria (122	Proteobacteria (2)	Bacteria (1)	99.90	99.60	WGS
287.7398	0.324	Pseudomonas aeruginosa (286)	Pseudomonas (135621)	Pseudomonadaceae (7227	Pseudomonadales (1236)	Gammaproteobacteria (122	Proteobacteria (2)	Bacteria (1)	99.80	99.60	WGS
287.7399	0.3636	Pseudomonas aeruginosa (286)	Pseudomonas (135621)	Pseudomonadaceae (7227	Pseudomonadales (1236)	Gammaproteobacteria (122	Proteobacteria (2)	Bacteria (1)	99.90	99.00	WGS
287.7400	0.324	Pseudomonas aeruginosa (286)	Pseudomonas (135621)	Pseudomonadaceae (7227	Pseudomonadales (1236)	Gammaproteobacteria (122	Proteobacteria (2)	Bacteria (1)	99.80	99.50	WGS
287.7401	0.3636	Pseudomonas aeruginosa (286)	Pseudomonas (135621)	Pseudomonadaceae (7227	Pseudomonadales (1236)	Gammaproteobacteria (122	Proteobacteria (2)	Bacteria (1)	99.90	99.60	WGS
287.7402	0.3646	Pseudomonas aeruginosa (286)	Pseudomonas (135621)	Pseudomonadaceae (7227	Pseudomonadales (1236)	Gammaproteobacteria (122	Proteobacteria (2)	Bacteria (1)	99.90	99.00	WGS
287.7403	0.3694	Pseudomonas aeruginosa (286)	Pseudomonas (135621)	Pseudomonadaceae (7227	Pseudomonadales (1236)	Gammaproteobacteria (122	Proteobacteria (2)	Bacteria (1)	99.90	99.30	WGS
287.7404	0.3376	Pseudomonas aeruginosa (286)	Pseudomonas (135621)	Pseudomonadaceae (7227	Pseudomonadales (1236)	Gammaproteobacteria (122	Proteobacteria (2)	Bacteria (1)	99.60	98.40	WGS
287.7405	0.3636	Pseudomonas aeruginosa (286)	Pseudomonas (135621)	Pseudomonadaceae (7227	Pseudomonadales (1236)	Gammaproteobacteria (122	Proteobacteria (2)	Bacteria (1)	100.00	99.70	WGS
287.7406	0.377	Pseudomonas aeruginosa (286)	Pseudomonas (135621)	Pseudomonadaceae (7227	Pseudomonadales (1236)	Gammaproteobacteria (122	Proteobacteria (2)	Bacteria (1)	99.90	98.70	WGS
287.7407	0.3376	Pseudomonas aeruginosa (286)	Pseudomonas (135621)	Pseudomonadaceae (7227	Pseudomonadales (1236)	Gammaproteobacteria (122	Proteobacteria (2)	Bacteria (1)	99.80	98.60	WGS
287.7408	0.324	Pseudomonas aeruginosa (286)	Pseudomonas (135621)	Pseudomonadaceae (7227	Pseudomonadales (1236)	Gammaproteobacteria (122	Proteobacteria (2)	Bacteria (1)	99.80	99.60	WGS
287.7409	0.368	Pseudomonas aeruginosa (286)	Pseudomonas (135621)	Pseudomonadaceae (7227	Pseudomonadales (1236)	Gammaproteobacteria (122	Proteobacteria (2)	Bacteria (1)	99.90	99.60	WGS
287.7410	0.3386	Pseudomonas aeruginosa (286)	Pseudomonas (135621)	Pseudomonadaceae (7227	Pseudomonadales (1236)	Gammaproteobacteria (122	Proteobacteria (2)	Bacteria (1)	99.90	98.00	WGS
287.7411	0.324	Pseudomonas aeruginosa (286)	Pseudomonas (135621)	Pseudomonadaceae (7227	Pseudomonadales (1236)	Gammaproteobacteria (122	Proteobacteria (2)	Bacteria (1)	99.90	99.10	WGS
287.7412	0.3652	Pseudomonas aeruginosa (286)	Pseudomonas (135621)	Pseudomonadaceae (7227	Pseudomonadales (1236)	Gammaproteobacteria (122	Proteobacteria (2)	Bacteria (1)	99.60	98.20	WGS
287.7413	0.3258	Pseudomonas aeruginosa (286)	Pseudomonas (135621)	Pseudomonadaceae (7227	Pseudomonadales (1236)	Gammaproteobacteria (122	Proteobacteria (2)	Bacteria (1)	99.80	98.70	WGS
287.7414	0.3324	Pseudomonas aeruginosa (286)	Pseudomonas (135621)	Pseudomonadaceae (7227	Pseudomonadales (1236)	Gammaproteobacteria (122	Proteobacteria (2)	Bacteria (1)	99.80	99.10	WGS
287.7415	0.3376	Pseudomonas aeruginosa (286)	Pseudomonas (135621)	Pseudomonadaceae (7227	Pseudomonadales (1236)	Gammaproteobacteria (122	Proteobacteria (2)	Bacteria (1)	99.90	99.00	WGS
287.7416	0.3636	Pseudomonas aeruginosa (286)	Pseudomonas (135621)	Pseudomonadaceae (7227	Pseudomonadales (1236)	Gammaproteobacteria (122	Proteobacteria (2)	Bacteria (1)	99.80	98.30	WGS
287.7417	0.3292	Pseudomonas aeruginosa (286)	Pseudomonas (135621)	Pseudomonadaceae (7227	Pseudomonadales (1236)	Gammaproteobacteria (122	Proteobacteria (2)	Bacteria (1)	99.80	98.30	WGS
287.7418	0.3258	Pseudomonas aeruginosa (286)	Pseudomonas (135621)	Pseudomonadaceae (7227	Pseudomonadales (1236)	Gammaproteobacteria (122	Proteobacteria (2)	Bacteria (1)	99.90	99.30	WGS
287.7419	0.3636	Pseudomonas aeruginosa (286)	Pseudomonas (135621)	Pseudomonadaceae (7227	Pseudomonadales (1236)	Gammaproteobacteria (122	Proteobacteria (2)	Bacteria (1)	99.80	99.00	WGS
287.7420	0.3738	Pseudomonas aeruginosa (286)	Pseudomonas (135621)	Pseudomonadaceae (7227	Pseudomonadales (1236)	Gammaproteobacteria (122	Proteobacteria (2)	Bacteria (1)	99.70	98.60	WGS
287.7421	0.3626	Pseudomonas aeruginosa (286)	Pseudomonas (135621)	Pseudomonadaceae (7227	Pseudomonadales (1236)	Gammaproteobacteria (122	Proteobacteria (2)	Bacteria (1)	99.80	99.00	WGS
287.7422	0.368	Pseudomonas aeruginosa (286)	Pseudomonas (135621)	Pseudomonadaceae (7227	Pseudomonadales (1236)	Gammaproteobacteria (122	Proteobacteria (2)	Bacteria (1)	99.80	99.00	WGS
287.7423	0.331	Pseudomonas aeruginosa (286)	Pseudomonas (135621)	Pseudomonadaceae (7227	Pseudomonadales (1236)	Gammaproteobacteria (122	Proteobacteria (2)	Bacteria (1)	99.70	97.20	WGS
287.7424	0.3324	Pseudomonas aeruginosa (286)	Pseudomonas (135621)	Pseudomonadaceae (7227	Pseudomonadales (1236)	Gammaproteobacteria (122	Proteobacteria (2)	Bacteria (1)	99.80	99.10	WGS
287.7425	0.3604	Pseudomonas aeruginosa (286)	Pseudomonas (135621)	Pseudomonadaceae (7227	Pseudomonadales (1236)	Gammaproteobacteria (122	Proteobacteria (2)	Bacteria (1)	99.80	97.90	WGS
287.7426	0.3694	Pseudomonas aeruginosa (286)	Pseudomonas (135621)	Pseudomonadaceae (7227	Pseudomonadales (1236)	Gammaproteobacteria (122	Proteobacteria (2)	Bacteria (1)	99.90	99.50	WGS
287.7427	0.325	Pseudomonas aeruginosa (286)	Pseudomonas (135621)	Pseudomonadaceae (7227	Pseudomonadales (1236)	Gammaproteobacteria (122	Proteobacteria (2)	Bacteria (1)	99.90	98.80	WGS
287.7428	0.369	Pseudomonas aeruginosa (286)	Pseudomonas (135621)	Pseudomonadaceae (7227	Pseudomonadales (1236)	Gammaproteobacteria (122	Proteobacteria (2)	Bacteria (1)	99.60	98.90	WGS
287.7429	0.3258	Pseudomonas aeruginosa (286)	Pseudomonas (135621)	Pseudomonadaceae (7227	Pseudomonadales (1236)	Gammaproteobacteria (122	Proteobacteria (2)	Bacteria (1)	99.80	99.10	WGS
287.7430	0.3184	Pseudomonas aeruginosa (286)	Pseudomonas (135621)	Pseudomonadaceae (7227	Pseudomonadales (1236)	Gammaproteobacteria (122	Proteobacteria (2)	Bacteria (1)	99.90	99.00	WGS
287.7431	0.3748	Pseudomonas aeruginosa (286)	Pseudomonas (135621)	Pseudomonadaceae (7227	Pseudomonadales (1236)	Gammaproteobacteria (122	Proteobacteria (2)	Bacteria (1)	99.90	99.50	WGS
287.7432	0.3292	Pseudomonas aeruginosa (286)	Pseudomonas (135621)	Pseudomonadaceae (7227	Pseudomonadales (1236)	Gammaproteobacteria (122	Proteobacteria (2)	Bacteria (1)	99.80	99.30	WGS
287.7433	0.3314	Pseudomonas aeruginosa (286)	Pseudomonas (135621)	Pseudomonadaceae (7227	Pseudomonadales (1236)	Gammaproteobacteria (122	Proteobacteria (2)	Bacteria (1)	99.90	98.70	WGS
287.7434	0.3336	Pseudomonas aeruginosa (286)	Pseudomonas (135621)	Pseudomonadaceae (7227	Pseudomonadales (1236)	Gammaproteobacteria (122	Proteobacteria (2)	Bacteria (1)	99.90	99.30	WGS
287.7435	0.3298	Pseudomonas aeruginosa (286)	Pseudomonas (135621)	Pseudomonadaceae (7227	Pseudomonadales (1236)	Gammaproteobacteria (122	Proteobacteria (2)	Bacteria (1)	99.90	99.10	WGS
287.7436	0.3738	Pseudomonas aeruginosa (286)	Pseudomonas (135621)	Pseudomonadaceae (7227	Pseudomonadales (1236)	Gammaproteobacteria (122	Proteobacteria (2)	Bacteria (1)	99.90	98.80	WGS
287.7437	0.3618	Pseudomonas aeruginosa (286)	Pseudomonas (135621)	Pseudomonadaceae (7227	Pseudomonadales (1236)	Gammaproteobacteria (122	Proteobacteria (2)	Bacteria (1)	99.80	99.20	WGS
287.7438	0.368	Pseudomonas aeruginosa (286)	Pseudomonas (135621)	Pseudomonadaceae (7227	Pseudomonadales (1236)	Gammaproteobacteria (122	Proteobacteria (2)	Bacteria (1)	99.90	99.40	WGS
287.7439	0.3814	Pseudomonas aeruginosa (286)	Pseudomonas (135621)	Pseudomonadaceae (7227	Pseudomonadales (1236)	Gammaproteobacteria (122	Proteobacteria (2)	Bacteria (1)	99.30	98.20	WGS
287.7440	0.3258	Pseudomonas aeruginosa (286)	Pseudomonas (135621)	Pseudomonadaceae (7227	Pseudomonadales (1236)	Gammaproteobacteria (122	Proteobacteria (2)	Bacteria (1)	99.80	99.10	WGS
287.7441	0.3738	Pseudomonas aeruginosa (286)	Pseudomonas (135621)	Pseudomonadaceae (7227	Pseudomonadales (1236)	Gammaproteobacteria (122	Proteobacteria (2)	Bacteria (1)	99.90	99.30	WGS
287.7442	0.3324	Pseudomonas aeruginosa (286)	Pseudomonas (135621)	Pseudomonadaceae (7227	Pseudomonadales (1236)	Gammaproteobacteria (122	Proteobacteria (2)	Bacteria (1)	99.90	98.90	WGS
287.7443	0.3258	Pseudomonas aeruginosa (286)	Pseudomonas (135621)	Pseudomonadaceae (7227	Pseudomonadales (1236)	Gammaproteobacteria (122	Proteobacteria (2)	Bacteria (1)	99.80	98.90	WGS
287.7444	0.3322	Pseudomonas aeruginosa (286)	Pseudomonas (135621)	Pseudomonadaceae (7227	Pseudomonadales (1236)	Gammaproteobacteria (122	Proteobacteria (2)	Bacteria (1)	99.90	99.20	WGS
287.7445	0.3604	Pseudomonas aeruginosa (286)	Pseudomonas (135621)	Pseudomonadaceae (7227	Pseudomonadales (1236)	Gammaproteobacteria (122	Proteobacteria (2)	Bacteria (1)	99.80	99.00	WGS
287.7446	0.324	Pseudomonas aeruginosa (286)	Pseudomonas (135621)	Pseudomonadaceae (7227	Pseudomonadales (1236)	Gammaproteobacteria (122	Proteobacteria (2)	Bacteria (1)	99.80	99.00	WGS
287.7447	0.3376	Pseudomonas aeruginosa (286)	Pseudomonas (135621)	Pseudomonadaceae (7227	Pseudomonadales (1236)	Gammaproteobacteria (122	Proteobacteria (2)	Bacteria (1)	99.80	99.10	WGS
287.7448	0.3738	Pseudomonas aeruginosa (286)	Pseudomonas (135621)	Pseudomonadaceae (7227	Pseudomonadales (1236)	Gammaproteobacteria (122	Proteobacteria (2)	Bacteria (1)	99.90	99.30	WGS
287.7449	0.3636	Pseudomonas aeruginosa (286)	Pseudomonas (135621)	Pseudomonadaceae (7227	Pseudomonadales (1236)	Gammaproteobacteria (122	Proteobacteria (2)	Bacteria (1)	99.90	99.60	WGS
287.7450	0.3226	Pseudomonas aeruginosa (286)	Pseudomonas (135621)	Pseudomonadaceae (7227	Pseudomonadales (1236)	Gammaproteobacteria (122	Proteobacteria (2)	Bacteria (1)	99.80	98.40	WGS
287.7451	0.3636	Pseudomonas aeruginosa (286)	Pseudomonas (135621)	Pseudomonadaceae (7227	Pseudomonadales (1236)	Gammaproteobacteria (122	Proteobacteria (2)	Bacteria (1)	99.90	99.60	WGS
287.7452	0.3324	Pseudomonas aeruginosa (286)	Pseudomonas (135621)	Pseudomonadaceae (7227	Pseudomonadales (1236)	Gammaproteobacteria (122	Proteobacteria (2)	Bacteria (1)	99.80	98.80	WGS
287.7453	0.3314	Pseudomonas aeruginosa (286)	Pseudomonas (135621)	Pseudomonadaceae (7227	Pseudomonadales (1236)	Gammaproteobacteria (122	Proteobacteria (2)	Bacteria (1)	99.90	98.60	WGS
287.7454	0.3738	Pseudomonas aeruginosa (286)	Pseudomonas (135621)	Pseudomonadaceae (7227	Pseudomonadales (1236)	Gammaproteobacteria (122	Proteobacteria (2)	Bacteria (1)	99.60	98.60	WGS
287.7455	0.3664	Pseudomonas aeruginosa (286)	Pseudomonas (135621)	Pseudomonadaceae (7227	Pseudomonadales (1236)	Gammaproteobacteria (122	Proteobacteria (2)	Bacteria (1)	99.70	98.70	WGS
287.7456	0.368	Pseudomonas aeruginosa (286)	Pseudomonas (135621)	Pseudomonadaceae (7227	Pseudomonadales (1236)	Gammaproteobacteria (122	Proteobacteria (2)	Bacteria (1)	99.90	99.80	WGS
287.7457	0.368	Pseudomonas aeruginosa (286)	Pseudomonas (135621)	Pseudomonadaceae (7227	Pseudomonadales (1236)	Gammaproteobacteria (122	Proteobacteria (2)	Bacteria (1)	99.80	97.60	WGS
287.7458	0.3618	Pseudomonas aeruginosa (286)	Pseudomonas (135621)	Pseudomonadaceae (7227	Pseudomonadales (1236)	Gammaproteobacteria (122	Proteobacteria (2)	Bacteria (1)	99.90	98.30	WGS
287.7459	0.368	Pseudomonas aeruginosa (286)	Pseudomonas (135621)	Pseudomonadaceae (7227	Pseudomonadales (1236)	Gammaproteobacteria (122	Proteobacteria (2)	Bacteria (1)	99.90	99.60	WGS
287.7460	0.324	Pseudomonas aeruginosa (286)	Pseudomonas (135621)	Pseudomonadaceae (7227	Pseudomonadales (1236)	Gammaproteobacteria (122	Proteobacteria (2)	Bacteria (1)	99.90	99.50	WGS
287.7461	0.397	Pseudomonas aeruginosa (286)	Pseudomonas (135621)	Pseudomonadaceae (7227	Pseudomonadales (1236)	Gammaproteobacteria (122	Proteobacteria (2)	Bacteria (1)	99.80	98.40	WGS
287.7462	0.3936	Pseudomonas aeruginosa (286)	Pseudomonas (135621)	Pseudomonadaceae (7227	Pseudomonadales (1236)	Gammaproteobacteria (122	Proteobacteria (2)	Bacteria (1)	99.60	98.60	WGS
287.7463	0.3376	Pseudomonas aeruginosa (286)	Pseudomonas (135621)	Pseudomonadaceae (7227	Pseudomonadales (1236)	Gammaproteobacteria (122	Proteobacteria (2)	Bacteria (1)	99.70	98.60	WGS
287.7464	0.3636	Pseudomonas aeruginosa (286)	Pseudomonas (135621)	Pseudomonadaceae (7227	Pseudomonadales (1236)	Gammaproteobacteria (122	Proteobacteria (2)	Bacteria (1)	99.70	98.90	WGS
287.7465	0.3292	Pseudomonas aeruginosa (286)	Pseudomonas (135621)	Pseudomonadaceae (7227	Pseudomonadales (1236)	Gammaproteobacteria (122	Proteobacteria (2)	Bacteria (1)	99.70	98.70	WGS
287.7466	0.3292	Pseudomonas aeruginosa (286)	Pseudomonas (135621)	Pseudomonadaceae (7227	Pseudomonadales (1236)	Gammaproteobacteria (122	Proteobacteria (2)	Bacteria (1)	99.70	98.70	WGS
287.7467	0.3258	Pseudomonas aeruginosa (286)	Pseudomonas (135621)	Pseudomonadaceae (7227	Pseudomonadales (1236)	Gammaproteobacteria (122	Proteobacteria (2)	Bacteria (1)	99.60	98.60	WGS
287.7468	0.3618	Pseudomonas aeruginosa (286)	Pseudomonas (135621)	Pseudomonadaceae (7227	Pseudomonadales (1236)	Gammaproteobacteria (122	Proteobacteria (2)	Bacteria (1)	99.90	98.30	WGS
287.7469	0.3618	Pseudomonas aeruginosa (286)	Pseudomonas (135621)	Pseudomonadaceae (7227	Pseudomonadales (1236)	Gammaproteobacteria (122	Proteobacteria (2)	Bacteria (1)	99.80	98.20	WGS
287.7470	0.331	Pseudomonas aeruginosa (286)	Pseudomonas (135621)	Pseudomonadaceae (7227	Pseudomonadales (1236)	Gammaproteobacteria (122	Proteobacteria (2)	Bacteria (1)	99.90	99.30	WGS
287.7471	0.3656	Pseudomonas aeruginosa (286)	Pseudomonas (135621)	Pseudomonadaceae (7227	Pseudomonadales (1236)	Gammaproteobacteria (122	Proteobacteria (2)	Bacteria (1)	99.80	99.00	WGS
287.7472	0.3534	Pseudomonas aeruginosa (286)	Pseudomonas (135621)	Pseudomonadaceae (7227	Pseudomonadales (1236)	Gammaproteobacteria (122	Proteobacteria (2)	Bacteria (1)	99.90	99.10	WGS
287.7473	0.3258	Pseudomonas aeruginosa (286)	Pseudomonas (135621)	Pseudomonadaceae (7227	Pseudomonadales (1236)	Gammaproteobacteria (122	Proteobacteria (2)	Bacteria (1)	99.80	99.30	WGS
287.7474	0.3314	Pseudomonas aeruginosa (286)	Pseudomonas (135621)	Pseudomonadaceae (7227	Pseudomonadales (1236)	Gammaproteobacteria (122	Proteobacteria (2)	Bacteria (1)	99.90	98.90	WGS
287.7475	0.3188	Pseudomonas aeruginosa (286)	Pseudomonas (135621)	Pseudomonadaceae (7227	Pseudomonadales (1236)	Gammaproteobacteria (122	Proteobacteria (2)	Bacteria (1)	99.60	98.70	WGS
287.7476	0.371	Pseudomonas aeruginosa (286)	Pseudomonas (135621)	Pseudomonadaceae (7227	Pseudomonadales (1236)	Gammaproteobacteria (122	Proteobacteria (2)	Bacteria (1)	99.90	98.90	WGS
287.7477	0.3322	Pseudomonas aeruginosa (286)	Pseudomonas (135621)	Pseudomonadaceae (7227	Pseudomonadales (1236)	Gammaproteobacteria (122	Proteobacteria (2)	Bacteria (1)	99.90	98.80	WGS
287.7478	0.3314	Pseudomonas aeruginosa (286)	Pseudomonas (135621)	Pseudomonadaceae (7227	Pseudomonadales (1236)	Gammaproteobacteria (122	Proteobacteria (2)	Bacteria (1)	99.90	98.70	WGS
287.7479	0.3662	Pseudomonas aeruginosa (286)	Pseudomonas (135621)	Pseudomonadaceae (7227	Pseudomonadales (1236)	Gammaproteobacteria (122	Proteobacteria (2)	Bacteria (1)	99.80	98.40	WGS
287.7480	0.3694	Pseudomonas aeruginosa (286)	Pseudomonas (135621)	Pseudomonadaceae (7227	Pseudomonadales (1236)	Gammaproteobacteria (122	Proteobacteria (2)	Bacteria (1)	99.80	99.20	WGS
287.7481	0.3636	Pseudomonas aeruginosa (286)	Pseudomonas (135621)	Pseudomonadaceae (7227	Pseudomonadales (1236)	Gammaproteobacteria (122	Proteobacteria (2)	Bacteria (1)	99.90	99.40	WGS
287.7482	0.3314	Pseudomonas aeruginosa (286)	Pseudomonas (135621)	Pseudomonadaceae (7227	Pseudomonadales (1236)	Gammaproteobacteria (122	Proteobacteria (2)	Bacteria (1)	99.90	98.90	WGS
287.7483	0.3376	Pseudomonas aeruginosa (286)	Pseudomonas (135621)	Pseudomonadaceae (7227	Pseudomonadales (1236)	Gammaproteobacteria (122	Proteobacteria (2)	Bacteria (1)	99.50	98.40	WGS
287.7484	0.3262	Pseudomonas aeruginosa (286)	Pseudomonas (135621)	Pseudomonadaceae (7227	Pseudomonadales (1236)	Gammaproteobacteria (122	Proteobacteria (2)	Bacteria (1)	99.90	98.60	WGS
287.7485	0.3304	Pseudomonas aeruginosa (286)	Pseudomonas (135621)	Pseudomonadaceae (7227	Pseudomonadales (1236)	Gammaproteobacteria (122	Proteobacteria (2)	Bacteria (1)	99.90	98.70	WGS
287.7486	0.3696	Pseudomonas aeruginosa (286)	Pseudomonas (135621)	Pseudomonadaceae (7227	Pseudomonadales (1236)	Gammaproteobacteria (122	Proteobacteria (2)	Bacteria (1)	99.80	99.00	WGS
287.7487	0.3748	Pseudomonas aeruginosa (286)	Pseudomonas (135621)	Pseudomonadaceae (7227	Pseudomonadales (1236)	Gammaproteobacteria (122	Proteobacteria (2)	Bacteria (1)	99.80	98.80	WGS
287.7488	0.3314	Pseudomonas aeruginosa (286)	Pseudomonas (135621)	Pseudomonadaceae (7227	Pseudomonadales (1236)	Gammaproteobacteria (122	Proteobacteria (2)	Bacteria (1)	99.90	98.90	WGS
287.7489	0.3258	Pseudomonas aeruginosa (286)	Pseudomonas (135621)	Pseudomonadaceae (7227	Pseudomonadales (1236)	Gammaproteobacteria (122	Proteobacteria (2)	Bacteria (1)	99.90	99.30	WGS
287.7490	0.332	Pseudomonas aeruginosa (286)	Pseudomonas (135621)	Pseudomonadaceae (7227	Pseudomonadales (1236)	Gammaproteobacteria (122	Proteobacteria (2)	Bacteria (1)	99.90	99.60	WGS
287.7491	0.3268	Pseudomonas aeruginosa (286)	Pseudomonas (135621)	Pseudomonadaceae (7227	Pseudomonadales (1236)	Gammaproteobacteria (122	Proteobacteria (2)	Bacteria (1)	99.90	99.10	WGS
287.7492	0.3376	Pseudomonas aeruginosa (286)	Pseudomonas (135621)	Pseudomonadaceae (7227	Pseudomonadales (1236)	Gammaproteobacteria (122	Proteobacteria (2)	Bacteria (1)	99.90	98.70	WGS
287.7493	0.3376	Pseudomonas aeruginosa (286)	Pseudomonas (135621)	Pseudomonadaceae (7227	Pseudomonadales (1236)	Gammaproteobacteria (122	Proteobacteria (2)	Bacteria (1)	99.70	99.00	WGS
287.7494	0.3184	Pseudomonas aeruginosa (286)	Pseudomonas (135621)	Pseudomonadaceae (7227	Pseudomonadales (1236)	Gammaproteobacteria (122	Proteobacteria (2)	Bacteria (1)	99.80	98.80	WGS
287.7495	0.3334	Pseudomonas aeruginosa (286)	Pseudomonas (135621)	Pseudomonadaceae (7227	Pseudomonadales (1236)	Gammaproteobacteria (122	Proteobacteria (2)	Bacteria (1)	99.90	99.20	WGS
287.7496	0.3174	Pseudomonas aeruginosa (286)	Pseudomonas (135621)	Pseudomonadaceae (7227	Pseudomonadales (1236)	Gammaproteobacteria (122	Proteobacteria (2)	Bacteria (1)	99.80	99.40	WGS
287.7497	0.3386	Pseudomonas aeruginosa (286)	Pseudomonas (135621)	Pseudomonadaceae (7227	Pseudomonadales (1236)	Gammaproteobacteria (122	Proteobacteria (2)	Bacteria (1)	99.90	98.40	WGS
287.7498	0.3636	Pseudomonas aeruginosa (286)	Pseudomonas (135621)	Pseudomonadaceae (7227	Pseudomonadales (1236)	Gammaproteobacteria (122	Proteobacteria (2)	Bacteria (1)	99.90	99.30	WGS
287.7499	0.3174	Pseudomonas aeruginosa (286)	Pseudomonas (135621)	Pseudomonadaceae (7227	Pseudomonadales (1236)	Gammaproteobacteria (122	Proteobacteria (2)	Bacteria (1)	99.80	99.00	WGS
287.7500	0.3258	Pseudomonas aeruginosa (286)	Pseudomonas (135621)	Pseudomonadaceae (7227	Pseudomonadales (1236)	Gammaproteobacteria (122	Proteobacteria (2)	Bacteria (1)	99.90	99.30	WGS
287.7501	0.3694	Pseudomonas aeruginosa (286)	Pseudomonas (135621)	Pseudomonadaceae (7227	Pseudomonadales (1236)	Gammaproteobacteria (122	Proteobacteria (2)	Bacteria (1)	99.80	99.20	WGS
287.7502	0.3738	Pseudomonas aeruginosa (286)	Pseudomonas (135621)	Pseudomonadaceae (7227	Pseudomonadales (1236)	Gammaproteobacteria (122	Proteobacteria (2)	Bacteria (1)	99.80	99.00	WGS
287.7503	0.3694	Pseudomonas aeruginosa (286)	Pseudomonas (135621)	Pseudomonadaceae (7227	Pseudomonadales (1236)	Gammaproteobacteria (122	Proteobacteria (2)	Bacteria (1)	100.00	99.40	WGS
287.7504	0.368	Pseudomonas aeruginosa (286)	Pseudomonas (135621)	Pseudomonadaceae (7227	Pseudomonadales (1236)	Gammaproteobacteria (122	Proteobacteria (2)	Bacteria (1)	99.90	98.80	WGS
287.7505	0.3738	Pseudomonas aeruginosa (286)	Pseudomonas (135621)	Pseudomonadaceae (7227	Pseudomonadales (1236)	Gammaproteobacteria (122	Proteobacteria (2)	Bacteria (1)	99.50	97.90	WGS
287.7506	0.368	Pseudomonas aeruginosa (286)	Pseudomonas (135621)	Pseudomonadaceae (7227	Pseudomonadales (1236)	Gammaproteobacteria (122	Proteobacteria (2)	Bacteria (1)	99.80	98.50	WGS
287.7507	0.369	Pseudomonas aeruginosa (286)	Pseudomonas (135621)	Pseudomonadaceae (7227	Pseudomonadales (1236)	Gammaproteobacteria (122	Proteobacteria (2)	Bacteria (1)	99.80	98.90	WGS
287.7508	0.3738	Pseudomonas aeruginosa (286)	Pseudomonas (135621)	Pseudomonadaceae (7227	Pseudomonadales (1236)	Gammaproteobacteria (122	Proteobacteria (2)	Bacteria (1)	99.80	98.80	WGS
287.7509	0.368	Pseudomonas aeruginosa (286)	Pseudomonas (135621)	Pseudomonadaceae (7227	Pseudomonadales (1236)	Gammaproteobacteria (122	Proteobacteria (2)	Bacteria (1)	99.80	97.60	WGS
287.7510	0.368	Pseudomonas aeruginosa (286)	Pseudomonas (135621)	Pseudomonadaceae (7227	Pseudomonadales (1236)	Gammaproteobacteria (122	Proteobacteria (2)	Bacteria (1)	99.80	98.70	WGS
287.7511	0.3174	Pseudomonas aeruginosa (286)	Pseudomonas (135621)	Pseudomonadaceae (7227	Pseudomonadales (1236)	Gammaproteobacteria (122	Proteobacteria (2)	Bacteria (1)	99.80	98.80	WGS
287.7512	0.3258	Pseudomonas aeruginosa (286)	Pseudomonas (135621)	Pseudomonadaceae (7227	Pseudomonadales (1236)	Gammaproteobacteria (122	Proteobacteria (2)	Bacteria (1)	99.90	99.00	WGS
287.7513	0.3738	Pseudomonas aeruginosa (286)	Pseudomonas (135621)	Pseudomonadaceae (7227	Pseudomonadales (1236)	Gammaproteobacteria (122	Proteobacteria (2)	Bacteria (1)	99.80	98.90	WGS
287.7514	0.332	Pseudomonas aeruginosa (286)	Pseudomonas (135621)	Pseudomonadaceae (7227	Pseudomonadales (1236)	Gammaproteobacteria (122	Proteobacteria (2)	Bacteria (1)	99.80	98.10	WGS
287.7515	0.3258	Pseudomonas aeruginosa (286)	Pseudomonas (135621)	Pseudomonadaceae (7227	Pseudomonadales (1236)	Gammaproteobacteria (122	Proteobacteria (2)	Bacteria (1)	99.80	98.70	WGS
287.7516	0.368	Pseudomonas aeruginosa (286)	Pseudomonas (135621)	Pseudomonadaceae (7227	Pseudomonadales (1236)	Gammaproteobacteria (122	Proteobacteria (2)	Bacteria (1)	99.90	98.70	WGS
287.7517	0.3636	Pseudomonas aeruginosa (286)	Pseudomonas (135621)	Pseudomonadaceae (7227	Pseudomonadales (1236)	Gammaproteobacteria (122	Proteobacteria (2)	Bacteria (1)	99.80	99.50	WGS
287.7518	0.3334	Pseudomonas aeruginosa (286)	Pseudomonas (135621)	Pseudomonadaceae (7227	Pseudomonadales (1236)	Gammaproteobacteria (122	Proteobacteria (2)	Bacteria (1)	99.10	98.10	WGS
287.7519	0.368	Pseudomonas aeruginosa (286)	Pseudomonas (135621)	Pseudomonadaceae (7227	Pseudomonadales (1236)	Gammaproteobacteria (122	Proteobacteria (2)	Bacteria (1)	99.80	99.50	WGS
287.7520	0.356	Pseudomonas aeruginosa (286)	Pseudomonas (135621)	Pseudomonadaceae (7227	Pseudomonadales (1236)	Gammaproteobacteria (122	Proteobacteria (2)	Bacteria (1)	99.80	99.10	WGS
287.7521	0.3268	Pseudomonas aeruginosa (286)	Pseudomonas (135621)	Pseudomonadaceae (7227	Pseudomonadales (1236)	Gammaproteobacteria (122	Proteobacteria (2)	Bacteria (1)	99.90	99.30	WGS
287.7522	0.356	Pseudomonas aeruginosa (286)	Pseudomonas (135621)	Pseudomonadaceae (7227	Pseudomonadales (1236)	Gammaproteobacteria (122	Proteobacteria (2)	Bacteria (1)	99.90	99.30	WGS
287.7523	0.327	Pseudomonas aeruginosa (286)	Pseudomonas (135621)	Pseudomonadaceae (7227	Pseudomonadales (1236)	Gammaproteobacteria (122	Proteobacteria (2)	Bacteria (1)	99.70	99.00	WGS
287.7524	0.3258	Pseudomonas aeruginosa (286)	Pseudomonas (135621)	Pseudomonadaceae (7227	Pseudomonadales (1236)	Gammaproteobacteria (122	Proteobacteria (2)	Bacteria (1)	99.90	99.50	WGS
287.7525	0.3652	Pseudomonas aeruginosa (286)	Pseudomonas (135621)	Pseudomonadaceae (7227	Pseudomonadales (1236)	Gammaproteobacteria (122	Proteobacteria (2)	Bacteria (1)	99.90	99.30	WGS
287.7526	0.3258	Pseudomonas aeruginosa (286)	Pseudomonas (135621)	Pseudomonadaceae (7227	Pseudomonadales (1236)	Gammaproteobacteria (122	Proteobacteria (2)	Bacteria (1)	99.80	99.10	WGS
287.7527	0.3694	Pseudomonas aeruginosa (286)	Pseudomonas (135621)	Pseudomonadaceae (7227	Pseudomonadales (1236)	Gammaproteobacteria (122	Proteobacteria (2)	Bacteria (1)	99.90	99.50	WGS
287.7528	0.3226	Pseudomonas aeruginosa (286)	Pseudomonas (135621)	Pseudomonadaceae (7227	Pseudomonadales (1236)	Gammaproteobacteria (122	Proteobacteria (2)	Bacteria (1)	99.90	98.40	WGS
287.7529	0.3758	Pseudomonas aeruginosa (286)	Pseudomonas (135621)	Pseudomonadaceae (7227	Pseudomonadales (1236)	Gammaproteobacteria (122	Proteobacteria (2)	Bacteria (1)	99.70	98.80	WGS
287.7530	0.3258	Pseudomonas aeruginosa (286)	Pseudomonas (135621)	Pseudomonadaceae (7227	Pseudomonadales (1236)	Gammaproteobacteria (122	Proteobacteria (2)	Bacteria (1)	99.90	99.60	WGS
287.7531	0.3636	Pseudomonas aeruginosa (286)	Pseudomonas (135621)	Pseudomonadaceae (7227	Pseudomonadales (1236)	Gammaproteobacteria (122	Proteobacteria (2)	Bacteria (1)	100.00	99.60	WGS
287.7532	0.3388	Pseudomonas aeruginosa (286)	Pseudomonas (135621)	Pseudomonadaceae (7227	Pseudomonadales (1236)	Gammaproteobacteria (122	Proteobacteria (2)	Bacteria (1)	99.90	98.80	WGS
287.7533	0.3738	Pseudomonas aeruginosa (286)	Pseudomonas (135621)	Pseudomonadaceae (7227	Pseudomonadales (1236)	Gammaproteobacteria (122	Proteobacteria (2)	Bacteria (1)	99.90	98.80	WGS
287.7534	0.3628	Pseudomonas aeruginosa (286)	Pseudomonas (135621)	Pseudomonadaceae (7227	Pseudomonadales (1236)	Gammaproteobacteria (122	Proteobacteria (2)	Bacteria (1)	99.70	98.30	WGS
287.7535	0.3324	Pseudomonas aeruginosa (286)	Pseudomonas (135621)	Pseudomonadaceae (7227	Pseudomonadales (1236)	Gammaproteobacteria (122	Proteobacteria (2)	Bacteria (1)	99.60	98.90	WGS
287.7536	0.371	Pseudomonas aeruginosa (286)	Pseudomonas (135621)	Pseudomonadaceae (7227	Pseudomonadales (1236)	Gammaproteobacteria (122	Proteobacteria (2)	Bacteria (1)	99.80	99.00	WGS
287.7537	0.3636	Pseudomonas aeruginosa (286)	Pseudomonas (135621)	Pseudomonadaceae (7227	Pseudomonadales (1236)	Gammaproteobacteria (122	Proteobacteria (2)	Bacteria (1)	99.50	98.00	WGS
287.7538	0.3258	Pseudomonas aeruginosa (286)	Pseudomonas (135621)	Pseudomonadaceae (7227	Pseudomonadales (1236)	Gammaproteobacteria (122	Proteobacteria (2)	Bacteria (1)	99.90	99.30	WGS
287.7539	0.3258	Pseudomonas aeruginosa (286)	Pseudomonas (135621)	Pseudomonadaceae (7227	Pseudomonadales (1236)	Gammaproteobacteria (122	Proteobacteria (2)	Bacteria (1)	99.80	99.30	WGS
287.7540	0.327	Pseudomonas aeruginosa (286)	Pseudomonas (135621)	Pseudomonadaceae (7227	Pseudomonadales (1236)	Gammaproteobacteria (122	Proteobacteria (2)	Bacteria (1)	99.60	98.20	WGS
287.7541	0.3268	Pseudomonas aeruginosa (286)	Pseudomonas (135621)	Pseudomonadaceae (7227	Pseudomonadales (1236)	Gammaproteobacteria (122	Proteobacteria (2)	Bacteria (1)	99.90	99.40	WGS
287.7542	0.3688	Pseudomonas aeruginosa (286)	Pseudomonas (135621)	Pseudomonadaceae (7227	Pseudomonadales (1236)	Gammaproteobacteria (122	Proteobacteria (2)	Bacteria (1)	99.80	98.90	WGS
287.7543	0.3738	Pseudomonas aeruginosa (286)	Pseudomonas (135621)	Pseudomonadaceae (7227	Pseudomonadales (1236)	Gammaproteobacteria (122	Proteobacteria (2)	Bacteria (1)	99.80	99.30	WGS
287.7544	0.3188	Pseudomonas aeruginosa (286)	Pseudomonas (135621)	Pseudomonadaceae (7227	Pseudomonadales (1236)	Gammaproteobacteria (122	Proteobacteria (2)	Bacteria (1)	99.90	99.10	WGS
287.7545	0.324	Pseudomonas aeruginosa (286)	Pseudomonas (135621)	Pseudomonadaceae (7227	Pseudomonadales (1236)	Gammaproteobacteria (122	Proteobacteria (2)	Bacteria (1)	99.90	99.40	WGS
287.7546	0.368	Pseudomonas aeruginosa (286)	Pseudomonas (135621)	Pseudomonadaceae (7227	Pseudomonadales (1236)	Gammaproteobacteria (122	Proteobacteria (2)	Bacteria (1)	99.70	98.40	WGS
287.7547	0.3652	Pseudomonas aeruginosa (286)	Pseudomonas (135621)	Pseudomonadaceae (7227	Pseudomonadales (1236)	Gammaproteobacteria (122	Proteobacteria (2)	Bacteria (1)	99.90	99.60	WGS
287.7548	0.3336	Pseudomonas aeruginosa (286)	Pseudomonas (135621)	Pseudomonadaceae (7227	Pseudomonadales (1236)	Gammaproteobacteria (122	Proteobacteria (2)	Bacteria (1)	99.70	98.50	WGS
287.7549	0.3376	Pseudomonas aeruginosa (286)	Pseudomonas (135621)	Pseudomonadaceae (7227	Pseudomonadales (1236)	Gammaproteobacteria (122	Proteobacteria (2)	Bacteria (1)	99.70	99.00	WGS
287.7550	0.331	Pseudomonas aeruginosa (286)	Pseudomonas (135621)	Pseudomonadaceae (7227	Pseudomonadales (1236)	Gammaproteobacteria (122	Proteobacteria (2)	Bacteria (1)	99.90	99.30	WGS
287.7551	0.371	Pseudomonas aeruginosa (286)	Pseudomonas (135621)	Pseudomonadaceae (7227	Pseudomonadales (1236)	Gammaproteobacteria (122	Proteobacteria (2)	Bacteria (1)	99.90	99.10	WGS
287.7552	0.3322	Pseudomonas aeruginosa (286)	Pseudomonas (135621)	Pseudomonadaceae (7227	Pseudomonadales (1236)	Gammaproteobacteria (122	Proteobacteria (2)	Bacteria (1)	99.80	99.30	WGS
287.7553	0.3696	Pseudomonas aeruginosa (286)	Pseudomonas (135621)	Pseudomonadaceae (7227	Pseudomonadales (1236)	Gammaproteobacteria (122	Proteobacteria (2)	Bacteria (1)	99.50	97.90	WGS
287.7554	0.3998	Pseudomonas aeruginosa (286)	Pseudomonas (135621)	Pseudomonadaceae (7227	Pseudomonadales (1236)	Gammaproteobacteria (122	Proteobacteria (2)	Bacteria (1)	99.70	98.50	WGS
287.7555	0.3636	Pseudomonas aeruginosa (286)	Pseudomonas (135621)	Pseudomonadaceae (7227	Pseudomonadales (1236)	Gammaproteobacteria (122	Proteobacteria (2)	Bacteria (1)	99.80	99.20	WGS
287.7556	0.3258	Pseudomonas aeruginosa (286)	Pseudomonas (135621)	Pseudomonadaceae (7227	Pseudomonadales (1236)	Gammaproteobacteria (122	Proteobacteria (2)	Bacteria (1)	99.90	98.80	WGS
287.7557	0.3672	Pseudomonas aeruginosa (286)	Pseudomonas (135621)	Pseudomonadaceae (7227	Pseudomonadales (1236)	Gammaproteobacteria (122	Proteobacteria (2)	Bacteria (1)	99.80	99.00	WGS
287.7558	0.3376	Pseudomonas aeruginosa (286)	Pseudomonas (135621)	Pseudomonadaceae (7227	Pseudomonadales (1236)	Gammaproteobacteria (122	Proteobacteria (2)	Bacteria (1)	99.80	99.00	WGS
287.7559	0.3322	Pseudomonas aeruginosa (286)	Pseudomonas (135621)	Pseudomonadaceae (7227	Pseudomonadales (1236)	Gammaproteobacteria (122	Proteobacteria (2)	Bacteria (1)	99.90	99.50	WGS
287.7560	0.371	Pseudomonas aeruginosa (286)	Pseudomonas (135621)	Pseudomonadaceae (7227	Pseudomonadales (1236)	Gammaproteobacteria (122	Proteobacteria (2)	Bacteria (1)	99.80	99.30	WGS
287.7561	0.3334	Pseudomonas aeruginosa (286)	Pseudomonas (135621)	Pseudomonadaceae (7227	Pseudomonadales (1236)	Gammaproteobacteria (122	Proteobacteria (2)	Bacteria (1)	99.90	98.70	WGS
287.7562	0.3316	Pseudomonas aeruginosa (286)	Pseudomonas (135621)	Pseudomonadaceae (7227	Pseudomonadales (1236)	Gammaproteobacteria (122	Proteobacteria (2)	Bacteria (1)	99.80	97.60	WGS
287.7563	0.3334	Pseudomonas aeruginosa (286)	Pseudomonas (135621)	Pseudomonadaceae (7227	Pseudomonadales (1236)	Gammaproteobacteria (122	Proteobacteria (2)	Bacteria (1)	99.90	98.70	WGS
287.7564	0.3694	Pseudomonas aeruginosa (286)	Pseudomonas (135621)	Pseudomonadaceae (7227	Pseudomonadales (1236)	Gammaproteobacteria (122	Proteobacteria (2)	Bacteria (1)	99.90	99.10	WGS
287.7565	0.3306	Pseudomonas aeruginosa (286)	Pseudomonas (135621)	Pseudomonadaceae (7227	Pseudomonadales (1236)	Gammaproteobacteria (122	Proteobacteria (2)	Bacteria (1)	99.90	99.60	WGS
287.7566	0.3258	Pseudomonas aeruginosa (286)	Pseudomonas (135621)	Pseudomonadaceae (7227	Pseudomonadales (1236)	Gammaproteobacteria (122	Proteobacteria (2)	Bacteria (1)	99.90	99.20	WGS
287.7567	0.3738	Pseudomonas aeruginosa (286)	Pseudomonas (135621)	Pseudomonadaceae (7227	Pseudomonadales (1236)	Gammaproteobacteria (122	Proteobacteria (2)	Bacteria (1)	99.90	99.20	WGS
287.7568	0.3662	Pseudomonas aeruginosa (286)	Pseudomonas (135621)	Pseudomonadaceae (7227	Pseudomonadales (1236)	Gammaproteobacteria (122	Proteobacteria (2)	Bacteria (1)	99.80	99.10	WGS
287.7569	0.3636	Pseudomonas aeruginosa (286)	Pseudomonas (135621)	Pseudomonadaceae (7227	Pseudomonadales (1236)	Gammaproteobacteria (122	Proteobacteria (2)	Bacteria (1)	99.70	99.00	WGS
287.7570	0.325	Pseudomonas aeruginosa (286)	Pseudomonas (135621)	Pseudomonadaceae (7227	Pseudomonadales (1236)	Gammaproteobacteria (122	Proteobacteria (2)	Bacteria (1)	99.40	98.80	WGS
287.7571	0.3376	Pseudomonas aeruginosa (286)	Pseudomonas (135621)	Pseudomonadaceae (7227	Pseudomonadales (1236)	Gammaproteobacteria (122	Proteobacteria (2)	Bacteria (1)	99.60	98.50	WGS
287.7572	0.3188	Pseudomonas aeruginosa (286)	Pseudomonas (135621)	Pseudomonadaceae (7227	Pseudomonadales (1236)	Gammaproteobacteria (122	Proteobacteria (2)	Bacteria (1)	99.80	99.20	WGS
287.7573	0.3258	Pseudomonas aeruginosa (286)	Pseudomonas (135621)	Pseudomonadaceae (7227	Pseudomonadales (1236)	Gammaproteobacteria (122	Proteobacteria (2)	Bacteria (1)	99.90	99.30	WGS
287.7574	0.3268	Pseudomonas aeruginosa (286)	Pseudomonas (135621)	Pseudomonadaceae (7227	Pseudomonadales (1236)	Gammaproteobacteria (122	Proteobacteria (2)	Bacteria (1)	99.90	99.10	WGS
287.7575	0.368	Pseudomonas aeruginosa (286)	Pseudomonas (135621)	Pseudomonadaceae (7227	Pseudomonadales (1236)	Gammaproteobacteria (122	Proteobacteria (2)	Bacteria (1)	99.80	98.50	WGS
287.7576	0.3174	Pseudomonas aeruginosa (286)	Pseudomonas (135621)	Pseudomonadaceae (7227	Pseudomonadales (1236)	Gammaproteobacteria (122	Proteobacteria (2)	Bacteria (1)	99.80	99.30	WGS
287.7577	0.3636	Pseudomonas aeruginosa (286)	Pseudomonas (135621)	Pseudomonadaceae (7227	Pseudomonadales (1236)	Gammaproteobacteria (122	Proteobacteria (2)	Bacteria (1)	99.90	99.30	WGS
287.7578	0.3694	Pseudomonas aeruginosa (286)	Pseudomonas (135621)	Pseudomonadaceae (7227	Pseudomonadales (1236)	Gammaproteobacteria (122	Proteobacteria (2)	Bacteria (1)	99.80	99.30	WGS
287.7579	0.3174	Pseudomonas aeruginosa (286)	Pseudomonas (135621)	Pseudomonadaceae (7227	Pseudomonadales (1236)	Gammaproteobacteria (122	Proteobacteria (2)	Bacteria (1)	100.00	99.70	WGS
287.7580	0.369	Pseudomonas aeruginosa (286)	Pseudomonas (135621)	Pseudomonadaceae (7227	Pseudomonadales (1236)	Gammaproteobacteria (122	Proteobacteria (2)	Bacteria (1)	99.80	99.20	WGS
287.7581	0.3258	Pseudomonas aeruginosa (286)	Pseudomonas (135621)	Pseudomonadaceae (7227	Pseudomonadales (1236)	Gammaproteobacteria (122	Proteobacteria (2)	Bacteria (1)	99.90	99.60	WGS
287.7582	0.3348	Pseudomonas aeruginosa (286)	Pseudomonas (135621)	Pseudomonadaceae (7227	Pseudomonadales (1236)	Gammaproteobacteria (122	Proteobacteria (2)	Bacteria (1)	99.80	99.40	WGS
287.7583	0.3268	Pseudomonas aeruginosa (286)	Pseudomonas (135621)	Pseudomonadaceae (7227	Pseudomonadales (1236)	Gammaproteobacteria (122	Proteobacteria (2)	Bacteria (1)	99.90	98.70	WGS
287.7584	0.331	Pseudomonas aeruginosa (286)	Pseudomonas (135621)	Pseudomonadaceae (7227	Pseudomonadales (1236)	Gammaproteobacteria (122	Proteobacteria (2)	Bacteria (1)	100.00	99.60	WGS
287.7585	0.3268	Pseudomonas aeruginosa (286)	Pseudomonas (135621)	Pseudomonadaceae (7227	Pseudomonadales (1236)	Gammaproteobacteria (122	Proteobacteria (2)	Bacteria (1)	99.90	99.00	WGS
287.7586	0.3226	Pseudomonas aeruginosa (286)	Pseudomonas (135621)	Pseudomonadaceae (7227	Pseudomonadales (1236)	Gammaproteobacteria (122	Proteobacteria (2)	Bacteria (1)	99.90	99.30	WGS
287.7587	0.3334	Pseudomonas aeruginosa (286)	Pseudomonas (135621)	Pseudomonadaceae (7227	Pseudomonadales (1236)	Gammaproteobacteria (122	Proteobacteria (2)	Bacteria (1)	99.90	99.60	WGS
287.7588	0.3636	Pseudomonas aeruginosa (286)	Pseudomonas (135621)	Pseudomonadaceae (7227	Pseudomonadales (1236)	Gammaproteobacteria (122	Proteobacteria (2)	Bacteria (1)	99.90	98.70	WGS

287.7589	0.4066	Pseudomonas aeruginosa (286)	Pseudomonas (135621)	Pseudomonadaceae (7227	Pseudomonadales (1236)	Gammaproteobacteria (122	Proteobacteria (2)	Bacteria (1)			99.80	99.30	WGS
287.7590	0.3636	Pseudomonas aeruginosa (286)	Pseudomonas (135621)	Pseudomonadaceae (7227	Pseudomonadales (1236)	Gammaproteobacteria (122	Proteobacteria (2)	Bacteria (1)			99.90	99.60	WGS
287.7591	0.3324	Pseudomonas aeruginosa (286)	Pseudomonas (135621)	Pseudomonadaceae (7227	Pseudomonadales (1236)	Gammaproteobacteria (122	Proteobacteria (2)	Bacteria (1)			99.90	99.30	WGS
287.7592	0.3174	Pseudomonas aeruginosa (286)	Pseudomonas (135621)	Pseudomonadaceae (7227	Pseudomonadales (1236)	Gammaproteobacteria (122	Proteobacteria (2)	Bacteria (1)			99.90	99.40	WGS
287.7593	0.3738	Pseudomonas aeruginosa (286)	Pseudomonas (135621)	Pseudomonadaceae (7227	Pseudomonadales (1236)	Gammaproteobacteria (122	Proteobacteria (2)	Bacteria (1)			99.70	99.50	WGS
287.7594	0.3258	Pseudomonas aeruginosa (286)	Pseudomonas (135621)	Pseudomonadaceae (7227	Pseudomonadales (1236)	Gammaproteobacteria (122	Proteobacteria (2)	Bacteria (1)			99.90	99.60	WGS
287.7595	0.3324	Pseudomonas aeruginosa (286)	Pseudomonas (135621)	Pseudomonadaceae (7227	Pseudomonadales (1236)	Gammaproteobacteria (122	Proteobacteria (2)	Bacteria (1)			99.70	98.80	WGS
287.7596	0.3694	Pseudomonas aeruginosa (286)	Pseudomonas (135621)	Pseudomonadaceae (7227	Pseudomonadales (1236)	Gammaproteobacteria (122	Proteobacteria (2)	Bacteria (1)			99.90	98.90	WGS
287.7597	0.3388	Pseudomonas aeruginosa (286)	Pseudomonas (135621)	Pseudomonadaceae (7227	Pseudomonadales (1236)	Gammaproteobacteria (122	Proteobacteria (2)	Bacteria (1)			99.90	99.10	WGS
287.7598	0.3258	Pseudomonas aeruginosa (286)	Pseudomonas (135621)	Pseudomonadaceae (7227	Pseudomonadales (1236)	Gammaproteobacteria (122	Proteobacteria (2)	Bacteria (1)			99.90	99.50	WGS
287.7599	0.3184	Pseudomonas aeruginosa (286)	Pseudomonas (135621)	Pseudomonadaceae (7227	Pseudomonadales (1236)	Gammaproteobacteria (122	Proteobacteria (2)	Bacteria (1)			99.90	99.60	WGS
287.7600	0.324	Pseudomonas aeruginosa (286)	Pseudomonas (135621)	Pseudomonadaceae (7227	Pseudomonadales (1236)	Gammaproteobacteria (122	Proteobacteria (2)	Bacteria (1)			99.60	98.90	WGS
287.7601	0.3936	Pseudomonas aeruginosa (286)	Pseudomonas (135621)	Pseudomonadaceae (7227	Pseudomonadales (1236)	Gammaproteobacteria (122	Proteobacteria (2)	Bacteria (1)			99.60	98.70	WGS
287.7602	0.3386	Pseudomonas aeruginosa (286)	Pseudomonas (135621)	Pseudomonadaceae (7227	Pseudomonadales (1236)	Gammaproteobacteria (122	Proteobacteria (2)	Bacteria (1)			99.90	99.00	WGS
287.7603	0.3636	Pseudomonas aeruginosa (286)	Pseudomonas (135621)	Pseudomonadaceae (7227	Pseudomonadales (1236)	Gammaproteobacteria (122	Proteobacteria (2)	Bacteria (1)			99.80	98.40	WGS
287.7604	0.3324	Pseudomonas aeruginosa (286)	Pseudomonas (135621)	Pseudomonadaceae (7227	Pseudomonadales (1236)	Gammaproteobacteria (122	Proteobacteria (2)	Bacteria (1)			99.80	99.40	WGS
287.7605	0.3258	Pseudomonas aeruginosa (286)	Pseudomonas (135621)	Pseudomonadaceae (7227	Pseudomonadales (1236)	Gammaproteobacteria (122	Proteobacteria (2)	Bacteria (1)			99.90	98.90	WGS
287.7606	0.331	Pseudomonas aeruginosa (286)	Pseudomonas (135621)	Pseudomonadaceae (7227	Pseudomonadales (1236)	Gammaproteobacteria (122	Proteobacteria (2)	Bacteria (1)			99.80	98.40	WGS
287.7607	0.3258	Pseudomonas aeruginosa (286)	Pseudomonas (135621)	Pseudomonadaceae (7227	Pseudomonadales (1236)	Gammaproteobacteria (122	Proteobacteria (2)	Bacteria (1)			99.80	99.20	WGS
287.7608	0.3652	Pseudomonas aeruginosa (286)	Pseudomonas (135621)	Pseudomonadaceae (7227	Pseudomonadales (1236)	Gammaproteobacteria (122	Proteobacteria (2)	Bacteria (1)			99.90	98.50	WGS
287.7609	0.3258	Pseudomonas aeruginosa (286)	Pseudomonas (135621)	Pseudomonadaceae (7227	Pseudomonadales (1236)	Gammaproteobacteria (122	Proteobacteria (2)	Bacteria (1)			99.90	98.70	WGS
287.7610	0.3258	Pseudomonas aeruginosa (286)	Pseudomonas (135621)	Pseudomonadaceae (7227	Pseudomonadales (1236)	Gammaproteobacteria (122	Proteobacteria (2)	Bacteria (1)			99.70	98.90	WGS
287.7611	0.3324	Pseudomonas aeruginosa (286)	Pseudomonas (135621)	Pseudomonadaceae (7227	Pseudomonadales (1236)	Gammaproteobacteria (122	Proteobacteria (2)	Bacteria (1)			99.90	99.00	WGS
287.7612	0.37	Pseudomonas aeruginosa (286)	Pseudomonas (135621)	Pseudomonadaceae (7227	Pseudomonadales (1236)	Gammaproteobacteria (122	Proteobacteria (2)	Bacteria (1)			99.90	98.90	WGS
287.7613	0.3376	Pseudomonas aeruginosa (286)	Pseudomonas (135621)	Pseudomonadaceae (7227	Pseudomonadales (1236)	Gammaproteobacteria (122	Proteobacteria (2)	Bacteria (1)			99.70	98.10	WGS
287.7614	0.3334	Pseudomonas aeruginosa (286)	Pseudomonas (135621)	Pseudomonadaceae (7227	Pseudomonadales (1236)	Gammaproteobacteria (122	Proteobacteria (2)	Bacteria (1)			99.90	98.40	WGS
287.7615	0.3324	Pseudomonas aeruginosa (286)	Pseudomonas (135621)	Pseudomonadaceae (7227	Pseudomonadales (1236)	Gammaproteobacteria (122	Proteobacteria (2)	Bacteria (1)			99.90	98.70	WGS
287.7616	0.3258	Pseudomonas aeruginosa (286)	Pseudomonas (135621)	Pseudomonadaceae (7227	Pseudomonadales (1236)	Gammaproteobacteria (122	Proteobacteria (2)	Bacteria (1)			99.80	99.30	WGS
287.7617	0.3314	Pseudomonas aeruginosa (286)	Pseudomonas (135621)	Pseudomonadaceae (7227	Pseudomonadales (1236)	Gammaproteobacteria (122	Proteobacteria (2)	Bacteria (1)			99.90	98.80	WGS
287.7618	0.3324	Pseudomonas aeruginosa (286)	Pseudomonas (135621)	Pseudomonadaceae (7227	Pseudomonadales (1236)	Gammaproteobacteria (122	Proteobacteria (2)	Bacteria (1)			99.80	99.20	WGS
287.7619	0.3324	Pseudomonas aeruginosa (286)	Pseudomonas (135621)	Pseudomonadaceae (7227	Pseudomonadales (1236)	Gammaproteobacteria (122	Proteobacteria (2)	Bacteria (1)			99.70	99.00	WGS
287.7620	0.3704	Pseudomonas aeruginosa (286)	Pseudomonas (135621)	Pseudomonadaceae (7227	Pseudomonadales (1236)	Gammaproteobacteria (122	Proteobacteria (2)	Bacteria (1)			99.90	98.40	WGS
287.7621	0.3268	Pseudomonas aeruginosa (286)	Pseudomonas (135621)	Pseudomonadaceae (7227	Pseudomonadales (1236)	Gammaproteobacteria (122	Proteobacteria (2)	Bacteria (1)			99.90	98.80	WGS
287.7622	0.3288	Pseudomonas aeruginosa (286)	Pseudomonas (135621)	Pseudomonadaceae (7227	Pseudomonadales (1236)	Gammaproteobacteria (122	Proteobacteria (2)	Bacteria (1)			99.50	97.60	WGS
287.7623	0.331	Pseudomonas aeruginosa (286)	Pseudomonas (135621)	Pseudomonadaceae (7227	Pseudomonadales (1236)	Gammaproteobacteria (122	Proteobacteria (2)	Bacteria (1)			99.90	98.20	WGS
287.7624	0.3336	Pseudomonas aeruginosa (286)	Pseudomonas (135621)	Pseudomonadaceae (7227	Pseudomonadales (1236)	Gammaproteobacteria (122	Proteobacteria (2)	Bacteria (1)			99.90	98.70	WGS
287.7625	0.3174	Pseudomonas aeruginosa (286)	Pseudomonas (135621)	Pseudomonadaceae (7227	Pseudomonadales (1236)	Gammaproteobacteria (122	Proteobacteria (2)	Bacteria (1)			99.90	99.10	WGS
287.7626	0.3636	Pseudomonas aeruginosa (286)	Pseudomonas (135621)	Pseudomonadaceae (7227	Pseudomonadales (1236)	Gammaproteobacteria (122	Proteobacteria (2)	Bacteria (1)			99.90	99.20	WGS
287.7627	0.3636	Pseudomonas aeruginosa (286)	Pseudomonas (135621)	Pseudomonadaceae (7227	Pseudomonadales (1236)	Gammaproteobacteria (122	Proteobacteria (2)	Bacteria (1)			99.90	99.20	WGS
287.7628	0.3174	Pseudomonas aeruginosa (286)	Pseudomonas (135621)	Pseudomonadaceae (7227	Pseudomonadales (1236)	Gammaproteobacteria (122	Proteobacteria (2)	Bacteria (1)			99.80	99.30	WGS
287.7629	0.3302	Pseudomonas aeruginosa (286)	Pseudomonas (135621)	Pseudomonadaceae (7227	Pseudomonadales (1236)	Gammaproteobacteria (122	Proteobacteria (2)	Bacteria (1)			99.70	98.80	WGS
287.7630	0.3376	Pseudomonas aeruginosa (286)	Pseudomonas (135621)	Pseudomonadaceae (7227	Pseudomonadales (1236)	Gammaproteobacteria (122	Proteobacteria (2)	Bacteria (1)			99.90	98.60	WGS
287.7631	0.3174	Pseudomonas aeruginosa (286)	Pseudomonas (135621)	Pseudomonadaceae (7227	Pseudomonadales (1236)	Gammaproteobacteria (122	Proteobacteria (2)	Bacteria (1)			99.90	99.00	WGS
287.7632	0.325	Pseudomonas aeruginosa (286)	Pseudomonas (135621)	Pseudomonadaceae (7227	Pseudomonadales (1236)	Gammaproteobacteria (122	Proteobacteria (2)	Bacteria (1)			99.70	99.00	WGS
287.7633	0.3694	Pseudomonas aeruginosa (286)	Pseudomonas (135621)	Pseudomonadaceae (7227	Pseudomonadales (1236)	Gammaproteobacteria (122	Proteobacteria (2)	Bacteria (1)			99.90	99.00	WGS
287.7634	0.3628	Pseudomonas aeruginosa (286)	Pseudomonas (135621)	Pseudomonadaceae (7227	Pseudomonadales (1236)	Gammaproteobacteria (122	Proteobacteria (2)	Bacteria (1)			99.90	99.10	WGS
287.7635	0.3376	Pseudomonas aeruginosa (286)	Pseudomonas (135621)	Pseudomonadaceae (7227	Pseudomonadales (1236)	Gammaproteobacteria (122	Proteobacteria (2)	Bacteria (1)			99.80	99.00	WGS
287.7636	0.3628	Pseudomonas aeruginosa (286)	Pseudomonas (135621)	Pseudomonadaceae (7227	Pseudomonadales (1236)	Gammaproteobacteria (122	Proteobacteria (2)	Bacteria (1)			99.90	99.10	WGS
287.7637	0.324	Pseudomonas aeruginosa (286)	Pseudomonas (135621)	Pseudomonadaceae (7227	Pseudomonadales (1236)	Gammaproteobacteria (122	Proteobacteria (2)	Bacteria (1)			99.90	99.30	WGS
287.7638	0.3258	Pseudomonas aeruginosa (286)	Pseudomonas (135621)	Pseudomonadaceae (7227	Pseudomonadales (1236)	Gammaproteobacteria (122	Proteobacteria (2)	Bacteria (1)			99.90	99.30	WGS
287.7639	0.368	Pseudomonas aeruginosa (286)	Pseudomonas (135621)	Pseudomonadaceae (7227	Pseudomonadales (1236)	Gammaproteobacteria (122	Proteobacteria (2)	Bacteria (1)			99.90	98.70	WGS
287.7640	0.3636	Pseudomonas aeruginosa (286)	Pseudomonas (135621)	Pseudomonadaceae (7227	Pseudomonadales (1236)	Gammaproteobacteria (122	Proteobacteria (2)	Bacteria (1)			99.90	99.50	WGS
287.7641	0.371	Pseudomonas aeruginosa (286)	Pseudomonas (135621)	Pseudomonadaceae (7227	Pseudomonadales (1236)	Gammaproteobacteria (122	Proteobacteria (2)	Bacteria (1)			99.90	97.50	WGS
287.7642	0.327	Pseudomonas aeruginosa (286)	Pseudomonas (135621)	Pseudomonadaceae (7227	Pseudomonadales (1236)	Gammaproteobacteria (122	Proteobacteria (2)	Bacteria (1)			99.90	98.70	WGS
287.7643	0.3684	Pseudomonas aeruginosa (286)	Pseudomonas (135621)	Pseudomonadaceae (7227	Pseudomonadales (1236)	Gammaproteobacteria (122	Proteobacteria (2)	Bacteria (1)			99.90	97.90	WGS
287.7644	0.3644	Pseudomonas aeruginosa (286)	Pseudomonas (135621)	Pseudomonadaceae (7227	Pseudomonadales (1236)	Gammaproteobacteria (122	Proteobacteria (2)	Bacteria (1)			94.80	79.70	WGS
287.7645	0.3646	Pseudomonas aeruginosa (286)	Pseudomonas (135621)	Pseudomonadaceae (7227	Pseudomonadales (1236)	Gammaproteobacteria (122	Proteobacteria (2)	Bacteria (1)			99.90	98.70	WGS
287.7646	0.3258	Pseudomonas aeruginosa (286)	Pseudomonas (135621)	Pseudomonadaceae (7227	Pseudomonadales (1236)	Gammaproteobacteria (122	Proteobacteria (2)	Bacteria (1)			99.90	99.10	WGS
287.7647	0.356	Pseudomonas aeruginosa (286)	Pseudomonas (135621)	Pseudomonadaceae (7227	Pseudomonadales (1236)	Gammaproteobacteria (122	Proteobacteria (2)	Bacteria (1)			99.90	98.90	WGS
287.7648	0.3694	Pseudomonas aeruginosa (286)	Pseudomonas (135621)	Pseudomonadaceae (7227	Pseudomonadales (1236)	Gammaproteobacteria (122	Proteobacteria (2)	Bacteria (1)			99.80	98.80	WGS
287.7649	0.3668	Pseudomonas aeruginosa (286)	Pseudomonas (135621)	Pseudomonadaceae (7227	Pseudomonadales (1236)	Gammaproteobacteria (122	Proteobacteria (2)	Bacteria (1)			99.90	98.40	WGS
287.7650	0.3694	Pseudomonas aeruginosa (286)	Pseudomonas (135621)	Pseudomonadaceae (7227	Pseudomonadales (1236)	Gammaproteobacteria (122	Proteobacteria (2)	Bacteria (1)			99.80	98.00	WGS
287.7651	0.3174	Pseudomonas aeruginosa (286)	Pseudomonas (135621)	Pseudomonadaceae (7227	Pseudomonadales (1236)	Gammaproteobacteria (122	Proteobacteria (2)	Bacteria (1)			99.90	99.20	WGS
287.7652	0.3306	Pseudomonas aeruginosa (286)	Pseudomonas (135621)	Pseudomonadaceae (7227	Pseudomonadales (1236)	Gammaproteobacteria (122	Proteobacteria (2)	Bacteria (1)			99.80	96.50	WGS
287.7653	0.324	Pseudomonas aeruginosa (286)	Pseudomonas (135621)	Pseudomonadaceae (7227	Pseudomonadales (1236)	Gammaproteobacteria (122	Proteobacteria (2)	Bacteria (1)			99.90	98.20	WGS
287.7654	0.3636	Pseudomonas aeruginosa (286)	Pseudomonas (135621)	Pseudomonadaceae (7227	Pseudomonadales (1236)	Gammaproteobacteria (122	Proteobacteria (2)	Bacteria (1)			99.90	98.20	WGS
287.7655	0.327	Pseudomonas aeruginosa (286)	Pseudomonas (135621)	Pseudomonadaceae (7227	Pseudomonadales (1236)	Gammaproteobacteria (122	Proteobacteria (2)	Bacteria (1)			99.90	98.80	WGS
287.7656	0.3174	Pseudomonas aeruginosa (286)	Pseudomonas (135621)	Pseudomonadaceae (7227	Pseudomonadales (1236)	Gammaproteobacteria (122	Proteobacteria (2)	Bacteria (1)			99.90	98.20	WGS
287.7657	0.3258	Pseudomonas aeruginosa (286)	Pseudomonas (135621)	Pseudomonadaceae (7227	Pseudomonadales (1236)	Gammaproteobacteria (122	Proteobacteria (2)	Bacteria (1)			99.90	98.30	WGS
287.7658	0.3694	Pseudomonas aeruginosa (286)	Pseudomonas (135621)	Pseudomonadaceae (7227	Pseudomonadales (1236)	Gammaproteobacteria (122	Proteobacteria (2)	Bacteria (1)			99.90	98.20	WGS
287.7659	0.3662	Pseudomonas aeruginosa (286)	Pseudomonas (135621)	Pseudomonadaceae (7227	Pseudomonadales (1236)	Gammaproteobacteria (122	Proteobacteria (2)	Bacteria (1)			99.90	98.20	WGS
287.7660	0.3662	Pseudomonas aeruginosa (286)	Pseudomonas (135621)	Pseudomonadaceae (7227	Pseudomonadales (1236)	Gammaproteobacteria (122	Proteobacteria (2)	Bacteria (1)			99.90	96.50	WGS
287.7661	0.331	Pseudomonas aeruginosa (286)	Pseudomonas (135621)	Pseudomonadaceae (7227	Pseudomonadales (1236)	Gammaproteobacteria (122	Proteobacteria (2)	Bacteria (1)			99.90	98.90	WGS
287.7662	0.3672	Pseudomonas aeruginosa (286)	Pseudomonas (135621)	Pseudomonadaceae (7227	Pseudomonadales (1236)	Gammaproteobacteria (122	Proteobacteria (2)	Bacteria (1)			99.90	97.60	WGS
287.7663	0.3636	Pseudomonas aeruginosa (286)	Pseudomonas (135621)	Pseudomonadaceae (7227	Pseudomonadales (1236)	Gammaproteobacteria (122	Proteobacteria (2)	Bacteria (1)			99.80	98.20	WGS
[illegible]	[illegible]	[illegible]	[illegible]	[illegible]	[illegible]	[illegible]	[illegible]	[illegible]	[illegible]	[illegible]	[illegible]	[illegible]	[illegible]
287.8521	0.368	Pseudomonas aeruginosa (286)	Pseudomonas (135621)	Pseudomonadaceae (7227	Pseudomonadales (1236)	Gammaproteobacteria (122	Proteobacteria (2)	Bacteria (1)			99.70	98.40	WGS
287.8522	0.368	Pseudomonas aeruginosa (286)	Pseudomonas (135621)	Pseudomonadaceae (7227	Pseudomonadales (1236)	Gammaproteobacteria (122	Proteobacteria (2)	Bacteria (1)			99.80	98.60	WGS
287.8523	0.3398	Pseudomonas aeruginosa (286)	Pseudomonas (135621)	Pseudomonadaceae (7227	Pseudomonadales (1236)	Gammaproteobacteria (122	Proteobacteria (2)	Bacteria (1)			99.80	77.50	WGS
287.8524	0.3226	Pseudomonas aeruginosa (286)	Pseudomonas (135621)	Pseudomonadaceae (7227	Pseudomonadales (1236)	Gammaproteobacteria (122	Proteobacteria (2)	Bacteria (1)			99.70	98.70	WGS
287.8525	0.3632	Pseudomonas aeruginosa (286)	Pseudomonas (135621)	Pseudomonadaceae (7227	Pseudomonadales (1236)	Gammaproteobacteria (122	Proteobacteria (2)	Bacteria (1)			99.80	98.40	WGS
287.8526	0.325	Pseudomonas aeruginosa (286)	Pseudomonas (135621)	Pseudomonadaceae (7227	Pseudomonadales (1236)	Gammaproteobacteria (122	Proteobacteria (2)	Bacteria (1)			99.90	98.40	WGS
287.8527	0.368	Pseudomonas aeruginosa (286)	Pseudomonas (135621)	Pseudomonadaceae (7227	Pseudomonadales (1236)	Gammaproteobacteria (122	Proteobacteria (2)	Bacteria (1)			99.80	98.70	WGS
287.8528	0.368	Pseudomonas aeruginosa (286)	Pseudomonas (135621)	Pseudomonadaceae (7227	Pseudomonadales (1236)	Gammaproteobacteria (122	Proteobacteria (2)	Bacteria (1)			99.90	98.90	WGS
287.8529	0.3632	Pseudomonas aeruginosa (286)	Pseudomonas (135621)	Pseudomonadaceae (7227	Pseudomonadales (1236)	Gammaproteobacteria (122	Proteobacteria (2)	Bacteria (1)			99.80	98.40	WGS
287.853	0.327	Pseudomonas aeruginosa (286)	Pseudomonas (135621)	Pseudomonadaceae (7227	Pseudomonadales (1236)	Gammaproteobacteria (122	Proteobacteria (2)	Bacteria (1)	99.70	0.00	99.70	99.40	WGS
287.8530	0.3258	Pseudomonas aeruginosa (286)	Pseudomonas (135621)	Pseudomonadaceae (7227	Pseudomonadales (1236)	Gammaproteobacteria (122	Proteobacteria (2)	Bacteria (1)			99.80	98.60	WGS
287.8531	0.356	Pseudomonas aeruginosa (286)	Pseudomonas (135621)	Pseudomonadaceae (7227	Pseudomonadales (1236)	Gammaproteobacteria (122	Proteobacteria (2)	Bacteria (1)			99.70	98.70	WGS
287.8532	0.325	Pseudomonas aeruginosa (286)	Pseudomonas (135621)	Pseudomonadaceae (7227	Pseudomonadales (1236)	Gammaproteobacteria (122	Proteobacteria (2)	Bacteria (1)			99.80	97.60	WGS
287.8533	0.369	Pseudomonas aeruginosa (286)	Pseudomonas (135621)	Pseudomonadaceae (7227	Pseudomonadales (1236)	Gammaproteobacteria (122	Proteobacteria (2)	Bacteria (1)			99.80	98.50	WGS
287.8534	0.369	Pseudomonas aeruginosa (286)	Pseudomonas (135621)	Pseudomonadaceae (7227	Pseudomonadales (1236)	Gammaproteobacteria (122	Proteobacteria (2)	Bacteria (1)			99.80	98.70	WGS
287.8535	0.3812	Pseudomonas aeruginosa (286)	Pseudomonas (135621)	Pseudomonadaceae (7227	Pseudomonadales (1236)	Gammaproteobacteria (122	Proteobacteria (2)	Bacteria (1)			99.90	98.50	WGS
287.8536	0.3748	Pseudomonas aeruginosa (286)	Pseudomonas (135621)	Pseudomonadaceae (7227	Pseudomonadales (1236)	Gammaproteobacteria (122	Proteobacteria (2)	Bacteria (1)			99.80	98.70	WGS
287.8537	0.3376	Pseudomonas aeruginosa (286)	Pseudomonas (135621)	Pseudomonadaceae (7227	Pseudomonadales (1236)	Gammaproteobacteria (122	Proteobacteria (2)	Bacteria (1)			99.80	98.60	WGS
287.8538	0.3236	Pseudomonas aeruginosa (286)	Pseudomonas (135621)	Pseudomonadaceae (7227	Pseudomonadales (1236)	Gammaproteobacteria (122	Proteobacteria (2)	Bacteria (1)			99.60	98.90	WGS
287.8539	0.3324	Pseudomonas aeruginosa (286)	Pseudomonas (135621)	Pseudomonadaceae (7227	Pseudomonadales (1236)	Gammaproteobacteria (122	Proteobacteria (2)	Bacteria (1)			99.80	99.40	WGS
287.8540	0.3268	Pseudomonas aeruginosa (286)	Pseudomonas (135621)	Pseudomonadaceae (7227	Pseudomonadales (1236)	Gammaproteobacteria (122	Proteobacteria (2)	Bacteria (1)			99.70	99.00	WGS
287.854	0.3636	Pseudomonas aeruginosa (286)	Pseudomonas (135621)	Pseudomonadaceae (7227	Pseudomonadales (1236)	Gammaproteobacteria (122	Proteobacteria (2)	Bacteria (1)	100.00	0.30	99.70	98.70	WGS
287.8541	0.3184	Pseudomonas aeruginosa (286)	Pseudomonas (135621)	Pseudomonadaceae (7227	Pseudomonadales (1236)	Gammaproteobacteria (122	Proteobacteria (2)	Bacteria (1)			99.90	98.40	WGS
287.8542	0.3376	Pseudomonas aeruginosa (286)	Pseudomonas (135621)	Pseudomonadaceae (7227	Pseudomonadales (1236)	Gammaproteobacteria (122	Proteobacteria (2)	Bacteria (1)			99.80	98.40	WGS
287.8543	0.3258	Pseudomonas aeruginosa (286)	Pseudomonas (135621)	Pseudomonadaceae (7227	Pseudomonadales (1236)	Gammaproteobacteria (122	Proteobacteria (2)	Bacteria (1)			99.60	98.30	WGS
287.8544	0.3174	Pseudomonas aeruginosa (286)	Pseudomonas (135621)	Pseudomonadaceae (7227	Pseudomonadales (1236)	Gammaproteobacteria (122	Proteobacteria (2)	Bacteria (1)			99.70	98.80	WGS
287.8545	0.325	Pseudomonas aeruginosa (286)	Pseudomonas (135621)	Pseudomonadaceae (7227	Pseudomonadales (1236)	Gammaproteobacteria (122	Proteobacteria (2)	Bacteria (1)			99.90	98.70	WGS
287.8546	0.3768	Pseudomonas aeruginosa (286)	Pseudomonas (135621)	Pseudomonadaceae (7227	Pseudomonadales (1236)	Gammaproteobacteria (122	Proteobacteria (2)	Bacteria (1)			99.90	98.60	WGS
287.8547	0.3184	Pseudomonas aeruginosa (286)	Pseudomonas (135621)	Pseudomonadaceae (7227	Pseudomonadales (1236)	Gammaproteobacteria (122	Proteobacteria (2)	Bacteria (1)			99.80	98.40	WGS
287.8548	0.325	Pseudomonas aeruginosa (286)	Pseudomonas (135621)	Pseudomonadaceae (7227	Pseudomonadales (1236)	Gammaproteobacteria (122	Proteobacteria (2)	Bacteria (1)			99.90	98.90	WGS
287.8549	0.3258	Pseudomonas aeruginosa (286)	Pseudomonas (135621)	Pseudomonadaceae (7227	Pseudomonadales (1236)	Gammaproteobacteria (122	Proteobacteria (2)	Bacteria (1)			99.70	99.30	WGS
287.855	0.3604	Pseudomonas aeruginosa (286)	Pseudomonas (135621)	Pseudomonadaceae (7227	Pseudomonadales (1236)	Gammaproteobacteria (122	Proteobacteria (2)	Bacteria (1)	100.00	0.00	99.60	98.90	WGS
287.8550	0.325	Pseudomonas aeruginosa (286)	Pseudomonas (135621)	Pseudomonadaceae (7227	Pseudomonadales (1236)	Gammaproteobacteria (122	Proteobacteria (2)	Bacteria (1)			99.80	98.80	WGS
287.8551	0.325	Pseudomonas aeruginosa (286)	Pseudomonas (135621)	Pseudomonadaceae (7227	Pseudomonadales (1236)	Gammaproteobacteria (122	Proteobacteria (2)	Bacteria (1)			99.80	98.70	WGS
287.856	0.331	Pseudomonas aeruginosa (286)	Pseudomonas (135621)	Pseudomonadaceae (7227	Pseudomonadales (1236)	Gammaproteobacteria (122	Proteobacteria (2)	Bacteria (1)	99.70	0.00	99.60	99.20	WGS
287.857	0.3622	Pseudomonas aeruginosa (286)	Pseudomonas (135621)	Pseudomonadaceae (7227	Pseudomonadales (1236)	Gammaproteobacteria (122	Proteobacteria (2)	Bacteria (1)	100.00	0.00	99.20	98.50	WGS
287.858	0.3398	Pseudomonas aeruginosa (286)	Pseudomonas (135621)	Pseudomonadaceae (7227	Pseudomonadales (1236)	Gammaproteobacteria (122	Proteobacteria (2)	Bacteria (1)	100.00	0.00	99.50	99.00	WGS
287.859	0.3376	Pseudomonas aeruginosa (286)	Pseudomonas (135621)	Pseudomonadaceae (7227	Pseudomonadales (1236)	Gammaproteobacteria (122	Proteobacteria (2)	Bacteria (1)	100.00	0.00	99.20	98.10	WGS
287.860	0.3618	Pseudomonas aeruginosa (286)	Pseudomonas (135621)	Pseudomonadaceae (7227	Pseudomonadales (1236)	Gammaproteobacteria (122	Proteobacteria (2)	Bacteria (1)	99.70	0.00	99.40	98.70	WGS
287.861	0.356	Pseudomonas aeruginosa (286)	Pseudomonas (135621)	Pseudomonadaceae (7227	Pseudomonadales (1236)	Gammaproteobacteria (122	Proteobacteria (2)	Bacteria (1)	100.00	0.00	99.40	99.20	WGS
287.862	0.3324	Pseudomonas aeruginosa (286)	Pseudomonas (135621)	Pseudomonadaceae (7227	Pseudomonadales (1236)	Gammaproteobacteria (122	Proteobacteria (2)	Bacteria (1)	99.10	0.30	98.70	98.40	WGS
287.863	0.3258	Pseudomonas aeruginosa (286)	Pseudomonas (135621)	Pseudomonadaceae (7227	Pseudomonadales (1236)	Gammaproteobacteria (122	Proteobacteria (2)	Bacteria (1)	100.00	0.00	99.80	99.60	WGS
287.864	0.3274	Pseudomonas aeruginosa (286)	Pseudomonas (135621)	Pseudomonadaceae (7227	Pseudomonadales (1236)	Gammaproteobacteria (122	Proteobacteria (2)	Bacteria (1)	98.80	0.00	99.50	98.60	WGS
287.865	0.324	Pseudomonas aeruginosa (286)	Pseudomonas (135621)	Pseudomonadaceae (7227	Pseudomonadales (1236)	Gammaproteobacteria (122	Proteobacteria (2)	Bacteria (1)	99.70	0.00	99.70	99.20	WGS
287.866	0.3188	Pseudomonas aeruginosa (286)	Pseudomonas (135621)	Pseudomonadaceae (7227	Pseudomonadales (1236)	Gammaproteobacteria (122	Proteobacteria (2)	Bacteria (1)	99.70	0.00	99.50	99.40	WGS

287.867	0.331	Pseudomonas aeruginosa (286)	Pseudomonas (135621)	Pseudomonadaceae (7227	Pseudomonadales (1236)	Gammaproteobacteria (122	Proteobacteria (2)	Bacteria (1)	99.70	0.00	99.50	99.00	WGS
287.868	0.332	Pseudomonas aeruginosa (286)	Pseudomonas (135621)	Pseudomonadaceae (7227	Pseudomonadales (1236)	Gammaproteobacteria (122	Proteobacteria (2)	Bacteria (1)	99.40	0.00	99.60	98.90	WGS
287.869	0.3258	Pseudomonas aeruginosa (286)	Pseudomonas (135621)	Pseudomonadaceae (7227	Pseudomonadales (1236)	Gammaproteobacteria (122	Proteobacteria (2)	Bacteria (1)	99.70	0.00	99.70	99.40	WGS
287.870	0.3184	Pseudomonas aeruginosa (286)	Pseudomonas (135621)	Pseudomonadaceae (7227	Pseudomonadales (1236)	Gammaproteobacteria (122	Proteobacteria (2)	Bacteria (1)	100.00	0.00	99.50	99.10	WGS
287.871	0.3376	Pseudomonas aeruginosa (286)	Pseudomonas (135621)	Pseudomonadaceae (7227	Pseudomonadales (1236)	Gammaproteobacteria (122	Proteobacteria (2)	Bacteria (1)	100.00	0.60	99.60	98.70	WGS
287.872	0.3268	Pseudomonas aeruginosa (286)	Pseudomonas (135621)	Pseudomonadaceae (7227	Pseudomonadales (1236)	Gammaproteobacteria (122	Proteobacteria (2)	Bacteria (1)	100.00	0.30	99.70	99.40	WGS
287.873	0.3268	Pseudomonas aeruginosa (286)	Pseudomonas (135621)	Pseudomonadaceae (7227	Pseudomonadales (1236)	Gammaproteobacteria (122	Proteobacteria (2)	Bacteria (1)	100.00	0.00	99.60	99.00	WGS
287.874	0.3388	Pseudomonas aeruginosa (286)	Pseudomonas (135621)	Pseudomonadaceae (7227	Pseudomonadales (1236)	Gammaproteobacteria (122	Proteobacteria (2)	Bacteria (1)	99.70	0.00	99.80	99.30	WGS
287.875	0.3334	Pseudomonas aeruginosa (286)	Pseudomonas (135621)	Pseudomonadaceae (7227	Pseudomonadales (1236)	Gammaproteobacteria (122	Proteobacteria (2)	Bacteria (1)	100.00	0.00	99.30	98.30	WGS
287.876	0.3738	Pseudomonas aeruginosa (286)	Pseudomonas (135621)	Pseudomonadaceae (7227	Pseudomonadales (1236)	Gammaproteobacteria (122	Proteobacteria (2)	Bacteria (1)	98.40	1.50	99.10	97.90	WGS
287.877	0.3748	Pseudomonas aeruginosa (286)	Pseudomonas (135621)	Pseudomonadaceae (7227	Pseudomonadales (1236)	Gammaproteobacteria (122	Proteobacteria (2)	Bacteria (1)	99.40	0.00	99.70	98.90	WGS
287.878	0.3334	Pseudomonas aeruginosa (286)	Pseudomonas (135621)	Pseudomonadaceae (7227	Pseudomonadales (1236)	Gammaproteobacteria (122	Proteobacteria (2)	Bacteria (1)	99.70	0.00	99.50	99.00	WGS
287.879	0.3302	Pseudomonas aeruginosa (286)	Pseudomonas (135621)	Pseudomonadaceae (7227	Pseudomonadales (1236)	Gammaproteobacteria (122	Proteobacteria (2)	Bacteria (1)	99.70	0.30	99.40	98.70	WGS
287.880	0.356	Pseudomonas aeruginosa (286)	Pseudomonas (135621)	Pseudomonadaceae (7227	Pseudomonadales (1236)	Gammaproteobacteria (122	Proteobacteria (2)	Bacteria (1)	100.00	0.00	99.60	99.30	WGS
287.881	0.3386	Pseudomonas aeruginosa (286)	Pseudomonas (135621)	Pseudomonadaceae (7227	Pseudomonadales (1236)	Gammaproteobacteria (122	Proteobacteria (2)	Bacteria (1)	100.00	0.00	99.60	98.60	WGS
287.882	0.356	Pseudomonas aeruginosa (286)	Pseudomonas (135621)	Pseudomonadaceae (7227	Pseudomonadales (1236)	Gammaproteobacteria (122	Proteobacteria (2)	Bacteria (1)	99.40	0.00	99.30	98.60	WGS
287.883	0.3694	Pseudomonas aeruginosa (286)	Pseudomonas (135621)	Pseudomonadaceae (7227	Pseudomonadales (1236)	Gammaproteobacteria (122	Proteobacteria (2)	Bacteria (1)	100.00	0.00	99.70	99.00	WGS
287.884	0.3376	Pseudomonas aeruginosa (286)	Pseudomonas (135621)	Pseudomonadaceae (7227	Pseudomonadales (1236)	Gammaproteobacteria (122	Proteobacteria (2)	Bacteria (1)	100.00	0.00	99.90	99.00	WGS
287.885	0.368	Pseudomonas aeruginosa (286)	Pseudomonas (135621)	Pseudomonadaceae (7227	Pseudomonadales (1236)	Gammaproteobacteria (122	Proteobacteria (2)	Bacteria (1)	99.70	0.00	99.70	99.20	WGS
287.886	0.332	Pseudomonas aeruginosa (286)	Pseudomonas (135621)	Pseudomonadaceae (7227	Pseudomonadales (1236)	Gammaproteobacteria (122	Proteobacteria (2)	Bacteria (1)	100.00	0.00	99.80	99.50	WGS
287.887	0.331	Pseudomonas aeruginosa (286)	Pseudomonas (135621)	Pseudomonadaceae (7227	Pseudomonadales (1236)	Gammaproteobacteria (122	Proteobacteria (2)	Bacteria (1)	100.00	0.00	99.50	99.00	WGS
287.8875	0.331	Pseudomonas aeruginosa (286)	Pseudomonas (135621)	Pseudomonadaceae (7227	Pseudomonadales (1236)	Gammaproteobacteria (122	Proteobacteria (2)	Bacteria (1)			100.00	99.50	WGS
287.8876	0.3322	Pseudomonas aeruginosa (286)	Pseudomonas (135621)	Pseudomonadaceae (7227	Pseudomonadales (1236)	Gammaproteobacteria (122	Proteobacteria (2)	Bacteria (1)			98.70	86.50	WGS
287.8877	0.3028	Pseudomonas aeruginosa (286)	Pseudomonas (135621)	Pseudomonadaceae (7227	Pseudomonadales (1236)	Gammaproteobacteria (122	Proteobacteria (2)	Bacteria (1)			98.50	83.40	WGS
287.8878	0.2978	Pseudomonas aeruginosa (286)	Pseudomonas (135621)	Pseudomonadaceae (7227	Pseudomonadales (1236)	Gammaproteobacteria (122	Proteobacteria (2)	Bacteria (1)			98.10	85.50	WGS
287.888	0.331	Pseudomonas aeruginosa (286)	Pseudomonas (135621)	Pseudomonadaceae (7227	Pseudomonadales (1236)	Gammaproteobacteria (122	Proteobacteria (2)	Bacteria (1)	100.00	0.00	99.60	98.50	WGS
287.8880	0.328	Pseudomonas aeruginosa (286)	Pseudomonas (135621)	Pseudomonadaceae (7227	Pseudomonadales (1236)	Gammaproteobacteria (122	Proteobacteria (2)	Bacteria (1)			99.60	92.80	WGS
287.8881	0.324	Pseudomonas aeruginosa (286)	Pseudomonas (135621)	Pseudomonadaceae (7227	Pseudomonadales (1236)	Gammaproteobacteria (122	Proteobacteria (2)	Bacteria (1)			99.30	93.90	WGS
287.8882	0.3604	Pseudomonas aeruginosa (286)	Pseudomonas (135621)	Pseudomonadaceae (7227	Pseudomonadales (1236)	Gammaproteobacteria (122	Proteobacteria (2)	Bacteria (1)			99.40	85.90	WGS
287.8883	0.3434	Pseudomonas aeruginosa (286)	Pseudomonas (135621)	Pseudomonadaceae (7227	Pseudomonadales (1236)	Gammaproteobacteria (122	Proteobacteria (2)	Bacteria (1)			99.30	72.10	WGS
287.8884	0.3606	Pseudomonas aeruginosa (286)	Pseudomonas (135621)	Pseudomonadaceae (7227	Pseudomonadales (1236)	Gammaproteobacteria (122	Proteobacteria (2)	Bacteria (1)			98.80	95.90	WGS
287.8885	0.3174	Pseudomonas aeruginosa (286)	Pseudomonas (135621)	Pseudomonadaceae (7227	Pseudomonadales (1236)	Gammaproteobacteria (122	Proteobacteria (2)	Bacteria (1)			99.80	98.70	WGS
287.8886	0.3738	Pseudomonas aeruginosa (286)	Pseudomonas (135621)	Pseudomonadaceae (7227	Pseudomonadales (1236)	Gammaproteobacteria (122	Proteobacteria (2)	Bacteria (1)			99.70	98.70	WGS
287.8887	0.324	Pseudomonas aeruginosa (286)	Pseudomonas (135621)	Pseudomonadaceae (7227	Pseudomonadales (1236)	Gammaproteobacteria (122	Proteobacteria (2)	Bacteria (1)			99.80	99.20	WGS
287.8888	0.3324	Pseudomonas aeruginosa (286)	Pseudomonas (135621)	Pseudomonadaceae (7227	Pseudomonadales (1236)	Gammaproteobacteria (122	Proteobacteria (2)	Bacteria (1)			99.70	98.80	WGS
287.8889	0.3184	Pseudomonas aeruginosa (286)	Pseudomonas (135621)	Pseudomonadaceae (7227	Pseudomonadales (1236)	Gammaproteobacteria (122	Proteobacteria (2)	Bacteria (1)			99.90	99.20	WGS
287.889	0.3632	Pseudomonas aeruginosa (286)	Pseudomonas (135621)	Pseudomonadaceae (7227	Pseudomonadales (1236)	Gammaproteobacteria (122	Proteobacteria (2)	Bacteria (1)	99.70	0.00	99.60	98.90	WGS
287.8890	0.324	Pseudomonas aeruginosa (286)	Pseudomonas (135621)	Pseudomonadaceae (7227	Pseudomonadales (1236)	Gammaproteobacteria (122	Proteobacteria (2)	Bacteria (1)			99.80	99.20	WGS
287.8891	0.3662	Pseudomonas aeruginosa (286)	Pseudomonas (135621)	Pseudomonadaceae (7227	Pseudomonadales (1236)	Gammaproteobacteria (122	Proteobacteria (2)	Bacteria (1)			99.70	98.70	WGS
287.8892	0.3226	Pseudomonas aeruginosa (286)	Pseudomonas (135621)	Pseudomonadaceae (7227	Pseudomonadales (1236)	Gammaproteobacteria (122	Proteobacteria (2)	Bacteria (1)			99.90	99.20	WGS
287.8893	0.2856	Pseudomonas aeruginosa (286)	Pseudomonas (135621)	Pseudomonadaceae (7227	Pseudomonadales (1236)	Gammaproteobacteria (122	Proteobacteria (2)	Bacteria (1)			97.60	95.90	WGS
287.8894	0.3188	Pseudomonas aeruginosa (286)	Pseudomonas (135621)	Pseudomonadaceae (7227	Pseudomonadales (1236)	Gammaproteobacteria (122	Proteobacteria (2)	Bacteria (1)			99.80	99.20	WGS
287.8895	0.3258	Pseudomonas aeruginosa (286)	Pseudomonas (135621)	Pseudomonadaceae (7227	Pseudomonadales (1236)	Gammaproteobacteria (122	Proteobacteria (2)	Bacteria (1)			99.80	99.00	WGS
287.8896	0.3636	Pseudomonas aeruginosa (286)	Pseudomonas (135621)	Pseudomonadaceae (7227	Pseudomonadales (1236)	Gammaproteobacteria (122	Proteobacteria (2)	Bacteria (1)			99.90	99.30	WGS
287.8897	0.3376	Pseudomonas aeruginosa (286)	Pseudomonas (135621)	Pseudomonadaceae (7227	Pseudomonadales (1236)	Gammaproteobacteria (122	Proteobacteria (2)	Bacteria (1)			99.70	98.90	WGS
287.8898	0.324	Pseudomonas aeruginosa (286)	Pseudomonas (135621)	Pseudomonadaceae (7227	Pseudomonadales (1236)	Gammaproteobacteria (122	Proteobacteria (2)	Bacteria (1)			100.00	99.40	Complete
287.8899	0.3306	Pseudomonas aeruginosa (286)	Pseudomonas (135621)	Pseudomonadaceae (7227	Pseudomonadales (1236)	Gammaproteobacteria (122	Proteobacteria (2)	Bacteria (1)			99.70	98.70	WGS
287.8900	0.3306	Pseudomonas aeruginosa (286)	Pseudomonas (135621)	Pseudomonadaceae (7227	Pseudomonadales (1236)	Gammaproteobacteria (122	Proteobacteria (2)	Bacteria (1)			99.70	98.60	WGS
287.890	0.3188	Pseudomonas aeruginosa (286)	Pseudomonas (135621)	Pseudomonadaceae (7227	Pseudomonadales (1236)	Gammaproteobacteria (122	Proteobacteria (2)	Bacteria (1)	99.70	0.00	99.10	98.70	WGS
287.8901	0.3306	Pseudomonas aeruginosa (286)	Pseudomonas (135621)	Pseudomonadaceae (7227	Pseudomonadales (1236)	Gammaproteobacteria (122	Proteobacteria (2)	Bacteria (1)			99.70	98.50	WGS
287.8902	0.356	Pseudomonas aeruginosa (286)	Pseudomonas (135621)	Pseudomonadaceae (7227	Pseudomonadales (1236)	Gammaproteobacteria (122	Proteobacteria (2)	Bacteria (1)			99.70	98.60	WGS
287.8903	0.356	Pseudomonas aeruginosa (286)	Pseudomonas (135621)	Pseudomonadaceae (7227	Pseudomonadales (1236)	Gammaproteobacteria (122	Proteobacteria (2)	Bacteria (1)			99.50	98.20	WGS
287.8904	0.356	Pseudomonas aeruginosa (286)	Pseudomonas (135621)	Pseudomonadaceae (7227	Pseudomonadales (1236)	Gammaproteobacteria (122	Proteobacteria (2)	Bacteria (1)			99.80	98.90	WGS
287.8905	0.356	Pseudomonas aeruginosa (286)	Pseudomonas (135621)	Pseudomonadaceae (7227	Pseudomonadales (1236)	Gammaproteobacteria (122	Proteobacteria (2)	Bacteria (1)			99.70	98.30	WGS
287.8906	0.356	Pseudomonas aeruginosa (286)	Pseudomonas (135621)	Pseudomonadaceae (7227	Pseudomonadales (1236)	Gammaproteobacteria (122	Proteobacteria (2)	Bacteria (1)			99.80	98.50	WGS
287.8907	0.356	Pseudomonas aeruginosa (286)	Pseudomonas (135621)	Pseudomonadaceae (7227	Pseudomonadales (1236)	Gammaproteobacteria (122	Proteobacteria (2)	Bacteria (1)			99.80	98.20	WGS
287.8908	0.356	Pseudomonas aeruginosa (286)	Pseudomonas (135621)	Pseudomonadaceae (7227	Pseudomonadales (1236)	Gammaproteobacteria (122	Proteobacteria (2)	Bacteria (1)			99.60	98.20	WGS
287.8909	0.3188	Pseudomonas aeruginosa (286)	Pseudomonas (135621)	Pseudomonadaceae (7227	Pseudomonadales (1236)	Gammaproteobacteria (122	Proteobacteria (2)	Bacteria (1)			100.00	99.30	WGS
287.891	0.3604	Pseudomonas aeruginosa (286)	Pseudomonas (135621)	Pseudomonadaceae (7227	Pseudomonadales (1236)	Gammaproteobacteria (122	Proteobacteria (2)	Bacteria (1)	99.70	0.00	99.70	99.30	WGS
287.8910	0.3306	Pseudomonas aeruginosa (286)	Pseudomonas (135621)	Pseudomonadaceae (7227	Pseudomonadales (1236)	Gammaproteobacteria (122	Proteobacteria (2)	Bacteria (1)			99.60	98.20	WGS
287.8911	0.3306	Pseudomonas aeruginosa (286)	Pseudomonas (135621)	Pseudomonadaceae (7227	Pseudomonadales (1236)	Gammaproteobacteria (122	Proteobacteria (2)	Bacteria (1)			99.70	97.80	WGS
287.8912	0.356	Pseudomonas aeruginosa (286)	Pseudomonas (135621)	Pseudomonadaceae (7227	Pseudomonadales (1236)	Gammaproteobacteria (122	Proteobacteria (2)	Bacteria (1)			99.60	98.00	WGS
287.8913	0.356	Pseudomonas aeruginosa (286)	Pseudomonas (135621)	Pseudomonadaceae (7227	Pseudomonadales (1236)	Gammaproteobacteria (122	Proteobacteria (2)	Bacteria (1)			99.60	98.50	WGS
287.8914	0.356	Pseudomonas aeruginosa (286)	Pseudomonas (135621)	Pseudomonadaceae (7227	Pseudomonadales (1236)	Gammaproteobacteria (122	Proteobacteria (2)	Bacteria (1)			99.80	98.40	WGS
287.8915	0.3306	Pseudomonas aeruginosa (286)	Pseudomonas (135621)	Pseudomonadaceae (7227	Pseudomonadales (1236)	Gammaproteobacteria (122	Proteobacteria (2)	Bacteria (1)			99.80	98.70	WGS
287.8916	0.3306	Pseudomonas aeruginosa (286)	Pseudomonas (135621)	Pseudomonadaceae (7227	Pseudomonadales (1236)	Gammaproteobacteria (122	Proteobacteria (2)	Bacteria (1)			99.80	98.90	WGS
287.8917	0.3306	Pseudomonas aeruginosa (286)	Pseudomonas (135621)	Pseudomonadaceae (7227	Pseudomonadales (1236)	Gammaproteobacteria (122	Proteobacteria (2)	Bacteria (1)			99.80	98.50	WGS
287.8918	0.3306	Pseudomonas aeruginosa (286)	Pseudomonas (135621)	Pseudomonadaceae (7227	Pseudomonadales (1236)	Gammaproteobacteria (122	Proteobacteria (2)	Bacteria (1)			99.80	98.80	WGS
287.8919	0.3198	Pseudomonas aeruginosa (286)	Pseudomonas (135621)	Pseudomonadaceae (7227	Pseudomonadales (1236)	Gammaproteobacteria (122	Proteobacteria (2)	Bacteria (1)			99.90	98.70	Complete
287.892	0.3258	Pseudomonas aeruginosa (286)	Pseudomonas (135621)	Pseudomonadaceae (7227	Pseudomonadales (1236)	Gammaproteobacteria (122	Proteobacteria (2)	Bacteria (1)	100.00	0.00	99.60	99.30	WGS
287.8920	0.3736	Pseudomonas aeruginosa (286)	Pseudomonas (135621)	Pseudomonadaceae (7227	Pseudomonadales (1236)	Gammaproteobacteria (122	Proteobacteria (2)	Bacteria (1)			99.90	98.20	WGS
287.8921	0.3324	Pseudomonas aeruginosa (286)	Pseudomonas (135621)	Pseudomonadaceae (7227	Pseudomonadales (1236)	Gammaproteobacteria (122	Proteobacteria (2)	Bacteria (1)			99.90	98.50	Complete
287.8922	0.3268	Pseudomonas aeruginosa (286)	Pseudomonas (135621)	Pseudomonadaceae (7227	Pseudomonadales (1236)	Gammaproteobacteria (122	Proteobacteria (2)	Bacteria (1)			100.00	99.30	Complete
287.8923	0.3334	Pseudomonas aeruginosa (286)	Pseudomonas (135621)	Pseudomonadaceae (7227	Pseudomonadales (1236)	Gammaproteobacteria (122	Proteobacteria (2)	Bacteria (1)			99.70	99.20	Complete
287.8924	0.3258	Pseudomonas aeruginosa (286)	Pseudomonas (135621)	Pseudomonadaceae (7227	Pseudomonadales (1236)	Gammaproteobacteria (122	Proteobacteria (2)	Bacteria (1)			99.70	99.00	Complete
287.8925	0.3622	Pseudomonas aeruginosa (286)	Pseudomonas (135621)	Pseudomonadaceae (7227	Pseudomonadales (1236)	Gammaproteobacteria (122	Proteobacteria (2)	Bacteria (1)			99.90	99.20	Complete
287.8926	0.3648	Pseudomonas aeruginosa (286)	Pseudomonas (135621)	Pseudomonadaceae (7227	Pseudomonadales (1236)	Gammaproteobacteria (122	Proteobacteria (2)	Bacteria (1)			99.20	91.30	WGS
287.8927	0.3662	Pseudomonas aeruginosa (286)	Pseudomonas (135621)	Pseudomonadaceae (7227	Pseudomonadales (1236)	Gammaproteobacteria (122	Proteobacteria (2)	Bacteria (1)			99.40	88.20	WGS
287.8928	0.3144	Pseudomonas aeruginosa (286)	Pseudomonas (135621)	Pseudomonadaceae (7227	Pseudomonadales (1236)	Gammaproteobacteria (122	Proteobacteria (2)	Bacteria (1)			99.00	86.30	WGS
287.8929	0.3264	Pseudomonas aeruginosa (286)	Pseudomonas (135621)	Pseudomonadaceae (7227	Pseudomonadales (1236)	Gammaproteobacteria (122	Proteobacteria (2)	Bacteria (1)			99.50	96.00	WGS
287.8930	0.3226	Pseudomonas aeruginosa (286)	Pseudomonas (135621)	Pseudomonadaceae (7227	Pseudomonadales (1236)	Gammaproteobacteria (122	Proteobacteria (2)	Bacteria (1)			99.80	97.40	WGS
287.893	0.3184	Pseudomonas aeruginosa (286)	Pseudomonas (135621)	Pseudomonadaceae (7227	Pseudomonadales (1236)	Gammaproteobacteria (122	Proteobacteria (2)	Bacteria (1)	100.00	0.00	99.90	99.00	WGS
287.8931	0.296	Pseudomonas aeruginosa (286)	Pseudomonas (135621)	Pseudomonadaceae (7227	Pseudomonadales (1236)	Gammaproteobacteria (122	Proteobacteria (2)	Bacteria (1)			99.20	96.40	WGS
287.8932	0.3636	Pseudomonas aeruginosa (286)	Pseudomonas (135621)	Pseudomonadaceae (7227	Pseudomonadales (1236)	Gammaproteobacteria (122	Proteobacteria (2)	Bacteria (1)			99.60	99.10	WGS
287.8933	0.324	Pseudomonas aeruginosa (286)	Pseudomonas (135621)	Pseudomonadaceae (7227	Pseudomonadales (1236)	Gammaproteobacteria (122	Proteobacteria (2)	Bacteria (1)			99.90	98.70	WGS
287.8934	0.325	Pseudomonas aeruginosa (286)	Pseudomonas (135621)	Pseudomonadaceae (7227	Pseudomonadales (1236)	Gammaproteobacteria (122	Proteobacteria (2)	Bacteria (1)			99.70	98.60	WGS
287.8935	0.324	Pseudomonas aeruginosa (286)	Pseudomonas (135621)	Pseudomonadaceae (7227	Pseudomonadales (1236)	Gammaproteobacteria (122	Proteobacteria (2)	Bacteria (1)			99.90	98.60	WGS
287.8936	0.3738	Pseudomonas aeruginosa (286)	Pseudomonas (135621)	Pseudomonadaceae (7227	Pseudomonadales (1236)	Gammaproteobacteria (122	Proteobacteria (2)	Bacteria (1)			99.80	97.60	WGS
287.8937	0.3628	Pseudomonas aeruginosa (286)	Pseudomonas (135621)	Pseudomonadaceae (7227	Pseudomonadales (1236)	Gammaproteobacteria (122	Proteobacteria (2)	Bacteria (1)			99.90	97.80	WGS
287.8938	0.3618	Pseudomonas aeruginosa (286)	Pseudomonas (135621)	Pseudomonadaceae (7227	Pseudomonadales (1236)	Gammaproteobacteria (122	Proteobacteria (2)	Bacteria (1)			99.90	98.70	WGS
287.8939	0.3174	Pseudomonas aeruginosa (286)	Pseudomonas (135621)	Pseudomonadaceae (7227	Pseudomonadales (1236)	Gammaproteobacteria (122	Proteobacteria (2)	Bacteria (1)			99.80	98.20	WGS
287.894	0.3268	Pseudomonas aeruginosa (286)	Pseudomonas (135621)	Pseudomonadaceae (7227	Pseudomonadales (1236)	Gammaproteobacteria (122	Proteobacteria (2)	Bacteria (1)	99.40	0.00	99.40	98.70	WGS
287.8940	0.3628	Pseudomonas aeruginosa (286)	Pseudomonas (135621)	Pseudomonadaceae (7227	Pseudomonadales (1236)	Gammaproteobacteria (122	Proteobacteria (2)	Bacteria (1)			99.80	97.10	WGS
287.8941	0.3324	Pseudomonas aeruginosa (286)	Pseudomonas (135621)	Pseudomonadaceae (7227	Pseudomonadales (1236)	Gammaproteobacteria (122	Proteobacteria (2)	Bacteria (1)			99.60	96.50	WGS
287.8942	0.3376	Pseudomonas aeruginosa (286)	Pseudomonas (135621)	Pseudomonadaceae (7227	Pseudomonadales (1236)	Gammaproteobacteria (122	Proteobacteria (2)	Bacteria (1)			99.90	98.80	WGS
287.8943	0.3738	Pseudomonas aeruginosa (286)	Pseudomonas (135621)	Pseudomonadaceae (7227	Pseudomonadales (1236)	Gammaproteobacteria (122	Proteobacteria (2)	Bacteria (1)			99.80	97.70	WGS
287.8944	0.298	Pseudomonas aeruginosa (286)	Pseudomonas (135621)	Pseudomonadaceae (7227	Pseudomonadales (1236)	Gammaproteobacteria (122	Proteobacteria (2)	Bacteria (1)			99.40	95.40	WGS
287.8945	0.3748	Pseudomonas aeruginosa (286)	Pseudomonas (135621)	Pseudomonadaceae (7227	Pseudomonadales (1236)	Gammaproteobacteria (122	Proteobacteria (2)	Bacteria (1)			99.70	96.60	WGS
287.8946	0.3628	Pseudomonas aeruginosa (286)	Pseudomonas (135621)	Pseudomonadaceae (7227	Pseudomonadales (1236)	Gammaproteobacteria (122	Proteobacteria (2)	Bacteria (1)			99.90	98.70	WGS
287.8947	0.3628	Pseudomonas aeruginosa (286)	Pseudomonas (135621)	Pseudomonadaceae (7227	Pseudomonadales (1236)	Gammaproteobacteria (122	Proteobacteria (2)	Bacteria (1)			99.90	98.80	WGS
287.8948	0.3628	Pseudomonas aeruginosa (286)	Pseudomonas (135621)	Pseudomonadaceae (7227	Pseudomonadales (1236)	Gammaproteobacteria (122	Proteobacteria (2)	Bacteria (1)			99.90	98.60	WGS
287.8949	0.333	Pseudomonas aeruginosa (286)	Pseudomonas (135621)	Pseudomonadaceae (7227	Pseudomonadales (1236)	Gammaproteobacteria (122	Proteobacteria (2)	Bacteria (1)			99.80	98.40	WGS
287.895	0.356	Pseudomonas aeruginosa (286)	Pseudomonas (135621)	Pseudomonadaceae (7227	Pseudomonadales (1236)	Gammaproteobacteria (122	Proteobacteria (2)	Bacteria (1)	100.00	0.00	99.60	99.30	WGS
287.8950	0.363	Pseudomonas aeruginosa (286)	Pseudomonas (135621)	Pseudomonadaceae (7227	Pseudomonadales (1236)	Gammaproteobacteria (122	Proteobacteria (2)	Bacteria (1)			99.90	97.60	WGS
287.8951	0.3324	Pseudomonas aeruginosa (286)	Pseudomonas (135621)	Pseudomonadaceae (7227	Pseudomonadales (1236)	Gammaproteobacteria (122	Proteobacteria (2)	Bacteria (1)			99.90	98.70	WGS
287.8952	0.3236	Pseudomonas aeruginosa (286)	Pseudomonas (135621)	Pseudomonadaceae (7227	Pseudomonadales (1236)	Gammaproteobacteria (122	Proteobacteria (2)	Bacteria (1)			99.50	90.50	WGS
287.8953	0.2976	Pseudomonas aeruginosa (286)	Pseudomonas (135621)	Pseudomonadaceae (7227	Pseudomonadales (1236)	Gammaproteobacteria (122	Proteobacteria (2)	Bacteria (1)			99.00	84.20	WGS
287.8954	0.3184	Pseudomonas aeruginosa (286)	Pseudomonas (135621)	Pseudomonadaceae (7227	Pseudomonadales (1236)	Gammaproteobacteria (122	Proteobacteria (2)	Bacteria (1)			99.50	94.80	WGS
287.8955	0.3174	Pseudomonas aeruginosa (286)	Pseudomonas (135621)	Pseudomonadaceae (7227	Pseudomonadales (1236)	Gammaproteobacteria (122	Proteobacteria (2)	Bacteria (1)			99.60	95.20	WGS
287.8956	0.3654	Pseudomonas aeruginosa (286)	Pseudomonas (135621)	Pseudomonadaceae (7227	Pseudomonadales (1236)	Gammaproteobacteria (122	Proteobacteria (2)	Bacteria (1)			99.60	95.60	WGS
287.8957	0.294	Pseudomonas aeruginosa (286)	Pseudomonas (135621)	Pseudomonadaceae (7227	Pseudomonadales (1236)	Gammaproteobacteria (122	Proteobacteria (2)	Bacteria (1)			96.20	78.60	WGS
287.8958	0.317	Pseudomonas aeruginosa (286)	Pseudomonas (135621)	Pseudomonadaceae (7227	Pseudomonadales (1236)	Gammaproteobacteria (122	Proteobacteria (2)	Bacteria (1)			98.30	82.30	WGS
287.8959	0.37	Pseudomonas aeruginosa (286)	Pseudomonas (135621)	Pseudomonadaceae (7227	Pseudomonadales (1236)	Gammaproteobacteria (122	Proteobacteria (2)	Bacteria (1)			99.70	97.90	WGS
287.896	0.3646	Pseudomonas aeruginosa (286)	Pseudomonas (135621)	Pseudomonadaceae (7227	Pseudomonadales (1236)	Gammaproteobacteria (122	Proteobacteria (2)	Bacteria (1)	99.70	0.30	99.60	99.30	WGS
287.8960	0.3628	Pseudomonas aeruginosa (286)	Pseudomonas (135621)	Pseudomonadaceae (7227	Pseudomonadales (1236)	Gammaproteobacteria (122	Proteobacteria (2)	Bacteria (1)			99.90	98.50	WGS
287.8961	0.325	Pseudomonas aeruginosa (286)	Pseudomonas (135621)	Pseudomonadaceae (7227	Pseudomonadales (1236)	Gammaproteobacteria (122	Proteobacteria (2)	Bacteria (1)			99.70	98.60	WGS
287.8962	0.3258	Pseudomonas aeruginosa (286)	Pseudomonas (135621)	Pseudomonadaceae (7227	Pseudomonadales (1236)	Gammaproteobacteria (122	Proteobacteria (2)	Bacteria (1)			99.90	99.20	WGS
287.8963	0.333	Pseudomonas aeruginosa (286)	Pseudomonas (135621)	Pseudomonadaceae (7227	Pseudomonadales (1236)	Gammaproteobacteria (122	Proteobacteria (2)	Bacteria (1)			99.70	95.80	WGS
287.8964	0.356	Pseudomonas aeruginosa (286)	Pseudomonas (135621)	Pseudomonadaceae (7227	Pseudomonadales (1236)	Gammaproteobacteria (122	Proteobacteria (2)	Bacteria (1)			99.70	99.00	WGS
287.8965	0.368	Pseudomonas aeruginosa (286)	Pseudomonas (135621)	Pseudomonadaceae (7227	Pseudomonadales (1236)	Gammaproteobacteria (122	Proteobacteria (2)	Bacteria (1)			100.00	99.00	WGS
287.8966	0.3662	Pseudomonas aeruginosa (286)	Pseudomonas (135621)	Pseudomonadaceae (7227	Pseudomonadales (1236)	Gammaproteobacteria (122	Proteobacteria (2)	Bacteria (1)			99.80	98.20	WGS
287.8967	0.4318	Pseudomonas aeruginosa (286)	Pseudomonas (135621)	Pseudomonadaceae (7227	Pseudomonadales (1236)	Gammaproteobacteria (122	Proteobacteria (2)	Bacteria (1)			98.80	96.60	WGS
287.8968	0.3376	Pseudomonas aeruginosa (286)	Pseudomonas (135621)	Pseudomonadaceae (7227	Pseudomonadales (1236)	Gammaproteobacteria (122	Proteobacteria (2)	Bacteria (1)			99.90	97.50	WGS
287.8969	0.3306	Pseudomonas aeruginosa (286)	Pseudomonas (135621)	Pseudomonadaceae (7227	Pseudomonadales (1236)	Gammaproteobacteria (122	Proteobacteria (2)	Bacteria (1)			99.80	97.40	WGS
287.8970	0.3662	Pseudomonas aeruginosa (286)	Pseudomonas (135621)	Pseudomonadaceae (7227	Pseudomonadales (1236)	Gammaproteobacteria (122	Proteobacteria (2)	Bacteria (1)			99.80	98.70	WGS
287.897	0.3286	Pseudomonas aeruginosa (286)	Pseudomonas (135621)	Pseudomonadaceae (7227	Pseudomonadales (1236)	Gammaproteobacteria (122	Proteobacteria (2)	Bacteria (1)	99.70	0.30	99.80	99.20	WGS
287.8971	0.3646	Pseudomonas aeruginosa (286)	Pseudomonas (135621)	Pseudomonadaceae (7227	Pseudomonadales (1236)	Gammaproteobacteria (122	Proteobacteria (2)	Bacteria (1)			97.70	82.20	WGS
287.8972	0.3572	Pseudomonas aeruginosa (286)	Pseudomonas (135621)	Pseudomonadaceae (7227	Pseudomonadales (1236)	Gammaproteobacteria (122	Proteobacteria (2)	Bacteria (1)			99.30	93.00	WGS
287.8973	0.3958	Pseudomonas aeruginosa (286)	Pseudomonas (135621)	Pseudomonadaceae (7227	Pseudomonadales (1236)	Gammaproteobacteria (122	Proteobacteria (2)	Bacteria (1)			98.40	81.70	WGS
287.8974	0.2928	Pseudomonas aeruginosa (286)	Pseudomonas (135621)	Pseudomonadaceae (7227	Pseudomonadales (1236)	Gammaproteobacteria (122	Proteobacteria (2)	Bacteria (1)			99.20	88.20	WGS
287.8975	0.2942	Pseudomonas aeruginosa (286)	Pseudomonas (135621)	Pseudomonadaceae (7227	Pseudomonadales (1236)	Gammaproteobacteria (122	Proteobacteria (2)	Bacteria (1)			97.90	81.40	WGS
287.8976	0.3374	Pseudomonas aeruginosa (286)	Pseudomonas (135621)	Pseudomonadaceae (7227	Pseudomonadales (1236)	Gammaproteobacteria (122	Proteobacteria (2)	Bacteria (1)			99.00	87.10	WGS
287.8977	0.3384	Pseudomonas aeruginosa (286)	Pseudomonas (135621)	Pseudomonadaceae (7227	Pseudomonadales (1236)	Gammaproteobacteria (122	Proteobacteria (2)	Bacteria (1)			98.70	80.90	WGS
287.8978	0.3254	Pseudomonas aeruginosa (286)	Pseudomonas (135621)	Pseudomonadaceae (7227	Pseudomonadales (1236)	Gammaproteobacteria (122	Proteobacteria (2)	Bacteria (1)			99.40	91.20	WGS
287.8979	0.308	Pseudomonas aeruginosa (286)	Pseudomonas (135621)	Pseudomonadaceae (7227	Pseudomonadales (1236)	Gammaproteobacteria (122	Proteobacteria (2)	Bacteria (1)			98.40	83.10	WGS
287.898	0.332	Pseudomonas aeruginosa (286)	Pseudomonas (135621)	Pseudomonadaceae (7227	Pseudomonadales (1236)	Gammaproteobacteria (122	Proteobacteria (2)	Bacteria (1)	99.70	0.00	99.30	98.30	WGS
287.8980	0.396	Pseudomonas aeruginosa (286)	Pseudomonas (135621)	Pseudomonadaceae (7227	Pseudomonadales (1236)	Gammaproteobacteria (122	Proteobacteria (2)	Bacteria (1)			97.90	84.90	WGS
287.8981	0.3696	Pseudomonas aeruginosa (286)	Pseudomonas (135621)	Pseudomonadaceae (7227	Pseudomonadales (1236)	Gammaproteobacteria (122	Proteobacteria (2)	Bacteria (1)			98.50	81.40	WGS
287.8982	0.3652	Pseudomonas aeruginosa (286)	Pseudomonas (135621)	Pseudomonadaceae (7227	Pseudomonadales (1236)	Gammaproteobacteria (122	Proteobacteria (2)	Bacteria (1)			99.90	96.50	WGS
287.8983	0.3628	Pseudomonas aeruginosa (286)	Pseudomonas (135621)	Pseudomonadaceae (7227	Pseudomonadales (1236)	Gammaproteobacteria (122	Proteobacteria (2)	Bacteria (1)			99.30	93.10	WGS
287.8984	0.331	Pseudomonas aeruginosa (286)	Pseudomonas (135621)	Pseudomonadaceae (7227	Pseudomonadales (1236)	Gammaproteobacteria (122	Proteobacteria (2)	Bacteria (1)			99.80	95.30	WGS
287.8985	0.3426	Pseudomonas aeruginosa (286)	Pseudomonas (135621)	Pseudomonadaceae (7227	Pseudomonadales (1236)	Gammaproteobacteria (122	Proteobacteria (2)	Bacteria (1)			99.60	91.80	WGS
287.8986	0.3174	Pseudomonas aeruginosa (286)	Pseudomonas (135621)	Pseudomonadaceae (7227	Pseudomonadales (1236)	Gammaproteobacteria (122	Proteobacteria (2)	Bacteria (1)			99.10	86.80	WGS
287.8987	0.3544	Pseudomonas aeruginosa (286)	Pseudomonas (135621)	Pseudomonadaceae (7227	Pseudomonadales (1236)	Gammaproteobacteria (122	Proteobacteria (2)	Bacteria (1)			99.00	83.80	WGS
287.8988	0.3646	Pseudomonas aeruginosa (286)	Pseudomonas (135621)	Pseudomonadaceae (7227	Pseudomonadales (1236)	Gammaproteobacteria (122	Proteobacteria (2)	Bacteria (1)			99.00	90.40	WGS
287.8989	0.3618	Pseudomonas aeruginosa (286)	Pseudomonas (135621)	Pseudomonadaceae (7227	Pseudomonadales (1236)	Gammaproteobacteria (122	Proteobacteria (2)	Bacteria (1)			99.70	89.60	WGS
287.8990	0.384	Pseudomonas aeruginosa (286)	Pseudomonas (135621)	Pseudomonadaceae (7227	Pseudomonadales (1236)	Gammaproteobacteria (122	Proteobacteria (2)	Bacteria (1)			99.60	95.20	WGS
287.899	0.3302	Pseudomonas aeruginosa (286)	Pseudomonas (135621)	Pseudomonadaceae (7227	Pseudomonadales (1236)	Gammaproteobacteria (122	Proteobacteria (2)	Bacteria (1)	100.00	0.60	99.30	98.40	WGS
287.8991	0.3046	Pseudomonas aeruginosa (286)	Pseudomonas (135621)	Pseudomonadaceae (7227	Pseudomonadales (1236)	Gammaproteobacteria (122	Proteobacteria (2)	Bacteria (1)			99.90	91.80	WGS
287.8992	0.3174	Pseudomonas aeruginosa (286)	Pseudomonas (135621)	Pseudomonadaceae (7227	Pseudomonadales (1236)	Gammaproteobacteria (122	Proteobacteria (2)	Bacteria (1)			99.50	92.20	WGS
287.8993	0.3278	Pseudomonas aeruginosa (286)	Pseudomonas (135621)	Pseudomonadaceae (7227	Pseudomonadales (1236)	Gammaproteobacteria (122	Proteobacteria (2)	Bacteria (1)			95.30	73.10	WGS
287.8994	0.3352	Pseudomonas aeruginosa (286)	Pseudomonas (135621)	Pseudomonadaceae (7227	Pseudomonadales (1236)	Gammaproteobacteria (122	Proteobacteria (2)	Bacteria (1)			97.90	80.20	WGS
287.8995	0.3238	Pseudomonas aeruginosa (286)	Pseudomonas (135621)	Pseudomonadaceae (7227	Pseudomonadales (1236)	Gammaproteobacteria (122	Proteobacteria (2)	Bacteria (1)			99.40	87.70	WGS
287.8996	0.3338	Pseudomonas aeruginosa (286)	Pseudomonas (135621)	Pseudomonadaceae (7227	Pseudomonadales (1236)	Gammaproteobacteria (122	Proteobacteria (2)	Bacteria (1)			99.90	92.60	WGS
287.8997	0.3422	Pseudomonas aeruginosa (286)	Pseudomonas (135621)	Pseudomonadaceae (7227	Pseudomonadales (1236)	Gammaproteobacteria (122	Proteobacteria (2)	Bacteria (1)			99.30	84.50	WGS
287.8998	0.3628	Pseudomonas aeruginosa (286)	Pseudomonas (135621)	Pseudomonadaceae (7227	Pseudomonadales (1236)	Gammaproteobacteria (122	Proteobacteria (2)	Bacteria (1)			99.70	97.10	WGS
287.8999	0.336	Pseudomonas aeruginosa (286)	Pseudomonas (135621)	Pseudomonadaceae (7227	Pseudomonadales (1236)	Gammaproteobacteria (122	Proteobacteria (2)	Bacteria (1)			99.30	92.70	WGS
287.9000	0.4168	Pseudomonas aeruginosa (286)	Pseudomonas (135621)	Pseudomonadaceae (7227	Pseudomonadales (1236)	Gammaproteobacteria (122	Proteobacteria (2)	Bacteria (1)			99.50	91.40	WGS
287.900	0.3646	Pseudomonas aeruginosa (286)	Pseudomonas (135621)	Pseudomonadaceae (7227	Pseudomonadales (1236)	Gammaproteobacteria (122	Proteobacteria (2)	Bacteria (1)	100.00	0.00	99.70	99.00	WGS
287.9001	0.3276	Pseudomonas aeruginosa (286)	Pseudomonas (135621)	Pseudomonadaceae (7227	Pseudomonadales (1236)	Gammaproteobacteria (122	Proteobacteria (2)	Bacteria (1)			99.60	93.60	WGS
287.9002	0.3446	Pseudomonas aeruginosa (286)	Pseudomonas (135621)	Pseudomonadaceae (7227	Pseudomonadales (1236)	Gammaproteobacteria (122	Proteobacteria (2)	Bacteria (1)			99.40	85.00	WGS
287.9003	0.3646	Pseudomonas aeruginosa (286)	Pseudomonas (135621)	Pseudomonadaceae (7227	Pseudomonadales (1236)	Gammaproteobacteria (122	Proteobacteria (2)	Bacteria (1)			99.40	88.30	WGS
287.9004	0.3336	Pseudomonas aeruginosa (286)	Pseudomonas (135621)	Pseudomonadaceae (7227	Pseudomonadales (1236)	Gammaproteobacteria (122	Proteobacteria (2)	Bacteria (1)			99.60	92.70	WGS
287.9005	0.313	Pseudomonas aeruginosa (286)	Pseudomonas (135621)	Pseudomonadaceae (7227	Pseudomonadales (1236)	Gammaproteobacteria (122	Proteobacteria (2)	Bacteria (1)			99.30	91.90	WGS
287.9006	0.3226	Pseudomonas aeruginosa (286)	Pseudomonas (135621)	Pseudomonadaceae (7227	Pseudomonadales (1236)	Gammaproteobacteria (122	Proteobacteria (2)	Bacteria (1)			99.50	93.50	WGS
287.9007	0.3526	Pseudomonas aeruginosa (286)	Pseudomonas (135621)	Pseudomonadaceae (7227	Pseudomonadales (1236)	Gammaproteobacteria (122	Proteobacteria (2)	Bacteria (1)			99.30	87.50	WGS
287.9008	0.3174	Pseudomonas aeruginosa (286)	Pseudomonas (135621)	Pseudomonadaceae (7227	Pseudomonadales (1236)	Gammaproteobacteria (122	Proteobacteria (2)	Bacteria (1)			99.40	95.10	WGS
287.9009	0.3574	Pseudomonas aeruginosa (286)	Pseudomonas (135621)	Pseudomonadaceae (7227	Pseudomonadales (1236)	Gammaproteobacteria (122	Proteobacteria (2)	Bacteria (1)			99.40	86.40	WGS
287.901	0.3598	Pseudomonas aeruginosa (286)	Pseudomonas (135621)	Pseudomonadaceae (7227	Pseudomonadales (1236)	Gammaproteobacteria (122	Proteobacteria (2)	Bacteria (1)	99.40	0.30	99.40	98.70	WGS
287.9010	0.3304	Pseudomonas aeruginosa (286)	Pseudomonas (135621)	Pseudomonadaceae (7227	Pseudomonadales (1236)	Gammaproteobacteria (122	Proteobacteria (2)	Bacteria (1)			98.90	85.10	WGS
287.9011	0.3308	Pseudomonas aeruginosa (286)	Pseudomonas (135621)	Pseudomonadaceae (7227	Pseudomonadales (1236)	Gammaproteobacteria (122	Proteobacteria (2)	Bacteria (1)			97.10	76.80	WGS
287.9012	0.3884	Pseudomonas aeruginosa (286)	Pseudomonas (135621)	Pseudomonadaceae (7227	Pseudomonadales (1236)	Gammaproteobacteria (122	Proteobacteria (2)	Bacteria (1)			99.50	94.00	WGS
287.9013	0.3588	Pseudomonas aeruginosa (286)	Pseudomonas (135621)	Pseudomonadaceae (7227	Pseudomonadales (1236)	Gammaproteobacteria (122	Proteobacteria (2)	Bacteria (1)			96.70	75.80	WGS
287.9014	0.364	Pseudomonas aeruginosa (286)	Pseudomonas (135621)	Pseudomonadaceae (7227	Pseudomonadales (1236)	Gammaproteobacteria (122	Proteobacteria (2)	Bacteria (1)			98.70	86.30	WGS
287.9015	0.3494	Pseudomonas aeruginosa (286)	Pseudomonas (135621)	Pseudomonadaceae (7227	Pseudomonadales (1236)	Gammaproteobacteria (122	Proteobacteria (2)	Bacteria (1)			93.50	73.70	WGS
287.9016	0.3626	Pseudomonas aeruginosa (286)	Pseudomonas (135621)	Pseudomonadaceae (7227	Pseudomonadales (1236)	Gammaproteobacteria (122	Proteobacteria (2)	Bacteria (1)			98.80	92.40	WGS
287.9017	0.324	Pseudomonas aeruginosa (286)	Pseudomonas (135621)	Pseudomonadaceae (7227	Pseudomonadales (1236)	Gammaproteobacteria (122	Proteobacteria (2)	Bacteria (1)			99.40	89.60	WGS
287.9018	0.3394	Pseudomonas aeruginosa (286)	Pseudomonas (135621)	Pseudomonadaceae (7227	Pseudomonadales (1236)	Gammaproteobacteria (122	Proteobacteria (2)	Bacteria (1)			99.30	95.00	WGS
287.9019	0.3406	Pseudomonas aeruginosa (286)	Pseudomonas (135621)	Pseudomonadaceae (7227	Pseudomonadales (1236)	Gammaproteobacteria (122	Proteobacteria (2)	Bacteria (1)			99.60	92.20	WGS
287.9020	0.2778	Pseudomonas aeruginosa (286)	Pseudomonas (135621)	Pseudomonadaceae (7227	Pseudomonadales (1236)	Gammaproteobacteria (122	Proteobacteria (2)	Bacteria (1)			99.30	83.80	WGS
287.902	0.3268	Pseudomonas aeruginosa (286)	Pseudomonas (135621)	Pseudomonadaceae (7227	Pseudomonadales (1236)	Gammaproteobacteria (122	Proteobacteria (2)	Bacteria (1)	99.10	0.30	99.50	98.70	WGS
287.9021	0.3226	Pseudomonas aeruginosa (286)	Pseudomonas (135621)	Pseudomonadaceae (7227	Pseudomonadales (1236)	Gammaproteobacteria (122	Proteobacteria (2)	Bacteria (1)			99.30	91.30	WGS
287.9022	0.315	Pseudomonas aeruginosa (286)	Pseudomonas (135621)	Pseudomonadaceae (7227	Pseudomonadales (1236)	Gammaproteobacteria (122	Proteobacteria (2)	Bacteria (1)			99.60	89.90	WGS
287.9023	0.3824	Pseudomonas aeruginosa (286)	Pseudomonas (135621)	Pseudomonadaceae (7227	Pseudomonadales (1236)	Gammaproteobacteria (122	Proteobacteria (2)	Bacteria (1)			99.60	94.00	WGS
287.9024	0.3836	Pseudomonas aeruginosa (286)	Pseudomonas (135621)	Pseudomonadaceae (7227	Pseudomonadales (1236)	Gammaproteobacteria (122	Proteobacteria (2)	Bacteria (1)			99.60	89.40	WGS
287.9025	0.3242	Pseudomonas aeruginosa (286)	Pseudomonas (135621)	Pseudomonadaceae (7227	Pseudomonadales (1236)	Gammaproteobacteria (122	Proteobacteria (2)	Bacteria (1)			99.30	87.70	WGS
287.9026	0.3014	Pseudomonas aeruginosa (286)	Pseudomonas (135621)	Pseudomonadaceae (7227	Pseudomonadales (1236)	Gammaproteobacteria (122	Proteobacteria (2)	Bacteria (1)			99.90	90.20	WGS
287.9027	0.3376	Pseudomonas aeruginosa (286)	Pseudomonas (135621)	Pseudomonadaceae (7227	Pseudomonadales (1236)	Gammaproteobacteria (122	Proteobacteria (2)	Bacteria (1)			99.60	92.70	WGS
287.9028	0.3558	Pseudomonas aeruginosa (286)	Pseudomonas (135621)	Pseudomonadaceae (7227	Pseudomonadales (1236)	Gammaproteobacteria (122	Proteobacteria (2)	Bacteria (1)			99.60	90.90	WGS
287.9029	0.3304	Pseudomonas aeruginosa (286)	Pseudomonas (135621)	Pseudomonadaceae (7227	Pseudomonadales (1236)	Gammaproteobacteria (122	Proteobacteria (2)	Bacteria (1)			99.60	93.30	WGS
287.9030	0.3618	Pseudomonas aeruginosa (286)	Pseudomonas (135621)	Pseudomonadaceae (7227	Pseudomonadales (1236)	Gammaproteobacteria (122	Proteobacteria (2)	Bacteria (1)			99.80	95.60	WGS
287.903	0.3604	Pseudomonas aeruginosa (286)	Pseudomonas (135621)	Pseudomonadaceae (7227	Pseudomonadales (1236)	Gammaproteobacteria (122	Proteobacteria (2)	Bacteria (1)	100.00	0.00	99.50	98.50	WGS
287.9031	0.3716	Pseudomonas aeruginosa (286)	Pseudomonas (135621)	Pseudomonadaceae (7227	Pseudomonadales (1236)	Gammaproteobacteria (122	Proteobacteria (2)	Bacteria (1)			99.80	96.90	WGS
287.9032	0.3206	Pseudomonas aeruginosa (286)	Pseudomonas (135621)	Pseudomonadaceae (7227	Pseudomonadales (1236)	Gammaproteobacteria (122	Proteobacteria (2)	Bacteria (1)			99.00	86.00	WGS
287.9033	0.3258	Pseudomonas aeruginosa (286)	Pseudomonas (135621)	Pseudomonadaceae (7227	Pseudomonadales (1236)	Gammaproteobacteria (122	Proteobacteria (2)	Bacteria (1)			99.70	97.30	WGS
287.9034	0.2348	Pseudomonas aeruginosa (286)	Pseudomonas (135621)	Pseudomonadaceae (7227	Pseudomonadales (1236)	Gammaproteobacteria (122	Proteobacteria (2)	Bacteria (1)			72.40	68.40	WGS
287.9035	0.2854	Pseudomonas aeruginosa (286)	Pseudomonas (135621)	Pseudomonadaceae (7227	Pseudomonadales (1236)	Gammaproteobacteria (122	Proteobacteria (2)	Bacteria (1)			89.70	70.70	WGS
287.9036	0.0958	Pseudomonas aeruginosa (286)	Pseudomonas (135621)	Pseudomonadaceae (7227	Pseudomonadales (1236)	Gammaproteobacteria (122	Proteobacteria (2)	Bacteria (1)			77.10	75.20	WGS
287.9037	0.3048	Pseudomonas aeruginosa (286)	Pseudomonas (135621)	Pseudomonadaceae (7227	Pseudomonadales (1236)	Gammaproteobacteria (122	Proteobacteria (2)	Bacteria (1)			92.30	72.90	WGS
287.9038	0.2992	Pseudomonas aeruginosa (286)	Pseudomonas (135621)	Pseudomonadaceae (7227	Pseudomonadales (1236)	Gammaproteobacteria (122	Proteobacteria (2)	Bacteria (1)			77.10	68.00	WGS
287.9039	0.3216	Pseudomonas aeruginosa (286)	Pseudomonas (135621)	Pseudomonadaceae (7227	Pseudomonadales (1236)	Gammaproteobacteria (122	Proteobacteria (2)	Bacteria (1)			94.00	74.40	WGS
287.9040	0.1858	Pseudomonas aeruginosa (286)	Pseudomonas (135621)	Pseudomonadaceae (7227	Pseudomonadales (1236)	Gammaproteobacteria (122	Proteobacteria (2)	Bacteria (1)			74.90	72.70	WGS
287.904	0.332	Pseudomonas aeruginosa (286)	Pseudomonas (135621)	Pseudomonadaceae (7227	Pseudomonadales (1236)	Gammaproteobacteria (122	Proteobacteria (2)	Bacteria (1)	99.10	0.00	99.60	99.30	WGS
287.9041	0.3166	Pseudomonas aeruginosa (286)	Pseudomonas (135621)	Pseudomonadaceae (7227	Pseudomonadales (1236)	Gammaproteobacteria (122	Proteobacteria (2)	Bacteria (1)			94.40	72.10	WGS
287.9042	0.1974	Pseudomonas aeruginosa (286)	Pseudomonas (135621)	Pseudomonadaceae (7227	Pseudomonadales (1236)	Gammaproteobacteria (122	Proteobacteria (2)	Bacteria (1)			71.10	67.10	WGS
287.9043	0.2424	Pseudomonas aeruginosa (286)	Pseudomonas (135621)	Pseudomonadaceae (7227	Pseudomonadales (1236)	Gammaproteobacteria (122	Proteobacteria (2)	Bacteria (1)			77.30	64.20	WGS
287.9044	0.2358	Pseudomonas aeruginosa (286)	Pseudomonas (135621)	Pseudomonadaceae (7227	Pseudomonadales (1236)	Gammaproteobacteria (122	Proteobacteria (2)	Bacteria (1)			72.20	63.90	WGS
287.9045	0.2738	Pseudomonas aeruginosa (286)	Pseudomonas (135621)	Pseudomonadaceae (7227	Pseudomonadales (1236)	Gammaproteobacteria (122	Proteobacteria (2)	Bacteria (1)			85.40	69.50	WGS
287.9046	0.3198	Pseudomonas aeruginosa (286)	Pseudomonas (135621)	Pseudomonadaceae (7227	Pseudomonadales (1236)	Gammaproteobacteria (122	Proteobacteria (2)	Bacteria (1)			76.90	66.00	WGS
287.9047	0.2338	Pseudomonas aeruginosa (286)	Pseudomonas (135621)	Pseudomonadaceae (7227	Pseudomonadales (1236)	Gammaproteobacteria (122	Proteobacteria (2)	Bacteria (1)			72.70	65.90	WGS
287.9048	0.184	Pseudomonas aeruginosa (286)	Pseudomonas (135621)	Pseudomonadaceae (7227	Pseudomonadales (1236)	Gammaproteobacteria (122	Proteobacteria (2)	Bacteria (1)			72.90	67.70	WGS
287.9049	0.259	Pseudomonas aeruginosa (286)	Pseudomonas (135621)	Pseudomonadaceae (7227	Pseudomonadales (1236)	Gammaproteobacteria (122	Proteobacteria (2)	Bacteria (1)			89.90	71.00	WGS
287.905	0.3174	Pseudomonas aeruginosa (286)	Pseudomonas (135621)	Pseudomonadaceae (7227	Pseudomonadales (1236)	Gammaproteobacteria (122	Proteobacteria (2)	Bacteria (1)	100.00	0.00	99.60	99.40	WGS
287.9050	0.3018	Pseudomonas aeruginosa (286)	Pseudomonas (135621)	Pseudomonadaceae (7227	Pseudomonadales (1236)	Gammaproteobacteria (122	Proteobacteria (2)	Bacteria (1)			93.40	70.80	WGS
287.9051	0.186	Pseudomonas aeruginosa (286)	Pseudomonas (135621)	Pseudomonadaceae (7227	Pseudomonadales (1236)	Gammaproteobacteria (122	Proteobacteria (2)	Bacteria (1)			76.90	76.30	WGS
287.9052	0.31	Pseudomonas aeruginosa (286)	Pseudomonas (135621)	Pseudomonadaceae (7227	Pseudomonadales (1236)	Gammaproteobacteria (122	Proteobacteria (2)	Bacteria (1)			83.30	67.50	WGS
287.9053	0.1446	Pseudomonas aeruginosa (286)	Pseudomonas (135621)	Pseudomonadaceae (7227	Pseudomonadales (1236)	Gammaproteobacteria (122	Proteobacteria (2)	Bacteria (1)			82.30	81.10	WGS
287.9054	0.306	Pseudomonas aeruginosa (286)	Pseudomonas (135621)	Pseudomonadaceae (7227	Pseudomonadales (1236)	Gammaproteobacteria (122	Proteobacteria (2)	Bacteria (1)			87.30	68.00	WGS
287.9055	0.0756	Pseudomonas aeruginosa (286)	Pseudomonas (135621)	Pseudomonadaceae (7227	Pseudomonadales (1236)	Gammaproteobacteria (122	Proteobacteria (2)	Bacteria (1)			82.20	81.90	WGS
287.9056	0.311	Pseudomonas aeruginosa (286)	Pseudomonas (135621)	Pseudomonadaceae (7227	Pseudomonadales (1236)	Gammaproteobacteria (122	Proteobacteria (2)	Bacteria (1)			93.00	72.10	WGS
287.9057	0.2986	Pseudomonas aeruginosa (286)	Pseudomonas (135621)	Pseudomonadaceae (7227	Pseudomonadales (1236)	Gammaproteobacteria (122	Proteobacteria (2)	Bacteria (1)			80.40	67.10	WGS
287.9058	0.313	Pseudomonas aeruginosa (286)	Pseudomonas (135621)	Pseudomonadaceae (7227	Pseudomonadales (1236)	Gammaproteobacteria (122	Proteobacteria (2)	Bacteria (1)			90.80	69.90	WGS
287.9059	0.3046	Pseudomonas aeruginosa (286)	Pseudomonas (135621)	Pseudomonadaceae (7227	Pseudomonadales (1236)	Gammaproteobacteria (122	Proteobacteria (2)	Bacteria (1)			96.20	73.10	WGS
287.9060	0.15	Pseudomonas aeruginosa (286)	Pseudomonas (135621)	Pseudomonadaceae (7227	Pseudomonadales (1236)	Gammaproteobacteria (122	Proteobacteria (2)	Bacteria (1)			78.30	77.50	WGS
287.906	0.3258	Pseudomonas aeruginosa (286)	Pseudomonas (135621)	Pseudomonadaceae (7227	Pseudomonadales (1236)	Gammaproteobacteria (122	Proteobacteria (2)	Bacteria (1)	99.70	0.30	99.90	99.20	WGS
287.9061	0.0712	Pseudomonas aeruginosa (286)	Pseudomonas (135621)	Pseudomonadaceae (7227	Pseudomonadales (1236)	Gammaproteobacteria (122	Proteobacteria (2)	Bacteria (1)			89.90	89.70	WGS
287.9062	0.115	Pseudomonas aeruginosa (286)	Pseudomonas (135621)	Pseudomonadaceae (7227	Pseudomonadales (1236)	Gammaproteobacteria (122	Proteobacteria (2)	Bacteria (1)			79.00	78.40	WGS
287.9063	0.175	Pseudomonas aeruginosa (286)	Pseudomonas (135621)	Pseudomonadaceae (7227	Pseudomonadales (1236)	Gammaproteobacteria (122	Proteobacteria (2)	Bacteria (1)			73.80	69.80	WGS
287.9064	0.3254	Pseudomonas aeruginosa (286)	Pseudomonas (135621)	Pseudomonadaceae (7227	Pseudomonadales (1236)	Gammaproteobacteria (122	Proteobacteria (2)	Bacteria (1)			80.20	67.40	WGS
287.9065	0.369	Pseudomonas aeruginosa (286)	Pseudomonas (135621)	Pseudomonadaceae (7227	Pseudomonadales (1236)	Gammaproteobacteria (122	Proteobacteria (2)	Bacteria (1)			89.20	70.50	WGS
287.9066	0.3168	Pseudomonas aeruginosa (286)	Pseudomonas (135621)	Pseudomonadaceae (7227	Pseudomonadales (1236)	Gammaproteobacteria (122	Proteobacteria (2)	Bacteria (1)			90.20	68.30	WGS
287.9067	0.2534	Pseudomonas aeruginosa (286)	Pseudomonas (135621)	Pseudomonadaceae (7227	Pseudomonadales (1236)	Gammaproteobacteria (122	Proteobacteria (2)	Bacteria (1)			74.10	66.30	WGS
287.9068	0.3206	Pseudomonas aeruginosa (286)	Pseudomonas (135621)	Pseudomonadaceae (7227	Pseudomonadales (1236)	Gammaproteobacteria (122	Proteobacteria (2)	Bacteria (1)			84.90	66.80	WGS
287.9069	0.2576	Pseudomonas aeruginosa (286)	Pseudomonas (135621)	Pseudomonadaceae (7227	Pseudomonadales (1236)	Gammaproteobacteria (122	Proteobacteria (2)	Bacteria (1)			74.50	69.30	WGS
287.907	0.332	Pseudomonas aeruginosa (286)	Pseudomonas (135621)	Pseudomonadaceae (7227	Pseudomonadales (1236)	Gammaproteobacteria (122	Proteobacteria (2)	Bacteria (1)	98.80	0.00	99.30	98.40	WGS
287.9070	0.2938	Pseudomonas aeruginosa (286)	Pseudomonas (135621)	Pseudomonadaceae (7227	Pseudomonadales (1236)	Gammaproteobacteria (122	Proteobacteria (2)	Bacteria (1)			85.70	69.50	WGS
287.9071	0.2786	Pseudomonas aeruginosa (286)	Pseudomonas (135621)	Pseudomonadaceae (7227	Pseudomonadales (1236)	Gammaproteobacteria (122	Proteobacteria (2)	Bacteria (1)			84.80	68.70	WGS
287.9072	0.3254	Pseudomonas aeruginosa (286)	Pseudomonas (135621)	Pseudomonadaceae (7227	Pseudomonadales (1236)	Gammaproteobacteria (122	Proteobacteria (2)	Bacteria (1)			99.90	93.10	WGS
287.9073	0.3324	Pseudomonas aeruginosa (286)	Pseudomonas (135621)	Pseudomonadaceae (7227	Pseudomonadales (1236)	Gammaproteobacteria (122	Proteobacteria (2)	Bacteria (1)			99.90	99.10	Complete
287.9074	0.3324	Pseudomonas aeruginosa (286)	Pseudomonas (135621)	Pseudomonadaceae (7227	Pseudomonadales (1236)	Gammaproteobacteria (122	Proteobacteria (2)	Bacteria (1)			99.90	98.90	Complete
287.9075	0.3334	Pseudomonas aeruginosa (286)	Pseudomonas (135621)	Pseudomonadaceae (7227	Pseudomonadales (1236)	Gammaproteobacteria (122	Proteobacteria (2)	Bacteria (1)			99.90	98.70	WGS
287.9076	0.3334	Pseudomonas aeruginosa (286)	Pseudomonas (135621)	Pseudomonadaceae (7227	Pseudomonadales (1236)	Gammaproteobacteria (122	Proteobacteria (2)	Bacteria (1)			99.90	98.70	WGS
287.9077	0.3636	Pseudomonas aeruginosa (286)	Pseudomonas (135621)	Pseudomonadaceae (7227	Pseudomonadales (1236)	Gammaproteobacteria (122	Proteobacteria (2)	Bacteria (1)			99.60	98.70	WGS
287.9078	0.3324	Pseudomonas aeruginosa (286)	Pseudomonas (135621)	Pseudomonadaceae (7227	Pseudomonadales (1236)	Gammaproteobacteria (122	Proteobacteria (2)	Bacteria (1)			99.90	99.10	WGS
287.9079	0.3636	Pseudomonas aeruginosa (286)	Pseudomonas (135621)	Pseudomonadaceae (7227	Pseudomonadales (1236)	Gammaproteobacteria (122	Proteobacteria (2)	Bacteria (1)			99.90	99.50	WGS
287.908	0.3372	Pseudomonas aeruginosa (286)	Pseudomonas (135621)	Pseudomonadaceae (7227	Pseudomonadales (1236)	Gammaproteobacteria (122	Proteobacteria (2)	Bacteria (1)	99.40	0.00	99.50	98.70	WGS
287.9080	0.3748	Pseudomonas aeruginosa (286)	Pseudomonas (135621)	Pseudomonadaceae (7227	Pseudomonadales (1236)	Gammaproteobacteria (122	Proteobacteria (2)	Bacteria (1)			99.90	97.30	WGS
287.9081	0.3322	Pseudomonas aeruginosa (286)	Pseudomonas (135621)	Pseudomonadaceae (7227	Pseudomonadales (1236)	Gammaproteobacteria (122	Proteobacteria (2)	Bacteria (1)			99.80	98.10	WGS
287.9082	0.3636	Pseudomonas aeruginosa (286)	Pseudomonas (135621)	Pseudomonadaceae (7227	Pseudomonadales (1236)	Gammaproteobacteria (122	Proteobacteria (2)	Bacteria (1)			99.60	98.20	WGS
287.9083	0.3322	Pseudomonas aeruginosa (286)	Pseudomonas (135621)	Pseudomonadaceae (7227	Pseudomonadales (1236)	Gammaproteobacteria (122	Proteobacteria (2)	Bacteria (1)			99.70	98.50	WGS
287.9084	0.3268	Pseudomonas aeruginosa (286)	Pseudomonas (135621)	Pseudomonadaceae (7227	Pseudomonadales (1236)	Gammaproteobacteria (122	Proteobacteria (2)	Bacteria (1)			99.70	98.70	WGS
287.9085	0.3268	Pseudomonas aeruginosa (286)	Pseudomonas (135621)	Pseudomonadaceae (7227	Pseudomonadales (1236)	Gammaproteobacteria (122	Proteobacteria (2)	Bacteria (1)			99.80	99.00	WGS
287.9086	0.3636	Pseudomonas aeruginosa (286)	Pseudomonas (135621)	Pseudomonadaceae (7227	Pseudomonadales (1236)	Gammaproteobacteria (122	Proteobacteria (2)	Bacteria (1)			99.60	98.00	WGS
287.9087	0.3662	Pseudomonas aeruginosa (286)	Pseudomonas (135621)	Pseudomonadaceae (7227	Pseudomonadales (1236)	Gammaproteobacteria (122	Proteobacteria (2)	Bacteria (1)			99.80	99.20	WGS
287.9088	0.37	Pseudomonas aeruginosa (286)	Pseudomonas (135621)	Pseudomonadaceae (7227	Pseudomonadales (1236)	Gammaproteobacteria (122	Proteobacteria (2)	Bacteria (1)			99.90	99.00	WGS
287.9089	0.3662	Pseudomonas aeruginosa (286)	Pseudomonas (135621)	Pseudomonadaceae (7227	Pseudomonadales (1236)	Gammaproteobacteria (122	Proteobacteria (2)	Bacteria (1)			99.80	99.30	WGS
287.9090	0.3278	Pseudomonas aeruginosa (286)	Pseudomonas (135621)	Pseudomonadaceae (7227	Pseudomonadales (1236)	Gammaproteobacteria (122	Proteobacteria (2)	Bacteria (1)			99.90	97.90	WGS
287.909	0.324	Pseudomonas aeruginosa (286)	Pseudomonas (135621)	Pseudomonadaceae (7227	Pseudomonadales (1236)	Gammaproteobacteria (122	Proteobacteria (2)	Bacteria (1)	100.00	0.00	99.60	98.90	WGS
287.9091	0.3694	Pseudomonas aeruginosa (286)	Pseudomonas (135621)	Pseudomonadaceae (7227	Pseudomonadales (1236)	Gammaproteobacteria (122	Proteobacteria (2)	Bacteria (1)			99.90	99.10	WGS
287.9092	0.3398	Pseudomonas aeruginosa (286)	Pseudomonas (135621)	Pseudomonadaceae (7227	Pseudomonadales (1236)	Gammaproteobacteria (122	Proteobacteria (2)	Bacteria (1)			99.80	98.70	WGS
287.9093	0.368	Pseudomonas aeruginosa (286)	Pseudomonas (135621)	Pseudomonadaceae (7227	Pseudomonadales (1236)	Gammaproteobacteria (122	Proteobacteria (2)	Bacteria (1)			99.80	98.40	WGS
287.9094	0.3634	Pseudomonas aeruginosa (286)	Pseudomonas (135621)	Pseudomonadaceae (7227	Pseudomonadales (1236)	Gammaproteobacteria (122	Proteobacteria (2)	Bacteria (1)			99.90	98.70	WGS
287.9095	0.3694	Pseudomonas aeruginosa (286)	Pseudomonas (135621)	Pseudomonadaceae (7227	Pseudomonadales (1236)	Gammaproteobacteria (122	Proteobacteria (2)	Bacteria (1)			99.80	98.00	WGS
287.9096	0.331	Pseudomonas aeruginosa (286)	Pseudomonas (135621)	Pseudomonadaceae (7227	Pseudomonadales (1236)	Gammaproteobacteria (122	Proteobacteria (2)	Bacteria (1)			99.80	98.70	WGS
287.9097	0.356	Pseudomonas aeruginosa (286)	Pseudomonas (135621)	Pseudomonadaceae (7227	Pseudomonadales (1236)	Gammaproteobacteria (122	Proteobacteria (2)	Bacteria (1)			99.80	99.10	WGS
287.9098	0.3258	Pseudomonas aeruginosa (286)	Pseudomonas (135621)	Pseudomonadaceae (7227	Pseudomonadales (1236)	Gammaproteobacteria (122	Proteobacteria (2)	Bacteria (1)			99.90	99.00	WGS
287.9099	0.3258	Pseudomonas aeruginosa (286)	Pseudomonas (135621)	Pseudomonadaceae (7227	Pseudomonadales (1236)	Gammaproteobacteria (122	Proteobacteria (2)	Bacteria (1)			99.90	99.00	WGS
287.910	0.3724	Pseudomonas aeruginosa (286)	Pseudomonas (135621)	Pseudomonadaceae (7227	Pseudomonadales (1236)	Gammaproteobacteria (122	Proteobacteria (2)	Bacteria (1)	99.10	0.00	99.70	99.00	WGS
287.9100	0.3662	Pseudomonas aeruginosa (286)	Pseudomonas (135621)	Pseudomonadaceae (7227	Pseudomonadales (1236)	Gammaproteobacteria (122	Proteobacteria (2)	Bacteria (1)			99.70	98.40	WGS
287.9101	0.3662	Pseudomonas aeruginosa (286)	Pseudomonas (135621)	Pseudomonadaceae (7227	Pseudomonadales (1236)	Gammaproteobacteria (122	Proteobacteria (2)	Bacteria (1)			99.80	98.20	WGS
287.9102	0.3662	Pseudomonas aeruginosa (286)	Pseudomonas (135621)	Pseudomonadaceae (7227	Pseudomonadales (1236)	Gammaproteobacteria (122	Proteobacteria (2)	Bacteria (1)			99.80	98.20	WGS
287.9103	0.3376	Pseudomonas aeruginosa (286)	Pseudomonas (135621)	Pseudomonadaceae (7227	Pseudomonadales (1236)	Gammaproteobacteria (122	Proteobacteria (2)	Bacteria (1)			99.50	98.30	WGS
287.9104	0.3636	Pseudomonas aeruginosa (286)	Pseudomonas (135621)	Pseudomonadaceae (7227	Pseudomonadales (1236)	Gammaproteobacteria (122	Proteobacteria (2)	Bacteria (1)			99.90	99.00	WGS
287.9105	0.331	Pseudomonas aeruginosa (286)	Pseudomonas (135621)	Pseudomonadaceae (7227	Pseudomonadales (1236)	Gammaproteobacteria (122	Proteobacteria (2)	Bacteria (1)			99.50	98.80	WGS
287.9106	0.371	Pseudomonas aeruginosa (286)	Pseudomonas (135621)	Pseudomonadaceae (7227	Pseudomonadales (1236)	Gammaproteobacteria (122	Proteobacteria (2)	Bacteria (1)			99.90	98.90	WGS
287.9107	0.3372	Pseudomonas aeruginosa (286)	Pseudomonas (135621)	Pseudomonadaceae (7227	Pseudomonadales (1236)	Gammaproteobacteria (122	Proteobacteria (2)	Bacteria (1)			100.00	98.90	WGS
287.9108	0.371	Pseudomonas aeruginosa (286)	Pseudomonas (135621)	Pseudomonadaceae (7227	Pseudomonadales (1236)	Gammaproteobacteria (122	Proteobacteria (2)	Bacteria (1)			99.90	99.00	WGS
287.9109	0.3738	Pseudomonas aeruginosa (286)	Pseudomonas (135621)	Pseudomonadaceae (7227	Pseudomonadales (1236)	Gammaproteobacteria (122	Proteobacteria (2)	Bacteria (1)			99.80	98.40	WGS
287.911	0.324	Pseudomonas aeruginosa (286)	Pseudomonas (135621)	Pseudomonadaceae (7227	Pseudomonadales (1236)	Gammaproteobacteria (122	Proteobacteria (2)	Bacteria (1)	100.00	0.00	99.40	98.40	WGS
287.9110	0.3704	Pseudomonas aeruginosa (286)	Pseudomonas (135621)	Pseudomonadaceae (7227	Pseudomonadales (1236)	Gammaproteobacteria (122	Proteobacteria (2)	Bacteria (1)			99.90	98.70	WGS
287.9111	0.3268	Pseudomonas aeruginosa (286)	Pseudomonas (135621)	Pseudomonadaceae (7227	Pseudomonadales (1236)	Gammaproteobacteria (122	Proteobacteria (2)	Bacteria (1)			99.90	99.30	WGS
287.9112	0.3824	Pseudomonas aeruginosa (286)	Pseudomonas (135621)	Pseudomonadaceae (7227	Pseudomonadales (1236)	Gammaproteobacteria (122	Proteobacteria (2)	Bacteria (1)			99.90	97.60	WGS
287.9113	0.368	Pseudomonas aeruginosa (286)	Pseudomonas (135621)	Pseudomonadaceae (7227	Pseudomonadales (1236)	Gammaproteobacteria (122	Proteobacteria (2)	Bacteria (1)			99.80	98.40	WGS
287.9114	0.3738	Pseudomonas aeruginosa (286)	Pseudomonas (135621)	Pseudomonadaceae (7227	Pseudomonadales (1236)	Gammaproteobacteria (122	Proteobacteria (2)	Bacteria (1)			99.80	98.70	WGS
287.9115	0.3386	Pseudomonas aeruginosa (286)	Pseudomonas (135621)	Pseudomonadaceae (7227	Pseudomonadales (1236)	Gammaproteobacteria (122	Proteobacteria (2)	Bacteria (1)			99.90	98.30	WGS
287.9116	0.3738	Pseudomonas aeruginosa (286)	Pseudomonas (135621)	Pseudomonadaceae (7227	Pseudomonadales (1236)	Gammaproteobacteria (122	Proteobacteria (2)	Bacteria (1)			99.80	98.40	WGS
287.9117	0.331	Pseudomonas aeruginosa (286)	Pseudomonas (135621)	Pseudomonadaceae (7227	Pseudomonadales (1236)	Gammaproteobacteria (122	Proteobacteria (2)	Bacteria (1)			99.90	99.80	WGS
287.9118	0.3386	Pseudomonas aeruginosa (286)	Pseudomonas (135621)	Pseudomonadaceae (7227	Pseudomonadales (1236)	Gammaproteobacteria (122	Proteobacteria (2)	Bacteria (1)			99.90	98.40	WGS
287.9119	0.3322	Pseudomonas aeruginosa (286)	Pseudomonas (135621)	Pseudomonadaceae (7227	Pseudomonadales (1236)	Gammaproteobacteria (122	Proteobacteria (2)	Bacteria (1)			99.80	98.10	WGS
287.912	0.3628	Pseudomonas aeruginosa (286)	Pseudomonas (135621)	Pseudomonadaceae (7227	Pseudomonadales (1236)	Gammaproteobacteria (122	Proteobacteria (2)	Bacteria (1)	99.10	0.60	99.20	98.40	WGS
287.9120	0.3324	Pseudomonas aeruginosa (286)	Pseudomonas (135621)	Pseudomonadaceae (7227	Pseudomonadales (1236)	Gammaproteobacteria (122	Proteobacteria (2)	Bacteria (1)			99.80	99.00	WGS
287.9121	0.3324	Pseudomonas aeruginosa (286)	Pseudomonas (135621)	Pseudomonadaceae (7227	Pseudomonadales (1236)	Gammaproteobacteria (122	Proteobacteria (2)	Bacteria (1)			99.80	99.30	WGS
287.9122	0.3324	Pseudomonas aeruginosa (286)	Pseudomonas (135621)	Pseudomonadaceae (7227	Pseudomonadales (1236)	Gammaproteobacteria (122	Proteobacteria (2)	Bacteria (1)			99.80	99.30	WGS
287.9123	0.331	Pseudomonas aeruginosa (286)	Pseudomonas (135621)	Pseudomonadaceae (7227	Pseudomonadales (1236)	Gammaproteobacteria (122	Proteobacteria (2)	Bacteria (1)			99.90	98.70	WGS
287.9124	0.3322	Pseudomonas aeruginosa (286)	Pseudomonas (135621)	Pseudomonadaceae (7227	Pseudomonadales (1236)	Gammaproteobacteria (122	Proteobacteria (2)	Bacteria (1)			99.70	98.60	WGS
287.9125	0.3662	Pseudomonas aeruginosa (286)	Pseudomonas (135621)	Pseudomonadaceae (7227	Pseudomonadales (1236)	Gammaproteobacteria (122	Proteobacteria (2)	Bacteria (1)			99.80	99.30	WGS
287.9126	0.3662	Pseudomonas aeruginosa (286)	Pseudomonas (135621)	Pseudomonadaceae (7227	Pseudomonadales (1236)	Gammaproteobacteria (122	Proteobacteria (2)	Bacteria (1)			99.80	98.30	WGS
287.9127	0.3174	Pseudomonas aeruginosa (286)	Pseudomonas (135621)	Pseudomonadaceae (7227	Pseudomonadales (1236)	Gammaproteobacteria (122	Proteobacteria (2)	Bacteria (1)			99.70	99.00	WGS
287.9128	0.371	Pseudomonas aeruginosa (286)	Pseudomonas (135621)	Pseudomonadaceae (7227	Pseudomonadales (1236)	Gammaproteobacteria (122	Proteobacteria (2)	Bacteria (1)			99.90	98.70	WGS
287.9129	0.3704	Pseudomonas aeruginosa (286)	Pseudomonas (135621)	Pseudomonadaceae (7227	Pseudomonadales (1236)	Gammaproteobacteria (122	Proteobacteria (2)	Bacteria (1)			99.90	98.70	WGS
287.9130	0.368	Pseudomonas aeruginosa (286)	Pseudomonas (135621)	Pseudomonadaceae (7227	Pseudomonadales (1236)	Gammaproteobacteria (122	Proteobacteria (2)	Bacteria (1)			99.90	98.90	WGS
287.913	0.3614	Pseudomonas aeruginosa (286)	Pseudomonas (135621)	Pseudomonadaceae (7227	Pseudomonadales (1236)	Gammaproteobacteria (122	Proteobacteria (2)	Bacteria (1)	99.40	0.00	99.00	98.20	WGS
287.9131	0.3322	Pseudomonas aeruginosa (286)	Pseudomonas (135621)	Pseudomonadaceae (7227	Pseudomonadales (1236)	Gammaproteobacteria (122	Proteobacteria (2)	Bacteria (1)			99.80	98.80	WGS
287.9132	0.3578	Pseudomonas aeruginosa (286)	Pseudomonas (135621)	Pseudomonadaceae (7227	Pseudomonadales (1236)	Gammaproteobacteria (122	Proteobacteria (2)	Bacteria (1)			98.80	97.90	WGS
287.9133	0.331	Pseudomonas aeruginosa (286)	Pseudomonas (135621)	Pseudomonadaceae (7227	Pseudomonadales (1236)	Gammaproteobacteria (122	Proteobacteria (2)	Bacteria (1)			99.80	98.60	WGS
287.9134	0.3662	Pseudomonas aeruginosa (286)	Pseudomonas (135621)	Pseudomonadaceae (7227	Pseudomonadales (1236)	Gammaproteobacteria (122	Proteobacteria (2)	Bacteria (1)			99.90	98.20	WGS
287.9135	0.3376	Pseudomonas aeruginosa (286)	Pseudomonas (135621)	Pseudomonadaceae (7227	Pseudomonadales (1236)	Gammaproteobacteria (122	Proteobacteria (2)	Bacteria (1)			99.90	99.00	WGS
287.9136	0.3376	Pseudomonas aeruginosa (286)	Pseudomonas (135621)	Pseudomonadaceae (7227	Pseudomonadales (1236)	Gammaproteobacteria (122	Proteobacteria (2)	Bacteria (1)			99.90	99.00	WGS
287.9137	0.3636	Pseudomonas aeruginosa (286)	Pseudomonas (135621)	Pseudomonadaceae (7227	Pseudomonadales (1236)	Gammaproteobacteria (122	Proteobacteria (2)	Bacteria (1)			99.90	99.00	WGS
287.9138	0.331	Pseudomonas aeruginosa (286)	Pseudomonas (135621)	Pseudomonadaceae (7227	Pseudomonadales (1236)	Gammaproteobacteria (122	Proteobacteria (2)	Bacteria (1)			99.90	98.70	WGS
287.9139	0.3336	Pseudomonas aeruginosa (286)	Pseudomonas (135621)	Pseudomonadaceae (7227	Pseudomonadales (1236)	Gammaproteobacteria (122	Proteobacteria (2)	Bacteria (1)			99.90	98.40	WGS
287.9140	0.3398	Pseudomonas aeruginosa (286)	Pseudomonas (135621)	Pseudomonadaceae (7227	Pseudomonadales (1236)	Gammaproteobacteria (122	Proteobacteria (2)	Bacteria (1)			99.90	98.80	WGS
287.914	0.3694	Pseudomonas aeruginosa (286)	Pseudomonas (135621)	Pseudomonadaceae (7227	Pseudomonadales (1236)	Gammaproteobacteria (122	Proteobacteria (2)	Bacteria (1)	100.00	0.00	99.60	99.30	WGS
287.9141	0.3824	Pseudomonas aeruginosa (286)	Pseudomonas (135621)	Pseudomonadaceae (7227	Pseudomonadales (1236)	Gammaproteobacteria (122	Proteobacteria (2)	Bacteria (1)			99.90	98.40	WGS
287.9142	0.3824	Pseudomonas aeruginosa (286)	Pseudomonas (135621)	Pseudomonadaceae (7227	Pseudomonadales (1236)	Gammaproteobacteria (122	Proteobacteria (2)	Bacteria (1)			99.90	97.80	WGS
287.9143	0.3614	Pseudomonas aeruginosa (286)	Pseudomonas (135621)	Pseudomonadaceae (7227	Pseudomonadales (1236)	Gammaproteobacteria (122	Proteobacteria (2)	Bacteria (1)			99.80	98.70	WGS
287.9144	0.3614	Pseudomonas aeruginosa (286)	Pseudomonas (135621)	Pseudomonadaceae (7227	Pseudomonadales (1236)	Gammaproteobacteria (122	Proteobacteria (2)	Bacteria (1)			99.80	98.70	WGS
287.9145	0.3738	Pseudomonas aeruginosa (286)	Pseudomonas (135621)	Pseudomonadaceae (7227	Pseudomonadales (1236)	Gammaproteobacteria (122	Proteobacteria (2)	Bacteria (1)			99.80	98.20	WGS
287.9146	0.3386	Pseudomonas aeruginosa (286)	Pseudomonas (135621)	Pseudomonadaceae (7227	Pseudomonadales (1236)	Gammaproteobacteria (122	Proteobacteria (2)	Bacteria (1)			100.00	98.30	WGS
287.9147	0.3258	Pseudomonas aeruginosa (286)	Pseudomonas (135621)	Pseudomonadaceae (7227	Pseudomonadales (1236)	Gammaproteobacteria (122	Proteobacteria (2)	Bacteria (1)			99.70	98.80	WGS
287.9148	0.3292	Pseudomonas aeruginosa (286)	Pseudomonas (135621)	Pseudomonadaceae (7227	Pseudomonadales (1236)	Gammaproteobacteria (122	Proteobacteria (2)	Bacteria (1)			99.40	98.20	WGS
287.9149	0.3376	Pseudomonas aeruginosa (286)	Pseudomonas (135621)	Pseudomonadaceae (7227	Pseudomonadales (1236)	Gammaproteobacteria (122	Proteobacteria (2)	Bacteria (1)			99.80	99.10	WGS
287.9150	0.356	Pseudomonas aeruginosa (286)	Pseudomonas (135621)	Pseudomonadaceae (7227	Pseudomonadales (1236)	Gammaproteobacteria (122	Proteobacteria (2)	Bacteria (1)			99.80	99.10	WGS
287.915	0.3796	Pseudomonas aeruginosa (286)	Pseudomonas (135621)	Pseudomonadaceae (7227	Pseudomonadales (1236)	Gammaproteobacteria (122	Proteobacteria (2)	Bacteria (1)	100.00	0.00	99.50	98.70	WGS
287.9151	0.356	Pseudomonas aeruginosa (286)	Pseudomonas (135621)	Pseudomonadaceae (7227	Pseudomonadales (1236)	Gammaproteobacteria (122	Proteobacteria (2)	Bacteria (1)			99.90	99.30	WGS
287.9152	0.3618	Pseudomonas aeruginosa (286)	Pseudomonas (135621)	Pseudomonadaceae (7227	Pseudomonadales (1236)	Gammaproteobacteria (122	Proteobacteria (2)	Bacteria (1)			99.90	98.20	WGS
287.9153	0.331	Pseudomonas aeruginosa (286)	Pseudomonas (135621)	Pseudomonadaceae (7227	Pseudomonadales (1236)	Gammaproteobacteria (122	Proteobacteria (2)	Bacteria (1)			99.90	99.20	WGS
287.9154	0.3148	Pseudomonas aeruginosa (286)	Pseudomonas (135621)	Pseudomonadaceae (7227	Pseudomonadales (1236)	Gammaproteobacteria (122	Proteobacteria (2)	Bacteria (1)			99.70	97.90	WGS
287.9155	0.3636	Pseudomonas aeruginosa (286)	Pseudomonas (135621)	Pseudomonadaceae (7227	Pseudomonadales (1236)	Gammaproteobacteria (122	Proteobacteria (2)	Bacteria (1)			100.00	99.10	WGS
287.9156	0.331	Pseudomonas aeruginosa (286)	Pseudomonas (135621)	Pseudomonadaceae (7227	Pseudomonadales (1236)	Gammaproteobacteria (122	Proteobacteria (2)	Bacteria (1)			99.90	99.10	WGS
287.9157	0.3694	Pseudomonas aeruginosa (286)	Pseudomonas (135621)	Pseudomonadaceae (7227	Pseudomonadales (1236)	Gammaproteobacteria (122	Proteobacteria (2)	Bacteria (1)			99.90	99.00	WGS
287.9158	0.371	Pseudomonas aeruginosa (286)	Pseudomonas (135621)	Pseudomonadaceae (7227	Pseudomonadales (1236)	Gammaproteobacteria (122	Proteobacteria (2)	Bacteria (1)			99.90	98.90	WGS
287.9159	0.333	Pseudomonas aeruginosa (286)	Pseudomonas (135621)	Pseudomonadaceae (7227	Pseudomonadales (1236)	Gammaproteobacteria (122	Proteobacteria (2)	Bacteria (1)			99.90	98.70	WGS
287.916	0.3236	Pseudomonas aeruginosa (286)	Pseudomonas (135621)	Pseudomonadaceae (7227	Pseudomonadales (1236)	Gammaproteobacteria (122	Proteobacteria (2)	Bacteria (1)	99.40	0.00	99.60	99.00	WGS
287.9160	0.369	Pseudomonas aeruginosa (286)	Pseudomonas (135621)	Pseudomonadaceae (7227	Pseudomonadales (1236)	Gammaproteobacteria (122	Proteobacteria (2)	Bacteria (1)			99.80	98.60	WGS
287.9161	0.369	Pseudomonas aeruginosa (286)	Pseudomonas (135621)	Pseudomonadaceae (7227	Pseudomonadales (1236)	Gammaproteobacteria (122	Proteobacteria (2)	Bacteria (1)			99.80	98.70	WGS
287.9162	0.3738	Pseudomonas aeruginosa (286)	Pseudomonas (135621)	Pseudomonadaceae (7227	Pseudomonadales (1236)	Gammaproteobacteria (122	Proteobacteria (2)	Bacteria (1)			99.80	98.80	WGS
287.9163	0.3738	Pseudomonas aeruginosa (286)	Pseudomonas (135621)	Pseudomonadaceae (7227	Pseudomonadales (1236)	Gammaproteobacteria (122	Proteobacteria (2)	Bacteria (1)			99.60	98.40	WGS
287.9164	0.3636	Pseudomonas aeruginosa (286)	Pseudomonas (135621)	Pseudomonadaceae (7227	Pseudomonadales (1236)	Gammaproteobacteria (122	Proteobacteria (2)	Bacteria (1)			99.90	99.40	WGS
287.9165	0.3634	Pseudomonas aeruginosa (286)	Pseudomonas (135621)	Pseudomonadaceae (7227	Pseudomonadales (1236)	Gammaproteobacteria (122	Proteobacteria (2)	Bacteria (1)			99.60	98.20	WGS
287.9166	0.316	Pseudomonas aeruginosa (286)	Pseudomonas (135621)	Pseudomonadaceae (7227	Pseudomonadales (1236)	Gammaproteobacteria (122	Proteobacteria (2)	Bacteria (1)			99.60	99.10	WGS
287.9167	0.3748	Pseudomonas aeruginosa (286)	Pseudomonas (135621)	Pseudomonadaceae (7227	Pseudomonadales (1236)	Gammaproteobacteria (122	Proteobacteria (2)	Bacteria (1)			99.80	98.50	WGS
287.9168	0.3634	Pseudomonas aeruginosa (286)	Pseudomonas (135621)	Pseudomonadaceae (7227	Pseudomonadales (1236)	Gammaproteobacteria (122	Proteobacteria (2)	Bacteria (1)			99.90	98.70	WGS
287.9169	0.3636	Pseudomonas aeruginosa (286)	Pseudomonas (135621)	Pseudomonadaceae (7227	Pseudomonadales (1236)	Gammaproteobacteria (122	Proteobacteria (2)	Bacteria (1)			99.90	99.30	WGS
287.9170	0.3258	Pseudomonas aeruginosa (286)	Pseudomonas (135621)	Pseudomonadaceae (7227	Pseudomonadales (1236)	Gammaproteobacteria (122	Proteobacteria (2)	Bacteria (1)			99.90	99.00	WGS
287.917	0.3324	Pseudomonas aeruginosa (286)	Pseudomonas (135621)	Pseudomonadaceae (7227	Pseudomonadales (1236)	Gammaproteobacteria (122	Proteobacteria (2)	Bacteria (1)	100.00	0.30	99.70	99.00	WGS
287.9171	0.37	Pseudomonas aeruginosa (286)	Pseudomonas (135621)	Pseudomonadaceae (7227	Pseudomonadales (1236)	Gammaproteobacteria (122	Proteobacteria (2)	Bacteria (1)			99.90	98.80	WGS
287.9172	0.324	Pseudomonas aeruginosa (286)	Pseudomonas (135621)	Pseudomonadaceae (7227	Pseudomonadales (1236)	Gammaproteobacteria (122	Proteobacteria (2)	Bacteria (1)			99.90	99.10	WGS
287.9173	0.3258	Pseudomonas aeruginosa (286)	Pseudomonas (135621)	Pseudomonadaceae (7227	Pseudomonadales (1236)	Gammaproteobacteria (122	Proteobacteria (2)	Bacteria (1)			99.90	99.40	WGS
287.9174	0.3758	Pseudomonas aeruginosa (286)	Pseudomonas (135621)	Pseudomonadaceae (7227	Pseudomonadales (1236)	Gammaproteobacteria (122	Proteobacteria (2)	Bacteria (1)			99.90	98.10	WGS
287.9175	0.3324	Pseudomonas aeruginosa (286)	Pseudomonas (135621)	Pseudomonadaceae (7227	Pseudomonadales (1236)	Gammaproteobacteria (122	Proteobacteria (2)	Bacteria (1)			99.90	98.90	WGS
287.9176	0.3634	Pseudomonas aeruginosa (286)	Pseudomonas (135621)	Pseudomonadaceae (7227	Pseudomonadales (1236)	Gammaproteobacteria (122	Proteobacteria (2)	Bacteria (1)			99.70	98.20	WGS
287.9177	0.368	Pseudomonas aeruginosa (286)	Pseudomonas (135621)	Pseudomonadaceae (7227	Pseudomonadales (1236)	Gammaproteobacteria (122	Proteobacteria (2)	Bacteria (1)			99.90	98.90	WGS
287.9178	0.3388	Pseudomonas aeruginosa (286)	Pseudomonas (135621)	Pseudomonadaceae (7227	Pseudomonadales (1236)	Gammaproteobacteria (122	Proteobacteria (2)	Bacteria (1)			99.90	98.80	WGS
287.9179	0.3388	Pseudomonas aeruginosa (286)	Pseudomonas (135621)	Pseudomonadaceae (7227	Pseudomonadales (1236)	Gammaproteobacteria (122	Proteobacteria (2)	Bacteria (1)			99.90	98.70	WGS
287.9180	0.356	Pseudomonas aeruginosa (286)	Pseudomonas (135621)	Pseudomonadaceae (7227	Pseudomonadales (1236)	Gammaproteobacteria (122	Proteobacteria (2)	Bacteria (1)			99.80	99.00	WGS
287.918	0.3738	Pseudomonas aeruginosa (286)	Pseudomonas (135621)	Pseudomonadaceae (7227	Pseudomonadales (1236)	Gammaproteobacteria (122	Proteobacteria (2)	Bacteria (1)	100.00	0.00	99.30	98.40	WGS
287.9181	0.3258	Pseudomonas aeruginosa (286)	Pseudomonas (135621)	Pseudomonadaceae (7227	Pseudomonadales (1236)	Gammaproteobacteria (122	Proteobacteria (2)	Bacteria (1)			99.90	99.30	WGS
287.9182	0.3386	Pseudomonas aeruginosa (286)	Pseudomonas (135621)	Pseudomonadaceae (7227	Pseudomonadales (1236)	Gammaproteobacteria (122	Proteobacteria (2)	Bacteria (1)			99.60	98.60	WGS
287.9183	0.3258	Pseudomonas aeruginosa (286)	Pseudomonas (135621)	Pseudomonadaceae (7227	Pseudomonadales (1236)	Gammaproteobacteria (122	Proteobacteria (2)	Bacteria (1)			99.60	98.20	WGS
287.9184	0.3738	Pseudomonas aeruginosa (286)	Pseudomonas (135621)	Pseudomonadaceae (7227	Pseudomonadales (1236)	Gammaproteobacteria (122	Proteobacteria (2)	Bacteria (1)			99.80	98.60	WGS
287.9185	0.3376	Pseudomonas aeruginosa (286)	Pseudomonas (135621)	Pseudomonadaceae (7227	Pseudomonadales (1236)	Gammaproteobacteria (122	Proteobacteria (2)	Bacteria (1)			99.90	98.40	WGS
287.9186	0.324	Pseudomonas aeruginosa (286)	Pseudomonas (135621)	Pseudomonadaceae (7227	Pseudomonadales (1236)	Gammaproteobacteria (122	Proteobacteria (2)	Bacteria (1)			99.90	99.00	WGS
287.9187	0.3258	Pseudomonas aeruginosa (286)	Pseudomonas (135621)	Pseudomonadaceae (7227	Pseudomonadales (1236)	Gammaproteobacteria (122	Proteobacteria (2)	Bacteria (1)			99.90	99.10	WGS
287.9188	0.3388	Pseudomonas aeruginosa (286)	Pseudomonas (135621)	Pseudomonadaceae (7227	Pseudomonadales (1236)	Gammaproteobacteria (122	Proteobacteria (2)	Bacteria (1)			99.90	98.80	WGS
287.9189	0.3388	Pseudomonas aeruginosa (286)	Pseudomonas (135621)	Pseudomonadaceae (7227	Pseudomonadales (1236)	Gammaproteobacteria (122	Proteobacteria (2)	Bacteria (1)			99.90	98.80	WGS
287.9190	0.3388	Pseudomonas aeruginosa (286)	Pseudomonas (135621)	Pseudomonadaceae (7227	Pseudomonadales (1236)	Gammaproteobacteria (122	Proteobacteria (2)	Bacteria (1)			99.90	98.80	WGS
287.919	0.369	Pseudomonas aeruginosa (286)	Pseudomonas (135621)	Pseudomonadaceae (7227	Pseudomonadales (1236)	Gammaproteobacteria (122	Proteobacteria (2)	Bacteria (1)	99.70	0.00	99.00	98.60	WGS
287.9191	0.3748	Pseudomonas aeruginosa (286)	Pseudomonas (135621)	Pseudomonadaceae (7227	Pseudomonadales (1236)	Gammaproteobacteria (122	Proteobacteria (2)	Bacteria (1)			99.80	98.80	WGS
287.9192	0.3388	Pseudomonas aeruginosa (286)	Pseudomonas (135621)	Pseudomonadaceae (7227	Pseudomonadales (1236)	Gammaproteobacteria (122	Proteobacteria (2)	Bacteria (1)			99.90	98.70	WGS
287.9193	0.3388	Pseudomonas aeruginosa (286)	Pseudomonas (135621)	Pseudomonadaceae (7227	Pseudomonadales (1236)	Gammaproteobacteria (122	Proteobacteria (2)	Bacteria (1)			99.90	98.80	WGS
287.9194	0.3738	Pseudomonas aeruginosa (286)	Pseudomonas (135621)	Pseudomonadaceae (7227	Pseudomonadales (1236)	Gammaproteobacteria (122	Proteobacteria (2)	Bacteria (1)			99.90	98.90	WGS
287.9195	0.3738	Pseudomonas aeruginosa (286)	Pseudomonas (135621)	Pseudomonadaceae (7227	Pseudomonadales (1236)	Gammaproteobacteria (122	Proteobacteria (2)	Bacteria (1)			99.80	98.90	WGS
287.9196	0.3738	Pseudomonas aeruginosa (286)	Pseudomonas (135621)	Pseudomonadaceae (7227	Pseudomonadales (1236)	Gammaproteobacteria (122	Proteobacteria (2)	Bacteria (1)			99.80	98.90	WGS
287.9197	0.3748	Pseudomonas aeruginosa (286)	Pseudomonas (135621)	Pseudomonadaceae (7227	Pseudomonadales (1236)	Gammaproteobacteria (122	Proteobacteria (2)	Bacteria (1)			99.80	98.80	WGS
287.9198	0.3748	Pseudomonas aeruginosa (286)	Pseudomonas (135621)	Pseudomonadaceae (7227	Pseudomonadales (1236)	Gammaproteobacteria (122	Proteobacteria (2)	Bacteria (1)			99.80	98.80	WGS
287.9199	0.3704	Pseudomonas aeruginosa (286)	Pseudomonas (135621)	Pseudomonadaceae (7227	Pseudomonadales (1236)	Gammaproteobacteria (122	Proteobacteria (2)	Bacteria (1)			99.90	98.70	WGS
287.920	0.3346	Pseudomonas aeruginosa (286)	Pseudomonas (135621)	Pseudomonadaceae (7227	Pseudomonadales (1236)	Gammaproteobacteria (122	Proteobacteria (2)	Bacteria (1)	100.00	0.00	99.90	99.30	WGS
287.9200	0.3704	Pseudomonas aeruginosa (286)	Pseudomonas (135621)	Pseudomonadaceae (7227	Pseudomonadales (1236)	Gammaproteobacteria (122	Proteobacteria (2)	Bacteria (1)			99.90	98.90	WGS
287.9201	0.3704	Pseudomonas aeruginosa (286)	Pseudomonas (135621)	Pseudomonadaceae (7227	Pseudomonadales (1236)	Gammaproteobacteria (122	Proteobacteria (2)	Bacteria (1)			99.80	98.90	WGS
287.9202	0.3704	Pseudomonas aeruginosa (286)	Pseudomonas (135621)	Pseudomonadaceae (7227	Pseudomonadales (1236)	Gammaproteobacteria (122	Proteobacteria (2)	Bacteria (1)			99.80	98.90	WGS
287.9203	0.3388	Pseudomonas aeruginosa (286)	Pseudomonas (135621)	Pseudomonadaceae (7227	Pseudomonadales (1236)	Gammaproteobacteria (122	Proteobacteria (2)	Bacteria (1)			99.90	98.70	WGS
287.9204	0.3388	Pseudomonas aeruginosa (286)	Pseudomonas (135621)	Pseudomonadaceae (7227	Pseudomonadales (1236)	Gammaproteobacteria (122	Proteobacteria (2)	Bacteria (1)			99.90	98.70	WGS
287.9205	0.3388	Pseudomonas aeruginosa (286)	Pseudomonas (135621)	Pseudomonadaceae (7227	Pseudomonadales (1236)	Gammaproteobacteria (122	Proteobacteria (2)	Bacteria (1)			99.90	98.60	WGS
287.9206	0.3388	Pseudomonas aeruginosa (286)	Pseudomonas (135621)	Pseudomonadaceae (7227	Pseudomonadales (1236)	Gammaproteobacteria (122	Proteobacteria (2)	Bacteria (1)			99.90	98.70	WGS
287.9207	0.3388	Pseudomonas aeruginosa (286)	Pseudomonas (135621)	Pseudomonadaceae (7227	Pseudomonadales (1236)	Gammaproteobacteria (122	Proteobacteria (2)	Bacteria (1)			99.90	98.70	WGS
287.9208	0.3388	Pseudomonas aeruginosa (286)	Pseudomonas (135621)	Pseudomonadaceae (7227	Pseudomonadales (1236)	Gammaproteobacteria (122	Proteobacteria (2)	Bacteria (1)			99.90	98.70	WGS
287.9209	0.3388	Pseudomonas aeruginosa (286)	Pseudomonas (135621)	Pseudomonadaceae (7227	Pseudomonadales (1236)	Gammaproteobacteria (122	Proteobacteria (2)	Bacteria (1)			99.90	98.70	WGS
287.921	0.331	Pseudomonas aeruginosa (286)	Pseudomonas (135621)	Pseudomonadaceae (7227	Pseudomonadales (1236)	Gammaproteobacteria (122	Proteobacteria (2)	Bacteria (1)	100.00	0.00	99.30	98.40	WGS
287.9210	0.3652	Pseudomonas aeruginosa (286)	Pseudomonas (135621)	Pseudomonadaceae (7227	Pseudomonadales (1236)	Gammaproteobacteria (122	Proteobacteria (2)	Bacteria (1)			99.70	99.00	WGS
287.9211	0.3652	Pseudomonas aeruginosa (286)	Pseudomonas (135621)	Pseudomonadaceae (7227	Pseudomonadales (1236)	Gammaproteobacteria (122	Proteobacteria (2)	Bacteria (1)			99.70	99.00	WGS
287.9212	0.3652	Pseudomonas aeruginosa (286)	Pseudomonas (135621)	Pseudomonadaceae (7227	Pseudomonadales (1236)	Gammaproteobacteria (122	Proteobacteria (2)	Bacteria (1)			99.70	99.00	WGS
287.9213	0.3652	Pseudomonas aeruginosa (286)	Pseudomonas (135621)	Pseudomonadaceae (7227	Pseudomonadales (1236)	Gammaproteobacteria (122	Proteobacteria (2)	Bacteria (1)			99.70	99.00	WGS
287.9214	0.3388	Pseudomonas aeruginosa (286)	Pseudomonas (135621)	Pseudomonadaceae (7227	Pseudomonadales (1236)	Gammaproteobacteria (122	Proteobacteria (2)	Bacteria (1)			99.90	98.80	WGS
287.9215	0.3388	Pseudomonas aeruginosa (286)	Pseudomonas (135621)	Pseudomonadaceae (7227	Pseudomonadales (1236)	Gammaproteobacteria (122	Proteobacteria (2)	Bacteria (1)			99.90	98.70	WGS
287.9216	0.3738	Pseudomonas aeruginosa (286)	Pseudomonas (135621)	Pseudomonadaceae (7227	Pseudomonadales (1236)	Gammaproteobacteria (122	Proteobacteria (2)	Bacteria (1)			99.80	98.70	WGS
287.9217	0.3748	Pseudomonas aeruginosa (286)	Pseudomonas (135621)	Pseudomonadaceae (7227	Pseudomonadales (1236)	Gammaproteobacteria (122	Proteobacteria (2)	Bacteria (1)			99.80	98.70	WGS
287.9218	0.3704	Pseudomonas aeruginosa (286)	Pseudomonas (135621)	Pseudomonadaceae (7227	Pseudomonadales (1236)	Gammaproteobacteria (122	Proteobacteria (2)	Bacteria (1)			99.90	98.60	WGS
287.9219	0.3748	Pseudomonas aeruginosa (286)	Pseudomonas (135621)	Pseudomonadaceae (7227	Pseudomonadales (1236)	Gammaproteobacteria (122	Proteobacteria (2)	Bacteria (1)			99.80	98.70	WGS
287.9220	0.3704	Pseudomonas aeruginosa (286)	Pseudomonas (135621)	Pseudomonadaceae (7227	Pseudomonadales (1236)	Gammaproteobacteria (122	Proteobacteria (2)	Bacteria (1)			99.90	98.70	WGS
287.922	0.3646	Pseudomonas aeruginosa (286)	Pseudomonas (135621)	Pseudomonadaceae (7227	Pseudomonadales (1236)	Gammaproteobacteria (122	Proteobacteria (2)	Bacteria (1)	100.00	0.00	99.50	99.10	WGS
287.9221	0.3268	Pseudomonas aeruginosa (286)	Pseudomonas (135621)	Pseudomonadaceae (7227	Pseudomonadales (1236)	Gammaproteobacteria (122	Proteobacteria (2)	Bacteria (1)			99.90	99.30	WGS
287.9222	0.3704	Pseudomonas aeruginosa (286)	Pseudomonas (135621)	Pseudomonadaceae (7227	Pseudomonadales (1236)	Gammaproteobacteria (122	Proteobacteria (2)	Bacteria (1)			99.80	98.90	WGS
287.9223	0.3652	Pseudomonas aeruginosa (286)	Pseudomonas (135621)	Pseudomonadaceae (7227	Pseudomonadales (1236)	Gammaproteobacteria (122	Proteobacteria (2)	Bacteria (1)			99.90	99.20	WGS
287.9224	0.3652	Pseudomonas aeruginosa (286)	Pseudomonas (135621)	Pseudomonadaceae (7227	Pseudomonadales (1236)	Gammaproteobacteria (122	Proteobacteria (2)	Bacteria (1)			99.70	98.90	WGS
287.9225	0.3652	Pseudomonas aeruginosa (286)	Pseudomonas (135621)	Pseudomonadaceae (7227	Pseudomonadales (1236)	Gammaproteobacteria (122	Proteobacteria (2)	Bacteria (1)			99.70	99.00	WGS
287.9226	0.3652	Pseudomonas aeruginosa (286)	Pseudomonas (135621)	Pseudomonadaceae (7227	Pseudomonadales (1236)	Gammaproteobacteria (122	Proteobacteria (2)	Bacteria (1)			99.70	99.00	WGS
287.9227	0.3604	Pseudomonas aeruginosa (286)	Pseudomonas (135621)	Pseudomonadaceae (7227	Pseudomonadales (1236)	Gammaproteobacteria (122	Proteobacteria (2)	Bacteria (1)			99.60	98.90	WGS
287.9228	0.356	Pseudomonas aeruginosa (286)	Pseudomonas (135621)	Pseudomonadaceae (7227	Pseudomonadales (1236)	Gammaproteobacteria (122	Proteobacteria (2)	Bacteria (1)			99.90	98.40	WGS
287.9229	0.356	Pseudomonas aeruginosa (286)	Pseudomonas (135621)	Pseudomonadaceae (7227	Pseudomonadales (1236)	Gammaproteobacteria (122	Proteobacteria (2)	Bacteria (1)			99.90	98.60	WGS
287.923	0.331	Pseudomonas aeruginosa (286)	Pseudomonas (135621)	Pseudomonadaceae (7227	Pseudomonadales (1236)	Gammaproteobacteria (122	Proteobacteria (2)	Bacteria (1)	99.40	0.00	99.20	98.70	WGS
287.9230	0.3738	Pseudomonas aeruginosa (286)	Pseudomonas (135621)	Pseudomonadaceae (7227	Pseudomonadales (1236)	Gammaproteobacteria (122	Proteobacteria (2)	Bacteria (1)			99.80	98.80	WGS
287.9231	0.3738	Pseudomonas aeruginosa (286)	Pseudomonas (135621)	Pseudomonadaceae (7227	Pseudomonadales (1236)	Gammaproteobacteria (122	Proteobacteria (2)	Bacteria (1)			99.90	98.90	WGS
287.9232	0.37	Pseudomonas aeruginosa (286)	Pseudomonas (135621)	Pseudomonadaceae (7227	Pseudomonadales (1236)	Gammaproteobacteria (122	Proteobacteria (2)	Bacteria (1)			99.90	99.00	WGS
287.9233	0.3322	Pseudomonas aeruginosa (286)	Pseudomonas (135621)	Pseudomonadaceae (7227	Pseudomonadales (1236)	Gammaproteobacteria (122	Proteobacteria (2)	Bacteria (1)			99.90	99.30	WGS
287.9234	0.3636	Pseudomonas aeruginosa (286)	Pseudomonas (135621)	Pseudomonadaceae (7227	Pseudomonadales (1236)	Gammaproteobacteria (122	Proteobacteria (2)	Bacteria (1)			99.90	99.60	WGS
287.9235	0.3636	Pseudomonas aeruginosa (286)	Pseudomonas (135621)	Pseudomonadaceae (7227	Pseudomonadales (1236)	Gammaproteobacteria (122	Proteobacteria (2)	Bacteria (1)			99.70	98.70	WGS
287.9236	0.368	Pseudomonas aeruginosa (286)	Pseudomonas (135621)	Pseudomonadaceae (7227	Pseudomonadales (1236)	Gammaproteobacteria (122	Proteobacteria (2)	Bacteria (1)			99.90	98.90	WGS
287.9237	0.328	Pseudomonas aeruginosa (286)	Pseudomonas (135621)	Pseudomonadaceae (7227	Pseudomonadales (1236)	Gammaproteobacteria (122	Proteobacteria (2)	Bacteria (1)			99.90	99.20	WGS
287.9238	0.3334	Pseudomonas aeruginosa (286)	Pseudomonas (135621)	Pseudomonadaceae (7227	Pseudomonadales (1236)	Gammaproteobacteria (122	Proteobacteria (2)	Bacteria (1)			99.90	98.80	WGS
287.9239	0.328	Pseudomonas aeruginosa (286)	Pseudomonas (135621)	Pseudomonadaceae (7227	Pseudomonadales (1236)	Gammaproteobacteria (122	Proteobacteria (2)	Bacteria (1)			99.90	99.20	WGS
287.9240	0.328	Pseudomonas aeruginosa (286)	Pseudomonas (135621)	Pseudomonadaceae (7227	Pseudomonadales (1236)	Gammaproteobacteria (122	Proteobacteria (2)	Bacteria (1)			99.90	99.20	WGS
287.924	0.327	Pseudomonas aeruginosa (286)	Pseudomonas (135621)	Pseudomonadaceae (7227	Pseudomonadales (1236)	Gammaproteobacteria (122	Proteobacteria (2)	Bacteria (1)	97.90	0.30	99.10	97.60	WGS
287.9241	0.328	Pseudomonas aeruginosa (286)	Pseudomonas (135621)	Pseudomonadaceae (7227	Pseudomonadales (1236)	Gammaproteobacteria (122	Proteobacteria (2)	Bacteria (1)			99.90	99.20	WGS
287.9242	0.328	Pseudomonas aeruginosa (286)	Pseudomonas (135621)	Pseudomonadaceae (7227	Pseudomonadales (1236)	Gammaproteobacteria (122	Proteobacteria (2)	Bacteria (1)			99.90	99.20	WGS
287.9243	0.371	Pseudomonas aeruginosa (286)	Pseudomonas (135621)	Pseudomonadaceae (7227	Pseudomonadales (1236)	Gammaproteobacteria (122	Proteobacteria (2)	Bacteria (1)			99.90	98.80	WGS
287.9244	0.371	Pseudomonas aeruginosa (286)	Pseudomonas (135621)	Pseudomonadaceae (7227	Pseudomonadales (1236)	Gammaproteobacteria (122	Proteobacteria (2)	Bacteria (1)			99.90	98.80	WGS
287.9245	0.371	Pseudomonas aeruginosa (286)	Pseudomonas (135621)	Pseudomonadaceae (7227	Pseudomonadales (1236)	Gammaproteobacteria (122	Proteobacteria (2)	Bacteria (1)			99.90	98.70	WGS
287.9246	0.371	Pseudomonas aeruginosa (286)	Pseudomonas (135621)	Pseudomonadaceae (7227	Pseudomonadales (1236)	Gammaproteobacteria (122	Proteobacteria (2)	Bacteria (1)			99.90	98.80	WGS
287.9247	0.3376	Pseudomonas aeruginosa (286)	Pseudomonas (135621)	Pseudomonadaceae (7227	Pseudomonadales (1236)	Gammaproteobacteria (122	Proteobacteria (2)	Bacteria (1)			99.90	98.60	WGS
287.9248	0.3636	Pseudomonas aeruginosa (286)	Pseudomonas (135621)	Pseudomonadaceae (7227	Pseudomonadales (1236)	Gammaproteobacteria (122	Proteobacteria (2)	Bacteria (1)			99.90	99.60	WGS
287.9249	0.3636	Pseudomonas aeruginosa (286)	Pseudomonas (135621)	Pseudomonadaceae (7227	Pseudomonadales (1236)	Gammaproteobacteria (122	Proteobacteria (2)	Bacteria (1)			99.90	99.60	WGS
287.9250	0.356	Pseudomonas aeruginosa (286)	Pseudomonas (135621)	Pseudomonadaceae (7227	Pseudomonadales (1236)	Gammaproteobacteria (122	Proteobacteria (2)	Bacteria (1)			99.90	98.50	WGS
287.925	0.377	Pseudomonas aeruginosa (286)	Pseudomonas (135621)	Pseudomonadaceae (7227	Pseudomonadales (1236)	Gammaproteobacteria (122	Proteobacteria (2)	Bacteria (1)	99.40	0.00	98.90	98.20	WGS
287.9251	0.356	Pseudomonas aeruginosa (286)	Pseudomonas (135621)	Pseudomonadaceae (7227	Pseudomonadales (1236)	Gammaproteobacteria (122	Proteobacteria (2)	Bacteria (1)			99.90	98.40	WGS
287.9252	0.356	Pseudomonas aeruginosa (286)	Pseudomonas (135621)	Pseudomonadaceae (7227	Pseudomonadales (1236)	Gammaproteobacteria (122	Proteobacteria (2)	Bacteria (1)			99.90	98.40	WGS
287.9253	0.356	Pseudomonas aeruginosa (286)	Pseudomonas (135621)	Pseudomonadaceae (7227	Pseudomonadales (1236)	Gammaproteobacteria (122	Proteobacteria (2)	Bacteria (1)			99.90	98.40	WGS
287.9254	0.368	Pseudomonas aeruginosa (286)	Pseudomonas (135621)	Pseudomonadaceae (7227	Pseudomonadales (1236)	Gammaproteobacteria (122	Proteobacteria (2)	Bacteria (1)			99.70	98.80	WGS
287.9255	0.368	Pseudomonas aeruginosa (286)	Pseudomonas (135621)	Pseudomonadaceae (7227	Pseudomonadales (1236)	Gammaproteobacteria (122	Proteobacteria (2)	Bacteria (1)			99.70	98.80	WGS
287.9256	0.3294	Pseudomonas aeruginosa (286)	Pseudomonas (135621)	Pseudomonadaceae (7227	Pseudomonadales (1236)	Gammaproteobacteria (122	Proteobacteria (2)	Bacteria (1)			99.60	97.90	WGS
287.9257	0.3294	Pseudomonas aeruginosa (286)	Pseudomonas (135621)	Pseudomonadaceae (7227	Pseudomonadales (1236)	Gammaproteobacteria (122	Proteobacteria (2)	Bacteria (1)			99.60	98.00	WGS
287.9258	0.3292	Pseudomonas aeruginosa (286)	Pseudomonas (135621)	Pseudomonadaceae (7227	Pseudomonadales (1236)	Gammaproteobacteria (122	Proteobacteria (2)	Bacteria (1)			99.90	98.90	WGS
287.9259	0.3636	Pseudomonas aeruginosa (286)	Pseudomonas (135621)	Pseudomonadaceae (7227	Pseudomonadales (1236)	Gammaproteobacteria (122	Proteobacteria (2)	Bacteria (1)			99.60	98.40	WGS
287.9260	0.3652	Pseudomonas aeruginosa (286)	Pseudomonas (135621)	Pseudomonadaceae (7227	Pseudomonadales (1236)	Gammaproteobacteria (122	Proteobacteria (2)	Bacteria (1)			99.70	99.00	WGS
287.926	0.371	Pseudomonas aeruginosa (286)	Pseudomonas (135621)	Pseudomonadaceae (7227	Pseudomonadales (1236)	Gammaproteobacteria (122	Proteobacteria (2)	Bacteria (1)	99.40	0.60	98.70	97.60	WGS
287.9261	0.3652	Pseudomonas aeruginosa (286)	Pseudomonas (135621)	Pseudomonadaceae (7227	Pseudomonadales (1236)	Gammaproteobacteria (122	Proteobacteria (2)	Bacteria (1)			99.70	99.00	WGS
287.9262	0.3738	Pseudomonas aeruginosa (286)	Pseudomonas (135621)	Pseudomonadaceae (7227	Pseudomonadales (1236)	Gammaproteobacteria (122	Proteobacteria (2)	Bacteria (1)			99.60	98.60	WGS
287.9263	0.368	Pseudomonas aeruginosa (286)	Pseudomonas (135621)	Pseudomonadaceae (7227	Pseudomonadales (1236)	Gammaproteobacteria (122	Proteobacteria (2)	Bacteria (1)			99.90	99.00	WGS
287.9264	0.368	Pseudomonas aeruginosa (286)	Pseudomonas (135621)	Pseudomonadaceae (7227	Pseudomonadales (1236)	Gammaproteobacteria (122	Proteobacteria (2)	Bacteria (1)			99.90	98.70	WGS
287.9265	0.328	Pseudomonas aeruginosa (286)	Pseudomonas (135621)	Pseudomonadaceae (7227	Pseudomonadales (1236)	Gammaproteobacteria (122	Proteobacteria (2)	Bacteria (1)			99.90	99.20	WGS
287.9266	0.3346	Pseudomonas aeruginosa (286)	Pseudomonas (135621)	Pseudomonadaceae (7227	Pseudomonadales (1236)	Gammaproteobacteria (122	Proteobacteria (2)	Bacteria (1)			99.90	99.00	WGS
287.9267	0.3346	Pseudomonas aeruginosa (286)	Pseudomonas (135621)	Pseudomonadaceae (7227	Pseudomonadales (1236)	Gammaproteobacteria (122	Proteobacteria (2)	Bacteria (1)			99.90	99.00	WGS
287.9268	0.371	Pseudomonas aeruginosa (286)	Pseudomonas (135621)	Pseudomonadaceae (7227	Pseudomonadales (1236)	Gammaproteobacteria (122	Proteobacteria (2)	Bacteria (1)			99.90	98.80	WGS
287.9269	0.3258	Pseudomonas aeruginosa (286)	Pseudomonas (135621)	Pseudomonadaceae (7227	Pseudomonadales (1236)	Gammaproteobacteria (122	Proteobacteria (2)	Bacteria (1)			99.60	98.40	WGS
287.9270	0.356	Pseudomonas aeruginosa (286)	Pseudomonas (135621)	Pseudomonadaceae (7227	Pseudomonadales (1236)	Gammaproteobacteria (122	Proteobacteria (2)	Bacteria (1)			99.90	98.60	WGS
287.927	0.3292	Pseudomonas aeruginosa (286)	Pseudomonas (135621)	Pseudomonadaceae (7227	Pseudomonadales (1236)	Gammaproteobacteria (122	Proteobacteria (2)	Bacteria (1)	99.70	0.00	99.00	98.40	WGS
287.9271	0.356	Pseudomonas aeruginosa (286)	Pseudomonas (135621)	Pseudomonadaceae (7227	Pseudomonadales (1236)	Gammaproteobacteria (122	Proteobacteria (2)	Bacteria (1)			99.90	98.60	WGS
287.9272	0.368	Pseudomonas aeruginosa (286)	Pseudomonas (135621)	Pseudomonadaceae (7227	Pseudomonadales (1236)	Gammaproteobacteria (122	Proteobacteria (2)	Bacteria (1)			99.70	98.80	WGS
287.9273	0.3636	Pseudomonas aeruginosa (286)	Pseudomonas (135621)	Pseudomonadaceae (7227	Pseudomonadales (1236)	Gammaproteobacteria (122	Proteobacteria (2)	Bacteria (1)			99.60	98.40	WGS
287.9274	0.3334	Pseudomonas aeruginosa (286)	Pseudomonas (135621)	Pseudomonadaceae (7227	Pseudomonadales (1236)	Gammaproteobacteria (122	Proteobacteria (2)	Bacteria (1)			99.70	99.00	WGS
287.9275	0.3226	Pseudomonas aeruginosa (286)	Pseudomonas (135621)	Pseudomonadaceae (7227	Pseudomonadales (1236)	Gammaproteobacteria (122	Proteobacteria (2)	Bacteria (1)			99.60	98.60	WGS
287.9276	0.3226	Pseudomonas aeruginosa (286)	Pseudomonas (135621)	Pseudomonadaceae (7227	Pseudomonadales (1236)	Gammaproteobacteria (122	Proteobacteria (2)	Bacteria (1)			99.70	98.70	WGS
287.9277	0.3226	Pseudomonas aeruginosa (286)	Pseudomonas (135621)	Pseudomonadaceae (7227	Pseudomonadales (1236)	Gammaproteobacteria (122	Proteobacteria (2)	Bacteria (1)			99.80	99.10	WGS
287.9278	0.37	Pseudomonas aeruginosa (286)	Pseudomonas (135621)	Pseudomonadaceae (7227	Pseudomonadales (1236)	Gammaproteobacteria (122	Proteobacteria (2)	Bacteria (1)			99.90	98.70	WGS
287.9279	0.3324	Pseudomonas aeruginosa (286)	Pseudomonas (135621)	Pseudomonadaceae (7227	Pseudomonadales (1236)	Gammaproteobacteria (122	Proteobacteria (2)	Bacteria (1)			99.70	99.00	WGS
287.9280	0.3334	Pseudomonas aeruginosa (286)	Pseudomonas (135621)	Pseudomonadaceae (7227	Pseudomonadales (1236)	Gammaproteobacteria (122	Proteobacteria (2)	Bacteria (1)			99.90	98.40	WGS
287.928	0.331	Pseudomonas aeruginosa (286)	Pseudomonas (135621)	Pseudomonadaceae (7227	Pseudomonadales (1236)	Gammaproteobacteria (122	Proteobacteria (2)	Bacteria (1)	100.00	0.00	99.30	98.70	WGS
287.9281	0.3334	Pseudomonas aeruginosa (286)	Pseudomonas (135621)	Pseudomonadaceae (7227	Pseudomonadales (1236)	Gammaproteobacteria (122	Proteobacteria (2)	Bacteria (1)			99.70	98.90	WGS
287.9282	0.3226	Pseudomonas aeruginosa (286)	Pseudomonas (135621)	Pseudomonadaceae (7227	Pseudomonadales (1236)	Gammaproteobacteria (122	Proteobacteria (2)	Bacteria (1)			99.70	98.60	WGS
287.9283	0.371	Pseudomonas aeruginosa (286)	Pseudomonas (135621)	Pseudomonadaceae (7227	Pseudomonadales (1236)	Gammaproteobacteria (122	Proteobacteria (2)	Bacteria (1)			99.90	98.70	WGS
287.9284	0.3268	Pseudomonas aeruginosa (286)	Pseudomonas (135621)	Pseudomonadaceae (7227	Pseudomonadales (1236)	Gammaproteobacteria (122	Proteobacteria (2)	Bacteria (1)			99.90	98.70	WGS
287.9285	0.3268	Pseudomonas aeruginosa (286)	Pseudomonas (135621)	Pseudomonadaceae (7227	Pseudomonadales (1236)	Gammaproteobacteria (122	Proteobacteria (2)	Bacteria (1)			99.90	98.70	WGS
287.9286	0.37	Pseudomonas aeruginosa (286)	Pseudomonas (135621)	Pseudomonadaceae (7227	Pseudomonadales (1236)	Gammaproteobacteria (122	Proteobacteria (2)	Bacteria (1)			99.90	98.70	WGS
287.9287	0.3268	Pseudomonas aeruginosa (286)	Pseudomonas (135621)	Pseudomonadaceae (7227	Pseudomonadales (1236)	Gammaproteobacteria (122	Proteobacteria (2)	Bacteria (1)			99.80	98.40	WGS
287.9288	0.3268	Pseudomonas aeruginosa (286)	Pseudomonas (135621)	Pseudomonadaceae (7227	Pseudomonadales (1236)	Gammaproteobacteria (122	Proteobacteria (2)	Bacteria (1)			99.90	98.30	WGS
287.9289	0.356	Pseudomonas aeruginosa (286)	Pseudomonas (135621)	Pseudomonadaceae (7227	Pseudomonadales (1236)	Gammaproteobacteria (122	Proteobacteria (2)	Bacteria (1)			99.90	98.60	WGS
287.9290	0.3386	Pseudomonas aeruginosa (286)	Pseudomonas (135621)	Pseudomonadaceae (7227	Pseudomonadales (1236)	Gammaproteobacteria (122	Proteobacteria (2)	Bacteria (1)			99.90	99.50	WGS
287.929	0.3636	Pseudomonas aeruginosa (286)	Pseudomonas (135621)	Pseudomonadaceae (7227	Pseudomonadales (1236)	Gammaproteobacteria (122	Proteobacteria (2)	Bacteria (1)	100.00	0.00	99.70	99.20	WGS
287.9291	0.3386	Pseudomonas aeruginosa (286)	Pseudomonas (135621)	Pseudomonadaceae (7227	Pseudomonadales (1236)	Gammaproteobacteria (122	Proteobacteria (2)	Bacteria (1)			99.90	99.00	WGS
287.9292	0.3376	Pseudomonas aeruginosa (286)	Pseudomonas (135621)	Pseudomonadaceae (7227	Pseudomonadales (1236)	Gammaproteobacteria (122	Proteobacteria (2)	Bacteria (1)			99.90	98.40	WGS
287.9293	0.3386	Pseudomonas aeruginosa (286)	Pseudomonas (135621)	Pseudomonadaceae (7227	Pseudomonadales (1236)	Gammaproteobacteria (122	Proteobacteria (2)	Bacteria (1)			99.90	99.10	WGS
287.9294	0.3738	Pseudomonas aeruginosa (286)	Pseudomonas (135621)	Pseudomonadaceae (7227	Pseudomonadales (1236)	Gammaproteobacteria (122	Proteobacteria (2)	Bacteria (1)			99.80	98.70	WGS
287.9295	0.3636	Pseudomonas aeruginosa (286)	Pseudomonas (135621)	Pseudomonadaceae (7227	Pseudomonadales (1236)	Gammaproteobacteria (122	Proteobacteria (2)	Bacteria (1)			99.80	99.00	WGS
287.9296	0.3636	Pseudomonas aeruginosa (286)	Pseudomonas (135621)	Pseudomonadaceae (7227	Pseudomonadales (1236)	Gammaproteobacteria (122	Proteobacteria (2)	Bacteria (1)			99.80	98.90	WGS
287.9297	0.3692	Pseudomonas aeruginosa (286)	Pseudomonas (135621)	Pseudomonadaceae (7227	Pseudomonadales (1236)	Gammaproteobacteria (122	Proteobacteria (2)	Bacteria (1)			99.90	98.50	WGS
287.9298	0.371	Pseudomonas aeruginosa (286)	Pseudomonas (135621)	Pseudomonadaceae (7227	Pseudomonadales (1236)	Gammaproteobacteria (122	Proteobacteria (2)	Bacteria (1)			99.90	98.80	WGS
287.9299	0.3258	Pseudomonas aeruginosa (286)	Pseudomonas (135621)	Pseudomonadaceae (7227	Pseudomonadales (1236)	Gammaproteobacteria (122	Proteobacteria (2)	Bacteria (1)			99.60	98.70	WGS
287.9300	0.3768	Pseudomonas aeruginosa (286)	Pseudomonas (135621)	Pseudomonadaceae (7227	Pseudomonadales (1236)	Gammaproteobacteria (122	Proteobacteria (2)	Bacteria (1)			99.80	98.50	WGS
287.930	0.3636	Pseudomonas aeruginosa (286)	Pseudomonas (135621)	Pseudomonadaceae (7227	Pseudomonadales (1236)	Gammaproteobacteria (122	Proteobacteria (2)	Bacteria (1)	100.00	0.00	99.60	99.10	WGS
287.9301	0.331	Pseudomonas aeruginosa (286)	Pseudomonas (135621)	Pseudomonadaceae (7227	Pseudomonadales (1236)	Gammaproteobacteria (122	Proteobacteria (2)	Bacteria (1)			99.90	98.70	WGS
287.9302	0.331	Pseudomonas aeruginosa (286)	Pseudomonas (135621)	Pseudomonadaceae (7227	Pseudomonadales (1236)	Gammaproteobacteria (122	Proteobacteria (2)	Bacteria (1)			99.90	98.70	WGS
287.9303	0.3268	Pseudomonas aeruginosa (286)	Pseudomonas (135621)	Pseudomonadaceae (7227	Pseudomonadales (1236)	Gammaproteobacteria (122	Proteobacteria (2)	Bacteria (1)			99.90	99.10	WGS
287.9304	0.3268	Pseudomonas aeruginosa (286)	Pseudomonas (135621)	Pseudomonadaceae (7227	Pseudomonadales (1236)	Gammaproteobacteria (122	Proteobacteria (2)	Bacteria (1)			99.90	98.20	WGS
287.9305	0.3268	Pseudomonas aeruginosa (286)	Pseudomonas (135621)	Pseudomonadaceae (7227	Pseudomonadales (1236)	Gammaproteobacteria (122	Proteobacteria (2)	Bacteria (1)			99.90	98.30	WGS
287.9306	0.3268	Pseudomonas aeruginosa (286)	Pseudomonas (135621)	Pseudomonadaceae (7227	Pseudomonadales (1236)	Gammaproteobacteria (122	Proteobacteria (2)	Bacteria (1)			99.90	98.70	WGS
287.9307	0.371	Pseudomonas aeruginosa (286)	Pseudomonas (135621)	Pseudomonadaceae (7227	Pseudomonadales (1236)	Gammaproteobacteria (122	Proteobacteria (2)	Bacteria (1)			99.70	98.70	WGS
287.9308	0.3376	Pseudomonas aeruginosa (286)	Pseudomonas (135621)	Pseudomonadaceae (7227	Pseudomonadales (1236)	Gammaproteobacteria (122	Proteobacteria (2)	Bacteria (1)			99.80	98.30	WGS
287.9309	0.3376	Pseudomonas aeruginosa (286)	Pseudomonas (135621)	Pseudomonadaceae (7227	Pseudomonadales (1236)	Gammaproteobacteria (122	Proteobacteria (2)	Bacteria (1)			99.80	98.30	WGS
287.9310	0.3386	Pseudomonas aeruginosa (286)	Pseudomonas (135621)	Pseudomonadaceae (7227	Pseudomonadales (1236)	Gammaproteobacteria (122	Proteobacteria (2)	Bacteria (1)			99.80	98.70	WGS
287.931	0.3324	Pseudomonas aeruginosa (286)	Pseudomonas (135621)	Pseudomonadaceae (7227	Pseudomonadales (1236)	Gammaproteobacteria (122	Proteobacteria (2)	Bacteria (1)	99.70	0.00	99.30	98.40	WGS
287.9311	0.3098	Pseudomonas aeruginosa (286)	Pseudomonas (135621)	Pseudomonadaceae (7227	Pseudomonadales (1236)	Gammaproteobacteria (122	Proteobacteria (2)	Bacteria (1)			99.80	97.70	WGS
287.9312	0.3738	Pseudomonas aeruginosa (286)	Pseudomonas (135621)	Pseudomonadaceae (7227	Pseudomonadales (1236)	Gammaproteobacteria (122	Proteobacteria (2)	Bacteria (1)			99.80	98.80	WGS
287.9313	0.3324	Pseudomonas aeruginosa (286)	Pseudomonas (135621)	Pseudomonadaceae (7227	Pseudomonadales (1236)	Gammaproteobacteria (122	Proteobacteria (2)	Bacteria (1)			99.90	98.70	WGS
287.9314	0.3258	Pseudomonas aeruginosa (286)	Pseudomonas (135621)	Pseudomonadaceae (7227	Pseudomonadales (1236)	Gammaproteobacteria (122	Proteobacteria (2)	Bacteria (1)			99.80	99.00	WGS
287.9315	0.3258	Pseudomonas aeruginosa (286)	Pseudomonas (135621)	Pseudomonadaceae (7227	Pseudomonadales (1236)	Gammaproteobacteria (122	Proteobacteria (2)	Bacteria (1)			99.80	97.80	WGS
287.9316	0.3768	Pseudomonas aeruginosa (286)	Pseudomonas (135621)	Pseudomonadaceae (7227	Pseudomonadales (1236)	Gammaproteobacteria (122	Proteobacteria (2)	Bacteria (1)			99.90	98.40	WGS
287.9317	0.371	Pseudomonas aeruginosa (286)	Pseudomonas (135621)	Pseudomonadaceae (7227	Pseudomonadales (1236)	Gammaproteobacteria (122	Proteobacteria (2)	Bacteria (1)			99.80	98.60	WGS
287.9318	0.3226	Pseudomonas aeruginosa (286)	Pseudomonas (135621)	Pseudomonadaceae (7227	Pseudomonadales (1236)	Gammaproteobacteria (122	Proteobacteria (2)	Bacteria (1)			99.80	98.20	WGS
287.9319	0.3376	Pseudomonas aeruginosa (286)	Pseudomonas (135621)	Pseudomonadaceae (7227	Pseudomonadales (1236)	Gammaproteobacteria (122	Proteobacteria (2)	Bacteria (1)			99.90	98.50	WGS
287.9320	0.368	Pseudomonas aeruginosa (286)	Pseudomonas (135621)	Pseudomonadaceae (7227	Pseudomonadales (1236)	Gammaproteobacteria (122	Proteobacteria (2)	Bacteria (1)			99.70	99.00	WGS
287.932	0.3324	Pseudomonas aeruginosa (286)	Pseudomonas (135621)	Pseudomonadaceae (7227	Pseudomonadales (1236)	Gammaproteobacteria (122	Proteobacteria (2)	Bacteria (1)	100.00	0.00	99.70	99.50	WGS
287.9321	0.368	Pseudomonas aeruginosa (286)	Pseudomonas (135621)	Pseudomonadaceae (7227	Pseudomonadales (1236)	Gammaproteobacteria (122	Proteobacteria (2)	Bacteria (1)			99.70	99.40	WGS
287.9322	0.368	Pseudomonas aeruginosa (286)	Pseudomonas (135621)	Pseudomonadaceae (7227	Pseudomonadales (1236)	Gammaproteobacteria (122	Proteobacteria (2)	Bacteria (1)			99.70	99.40	WGS
287.9323	0.3636	Pseudomonas aeruginosa (286)	Pseudomonas (135621)	Pseudomonadaceae (7227	Pseudomonadales (1236)	Gammaproteobacteria (122	Proteobacteria (2)	Bacteria (1)			99.80	99.00	WGS
287.9324	0.3636	Pseudomonas aeruginosa (286)	Pseudomonas (135621)	Pseudomonadaceae (7227	Pseudomonadales (1236)	Gammaproteobacteria (122	Proteobacteria (2)	Bacteria (1)			99.80	99.20	WGS
287.9325	0.329	Pseudomonas aeruginosa (286)	Pseudomonas (135621)	Pseudomonadaceae (7227	Pseudomonadales (1236)	Gammaproteobacteria (122	Proteobacteria (2)	Bacteria (1)			99.90	98.90	WGS
287.9326	0.3738	Pseudomonas aeruginosa (286)	Pseudomonas (135621)	Pseudomonadaceae (7227	Pseudomonadales (1236)	Gammaproteobacteria (122	Proteobacteria (2)	Bacteria (1)			100.00	99.00	WGS
287.9327	0.368	Pseudomonas aeruginosa (286)	Pseudomonas (135621)	Pseudomonadaceae (7227	Pseudomonadales (1236)	Gammaproteobacteria (122	Proteobacteria (2)	Bacteria (1)			99.70	99.30	WGS
287.9328	0.3738	Pseudomonas aeruginosa (286)	Pseudomonas (135621)	Pseudomonadaceae (7227	Pseudomonadales (1236)	Gammaproteobacteria (122	Proteobacteria (2)	Bacteria (1)			100.00	99.20	WGS
287.9329	0.371	Pseudomonas aeruginosa (286)	Pseudomonas (135621)	Pseudomonadaceae (7227	Pseudomonadales (1236)	Gammaproteobacteria (122	Proteobacteria (2)	Bacteria (1)			99.80	98.30	WGS
287.9330	0.368	Pseudomonas aeruginosa (286)	Pseudomonas (135621)	Pseudomonadaceae (7227	Pseudomonadales (1236)	Gammaproteobacteria (122	Proteobacteria (2)	Bacteria (1)			99.70	99.20	WGS
287.933	0.3636	Pseudomonas aeruginosa (286)	Pseudomonas (135621)	Pseudomonadaceae (7227	Pseudomonadales (1236)	Gammaproteobacteria (122	Proteobacteria (2)	Bacteria (1)	100.00	0.00	99.40	98.70	WGS
287.9331	0.368	Pseudomonas aeruginosa (286)	Pseudomonas (135621)	Pseudomonadaceae (7227	Pseudomonadales (1236)	Gammaproteobacteria (122	Proteobacteria (2)	Bacteria (1)			99.70	99.40	WGS
287.9332	0.3376	Pseudomonas aeruginosa (286)	Pseudomonas (135621)	Pseudomonadaceae (7227	Pseudomonadales (1236)	Gammaproteobacteria (122	Proteobacteria (2)	Bacteria (1)			99.90	98.50	WGS
287.9333	0.3604	Pseudomonas aeruginosa (286)	Pseudomonas (135621)	Pseudomonadaceae (7227	Pseudomonadales (1236)	Gammaproteobacteria (122	Proteobacteria (2)	Bacteria (1)			99.70	99.10	WGS
287.9334	0.356	Pseudomonas aeruginosa (286)	Pseudomonas (135621)	Pseudomonadaceae (7227	Pseudomonadales (1236)	Gammaproteobacteria (122	Proteobacteria (2)	Bacteria (1)			99.80	98.20	WGS
287.9335	0.3226	Pseudomonas aeruginosa (286)	Pseudomonas (135621)	Pseudomonadaceae (7227	Pseudomonadales (1236)	Gammaproteobacteria (122	Proteobacteria (2)	Bacteria (1)			99.80	98.10	WGS
287.9336	0.3636	Pseudomonas aeruginosa (286)	Pseudomonas (135621)	Pseudomonadaceae (7227	Pseudomonadales (1236)	Gammaproteobacteria (122	Proteobacteria (2)	Bacteria (1)					WGS
287.9337	0.3268	Pseudomonas aeruginosa (286)	Pseudomonas (135621)	Pseudomonadaceae (7227	Pseudomonadales (1236)	Gammaproteobacteria (122	Proteobacteria (2)	Bacteria (1)			99.90	98.70	WGS
287.9338	0.3636	Pseudomonas aeruginosa (286)	Pseudomonas (135621)	Pseudomonadaceae (7227	Pseudomonadales (1236)	Gammaproteobacteria (122	Proteobacteria (2)	Bacteria (1)			99.80	99.20	WGS
287.9339	0.368	Pseudomonas aeruginosa (286)	Pseudomonas (135621)	Pseudomonadaceae (7227	Pseudomonadales (1236)	Gammaproteobacteria (122	Proteobacteria (2)	Bacteria (1)			99.70	99.40	WGS
287.9340	0.3376	Pseudomonas aeruginosa (286)	Pseudomonas (135621)	Pseudomonadaceae (7227	Pseudomonadales (1236)	Gammaproteobacteria (122	Proteobacteria (2)	Bacteria (1)			99.90	99.10	WGS
287.934	0.3588	Pseudomonas aeruginosa (286)	Pseudomonas (135621)	Pseudomonadaceae (7227	Pseudomonadales (1236)	Gammaproteobacteria (122	Proteobacteria (2)	Bacteria (1)	100.00	0.00	99.40	99.00	WGS
287.9341	0.3376	Pseudomonas aeruginosa (286)	Pseudomonas (135621)	Pseudomonadaceae (7227	Pseudomonadales (1236)	Gammaproteobacteria (122	Proteobacteria (2)	Bacteria (1)			99.90	99.10	WGS
287.9342	0.3652	Pseudomonas aeruginosa (286)	Pseudomonas (135621)	Pseudomonadaceae (7227	Pseudomonadales (1236)	Gammaproteobacteria (122	Proteobacteria (2)	Bacteria (1)			99.70	99.00	WGS
287.9343	0.3748	Pseudomonas aeruginosa (286)	Pseudomonas (135621)	Pseudomonadaceae (7227	Pseudomonadales (1236)	Gammaproteobacteria (122	Proteobacteria (2)	Bacteria (1)			99.90	98.80	WGS
287.9344	0.328	Pseudomonas aeruginosa (286)	Pseudomonas (135621)	Pseudomonadaceae (7227	Pseudomonadales (1236)	Gammaproteobacteria (122	Proteobacteria (2)	Bacteria (1)			99.90	99.20	WGS
287.9345	0.371	Pseudomonas aeruginosa (286)	Pseudomonas (135621)	Pseudomonadaceae (7227	Pseudomonadales (1236)	Gammaproteobacteria (122	Proteobacteria (2)	Bacteria (1)			99.90	98.80	WGS
287.9346	0.371	Pseudomonas aeruginosa (286)	Pseudomonas (135621)	Pseudomonadaceae (7227	Pseudomonadales (1236)	Gammaproteobacteria (122	Proteobacteria (2)	Bacteria (1)			99.90	98.60	WGS
287.9347	0.368	Pseudomonas aeruginosa (286)	Pseudomonas (135621)	Pseudomonadaceae (7227	Pseudomonadales (1236)	Gammaproteobacteria (122	Proteobacteria (2)	Bacteria (1)			99.80	99.10	WGS
287.9348	0.3388	Pseudomonas aeruginosa (286)	Pseudomonas (135621)	Pseudomonadaceae (7227	Pseudomonadales (1236)	Gammaproteobacteria (122	Proteobacteria (2)	Bacteria (1)			99.90	98.80	WGS
287.9349	0.3236	Pseudomonas aeruginosa (286)	Pseudomonas (135621)	Pseudomonadaceae (7227	Pseudomonadales (1236)	Gammaproteobacteria (122	Proteobacteria (2)	Bacteria (1)			99.70	98.10	WGS
287.9350	0.3632	Pseudomonas aeruginosa (286)	Pseudomonas (135621)	Pseudomonadaceae (7227	Pseudomonadales (1236)	Gammaproteobacteria (122	Proteobacteria (2)	Bacteria (1)			99.70	98.40	WGS
287.9351	0.3416	Pseudomonas aeruginosa (286)	Pseudomonas (135621)	Pseudomonadaceae (7227	Pseudomonadales (1236)	Gammaproteobacteria (122	Proteobacteria (2)	Bacteria (1)			98.70	81.20	WGS
287.9352	0.3782	Pseudomonas aeruginosa (286)	Pseudomonas (135621)	Pseudomonadaceae (7227	Pseudomonadales (1236)	Gammaproteobacteria (122	Proteobacteria (2)	Bacteria (1)			98.90	80.70	WGS
287.9353	0.3542	Pseudomonas aeruginosa (286)	Pseudomonas (135621)	Pseudomonadaceae (7227	Pseudomonadales (1236)	Gammaproteobacteria (122	Proteobacteria (2)	Bacteria (1)			98.70	87.40	WGS
287.9354	0.3086	Pseudomonas aeruginosa (286)	Pseudomonas (135621)	Pseudomonadaceae (7227	Pseudomonadales (1236)	Gammaproteobacteria (122	Proteobacteria (2)	Bacteria (1)			97.30	79.20	WGS
287.9355	0.327	Pseudomonas aeruginosa (286)	Pseudomonas (135621)	Pseudomonadaceae (7227	Pseudomonadales (1236)	Gammaproteobacteria (122	Proteobacteria (2)	Bacteria (1)			100.00	99.30	Complete
287.9356	0.3306	Pseudomonas aeruginosa (286)	Pseudomonas (135621)	Pseudomonadaceae (7227	Pseudomonadales (1236)	Gammaproteobacteria (122	Proteobacteria (2)	Bacteria (1)			99.80	98.70	WGS
287.9357	0.3306	Pseudomonas aeruginosa (286)	Pseudomonas (135621)	Pseudomonadaceae (7227	Pseudomonadales (1236)	Gammaproteobacteria (122	Proteobacteria (2)	Bacteria (1)			99.80	98.70	WGS
287.9358	0.324	Pseudomonas aeruginosa (286)	Pseudomonas (135621)	Pseudomonadaceae (7227	Pseudomonadales (1236)	Gammaproteobacteria (122	Proteobacteria (2)	Bacteria (1)			99.90	99.20	WGS
287.9359	0.3314	Pseudomonas aeruginosa (286)	Pseudomonas (135621)	Pseudomonadaceae (7227	Pseudomonadales (1236)	Gammaproteobacteria (122	Proteobacteria (2)	Bacteria (1)			99.90	98.80	WGS
287.9360	0.3262	Pseudomonas aeruginosa (286)	Pseudomonas (135621)	Pseudomonadaceae (7227	Pseudomonadales (1236)	Gammaproteobacteria (122	Proteobacteria (2)	Bacteria (1)			99.90	98.70	WGS
287.9361	0.3314	Pseudomonas aeruginosa (286)	Pseudomonas (135621)	Pseudomonadaceae (7227	Pseudomonadales (1236)	Gammaproteobacteria (122	Proteobacteria (2)	Bacteria (1)			99.90	98.80	WGS
287.9362	0.3824	Pseudomonas aeruginosa (286)	Pseudomonas (135621)	Pseudomonadaceae (7227	Pseudomonadales (1236)	Gammaproteobacteria (122	Proteobacteria (2)	Bacteria (1)			98.40	88.00	WGS
287.9363	0.3316	Pseudomonas aeruginosa (286)	Pseudomonas (135621)	Pseudomonadaceae (7227	Pseudomonadales (1236)	Gammaproteobacteria (122	Proteobacteria (2)	Bacteria (1)			98.40	81.20	WGS
287.9364	0.347	Pseudomonas aeruginosa (286)	Pseudomonas (135621)	Pseudomonadaceae (7227	Pseudomonadales (1236)	Gammaproteobacteria (122	Proteobacteria (2)	Bacteria (1)			98.40	81.70	WGS
287.9365	0.333	Pseudomonas aeruginosa (286)	Pseudomonas (135621)	Pseudomonadaceae (7227	Pseudomonadales (1236)	Gammaproteobacteria (122	Proteobacteria (2)	Bacteria (1)			99.00	84.80	WGS
287.9366	0.3074	Pseudomonas aeruginosa (286)	Pseudomonas (135621)	Pseudomonadaceae (7227	Pseudomonadales (1236)	Gammaproteobacteria (122	Proteobacteria (2)	Bacteria (1)			98.60	82.80	WGS
287.9367	0.3424	Pseudomonas aeruginosa (286)	Pseudomonas (135621)	Pseudomonadaceae (7227	Pseudomonadales (1236)	Gammaproteobacteria (122	Proteobacteria (2)	Bacteria (1)			99.70	92.00	WGS
287.9368	0.3278	Pseudomonas aeruginosa (286)	Pseudomonas (135621)	Pseudomonadaceae (7227	Pseudomonadales (1236)	Gammaproteobacteria (122	Proteobacteria (2)	Bacteria (1)			99.50	91.20	WGS
287.9369	0.3474	Pseudomonas aeruginosa (286)	Pseudomonas (135621)	Pseudomonadaceae (7227	Pseudomonadales (1236)	Gammaproteobacteria (122	Proteobacteria (2)	Bacteria (1)			92.80	75.80	WGS
287.9370	0.3818	Pseudomonas aeruginosa (286)	Pseudomonas (135621)	Pseudomonadaceae (7227	Pseudomonadales (1236)	Gammaproteobacteria (122	Proteobacteria (2)	Bacteria (1)			97.30	80.30	WGS
287.9371	0.3384	Pseudomonas aeruginosa (286)	Pseudomonas (135621)	Pseudomonadaceae (7227	Pseudomonadales (1236)	Gammaproteobacteria (122	Proteobacteria (2)	Bacteria (1)			97.40	82.40	WGS

287.9372	0.3578	Pseudomonas aeruginosa (286)	Pseudomonas (135621)	Pseudomonadaceae (7227	Pseudomonadales (1236)	Gammaproteobacteria (122	Proteobacteria (2)	Bacteria (1)			99.30	89.10	WGS
287.9373	0.3426	Pseudomonas aeruginosa (286)	Pseudomonas (135621)	Pseudomonadaceae (7227	Pseudomonadales (1236)	Gammaproteobacteria (122	Proteobacteria (2)	Bacteria (1)			96.00	75.10	WGS
287.9374	0.3248	Pseudomonas aeruginosa (286)	Pseudomonas (135621)	Pseudomonadaceae (7227	Pseudomonadales (1236)	Gammaproteobacteria (122	Proteobacteria (2)	Bacteria (1)			99.90	90.80	WGS
287.9375	0.3604	Pseudomonas aeruginosa (286)	Pseudomonas (135621)	Pseudomonadaceae (7227	Pseudomonadales (1236)	Gammaproteobacteria (122	Proteobacteria (2)	Bacteria (1)			99.80	98.20	WGS
287.9376	0.3664	Pseudomonas aeruginosa (286)	Pseudomonas (135621)	Pseudomonadaceae (7227	Pseudomonadales (1236)	Gammaproteobacteria (122	Proteobacteria (2)	Bacteria (1)			99.90	98.70	WGS
287.9377	0.3614	Pseudomonas aeruginosa (286)	Pseudomonas (135621)	Pseudomonadaceae (7227	Pseudomonadales (1236)	Gammaproteobacteria (122	Proteobacteria (2)	Bacteria (1)			99.30	96.60	WGS
287.9378	0.3376	Pseudomonas aeruginosa (286)	Pseudomonas (135621)	Pseudomonadaceae (7227	Pseudomonadales (1236)	Gammaproteobacteria (122	Proteobacteria (2)	Bacteria (1)			99.90	97.00	Complete
287.9379	0.331	Pseudomonas aeruginosa (286)	Pseudomonas (135621)	Pseudomonadaceae (7227	Pseudomonadales (1236)	Gammaproteobacteria (122	Proteobacteria (2)	Bacteria (1)			99.70	98.50	Complete
287.9380	0.3314	Pseudomonas aeruginosa (286)	Pseudomonas (135621)	Pseudomonadaceae (7227	Pseudomonadales (1236)	Gammaproteobacteria (122	Proteobacteria (2)	Bacteria (1)			99.90	97.60	WGS
287.9381	0.3314	Pseudomonas aeruginosa (286)	Pseudomonas (135621)	Pseudomonadaceae (7227	Pseudomonadales (1236)	Gammaproteobacteria (122	Proteobacteria (2)	Bacteria (1)			99.90	98.40	WGS
287.9382	0.3314	Pseudomonas aeruginosa (286)	Pseudomonas (135621)	Pseudomonadaceae (7227	Pseudomonadales (1236)	Gammaproteobacteria (122	Proteobacteria (2)	Bacteria (1)			99.90	98.50	WGS
287.9383	0.3244	Pseudomonas aeruginosa (286)	Pseudomonas (135621)	Pseudomonadaceae (7227	Pseudomonadales (1236)	Gammaproteobacteria (122	Proteobacteria (2)	Bacteria (1)			99.90	98.20	WGS
287.9384	0.3314	Pseudomonas aeruginosa (286)	Pseudomonas (135621)	Pseudomonadaceae (7227	Pseudomonadales (1236)	Gammaproteobacteria (122	Proteobacteria (2)	Bacteria (1)			99.90	98.70	WGS
287.9385	0.3314	Pseudomonas aeruginosa (286)	Pseudomonas (135621)	Pseudomonadaceae (7227	Pseudomonadales (1236)	Gammaproteobacteria (122	Proteobacteria (2)	Bacteria (1)			99.90	98.60	WGS
287.9386	0.3314	Pseudomonas aeruginosa (286)	Pseudomonas (135621)	Pseudomonadaceae (7227	Pseudomonadales (1236)	Gammaproteobacteria (122	Proteobacteria (2)	Bacteria (1)			99.90	98.20	WGS
287.9387	0.3314	Pseudomonas aeruginosa (286)	Pseudomonas (135621)	Pseudomonadaceae (7227	Pseudomonadales (1236)	Gammaproteobacteria (122	Proteobacteria (2)	Bacteria (1)			99.90	97.90	WGS
287.9388	0.3314	Pseudomonas aeruginosa (286)	Pseudomonas (135621)	Pseudomonadaceae (7227	Pseudomonadales (1236)	Gammaproteobacteria (122	Proteobacteria (2)	Bacteria (1)			99.90	98.40	WGS
287.9389	0.3314	Pseudomonas aeruginosa (286)	Pseudomonas (135621)	Pseudomonadaceae (7227	Pseudomonadales (1236)	Gammaproteobacteria (122	Proteobacteria (2)	Bacteria (1)			99.90	98.10	WGS
287.9390	0.3314	Pseudomonas aeruginosa (286)	Pseudomonas (135621)	Pseudomonadaceae (7227	Pseudomonadales (1236)	Gammaproteobacteria (122	Proteobacteria (2)	Bacteria (1)			99.90	98.10	WGS
287.9391	0.3314	Pseudomonas aeruginosa (286)	Pseudomonas (135621)	Pseudomonadaceae (7227	Pseudomonadales (1236)	Gammaproteobacteria (122	Proteobacteria (2)	Bacteria (1)			99.90	98.20	WGS
287.9392	0.3314	Pseudomonas aeruginosa (286)	Pseudomonas (135621)	Pseudomonadaceae (7227	Pseudomonadales (1236)	Gammaproteobacteria (122	Proteobacteria (2)	Bacteria (1)			99.90	98.50	WGS
287.9393	0.3314	Pseudomonas aeruginosa (286)	Pseudomonas (135621)	Pseudomonadaceae (7227	Pseudomonadales (1236)	Gammaproteobacteria (122	Proteobacteria (2)	Bacteria (1)			99.90	98.50	WGS
287.9394	0.3314	Pseudomonas aeruginosa (286)	Pseudomonas (135621)	Pseudomonadaceae (7227	Pseudomonadales (1236)	Gammaproteobacteria (122	Proteobacteria (2)	Bacteria (1)			99.90	98.70	WGS
287.9395	0.3314	Pseudomonas aeruginosa (286)	Pseudomonas (135621)	Pseudomonadaceae (7227	Pseudomonadales (1236)	Gammaproteobacteria (122	Proteobacteria (2)	Bacteria (1)			99.90	98.60	WGS
287.9396	0.3314	Pseudomonas aeruginosa (286)	Pseudomonas (135621)	Pseudomonadaceae (7227	Pseudomonadales (1236)	Gammaproteobacteria (122	Proteobacteria (2)	Bacteria (1)			99.90	98.60	WGS
287.9397	0.3314	Pseudomonas aeruginosa (286)	Pseudomonas (135621)	Pseudomonadaceae (7227	Pseudomonadales (1236)	Gammaproteobacteria (122	Proteobacteria (2)	Bacteria (1)			99.90	98.70	WGS
287.9398	0.3314	Pseudomonas aeruginosa (286)	Pseudomonas (135621)	Pseudomonadaceae (7227	Pseudomonadales (1236)	Gammaproteobacteria (122	Proteobacteria (2)	Bacteria (1)			99.90	98.30	WGS
287.9399	0.3314	Pseudomonas aeruginosa (286)	Pseudomonas (135621)	Pseudomonadaceae (7227	Pseudomonadales (1236)	Gammaproteobacteria (122	Proteobacteria (2)	Bacteria (1)			99.90	98.50	WGS
287.9400	0.3314	Pseudomonas aeruginosa (286)	Pseudomonas (135621)	Pseudomonadaceae (7227	Pseudomonadales (1236)	Gammaproteobacteria (122	Proteobacteria (2)	Bacteria (1)			99.90	98.30	WGS
287.9401	0.3314	Pseudomonas aeruginosa (286)	Pseudomonas (135621)	Pseudomonadaceae (7227	Pseudomonadales (1236)	Gammaproteobacteria (122	Proteobacteria (2)	Bacteria (1)			99.90	98.30	WGS
287.9402	0.3314	Pseudomonas aeruginosa (286)	Pseudomonas (135621)	Pseudomonadaceae (7227	Pseudomonadales (1236)	Gammaproteobacteria (122	Proteobacteria (2)	Bacteria (1)			99.90	98.30	WGS
287.9403	0.3314	Pseudomonas aeruginosa (286)	Pseudomonas (135621)	Pseudomonadaceae (7227	Pseudomonadales (1236)	Gammaproteobacteria (122	Proteobacteria (2)	Bacteria (1)			99.90	98.50	WGS
287.9404	0.3314	Pseudomonas aeruginosa (286)	Pseudomonas (135621)	Pseudomonadaceae (7227	Pseudomonadales (1236)	Gammaproteobacteria (122	Proteobacteria (2)	Bacteria (1)			99.90	98.00	WGS
287.9405	0.3314	Pseudomonas aeruginosa (286)	Pseudomonas (135621)	Pseudomonadaceae (7227	Pseudomonadales (1236)	Gammaproteobacteria (122	Proteobacteria (2)	Bacteria (1)			99.90	98.40	WGS
287.9406	0.3314	Pseudomonas aeruginosa (286)	Pseudomonas (135621)	Pseudomonadaceae (7227	Pseudomonadales (1236)	Gammaproteobacteria (122	Proteobacteria (2)	Bacteria (1)			99.90	98.40	WGS
287.9407	0.0294	Pseudomonas aeruginosa (286)	Pseudomonas (135621)	Pseudomonadaceae (7227	Pseudomonadales (1236)	Gammaproteobacteria (122	Proteobacteria (2)	Bacteria (1)			96.30	96.30	Complete
287.9408	0.3614	Pseudomonas aeruginosa (286)	Pseudomonas (135621)	Pseudomonadaceae (7227	Pseudomonadales (1236)	Gammaproteobacteria (122	Proteobacteria (2)	Bacteria (1)			98.70	98.20	WGS
287.9409	0.0294	Pseudomonas aeruginosa (286)	Pseudomonas (135621)	Pseudomonadaceae (7227	Pseudomonadales (1236)	Gammaproteobacteria (122	Proteobacteria (2)	Bacteria (1)			96.30	96.30	Complete
287.9410	0.0294	Pseudomonas aeruginosa (286)	Pseudomonas (135621)	Pseudomonadaceae (7227	Pseudomonadales (1236)	Gammaproteobacteria (122	Proteobacteria (2)	Bacteria (1)			96.10	96.10	Complete
287.9411	0.3252	Pseudomonas aeruginosa (286)	Pseudomonas (135621)	Pseudomonadaceae (7227	Pseudomonadales (1236)	Gammaproteobacteria (122	Proteobacteria (2)	Bacteria (1)			99.60	97.60	WGS
287.9412	0.3376	Pseudomonas aeruginosa (286)	Pseudomonas (135621)	Pseudomonadaceae (7227	Pseudomonadales (1236)	Gammaproteobacteria (122	Proteobacteria (2)	Bacteria (1)			99.00	97.00	WGS
287.9413	0.331	Pseudomonas aeruginosa (286)	Pseudomonas (135621)	Pseudomonadaceae (7227	Pseudomonadales (1236)	Gammaproteobacteria (122	Proteobacteria (2)	Bacteria (1)			98.70	95.90	WGS
287.9414	0.3604	Pseudomonas aeruginosa (286)	Pseudomonas (135621)	Pseudomonadaceae (7227	Pseudomonadales (1236)	Gammaproteobacteria (122	Proteobacteria (2)	Bacteria (1)			99.30	98.60	WGS
287.9415	0.3684	Pseudomonas aeruginosa (286)	Pseudomonas (135621)	Pseudomonadaceae (7227	Pseudomonadales (1236)	Gammaproteobacteria (122	Proteobacteria (2)	Bacteria (1)			99.80	98.10	WGS
287.9416	0.3268	Pseudomonas aeruginosa (286)	Pseudomonas (135621)	Pseudomonadaceae (7227	Pseudomonadales (1236)	Gammaproteobacteria (122	Proteobacteria (2)	Bacteria (1)			99.80	98.50	WGS
287.9417	0.3672	Pseudomonas aeruginosa (286)	Pseudomonas (135621)	Pseudomonadaceae (7227	Pseudomonadales (1236)	Gammaproteobacteria (122	Proteobacteria (2)	Bacteria (1)			99.60	98.40	WGS
287.9418	0.3656	Pseudomonas aeruginosa (286)	Pseudomonas (135621)	Pseudomonadaceae (7227	Pseudomonadales (1236)	Gammaproteobacteria (122	Proteobacteria (2)	Bacteria (1)			99.60	99.10	WGS
287.9419	0.3314	Pseudomonas aeruginosa (286)	Pseudomonas (135621)	Pseudomonadaceae (7227	Pseudomonadales (1236)	Gammaproteobacteria (122	Proteobacteria (2)	Bacteria (1)			99.40	98.20	WGS
287.9420	0.3306	Pseudomonas aeruginosa (286)	Pseudomonas (135621)	Pseudomonadaceae (7227	Pseudomonadales (1236)	Gammaproteobacteria (122	Proteobacteria (2)	Bacteria (1)			99.30	97.90	WGS
287.9421	0.331	Pseudomonas aeruginosa (286)	Pseudomonas (135621)	Pseudomonadaceae (7227	Pseudomonadales (1236)	Gammaproteobacteria (122	Proteobacteria (2)	Bacteria (1)			99.90	99.00	WGS
287.9422	0.3314	Pseudomonas aeruginosa (286)	Pseudomonas (135621)	Pseudomonadaceae (7227	Pseudomonadales (1236)	Gammaproteobacteria (122	Proteobacteria (2)	Bacteria (1)			99.90	98.80	Complete
287.9423	0.3422	Pseudomonas aeruginosa (286)	Pseudomonas (135621)	Pseudomonadaceae (7227	Pseudomonadales (1236)	Gammaproteobacteria (122	Proteobacteria (2)	Bacteria (1)			99.80	86.10	WGS
287.9424	0.363	Pseudomonas aeruginosa (286)	Pseudomonas (135621)	Pseudomonadaceae (7227	Pseudomonadales (1236)	Gammaproteobacteria (122	Proteobacteria (2)	Bacteria (1)			99.40	88.30	WGS
287.9425	0.3226	Pseudomonas aeruginosa (286)	Pseudomonas (135621)	Pseudomonadaceae (7227	Pseudomonadales (1236)	Gammaproteobacteria (122	Proteobacteria (2)	Bacteria (1)			99.90	92.60	WGS
287.9426	0.36	Pseudomonas aeruginosa (286)	Pseudomonas (135621)	Pseudomonadaceae (7227	Pseudomonadales (1236)	Gammaproteobacteria (122	Proteobacteria (2)	Bacteria (1)			99.40	94.20	WGS
287.9427	0.3676	Pseudomonas aeruginosa (286)	Pseudomonas (135621)	Pseudomonadaceae (7227	Pseudomonadales (1236)	Gammaproteobacteria (122	Proteobacteria (2)	Bacteria (1)			99.30	94.50	WGS
287.9428	0.3342	Pseudomonas aeruginosa (286)	Pseudomonas (135621)	Pseudomonadaceae (7227	Pseudomonadales (1236)	Gammaproteobacteria (122	Proteobacteria (2)	Bacteria (1)			98.50	88.60	WGS
287.9429	0.4078	Pseudomonas aeruginosa (286)	Pseudomonas (135621)	Pseudomonadaceae (7227	Pseudomonadales (1236)	Gammaproteobacteria (122	Proteobacteria (2)	Bacteria (1)			99.00	87.90	WGS
287.9430	0.3696	Pseudomonas aeruginosa (286)	Pseudomonas (135621)	Pseudomonadaceae (7227	Pseudomonadales (1236)	Gammaproteobacteria (122	Proteobacteria (2)	Bacteria (1)			99.60	97.30	WGS
287.9431	0.331	Pseudomonas aeruginosa (286)	Pseudomonas (135621)	Pseudomonadaceae (7227	Pseudomonadales (1236)	Gammaproteobacteria (122	Proteobacteria (2)	Bacteria (1)			99.40	95.30	WGS
287.9432	0.3454	Pseudomonas aeruginosa (286)	Pseudomonas (135621)	Pseudomonadaceae (7227	Pseudomonadales (1236)	Gammaproteobacteria (122	Proteobacteria (2)	Bacteria (1)			99.60	94.10	WGS
287.9433	0.3376	Pseudomonas aeruginosa (286)	Pseudomonas (135621)	Pseudomonadaceae (7227	Pseudomonadales (1236)	Gammaproteobacteria (122	Proteobacteria (2)	Bacteria (1)			99.90	86.90	WGS
287.9434	0.3226	Pseudomonas aeruginosa (286)	Pseudomonas (135621)	Pseudomonadaceae (7227	Pseudomonadales (1236)	Gammaproteobacteria (122	Proteobacteria (2)	Bacteria (1)			99.60	91.60	WGS
287.9435	0.3944	Pseudomonas aeruginosa (286)	Pseudomonas (135621)	Pseudomonadaceae (7227	Pseudomonadales (1236)	Gammaproteobacteria (122	Proteobacteria (2)	Bacteria (1)			99.70	91.90	WGS
287.9436	0.3364	Pseudomonas aeruginosa (286)	Pseudomonas (135621)	Pseudomonadaceae (7227	Pseudomonadales (1236)	Gammaproteobacteria (122	Proteobacteria (2)	Bacteria (1)			99.80	86.30	WGS
287.9437	0.3696	Pseudomonas aeruginosa (286)	Pseudomonas (135621)	Pseudomonadaceae (7227	Pseudomonadales (1236)	Gammaproteobacteria (122	Proteobacteria (2)	Bacteria (1)			99.40	83.10	WGS
287.9438	0.3672	Pseudomonas aeruginosa (286)	Pseudomonas (135621)	Pseudomonadaceae (7227	Pseudomonadales (1236)	Gammaproteobacteria (122	Proteobacteria (2)	Bacteria (1)			99.50	87.80	WGS
287.9439	0.3378	Pseudomonas aeruginosa (286)	Pseudomonas (135621)	Pseudomonadaceae (7227	Pseudomonadales (1236)	Gammaproteobacteria (122	Proteobacteria (2)	Bacteria (1)			98.60	76.70	WGS
287.9440	0.3738	Pseudomonas aeruginosa (286)	Pseudomonas (135621)	Pseudomonadaceae (7227	Pseudomonadales (1236)	Gammaproteobacteria (122	Proteobacteria (2)	Bacteria (1)			99.30	85.10	WGS
287.9441	0.369	Pseudomonas aeruginosa (286)	Pseudomonas (135621)	Pseudomonadaceae (7227	Pseudomonadales (1236)	Gammaproteobacteria (122	Proteobacteria (2)	Bacteria (1)			99.60	92.20	WGS
287.9442	0.3738	Pseudomonas aeruginosa (286)	Pseudomonas (135621)	Pseudomonadaceae (7227	Pseudomonadales (1236)	Gammaproteobacteria (122	Proteobacteria (2)	Bacteria (1)			99.60	92.00	WGS
287.9443	0.327	Pseudomonas aeruginosa (286)	Pseudomonas (135621)	Pseudomonadaceae (7227	Pseudomonadales (1236)	Gammaproteobacteria (122	Proteobacteria (2)	Bacteria (1)			99.70	84.60	WGS
287.9444	0.3346	Pseudomonas aeruginosa (286)	Pseudomonas (135621)	Pseudomonadaceae (7227	Pseudomonadales (1236)	Gammaproteobacteria (122	Proteobacteria (2)	Bacteria (1)			99.60	86.90	WGS
287.9445	0.3614	Pseudomonas aeruginosa (286)	Pseudomonas (135621)	Pseudomonadaceae (7227	Pseudomonadales (1236)	Gammaproteobacteria (122	Proteobacteria (2)	Bacteria (1)			99.40	90.20	WGS
287.9446	0.3284	Pseudomonas aeruginosa (286)	Pseudomonas (135621)	Pseudomonadaceae (7227	Pseudomonadales (1236)	Gammaproteobacteria (122	Proteobacteria (2)	Bacteria (1)			99.30	88.10	WGS
287.9447	0.3618	Pseudomonas aeruginosa (286)	Pseudomonas (135621)	Pseudomonadaceae (7227	Pseudomonadales (1236)	Gammaproteobacteria (122	Proteobacteria (2)	Bacteria (1)			99.50	93.10	WGS
287.9448	0.333	Pseudomonas aeruginosa (286)	Pseudomonas (135621)	Pseudomonadaceae (7227	Pseudomonadales (1236)	Gammaproteobacteria (122	Proteobacteria (2)	Bacteria (1)			99.80	87.30	WGS
287.9449	0.37	Pseudomonas aeruginosa (286)	Pseudomonas (135621)	Pseudomonadaceae (7227	Pseudomonadales (1236)	Gammaproteobacteria (122	Proteobacteria (2)	Bacteria (1)			99.50	84.60	WGS
287.9450	0.3308	Pseudomonas aeruginosa (286)	Pseudomonas (135621)	Pseudomonadaceae (7227	Pseudomonadales (1236)	Gammaproteobacteria (122	Proteobacteria (2)	Bacteria (1)			99.60	90.30	WGS
287.9451	0.3628	Pseudomonas aeruginosa (286)	Pseudomonas (135621)	Pseudomonadaceae (7227	Pseudomonadales (1236)	Gammaproteobacteria (122	Proteobacteria (2)	Bacteria (1)			99.80	87.50	WGS
287.9452	0.3174	Pseudomonas aeruginosa (286)	Pseudomonas (135621)	Pseudomonadaceae (7227	Pseudomonadales (1236)	Gammaproteobacteria (122	Proteobacteria (2)	Bacteria (1)			99.50	86.70	WGS
287.9453	0.3174	Pseudomonas aeruginosa (286)	Pseudomonas (135621)	Pseudomonadaceae (7227	Pseudomonadales (1236)	Gammaproteobacteria (122	Proteobacteria (2)	Bacteria (1)			99.60	92.70	WGS
287.9454	0.3262	Pseudomonas aeruginosa (286)	Pseudomonas (135621)	Pseudomonadaceae (7227	Pseudomonadales (1236)	Gammaproteobacteria (122	Proteobacteria (2)	Bacteria (1)			99.60	88.00	WGS
287.9455	0.2896	Pseudomonas aeruginosa (286)	Pseudomonas (135621)	Pseudomonadaceae (7227	Pseudomonadales (1236)	Gammaproteobacteria (122	Proteobacteria (2)	Bacteria (1)			99.30	85.70	WGS
287.9456	0.3174	Pseudomonas aeruginosa (286)	Pseudomonas (135621)	Pseudomonadaceae (7227	Pseudomonadales (1236)	Gammaproteobacteria (122	Proteobacteria (2)	Bacteria (1)			99.40	80.90	WGS
287.9457	0.3618	Pseudomonas aeruginosa (286)	Pseudomonas (135621)	Pseudomonadaceae (7227	Pseudomonadales (1236)	Gammaproteobacteria (122	Proteobacteria (2)	Bacteria (1)			99.60	87.70	WGS
287.9458	0.3386	Pseudomonas aeruginosa (286)	Pseudomonas (135621)	Pseudomonadaceae (7227	Pseudomonadales (1236)	Gammaproteobacteria (122	Proteobacteria (2)	Bacteria (1)			99.10	83.40	WGS
287.9459	0.3284	Pseudomonas aeruginosa (286)	Pseudomonas (135621)	Pseudomonadaceae (7227	Pseudomonadales (1236)	Gammaproteobacteria (122	Proteobacteria (2)	Bacteria (1)			99.30	79.20	WGS
287.9460	0.4048	Pseudomonas aeruginosa (286)	Pseudomonas (135621)	Pseudomonadaceae (7227	Pseudomonadales (1236)	Gammaproteobacteria (122	Proteobacteria (2)	Bacteria (1)			99.30	88.10	WGS
287.9461	0.3696	Pseudomonas aeruginosa (286)	Pseudomonas (135621)	Pseudomonadaceae (7227	Pseudomonadales (1236)	Gammaproteobacteria (122	Proteobacteria (2)	Bacteria (1)			99.60	89.20	WGS
287.9462	0.3236	Pseudomonas aeruginosa (286)	Pseudomonas (135621)	Pseudomonadaceae (7227	Pseudomonadales (1236)	Gammaproteobacteria (122	Proteobacteria (2)	Bacteria (1)			99.50	86.50	WGS
287.9463	0.3248	Pseudomonas aeruginosa (286)	Pseudomonas (135621)	Pseudomonadaceae (7227	Pseudomonadales (1236)	Gammaproteobacteria (122	Proteobacteria (2)	Bacteria (1)			99.50	91.40	WGS
287.9464	0.3372	Pseudomonas aeruginosa (286)	Pseudomonas (135621)	Pseudomonadaceae (7227	Pseudomonadales (1236)	Gammaproteobacteria (122	Proteobacteria (2)	Bacteria (1)			99.70	87.30	WGS
287.9465	0.3424	Pseudomonas aeruginosa (286)	Pseudomonas (135621)	Pseudomonadaceae (7227	Pseudomonadales (1236)	Gammaproteobacteria (122	Proteobacteria (2)	Bacteria (1)			99.50	92.20	WGS
287.9466	0.3466	Pseudomonas aeruginosa (286)	Pseudomonas (135621)	Pseudomonadaceae (7227	Pseudomonadales (1236)	Gammaproteobacteria (122	Proteobacteria (2)	Bacteria (1)			98.40	79.50	WGS
287.9467	0.3604	Pseudomonas aeruginosa (286)	Pseudomonas (135621)	Pseudomonadaceae (7227	Pseudomonadales (1236)	Gammaproteobacteria (122	Proteobacteria (2)	Bacteria (1)			99.70	93.30	WGS
287.9468	0.3556	Pseudomonas aeruginosa (286)	Pseudomonas (135621)	Pseudomonadaceae (7227	Pseudomonadales (1236)	Gammaproteobacteria (122	Proteobacteria (2)	Bacteria (1)			99.50	82.90	WGS
287.9469	0.3814	Pseudomonas aeruginosa (286)	Pseudomonas (135621)	Pseudomonadaceae (7227	Pseudomonadales (1236)	Gammaproteobacteria (122	Proteobacteria (2)	Bacteria (1)			99.70	92.80	WGS
287.9470	0.3174	Pseudomonas aeruginosa (286)	Pseudomonas (135621)	Pseudomonadaceae (7227	Pseudomonadales (1236)	Gammaproteobacteria (122	Proteobacteria (2)	Bacteria (1)			99.60	87.60	WGS
287.9471	0.3464	Pseudomonas aeruginosa (286)	Pseudomonas (135621)	Pseudomonadaceae (7227	Pseudomonadales (1236)	Gammaproteobacteria (122	Proteobacteria (2)	Bacteria (1)			99.70	86.80	WGS
287.9472	0.394	Pseudomonas aeruginosa (286)	Pseudomonas (135621)	Pseudomonadaceae (7227	Pseudomonadales (1236)	Gammaproteobacteria (122	Proteobacteria (2)	Bacteria (1)			99.70	93.90	WGS
287.9473	0.3324	Pseudomonas aeruginosa (286)	Pseudomonas (135621)	Pseudomonadaceae (7227	Pseudomonadales (1236)	Gammaproteobacteria (122	Proteobacteria (2)	Bacteria (1)			99.80	91.40	WGS
287.9474	0.3324	Pseudomonas aeruginosa (286)	Pseudomonas (135621)	Pseudomonadaceae (7227	Pseudomonadales (1236)	Gammaproteobacteria (122	Proteobacteria (2)	Bacteria (1)			99.80	93.60	WGS
287.9475	0.3636	Pseudomonas aeruginosa (286)	Pseudomonas (135621)	Pseudomonadaceae (7227	Pseudomonadales (1236)	Gammaproteobacteria (122	Proteobacteria (2)	Bacteria (1)			99.80	94.80	WGS
287.9476	0.3662	Pseudomonas aeruginosa (286)	Pseudomonas (135621)	Pseudomonadaceae (7227	Pseudomonadales (1236)	Gammaproteobacteria (122	Proteobacteria (2)	Bacteria (1)			99.60	93.00	WGS
287.9477	0.3754	Pseudomonas aeruginosa (286)	Pseudomonas (135621)	Pseudomonadaceae (7227	Pseudomonadales (1236)	Gammaproteobacteria (122	Proteobacteria (2)	Bacteria (1)			99.70	96.40	WGS
287.9478	0.3898	Pseudomonas aeruginosa (286)	Pseudomonas (135621)	Pseudomonadaceae (7227	Pseudomonadales (1236)	Gammaproteobacteria (122	Proteobacteria (2)	Bacteria (1)			99.80	90.40	WGS
287.9479	0.3174	Pseudomonas aeruginosa (286)	Pseudomonas (135621)	Pseudomonadaceae (7227	Pseudomonadales (1236)	Gammaproteobacteria (122	Proteobacteria (2)	Bacteria (1)			99.70	89.20	WGS
287.9480	0.3334	Pseudomonas aeruginosa (286)	Pseudomonas (135621)	Pseudomonadaceae (7227	Pseudomonadales (1236)	Gammaproteobacteria (122	Proteobacteria (2)	Bacteria (1)			99.50	88.70	WGS
287.9481	0.3424	Pseudomonas aeruginosa (286)	Pseudomonas (135621)	Pseudomonadaceae (7227	Pseudomonadales (1236)	Gammaproteobacteria (122	Proteobacteria (2)	Bacteria (1)			99.30	84.80	WGS
287.9482	0.3174	Pseudomonas aeruginosa (286)	Pseudomonas (135621)	Pseudomonadaceae (7227	Pseudomonadales (1236)	Gammaproteobacteria (122	Proteobacteria (2)	Bacteria (1)			99.60	86.20	WGS
287.9483	0.3028	Pseudomonas aeruginosa (286)	Pseudomonas (135621)	Pseudomonadaceae (7227	Pseudomonadales (1236)	Gammaproteobacteria (122	Proteobacteria (2)	Bacteria (1)			99.70	92.40	WGS
287.9484	0.3636	Pseudomonas aeruginosa (286)	Pseudomonas (135621)	Pseudomonadaceae (7227	Pseudomonadales (1236)	Gammaproteobacteria (122	Proteobacteria (2)	Bacteria (1)			99.40	83.90	WGS
287.9485	0.369	Pseudomonas aeruginosa (286)	Pseudomonas (135621)	Pseudomonadaceae (7227	Pseudomonadales (1236)	Gammaproteobacteria (122	Proteobacteria (2)	Bacteria (1)			99.70	91.10	WGS
287.9486	0.3636	Pseudomonas aeruginosa (286)	Pseudomonas (135621)	Pseudomonadaceae (7227	Pseudomonadales (1236)	Gammaproteobacteria (122	Proteobacteria (2)	Bacteria (1)			99.50	93.30	WGS
287.9487	0.2978	Pseudomonas aeruginosa (286)	Pseudomonas (135621)	Pseudomonadaceae (7227	Pseudomonadales (1236)	Gammaproteobacteria (122	Proteobacteria (2)	Bacteria (1)			99.60	91.00	WGS
287.9488	0.3226	Pseudomonas aeruginosa (286)	Pseudomonas (135621)	Pseudomonadaceae (7227	Pseudomonadales (1236)	Gammaproteobacteria (122	Proteobacteria (2)	Bacteria (1)			99.60	94.60	WGS
287.9489	0.2896	Pseudomonas aeruginosa (286)	Pseudomonas (135621)	Pseudomonadaceae (7227	Pseudomonadales (1236)	Gammaproteobacteria (122	Proteobacteria (2)	Bacteria (1)			99.60	89.80	WGS
287.9490	0.3268	Pseudomonas aeruginosa (286)	Pseudomonas (135621)	Pseudomonadaceae (7227	Pseudomonadales (1236)	Gammaproteobacteria (122	Proteobacteria (2)	Bacteria (1)			99.60	86.80	WGS
287.9491	0.3174	Pseudomonas aeruginosa (286)	Pseudomonas (135621)	Pseudomonadaceae (7227	Pseudomonadales (1236)	Gammaproteobacteria (122	Proteobacteria (2)	Bacteria (1)			99.70	92.30	WGS
287.9492	0.328	Pseudomonas aeruginosa (286)	Pseudomonas (135621)	Pseudomonadaceae (7227	Pseudomonadales (1236)	Gammaproteobacteria (122	Proteobacteria (2)	Bacteria (1)			99.80	91.10	WGS
287.9493	0.3424	Pseudomonas aeruginosa (286)	Pseudomonas (135621)	Pseudomonadaceae (7227	Pseudomonadales (1236)	Gammaproteobacteria (122	Proteobacteria (2)	Bacteria (1)			99.60	86.00	WGS
287.9494	0.3174	Pseudomonas aeruginosa (286)	Pseudomonas (135621)	Pseudomonadaceae (7227	Pseudomonadales (1236)	Gammaproteobacteria (122	Proteobacteria (2)	Bacteria (1)			99.60	93.50	WGS
287.9495	0.3314	Pseudomonas aeruginosa (286)	Pseudomonas (135621)	Pseudomonadaceae (7227	Pseudomonadales (1236)	Gammaproteobacteria (122	Proteobacteria (2)	Bacteria (1)			99.90	94.20	WGS
287.9496	0.3174	Pseudomonas aeruginosa (286)	Pseudomonas (135621)	Pseudomonadaceae (7227	Pseudomonadales (1236)	Gammaproteobacteria (122	Proteobacteria (2)	Bacteria (1)			99.70	88.20	WGS
287.9497	0.3172	Pseudomonas aeruginosa (286)	Pseudomonas (135621)	Pseudomonadaceae (7227	Pseudomonadales (1236)	Gammaproteobacteria (122	Proteobacteria (2)	Bacteria (1)			99.60	93.30	WGS
287.9498	0.3174	Pseudomonas aeruginosa (286)	Pseudomonas (135621)	Pseudomonadaceae (7227	Pseudomonadales (1236)	Gammaproteobacteria (122	Proteobacteria (2)	Bacteria (1)			99.60	93.70	WGS
287.9499	0.3174	Pseudomonas aeruginosa (286)	Pseudomonas (135621)	Pseudomonadaceae (7227	Pseudomonadales (1236)	Gammaproteobacteria (122	Proteobacteria (2)	Bacteria (1)			99.60	81.80	WGS
287.9500	0.3174	Pseudomonas aeruginosa (286)	Pseudomonas (135621)	Pseudomonadaceae (7227	Pseudomonadales (1236)	Gammaproteobacteria (122	Proteobacteria (2)	Bacteria (1)			99.90	91.50	WGS
287.9501	0.3226	Pseudomonas aeruginosa (286)	Pseudomonas (135621)	Pseudomonadaceae (7227	Pseudomonadales (1236)	Gammaproteobacteria (122	Proteobacteria (2)	Bacteria (1)			99.30	88.50	WGS
287.9502	0.356	Pseudomonas aeruginosa (286)	Pseudomonas (135621)	Pseudomonadaceae (7227	Pseudomonadales (1236)	Gammaproteobacteria (122	Proteobacteria (2)	Bacteria (1)			99.80	93.10	WGS
287.9503	0.3174	Pseudomonas aeruginosa (286)	Pseudomonas (135621)	Pseudomonadaceae (7227	Pseudomonadales (1236)	Gammaproteobacteria (122	Proteobacteria (2)	Bacteria (1)			99.60	94.40	WGS
287.9504	0.313	Pseudomonas aeruginosa (286)	Pseudomonas (135621)	Pseudomonadaceae (7227	Pseudomonadales (1236)	Gammaproteobacteria (122	Proteobacteria (2)	Bacteria (1)			99.70	92.20	WGS
287.9505	0.328	Pseudomonas aeruginosa (286)	Pseudomonas (135621)	Pseudomonadaceae (7227	Pseudomonadales (1236)	Gammaproteobacteria (122	Proteobacteria (2)	Bacteria (1)			99.80	91.60	WGS
287.9506	0.3888	Pseudomonas aeruginosa (286)	Pseudomonas (135621)	Pseudomonadaceae (7227	Pseudomonadales (1236)	Gammaproteobacteria (122	Proteobacteria (2)	Bacteria (1)			99.10	87.00	WGS
287.9507	0.3416	Pseudomonas aeruginosa (286)	Pseudomonas (135621)	Pseudomonadaceae (7227	Pseudomonadales (1236)	Gammaproteobacteria (122	Proteobacteria (2)	Bacteria (1)			99.20	78.90	WGS
287.9508	0.3174	Pseudomonas aeruginosa (286)	Pseudomonas (135621)	Pseudomonadaceae (7227	Pseudomonadales (1236)	Gammaproteobacteria (122	Proteobacteria (2)	Bacteria (1)			99.60	93.30	WGS
287.9509	0.356	Pseudomonas aeruginosa (286)	Pseudomonas (135621)	Pseudomonadaceae (7227	Pseudomonadales (1236)	Gammaproteobacteria (122	Proteobacteria (2)	Bacteria (1)			99.60	94.40	WGS
287.951	0.3236	Pseudomonas aeruginosa (286)	Pseudomonas (135621)	Pseudomonadaceae (7227	Pseudomonadales (1236)	Gammaproteobacteria (122	Proteobacteria (2)	Bacteria (1)	100.00	0.00	99.70	99.00	WGS
287.9510	0.3226	Pseudomonas aeruginosa (286)	Pseudomonas (135621)	Pseudomonadaceae (7227	Pseudomonadales (1236)	Gammaproteobacteria (122	Proteobacteria (2)	Bacteria (1)			99.40	87.10	WGS
287.9511	0.312	Pseudomonas aeruginosa (286)	Pseudomonas (135621)	Pseudomonadaceae (7227	Pseudomonadales (1236)	Gammaproteobacteria (122	Proteobacteria (2)	Bacteria (1)			99.60	87.20	WGS
287.9512	0.2474	Pseudomonas aeruginosa (286)	Pseudomonas (135621)	Pseudomonadaceae (7227	Pseudomonadales (1236)	Gammaproteobacteria (122	Proteobacteria (2)	Bacteria (1)			94.20	76.60	WGS
287.9513	0.3314	Pseudomonas aeruginosa (286)	Pseudomonas (135621)	Pseudomonadaceae (7227	Pseudomonadales (1236)	Gammaproteobacteria (122	Proteobacteria (2)	Bacteria (1)			99.90	94.20	WGS
287.9514	0.328	Pseudomonas aeruginosa (286)	Pseudomonas (135621)	Pseudomonadaceae (7227	Pseudomonadales (1236)	Gammaproteobacteria (122	Proteobacteria (2)	Bacteria (1)			99.80	93.90	WGS
287.9515	0.3624	Pseudomonas aeruginosa (286)	Pseudomonas (135621)	Pseudomonadaceae (7227	Pseudomonadales (1236)	Gammaproteobacteria (122	Proteobacteria (2)	Bacteria (1)			99.60	93.10	WGS
287.9516	0.3468	Pseudomonas aeruginosa (286)	Pseudomonas (135621)	Pseudomonadaceae (7227	Pseudomonadales (1236)	Gammaproteobacteria (122	Proteobacteria (2)	Bacteria (1)			96.10	80.90	WGS
287.9517	0.3258	Pseudomonas aeruginosa (286)	Pseudomonas (135621)	Pseudomonadaceae (7227	Pseudomonadales (1236)	Gammaproteobacteria (122	Proteobacteria (2)	Bacteria (1)			99.60	89.90	WGS
287.9518	0.3226	Pseudomonas aeruginosa (286)	Pseudomonas (135621)	Pseudomonadaceae (7227	Pseudomonadales (1236)	Gammaproteobacteria (122	Proteobacteria (2)	Bacteria (1)			99.60	94.50	WGS
287.9519	0.332	Pseudomonas aeruginosa (286)	Pseudomonas (135621)	Pseudomonadaceae (7227	Pseudomonadales (1236)	Gammaproteobacteria (122	Proteobacteria (2)	Bacteria (1)			99.90	94.20	WGS
287.9520	0.2948	Pseudomonas aeruginosa (286)	Pseudomonas (135621)	Pseudomonadaceae (7227	Pseudomonadales (1236)	Gammaproteobacteria (122	Proteobacteria (2)	Bacteria (1)			99.20	84.60	WGS
287.952	0.3636	Pseudomonas aeruginosa (286)	Pseudomonas (135621)	Pseudomonadaceae (7227	Pseudomonadales (1236)	Gammaproteobacteria (122	Proteobacteria (2)	Bacteria (1)	99.70	0.00	99.30	99.00	WGS
287.9521	0.3778	Pseudomonas aeruginosa (286)	Pseudomonas (135621)	Pseudomonadaceae (7227	Pseudomonadales (1236)	Gammaproteobacteria (122	Proteobacteria (2)	Bacteria (1)			95.30	69.90	WGS
287.9522	0.368	Pseudomonas aeruginosa (286)	Pseudomonas (135621)	Pseudomonadaceae (7227	Pseudomonadales (1236)	Gammaproteobacteria (122	Proteobacteria (2)	Bacteria (1)			99.70	92.20	WGS
287.9523	0.3298	Pseudomonas aeruginosa (286)	Pseudomonas (135621)	Pseudomonadaceae (7227	Pseudomonadales (1236)	Gammaproteobacteria (122	Proteobacteria (2)	Bacteria (1)			99.80	94.60	WGS
287.9524	0.3222	Pseudomonas aeruginosa (286)	Pseudomonas (135621)	Pseudomonadaceae (7227	Pseudomonadales (1236)	Gammaproteobacteria (122	Proteobacteria (2)	Bacteria (1)			99.50	94.40	WGS
287.9525	0.3704	Pseudomonas aeruginosa (286)	Pseudomonas (135621)	Pseudomonadaceae (7227	Pseudomonadales (1236)	Gammaproteobacteria (122	Proteobacteria (2)	Bacteria (1)			99.60	95.30	WGS
287.9526	0.341	Pseudomonas aeruginosa (286)	Pseudomonas (135621)	Pseudomonadaceae (7227	Pseudomonadales (1236)	Gammaproteobacteria (122	Proteobacteria (2)	Bacteria (1)			99.50	89.70	WGS
287.9527	0.3174	Pseudomonas aeruginosa (286)	Pseudomonas (135621)	Pseudomonadaceae (7227	Pseudomonadales (1236)	Gammaproteobacteria (122	Proteobacteria (2)	Bacteria (1)			99.70	95.30	WGS
287.9528	0.3324	Pseudomonas aeruginosa (286)	Pseudomonas (135621)	Pseudomonadaceae (7227	Pseudomonadales (1236)	Gammaproteobacteria (122	Proteobacteria (2)	Bacteria (1)			99.60	96.10	WGS
287.9529	0.3174	Pseudomonas aeruginosa (286)	Pseudomonas (135621)	Pseudomonadaceae (7227	Pseudomonadales (1236)	Gammaproteobacteria (122	Proteobacteria (2)	Bacteria (1)			99.60	92.10	WGS
287.9530	0.3372	Pseudomonas aeruginosa (286)	Pseudomonas (135621)	Pseudomonadaceae (7227	Pseudomonadales (1236)	Gammaproteobacteria (122	Proteobacteria (2)	Bacteria (1)			99.40	79.30	WGS
287.953	0.3378	Pseudomonas aeruginosa (286)	Pseudomonas (135621)	Pseudomonadaceae (7227	Pseudomonadales (1236)	Gammaproteobacteria (122	Proteobacteria (2)	Bacteria (1)	99.10	0.00	99.20	99.00	WGS
287.9531	0.3618	Pseudomonas aeruginosa (286)	Pseudomonas (135621)	Pseudomonadaceae (7227	Pseudomonadales (1236)	Gammaproteobacteria (122	Proteobacteria (2)	Bacteria (1)			99.80	91.90	WGS
287.9532	0.3272	Pseudomonas aeruginosa (286)	Pseudomonas (135621)	Pseudomonadaceae (7227	Pseudomonadales (1236)	Gammaproteobacteria (122	Proteobacteria (2)	Bacteria (1)			99.50	90.70	WGS
287.9533	0.354	Pseudomonas aeruginosa (286)	Pseudomonas (135621)	Pseudomonadaceae (7227	Pseudomonadales (1236)	Gammaproteobacteria (122	Proteobacteria (2)	Bacteria (1)			99.60	94.00	WGS
287.9534	0.3628	Pseudomonas aeruginosa (286)	Pseudomonas (135621)	Pseudomonadaceae (7227	Pseudomonadales (1236)	Gammaproteobacteria (122	Proteobacteria (2)	Bacteria (1)			99.90	93.40	WGS
287.9535	0.3694	Pseudomonas aeruginosa (286)	Pseudomonas (135621)	Pseudomonadaceae (7227	Pseudomonadales (1236)	Gammaproteobacteria (122	Proteobacteria (2)	Bacteria (1)			99.40	90.20	WGS
287.9536	0.3662	Pseudomonas aeruginosa (286)	Pseudomonas (135621)	Pseudomonadaceae (7227	Pseudomonadales (1236)	Gammaproteobacteria (122	Proteobacteria (2)	Bacteria (1)			99.70	94.70	WGS
287.9537	0.3482	Pseudomonas aeruginosa (286)	Pseudomonas (135621)	Pseudomonadaceae (7227	Pseudomonadales (1236)	Gammaproteobacteria (122	Proteobacteria (2)	Bacteria (1)			99.00	94.30	WGS
287.9538	0.3434	Pseudomonas aeruginosa (286)	Pseudomonas (135621)	Pseudomonadaceae (7227	Pseudomonadales (1236)	Gammaproteobacteria (122	Proteobacteria (2)	Bacteria (1)			99.20	76.90	WGS
287.9539	0.3174	Pseudomonas aeruginosa (286)	Pseudomonas (135621)	Pseudomonadaceae (7227	Pseudomonadales (1236)	Gammaproteobacteria (122	Proteobacteria (2)	Bacteria (1)			99.60	93.30	WGS
287.954	0.3258	Pseudomonas aeruginosa (286)	Pseudomonas (135621)	Pseudomonadaceae (7227	Pseudomonadales (1236)	Gammaproteobacteria (122	Proteobacteria (2)	Bacteria (1)	100.00	0.60	99.90	98.80	WGS
287.9540	0.3372	Pseudomonas aeruginosa (286)	Pseudomonas (135621)	Pseudomonadaceae (7227	Pseudomonadales (1236)	Gammaproteobacteria (122	Proteobacteria (2)	Bacteria (1)			99.70	93.10	WGS
287.9541	0.3226	Pseudomonas aeruginosa (286)	Pseudomonas (135621)	Pseudomonadaceae (7227	Pseudomonadales (1236)	Gammaproteobacteria (122	Proteobacteria (2)	Bacteria (1)			99.90	93.50	WGS
287.9542	0.32	Pseudomonas aeruginosa (286)	Pseudomonas (135621)	Pseudomonadaceae (7227	Pseudomonadales (1236)	Gammaproteobacteria (122	Proteobacteria (2)	Bacteria (1)			99.50	89.50	WGS
287.9543	0.3346	Pseudomonas aeruginosa (286)	Pseudomonas (135621)	Pseudomonadaceae (7227	Pseudomonadales (1236)	Gammaproteobacteria (122	Proteobacteria (2)	Bacteria (1)			99.40	86.30	WGS
287.9544	0.3658	Pseudomonas aeruginosa (286)	Pseudomonas (135621)	Pseudomonadaceae (7227	Pseudomonadales (1236)	Gammaproteobacteria (122	Proteobacteria (2)	Bacteria (1)			99.80	88.10	WGS
287.9545	0.3258	Pseudomonas aeruginosa (286)	Pseudomonas (135621)	Pseudomonadaceae (7227	Pseudomonadales (1236)	Gammaproteobacteria (122	Proteobacteria (2)	Bacteria (1)			99.90	92.80	WGS
287.9546	0.371	Pseudomonas aeruginosa (286)	Pseudomonas (135621)	Pseudomonadaceae (7227	Pseudomonadales (1236)	Gammaproteobacteria (122	Proteobacteria (2)	Bacteria (1)					WGS
287.9547	0.3266	Pseudomonas aeruginosa (286)	Pseudomonas (135621)	Pseudomonadaceae (7227	Pseudomonadales (1236)	Gammaproteobacteria (122	Proteobacteria (2)	Bacteria (1)			99.60	82.60	WGS
287.9548	0.3656	Pseudomonas aeruginosa (286)	Pseudomonas (135621)	Pseudomonadaceae (7227	Pseudomonadales (1236)	Gammaproteobacteria (122	Proteobacteria (2)	Bacteria (1)			99.30	79.70	WGS
287.9549	0.3556	Pseudomonas aeruginosa (286)	Pseudomonas (135621)	Pseudomonadaceae (7227	Pseudomonadales (1236)	Gammaproteobacteria (122	Proteobacteria (2)	Bacteria (1)			98.30	77.20	WGS
287.9550	0.3344	Pseudomonas aeruginosa (286)	Pseudomonas (135621)	Pseudomonadaceae (7227	Pseudomonadales (1236)	Gammaproteobacteria (122	Proteobacteria (2)	Bacteria (1)			99.00	72.10	WGS
287.955	0.3662	Pseudomonas aeruginosa (286)	Pseudomonas (135621)	Pseudomonadaceae (7227	Pseudomonadales (1236)	Gammaproteobacteria (122	Proteobacteria (2)	Bacteria (1)	100.00	0.30	99.10	98.10	WGS
287.9551	0.368	Pseudomonas aeruginosa (286)	Pseudomonas (135621)	Pseudomonadaceae (7227	Pseudomonadales (1236)	Gammaproteobacteria (122	Proteobacteria (2)	Bacteria (1)			99.80	96.00	WGS
287.9552	0.3378	Pseudomonas aeruginosa (286)	Pseudomonas (135621)	Pseudomonadaceae (7227	Pseudomonadales (1236)	Gammaproteobacteria (122	Proteobacteria (2)	Bacteria (1)			99.50	79.50	WGS
287.9553	0.357	Pseudomonas aeruginosa (286)	Pseudomonas (135621)	Pseudomonadaceae (7227	Pseudomonadales (1236)	Gammaproteobacteria (122	Proteobacteria (2)	Bacteria (1)			99.80	96.20	WGS
287.9554	0.3292	Pseudomonas aeruginosa (286)	Pseudomonas (135621)	Pseudomonadaceae (7227	Pseudomonadales (1236)	Gammaproteobacteria (122	Proteobacteria (2)	Bacteria (1)			99.80	95.00	WGS
287.9555	0.3256	Pseudomonas aeruginosa (286)	Pseudomonas (135621)	Pseudomonadaceae (7227	Pseudomonadales (1236)	Gammaproteobacteria (122	Proteobacteria (2)	Bacteria (1)			87.60	73.50	WGS
287.9556	0.332	Pseudomonas aeruginosa (286)	Pseudomonas (135621)	Pseudomonadaceae (7227	Pseudomonadales (1236)	Gammaproteobacteria (122	Proteobacteria (2)	Bacteria (1)			99.60	94.20	WGS
287.9557	0.3376	Pseudomonas aeruginosa (286)	Pseudomonas (135621)	Pseudomonadaceae (7227	Pseudomonadales (1236)	Gammaproteobacteria (122	Proteobacteria (2)	Bacteria (1)			99.60	95.90	WGS
287.9558	0.3694	Pseudomonas aeruginosa (286)	Pseudomonas (135621)	Pseudomonadaceae (7227	Pseudomonadales (1236)	Gammaproteobacteria (122	Proteobacteria (2)	Bacteria (1)			99.60	92.90	WGS
287.9559	0.3388	Pseudomonas aeruginosa (286)	Pseudomonas (135621)	Pseudomonadaceae (7227	Pseudomonadales (1236)	Gammaproteobacteria (122	Proteobacteria (2)	Bacteria (1)			99.60	92.90	WGS
287.9560	0.3636	Pseudomonas aeruginosa (286)	Pseudomonas (135621)	Pseudomonadaceae (7227	Pseudomonadales (1236)	Gammaproteobacteria (122	Proteobacteria (2)	Bacteria (1)			99.70	93.50	WGS
287.956	0.3174	Pseudomonas aeruginosa (286)	Pseudomonas (135621)	Pseudomonadaceae (7227	Pseudomonadales (1236)	Gammaproteobacteria (122	Proteobacteria (2)	Bacteria (1)	99.70	0.30	99.50	98.30	WGS
287.9561	0.3322	Pseudomonas aeruginosa (286)	Pseudomonas (135621)	Pseudomonadaceae (7227	Pseudomonadales (1236)	Gammaproteobacteria (122	Proteobacteria (2)	Bacteria (1)			99.10	86.60	WGS
287.9562	0.3038	Pseudomonas aeruginosa (286)	Pseudomonas (135621)	Pseudomonadaceae (7227	Pseudomonadales (1236)	Gammaproteobacteria (122	Proteobacteria (2)	Bacteria (1)			99.30	84.30	WGS
287.9563	0.3388	Pseudomonas aeruginosa (286)	Pseudomonas (135621)	Pseudomonadaceae (7227	Pseudomonadales (1236)	Gammaproteobacteria (122	Proteobacteria (2)	Bacteria (1)			99.60	92.80	WGS
287.9564	0.2948	Pseudomonas aeruginosa (286)	Pseudomonas (135621)	Pseudomonadaceae (7227	Pseudomonadales (1236)	Gammaproteobacteria (122	Proteobacteria (2)	Bacteria (1)			99.50	90.20	WGS
287.9565	0.342	Pseudomonas aeruginosa (286)	Pseudomonas (135621)	Pseudomonadaceae (7227	Pseudomonadales (1236)	Gammaproteobacteria (122	Proteobacteria (2)	Bacteria (1)			98.80	91.50	WGS
287.9566	0.3174	Pseudomonas aeruginosa (286)	Pseudomonas (135621)	Pseudomonadaceae (7227	Pseudomonadales (1236)	Gammaproteobacteria (122	Proteobacteria (2)	Bacteria (1)			99.60	95.30	WGS
287.9567	0.3146	Pseudomonas aeruginosa (286)	Pseudomonas (135621)	Pseudomonadaceae (7227	Pseudomonadales (1236)	Gammaproteobacteria (122	Proteobacteria (2)	Bacteria (1)			99.70	89.20	WGS
287.9568	0.3634	Pseudomonas aeruginosa (286)	Pseudomonas (135621)	Pseudomonadaceae (7227	Pseudomonadales (1236)	Gammaproteobacteria (122	Proteobacteria (2)	Bacteria (1)			99.50	93.60	WGS
287.9569	0.3684	Pseudomonas aeruginosa (286)	Pseudomonas (135621)	Pseudomonadaceae (7227	Pseudomonadales (1236)	Gammaproteobacteria (122	Proteobacteria (2)	Bacteria (1)			97.90	83.90	WGS
287.957	0.3258	Pseudomonas aeruginosa (286)	Pseudomonas (135621)	Pseudomonadaceae (7227	Pseudomonadales (1236)	Gammaproteobacteria (122	Proteobacteria (2)	Bacteria (1)	100.00	0.00	99.60	99.20	WGS
287.9570	0.328	Pseudomonas aeruginosa (286)	Pseudomonas (135621)	Pseudomonadaceae (7227	Pseudomonadales (1236)	Gammaproteobacteria (122	Proteobacteria (2)	Bacteria (1)			99.40	88.20	WGS
287.9571	0.3174	Pseudomonas aeruginosa (286)	Pseudomonas (135621)	Pseudomonadaceae (7227	Pseudomonadales (1236)	Gammaproteobacteria (122	Proteobacteria (2)	Bacteria (1)			99.60	94.00	WGS
287.9572	0.3292	Pseudomonas aeruginosa (286)	Pseudomonas (135621)	Pseudomonadaceae (7227	Pseudomonadales (1236)	Gammaproteobacteria (122	Proteobacteria (2)	Bacteria (1)			99.60	94.80	WGS
287.9573	0.349	Pseudomonas aeruginosa (286)	Pseudomonas (135621)	Pseudomonadaceae (7227	Pseudomonadales (1236)	Gammaproteobacteria (122	Proteobacteria (2)	Bacteria (1)			99.90	92.80	WGS
287.9574	0.3706	Pseudomonas aeruginosa (286)	Pseudomonas (135621)	Pseudomonadaceae (7227	Pseudomonadales (1236)	Gammaproteobacteria (122	Proteobacteria (2)	Bacteria (1)			99.20	90.10	WGS
287.9575	0.3614	Pseudomonas aeruginosa (286)	Pseudomonas (135621)	Pseudomonadaceae (7227	Pseudomonadales (1236)	Gammaproteobacteria (122	Proteobacteria (2)	Bacteria (1)			98.40	88.70	WGS
287.9576	0.3226	Pseudomonas aeruginosa (286)	Pseudomonas (135621)	Pseudomonadaceae (7227	Pseudomonadales (1236)	Gammaproteobacteria (122	Proteobacteria (2)	Bacteria (1)			99.60	92.40	WGS
287.9577	0.371	Pseudomonas aeruginosa (286)	Pseudomonas (135621)	Pseudomonadaceae (7227	Pseudomonadales (1236)	Gammaproteobacteria (122	Proteobacteria (2)	Bacteria (1)			99.80	90.20	WGS
287.9578	0.3852	Pseudomonas aeruginosa (286)	Pseudomonas (135621)	Pseudomonadaceae (7227	Pseudomonadales (1236)	Gammaproteobacteria (122	Proteobacteria (2)	Bacteria (1)			99.80	85.60	WGS
287.9579	0.3814	Pseudomonas aeruginosa (286)	Pseudomonas (135621)	Pseudomonadaceae (7227	Pseudomonadales (1236)	Gammaproteobacteria (122	Proteobacteria (2)	Bacteria (1)			99.80	86.80	WGS
287.9580	0.3614	Pseudomonas aeruginosa (286)	Pseudomonas (135621)	Pseudomonadaceae (7227	Pseudomonadales (1236)	Gammaproteobacteria (122	Proteobacteria (2)	Bacteria (1)			99.80	92.20	WGS
287.958	0.3236	Pseudomonas aeruginosa (286)	Pseudomonas (135621)	Pseudomonadaceae (7227	Pseudomonadales (1236)	Gammaproteobacteria (122	Proteobacteria (2)	Bacteria (1)	99.70	0.00	99.60	98.80	WGS
287.9581	0.3226	Pseudomonas aeruginosa (286)	Pseudomonas (135621)	Pseudomonadaceae (7227	Pseudomonadales (1236)	Gammaproteobacteria (122	Proteobacteria (2)	Bacteria (1)			99.60	89.90	WGS
287.9582	0.3478	Pseudomonas aeruginosa (286)	Pseudomonas (135621)	Pseudomonadaceae (7227	Pseudomonadales (1236)	Gammaproteobacteria (122	Proteobacteria (2)	Bacteria (1)			99.70	89.40	WGS
287.9583	0.3226	Pseudomonas aeruginosa (286)	Pseudomonas (135621)	Pseudomonadaceae (7227	Pseudomonadales (1236)	Gammaproteobacteria (122	Proteobacteria (2)	Bacteria (1)			99.60	93.50	WGS
287.9584	0.385	Pseudomonas aeruginosa (286)	Pseudomonas (135621)	Pseudomonadaceae (7227	Pseudomonadales (1236)	Gammaproteobacteria (122	Proteobacteria (2)	Bacteria (1)			99.60	87.80	WGS
287.9585	0.3738	Pseudomonas aeruginosa (286)	Pseudomonas (135621)	Pseudomonadaceae (7227	Pseudomonadales (1236)	Gammaproteobacteria (122	Proteobacteria (2)	Bacteria (1)			99.60	90.00	WGS
287.9586	0.348	Pseudomonas aeruginosa (286)	Pseudomonas (135621)	Pseudomonadaceae (7227	Pseudomonadales (1236)	Gammaproteobacteria (122	Proteobacteria (2)	Bacteria (1)			90.00	76.30	WGS
287.9587	0.314	Pseudomonas aeruginosa (286)	Pseudomonas (135621)	Pseudomonadaceae (7227	Pseudomonadales (1236)	Gammaproteobacteria (122	Proteobacteria (2)	Bacteria (1)			98.40	85.40	WGS
287.9588	0.3334	Pseudomonas aeruginosa (286)	Pseudomonas (135621)	Pseudomonadaceae (7227	Pseudomonadales (1236)	Gammaproteobacteria (122	Proteobacteria (2)	Bacteria (1)			99.70	92.30	WGS
287.9589	0.3674	Pseudomonas aeruginosa (286)	Pseudomonas (135621)	Pseudomonadaceae (7227	Pseudomonadales (1236)	Gammaproteobacteria (122	Proteobacteria (2)	Bacteria (1)			99.60	88.80	WGS
287.9590	0.3302	Pseudomonas aeruginosa (286)	Pseudomonas (135621)	Pseudomonadaceae (7227	Pseudomonadales (1236)	Gammaproteobacteria (122	Proteobacteria (2)	Bacteria (1)			99.60	93.10	WGS
287.959	0.3636	Pseudomonas aeruginosa (286)	Pseudomonas (135621)	Pseudomonadaceae (7227	Pseudomonadales (1236)	Gammaproteobacteria (122	Proteobacteria (2)	Bacteria (1)	99.10	0.00	99.20	99.00	WGS
287.9591	0.3284	Pseudomonas aeruginosa (286)	Pseudomonas (135621)	Pseudomonadaceae (7227	Pseudomonadales (1236)	Gammaproteobacteria (122	Proteobacteria (2)	Bacteria (1)			99.60	87.60	WGS
287.9592	0.3336	Pseudomonas aeruginosa (286)	Pseudomonas (135621)	Pseudomonadaceae (7227	Pseudomonadales (1236)	Gammaproteobacteria (122	Proteobacteria (2)	Bacteria (1)			99.60	93.40	WGS
287.9593	0.331	Pseudomonas aeruginosa (286)	Pseudomonas (135621)	Pseudomonadaceae (7227	Pseudomonadales (1236)	Gammaproteobacteria (122	Proteobacteria (2)	Bacteria (1)			99.50	86.90	WGS
287.9594	0.3424	Pseudomonas aeruginosa (286)	Pseudomonas (135621)	Pseudomonadaceae (7227	Pseudomonadales (1236)	Gammaproteobacteria (122	Proteobacteria (2)	Bacteria (1)			99.30	87.50	WGS
287.9595	0.3738	Pseudomonas aeruginosa (286)	Pseudomonas (135621)	Pseudomonadaceae (7227	Pseudomonadales (1236)	Gammaproteobacteria (122	Proteobacteria (2)	Bacteria (1)			99.60	94.50	WGS
287.9596	0.3268	Pseudomonas aeruginosa (286)	Pseudomonas (135621)	Pseudomonadaceae (7227	Pseudomonadales (1236)	Gammaproteobacteria (122	Proteobacteria (2)	Bacteria (1)			99.80	90.30	WGS
287.9597	0.3274	Pseudomonas aeruginosa (286)	Pseudomonas (135621)	Pseudomonadaceae (7227	Pseudomonadales (1236)	Gammaproteobacteria (122	Proteobacteria (2)	Bacteria (1)			99.70	89.30	WGS
287.9598	0.3372	Pseudomonas aeruginosa (286)	Pseudomonas (135621)	Pseudomonadaceae (7227	Pseudomonadales (1236)	Gammaproteobacteria (122	Proteobacteria (2)	Bacteria (1)			99.70	88.20	WGS
287.9599	0.3268	Pseudomonas aeruginosa (286)	Pseudomonas (135621)	Pseudomonadaceae (7227	Pseudomonadales (1236)	Gammaproteobacteria (122	Proteobacteria (2)	Bacteria (1)			99.80	86.10	WGS
287.960	0.3512	Pseudomonas aeruginosa (286)	Pseudomonas (135621)	Pseudomonadaceae (7227	Pseudomonadales (1236)	Gammaproteobacteria (122	Proteobacteria (2)	Bacteria (1)	99.70	0.00	98.90	97.60	WGS
287.9600	0.32	Pseudomonas aeruginosa (286)	Pseudomonas (135621)	Pseudomonadaceae (7227	Pseudomonadales (1236)	Gammaproteobacteria (122	Proteobacteria (2)	Bacteria (1)			99.60	87.40	WGS
287.9601	0.3346	Pseudomonas aeruginosa (286)	Pseudomonas (135621)	Pseudomonadaceae (7227	Pseudomonadales (1236)	Gammaproteobacteria (122	Proteobacteria (2)	Bacteria (1)			99.60	84.80	WGS
287.9602	0.3636	Pseudomonas aeruginosa (286)	Pseudomonas (135621)	Pseudomonadaceae (7227	Pseudomonadales (1236)	Gammaproteobacteria (122	Proteobacteria (2)	Bacteria (1)			99.70	86.50	WGS
287.9603	0.3174	Pseudomonas aeruginosa (286)	Pseudomonas (135621)	Pseudomonadaceae (7227	Pseudomonadales (1236)	Gammaproteobacteria (122	Proteobacteria (2)	Bacteria (1)			99.60	88.20	WGS
287.9604	0.342	Pseudomonas aeruginosa (286)	Pseudomonas (135621)	Pseudomonadaceae (7227	Pseudomonadales (1236)	Gammaproteobacteria (122	Proteobacteria (2)	Bacteria (1)			99.70	90.90	WGS
287.9605	0.3174	Pseudomonas aeruginosa (286)	Pseudomonas (135621)	Pseudomonadaceae (7227	Pseudomonadales (1236)	Gammaproteobacteria (122	Proteobacteria (2)	Bacteria (1)			99.60	91.40	WGS
287.9606	0.3174	Pseudomonas aeruginosa (286)	Pseudomonas (135621)	Pseudomonadaceae (7227	Pseudomonadales (1236)	Gammaproteobacteria (122	Proteobacteria (2)	Bacteria (1)			99.70	95.10	WGS
287.9607	0.285	Pseudomonas aeruginosa (286)	Pseudomonas (135621)	Pseudomonadaceae (7227	Pseudomonadales (1236)	Gammaproteobacteria (122	Proteobacteria (2)	Bacteria (1)			99.50	83.00	WGS
287.9608	0.3328	Pseudomonas aeruginosa (286)	Pseudomonas (135621)	Pseudomonadaceae (7227	Pseudomonadales (1236)	Gammaproteobacteria (122	Proteobacteria (2)	Bacteria (1)			99.60	84.40	WGS
287.9609	0.3304	Pseudomonas aeruginosa (286)	Pseudomonas (135621)	Pseudomonadaceae (7227	Pseudomonadales (1236)	Gammaproteobacteria (122	Proteobacteria (2)	Bacteria (1)			99.70	89.70	WGS
287.961	0.3306	Pseudomonas aeruginosa (286)	Pseudomonas (135621)	Pseudomonadaceae (7227	Pseudomonadales (1236)	Gammaproteobacteria (122	Proteobacteria (2)	Bacteria (1)	99.70	0.00	99.80	98.60	WGS
287.9610	0.357	Pseudomonas aeruginosa (286)	Pseudomonas (135621)	Pseudomonadaceae (7227	Pseudomonadales (1236)	Gammaproteobacteria (122	Proteobacteria (2)	Bacteria (1)			99.60	93.60	WGS
287.9611	0.3226	Pseudomonas aeruginosa (286)	Pseudomonas (135621)	Pseudomonadaceae (7227	Pseudomonadales (1236)	Gammaproteobacteria (122	Proteobacteria (2)	Bacteria (1)			99.70	91.90	WGS
287.9612	0.3498	Pseudomonas aeruginosa (286)	Pseudomonas (135621)	Pseudomonadaceae (7227	Pseudomonadales (1236)	Gammaproteobacteria (122	Proteobacteria (2)	Bacteria (1)			99.60	84.30	WGS
287.9613	0.3376	Pseudomonas aeruginosa (286)	Pseudomonas (135621)	Pseudomonadaceae (7227	Pseudomonadales (1236)	Gammaproteobacteria (122	Proteobacteria (2)	Bacteria (1)			99.60	96.20	Complete
287.9614	0.338	Pseudomonas aeruginosa (286)	Pseudomonas (135621)	Pseudomonadaceae (7227	Pseudomonadales (1236)	Gammaproteobacteria (122	Proteobacteria (2)	Bacteria (1)			99.60	89.40	WGS
287.9615	0.3758	Pseudomonas aeruginosa (286)	Pseudomonas (135621)	Pseudomonadaceae (7227	Pseudomonadales (1236)	Gammaproteobacteria (122	Proteobacteria (2)	Bacteria (1)			99.90	97.00	Complete
287.9616	0.3376	Pseudomonas aeruginosa (286)	Pseudomonas (135621)	Pseudomonadaceae (7227	Pseudomonadales (1236)	Gammaproteobacteria (122	Proteobacteria (2)	Bacteria (1)			99.60	97.40	Complete
287.9617	0.0604	Pseudomonas aeruginosa (286)	Pseudomonas (135621)	Pseudomonadaceae (7227	Pseudomonadales (1236)	Gammaproteobacteria (122	Proteobacteria (2)	Bacteria (1)			99.20	98.80	Complete
287.962	0.3738	Pseudomonas aeruginosa (286)	Pseudomonas (135621)	Pseudomonadaceae (7227	Pseudomonadales (1236)	Gammaproteobacteria (122	Proteobacteria (2)	Bacteria (1)	98.50	0.00	99.00	98.20	WGS
287.963	0.3636	Pseudomonas aeruginosa (286)	Pseudomonas (135621)	Pseudomonadaceae (7227	Pseudomonadales (1236)	Gammaproteobacteria (122	Proteobacteria (2)	Bacteria (1)	96.40	0.00	99.00	98.40	WGS
287.964	0.3614	Pseudomonas aeruginosa (286)	Pseudomonas (135621)	Pseudomonadaceae (7227	Pseudomonadales (1236)	Gammaproteobacteria (122	Proteobacteria (2)	Bacteria (1)	99.10	0.00	98.90	98.10	WGS
287.965	0.3614	Pseudomonas aeruginosa (286)	Pseudomonas (135621)	Pseudomonadaceae (7227	Pseudomonadales (1236)	Gammaproteobacteria (122	Proteobacteria (2)	Bacteria (1)	98.50	0.00	98.40	97.60	WGS
287.966	0.3628	Pseudomonas aeruginosa (286)	Pseudomonas (135621)	Pseudomonadaceae (7227	Pseudomonadales (1236)	Gammaproteobacteria (122	Proteobacteria (2)	Bacteria (1)	99.10	0.30	99.10	98.40	WGS
287.967	0.331	Pseudomonas aeruginosa (286)	Pseudomonas (135621)	Pseudomonadaceae (7227	Pseudomonadales (1236)	Gammaproteobacteria (122	Proteobacteria (2)	Bacteria (1)	100.00	0.00	99.60	99.10	WGS
287.968	0.331	Pseudomonas aeruginosa (286)	Pseudomonas (135621)	Pseudomonadaceae (7227	Pseudomonadales (1236)	Gammaproteobacteria (122	Proteobacteria (2)	Bacteria (1)	99.70	0.00	99.50	98.70	WGS
287.969	0.331	Pseudomonas aeruginosa (286)	Pseudomonas (135621)	Pseudomonadaceae (7227	Pseudomonadales (1236)	Gammaproteobacteria (122	Proteobacteria (2)	Bacteria (1)	100.00	0.00	99.60	98.70	WGS
287.970	0.3704	Pseudomonas aeruginosa (286)	Pseudomonas (135621)	Pseudomonadaceae (7227	Pseudomonadales (1236)	Gammaproteobacteria (122	Proteobacteria (2)	Bacteria (1)	100.00	0.00	99.50	98.90	WGS
287.971	0.368	Pseudomonas aeruginosa (286)	Pseudomonas (135621)	Pseudomonadaceae (7227	Pseudomonadales (1236)	Gammaproteobacteria (122	Proteobacteria (2)	Bacteria (1)	100.00	0.00	99.50	99.00	WGS
287.972	0.369	Pseudomonas aeruginosa (286)	Pseudomonas (135621)	Pseudomonadaceae (7227	Pseudomonadales (1236)	Gammaproteobacteria (122	Proteobacteria (2)	Bacteria (1)	99.70	0.00	99.60	99.00	WGS
287.973	0.3242	Pseudomonas aeruginosa (286)	Pseudomonas (135621)	Pseudomonadaceae (7227	Pseudomonadales (1236)	Gammaproteobacteria (122	Proteobacteria (2)	Bacteria (1)	99.10	0.00	99.60	99.40	WGS
287.974	0.3622	Pseudomonas aeruginosa (286)	Pseudomonas (135621)	Pseudomonadaceae (7227	Pseudomonadales (1236)	Gammaproteobacteria (122	Proteobacteria (2)	Bacteria (1)	100.00	0.30	99.60	99.00	WGS
287.975	0.3174	Pseudomonas aeruginosa (286)	Pseudomonas (135621)	Pseudomonadaceae (7227	Pseudomonadales (1236)	Gammaproteobacteria (122	Proteobacteria (2)	Bacteria (1)	99.70	0.30	99.50	98.30	WGS
287.976	0.327	Pseudomonas aeruginosa (286)	Pseudomonas (135621)	Pseudomonadaceae (7227	Pseudomonadales (1236)	Gammaproteobacteria (122	Proteobacteria (2)	Bacteria (1)	98.50	0.90	98.90	97.80	WGS
287.977	0.3154	Pseudomonas aeruginosa (286)	Pseudomonas (135621)	Pseudomonadaceae (7227	Pseudomonadales (1236)	Gammaproteobacteria (122	Proteobacteria (2)	Bacteria (1)	96.70	0.30	97.90	96.10	WGS
287.978	0.3578	Pseudomonas aeruginosa (286)	Pseudomonas (135621)	Pseudomonadaceae (7227	Pseudomonadales (1236)	Gammaproteobacteria (122	Proteobacteria (2)	Bacteria (1)	100.00	0.00	99.60	99.40	WGS
287.979	0.331	Pseudomonas aeruginosa (286)	Pseudomonas (135621)	Pseudomonadaceae (7227	Pseudomonadales (1236)	Gammaproteobacteria (122	Proteobacteria (2)	Bacteria (1)	100.00	0.00	99.50	99.00	WGS
287.980	0.3604	Pseudomonas aeruginosa (286)	Pseudomonas (135621)	Pseudomonadaceae (7227	Pseudomonadales (1236)	Gammaproteobacteria (122	Proteobacteria (2)	Bacteria (1)	100.00	0.00	99.50	98.80	WGS
287.981	0.3258	Pseudomonas aeruginosa (286)	Pseudomonas (135621)	Pseudomonadaceae (7227	Pseudomonadales (1236)	Gammaproteobacteria (122	Proteobacteria (2)	Bacteria (1)	99.40	0.00	99.40	98.70	WGS
287.982	0.331	Pseudomonas aeruginosa (286)	Pseudomonas (135621)	Pseudomonadaceae (7227	Pseudomonadales (1236)	Gammaproteobacteria (122	Proteobacteria (2)	Bacteria (1)	99.10	0.00	99.60	99.00	WGS
287.983	0.3226	Pseudomonas aeruginosa (286)	Pseudomonas (135621)	Pseudomonadaceae (7227	Pseudomonadales (1236)	Gammaproteobacteria (122	Proteobacteria (2)	Bacteria (1)	99.10	0.00	99.00	98.50	WGS
287.984	0.356	Pseudomonas aeruginosa (286)	Pseudomonas (135621)	Pseudomonadaceae (7227	Pseudomonadales (1236)	Gammaproteobacteria (122	Proteobacteria (2)	Bacteria (1)	99.70	0.00	99.00	98.20	WGS
287.985	0.3268	Pseudomonas aeruginosa (286)	Pseudomonas (135621)	Pseudomonadaceae (7227	Pseudomonadales (1236)	Gammaproteobacteria (122	Proteobacteria (2)	Bacteria (1)	100.00	0.00	99.30	98.40	WGS
287.986	0.327	Pseudomonas aeruginosa (286)	Pseudomonas (135621)	Pseudomonadaceae (7227	Pseudomonadales (1236)	Gammaproteobacteria (122	Proteobacteria (2)	Bacteria (1)	100.00	0.30	99.60	99.20	WGS
287.987	0.3324	Pseudomonas aeruginosa (286)	Pseudomonas (135621)	Pseudomonadaceae (7227	Pseudomonadales (1236)	Gammaproteobacteria (122	Proteobacteria (2)	Bacteria (1)	100.00	0.00	99.70	99.00	WGS
287.988	0.3618	Pseudomonas aeruginosa (286)	Pseudomonas (135621)	Pseudomonadaceae (7227	Pseudomonadales (1236)	Gammaproteobacteria (122	Proteobacteria (2)	Bacteria (1)	100.00	0.60	99.80	99.10	WGS
287.989	0.357	Pseudomonas aeruginosa (286)	Pseudomonas (135621)	Pseudomonadaceae (7227	Pseudomonadales (1236)	Gammaproteobacteria (122	Proteobacteria (2)	Bacteria (1)	99.10	0.00	99.50	98.70	WGS
287.990	0.3258	Pseudomonas aeruginosa (286)	Pseudomonas (135621)	Pseudomonadaceae (7227	Pseudomonadales (1236)	Gammaproteobacteria (122	Proteobacteria (2)	Bacteria (1)	99.40	0.00	99.30	99.10	WGS
287.991	0.3636	Pseudomonas aeruginosa (286)	Pseudomonas (135621)	Pseudomonadaceae (7227	Pseudomonadales (1236)	Gammaproteobacteria (122	Proteobacteria (2)	Bacteria (1)	99.10	0.00	99.70	99.20	WGS
287.992	0.368	Pseudomonas aeruginosa (286)	Pseudomonas (135621)	Pseudomonadaceae (7227	Pseudomonadales (1236)	Gammaproteobacteria (122	Proteobacteria (2)	Bacteria (1)	97.80	0.90	98.90	97.50	WGS
287.993	0.3398	Pseudomonas aeruginosa (286)	Pseudomonas (135621)	Pseudomonadaceae (7227	Pseudomonadales (1236)	Gammaproteobacteria (122	Proteobacteria (2)	Bacteria (1)	99.40	0.00	99.60	98.70	WGS
287.994	0.3636	Pseudomonas aeruginosa (286)	Pseudomonas (135621)	Pseudomonadaceae (7227	Pseudomonadales (1236)	Gammaproteobacteria (122	Proteobacteria (2)	Bacteria (1)	99.40	0.00	99.20	98.60	WGS
287.995	0.3618	Pseudomonas aeruginosa (286)	Pseudomonas (135621)	Pseudomonadaceae (7227	Pseudomonadales (1236)	Gammaproteobacteria (122	Proteobacteria (2)	Bacteria (1)	99.70	0.00	99.40	98.10	WGS
287.996	0.331	Pseudomonas aeruginosa (286)	Pseudomonas (135621)	Pseudomonadaceae (7227	Pseudomonadales (1236)	Gammaproteobacteria (122	Proteobacteria (2)	Bacteria (1)	100.00	0.00	99.90	99.30	WGS
287.997	0.2952	Pseudomonas aeruginosa (286)	Pseudomonas (135621)	Pseudomonadaceae (7227	Pseudomonadales (1236)	Gammaproteobacteria (122	Proteobacteria (2)	Bacteria (1)	98.50	0.00	99.10	98.70	WGS
287.998	0.3568	Pseudomonas aeruginosa (286)	Pseudomonas (135621)	Pseudomonadaceae (7227	Pseudomonadales (1236)	Gammaproteobacteria (122	Proteobacteria (2)	Bacteria (1)	99.70	0.00	99.60	99.00	WGS
287.999	0.3258	Pseudomonas aeruginosa (286)	Pseudomonas (135621)	Pseudomonadaceae (7227	Pseudomonadales (1236)	Gammaproteobacteria (122	Proteobacteria (2)	Bacteria (1)	100.00	0.00	99.60	99.00	WGS
288.4	0.3972	Pseudoalteromonas atlantica (532	Pseudoalteromonas (26788	Pseudoalteromonadaceae (	Alteromonadales (1236)	Gammaproteobacteria (122	Proteobacteria (2)	Bacteria (1)	96.00	0.00	97.50	96.70	Complete
292.100	0.3946	Burkholderia cepacia (32008)	Burkholderia (119060)	Burkholderiaceae (80840)	Burkholderiales (28216)	Betaproteobacteria (1224)	Proteobacteria (2)	Bacteria (1)	100.00	0.00	99.60	94.80	WGS
292.101	0.3874	Burkholderia cepacia (32008)	Burkholderia (119060)	Burkholderiaceae (80840)	Burkholderiales (28216)	Betaproteobacteria (1224)	Proteobacteria (2)	Bacteria (1)	100.00	0.00	99.60	95.00	WGS
292.102	0.3938	Burkholderia cepacia (32008)	Burkholderia (119060)	Burkholderiaceae (80840)	Burkholderiales (28216)	Betaproteobacteria (1224)	Proteobacteria (2)	Bacteria (1)	100.00	0.30	99.40	94.90	WGS
292.103	0.355	Burkholderia cepacia (32008)	Burkholderia (119060)	Burkholderiaceae (80840)	Burkholderiales (28216)	Betaproteobacteria (1224)	Proteobacteria (2)	Bacteria (1)	100.00	0.70	99.60	95.20	WGS
292.104	0.3582	Burkholderia cepacia (32008)	Burkholderia (119060)	Burkholderiaceae (80840)	Burkholderiales (28216)	Betaproteobacteria (1224)	Proteobacteria (2)	Bacteria (1)	100.00	0.00	99.70	95.10	WGS
292.105	0.355	Burkholderia cepacia (32008)	Burkholderia (119060)	Burkholderiaceae (80840)	Burkholderiales (28216)	Betaproteobacteria (1224)	Proteobacteria (2)	Bacteria (1)	100.00	0.00	99.30	94.80	WGS
292.11	0.355	Burkholderia cepacia (32008)	Burkholderia (119060)	Burkholderiaceae (80840)	Burkholderiales (28216)	Betaproteobacteria (1224)	Proteobacteria (2)	Bacteria (1)	100.00	0.00	99.40	95.90	WGS
292.113	0.354	Burkholderia cepacia (32008)	Burkholderia (119060)	Burkholderiaceae (80840)	Burkholderiales (28216)	Betaproteobacteria (1224)	Proteobacteria (2)	Bacteria (1)	100.00	0.00	99.30	95.00	Complete
292.114	0.3274	Burkholderia cepacia (32008)	Burkholderia (119060)	Burkholderiaceae (80840)	Burkholderiales (28216)	Betaproteobacteria (1224)	Proteobacteria (2)	Bacteria (1)	100.00	0.00	99.30	92.60	Complete
292.12	0.4006	Burkholderia cepacia (32008)	Burkholderia (119060)	Burkholderiaceae (80840)	Burkholderiales (28216)	Betaproteobacteria (1224)	Proteobacteria (2)	Bacteria (1)	100.00	0.70	99.10	95.00	WGS
292.125	0.3672	Burkholderia cepacia (32008)	Burkholderia (119060)	Burkholderiaceae (80840)	Burkholderiales (28216)	Betaproteobacteria (1224)	Proteobacteria (2)	Bacteria (1)	100.00	0.70	99.50	94.80	Complete
292.127	0.3672	Burkholderia cepacia (32008)	Burkholderia (119060)	Burkholderiaceae (80840)	Burkholderiales (28216)	Betaproteobacteria (1224)	Proteobacteria (2)	Bacteria (1)	100.00	0.00	99.50	95.00	Complete
292.129	0.031	Burkholderia cepacia (32008)	Burkholderia (119060)	Burkholderiaceae (80840)	Burkholderiales (28216)	Betaproteobacteria (1224)	Proteobacteria (2)	Bacteria (1)					Plasmid
292.130	0.031	Burkholderia cepacia (32008)	Burkholderia (119060)	Burkholderiaceae (80840)	Burkholderiales (28216)	Betaproteobacteria (1224)	Proteobacteria (2)	Bacteria (1)					Plasmid
292.131	0.0294	Burkholderia cepacia (32008)	Burkholderia (119060)	Burkholderiaceae (80840)	Burkholderiales (28216)	Betaproteobacteria (1224)	Proteobacteria (2)	Bacteria (1)					Plasmid
292.132	0.0294	Burkholderia cepacia (32008)	Burkholderia (119060)	Burkholderiaceae (80840)	Burkholderiales (28216)	Betaproteobacteria (1224)	Proteobacteria (2)	Bacteria (1)					Plasmid
292.133	0.0294	Burkholderia cepacia (32008)	Burkholderia (119060)	Burkholderiaceae (80840)	Burkholderiales (28216)	Betaproteobacteria (1224)	Proteobacteria (2)	Bacteria (1)					Plasmid
292.135	0.354	Burkholderia cepacia (32008)	Burkholderia (119060)	Burkholderiaceae (80840)	Burkholderiales (28216)	Betaproteobacteria (1224)	Proteobacteria (2)	Bacteria (1)	100.00	7.60	99.30	88.90	WGS
292.136	0.3708	Burkholderia cepacia (32008)	Burkholderia (119060)	Burkholderiaceae (80840)	Burkholderiales (28216)	Betaproteobacteria (1224)	Proteobacteria (2)	Bacteria (1)	100.00	0.00	99.50	94.30	WGS
292.137	0.4122	Burkholderia cepacia (32008)	Burkholderia (119060)	Burkholderiaceae (80840)	Burkholderiales (28216)	Betaproteobacteria (1224)	Proteobacteria (2)	Bacteria (1)	100.00	0.00	99.00	94.20	WGS
292.138	0.4062	Burkholderia cepacia (32008)	Burkholderia (119060)	Burkholderiaceae (80840)	Burkholderiales (28216)	Betaproteobacteria (1224)	Proteobacteria (2)	Bacteria (1)	100.00	2.00	99.70	94.80	WGS
292.148	0.3716	Burkholderia cepacia (32008)	Burkholderia (119060)	Burkholderiaceae (80840)	Burkholderiales (28216)	Betaproteobacteria (1224)	Proteobacteria (2)	Bacteria (1)	100.00	2.60	99.30	94.70	WGS
292.149	0.3492	Burkholderia cepacia (32008)	Burkholderia (119060)	Burkholderiaceae (80840)	Burkholderiales (28216)	Betaproteobacteria (1224)	Proteobacteria (2)	Bacteria (1)	99.30	2.70	98.70	93.40	WGS
292.162	0.3674	Burkholderia cepacia (32008)	Burkholderia (119060)	Burkholderiaceae (80840)	Burkholderiales (28216)	Betaproteobacteria (1224)	Proteobacteria (2)	Bacteria (1)			99.50	94.70	WGS
292.163	0.3836	Burkholderia cepacia (32008)	Burkholderia (119060)	Burkholderiaceae (80840)	Burkholderiales (28216)	Betaproteobacteria (1224)	Proteobacteria (2)	Bacteria (1)			99.30	94.50	WGS
292.164	0.3608	Burkholderia cepacia (32008)	Burkholderia (119060)	Burkholderiaceae (80840)	Burkholderiales (28216)	Betaproteobacteria (1224)	Proteobacteria (2)	Bacteria (1)			99.40	95.20	WGS
292.165	0.3906	Burkholderia cepacia (32008)	Burkholderia (119060)	Burkholderiaceae (80840)	Burkholderiales (28216)	Betaproteobacteria (1224)	Proteobacteria (2)	Bacteria (1)			99.40	95.30	WGS
292.166	0.3836	Burkholderia cepacia (32008)	Burkholderia (119060)	Burkholderiaceae (80840)	Burkholderiales (28216)	Betaproteobacteria (1224)	Proteobacteria (2)	Bacteria (1)			99.30	94.50	WGS
292.167	0.3688	Burkholderia cepacia (32008)	Burkholderia (119060)	Burkholderiaceae (80840)	Burkholderiales (28216)	Betaproteobacteria (1224)	Proteobacteria (2)	Bacteria (1)			99.60	94.30	WGS
292.168	0.4024	Burkholderia cepacia (32008)	Burkholderia (119060)	Burkholderiaceae (80840)	Burkholderiales (28216)	Betaproteobacteria (1224)	Proteobacteria (2)	Bacteria (1)			99.60	94.80	WGS
292.169	0.3928	Burkholderia cepacia (32008)	Burkholderia (119060)	Burkholderiaceae (80840)	Burkholderiales (28216)	Betaproteobacteria (1224)	Proteobacteria (2)	Bacteria (1)			99.40	94.80	WGS
292.170	0.38	Burkholderia cepacia (32008)	Burkholderia (119060)	Burkholderiaceae (80840)	Burkholderiales (28216)	Betaproteobacteria (1224)	Proteobacteria (2)	Bacteria (1)			99.30	94.30	WGS
292.171	0.3932	Burkholderia cepacia (32008)	Burkholderia (119060)	Burkholderiaceae (80840)	Burkholderiales (28216)	Betaproteobacteria (1224)	Proteobacteria (2)	Bacteria (1)			99.60	94.70	WGS
292.172	0.3836	Burkholderia cepacia (32008)	Burkholderia (119060)	Burkholderiaceae (80840)	Burkholderiales (28216)	Betaproteobacteria (1224)	Proteobacteria (2)	Bacteria (1)			99.40	94.60	WGS
292.173	0.378	Burkholderia cepacia (32008)	Burkholderia (119060)	Burkholderiaceae (80840)	Burkholderiales (28216)	Betaproteobacteria (1224)	Proteobacteria (2)	Bacteria (1)			99.40	94.50	WGS
292.174	0.378	Burkholderia cepacia (32008)	Burkholderia (119060)	Burkholderiaceae (80840)	Burkholderiales (28216)	Betaproteobacteria (1224)	Proteobacteria (2)	Bacteria (1)			99.40	94.20	WGS
292.175	0.397	Burkholderia cepacia (32008)	Burkholderia (119060)	Burkholderiaceae (80840)	Burkholderiales (28216)	Betaproteobacteria (1224)	Proteobacteria (2)	Bacteria (1)			99.50	95.00	WGS
292.176	0.3688	Burkholderia cepacia (32008)	Burkholderia (119060)	Burkholderiaceae (80840)	Burkholderiales (28216)	Betaproteobacteria (1224)	Proteobacteria (2)	Bacteria (1)			99.80	95.10	WGS
292.196	0.3834	Burkholderia cepacia (32008)	Burkholderia (119060)	Burkholderiaceae (80840)	Burkholderiales (28216)	Betaproteobacteria (1224)	Proteobacteria (2)	Bacteria (1)			99.50	94.50	WGS
292.197	0.402	Burkholderia cepacia (32008)	Burkholderia (119060)	Burkholderiaceae (80840)	Burkholderiales (28216)	Betaproteobacteria (1224)	Proteobacteria (2)	Bacteria (1)			99.30	92.40	WGS
292.198	0.402	Burkholderia cepacia (32008)	Burkholderia (119060)	Burkholderiaceae (80840)	Burkholderiales (28216)	Betaproteobacteria (1224)	Proteobacteria (2)	Bacteria (1)			99.10	92.60	WGS
292.199	0.3992	Burkholderia cepacia (32008)	Burkholderia (119060)	Burkholderiaceae (80840)	Burkholderiales (28216)	Betaproteobacteria (1224)	Proteobacteria (2)	Bacteria (1)			99.10	93.50	WGS
292.200	0.402	Burkholderia cepacia (32008)	Burkholderia (119060)	Burkholderiaceae (80840)	Burkholderiales (28216)	Betaproteobacteria (1224)	Proteobacteria (2)	Bacteria (1)			99.00	93.00	WGS
292.201	0.3992	Burkholderia cepacia (32008)	Burkholderia (119060)	Burkholderiaceae (80840)	Burkholderiales (28216)	Betaproteobacteria (1224)	Proteobacteria (2)	Bacteria (1)			99.10	93.30	WGS
292.202	0.3992	Burkholderia cepacia (32008)	Burkholderia (119060)	Burkholderiaceae (80840)	Burkholderiales (28216)	Betaproteobacteria (1224)	Proteobacteria (2)	Bacteria (1)			99.10	93.60	WGS
292.203	0.402	Burkholderia cepacia (32008)	Burkholderia (119060)	Burkholderiaceae (80840)	Burkholderiales (28216)	Betaproteobacteria (1224)	Proteobacteria (2)	Bacteria (1)			99.00	92.90	WGS
292.204	0.3992	Burkholderia cepacia (32008)	Burkholderia (119060)	Burkholderiaceae (80840)	Burkholderiales (28216)	Betaproteobacteria (1224)	Proteobacteria (2)	Bacteria (1)			99.10	93.80	WGS
292.205	0.3992	Burkholderia cepacia (32008)	Burkholderia (119060)	Burkholderiaceae (80840)	Burkholderiales (28216)	Betaproteobacteria (1224)	Proteobacteria (2)	Bacteria (1)			99.20	92.70	WGS
292.206	0.3806	Burkholderia cepacia (32008)	Burkholderia (119060)	Burkholderiaceae (80840)	Burkholderiales (28216)	Betaproteobacteria (1224)	Proteobacteria (2)	Bacteria (1)			99.50	95.00	WGS
292.207	0.402	Burkholderia cepacia (32008)	Burkholderia (119060)	Burkholderiaceae (80840)	Burkholderiales (28216)	Betaproteobacteria (1224)	Proteobacteria (2)	Bacteria (1)			99.10	93.00	WGS
292.208	0.402	Burkholderia cepacia (32008)	Burkholderia (119060)	Burkholderiaceae (80840)	Burkholderiales (28216)	Betaproteobacteria (1224)	Proteobacteria (2)	Bacteria (1)			99.20	93.10	WGS
292.209	0.402	Burkholderia cepacia (32008)	Burkholderia (119060)	Burkholderiaceae (80840)	Burkholderiales (28216)	Betaproteobacteria (1224)	Proteobacteria (2)	Bacteria (1)			99.20	92.90	WGS
292.210	0.3992	Burkholderia cepacia (32008)	Burkholderia (119060)	Burkholderiaceae (80840)	Burkholderiales (28216)	Betaproteobacteria (1224)	Proteobacteria (2)	Bacteria (1)			99.10	93.40	WGS
292.23	0.4374	Burkholderia cepacia (32008)	Burkholderia (119060)	Burkholderiaceae (80840)	Burkholderiales (28216)	Betaproteobacteria (1224)	Proteobacteria (2)	Bacteria (1)	100.00	3.90	99.30	94.50	WGS
292.24	0.4006	Burkholderia cepacia (32008)	Burkholderia (119060)	Burkholderiaceae (80840)	Burkholderiales (28216)	Betaproteobacteria (1224)	Proteobacteria (2)	Bacteria (1)	100.00	0.70	99.20	94.90	Complete
292.25	0.3862	Burkholderia cepacia (32008)	Burkholderia (119060)	Burkholderiaceae (80840)	Burkholderiales (28216)	Betaproteobacteria (1224)	Proteobacteria (2)	Bacteria (1)	100.00	0.70	98.90	94.80	WGS
292.26	0.3362	Burkholderia cepacia (32008)	Burkholderia (119060)	Burkholderiaceae (80840)	Burkholderiales (28216)	Betaproteobacteria (1224)	Proteobacteria (2)	Bacteria (1)	99.30	0.00	99.50	96.10	Complete
292.27	0.4276	Burkholderia cepacia (32008)	Burkholderia (119060)	Burkholderiaceae (80840)	Burkholderiales (28216)	Betaproteobacteria (1224)	Proteobacteria (2)	Bacteria (1)	100.00	0.00	99.20	93.90	WGS
292.28	0.381	Burkholderia cepacia (32008)	Burkholderia (119060)	Burkholderiaceae (80840)	Burkholderiales (28216)	Betaproteobacteria (1224)	Proteobacteria (2)	Bacteria (1)	100.00	0.00	99.50	94.10	WGS
292.29	0.3632	Burkholderia cepacia (32008)	Burkholderia (119060)	Burkholderiaceae (80840)	Burkholderiales (28216)	Betaproteobacteria (1224)	Proteobacteria (2)	Bacteria (1)	100.00	0.70	99.70	95.00	WGS
292.30	0.4044	Burkholderia cepacia (32008)	Burkholderia (119060)	Burkholderiaceae (80840)	Burkholderiales (28216)	Betaproteobacteria (1224)	Proteobacteria (2)	Bacteria (1)	100.00	0.70	99.60	94.70	WGS
292.31	0.3812	Burkholderia cepacia (32008)	Burkholderia (119060)	Burkholderiaceae (80840)	Burkholderiales (28216)	Betaproteobacteria (1224)	Proteobacteria (2)	Bacteria (1)	100.00	0.00	99.70	94.70	WGS
292.32	0.381	Burkholderia cepacia (32008)	Burkholderia (119060)	Burkholderiaceae (80840)	Burkholderiales (28216)	Betaproteobacteria (1224)	Proteobacteria (2)	Bacteria (1)	100.00	0.00	99.50	93.90	WGS
292.33	0.4044	Burkholderia cepacia (32008)	Burkholderia (119060)	Burkholderiaceae (80840)	Burkholderiales (28216)	Betaproteobacteria (1224)	Proteobacteria (2)	Bacteria (1)	100.00	0.70	99.60	94.70	WGS
292.34	0.3668	Burkholderia cepacia (32008)	Burkholderia (119060)	Burkholderiaceae (80840)	Burkholderiales (28216)	Betaproteobacteria (1224)	Proteobacteria (2)	Bacteria (1)	100.00	0.00	99.30	94.60	WGS
292.35	0.381	Burkholderia cepacia (32008)	Burkholderia (119060)	Burkholderiaceae (80840)	Burkholderiales (28216)	Betaproteobacteria (1224)	Proteobacteria (2)	Bacteria (1)	100.00	0.00	99.50	93.90	WGS
292.36	0.381	Burkholderia cepacia (32008)	Burkholderia (119060)	Burkholderiaceae (80840)	Burkholderiales (28216)	Betaproteobacteria (1224)	Proteobacteria (2)	Bacteria (1)	100.00	0.00	99.50	94.10	WGS
292.37	0.381	Burkholderia cepacia (32008)	Burkholderia (119060)	Burkholderiaceae (80840)	Burkholderiales (28216)	Betaproteobacteria (1224)	Proteobacteria (2)	Bacteria (1)	100.00	0.00	99.50	94.20	WGS
292.38	0.378	Burkholderia cepacia (32008)	Burkholderia (119060)	Burkholderiaceae (80840)	Burkholderiales (28216)	Betaproteobacteria (1224)	Proteobacteria (2)	Bacteria (1)	100.00	0.00	99.50	94.10	WGS
292.39	0.381	Burkholderia cepacia (32008)	Burkholderia (119060)	Burkholderiaceae (80840)	Burkholderiales (28216)	Betaproteobacteria (1224)	Proteobacteria (2)	Bacteria (1)	100.00	0.00	99.50	93.90	WGS
292.40	0.381	Burkholderia cepacia (32008)	Burkholderia (119060)	Burkholderiaceae (80840)	Burkholderiales (28216)	Betaproteobacteria (1224)	Proteobacteria (2)	Bacteria (1)	100.00	0.00	99.50	94.10	WGS
292.41	0.381	Burkholderia cepacia (32008)	Burkholderia (119060)	Burkholderiaceae (80840)	Burkholderiales (28216)	Betaproteobacteria (1224)	Proteobacteria (2)	Bacteria (1)	100.00	0.00	99.50	94.20	WGS
292.42	0.381	Burkholderia cepacia (32008)	Burkholderia (119060)	Burkholderiaceae (80840)	Burkholderiales (28216)	Betaproteobacteria (1224)	Proteobacteria (2)	Bacteria (1)	100.00	0.00	99.50	93.90	WGS
292.43	0.381	Burkholderia cepacia (32008)	Burkholderia (119060)	Burkholderiaceae (80840)	Burkholderiales (28216)	Betaproteobacteria (1224)	Proteobacteria (2)	Bacteria (1)	100.00	0.00	99.50	93.90	WGS
292.44	0.378	Burkholderia cepacia (32008)	Burkholderia (119060)	Burkholderiaceae (80840)	Burkholderiales (28216)	Betaproteobacteria (1224)	Proteobacteria (2)	Bacteria (1)	100.00	0.00	99.50	94.10	WGS
292.45	0.381	Burkholderia cepacia (32008)	Burkholderia (119060)	Burkholderiaceae (80840)	Burkholderiales (28216)	Betaproteobacteria (1224)	Proteobacteria (2)	Bacteria (1)	100.00	0.00	99.50	93.90	WGS
292.46	0.3668	Burkholderia cepacia (32008)	Burkholderia (119060)	Burkholderiaceae (80840)	Burkholderiales (28216)	Betaproteobacteria (1224)	Proteobacteria (2)	Bacteria (1)	100.00	0.00	99.60	94.60	WGS
292.47	0.3658	Burkholderia cepacia (32008)	Burkholderia (119060)	Burkholderiaceae (80840)	Burkholderiales (28216)	Betaproteobacteria (1224)	Proteobacteria (2)	Bacteria (1)	100.00	0.70	99.80	94.90	WGS
292.48	0.3916	Burkholderia cepacia (32008)	Burkholderia (119060)	Burkholderiaceae (80840)	Burkholderiales (28216)	Betaproteobacteria (1224)	Proteobacteria (2)	Bacteria (1)	100.00	0.00	99.60	94.90	WGS
292.49	0.3558	Burkholderia cepacia (32008)	Burkholderia (119060)	Burkholderiaceae (80840)	Burkholderiales (28216)	Betaproteobacteria (1224)	Proteobacteria (2)	Bacteria (1)	100.00	0.70	99.70	95.20	WGS
292.50	0.3988	Burkholderia cepacia (32008)	Burkholderia (119060)	Burkholderiaceae (80840)	Burkholderiales (28216)	Betaproteobacteria (1224)	Proteobacteria (2)	Bacteria (1)	100.00	0.00	99.60	95.70	WGS
292.51	0.4028	Burkholderia cepacia (32008)	Burkholderia (119060)	Burkholderiaceae (80840)	Burkholderiales (28216)	Betaproteobacteria (1224)	Proteobacteria (2)	Bacteria (1)	100.00	0.70	99.50	94.90	WGS
292.52	0.3608	Burkholderia cepacia (32008)	Burkholderia (119060)	Burkholderiaceae (80840)	Burkholderiales (28216)	Betaproteobacteria (1224)	Proteobacteria (2)	Bacteria (1)	100.00	0.70	99.70	95.00	WGS
292.53	0.378	Burkholderia cepacia (32008)	Burkholderia (119060)	Burkholderiaceae (80840)	Burkholderiales (28216)	Betaproteobacteria (1224)	Proteobacteria (2)	Bacteria (1)	100.00	0.00	99.50	93.90	WGS
292.54	0.381	Burkholderia cepacia (32008)	Burkholderia (119060)	Burkholderiaceae (80840)	Burkholderiales (28216)	Betaproteobacteria (1224)	Proteobacteria (2)	Bacteria (1)	100.00	0.00	99.40	94.20	WGS
292.55	0.381	Burkholderia cepacia (32008)	Burkholderia (119060)	Burkholderiaceae (80840)	Burkholderiales (28216)	Betaproteobacteria (1224)	Proteobacteria (2)	Bacteria (1)	100.00	0.00	99.50	94.10	WGS
292.56	0.381	Burkholderia cepacia (32008)	Burkholderia (119060)	Burkholderiaceae (80840)	Burkholderiales (28216)	Betaproteobacteria (1224)	Proteobacteria (2)	Bacteria (1)	100.00	0.00	99.50	94.20	WGS
292.57	0.381	Burkholderia cepacia (32008)	Burkholderia (119060)	Burkholderiaceae (80840)	Burkholderiales (28216)	Betaproteobacteria (1224)	Proteobacteria (2)	Bacteria (1)	100.00	0.00	99.50	94.20	WGS
292.58	0.381	Burkholderia cepacia (32008)	Burkholderia (119060)	Burkholderiaceae (80840)	Burkholderiales (28216)	Betaproteobacteria (1224)	Proteobacteria (2)	Bacteria (1)	100.00	0.00	99.50	93.90	WGS
292.59	0.381	Burkholderia cepacia (32008)	Burkholderia (119060)	Burkholderiaceae (80840)	Burkholderiales (28216)	Betaproteobacteria (1224)	Proteobacteria (2)	Bacteria (1)	100.00	0.00	99.50	94.10	WGS
292.60	0.3912	Burkholderia cepacia (32008)	Burkholderia (119060)	Burkholderiaceae (80840)	Burkholderiales (28216)	Betaproteobacteria (1224)	Proteobacteria (2)	Bacteria (1)	100.00	0.70	99.60	95.50	WGS
292.61	0.4524	Burkholderia cepacia (32008)	Burkholderia (119060)	Burkholderiaceae (80840)	Burkholderiales (28216)	Betaproteobacteria (1224)	Proteobacteria (2)	Bacteria (1)	100.00	0.70	99.40	95.00	WGS
292.62	0.3514	Burkholderia cepacia (32008)	Burkholderia (119060)	Burkholderiaceae (80840)	Burkholderiales (28216)	Betaproteobacteria (1224)	Proteobacteria (2)	Bacteria (1)	100.00	0.00	99.60	95.10	WGS
292.63	0.4044	Burkholderia cepacia (32008)	Burkholderia (119060)	Burkholderiaceae (80840)	Burkholderiales (28216)	Betaproteobacteria (1224)	Proteobacteria (2)	Bacteria (1)	100.00	0.70	99.50	95.00	WGS
292.64	0.3812	Burkholderia cepacia (32008)	Burkholderia (119060)	Burkholderiaceae (80840)	Burkholderiales (28216)	Betaproteobacteria (1224)	Proteobacteria (2)	Bacteria (1)	100.00	0.00	99.60	94.80	WGS
292.65	0.3514	Burkholderia cepacia (32008)	Burkholderia (119060)	Burkholderiaceae (80840)	Burkholderiales (28216)	Betaproteobacteria (1224)	Proteobacteria (2)	Bacteria (1)	100.00	0.00	99.60	95.10	WGS
292.66	0.3688	Burkholderia cepacia (32008)	Burkholderia (119060)	Burkholderiaceae (80840)	Burkholderiales (28216)	Betaproteobacteria (1224)	Proteobacteria (2)	Bacteria (1)	100.00	0.00	99.30	95.20	WGS
292.67	0.3658	Burkholderia cepacia (32008)	Burkholderia (119060)	Burkholderiaceae (80840)	Burkholderiales (28216)	Betaproteobacteria (1224)	Proteobacteria (2)	Bacteria (1)	100.00	0.00	99.40	95.40	WGS
292.68	0.4304	Burkholderia cepacia (32008)	Burkholderia (119060)	Burkholderiaceae (80840)	Burkholderiales (28216)	Betaproteobacteria (1224)	Proteobacteria (2)	Bacteria (1)	100.00	0.70	99.60	95.00	WGS
292.69	0.3806	Burkholderia cepacia (32008)	Burkholderia (119060)	Burkholderiaceae (80840)	Burkholderiales (28216)	Betaproteobacteria (1224)	Proteobacteria (2)	Bacteria (1)	100.00	0.70	99.40	94.50	WGS
292.70	0.3662	Burkholderia cepacia (32008)	Burkholderia (119060)	Burkholderiaceae (80840)	Burkholderiales (28216)	Betaproteobacteria (1224)	Proteobacteria (2)	Bacteria (1)	100.00	0.00	99.60	95.30	WGS
292.71	0.3776	Burkholderia cepacia (32008)	Burkholderia (119060)	Burkholderiaceae (80840)	Burkholderiales (28216)	Betaproteobacteria (1224)	Proteobacteria (2)	Bacteria (1)	100.00	0.00	99.50	94.80	WGS
292.72	0.3658	Burkholderia cepacia (32008)	Burkholderia (119060)	Burkholderiaceae (80840)	Burkholderiales (28216)	Betaproteobacteria (1224)	Proteobacteria (2)	Bacteria (1)	100.00	0.00	99.60	95.00	WGS
292.73	0.3602	Burkholderia cepacia (32008)	Burkholderia (119060)	Burkholderiaceae (80840)	Burkholderiales (28216)	Betaproteobacteria (1224)	Proteobacteria (2)	Bacteria (1)	100.00	0.00	99.50	95.00	WGS
292.74	0.3806	Burkholderia cepacia (32008)	Burkholderia (119060)	Burkholderiaceae (80840)	Burkholderiales (28216)	Betaproteobacteria (1224)	Proteobacteria (2)	Bacteria (1)	100.00	0.00	99.30	95.30	WGS
292.75	0.3602	Burkholderia cepacia (32008)	Burkholderia (119060)	Burkholderiaceae (80840)	Burkholderiales (28216)	Betaproteobacteria (1224)	Proteobacteria (2)	Bacteria (1)	100.00	0.00	99.70	95.00	WGS
292.76	0.3688	Burkholderia cepacia (32008)	Burkholderia (119060)	Burkholderiaceae (80840)	Burkholderiales (28216)	Betaproteobacteria (1224)	Proteobacteria (2)	Bacteria (1)	100.00	1.30	99.60	94.60	WGS
292.77	0.3602	Burkholderia cepacia (32008)	Burkholderia (119060)	Burkholderiaceae (80840)	Burkholderiales (28216)	Betaproteobacteria (1224)	Proteobacteria (2)	Bacteria (1)	100.00	0.00	99.50	95.00	WGS
292.78	0.3602	Burkholderia cepacia (32008)	Burkholderia (119060)	Burkholderiaceae (80840)	Burkholderiales (28216)	Betaproteobacteria (1224)	Proteobacteria (2)	Bacteria (1)	100.00	0.00	99.70	95.00	WGS
292.79	0.3602	Burkholderia cepacia (32008)	Burkholderia (119060)	Burkholderiaceae (80840)	Burkholderiales (28216)	Betaproteobacteria (1224)	Proteobacteria (2)	Bacteria (1)	100.00	0.00	99.70	95.00	WGS
292.80	0.3602	Burkholderia cepacia (32008)	Burkholderia (119060)	Burkholderiaceae (80840)	Burkholderiales (28216)	Betaproteobacteria (1224)	Proteobacteria (2)	Bacteria (1)	98.00	0.00	99.70	94.80	WGS
292.81	0.3602	Burkholderia cepacia (32008)	Burkholderia (119060)	Burkholderiaceae (80840)	Burkholderiales (28216)	Betaproteobacteria (1224)	Proteobacteria (2)	Bacteria (1)	100.00	0.00	99.70	95.00	WGS
292.82	0.3602	Burkholderia cepacia (32008)	Burkholderia (119060)	Burkholderiaceae (80840)	Burkholderiales (28216)	Betaproteobacteria (1224)	Proteobacteria (2)	Bacteria (1)	100.00	0.00	99.70	95.00	WGS
292.83	0.4264	Burkholderia cepacia (32008)	Burkholderia (119060)	Burkholderiaceae (80840)	Burkholderiales (28216)	Betaproteobacteria (1224)	Proteobacteria (2)	Bacteria (1)	100.00	0.30	99.40	94.60	WGS
292.84	0.3632	Burkholderia cepacia (32008)	Burkholderia (119060)	Burkholderiaceae (80840)	Burkholderiales (28216)	Betaproteobacteria (1224)	Proteobacteria (2)	Bacteria (1)	100.00	0.70	99.60	95.60	WGS
292.85	0.4264	Burkholderia cepacia (32008)	Burkholderia (119060)	Burkholderiaceae (80840)	Burkholderiales (28216)	Betaproteobacteria (1224)	Proteobacteria (2)	Bacteria (1)	100.00	0.30	99.50	94.80	WGS
292.86	0.3754	Burkholderia cepacia (32008)	Burkholderia (119060)	Burkholderiaceae (80840)	Burkholderiales (28216)	Betaproteobacteria (1224)	Proteobacteria (2)	Bacteria (1)	100.00	0.00	99.50	95.10	WGS
292.87	0.3602	Burkholderia cepacia (32008)	Burkholderia (119060)	Burkholderiaceae (80840)	Burkholderiales (28216)	Betaproteobacteria (1224)	Proteobacteria (2)	Bacteria (1)	100.00	0.00	99.70	95.00	WGS
292.88	0.3602	Burkholderia cepacia (32008)	Burkholderia (119060)	Burkholderiaceae (80840)	Burkholderiales (28216)	Betaproteobacteria (1224)	Proteobacteria (2)	Bacteria (1)	100.00	0.00	99.50	95.10	WGS
292.89	0.3658	Burkholderia cepacia (32008)	Burkholderia (119060)	Burkholderiaceae (80840)	Burkholderiales (28216)	Betaproteobacteria (1224)	Proteobacteria (2)	Bacteria (1)	100.00	0.00	99.50	95.20	WGS
292.90	0.3582	Burkholderia cepacia (32008)	Burkholderia (119060)	Burkholderiaceae (80840)	Burkholderiales (28216)	Betaproteobacteria (1224)	Proteobacteria (2)	Bacteria (1)	100.00	0.00	99.60	95.10	WGS
292.91	0.3638	Burkholderia cepacia (32008)	Burkholderia (119060)	Burkholderiaceae (80840)	Burkholderiales (28216)	Betaproteobacteria (1224)	Proteobacteria (2)	Bacteria (1)	100.00	0.00	99.70	95.30	WGS
292.92	0.4028	Burkholderia cepacia (32008)	Burkholderia (119060)	Burkholderiaceae (80840)	Burkholderiales (28216)	Betaproteobacteria (1224)	Proteobacteria (2)	Bacteria (1)	100.00	0.70	99.50	95.00	WGS
292.93	0.3612	Burkholderia cepacia (32008)	Burkholderia (119060)	Burkholderiaceae (80840)	Burkholderiales (28216)	Betaproteobacteria (1224)	Proteobacteria (2)	Bacteria (1)	100.00	0.70	99.70	94.80	WGS
292.94	0.3908	Burkholderia cepacia (32008)	Burkholderia (119060)	Burkholderiaceae (80840)	Burkholderiales (28216)	Betaproteobacteria (1224)	Proteobacteria (2)	Bacteria (1)	100.00	0.00	99.30	95.30	WGS
292.95	0.3492	Burkholderia cepacia (32008)	Burkholderia (119060)	Burkholderiaceae (80840)	Burkholderiales (28216)	Betaproteobacteria (1224)	Proteobacteria (2)	Bacteria (1)	100.00	0.00	99.60	94.80	WGS
292.96	0.346	Burkholderia cepacia (32008)	Burkholderia (119060)	Burkholderiaceae (80840)	Burkholderiales (28216)	Betaproteobacteria (1224)	Proteobacteria (2)	Bacteria (1)	100.00	0.00	99.70	94.50	WGS
292.97	0.3754	Burkholderia cepacia (32008)	Burkholderia (119060)	Burkholderiaceae (80840)	Burkholderiales (28216)	Betaproteobacteria (1224)	Proteobacteria (2)	Bacteria (1)	100.00	0.00	99.40	95.10	WGS
292.98	0.3754	Burkholderia cepacia (32008)	Burkholderia (119060)	Burkholderiaceae (80840)	Burkholderiales (28216)	Betaproteobacteria (1224)	Proteobacteria (2)	Bacteria (1)	100.00	0.00	99.40	95.20	WGS
292.99	0.391	Burkholderia cepacia (32008)	Burkholderia (119060)	Burkholderiaceae (80840)	Burkholderiales (28216)	Betaproteobacteria (1224)	Proteobacteria (2)	Bacteria (1)	100.00	0.00	99.40	94.50	WGS
293.10	0.0462	Brevundimonas diminuta (41275)	Brevundimonas (76892)	Caulobacteraceae (204458	Caulobacterales (28211)	Alphaproteobacteria (1224)	Proteobacteria (2)	Bacteria (1)	100.00	0.00	98.70	97.40	WGS
293.11	0.0488	Brevundimonas diminuta (41275)	Brevundimonas (76892)	Caulobacteraceae (204458	Caulobacterales (28211)	Alphaproteobacteria (1224)	Proteobacteria (2)	Bacteria (1)	86.30	15.30	93.60	84.60	WGS
293.14	0.0598	Brevundimonas diminuta (41275)	Brevundimonas (76892)	Caulobacteraceae (204458	Caulobacterales (28211)	Alphaproteobacteria (1224)	Proteobacteria (2)	Bacteria (1)	100.00	5.50	98.70	96.00	WGS
293.15	0.0468	Brevundimonas diminuta (41275)	Brevundimonas (76892)	Caulobacteraceae (204458	Caulobacterales (28211)	Alphaproteobacteria (1224)	Proteobacteria (2)	Bacteria (1)	100.00	0.00	98.70	97.60	WGS
293.16	0.0662	Brevundimonas diminuta (41275)	Brevundimonas (76892)	Caulobacteraceae (204458	Caulobacterales (28211)	Alphaproteobacteria (1224)	Proteobacteria (2)	Bacteria (1)	86.20	6.50	88.20	82.50	WGS
293.17	0.0462	Brevundimonas diminuta (41275)	Brevundimonas (76892)	Caulobacteraceae (204458	Caulobacterales (28211)	Alphaproteobacteria (1224)	Proteobacteria (2)	Bacteria (1)	100.00	0.00	98.80	97.60	WGS
293.21	0.13	Brevundimonas diminuta (41275)	Brevundimonas (76892)	Caulobacteraceae (204458	Caulobacterales (28211)	Alphaproteobacteria (1224)	Proteobacteria (2)	Bacteria (1)			98.70	97.30	WGS
293.22	0.0646	Brevundimonas diminuta (41275)	Brevundimonas (76892)	Caulobacteraceae (204458	Caulobacterales (28211)	Alphaproteobacteria (1224)	Proteobacteria (2)	Bacteria (1)			99.10	98.00	Complete
293.7	0.0494	Brevundimonas diminuta (41275)	Brevundimonas (76892)	Caulobacteraceae (204458	Caulobacterales (28211)	Alphaproteobacteria (1224)	Proteobacteria (2)	Bacteria (1)	100.00	0.00	98.20	97.30	WGS
293.9	0.1266	Brevundimonas diminuta (41275)	Brevundimonas (76892)	Caulobacteraceae (204458	Caulobacterales (28211)	Alphaproteobacteria (1224)	Proteobacteria (2)	Bacteria (1)	100.00	0.00	98.80	97.30	WGS
294.122	0.3844	Pseudomonas fluorescens (286)	Pseudomonas (135621)	Pseudomonadaceae (7227	Pseudomonadales (1236)	Gammaproteobacteria (122	Proteobacteria (2)	Bacteria (1)	100.00	0.70	97.90	95.60	WGS
294.123	0.4208	Pseudomonas fluorescens (286)	Pseudomonas (135621)	Pseudomonadaceae (7227	Pseudomonadales (1236)	Gammaproteobacteria (122	Proteobacteria (2)	Bacteria (1)	100.00	0.00	97.90	95.90	Complete
294.124	0.3862	Pseudomonas fluorescens (286)	Pseudomonas (135621)	Pseudomonadaceae (7227	Pseudomonadales (1236)	Gammaproteobacteria (122	Proteobacteria (2)	Bacteria (1)	98.20	2.30	98.20	93.50	WGS
294.125	0.3594	Pseudomonas fluorescens (286)	Pseudomonas (135621)	Pseudomonadaceae (7227	Pseudomonadales (1236)	Gammaproteobacteria (122	Proteobacteria (2)	Bacteria (1)	100.00	0.20	98.40	96.00	WGS
294.126	0.399	Pseudomonas fluorescens (286)	Pseudomonas (135621)	Pseudomonadaceae (7227	Pseudomonadales (1236)	Gammaproteobacteria (122	Proteobacteria (2)	Bacteria (1)	99.60	0.00	98.40	95.80	WGS
294.127	0.3896	Pseudomonas fluorescens (286)	Pseudomonas (135621)	Pseudomonadaceae (7227	Pseudomonadales (1236)	Gammaproteobacteria (122	Proteobacteria (2)	Bacteria (1)	100.00	0.00	98.20	95.30	Complete
294.128	0.3992	Pseudomonas fluorescens (286)	Pseudomonas (135621)	Pseudomonadaceae (7227	Pseudomonadales (1236)	Gammaproteobacteria (122	Proteobacteria (2)	Bacteria (1)	100.00	0.50	98.80	96.00	WGS
294.129	0.3922	Pseudomonas fluorescens (286)	Pseudomonas (135621)	Pseudomonadaceae (7227	Pseudomonadales (1236)	Gammaproteobacteria (122	Proteobacteria (2)	Bacteria (1)	100.00	0.00	98.70	95.10	WGS
294.130	0.3696	Pseudomonas fluorescens (286)	Pseudomonas (135621)	Pseudomonadaceae (7227	Pseudomonadales (1236)	Gammaproteobacteria (122	Proteobacteria (2)	Bacteria (1)	100.00	1.10	99.50	96.90	WGS
294.131	0.3916	Pseudomonas fluorescens (286)	Pseudomonas (135621)	Pseudomonadaceae (7227	Pseudomonadales (1236)	Gammaproteobacteria (122	Proteobacteria (2)	Bacteria (1)	99.60	0.00	99.30	96.80	WGS
294.132	0.3414	Pseudomonas fluorescens (286)	Pseudomonas (135621)	Pseudomonadaceae (7227	Pseudomonadales (1236)	Gammaproteobacteria (122	Proteobacteria (2)	Bacteria (1)	99.60	0.00	99.00	96.40	WGS
294.133	0.3918	Pseudomonas fluorescens (286)	Pseudomonas (135621)	Pseudomonadaceae (7227	Pseudomonadales (1236)	Gammaproteobacteria (122	Proteobacteria (2)	Bacteria (1)	99.60	0.00	99.30	97.20	WGS
294.134	0.3922	Pseudomonas fluorescens (286)	Pseudomonas (135621)	Pseudomonadaceae (7227	Pseudomonadales (1236)	Gammaproteobacteria (122	Proteobacteria (2)	Bacteria (1)	100.00	0.00	98.70	95.00	WGS
294.136	0.344	Pseudomonas fluorescens (286)	Pseudomonas (135621)	Pseudomonadaceae (7227	Pseudomonadales (1236)	Gammaproteobacteria (122	Proteobacteria (2)	Bacteria (1)	100.00	41.10	97.30	63.80	Complete
294.160	0.3462	Pseudomonas fluorescens (286)	Pseudomonas (135621)	Pseudomonadaceae (7227	Pseudomonadales (1236)	Gammaproteobacteria (122	Proteobacteria (2)	Bacteria (1)	99.30	0.10	99.00	96.00	Complete
294.161	0.4292	Pseudomonas fluorescens (286)	Pseudomonas (135621)	Pseudomonadaceae (7227	Pseudomonadales (1236)	Gammaproteobacteria (122	Proteobacteria (2)	Bacteria (1)	100.00	0.00	98.70	96.40	Complete
294.162	0.3644	Pseudomonas fluorescens (286)	Pseudomonas (135621)	Pseudomonadaceae (7227	Pseudomonadales (1236)	Gammaproteobacteria (122	Proteobacteria (2)	Bacteria (1)	100.00	0.00	99.00	96.20	WGS
294.165	0.3406	Pseudomonas fluorescens (286)	Pseudomonas (135621)	Pseudomonadaceae (7227	Pseudomonadales (1236)	Gammaproteobacteria (122	Proteobacteria (2)	Bacteria (1)	100.00	0.00	99.20	97.50	WGS
294.166	0.3954	Pseudomonas fluorescens (286)	Pseudomonas (135621)	Pseudomonadaceae (7227	Pseudomonadales (1236)	Gammaproteobacteria (122	Proteobacteria (2)	Bacteria (1)	100.00	0.40	98.20	96.20	WGS
294.167	0.3884	Pseudomonas fluorescens (286)	Pseudomonas (135621)	Pseudomonadaceae (7227	Pseudomonadales (1236)	Gammaproteobacteria (122	Proteobacteria (2)	Bacteria (1)	98.60	3.70	97.60	91.10	WGS
294.168	0.356	Pseudomonas fluorescens (286)	Pseudomonas (135621)	Pseudomonadaceae (7227	Pseudomonadales (1236)	Gammaproteobacteria (122	Proteobacteria (2)	Bacteria (1)	97.80	19.50	96.60	83.70	WGS
294.169	0.3424	Pseudomonas fluorescens (286)	Pseudomonas (135621)	Pseudomonadaceae (7227	Pseudomonadales (1236)	Gammaproteobacteria (122	Proteobacteria (2)	Bacteria (1)	99.60	0.40	98.90	96.90	WGS
294.170	0.4244	Pseudomonas fluorescens (286)	Pseudomonas (135621)	Pseudomonadaceae (7227	Pseudomonadales (1236)	Gammaproteobacteria (122	Proteobacteria (2)	Bacteria (1)	99.60	0.00	98.20	95.00	WGS
294.171	0.4102	Pseudomonas fluorescens (286)	Pseudomonas (135621)	Pseudomonadaceae (7227	Pseudomonadales (1236)	Gammaproteobacteria (122	Proteobacteria (2)	Bacteria (1)	100.00	0.00	99.30	98.10	WGS
294.172	0.401	Pseudomonas fluorescens (286)	Pseudomonas (135621)	Pseudomonadaceae (7227	Pseudomonadales (1236)	Gammaproteobacteria (122	Proteobacteria (2)	Bacteria (1)	100.00	0.40	99.60	98.70	WGS
294.173	0.4236	Pseudomonas fluorescens (286)	Pseudomonas (135621)	Pseudomonadaceae (7227	Pseudomonadales (1236)	Gammaproteobacteria (122	Proteobacteria (2)	Bacteria (1)	99.60	0.00	98.30	95.10	WGS
294.174	0.313	Pseudomonas fluorescens (286)	Pseudomonas (135621)	Pseudomonadaceae (7227	Pseudomonadales (1236)	Gammaproteobacteria (122	Proteobacteria (2)	Bacteria (1)	100.00	0.00	98.80	97.30	WGS
294.175	0.3062	Pseudomonas fluorescens (286)	Pseudomonas (135621)	Pseudomonadaceae (7227	Pseudomonadales (1236)	Gammaproteobacteria (122	Proteobacteria (2)	Bacteria (1)	100.00	0.00	98.40	96.70	WGS
294.176	0.3196	Pseudomonas fluorescens (286)	Pseudomonas (135621)	Pseudomonadaceae (7227	Pseudomonadales (1236)	Gammaproteobacteria (122	Proteobacteria (2)	Bacteria (1)	100.00	0.00	98.80	97.30	WGS
294.178	0.3028	Pseudomonas fluorescens (286)	Pseudomonas (135621)	Pseudomonadaceae (7227	Pseudomonadales (1236)	Gammaproteobacteria (122	Proteobacteria (2)	Bacteria (1)	100.00	0.00	98.90	97.20	WGS
294.179	0.3916	Pseudomonas fluorescens (286)	Pseudomonas (135621)	Pseudomonadaceae (7227	Pseudomonadales (1236)	Gammaproteobacteria (122	Proteobacteria (2)	Bacteria (1)	99.60	0.70	98.10	95.20	WGS
294.180	0.3028	Pseudomonas fluorescens (286)	Pseudomonas (135621)	Pseudomonadaceae (7227	Pseudomonadales (1236)	Gammaproteobacteria (122	Proteobacteria (2)	Bacteria (1)	100.00	0.00	98.80	97.00	WGS
294.181	0.313	Pseudomonas fluorescens (286)	Pseudomonas (135621)	Pseudomonadaceae (7227	Pseudomonadales (1236)	Gammaproteobacteria (122	Proteobacteria (2)	Bacteria (1)	100.00	0.00	98.80	97.30	WGS
294.182	0.313	Pseudomonas fluorescens (286)	Pseudomonas (135621)	Pseudomonadaceae (7227	Pseudomonadales (1236)	Gammaproteobacteria (122	Proteobacteria (2)	Bacteria (1)	100.00	0.00	98.80	97.30	WGS
294.183	0.313	Pseudomonas fluorescens (286)	Pseudomonas (135621)	Pseudomonadaceae (7227	Pseudomonadales (1236)	Gammaproteobacteria (122	Proteobacteria (2)	Bacteria (1)	100.00	0.40	98.80	97.30	WGS
294.184	0.313	Pseudomonas fluorescens (286)	Pseudomonas (135621)	Pseudomonadaceae (7227	Pseudomonadales (1236)	Gammaproteobacteria (122	Proteobacteria (2)	Bacteria (1)	100.00	0.00	98.80	97.30	WGS
294.185	0.4054	Pseudomonas fluorescens (286)	Pseudomonas (135621)	Pseudomonadaceae (7227	Pseudomonadales (1236)	Gammaproteobacteria (122	Proteobacteria (2)	Bacteria (1)	100.00	0.40	98.60	95.30	WGS
294.186	0.4188	Pseudomonas fluorescens (286)	Pseudomonas (135621)	Pseudomonadaceae (7227	Pseudomonadales (1236)	Gammaproteobacteria (122	Proteobacteria (2)	Bacteria (1)	100.00	1.40	98.50	93.40	WGS
294.191	0.3784	Pseudomonas fluorescens (286)	Pseudomonas (135621)	Pseudomonadaceae (7227	Pseudomonadales (1236)	Gammaproteobacteria (122	Proteobacteria (2)	Bacteria (1)	100.00	0.00	98.70	95.90	WGS
294.192	0.2992	Pseudomonas fluorescens (286)	Pseudomonas (135621)	Pseudomonadaceae (7227	Pseudomonadales (1236)	Gammaproteobacteria (122	Proteobacteria (2)	Bacteria (1)	100.00	0.00	97.90	95.60	WGS
294.193	0.3038	Pseudomonas fluorescens (286)	Pseudomonas (135621)	Pseudomonadaceae (7227	Pseudomonadales (1236)	Gammaproteobacteria (122	Proteobacteria (2)	Bacteria (1)	100.00	0.00	98.20	95.10	WGS
294.200	0.3956	Pseudomonas fluorescens (286)	Pseudomonas (135621)	Pseudomonadaceae (7227	Pseudomonadales (1236)	Gammaproteobacteria (122	Proteobacteria (2)	Bacteria (1)	100.00	0.00	98.60	95.30	WGS
294.201	0.3542	Pseudomonas fluorescens (286)	Pseudomonas (135621)	Pseudomonadaceae (7227	Pseudomonadales (1236)	Gammaproteobacteria (122	Proteobacteria (2)	Bacteria (1)	100.00	0.20	98.70	96.20	Complete
294.202	0.3474	Pseudomonas fluorescens (286)	Pseudomonas (135621)	Pseudomonadaceae (7227	Pseudomonadales (1236)	Gammaproteobacteria (122	Proteobacteria (2)	Bacteria (1)	100.00	40.80	97.10	62.90	Complete
294.203	0.4048	Pseudomonas fluorescens (286)	Pseudomonas (135621)	Pseudomonadaceae (7227	Pseudomonadales (1236)	Gammaproteobacteria (122	Proteobacteria (2)	Bacteria (1)	100.00	0.40	98.20	95.90	Complete
294.204	0.436	Pseudomonas fluorescens (286)	Pseudomonas (135621)	Pseudomonadaceae (7227	Pseudomonadales (1236)	Gammaproteobacteria (122	Proteobacteria (2)	Bacteria (1)	100.00	0.70	99.20	96.40	WGS
294.208	0.355	Pseudomonas fluorescens (286)	Pseudomonas (135621)	Pseudomonadaceae (7227	Pseudomonadales (1236)	Gammaproteobacteria (122	Proteobacteria (2)	Bacteria (1)	100.00	0.40	99.60	98.30	WGS
294.210	0.3658	Pseudomonas fluorescens (286)	Pseudomonas (135621)	Pseudomonadaceae (7227	Pseudomonadales (1236)	Gammaproteobacteria (122	Proteobacteria (2)	Bacteria (1)	100.00	1.40	99.60	97.20	WGS
294.211	0.3478	Pseudomonas fluorescens (286)	Pseudomonas (135621)	Pseudomonadaceae (7227	Pseudomonadales (1236)	Gammaproteobacteria (122	Proteobacteria (2)	Bacteria (1)	100.00	0.00	98.20	94.00	Complete
294.212	0.3478	Pseudomonas fluorescens (286)	Pseudomonas (135621)	Pseudomonadaceae (7227	Pseudomonadales (1236)	Gammaproteobacteria (122	Proteobacteria (2)	Bacteria (1)	99.60	0.00	98.00	93.90	Complete
294.224	0.3124	Pseudomonas fluorescens (286)	Pseudomonas (135621)	Pseudomonadaceae (7227	Pseudomonadales (1236)	Gammaproteobacteria (122	Proteobacteria (2)	Bacteria (1)	100.00	0.00	98.40	96.10	Complete
294.232	0.3658	Pseudomonas fluorescens (286)	Pseudomonas (135621)	Pseudomonadaceae (7227	Pseudomonadales (1236)	Gammaproteobacteria (122	Proteobacteria (2)	Bacteria (1)	94.60	0.40	96.20	94.70	Complete
294.233	0.3208	Pseudomonas fluorescens (286)	Pseudomonas (135621)	Pseudomonadaceae (7227	Pseudomonadales (1236)	Gammaproteobacteria (122	Proteobacteria (2)	Bacteria (1)	100.00	1.10	98.40	95.60	WGS
294.234	0.3132	Pseudomonas fluorescens (286)	Pseudomonas (135621)	Pseudomonadaceae (7227	Pseudomonadales (1236)	Gammaproteobacteria (122	Proteobacteria (2)	Bacteria (1)	100.00	0.40	98.40	95.10	WGS
294.236	0.3098	Pseudomonas fluorescens (286)	Pseudomonas (135621)	Pseudomonadaceae (7227	Pseudomonadales (1236)	Gammaproteobacteria (122	Proteobacteria (2)	Bacteria (1)	100.00	1.30	98.40	94.90	WGS
294.254	0.371	Pseudomonas fluorescens (286)	Pseudomonas (135621)	Pseudomonadaceae (7227	Pseudomonadales (1236)	Gammaproteobacteria (122	Proteobacteria (2)	Bacteria (1)	100.00	0.00	98.60	96.50	WGS
294.255	0.3682	Pseudomonas fluorescens (286)	Pseudomonas (135621)	Pseudomonadaceae (7227	Pseudomonadales (1236)	Gammaproteobacteria (122	Proteobacteria (2)	Bacteria (1)	100.00	0.00	98.70	96.70	WGS
294.256	0.3382	Pseudomonas fluorescens (286)	Pseudomonas (135621)	Pseudomonadaceae (7227	Pseudomonadales (1236)	Gammaproteobacteria (122	Proteobacteria (2)	Bacteria (1)	100.00	1.10	98.70	94.90	WGS
294.257	0.3958	Pseudomonas fluorescens (286)	Pseudomonas (135621)	Pseudomonadaceae (7227	Pseudomonadales (1236)	Gammaproteobacteria (122	Proteobacteria (2)	Bacteria (1)	100.00	0.40	98.70	95.60	WGS
294.258	0.371	Pseudomonas fluorescens (286)	Pseudomonas (135621)	Pseudomonadaceae (7227	Pseudomonadales (1236)	Gammaproteobacteria (122	Proteobacteria (2)	Bacteria (1)	100.00	0.00	98.50	96.60	WGS
294.259	0.371	Pseudomonas fluorescens (286)	Pseudomonas (135621)	Pseudomonadaceae (7227	Pseudomonadales (1236)	Gammaproteobacteria (122	Proteobacteria (2)	Bacteria (1)	100.00	0.00	98.70	96.70	WGS
294.260	0.3094	Pseudomonas fluorescens (286)	Pseudomonas (135621)	Pseudomonadaceae (7227	Pseudomonadales (1236)	Gammaproteobacteria (122	Proteobacteria (2)	Bacteria (1)	100.00	0.00	98.70	96.20	WGS
294.273	0.309	Pseudomonas fluorescens (286)	Pseudomonas (135621)	Pseudomonadaceae (7227	Pseudomonadales (1236)	Gammaproteobacteria (122	Proteobacteria (2)	Bacteria (1)	100.00	0.00	98.20	95.50	WGS
294.281	0.3622	Pseudomonas fluorescens (286)	Pseudomonas (135621)	Pseudomonadaceae (7227	Pseudomonadales (1236)	Gammaproteobacteria (122	Proteobacteria (2)	Bacteria (1)	100.00	10.70	98.20	92.20	WGS
294.282	0.3488	Pseudomonas fluorescens (286)	Pseudomonas (135621)	Pseudomonadaceae (7227	Pseudomonadales (1236)	Gammaproteobacteria (122	Proteobacteria (2)	Bacteria (1)	89.50	5.40	92.00	88.60	WGS
294.283	0.301	Pseudomonas fluorescens (286)	Pseudomonas (135621)	Pseudomonadaceae (7227	Pseudomonadales (1236)	Gammaproteobacteria (122	Proteobacteria (2)	Bacteria (1)	100.00	0.00	98.40	95.60	WGS
294.284	0.3976	Pseudomonas fluorescens (286)	Pseudomonas (135621)	Pseudomonadaceae (7227	Pseudomonadales (1236)	Gammaproteobacteria (122	Proteobacteria (2)	Bacteria (1)	100.00	0.00	98.70	95.40	Complete
294.286	0.38	Pseudomonas fluorescens (286)	Pseudomonas (135621)	Pseudomonadaceae (7227	Pseudomonadales (1236)	Gammaproteobacteria (122	Proteobacteria (2)	Bacteria (1)	100.00	7.10	98.40	92.60	WGS
294.287	0.38	Pseudomonas fluorescens (286)	Pseudomonas (135621)	Pseudomonadaceae (7227	Pseudomonadales (1236)	Gammaproteobacteria (122	Proteobacteria (2)	Bacteria (1)	100.00	7.10	98.40	92.60	WGS
294.333	0.3956	Pseudomonas fluorescens (286)	Pseudomonas (135621)	Pseudomonadaceae (7227	Pseudomonadales (1236)	Gammaproteobacteria (122	Proteobacteria (2)	Bacteria (1)	100.00	0.40	99.90	98.60	WGS
294.334	0.3552	Pseudomonas fluorescens (286)	Pseudomonas (135621)	Pseudomonadaceae (7227	Pseudomonadales (1236)	Gammaproteobacteria (122	Proteobacteria (2)	Bacteria (1)	100.00	0.00	99.70	98.70	WGS
294.362	0.0294	Pseudomonas fluorescens (286)	Pseudomonas (135621)	Pseudomonadaceae (7227	Pseudomonadales (1236)	Gammaproteobacteria (122	Proteobacteria (2)	Bacteria (1)					Plasmid
294.363	0.03	Pseudomonas fluorescens (286)	Pseudomonas (135621)	Pseudomonadaceae (7227	Pseudomonadales (1236)	Gammaproteobacteria (122	Proteobacteria (2)	Bacteria (1)					Plasmid
294.364	0.3592	Pseudomonas fluorescens (286)	Pseudomonas (135621)	Pseudomonadaceae (7227	Pseudomonadales (1236)	Gammaproteobacteria (122	Proteobacteria (2)	Bacteria (1)	100.00	0.00	98.60	95.90	WGS
294.390	0.434	Pseudomonas fluorescens (286)	Pseudomonas (135621)	Pseudomonadaceae (7227	Pseudomonadales (1236)	Gammaproteobacteria (122	Proteobacteria (2)	Bacteria (1)	100.00	3.20	98.40	94.30	
294.391	0.425	Pseudomonas fluorescens (286)	Pseudomonas (135621)	Pseudomonadaceae (7227	Pseudomonadales (1236)	Gammaproteobacteria (122	Proteobacteria (2)	Bacteria (1)	100.00	0.00	98.80	94.50	Complete
294.392	0.3166	Pseudomonas fluorescens (286)	Pseudomonas (135621)	Pseudomonadaceae (7227	Pseudomonadales (1236)	Gammaproteobacteria (122	Proteobacteria (2)	Bacteria (1)	100.00	0.00	98.40	96.00	WGS
294.393	0.3104	Pseudomonas fluorescens (286)	Pseudomonas (135621)	Pseudomonadaceae (7227	Pseudomonadales (1236)	Gammaproteobacteria (122	Proteobacteria (2)	Bacteria (1)	100.00	0.00	98.30	95.40	WGS
294.394	0.3976	Pseudomonas fluorescens (286)	Pseudomonas (135621)	Pseudomonadaceae (7227	Pseudomonadales (1236)	Gammaproteobacteria (122	Proteobacteria (2)	Bacteria (1)	100.00	0.40	98.80	95.30	WGS
294.395	0.3476	Pseudomonas fluorescens (286)	Pseudomonas (135621)	Pseudomonadaceae (7227	Pseudomonadales (1236)	Gammaproteobacteria (122	Proteobacteria (2)	Bacteria (1)	100.00	0.00	98.20	95.40	WGS
294.396	0.3476	Pseudomonas fluorescens (286)	Pseudomonas (135621)	Pseudomonadaceae (7227	Pseudomonadales (1236)	Gammaproteobacteria (122	Proteobacteria (2)	Bacteria (1)	100.00	0.00	98.30	95.50	WGS
294.397	0.3476	Pseudomonas fluorescens (286)	Pseudomonas (135621)	Pseudomonadaceae (7227	Pseudomonadales (1236)	Gammaproteobacteria (122	Proteobacteria (2)	Bacteria (1)	100.00	0.00	98.30	95.50	WGS
294.398	0.302	Pseudomonas fluorescens (286)	Pseudomonas (135621)	Pseudomonadaceae (7227	Pseudomonadales (1236)	Gammaproteobacteria (122	Proteobacteria (2)	Bacteria (1)	100.00	0.40	98.50	95.50	WGS
294.399	0.3676	Pseudomonas fluorescens (286)	Pseudomonas (135621)	Pseudomonadaceae (7227	Pseudomonadales (1236)	Gammaproteobacteria (122	Proteobacteria (2)	Bacteria (1)	100.00	2.10	98.10	94.20	WGS
294.400	0.312	Pseudomonas fluorescens (286)	Pseudomonas (135621)	Pseudomonadaceae (7227	Pseudomonadales (1236)	Gammaproteobacteria (122	Proteobacteria (2)	Bacteria (1)	100.00	0.70	98.40	95.30	WGS
294.401	0.302	Pseudomonas fluorescens (286)	Pseudomonas (135621)	Pseudomonadaceae (7227	Pseudomonadales (1236)	Gammaproteobacteria (122	Proteobacteria (2)	Bacteria (1)	100.00	0.00	98.50	95.60	WGS
294.402	0.3688	Pseudomonas fluorescens (286)	Pseudomonas (135621)	Pseudomonadaceae (7227	Pseudomonadales (1236)	Gammaproteobacteria (122	Proteobacteria (2)	Bacteria (1)	100.00	0.40	99.90	99.30	Complete
294.425	0.0352	Pseudomonas fluorescens (286)	Pseudomonas (135621)	Pseudomonadaceae (7227	Pseudomonadales (1236)	Gammaproteobacteria (122	Proteobacteria (2)	Bacteria (1)	22.70	8.70	90.20	88.80	WGS
294.426	0.3664	Pseudomonas fluorescens (286)	Pseudomonas (135621)	Pseudomonadaceae (7227	Pseudomonadales (1236)	Gammaproteobacteria (122	Proteobacteria (2)	Bacteria (1)	100.00	1.80	100.00	99.90	WGS
294.427	0.3824	Pseudomonas fluorescens (286)	Pseudomonas (135621)	Pseudomonadaceae (7227	Pseudomonadales (1236)	Gammaproteobacteria (122	Proteobacteria (2)	Bacteria (1)	99.30	1.10	99.20	95.70	WGS
294.428	0.3956	Pseudomonas fluorescens (286)	Pseudomonas (135621)	Pseudomonadaceae (7227	Pseudomonadales (1236)	Gammaproteobacteria (122	Proteobacteria (2)	Bacteria (1)	99.60	1.80	98.60	94.50	Complete
294.451	0.4238	Pseudomonas fluorescens (286)	Pseudomonas (135621)	Pseudomonadaceae (7227	Pseudomonadales (1236)	Gammaproteobacteria (122	Proteobacteria (2)	Bacteria (1)	100.00	1.40	98.40	96.30	WGS
294.452	0.3572	Pseudomonas fluorescens (286)	Pseudomonas (135621)	Pseudomonadaceae (7227	Pseudomonadales (1236)	Gammaproteobacteria (122	Proteobacteria (2)	Bacteria (1)	100.00	1.40	99.60	98.70	WGS
294.453	0.3544	Pseudomonas fluorescens (286)	Pseudomonas (135621)	Pseudomonadaceae (7227	Pseudomonadales (1236)	Gammaproteobacteria (122	Proteobacteria (2)	Bacteria (1)	100.00	1.80	98.60	95.10	WGS
294.454	0.4666	Pseudomonas fluorescens (286)	Pseudomonas (135621)	Pseudomonadaceae (7227	Pseudomonadales (1236)	Gammaproteobacteria (122	Proteobacteria (2)	Bacteria (1)	100.00	0.70	98.50	95.80	Complete
294.455	0.3976	Pseudomonas fluorescens (286)	Pseudomonas (135621)	Pseudomonadaceae (7227	Pseudomonadales (1236)	Gammaproteobacteria (122	Proteobacteria (2)	Bacteria (1)	100.00	12.20	98.70	88.50	WGS
294.456	0.385	Pseudomonas fluorescens (286)	Pseudomonas (135621)	Pseudomonadaceae (7227	Pseudomonadales (1236)	Gammaproteobacteria (122	Proteobacteria (2)	Bacteria (1)	100.00	0.70	97.60	94.80	WGS
294.457	0.4066	Pseudomonas fluorescens (286)	Pseudomonas (135621)	Pseudomonadaceae (7227	Pseudomonadales (1236)	Gammaproteobacteria (122	Proteobacteria (2)	Bacteria (1)	100.00	0.40	98.20	95.90	WGS
294.463	0.3156	Pseudomonas fluorescens (286)	Pseudomonas (135621)	Pseudomonadaceae (7227	Pseudomonadales (1236)	Gammaproteobacteria (122	Proteobacteria (2)	Bacteria (1)			98.60	95.30	Complete
294.464	0.387	Pseudomonas fluorescens (286)	Pseudomonas (135621)	Pseudomonadaceae (7227	Pseudomonadales (1236)	Gammaproteobacteria (122	Proteobacteria (2)	Bacteria (1)			98.60	95.90	Complete
294.465	0.3506	Pseudomonas fluorescens (286)	Pseudomonas (135621)	Pseudomonadaceae (7227	Pseudomonadales (1236)	Gammaproteobacteria (122	Proteobacteria (2)	Bacteria (1)			98.70	93.60	WGS
294.466	0.3956	Pseudomonas fluorescens (286)	Pseudomonas (135621)	Pseudomonadaceae (7227	Pseudomonadales (1236)	Gammaproteobacteria (122	Proteobacteria (2)	Bacteria (1)			98.50	94.90	WGS
294.467	0.3124	Pseudomonas fluorescens (286)	Pseudomonas (135621)	Pseudomonadaceae (7227	Pseudomonadales (1236)	Gammaproteobacteria (122	Proteobacteria (2)	Bacteria (1)			98.50	94.40	WGS
294.468	0.3802	Pseudomonas fluorescens (286)	Pseudomonas (135621)	Pseudomonadaceae (7227	Pseudomonadales (1236)	Gammaproteobacteria (122	Proteobacteria (2)	Bacteria (1)			99.20	97.90	WGS
294.469	0.359	Pseudomonas fluorescens (286)	Pseudomonas (135621)	Pseudomonadaceae (7227	Pseudomonadales (1236)	Gammaproteobacteria (122	Proteobacteria (2)	Bacteria (1)			99.30	93.00	WGS
294.470	0.3754	Pseudomonas fluorescens (286)	Pseudomonas (135621)	Pseudomonadaceae (7227	Pseudomonadales (1236)	Gammaproteobacteria (122	Proteobacteria (2)	Bacteria (1)			99.20	94.50	WGS
294.471	0.3702	Pseudomonas fluorescens (286)	Pseudomonas (135621)	Pseudomonadaceae (7227	Pseudomonadales (1236)	Gammaproteobacteria (122	Proteobacteria (2)	Bacteria (1)			99.60	98.40	WGS
294.472	0.4052	Pseudomonas fluorescens (286)	Pseudomonas (135621)	Pseudomonadaceae (7227	Pseudomonadales (1236)	Gammaproteobacteria (122	Proteobacteria (2)	Bacteria (1)			99.50	98.50	WGS
294.473	0.3486	Pseudomonas fluorescens (286)	Pseudomonas (135621)	Pseudomonadaceae (7227	Pseudomonadales (1236)	Gammaproteobacteria (122	Proteobacteria (2)	Bacteria (1)			99.30	97.90	WGS
294.474	0.3574	Pseudomonas fluorescens (286)	Pseudomonas (135621)	Pseudomonadaceae (7227	Pseudomonadales (1236)	Gammaproteobacteria (122	Proteobacteria (2)	Bacteria (1)			99.40	98.30	WGS
294.475	0.3802	Pseudomonas fluorescens (286)	Pseudomonas (135621)	Pseudomonadaceae (7227	Pseudomonadales (1236)	Gammaproteobacteria (122	Proteobacteria (2)	Bacteria (1)			99.00	95.20	WGS
294.476	0.3802	Pseudomonas fluorescens (286)	Pseudomonas (135621)	Pseudomonadaceae (7227	Pseudomonadales (1236)	Gammaproteobacteria (122	Proteobacteria (2)	Bacteria (1)			99.30	95.30	WGS
294.492	0.3856	Pseudomonas fluorescens (286)	Pseudomonas (135621)	Pseudomonadaceae (7227	Pseudomonadales (1236)	Gammaproteobacteria (122	Proteobacteria (2)	Bacteria (1)			99.30	98.00	WGS
294.493	0.3752	Pseudomonas fluorescens (286)	Pseudomonas (135621)	Pseudomonadaceae (7227	Pseudomonadales (1236)	Gammaproteobacteria (122	Proteobacteria (2)	Bacteria (1)			99.20	97.30	Complete
294.494	0.3592	Pseudomonas fluorescens (286)	Pseudomonas (135621)	Pseudomonadaceae (7227	Pseudomonadales (1236)	Gammaproteobacteria (122	Proteobacteria (2)	Bacteria (1)			99.40	97.40	Complete
294.545	0.304	Pseudomonas fluorescens (286)	Pseudomonas (135621)	Pseudomonadaceae (7227	Pseudomonadales (1236)	Gammaproteobacteria (122	Proteobacteria (2)	Bacteria (1)			97.60	88.80	WGS
294.546	0.3316	Pseudomonas fluorescens (286)	Pseudomonas (135621)	Pseudomonadaceae (7227	Pseudomonadales (1236)	Gammaproteobacteria (122	Proteobacteria (2)	Bacteria (1)			98.60	95.30	WGS
294.547	0.4292	Pseudomonas fluorescens (286)	Pseudomonas (135621)	Pseudomonadaceae (7227	Pseudomonadales (1236)	Gammaproteobacteria (122	Proteobacteria (2)	Bacteria (1)			96.20	93.40	WGS
294.548	0.3814	Pseudomonas fluorescens (286)	Pseudomonas (135621)	Pseudomonadaceae (7227	Pseudomonadales (1236)	Gammaproteobacteria (122	Proteobacteria (2)	Bacteria (1)			99.40	98.10	WGS
294.549	0.3776	Pseudomonas fluorescens (286)	Pseudomonas (135621)	Pseudomonadaceae (7227	Pseudomonadales (1236)	Gammaproteobacteria (122	Proteobacteria (2)	Bacteria (1)			98.30	93.50	WGS
294.550	0.3818	Pseudomonas fluorescens (286)	Pseudomonas (135621)	Pseudomonadaceae (7227	Pseudomonadales (1236)	Gammaproteobacteria (122	Proteobacteria (2)	Bacteria (1)			99.90	98.90	Complete
294.551	0.3776	Pseudomonas fluorescens (286)	Pseudomonas (135621)	Pseudomonadaceae (7227	Pseudomonadales (1236)	Gammaproteobacteria (122	Proteobacteria (2)	Bacteria (1)			98.70	96.50	WGS
294.552	0.32	Pseudomonas fluorescens (286)	Pseudomonas (135621)	Pseudomonadaceae (7227	Pseudomonadales (1236)	Gammaproteobacteria (122	Proteobacteria (2)	Bacteria (1)			98.50	95.90	WGS
294.553	0.347	Pseudomonas fluorescens (286)	Pseudomonas (135621)	Pseudomonadaceae (7227	Pseudomonadales (1236)	Gammaproteobacteria (122	Proteobacteria (2)	Bacteria (1)			98.70	95.50	WGS
294.554	0.3844	Pseudomonas fluorescens (286)	Pseudomonas (135621)	Pseudomonadaceae (7227	Pseudomonadales (1236)	Gammaproteobacteria (122	Proteobacteria (2)	Bacteria (1)			98.40	95.30	WGS
294.555	0.3612	Pseudomonas fluorescens (286)	Pseudomonas (135621)	Pseudomonadaceae (7227	Pseudomonadales (1236)	Gammaproteobacteria (122	Proteobacteria (2)	Bacteria (1)			99.70	98.20	WGS
294.76	0.3914	Pseudomonas fluorescens (286)	Pseudomonas (135621)	Pseudomonadaceae (7227	Pseudomonadales (1236)	Gammaproteobacteria (122	Proteobacteria (2)	Bacteria (1)	99.30	0.00	99.00	97.90	WGS
294.77	0.343	Pseudomonas fluorescens (286)	Pseudomonas (135621)	Pseudomonadaceae (7227	Pseudomonadales (1236)	Gammaproteobacteria (122	Proteobacteria (2)	Bacteria (1)	100.00	0.00	98.80	97.10	WGS

294.78	0.35	Pseudomonas fluorescens (286)	Pseudomonas (135621)	Pseudomonadaceae (7227	Pseudomonadales (1236)	Gammaproteobacteria (122	Proteobacteria (2)	Bacteria (1)	100.00	0.40	98.30	96.50	WGS
294.79	0.3016	Pseudomonas fluorescens (286)	Pseudomonas (135621)	Pseudomonadaceae (7227	Pseudomonadales (1236)	Gammaproteobacteria (122	Proteobacteria (2)	Bacteria (1)	100.00	0.00	97.90	95.30	WGS
294.80	0.351	Pseudomonas fluorescens (286)	Pseudomonas (135621)	Pseudomonadaceae (7227	Pseudomonadales (1236)	Gammaproteobacteria (122	Proteobacteria (2)	Bacteria (1)	100.00	0.00	98.00	95.20	WGS
294.81	0.3954	Pseudomonas fluorescens (286)	Pseudomonas (135621)	Pseudomonadaceae (7227	Pseudomonadales (1236)	Gammaproteobacteria (122	Proteobacteria (2)	Bacteria (1)	100.00	0.70	98.90	96.50	WGS
294.82	0.3108	Pseudomonas fluorescens (286)	Pseudomonas (135621)	Pseudomonadaceae (7227	Pseudomonadales (1236)	Gammaproteobacteria (122	Proteobacteria (2)	Bacteria (1)	100.00	0.40	98.70	95.50	WGS
294.83	0.3512	Pseudomonas fluorescens (286)	Pseudomonas (135621)	Pseudomonadaceae (7227	Pseudomonadales (1236)	Gammaproteobacteria (122	Proteobacteria (2)	Bacteria (1)	100.00	0.00	98.20	95.60	WGS
294.84	0.3016	Pseudomonas fluorescens (286)	Pseudomonas (135621)	Pseudomonadaceae (7227	Pseudomonadales (1236)	Gammaproteobacteria (122	Proteobacteria (2)	Bacteria (1)	100.00	0.00	98.00	95.50	WGS
294.85	0.3206	Pseudomonas fluorescens (286)	Pseudomonas (135621)	Pseudomonadaceae (7227	Pseudomonadales (1236)	Gammaproteobacteria (122	Proteobacteria (2)	Bacteria (1)	100.00	0.40	98.40	95.60	WGS
294.86	0.3686	Pseudomonas fluorescens (286)	Pseudomonas (135621)	Pseudomonadaceae (7227	Pseudomonadales (1236)	Gammaproteobacteria (122	Proteobacteria (2)	Bacteria (1)	100.00	0.00	98.80	96.70	WGS
294.87	0.3354	Pseudomonas fluorescens (286)	Pseudomonas (135621)	Pseudomonadaceae (7227	Pseudomonadales (1236)	Gammaproteobacteria (122	Proteobacteria (2)	Bacteria (1)	100.00	0.40	99.30	97.60	WGS
294.88	0.3838	Pseudomonas fluorescens (286)	Pseudomonas (135621)	Pseudomonadaceae (7227	Pseudomonadales (1236)	Gammaproteobacteria (122	Proteobacteria (2)	Bacteria (1)	100.00	0.00	99.30	98.20	WGS
294.89	0.3726	Pseudomonas fluorescens (286)	Pseudomonas (135621)	Pseudomonadaceae (7227	Pseudomonadales (1236)	Gammaproteobacteria (122	Proteobacteria (2)	Bacteria (1)	100.00	0.00	99.00	97.70	WGS
294.90	0.3272	Pseudomonas fluorescens (286)	Pseudomonas (135621)	Pseudomonadaceae (7227	Pseudomonadales (1236)	Gammaproteobacteria (122	Proteobacteria (2)	Bacteria (1)	100.00	0.40	99.40	98.50	WGS
294.93	0.3772	Pseudomonas fluorescens (286)	Pseudomonas (135621)	Pseudomonadaceae (7227	Pseudomonadales (1236)	Gammaproteobacteria (122	Proteobacteria (2)	Bacteria (1)	100.00	0.00	98.80	97.80	WGS
296.11	0.2756	Pseudomonas fragi (286)	Pseudomonas (135621)	Pseudomonadaceae (7227	Pseudomonadales (1236)	Gammaproteobacteria (122	Proteobacteria (2)	Bacteria (1)	100.00	0.80	98.60	95.30	Complete
296.15	0.2772	Pseudomonas fragi (286)	Pseudomonas (135621)	Pseudomonadaceae (7227	Pseudomonadales (1236)	Gammaproteobacteria (122	Proteobacteria (2)	Bacteria (1)	100.00	0.00	98.60	96.00	WGS
296.17	0.332	Pseudomonas fragi (286)	Pseudomonas (135621)	Pseudomonadaceae (7227	Pseudomonadales (1236)	Gammaproteobacteria (122	Proteobacteria (2)	Bacteria (1)	100.00	0.00	98.70	95.90	WGS
296.18	0.3162	Pseudomonas fragi (286)	Pseudomonas (135621)	Pseudomonadaceae (7227	Pseudomonadales (1236)	Gammaproteobacteria (122	Proteobacteria (2)	Bacteria (1)	100.00	0.40	98.70	96.80	WGS
296.19	0.3504	Pseudomonas fragi (286)	Pseudomonas (135621)	Pseudomonadaceae (7227	Pseudomonadales (1236)	Gammaproteobacteria (122	Proteobacteria (2)	Bacteria (1)	100.00	3.30	98.70	95.30	WGS
296.20	0.0294	Pseudomonas fragi (286)	Pseudomonas (135621)	Pseudomonadaceae (7227	Pseudomonadales (1236)	Gammaproteobacteria (122	Proteobacteria (2)	Bacteria (1)	100.00	0.40	98.60	96.70	WGS
296.21	0.326	Pseudomonas fragi (286)	Pseudomonas (135621)	Pseudomonadaceae (7227	Pseudomonadales (1236)	Gammaproteobacteria (122	Proteobacteria (2)	Bacteria (1)	100.00	0.00	98.70	96.70	WGS
296.22	0.3228	Pseudomonas fragi (286)	Pseudomonas (135621)	Pseudomonadaceae (7227	Pseudomonadales (1236)	Gammaproteobacteria (122	Proteobacteria (2)	Bacteria (1)	100.00	0.80	98.70	96.50	WGS
296.23	0.3268	Pseudomonas fragi (286)	Pseudomonas (135621)	Pseudomonadaceae (7227	Pseudomonadales (1236)	Gammaproteobacteria (122	Proteobacteria (2)	Bacteria (1)	100.00	0.40	98.60	95.90	WGS
296.24	0.2834	Pseudomonas fragi (286)	Pseudomonas (135621)	Pseudomonadaceae (7227	Pseudomonadales (1236)	Gammaproteobacteria (122	Proteobacteria (2)	Bacteria (1)	100.00	0.80	98.50	96.50	WGS
296.25	0.3154	Pseudomonas fragi (286)	Pseudomonas (135621)	Pseudomonadaceae (7227	Pseudomonadales (1236)	Gammaproteobacteria (122	Proteobacteria (2)	Bacteria (1)	100.00	1.70	98.70	96.50	WGS
296.26	0.2886	Pseudomonas fragi (286)	Pseudomonas (135621)	Pseudomonadaceae (7227	Pseudomonadales (1236)	Gammaproteobacteria (122	Proteobacteria (2)	Bacteria (1)	100.00	0.40	98.70	95.80	WGS
296.27	0.3038	Pseudomonas fragi (286)	Pseudomonas (135621)	Pseudomonadaceae (7227	Pseudomonadales (1236)	Gammaproteobacteria (122	Proteobacteria (2)	Bacteria (1)	100.00	0.80	98.70	96.40	WGS
296.28	0.2746	Pseudomonas fragi (286)	Pseudomonas (135621)	Pseudomonadaceae (7227	Pseudomonadales (1236)	Gammaproteobacteria (122	Proteobacteria (2)	Bacteria (1)	100.00	0.40	98.40	95.60	WGS
296.32	0.0294	Pseudomonas fragi (286)	Pseudomonas (135621)	Pseudomonadaceae (7227	Pseudomonadales (1236)	Gammaproteobacteria (122	Proteobacteria (2)	Bacteria (1)					Plasmid
296.33	0.0298	Pseudomonas fragi (286)	Pseudomonas (135621)	Pseudomonadaceae (7227	Pseudomonadales (1236)	Gammaproteobacteria (122	Proteobacteria (2)	Bacteria (1)					Plasmid
296.34	0.0294	Pseudomonas fragi (286)	Pseudomonas (135621)	Pseudomonadaceae (7227	Pseudomonadales (1236)	Gammaproteobacteria (122	Proteobacteria (2)	Bacteria (1)					Plasmid
296.39	0.3282	Pseudomonas fragi (286)	Pseudomonas (135621)	Pseudomonadaceae (7227	Pseudomonadales (1236)	Gammaproteobacteria (122	Proteobacteria (2)	Bacteria (1)	100.00	10.30	98.10	89.90	WGS
296.44	0.318	Pseudomonas fragi (286)	Pseudomonas (135621)	Pseudomonadaceae (7227	Pseudomonadales (1236)	Gammaproteobacteria (122	Proteobacteria (2)	Bacteria (1)			98.40	93.70	WGS
296.7	0.3364	Pseudomonas fragi (286)	Pseudomonas (135621)	Pseudomonadaceae (7227	Pseudomonadales (1236)	Gammaproteobacteria (122	Proteobacteria (2)	Bacteria (1)	99.60	3.10	98.20	93.90	Complete
296.9	0.2772	Pseudomonas fragi (286)	Pseudomonas (135621)	Pseudomonadaceae (7227	Pseudomonadales (1236)	Gammaproteobacteria (122	Proteobacteria (2)	Bacteria (1)	100.00	0.40	98.70	95.90	Complete
297.17	0.1854	Hydrogenophilus thermoluteolus (7	Hydrogenophilus (206349)	Hydrogenophilaceae (1190	Hydrogenophilales (200878	Hydrogenophilalia (1224)	Proteobacteria (2)	Bacteria (1)	100.00	1.00	93.10	92.80	WGS
298.4	0.3634	Pseudomonas marginalis (286)	Pseudomonas (135621)	Pseudomonadaceae (7227	Pseudomonadales (1236)	Gammaproteobacteria (122	Proteobacteria (2)	Bacteria (1)	100.00	0.30	98.70	96.10	WGS
298.8	0.3692	Pseudomonas marginalis (286)	Pseudomonas (135621)	Pseudomonadaceae (7227	Pseudomonadales (1236)	Gammaproteobacteria (122	Proteobacteria (2)	Bacteria (1)			98.40	94.50	WGS
300.11	0.3698	Pseudomonas mendocina (286)	Pseudomonas (135621)	Pseudomonadaceae (7227	Pseudomonadales (1236)	Gammaproteobacteria (122	Proteobacteria (2)	Bacteria (1)	76.80	5.20	97.50	94.50	WGS
300.12	0.37	Pseudomonas mendocina (286)	Pseudomonas (135621)	Pseudomonadaceae (7227	Pseudomonadales (1236)	Gammaproteobacteria (122	Proteobacteria (2)	Bacteria (1)	100.00	2.20	99.80	96.70	WGS
300.13	0.3636	Pseudomonas mendocina (286)	Pseudomonas (135621)	Pseudomonadaceae (7227	Pseudomonadales (1236)	Gammaproteobacteria (122	Proteobacteria (2)	Bacteria (1)	100.00	13.80	99.70	87.70	WGS
300.18	0.4754	Pseudomonas mendocina (286)	Pseudomonas (135621)	Pseudomonadaceae (7227	Pseudomonadales (1236)	Gammaproteobacteria (122	Proteobacteria (2)	Bacteria (1)	84.00	3.70	97.00	94.80	WGS
300.19	0.4936	Pseudomonas mendocina (286)	Pseudomonas (135621)	Pseudomonadaceae (7227	Pseudomonadales (1236)	Gammaproteobacteria (122	Proteobacteria (2)	Bacteria (1)	84.30	4.00	97.80	96.20	Complete
300.43	0.518	Pseudomonas mendocina (286)	Pseudomonas (135621)	Pseudomonadaceae (7227	Pseudomonadales (1236)	Gammaproteobacteria (122	Proteobacteria (2)	Bacteria (1)	86.50	3.60	97.80	95.40	Complete
300.56	0.4616	Pseudomonas mendocina (286)	Pseudomonas (135621)	Pseudomonadaceae (7227	Pseudomonadales (1236)	Gammaproteobacteria (122	Proteobacteria (2)	Bacteria (1)	87.20	2.90	98.30	96.60	WGS
300.57	0.4812	Pseudomonas mendocina (286)	Pseudomonas (135621)	Pseudomonadaceae (7227	Pseudomonadales (1236)	Gammaproteobacteria (122	Proteobacteria (2)	Bacteria (1)	86.90	2.90	98.40	96.30	WGS
300.59	0.5072	Pseudomonas mendocina (286)	Pseudomonas (135621)	Pseudomonadaceae (7227	Pseudomonadales (1236)	Gammaproteobacteria (122	Proteobacteria (2)	Bacteria (1)			97.80	95.90	Complete
300.68	0.0972	Pseudomonas mendocina (286)	Pseudomonas (135621)	Pseudomonadaceae (7227	Pseudomonadales (1236)	Gammaproteobacteria (122	Proteobacteria (2)	Bacteria (1)			86.30	86.10	WGS
300.69	0.2354	Pseudomonas mendocina (286)	Pseudomonas (135621)	Pseudomonadaceae (7227	Pseudomonadales (1236)	Gammaproteobacteria (122	Proteobacteria (2)	Bacteria (1)			77.30	76.50	WGS
300.7	0.5116	Pseudomonas mendocina (286)	Pseudomonas (135621)	Pseudomonadaceae (7227	Pseudomonadales (1236)	Gammaproteobacteria (122	Proteobacteria (2)	Bacteria (1)	86.50	3.90	97.90	95.90	WGS
301.11	0.4898	Pseudomonas oleovorans (286)	Pseudomonas (135621)	Pseudomonadaceae (7227	Pseudomonadales (1236)	Gammaproteobacteria (122	Proteobacteria (2)	Bacteria (1)	67.60	8.50	86.30	82.10	WGS
301.12	0.5422	Pseudomonas oleovorans (286)	Pseudomonas (135621)	Pseudomonadaceae (7227	Pseudomonadales (1236)	Gammaproteobacteria (122	Proteobacteria (2)	Bacteria (1)	85.90	2.70	95.20	92.90	WGS
301.18	0.3468	Pseudomonas oleovorans (286)	Pseudomonas (135621)	Pseudomonadaceae (7227	Pseudomonadales (1236)	Gammaproteobacteria (122	Proteobacteria (2)	Bacteria (1)	79.20	5.70	97.80	95.60	WGS
301.20	0.4362	Pseudomonas oleovorans (286)	Pseudomonas (135621)	Pseudomonadaceae (7227	Pseudomonadales (1236)	Gammaproteobacteria (122	Proteobacteria (2)	Bacteria (1)	83.20	8.60	97.90	93.30	WGS
301.21	0.4382	Pseudomonas oleovorans (286)	Pseudomonas (135621)	Pseudomonadaceae (7227	Pseudomonadales (1236)	Gammaproteobacteria (122	Proteobacteria (2)	Bacteria (1)			98.20	95.20	WGS
301.22	0.4362	Pseudomonas oleovorans (286)	Pseudomonas (135621)	Pseudomonadaceae (7227	Pseudomonadales (1236)	Gammaproteobacteria (122	Proteobacteria (2)	Bacteria (1)			98.10	94.80	WGS
301.9	0.5222	Pseudomonas oleovorans (286)	Pseudomonas (135621)	Pseudomonadaceae (7227	Pseudomonadales (1236)	Gammaproteobacteria (122	Proteobacteria (2)	Bacteria (1)	86.90	2.40	98.20	96.50	WGS
303.139	0.4706	Pseudomonas putida (286)	Pseudomonas (135621)	Pseudomonadaceae (7227	Pseudomonadales (1236)	Gammaproteobacteria (122	Proteobacteria (2)	Bacteria (1)	100.00	0.00	99.30	97.40	WGS
303.140	0.299	Pseudomonas putida (286)	Pseudomonas (135621)	Pseudomonadaceae (7227	Pseudomonadales (1236)	Gammaproteobacteria (122	Proteobacteria (2)	Bacteria (1)	99.60	0.80	98.20	95.30	WGS
303.141	0.4682	Pseudomonas putida (286)	Pseudomonas (135621)	Pseudomonadaceae (7227	Pseudomonadales (1236)	Gammaproteobacteria (122	Proteobacteria (2)	Bacteria (1)	96.10	2.80	96.90	93.90	WGS
303.142	0.4584	Pseudomonas putida (286)	Pseudomonas (135621)	Pseudomonadaceae (7227	Pseudomonadales (1236)	Gammaproteobacteria (122	Proteobacteria (2)	Bacteria (1)	100.00	0.00	99.00	96.90	WGS
303.167	0.4576	Pseudomonas putida (286)	Pseudomonas (135621)	Pseudomonadaceae (7227	Pseudomonadales (1236)	Gammaproteobacteria (122	Proteobacteria (2)	Bacteria (1)	100.00	11.70	99.40	90.80	Complete
303.168	0.3686	Pseudomonas putida (286)	Pseudomonas (135621)	Pseudomonadaceae (7227	Pseudomonadales (1236)	Gammaproteobacteria (122	Proteobacteria (2)	Bacteria (1)	100.00	1.20	99.40	95.50	WGS
303.169	0.4236	Pseudomonas putida (286)	Pseudomonas (135621)	Pseudomonadaceae (7227	Pseudomonadales (1236)	Gammaproteobacteria (122	Proteobacteria (2)	Bacteria (1)	100.00	1.00	99.40	96.90	WGS
303.170	0.3456	Pseudomonas putida (286)	Pseudomonas (135621)	Pseudomonadaceae (7227	Pseudomonadales (1236)	Gammaproteobacteria (122	Proteobacteria (2)	Bacteria (1)	100.00	1.50	97.90	93.90	WGS
303.171	0.4566	Pseudomonas putida (286)	Pseudomonas (135621)	Pseudomonadaceae (7227	Pseudomonadales (1236)	Gammaproteobacteria (122	Proteobacteria (2)	Bacteria (1)	100.00	1.50	98.40	95.30	WGS
303.172	0.3908	Pseudomonas putida (286)	Pseudomonas (135621)	Pseudomonadaceae (7227	Pseudomonadales (1236)	Gammaproteobacteria (122	Proteobacteria (2)	Bacteria (1)	100.00	0.00	98.90	95.70	WGS
303.173	0.451	Pseudomonas putida (286)	Pseudomonas (135621)	Pseudomonadaceae (7227	Pseudomonadales (1236)	Gammaproteobacteria (122	Proteobacteria (2)	Bacteria (1)	100.00	0.40	99.40	96.80	WGS
303.174	0.4192	Pseudomonas putida (286)	Pseudomonas (135621)	Pseudomonadaceae (7227	Pseudomonadales (1236)	Gammaproteobacteria (122	Proteobacteria (2)	Bacteria (1)	100.00	3.40	99.40	93.90	WGS
303.175	0.4054	Pseudomonas putida (286)	Pseudomonas (135621)	Pseudomonadaceae (7227	Pseudomonadales (1236)	Gammaproteobacteria (122	Proteobacteria (2)	Bacteria (1)	100.00	0.80	99.30	97.90	WGS
303.176	0.4338	Pseudomonas putida (286)	Pseudomonas (135621)	Pseudomonadaceae (7227	Pseudomonadales (1236)	Gammaproteobacteria (122	Proteobacteria (2)	Bacteria (1)	100.00	1.30	99.50	97.30	WGS
303.177	0.4548	Pseudomonas putida (286)	Pseudomonas (135621)	Pseudomonadaceae (7227	Pseudomonadales (1236)	Gammaproteobacteria (122	Proteobacteria (2)	Bacteria (1)	100.00	0.00	99.30	97.80	WGS
303.178	0.4554	Pseudomonas putida (286)	Pseudomonas (135621)	Pseudomonadaceae (7227	Pseudomonadales (1236)	Gammaproteobacteria (122	Proteobacteria (2)	Bacteria (1)	100.00	0.00	99.10	96.70	WGS
303.179	0.426	Pseudomonas putida (286)	Pseudomonas (135621)	Pseudomonadaceae (7227	Pseudomonadales (1236)	Gammaproteobacteria (122	Proteobacteria (2)	Bacteria (1)	100.00	0.80	99.30	97.20	WGS
303.180	0.4004	Pseudomonas putida (286)	Pseudomonas (135621)	Pseudomonadaceae (7227	Pseudomonadales (1236)	Gammaproteobacteria (122	Proteobacteria (2)	Bacteria (1)	100.00	0.50	99.50	98.70	WGS
303.186	0.329	Pseudomonas putida (286)	Pseudomonas (135621)	Pseudomonadaceae (7227	Pseudomonadales (1236)	Gammaproteobacteria (122	Proteobacteria (2)	Bacteria (1)	98.80	45.50	98.20	69.10	WGS
303.187	0.355	Pseudomonas putida (286)	Pseudomonas (135621)	Pseudomonadaceae (7227	Pseudomonadales (1236)	Gammaproteobacteria (122	Proteobacteria (2)	Bacteria (1)	100.00	0.00	98.20	96.10	WGS
303.188	0.3078	Pseudomonas putida (286)	Pseudomonas (135621)	Pseudomonadaceae (7227	Pseudomonadales (1236)	Gammaproteobacteria (122	Proteobacteria (2)	Bacteria (1)	98.80	18.50	99.30	86.60	WGS
303.189	0.347	Pseudomonas putida (286)	Pseudomonas (135621)	Pseudomonadaceae (7227	Pseudomonadales (1236)	Gammaproteobacteria (122	Proteobacteria (2)	Bacteria (1)	100.00	0.40	99.00	97.80	WGS
303.192	0.3668	Pseudomonas putida (286)	Pseudomonas (135621)	Pseudomonadaceae (7227	Pseudomonadales (1236)	Gammaproteobacteria (122	Proteobacteria (2)	Bacteria (1)	100.00	0.00	99.00	97.90	WGS
303.199	0.4106	Pseudomonas putida (286)	Pseudomonas (135621)	Pseudomonadaceae (7227	Pseudomonadales (1236)	Gammaproteobacteria (122	Proteobacteria (2)	Bacteria (1)	100.00	0.00	99.00	95.90	WGS
303.200	0.396	Pseudomonas putida (286)	Pseudomonas (135621)	Pseudomonadaceae (7227	Pseudomonadales (1236)	Gammaproteobacteria (122	Proteobacteria (2)	Bacteria (1)	100.00	0.00	99.00	97.80	Complete
303.203	0.3764	Pseudomonas putida (286)	Pseudomonas (135621)	Pseudomonadaceae (7227	Pseudomonadales (1236)	Gammaproteobacteria (122	Proteobacteria (2)	Bacteria (1)	99.60	0.80	98.40	96.30	WGS
303.204	0.3894	Pseudomonas putida (286)	Pseudomonas (135621)	Pseudomonadaceae (7227	Pseudomonadales (1236)	Gammaproteobacteria (122	Proteobacteria (2)	Bacteria (1)	100.00	0.00	99.30	97.60	WGS
303.205	0.442	Pseudomonas putida (286)	Pseudomonas (135621)	Pseudomonadaceae (7227	Pseudomonadales (1236)	Gammaproteobacteria (122	Proteobacteria (2)	Bacteria (1)	100.00	0.00	99.60	96.70	WGS
303.209	0.3904	Pseudomonas putida (286)	Pseudomonas (135621)	Pseudomonadaceae (7227	Pseudomonadales (1236)	Gammaproteobacteria (122	Proteobacteria (2)	Bacteria (1)	100.00	0.00	99.30	97.00	WGS
303.212	0.3904	Pseudomonas putida (286)	Pseudomonas (135621)	Pseudomonadaceae (7227	Pseudomonadales (1236)	Gammaproteobacteria (122	Proteobacteria (2)	Bacteria (1)	100.00	0.00	99.30	97.30	WGS
303.214	0.3904	Pseudomonas putida (286)	Pseudomonas (135621)	Pseudomonadaceae (7227	Pseudomonadales (1236)	Gammaproteobacteria (122	Proteobacteria (2)	Bacteria (1)	100.00	0.00	99.30	97.00	WGS
303.215	0.3904	Pseudomonas putida (286)	Pseudomonas (135621)	Pseudomonadaceae (7227	Pseudomonadales (1236)	Gammaproteobacteria (122	Proteobacteria (2)	Bacteria (1)	100.00	0.00	99.30	96.90	WGS
303.234	0.4384	Pseudomonas putida (286)	Pseudomonas (135621)	Pseudomonadaceae (7227	Pseudomonadales (1236)	Gammaproteobacteria (122	Proteobacteria (2)	Bacteria (1)	100.00	3.40	99.00	95.80	WGS
303.397	0.433	Pseudomonas putida (286)	Pseudomonas (135621)	Pseudomonadaceae (7227	Pseudomonadales (1236)	Gammaproteobacteria (122	Proteobacteria (2)	Bacteria (1)	100.00	0.00	98.90	97.00	Complete
303.398	0.3732	Pseudomonas putida (286)	Pseudomonas (135621)	Pseudomonadaceae (7227	Pseudomonadales (1236)	Gammaproteobacteria (122	Proteobacteria (2)	Bacteria (1)	94.10	4.00	95.80	90.60	WGS
303.409	0.3634	Pseudomonas putida (286)	Pseudomonas (135621)	Pseudomonadaceae (7227	Pseudomonadales (1236)	Gammaproteobacteria (122	Proteobacteria (2)	Bacteria (1)	100.00	0.00	98.90	95.30	WGS
303.410	0.3354	Pseudomonas putida (286)	Pseudomonas (135621)	Pseudomonadaceae (7227	Pseudomonadales (1236)	Gammaproteobacteria (122	Proteobacteria (2)	Bacteria (1)	100.00	1.20	99.40	96.70	Complete
303.411	0.4824	Pseudomonas putida (286)	Pseudomonas (135621)	Pseudomonadaceae (7227	Pseudomonadales (1236)	Gammaproteobacteria (122	Proteobacteria (2)	Bacteria (1)	100.00	0.00	99.60	98.30	Complete
303.424	0.462	Pseudomonas putida (286)	Pseudomonas (135621)	Pseudomonadaceae (7227	Pseudomonadales (1236)	Gammaproteobacteria (122	Proteobacteria (2)	Bacteria (1)	100.00	0.80	99.60	98.00	WGS
303.425	0.3888	Pseudomonas putida (286)	Pseudomonas (135621)	Pseudomonadaceae (7227	Pseudomonadales (1236)	Gammaproteobacteria (122	Proteobacteria (2)	Bacteria (1)	100.00	0.40	99.40	96.70	WGS
303.426	0.3426	Pseudomonas putida (286)	Pseudomonas (135621)	Pseudomonadaceae (7227	Pseudomonadales (1236)	Gammaproteobacteria (122	Proteobacteria (2)	Bacteria (1)	100.00	0.40	99.10	97.30	WGS
303.427	0.4078	Pseudomonas putida (286)	Pseudomonas (135621)	Pseudomonadaceae (7227	Pseudomonadales (1236)	Gammaproteobacteria (122	Proteobacteria (2)	Bacteria (1)	100.00	1.30	99.60	98.20	Complete
303.429	0.483	Pseudomonas putida (286)	Pseudomonas (135621)	Pseudomonadaceae (7227	Pseudomonadales (1236)	Gammaproteobacteria (122	Proteobacteria (2)	Bacteria (1)	100.00	0.00	99.00	95.40	WGS
303.430	0.5026	Pseudomonas putida (286)	Pseudomonas (135621)	Pseudomonadaceae (7227	Pseudomonadales (1236)	Gammaproteobacteria (122	Proteobacteria (2)	Bacteria (1)	100.00	0.00	99.50	98.00	WGS
303.431	0.5026	Pseudomonas putida (286)	Pseudomonas (135621)	Pseudomonadaceae (7227	Pseudomonadales (1236)	Gammaproteobacteria (122	Proteobacteria (2)	Bacteria (1)	100.00	0.00	99.50	97.90	WGS
303.432	0.4772	Pseudomonas putida (286)	Pseudomonas (135621)	Pseudomonadaceae (7227	Pseudomonadales (1236)	Gammaproteobacteria (122	Proteobacteria (2)	Bacteria (1)	100.00	0.00	99.20	97.40	WGS
303.447	0.364	Pseudomonas putida (286)	Pseudomonas (135621)	Pseudomonadaceae (7227	Pseudomonadales (1236)	Gammaproteobacteria (122	Proteobacteria (2)	Bacteria (1)	100.00	0.40	98.70	95.90	Complete
303.449	0.4772	Pseudomonas putida (286)	Pseudomonas (135621)	Pseudomonadaceae (7227	Pseudomonadales (1236)	Gammaproteobacteria (122	Proteobacteria (2)	Bacteria (1)	100.00	0.00	99.40	97.70	WGS
303.450	0.385	Pseudomonas putida (286)	Pseudomonas (135621)	Pseudomonadaceae (7227	Pseudomonadales (1236)	Gammaproteobacteria (122	Proteobacteria (2)	Bacteria (1)	100.00	1.20	99.90	98.40	WGS
303.451	0.4356	Pseudomonas putida (286)	Pseudomonas (135621)	Pseudomonadaceae (7227	Pseudomonadales (1236)	Gammaproteobacteria (122	Proteobacteria (2)	Bacteria (1)	100.00	0.00	99.00	96.90	Complete
303.452	0.3916	Pseudomonas putida (286)	Pseudomonas (135621)	Pseudomonadaceae (7227	Pseudomonadales (1236)	Gammaproteobacteria (122	Proteobacteria (2)	Bacteria (1)	100.00	0.80	98.70	96.50	Complete
303.453	0.3946	Pseudomonas putida (286)	Pseudomonas (135621)	Pseudomonadaceae (7227	Pseudomonadales (1236)	Gammaproteobacteria (122	Proteobacteria (2)	Bacteria (1)	100.00	0.20	99.00	97.30	Complete
303.458	0.0394	Pseudomonas putida (286)	Pseudomonas (135621)	Pseudomonadaceae (7227	Pseudomonadales (1236)	Gammaproteobacteria (122	Proteobacteria (2)	Bacteria (1)					Plasmid
303.459	0.0322	Pseudomonas putida (286)	Pseudomonas (135621)	Pseudomonadaceae (7227	Pseudomonadales (1236)	Gammaproteobacteria (122	Proteobacteria (2)	Bacteria (1)					Plasmid
303.460	0.0294	Pseudomonas putida (286)	Pseudomonas (135621)	Pseudomonadaceae (7227	Pseudomonadales (1236)	Gammaproteobacteria (122	Proteobacteria (2)	Bacteria (1)					Plasmid
303.461	0.0294	Pseudomonas putida (286)	Pseudomonas (135621)	Pseudomonadaceae (7227	Pseudomonadales (1236)	Gammaproteobacteria (122	Proteobacteria (2)	Bacteria (1)					Plasmid
303.462	0.0294	Pseudomonas putida (286)	Pseudomonas (135621)	Pseudomonadaceae (7227	Pseudomonadales (1236)	Gammaproteobacteria (122	Proteobacteria (2)	Bacteria (1)					Plasmid
303.463	0.0294	Pseudomonas putida (286)	Pseudomonas (135621)	Pseudomonadaceae (7227	Pseudomonadales (1236)	Gammaproteobacteria (122	Proteobacteria (2)	Bacteria (1)					Plasmid
303.464	0.0294	Pseudomonas putida (286)	Pseudomonas (135621)	Pseudomonadaceae (7227	Pseudomonadales (1236)	Gammaproteobacteria (122	Proteobacteria (2)	Bacteria (1)					Plasmid
303.465	0.0294	Pseudomonas putida (286)	Pseudomonas (135621)	Pseudomonadaceae (7227	Pseudomonadales (1236)	Gammaproteobacteria (122	Proteobacteria (2)	Bacteria (1)					Plasmid
303.466	0.0294	Pseudomonas putida (286)	Pseudomonas (135621)	Pseudomonadaceae (7227	Pseudomonadales (1236)	Gammaproteobacteria (122	Proteobacteria (2)	Bacteria (1)					Plasmid
303.467	0.0294	Pseudomonas putida (286)	Pseudomonas (135621)	Pseudomonadaceae (7227	Pseudomonadales (1236)	Gammaproteobacteria (122	Proteobacteria (2)	Bacteria (1)					Plasmid
303.468	0.0294	Pseudomonas putida (286)	Pseudomonas (135621)	Pseudomonadaceae (7227	Pseudomonadales (1236)	Gammaproteobacteria (122	Proteobacteria (2)	Bacteria (1)					Plasmid
303.469	0.0322	Pseudomonas putida (286)	Pseudomonas (135621)	Pseudomonadaceae (7227	Pseudomonadales (1236)	Gammaproteobacteria (122	Proteobacteria (2)	Bacteria (1)					Plasmid
303.470	0.0322	Pseudomonas putida (286)	Pseudomonas (135621)	Pseudomonadaceae (7227	Pseudomonadales (1236)	Gammaproteobacteria (122	Proteobacteria (2)	Bacteria (1)					Plasmid
303.471	0.0292	Pseudomonas putida (286)	Pseudomonas (135621)	Pseudomonadaceae (7227	Pseudomonadales (1236)	Gammaproteobacteria (122	Proteobacteria (2)	Bacteria (1)					Plasmid
303.472	0.0294	Pseudomonas putida (286)	Pseudomonas (135621)	Pseudomonadaceae (7227	Pseudomonadales (1236)	Gammaproteobacteria (122	Proteobacteria (2)	Bacteria (1)					Plasmid
303.473	0.4776	Pseudomonas putida (286)	Pseudomonas (135621)	Pseudomonadaceae (7227	Pseudomonadales (1236)	Gammaproteobacteria (122	Proteobacteria (2)	Bacteria (1)	100.00	0.40	99.60	98.30	WGS
303.486	0.4406	Pseudomonas putida (286)	Pseudomonas (135621)	Pseudomonadaceae (7227	Pseudomonadales (1236)	Gammaproteobacteria (122	Proteobacteria (2)	Bacteria (1)	99.60	0.00	99.30	97.30	WGS
303.487	0.3074	Pseudomonas putida (286)	Pseudomonas (135621)	Pseudomonadaceae (7227	Pseudomonadales (1236)	Gammaproteobacteria (122	Proteobacteria (2)	Bacteria (1)	100.00	0.00	99.00	97.80	WGS
303.488	0.4548	Pseudomonas putida (286)	Pseudomonas (135621)	Pseudomonadaceae (7227	Pseudomonadales (1236)	Gammaproteobacteria (122	Proteobacteria (2)	Bacteria (1)	100.00	0.00	99.30	97.60	WGS
303.489	0.4642	Pseudomonas putida (286)	Pseudomonas (135621)	Pseudomonadaceae (7227	Pseudomonadales (1236)	Gammaproteobacteria (122	Proteobacteria (2)	Bacteria (1)	100.00	0.00	99.30	98.30	WGS
303.490	0.465	Pseudomonas putida (286)	Pseudomonas (135621)	Pseudomonadaceae (7227	Pseudomonadales (1236)	Gammaproteobacteria (122	Proteobacteria (2)	Bacteria (1)	100.00	0.00	99.40	97.30	WGS
303.491	0.3976	Pseudomonas putida (286)	Pseudomonas (135621)	Pseudomonadaceae (7227	Pseudomonadales (1236)	Gammaproteobacteria (122	Proteobacteria (2)	Bacteria (1)	100.00	0.00	99.20	97.30	WGS
303.492	0.407	Pseudomonas putida (286)	Pseudomonas (135621)	Pseudomonadaceae (7227	Pseudomonadales (1236)	Gammaproteobacteria (122	Proteobacteria (2)	Bacteria (1)	100.00	0.40	99.30	97.30	WGS
303.493	0.4672	Pseudomonas putida (286)	Pseudomonas (135621)	Pseudomonadaceae (7227	Pseudomonadales (1236)	Gammaproteobacteria (122	Proteobacteria (2)	Bacteria (1)	100.00	0.40	99.50	98.00	WGS
303.494	0.4772	Pseudomonas putida (286)	Pseudomonas (135621)	Pseudomonadaceae (7227	Pseudomonadales (1236)	Gammaproteobacteria (122	Proteobacteria (2)	Bacteria (1)	100.00	0.40	99.20	96.20	WGS
303.506	0.3972	Pseudomonas putida (286)	Pseudomonas (135621)	Pseudomonadaceae (7227	Pseudomonadales (1236)	Gammaproteobacteria (122	Proteobacteria (2)	Bacteria (1)	100.00	1.50	98.20	95.70	Complete
303.507	0.3804	Pseudomonas putida (286)	Pseudomonas (135621)	Pseudomonadaceae (7227	Pseudomonadales (1236)	Gammaproteobacteria (122	Proteobacteria (2)	Bacteria (1)	98.80	4.00	98.50	93.00	WGS
303.508	0.44	Pseudomonas putida (286)	Pseudomonas (135621)	Pseudomonadaceae (7227	Pseudomonadales (1236)	Gammaproteobacteria (122	Proteobacteria (2)	Bacteria (1)	100.00	0.40	99.00	96.70	Complete
303.509	0.398	Pseudomonas putida (286)	Pseudomonas (135621)	Pseudomonadaceae (7227	Pseudomonadales (1236)	Gammaproteobacteria (122	Proteobacteria (2)	Bacteria (1)	100.00	0.40	98.70	97.20	Complete
303.529	0.4186	Pseudomonas putida (286)	Pseudomonas (135621)	Pseudomonadaceae (7227	Pseudomonadales (1236)	Gammaproteobacteria (122	Proteobacteria (2)	Bacteria (1)	100.00	0.80	99.30	96.50	WGS
303.530	0.4278	Pseudomonas putida (286)	Pseudomonas (135621)	Pseudomonadaceae (7227	Pseudomonadales (1236)	Gammaproteobacteria (122	Proteobacteria (2)	Bacteria (1)	100.00	16.30	99.00	89.00	WGS
303.531	0.4294	Pseudomonas putida (286)	Pseudomonas (135621)	Pseudomonadaceae (7227	Pseudomonadales (1236)	Gammaproteobacteria (122	Proteobacteria (2)	Bacteria (1)	100.00	0.00	98.60	96.50	WGS
303.532	0.4524	Pseudomonas putida (286)	Pseudomonas (135621)	Pseudomonadaceae (7227	Pseudomonadales (1236)	Gammaproteobacteria (122	Proteobacteria (2)	Bacteria (1)	100.00	0.40	99.50	98.20	WGS
303.533	0.4392	Pseudomonas putida (286)	Pseudomonas (135621)	Pseudomonadaceae (7227	Pseudomonadales (1236)	Gammaproteobacteria (122	Proteobacteria (2)	Bacteria (1)	100.00	0.80	99.20	97.30	WGS
303.534	0.4122	Pseudomonas putida (286)	Pseudomonas (135621)	Pseudomonadaceae (7227	Pseudomonadales (1236)	Gammaproteobacteria (122	Proteobacteria (2)	Bacteria (1)	100.00	2.30	99.00	95.20	WGS
303.535	0.434	Pseudomonas putida (286)	Pseudomonas (135621)	Pseudomonadaceae (7227	Pseudomonadales (1236)	Gammaproteobacteria (122	Proteobacteria (2)	Bacteria (1)	100.00	0.00	98.70	97.10	WGS
303.546	0.4684	Pseudomonas putida (286)	Pseudomonas (135621)	Pseudomonadaceae (7227	Pseudomonadales (1236)	Gammaproteobacteria (122	Proteobacteria (2)	Bacteria (1)			99.50	97.70	Complete
303.547	0.439	Pseudomonas putida (286)	Pseudomonas (135621)	Pseudomonadaceae (7227	Pseudomonadales (1236)	Gammaproteobacteria (122	Proteobacteria (2)	Bacteria (1)			99.00	95.60	WGS
303.548	0.4264	Pseudomonas putida (286)	Pseudomonas (135621)	Pseudomonadaceae (7227	Pseudomonadales (1236)	Gammaproteobacteria (122	Proteobacteria (2)	Bacteria (1)			99.40	95.50	WGS
303.549	0.3704	Pseudomonas putida (286)	Pseudomonas (135621)	Pseudomonadaceae (7227	Pseudomonadales (1236)	Gammaproteobacteria (122	Proteobacteria (2)	Bacteria (1)			98.30	95.90	WGS
303.563	0.3926	Pseudomonas putida (286)	Pseudomonas (135621)	Pseudomonadaceae (7227	Pseudomonadales (1236)	Gammaproteobacteria (122	Proteobacteria (2)	Bacteria (1)			99.00	97.00	WGS
303.564	0.4054	Pseudomonas putida (286)	Pseudomonas (135621)	Pseudomonadaceae (7227	Pseudomonadales (1236)	Gammaproteobacteria (122	Proteobacteria (2)	Bacteria (1)			99.30	99.00	Complete
303.604	0.3452	Pseudomonas putida (286)	Pseudomonas (135621)	Pseudomonadaceae (7227	Pseudomonadales (1236)	Gammaproteobacteria (122	Proteobacteria (2)	Bacteria (1)			98.30	70.90	WGS
303.605	0.3728	Pseudomonas putida (286)	Pseudomonas (135621)	Pseudomonadaceae (7227	Pseudomonadales (1236)	Gammaproteobacteria (122	Proteobacteria (2)	Bacteria (1)			99.20	96.40	WGS
303.606	0.4486	Pseudomonas putida (286)	Pseudomonas (135621)	Pseudomonadaceae (7227	Pseudomonadales (1236)	Gammaproteobacteria (122	Proteobacteria (2)	Bacteria (1)			99.00	97.20	WGS
303.607	0.396	Pseudomonas putida (286)	Pseudomonas (135621)	Pseudomonadaceae (7227	Pseudomonadales (1236)	Gammaproteobacteria (122	Proteobacteria (2)	Bacteria (1)			98.30	94.80	WGS
303.608	0.4398	Pseudomonas putida (286)	Pseudomonas (135621)	Pseudomonadaceae (7227	Pseudomonadales (1236)	Gammaproteobacteria (122	Proteobacteria (2)	Bacteria (1)			99.40	97.60	WGS
303.609	0.4676	Pseudomonas putida (286)	Pseudomonas (135621)	Pseudomonadaceae (7227	Pseudomonadales (1236)	Gammaproteobacteria (122	Proteobacteria (2)	Bacteria (1)			99.00	96.80	WGS
303.610	0.3846	Pseudomonas putida (286)	Pseudomonas (135621)	Pseudomonadaceae (7227	Pseudomonadales (1236)	Gammaproteobacteria (122	Proteobacteria (2)	Bacteria (1)			98.70	95.60	Complete
303.611	0.3698	Pseudomonas putida (286)	Pseudomonas (135621)	Pseudomonadaceae (7227	Pseudomonadales (1236)	Gammaproteobacteria (122	Proteobacteria (2)	Bacteria (1)			99.00	94.80	WGS
303.612	0.3698	Pseudomonas putida (286)	Pseudomonas (135621)	Pseudomonadaceae (7227	Pseudomonadales (1236)	Gammaproteobacteria (122	Proteobacteria (2)	Bacteria (1)			99.00	94.80	WGS
303.613	0.3368	Pseudomonas putida (286)	Pseudomonas (135621)	Pseudomonadaceae (7227	Pseudomonadales (1236)	Gammaproteobacteria (122	Proteobacteria (2)	Bacteria (1)			99.80	99.00	WGS
304.35	0.3614	Pelomonas saccharophila (335058	Pelomonas (80864)	Comamonadaceae (80840)	Burkholderiales (28216)	Betaproteobacteria (1224)	Proteobacteria (2)	Bacteria (1)			95.70	93.30	WGS
305.100	0.4856	Ralstonia solanacearum (48736)	Ralstonia (119060)	Burkholderiaceae (80840)	Burkholderiales (28216)	Betaproteobacteria (1224)	Proteobacteria (2)	Bacteria (1)	100.00	0.00	99.30	98.20	Complete
305.101	0.3642	Ralstonia solanacearum (48736)	Ralstonia (119060)	Burkholderiaceae (80840)	Burkholderiales (28216)	Betaproteobacteria (1224)	Proteobacteria (2)	Bacteria (1)	100.00	0.00	99.20	97.80	Complete
305.102	0.3642	Ralstonia solanacearum (48736)	Ralstonia (119060)	Burkholderiaceae (80840)	Burkholderiales (28216)	Betaproteobacteria (1224)	Proteobacteria (2)	Bacteria (1)	100.00	0.00	99.30	97.60	Complete
305.103	0.404	Ralstonia solanacearum (48736)	Ralstonia (119060)	Burkholderiaceae (80840)	Burkholderiales (28216)	Betaproteobacteria (1224)	Proteobacteria (2)	Bacteria (1)	100.00	0.00	99.30	97.10	WGS
305.104	0.4066	Ralstonia solanacearum (48736)	Ralstonia (119060)	Burkholderiaceae (80840)	Burkholderiales (28216)	Betaproteobacteria (1224)	Proteobacteria (2)	Bacteria (1)	100.00	1.70	99.30	94.50	WGS
305.105	0.349	Ralstonia solanacearum (48736)	Ralstonia (119060)	Burkholderiaceae (80840)	Burkholderiales (28216)	Betaproteobacteria (1224)	Proteobacteria (2)	Bacteria (1)	100.00	0.40	99.30	96.50	WGS
305.106	0.445	Ralstonia solanacearum (48736)	Ralstonia (119060)	Burkholderiaceae (80840)	Burkholderiales (28216)	Betaproteobacteria (1224)	Proteobacteria (2)	Bacteria (1)	100.00	1.70	99.50	95.90	WGS
305.107	0.4318	Ralstonia solanacearum (48736)	Ralstonia (119060)	Burkholderiaceae (80840)	Burkholderiales (28216)	Betaproteobacteria (1224)	Proteobacteria (2)	Bacteria (1)	100.00	0.40	99.80	97.50	WGS
305.108	0.4302	Ralstonia solanacearum (48736)	Ralstonia (119060)	Burkholderiaceae (80840)	Burkholderiales (28216)	Betaproteobacteria (1224)	Proteobacteria (2)	Bacteria (1)	100.00	0.90	99.60	98.90	WGS
305.109	0.4362	Ralstonia solanacearum (48736)	Ralstonia (119060)	Burkholderiaceae (80840)	Burkholderiales (28216)	Betaproteobacteria (1224)	Proteobacteria (2)	Bacteria (1)	100.00	0.00	99.40	98.40	Complete
305.118	0.4116	Ralstonia solanacearum (48736)	Ralstonia (119060)	Burkholderiaceae (80840)	Burkholderiales (28216)	Betaproteobacteria (1224)	Proteobacteria (2)	Bacteria (1)	100.00	0.40	99.40	98.50	Complete
305.332	0.422	Ralstonia solanacearum (48736)	Ralstonia (119060)	Burkholderiaceae (80840)	Burkholderiales (28216)	Betaproteobacteria (1224)	Proteobacteria (2)	Bacteria (1)	100.00	0.00	99.60	99.20	WGS
305.333	0.406	Ralstonia solanacearum (48736)	Ralstonia (119060)	Burkholderiaceae (80840)	Burkholderiales (28216)	Betaproteobacteria (1224)	Proteobacteria (2)	Bacteria (1)	100.00	0.00	99.50	99.00	WGS
305.334	0.415	Ralstonia solanacearum (48736)	Ralstonia (119060)	Burkholderiaceae (80840)	Burkholderiales (28216)	Betaproteobacteria (1224)	Proteobacteria (2)	Bacteria (1)	100.00	0.00	99.60	99.10	Complete
305.335	0.3274	Ralstonia solanacearum (48736)	Ralstonia (119060)	Burkholderiaceae (80840)	Burkholderiales (28216)	Betaproteobacteria (1224)	Proteobacteria (2)	Bacteria (1)	89.20	11.60	89.40	84.00	WGS
305.336	0.3282	Ralstonia solanacearum (48736)	Ralstonia (119060)	Burkholderiaceae (80840)	Burkholderiales (28216)	Betaproteobacteria (1224)	Proteobacteria (2)	Bacteria (1)	83.00	6.00	87.60	83.00	WGS
305.337	0.4148	Ralstonia solanacearum (48736)	Ralstonia (119060)	Burkholderiaceae (80840)	Burkholderiales (28216)	Betaproteobacteria (1224)	Proteobacteria (2)	Bacteria (1)	87.00	7.10	89.30	83.80	WGS
305.338	0.4034	Ralstonia solanacearum (48736)	Ralstonia (119060)	Burkholderiaceae (80840)	Burkholderiales (28216)	Betaproteobacteria (1224)	Proteobacteria (2)	Bacteria (1)	91.60	5.70	90.80	86.70	WGS
305.339	0.3882	Ralstonia solanacearum (48736)	Ralstonia (119060)	Burkholderiaceae (80840)	Burkholderiales (28216)	Betaproteobacteria (1224)	Proteobacteria (2)	Bacteria (1)	100.00	0.00	99.20	97.70	WGS
305.340	0.3882	Ralstonia solanacearum (48736)	Ralstonia (119060)	Burkholderiaceae (80840)	Burkholderiales (28216)	Betaproteobacteria (1224)	Proteobacteria (2)	Bacteria (1)	100.00	0.00	99.20	97.80	WGS
305.341	0.3882	Ralstonia solanacearum (48736)	Ralstonia (119060)	Burkholderiaceae (80840)	Burkholderiales (28216)	Betaproteobacteria (1224)	Proteobacteria (2)	Bacteria (1)	100.00	0.00	99.20	97.90	WGS
305.342	0.4148	Ralstonia solanacearum (48736)	Ralstonia (119060)	Burkholderiaceae (80840)	Burkholderiales (28216)	Betaproteobacteria (1224)	Proteobacteria (2)	Bacteria (1)	100.00	0.00	99.60	98.70	Complete
305.391	0.242	Ralstonia solanacearum (48736)	Ralstonia (119060)	Burkholderiaceae (80840)	Burkholderiales (28216)	Betaproteobacteria (1224)	Proteobacteria (2)	Bacteria (1)	95.60	29.90	94.60	79.70	WGS
305.392	0.3882	Ralstonia solanacearum (48736)	Ralstonia (119060)	Burkholderiaceae (80840)	Burkholderiales (28216)	Betaproteobacteria (1224)	Proteobacteria (2)	Bacteria (1)	100.00	0.40	99.20	97.60	WGS
305.393	0.3746	Ralstonia solanacearum (48736)	Ralstonia (119060)	Burkholderiaceae (80840)	Burkholderiales (28216)	Betaproteobacteria (1224)	Proteobacteria (2)	Bacteria (1)	100.00	0.40	98.80	97.30	WGS
305.394	0.4088	Ralstonia solanacearum (48736)	Ralstonia (119060)	Burkholderiaceae (80840)	Burkholderiales (28216)	Betaproteobacteria (1224)	Proteobacteria (2)	Bacteria (1)	100.00	2.10	99.60	98.00	Complete
305.395	0.3882	Ralstonia solanacearum (48736)	Ralstonia (119060)	Burkholderiaceae (80840)	Burkholderiales (28216)	Betaproteobacteria (1224)	Proteobacteria (2)	Bacteria (1)	100.00	0.00	98.80	97.50	Complete
305.396	0.4048	Ralstonia solanacearum (48736)	Ralstonia (119060)	Burkholderiaceae (80840)	Burkholderiales (28216)	Betaproteobacteria (1224)	Proteobacteria (2)	Bacteria (1)	100.00	0.00	98.70	97.10	Complete
305.397	0.0294	Ralstonia solanacearum (48736)	Ralstonia (119060)	Burkholderiaceae (80840)	Burkholderiales (28216)	Betaproteobacteria (1224)	Proteobacteria (2)	Bacteria (1)					Plasmid
305.398	0.1364	Ralstonia solanacearum (48736)	Ralstonia (119060)	Burkholderiaceae (80840)	Burkholderiales (28216)	Betaproteobacteria (1224)	Proteobacteria (2)	Bacteria (1)					Plasmid
305.399	0.137	Ralstonia solanacearum (48736)	Ralstonia (119060)	Burkholderiaceae (80840)	Burkholderiales (28216)	Betaproteobacteria (1224)	Proteobacteria (2)	Bacteria (1)					Plasmid
305.400	0.1238	Ralstonia solanacearum (48736)	Ralstonia (119060)	Burkholderiaceae (80840)	Burkholderiales (28216)	Betaproteobacteria (1224)	Proteobacteria (2)	Bacteria (1)					Plasmid
305.401	0.0294	Ralstonia solanacearum (48736)	Ralstonia (119060)	Burkholderiaceae (80840)	Burkholderiales (28216)	Betaproteobacteria (1224)	Proteobacteria (2)	Bacteria (1)					Plasmid
305.402	0.0294	Ralstonia solanacearum (48736)	Ralstonia (119060)	Burkholderiaceae (80840)	Burkholderiales (28216)	Betaproteobacteria (1224)	Proteobacteria (2)	Bacteria (1)					Plasmid
305.403	0.1422	Ralstonia solanacearum (48736)	Ralstonia (119060)	Burkholderiaceae (80840)	Burkholderiales (28216)	Betaproteobacteria (1224)	Proteobacteria (2)	Bacteria (1)					Plasmid
305.404	0.1348	Ralstonia solanacearum (48736)	Ralstonia (119060)	Burkholderiaceae (80840)	Burkholderiales (28216)	Betaproteobacteria (1224)	Proteobacteria (2)	Bacteria (1)					Plasmid
305.405	0.127	Ralstonia solanacearum (48736)	Ralstonia (119060)	Burkholderiaceae (80840)	Burkholderiales (28216)	Betaproteobacteria (1224)	Proteobacteria (2)	Bacteria (1)					Plasmid
305.406	0.1048	Ralstonia solanacearum (48736)	Ralstonia (119060)	Burkholderiaceae (80840)	Burkholderiales (28216)	Betaproteobacteria (1224)	Proteobacteria (2)	Bacteria (1)					Plasmid
305.407	0.1508	Ralstonia solanacearum (48736)	Ralstonia (119060)	Burkholderiaceae (80840)	Burkholderiales (28216)	Betaproteobacteria (1224)	Proteobacteria (2)	Bacteria (1)					Plasmid
305.408	0.1236	Ralstonia solanacearum (48736)	Ralstonia (119060)	Burkholderiaceae (80840)	Burkholderiales (28216)	Betaproteobacteria (1224)	Proteobacteria (2)	Bacteria (1)					Plasmid
305.409	0.127	Ralstonia solanacearum (48736)	Ralstonia (119060)	Burkholderiaceae (80840)	Burkholderiales (28216)	Betaproteobacteria (1224)	Proteobacteria (2)	Bacteria (1)					Plasmid
305.410	0.137	Ralstonia solanacearum (48736)	Ralstonia (119060)	Burkholderiaceae (80840)	Burkholderiales (28216)	Betaproteobacteria (1224)	Proteobacteria (2)	Bacteria (1)					Plasmid
305.411	0.1414	Ralstonia solanacearum (48736)	Ralstonia (119060)	Burkholderiaceae (80840)	Burkholderiales (28216)	Betaproteobacteria (1224)	Proteobacteria (2)	Bacteria (1)					Plasmid
305.42	0.3508	Ralstonia solanacearum (48736)	Ralstonia (119060)	Burkholderiaceae (80840)	Burkholderiales (28216)	Betaproteobacteria (1224)	Proteobacteria (2)	Bacteria (1)	100.00	0.40	99.10	97.60	WGS
305.43	0.3926	Ralstonia solanacearum (48736)	Ralstonia (119060)	Burkholderiaceae (80840)	Burkholderiales (28216)	Betaproteobacteria (1224)	Proteobacteria (2)	Bacteria (1)	100.00	0.90	98.70	96.90	WGS
305.44	0.3782	Ralstonia solanacearum (48736)	Ralstonia (119060)	Burkholderiaceae (80840)	Burkholderiales (28216)	Betaproteobacteria (1224)	Proteobacteria (2)	Bacteria (1)	99.60	0.00	98.40	96.30	WGS
305.441	0.423	Ralstonia solanacearum (48736)	Ralstonia (119060)	Burkholderiaceae (80840)	Burkholderiales (28216)	Betaproteobacteria (1224)	Proteobacteria (2)	Bacteria (1)	100.00	0.00	99.30	98.60	Complete
305.442	0.3882	Ralstonia solanacearum (48736)	Ralstonia (119060)	Burkholderiaceae (80840)	Burkholderiales (28216)	Betaproteobacteria (1224)	Proteobacteria (2)	Bacteria (1)	100.00	0.00	98.60	97.30	WGS
305.443	0.3882	Ralstonia solanacearum (48736)	Ralstonia (119060)	Burkholderiaceae (80840)	Burkholderiales (28216)	Betaproteobacteria (1224)	Proteobacteria (2)	Bacteria (1)	100.00	0.00	99.00	97.50	WGS
305.444	0.3734	Ralstonia solanacearum (48736)	Ralstonia (119060)	Burkholderiaceae (80840)	Burkholderiales (28216)	Betaproteobacteria (1224)	Proteobacteria (2)	Bacteria (1)	100.00	0.00	99.30	97.90	WGS
305.445	0.3994	Ralstonia solanacearum (48736)	Ralstonia (119060)	Burkholderiaceae (80840)	Burkholderiales (28216)	Betaproteobacteria (1224)	Proteobacteria (2)	Bacteria (1)	88.60	0.00	93.90	92.50	WGS
305.446	0.3882	Ralstonia solanacearum (48736)	Ralstonia (119060)	Burkholderiaceae (80840)	Burkholderiales (28216)	Betaproteobacteria (1224)	Proteobacteria (2)	Bacteria (1)	100.00	0.00	99.10	97.40	WGS
305.447	0.4262	Ralstonia solanacearum (48736)	Ralstonia (119060)	Burkholderiaceae (80840)	Burkholderiales (28216)	Betaproteobacteria (1224)	Proteobacteria (2)	Bacteria (1)	100.00	6.60	99.70	90.00	WGS
305.45	0.3546	Ralstonia solanacearum (48736)	Ralstonia (119060)	Burkholderiaceae (80840)	Burkholderiales (28216)	Betaproteobacteria (1224)	Proteobacteria (2)	Bacteria (1)	100.00	0.00	99.00	97.60	WGS
305.46	0.3546	Ralstonia solanacearum (48736)	Ralstonia (119060)	Burkholderiaceae (80840)	Burkholderiales (28216)	Betaproteobacteria (1224)	Proteobacteria (2)	Bacteria (1)	100.00	0.00	98.80	97.50	WGS
305.47	0.4326	Ralstonia solanacearum (48736)	Ralstonia (119060)	Burkholderiaceae (80840)	Burkholderiales (28216)	Betaproteobacteria (1224)	Proteobacteria (2)	Bacteria (1)	99.60	0.00	98.20	97.10	WGS
305.48	0.3546	Ralstonia solanacearum (48736)	Ralstonia (119060)	Burkholderiaceae (80840)	Burkholderiales (28216)	Betaproteobacteria (1224)	Proteobacteria (2)	Bacteria (1)	100.00	0.00	98.80	97.40	WGS
305.489	0.3494	Ralstonia solanacearum (48736)	Ralstonia (119060)	Burkholderiaceae (80840)	Burkholderiales (28216)	Betaproteobacteria (1224)	Proteobacteria (2)	Bacteria (1)	81.50	0.00	90.50	89.20	WGS
305.490	0.4856	Ralstonia solanacearum (48736)	Ralstonia (119060)	Burkholderiaceae (80840)	Burkholderiales (28216)	Betaproteobacteria (1224)	Proteobacteria (2)	Bacteria (1)	100.00	0.00	99.30	97.90	Complete
305.49	0.391	Ralstonia solanacearum (48736)	Ralstonia (119060)	Burkholderiaceae (80840)	Burkholderiales (28216)	Betaproteobacteria (1224)	Proteobacteria (2)	Bacteria (1)	100.00	0.00	98.70	97.00	WGS
305.491	0.4856	Ralstonia solanacearum (48736)	Ralstonia (119060)	Burkholderiaceae (80840)	Burkholderiales (28216)	Betaproteobacteria (1224)	Proteobacteria (2)	Bacteria (1)	100.00	0.00	99.30	97.60	Complete
305.492	0.4838	Ralstonia solanacearum (48736)	Ralstonia (119060)	Burkholderiaceae (80840)	Burkholderiales (28216)	Betaproteobacteria (1224)	Proteobacteria (2)	Bacteria (1)	100.00	0.40	99.30	97.90	Complete
305.493	0.4292	Ralstonia solanacearum (48736)	Ralstonia (119060)	Burkholderiaceae (80840)	Burkholderiales (28216)	Betaproteobacteria (1224)	Proteobacteria (2)	Bacteria (1)	100.00	1.30	99.50	98.10	Complete
305.494	0.4284	Ralstonia solanacearum (48736)	Ralstonia (119060)	Burkholderiaceae (80840)	Burkholderiales (28216)	Betaproteobacteria (1224)	Proteobacteria (2)	Bacteria (1)	100.00	1.30	99.80	99.10	Complete
305.495	0.4318	Ralstonia solanacearum (48736)	Ralstonia (119060)	Burkholderiaceae (80840)	Burkholderiales (28216)	Betaproteobacteria (1224)	Proteobacteria (2)	Bacteria (1)	100.00	0.40	99.60	98.90	Complete
305.496	0.4838	Ralstonia solanacearum (48736)	Ralstonia (119060)	Burkholderiaceae (80840)	Burkholderiales (28216)	Betaproteobacteria (1224)	Proteobacteria (2)	Bacteria (1)	100.00	0.00	99.30	97.70	Complete
305.497	0.4324	Ralstonia solanacearum (48736)	Ralstonia (119060)	Burkholderiaceae (80840)	Burkholderiales (28216)	Betaproteobacteria (1224)	Proteobacteria (2)	Bacteria (1)	100.00	0.00	99.50	98.80	Complete
305.498	0.4284	Ralstonia solanacearum (48736)	Ralstonia (119060)	Burkholderiaceae (80840)	Burkholderiales (28216)	Betaproteobacteria (1224)	Proteobacteria (2)	Bacteria (1)	100.00	6.20	99.60	93.80	Complete
305.499	0.4572	Ralstonia solanacearum (48736)	Ralstonia (119060)	Burkholderiaceae (80840)	Burkholderiales (28216)	Betaproteobacteria (1224)	Proteobacteria (2)	Bacteria (1)	100.00	2.20	99.30	96.20	Complete
305.500	0.4316	Ralstonia solanacearum (48736)	Ralstonia (119060)	Burkholderiaceae (80840)	Burkholderiales (28216)	Betaproteobacteria (1224)	Proteobacteria (2)	Bacteria (1)	100.00	0.90	99.80	98.80	Complete
305.501	0.435	Ralstonia solanacearum (48736)	Ralstonia (119060)	Burkholderiaceae (80840)	Burkholderiales (28216)	Betaproteobacteria (1224)	Proteobacteria (2)	Bacteria (1)	100.00	3.20	99.60	98.20	Complete
305.502	0.4916	Ralstonia solanacearum (48736)	Ralstonia (119060)	Burkholderiaceae (80840)	Burkholderiales (28216)	Betaproteobacteria (1224)	Proteobacteria (2)	Bacteria (1)	100.00	5.20	99.20	94.50	Complete
305.503	0.435	Ralstonia solanacearum (48736)	Ralstonia (119060)	Burkholderiaceae (80840)	Burkholderiales (28216)	Betaproteobacteria (1224)	Proteobacteria (2)	Bacteria (1)	100.00	0.40	99.60	99.00	Complete
305.504	0.4838	Ralstonia solanacearum (48736)	Ralstonia (119060)	Burkholderiaceae (80840)	Burkholderiales (28216)	Betaproteobacteria (1224)	Proteobacteria (2)	Bacteria (1)	100.00	0.40	99.20	96.80	Complete
305.505	0.4856	Ralstonia solanacearum (48736)	Ralstonia (119060)	Burkholderiaceae (80840)	Burkholderiales (28216)	Betaproteobacteria (1224)	Proteobacteria (2)	Bacteria (1)	100.00	0.00	99.30	97.90	Complete
305.506	0.4124	Ralstonia solanacearum (48736)	Ralstonia (119060)	Burkholderiaceae (80840)	Burkholderiales (28216)	Betaproteobacteria (1224)	Proteobacteria (2)	Bacteria (1)	98.70	24.40	99.00	81.80	Complete
305.507	0.4572	Ralstonia solanacearum (48736)	Ralstonia (119060)	Burkholderiaceae (80840)	Burkholderiales (28216)	Betaproteobacteria (1224)	Proteobacteria (2)	Bacteria (1)	100.00	0.40	99.30	97.20	Complete
305.508	0.4856	Ralstonia solanacearum (48736)	Ralstonia (119060)	Burkholderiaceae (80840)	Burkholderiales (28216)	Betaproteobacteria (1224)	Proteobacteria (2)	Bacteria (1)	100.00	0.00	99.30	97.80	Complete
305.509	0.4838	Ralstonia solanacearum (48736)	Ralstonia (119060)	Burkholderiaceae (80840)	Burkholderiales (28216)	Betaproteobacteria (1224)	Proteobacteria (2)	Bacteria (1)	100.00	0.40	99.30	97.00	Complete
305.510	0.4316	Ralstonia solanacearum (48736)	Ralstonia (119060)	Burkholderiaceae (80840)	Burkholderiales (28216)	Betaproteobacteria (1224)	Proteobacteria (2)	Bacteria (1)	100.00	0.70	99.60	98.80	Complete
305.511	0.4416	Ralstonia solanacearum (48736)	Ralstonia (119060)	Burkholderiaceae (80840)	Burkholderiales (28216)	Betaproteobacteria (1224)	Proteobacteria (2)	Bacteria (1)	100.00	0.00	98.80	98.10	Complete
305.512	0.4316	Ralstonia solanacearum (48736)	Ralstonia (119060)	Burkholderiaceae (80840)	Burkholderiales (28216)	Betaproteobacteria (1224)	Proteobacteria (2)	Bacteria (1)	100.00	0.00	99.60	99.00	Complete
305.513	0.4284	Ralstonia solanacearum (48736)	Ralstonia (119060)	Burkholderiaceae (80840)	Burkholderiales (28216)	Betaproteobacteria (1224)	Proteobacteria (2)	Bacteria (1)	100.00	0.00	99.60	99.00	Complete
305.514	0.4316	Ralstonia solanacearum (48736)	Ralstonia (119060)	Burkholderiaceae (80840)	Burkholderiales (28216)	Betaproteobacteria (1224)	Proteobacteria (2)	Bacteria (1)	100.00	0.40	99.60	98.80	Complete
305.515	0.3722	Ralstonia solanacearum (48736)	Ralstonia (119060)	Burkholderiaceae (80840)	Burkholderiales (28216)	Betaproteobacteria (1224)	Proteobacteria (2)	Bacteria (1)	100.00	0.00	98.70	97.50	WGS
305.516	0.4118	Ralstonia solanacearum (48736)	Ralstonia (119060)	Burkholderiaceae (80840)	Burkholderiales (28216)	Betaproteobacteria (1224)	Proteobacteria (2)	Bacteria (1)	100.00	0.00	99.60	99.00	Complete
305.517	0.358	Ralstonia solanacearum (48736)	Ralstonia (119060)	Burkholderiaceae (80840)	Burkholderiales (28216)	Betaproteobacteria (1224)	Proteobacteria (2)	Bacteria (1)	100.00	0.00	98.70	97.00	Complete
305.518	0.3642	Ralstonia solanacearum (48736)	Ralstonia (119060)	Burkholderiaceae (80840)	Burkholderiales (28216)	Betaproteobacteria (1224)	Proteobacteria (2)	Bacteria (1)	100.00	0.40	98.70	97.30	Complete
305.519	0.3642	Ralstonia solanacearum (48736)	Ralstonia (119060)	Burkholderiaceae (80840)	Burkholderiales (28216)	Betaproteobacteria (1224)	Proteobacteria (2)	Bacteria (1)	100.00	0.90	98.70	96.70	Complete
305.554	0.406	Ralstonia solanacearum (48736)	Ralstonia (119060)	Burkholderiaceae (80840)	Burkholderiales (28216)	Betaproteobacteria (1224)	Proteobacteria (2)	Bacteria (1)			99.40	98.90	Complete
305.561	0.4094	Ralstonia solanacearum (48736)	Ralstonia (119060)	Burkholderiaceae (80840)	Burkholderiales (28216)	Betaproteobacteria (1224)	Proteobacteria (2)	Bacteria (1)			98.60	97.00	Complete
305.562	0.4094	Ralstonia solanacearum (48736)	Ralstonia (119060)	Burkholderiaceae (80840)	Burkholderiales (28216)	Betaproteobacteria (1224)	Proteobacteria (2)	Bacteria (1)			99.00	97.00	Complete
305.563	0.3824	Ralstonia solanacearum (48736)	Ralstonia (119060)	Burkholderiaceae (80840)	Burkholderiales (28216)	Betaproteobacteria (1224)	Proteobacteria (2)	Bacteria (1)			99.30	97.90	Complete
305.564	0.384	Ralstonia solanacearum (48736)	Ralstonia (119060)	Burkholderiaceae (80840)	Burkholderiales (28216)	Betaproteobacteria (1224)	Proteobacteria (2)	Bacteria (1)			99.20	97.60	Complete
305.565	0.3642	Ralstonia solanacearum (48736)	Ralstonia (119060)	Burkholderiaceae (80840)	Burkholderiales (28216)	Betaproteobacteria (1224)	Proteobacteria (2)	Bacteria (1)			99.30	97.80	Complete
305.566	0.384	Ralstonia solanacearum (48736)	Ralstonia (119060)	Burkholderiaceae (80840)	Burkholderiales (28216)	Betaproteobacteria (1224)	Proteobacteria (2)	Bacteria (1)			99.20	97.60	Complete
305.568	0.4228	Ralstonia solanacearum (48736)	Ralstonia (119060)	Burkholderiaceae (80840)	Burkholderiales (28216)	Betaproteobacteria (1224)	Proteobacteria (2)	Bacteria (1)			99.60	99.00	Complete
305.745	0.4066	Ralstonia solanacearum (48736)	Ralstonia (119060)	Burkholderiaceae (80840)	Burkholderiales (28216)	Betaproteobacteria (1224)	Proteobacteria (2)	Bacteria (1)			98.70	95.60	WGS
305.746	0.388	Ralstonia solanacearum (48736)	Ralstonia (119060)	Burkholderiaceae (80840)	Burkholderiales (28216)	Betaproteobacteria (1224)	Proteobacteria (2)	Bacteria (1)			98.80	96.50	WGS
305.747	0.418	Ralstonia solanacearum (48736)	Ralstonia (119060)	Burkholderiaceae (80840)	Burkholderiales (28216)	Betaproteobacteria (1224)	Proteobacteria (2)	Bacteria (1)			98.70	95.80	WGS
305.748	0.4032	Ralstonia solanacearum (48736)	Ralstonia (119060)	Burkholderiaceae (80840)	Burkholderiales (28216)	Betaproteobacteria (1224)	Proteobacteria (2)	Bacteria (1)			98.70	95.80	WGS
305.749	0.3882	Ralstonia solanacearum (48736)	Ralstonia (119060)	Burkholderiaceae (80840)	Burkholderiales (28216)	Betaproteobacteria (1224)	Proteobacteria (2)	Bacteria (1)			99.00	97.50	WGS
305.750	0.3718	Ralstonia solanacearum (48736)	Ralstonia (119060)	Burkholderiaceae (80840)	Burkholderiales (28216)	Betaproteobacteria (1224)	Proteobacteria (2)	Bacteria (1)			99.30	97.90	WGS
305.751	0.3882	Ralstonia solanacearum (48736)	Ralstonia (119060)	Burkholderiaceae (80840)	Burkholderiales (28216)	Betaproteobacteria (1224)	Proteobacteria (2)	Bacteria (1)			99.30	97.80	WGS
305.752	0.3882	Ralstonia solanacearum (48736)	Ralstonia (119060)	Burkholderiaceae (80840)	Burkholderiales (28216)	Betaproteobacteria (1224)	Proteobacteria (2)	Bacteria (1)			99.30	97.90	WGS
305.753	0.4126	Ralstonia solanacearum (48736)	Ralstonia (119060)	Burkholderiaceae (80840)	Burkholderiales (28216)	Betaproteobacteria (1224)	Proteobacteria (2)	Bacteria (1)			99.80	99.30	WGS
305.754	0.415	Ralstonia solanacearum (48736)	Ralstonia (119060)	Burkholderiaceae (80840)	Burkholderiales (28216)	Betaproteobacteria (1224)	Proteobacteria (2)	Bacteria (1)			99.60	99.30	WGS
305.755	0.4326	Ralstonia solanacearum (48736)	Ralstonia (119060)	Burkholderiaceae (80840)	Burkholderiales (28216)	Betaproteobacteria (1224)	Proteobacteria (2)	Bacteria (1)			99.20	98.40	WGS
305.756	0.4746	Ralstonia solanacearum (48736)	Ralstonia (119060)	Burkholderiaceae (80840)	Burkholderiales (28216)	Betaproteobacteria (1224)	Proteobacteria (2)	Bacteria (1)			99.30	98.20	Complete
305.86	0.3882	Ralstonia solanacearum (48736)	Ralstonia (119060)	Burkholderiaceae (80840)	Burkholderiales (28216)	Betaproteobacteria (1224)	Proteobacteria (2)	Bacteria (1)	100.00	0.40	99.30	97.90	WGS
305.87	0.3822	Ralstonia solanacearum (48736)	Ralstonia (119060)	Burkholderiaceae (80840)	Burkholderiales (28216)	Betaproteobacteria (1224)	Proteobacteria (2)	Bacteria (1)	100.00	0.00	99.30	97.50	WGS
305.88	0.3908	Ralstonia solanacearum (48736)	Ralstonia (119060)	Burkholderiaceae (80840)	Burkholderiales (28216)	Betaproteobacteria (1224)	Proteobacteria (2)	Bacteria (1)	100.00	0.00	99.00	97.30	WGS
305.89	0.3596	Ralstonia solanacearum (48736)	Ralstonia (119060)	Burkholderiaceae (80840)	Burkholderiales (28216)	Betaproteobacteria (1224)	Proteobacteria (2)	Bacteria (1)	100.00	0.00	98.90	97.00	WGS
305.90	0.4098	Ralstonia solanacearum (48736)	Ralstonia (119060)	Burkholderiaceae (80840)	Burkholderiales (28216)	Betaproteobacteria (1224)	Proteobacteria (2)	Bacteria (1)	100.00	1.30	99.20	97.30	WGS
305.91	0.423	Ralstonia solanacearum (48736)	Ralstonia (119060)	Burkholderiaceae (80840)	Burkholderiales (28216)	Betaproteobacteria (1224)	Proteobacteria (2)	Bacteria (1)	100.00	1.30	98.90	95.30	WGS
305.92	0.415	Ralstonia solanacearum (48736)	Ralstonia (119060)	Burkholderiaceae (80840)	Burkholderiales (28216)	Betaproteobacteria (1224)	Proteobacteria (2)	Bacteria (1)	100.00	0.40	99.50	96.50	Complete
305.93	0.3882	Ralstonia solanacearum (48736)	Ralstonia (119060)	Burkholderiaceae (80840)	Burkholderiales (28216)	Betaproteobacteria (1224)	Proteobacteria (2)	Bacteria (1)	100.00	0.00	98.80	97.50	Complete
305.96	0.3812	Ralstonia solanacearum (48736)	Ralstonia (119060)	Burkholderiaceae (80840)	Burkholderiales (28216)	Betaproteobacteria (1224)	Proteobacteria (2)	Bacteria (1)	100.00	0.00	99.00	98.00	Complete
305.99	0.4168	Ralstonia solanacearum (48736)	Ralstonia (119060)	Burkholderiaceae (80840)	Burkholderiales (28216)	Betaproteobacteria (1224)	Proteobacteria (2)	Bacteria (1)	100.00	0.00	99.50	99.00	WGS
306.135	0.297	Pseudomonas sp. (286)	Pseudomonas (135621)	Pseudomonadaceae (7227	Pseudomonadales (1236)	Gammaproteobacteria (122	Proteobacteria (2)	Bacteria (1)	82.60	4.00	88.50	83.80	WGS
306.136	0.3354	Pseudomonas sp. (286)	Pseudomonas (135621)	Pseudomonadaceae (7227	Pseudomonadales (1236)	Gammaproteobacteria (122	Proteobacteria (2)	Bacteria (1)	70.90	6.70	78.90	73.20	WGS
306.137	0.3636	Pseudomonas sp. (286)	Pseudomonas (135621)	Pseudomonadaceae (7227	Pseudomonadales (1236)	Gammaproteobacteria (122	Proteobacteria (2)	Bacteria (1)	67.60	16.50	77.40	71.00	WGS
306.138	0.2408	Pseudomonas sp. (286)	Pseudomonas (135621)	Pseudomonadaceae (7227	Pseudomonadales (1236)	Gammaproteobacteria (122	Proteobacteria (2)	Bacteria (1)	62.30	10.40	80.90	76.10	WGS
306.139	0.3608	Pseudomonas sp. (286)	Pseudomonas (135621)	Pseudomonadaceae (7227	Pseudomonadales (1236)	Gammaproteobacteria (122	Proteobacteria (2)	Bacteria (1)	90.20	16.00	91.60	80.60	WGS
306.140	0.1156	Pseudomonas sp. (286)	Pseudomonas (135621)	Pseudomonadaceae (7227	Pseudomonadales (1236)	Gammaproteobacteria (122	Proteobacteria (2)	Bacteria (1)	77.90	21.90	88.70	79.70	WGS
306.141	0.226	Pseudomonas sp. (286)	Pseudomonas (135621)	Pseudomonadaceae (7227	Pseudomonadales (1236)	Gammaproteobacteria (122	Proteobacteria (2)	Bacteria (1)	81.00	16.00	88.20	80.50	WGS
306.142	0.2246	Pseudomonas sp. (286)	Pseudomonas (135621)	Pseudomonadaceae (7227	Pseudomonadales (1236)	Gammaproteobacteria (122	Proteobacteria (2)	Bacteria (1)	87.70	27.70	90.20	77.60	WGS
306.143	0.4058	Pseudomonas sp. (286)	Pseudomonas (135621)	Pseudomonadaceae (7227	Pseudomonadales (1236)	Gammaproteobacteria (122	Proteobacteria (2)	Bacteria (1)	91.90	16.70	92.60	80.90	WGS
306.144	0.1774	Pseudomonas sp. (286)	Pseudomonas (135621)	Pseudomonadaceae (7227	Pseudomonadales (1236)	Gammaproteobacteria (122	Proteobacteria (2)	Bacteria (1)	66.80	11.00	80.60	76.90	WGS
306.145	0.4004	Pseudomonas sp. (286)	Pseudomonas (135621)	Pseudomonadaceae (7227	Pseudomonadales (1236)	Gammaproteobacteria (122	Proteobacteria (2)	Bacteria (1)	87.00	17.50	89.60	81.10	WGS
306.146	0.3032	Pseudomonas sp. (286)	Pseudomonas (135621)	Pseudomonadaceae (7227	Pseudomonadales (1236)	Gammaproteobacteria (122	Proteobacteria (2)	Bacteria (1)	88.30	21.80	87.70	76.40	WGS
306.147	0.3024	Pseudomonas sp. (286)	Pseudomonas (135621)	Pseudomonadaceae (7227	Pseudomonadales (1236)	Gammaproteobacteria (122	Proteobacteria (2)	Bacteria (1)	93.80	22.00	91.10	78.80	WGS
306.148	0.368	Pseudomonas sp. (286)	Pseudomonas (135621)	Pseudomonadaceae (7227	Pseudomonadales (1236)	Gammaproteobacteria (122	Proteobacteria (2)	Bacteria (1)	88.80	12.60	89.60	82.30	WGS
306.149	0.352	Pseudomonas sp. (286)	Pseudomonas (135621)	Pseudomonadaceae (7227	Pseudomonadales (1236)	Gammaproteobacteria (122	Proteobacteria (2)	Bacteria (1)	92.10	16.90	91.20	81.50	WGS
306.150	0.208	Pseudomonas sp. (286)	Pseudomonas (135621)	Pseudomonadaceae (7227	Pseudomonadales (1236)	Gammaproteobacteria (122	Proteobacteria (2)	Bacteria (1)	69.30	10.90	84.70	80.00	WGS
306.151	0.291	Pseudomonas sp. (286)	Pseudomonas (135621)	Pseudomonadaceae (7227	Pseudomonadales (1236)	Gammaproteobacteria (122	Proteobacteria (2)	Bacteria (1)	66.20	8.30	82.60	79.30	WGS
306.152	0.3358	Pseudomonas sp. (286)	Pseudomonas (135621)	Pseudomonadaceae (7227	Pseudomonadales (1236)	Gammaproteobacteria (122	Proteobacteria (2)	Bacteria (1)	90.70	29.70	90.40	72.70	WGS
306.153	0.3232	Pseudomonas sp. (286)	Pseudomonas (135621)	Pseudomonadaceae (7227	Pseudomonadales (1236)	Gammaproteobacteria (122	Proteobacteria (2)	Bacteria (1)	88.70	17.20	87.50	76.60	WGS
306.154	0.2888	Pseudomonas sp. (286)	Pseudomonas (135621)	Pseudomonadaceae (7227	Pseudomonadales (1236)	Gammaproteobacteria (122	Proteobacteria (2)	Bacteria (1)	84.40	18.20	90.40	79.50	WGS
306.155	0.3226	Pseudomonas sp. (286)	Pseudomonas (135621)	Pseudomonadaceae (7227	Pseudomonadales (1236)	Gammaproteobacteria (122	Proteobacteria (2)	Bacteria (1)	87.10	20.20	89.50	78.80	WGS
306.156	0.3652	Pseudomonas sp. (286)	Pseudomonas (135621)	Pseudomonadaceae (7227	Pseudomonadales (1236)	Gammaproteobacteria (122	Proteobacteria (2)	Bacteria (1)	99.60	17.90	94.80	82.30	WGS
306.157	0.3702	Pseudomonas sp. (286)	Pseudomonas (135621)	Pseudomonadaceae (7227	Pseudomonadales (1236)	Gammaproteobacteria (122	Proteobacteria (2)	Bacteria (1)	79.50	16.30	84.80	77.40	WGS
306.158	0.2298	Pseudomonas sp. (286)	Pseudomonas (135621)	Pseudomonadaceae (7227	Pseudomonadales (1236)	Gammaproteobacteria (122	Proteobacteria (2)	Bacteria (1)	88.30	24.50	88.90	74.00	WGS
306.159	0.3024	Pseudomonas sp. (286)	Pseudomonas (135621)	Pseudomonadaceae (7227	Pseudomonadales (1236)	Gammaproteobacteria (122	Proteobacteria (2)	Bacteria (1)	94.00	20.80	94.00	80.30	WGS
306.160	0.3298	Pseudomonas sp. (286)	Pseudomonas (135621)	Pseudomonadaceae (7227	Pseudomonadales (1236)	Gammaproteobacteria (122	Proteobacteria (2)	Bacteria (1)	77.90	12.00	82.90	77.10	WGS
306.161	0.4616	Pseudomonas sp. (286)	Pseudomonas (135621)	Pseudomonadaceae (7227	Pseudomonadales (1236)	Gammaproteobacteria (122	Proteobacteria (2)	Bacteria (1)	97.90	12.00	97.00	88.10	WGS
306.162	0.4006	Pseudomonas sp. (286)	Pseudomonas (135621)	Pseudomonadaceae (7227	Pseudomonadales (1236)	Gammaproteobacteria (122	Proteobacteria (2)	Bacteria (1)	87.50	22.70	89.10	76.90	WGS
306.163	0.4132	Pseudomonas sp. (286)	Pseudomonas (135621)	Pseudomonadaceae (7227	Pseudomonadales (1236)	Gammaproteobacteria (122	Proteobacteria (2)	Bacteria (1)	83.20	11.80	87.30	81.00	WGS
306.164	0.4022	Pseudomonas sp. (286)	Pseudomonas (135621)	Pseudomonadaceae (7227	Pseudomonadales (1236)	Gammaproteobacteria (122	Proteobacteria (2)	Bacteria (1)	90.60	18.30	95.00	86.80	WGS
306.165	0.3558	Pseudomonas sp. (286)	Pseudomonas (135621)	Pseudomonadaceae (7227	Pseudomonadales (1236)	Gammaproteobacteria (122	Proteobacteria (2)	Bacteria (1)	86.70	18.90	90.80	78.40	WGS
306.166	0.3482	Pseudomonas sp. (286)	Pseudomonas (135621)	Pseudomonadaceae (7227	Pseudomonadales (1236)	Gammaproteobacteria (122	Proteobacteria (2)	Bacteria (1)	86.80	21.40	90.90	82.60	WGS
306.167	0.3782	Pseudomonas sp. (286)	Pseudomonas (135621)	Pseudomonadaceae (7227	Pseudomonadales (1236)	Gammaproteobacteria (122	Proteobacteria (2)	Bacteria (1)	95.70	15.30	92.50	82.00	WGS
306.168	0.434	Pseudomonas sp. (286)	Pseudomonas (135621)	Pseudomonadaceae (7227	Pseudomonadales (1236)	Gammaproteobacteria (122	Proteobacteria (2)	Bacteria (1)	89.80	20.40	92.40	78.60	WGS
306.169	0.5218	Pseudomonas sp. (286)	Pseudomonas (135621)	Pseudomonadaceae (7227	Pseudomonadales (1236)	Gammaproteobacteria (122	Proteobacteria (2)	Bacteria (1)	80.90	10.00	88.50	83.10	WGS
306.170	0.3994	Pseudomonas sp. (286)	Pseudomonas (135621)	Pseudomonadaceae (7227	Pseudomonadales (1236)	Gammaproteobacteria (122	Proteobacteria (2)	Bacteria (1)	94.40	23.80	92.90	80.80	WGS
306.171	0.4928	Pseudomonas sp. (286)	Pseudomonas (135621)	Pseudomonadaceae (7227	Pseudomonadales (1236)	Gammaproteobacteria (122	Proteobacteria (2)	Bacteria (1)	79.10	12.70	88.00	82.40	WGS
306.172	0.2822	Pseudomonas sp. (286)	Pseudomonas (135621)	Pseudomonadaceae (7227	Pseudomonadales (1236)	Gammaproteobacteria (122	Proteobacteria (2)	Bacteria (1)	82.50	27.50	87.10	71.70	WGS
306.173	0.4232	Pseudomonas sp. (286)	Pseudomonas (135621)	Pseudomonadaceae (7227	Pseudomonadales (1236)	Gammaproteobacteria (122	Proteobacteria (2)	Bacteria (1)	85.60	8.20	90.90	84.10	WGS
306.174	0.3338	Pseudomonas sp. (286)	Pseudomonas (135621)	Pseudomonadaceae (7227	Pseudomonadales (1236)	Gammaproteobacteria (122	Proteobacteria (2)	Bacteria (1)	88.60	23.80	92.00	79.00	WGS
306.175	0.2888	Pseudomonas sp. (286)	Pseudomonas (135621)	Pseudomonadaceae (7227	Pseudomonadales (1236)	Gammaproteobacteria (122	Proteobacteria (2)	Bacteria (1)	70.40	12.80	81.30	75.50	WGS
306.176	0.3204	Pseudomonas sp. (286)	Pseudomonas (135621)	Pseudomonadaceae (7227	Pseudomonadales (1236)	Gammaproteobacteria (122	Proteobacteria (2)	Bacteria (1)	79.40	13.60	86.00	76.50	WGS
306.177	0.3138	Pseudomonas sp. (286)	Pseudomonas (135621)	Pseudomonadaceae (7227	Pseudomonadales (1236)	Gammaproteobacteria (122	Proteobacteria (2)	Bacteria (1)	94.90	25.80	94.90	82.30	WGS
306.178	0.4208	Pseudomonas sp. (286)	Pseudomonas (135621)	Pseudomonadaceae (7227	Pseudomonadales (1236)	Gammaproteobacteria (122	Proteobacteria (2)	Bacteria (1)	85.40	23.00	87.40	78.00	WGS
306.179	0.41	Pseudomonas sp. (286)	Pseudomonas (135621)	Pseudomonadaceae (7227	Pseudomonadales (1236)	Gammaproteobacteria (122	Proteobacteria (2)	Bacteria (1)	90.60	16.10	92.30	84.00	WGS
306.180	0.4166	Pseudomonas sp. (286)	Pseudomonas (135621)	Pseudomonadaceae (7227	Pseudomonadales (1236)	Gammaproteobacteria (122	Proteobacteria (2)	Bacteria (1)	83.00	12.90	85.70	79.30	WGS
306.181	0.2986	Pseudomonas sp. (286)	Pseudomonas (135621)	Pseudomonadaceae (7227	Pseudomonadales (1236)	Gammaproteobacteria (122	Proteobacteria (2)	Bacteria (1)	75.90	15.00	82.50	74.70	WGS
306.182	0.1566	Pseudomonas sp. (286)	Pseudomonas (135621)	Pseudomonadaceae (7227	Pseudomonadales (1236)	Gammaproteobacteria (122	Proteobacteria (2)	Bacteria (1)	49.60	13.00	86.00	82.30	WGS
306.183	0.3582	Pseudomonas sp. (286)	Pseudomonas (135621)	Pseudomonadaceae (7227	Pseudomonadales (1236)	Gammaproteobacteria (122	Proteobacteria (2)	Bacteria (1)	90.80	15.90	92.10	82.90	WGS
306.184	0.3622	Pseudomonas sp. (286)	Pseudomonas (135621)	Pseudomonadaceae (7227	Pseudomonadales (1236)	Gammaproteobacteria (122	Proteobacteria (2)	Bacteria (1)	96.50	14.80	95.30	84.80	WGS
306.185	0.4296	Pseudomonas sp. (286)	Pseudomonas (135621)	Pseudomonadaceae (7227	Pseudomonadales (1236)	Gammaproteobacteria (122	Proteobacteria (2)	Bacteria (1)	61.90	21.50	79.70	74.20	WGS
306.186	0.3942	Pseudomonas sp. (286)	Pseudomonas (135621)	Pseudomonadaceae (7227	Pseudomonadales (1236)	Gammaproteobacteria (122	Proteobacteria (2)	Bacteria (1)	86.80	2.40	93.00	90.20	WGS
306.187	0.3142	Pseudomonas sp. (286)	Pseudomonas (135621)	Pseudomonadaceae (7227	Pseudomonadales (1236)	Gammaproteobacteria (122	Proteobacteria (2)	Bacteria (1)	95.30	1.30	94.50	91.60	WGS
306.188	0.3154	Pseudomonas sp. (286)	Pseudomonas (135621)	Pseudomonadaceae (7227	Pseudomonadales (1236)	Gammaproteobacteria (122	Proteobacteria (2)	Bacteria (1)	99.20	2.50	98.20	94.20	WGS
306.189	0.3332	Pseudomonas sp. (286)	Pseudomonas (135621)	Pseudomonadaceae (7227	Pseudomonadales (1236)	Gammaproteobacteria (122	Proteobacteria (2)	Bacteria (1)	86.80	9.00	89.60	82.90	WGS
306.190	0.3532	Pseudomonas sp. (286)	Pseudomonas (135621)	Pseudomonadaceae (7227	Pseudomonadales (1236)	Gammaproteobacteria (122	Proteobacteria (2)	Bacteria (1)	98.30	1.30	97.60	94.50	WGS
306.191	0.182	Pseudomonas sp. (286)	Pseudomonas (135621)	Pseudomonadaceae (7227	Pseudomonadales (1236)	Gammaproteobacteria (122	Proteobacteria (2)	Bacteria (1)	77.00	11.40	85.10	80.00	WGS
306.192	0.3304	Pseudomonas sp. (286)	Pseudomonas (135621)	Pseudomonadaceae (7227	Pseudomonadales (1236)	Gammaproteobacteria (122	Proteobacteria (2)	Bacteria (1)	88.70	16.40	87.40	80.00	WGS
306.193	0.3498	Pseudomonas sp. (286)	Pseudomonas (135621)	Pseudomonadaceae (7227	Pseudomonadales (1236)	Gammaproteobacteria (122	Proteobacteria (2)	Bacteria (1)	83.40	15.20	88.60	79.60	WGS
306.194	0.2726	Pseudomonas sp. (286)	Pseudomonas (135621)	Pseudomonadaceae (7227	Pseudomonadales (1236)	Gammaproteobacteria (122	Proteobacteria (2)	Bacteria (1)	94.40	12.10	91.90	84.70	WGS
306.195	0.3258	Pseudomonas sp. (286)	Pseudomonas (135621)	Pseudomonadaceae (7227	Pseudomonadales (1236)	Gammaproteobacteria (122	Proteobacteria (2)	Bacteria (1)	93.20	4.40	97.40	94.60	WGS
306.204	0.0364	Pseudomonas sp. (286)	Pseudomonas (135621)	Pseudomonadaceae (7227	Pseudomonadales (1236)	Gammaproteobacteria (122	Proteobacteria (2)	Bacteria (1)			91.10	89.90	WGS
306.205	0.0556	Pseudomonas sp. (286)	Pseudomonas (135621)	Pseudomonadaceae (7227	Pseudomonadales (1236)	Gammaproteobacteria (122	Proteobacteria (2)	Bacteria (1)			91.00	90.50	WGS
306.206	0.3506	Pseudomonas sp. (286)	Pseudomonas (135621)	Pseudomonadaceae (7227	Pseudomonadales (1236)	Gammaproteobacteria (122	Proteobacteria (2)	Bacteria (1)			96.30	80.30	WGS
306.207	0.3396	Pseudomonas sp. (286)	Pseudomonas (135621)	Pseudomonadaceae (7227	Pseudomonadales (1236)	Gammaproteobacteria (122	Proteobacteria (2)	Bacteria (1)			93.30	82.60	WGS
306.51	0.2262	Pseudomonas sp. (286)	Pseudomonas (135621)	Pseudomonadaceae (7227	Pseudomonadales (1236)	Gammaproteobacteria (122	Proteobacteria (2)	Bacteria (1)	52.10	8.40	77.40	74.40	WGS
306.52	0.385	Pseudomonas sp. (286)	Pseudomonas (135621)	Pseudomonadaceae (7227	Pseudomonadales (1236)	Gammaproteobacteria (122	Proteobacteria (2)	Bacteria (1)	69.80	3.50	85.90	83.90	WGS
306.53	0.37	Pseudomonas sp. (286)	Pseudomonas (135621)	Pseudomonadaceae (7227	Pseudomonadales (1236)	Gammaproteobacteria (122	Proteobacteria (2)	Bacteria (1)	69.40	7.40	83.60	80.30	WGS
306.54	0.4138	Pseudomonas sp. (286)	Pseudomonas (135621)	Pseudomonadaceae (7227	Pseudomonadales (1236)	Gammaproteobacteria (122	Proteobacteria (2)	Bacteria (1)	83.30	8.20	86.50	82.10	WGS
306.55	0.4714	Pseudomonas sp. (286)	Pseudomonas (135621)	Pseudomonadaceae (7227	Pseudomonadales (1236)	Gammaproteobacteria (122	Proteobacteria (2)	Bacteria (1)	85.10	6.10	87.60	83.70	WGS
306.56	0.3244	Pseudomonas sp. (286)	Pseudomonas (135621)	Pseudomonadaceae (7227	Pseudomonadales (1236)	Gammaproteobacteria (122	Proteobacteria (2)	Bacteria (1)	51.00	4.00	79.50	78.60	WGS
306.57	0.5744	Pseudomonas sp. (286)	Pseudomonas (135621)	Pseudomonadaceae (7227	Pseudomonadales (1236)	Gammaproteobacteria (122	Proteobacteria (2)	Bacteria (1)	87.20	2.40	92.80	90.30	WGS
306.58	0.4554	Pseudomonas sp. (286)	Pseudomonas (135621)	Pseudomonadaceae (7227	Pseudomonadales (1236)	Gammaproteobacteria (122	Proteobacteria (2)	Bacteria (1)	99.60	4.50	97.20	93.20	WGS
306.59	0.3438	Pseudomonas sp. (286)	Pseudomonas (135621)	Pseudomonadaceae (7227	Pseudomonadales (1236)	Gammaproteobacteria (122	Proteobacteria (2)	Bacteria (1)	97.00	2.10	97.60	95.30	WGS
306.60	0.3966	Pseudomonas sp. (286)	Pseudomonas (135621)	Pseudomonadaceae (7227	Pseudomonadales (1236)	Gammaproteobacteria (122	Proteobacteria (2)	Bacteria (1)	95.20	10.70	95.00	88.20	WGS
306.61	0.3432	Pseudomonas sp. (286)	Pseudomonas (135621)	Pseudomonadaceae (7227	Pseudomonadales (1236)	Gammaproteobacteria (122	Proteobacteria (2)	Bacteria (1)	66.00	4.90	81.40	80.00	WGS
306.62	0.2714	Pseudomonas sp. (286)	Pseudomonas (135621)	Pseudomonadaceae (7227	Pseudomonadales (1236)	Gammaproteobacteria (122	Proteobacteria (2)	Bacteria (1)	75.30	44.30	90.40	61.90	WGS
306.63	0.4384	Pseudomonas sp. (286)	Pseudomonas (135621)	Pseudomonadaceae (7227	Pseudomonadales (1236)	Gammaproteobacteria (122	Proteobacteria (2)	Bacteria (1)	99.60	1.50	98.20	96.70	WGS
306.64	0.4268	Pseudomonas sp. (286)	Pseudomonas (135621)	Pseudomonadaceae (7227	Pseudomonadales (1236)	Gammaproteobacteria (122	Proteobacteria (2)	Bacteria (1)	100.00	0.80	98.40	96.30	WGS
306.67	0.512	Pseudomonas sp. (286)	Pseudomonas (135621)	Pseudomonadaceae (7227	Pseudomonadales (1236)	Gammaproteobacteria (122	Proteobacteria (2)	Bacteria (1)	97.60	6.60	95.90	88.50	
306.68	0.3454	Pseudomonas sp. (286)	Pseudomonas (135621)	Pseudomonadaceae (7227	Pseudomonadales (1236)	Gammaproteobacteria (122	Proteobacteria (2)	Bacteria (1)	93.20	57.80	91.30	51.40	WGS
316.100	0.3716	Pseudomonas stutzeri (286)	Pseudomonas (135621)	Pseudomonadaceae (7227	Pseudomonadales (1236)	Gammaproteobacteria (122	Proteobacteria (2)	Bacteria (1)	100.00	0.00	98.00	95.60	WGS
316.101	0.45	Pseudomonas stutzeri (286)	Pseudomonas (135621)	Pseudomonadaceae (7227	Pseudomonadales (1236)	Gammaproteobacteria (122	Proteobacteria (2)	Bacteria (1)	100.00	0.00	97.60	95.20	WGS
316.104	0.4262	Pseudomonas stutzeri (286)	Pseudomonas (135621)	Pseudomonadaceae (7227	Pseudomonadales (1236)	Gammaproteobacteria (122	Proteobacteria (2)	Bacteria (1)	100.00	0.00	98.20	95.50	WGS
316.105	0.4128	Pseudomonas stutzeri (286)	Pseudomonas (135621)	Pseudomonadaceae (7227	Pseudomonadales (1236)	Gammaproteobacteria (122	Proteobacteria (2)	Bacteria (1)	99.10	0.90	97.60	94.30	Complete
316.106	0.4176	Pseudomonas stutzeri (286)	Pseudomonas (135621)	Pseudomonadaceae (7227	Pseudomonadales (1236)	Gammaproteobacteria (122	Proteobacteria (2)	Bacteria (1)	100.00	0.90	97.70	94.20	WGS
316.107	0.3626	Pseudomonas stutzeri (286)	Pseudomonas (135621)	Pseudomonadaceae (7227	Pseudomonadales (1236)	Gammaproteobacteria (122	Proteobacteria (2)	Bacteria (1)	100.00	0.00	97.60	93.90	WGS
316.108	0.3786	Pseudomonas stutzeri (286)	Pseudomonas (135621)	Pseudomonadaceae (7227	Pseudomonadales (1236)	Gammaproteobacteria (122	Proteobacteria (2)	Bacteria (1)	100.00	0.00	97.80	95.50	WGS
316.110	0.4404	Pseudomonas stutzeri (286)	Pseudomonas (135621)	Pseudomonadaceae (7227	Pseudomonadales (1236)	Gammaproteobacteria (122	Proteobacteria (2)	Bacteria (1)	100.00	1.80	98.30	94.20	WGS
316.115	0.3726	Pseudomonas stutzeri (286)	Pseudomonas (135621)	Pseudomonadaceae (7227	Pseudomonadales (1236)	Gammaproteobacteria (122	Proteobacteria (2)	Bacteria (1)	100.00	0.90	97.80	95.20	WGS
316.119	0.4424	Pseudomonas stutzeri (286)	Pseudomonas (135621)	Pseudomonadaceae (7227	Pseudomonadales (1236)	Gammaproteobacteria (122	Proteobacteria (2)	Bacteria (1)	100.00	0.90	97.90	94.10	WGS
316.120	0.4716	Pseudomonas stutzeri (286)	Pseudomonas (135621)	Pseudomonadaceae (7227	Pseudomonadales (1236)	Gammaproteobacteria (122	Proteobacteria (2)	Bacteria (1)	100.00	1.80	97.60	94.80	Complete
316.146	0.3506	Pseudomonas stutzeri (286)	Pseudomonas (135621)	Pseudomonadaceae (7227	Pseudomonadales (1236)	Gammaproteobacteria (122	Proteobacteria (2)	Bacteria (1)	100.00	0.00	97.90	95.30	WGS
316.154	0.3724	Pseudomonas stutzeri (286)	Pseudomonas (135621)	Pseudomonadaceae (7227	Pseudomonadales (1236)	Gammaproteobacteria (122	Proteobacteria (2)	Bacteria (1)	100.00	0.90	97.90	94.50	WGS
316.155	0.3594	Pseudomonas stutzeri (286)	Pseudomonas (135621)	Pseudomonadaceae (7227	Pseudomonadales (1236)	Gammaproteobacteria (122	Proteobacteria (2)	Bacteria (1)	100.00	2.60	97.90	92.50	WGS
316.159	0.3654	Pseudomonas stutzeri (286)	Pseudomonas (135621)	Pseudomonadaceae (7227	Pseudomonadales (1236)	Gammaproteobacteria (122	Proteobacteria (2)	Bacteria (1)	100.00	0.00	98.10	93.20	WGS
316.162	0.3762	Pseudomonas stutzeri (286)	Pseudomonas (135621)	Pseudomonadaceae (7227	Pseudomonadales (1236)	Gammaproteobacteria (122	Proteobacteria (2)	Bacteria (1)	100.00	0.00	97.90	95.50	WGS
316.163	0.2382	Pseudomonas stutzeri (286)	Pseudomonas (135621)	Pseudomonadaceae (7227	Pseudomonadales (1236)	Gammaproteobacteria (122	Proteobacteria (2)	Bacteria (1)	58.60	1.50	86.30	83.80	WGS
316.164	0.4058	Pseudomonas stutzeri (286)	Pseudomonas (135621)	Pseudomonadaceae (7227	Pseudomonadales (1236)	Gammaproteobacteria (122	Proteobacteria (2)	Bacteria (1)	100.00	0.00	94.60	92.00	WGS
316.165	0.3964	Pseudomonas stutzeri (286)	Pseudomonas (135621)	Pseudomonadaceae (7227	Pseudomonadales (1236)	Gammaproteobacteria (122	Proteobacteria (2)	Bacteria (1)	99.10	0.00	97.70	95.30	WGS
316.166	0.3922	Pseudomonas stutzeri (286)	Pseudomonas (135621)	Pseudomonadaceae (7227	Pseudomonadales (1236)	Gammaproteobacteria (122	Proteobacteria (2)	Bacteria (1)	100.00	0.00	96.10	93.70	WGS
316.167	0.3038	Pseudomonas stutzeri (286)	Pseudomonas (135621)	Pseudomonadaceae (7227	Pseudomonadales (1236)	Gammaproteobacteria (122	Proteobacteria (2)	Bacteria (1)	99.10	0.00	95.90	92.50	WGS
316.168	0.4372	Pseudomonas stutzeri (286)	Pseudomonas (135621)	Pseudomonadaceae (7227	Pseudomonadales (1236)	Gammaproteobacteria (122	Proteobacteria (2)	Bacteria (1)	91.90	3.80	92.20	87.80	WGS
316.169	0.3414	Pseudomonas stutzeri (286)	Pseudomonas (135621)	Pseudomonadaceae (7227	Pseudomonadales (1236)	Gammaproteobacteria (122	Proteobacteria (2)	Bacteria (1)	95.50	1.80	92.70	90.30	WGS
316.170	0.366	Pseudomonas stutzeri (286)	Pseudomonas (135621)	Pseudomonadaceae (7227	Pseudomonadales (1236)	Gammaproteobacteria (122	Proteobacteria (2)	Bacteria (1)	98.20	0.90	96.10	93.60	WGS
316.171	0.3714	Pseudomonas stutzeri (286)	Pseudomonas (135621)	Pseudomonadaceae (7227	Pseudomonadales (1236)	Gammaproteobacteria (122	Proteobacteria (2)	Bacteria (1)	91.00	3.80	89.00	86.10	WGS
316.172	0.3014	Pseudomonas stutzeri (286)	Pseudomonas (135621)	Pseudomonadaceae (7227	Pseudomonadales (1236)	Gammaproteobacteria (122	Proteobacteria (2)	Bacteria (1)	97.30	2.70	94.80	89.70	WGS
316.173	0.397	Pseudomonas stutzeri (286)	Pseudomonas (135621)	Pseudomonadaceae (7227	Pseudomonadales (1236)	Gammaproteobacteria (122	Proteobacteria (2)	Bacteria (1)	100.00	0.00	97.30	94.20	WGS
316.174	0.3602	Pseudomonas stutzeri (286)	Pseudomonas (135621)	Pseudomonadaceae (7227	Pseudomonadales (1236)	Gammaproteobacteria (122	Proteobacteria (2)	Bacteria (1)	100.00	1.80	96.80	94.60	WGS
316.175	0.4514	Pseudomonas stutzeri (286)	Pseudomonas (135621)	Pseudomonadaceae (7227	Pseudomonadales (1236)	Gammaproteobacteria (122	Proteobacteria (2)	Bacteria (1)	100.00	0.00	97.10	94.70	WGS
316.176	0.335	Pseudomonas stutzeri (286)	Pseudomonas (135621)	Pseudomonadaceae (7227	Pseudomonadales (1236)	Gammaproteobacteria (122	Proteobacteria (2)	Bacteria (1)	100.00	0.00	96.90	94.80	WGS
316.177	0.4432	Pseudomonas stutzeri (286)	Pseudomonas (135621)	Pseudomonadaceae (7227	Pseudomonadales (1236)	Gammaproteobacteria (122	Proteobacteria (2)	Bacteria (1)	92.80	0.00	90.80	88.80	WGS
316.178	0.3408	Pseudomonas stutzeri (286)	Pseudomonas (135621)	Pseudomonadaceae (7227	Pseudomonadales (1236)	Gammaproteobacteria (122	Proteobacteria (2)	Bacteria (1)	81.10	0.00	91.80	90.10	WGS
316.179	0.368	Pseudomonas stutzeri (286)	Pseudomonas (135621)	Pseudomonadaceae (7227	Pseudomonadales (1236)	Gammaproteobacteria (122	Proteobacteria (2)	Bacteria (1)	100.00	0.00	96.70	93.40	WGS
316.180	0.4108	Pseudomonas stutzeri (286)	Pseudomonas (135621)	Pseudomonadaceae (7227	Pseudomonadales (1236)	Gammaproteobacteria (122	Proteobacteria (2)	Bacteria (1)	91.00	0.00	92.70	90.50	WGS
316.181	0.333	Pseudomonas stutzeri (286)	Pseudomonas (135621)	Pseudomonadaceae (7227	Pseudomonadales (1236)	Gammaproteobacteria (122	Proteobacteria (2)	Bacteria (1)	87.40	8.50	90.80	86.10	WGS
316.182	0.4184	Pseudomonas stutzeri (286)	Pseudomonas (135621)	Pseudomonadaceae (7227	Pseudomonadales (1236)	Gammaproteobacteria (122	Proteobacteria (2)	Bacteria (1)	100.00	0.00	94.50	91.90	WGS
316.183	0.3256	Pseudomonas stutzeri (286)	Pseudomonas (135621)	Pseudomonadaceae (7227	Pseudomonadales (1236)	Gammaproteobacteria (122	Proteobacteria (2)	Bacteria (1)	100.00	0.00	96.50	94.20	WGS
316.184	0.3962	Pseudomonas stutzeri (286)	Pseudomonas (135621)	Pseudomonadaceae (7227	Pseudomonadales (1236)	Gammaproteobacteria (122	Proteobacteria (2)	Bacteria (1)	99.10	0.90	96.80	92.90	WGS
316.185	0.3086	Pseudomonas stutzeri (286)	Pseudomonas (135621)	Pseudomonadaceae (7227	Pseudomonadales (1236)	Gammaproteobacteria (122	Proteobacteria (2)	Bacteria (1)	98.20	1.80	94.80	92.20	WGS
316.186	0.4016	Pseudomonas stutzeri (286)	Pseudomonas (135621)	Pseudomonadaceae (7227	Pseudomonadales (1236)	Gammaproteobacteria (122	Proteobacteria (2)	Bacteria (1)	100.00	0.00	96.70	93.60	WGS
316.187	0.2892	Pseudomonas stutzeri (286)	Pseudomonas (135621)	Pseudomonadaceae (7227	Pseudomonadales (1236)	Gammaproteobacteria (122	Proteobacteria (2)	Bacteria (1)	84.70	1.10	87.00	82.30	WGS
316.188	0.3962	Pseudomonas stutzeri (286)	Pseudomonas (135621)	Pseudomonadaceae (7227	Pseudomonadales (1236)	Gammaproteobacteria (122	Proteobacteria (2)	Bacteria (1)	96.40	1.90	93.30	87.70	WGS
316.189	0.398	Pseudomonas stutzeri (286)	Pseudomonas (135621)	Pseudomonadaceae (7227	Pseudomonadales (1236)	Gammaproteobacteria (122	Proteobacteria (2)	Bacteria (1)	100.00	0.00	96.70	94.10	WGS
316.190	0.3942	Pseudomonas stutzeri (286)	Pseudomonas (135621)	Pseudomonadaceae (7227	Pseudomonadales (1236)	Gammaproteobacteria (122	Proteobacteria (2)	Bacteria (1)	100.00	5.10	92.20	89.30	WGS
316.191	0.3874	Pseudomonas stutzeri (286)	Pseudomonas (135621)	Pseudomonadaceae (7227	Pseudomonadales (1236)	Gammaproteobacteria (122	Proteobacteria (2)	Bacteria (1)	99.10	1.80	94.30	91.60	WGS
316.192	0.2358	Pseudomonas stutzeri (286)	Pseudomonas (135621)	Pseudomonadaceae (7227	Pseudomonadales (1236)	Gammaproteobacteria (122	Proteobacteria (2)	Bacteria (1)	74.80	2.40	85.10	80.90	WGS
316.193	0.3316	Pseudomonas stutzeri (286)	Pseudomonas (135621)	Pseudomonadaceae (7227	Pseudomonadales (1236)	Gammaproteobacteria (122	Proteobacteria (2)	Bacteria (1)	94.50	5.40	89.40	84.00	WGS
316.194	0.3774	Pseudomonas stutzeri (286)	Pseudomonas (135621)	Pseudomonadaceae (7227	Pseudomonadales (1236)	Gammaproteobacteria (122	Proteobacteria (2)	Bacteria (1)	100.00	0.00	97.00	94.80	WGS
316.195	0.5066	Pseudomonas stutzeri (286)	Pseudomonas (135621)	Pseudomonadaceae (7227	Pseudomonadales (1236)	Gammaproteobacteria (122	Proteobacteria (2)	Bacteria (1)	96.40	1.80	92.70	87.80	WGS
316.196	0.4218	Pseudomonas stutzeri (286)	Pseudomonas (135621)	Pseudomonadaceae (7227	Pseudomonadales (1236)	Gammaproteobacteria (122	Proteobacteria (2)	Bacteria (1)	93.70	3.70	90.60	86.50	WGS
316.197	0.3512	Pseudomonas stutzeri (286)	Pseudomonas (135621)	Pseudomonadaceae (7227	Pseudomonadales (1236)	Gammaproteobacteria (122	Proteobacteria (2)	Bacteria (1)	100.00	6.70	94.00	89.10	WGS
316.198	0.4184	Pseudomonas stutzeri (286)	Pseudomonas (135621)	Pseudomonadaceae (7227	Pseudomonadales (1236)	Gammaproteobacteria (122	Proteobacteria (2)	Bacteria (1)	98.20	0.00	96.70	94.60	WGS
316.199	0.3806	Pseudomonas stutzeri (286)	Pseudomonas (135621)	Pseudomonadaceae (7227	Pseudomonadales (1236)	Gammaproteobacteria (122	Proteobacteria (2)	Bacteria (1)	100.00	0.00	96.60	94.40	WGS
316.200	0.3864	Pseudomonas stutzeri (286)	Pseudomonas (135621)	Pseudomonadaceae (7227	Pseudomonadales (1236)	Gammaproteobacteria (122	Proteobacteria (2)	Bacteria (1)	92.80	1.90	91.50	86.00	WGS
316.201	0.3874	Pseudomonas stutzeri (286)	Pseudomonas (135621)	Pseudomonadaceae (7227	Pseudomonadales (1236)	Gammaproteobacteria (122	Proteobacteria (2)	Bacteria (1)	100.00	0.00	95.00	93.30	WGS
316.202	0.3406	Pseudomonas stutzeri (286)	Pseudomonas (135621)	Pseudomonadaceae (7227	Pseudomonadales (1236)	Gammaproteobacteria (122	Proteobacteria (2)	Bacteria (1)	96.40	0.00	95.10	92.30	WGS
316.203	0.4152	Pseudomonas stutzeri (286)	Pseudomonas (135621)	Pseudomonadaceae (7227	Pseudomonadales (1236)	Gammaproteobacteria (122	Proteobacteria (2)	Bacteria (1)	95.50	0.90	95.00	93.00	WGS
316.204	0.3424	Pseudomonas stutzeri (286)	Pseudomonas (135621)	Pseudomonadaceae (7227	Pseudomonadales (1236)	Gammaproteobacteria (122	Proteobacteria (2)	Bacteria (1)	95.50	11.70	91.60	82.70	WGS
316.205	0.448	Pseudomonas stutzeri (286)	Pseudomonas (135621)	Pseudomonadaceae (7227	Pseudomonadales (1236)	Gammaproteobacteria (122	Proteobacteria (2)	Bacteria (1)	100.00	0.00	97.30	95.40	WGS
316.206	0.424	Pseudomonas stutzeri (286)	Pseudomonas (135621)	Pseudomonadaceae (7227	Pseudomonadales (1236)	Gammaproteobacteria (122	Proteobacteria (2)	Bacteria (1)	100.00	0.00	96.70	94.20	WGS
316.208	0.4094	Pseudomonas stutzeri (286)	Pseudomonas (135621)	Pseudomonadaceae (7227	Pseudomonadales (1236)	Gammaproteobacteria (122	Proteobacteria (2)	Bacteria (1)	97.30	9.20	95.40	88.50	WGS
316.209	0.43	Pseudomonas stutzeri (286)	Pseudomonas (135621)	Pseudomonadaceae (7227	Pseudomonadales (1236)	Gammaproteobacteria (122	Proteobacteria (2)	Bacteria (1)	100.00	0.00	97.60	94.70	WGS
316.210	0.3488	Pseudomonas stutzeri (286)	Pseudomonas (135621)	Pseudomonadaceae (7227	Pseudomonadales (1236)	Gammaproteobacteria (122	Proteobacteria (2)	Bacteria (1)	100.00	0.00	95.20	92.50	WGS
316.211	0.387	Pseudomonas stutzeri (286)	Pseudomonas (135621)	Pseudomonadaceae (7227	Pseudomonadales (1236)	Gammaproteobacteria (122	Proteobacteria (2)	Bacteria (1)	98.20	0.00	96.00	93.70	WGS
316.212	0.34	Pseudomonas stutzeri (286)	Pseudomonas (135621)	Pseudomonadaceae (7227	Pseudomonadales (1236)	Gammaproteobacteria (122	Proteobacteria (2)	Bacteria (1)	100.00	0.90	96.40	93.10	WGS
316.213	0.3682	Pseudomonas stutzeri (286)	Pseudomonas (135621)	Pseudomonadaceae (7227	Pseudomonadales (1236)	Gammaproteobacteria (122	Proteobacteria (2)	Bacteria (1)	97.30	0.00	95.90	93.40	WGS
316.214	0.3716	Pseudomonas stutzeri (286)	Pseudomonas (135621)	Pseudomonadaceae (7227	Pseudomonadales (1236)	Gammaproteobacteria (122	Proteobacteria (2)	Bacteria (1)	99.10	0.00	94.20	92.00	WGS
316.215	0.4368	Pseudomonas stutzeri (286)	Pseudomonas (135621)	Pseudomonadaceae (7227	Pseudomonadales (1236)	Gammaproteobacteria (122	Proteobacteria (2)	Bacteria (1)	100.00	0.90	96.90	94.20	WGS
316.216	0.3642	Pseudomonas stutzeri (286)	Pseudomonas (135621)	Pseudomonadaceae (7227	Pseudomonadales (1236)	Gammaproteobacteria (122	Proteobacteria (2)	Bacteria (1)	100.00	0.00	96.50	94.60	WGS
316.217	0.425	Pseudomonas stutzeri (286)	Pseudomonas (135621)	Pseudomonadaceae (7227	Pseudomonadales (1236)	Gammaproteobacteria (122	Proteobacteria (2)	Bacteria (1)	100.00	0.00	98.00	95.90	WGS
316.218	0.3404	Pseudomonas stutzeri (286)	Pseudomonas (135621)	Pseudomonadaceae (7227	Pseudomonadales (1236)	Gammaproteobacteria (122	Proteobacteria (2)	Bacteria (1)	97.30	0.00	95.50	92.80	WGS
316.219	0.3926	Pseudomonas stutzeri (286)	Pseudomonas (135621)	Pseudomonadaceae (7227	Pseudomonadales (1236)	Gammaproteobacteria (122	Proteobacteria (2)	Bacteria (1)	100.00	0.00	96.10	93.80	WGS
316.220	0.3898	Pseudomonas stutzeri (286)	Pseudomonas (135621)	Pseudomonadaceae (7227	Pseudomonadales (1236)	Gammaproteobacteria (122	Proteobacteria (2)	Bacteria (1)	100.00	0.00	98.20	95.60	WGS
316.221	0.4314	Pseudomonas stutzeri (286)	Pseudomonas (135621)	Pseudomonadaceae (7227	Pseudomonadales (1236)	Gammaproteobacteria (122	Proteobacteria (2)	Bacteria (1)	97.30	0.00	95.10	92.50	WGS
316.222	0.4384	Pseudomonas stutzeri (286)	Pseudomonas (135621)	Pseudomonadaceae (7227	Pseudomonadales (1236)	Gammaproteobacteria (122	Proteobacteria (2)	Bacteria (1)	99.00	2.70	91.10	86.70	WGS
316.223	0.357	Pseudomonas stutzeri (286)	Pseudomonas (135621)	Pseudomonadaceae (7227	Pseudomonadales (1236)	Gammaproteobacteria (122	Proteobacteria (2)	Bacteria (1)	100.00	0.00	96.70	94.30	WGS
316.224	0.2848	Pseudomonas stutzeri (286)	Pseudomonas (135621)	Pseudomonadaceae (7227	Pseudomonadales (1236)	Gammaproteobacteria (122	Proteobacteria (2)	Bacteria (1)	82.90	1.10	86.50	82.60	WGS
316.225	0.3552	Pseudomonas stutzeri (286)	Pseudomonas (135621)	Pseudomonadaceae (7227	Pseudomonadales (1236)	Gammaproteobacteria (122	Proteobacteria (2)	Bacteria (1)	97.30	1.80	92.00	89.30	WGS
316.226	0.3396	Pseudomonas stutzeri (286)	Pseudomonas (135621)	Pseudomonadaceae (7227	Pseudomonadales (1236)	Gammaproteobacteria (122	Proteobacteria (2)	Bacteria (1)	83.80	0.00	90.90	89.40	WGS
316.227	0.3814	Pseudomonas stutzeri (286)	Pseudomonas (135621)	Pseudomonadaceae (7227	Pseudomonadales (1236)	Gammaproteobacteria (122	Proteobacteria (2)	Bacteria (1)	100.00	0.00	96.50	94.10	WGS
316.228	0.3632	Pseudomonas stutzeri (286)	Pseudomonas (135621)	Pseudomonadaceae (7227	Pseudomonadales (1236)	Gammaproteobacteria (122	Proteobacteria (2)	Bacteria (1)	100.00	0.00	96.30	94.20	WGS
316.229	0.405	Pseudomonas stutzeri (286)	Pseudomonas (135621)	Pseudomonadaceae (7227	Pseudomonadales (1236)	Gammaproteobacteria (122	Proteobacteria (2)	Bacteria (1)	99.10	0.00	96.10	92.80	WGS
316.230	0.3004	Pseudomonas stutzeri (286)	Pseudomonas (135621)	Pseudomonadaceae (7227	Pseudomonadales (1236)	Gammaproteobacteria (122	Proteobacteria (2)	Bacteria (1)	98.20	2.70	92.30	88.60	WGS
316.231	0.427	Pseudomonas stutzeri (286)	Pseudomonas (135621)	Pseudomonadaceae (7227	Pseudomonadales (1236)	Gammaproteobacteria (122	Proteobacteria (2)	Bacteria (1)	100.00	0.00	97.30	95.20	WGS
316.232	0.4008	Pseudomonas stutzeri (286)	Pseudomonas (135621)	Pseudomonadaceae (7227	Pseudomonadales (1236)	Gammaproteobacteria (122	Proteobacteria (2)	Bacteria (1)	100.00	0.00	94.30	92.10	WGS
316.233	0.3776	Pseudomonas stutzeri (286)	Pseudomonas (135621)	Pseudomonadaceae (7227	Pseudomonadales (1236)	Gammaproteobacteria (122	Proteobacteria (2)	Bacteria (1)	100.00	3.50	93.10	89.40	WGS
316.234	0.3466	Pseudomonas stutzeri (286)	Pseudomonas (135621)	Pseudomonadaceae (7227	Pseudomonadales (1236)	Gammaproteobacteria (122	Proteobacteria (2)	Bacteria (1)	100.00	2.60	94.60	90.30	WGS
316.235	0.3614	Pseudomonas stutzeri (286)	Pseudomonas (135621)	Pseudomonadaceae (7227	Pseudomonadales (1236)	Gammaproteobacteria (122	Proteobacteria (2)	Bacteria (1)	87.40	4.90	87.20	83.80	WGS
316.236	0.4062	Pseudomonas stutzeri (286)	Pseudomonas (135621)	Pseudomonadaceae (7227	Pseudomonadales (1236)	Gammaproteobacteria (122	Proteobacteria (2)	Bacteria (1)	82.90	0.00	87.60	84.60	WGS
316.237	0.3638	Pseudomonas stutzeri (286)	Pseudomonas (135621)	Pseudomonadaceae (7227	Pseudomonadales (1236)	Gammaproteobacteria (122	Proteobacteria (2)	Bacteria (1)	92.80	3.80	92.80	87.60	WGS
316.238	0.4494	Pseudomonas stutzeri (286)	Pseudomonas (135621)	Pseudomonadaceae (7227	Pseudomonadales (1236)	Gammaproteobacteria (122	Proteobacteria (2)	Bacteria (1)	99.10	0.00	95.40	93.20	WGS
316.239	0.3314	Pseudomonas stutzeri (286)	Pseudomonas (135621)	Pseudomonadaceae (7227	Pseudomonadales (1236)	Gammaproteobacteria (122	Proteobacteria (2)	Bacteria (1)	91.00	0.00	94.20	91.70	WGS
316.240	0.3728	Pseudomonas stutzeri (286)	Pseudomonas (135621)	Pseudomonadaceae (7227	Pseudomonadales (1236)	Gammaproteobacteria (122	Proteobacteria (2)	Bacteria (1)	100.00	0.00	96.00	93.10	WGS
316.241	0.3808	Pseudomonas stutzeri (286)	Pseudomonas (135621)	Pseudomonadaceae (7227	Pseudomonadales (1236)	Gammaproteobacteria (122	Proteobacteria (2)	Bacteria (1)	100.00	0.00	97.20	94.90	WGS
316.242	0.4458	Pseudomonas stutzeri (286)	Pseudomonas (135621)	Pseudomonadaceae (7227	Pseudomonadales (1236)	Gammaproteobacteria (122	Proteobacteria (2)	Bacteria (1)	100.00	0.00	96.20	93.40	WGS
316.243	0.3546	Pseudomonas stutzeri (286)	Pseudomonas (135621)	Pseudomonadaceae (7227	Pseudomonadales (1236)	Gammaproteobacteria (122	Proteobacteria (2)	Bacteria (1)	99.10	0.00	96.70	93.90	WGS
316.244	0.4068	Pseudomonas stutzeri (286)	Pseudomonas (135621)	Pseudomonadaceae (7227	Pseudomonadales (1236)	Gammaproteobacteria (122	Proteobacteria (2)	Bacteria (1)	100.00	0.00	97.50	94.90	WGS
316.245	0.3138	Pseudomonas stutzeri (286)	Pseudomonas (135621)	Pseudomonadaceae (7227	Pseudomonadales (1236)	Gammaproteobacteria (122	Proteobacteria (2)	Bacteria (1)	76.50	1.20	86.30	84.90	WGS
316.246	0.37	Pseudomonas stutzeri (286)	Pseudomonas (135621)	Pseudomonadaceae (7227	Pseudomonadales (1236)	Gammaproteobacteria (122	Proteobacteria (2)	Bacteria (1)	98.20	0.00	95.30	93.10	WGS
316.247	0.2772	Pseudomonas stutzeri (286)	Pseudomonas (135621)	Pseudomonadaceae (7227	Pseudomonadales (1236)	Gammaproteobacteria (122	Proteobacteria (2)	Bacteria (1)	80.20	2.20	90.20	83.90	WGS
316.248	0.3602	Pseudomonas stutzeri (286)	Pseudomonas (135621)	Pseudomonadaceae (7227	Pseudomonadales (1236)	Gammaproteobacteria (122	Proteobacteria (2)	Bacteria (1)	100.00	0.00	97.20	94.90	WGS
316.249	0.3998	Pseudomonas stutzeri (286)	Pseudomonas (135621)	Pseudomonadaceae (7227	Pseudomonadales (1236)	Gammaproteobacteria (122	Proteobacteria (2)	Bacteria (1)	97.30	0.90	93.80	91.10	WGS
316.250	0.4108	Pseudomonas stutzeri (286)	Pseudomonas (135621)	Pseudomonadaceae (7227	Pseudomonadales (1236)	Gammaproteobacteria (122	Proteobacteria (2)	Bacteria (1)	100.00	0.00	96.80	94.50	WGS
316.251	0.4188	Pseudomonas stutzeri (286)	Pseudomonas (135621)	Pseudomonadaceae (7227	Pseudomonadales (1236)	Gammaproteobacteria (122	Proteobacteria (2)	Bacteria (1)	99.10	0.00	96.90	94.30	WGS
316.252	0.4622	Pseudomonas stutzeri (286)	Pseudomonas (135621)	Pseudomonadaceae (7227	Pseudomonadales (1236)	Gammaproteobacteria (122	Proteobacteria (2)	Bacteria (1)	82.00	0.00	88.80	87.10	WGS
316.253	0.391	Pseudomonas stutzeri (286)	Pseudomonas (135621)	Pseudomonadaceae (7227	Pseudomonadales (1236)	Gammaproteobacteria (122	Proteobacteria (2)	Bacteria (1)	96.40	0.00	96.20	94.20	WGS
316.254	0.4532	Pseudomonas stutzeri (286)	Pseudomonas (135621)	Pseudomonadaceae (7227	Pseudomonadales (1236)	Gammaproteobacteria (122	Proteobacteria (2)	Bacteria (1)	100.00	0.00	97.20	94.90	WGS
316.255	0.401	Pseudomonas stutzeri (286)	Pseudomonas (135621)	Pseudomonadaceae (7227	Pseudomonadales (1236)	Gammaproteobacteria (122	Proteobacteria (2)	Bacteria (1)	100.00	0.90	96.50	94.20	WGS
316.256	0.305	Pseudomonas stutzeri (286)	Pseudomonas (135621)	Pseudomonadaceae (7227	Pseudomonadales (1236)	Gammaproteobacteria (122	Proteobacteria (2)	Bacteria (1)	89.20	0.00	91.40	89.20	WGS
316.257	0.4282	Pseudomonas stutzeri (286)	Pseudomonas (135621)	Pseudomonadaceae (7227	Pseudomonadales (1236)	Gammaproteobacteria (122	Proteobacteria (2)	Bacteria (1)	100.00	0.00	93.60	91.50	WGS
316.258	0.356	Pseudomonas stutzeri (286)	Pseudomonas (135621)	Pseudomonadaceae (7227	Pseudomonadales (1236)	Gammaproteobacteria (122	Proteobacteria (2)	Bacteria (1)	90.10	2.90	89.40	85.10	WGS
316.259	0.3834	Pseudomonas stutzeri (286)	Pseudomonas (135621)	Pseudomonadaceae (7227	Pseudomonadales (1236)	Gammaproteobacteria (122	Proteobacteria (2)	Bacteria (1)	98.20	0.00	96.20	93.70	WGS
316.260	0.4178	Pseudomonas stutzeri (286)	Pseudomonas (135621)	Pseudomonadaceae (7227	Pseudomonadales (1236)	Gammaproteobacteria (122	Proteobacteria (2)	Bacteria (1)	100.00	0.00	97.10	94.70	WGS
316.261	0.3772	Pseudomonas stutzeri (286)	Pseudomonas (135621)	Pseudomonadaceae (7227	Pseudomonadales (1236)	Gammaproteobacteria (122	Proteobacteria (2)	Bacteria (1)	100.00	0.90	96.70	94.10	WGS
316.262	0.3812	Pseudomonas stutzeri (286)	Pseudomonas (135621)	Pseudomonadaceae (7227	Pseudomonadales (1236)	Gammaproteobacteria (122	Proteobacteria (2)	Bacteria (1)	97.30	1.80	97.30	93.00	WGS
316.263	0.3868	Pseudomonas stutzeri (286)	Pseudomonas (135621)	Pseudomonadaceae (7227	Pseudomonadales (1236)	Gammaproteobacteria (122	Proteobacteria (2)	Bacteria (1)	94.60	0.90	93.80	89.00	WGS
316.264	0.3526	Pseudomonas stutzeri (286)	Pseudomonas (135621)	Pseudomonadaceae (7227	Pseudomonadales (1236)	Gammaproteobacteria (122	Proteobacteria (2)	Bacteria (1)	98.20	0.90	95.30	89.90	WGS
316.265	0.4068	Pseudomonas stutzeri (286)	Pseudomonas (135621)	Pseudomonadaceae (7227	Pseudomonadales (1236)	Gammaproteobacteria (122	Proteobacteria (2)	Bacteria (1)	100.00	0.00	96.50	93.90	WGS
316.266	0.4326	Pseudomonas stutzeri (286)	Pseudomonas (135621)	Pseudomonadaceae (7227	Pseudomonadales (1236)	Gammaproteobacteria (122	Proteobacteria (2)	Bacteria (1)	100.00	0.90	97.80	94.30	WGS
316.267	0.3316	Pseudomonas stutzeri (286)	Pseudomonas (135621)	Pseudomonadaceae (7227	Pseudomonadales (1236)	Gammaproteobacteria (122	Proteobacteria (2)	Bacteria (1)	99.10	0.00	93.50	90.30	WGS
316.268	0.2812	Pseudomonas stutzeri (286)	Pseudomonas (135621)	Pseudomonadaceae (7227	Pseudomonadales (1236)	Gammaproteobacteria (122	Proteobacteria (2)	Bacteria (1)	79.30	1.10	85.70	84.30	WGS
316.269	0.4262	Pseudomonas stutzeri (286)	Pseudomonas (135621)	Pseudomonadaceae (7227	Pseudomonadales (1236)	Gammaproteobacteria (122	Proteobacteria (2)	Bacteria (1)	100.00	1.80	95.90	94.20	WGS
316.270	0.4294	Pseudomonas stutzeri (286)	Pseudomonas (135621)	Pseudomonadaceae (7227	Pseudomonadales (1236)	Gammaproteobacteria (122	Proteobacteria (2)	Bacteria (1)	100.00	0.00	97.10	94.80	WGS
316.271	0.4224	Pseudomonas stutzeri (286)	Pseudomonas (135621)	Pseudomonadaceae (7227	Pseudomonadales (1236)	Gammaproteobacteria (122	Proteobacteria (2)	Bacteria (1)	100.00	0.00	96.50	93.90	WGS
316.272	0.38	Pseudomonas stutzeri (286)	Pseudomonas (135621)	Pseudomonadaceae (7227	Pseudomonadales (1236)	Gammaproteobacteria (122	Proteobacteria (2)	Bacteria (1)	100.00	0.00	97.60	95.00	WGS
316.273	0.4174	Pseudomonas stutzeri (286)	Pseudomonas (135621)	Pseudomonadaceae (7227	Pseudomonadales (1236)	Gammaproteobacteria (122	Proteobacteria (2)	Bacteria (1)	100.00	0.00	96.80	94.50	WGS
316.274	0.36	Pseudomonas stutzeri (286)	Pseudomonas (135621)	Pseudomonadaceae (7227	Pseudomonadales (1236)	Gammaproteobacteria (122	Proteobacteria (2)	Bacteria (1)	100.00	0.00	97.00	94.80	WGS
316.275	0.3228	Pseudomonas stutzeri (286)	Pseudomonas (135621)	Pseudomonadaceae (7227	Pseudomonadales (1236)	Gammaproteobacteria (122	Proteobacteria (2)	Bacteria (1)	96.40	11.50	94.50	86.00	WGS
316.276	0.3508	Pseudomonas stutzeri (286)	Pseudomonas (135621)	Pseudomonadaceae (7227	Pseudomonadales (1236)	Gammaproteobacteria (122	Proteobacteria (2)	Bacteria (1)	83.80	0.00	88.20	86.10	WGS
316.277	0.3188	Pseudomonas stutzeri (286)	Pseudomonas (135621)	Pseudomonadaceae (7227	Pseudomonadales (1236)	Gammaproteobacteria (122	Proteobacteria (2)	Bacteria (1)	64.00	0.00	82.70	81.80	WGS
316.278	0.3656	Pseudomonas stutzeri (286)	Pseudomonas (135621)	Pseudomonadaceae (7227	Pseudomonadales (1236)	Gammaproteobacteria (122	Proteobacteria (2)	Bacteria (1)	100.00	1.80	96.80	93.90	WGS
316.279	0.429	Pseudomonas stutzeri (286)	Pseudomonas (135621)	Pseudomonadaceae (7227	Pseudomonadales (1236)	Gammaproteobacteria (122	Proteobacteria (2)	Bacteria (1)	100.00	0.00	96.60	93.90	WGS
316.280	0.476	Pseudomonas stutzeri (286)	Pseudomonas (135621)	Pseudomonadaceae (7227	Pseudomonadales (1236)	Gammaproteobacteria (122	Proteobacteria (2)	Bacteria (1)	94.60	0.00	92.50	89.70	WGS
316.281	0.4142	Pseudomonas stutzeri (286)	Pseudomonas (135621)	Pseudomonadaceae (7227	Pseudomonadales (1236)	Gammaproteobacteria (122	Proteobacteria (2)	Bacteria (1)	100.00	0.00	96.90	94.50	WGS
316.282	0.3034	Pseudomonas stutzeri (286)	Pseudomonas (135621)	Pseudomonadaceae (7227	Pseudomonadales (1236)	Gammaproteobacteria (122	Proteobacteria (2)	Bacteria (1)	96.40	10.10	93.90	87.50	WGS
316.283	0.3454	Pseudomonas stutzeri (286)	Pseudomonas (135621)	Pseudomonadaceae (7227	Pseudomonadales (1236)	Gammaproteobacteria (122	Proteobacteria (2)	Bacteria (1)	93.70	0.00	93.00	91.50	WGS
316.284	0.435	Pseudomonas stutzeri (286)	Pseudomonas (135621)	Pseudomonadaceae (7227	Pseudomonadales (1236)	Gammaproteobacteria (122	Proteobacteria (2)	Bacteria (1)	100.00	0.00	96.70	93.90	WGS
316.285	0.415	Pseudomonas stutzeri (286)	Pseudomonas (135621)	Pseudomonadaceae (7227	Pseudomonadales (1236)	Gammaproteobacteria (122	Proteobacteria (2)	Bacteria (1)	93.70	0.90	94.00	91.20	WGS
316.286	0.4128	Pseudomonas stutzeri (286)	Pseudomonas (135621)	Pseudomonadaceae (7227	Pseudomonadales (1236)	Gammaproteobacteria (122	Proteobacteria (2)	Bacteria (1)	94.60	0.90	93.00	88.30	WGS
316.287	0.3608	Pseudomonas stutzeri (286)	Pseudomonas (135621)	Pseudomonadaceae (7227	Pseudomonadales (1236)	Gammaproteobacteria (122	Proteobacteria (2)	Bacteria (1)	91.90	0.00	91.30	90.10	WGS
316.288	0.3446	Pseudomonas stutzeri (286)	Pseudomonas (135621)	Pseudomonadaceae (7227	Pseudomonadales (1236)	Gammaproteobacteria (122	Proteobacteria (2)	Bacteria (1)	100.00	0.00	96.90	94.60	WGS
316.289	0.4424	Pseudomonas stutzeri (286)	Pseudomonas (135621)	Pseudomonadaceae (7227	Pseudomonadales (1236)	Gammaproteobacteria (122	Proteobacteria (2)	Bacteria (1)	99.10	0.90	95.20	92.50	WGS
316.290	0.3672	Pseudomonas stutzeri (286)	Pseudomonas (135621)	Pseudomonadaceae (7227	Pseudomonadales (1236)	Gammaproteobacteria (122	Proteobacteria (2)	Bacteria (1)	100.00	0.00	97.30	95.20	WGS
316.291	0.3372	Pseudomonas stutzeri (286)	Pseudomonas (135621)	Pseudomonadaceae (7227	Pseudomonadales (1236)	Gammaproteobacteria (122	Proteobacteria (2)	Bacteria (1)	98.20	3.50	95.40	90.20	WGS
316.292	0.2714	Pseudomonas stutzeri (286)	Pseudomonas (135621)	Pseudomonadaceae (7227	Pseudomonadales (1236)	Gammaproteobacteria (122	Proteobacteria (2)	Bacteria (1)	93.70	5.50	91.10	83.60	WGS
316.293	0.131	Pseudomonas stutzeri (286)	Pseudomonas (135621)	Pseudomonadaceae (7227	Pseudomonadales (1236)	Gammaproteobacteria (122	Proteobacteria (2)	Bacteria (1)	71.20	2.50	80.60	79.20	WGS
316.294	0.3398	Pseudomonas stutzeri (286)	Pseudomonas (135621)	Pseudomonadaceae (7227	Pseudomonadales (1236)	Gammaproteobacteria (122	Proteobacteria (2)	Bacteria (1)	72.10	2.40	86.20	85.20	WGS
316.295	0.3192	Pseudomonas stutzeri (286)	Pseudomonas (135621)	Pseudomonadaceae (7227	Pseudomonadales (1236)	Gammaproteobacteria (122	Proteobacteria (2)	Bacteria (1)	97.30	5.30	92.60	85.90	WGS
316.296	0.3826	Pseudomonas stutzeri (286)	Pseudomonas (135621)	Pseudomonadaceae (7227	Pseudomonadales (1236)	Gammaproteobacteria (122	Proteobacteria (2)	Bacteria (1)	99.10	2.70	96.00	90.50	WGS
316.297	0.363	Pseudomonas stutzeri (286)	Pseudomonas (135621)	Pseudomonadaceae (7227	Pseudomonadales (1236)	Gammaproteobacteria (122	Proteobacteria (2)	Bacteria (1)	89.20	9.10	90.50	84.80	WGS
316.298	0.3244	Pseudomonas stutzeri (286)	Pseudomonas (135621)	Pseudomonadaceae (7227	Pseudomonadales (1236)	Gammaproteobacteria (122	Proteobacteria (2)	Bacteria (1)	99.10	1.80	94.00	91.00	WGS
316.299	0.362	Pseudomonas stutzeri (286)	Pseudomonas (135621)	Pseudomonadaceae (7227	Pseudomonadales (1236)	Gammaproteobacteria (122	Proteobacteria (2)	Bacteria (1)	98.20	0.00	92.50	90.50	WGS
316.300	0.4008	Pseudomonas stutzeri (286)	Pseudomonas (135621)	Pseudomonadaceae (7227	Pseudomonadales (1236)	Gammaproteobacteria (122	Proteobacteria (2)	Bacteria (1)	92.80	8.80	91.60	86.30	WGS
316.301	0.3356	Pseudomonas stutzeri (286)	Pseudomonas (135621)	Pseudomonadaceae (7227	Pseudomonadales (1236)	Gammaproteobacteria (122	Proteobacteria (2)	Bacteria (1)	91.90	2.80	92.80	89.70	WGS
316.302	0.2826	Pseudomonas stutzeri (286)	Pseudomonas (135621)	Pseudomonadaceae (7227	Pseudomonadales (1236)	Gammaproteobacteria (122	Proteobacteria (2)	Bacteria (1)	86.50	11.90	88.20	82.50	WGS
316.303	0.4062	Pseudomonas stutzeri (286)	Pseudomonas (135621)	Pseudomonadaceae (7227	Pseudomonadales (1236)	Gammaproteobacteria (122	Proteobacteria (2)	Bacteria (1)	99.10	0.00	96.50	94.20	WGS
316.304	0.3958	Pseudomonas stutzeri (286)	Pseudomonas (135621)	Pseudomonadaceae (7227	Pseudomonadales (1236)	Gammaproteobacteria (122	Proteobacteria (2)	Bacteria (1)	91.90	5.60	89.70	83.90	WGS
316.305	0.3272	Pseudomonas stutzeri (286)	Pseudomonas (135621)	Pseudomonadaceae (7227	Pseudomonadales (1236)	Gammaproteobacteria (122	Proteobacteria (2)	Bacteria (1)	97.30	8.40	94.40	88.30	WGS
316.306	0.4152	Pseudomonas stutzeri (286)	Pseudomonas (135621)	Pseudomonadaceae (7227	Pseudomonadales (1236)	Gammaproteobacteria (122	Proteobacteria (2)	Bacteria (1)	93.70	0.00	95.60	92.60	WGS
316.307	0.3542	Pseudomonas stutzeri (286)	Pseudomonas (135621)	Pseudomonadaceae (7227	Pseudomonadales (1236)	Gammaproteobacteria (122	Proteobacteria (2)	Bacteria (1)	100.00	0.00	96.60	93.70	WGS
316.308	0.4476	Pseudomonas stutzeri (286)	Pseudomonas (135621)	Pseudomonadaceae (7227	Pseudomonadales (1236)	Gammaproteobacteria (122	Proteobacteria (2)	Bacteria (1)	98.20	5.20	93.70	89.10	WGS
316.309	0.4348	Pseudomonas stutzeri (286)	Pseudomonas (135621)	Pseudomonadaceae (7227	Pseudomonadales (1236)	Gammaproteobacteria (122	Proteobacteria (2)	Bacteria (1)	100.00	3.50	93.60	89.00	WGS
316.310	0.4114	Pseudomonas stutzeri (286)	Pseudomonas (135621)	Pseudomonadaceae (7227	Pseudomonadales (1236)	Gammaproteobacteria (122	Proteobacteria (2)	Bacteria (1)	99.10	1.80	94.70	90.40	WGS
316.311	0.4374	Pseudomonas stutzeri (286)	Pseudomonas (135621)	Pseudomonadaceae (7227	Pseudomonadales (1236)	Gammaproteobacteria (122	Proteobacteria (2)	Bacteria (1)	100.00	0.00	96.70	94.80	WGS
316.312	0.3706	Pseudomonas stutzeri (286)	Pseudomonas (135621)	Pseudomonadaceae (7227	Pseudomonadales (1236)	Gammaproteobacteria (122	Proteobacteria (2)	Bacteria (1)	73.00	3.60	84.80	80.80	WGS
316.313	0.4456	Pseudomonas stutzeri (286)	Pseudomonas (135621)	Pseudomonadaceae (7227	Pseudomonadales (1236)	Gammaproteobacteria (122	Proteobacteria (2)	Bacteria (1)	100.00	0.90	96.50	91.80	WGS
316.314	0.359	Pseudomonas stutzeri (286)	Pseudomonas (135621)	Pseudomonadaceae (7227	Pseudomonadales (1236)	Gammaproteobacteria (122	Proteobacteria (2)	Bacteria (1)	100.00	0.00	95.80	93.70	WGS
316.315	0.4158	Pseudomonas stutzeri (286)	Pseudomonas (135621)	Pseudomonadaceae (7227	Pseudomonadales (1236)	Gammaproteobacteria (122	Proteobacteria (2)	Bacteria (1)	100.00	0.00	96.80	93.60	WGS
316.316	0.3542	Pseudomonas stutzeri (286)	Pseudomonas (135621)	Pseudomonadaceae (7227	Pseudomonadales (1236)	Gammaproteobacteria (122	Proteobacteria (2)	Bacteria (1)	100.00	0.00	96.40	94.50	WGS
316.317	0.4118	Pseudomonas stutzeri (286)	Pseudomonas (135621)	Pseudomonadaceae (7227	Pseudomonadales (1236)	Gammaproteobacteria (122	Proteobacteria (2)	Bacteria (1)	91.90	1.00	90.60	89.10	WGS
316.318	0.4044	Pseudomonas stutzeri (286)	Pseudomonas (135621)	Pseudomonadaceae (7227	Pseudomonadales (1236)	Gammaproteobacteria (122	Proteobacteria (2)	Bacteria (1)	100.00	0.00	97.00	94.80	WGS
316.319	0.4016	Pseudomonas stutzeri (286)	Pseudomonas (135621)	Pseudomonadaceae (7227	Pseudomonadales (1236)	Gammaproteobacteria (122	Proteobacteria (2)	Bacteria (1)	99.10	2.60	97.00	92.20	WGS
316.320	0.3348	Pseudomonas stutzeri (286)	Pseudomonas (135621)	Pseudomonadaceae (7227	Pseudomonadales (1236)	Gammaproteobacteria (122	Proteobacteria (2)	Bacteria (1)	81.10	2.20	89.40	86.00	WGS
316.321	0.4424	Pseudomonas stutzeri (286)	Pseudomonas (135621)	Pseudomonadaceae (7227	Pseudomonadales (1236)	Gammaproteobacteria (122	Proteobacteria (2)	Bacteria (1)	100.00	0.00	95.60	93.30	WGS
316.322	0.4396	Pseudomonas stutzeri (286)	Pseudomonas (135621)	Pseudomonadaceae (7227	Pseudomonadales (1236)	Gammaproteobacteria (122	Proteobacteria (2)	Bacteria (1)	100.00	0.00	96.80	94.30	WGS
316.323	0.3574	Pseudomonas stutzeri (286)	Pseudomonas (135621)	Pseudomonadaceae (7227	Pseudomonadales (1236)	Gammaproteobacteria (122	Proteobacteria (2)	Bacteria (1)	98.20	0.00	94.90	92.30	WGS
316.324	0.4854	Pseudomonas stutzeri (286)	Pseudomonas (135621)	Pseudomonadaceae (7227	Pseudomonadales (1236)	Gammaproteobacteria (122	Proteobacteria (2)	Bacteria (1)	100.00	0.00	97.00	94.90	WGS
316.325	0.4582	Pseudomonas stutzeri (286)	Pseudomonas (135621)	Pseudomonadaceae (7227	Pseudomonadales (1236)	Gammaproteobacteria (122	Proteobacteria (2)	Bacteria (1)	100.00	0.00	98.20	96.20	WGS
316.326	0.4356	Pseudomonas stutzeri (286)	Pseudomonas (135621)	Pseudomonadaceae (7227	Pseudomonadales (1236)	Gammaproteobacteria (122	Proteobacteria (2)	Bacteria (1)	99.10	1.80	97.60	94.90	WGS
316.327	0.3388	Pseudomonas stutzeri (286)	Pseudomonas (135621)	Pseudomonadaceae (7227	Pseudomonadales (1236)	Gammaproteobacteria (122	Proteobacteria (2)	Bacteria (1)	99.10	0.00	96.70	94.10	WGS
316.328	0.2606	Pseudomonas stutzeri (286)	Pseudomonas (135621)	Pseudomonadaceae (7227	Pseudomonadales (1236)	Gammaproteobacteria (122	Proteobacteria (2)	Bacteria (1)	99.10	4.40	93.60	87.70	WGS
316.329	0.2968	Pseudomonas stutzeri (286)	Pseudomonas (135621)	Pseudomonadaceae (7227	Pseudomonadales (1236)	Gammaproteobacteria (122	Proteobacteria (2)	Bacteria (1)	72.10	0.00	87.00	86.00	WGS
316.330	0.3918	Pseudomonas stutzeri (286)	Pseudomonas (135621)	Pseudomonadaceae (7227	Pseudomonadales (1236)	Gammaproteobacteria (122	Proteobacteria (2)	Bacteria (1)	99.10	0.00	96.20	93.90	WGS
316.331	0.395	Pseudomonas stutzeri (286)	Pseudomonas (135621)	Pseudomonadaceae (7227	Pseudomonadales (1236)	Gammaproteobacteria (122	Proteobacteria (2)	Bacteria (1)	93.70	1.90	91.20	86.20	WGS
316.332	0.2306	Pseudomonas stutzeri (286)	Pseudomonas (135621)	Pseudomonadaceae (7227	Pseudomonadales (1236)	Gammaproteobacteria (122	Proteobacteria (2)	Bacteria (1)	76.70	11.50	86.80	80.60	WGS
316.333	0.3698	Pseudomonas stutzeri (286)	Pseudomonas (135621)	Pseudomonadaceae (7227	Pseudomonadales (1236)	Gammaproteobacteria (122	Proteobacteria (2)	Bacteria (1)	100.00	0.00	96.90	94.60	WGS
316.334	0.4342	Pseudomonas stutzeri (286)	Pseudomonas (135621)	Pseudomonadaceae (7227	Pseudomonadales (1236)	Gammaproteobacteria (122	Proteobacteria (2)	Bacteria (1)	100.00	0.00	97.30	95.00	WGS
316.335	0.3602	Pseudomonas stutzeri (286)	Pseudomonas (135621)	Pseudomonadaceae (7227	Pseudomonadales (1236)	Gammaproteobacteria (122	Proteobacteria (2)	Bacteria (1)	98.20	1.80	96.40	93.30	WGS
316.336	0.45	Pseudomonas stutzeri (286)	Pseudomonas (135621)	Pseudomonadaceae (7227	Pseudomonadales (1236)	Gammaproteobacteria (122	Proteobacteria (2)	Bacteria (1)	98.20	0.00	93.10	91.10	WGS
316.337	0.3542	Pseudomonas stutzeri (286)	Pseudomonas (135621)	Pseudomonadaceae (7227	Pseudomonadales (1236)	Gammaproteobacteria (122	Proteobacteria (2)	Bacteria (1)	100.00	0.00	94.00	91.80	WGS
316.338	0.3754	Pseudomonas stutzeri (286)	Pseudomonas (135621)	Pseudomonadaceae (7227	Pseudomonadales (1236)	Gammaproteobacteria (122	Proteobacteria (2)	Bacteria (1)	94.60	7.10	91.70	86.40	WGS
316.339	0.369	Pseudomonas stutzeri (286)	Pseudomonas (135621)	Pseudomonadaceae (7227	Pseudomonadales (1236)	Gammaproteobacteria (122	Proteobacteria (2)	Bacteria (1)	100.00	0.00	97.30	94.80	WGS
316.340	0.375	Pseudomonas stutzeri (286)	Pseudomonas (135621)	Pseudomonadaceae (7227	Pseudomonadales (1236)	Gammaproteobacteria (122	Proteobacteria (2)	Bacteria (1)	98.20	0.90	95.20	91.90	WGS
316.341	0.3616	Pseudomonas stutzeri (286)	Pseudomonas (135621)	Pseudomonadaceae (7227	Pseudomonadales (1236)	Gammaproteobacteria (122	Proteobacteria (2)	Bacteria (1)	92.80	1.00	94.10	92.10	WGS
[truncated: 9,514,502 more chars]
