# Supplementary material for: Structural color in the bacterial domain: The ecogenomics of a 2-dimensional optical phenotype
Source: Proc Natl Acad Sci U S A. 2024 Jul 11;121(29):e2309757121. doi: 10.1073/pnas.2309757121 (PMC11260094; doi:10.1073/pnas.2309757121)
Supplement: Supplementary file 9 — Dataset S03 (PDF) [file pnas.2309757121.sd03.pdf]

Species	Average score of bottom 5 strains	Average score of top 5 strains
Acaryochloris marina	0.03028	0.10
Acetobacter aceti	0.165	0.29
Acetobacter pasteurianus	0.02916	0.29
Acetobacteraceae bacterium	0.16324	0.48
Acholeplasma laidlawii	0.034	0.04
Acholeplasmataceae bacterium	0.04572	0.11
Achromobacter denitrificans	0.17824	0.36
Achromobacter sp	0.22444	0.46
Achromobacter xylosoxidans	0.02964	0.43
Acidiferrobacteraceae bacterium	0.09448	0.29
Acidimicrobiaceae bacterium	0.0608	0.33
Acidimicrobiia bacterium	0.09684	0.33
Acidiphilium multivorum	0.0294	0.15
Acidipropionibacterium acidipropionici	0.07016	0.14
Acidithiobacillus caldus	0.0294	0.23
Acidithiobacillus thiooxidans	0.25408	0.29
Acidobacteria bacterium	0.03528	0.46
Acidobacteriaceae bacterium	0.1014	0.43
Acidobacterium sp	0.32436	0.39
Acidovorax avenae	0.26312	0.46
Acidovorax citrulli	0.421	0.44
Acinetobacter baumannii	0.02856	0.43
Acinetobacter calcoaceticus	0.0298	0.39
Acinetobacter haemolyticus	0.09668	0.23
Acinetobacter indicus	0.17568	0.20
Acinetobacter johnsonii	0.02944	0.23
Acinetobacter junii	0.2064	0.25
Acinetobacter lwoffii	0.0294	0.21
Acinetobacter nosocomialis	0.02928	0.38
Acinetobacter pittii	0.0294	0.42
Acinetobacter radioresistens	0.05108	0.20
Acinetobacter schindleri	0.0294	0.20
Acinetobacter soli	0.02964	0.32
Acinetobacter sp	0.0294	0.41
Acinetobacter ursingii	0.20036	0.31
Acinetobacter venetianus	0.10116	0.25
Actinobacillus pleuropneumoniae	0.02868	0.19
Actinobacteria bacterium	0.03496	0.39
Actinomyces naeslundii	0.15568	0.18
Actinomyces oris	0.13548	0.17
Aequorivita sp	0.04176	0.10
Aerococcus urinae	0.0676	0.11
Aeromonas caviae	0.10136	0.20
Aeromonas dhakensis	0.2188	0.33
Aeromonas hydrophila	0.02912	0.37
Aeromonas salmonicida	0.02852	0.22
Aeromonas veronii	0.19324	0.31
Aggregatibacter actinomycetemcomitans	0.04708	0.21
Aggregatibacter aphrophilus	0.18404	0.20
Agrobacterium rhizogenes	0.13936	0.33
Agrobacterium tumefaciens	0.03064	0.40
Agrobacterium vitis	0.03692	0.33
Akkermansia muciniphila	0.06356	0.09
Alcaligenes faecalis	0.21568	0.41
Alcanivorax sp	0.0432	0.58
Algoriphagus sp	0.30188	0.42
Aliivibrio finisterrensis	0.2058	0.21
Aliivibrio fischeri	0.02948	0.23
Alistipes sp	0.02884	0.08
Alphaproteobacteria bacterium	0.02908	0.51
Alteromonadaceae bacterium	0.18132	0.60
Alteromonas australica	0.30524	0.48
Alteromonas macleodii	0.16864	0.53
Alteromonas mediterranea	0.03596	0.55
Alteromonas sp	0.1956	0.55
Anabaena cylindrica	0.0294	0.08
Anaerobutyricum hallii	0.19744	0.23
Anaerolineaceae bacterium	0.08064	0.34
Anaerolineae bacterium	0.0766	0.37
Anaerolineales bacterium	0.1126	0.37
Anaerostipes hadrus	0.15904	0.17
Anaplasma marginale	0.0372	0.04
Anaplasma phagocytophilum	0.035	0.04
Anoxybacillus flavithermus	0.09216	0.23
Arcobacter butzleri	0.0294	0.18
Arcobacter cryaerophilus	0.10624	0.15
Arcobacter skirrowii	0.10556	0.12
Arcobacter thereius	0.10592	0.11
Armatimonadetes bacterium	0.0494	0.27
Aureimonas sp AU20	0.02988	0.21
Avibacterium paragallinarum	0.1004	0.18
Azospirillum brasilense	0.034	0.45
Azospirillum lipoferum	0.03832	0.30
Azotobacter chroococcum	0.0294	0.35
Bacillus altitudinis	0.2036	0.26
Bacillus amyloliquefaciens	0.0294	0.39
Bacillus anthracis	0.0294	0.35
Bacillus aryabhattai	0.09036	0.42
Bacillus atrophaeus	0.25052	0.27
Bacillus cereus	0.02912	0.47
Bacillus clausii	0.22012	0.42
Bacillus coagulans	0.14636	0.25
Bacillus cytotoxicus	0.18896	0.26
Bacillus endophyticus	0.0294	0.40
Bacillus flexus	0.10668	0.24
Bacillus halotolerans	0.34712	0.36
Bacillus licheniformis	0.0294	0.31
Bacillus megaterium	0.02896	0.48
Bacillus mycoides	0.02936	0.41
Bacillus paralicheniformis	0.19532	0.26
Bacillus pseudomycoides	0.08008	0.43
Bacillus pumilus	0.0294	0.26
Bacillus safensis	0.0294	0.26
Bacillus simplex	0.336	0.48
Bacillus stratosphericus	0.19856	0.25
Bacillus subtilis	0.0294	0.42
Bacillus thuringiensis	0.02928	0.47
Bacillus toyonensis	0.0294	0.47
Bacillus velezensis	0.0294	0.40
Bacillus wiedmannii	0.22184	0.38
Bacteriodetes bacterium	0.01836	0.29
bacterium	0.02724	0.50
Bacteroidales bacterium	0.02008	0.34
Bacteroides caccae	0.05164	0.07
Bacteroides cellulosilyticus	0.06296	0.10
Bacteroides dorei	0.06468	0.09
Bacteroides eggerthii	0.05888	0.07
Bacteroides fragilis	0.0294	0.14
Bacteroides intestinalis	0.08024	0.11
Bacteroides ovatus	0.05516	0.11
Bacteroides plebeius	0.04196	0.07
Bacteroides sp	0.03416	0.14
Bacteroides stercoris	0.04976	0.07
Bacteroides thetaiotaomicron	0.0478	0.08
Bacteroides uniformis	0.06396	0.11
Bacteroides vulgatus	0.05332	0.09
Bacteroides xylanisolvens	0.05044	0.09
Bacteroidetes bacterium	0.01124	0.48
Bacteroidia bacterium	0.03096	0.23
Balneola sp	0.0928	0.14
Balneolaceae bacterium	0.06972	0.19
Balneolia bacterium	0.03564	0.21
Bartonella bacilliformis	0.035	0.04
Bartonella henselae	0.0478	0.05
Bartonella quintana	0.0356	0.04
Bdellovibrionales bacterium	0.036	0.24
Betaproteobacteria bacterium	0.07072	0.48
Bifidobacterium adolescentis	0.06768	0.08
Bifidobacterium animalis	0.03744	0.09
Bifidobacterium asteroides	0.05136	0.07
Bifidobacterium bifidum	0.05308	0.07
Bifidobacterium breve	0.03764	0.07
Bifidobacterium catenulatum	0.04524	0.07
Bifidobacterium dentium	0.06948	0.08
Bifidobacterium longum	0.0294	0.07
Bifidobacterium moukalabense	0.06632	0.07
Bifidobacterium pseudocatenulatum	0.0564	0.08
Bifidobacterium pseudolongum	0.05468	0.07
Blastocatellia bacterium	0.18148	0.36
Blattabacterium punctulatus	0.0326	0.04
Blautia obeum	0.19116	0.23
Bordetella avium	0.20912	0.24
Bordetella bronchiseptica	0.20144	0.29
Bordetella hinzii	0.29516	0.34
Bordetella holmesii	0.16092	0.17
Bordetella parapertussis	0.19084	0.24
Bordetella pertussis	0.04952	0.28
Bordetella trematum	0.19428	0.24
Borrelia crocidurae	0.0294	0.04
Borrelia duttonii	0.0294	0.04
Borrelia hermsii	0.0294	0.05
Borrelia mayonii	0.0294	0.03
Borrelia miyamotoi	0.0294	0.05
Borrelia turicatae	0.0294	0.04
Borreliella afzelii	0.0294	0.04
Borreliella bissettii	0.0294	0.03
Borreliella burgdorferi	0.0294	0.04
Borreliella finlandensis	0.0294	0.03
Borreliella garinii	0.029	0.04
Borreliella valaisiana	0.0294	0.03
Brachyspira hyodysenteriae	0.12052	0.19
Bradyrhizobium canariense	0.34352	0.47
Bradyrhizobium diazoefficiens	0.03164	0.42
Bradyrhizobium elkanii	0.42696	0.47
Bradyrhizobium japonicum	0.42072	0.47
Brevibacillus brevis	0.22132	0.24
Brevibacillus laterosporus	0.14088	0.35
Brevibacterium aurantiacum	0.3364	0.36
Brevibacterium linens	0.24252	0.36
Brevundimonas diminuta	0.04684	0.09
Brevundimonas naejangsanensis	0.05292	0.07
Brevundimonas sp	0.04472	0.22
Brochothrix thermosphacta	0.0842	0.10
Brucella abortus	0.21032	0.28
Brucella canis	0.25468	0.26
Brucella melitensis	0.24196	0.27
Brucella ovis	0.2132	0.21
Brucella suis	0.23508	0.27
Bryobacterales bacterium	0.2206	0.44
Buchnera aphidicola	0.0294	0.06
Burkholderia ambifaria	0.177	0.42
Burkholderia anthina	0.34448	0.38
Burkholderia cenocepacia	0.0292	0.44
Burkholderia cepacia	0.03004	0.44
Burkholderia contaminans	0.41264	0.45
Burkholderia diffusa	0.37976	0.39
Burkholderia gladioli	0.0294	0.49
Burkholderia glumae	0.031	0.46
Burkholderia mallei	0.36448	0.39
Burkholderia multivorans	0.03044	0.43
Burkholderia pseudomallei	0.02936	0.41
Burkholderia pseudomultivorans	0.34776	0.45
Burkholderia pyrrocinia	0.32048	0.43
Burkholderia sp	0.02936	0.55
Burkholderia stagnalis	0.41036	0.44
Burkholderia territorii	0.37844	0.45
Burkholderia thailandensis	0.22564	0.40
Burkholderia ubonensis	0.14844	0.41
Burkholderia vietnamiensis	0.03132	0.38
Burkholderiales bacterium	0.03444	0.51
Butyrivibrio fibrisolvens	0.05592	0.10
Caldicellulosiruptor bescii	0.10368	0.15
Caldiserica bacterium	0.06732	0.18
Calditrichaeota bacterium	0.12504	0.29
Campylobacter coli	0.0294	0.12
Campylobacter concisus	0.037	0.15
Campylobacter fetus	0.0294	0.13
Campylobacter helveticus	0.0294	0.11
Campylobacter hepaticus	0.123	0.12
Campylobacter hyointestinalis	0.11232	0.14
Campylobacter jejuni	0.0294	0.23
Campylobacter lanienae	0.1168	0.12
Campylobacter lari	0.0522	0.10
Campylobacter showae	0.14124	0.15
candidate division KSB1 bacterium	0.0592	0.21
candidate division WWE3 bacterium	0.0288	0.05
candidate division Zixibacteria bacterium	0.0372	0.21
Candidatus Accumulibacter phosphatis	0.10192	0.29
Candidatus Acetothermia bacterium	0.07208	0.22
Candidatus Aerophobetes bacterium	0.05164	0.12
Candidatus Aminicenantes bacterium	0.06912	0.24
Candidatus Atribacteria bacterium	0.06164	0.22
Candidatus Azambacteria bacterium	0.02864	0.10
Candidatus Azobacteroides pseudotrichonymp	0.0294	0.04
Candidatus Carsonella ruddii	0.04764	0.05
Candidatus Cloacimonetes bacterium	0.0492	0.16
Candidatus Collierbacteria bacterium	0.0294	0.07
Candidatus Dadabacteria bacterium	0.05208	0.21
Candidatus Dependentiae bacterium	0.03252	0.05
Candidatus Dormibacteraeota bacterium	0.05596	0.37
Candidatus Eremiobacteraeota bacterium	0.16628	0.36
Candidatus Erwinia haradaeae	0.0366	0.04
Candidatus Falkowbacteria bacterium	0.02568	0.06
Candidatus Hamiltonella defensa	0.03052	0.03
Candidatus Hodgkinia cicadicola	0.0294	0.04
Candidatus Hydrogenedentes bacterium	0.11656	0.25
Candidatus Kaiserbacteria bacterium	0.0274	0.06
Candidatus Kryptonium thompsoni	0.1234	0.17
Candidatus Latescibacteria bacterium	0.05284	0.36
Candidatus Liberibacter asiaticus	0.03964	0.04
Candidatus Magasanikbacteria bacterium	0.02932	0.07
Candidatus Marinimicrobia bacterium	0.03112	0.34
Candidatus Melainabacteria bacterium	0.04072	0.31
Candidatus Moranbacteria bacterium	0.0296	0.06
Candidatus Nealsonbacteria bacterium	0.02888	0.06
Candidatus Nomurabacteria bacterium	0.0286	0.05
Candidatus Omnitrophica bacterium	0.03548	0.21
Candidatus Pacebacteria bacterium	0.02988	0.05
Candidatus Parcubacteria bacterium	0.02964	0.06
Candidatus Pelagibacter sp	0.02896	0.24
Candidatus Pelagibacter ubique	0.03656	0.23
Candidatus Peregrinibacteria bacterium	0.0314	0.06
Candidatus Poribacteria bacterium	0.0746	0.45
Candidatus Portiera aleyrodidarum	0.04996	0.05
Candidatus Rokubacteria bacterium	0.14296	0.37
Candidatus Saccharibacteria bacterium	0.02832	0.07
Candidatus Sulcia muelleri	0.0324	0.03
Candidatus Thioglobus sp	0.10244	0.23
Candidatus Uhrbacteria bacterium	0.029	0.06
Candidatus Wolfebacteria bacterium	0.02852	0.04
Candidatus Zambryskibacteria bacterium	0.02876	0.04
Capnocytophaga canimorsus	0.0538	0.10
Carnobacterium divergens	0.05548	0.06
Carnobacterium maltaromaticum	0.07564	0.09
Caulobacter vibrioides	0.3148	0.34
Chitinophagaceae bacterium	0.0462	0.47
Chlamydia abortus	0.02992	0.10
Chlamydia muridarum	0.03108	0.03
Chlamydia pecorum	0.03132	0.03
Chlamydia pneumoniae	0.04116	0.05
Chlamydia psittaci	0.0294	0.03
Chlamydia suis	0.02952	0.03
Chlamydia trachomatis	0.02808	0.14
Chloroflexi bacterium	0.03148	0.43
Chromobacterium violaceum	0.25776	0.32
Chryseobacterium indologenes	0.2536	0.41
Chryseobacterium sp	0.0272	0.39
Citrobacter amalonaticus	0.12908	0.27
Citrobacter braakii	0.14352	0.21
Citrobacter freundii	0.02916	0.24
Citrobacter koseri	0.0294	0.21
Citromicrobium sp	0.21348	0.37
Clavibacter michiganensis	0.02936	0.23
Clostridia bacterium	0.04188	0.24
Clostridiaceae bacterium	0.05068	0.23
Clostridiales bacterium	0.031	0.27
Clostridioides difficile	0.0294	0.26
Clostridium acetobutylicum	0.031	0.12
Clostridium baratii	0.09552	0.15
Clostridium beijerinckii	0.24016	0.29
Clostridium botulinum	0.0294	0.28
Clostridium butyricum	0.0294	0.30
Clostridium novyi	0.0294	0.14
Clostridium paraputrificum	0.21068	0.25
Clostridium pasteurianum	0.17232	0.25
Clostridium perfringens	0.02924	0.27
Clostridium sp	0.02948	0.30
Clostridium sporogenes	0.08044	0.25
Clostridium tetani	0.10124	0.16
Clostridium tyrobutyricum	0.14056	0.26
Collinsella aerofaciens	0.18344	0.21
Comamonas testosteroni	0.03132	0.50
Coprococcus comes	0.16808	0.19
Coriobacteriaceae bacterium	0.07976	0.18
Corynebacterium aurimucosum	0.1176	0.20
Corynebacterium belfantii	0.12804	0.14
Corynebacterium bovis	0.13152	0.15
Corynebacterium diphtheriae	0.0418	0.15
Corynebacterium glutamicum	0.02892	0.16
Corynebacterium jeikeium	0.0294	0.16
Corynebacterium pseudotuberculosis	0.08564	0.14
Corynebacterium striatum	0.07148	0.18
Corynebacterium ulcerans	0.06412	0.09
Coxiella burnetii	0.0292	0.09
Crocinitomicaceae bacterium	0.02912	0.21
Cronobacter dublinensis	0.17196	0.24
Cronobacter malonaticus	0.061	0.25
Cronobacter sakazakii	0.02812	0.26
Cronobacter turicensis	0.09272	0.24
Cryomorphaceae bacterium	0.01176	0.16
Cupriavidus metallidurans	0.04964	0.48
Cupriavidus necator	0.21932	0.50
Cupriavidus taiwanensis	0.17816	0.49
Cutibacterium acnes	0.0294	0.13
Cutibacterium avidum	0.06844	0.10
Cylindrospermopsis raciborskii	0.13768	0.15
Cytophagales bacterium	0.05816	0.58
Dehalococcoides mccartyi	0.03556	0.06
Dehalococcoidia bacterium	0.03752	0.37
Delftia acidovorans	0.0744	0.47
Deltaproteobacteria bacterium	0.03028	0.47
Desulfobacteraceae bacterium	0.06232	0.32
Desulfobacterales bacterium	0.04888	0.23
Desulfobulbaceae bacterium	0.07148	0.24
Desulfobulbus propionicus	0.07932	0.15
Desulfonatronospira sp	0.09276	0.14
Desulfovibrio sp	0.08792	0.24
Desulfuromonas sp	0.11652	0.18
Dickeya dianthicola	0.2388	0.27
Dickeya solani	0.15804	0.27
Dickeya zeae	0.25592	0.27
Dolosigranulum pigrum	0.07176	0.08
Dorea formicigenerans	0.2162	0.24
Dorea longicatena	0.22548	0.25
Edwardsiella ictaluri	0.0294	0.25
Edwardsiella piscicida	0.14424	0.24
Edwardsiella tarda	0.05932	0.27
Eggerthella lenta	0.09956	0.19
Ehrlichia ruminantium	0.03824	0.04
Eikenella corrodens	0.15508	0.20
Elizabethkingia anophelis	0.23392	0.32
Elizabethkingia meningoseptica	0.13744	0.31
Elizabethkingia miricola	0.26336	0.35
Elusimicrobia bacterium	0.03676	0.22
Emticicia oligotrophica	0.03372	0.26
Ensifer adhaerens	0.14504	0.36
Enterobacter asburiae	0.02908	0.22
Enterobacter bugandensis	0.05892	0.19
Enterobacter cancerogenus	0.1384	0.21
Enterobacter cloacae	0.02928	0.37
Enterobacter hormaechei	0.0294	0.19
Enterobacter kobei	0.13084	0.20
Enterobacter ludwigii	0.154	0.23
Enterobacter roggenkampii	0.14424	0.19
Enterococcus avium	0.0766	0.10
Enterococcus casseliflavus	0.06064	0.11
Enterococcus cecorum	0.07764	0.10
Enterococcus durans	0.0294	0.08
Enterococcus faecalis	0.02916	0.28
Enterococcus faecium	0.02844	0.25
Enterococcus gallinarum	0.06884	0.13
Enterococcus hirae	0.05004	0.10
Enterococcus mundtii	0.0294	0.10
Enterococcus sp	0.02984	0.21
Enterovibrio norvegicus	0.39908	0.43
Epibacterium mobile	0.13256	0.39

Epsilonproteobacteria bacterium	0.03972	0.16
Erwinia amylovora	0.0294	0.14
Erwinia persicina	0.23216	0.26
Erwinia pyrifoliae	0.0294	0.10
Erysipelatoclostridium ramosum	0.08064	0.08
Erysipelothrix rhusiopathiae	0.05148	0.06
Erysipelotrichaceae bacterium	0.03988	0.16
Erythrobacter sp	0.14272	0.37
Escherichia albertii	0.0294	0.27
Escherichia coli	0.02656	0.39
Escherichia fergusonii	0.02928	0.26
Escherichia marmotae	0.26032	0.26
Eubacterium sp	0.05252	0.24
Eubacterium ventriosum	0.06232	0.08
Faecalibacterium prausnitzii	0.09988	0.20
Finegoldia magna	0.15004	0.19
Firmicutes bacterium	0.02964	0.29
Fischerella thermalis	0.20924	0.26
Flammeovirgaceae bacterium	0.05772	0.56
Flavobacteriaceae bacterium	0.01492	0.86
Flavobacteriales bacterium	0.01396	0.45
Flavobacterium columnare	0.21028	0.26
Flavobacterium johnsoniae	0.47928	0.87
Flavobacterium psychrophilum	0.02424	0.09
Flavobacterium sp	0.02468	0.84
Flavonifractor plautii	0.12792	0.16
Francisella philomiragia	0.0294	0.08
Francisella tularensis	0.02916	0.08
Frankia symbiont of Datisca glomerata	0.15288	0.26
Fusobacterium hwasookii	0.03608	0.07
Fusobacterium necrophorum	0.05136	0.06
Fusobacterium nucleatum	0.0294	0.10
Fusobacterium periodonticum	0.0422	0.06
Gallibacterium anatis	0.13676	0.19
gamma proteobacterium symbiont of Ctena or	0.27824	0.32
Gammaproteobacteria bacterium	0.02976	0.56
Gardnerella vaginalis	0.04288	0.08
Gemmatimonadetes bacterium	0.02992	0.38
Geobacillus stearothermophilus	0.0668	0.32
Geobacillus thermoleovorans	0.19984	0.33
Geobacter sp	0.09132	0.23
Gilliamella apicola	0.08644	0.16
Glaesserella parasuis	0.02892	0.19
Gluconobacter oxydans	0.0294	0.37
Granulibacter bethesdensis	0.33688	0.36
Granulicella sp	0.09688	0.36
Haemophilus haemolyticus	0.14972	0.20
Haemophilus influenzae	0.02916	0.19
Haemophilus parainfluenzae	0.1244	0.19
Hafnia alvei	0.1132	0.29
Hafnia paralvei	0.29624	0.31
Halanaerobiaceae bacterium	0.10188	0.20
Halanaerobium congolense	0.11916	0.14
Haliea sp	0.34944	0.42
Halieaceae bacterium	0.13312	0.45
Halomonas sp	0.04884	0.54
Helicobacter bilis	0.06296	0.07
Helicobacter bizzozeronii	0.09196	0.11
Helicobacter cinaedi	0.10068	0.12
Helicobacter felis	0.1042	0.11
Helicobacter pullorum	0.05272	0.10
Helicobacter pylori	0.0294	0.11
Helicobacter suis	0.1066	0.11
Histophilus somni	0.06116	0.17
Hungateiclostridium thermocellum	0.05824	0.11
Hungatella hathewayi	0.19132	0.25
Hyphomonadaceae bacterium	0.09272	0.34
Hyphomonas sp	0.11196	0.42
Idiomarina abyssalis	0.12644	0.16
Idiomarina sp	0.0498	0.35
Ignavibacteria bacterium	0.03028	0.29
Ignavibacteriae bacterium	0.05368	0.30
Ignavibacteriales bacterium	0.0738	0.31
Janthinobacterium lividum	0.4054	0.46
Kandleria vitulina	0.05916	0.07
Ketogulonicigenium vulgare	0.04268	0.27
Kingella kingae	0.0838	0.11
Klebsiella aerogenes	0.0294	0.35
Klebsiella michiganensis	0.0294	0.34
Klebsiella oxytoca	0.02928	0.37
Klebsiella pneumoniae	0.0282	0.42
Klebsiella quasipneumoniae	0.02956	0.24
Klebsiella variicola	0.02956	0.25
Kocuria rhizophila	0.11084	0.22
Kocuria rosea	0.17908	0.25
Komagataeibacter europaeus	0.0294	0.32
Komagataeibacter xylinus	0.04056	0.33
Kozakia baliensis	0.02972	0.15
Lachnospiraceae bacterium	0.04936	0.25
Lactobacillus acetotolerans	0.046	0.06
Lactobacillus acidophilus	0.0294	0.08
Lactobacillus agilis	0.0716	0.07
Lactobacillus amylovorus	0.02948	0.08
Lactobacillus aviarius	0.04932	0.05
Lactobacillus backii	0.0294	0.12
Lactobacillus brevis	0.0294	0.09
Lactobacillus buchneri	0.0296	0.08
Lactobacillus casei	0.0294	0.09
Lactobacillus coryniformis	0.07252	0.25
Lactobacillus crispatus	0.0562	0.08
Lactobacillus curvatus	0.04032	0.07
Lactobacillus delbrueckii	0.0294	0.09
Lactobacillus farciminis	0.03892	0.10
Lactobacillus fermentum	0.0294	0.18
Lactobacillus gallinarum	0.0294	0.08
Lactobacillus gasseri	0.04628	0.08
Lactobacillus helveticus	0.0294	0.08
Lactobacillus iners	0.0494	0.06
Lactobacillus jensenii	0.05296	0.06
Lactobacillus johnsonii	0.0294	0.08
Lactobacillus kefiranofaciens	0.05684	0.08
Lactobacillus kunkeei	0.05876	0.08
Lactobacillus lindneri	0.03536	0.06
Lactobacillus mucosae	0.06676	0.08
Lactobacillus murinus	0.06248	0.08
Lactobacillus parabuchneri	0.06432	0.11
Lactobacillus paracasei	0.02916	0.08
Lactobacillus paracollinoides	0.02932	0.11
Lactobacillus paraplantarum	0.107	0.15
Lactobacillus pentosus	0.09872	0.16
Lactobacillus plantarum	0.02936	0.16
Lactobacillus reuteri	0.0294	0.16
Lactobacillus rhamnosus	0.03004	0.09
Lactobacillus ruminis	0.0674	0.08
Lactobacillus sakei	0.02904	0.06
Lactobacillus salivarius	0.0286	0.08
Lactobacillus sanfranciscensis	0.04648	0.06
Lactobacillus sp	0.0408	0.22
Lactobacillus taiwanensis	0.071	0.08
Lactococcus garvieae	0.0294	0.07
Lactococcus lactis	0.0292	0.09
Lawsonia intracellularis	0.0294	0.04
Leclercia adecarboxylata	0.17088	0.37
Leeuwenhoekiella sp	0.32688	0.48
Legionella pneumophila	0.02916	0.13
Legionella taurinensis	0.1042	0.10
Legionellales bacterium	0.03272	0.15
Lentisphaeria bacterium	0.04548	0.15
Leptolyngbya boryana	0.0294	0.25
Leptospira borgpetersenii	0.0766	0.12
Leptospira interrogans	0.0292	0.19
Leptospira kirschneri	0.11016	0.13
Leptospira levettii	0.24704	0.25
Leptospira noguchii	0.11976	0.13
Leptospira santarosai	0.09968	0.13
Leptospira weilii	0.09812	0.12
Leptospirillum ferriphilum	0.0674	0.13
Leuconostoc citreum	0.02936	0.09
Leuconostoc gelidum	0.05112	0.09
Leuconostoc lactis	0.04536	0.08
Leuconostoc mesenteroides	0.02916	0.08
Leuconostoc pseudomesenteroides	0.08912	0.09
Listeria innocua	0.11964	0.19
Listeria ivanovii	0.20312	0.23
Listeria monocytogenes	0.0294	0.21
Lonsdalea populi	0.1432	0.23
Lysinibacillus fusiformis	0.2902	0.34
Lysinibacillus sphaericus	0.03064	0.33
Macrococcus caseolyticus	0.0294	0.20
Mannheimia haemolytica	0.05292	0.20
Marinilabiliales bacterium	0.1352	0.27
Marinobacter adhaerens	0.33496	0.78
Marinobacter hydrocarbonoclasticus	0.35176	0.78
Marinobacter sp	0.13736	0.88
Marinovum algicola	0.0292	0.16
Marinovum sp	0.15796	0.34
Megasphaera elsdenii	0.16072	0.19
Melissococcus plutonius	0.03308	0.06
Mesoplasma florum	0.02876	0.03
Mesorhizobium ciceri	0.14124	0.37
Mesorhizobium loti	0.04376	0.39
Mesorhizobium sp	0.0332	0.46
Methylobacterium aquaticum	0.05388	0.29
Methylobacterium radiotolerans	0.0294	0.54
Methylococcaceae bacterium	0.1186	0.32
Methylocystaceae bacterium	0.04696	0.35
Methylophaga sp	0.17208	0.35
Methylophilaceae bacterium	0.07368	0.27
Methylorubrum extorquens	0.0294	0.32
Methylorubrum populi	0.0294	0.23
Methylotenera sp	0.08896	0.33
Microbacterium sp	0.03004	0.30
Microchaete diplosiphon	0.0294	0.09
Micrococcales bacterium	0.04924	0.22
Micrococcus luteus	0.05584	0.21
Microcystis aeruginosa	0.06412	0.14
Mollicutes bacterium	0.0346	0.15
Moorella thermoacetica	0.18908	0.19
Moraxella catarrhalis	0.05356	0.18
Moraxella osloensis	0.02908	0.27
Morganella morganii	0.0294	0.25
Moritella viscosa	0.40424	0.42
Muribaculaceae bacterium	0.02892	0.10
Mycobacterium asiaticum	0.2406	0.28
Mycobacterium avium	0.05316	0.20
Mycobacterium canettii	0.28328	0.28
Mycobacterium chimaera	0.0294	0.19
Mycobacterium colombiense	0.16352	0.20
Mycobacterium intracellulare	0.08884	0.19
Mycobacterium kansasii	0.20776	0.28
Mycobacterium leprae	0.08628	0.11
Mycobacterium marinum	0.11704	0.27
Mycobacterium sp	0.0294	0.33
Mycobacterium tuberculosis	0.02996	0.53
Mycobacteroides abscessus	0.0294	0.36
Mycobacteroides chelonae	0.23828	0.31
Mycobacteroides franklinii	0.2836	0.29
Mycobacteroides immunogenum	0.2874	0.30
Mycobacteroides salmoniphilum	0.287	0.30
Mycolicibacterium conceptionense	0.20624	0.23
Mycolicibacterium fortuitum	0.22388	0.25
Mycolicibacterium smegmatis	0.2364	0.24
Mycoplasma bovis	0.04096	0.04
Mycoplasma capricolum	0.03236	0.04
Mycoplasma gallisepticum	0.0294	0.05
Mycoplasma hominis	0.0298	0.03
Mycoplasma hyopneumoniae	0.0404	0.04
Mycoplasma mycoides	0.02928	0.04
Mycoplasma ovipneumoniae	0.03616	0.04
Mycoplasma pneumoniae	0.04784	0.05
Myroides odoratimimus	0.08392	0.11
Myxococcales bacterium	0.0742	0.42
Neisseria elongata	0.12088	0.19
Neisseria gonorrhoeae	0.0294	0.15
Neisseria lactamica	0.0294	0.14
Neisseria meningitidis	0.0294	0.20
Neisseria sicca	0.12856	0.19
Neisseria subflava	0.104	0.13
Neorhizobium galegae	0.18568	0.38
Nitriliruptoraceae bacterium	0.14188	0.28
Nitrosospira multiformis	0.074	0.16
Nitrospina sp	0.09304	0.15
Nitrospira sp	0.0906	0.27
Nitrospiraceae bacterium	0.0722	0.25
Nitrospirae bacterium	0.02976	0.25
Nocardia farcinica	0.0292	0.25
Nostoc linckia	0.10624	0.39
Novosphingobium pentaromativorans	0.03108	0.20
Oceanicaulis sp	0.0886	0.17
Oceanospirillaceae bacterium	0.1444	0.39
Oceanospirillales bacterium	0.09528	0.44
Ochrobactrum anthropi	0.0302	0.32
Ochrobactrum intermedium	0.22096	0.29
Odoribacter splanchnicus	0.06592	0.08
Oenococcus oeni	0.0294	0.08
Oligotropha carboxidovorans	0.1024	0.32
Onion yellows phytoplasma	0.0294	0.03
Opitutaceae bacterium	0.06668	0.42
Opitutae bacterium	0.03268	0.40
Orientia tsutsugamushi	0.04368	0.05
Oscillatoriales cyanobacterium	0.13788	0.24
Oscillibacter sp	0.11148	0.22
Paenibacillus amylolyticus	0.32076	0.34
Paenibacillus larvae	0.02844	0.21
Paenibacillus odorifer	0.29364	0.32
Paenibacillus polymyxa	0.03504	0.28
Paeniclostridium sordellii	0.14056	0.21
Pandoraea apista	0.33072	0.42
Pandoraea pnomenusa	0.18604	0.43
Pantoea agglomerans	0.03004	0.25
Pantoea ananatis	0.04888	0.35
Pantoea dispersa	0.22444	0.24
Pantoea stewartii	0.0292	0.26
Pantoea vagans	0.126	0.24
Parabacteroides distasonis	0.05904	0.10
Parabacteroides goldsteinii	0.06108	0.08
Parabacteroides merdae	0.06276	0.08
Paracoccus denitrificans	0.22124	0.43
Paracoccus yeei	0.03684	0.37
Parageobacillus thermoglucosidasius	0.0294	0.39
Parcubacteria group bacterium	0.02872	0.09
Pasteurella multocida	0.02916	0.20
Patescibacteria group bacterium	0.02572	0.06
Pectobacterium atrosepticum	0.06036	0.20
Pectobacterium brasiliense	0.20088	0.23
Pectobacterium carotovorum	0.1744	0.23
Pectobacterium odoriferum	0.18944	0.21
Pectobacterium parmentieri	0.19112	0.20
Pediococcus acidilactici	0.02916	0.08
Pediococcus claussenii	0.0294	0.08
Pediococcus damnosus	0.0294	0.08
Pediococcus pentosaceus	0.0294	0.08
Pedobacter sp	0.03096	0.53
Pelagibacteraceae bacterium	0.0282	0.24
Pelagibacterales bacterium	0.03536	0.27
Peptococcaceae bacterium	0.05452	0.25
Phaeobacter gallaeciensis	0.0294	0.37
Phaeobacter inhibens	0.03012	0.37
Phaeobacter piscinae	0.0294	0.34
Photobacterium angustum	0.25584	0.28
Photobacterium damselae	0.02852	0.42
Photobacterium kishitanii	0.3816	0.51
Photobacterium leiognathi	0.2282	0.25
Photobacterium phosphoreum	0.3272	0.50
Photorhabdus luminescens	0.18676	0.25
Phycisphaerae bacterium	0.03956	0.22
Phycisphaerales bacterium	0.04364	0.26
Piscirickettsia salmonis	0.02868	0.05
Planctomyces sp	0.03104	0.35
Planctomycetaceae bacterium	0.04804	0.37
Planctomycetes bacterium	0.02528	0.35
Planctomycetia bacterium	0.02656	0.26
Planktothrix agardhii	0.02948	0.24
Plesiomonas shigelloides	0.18728	0.21
Pluralibacter gergoviae	0.29664	0.31
Polynucleobacter asymbioticus	0.30272	0.36
Polynucleobacter necessarius	0.16772	0.25
Porphyrobacter donghaensis	0.27752	0.37
Porphyromonadaceae bacterium	0.0212	0.11
Porphyromonas gingivalis	0.02216	0.04
Porphyromonas gulae	0.0234	0.03
Porticoccaceae bacterium	0.08068	0.41
Prevotella copri	0.04656	0.08
Prevotella denticola	0.06248	0.08
Prevotella intermedia	0.02512	0.04
Prevotella ruminicola	0.05348	0.09
Prevotella sp	0.02488	0.11
Prochlorococcus marinus	0.04256	0.09
Propionibacterium freudenreichii	0.04288	0.14
Proteobacteria bacterium	0.02888	0.45
Proteus mirabilis	0.02936	0.19
Proteus vulgaris	0.0538	0.21
Providencia alcalifaciens	0.20484	0.28
Providencia rettgeri	0.0294	0.34
Providencia stuartii	0.02948	0.24
Pseudoalteromonas luteoviolacea	0.1696	0.19
Pseudoalteromonas piscicida	0.17068	0.20
Pseudoalteromonas rubra	0.1304	0.26
Pseudoalteromonas ruthenica	0.17196	0.18
Pseudoalteromonas sp	0.0304	0.59
Pseudomonadales bacterium	0.10676	0.53
Pseudomonas aeruginosa	0.0292	0.43
Pseudomonas amygdali	0.24744	0.39
Pseudomonas avellanae	0.27656	0.37
Pseudomonas brassicacearum	0.3758	0.44
Pseudomonas chlororaphis	0.27564	0.40
Pseudomonas cichorii	0.43176	0.47
Pseudomonas citronellolis	0.31896	0.40
Pseudomonas coronafaciens	0.33416	0.37
Pseudomonas fluorescens	0.02964	0.44
Pseudomonas fragi	0.0728	0.34
Pseudomonas frederiksbergensis	0.2316	0.42
Pseudomonas fulva	0.36456	0.40
Pseudomonas koreensis	0.34932	0.40
Pseudomonas lundensis	0.30092	0.31
Pseudomonas mendocina	0.2192	0.51
Pseudomonas monteilii	0.37136	0.47
Pseudomonas oleovorans	0.39596	0.50
Pseudomonas oryzihabitans	0.29312	0.34
Pseudomonas plecoglossicida	0.26552	0.46
Pseudomonas poae	0.30744	0.35
Pseudomonas protegens	0.3362	0.39
Pseudomonas psychrotolerans	0.275	0.37
Pseudomonas putida	0.02936	0.49
Pseudomonas savastanoi	0.0748	0.37
Pseudomonas sp	0.0294	0.55
Pseudomonas sp 286	0.22552	0.44
Pseudomonas stutzeri	0.02952	0.49
Pseudomonas synxantha	0.33852	0.37
Pseudomonas syringae	0.02876	0.46
Pseudomonas syringae group genomosp 3	0.0292	0.45
Pseudomonas veronii	0.33684	0.35
Pseudomonas viridiflava	0.34776	0.41
Psychrobacter sp	0.0294	0.40
Puniceicoccaceae bacterium	0.04184	0.18
PVC group bacterium	0.05204	0.22
Rahnella aquatilis	0.0294	0.25
Ralstonia mannitolilytica	0.3418	0.44
Ralstonia pickettii	0.0294	0.46
Ralstonia solanacearum	0.04456	0.49
Raoultella ornithinolytica	0.0296	0.32
Raoultella planticola	0.09464	0.30
Rathayibacter rathayi	0.2242	0.24
Rathayibacter toxicus	0.2246	0.22
Rhizobiales bacterium	0.076	0.48
Rhizobium etli	0.03196	0.35
Rhizobium gallicum	0.04196	0.26
Rhizobium leguminosarum	0.02924	0.43
Rhizobium phaseoli	0.0294	0.32
Rhizobium tropici	0.18276	0.38
Rhodanobacter sp	0.1194	0.37
Rhodobacter capsulatus	0.19656	0.25
Rhodobacter sphaeroides	0.0278	0.36
Rhodobacteraceae bacterium	0.06956	0.53
Rhodobacterales bacterium	0.04324	0.42
Rhodobiaceae bacterium	0.07664	0.44
Rhodococcus erythropolis	0.0294	0.31

Rhodococcus fascians	0.09572	0.22
Rhodococcus hoagii	0.0294	0.27
Rhodococcus opacus	0.02828	0.27
Rhodococcus pyridinivorans	0.12824	0.27
Rhodococcus qingshengii	0.27692	0.31
Rhodococcus rhodochrous	0.17128	0.27
Rhodococcus ruber	0.186	0.23
Rhodocyclaceae bacterium	0.16028	0.39
Rhodopirellula sp	0.1674	0.33
Rhodopseudomonas palustris	0.1438	0.40
Rhodospirillaceae bacterium	0.04316	0.43
Rhodospirillales bacterium	0.05048	0.47
Rhodovulum sulfidophilum	0.03236	0.35
Rickettsia amblyommatis	0.0294	0.04
Rickettsia japonica	0.03556	0.04
Rickettsia prowazekii	0.0314	0.03
Rickettsia rickettsii	0.0314	0.03
Rickettsiales bacterium	0.03088	0.37
Riemerella anatipestifer	0.0294	0.05
Rikenellaceae bacterium	0.03912	0.15
Robiginitomaculum sp	0.24964	0.36
Rodentibacter pneumotropicus	0.15896	0.18
Roseburia intestinalis	0.1562	0.16
Roseburia inulinivorans	0.16012	0.18
Roseburia sp	0.05932	0.21
Rossibacillus sp	0.14668	0.18
Rothia dentocariosa	0.13628	0.15
Rothia kristinae	0.07184	0.09
Rothia mucilaginosa	0.11092	0.16
Ruminococcaceae bacterium	0.03884	0.16
Ruminococcus albus	0.04316	0.12
Ruminococcus bromii	0.07212	0.08
Ruminococcus flavefaciens	0.07088	0.13
Ruminococcus sp	0.04132	0.26
Runella slithyformis	0.03756	0.34
Salinibacter ruber	0.02948	0.14
Salinispora arenicola	0.17368	0.25
Salinispora pacifica	0.1434	0.22
Salinispora tropica	0.15668	0.16
Salmonella bongori	0.10932	0.16
Salmonella enterica	0.0274	0.32
Saprospirales bacterium	0.0598	0.26
SAR116 cluster bacterium	0.12496	0.30
SAR86 cluster bacterium	0.03604	0.34
Selenomonas ruminantium	0.0294	0.15
Serratia fonticola	0.15868	0.27
Serratia liquefaciens	0.2014	0.27
Serratia marcescens	0.02936	0.32
Serratia plymuthica	0.19244	0.28
Shewanella algae	0.37932	0.40
Shewanella baltica	0.0294	0.43
Shewanella putrefaciens	0.33944	0.43
Shigella boydii	0.0294	0.31
Shigella dysenteriae	0.02984	0.30
Shigella flexneri	0.0288	0.31
Shigella sonnei	0.02844	0.35
Shigella sp	0.09388	0.31
Shinella sp HZN7	0.03124	0.12
Sinorhizobium americanum	0.06892	0.32
Sinorhizobium fredii	0.02988	0.35
Sinorhizobium medicae	0.11088	0.33
Sinorhizobium meliloti	0.0294	0.33
Snodgrassella alvi	0.1076	0.16
Sodalis glossinidius	0.02844	0.06
Solemya velum gill symbiont	0.21132	0.24
Solirubrobacterales bacterium	0.08628	0.28
Sorangium cellulosum	0.28984	0.49
Spartobacteria bacterium	0.04312	0.30
Sphingobacteriales bacterium	0.083	0.39
Sphingobacterium sp	0.0978	0.57
Sphingobium baderi	0.0294	0.19
Sphingobium sp	0.02876	0.38
Sphingobium sp TKS	0.02908	0.09
Sphingobium yanoikuyae	0.19036	0.39
Sphingomonadaceae bacterium	0.17468	0.37
Sphingomonadales bacterium	0.05616	0.38
Sphingomonas koreensis	0.243	0.32
Sphingomonas sp	0.02844	0.40
Sphingomonas wittichii	0.0294	0.25
Sphingopyxis macrogoltabida	0.02924	0.28
Spirochaetaceae bacterium	0.05952	0.31
Spirochaetales bacterium	0.08068	0.19
Spirochaetes bacterium	0.06428	0.26
Spiroplasma citri	0.0294	0.03
Staphylococcus agnetis	0.16808	0.21
Staphylococcus argenteus	0.15444	0.20
Staphylococcus arlettae	0.16692	0.21
Staphylococcus aureus	0.0282	0.36
Staphylococcus capitis	0.0294	0.21
Staphylococcus chromogenes	0.0294	0.19
Staphylococcus cohnii	0.14132	0.26
Staphylococcus delphini	0.14564	0.17
Staphylococcus devriesei	0.19564	0.20
Staphylococcus epidermidis	0.02892	0.22
Staphylococcus equorum	0.0294	0.23
Staphylococcus felis	0.15792	0.16
Staphylococcus gallinarum	0.19424	0.21
Staphylococcus haemolyticus	0.02916	0.24
Staphylococcus hominis	0.10492	0.19
Staphylococcus hyicus	0.0294	0.21
Staphylococcus lugdunensis	0.0294	0.20
Staphylococcus nepalensis	0.06696	0.22
Staphylococcus pasteuri	0.0742	0.20
Staphylococcus petrasii	0.18544	0.19
Staphylococcus pseudintermedius	0.1016	0.18
Staphylococcus saccharolyticus	0.13872	0.18
Staphylococcus saprophyticus	0.0294	0.22
Staphylococcus schleiferi	0.1896	0.21
Staphylococcus sciuri	0.0608	0.22
Staphylococcus simulans	0.0294	0.23
Staphylococcus succinus	0.20944	0.25
Staphylococcus warneri	0.0294	0.20
Staphylococcus xylosus	0.17176	0.23
Stenotrophomonas maltophilia	0.0294	0.41
Stenotrophomonas sp	0.08456	0.38
Streptobacillus moniliformis	0.0366	0.04
Streptococcus agalactiae	0.02916	0.13
Streptococcus anginosus	0.06152	0.09
Streptococcus constellatus	0.07316	0.08
Streptococcus cristatus	0.07	0.08
Streptococcus dysgalactiae	0.03732	0.07
Streptococcus equi	0.0462	0.11
Streptococcus equinus	0.05888	0.08
Streptococcus gallolyticus	0.06352	0.09
Streptococcus gordonii	0.07532	0.09
Streptococcus infantis	0.04836	0.07
Streptococcus iniae	0.049	0.06
Streptococcus intermedius	0.0746	0.10
Streptococcus mitis	0.05412	0.09
Streptococcus mutans	0.03816	0.10
Streptococcus oralis	0.03552	0.08
Streptococcus parasanguinis	0.05448	0.09
Streptococcus parauberis	0.05796	0.08
Streptococcus pneumoniae	0.0294	0.37
Streptococcus pseudopneumoniae	0.05988	0.10
Streptococcus pyogenes	0.0294	0.07
Streptococcus salivarius	0.04168	0.11
Streptococcus sanguinis	0.07648	0.10
Streptococcus sobrinus	0.06752	0.14
Streptococcus suis	0.0358	0.11
Streptococcus thermophilus	0.0294	0.09
Streptococcus uberis	0.0542	0.07
Streptomyces acidiscabies	0.32272	0.34
Streptomyces albidoflavus	0.28184	0.30
Streptomyces albus	0.27628	0.35
Streptomyces griseus	0.27528	0.35
Streptomyces hygroscopicus	0.086	0.34
Streptomyces lavendulae	0.15028	0.35
Streptomyces rimosus	0.33104	0.43
Streptomyces scabiei	0.29388	0.35
Sulfitobacter pseudonitzschiae	0.04404	0.24
Sulfurovum sp	0.09092	0.19
Synechococcus sp P1	0.10676	0.12
Synechocystis sp PCC 6803	0.02956	0.23
Synergistaceae bacterium	0.05952	0.17
Syntrophaceae bacterium	0.06772	0.27
Syntrophomonadaceae bacterium	0.07232	0.28
Tannerella forsythia	0.04864	0.06
Taylorella equigenitalis	0.07144	0.08
Terribacillus saccharophilus	0.10644	0.15
Tetragenococcus halophilus	0.0294	0.25
Thalassospira sp	0.13048	0.34
Thalassospira xiamenensis	0.22044	0.34
Thermus scotoductus	0.14964	0.25
Thermus thermophilus	0.0294	0.21
Thioalkalivibrio sp	0.07668	0.19
Thiotrichales bacterium	0.04204	0.25
Treponema denticola	0.06452	0.11
Treponema pallidum	0.04296	0.04
Treponema sp	0.04652	0.20
Trichormus variabilis	0.0294	0.18
Tropheryma whipplei	0.0524	0.05
Trueperaceae bacterium	0.05616	0.16
Trueperella pyogenes	0.07896	0.12
uncultured actinobacterium	0.07024	0.14
uncultured Actinomyces sp	0.07936	0.11
uncultured Alistipes sp	0.03332	0.07
uncultured bacterium	0.02832	0.10
uncultured Bacteroidales bacterium	0.027	0.11
uncultured Bacteroides sp	0.0376	0.10
uncultured Blautia sp	0.0892	0.24
uncultured Clostridia bacterium	0.04264	0.08
uncultured Clostridiales bacterium	0.03264	0.26
uncultured Clostridium sp	0.03444	0.26
uncultured Collinsella sp	0.05864	0.21
uncultured Coriobacteriaceae bacterium	0.06604	0.18
uncultured Desulfovibrio sp	0.13228	0.22
uncultured Dialister sp	0.0428	0.07
uncultured Eggerthella sp	0.06688	0.19
uncultured Erysipelotrichaceae bacterium	0.04312	0.16
uncultured Eubacterium sp	0.05556	0.23
uncultured Faecalibacterium sp	0.11232	0.17
uncultured Firmicutes bacterium	0.0328	0.07
uncultured Fusobacterium sp	0.1012	0.15
uncultured Lachnobacterium sp	0.09416	0.21
uncultured Lachnospiraceae bacterium	0.05396	0.24
uncultured Megasphaera sp	0.13744	0.19
uncultured Mollicutes bacterium	0.03536	0.07
uncultured Olsenella sp	0.08168	0.17
uncultured Opitutales bacterium	0.04296	0.05
uncultured Oscillibacter sp	0.14468	0.21
uncultured Parabacteroides sp	0.05352	0.09
uncultured Phascolarctobacterium sp	0.0456	0.14
uncultured Porphyromonadaceae bacterium	0.0282	0.08
uncultured Porphyromonas sp	0.02376	0.04
uncultured Prevotella sp	0.02252	0.10
uncultured Prevotellaceae bacterium	0.02624	0.10
uncultured proteobacterium	0.03508	0.17
uncultured Roseburia sp	0.08964	0.18
uncultured Ruminococcaceae bacterium	0.0408	0.16
uncultured Ruminococcus sp	0.044	0.25
uncultured Selenomonadales bacterium	0.0466	0.07
uncultured Solobacterium sp	0.05036	0.17
uncultured Sutterella sp	0.04456	0.17
uncultured Tenericutes bacterium	0.03152	0.06
uncultured Treponema sp	0.10696	0.15
uncultured Veillonella sp	0.14808	0.24
uncultured Veillonellaceae bacterium	0.04868	0.12
Ureaplasma parvum	0.02972	0.03
Ureaplasma urealyticum	0.02988	0.03
Variovorax paradoxus	0.42256	0.03
Veillonella atypica	0.1978	0.21
Veillonella parvula	0.19136	0.24
Verrucomicrobia bacterium	0.03044	0.37
Verrucomicrobia subdivision 3 bacterium	0.05248	0.35
Verrucomicrobiaceae bacterium	0.05692	0.28
Verrucomicrobiales bacterium	0.03748	0.36
Vibrio alginolyticus	0.02976	0.26
Vibrio anguillarum	0.0294	0.25
Vibrio breoganii	0.16	0.24
Vibrio campbellii	0.02884	0.25
Vibrio cholerae	0.0294	0.32
Vibrio coralliilyticus	0.03188	0.41
Vibrio crassostreae	0.2808	0.35
Vibrio cyclitrophicus	0.30632	0.34
Vibrio diazotrophicus	0.27596	0.42
Vibrio fluvialis	0.29016	0.45
Vibrio harveyi	0.06744	0.27
Vibrio jasicida	0.23796	0.26
Vibrio lentus	0.13592	0.37
Vibrio metoecus	0.19772	0.25
Vibrio mimicus	0.2182	0.26
Vibrio nigripulchritudo	0.15488	0.37
Vibrio owensii	0.23792	0.28
Vibrio parahaemolyticus	0.02916	0.28
Vibrio rotiferianus	0.18984	0.22
Vibrio sp F13	0.32244	0.34
Vibrio splendidus	0.28872	0.38
Vibrio tasmaniensis	0.19948	0.32
Vibrio vulnificus	0.0294	0.30
Weissella cibaria	0.0294	0.08
Weissella confusa	0.06896	0.08
Wohlfahrtiimonas chitiniclastica	0.20372	0.22
Wolbachia pipientis	0.03688	0.05
Xanthomonadaceae bacterium	0.04088	0.37
Xanthomonadales bacterium	0.1538	0.38
Xanthomonas albilineans	0.11508	0.27
Xanthomonas arboricola	0.21364	0.41
Xanthomonas axonopodis	0.12528	0.40
Xanthomonas campestris	0.0294	0.41
Xanthomonas citri	0.0294	0.42
Xanthomonas euvesicatoria	0.0294	0.37
Xanthomonas fragariae	0.0294	0.36
Xanthomonas gardneri	0.0294	0.37
Xanthomonas oryzae	0.0294	0.37
Xanthomonas perforans	0.09016	0.41
Xanthomonas phaseoli	0.0294	0.40
Xanthomonas translucens	0.19796	0.39
Xanthomonas vasicola	0.20372	0.39
Xanthomonas vesicatoria	0.0294	0.40
Xenorhabdus bovienii	0.12828	0.18
Xylella fastidiosa	0.0294	0.10
Yersinia enterocolitica	0.0294	0.20
Yersinia frederiksenii	0.1064	0.21
Yersinia intermedia	0.16208	0.19
Yersinia kristensenii	0.2014	0.25
Yersinia mollaretii	0.15016	0.16
Yersinia pestis	0.0292	0.15
Yersinia pseudotuberculosis	0.0294	0.18
Yersinia ruckeri	0.12704	0.17
Zetaproteobacteria bacterium	0.04584	0.21
Zymomonas mobilis	0.02916	0.11
